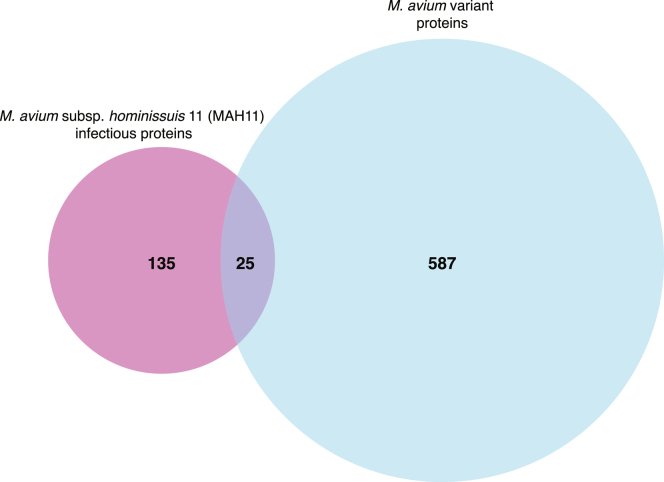# Development of a Spectral Library for the Discovery of Altered Genomic Events in *Mycobacterium avium* Associated With Virulence Using Mass Spectrometry–Based Proteogenomic Analysis

**DOI:** 10.1016/j.mcpro.2023.100533

**Published:** 2023-03-21

**Authors:** Chinmaya Narayana Kotimoole, Neelam Antil, Sandeep Kasaragod, Santosh Kumar Behera, Anjana Aravind, Norbert Reiling, Trude Helen Flo, Thottethodi Subrahmanya Keshava Prasad

**Affiliations:** 1 Center for Systems Biology and Molecular Medicine, Yenepoya Research Centre, Yenepoya (Deemed to Be University), Mangalore, India; 2 Institute of Bioinformatics, International Technology Park, Bangalore, India; 3 Microbial Interface Biology, Research Center Borstel, Leibniz Lung Center, Borstel, Germany; 4 German Center for Infection Research (DZIF), Site Hamburg-Lübeck-Borstel-Riems, Borstel, Germany; 5 Department of Clinical and Molecular Medicine Faculty of Medicine and Health Sciences, Centre of Molecular Inflammation Research, Norwegian University of Science and Technology, Øya, Norway

**Keywords:** *Mycobacterium avium*, proteogenomics, variant calling, DDA, DIA

## Abstract

*Mycobacterium avium* is one of the prominent disease-causing bacteria in humans. It causes lymphadenitis, chronic and extrapulmonary, and disseminated infections in adults, children, and immunocompromised patients. *M. avium* has ∼4500 predicted protein-coding regions on average, which can help discover several variants at the proteome level. Many of them are potentially associated with virulence; thus, identifying such proteins can be a helpful feature in developing panel-based theranostics. In line with such a long-term goal, we carried out an in-depth proteomic analysis of *M. avium* with both data-dependent and data-independent acquisition methods. Further, a set of proteogenomic investigations were carried out using (i) a protein database for *Mycobacterium tuberculosis,* (ii) an *M. avium* genome six-frame–translated database, and (iii) a variant protein database of *M. avium*. A search of mass spectrometry data against *M. avium* protein database resulted in identifying 2954 proteins. Further, proteogenomic analyses aided in identifying 1301 novel peptide sequences and correcting translation start sites for 15 proteins. Ultimately, we created a spectral library of *M. avium* proteins, including novel genome search-specific peptides and variant peptides detected in this study. We validated the spectral library by a data-independent acquisition of the *M. avium* proteome. Thus, we present an *M. avium* spectral library of 29,033 peptide precursors supported by 0.4 million fragment ions for further use by the biomedical community.

## Highlights

• Generated a *Mycobacterium avium* spectral library consisting of 29,033 peptide precursors.
• Discovered several novel proteins, and variants of *M. avium* by proteogenomic analysis.
• Demonstrated a cost effective DIA-based approach for future research on NTM’s.
• SNVs in *M. avium* proteome can act as species-specific for a future multispecies detection assay.

## Graphical Abstract

**Figure:**
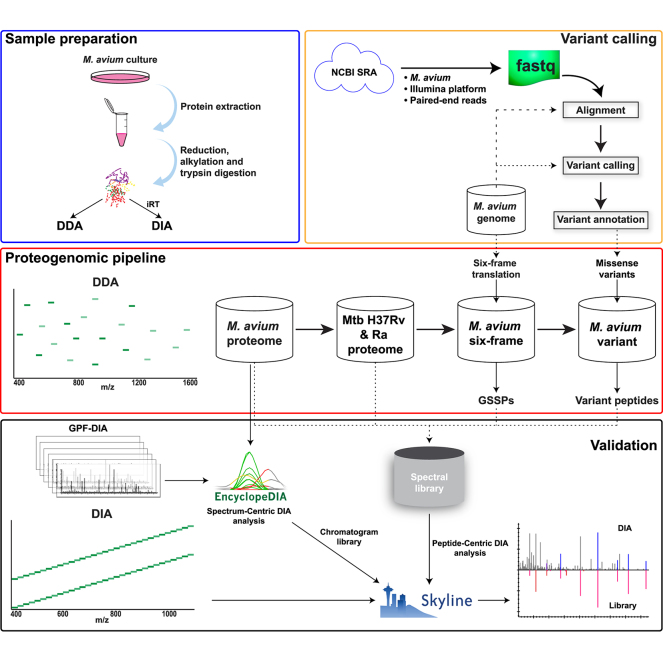

## In Brief

Detection of coinfecting pathogens in clinical samples is challenging owing to lack of tools and molecular data. Through a combination of in-depth proteomics and integrated genomic analysis, we identified several unique molecular leads in *Mycobacterium avium* with a potential to distinguish it from other coinfecting pathogens in a unique mass spectrometry–based data-independent acquisition assay. As and when more molecular data emanate from other coinfecting pathogens are available, this assay can be expanded for the detection of multiple pathogens.

Among 172 different non-tuberculous mycobacteria (NTM) species, *Mycobacterium avium* is one of the significant disease-causing bacteria in humans (1). *M. avium* Complex (MAC) is globally responsible for more than 50% of NTM infections in humans (2). The incidences of MAC infection are rapidly increasing worldwide. MAC is known to cause lymphadenitis, chronic pulmonary, extrapulmonary, and disseminated infections among adults, children, and immunocompromised patients (3, 4, 5, 6, 7). *M. avium* has been divided into four subspecies based on their average nucleotide identity and genome-to-genome distance pairwise values (8). Out of the four subspecies, *M. avium* subsp. *hominissuis* (MAH) infects mainly pigs and humans (9, 10, 11). Currently, diagnosis of *M. avium* in patients with pulmonary diseases is limited to a multiplexed PCR, growth rate, and staining (12, 13).

Mass spectrometry–based proteomic methods have been used to investigate the biology, pathophysiology, and virulence of infectious mycobacteria such as *M. tu*berculosis (*Mtb*), *Mycobacterium abscessus*, *M. avium*, *Mycobacterium bovis*, *Mycobacterium marinum*, *Mycobacterium smegmatis*, and *Mycobacterium vaccae* (14, 15, 16, 17, 18, 19). Numerous drug sensitive/resistant proteins, posttranslational modifications, and variants in *Mtb* have been identified by a combination of genomic and proteomic approaches, which can have ramifications in the development of newer diagnostic/prognostic tools to detect presence of tuberculosis and monitor treatment outcome (20). Such sort of experimental approach is yet to be executed for *M. avium*. Therefore, combining proteomic data with proteogenomic and variant proteins will be helpful for the in-depth mapping of the *M. avium* proteome.

Annotation of the *M. avium* genome is based on various computational pipelines such as Rapid Annotations Subsystem Technology, Prokka, and EMBOSS (21, 22). There are 36 complete genome assemblies of *M. avium* with an average of 4533 (SD ± 280) protein-coding regions (CDS) resulting from various gene model predictions. However, as is the case of any draft map of the genome, not all the predicted CDS are experimentally verified, leaving a scope open for further revisions (23). In the past, proteogenomics has emerged as a valuable tool in validating and correcting the genome annotation of various organisms, from bacteria to humans (24, 25, 26, 27, 28, 29). Proteogenomics was used to reannotate several mycobacteria species such as *Mtb*, *M. abscessus*, and *M. vaccae* (19, 30, 31, 32). Genome variation among *M. avium* subspecies is evident from several published reports. A study by Uchiya *et al.* (2017) (33) resulted in the identification of 101,139 single nucleotide variants (SNVs) from 79 *M. avium* isolates. In another study, variant calling from several genomes of MAH, *M. avium* subsp. *avium*, and *M. avium* subsp. *paratuberculosis* has shown that there were more SNVs (>11,500) in MAH and *M. avium* subsp. *avium* strains than the *M. avium* subsp. *paratuberculosis* K-10 strain (34). However, there are no reports of any pipeline that will detect these *M. avium* variants at the proteome level.

In the present study, we developed and executed a proteomic pipeline with a variant calling protocol, which can search nonsynonymous variants of *M. avium* proteins. This library of variants, ‘*M. avium* variant protein database,’ at the proteomic level was conceptually translated from genomic variants gathered from over 239 *M. avium* genomes from the NCBI database. Next, we performed shotgun proteomics for *M. avium* cultures by acquiring samples in the data-dependent acquisition (DDA) method. The resulting raw data were subjected to a proteogenomics pipeline using protein databases of *M. avium* and *Mtb* (H37Rv and H37Ra), an *M. avium* genome six-frame–translated database, and an *M. avium* variant protein database, sequentially. All four search results consisting of peptide spectrum matches (PSMs) were converted into a large spectral library of the *M. avium* proteome, comprising mass spectra of 29,033 peptide precursors. Besides several novel, revised, variant peptides, the spectral library contains peptide evidence for >62% of the *M. avium* proteome.

To further demonstrate the usability of spectral library for developing detection assay, we performed data-independent acquisition (DIA) of *M. avium* proteins followed by a spectral library search of the DIA-MS–derived data. DIA in proteomics is a very promising method, due to its dynamic quantification range and ability to detect peptides in their attomoles level with high reproducibility owing to its unbiased acquisition approach (35). A single DIA run can also identify more or equal numbers of proteins than the DDA of the same sample with offline fractionation (36). To our knowledge, this is the first such a library comprising novel and variant peptide spectral evidence for *M. avium*. Hence, the *M. avium* peptide spectral library generated in this study combined with the DIA methodology provides an exquisite and beneficial resource to drug mode-of-action studies, identification of novel therapeutic targets, virulence and pathogenicity, and studies aimed at identifying diagnostic or prognostic markers for *M. avium* infection.

## Experimental Procedures

### Experimental Design and Statistical Rationale

All the proteomics analysis was performed with *M. avium* strain 104 cultured in three biological replicates. All the biological replicates were processed together, and proteins were digested using trypsin. Post in-solution digestion, a pool of all the replicates, was fractionated using C18 StageTip and subjected to DDA in technical triplicates for in-depth proteomics. The unfractionated *M. avium* protein digest replicates were acquired in DIA mode in technical triplicates. Commercially available retention time peptide standards were spiked to each sample before acquiring them in the DIA method. Meanwhile, *M. avium* genome variants were detected by performing variant calling using raw paired-end reads of *M. avium* from the Illumina platform. Single amino acid variants, categorized as “missense variants,” were incorporated into the *M. avium* protein database. This database was searched with DDA-derived proteomics data, followed by proteogenomics analysis to identify novel protein sequences. The database search results were converted into spectral libraries and used for DIA data analysis in peptide-centric analysis. Peptides were identified with 1% false discovery rate (FDR, q-value < 0.01) in both database and spectral library searches of DDA and DIA, respectively. The *M. avium* representative reference database *M. avium* subsp. *hominissuis* strain OCU464 was downloaded from NCBI RefSeq (GCF_001865635.4_ASM186563v4, Release Date: 17-July-2020) and used in proteomics, proteogenomics, and variant calling experiments.

### Quality Check and Alignment of *M. avium* Genomes

The paired-end reads from the Illumina platform were subjected to quality check (QC) by running FastQC; due to the larger number of samples, MultiQC was used to visualize the QC results in a combined single plot (37). From the MultiQC analysis, a list of raw files with poor-quality reads was extracted and subjected to trimming using FASTX-Toolkit (38). The low-quality bases flagged by the Phred score ≤20 were subjected to trimming.

### Variant Calling Analysis

**Fig. 1:**
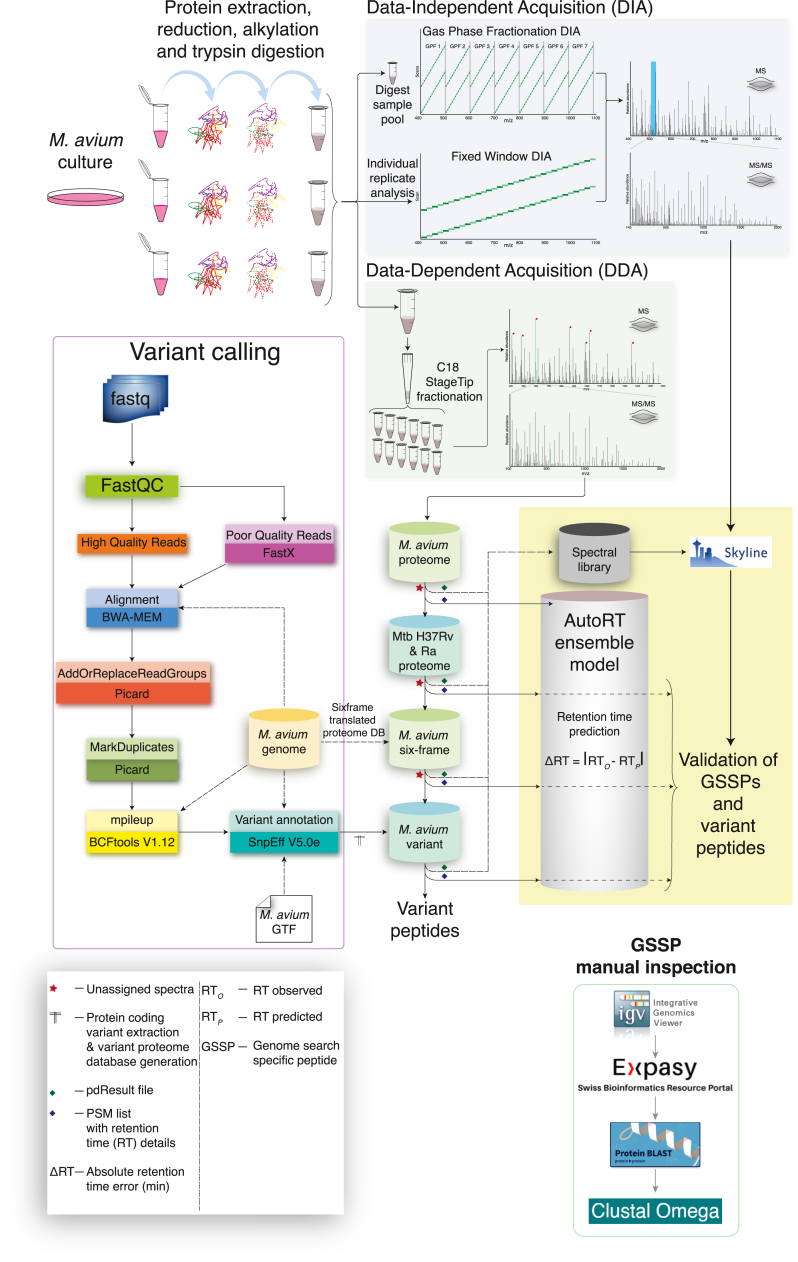
**A detailed workflow of the methodology.***Mycobacterium avium* cultures were processed for the bottom-up proteomics by reduction, alkylation, and overnight trypsin digestion. Digested samples were pooled and fractionated into 12 using C18 StageTips, followed by LC-MS/MS acquisition by 120 min data-dependent acquisition (DDA) method. Later, individual replicates of *M. avium* were desalted using C18 StageTips and acquired using the 120 min fixed isolation window (25 Da) data-independent acquisition (DIA) method. A pooled sample was acquired in-between fixed isolation window DIA method by gas-phase fractionation technique-based DIA method of overlapping 4 Da isolation scheme. Meanwhile, *M. avium*–specific genome sequence reads from the Illumina platform were subjected for variant calling pipeline using FastQC, BWA-MEM, AddOrReplaceReadGroups (Picard), MarkDuplicates (Picard), mpileup (BCFtools V1.12), and variant annotation using SnpEff V5.0e packages. Finally, raw DDA data was subjected for proteogenomics analysis by searching against the protein databases of *M. avium*, *Mycobacterium tuberculosis* (*Mtb*) H37Rv and H37Ra, *M. avium* six-frame–translated genome database, and a *M. avium* variant proteins database sequentially. All the database search results were converted into Skyline compatible spectral library, and raw DIA data was searched against the combined spectral library in Skyline as part of validation. The genome search-specific peptides from six-frame–translated genome database search were inspected manually and categorized. BWA, Burrows-Wheeler Aligner; MS/MS, tandem mass spectrometry.

Processed raw reads were aligned to the *M. avium* representative reference genome GCF_001865635.4_ASM186563v4 (Release Date: 17-July-2020, RefSeq, NCBI) using Burrows-Wheeler Aligner v 0.7.17 and converted to bam files using samtools v1.9 (39, 40). The genome-aligned bam files were subjected to “AddOrReplaceReadGroups” and “MarkDuplicates” analysis using Picard (https://broadinstitute.github.io/picard/). Finally, the variant calling was performed for all bam files from the previous step using the “mpileup” algorithm in BCFtools V1.12 (41). The resulting vcf files from variant calling analysis were annotated with the help of SnpEff V5.0e (42). The protein-coding variants were extracted from the SnpEff annotated vcf files, and those variants annotated as “missense variants” were conceptually translated into a protein database using an in-house Python script (https://github.com/chinmayaNK22/Variant-Proteome-DB-Generator) (Fig. 1).

### Proteomic Analysis

#### *M. avium* Culture and Sample Preparation

*M. avium* strain 104 (Taxon ID: 243243) carrying a cyan-fluorescent-protein–expressing plasmid (43) was cultured in 7H9 medium (BD Difco; Becton Dickinson) supplemented with 10% albumin dextrose-catalase (Becton Dickinson), 0.2% glycerol (Applichem), 0.05% Tween80 (Sigma Aldrich), and 20 μg/ml Kanamycin (Sigma Aldrich) to mid-log phase (OD600: 0.4–0.5) (43).

Cultures (28 ml ≥ 4 × 7 ml per experiment, n = 3) were centrifuged at 10,000 rpm for 10 min at 4 °C room temperature, and the supernatant was discarded. Sterility controls (blood agar, brain heart infusion, and LB medium) were performed for all precultures and main cultures. The pellet was resuspended in 500 μl cell lysis buffer (4% SDS, 1 mM of sodium orthovanadate, 2.5 mM of sodium pyrophosphate, and 1 mM of ß-glycerophosphate in 50 mM of triethylammonium bicarbonate (TEABC) and vortexed for 5 min). Cells were disrupted by sonication two times (with 5 min intermission on ice) in closed screw caps using a Branson Sonifier 450 II Classic (Duty cycle 50, Output Control constant; G. Heinemann Ultraschall-und Labortechnik) and a closed cylinder (Cup Horn ‘High Intensity’) device. A 1:1 ethanol/water mixture (0.1% dishwashing liquid) acted as an ultrasound transmitter and cooling medium at the same time and was constantly kept at 4 °C. This was followed by a 10 min incubation step at 90 °C. After centrifugation (20,000*g* for 10 min at 4 °C), the resulting supernatant was transferred to a new microcentrifuge tube, and the samples were lyophilized and stored at −80 °C.

The lyophilized *M. avium* lysate samples were resuspended with 100 mM of TEABC buffer and subjected to protein estimation by bicinchoninic acid–based colorimetric assay (Pierce, Thermo Fisher Scientific). Later, an equal amount of protein samples of *M. avium* were reduced with 10 mM of DTT at 60 °C for 45 min and alkylated with 20 mM of iodoacetamide with 30 min of incubation under the dark condition at room temperature. Finally, protein samples were subjected to overnight digestion with trypsin at a 1:20 enzyme-to-protein ratio. Post trypsin digestion, *M. avium* samples were desalted using C18 StageTips, where peptides bound to the C18 were eluted by passing 0.1% formic acid (FA) in 40% acetonitrile (ACN).

#### Basic Reverse-Phase Liquid Chromatography and DDA

The desalted *M. avium* peptide samples were loaded onto a C18 StageTip and fractioned into 12 fractions by passing elution buffers containing varying concentrations of ACN in 10 mM TEABC. All the 12 fractions were vacuum evaporated and subjected to DDA analysis using EASY-nLC 1200 liquid chromatography system connected to Orbitrap Fusion Tribrid (Thermo Fisher Scientific) mass spectrometer. Each fraction was resuspended with 0.1% FA (Mobile Phase A) and loaded onto a 96-well plate. The peptide mixture sample in the well was loaded onto a trap column (Acclaim PepMap 100, 75 μm × 2 cm, nanoViper, C18, 3 μm, 100 Å) and then made to elute out by passing 0.1% FA in 80% ACN (Mobile Phase B). The separation of peptides based on their hydrophobicity was performed in the analytical column (PepMap RSLC C18, 2 μm, 100 Å, 50 μm × 15 cm) by increasing the Mobile Phase B in gradient mode for 120 min at 300 nl/min flow rate. The column's equilibration before each run and sample loading was performed by passing mobile phase A (0.1% FA). The percentage of mobile phase B was increased gradually from 5% at 0 min to 30% in 100 min, and this was further increased to 60% in 4 min and 100% in another 7 min. Finally, the percentage of mobile phase B was kept at 100% for 9 min. The analytical column temperature was set to 45 °C during the complete sample acquisition process.

Peptides eluted from the column were ionized in positive ion mode at EASY-Spray source with spray voltage and ion transfer tube temperature of 1.9 kV and 275 °C, respectively. Ionized peptides were acquired in DDA mode with a 3 s cycle time. Under a FullMS scan, peptide precursors ranging between 400 and 1600 m/z were acquired in an Orbitrap mass analyzer with a mass resolution of 120,000. The automatic gain control (AGC) and maximum ion injection time (Max. IT) for the precursor scan were set at 2e5 and 50 ms, respectively. Precursors with charge state (z) between 2 and 7 and intensity ≥5e3 were separated using a Quadrupole with an isolation window of 1 m/z and isolation offset of 0.5 m/z. Quadrupole isolated precursors were fragmented under high-energy collision-induced dissociation (HCD) with normalized collision energy of 35 ± 3%. Finally, the fragment ions were acquired on Orbitrap mass analyzer at 60,000 resolutions. Tandem mass spectrometry (MS/MS) scan range mode was set to “Auto” with AGC and Max. IT being 1e5 and 200 ms, respectively.

#### Database Search

The raw files were searched against the *M. avium* protein database (*M. avium hominissuis* OCU464, Release Date: 17-July-2020, RefSeq, NCBI: 4757 sequences) using Sequest HT and MSAmanda search engines in Proteome Discoverer v2.2 (Thermo Fisher Scientific). A common contaminant protein database (116 sequences) was also included in the search. Trypsin was set as a protease enzyme with maximum missed cleavage of 2 and a minimum peptide length of 7 aa. The PSMs with precursor tolerance ≤10 ppm and fragment tolerance ≤0.02 Da were subjected to FDR calculation using the Percolator module. Commonly found modifications such as carbamidomethylation of cysteine were set as fixed modifications, whereas oxidation of methionine and acetylation of protein N-terminus were set as variable modifications. Finally, PSMs with a 1% FDR cutoff (q-value < 0.01) were considered true identifications and used for further analysis.

### Proteogenomic Analysis

The proteogenomic analysis was performed, as mentioned in Figure 1. The unassigned spectra files from the previous search against *M. avium* protein database were extracted and searched sequentially against (i) a nonredundant protein database of *Mtb* H37Rv and H37Ra strains (5745 sequences); (ii) a *M. avium* genome (GCF_001865635.4_ASM186563v4) six-frame–translated database (121,811 sequences); and (iii) a variant proteins database of *M. avium* (50,774 sequences) generated using missense variants identified from variant calling approach. The workflow and parameters as mentioned in the first database search was extended to this as well.

#### Genome Search-Specific Peptide Analysis

The peptides identified from the six-frame–translated genome and variant database search were used for genome annotation by computational and manual categorization. Firstly, peptides were mapped to the *M. avium* protein database and those peptides which did not map to the proteome were considered as genome search-specific peptides (GSSPs). By using the genome coordinates of GSSPs, we generated a GTF file for visualization in Integrative Genomics Viewer (44).

All the GSSPs were inspected manually using Integrative Genomics Viewer. The ORFs of each GSSP were extracted, translated into amino acid sequences, and subjected to protein-protein BLAST analysis against NCBI nonredundant database tool (https://blast.ncbi.nlm.nih.gov/Blast.cgi). Meanwhile, GSSPs were categorized into novel exons/N-terminal extensions/Alternative frames of translations based on the genome region in which the GSSP was falling. The MS/MS spectra of these GSSPs were manually inspected to ensure the correctness of peptide assignments based on the assignment of b and y ions for more than 60% of peptide sequence (including b-H2O, b-NH3, y-H2O, and y-NH3) (45).

### Variants in Virulence Factor

The search for virulence factors of *M. avium* was performed using an online tool VFanalyzer available in virulence factor database (VFDB) (46). *M. avium* representative reference genome file (*M. avium* subsp. *hominissuis* strain OCU464) was searched against the closely related genome, that is, the *M. avium* 104 strain available from the VFDB database. Genome coordinates were extracted for the ORFs of virulent factors, and the variants of peptides falling within these ORFs were fetched. Virulent factor class (VFclass), virulence factors, and related genes details were extracted for all the matched variants from the result file using in-house python scripts (https://github.com/chinmayaNK22/VFDB_result_fetcher).

### Quality Control of GSSPs and Variant Peptides by Retention Time Prediction

Retention time (RT; measured in minutes) for PSMs obtained from the search against the *M. avium* protein database were used for training an ensemble AutoRT v1.0 model (47). The peptide RT model was trained with 100 epochs, batch size of 64. The scaling method for RT transformation and early stop patience was set to “min_max” and 20, respectively. The option to reduce the learning rate was set when the metrics stopped improving. Finally, using the trained ensemble model, we predicted the RT of GSSPs and variant peptides. The median absolute RT error (MAE), that is, the median of the absolute differences between observed and predicted RT of a peptide sequence was considered a quality control metric.

Variant peptides detected from the search against a variant protein database were further subjected to class-FDR calculation based on transferred FDR strategy as reported by Fu and Qian (2014) (48). As mentioned in the original study by Fu and Qian (2014), all target and decoy peptide hits from the Sequest HT search were considered with the search engine score, that is, Xcorr. Further, we measured transfer-FDR using global FDR calculated for each peptide. Finally, a transfer-FDR cutoff of 1% (q-value < 0.01) was applied to calculate the false discovery/positive proportion (FDP) calculation and class-FDR error estimation for each variant peptide.

### Data-Independent Acquisition

Mass spectrometry data for three biological replicates of digested *M. avium* peptides were acquired in technical triplicates using a 120 min fixed window DIA method on an Orbitrap Fusion Tribrid mass spectrometer (Thermo Fisher Scientific) coupled with an EASY-nLC 1200 liquid chromatogram system. Columns, LC parameters, and conditions used in the DDA experiment were used for this acquisition. To acquire the samples in the DIA method, a fixed window isolation scheme was generated in Skyline v20.2 for the m/z range 400 to 1100 m/z with a 0.5 m/z margin width (49). An isolation scheme of 30 × 25 Da isolation windows (400–1121 m/z) was created, which was exported from Skyline in Thermo Fusion compatible format. The same was used to generate a method in, the Orbitrap Fusion Tribrid mass spectrometer and other parameters. Peptides were ionized in positive ionization mode with other source parameters such as spray voltage of 2.1 kV, ion transfer tube temperature of 275 °C, and S-Lens radio frequency of 55. Peptide precursors (MS1) falling in the range of 350 to 1500 m/z were acquired in the Orbitrap mass analyzer at a mass resolution of 120,000 in the “Profile” mode. Max. IT of 50 ms and AGC of 1e6 was set for the precursor scan. The precursor scan was followed by 30 MS/MS scans, where precursors within a 25 Da isolation window were subjected to fragmentation (MS/MS). The Max. IT of 60 ms, AGC of 1e6, and HCD Collision energy of 30% was set for the MS/MS scan. MS/MS fragments within the m/z range of 145 to 2000 m/z were acquired in the Orbitrap mass analyzer at a mass resolution of 30,000 in the “Centroid” mode.

#### Gas-Phase Fractionation of Pooled *M. avium* Digest Sample

A pool of digested *M. avium* replicates was acquired seven times as part of gas-phase fractionation (GPF) in between the fixed window DIA runs, as mentioned by Searle *et al.* (2020) and Pino *et al.* (2020) (50, 51). Here, the LC-MS/MS parameters from the fixed window DIA method were used except for the precursor isolation window. A total of seven precursor isolation schemes were generated in Skyline v20.2 for the MS range 400 to 1100 m/z with a staggered/overlapping isolation window of 4 Da. In total, seven DIA methods were generated and each GPF acquisition was used with a particular method.

#### Generation of Chromatogram Library From GPF Data

The GPF raw files were converted to mzML format using ProteoWizard. Filters such as demultiplex (Overlap only) with 10 ppm mass error and peak picking at MS1 and 2 levels were used. All the seven mzML files were searched against the Prosit generated predicted peptide spectral library and *M. avium* protein database using the EncyclopeDIA and Walnut search algorithms present in EncylopedDIA (version 0.9.5) (52, 53). The search was performed with default parameters in Target/Decoy approach. Trypsin was set as an enzyme with maximum missed cleavage set to 1. Peptide precursors and fragments with mass tolerance cutoff of 10 ppm were searched with carbamidomethylation (C+57) as a fixed modification. HCD (Y-only) fragmentation model was used in both searches. PSMs from both searches were validated using Percolator (version v3-01). Peptide precursors with charge state 2 to 3 and 5 quantitative ions were searched in the spectral library search. Finally, the PSMs identified with 1% FDR from individual GPF files were merged and saved as a chromatogram library.

#### The Generation of *M. avium* Peptide Spectral Library

All four database search result files from Proteome Discoverer (pdResult) were converted into Skyline v20.2 compatible spectral library format (blib) using the standalone BlibBuild algorithm from the Bibliospec package (54). Individual spectral library files (blib format) corresponding to each database search were merged, and a single spectral library was generated using the BlibBuild algorithm. To check the quality and features of this library, the library was converted to OpenSwath compatible tsv format using EncyclopeDIA (53), and the library characteristics were assessed using the DIALib-QC v1.2 tool (55).

#### DIA Data Analysis by Spectral Library Search

Raw DIA files were searched against all four spectral libraries simultaneously in Skyline (Fig. 1). Peptides identified in the chromatogram library were used to calibrate an indexed retention time model consisting of 14 retention time standard peptides. Peptide precursors from spectral libraries were associated with a combined protein database (*M. avium* proteogenomic databases). This database was prepared by merging the *M. avium* protein database with the other three protein databases used earlier in the proteogenomic analysis. In the peptide settings, trypsin [KR|P] was set as an enzyme with two maximum missed cleavages. Enforce peptide uniqueness was set to None. Peptides with a minimum of 5 to a maximum of 80 amino acids were used for the search. Carbamidomethylation of cysteine was set as fixed modification, whereas oxidation of methionine and acetylation of protein N-termini were set as variable modifications. An equal number of decoy peptide precursors were generated by reversing the target peptide sequences and incorporating them into the Skyline document.

Peptide precursors with charge state 2 to 7 and fragment ions (b, y, and p) from “ion 3” to “last ion” with charge state 1 and 2 were set under the filter tab of transition settings. N-terminal to proline as a special ion and use DIA precursor window for exclusion were also selected. For peptide precursors found to have a MS/MS, library spectrum were searched with ion match tolerance cutoff of 0.02 Da. A minimum of three to a maximum of six most intense product ions were selected from the filtered product ions. Fragment ions within the m/z range of 50 to 2000 m/z were considered. Under the Full-Scan tab, MS and MS/MS filtering parameters were set such as the following: isotope peaks included: count, precursor mass analyzer: Orbitrap, peaks: 3, resolving power: 120,000 at 200 m/z, acquisition method: DIA, product mass analyzer: centroided, isolation schema: 400 to 1100 m/z with 25 m/z isolation window and 0.5 m/z margins, mass accuracy: 20 ppm, use high-selectivity extraction and use only scans within 5 min of predicted RT were set. Peptide precursors extracted with less than six transitions (three precursors + three fragments) were discarded. Finally, peptide precursors were reintegrated using a decoy-trained mProphet peak scoring model. Peptide precursors with 1% FDR (q-values < 0.01) were considered true identifications.

## Results

### *M. avium* Genome Variants

To identify the genomic variants of *M. avium*, we downloaded 497 paired-end fastq files sequenced using the Illumina platform for *M. avium* from NCBI Sequence Read Archive (SRA: https://www.ncbi.nlm.nih.gov/sra). A sequence QC was performed on all the raw reads (fastq) and those raw read sequences having per base sequence quality (Phred score) ≤20 in their lower quartile range were trimmed out using the *fastx_trimmer* command. Finally, 928 raw files were subjected to Burrows-Wheeler Aligner alignment, and based on the alignment, we have considered only 239 genomes (bam) with mapping coverage of >80% for variant calling and annotation.

**Supplemental Figure S1:**
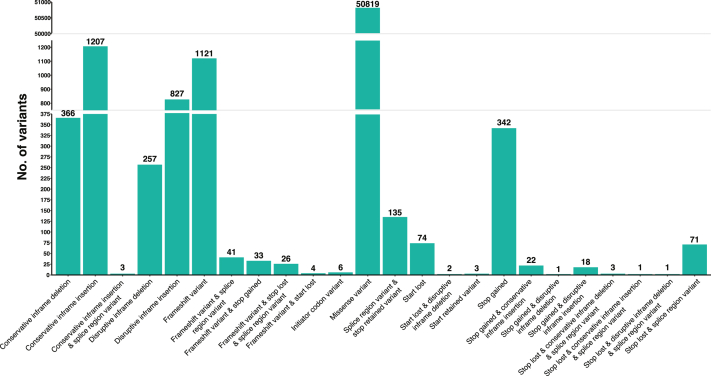


Variant calling using bcf tools, followed by protein annotation and impact prediction using SnpEff, resulted in the identification of 24 different types of nonsynonymous variants consisting of 55,383 SNV in total, with 50,819 representing missense variants. We have also observed indels and other variant types apart from missense variants. The bar plot in supplemental Fig. S1 represents the number of variants found in each type. SNVs identified from the SnpEff analysis were found to annotate 3897 *M. avium* proteins. The putative variant impact prediction of each variant has helped in cataloging 1760 high, 53,479 moderate, and 144 low impact nonsynonymous variants. Supplemental File S1 consists of annotated variants detected from the variant calling pipeline and the percentage alignment of input reference genomes.

### Database Search Results of *M. avium* DDA Data

**Fig. 2:**
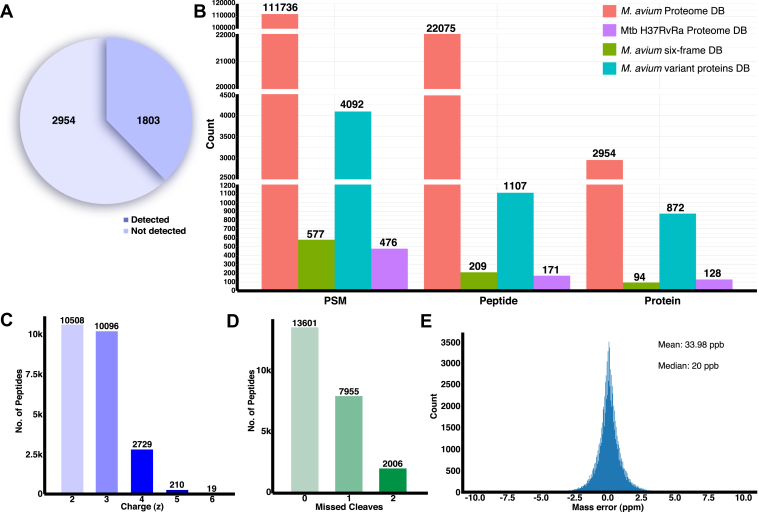
**Summary of database search results and quality features.***A*, grouped bar plot shows the number of peptide spectrum matches (PSMs), peptides, and proteins identified in each database search of the proteogenomic pipeline. *B*, a pie chart shows that 62.09% of the proteins were detected (2654) and 37.91% of the proteins were not detected (1803) from the *Mycobacterium avium* database search. *C*, peptides identified with different charge states were depicted in a bar plot, which shows that 87.44% of the peptides were detected with z = 2 or 3. *D*, bar plot shows that 57.72% (13,601) of the peptides were identified with zero missed cleavages, whereas remaining peptides were detected with either one or two missed cleavages. *E*, mass error between theoretical and experimental peptide precursor m/z were summarized using a histogram, which shows that a good number of peptides were identified with a mass error of ±2.5 ppm with mean and median being 33.98 ppb (0.03398 ppm) and 20 ppb (0.02 ppm), respectively.

A search of DDA-derived MS/MS data against the *M. avium* protein database has resulted in the identification of 111,736 PSMs, 22,075 peptides corresponding to 2954 proteins. It has covered 62.09% of the current proteome of *M. avium*, with at least one unique peptide supporting each of the 2954 proteins (Fig. 2*A*). Peptides corresponding to those proteins identified with single unique peptides were annotated and provided in supplemental Document 1. The subsequent search of unassigned spectra against a nonredundant protein database of *Mtb* H37Rv and H37Ra strain resulted in identifying 171 peptides (476 PSMs) corresponding to 128 proteins. Later, search of the unassigned spectra against the *M. avium* genome six-frame–translated database from the previous search against *Mtb* database has resulted in 209 peptides supported by 577 PSMs. The final search of the pipeline, where unassigned spectra from the search against *M. avium* genome six-frame–translated database, was then searched against the variant protein database. This resulted in the identification of 4092 PSMs for 1107 peptides corresponding to 872 proteins. Peptide sequences identified from the sequential database searches of unassigned spectra were mapped to the *M. avium* proteome to check for their specificity to the database. A grouped bar plot (Fig. 2*B*) depicts the identifications from database searches. Supplemental File S2 consists of the proteins and peptides identified from the *M. avium*, *Mtb* H37Rv and H37Ra, *M. avium* six-frame–translated genome, and *M. avium* variant proteins database searches, respectively.

### Peptide Quality Metrics

**Supplemental Figure S2:**
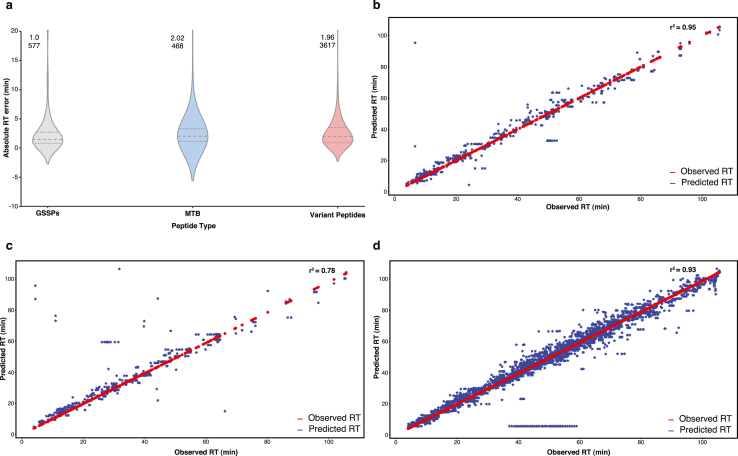


The accuracy of identified peptides was evaluated based on the maximum number of missed cleavages, charge states, number of PSMs supporting each peptide, mass error, and MAE of RT. We identified 116,881 PSMs from the Sequest HT search engine with a median and mean mass error of 20 and 33.98 ppb, respectively (Fig. 2*E*). Out of 23,562 peptides, 78.8% (18,586) were identified with two or more PSMs. Summarization of peptides identified with different charge states (z) shows that 87.4% of peptides were identified with z = 2 (10,508) and/or z = 3 (10,096). Similarly, peptides with zero, one, and two missed cleavages were observed at 57.7%, 33.8%, and 8.5%, respectively. Figure 2, *C* and *D* show the number of peptides identified with different charge states and missed cleavages. The MAE for peptides from *Mtb* protein database (468 peptides), *M. avium* six-frame–translated genome database (577 peptides), and *M. avium* variant proteins database (3617 peptides) searches were found to be 2.02 min, 1.0 min, and 1.96 min, respectively (supplemental Fig. S2).

### Novel Peptides Identified in *M. avium*

As part of the proteogenomic analysis, the database searches resulted in identifying 1301 novel peptide sequences and correcting translation sites of 15 proteins. Of these, 171, 143 (GSSPs), and 987 peptides were observed from the searches against *Mtb* protein database, *M. avium* genome six-frame–translated database, and *M. avium* variant protein database, respectively. These novel peptides corresponded to 771 *M. avium* protein sequences. These peptides with their annotated spectra are provided in pdf format in a compressed Supplemental Document 1.

### GSSPs and Their Categories From Proteogenomic Analysis

**Fig. 3:**
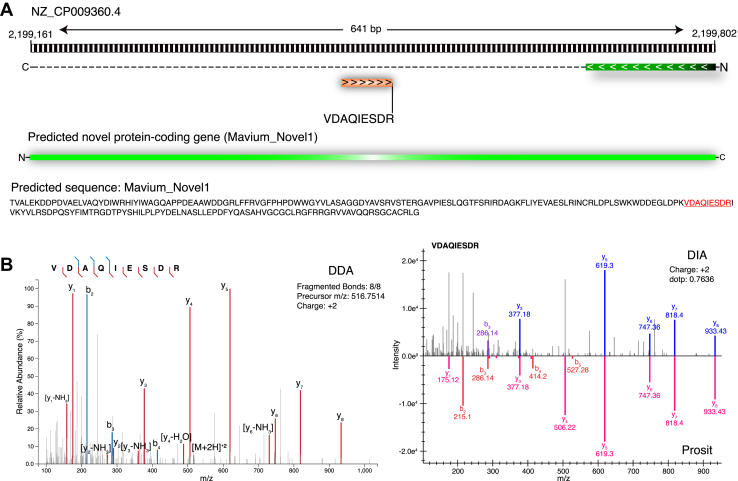
**A genome search-specific peptide “VDAQIESDR” was identified in the genome region NZ_CP009360.4: 2,199,161-2,199,802 and the manual inspection shows the presence of a novel gene.***A*, the genome region in which the novel exon identified has been depicted with the novel gene-coding protein sequence. *B*, the supporting spectral evidence from database search of data-dependent acquisition (DDA) and spectral library search of data-independent acquisition (DIA) has been shown with peptide precursor m/z, charge state, and number of fragments supporting the peptide sequence. The mirror spectrum match plot from the spectral library search of DIA data shows the number of fragments matching to the spectral library (*blue*) and the predicted spectra for the same peptide from Prosit (*red*).

Among 209 identified peptides from the *M. avium* genome six-frame–translated database, we observed 174 peptide sequences exclusive to the current search, mapping at least to a single locus of the translated protein database. These peptide sequences were considered GSSPs and subjected to manual inspection and categorization. From the manual categorization, we observed a single GSSP for a novel gene, 142 GSSPs providing coding evidence for 30 pseudogenes, and 23 GSSPs for N-terminal extensions in 15 proteins/genes. For example, the novel GSSP *VDAQIESDR* (516.7517 m/z, z = 2) was found lying in the genomic region (NZ_CP009360.4: 2,199,161-2,199,802). This GSSP was supported by three PSMs with highest *Xcorr* of 2.82 (Fig. 3). The BLAST analysis of the corresponding ORF (NZ_CP009360.4: 2,199,161-2,199,802) covering this novel protein-coding region was found matching to a “hypothetical protein” with two paralogous pieces of evidence within the strains of *M. avium* (WP_134799608.1, WP_134797029.1) and two orthologous pieces of evidence (*M. sp. UM_CSW*: WP_152531577.1, *Mycobacterium colombiense*: WP_139334793.1). The identification of GSSP *VDAQIESDR* in the spectral library search against the DIA (q-value: 0.005) and the absolute RT error of 1.5 min validate the same and show the quality of identification.

**Supplemental Figure S3:**
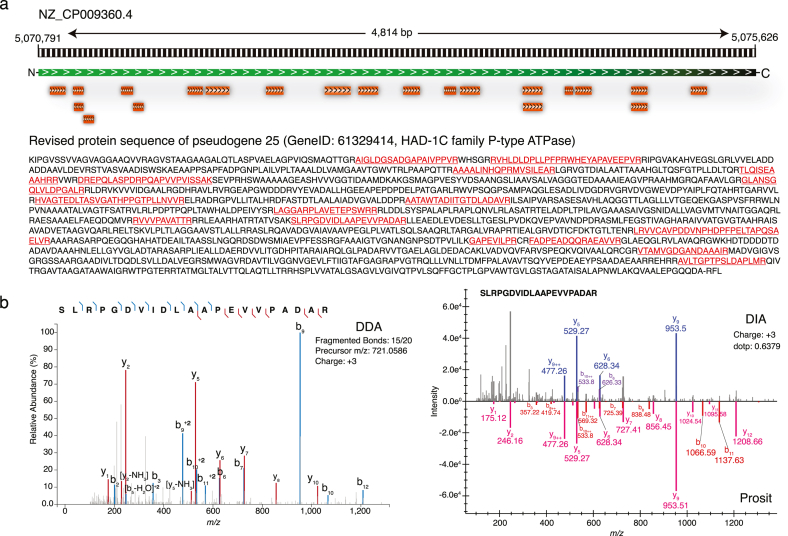


The translated CDS (predicted protein-coding regions) sequence file for the *M. avium* reference genome assembly (GCF_001865635.4) used in the current study has 4936 CSDs. Of these, 106 CSDs are categorized as pseudogenes, most probably because of incomplete sequencing or missing amino acids in their sequences. However, our proteogenomic analysis, followed by identifying GSSPs and their categorization, has shown that 30 of these translated CSDs, tagged as pseudogenes, have protein-coding evidence. Out of these 30 proteins identified, 21 were supported by ≥2 GSSPs and the remaining nine were supported by only one GSSP. For example, 21 GSSPs were aligned to the genome region between NZ_CP009360.4: 5,070,791-5,075,626 and this region was predicted and categorized as a pseudogene with locus tag KV38_RS23765. From the BLAST analysis, we found paralogous evidence from other strains of *M. avium* and orthologous evidence from other NTMs (ORB80990.1: *Mycobacterium timonense*; ATA27309.1: *Mycobacterium lepraemurium*) for the same. All the 142 GSSPs coding for pseudogenes were found to have absolute RT differences ranging from 0.05 to 10.73 min, and their *Xcorr* range was between 1.53 and 5.27. The DIA spectral library search also could detect 96 GSSPs with q-value <0.01 out of 142 GSSPs observed. Out of the remaining 46 GSSPs, five each were detected with q-value in the range of ≥0.01 to <0.02 and ≥0.02 to <0.05, respectively, whereas there were 17 GSSPs within ≥0.05 and <0.1 and 19 GSSPs with q-value ≥0.1, respectively. Supplemental File S3 contains all the GSSPs identified and details of their categorization from *M. avium* genome six-frame–translated database search. Their identification in DIA spectral library search also mentioned with q-value. As an example, supplemental Fig. S3 depicts the protein-coding evidence with revised sequence and spectral evidence from DDA and DIA experiments for pseudogene 25.

### Correction of the Translation Start Sites

**Fig. 4:**
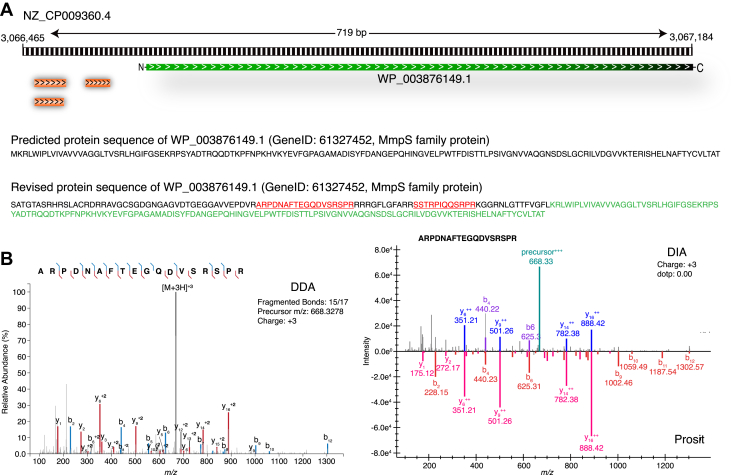
**An example of N-terminal extension for the MmpS family protein was observed (WP_003876149.1, GeneID: 61327452) with the identification of three genome search-specific peptides.***A*, the figure contains the protein sequence of MmpS family protein in the *Mycobacterium avium* protein database and the revised protein sequence after the identification of GSSPs in the N-terminus. *B*, peptide spectrum match and spectral library match evidence for the GSSP: ARPDNAFTEGQDVSRSPR from data-dependent acquisition (DDA) and data-independent acquisition (DIA) were depicted. From the database search result of DDA raw data, we could fetch the information on the amino acids supported by the identification of b and y ions. GSSP, genome search-specific peptide.

From the manual inspection of identified GSSPs, we have also observed 23 GSSPs supporting N-terminal extensions of 15 predicted CDSs. Of 23 GSSPs, 16 were supported by ≥2 PSMs, whereas the remaining seven GSSPs were supported by one PSM. The *Xcorr* of these 23 GSSPs ranged from 1.83 to 4.67. Spectral library search of DIA data shows that there were 17 GSSPs observed with q-value <0.01, three GSSPs within ≥0.02 and <0.05, two GSSPs within ≥0.05 and <0.1, and one GSSP with q-value ≥0.1, respectively. For example, the genome coordinates for the MmpS family protein from the predicted gene model was NZ_CP009360.4: 3,066,764-3,067,184, whereas the identification of GSSP *SSTRPIQQSRPR* (11 PSMs), *ARPDNAFTEGQDVSRSPR* (10 PSMs), and *ARPDNAFTEGQDVSR* (1 PSMs) in the same frame has shifted the translating start site to 3,066,465 from 3,066,765 and the revised genome coordinates for the same is NZ_CP009360.4: 3,066,465-3,067,184 (Fig. 4). This extension has resulted in the addition of 99 amino acids at the upstream region of the MmpS family protein. The N-terminal extensions of all the 15 genes were revised, and Table 1 lists all the N-terminal extensions, gene ID, gene description, and revised genome coordinates.

**Table 1:** List of N-terminal extensions observed from the manual inspection and annotation of genome search-specific peptides

Categorization	No. of GSSPs supporting	Gene ID	Description	Revised genome coordinates	Paralogous evidence	Orthologous evidence
5′ Extension	1	61425681	Pas domain-containing protein/histidine kinase	NZ_CP009360.4:1,022,782-1,023,180	ANR90392.1, AJK76050.1	AFC42246.1, ORA91558.1
5′ Extension	1	61425964	Paal family thioesterase	NZ_CP009360.4:1,337,739-1,338,185	AAS04803.1, ETB11634.1	ORB78349.1, ATA28052.1
5′ Extension	1	61426200	HAD-IB family hydrolase	NZ_CP009360.4:1,585,254-1,587,083	ANR89967.1, AGL36453.1	ATA29005.1
5′ Extension	1	61426320	Hypothetical protein	NZ_CP009360.4:1,731,347-1,731,850	ETA93188.1, ETA92417.1	ETA92417.1, TPW14379.1
5′ Extension	2	61426620	Cell division protein Ftsz	NZ_CP009360.4:2,061,228-2,062,393	WP_003872223.1, ELP46292.1	ATA28514.1, BBX43184.1
5′ Extension	3	61427452	MmpS family protein	NZ_CP009360.4:3,066,465-3,067,184	AAD44232.1	AFC44300.1
5′ Extension	2	61427635	Secondary thiamine-phosphate synthase enzyme YjbQ	NZ_CP009360.4:3,268,080-3,268,481	ABK64853.1, AAS03393.1	ORA14910.1, KKC02471.1
5′ Extension	1	61425057	SpoIIE family protein phosphatase	NZ_CP009360.4:400,971-401,597	ETZ49082.1, ANR91639.1	
5′ Extension	1	61428555	Hypothetical protein	NZ_CP009360.4:4,182,300-4,182,818	ETA95479.1, AAS05957.1	BBY68684.1, BCO64283.1
5′ Extension	4	61425081	Endonuclease III	NZ_CP009360.4:418,921-419,718	ETA94536.1, ETB21651.1	ATA27549.1, ATA27549.1
5′ Extension	2	61428740	50S ribosomal protein L18	NZ_CP009360.4:4,374,590-4,374,997	Q73S93.1, ETB47533.1	OSC25636.1, OBH44735.1
5′ Extension	1	61425172	Cholesterol catabolism transcriptional regulator K	NZ_CP009360.4:514,367-514,114	AAS02808.1, ETA91460.1	ASL07318.1, AGZ50936.1
5′ Extension	1	61429502	ParA family protein	NZ_CP009360.4:5,173,523-5,174,608	PBA23989.1, AYJ07438.1	
5′ Extension	1	61425597	M23 family metallopeptidase	NZ_CP009360.4:932,114-933,239	AAS03214.1, ETA91451.1	ATA27836.1
5′ Extension	1	61425648	GNAT family N-acetyltransferase	NZ_CP009360.4:982,760-983,849	OUZ02391.1, KBR64521.1	ATA27865.1, ORA54110.1

It also consists of Gene ID, gene description, revised genome coordinates with paralogous and orthologous protein evidence found from BLAST analysis.

### SNVs Expressed at the Proteome Level of *M. avium*

**Fig. 5:**
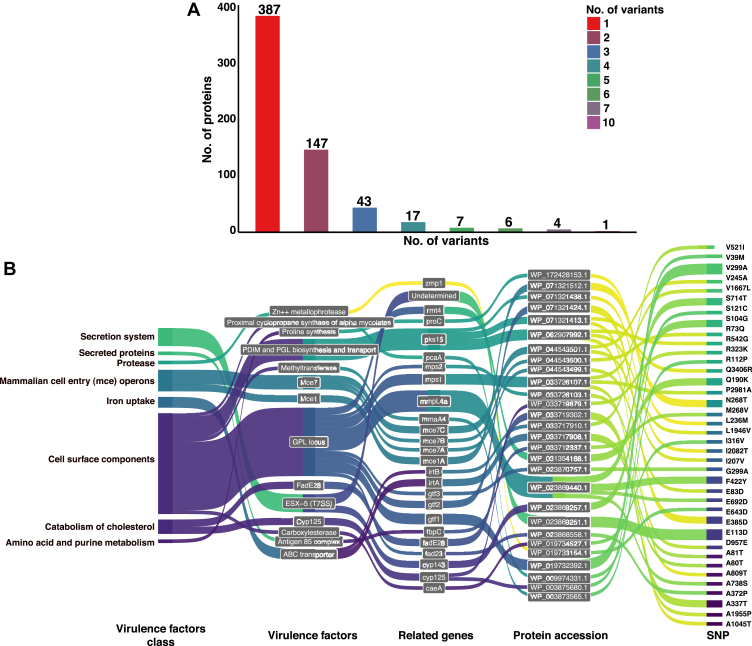
**Proteome level evidence for several variants of proteins was observed from the variant proteins database search as part of proteogenomic pipeline.***A*, number of *Mycobacterium avium* proteins identified (612) with the different number of missense variants after the variant proteins database search has been plotted using a bar graph. *B*, Sankey plot represents the correlation between 38 SNVs in 25 proteins known to be a part of 14 virulent factors of *M. avium*. This virulent factor search was performed using virulent factor database (VFDB). SNV, single nucleotide variant.

Out of 1107 peptides from the variant database search, we found 987 variant peptide sequences corresponding to 795 SNVs on 612 proteins of *M. avium*. The uniqueness of the variant peptide was checked by matching it with *M. avium* protein and variant protein databases using an in-house python script (https://github.com/chinmayaNK22/Variant-Proteome-DB-Generator). By performing BLAST analysis of 612 variant proteins, we fetched the corresponding protein sequence in *Mtb* H37Rv (Release 4, Mycobrowser, https://mycobrowser.epfl.ch/), MAH11 (GCA_003122745.2), and MAH104 (GCF_000014985.1) strains. From the BLAST analysis, we observed 539, 611, and 607 variant proteins matching to the *Mtb* H37Rv, MAH11, and MAH104 proteins with e-value <1e-10, respectively. In Figure 5*A*, the number of *M. avium* proteins identified with the different missense variants has been plotted using a bar plot. From the class-FDR calculation for variant peptides, we found 2241 PSMs corresponding to 700 peptides (572 variants) with class-FDR <0.01. There were another 753 PSMs corresponding to 487 peptides (440 variants) observed within 0.01> class-FDR <0.05. The mean class-FDR error (class-FDR < 0.01) was found to be 0.001767% with SD of ±0.0025%. PSMs corresponding to variant peptides detected by the percolator (q-value < 0.01) and their class-FDR values, FDP, and class-FDR error is available in supplemental File S6. The least squares regression curve was plotted using the proportion of variant peptides among decoy peptides at varying Xcorr thresholds (Supplemental Document 2). The tail generated in the high-score region was excluded as mentioned by Fu and Qian (2014). Coefficient correlation (r) was calculated for decoy peptides within 0.4 > Xcorr < 2.0.

The VFDB search shows that there were 38 SNVs found in 25 genes corresponding to 14 various virulent factors (Fig. 5*B*). These variant proteins known to have virulent factors were part of eight different virulent factor classes; cell surface components, catabolism of cholesterol, mammalian cell entry operons, iron uptake, secretion system, amino acid and purine metabolism, protease, secreted proteins. Variant peptides detected with information on their observed RT, experimental RT, genome-level variation information, variant annotation impact identified in several raw genome sequences and variant peptides identified in *M. avium* virulence factors are available in supplemental File S4.

### Quality Features of the Spectral Library Created for *M. avium* Proteome

**Fig. 6:**
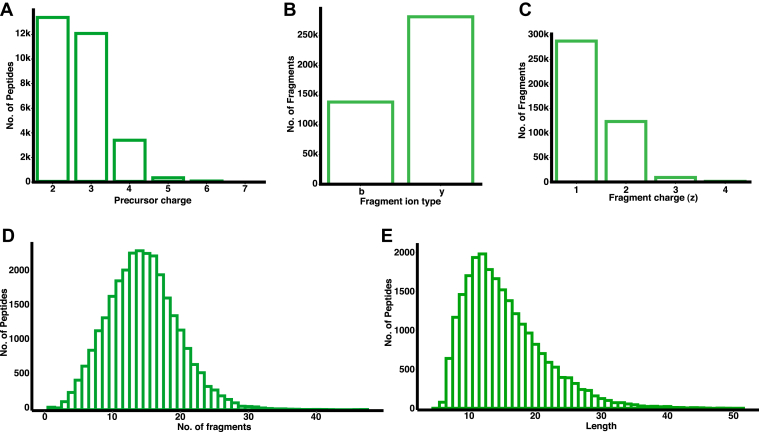
***Mycobacterium avium*****spectral library features.** Such as peptide precursor charge state (*A*), total number of b and y fragment ion detected (*B*), charge state distribution at the fragment ion level (*C*) are represented using the bar plots. Distribution of number of peptides with fragment detected (*D*) and peptide length (*E*) are depicted using histograms.

The evaluation of the quality and features of the final *M. avium* peptide spectral library using DIALib-QC analysis has led to the summarization of 41 various characteristics. The library was found to have 29,033 peptide precursor ions supported by 420,510 fragment ions with 1% FDR. The precursor charge distribution shows that 84% of the peptides were detected with z = +2 or +3. Among all the peptide precursors available in the library, 28,175 (97%) of the precursors were detected with ≥6 fragment transitions. Retention time characteristics of peptide precursors show the minimum and maximum RT in the library are 3.90 and 114.28 min, respectively. A number of y fragment ions were detected more in number than b fragment ions with 67.1% and 32.9%, respectively. More than 97% of the fragment ions were detected with charge state +1 or +2, out of which a greater number of +1 (68.3%) than +2 (29.3%) charge state ions were observed. From the fragment ion charge state summary, various library features are plotted using ggplot2, and seaborn packages in R and Python are available in Figure 6, respectively.

### Reproducibility of GSSPs and Variant Peptides by DIA

**Fig. 7:**
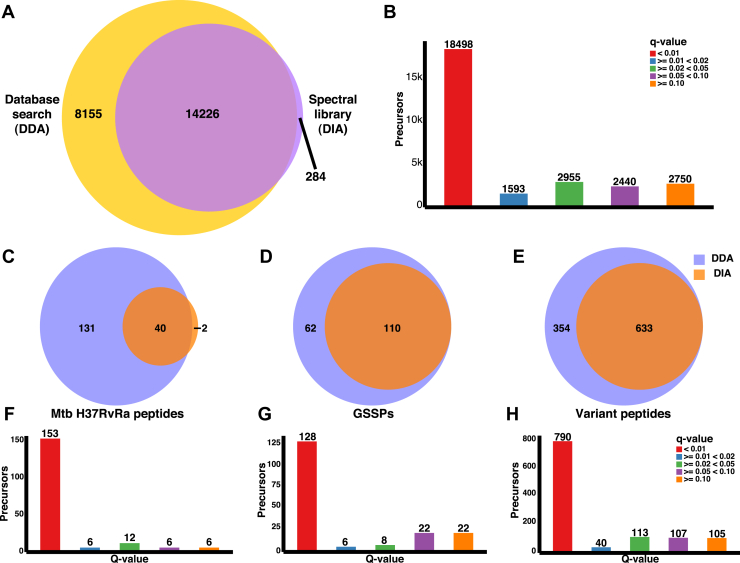
**Comparison of data-independent acquisition and data-dependent acquisition search results.***A*, the Venn diagram shows the number of common and experiment-specific nonredundant peptide sequences identified in both DDA and DIA analysis with 1% false discovery rate (FDR) (q-value < 0.01). *B*, a bar plot shows the number of peptide precursors identified in different q-value bins after the spectral library search of DIA data. *C*, common and experiment-specific (*C*) *Mycobacterium tuberculosis* (*Mtb*) H37RvRa peptides, (*D*) genome search-specific peptides (GSSPs), (*E*) variant peptides identified with 1% FDR from DDA and DIA data analysis. Number of peptide precursors specific to (*F*) *Mtb* H37Rv and H37Ra protein database, (*G*) *Mycobacterium avium* (*M. avium*) six-frame–translated genome database, (*H*) *M. avium* variant proteins database identified with different q-values after the spectral library searches were falling in different q-value bins were plotted. DDA, data-dependent acquisition; DIA, data-independent acquisition.

Spectral library search of *M. avium* DIA data has identified 18,498 peptide precursors with q-value <0.01 corresponding to 14,510 nonredundant peptide sequences. The detailed list of all the transitions and peptides identified in the DIA analysis is available in supplemental File S5. When we compared the 22,381 nonredundant peptide sequences identified from the DDA search with the 14,510 peptide sequences from the DIA analysis, we observed 14,226 common to both searches (Fig. 7*A*), that is, 63.56% of all the peptide sequences identified in DDA. The remaining 8155 peptides were not detected in DIA with 1% FDR (q-value < 0.01). There were 284 peptide precursors in the library, which were categorized as low rank/confident hits in the DDA database search. The same was observed in the spectral library search DIA data with q-value <0.01. Similarly, we compared the peptide sequences identified in *Mtb* H37Rv and Ra protein database, six-frame–translated genome, and variant protein database searches with the results from spectral library search of DIA data to check the common and search-specific peptides (Fig. 7, *C*–*E*).

Eventhough DIA is a highly reproducible targeted acquisition approach and RT-calibrated spectral library search is the sensitive method for detecting low abundant peptides, many peptide sequences get detected with higher q-value. Such occurrences in DIA analysis could be because of factors such as low peptide amount in the sample, unfractionated sample, co-elution followed by ion suppression, or poor fragmentation resulting in fragment spectra in the noise level. Therefore, peptides with q-value >0.01 cannot be ruled out as complete false identification or not present in the raw data acquired for the sample. Because true peptides can get identified with q-value >0.01, we have plotted bar graphs for peptide precursors placed in five different q-value bins, that is, <0.01, ≥0.01 and <0.02, ≥0.02 and <0.05, ≥0.05 and <0.1, and ≥0.1. The bar plot was generated for all the peptide precursors searched in DIA (Fig. 7*B*), *Mtb*-specific peptides (Fig. 7*F*), GSSPs (Fig. 7*G*), and variant peptides (Fig. 7*H*).

## Discussion

The high throughput sequencing of several strains of *M. avium* provides the base for identifying genomic features and detecting variants in the genome. The protein database search, proteogenomic analysis, and variant calling were performed using a representative *M. avium* genome and protein database from the assembly GCF_000007865.1 from the strain OCU464. This complete genome assembly is made of 5,275,873 bp (Size = 5.28 Mb) consisting of 3 scaffolds (Chromosome: NZ_CP009360.4/CP009360.4; plasmid p18K: NZ_CP009406.2/CP009406.2; plasmid p78K: NZ_CP009405.2/CP009405.2) with 68.94 GC%. There were 4797 CDS, which were found along with rRNAs, tRNAs, noncoding ribonucleic acids, repeat region, and pseudogenes.

The DDA of C18 fractions of the *M. avium* proteomics sample resulted in the generation of 0.76 million (762,373) MS/MS scans. The first search of MS/MS data against the *M. avium* proteome helped to annotate only 14.65% of the MS/MS scans. The remaining were considered unassigned. The presence of mass spectra without any peptide assignment is a common phenomenon in mass spectrometry–based proteomics. The lack of assignment of spectra can be due to several factors such as expression of novel protein sequences, errors in sequence annotation, single amino acid variants, and/or posttranslational modifications (56). To overcome this challenge and extract maximum information from the unassigned spectra, we executed proteogenomic analysis after the search against *M. avium* protein database. The proteogenomic analysis pipeline consisted of iterative searches of unassigned MS/MS spectra against three custom generated hypothetical protein databases. The first search was against the protein database of *Mycobacterium tuberculosis*, a well annotated organism among closely related species. The second one was against a database of hypothetical protein sequences obtained by conceptual translation of the *M. avium* genome in six-frames. As it is known, this search has aided in identifying/extending the protein start sites and novel proteins (57). Finally, the third database search was performed against a variant protein database as it will help identify protein variants that could cause diseases or virulence, identify species-specific proteins, and study genome variation in *M. avium*.

Genes or groups of genes encode virulence factors and are known to be the reason behind the virulence of bacterial pathogens such as *M. avium* (58). VFDB is a repository consisting of pathogenic species-specific virulent factors and genes. The VFDB search of current genome assembly GCF_000007865.1 against the *M. avium* strain 104 predicts the ORFs corresponding to 186 virulent genes, out of which 25 genes were found with one or more variants in our variant database search (supplemental File S4). For example, among these variants identified in virulent genes, the detection of variants “E113D” and “S104G” in protein EspG (WP_023869251.1) and cytochrome P450 (WP_023869257.1) belonging to the ESX-5 (type VII secretion system - T7SS) virulence factor may have a significant role in the biology and pathogenesis of *M. avium*. The type VII secretion systems are known as the 6 kDa early secretory antigenic target (ESAT-6) secretion systems (ESX systems). In *M. avium*, the ESX system secrets PPE (PPE25-MAV) and PE-PGRS proteins, known to promote intracellular survival, host–pathogen interactions, and virulence (59, 60). Similarly, four variants in PPE domain-containing protein, that is, WP_031354086.1 (P182Q, N89D) and PPE family protein, that is, WP_062887804.1 (Y97F) and WP_033719961.1 (S327T) were also observed. Phylogenetic analysis of *M. avium* subspecies based on SNVs by Uchiya *et al.* (2017) (33) has shown that there is a high percentage of polymorphic sites in CDS corresponding to ESX systems (ESX-1 to ESX-5) and which is considered as one of the “hot spot regions”. A transposon mutagenesis study by Dragset *et al.* (2019) (61) on MAH strains identified 160 genes of MAH11 required for infection in mice. We have also observed that there were 25 variant proteins identified in our current study that were considered as infection-specific genes of MAH (supplemental Fig. S4).

Glycopeptidolipids (GPL) are mycobacterial cell wall components. In *M. avium*, based on the presence and absence of the GPL layer on the cell wall surface, they are categorized into smooth opaque, smooth transparent, and rough (62, 63). The GPL locus is one of the virulence factors considered for identifying the virulence of the NTMs based on the presence and absence of the GPL layer. In our study, we have identified variations in proteins encoding the GPL locus. Proteins encoded in this locus are involved in GPL modifications and transportation through the membrane. For example, gtf1 (WP_019732392.1: V299A), gtf2 (WP_071321424.1: E385D), gtf3 (WP_023870757.1: G299A) were found to be involve in glycosylation, rmt4 (WP_031354168.1: Q190K) involves in methylation, mps1 (WP_033726107.1: P2981A, A1955P, V1667L), mps2 (WP_033726103.1: D957E), and fad23 (WP_023868558.1: A372P) are involved in lipid biosynthesis, and mmpL4a (WP_023869440.1: F422Y, E692D, A738S, S714T) was found to be involved in transportation (64).

Validation of GSSPs was performed either by enrichment approaches such as Western blot, ELISA or by performing the LC-MS/MS analysis of synthetic peptides (27, 28). In the case of LC-MS/MS analysis, targeted acquisition approaches such as selected/multiple reaction monitoring or parallel reaction monitoring assays were used for the validation of peptides identified in the proteogenomics pipeline (65, 66). The resulting tandem mass spectra of synthetic peptides were correlated or compared with annotated peptide spectra from database searches with the help of tools such as Skyline or manually to confirm its presence and to reduce any false positive identifications. Although manual inspection is the best resort in validating MS/MS spectra-based identifications, it is laborious when a large number of peptides are to be inspected. Moreover, the quality of such an interpretation of peptide spectral annotation differs among individuals. Thus, we employed DIA, another targeted acquisition method, which is considered to provide more accurate and reproducible results with unbiased isolation of precursors, fragmentation, and acquisition of fragments (67).

We performed DIA analysis of the unfractionated *M. avium* lysate and subjected the MS/MS data to a spectral library search for re-identification of peptides. A similar approach was employed earlier by Schubert *et al.* (2013) (36) on *Mtb* proteogenomic results with the help of selected reaction monitoring as a validation method. The peptide-centric analysis of DIA data against the peptide spectral library is considered more accurate since the peptide transitions are extracted and validated within the stipulated RT window predicted using high-precision calibrated indexed retention time peptides (68). Later, features such as library intensity dot-product, relative intensities, co-elution, peak shape similarity mass error, and other information accessible from a spectral library search were fetched for extracted ion chromatograms within the stipulated RT window. These feature scores were used for statistical validation using the mProphet algorithm and to confirm the presence of peptides by removing any false positive hits based on the q-value cutoff (69). The reproducible identification of peptides in DIA over DDA was observed in the current study as well. Our comparison of peptide precursors and their RTs across technical replicates in DDA and DIA shows a similar trend. The DIA spectral library search has resulted in the consistent detection of 98.8% (28,004/28,317) peptide precursors over 87.5% (15,873/18,143) from DDA with RT percentage coefficient of variance (%CV) <2%. Thus, the development of spectral library and its use in DIA data analysis have been found advantageous over carrying out the DDA of fractionated samples (70).

NTMs are often found to be codetected with *Mtb* (13). Several SNVs have been reported to cause drug resistance; at least more direct evidence is available in the context of *Mtb* (20). Genomic analysis of clinical samples to simultaneously detect *Mtb* and NTMs is still developing. DIA-based data acquisition and analysis to identify specific peptides of NTMs and *Mtb* will provide a lucrative alternative platform in the management of mycobacterial infections. Towards this long-term goal, we generated a spectral library for a critical NTM – *M. avium*.

## Conclusion

A systematic in-depth proteomics analysis followed by a proteogenomic analysis provided protein level evidence for several well-known, hypothetical, and novel proteins of *M. avium*. In addition, a search of mass spectrometry data against an SNV-based conceptual protein variant database identified 795 SNVs at the proteome level, which can further provide a list of candidates of proteotypic peptides specific to *M. avium* and can be effective in the development of a future multispecies detection assay. The presence of novel and variant peptides from proteogenomic analysis in the library and the use of DIA will have a synergistic effect as this resource, with a high throughput acquisition technique, can be used in diagnostics, drug development, or for a deeper understanding of the biology of *M. avium*.

## Data Availability

All the proteomic RAW files and database search result files are available at the PRoteomics IDEntifications (PRIDE) repository under the ID PXD032067. Spectral libraries generated from protein database searches, SnpEff outputs, in-house scripts used in the current study are available in Zenodo (https://doi.org/10.5281/zenodo.7321904).

## Supplemental data

This article contains supplemental data.

## Conflict of interest

The authors declare no competing interests.

## Figures and Tables

**Supplemental Figure S4:**