# Supplementary figures and images for: Development of a Spectral Library for the Discovery of Altered Genomic Events in Mycobacterium avium Associated With Virulence Using Mass Spectrometry–Based Proteogenomic Analysis
Source: Mol Cell Proteomics. 2023 Mar 21;22(5):100533. doi: 10.1016/j.mcpro.2023.100533 (PMC10149365; doi:10.1016/j.mcpro.2023.100533)

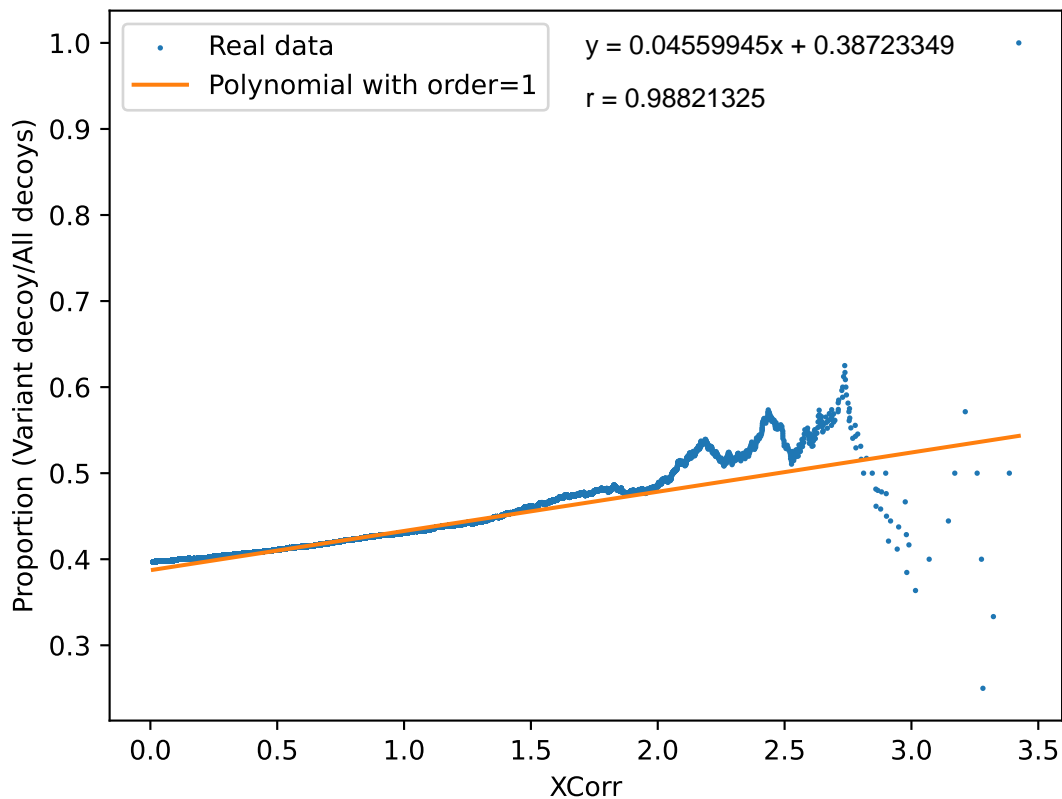

Supplement: Supplemental Data 1 [file mmc7.pdf]

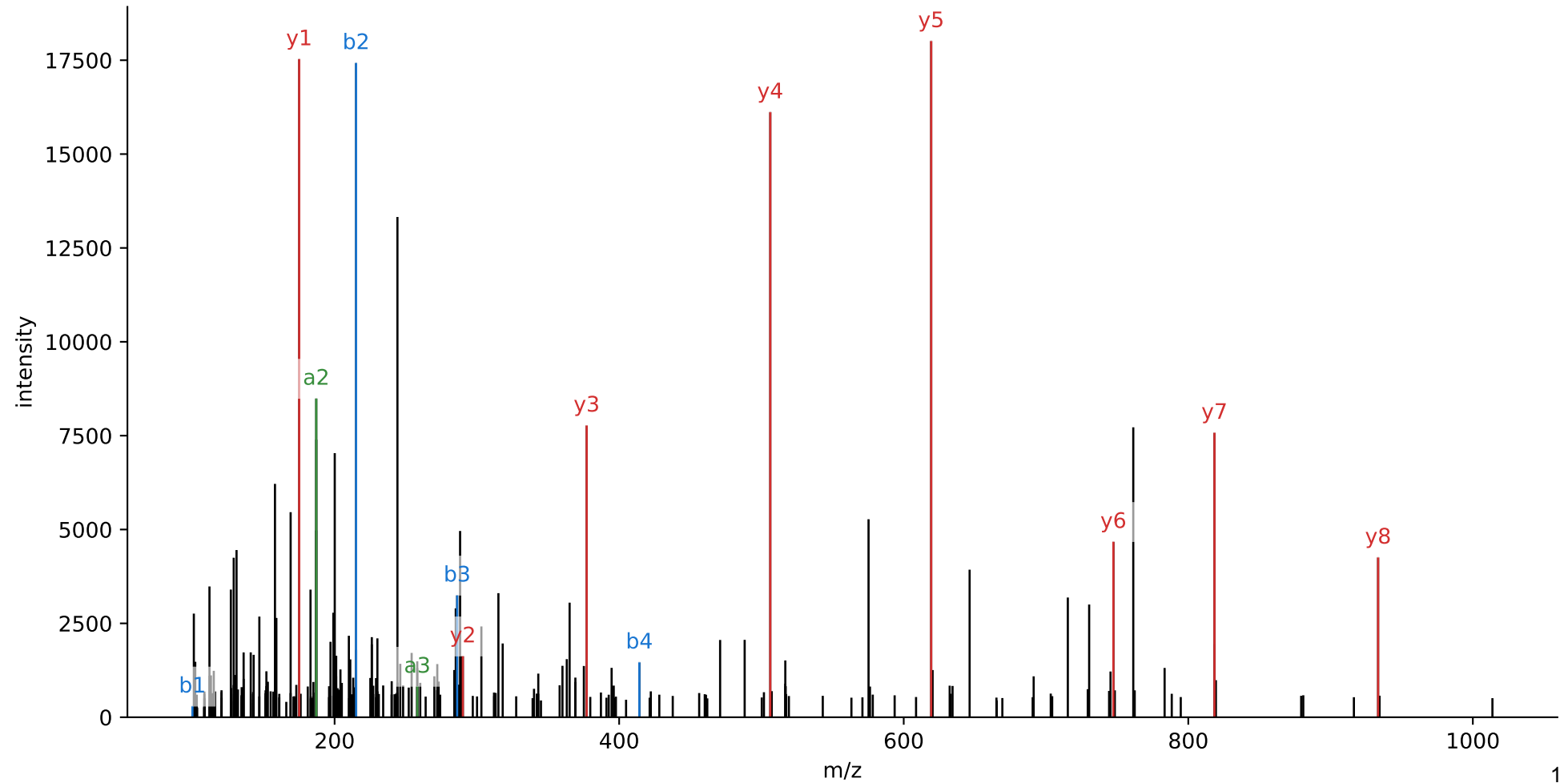

Supplement: Supplemental Data 5 [file mmc11.pdf]

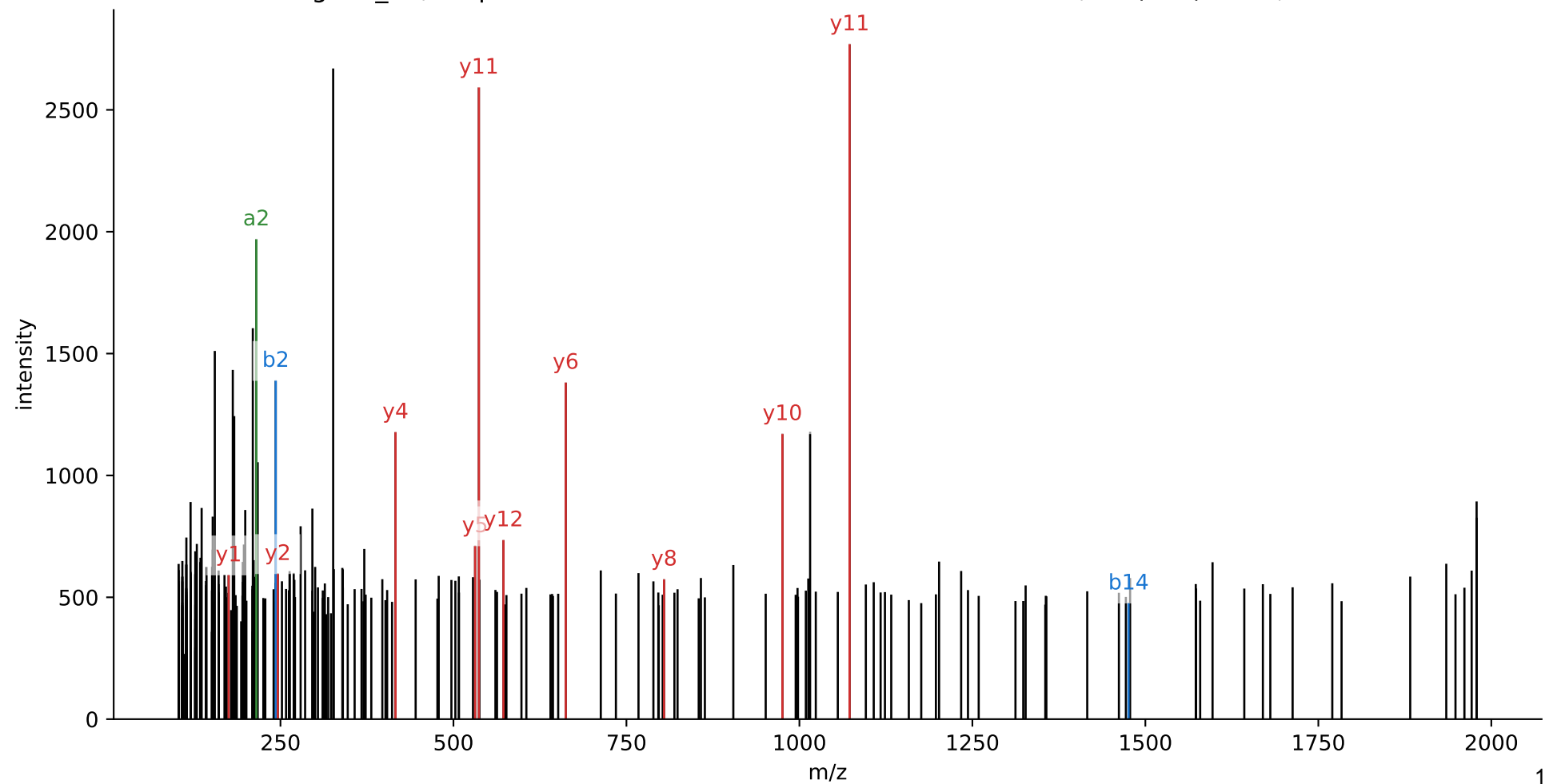

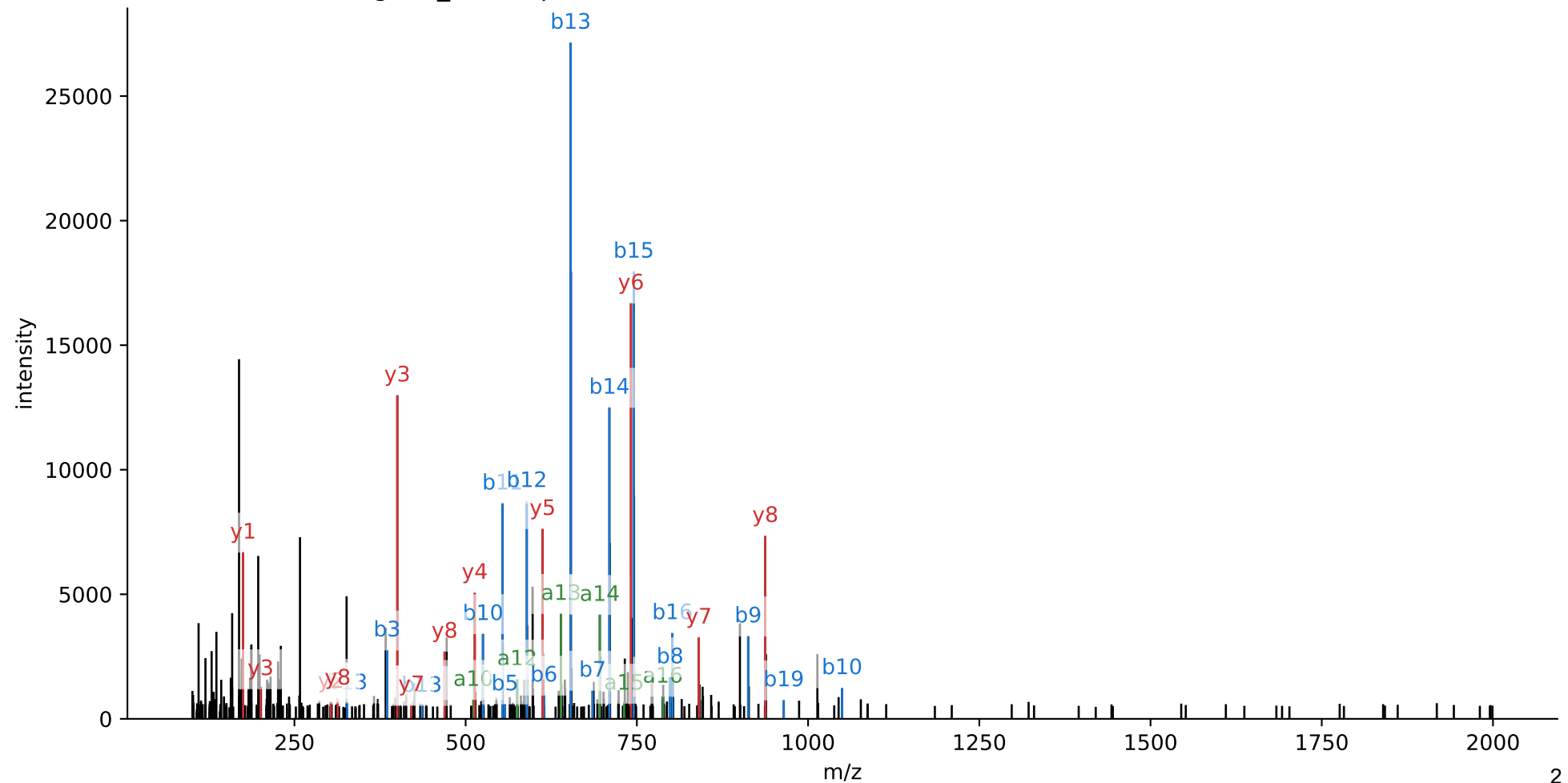

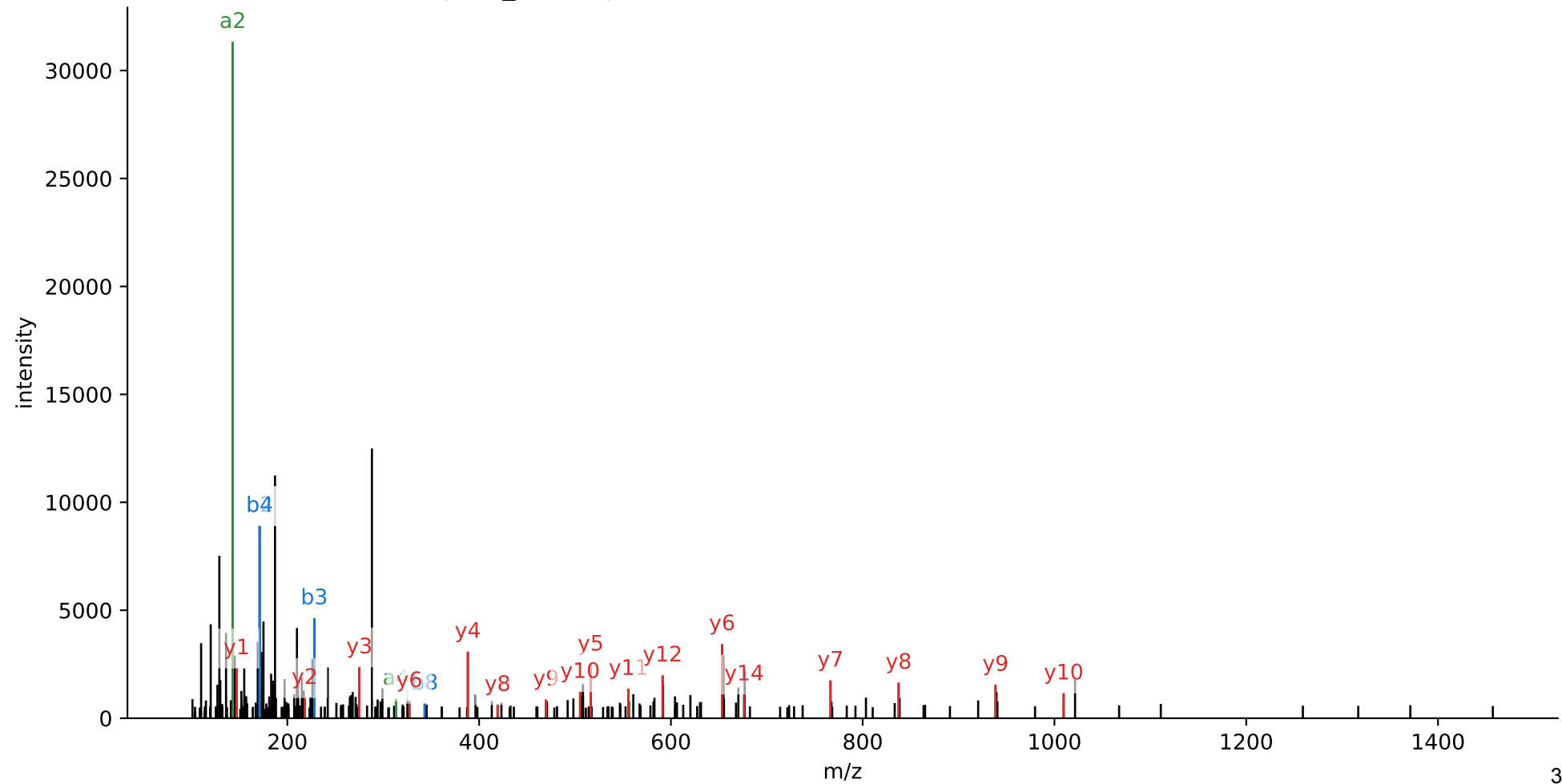

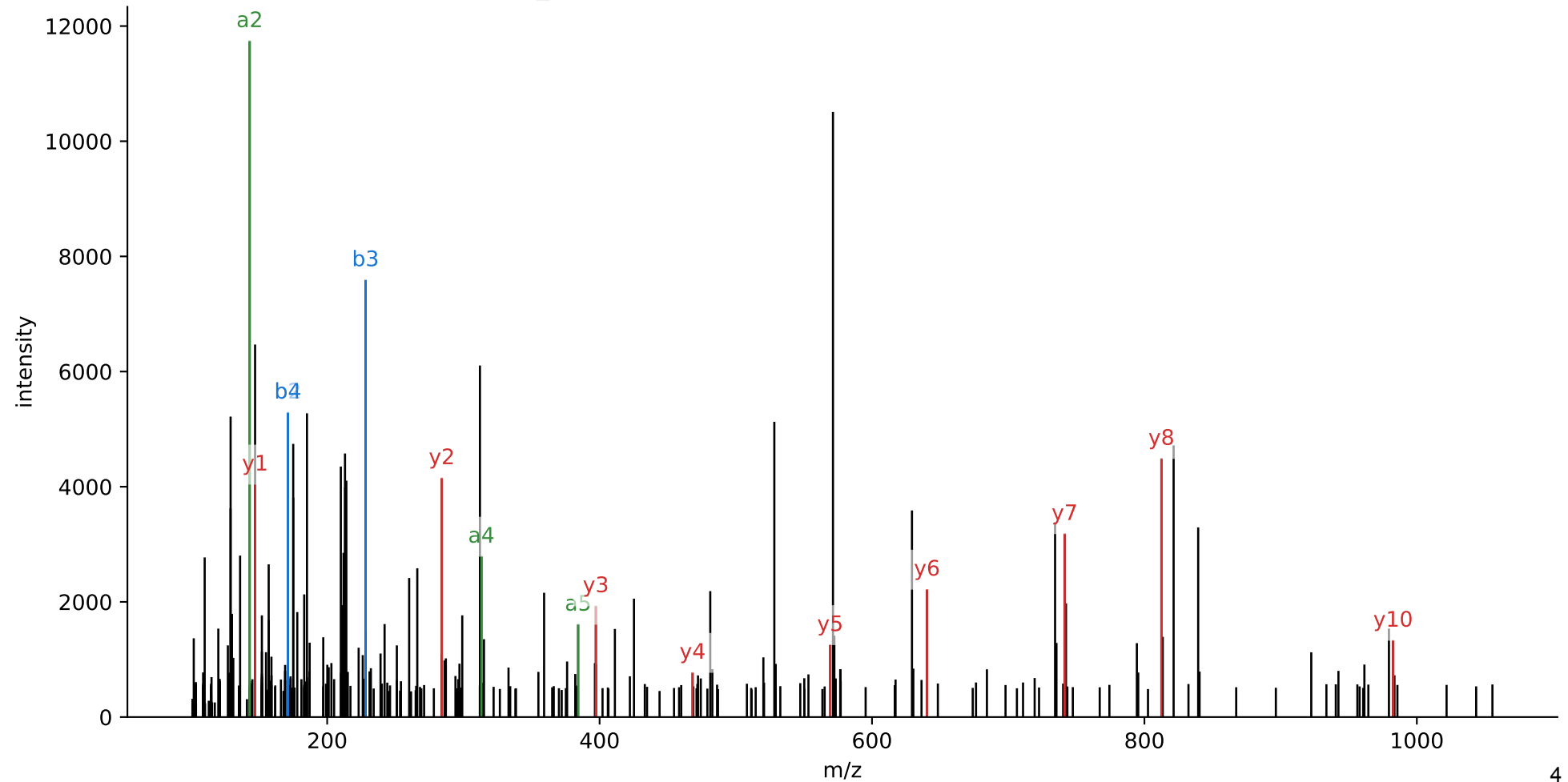

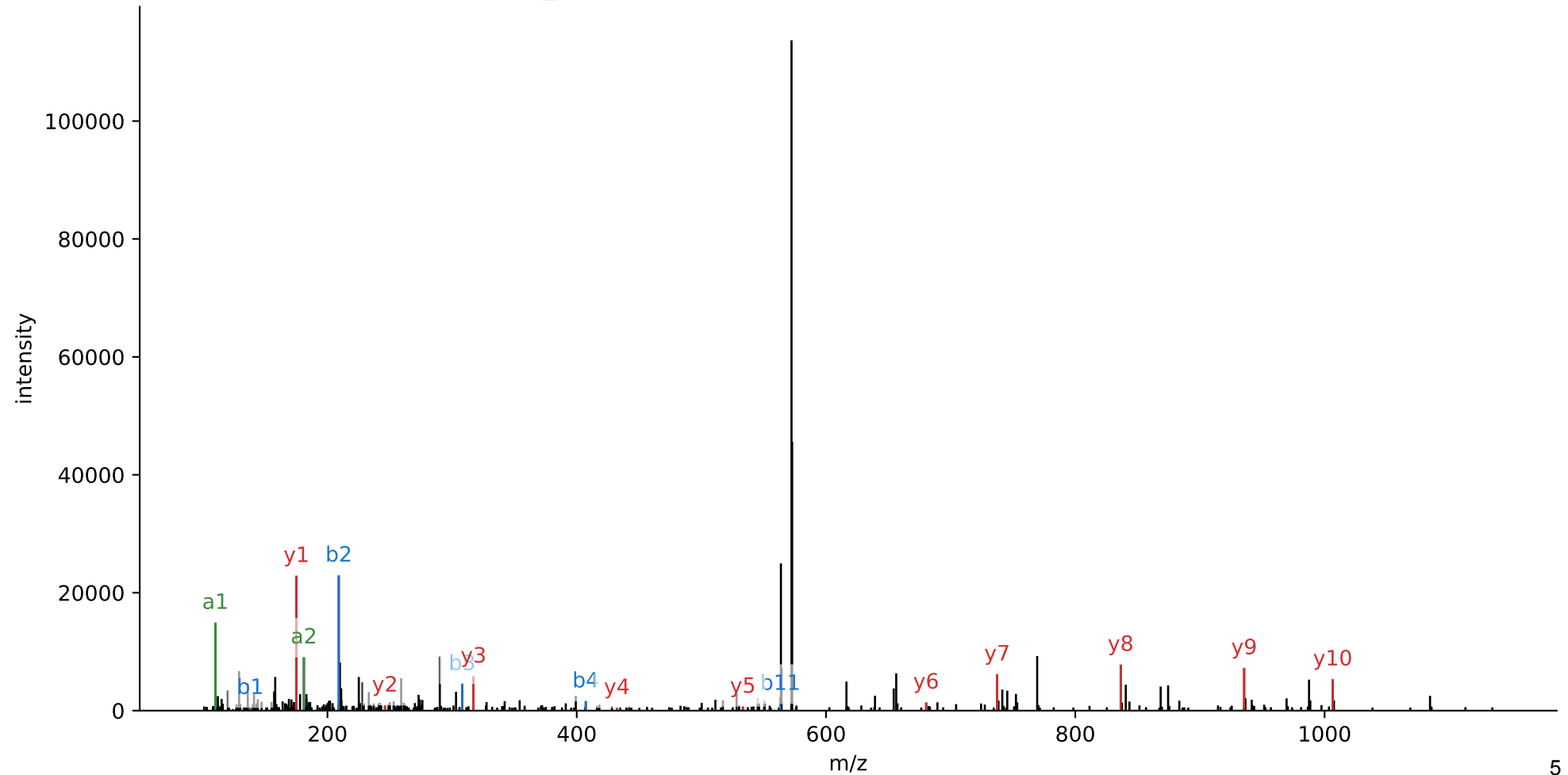

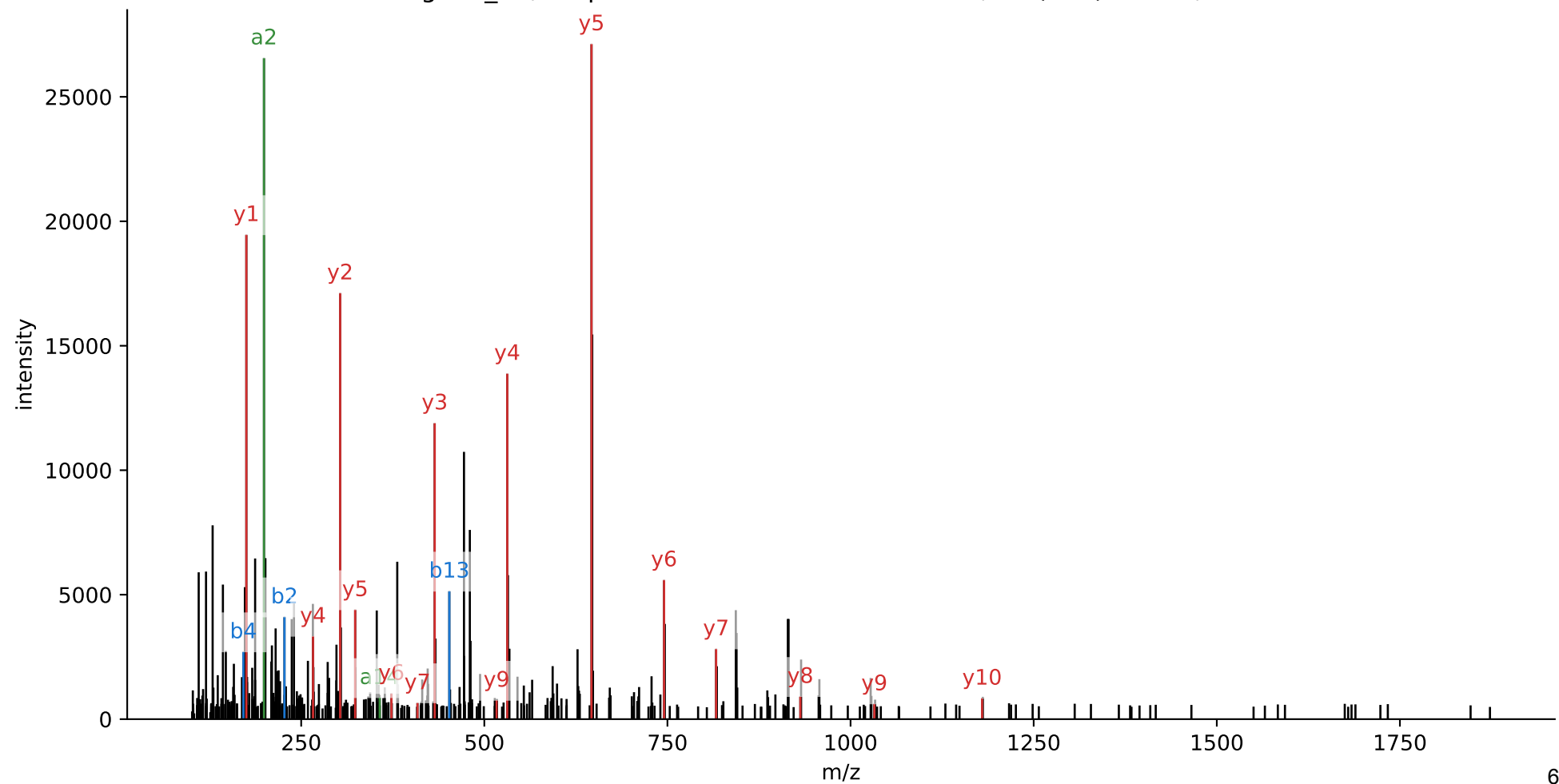

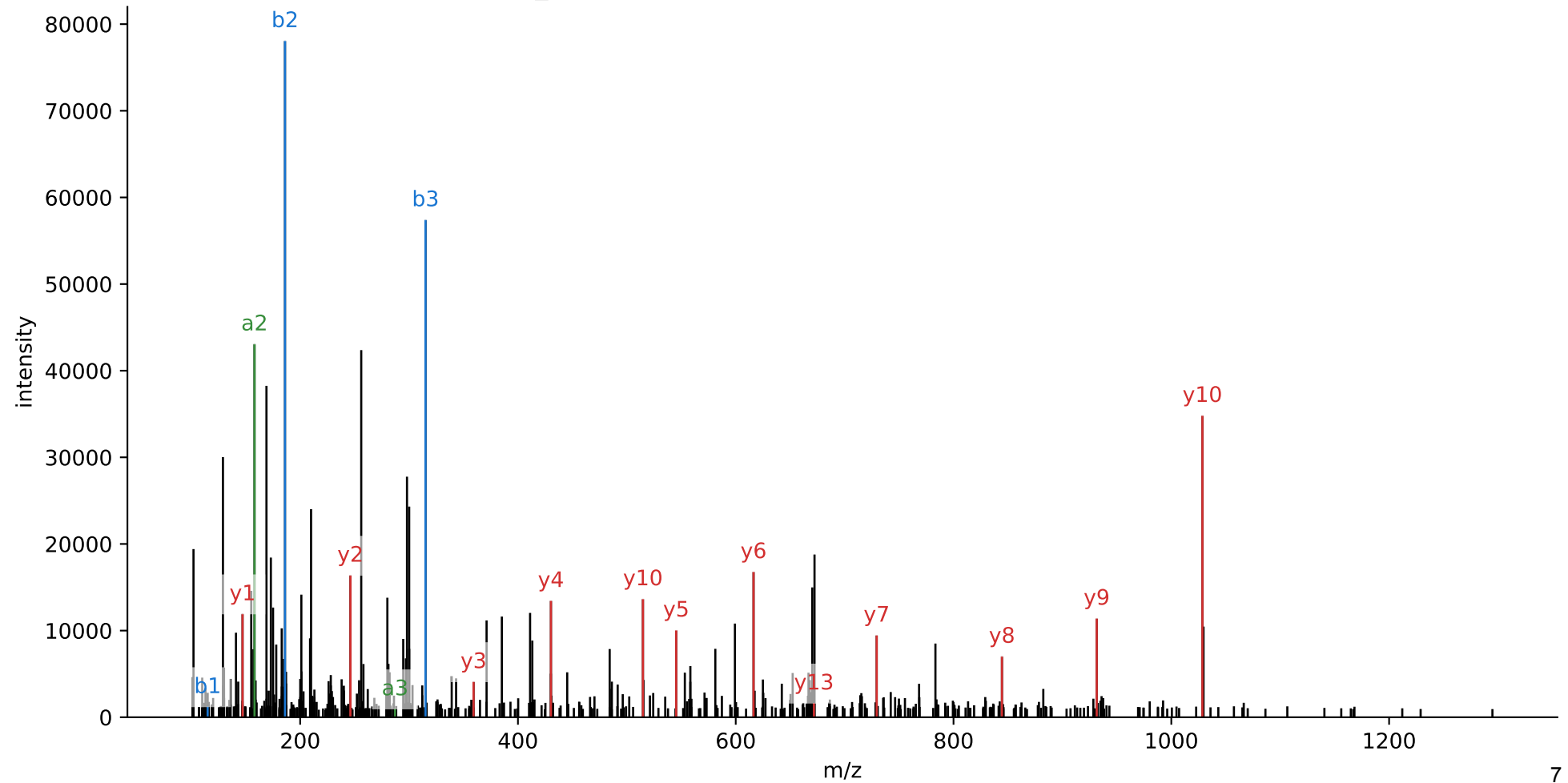

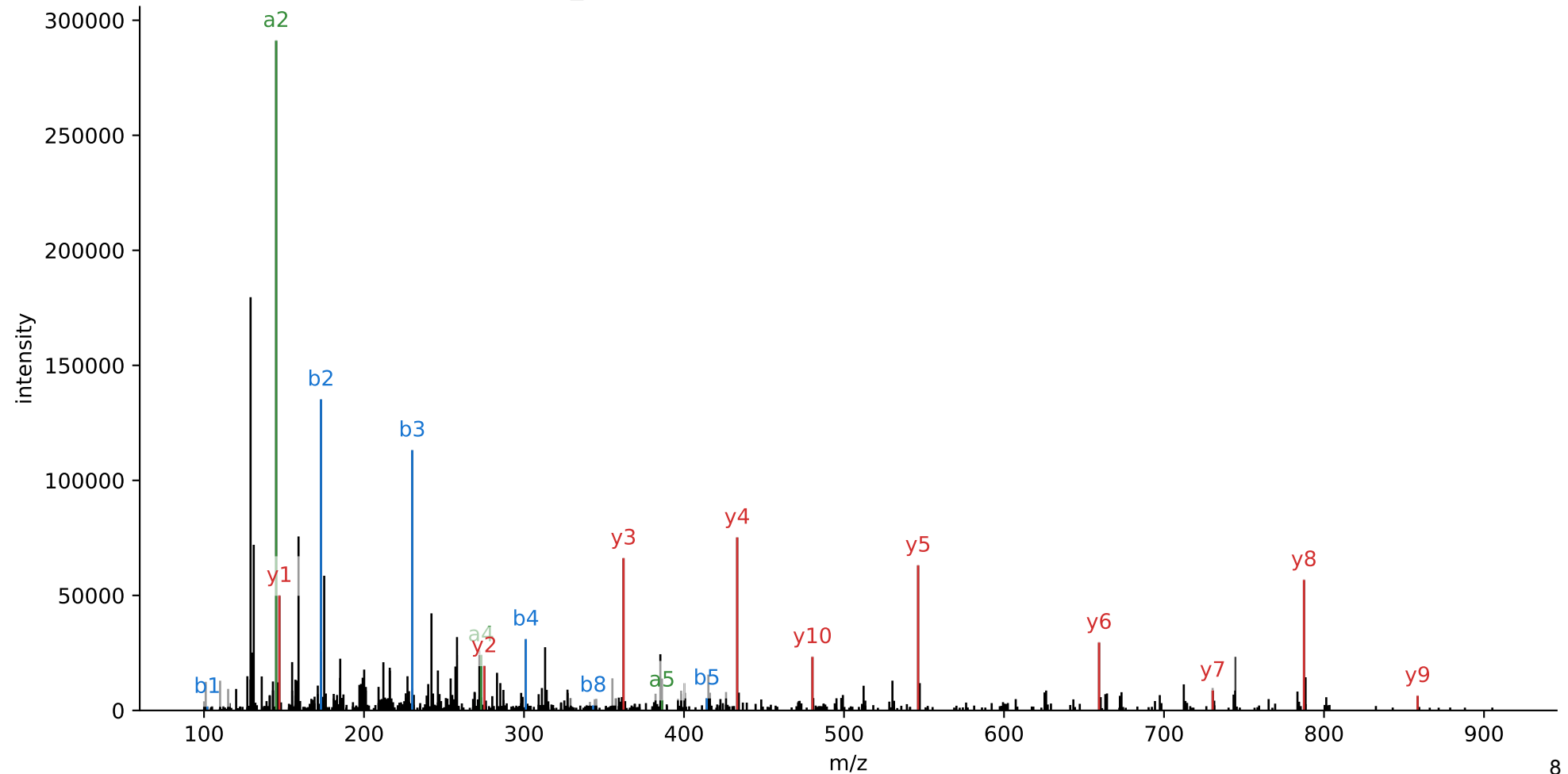

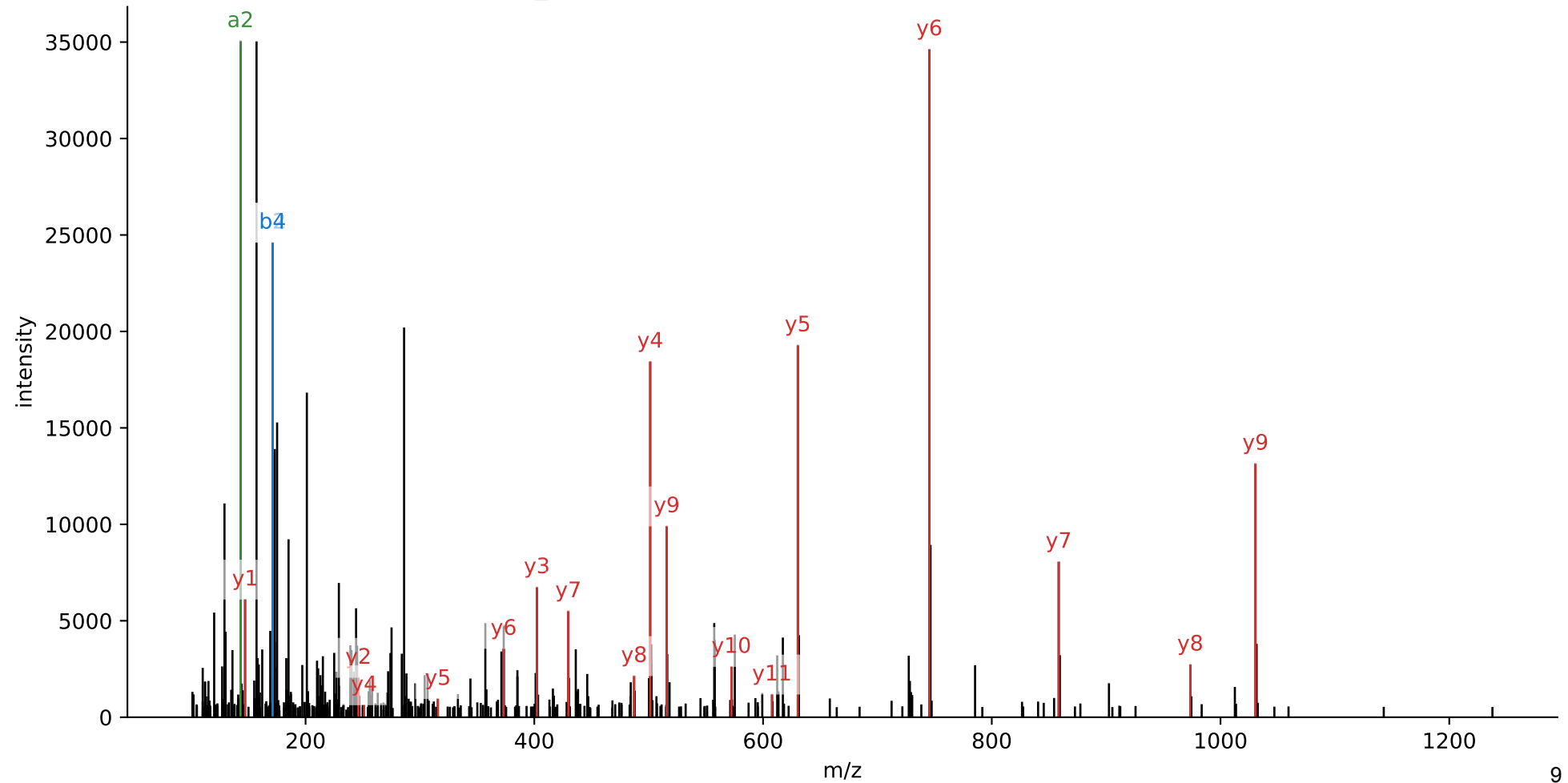

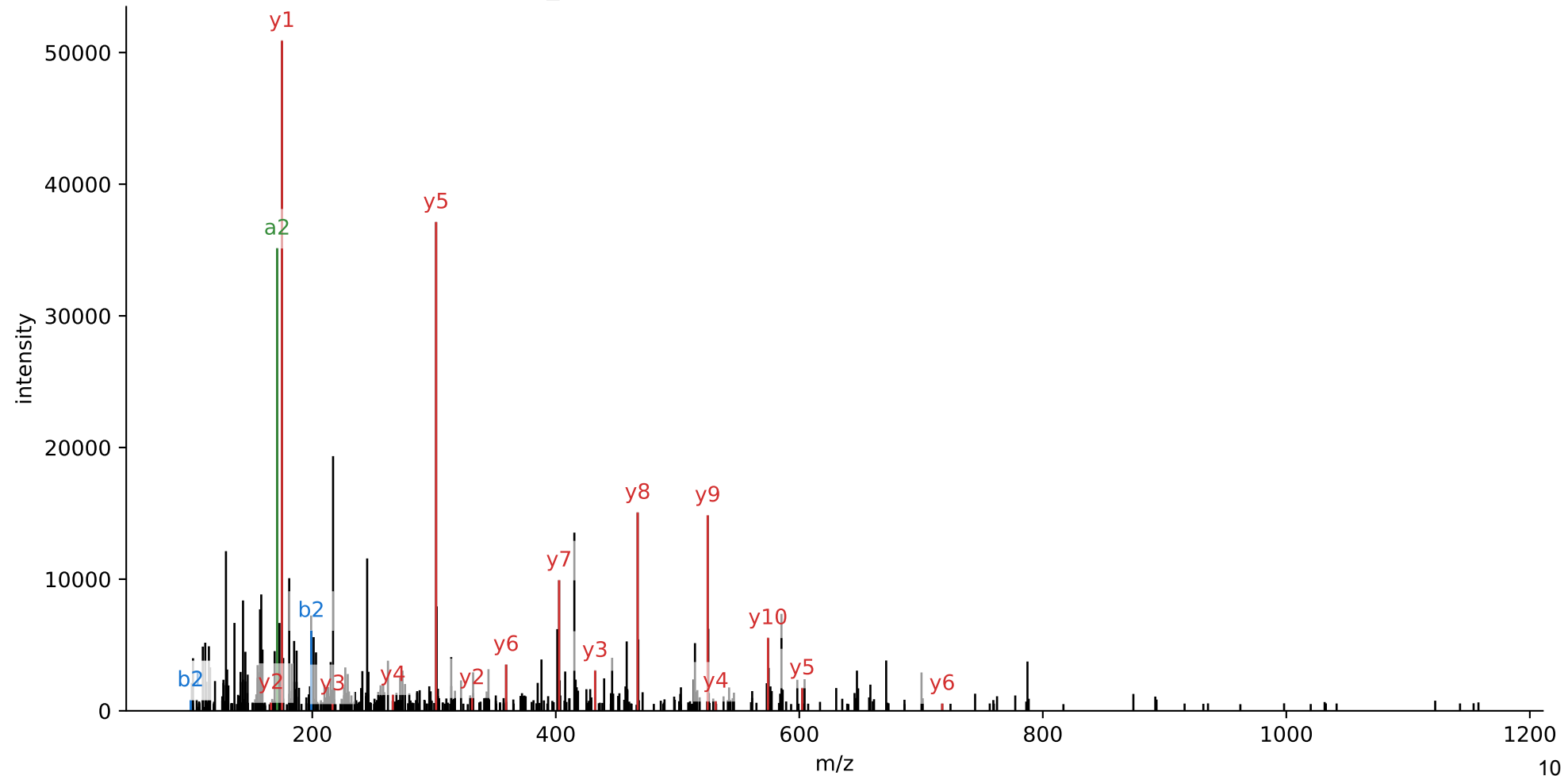

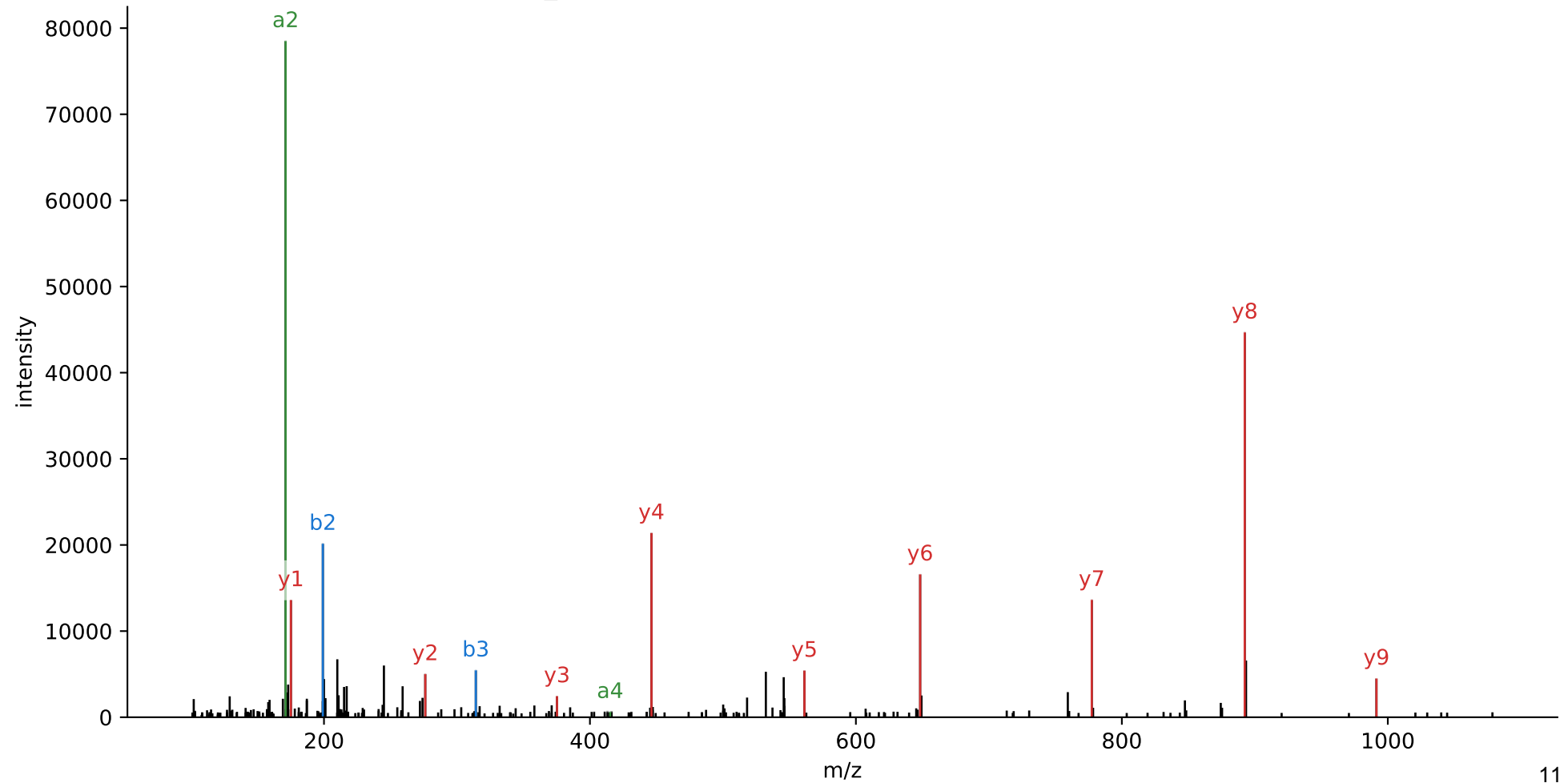

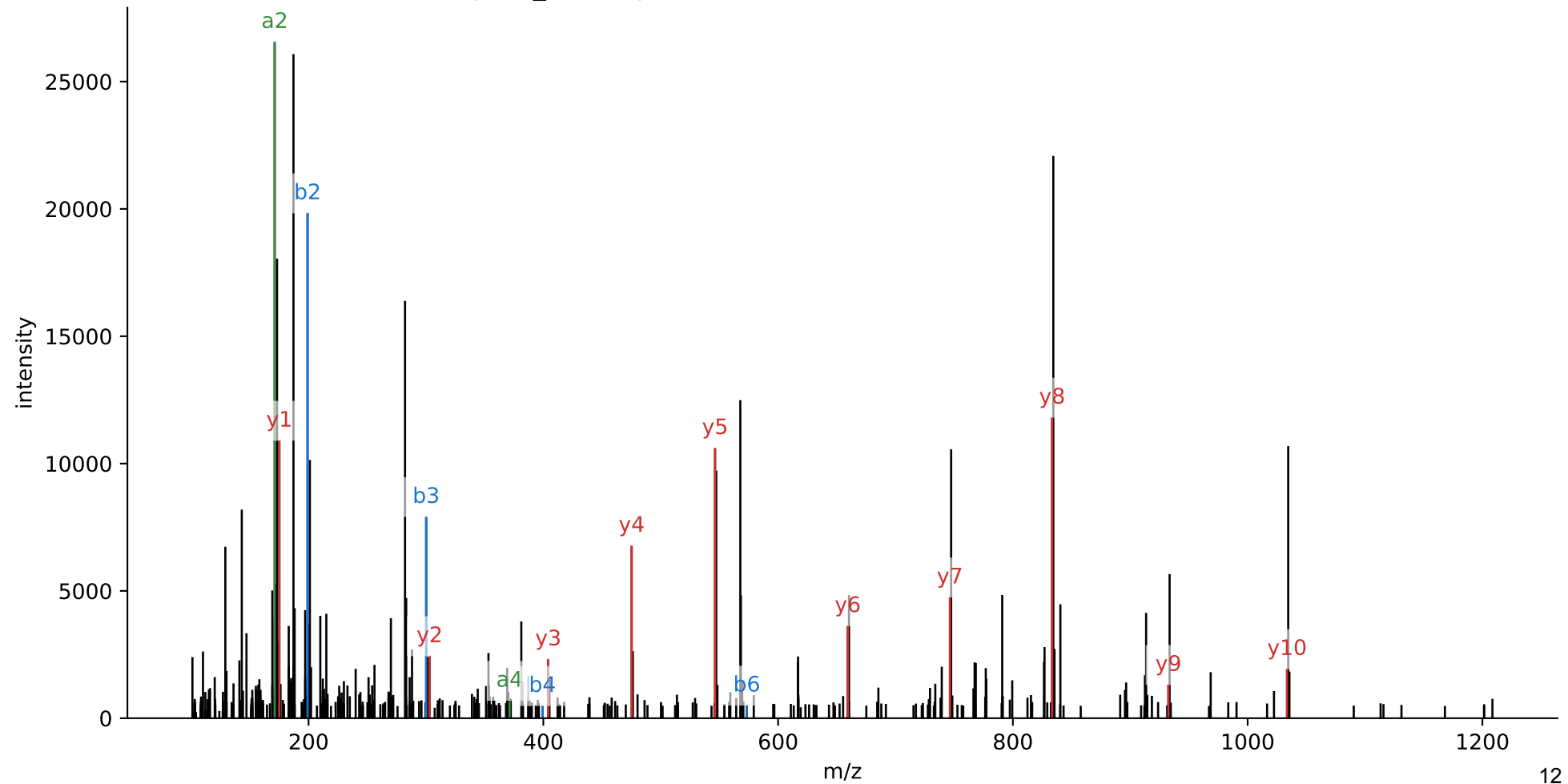

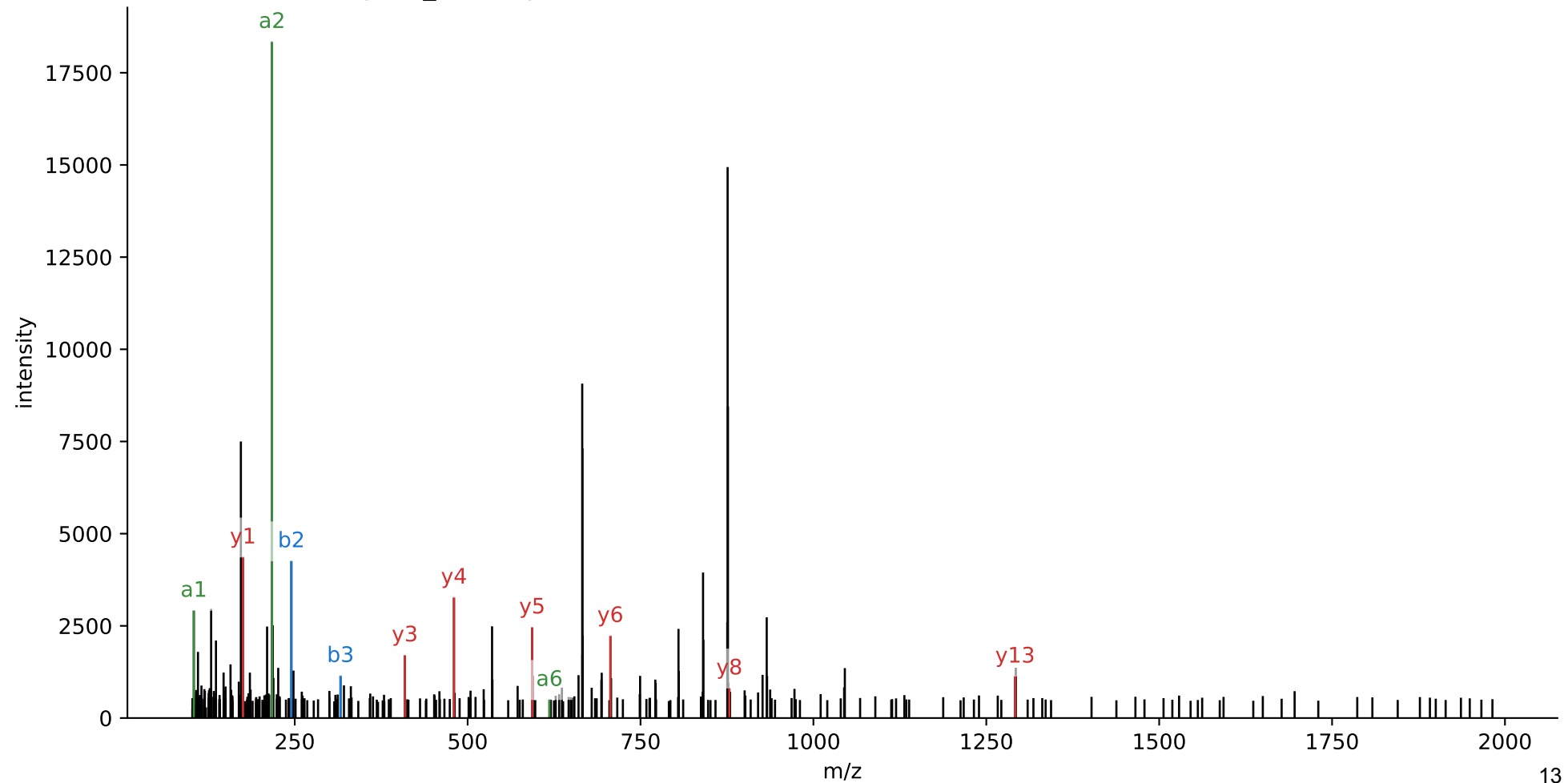

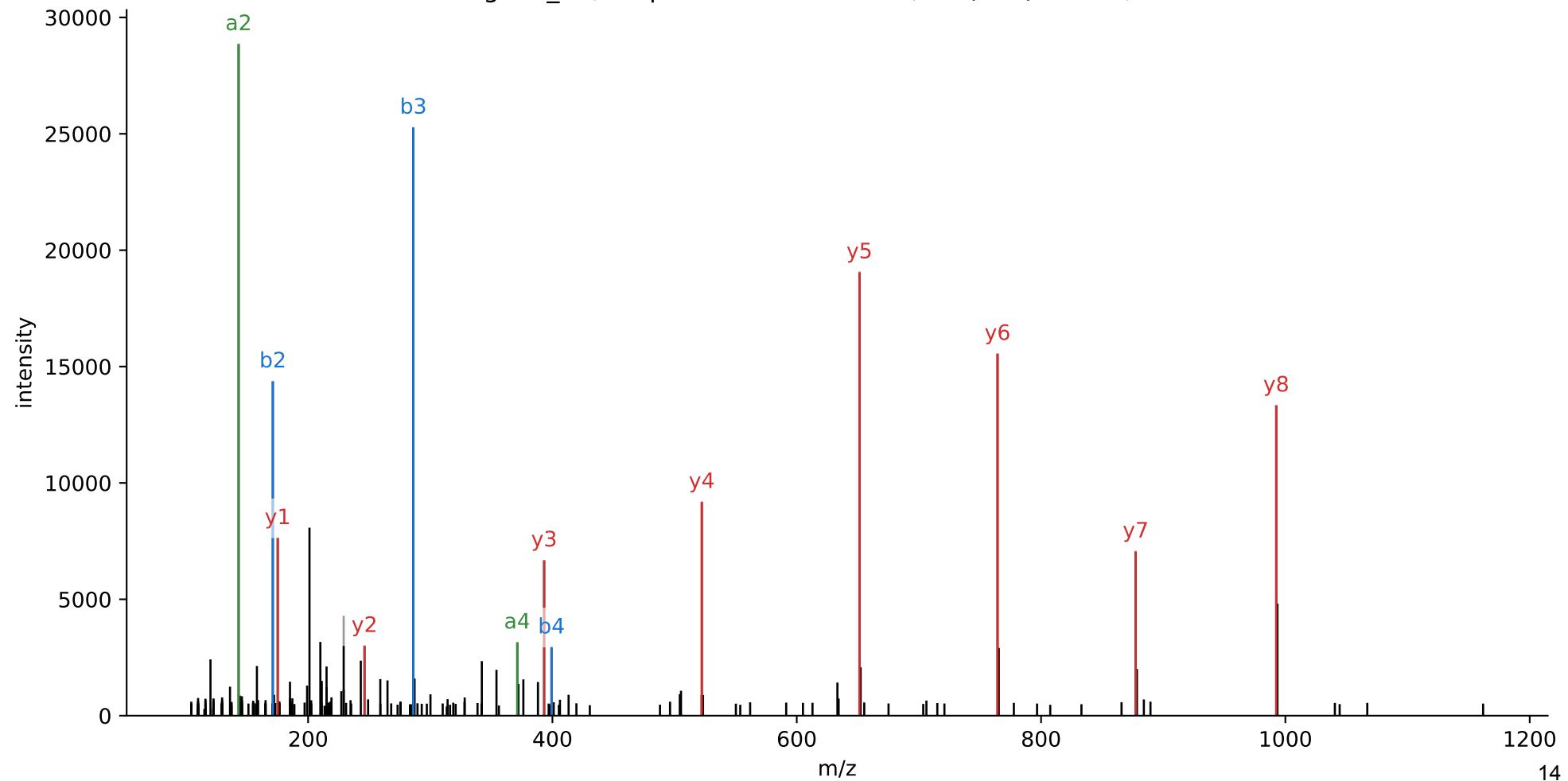

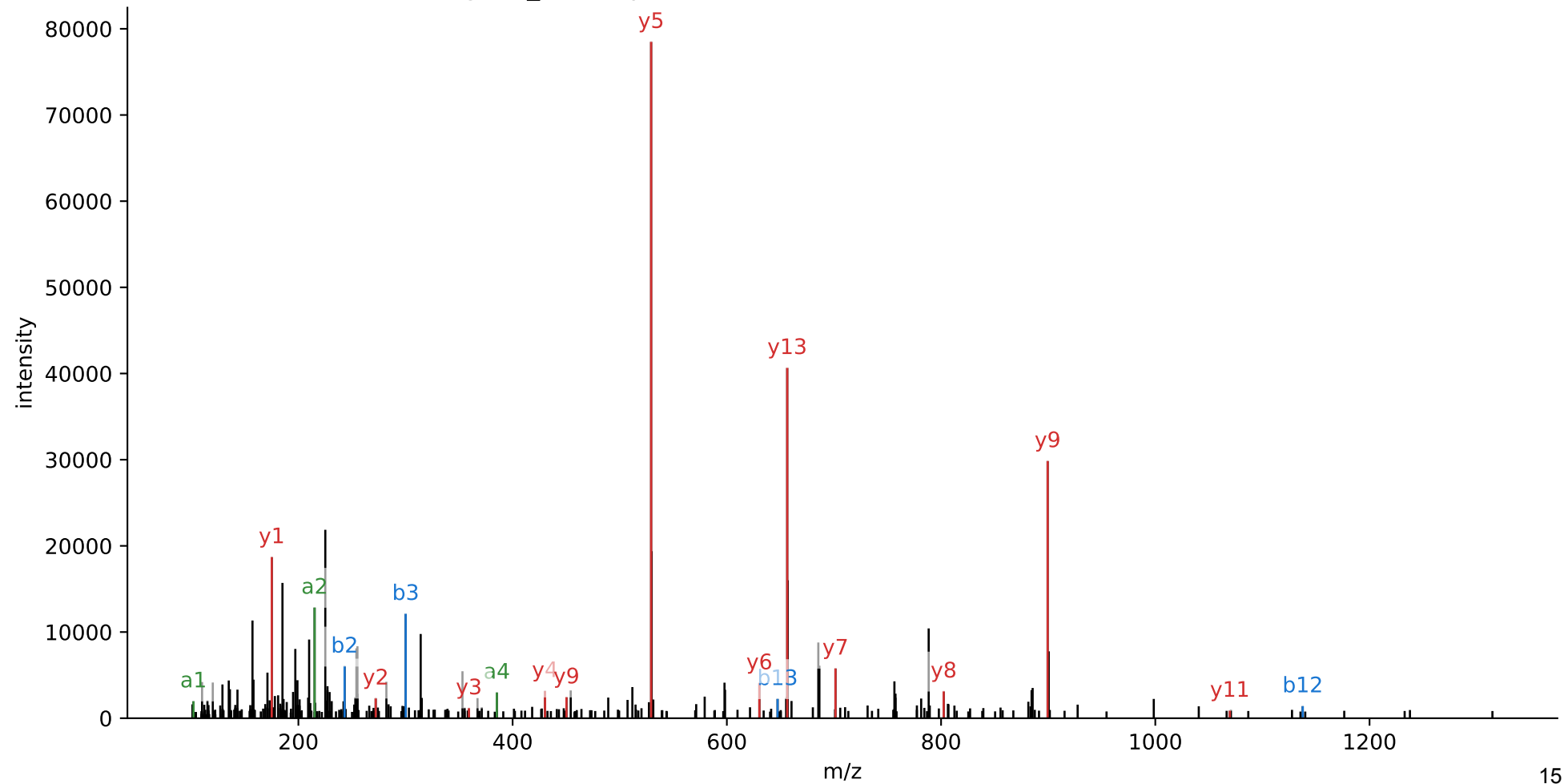

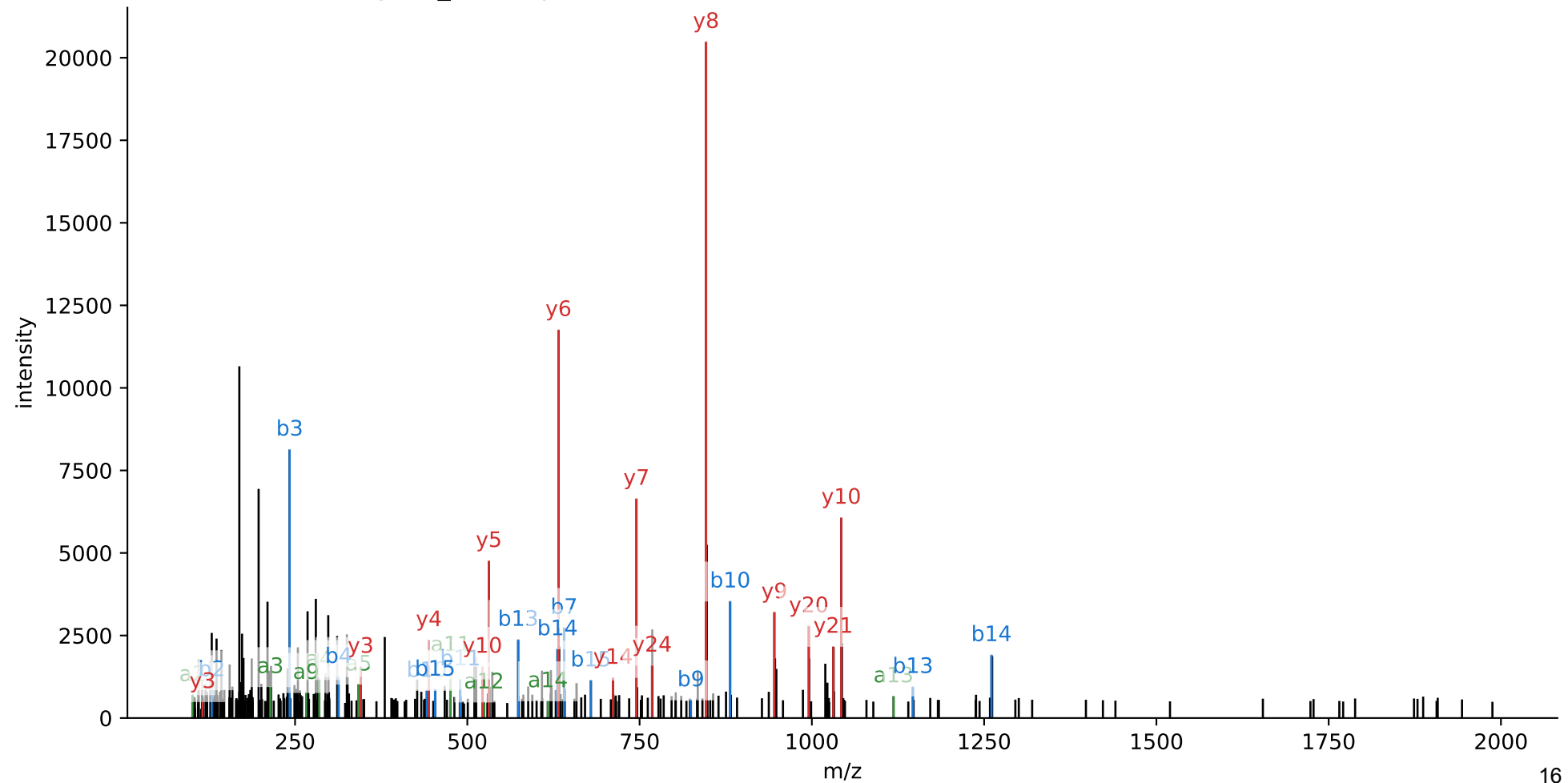

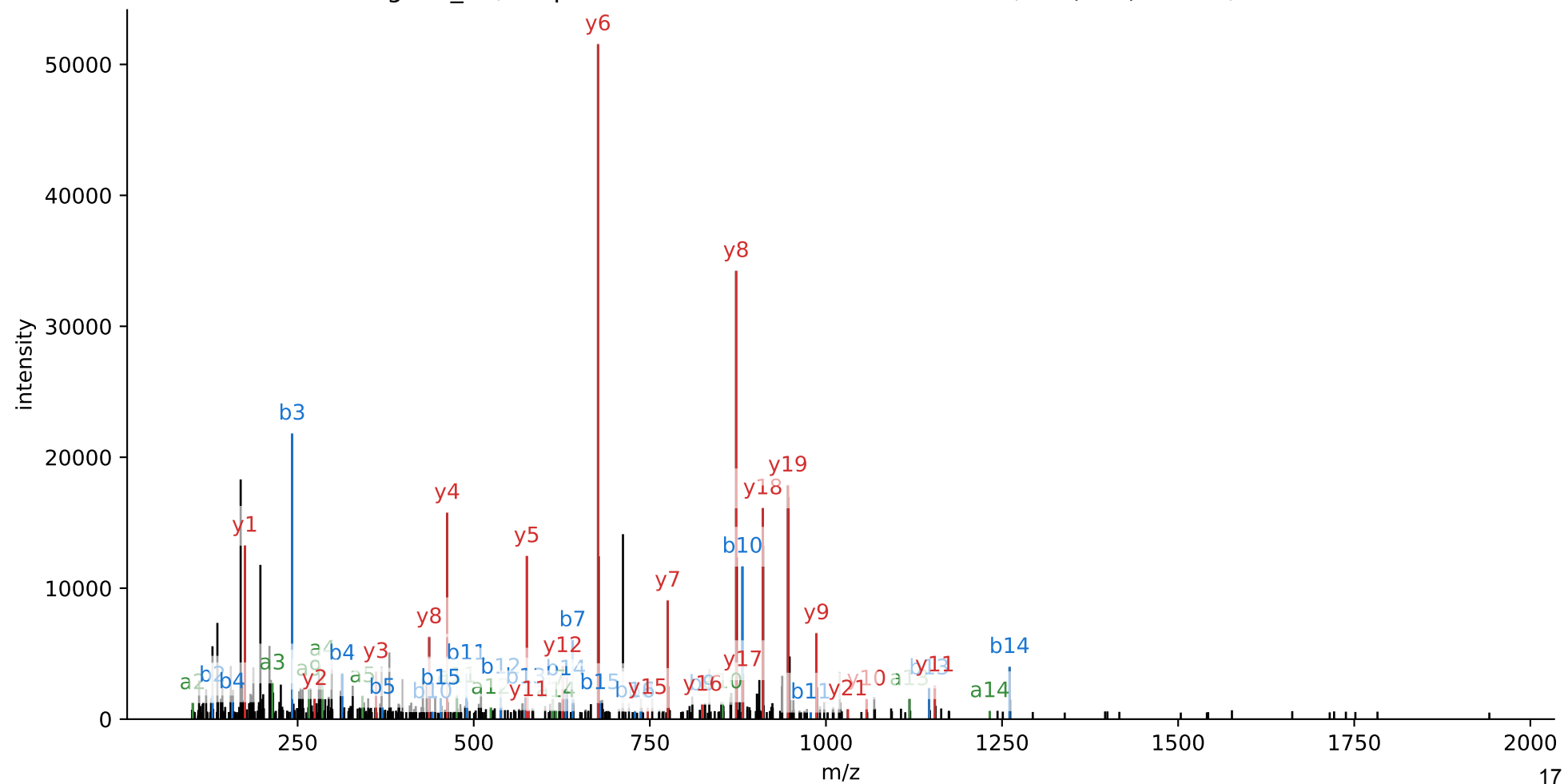

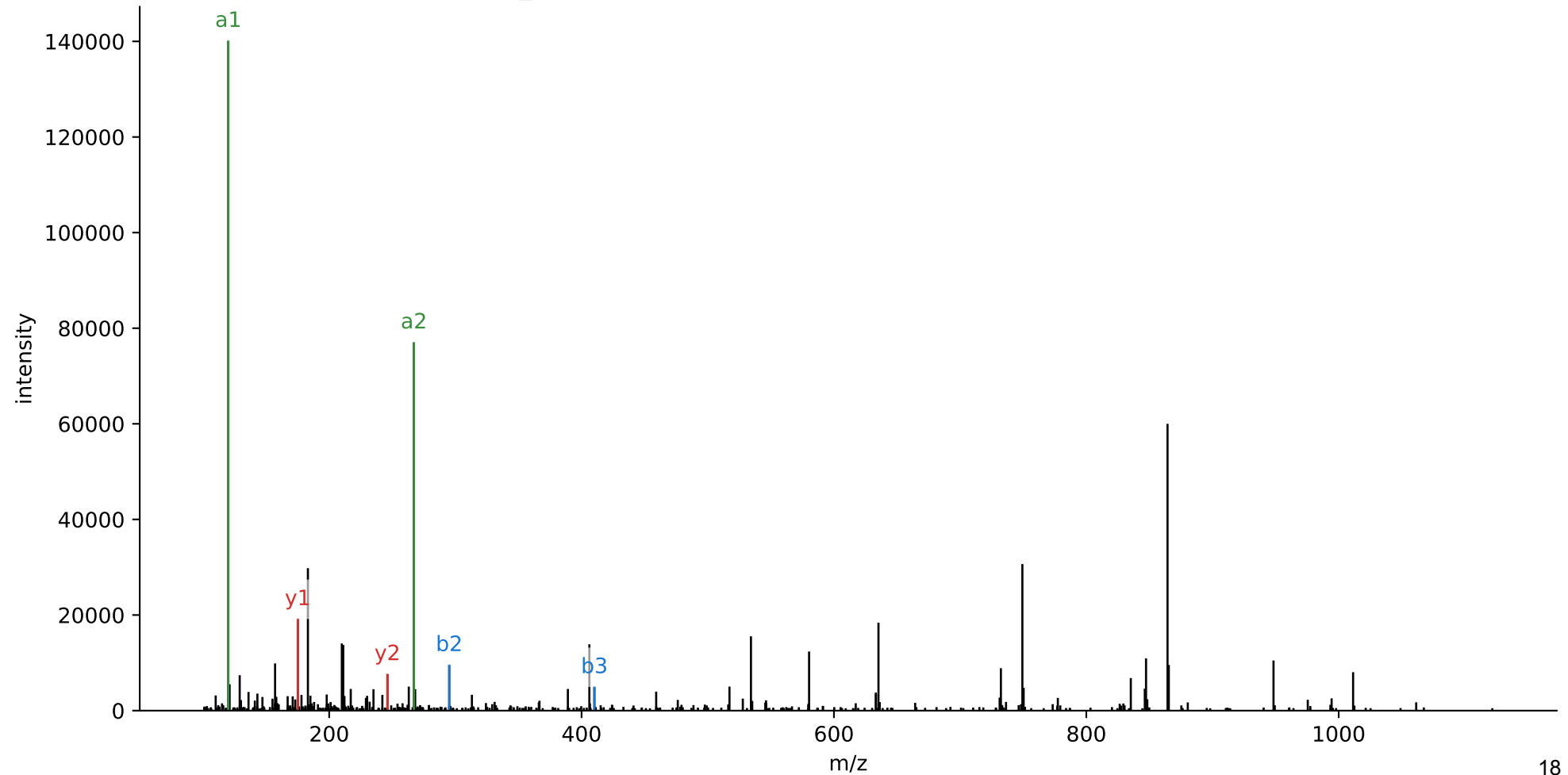

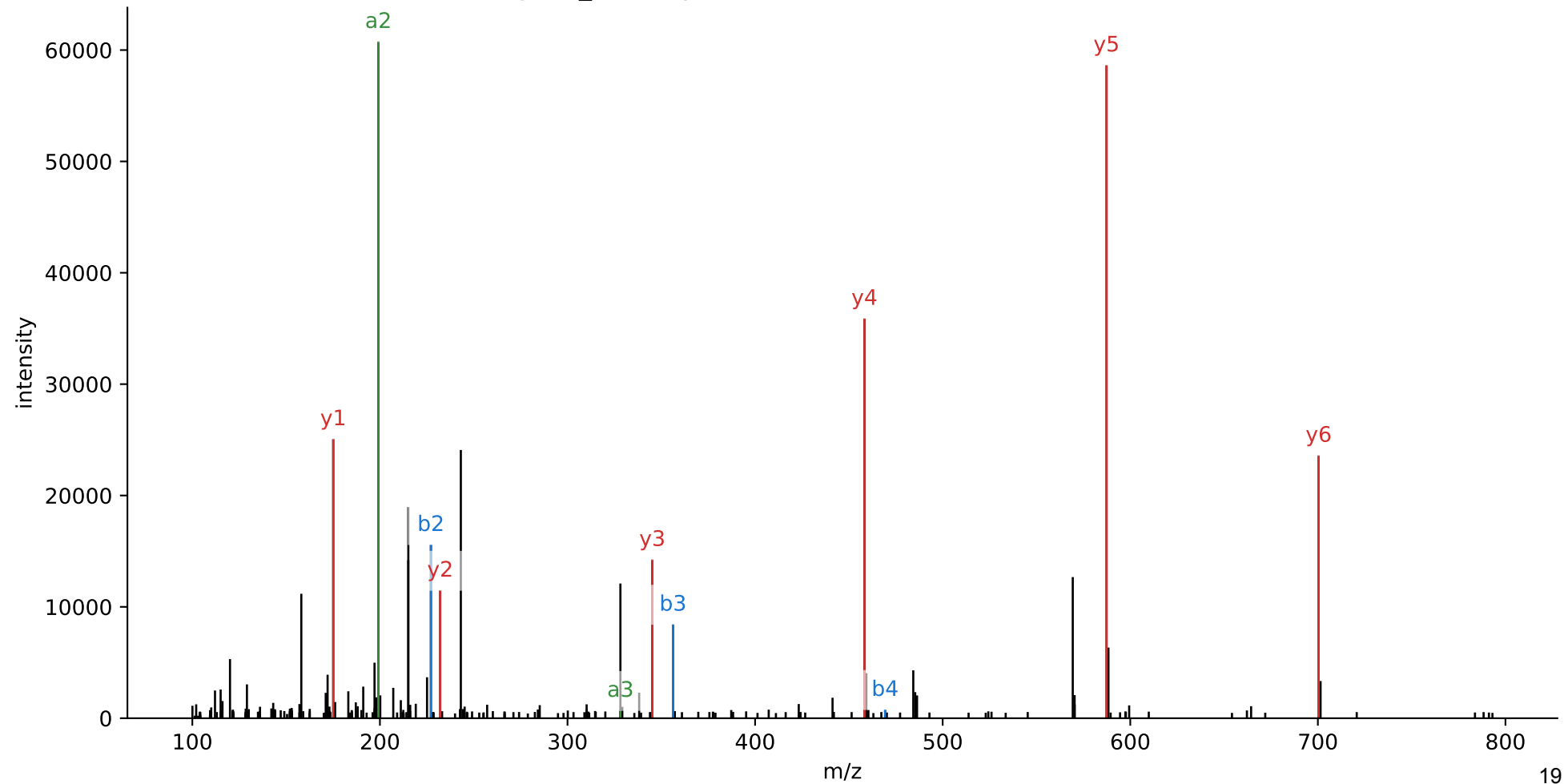

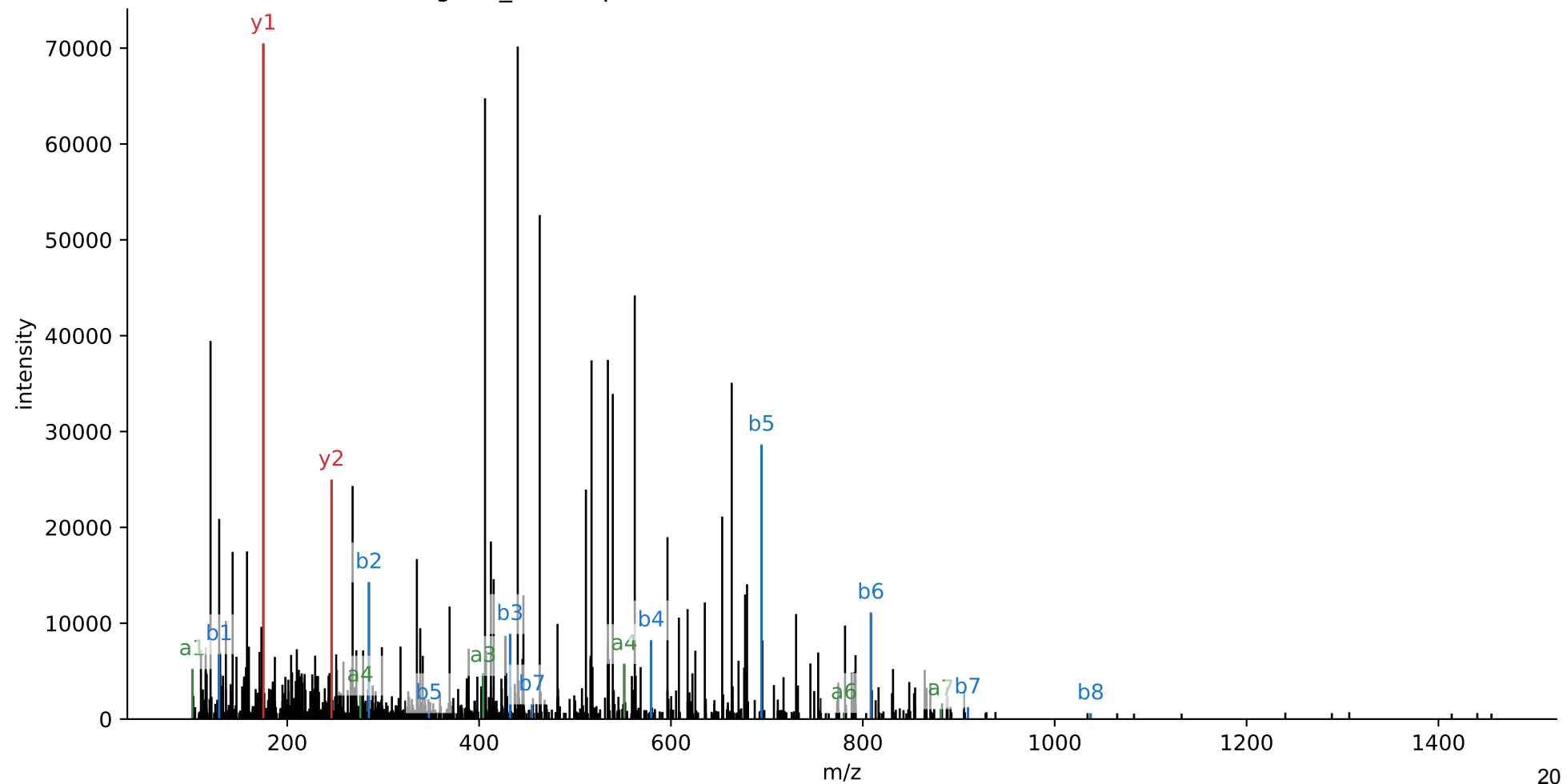

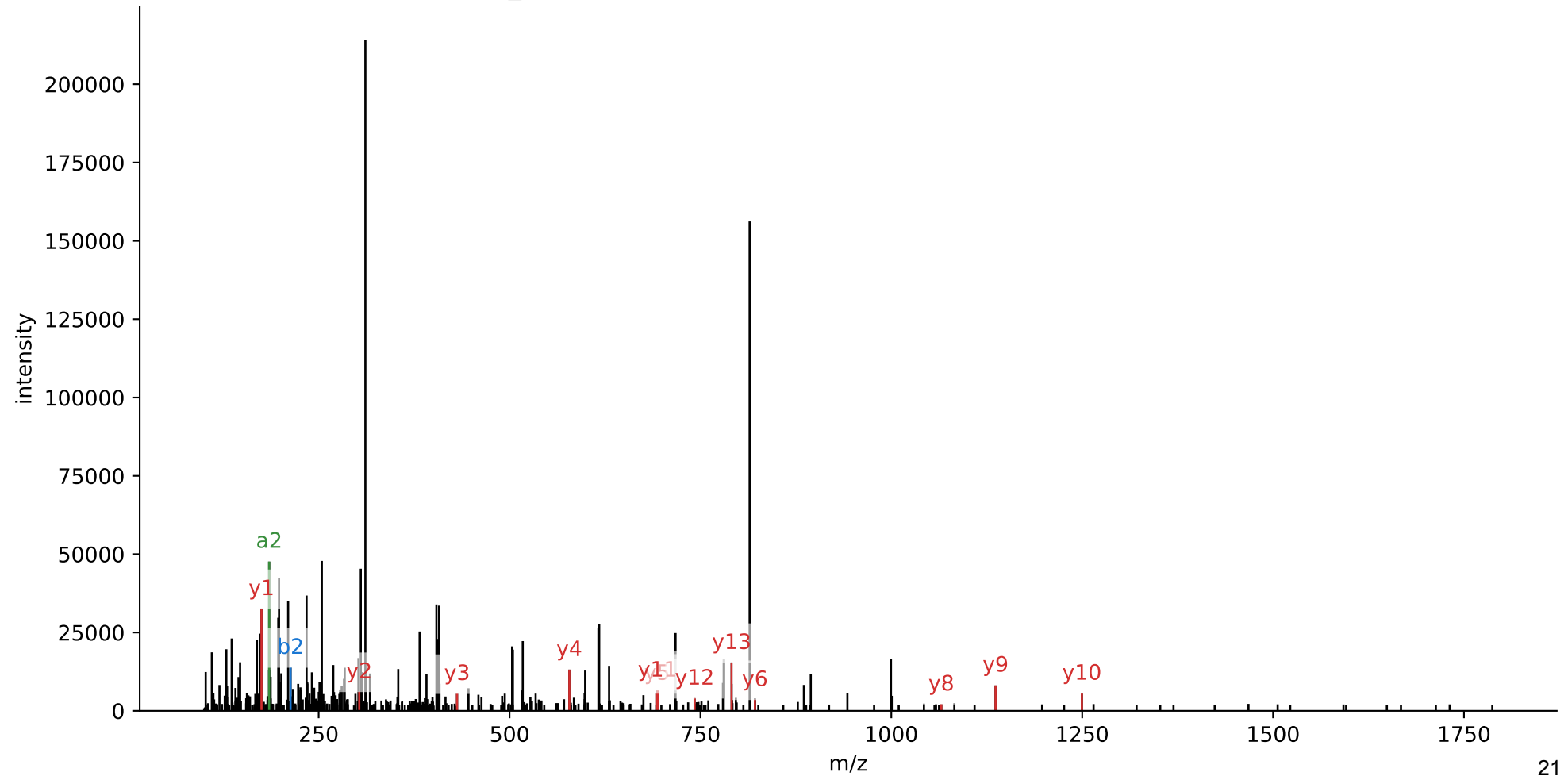

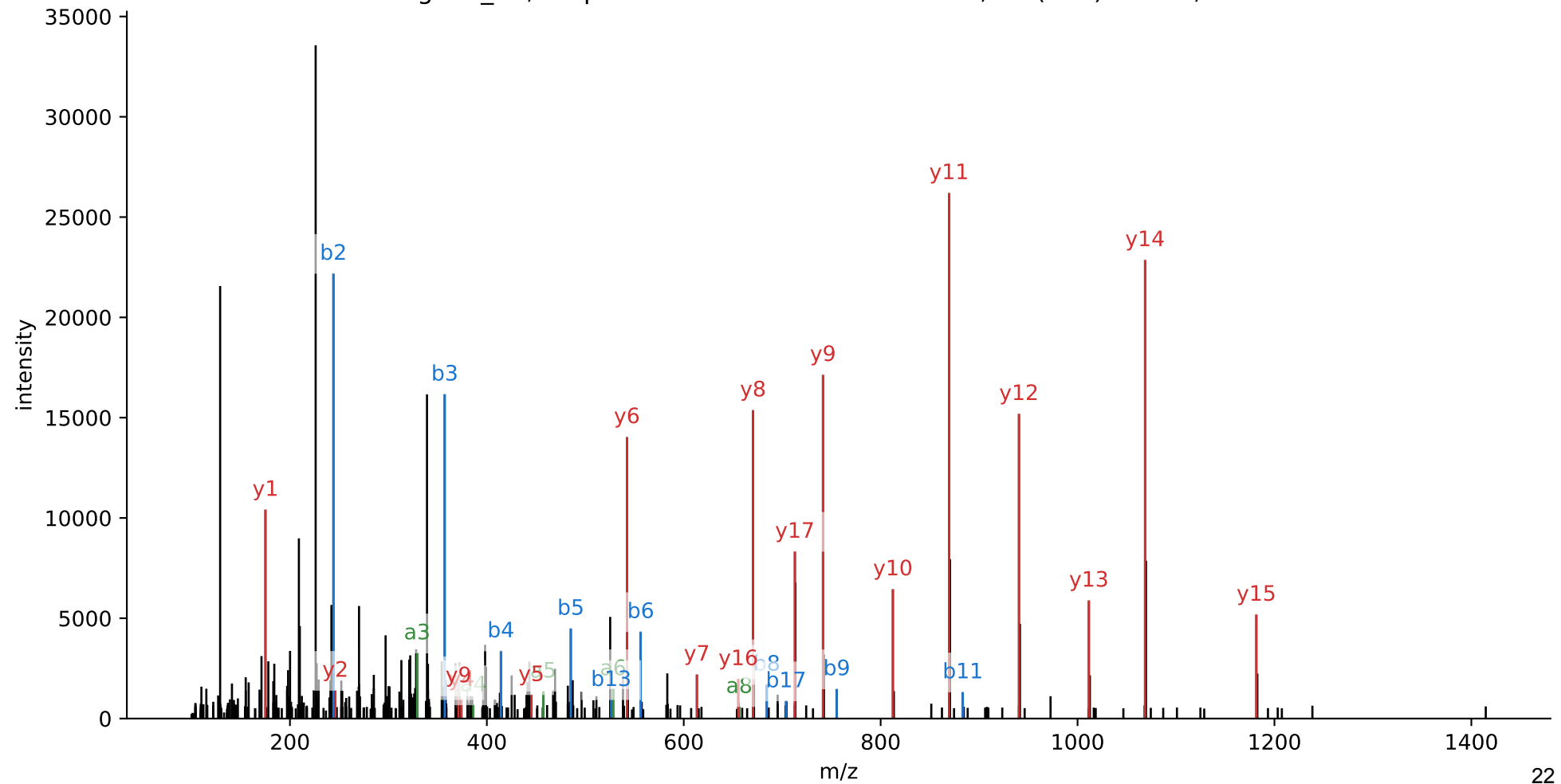

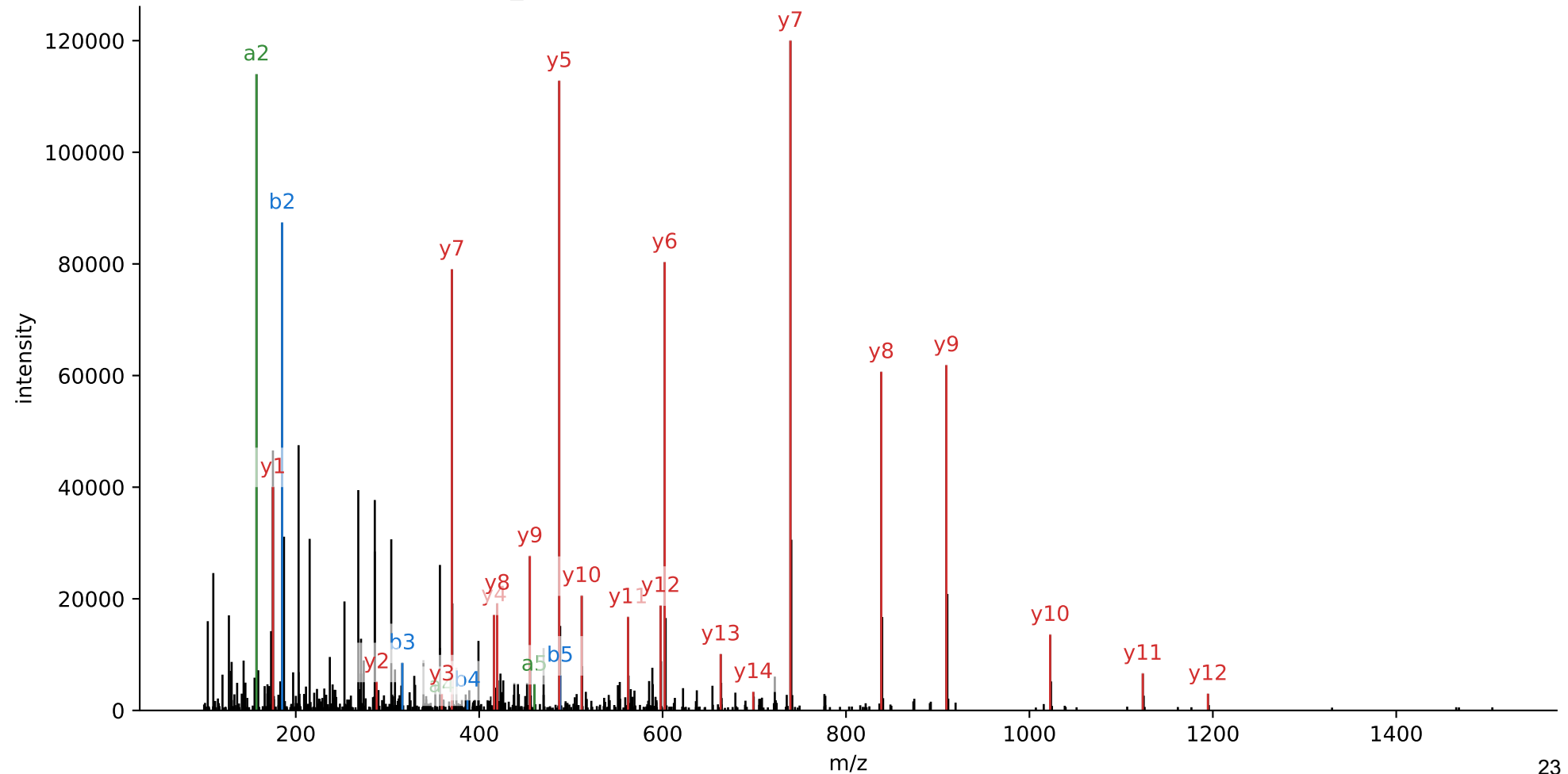

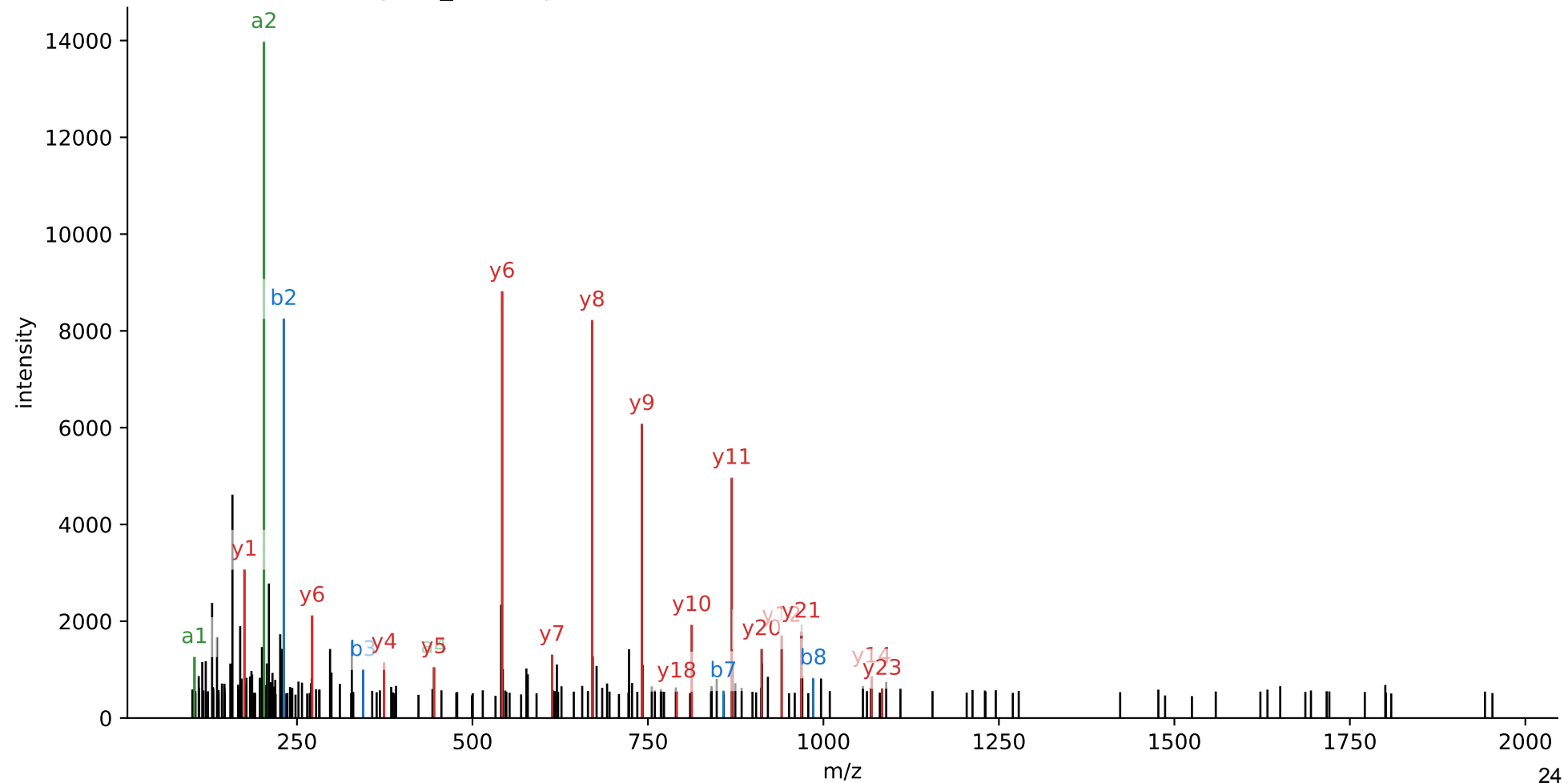

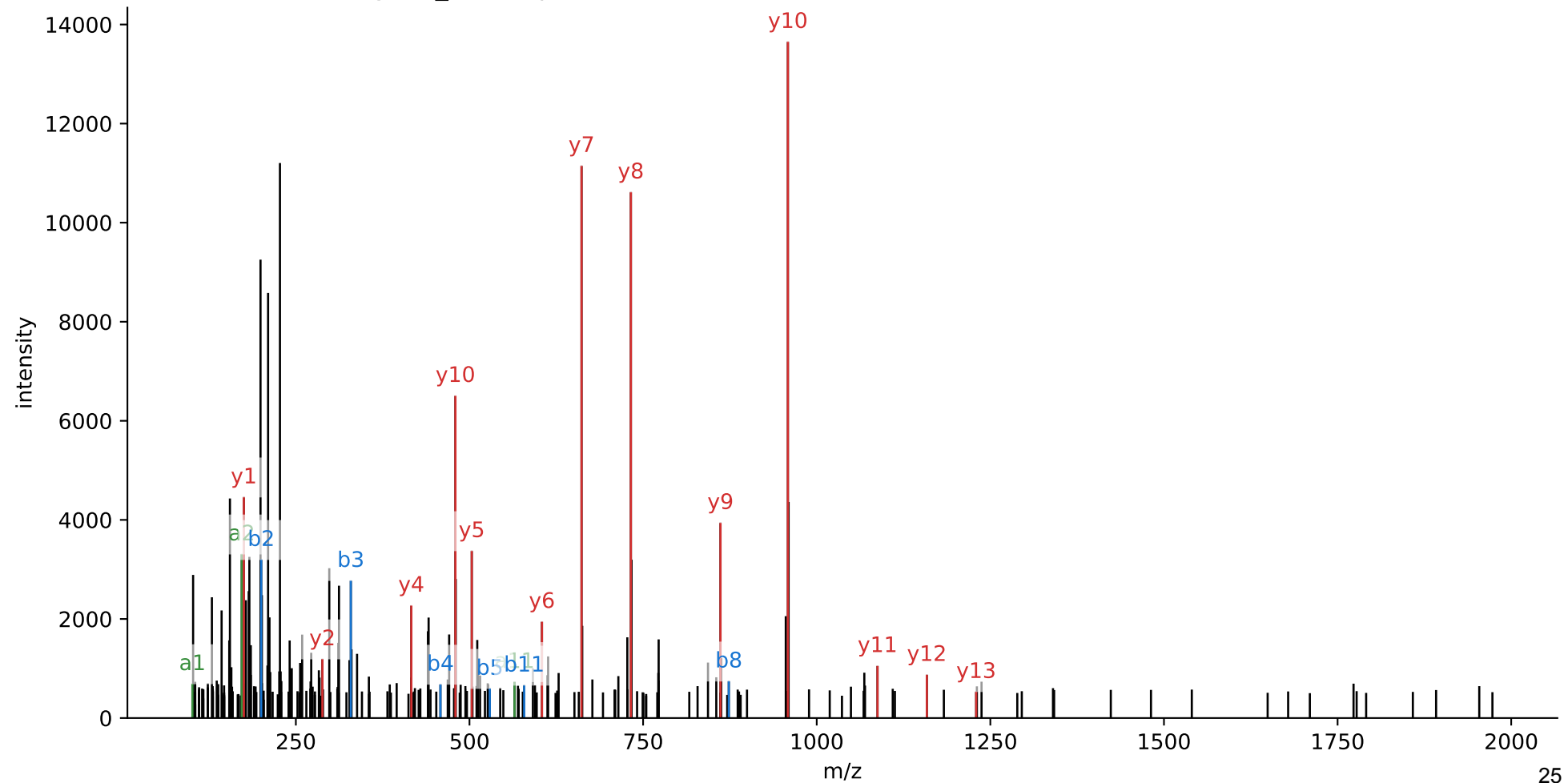

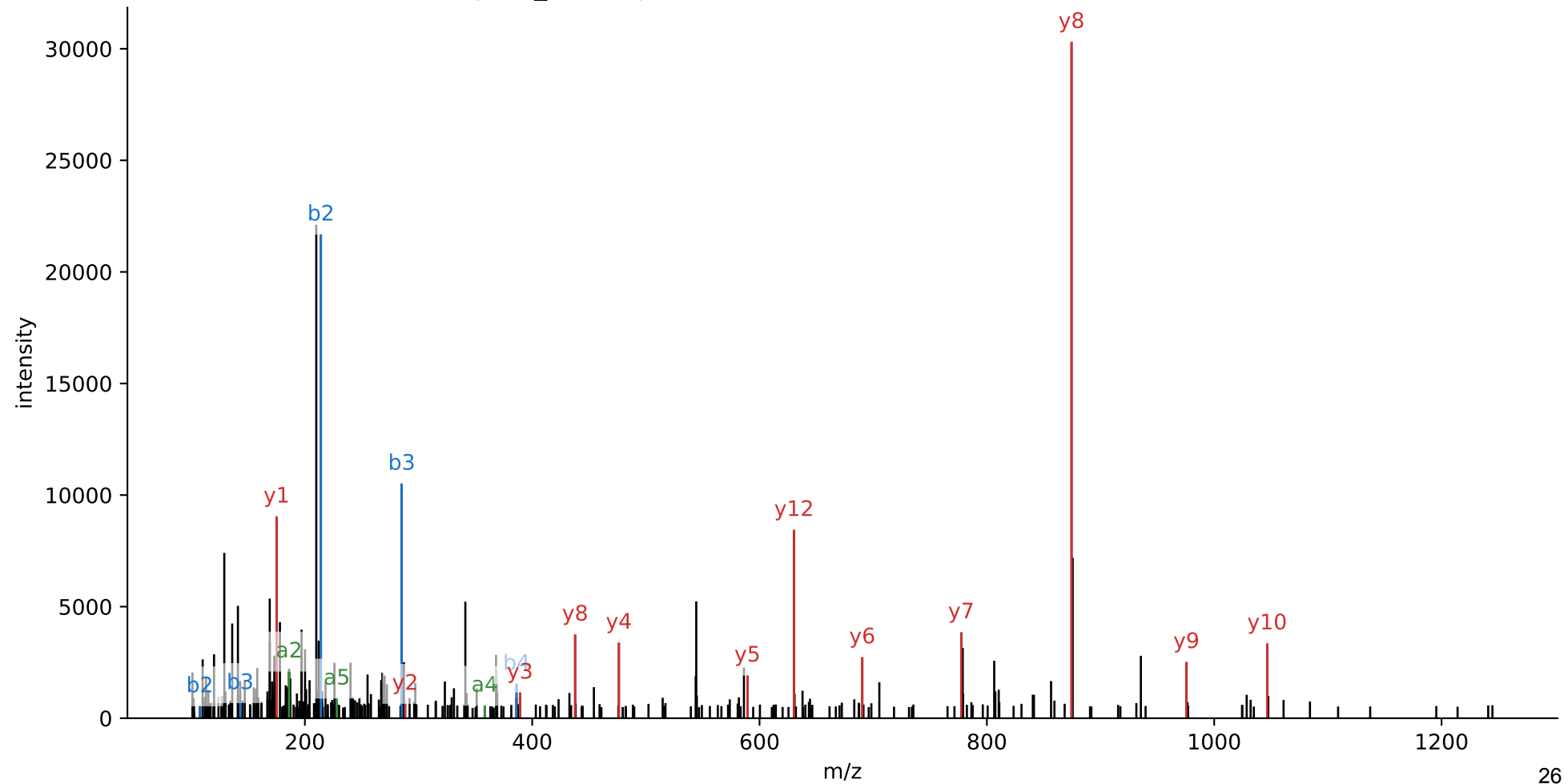

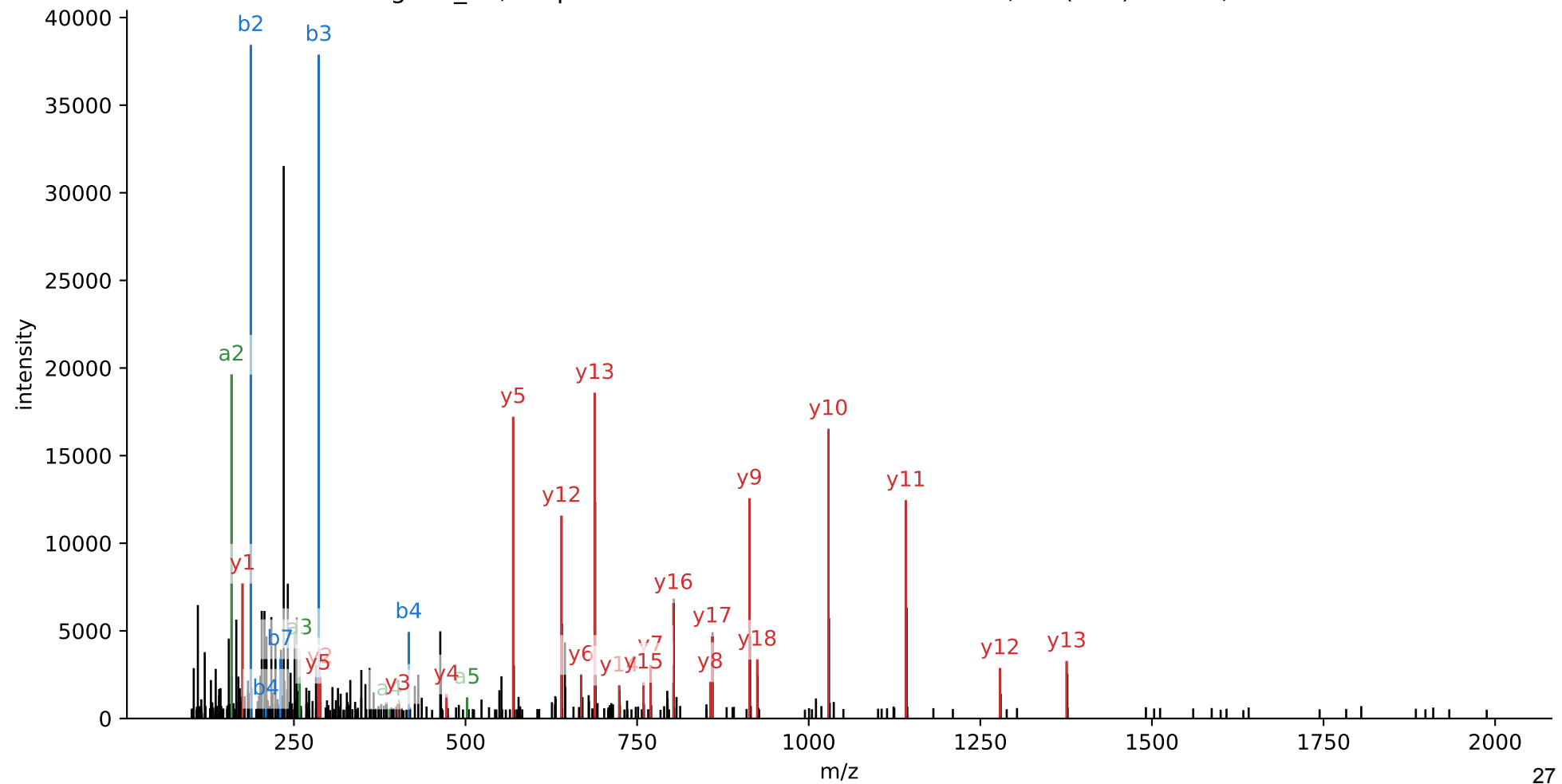

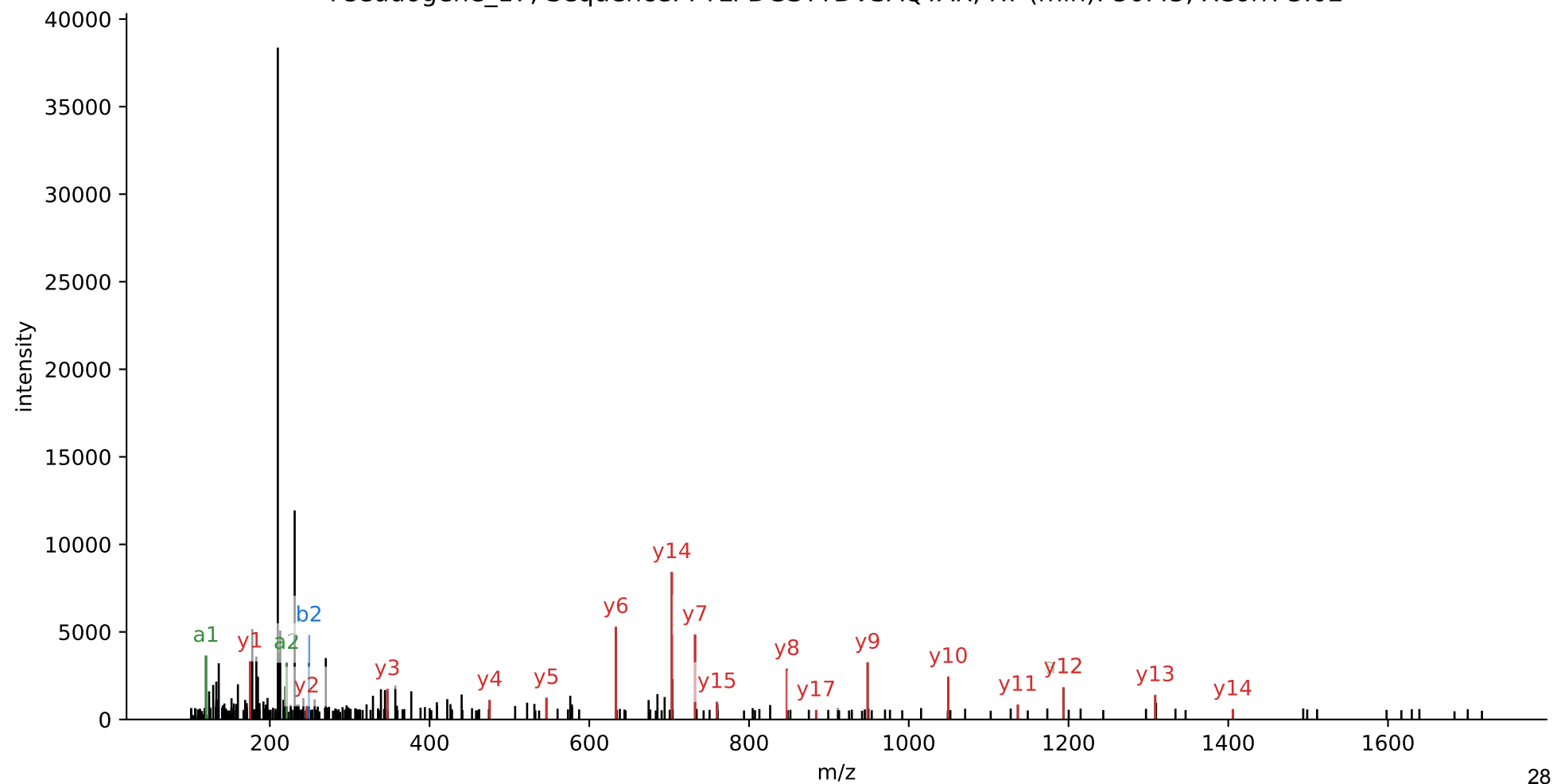

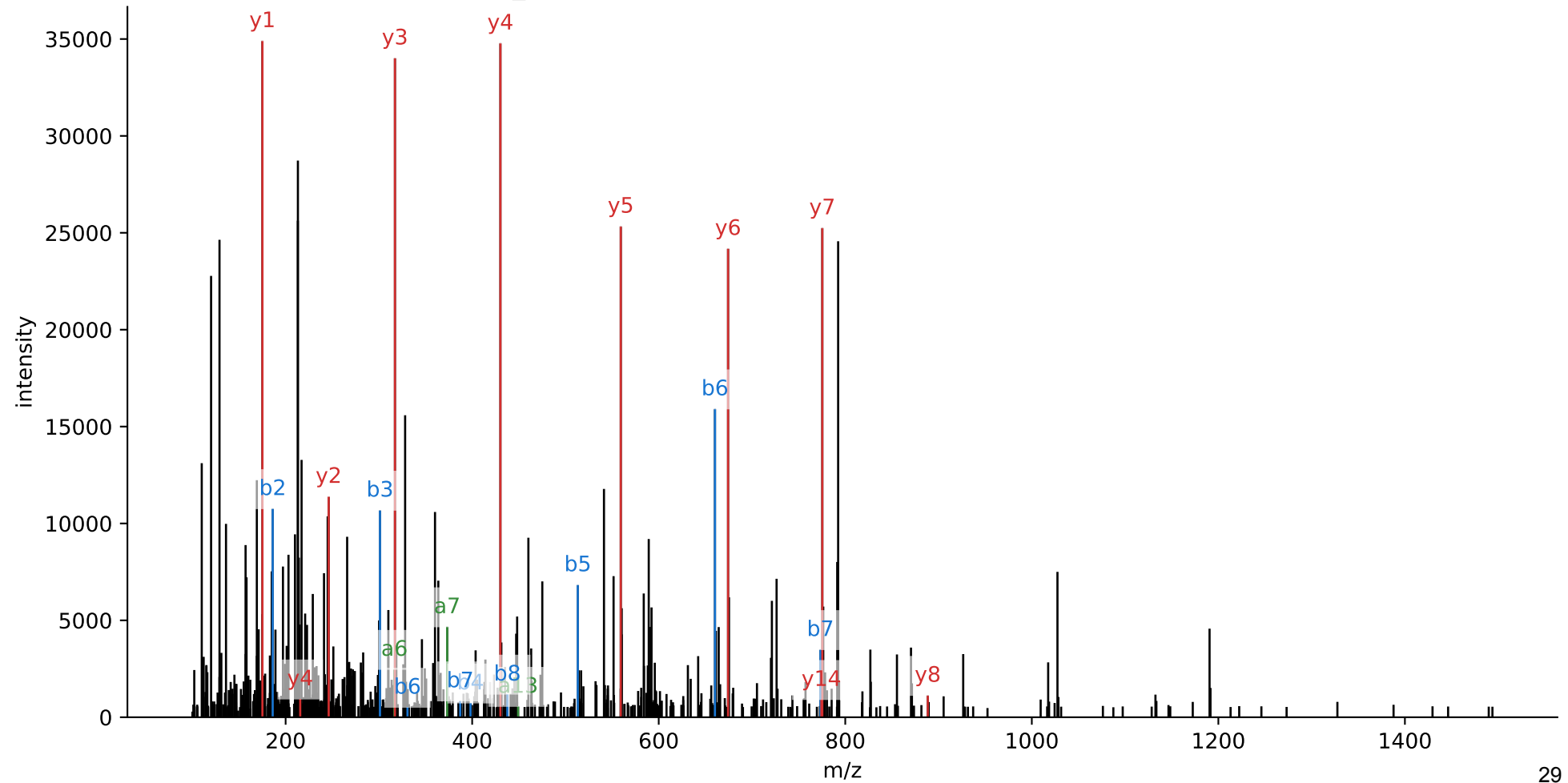

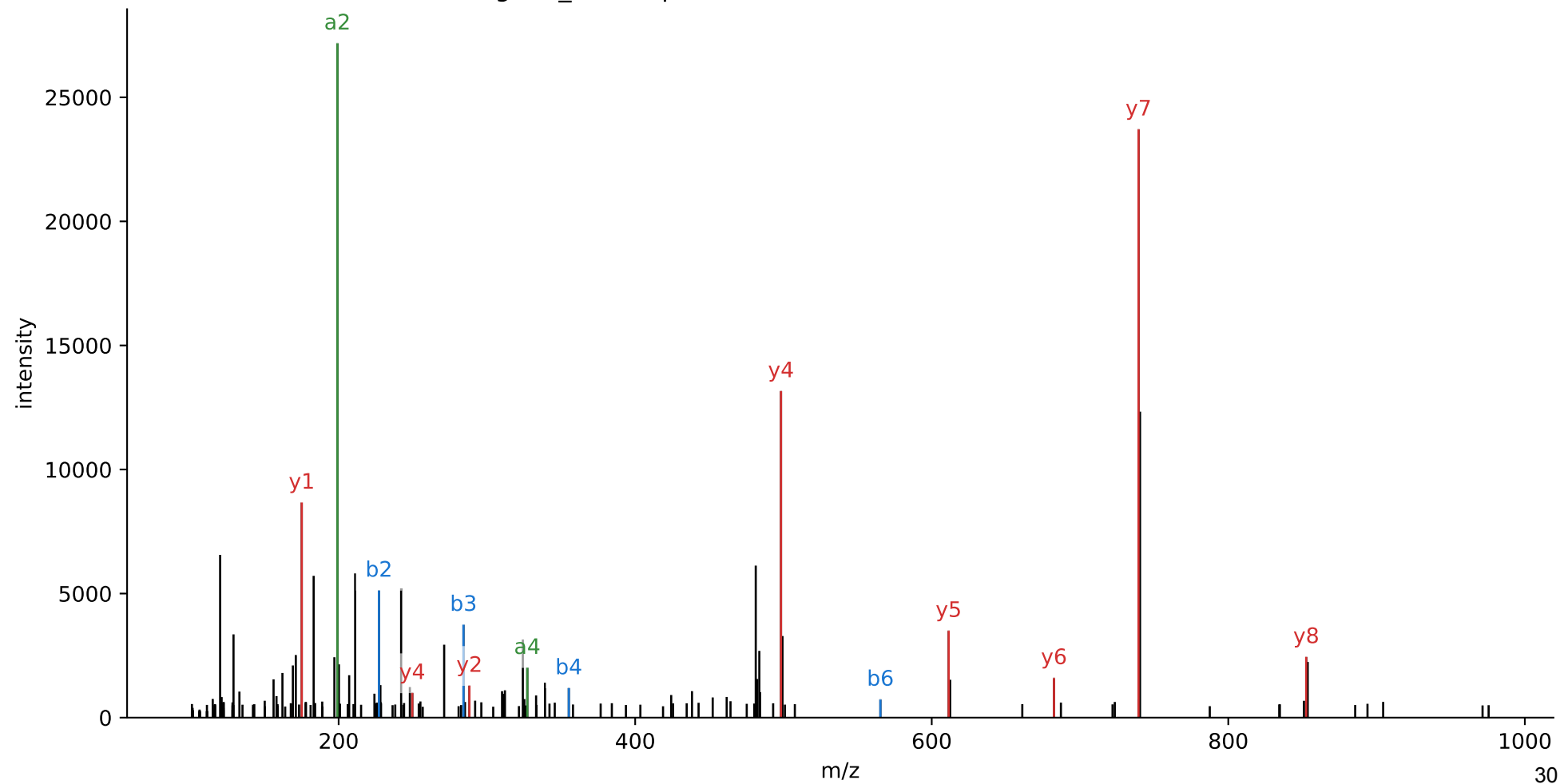

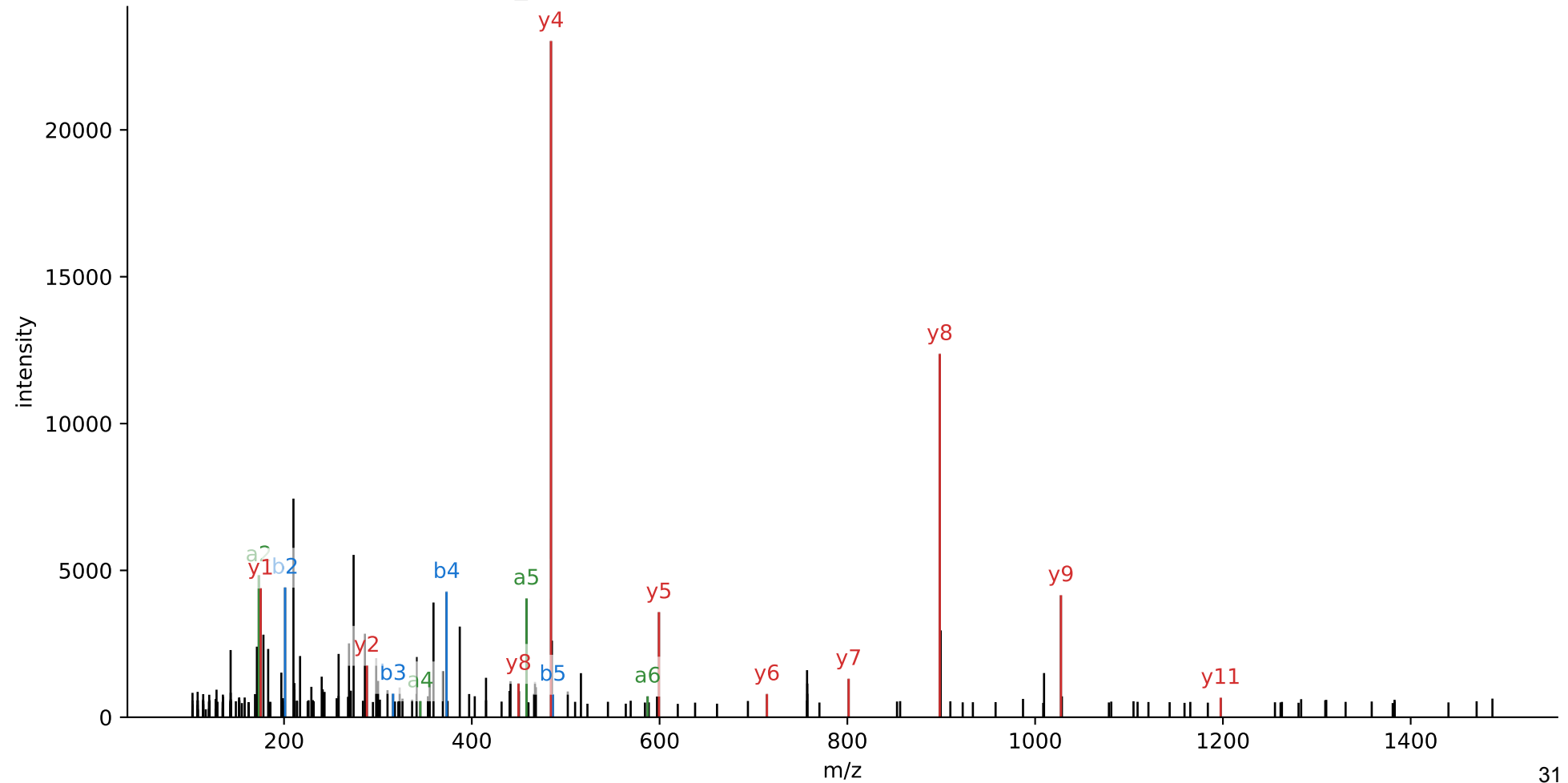

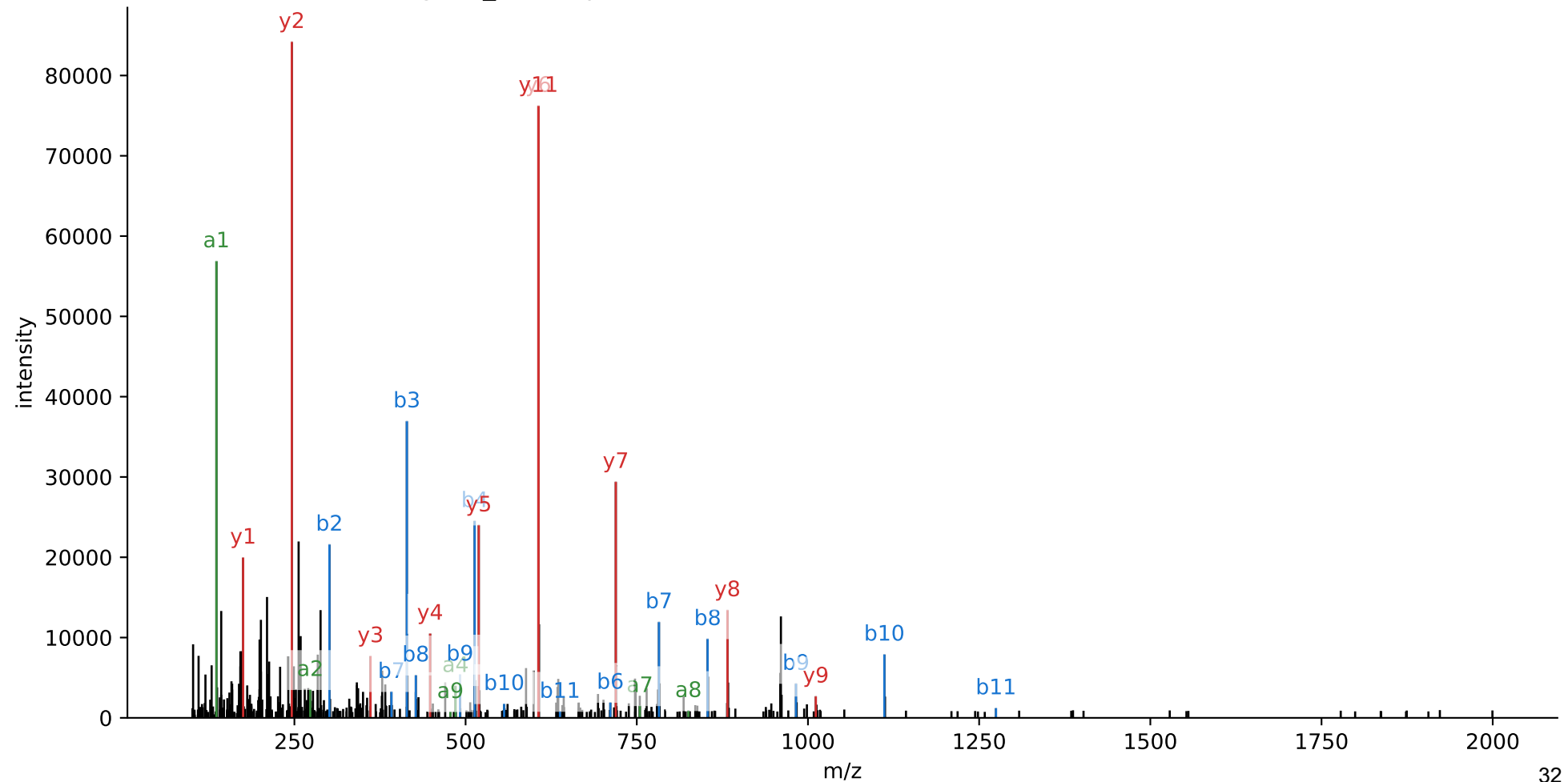

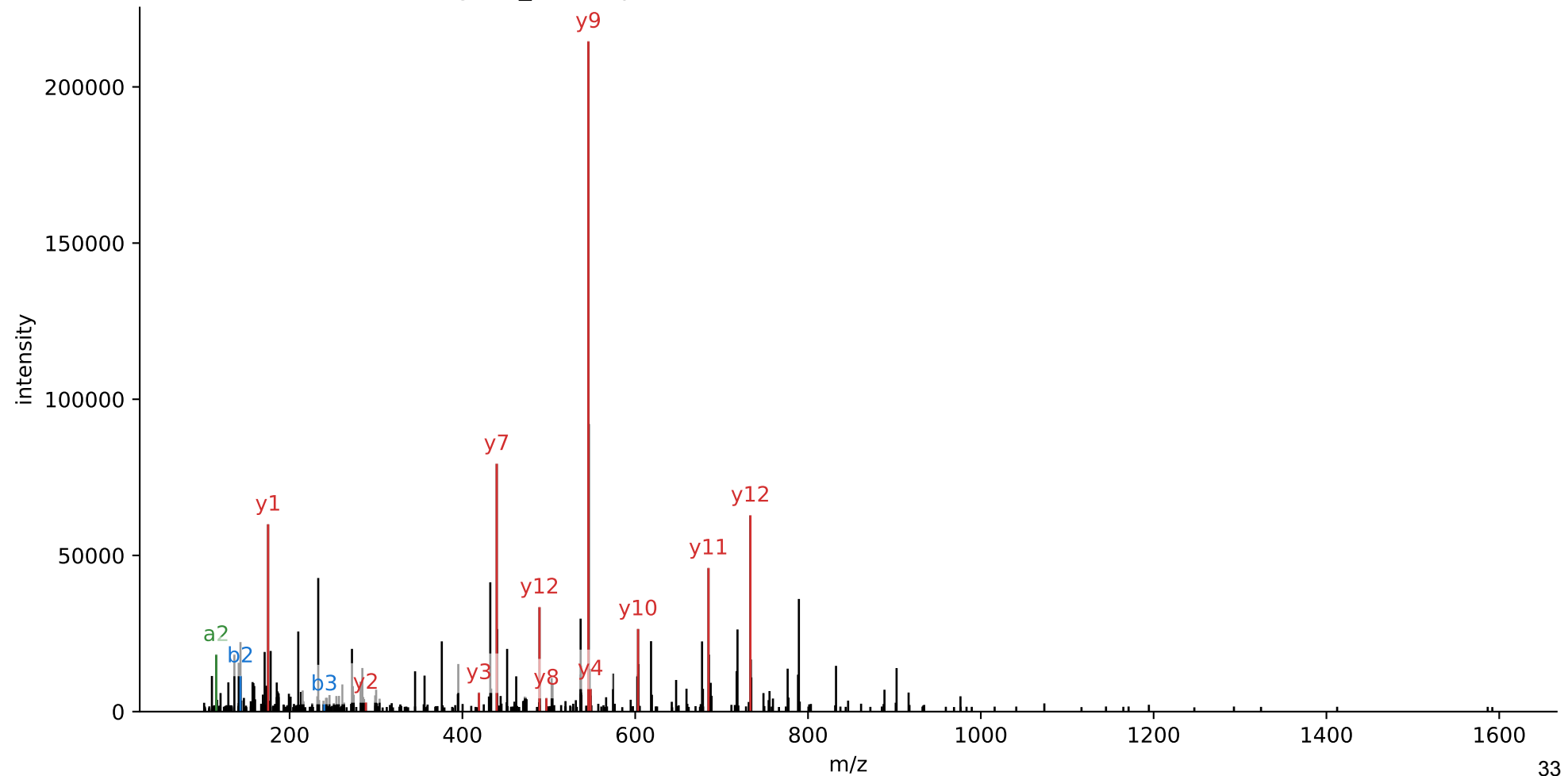

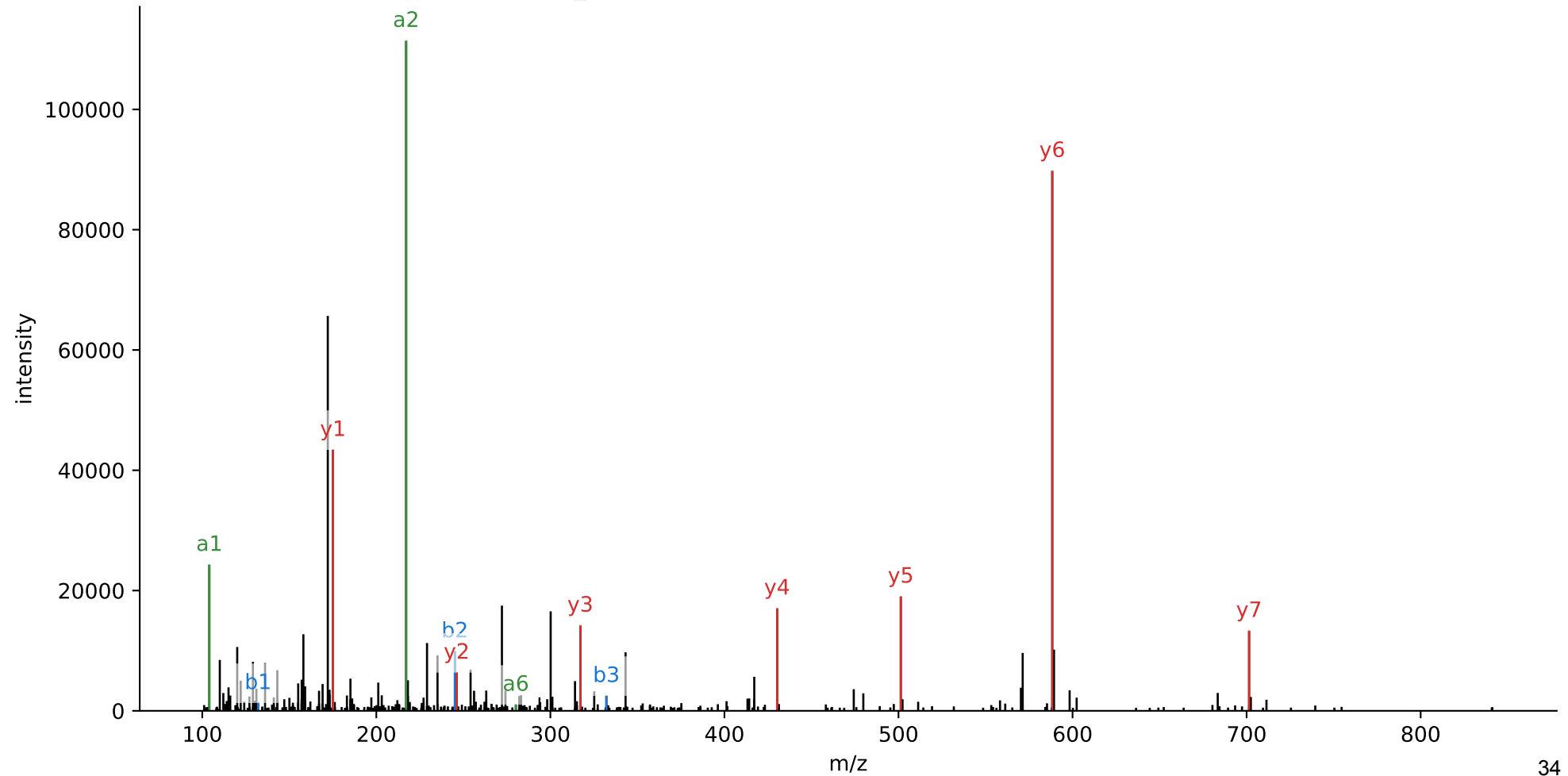

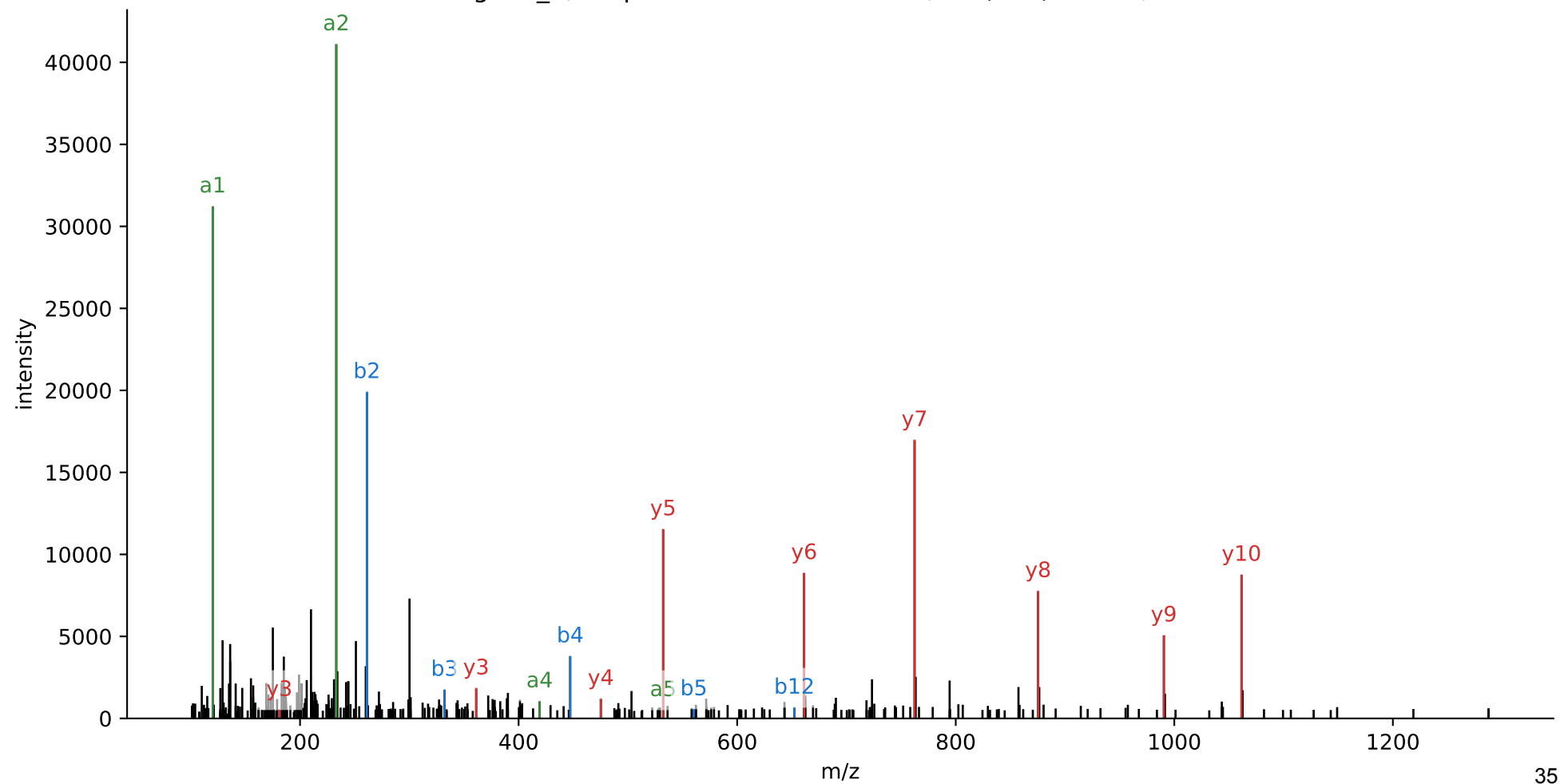

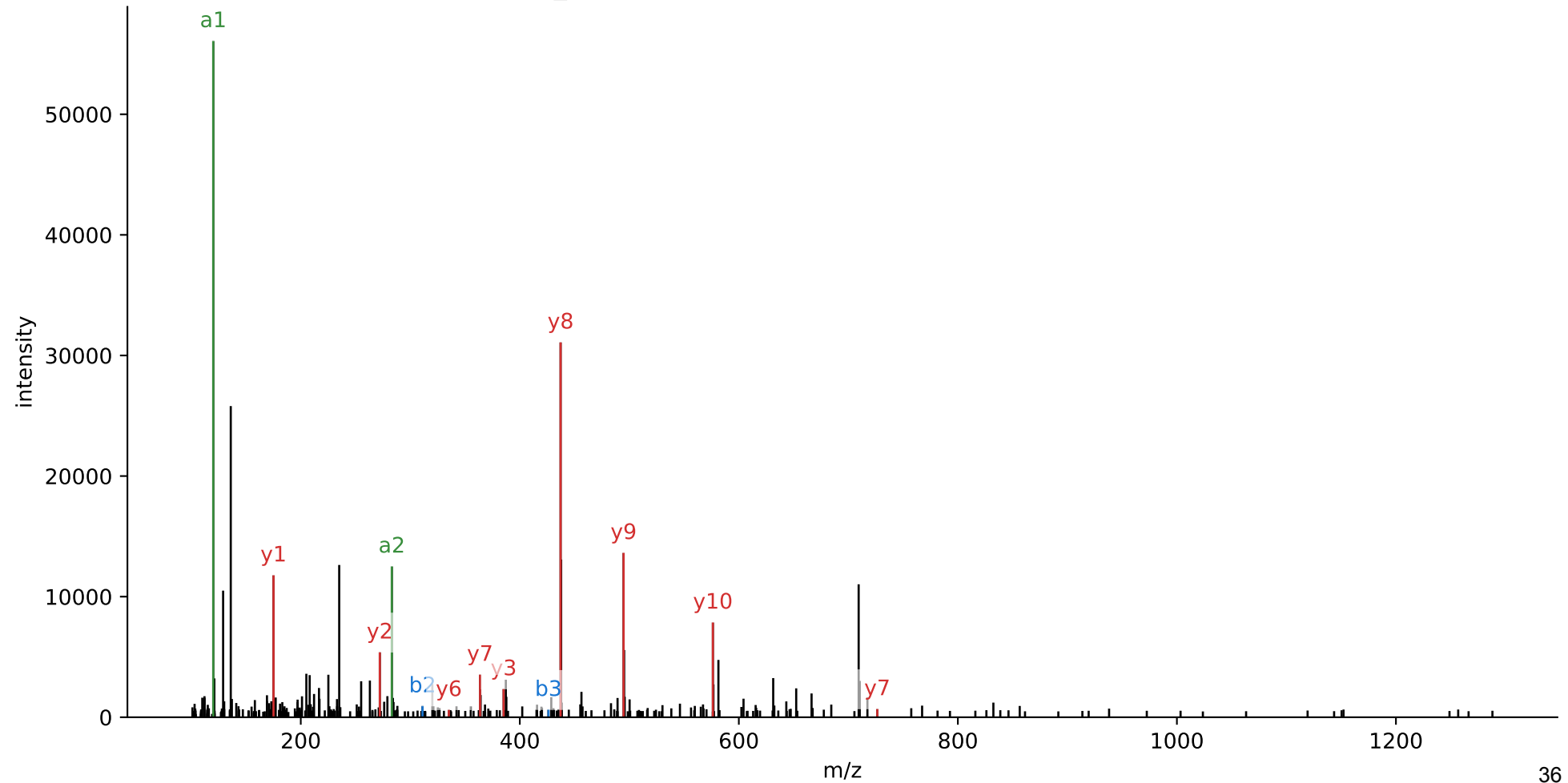

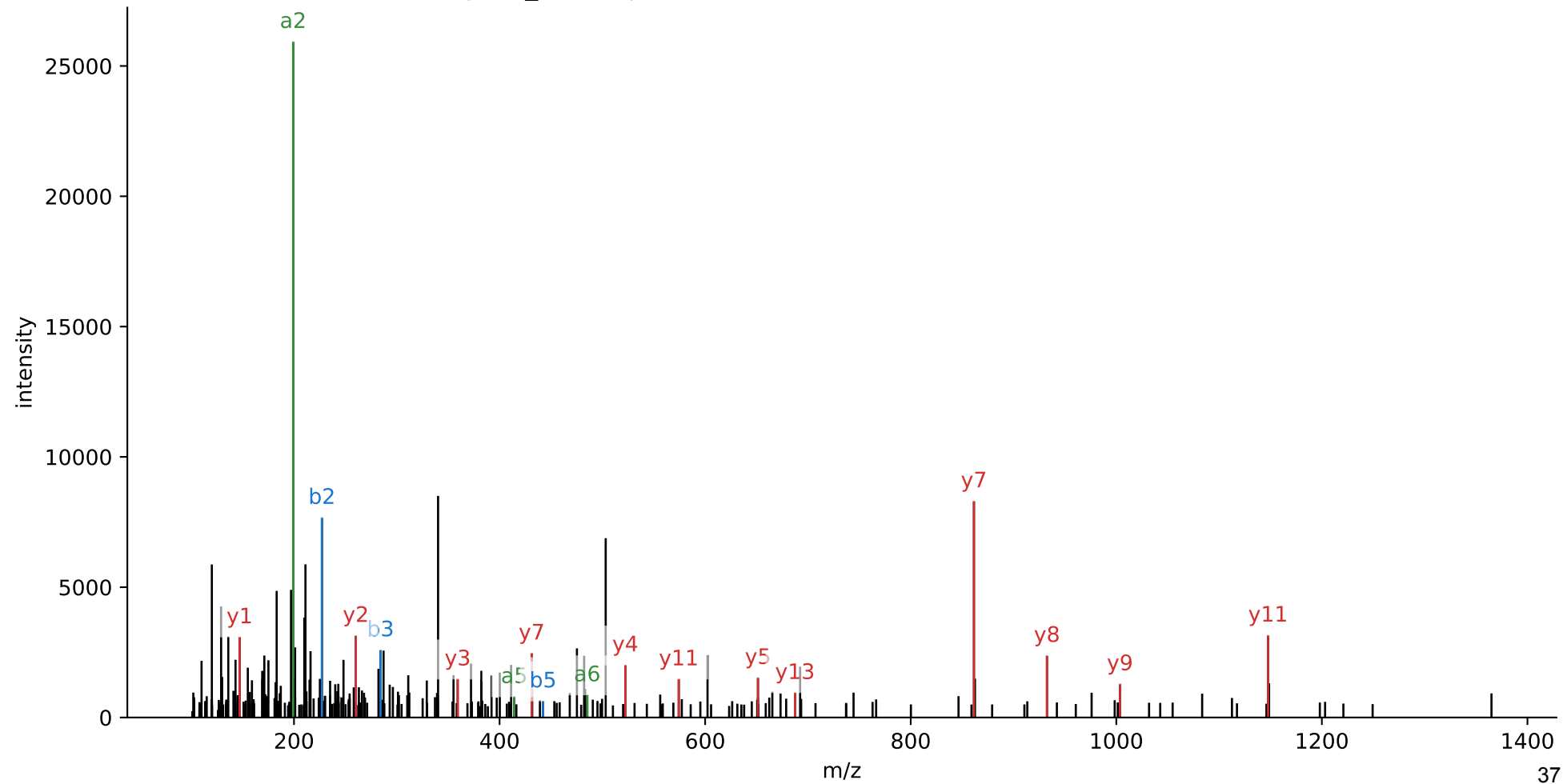

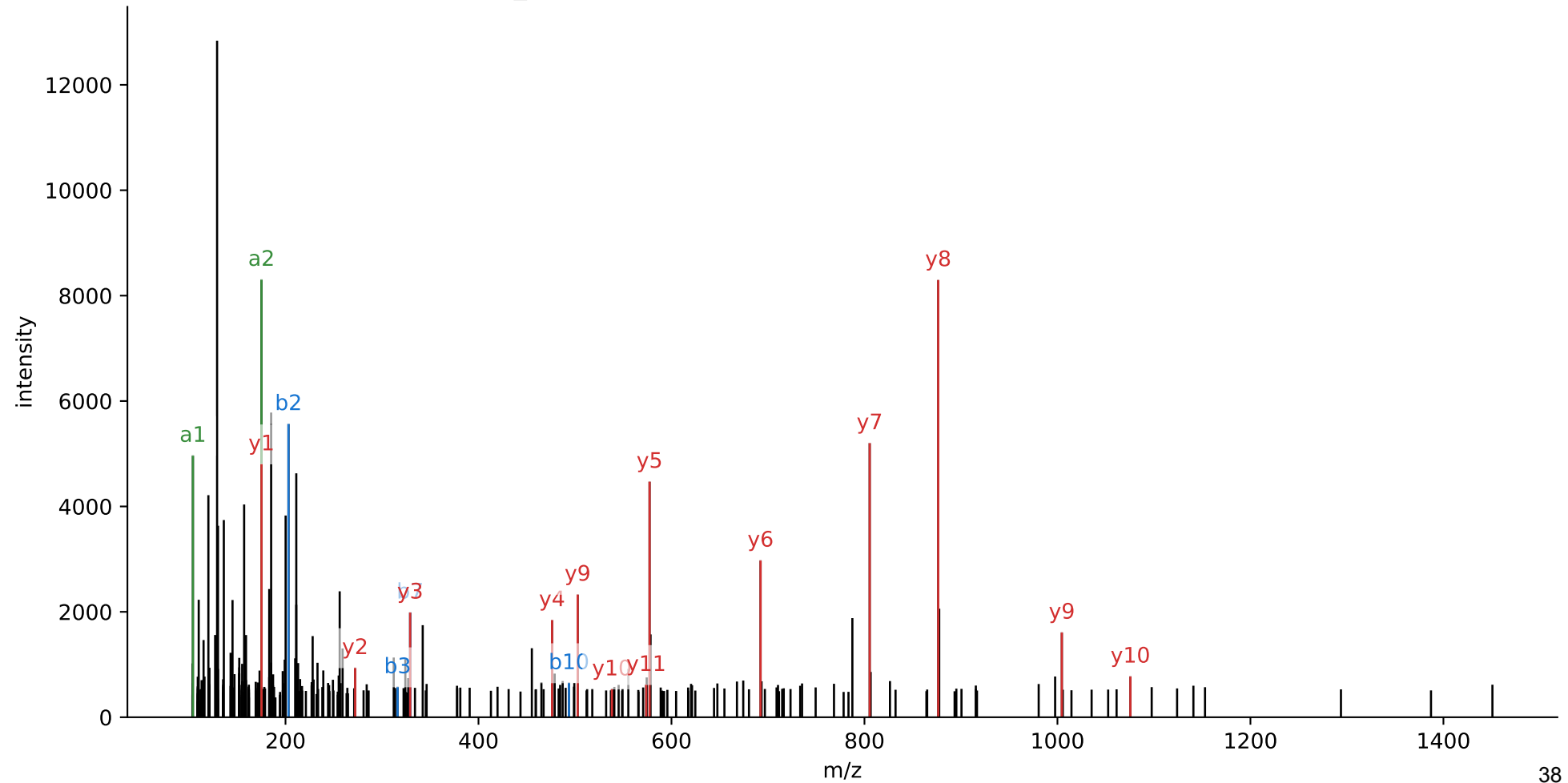

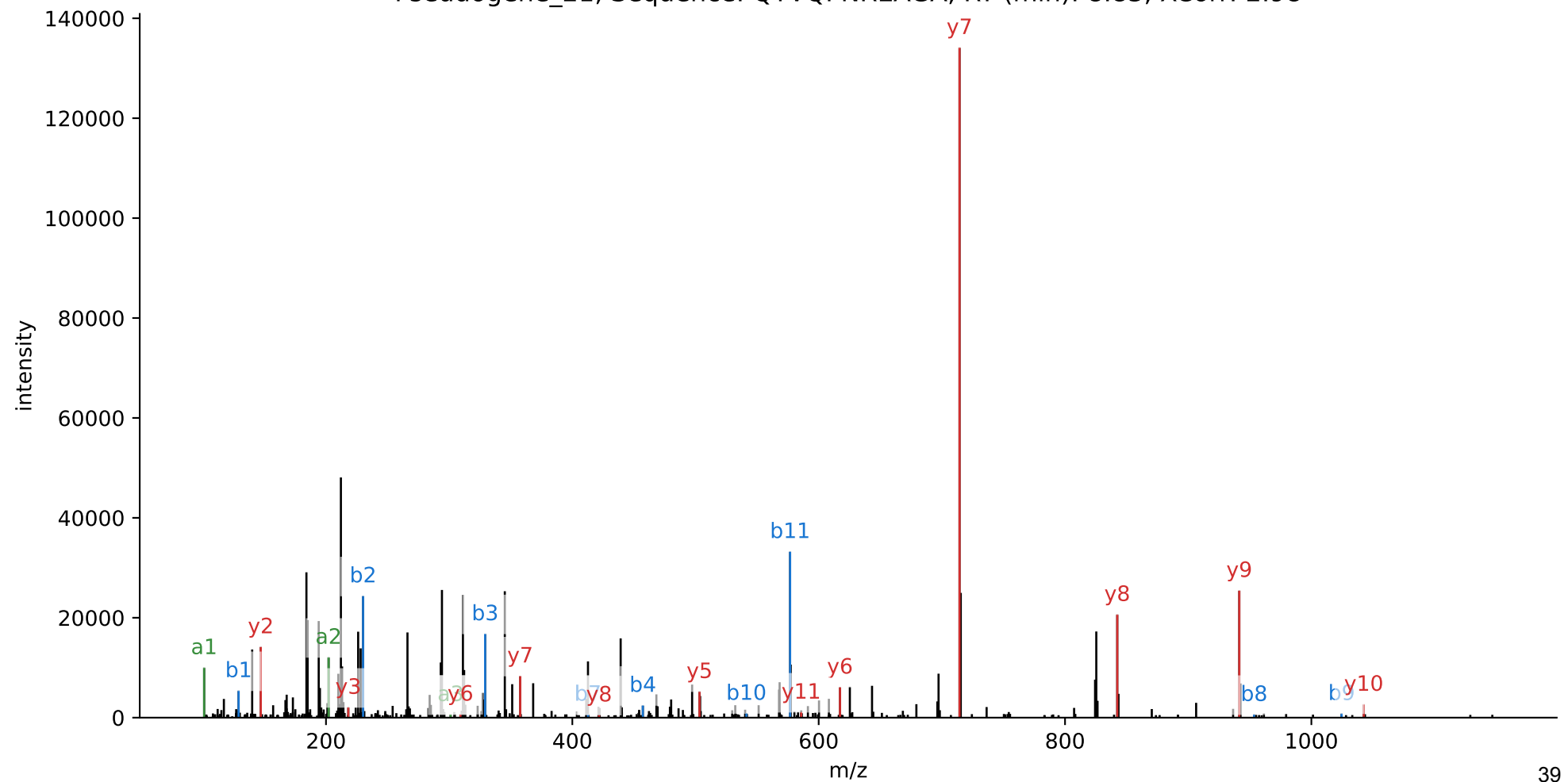

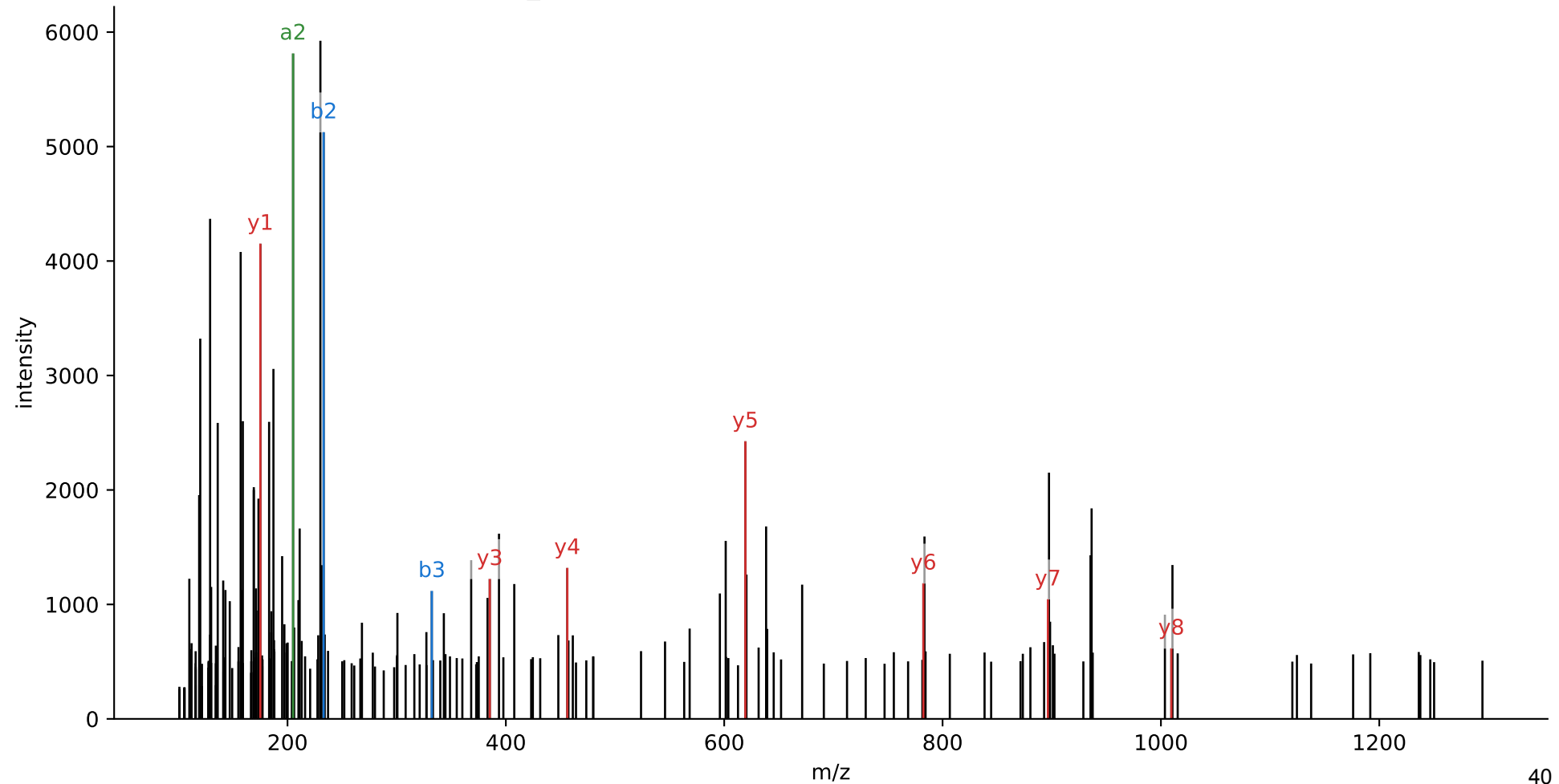

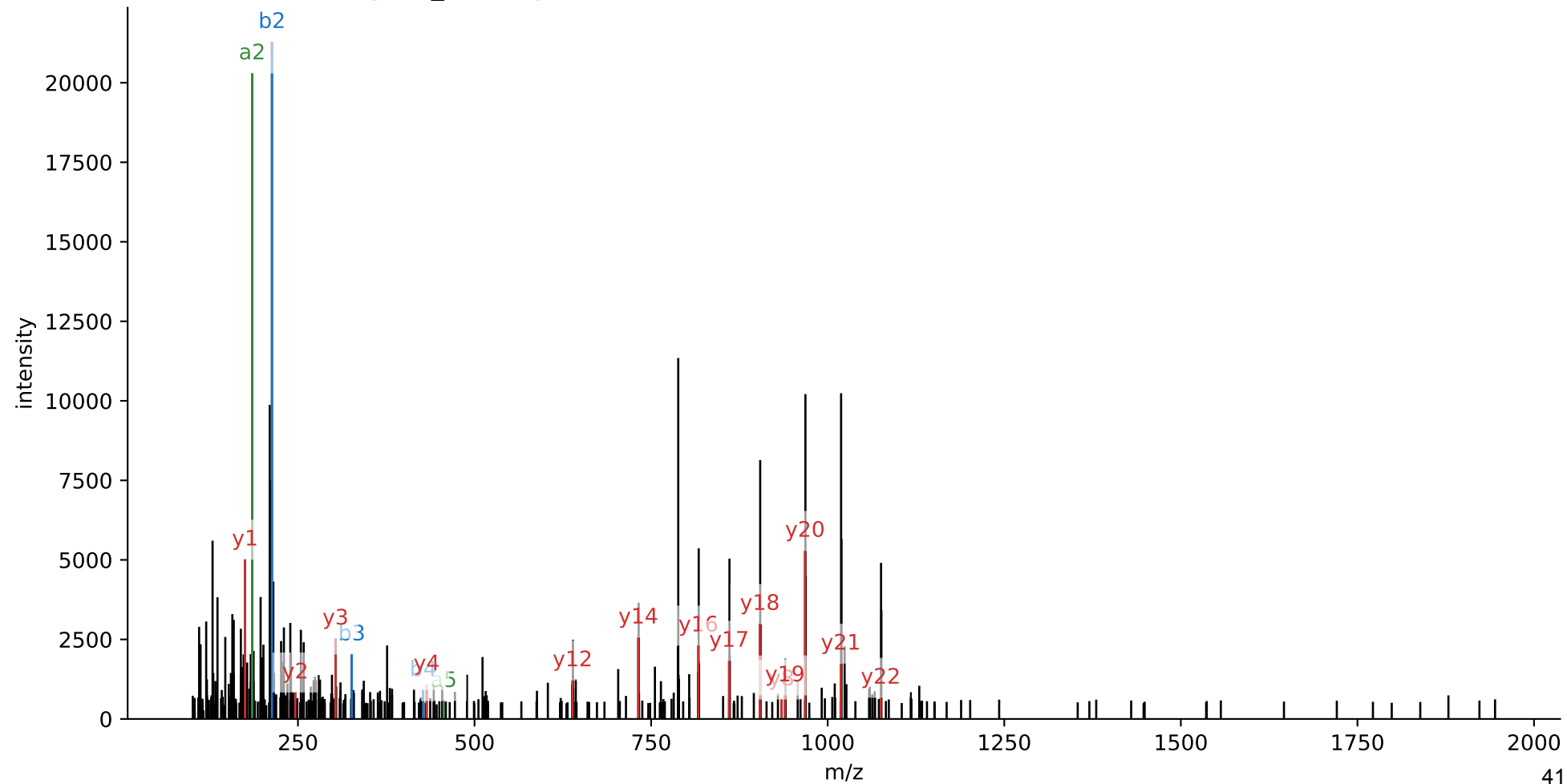

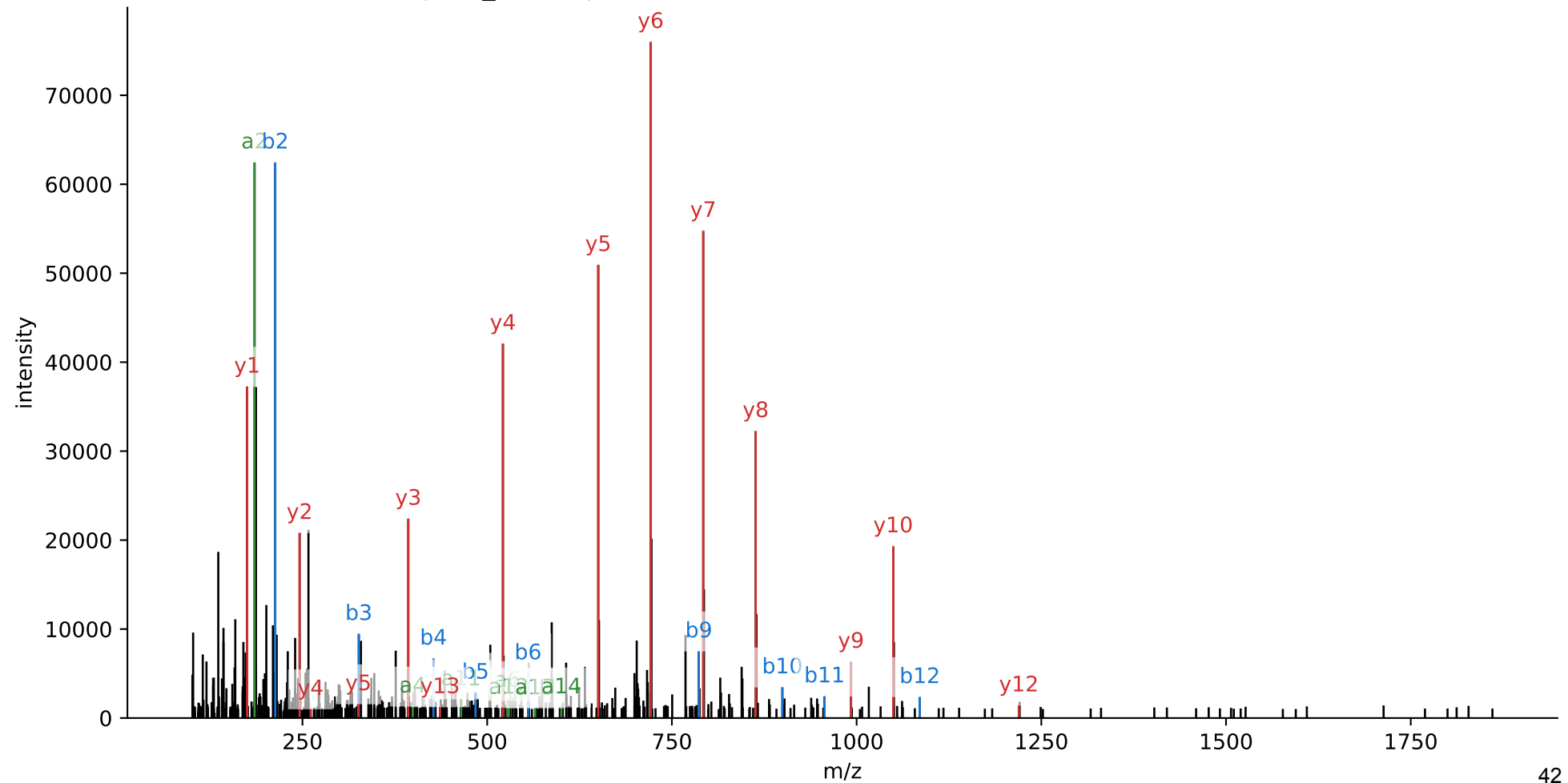

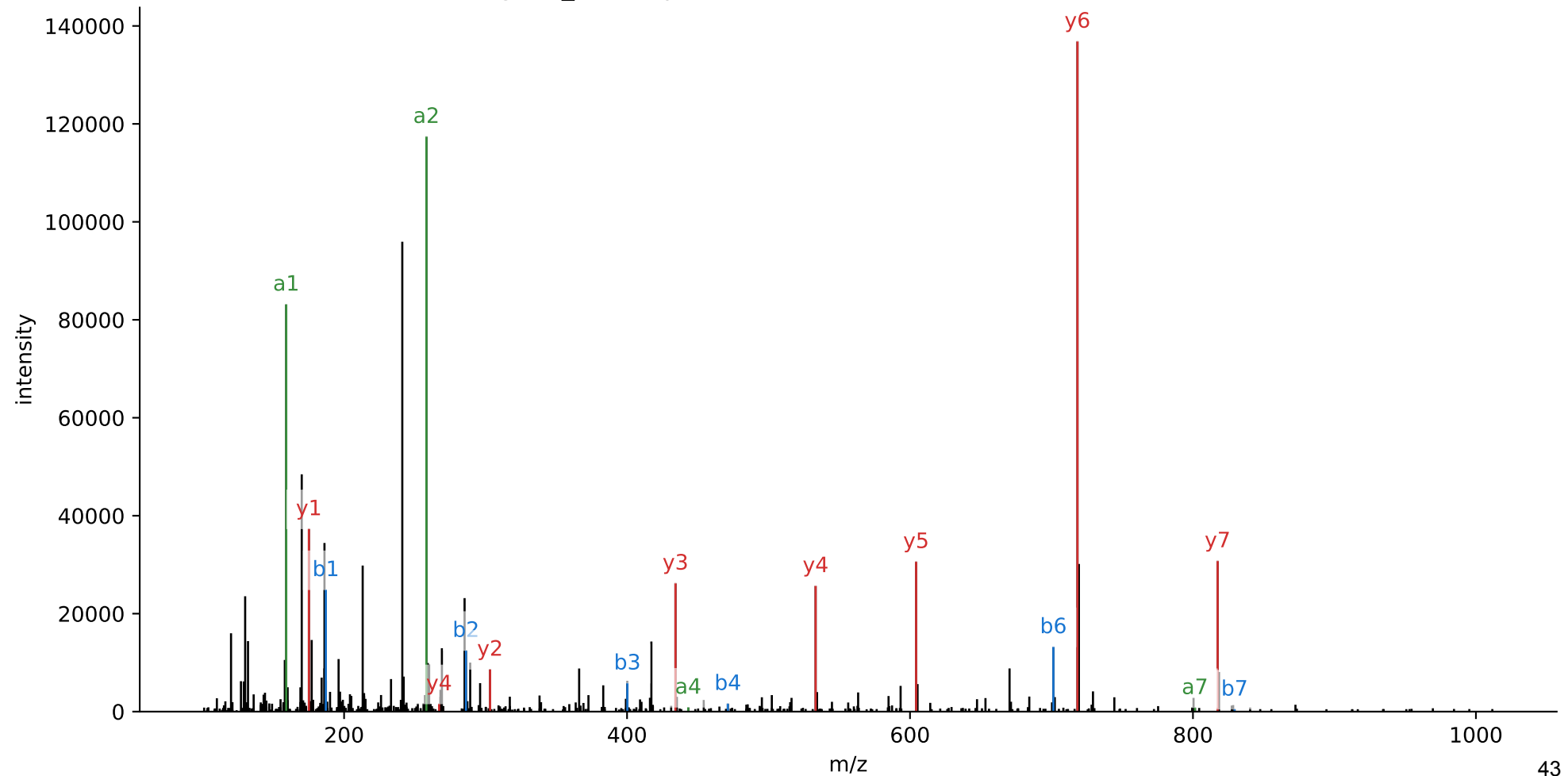

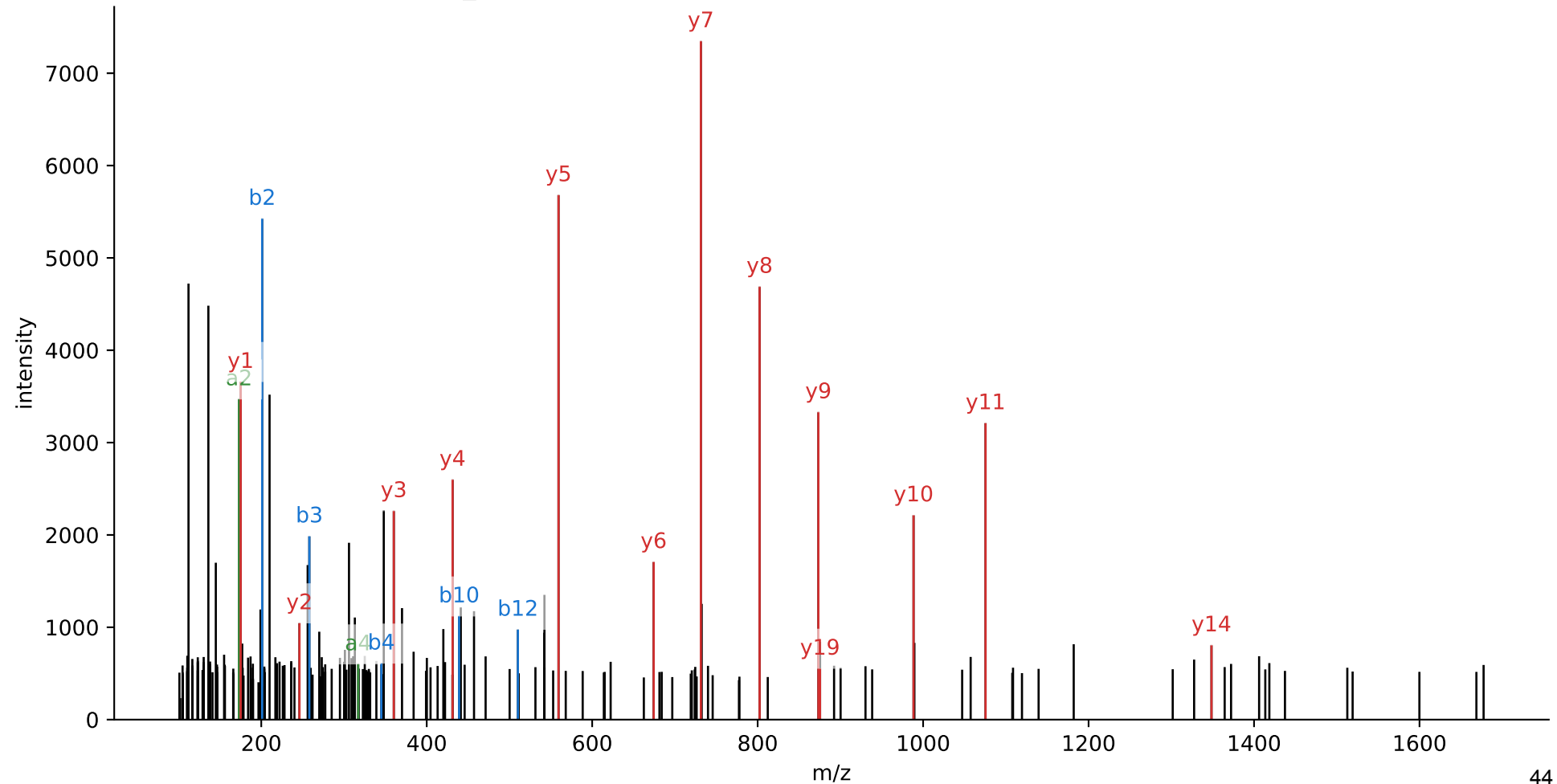

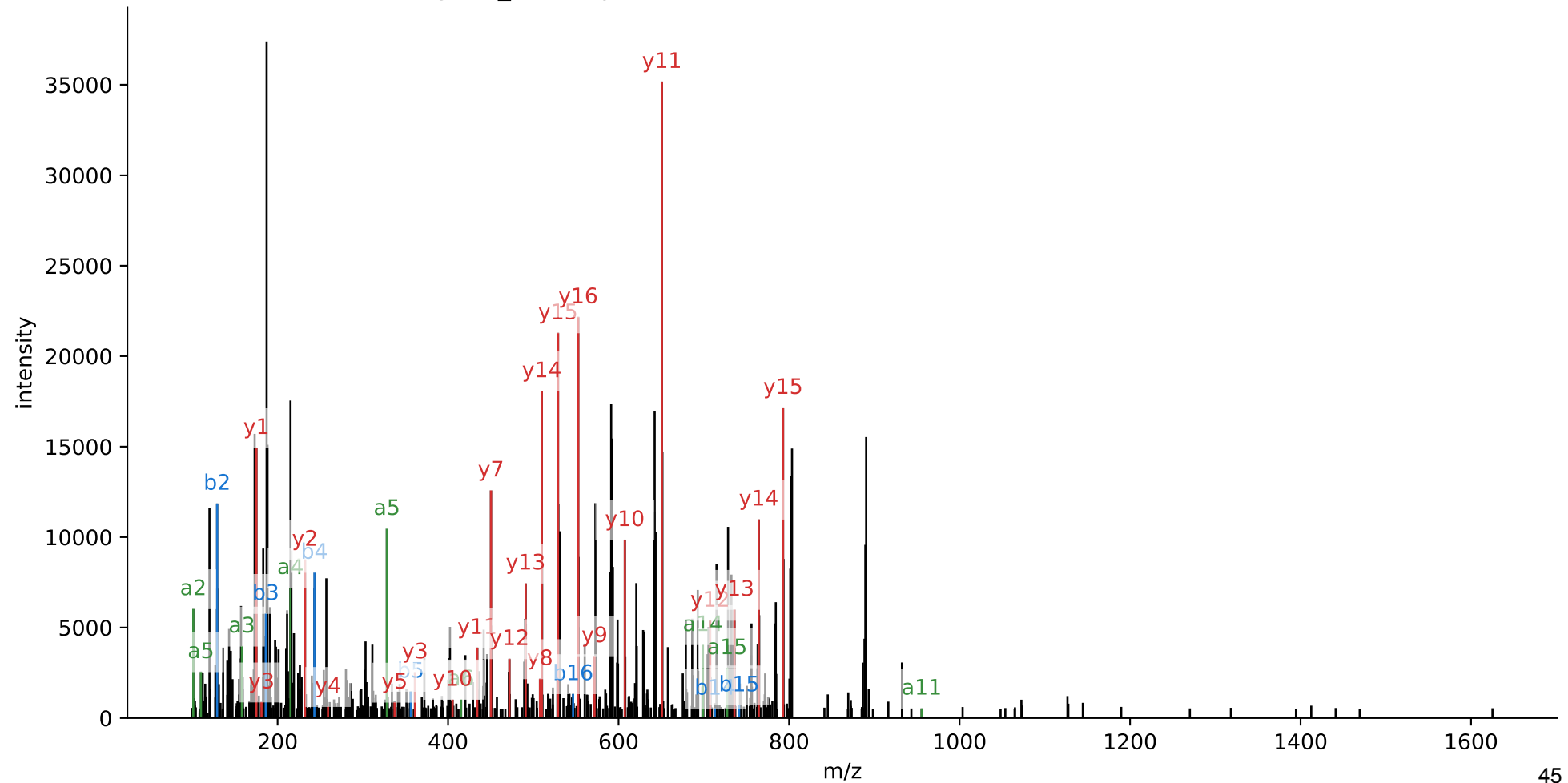

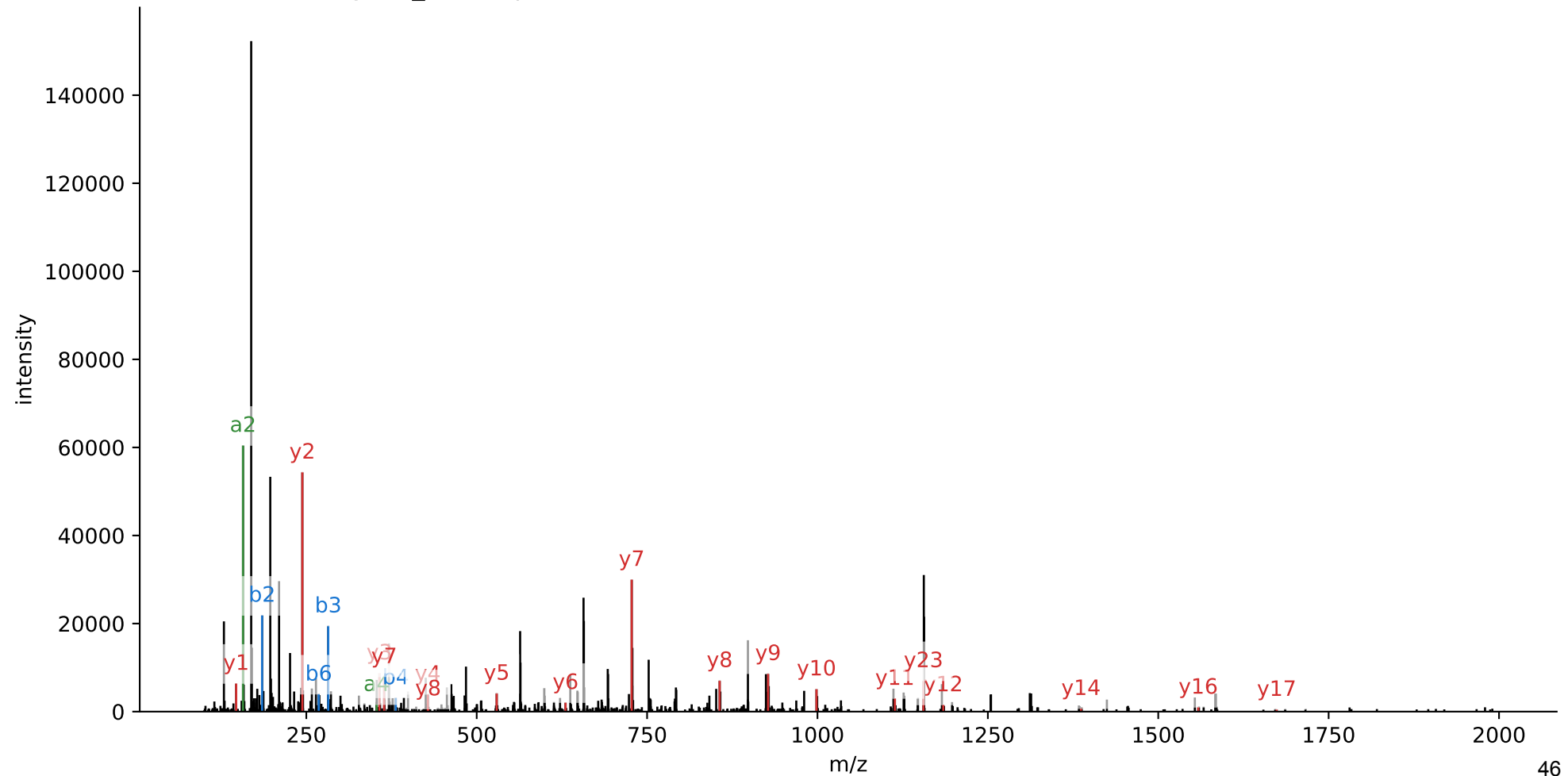

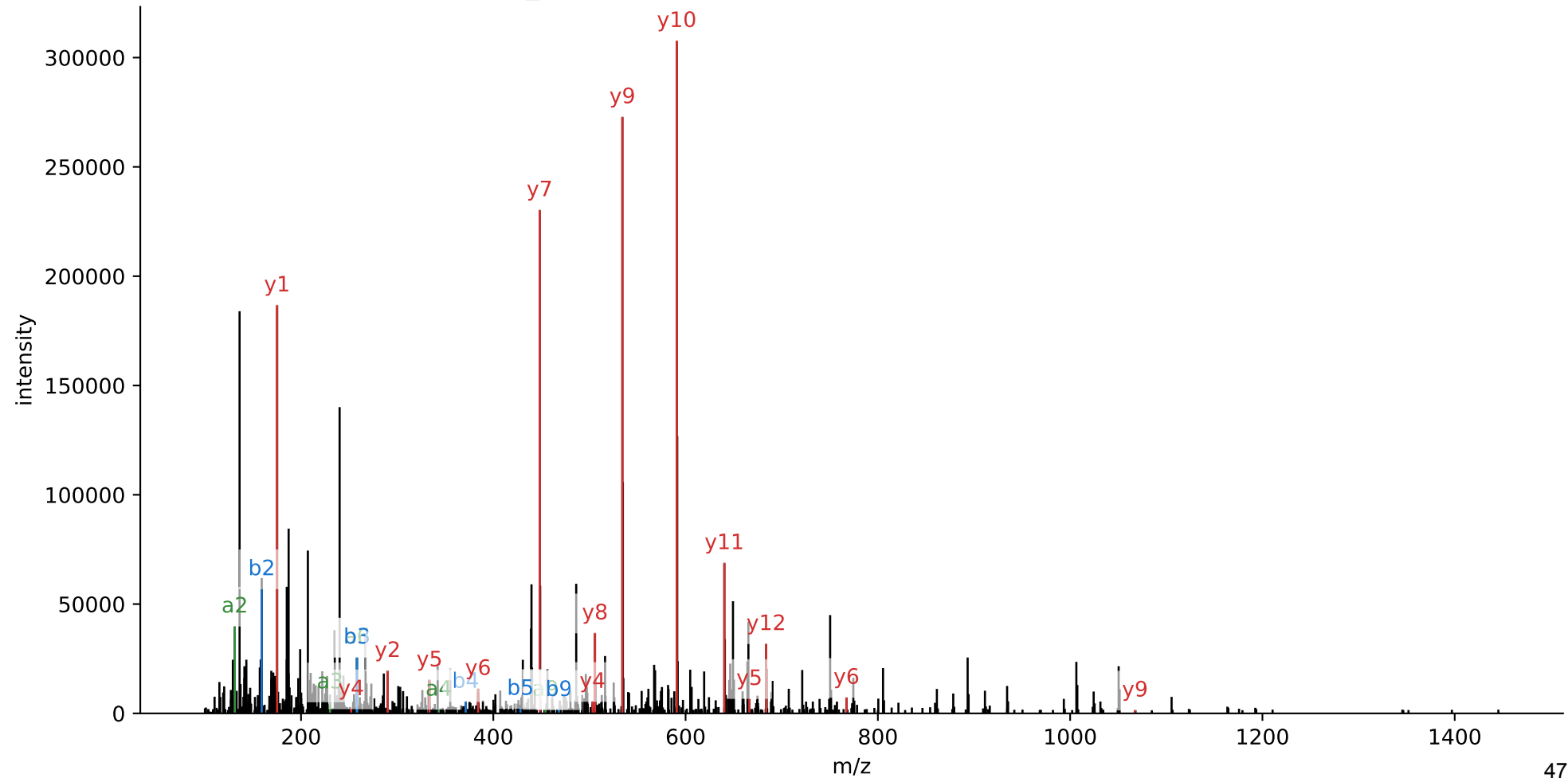

Pseudogene\_22, Sequence: ASVLGDQTYGR, RT (min): 20.98, XCorr: 2.81

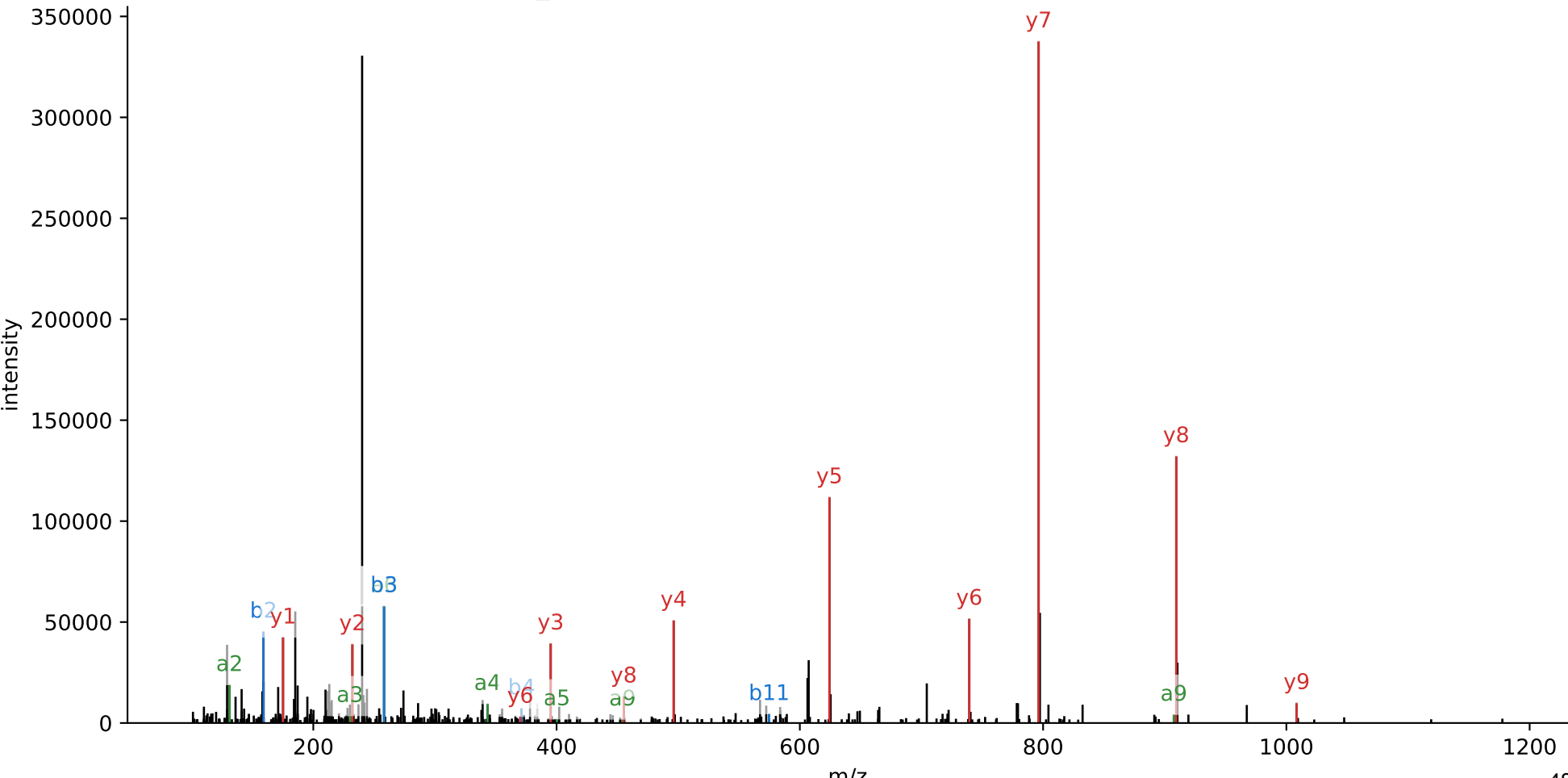

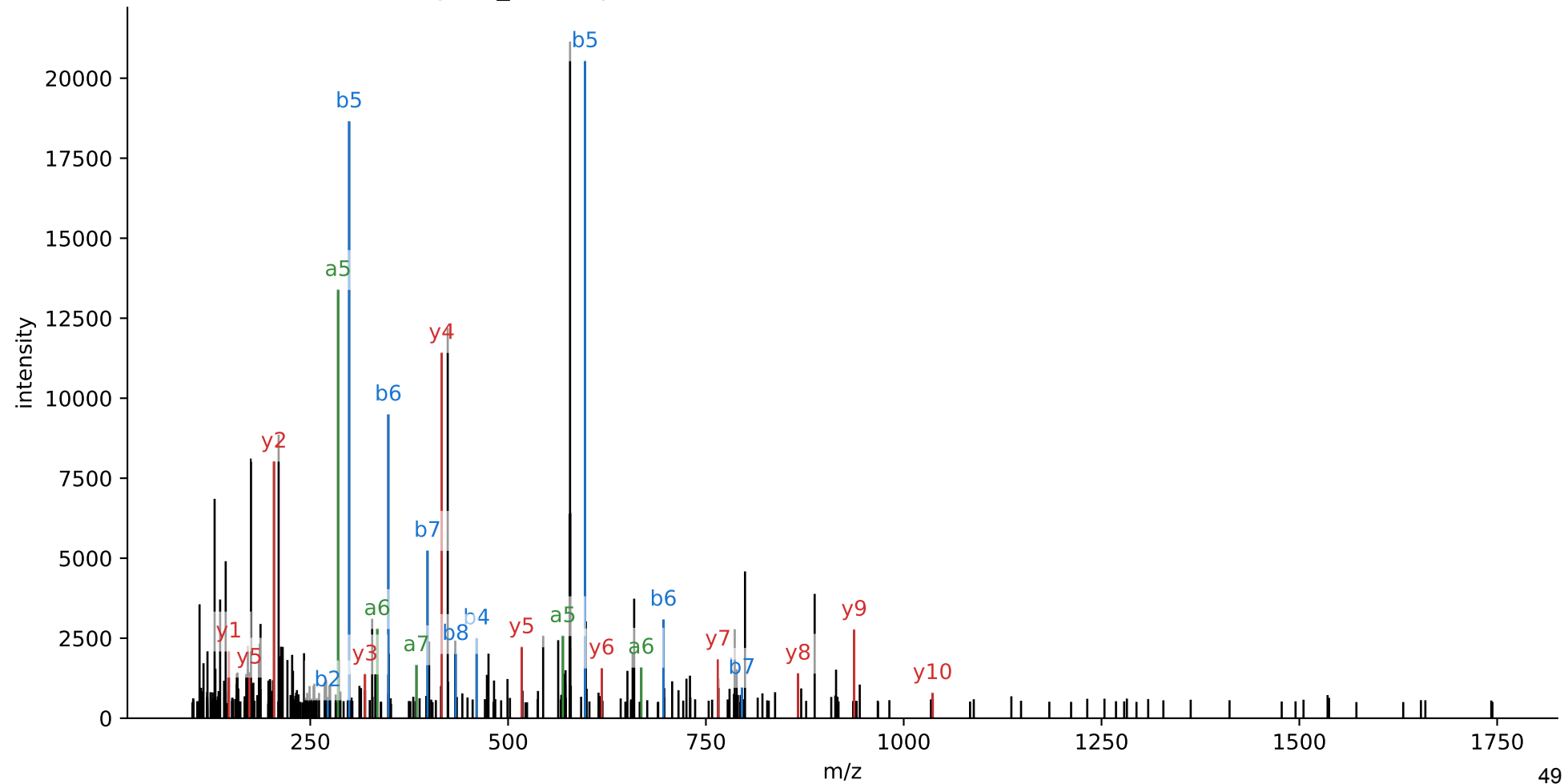

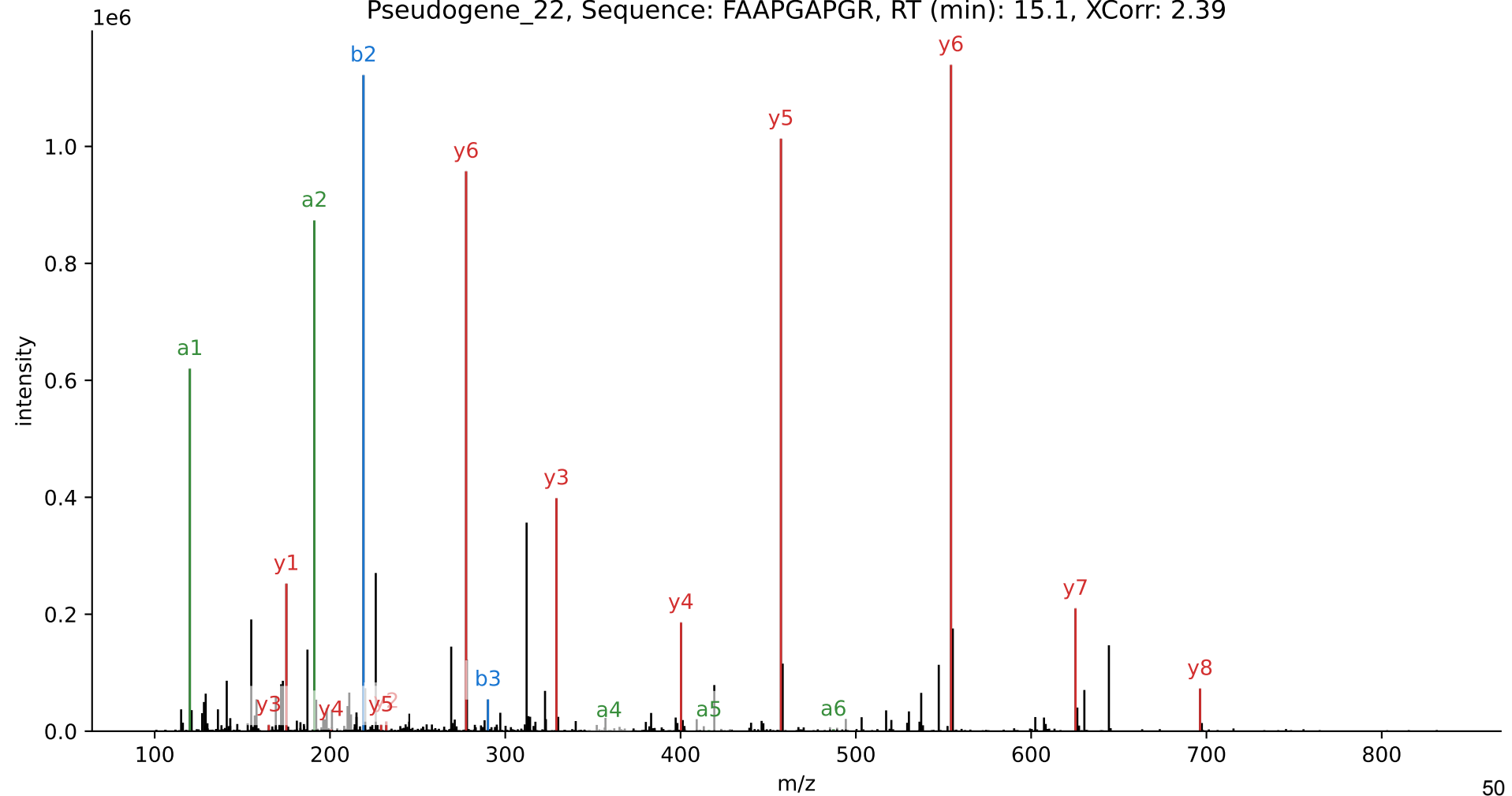

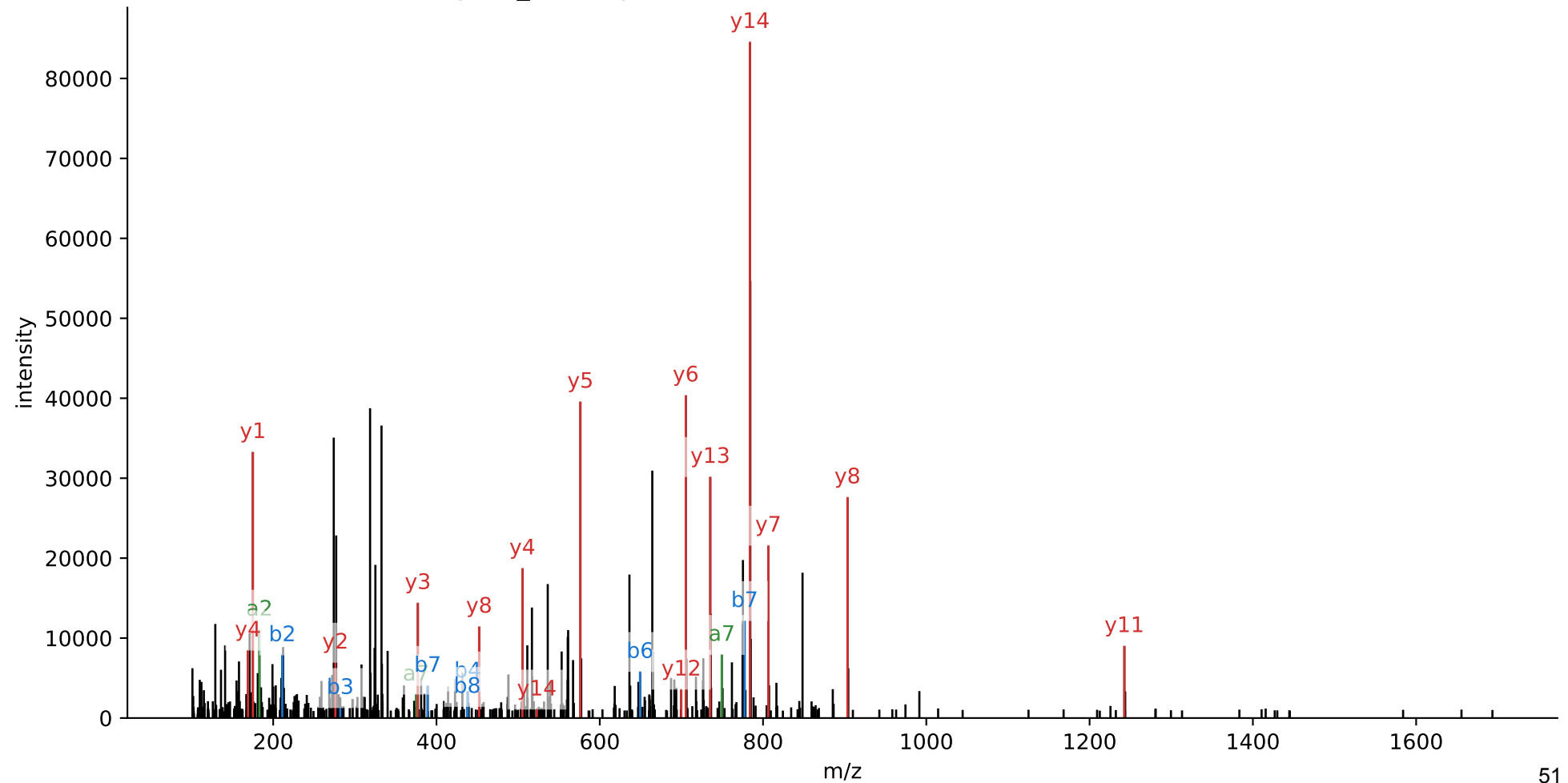

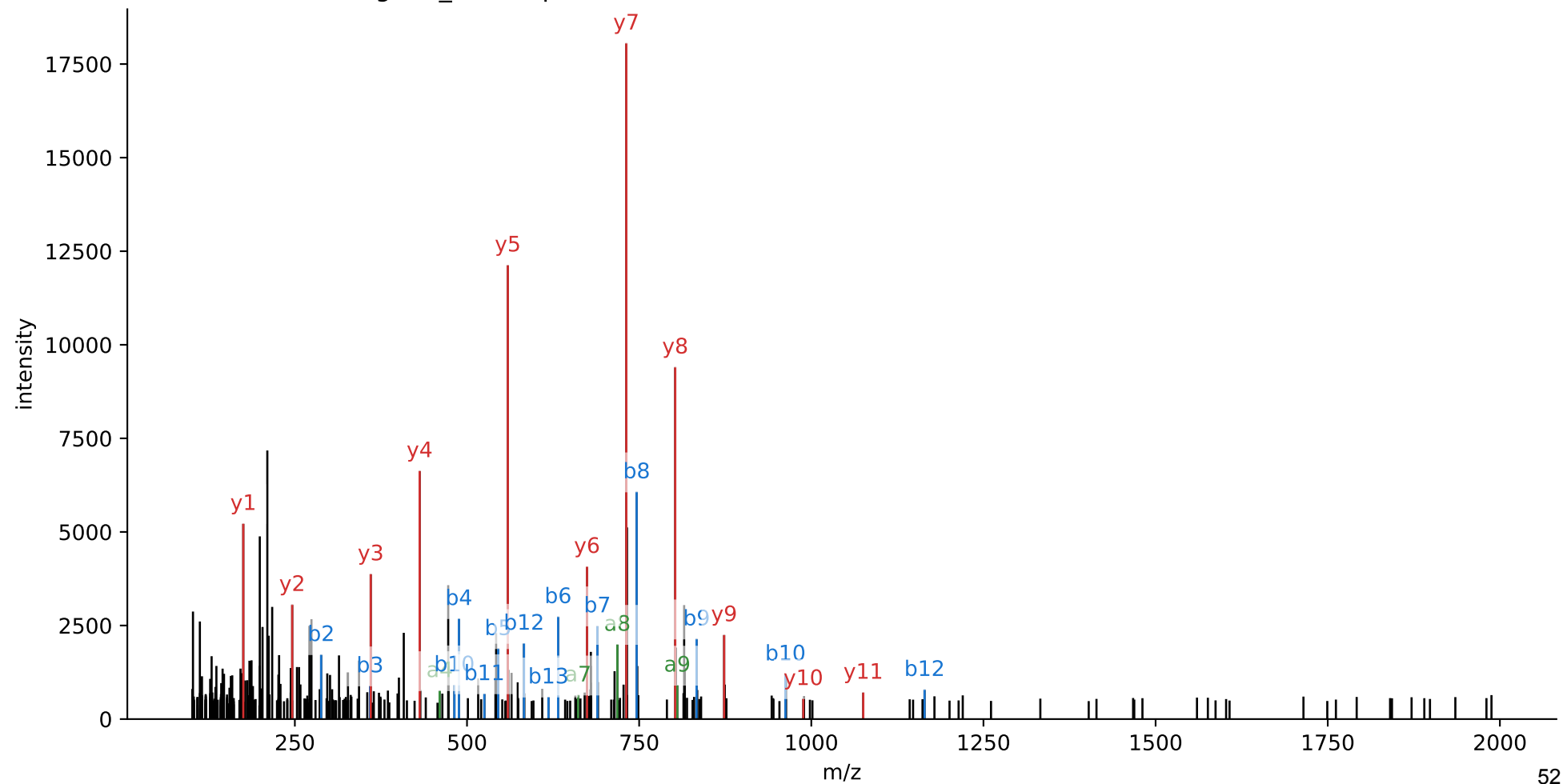

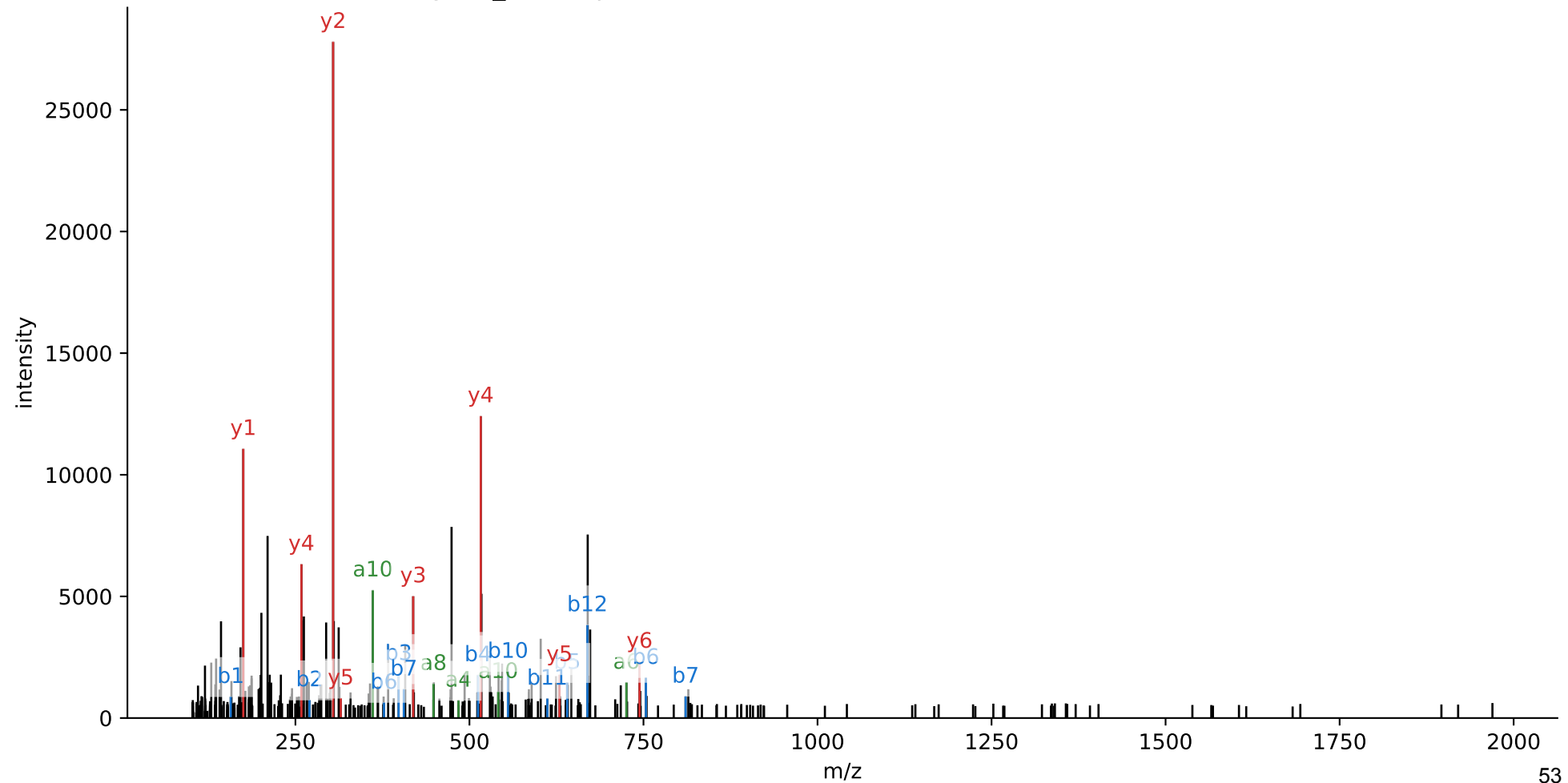

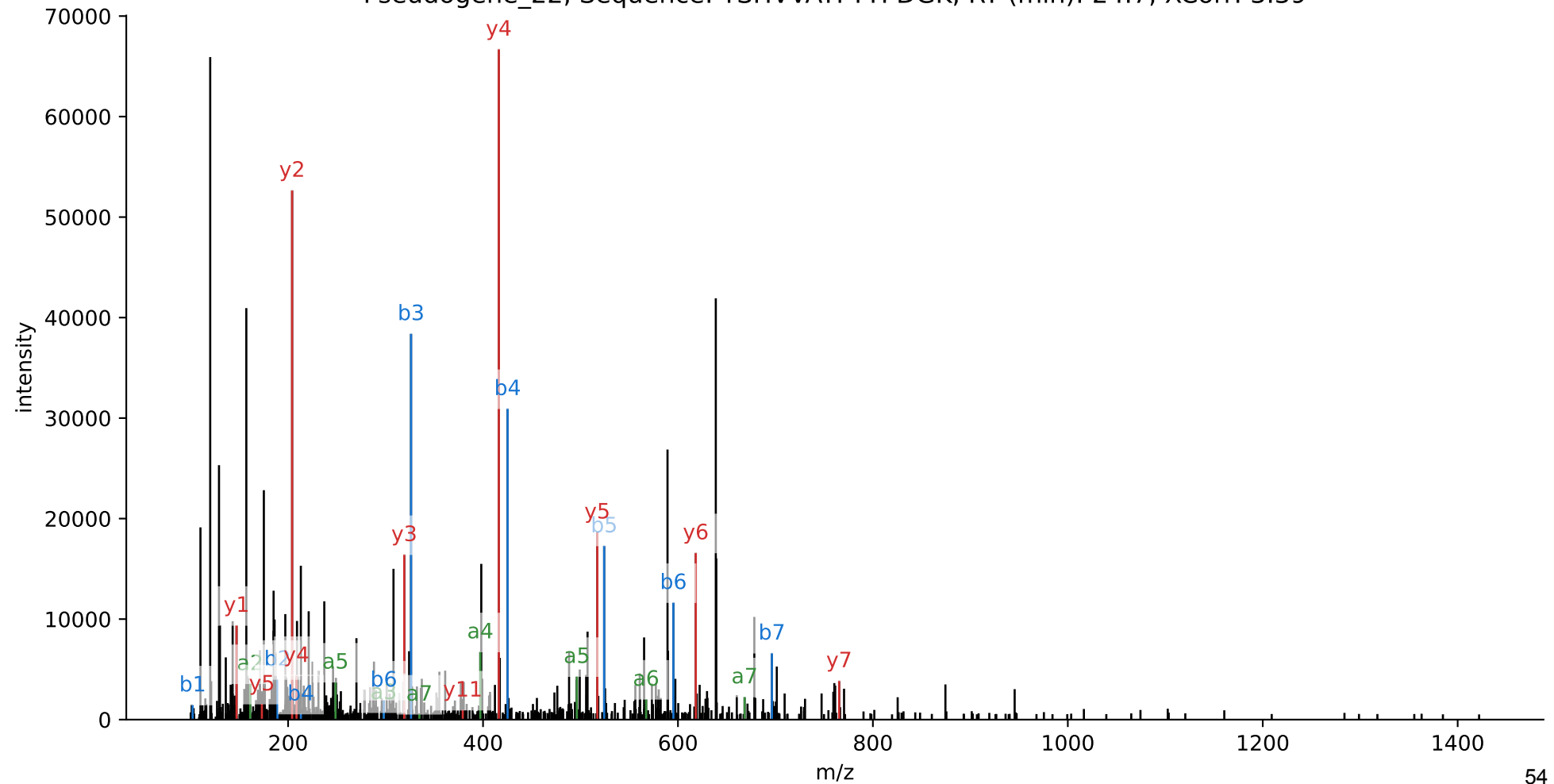

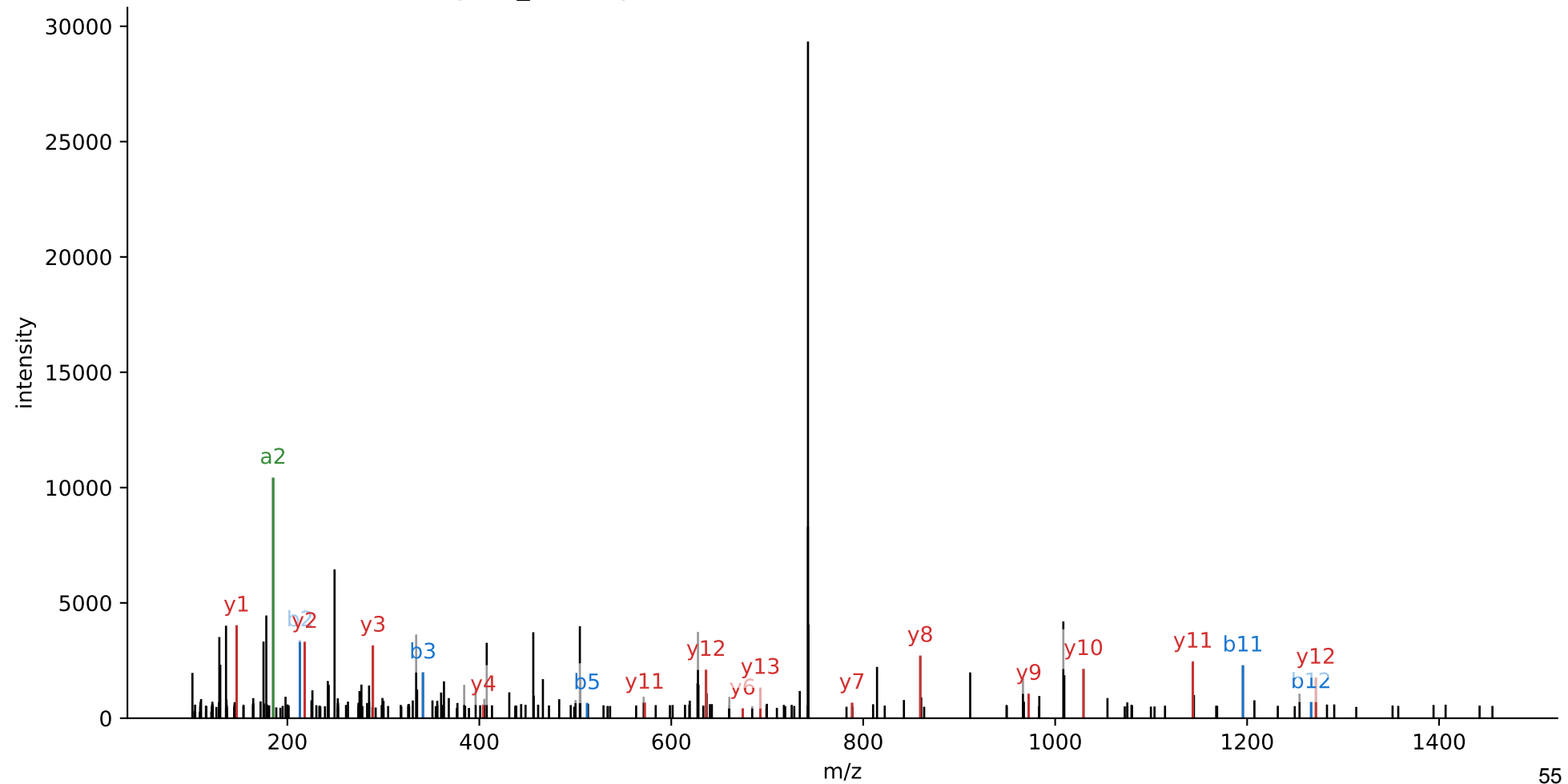

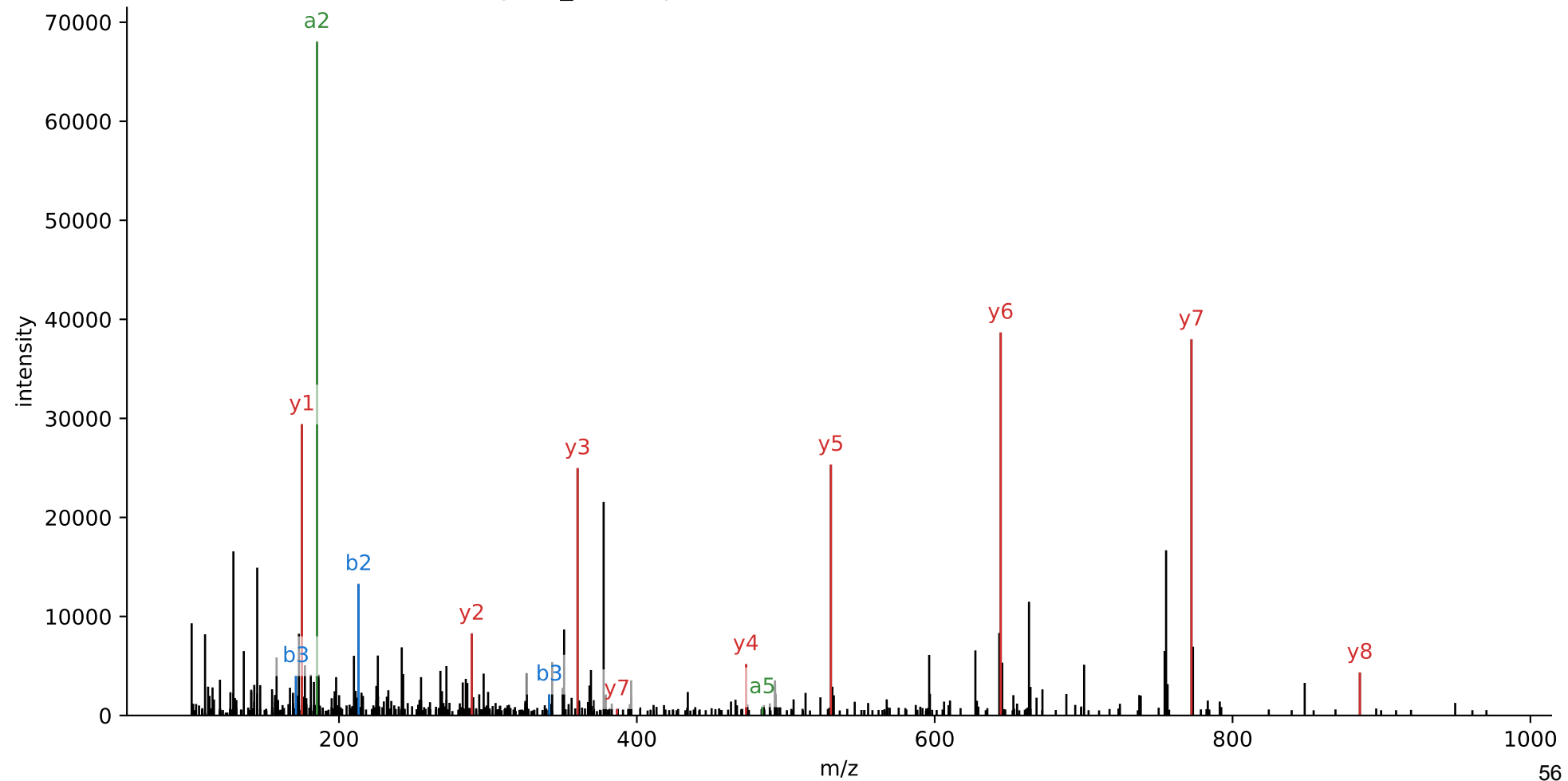

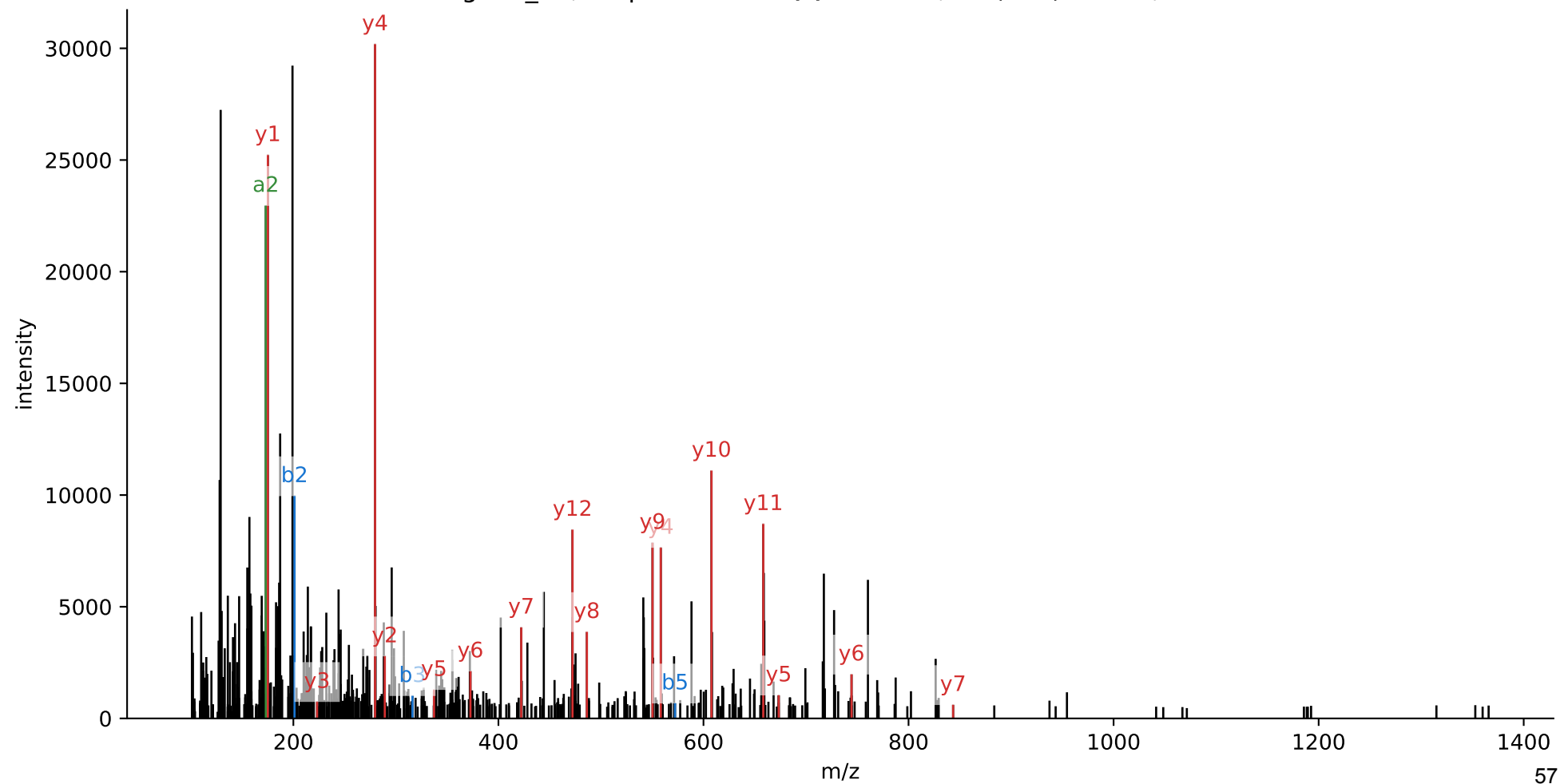

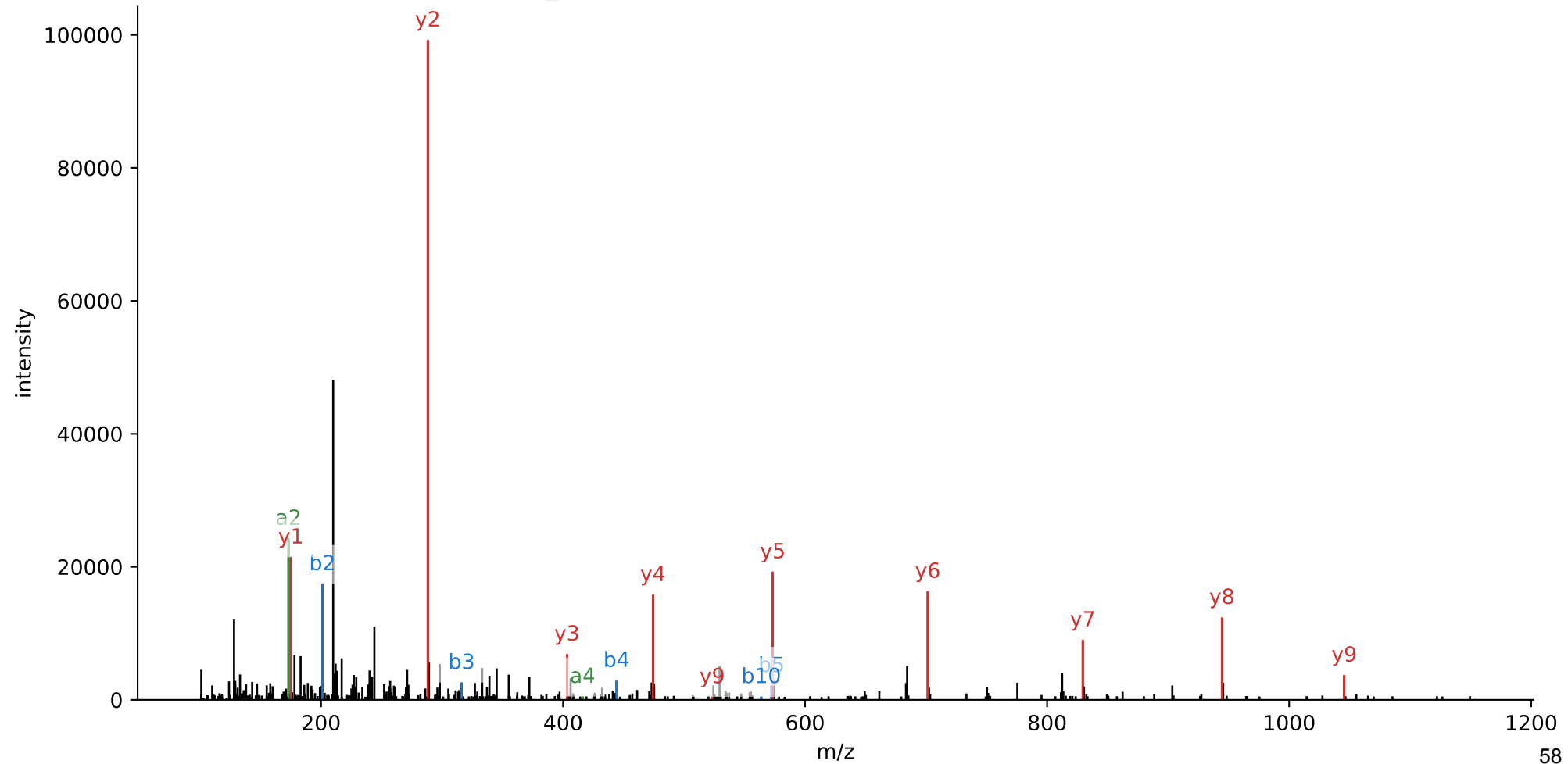

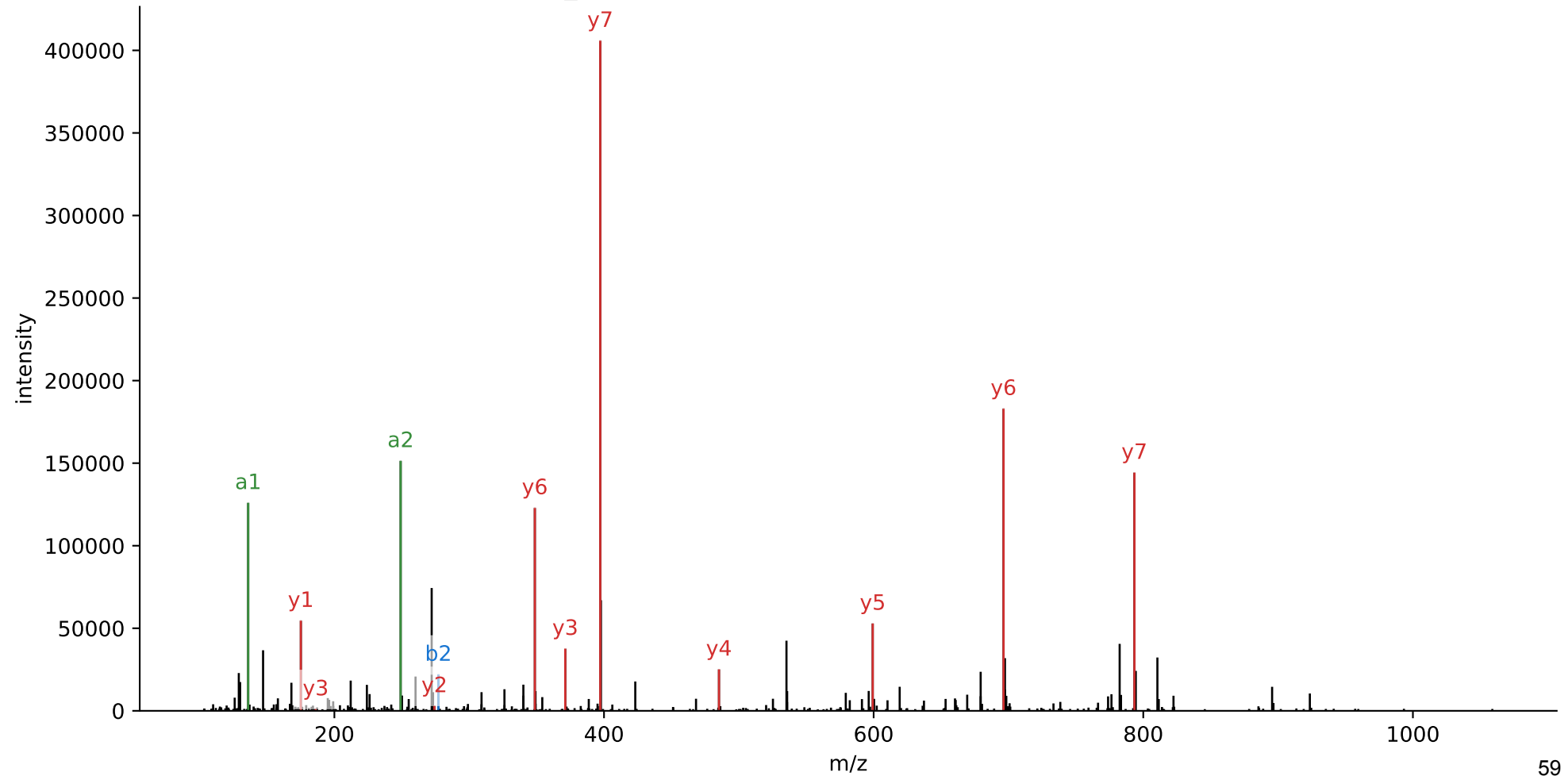

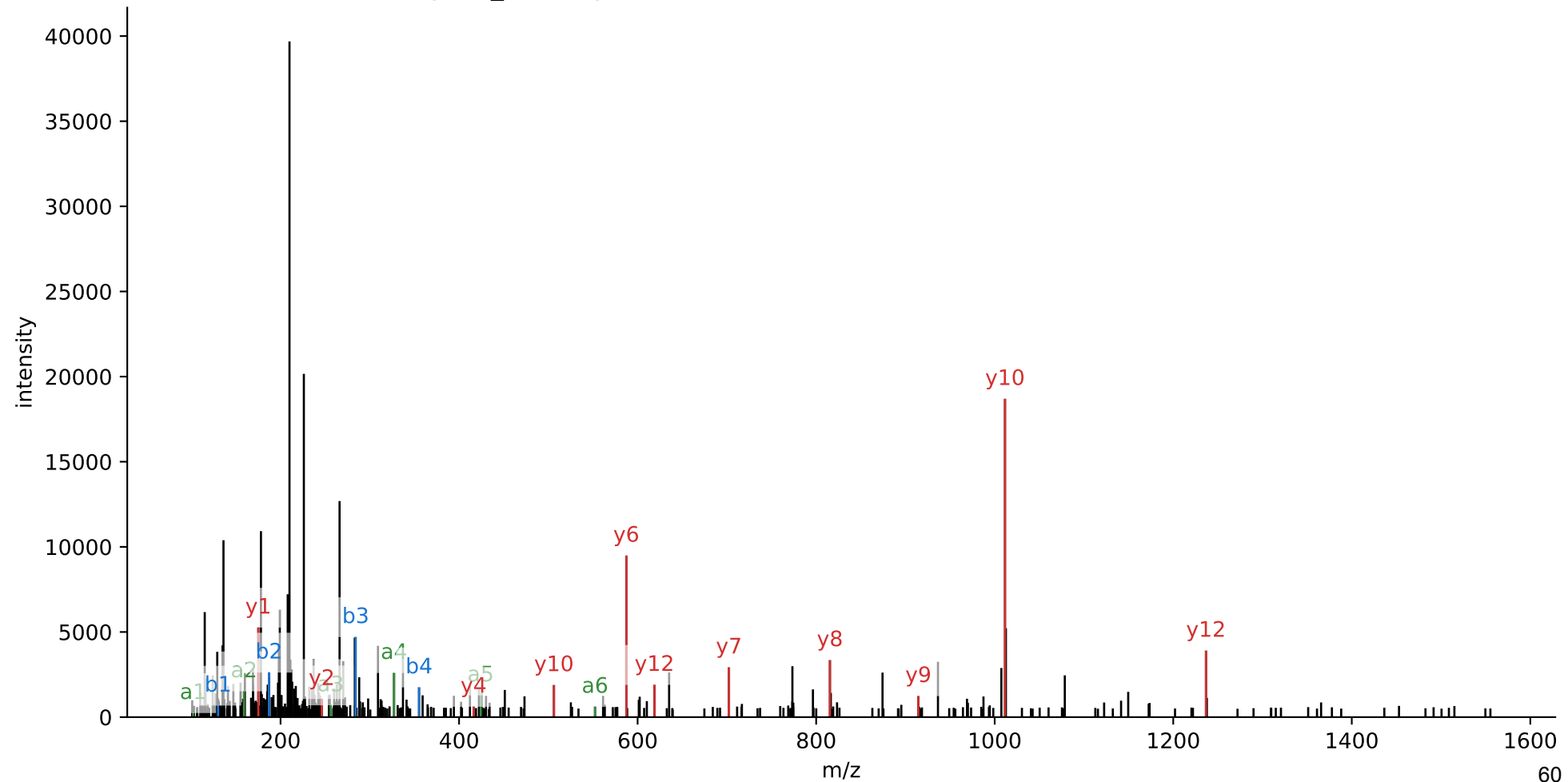

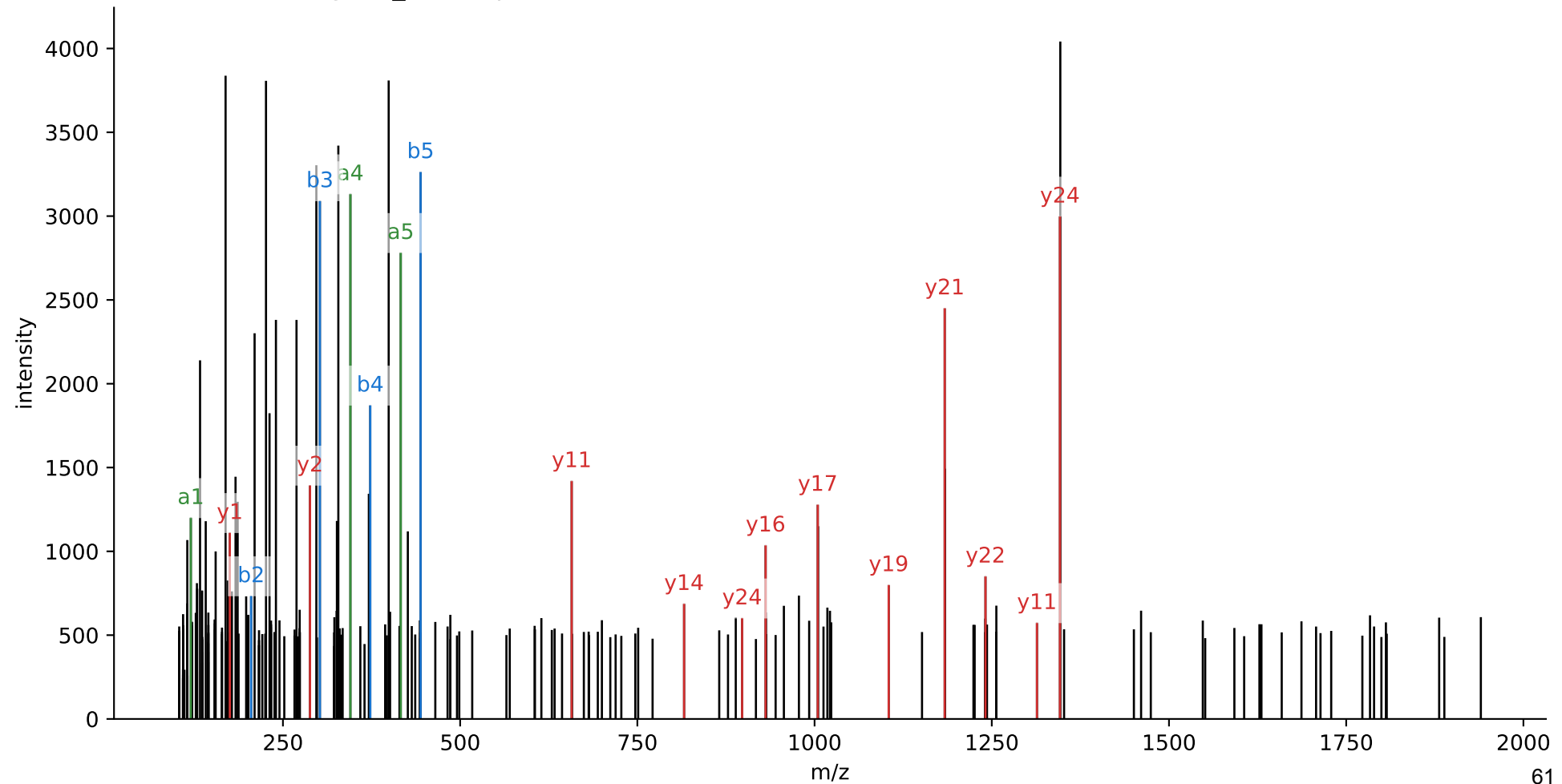

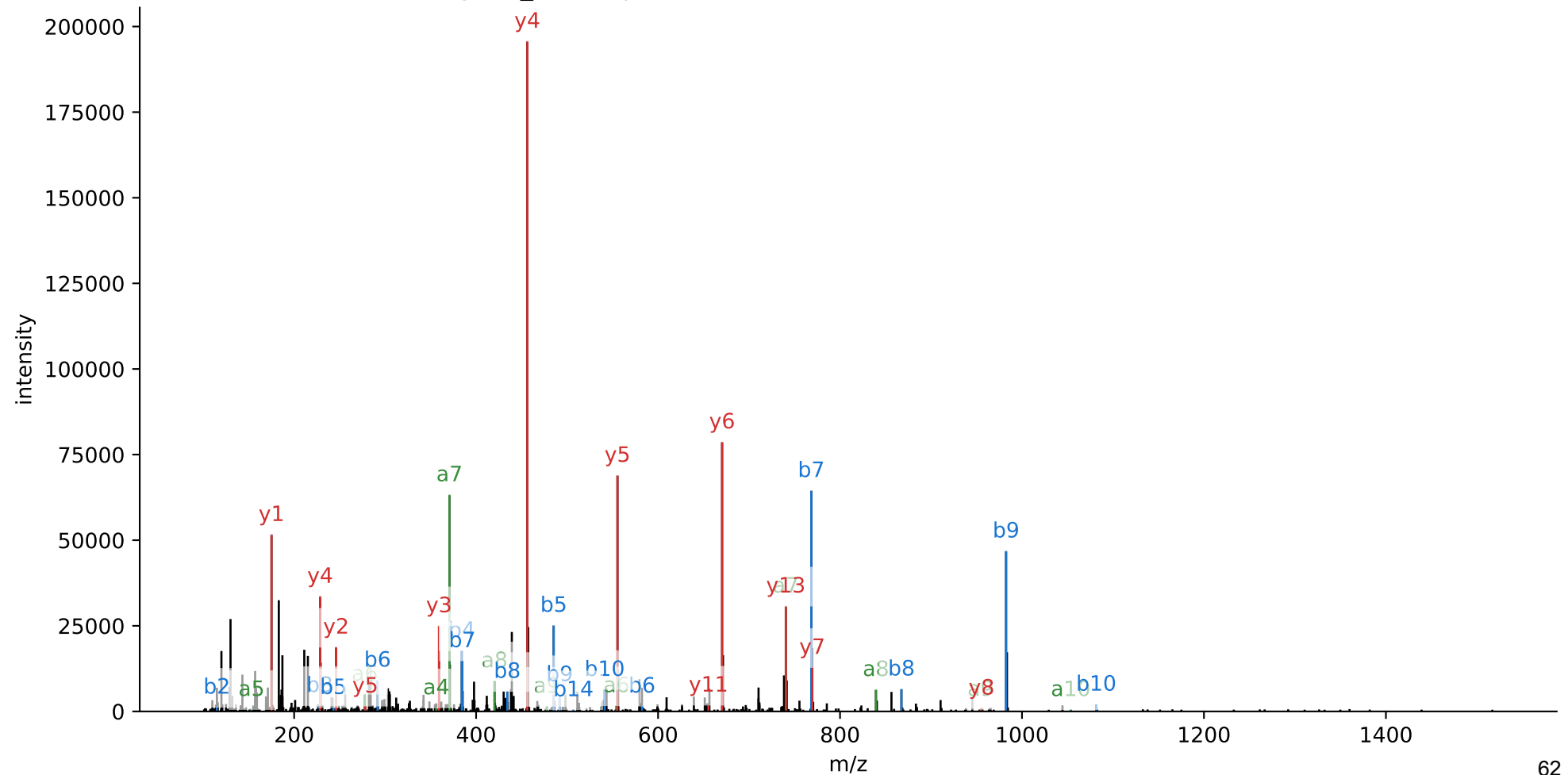

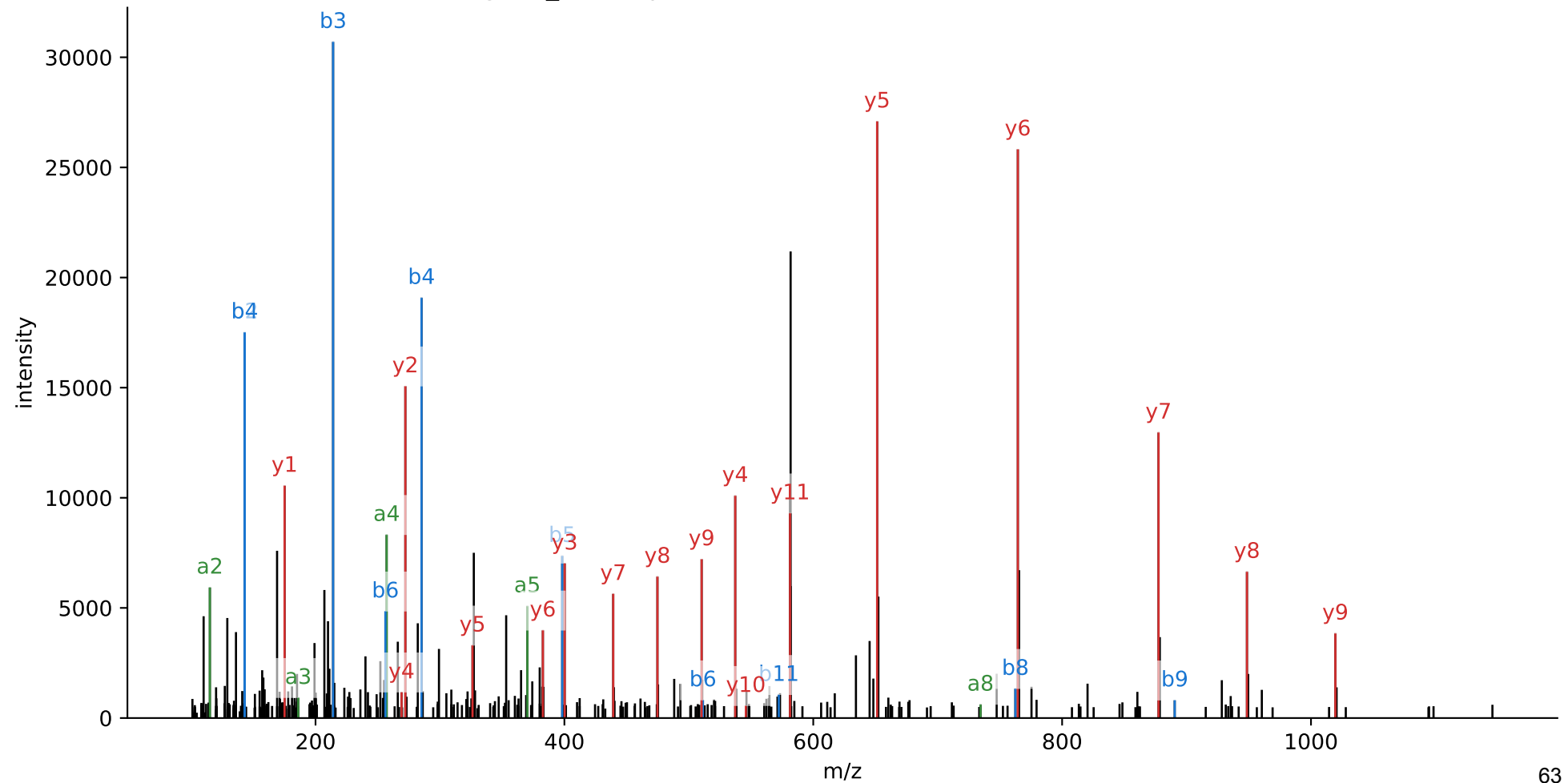

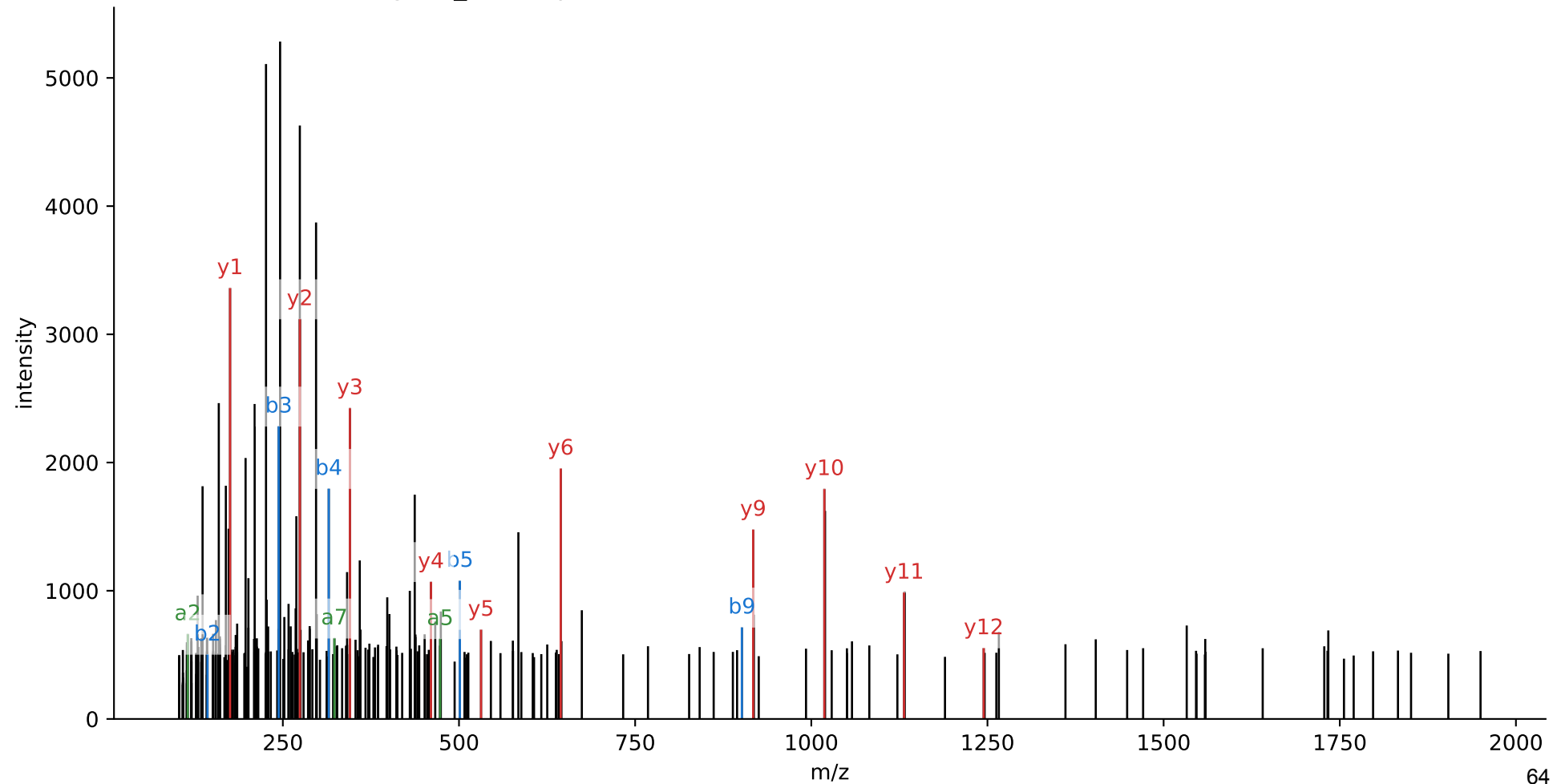

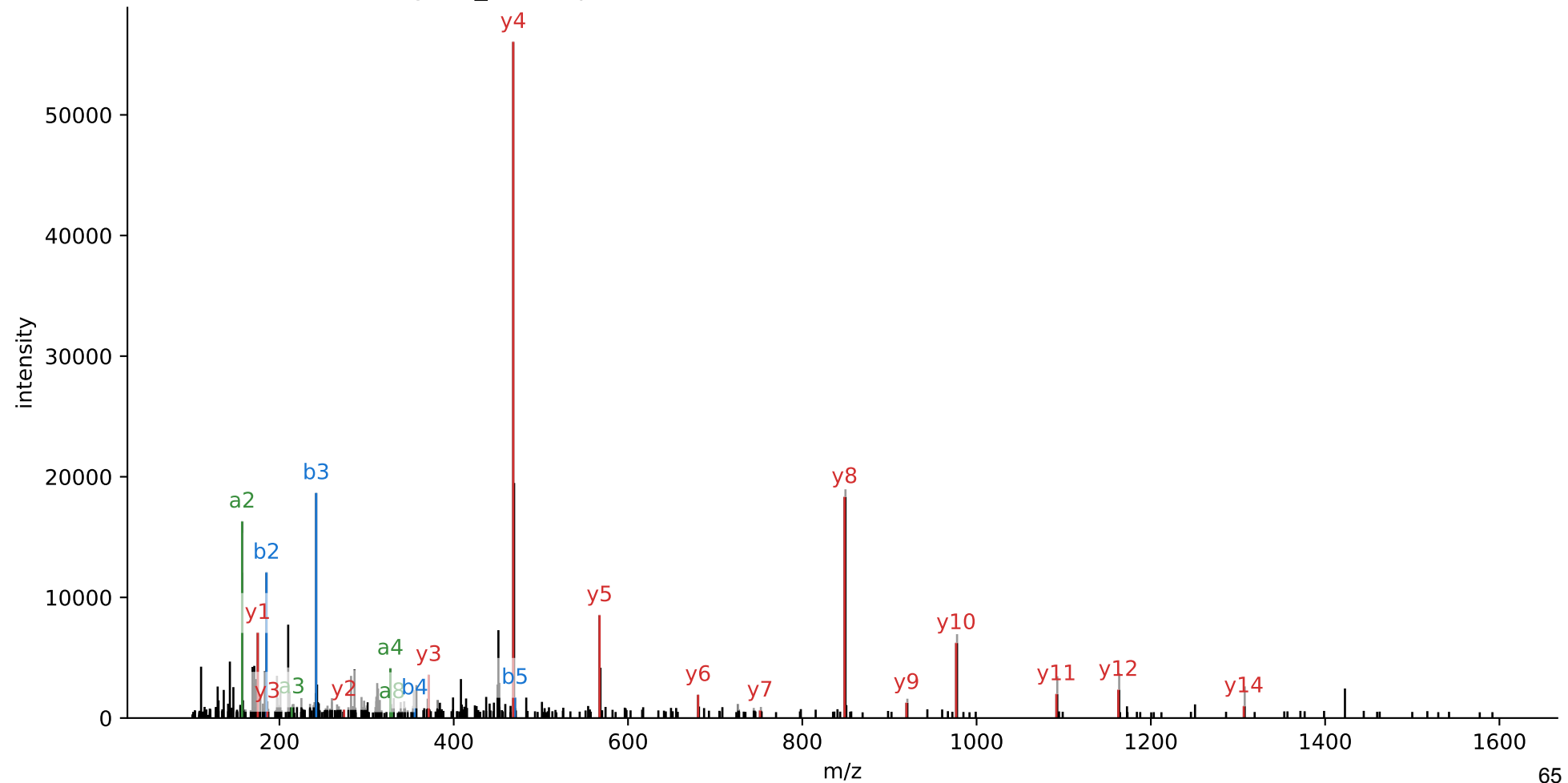

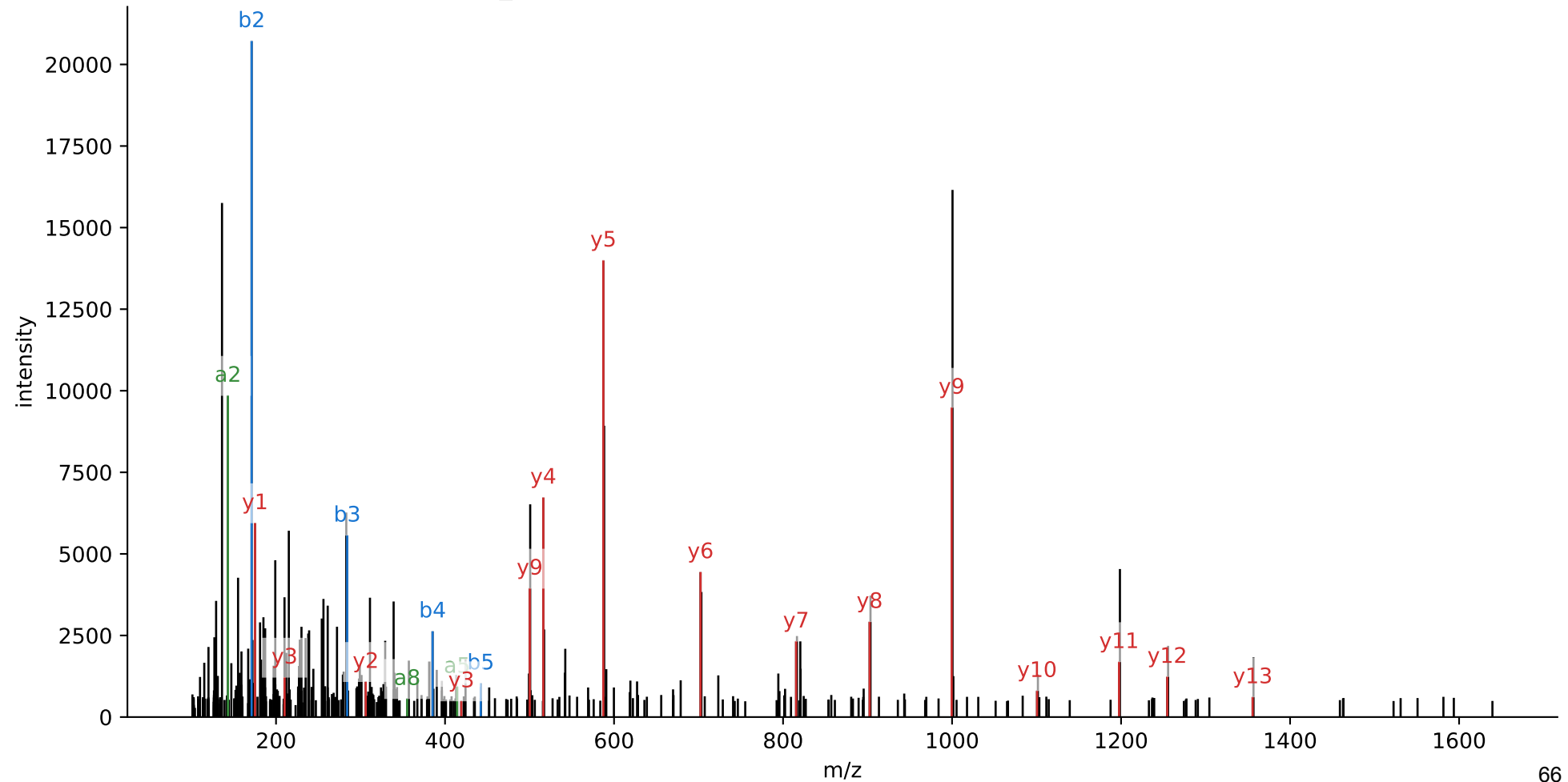

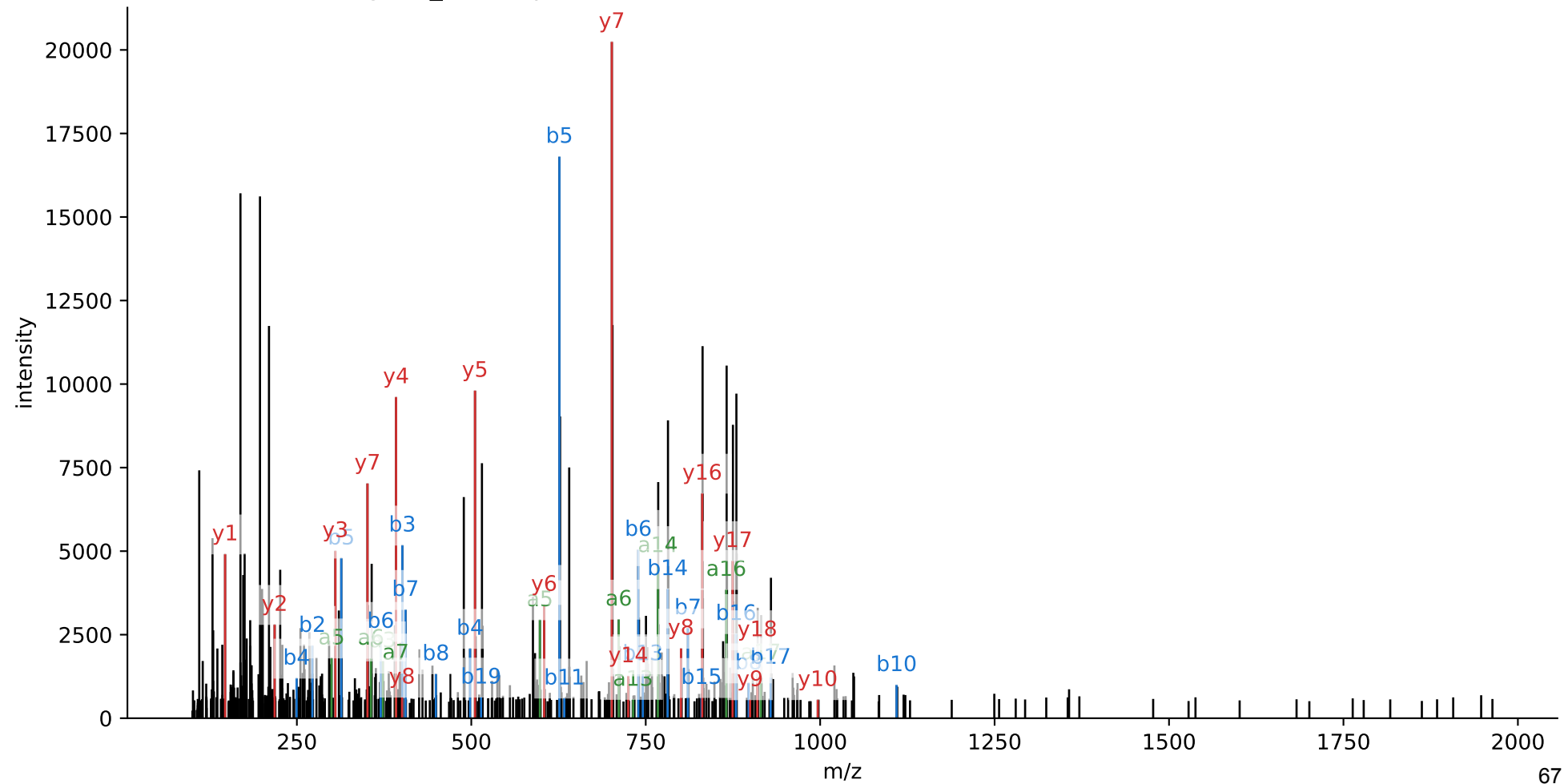

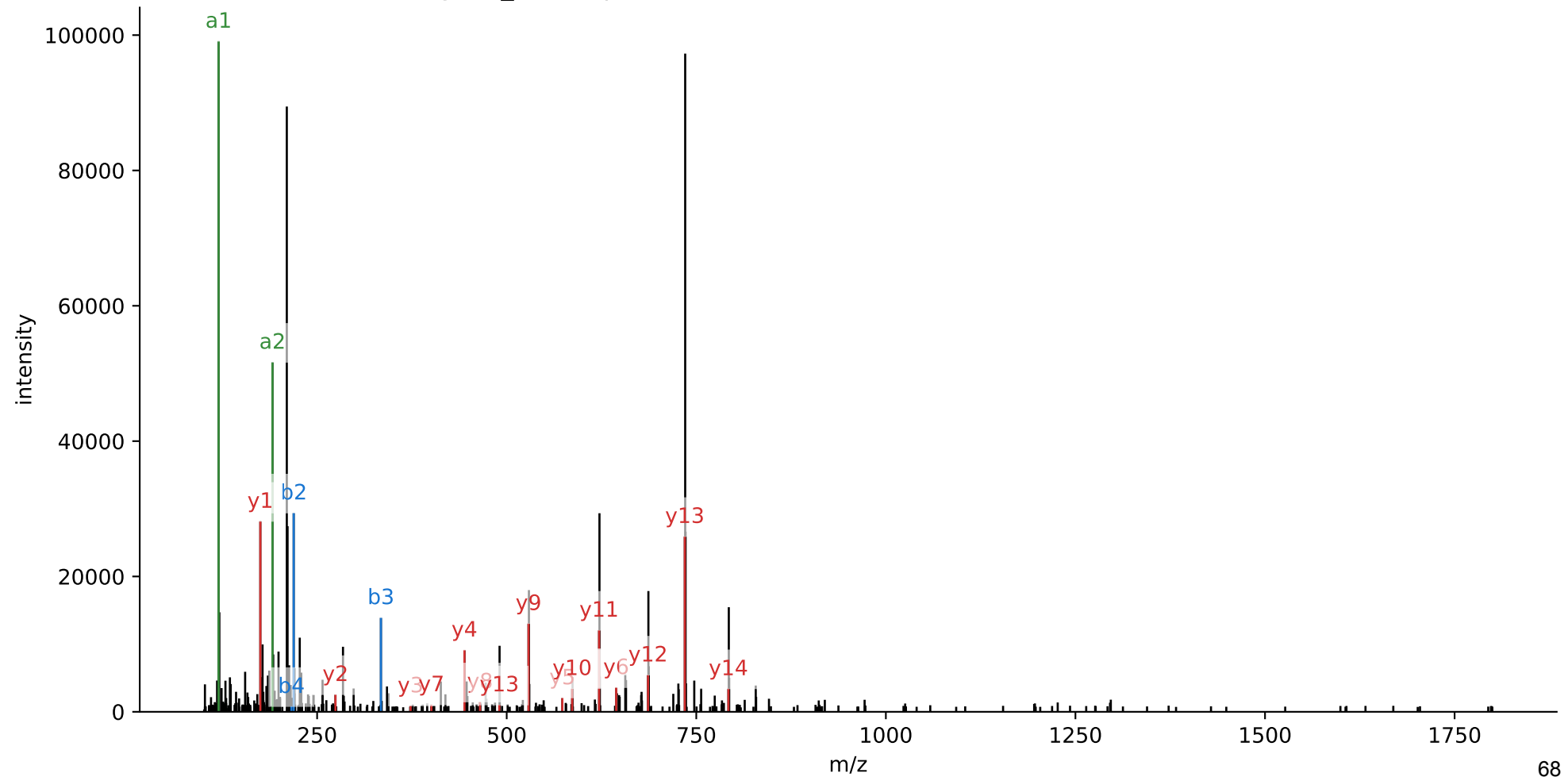

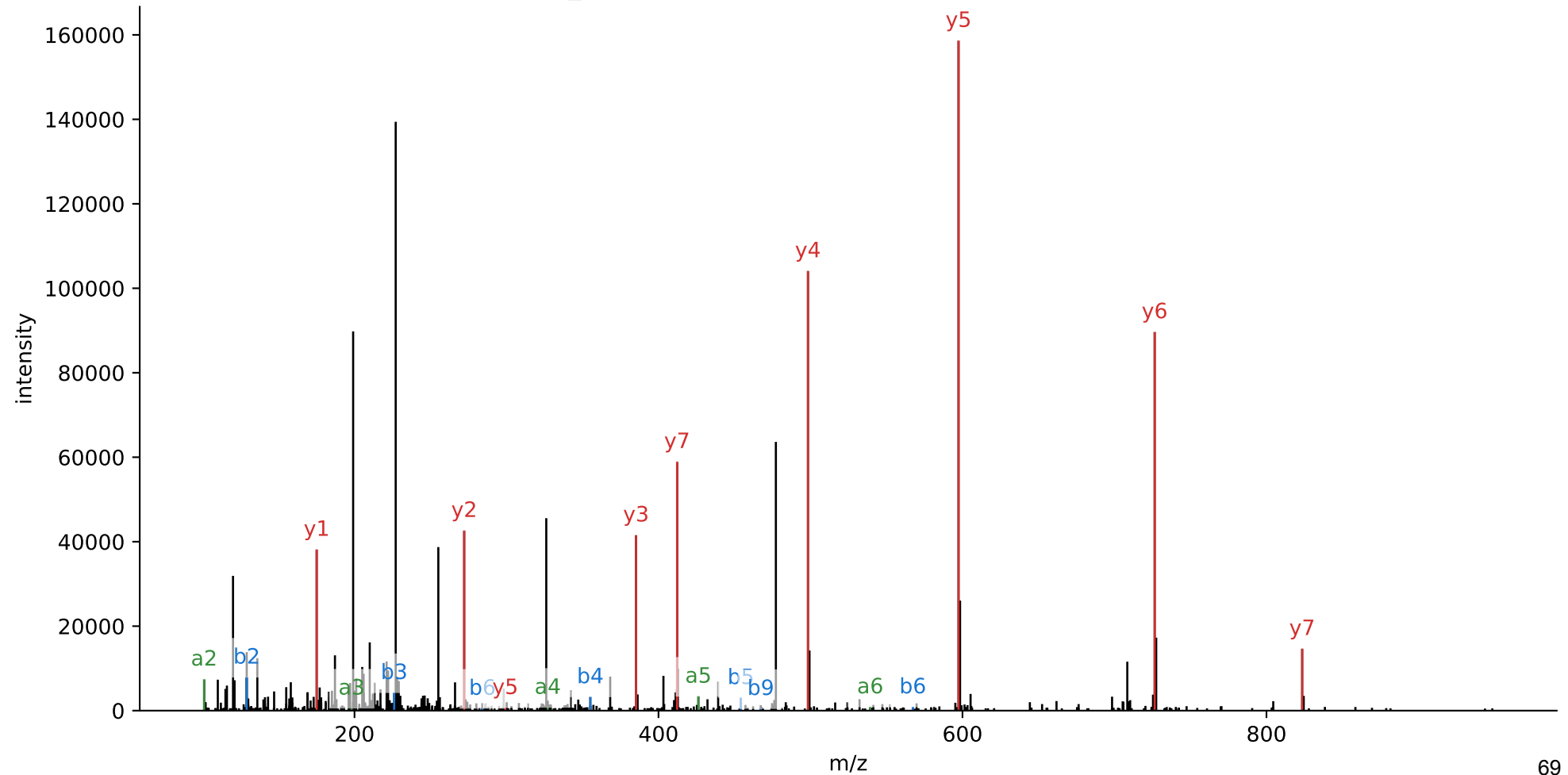

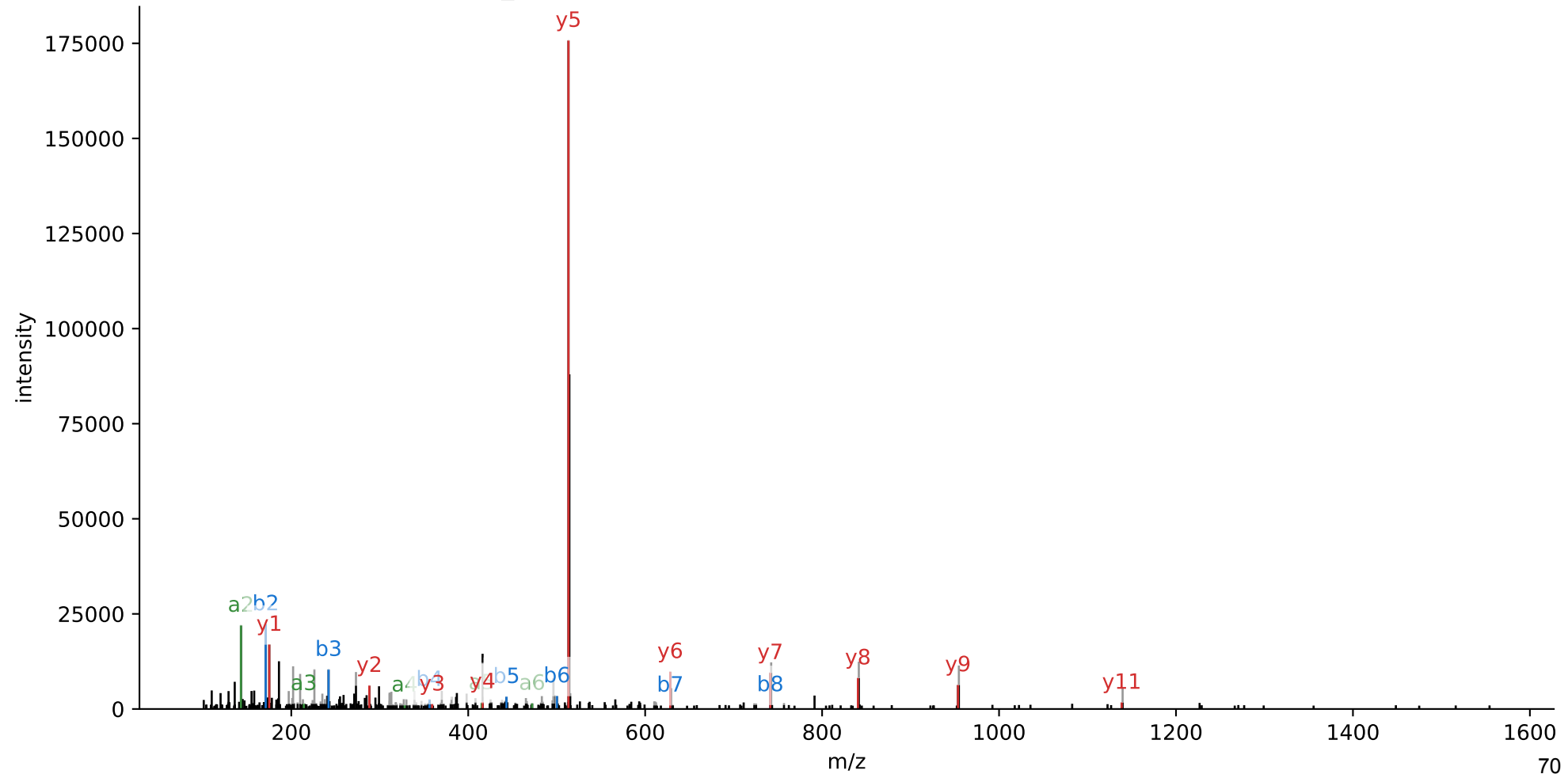

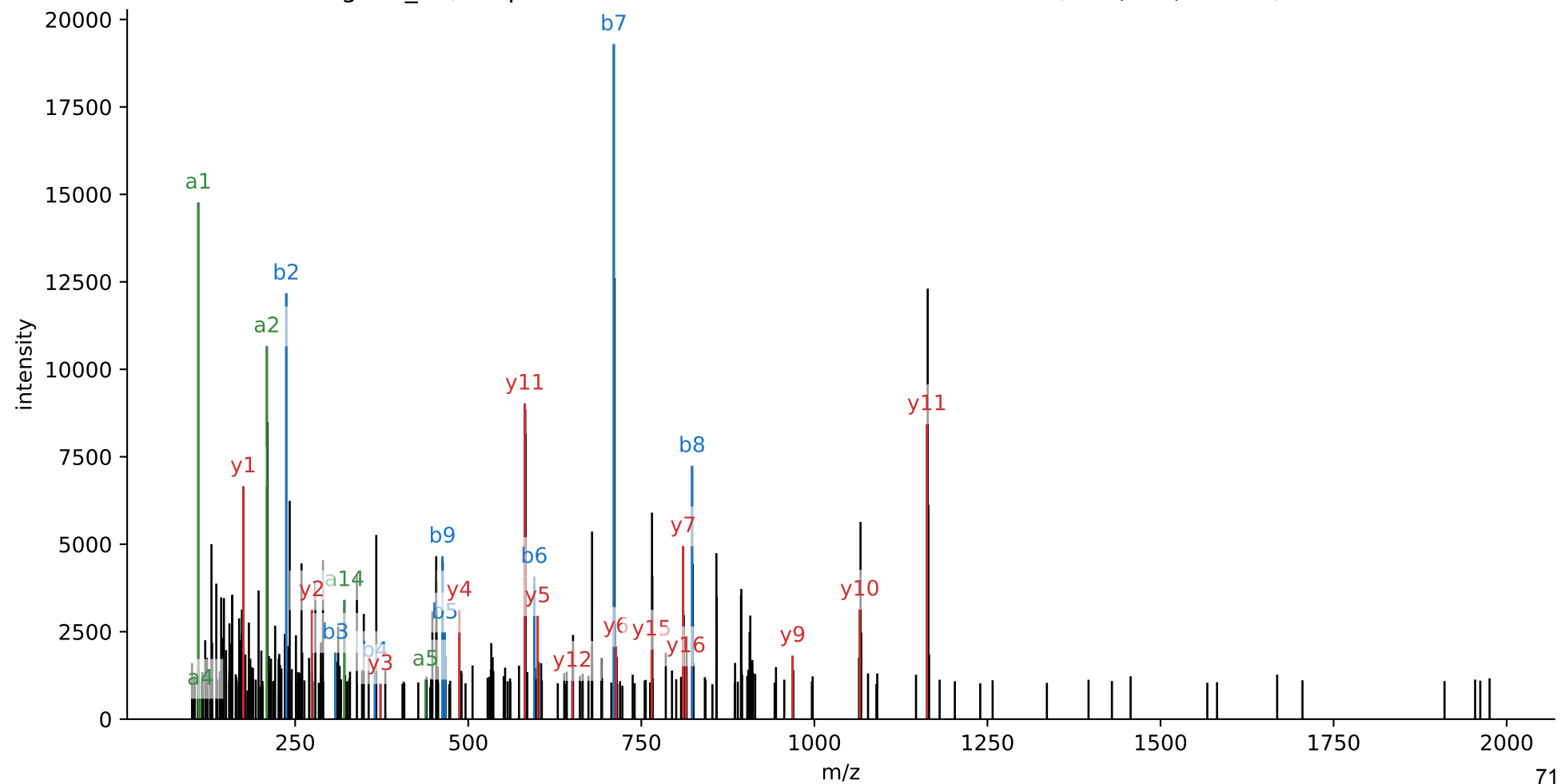

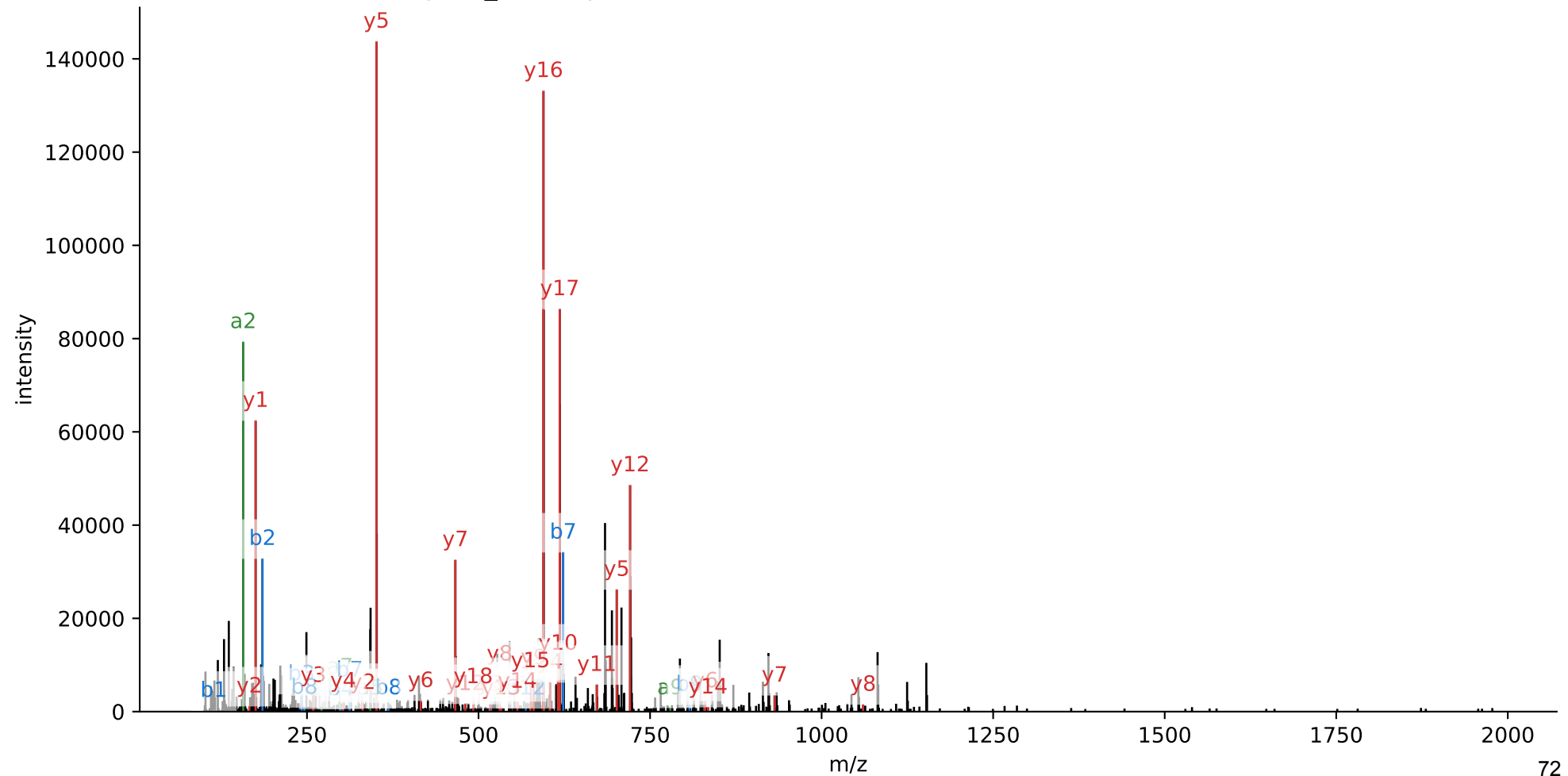

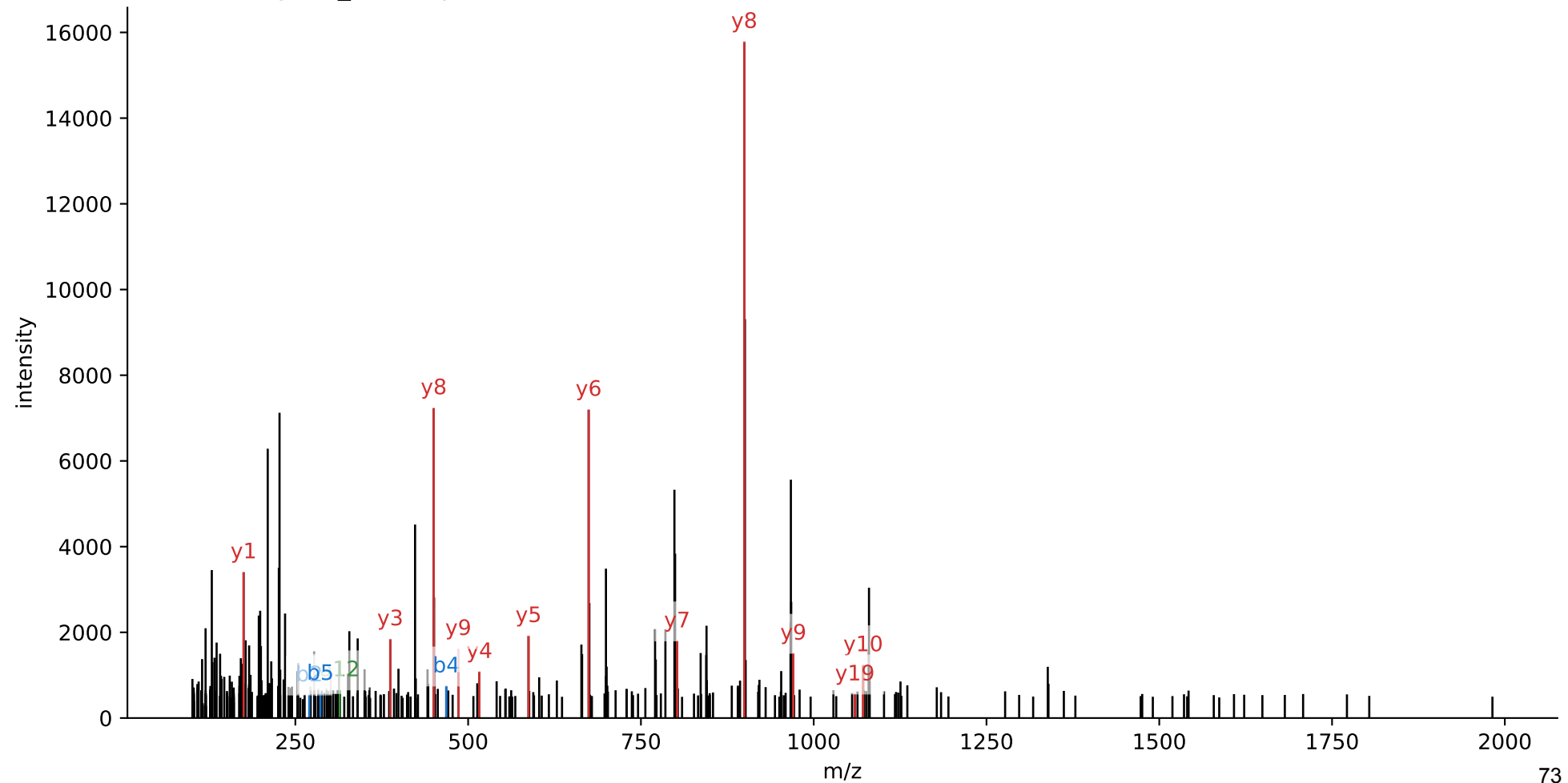

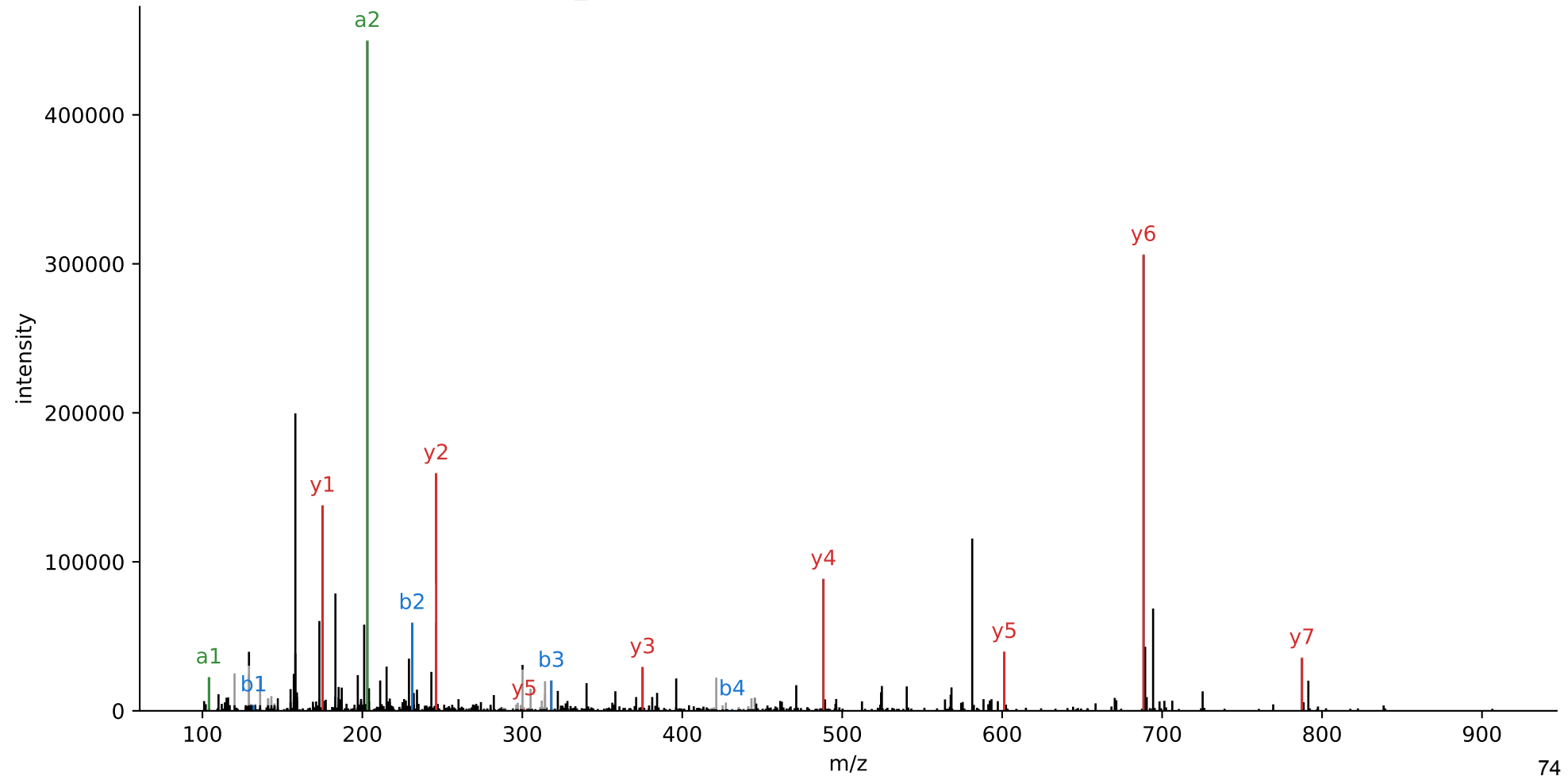

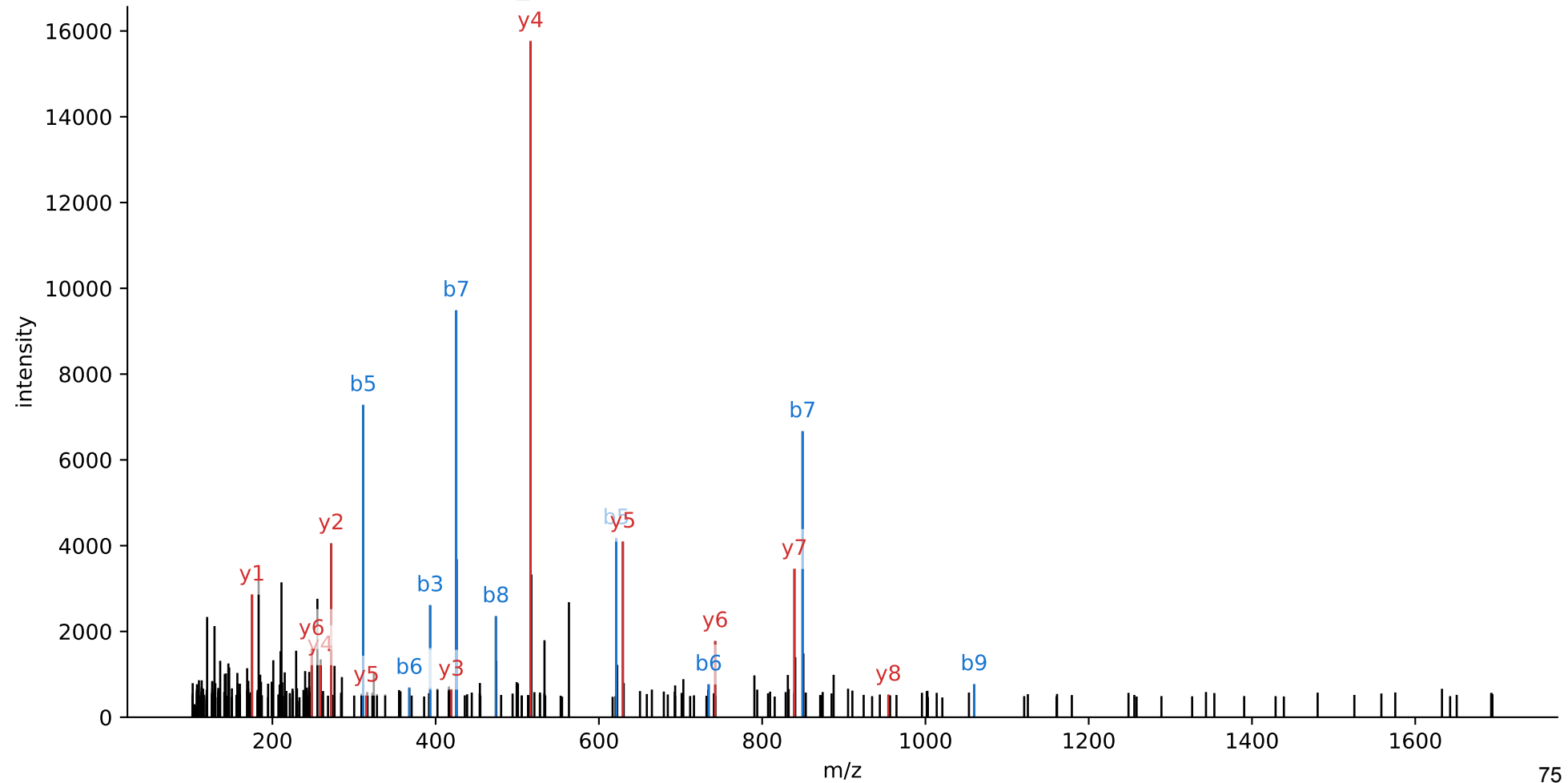

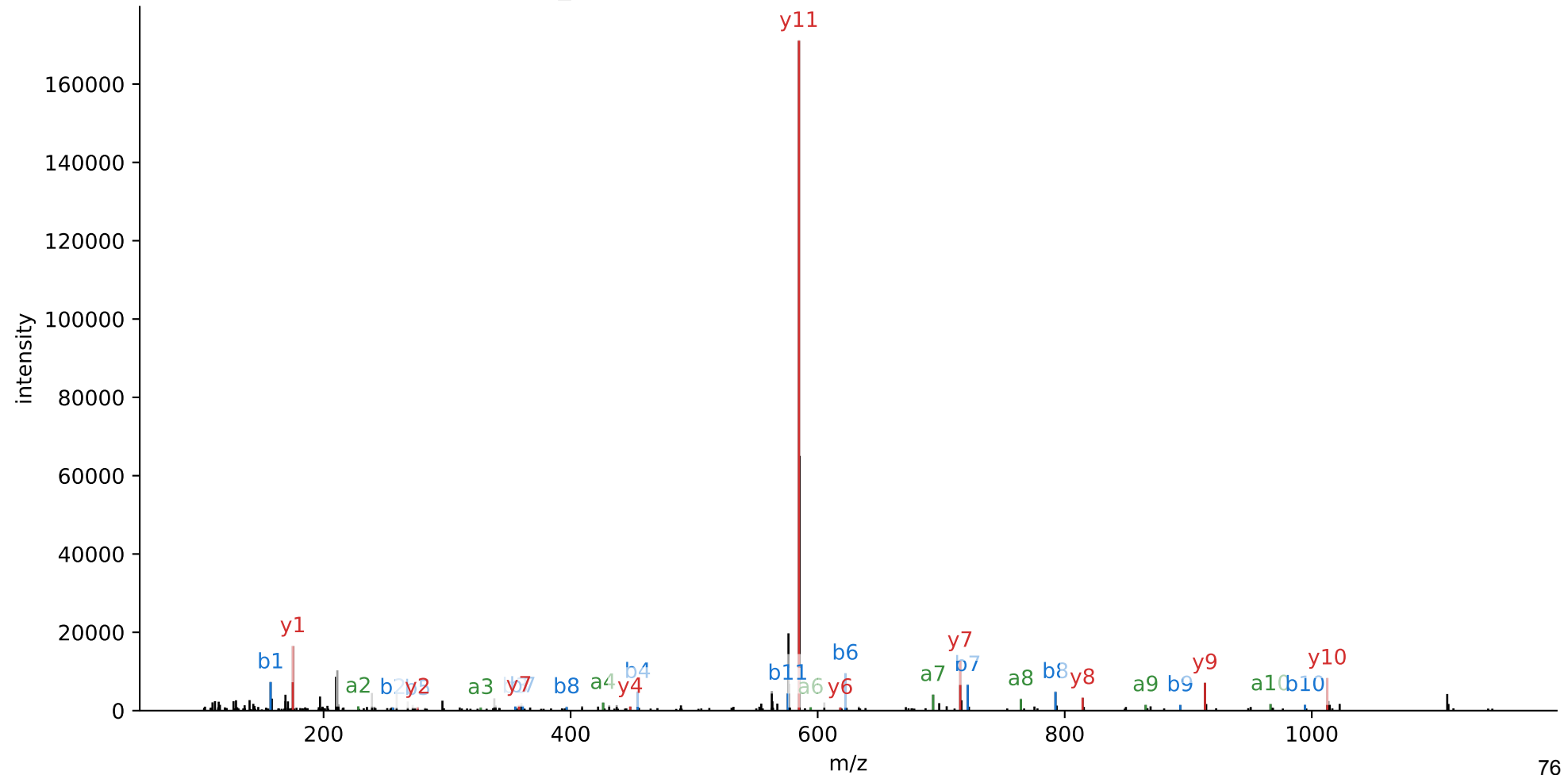

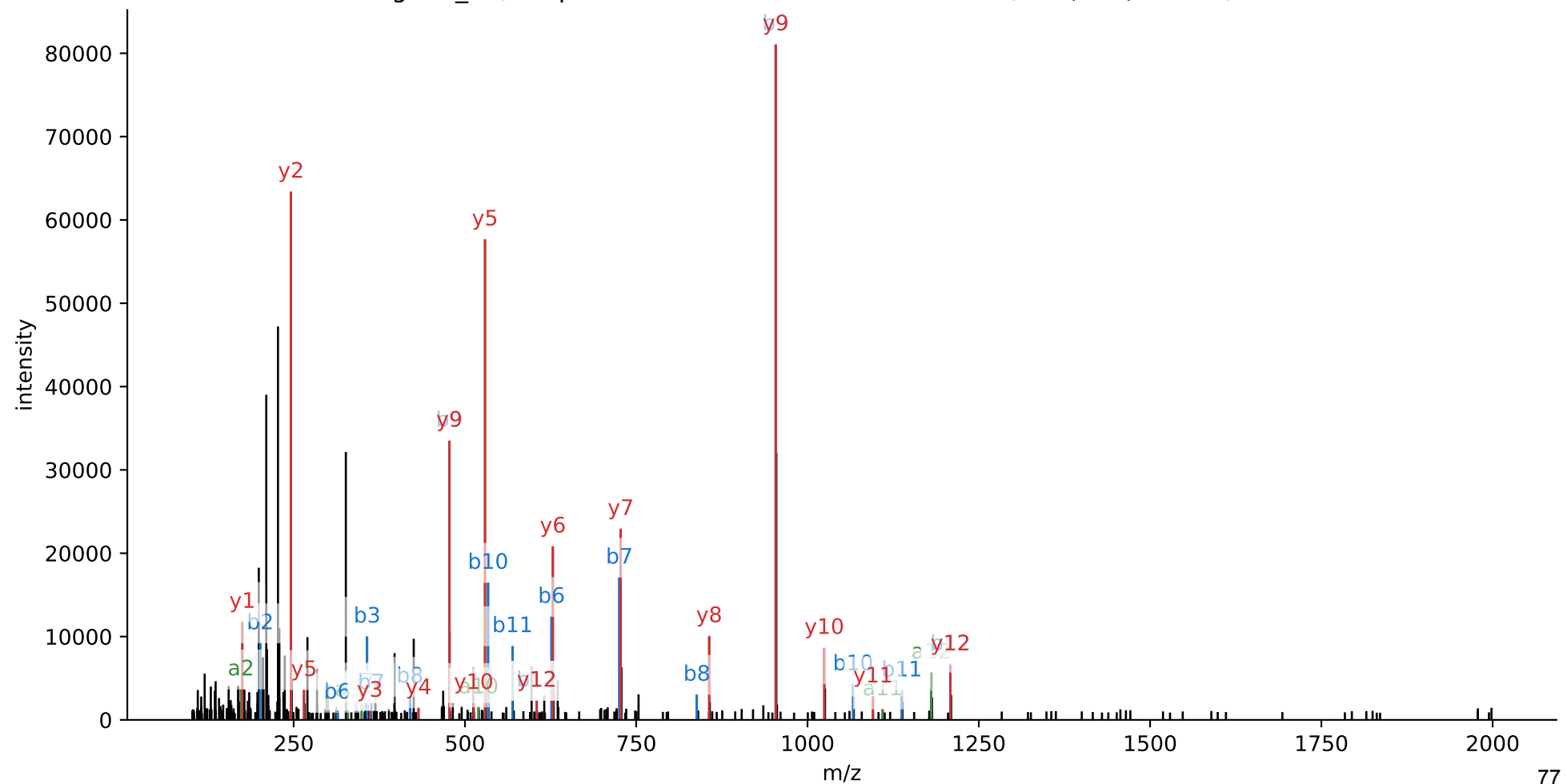

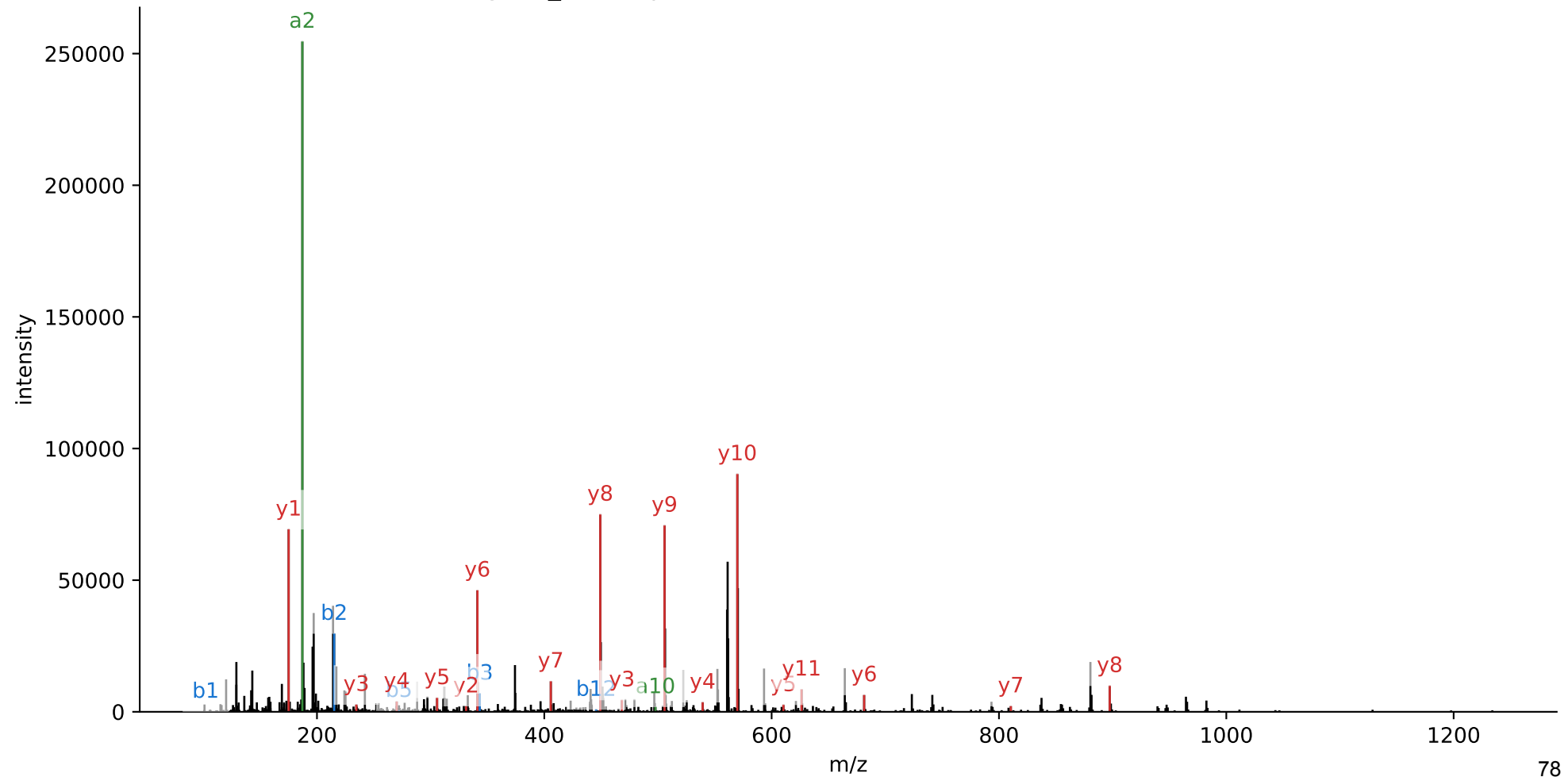

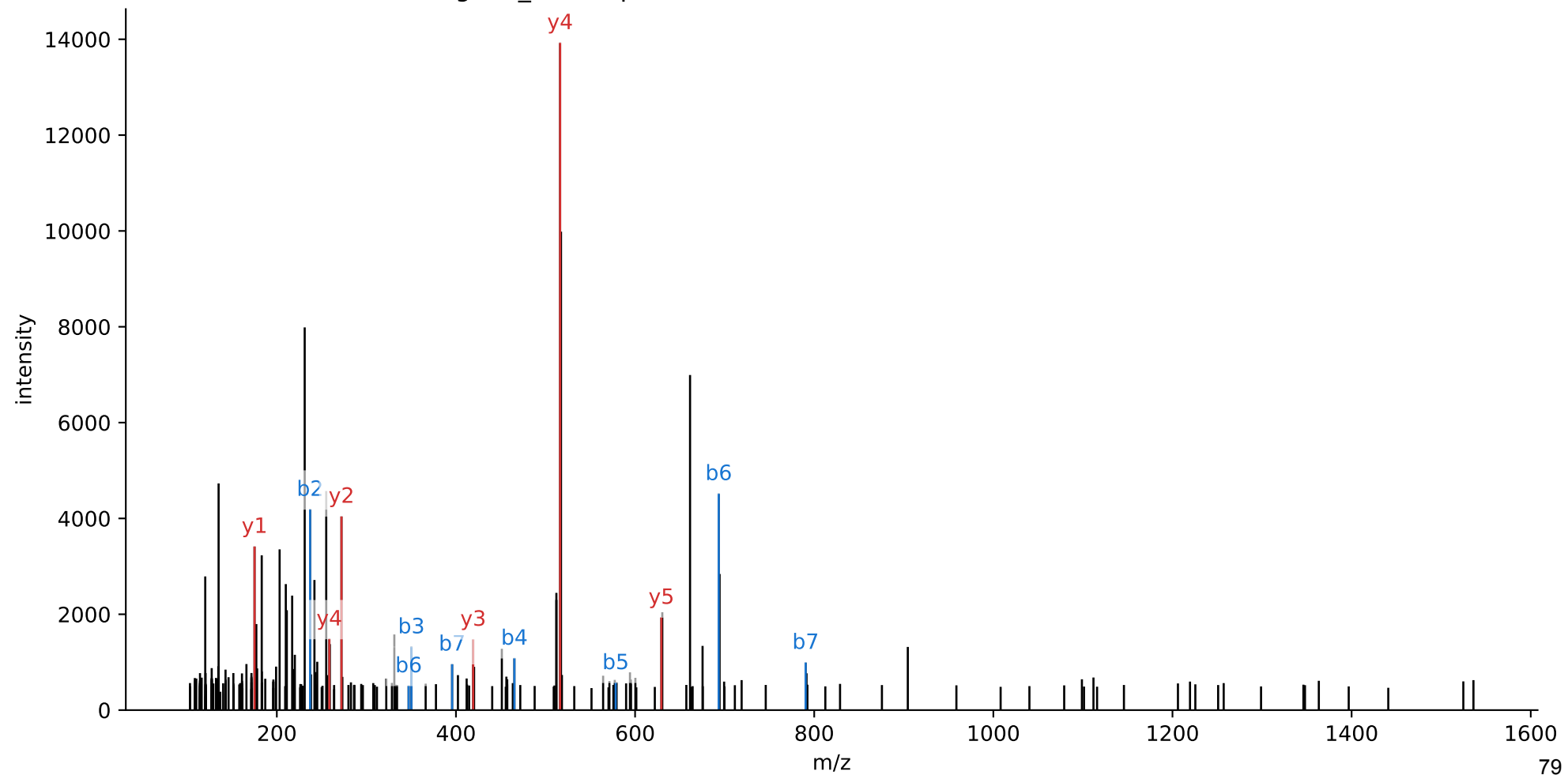

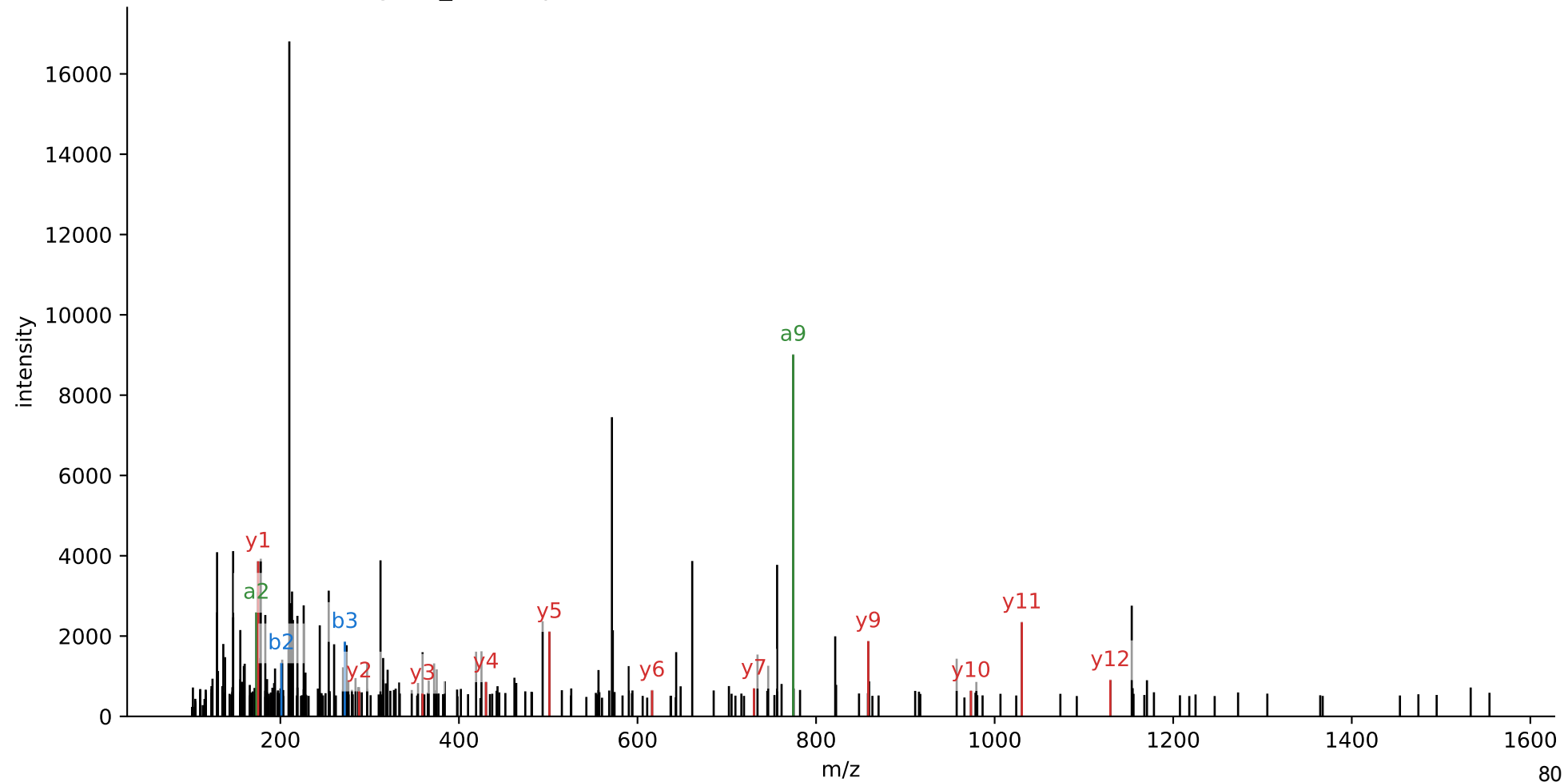

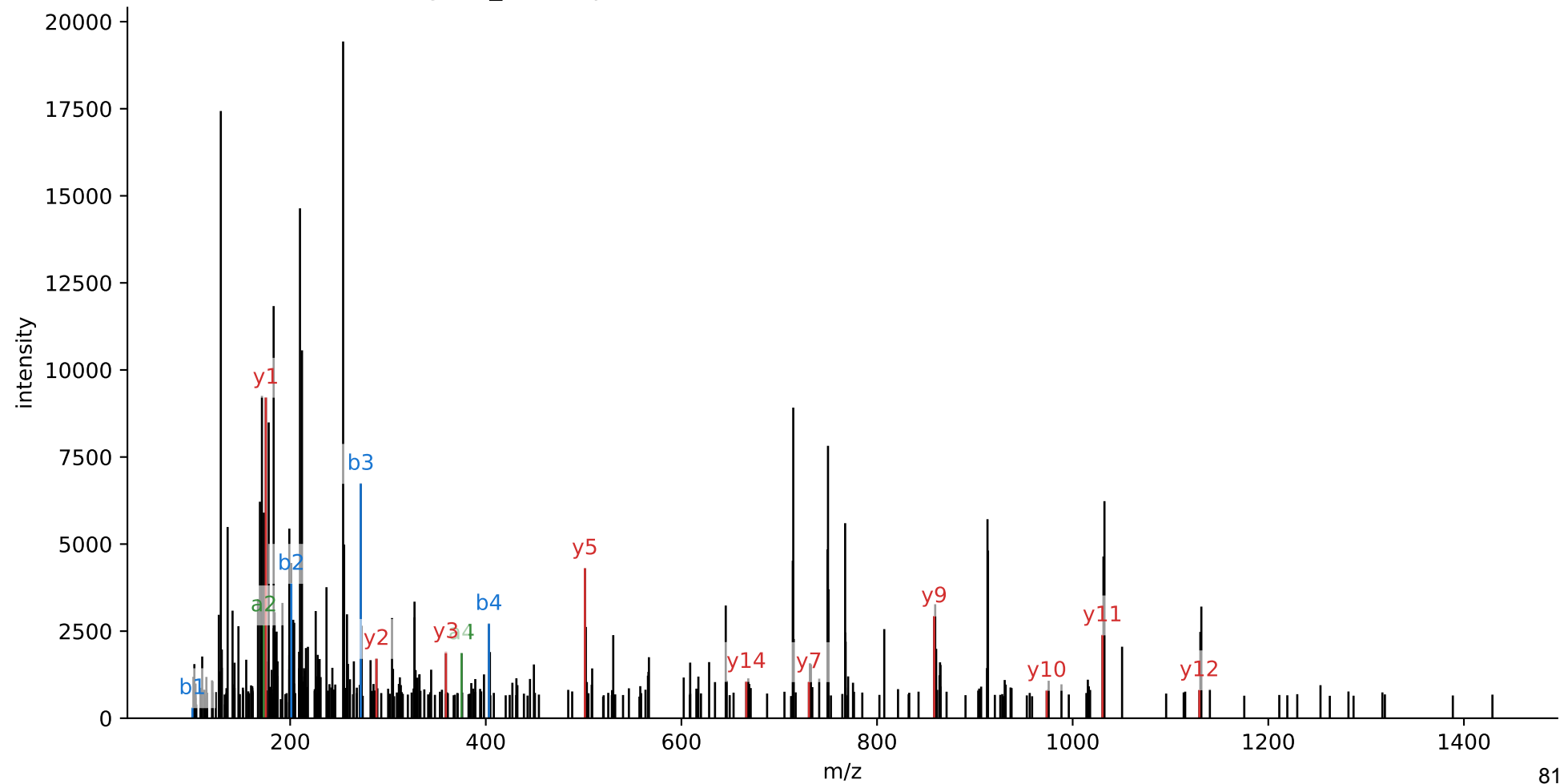

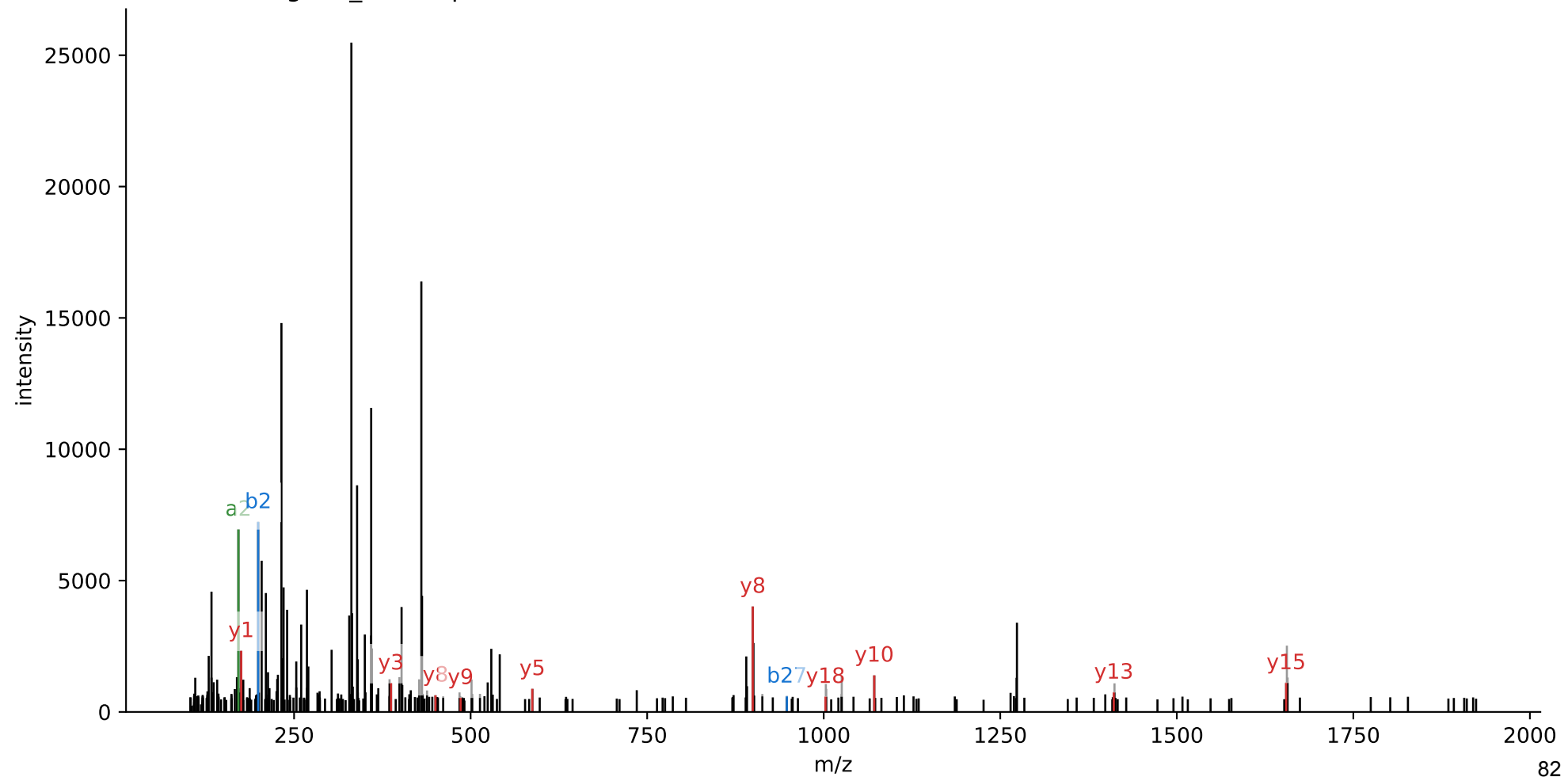

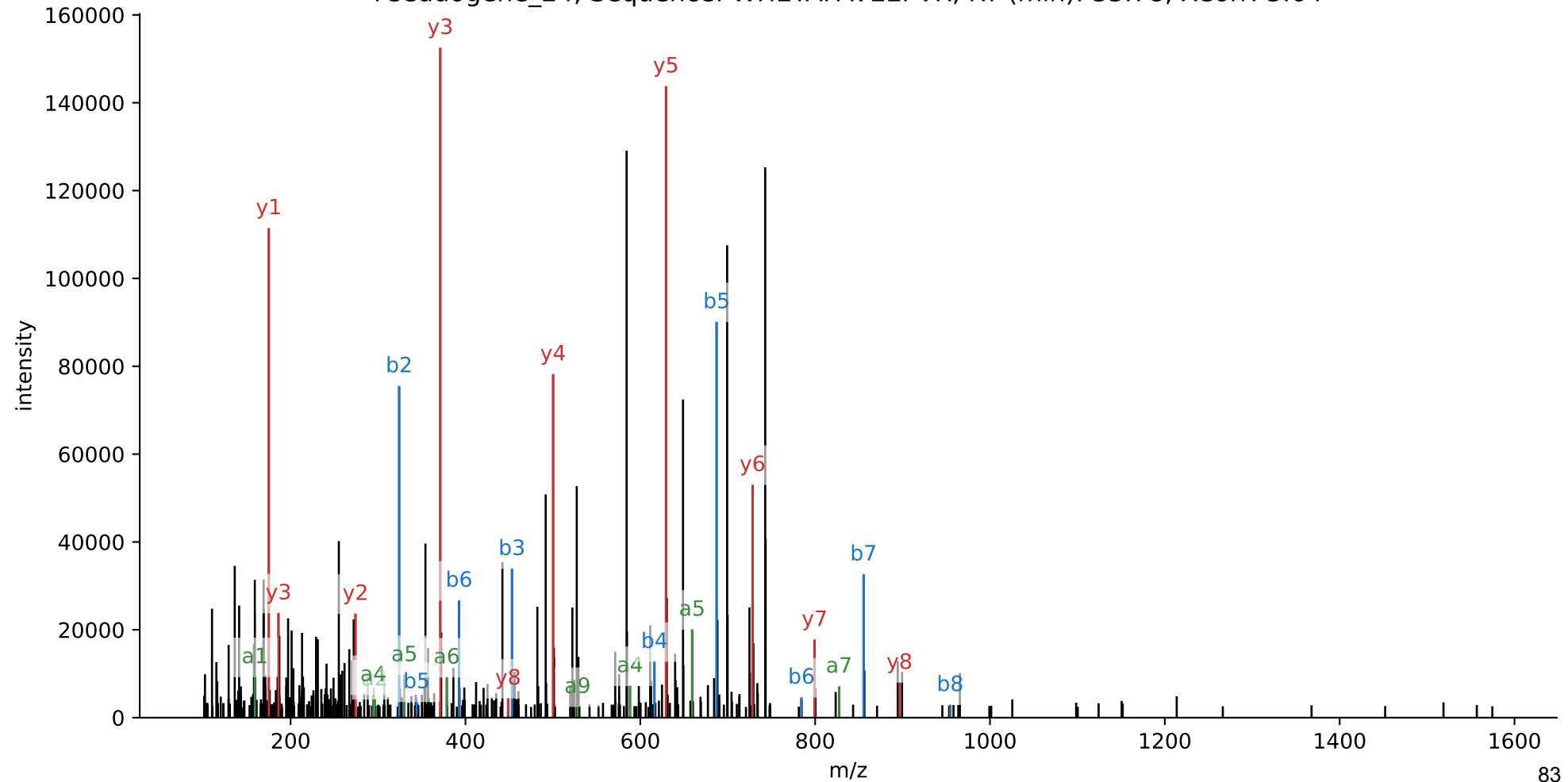

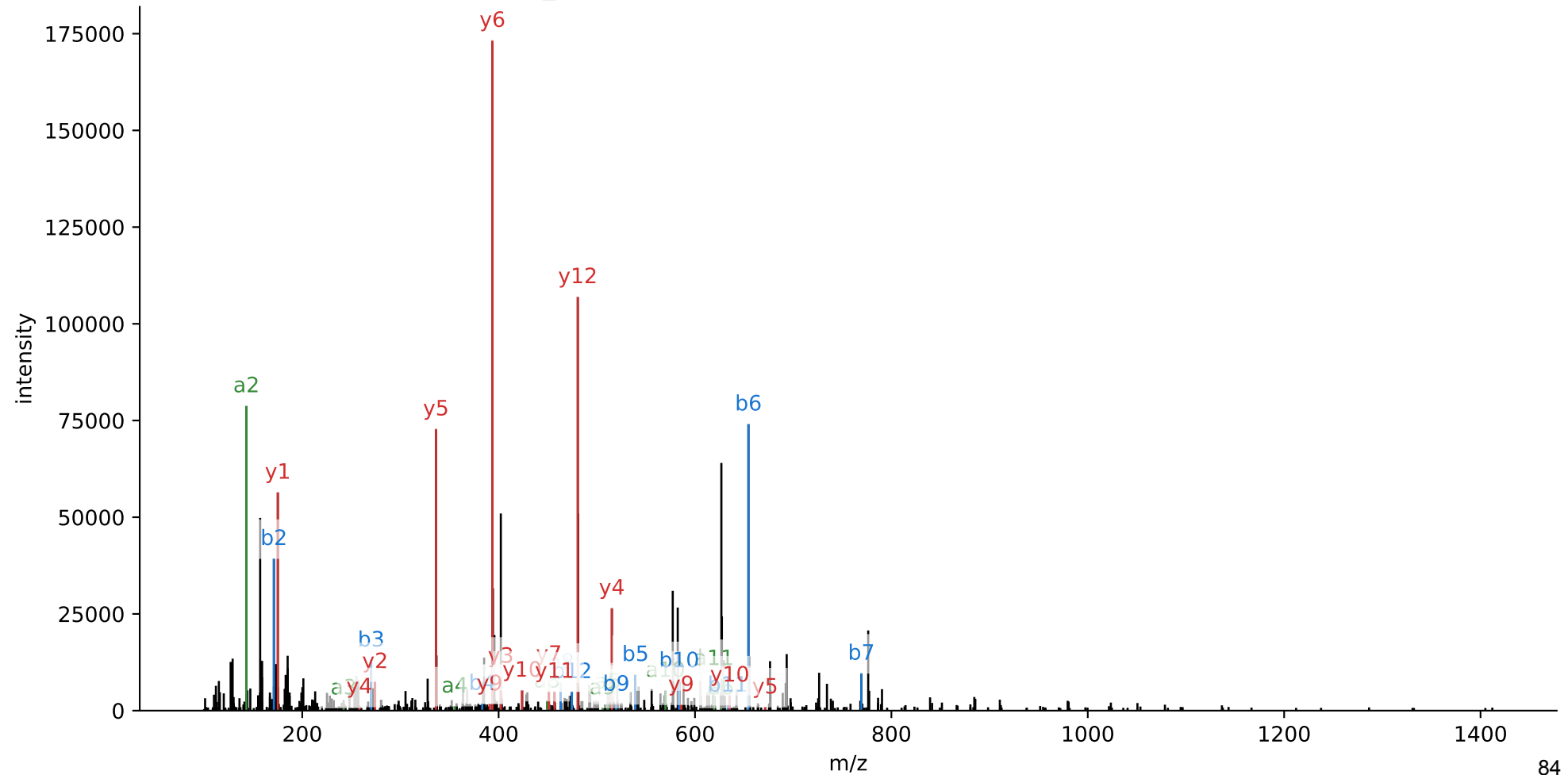

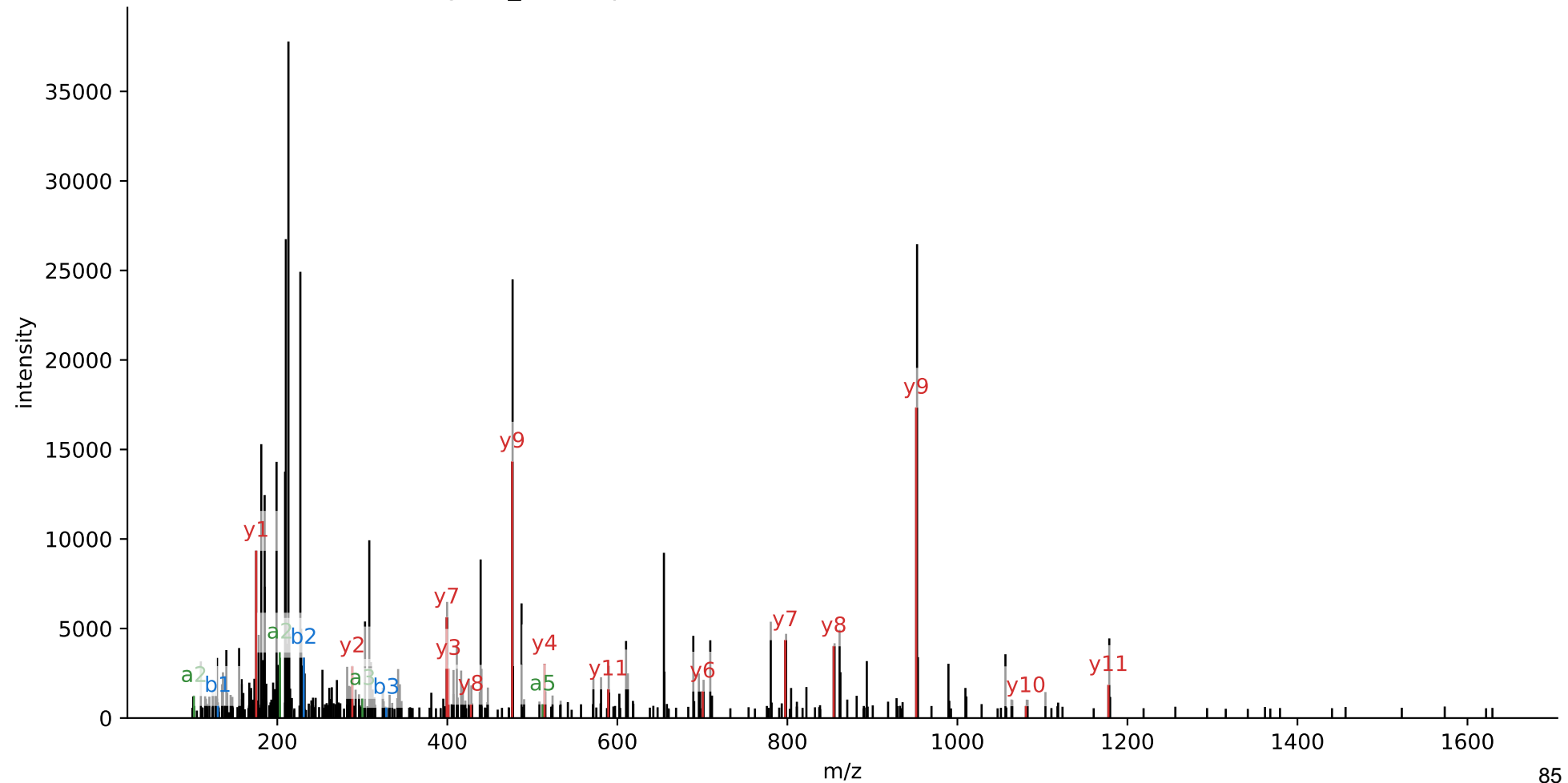

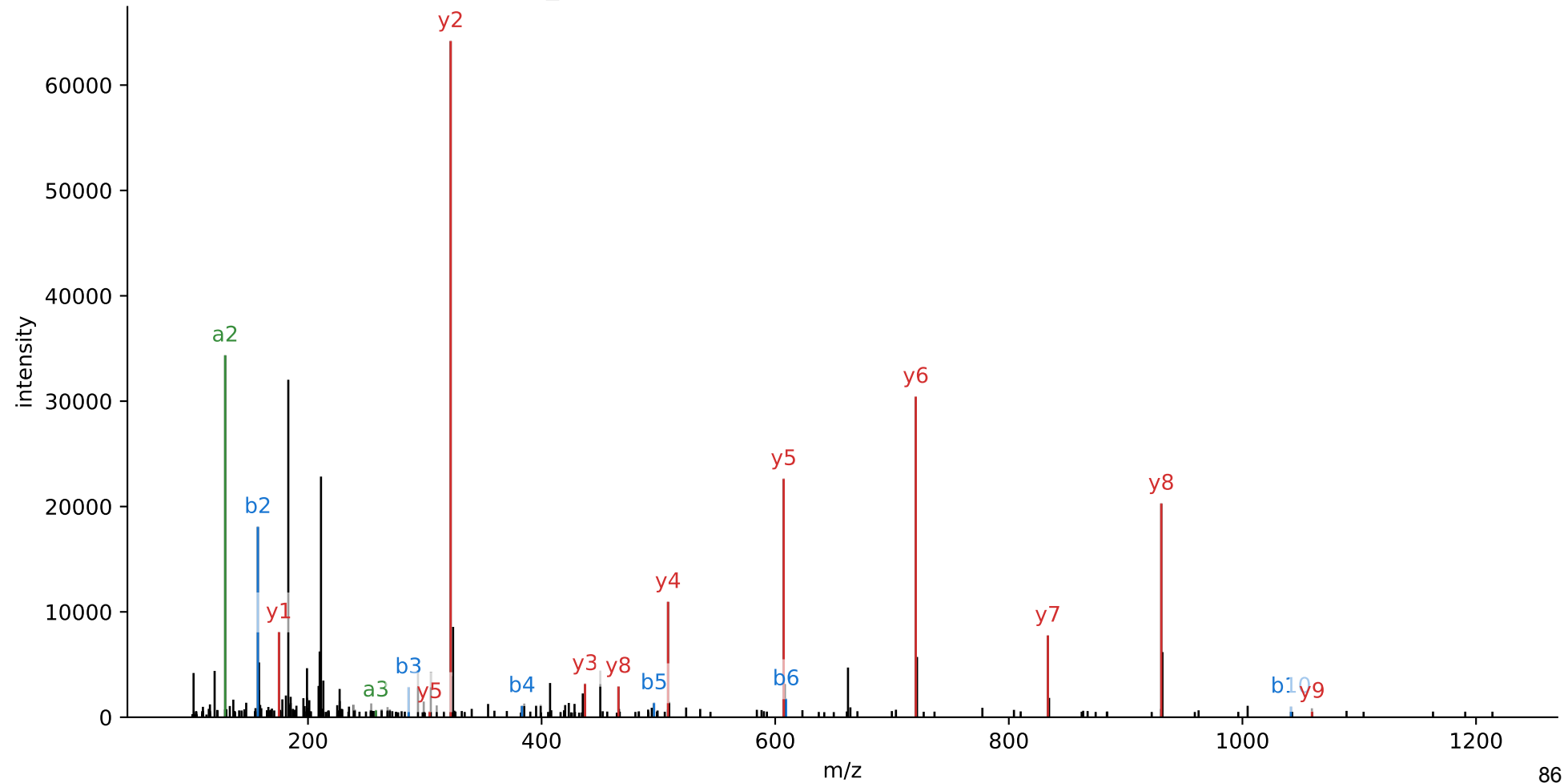

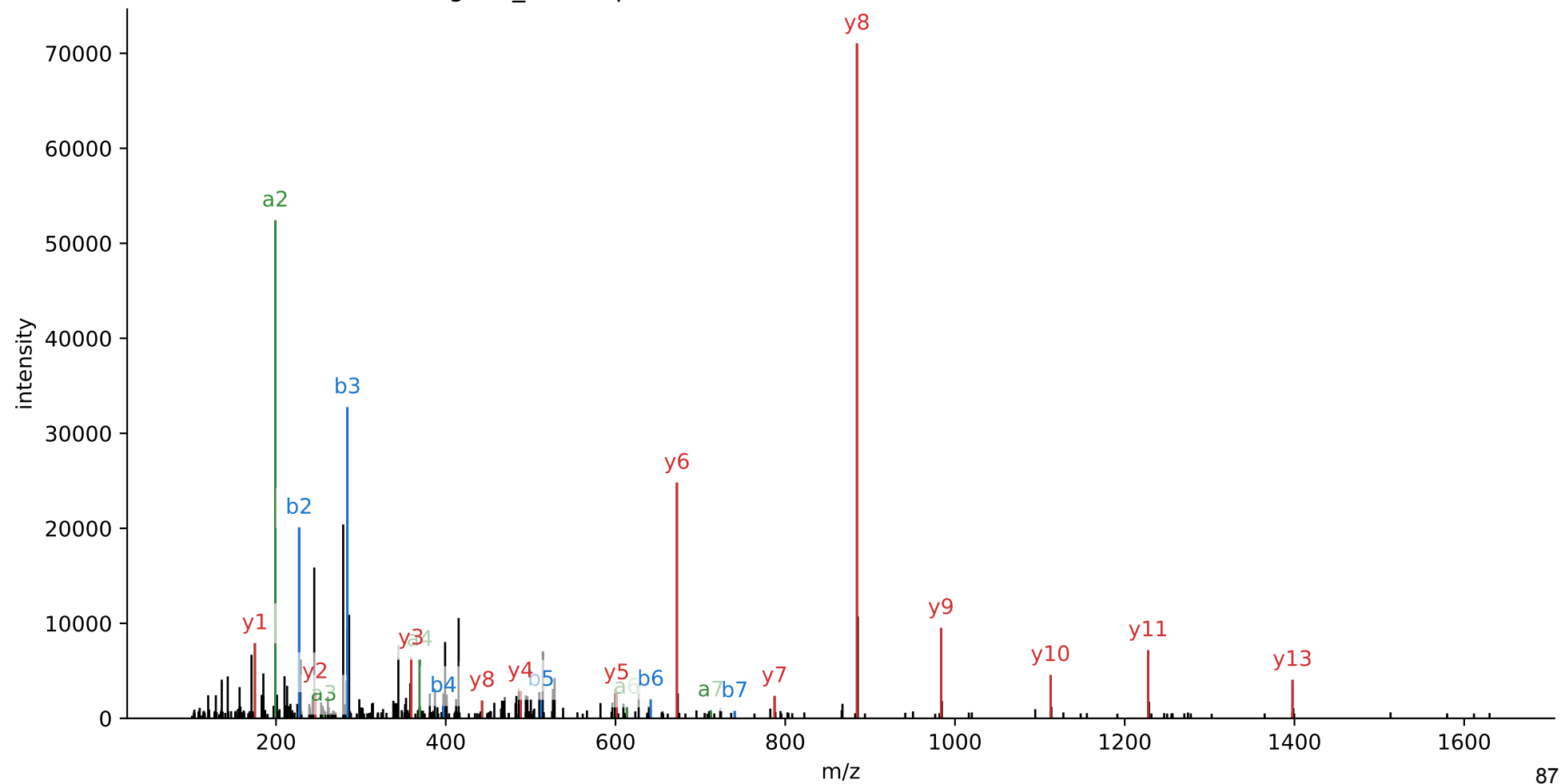

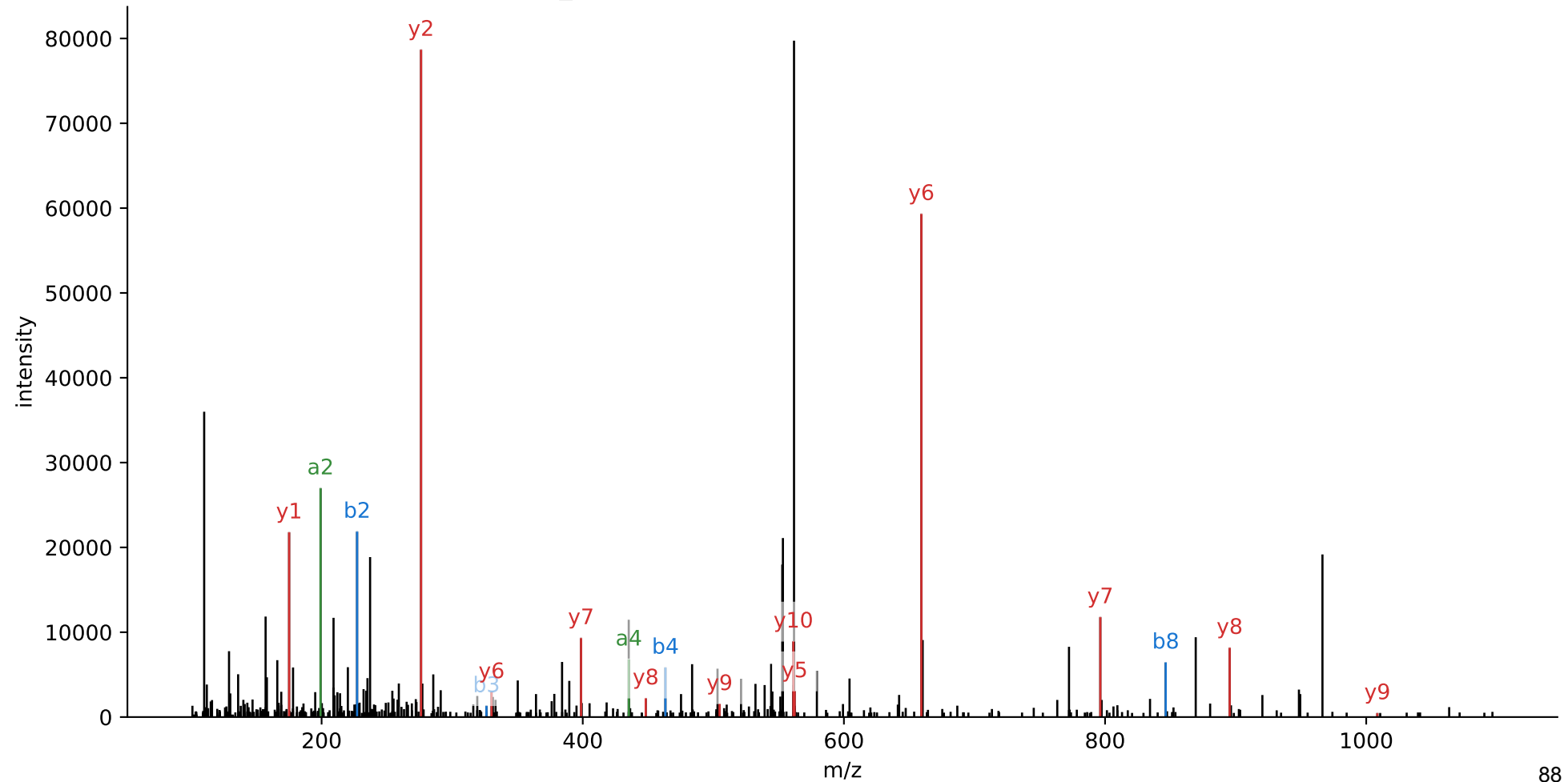

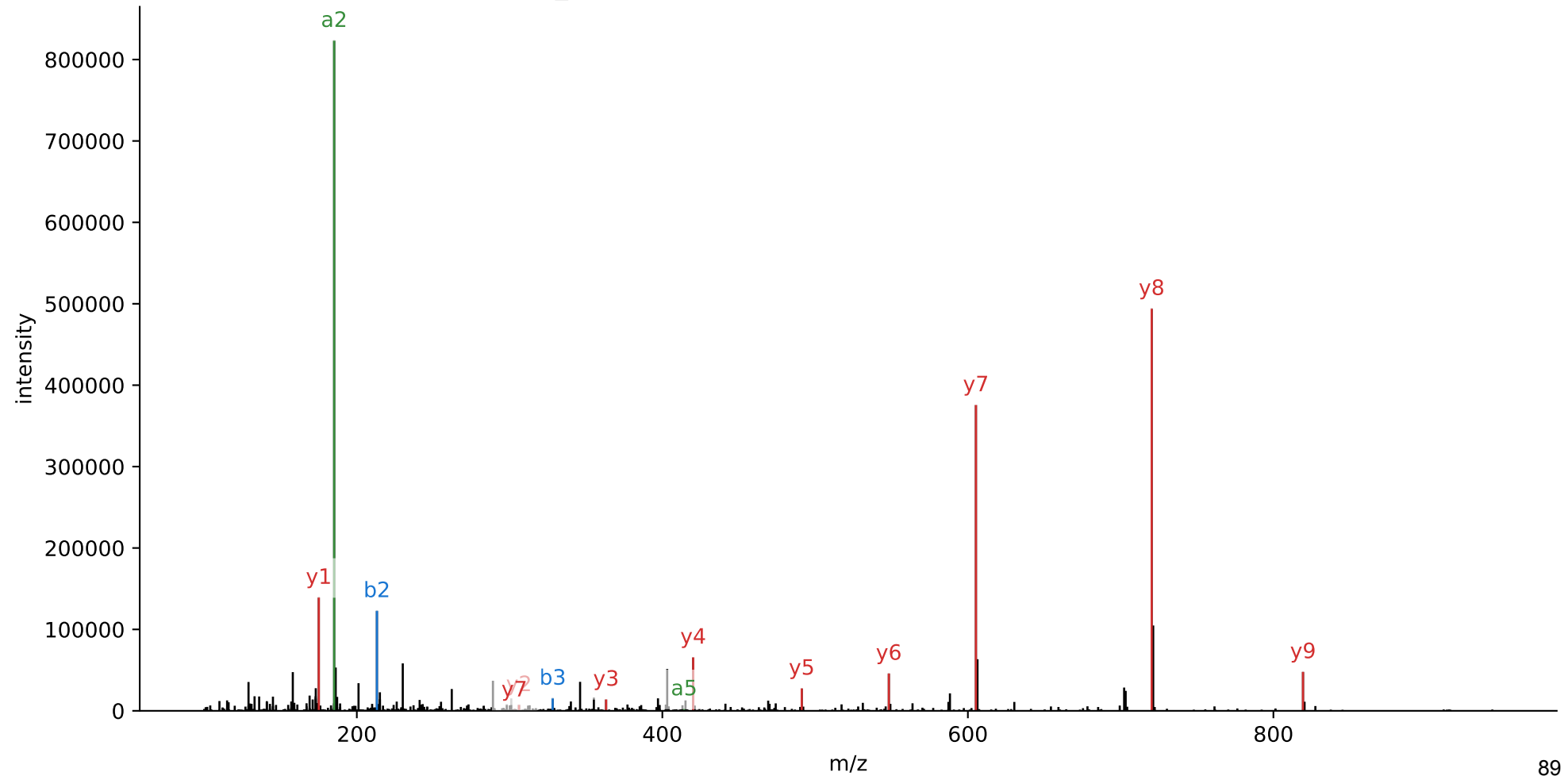

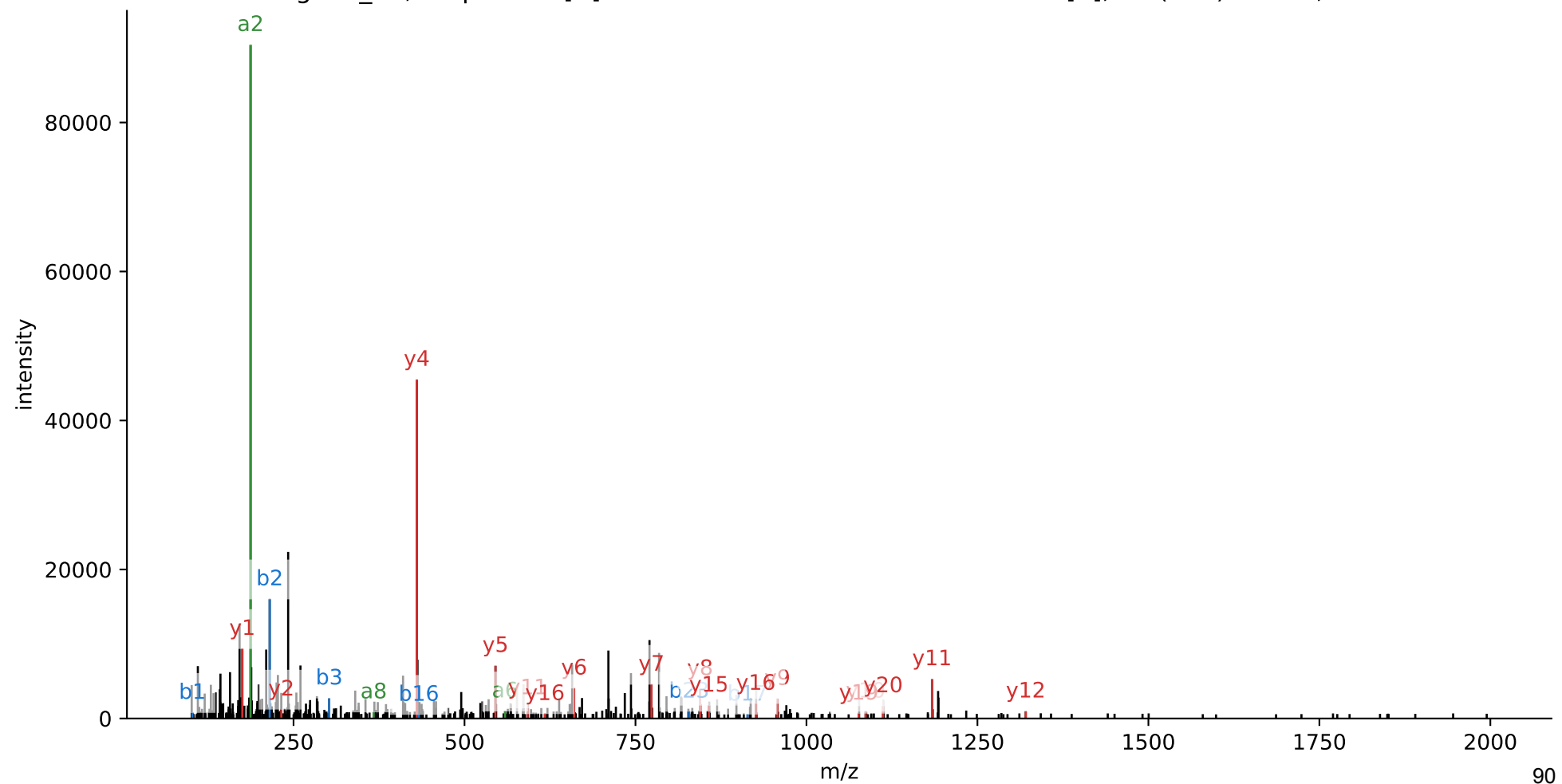

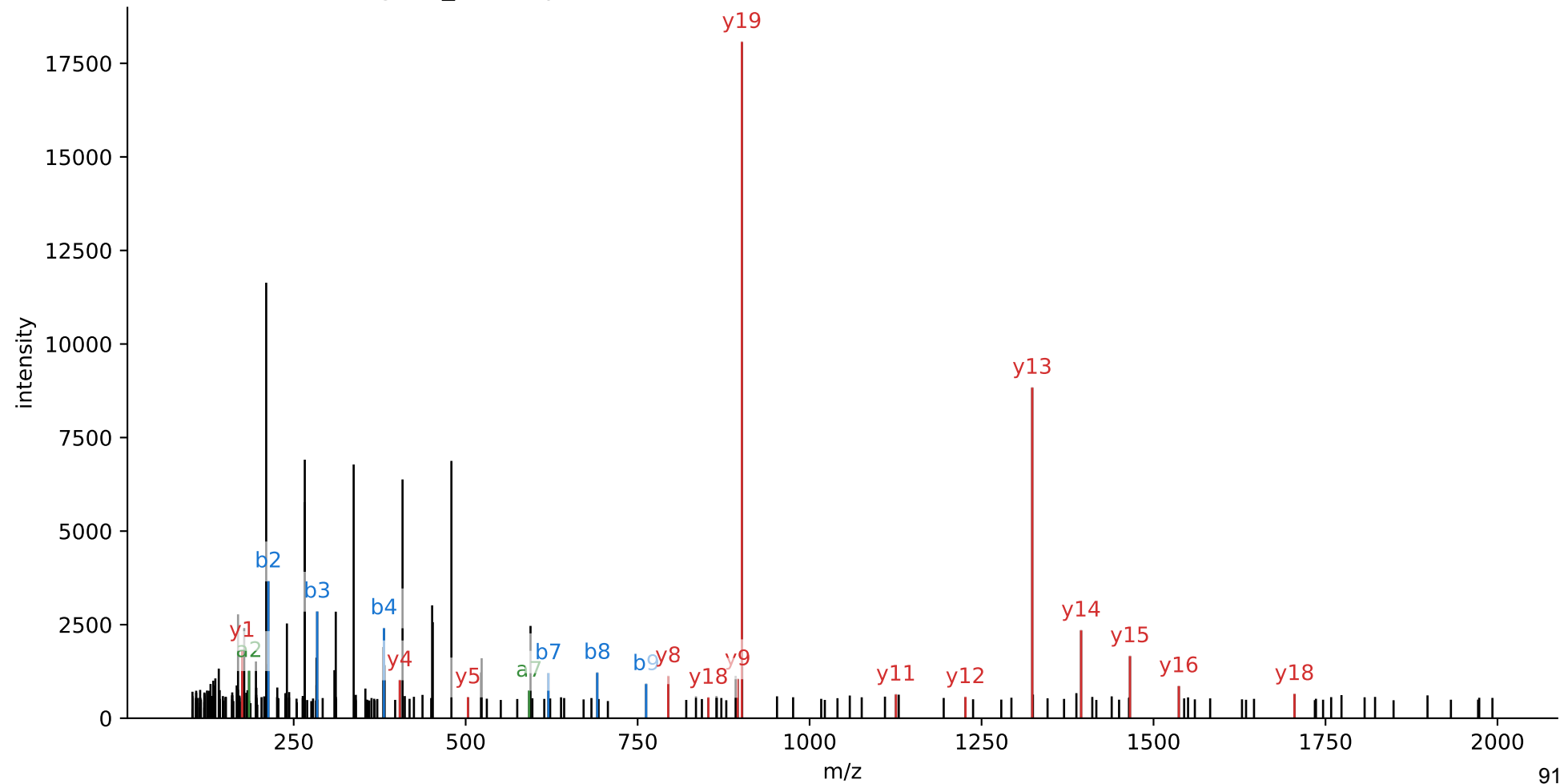

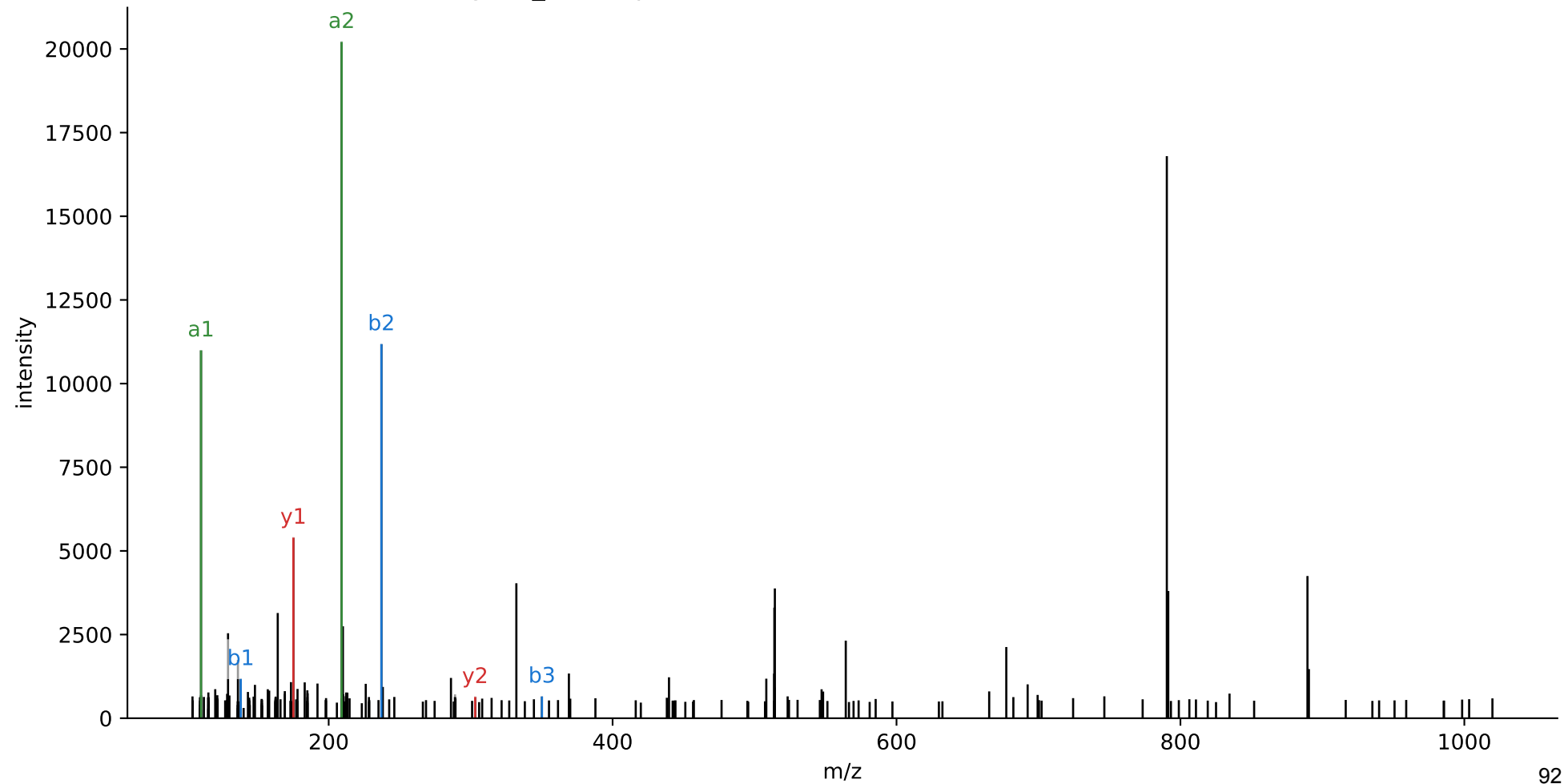

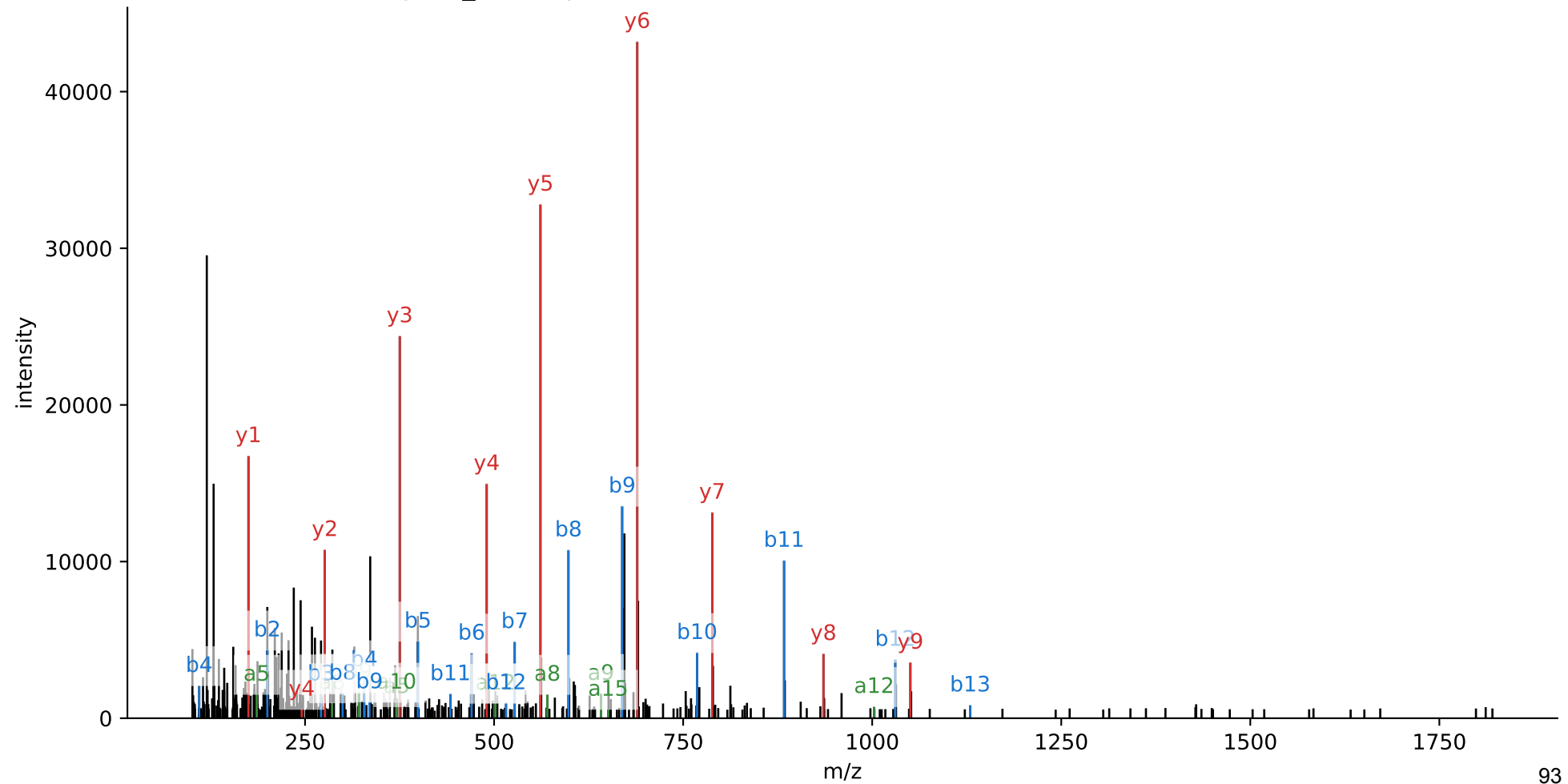

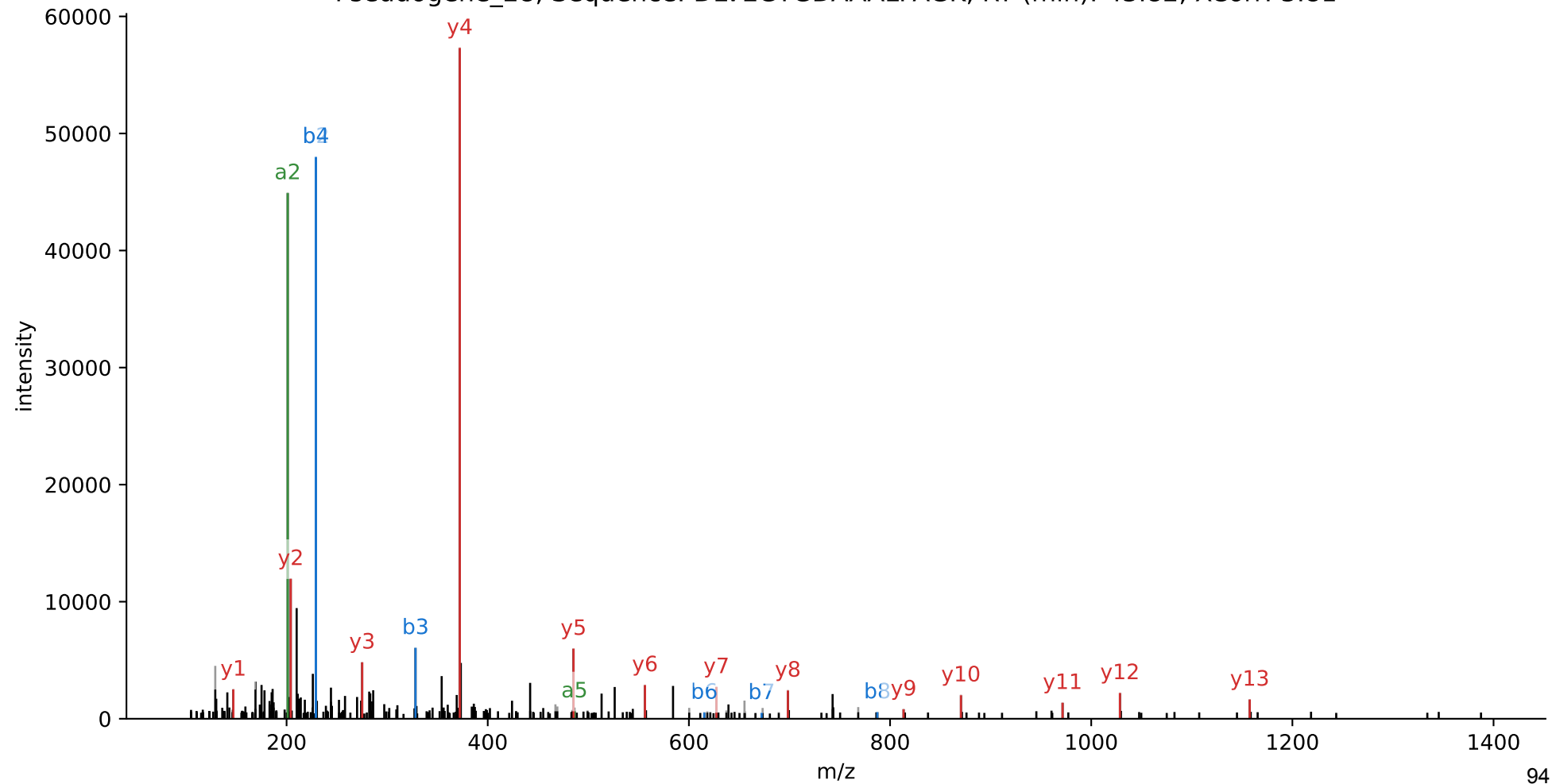

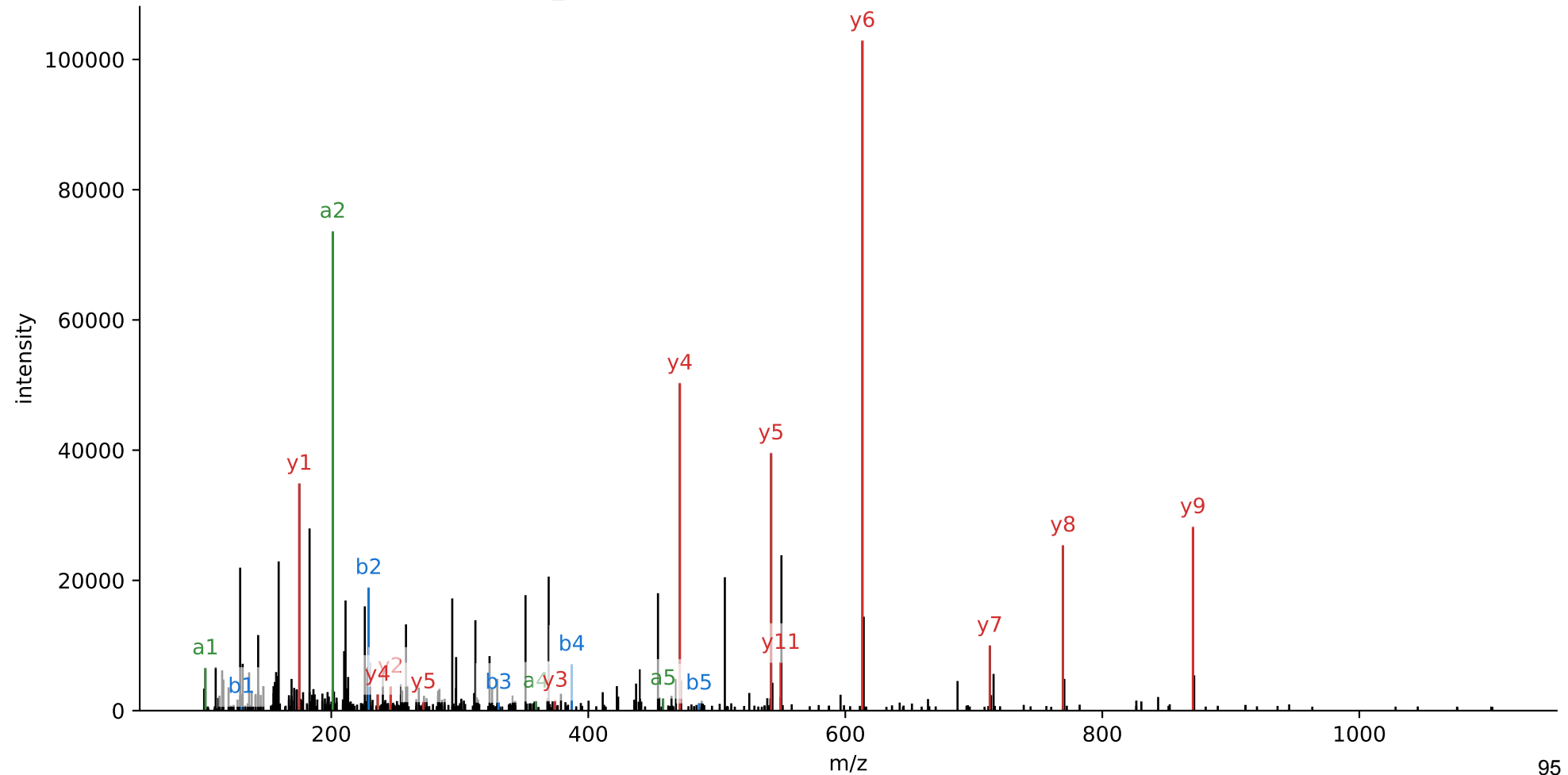

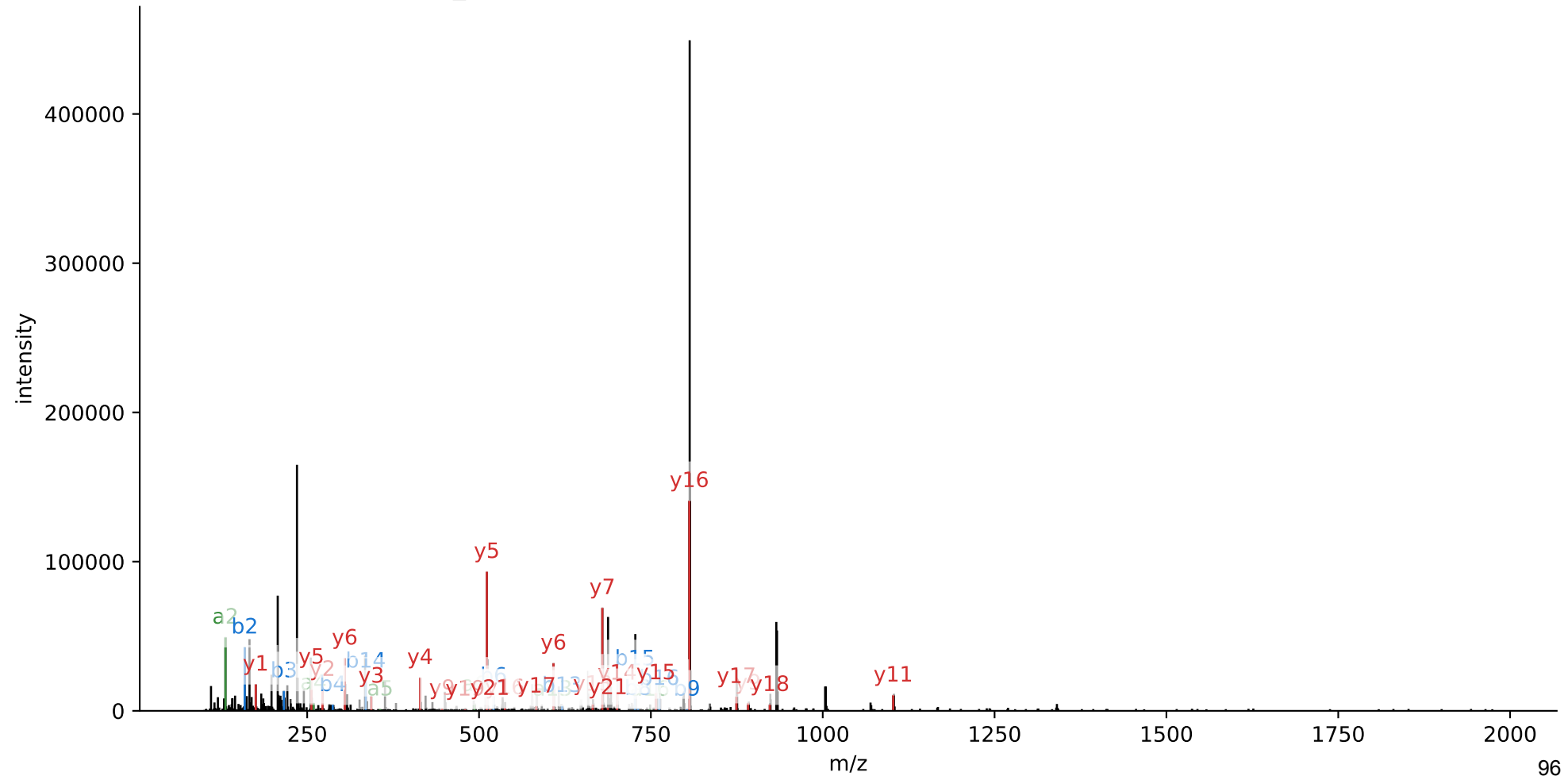

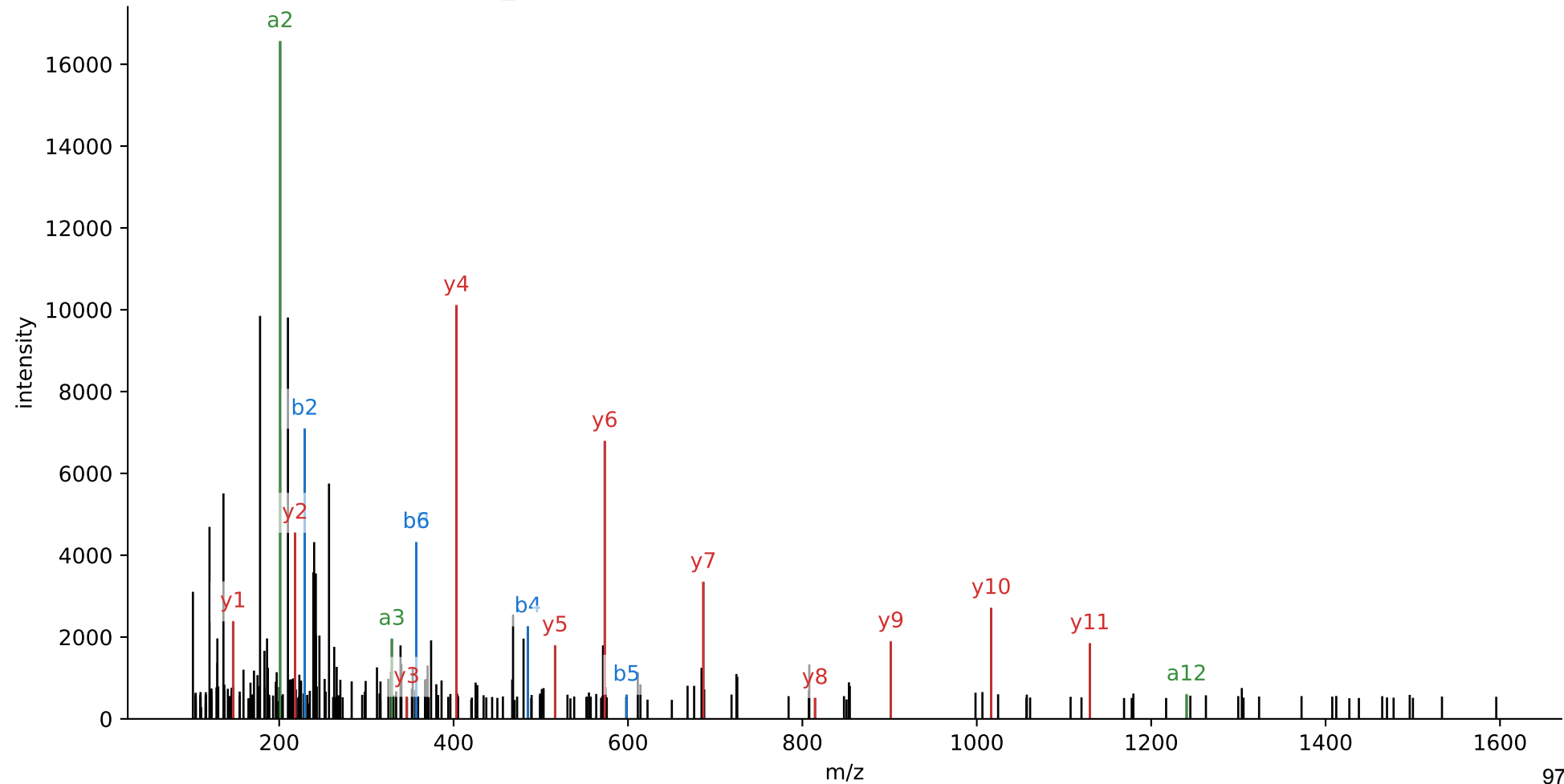

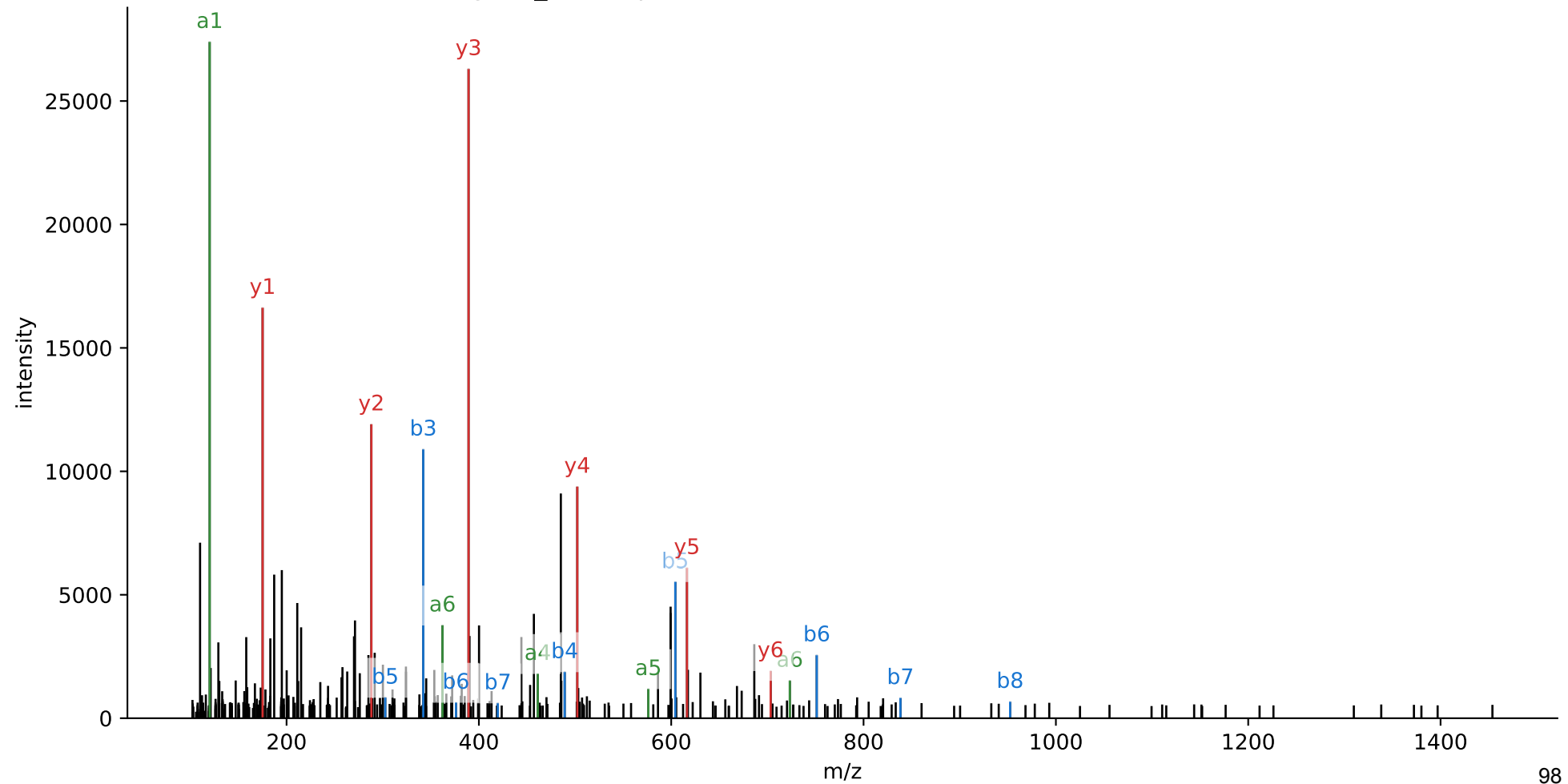

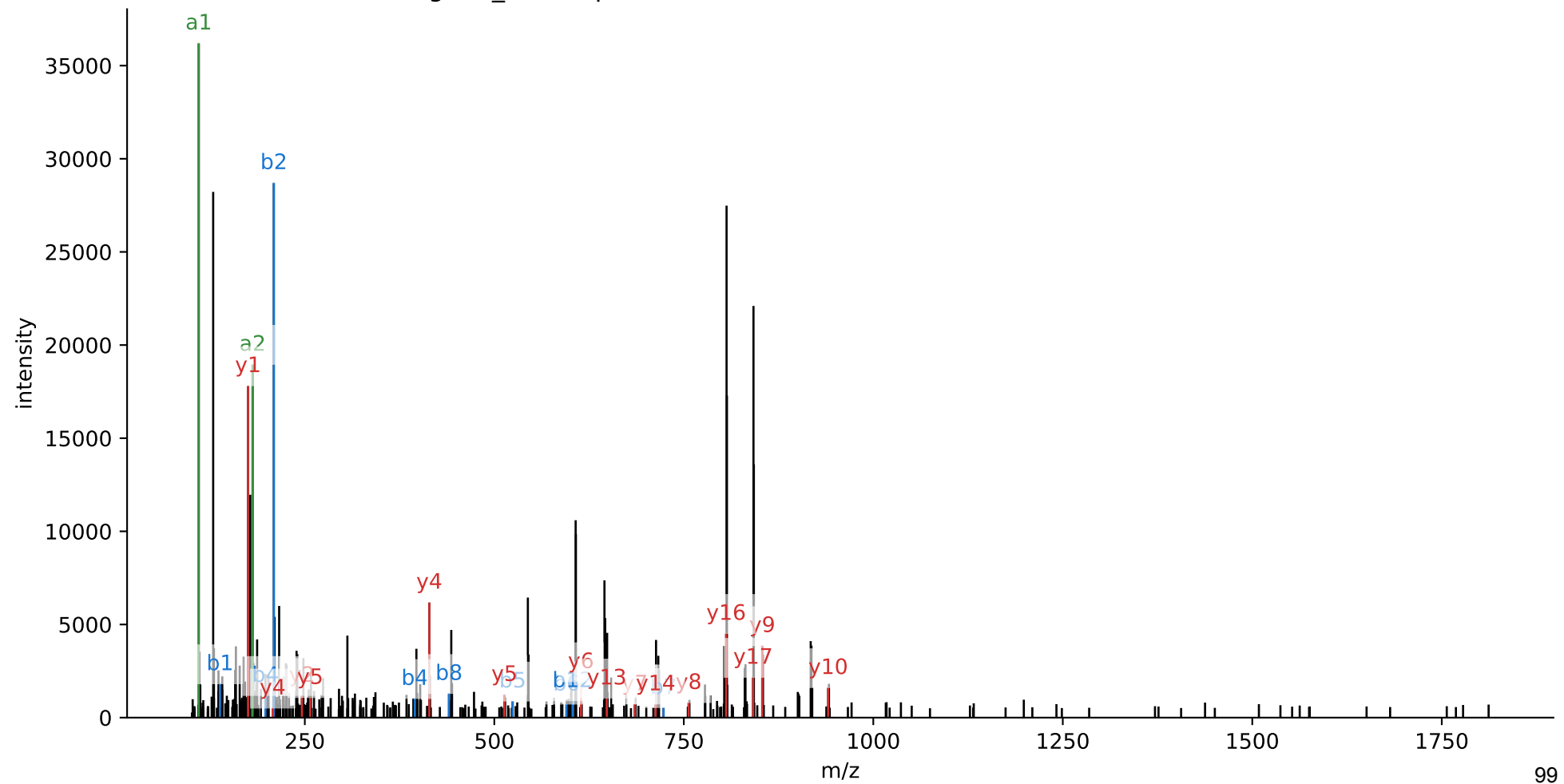

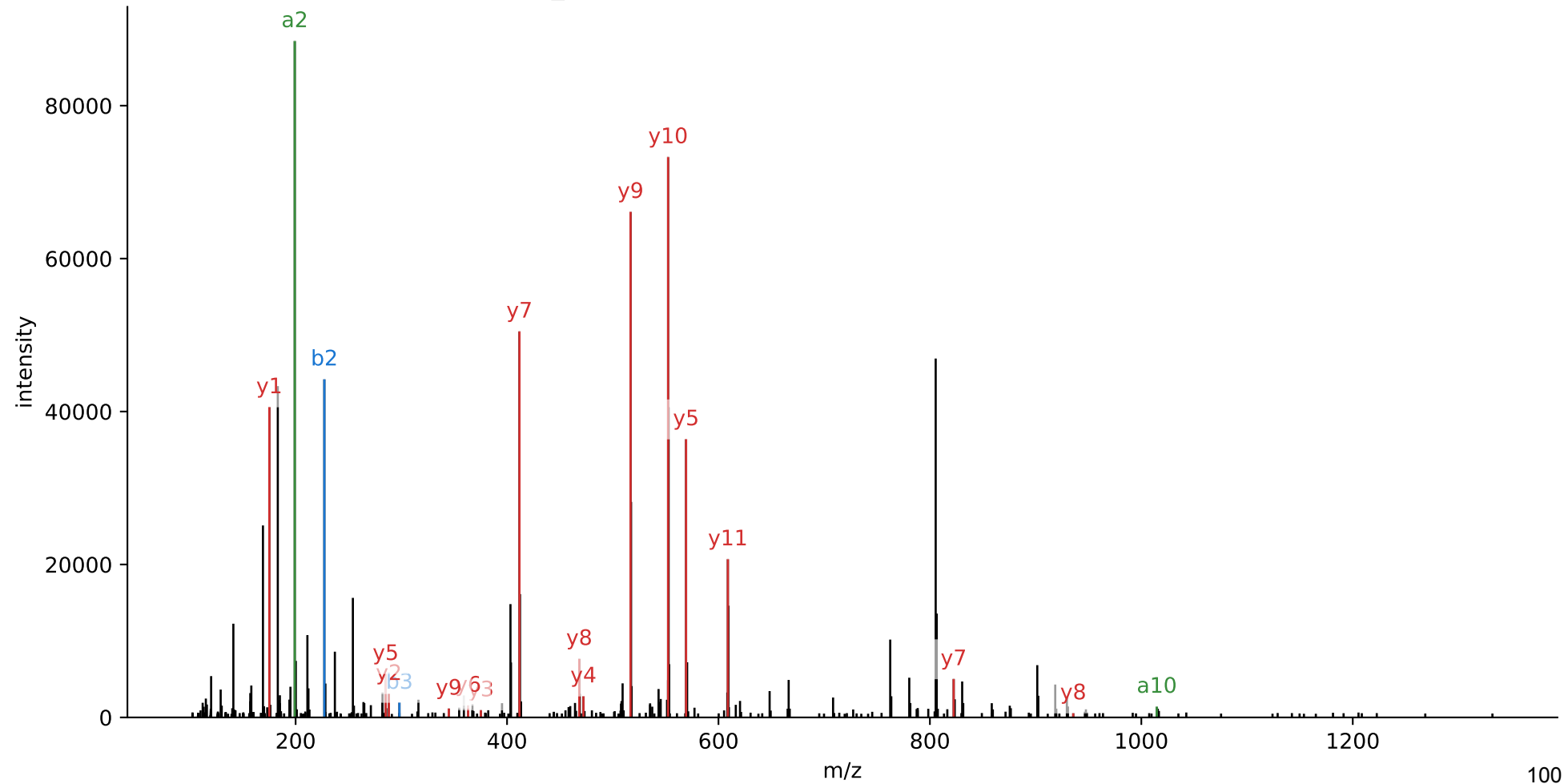

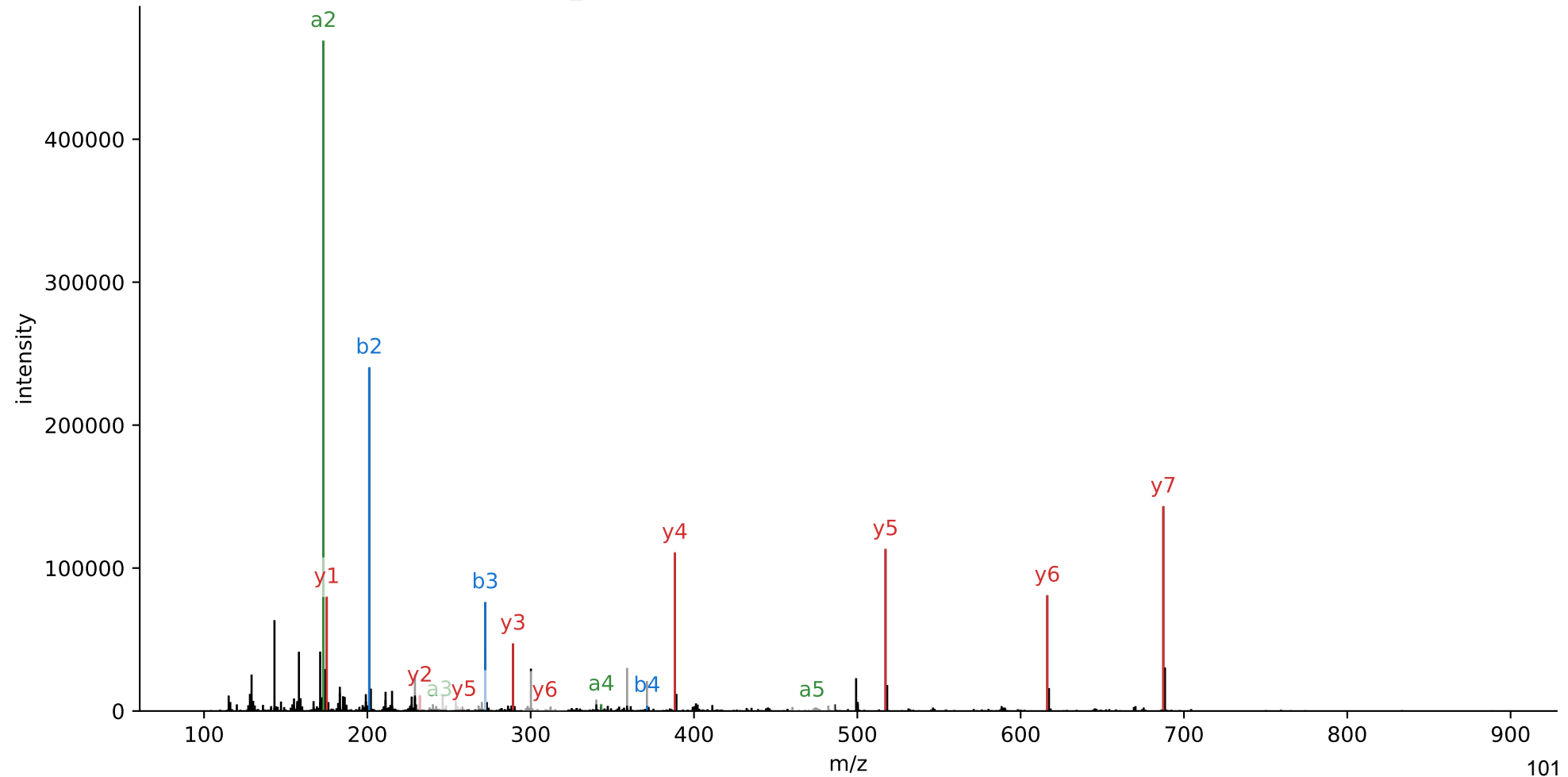

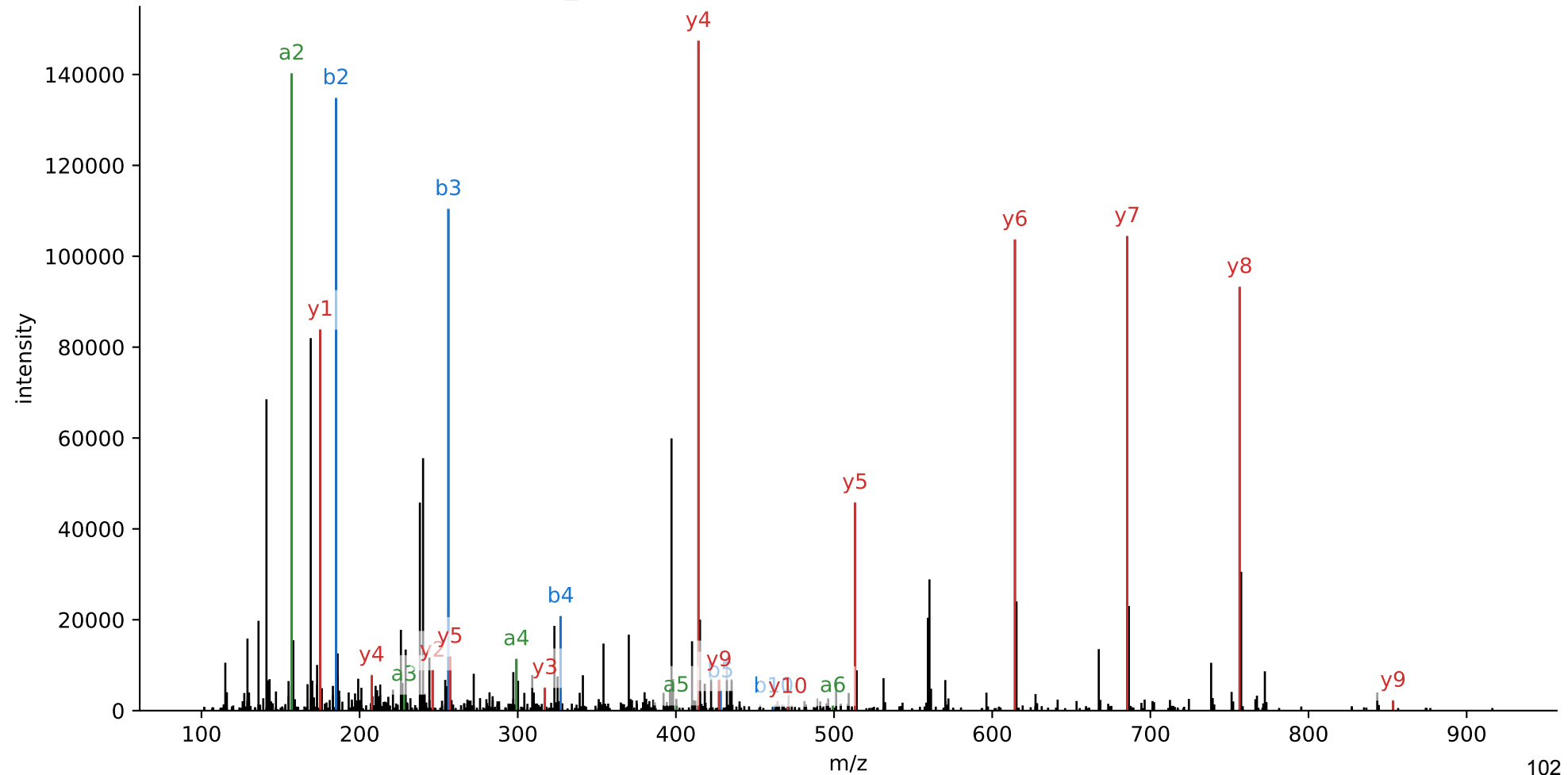

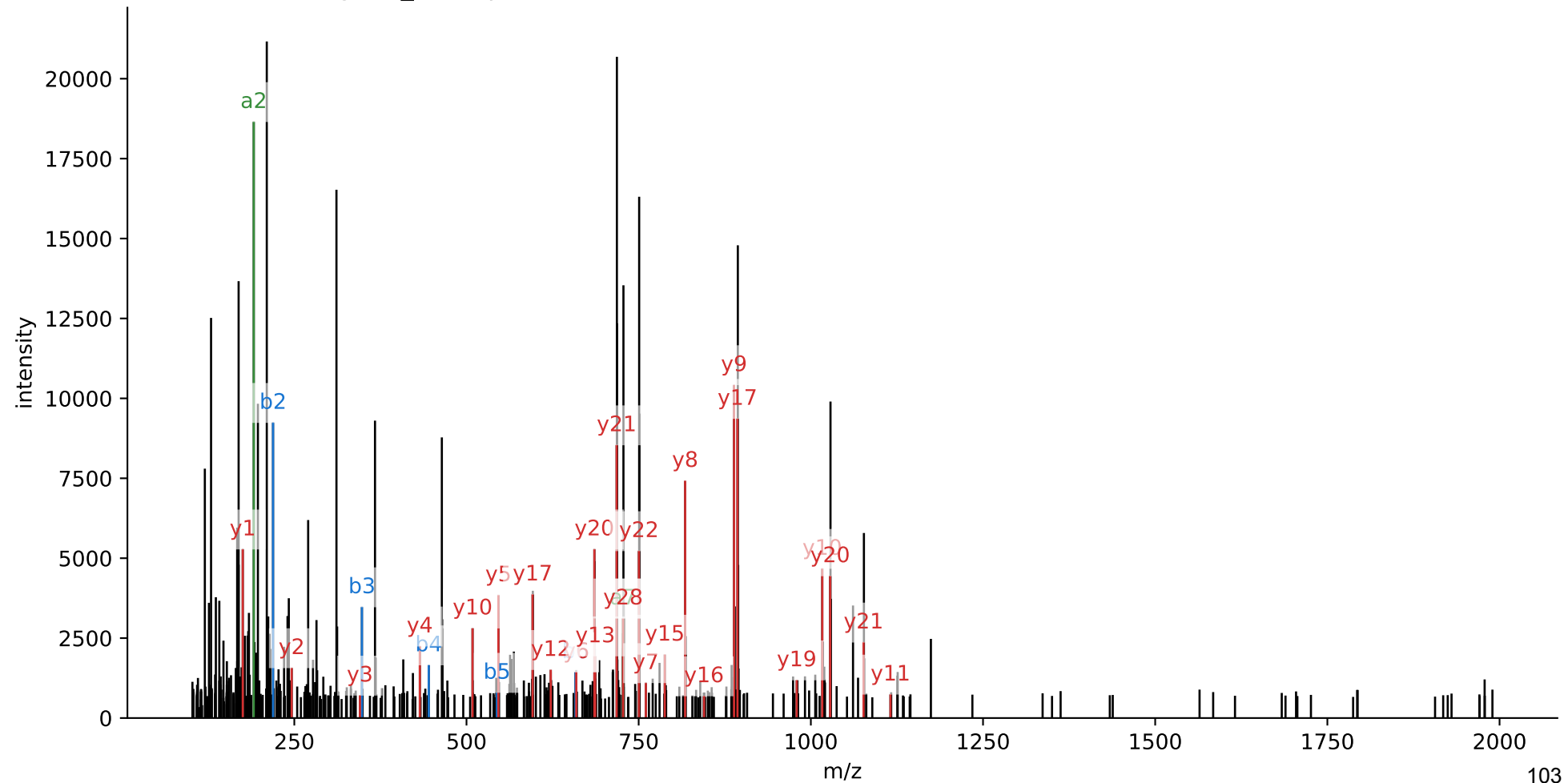

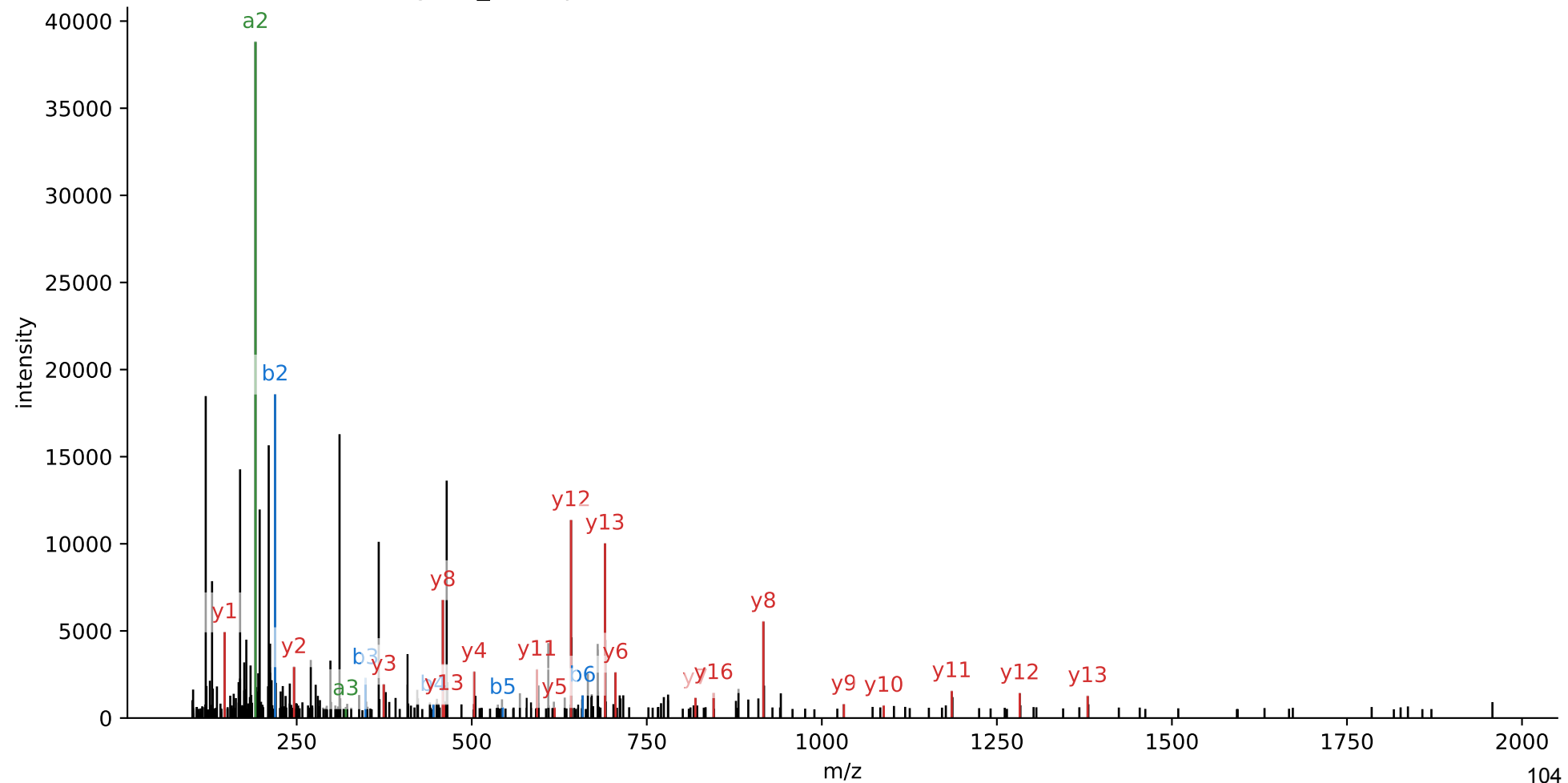

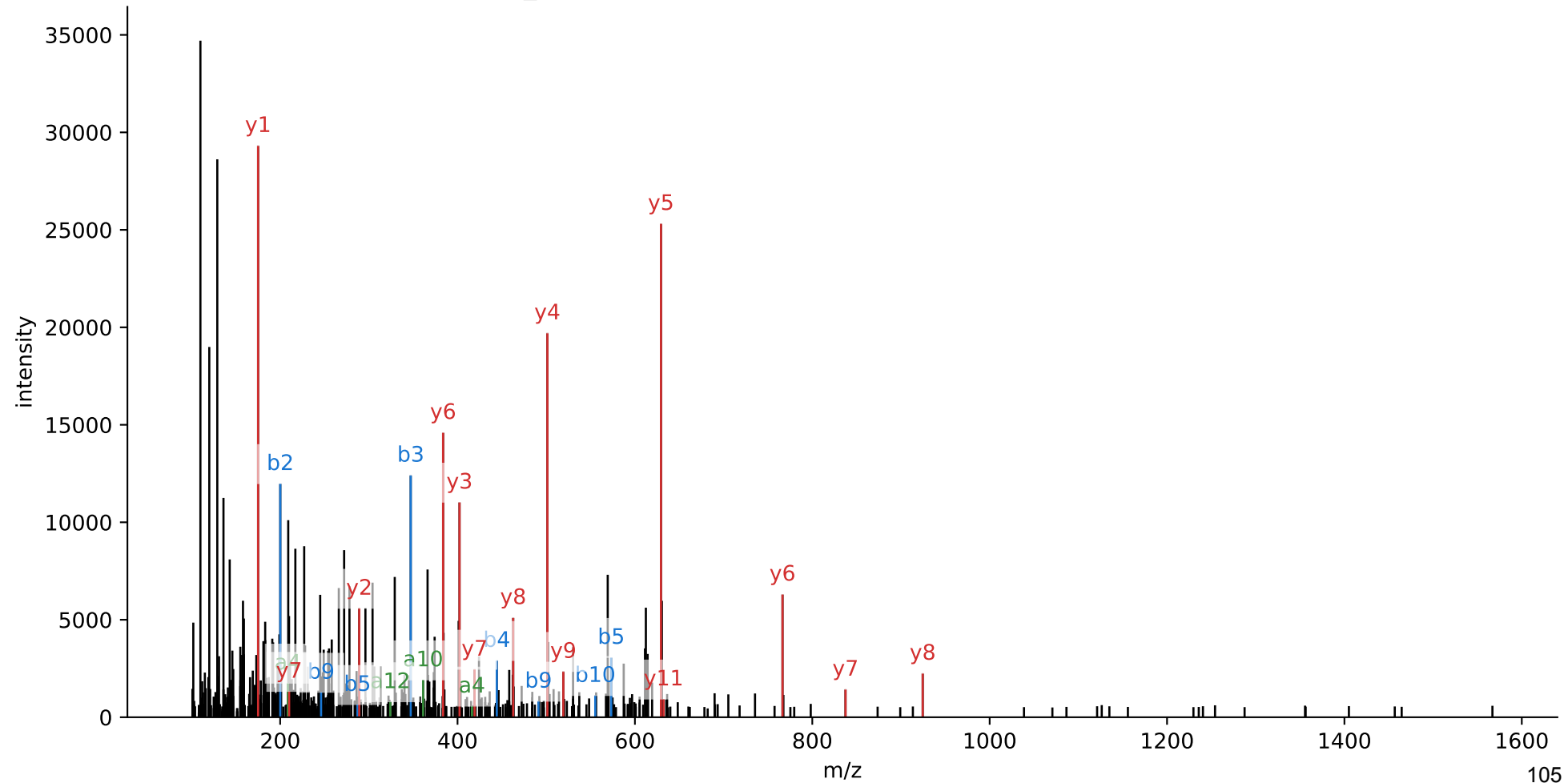

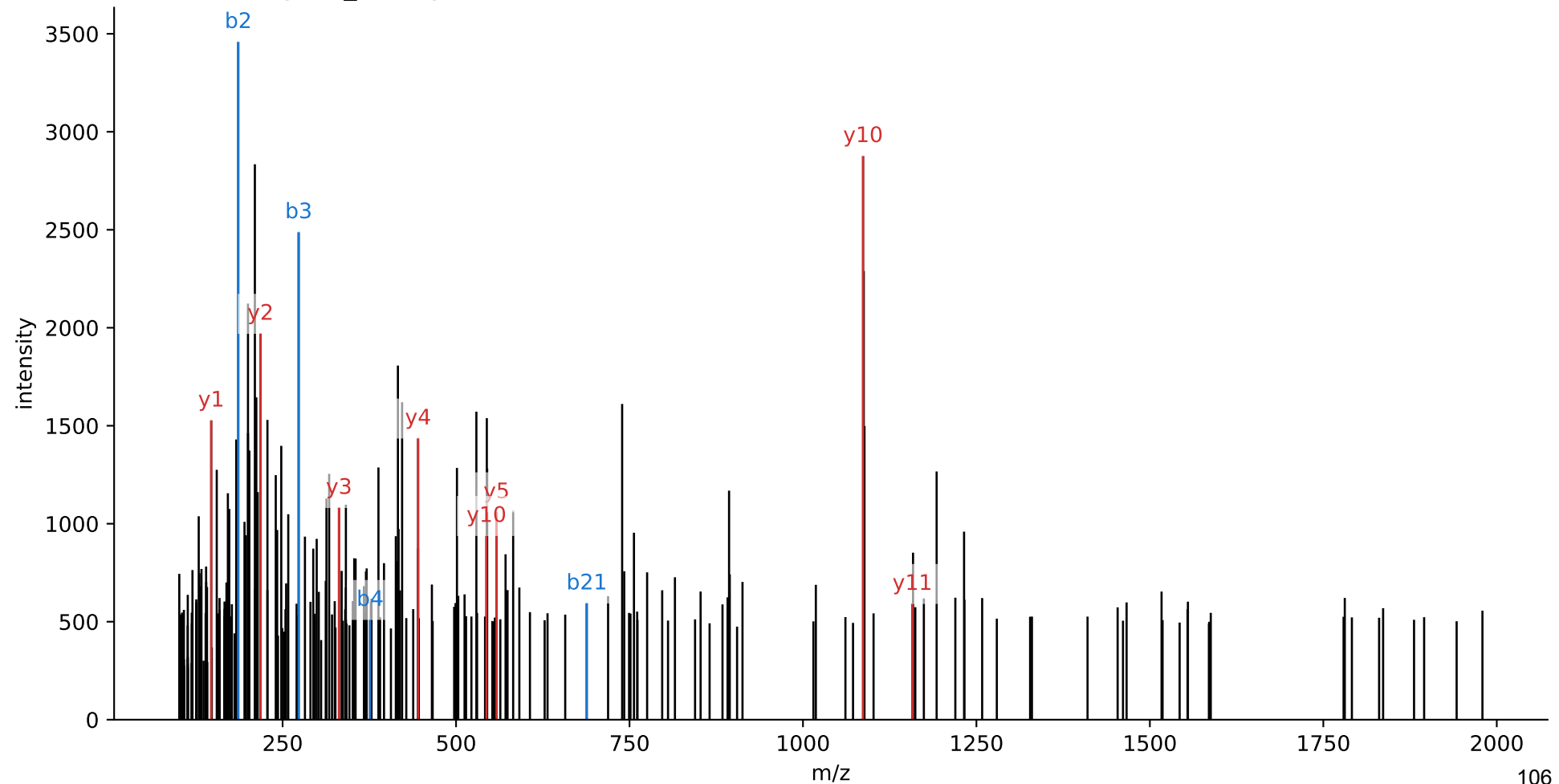

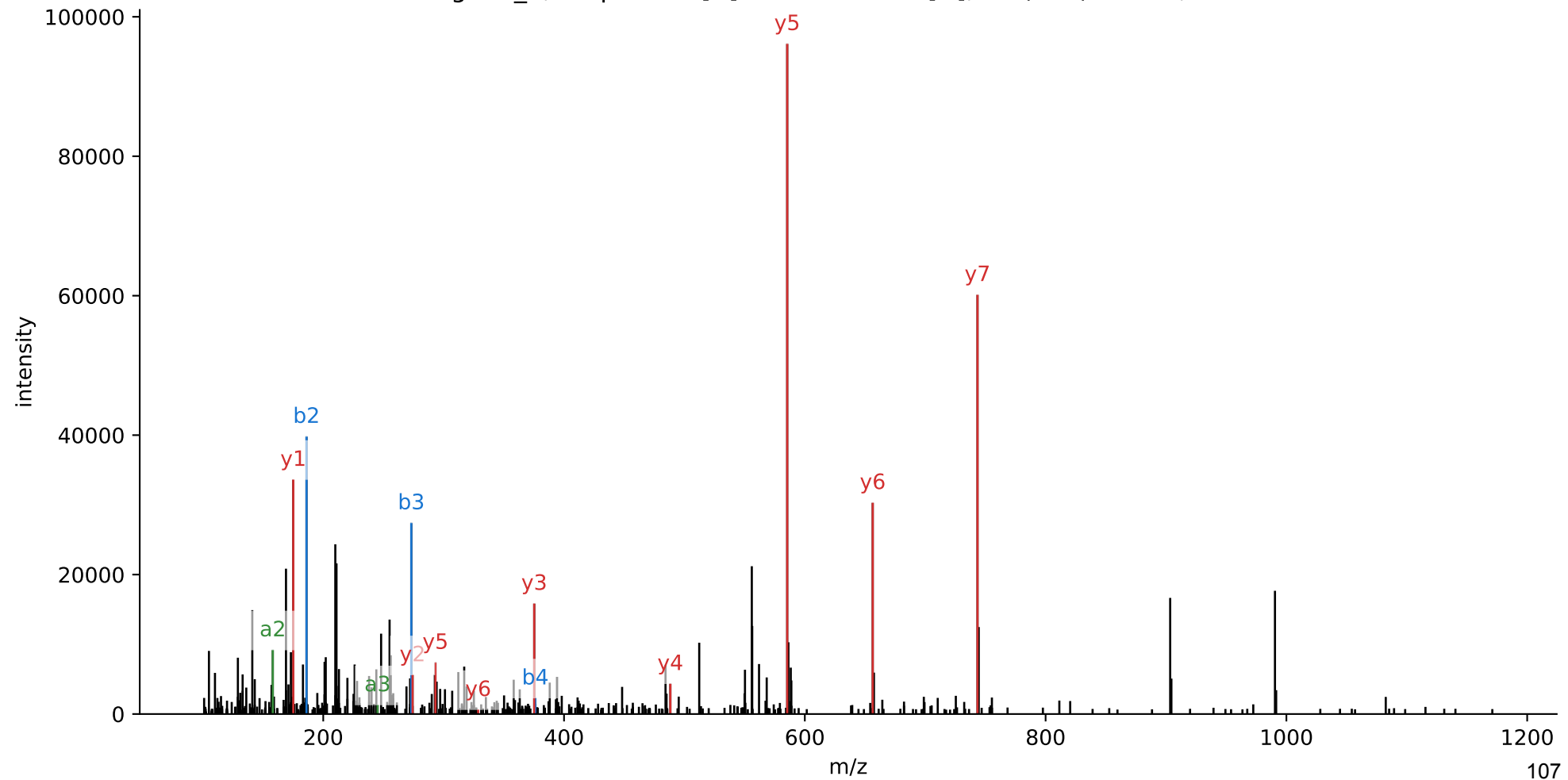

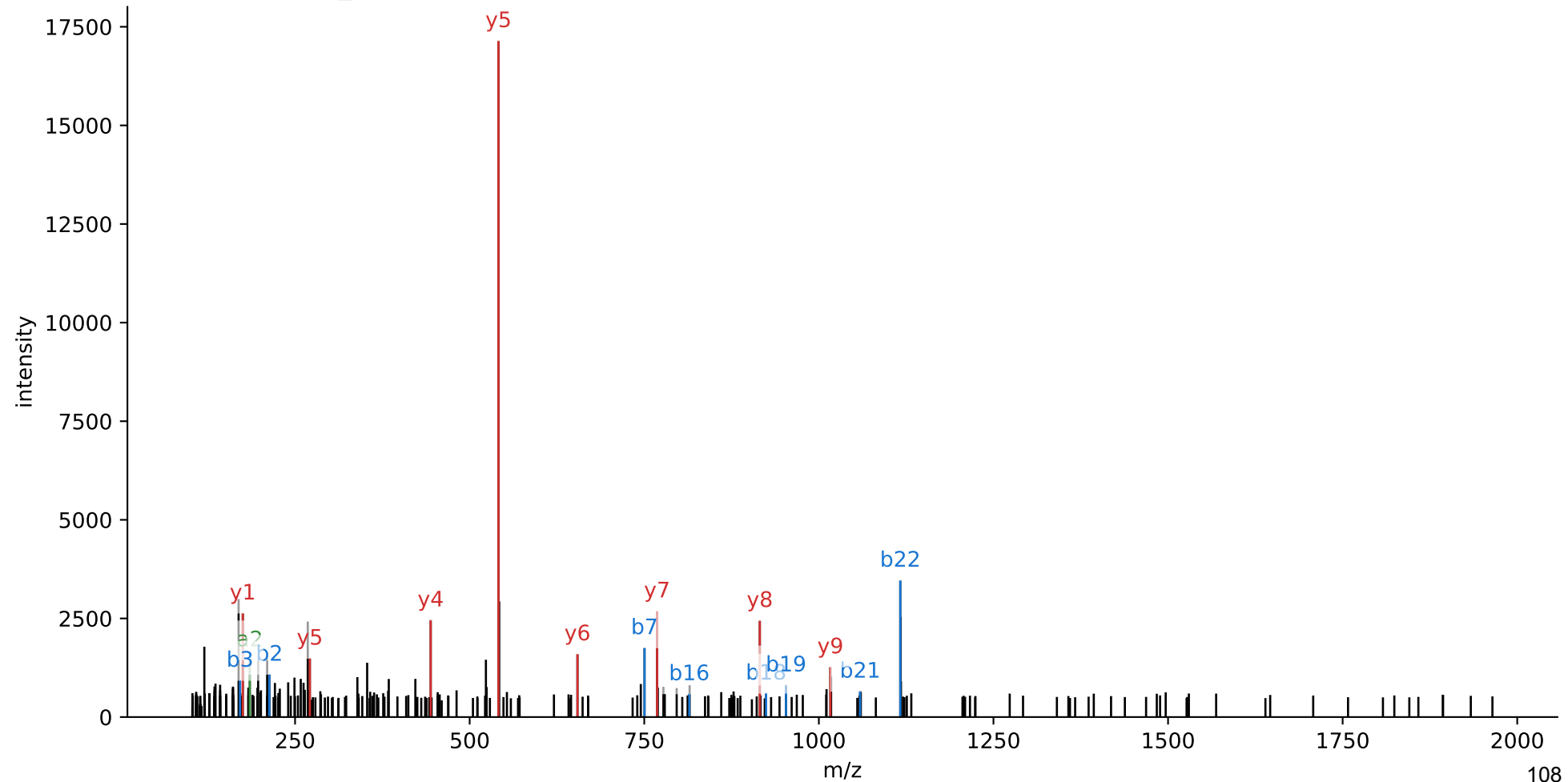

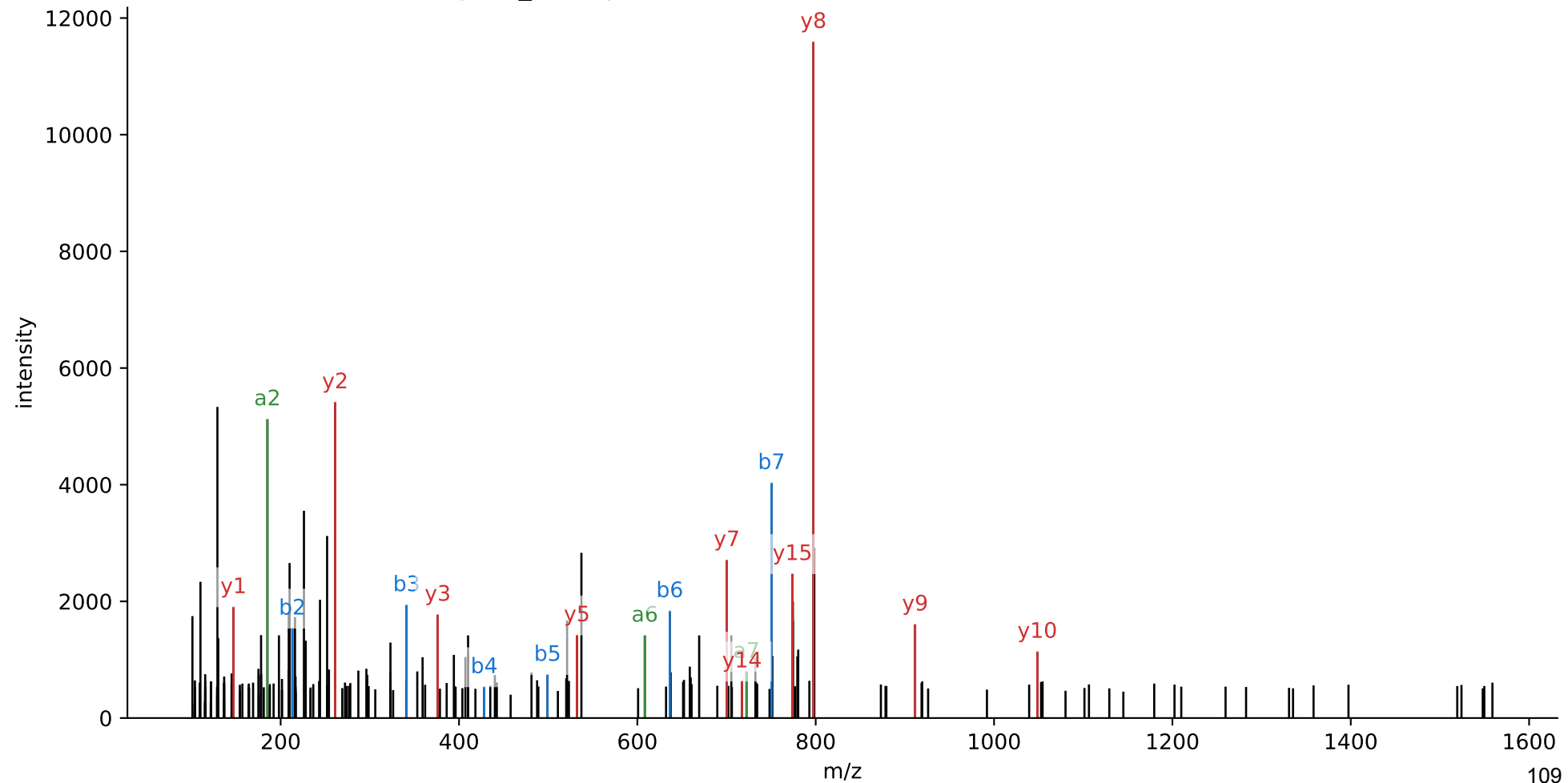

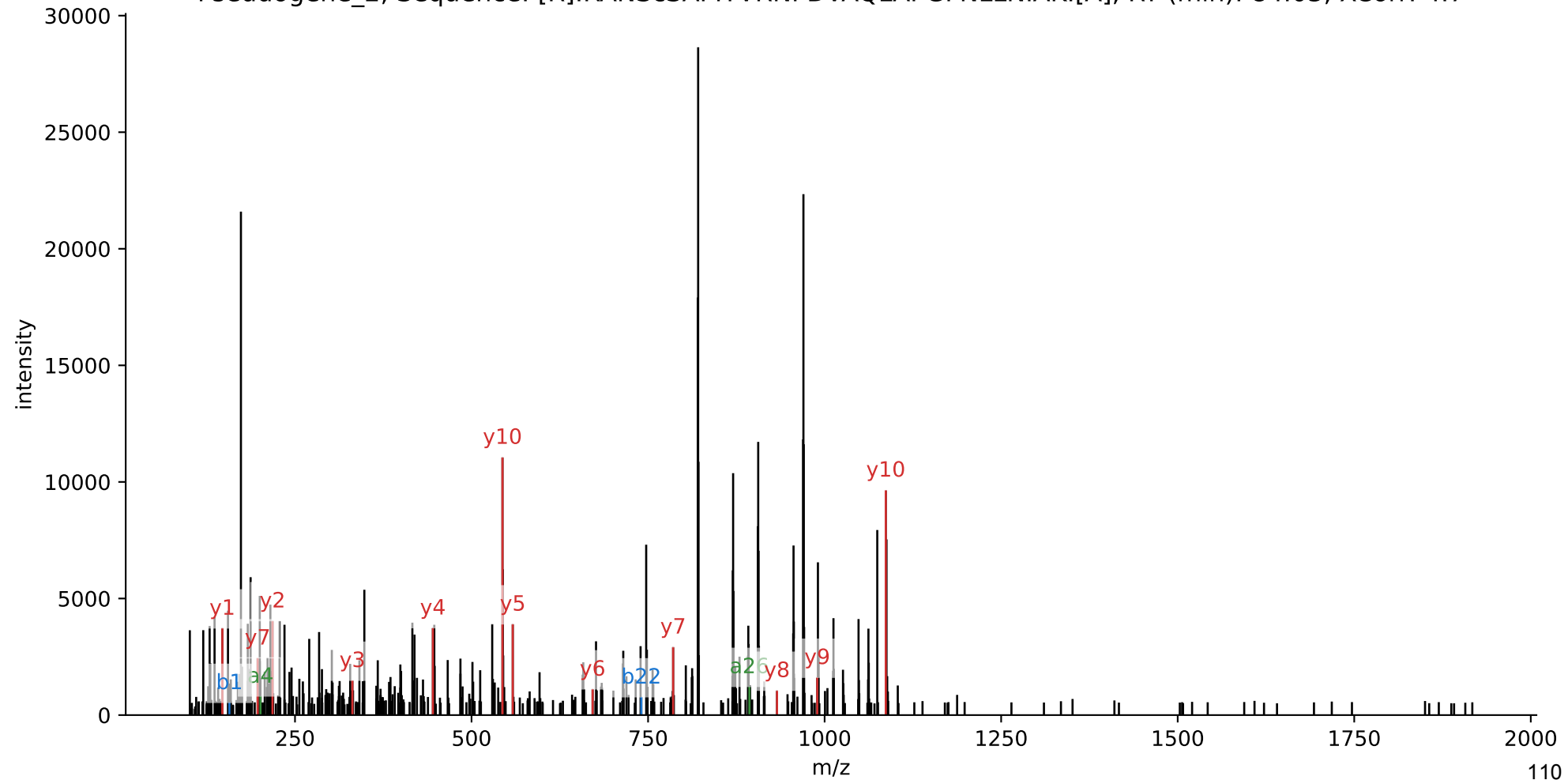

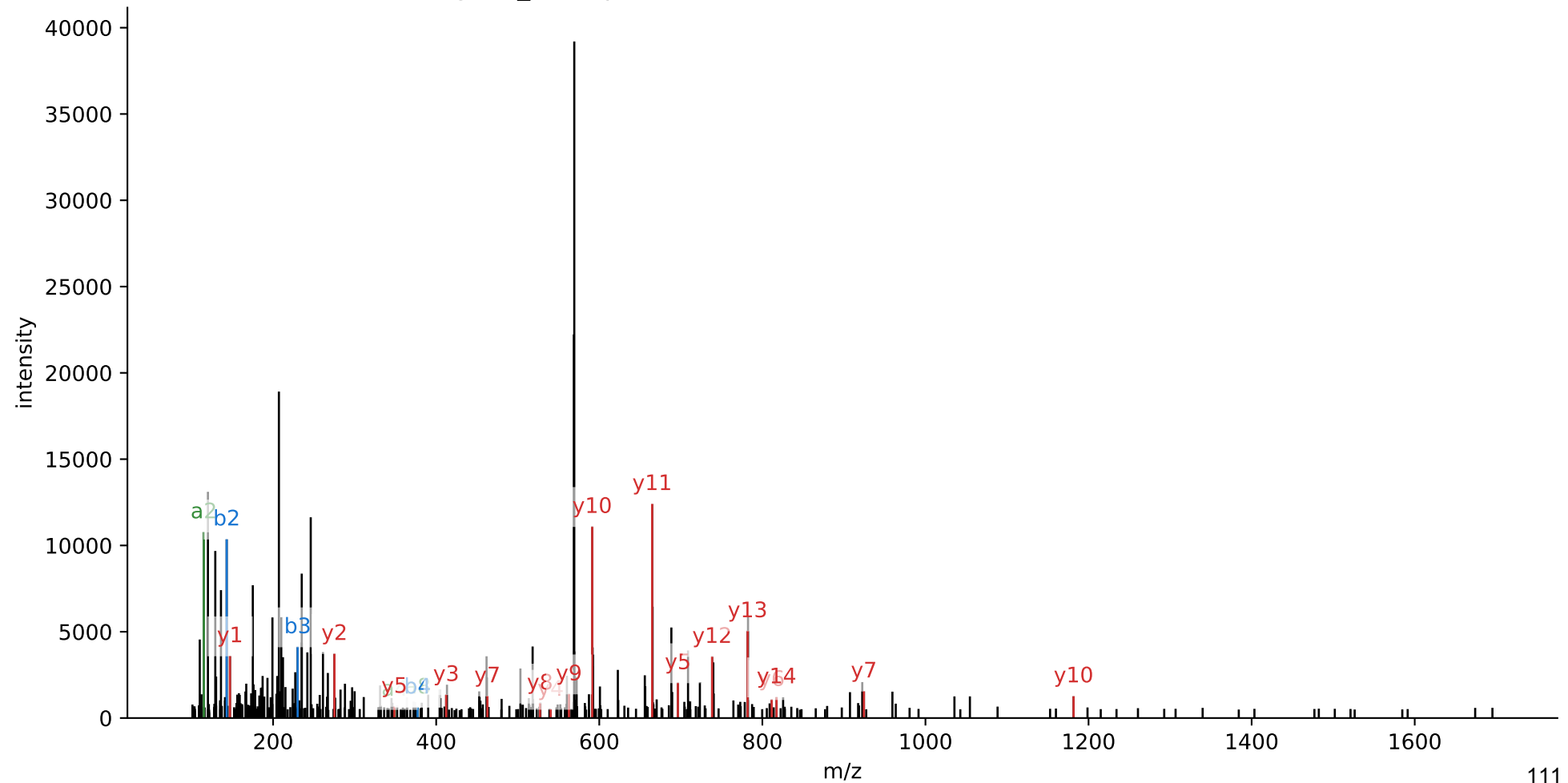

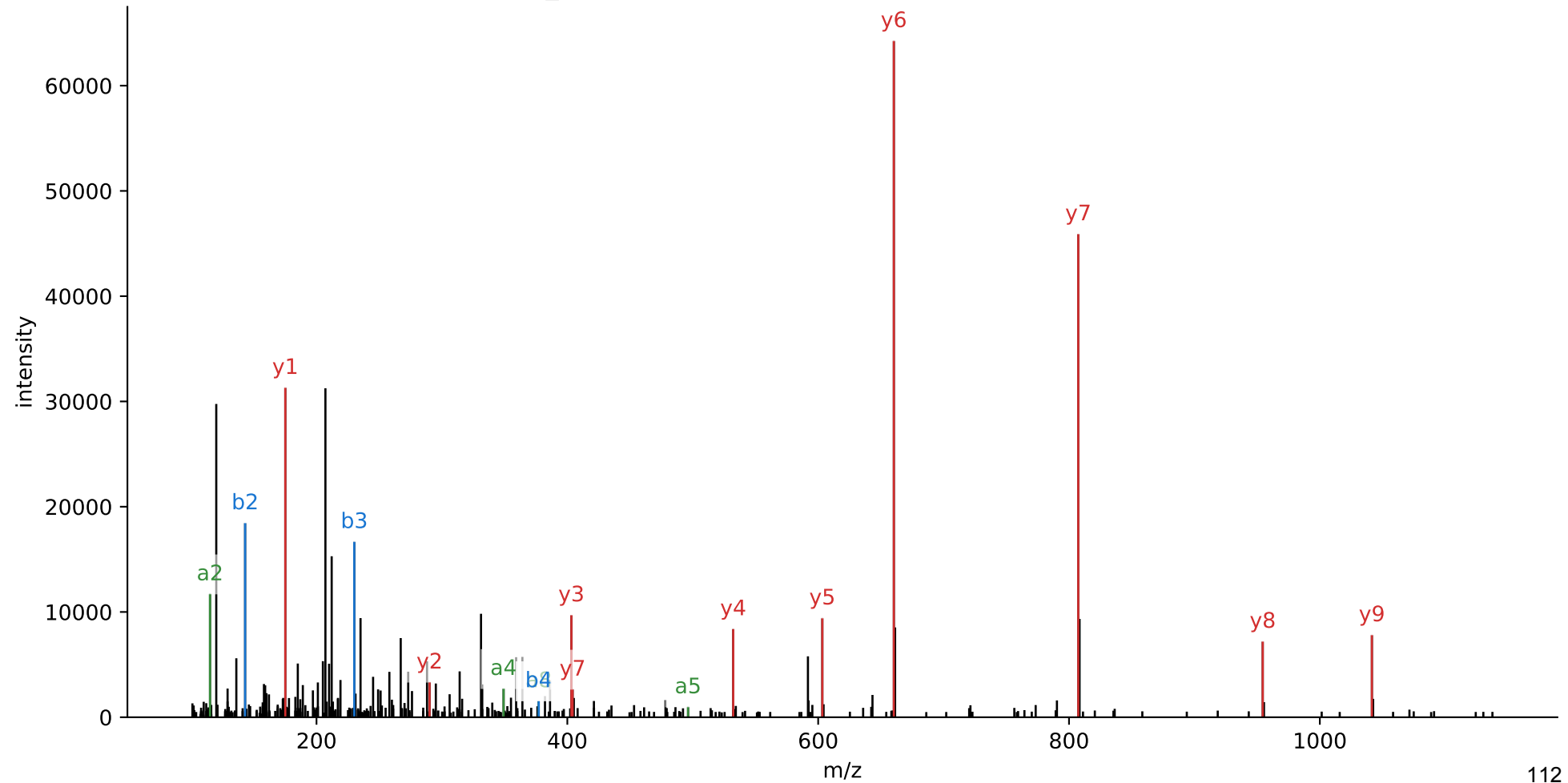

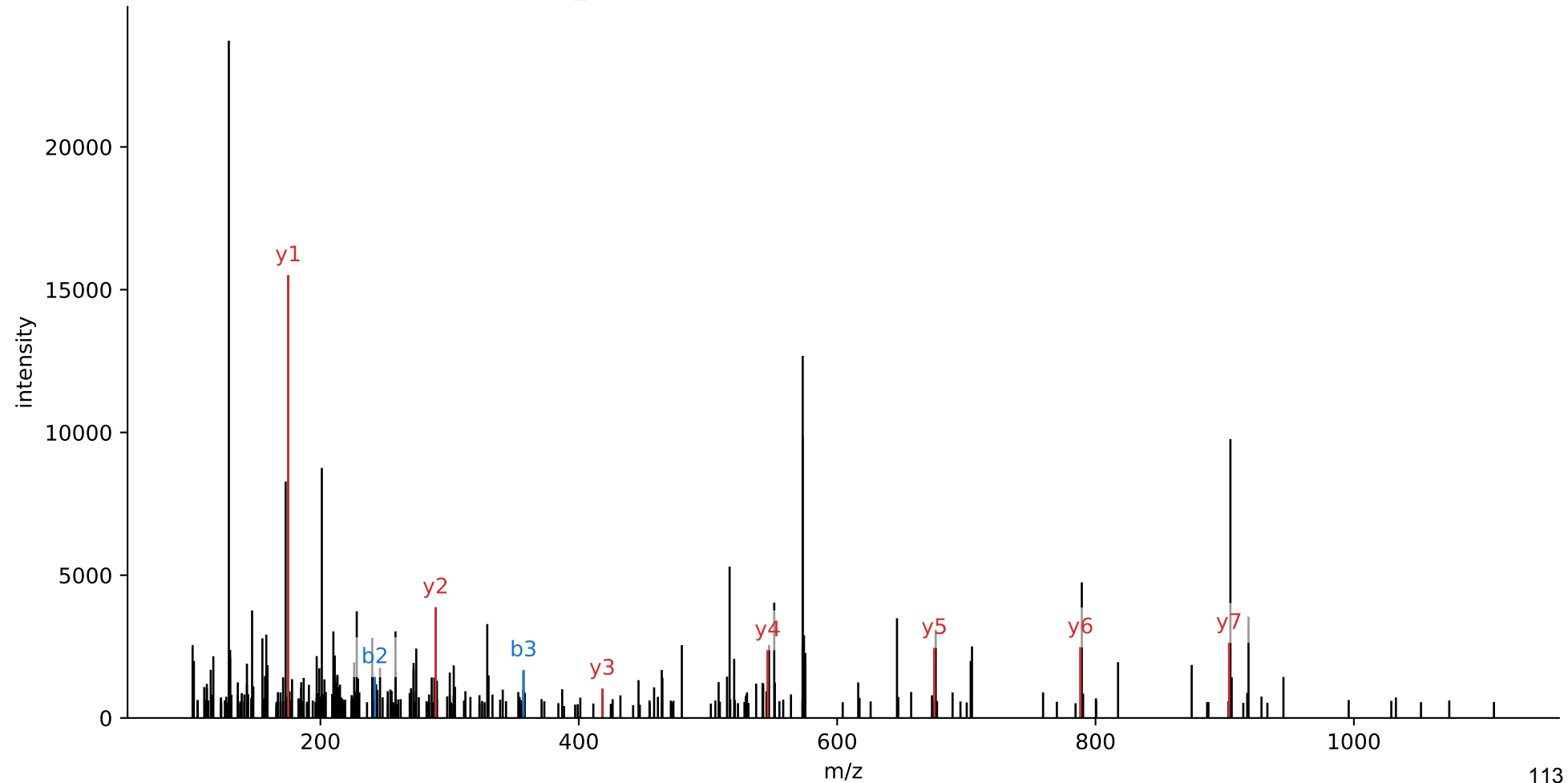

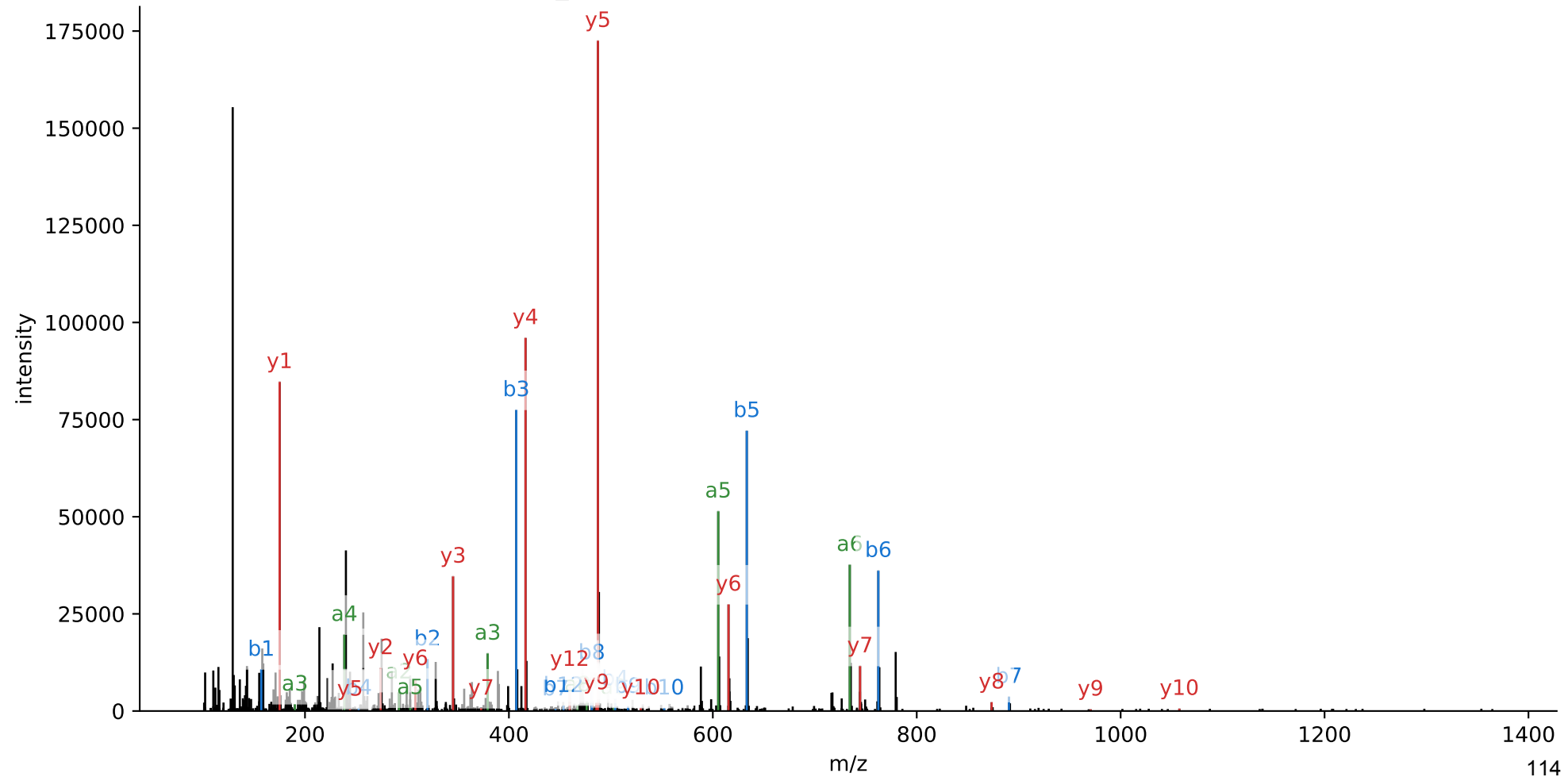

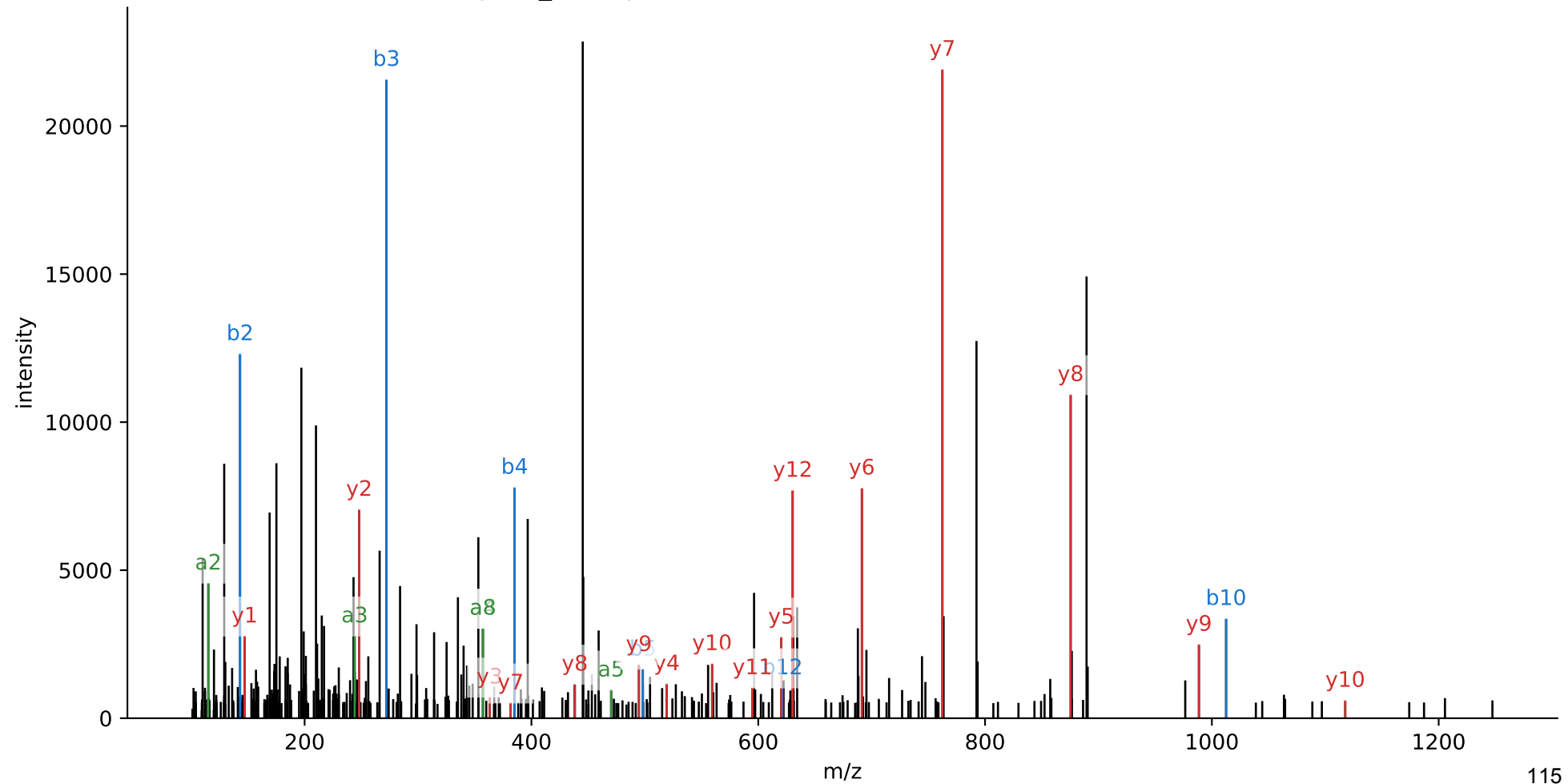

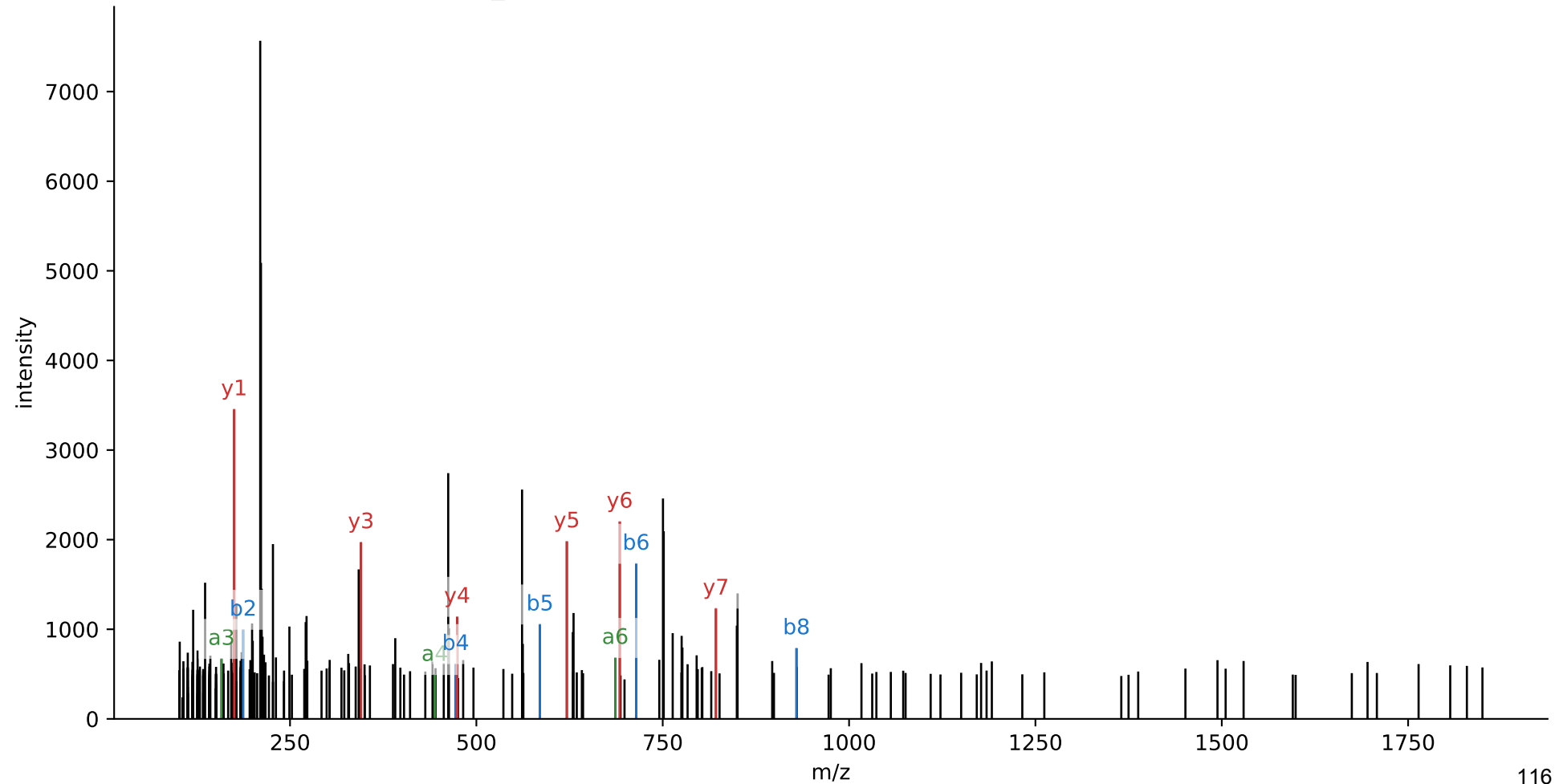

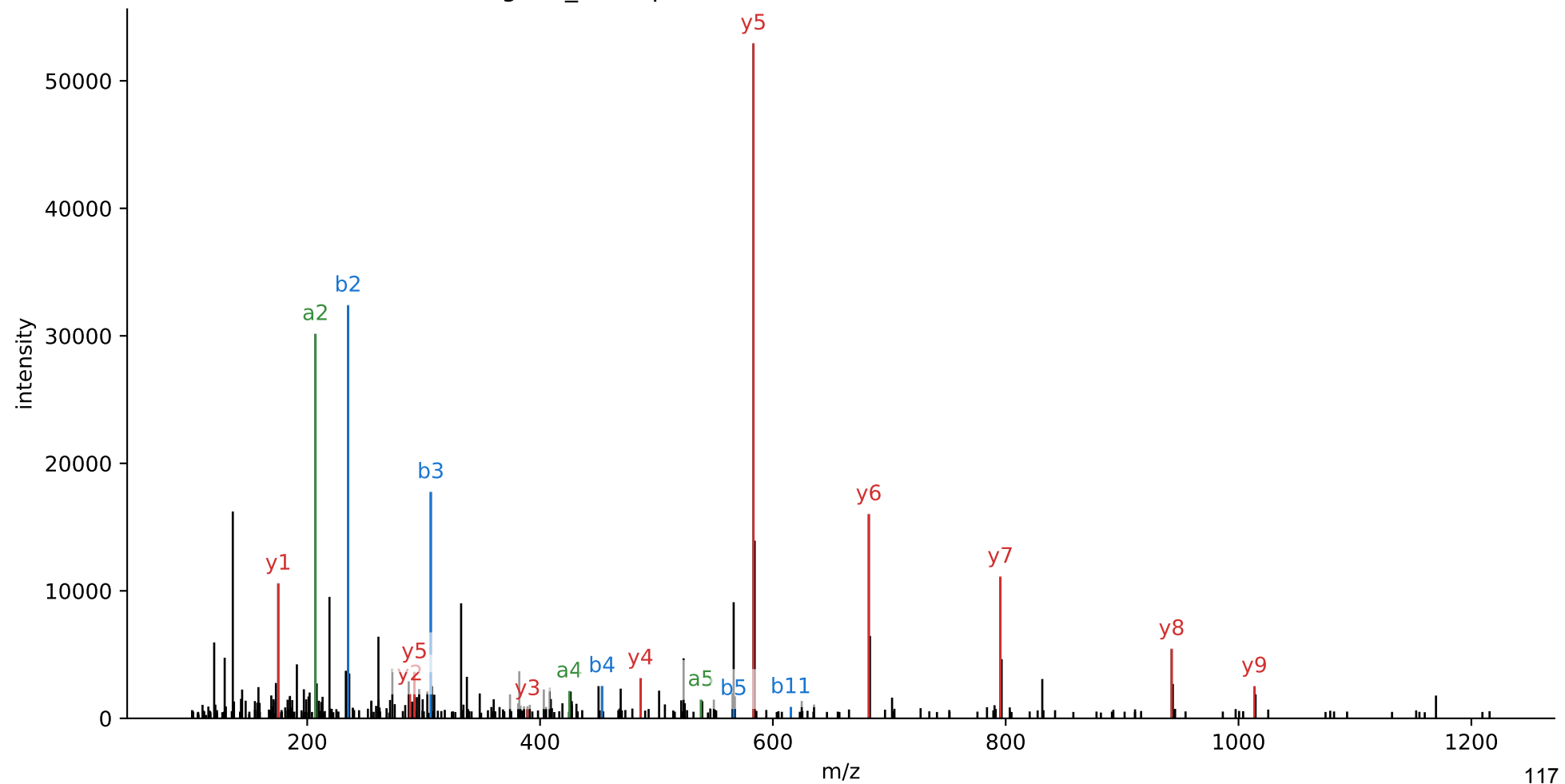

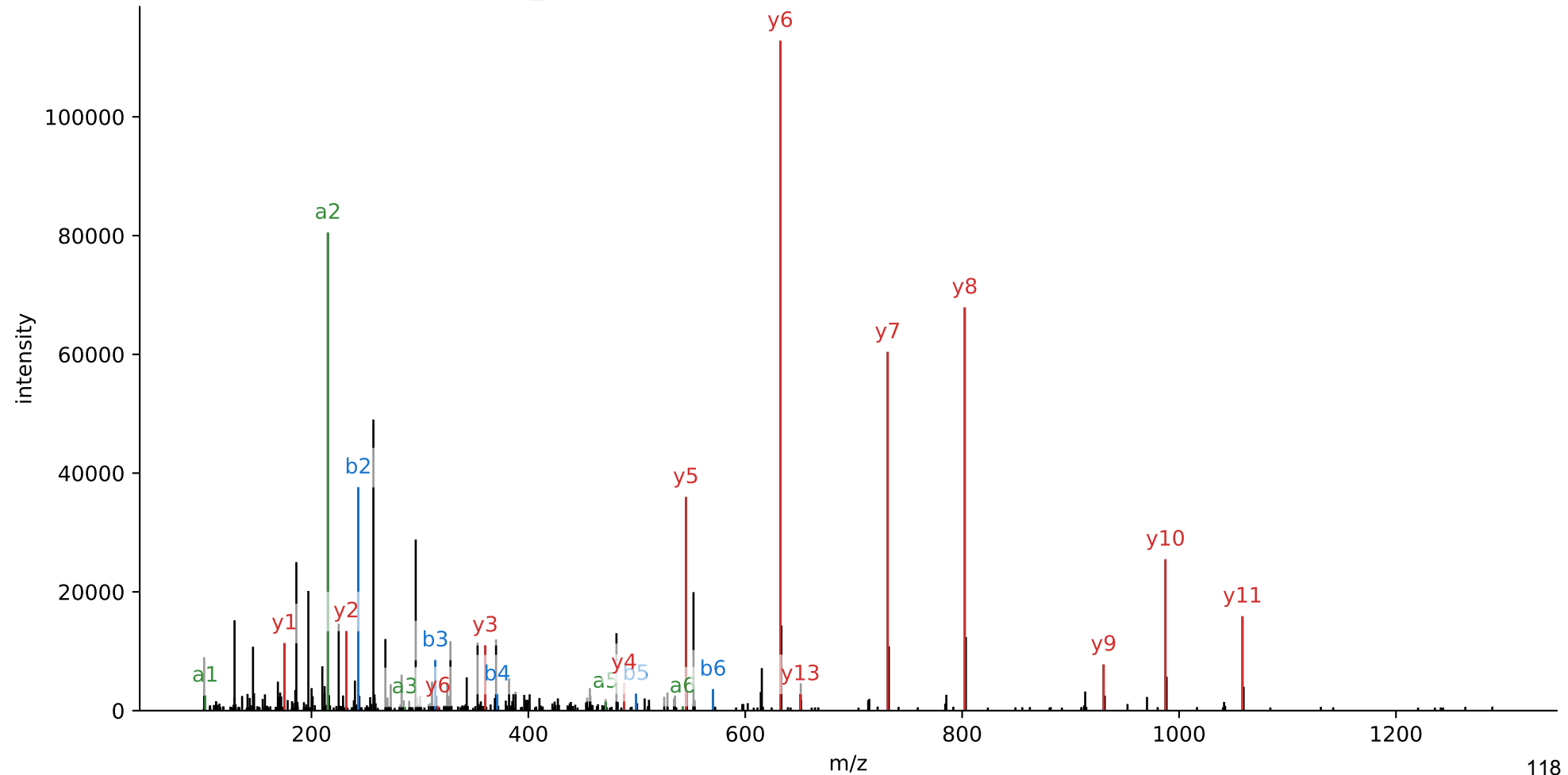

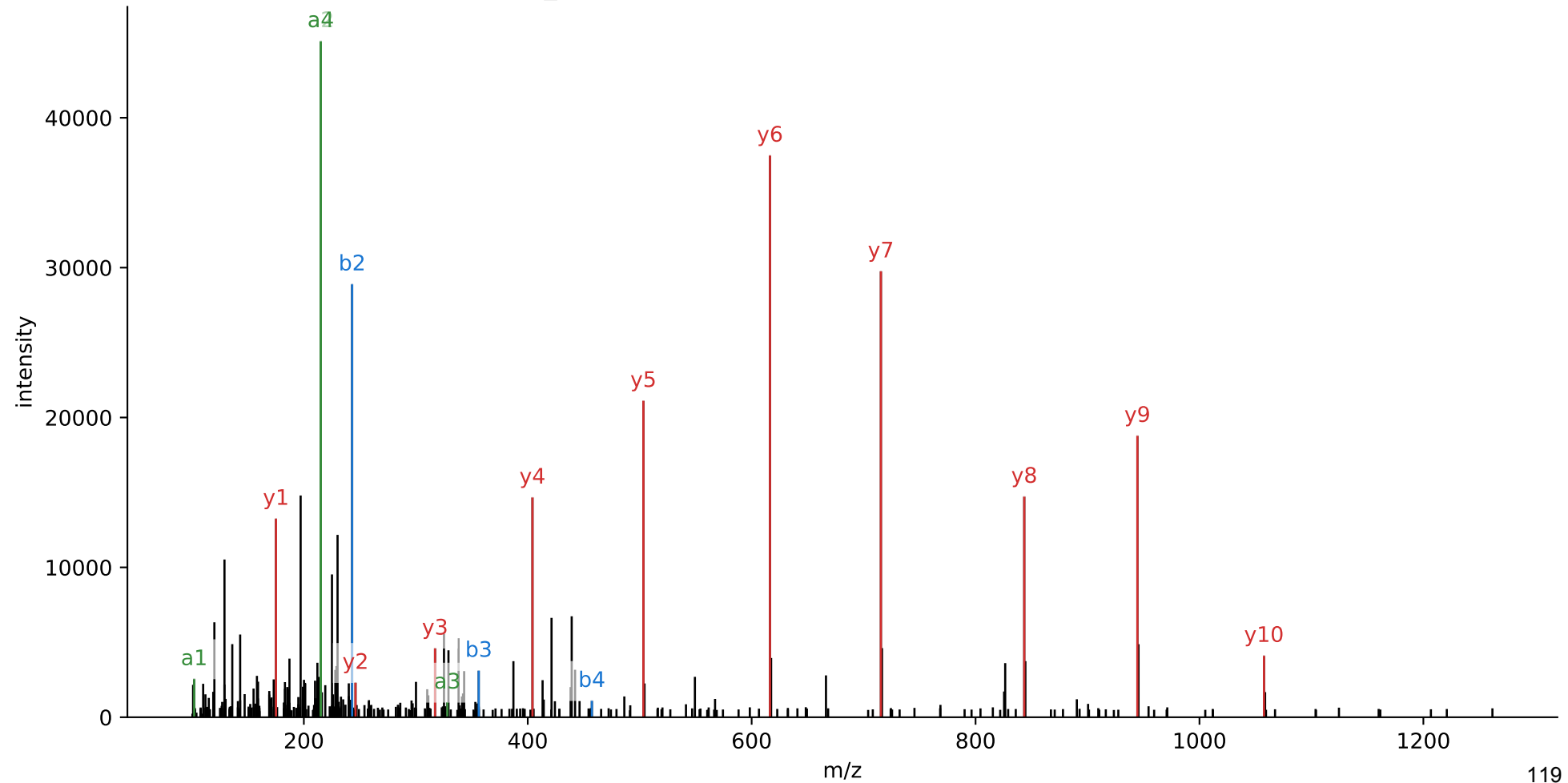

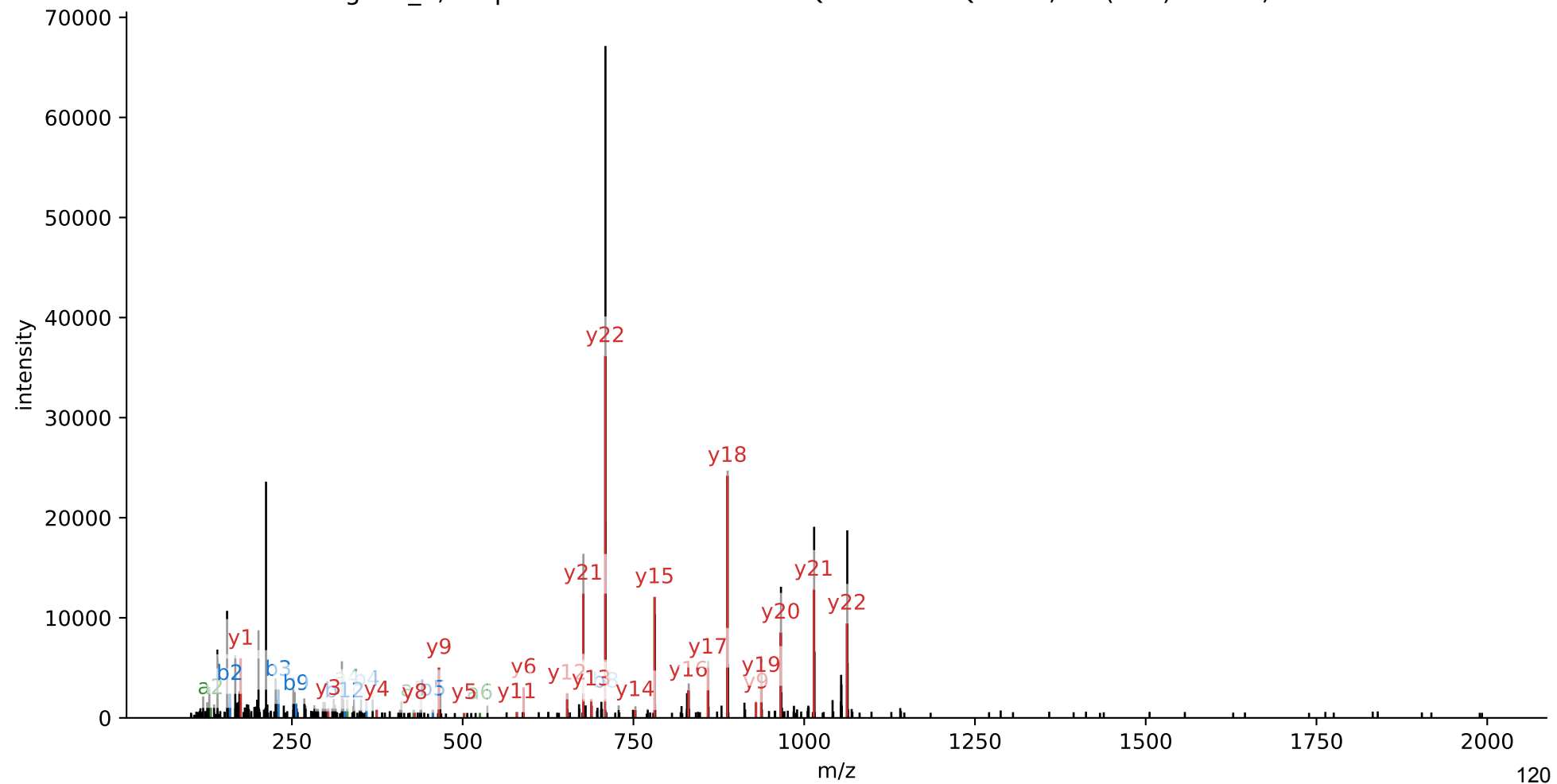

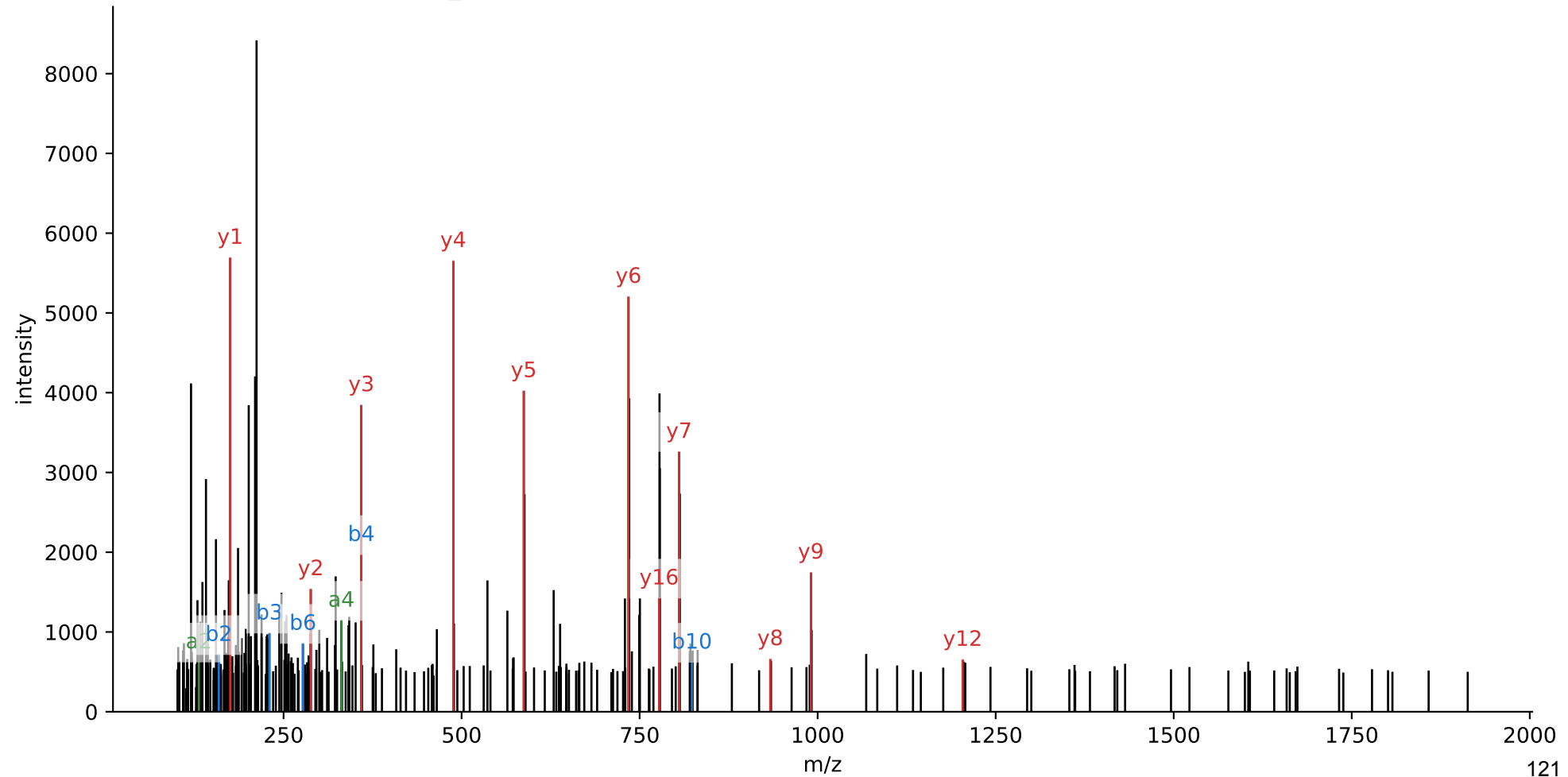

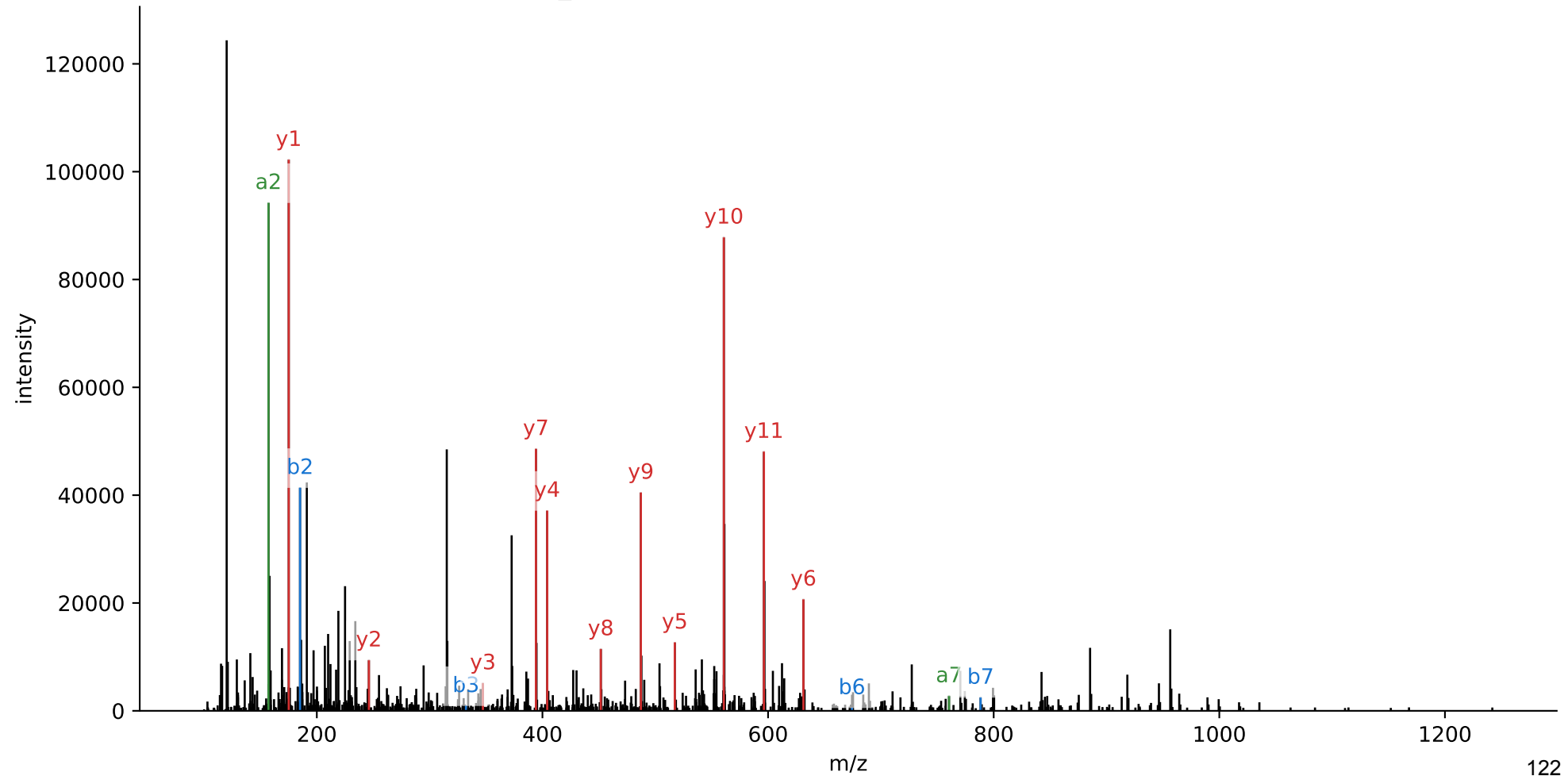

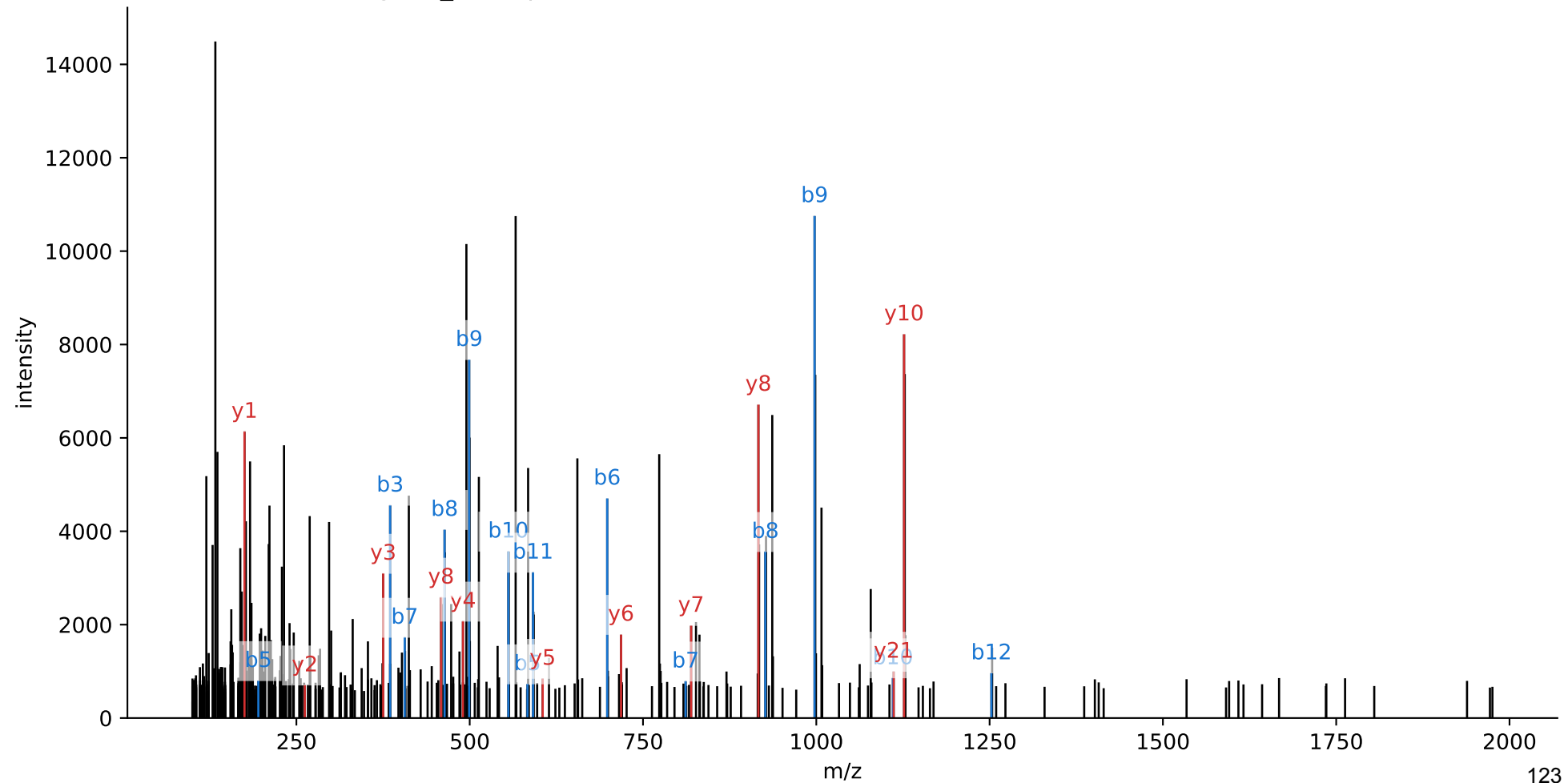

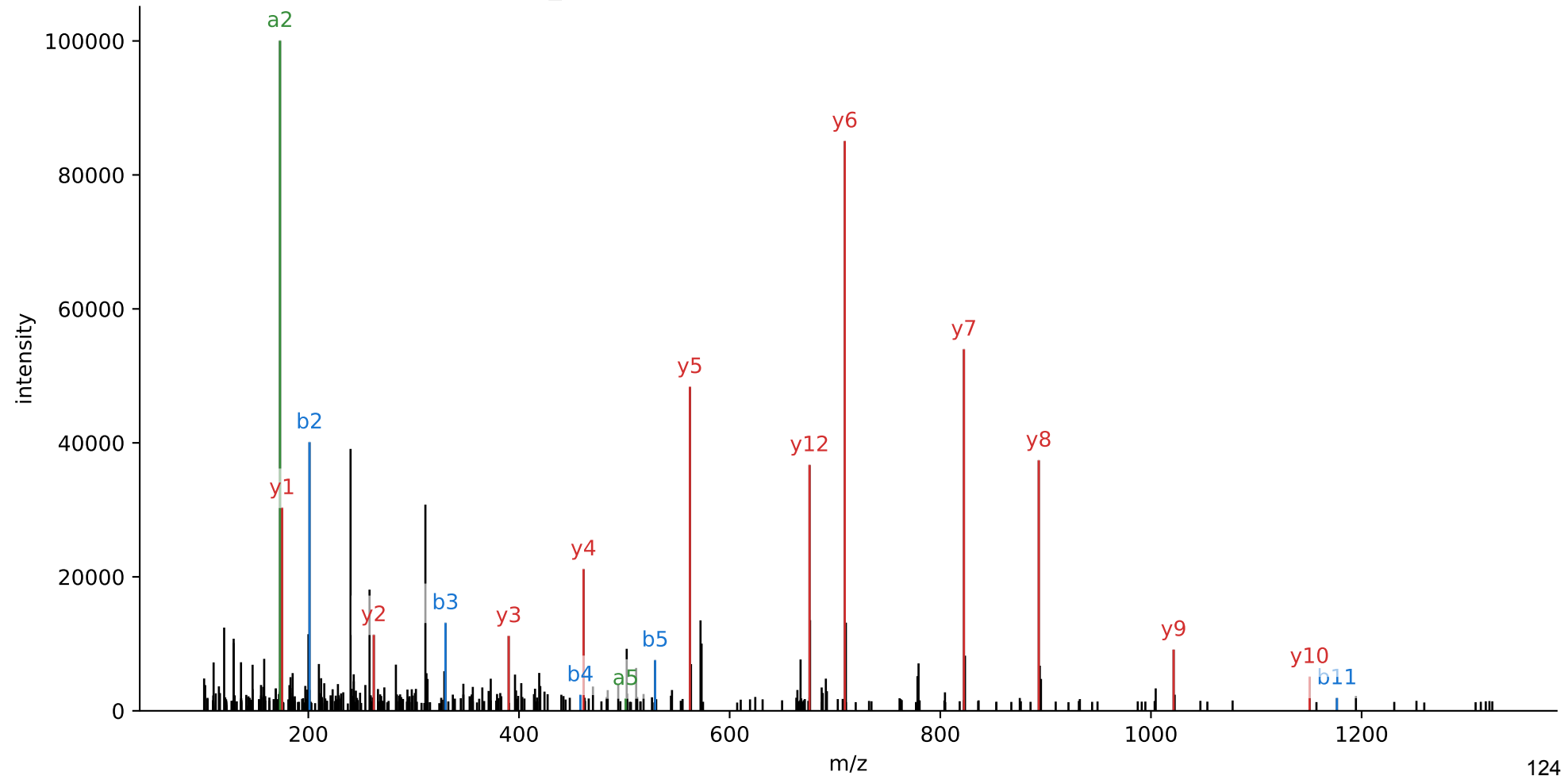

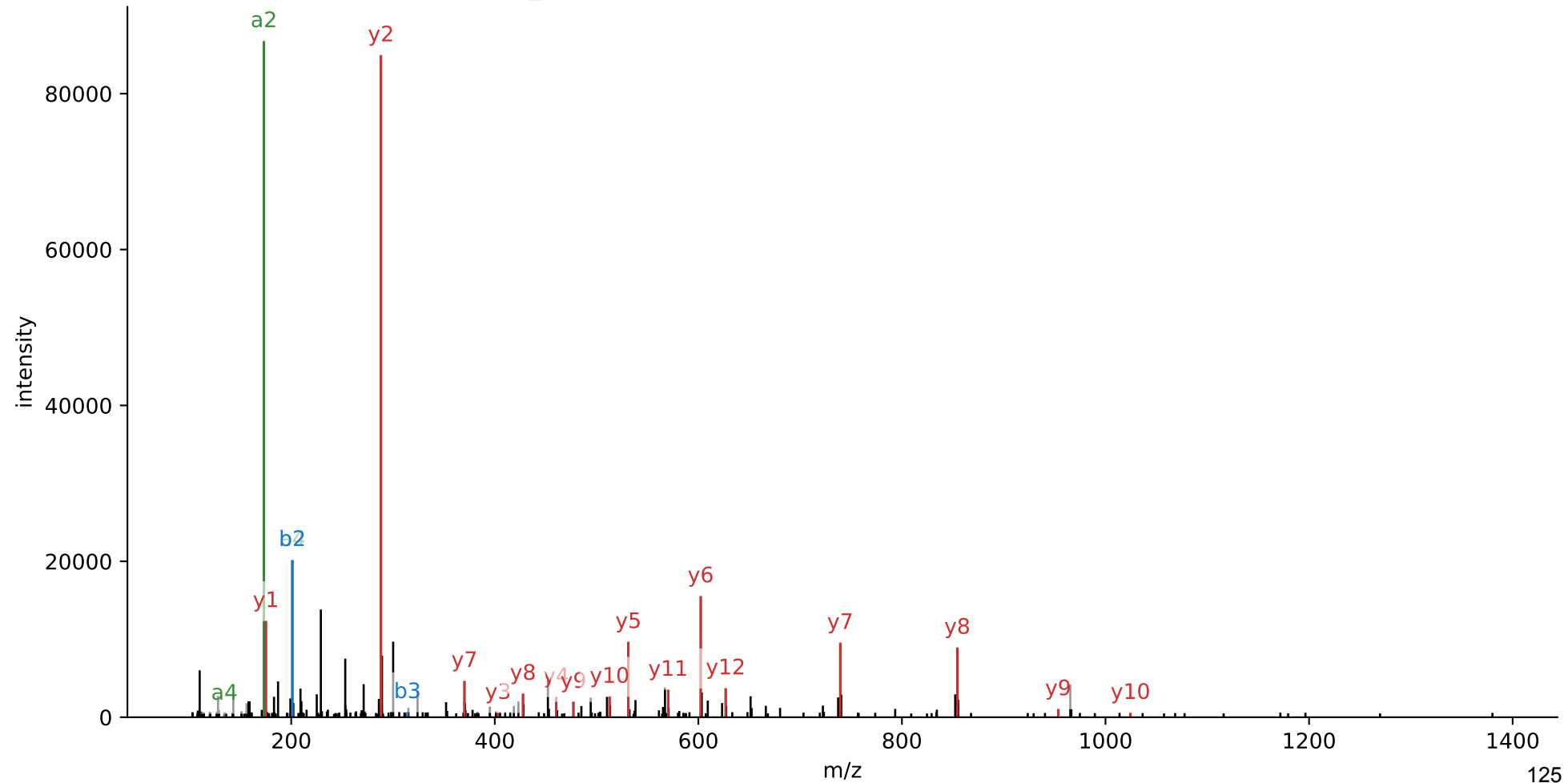

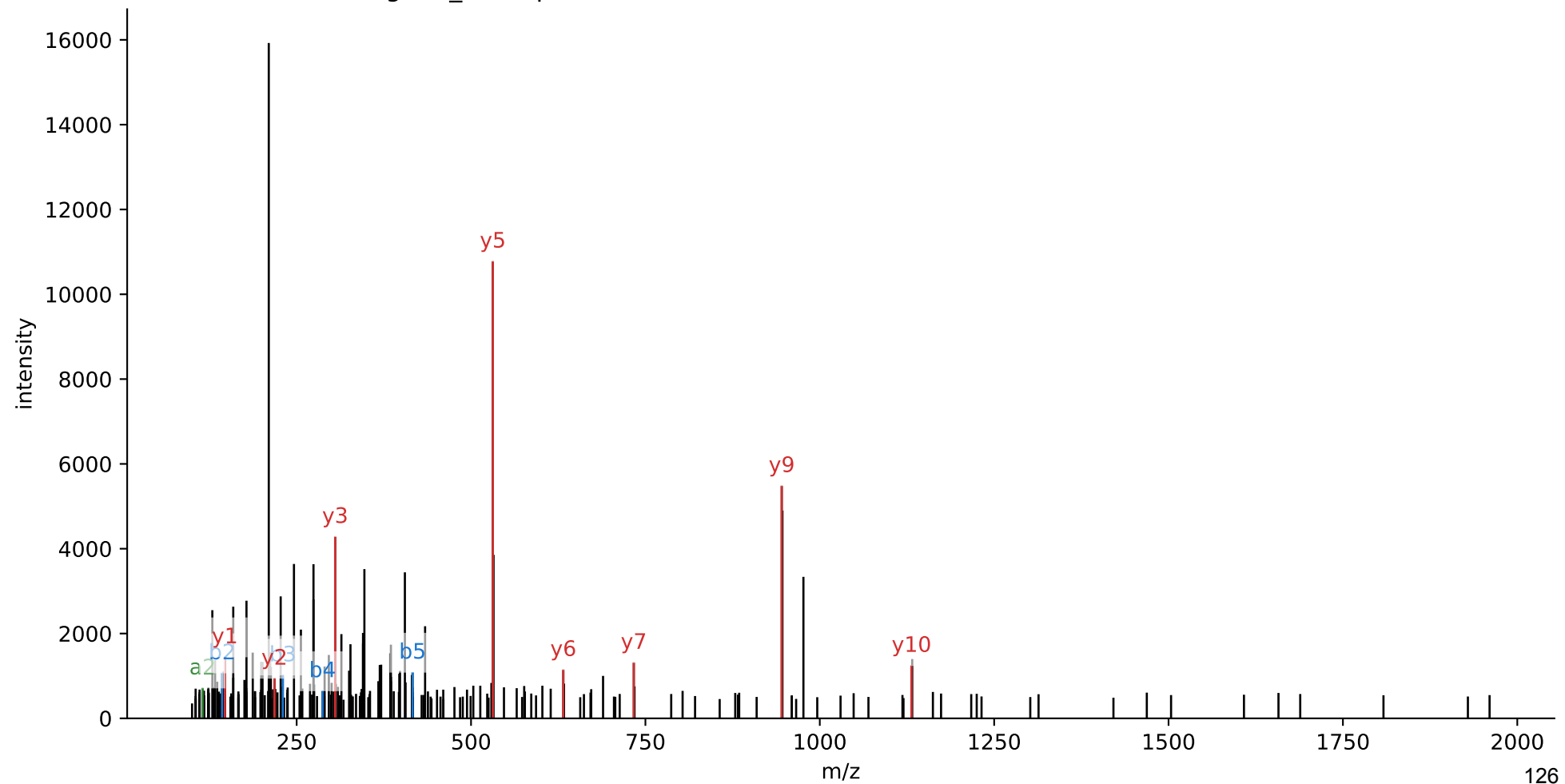

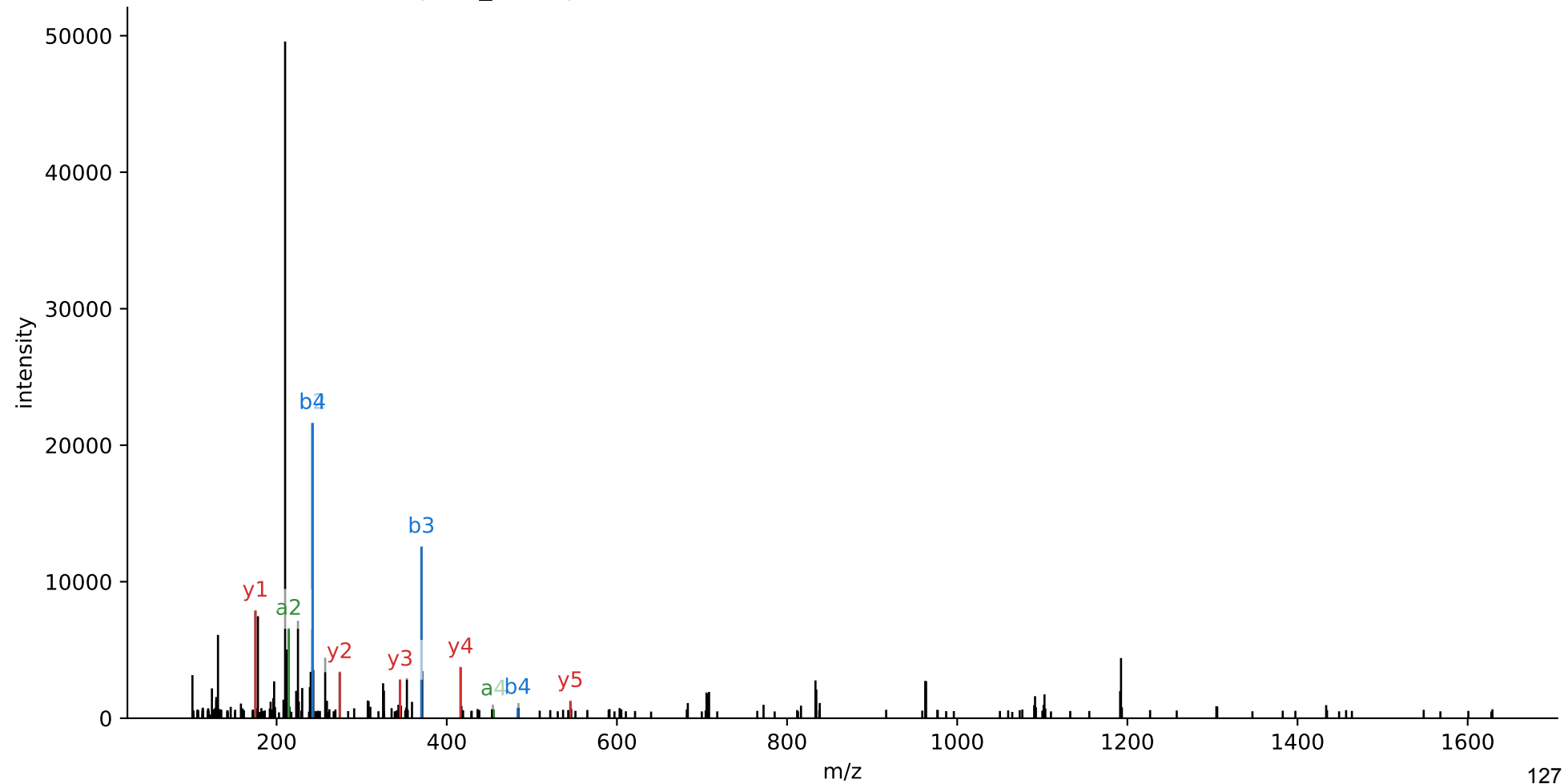

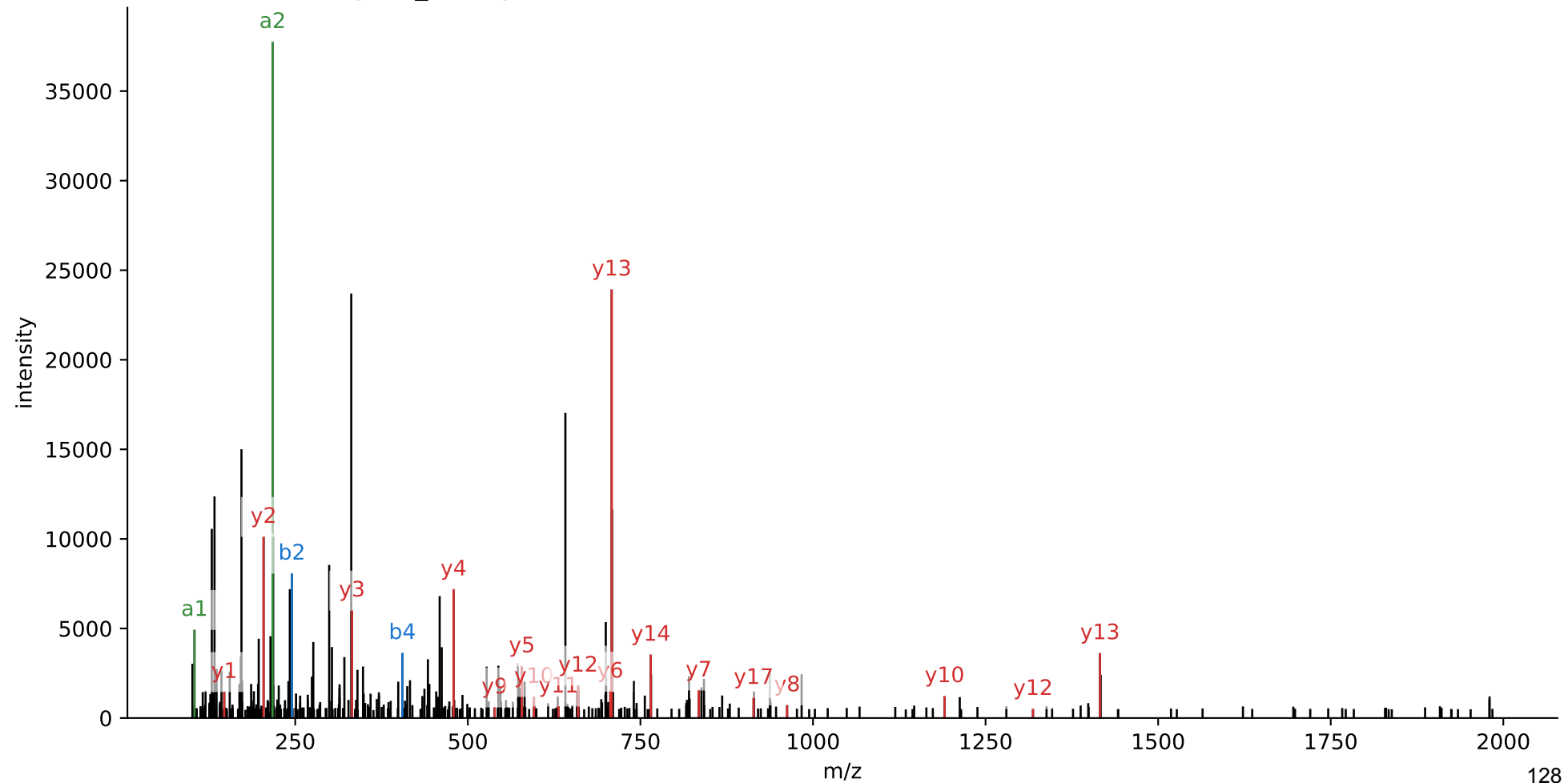

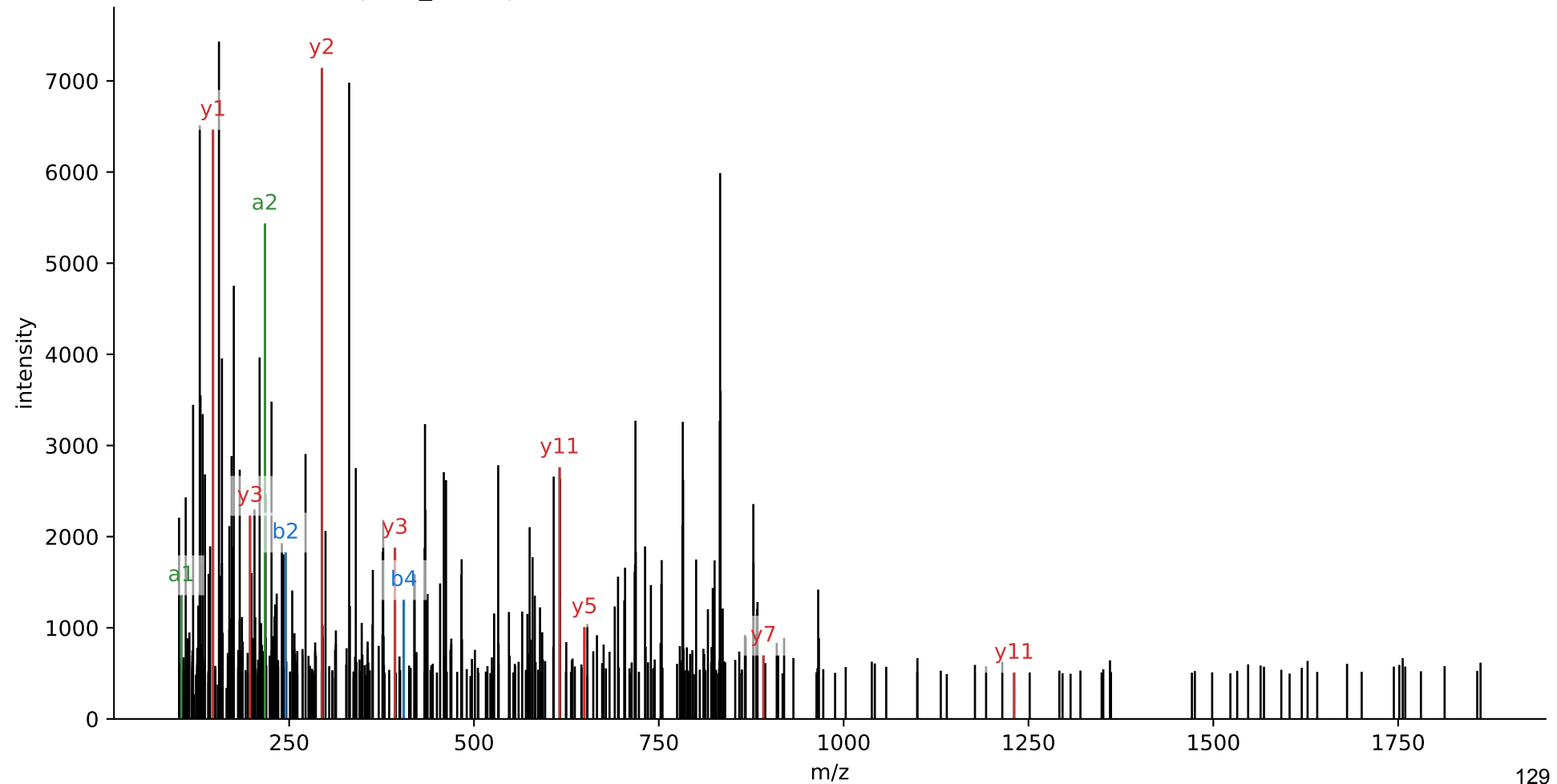

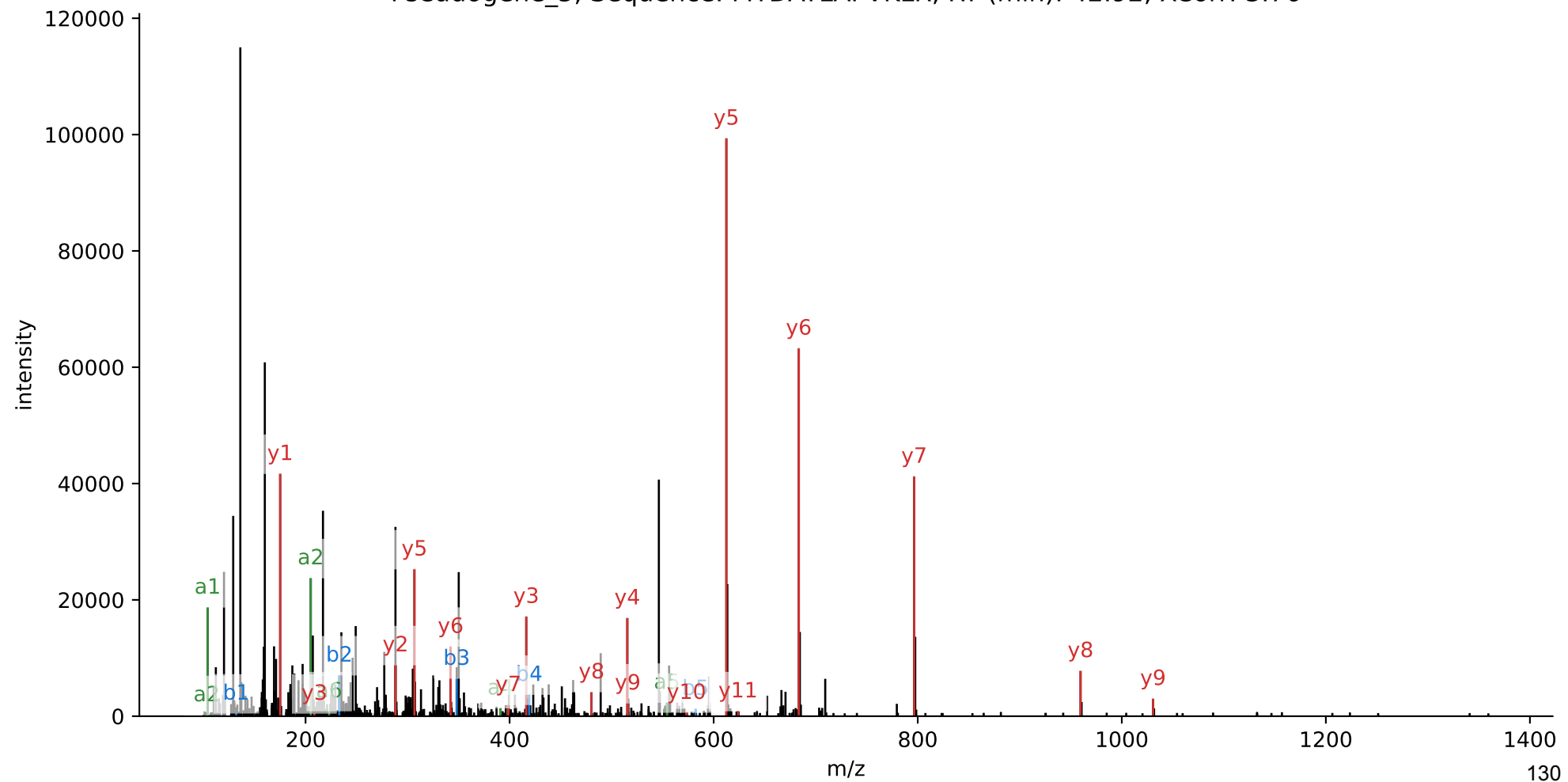

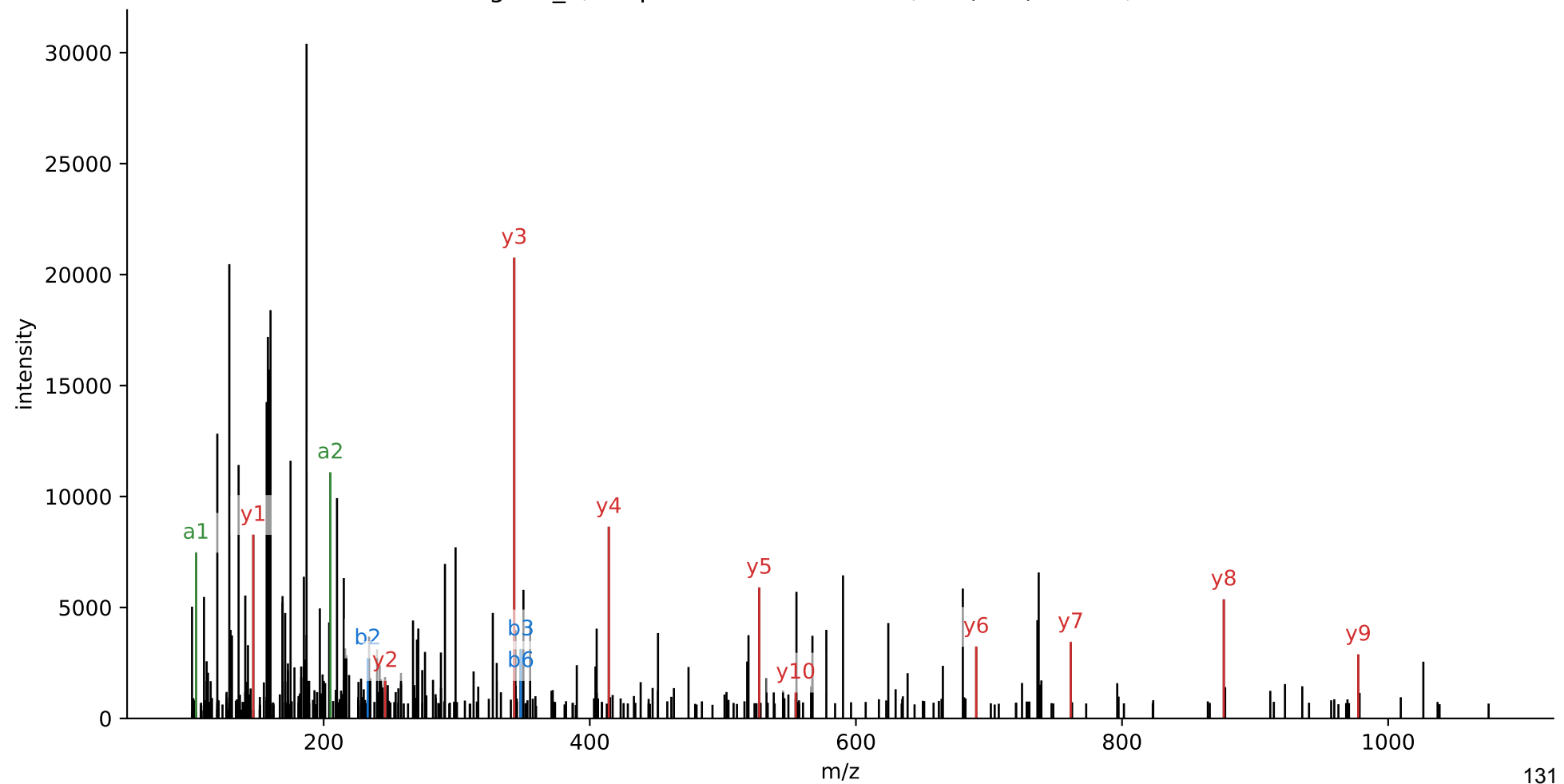

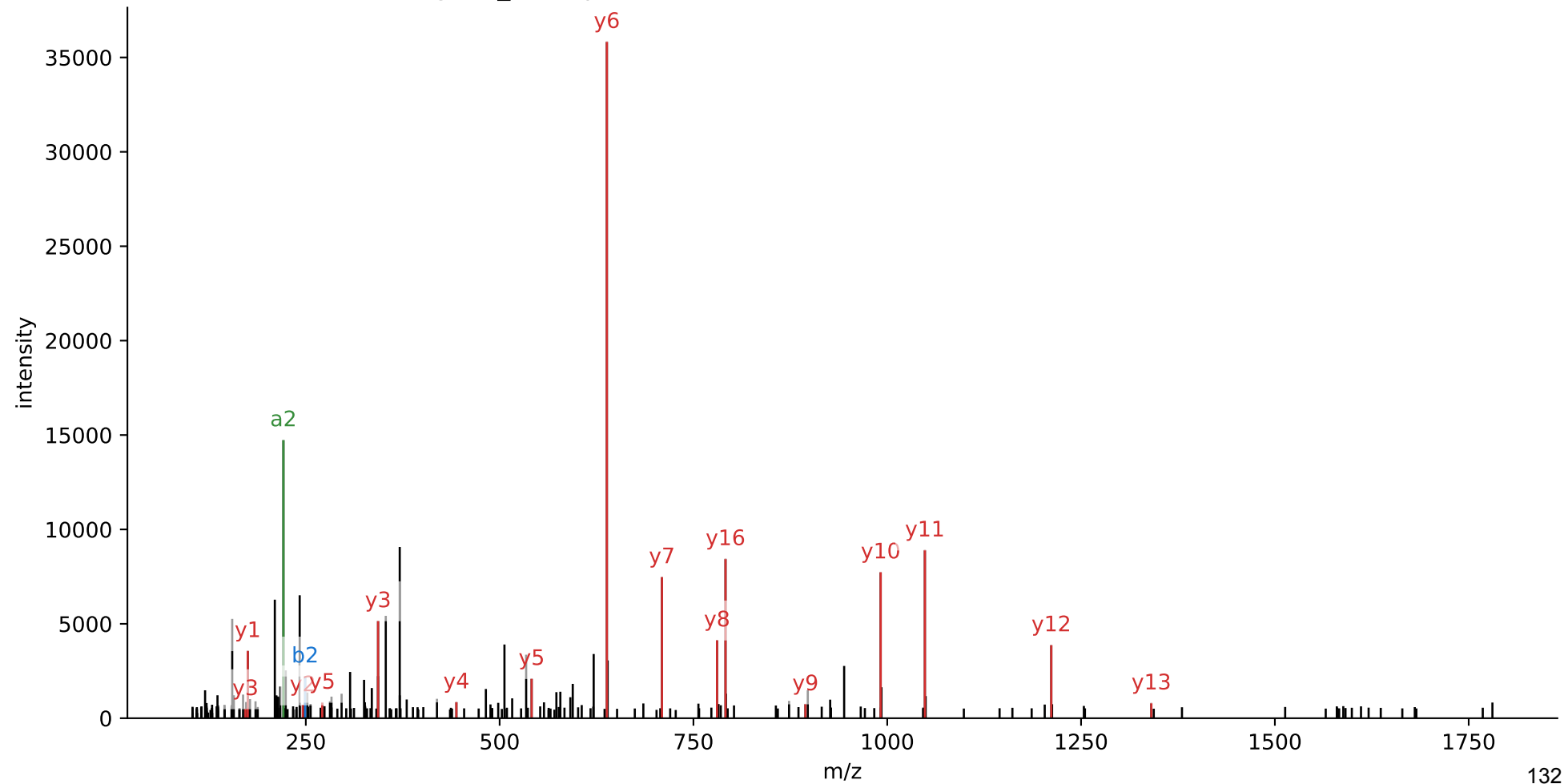

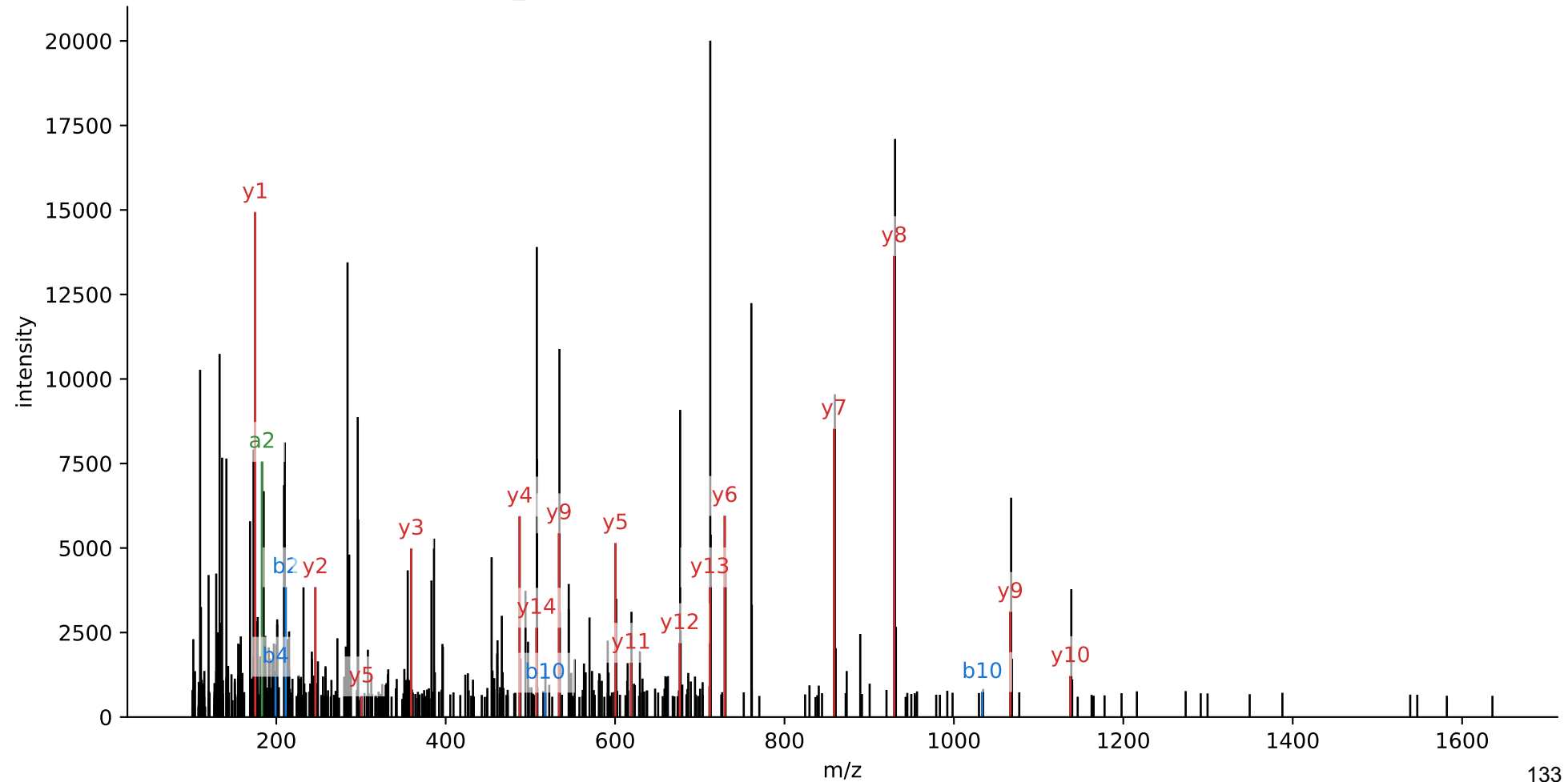

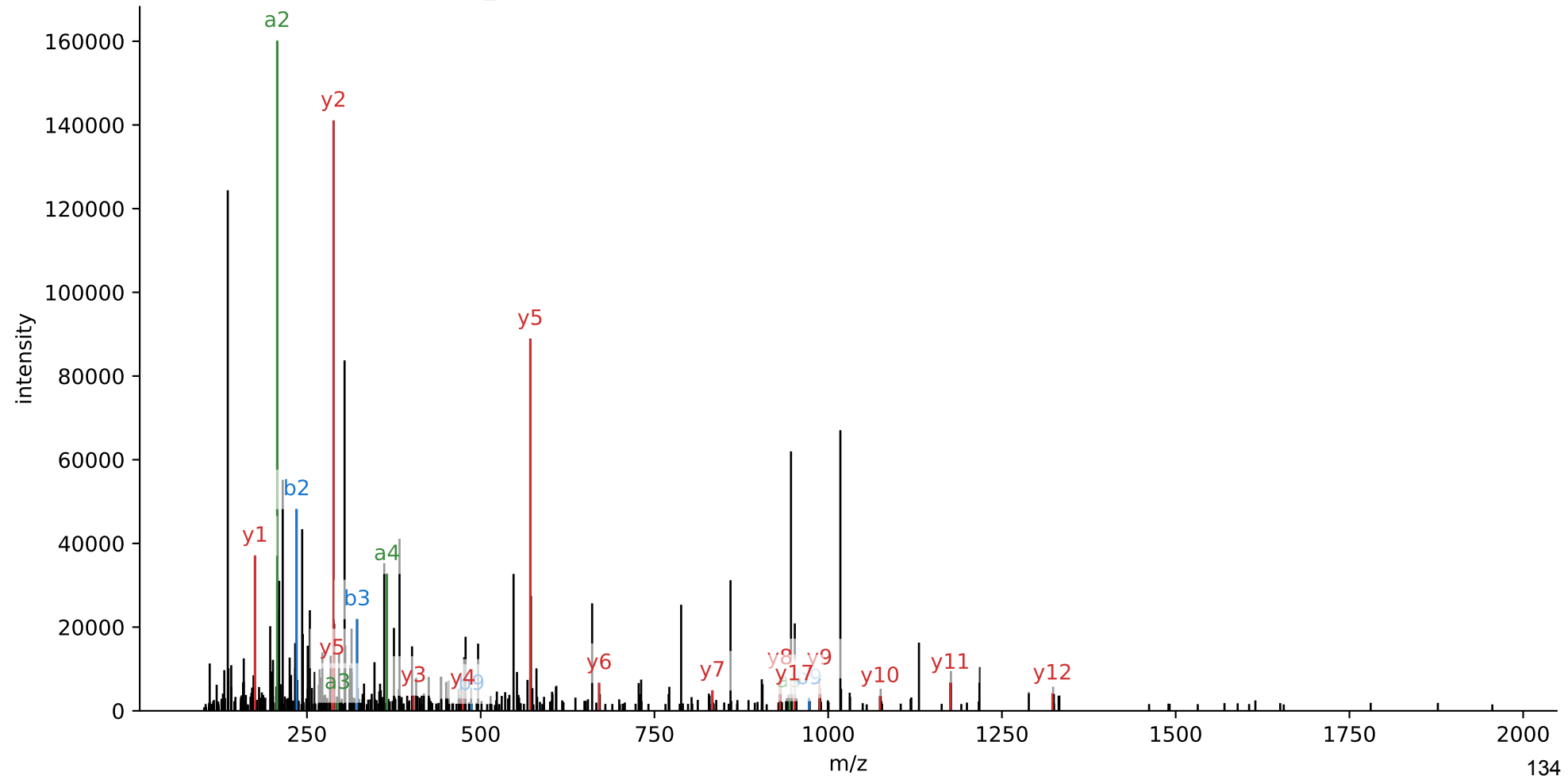

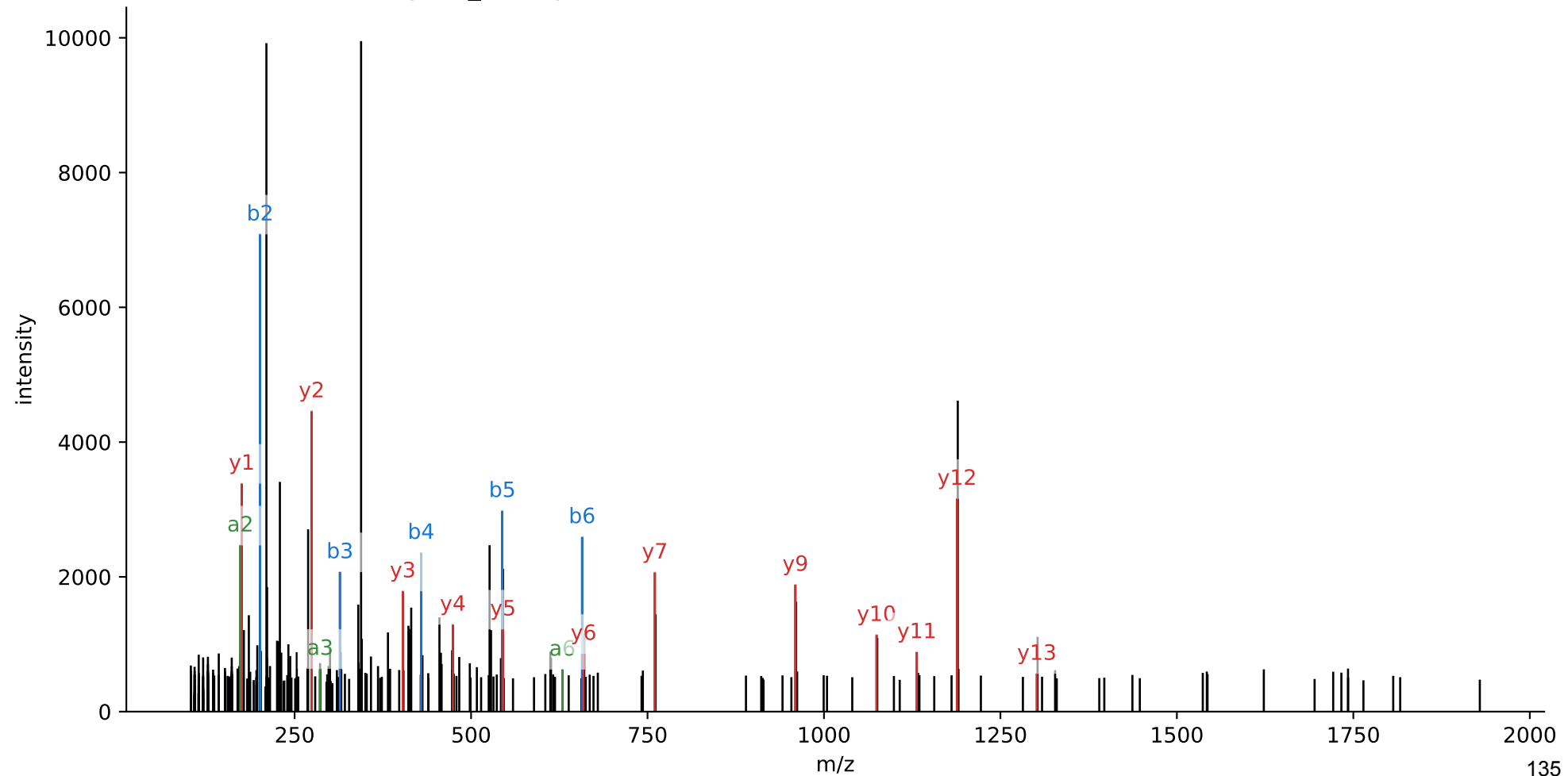

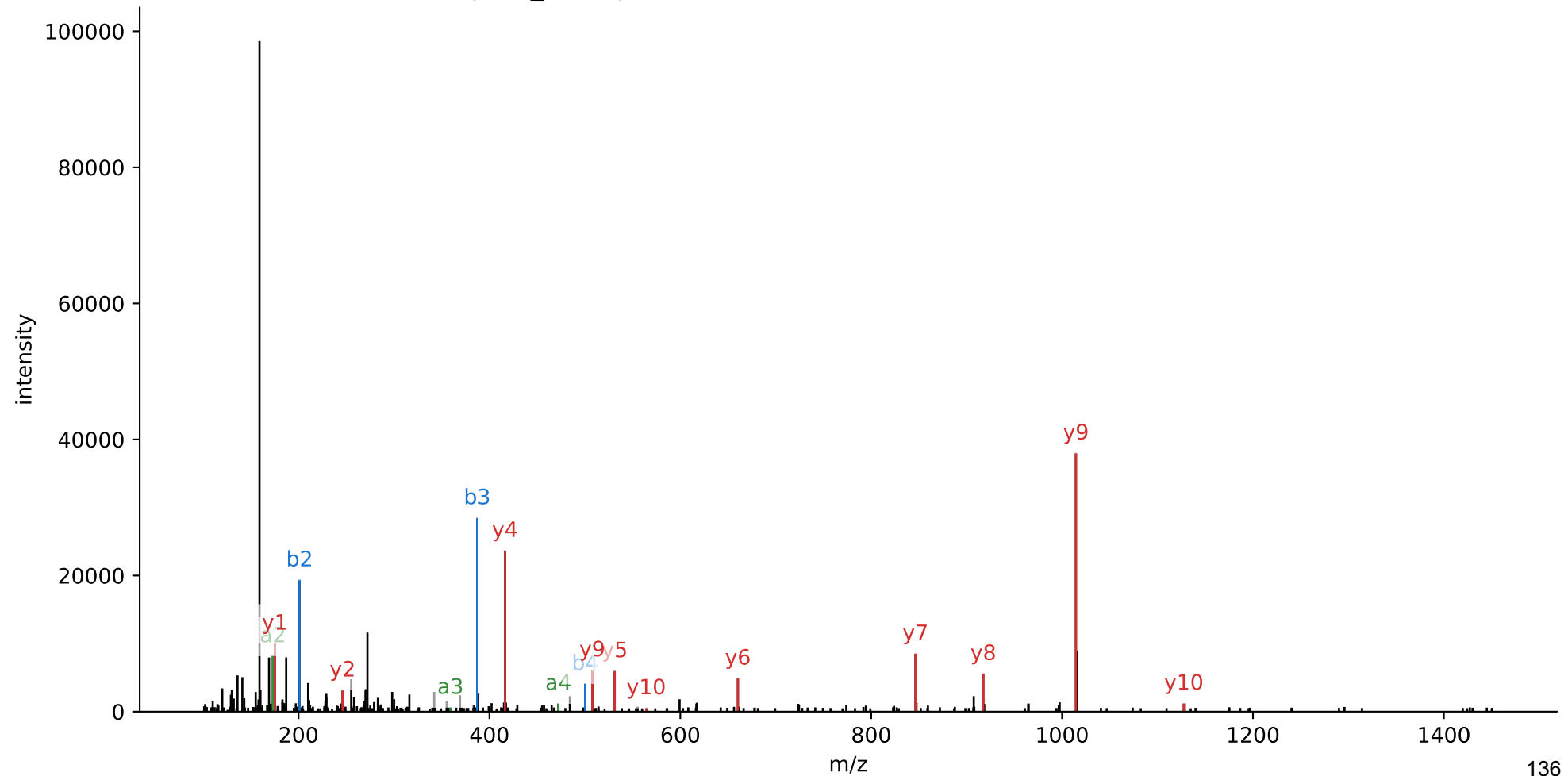

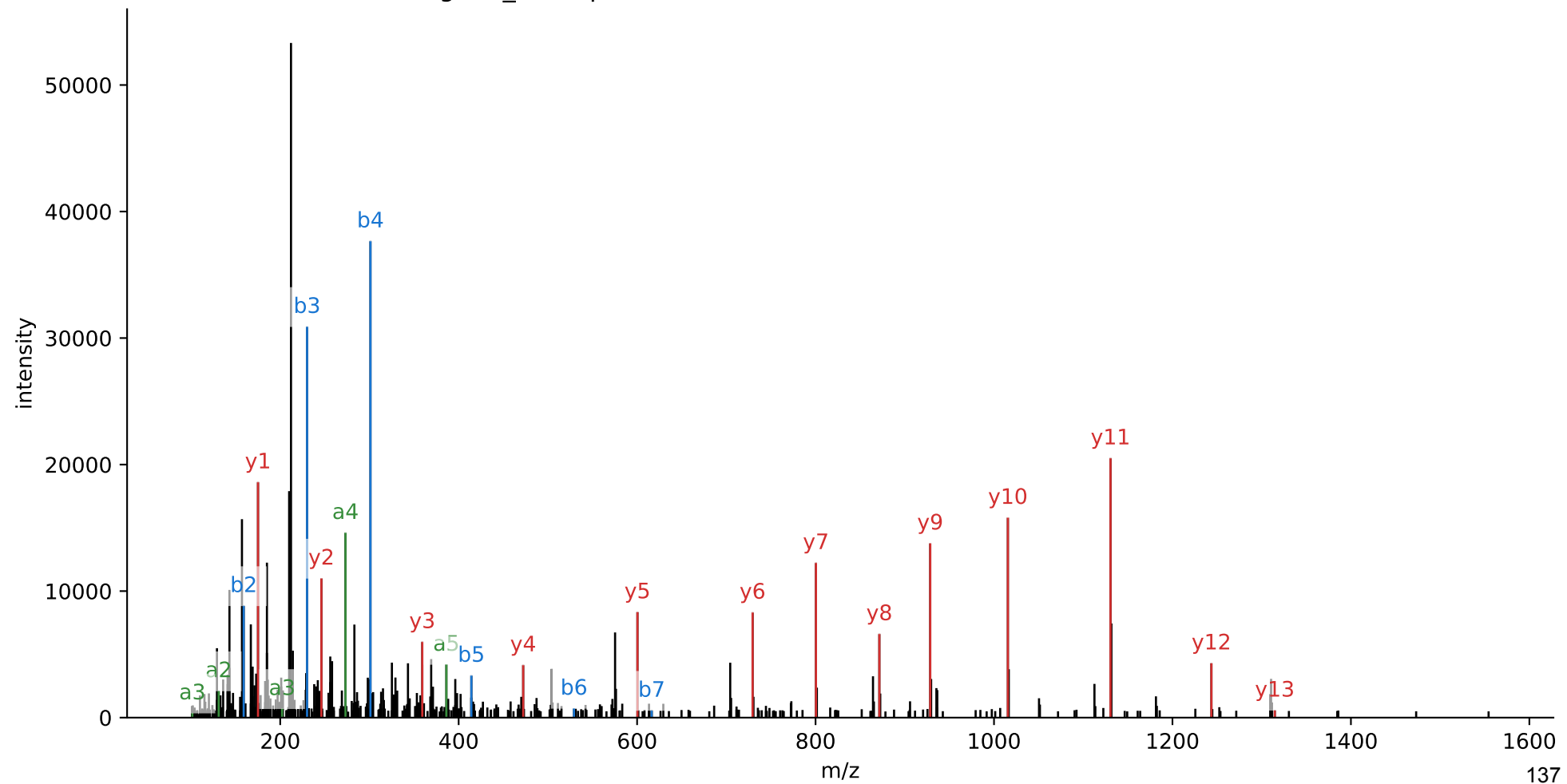

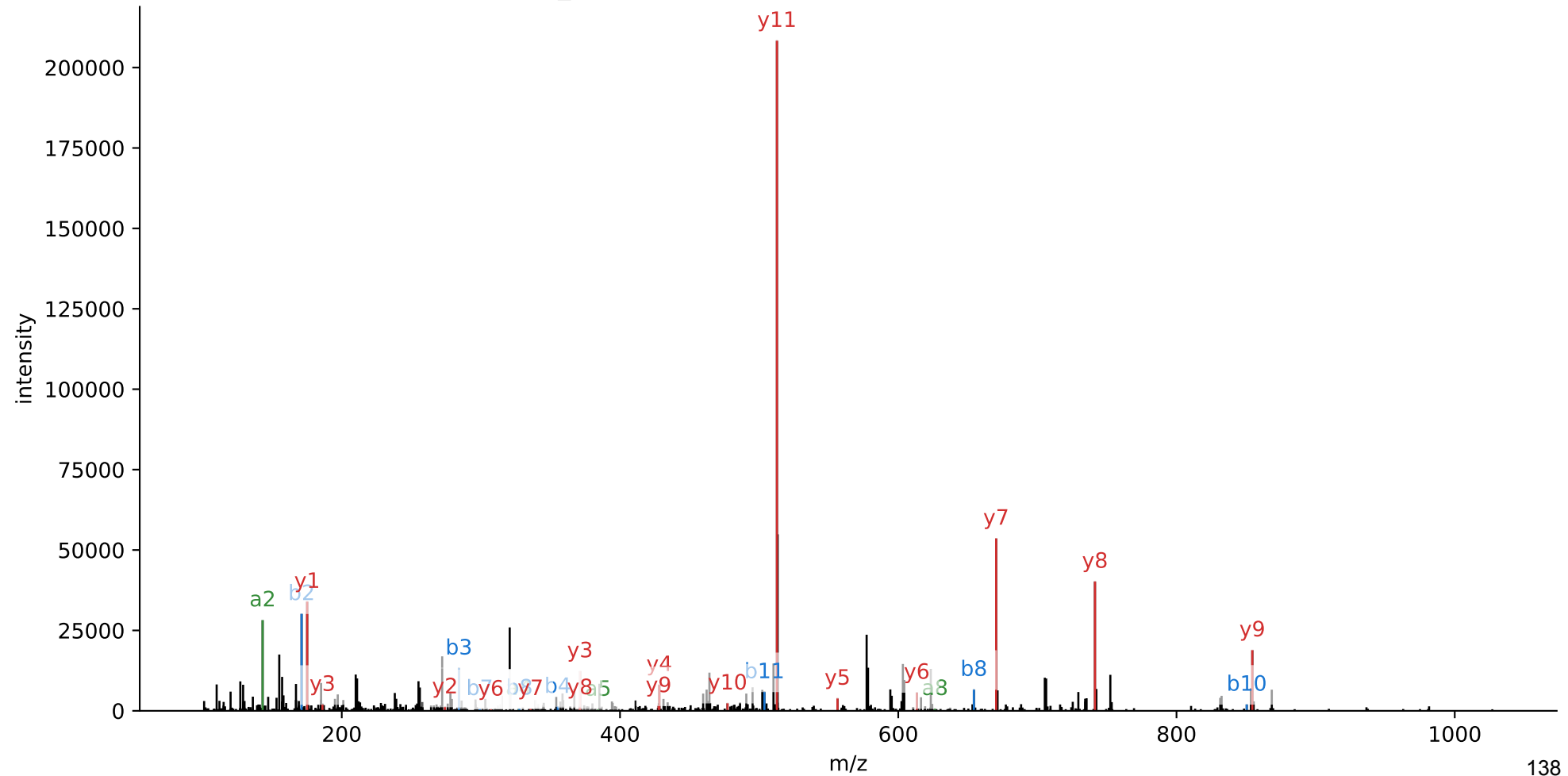

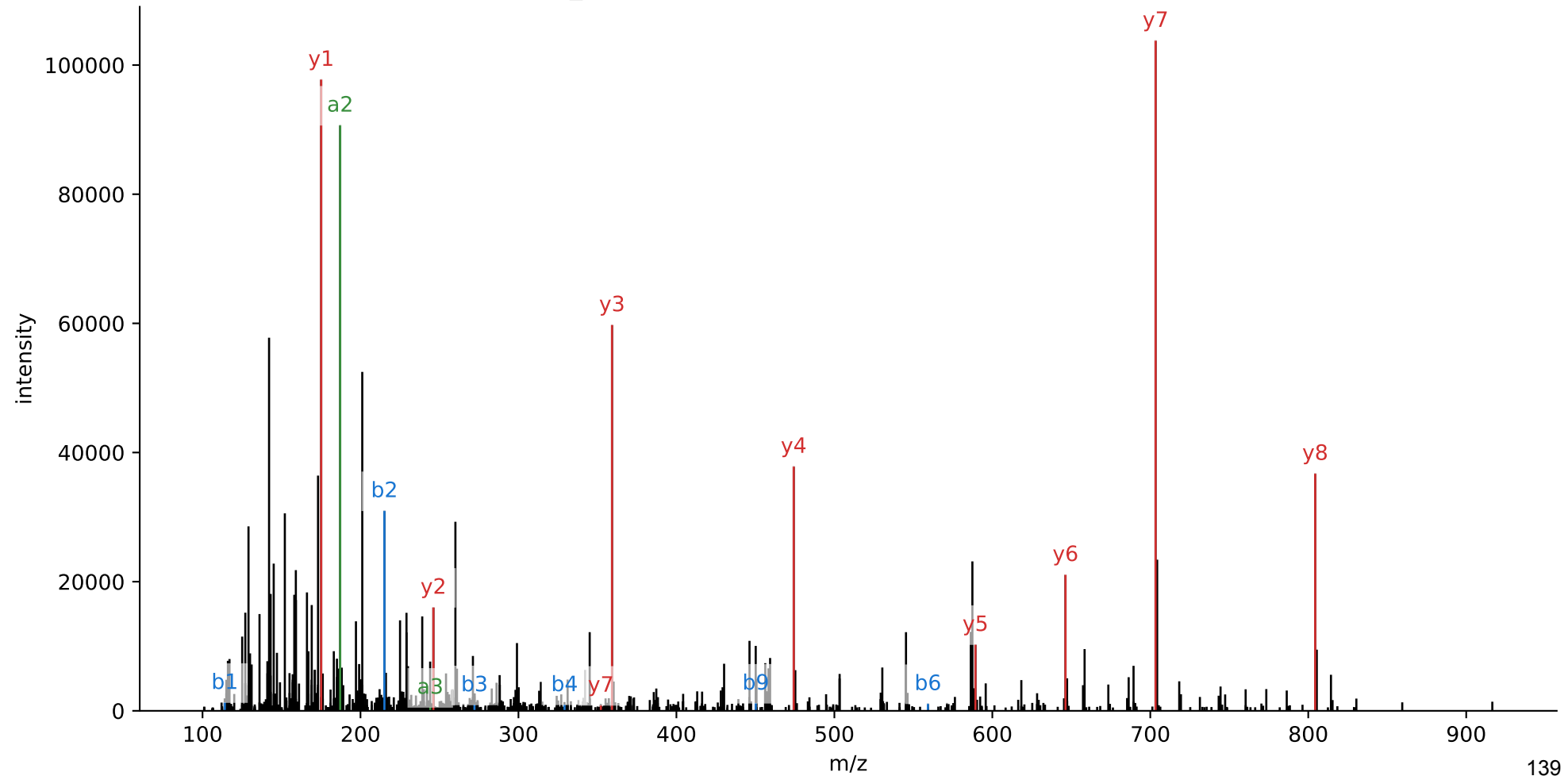

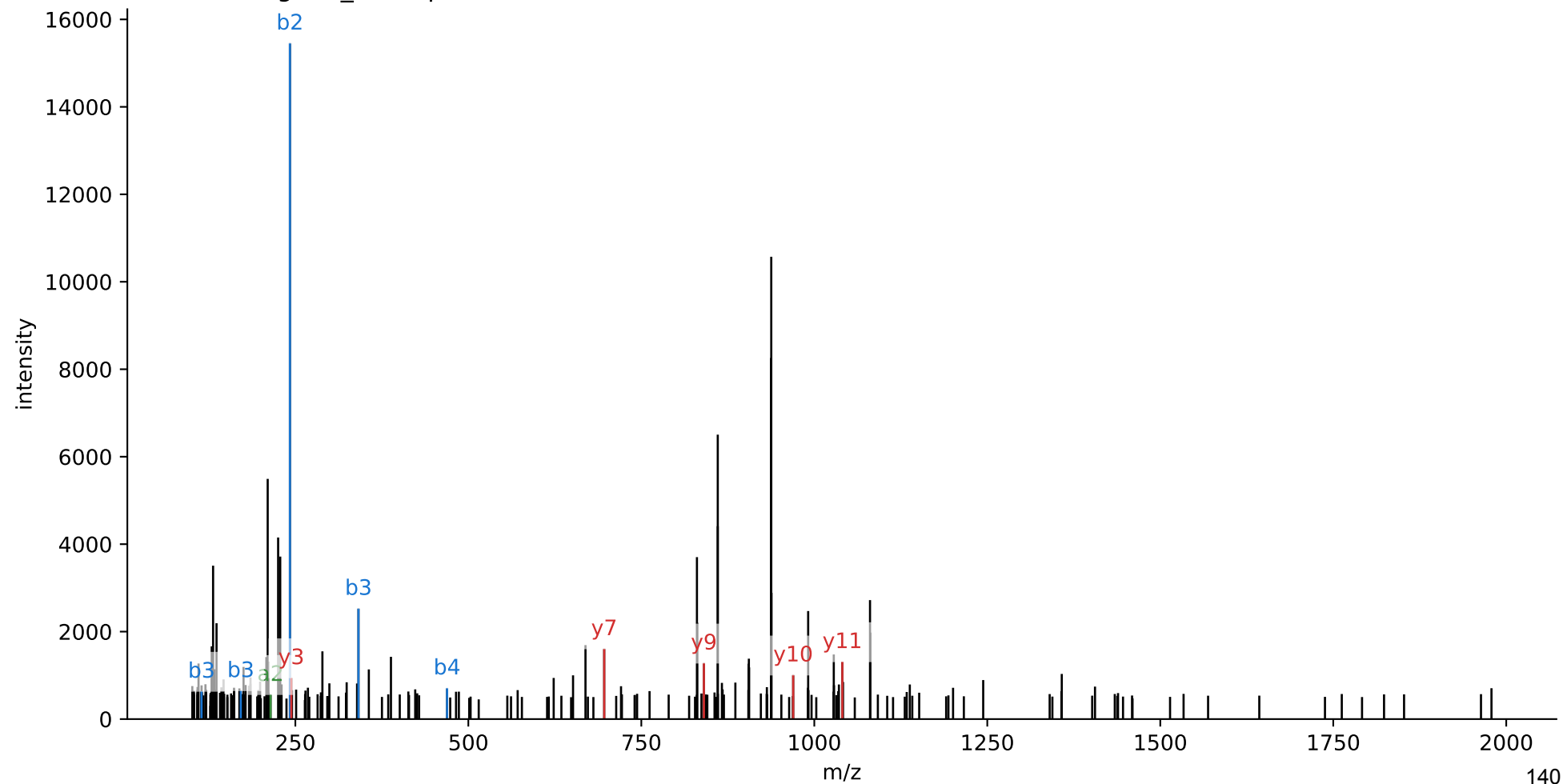

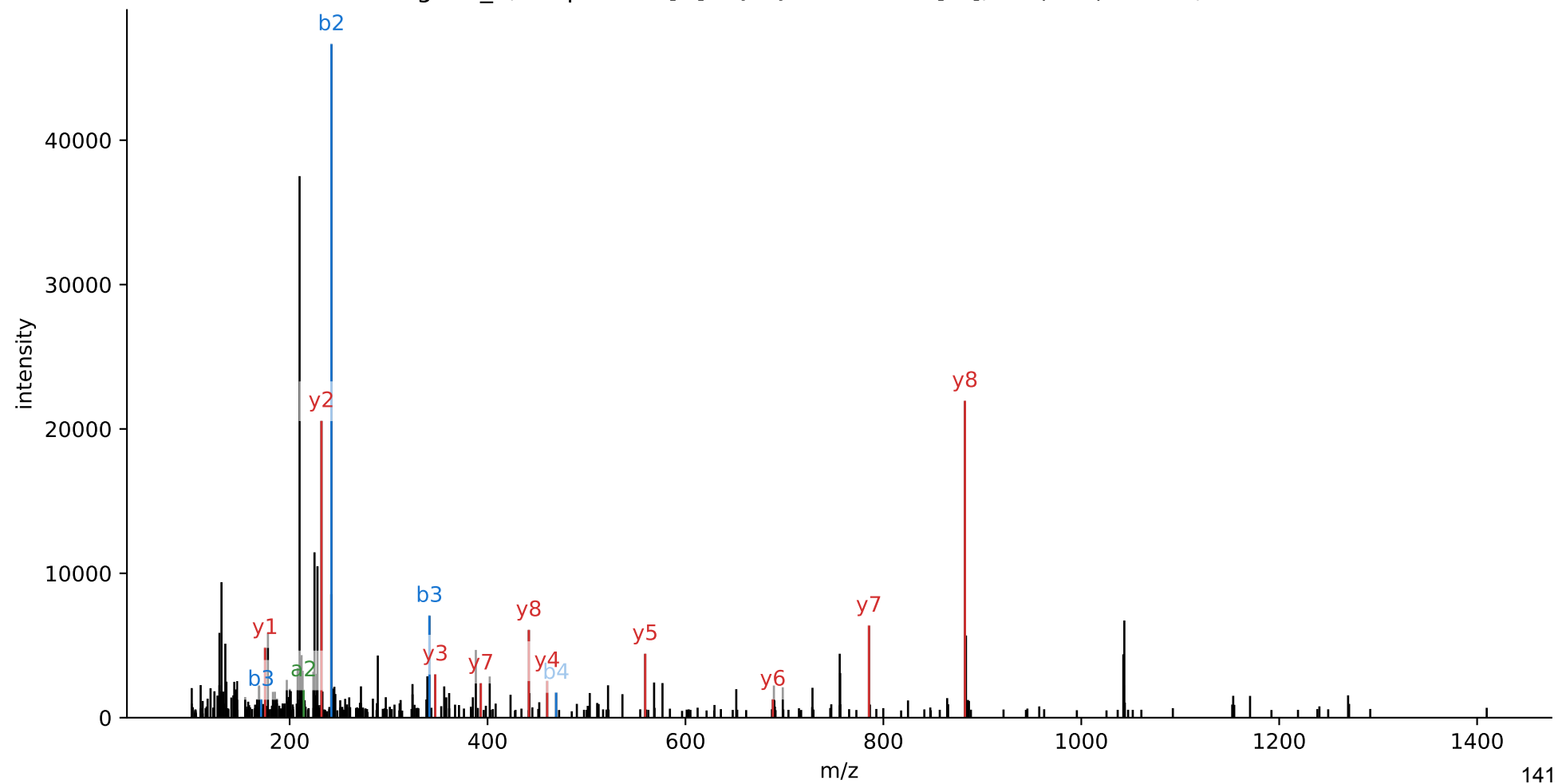

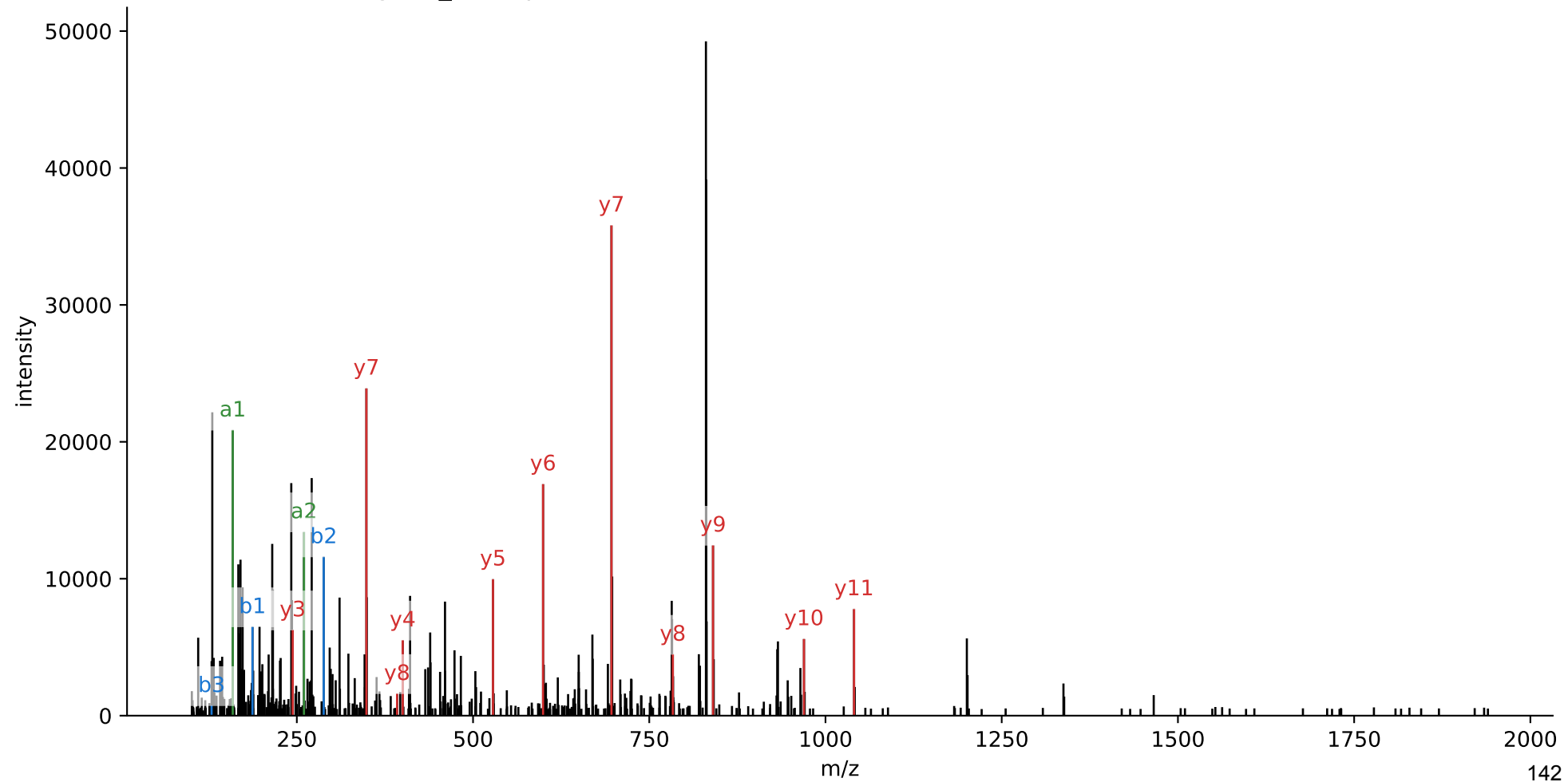

Supplement: Supplemental Data 7 [file mmc13.pdf]
