# Supplementary material for: Development of a Spectral Library for the Discovery of Altered Genomic Events in Mycobacterium avium Associated With Virulence Using Mass Spectrometry–Based Proteogenomic Analysis
Source: Mol Cell Proteomics. 2023 Mar 21;22(5):100533. doi: 10.1016/j.mcpro.2023.100533 (PMC10149365; doi:10.1016/j.mcpro.2023.100533)
Supplement: Supplemental Data 2 [file mmc8.pdf]

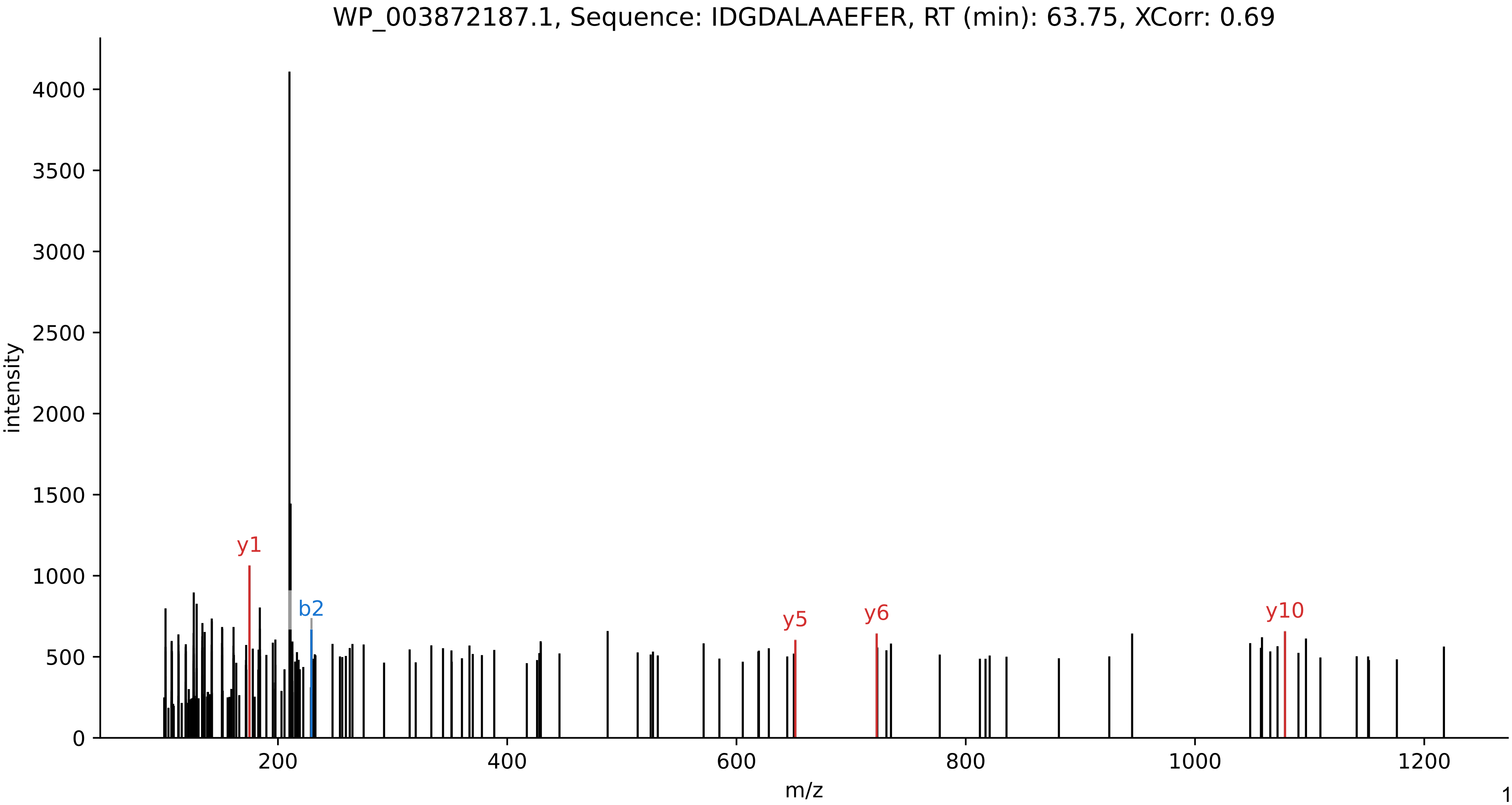
WP_003872187.1, Sequence: IDGDALAAEFER, RT (min): 63.75, XCorr: 0.69
intensity
m/z
y1
b2
y5
y6
y10
0
500
1000
1500
2000
2500
3000
3500
4000
200
400
600
800
1000
1200

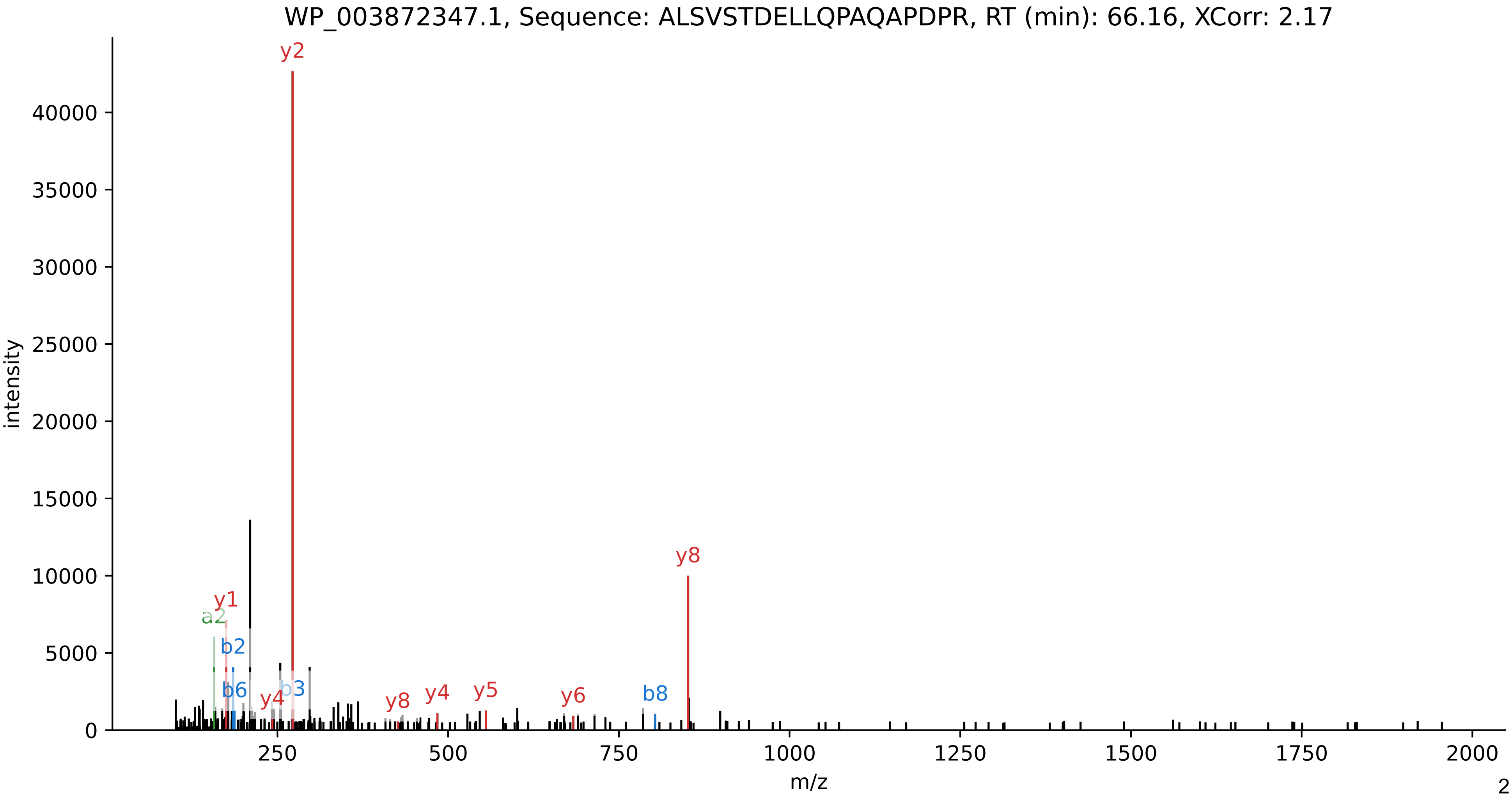

WP_003872347.1, Sequence: ALSVSTDELLQPAQAPDPR, RT (min): 66.16, XCorr: 2.17
y2
y8
y1
a2
b2
b6
b3
y4
y8
y4
y5
y6
b8
intensity
m/z

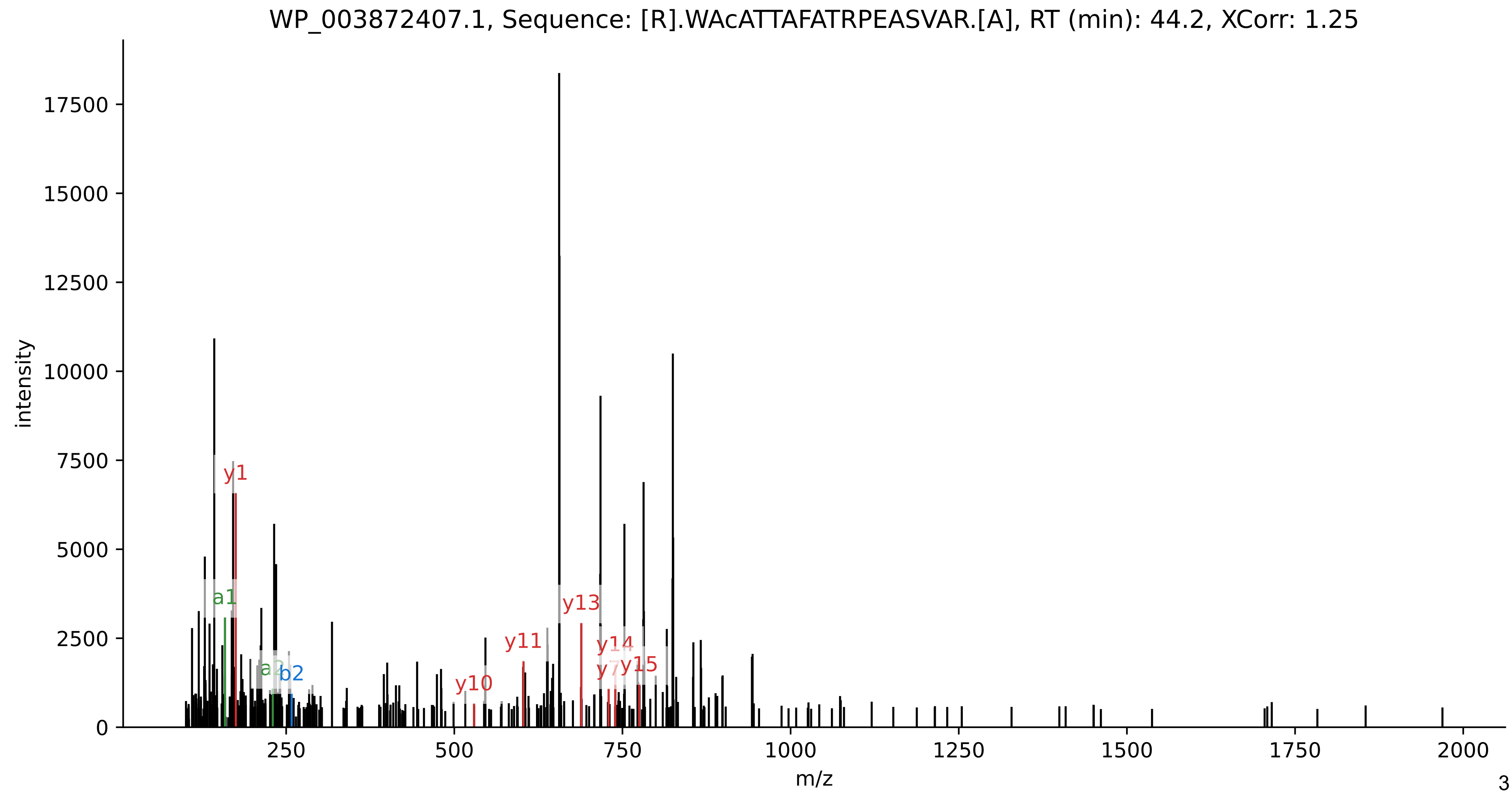
WP_003872407.1, Sequence: [R].WAcATTAFATRPEASVAR.[A], RT (min): 44.2, XCorr: 1.25
intensity
17500
15000
12500
10000
7500
5000
2500
0
250
500
750
1000
1250
1500
1750
2000
m/z
y1
a1
a2
b2
y10
y11
y13
y14
y7
y15

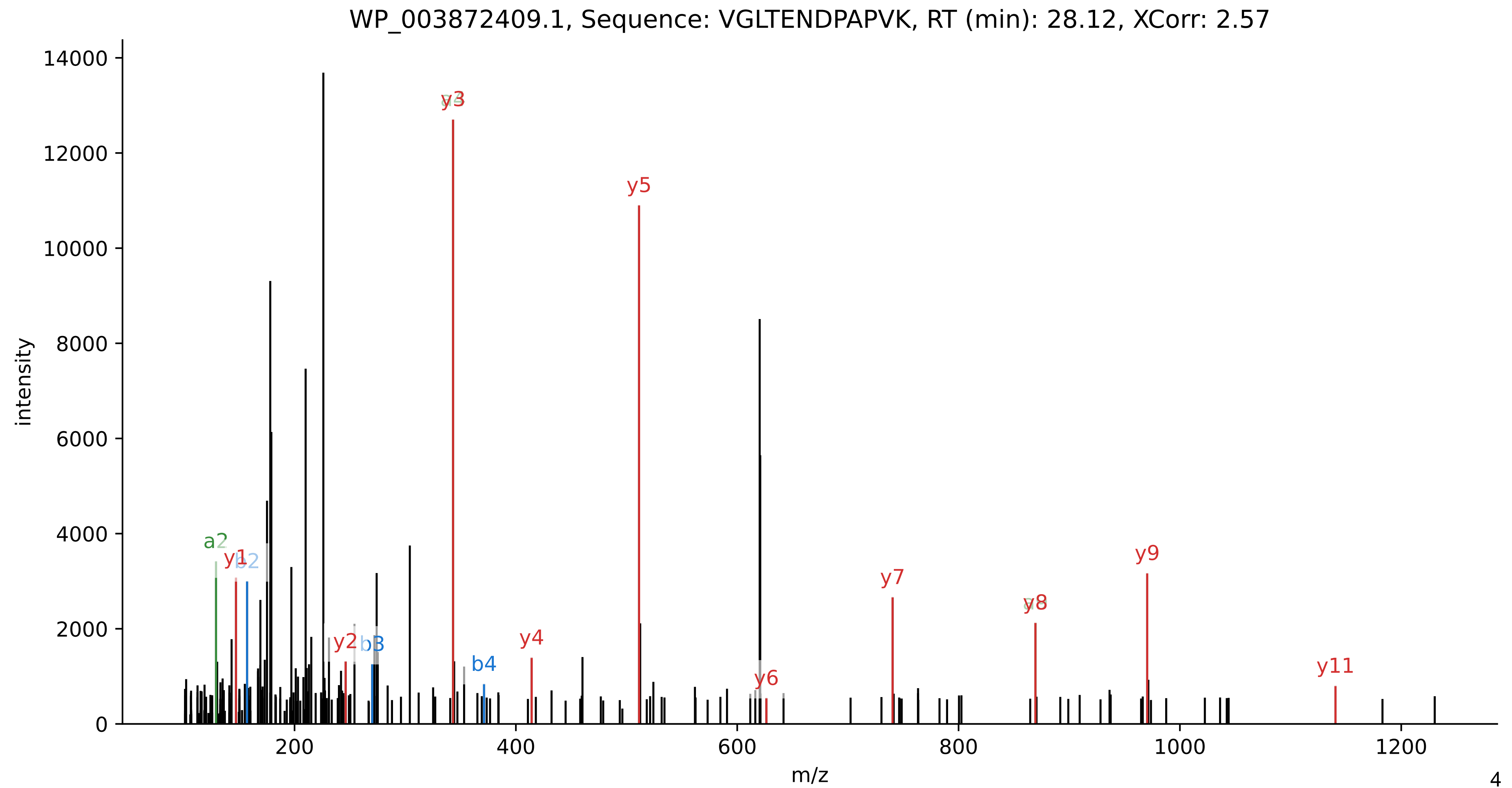
WP_003872409.1, Sequence: VGLTENDPAPVK, RT (min): 28.12, XCorr: 2.57
intensity
m/z
a2
y1
b2
y2
b3
y3
b4
y4
y5
y6
y7
y8
y9
y11

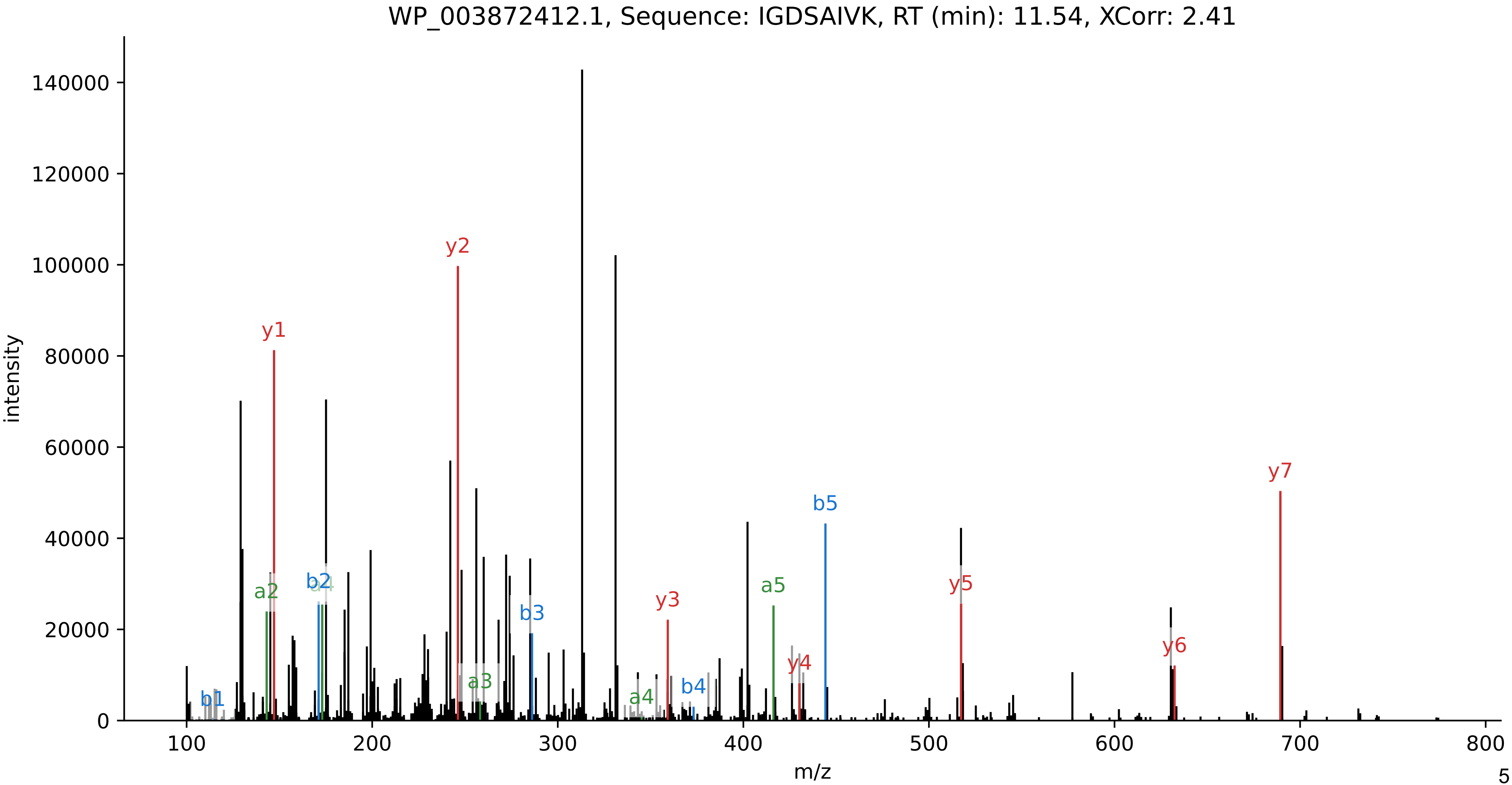

WP_003872412.1, Sequence: IGDSAIVK, RT (min): 11.54, XCorr: 2.41
intensity
m/z
y1
y2
y3
y4
y5
y6
y7
b1
b2
b3
b4
b5
a2
a3
a4
a5

WP_003872429.1, Sequence: [K].LcDPNcATGSQLTEPVVVHAWNPQPPRR.[E], RT (min): 58.18, XCorr: 1.77

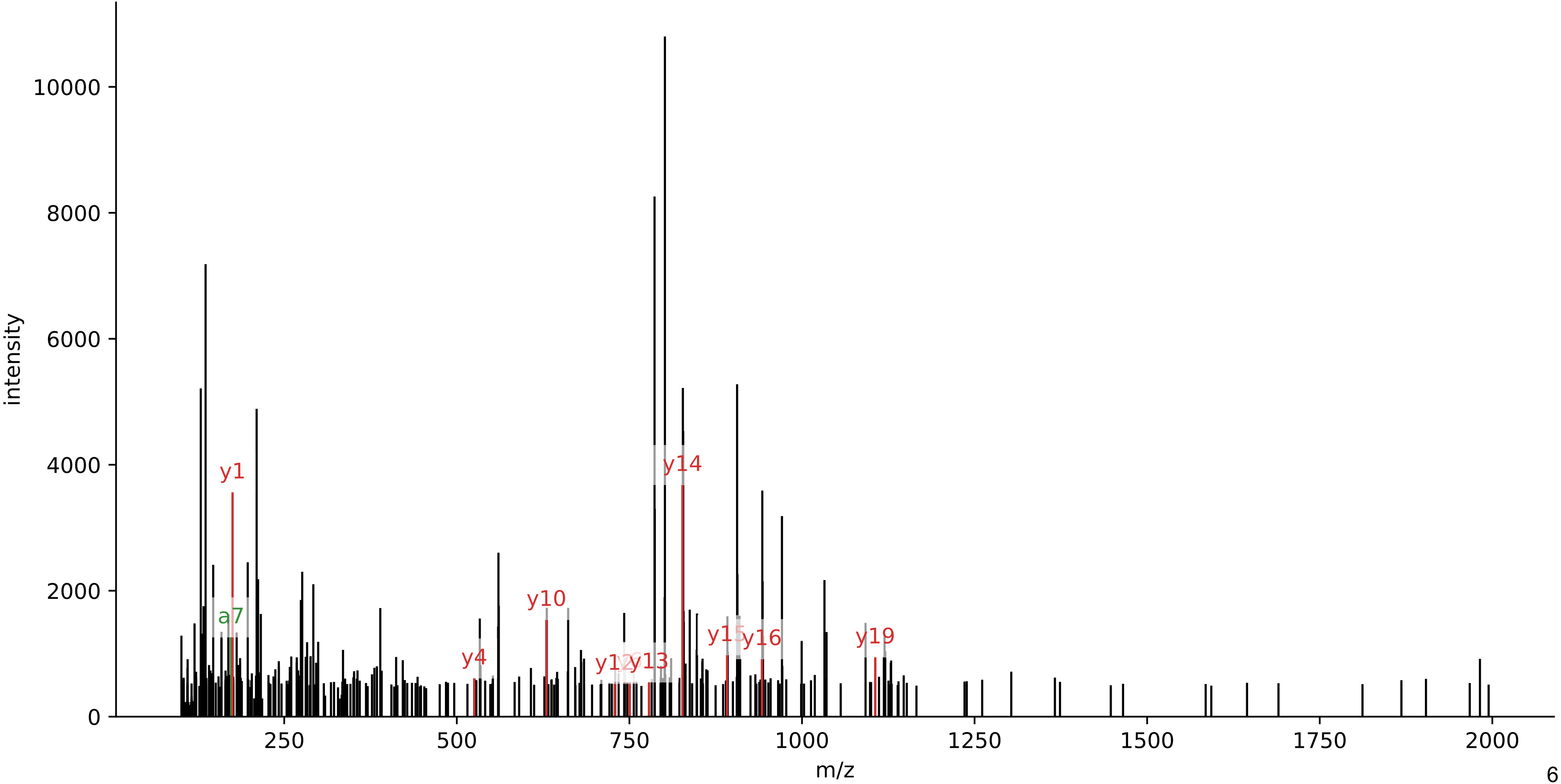

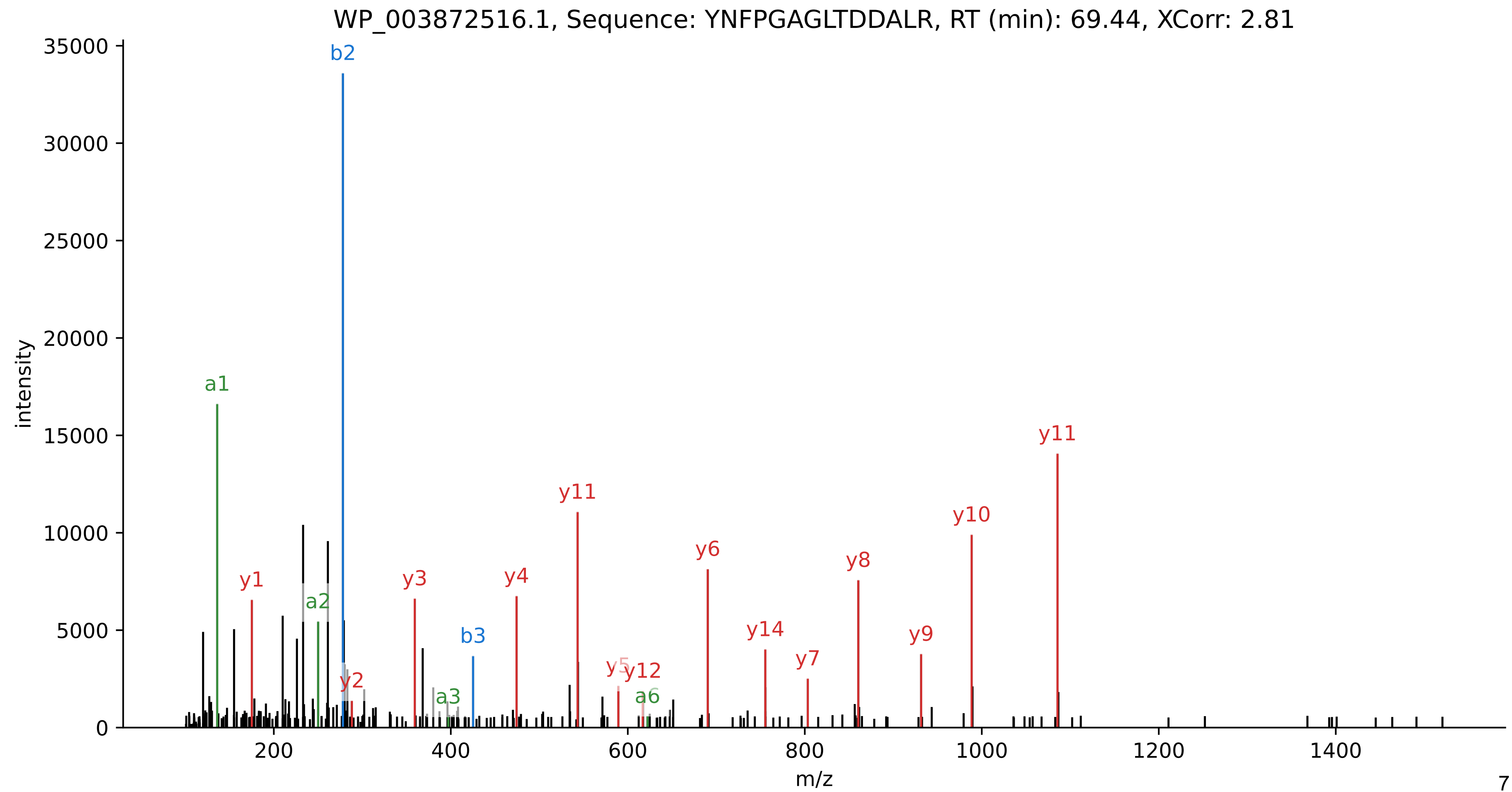

WP_003872516.1, Sequence: YNFPGAGLTDDALR, RT (min): 69.44, XCorr: 2.81
b2
a1
y11
y10
y11
y6
y8
y1
y3
y4
a2
y14
b3
y9
y7
y5
y12
y2
a3
a6
intensity
m/z
0
5000
10000
15000
20000
25000
30000
35000
200
400
600
800
1000
1200
1400

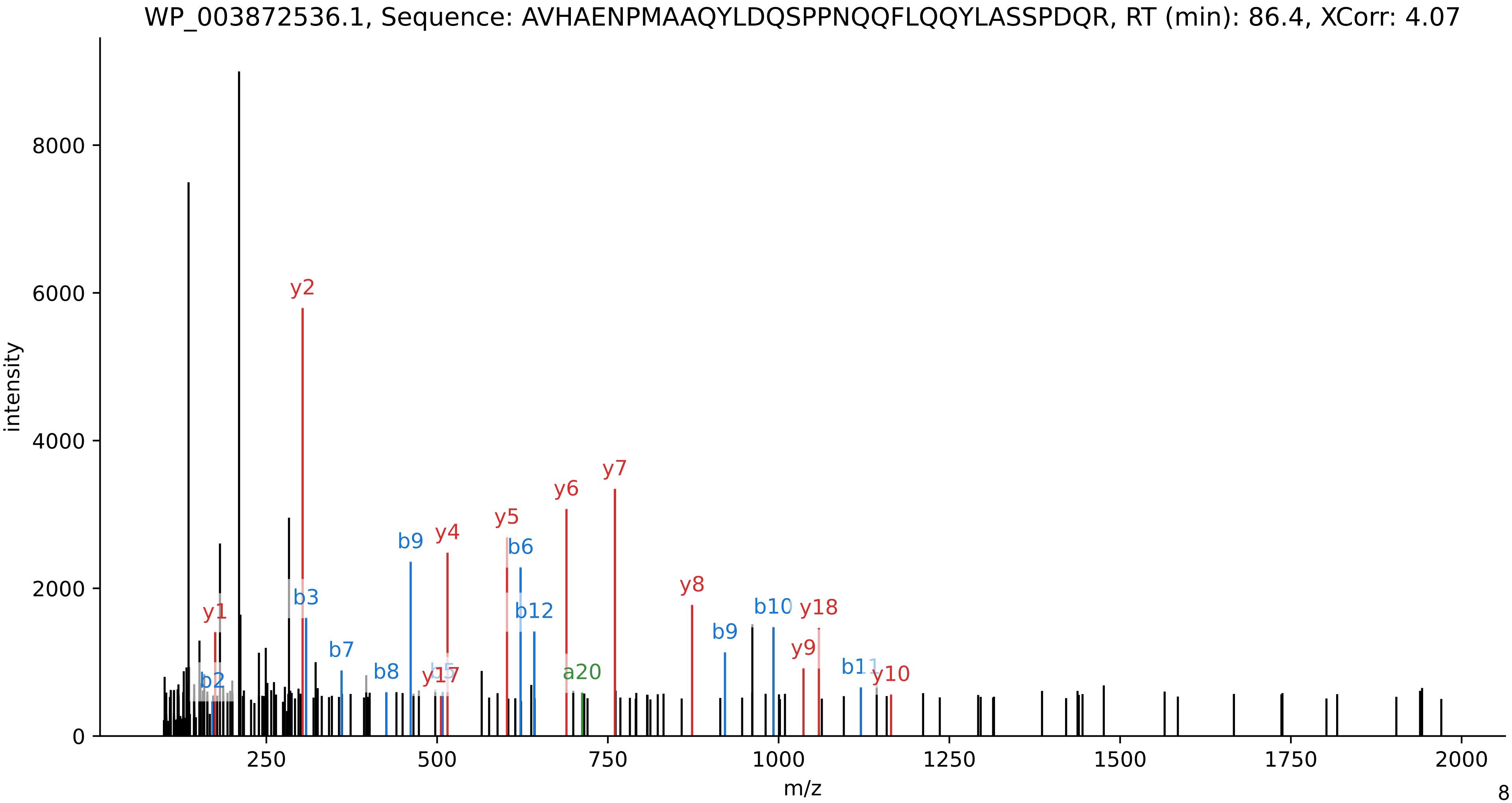

WP_003872536.1, Sequence: AVHAENPMAAQYLDQSPPNQQFLQQYLASSPDQR, RT (min): 86.4, XCorr: 4.07
intensity
m/z
y1
b2
y2
b3
b7
b8
b9
y4
y17
b5
y5
b6
b12
y6
a20
y7
y8
b9
b10
y18
y9
b11
y10

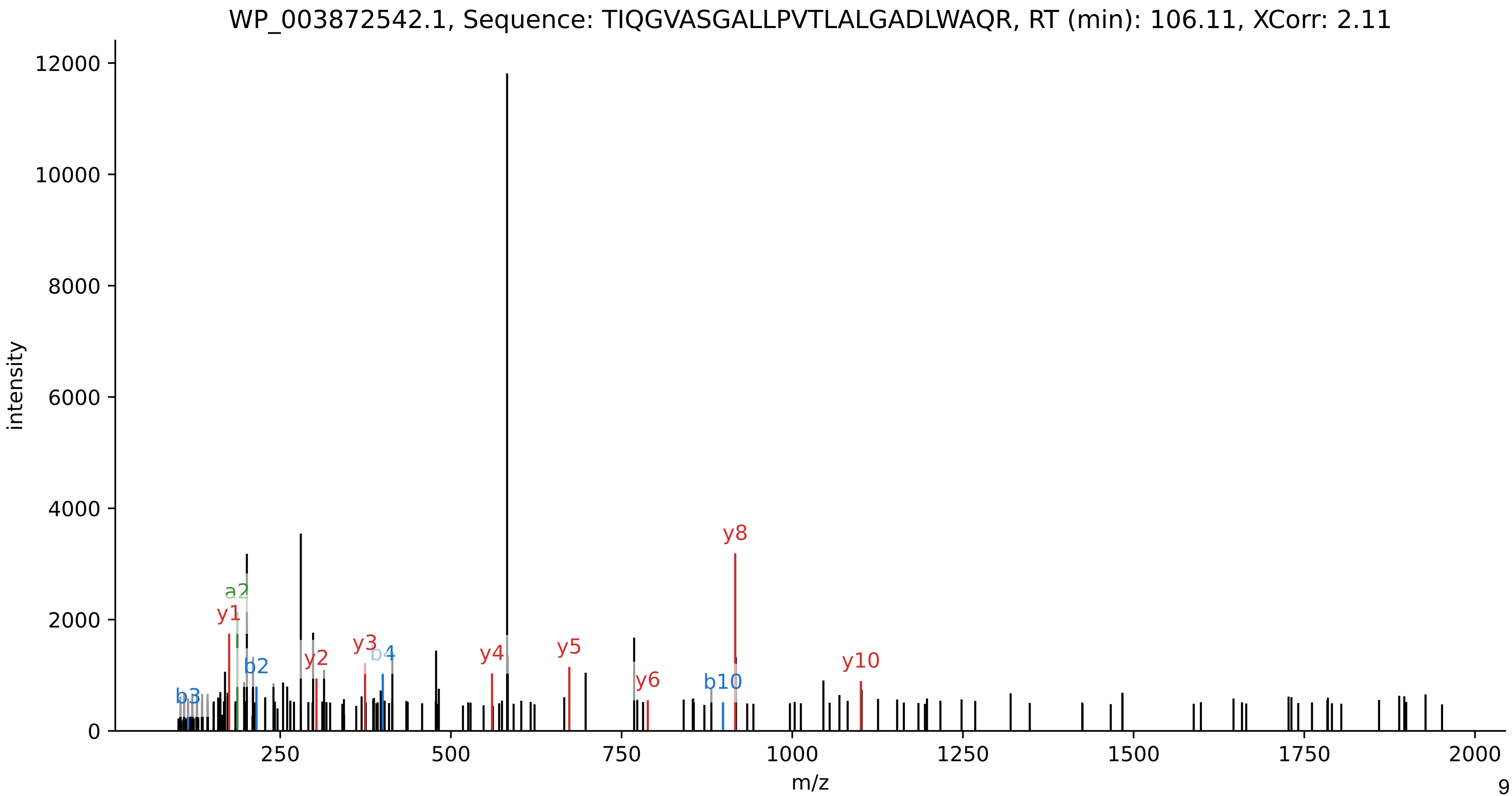
WP_003872542.1, Sequence: TIQGVASGALLPVTLALGADLWAQR, RT (min): 106.11, XCorr: 2.11
intensity
m/z
0
2000
4000
6000
8000
10000
12000
250
500
750
1000
1250
1500
1750
2000
b3
a2
y1
b2
y2
y3
b4
y4
y5
y6
b10
y8
y10

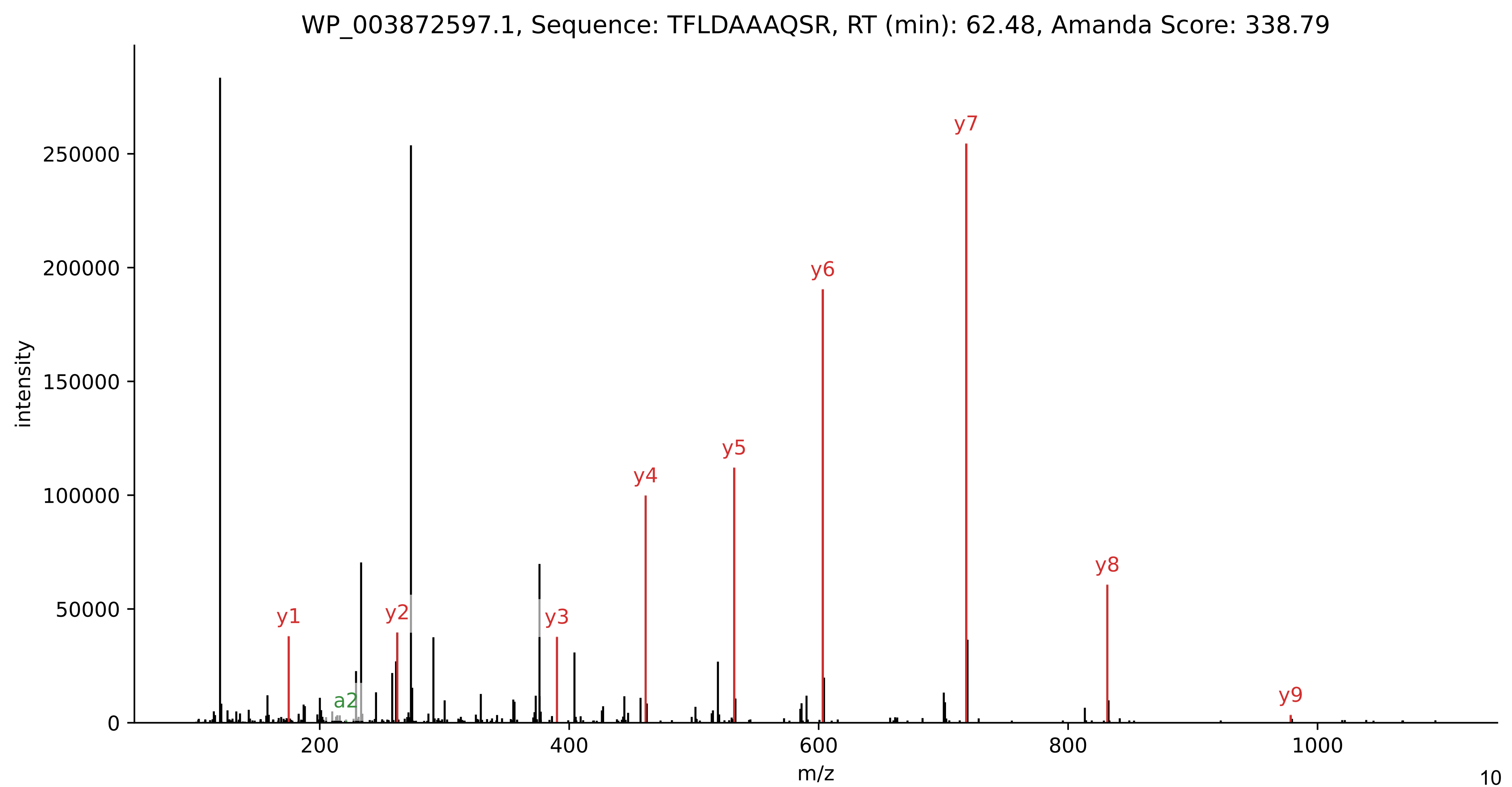
WP_003872597.1, Sequence: TFLDAAAQSR, RT (min): 62.48, Amanda Score: 338.79
intensity
m/z
y1
y2
y3
y4
y5
y6
y7
y8
y9
a2
0
50000
100000
150000
200000
250000
200
400
600
800
1000

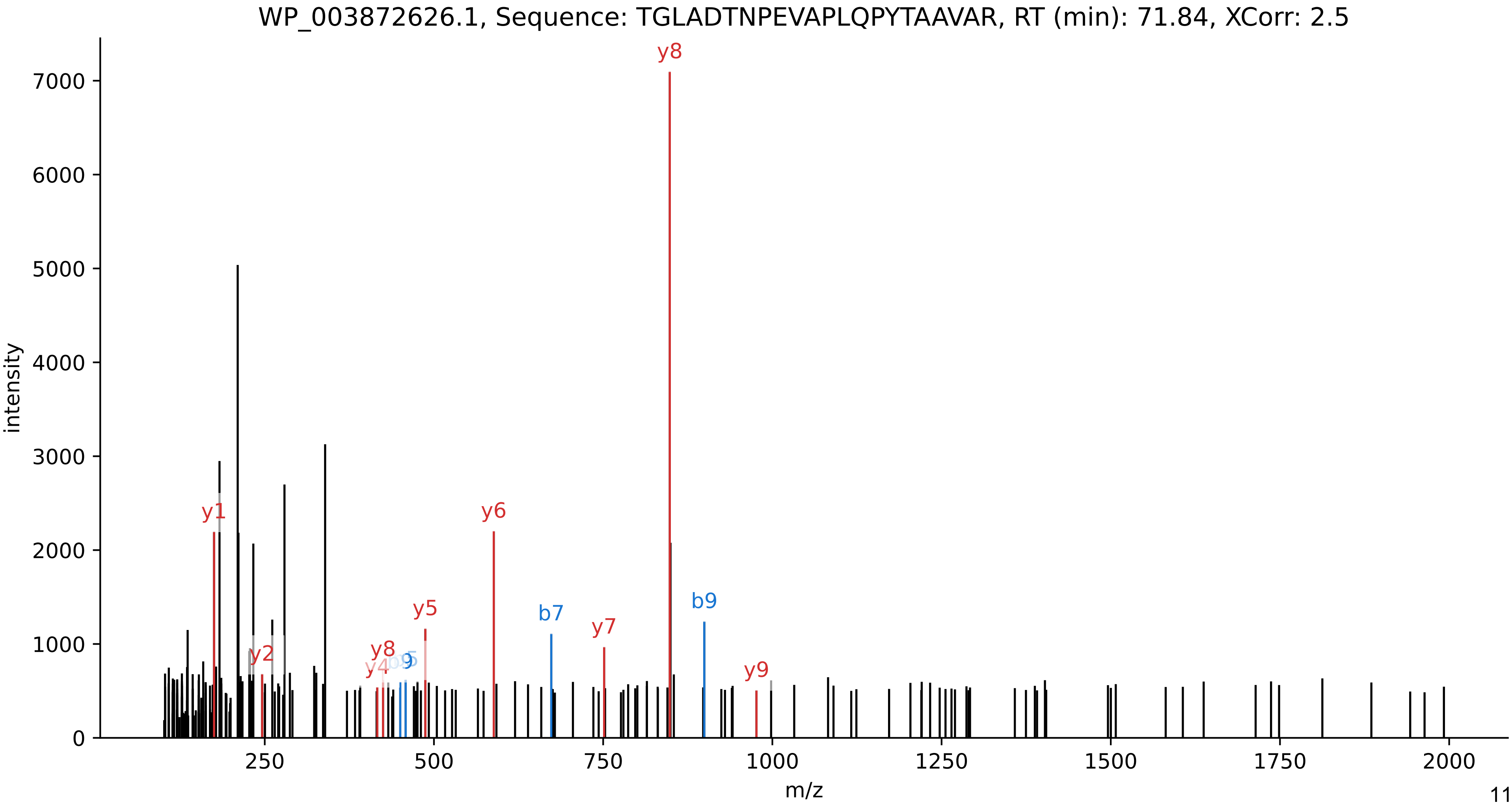
WP_003872626.1, Sequence: TGLADTNPEVAPLQPYTAAVAR, RT (min): 71.84, XCorr: 2.5
intensity
m/z
0
1000
2000
3000
4000
5000
6000
7000
250
500
750
1000
1250
1500
1750
2000
y1
y2
y4
y8
y5
y6
b7
y7
y8
b9
y9

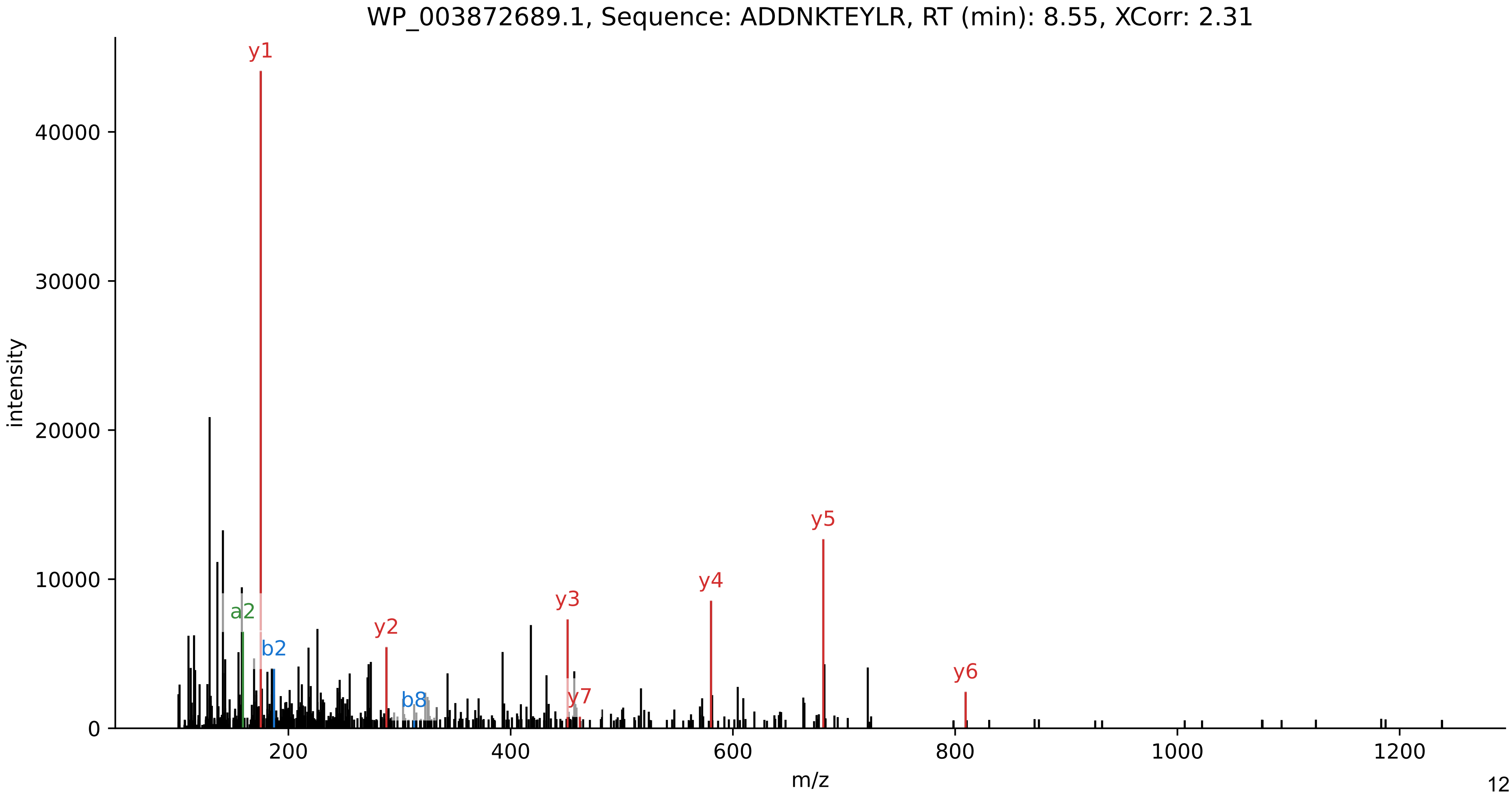

WP_003872689.1, Sequence: ADDNKTEYLR, RT (min): 8.55, XCorr: 2.31
intensity
m/z
y1
y2
y3
y4
y5
y6
y7
a2
b2
b8
0
10000
20000
30000
40000
200
400
600
800
1000
1200

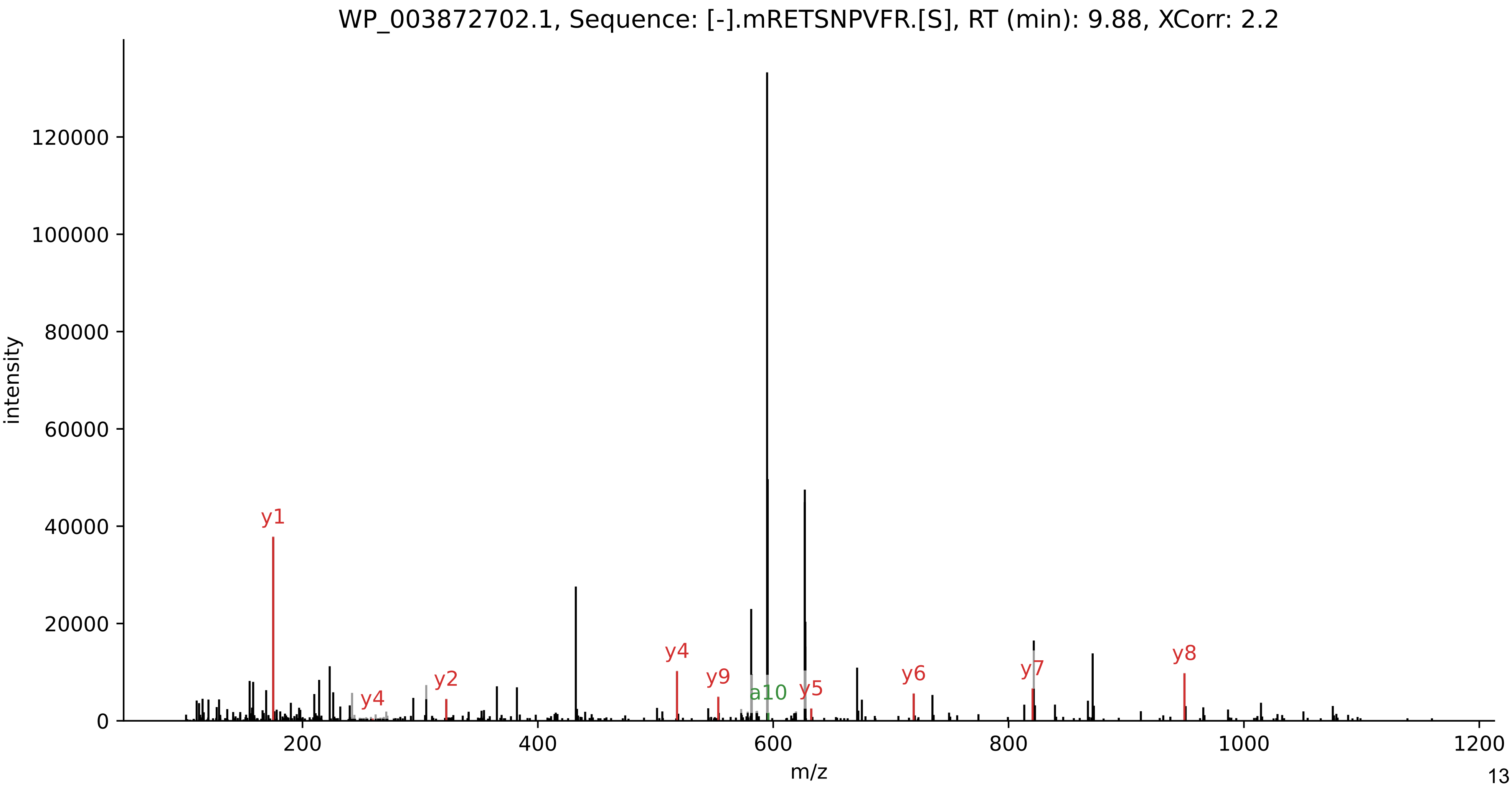
WP_003872702.1, Sequence: [-].mRETSNPVFR.[S], RT (min): 9.88, XCorr: 2.2
intensity
m/z
y1
y4
y2
y4
y9
a10
y5
y6
y7
y8

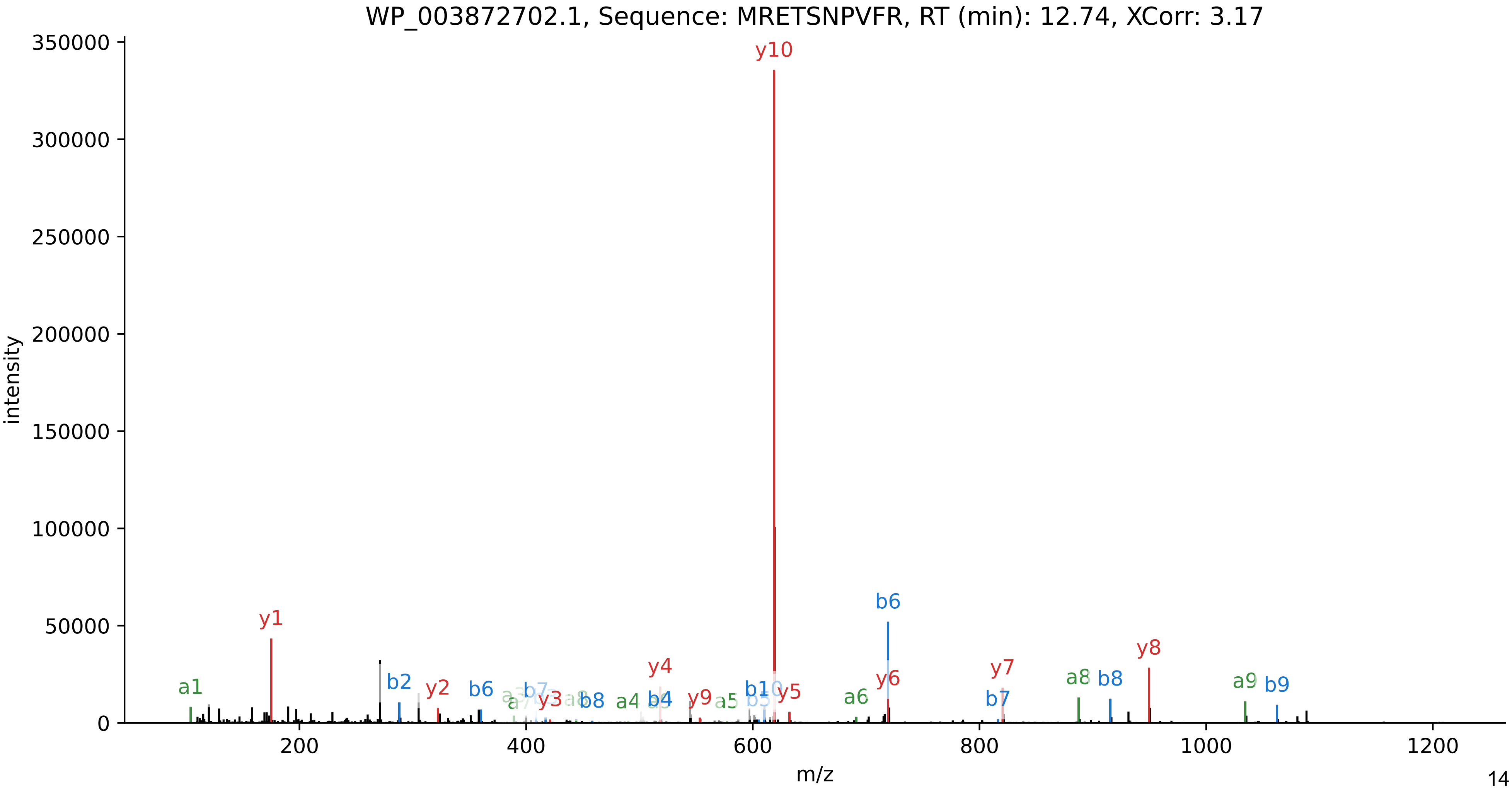
WP_003872702.1, Sequence: MRETSNPVFR, RT (min): 12.74, XCorr: 3.17
intensity
m/z
y10
b6
y1
y8
y7
y6
y4
y2
y3
y5
y9
a1
b2
b6
b4
b8
b7
b10
a4
a5
a6
a8
b8
a9
b9
b7
0
50000
100000
150000
200000
250000
300000
350000
200
400
600
800
1000
1200

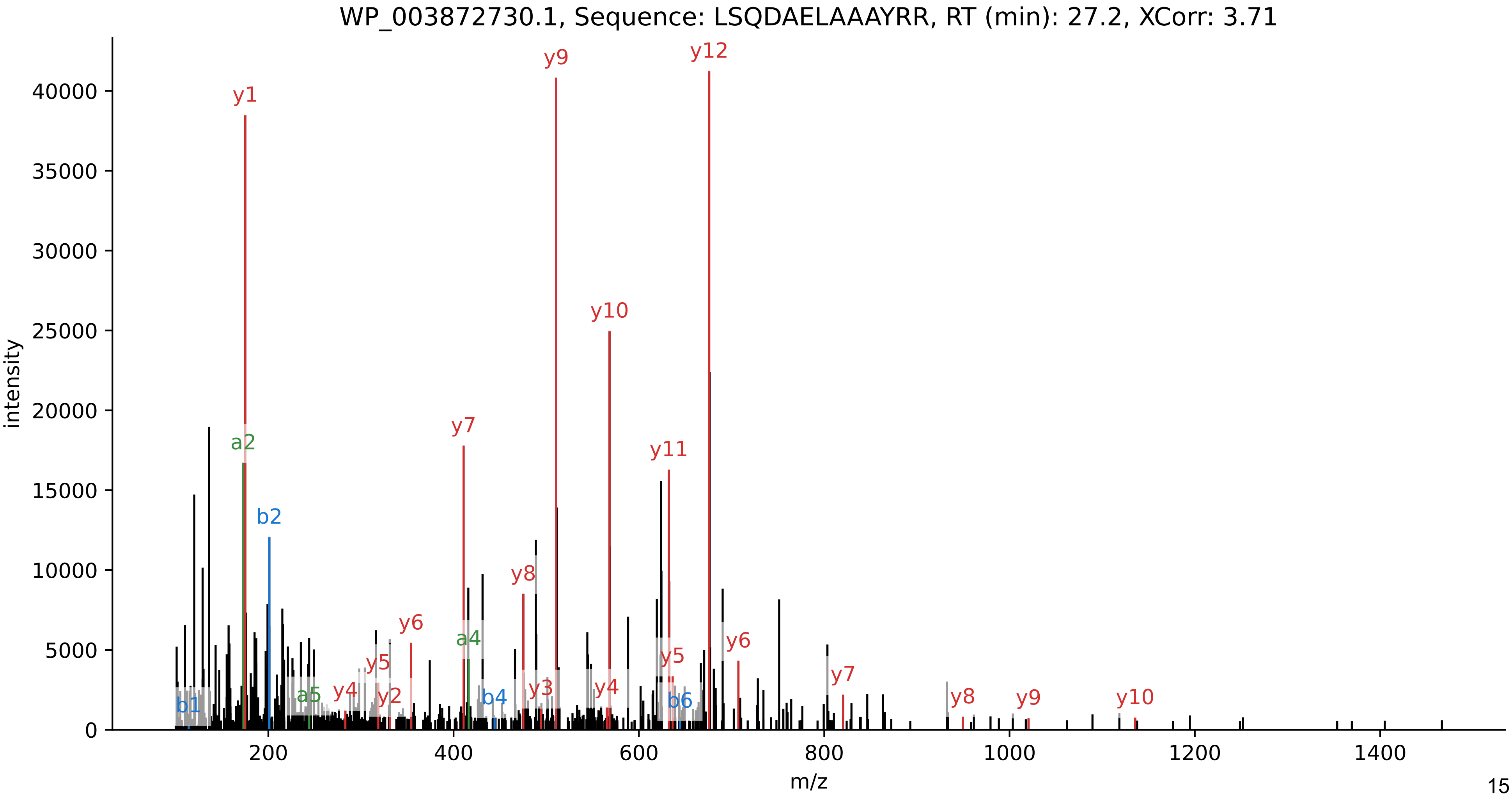
WP_003872730.1, Sequence: LSQDAELAAAYRR, RT (min): 27.2, XCorr: 3.71
intensity
m/z
y1
y9
y12
y10
y7
y11
a2
b2
y8
y6
a4
y5
y4
a5
y2
b1
y3
b4
y4
y5
b6
y6
y7
y8
y9
y10

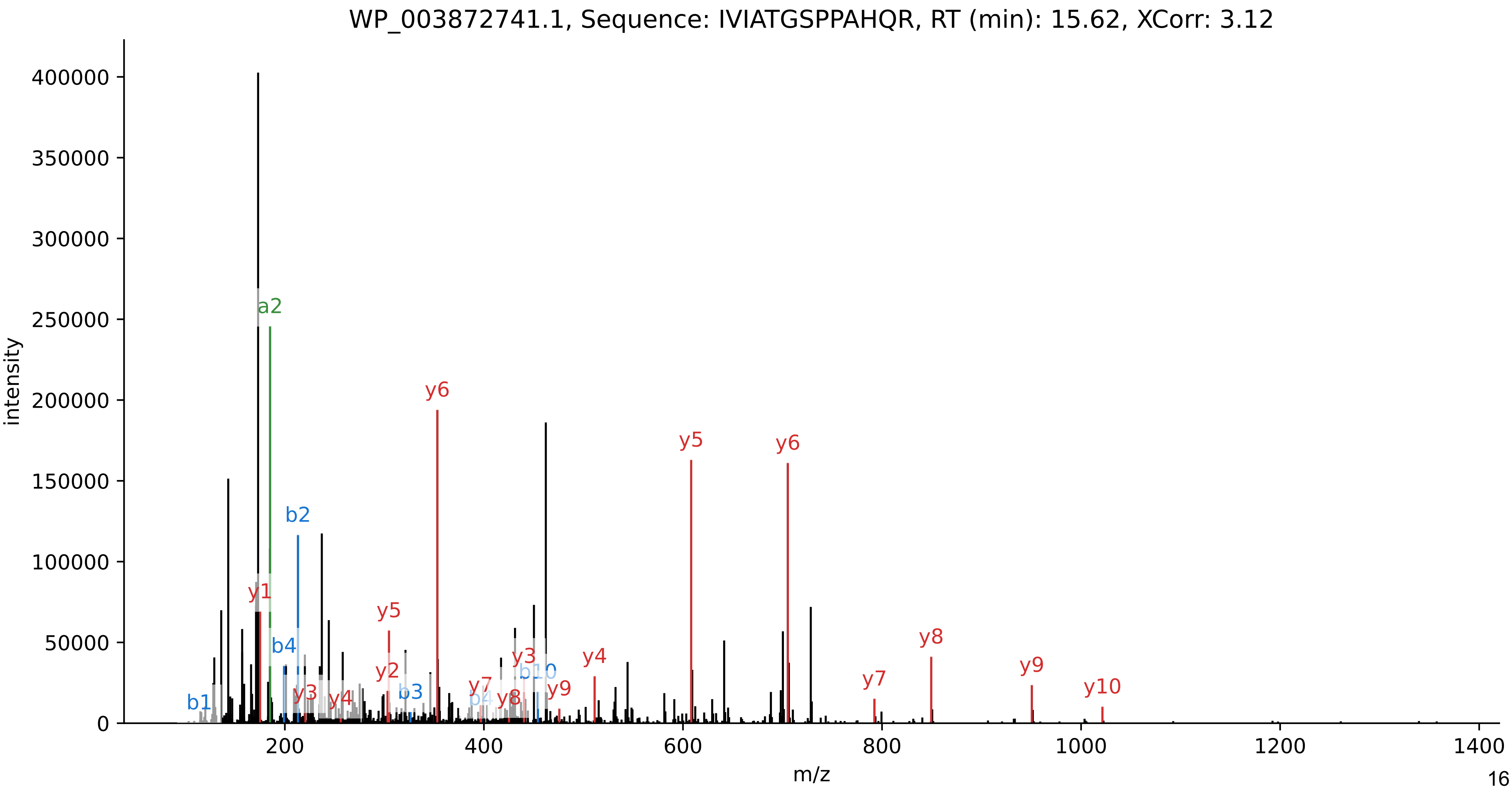

WP_003872741.1, Sequence: IVIATGSPPAHQR, RT (min): 15.62, XCorr: 3.12
intensity
m/z
a2
y6
y5
y6
b2
y1
y5
b4
y2
y3
y4
b1
b3
y7
b4
y8
y3
b10
y9
y4
y7
y8
y9
y10

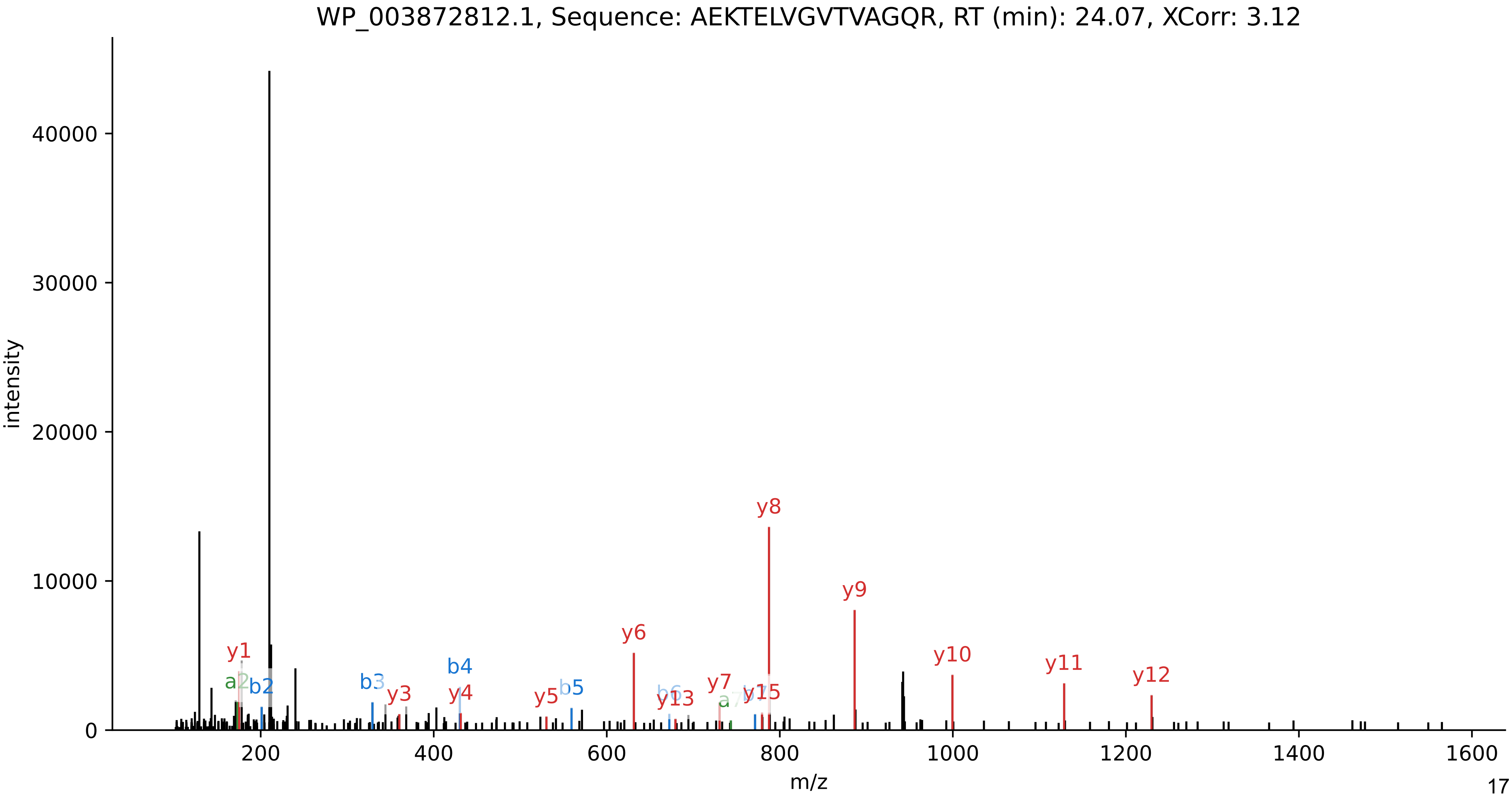
WP_003872812.1, Sequence: AEKTELVGVTVAGQR, RT (min): 24.07, XCorr: 3.12
intensity
m/z
0
10000
20000
30000
40000
200
400
600
800
1000
1200
1400
1600
y1
a2
b2
b3
y3
b4
y4
y5
b5
y6
b6
y13
y7
y15
y8
y9
y10
y11
y12

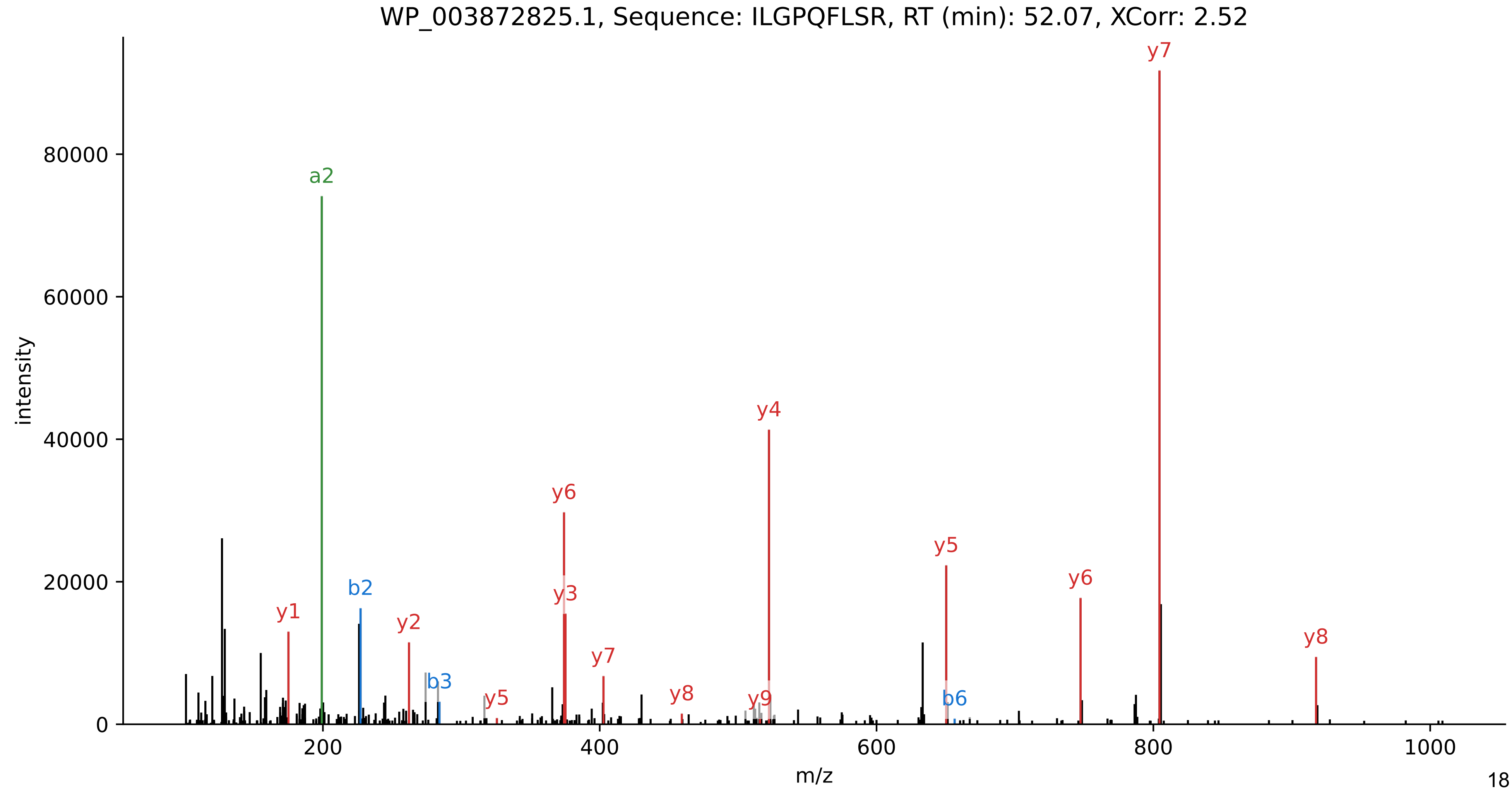
WP_003872825.1, Sequence: ILGPQFLSR, RT (min): 52.07, XCorr: 2.52
intensity
m/z
a2
b2
b3
b6
y1
y2
y3
y4
y5
y6
y7
y8
y9
0
20000
40000
60000
80000
200
400
600
800
1000

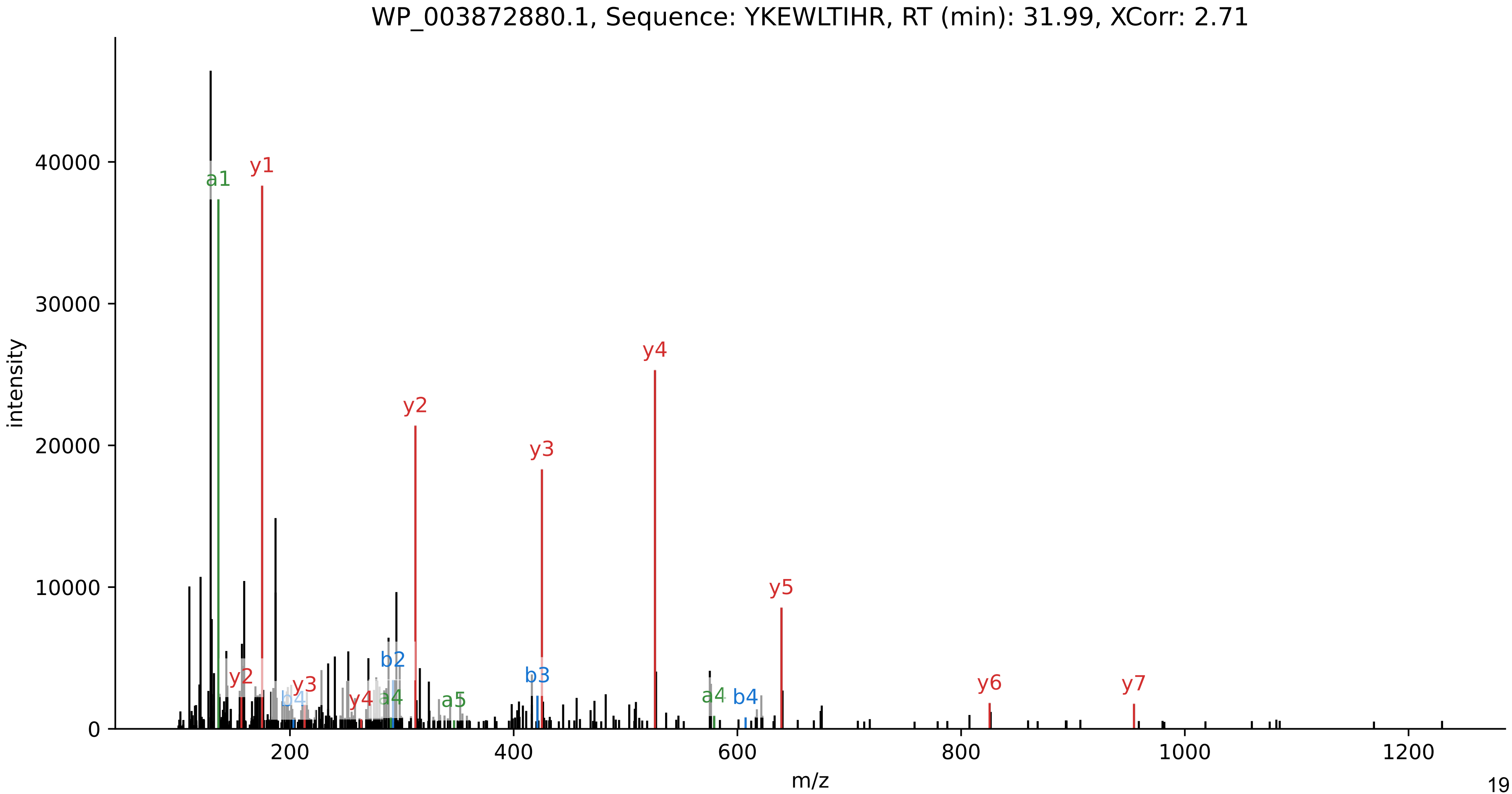
WP_003872880.1, Sequence: YKEWLTIHR, RT (min): 31.99, XCorr: 2.71
intensity
m/z
0
10000
20000
30000
40000
200
400
600
800
1000
1200
a1
y1
y2
y3
y4
y5
y6
y7
b2
b3
b4
a4
a5

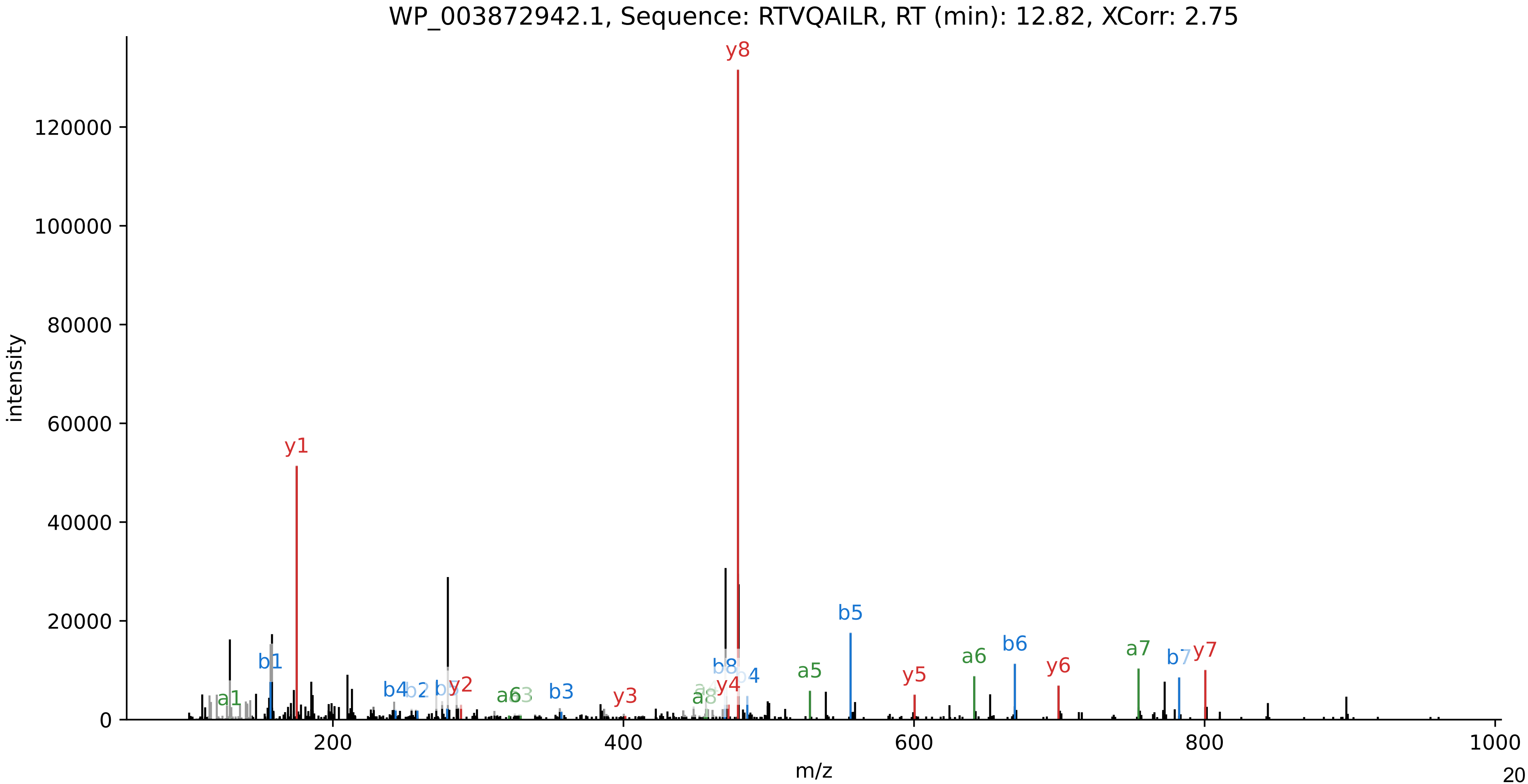
WP_003872942.1, Sequence: RTVQAILR, RT (min): 12.82, XCorr: 2.75
intensity
m/z
0
20000
40000
60000
80000
100000
120000
200
400
600
800
1000
y8
y1
b1
a1
b4
b2
y2
b3
y3
a8
y4
b8
a5
b5
y5
a6
b6
y6
a7
b7
y7

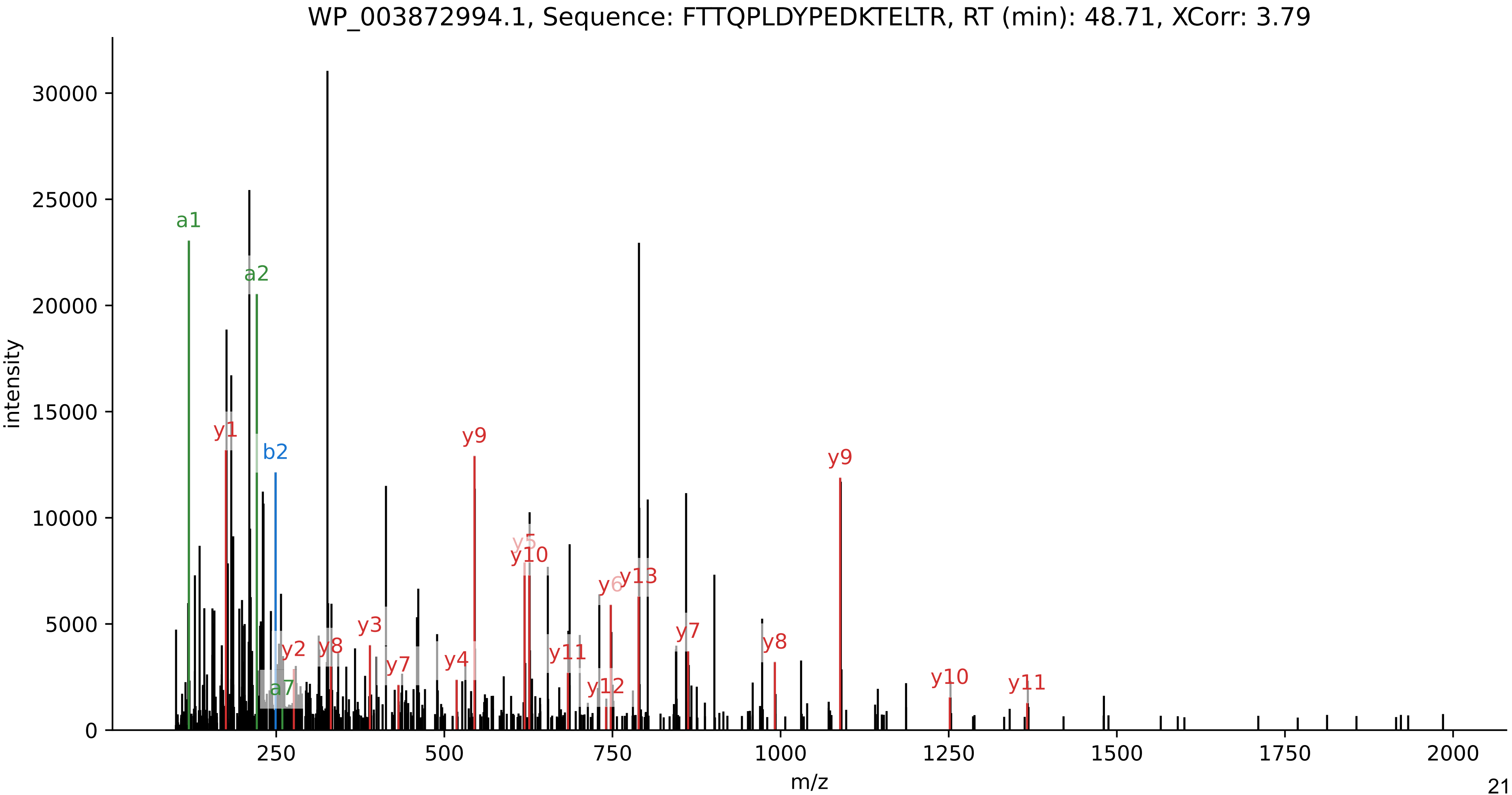
WP_003872994.1, Sequence: FTTQPLDYPEDKTELTR, RT (min): 48.71, XCorr: 3.79
intensity
m/z
a1
a2
b2
a7
y1
y2
y8
y3
y7
y4
y9
y5
y10
y11
y6
y12
y13
y7
y8
y9
y10
y11
30000
25000
20000
15000
10000
5000
0
250
500
750
1000
1250
1500
1750
2000

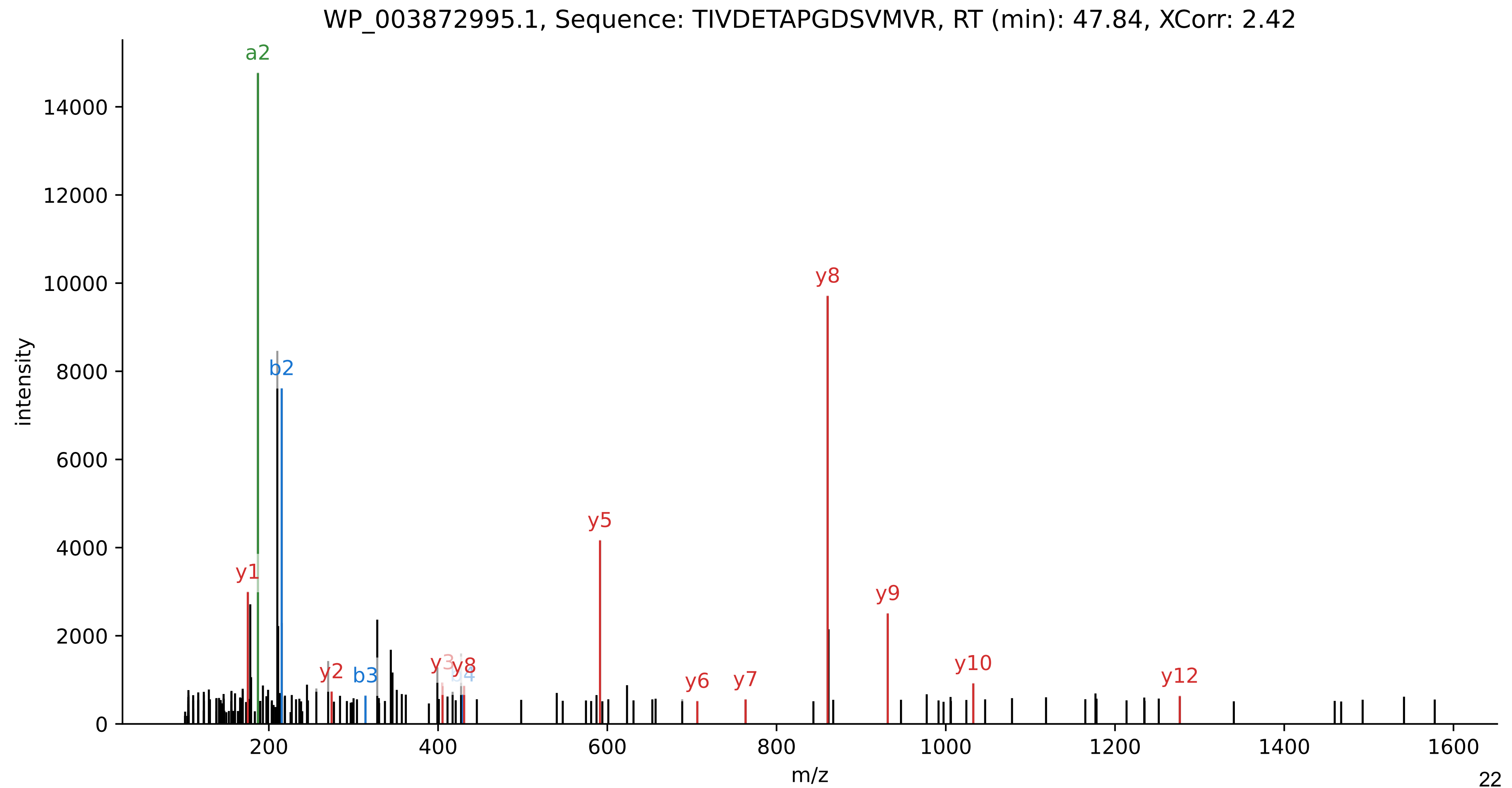

WP_003872995.1, Sequence: TIVDETAPGDSVMVR, RT (min): 47.84, XCorr: 2.42
intensity
m/z
a2
b2
y8
y5
y1
y9
y2
b3
y3
y8
b4
y10
y6
y7
y12

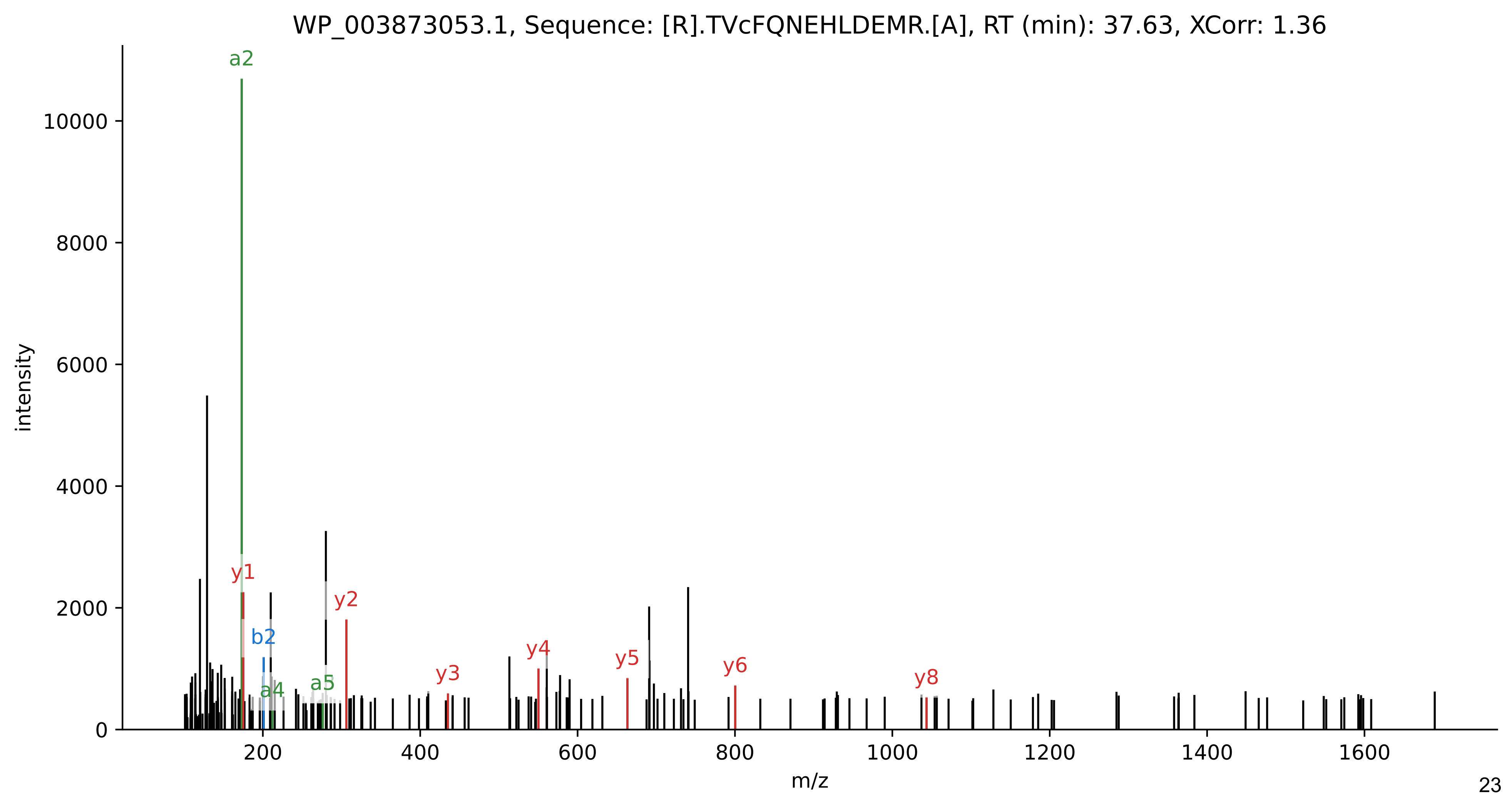
WP_003873053.1, Sequence: [R].TVcFQNEHLDEMR.[A], RT (min): 37.63, XCorr: 1.36
a2
y1
b2
a4
a5
y2
y3
y4
y5
y6
y8
intensity
m/z
0
2000
4000
6000
8000
10000
200
400
600
800
1000
1200
1400
1600

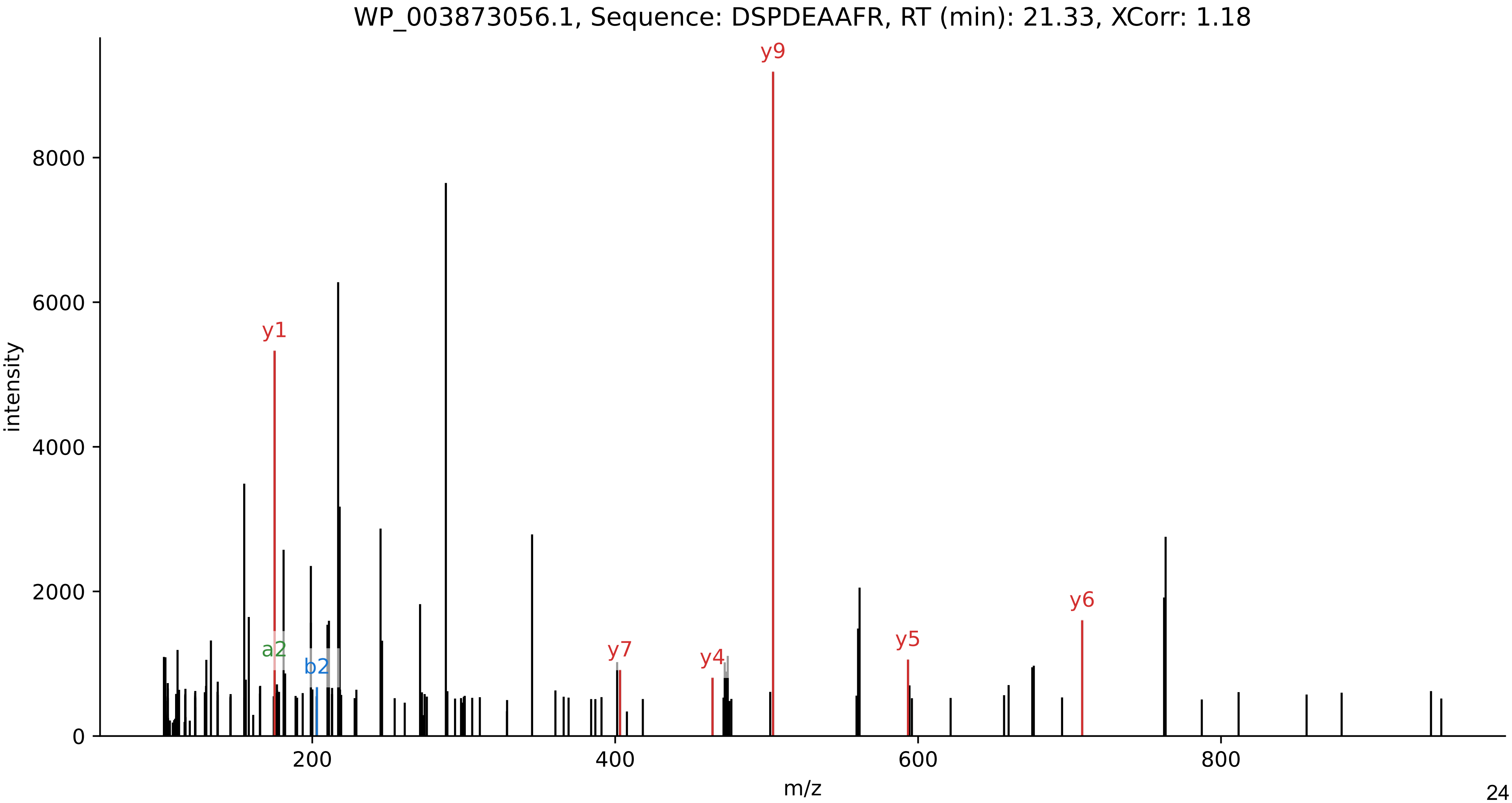
WP_003873056.1, Sequence: DSPDEAAFR, RT (min): 21.33, XCorr: 1.18
intensity
m/z
y9
y1
a2
b2
y7
y4
y5
y6
0
2000
4000
6000
8000
200
400
600
800

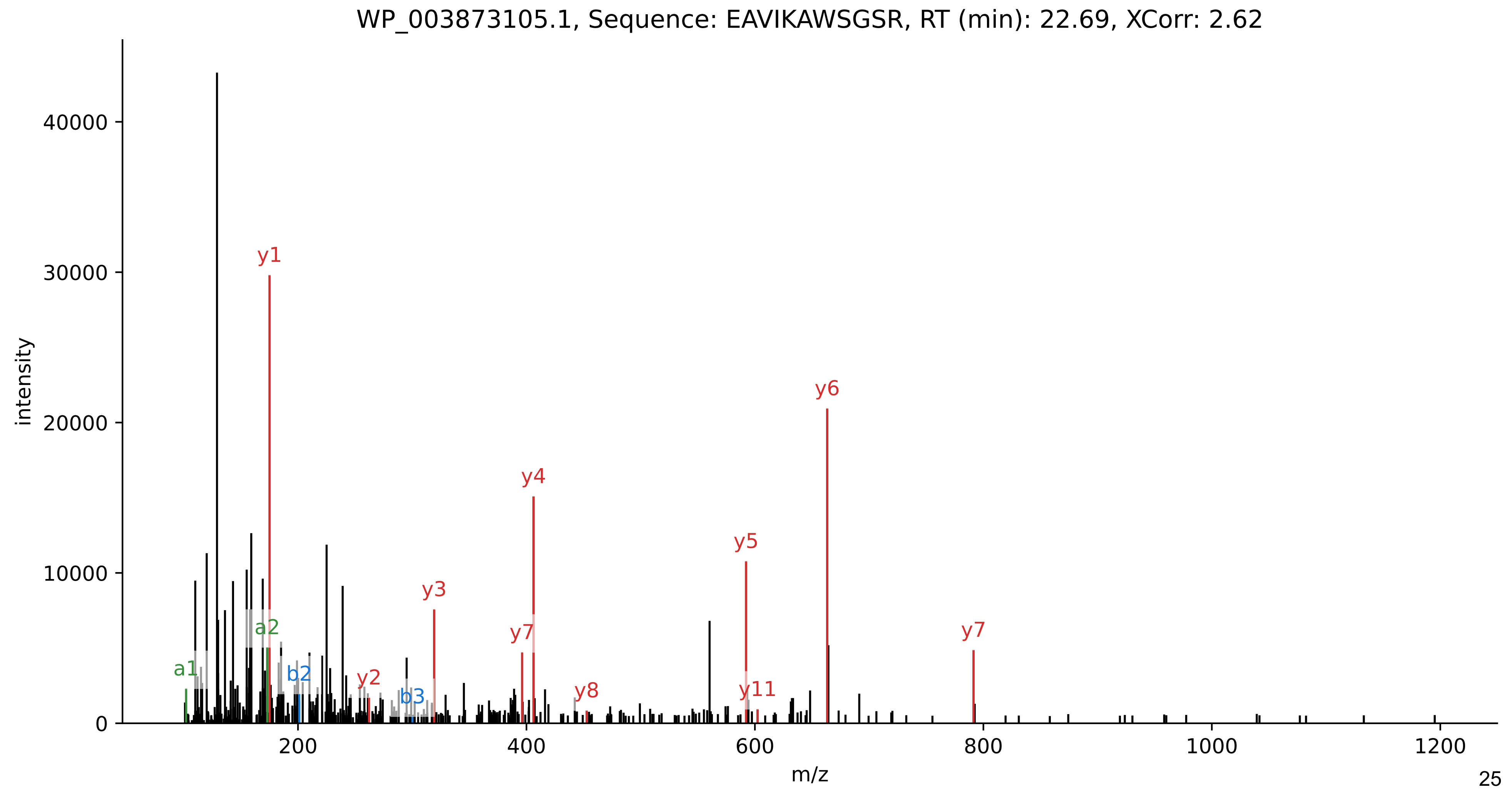
WP_003873105.1, Sequence: EAVIKAWSGSR, RT (min): 22.69, XCorr: 2.62
intensity
m/z
y1
y6
y4
y5
y3
y7
y7
a2
a1
b2
y2
b3
y8
y11
0
10000
20000
30000
40000
200
400
600
800
1000
1200

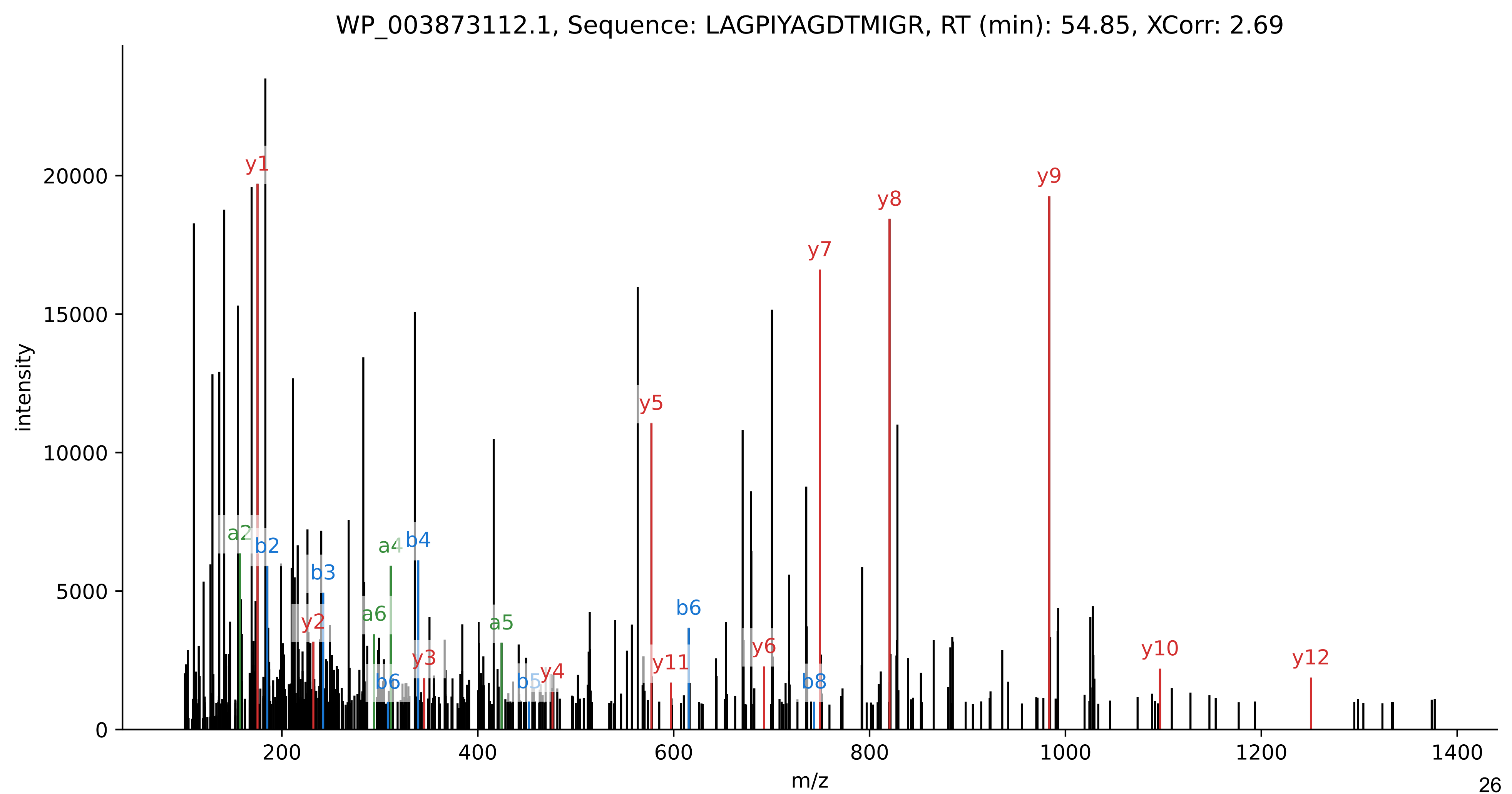

WP_003873112.1, Sequence: LAGPIYAGDTMIGR, RT (min): 54.85, XCorr: 2.69
intensity
m/z
y1
y2
y3
y4
y5
y6
y7
y8
y9
y10
y11
y12
a2
a4
a5
a6
b2
b3
b4
b5
b6
b6
b8

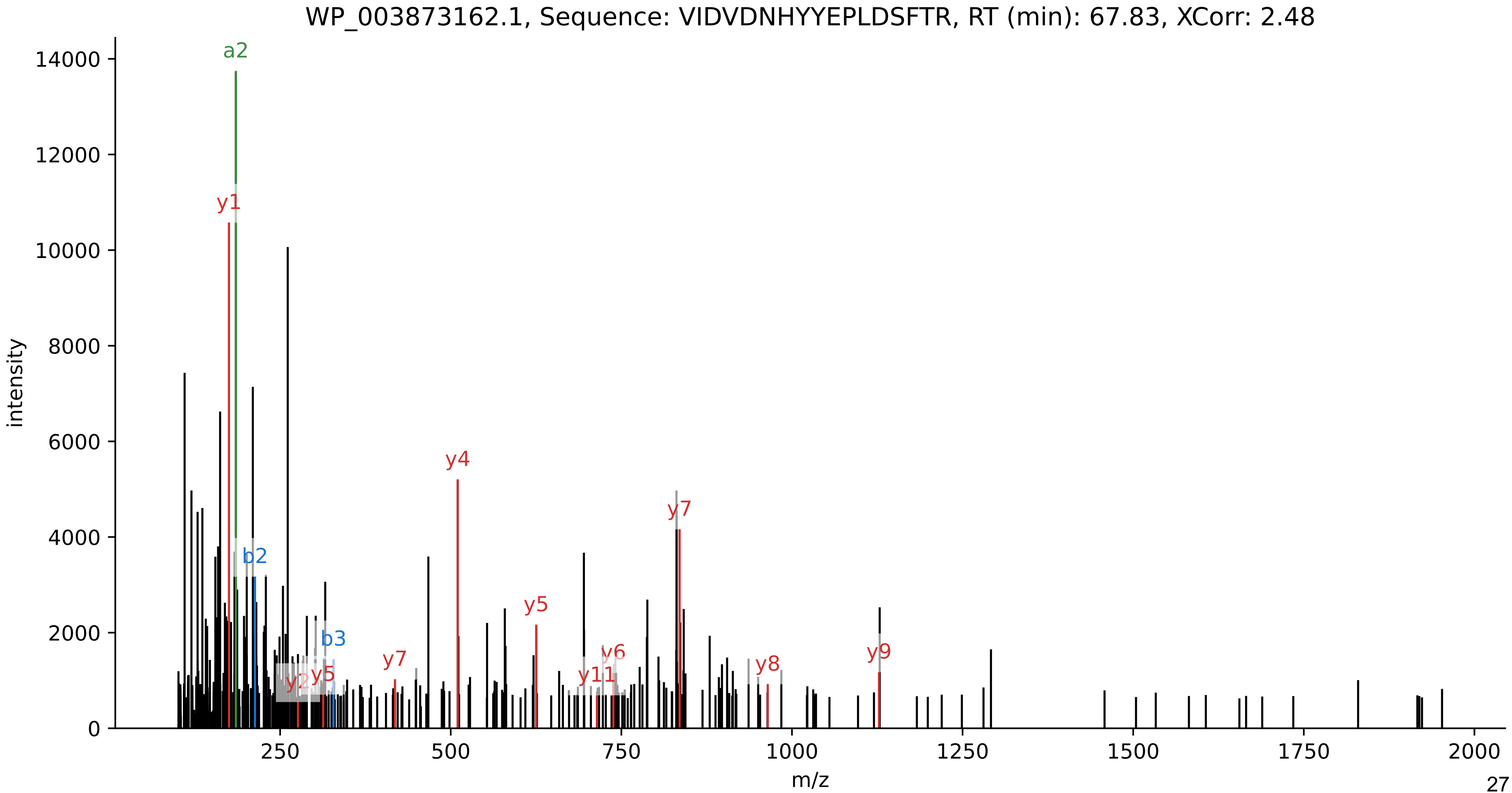
WP_003873162.1, Sequence: VIDVDNHYYEPLDSFTR, RT (min): 67.83, XCorr: 2.48
intensity
m/z
0
2000
4000
6000
8000
10000
12000
14000
250
500
750
1000
1250
1500
1750
2000
a2
y1
b2
b3
y2
y5
y7
y4
y5
y11
y6
y7
y8
y9

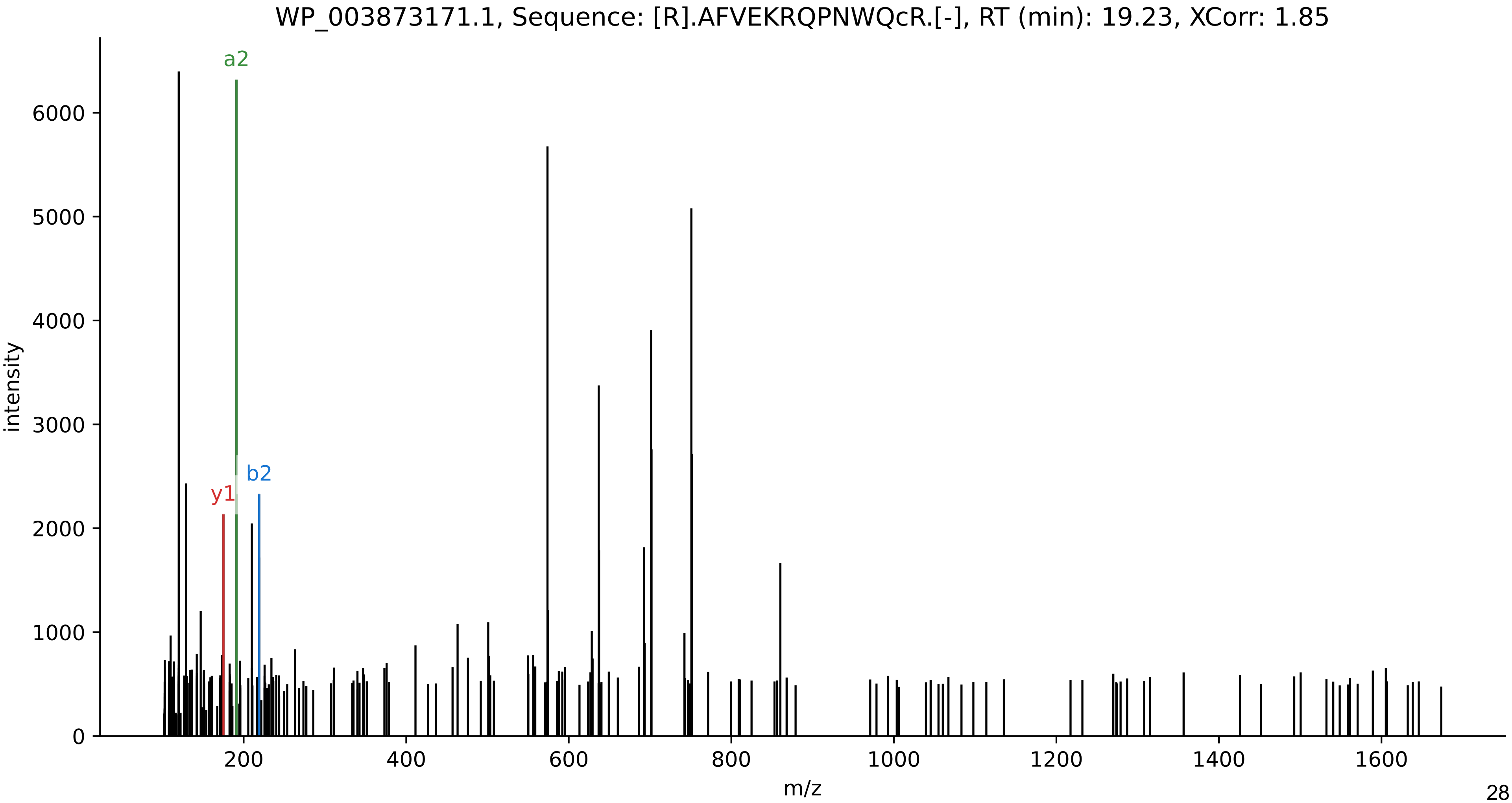

WP_003873171.1, Sequence: [R].AFVEKRQPNWQcR.[-], RT (min): 19.23, XCorr: 1.85
a2
b2
y1
intensity
m/z
0
1000
2000
3000
4000
5000
6000
200
400
600
800
1000
1200
1400
1600

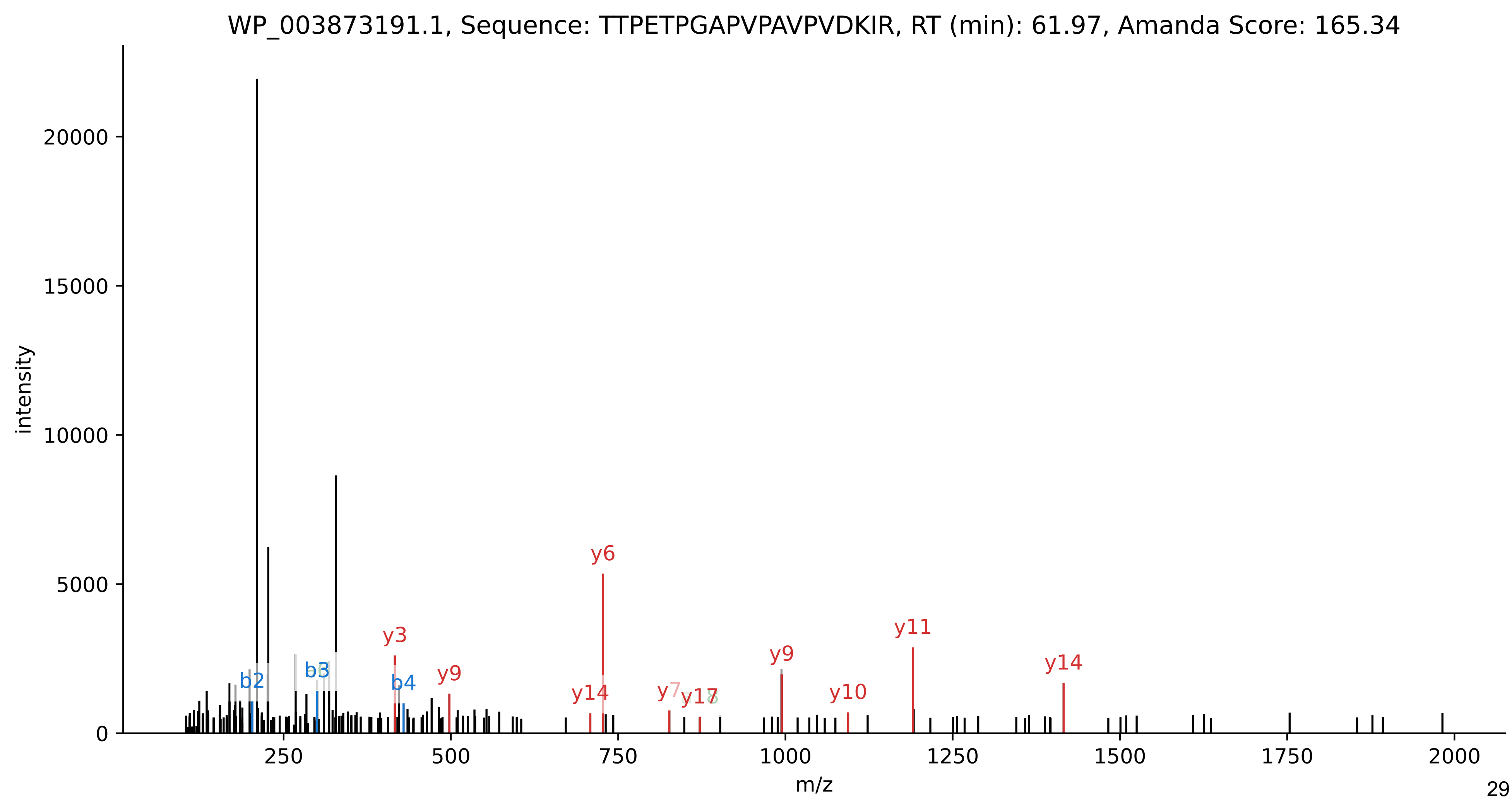
WP_003873191.1, Sequence: TTPETPGAPVPAVPVDKIR, RT (min): 61.97, Amanda Score: 165.34
intensity
m/z
b2
b3
b4
y3
y9
y14
y6
y7
y17
y9
y10
y11
y14

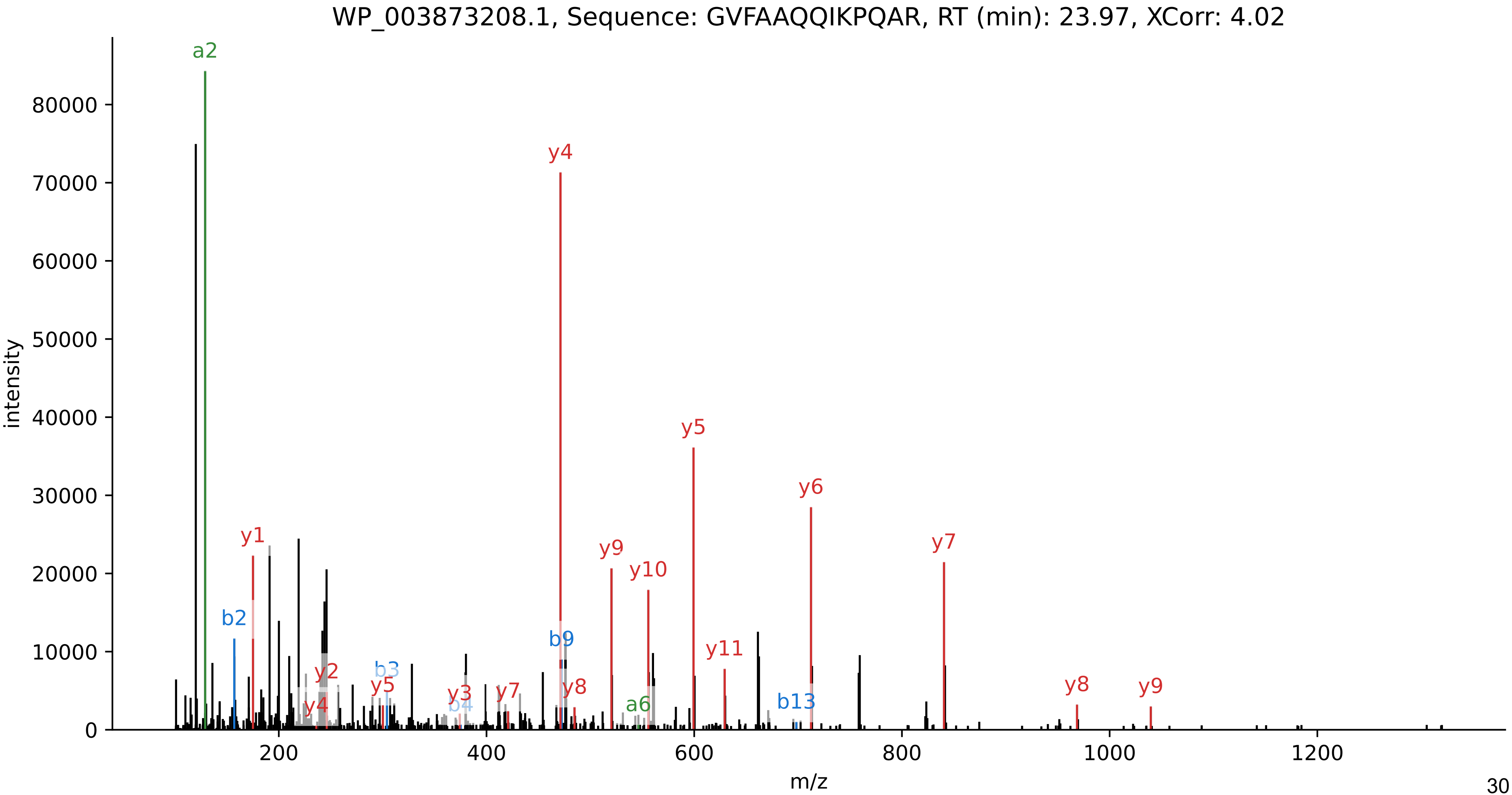
WP_003873208.1, Sequence: GVFAAQQIKPQAR, RT (min): 23.97, XCorr: 4.02
intensity
m/z
0
10000
20000
30000
40000
50000
60000
70000
80000
200
400
600
800
1000
1200
a2
y4
y5
y6
y7
y1
y9
y10
b2
b9
y11
y2
b3
y5
y3
y7
y8
a6
b13
y8
y9
y4
b4

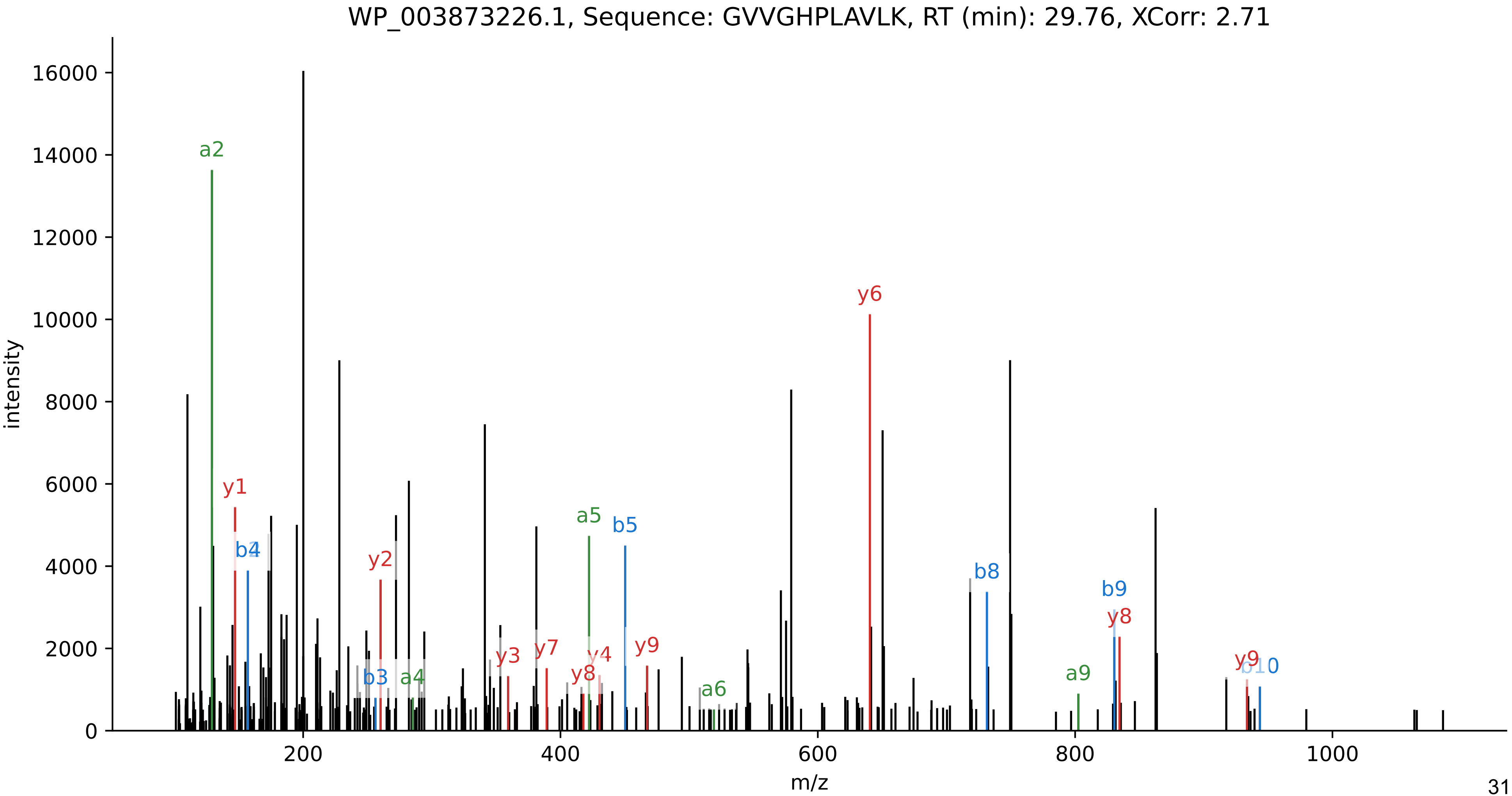
WP_003873226.1, Sequence: GVVGHPLAVLK, RT (min): 29.76, XCorr: 2.71
intensity
m/z
a2
y1
b4
y2
b3
a4
y3
y7
y8
a5
y4
b5
y9
a6
y6
b8
b9
y8
a9
y9
b10

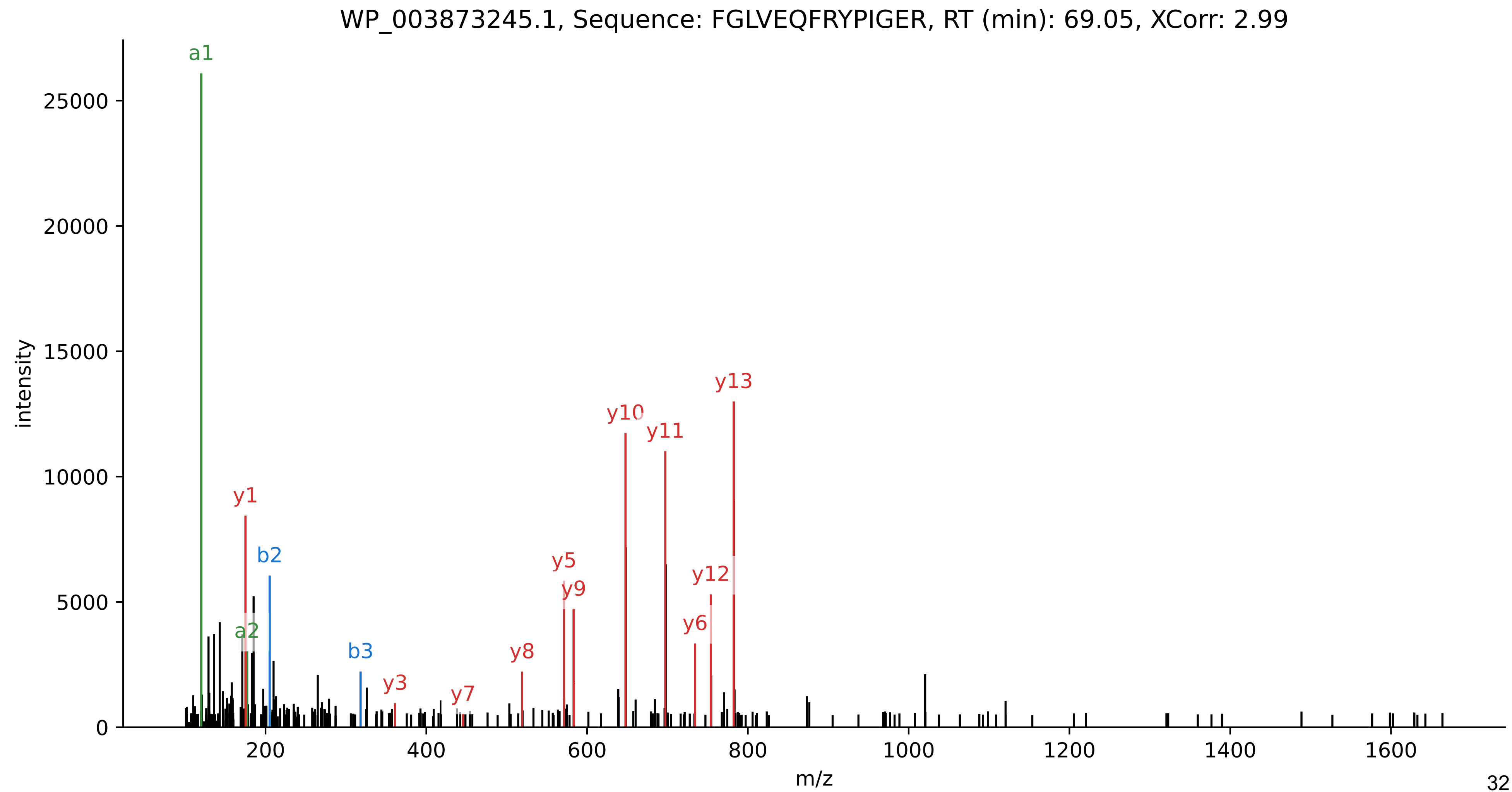

WP_003873245.1, Sequence: FGLVEQFRYPIGER, RT (min): 69.05, XCorr: 2.99
a1
y1
b2
a2
b3
y3
y7
y8
y5
y9
y10
y11
y6
y12
y13
intensity
m/z
0
5000
10000
15000
20000
25000
200
400
600
800
1000
1200
1400
1600

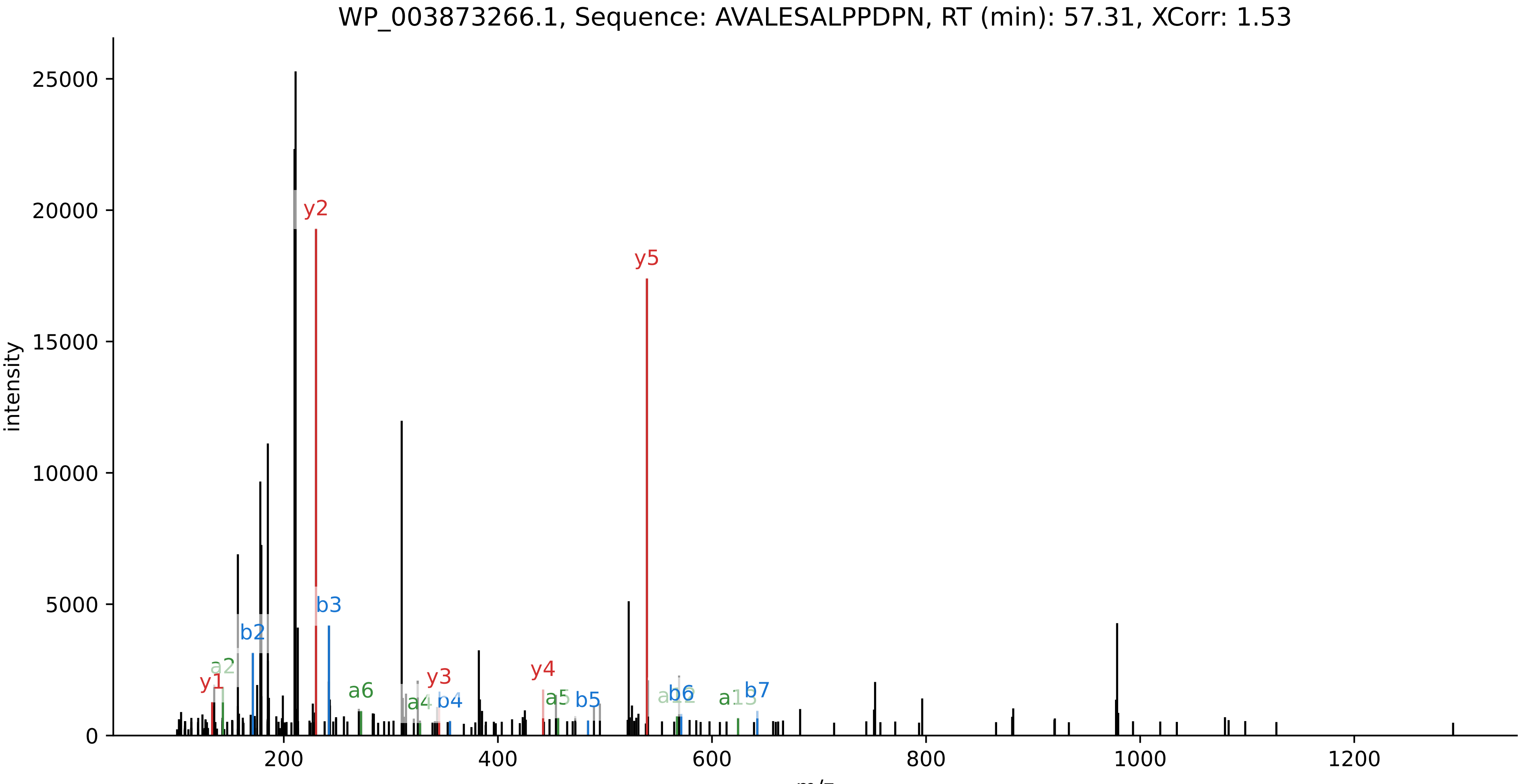
WP_003873266.1, Sequence: AVALESALPPDPN, RT (min): 57.31, XCorr: 1.53
intensity
m/z
0
5000
10000
15000
20000
25000
200
400
600
800
1000
1200
y1
a2
b2
y2
b3
y5
a6
y3
a4
b4
y4
a5
b5
b6
b7

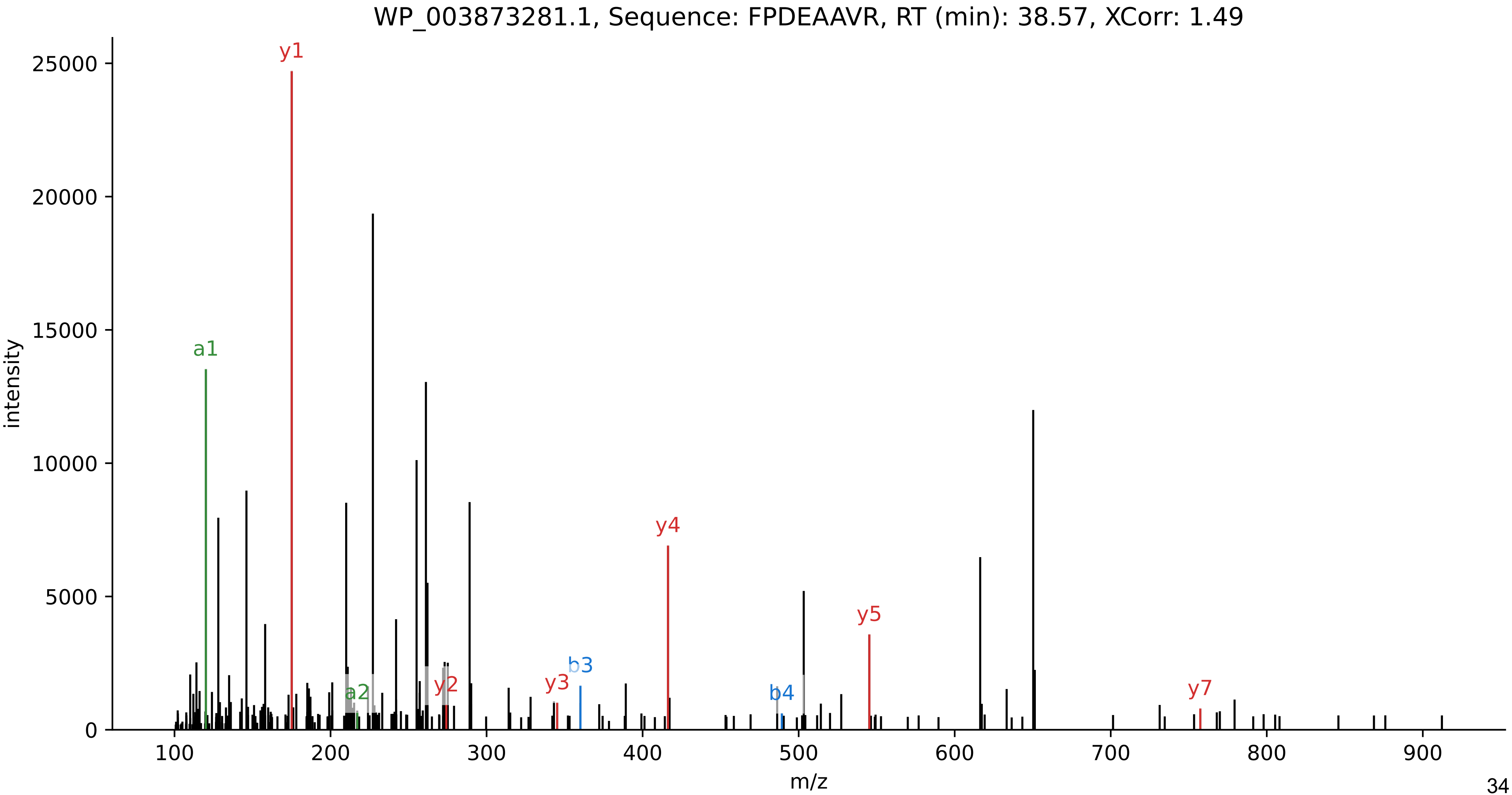
WP_003873281.1, Sequence: FPDEAAVR, RT (min): 38.57, XCorr: 1.49
intensity
m/z
y1
a1
a2
y2
y3
b3
y4
b4
y5
y7
25000
20000
15000
10000
5000
0
100
200
300
400
500
600
700
800
900

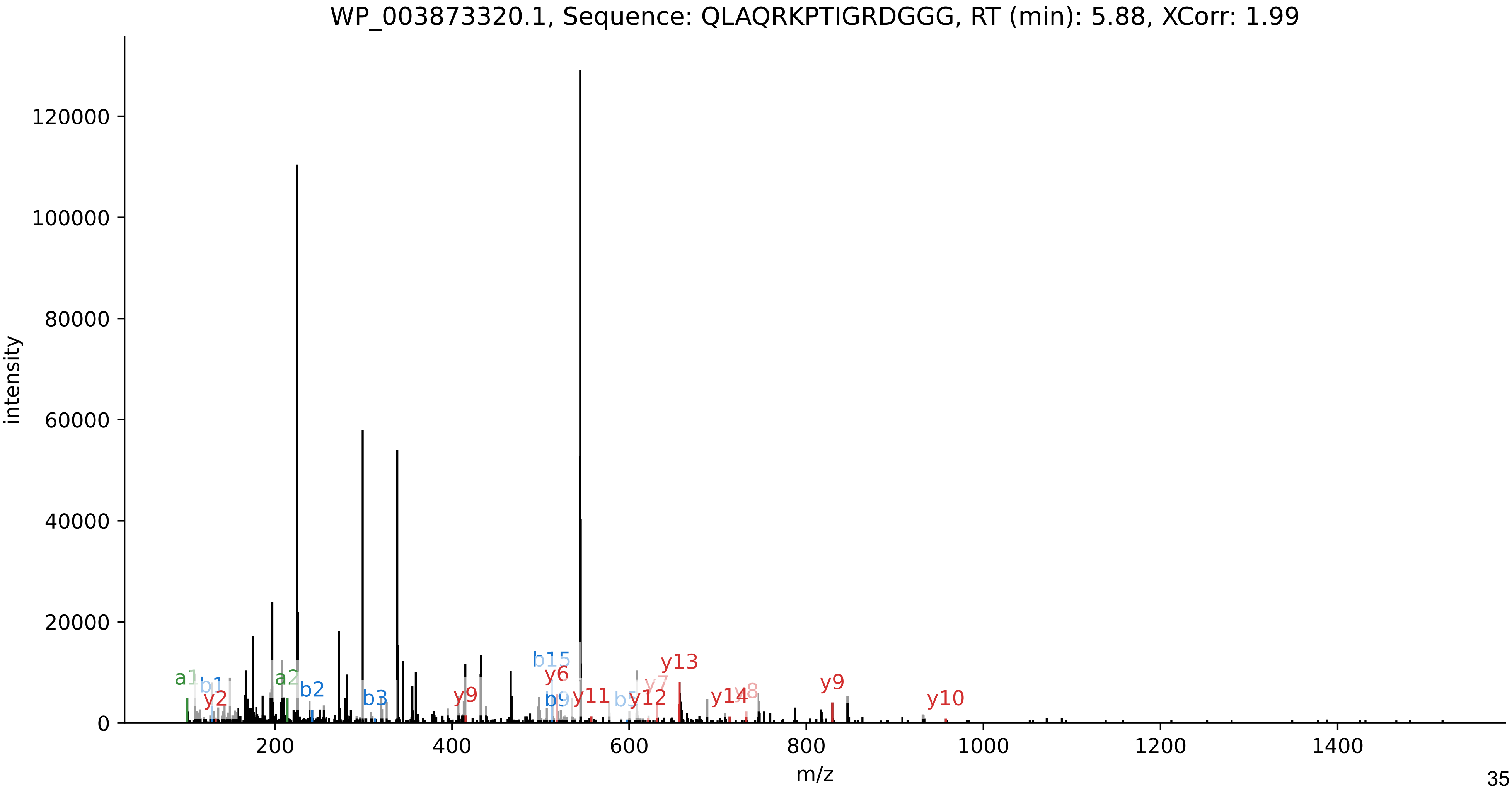

WP_003873320.1, Sequence: QLAQRKPTIGRDGGG, RT (min): 5.88, XCorr: 1.99
intensity
m/z
0
20000
40000
60000
80000
100000
120000
200
400
600
800
1000
1200
1400
a1
b1
y2
a2
b2
b3
y9
b15
y6
b9
y11
y12
y7
y13
y14
y8
y9
y10

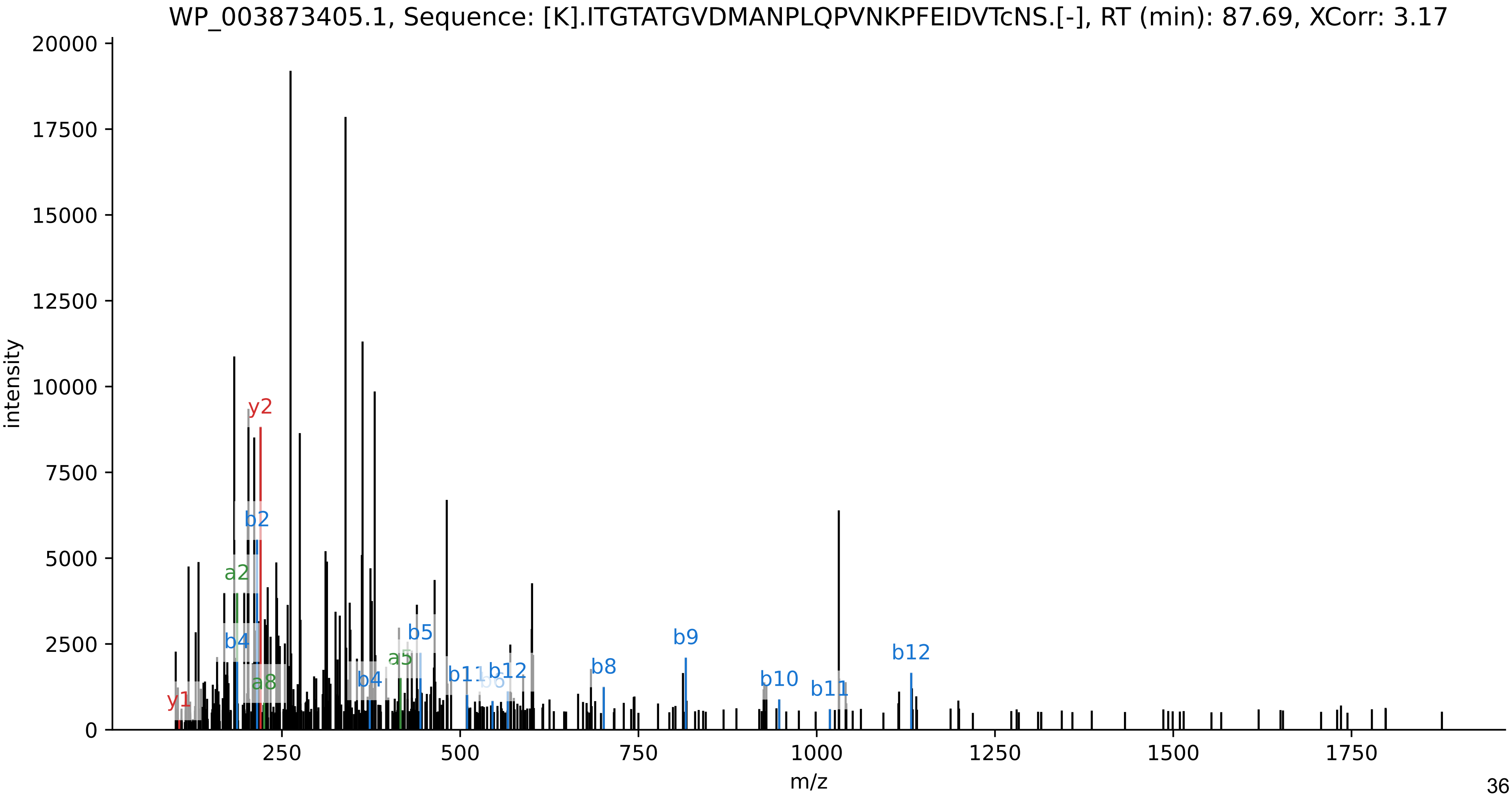
WP_003873405.1, Sequence: [K].ITGTATGVDMANPLQPVNKPFEIDVTcNS.[-], RT (min): 87.69, XCorr: 3.17
intensity
m/z
0
2500
5000
7500
10000
12500
15000
17500
20000
250
500
750
1000
1250
1500
1750
y1
y2
a2
a5
a8
b2
b4
b5
b8
b9
b10
b11
b12

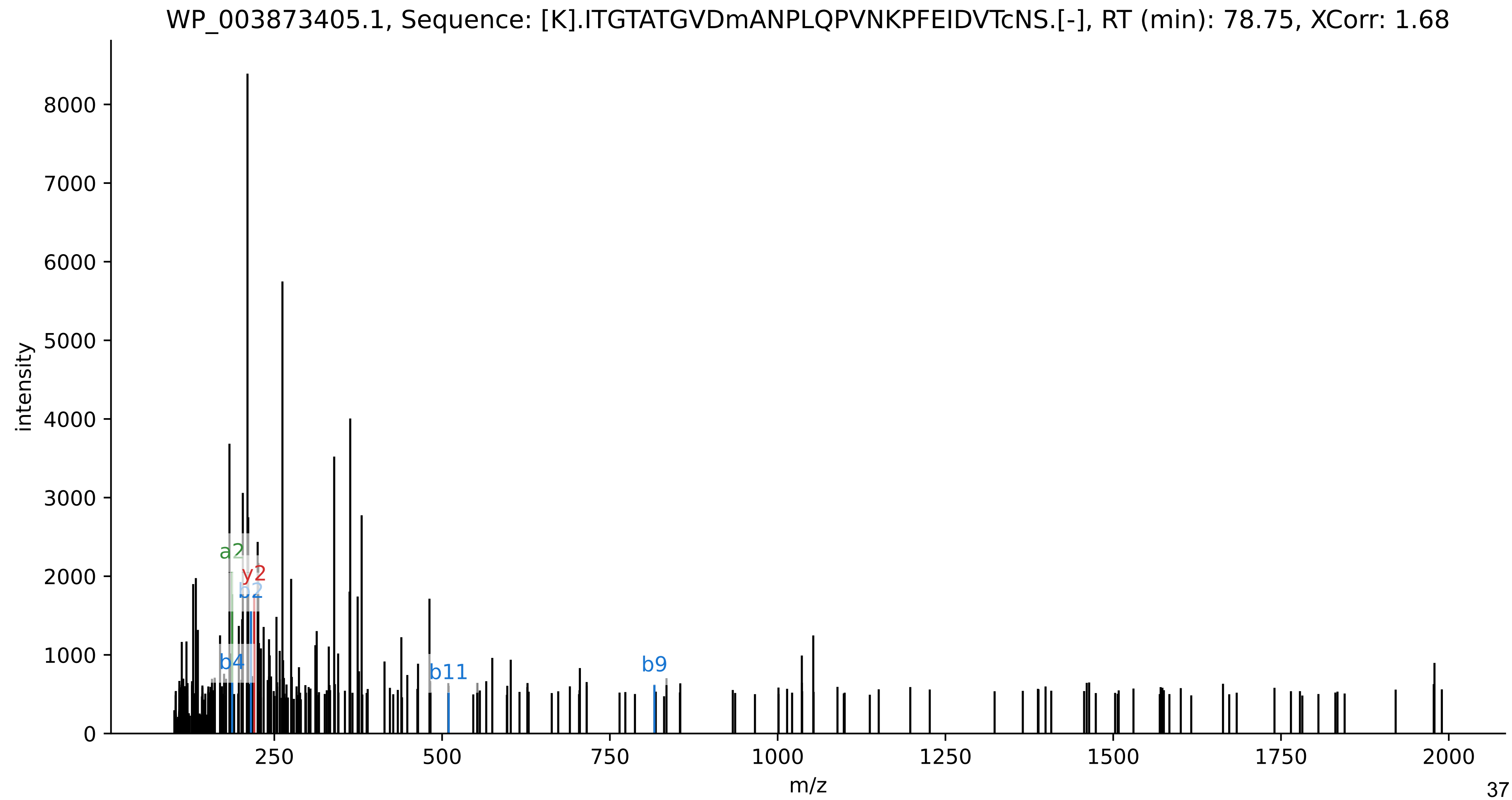
WP_003873405.1, Sequence: [K].ITGTATGVDmANPLQPVNKPFEIDVTcNS.[-], RT (min): 78.75, XCorr: 1.68
intensity
m/z
a2
y2
b2
b4
b11
b9
0
1000
2000
3000
4000
5000
6000
7000
8000
250
500
750
1000
1250
1500
1750
2000

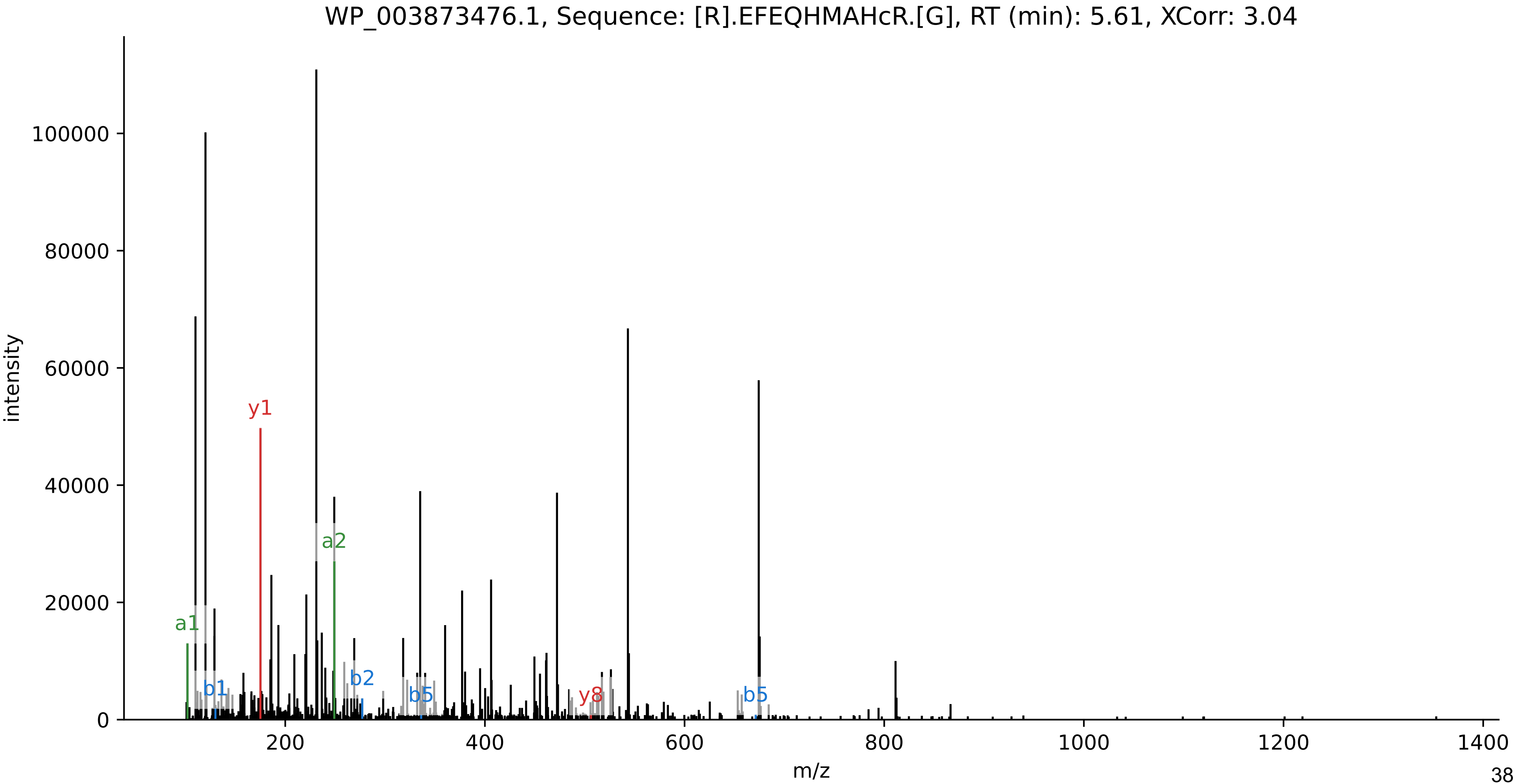
WP_003873476.1, Sequence: [R].EFEQHMAHcR.[G], RT (min): 5.61, XCorr: 3.04
intensity
m/z
0
20000
40000
60000
80000
100000
200
400
600
800
1000
1200
1400
y1
a1
a2
b1
b2
b5
y8
b5

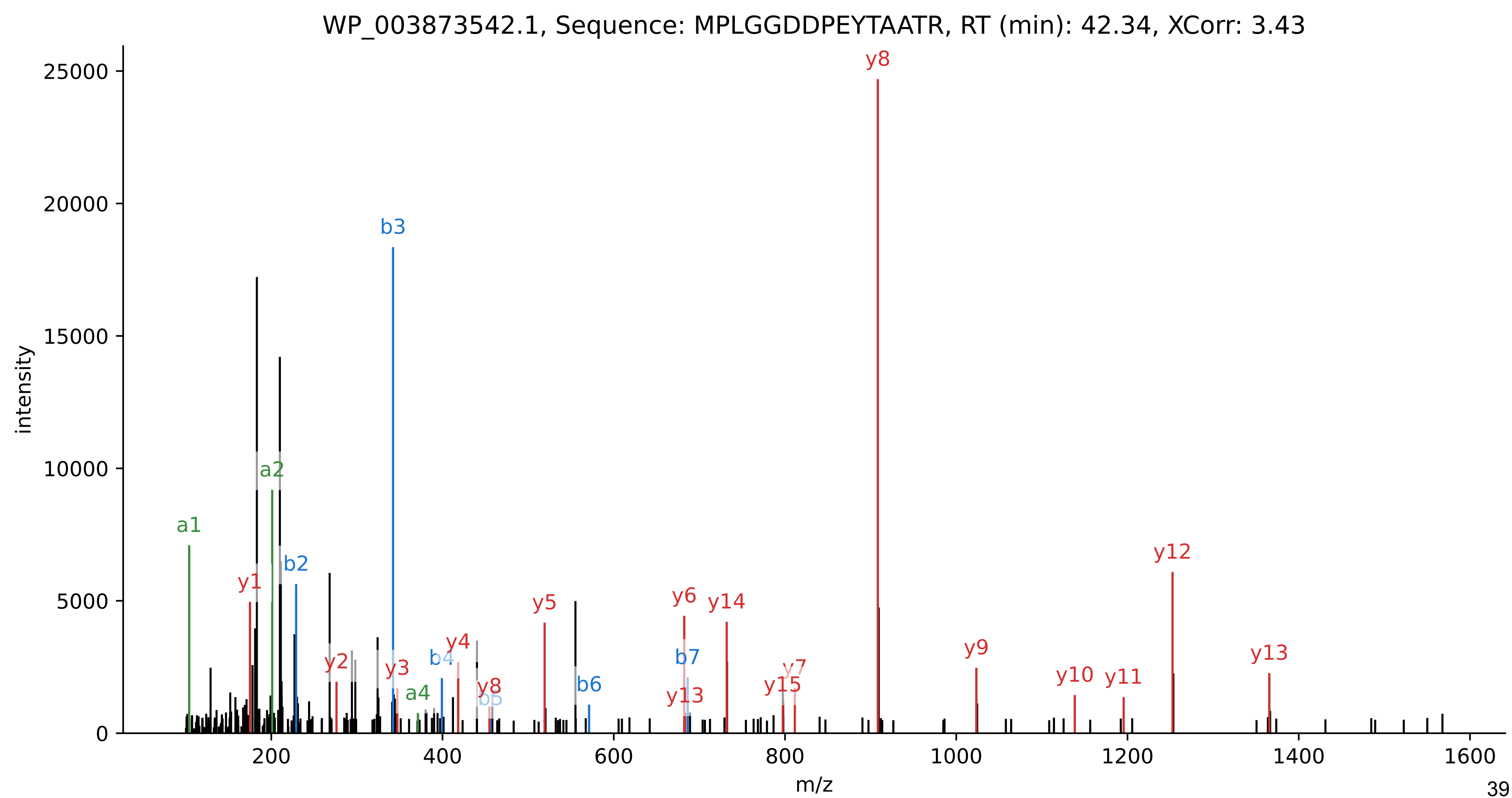

WP_003873542.1, Sequence: MPLGGDDPEYTAATR, RT (min): 42.34, XCorr: 3.43
intensity
m/z
y8
b3
a2
a1
b2
y1
y12
y5
y6
y14
y4
b4
y2
y3
a4
y8
b5
b6
b7
y13
y15
y7
y9
y10
y11
y13

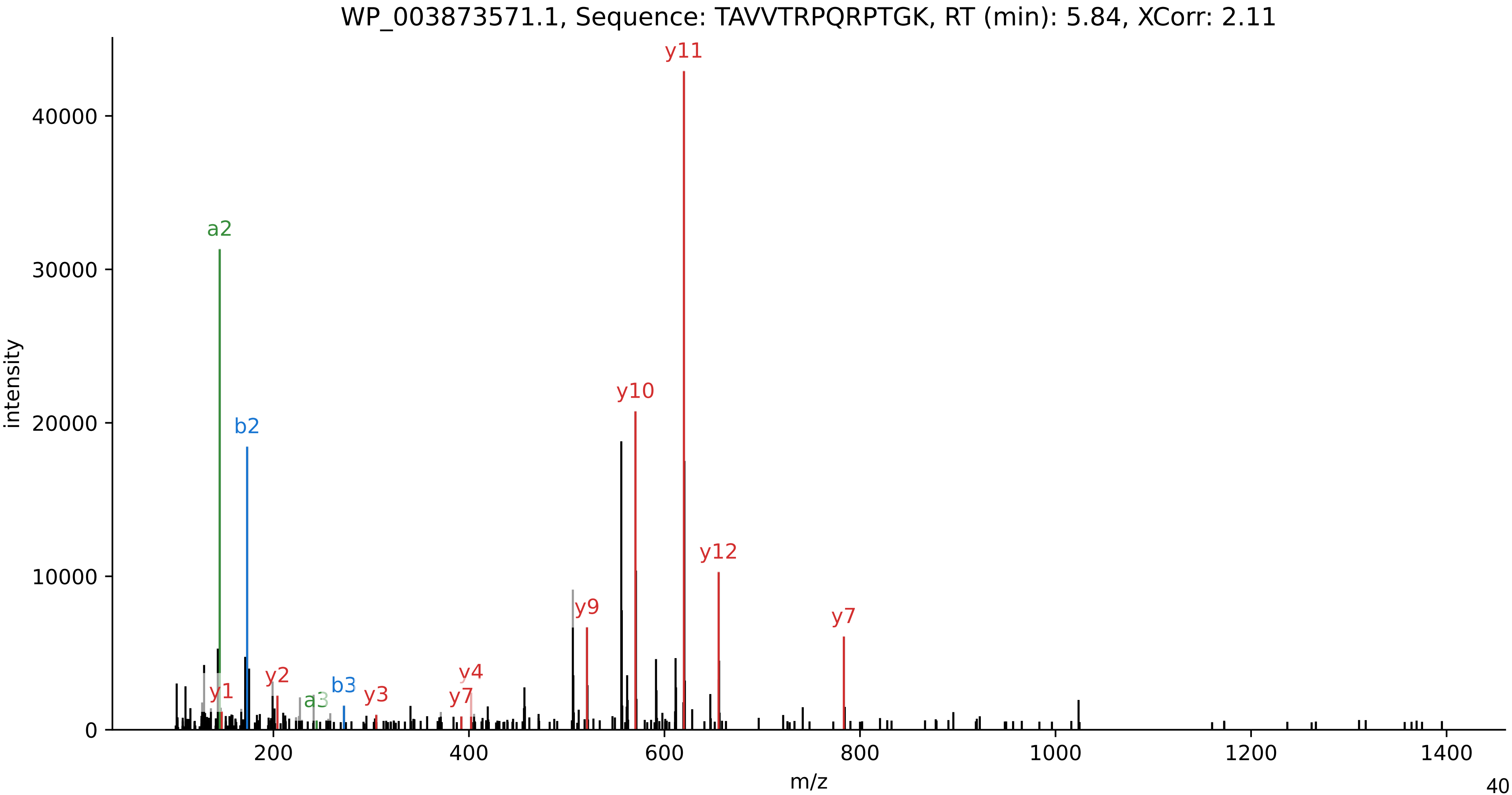
WP_003873571.1, Sequence: TAVVTRPQRPTGK, RT (min): 5.84, XCorr: 2.11
intensity
m/z
y11
a2
y10
b2
y12
y9
y7
y4
y2
y1
a3
b3
y3
y7
0
10000
20000
30000
40000
200
400
600
800
1000
1200
1400

WP_003873622.1, Sequence: RDEPQVEAEMPGAAA, RT (min): 39.24, XCorr: 2.93

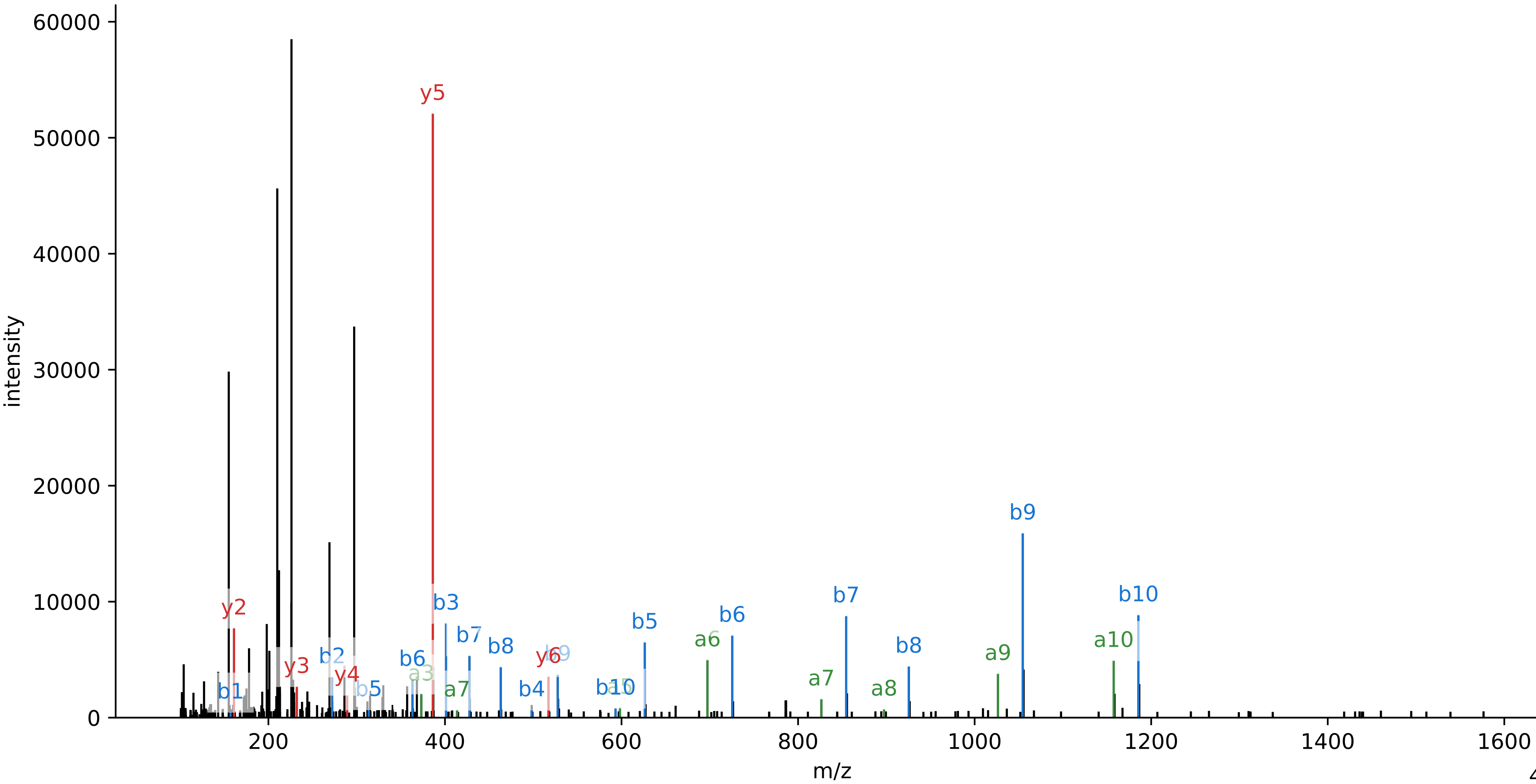

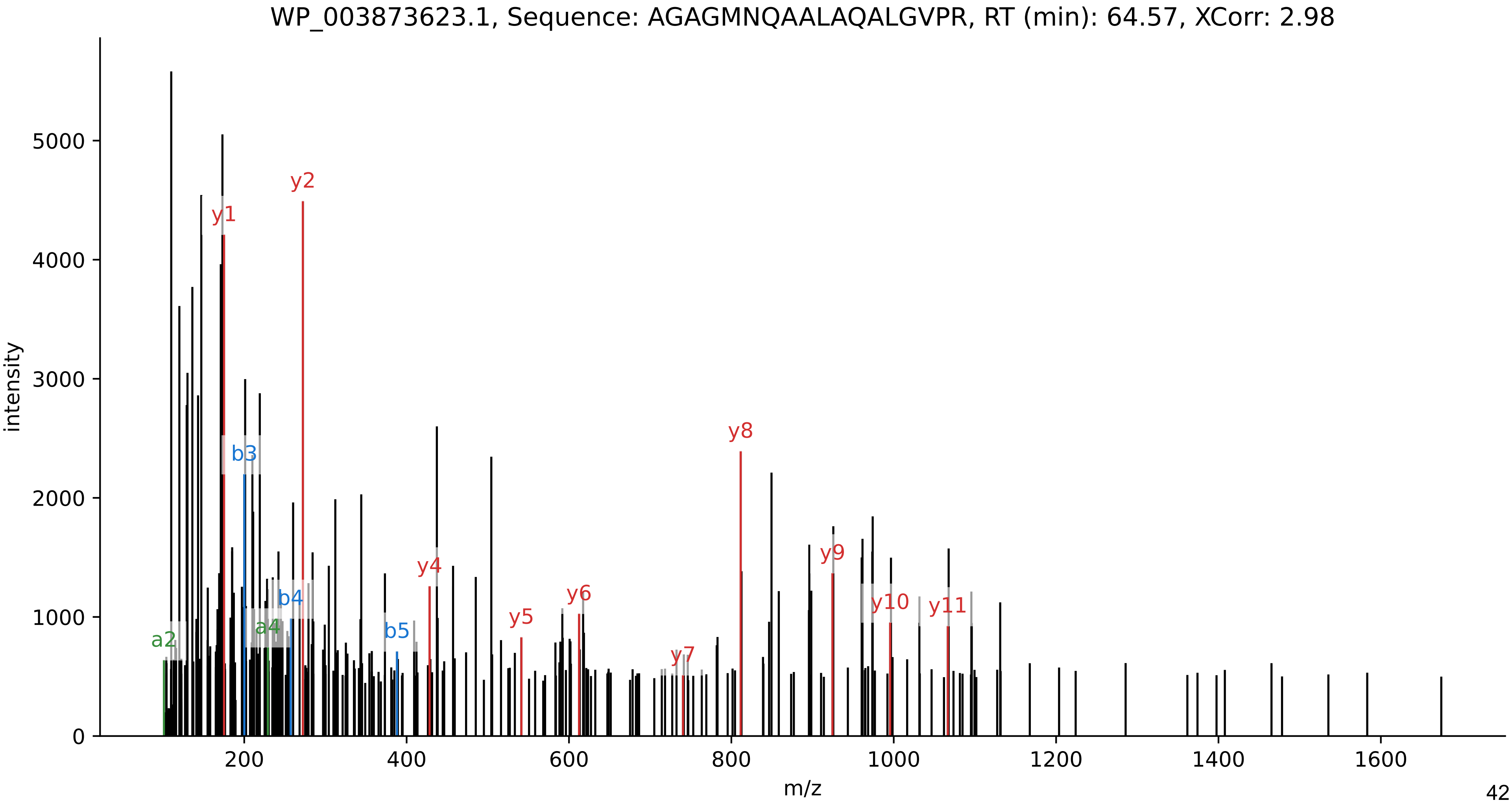
WP_003873623.1, Sequence: AGAGMNQAALAQALGVPR, RT (min): 64.57, XCorr: 2.98
intensity
m/z
y1
y2
b3
b4
a2
a4
b5
y4
y5
y6
y7
y8
y9
y10
y11
0
1000
2000
3000
4000
5000
200
400
600
800
1000
1200
1400
1600

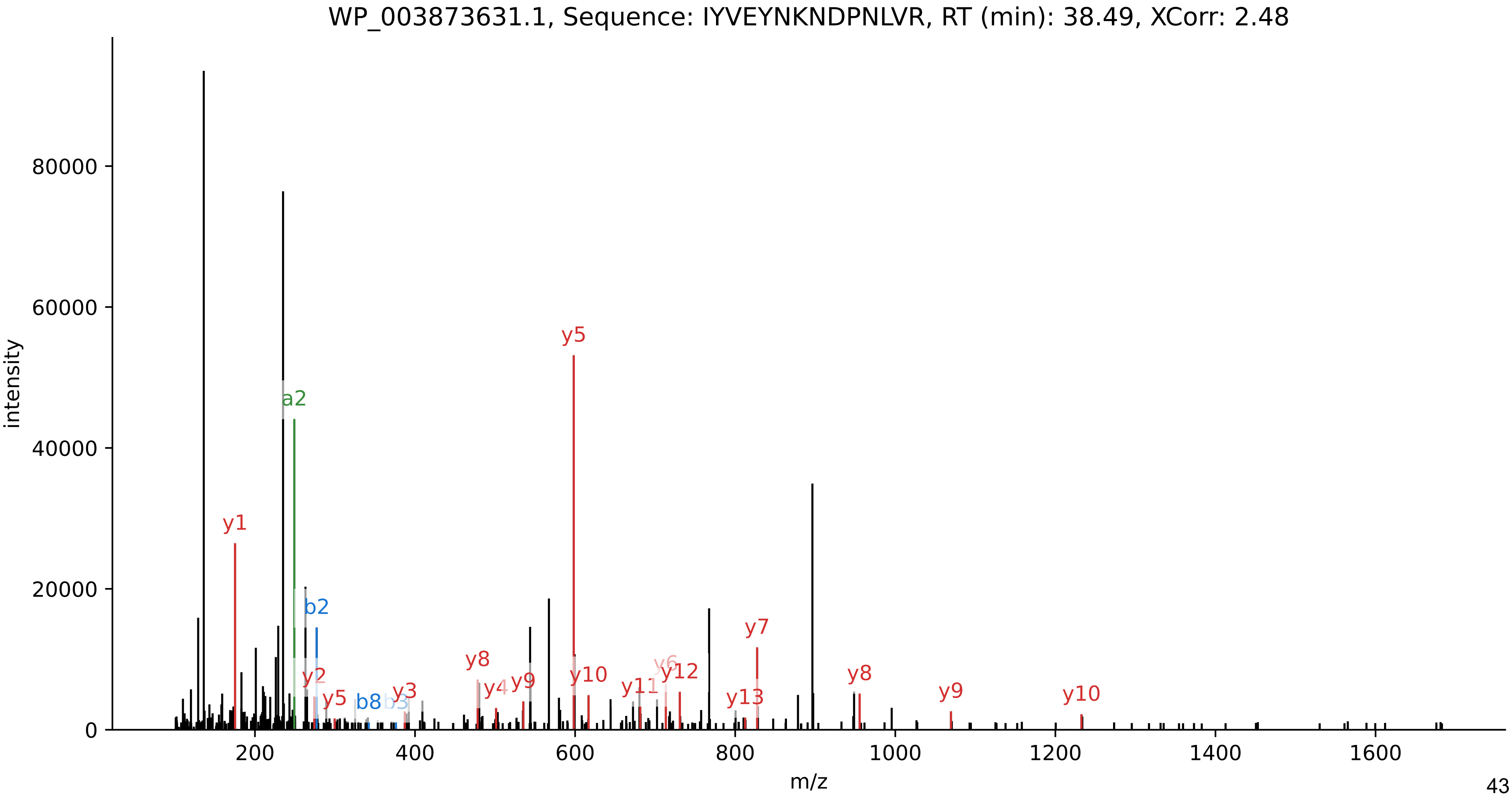

WP_003873631.1, Sequence: IYVEYNKNDPNLVR, RT (min): 38.49, XCorr: 2.48
intensity
m/z
80000
60000
40000
20000
0
200
400
600
800
1000
1200
1400
1600
y1
a2
b2
y2
y5
b8
b3
y3
y8
y4
y9
y5
y10
y11
y6
y12
y13
y7
y8
y9
y10

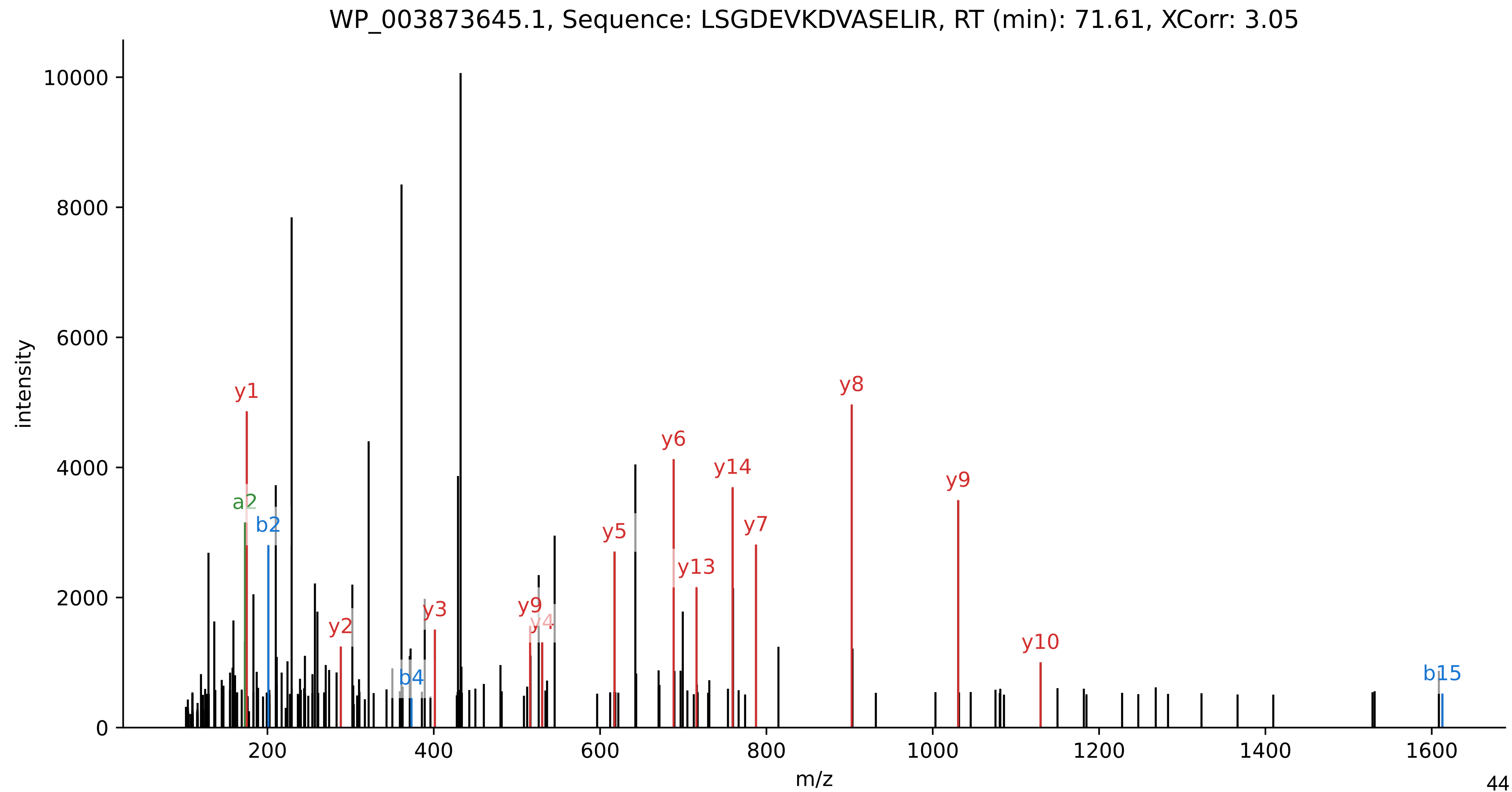
WP_003873645.1, Sequence: LSGDEVKDVASELIR, RT (min): 71.61, XCorr: 3.05
intensity
m/z
y1
a2
b2
y2
b4
y3
y9
y4
y5
y6
y13
y14
y7
y8
y9
y10
b15

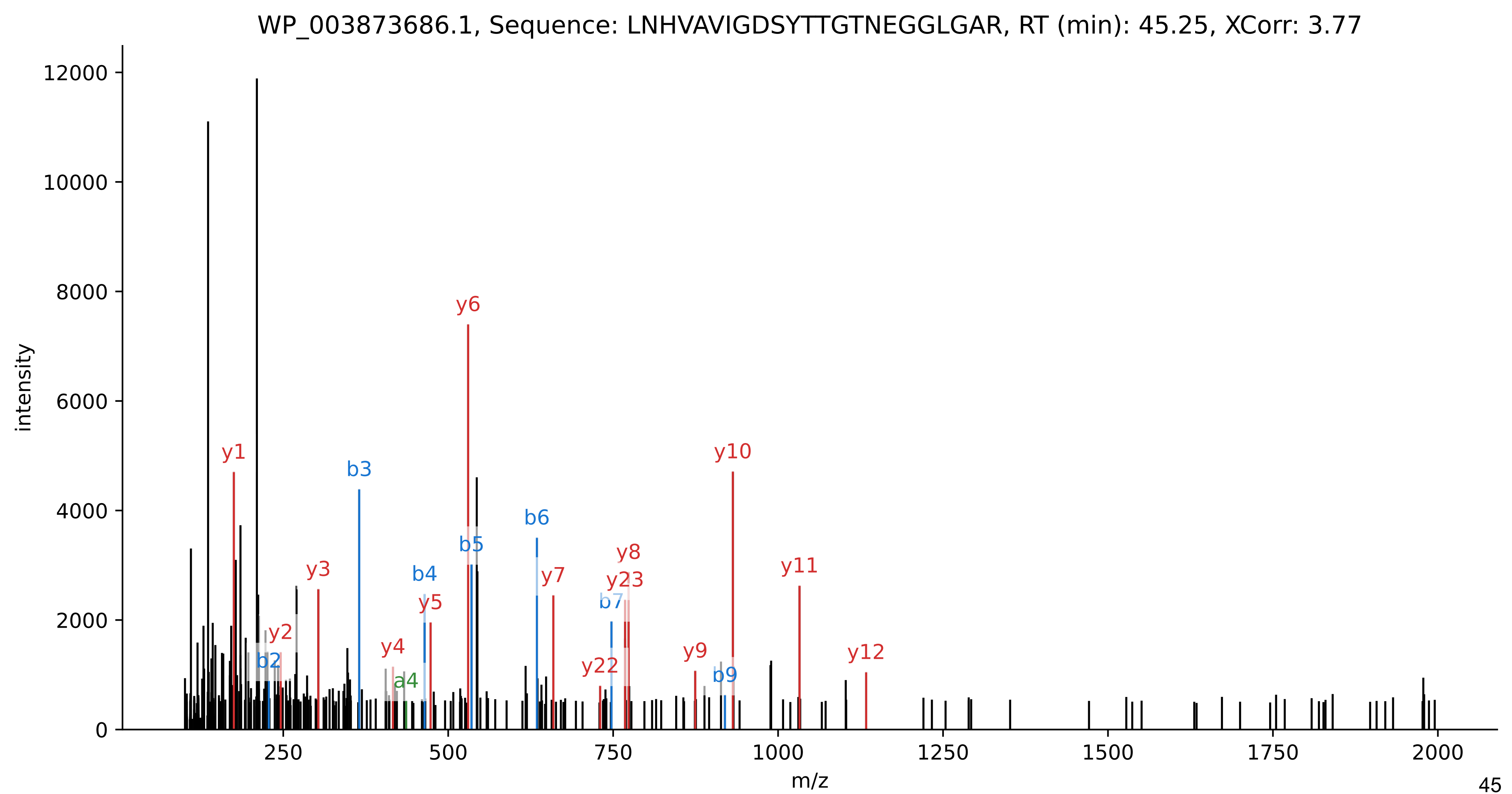
WP_003873686.1, Sequence: LNHVAVIGDSYTTGTNEGGLGAR, RT (min): 45.25, XCorr: 3.77
intensity
m/z
0
2000
4000
6000
8000
10000
12000
250
500
750
1000
1250
1500
1750
2000
y1
y2
y3
y4
y5
y6
y7
y8
y9
y10
y11
y12
y22
y23
b2
b3
b4
b5
b6
b7
b9
a4

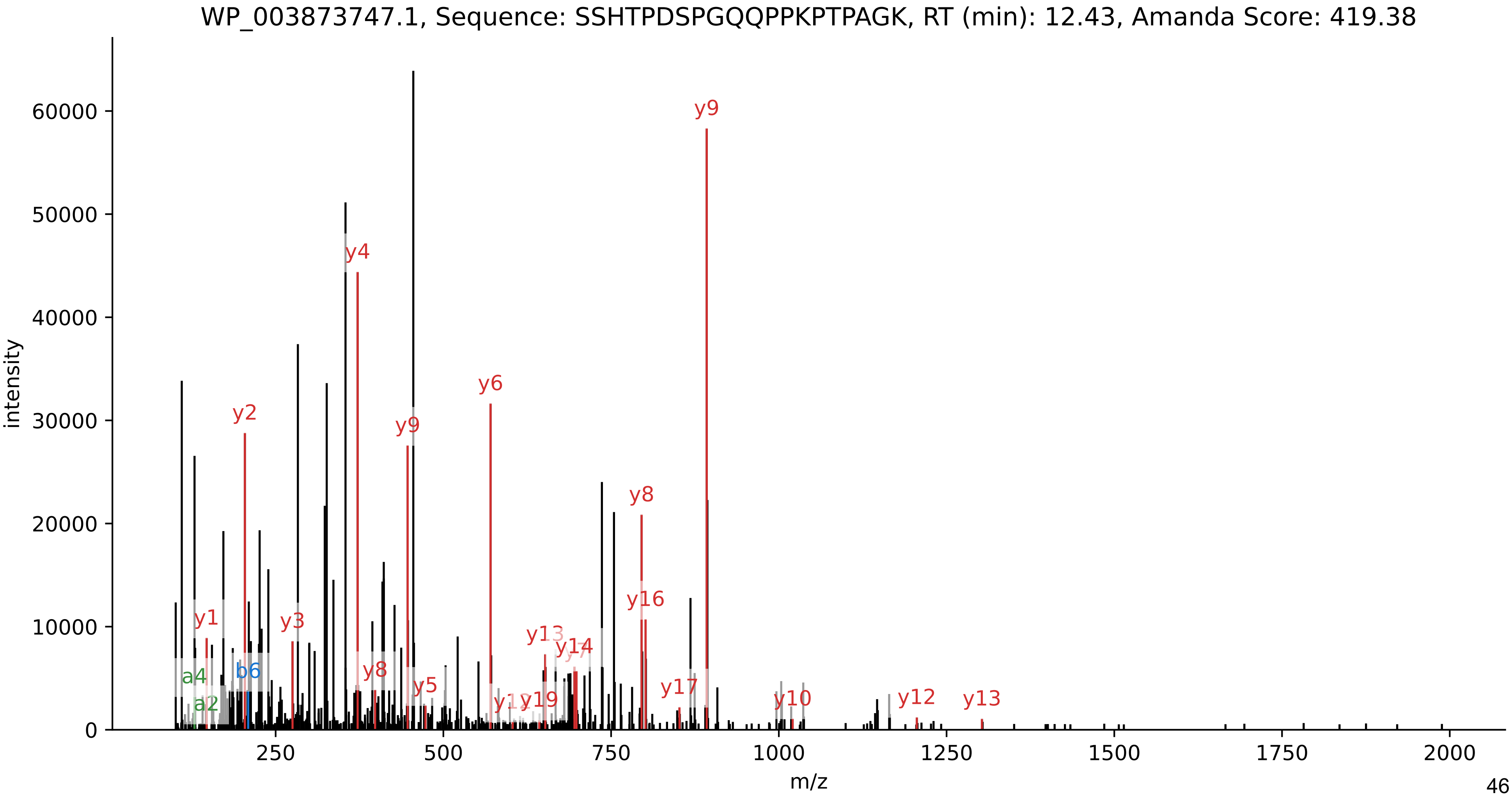
WP_003873747.1, Sequence: SSHTPDSPGQQPPKPTPAGK, RT (min): 12.43, Amanda Score: 419.38
intensity
m/z
y9
y4
y6
y9
y2
y8
y16
y1
y3
y13
y14
y8
b6
a4
y5
y17
a2
y19
y10
y12
y13
0
10000
20000
30000
40000
50000
60000
250
500
750
1000
1250
1500
1750
2000

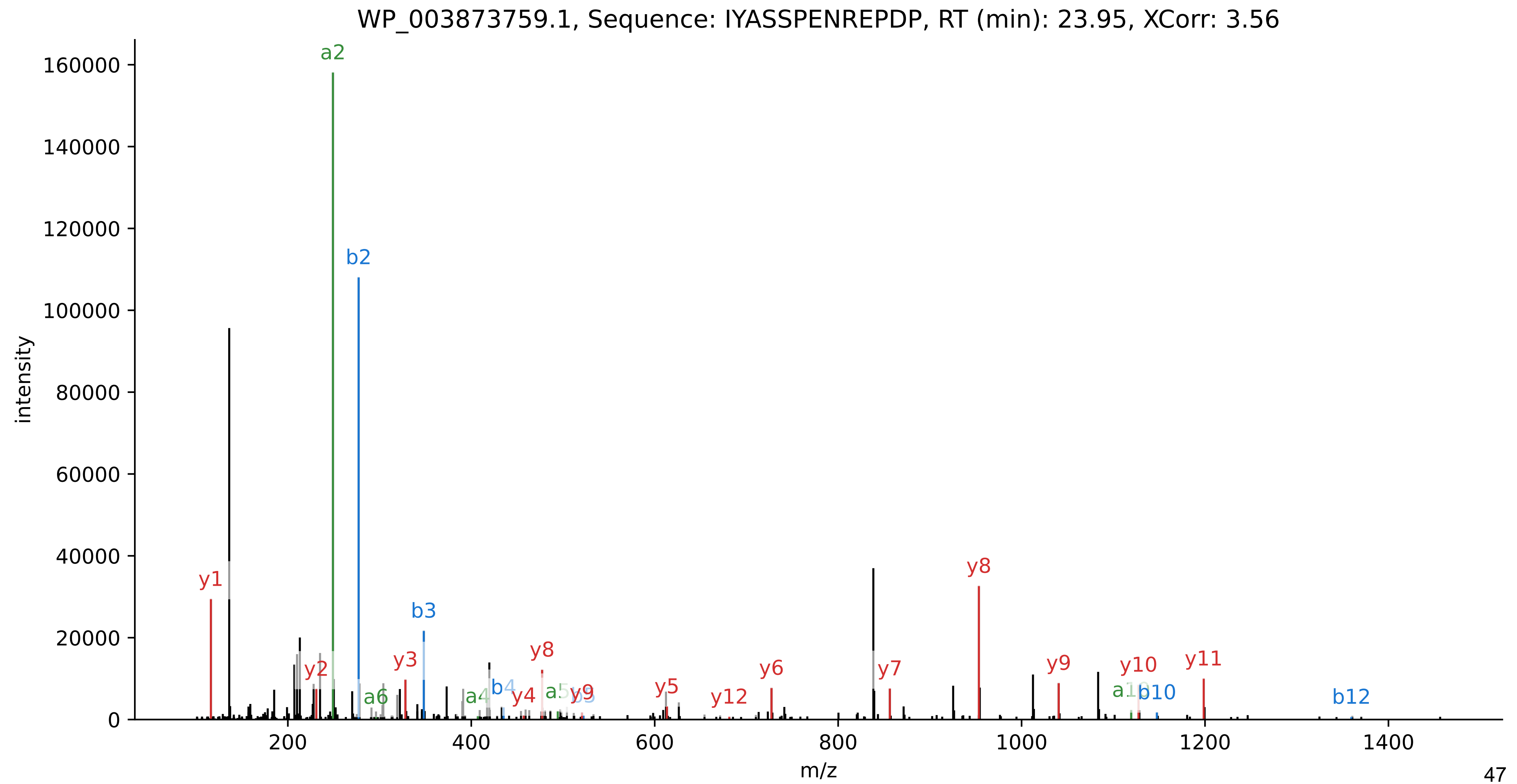

WP_003873759.1, Sequence: IYASSPENREPDP, RT (min): 23.95, XCorr: 3.56
intensity
m/z
a2
b2
y1
b3
y2
y3
y8
a6
a4
b4
y4
y9
y5
y12
y6
y7
y8
y9
y10
y11
b10
b12

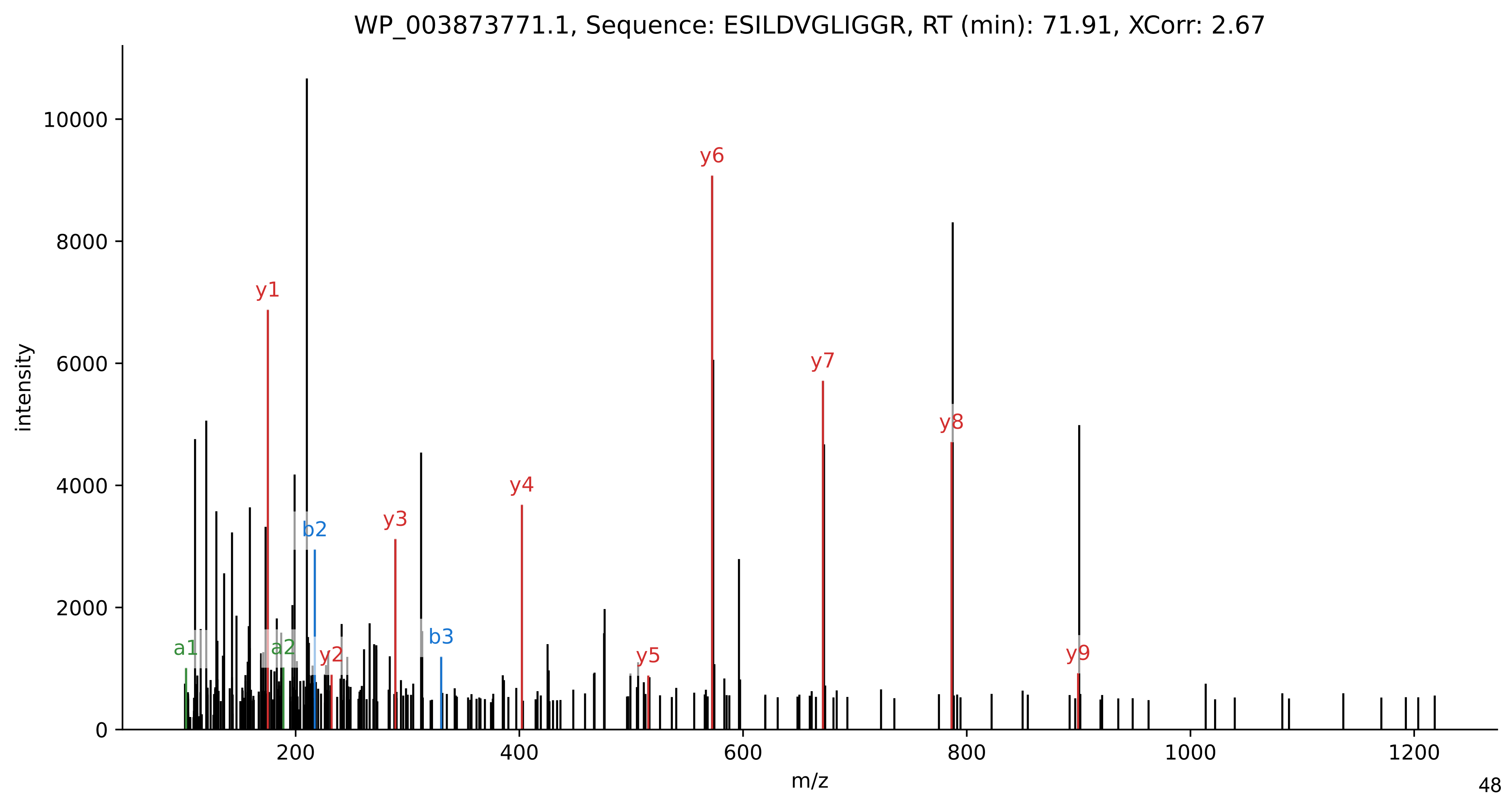
WP_003873771.1, Sequence: ESILDVGLIGGR, RT (min): 71.91, XCorr: 2.67
intensity
m/z
0
2000
4000
6000
8000
10000
200
400
600
800
1000
1200
y1
y2
y3
y4
y5
y6
y7
y8
y9
b2
b3
a1
a2

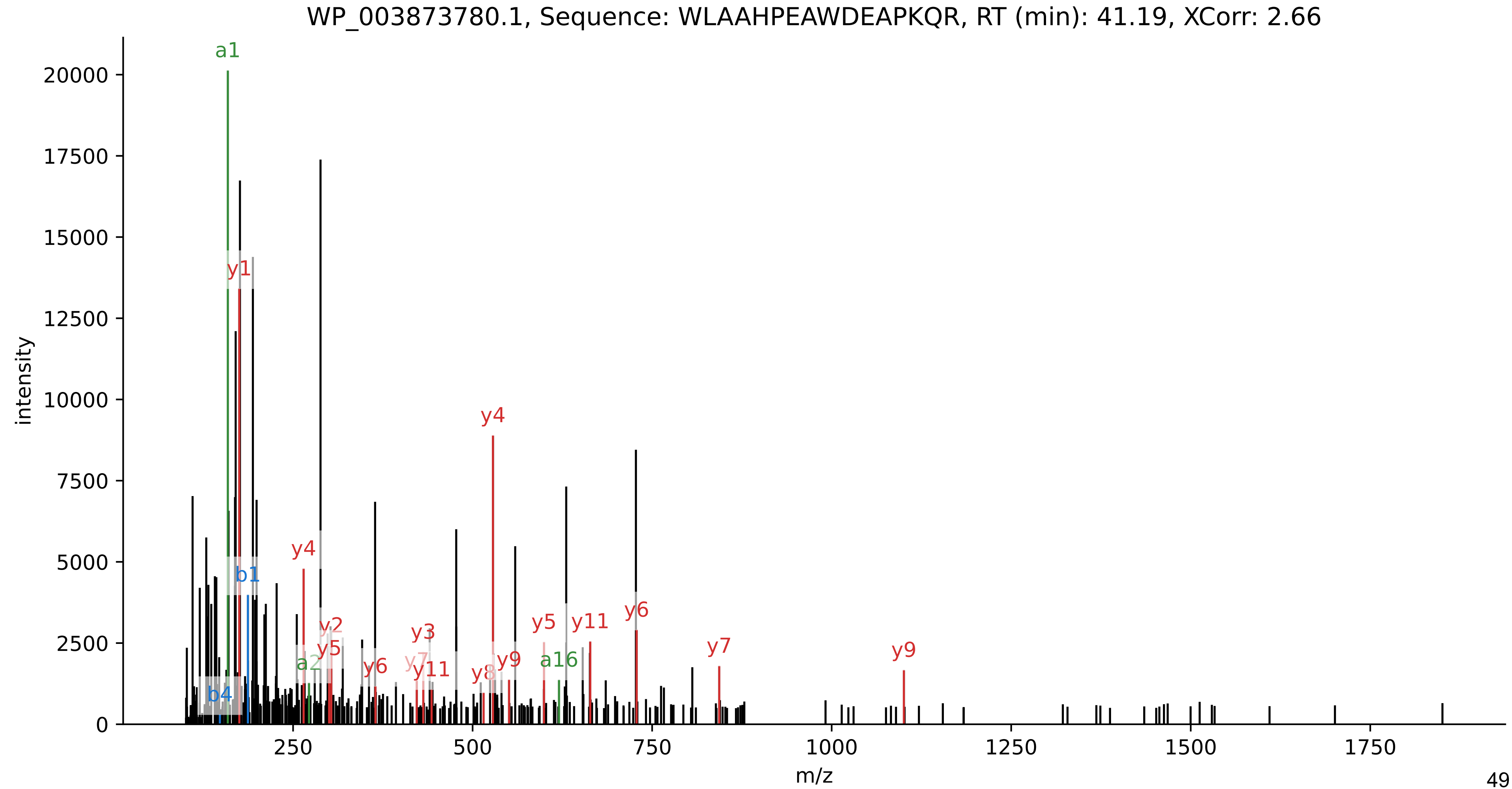
WP_003873780.1, Sequence: WLAAHPEAWDEAPKQR, RT (min): 41.19, XCorr: 2.66
intensity
m/z
a1
y1
b1
y4
y2
y5
a2
b4
y6
y3
y7
y11
y8
y9
a16
y5
y11
y6
y7
y9
0
2500
5000
7500
10000
12500
15000
17500
20000
250
500
750
1000
1250
1500
1750

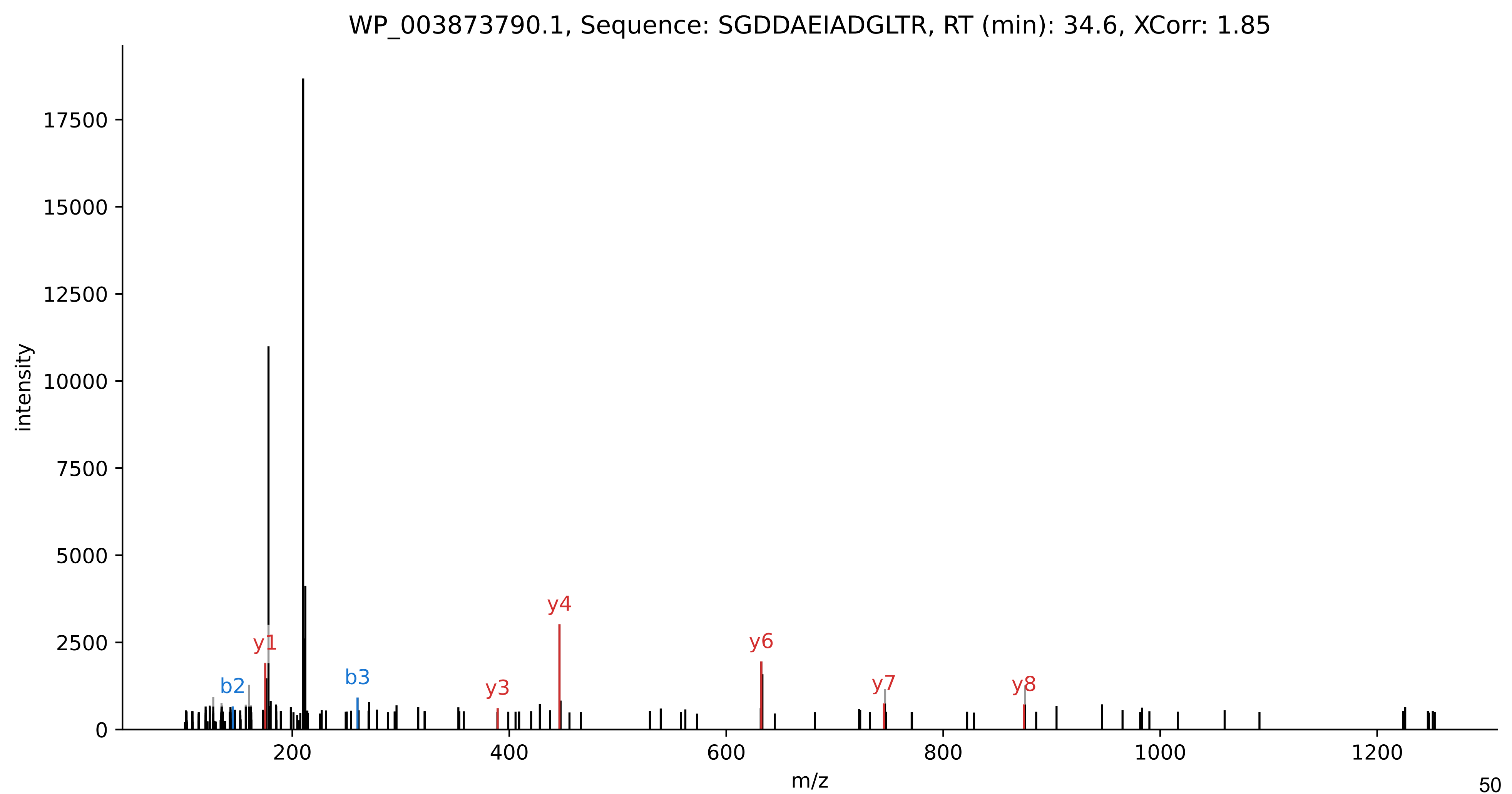
WP_003873790.1, Sequence: SGDDAEIADGLTR, RT (min): 34.6, XCorr: 1.85
intensity
m/z
b2
b3
y1
y3
y4
y6
y7
y8
0
2500
5000
7500
10000
12500
15000
17500
200
400
600
800
1000
1200

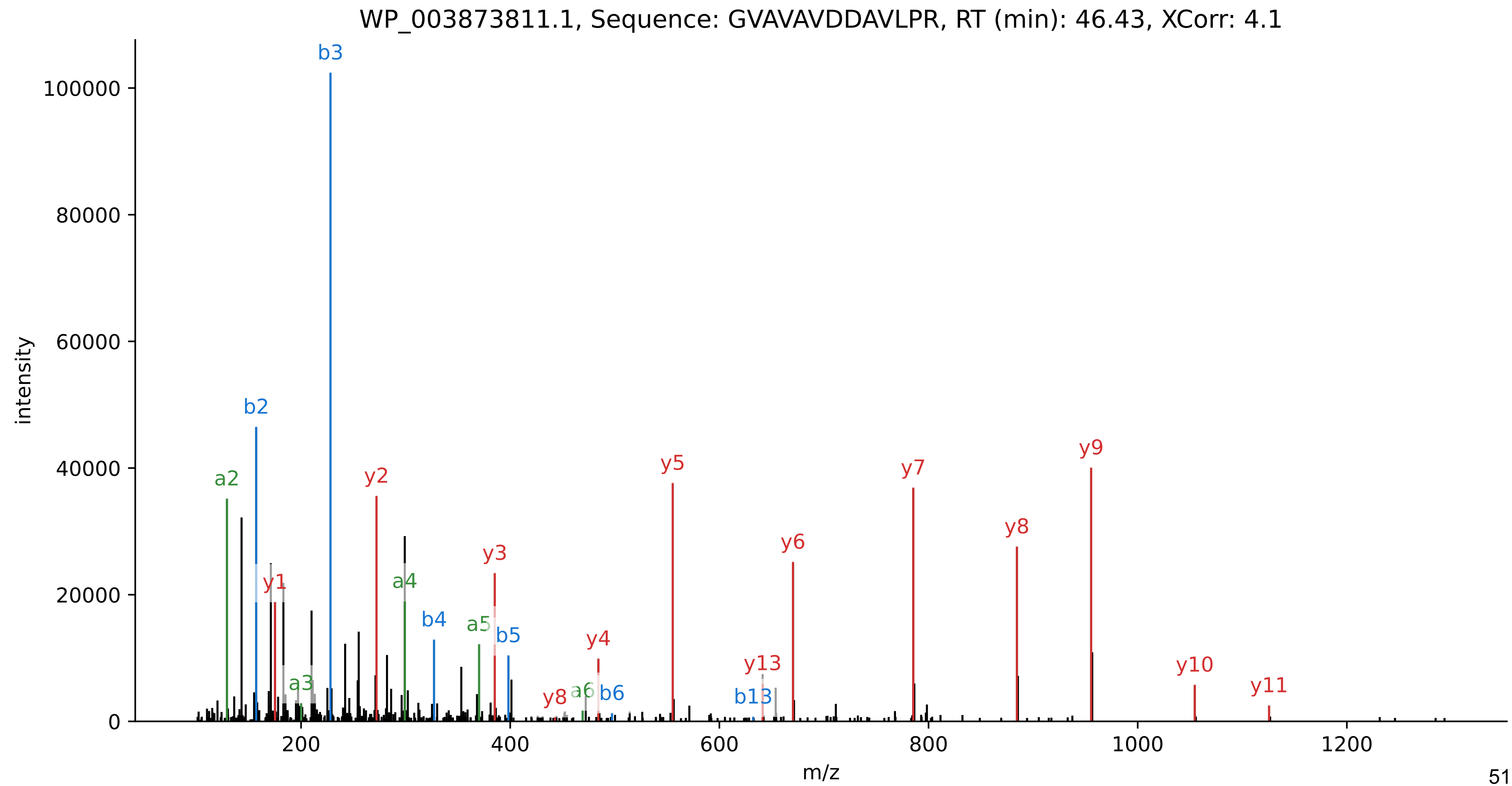
WP_003873811.1, Sequence: GVAVAVDDAVLPR, RT (min): 46.43, XCorr: 4.1
intensity
m/z
b3
b2
a2
y2
y1
a3
a4
b4
a5
b5
y3
y4
y8
a6
b6
y5
b13
y13
y6
y7
y8
y9
y10
y11

WP_003873848.1, Sequence: HTIGNFDAAIQR, RT (min): 33.06, XCorr: 3.25

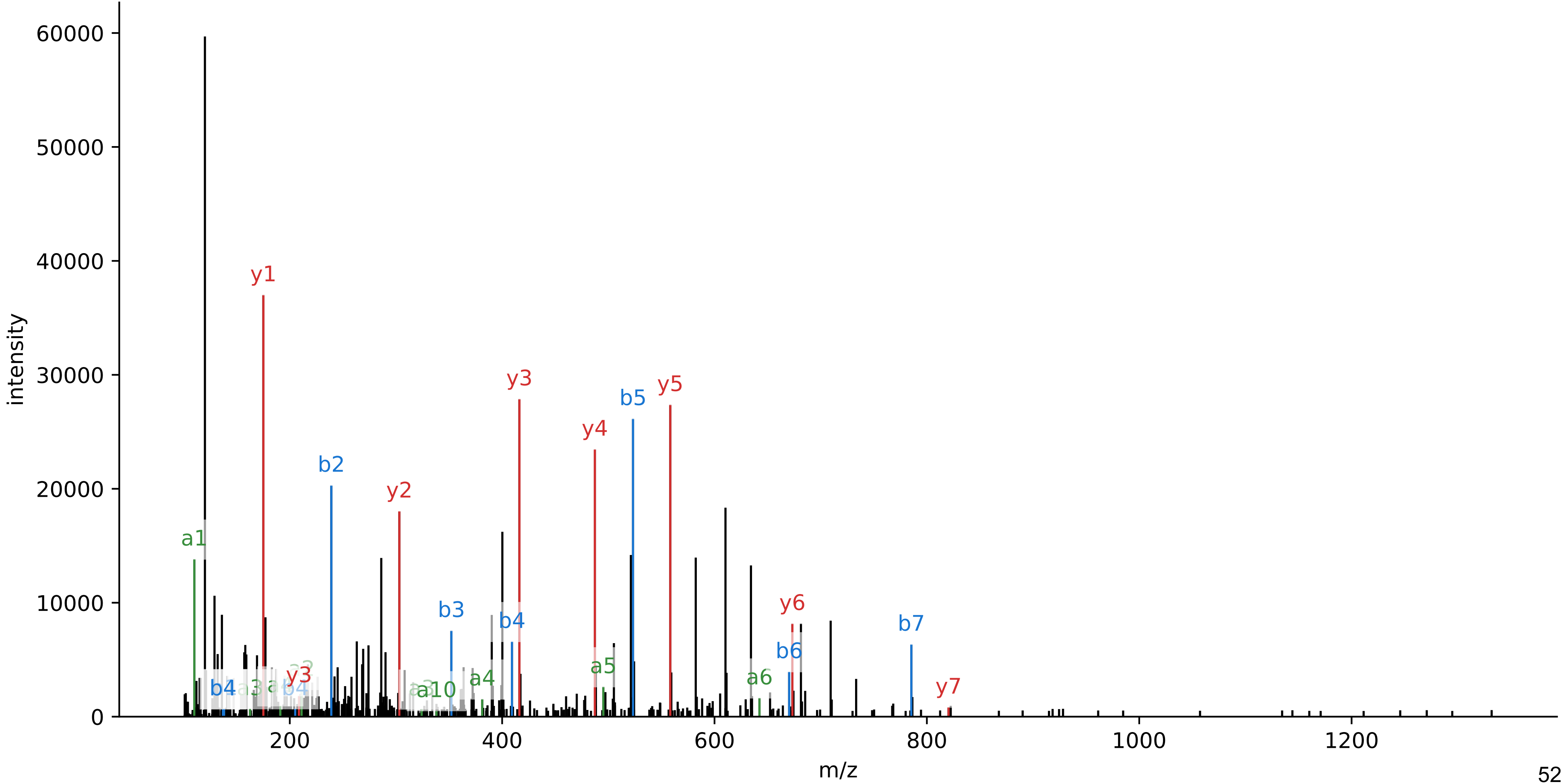

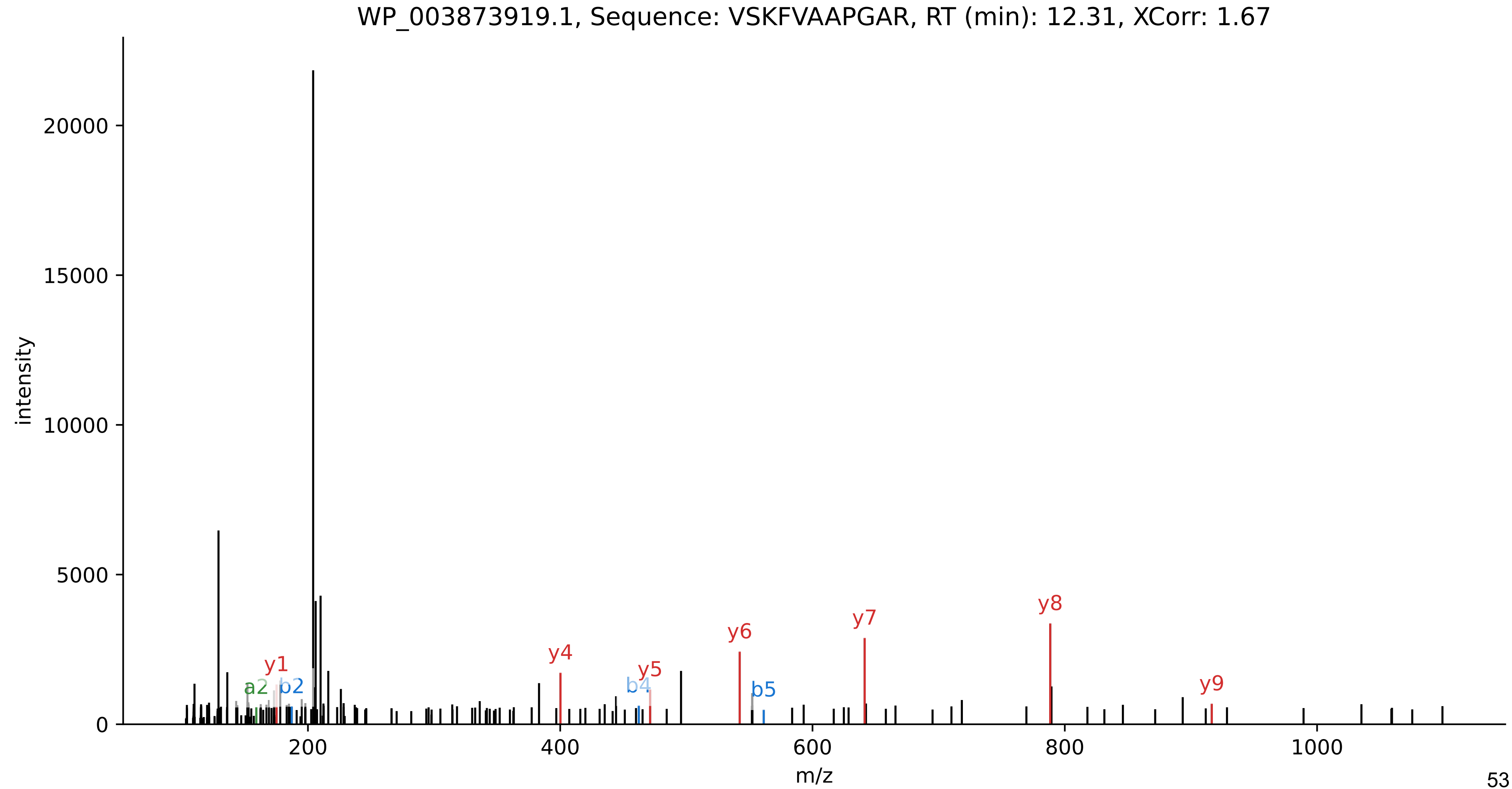
WP_003873919.1, Sequence: VSKFVAAPGAR, RT (min): 12.31, XCorr: 1.67
intensity
m/z
0
5000
10000
15000
20000
200
400
600
800
1000
y1
a2
b2
y4
b4
y5
y6
b5
y7
y8
y9

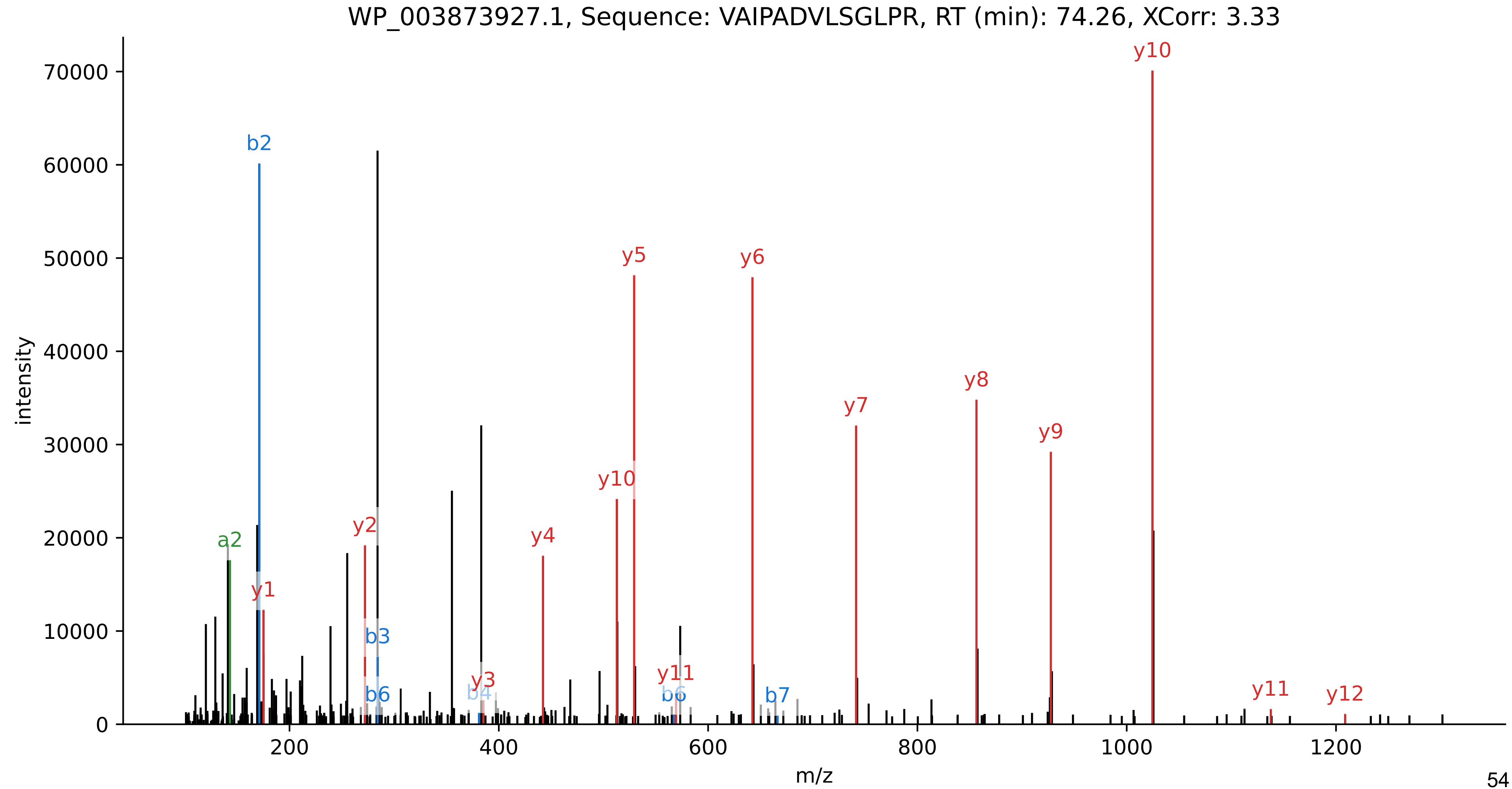
WP_003873927.1, Sequence: VAIPADVLSGLPR, RT (min): 74.26, XCorr: 3.33
intensity
m/z
b2
y10
y5
y6
y8
y7
y9
y10
y4
y2
a2
y1
b3
y3
b4
b6
y11
b6
b7
y11
y12

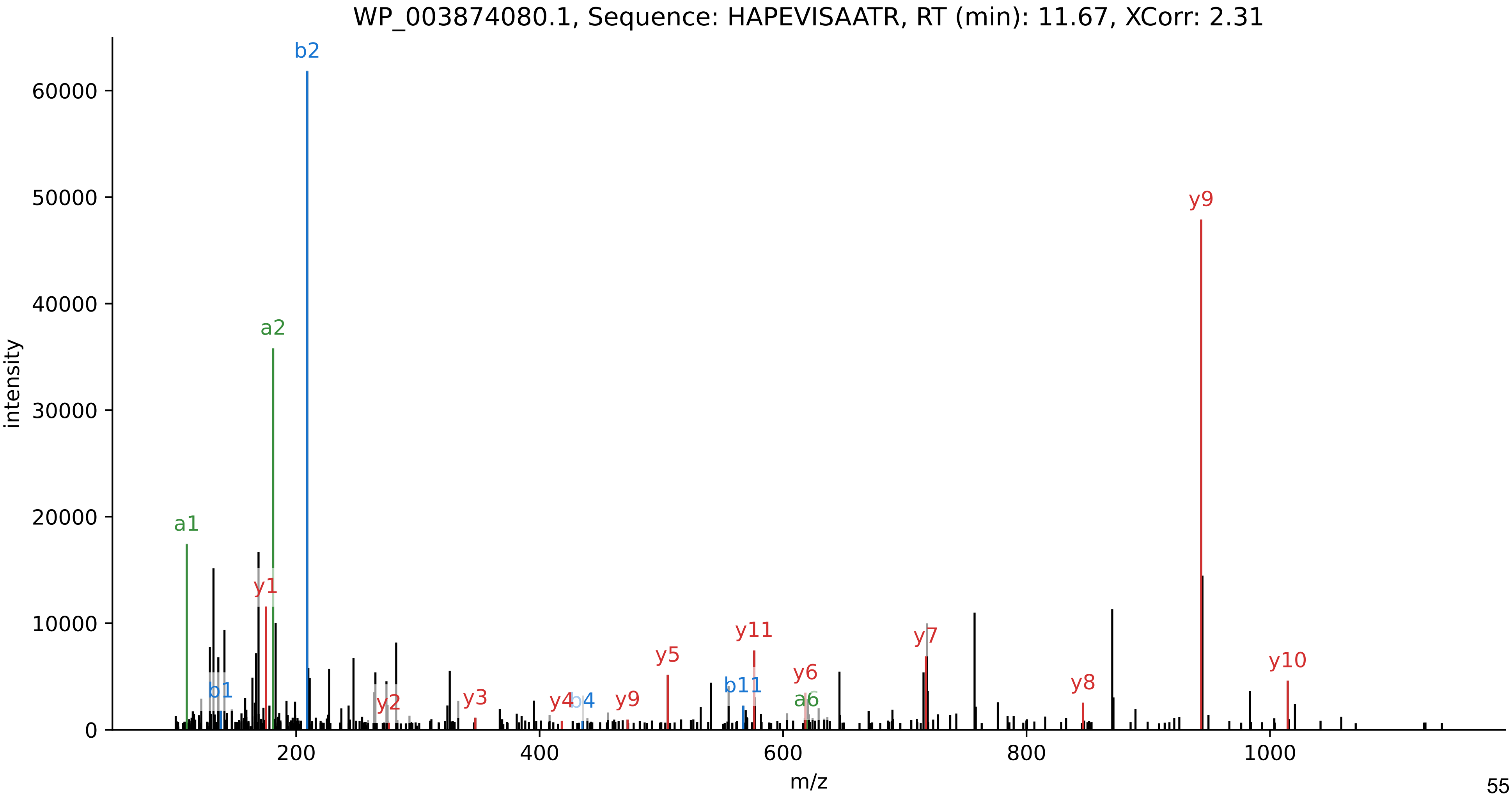
WP_003874080.1, Sequence: HAPEVISAATR, RT (min): 11.67, XCorr: 2.31
intensity
m/z
0
10000
20000
30000
40000
50000
60000
200
400
600
800
1000
b2
y9
a2
a1
y1
b1
y2
y3
y4
b4
y9
y5
b11
y11
y6
a6
y7
y8
y10

WP_003874106.1, Sequence: STVHSSIDHHPDLLALR, RT (min): 45.02, Amanda Score: 328.83

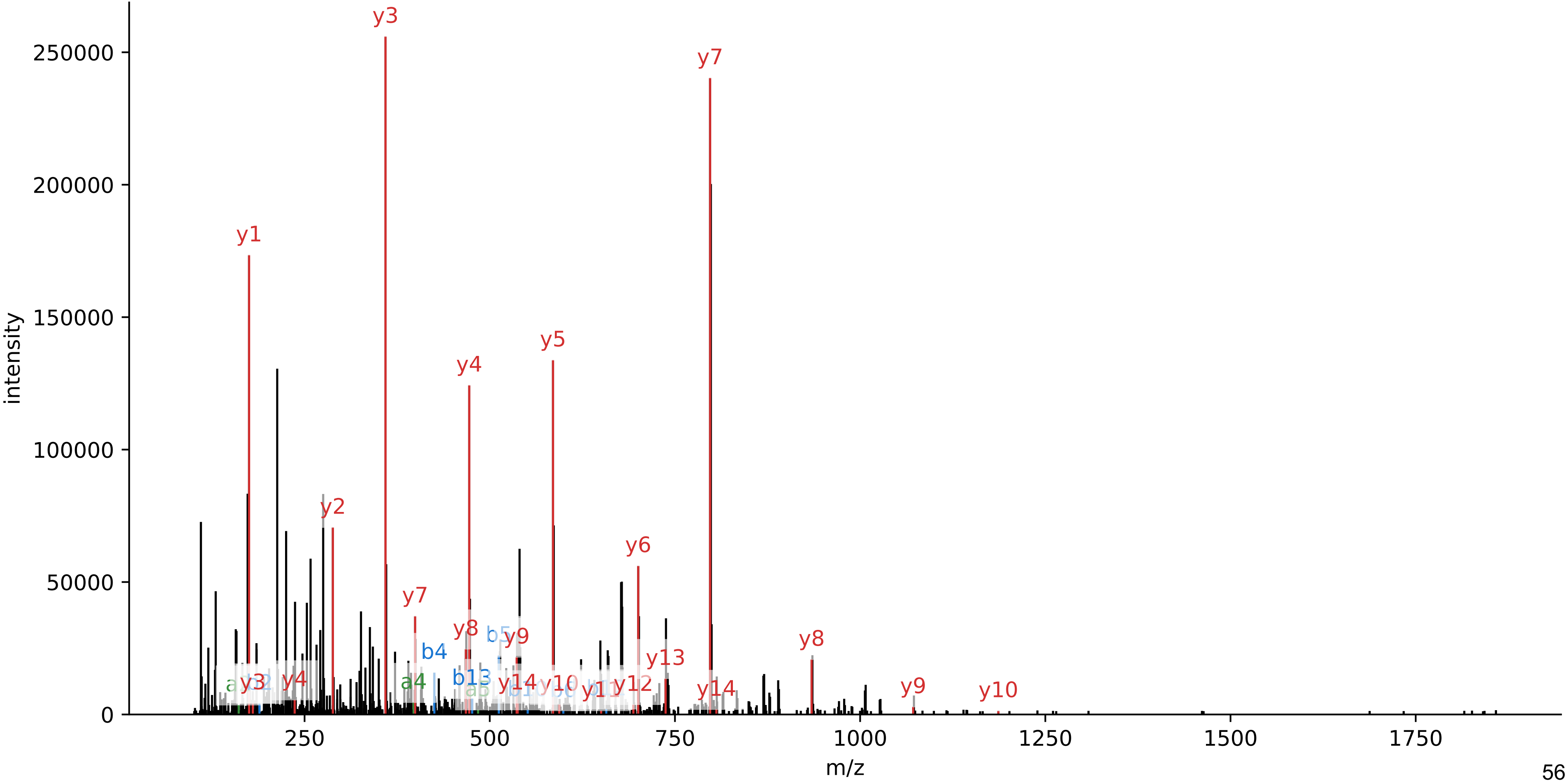

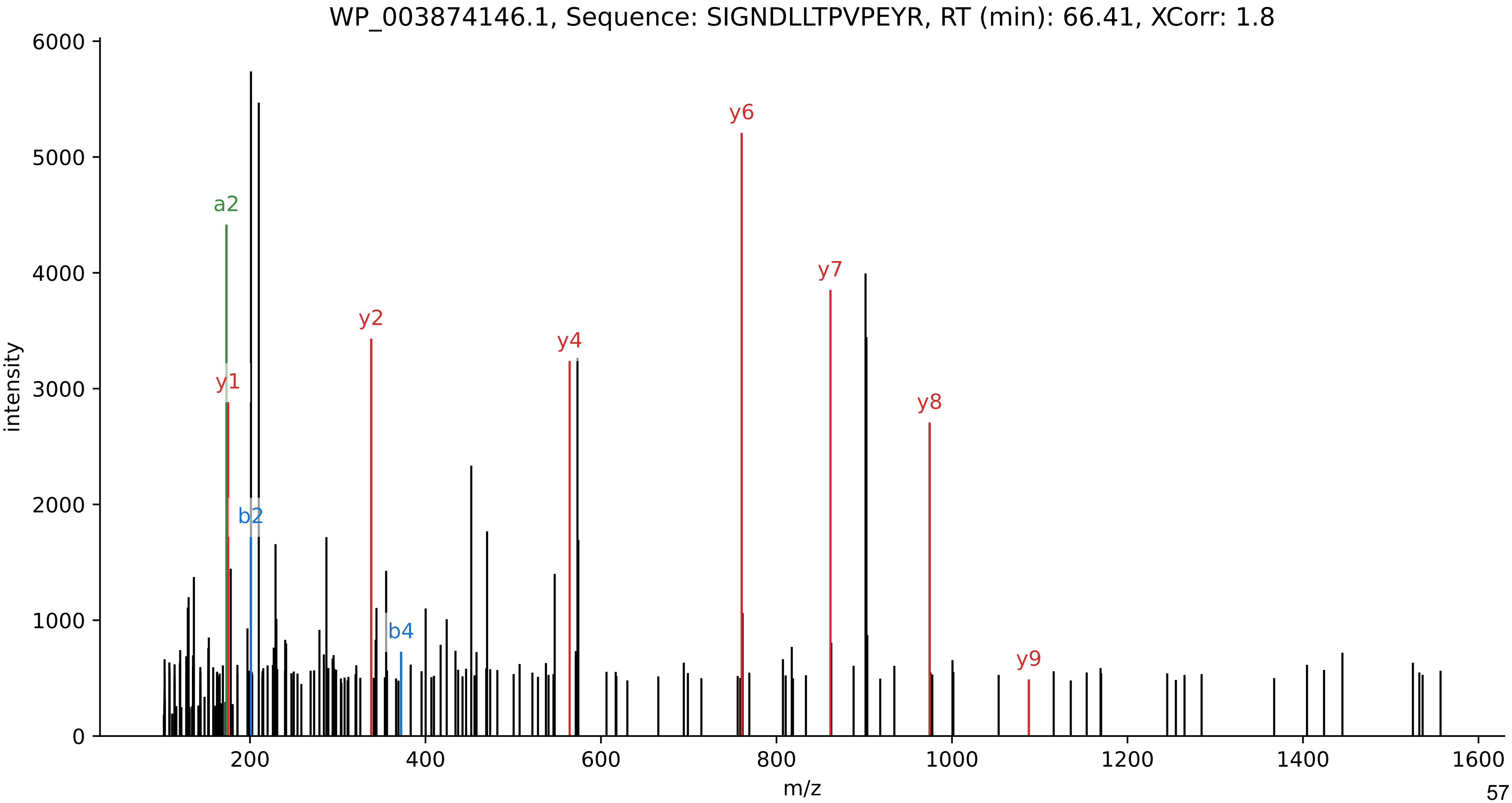
WP_003874146.1, Sequence: SIGNDLLTPVPEYR, RT (min): 66.41, XCorr: 1.8
a2
y1
b2
y2
b4
y4
y6
y7
y8
y9
intensity
m/z

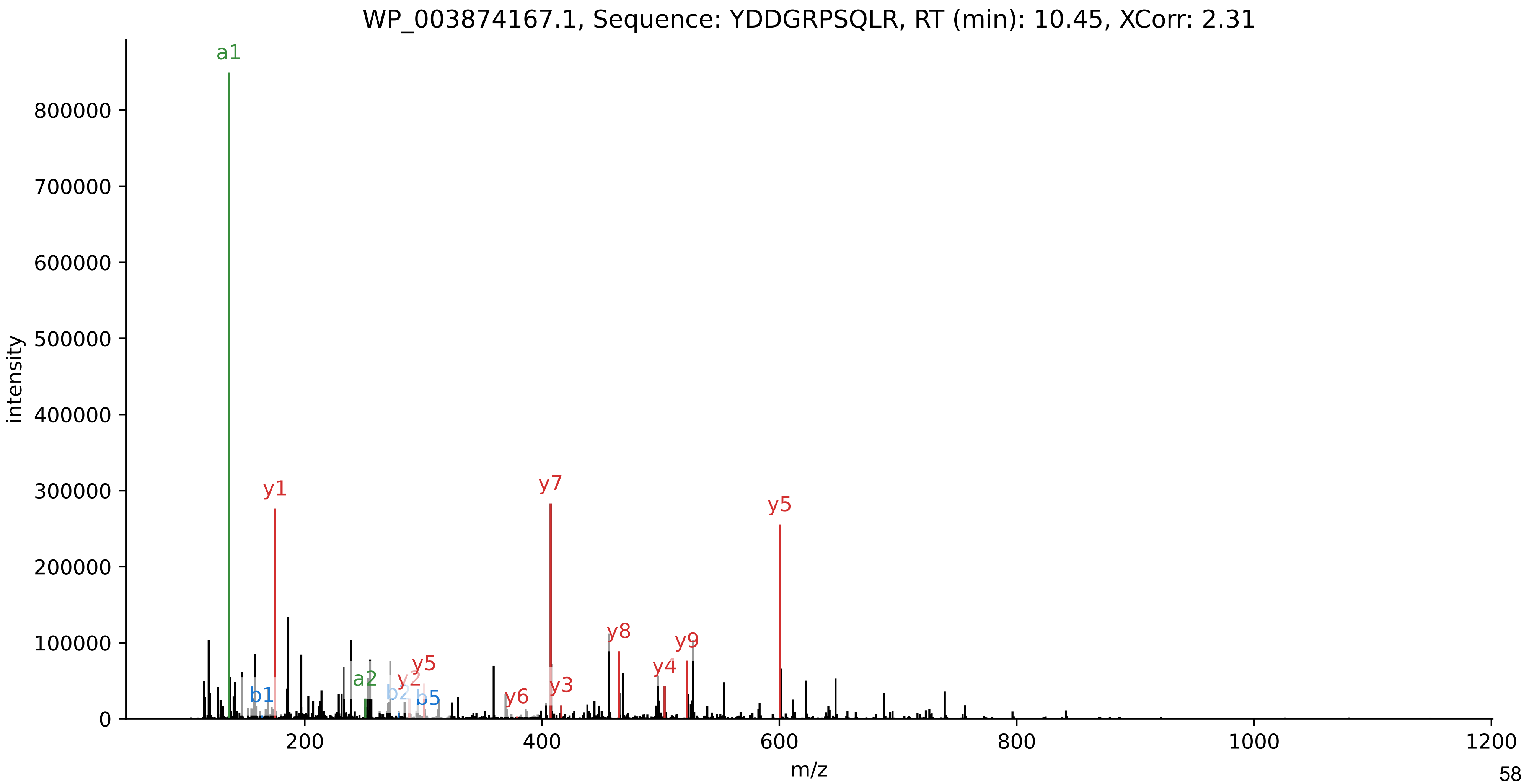
WP_003874167.1, Sequence: YDDGRPSQLR, RT (min): 10.45, XCorr: 2.31
a1
y1
y7
y5
y8
y9
y4
y3
y6
a2
y2
y5
b1
b2
b5
intensity
m/z
0
100000
200000
300000
400000
500000
600000
700000
800000
200
400
600
800
1000
1200

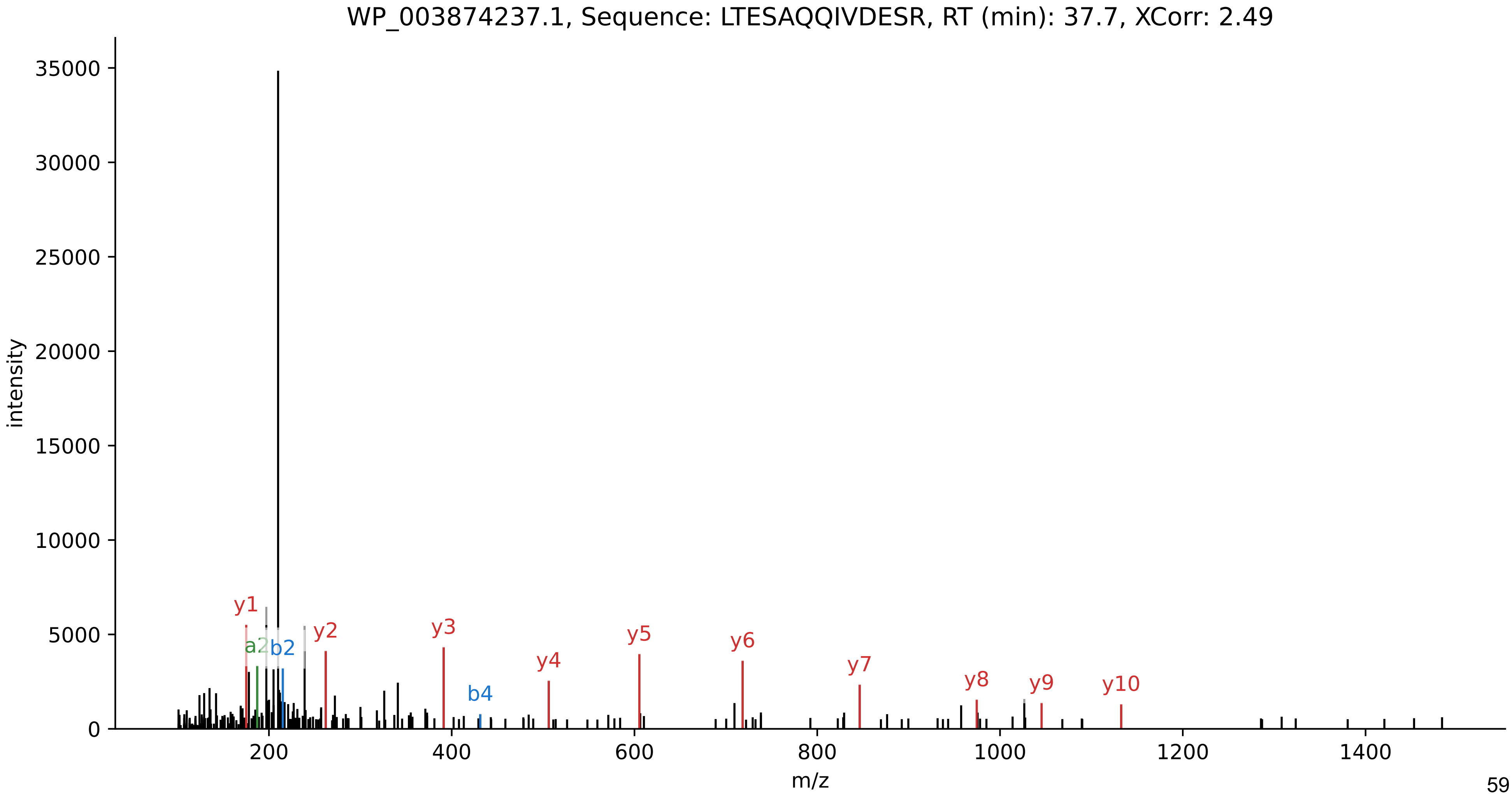

WP_003874237.1, Sequence: LTESAQQIVDESR, RT (min): 37.7, XCorr: 2.49
intensity
m/z
y1
a2
b2
y2
y3
b4
y4
y5
y6
y7
y8
y9
y10
35000
30000
25000
20000
15000
10000
5000
0
200
400
600
800
1000
1200
1400

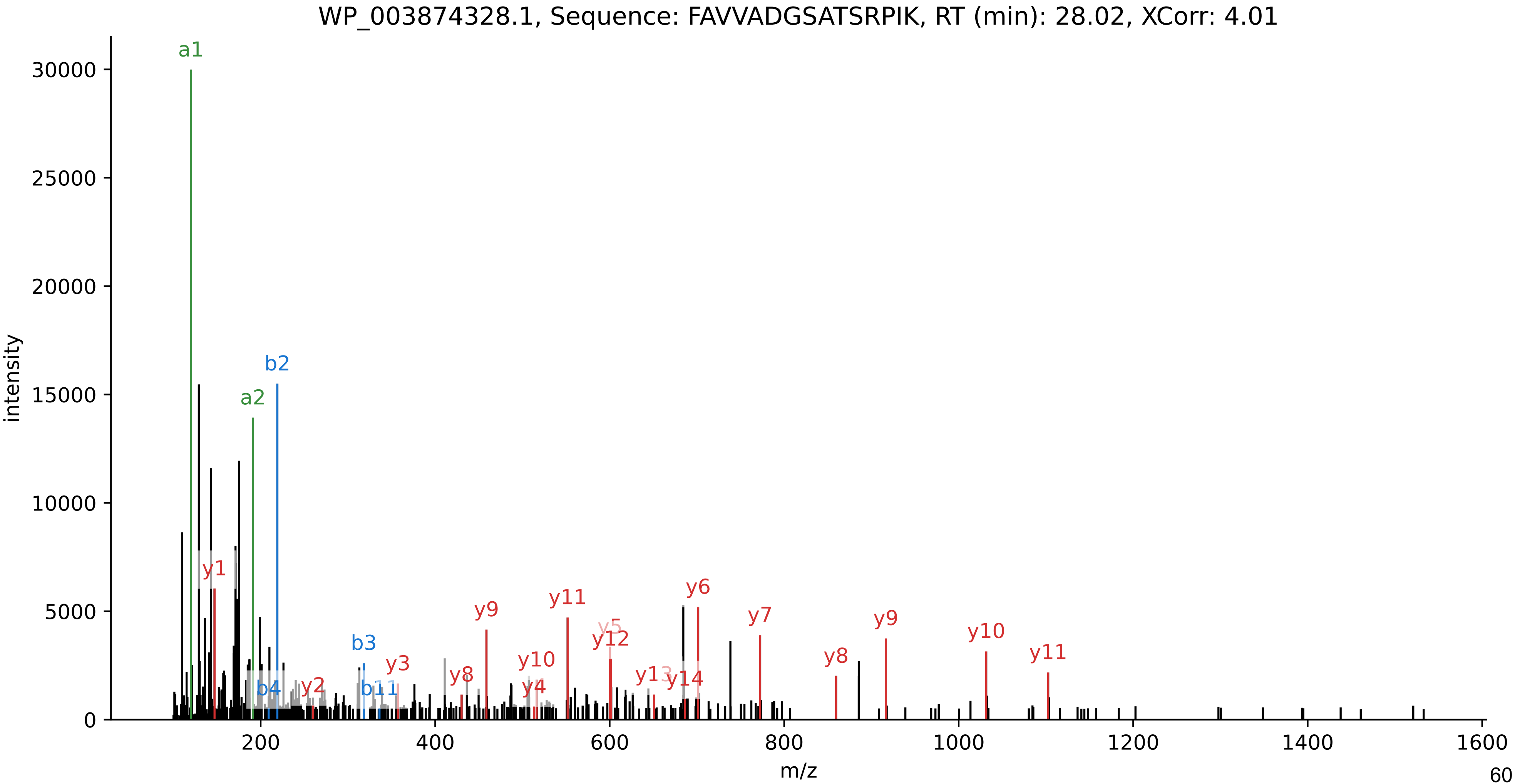
WP_003874328.1, Sequence: FAVVADGSATSRPIK, RT (min): 28.02, XCorr: 4.01
intensity
m/z
a1
a2
b2
b3
b4
b11
y1
y2
y3
y8
y9
y10
y4
y11
y5
y12
y13
y14
y6
y7
y8
y9
y10
y11

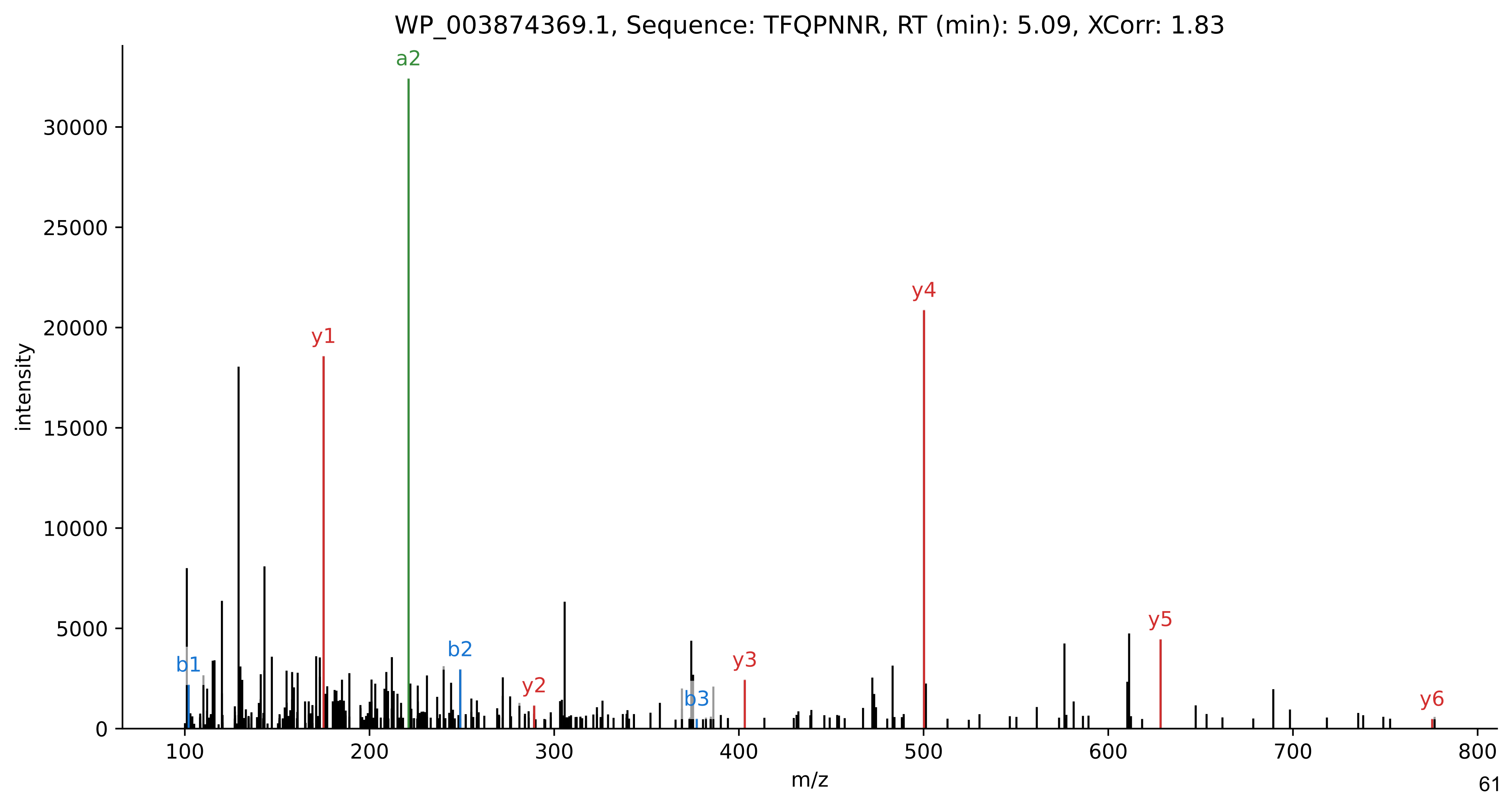

WP_003874369.1, Sequence: TFQPNNR, RT (min): 5.09, XCorr: 1.83
a2
y4
y1
y5
b2
b1
y3
y2
b3
y6
intensity
m/z
0
5000
10000
15000
20000
25000
30000
100
200
300
400
500
600
700
800

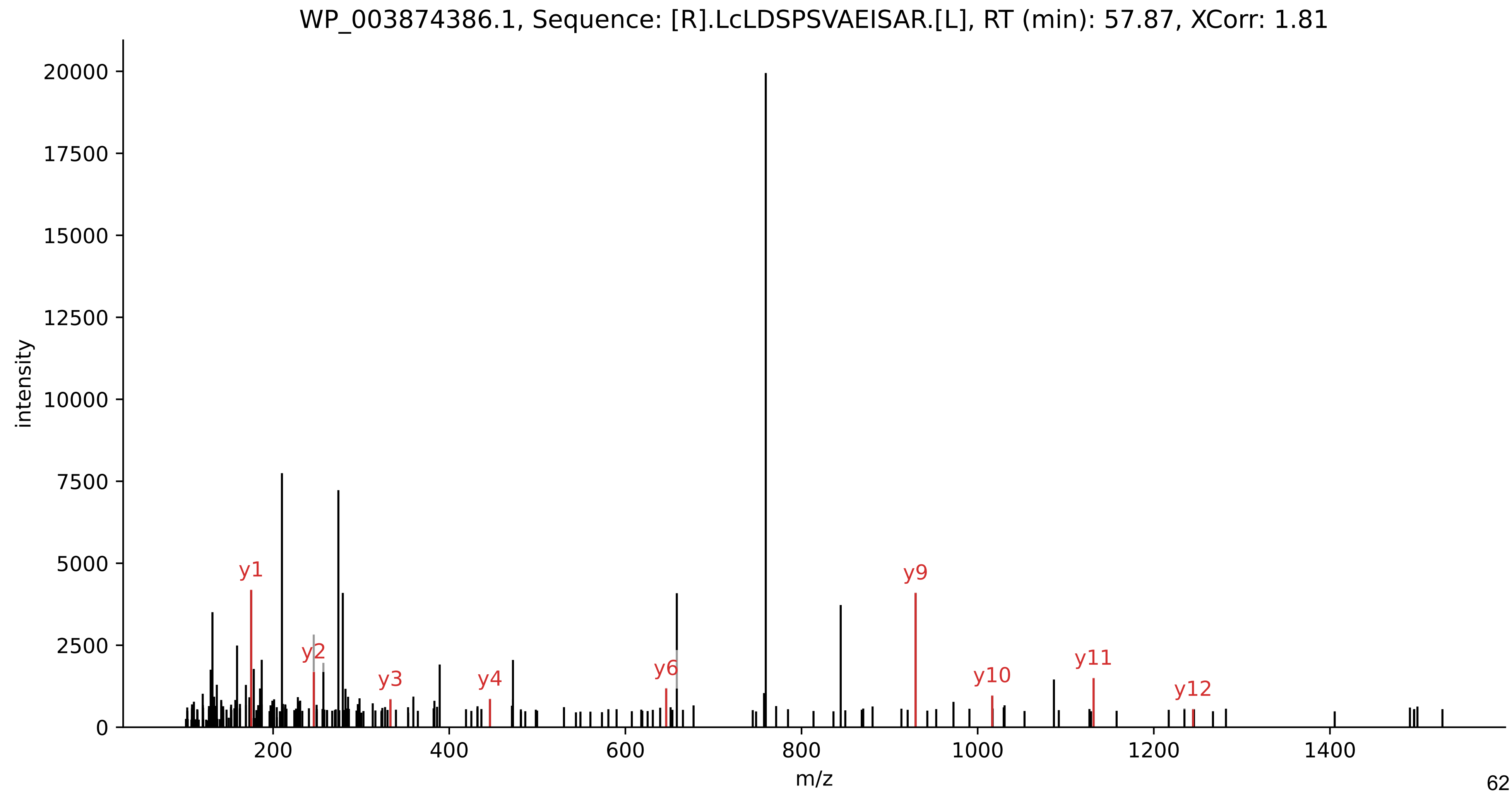
WP_003874386.1, Sequence: [R].LcLDSPSVAEISAR.[L], RT (min): 57.87, XCorr: 1.81
intensity
m/z
y1
y2
y3
y4
y6
y9
y10
y11
y12

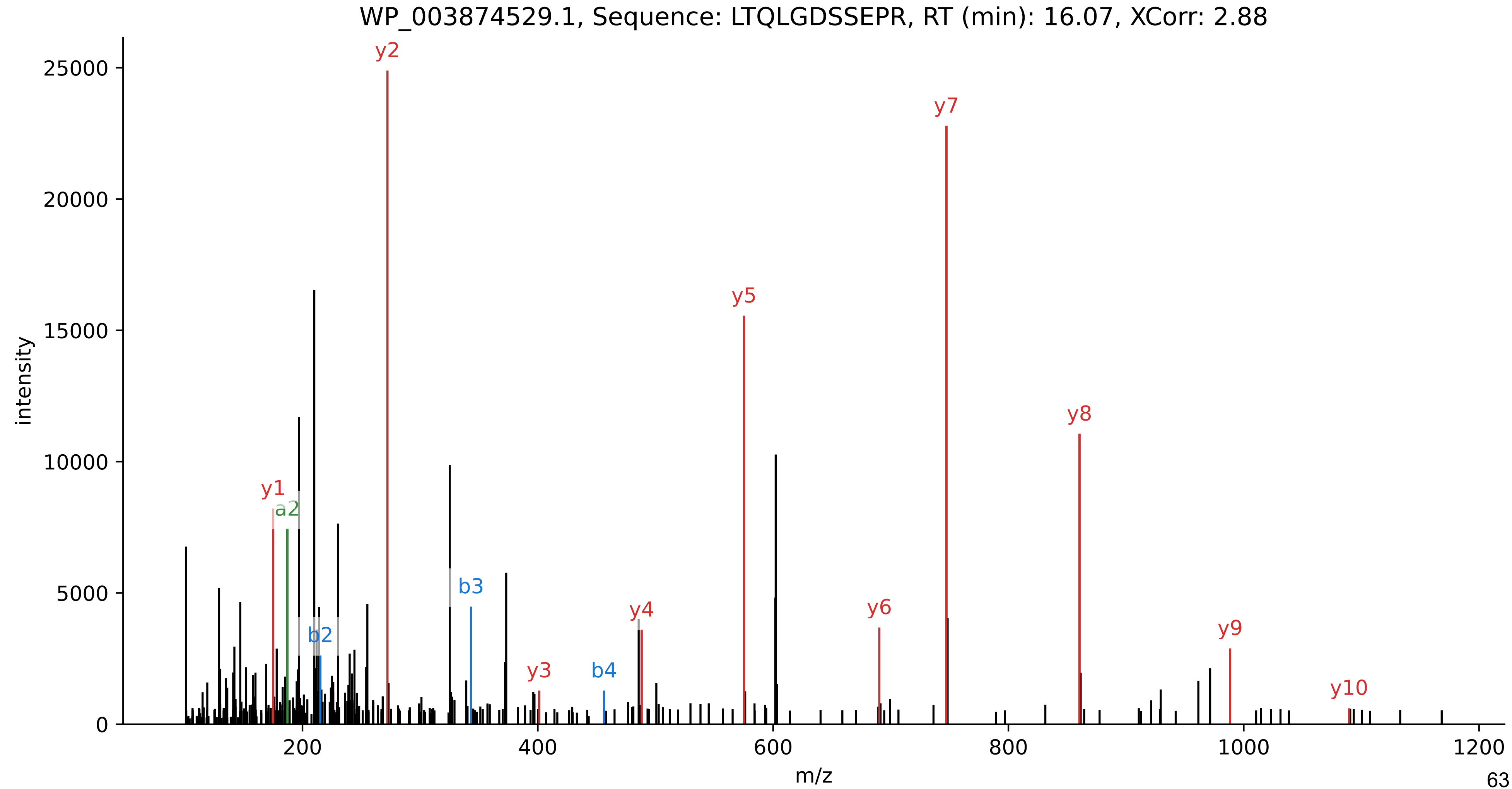
WP_003874529.1, Sequence: LTQLGDSSEPR, RT (min): 16.07, XCorr: 2.88
intensity
m/z
y1
a2
b2
y2
b3
y3
b4
y4
y5
y6
y7
y8
y9
y10
0
5000
10000
15000
20000
25000
200
400
600
800
1000
1200

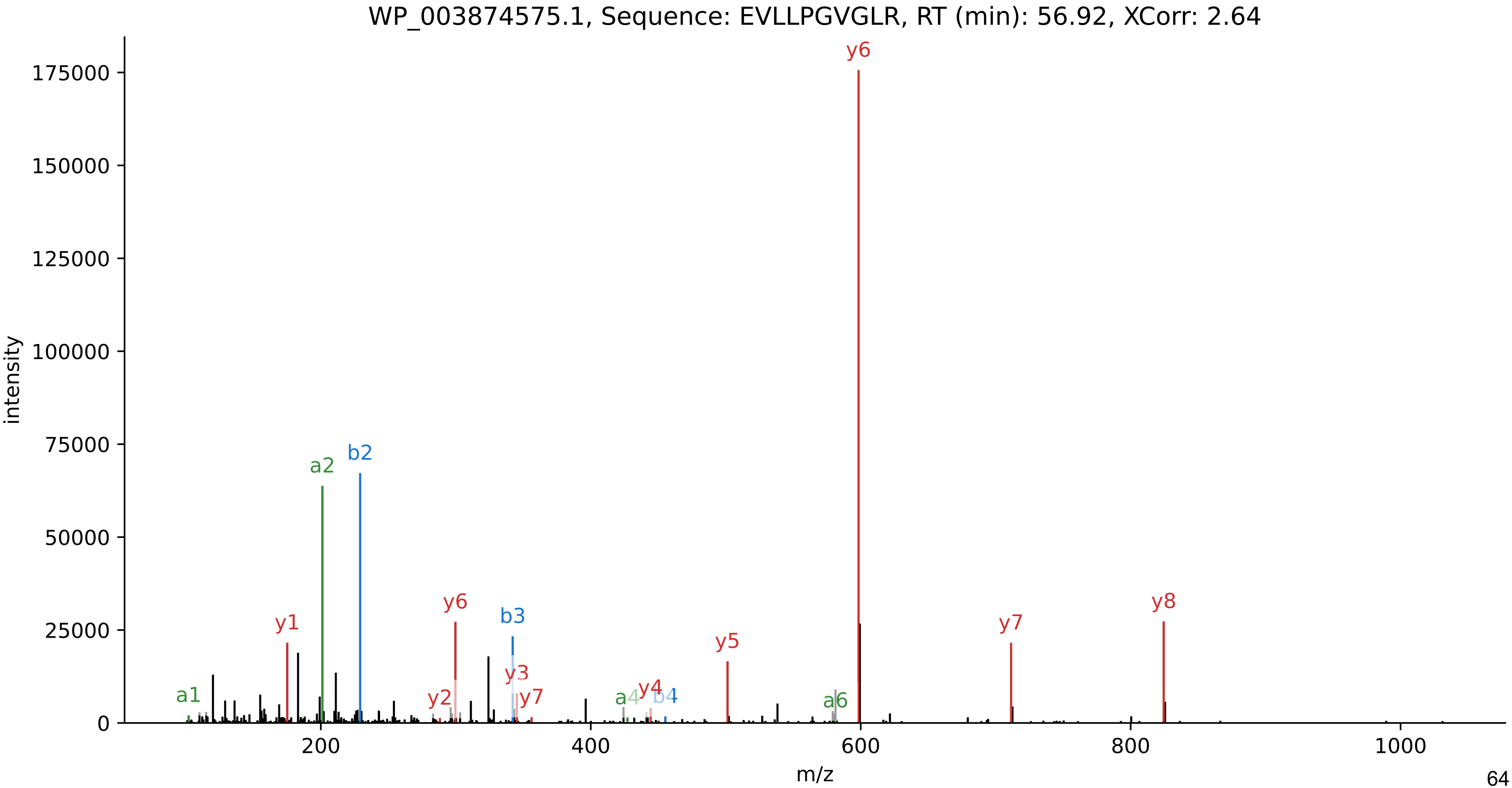
WP_003874575.1, Sequence: EVLLPGVGLR, RT (min): 56.92, XCorr: 2.64
intensity
m/z
y6
a2
b2
y1
y6
b3
y3
y7
y2
a1
a4
y4
b4
y5
a6
y7
y8
175000
150000
125000
100000
75000
50000
25000
0
200
400
600
800
1000

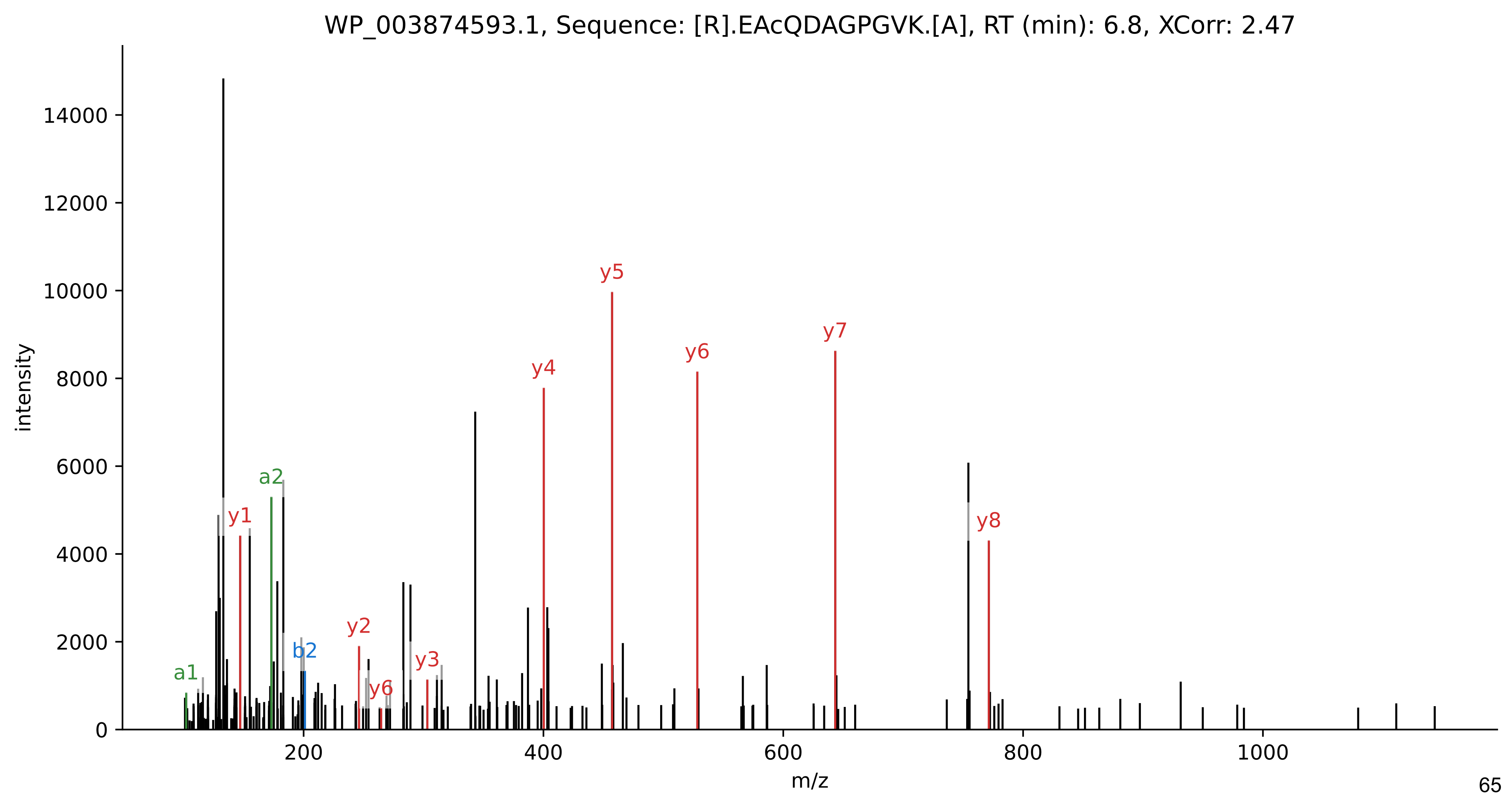

WP_003874593.1, Sequence: [R].EAcQDAGPGVK.[A], RT (min): 6.8, XCorr: 2.47
intensity
m/z
0
2000
4000
6000
8000
10000
12000
14000
200
400
600
800
1000
a1
a2
b2
y1
y2
y6
y3
y4
y5
y6
y7
y8

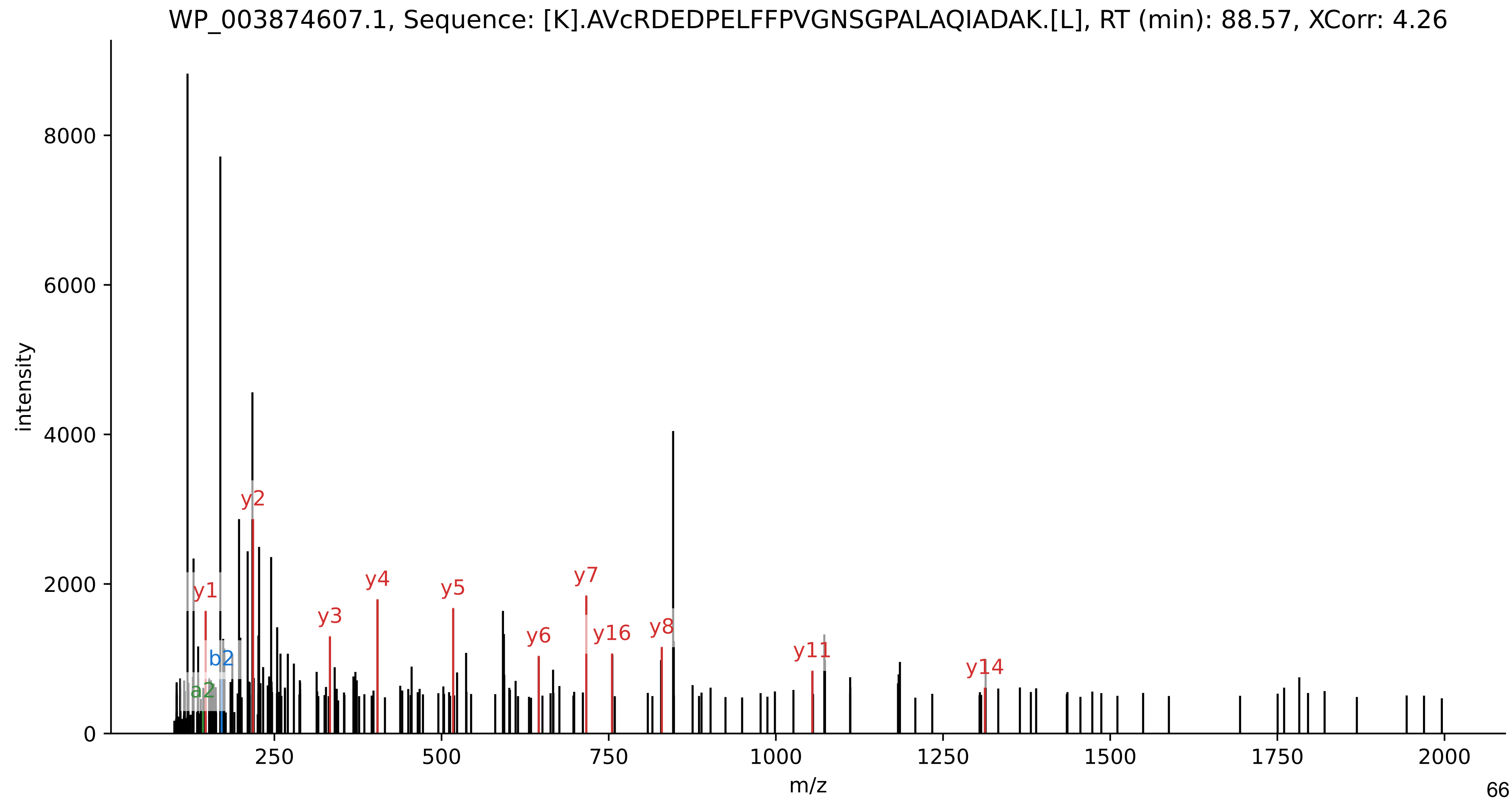

WP_003874607.1, Sequence: [K].AVcRDEDPELFFPVGNSGPALAQIADAK.[L], RT (min): 88.57, XCorr: 4.26
intensity
m/z
y1
a2
b2
y2
y3
y4
y5
y6
y7
y16
y8
y11
y14

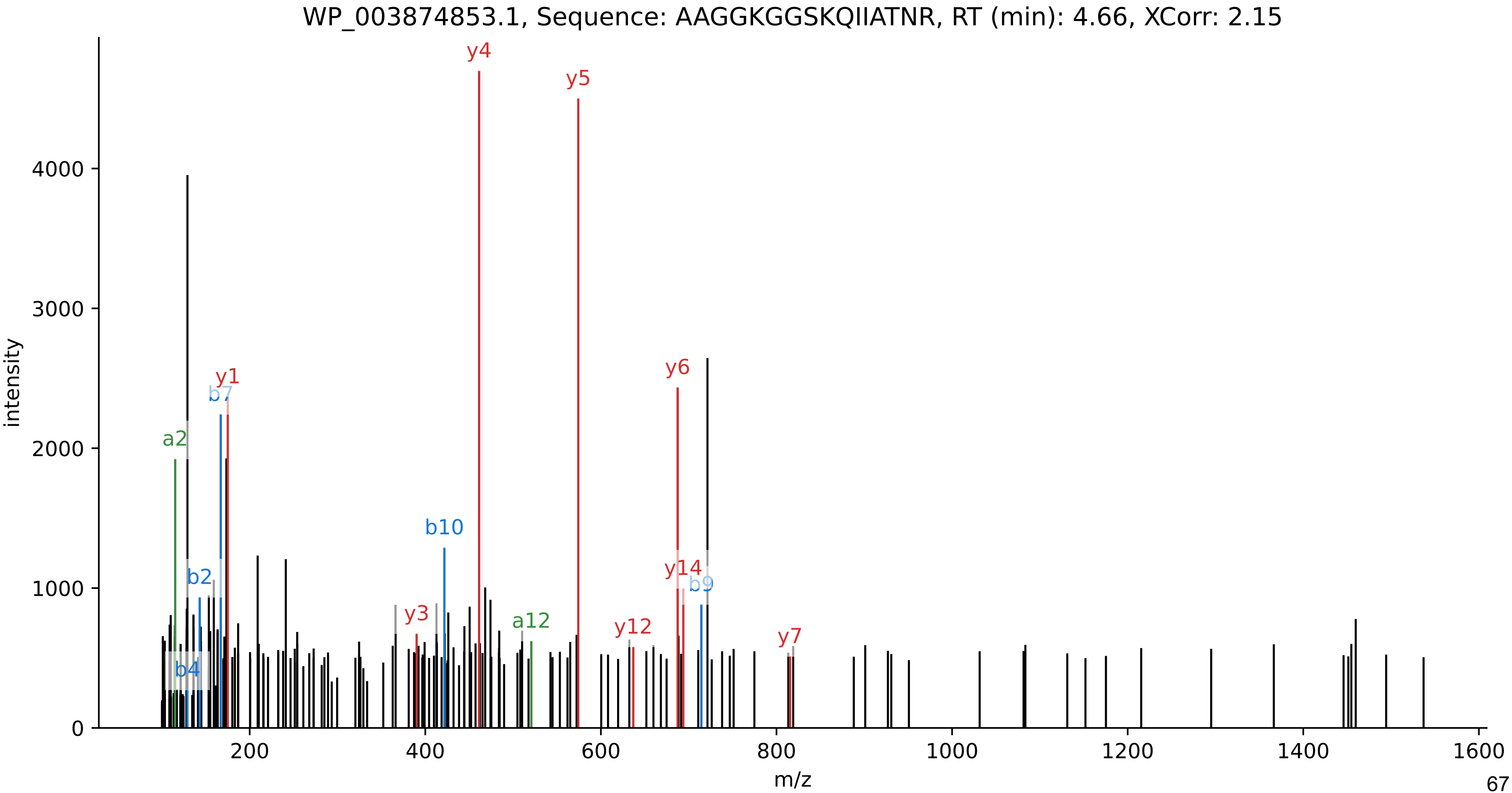
WP_003874853.1, Sequence: AAGGKGGSKQIIATNR, RT (min): 4.66, XCorr: 2.15
intensity
m/z
y4
y5
y6
y1
b7
a2
b10
b2
y14
b9
a12
y3
y12
y7
b4
0
1000
2000
3000
4000
200
400
600
800
1000
1200
1400
1600

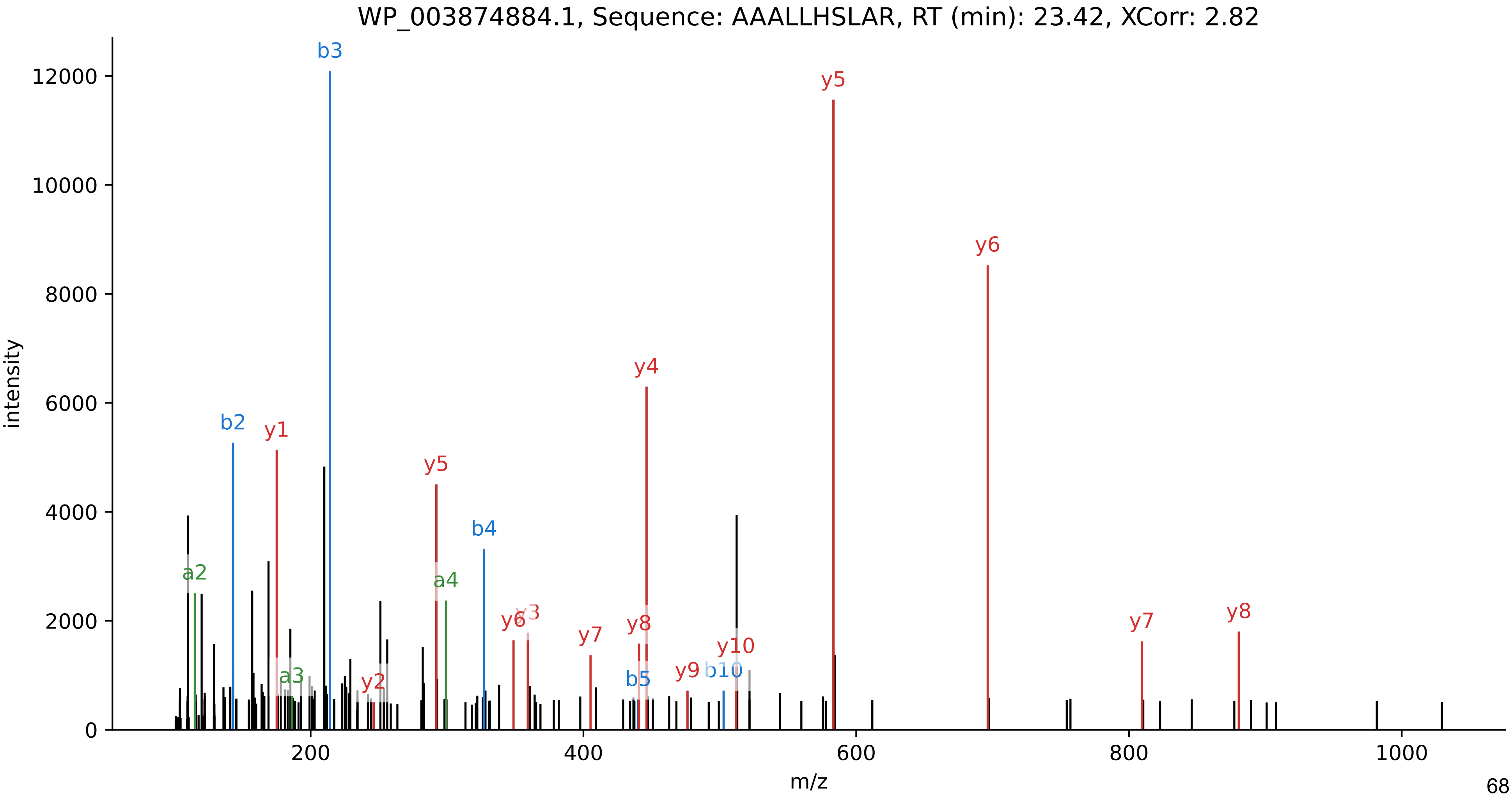
WP_003874884.1, Sequence: AAALLHSLAR, RT (min): 23.42, XCorr: 2.82
intensity
m/z
0
2000
4000
6000
8000
10000
12000
200
400
600
800
1000
b3
y5
y6
y4
b2
y1
y5
b4
a2
a4
y6
y3
y7
y8
y9
b10
y10
b5
a3
y2
y7
y8

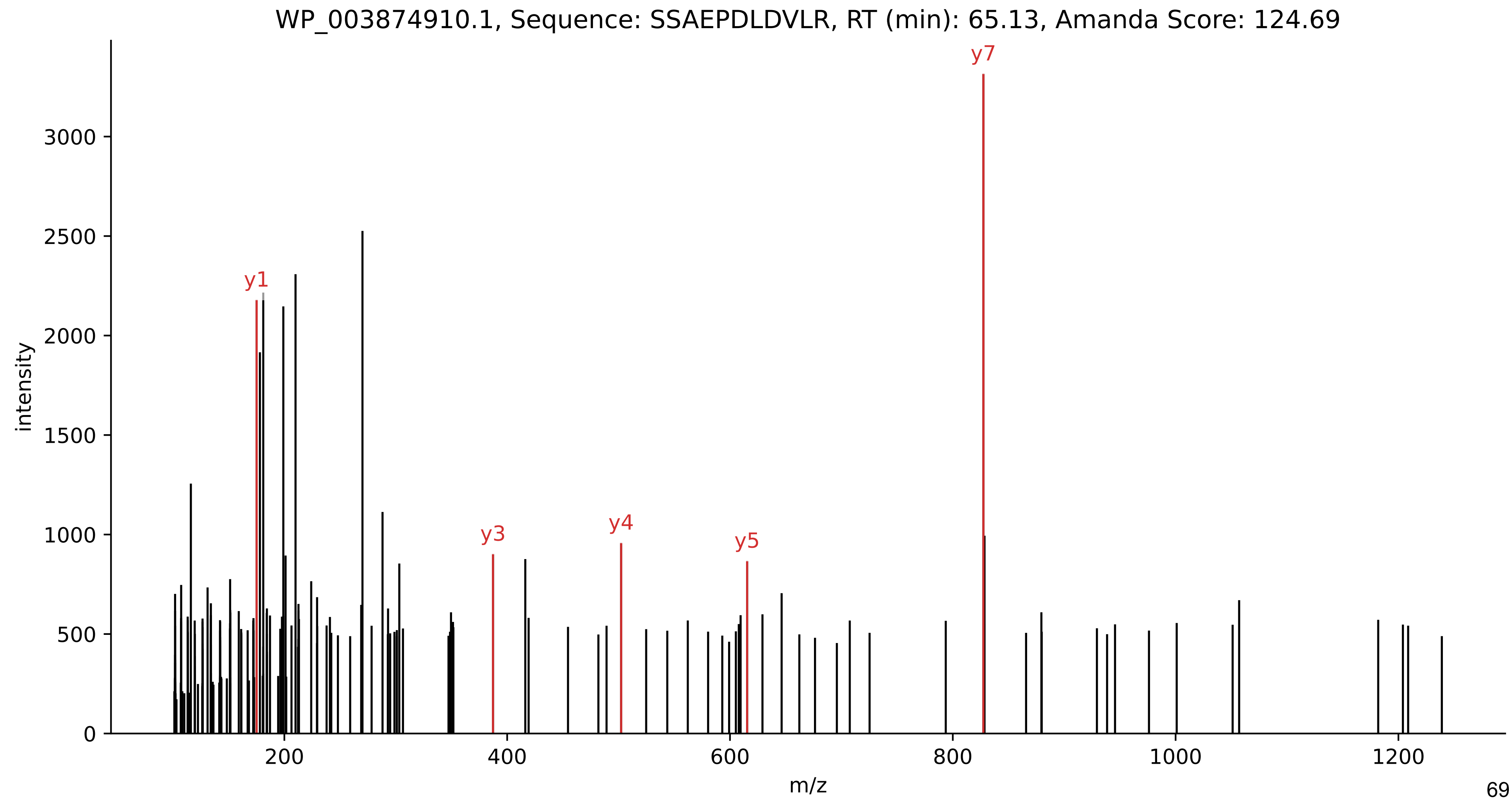

WP_003874910.1, Sequence: SSAEPDLDVLR, RT (min): 65.13, Amanda Score: 124.69
intensity
m/z
y1
y3
y4
y5
y7
0
500
1000
1500
2000
2500
3000
200
400
600
800
1000
1200

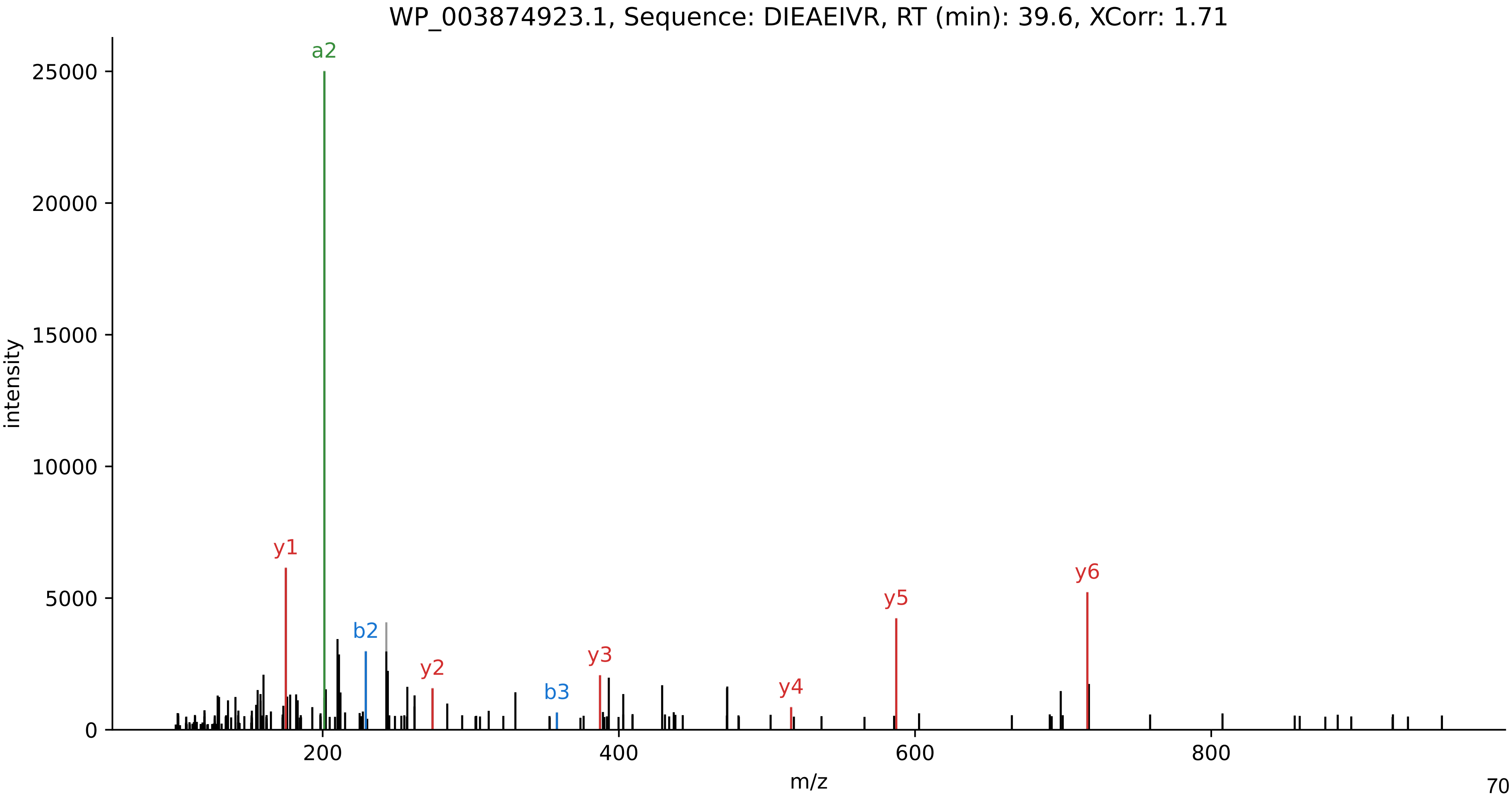
WP_003874923.1, Sequence: DIEAEIVR, RT (min): 39.6, XCorr: 1.71
a2
y1
b2
y2
b3
y3
y4
y5
y6
intensity
0
5000
10000
15000
20000
25000
200
400
600
800
m/z

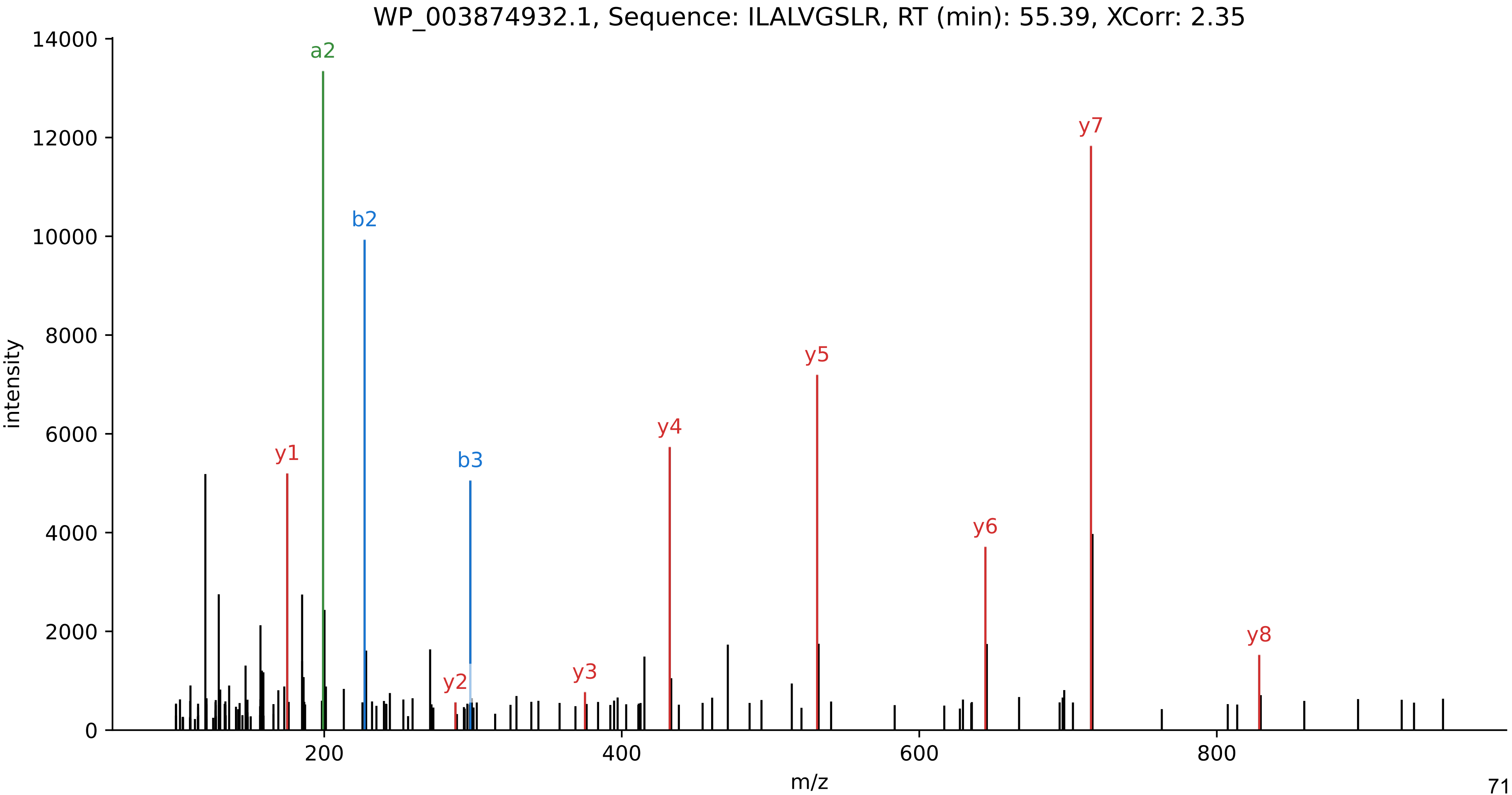

WP_003874932.1, Sequence: ILALVGSLR, RT (min): 55.39, XCorr: 2.35
intensity
m/z
a2
b2
b3
y1
y2
y3
y4
y5
y6
y7
y8
0
2000
4000
6000
8000
10000
12000
14000
200
400
600
800

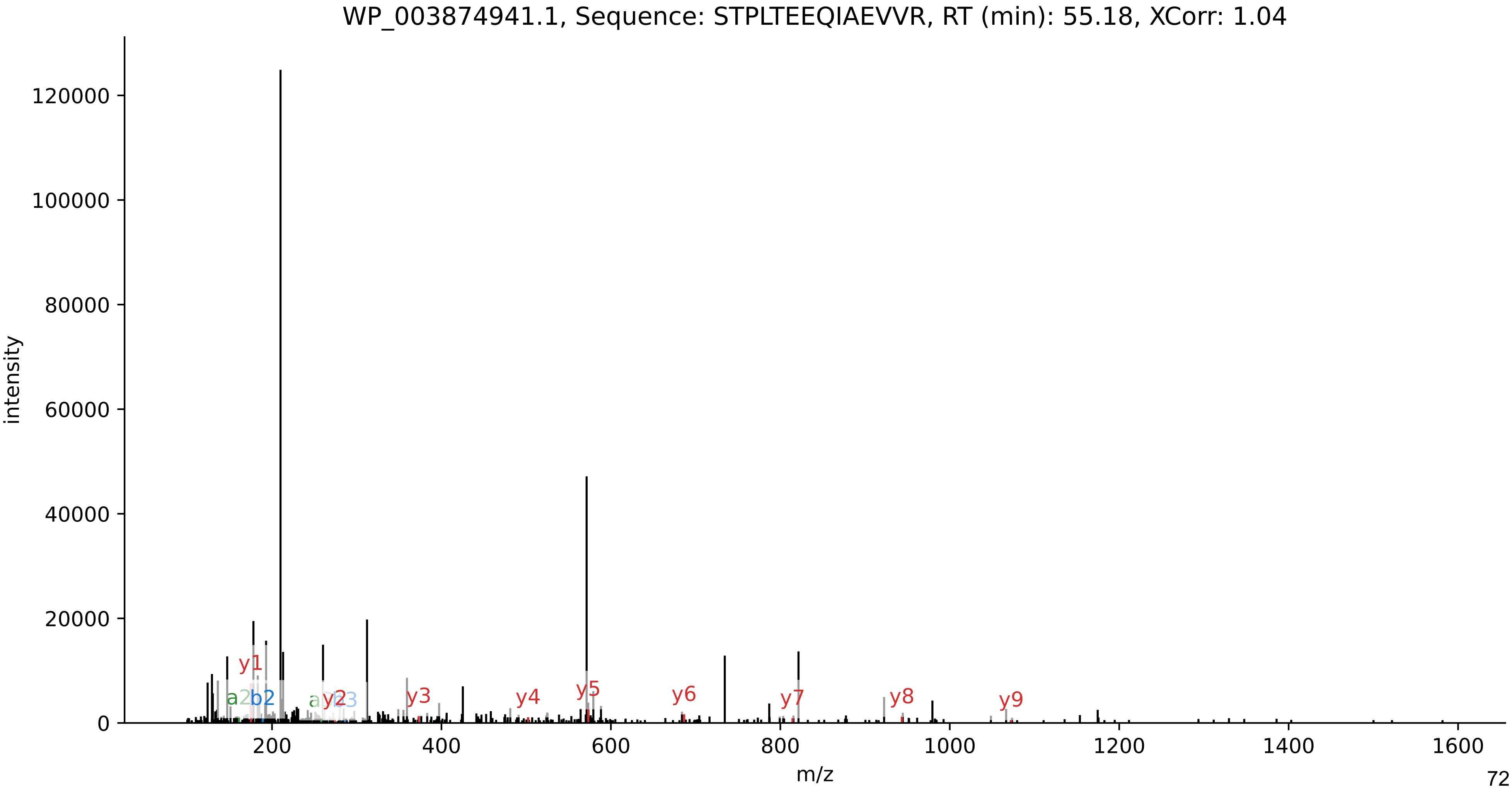
WP_003874941.1, Sequence: STPLTEEQIAEVVR, RT (min): 55.18, XCorr: 1.04
intensity
m/z
y1
a2
b2
y2
y3
y4
y5
y6
y7
y8
y9
0
20000
40000
60000
80000
100000
120000
200
400
600
800
1000
1200
1400
1600

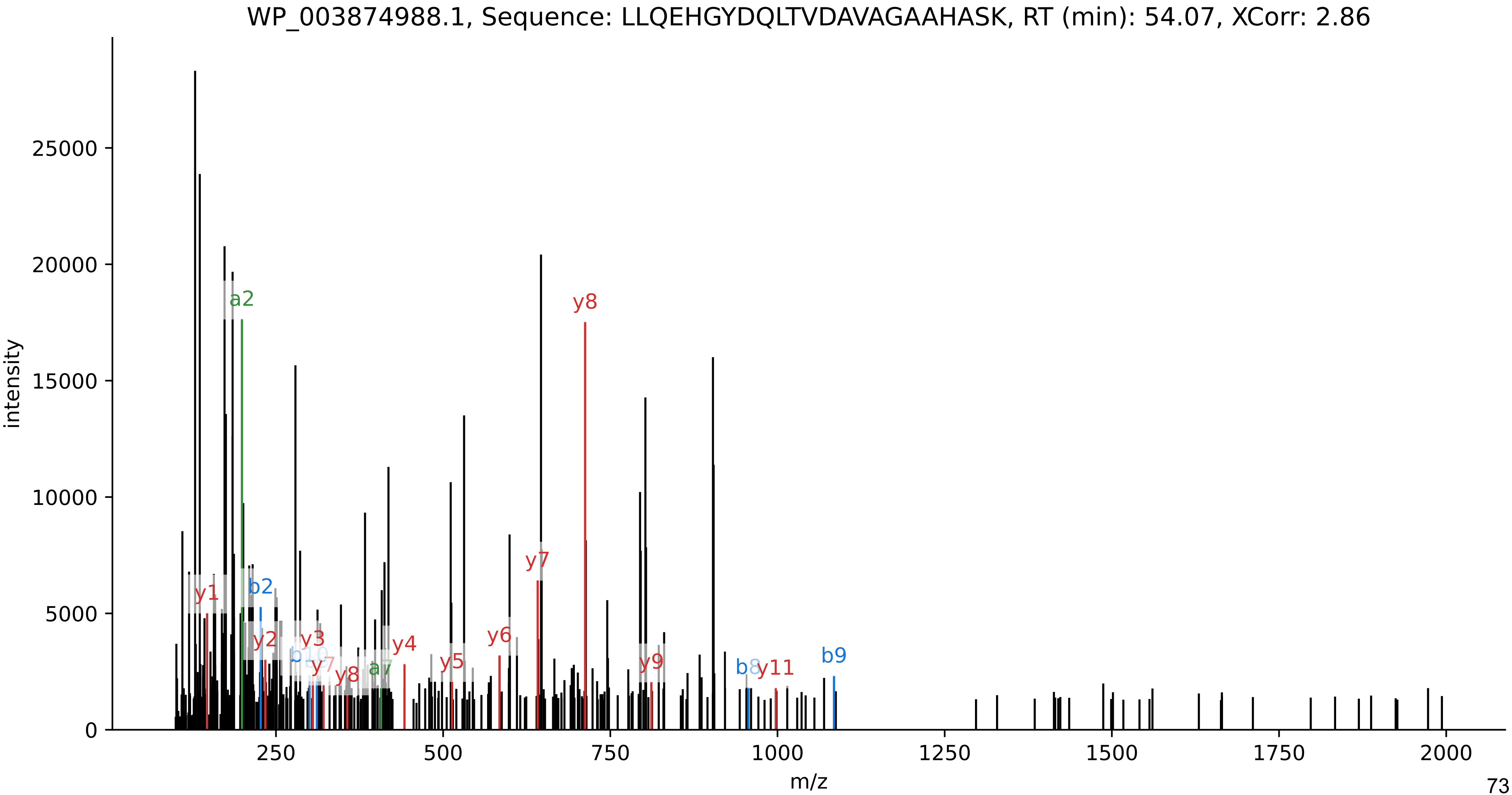

WP_003874988.1, Sequence: LLQEHGYDQLTVDAVAGAAHASK, RT (min): 54.07, XCorr: 2.86
intensity
m/z
a2
y8
y7
y1
b2
y2
y3
y4
y6
y5
y9
b8
y11
b9
a7
y8

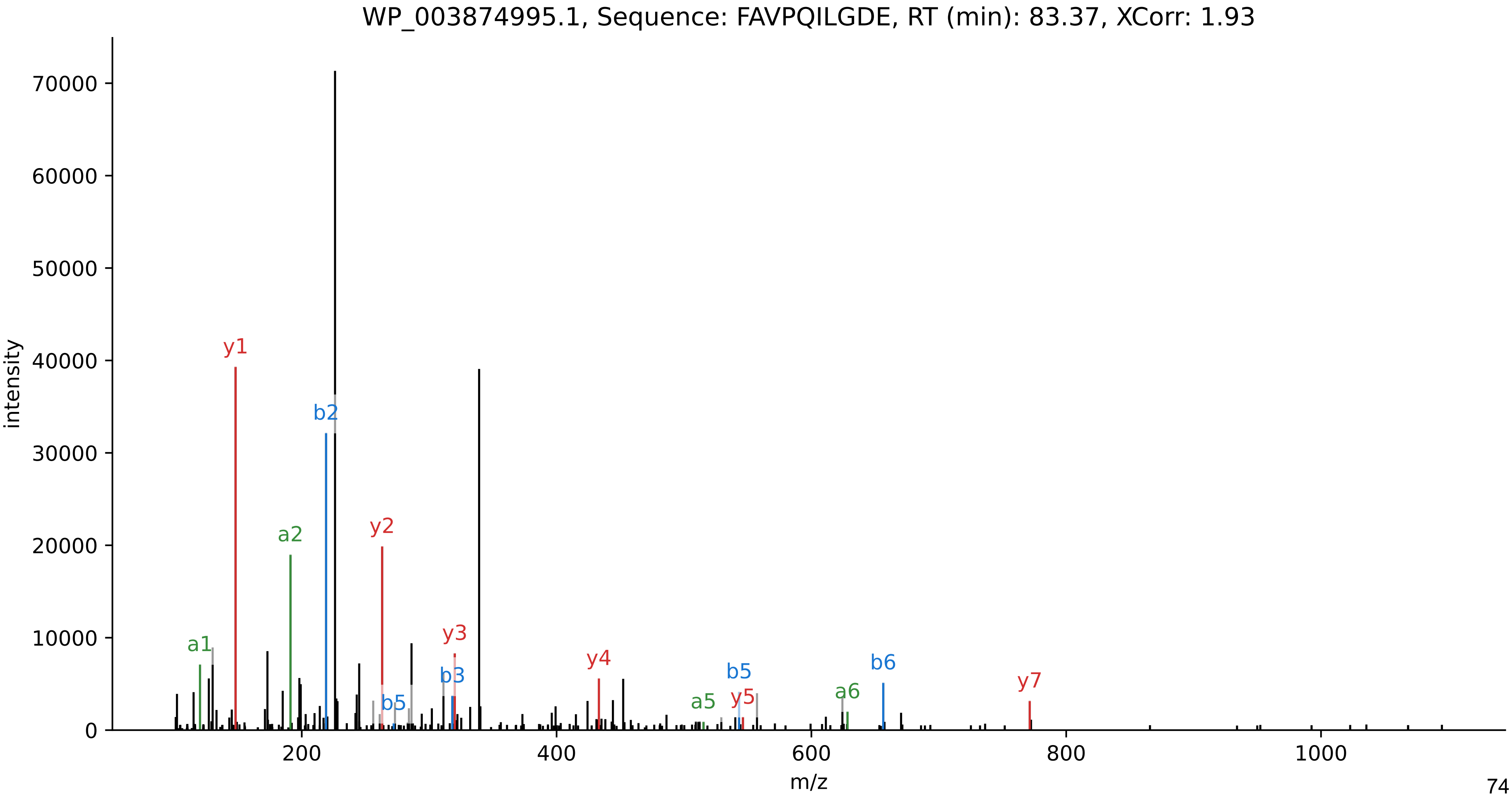
WP_003874995.1, Sequence: FAVPQILGDE, RT (min): 83.37, XCorr: 1.93
intensity
m/z
y1
b2
a2
y2
a1
b5
b3
y3
y4
a5
b5
y5
a6
b6
y7

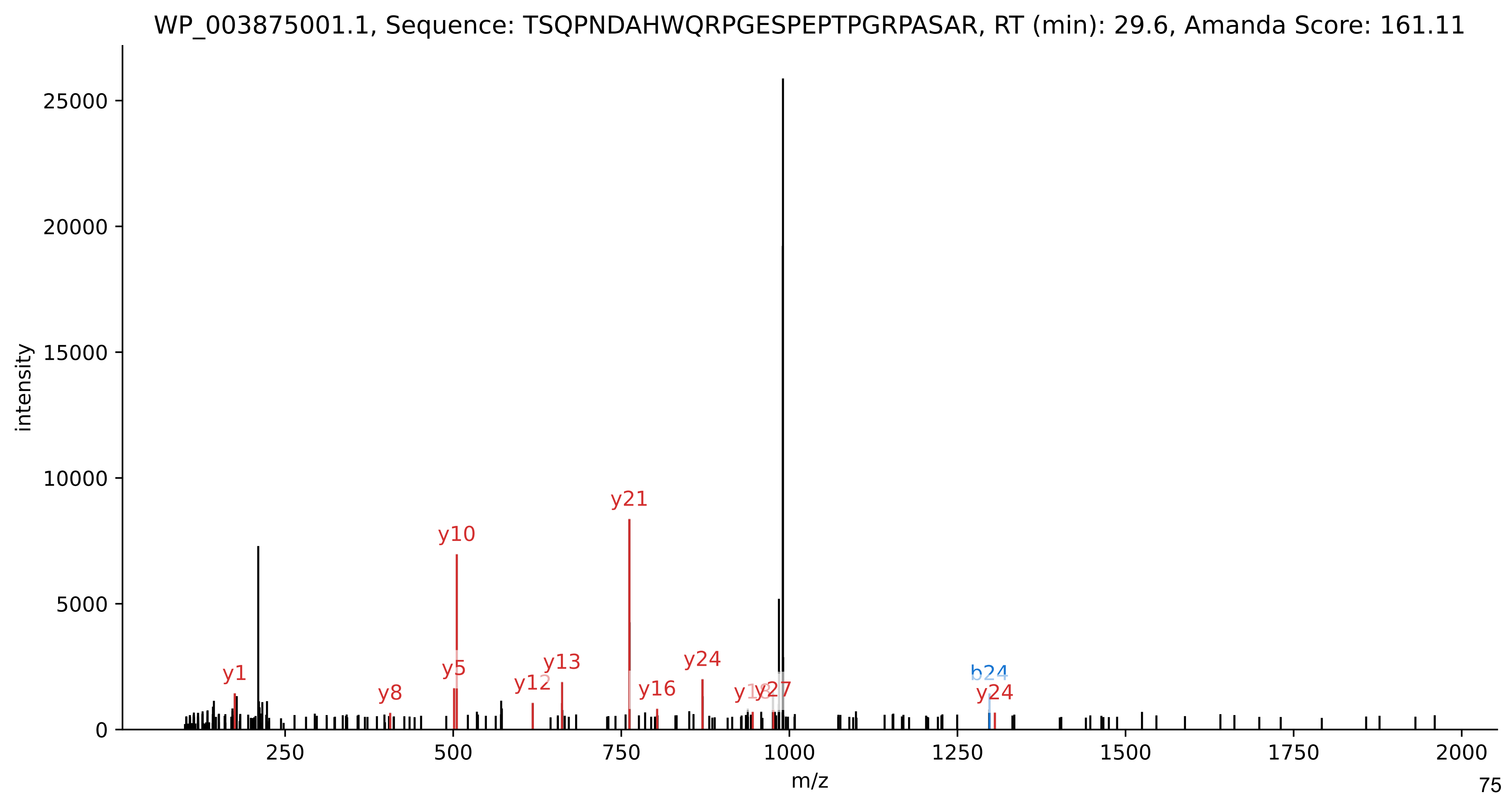
WP_003875001.1, Sequence: TSQPNDAHWQRPGESPEPTPGRPASAR, RT (min): 29.6, Amanda Score: 161.11
intensity
m/z
y1
y5
y8
y10
y12
y13
y16
y21
y24
y27
b24
y24

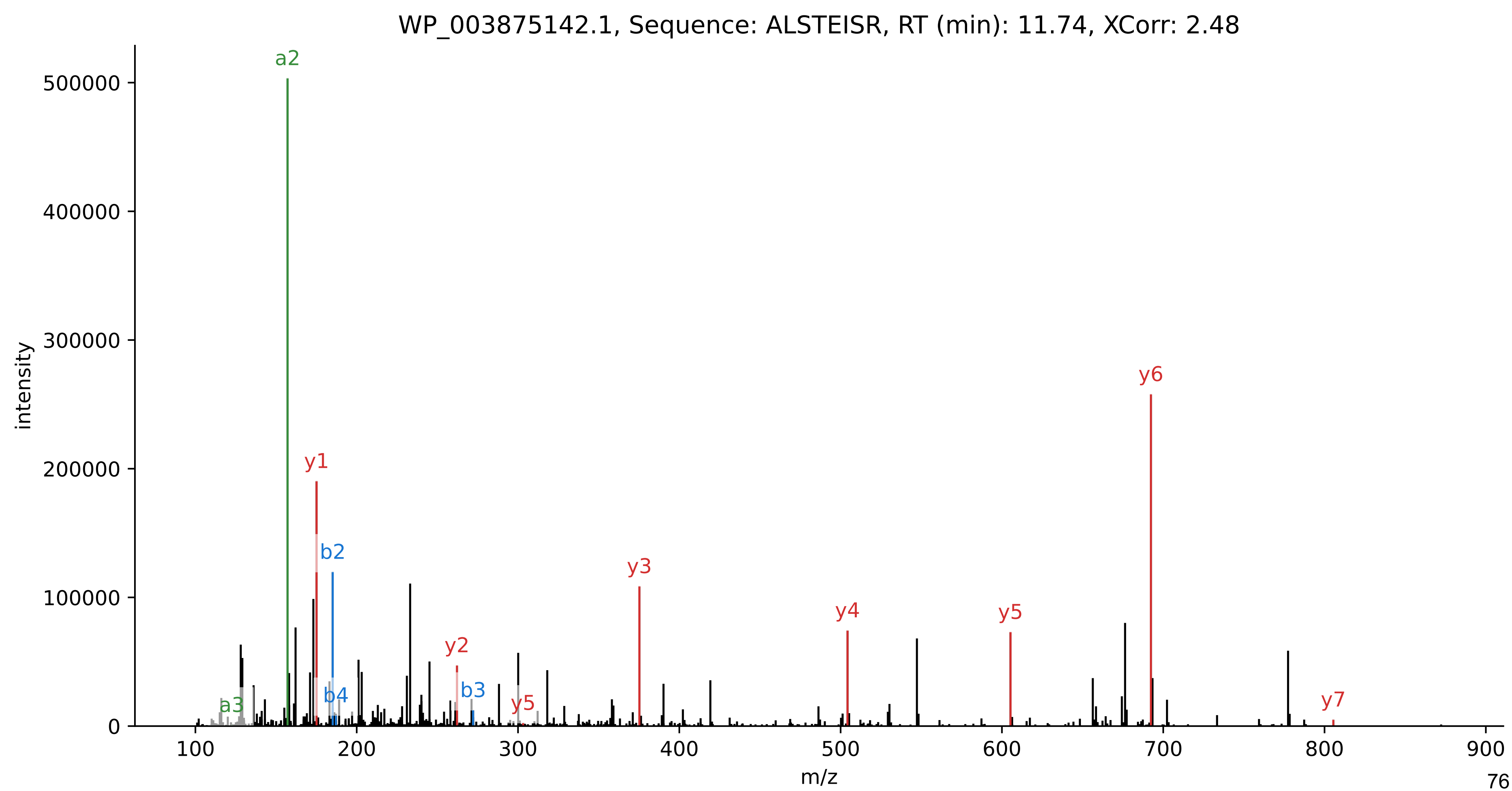
WP_003875142.1, Sequence: ALSTEISR, RT (min): 11.74, XCorr: 2.48
intensity
m/z
a2
y1
b2
y3
y2
b3
b4
a3
y5
y4
y5
y6
y7
0
100000
200000
300000
400000
500000
100
200
300
400
500
600
700
800
900

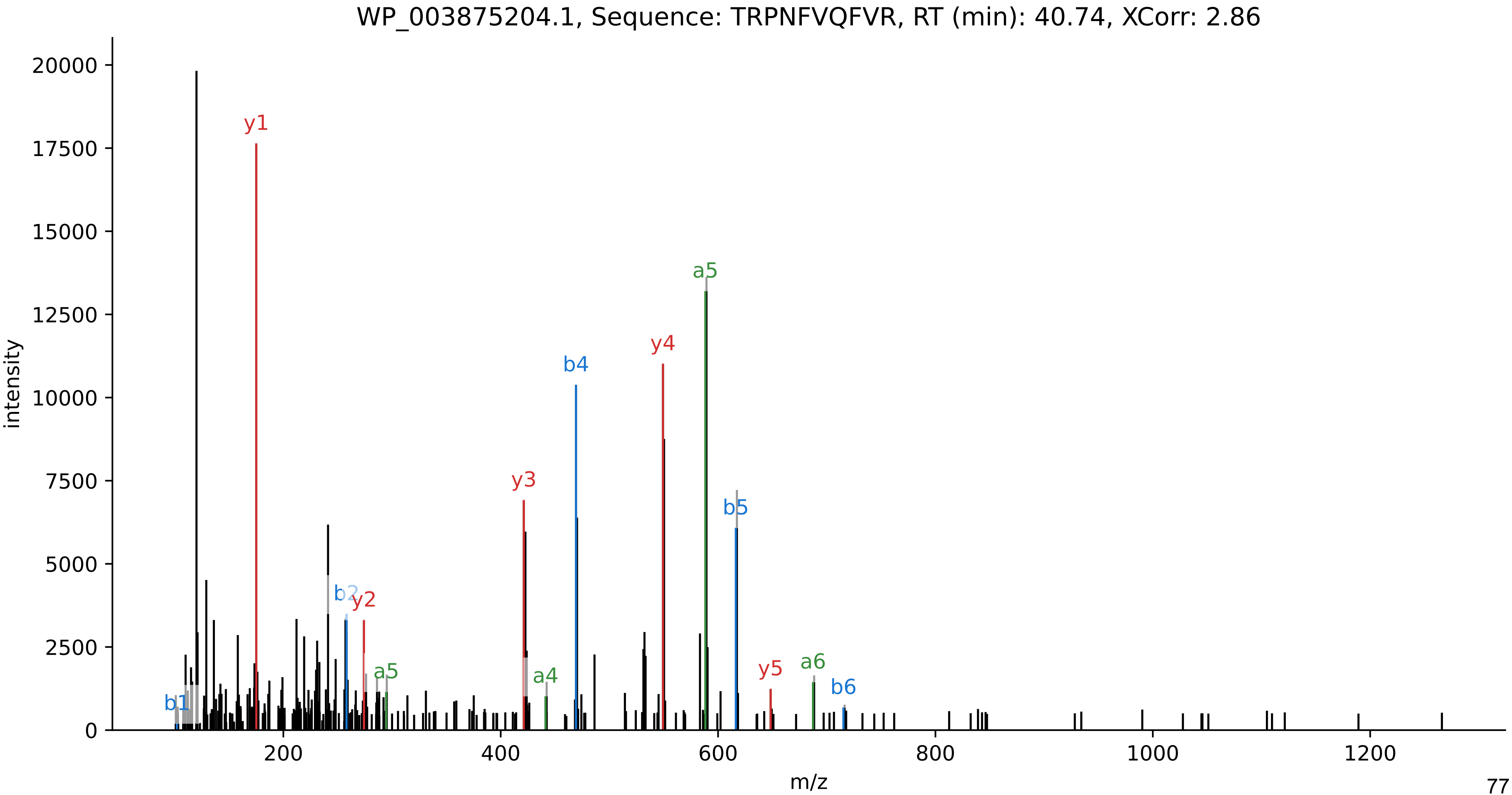

WP_003875204.1, Sequence: TRPNFVQFVR, RT (min): 40.74, XCorr: 2.86
intensity
m/z
0
2500
5000
7500
10000
12500
15000
17500
20000
200
400
600
800
1000
1200
y1
y2
y3
y4
y5
b1
b2
b4
b5
b6
a4
a5
a5
a6

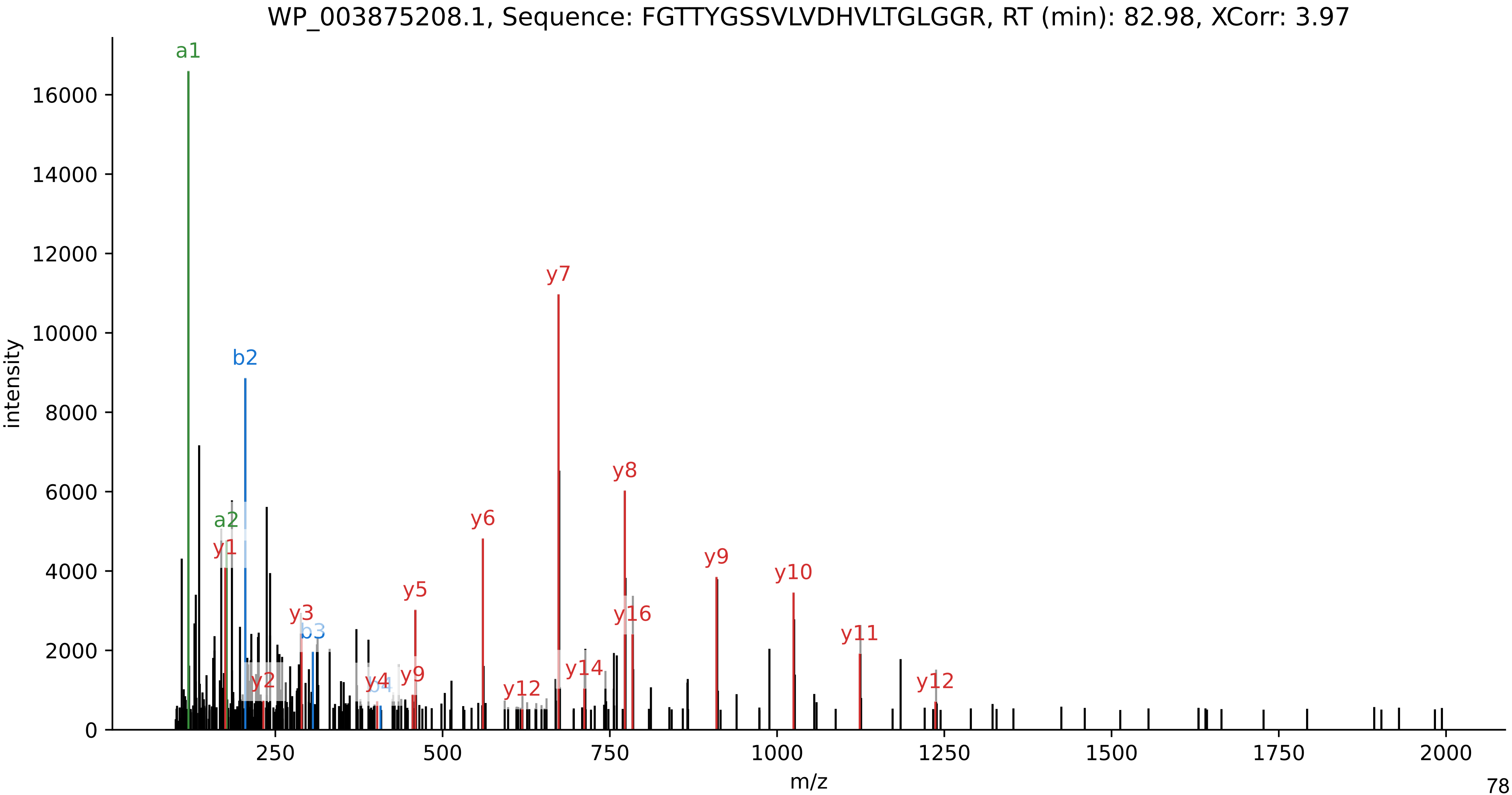
WP_003875208.1, Sequence: FGTTYGSSVLVDHVLTGLGGR, RT (min): 82.98, XCorr: 3.97
intensity
m/z
a1
b2
a2
y1
y2
y3
b3
y4
y9
y5
y6
y7
y8
y16
y14
y12
y9
y10
y11
y12
0
2000
4000
6000
8000
10000
12000
14000
16000
250
500
750
1000
1250
1500
1750
2000

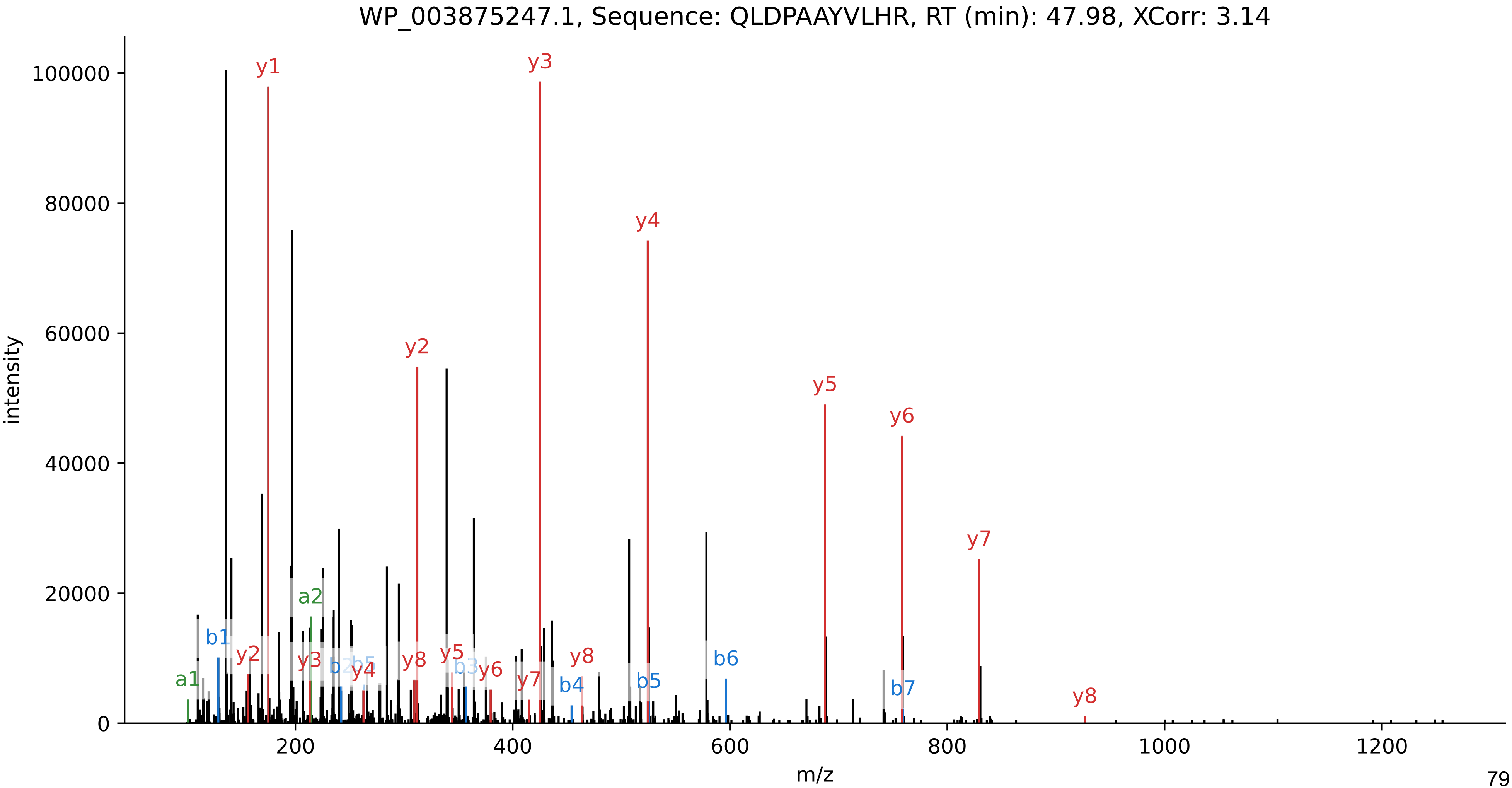
WP_003875247.1, Sequence: QLDPAAYVLHR, RT (min): 47.98, XCorr: 3.14
intensity
m/z
y1
y3
y4
y2
y5
y6
y7
y8
a1
a2
b1
b4
b5
b6
b7
0
20000
40000
60000
80000
100000
200
400
600
800
1000
1200

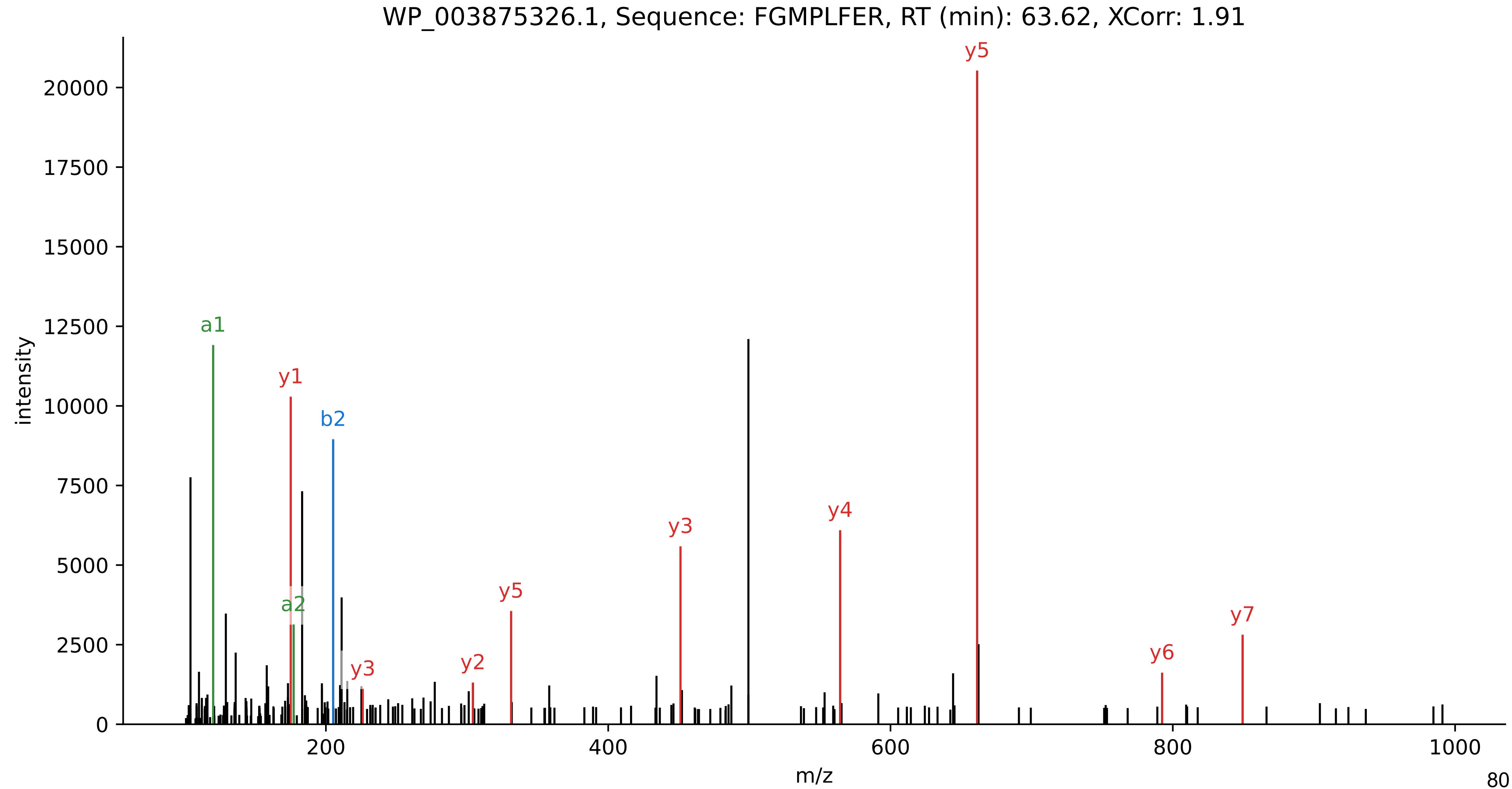

WP_003875326.1, Sequence: FGMPLFER, RT (min): 63.62, XCorr: 1.91
intensity
m/z
a1
y1
b2
a2
y3
y2
y5
y3
y4
y5
y6
y7
0
2500
5000
7500
10000
12500
15000
17500
20000
200
400
600
800
1000

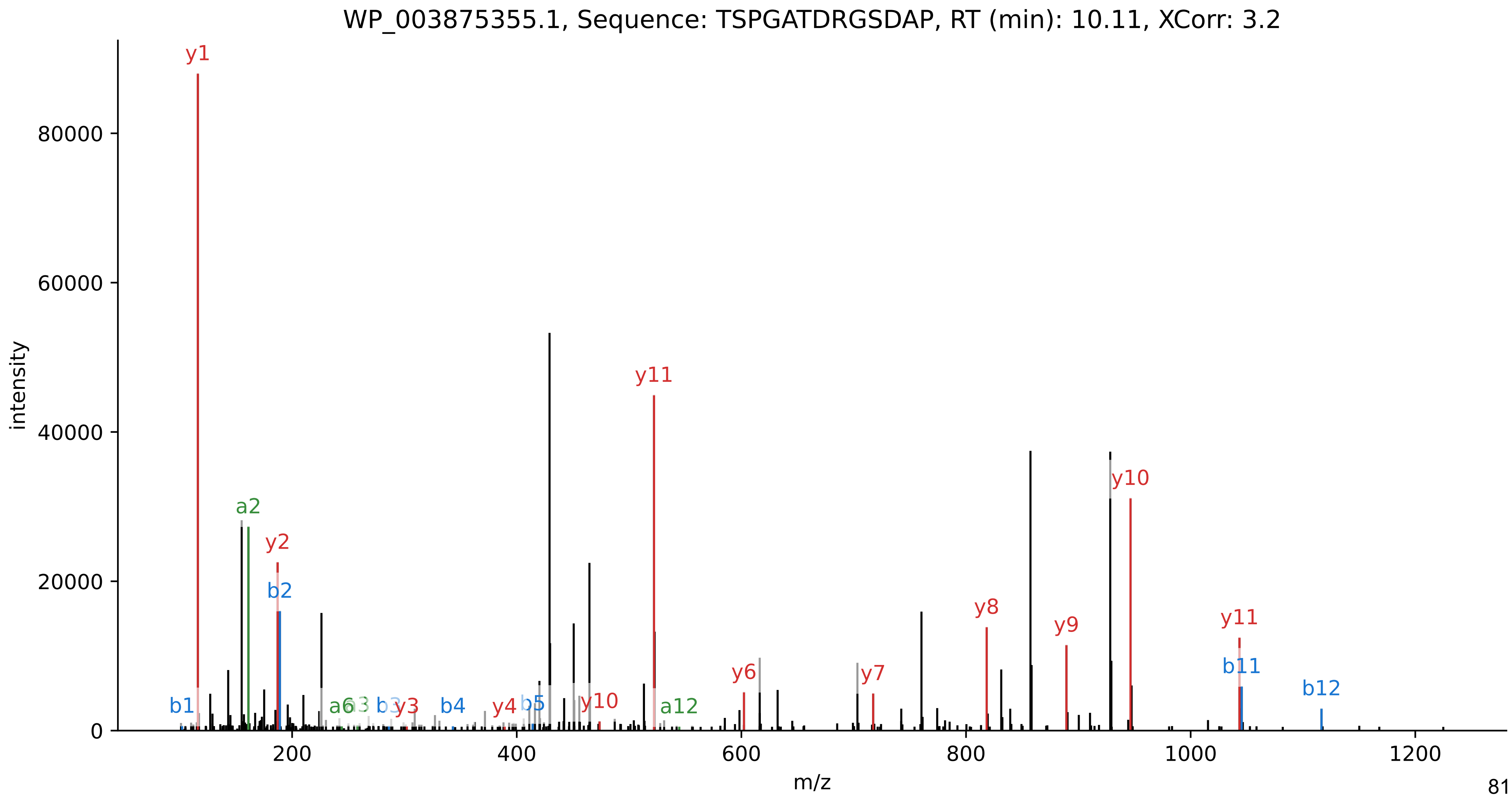
WP_003875355.1, Sequence: TSPGATDRGSDAP, RT (min): 10.11, XCorr: 3.2
intensity
m/z
0
20000
40000
60000
80000
200
400
600
800
1000
1200
y1
a2
y2
b2
b1
a6
y3
b4
y4
b5
y10
y11
a12
y6
y7
y8
y9
y10
y11
b11
b12

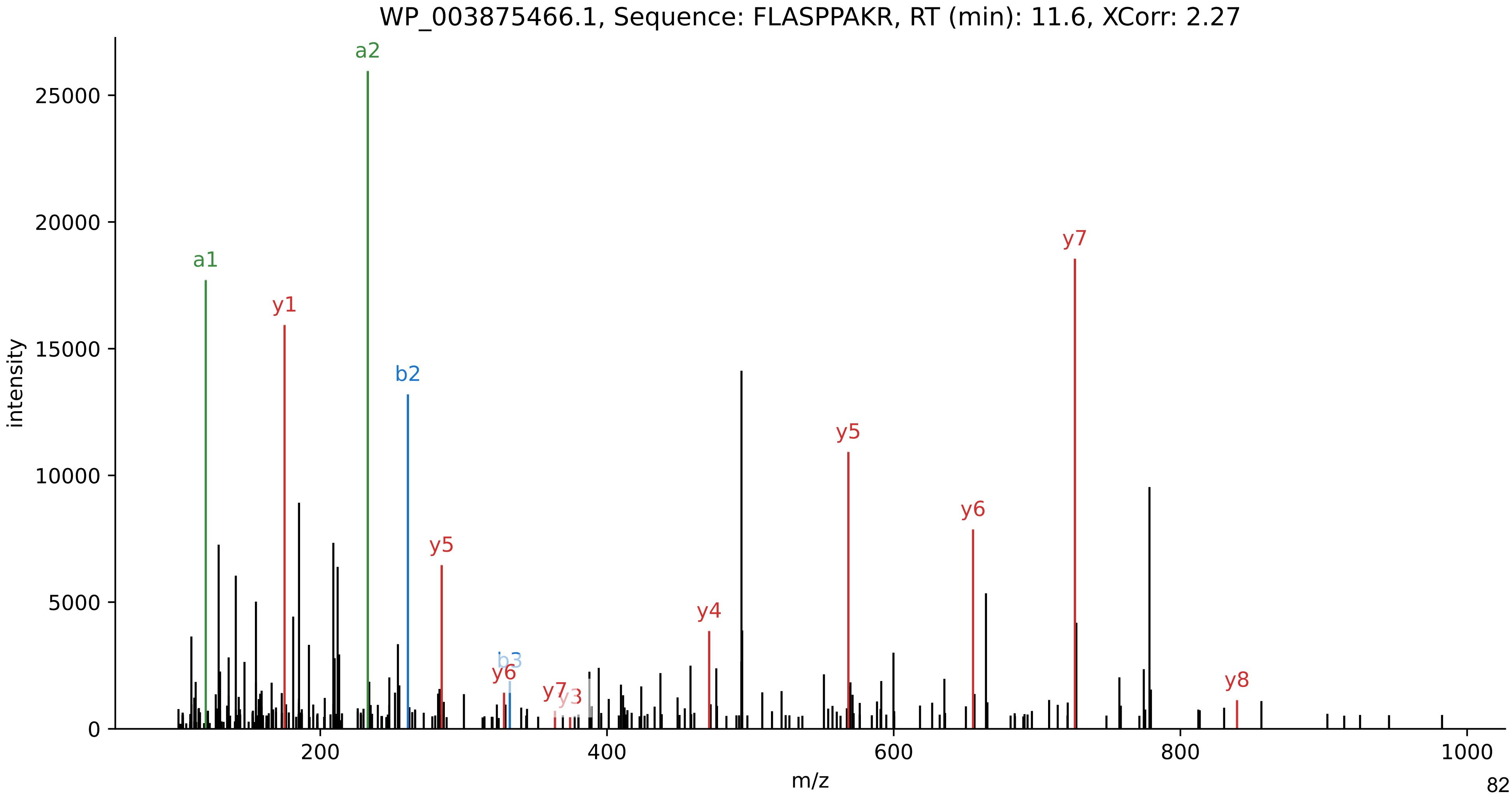

WP_003875466.1, Sequence: FLASPPAKR, RT (min): 11.6, XCorr: 2.27
intensity
m/z
a1
a2
y1
b2
y5
b3
y6
y7
y3
y4
y5
y6
y7
y8

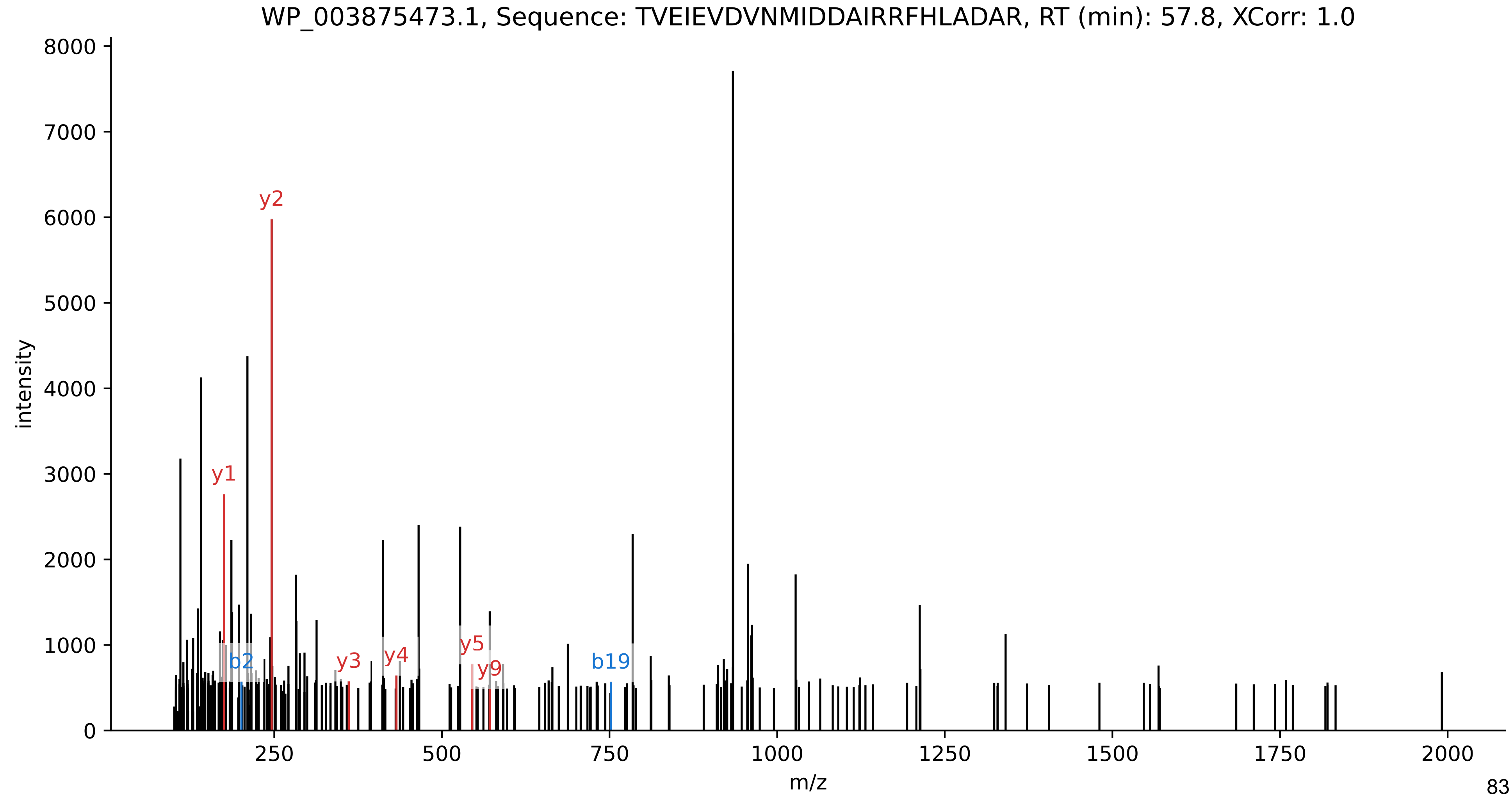
WP_003875473.1, Sequence: TVEIEVDVNMIDDAIRRFHLADAR, RT (min): 57.8, XCorr: 1.0
intensity
m/z
y1
y2
y3
y4
y5
y9
b2
b19
0
1000
2000
3000
4000
5000
6000
7000
8000
250
500
750
1000
1250
1500
1750
2000

WP_003875593.1, Sequence: AHSLGLDEPIPIR, RT (min): 48.87, XCorr: 1.85

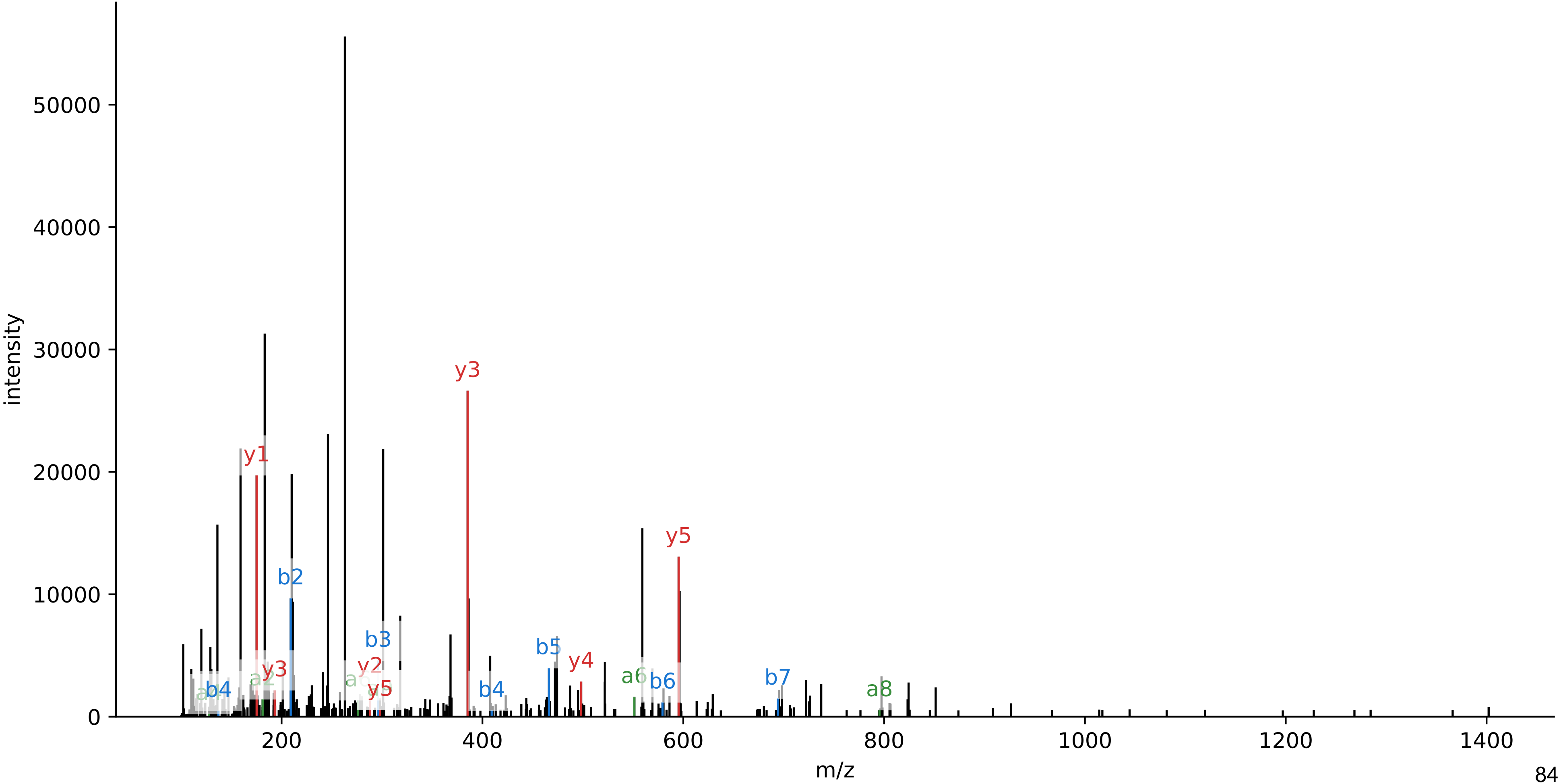

WP_003875613.1, Sequence: LVTYAITGER, RT (min): 33.94, XCorr: 2.54

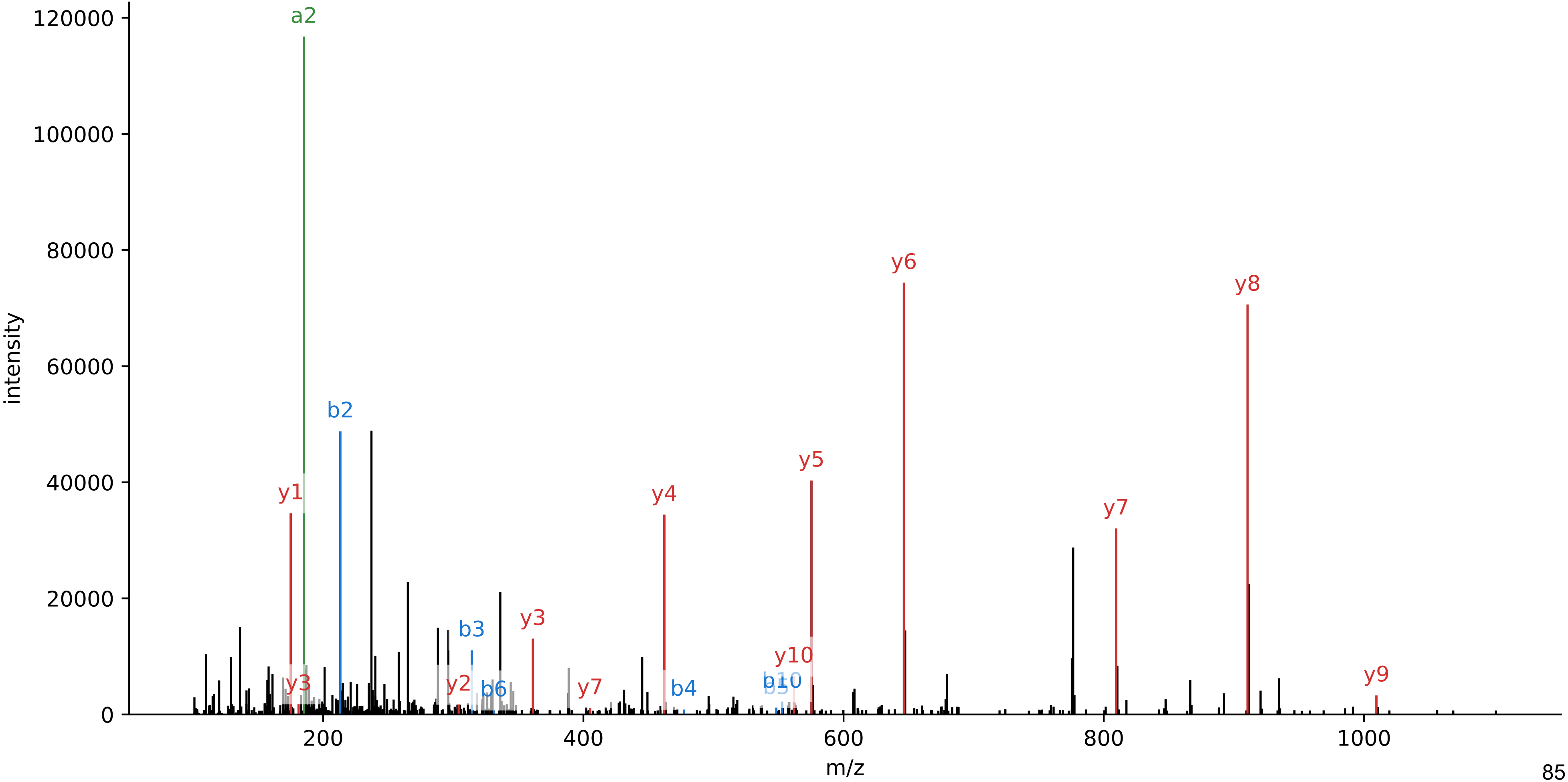

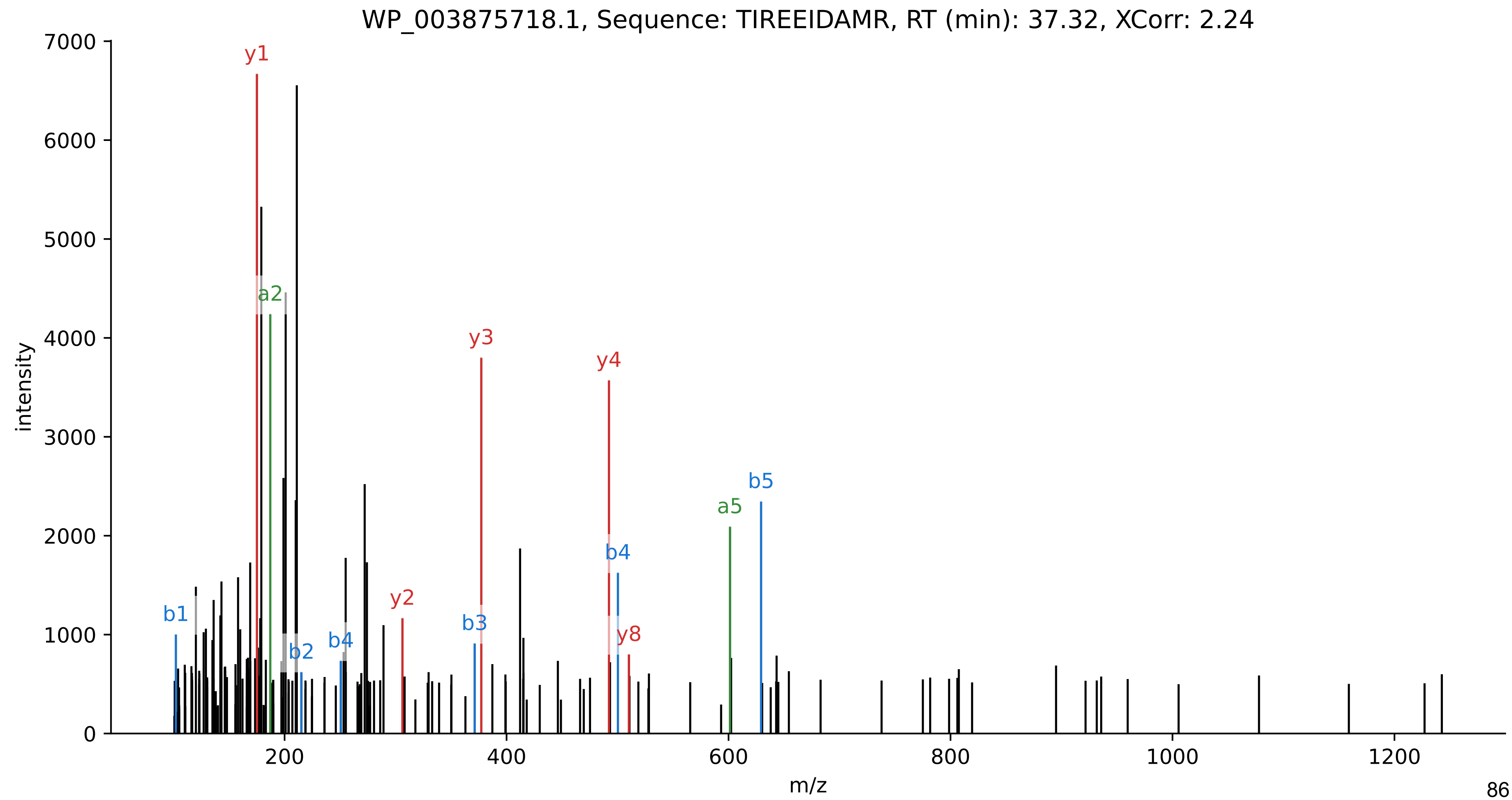
WP_003875718.1, Sequence: TIREEIDAMR, RT (min): 37.32, XCorr: 2.24
intensity
m/z
y1
a2
y3
y4
b5
a5
b4
y2
b1
b3
y8
b2
b4
0
1000
2000
3000
4000
5000
6000
7000
200
400
600
800
1000
1200

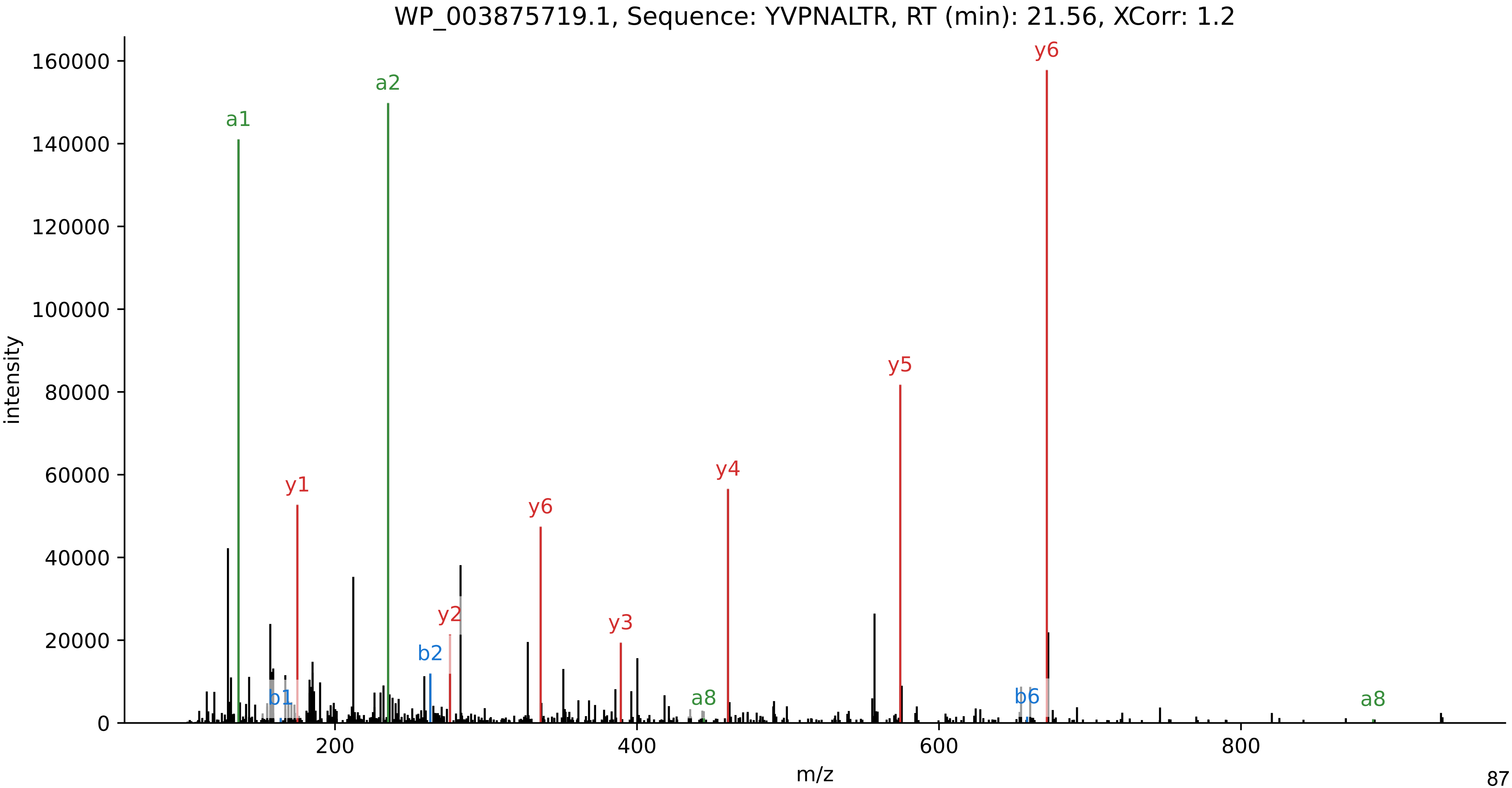

WP_003875719.1, Sequence: YVPNALTR, RT (min): 21.56, XCorr: 1.2
intensity
m/z
a1
a2
y1
y6
y2
b2
b1
y3
a8
y4
y5
y6
b6
a8
160000
140000
120000
100000
80000
60000
40000
20000
0
200
400
600
800

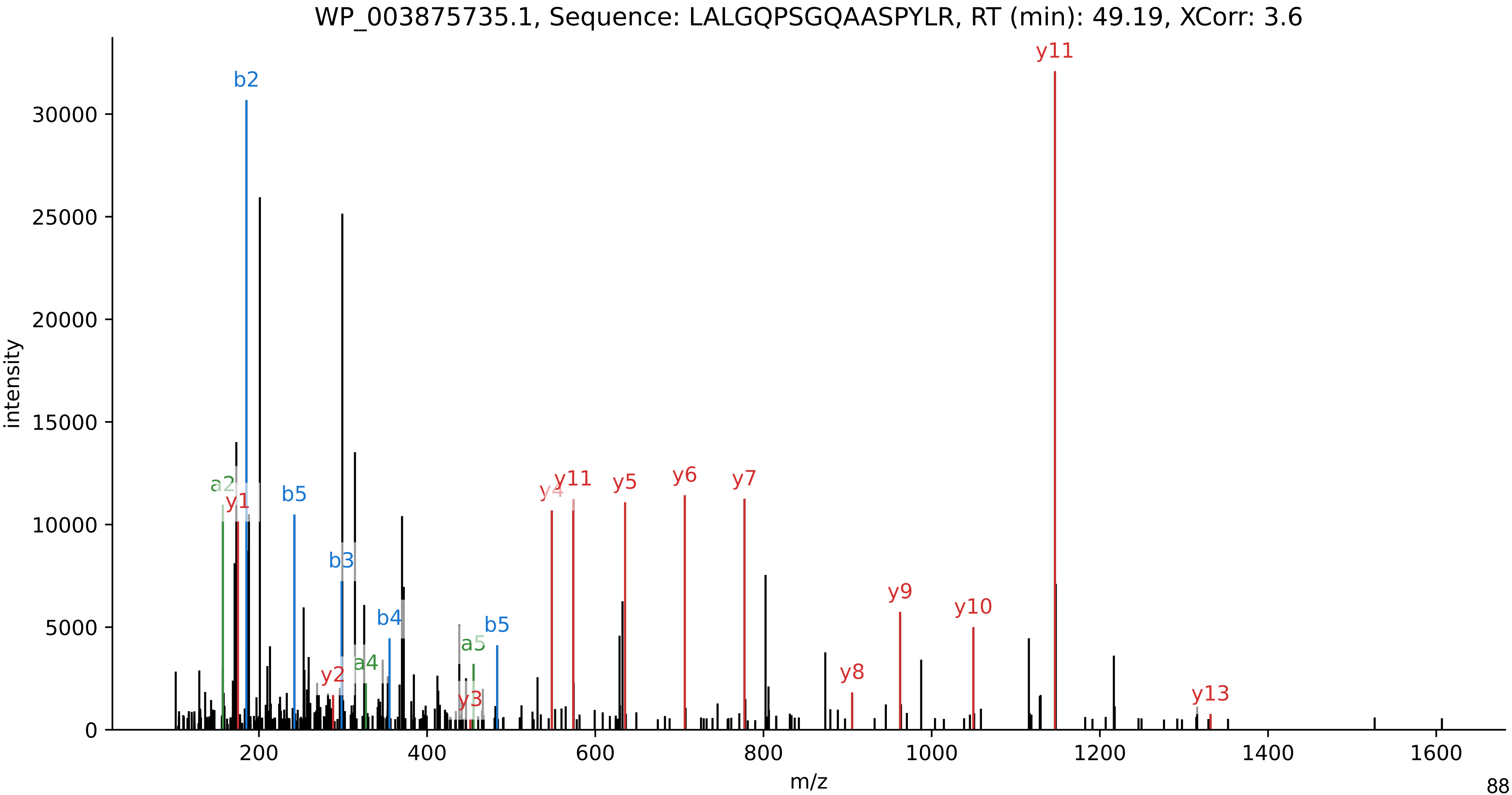

WP_003875735.1, Sequence: LALGQPSGQAASPYLR, RT (min): 49.19, XCorr: 3.6
intensity
m/z
y11
b2
a2
y1
b5
y4
y11
y5
y6
y7
b3
b4
b5
a5
a4
y2
y3
y9
y10
y8
y13
30000
25000
20000
15000
10000
5000
0
200
400
600
800
1000
1200
1400
1600

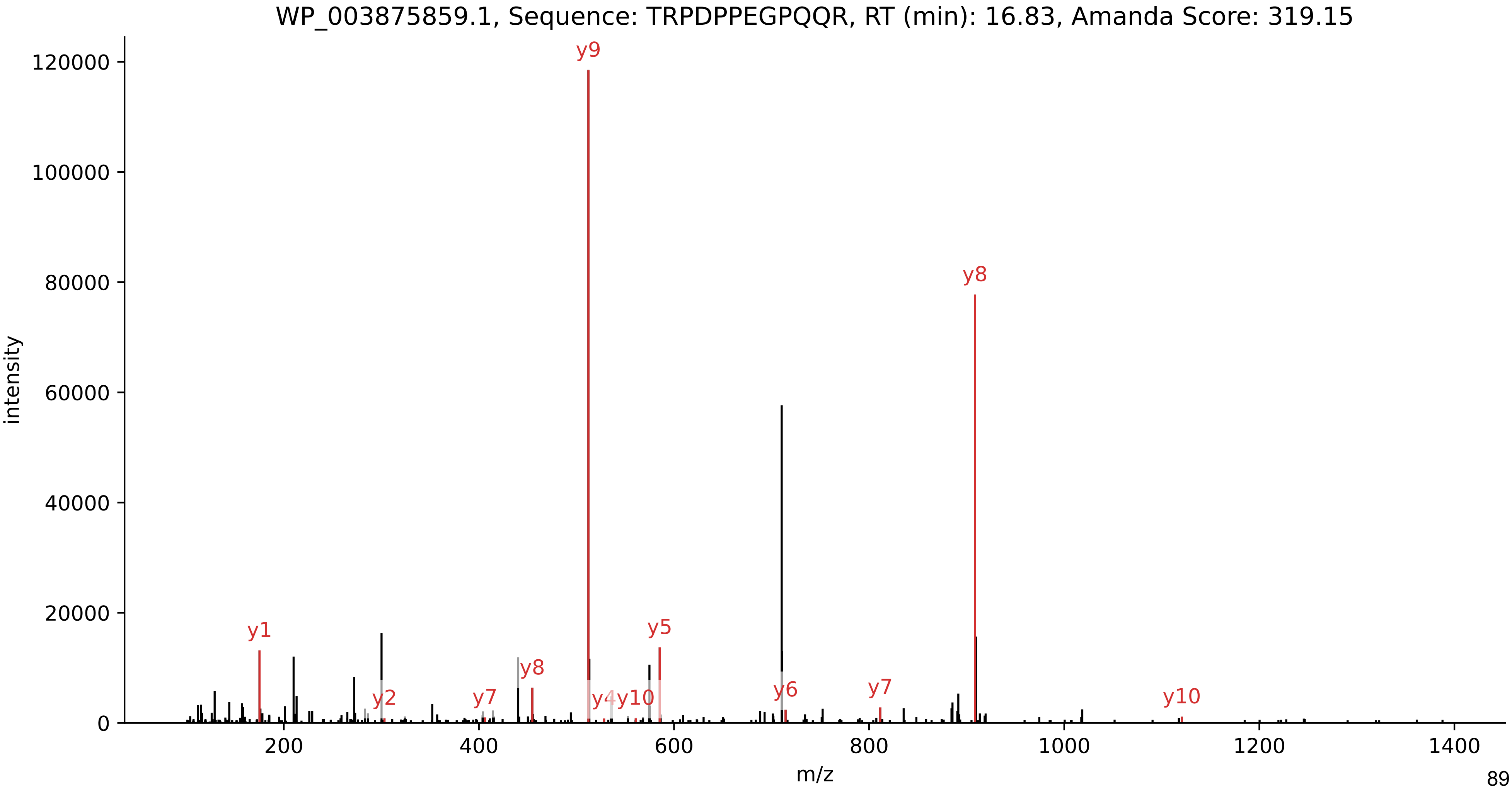

WP_003875859.1, Sequence: TRPDPPEGPQQR, RT (min): 16.83, Amanda Score: 319.15
intensity
m/z
y9
y8
y1
y5
y8
y2
y7
y4
y10
y6
y7
y10
0
20000
40000
60000
80000
100000
120000
200
400
600
800
1000
1200
1400

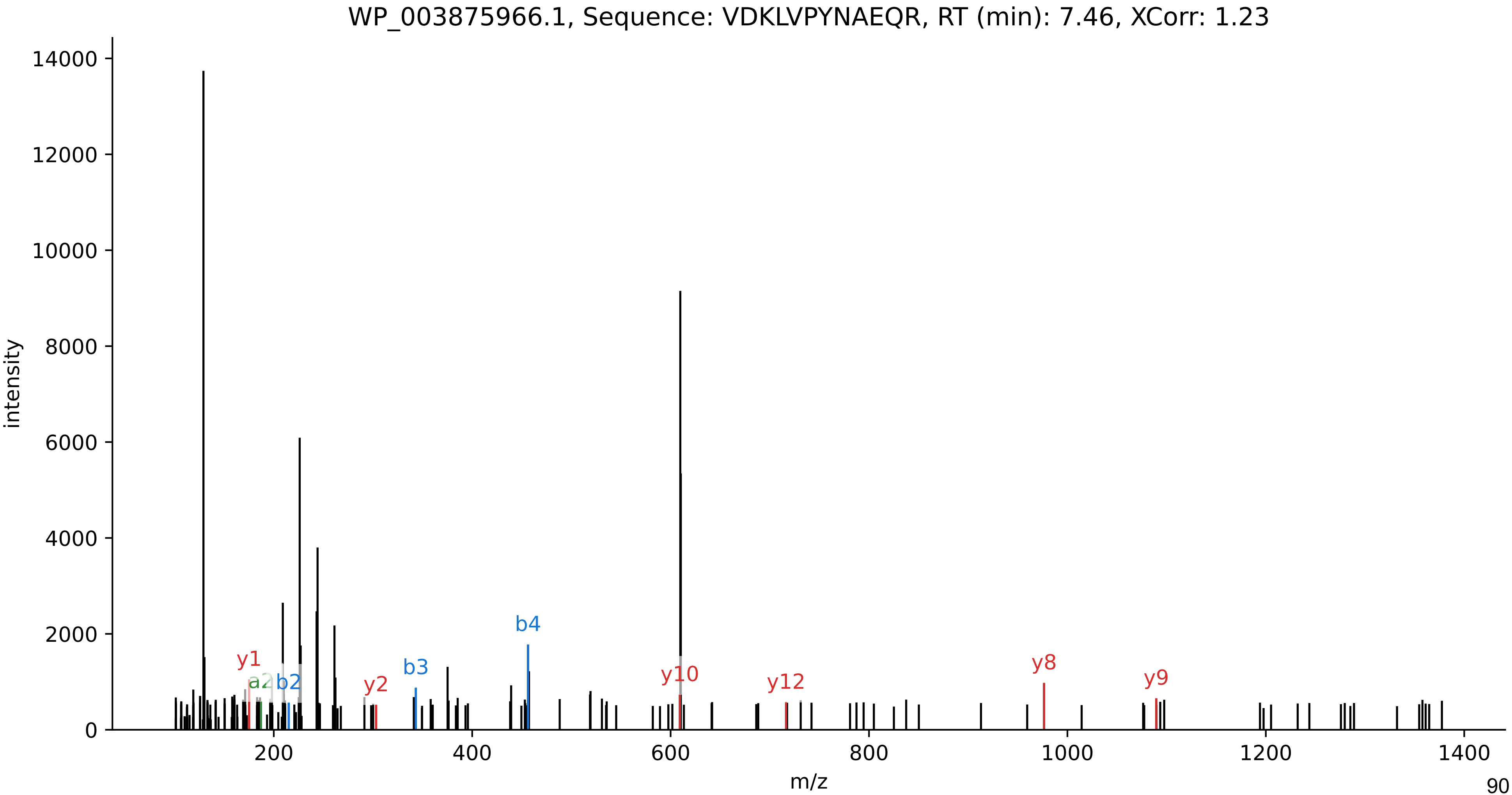
WP_003875966.1, Sequence: VDKLVPYNAEQR, RT (min): 7.46, XCorr: 1.23
intensity
m/z
y1
a2
b2
y2
b3
b4
y10
y12
y8
y9
0
2000
4000
6000
8000
10000
12000
14000
200
400
600
800
1000
1200
1400

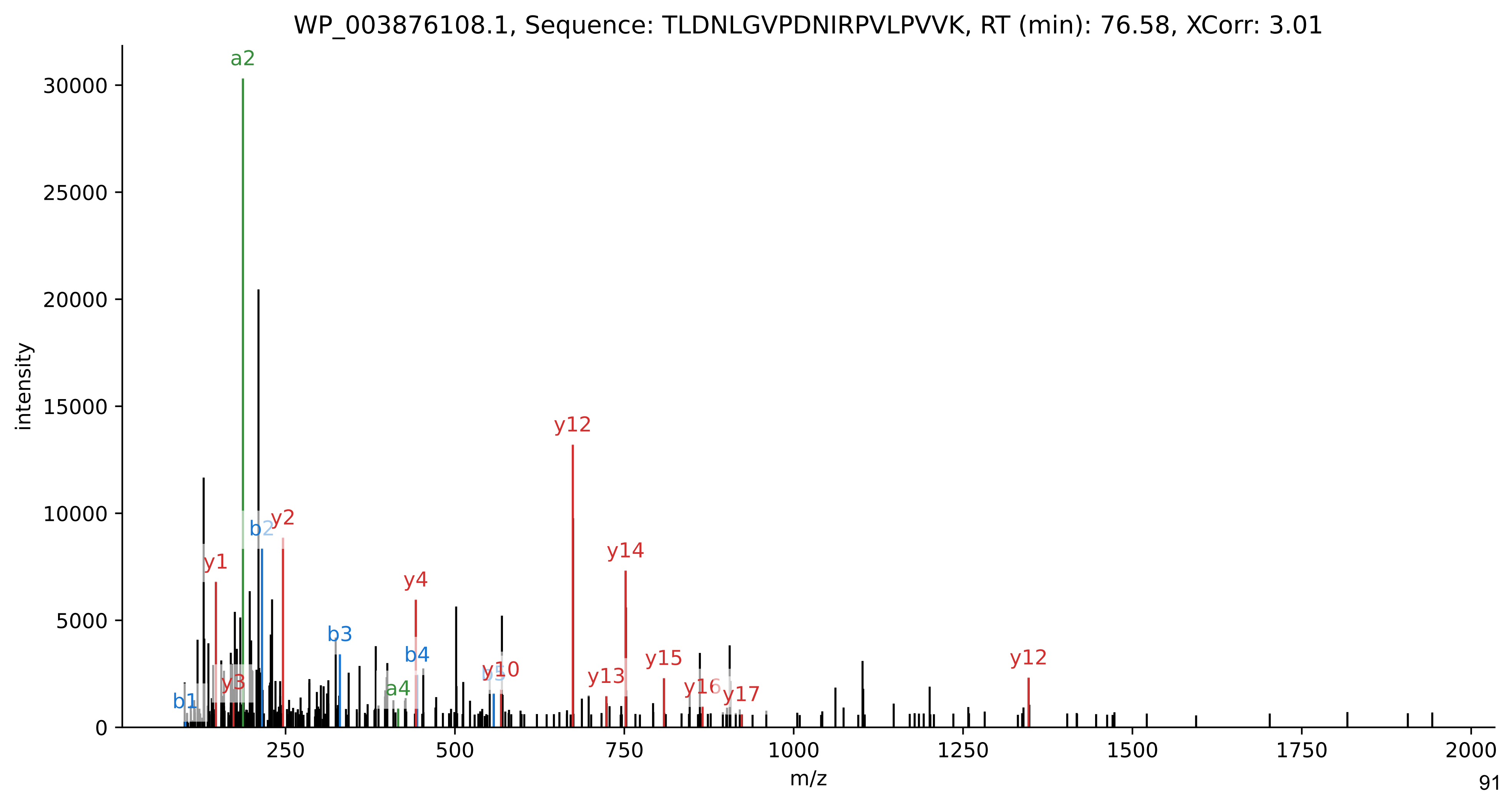
WP_003876108.1, Sequence: TLDNLGVPDNIRPVLPVVK, RT (min): 76.58, XCorr: 3.01
intensity
m/z
a2
y12
y2
b2
y1
y14
y4
b3
b4
y10
b5
y3
a4
b1
y13
y15
y16
y17
y12
0
5000
10000
15000
20000
25000
30000
250
500
750
1000
1250
1500
1750
2000

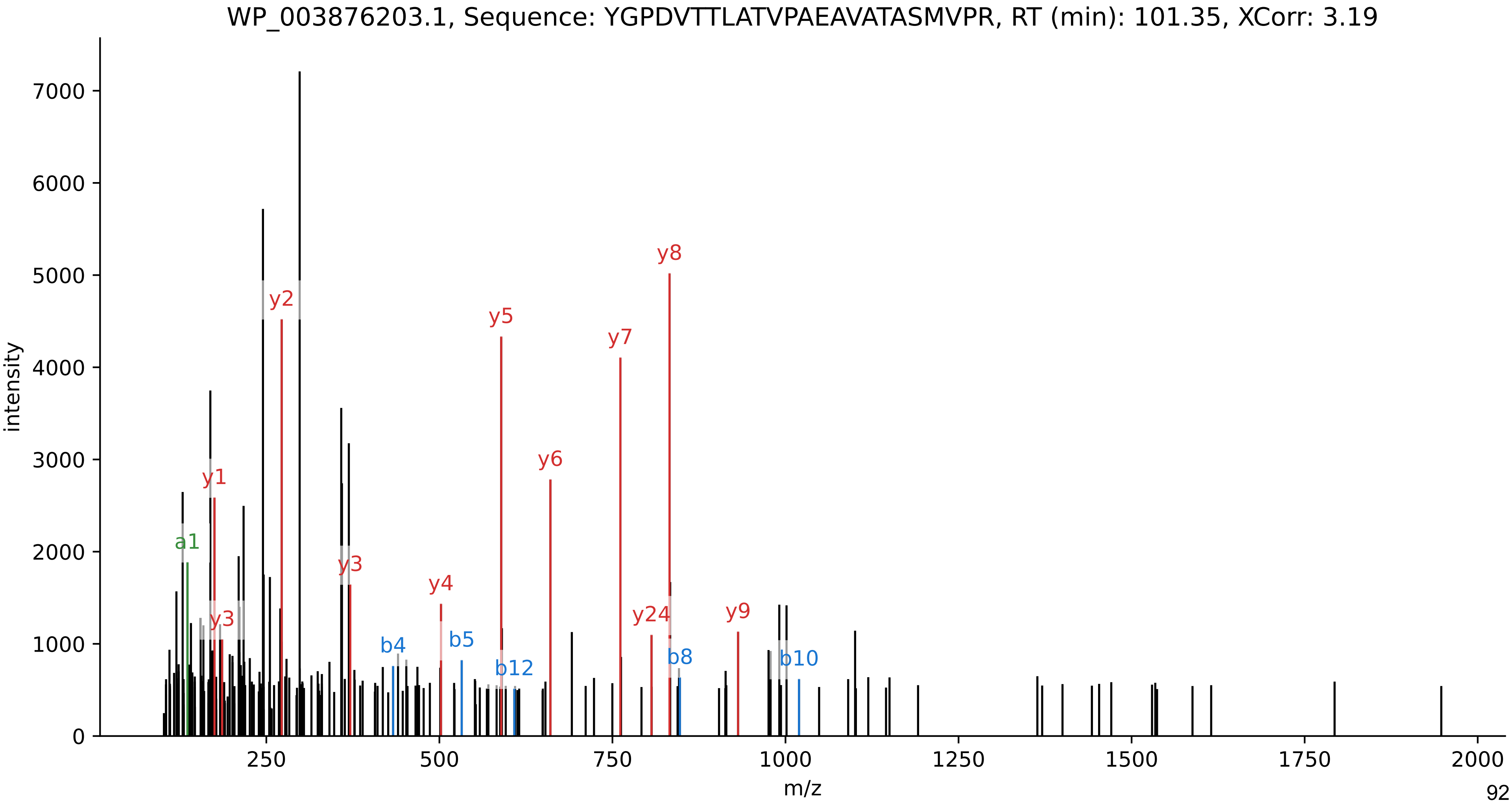
WP_003876203.1, Sequence: YGPDVTTLATVPAEAVATASMVPR, RT (min): 101.35, XCorr: 3.19
intensity
m/z
a1
y1
y3
y2
y3
y4
b4
b5
y5
b12
y6
y7
y24
y8
b8
y9
b10
0
1000
2000
3000
4000
5000
6000
7000
250
500
750
1000
1250
1500
1750
2000

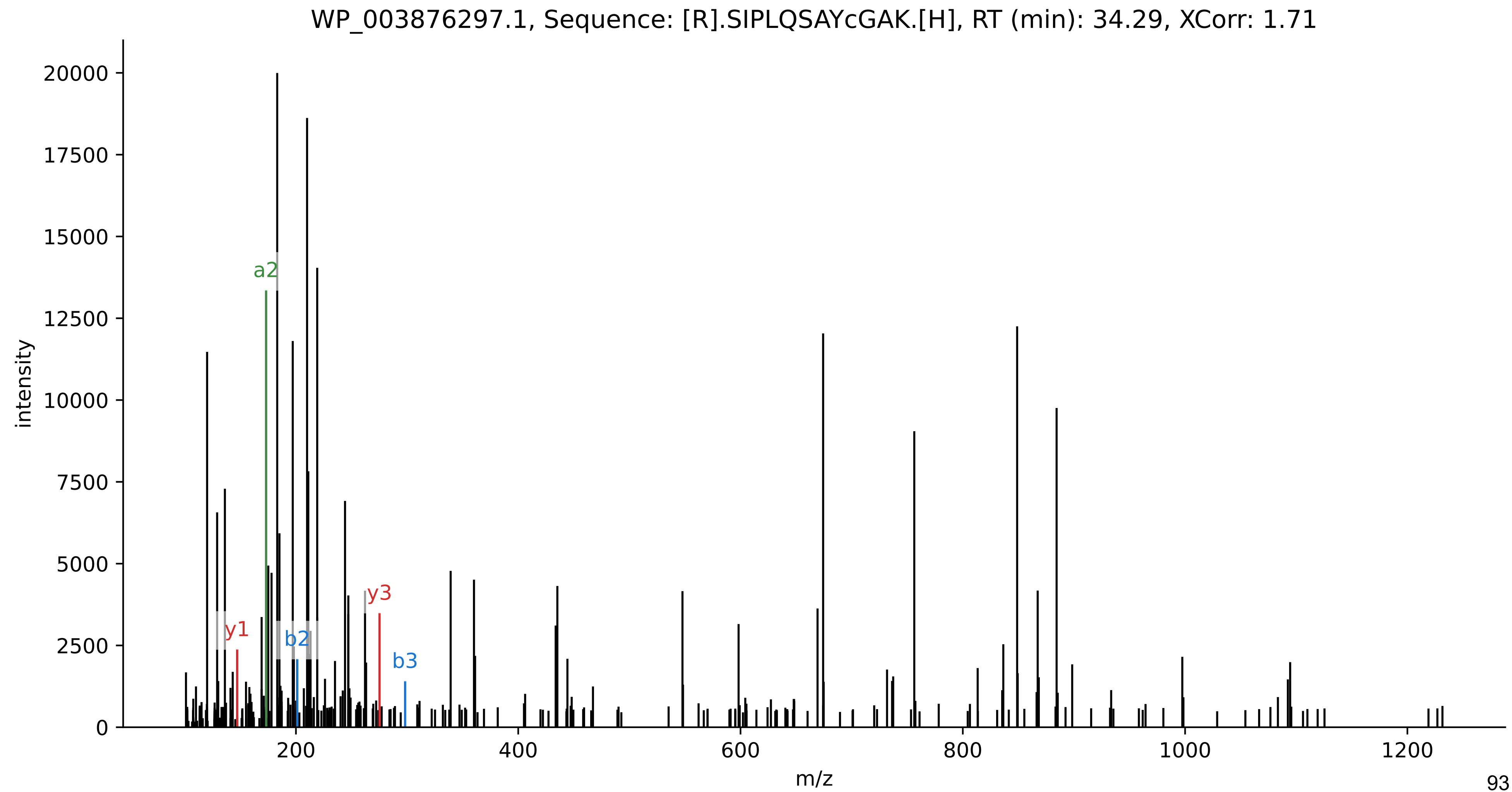
WP_003876297.1, Sequence: [R].SIPLQSAYcGAK.[H], RT (min): 34.29, XCorr: 1.71
intensity
m/z
a2
y1
b2
y3
b3
0
2500
5000
7500
10000
12500
15000
17500
20000
200
400
600
800
1000
1200

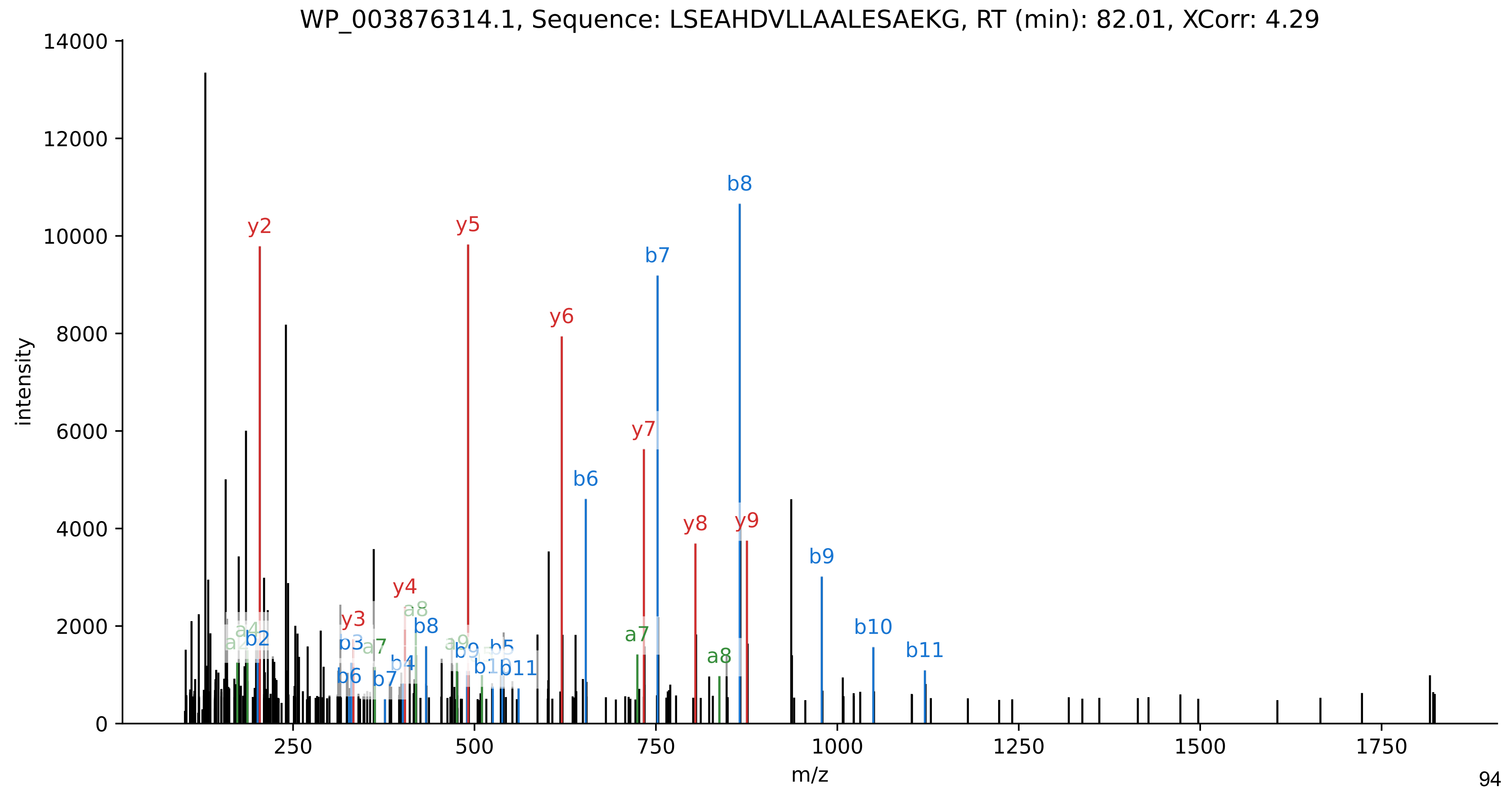

WP_003876314.1, Sequence: LSEAHDVLLAALESAEKG, RT (min): 82.01, XCorr: 4.29
intensity
m/z
y2
y5
y6
y7
y8
y9
y4
y3
b2
b6
b7
b8
b9
b10
b11
a7
a8

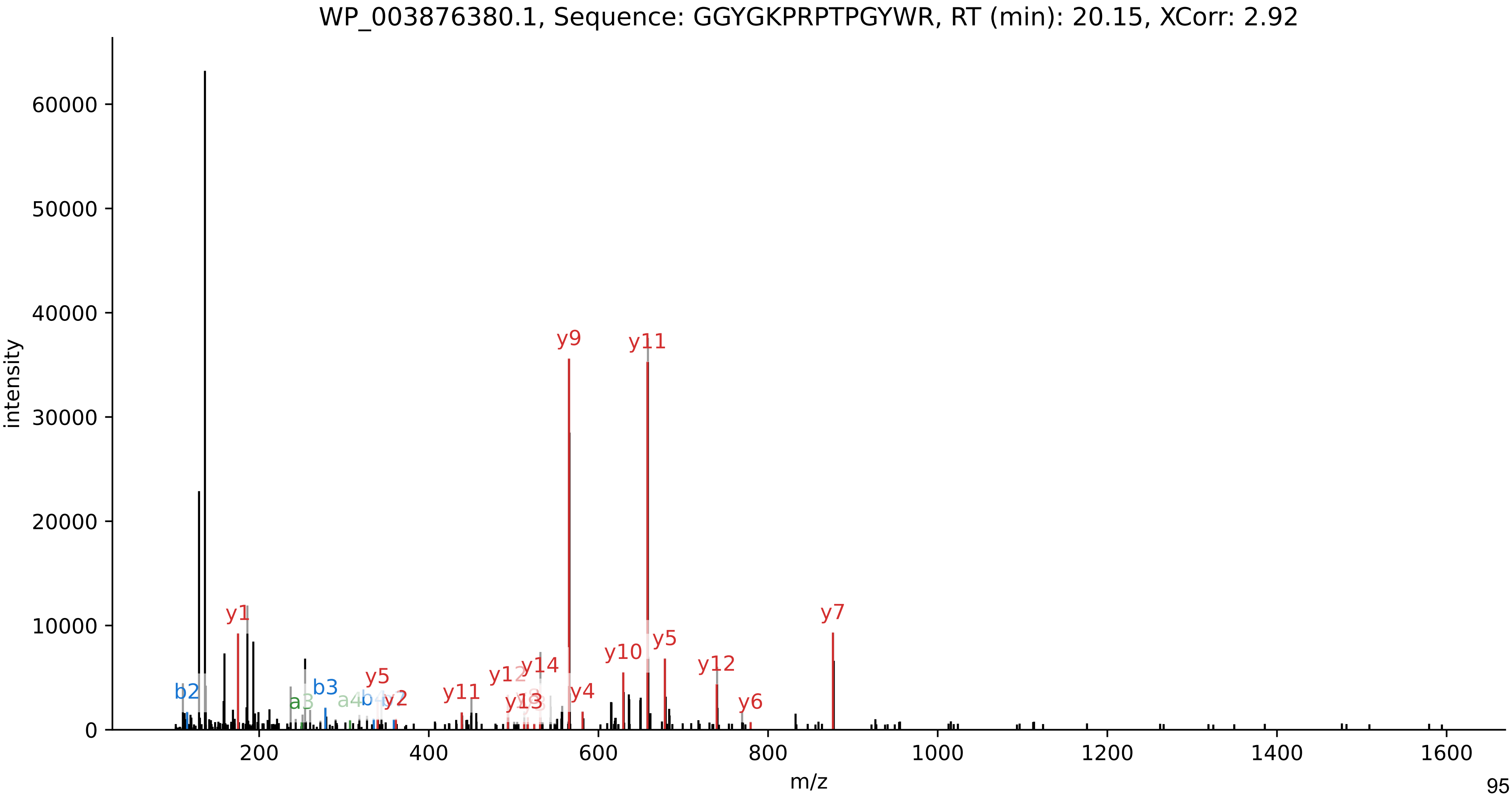
WP_003876380.1, Sequence: GGYGKPRPTPGYWR, RT (min): 20.15, XCorr: 2.92
intensity
m/z
y9
y11
y1
y7
y10
y5
y12
y14
y12
y13
y4
y6
y11
y5
y2
b2
b3
a3
a4
b4
0
10000
20000
30000
40000
50000
60000
200
400
600
800
1000
1200
1400
1600

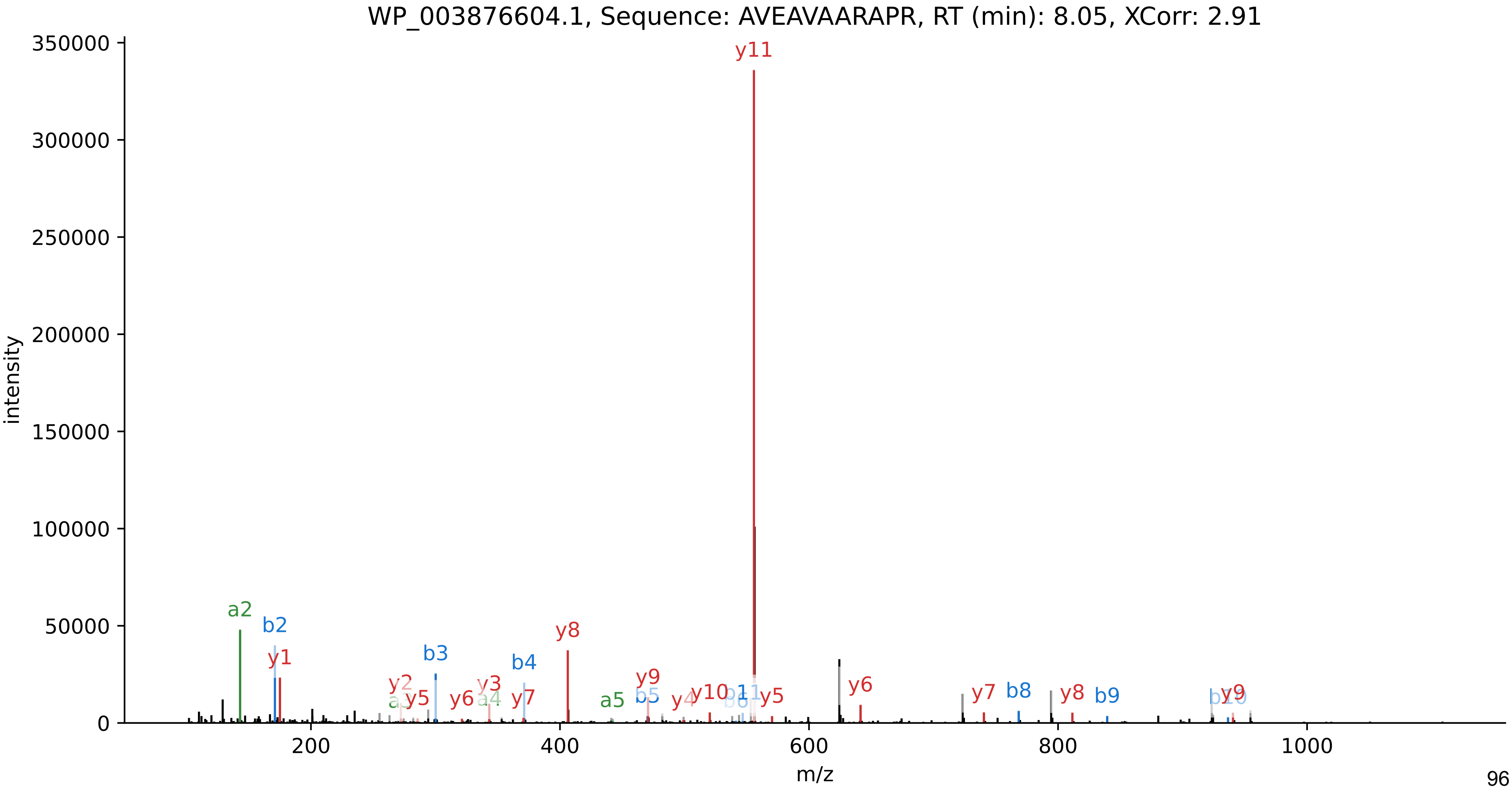

WP_003876604.1, Sequence: AVEAVAARAPR, RT (min): 8.05, XCorr: 2.91
intensity
m/z
y11
a2
b2
y1
b3
b4
y8
y2
y3
y5
y6
a4
y7
a5
y9
b5
y4
y10
y5
y6
y7
b8
y8
b9
y9

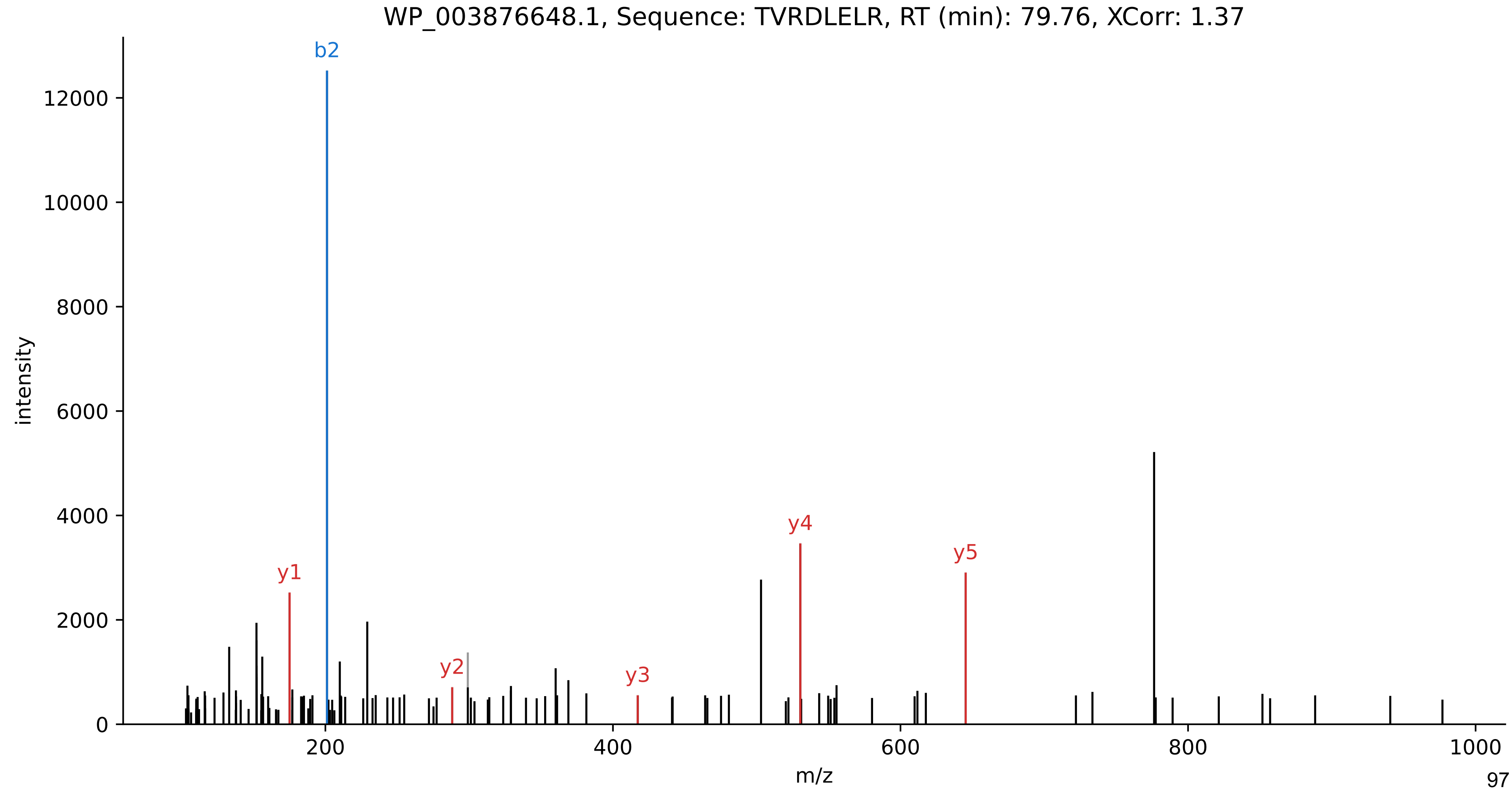

WP_003876648.1, Sequence: TVRDLELR, RT (min): 79.76, XCorr: 1.37
b2
y1
y2
y3
y4
y5
intensity
m/z
0
2000
4000
6000
8000
10000
12000
200
400
600
800
1000

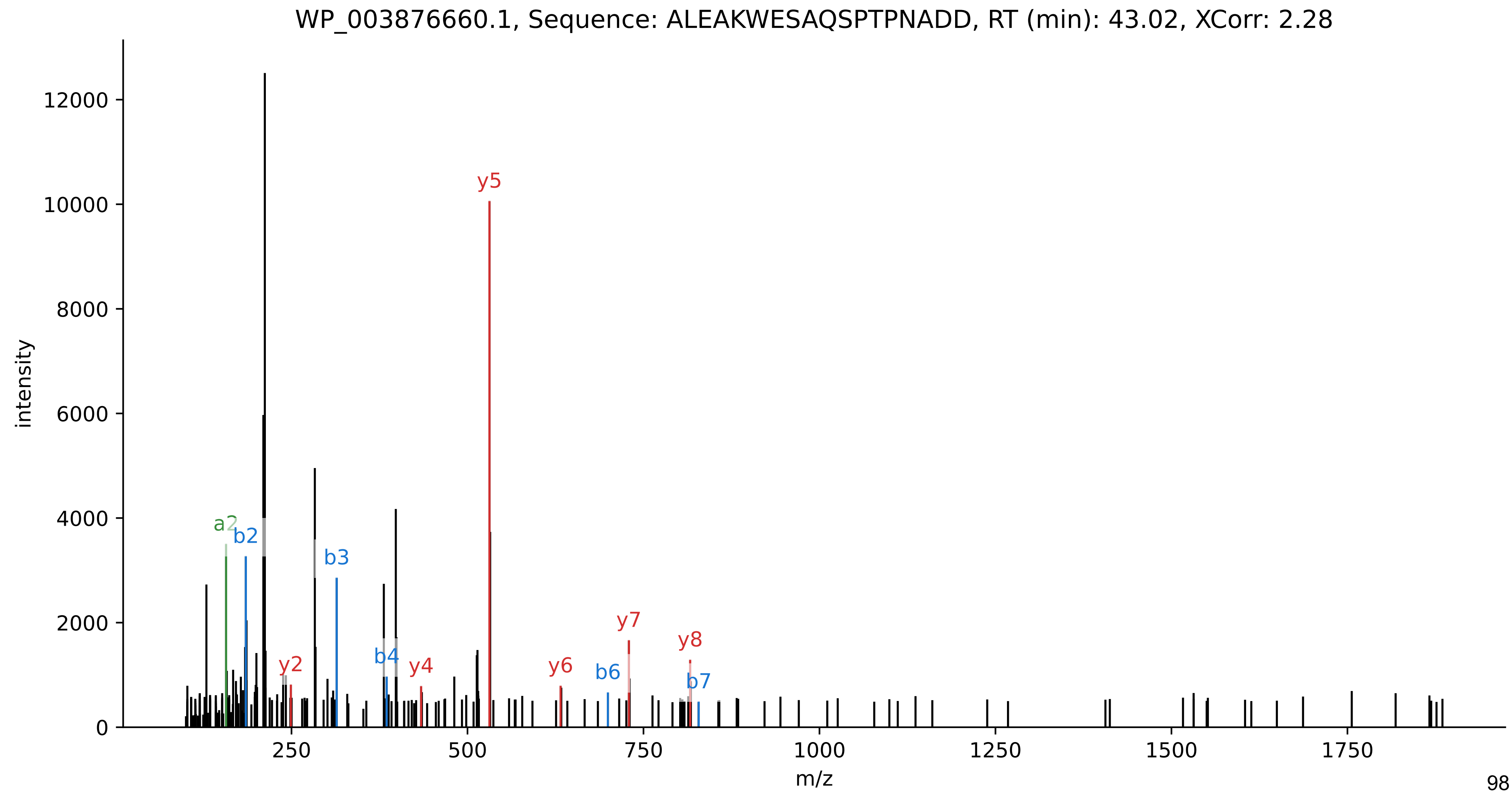
WP_003876660.1, Sequence: ALEAKWESAQSPTPNADD, RT (min): 43.02, XCorr: 2.28
intensity
m/z
a2
b2
b3
b4
b6
b7
y2
y4
y5
y6
y7
y8
0
2000
4000
6000
8000
10000
12000
250
500
750
1000
1250
1500
1750

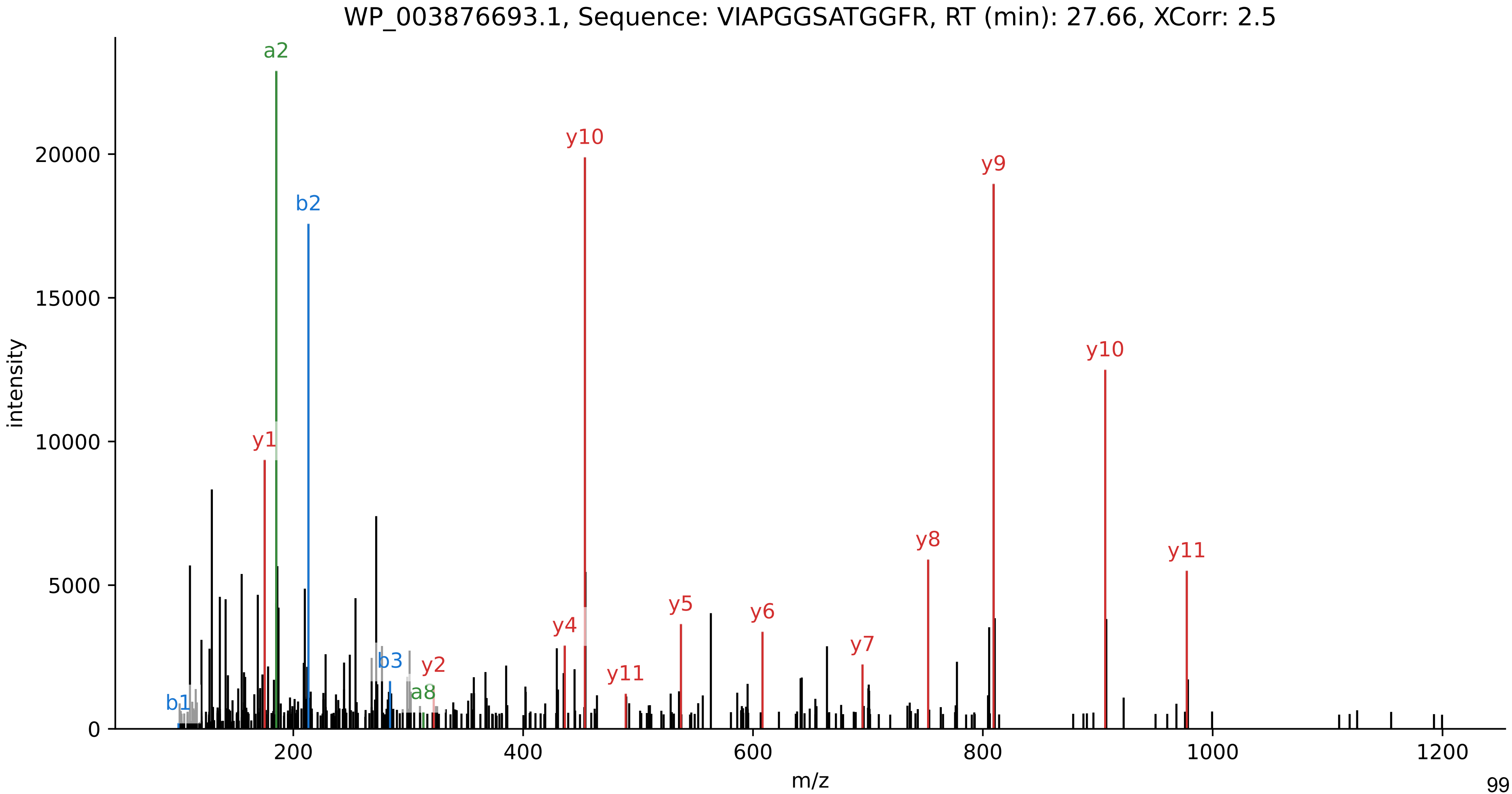
WP_003876693.1, Sequence: VIAPGGSATGGFR, RT (min): 27.66, XCorr: 2.5
intensity
m/z
a2
b2
y10
y9
y10
y1
y8
y11
y5
y6
y4
y7
y11
b3
y2
a8
b1
0
5000
10000
15000
20000
200
400
600
800
1000
1200

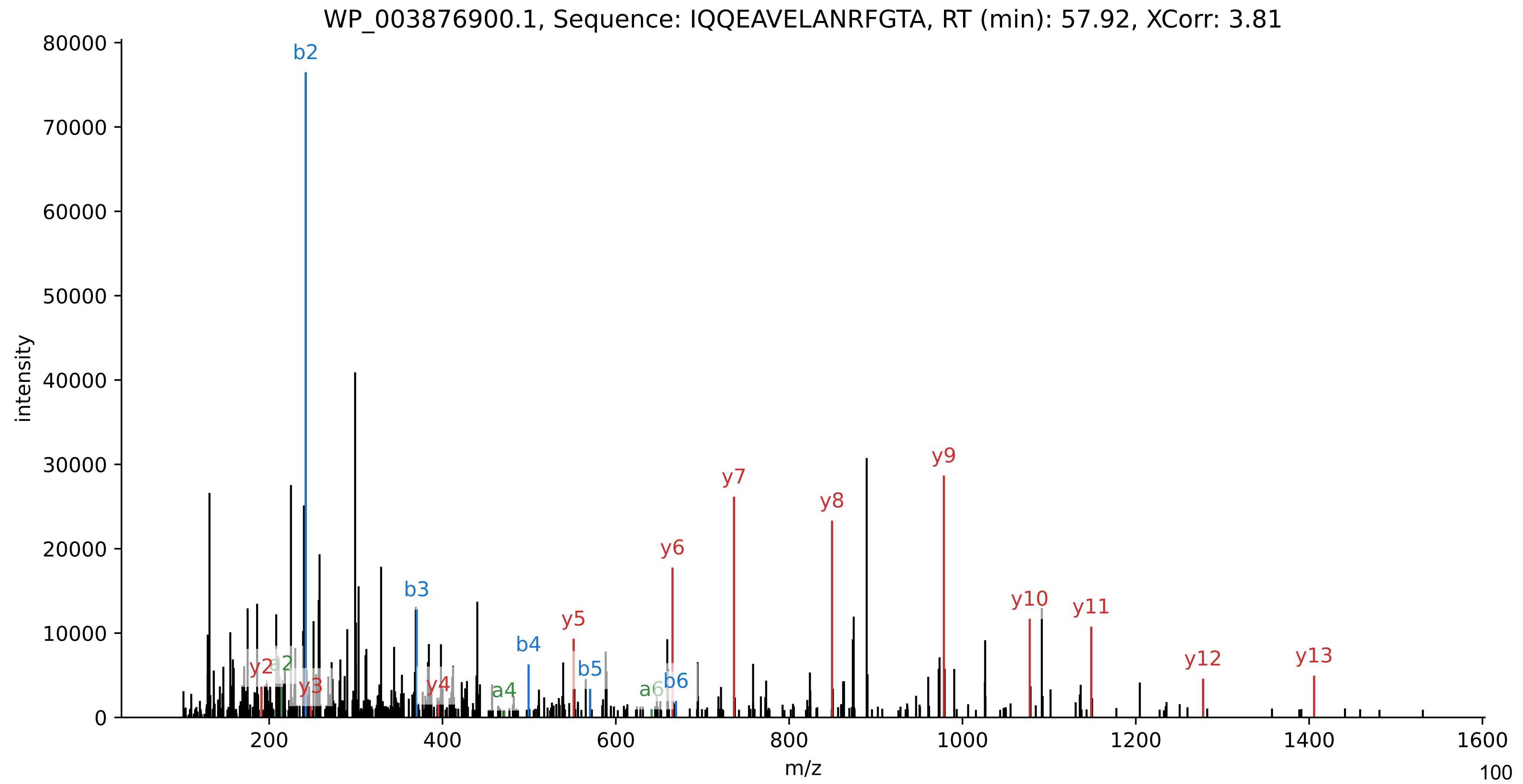

WP_003876900.1, Sequence: IQQEAVELANRFGTA, RT (min): 57.92, XCorr: 3.81
intensity
m/z
b2
b3
b4
b5
b6
a2
a4
a6
y2
y3
y4
y5
y6
y7
y8
y9
y10
y11
y12
y13
0
10000
20000
30000
40000
50000
60000
70000
80000
200
400
600
800
1000
1200
1400
1600

WP_003876987.1, Sequence: IVAAATDANPAFGAYYDLQTDETGATKR, RT (min): 68.71, XCorr: 5.32

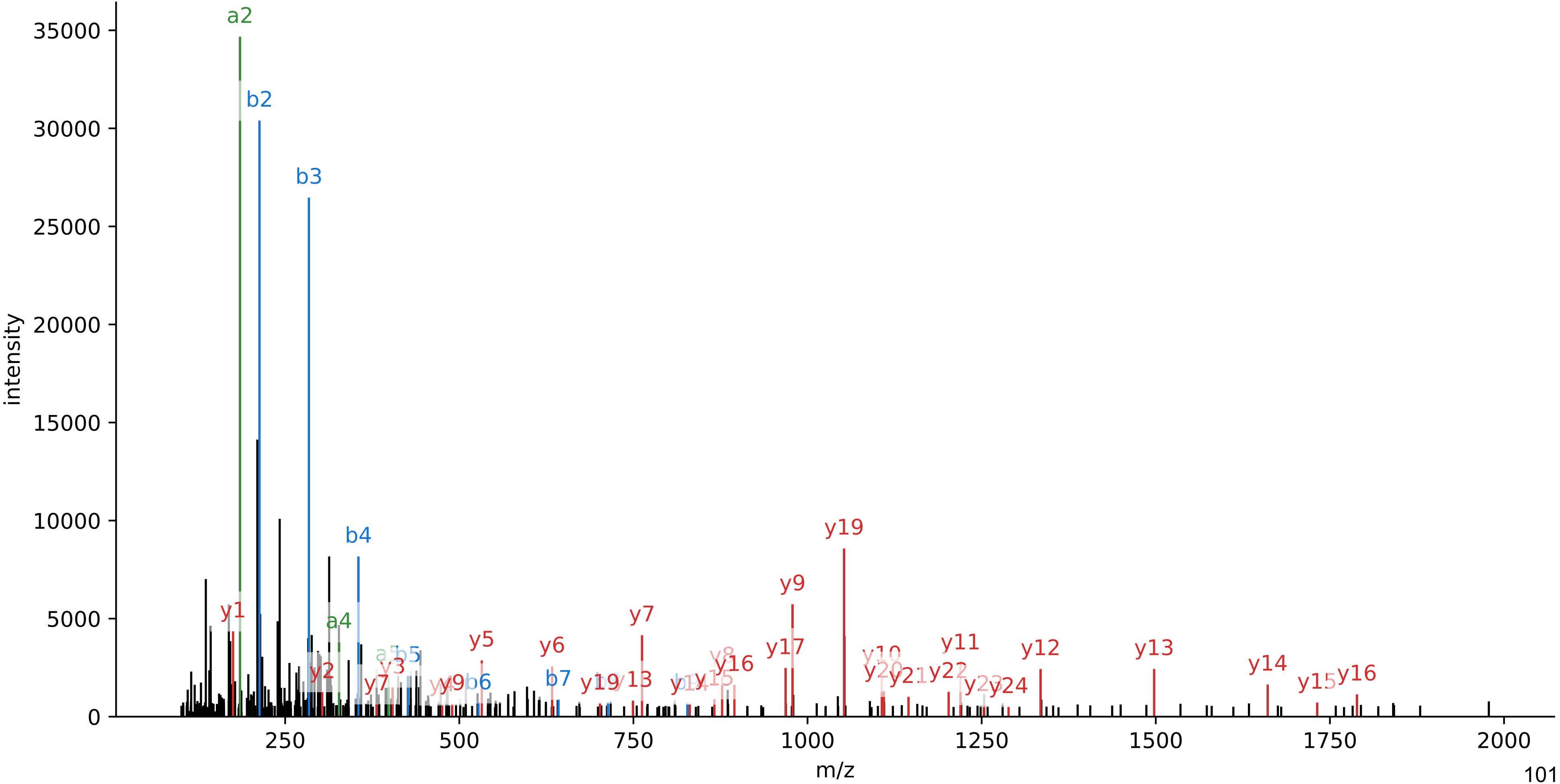

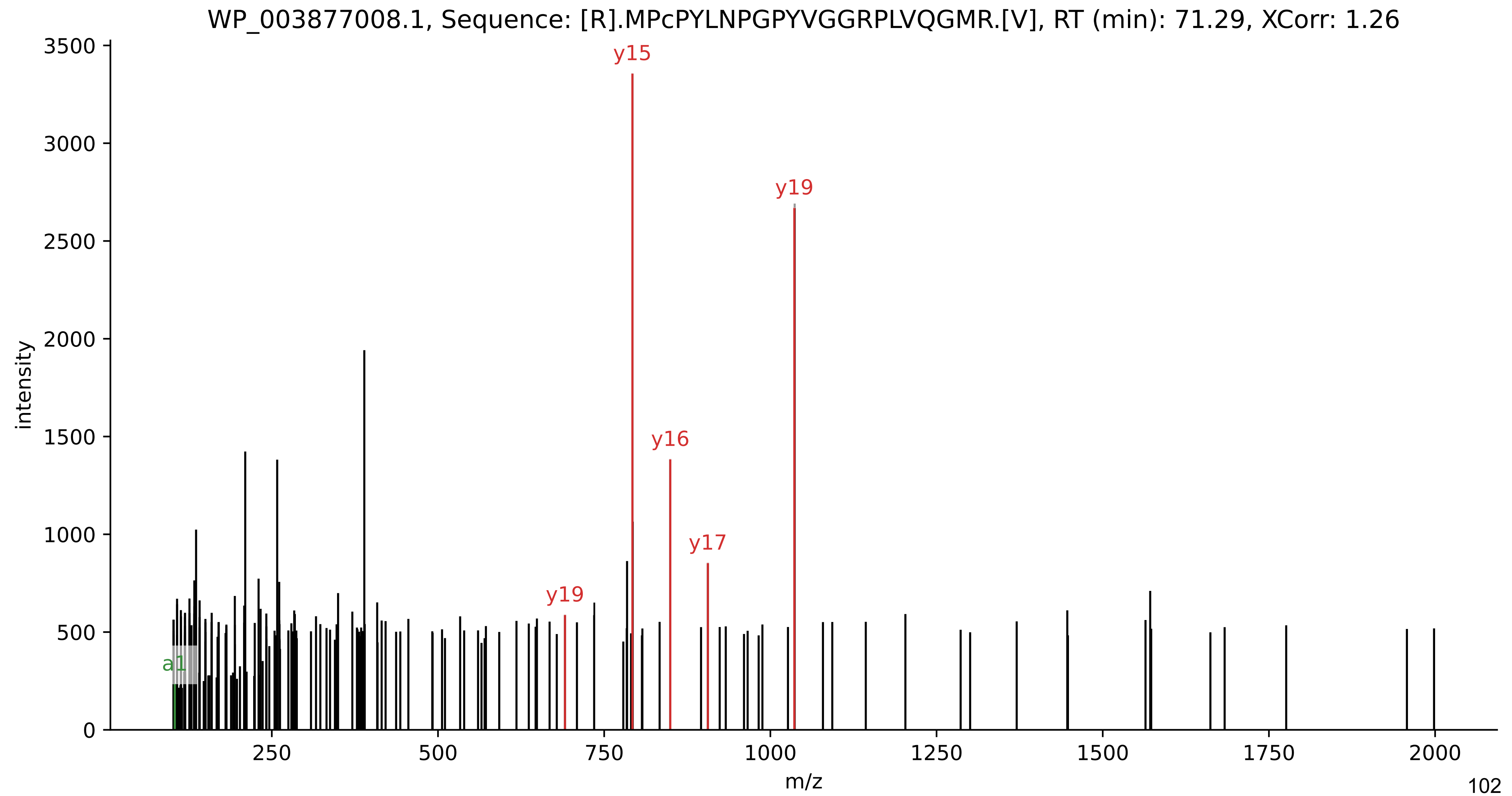
WP_003877008.1, Sequence: [R].MPcPYLNPGPYVGGRPLVQGMR.[V], RT (min): 71.29, XCorr: 1.26
y15
y19
y16
y17
y19
a1
intensity
m/z
0
500
1000
1500
2000
2500
3000
3500
250
500
750
1000
1250
1500
1750
2000

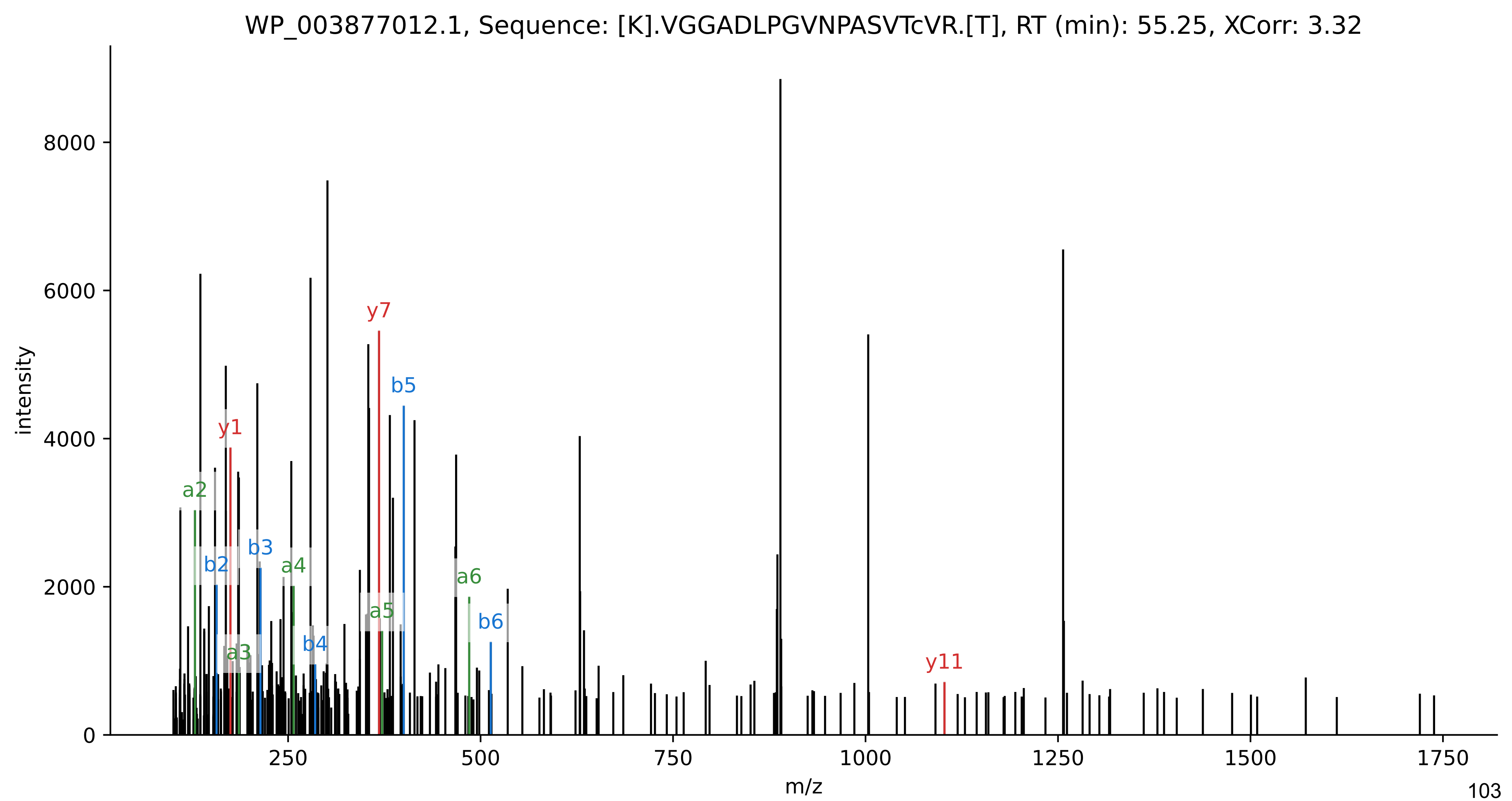
WP_003877012.1, Sequence: [K].VGGADLPGVNPASVTcVR.[T], RT (min): 55.25, XCorr: 3.32
intensity
m/z
0
2000
4000
6000
8000
250
500
750
1000
1250
1500
1750
y1
y7
y11
a2
a3
a4
a5
a6
b2
b3
b4
b5
b6

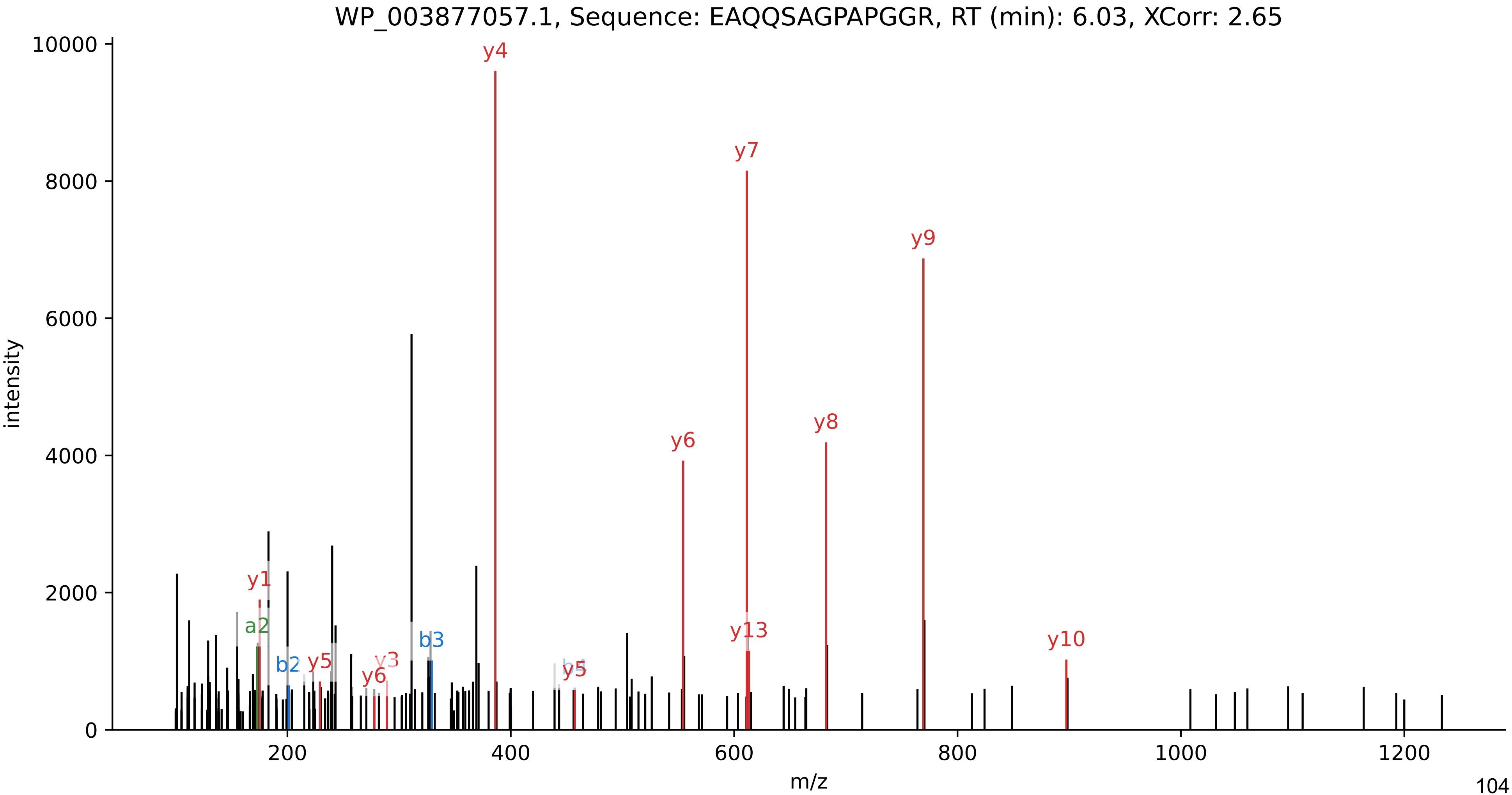
WP_003877057.1, Sequence: EAQQSAGPAPGGR, RT (min): 6.03, XCorr: 2.65
intensity
m/z
y4
y7
y9
y8
y6
y13
y10
y1
a2
b2
y5
y3
y6
b3
y5
10000
8000
6000
4000
2000
0
200
400
600
800
1000
1200

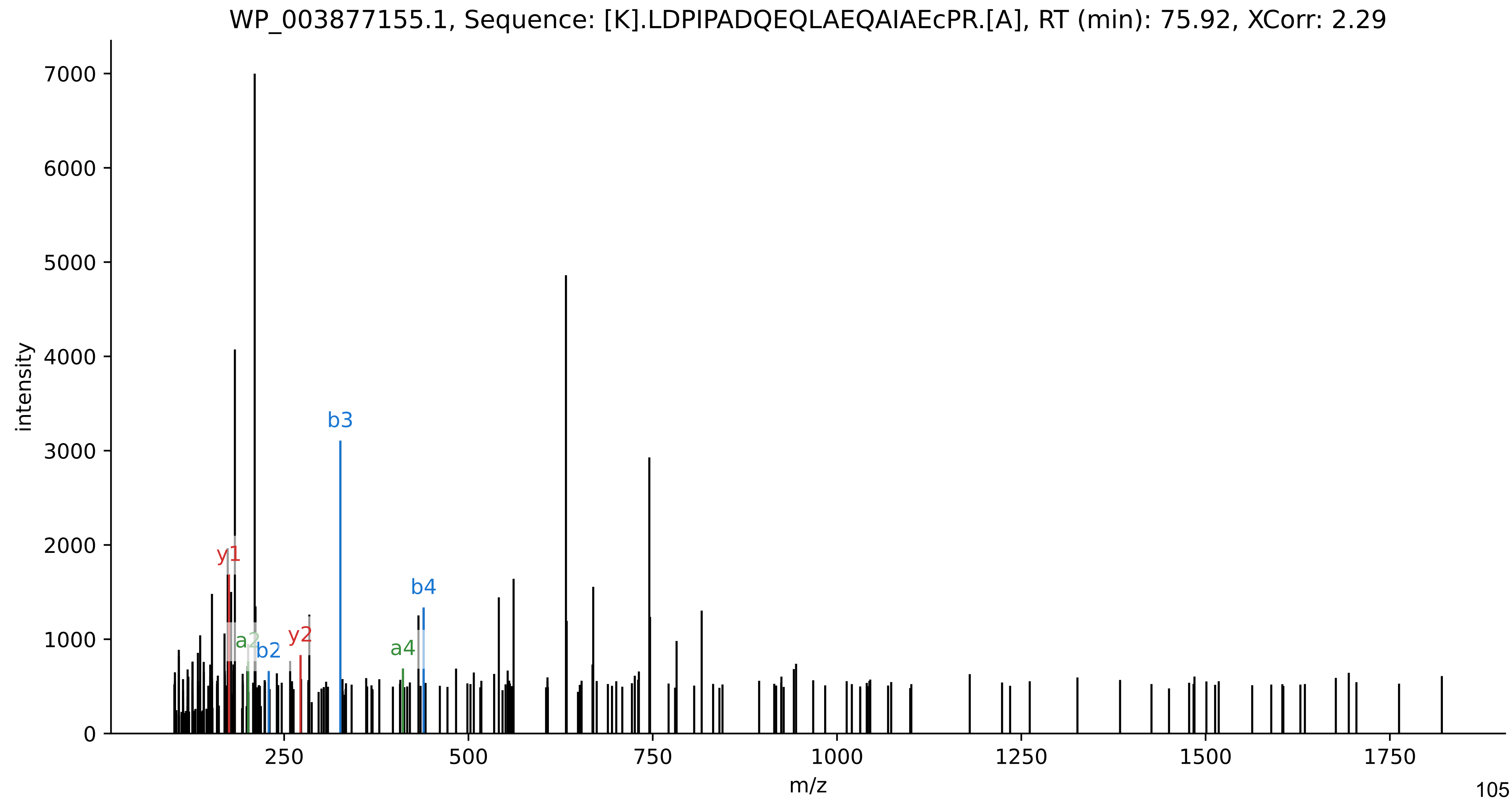

WP_003877155.1, Sequence: [K].LDPIPADQEQLAEQAIAEcPR.[A], RT (min): 75.92, XCorr: 2.29
intensity
m/z
0
1000
2000
3000
4000
5000
6000
7000
250
500
750
1000
1250
1500
1750
y1
a2
b2
y2
b3
a4
b4

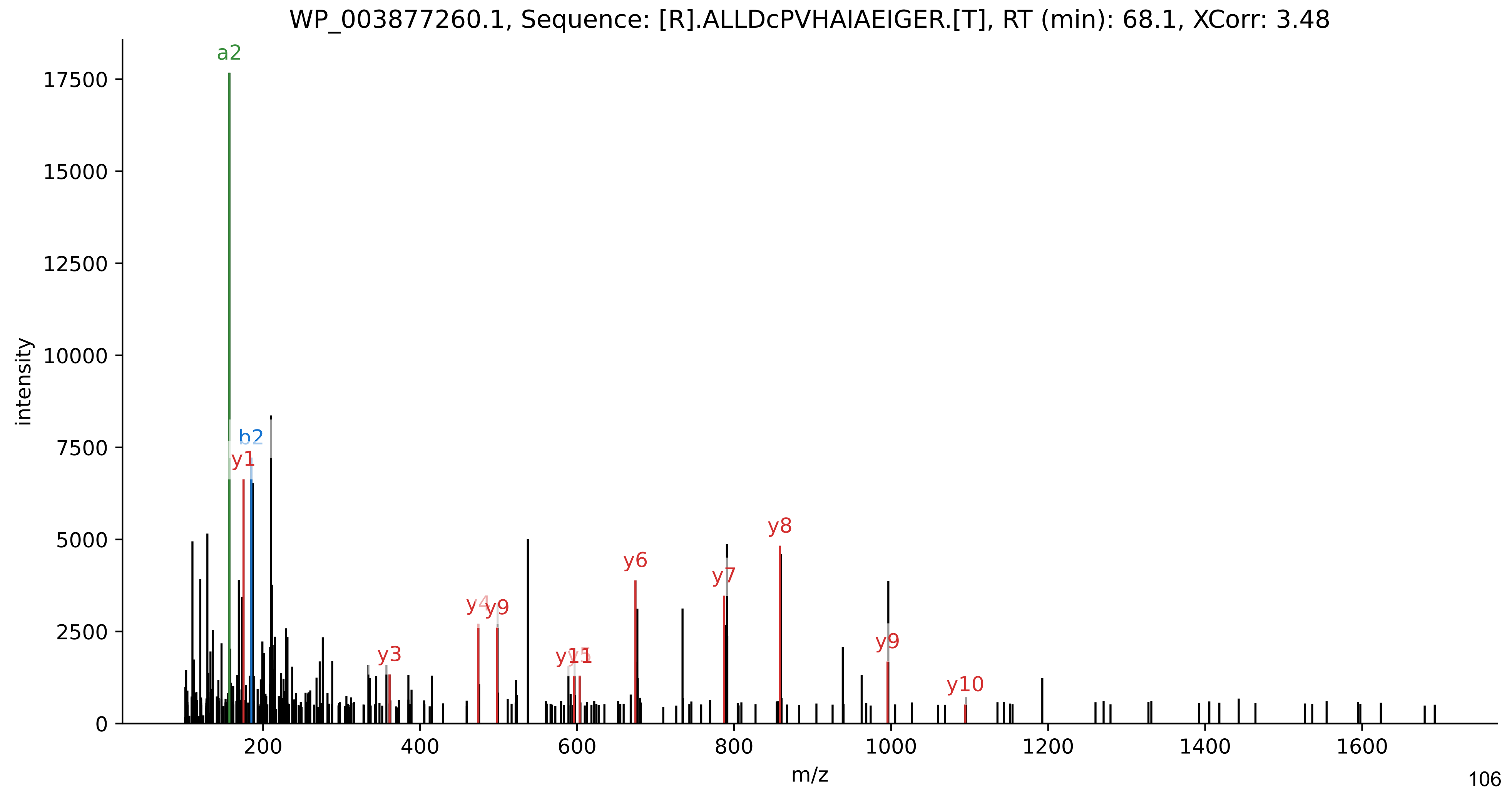
WP_003877260.1, Sequence: [R].ALLDcPVHAIAEIGER.[T], RT (min): 68.1, XCorr: 3.48
intensity
m/z
a2
b2
y1
y3
y4
y9
y11
y6
y7
y8
y9
y10

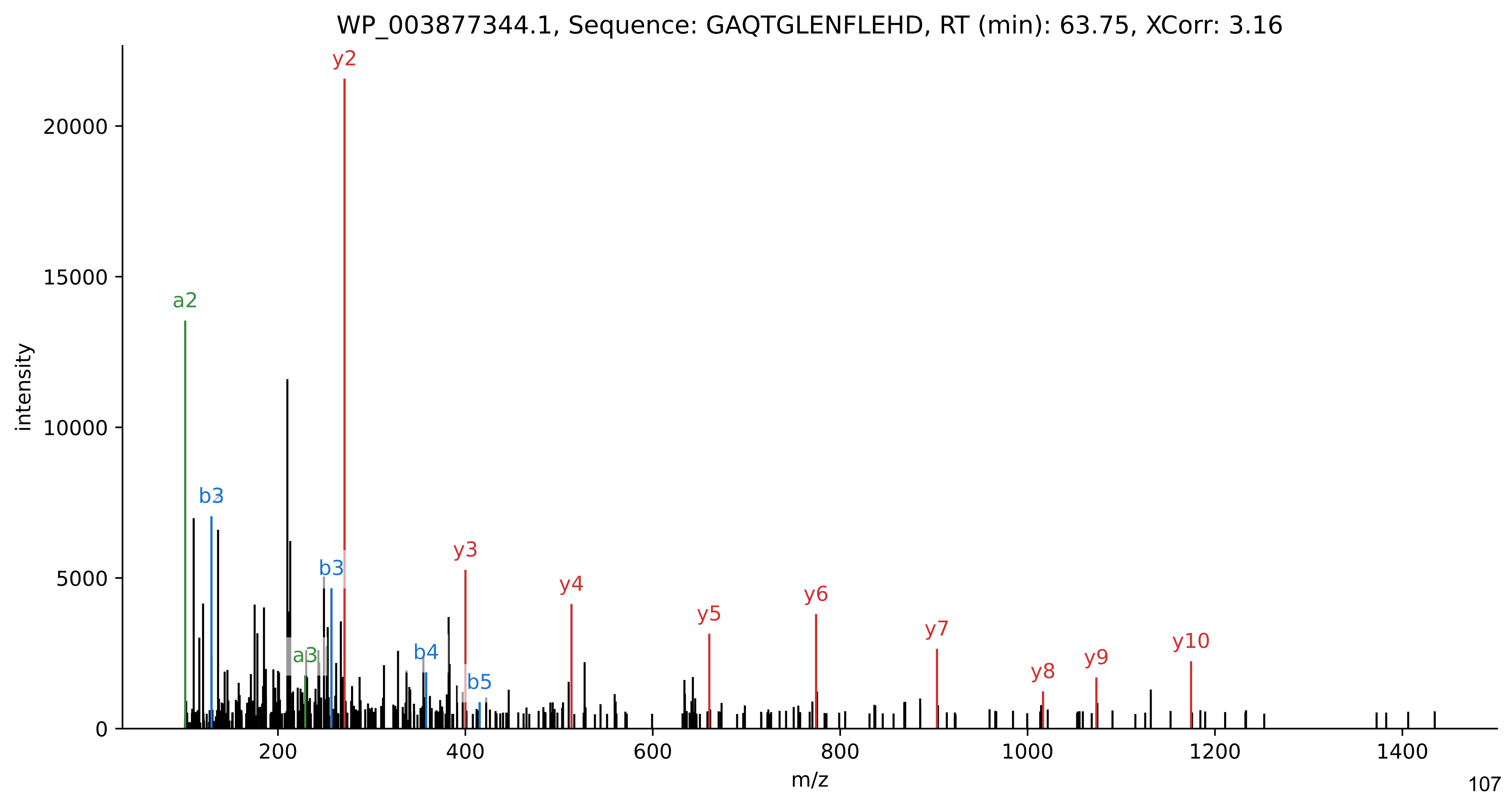
WP_003877344.1, Sequence: GAQTGLENFLEHD, RT (min): 63.75, XCorr: 3.16
intensity
m/z
a2
b3
y2
b3
y3
a3
b4
b5
y4
y5
y6
y7
y8
y9
y10

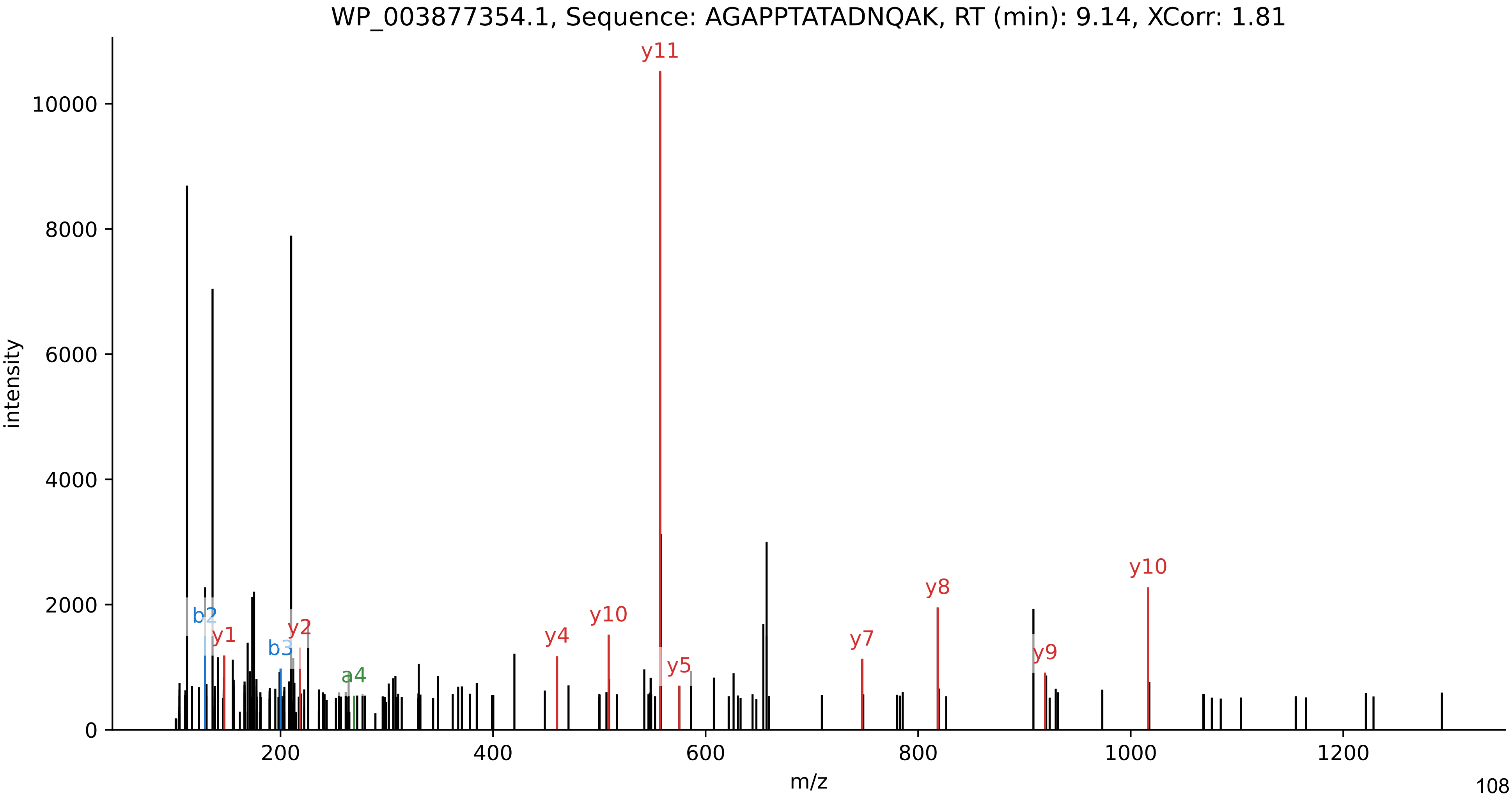

WP_003877354.1, Sequence: AGAPPTATADNQAK, RT (min): 9.14, XCorr: 1.81
intensity
m/z
y11
b2
y1
b3
y2
a4
y4
y10
y5
y7
y8
y9
y10
0
2000
4000
6000
8000
10000
200
400
600
800
1000
1200

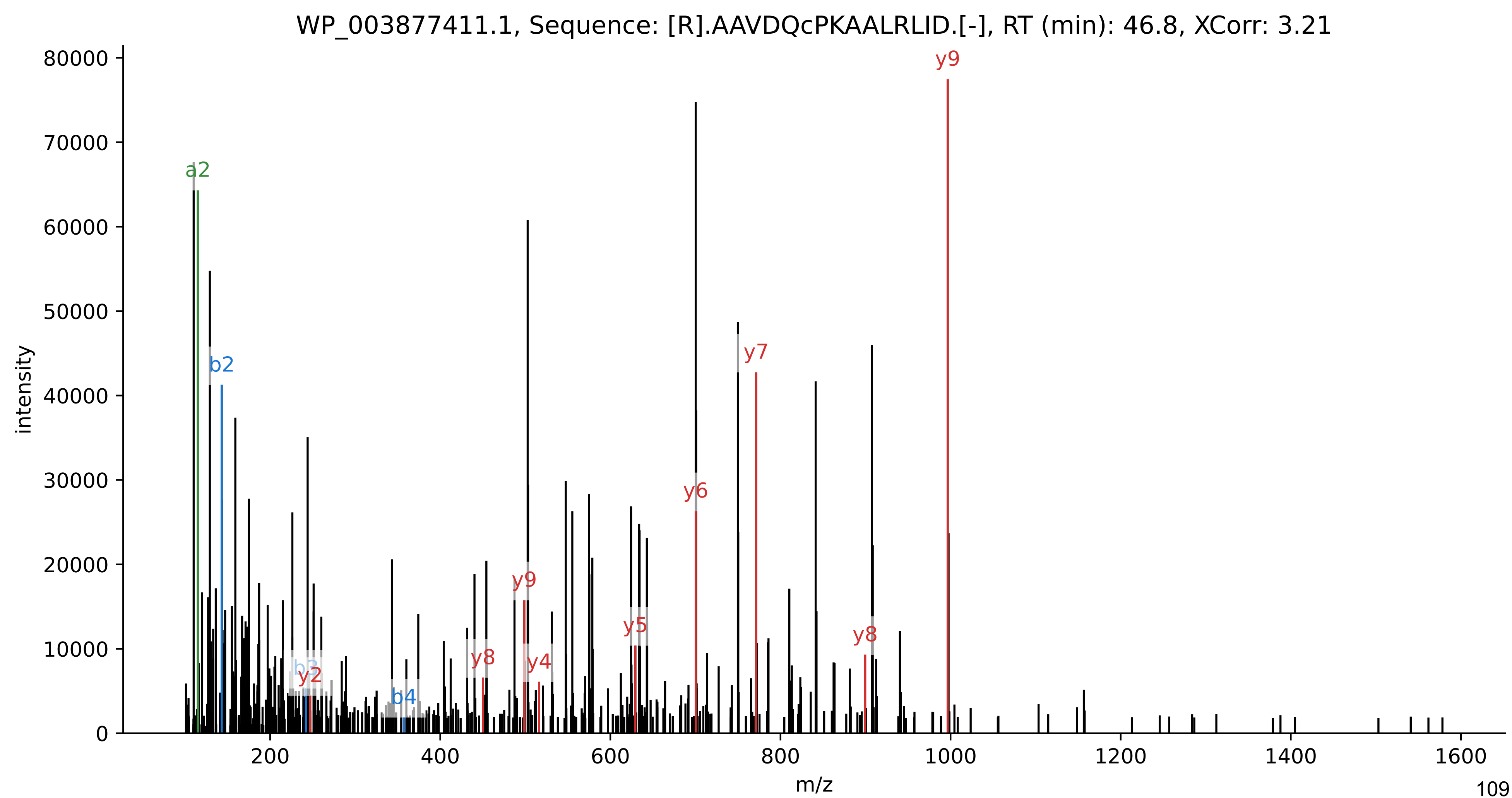
WP_003877411.1, Sequence: [R].AAVDQcPKAALRLID.[-], RT (min): 46.8, XCorr: 3.21
intensity
m/z
0
10000
20000
30000
40000
50000
60000
70000
80000
200
400
600
800
1000
1200
1400
1600
a2
b2
b4
y2
y4
y5
y6
y7
y8
y8
y9
y9

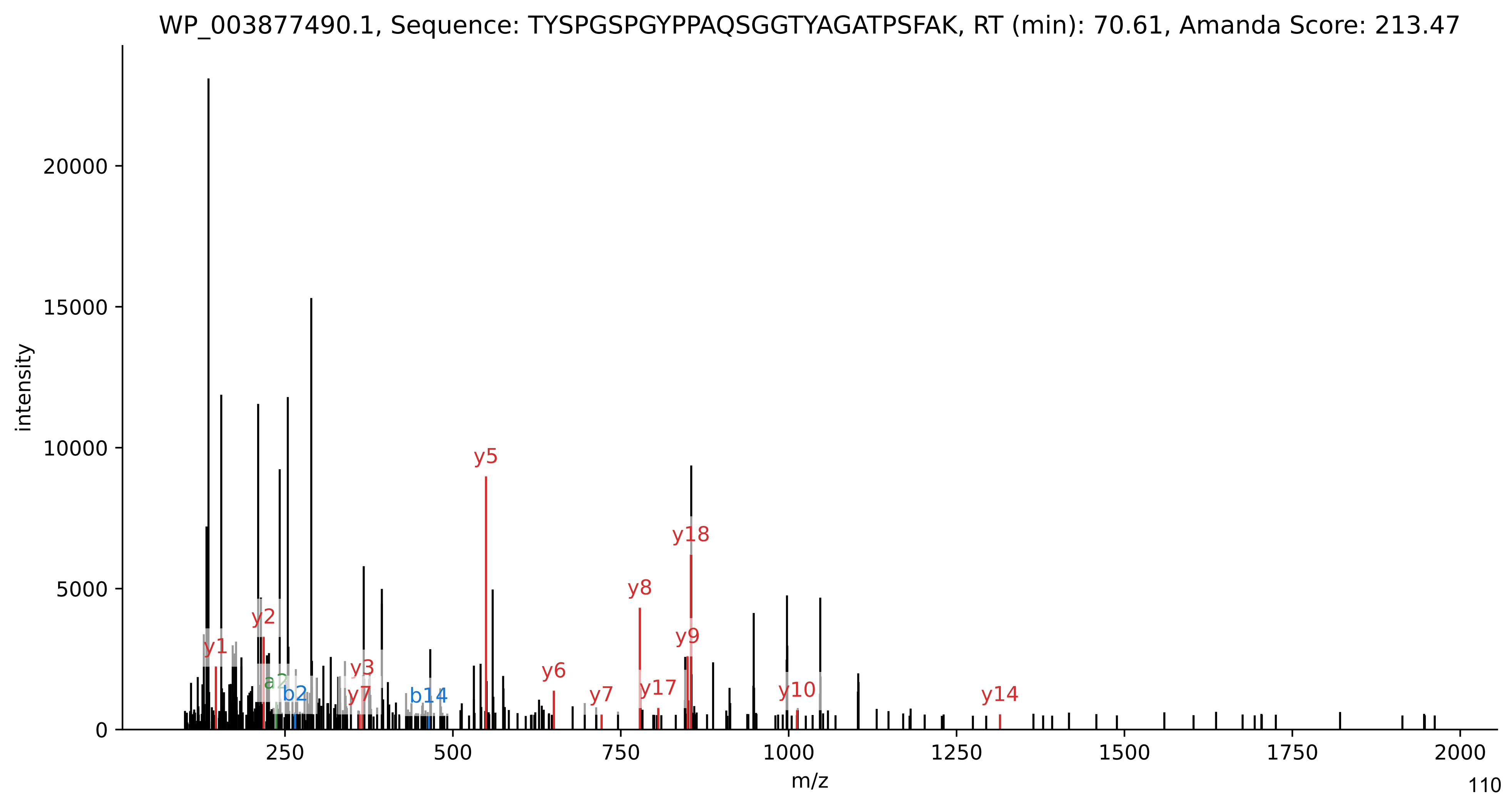

WP_003877490.1, Sequence: TYSPGSPGYPPAQSGGTYAGATPSFAK, RT (min): 70.61, Amanda Score: 213.47
intensity
m/z
y1
y2
a2
b2
y3
y7
b14
y5
y6
y7
y8
y17
y18
y9
y10
y14

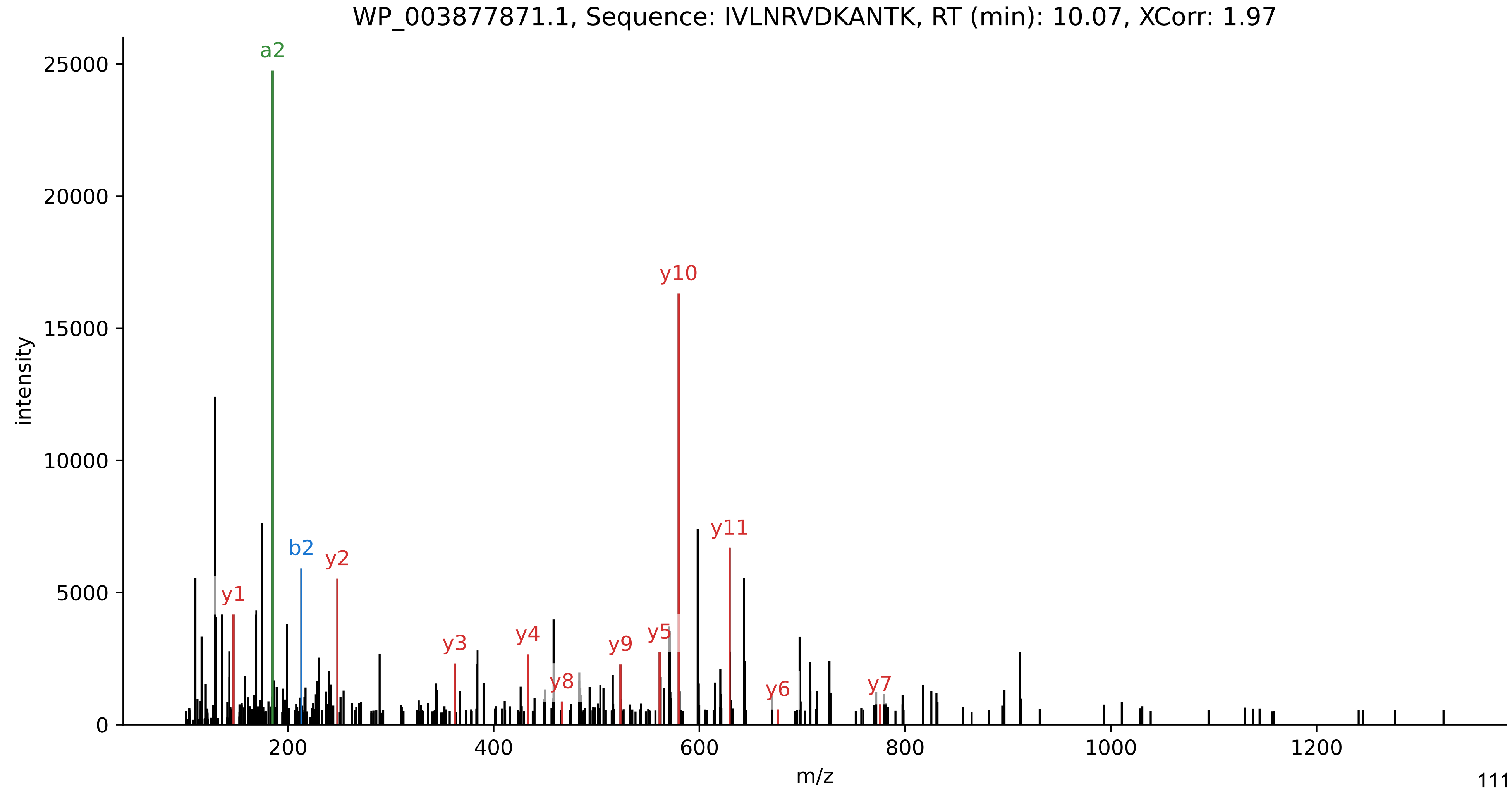
WP_003877871.1, Sequence: IVLNRVDKANTK, RT (min): 10.07, XCorr: 1.97
a2
b2
y1
y2
y3
y4
y8
y9
y5
y10
y11
y6
y7
intensity
m/z
0
5000
10000
15000
20000
25000
200
400
600
800
1000
1200

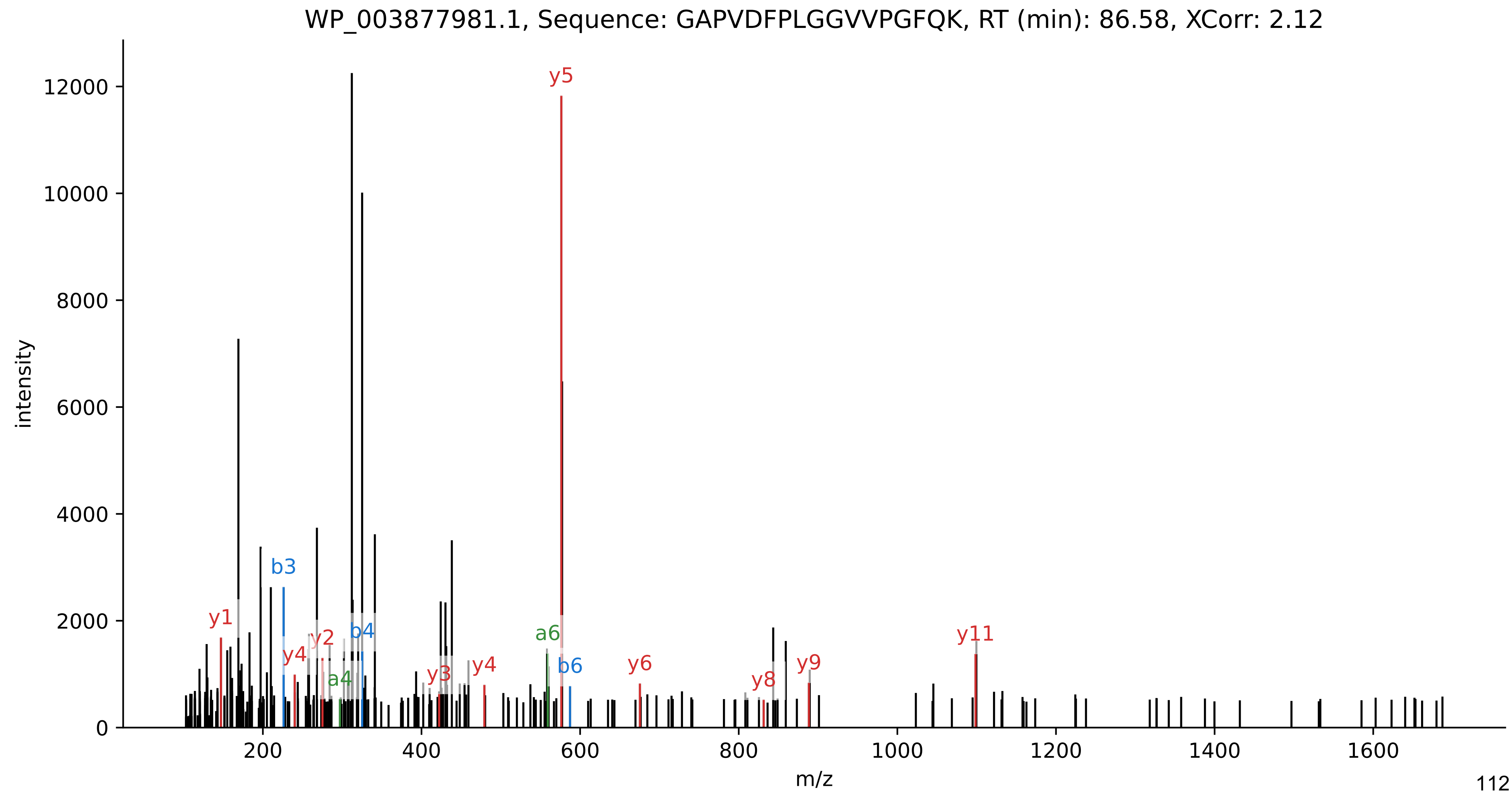

WP_003877981.1, Sequence: GAPVDFPLGGVVPGFQK, RT (min): 86.58, XCorr: 2.12
y5
b3
y1
y2
b4
y4
a4
y3
y4
a6
b6
y6
y8
y9
y11
intensity
m/z

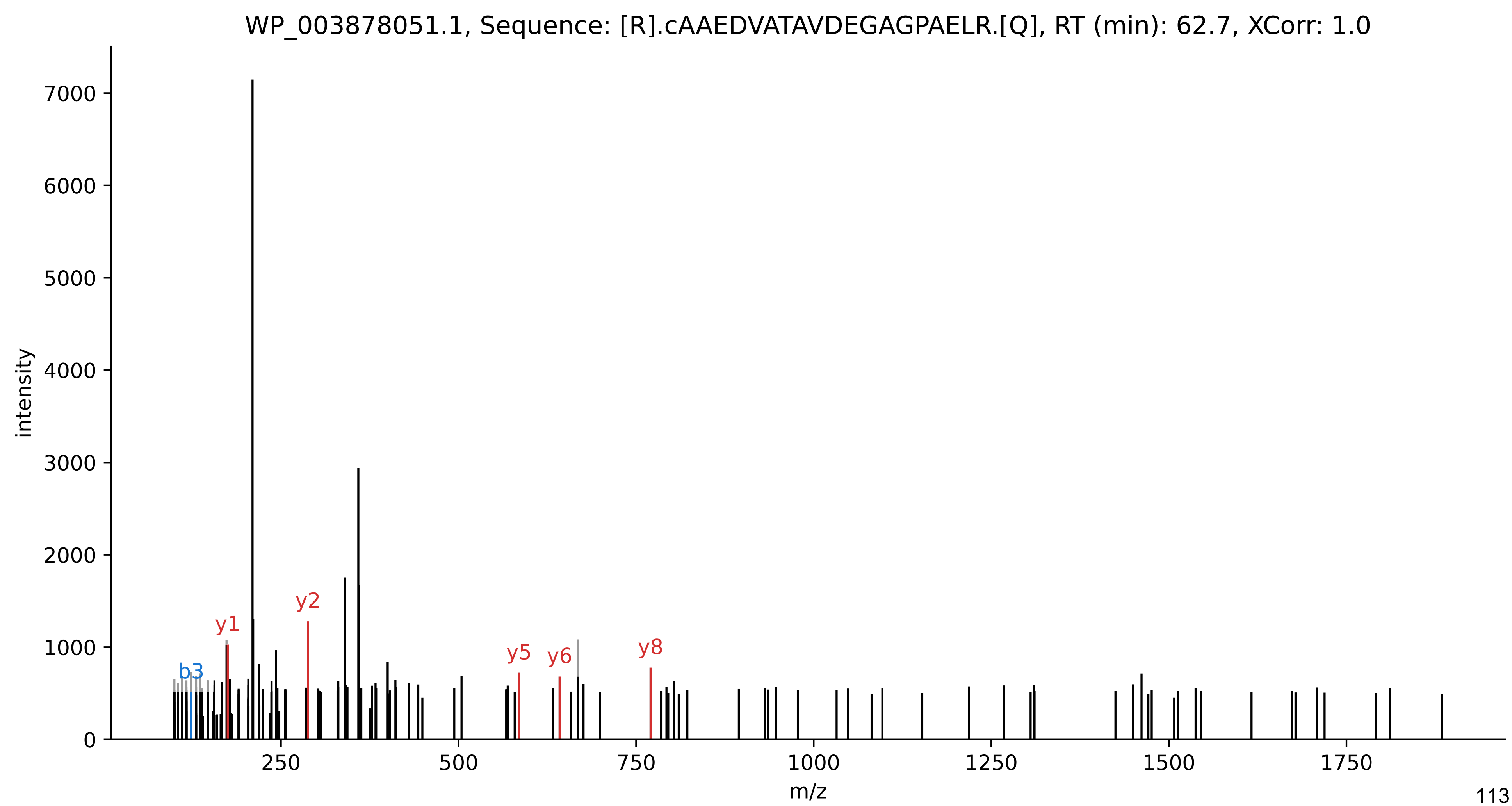
WP_003878051.1, Sequence: [R].cAAEDVATAVDEGAGPAELR.[Q], RT (min): 62.7, XCorr: 1.0
intensity
m/z
0
1000
2000
3000
4000
5000
6000
7000
250
500
750
1000
1250
1500
1750
b3
y1
y2
y5
y6
y8

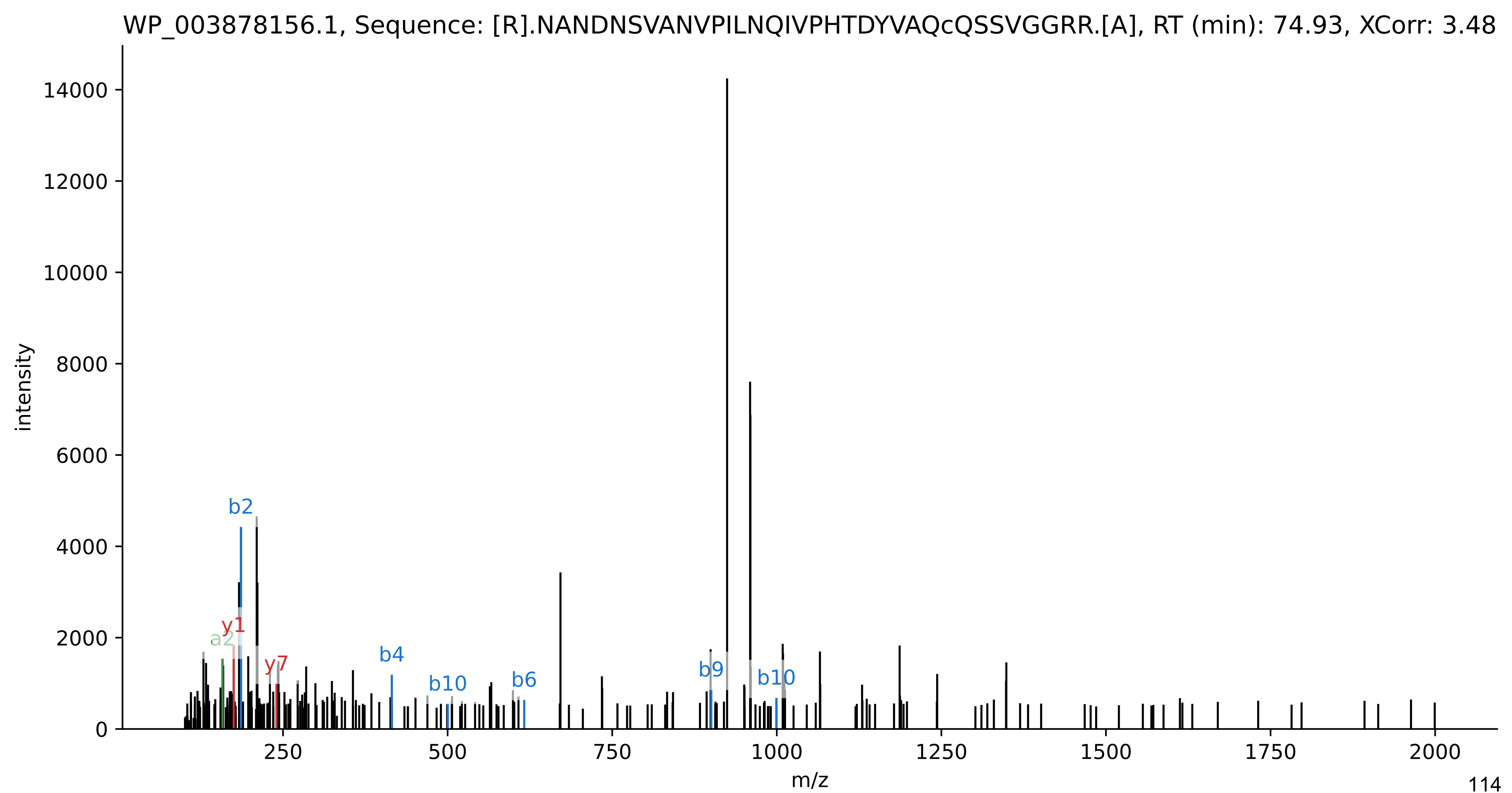

WP_003878156.1, Sequence: [R].NANDNSVANVPILNQIVPHTDYVAQcQSSVGGRR.[A], RT (min): 74.93, XCorr: 3.48
intensity
m/z
b2
y1
a2
y7
b4
b10
b6
b9
b10

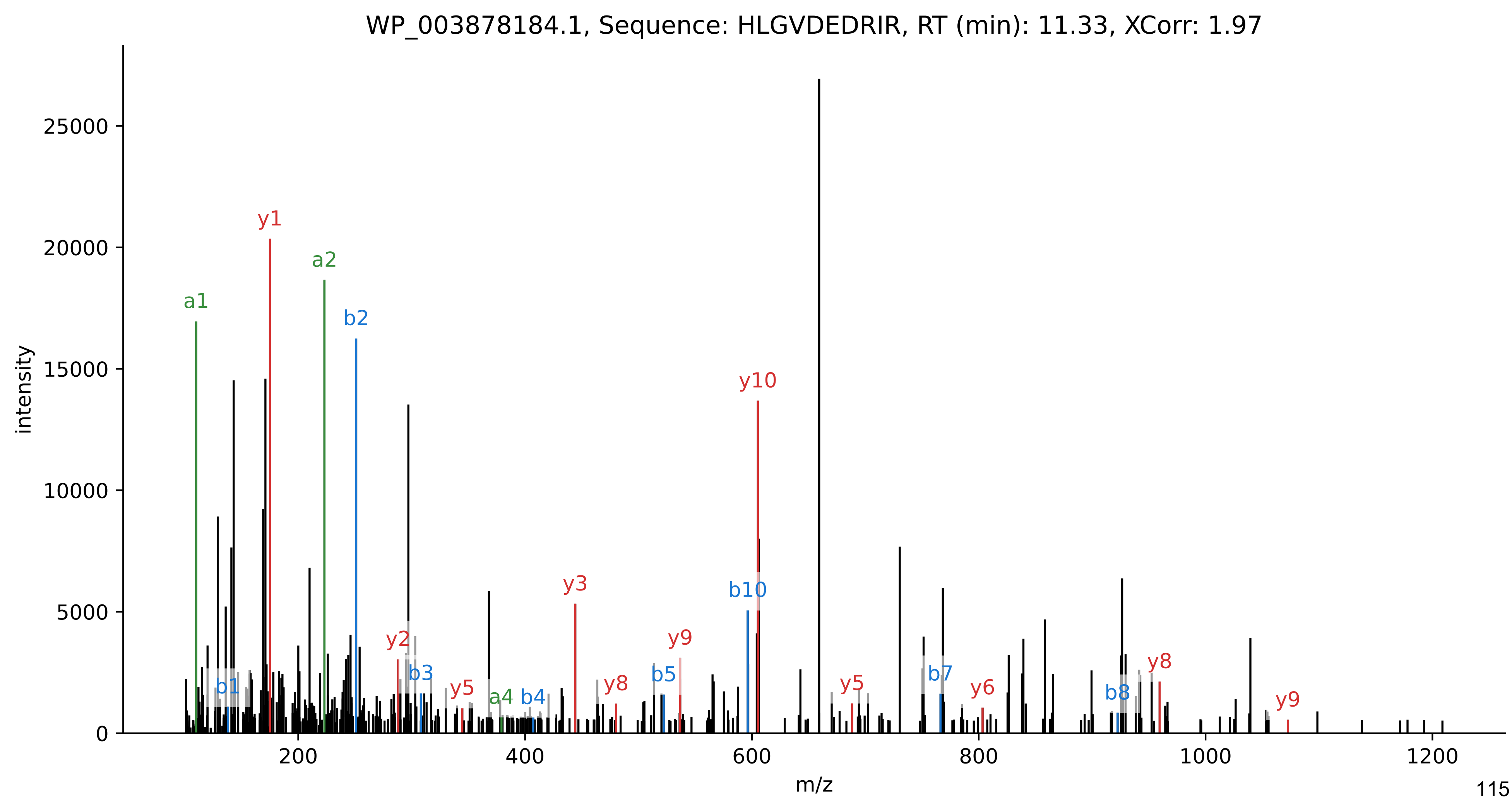

WP_003878184.1, Sequence: HLGVDEDRIR, RT (min): 11.33, XCorr: 1.97
intensity
m/z
25000
20000
15000
10000
5000
0
200
400
600
800
1000
1200
y1
a2
a1
b2
y10
y3
b10
y2
y9
b3
b1
y5
a4
b4
y8
b5
y5
b7
y6
b8
y8
y9

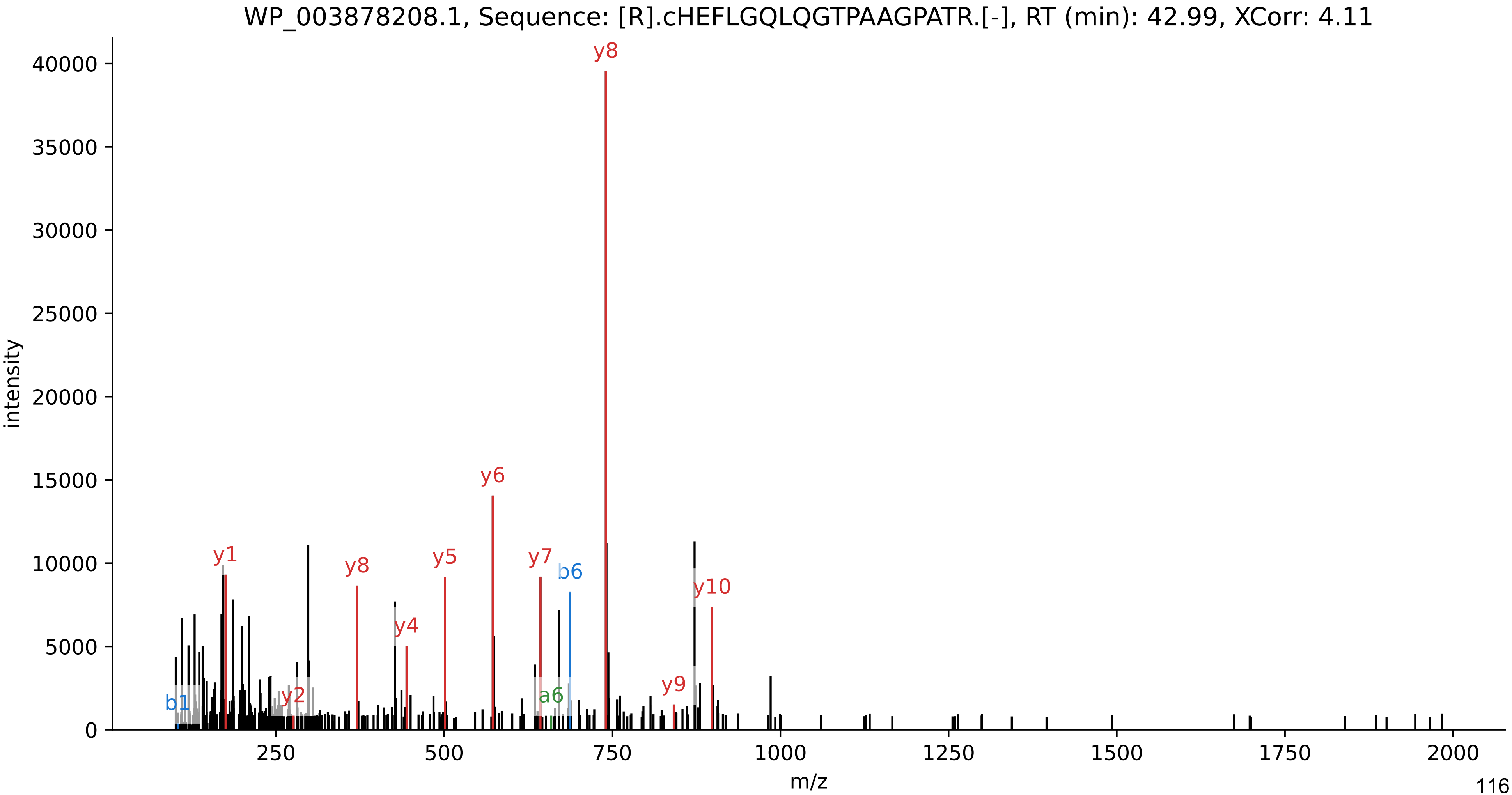

WP_003878208.1, Sequence: [R].cHEFLGQLQGTPAAGPATR.[-], RT (min): 42.99, XCorr: 4.11
intensity
m/z
y8
y6
y1
y8
y5
y7
b6
y10
y4
y9
a6
b1
y2

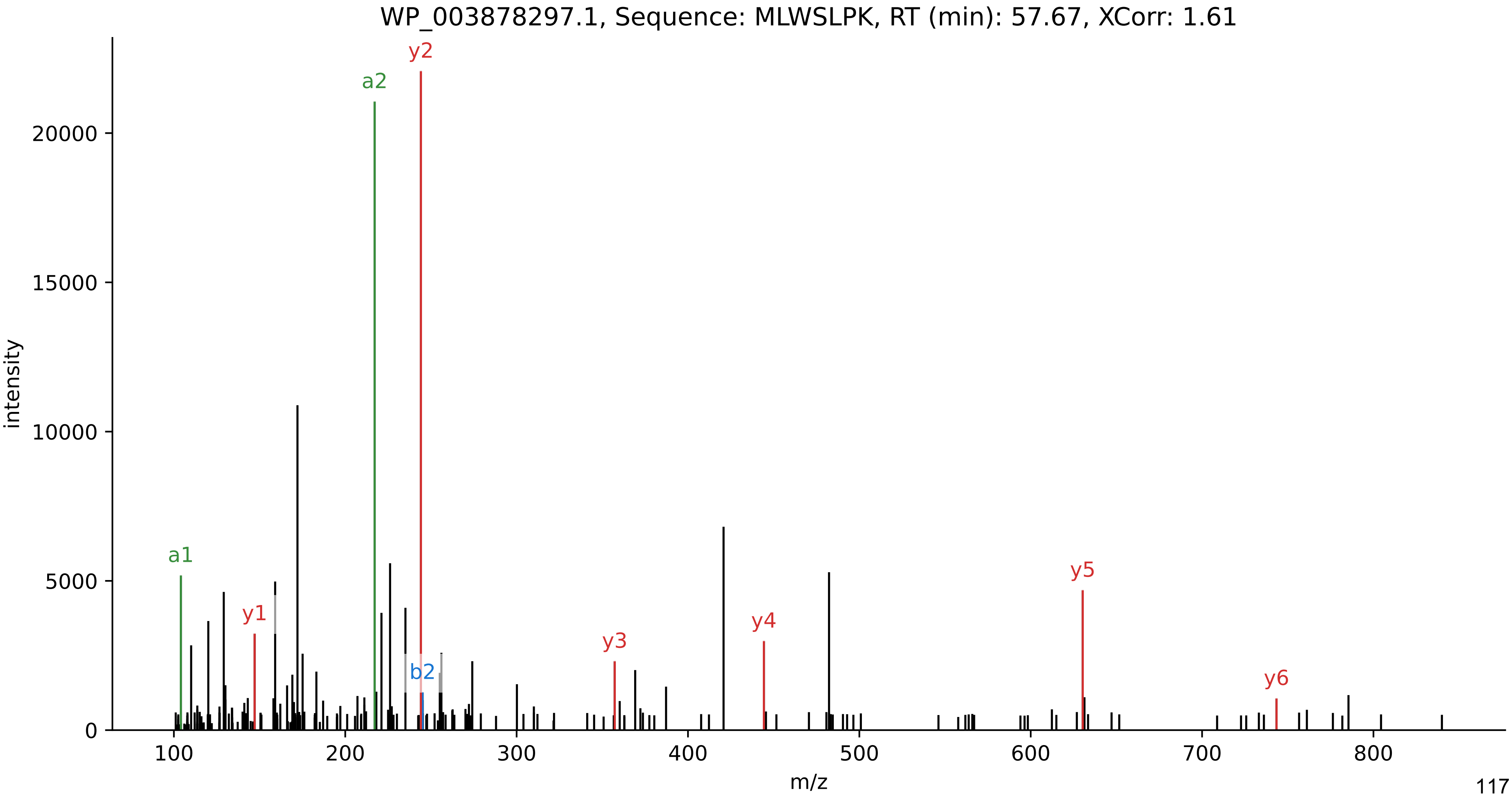

WP_003878297.1, Sequence: MLWSLPK, RT (min): 57.67, XCorr: 1.61
y2
a2
a1
y1
b2
y3
y4
y5
y6
intensity
m/z
0
5000
10000
15000
20000
100
200
300
400
500
600
700
800

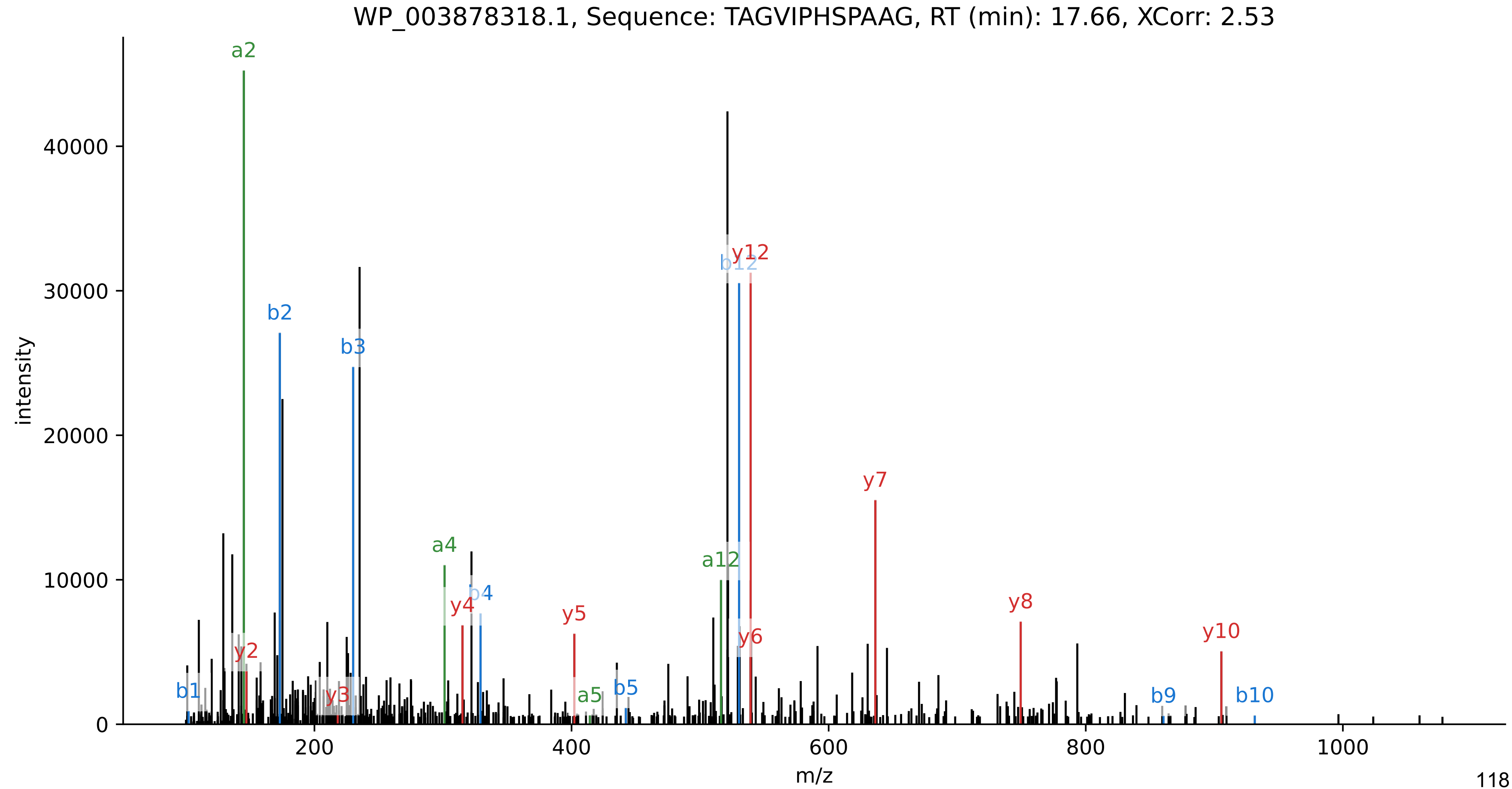

WP_003878318.1, Sequence: TAGVIPHSPAAG, RT (min): 17.66, XCorr: 2.53
intensity
m/z
0
10000
20000
30000
40000
200
400
600
800
1000
a2
b2
b3
a4
b4
y4
y5
a5
b5
a12
b12
y12
y6
y7
y8
y10
b9
b10
b1
y2
y3

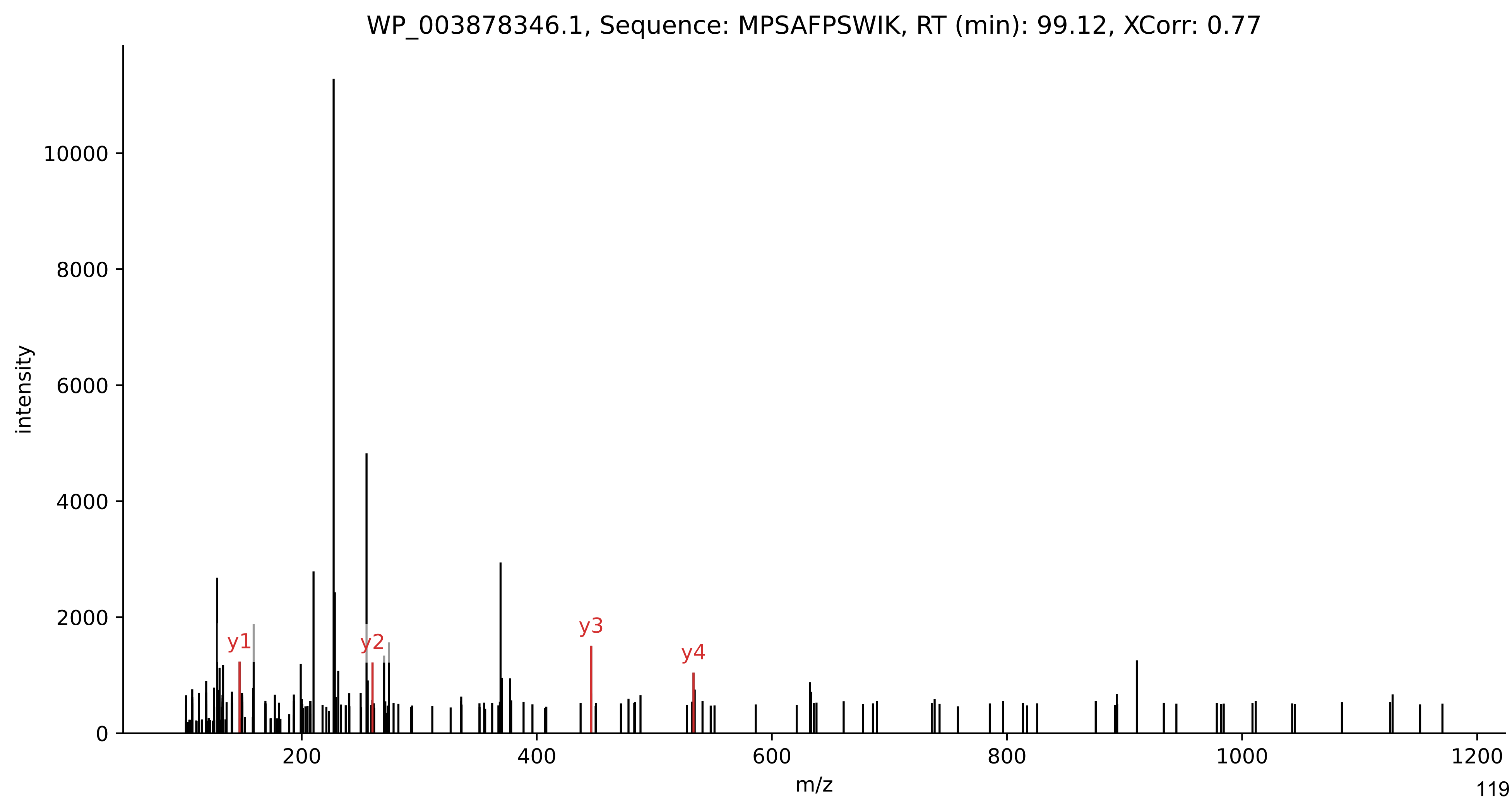
WP_003878346.1, Sequence: MPSAFPSWIK, RT (min): 99.12, XCorr: 0.77
intensity
m/z
y1
y2
y3
y4
0
2000
4000
6000
8000
10000
200
400
600
800
1000
1200

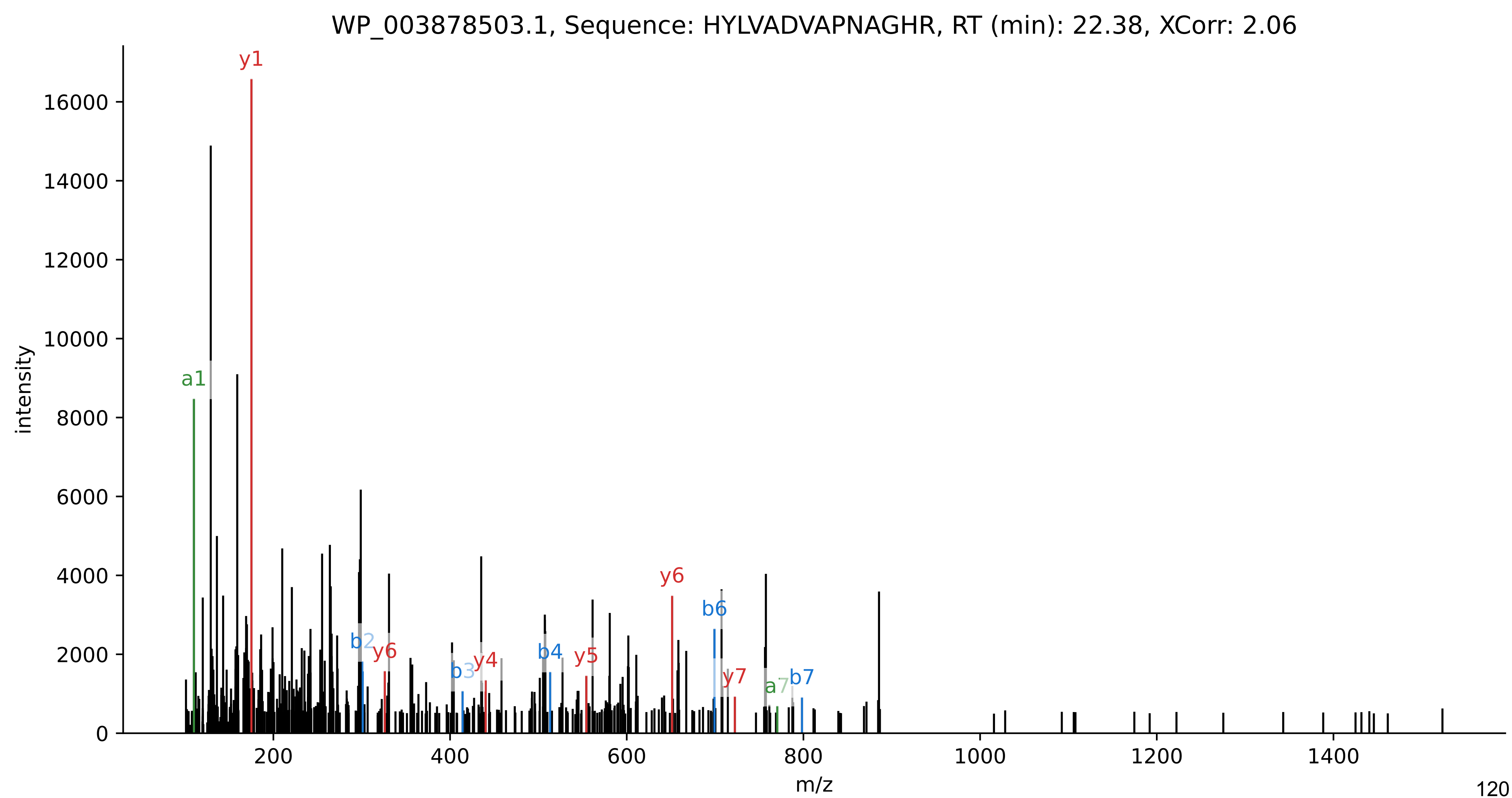
WP_003878503.1, Sequence: HYLVADVAPNAGHR, RT (min): 22.38, XCorr: 2.06
intensity
m/z
y1
a1
b2
y6
b3
y4
b4
y5
y6
b6
y7
a7
b7
0
2000
4000
6000
8000
10000
12000
14000
16000
200
400
600
800
1000
1200
1400

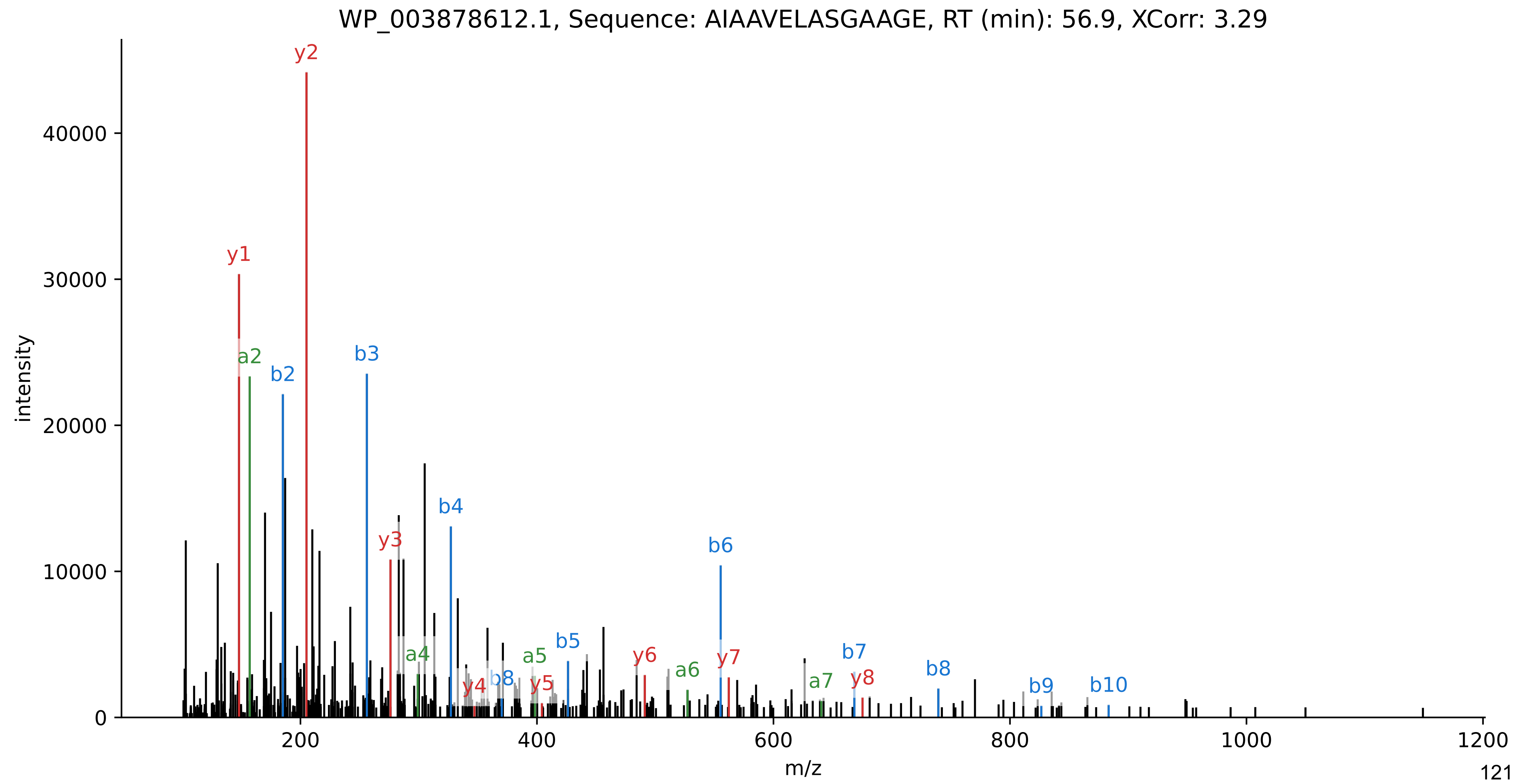

WP_003878612.1, Sequence: AIAAVELASGAAGE, RT (min): 56.9, XCorr: 3.29
intensity
m/z
y1
y2
a2
b2
b3
b4
y3
a4
y4
b8
a5
y5
b5
y6
a6
b6
y7
a7
b7
y8
b8
b9
b10

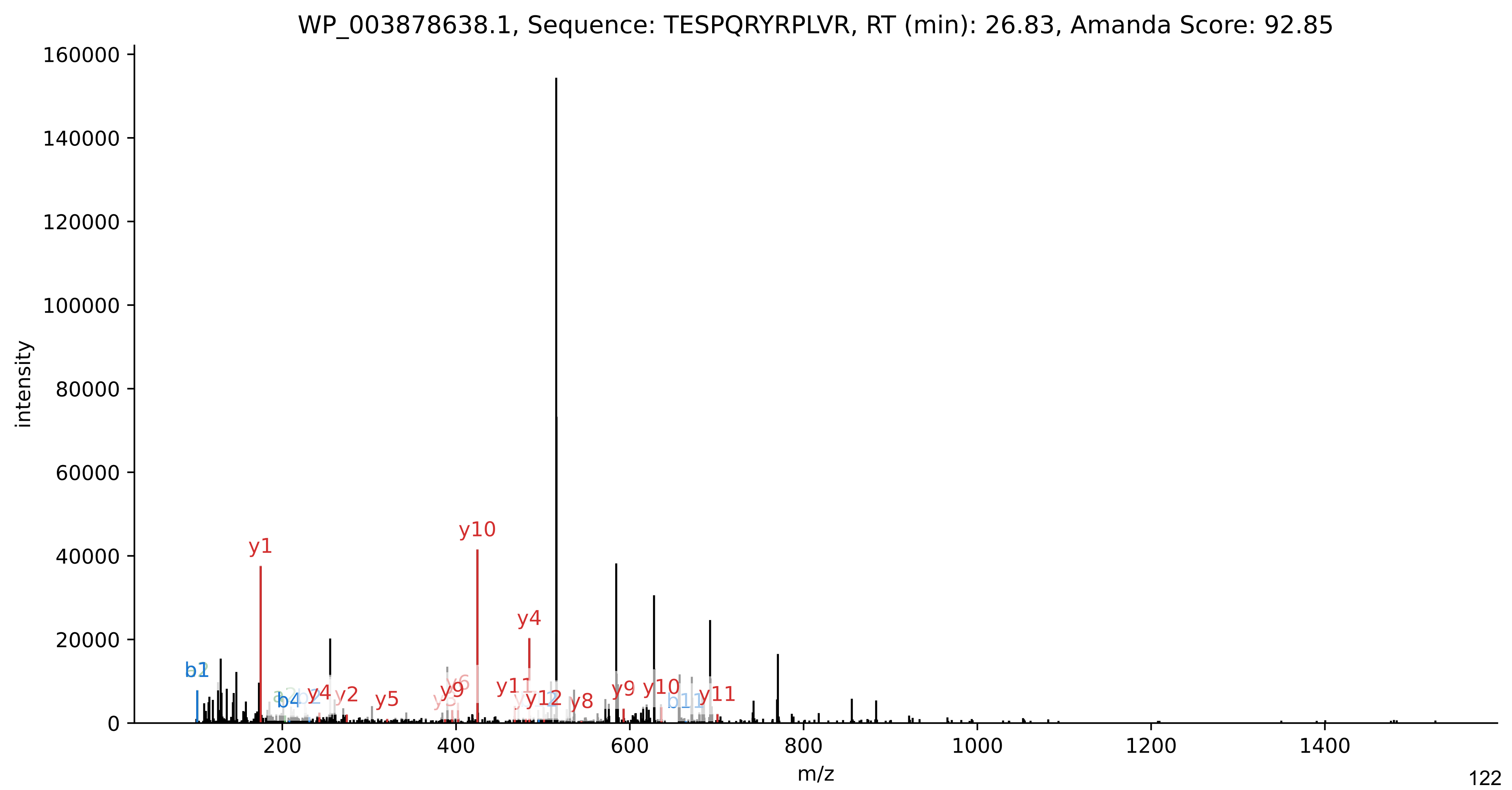

WP_003878638.1, Sequence: TESPQRYRPLVR, RT (min): 26.83, Amanda Score: 92.85
intensity
m/z
y1
y10
y4
b1
b4
y4
y2
y5
y9
y6
y11
y12
y8
y9
y10
b11
y11

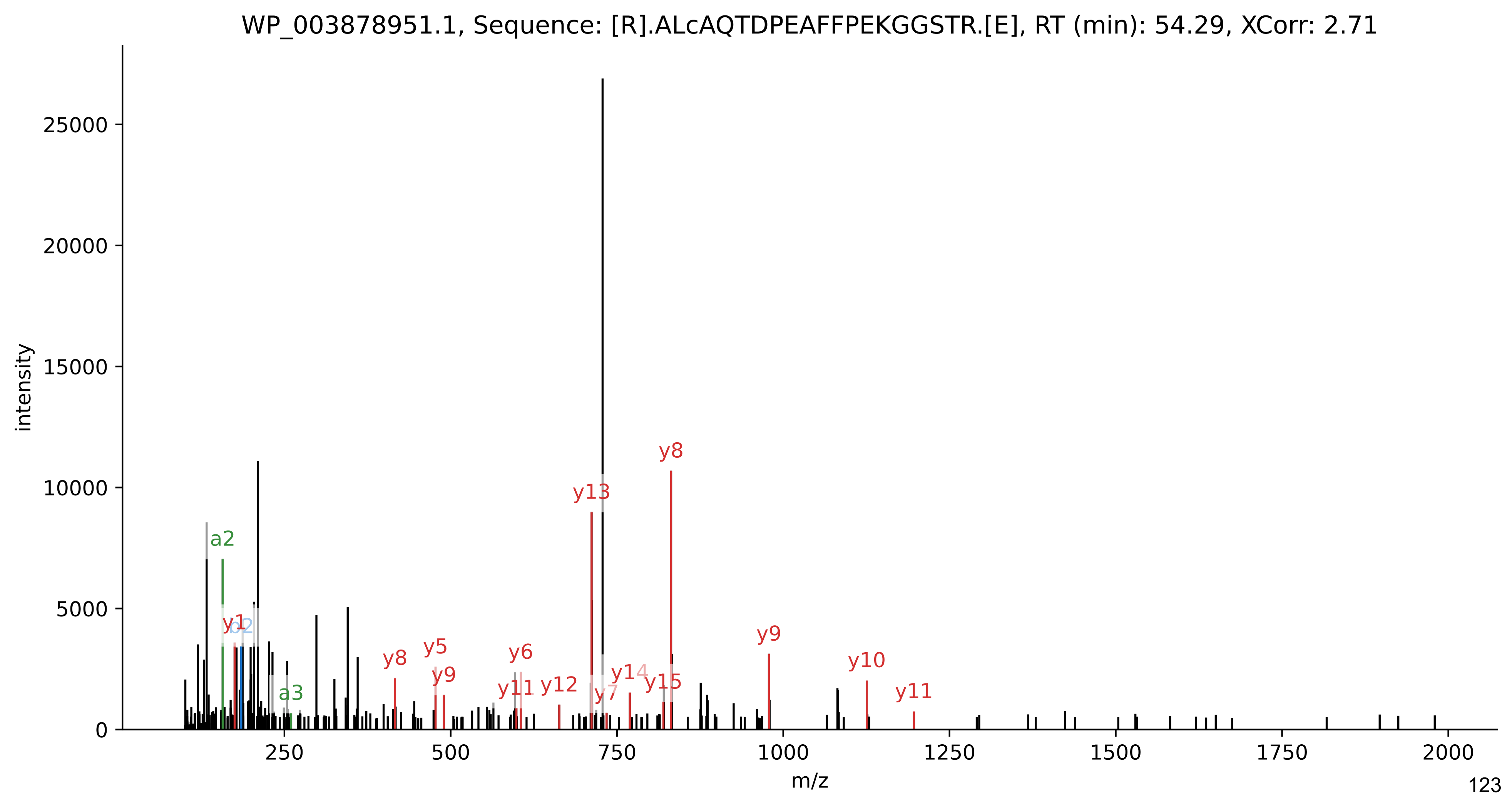
WP_003878951.1, Sequence: [R].ALcAQTDPEAFFPEKGGSTR.[E], RT (min): 54.29, XCorr: 2.71
intensity
m/z
0
5000
10000
15000
20000
25000
250
500
750
1000
1250
1500
1750
2000
a2
a3
y1
y5
y6
y7
y8
y8
y9
y9
y10
y11
y11
y12
y13
y14
y15

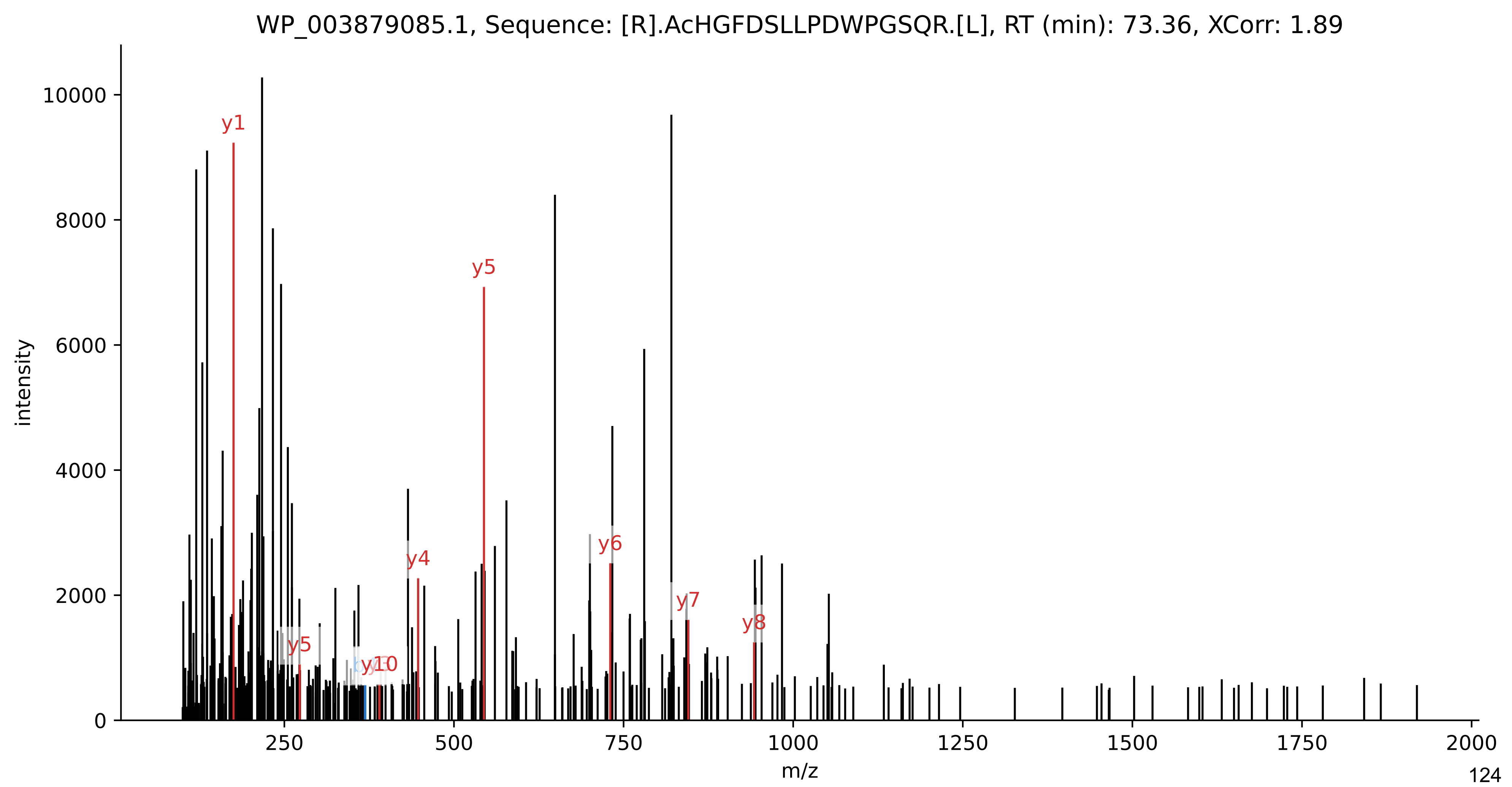
WP_003879085.1, Sequence: [R].AcHGFDSLLPDWPGSQR.[L], RT (min): 73.36, XCorr: 1.89
intensity
m/z
y1
y5
y4
y6
y7
y8
y10
0
2000
4000
6000
8000
10000
250
500
750
1000
1250
1500
1750
2000

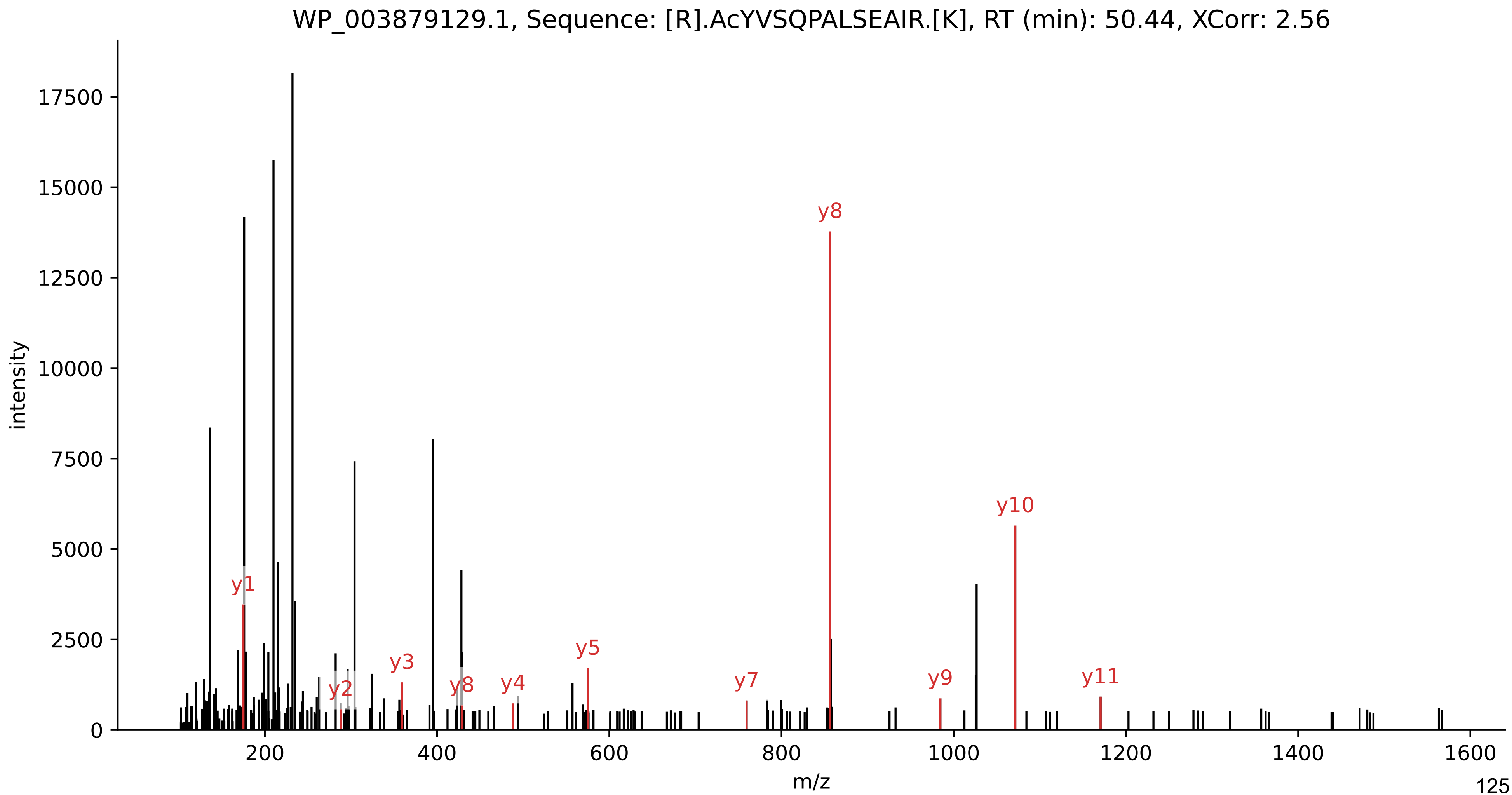
WP_003879129.1, Sequence: [R].AcYVSQPALSEAIR.[K], RT (min): 50.44, XCorr: 2.56
intensity
m/z
0
2500
5000
7500
10000
12500
15000
17500
200
400
600
800
1000
1200
1400
1600
y1
y2
y3
y8
y4
y5
y7
y8
y9
y10
y11

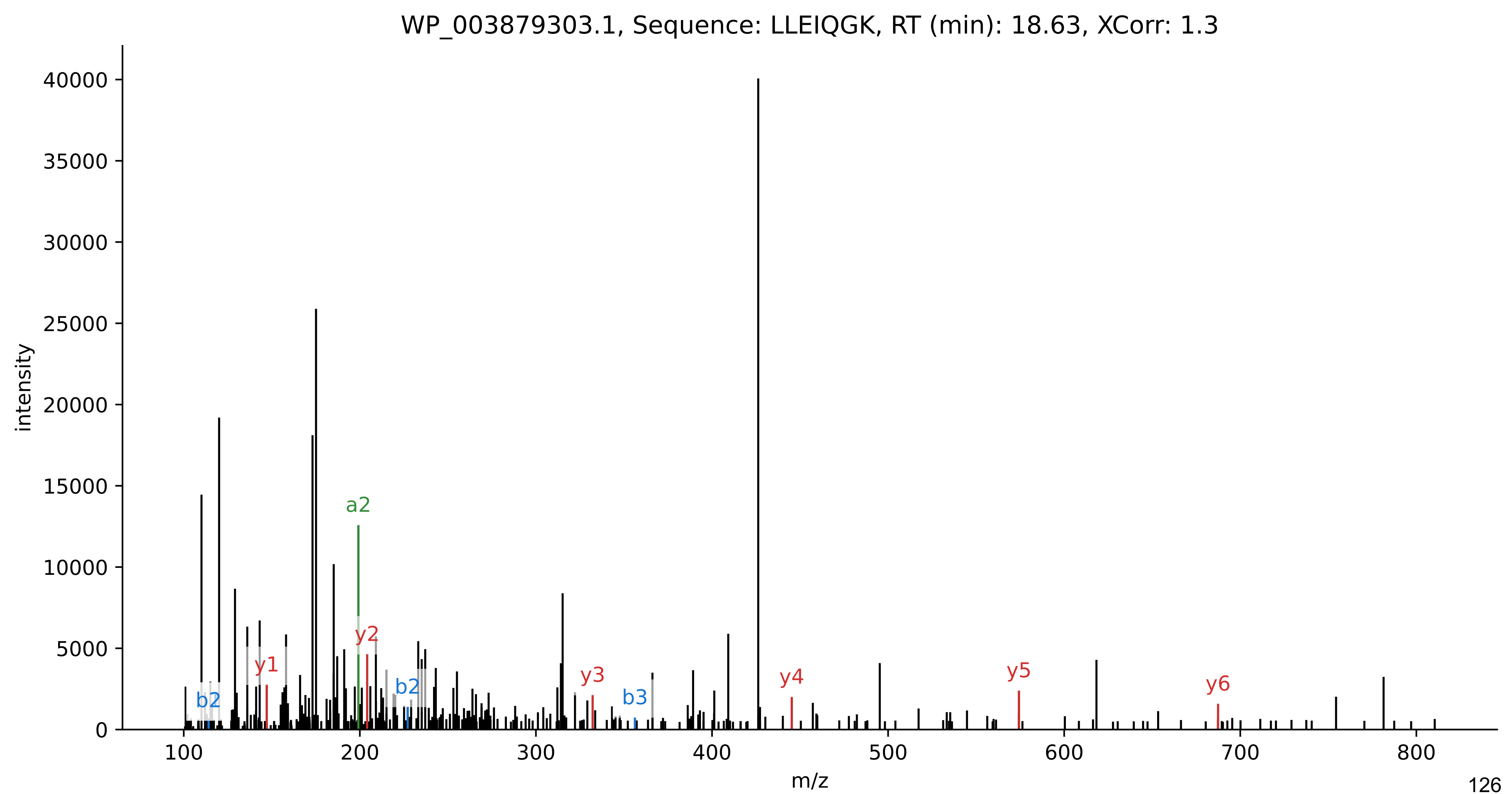
WP_003879303.1, Sequence: LLEIQGK, RT (min): 18.63, XCorr: 1.3
intensity
m/z
a2
y1
y2
y3
y4
y5
y6
b2
b2
b3

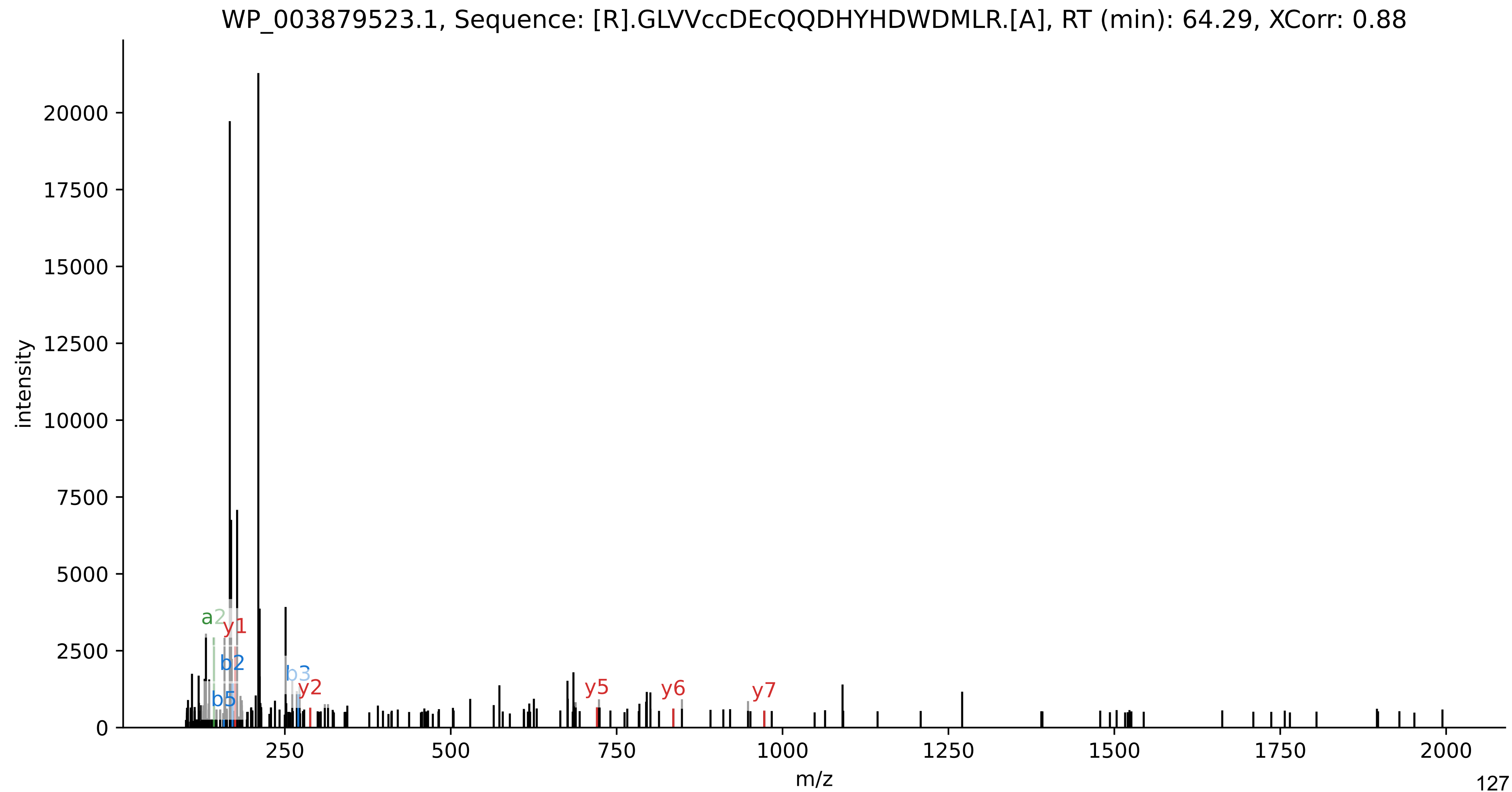
WP_003879523.1, Sequence: [R].GLVVccDEcQQDHYHDWDMLR.[A], RT (min): 64.29, XCorr: 0.88
intensity
m/z
a2
y1
b2
b5
b3
y2
y5
y6
y7

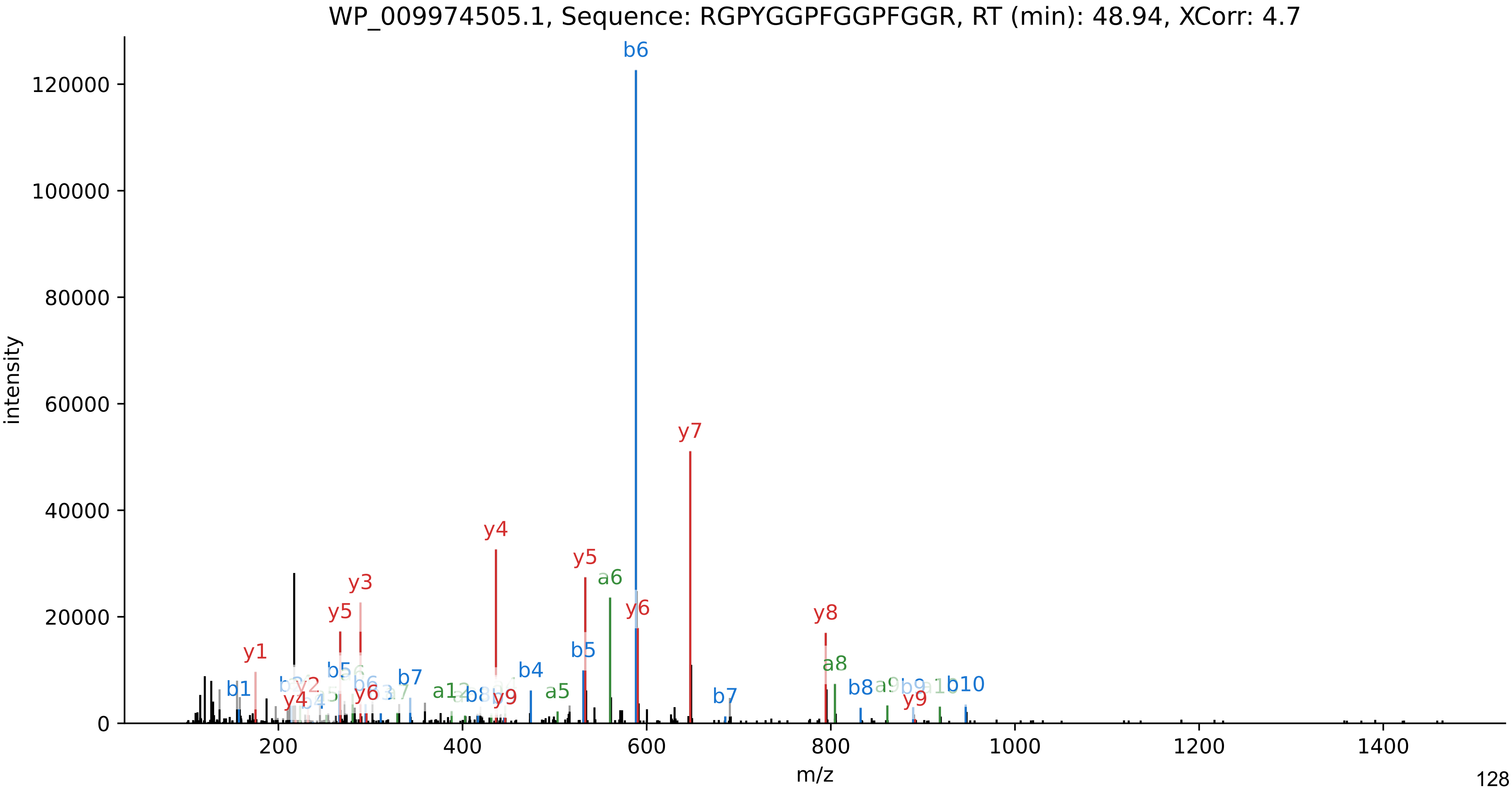
WP_009974505.1, Sequence: RGPYGGPFGGPFGGR, RT (min): 48.94, XCorr: 4.7
intensity
m/z
0
20000
40000
60000
80000
100000
120000
200
400
600
800
1000
1200
1400
b6
y7
y4
y5
a6
y3
y6
y5
y8
y1
b5
b4
b5
b7
a8
b1
a5
b7
b8
b10
y2
y4
y6
a12
b8
y9
y9

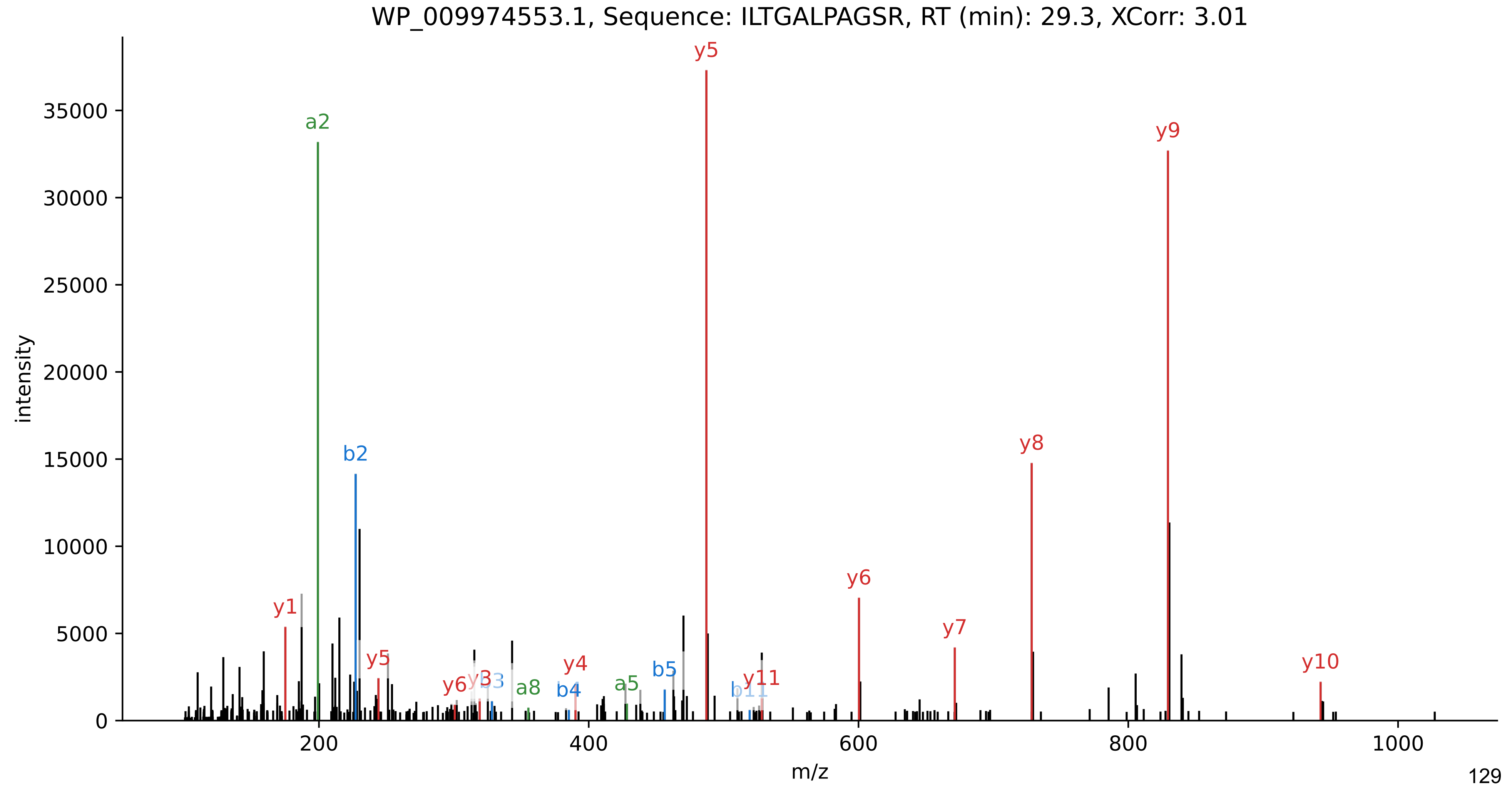
WP_009974553.1, Sequence: ILTGALPAGSR, RT (min): 29.3, XCorr: 3.01
intensity
m/z
0
5000
10000
15000
20000
25000
30000
35000
200
400
600
800
1000
a2
b2
y1
y5
y6
y3
b3
a8
y4
b4
a5
b5
b11
y11
y5
y6
y7
y8
y9
y10

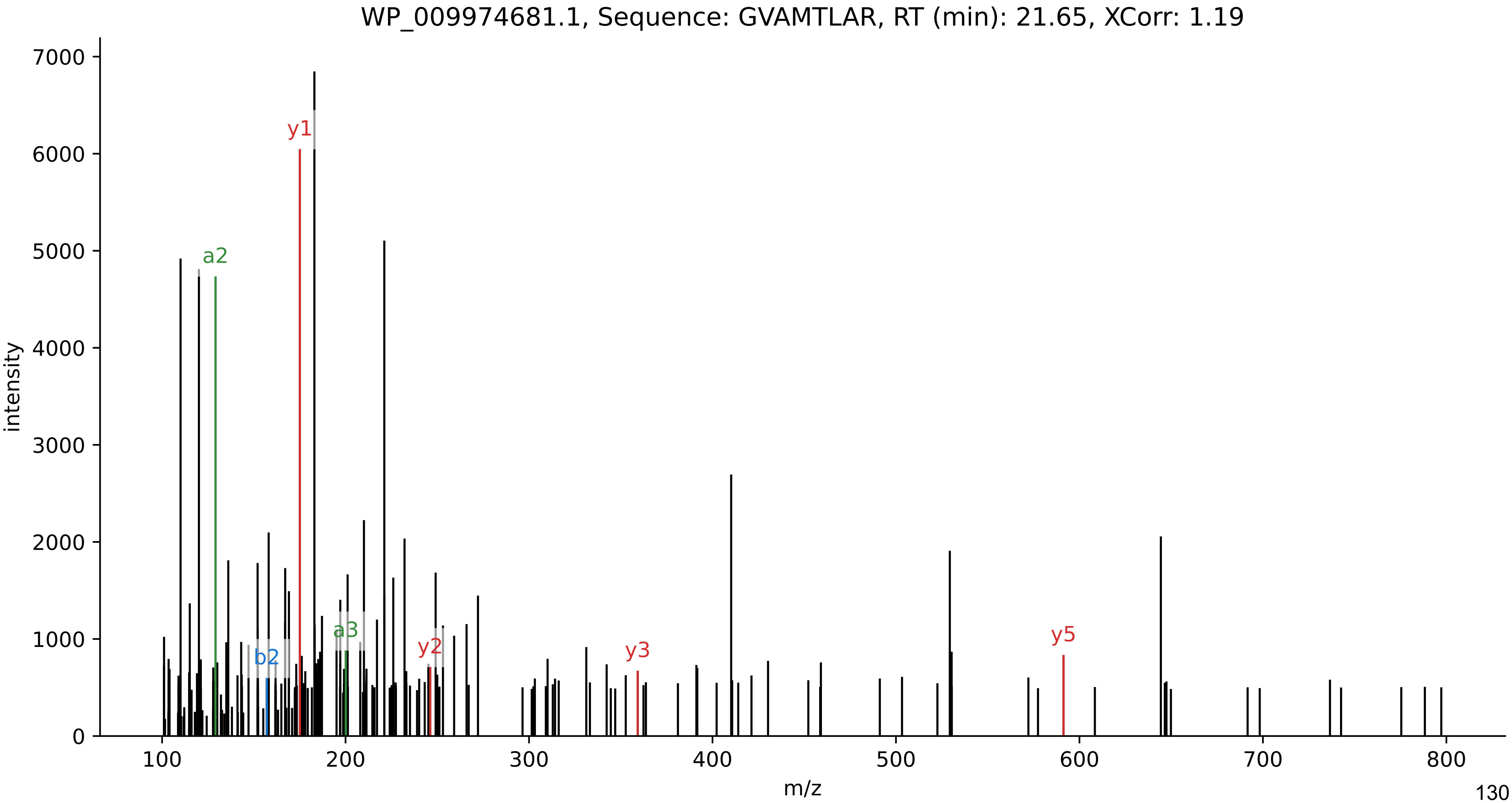
WP_009974681.1, Sequence: GVAMTLAR, RT (min): 21.65, XCorr: 1.19
intensity
m/z
y1
a2
a3
b2
y2
y3
y5
0
1000
2000
3000
4000
5000
6000
7000
100
200
300
400
500
600
700
800

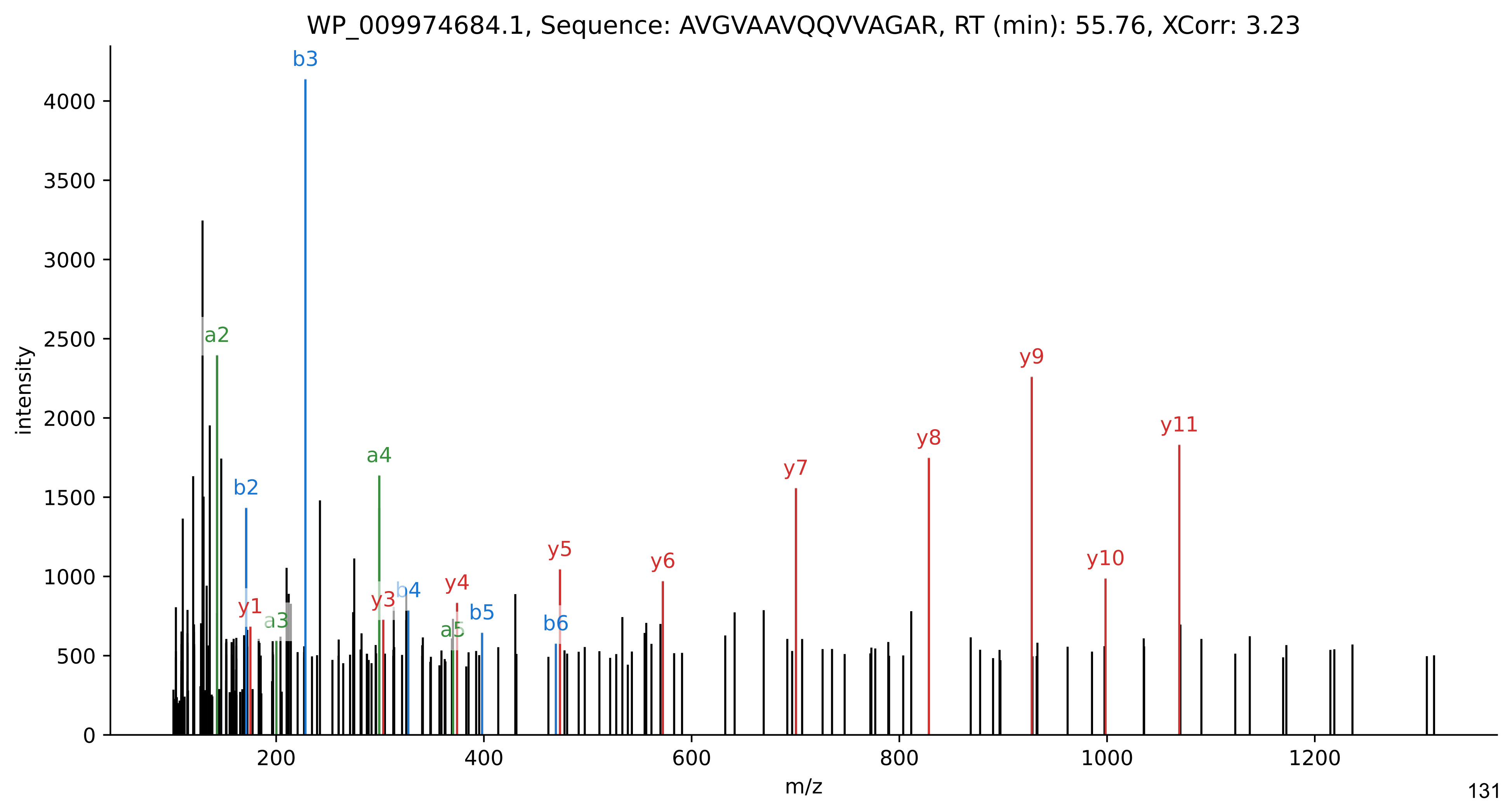

WP_009974684.1, Sequence: AVGVAAVQQVVAGAR, RT (min): 55.76, XCorr: 3.23
intensity
m/z
0
500
1000
1500
2000
2500
3000
3500
4000
200
400
600
800
1000
1200
b3
a2
b2
a4
y1
a3
y3
b4
y4
a5
b5
b6
y5
y6
y7
y8
y9
y10
y11

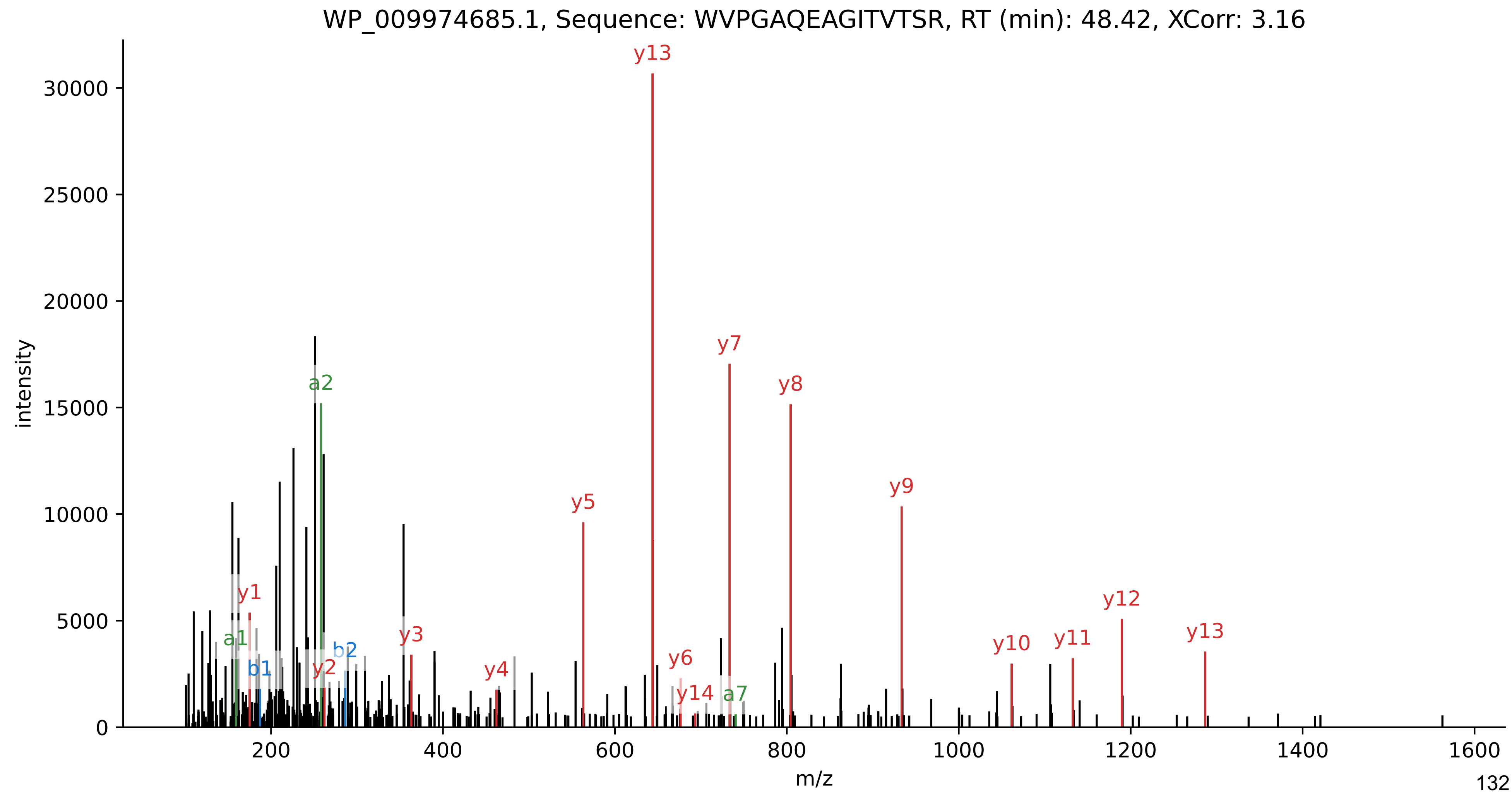
WP_009974685.1, Sequence: WVPGAQEAGITVTSR, RT (min): 48.42, XCorr: 3.16
intensity
m/z
y13
y7
y8
a2
y9
y5
y1
y12
y3
y13
y11
y10
a1
b2
y6
b1
y2
y4
y14
a7

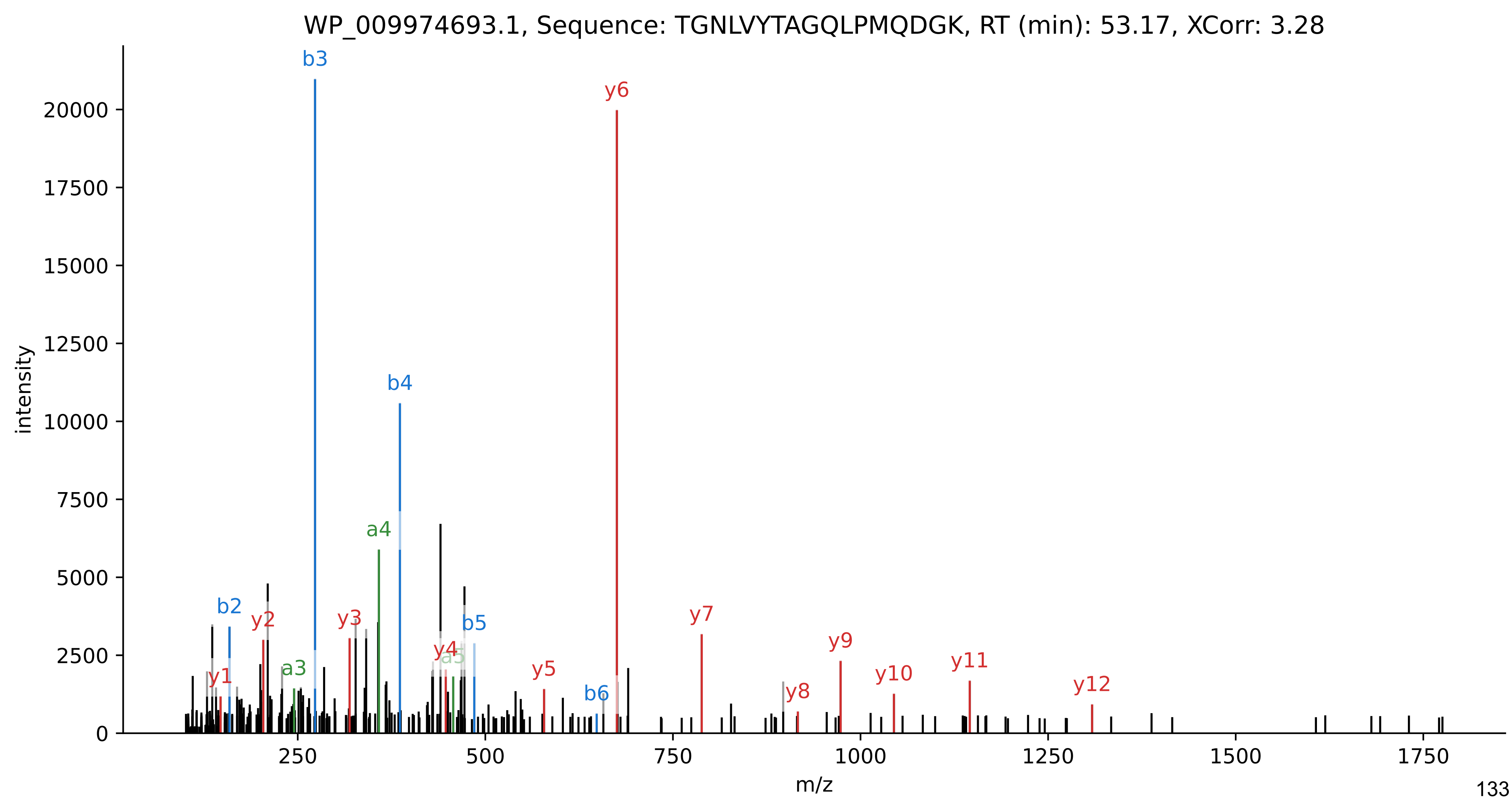
WP_009974693.1, Sequence: TGNLVYTAGQLPMQDGK, RT (min): 53.17, XCorr: 3.28
intensity
m/z
b2
b3
b4
b5
b6
y1
y2
y3
y4
y5
y6
y7
y8
y9
y10
y11
y12
a3
a4
a5

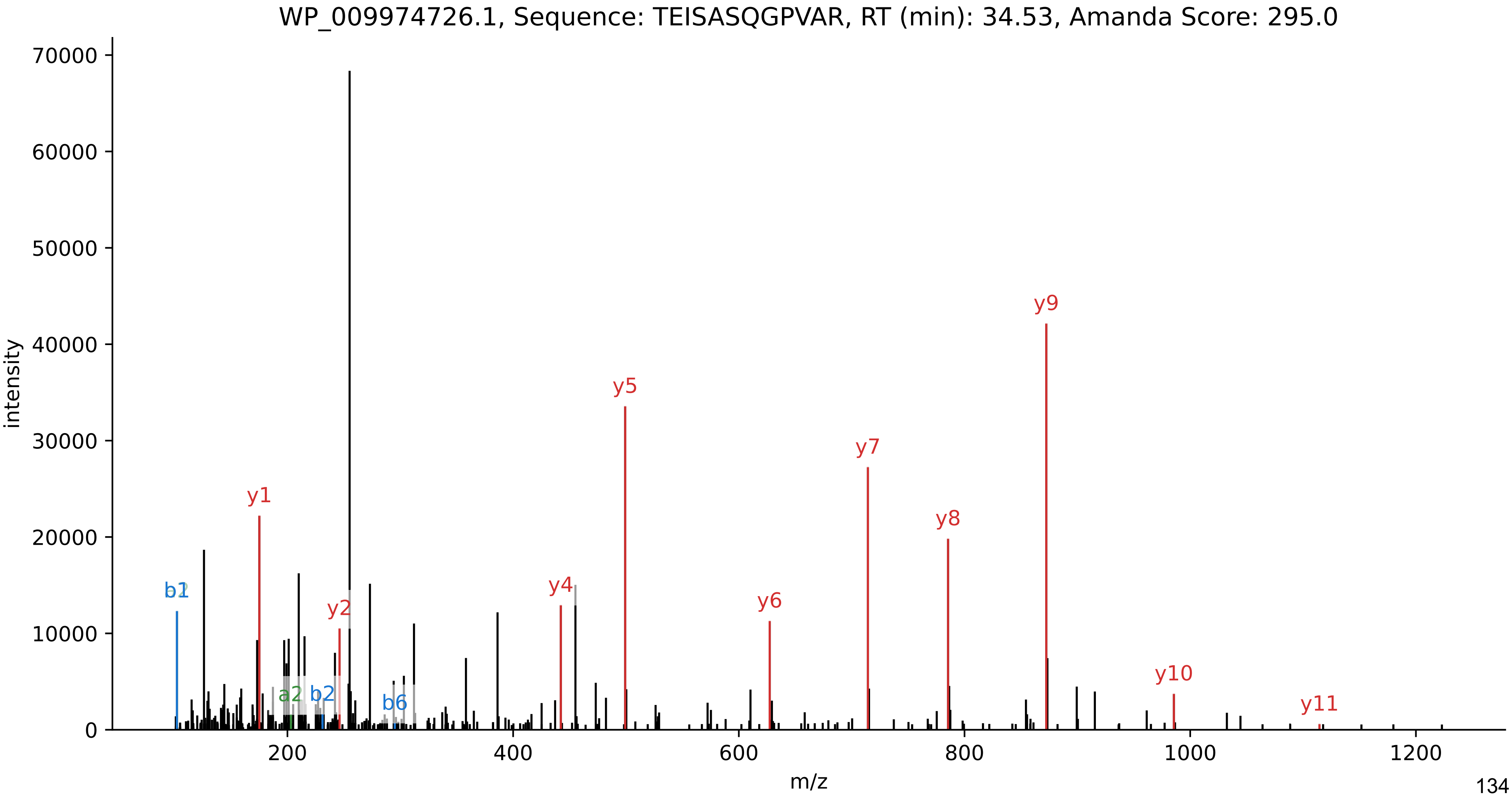

WP_009974726.1, Sequence: TEISASQGPVAR, RT (min): 34.53, Amanda Score: 295.0
intensity
m/z
y1
y2
y4
y5
y6
y7
y8
y9
y10
y11
b1
a2
b2
b6

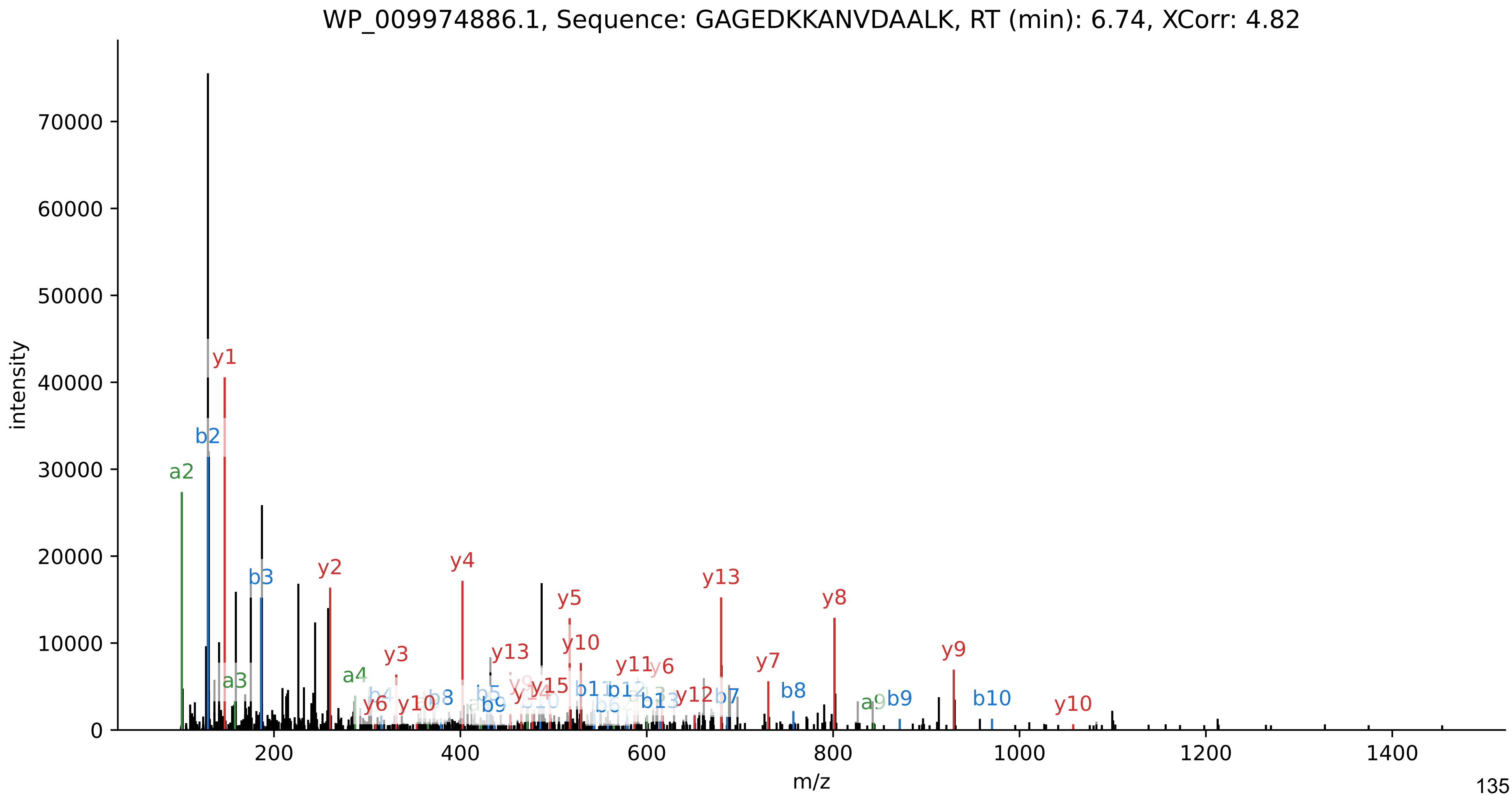
WP_009974886.1, Sequence: GAGEDKKANVDAALK, RT (min): 6.74, XCorr: 4.82
intensity
m/z
70000
60000
50000
40000
30000
20000
10000
0
200
400
600
800
1000
1200
1400
a2
b2
y1
b3
y2
y4
a3
a4
y3
y6
y10
b9
y13
y5
y10
y15
b11
b12
y11
y6
b13
y12
y13
b7
y7
b8
y8
b9
y9
b10
y10

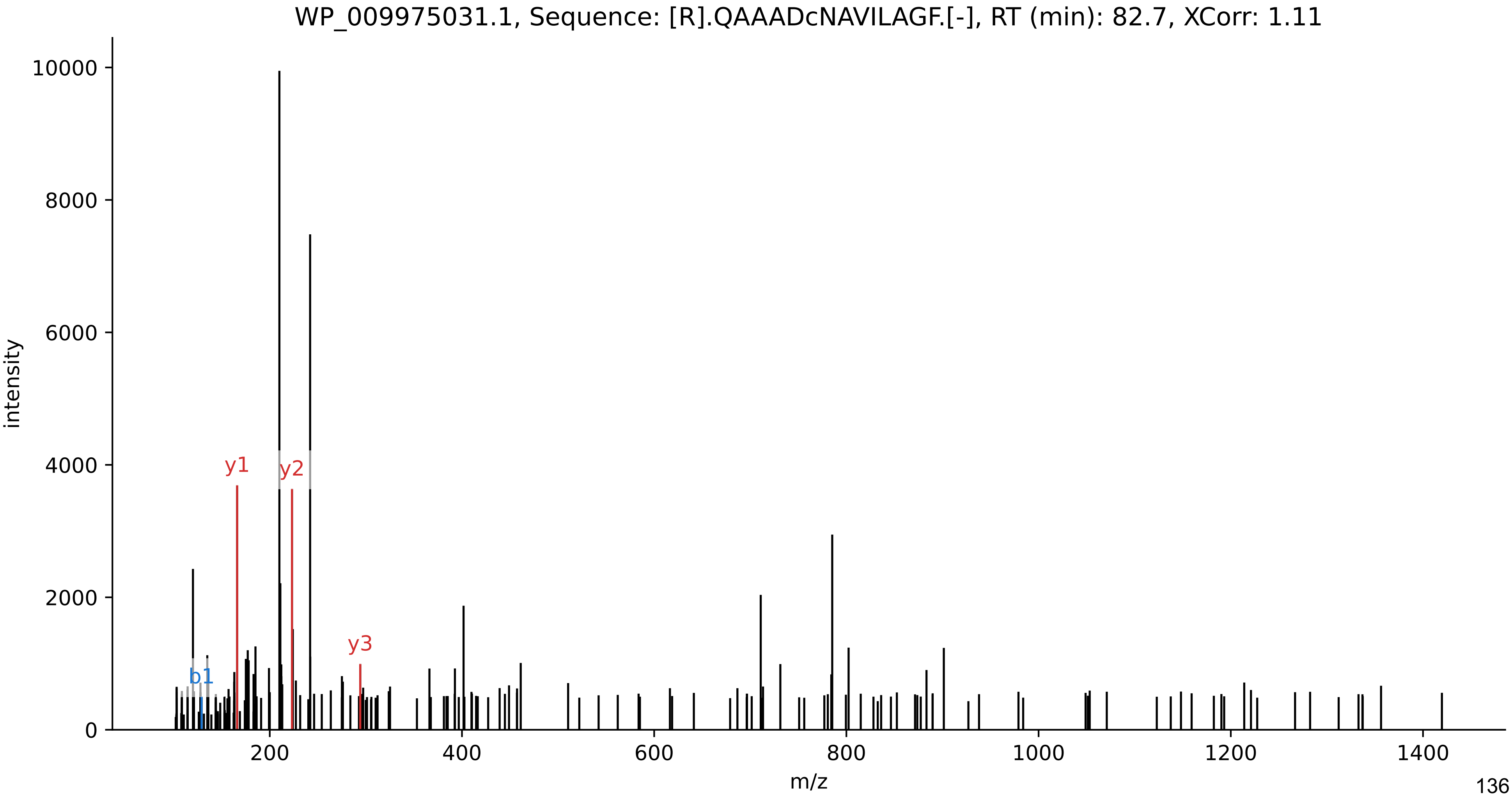
WP_009975031.1, Sequence: [R].QAAADcNAVILAGF.[-], RT (min): 82.7, XCorr: 1.11
intensity
m/z
y1
y2
y3
b1
0
2000
4000
6000
8000
10000
200
400
600
800
1000
1200
1400

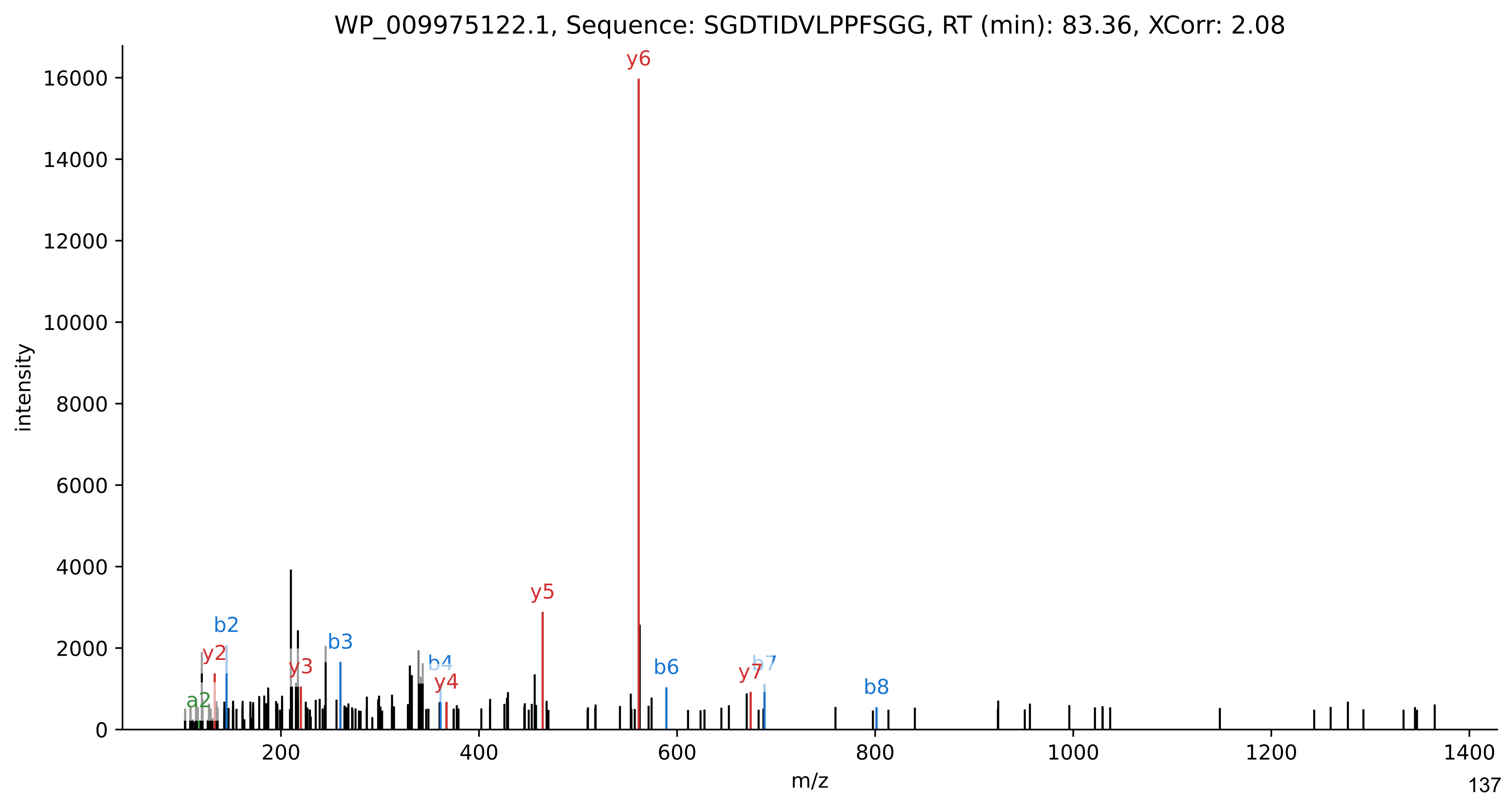
WP_009975122.1, Sequence: SGDTIDVLPPFSGG, RT (min): 83.36, XCorr: 2.08
intensity
m/z
y6
y5
b2
y2
a2
y3
b3
b4
y4
b6
y7
b7
b8
0
2000
4000
6000
8000
10000
12000
14000
16000
200
400
600
800
1000
1200
1400

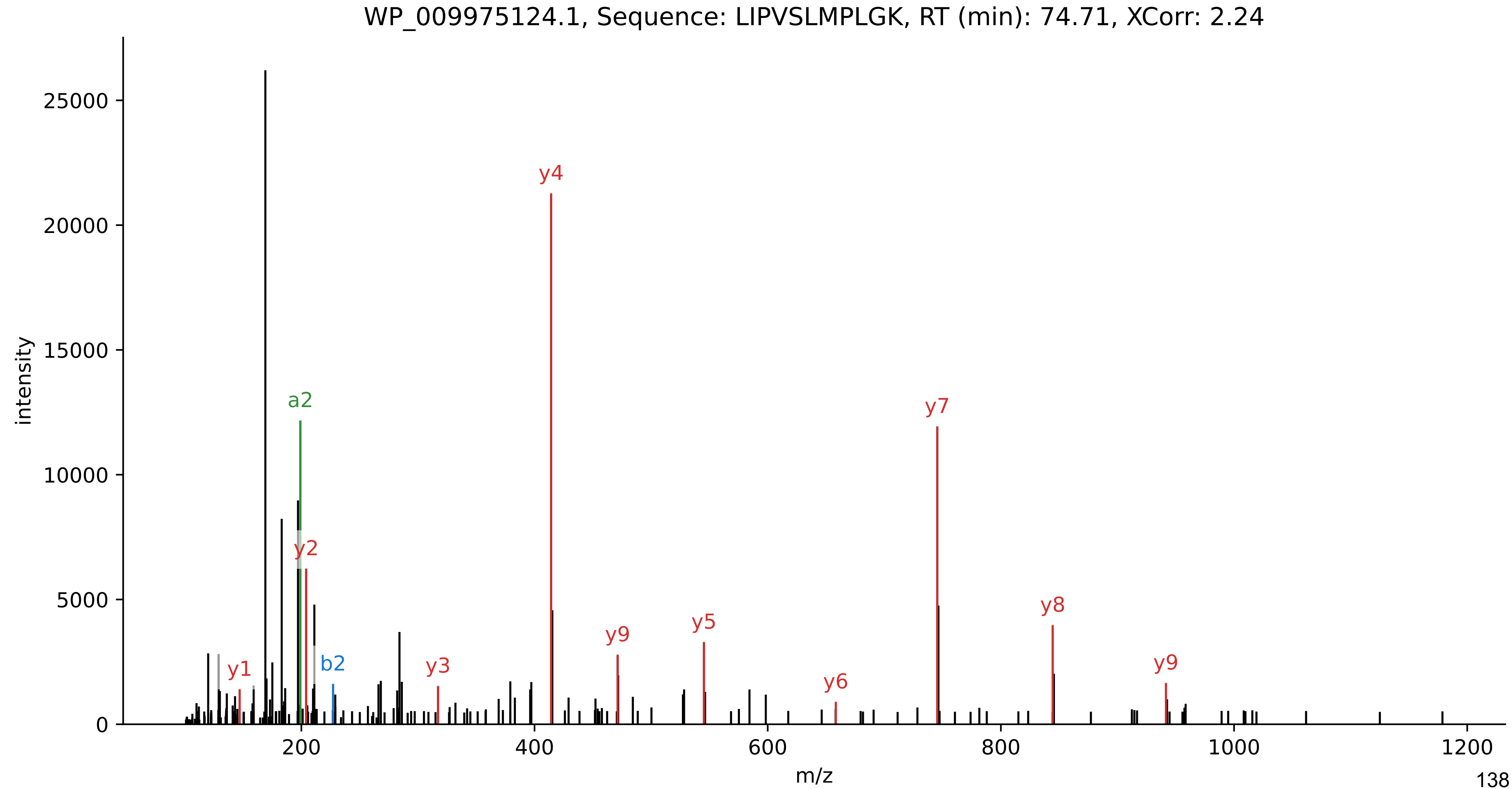
WP_009975124.1, Sequence: LIPVSLMPLGK, RT (min): 74.71, XCorr: 2.24
intensity
m/z
0
5000
10000
15000
20000
25000
200
400
600
800
1000
1200
a2
b2
y1
y2
y3
y4
y5
y6
y7
y8
y9
y9

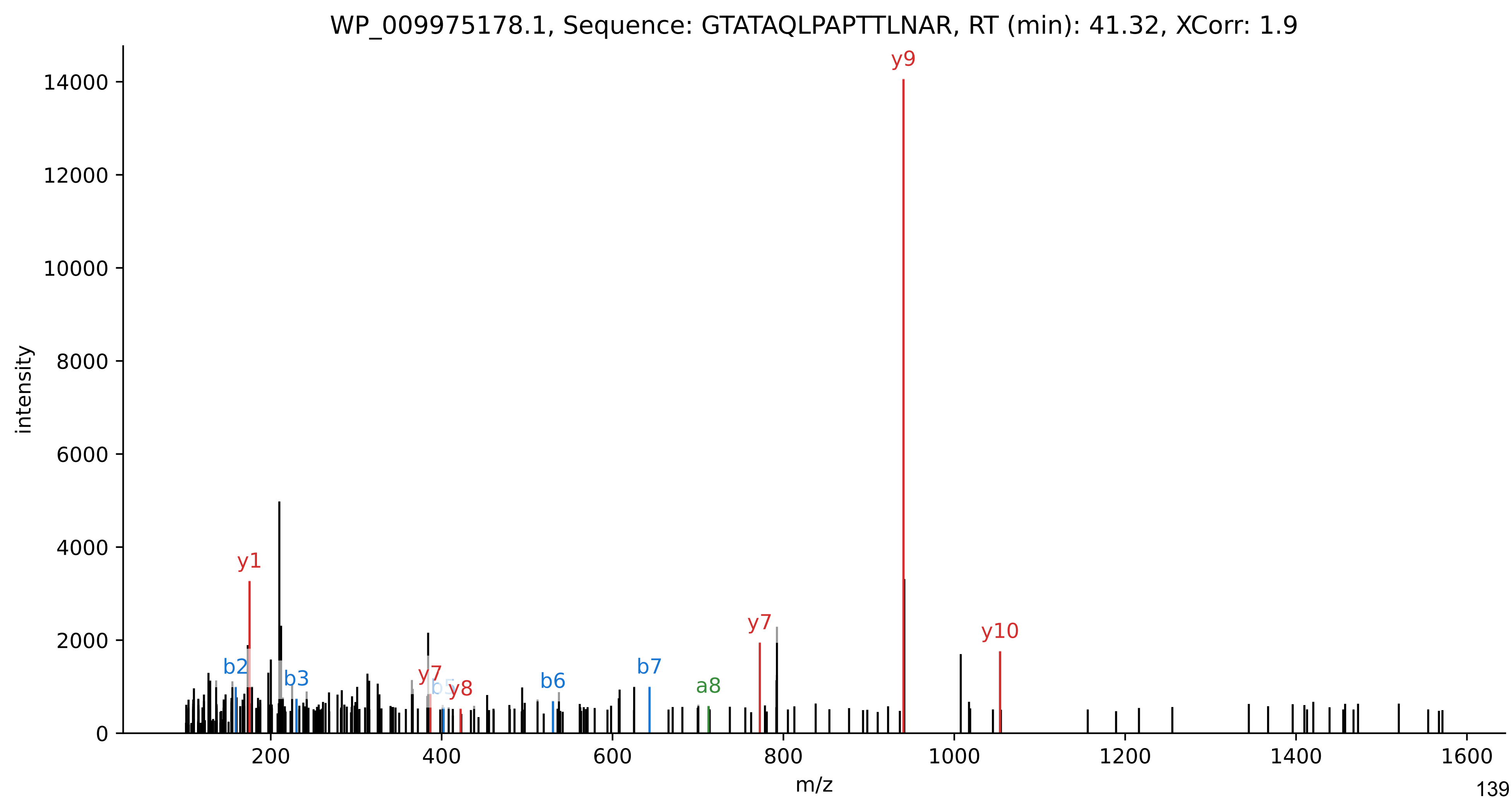
WP_009975178.1, Sequence: GTATAQLPAPTTLNAR, RT (min): 41.32, XCorr: 1.9
intensity
m/z
y9
y1
b2
b3
y7
y8
b6
b7
a8
y7
y10
0
2000
4000
6000
8000
10000
12000
14000
200
400
600
800
1000
1200
1400
1600

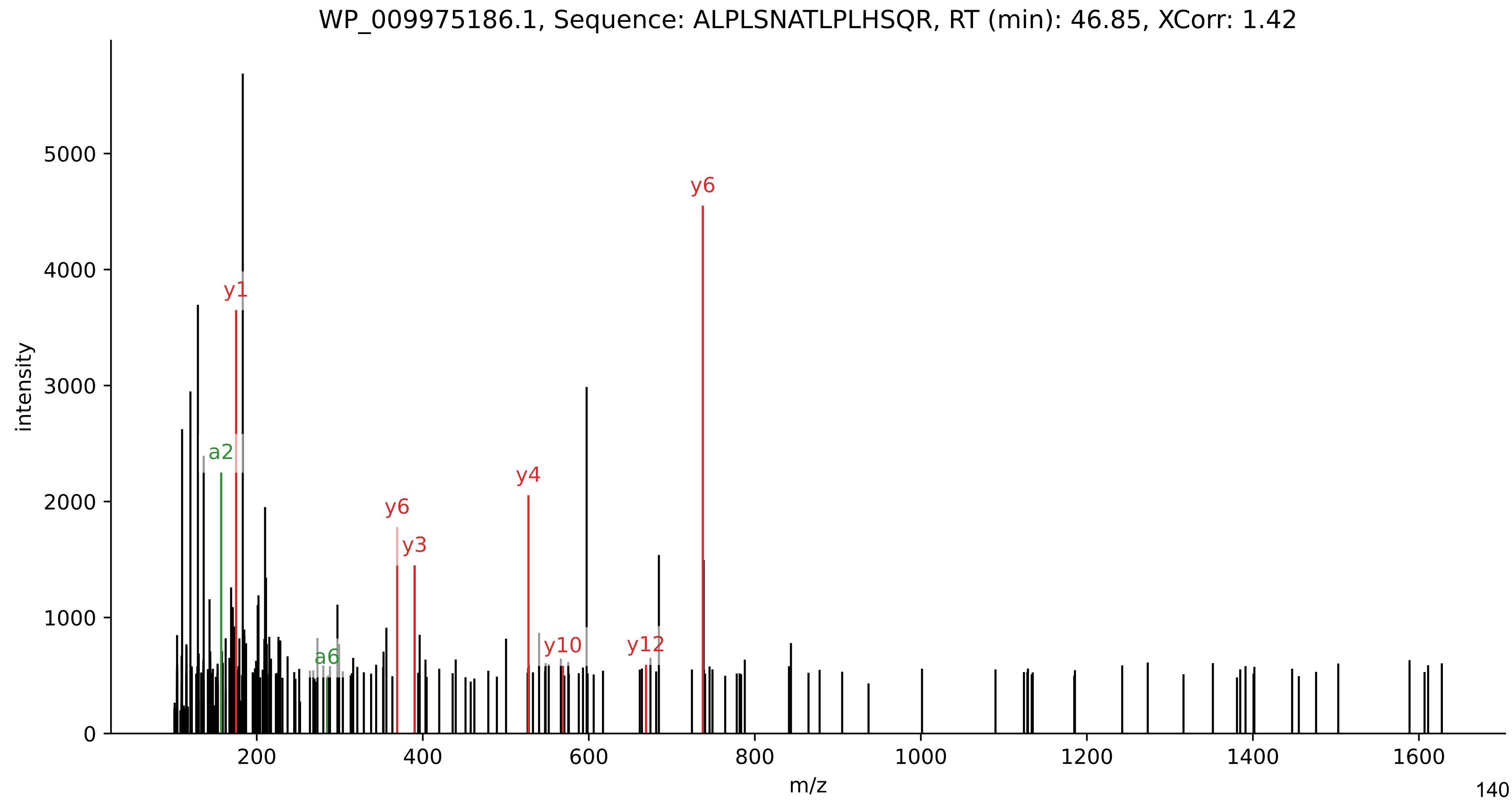

WP_009975186.1, Sequence: ALPLSNATLPLHSQR, RT (min): 46.85, XCorr: 1.42
intensity
m/z
y1
a2
y6
y3
y4
y10
y12
y6
a6
0
1000
2000
3000
4000
5000
200
400
600
800
1000
1200
1400
1600

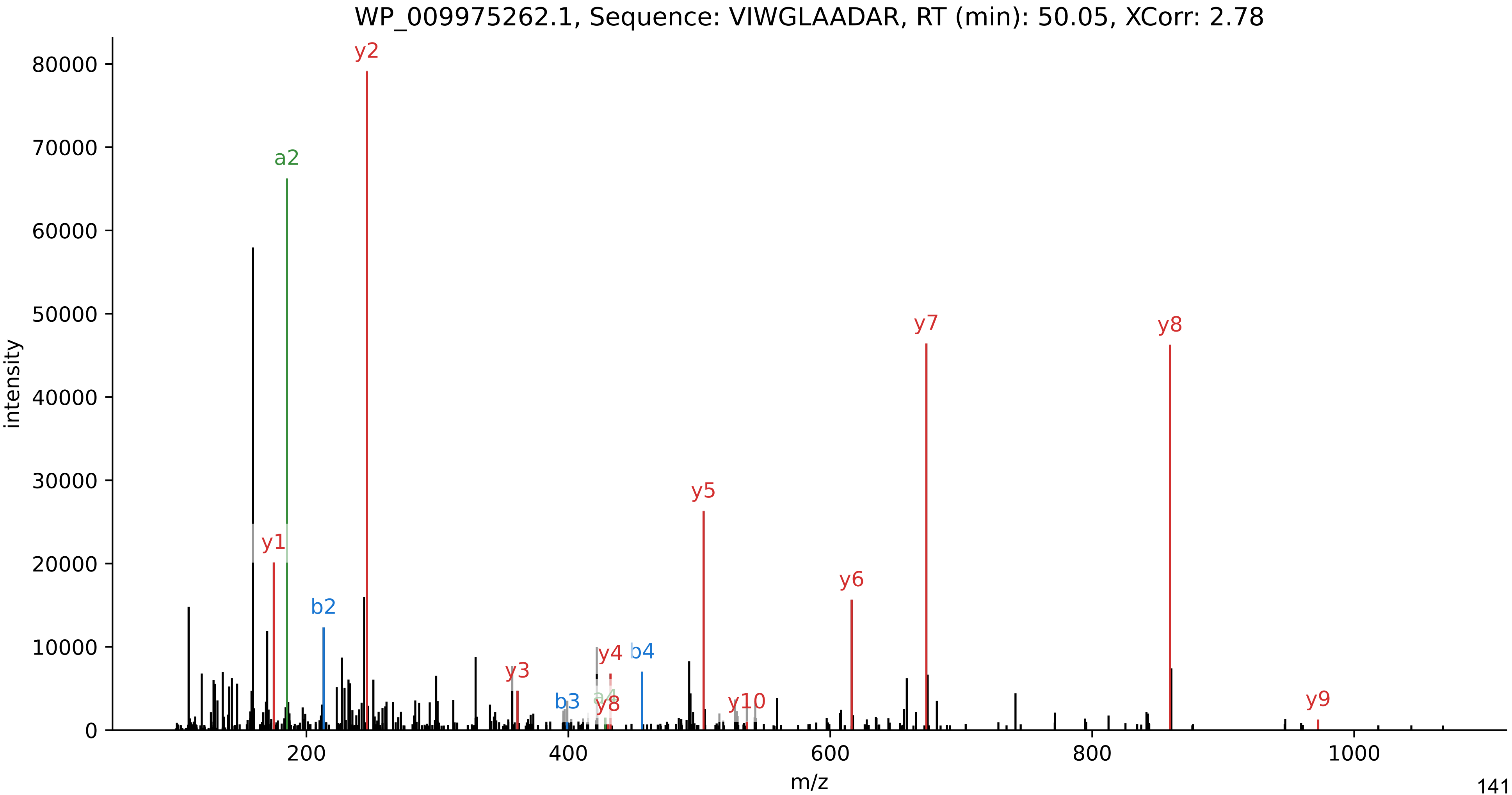
WP_009975262.1, Sequence: VIWGLAADAR, RT (min): 50.05, XCorr: 2.78
intensity
m/z
y2
a2
y1
b2
y3
b3
y4
b4
y8
y5
y10
y6
y7
y8
y9
80000
70000
60000
50000
40000
30000
20000
10000
0
200
400
600
800
1000

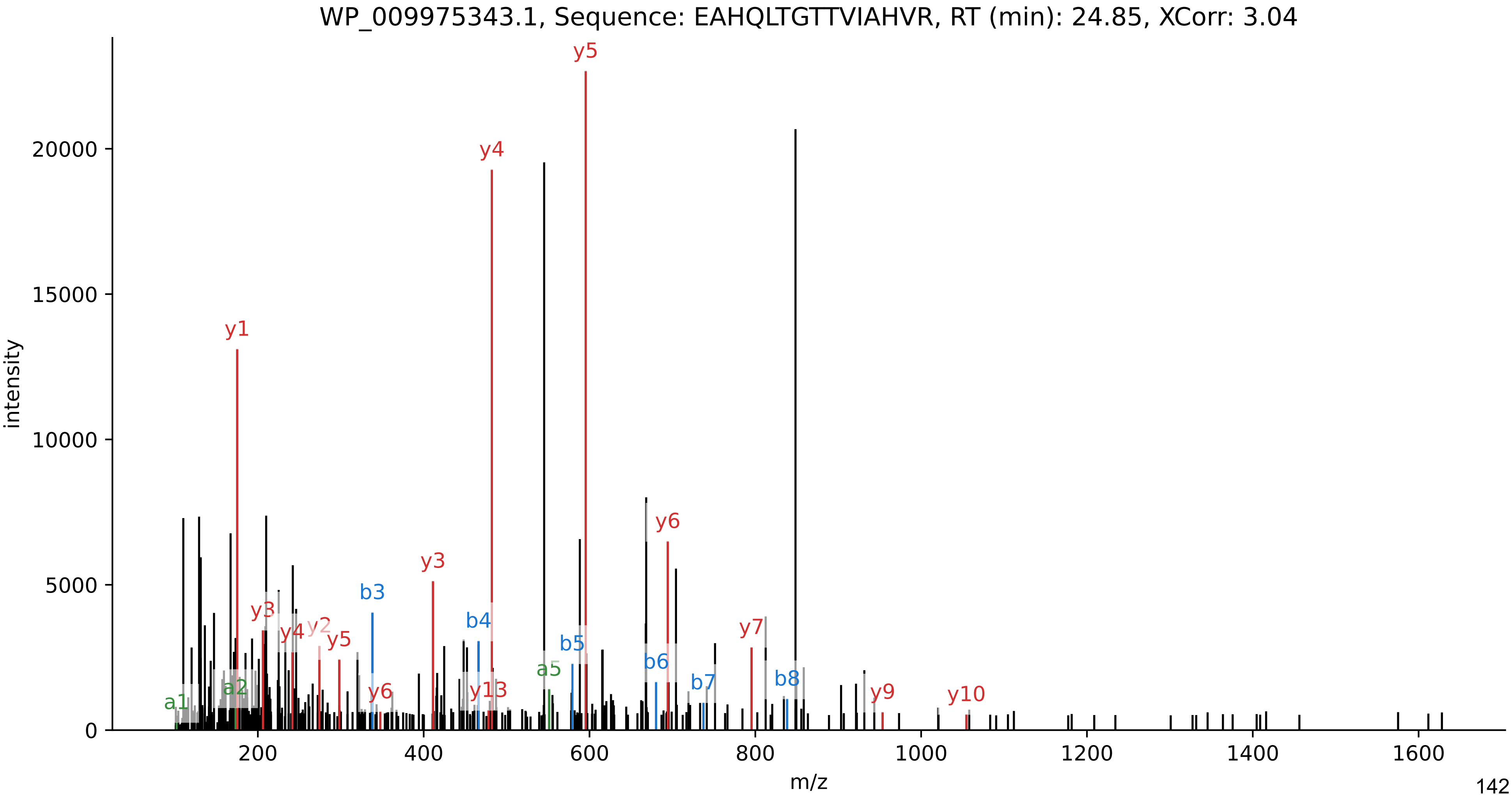
WP_009975343.1, Sequence: EAHQLTGTTVIAHVR, RT (min): 24.85, XCorr: 3.04
intensity
m/z
0
5000
10000
15000
20000
200
400
600
800
1000
1200
1400
1600
y5
y4
y1
y6
y3
b3
y3
y4
y2
y5
b4
b5
a5
b6
y7
b7
b8
a1
a2
y6
y13
y9
y10

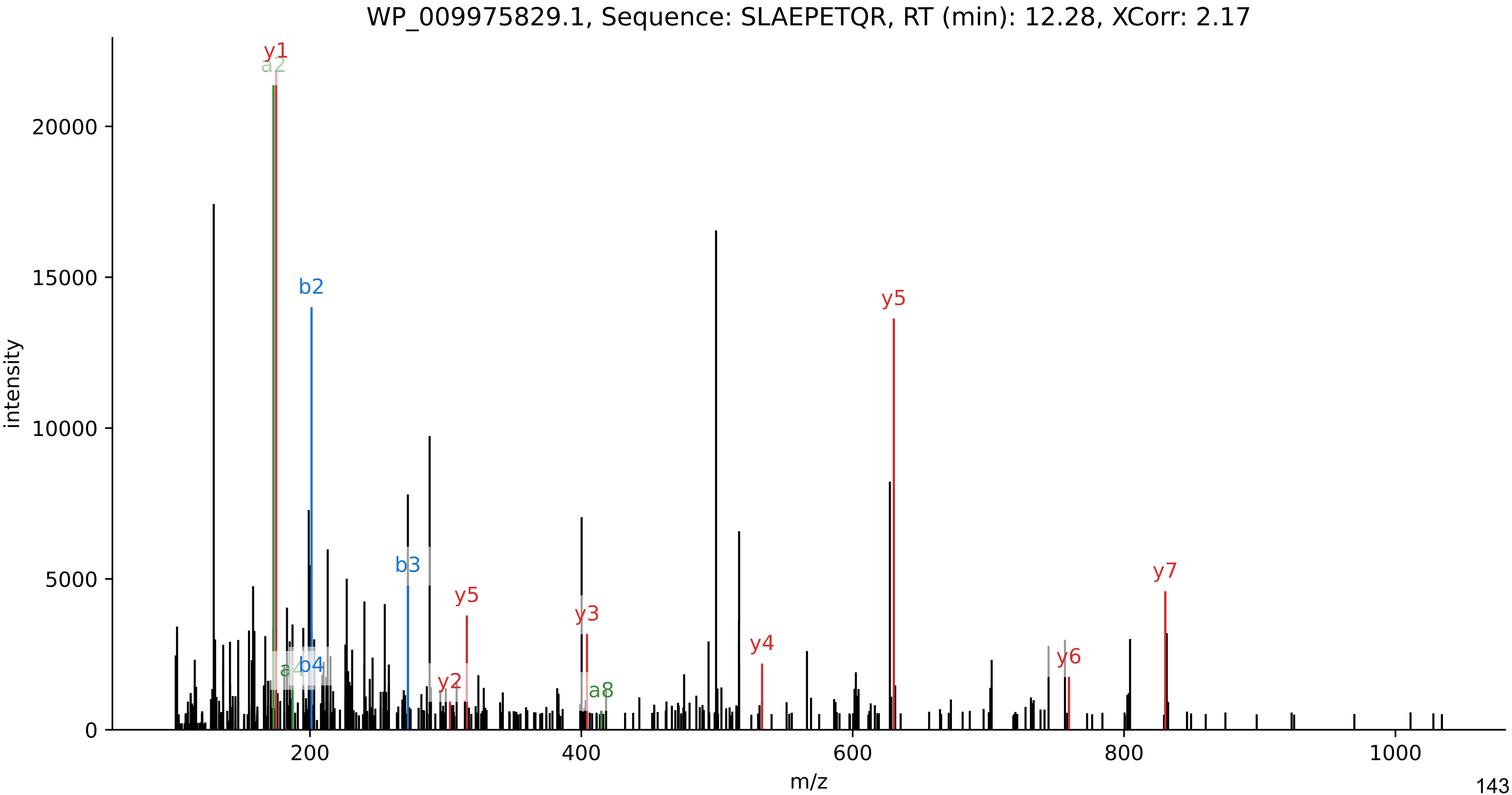
WP_009975829.1, Sequence: SLAEPETQR, RT (min): 12.28, XCorr: 2.17
intensity
m/z
y1
a2
b2
y5
b3
y5
y3
y4
y7
y6
y2
a8
b4
20000
15000
10000
5000
0
200
400
600
800
1000

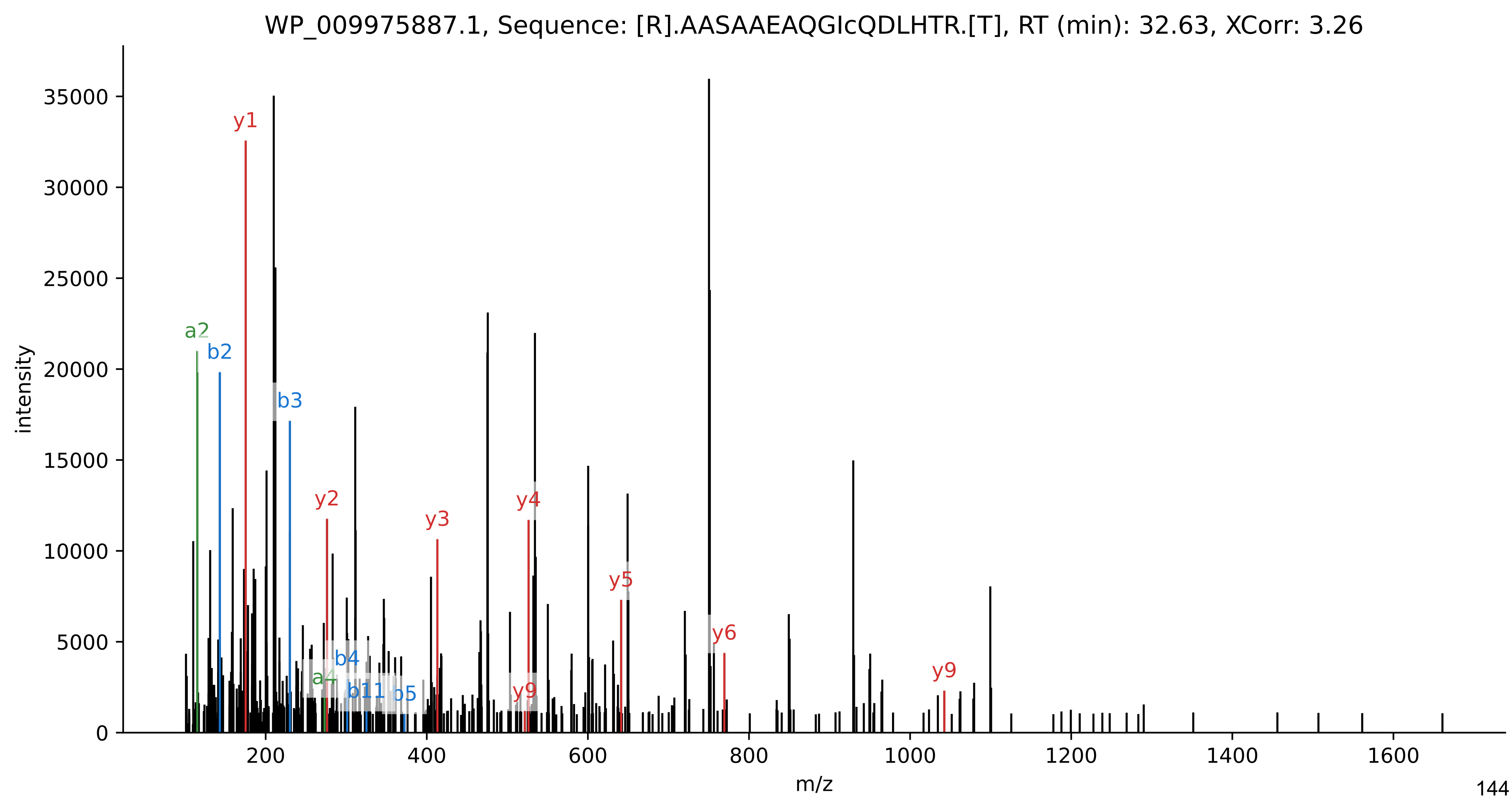
WP_009975887.1, Sequence: [R].AASAAEAQGIcQDLHTR.[T], RT (min): 32.63, XCorr: 3.26
intensity
m/z
a2
b2
y1
b3
y2
y3
y4
y5
y6
y9
y9
b4
a4
b11
b5
0
5000
10000
15000
20000
25000
30000
35000
200
400
600
800
1000
1200
1400
1600

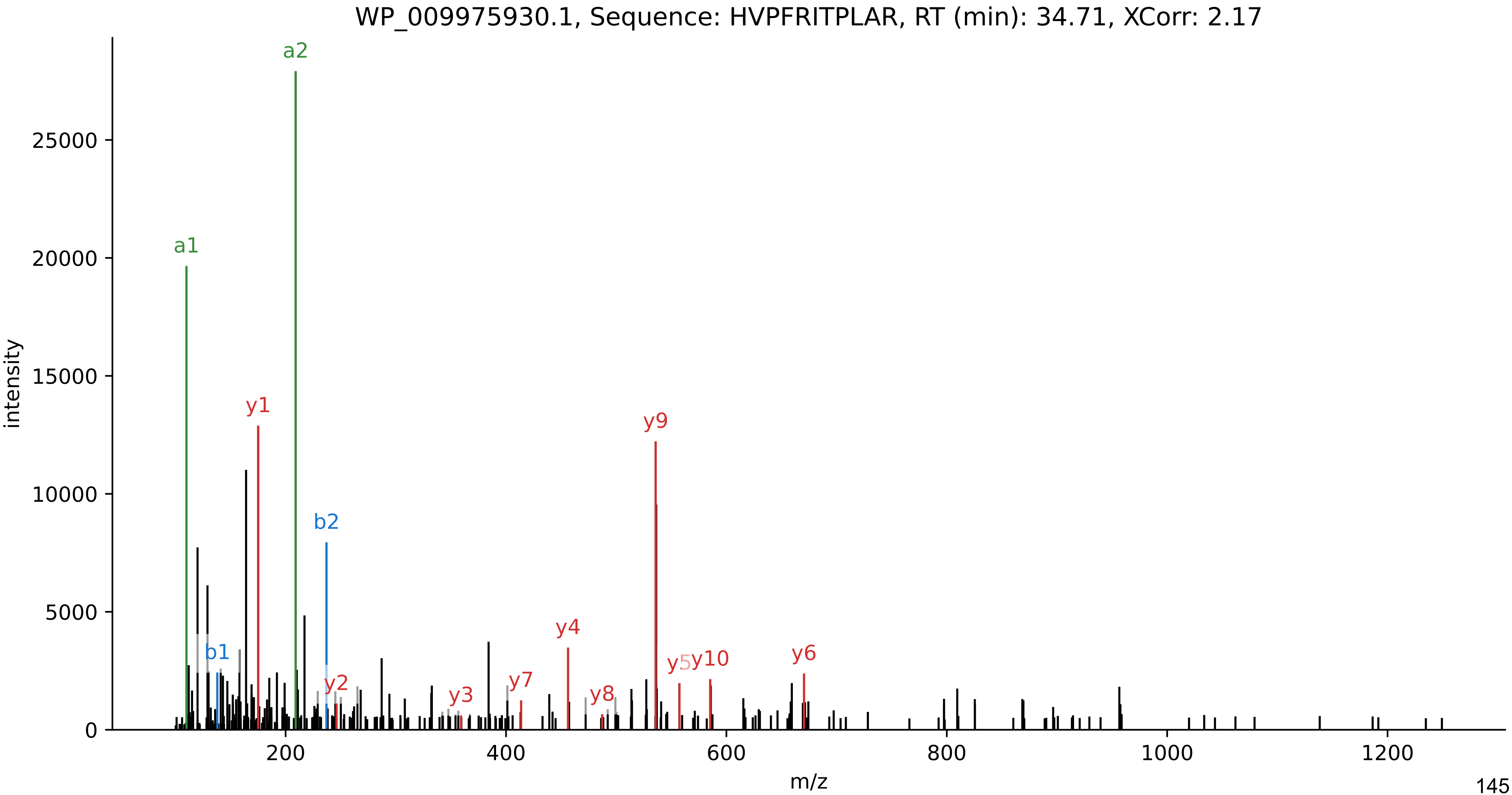
WP_009975930.1, Sequence: HVPFRITPLAR, RT (min): 34.71, XCorr: 2.17
intensity
m/z
a1
a2
b1
b2
y1
y2
y3
y4
y5
y6
y7
y8
y9
y10
0
5000
10000
15000
20000
25000
200
400
600
800
1000
1200

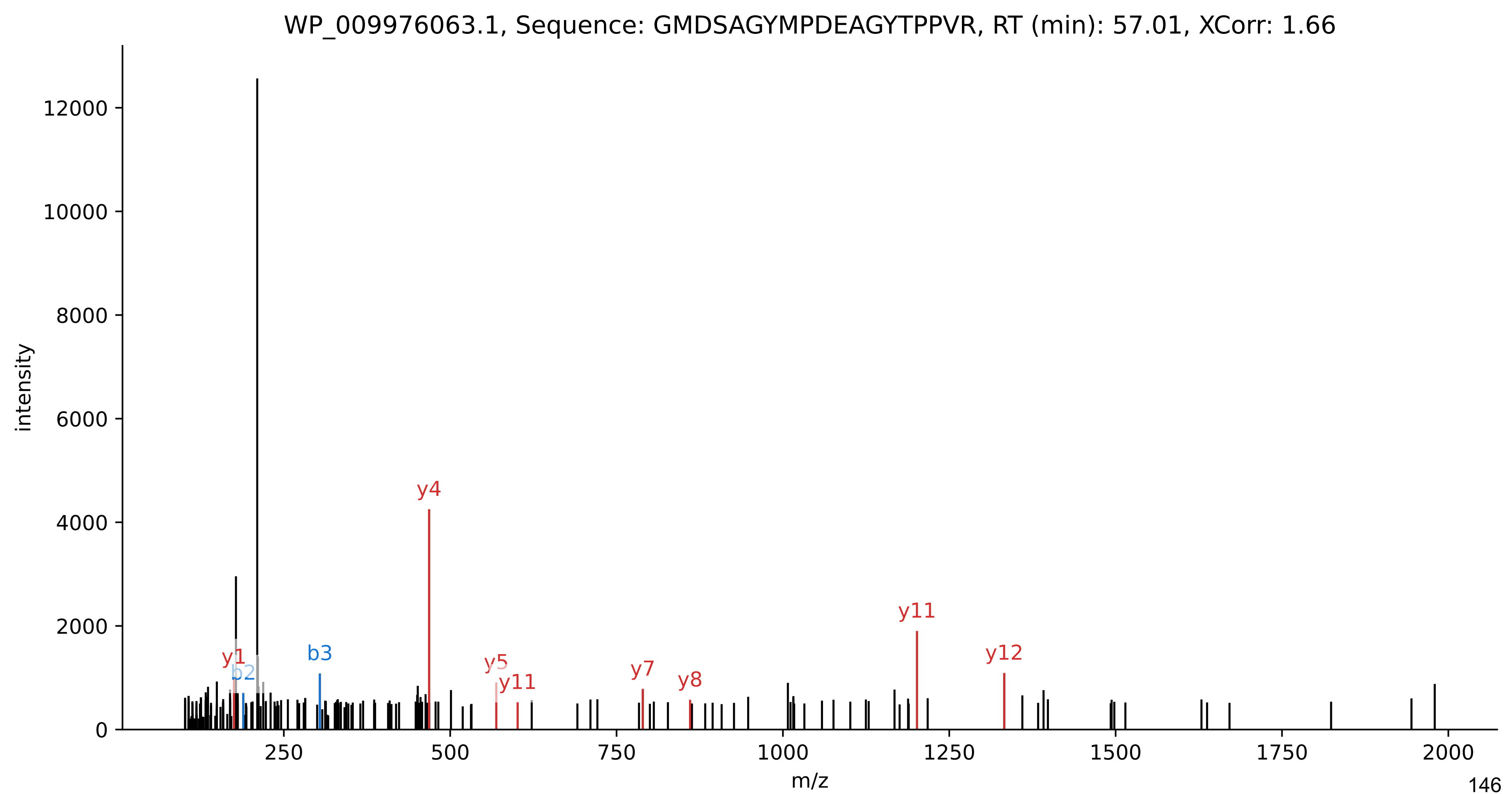
WP_009976063.1, Sequence: GMDSAGYMPDEAGYTPPVR, RT (min): 57.01, XCorr: 1.66
intensity
m/z
y1
b2
b3
y4
y5
y11
y7
y8
y11
y12
0
2000
4000
6000
8000
10000
12000
250
500
750
1000
1250
1500
1750
2000

WP_009976539.1, Sequence: ANVLPKLPPPASQLWR, RT (min): 71.27, XCorr: 4.13

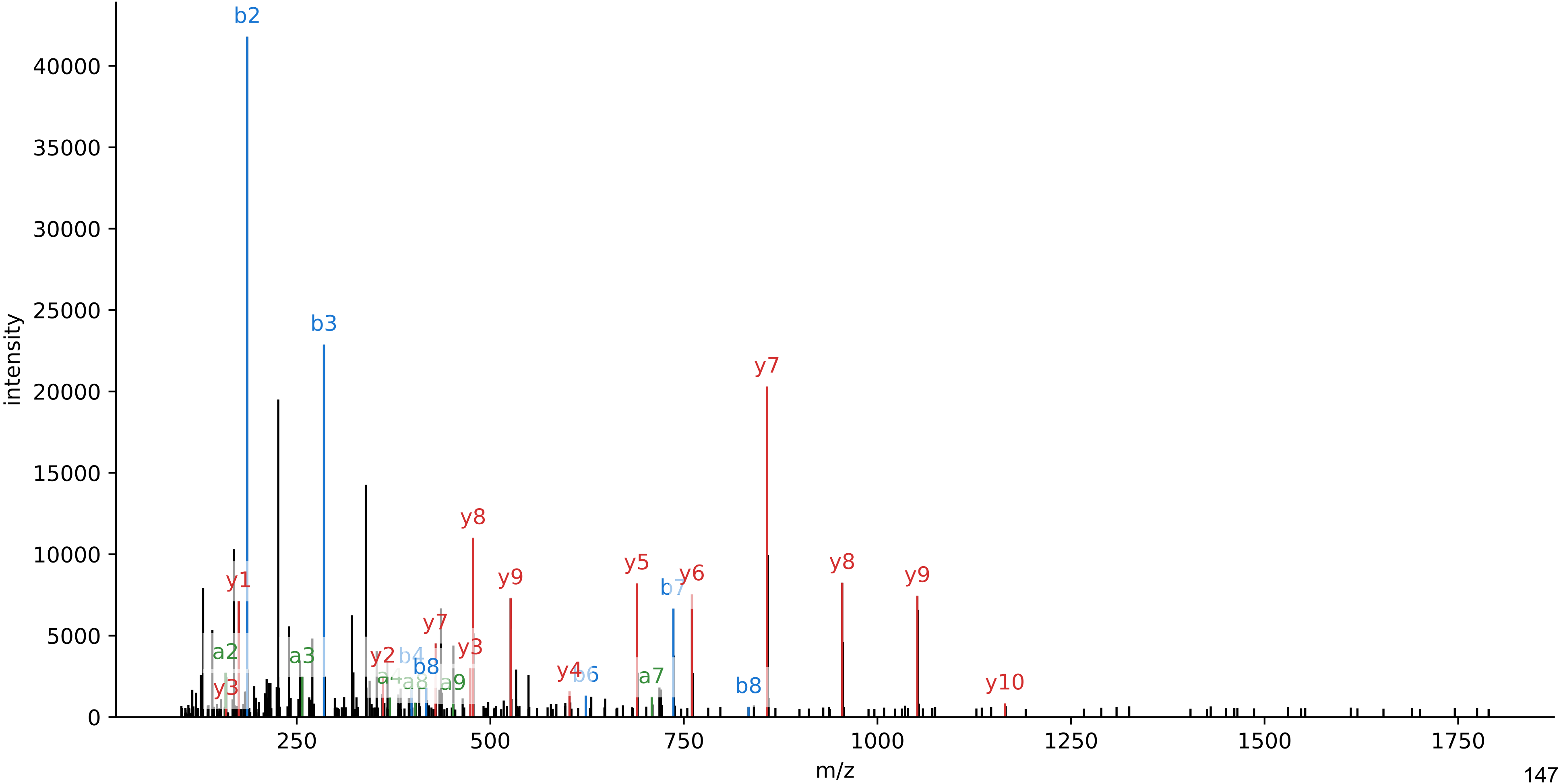

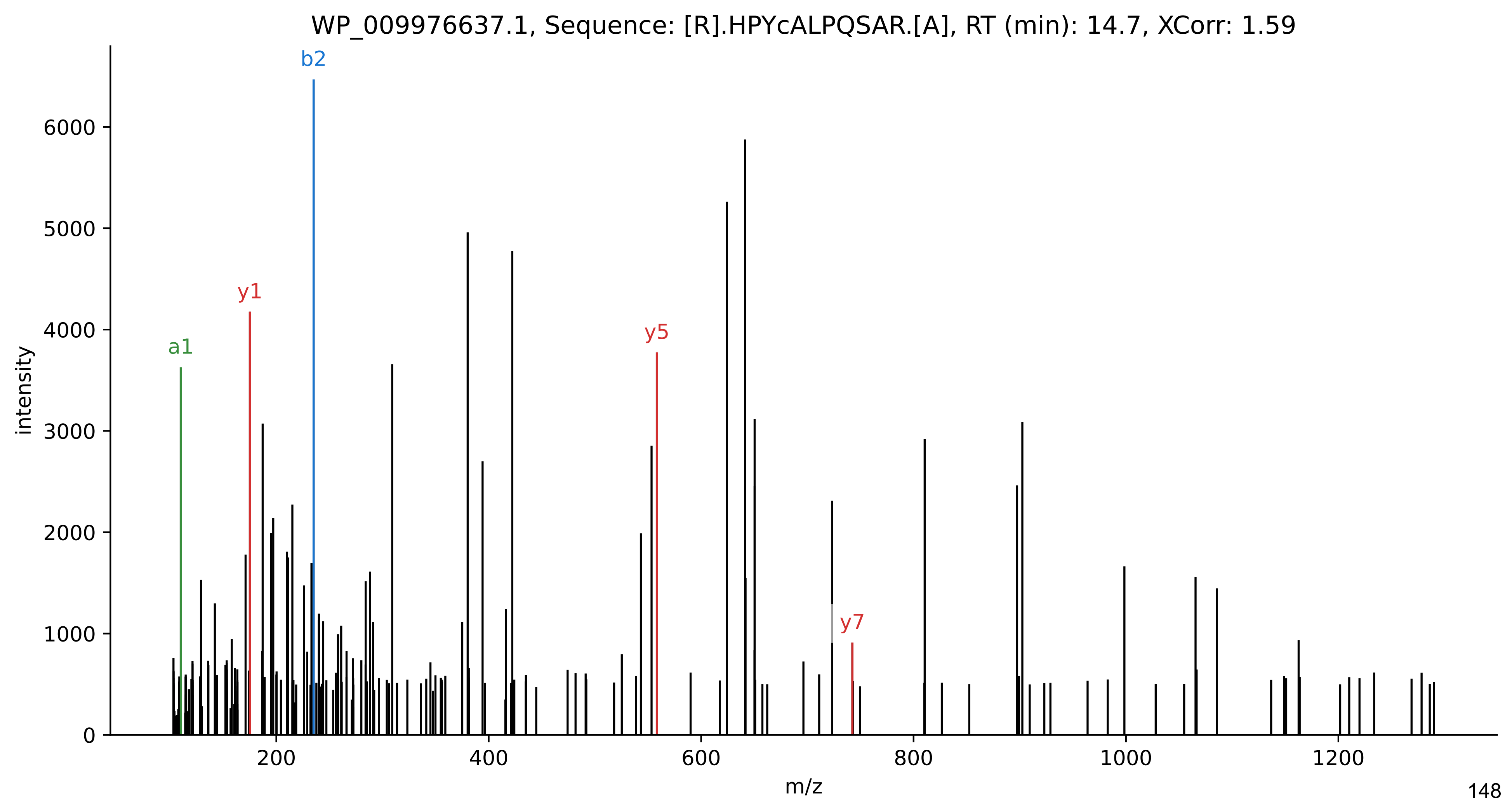
WP_009976637.1, Sequence: [R].HPYcALPQSAR.[A], RT (min): 14.7, XCorr: 1.59
intensity
m/z
a1
y1
b2
y5
y7
0
1000
2000
3000
4000
5000
6000
200
400
600
800
1000
1200

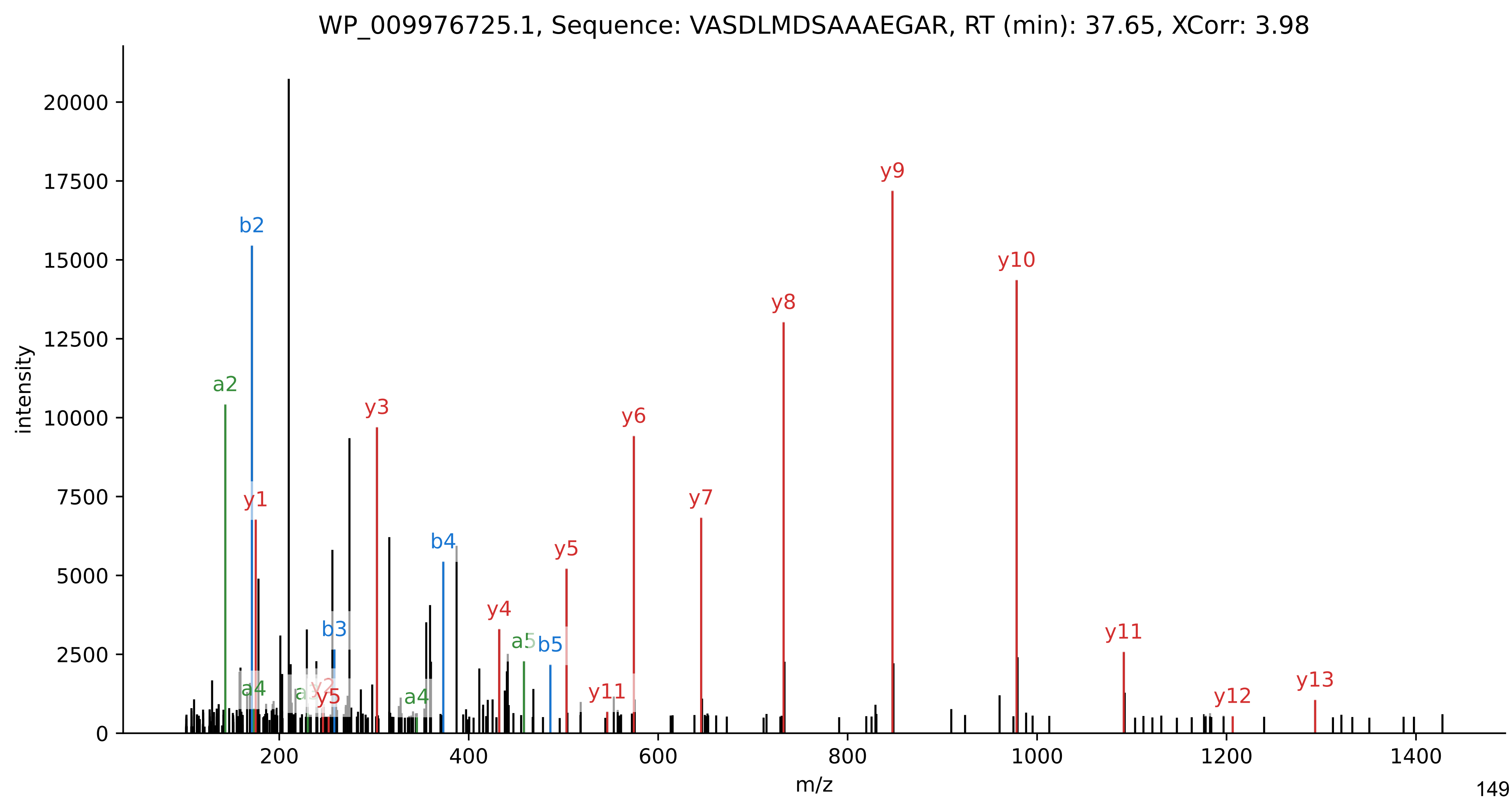

WP_009976725.1, Sequence: VASDLMDSAAAEGAR, RT (min): 37.65, XCorr: 3.98
intensity
m/z
b2
a2
y1
y3
b4
b3
y4
a5
b5
y5
y6
y7
y8
y9
y10
y11
y12
y13
a4
y5
y11

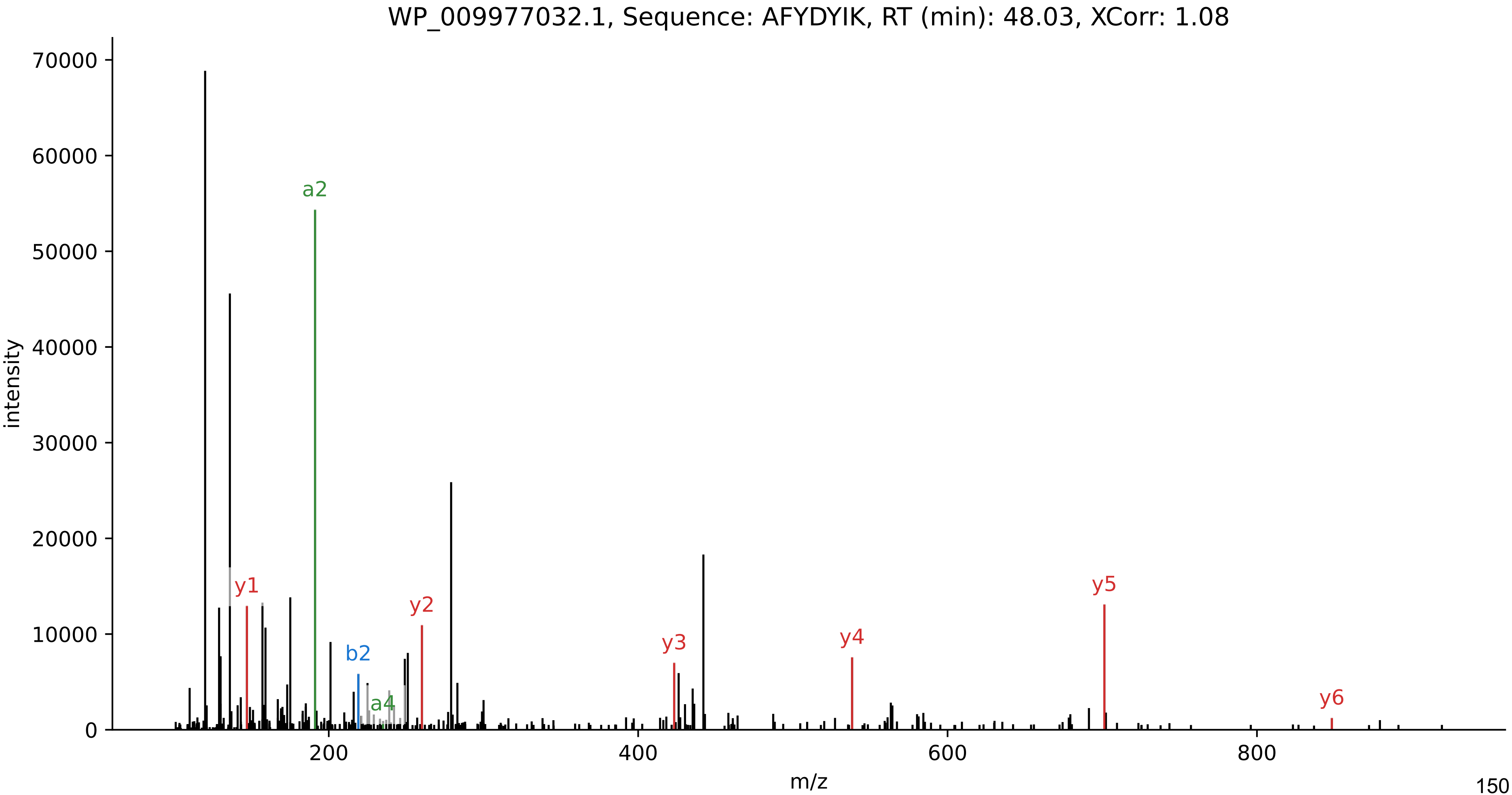
WP_009977032.1, Sequence: AFYDYIK, RT (min): 48.03, XCorr: 1.08
intensity
m/z
a2
y1
y2
b2
a4
y3
y4
y5
y6
70000
60000
50000
40000
30000
20000
10000
0
200
400
600
800

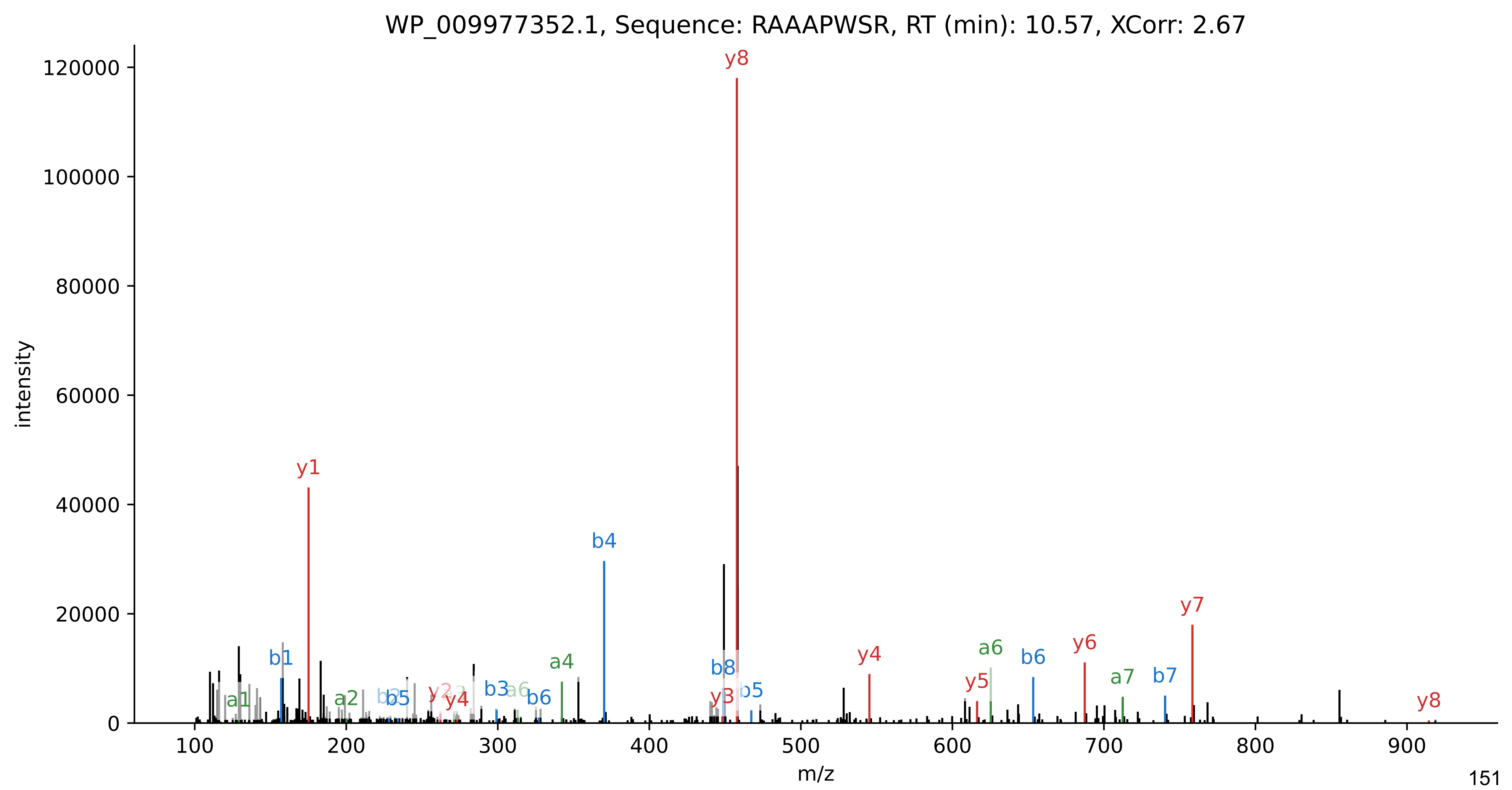

WP_009977352.1, Sequence: RAAAPWSR, RT (min): 10.57, XCorr: 2.67
intensity
m/z
y8
y1
b4
y7
y6
a6
b6
y4
b8
b1
a4
y5
a7
b7
y8
b3
b5
b6
y3
a1
a2
y4

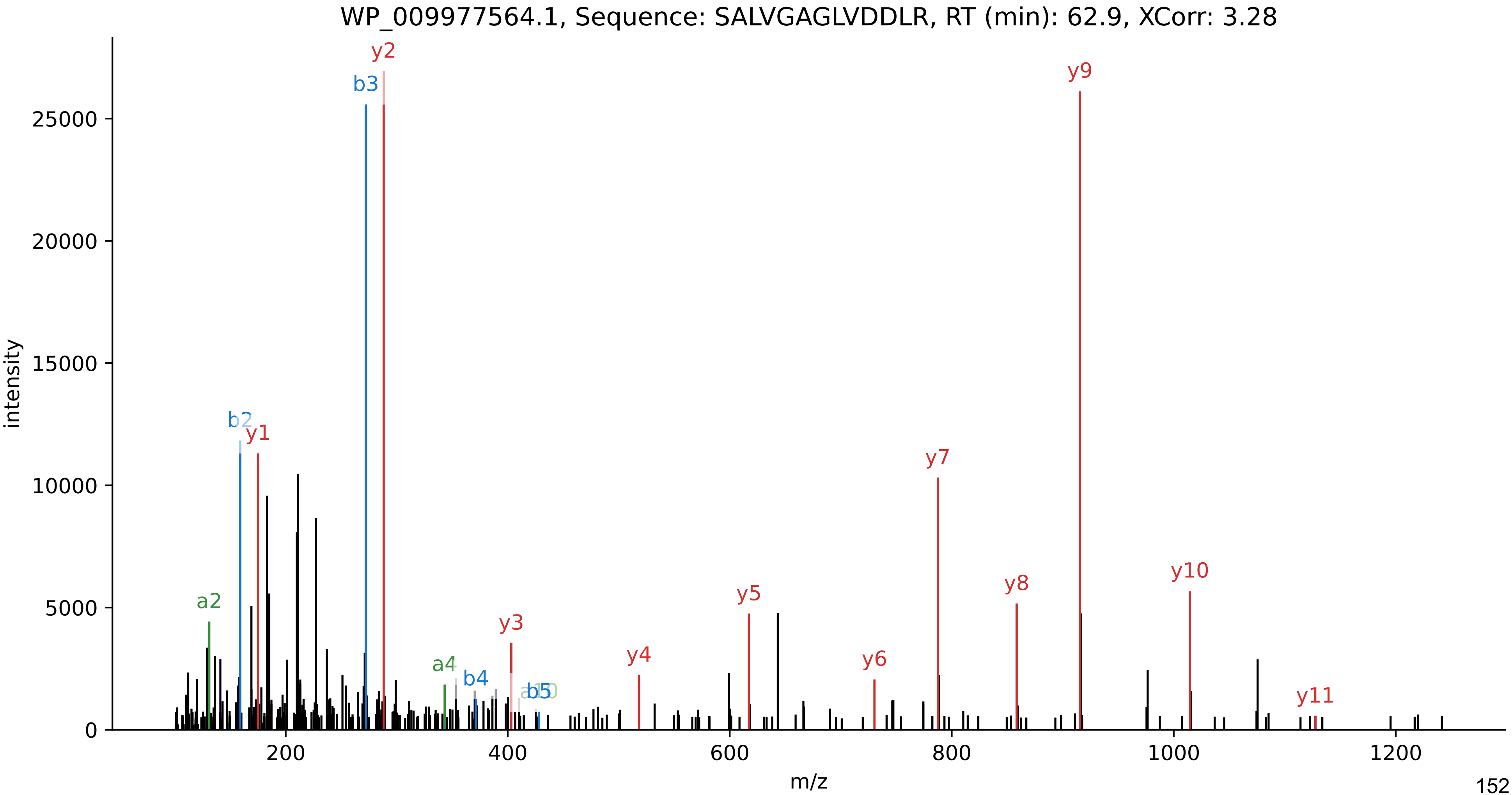
WP_009977564.1, Sequence: SALVGAGLVDDLR, RT (min): 62.9, XCorr: 3.28
intensity
m/z
0
5000
10000
15000
20000
25000
200
400
600
800
1000
1200
a2
b2
y1
b3
y2
a4
b4
y3
b5
y4
y5
y6
y7
y8
y9
y10
y11

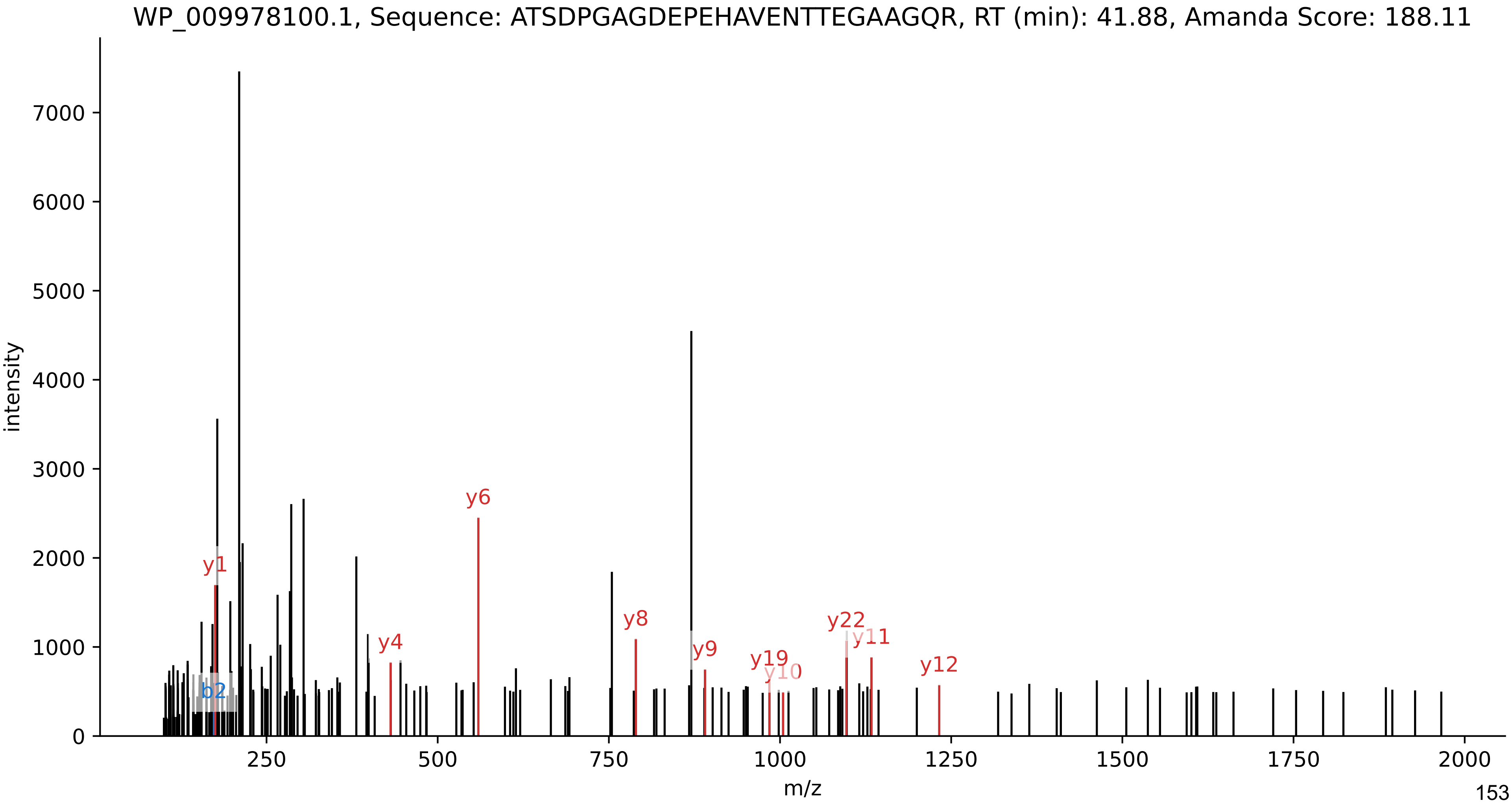
WP_009978100.1, Sequence: ATSDPGAGDEPEHAVENTTEGAAGQR, RT (min): 41.88, Amanda Score: 188.11
intensity
m/z
y1
b2
y4
y6
y8
y9
y19
y10
y22
y11
y12
0
1000
2000
3000
4000
5000
6000
7000
250
500
750
1000
1250
1500
1750
2000

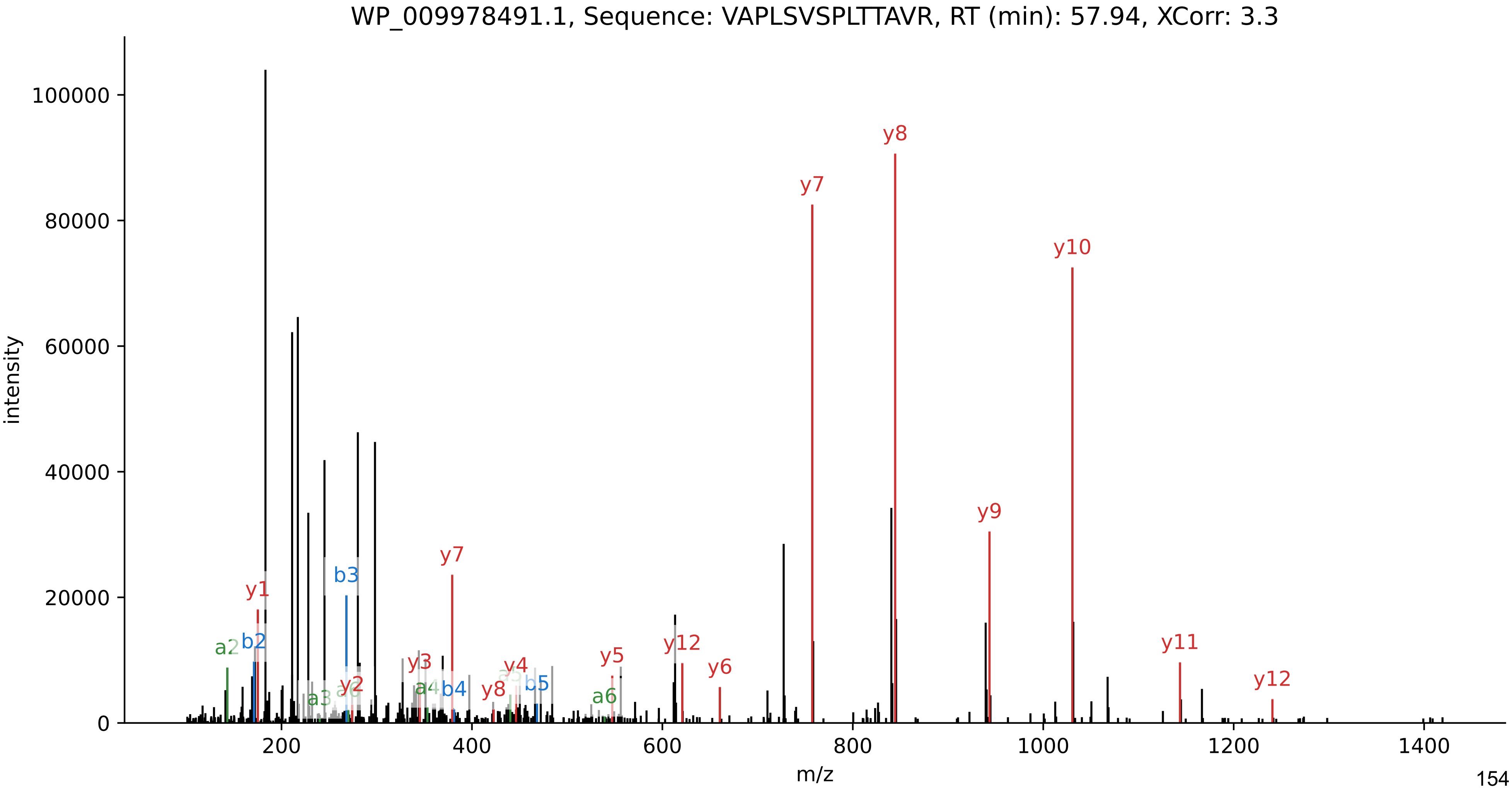

WP_009978491.1, Sequence: VAPLSVSPLTTAVR, RT (min): 57.94, XCorr: 3.3
intensity
m/z
100000
80000
60000
40000
20000
0
200
400
600
800
1000
1200
1400
y1
a2
b2
b3
y7
y3
y2
a3
a4
b4
y8
y4
b5
y5
a6
y12
y6
y7
y8
y9
y10
y11
y12

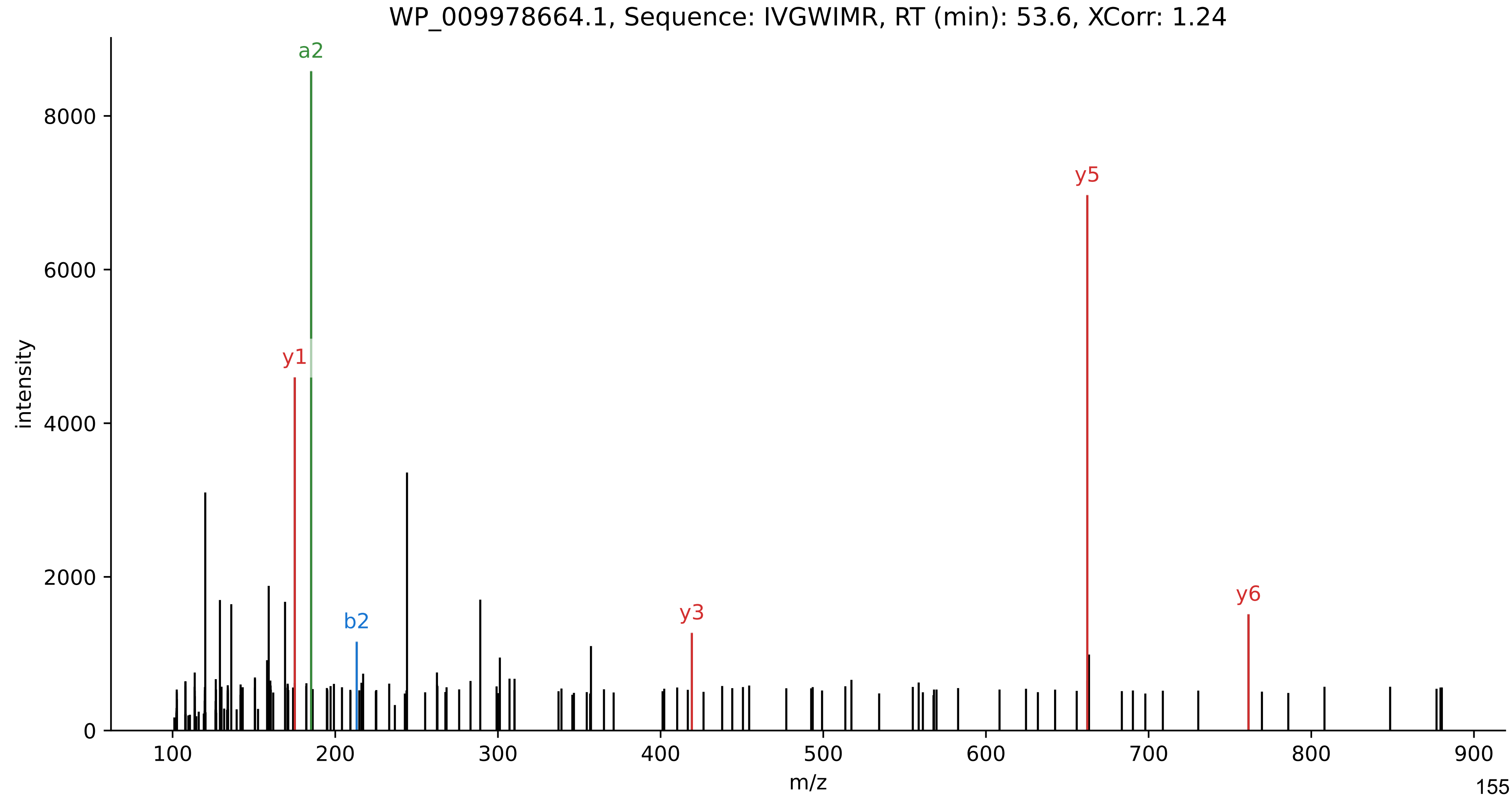
WP_009978664.1, Sequence: IVGWIMR, RT (min): 53.6, XCorr: 1.24
a2
y1
y5
y3
y6
b2
intensity
m/z
0
2000
4000
6000
8000
100
200
300
400
500
600
700
800
900

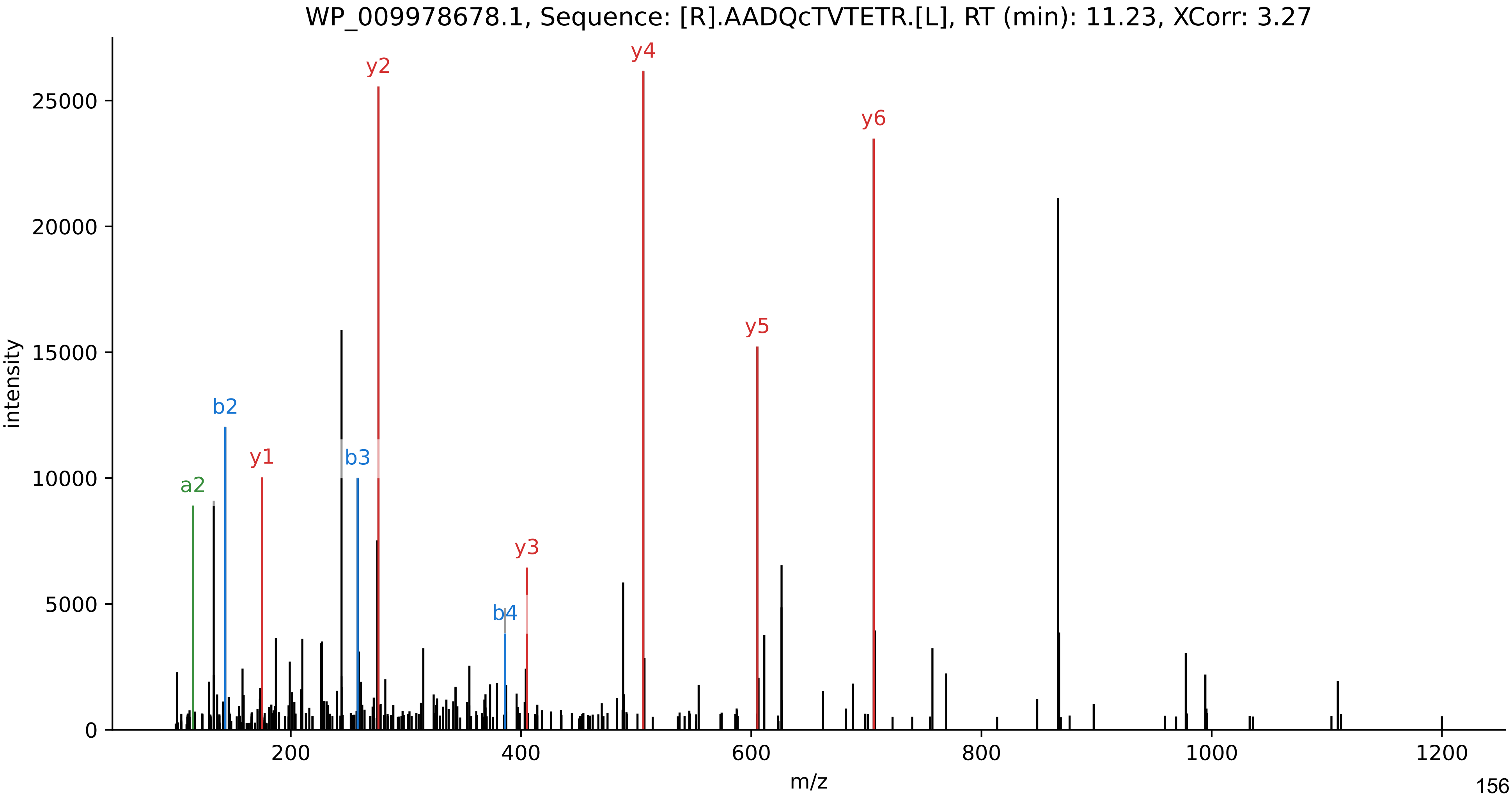
WP_009978678.1, Sequence: [R].AADQcTVTETR.[L], RT (min): 11.23, XCorr: 3.27
intensity
m/z
0
5000
10000
15000
20000
25000
200
400
600
800
1000
1200
a2
b2
y1
b3
y2
b4
y3
y4
y5
y6

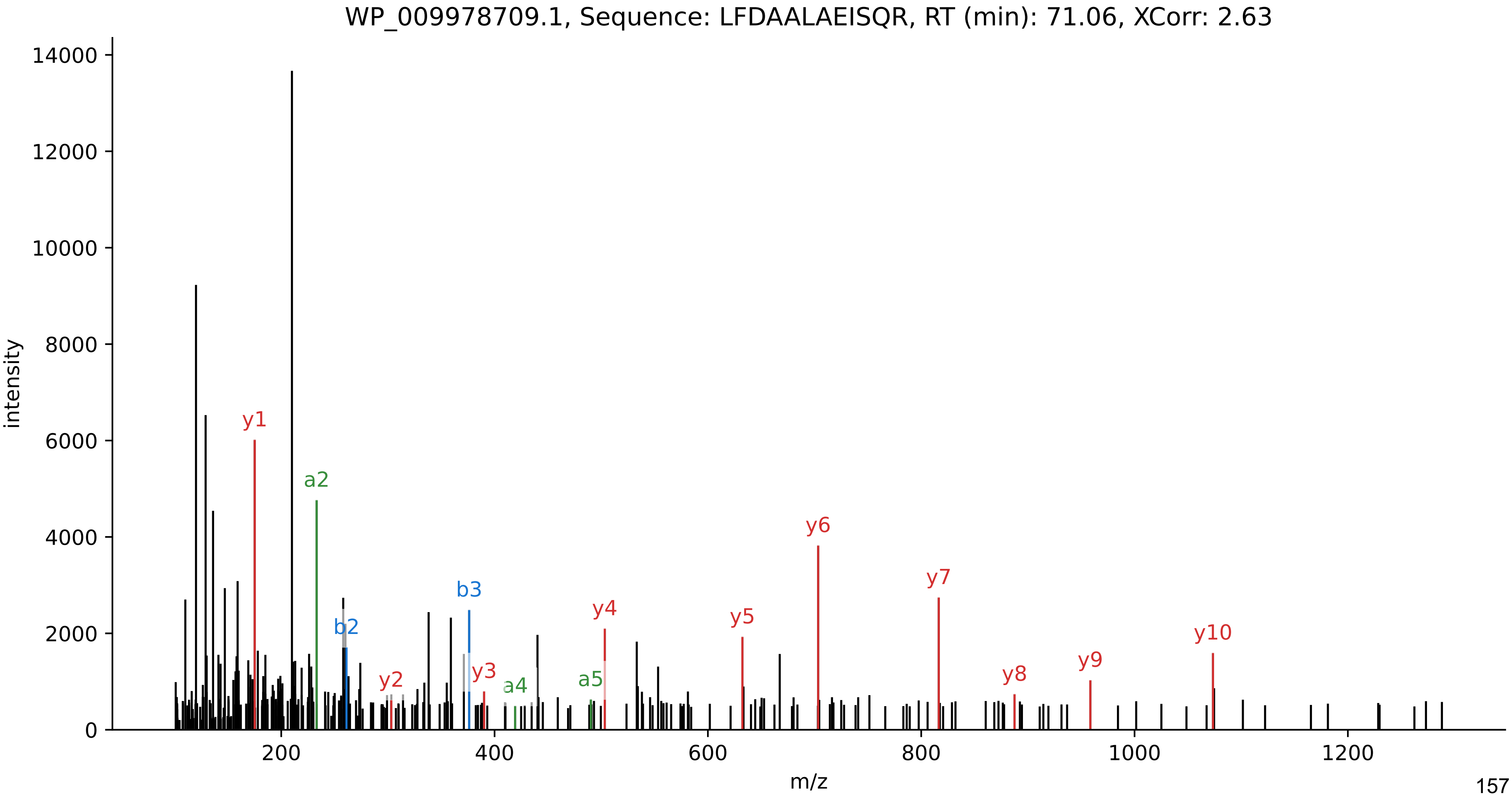
WP_009978709.1, Sequence: LFDAALAEISQR, RT (min): 71.06, XCorr: 2.63
intensity
m/z
y1
a2
b2
b3
y2
y3
a4
a5
y4
y5
y6
y7
y8
y9
y10
0
2000
4000
6000
8000
10000
12000
14000
200
400
600
800
1000
1200

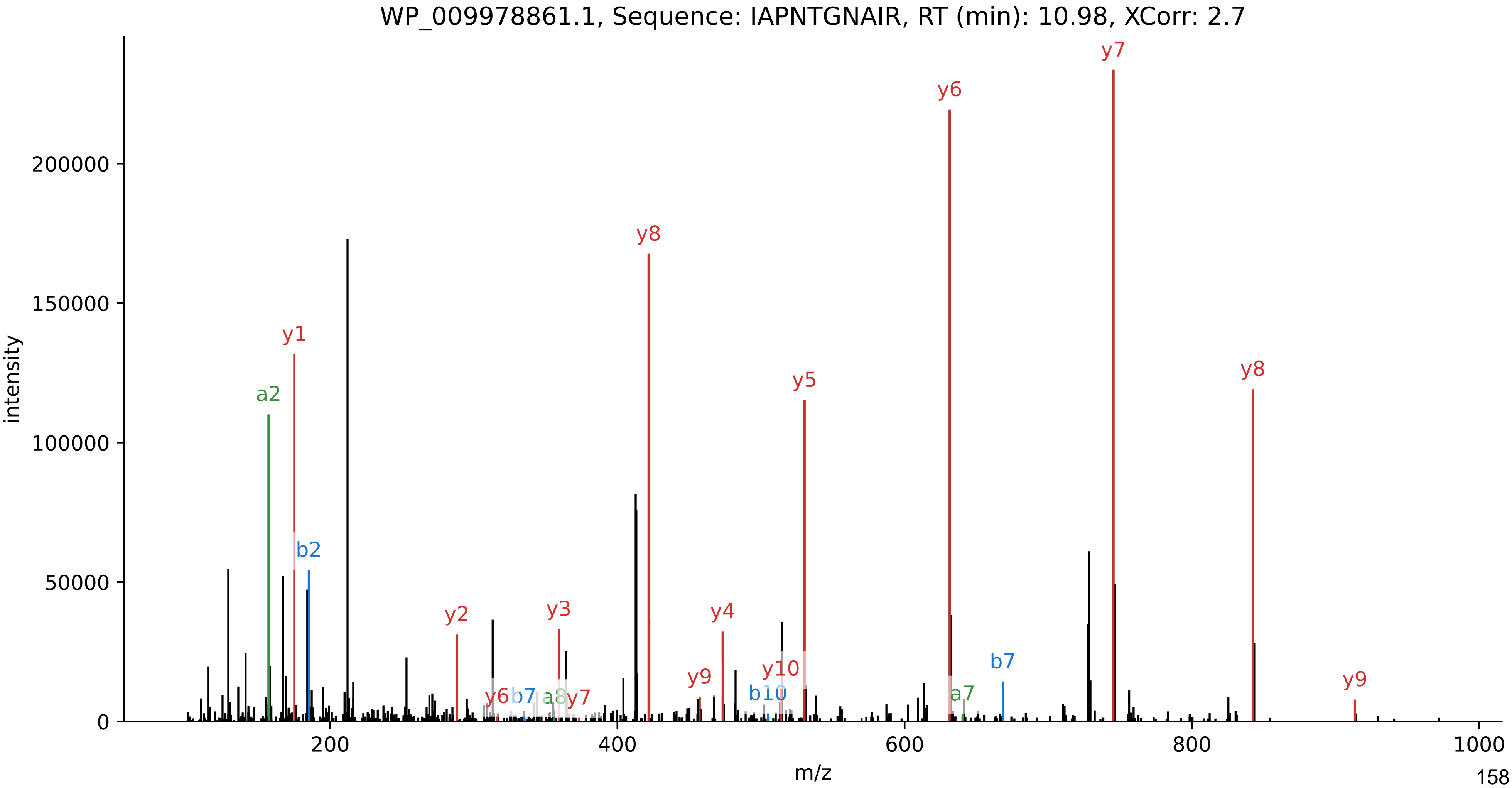
WP_009978861.1, Sequence: IAPNTGNAIR, RT (min): 10.98, XCorr: 2.7
intensity
m/z
y7
y6
y8
y1
a2
y5
y8
b2
y2
y3
y4
y9
y10
b10
b7
a7
y9
y6
b7
a8
y7

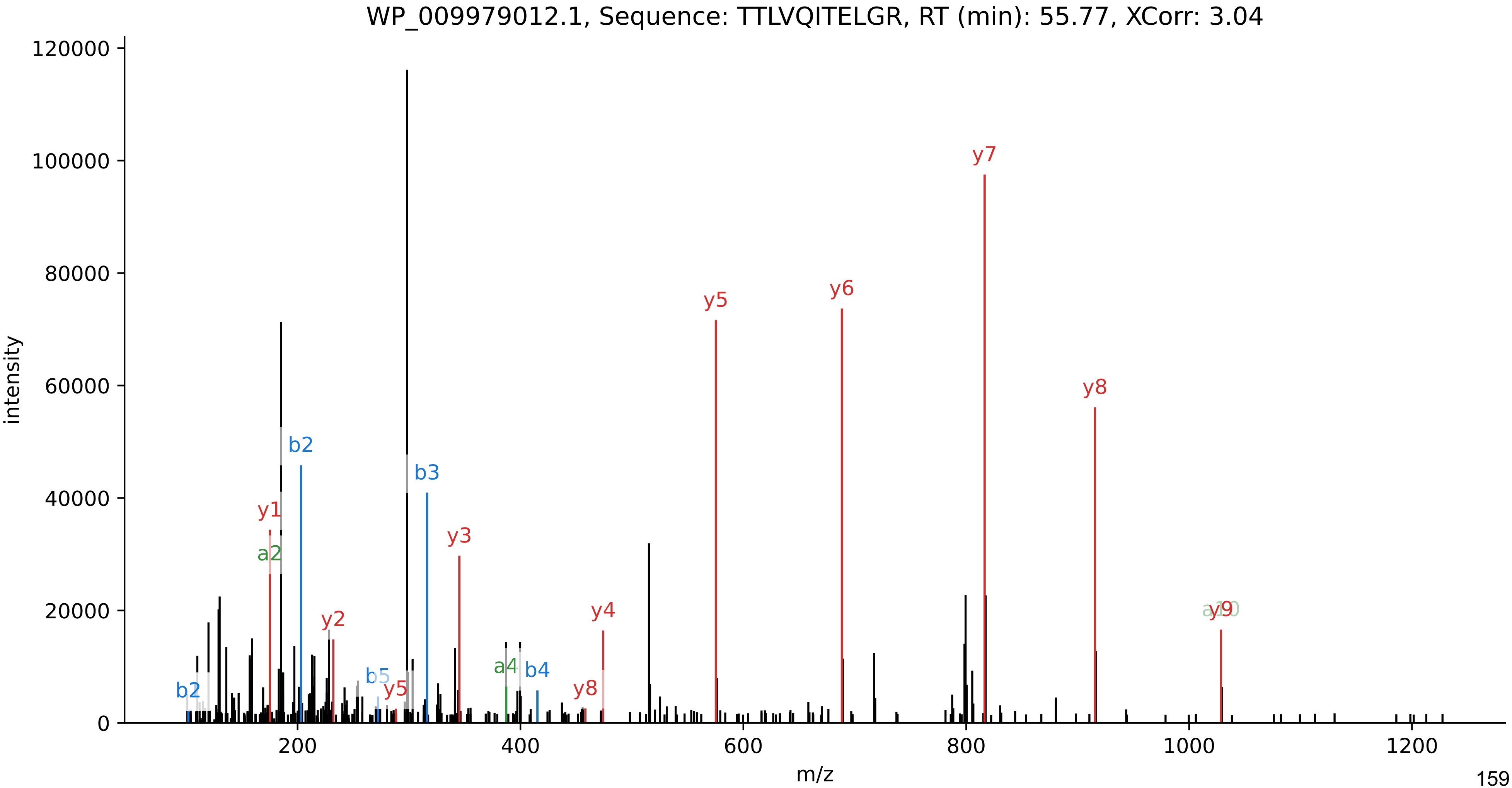
WP_009979012.1, Sequence: TTLVQITELGR, RT (min): 55.77, XCorr: 3.04
intensity
m/z
120000
100000
80000
60000
40000
20000
0
200
400
600
800
1000
1200
y7
y6
y5
y8
b2
b3
y1
a2
y3
y2
y4
y9
a10
b5
b4
a4
y5
y8
b2

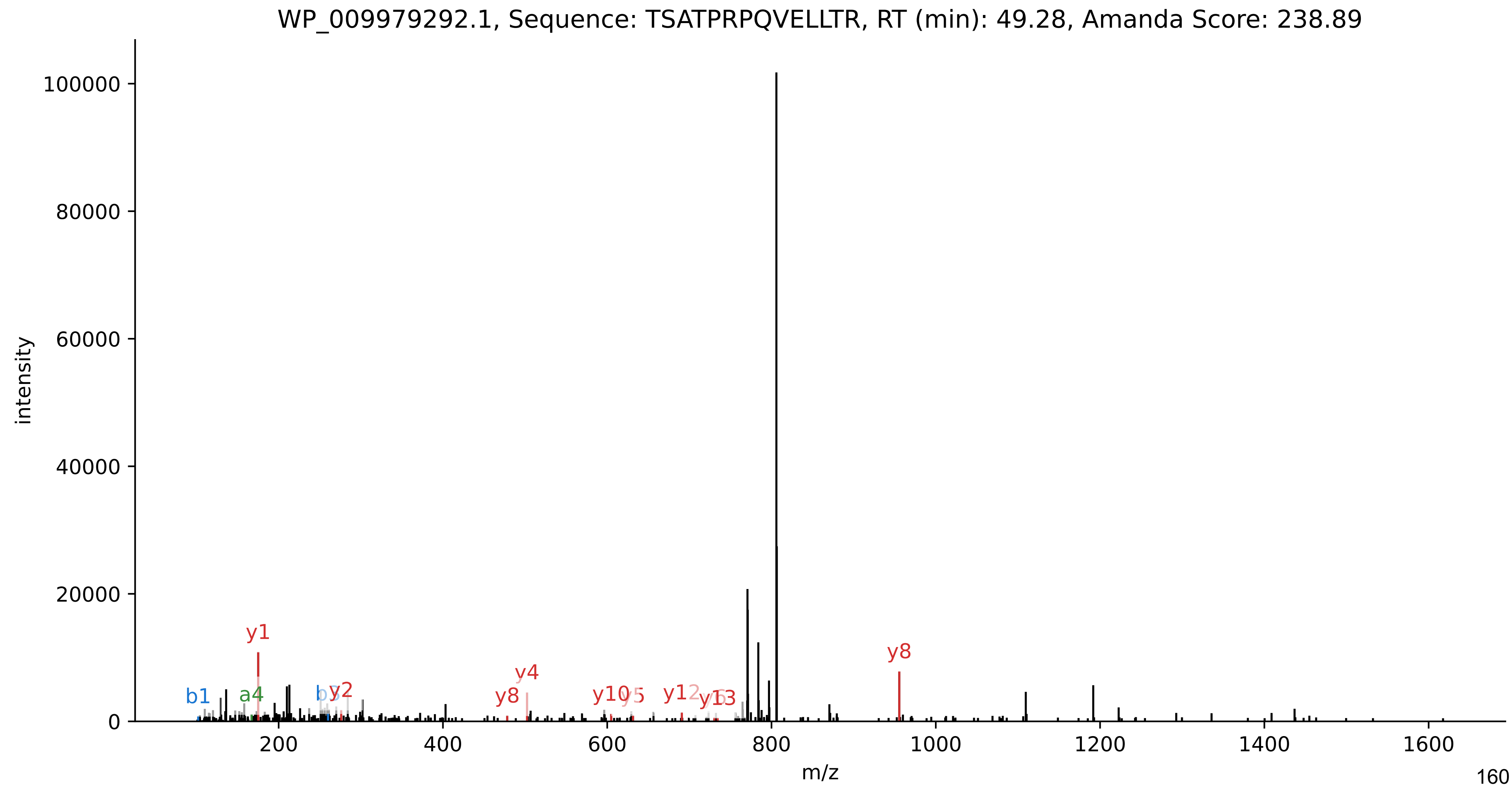
WP_009979292.1, Sequence: TSATPRPQVELLTR, RT (min): 49.28, Amanda Score: 238.89
intensity
m/z
y1
y8
y4
y2
b1
a4
y10
y5
y12
y13
y8

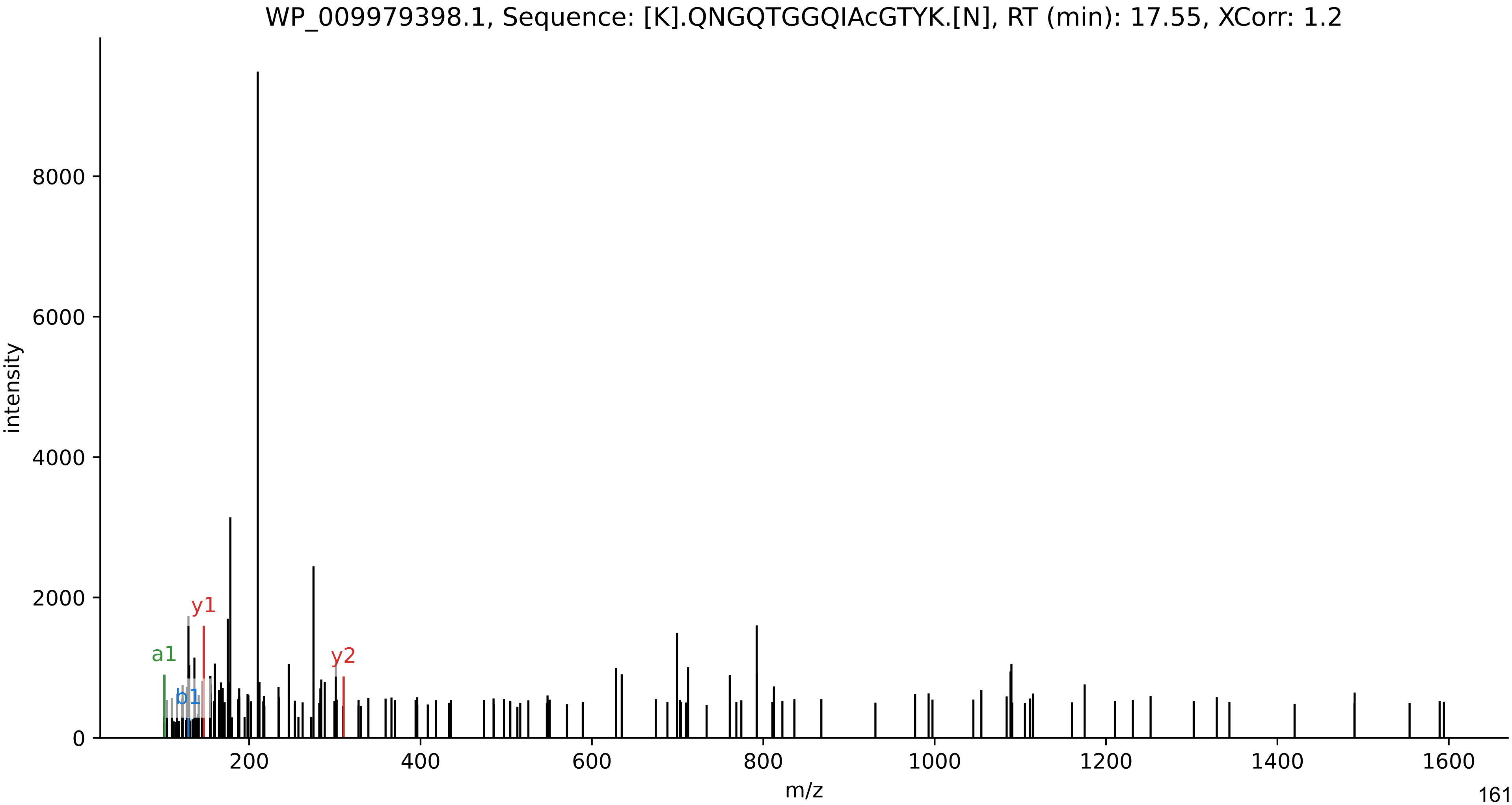
WP_009979398.1, Sequence: [K].QNGQTGGQIAcGTYK.[N], RT (min): 17.55, XCorr: 1.2
intensity
m/z
0
2000
4000
6000
8000
200
400
600
800
1000
1200
1400
1600
a1
b1
y1
y2

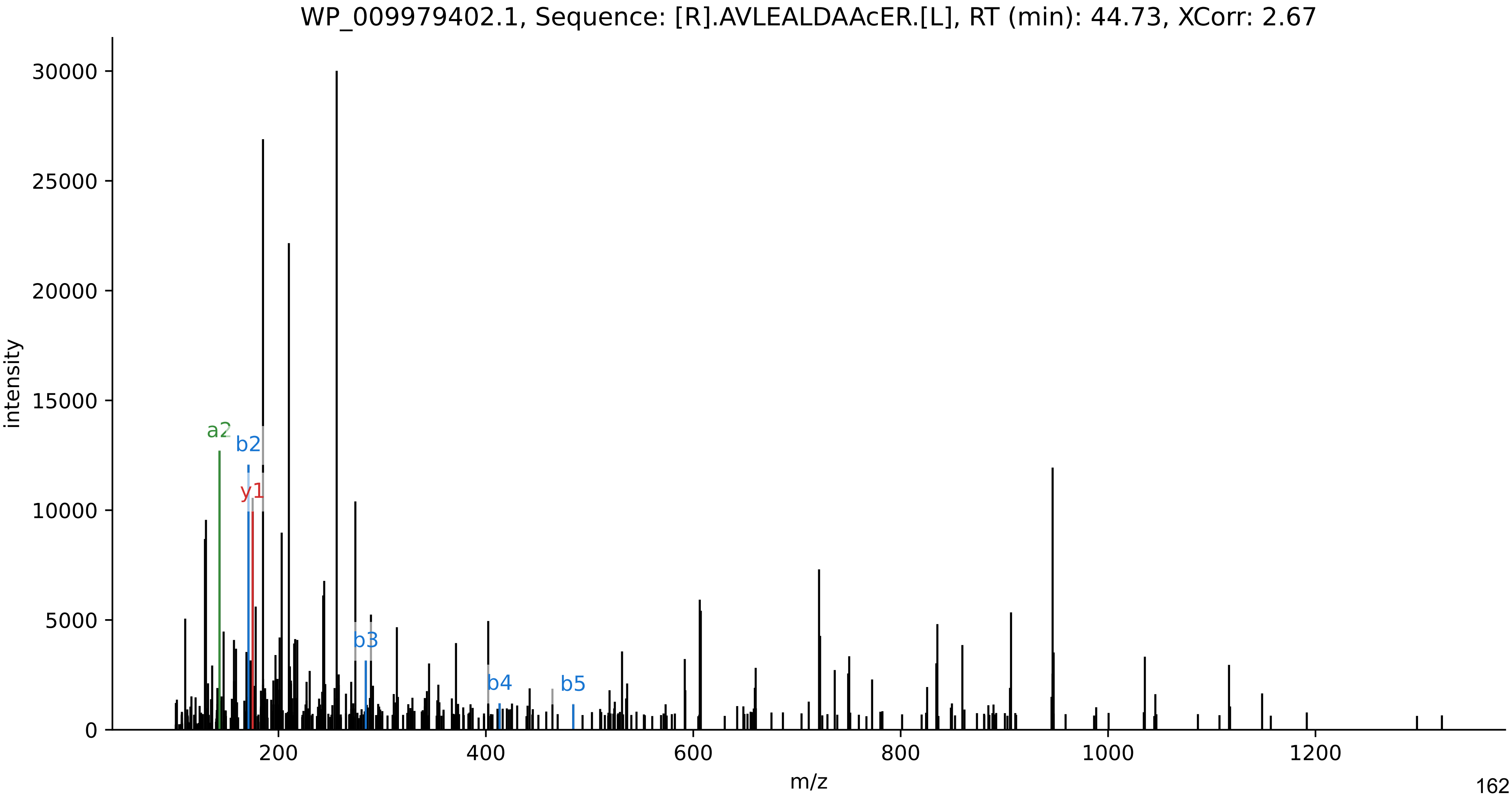
WP_009979402.1, Sequence: [R].AVLEALDAAcER.[L], RT (min): 44.73, XCorr: 2.67
intensity
m/z
a2
b2
y1
b3
b4
b5
0
5000
10000
15000
20000
25000
30000
200
400
600
800
1000
1200

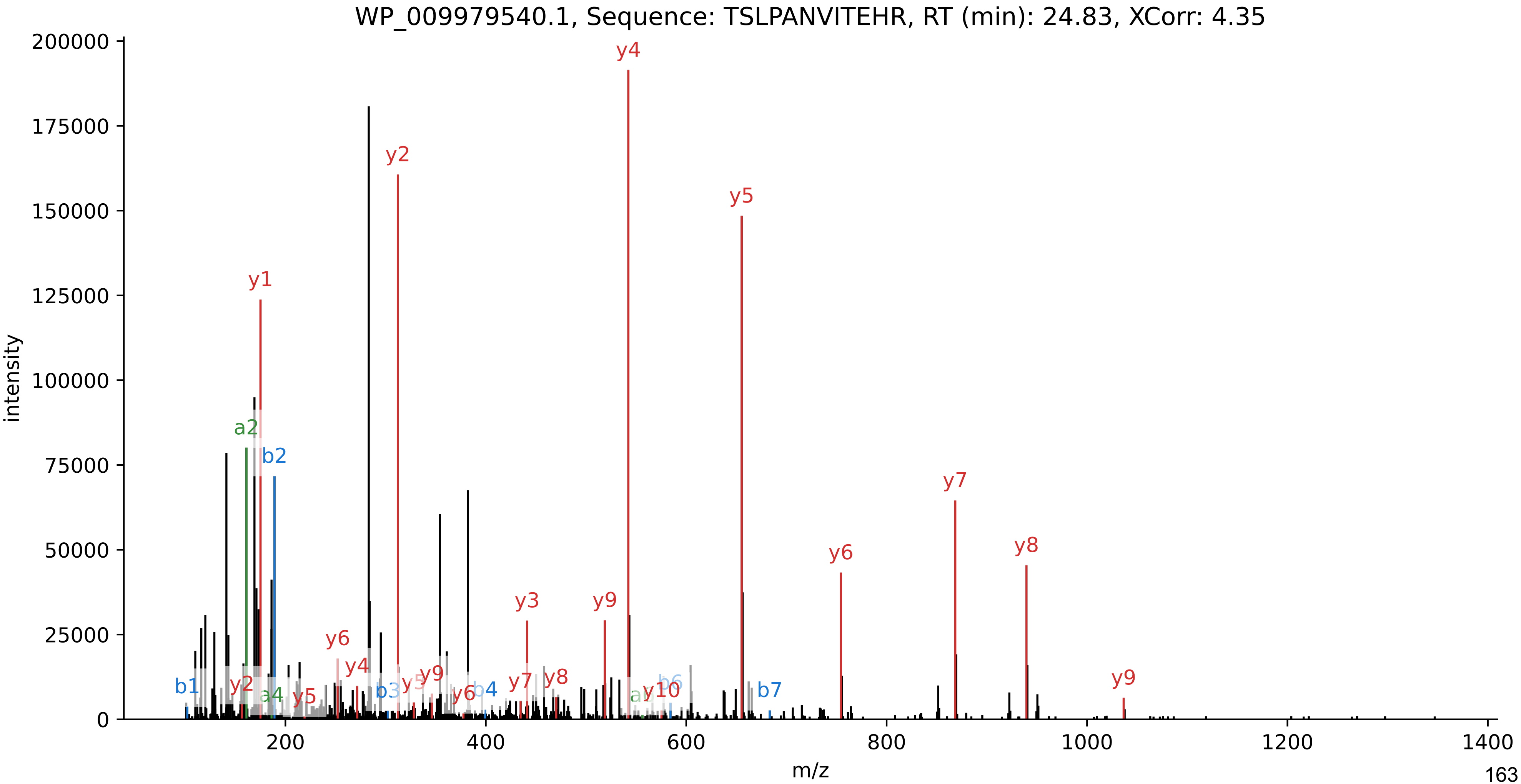
WP_009979540.1, Sequence: TSLPANVITEHR, RT (min): 24.83, XCorr: 4.35
intensity
m/z
200000
175000
150000
125000
100000
75000
50000
25000
0
200
400
600
800
1000
1200
1400
y4
y2
y5
y1
a2
b2
y7
y6
y8
y3
y9
y9
b1
y2
a4
y5
y6
y4
b3
y5
y9
y6
b4
y7
y8
y10
b6
b7

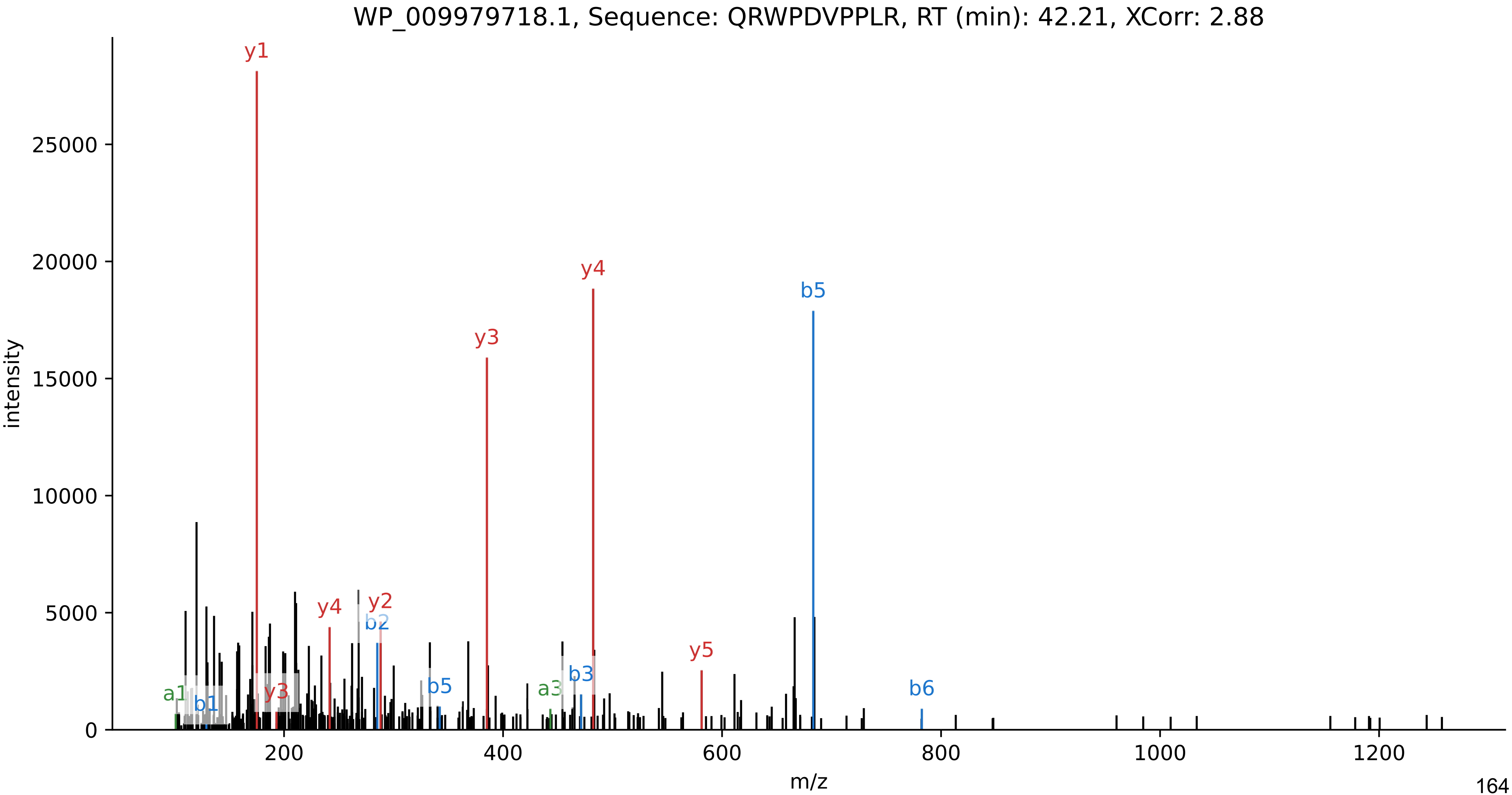
WP_009979718.1, Sequence: QRWPDVPPLR, RT (min): 42.21, XCorr: 2.88
intensity
m/z
y1
y4
b5
y3
y4
y2
b2
y5
b3
a3
b5
b6
a1
b1
y3
0
5000
10000
15000
20000
25000
200
400
600
800
1000
1200

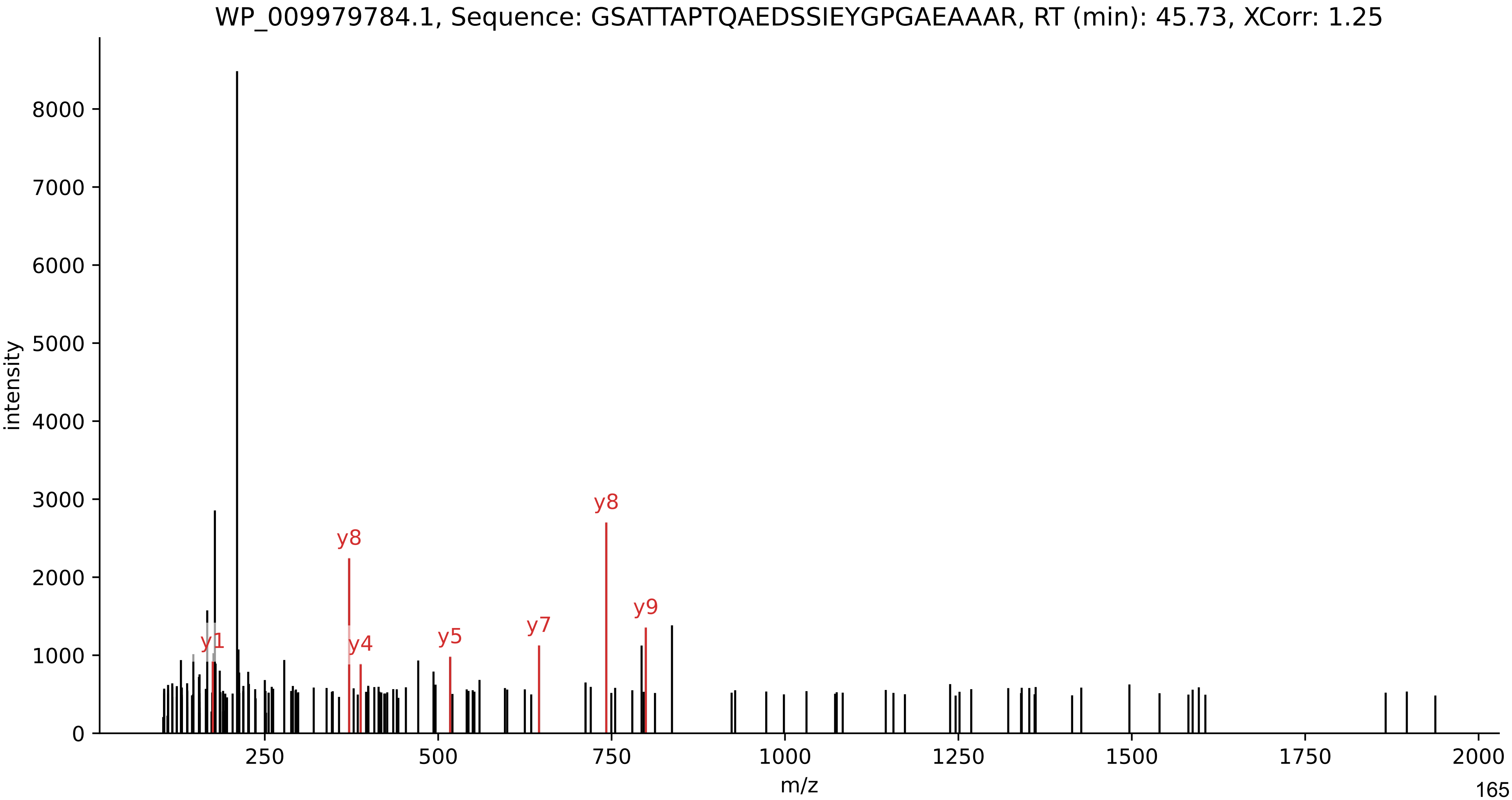

WP_009979784.1, Sequence: GSATTAPTQAEDSSIEYGPGAEAAAR, RT (min): 45.73, XCorr: 1.25
intensity
m/z
y1
y8
y4
y5
y7
y8
y9

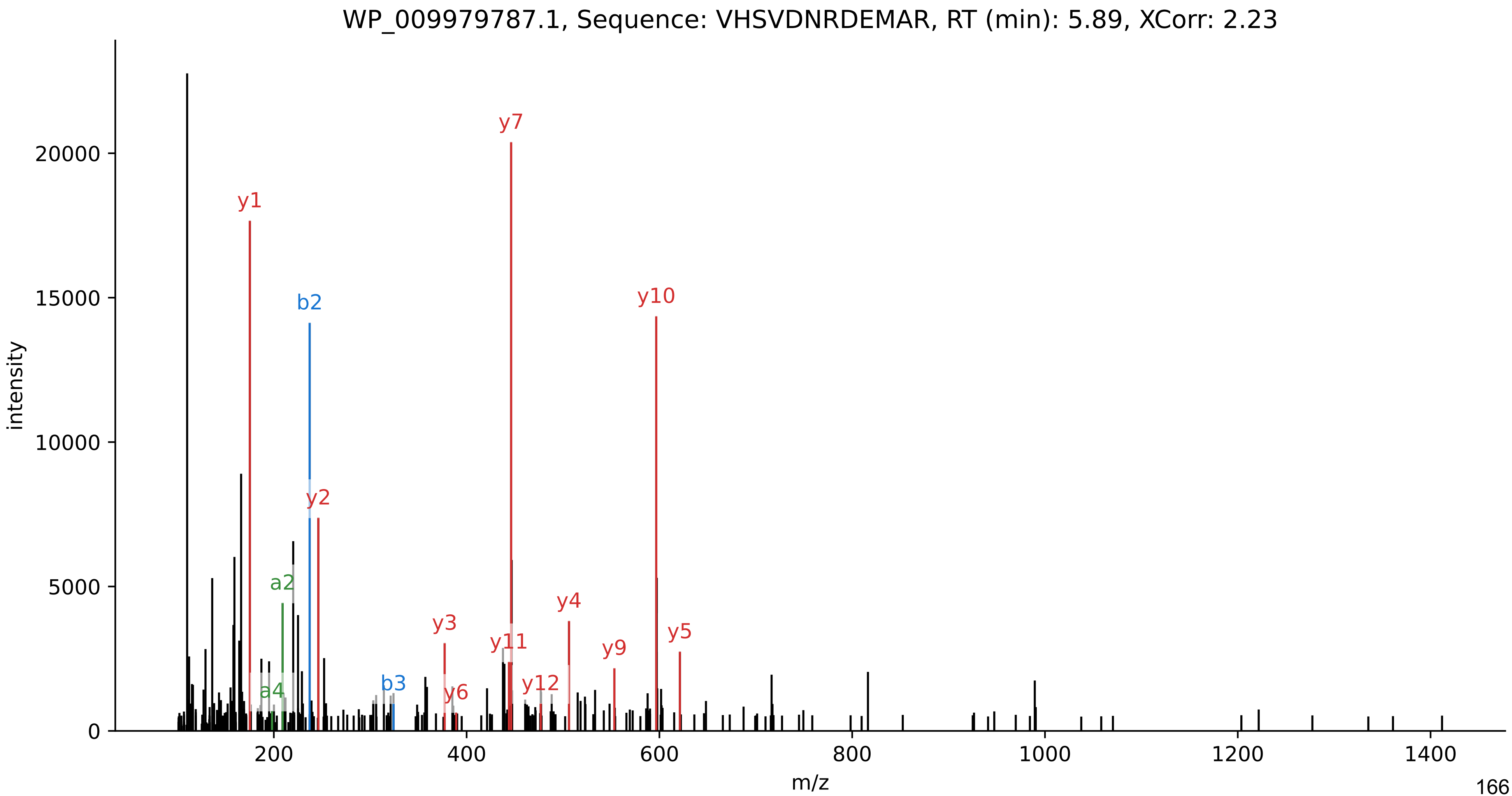
WP_009979787.1, Sequence: VHSVDNRDEMAR, RT (min): 5.89, XCorr: 2.23
intensity
m/z
y7
y1
b2
y10
y2
a2
y4
y3
y5
y11
y9
y12
b3
a4
y6
0
5000
10000
15000
20000
200
400
600
800
1000
1200
1400

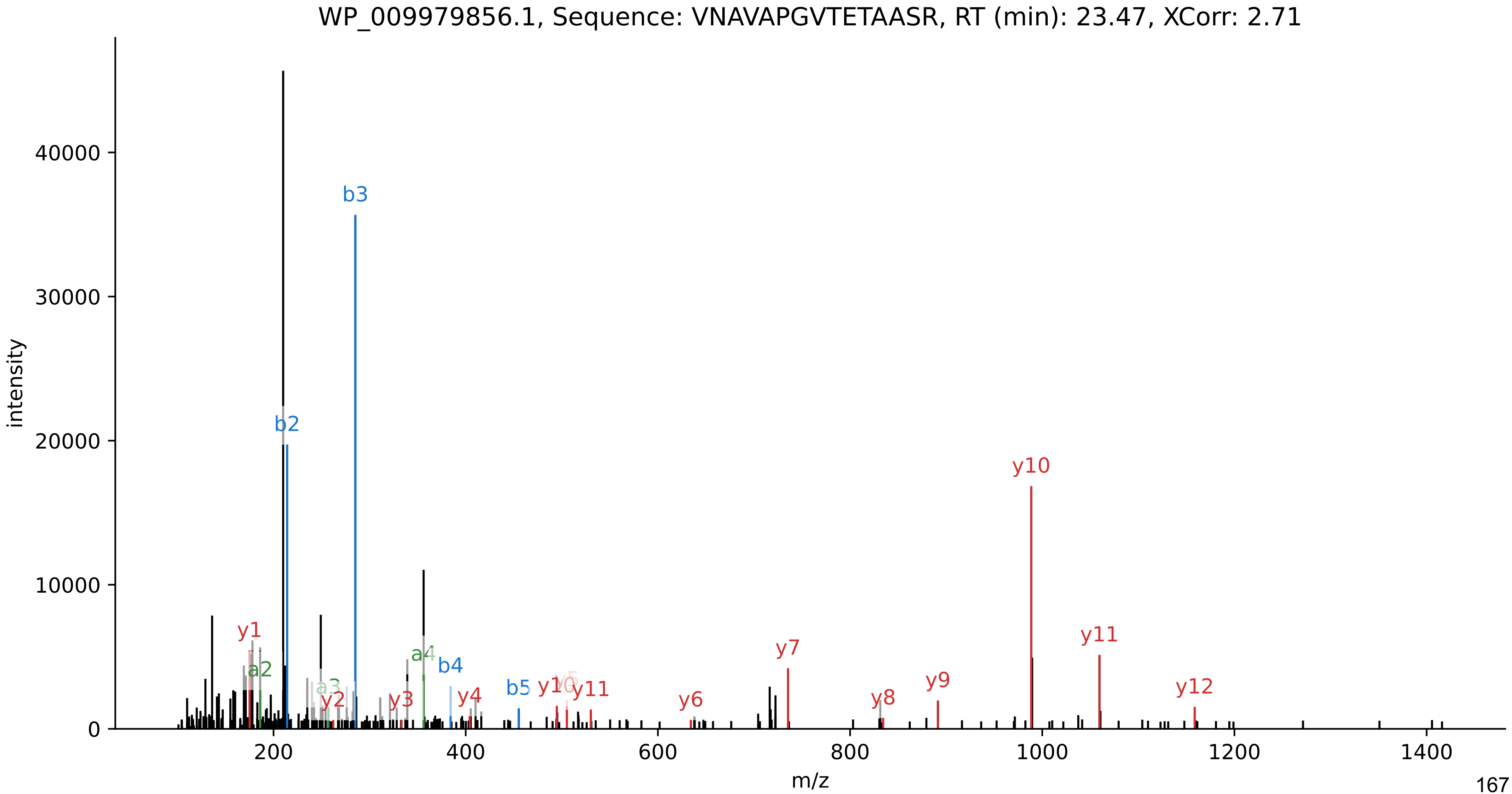

WP_009979856.1, Sequence: VNAVAPGVTETAASR, RT (min): 23.47, XCorr: 2.71
intensity
m/z
b2
b3
b4
b5
a2
a3
a4
y1
y2
y3
y4
y6
y7
y8
y9
y10
y11
y12

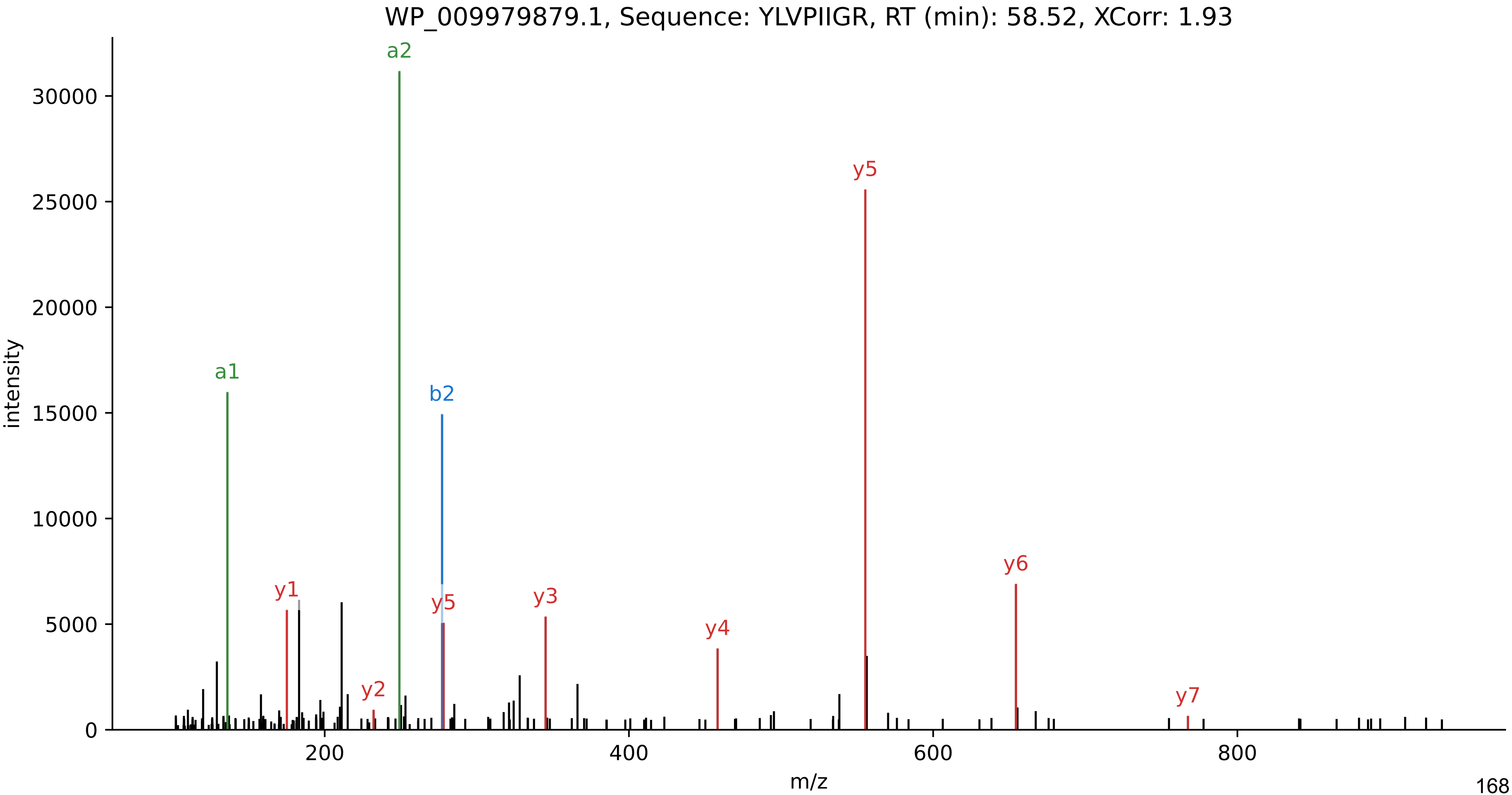
WP_009979879.1, Sequence: YLVPIIGR, RT (min): 58.52, XCorr: 1.93
a2
y5
a1
b2
y6
y1
y5
y3
y4
y2
y7
intensity
30000
25000
20000
15000
10000
5000
0
200
400
600
800
m/z

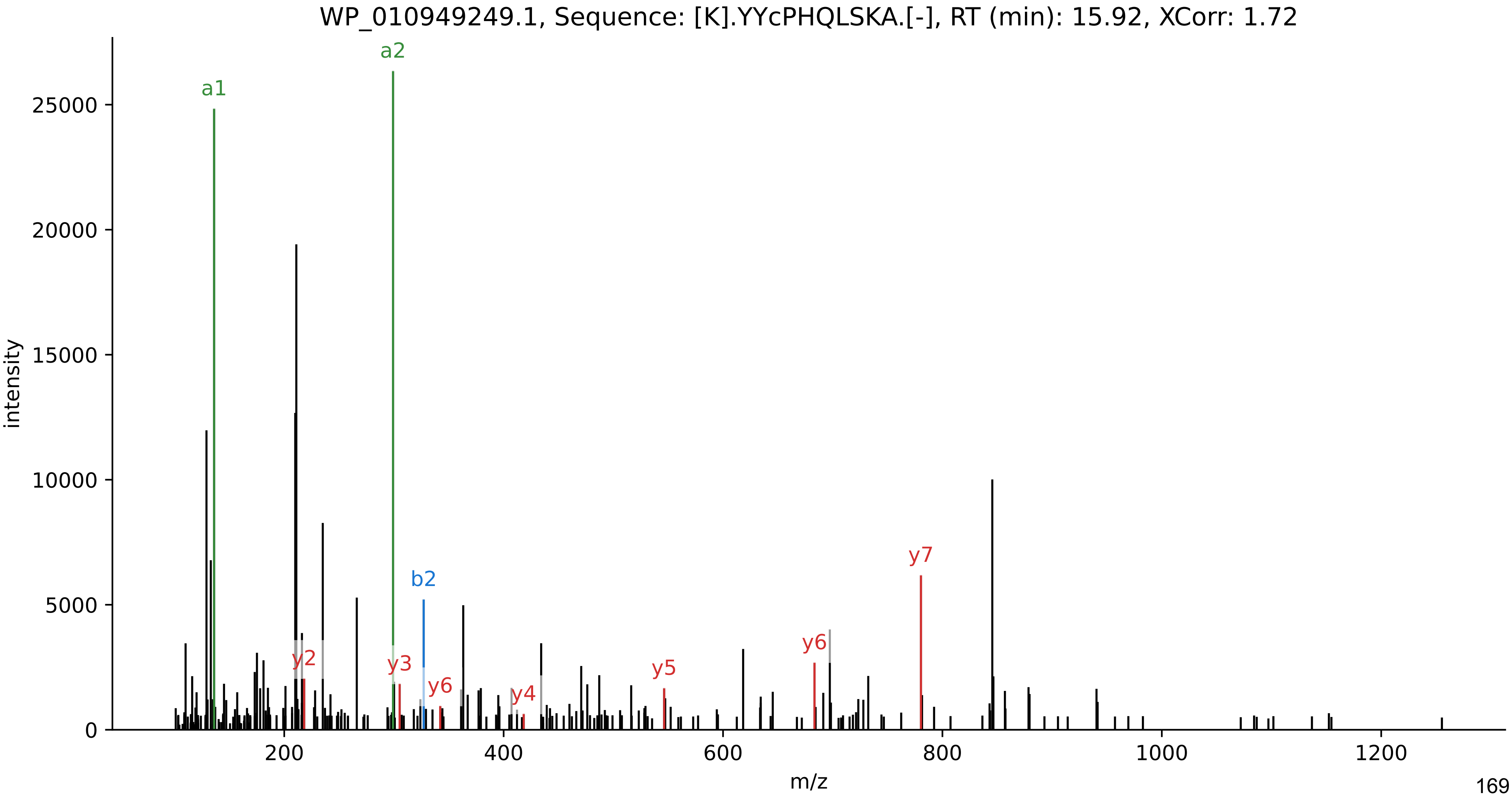
WP_010949249.1, Sequence: [K].YYcPHQLSKA.[-], RT (min): 15.92, XCorr: 1.72
a1
a2
b2
y2
y3
y6
y4
y5
y6
y7
intensity
m/z
0
5000
10000
15000
20000
25000
200
400
600
800
1000
1200

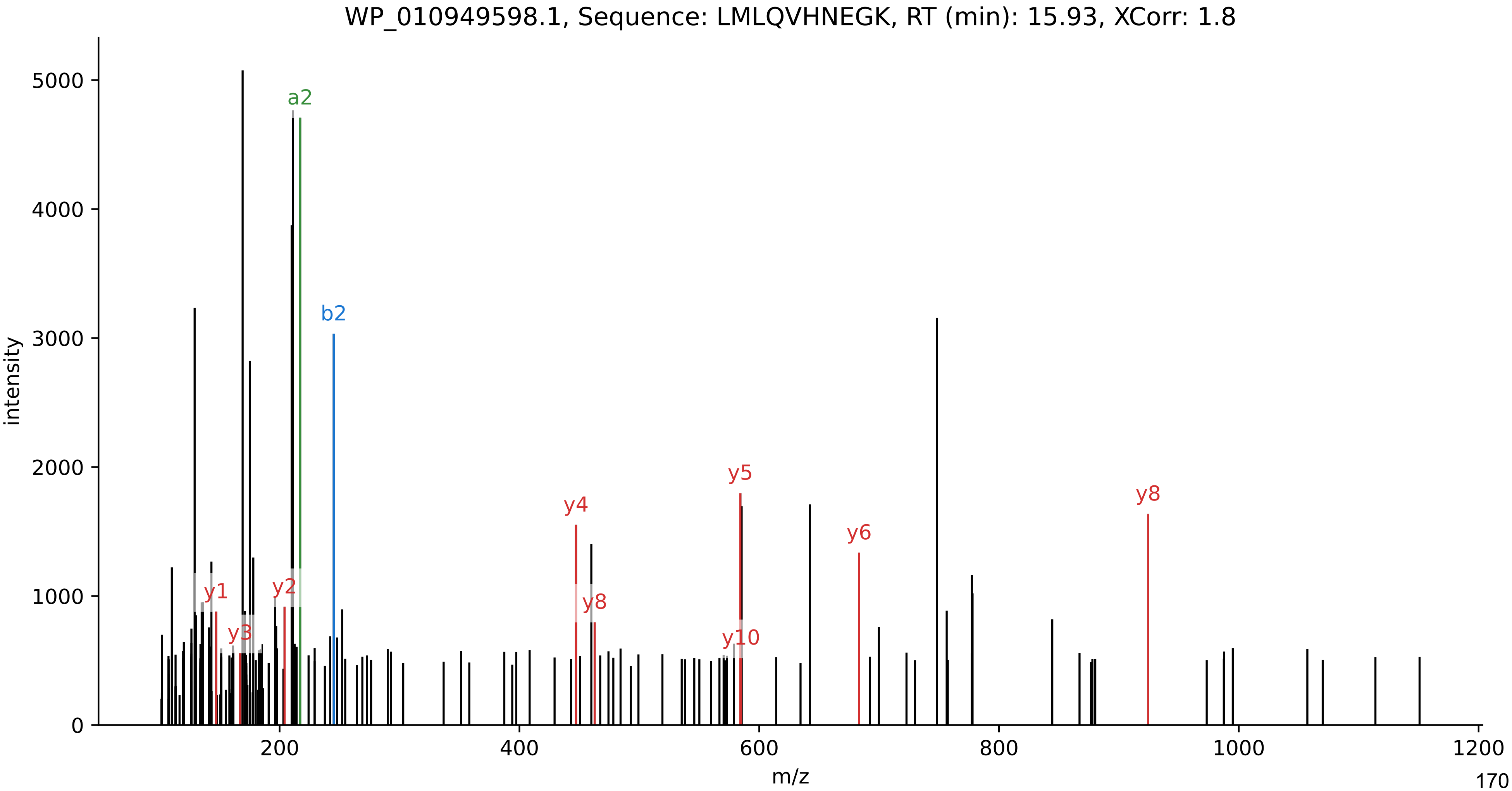
WP_010949598.1, Sequence: LMLQVHNEGK, RT (min): 15.93, XCorr: 1.8
intensity
m/z
a2
b2
y1
y2
y3
y4
y5
y6
y8
y8
y10
0
1000
2000
3000
4000
5000
200
400
600
800
1000
1200

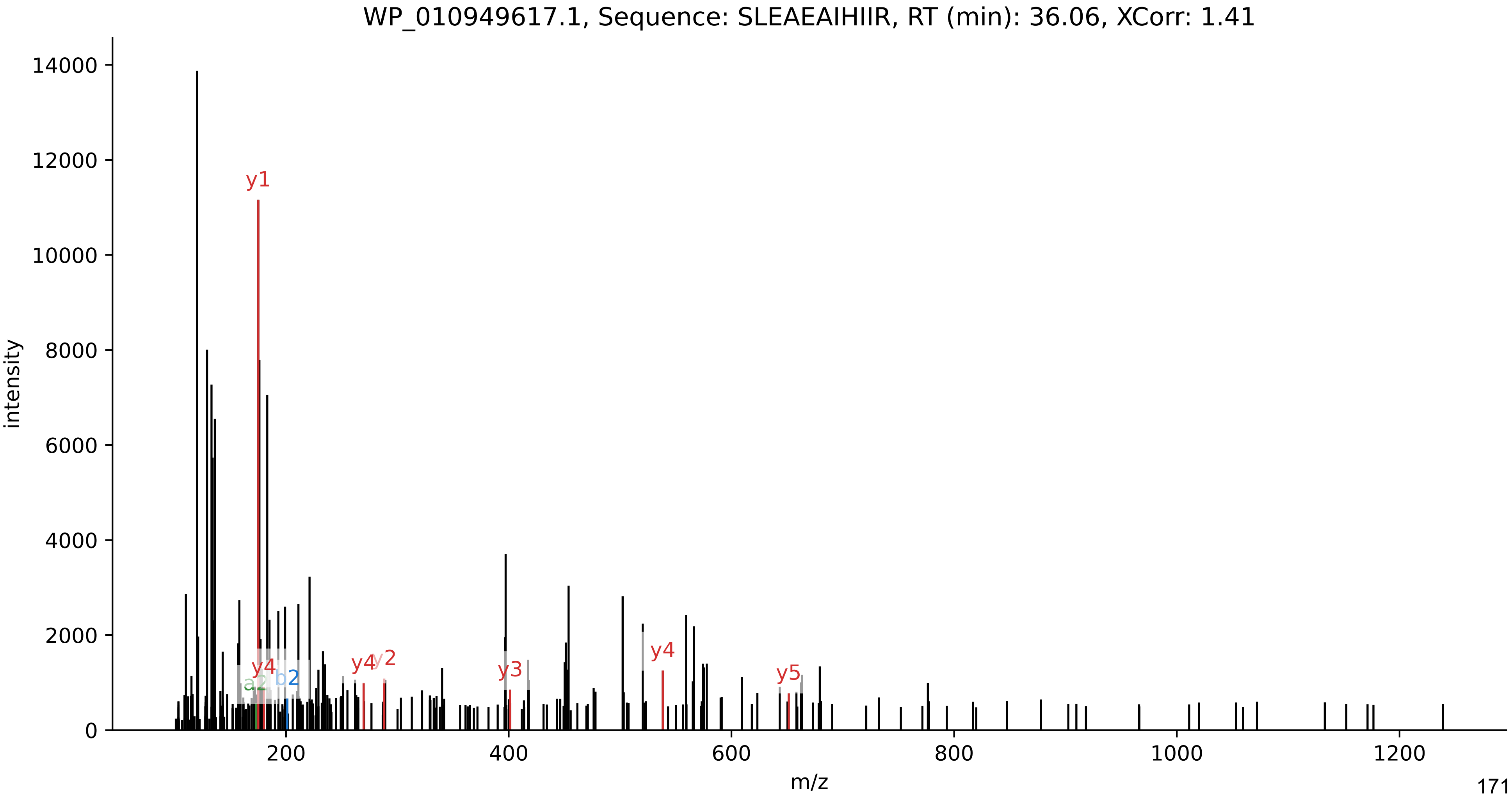
WP_010949617.1, Sequence: SLEAEAIHIIR, RT (min): 36.06, XCorr: 1.41
intensity
m/z
y1
y4
a2
b2
y4
y2
y3
y4
y5

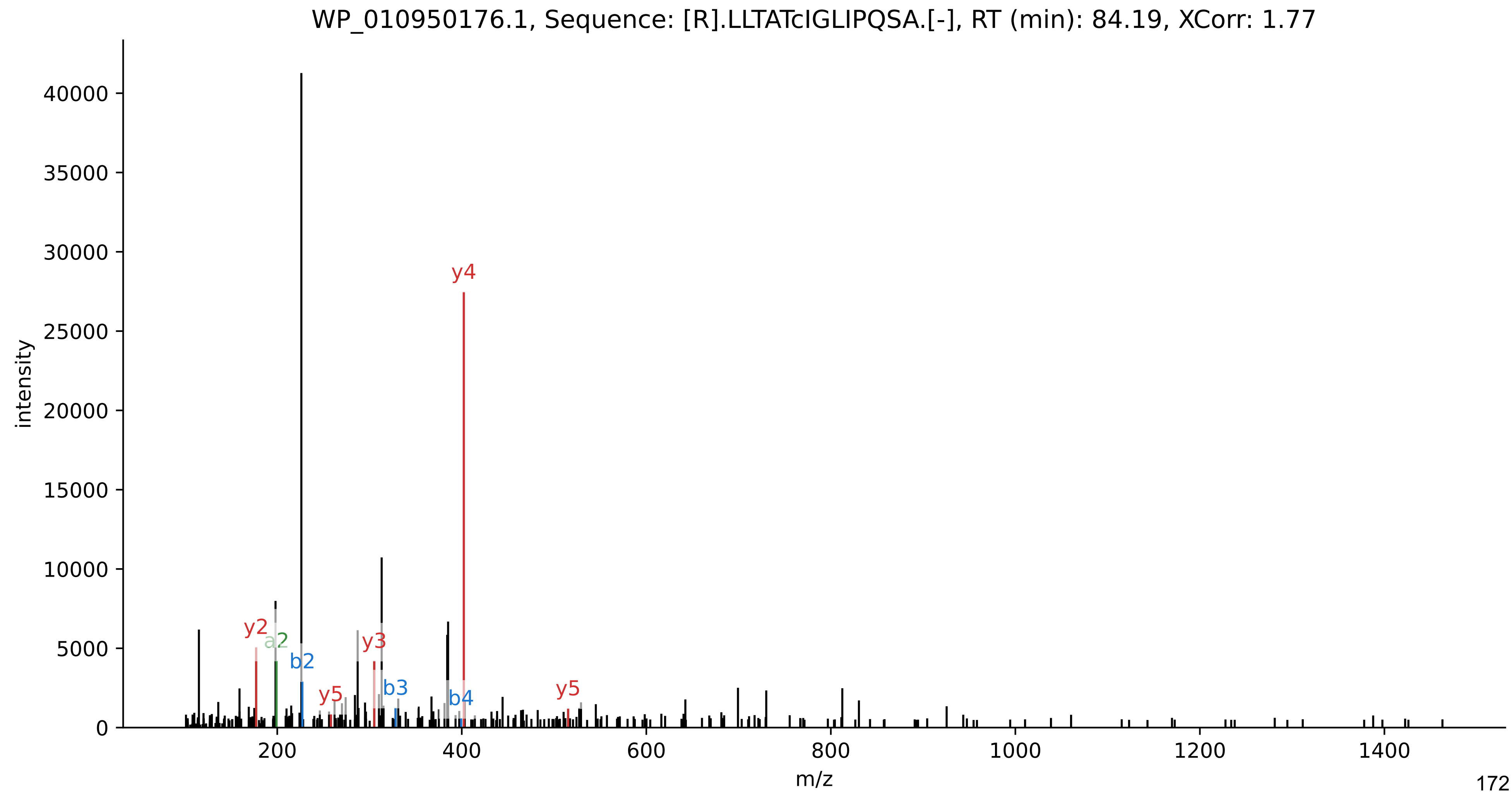
WP_010950176.1, Sequence: [R].LLTATcIGLIPQSA.[-], RT (min): 84.19, XCorr: 1.77
intensity
m/z
y2
a2
b2
y5
y3
b3
b4
y4
y5
0
5000
10000
15000
20000
25000
30000
35000
40000
200
400
600
800
1000
1200
1400

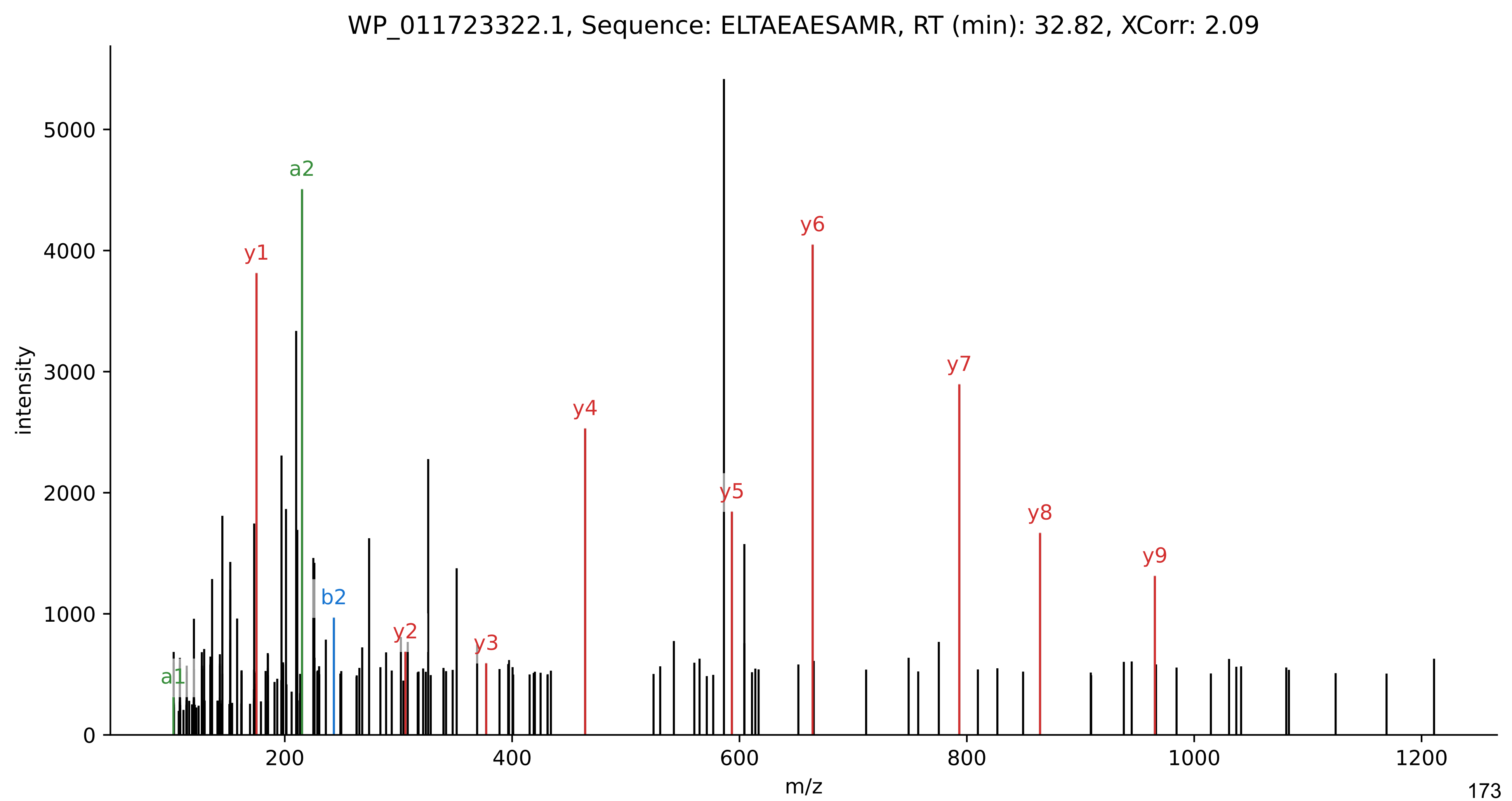
WP_011723322.1, Sequence: ELTAEAESAMR, RT (min): 32.82, XCorr: 2.09
intensity
m/z
a1
a2
b2
y1
y2
y3
y4
y5
y6
y7
y8
y9
0
1000
2000
3000
4000
5000
200
400
600
800
1000
1200

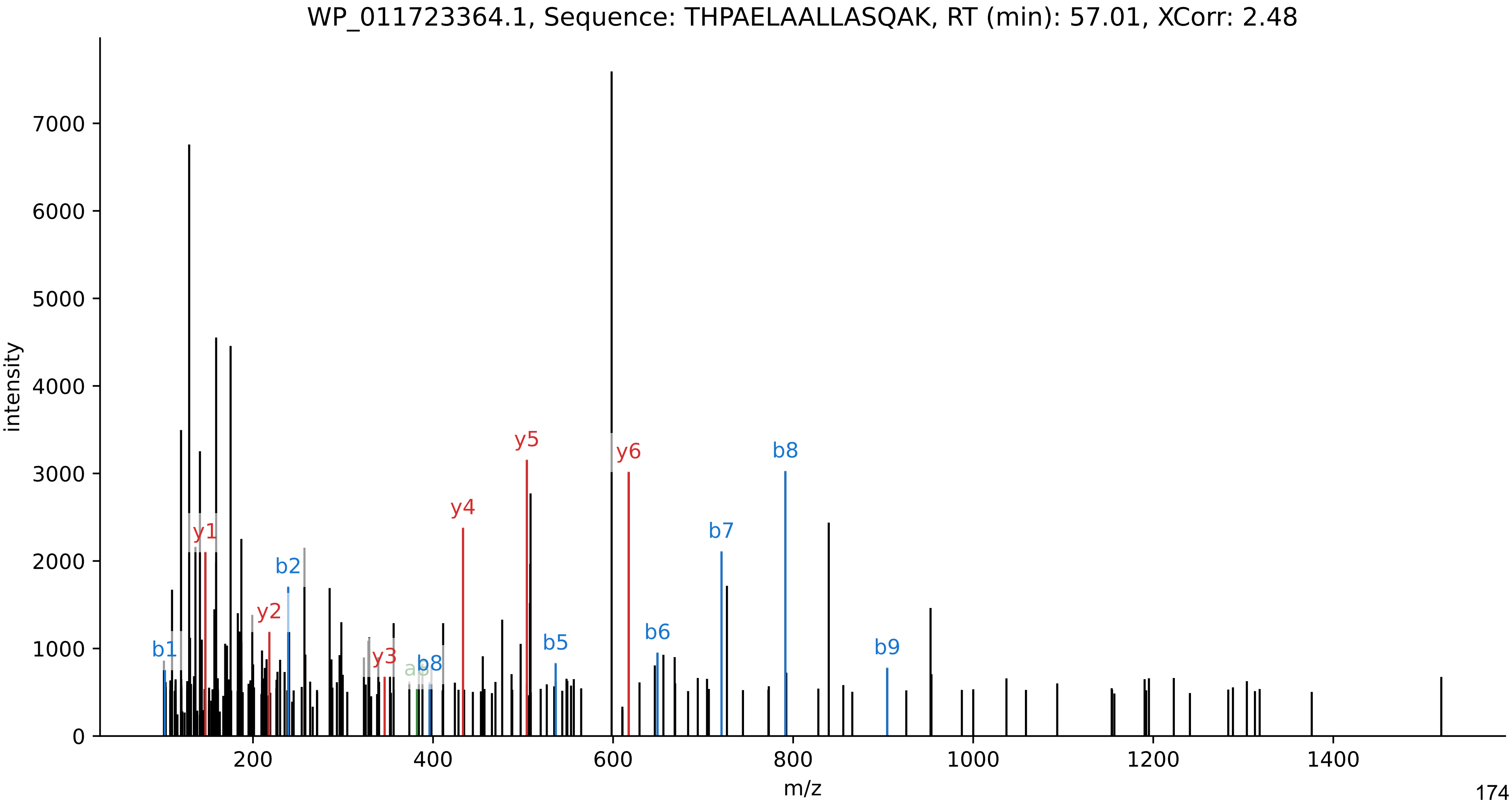
WP_011723364.1, Sequence: THPAELAALLASQAK, RT (min): 57.01, XCorr: 2.48
intensity
m/z
0
1000
2000
3000
4000
5000
6000
7000
200
400
600
800
1000
1200
1400
b1
y1
b2
y2
y3
b8
y4
y5
b5
y6
b6
b7
b8
b9

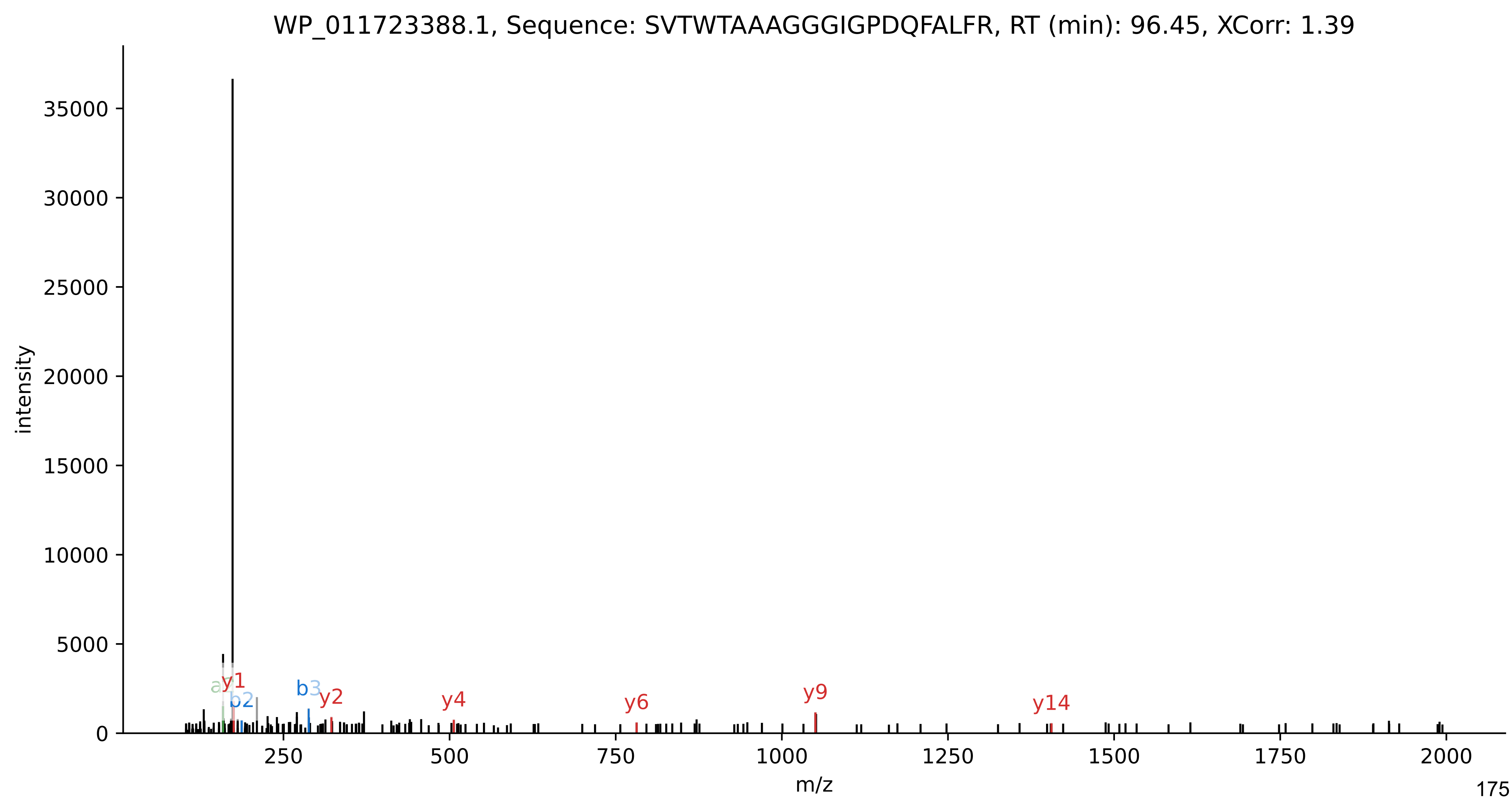

WP_011723388.1, Sequence: SVTWTAAAGGGIGPDQFALFR, RT (min): 96.45, XCorr: 1.39
intensity
m/z
y1
b2
b3
y2
y4
y6
y9
y14

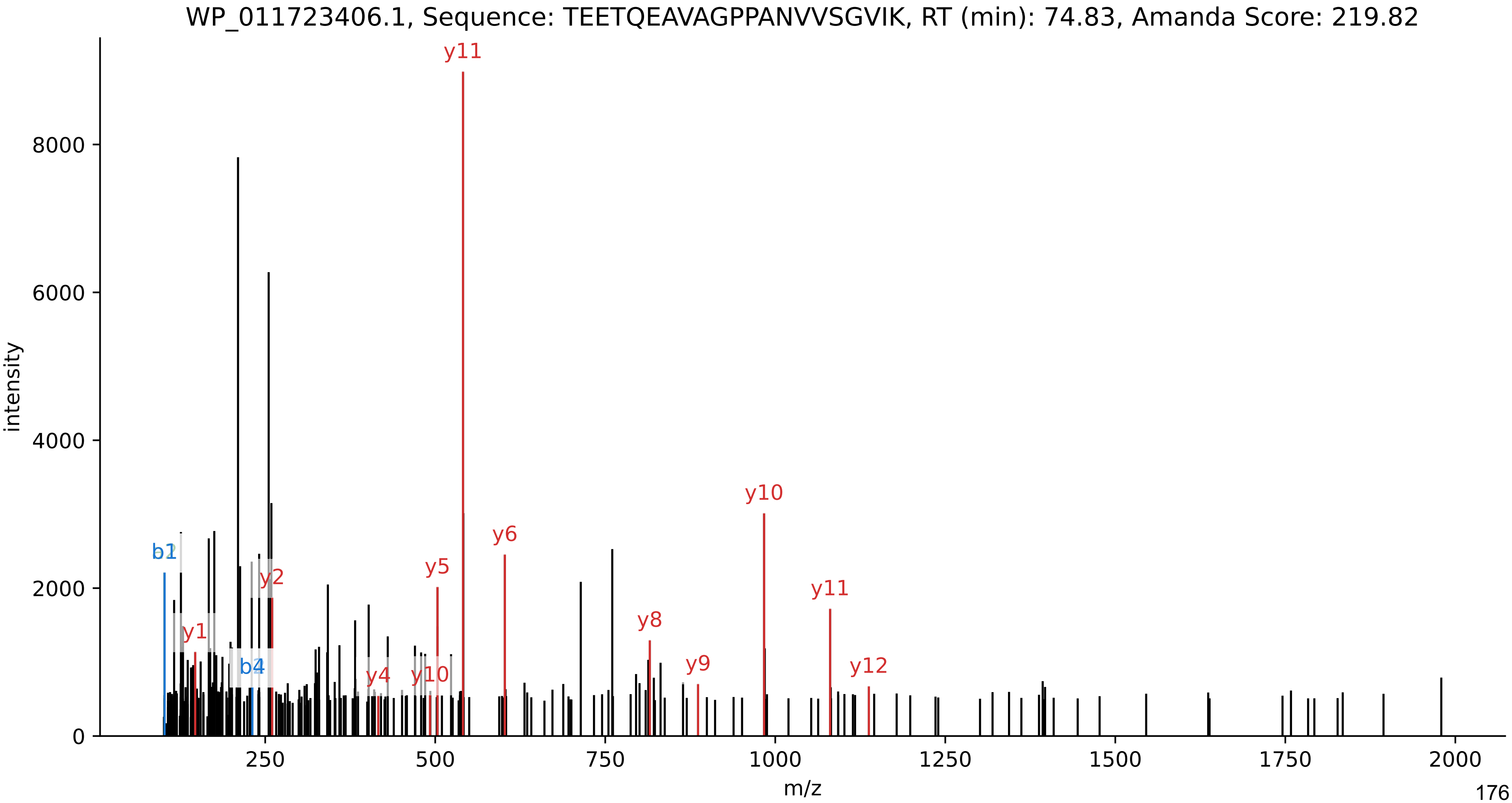
WP_011723406.1, Sequence: TEETQEAVAGPPANVVSGVIK, RT (min): 74.83, Amanda Score: 219.82
intensity
m/z
y11
y10
y6
y5
y2
b1
y1
b4
y4
y10
y8
y9
y11
y12
0
2000
4000
6000
8000
250
500
750
1000
1250
1500
1750
2000

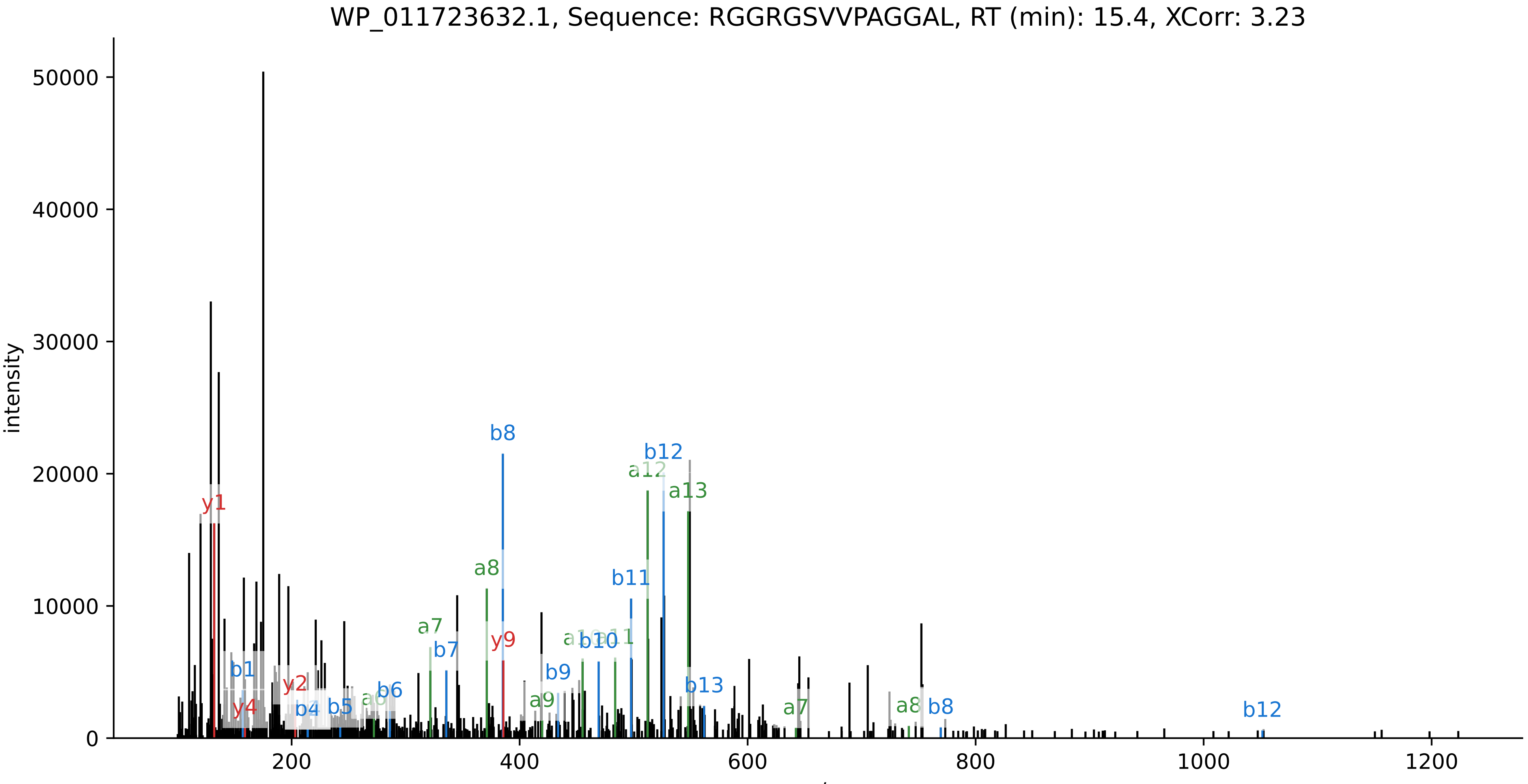
WP_011723632.1, Sequence: RGGRGSVVPAGGAL, RT (min): 15.4, XCorr: 3.23
intensity
m/z
50000
40000
30000
20000
10000
0
200
400
600
800
1000
1200
y1
y2
y4
y9
b1
b4
b5
b6
b7
b8
b9
b10
b11
b12
b13
b8
b12
a7
a8
a9
a12
a13
a7
a8

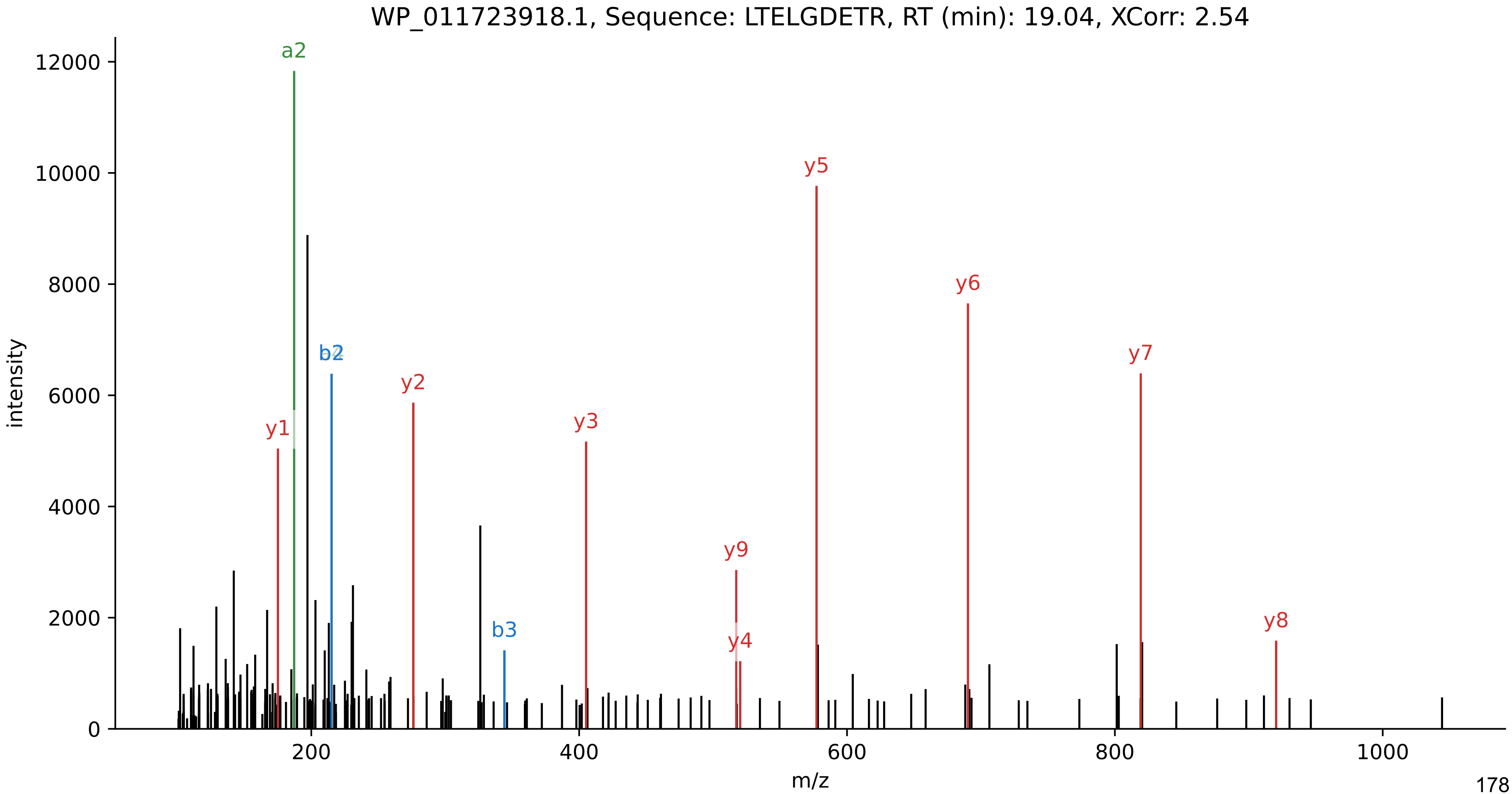
WP_011723918.1, Sequence: LTELGDETR, RT (min): 19.04, XCorr: 2.54
a2
b2
y1
y2
y3
y9
y4
b3
y5
y6
y7
y8
intensity
m/z
0
2000
4000
6000
8000
10000
12000
200
400
600
800
1000

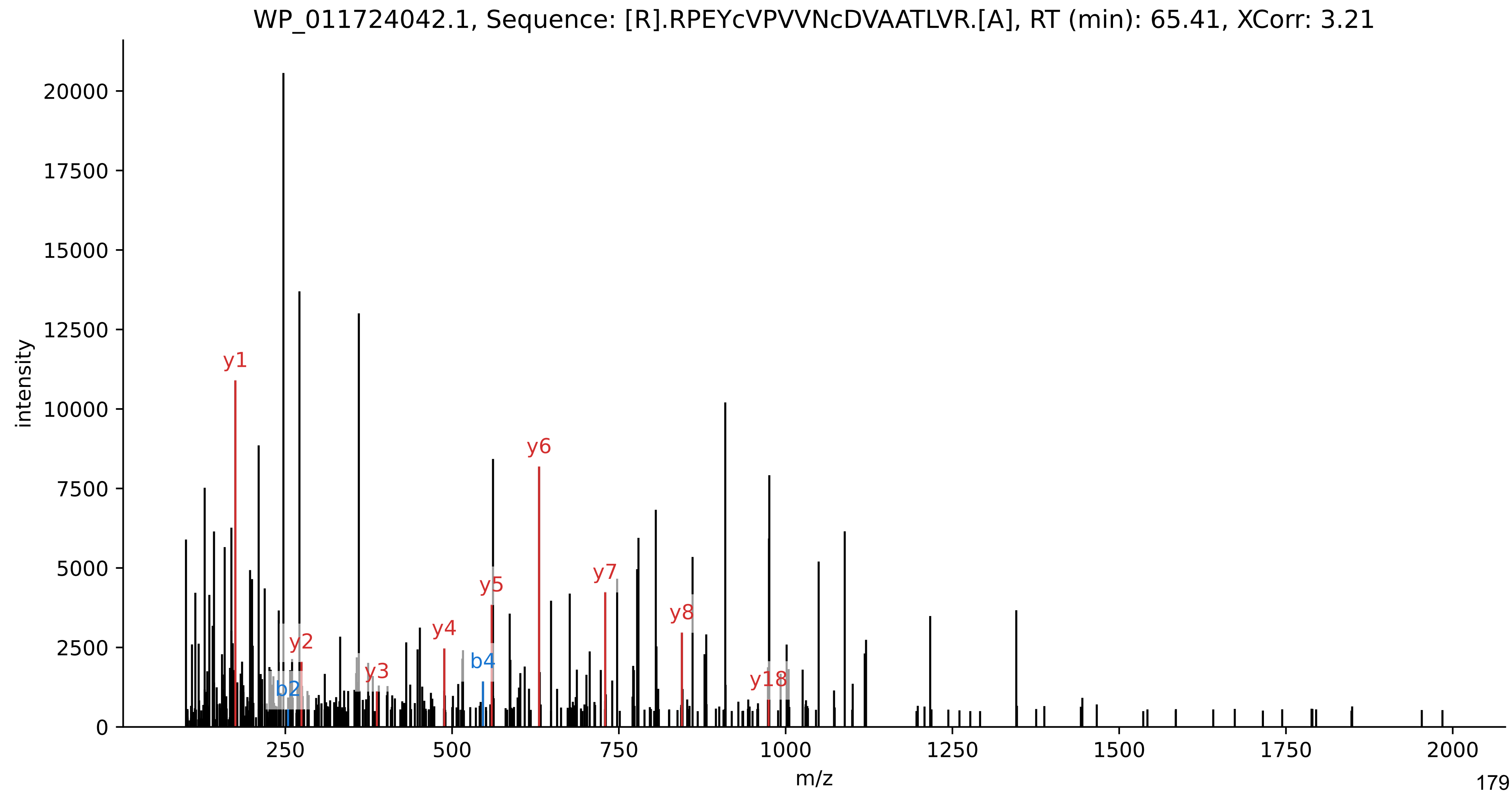
WP_011724042.1, Sequence: [R].RPEYcVPVVNcDVAATLVR.[A], RT (min): 65.41, XCorr: 3.21
intensity
m/z
y1
y2
y3
y4
y5
y6
y7
y8
y18
b2
b4
0
2500
5000
7500
10000
12500
15000
17500
20000
250
500
750
1000
1250
1500
1750
2000

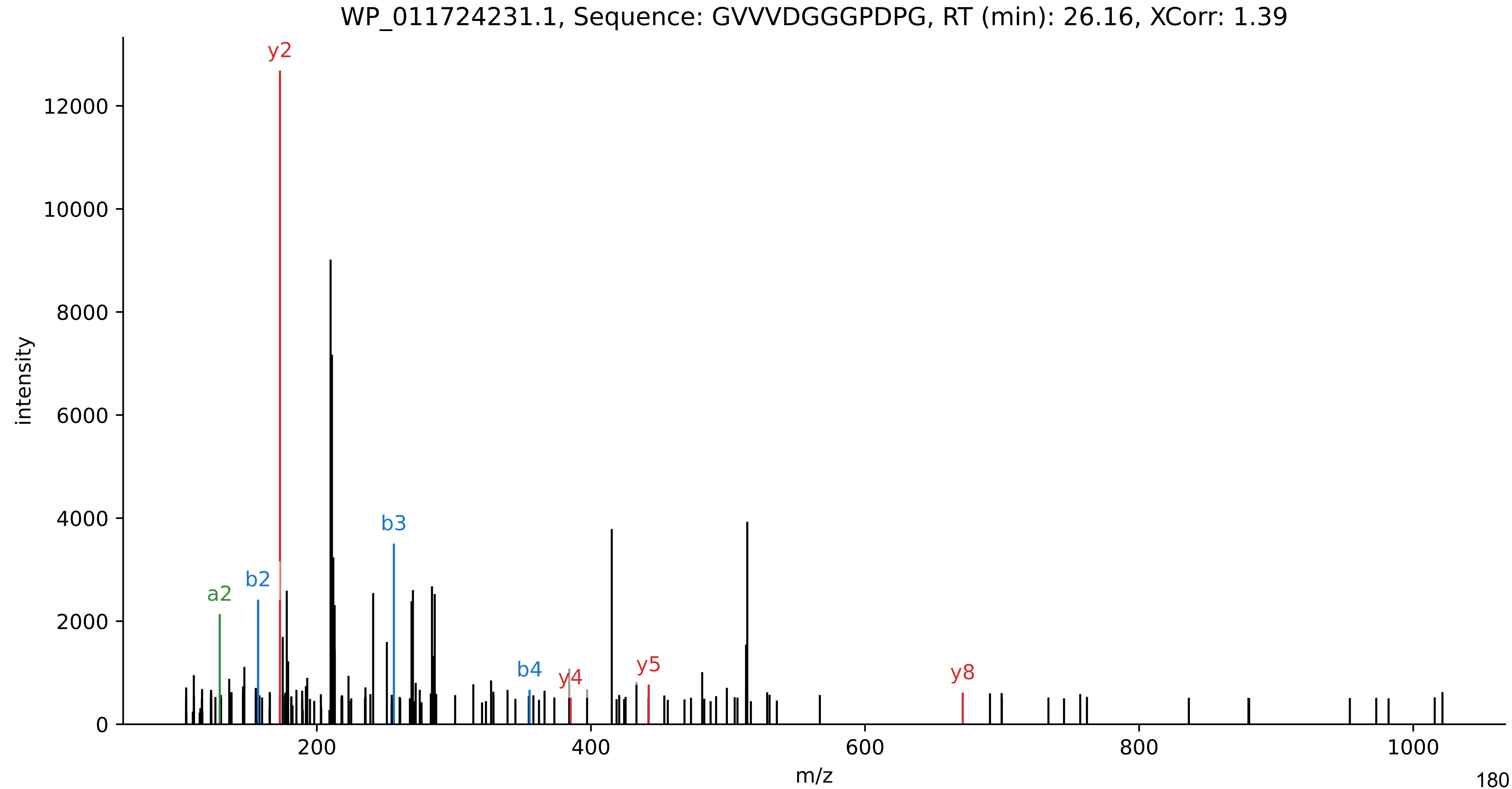
WP_011724231.1, Sequence: GVVVDGGGPDPG, RT (min): 26.16, XCorr: 1.39
y2
b3
b2
a2
b4
y4
y5
y8
intensity
m/z
0
2000
4000
6000
8000
10000
12000
200
400
600
800
1000

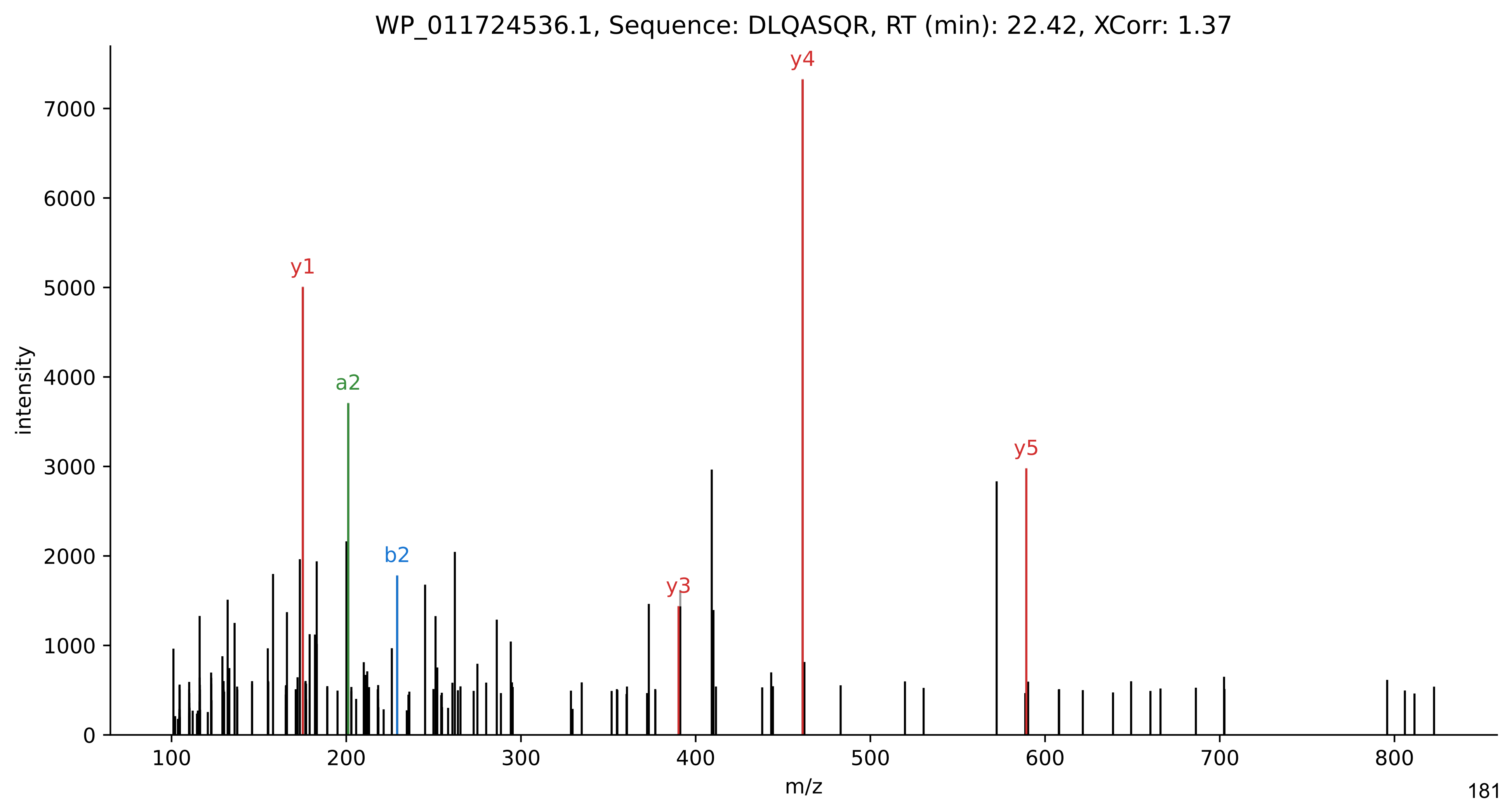
WP_011724536.1, Sequence: DLQASQR, RT (min): 22.42, XCorr: 1.37
y4
y1
a2
y5
b2
y3
intensity
m/z
0
1000
2000
3000
4000
5000
6000
7000
100
200
300
400
500
600
700
800

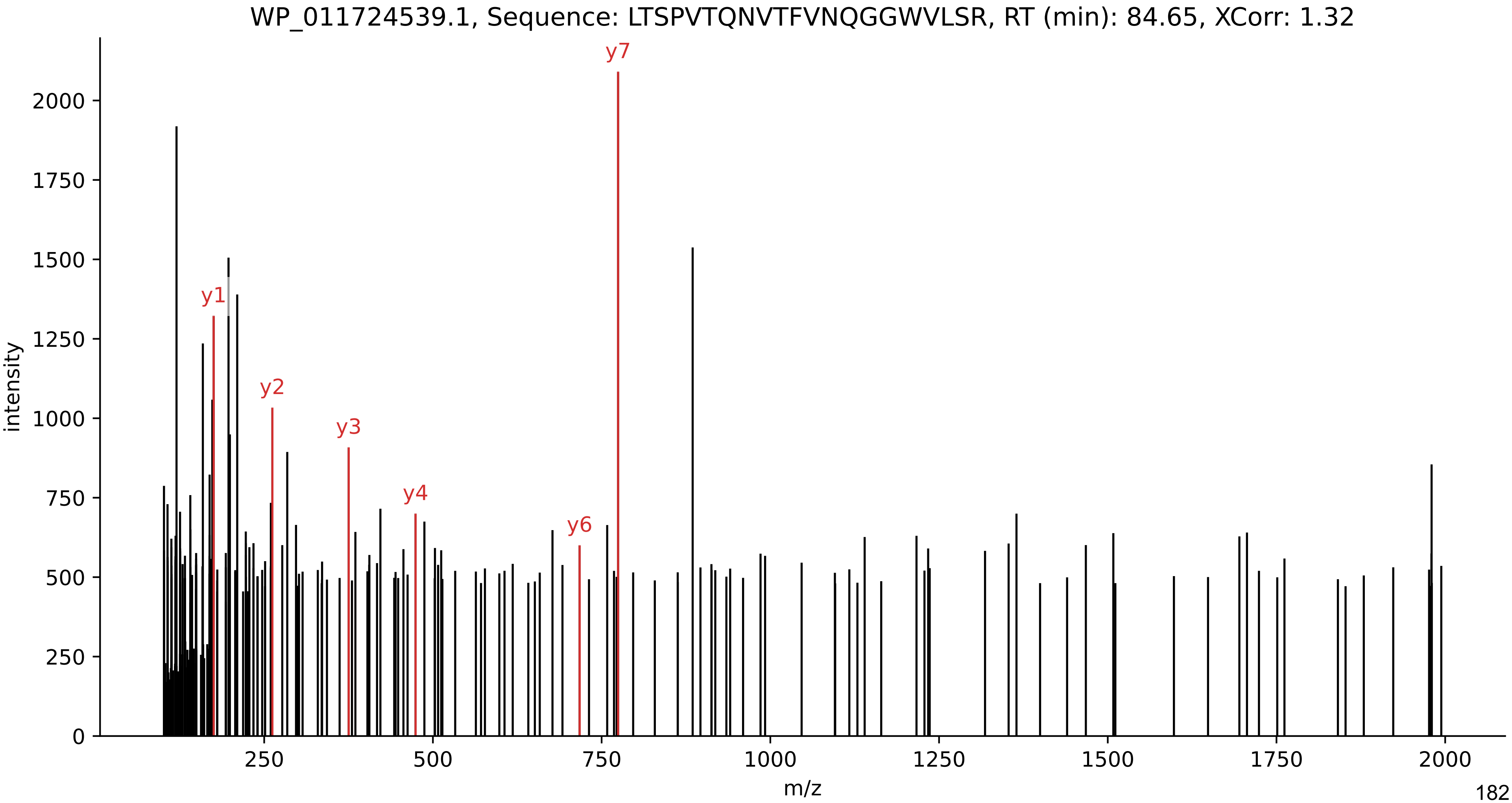
WP_011724539.1, Sequence: LTSPVTQNVTFVNQGGWVLSR, RT (min): 84.65, XCorr: 1.32
y7
y1
y2
y3
y4
y6
intensity
m/z
0
250
500
750
1000
1250
1500
1750
2000

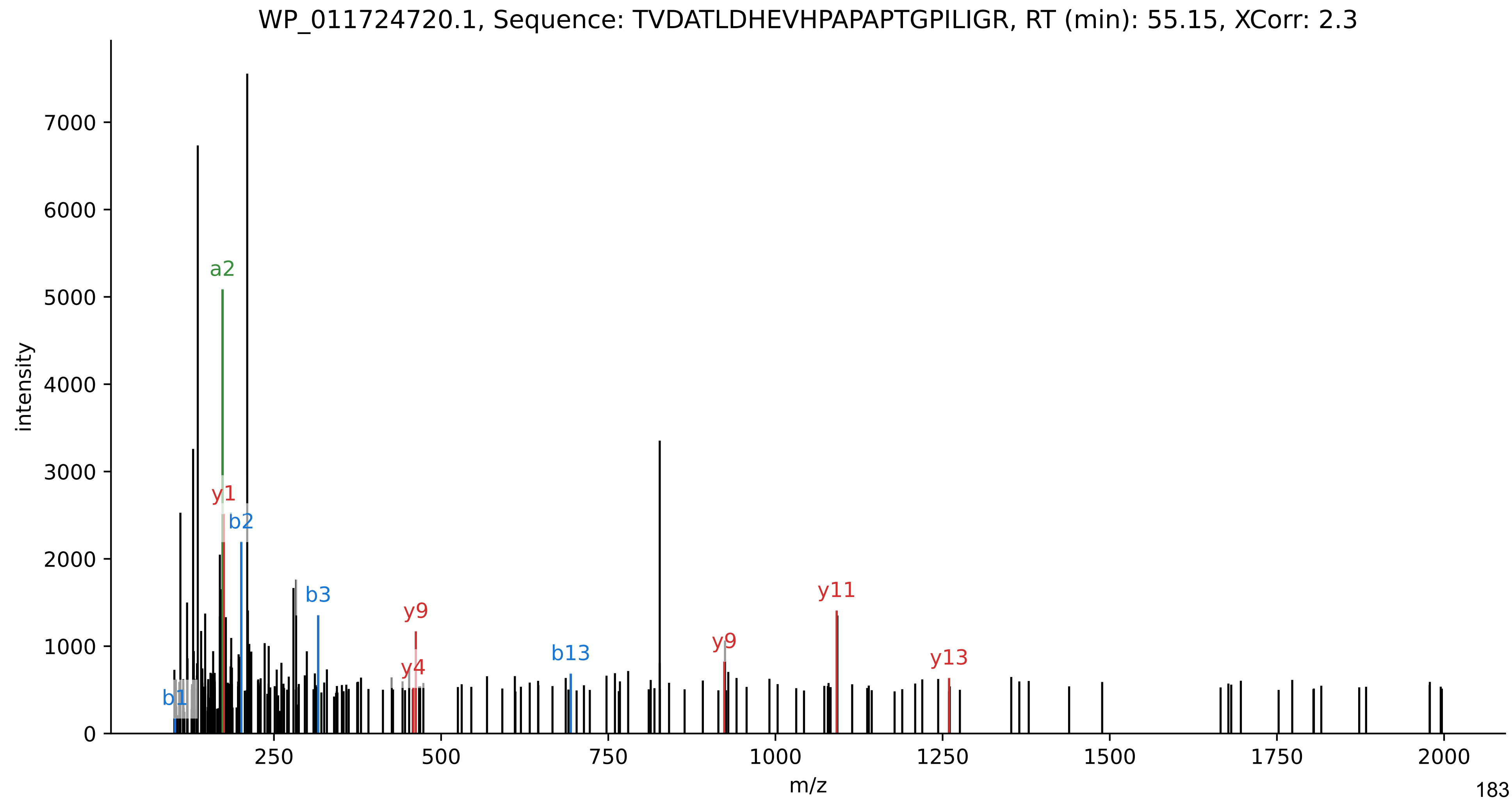
WP_011724720.1, Sequence: TVDATLDHEVHPAPAPTGPILIGR, RT (min): 55.15, XCorr: 2.3
intensity
m/z
a2
y1
b2
b3
y9
y4
b13
y9
y11
y13
b1

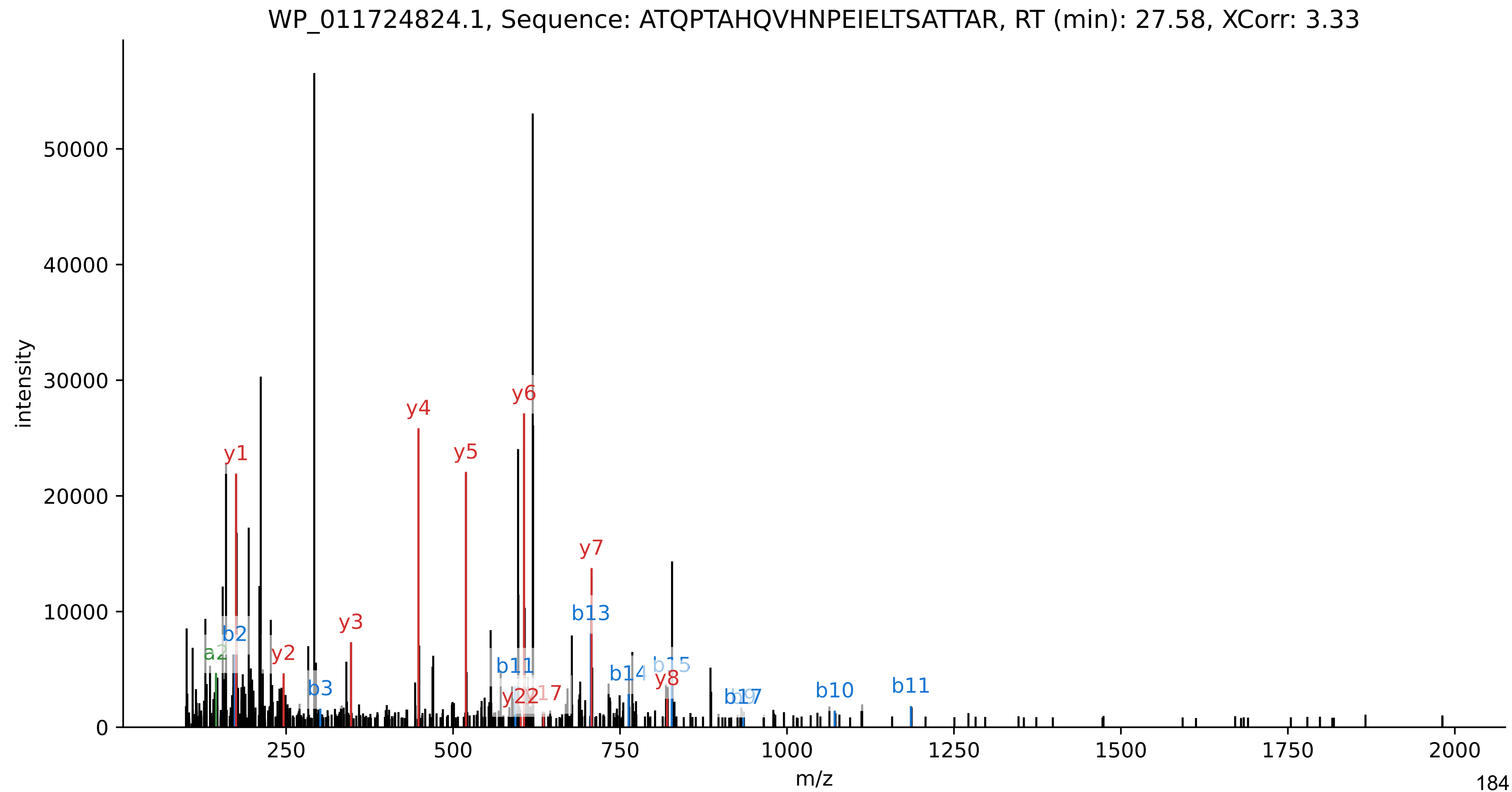
WP_011724824.1, Sequence: ATQPTAHQVHNPEIELTSATTAR, RT (min): 27.58, XCorr: 3.33
intensity
m/z
y1
y2
y3
y4
y5
y6
y7
y8
y22
a2
b2
b3
b10
b11
b13
b14
b15
b17

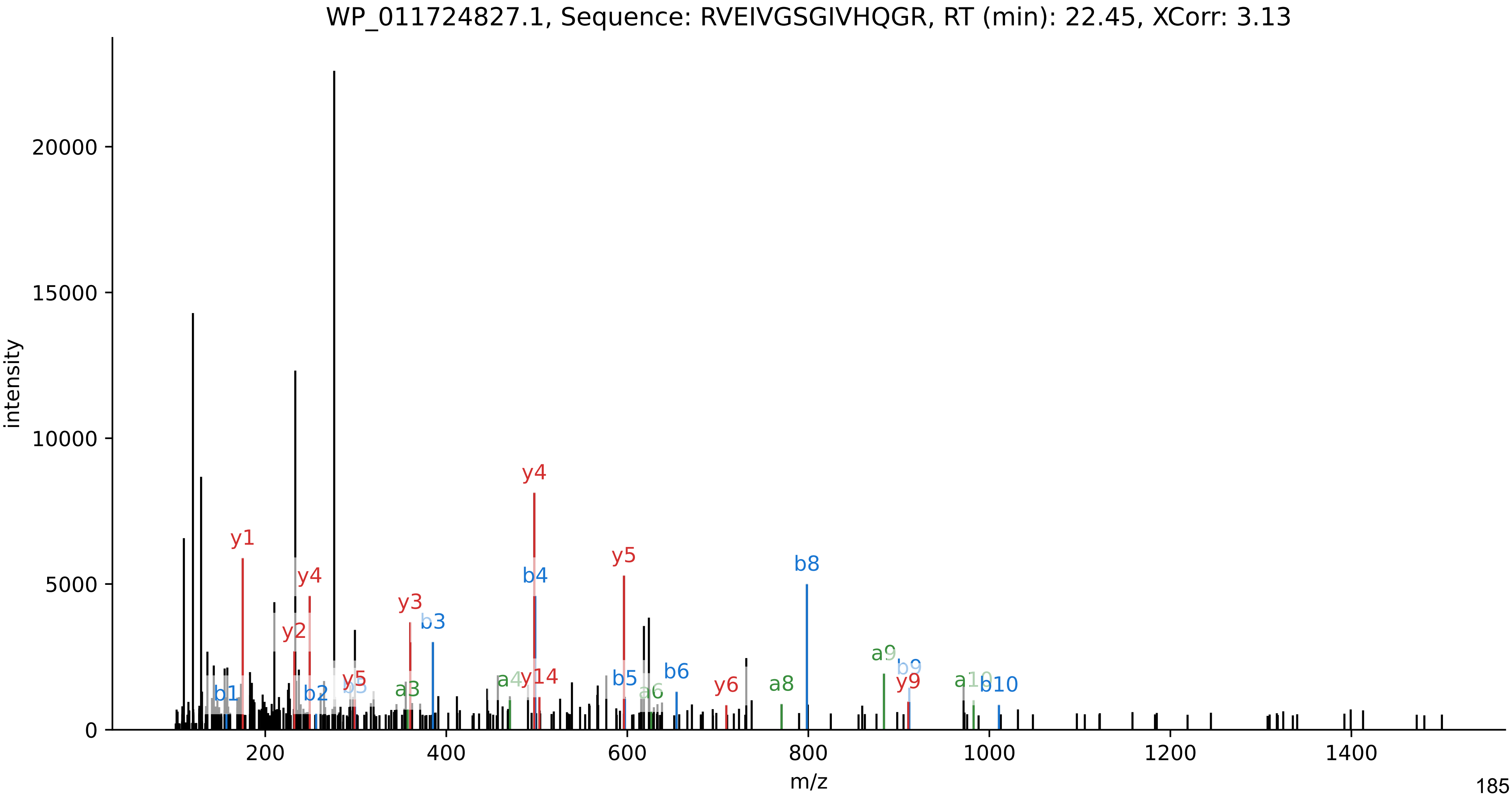

WP_011724827.1, Sequence: RVEIVGSGIVHQGR, RT (min): 22.45, XCorr: 3.13
intensity
m/z
y1
y2
y3
y4
y5
y6
y9
y14
b1
b2
b3
b4
b5
b6
b8
b9
b10
a3
a4
a6
a8
a9
a10

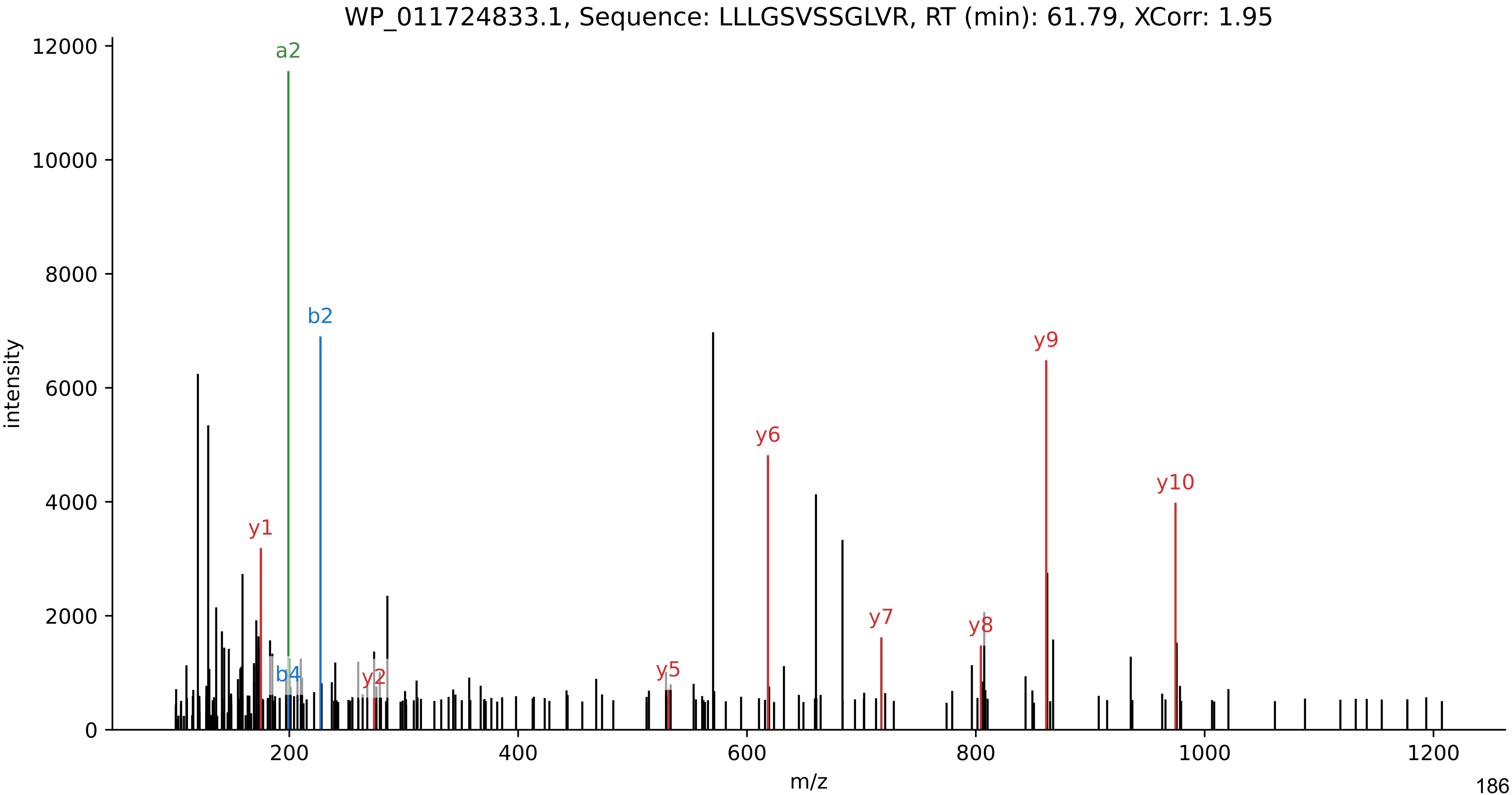

WP_011724833.1, Sequence: LLLGSVSSGLVR, RT (min): 61.79, XCorr: 1.95
a2
b2
b4
y1
y2
y5
y6
y7
y8
y9
y10
intensity
m/z

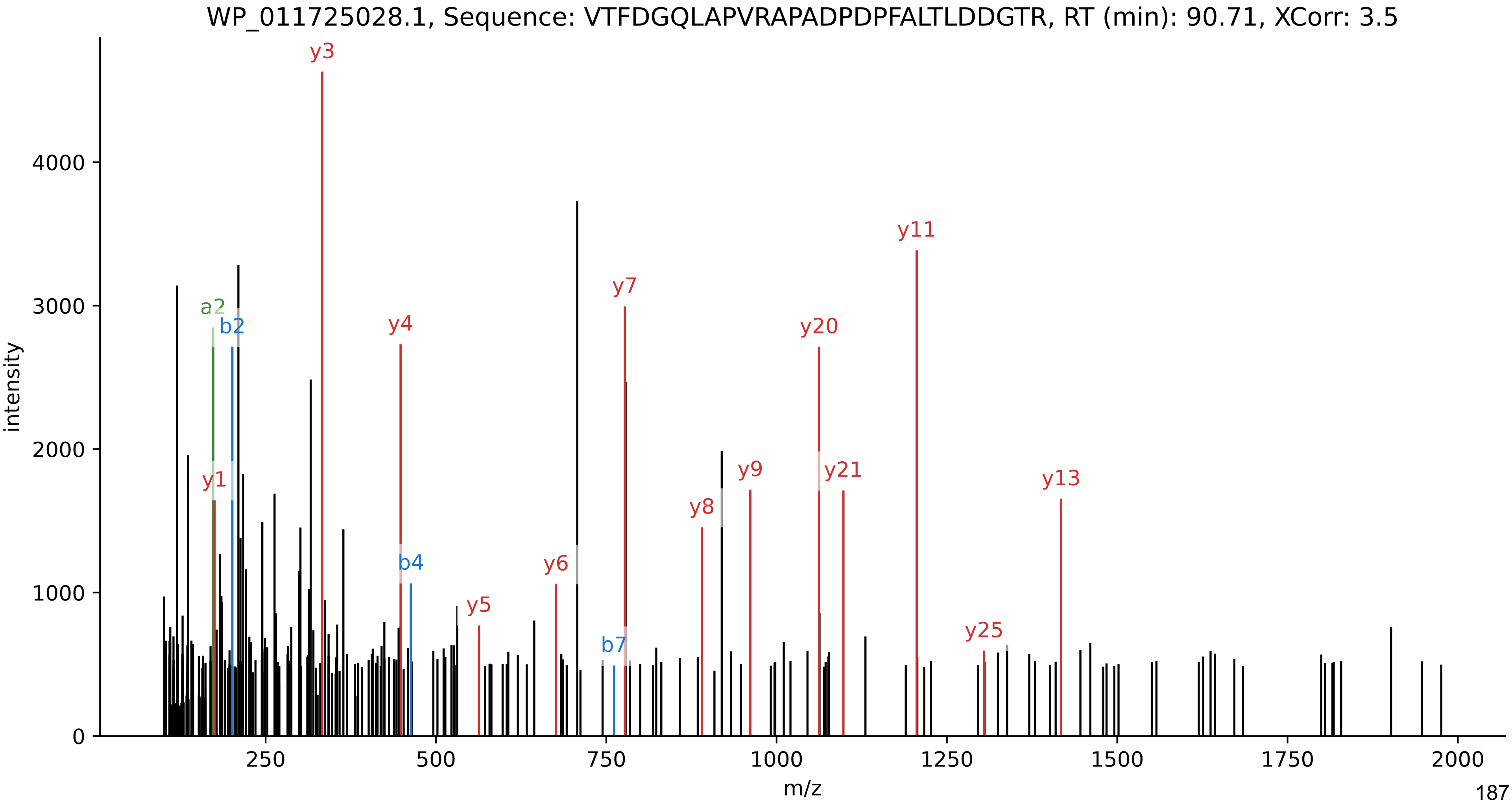
WP_011725028.1, Sequence: VTFDGQLAPVRAPADPDPFALTLDDGTR, RT (min): 90.71, XCorr: 3.5
intensity
m/z
y3
y11
y7
y4
y20
a2
b2
y1
y9
y21
y13
y8
b4
y6
y5
y25
b7
0
1000
2000
3000
4000
250
500
750
1000
1250
1500
1750
2000

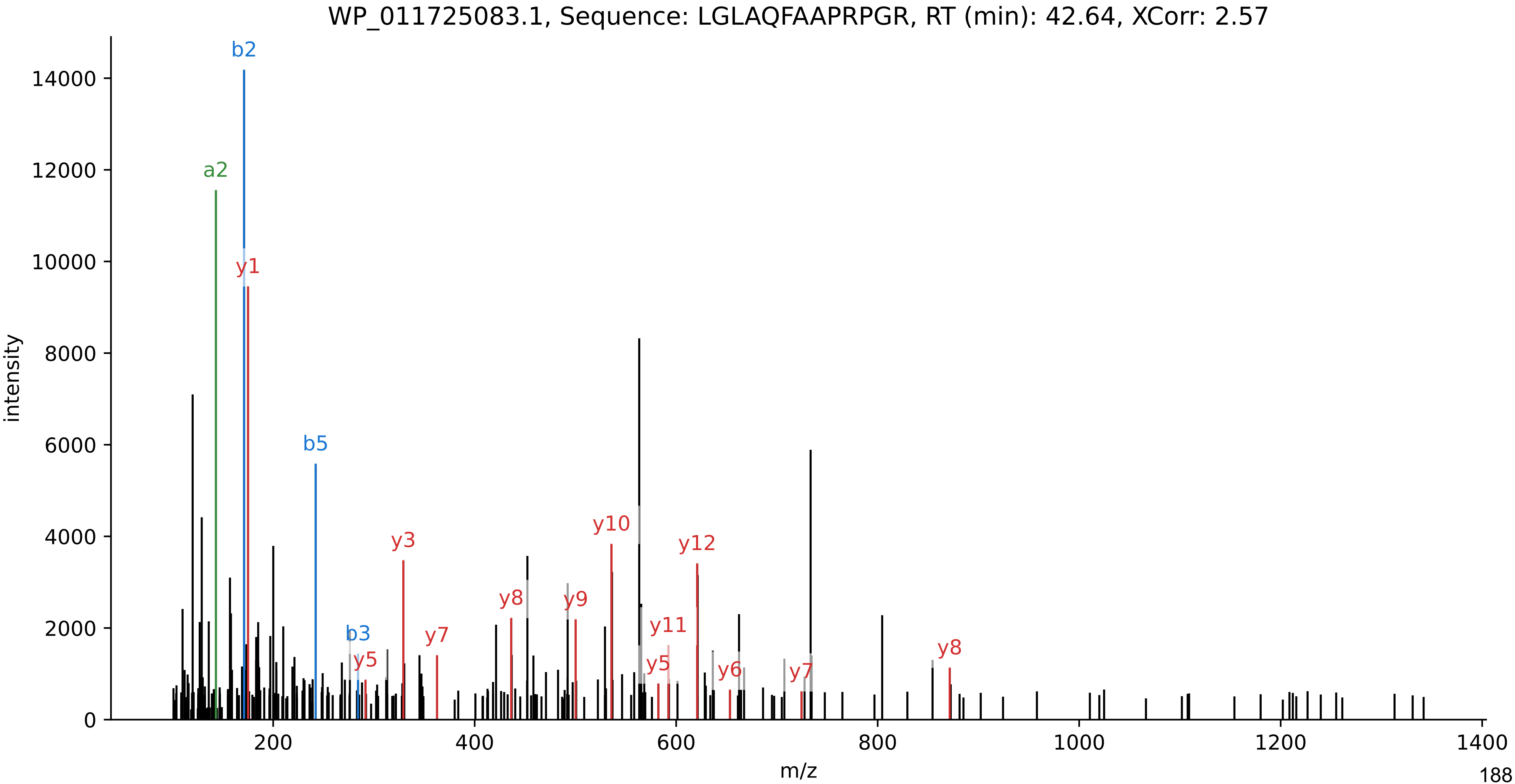

WP_011725083.1, Sequence: LGLAQFAAPRPGR, RT (min): 42.64, XCorr: 2.57
intensity
m/z
b2
a2
y1
b5
b3
y5
y3
y7
y8
y9
y10
y11
y5
y12
y6
y7
y8
0
2000
4000
6000
8000
10000
12000
14000
200
400
600
800
1000
1200
1400

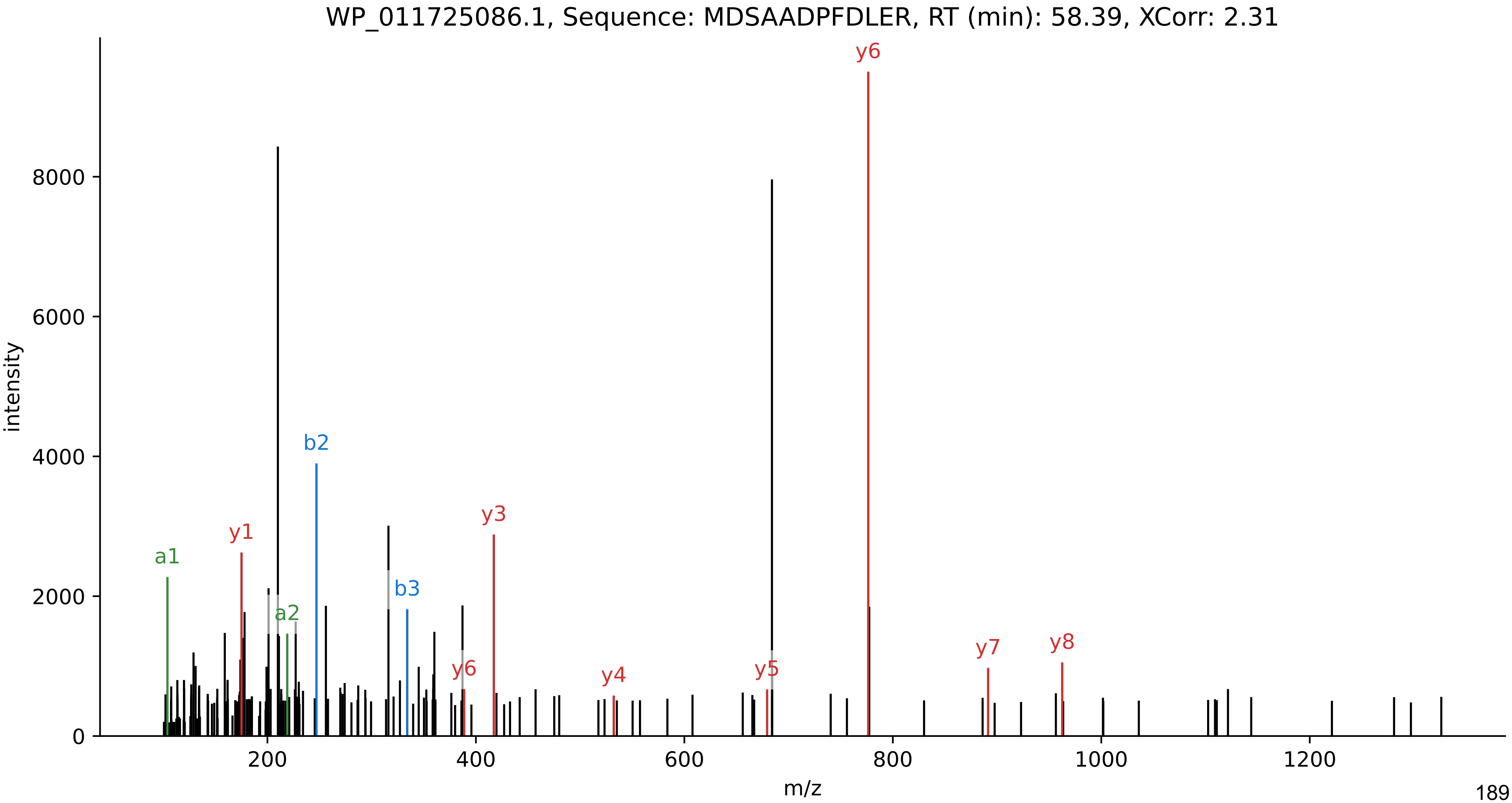

WP_011725086.1, Sequence: MDSAADPFDLER, RT (min): 58.39, XCorr: 2.31
y6
b2
y1
y3
a1
b3
a2
y6
y4
y5
y7
y8
intensity
m/z
0
2000
4000
6000
8000
200
400
600
800
1000
1200

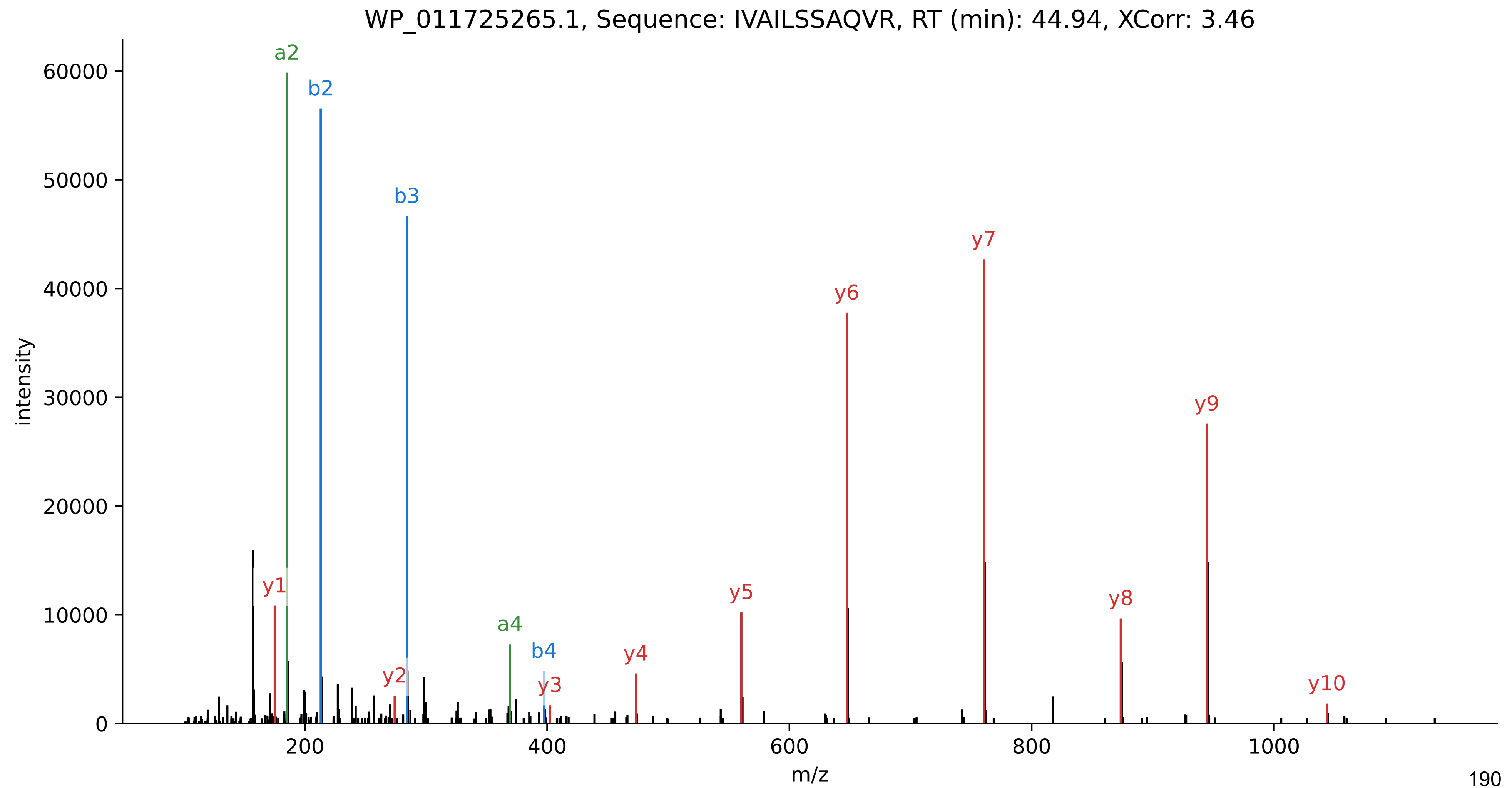
WP_011725265.1, Sequence: IVAILSSAQVR, RT (min): 44.94, XCorr: 3.46
intensity
m/z
a2
b2
b3
y7
y6
y9
y1
y5
y8
a4
b4
y4
y2
y3
y10
0
10000
20000
30000
40000
50000
60000
200
400
600
800
1000

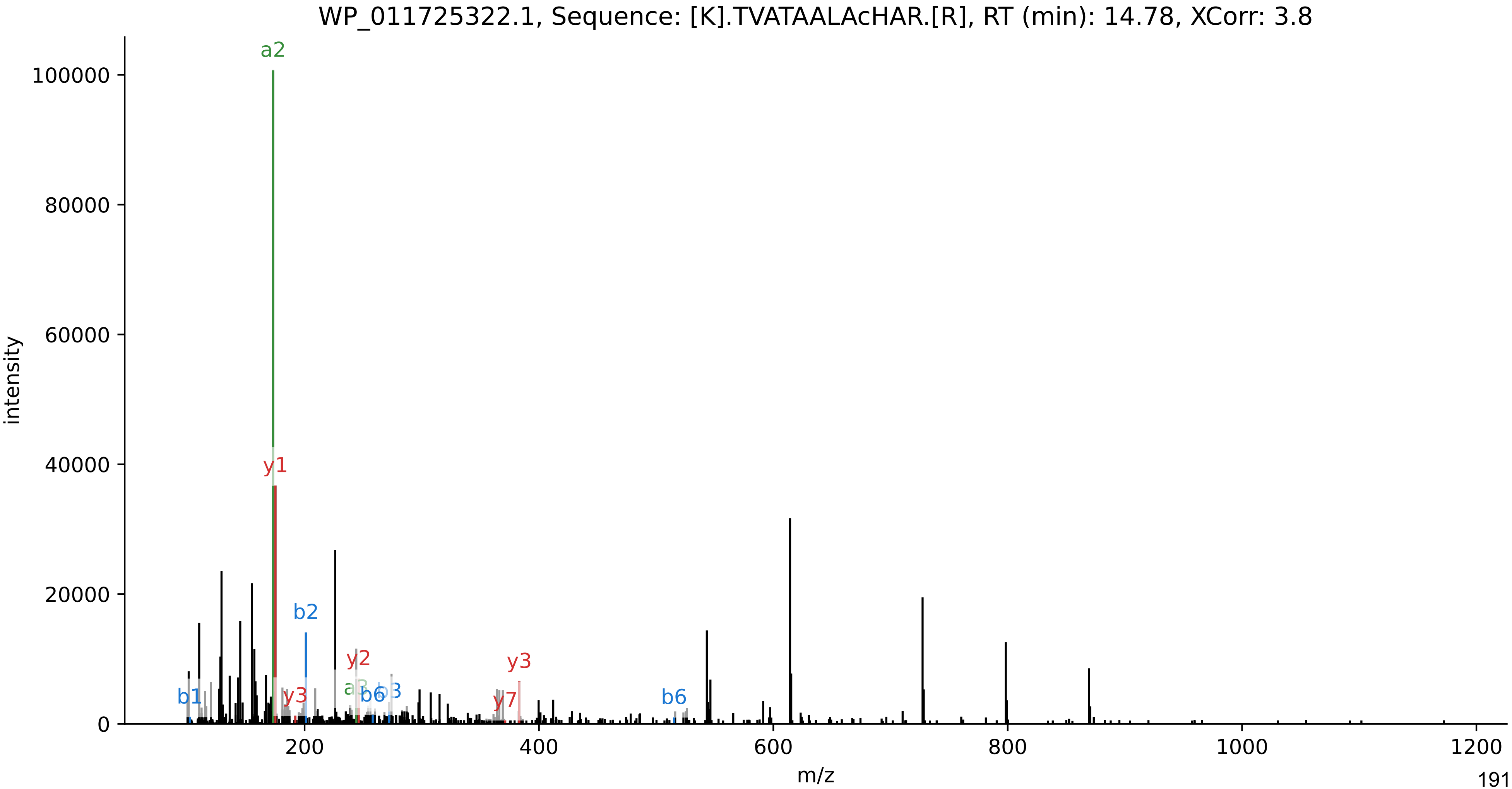

WP_011725322.1, Sequence: [K].TVATAALAcHAR.[R], RT (min): 14.78, XCorr: 3.8
intensity
m/z
a2
y1
b2
y2
y3
a3
b6
b3
b1
y3
y7
b6
0
20000
40000
60000
80000
100000
200
400
600
800
1000
1200

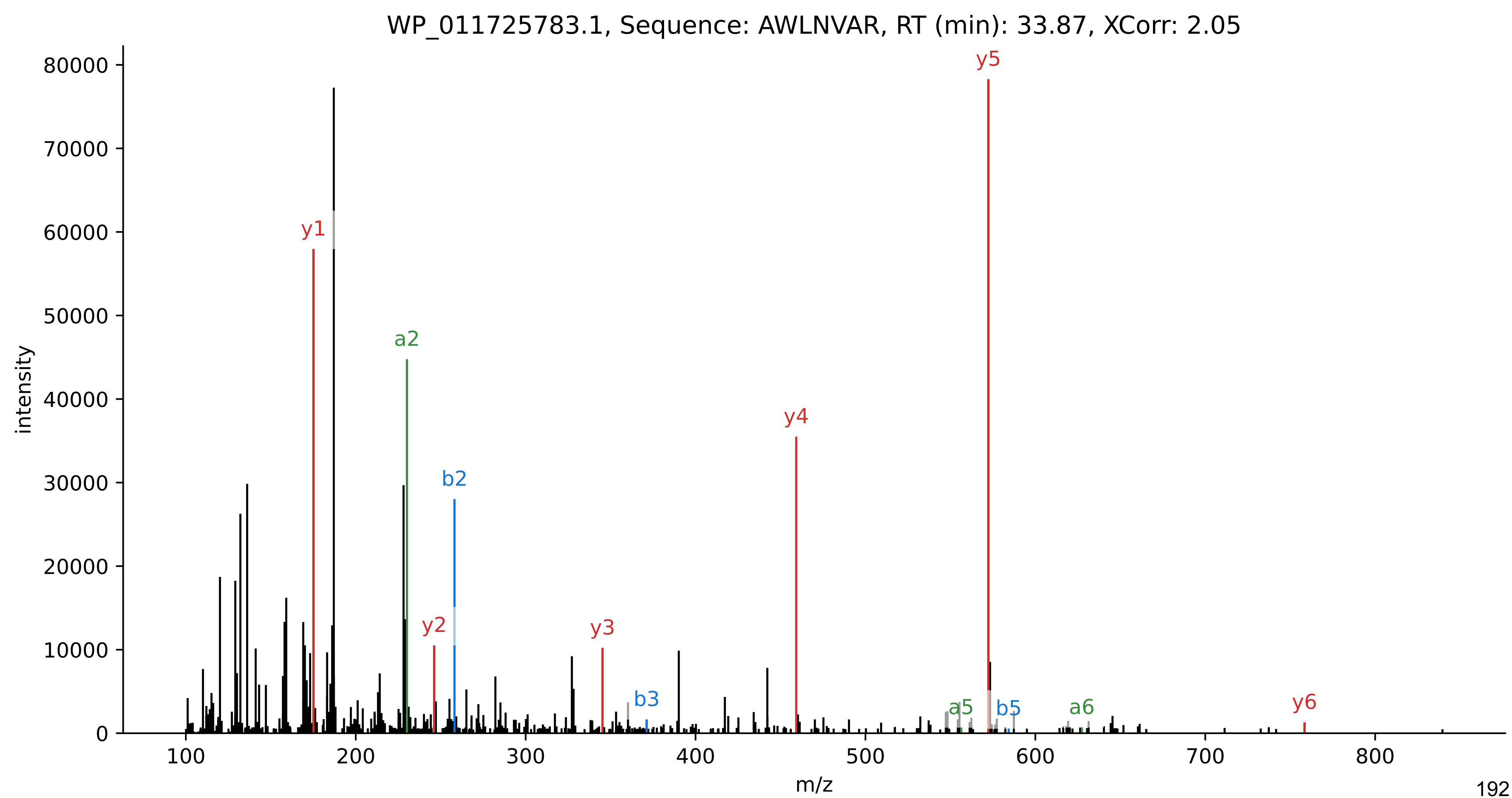

WP_011725783.1, Sequence: AWLNVAR, RT (min): 33.87, XCorr: 2.05
intensity
m/z
y1
y2
y3
y4
y5
y6
a2
a5
a6
b2
b3
b5

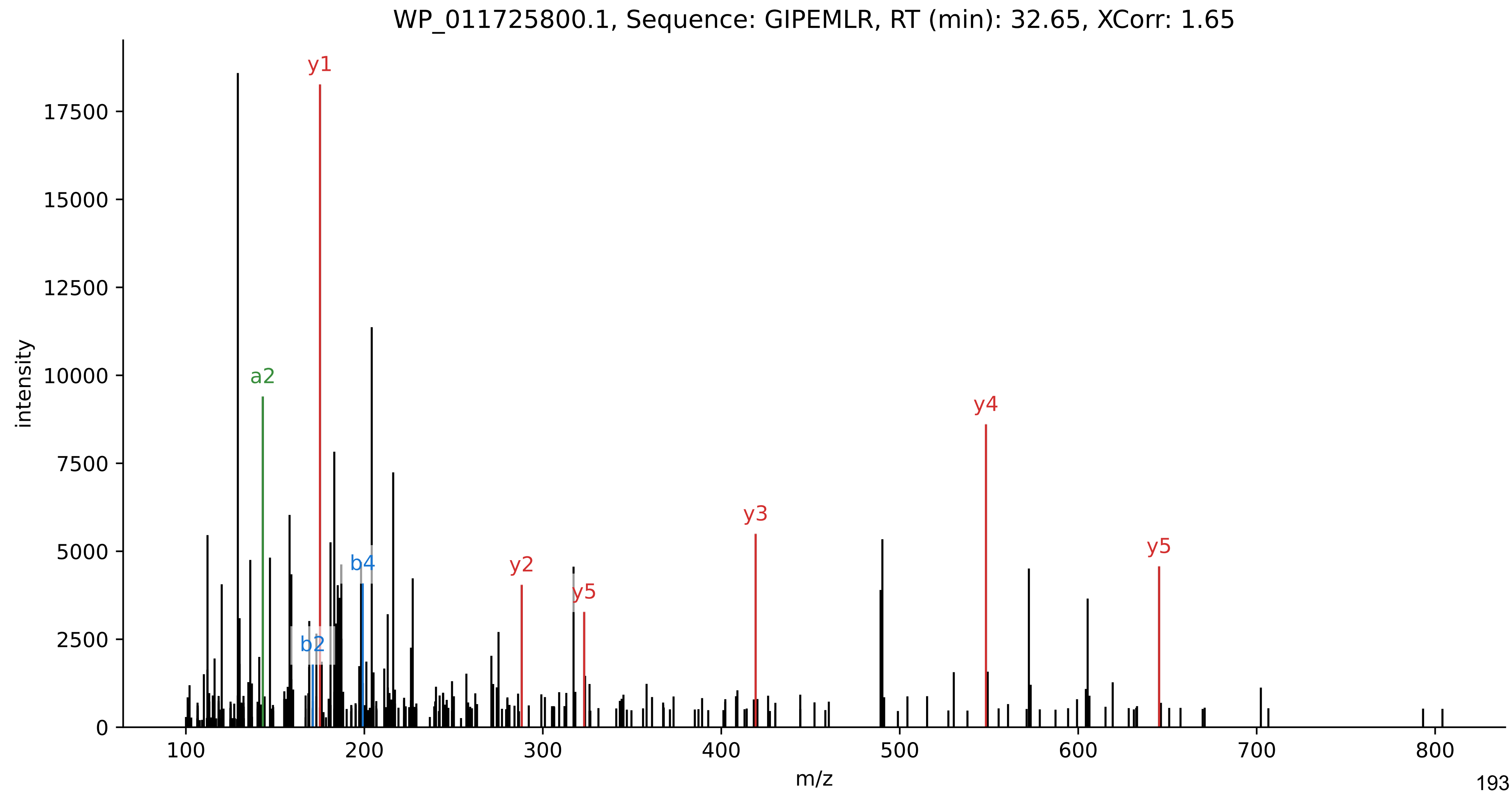

WP_011725800.1, Sequence: GIPEMLR, RT (min): 32.65, XCorr: 1.65
intensity
m/z
y1
a2
b2
b4
y2
y5
y3
y4
y5

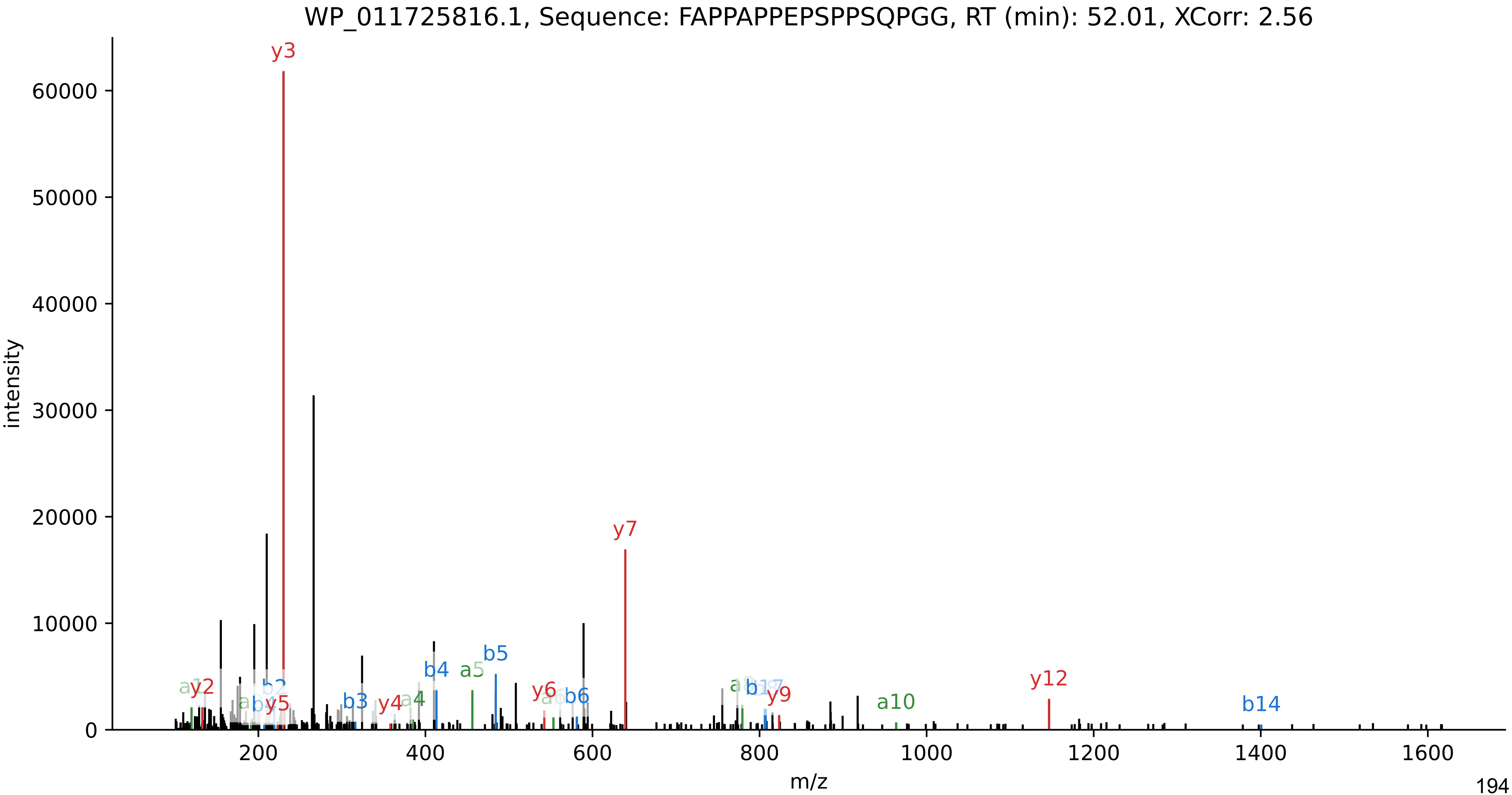
WP_011725816.1, Sequence: FAPPAPPEPSPPSQPGG, RT (min): 52.01, XCorr: 2.56
intensity
m/z
y3
y7
y12
b4
b5
a5
b3
y4
a4
y2
y5
y6
b6
y9
a10
b14
0
10000
20000
30000
40000
50000
60000
200
400
600
800
1000
1200
1400
1600

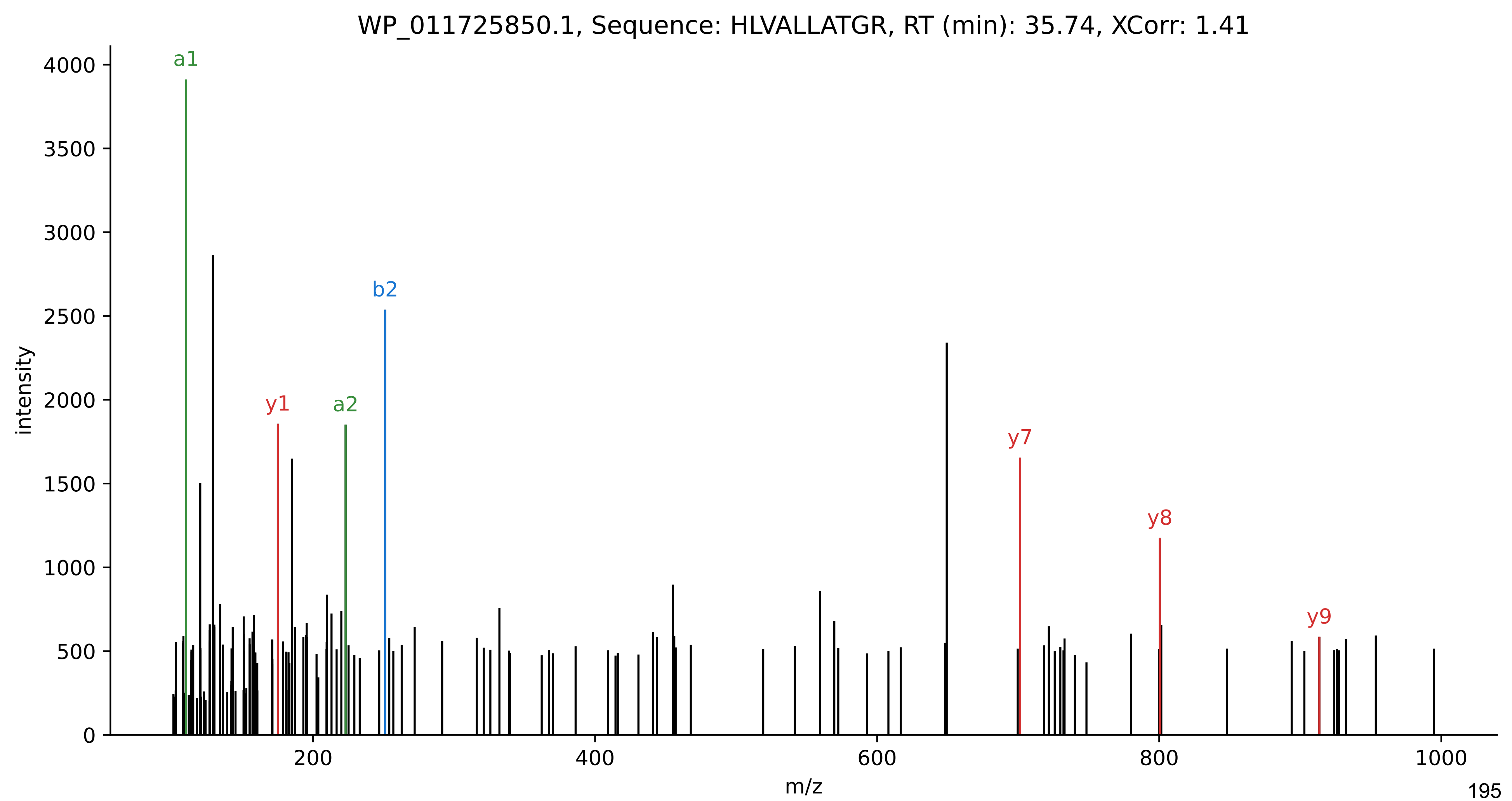
WP_011725850.1, Sequence: HLVALLATGR, RT (min): 35.74, XCorr: 1.41
a1
b2
y1
a2
y7
y8
y9
intensity
m/z
0
500
1000
1500
2000
2500
3000
3500
4000
200
400
600
800
1000

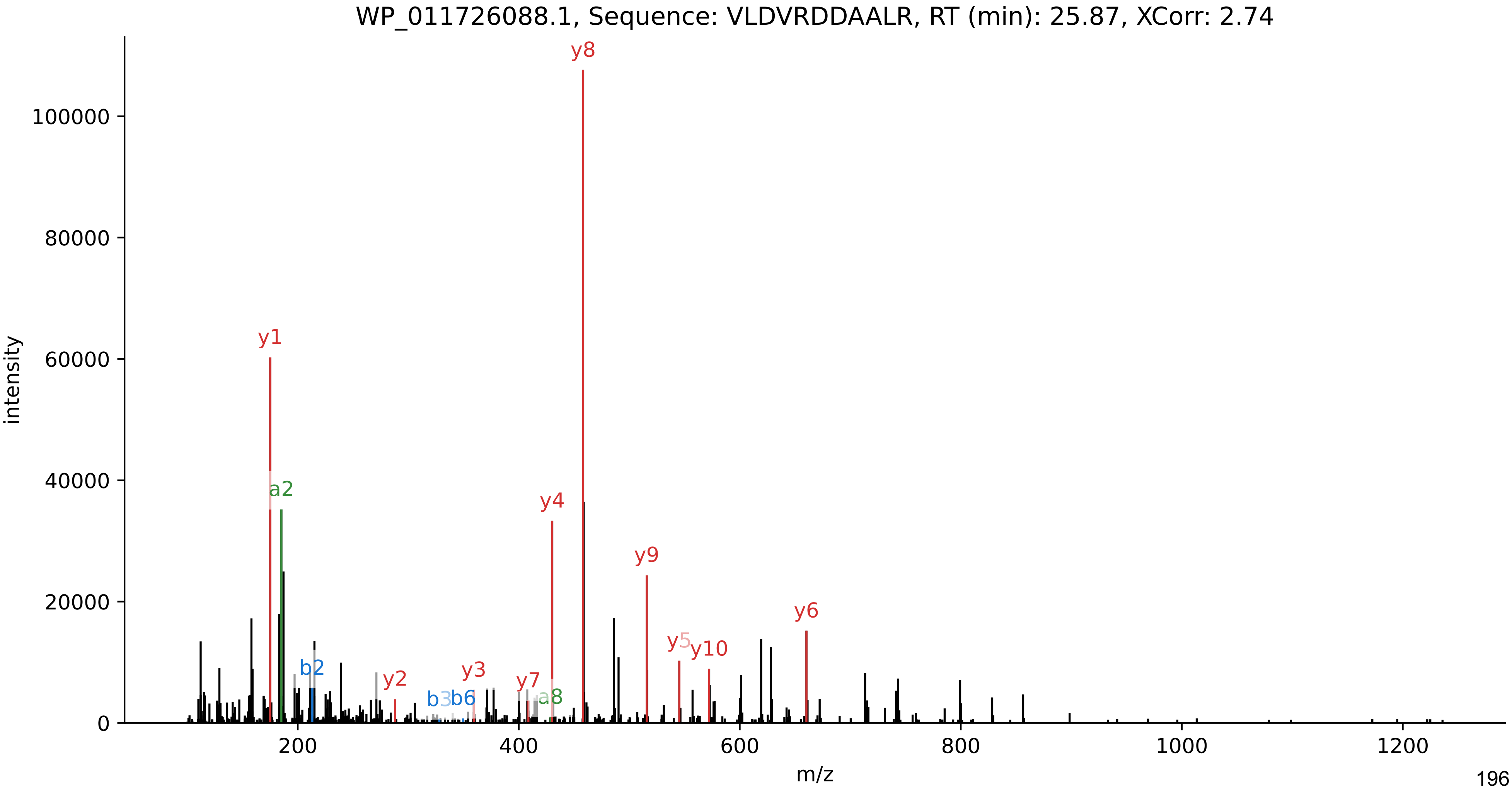

WP_011726088.1, Sequence: VLDVRDDAALR, RT (min): 25.87, XCorr: 2.74
intensity
m/z
y8
y1
a2
y4
y9
y6
y5
y10
b2
y2
y3
y7
b3
b6
a8
0
20000
40000
60000
80000
100000
200
400
600
800
1000
1200

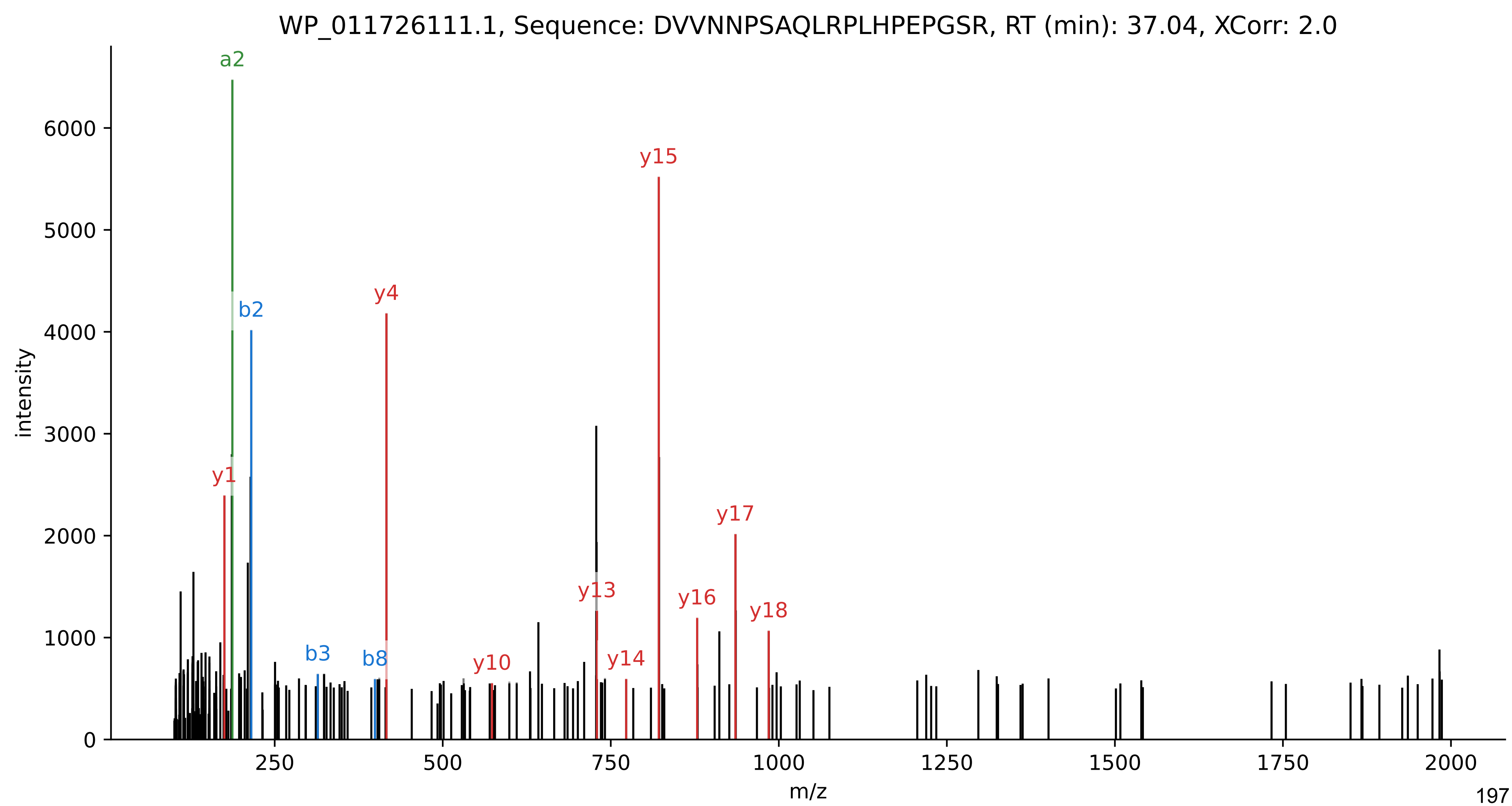
WP_011726111.1, Sequence: DVVNNPSAQLRPLHPEPGSR, RT (min): 37.04, XCorr: 2.0
intensity
m/z
a2
b2
y1
y4
y15
y17
y13
y16
y18
y14
y10
b3
b8
0
1000
2000
3000
4000
5000
6000
250
500
750
1000
1250
1500
1750
2000

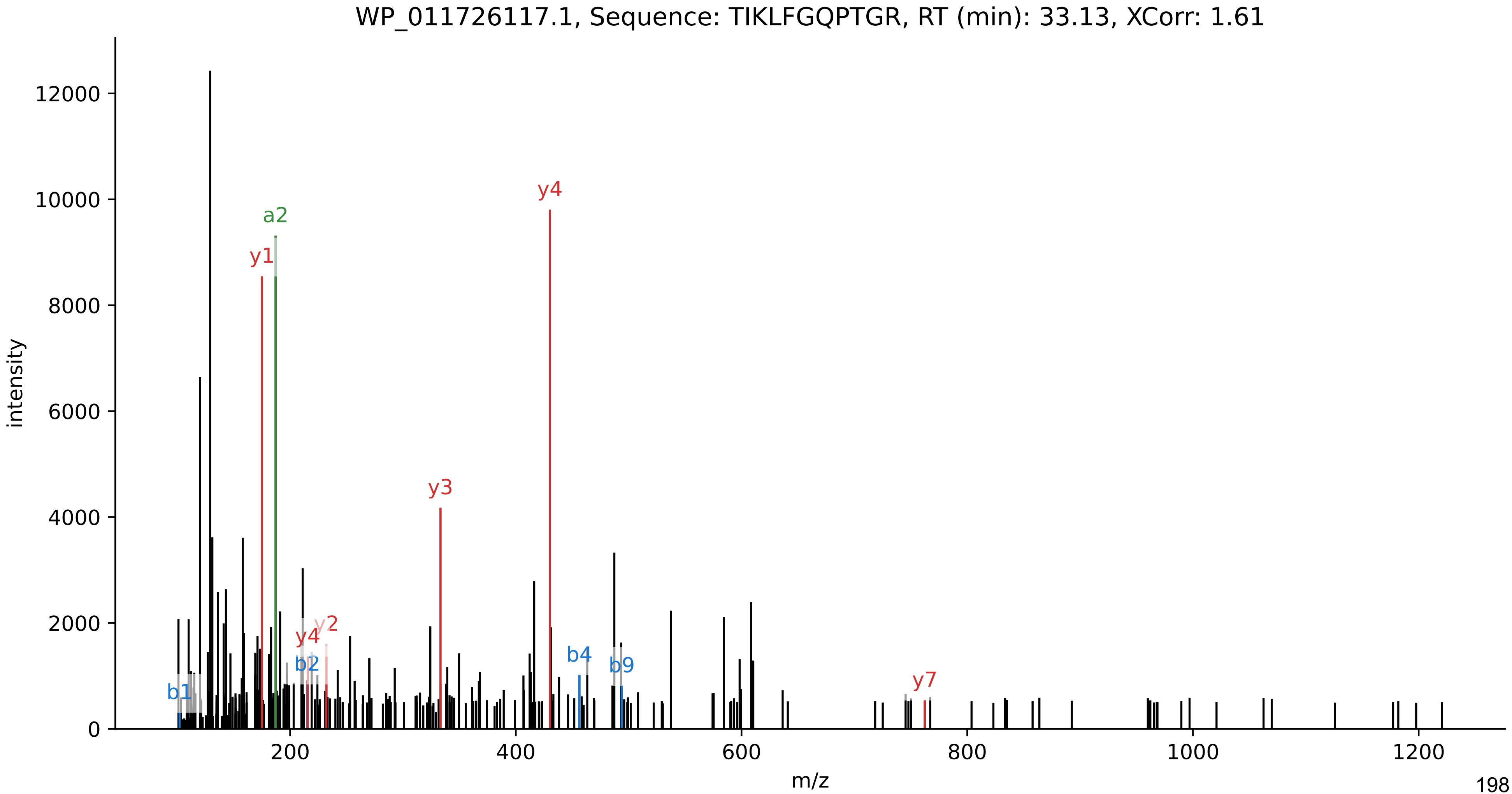
WP_011726117.1, Sequence: TIKLFGQPTGR, RT (min): 33.13, XCorr: 1.61
intensity
m/z
0
2000
4000
6000
8000
10000
12000
200
400
600
800
1000
1200
a2
y1
y4
y3
y4
y2
b1
b2
b4
b9
y7

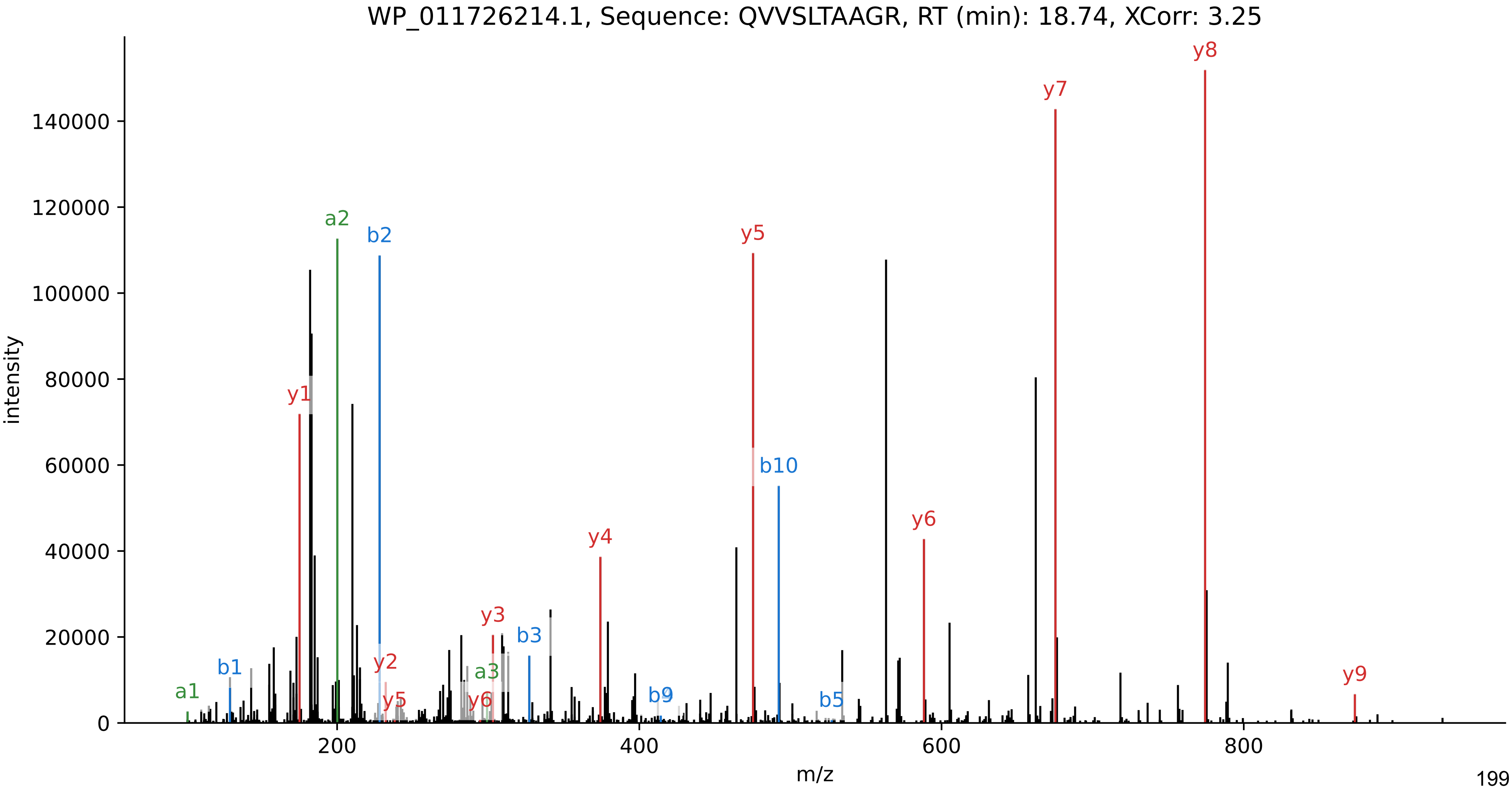
WP_011726214.1, Sequence: QVVSLTAAGR, RT (min): 18.74, XCorr: 3.25
intensity
m/z
0
20000
40000
60000
80000
100000
120000
140000
200
400
600
800
a1
b1
y1
a2
b2
y2
y5
y6
y3
a3
b3
y4
b9
y5
b10
b5
y6
y7
y8
y9

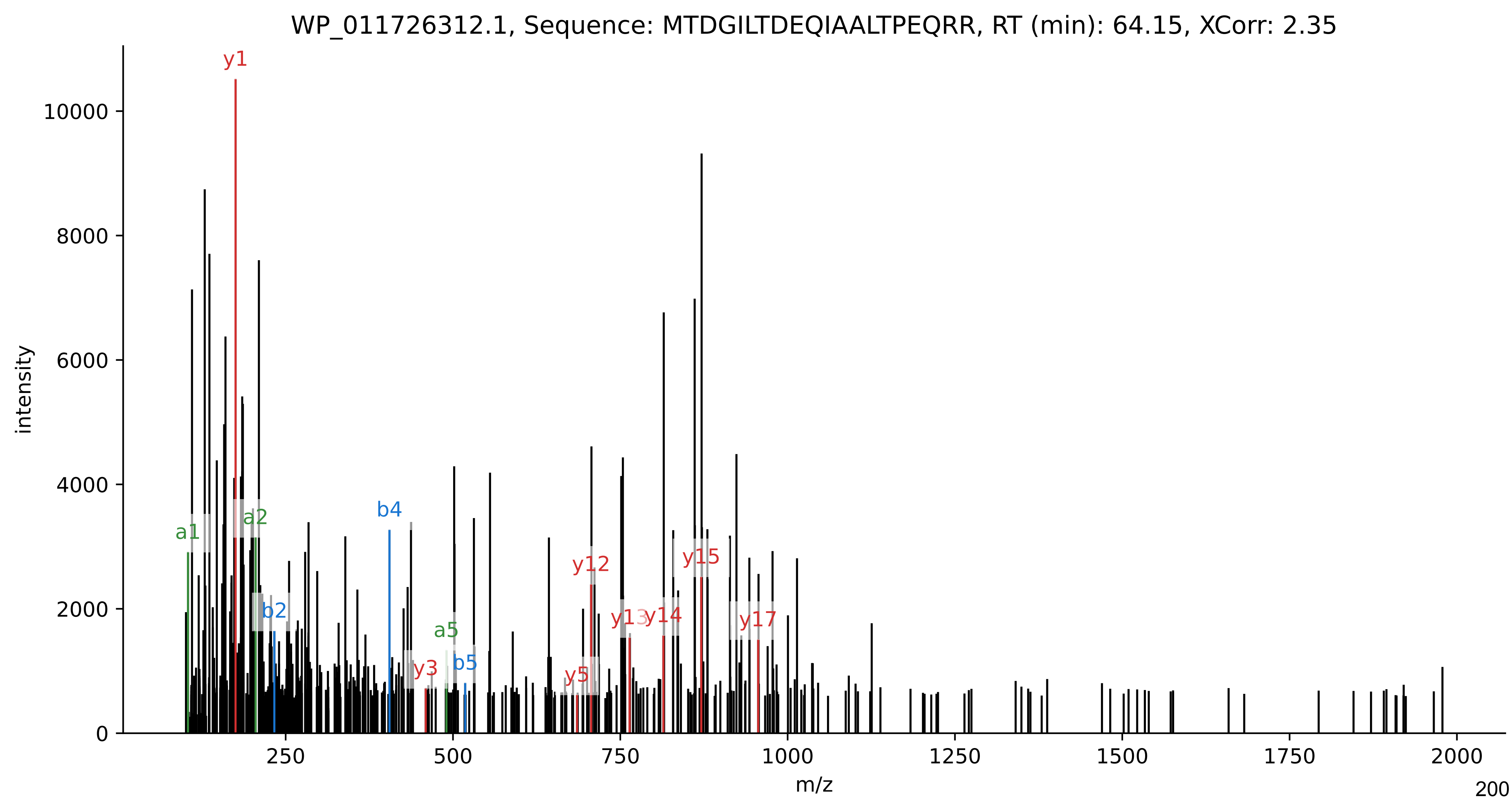
WP_011726312.1, Sequence: MTDGILTDEQIAALTPEQRR, RT (min): 64.15, XCorr: 2.35
intensity
m/z
y1
a1
a2
b2
b4
a5
y3
b5
y5
y12
y13
y14
y15
y17
0
2000
4000
6000
8000
10000
250
500
750
1000
1250
1500
1750
2000

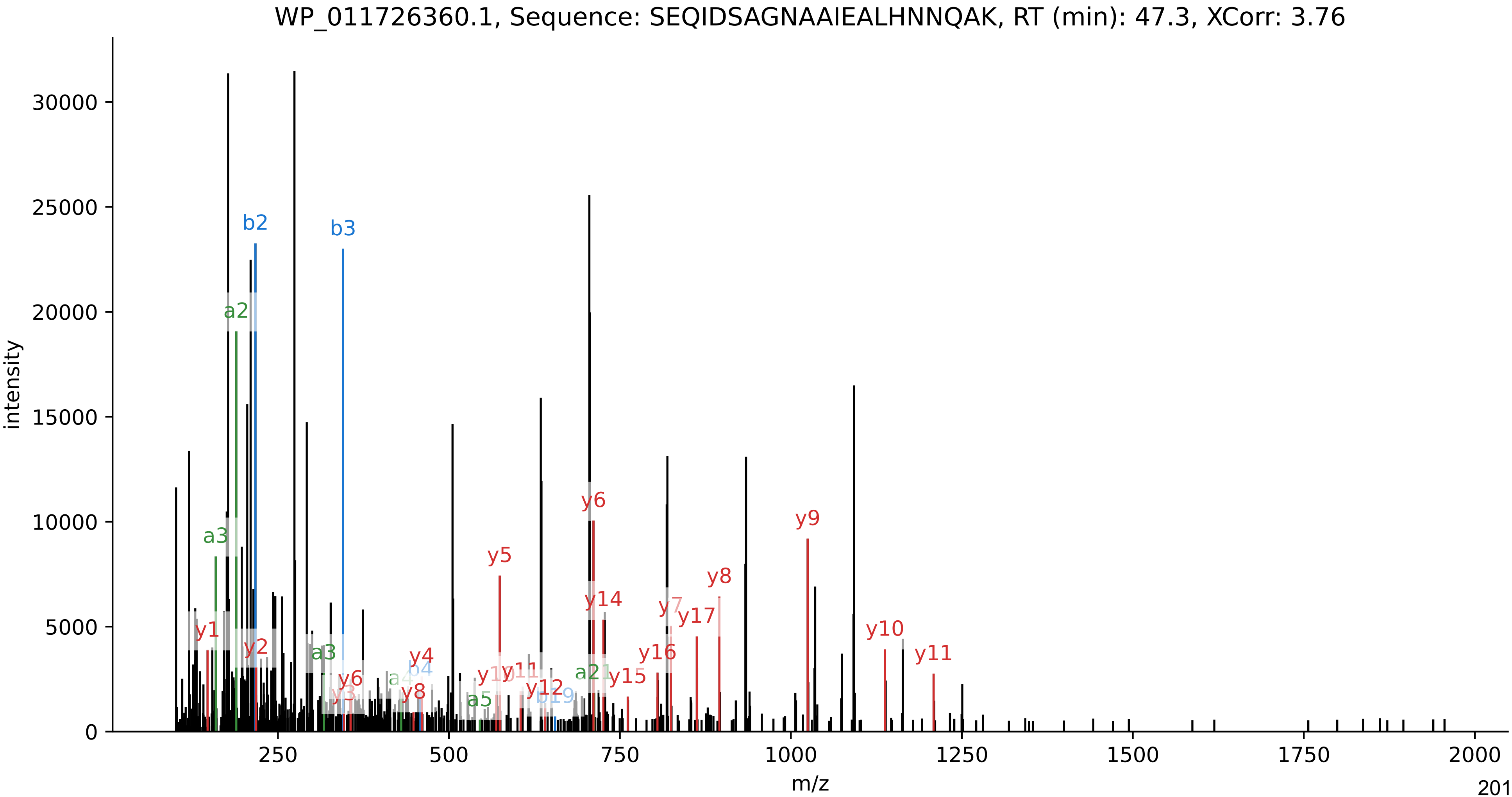

WP_011726360.1, Sequence: SEQIDSAGNAAIEALHNNQAK, RT (min): 47.3, XCorr: 3.76
intensity
m/z
b2
b3
a2
a3
y1
y2
y4
y5
y6
y8
y9
y10
y11
y12
y14
y15
y16
y17
a5

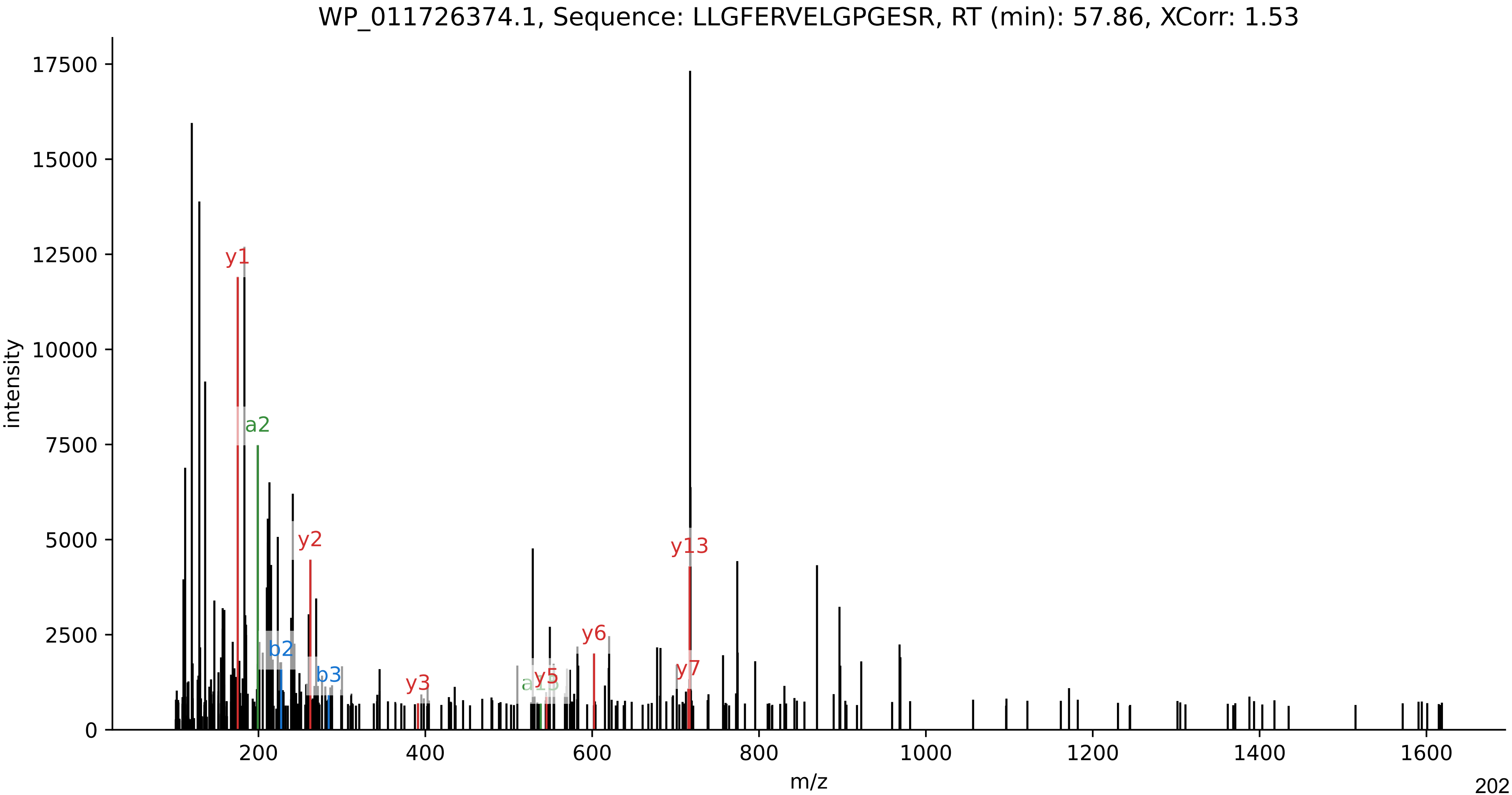

WP_011726374.1, Sequence: LLGFERVELGPGESR, RT (min): 57.86, XCorr: 1.53
intensity
m/z
y1
a2
y2
b2
b3
y3
y5
y6
y7
y13

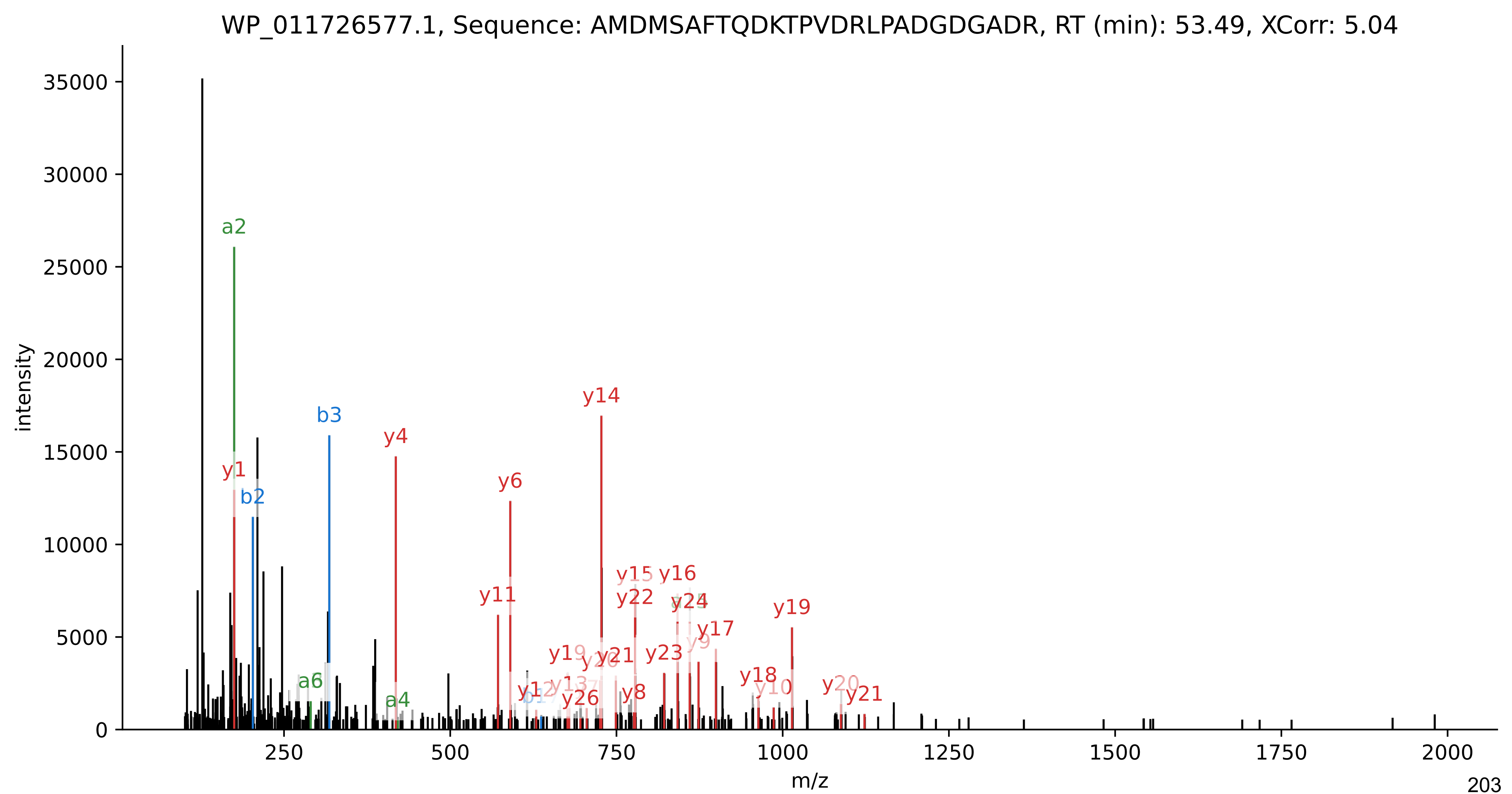

WP_011726577.1, Sequence: AMDMSAFTQDKTPVDRLPADGDGADR, RT (min): 53.49, XCorr: 5.04
intensity
m/z
a2
b3
y4
y14
y1
b2
y6
y11
y15
y16
y22
y24
y19
y17
y23
y21
y8
y26
y18
y10
y20
a6
a4

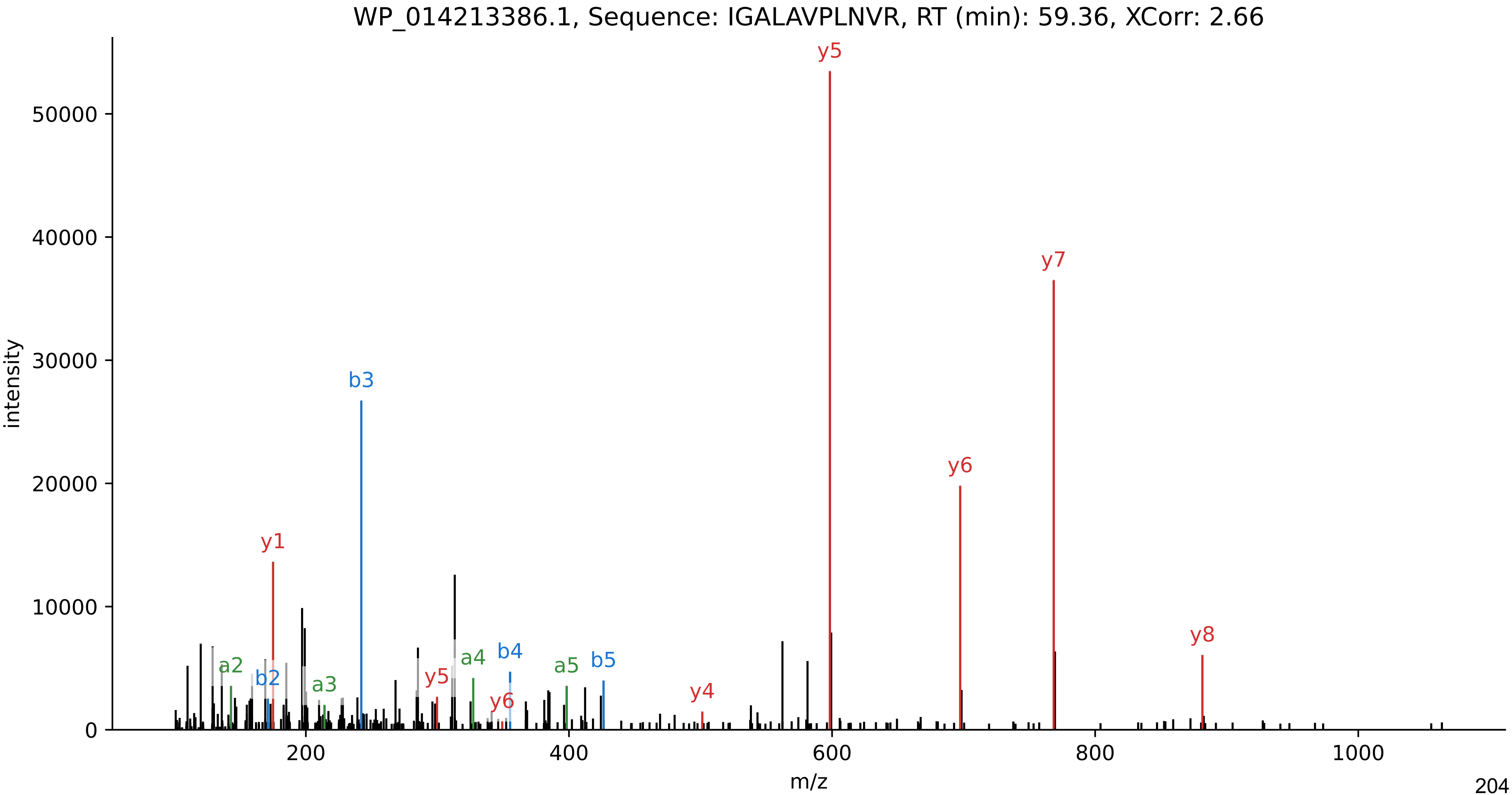
WP_014213386.1, Sequence: IGALAVPLNVR, RT (min): 59.36, XCorr: 2.66
intensity
m/z
y5
y7
y6
b3
y1
y8
a2
b2
a3
y5
a4
b4
y6
a5
b5
y4
50000
40000
30000
20000
10000
0
200
400
600
800
1000

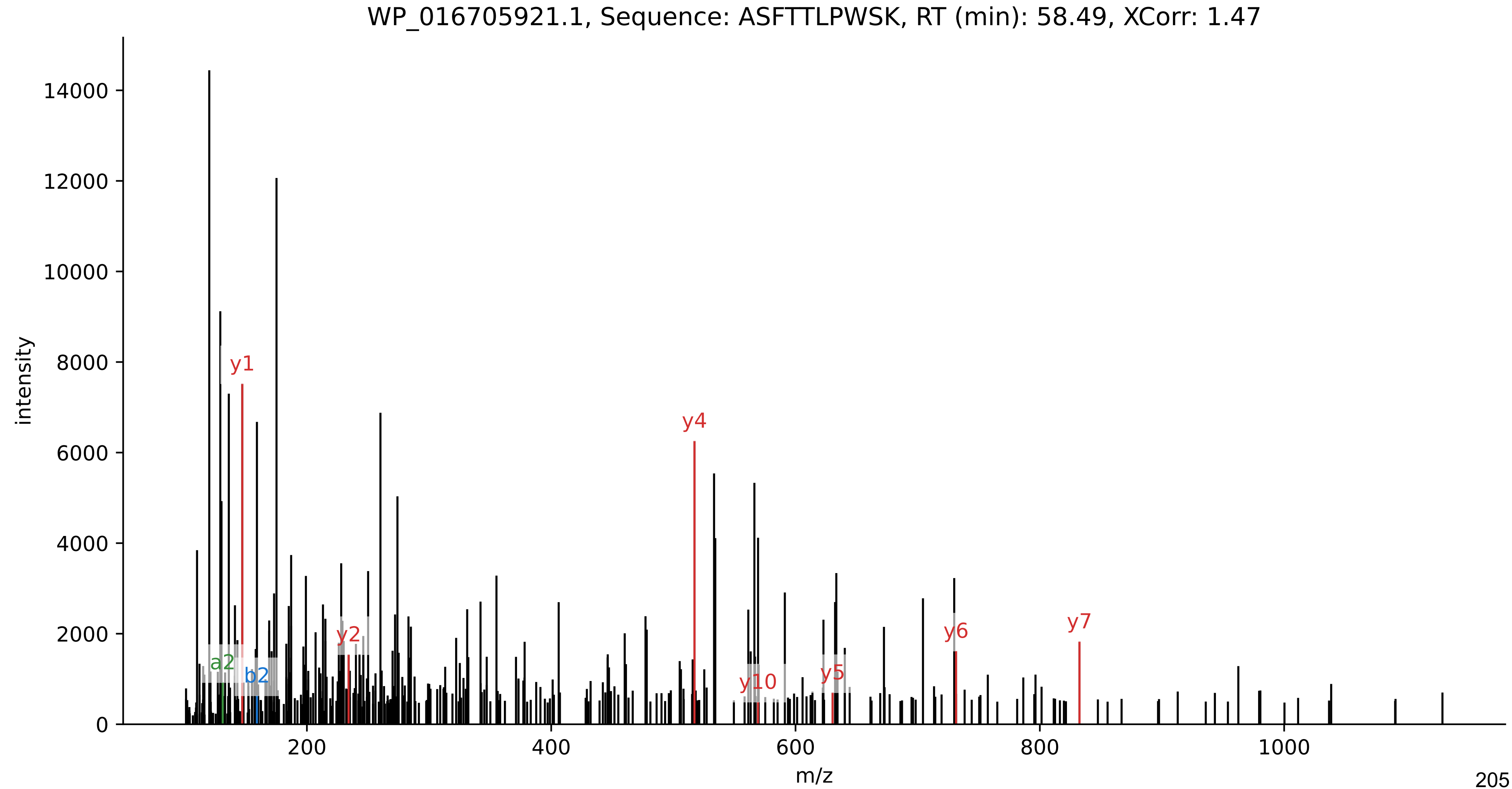

WP_016705921.1, Sequence: ASFTTLPWSK, RT (min): 58.49, XCorr: 1.47
intensity
m/z
y1
y2
a2
b2
y4
y10
y5
y6
y7

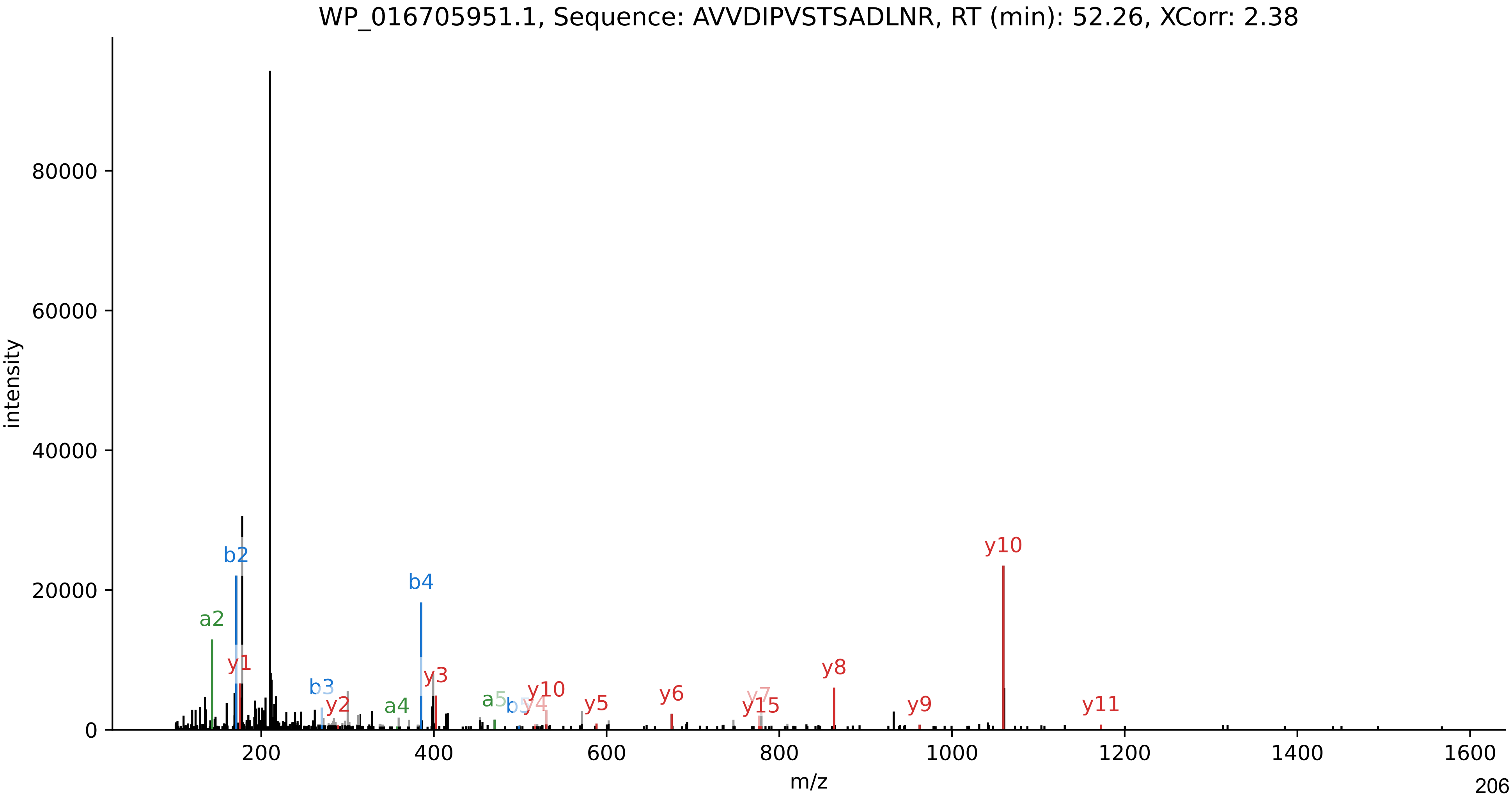
WP_016705951.1, Sequence: AVVDIPVSTSADLNR, RT (min): 52.26, XCorr: 2.38
intensity
m/z
80000
60000
40000
20000
0
200
400
600
800
1000
1200
1400
1600
b2
a2
y1
b4
b3
y2
y3
a4
a5
b5
y4
y10
y5
y6
y7
y15
y8
y9
y10
y11

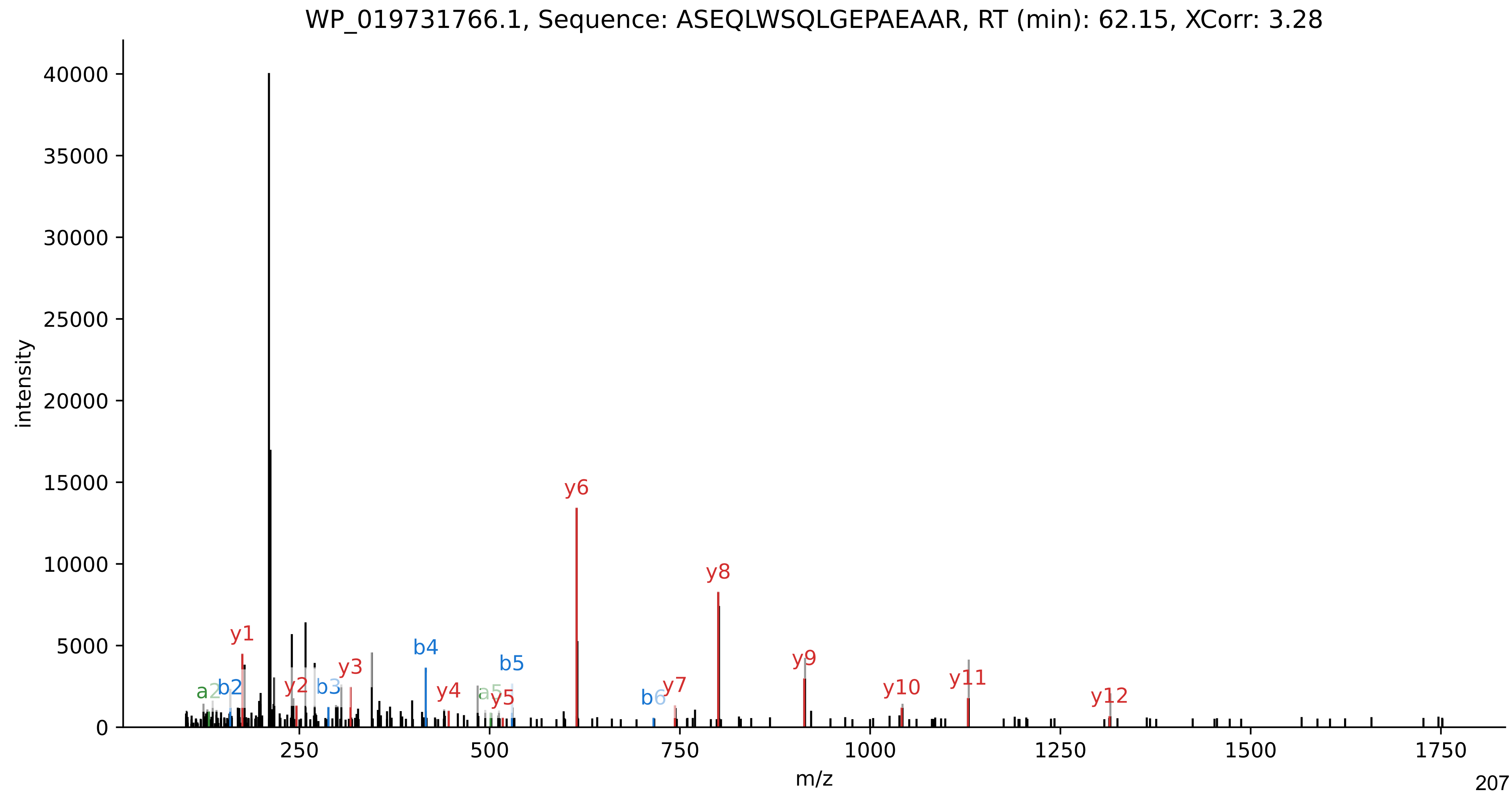

WP_019731766.1, Sequence: ASEQLWSQLGEPAEAAR, RT (min): 62.15, XCorr: 3.28
intensity
m/z
y1
y2
y3
y4
y5
y6
y7
y8
y9
y10
y11
y12
a2
a5
b2
b3
b4
b5
b6

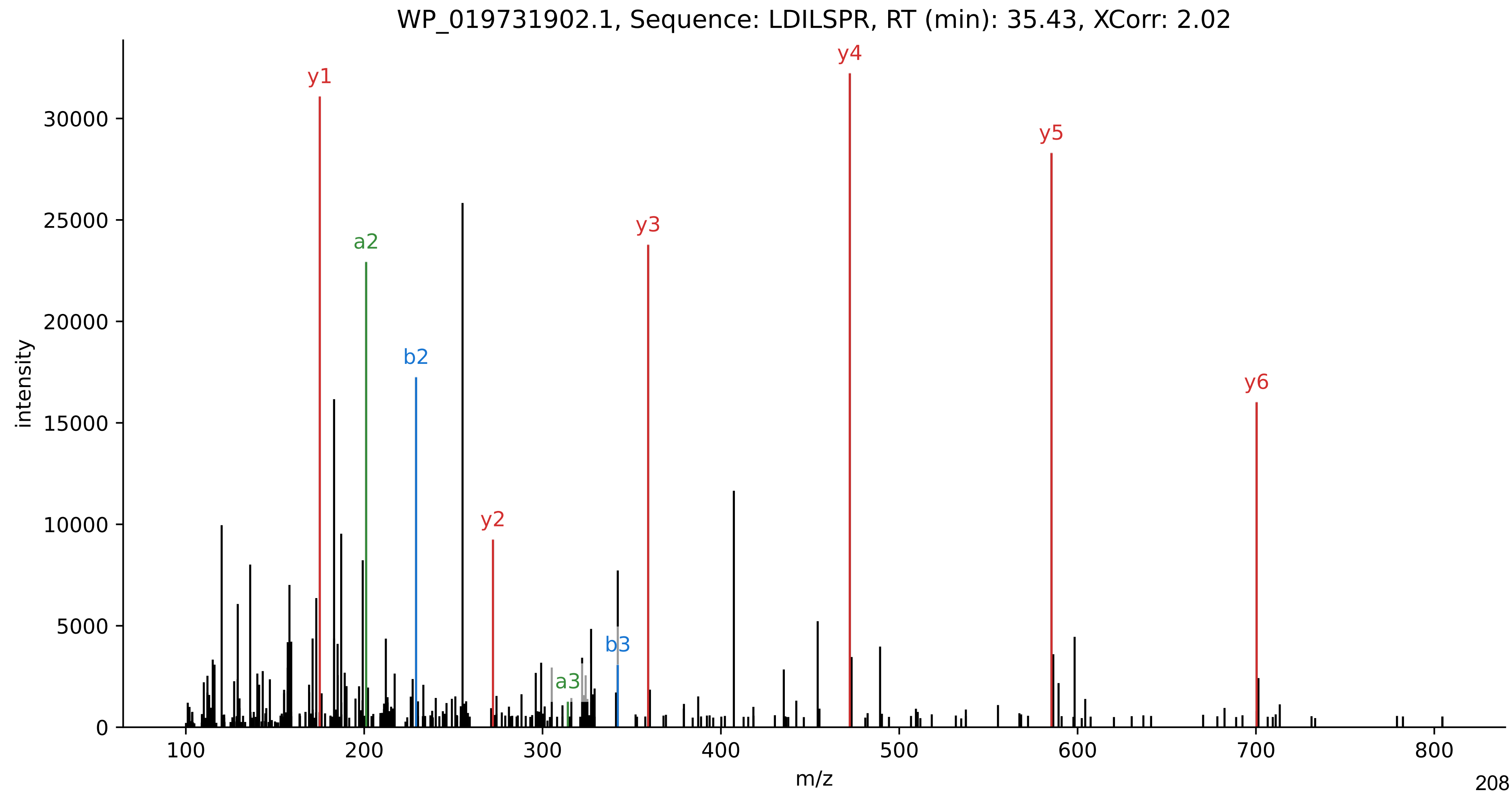
WP_019731902.1, Sequence: LDILSPR, RT (min): 35.43, XCorr: 2.02
intensity
m/z
y1
y2
y3
y4
y5
y6
a2
a3
b2
b3
0
5000
10000
15000
20000
25000
30000
100
200
300
400
500
600
700
800

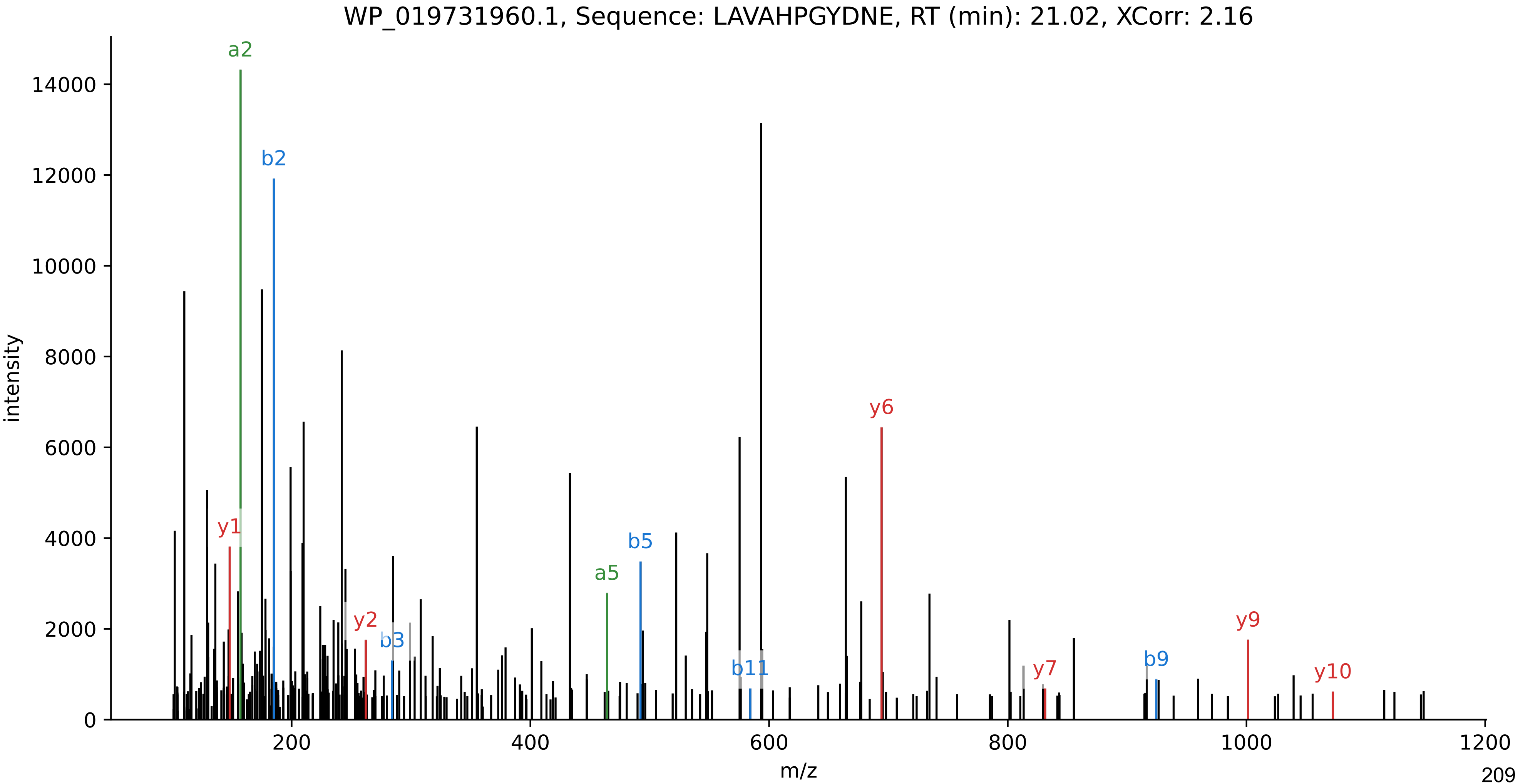
WP_019731960.1, Sequence: LAVAHPGYDNE, RT (min): 21.02, XCorr: 2.16
intensity
m/z
a2
b2
y1
y6
b5
a5
y2
b3
b11
y9
b9
y7
y10
0
2000
4000
6000
8000
10000
12000
14000
200
400
600
800
1000
1200

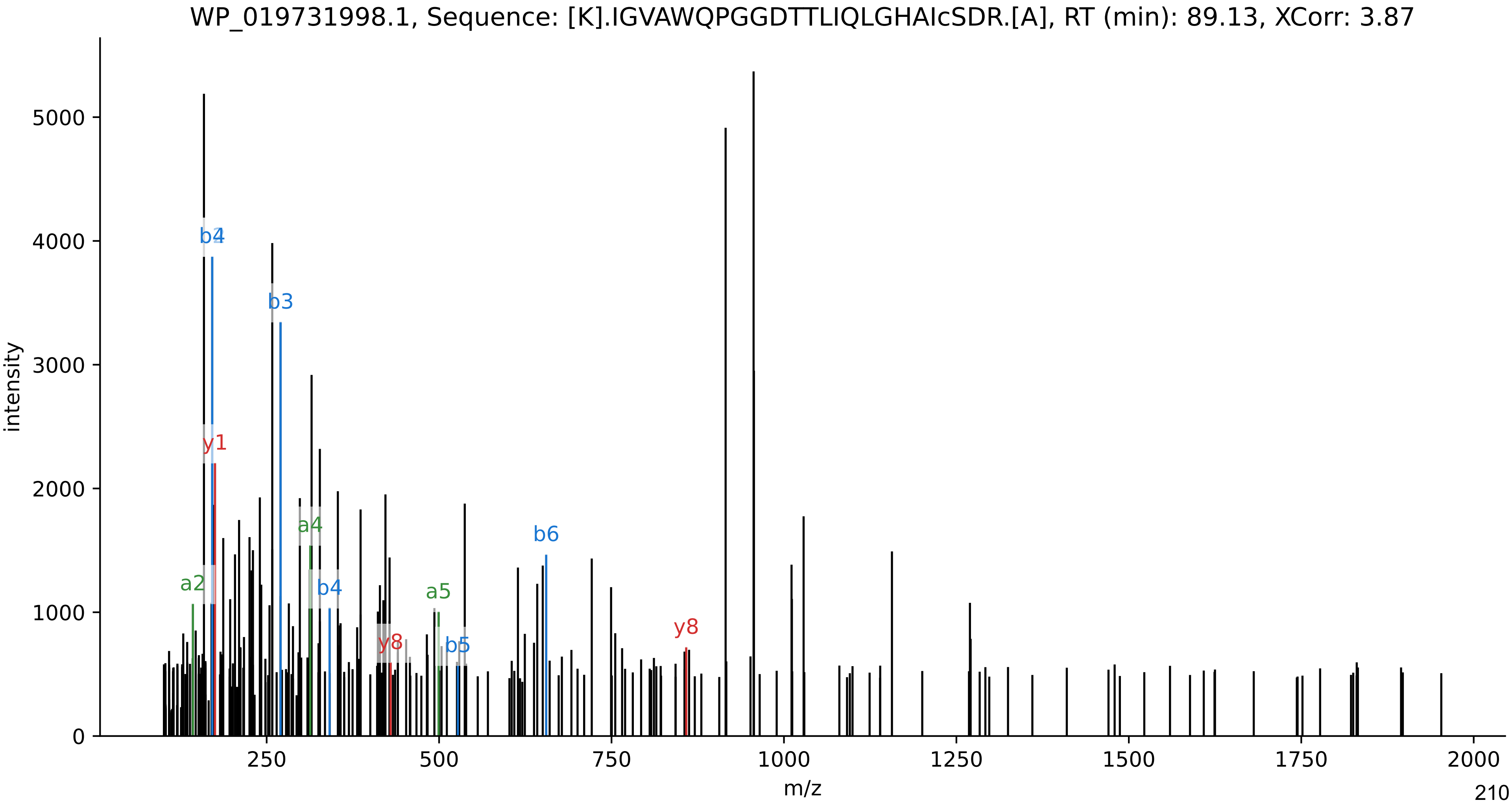

WP_019731998.1, Sequence: [K].IGVAWQPGGDTTLIQLGHAIcSDR.[A], RT (min): 89.13, XCorr: 3.87
intensity
m/z
b4
b3
y1
a4
a2
b4
a5
y8
b5
b6
y8

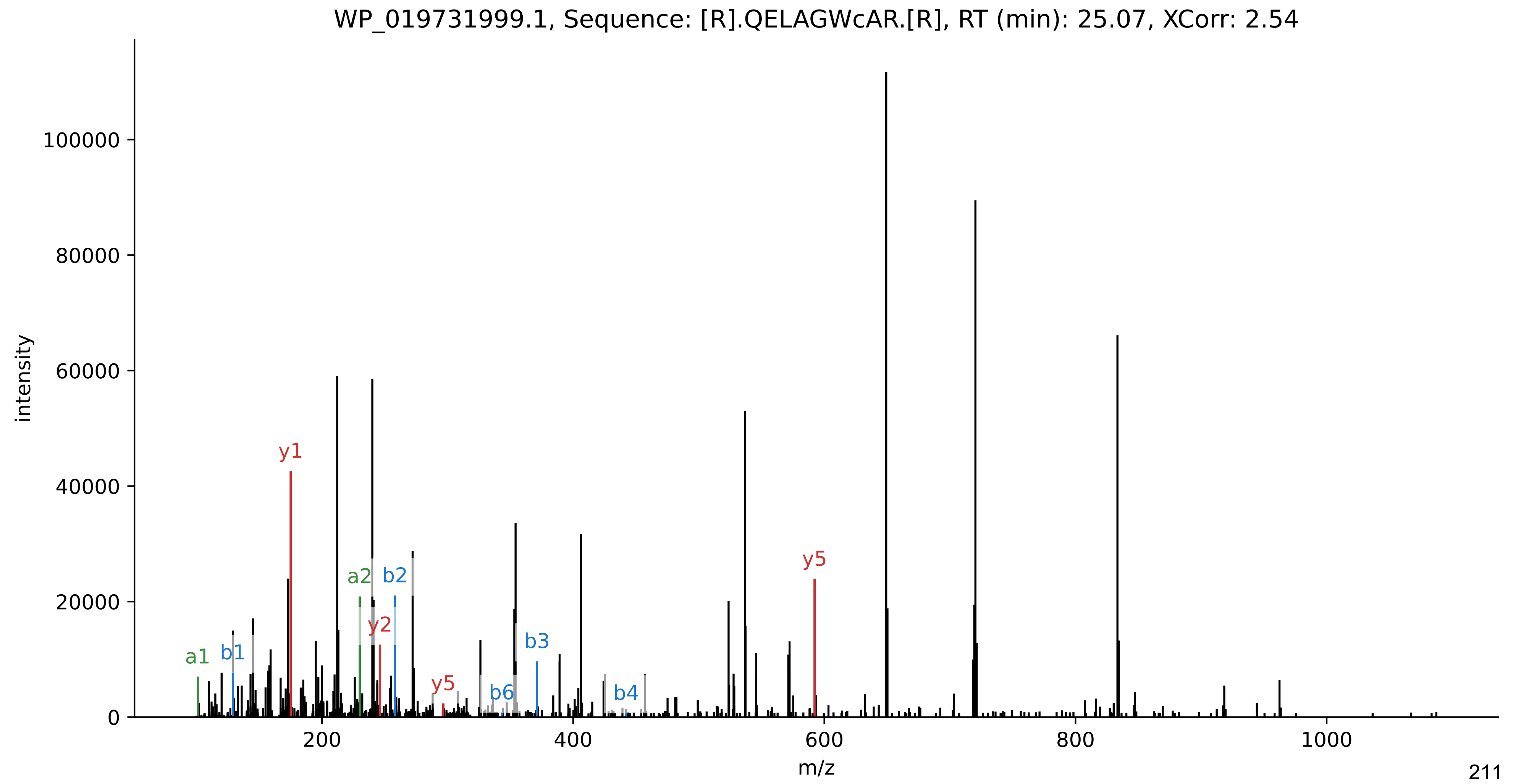

WP_019731999.1, Sequence: [R].QELAGWcAR.[R], RT (min): 25.07, XCorr: 2.54
intensity
m/z
a1
b1
y1
a2
b2
y2
y5
b6
b3
b4
y5
0
20000
40000
60000
80000
100000
200
400
600
800
1000

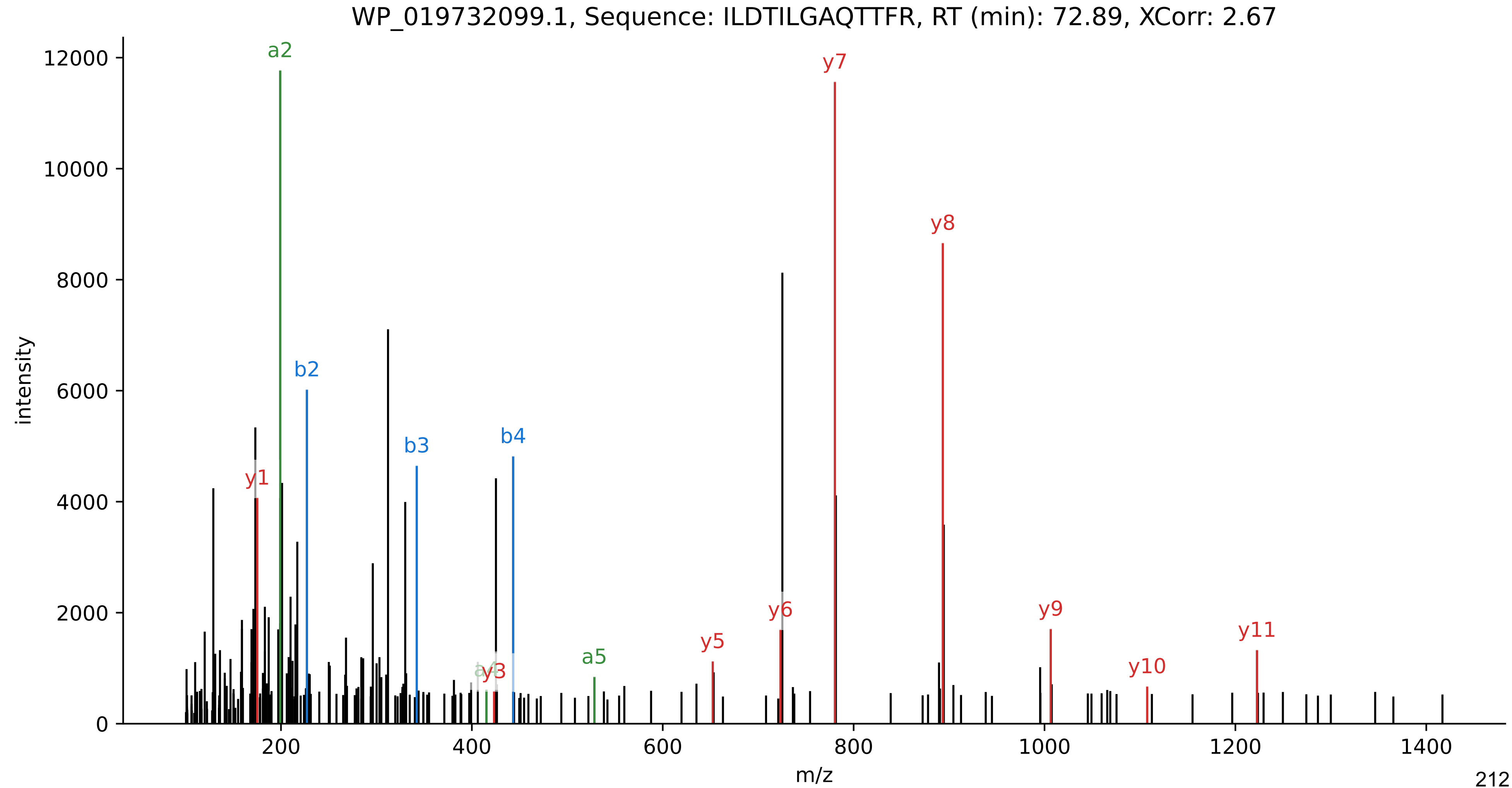
WP_019732099.1, Sequence: ILDTILGAQTTFR, RT (min): 72.89, XCorr: 2.67
intensity
m/z
a2
b2
b3
b4
y1
y3
a5
y5
y6
y7
y8
y9
y10
y11

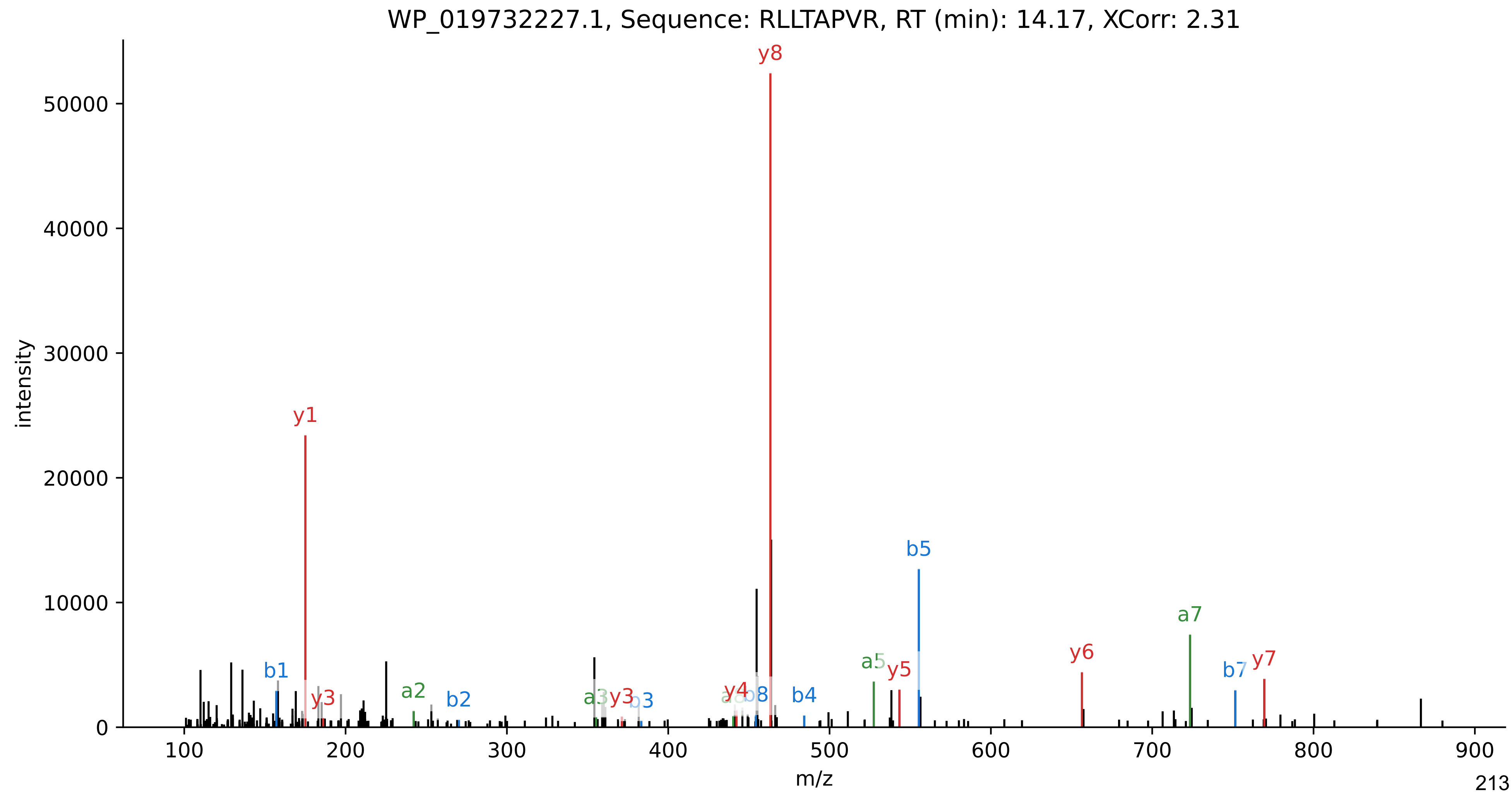
WP_019732227.1, Sequence: RLLTAPVR, RT (min): 14.17, XCorr: 2.31
intensity
m/z
y8
y1
b5
a7
y6
y7
b7
a5
y5
b1
y3
a2
b2
a3
y3
b3
y4
b8
b4
0
10000
20000
30000
40000
50000
100
200
300
400
500
600
700
800
900

WP_019732306.1, Sequence: [R].NDGPAmSIVYYIPTDNDELLVSTMAAR.[G], RT (min): 97.03, XCorr: 1.42

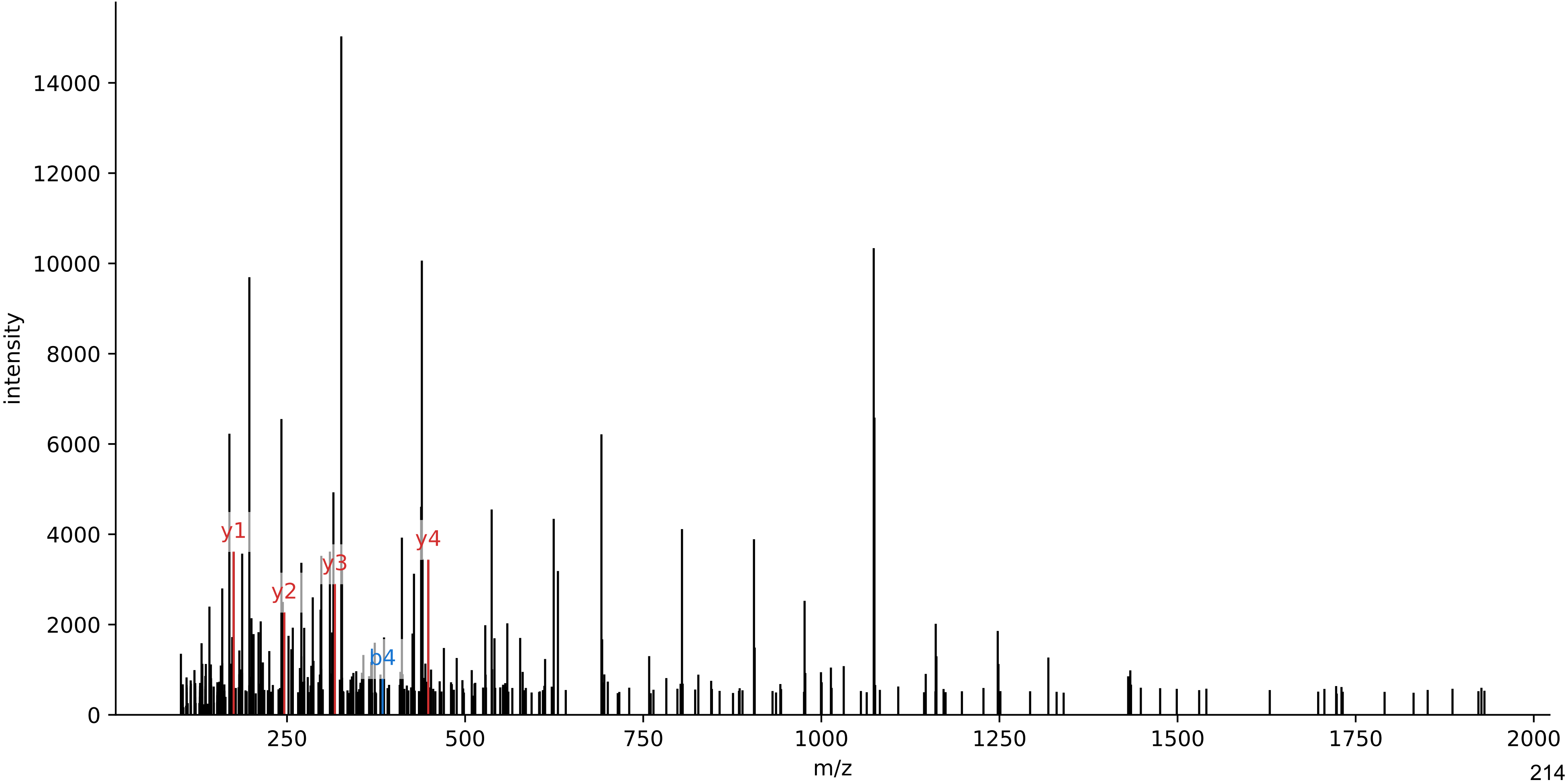

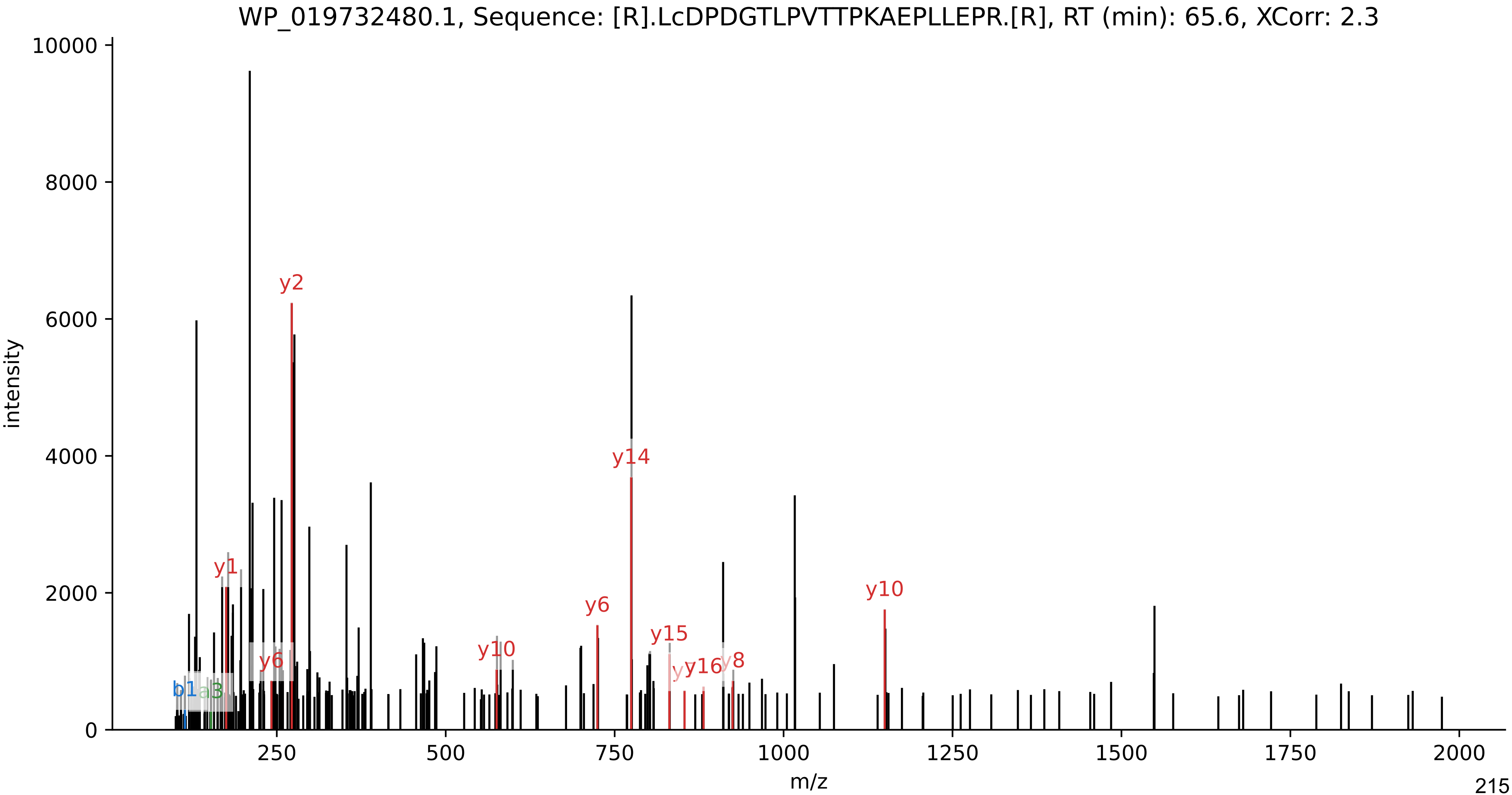
WP_019732480.1, Sequence: [R].LcDPDGTLPVTTPKAEPLLEPR.[R], RT (min): 65.6, XCorr: 2.3
intensity
m/z
0
2000
4000
6000
8000
10000
250
500
750
1000
1250
1500
1750
2000
y2
y14
y1
y6
y10
y6
y15
y16
y8
y10
b1
a3

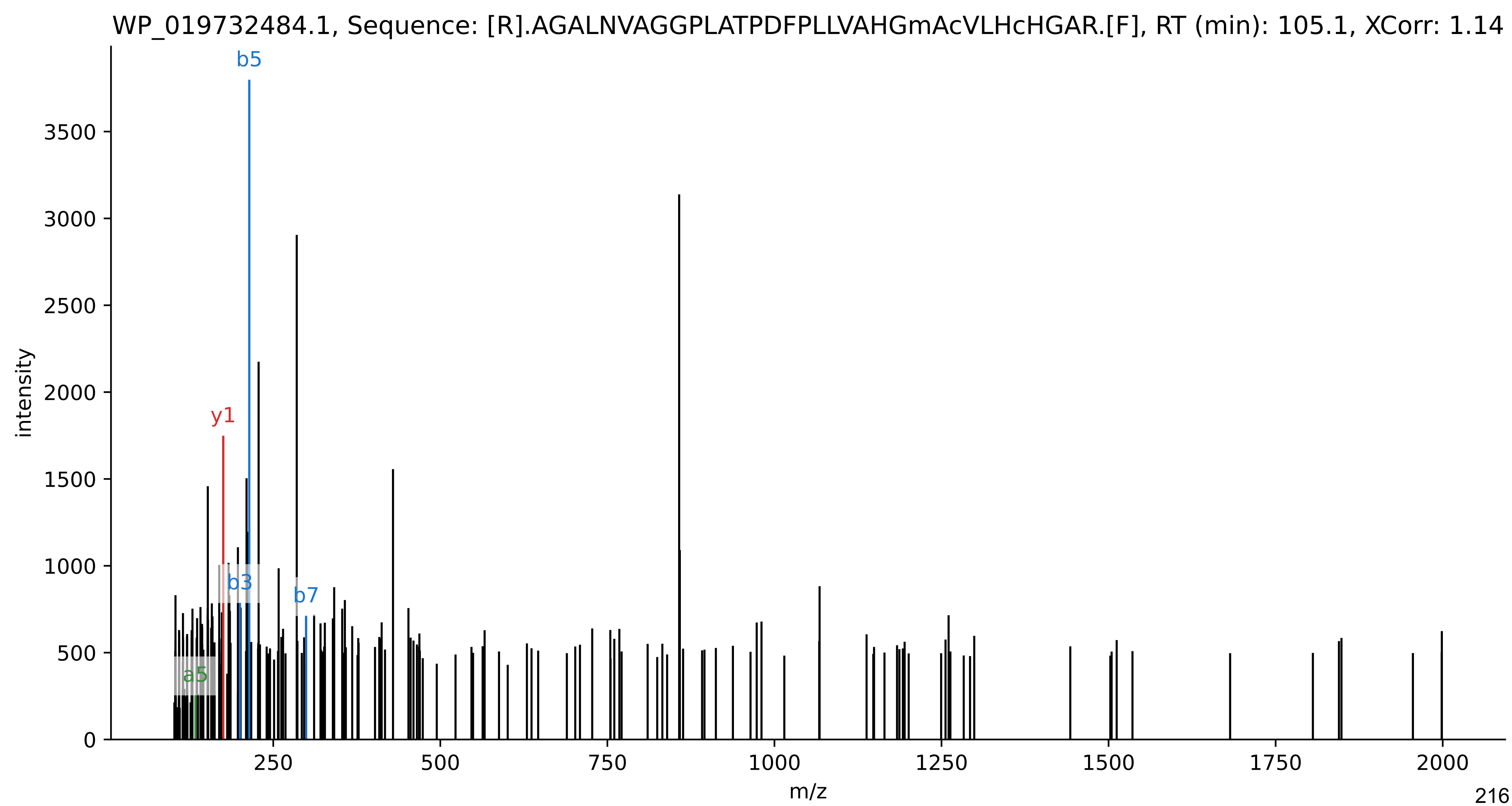

WP_019732484.1, Sequence: [R].AGALNVAGGPLATPDFPLLVAHGmAcVLHcHGAR.[F], RT (min): 105.1, XCorr: 1.14
b5
y1
b3
b7
a5
intensity
m/z
0
500
1000
1500
2000
2500
3000
3500
250
500
750
1000
1250
1500
1750
2000

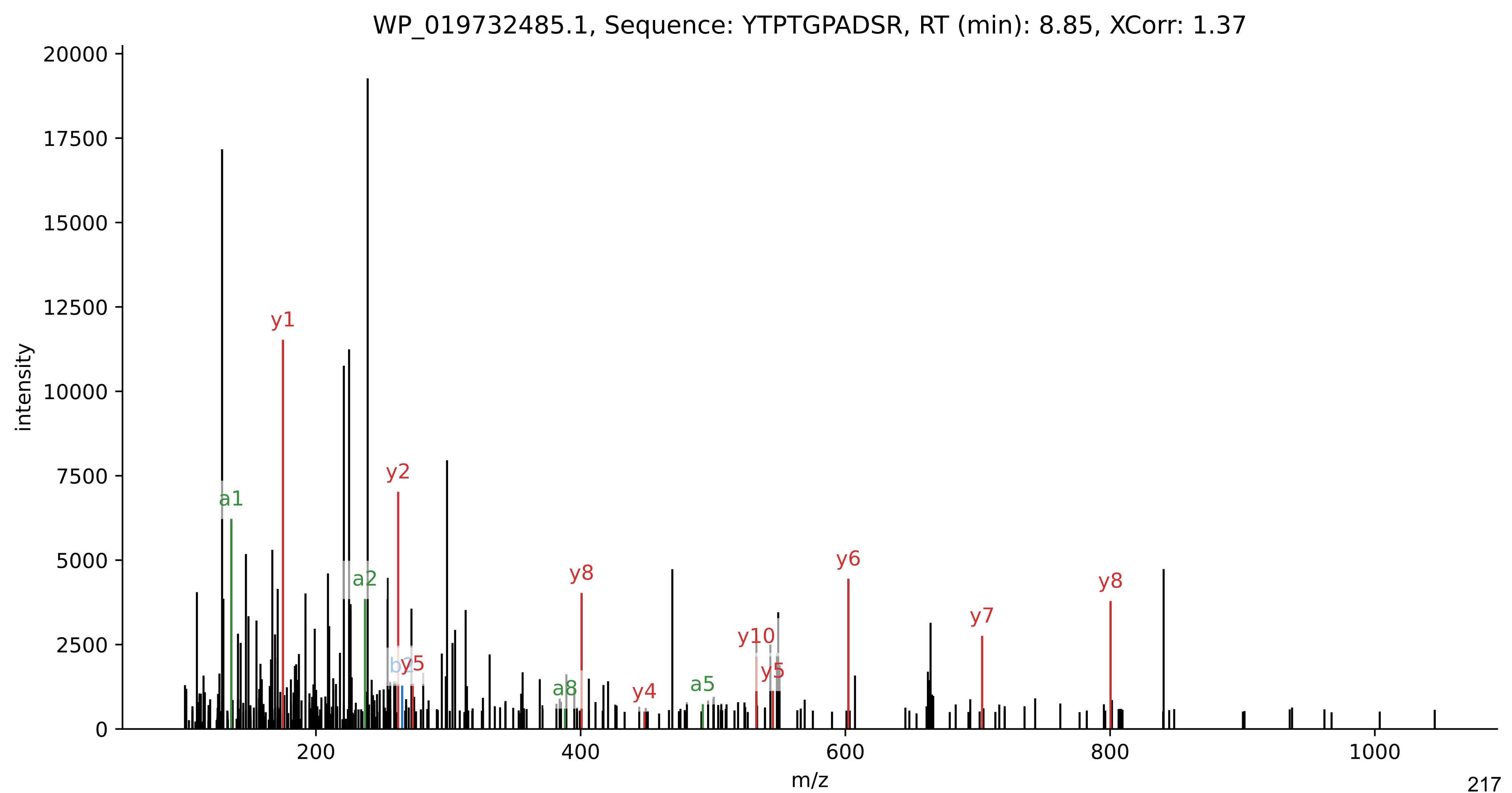
WP_019732485.1, Sequence: YTPTGPADSR, RT (min): 8.85, XCorr: 1.37
intensity
m/z
a1
y1
y2
a2
b2
y5
a8
y8
y4
a5
y10
y5
y6
y7
y8

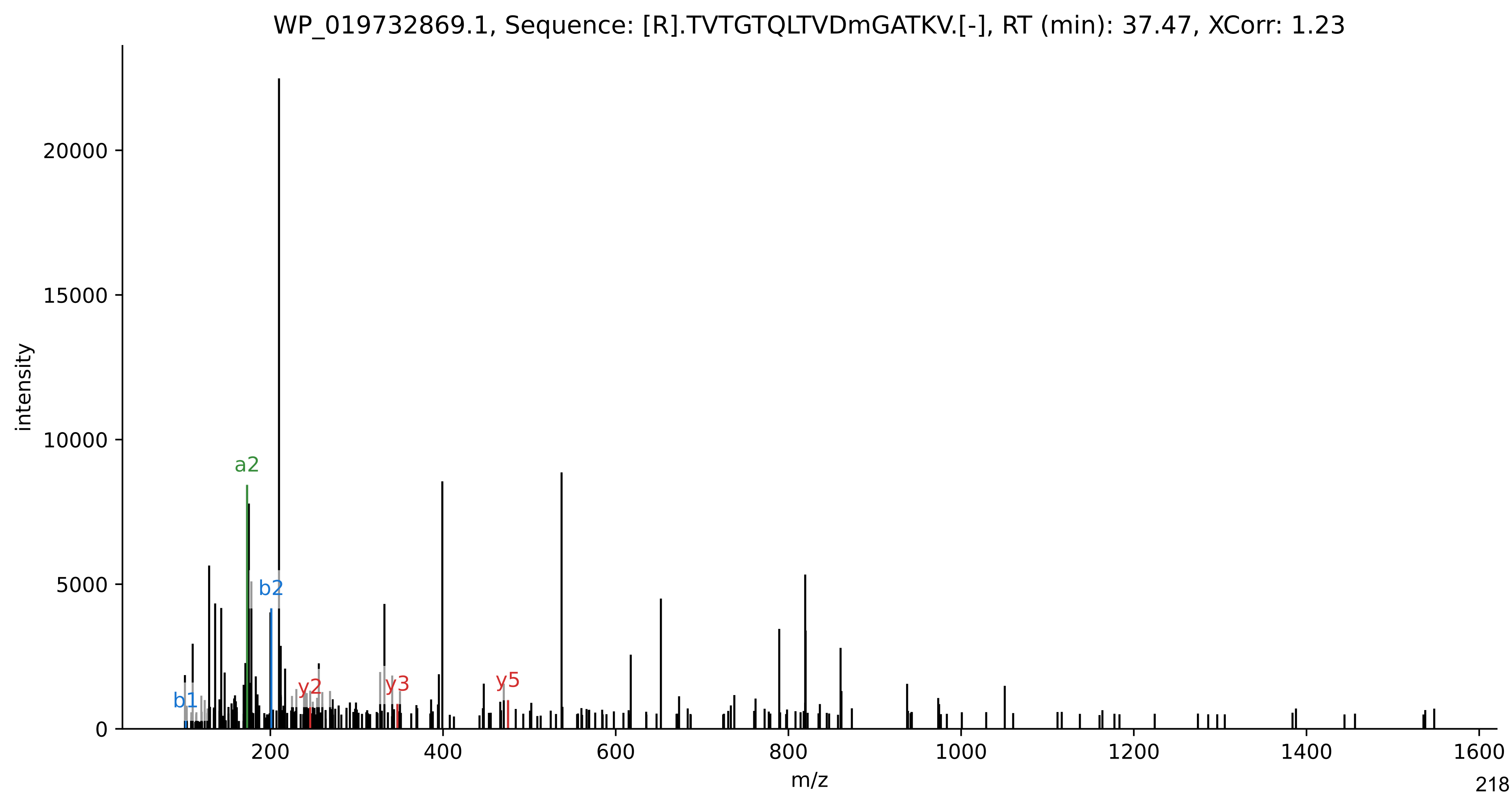
WP_019732869.1, Sequence: [R].TVTGTQLTVDmGATKV.[-], RT (min): 37.47, XCorr: 1.23
intensity
m/z
a2
b1
b2
y2
y3
y5
0
5000
10000
15000
20000
200
400
600
800
1000
1200
1400
1600

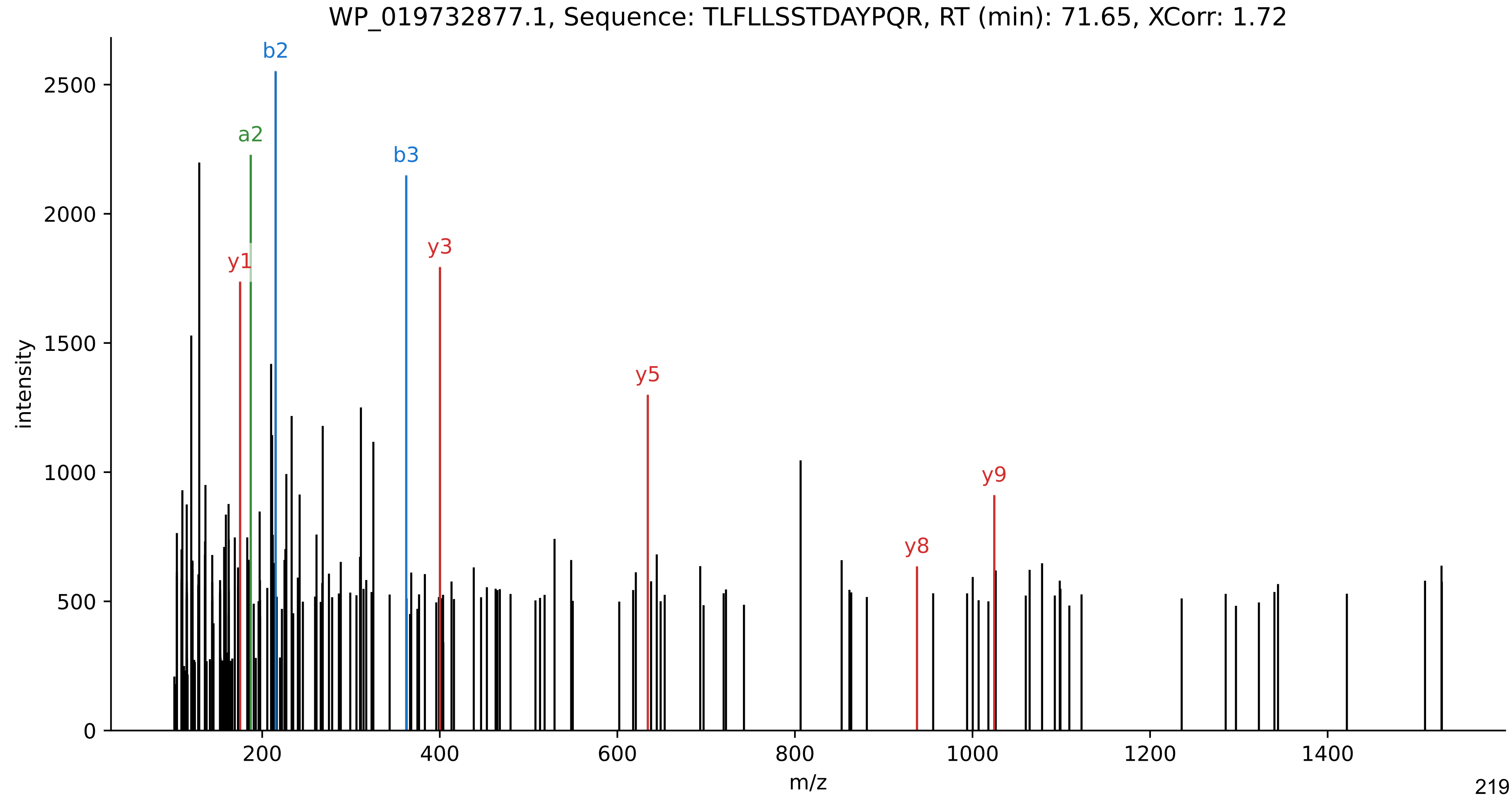
WP_019732877.1, Sequence: TLFLLSSTDAYPQR, RT (min): 71.65, XCorr: 1.72
intensity
m/z
b2
a2
b3
y1
y3
y5
y9
y8
0
500
1000
1500
2000
2500
200
400
600
800
1000
1200
1400

WP_019732919.1, Sequence: ILSESSPTHIDVEELR, RT (min): 45.32, XCorr: 3.78

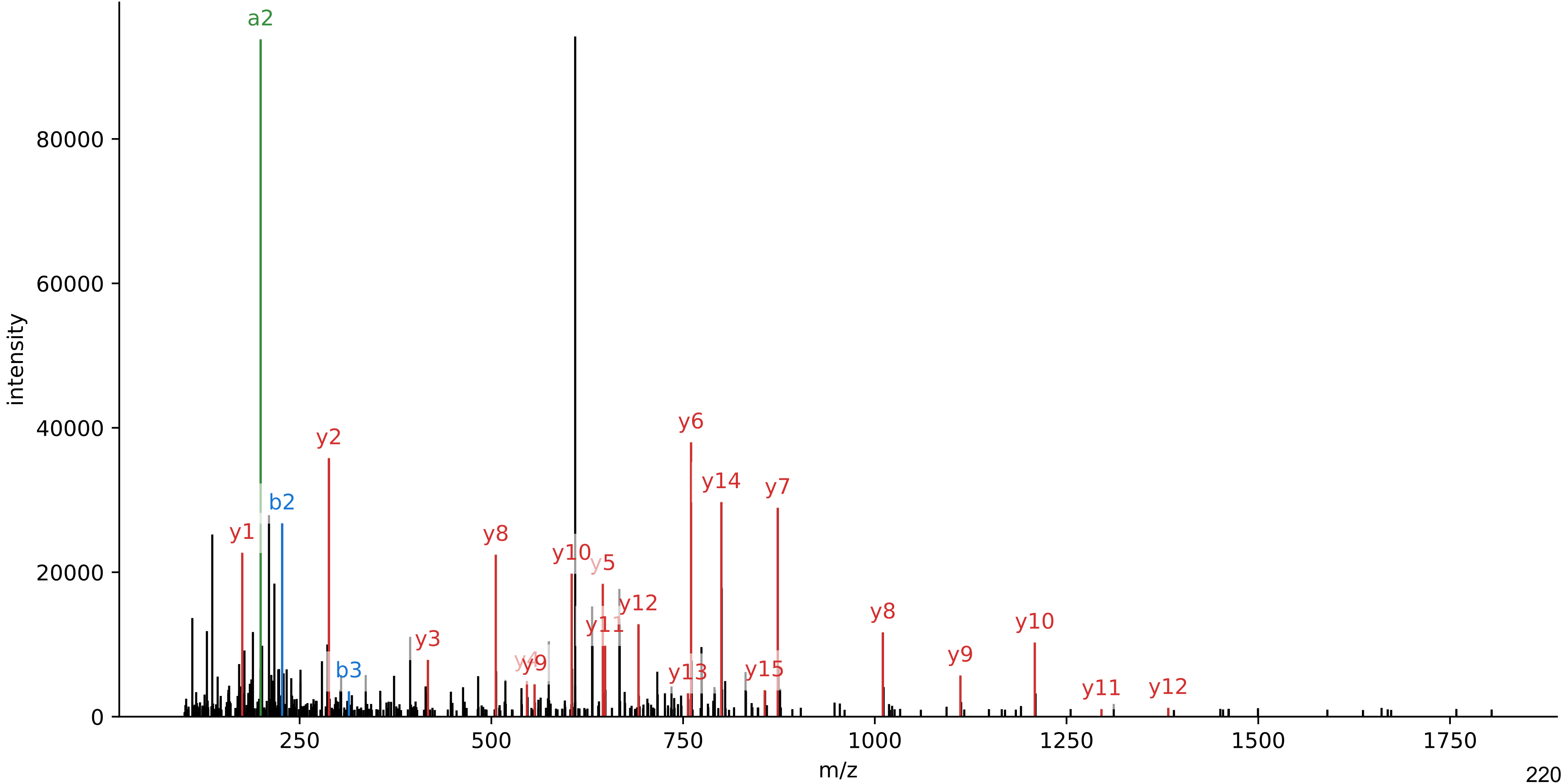

WP_019732929.1, Sequence: WLDANDAPGSGEETR, RT (min): 37.06, XCorr: 1.38

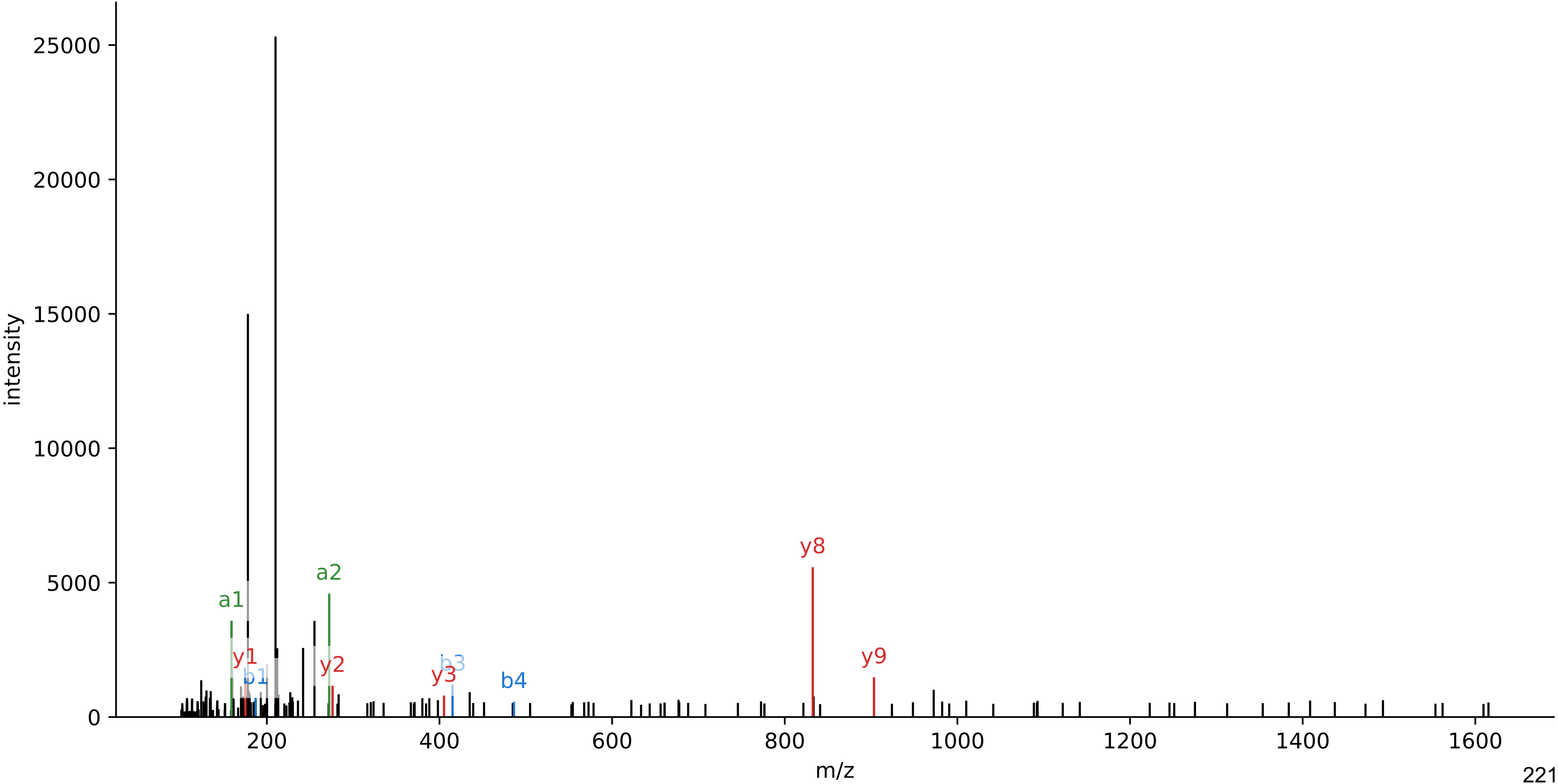

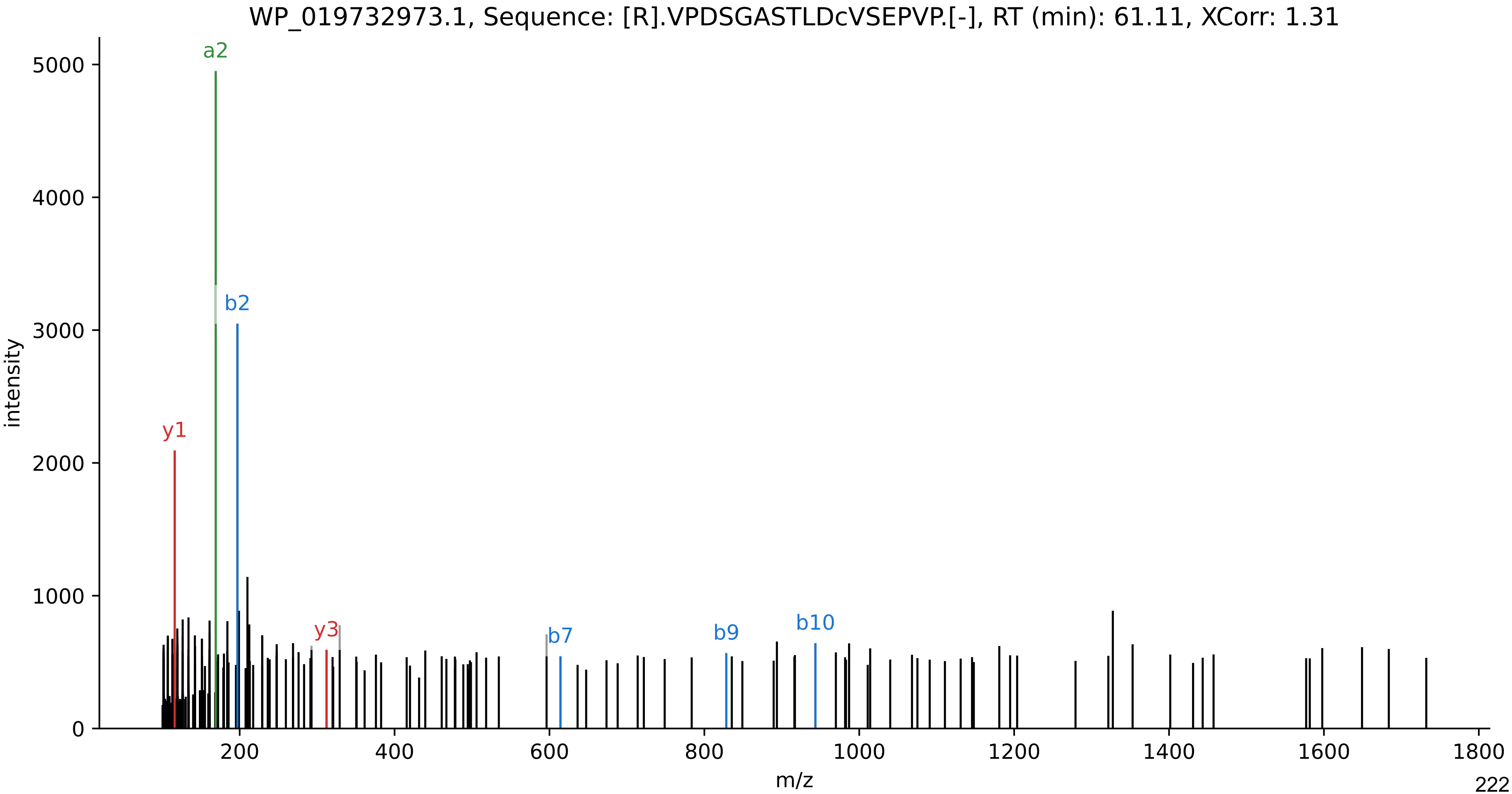

WP_019732973.1, Sequence: [R].VPDSGASTLDcVSEPVP.[-], RT (min): 61.11, XCorr: 1.31
a2
b2
y1
y3
b7
b9
b10
intensity
m/z
0
1000
2000
3000
4000
5000
200
400
600
800
1000
1200
1400
1600
1800

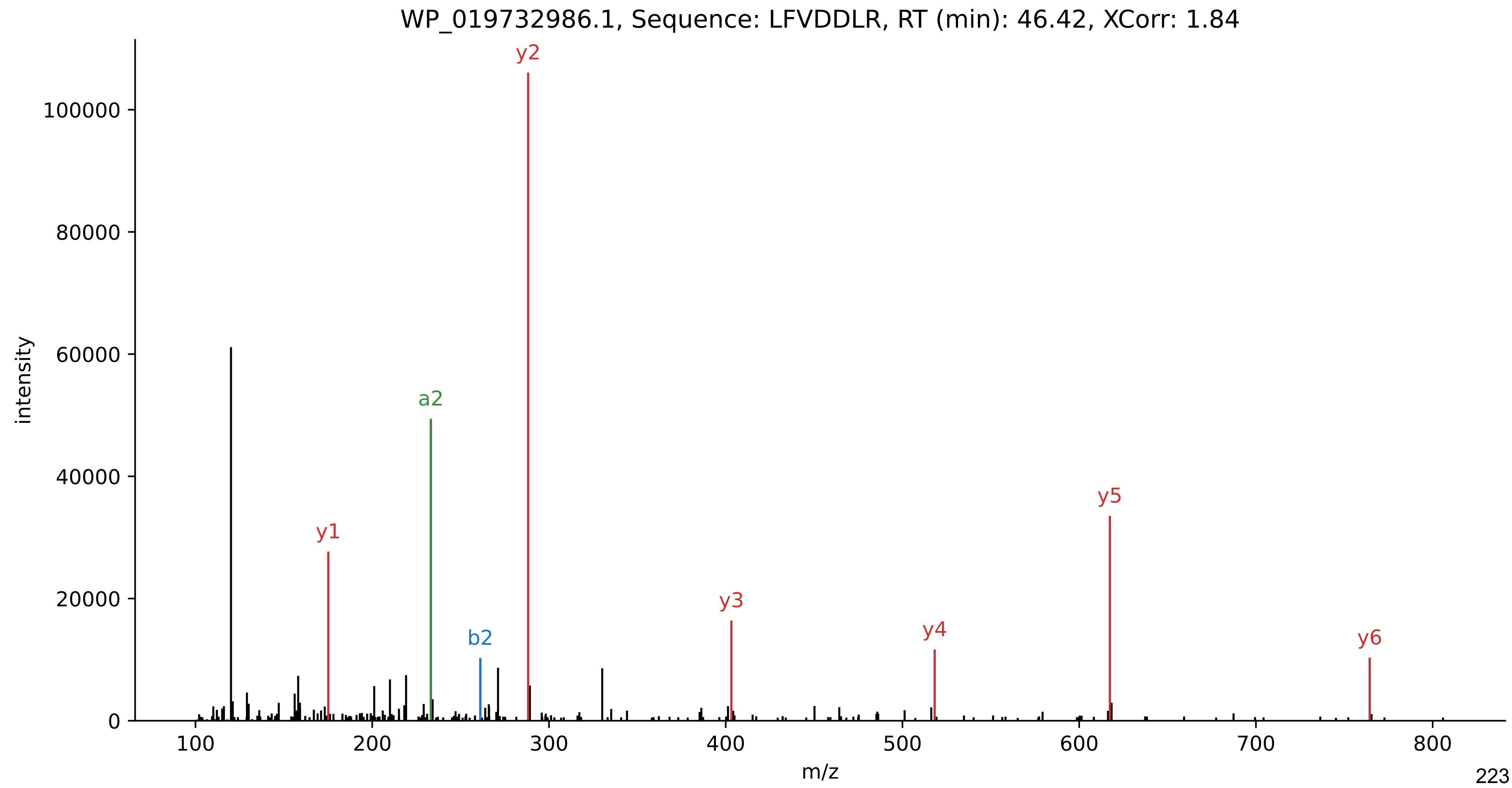
WP_019732986.1, Sequence: LFVDDLR, RT (min): 46.42, XCorr: 1.84
intensity
m/z
0
20000
40000
60000
80000
100000
100
200
300
400
500
600
700
800
y1
a2
b2
y2
y3
y4
y5
y6

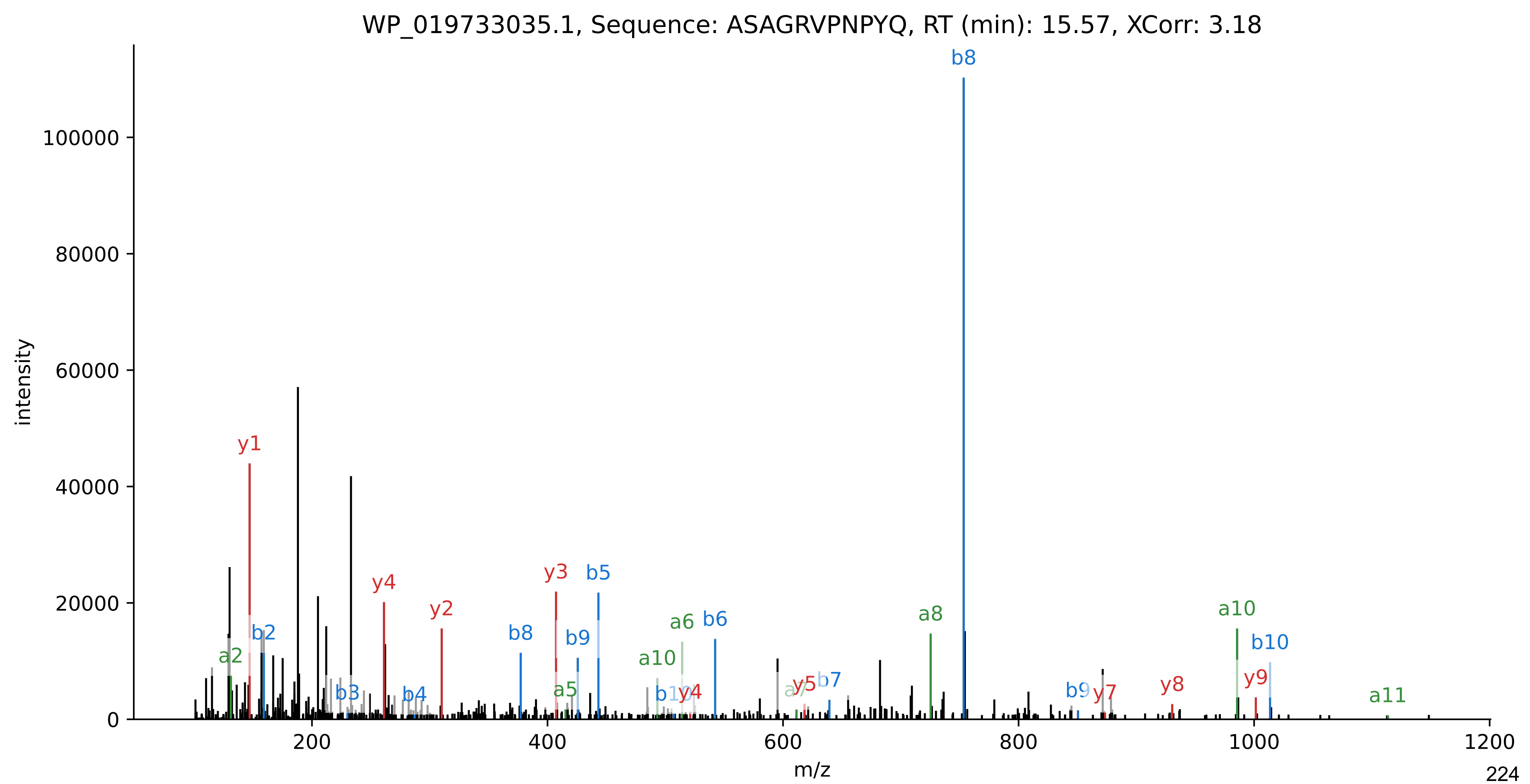

WP_019733035.1, Sequence: ASAGRVPNPYQ, RT (min): 15.57, XCorr: 3.18
intensity
m/z
b8
y1
y4
y2
y3
b5
b2
a2
b8
b9
a6
b6
a10
a5
b3
b4
y4
y5
b7
a8
a10
b10
y9
b9
y7
y8
a11

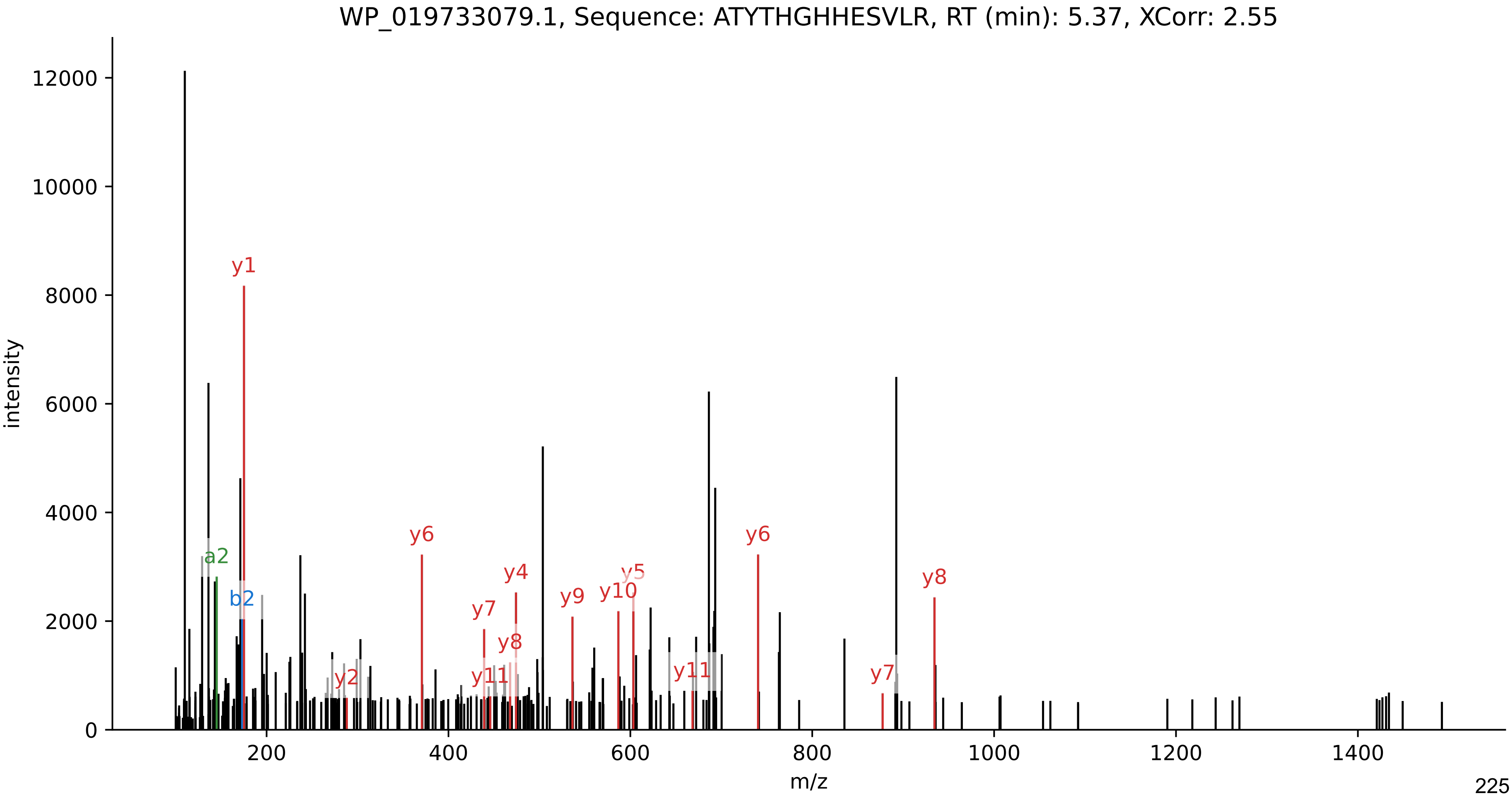
WP_019733079.1, Sequence: ATYTHGHHESVLR, RT (min): 5.37, XCorr: 2.55
intensity
m/z
y1
y6
a2
b2
y2
y7
y4
y8
y11
y9
y10
y5
y11
y6
y7
y8
0
2000
4000
6000
8000
10000
12000
200
400
600
800
1000
1200
1400

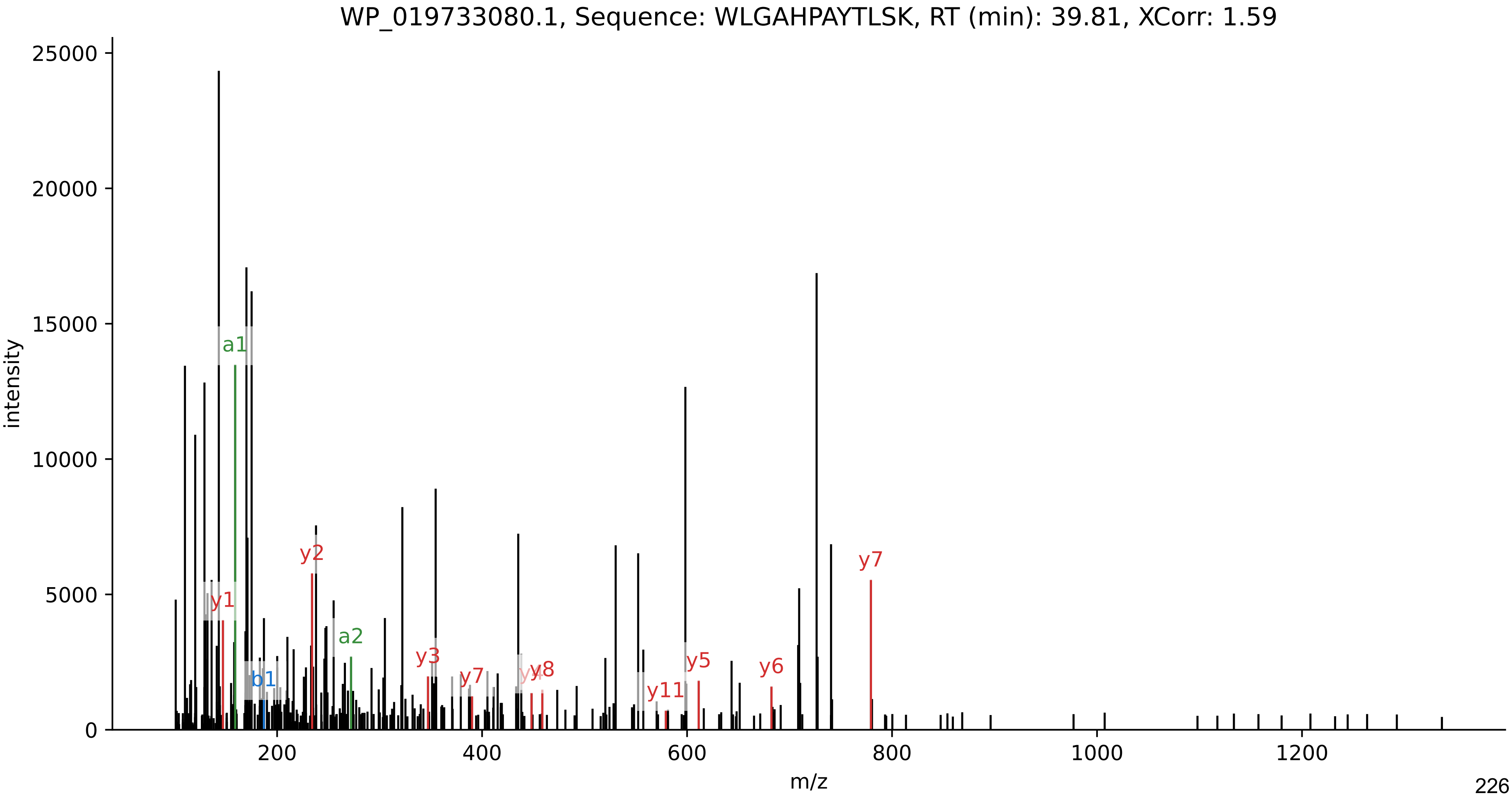

WP_019733080.1, Sequence: WLGAHPAYTLSK, RT (min): 39.81, XCorr: 1.59
intensity
m/z
a1
y1
b1
y2
a2
y3
y7
y8
y11
y5
y6
y7

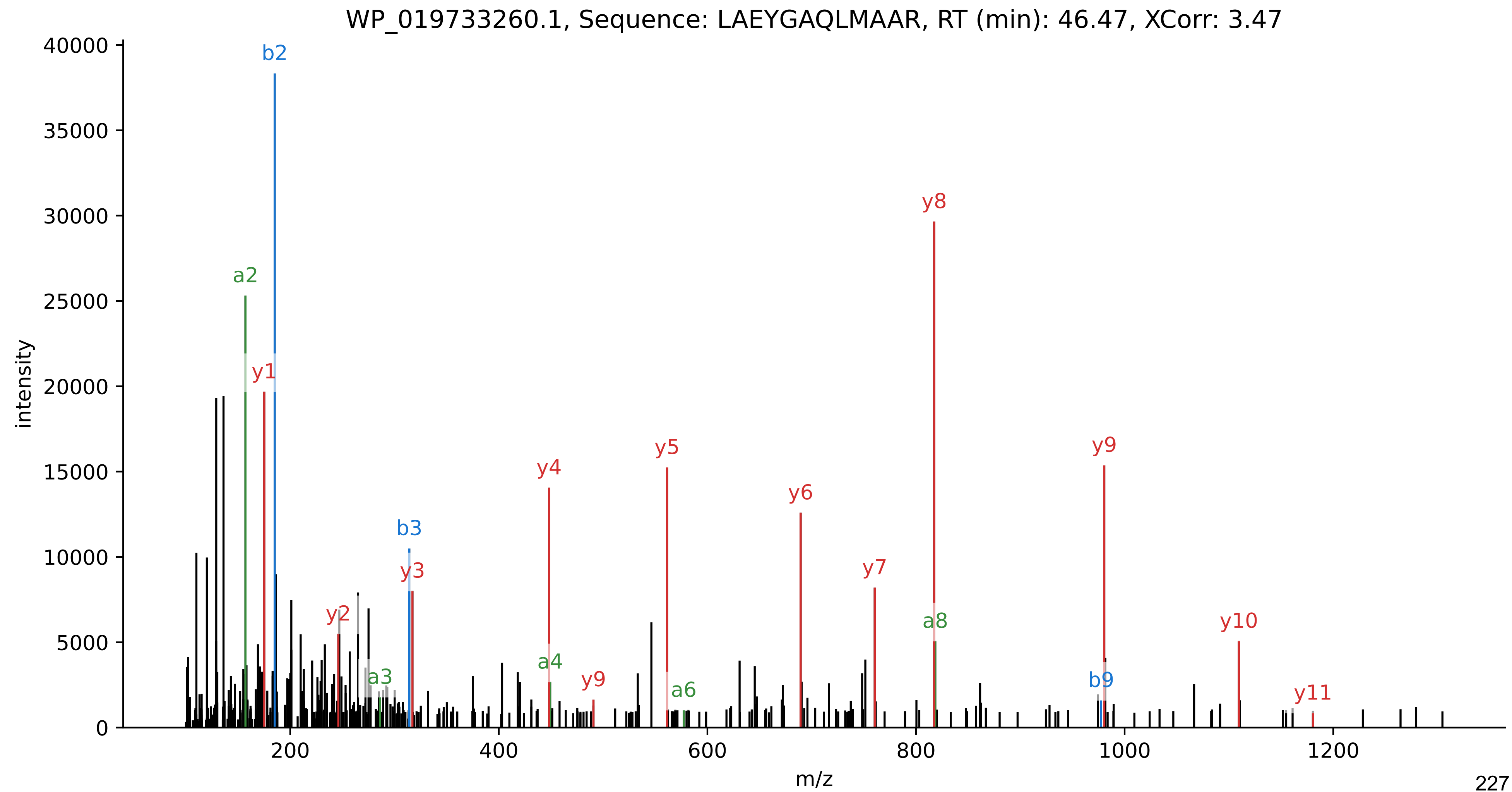

WP_019733260.1, Sequence: LAEYGAQLMAAR, RT (min): 46.47, XCorr: 3.47
intensity
m/z
b2
a2
y1
b3
y3
y2
a3
y4
a4
y9
y5
a6
y6
y7
y8
a8
y9
b9
y10
y11

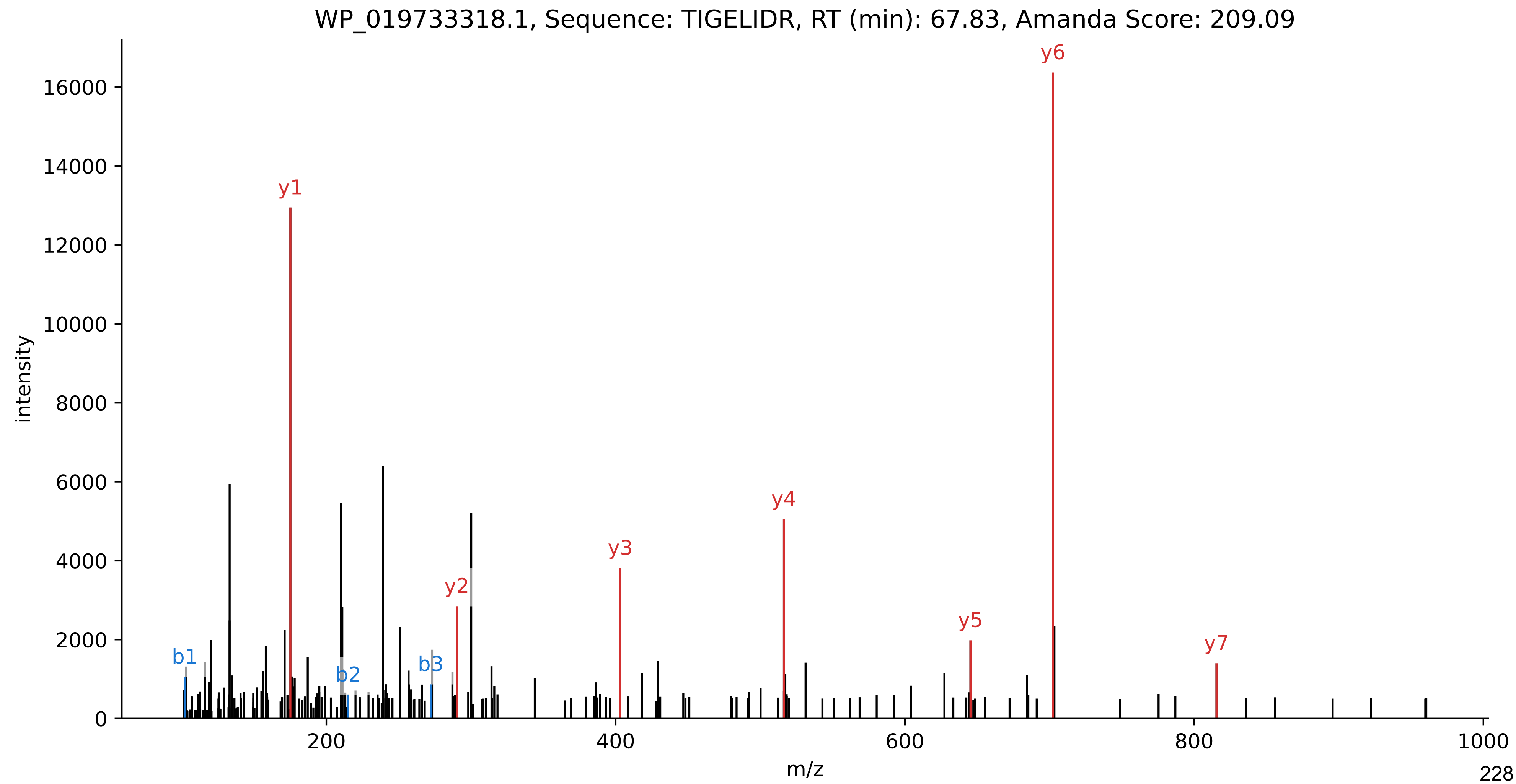
WP_019733318.1, Sequence: TIGELIDR, RT (min): 67.83, Amanda Score: 209.09
intensity
m/z
y1
y2
y3
y4
y5
y6
y7
b1
b2
b3
0
2000
4000
6000
8000
10000
12000
14000
16000
200
400
600
800
1000

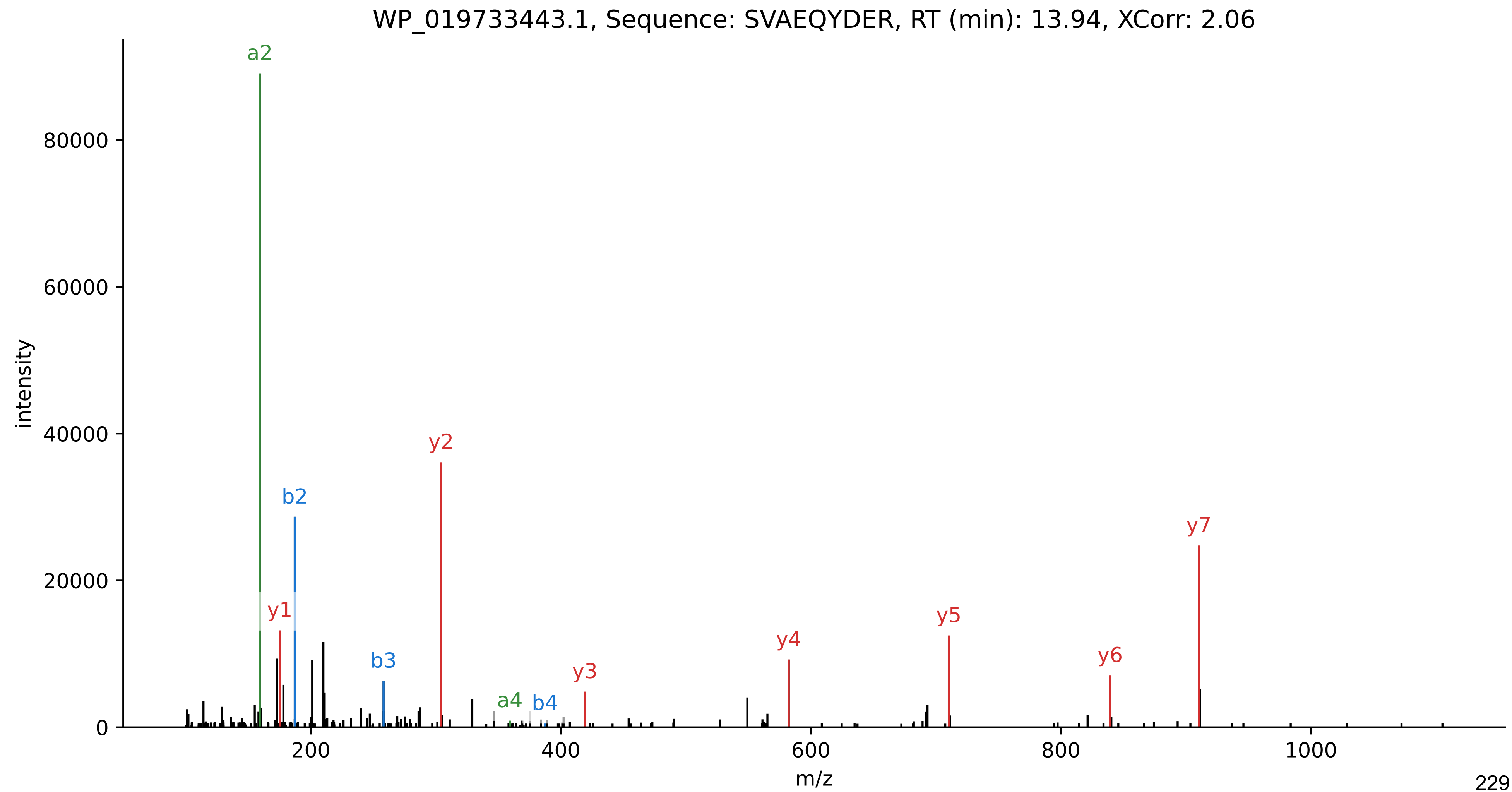
WP_019733443.1, Sequence: SVAEQYDER, RT (min): 13.94, XCorr: 2.06
a2
b2
y1
y2
b3
a4
b4
y3
y4
y5
y6
y7
intensity
0
20000
40000
60000
80000
200
400
600
800
1000
m/z

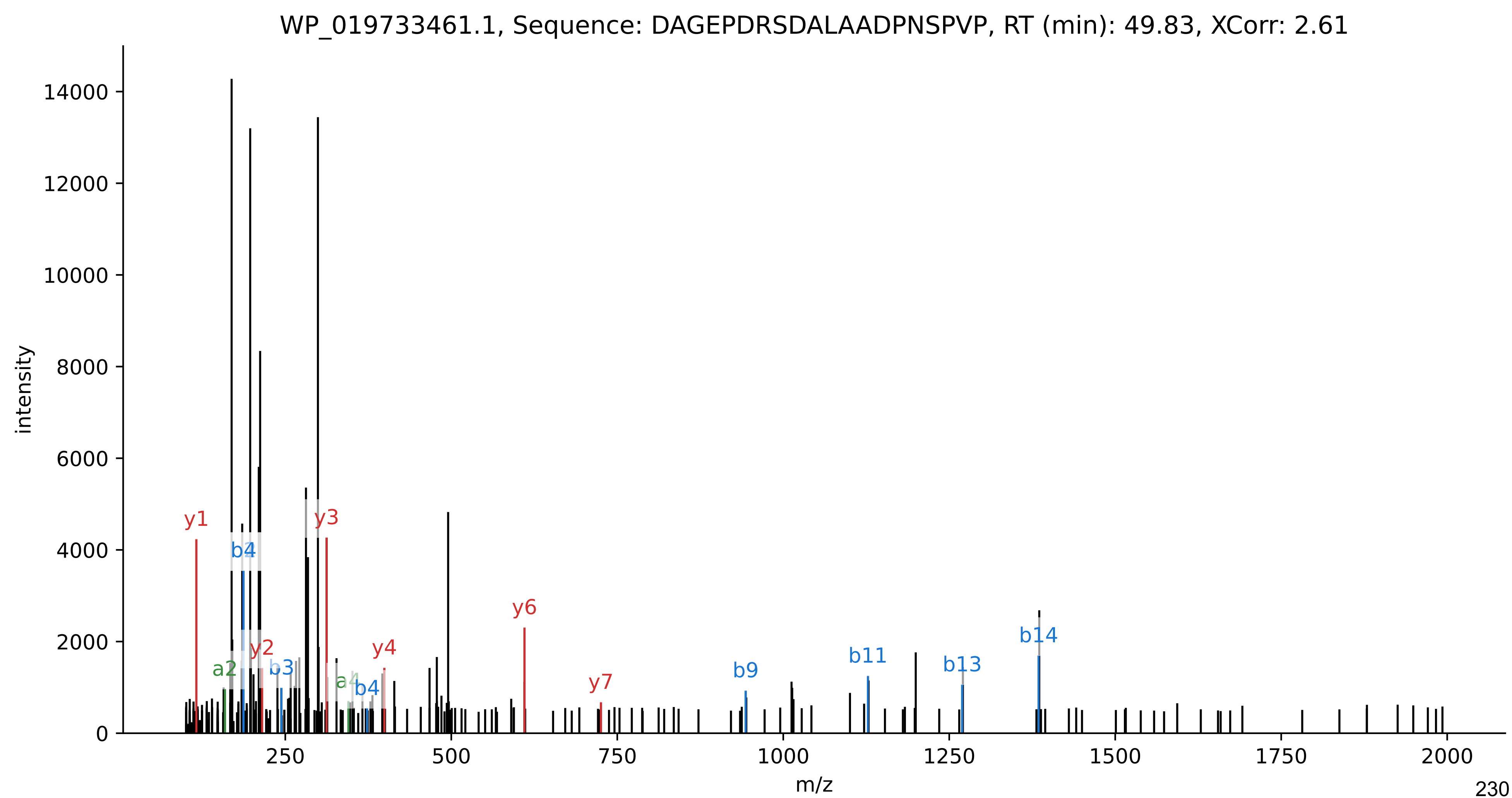
WP_019733461.1, Sequence: DAGEPDRSDALAADPNSPVP, RT (min): 49.83, XCorr: 2.61
intensity
m/z
0
2000
4000
6000
8000
10000
12000
14000
250
500
750
1000
1250
1500
1750
2000
y1
b4
y3
a2
y2
b3
a4
b4
y4
y6
y7
b9
b11
b13
b14

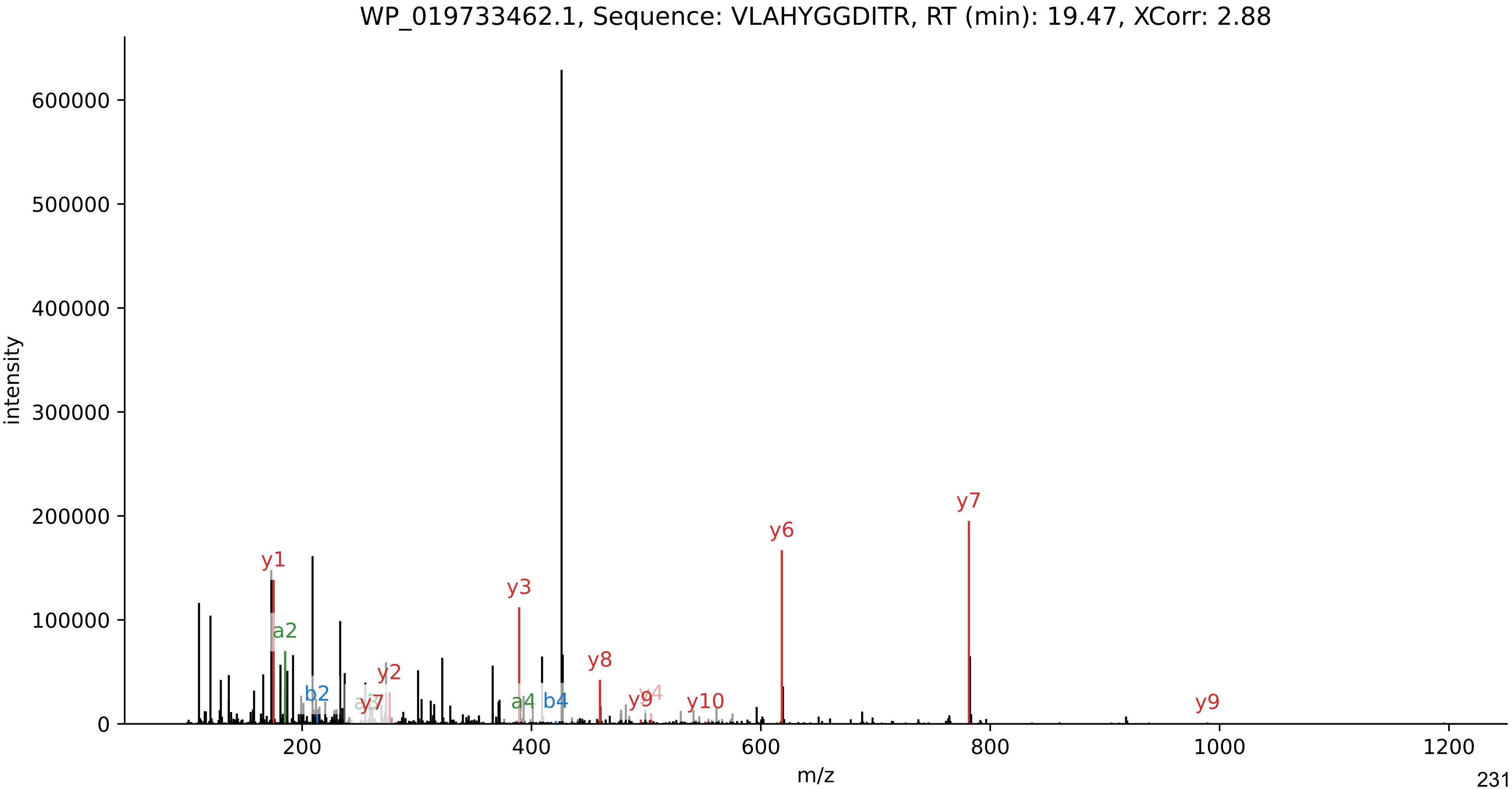

WP_019733462.1, Sequence: VLAHYGGDITR, RT (min): 19.47, XCorr: 2.88
intensity
m/z
y1
a2
b2
y2
y3
a4
b4
y8
y9
y10
y6
y7
y9

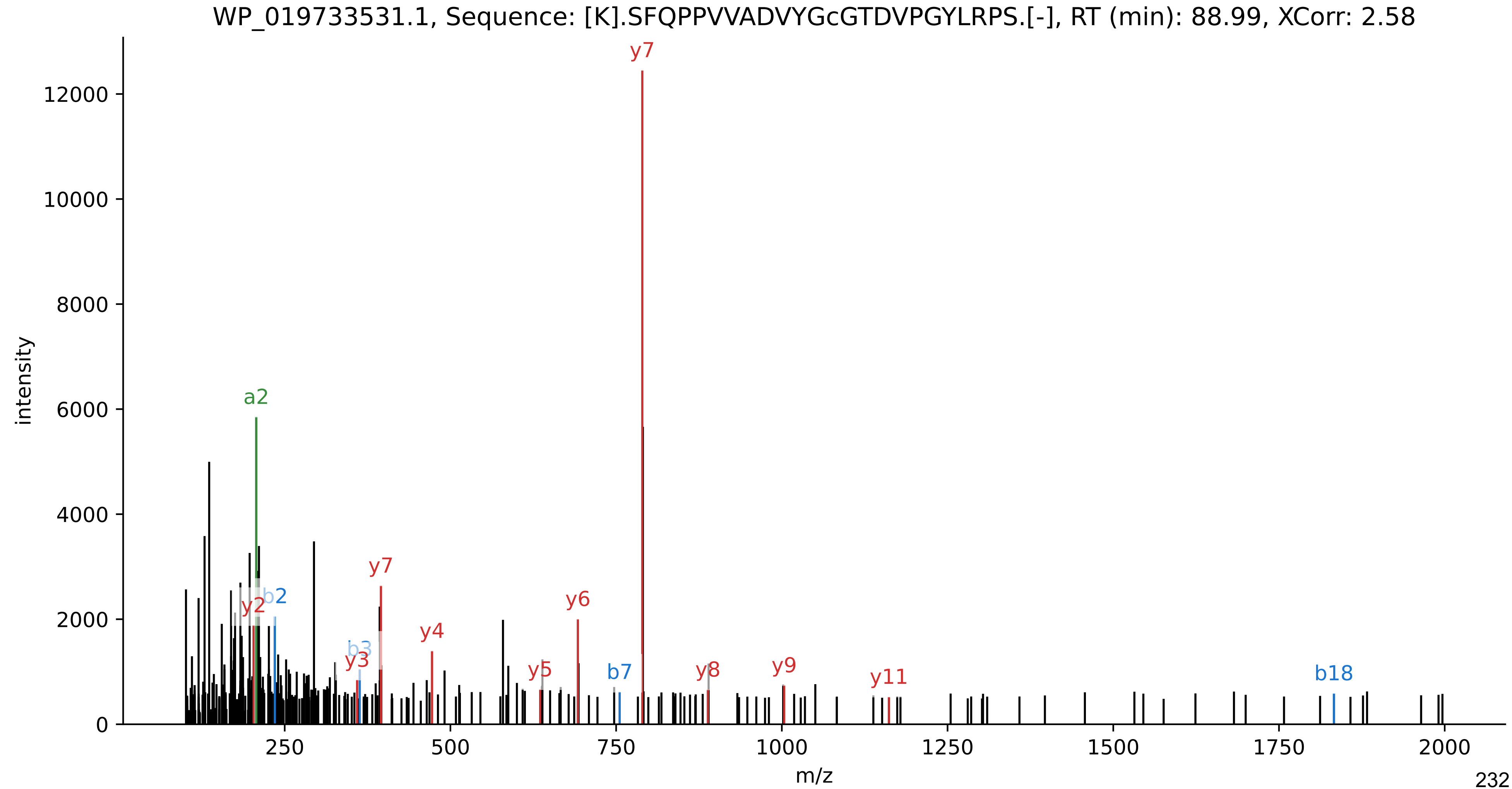
WP_019733531.1, Sequence: [K].SFQPPVVADVYGcGTDVPGYLRPS.[-], RT (min): 88.99, XCorr: 2.58
intensity
m/z
y7
a2
y2
b2
b3
y3
y7
y4
y6
y5
b7
y8
y9
y11
b18
0
2000
4000
6000
8000
10000
12000
250
500
750
1000
1250
1500
1750
2000

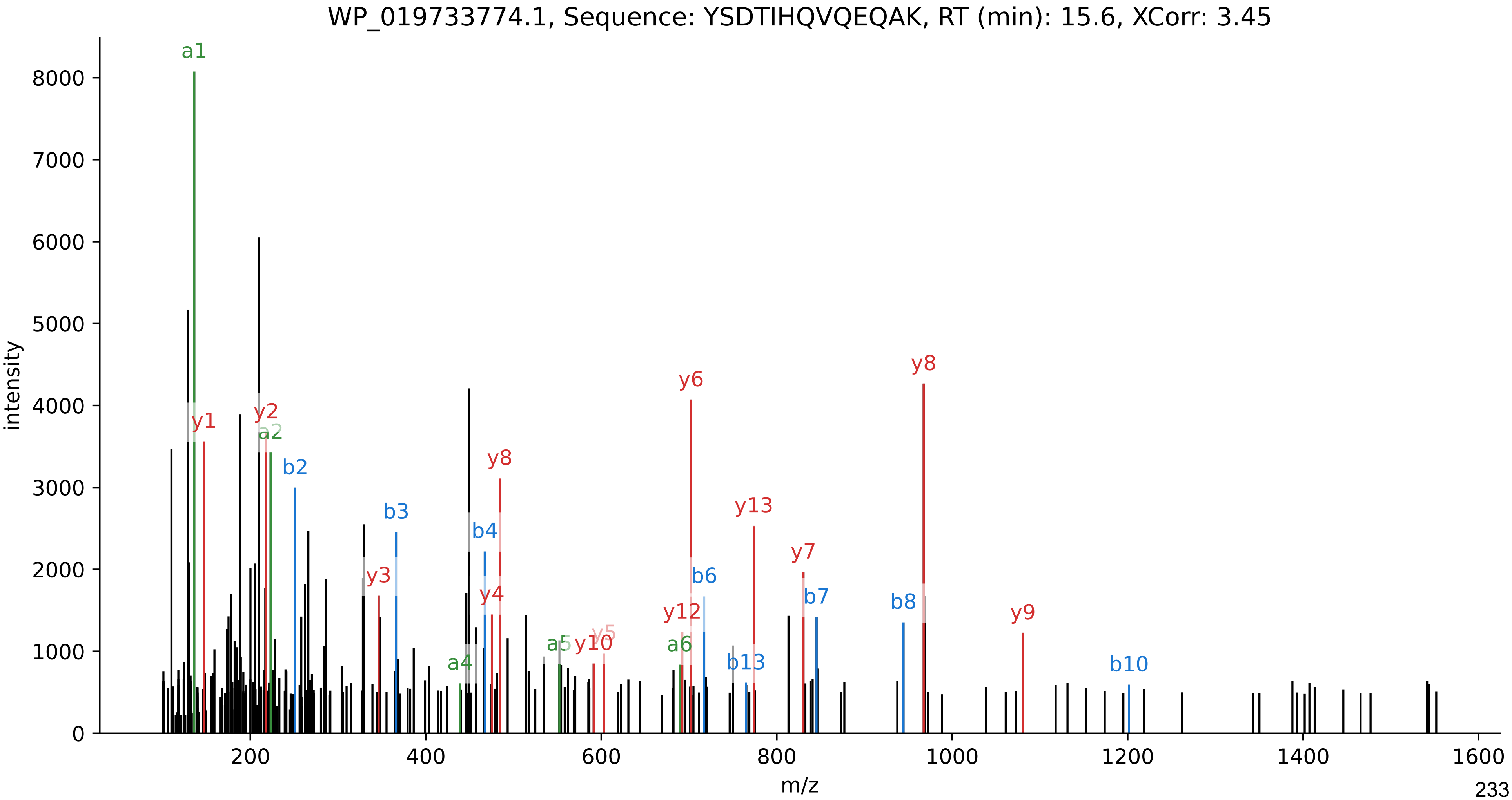
WP_019733774.1, Sequence: YSDTIHQVQEQAK, RT (min): 15.6, XCorr: 3.45
intensity
m/z
a1
y1
y2
a2
b2
b3
y3
b4
y4
y8
a4
a5
y10
y5
y12
a6
y6
b6
b13
y13
y7
b7
b8
y8
y9
b10

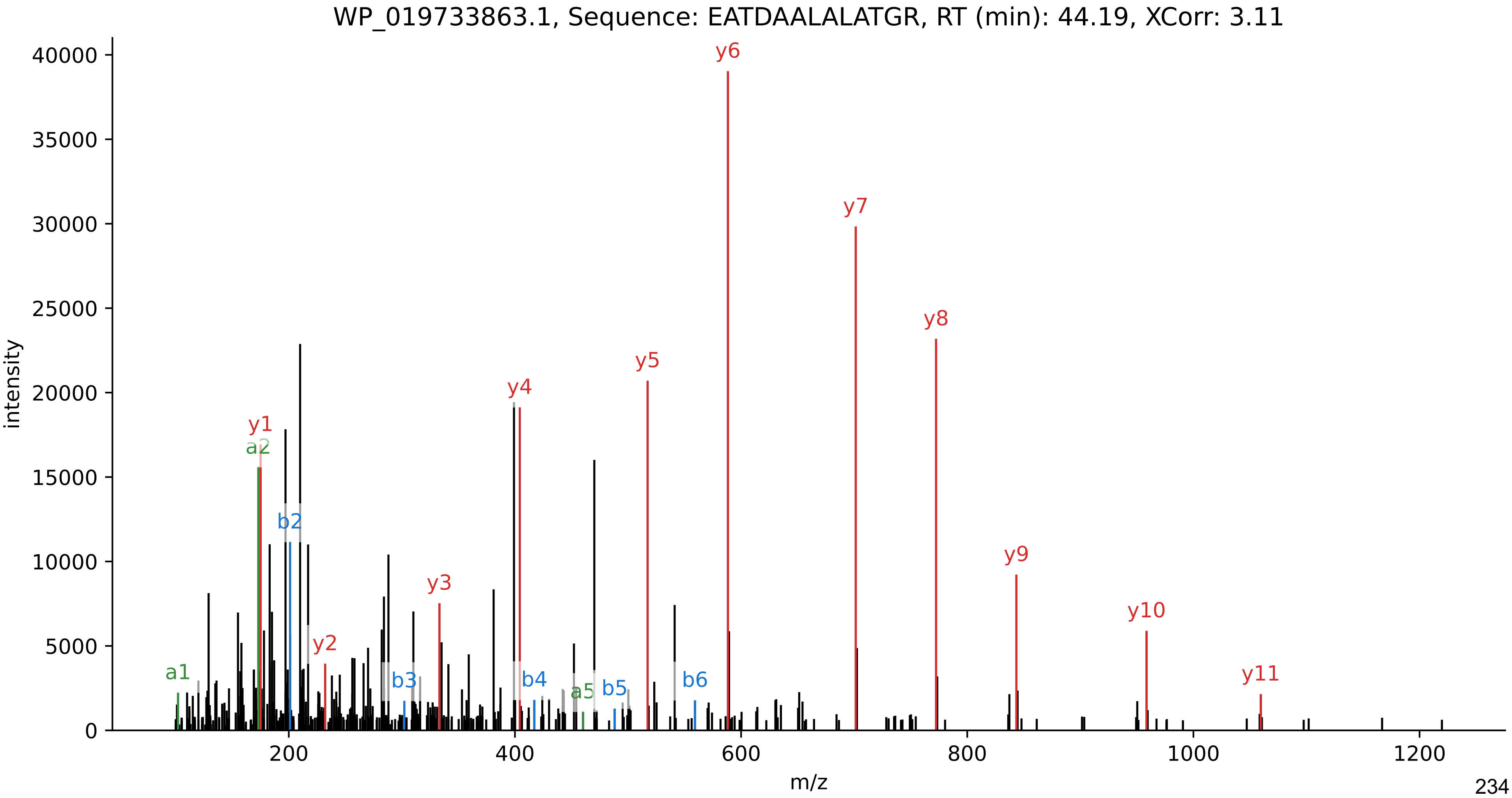
WP_019733863.1, Sequence: EATDAALALATGR, RT (min): 44.19, XCorr: 3.11
intensity
m/z
y1
a2
b2
y2
a1
y3
b3
y4
b4
a5
b5
y5
b6
y6
y7
y8
y9
y10
y11
0
5000
10000
15000
20000
25000
30000
35000
40000
200
400
600
800
1000
1200

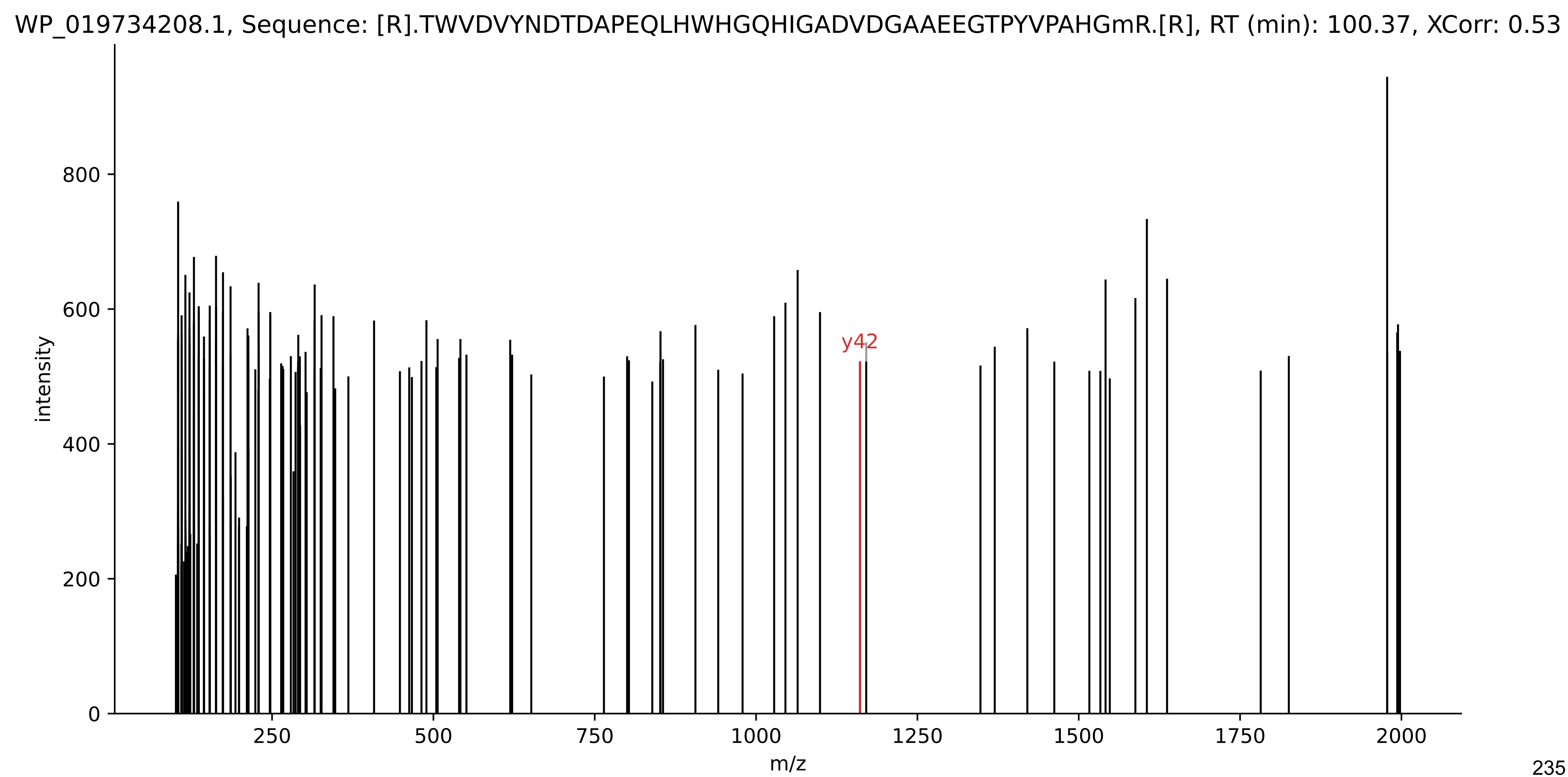
WP_019734208.1, Sequence: [R].TWVDVYNDTDAPEQLHWHGQHIGADVDGAAEEGTPYVPAHGmR.[R], RT (min): 100.37, XCorr: 0.53
intensity
m/z
y42
0
200
400
600
800
250
500
750
1000
1250
1500
1750
2000

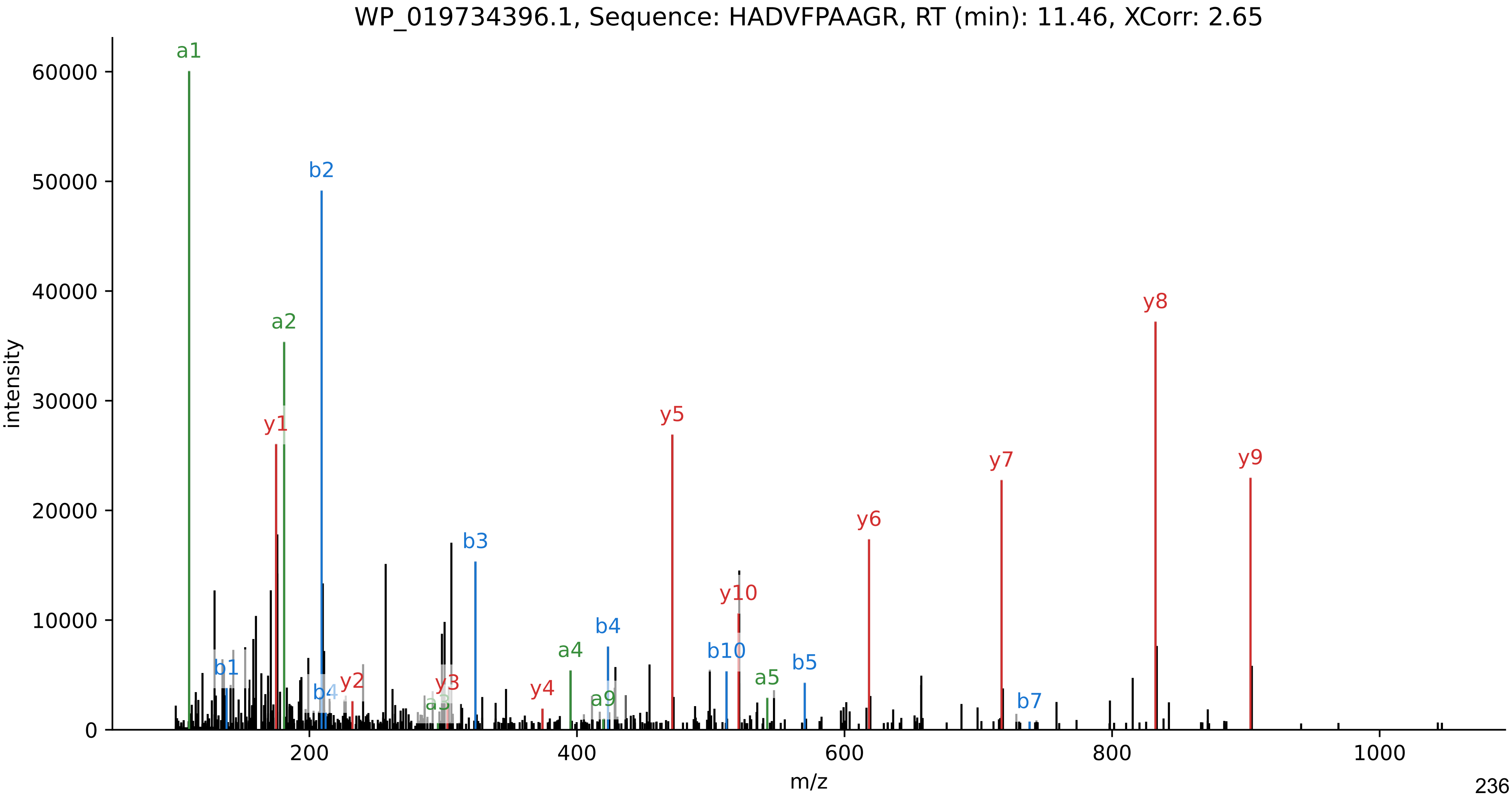
WP_019734396.1, Sequence: HADVFPAAGR, RT (min): 11.46, XCorr: 2.65
intensity
m/z
60000
50000
40000
30000
20000
10000
0
200
400
600
800
1000
a1
b2
a2
y1
y5
y8
y7
y9
y6
b3
y10
b4
a4
b10
b5
a5
b1
y2
y3
y4
a9
b7

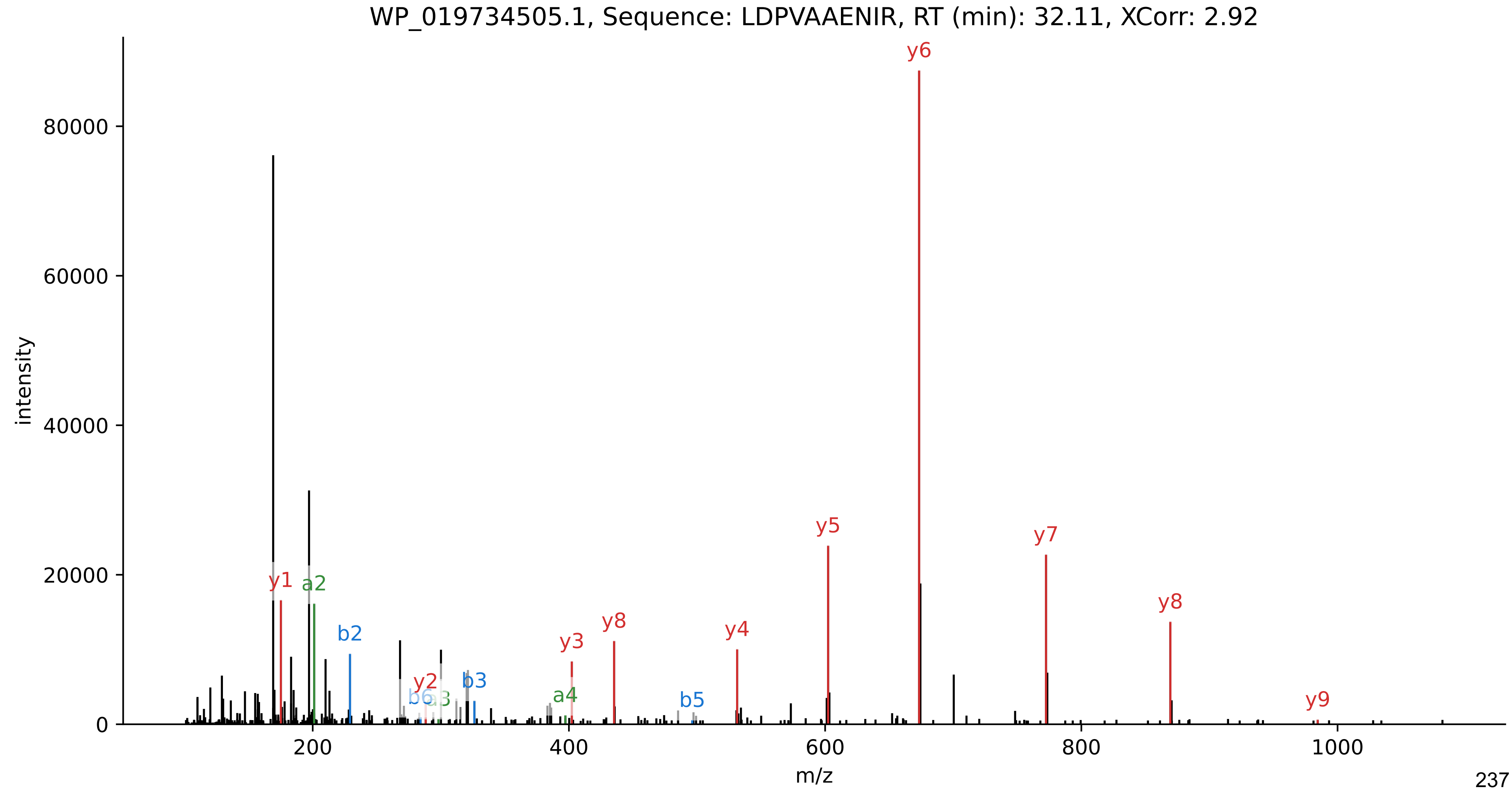
WP_019734505.1, Sequence: LDPVAAENIR, RT (min): 32.11, XCorr: 2.92
intensity
m/z
0
20000
40000
60000
80000
200
400
600
800
1000
y6
y5
y7
y8
y1
a2
b2
y8
y4
y3
y2
b3
b6
a3
a4
b5
y9

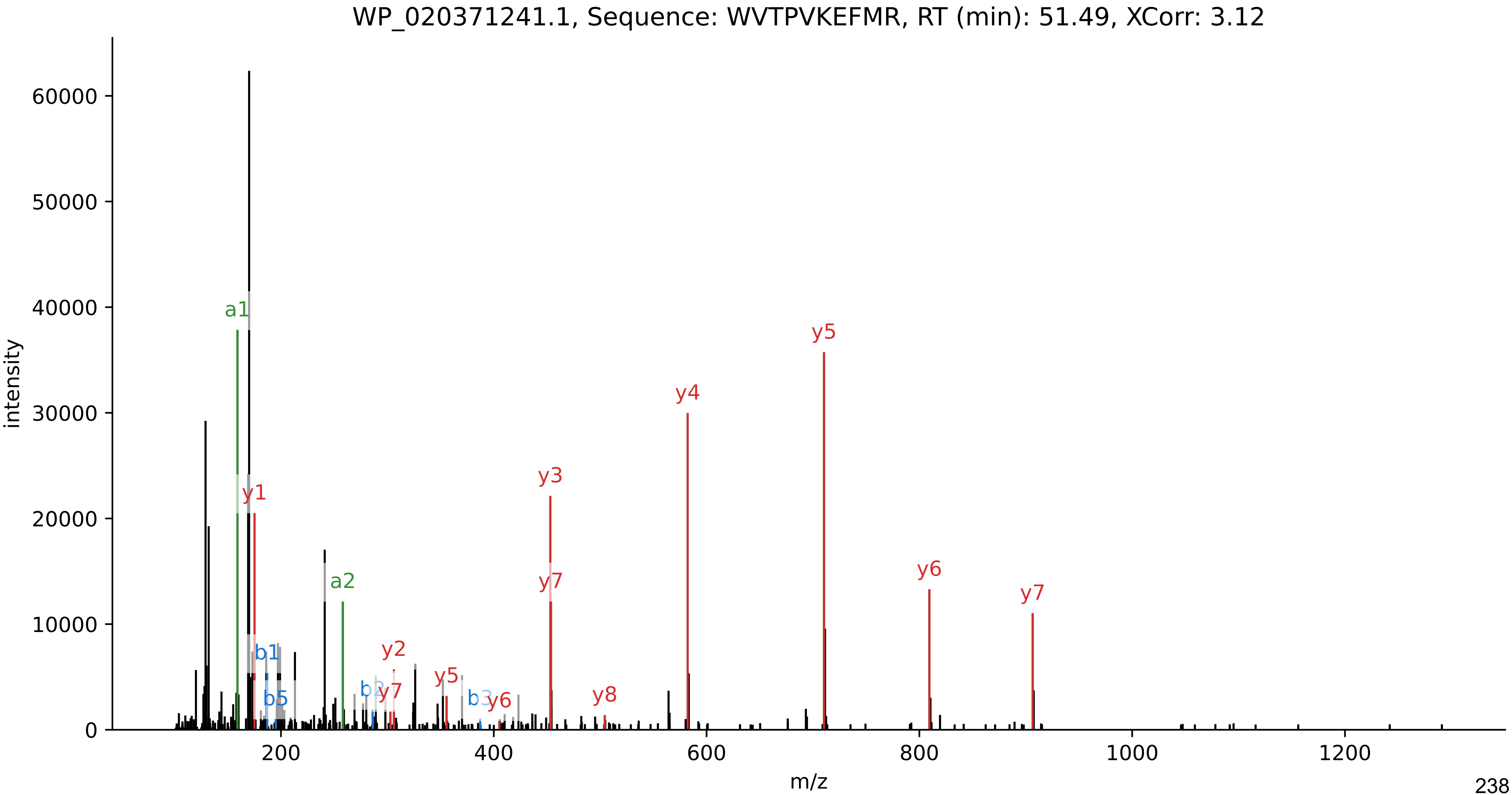
WP_020371241.1, Sequence: WVTPVKEFMR, RT (min): 51.49, XCorr: 3.12
intensity
m/z
a1
y1
a2
b1
b5
b2
y7
y2
y5
b3
y6
y3
y7
y8
y4
y5
y6
y7
0
10000
20000
30000
40000
50000
60000
200
400
600
800
1000
1200

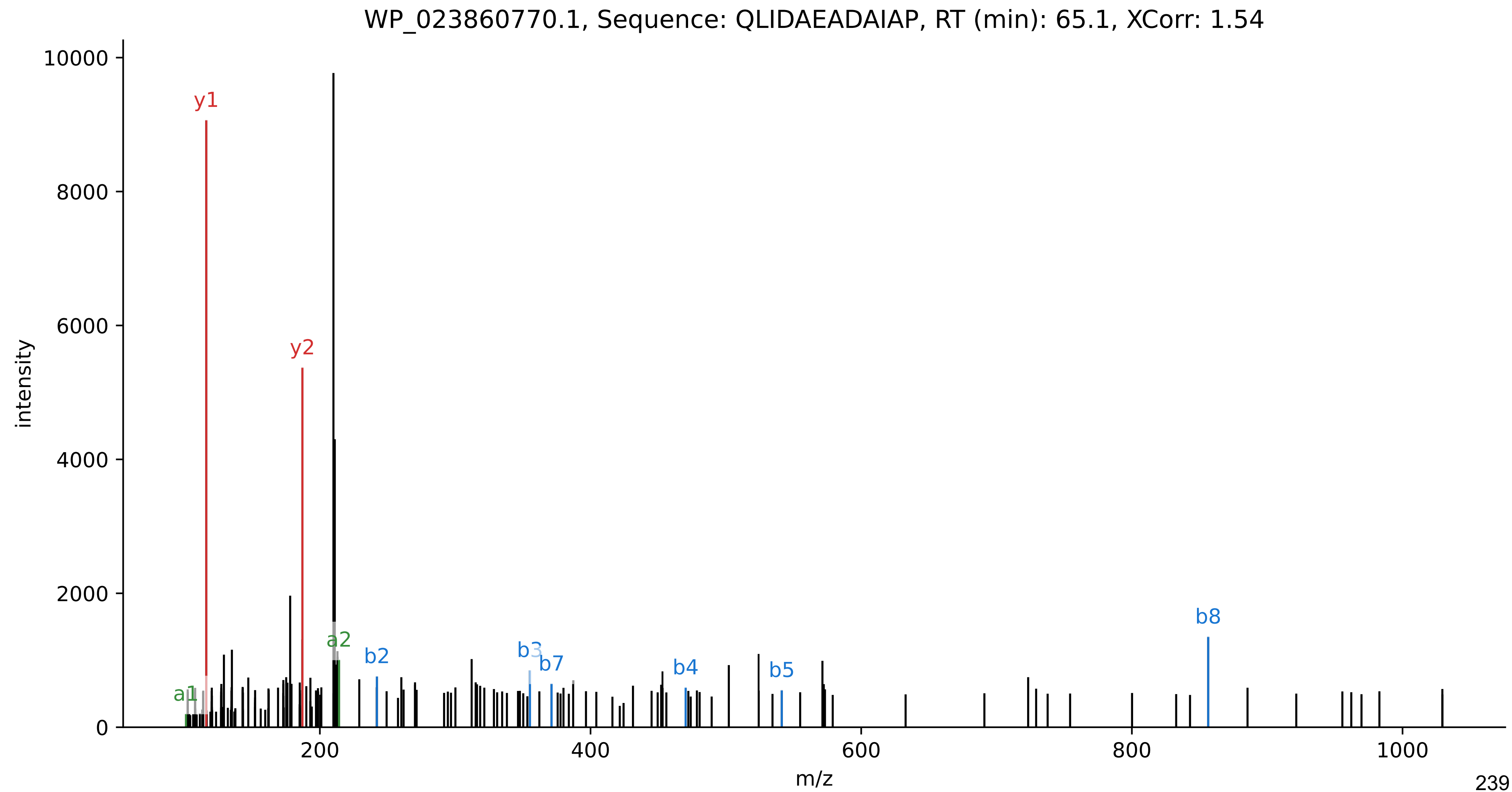

WP_023860770.1, Sequence: QLIDAEADAIAP, RT (min): 65.1, XCorr: 1.54
intensity
m/z
10000
8000
6000
4000
2000
0
200
400
600
800
1000
y1
y2
a1
a2
b2
b3
b7
b4
b5
b8

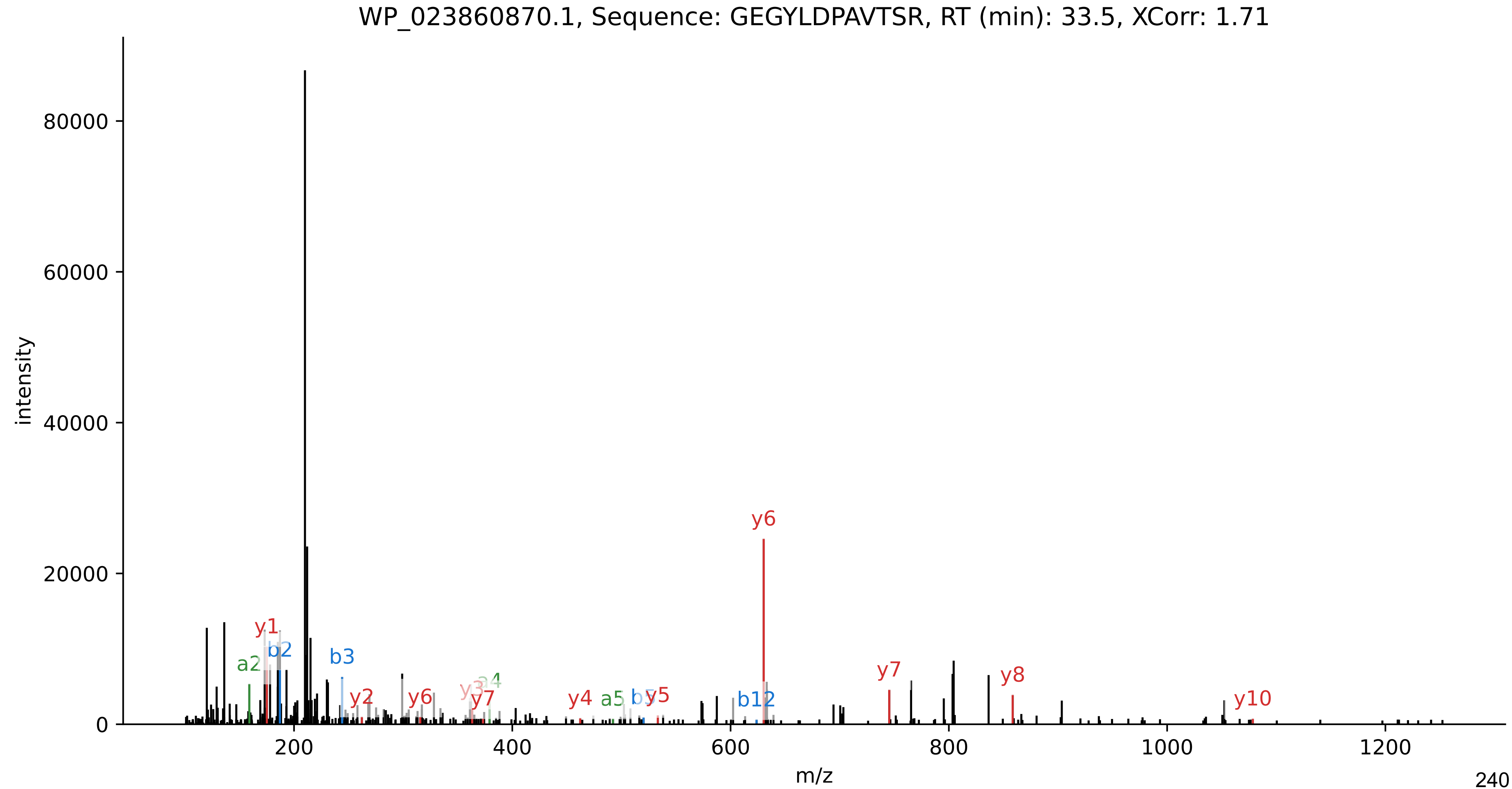

WP_023860870.1, Sequence: GEGYLDPAVTSR, RT (min): 33.5, XCorr: 1.71
intensity
m/z
0
20000
40000
60000
80000
200
400
600
800
1000
1200
y1
b2
a2
b3
y2
y6
y3
a4
y7
y4
a5
y5
y6
b12
y7
y8
y10

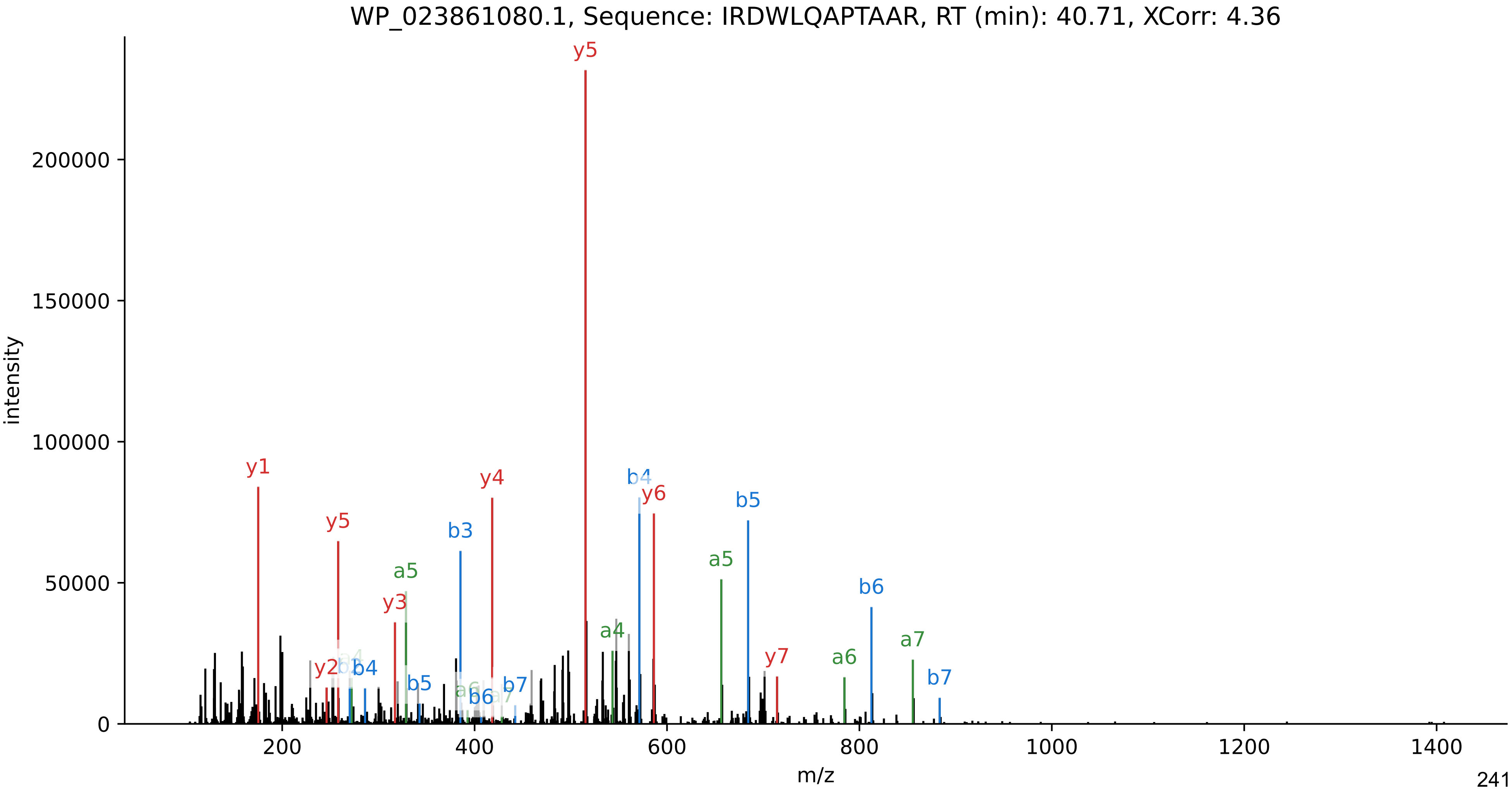
WP_023861080.1, Sequence: IRDWLQAPTAAR, RT (min): 40.71, XCorr: 4.36
intensity
m/z
y5
y1
y4
b4
y6
b5
y5
b3
a5
a5
y3
b6
a4
a7
y7
a6
y2
b4
b7
b5
b6
b7
0
50000
100000
150000
200000
200
400
600
800
1000
1200
1400

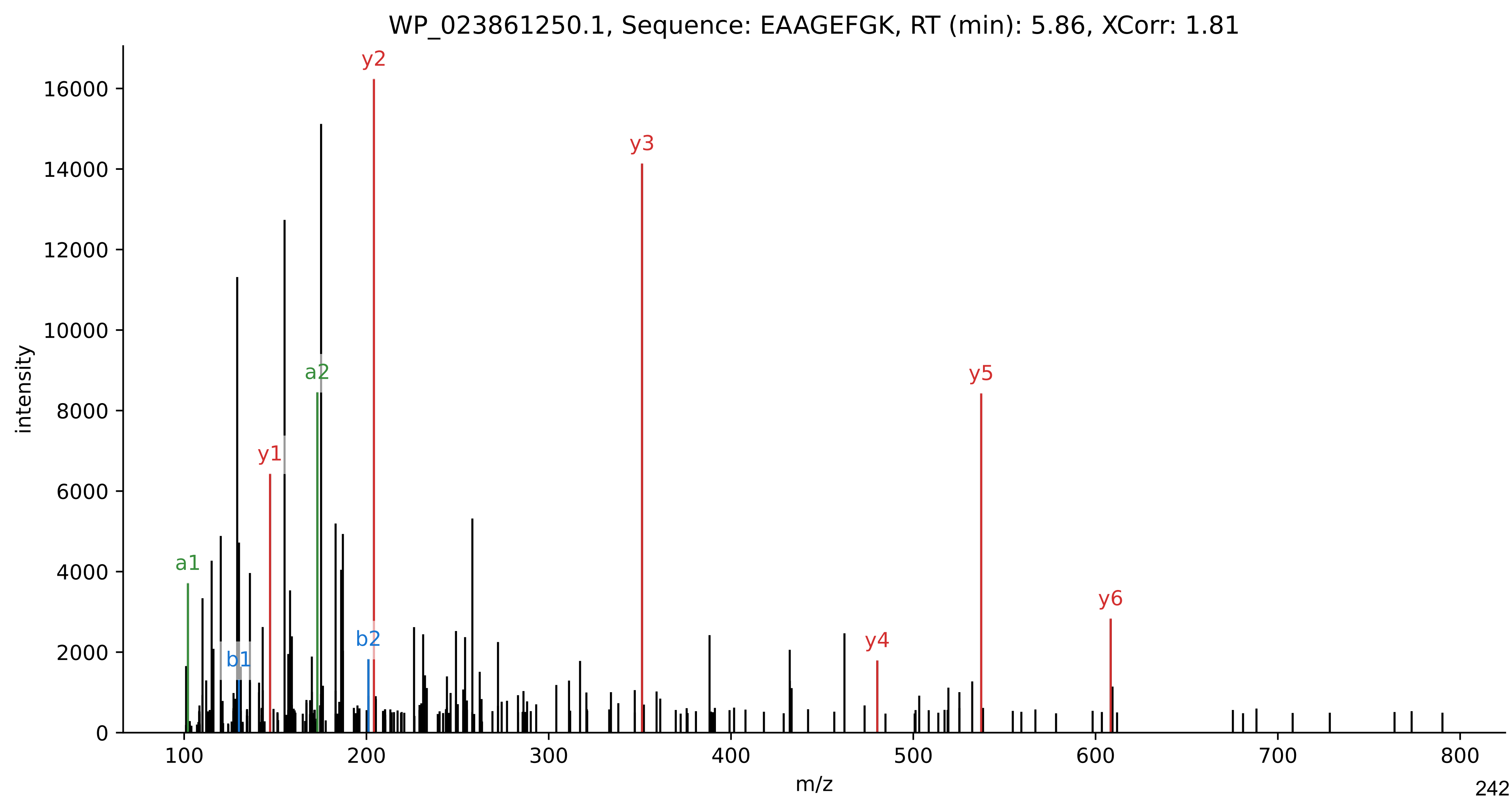
WP_023861250.1, Sequence: EAAGEFGK, RT (min): 5.86, XCorr: 1.81
intensity
m/z
a1
a2
b1
b2
y1
y2
y3
y4
y5
y6

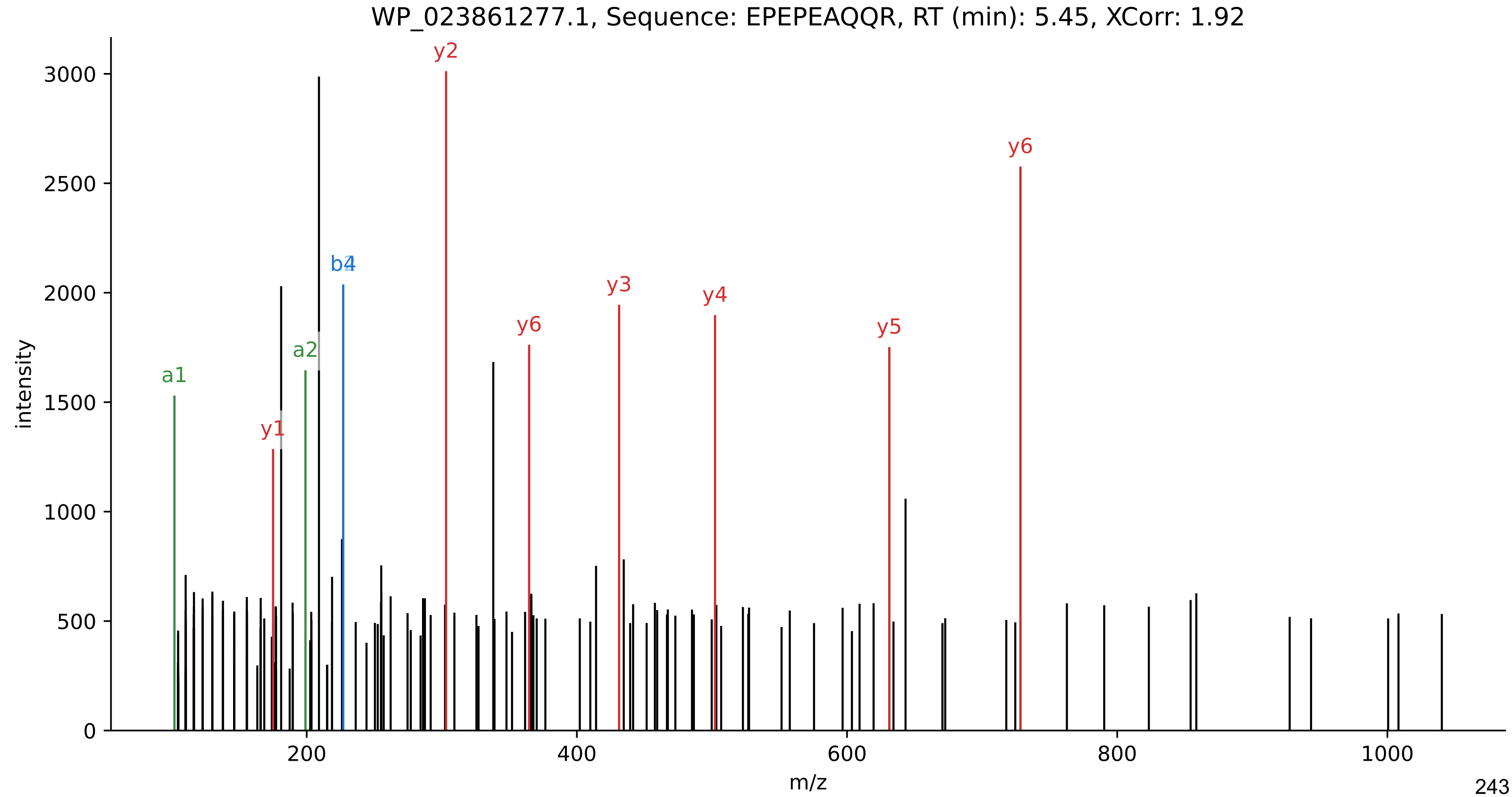
WP_023861277.1, Sequence: EPEPEAQQR, RT (min): 5.45, XCorr: 1.92
intensity
m/z
3000
2500
2000
1500
1000
500
0
200
400
600
800
1000
a1
a2
b4
y1
y2
y6
y3
y4
y5
y6

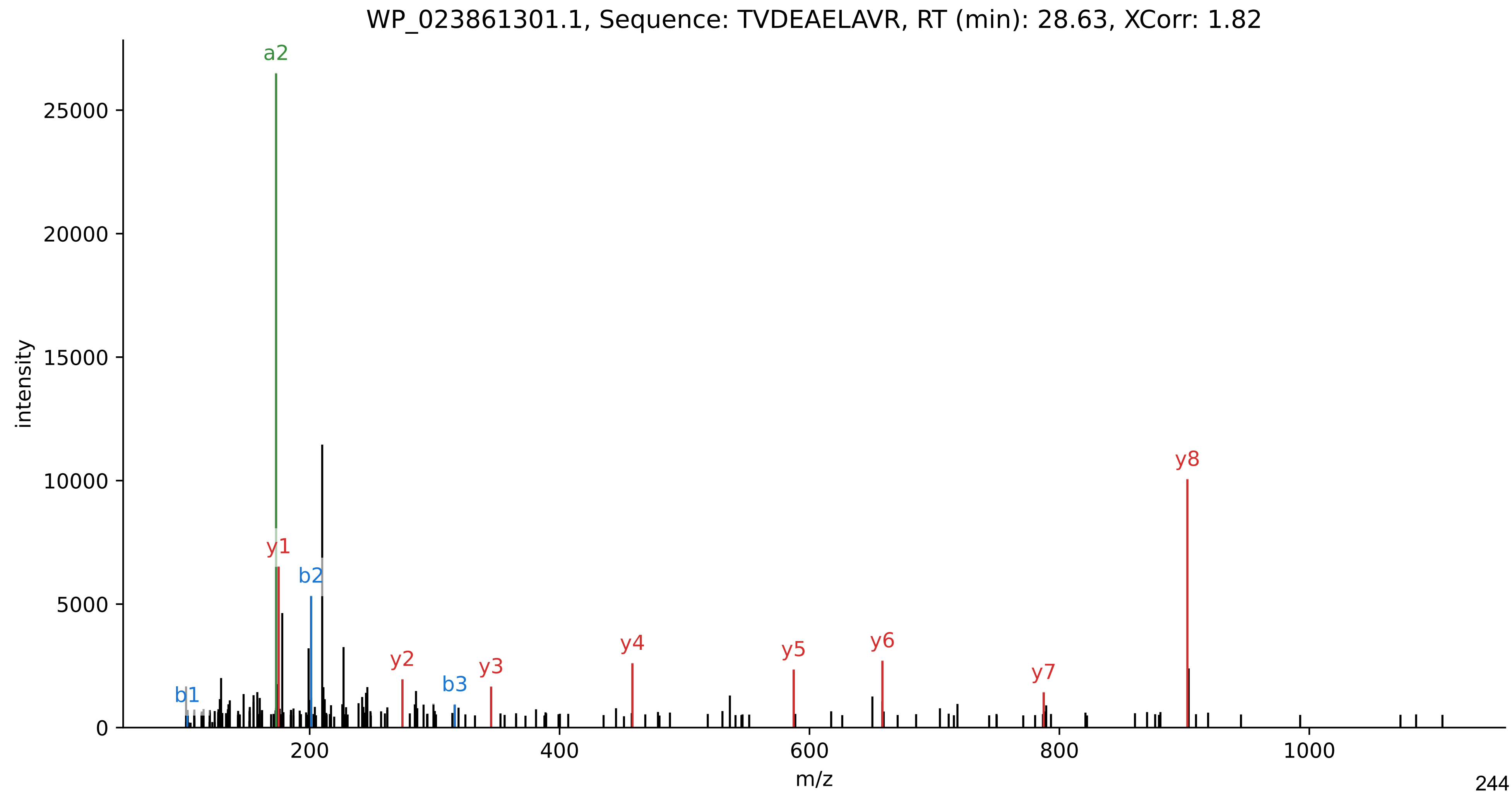

WP_023861301.1, Sequence: TVDEAELAVR, RT (min): 28.63, XCorr: 1.82
a2
y1
b2
b1
y2
b3
y3
y4
y5
y6
y7
y8
intensity
m/z
0
5000
10000
15000
20000
25000
200
400
600
800
1000

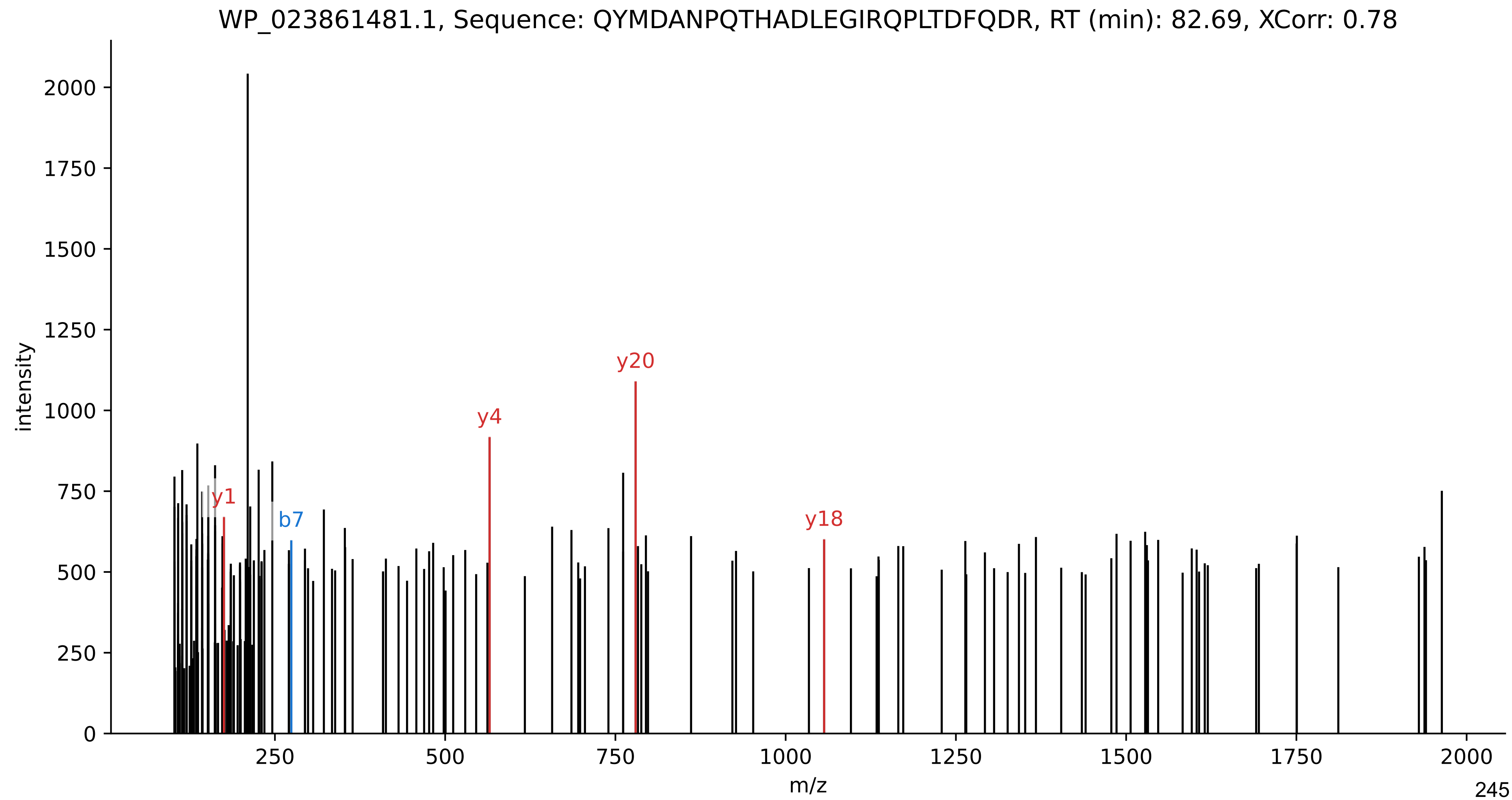

WP_023861481.1, Sequence: QYMDANPQTHADLEGIRQPLTDFQDR, RT (min): 82.69, XCorr: 0.78
intensity
m/z
y1
b7
y4
y20
y18
0
250
500
750
1000
1250
1500
1750
2000

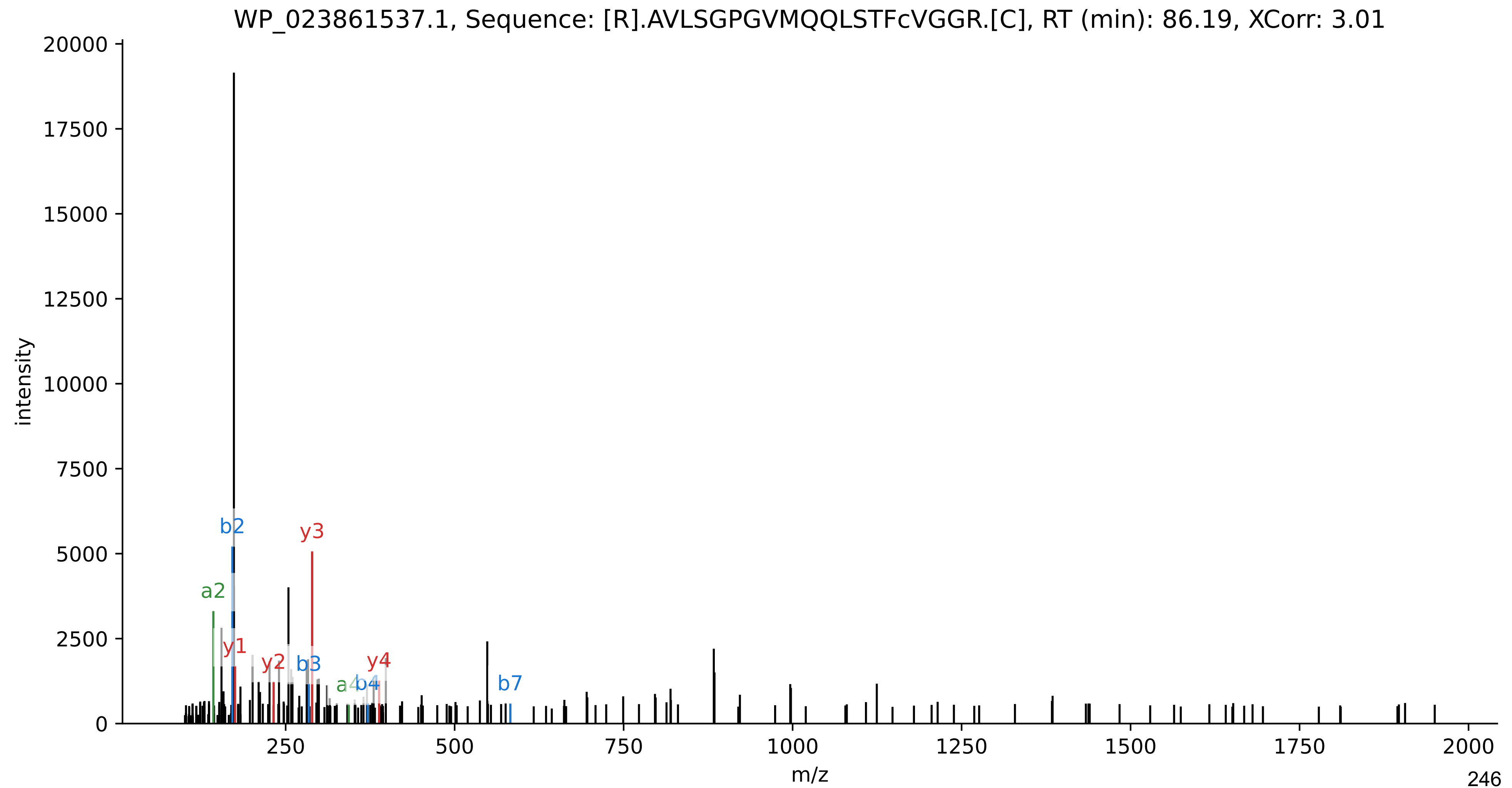

WP_023861537.1, Sequence: [R].AVLSGPGVMQQLSTFcVGGR.[C], RT (min): 86.19, XCorr: 3.01
intensity
m/z
b2
y3
a2
y1
y2
b3
y4
a4
b4
b7

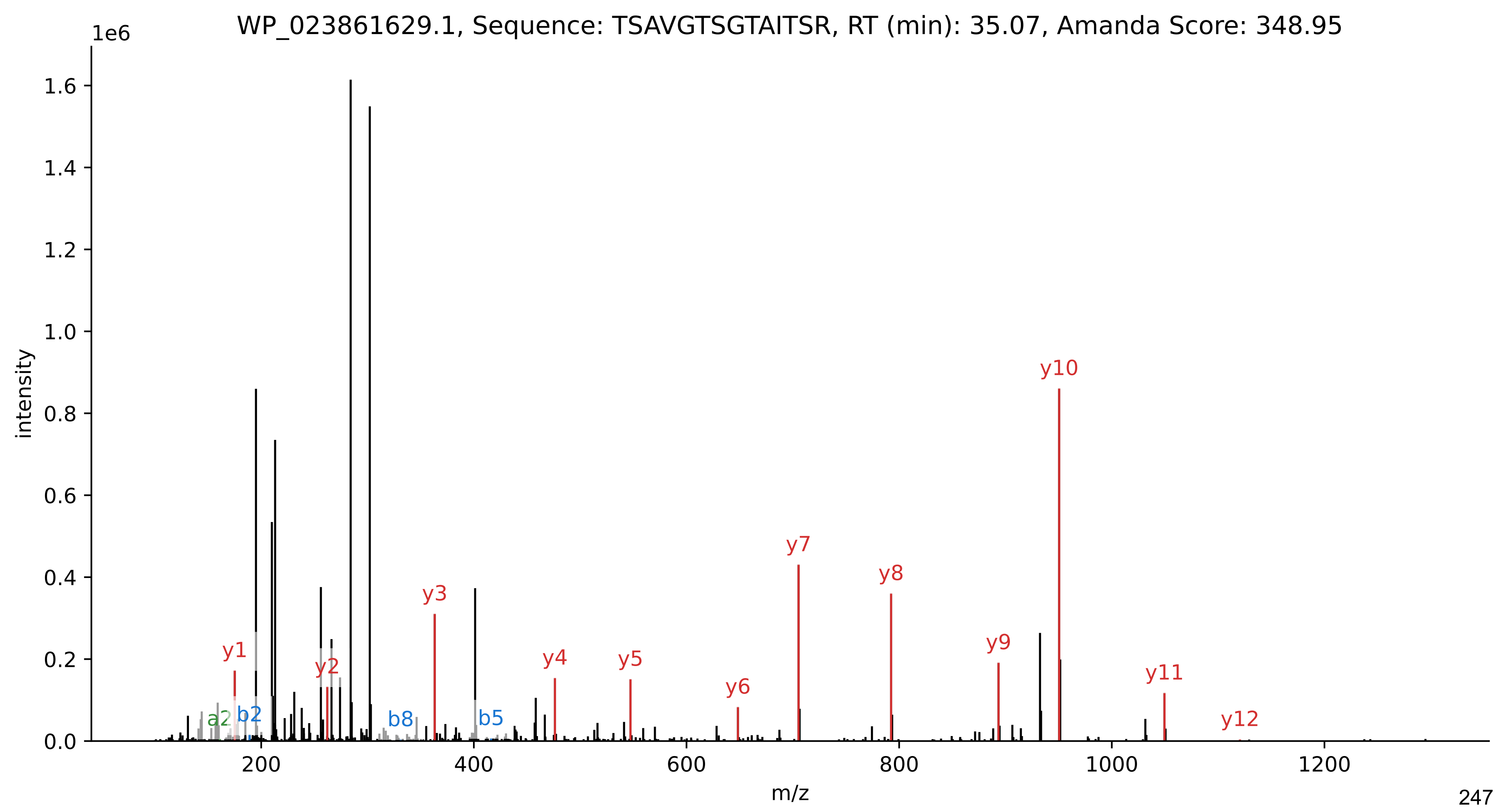
WP_023861629.1, Sequence: TSAVGTSGTAITSR, RT (min): 35.07, Amanda Score: 348.95
1e6
intensity
m/z
y1
y2
y3
y4
y5
y6
y7
y8
y9
y10
y11
y12
a2
b2
b8
b5

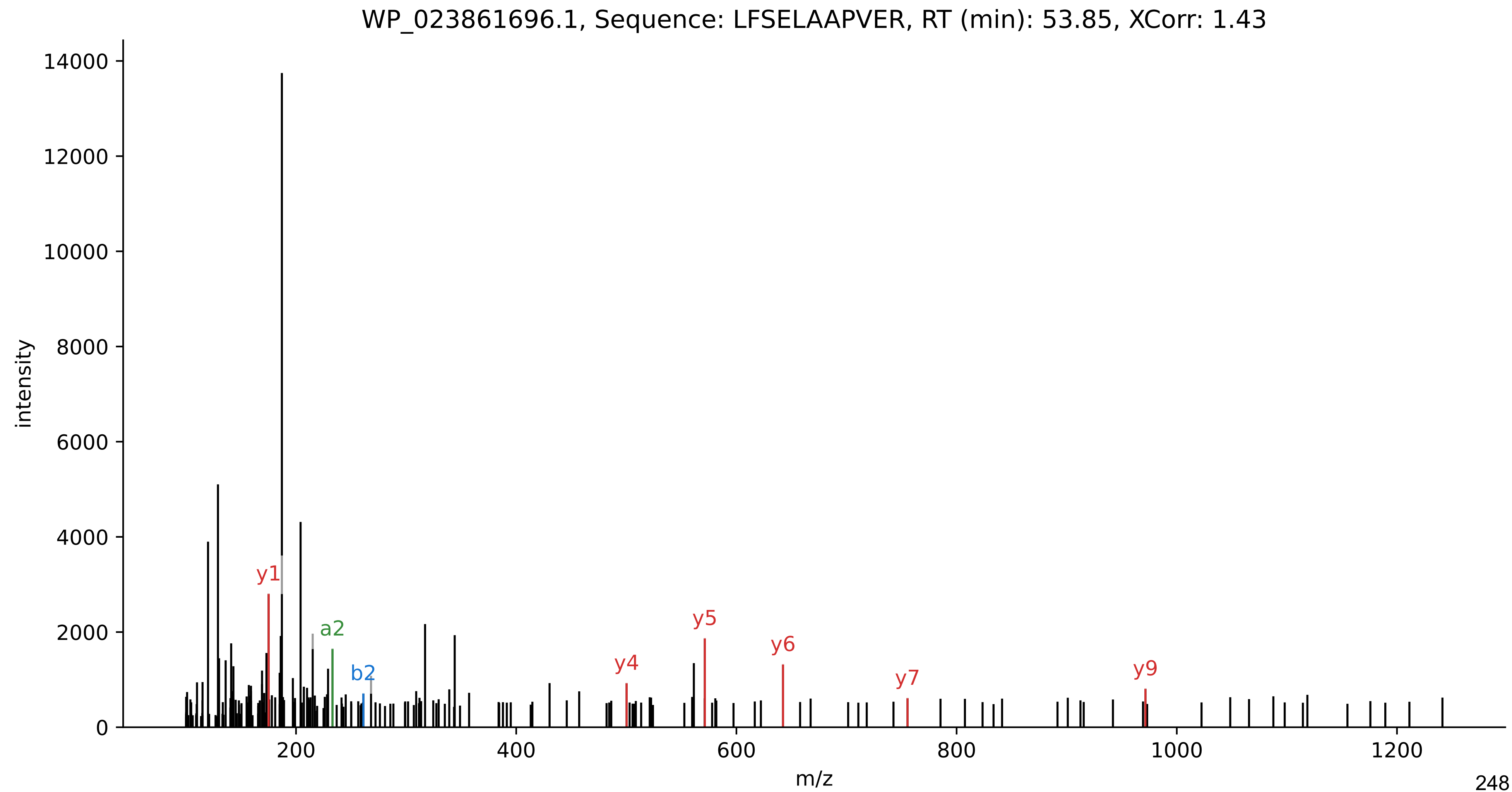
WP_023861696.1, Sequence: LFSELAAPVER, RT (min): 53.85, XCorr: 1.43
intensity
m/z
y1
a2
b2
y4
y5
y6
y7
y9
0
2000
4000
6000
8000
10000
12000
14000
200
400
600
800
1000
1200

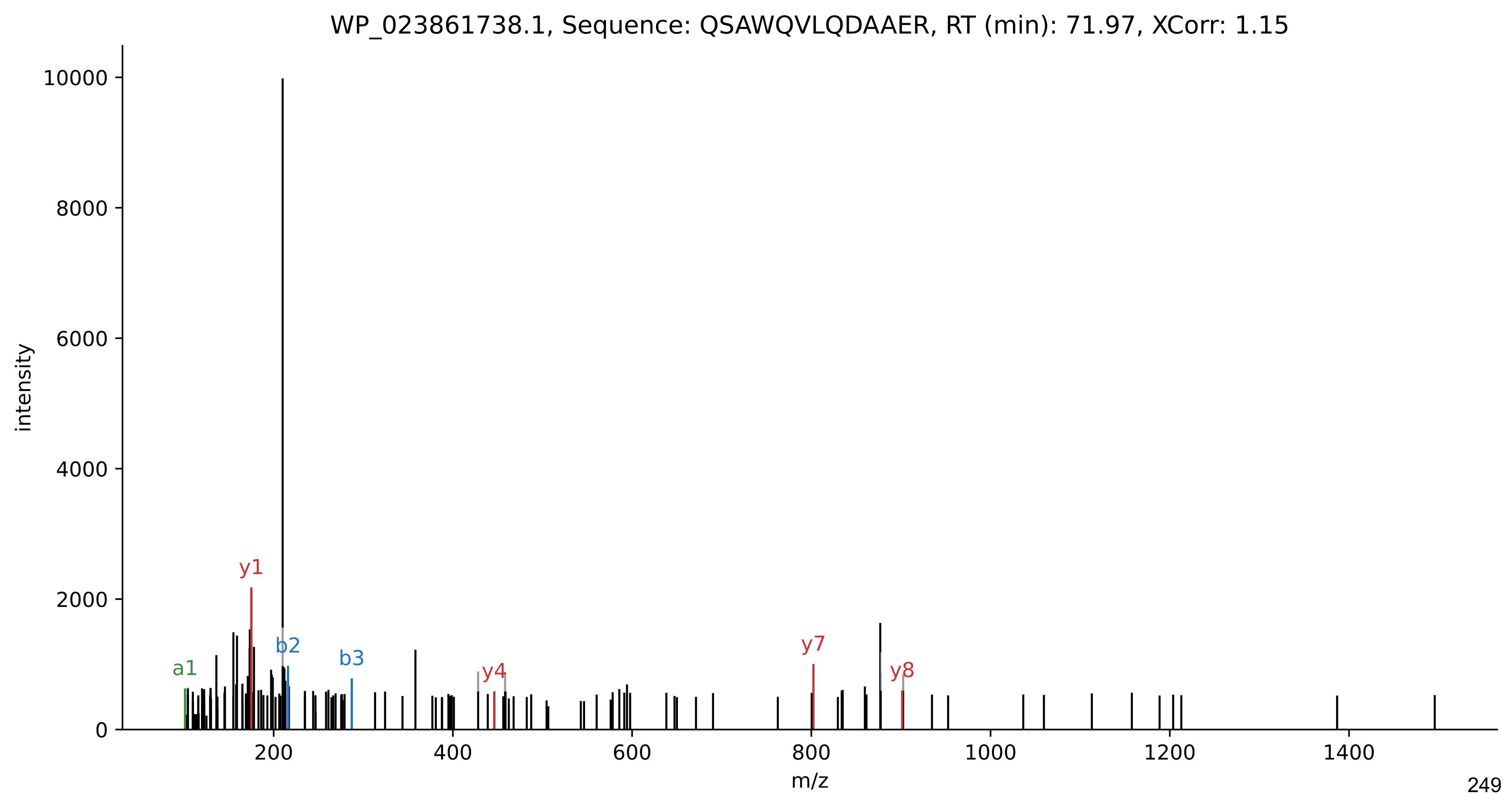
WP_023861738.1, Sequence: QSAWQVLQDAAER, RT (min): 71.97, XCorr: 1.15
intensity
m/z
10000
8000
6000
4000
2000
0
200
400
600
800
1000
1200
1400
a1
y1
b2
b3
y4
y7
y8

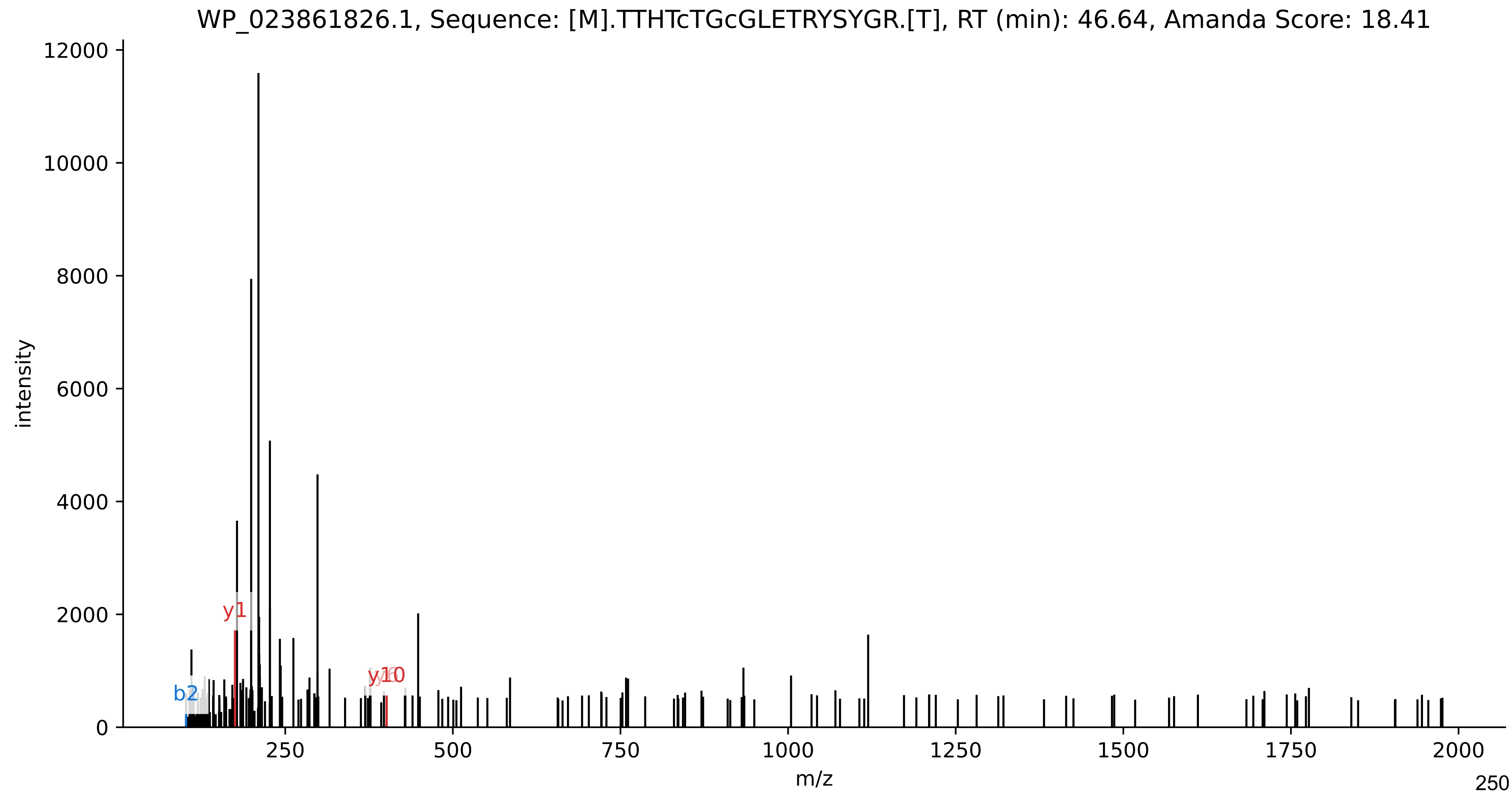

WP_023861826.1, Sequence: [M].TTHTcTGcGLETRYSYGR.[T], RT (min): 46.64, Amanda Score: 18.41
intensity
m/z
b2
y1
y10

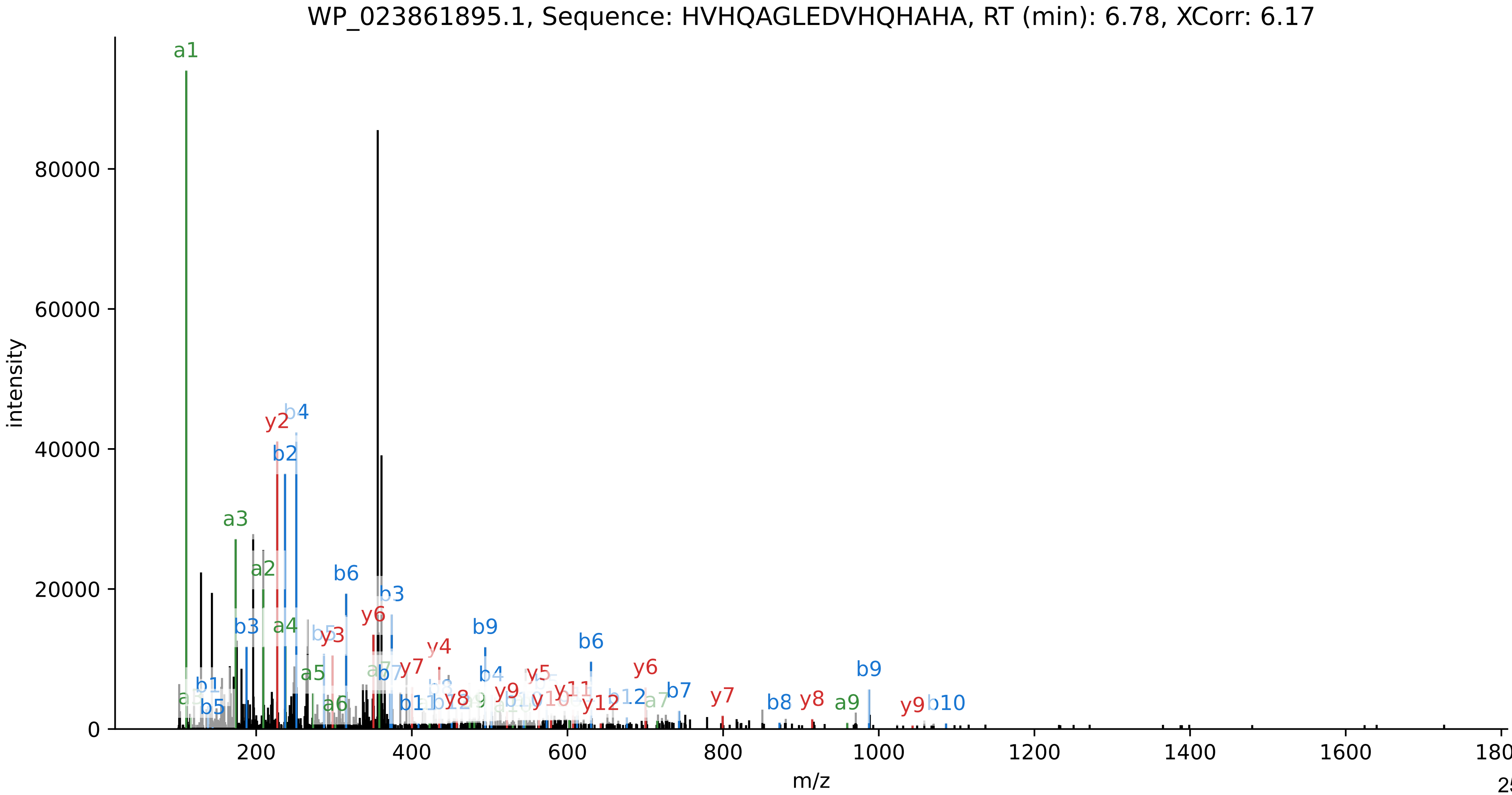
WP_023861895.1, Sequence: HVHQAGLEDVHQHAHA, RT (min): 6.78, XCorr: 6.17
intensity
m/z
0
20000
40000
60000
80000
200
400
600
800
1000
1200
1400
1600
1800
a1
y2
b4
b2
a3
a2
b6
b3
b3
a4
y6
b5
y3
y4
b9
b6
a5
b7
y7
b4
y6
y5
b1
y9
y11
b7
y7
a6
b11
y8
y10
y12
b12
a7
b8
y8
a9
b9
y9
b10

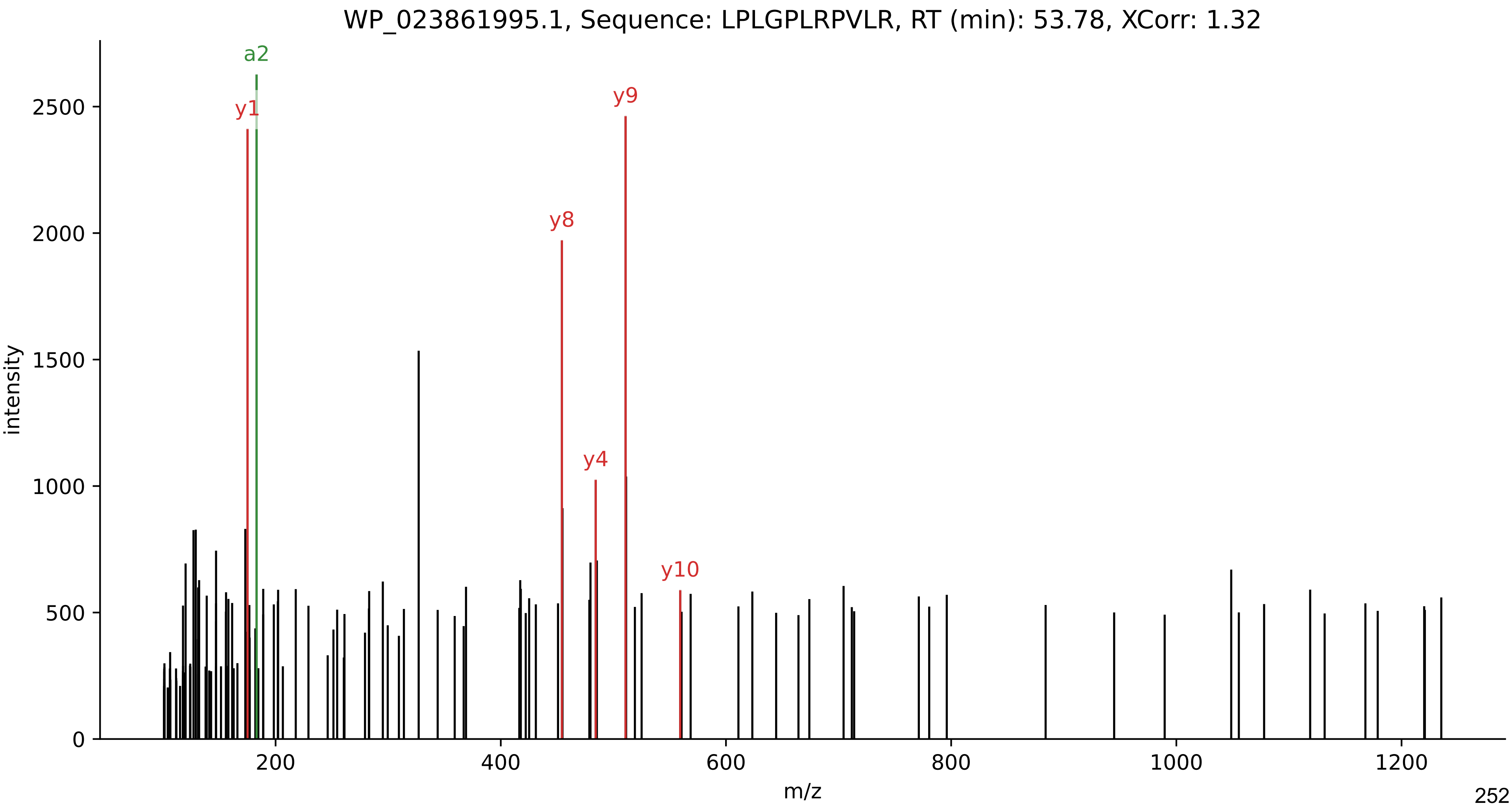

WP_023861995.1, Sequence: LPLGPLRPVLR, RT (min): 53.78, XCorr: 1.32
a2
y1
y9
y8
y4
y10
intensity
m/z
0
500
1000
1500
2000
2500
200
400
600
800
1000
1200

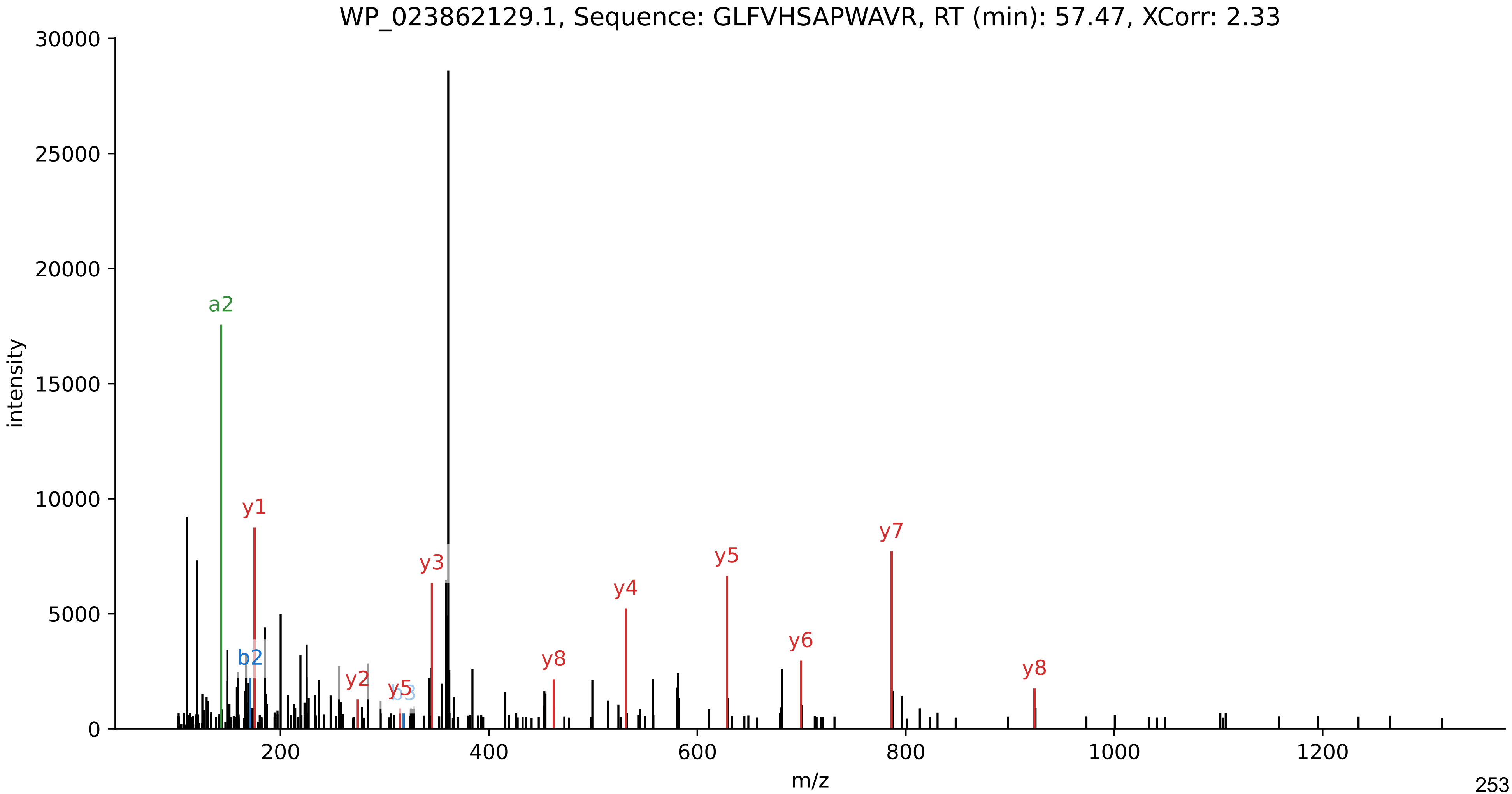
WP_023862129.1, Sequence: GLFVHSAPWAVR, RT (min): 57.47, XCorr: 2.33
intensity
m/z
30000
25000
20000
15000
10000
5000
0
200
400
600
800
1000
1200
a2
y1
b2
y2
y5
b3
y3
y8
y4
y5
y6
y7
y8

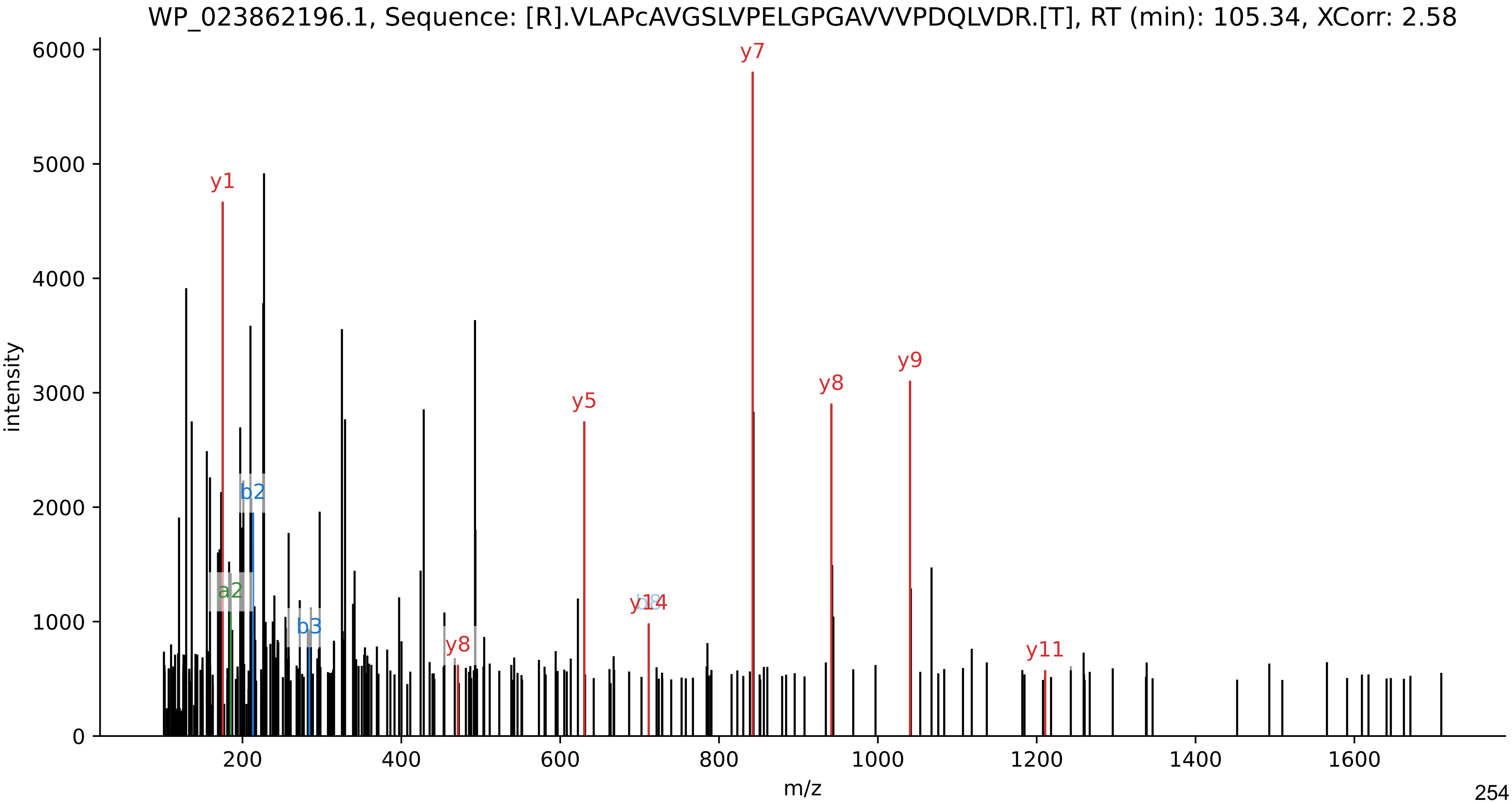
WP_023862196.1, Sequence: [R].VLAPcAVGSLVPELGPGAVVVPDQLVDR.[T], RT (min): 105.34, XCorr: 2.58
intensity
m/z
y1
b2
a2
b3
y8
y5
y14
y7
y8
y9
y11
0
1000
2000
3000
4000
5000
6000
200
400
600
800
1000
1200
1400
1600

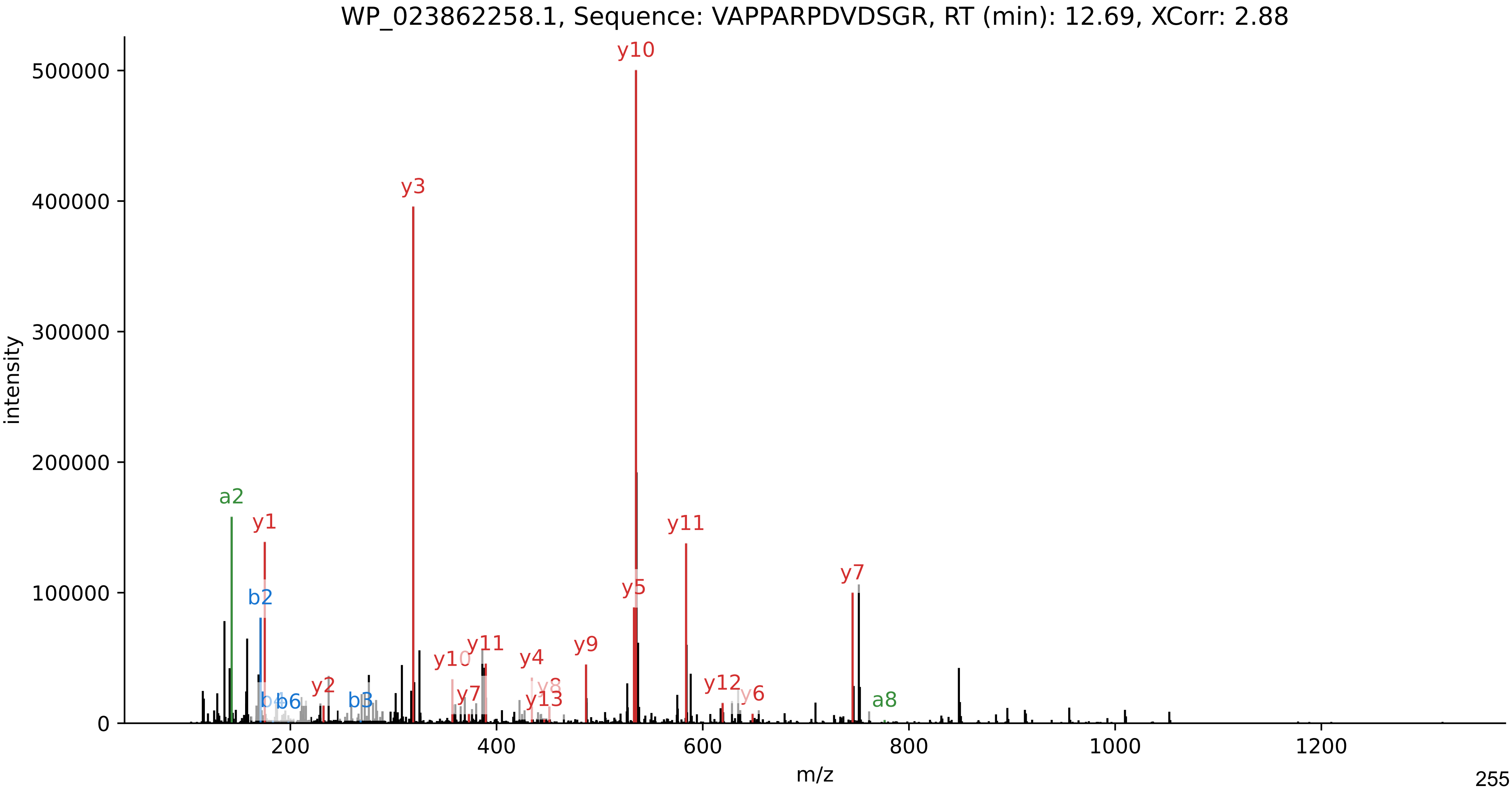

WP_023862258.1, Sequence: VAPPARPDVDSGR, RT (min): 12.69, XCorr: 2.88
intensity
m/z
y10
y3
a2
y1
y11
b2
y5
y7
y11
y10
y4
y9
y2
y7
y13
y12
y6
b6
b3
a8

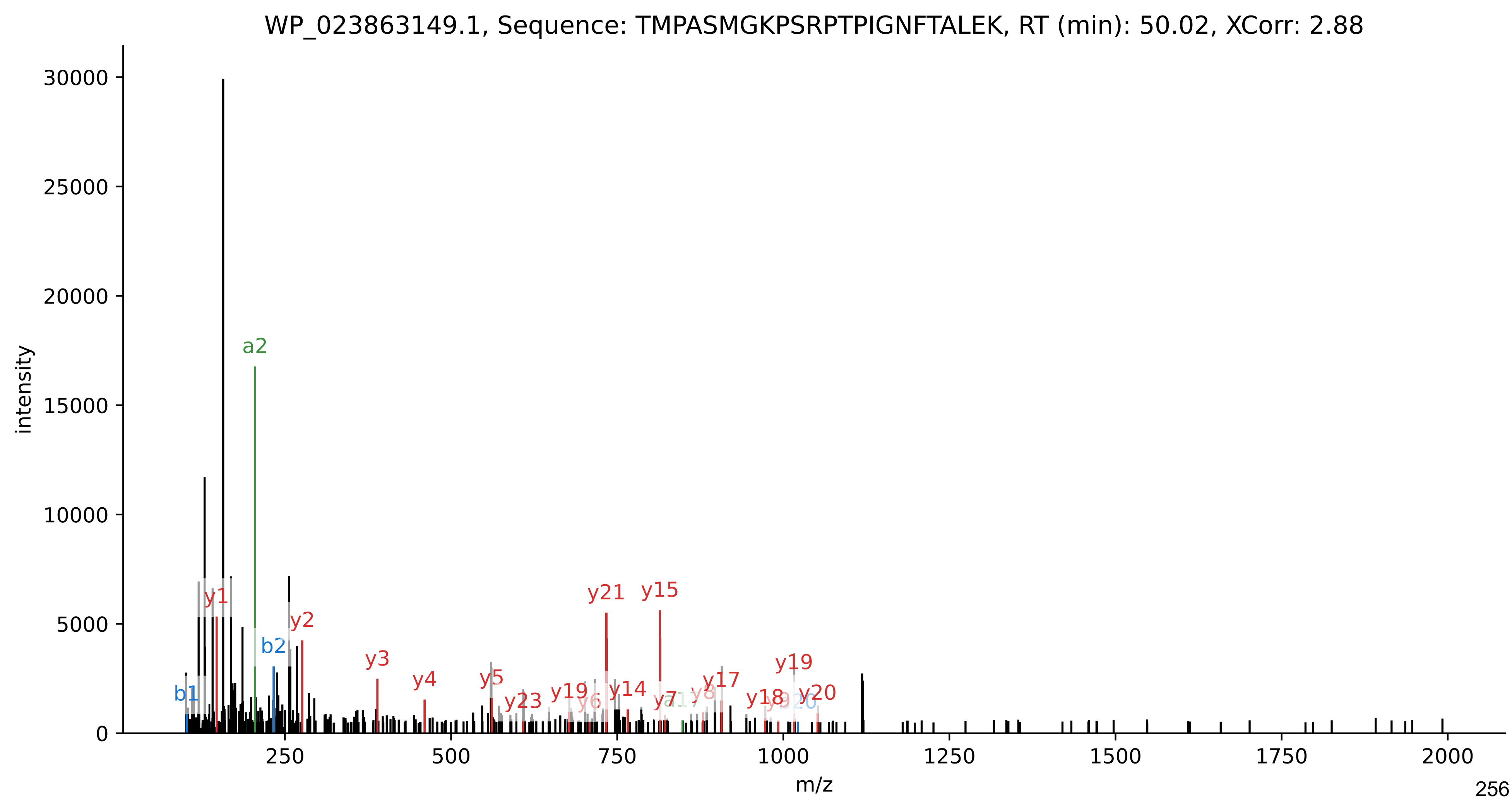
WP_023863149.1, Sequence: TMPASMGKPSRPTPIGNFTALEK, RT (min): 50.02, XCorr: 2.88
intensity
m/z
a2
y1
y2
b2
b1
y3
y4
y5
y21
y15
y23
y19
y14
y17
y19
y18
y20
0
5000
10000
15000
20000
25000
30000
250
500
750
1000
1250
1500
1750
2000

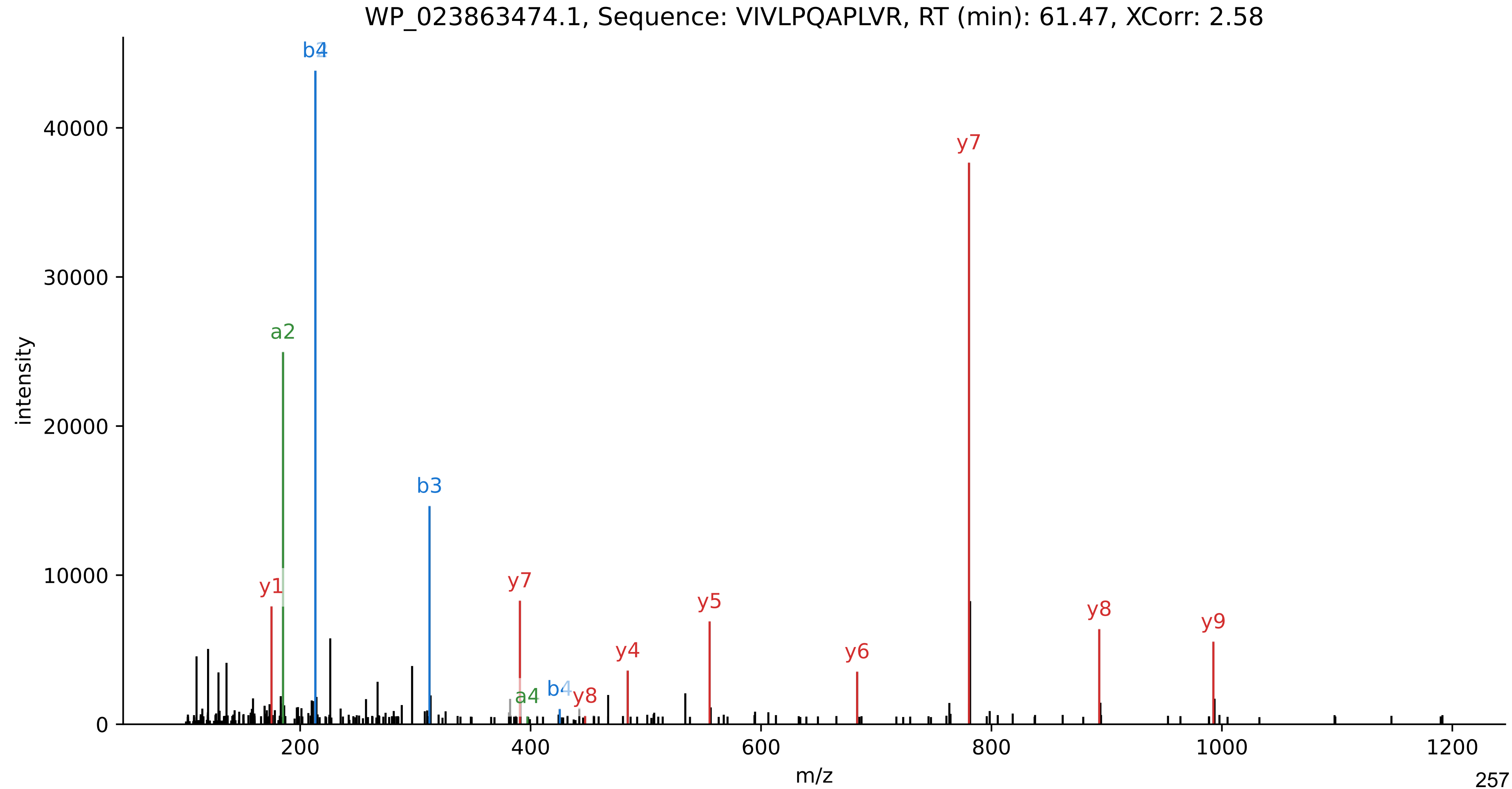
WP_023863474.1, Sequence: VIVLPQAPLVR, RT (min): 61.47, XCorr: 2.58
intensity
m/z
b4
y7
a2
b3
y1
y7
y5
y4
y6
y8
y9
a4
b4
y8
0
10000
20000
30000
40000
200
400
600
800
1000
1200

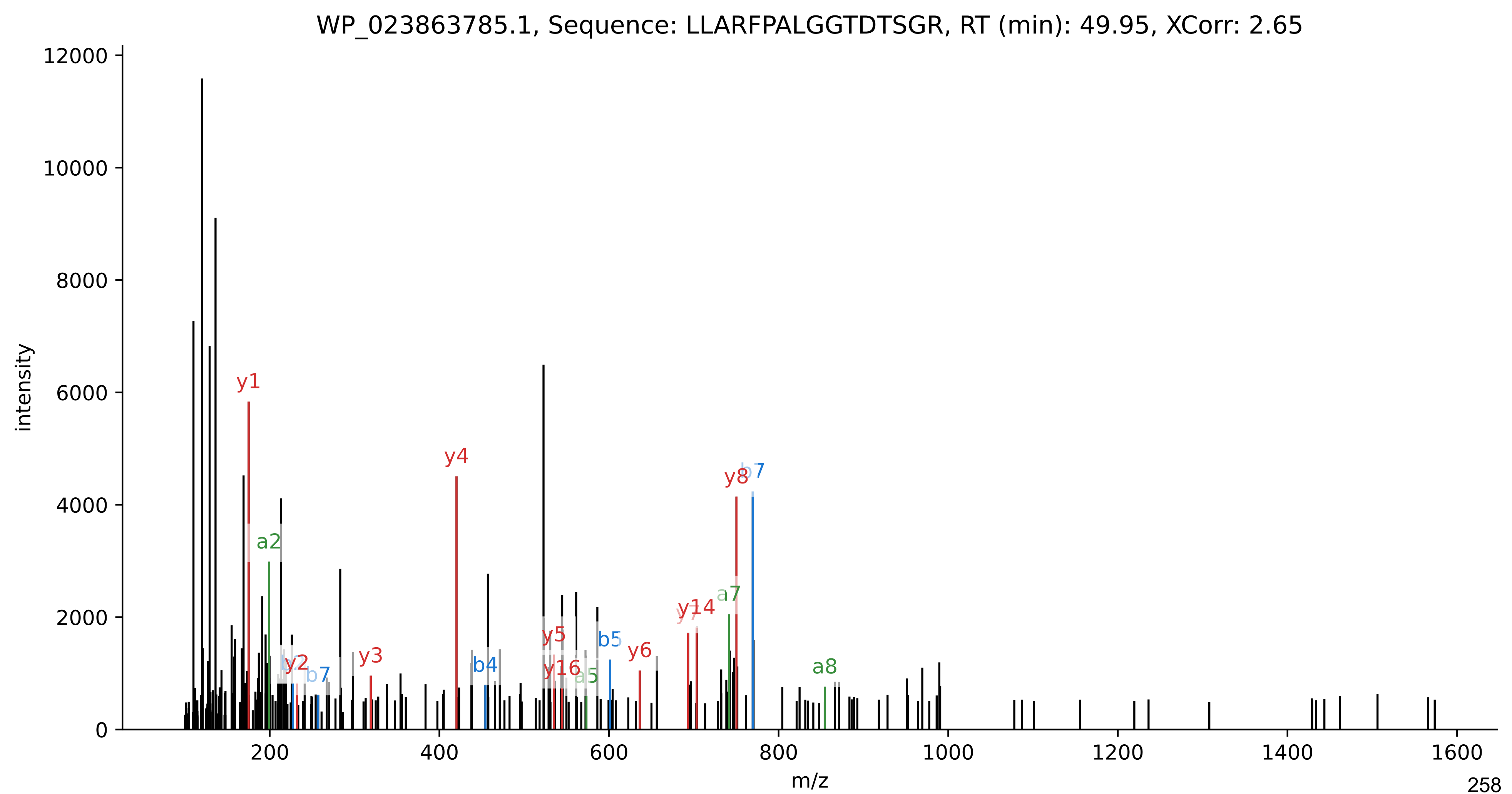
WP_023863785.1, Sequence: LLARFPALGGTDTSGR, RT (min): 49.95, XCorr: 2.65
intensity
m/z
y1
a2
y2
b7
y3
y4
b4
y5
y16
a5
b5
y6
y14
a7
y8
b7
a8

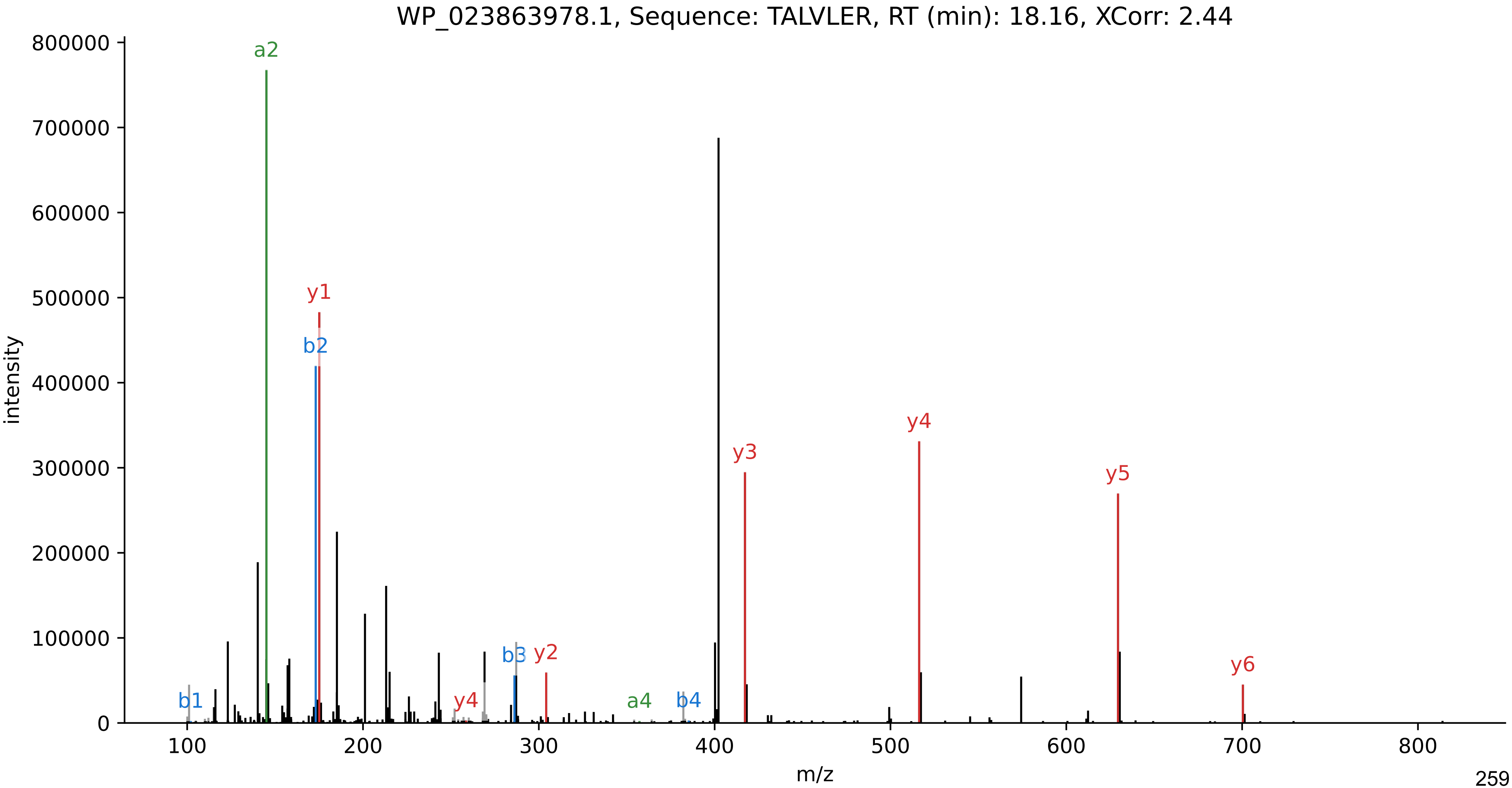
WP_023863978.1, Sequence: TALVLER, RT (min): 18.16, XCorr: 2.44
800000
700000
600000
500000
400000
300000
200000
100000
0
intensity
100
200
300
400
500
600
700
800
m/z
a2
y1
b2
y4
y3
y5
y6
b3
y2
b1
y4
a4
b4

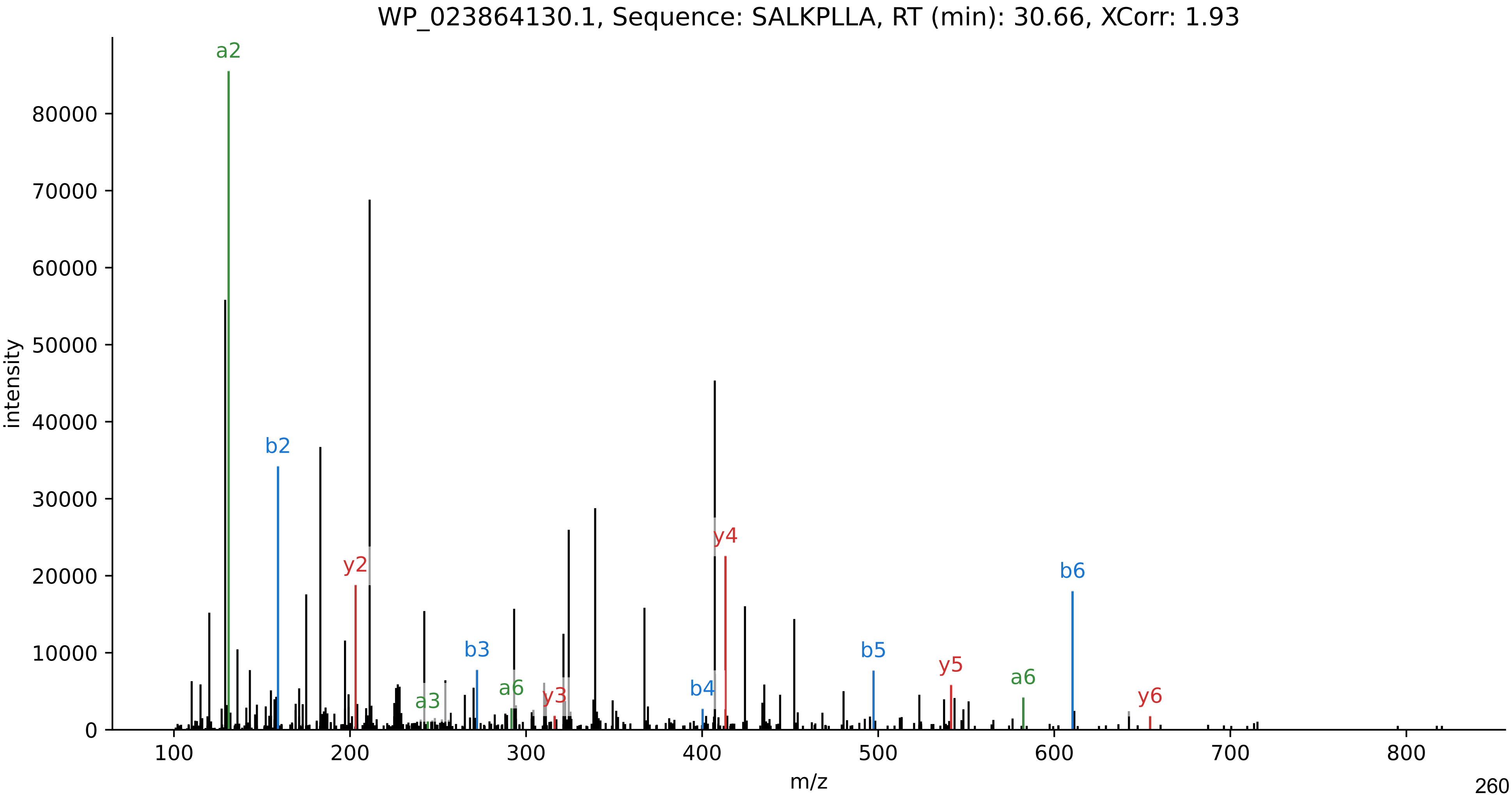
WP_023864130.1, Sequence: SALKPLLA, RT (min): 30.66, XCorr: 1.93
intensity
m/z
a2
b2
y2
a3
b3
a6
y3
b4
y4
b5
y5
a6
b6
y6

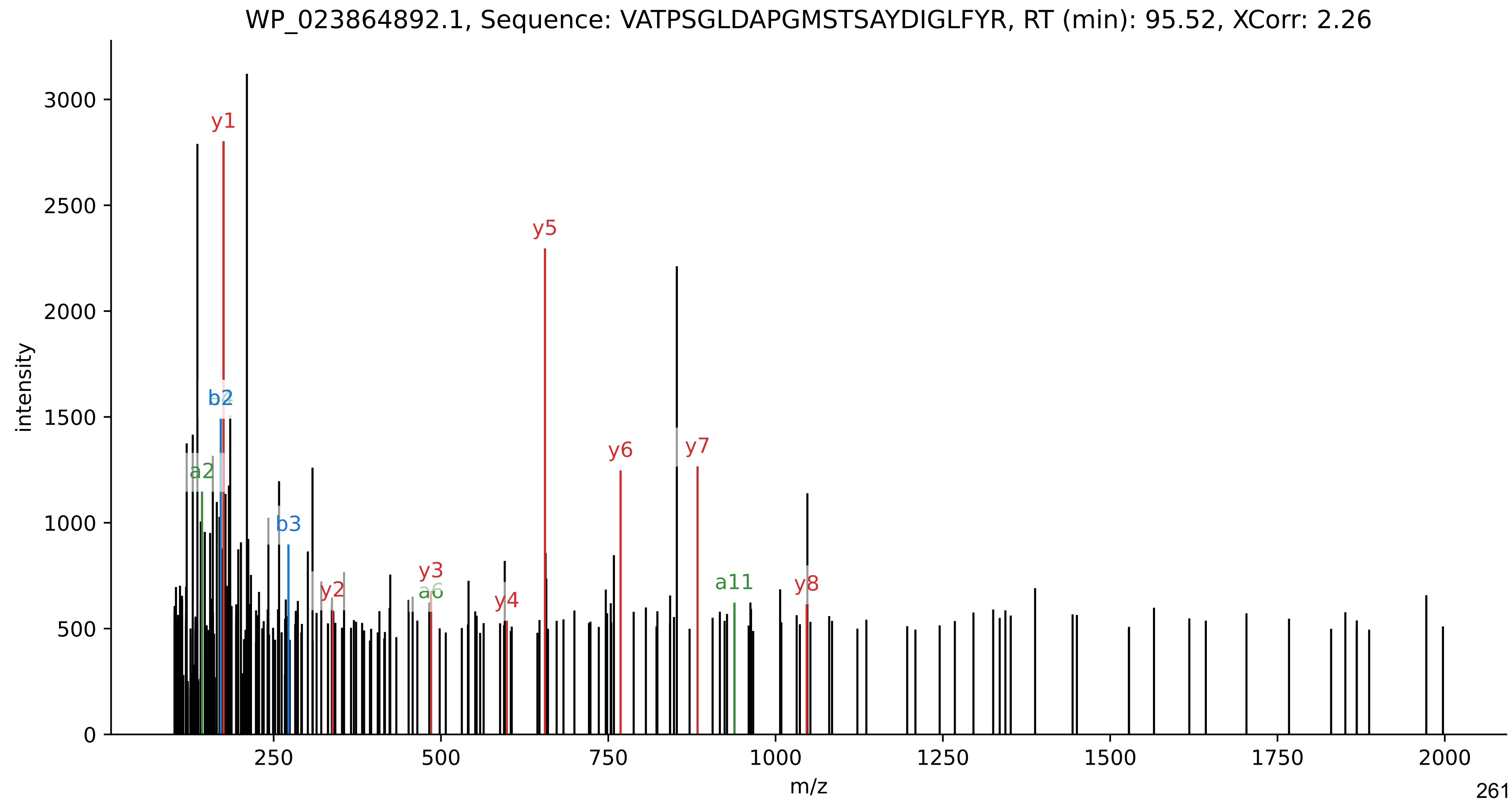

WP_023864892.1, Sequence: VATPSGLDAPGMSTSAYDIGLFYR, RT (min): 95.52, XCorr: 2.26
intensity
m/z
y1
b2
a2
b3
y2
y3
a6
y4
y5
y6
y7
a11
y8

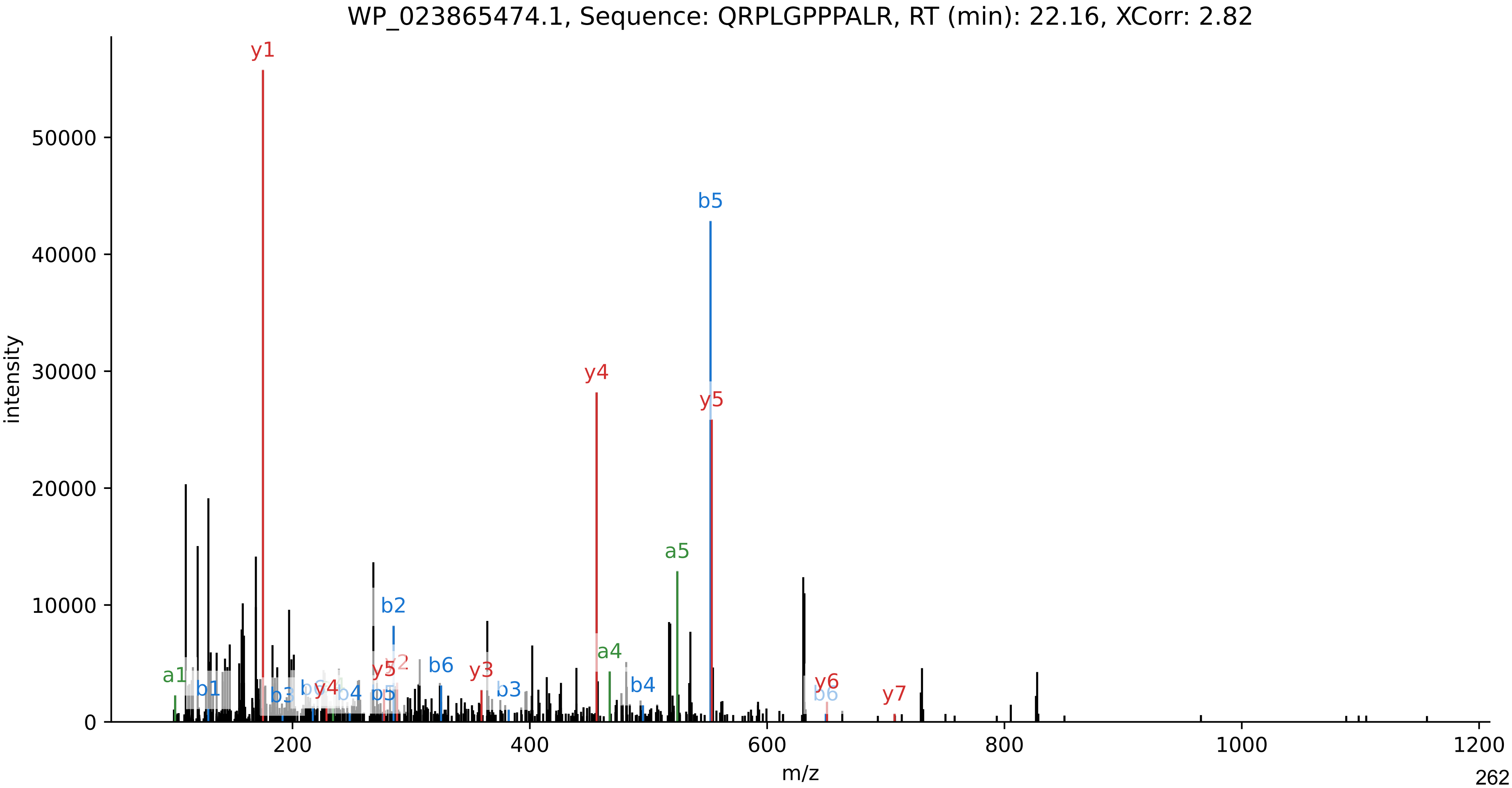

WP_023865474.1, Sequence: QRPLGPPPALR, RT (min): 22.16, XCorr: 2.82
intensity
m/z
y1
b5
y4
y5
a5
b2
a4
a1
b1
b3
y4
b4
y5
b6
y3
b3
b4
y6
b6
y7
0
10000
20000
30000
40000
50000
200
400
600
800
1000
1200

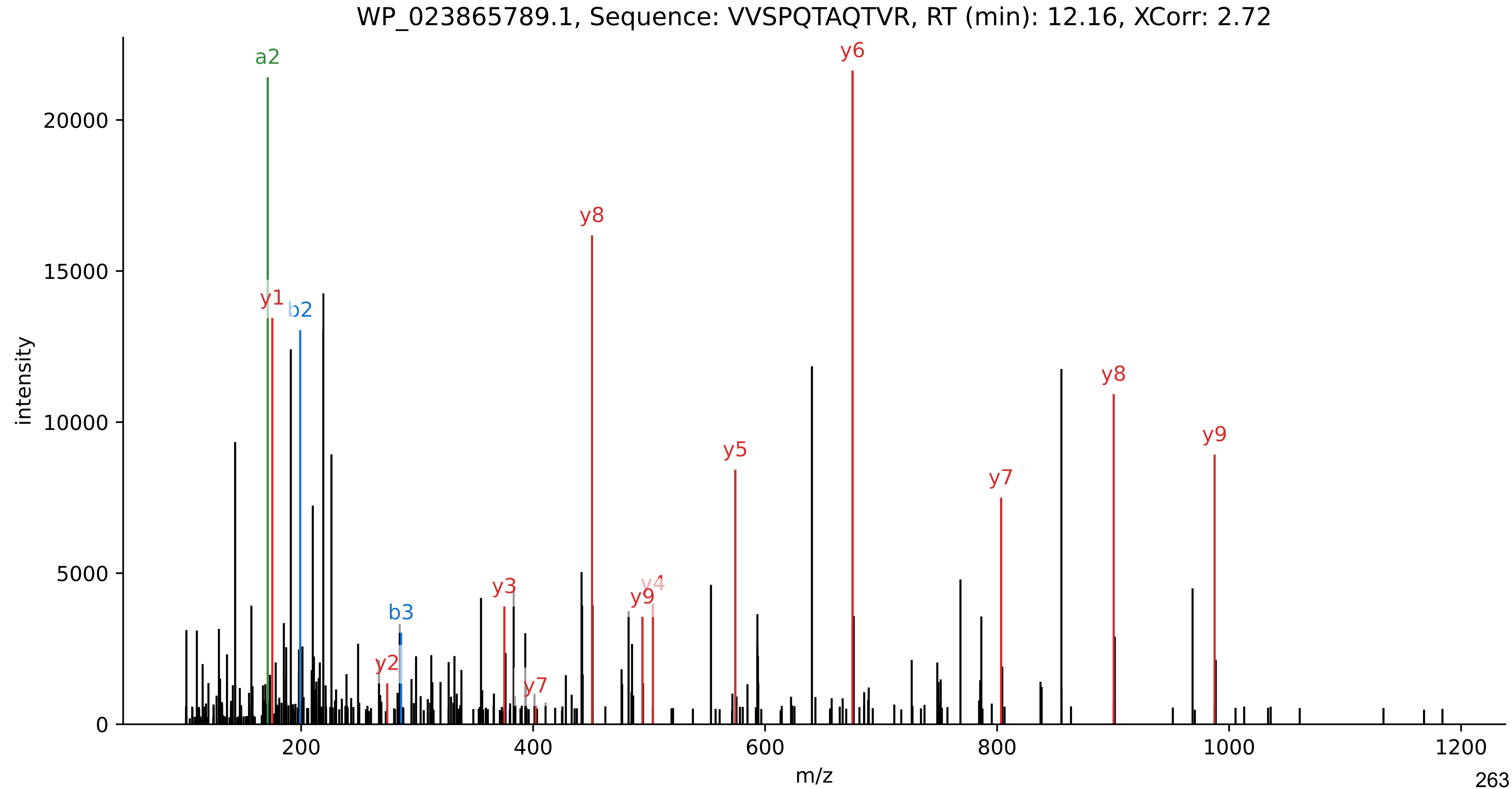

WP_023865789.1, Sequence: VVSPQTAQTVR, RT (min): 12.16, XCorr: 2.72
intensity
m/z
a2
y1
b2
b3
y2
y3
y7
y8
y9
y4
y5
y6
y7
y8
y9
0
5000
10000
15000
20000
200
400
600
800
1000
1200

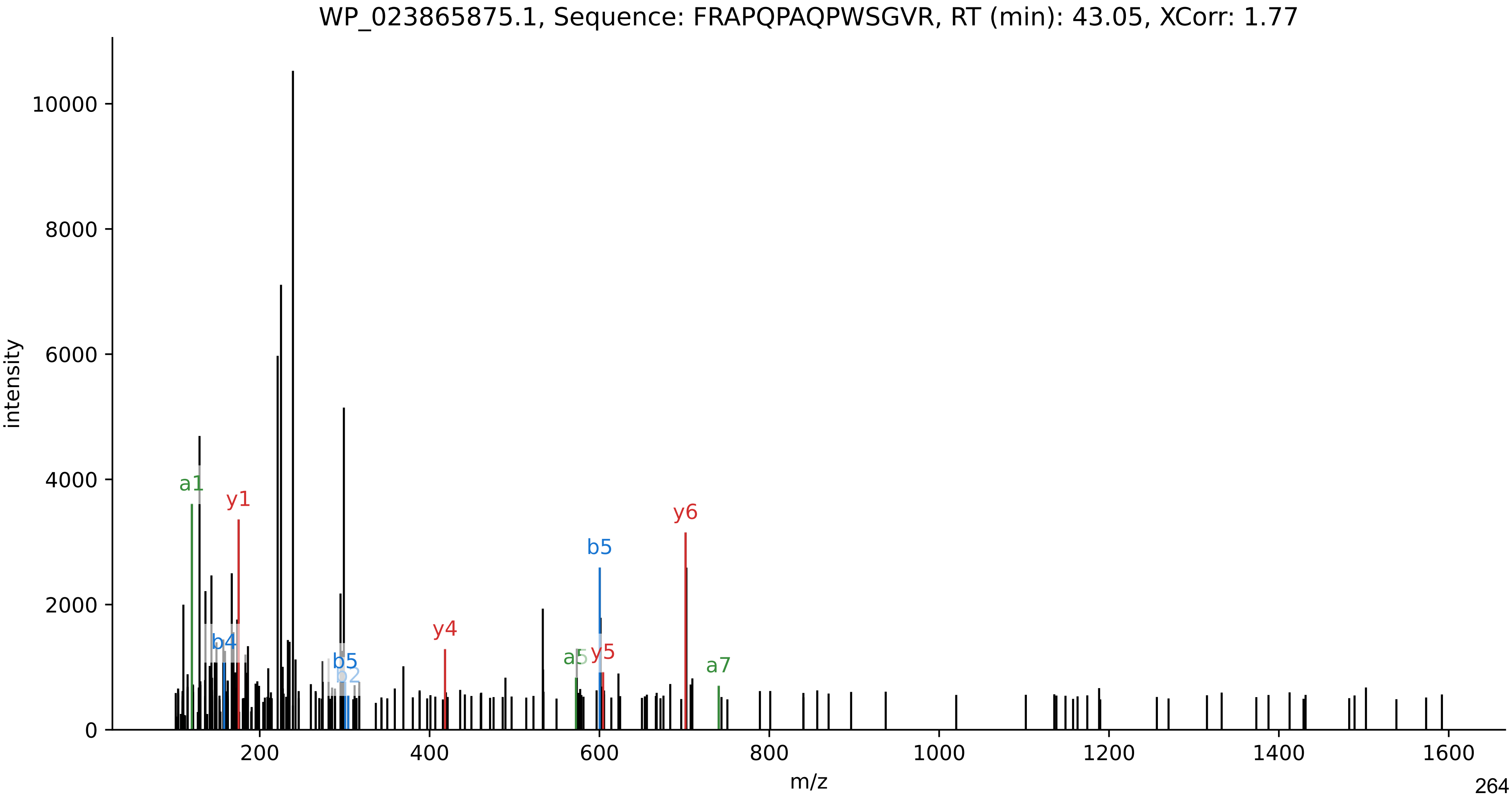

WP_023865875.1, Sequence: FRAPQPAQPWSGVR, RT (min): 43.05, XCorr: 1.77
intensity
m/z
a1
y1
b4
b5
b2
y4
a5
y5
b5
y6
a7

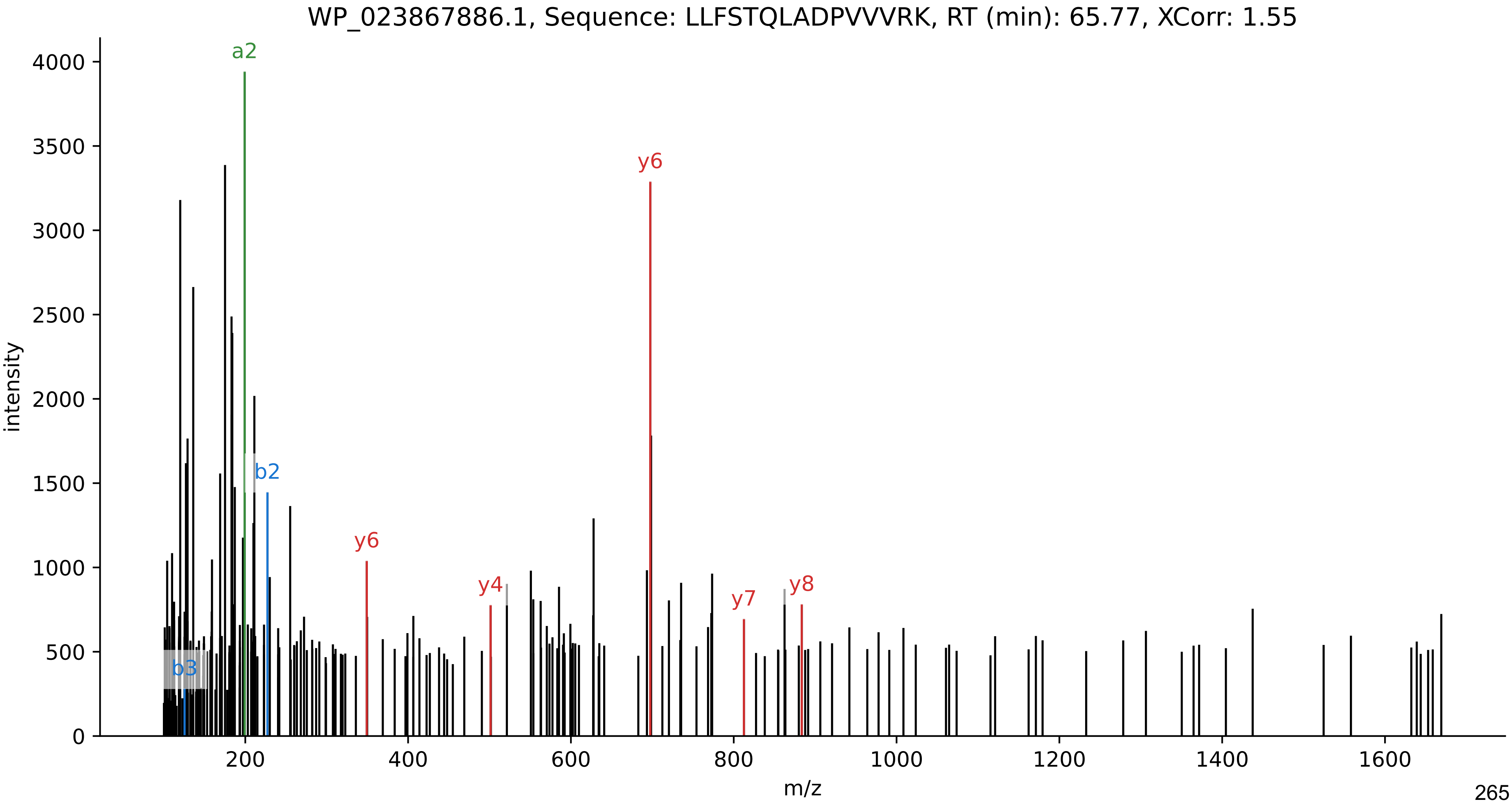
WP_023867886.1, Sequence: LLFSTQLADPVVVRK, RT (min): 65.77, XCorr: 1.55
intensity
m/z
a2
b2
b3
y6
y6
y4
y7
y8
0
500
1000
1500
2000
2500
3000
3500
4000
200
400
600
800
1000
1200
1400
1600

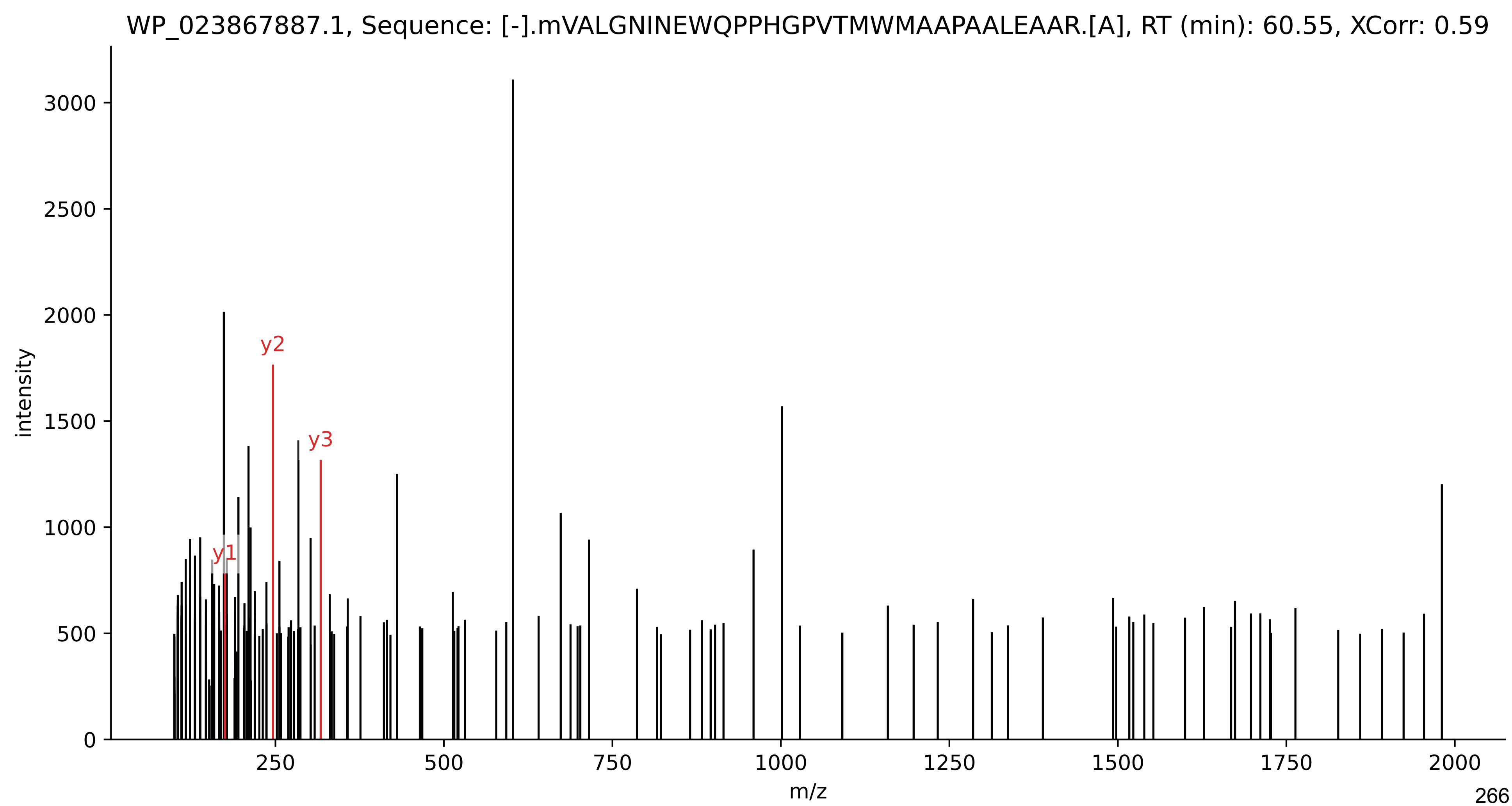
WP_023867887.1, Sequence: [-].mVALGNINEWQPPHGPVTMWMAAPAALEAAR.[A], RT (min): 60.55, XCorr: 0.59
intensity
m/z
y1
y2
y3
0
500
1000
1500
2000
2500
3000
250
500
750
1000
1250
1500
1750
2000

WP_023868373.1, Sequence: [R].SDGGVVTPmVHDFIAR.[G], RT (min): 49.53, XCorr: 2.15

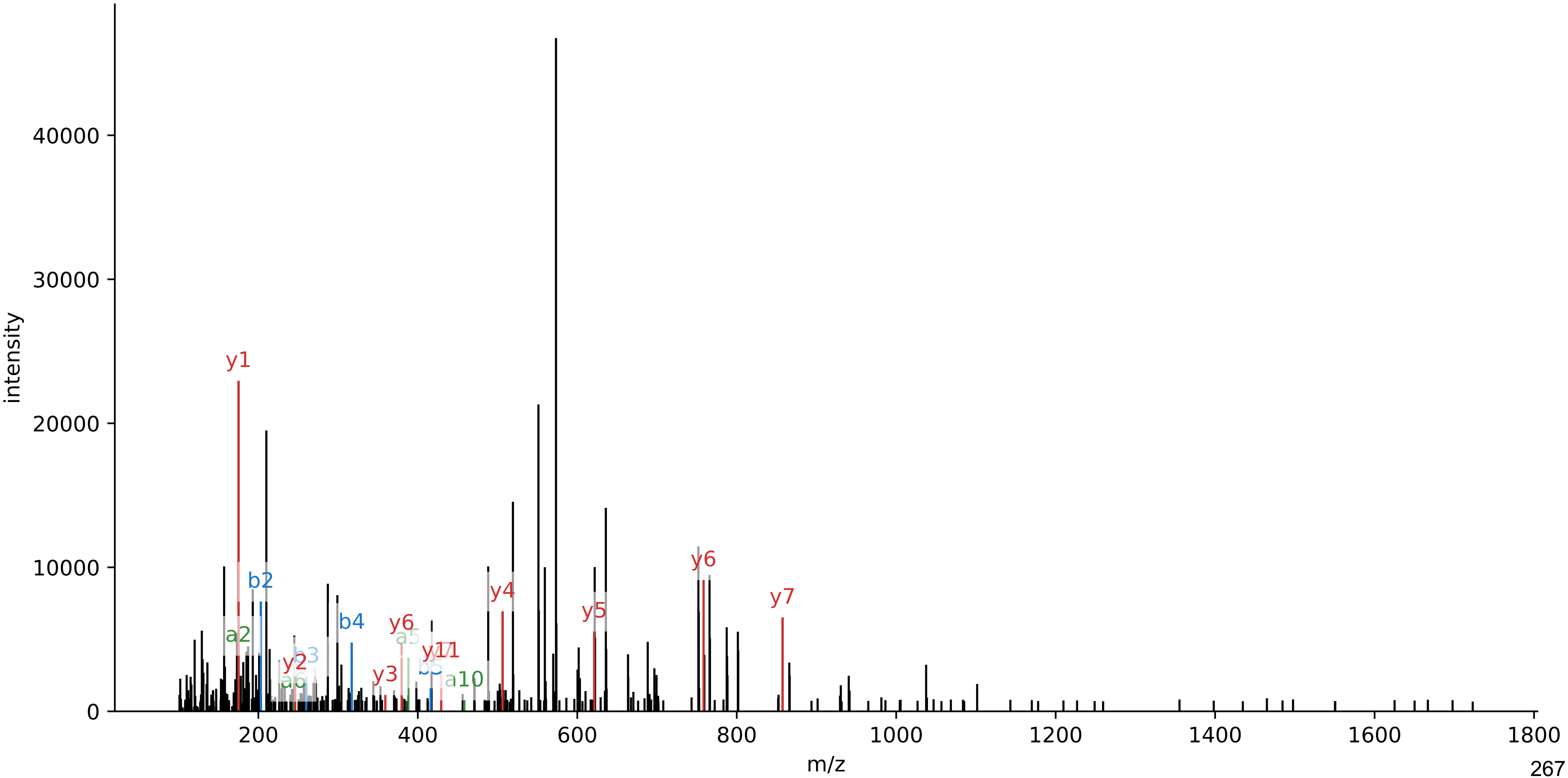

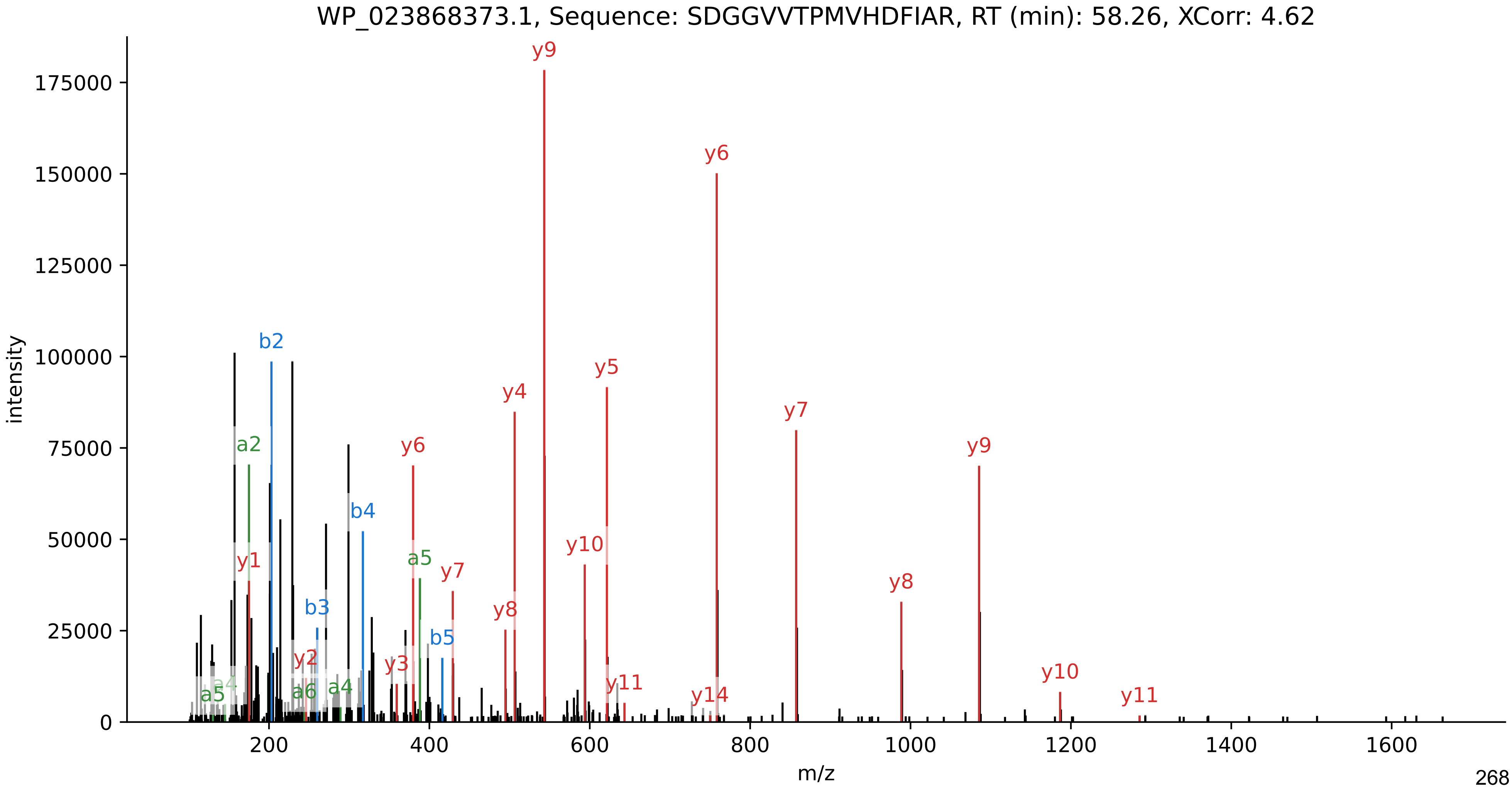
WP_023868373.1, Sequence: SDGGVVTPMVHDFIAR, RT (min): 58.26, XCorr: 4.62
intensity
m/z
0
25000
50000
75000
100000
125000
150000
175000
200
400
600
800
1000
1200
1400
1600
y9
y6
b2
y5
y4
y7
y9
a2
y6
b4
y10
a5
y1
y7
y8
y8
b3
b5
y2
y3
y10
y11
a4
a5
a6
a4
y14
y11

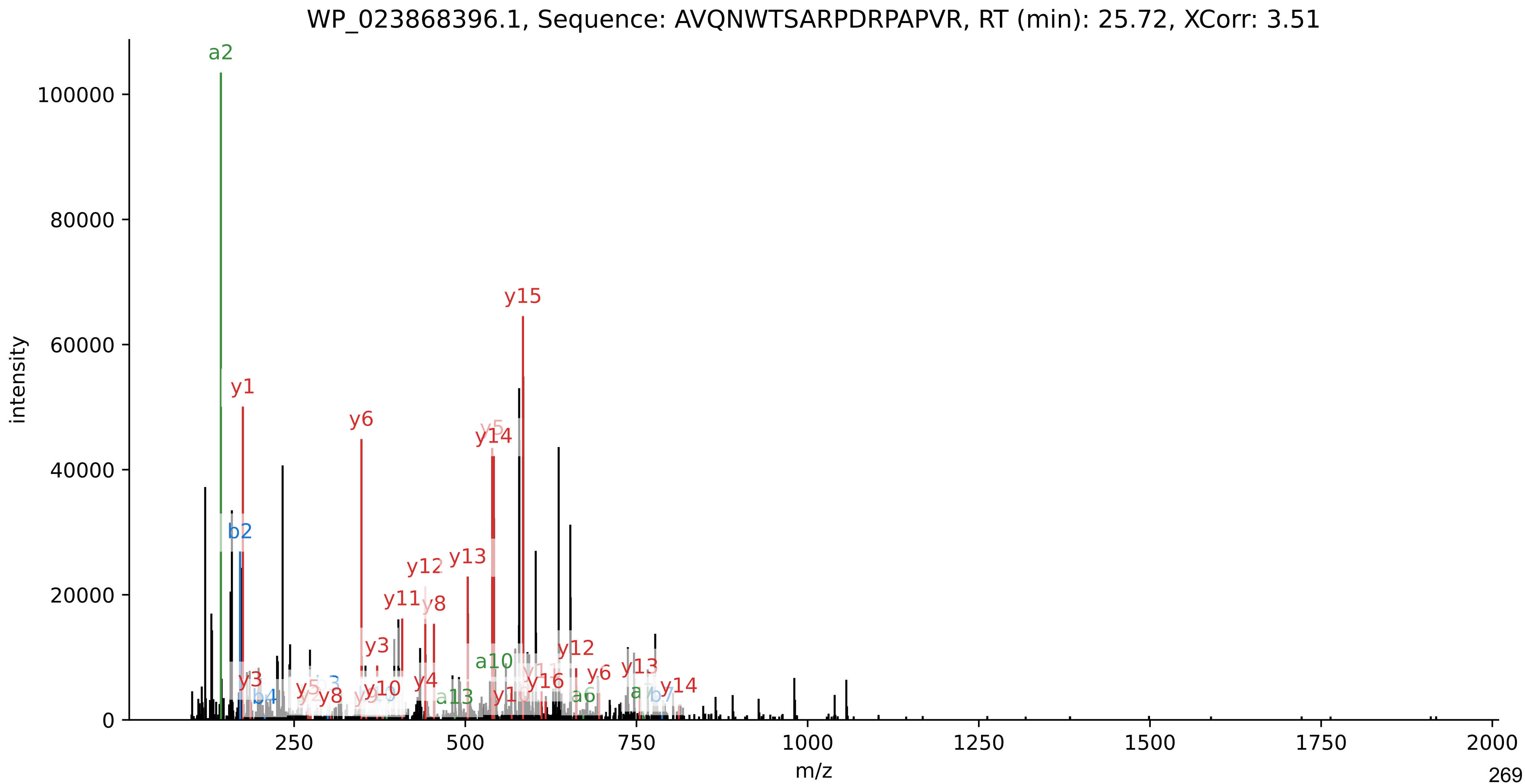
WP_023868396.1, Sequence: AVQNWTSARPDRPAPVR, RT (min): 25.72, XCorr: 3.51
intensity
m/z
0
20000
40000
60000
80000
100000
250
500
750
1000
1250
1500
1750
2000
a2
y1
b2
y6
y14
y15
y13
y12
y11
y8
y3
y4
a10
y12
y16
y6
y13
y14
y10
y3
y8
b4
a13
a6

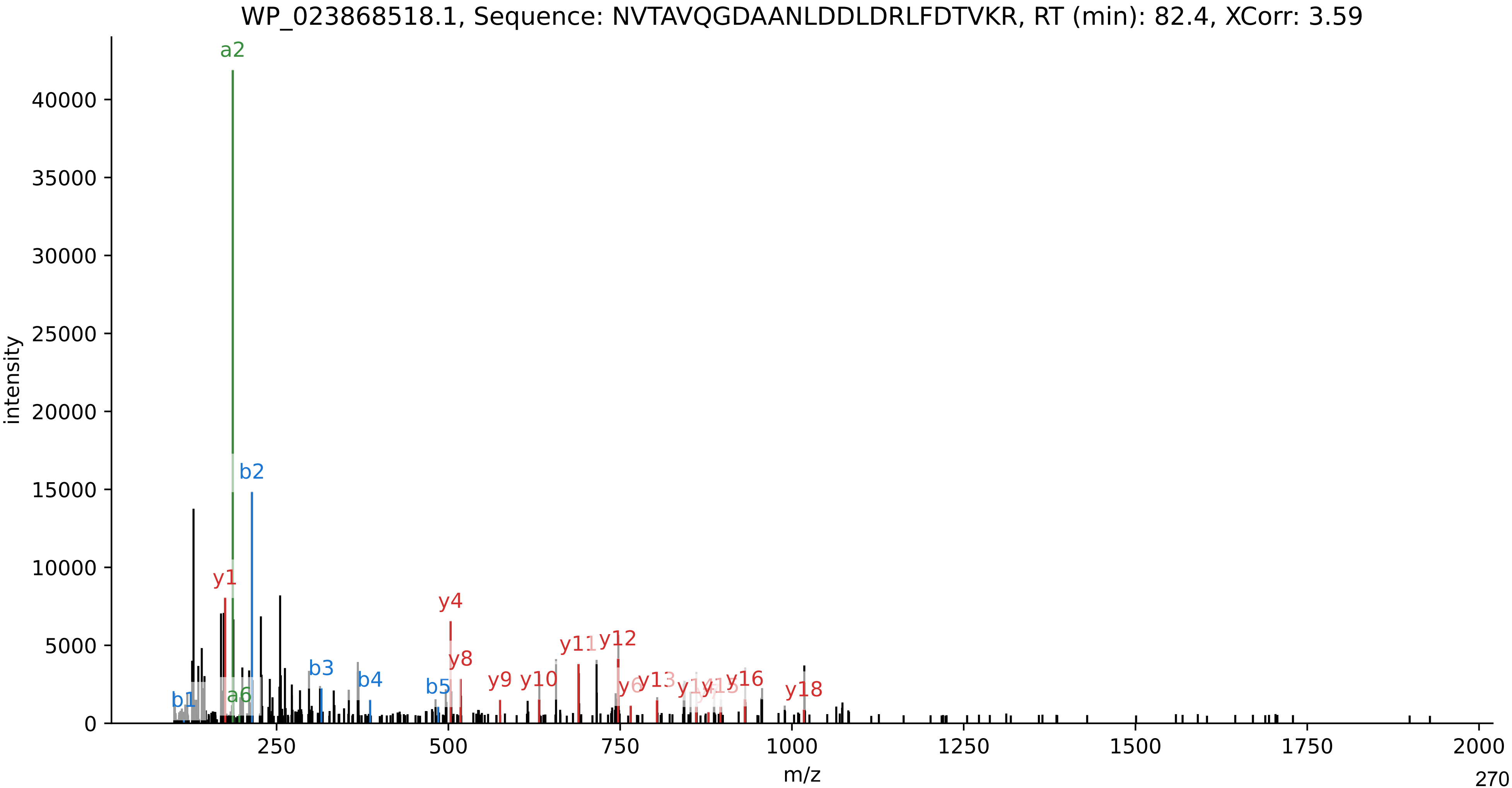
WP_023868518.1, Sequence: NVTAVQGDAANLDDLDRLFDTVKR, RT (min): 82.4, XCorr: 3.59
a2
b2
y1
y4
y8
y11
y12
y9
y10
y13
y16
y18
b1
a6
b3
b4
b5
intensity
m/z
0
5000
10000
15000
20000
25000
30000
35000
40000
250
500
750
1000
1250
1500
1750
2000

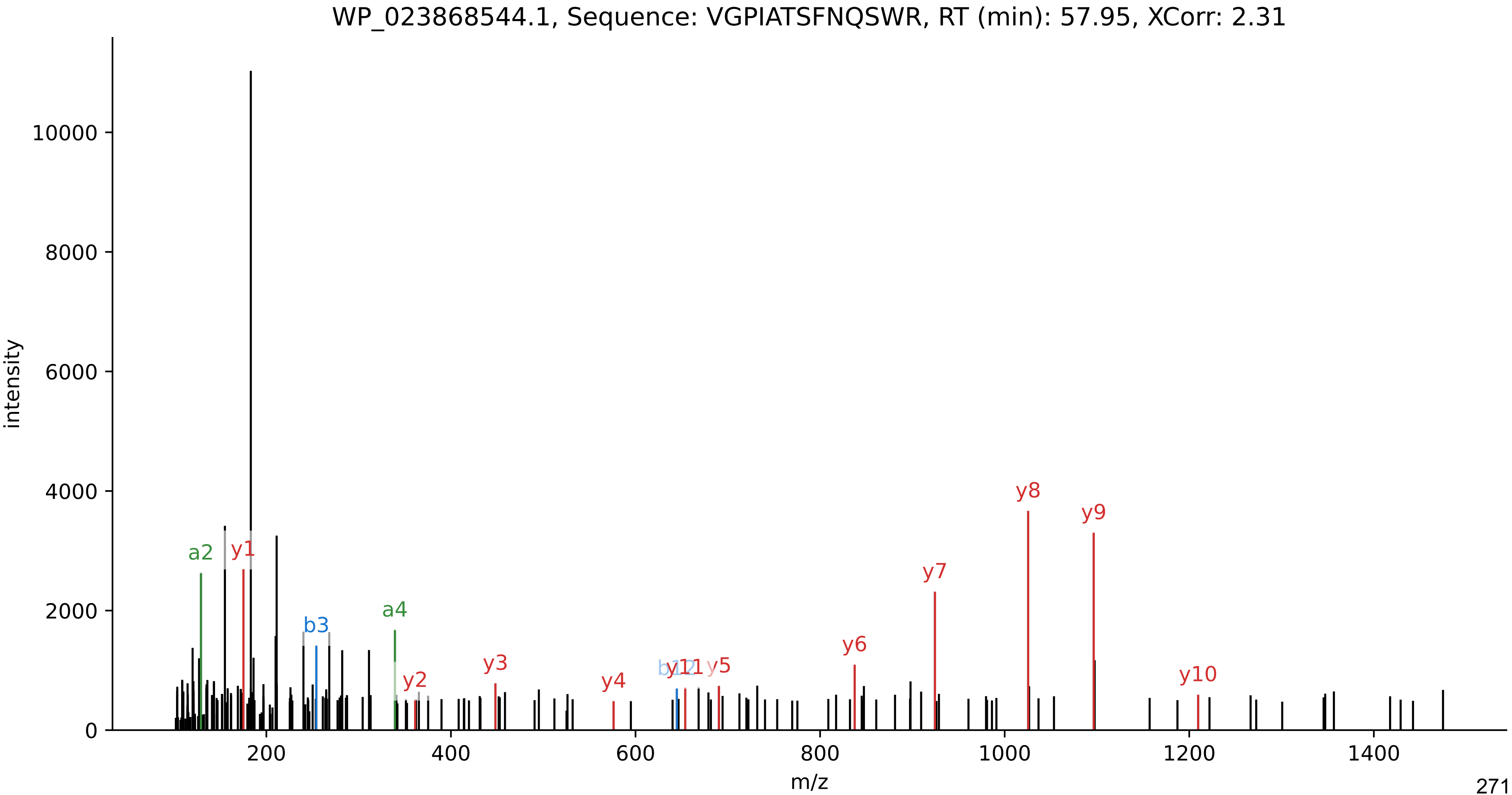

WP_023868544.1, Sequence: VGPIATSFNQSWR, RT (min): 57.95, XCorr: 2.31
intensity
m/z
a2
y1
b3
a4
y2
y3
y4
y11
y5
y6
y7
y8
y9
y10

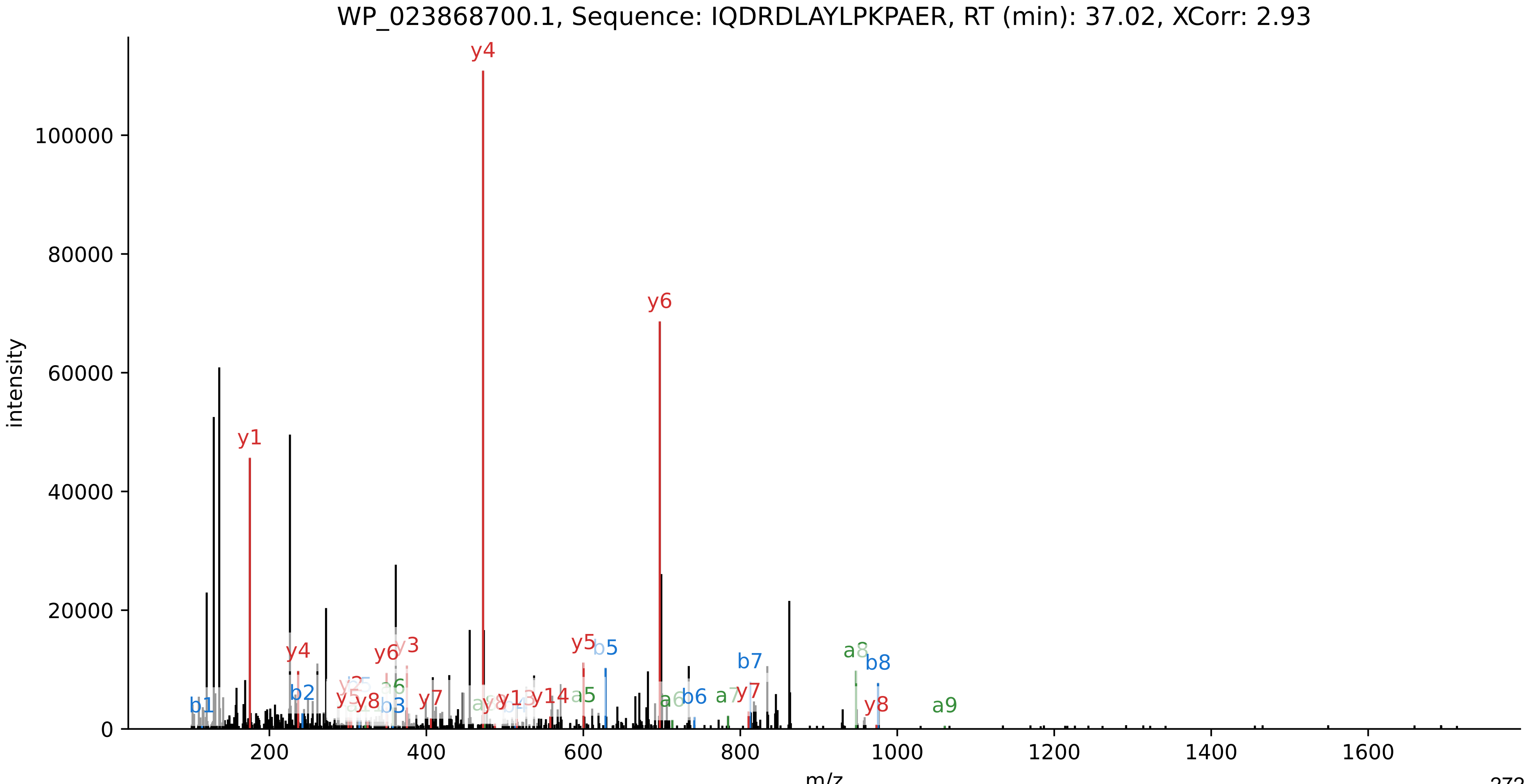
WP_023868700.1, Sequence: IQDRDLAYLPKPAER, RT (min): 37.02, XCorr: 2.93
intensity
m/z
0
20000
40000
60000
80000
100000
200
400
600
800
1000
1200
1400
1600
y4
y6
y1
y4
y6
y3
y5
b5
b7
a8
b8
b1
b2
y7
y14
a5
b6
a7
y7
y8
a9

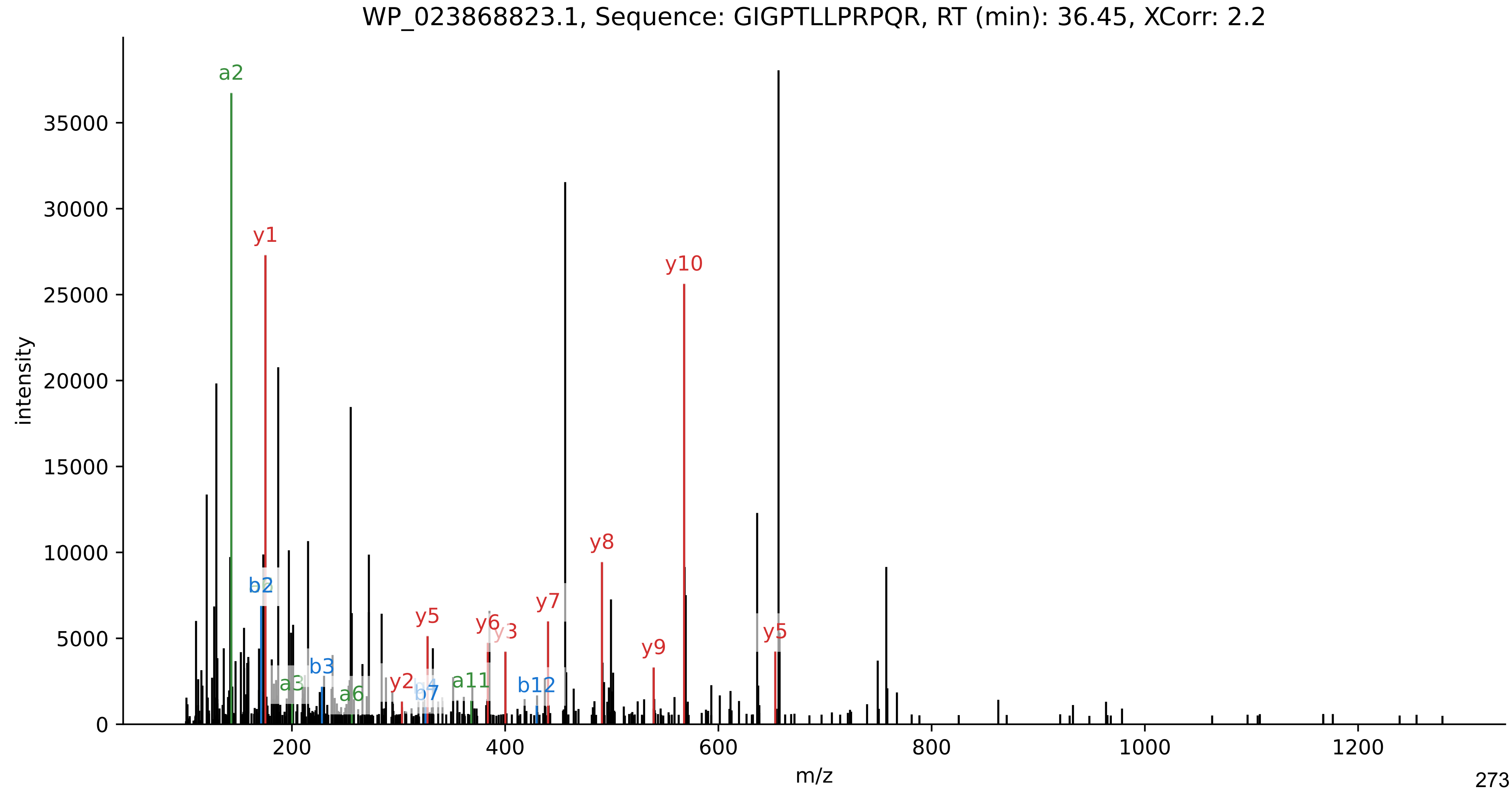
WP_023868823.1, Sequence: GIGPTLLPRPQR, RT (min): 36.45, XCorr: 2.2
intensity
m/z
a2
y1
y10
y8
y7
y5
y6
y3
y9
y5
b2
b3
a3
a6
y2
b7
a11
b12
0
5000
10000
15000
20000
25000
30000
35000
200
400
600
800
1000
1200

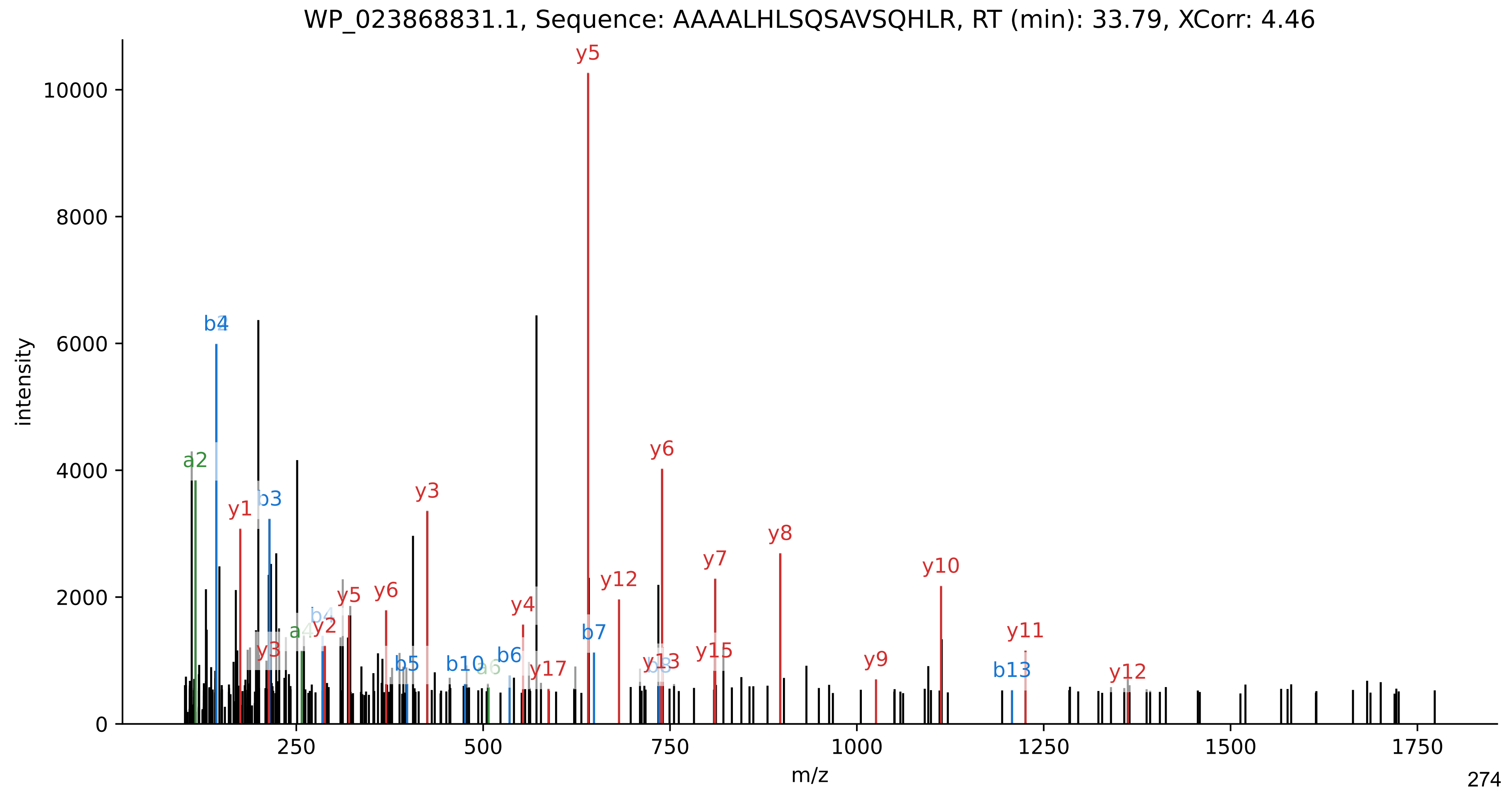
WP_023868831.1, Sequence: AAAALHLSQSAVSQHLR, RT (min): 33.79, XCorr: 4.46
intensity
m/z
y5
b4
a2
y1
b3
y3
y6
y8
y7
y10
y12
y11
y4
b7
y13
y15
y9
b13
y17
b6
b10
b5
y2
a4
b4
y5
y6
y3
y12
a6
0
2000
4000
6000
8000
10000
250
500
750
1000
1250
1500
1750

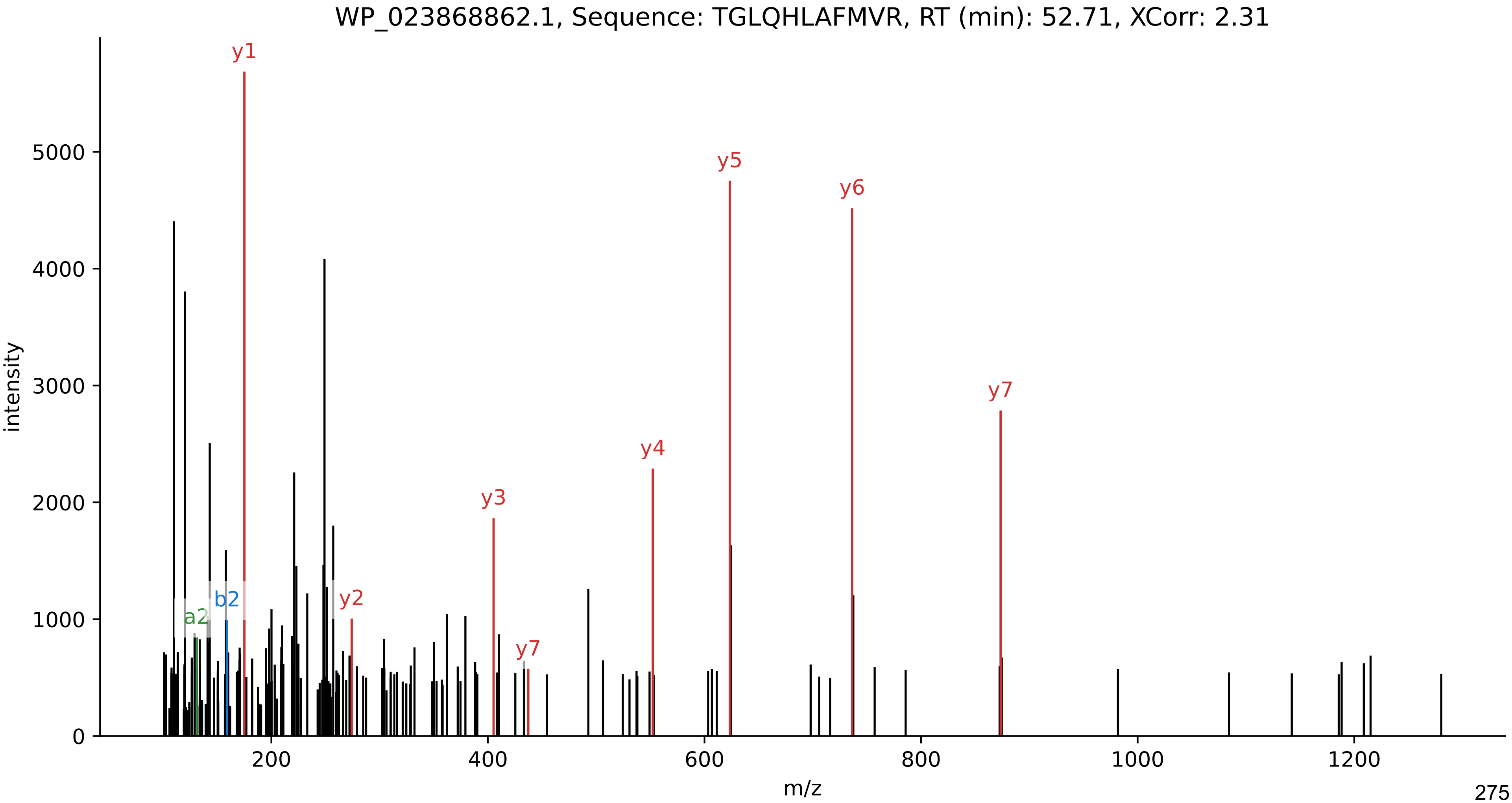

WP_023868862.1, Sequence: TGLQHLAFMVR, RT (min): 52.71, XCorr: 2.31
y1
y5
y6
y7
y4
y3
y2
b2
a2
y7
intensity
m/z
0
1000
2000
3000
4000
5000
200
400
600
800
1000
1200

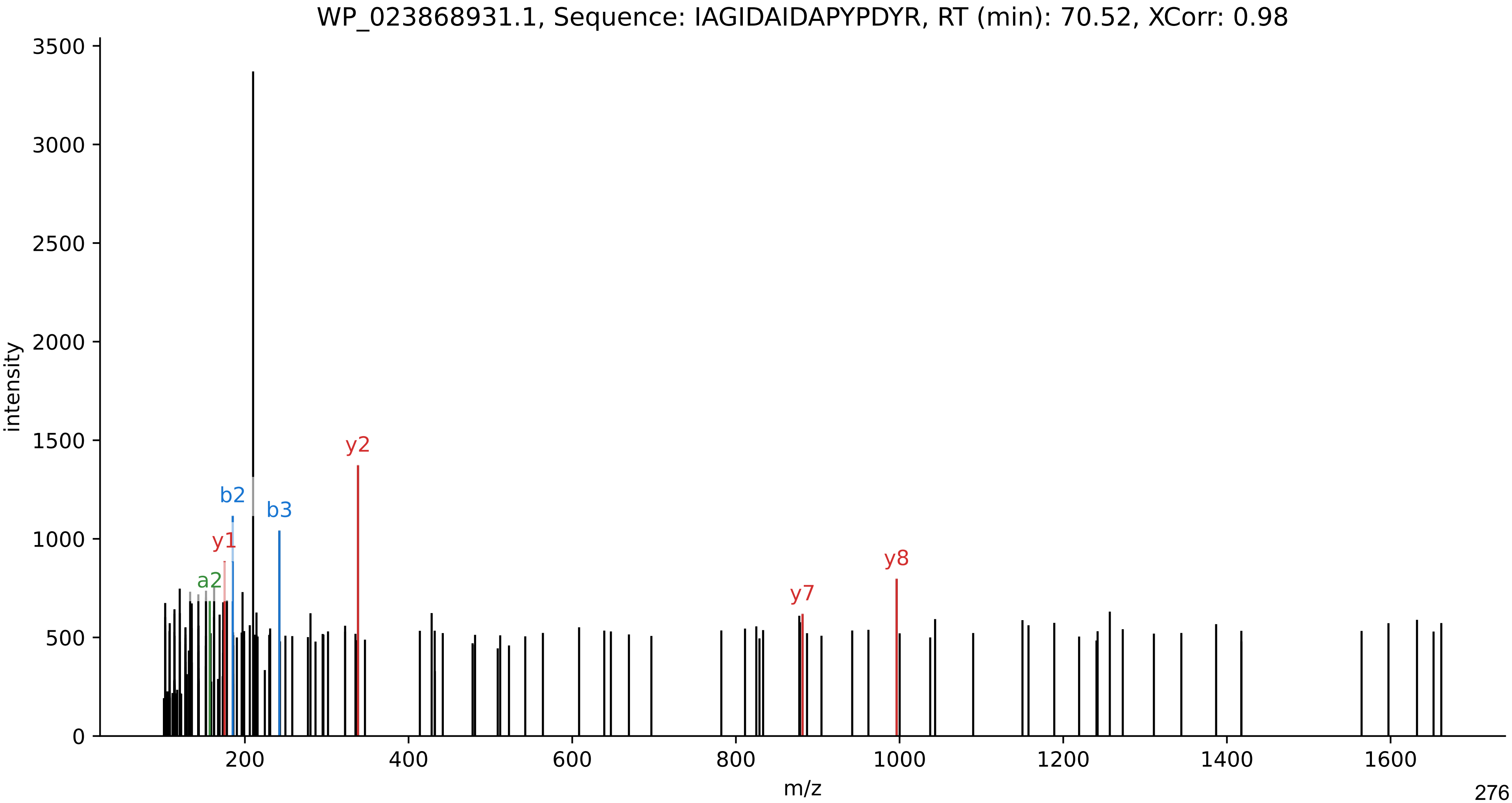
WP_023868931.1, Sequence: IAGIDAIDAPYPDYR, RT (min): 70.52, XCorr: 0.98
intensity
m/z
y2
b2
b3
y1
a2
y7
y8
0
500
1000
1500
2000
2500
3000
3500
200
400
600
800
1000
1200
1400
1600

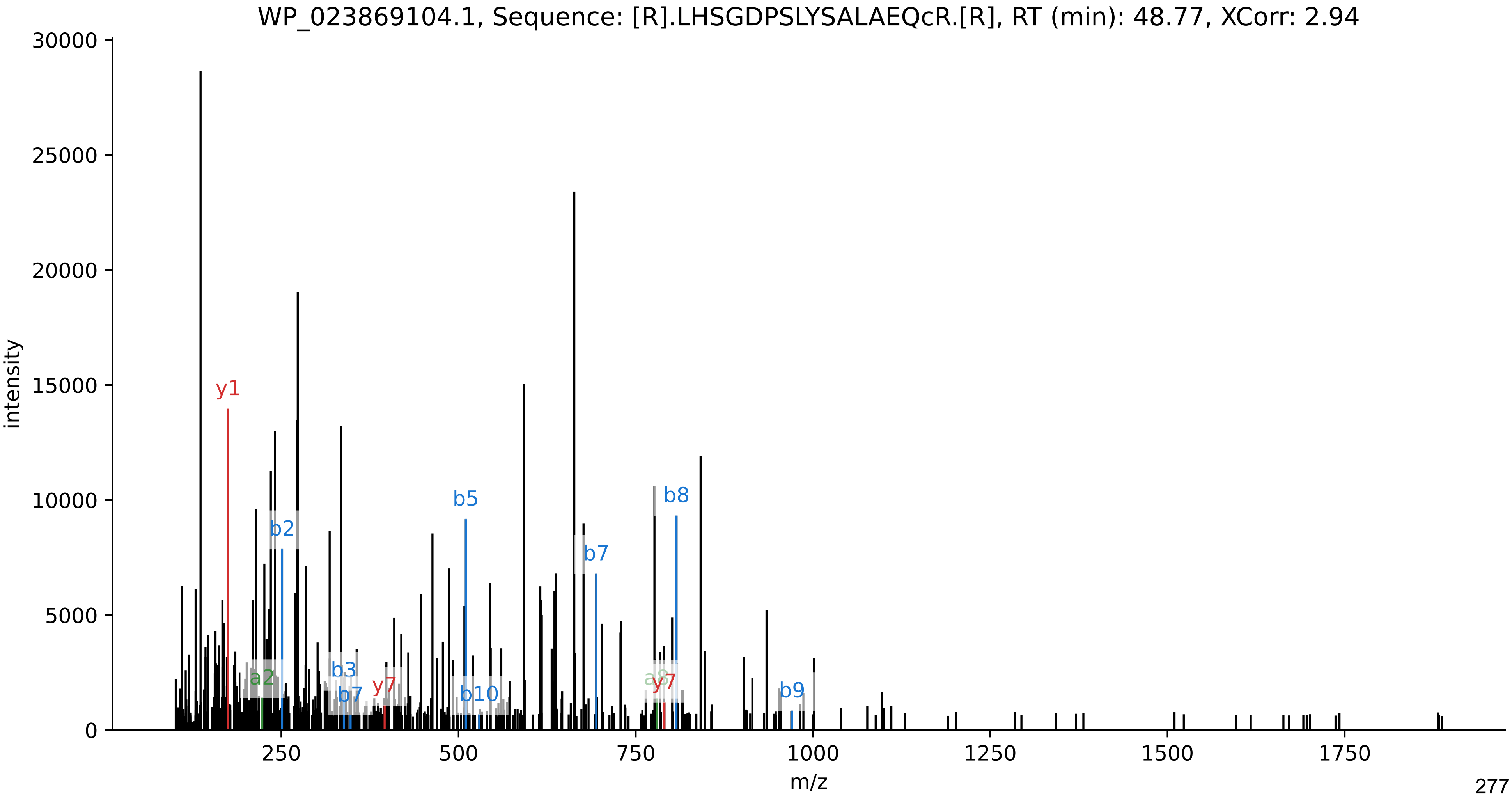
WP_023869104.1, Sequence: [R].LHSGDPSLYSALAEQcR.[R], RT (min): 48.77, XCorr: 2.94
intensity
m/z
y1
b2
a2
b3
b7
y7
b5
b10
b7
b8
y7
b9
30000
25000
20000
15000
10000
5000
0
250
500
750
1000
1250
1500
1750

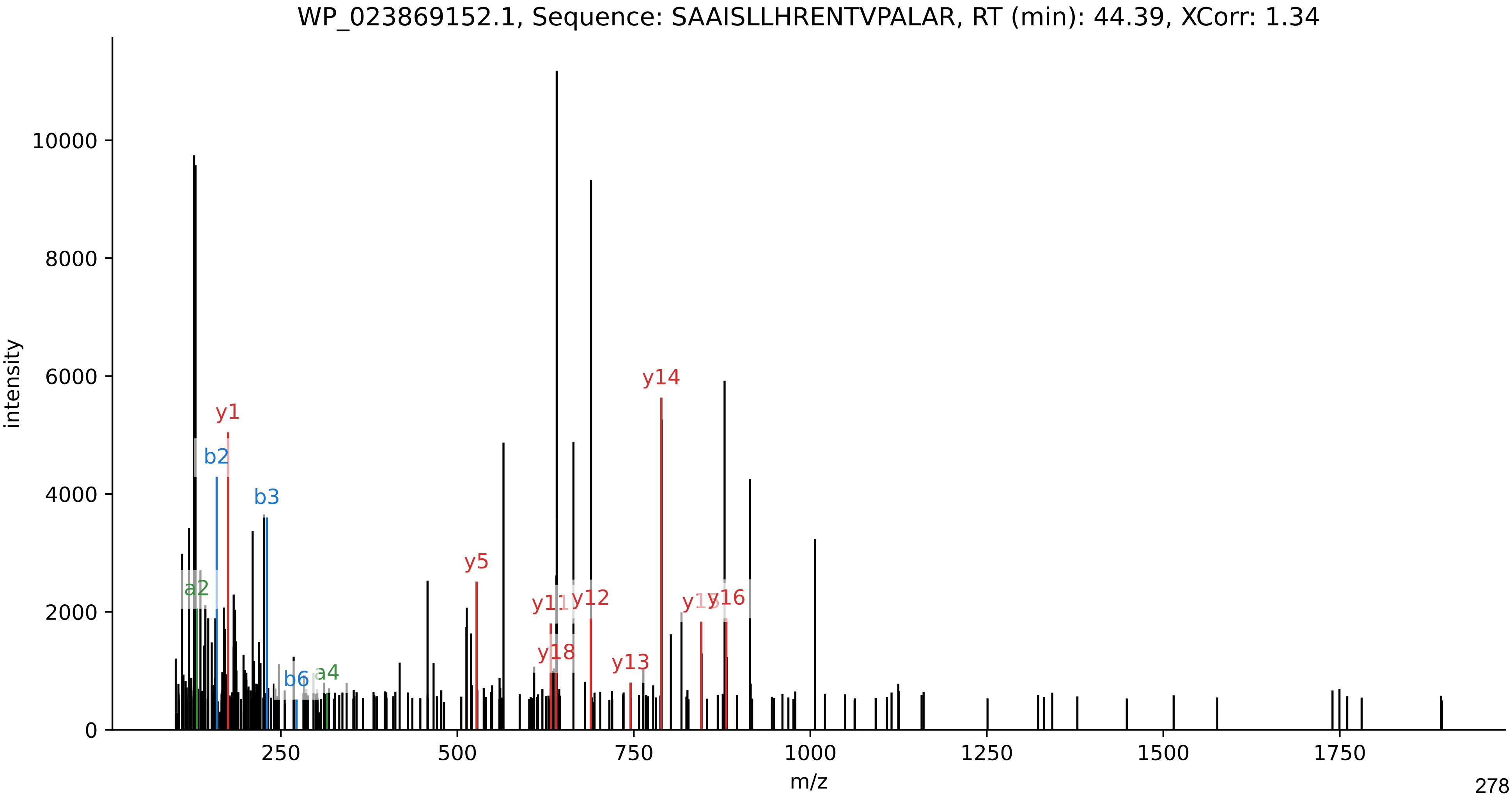
WP_023869152.1, Sequence: SAAISLLHRENTVPALAR, RT (min): 44.39, XCorr: 1.34
intensity
m/z
y1
b2
b3
a2
b6
a4
y5
y11
y12
y18
y13
y14
y16
0
2000
4000
6000
8000
10000
250
500
750
1000
1250
1500
1750

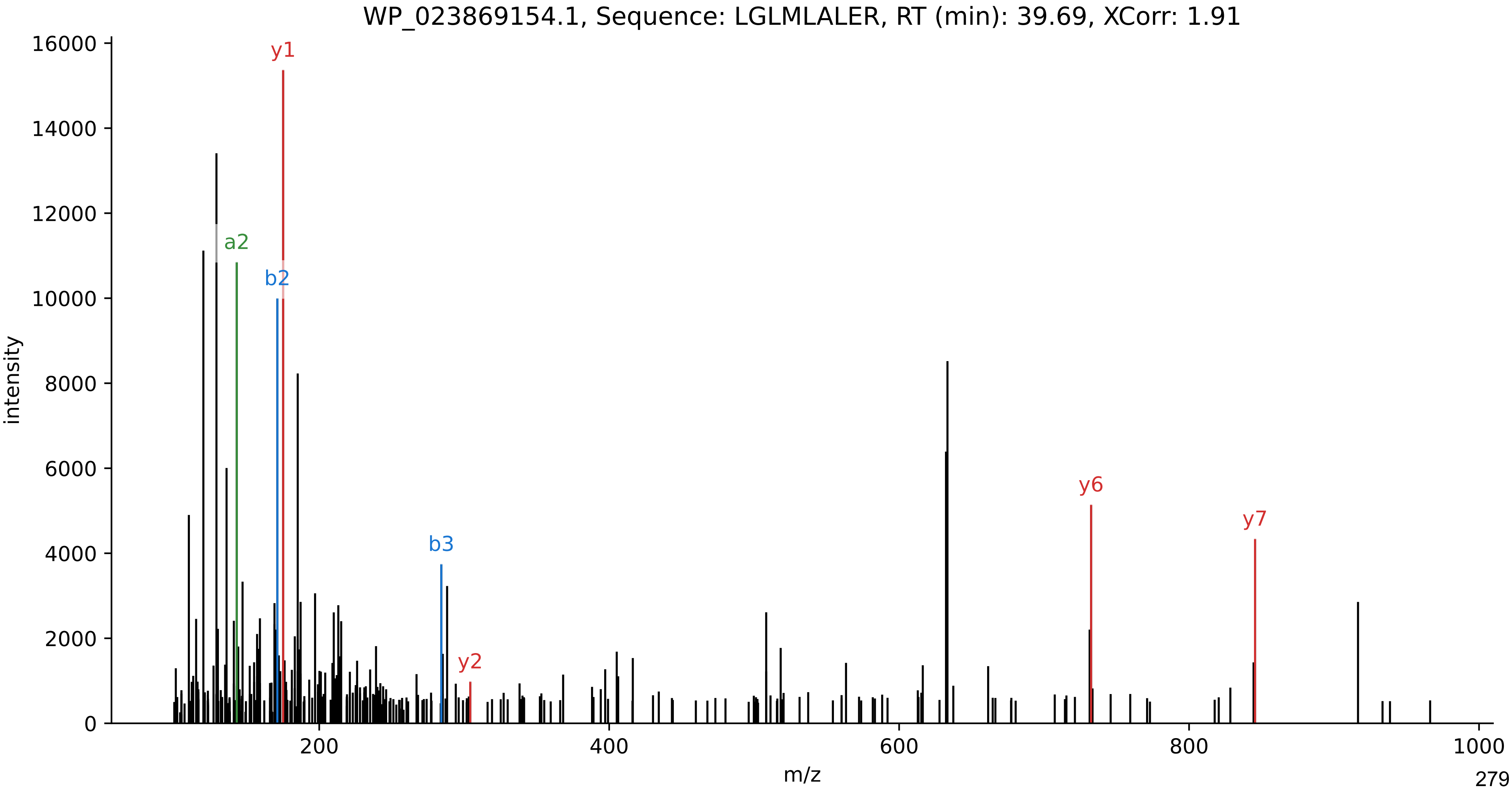

WP_023869154.1, Sequence: LGLMLALER, RT (min): 39.69, XCorr: 1.91
y1
a2
b2
b3
y2
y6
y7
intensity
m/z
16000
14000
12000
10000
8000
6000
4000
2000
0
200
400
600
800
1000

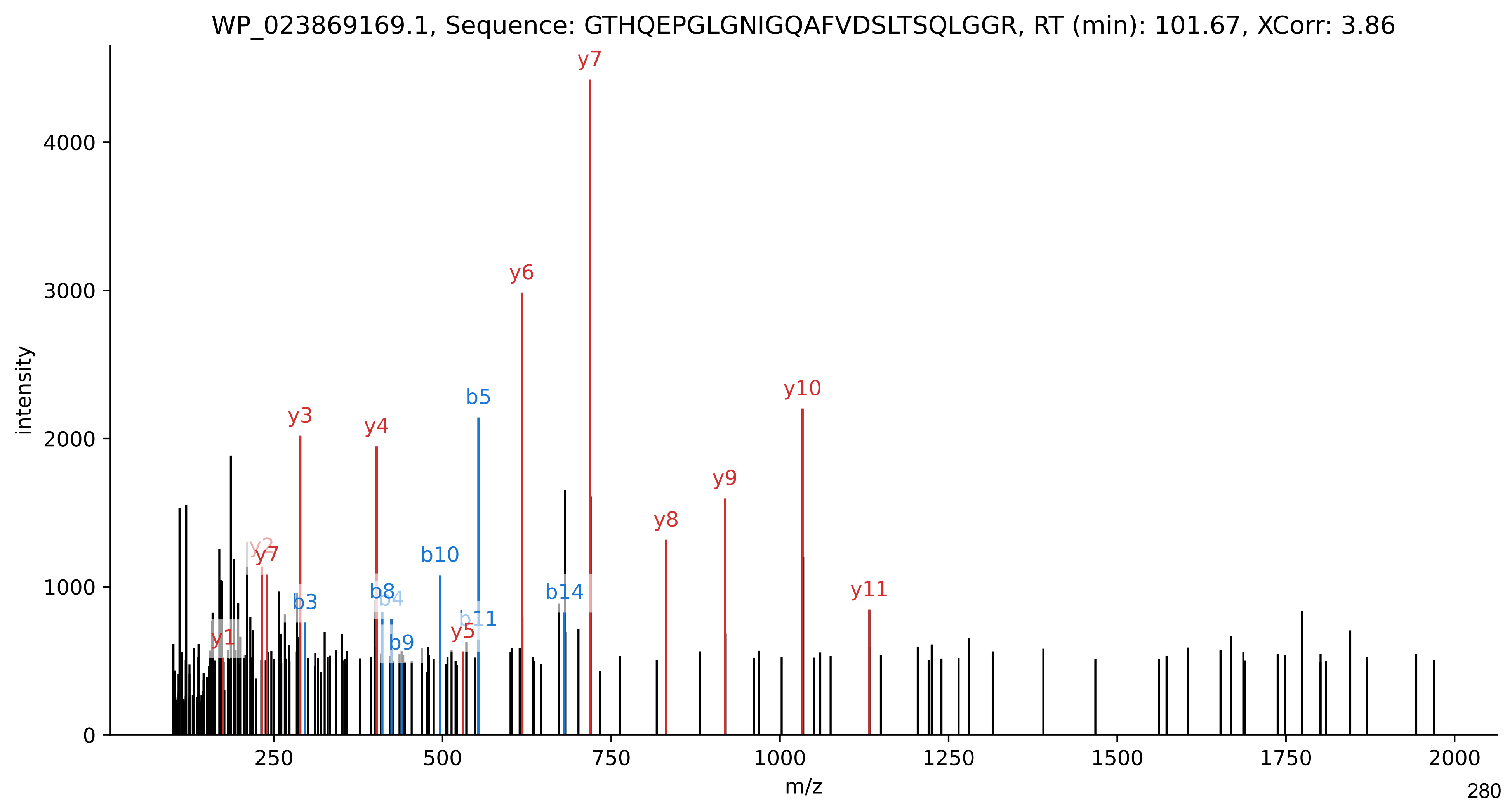
WP_023869169.1, Sequence: GTHQEPGLGNIGQAFVDSLTSQLGGR, RT (min): 101.67, XCorr: 3.86
intensity
m/z
y1
y2
y7
y3
b3
y4
b8
b4
b9
b10
b11
y5
b5
y6
b14
y7
y8
y9
y10
y11
0
1000
2000
3000
4000
250
500
750
1000
1250
1500
1750
2000

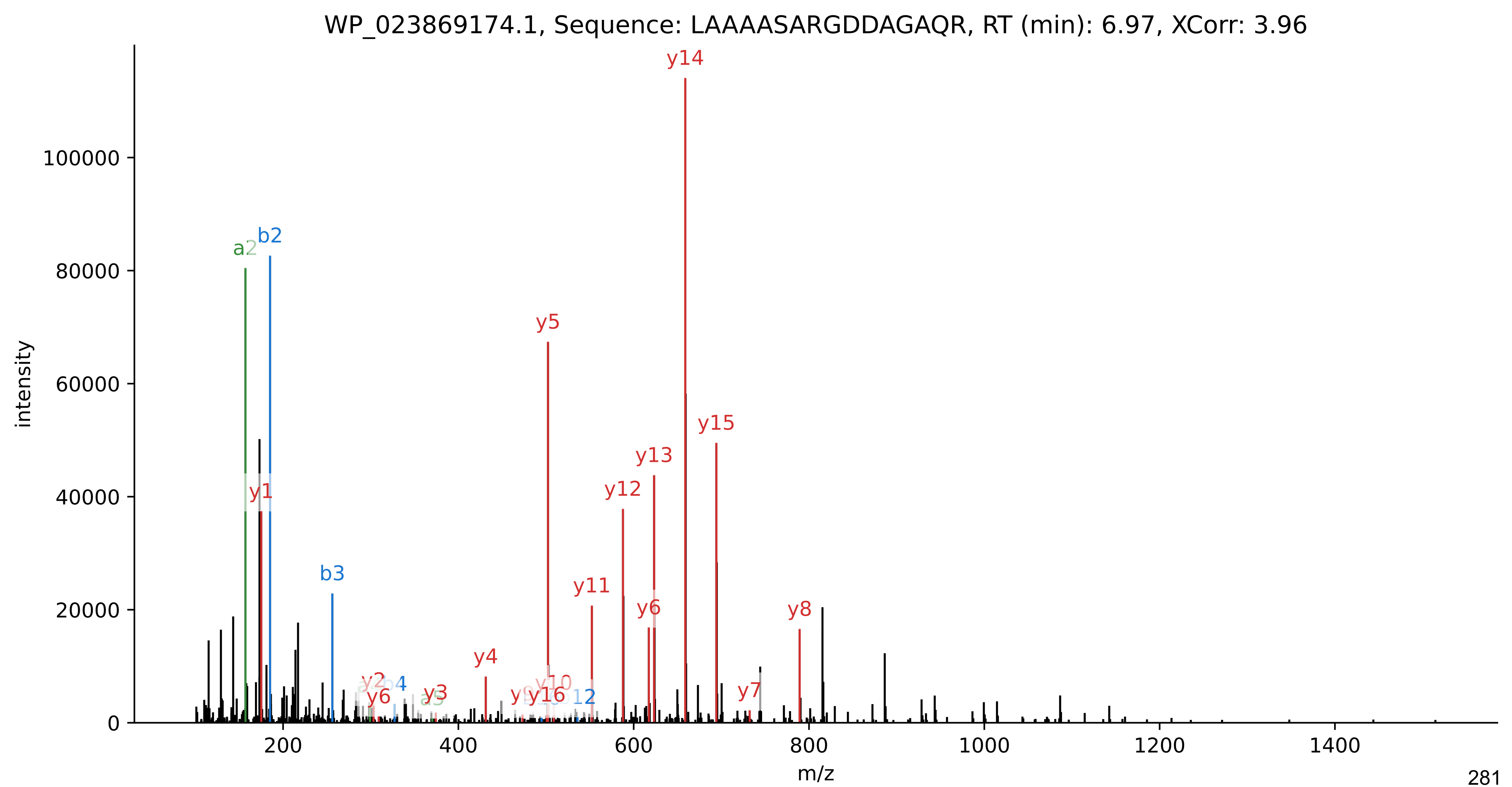

WP_023869174.1, Sequence: LAAAASARGDDAGAQR, RT (min): 6.97, XCorr: 3.96
intensity
m/z
y14
a2
b2
y5
y15
y13
y12
y1
b3
y11
y6
y8
y4
y2
y6
y3
y7
y10
y16
0
20000
40000
60000
80000
100000
200
400
600
800
1000
1200
1400

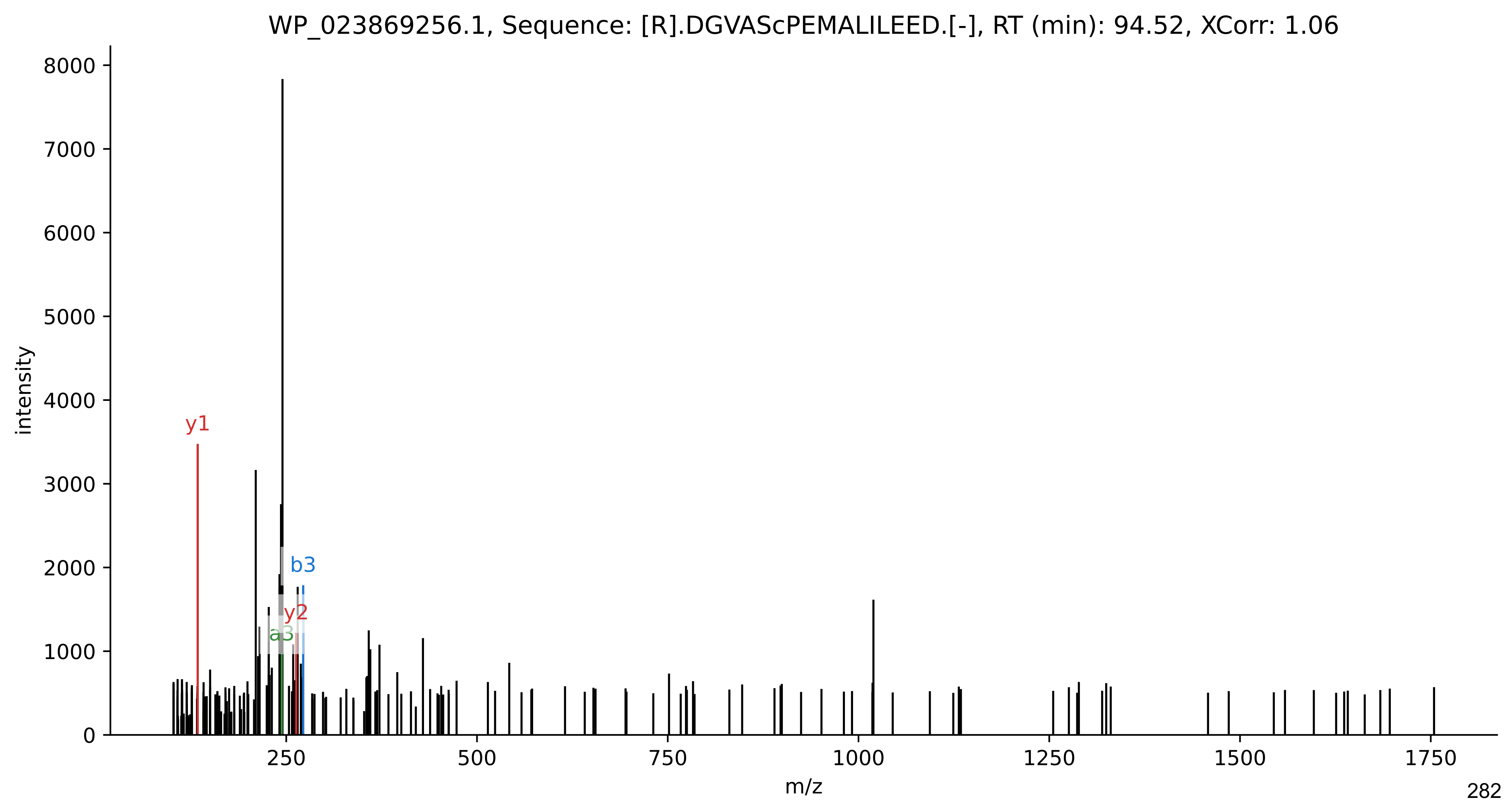
WP_023869256.1, Sequence: [R].DGVAScPEMALILEED.[-], RT (min): 94.52, XCorr: 1.06
intensity
m/z
y1
b3
y2
a3
0
1000
2000
3000
4000
5000
6000
7000
8000
250
500
750
1000
1250
1500
1750

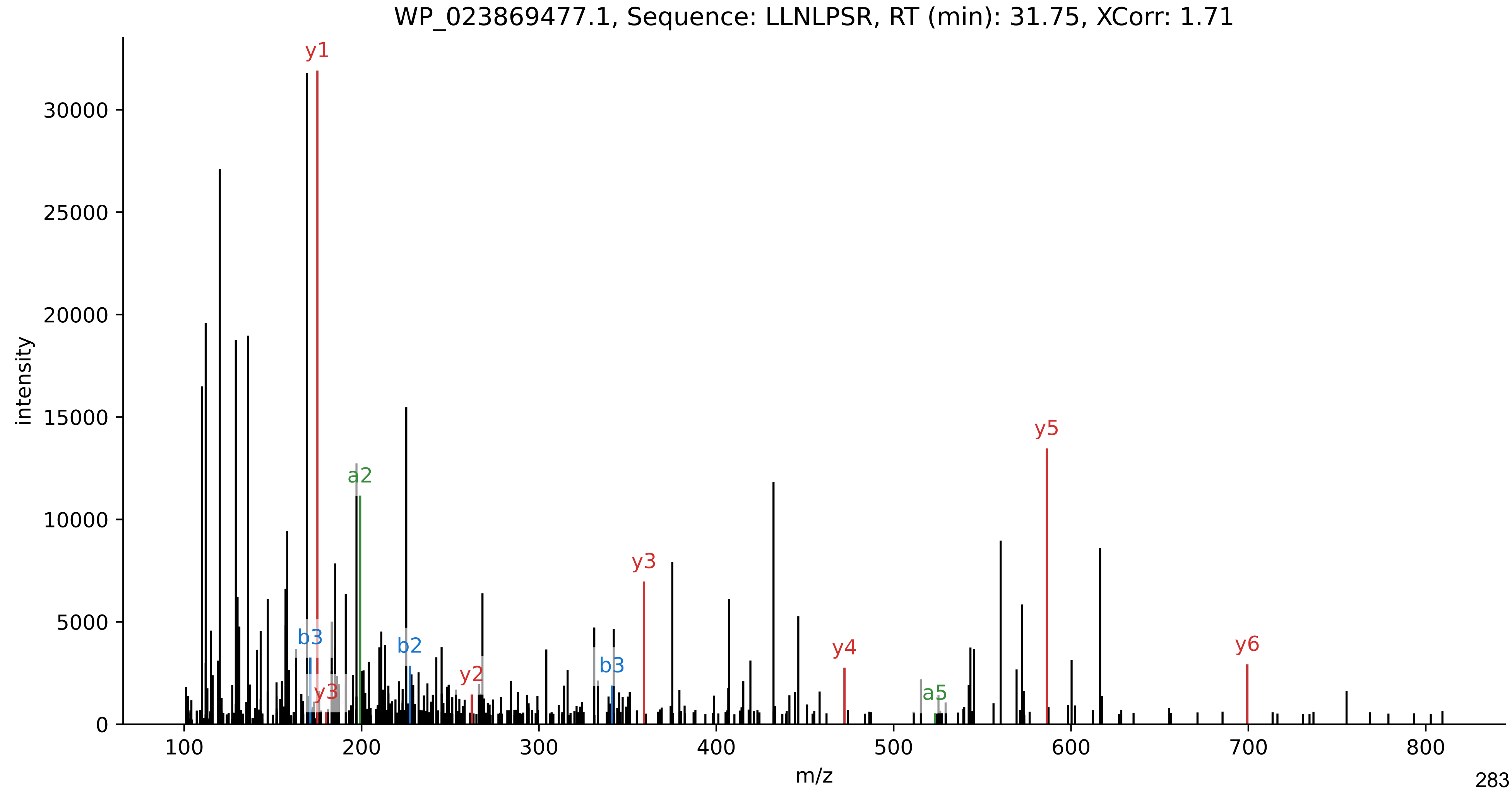
WP_023869477.1, Sequence: LLNLPSR, RT (min): 31.75, XCorr: 1.71
intensity
m/z
y1
a2
b3
y3
b2
y2
b3
y3
y4
a5
y5
y6
0
5000
10000
15000
20000
25000
30000
100
200
300
400
500
600
700
800

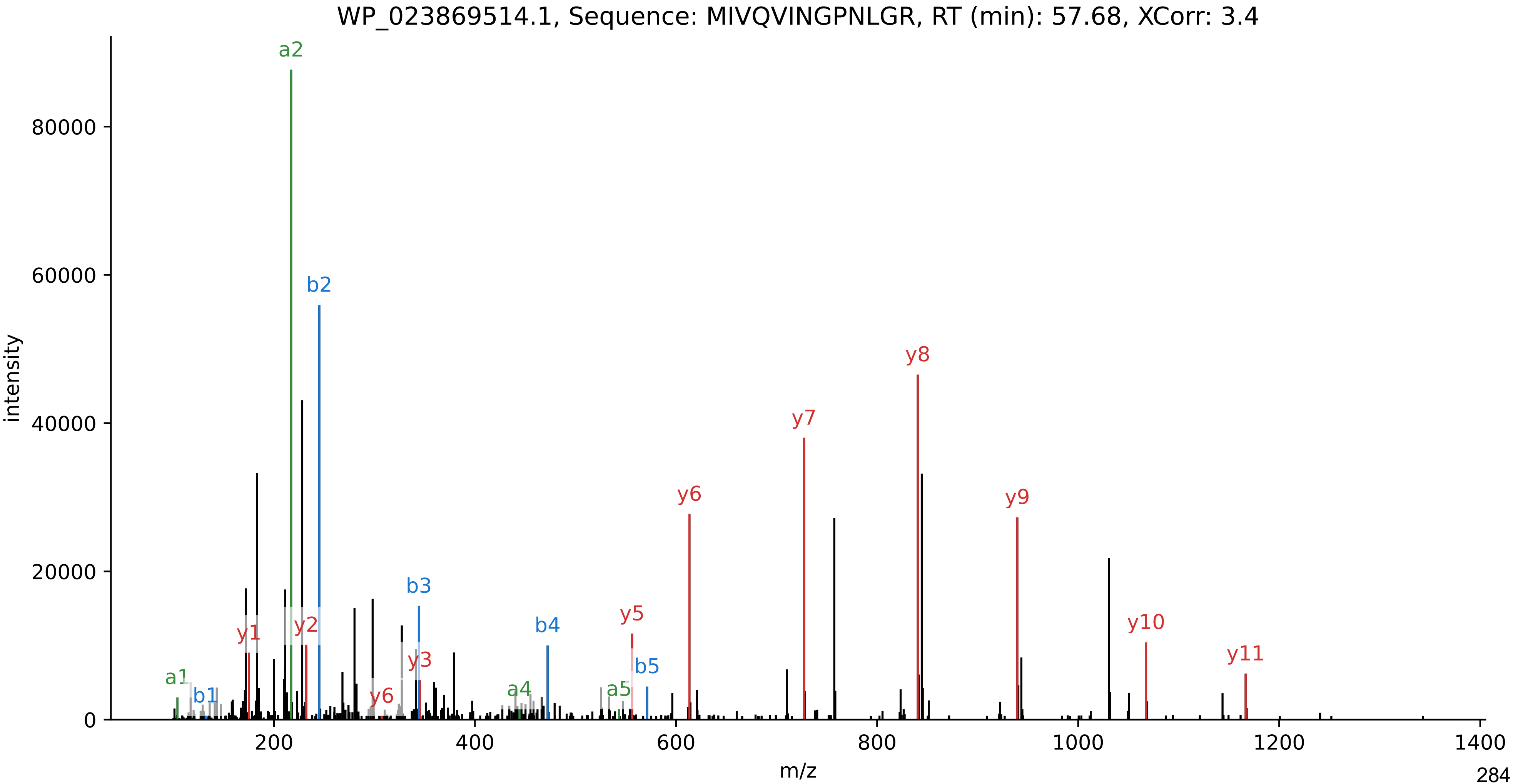

WP_023869514.1, Sequence: MIVQVINGPNLGR, RT (min): 57.68, XCorr: 3.4
intensity
m/z
a2
b2
y8
y7
y6
y9
y10
y11
y5
b5
b4
b3
y3
y2
y1
a1
b1
y6
a4
a5
0
20000
40000
60000
80000
200
400
600
800
1000
1200
1400

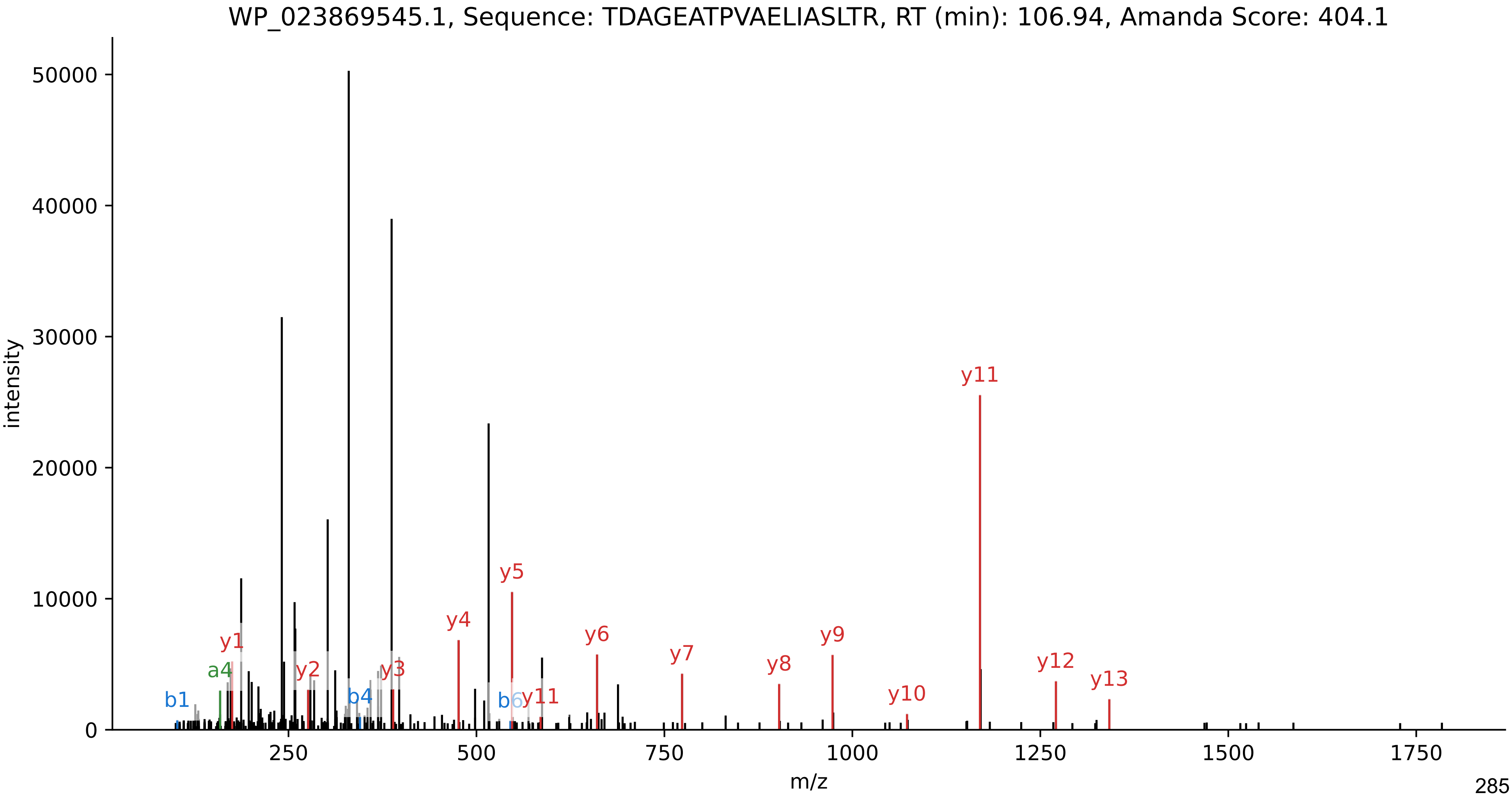
WP_023869545.1, Sequence: TDAGEATPVAELIASLTR, RT (min): 106.94, Amanda Score: 404.1
intensity
m/z
0
10000
20000
30000
40000
50000
250
500
750
1000
1250
1500
1750
b1
a4
y1
y2
b4
y3
y4
y5
b6
y11
y6
y7
y8
y9
y10
y11
y12
y13

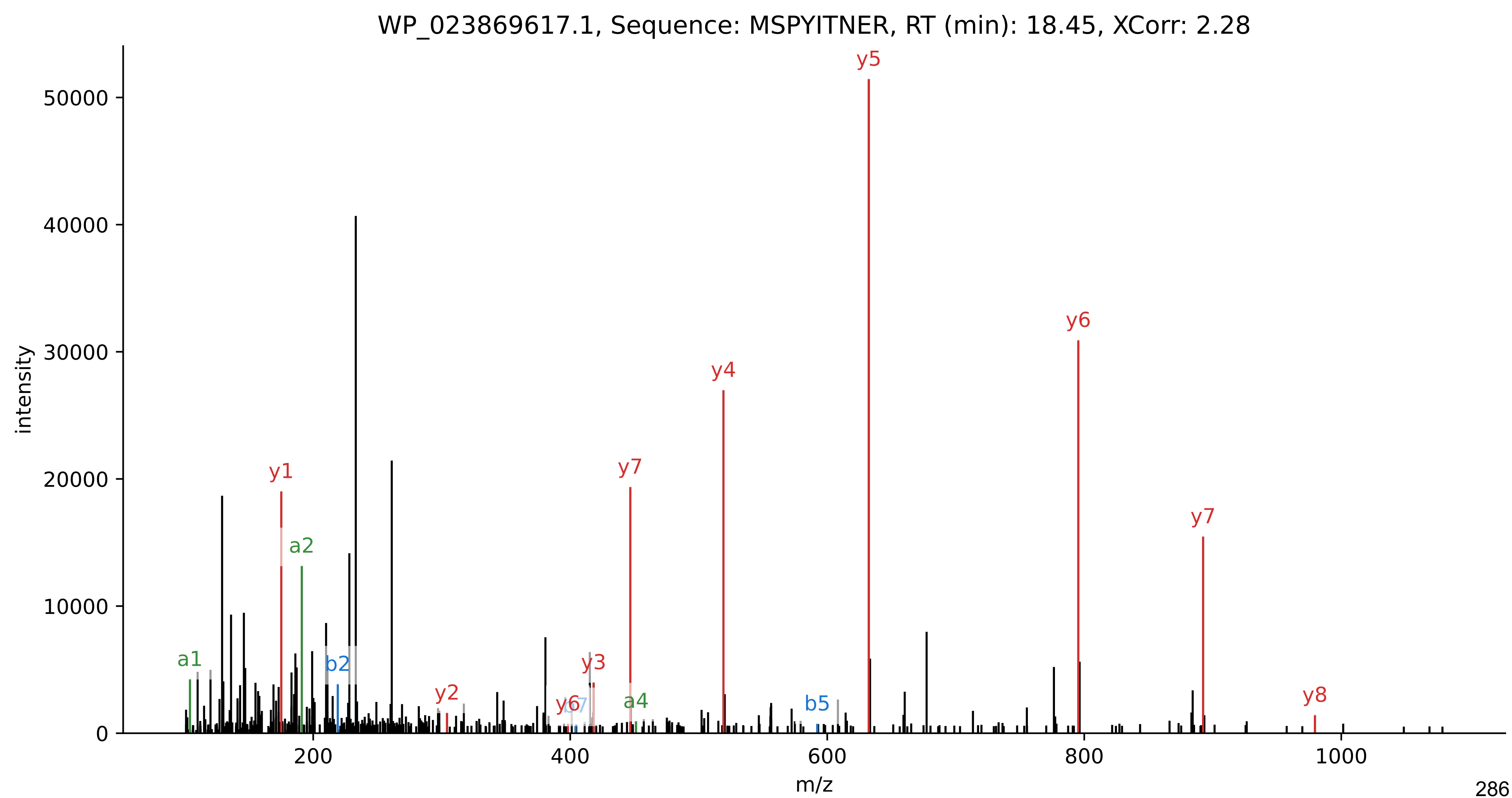
WP_023869617.1, Sequence: MSPYITNER, RT (min): 18.45, XCorr: 2.28
intensity
m/z
50000
40000
30000
20000
10000
0
200
400
600
800
1000
y5
y6
y4
y7
y1
y7
a2
a1
b2
y2
y6
b7
y3
a4
b5
y8

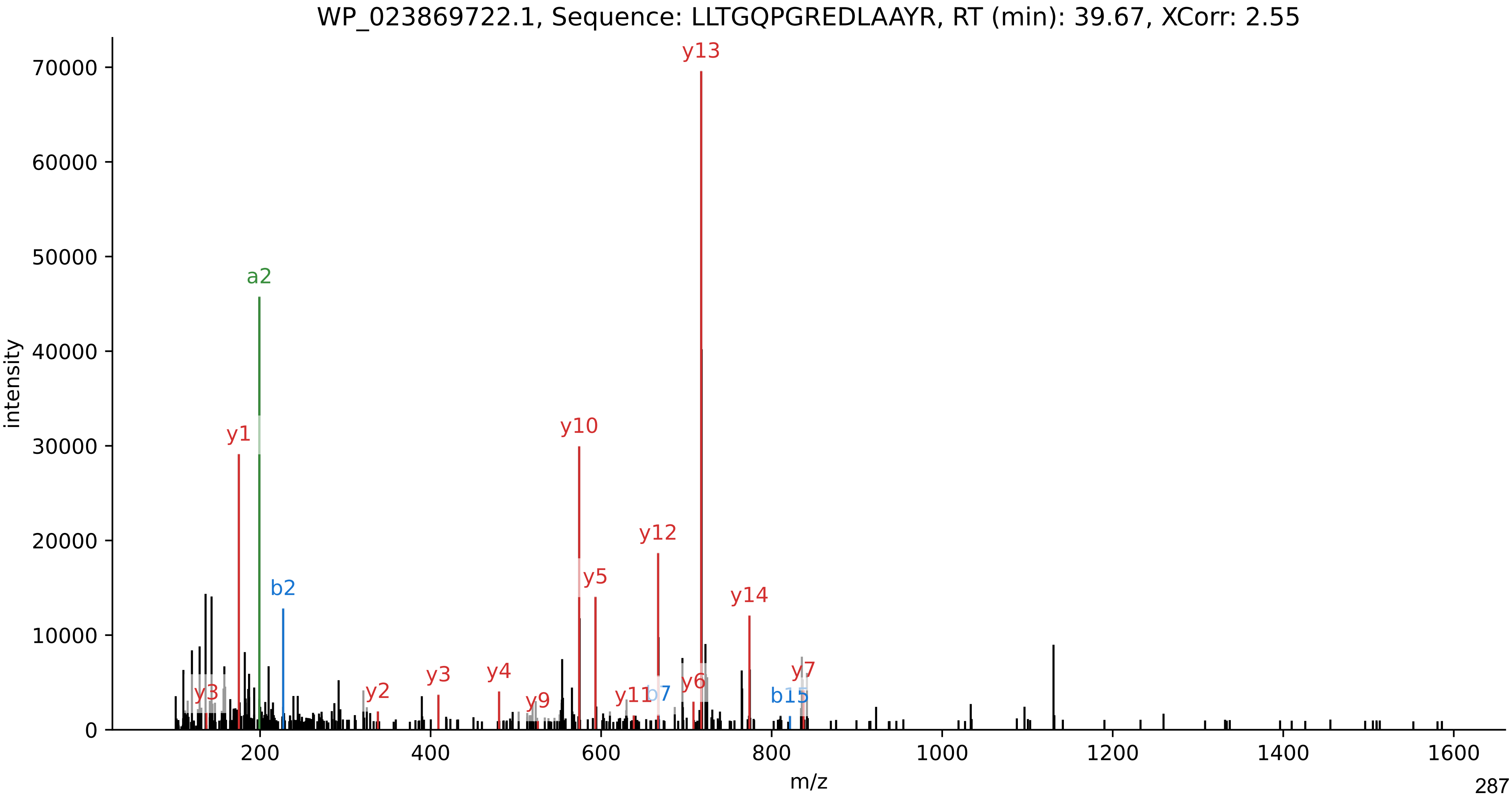
WP_023869722.1, Sequence: LLTGQPGREDLAAYR, RT (min): 39.67, XCorr: 2.55
intensity
m/z
y13
a2
y1
y10
y12
y5
b2
y14
y7
y3
y2
y3
y4
y9
y11
b7
y6
b15
0
10000
20000
30000
40000
50000
60000
70000
200
400
600
800
1000
1200
1400
1600

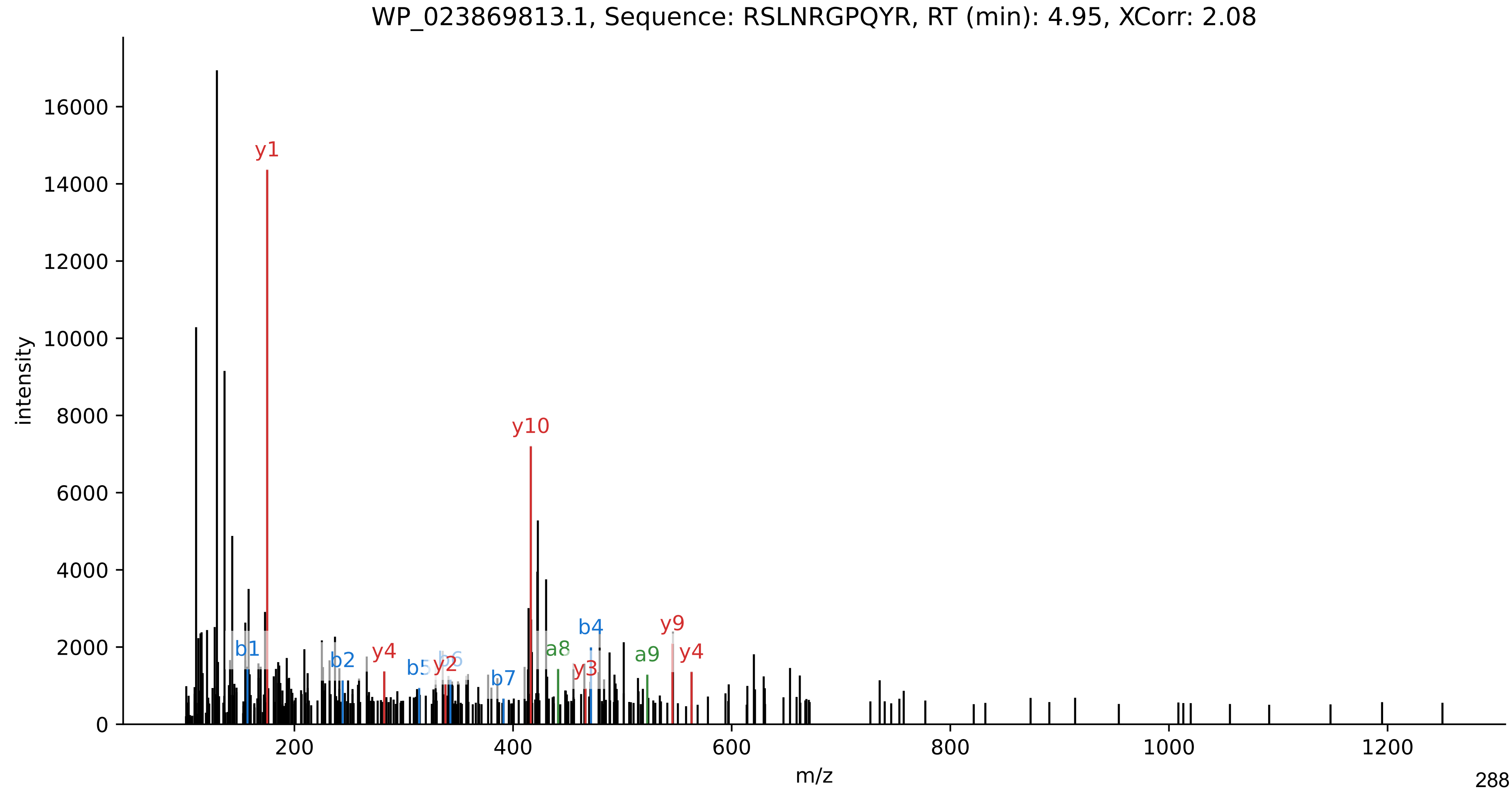

WP_023869813.1, Sequence: RSLNRGPQYR, RT (min): 4.95, XCorr: 2.08
intensity
m/z
y1
y10
b1
b2
y4
b5
y2
b6
b7
a8
y3
b4
a9
y9
y4

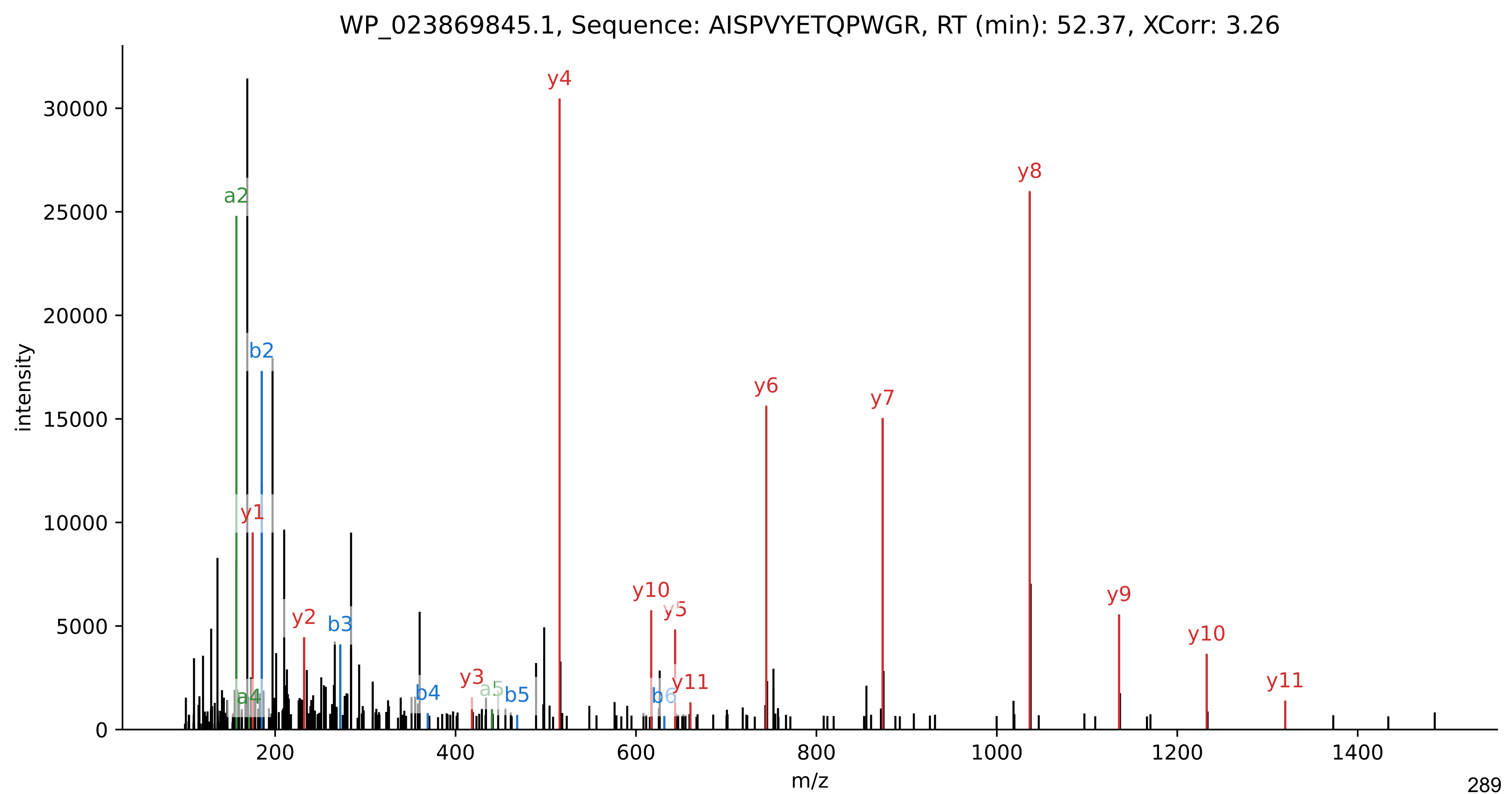
WP_023869845.1, Sequence: AISPVYETQPWGR, RT (min): 52.37, XCorr: 3.26
intensity
m/z
y4
y8
a2
b2
y6
y7
y1
y10
y5
y9
y2
b3
y10
y3
a5
b4
b5
b6
y11
y11
a4

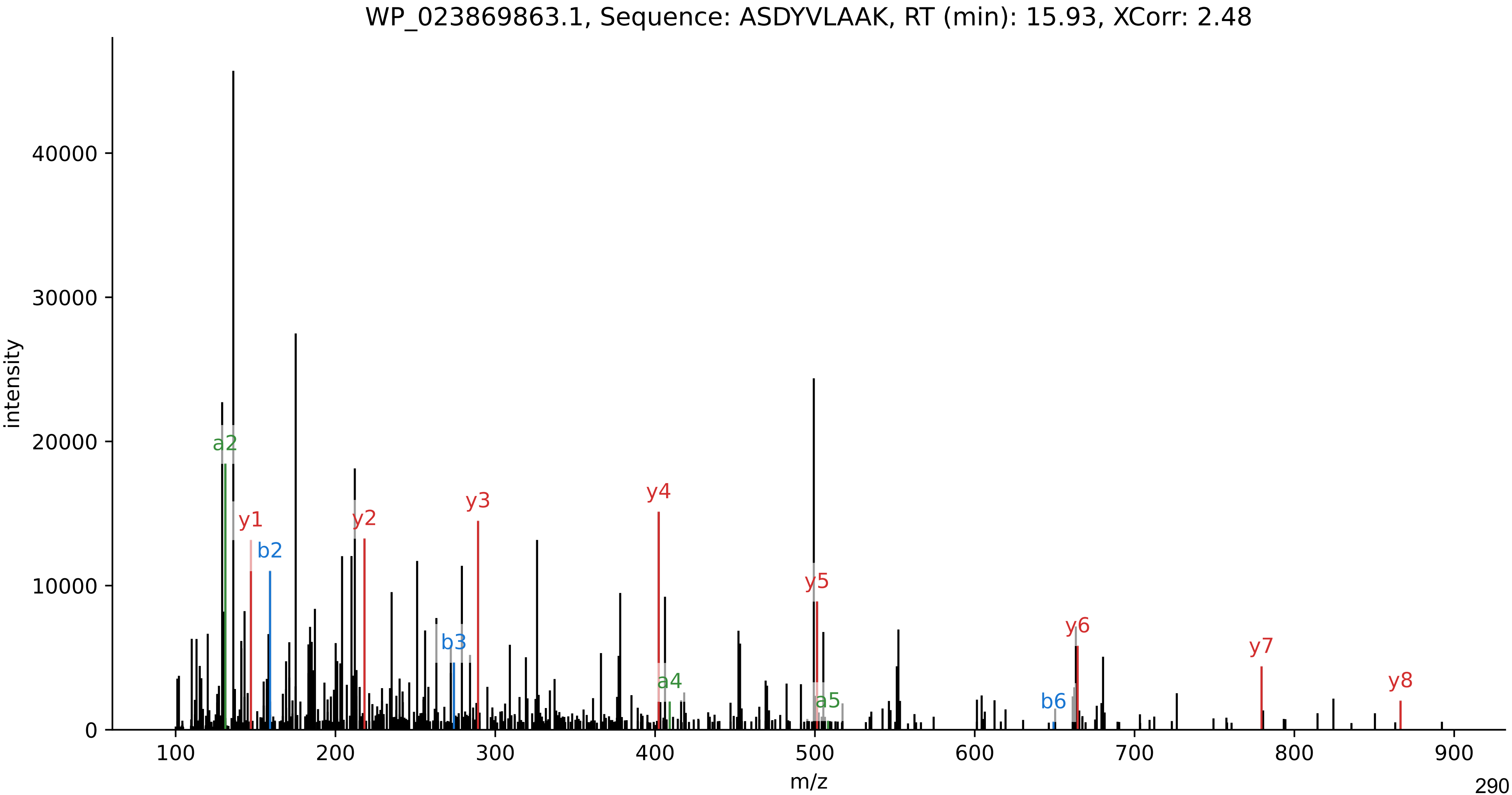

WP_023869863.1, Sequence: ASDYVLAAK, RT (min): 15.93, XCorr: 2.48
intensity
m/z
a2
y1
b2
y2
y3
y4
b3
a4
y5
a5
y6
b6
y7
y8

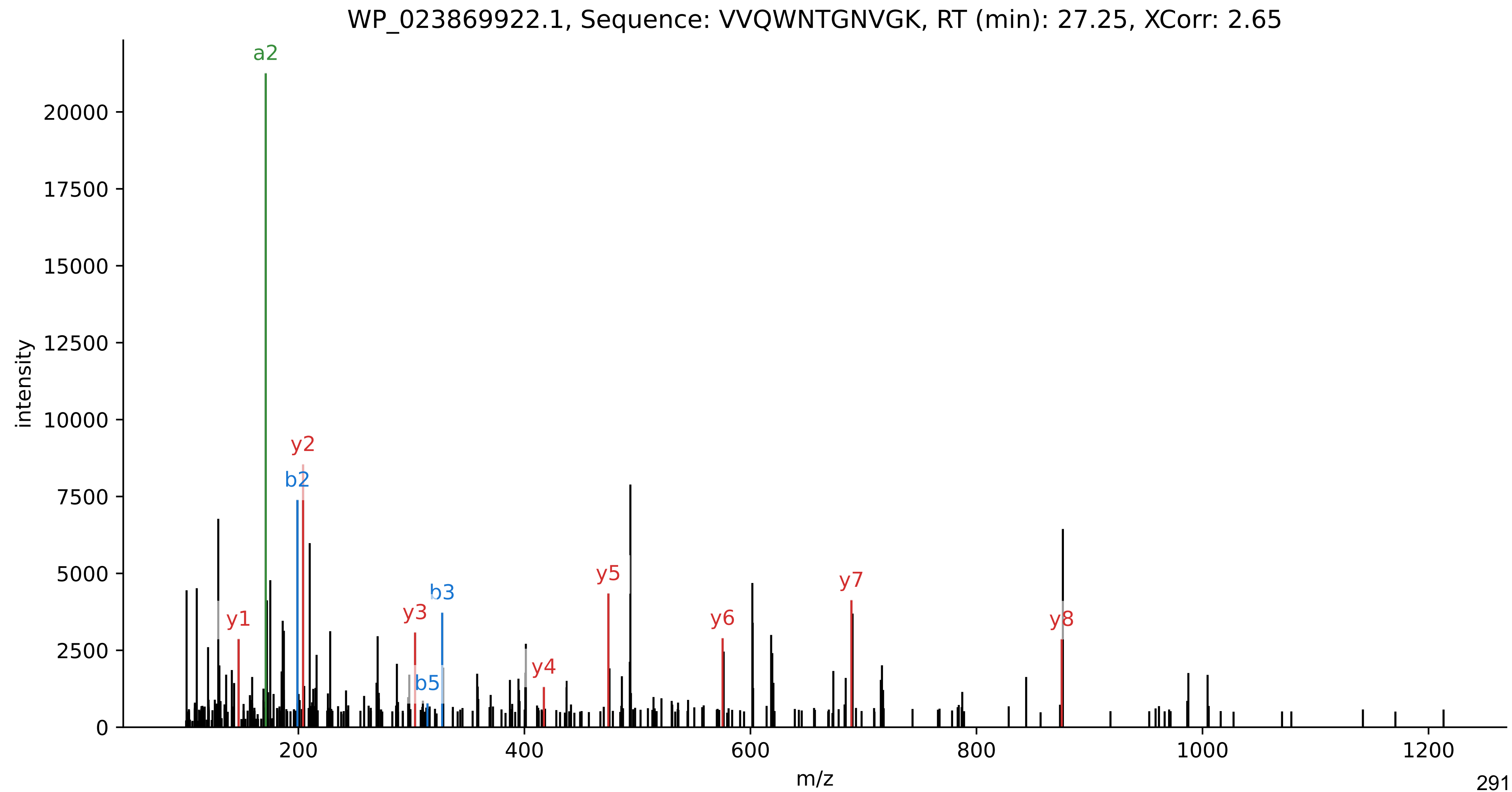
WP_023869922.1, Sequence: VVQWNTGNVGK, RT (min): 27.25, XCorr: 2.65
intensity
m/z
a2
y2
b2
y1
y3
b3
b5
y4
y5
y6
y7
y8
0
2500
5000
7500
10000
12500
15000
17500
20000
200
400
600
800
1000
1200

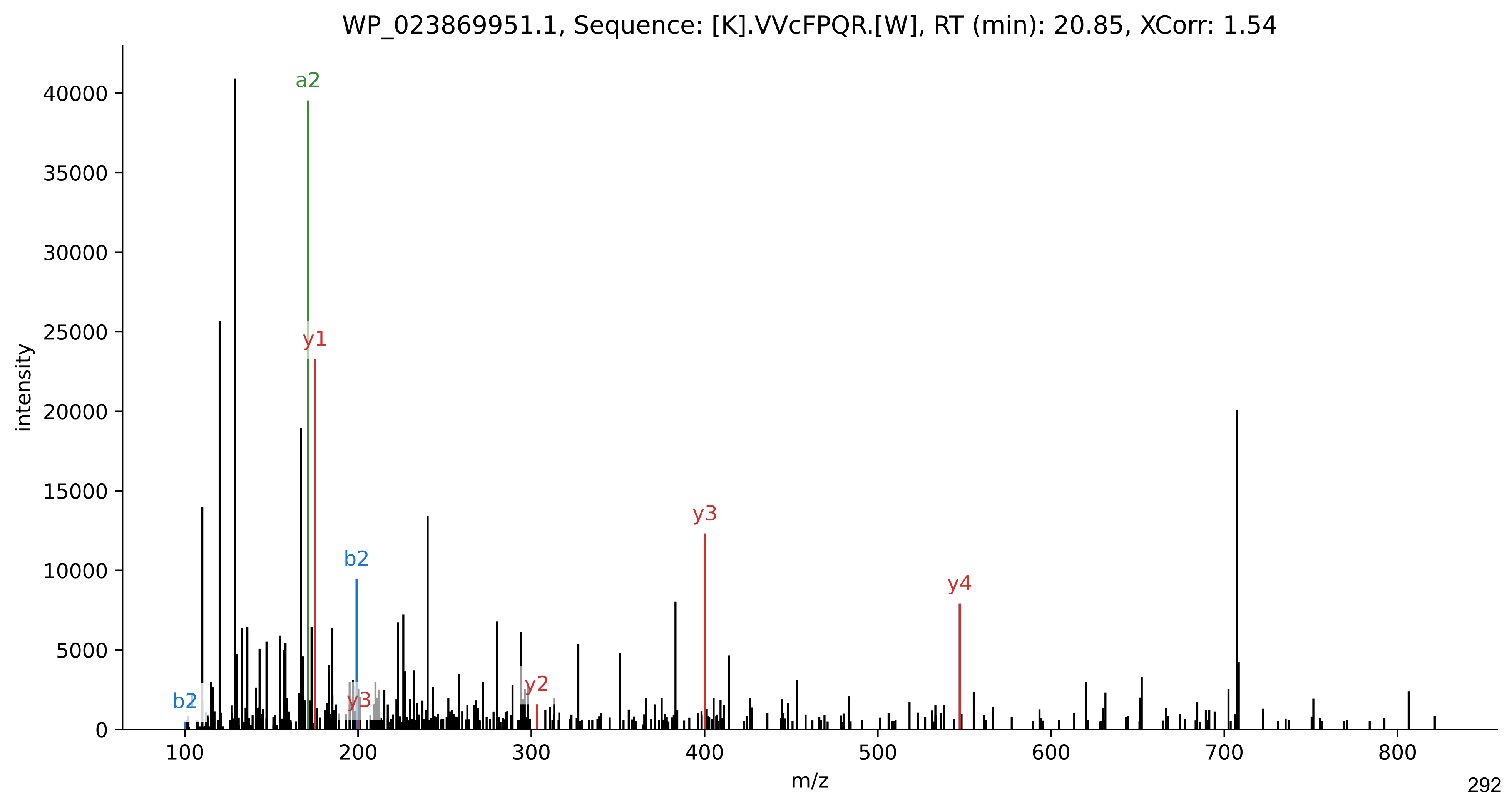
WP_023869951.1, Sequence: [K].VVcFPQR.[W], RT (min): 20.85, XCorr: 1.54
a2
y1
b2
y3
y4
b2
y3
y2
intensity
m/z
0
5000
10000
15000
20000
25000
30000
35000
40000
100
200
300
400
500
600
700
800

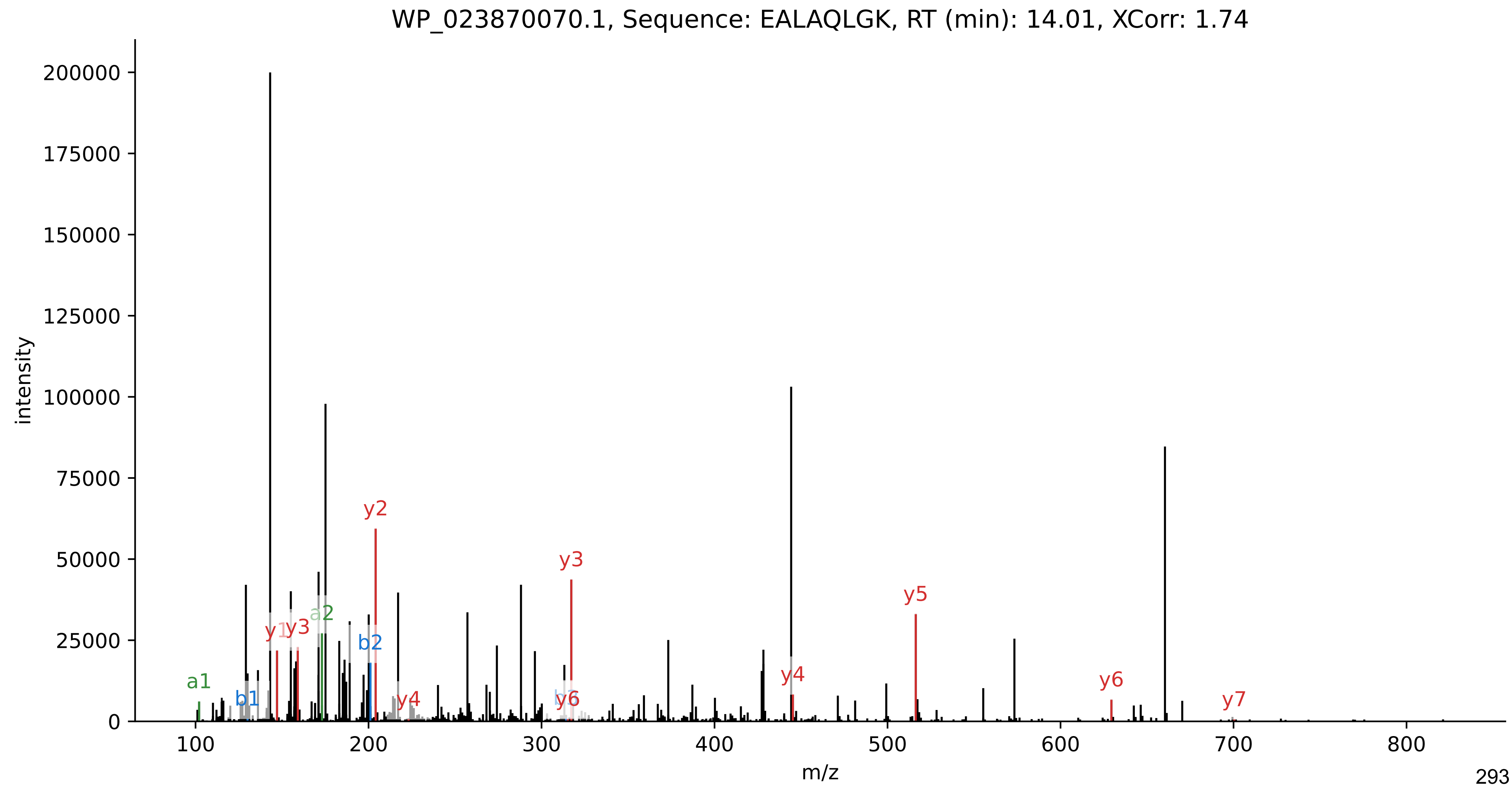
WP_023870070.1, Sequence: EALAQLGK, RT (min): 14.01, XCorr: 1.74
intensity
m/z
200000
175000
150000
125000
100000
75000
50000
25000
0
100
200
300
400
500
600
700
800
a1
b1
y1
y3
a2
b2
y2
y4
y3
y6
y4
y5
y6
y7

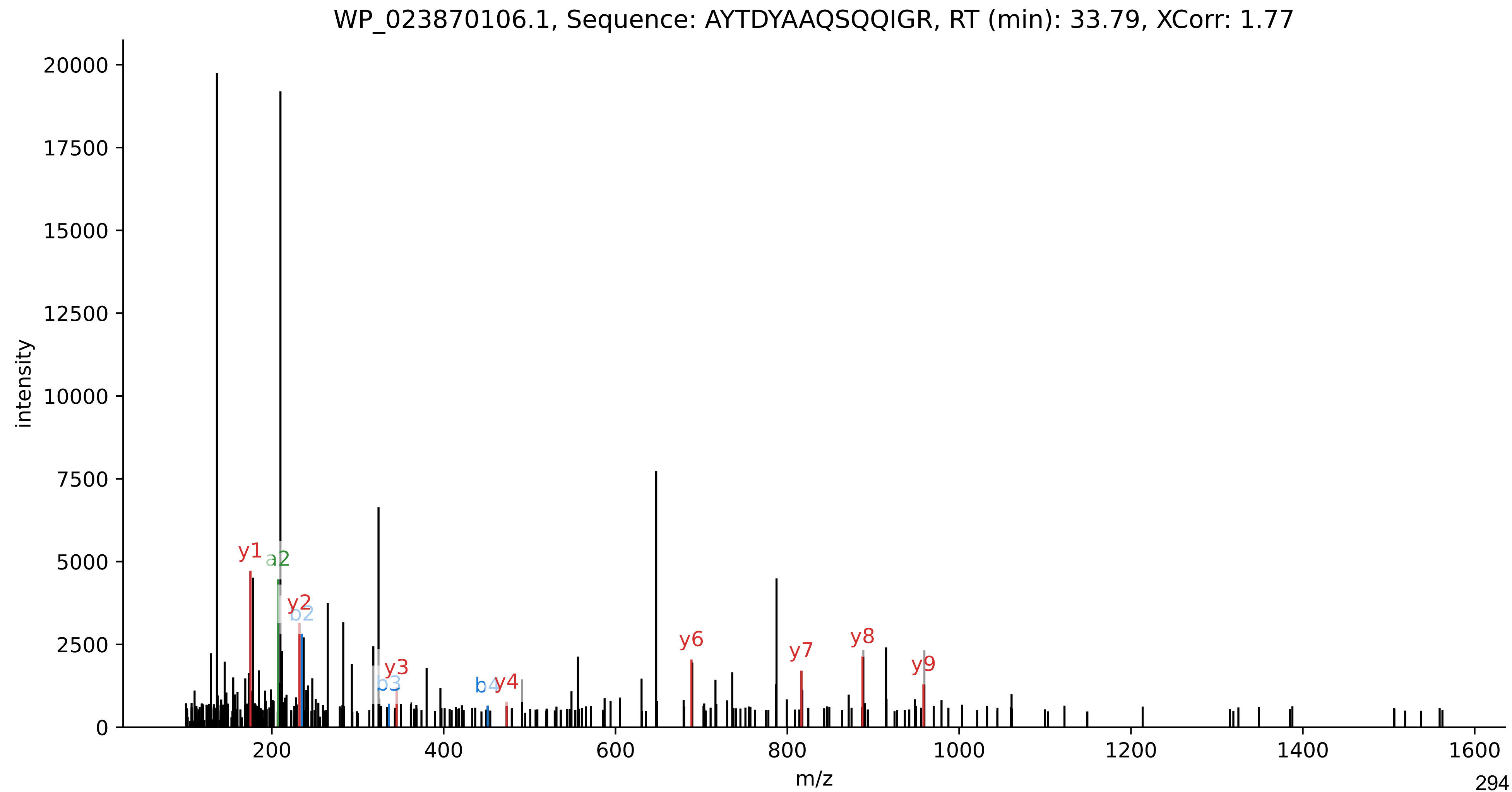
WP_023870106.1, Sequence: AYTDYAAQSQQIGR, RT (min): 33.79, XCorr: 1.77
intensity
m/z
y1
a2
y2
b2
y3
b3
b4
y4
y6
y7
y8
y9
0
2500
5000
7500
10000
12500
15000
17500
20000
200
400
600
800
1000
1200
1400
1600

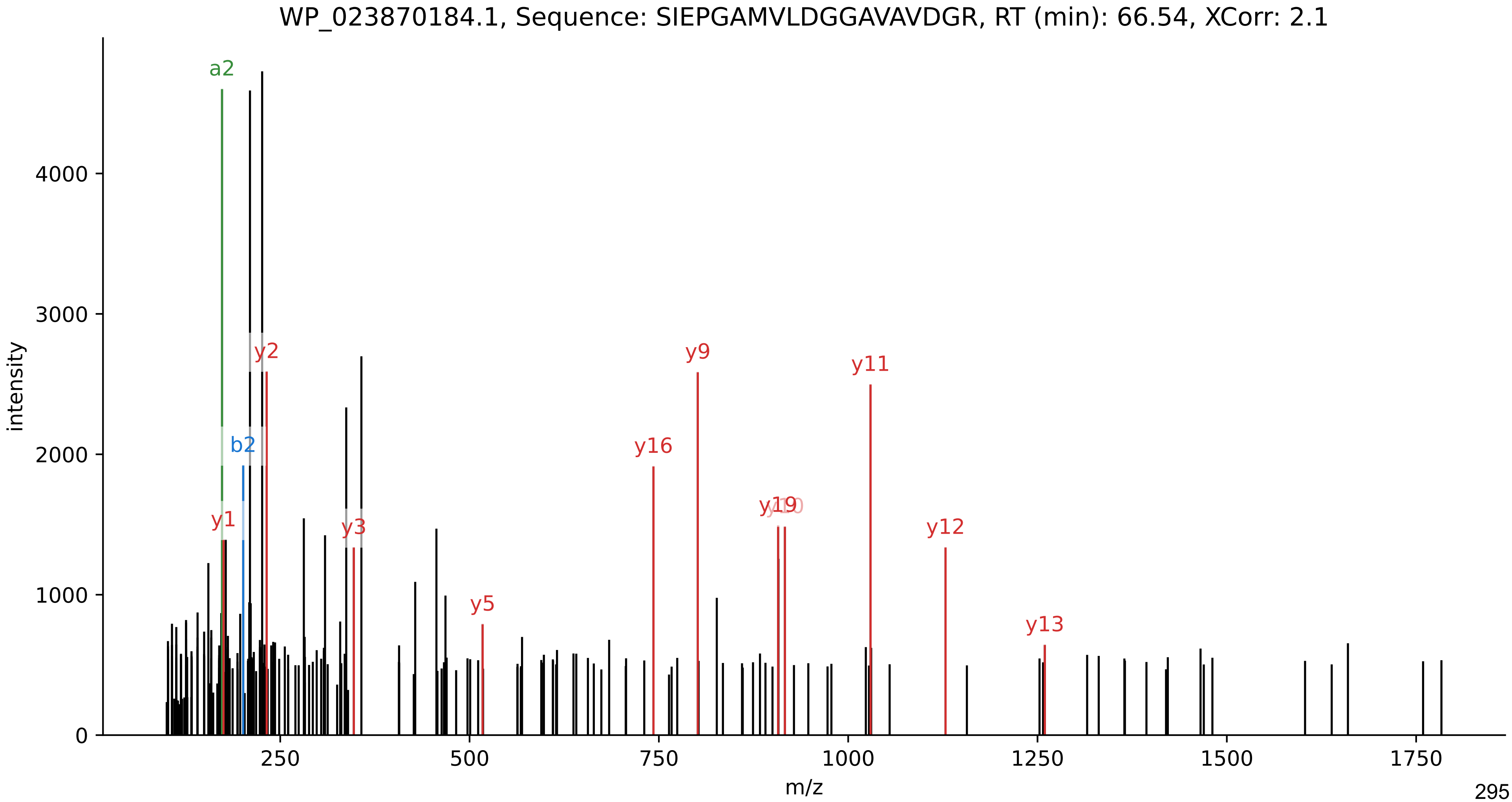
WP_023870184.1, Sequence: SIEPGAMVLDGGAVAVDGR, RT (min): 66.54, XCorr: 2.1
a2
y2
b2
y1
y3
y5
y16
y9
y19
y11
y12
y13
intensity
0
1000
2000
3000
4000
m/z
250
500
750
1000
1250
1500
1750

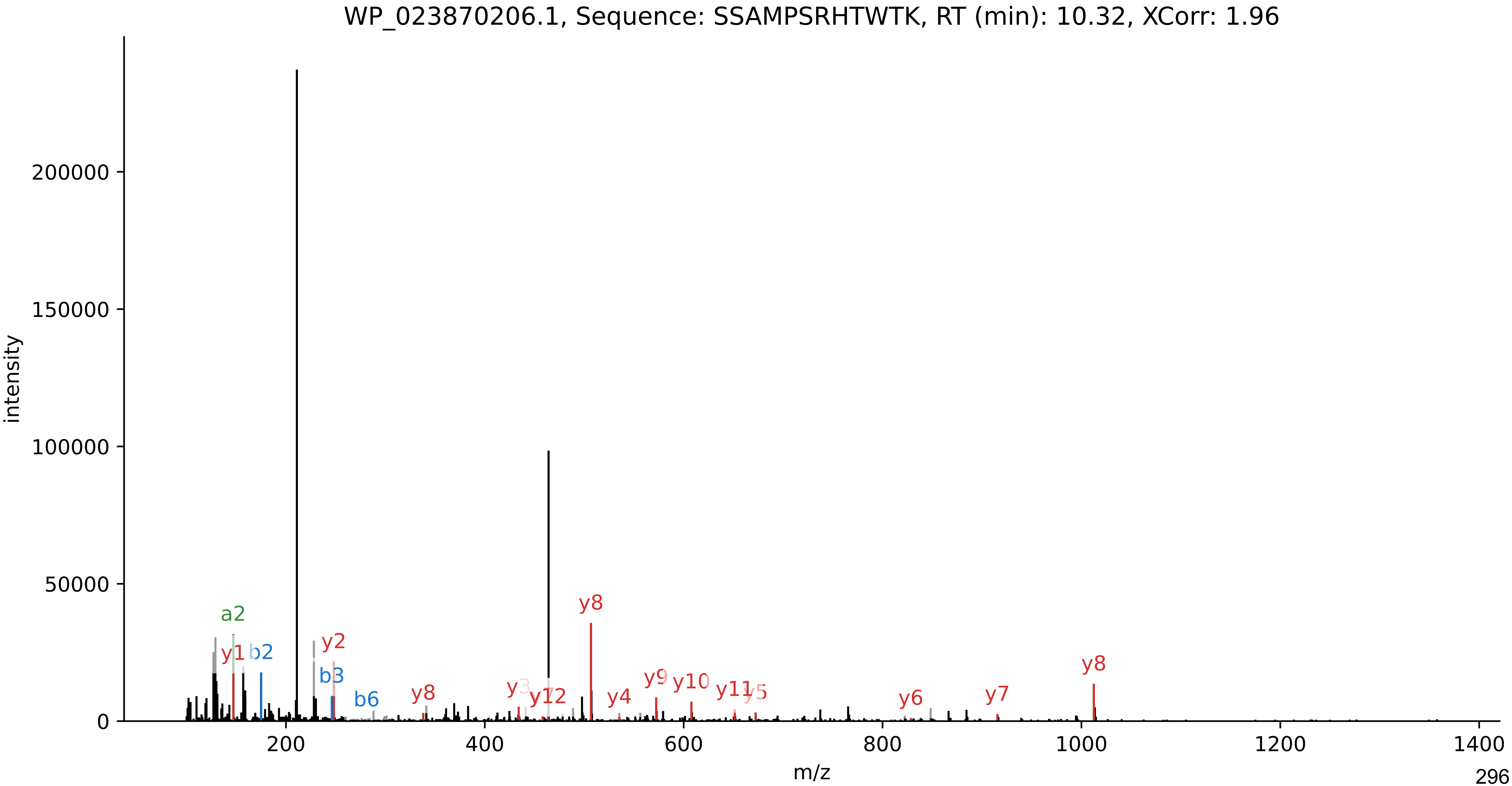

WP_023870206.1, Sequence: SSAMPSRHTWTK, RT (min): 10.32, XCorr: 1.96
intensity
m/z
0
50000
100000
150000
200000
200
400
600
800
1000
1200
1400
a2
y1
b2
y2
b3
b6
y8
y3
y12
y8
y4
y9
y10
y11
y5
y6
y7
y8

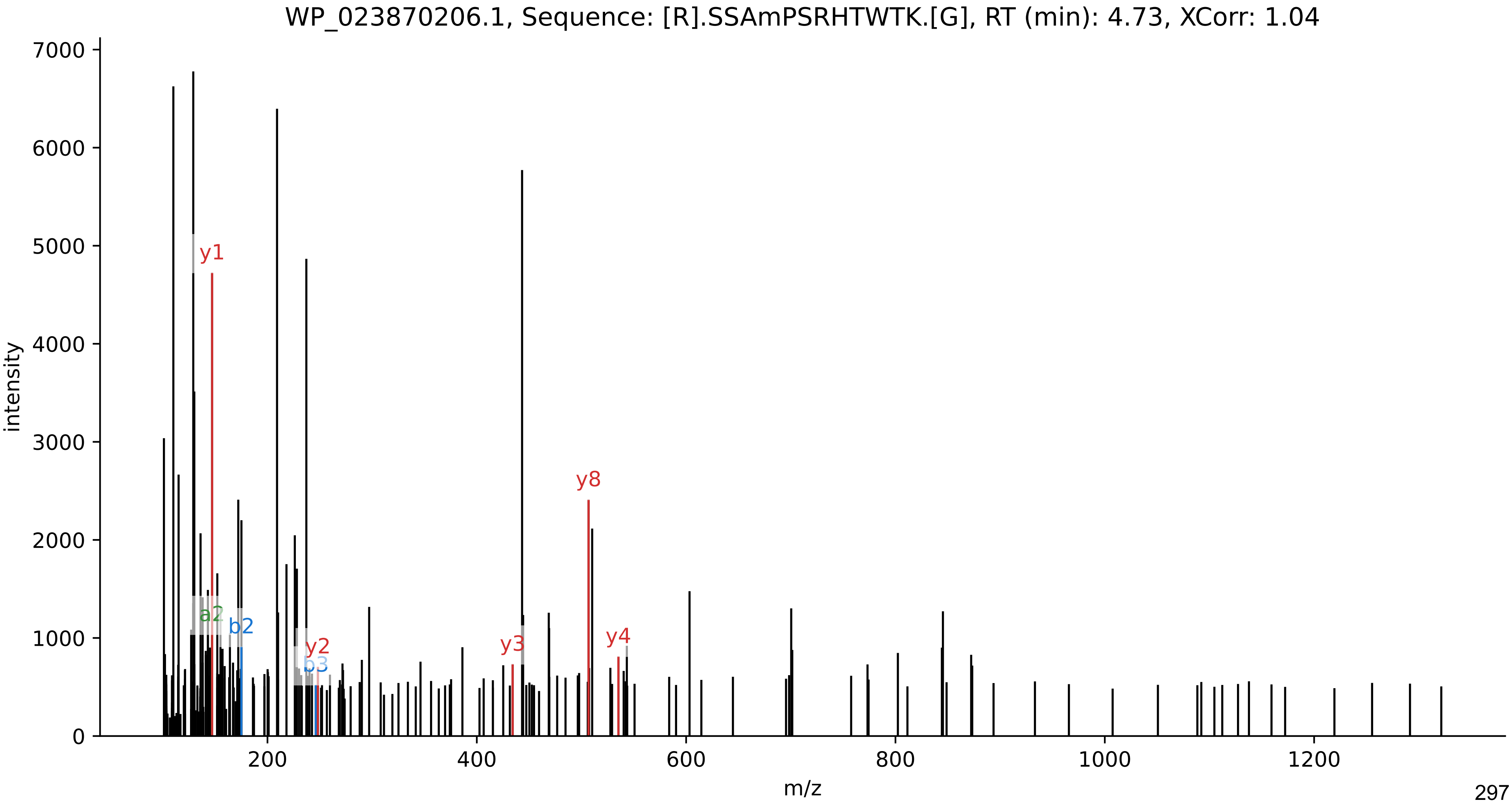
WP_023870206.1, Sequence: [R].SSAmPSRHTWTK.[G], RT (min): 4.73, XCorr: 1.04
intensity
m/z
y1
a2
b2
y2
b3
y3
y8
y4
0
1000
2000
3000
4000
5000
6000
7000
200
400
600
800
1000
1200

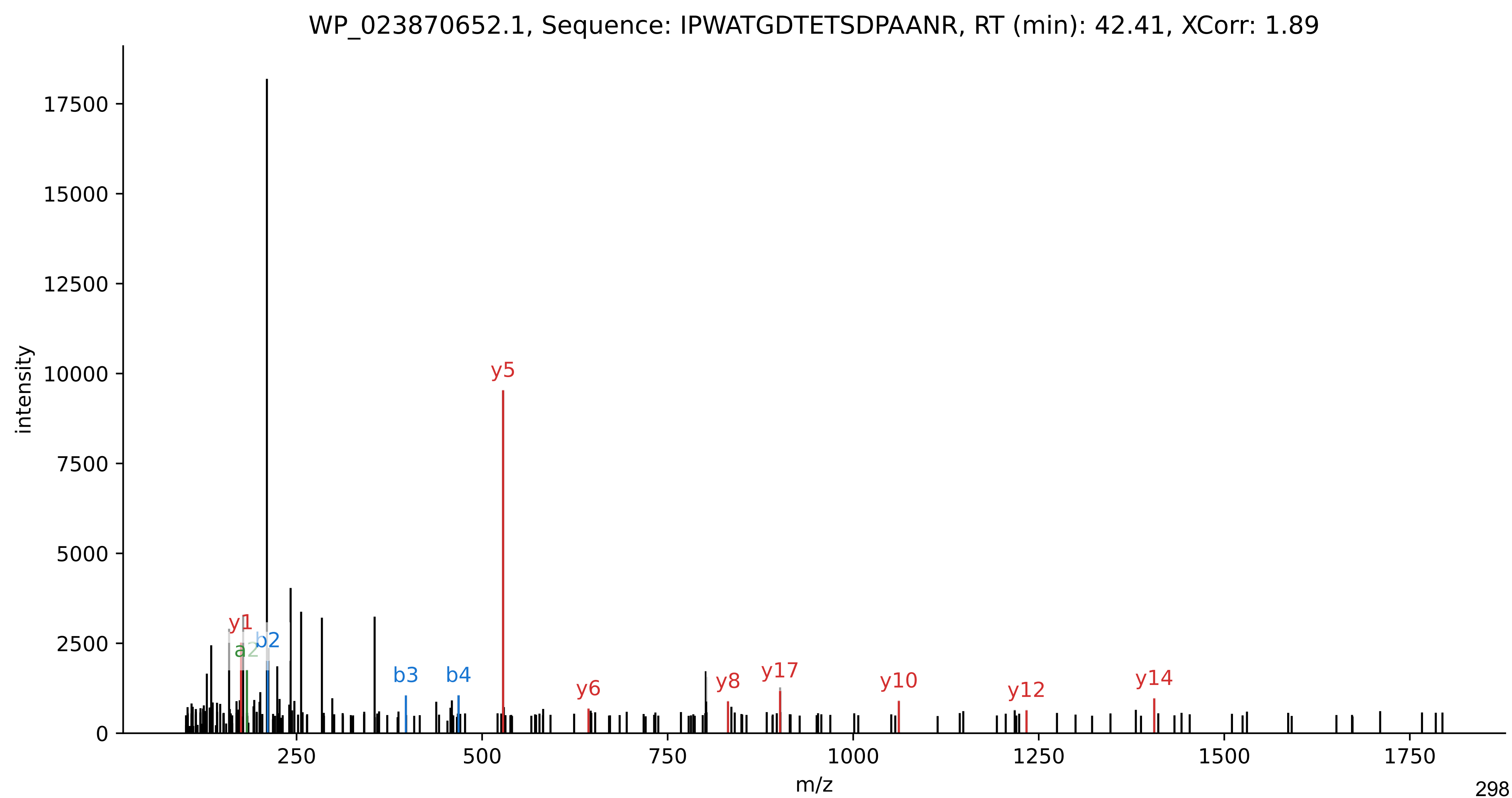

WP_023870652.1, Sequence: IPWATGDTETSDPAANR, RT (min): 42.41, XCorr: 1.89
intensity
m/z
y1
a2
b2
b3
b4
y5
y6
y8
y17
y10
y12
y14

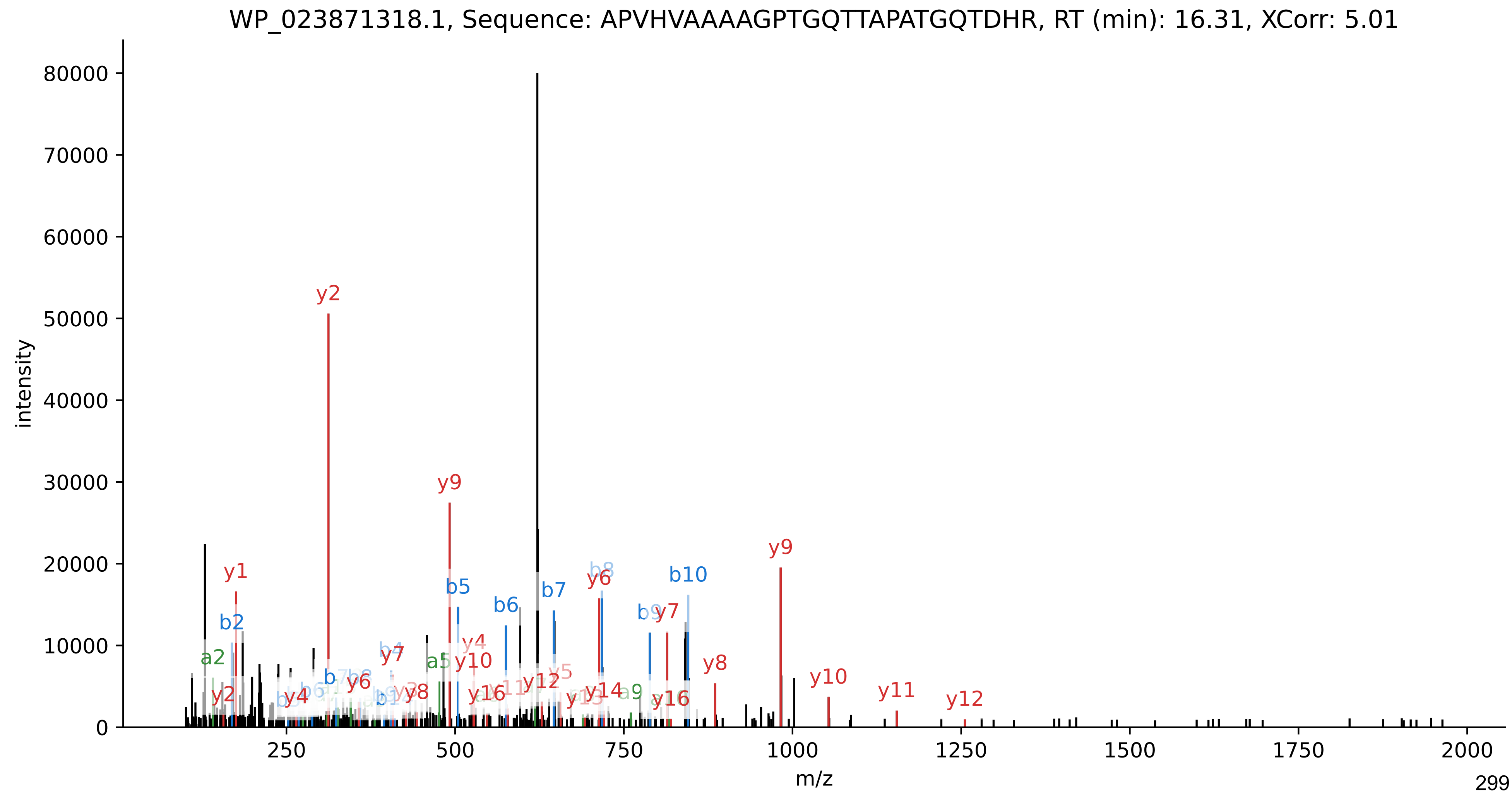
WP_023871318.1, Sequence: APVHVAAAAGPTGQTTAPATGQTDHR, RT (min): 16.31, XCorr: 5.01
intensity
m/z
80000
70000
60000
50000
40000
30000
20000
10000
0
250
500
750
1000
1250
1500
1750
2000
y2
y9
y1
b2
a2
b5
b6
b7
y6
b10
y7
y9
y8
y10
y11
y12
y4
y10
a5
y12
y14
a9
y16

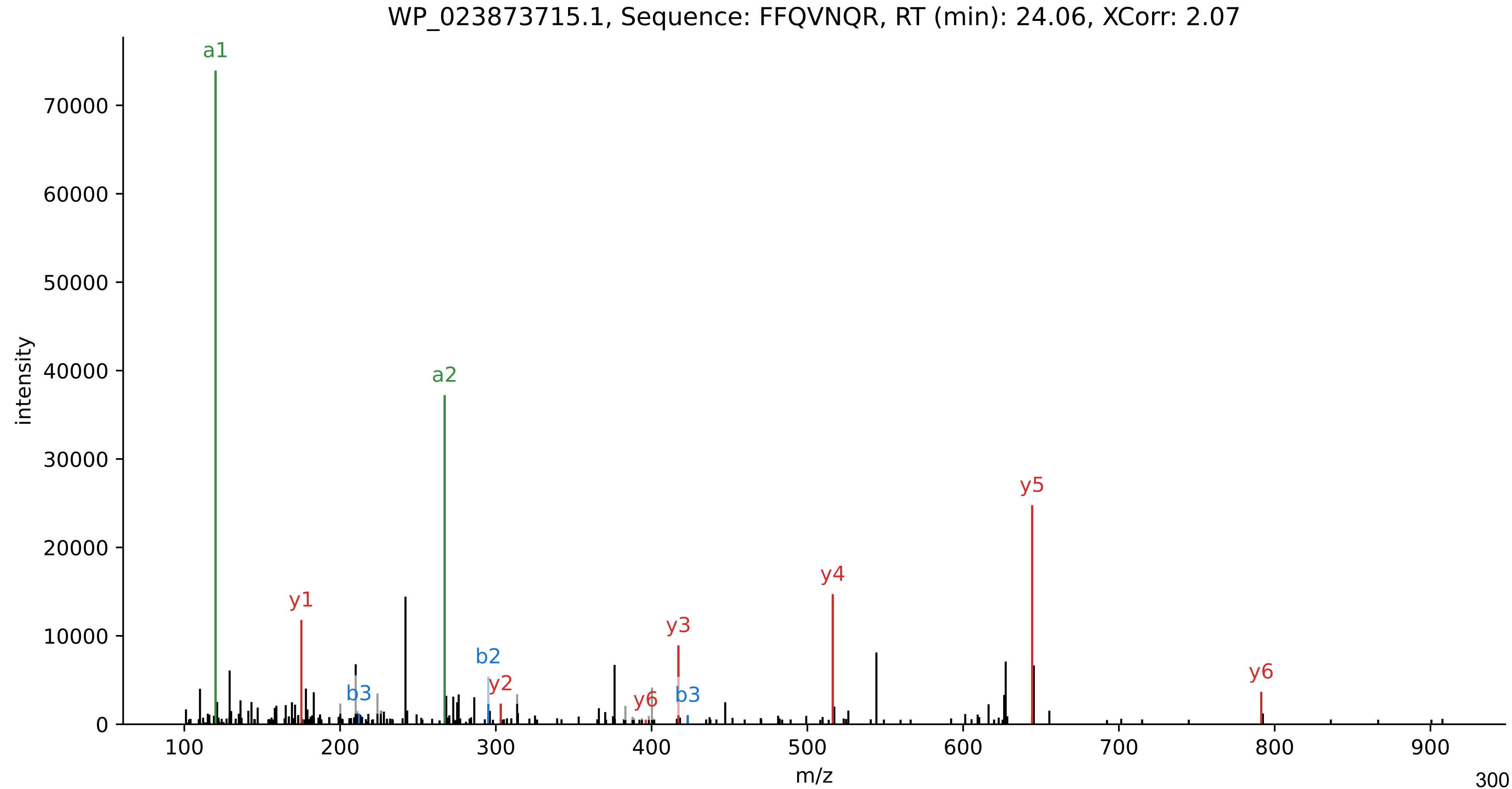

WP_023873715.1, Sequence: FFQVNQR, RT (min): 24.06, XCorr: 2.07
intensity
m/z
a1
a2
y1
y2
y3
y4
y5
y6
y6
b2
b3
b3

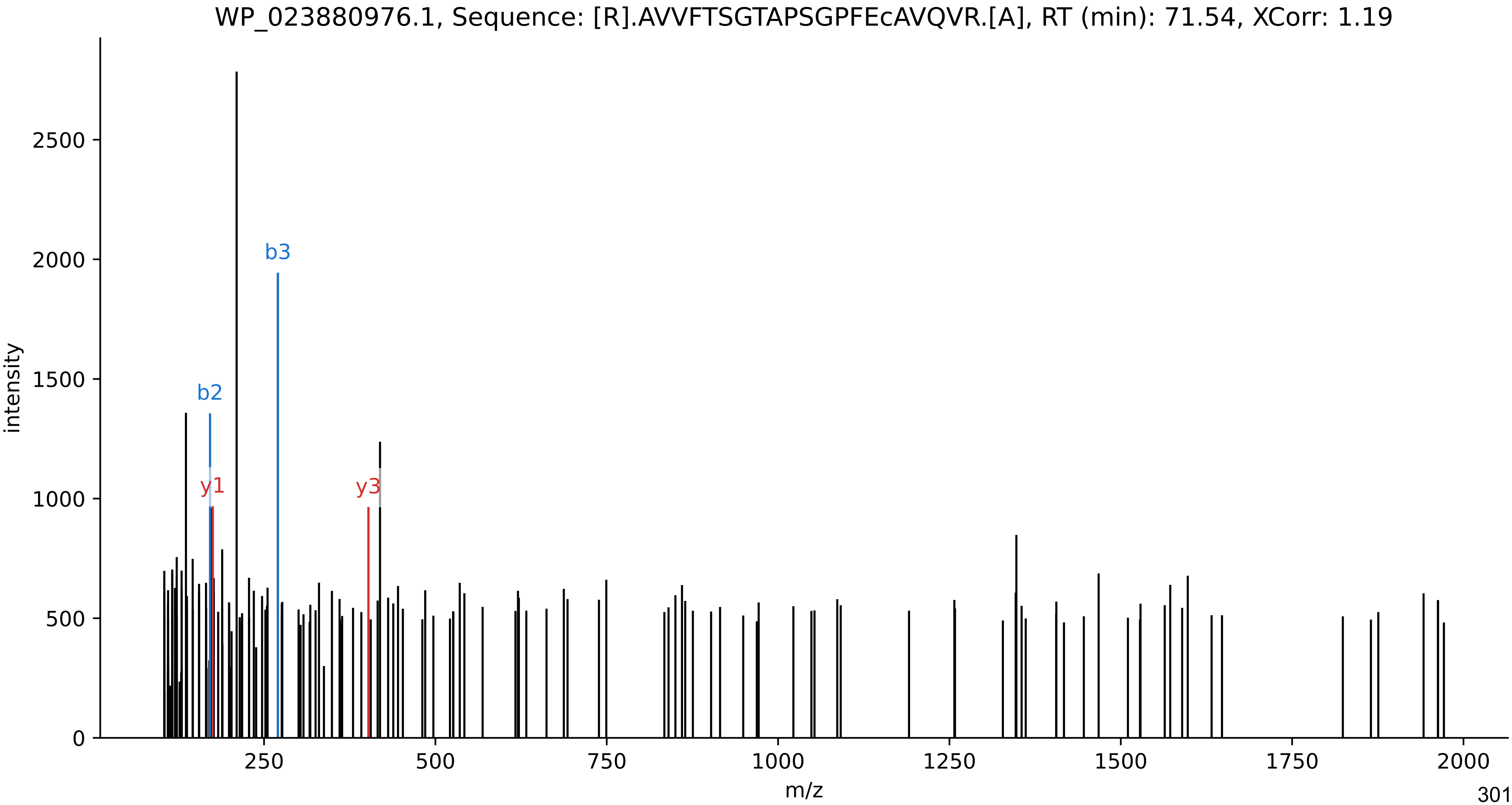
WP_023880976.1, Sequence: [R].AVVFTSGTAPSGPFEcAVQVR.[A], RT (min): 71.54, XCorr: 1.19
intensity
m/z
b2
b3
y1
y3
0
500
1000
1500
2000
2500
250
500
750
1000
1250
1500
1750
2000

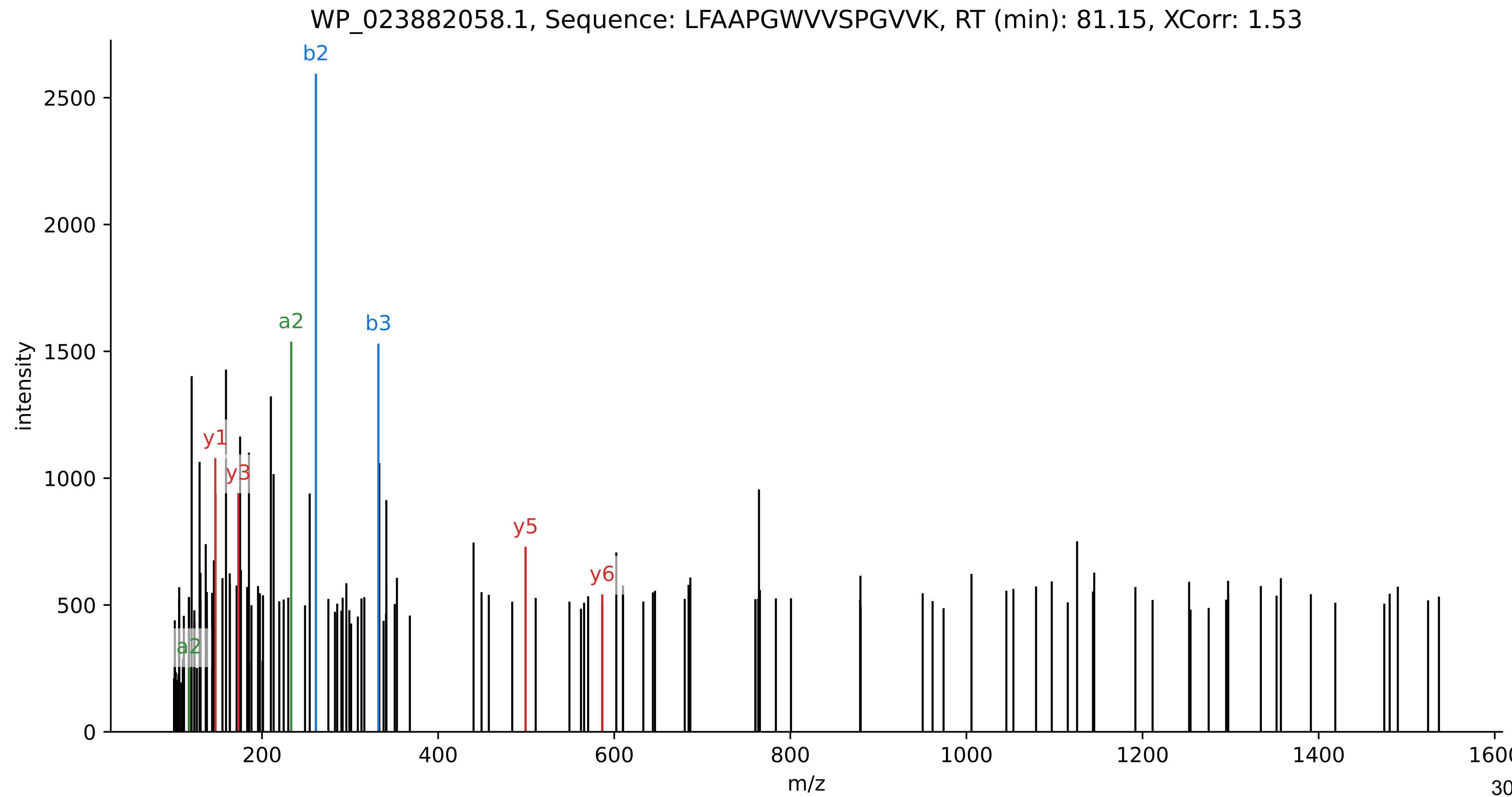
WP_023882058.1, Sequence: LFAAPGWVVSPGVVK, RT (min): 81.15, XCorr: 1.53
intensity
m/z
b2
b3
a2
y1
y3
y5
y6
a2
0
500
1000
1500
2000
2500
200
400
600
800
1000
1200
1400
1600

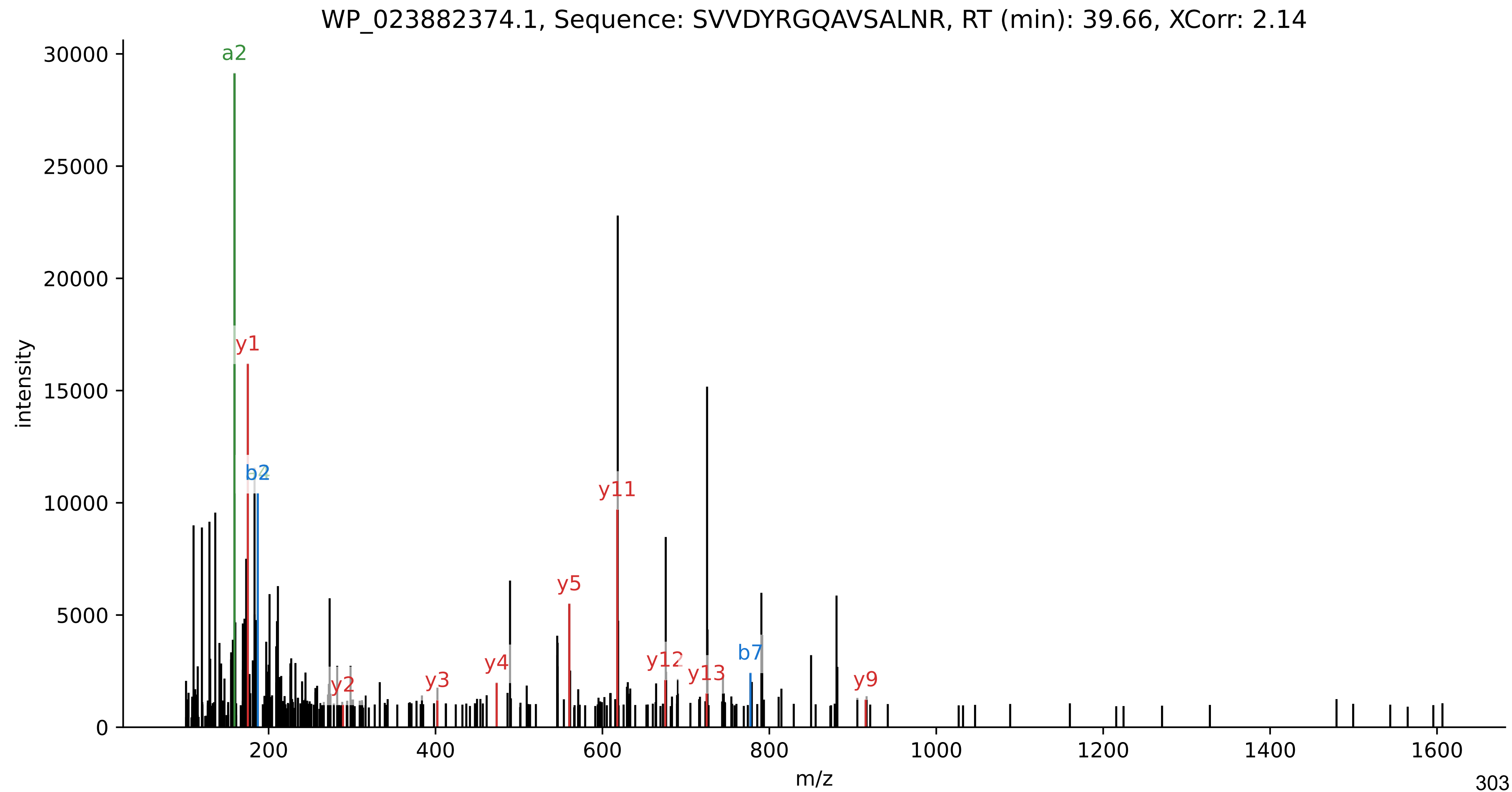
WP_023882374.1, Sequence: SVVDYRGQAVSALNR, RT (min): 39.66, XCorr: 2.14
intensity
m/z
a2
y1
b2
y11
y5
y4
y3
y2
y12
y13
b7
y9
30000
25000
20000
15000
10000
5000
0
200
400
600
800
1000
1200
1400
1600

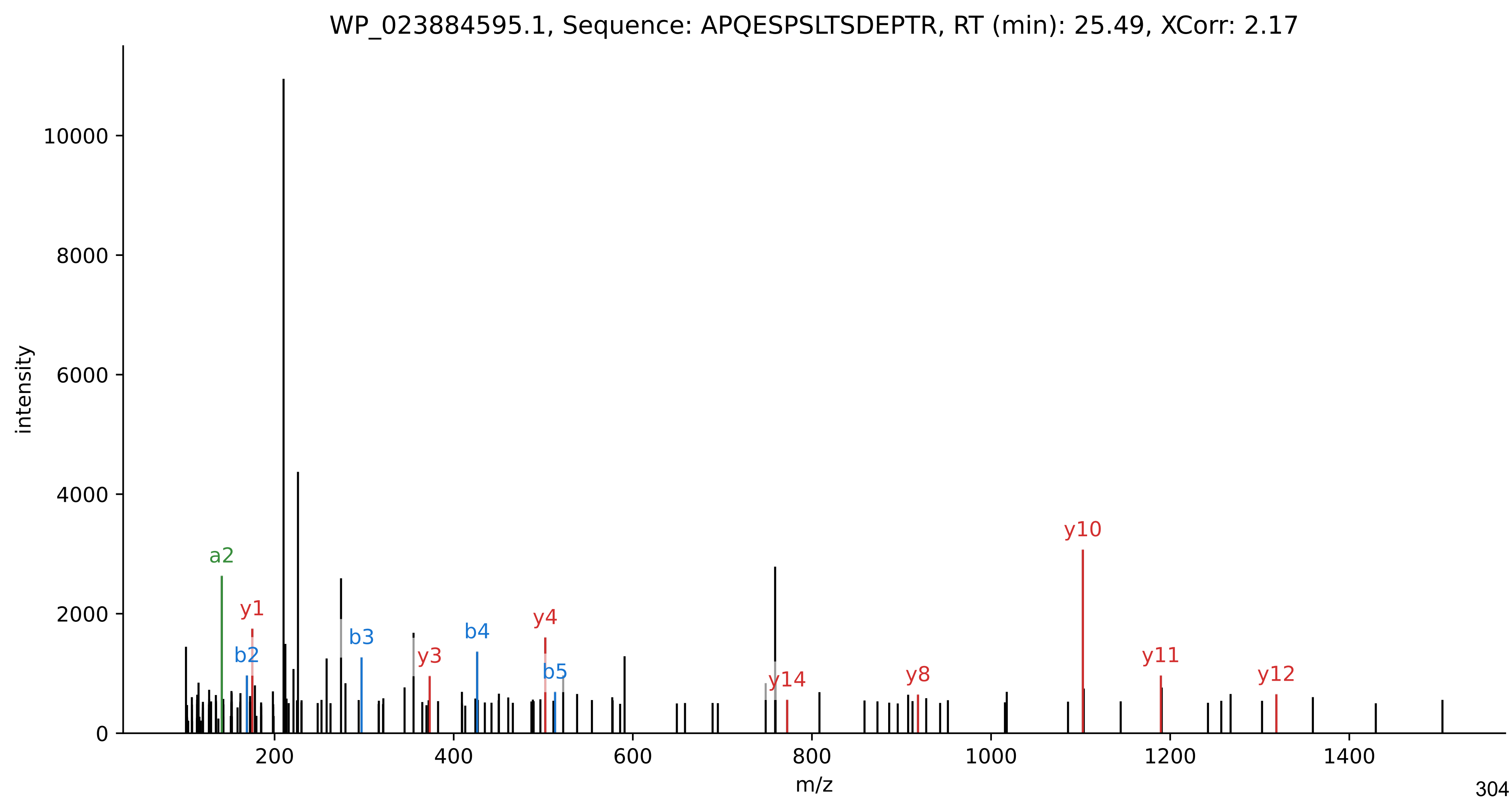
WP_023884595.1, Sequence: APQESPSLTSDEPTR, RT (min): 25.49, XCorr: 2.17
intensity
m/z
a2
b2
y1
b3
y3
b4
y4
b5
y14
y8
y10
y11
y12

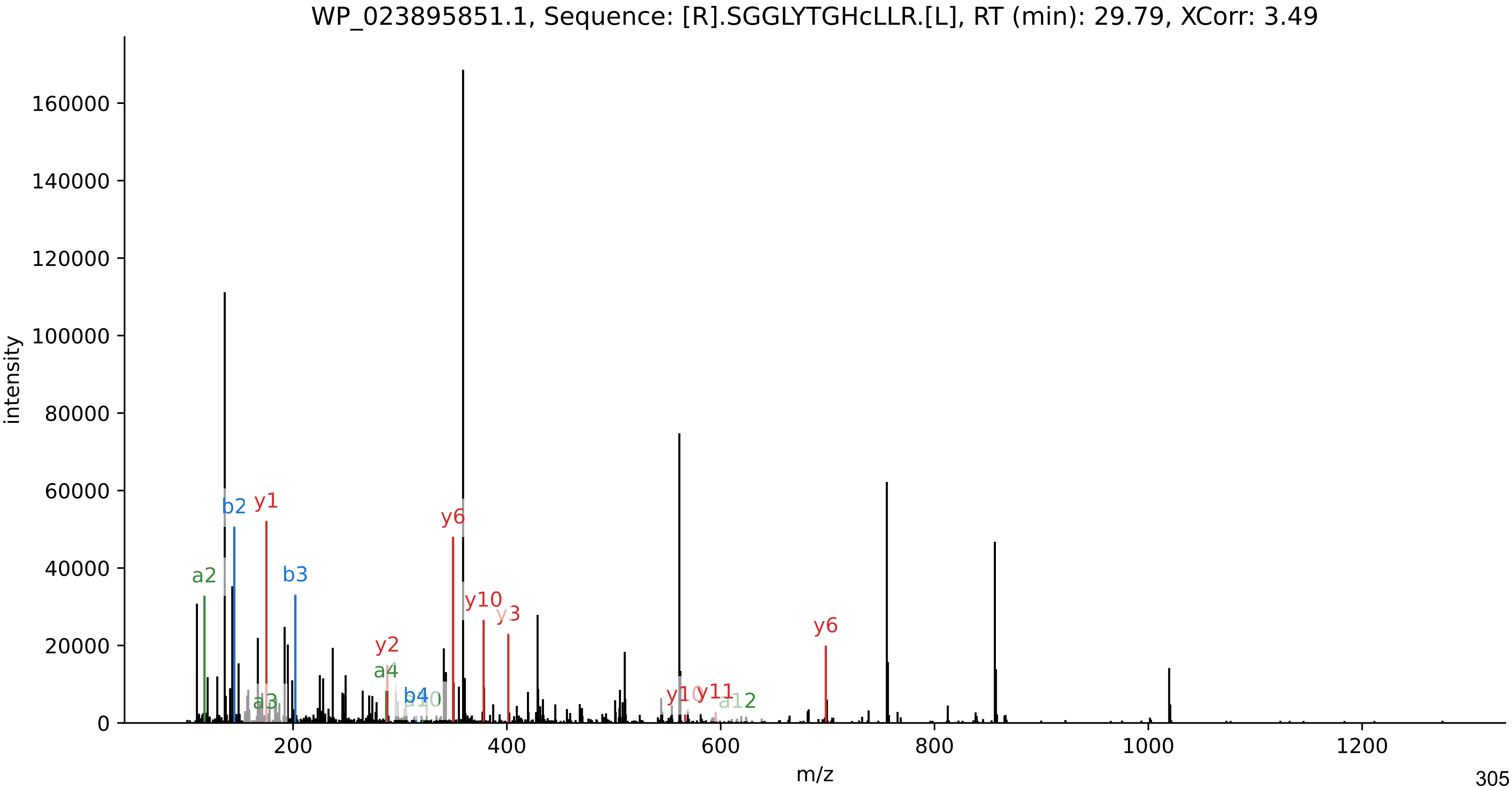

WP_023895851.1, Sequence: [R].SGGLYTGHcLLR.[L], RT (min): 29.79, XCorr: 3.49
intensity
m/z
a2
b2
y1
b3
a3
y2
a4
b4
y6
y10
y3
y10
y11
a12
y6

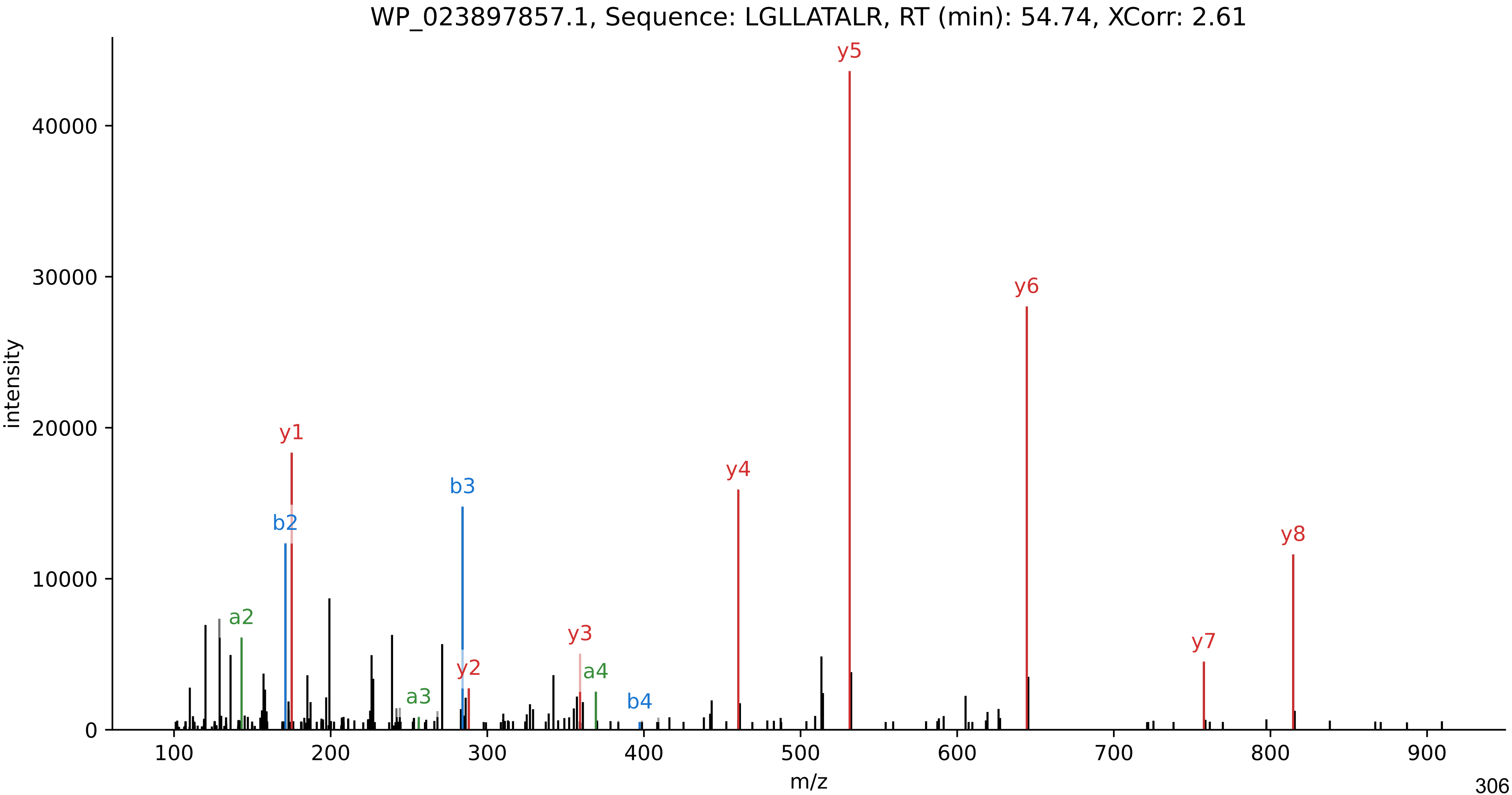

WP_023897857.1, Sequence: LGLLATALR, RT (min): 54.74, XCorr: 2.61
intensity
m/z
y5
y6
y1
y4
b3
b2
y8
a2
y3
y7
y2
a4
a3
b4
0
10000
20000
30000
40000
100
200
300
400
500
600
700
800
900

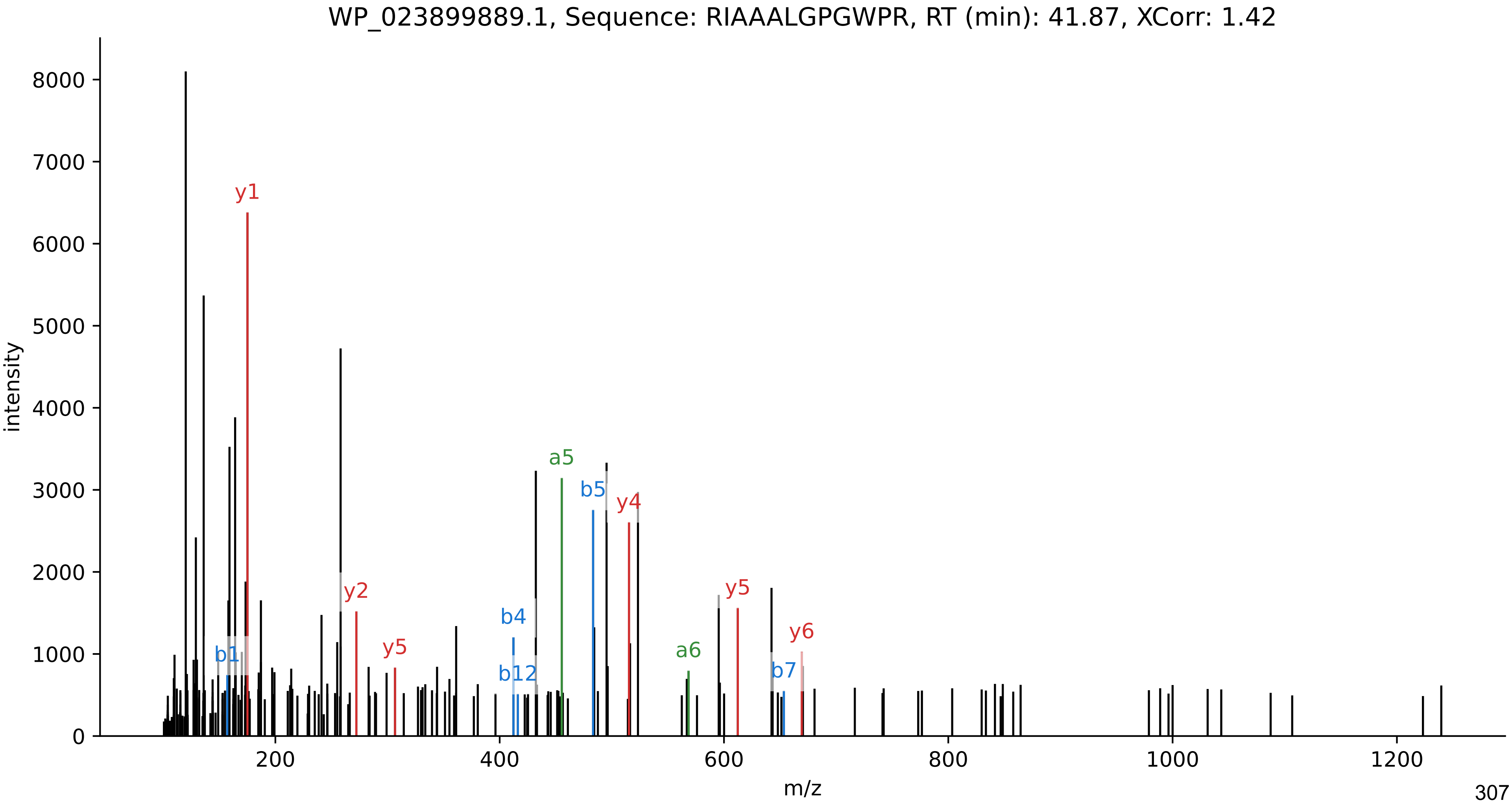

WP_023899889.1, Sequence: RIAAALGPGWPR, RT (min): 41.87, XCorr: 1.42
intensity
m/z
y1
y2
y5
b1
b4
b12
a5
b5
y4
a6
y5
b7
y6

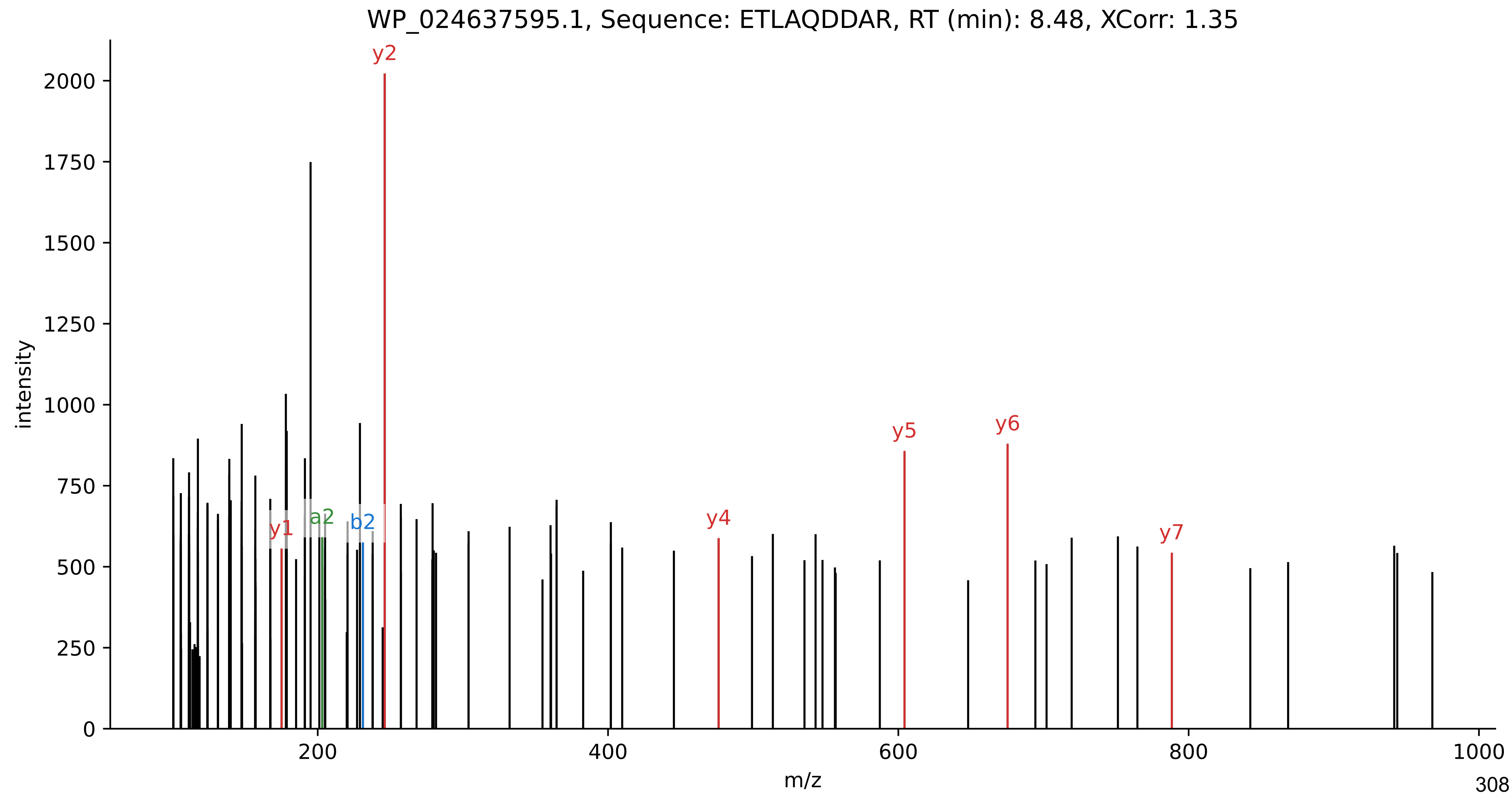
WP_024637595.1, Sequence: ETLAQDDAR, RT (min): 8.48, XCorr: 1.35
intensity
m/z
y1
a2
b2
y2
y4
y5
y6
y7
0
250
500
750
1000
1250
1500
1750
2000
200
400
600
800
1000

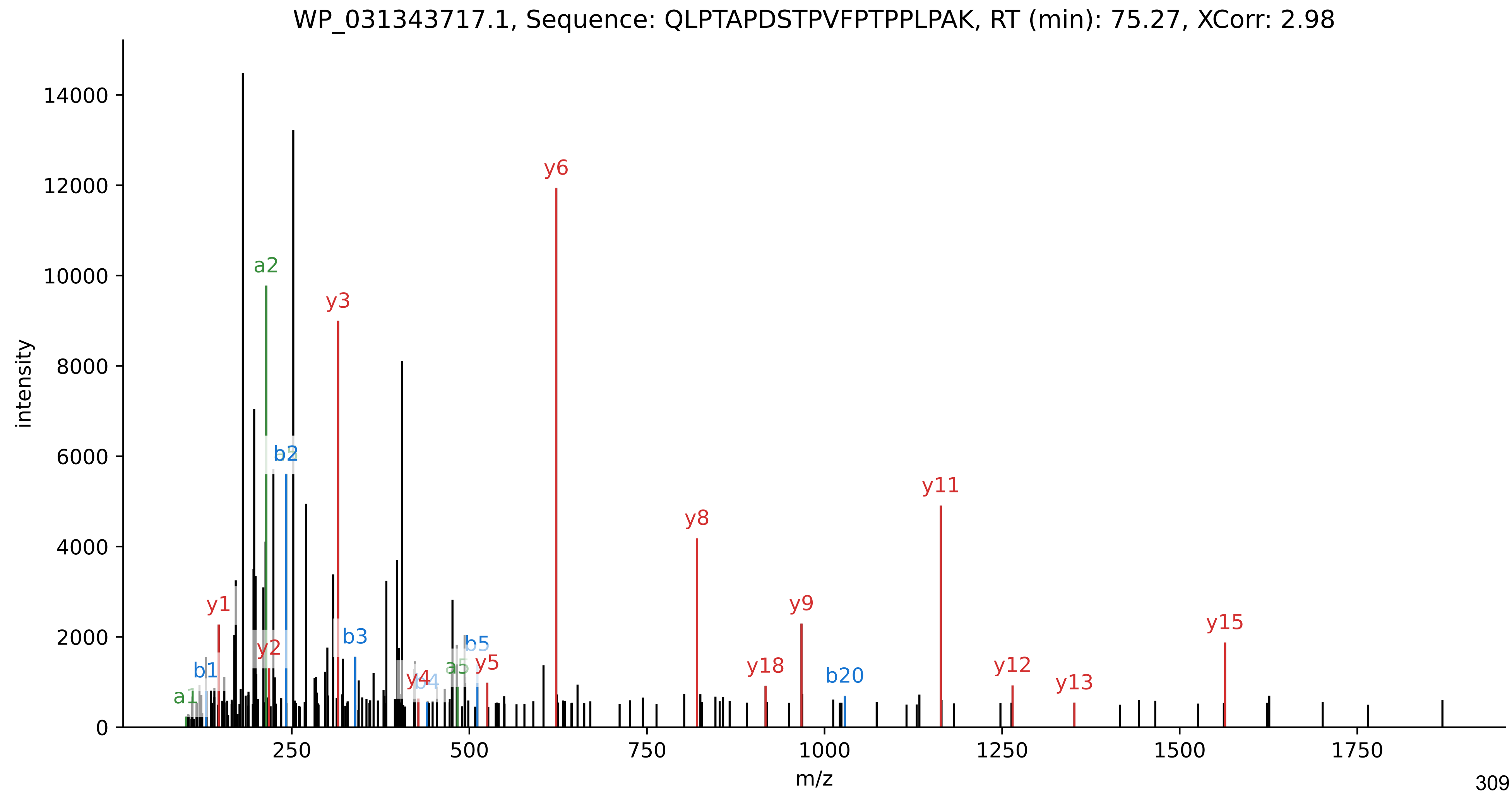
WP_031343717.1, Sequence: QLPTAPDSTPVFPTPPLPAK, RT (min): 75.27, XCorr: 2.98
intensity
m/z
y6
a2
y3
b2
y11
y8
y9
y15
y1
b3
b5
y2
a5
y5
b1
y4
b4
y18
b20
y12
y13
a1

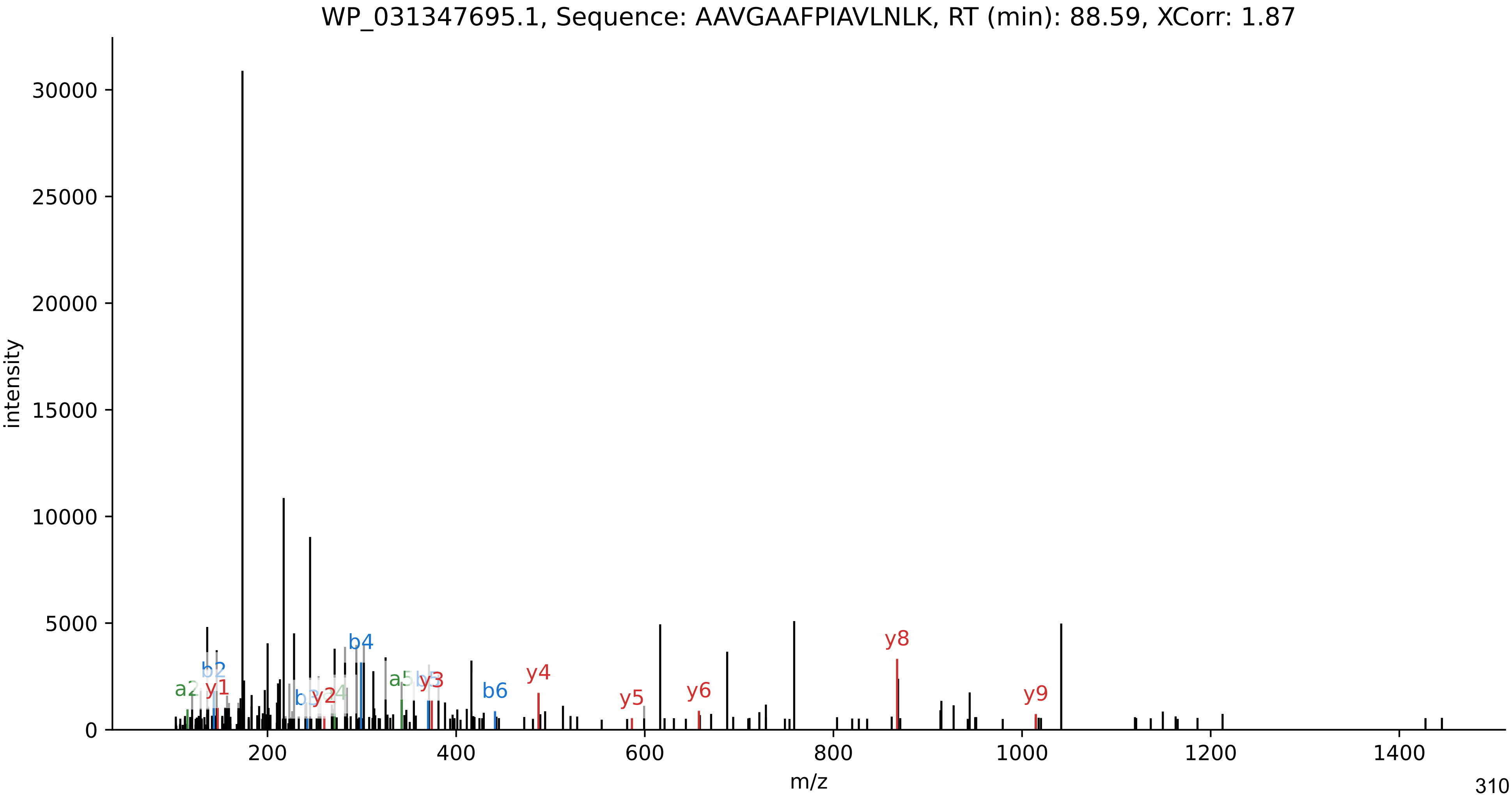

WP_031347695.1, Sequence: AAVGAAFPIAVLNLK, RT (min): 88.59, XCorr: 1.87
intensity
m/z
b4
b2
a2
y1
y2
b3
a5
y3
b6
y4
y5
y6
y8
y9

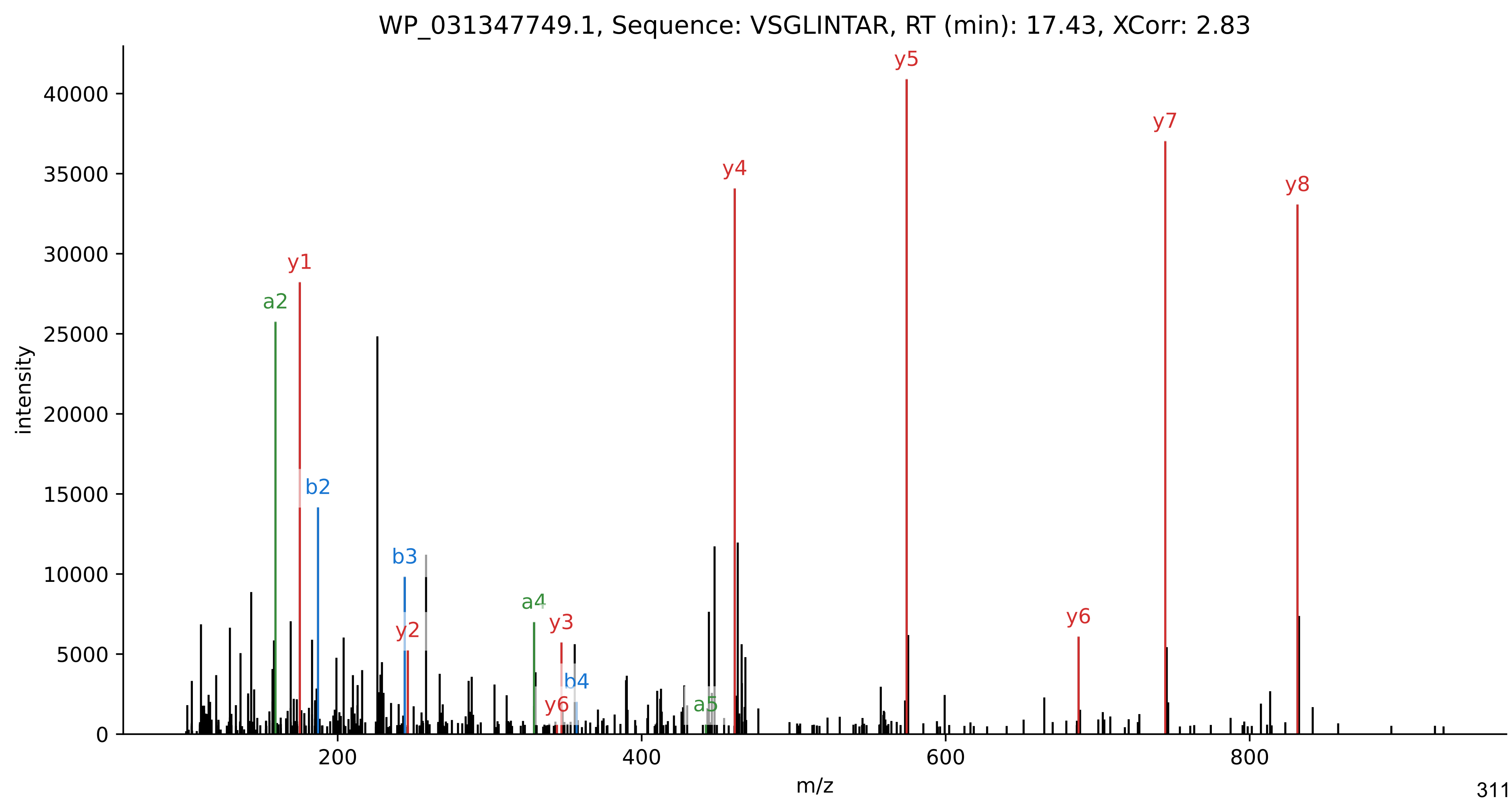

WP_031347749.1, Sequence: VSGLINTAR, RT (min): 17.43, XCorr: 2.83
intensity
m/z
y1
a2
b2
b3
y2
a4
y3
y6
b4
a5
y4
y5
y6
y7
y8
0
5000
10000
15000
20000
25000
30000
35000
40000
200
400
600
800

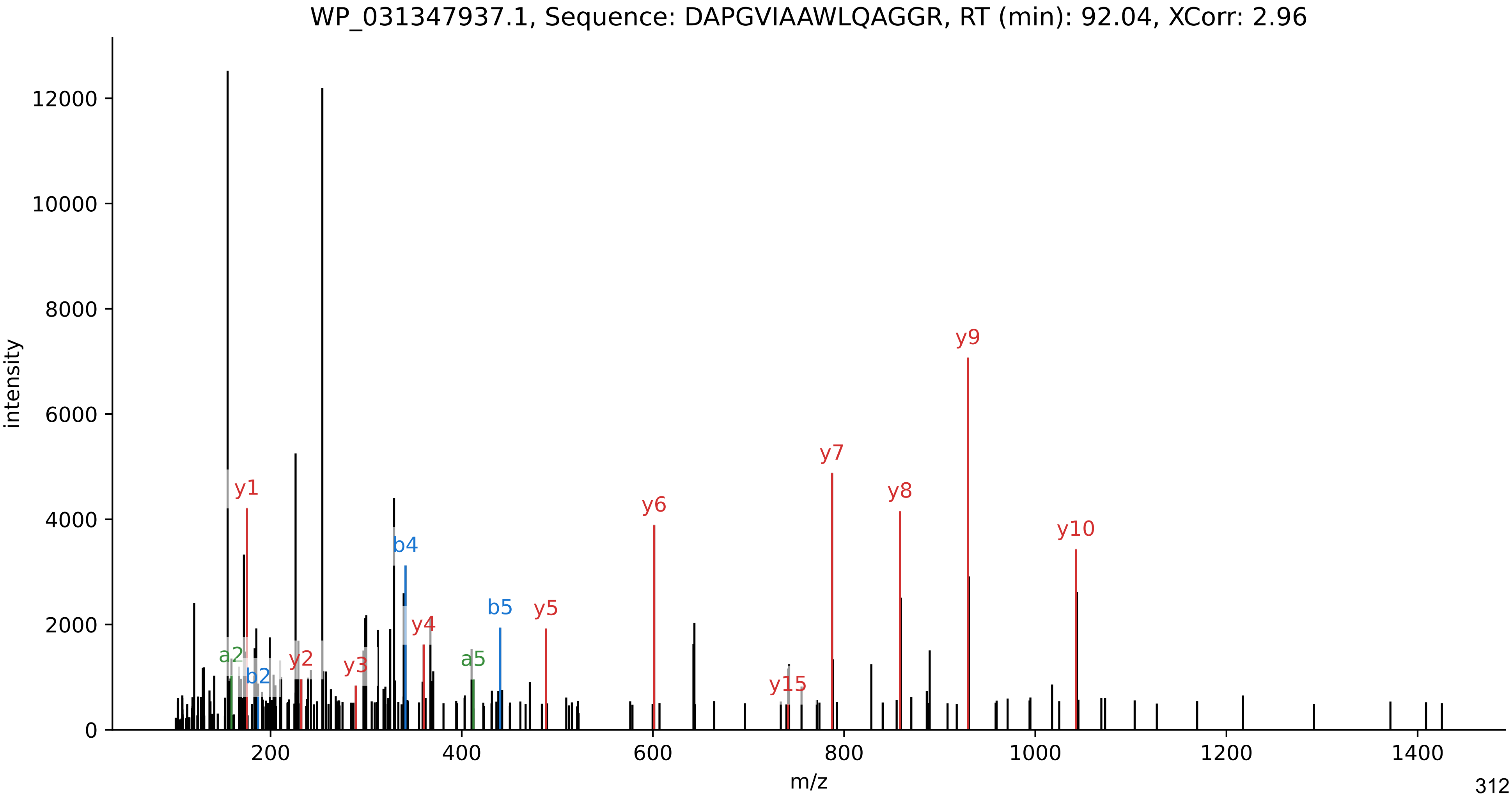
WP_031347937.1, Sequence: DAPGVIAAWLQAGGR, RT (min): 92.04, XCorr: 2.96
intensity
m/z
y1
y2
y3
y4
y5
y6
y7
y8
y9
y10
y15
b2
b4
b5
a2
a5
0
2000
4000
6000
8000
10000
12000
200
400
600
800
1000
1200
1400

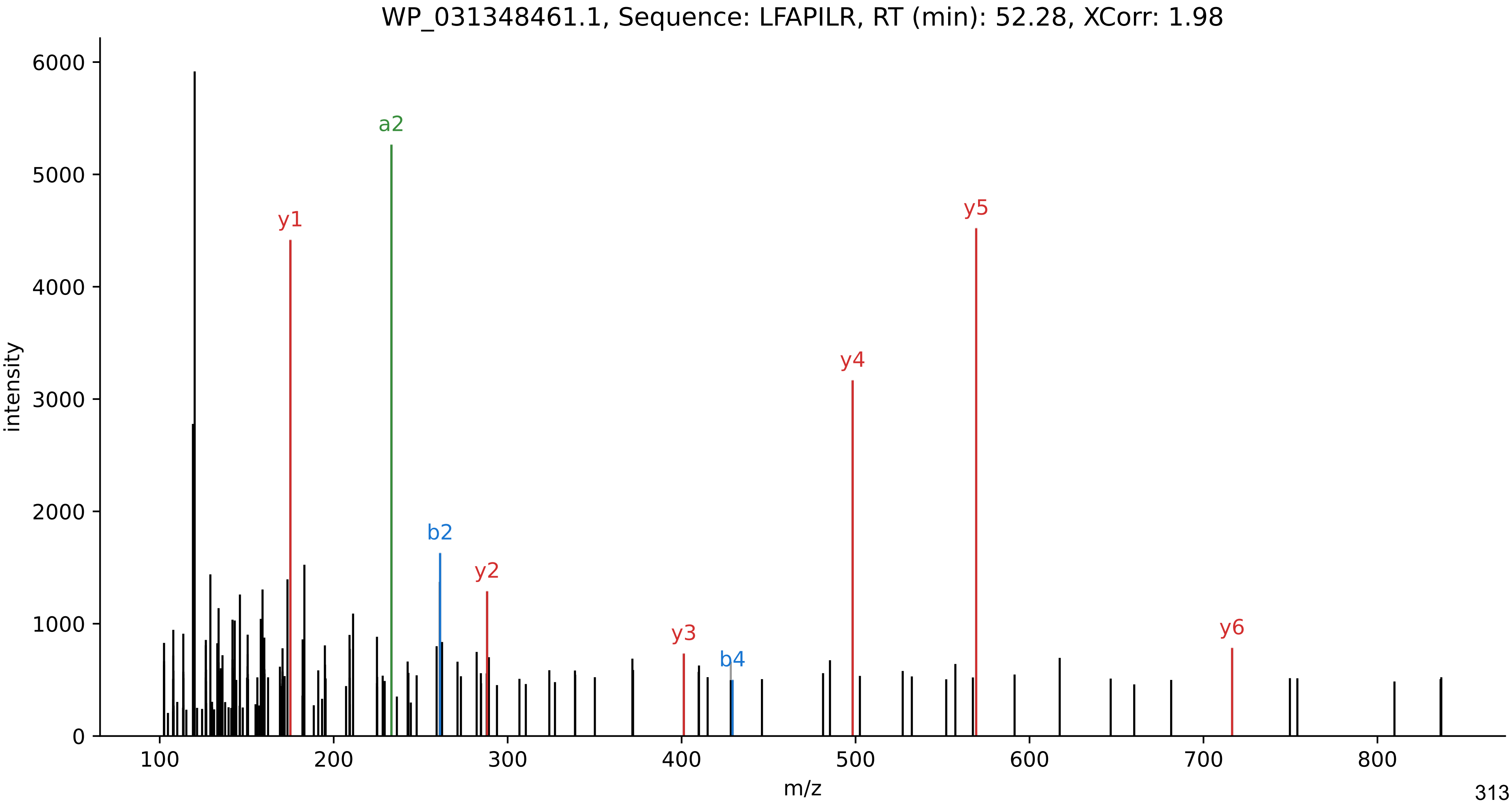
WP_031348461.1, Sequence: LFAPILR, RT (min): 52.28, XCorr: 1.98
intensity
m/z
a2
y1
y5
y4
b2
y2
y3
b4
y6
6000
5000
4000
3000
2000
1000
0
100
200
300
400
500
600
700
800

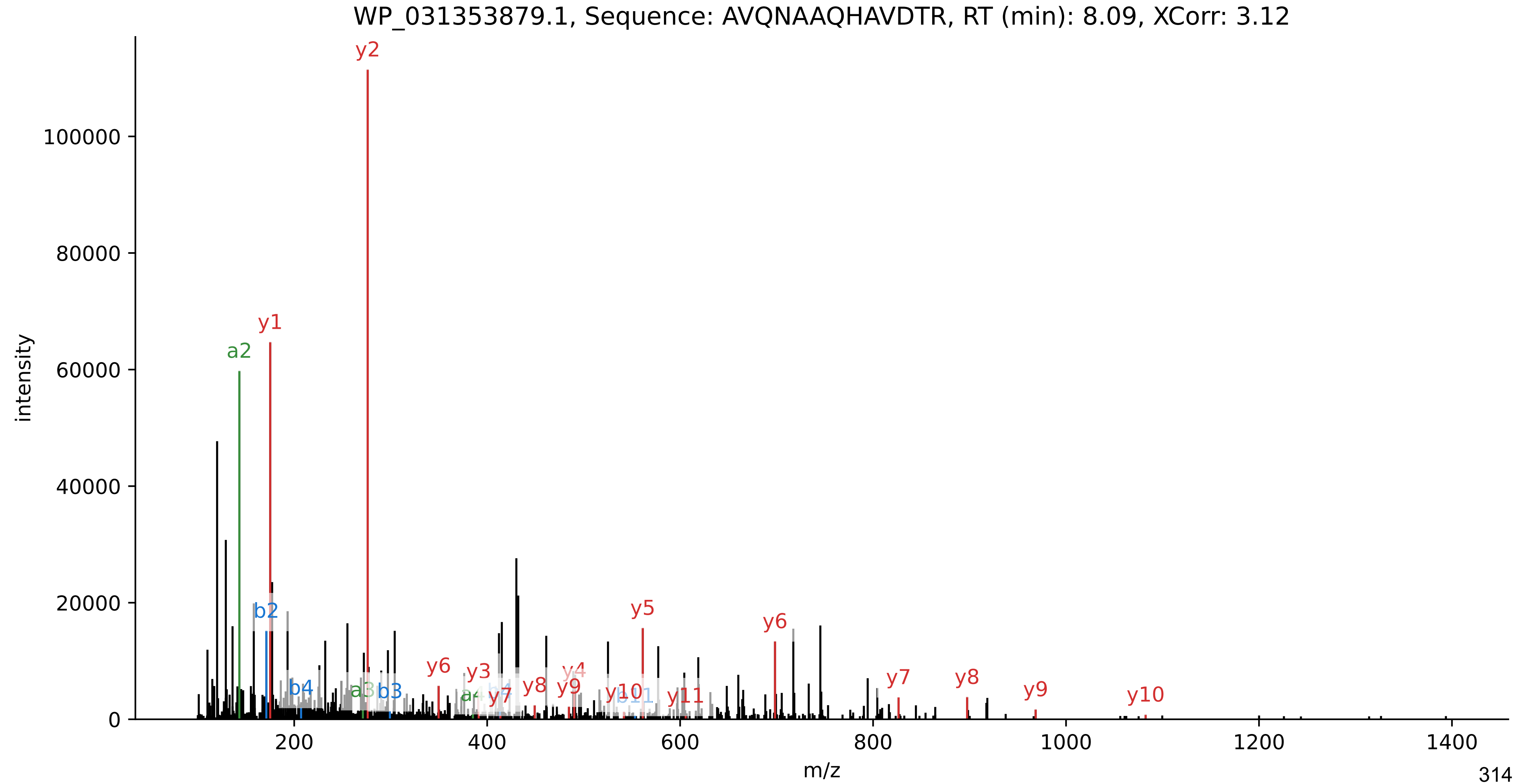

WP_031353879.1, Sequence: AVQNAAQHAVDTR, RT (min): 8.09, XCorr: 3.12
intensity
m/z
0
20000
40000
60000
80000
100000
200
400
600
800
1000
1200
1400
y2
y1
a2
b2
b4
a3
b3
y6
y3
y7
y8
y9
y4
y10
y11
y5
y6
y7
y8
y9
y10

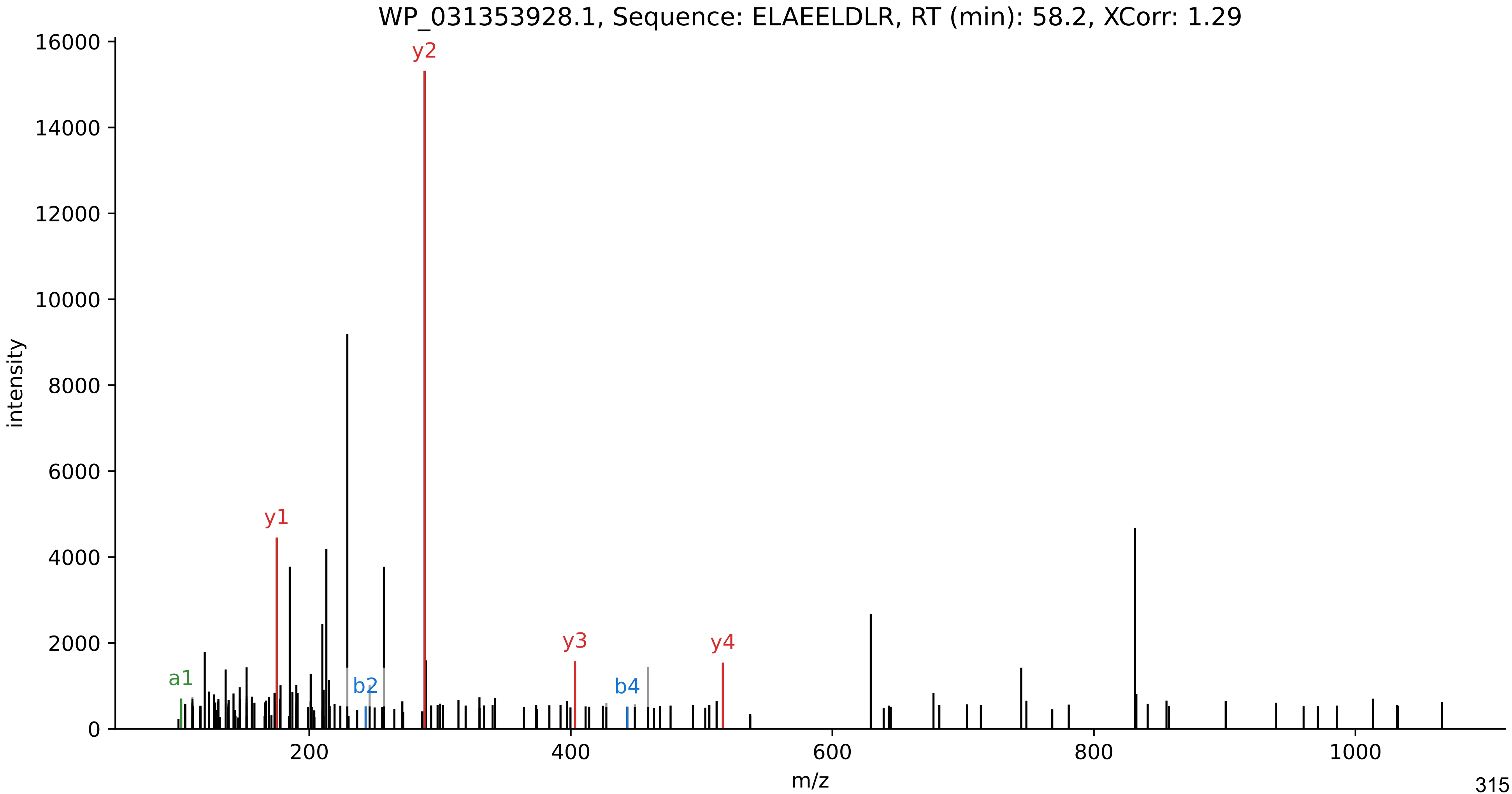
WP_031353928.1, Sequence: ELAEELDLR, RT (min): 58.2, XCorr: 1.29
intensity
m/z
y2
y1
y3
y4
a1
b2
b4
16000
14000
12000
10000
8000
6000
4000
2000
0
200
400
600
800
1000

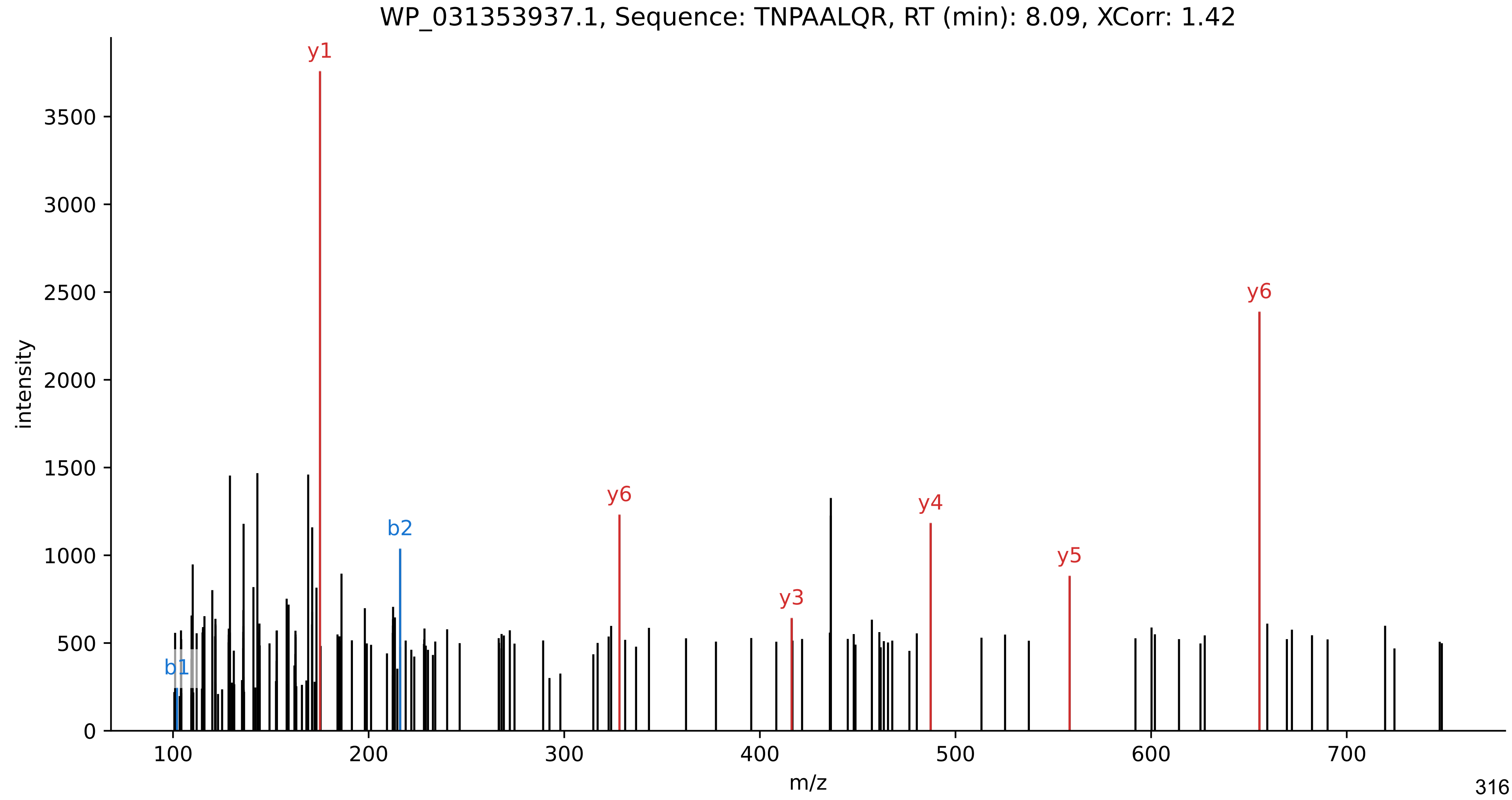

WP_031353937.1, Sequence: TNPAALQR, RT (min): 8.09, XCorr: 1.42
y1
y6
y6
b2
y4
y5
y3
b1
intensity
m/z
0
500
1000
1500
2000
2500
3000
3500
100
200
300
400
500
600
700

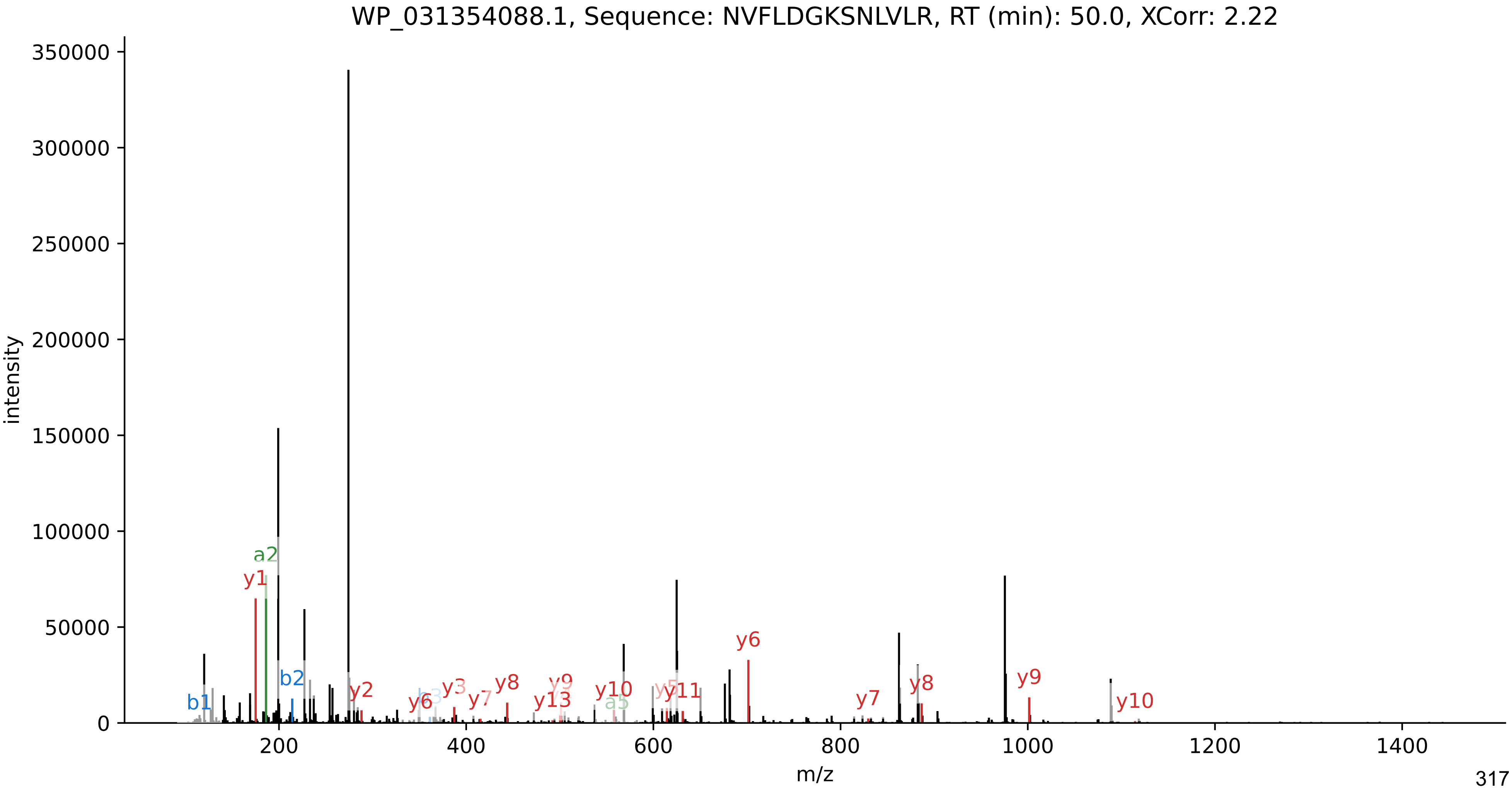
WP_031354088.1, Sequence: NVFLDGKSNLVLR, RT (min): 50.0, XCorr: 2.22
intensity
m/z
350000
300000
250000
200000
150000
100000
50000
0
200
400
600
800
1000
1200
1400
a2
y1
b1
b2
y2
y6
y3
y7
y8
y9
y13
y10
a5
y11
y6
y7
y8
y9
y10

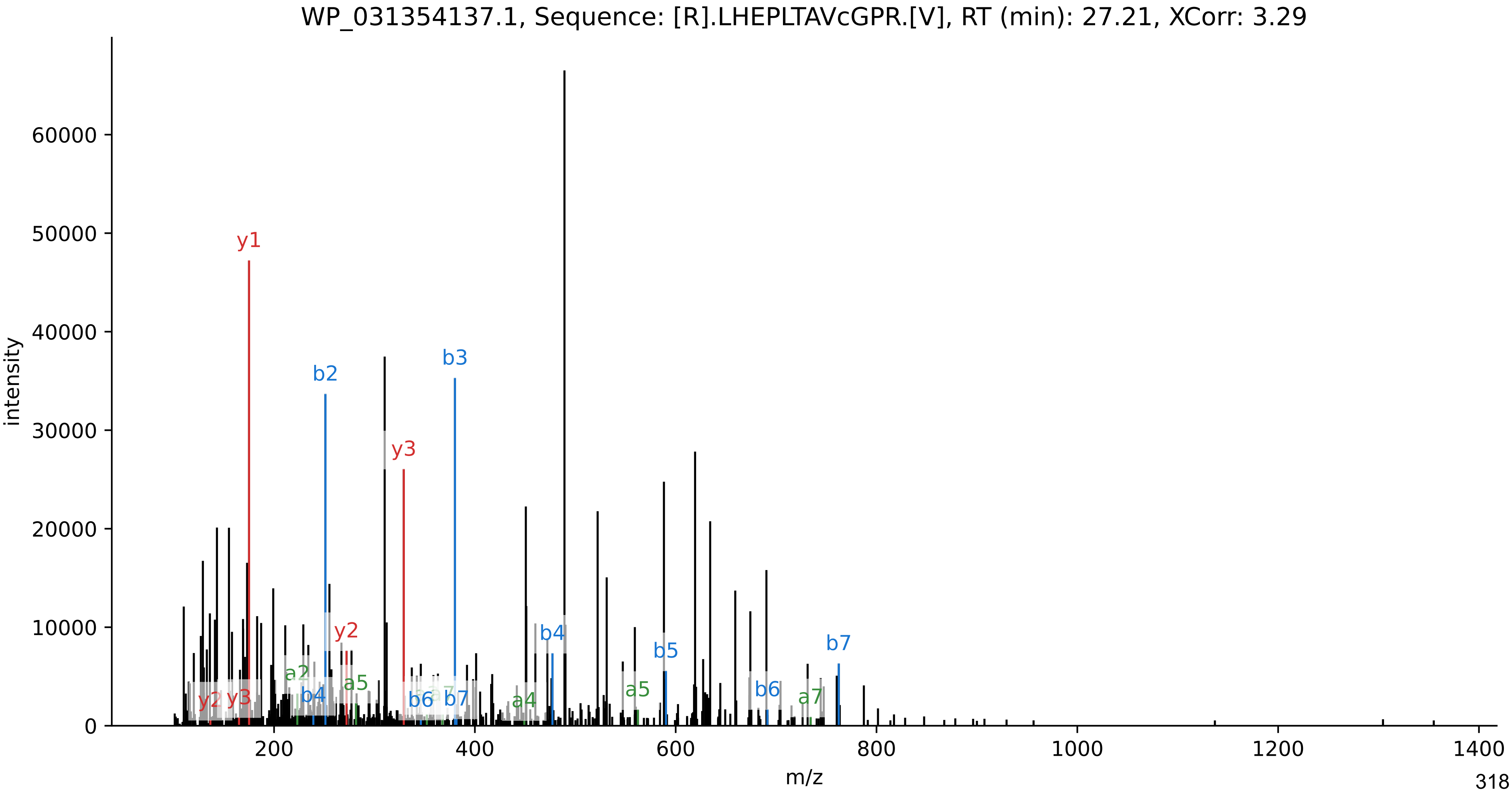
WP_031354137.1, Sequence: [R].LHEPLTAVcGPR.[V], RT (min): 27.21, XCorr: 3.29
intensity
m/z
y1
b2
b3
y3
y2
a2
a5
b4
y2
y3
b6
b7
a4
b4
b5
a5
b6
a7
b7
0
10000
20000
30000
40000
50000
60000
200
400
600
800
1000
1200
1400

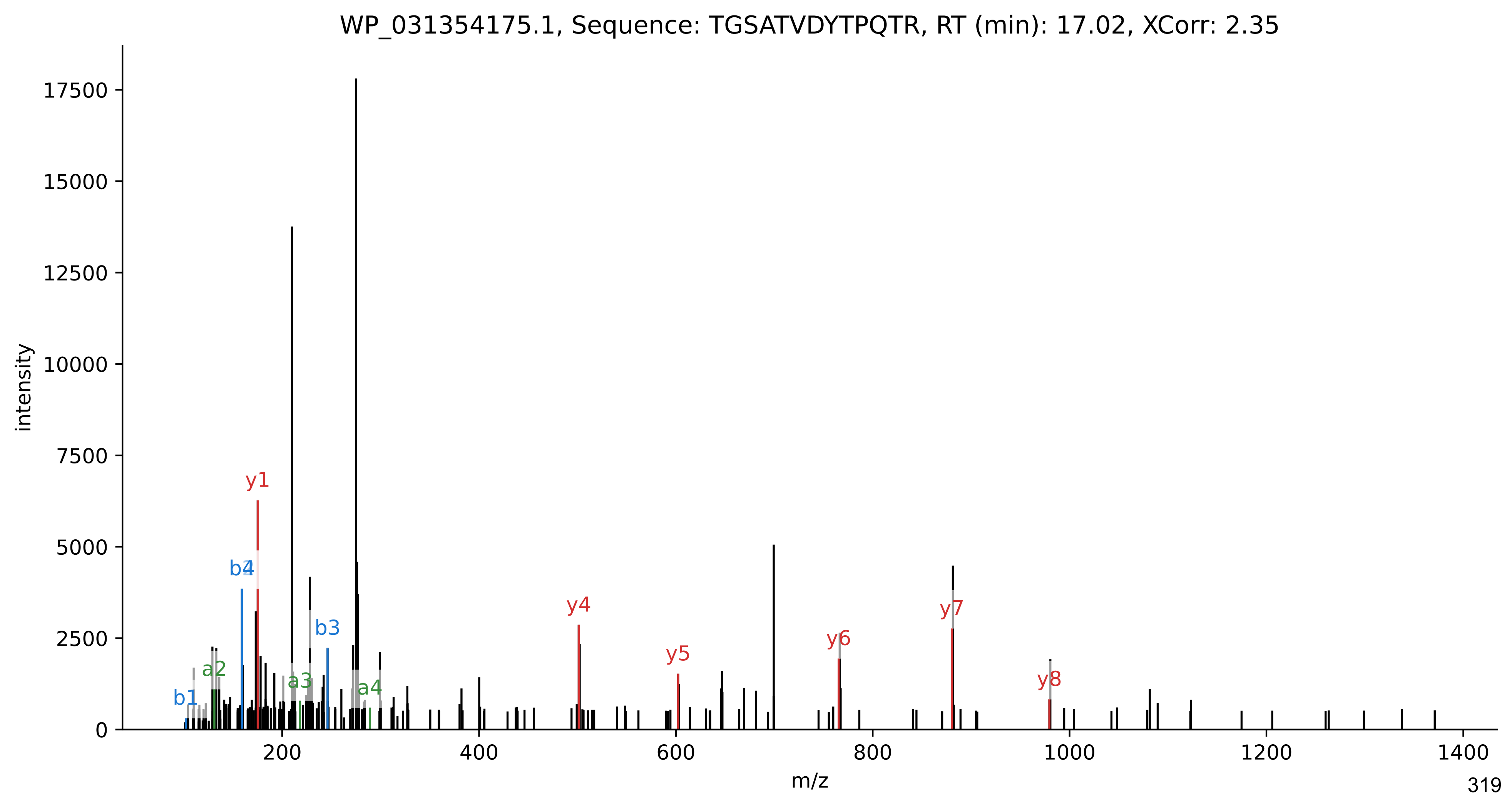

WP_031354175.1, Sequence: TGSATVDYTPQTR, RT (min): 17.02, XCorr: 2.35
intensity
m/z
y1
b4
b3
a2
a3
a4
b1
y4
y5
y6
y7
y8

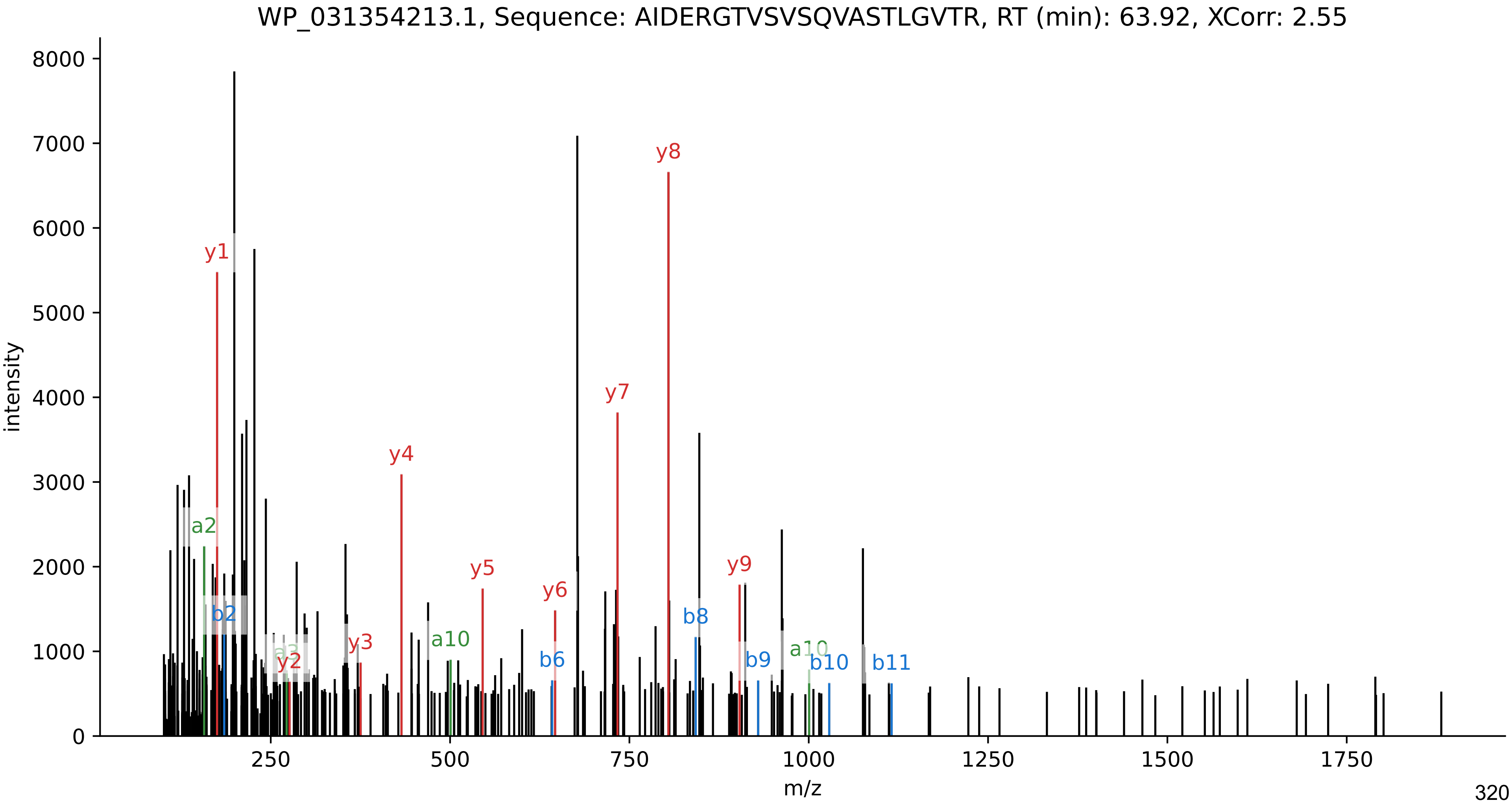
WP_031354213.1, Sequence: AIDERGTVSVSQVASTLGVTR, RT (min): 63.92, XCorr: 2.55
intensity
m/z
y1
y2
y3
y4
y5
y6
y7
y8
y9
a2
a3
a10
a10
b2
b6
b8
b9
b10
b11
0
1000
2000
3000
4000
5000
6000
7000
8000
250
500
750
1000
1250
1500
1750

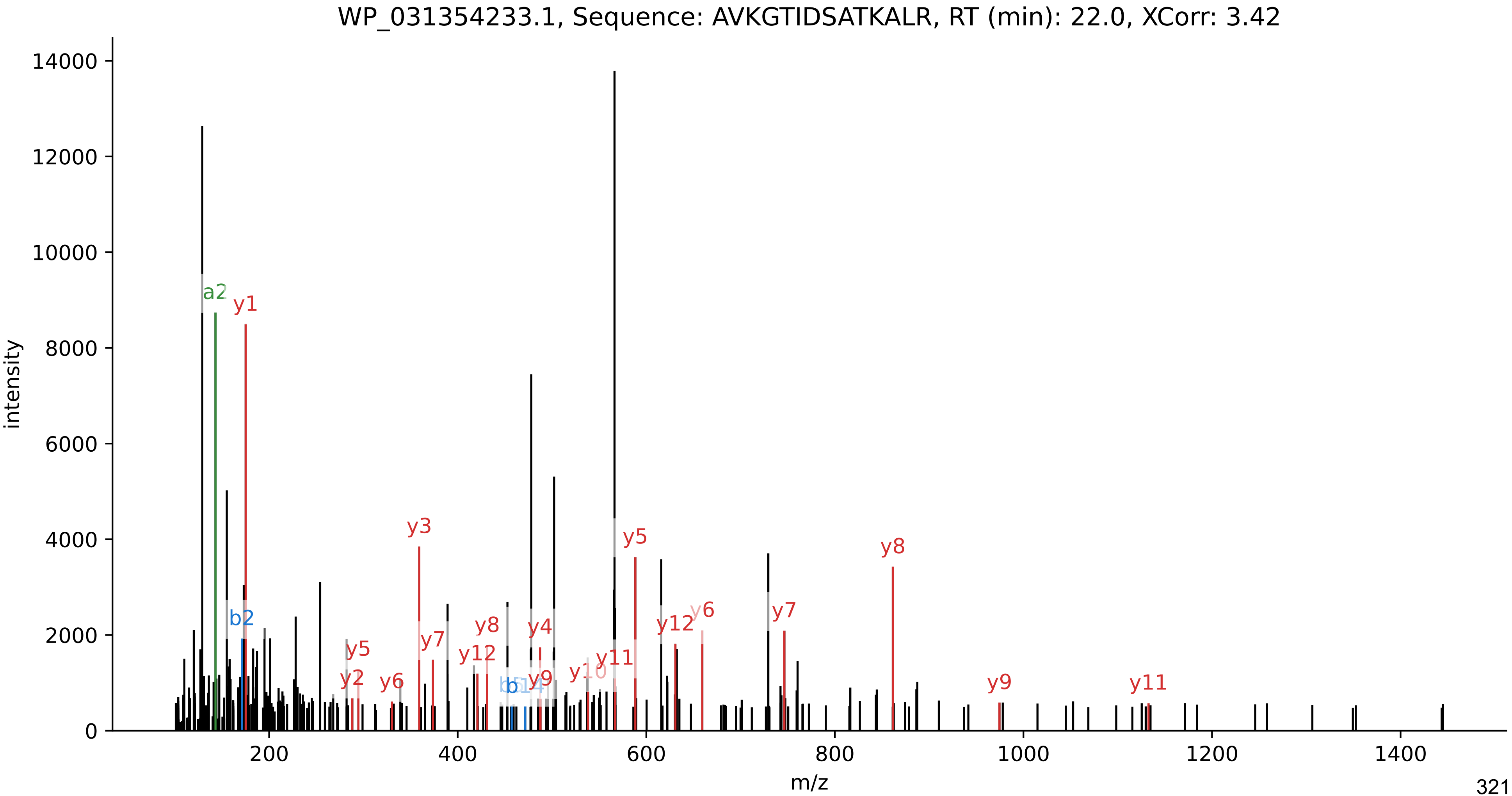
WP_031354233.1, Sequence: AVKGTIDSATKALR, RT (min): 22.0, XCorr: 3.42
intensity
m/z
a2
y1
b2
y3
y5
y8
y7
y12
y6
y4
y9
y10
y11
y2
b
y5
y7
y8
y12
y6
y7
y9
y11
0
2000
4000
6000
8000
10000
12000
14000
200
400
600
800
1000
1200
1400

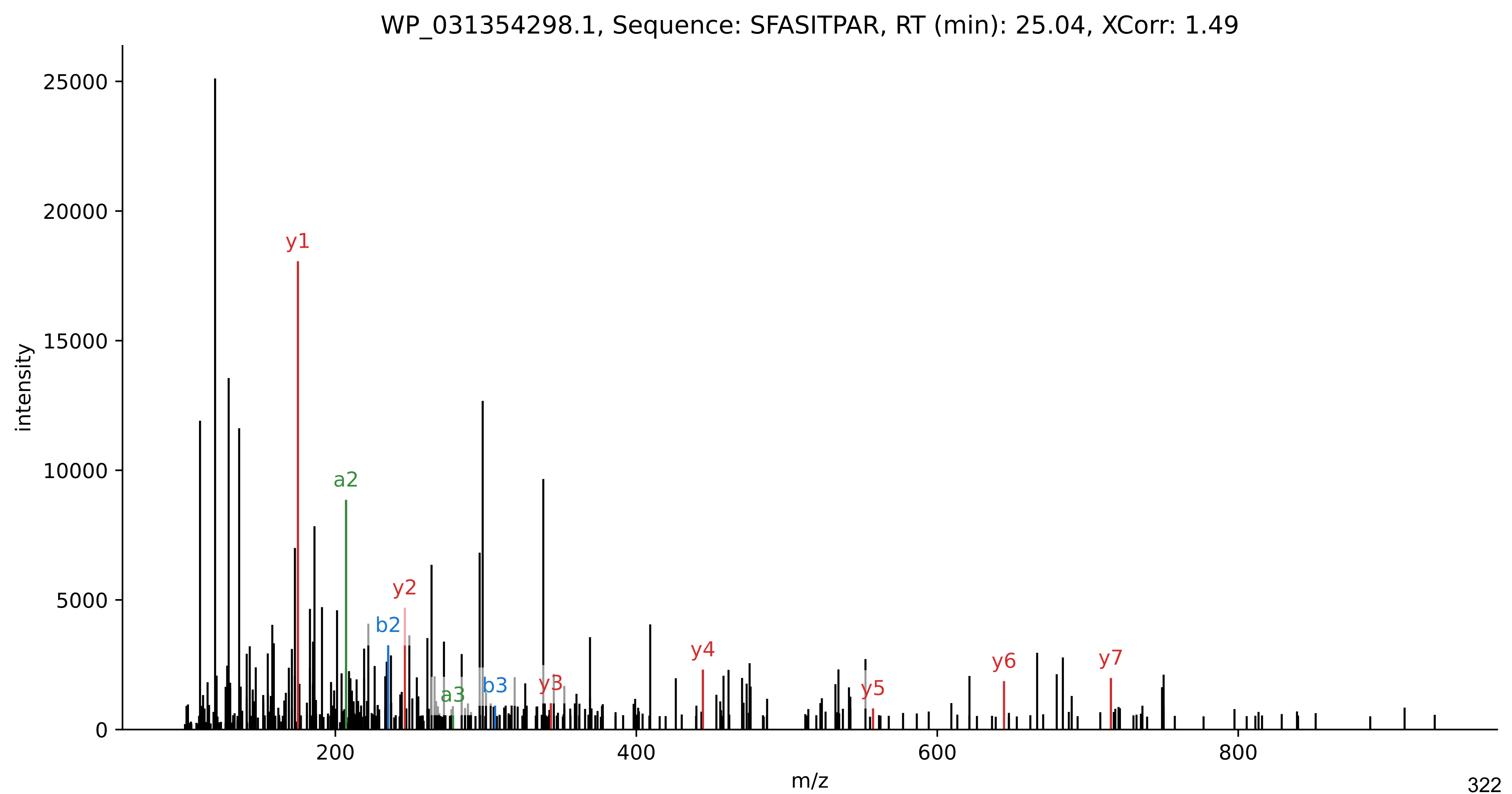
WP_031354298.1, Sequence: SFASITPAR, RT (min): 25.04, XCorr: 1.49
intensity
m/z
y1
a2
y2
b2
a3
b3
y3
y4
y5
y6
y7
25000
20000
15000
10000
5000
0
200
400
600
800

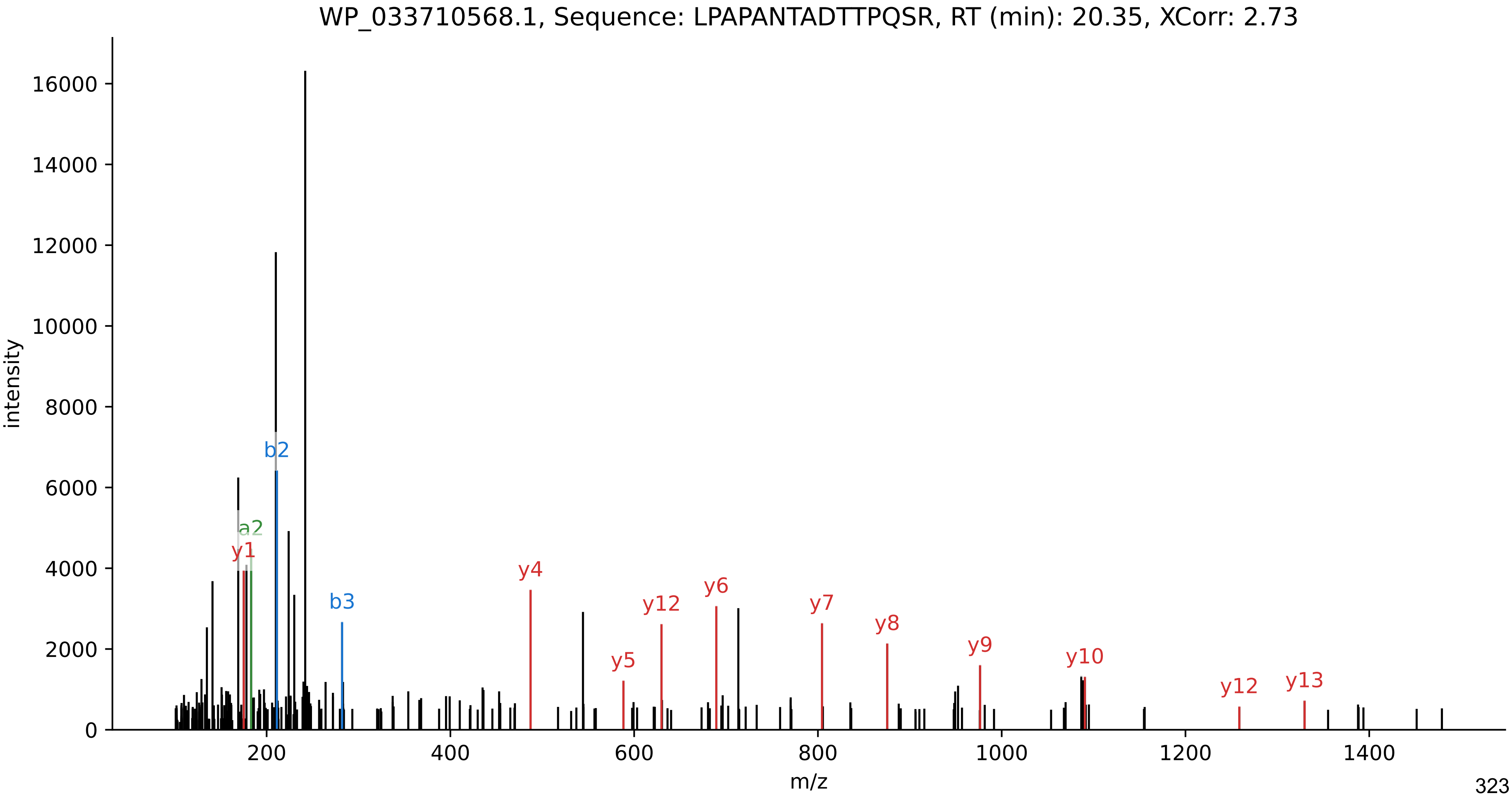

WP_033710568.1, Sequence: LPAPANTADTTPQSR, RT (min): 20.35, XCorr: 2.73
intensity
m/z
b2
a2
y1
b3
y4
y5
y12
y6
y7
y8
y9
y10
y12
y13

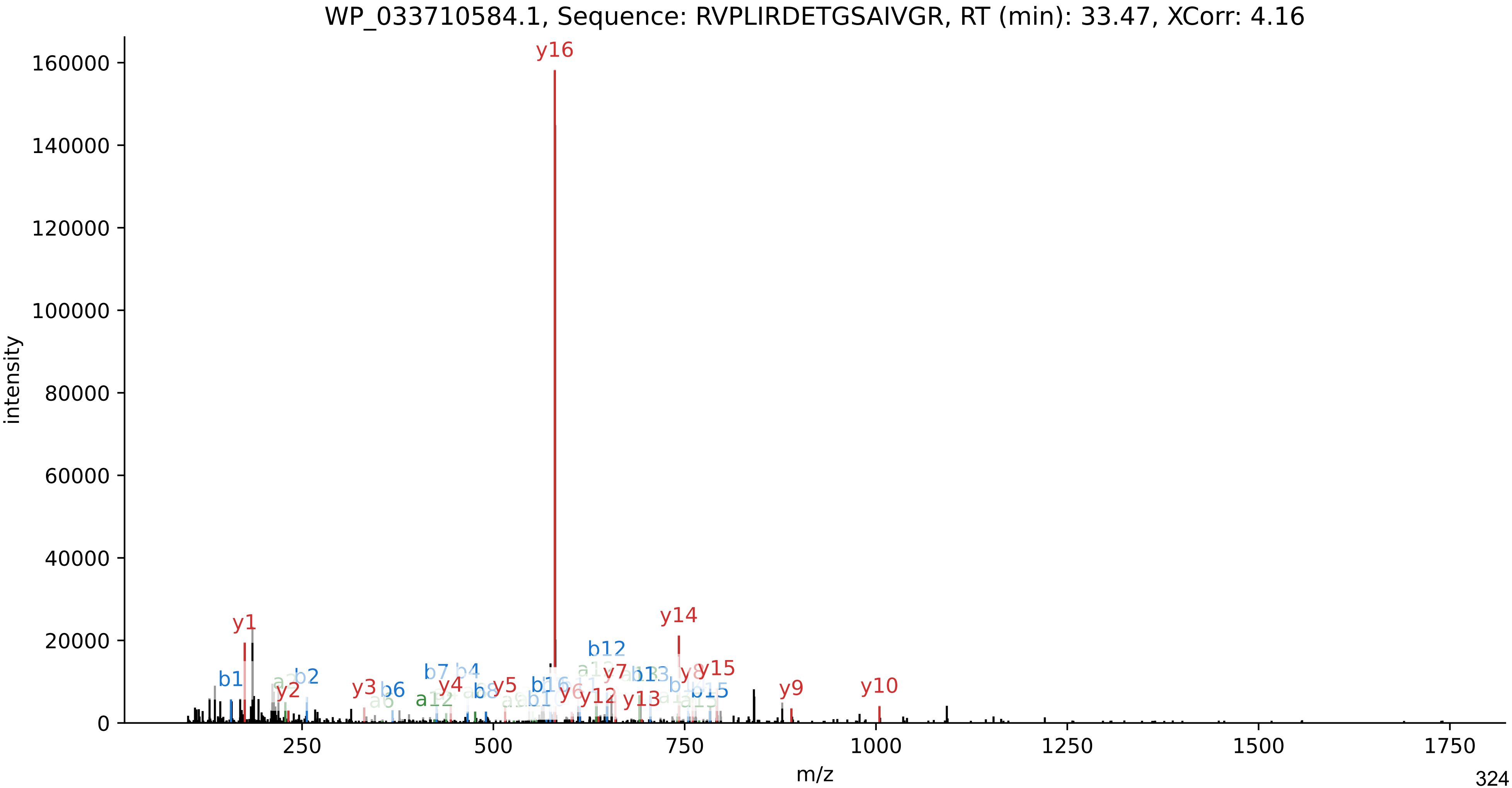

WP_033710584.1, Sequence: RVPLIRDETGSAIVGR, RT (min): 33.47, XCorr: 4.16
intensity
m/z
y16
y14
y1
b12
y15
y9
y10
b1
b2
y2
y3
b6
b7
b4
y4
a12
b8
y5
b16
y7
y12
y13
b15
a6

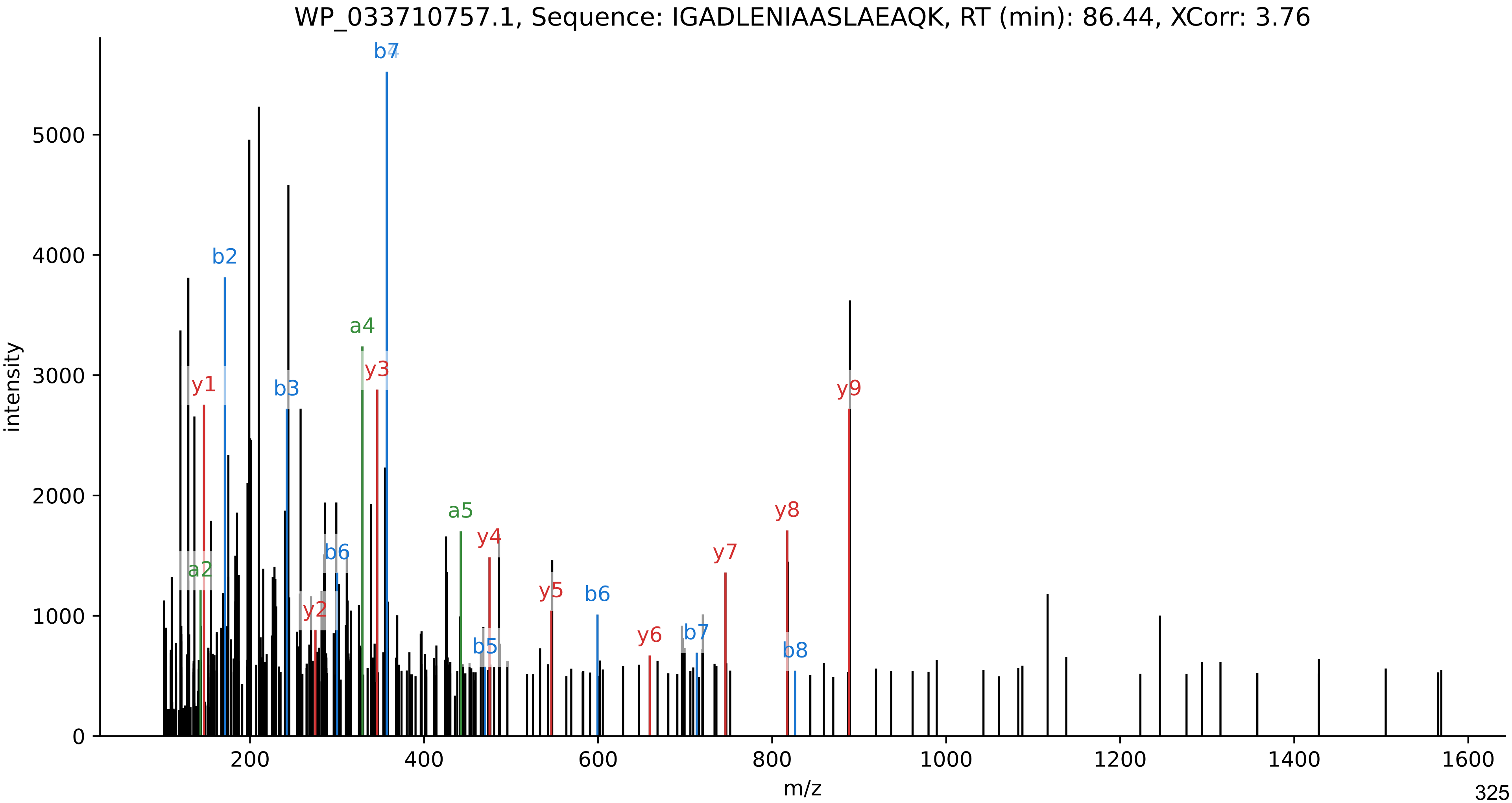

WP_033710757.1, Sequence: IGADLENIAASLAEAQK, RT (min): 86.44, XCorr: 3.76
intensity
m/z
b2
b3
b6
b7
a2
a4
a5
y1
y2
y3
y4
y5
y6
y7
y8
y9
b5
b6
b7
b8

WP_033711483.1, Sequence: NADGLVTAVVQER, RT (min): 48.68, XCorr: 1.77

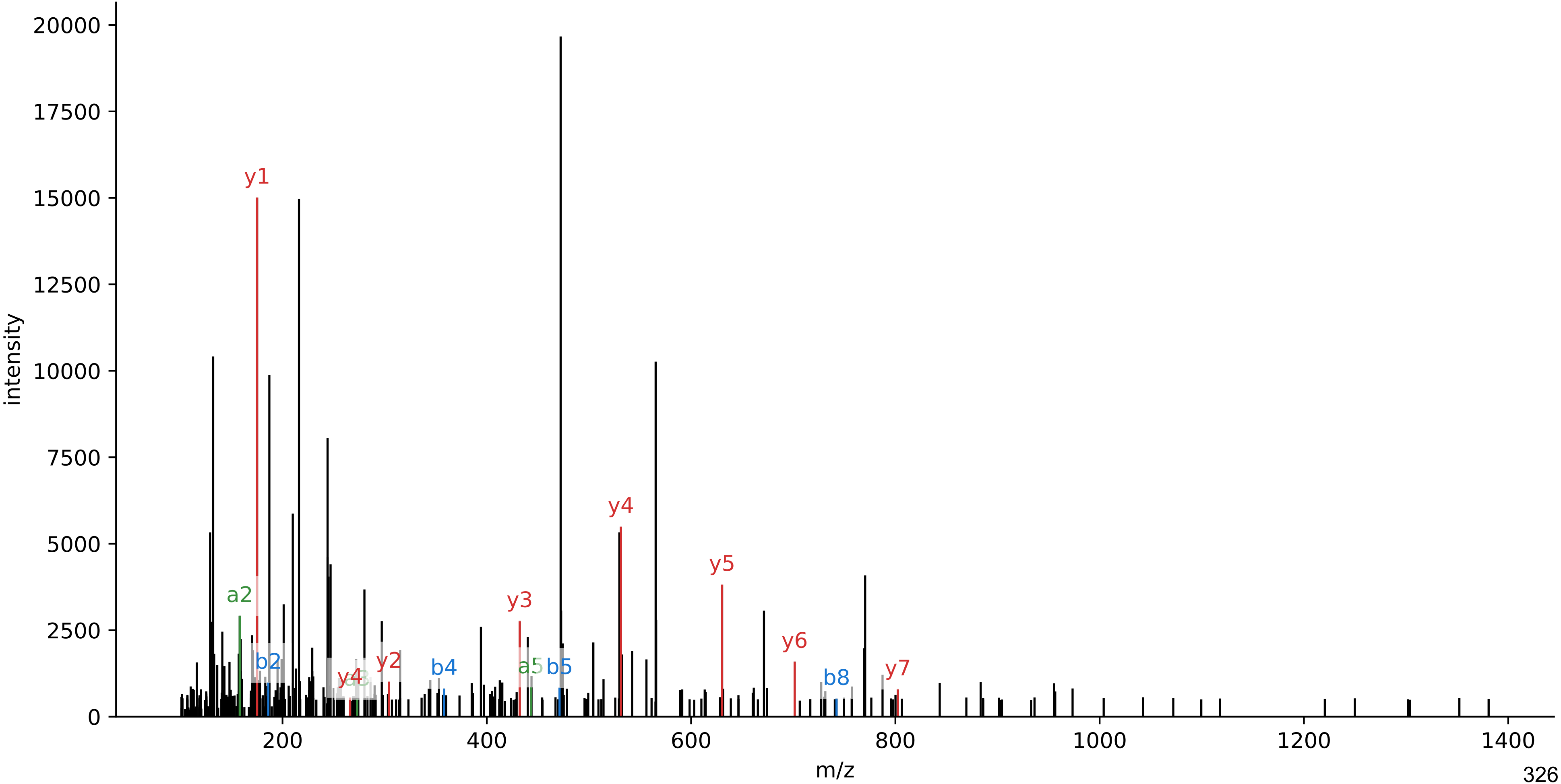

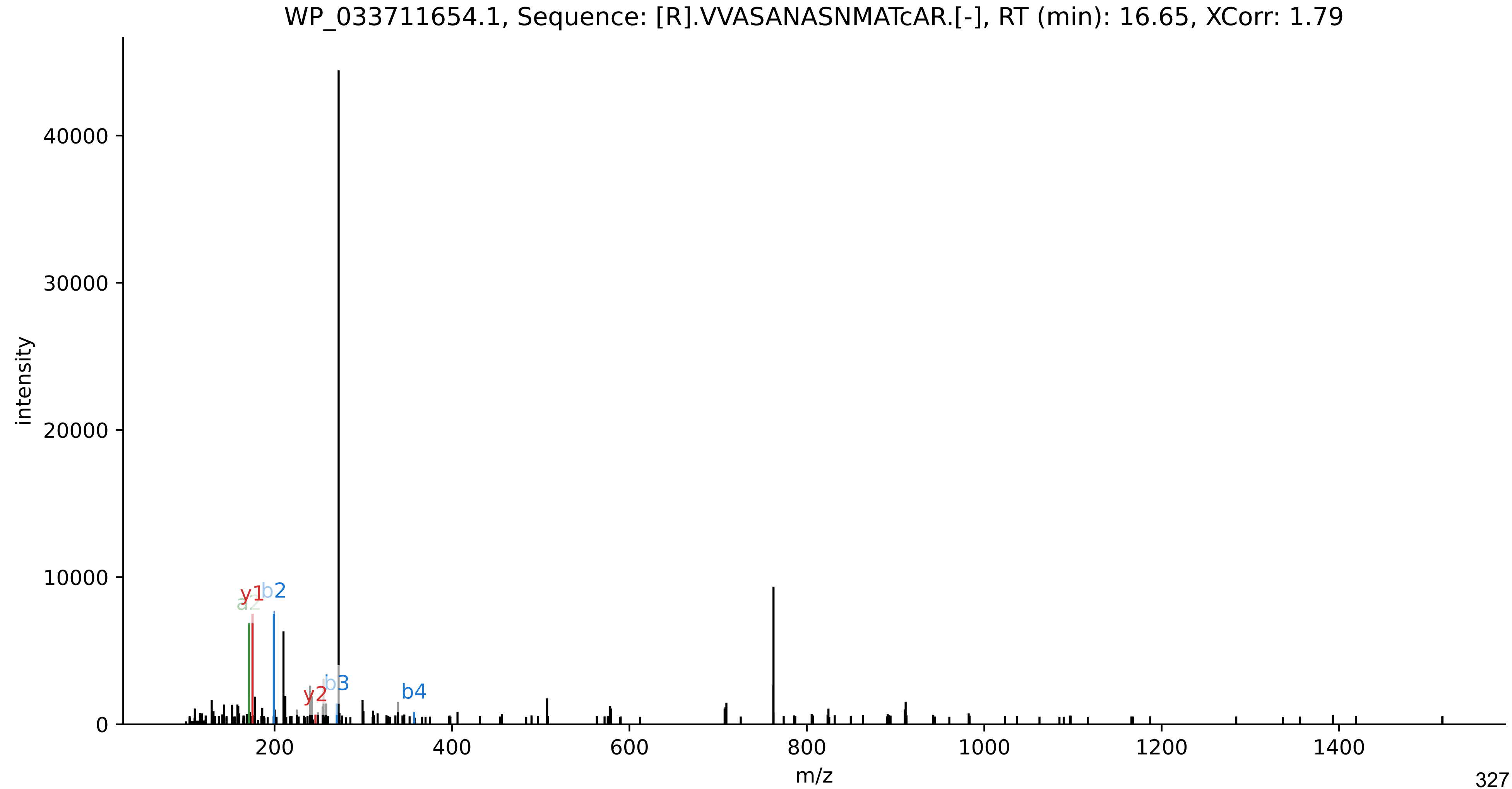

WP_033711654.1, Sequence: [R].VVASANASNMATcAR.[-], RT (min): 16.65, XCorr: 1.79
intensity
m/z
y1
a2
b2
y2
b3
b4

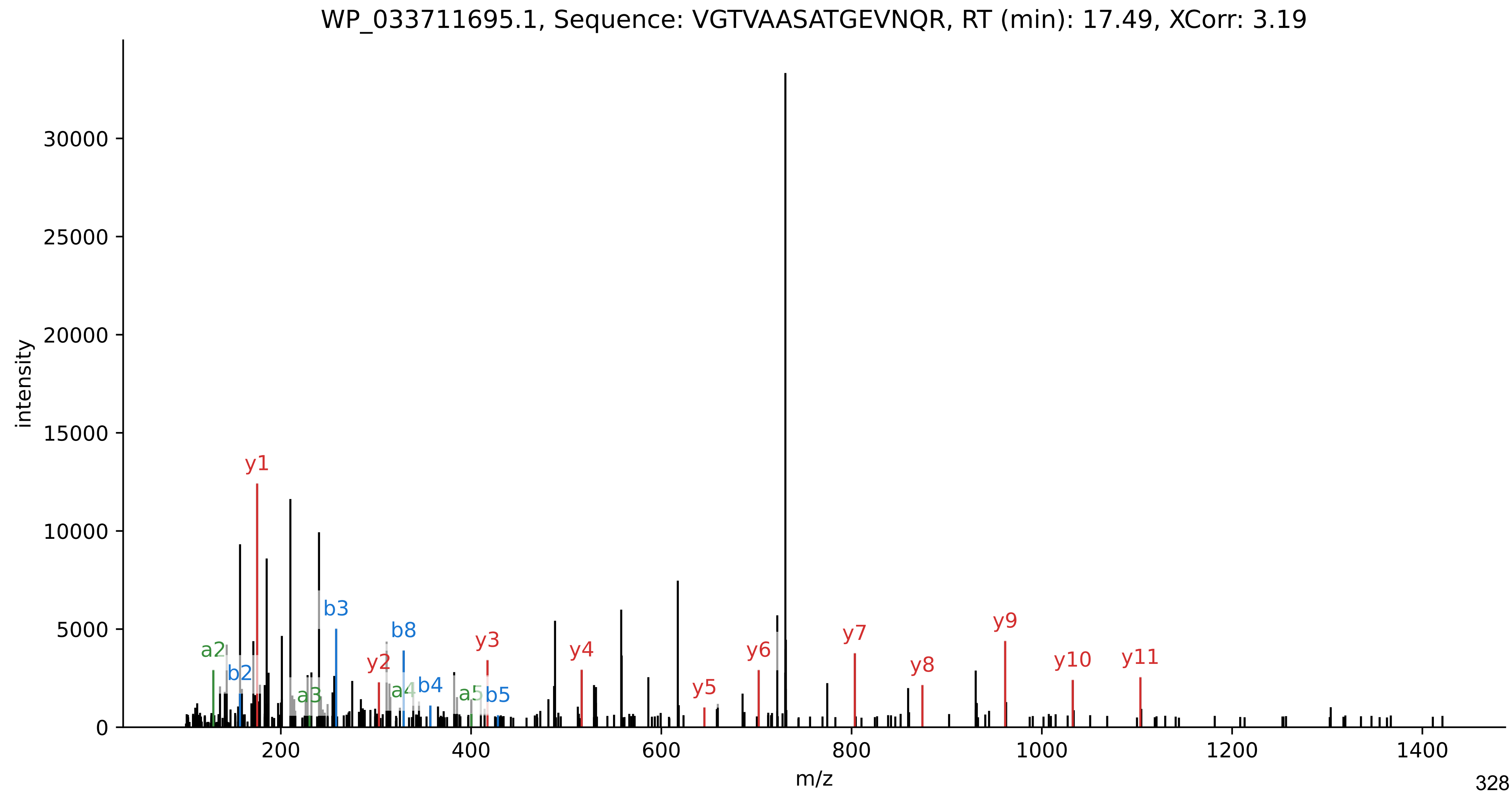

WP_033711695.1, Sequence: VGTVAASATGEVNQR, RT (min): 17.49, XCorr: 3.19
intensity
m/z
y1
b3
b8
y2
y3
y4
y5
y6
y7
y8
y9
y10
y11
a2
b2
a3
a4
b4
a5
b5

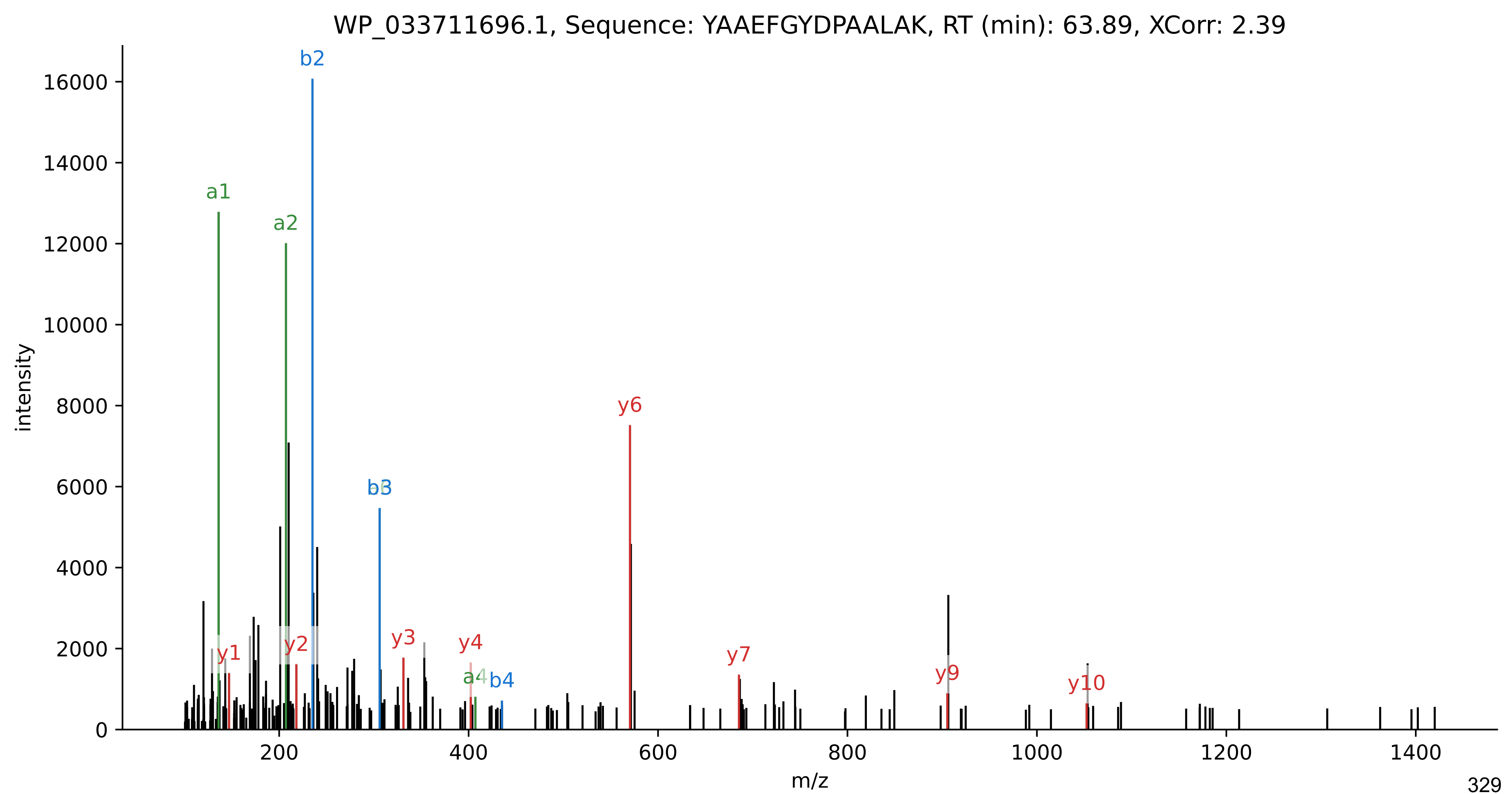
WP_033711696.1, Sequence: YAAEFGYDPAALAK, RT (min): 63.89, XCorr: 2.39
intensity
m/z
b2
a1
a2
y6
b3
y1
y2
y3
y4
a4
b4
y7
y9
y10

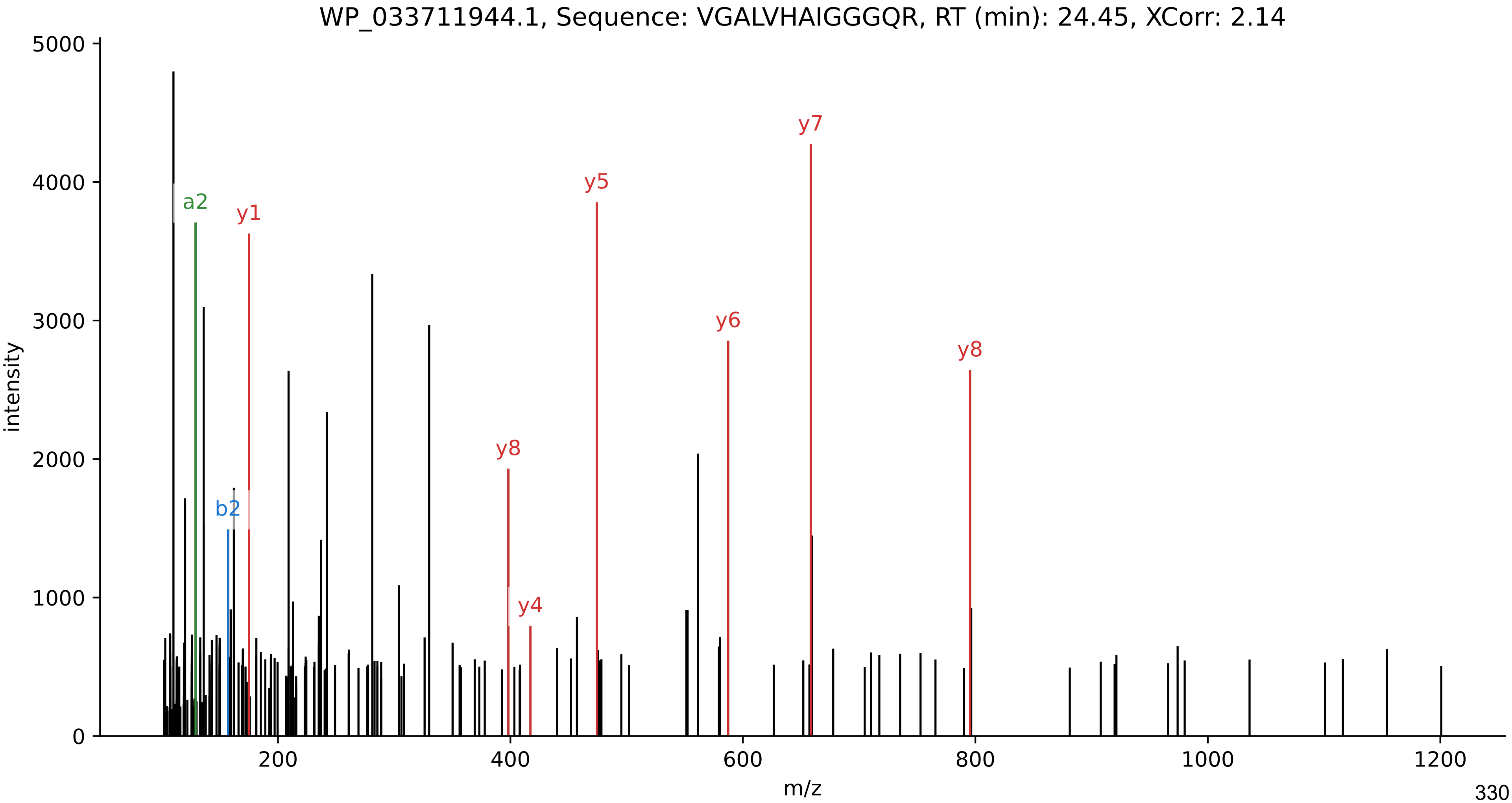

WP_033711944.1, Sequence: VGALVHAIGGGQR, RT (min): 24.45, XCorr: 2.14
intensity
m/z
a2
b2
y1
y4
y5
y6
y7
y8
y8
0
1000
2000
3000
4000
5000
200
400
600
800
1000
1200

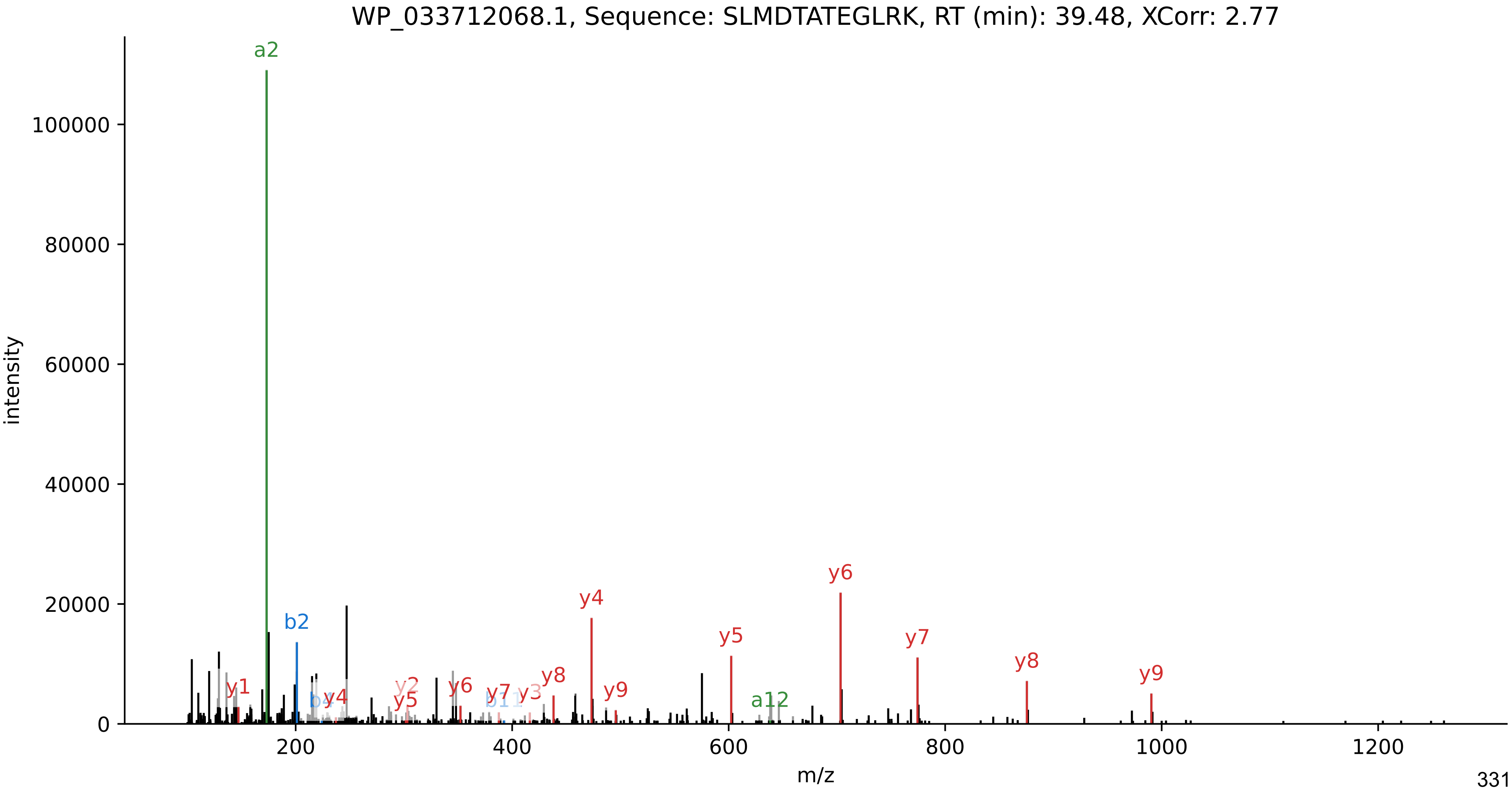
WP_033712068.1, Sequence: SLMDTATEGLRK, RT (min): 39.48, XCorr: 2.77
intensity
m/z
a2
b2
y1
y4
y2
y5
y6
y7
y3
y8
y9
b11
y4
y5
y6
y7
y8
y9
a12
0
20000
40000
60000
80000
100000
200
400
600
800
1000
1200

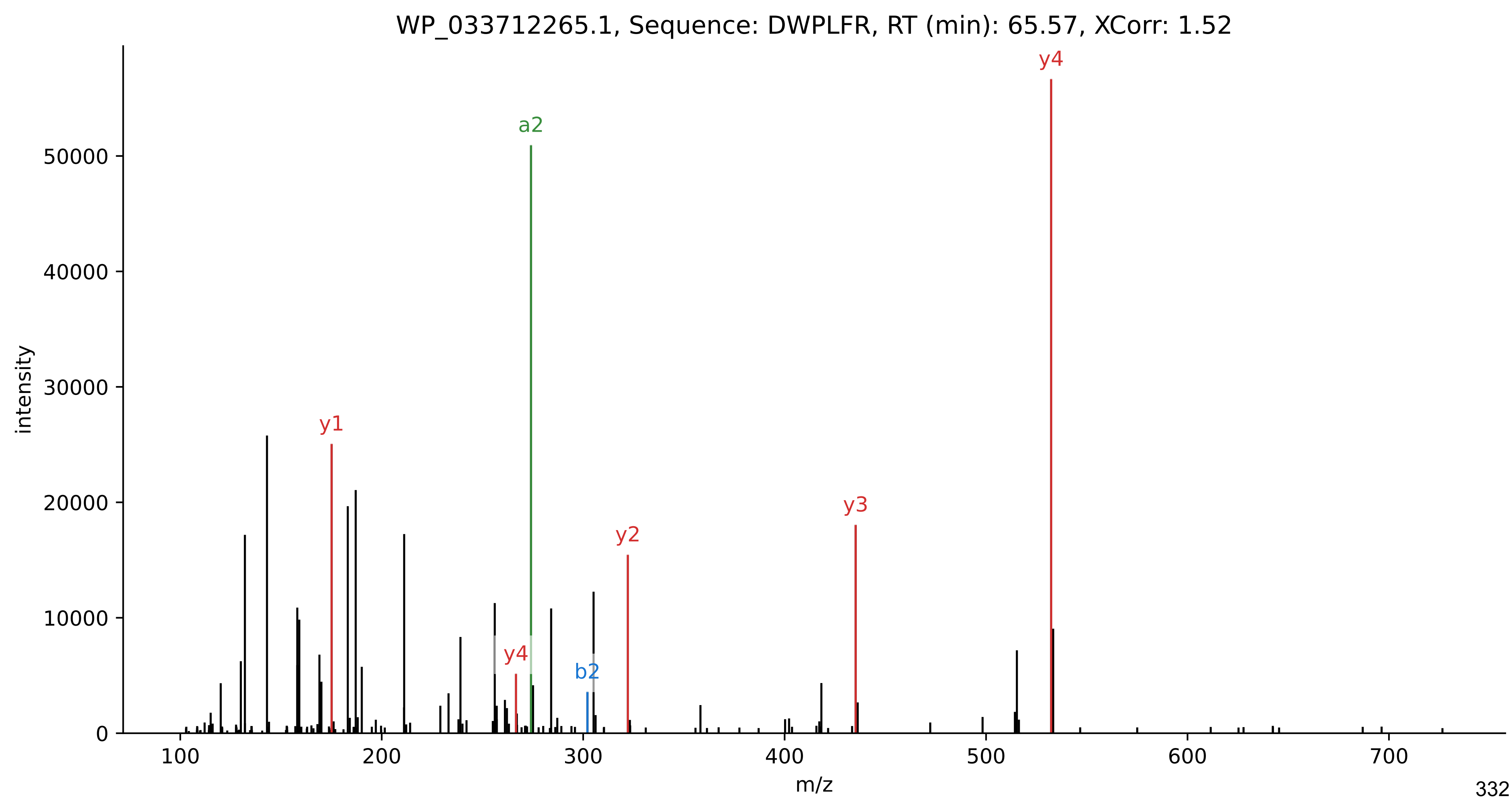

WP_033712265.1, Sequence: DWPLFR, RT (min): 65.57, XCorr: 1.52
intensity
m/z
y4
a2
y1
y4
b2
y2
y3
0
10000
20000
30000
40000
50000
100
200
300
400
500
600
700

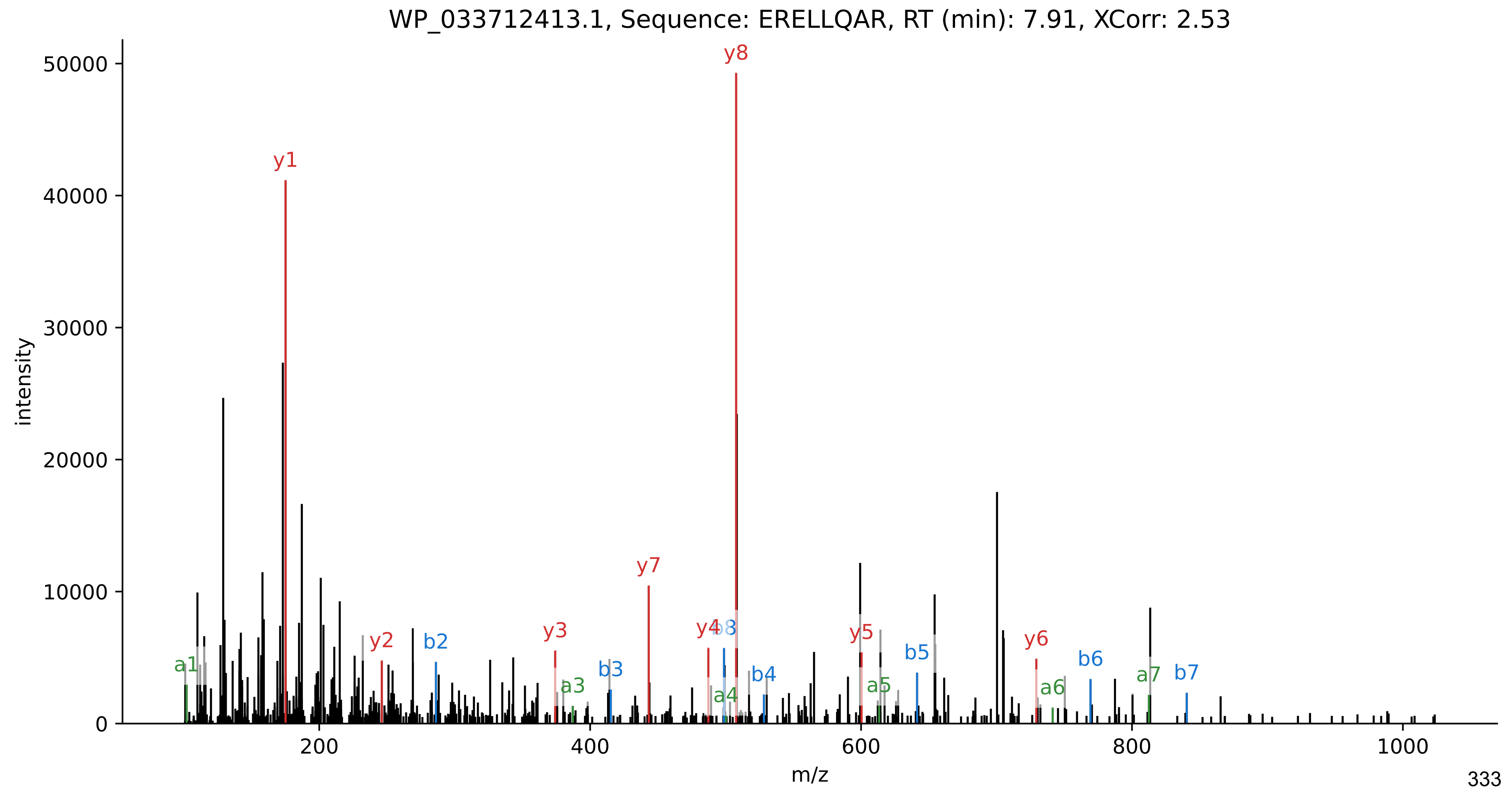
WP_033712413.1, Sequence: ERELLQAR, RT (min): 7.91, XCorr: 2.53
intensity
m/z
50000
40000
30000
20000
10000
0
200
400
600
800
1000
y1
y2
y3
y4
y5
y6
y7
y8
b2
b3
b4
b5
b6
b7
b8
a1
a3
a4
a5
a6
a7

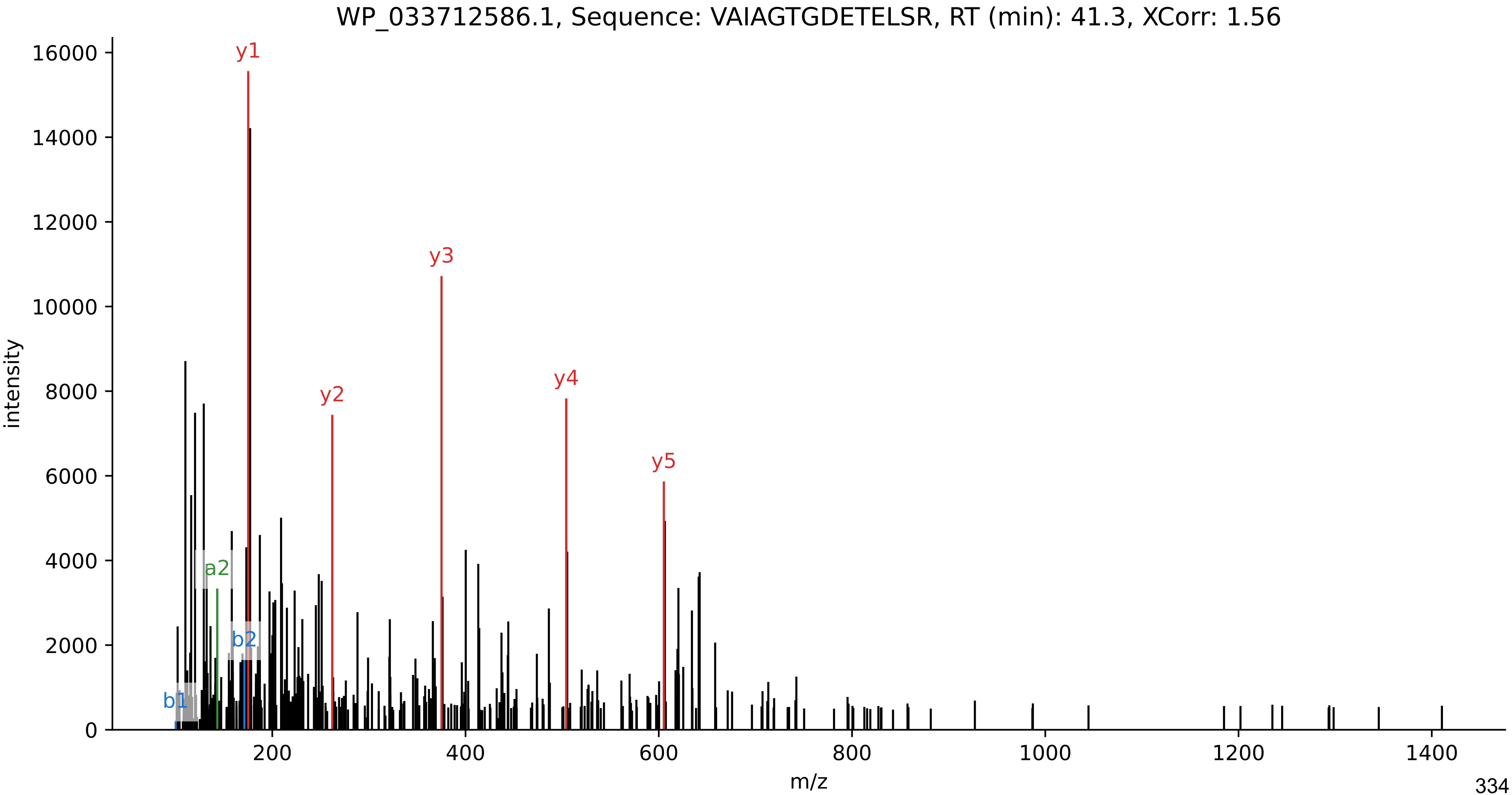

WP_033712586.1, Sequence: VAIAGTGDETELSR, RT (min): 41.3, XCorr: 1.56
y1
y2
y3
y4
y5
a2
b1
b2
intensity
m/z
0
2000
4000
6000
8000
10000
12000
14000
16000
200
400
600
800
1000
1200
1400

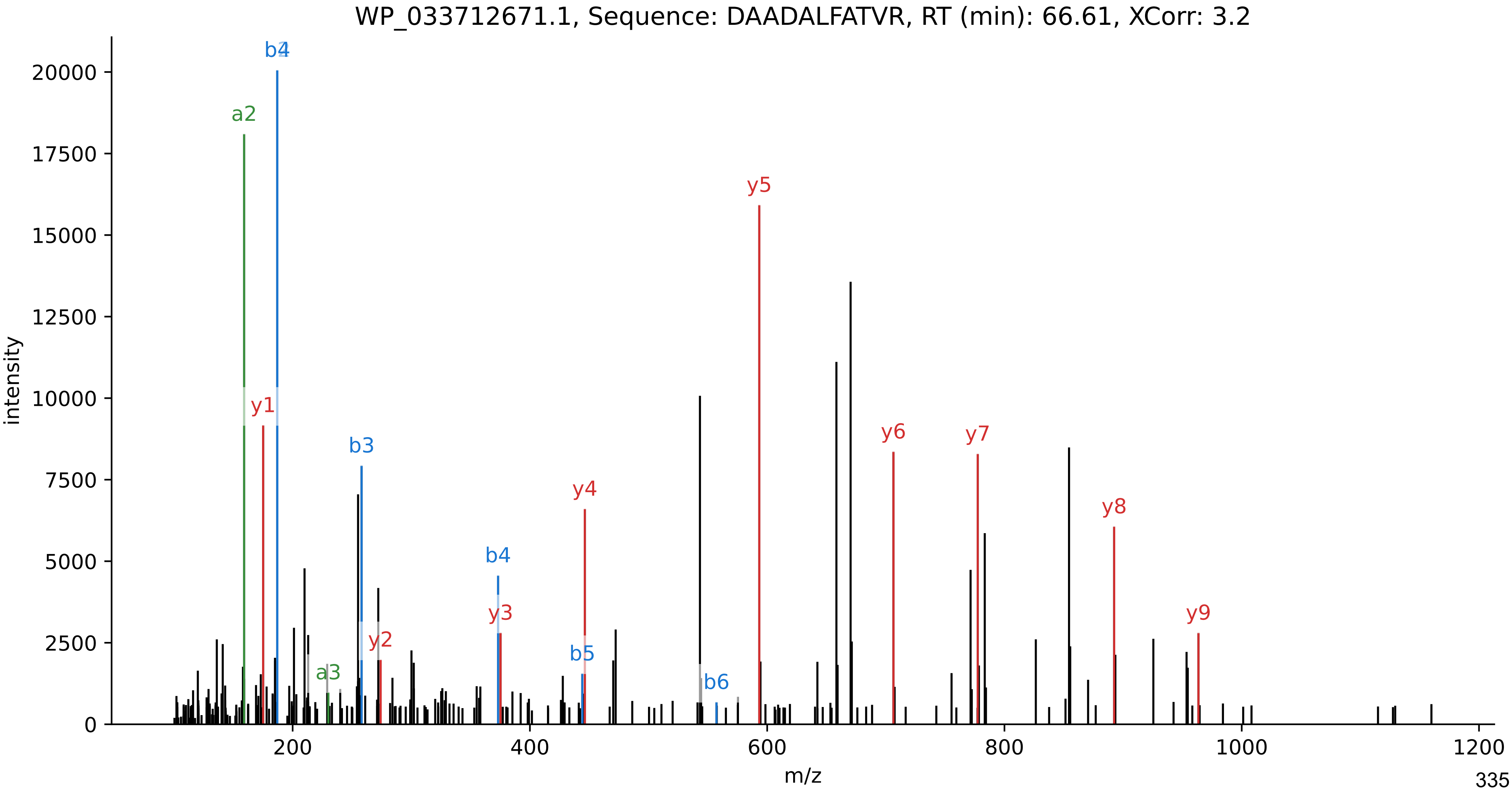
WP_033712671.1, Sequence: DAADALFATVR, RT (min): 66.61, XCorr: 3.2
intensity
m/z
0
2500
5000
7500
10000
12500
15000
17500
20000
200
400
600
800
1000
1200
a2
b4
y1
b3
y4
y5
y6
y7
y8
y9
b4
y3
y2
a3
b5
b6

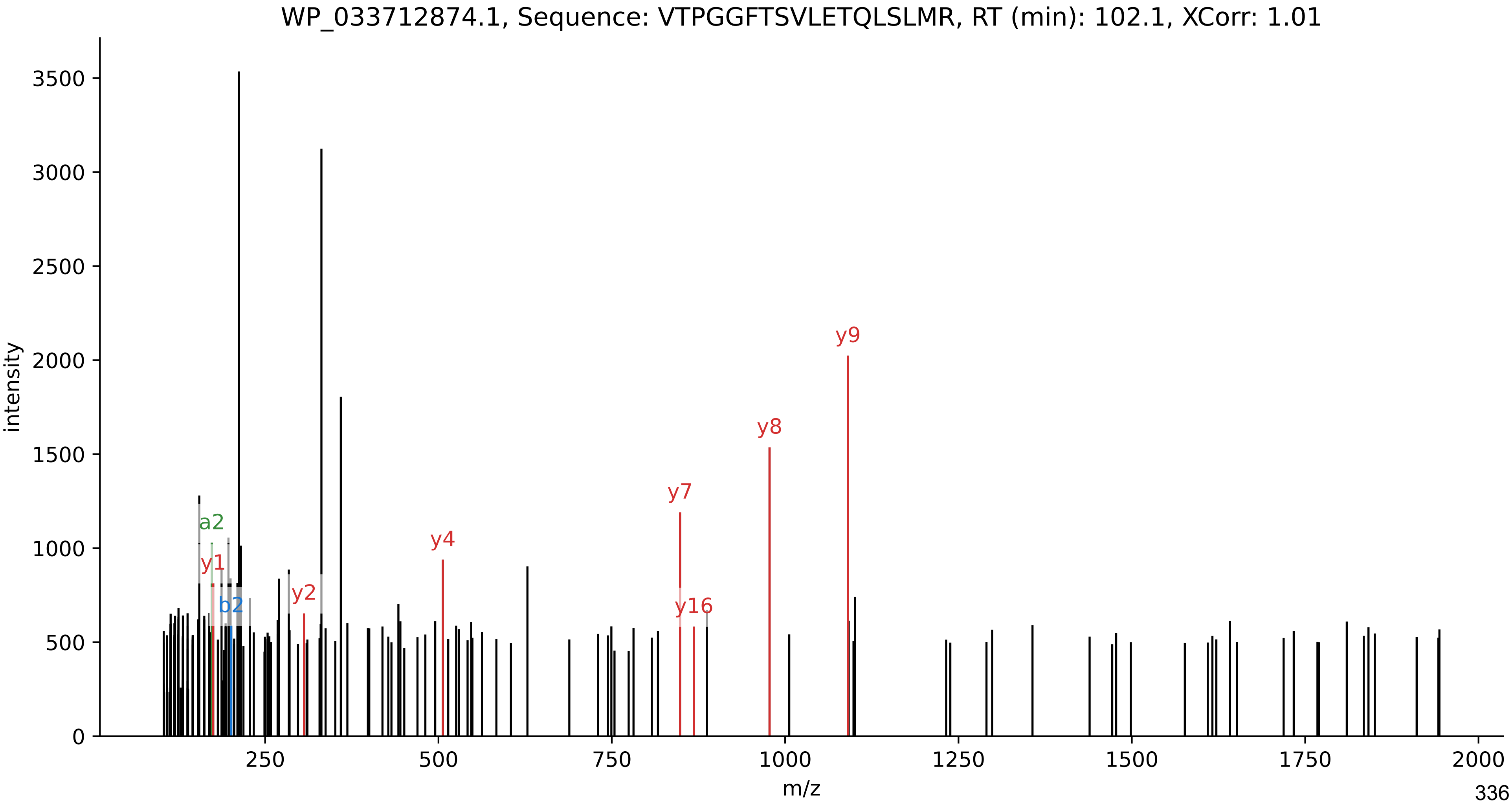
WP_033712874.1, Sequence: VTPGGFTSVLETQLSLMR, RT (min): 102.1, XCorr: 1.01
intensity
m/z
a2
y1
b2
y2
y4
y7
y16
y8
y9
3500
3000
2500
2000
1500
1000
500
0
250
500
750
1000
1250
1500
1750
2000

WP_033713005.1, Sequence: SVSAERPHAAPQYR, RT (min): 8.28, XCorr: 2.95

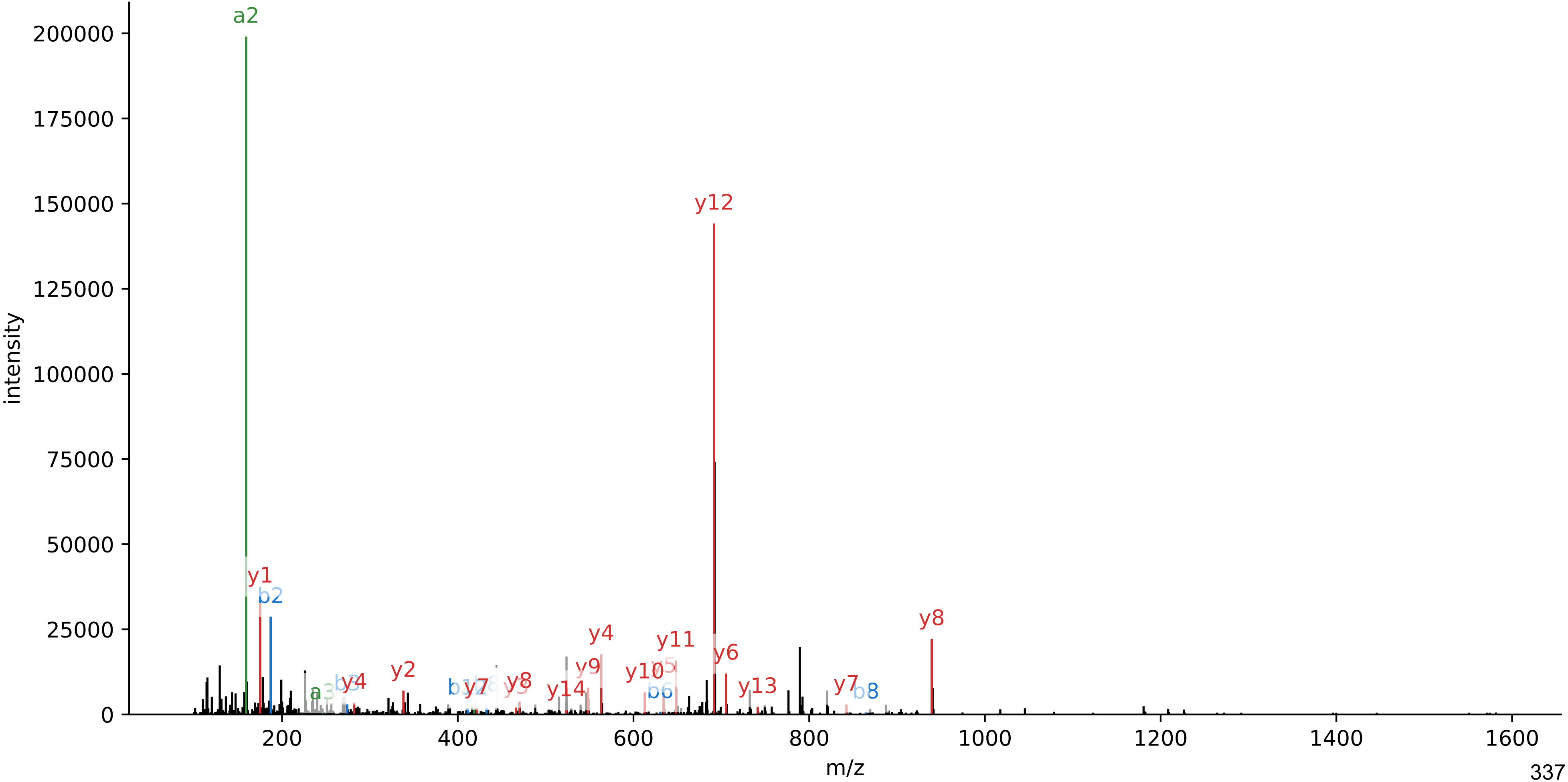

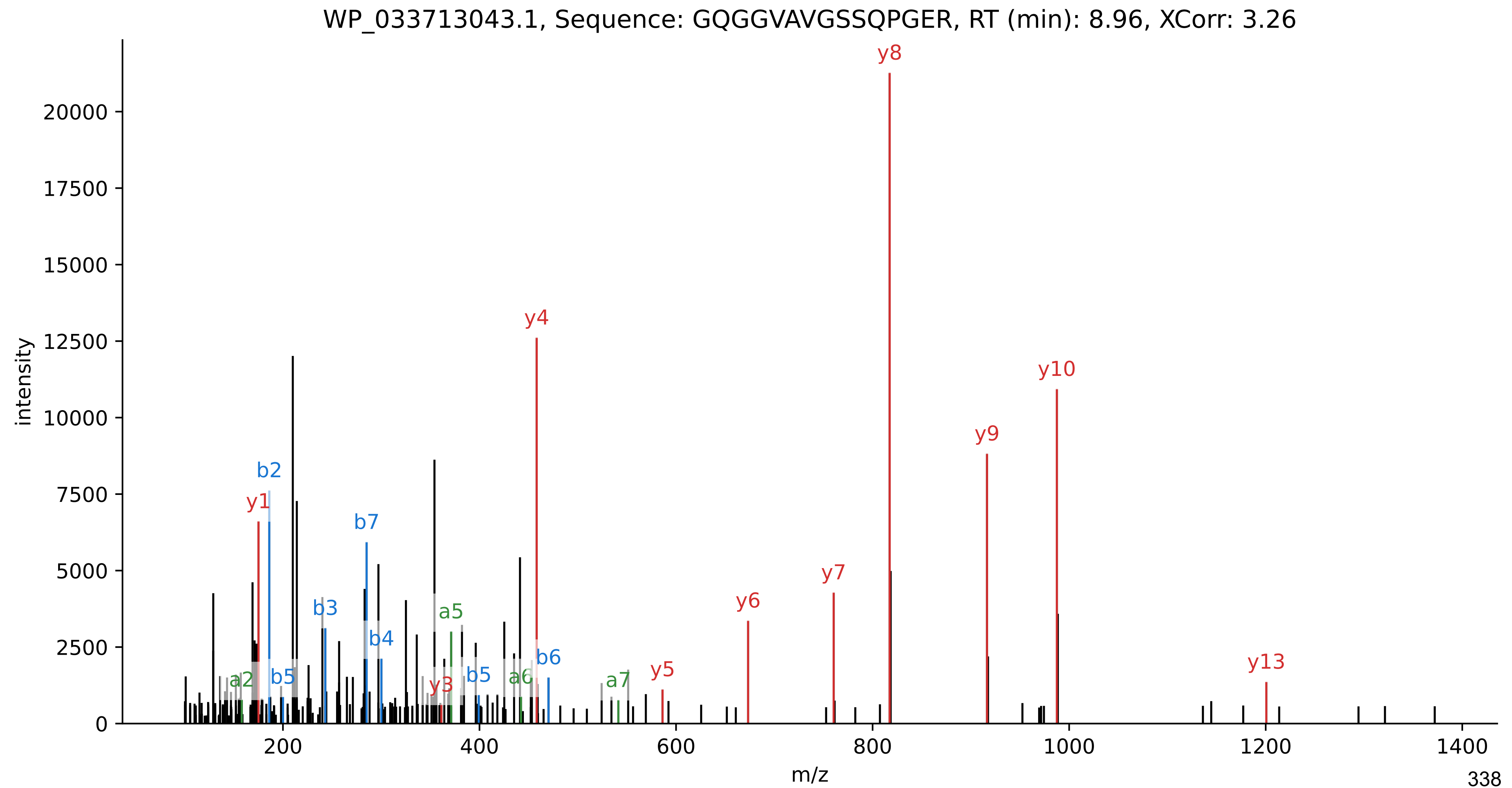
WP_033713043.1, Sequence: GQGGVAVGSSQPGER, RT (min): 8.96, XCorr: 3.26
intensity
m/z
0
2500
5000
7500
10000
12500
15000
17500
20000
200
400
600
800
1000
1200
1400
y8
y4
y10
y9
b2
y1
b7
y7
y6
b3
a5
b4
b6
y5
y13
a2
b5
y3
b5
a6
a7

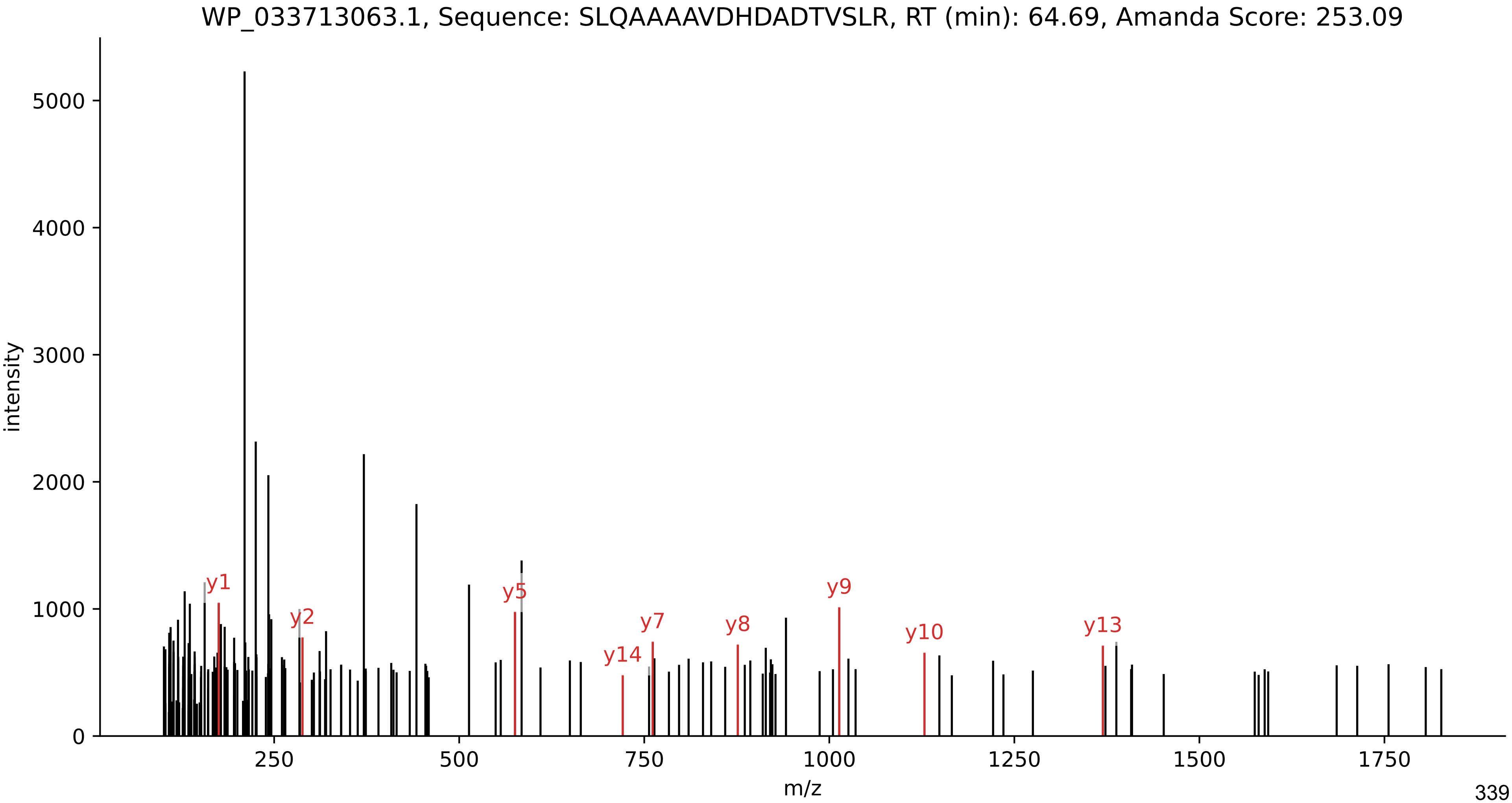
WP_033713063.1, Sequence: SLQAAAAVDHDADTVSLR, RT (min): 64.69, Amanda Score: 253.09
intensity
m/z
0
1000
2000
3000
4000
5000
250
500
750
1000
1250
1500
1750
y1
y2
y5
y7
y8
y9
y10
y13
y14

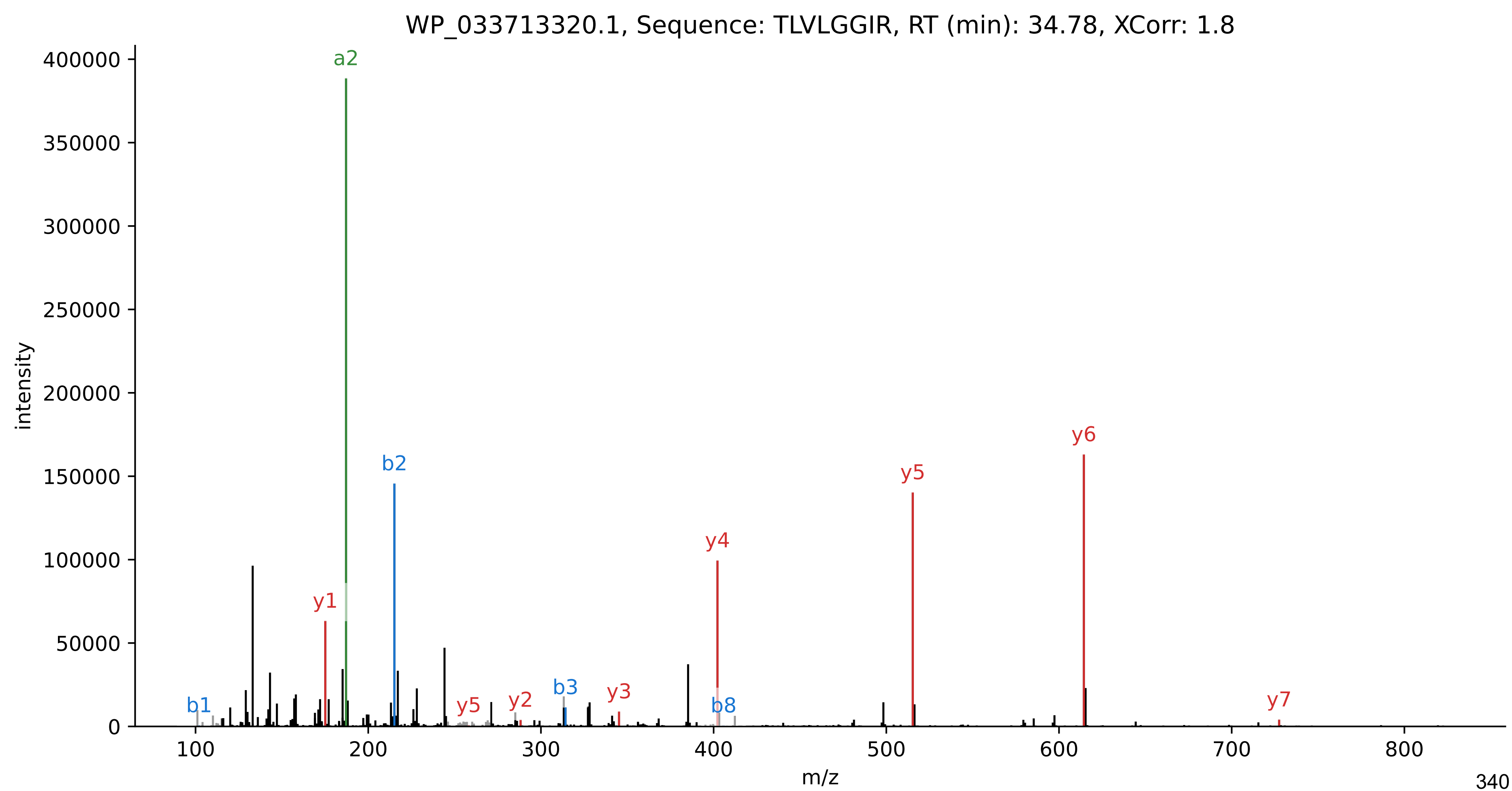
WP_033713320.1, Sequence: TLVLGGIR, RT (min): 34.78, XCorr: 1.8
intensity
m/z
a2
b1
b2
b3
b8
y1
y2
y3
y4
y5
y5
y6
y7
0
50000
100000
150000
200000
250000
300000
350000
400000
100
200
300
400
500
600
700
800

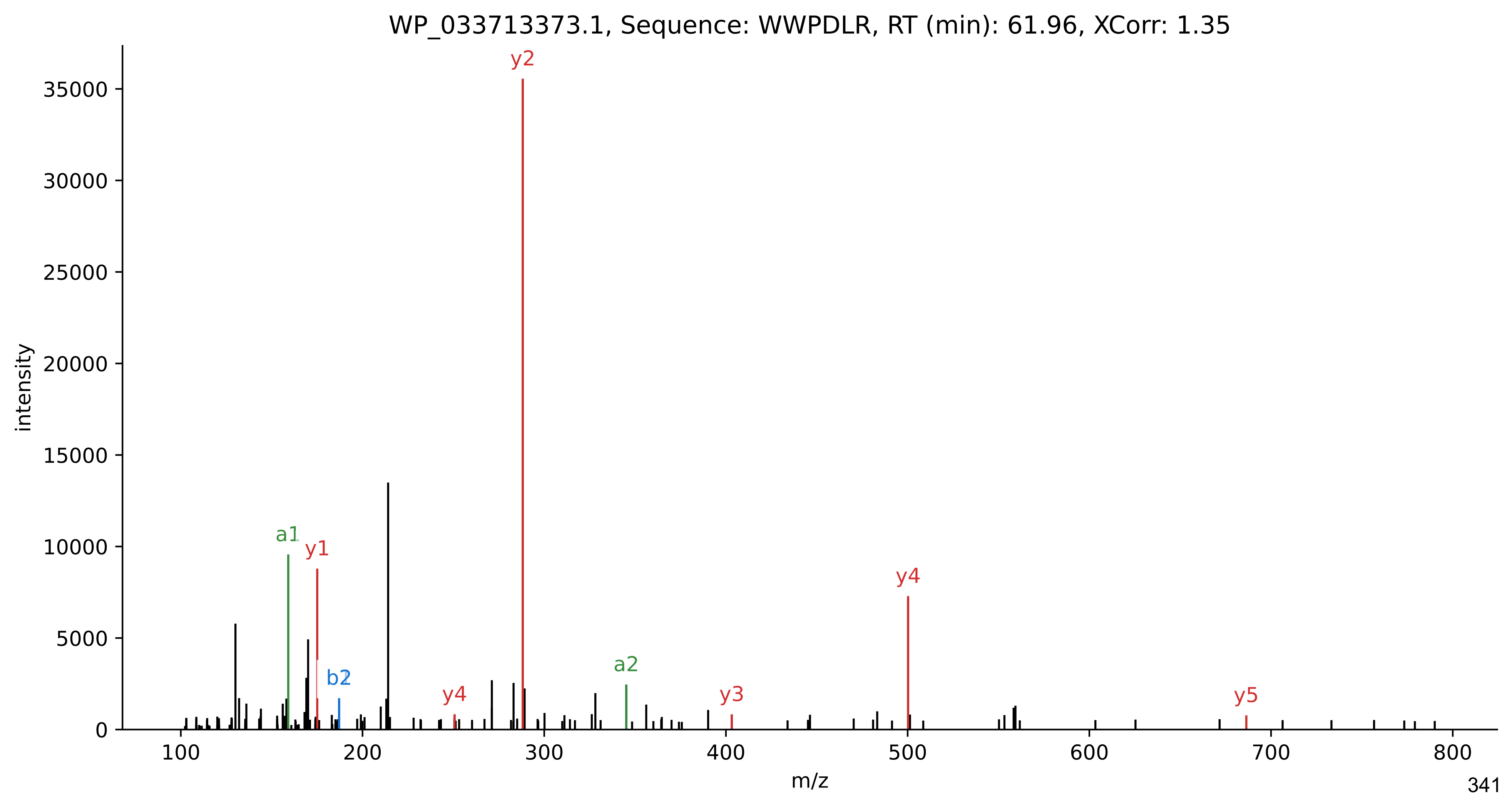

WP_033713373.1, Sequence: WWPDLR, RT (min): 61.96, XCorr: 1.35
y2
a1
y1
b2
y4
a2
y3
y4
y5
intensity
m/z

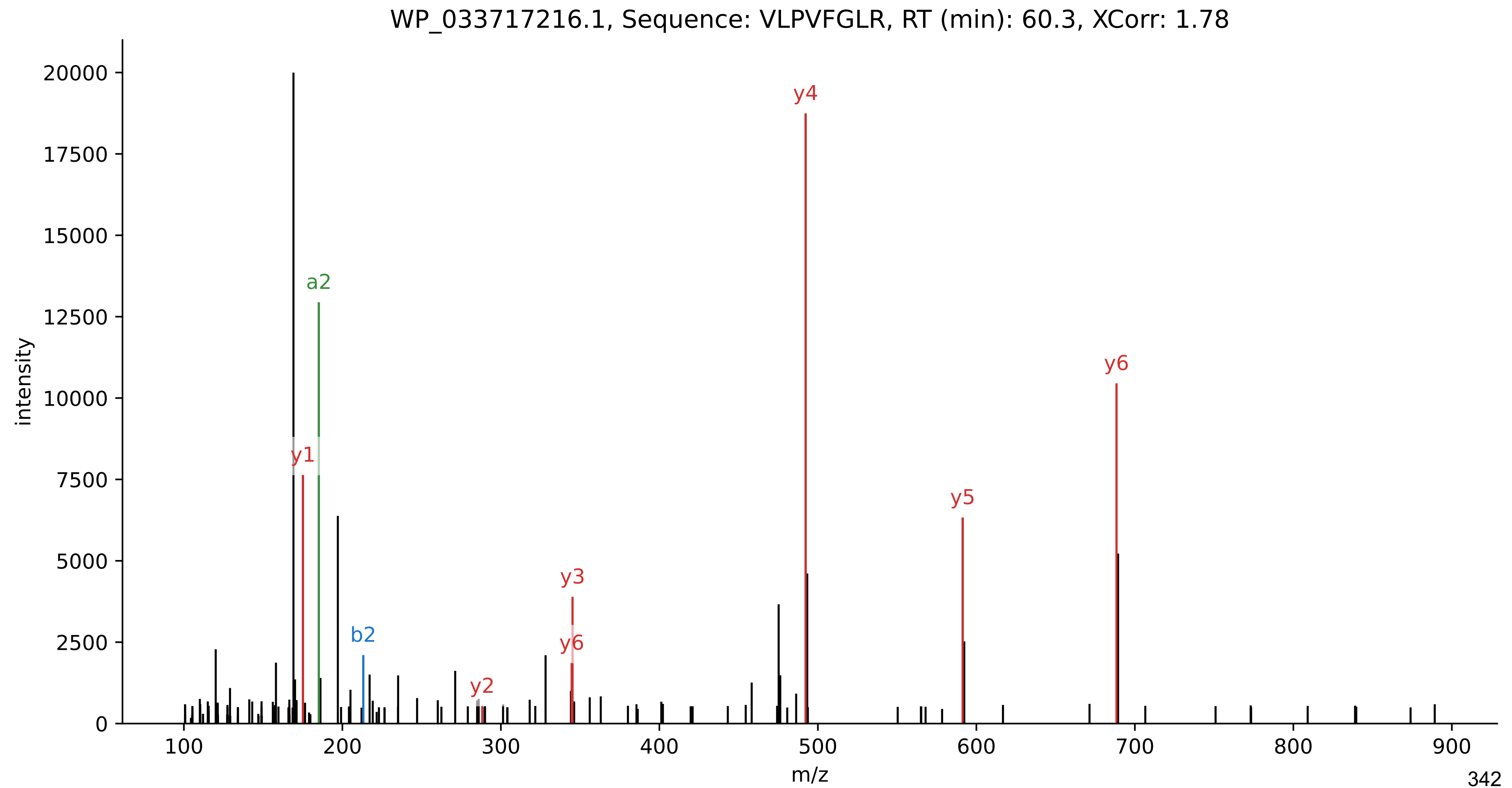
WP_033717216.1, Sequence: VLPVFGLR, RT (min): 60.3, XCorr: 1.78
intensity
m/z
0
2500
5000
7500
10000
12500
15000
17500
20000
100
200
300
400
500
600
700
800
900
a2
y1
b2
y2
y3
y6
y4
y5
y6

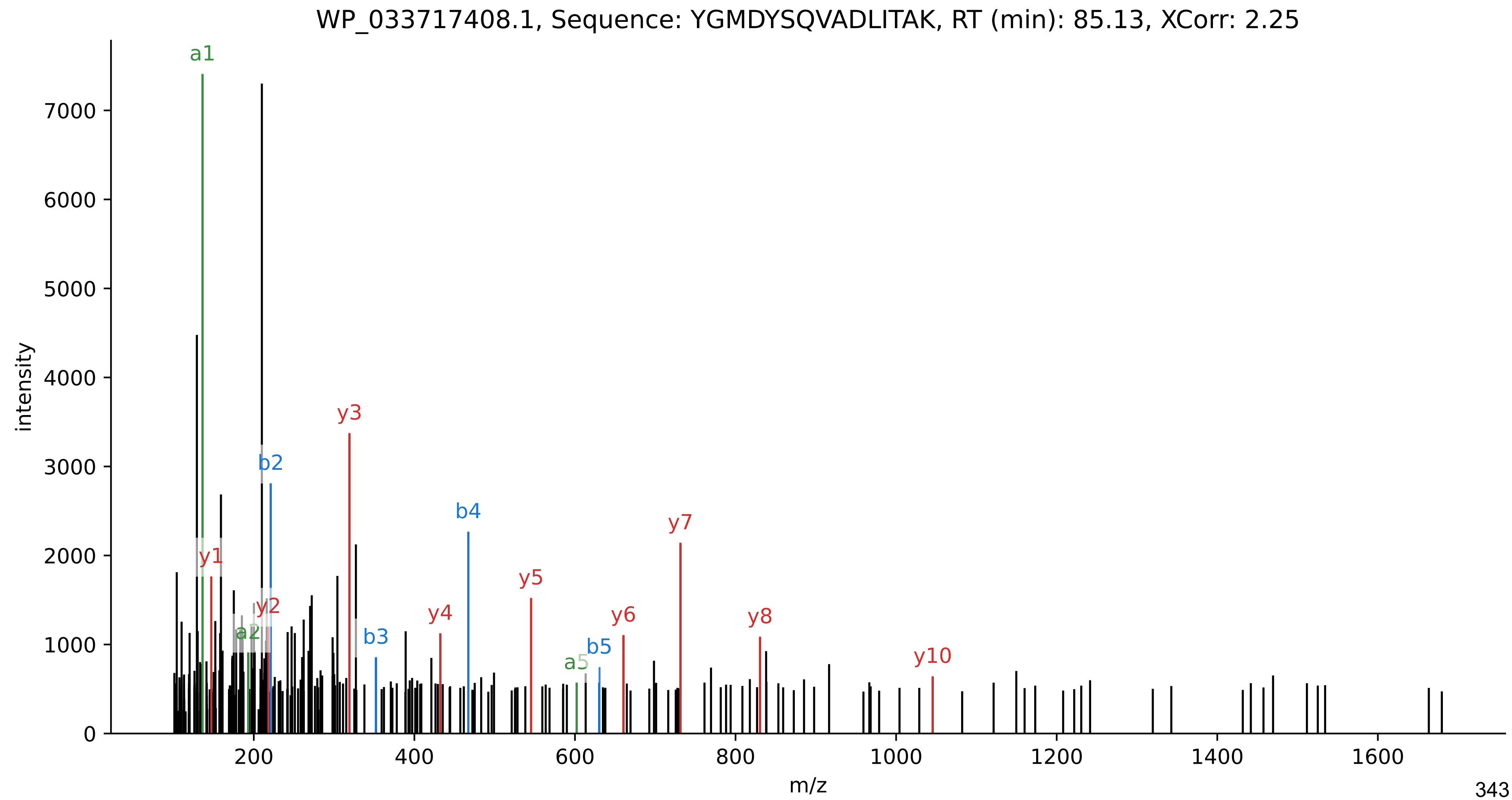
WP_033717408.1, Sequence: YGMDYSQVADLITAK, RT (min): 85.13, XCorr: 2.25
intensity
m/z
a1
y1
a2
y2
b2
y3
b3
b4
y4
y5
a5
b5
y6
y7
y8
y10
0
1000
2000
3000
4000
5000
6000
7000
200
400
600
800
1000
1200
1400
1600

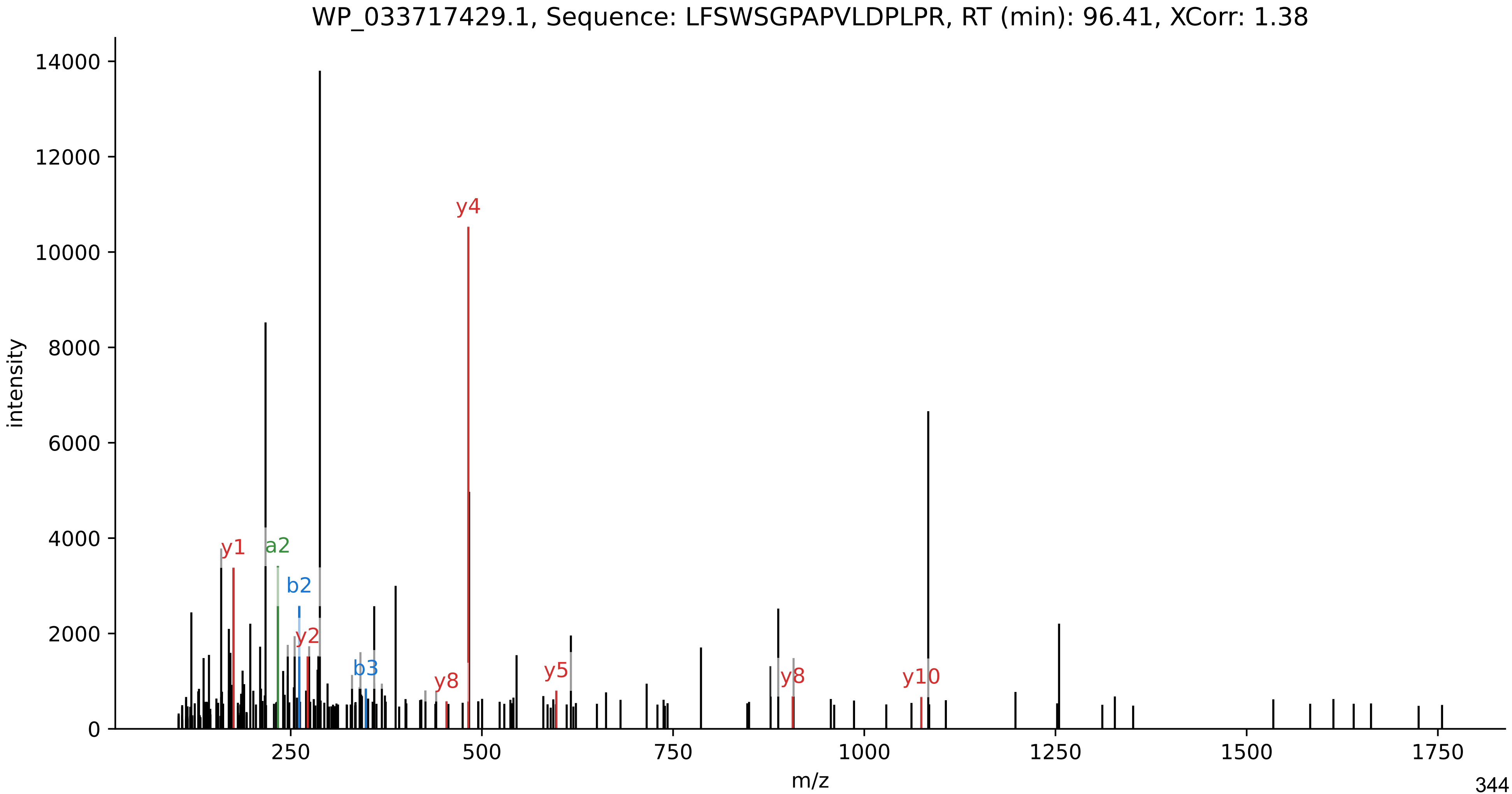

WP_033717429.1, Sequence: LFSWSGPAPVLDPLPR, RT (min): 96.41, XCorr: 1.38
intensity
m/z
y1
a2
b2
y2
b3
y8
y4
y5
y8
y10

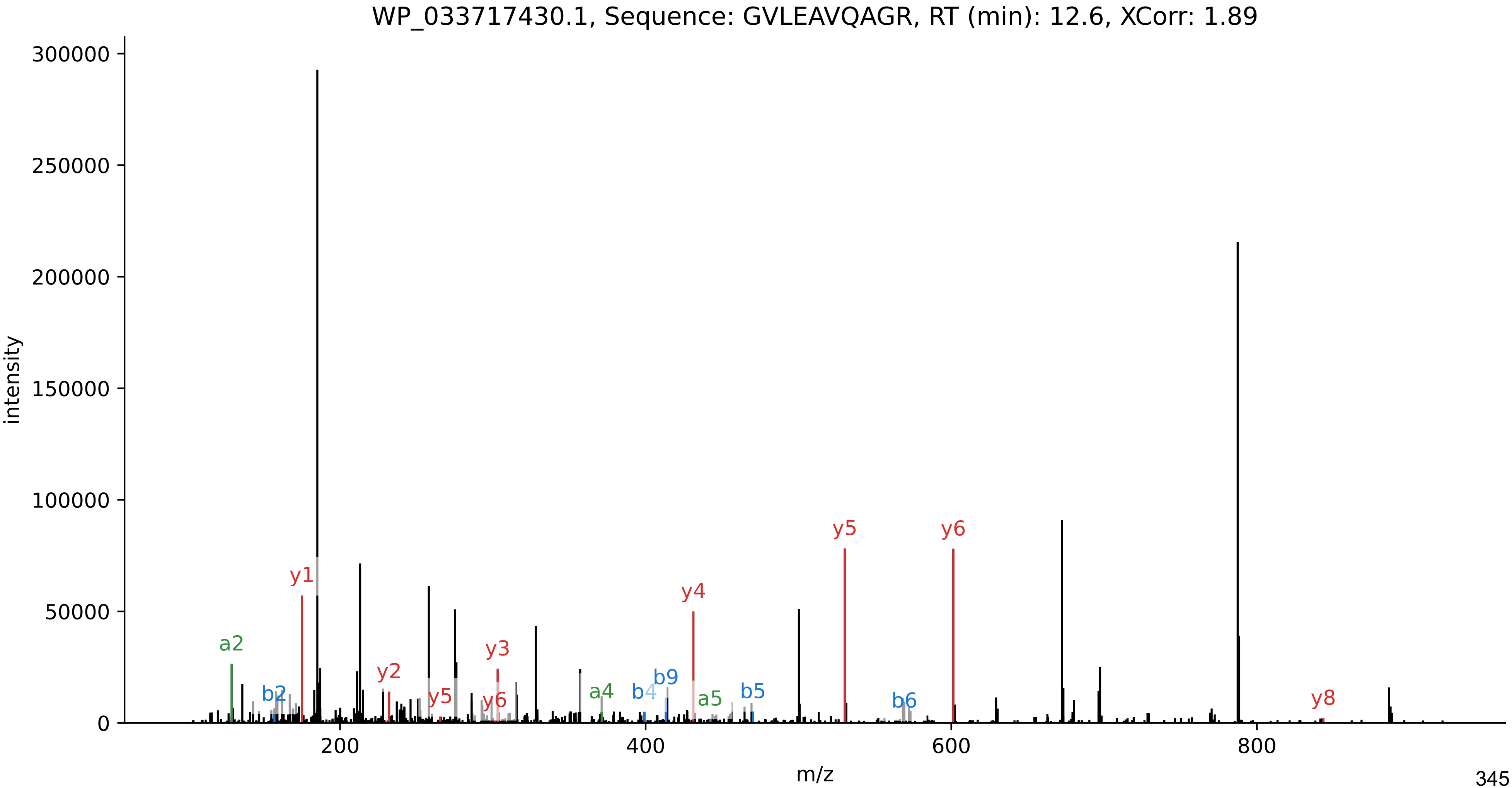

WP_033717430.1, Sequence: GVLEAVQAGR, RT (min): 12.6, XCorr: 1.89
intensity
m/z
300000
250000
200000
150000
100000
50000
0
200
400
600
800
a2
b2
y1
y2
y5
y6
y3
a4
b4
b9
y4
a5
b5
y5
b6
y6
y8

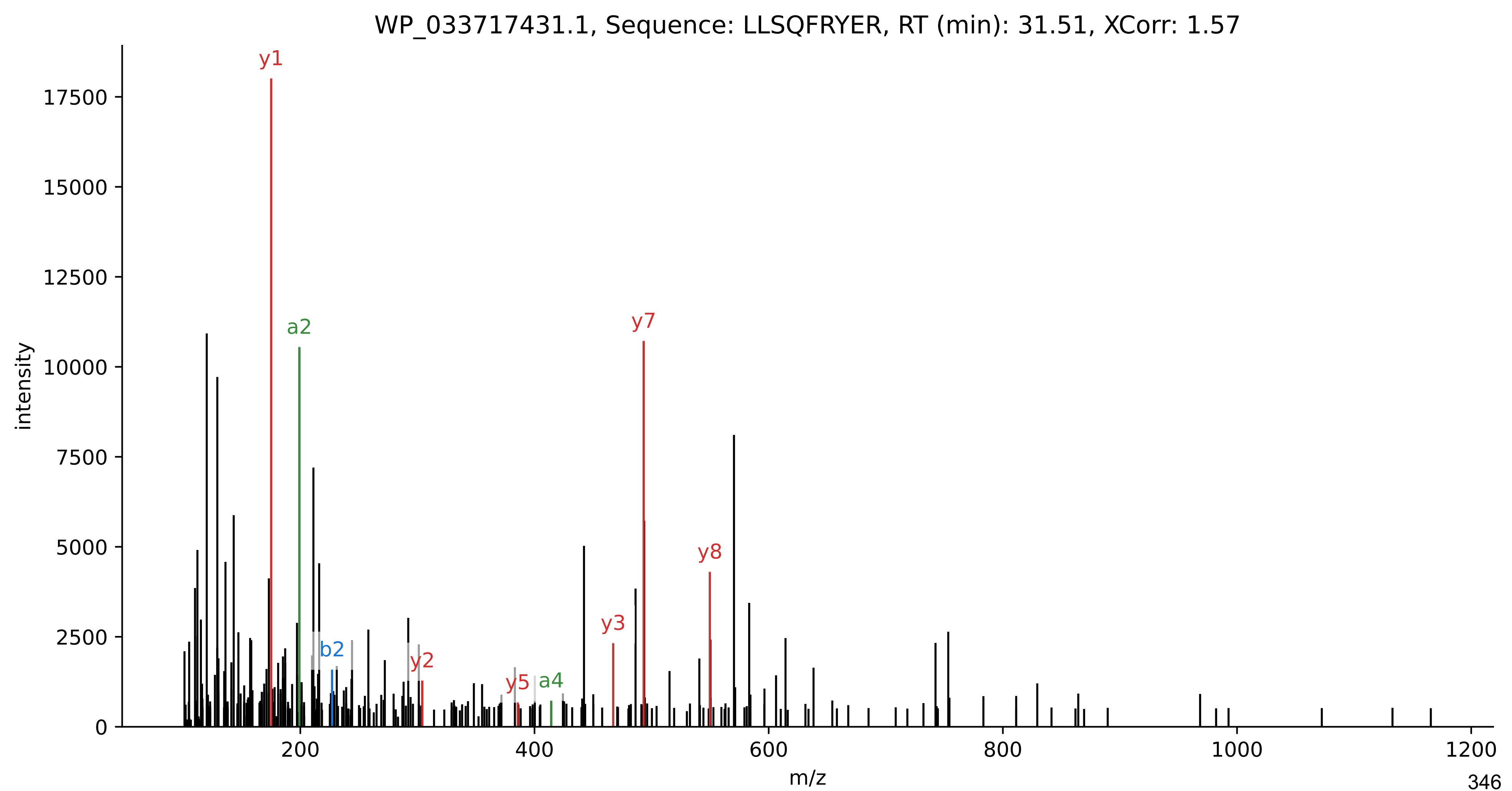

WP_033717431.1, Sequence: LLSQFRYER, RT (min): 31.51, XCorr: 1.57
intensity
m/z
y1
a2
y7
y8
y3
b2
y2
y5
a4
0
2500
5000
7500
10000
12500
15000
17500
200
400
600
800
1000
1200

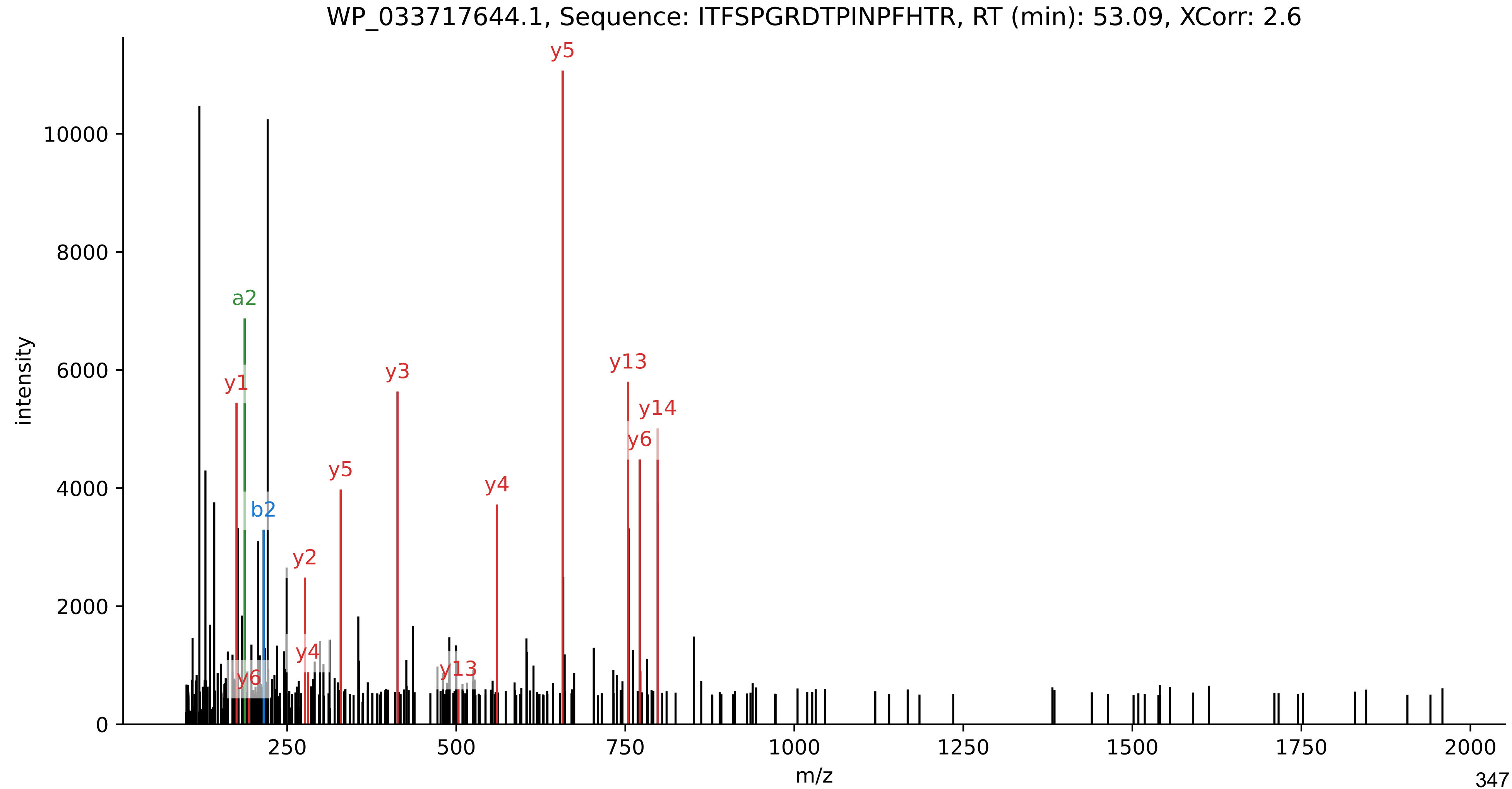
WP_033717644.1, Sequence: ITFSPGRDTPINPFHTR, RT (min): 53.09, XCorr: 2.6
intensity
m/z
0
2000
4000
6000
8000
10000
250
500
750
1000
1250
1500
1750
2000
y5
a2
y1
y3
y13
y14
y6
y5
y4
b2
y2
y4
y6
y13

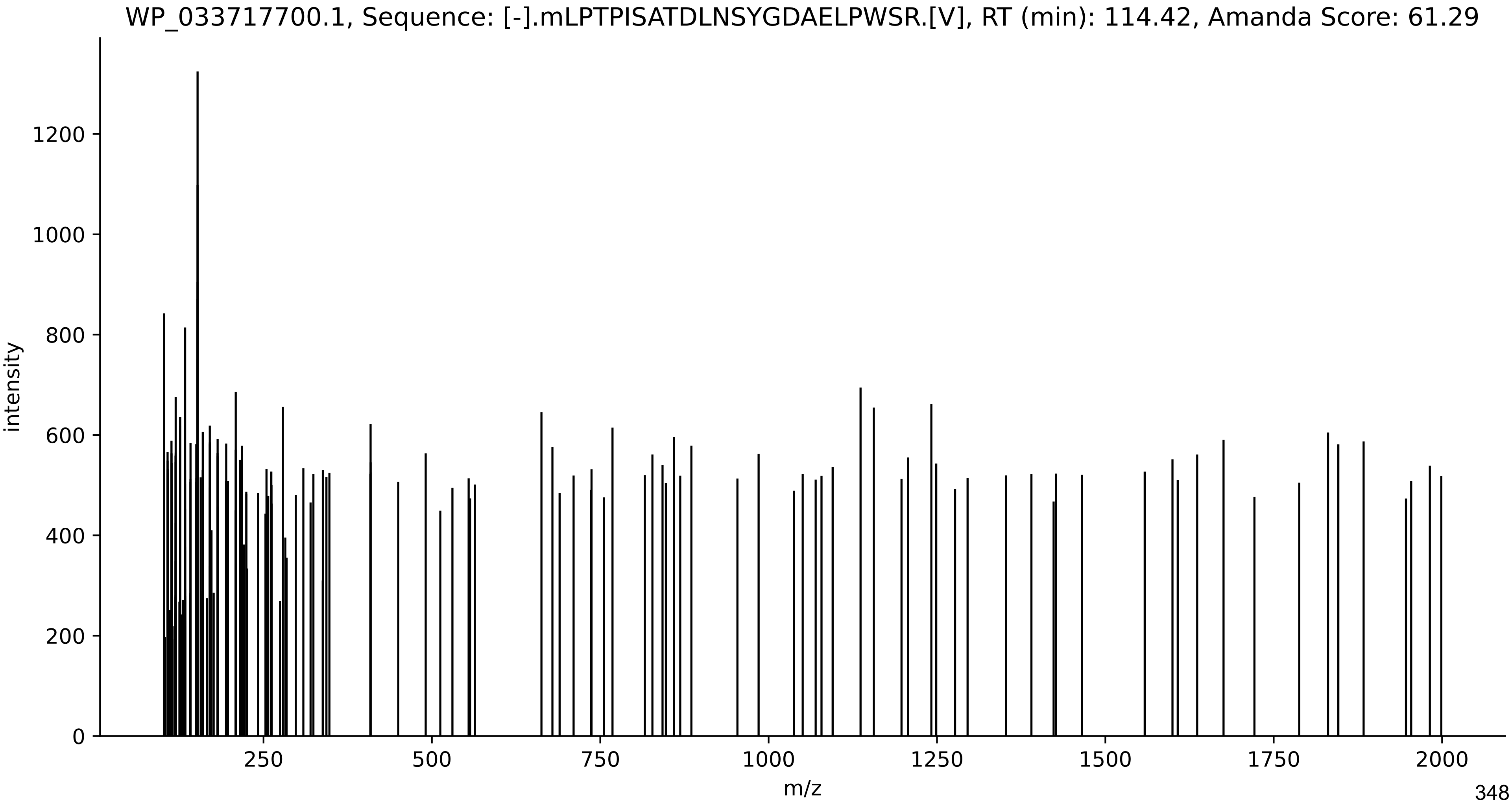

WP_033717700.1, Sequence: [-].mLPTPISATDLNSYGDAELPWSR.[V], RT (min): 114.42, Amanda Score: 61.29
intensity
m/z
0
200
400
600
800
1000
1200
250
500
750
1000
1250
1500
1750
2000

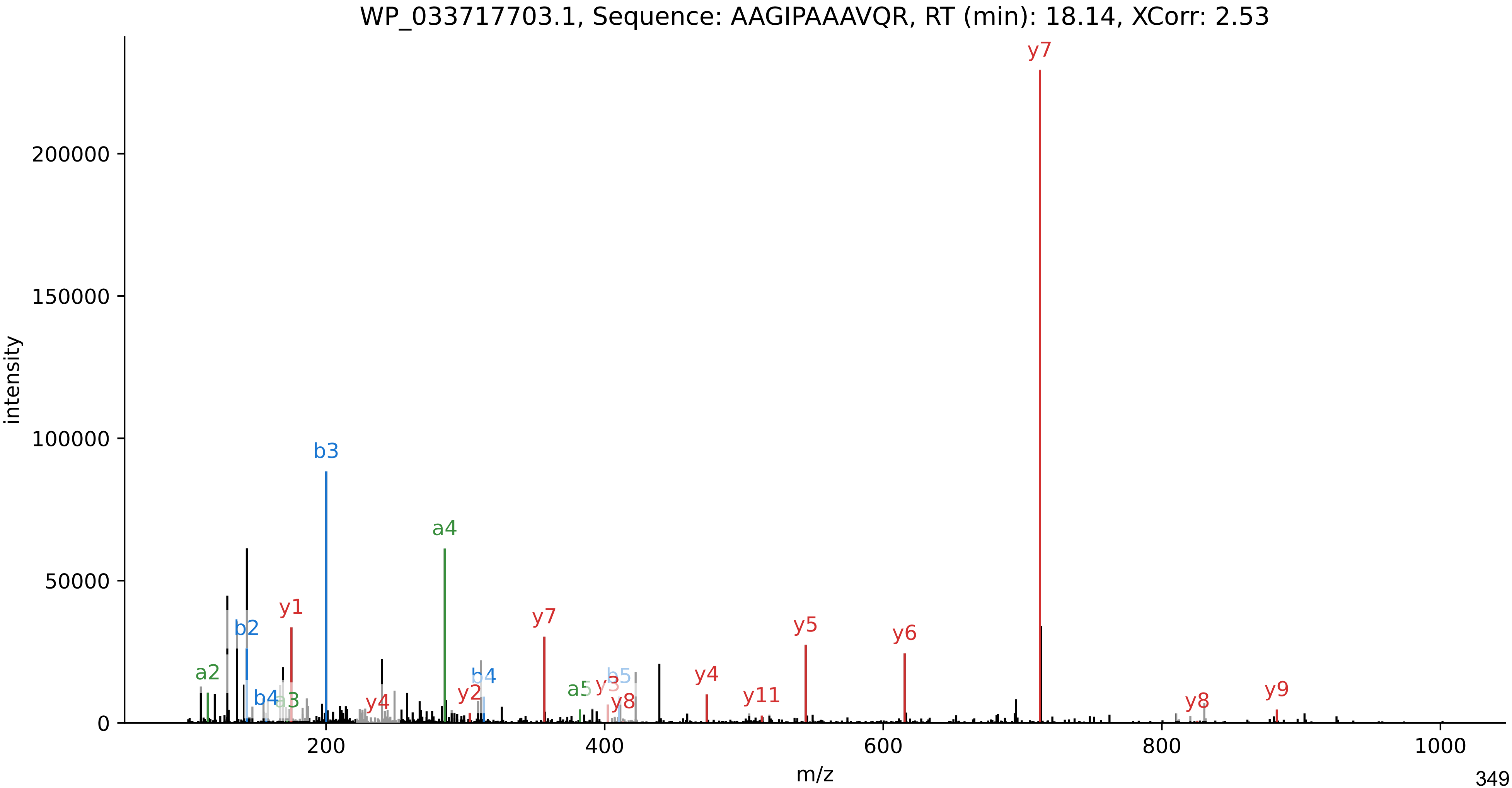
WP_033717703.1, Sequence: AAGIPAAAVQR, RT (min): 18.14, XCorr: 2.53
intensity
m/z
0
50000
100000
150000
200000
200
400
600
800
1000
y7
b3
a4
y1
b2
a2
b4
a3
y4
y7
y2
b4
a5
y3
b5
y8
y4
y11
y5
y6
y8
y9

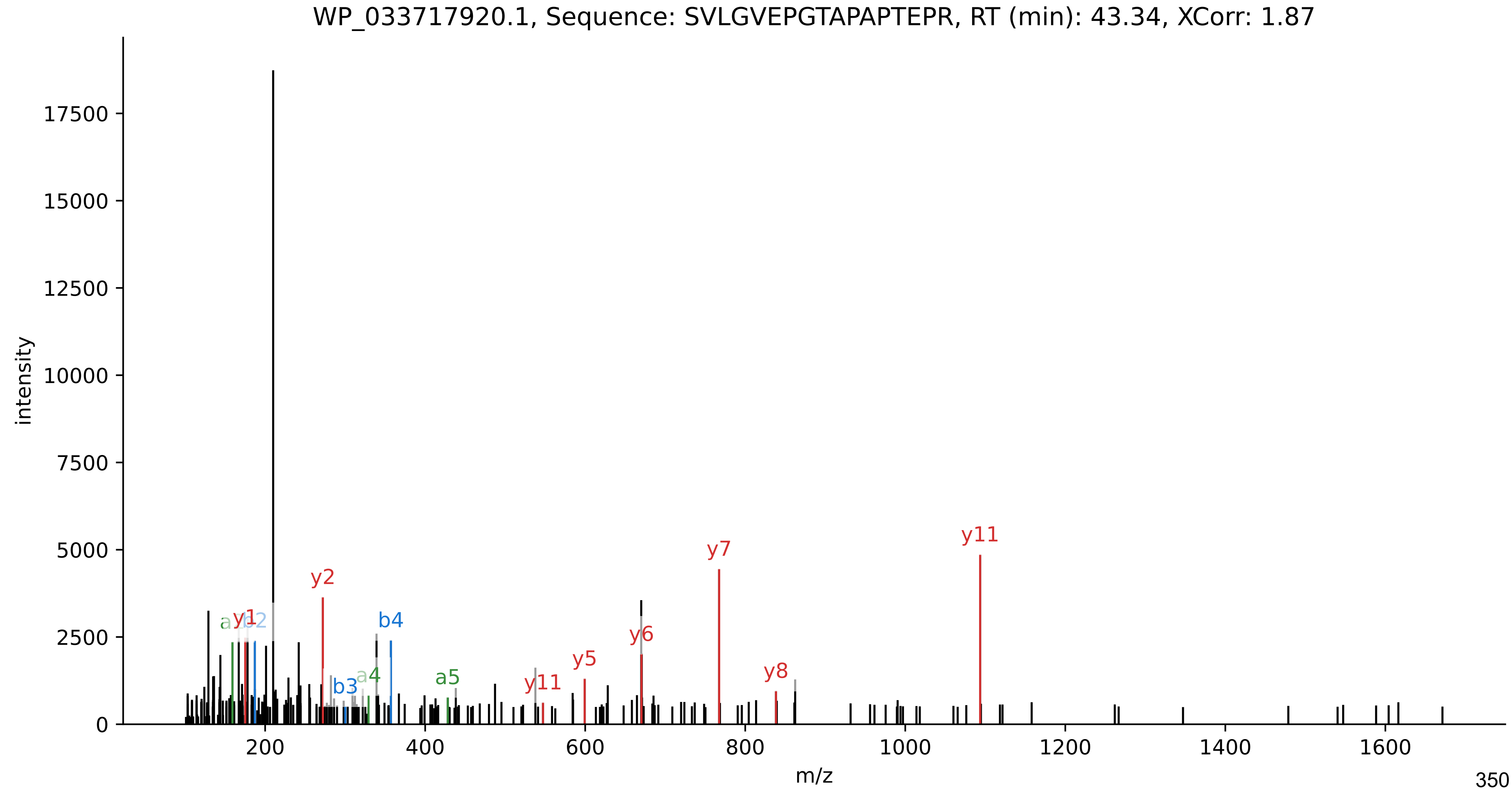

WP_033717920.1, Sequence: SVLGVEPGTAPAPTEPR, RT (min): 43.34, XCorr: 1.87
intensity
m/z
y1
b2
y2
b3
a4
b4
a5
y11
y5
y6
y7
y8
y11

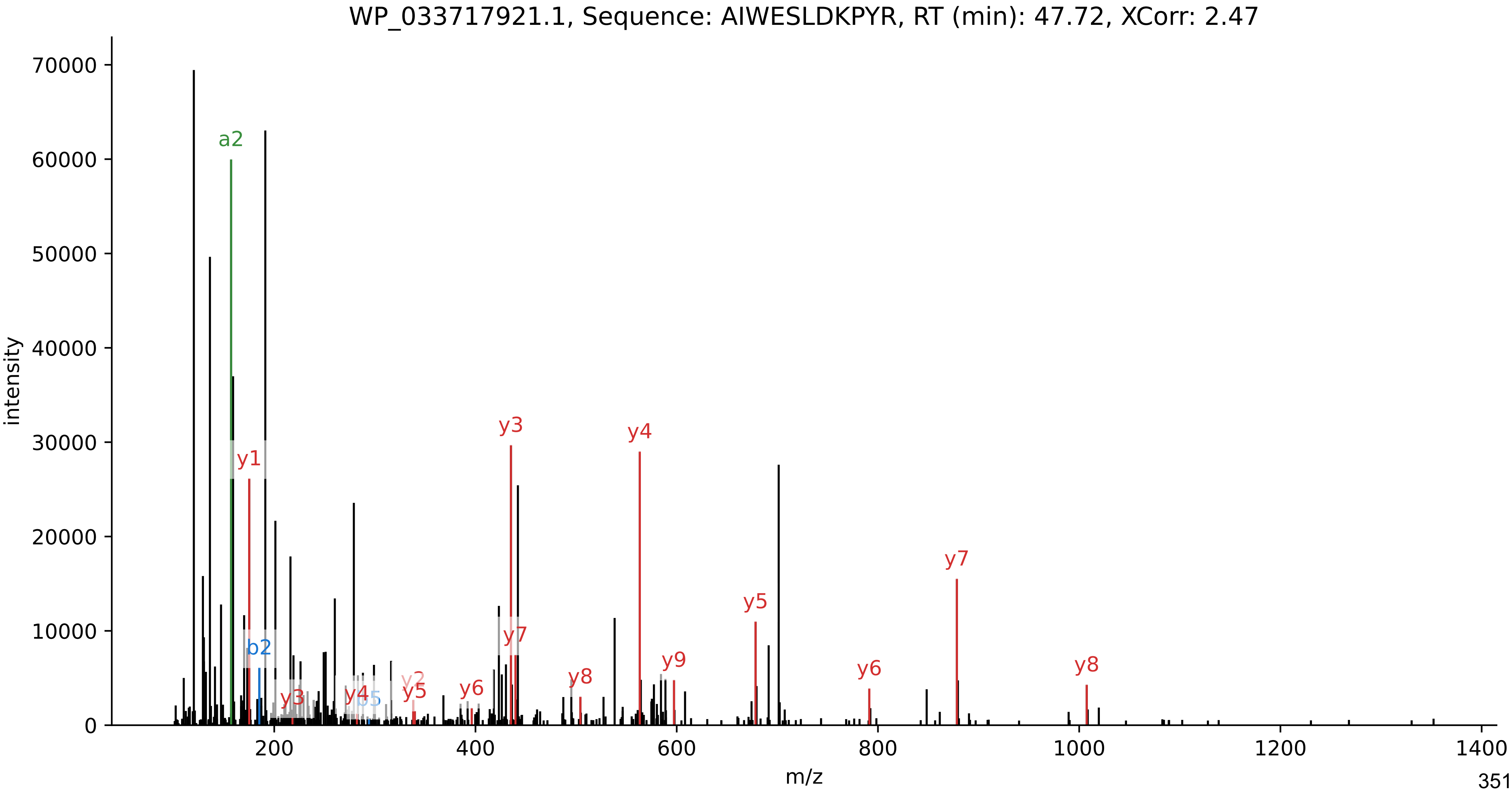

WP_033717921.1, Sequence: AIWESLDKPYR, RT (min): 47.72, XCorr: 2.47
intensity
m/z
a2
y1
b2
y3
y4
b5
y2
y5
y6
y3
y7
y8
y4
y9
y5
y6
y7
y8

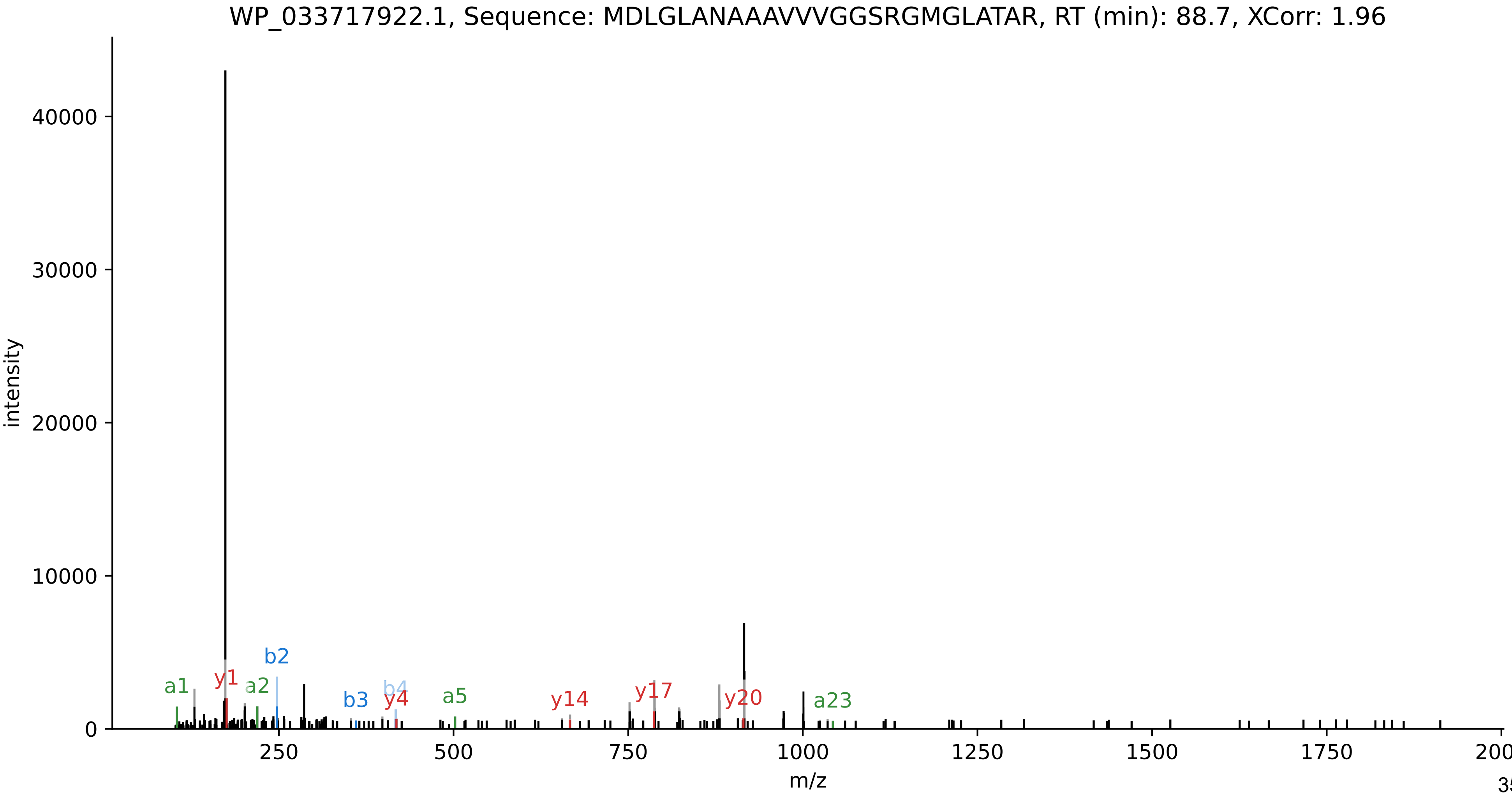
WP_033717922.1, Sequence: MDLGLANAAAVVVGGSRGMGLATAR, RT (min): 88.7, XCorr: 1.96
intensity
m/z
a1
y1
a2
b2
b3
b4
y4
a5
y14
y17
y20
a23

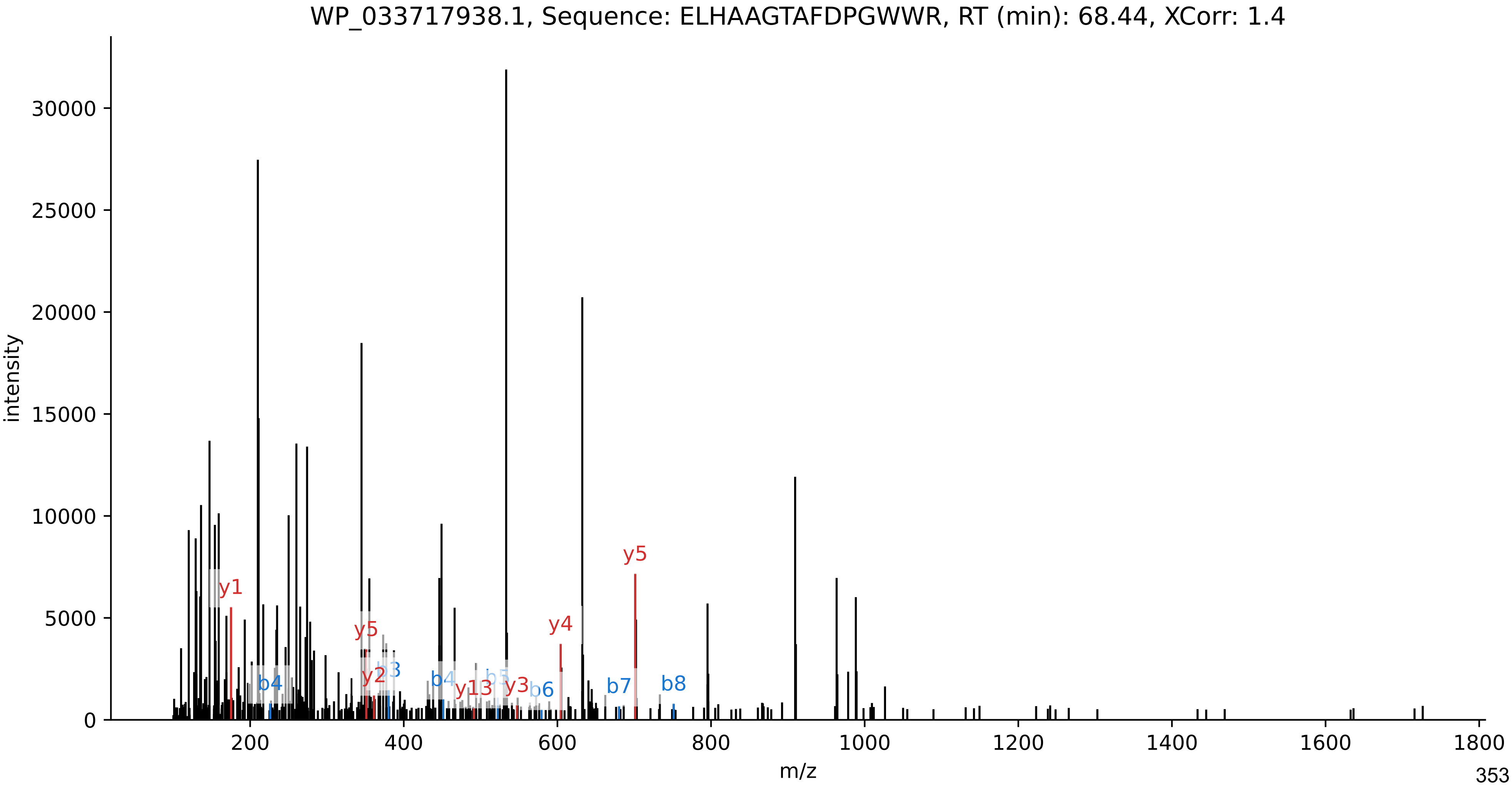
WP_033717938.1, Sequence: ELHAAGTAFDPGWWR, RT (min): 68.44, XCorr: 1.4
intensity
m/z
y1
y5
y2
b3
b4
b4
y13
b5
y3
b6
y4
b7
y5
b8
0
5000
10000
15000
20000
25000
30000
200
400
600
800
1000
1200
1400
1600
1800

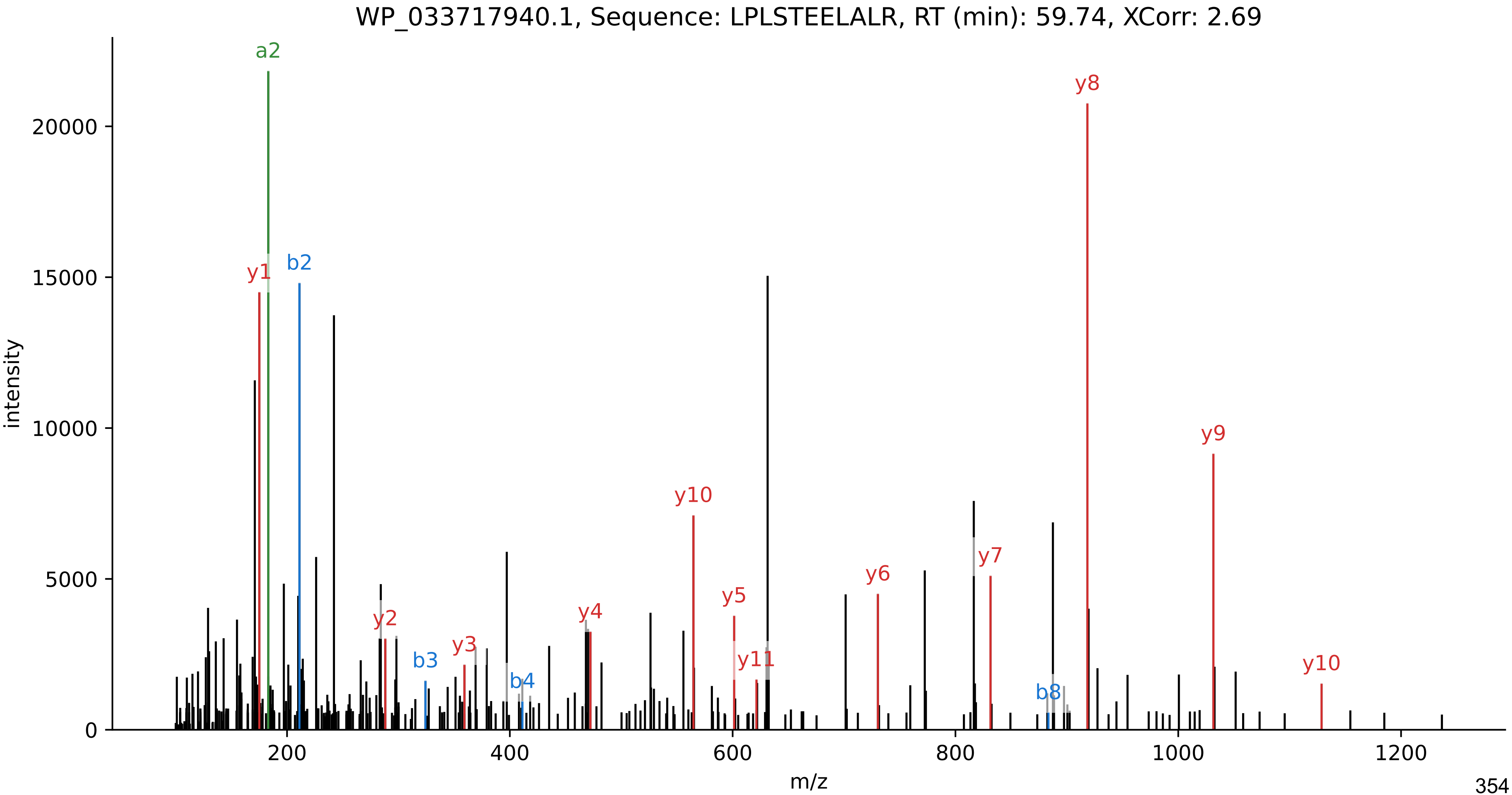
WP_033717940.1, Sequence: LPLSTEELALR, RT (min): 59.74, XCorr: 2.69
intensity
m/z
a2
y1
b2
y2
b3
y3
b4
y4
y10
y5
y11
y6
y7
b8
y8
y9
y10
0
5000
10000
15000
20000
200
400
600
800
1000
1200

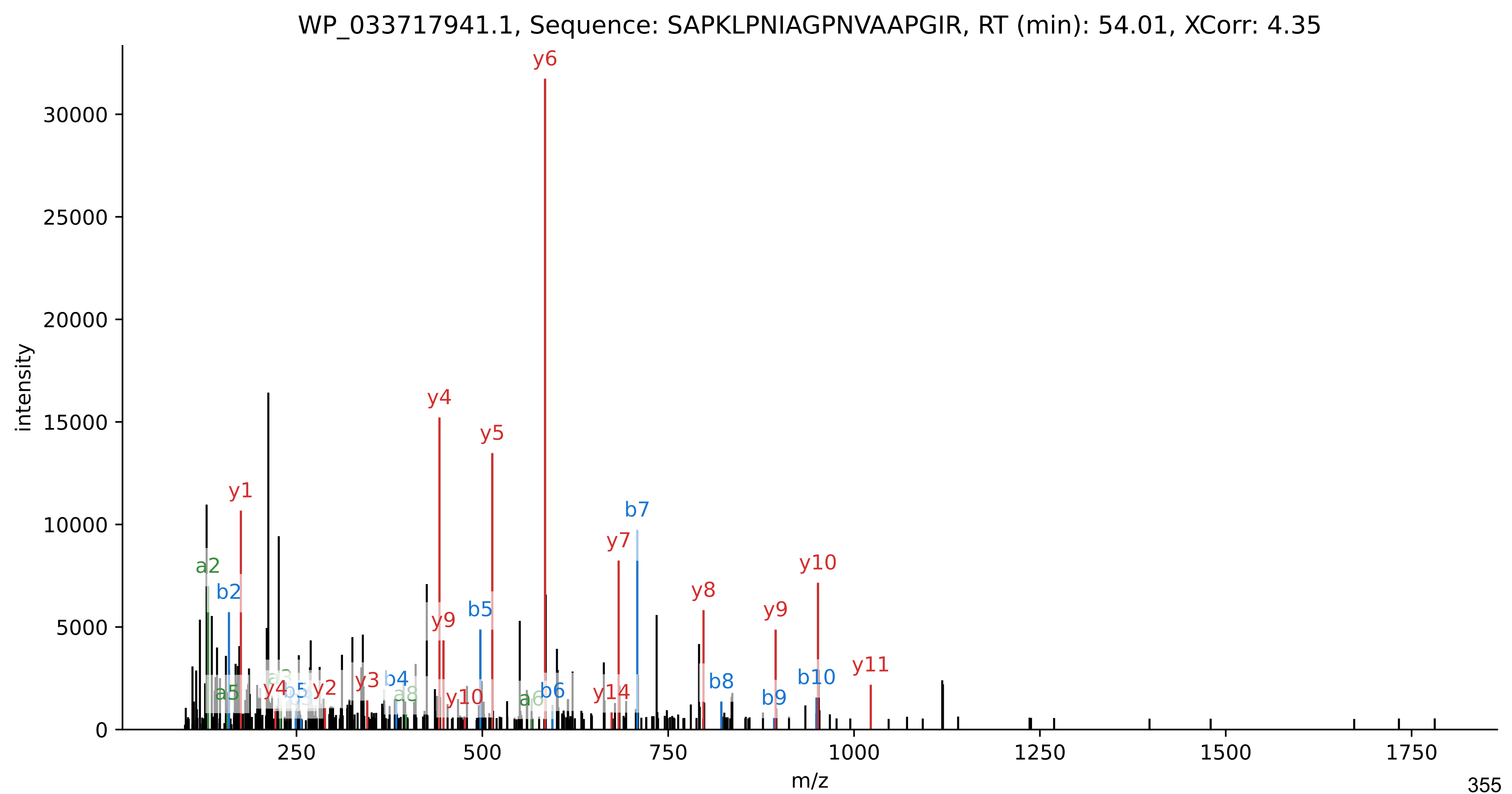
WP_033717941.1, Sequence: SAPKLPNIAGPNVAAPGIR, RT (min): 54.01, XCorr: 4.35
intensity
m/z
y6
y4
y5
y1
b7
y7
y10
a2
b2
y8
y9
b5
y9
y11
b8
b10
b4
y3
y2
y4
a5
a8
y10
a6
b6
y14
b9

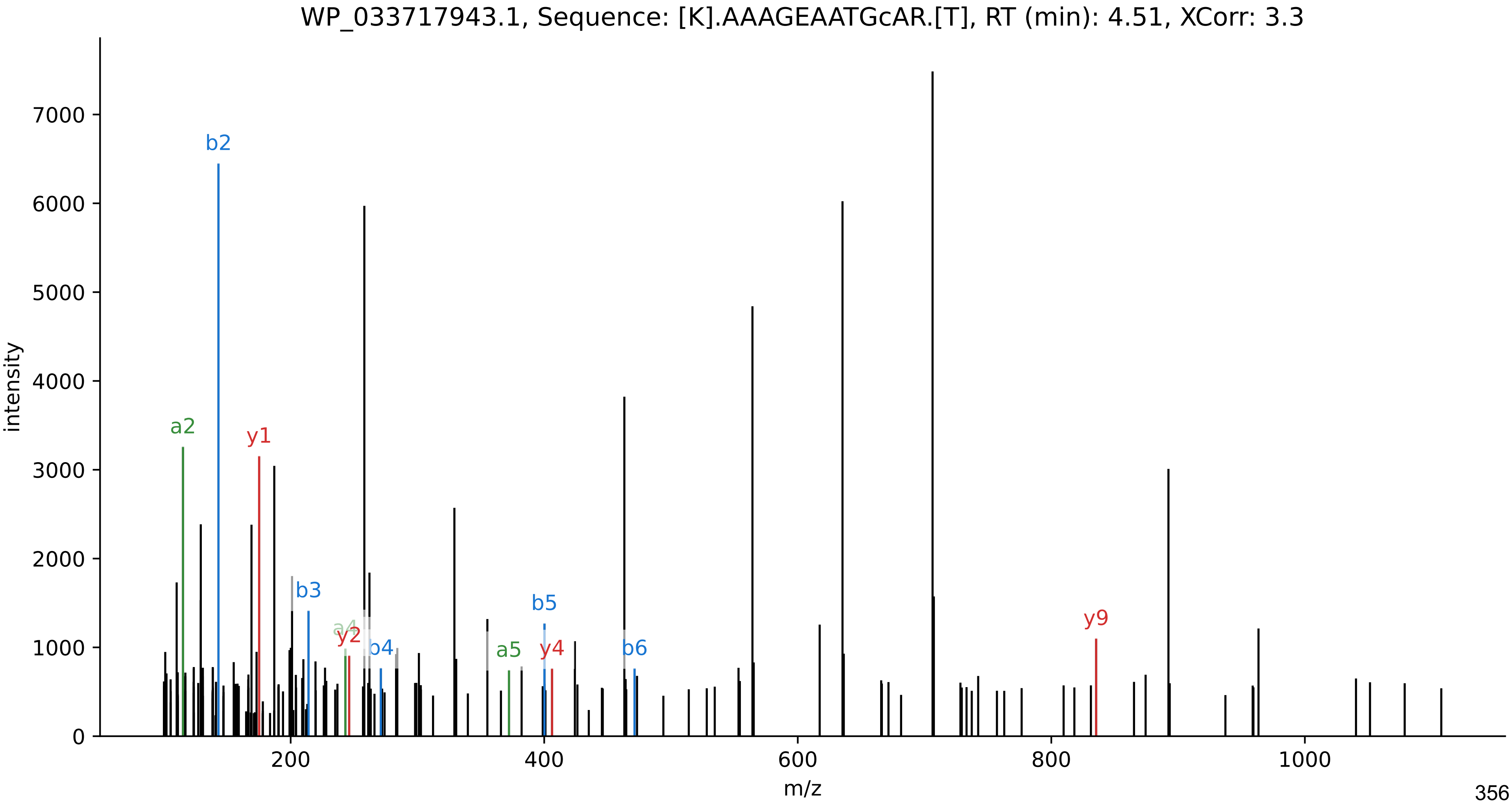
WP_033717943.1, Sequence: [K].AAAGEAATGcAR.[T], RT (min): 4.51, XCorr: 3.3
intensity
m/z
0
1000
2000
3000
4000
5000
6000
7000
200
400
600
800
1000
b2
a2
y1
b3
a4
y2
b4
a5
b5
y4
b6
y9

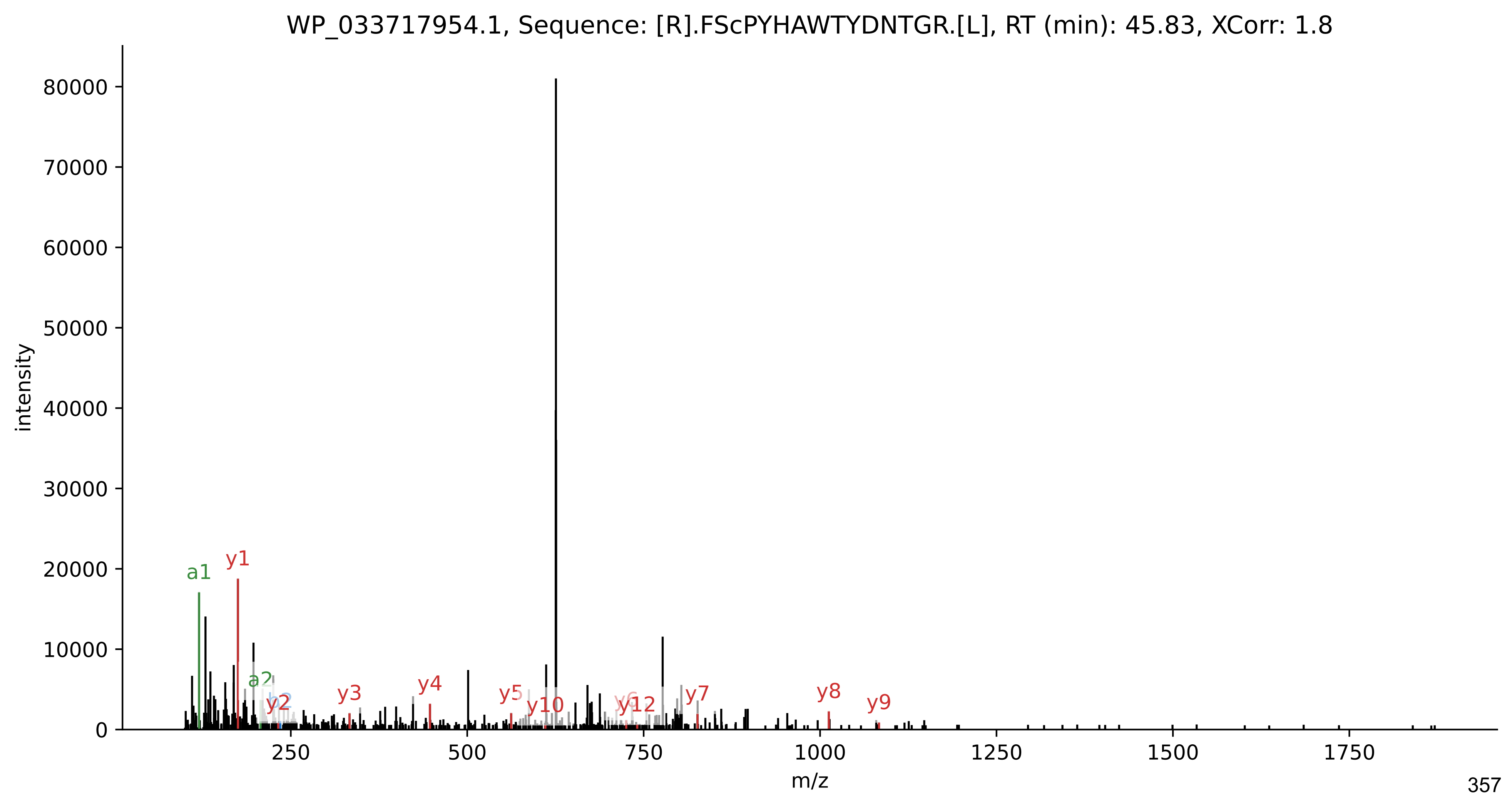

WP_033717954.1, Sequence: [R].FScPYHAWTYDNTGR.[L], RT (min): 45.83, XCorr: 1.8
intensity
m/z
a1
y1
a2
y2
y3
y4
y5
y10
y12
y7
y8
y9

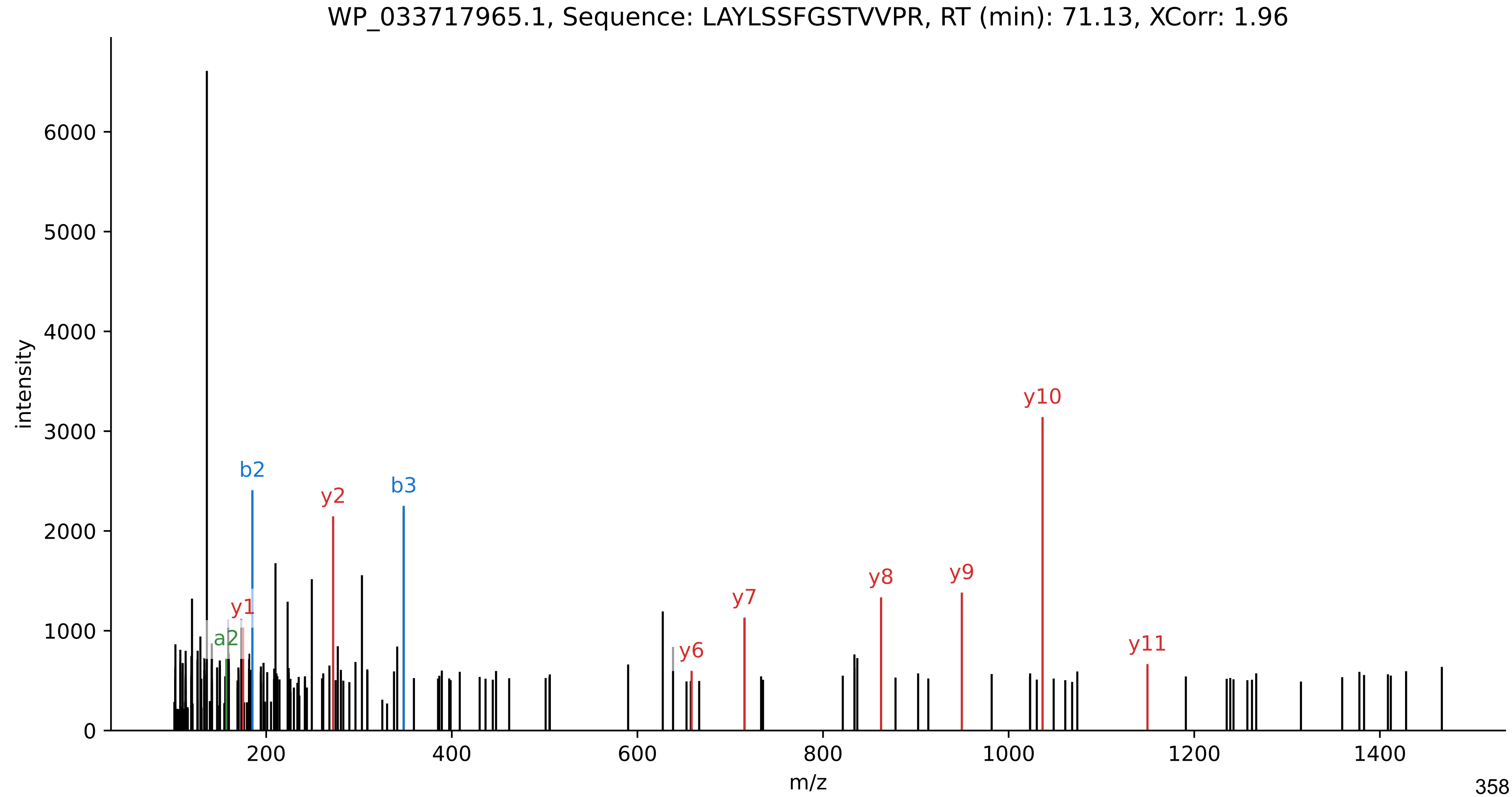

WP_033717965.1, Sequence: LAYLSSFGSTVVPR, RT (min): 71.13, XCorr: 1.96
intensity
m/z
b2
b3
y1
y2
a2
y6
y7
y8
y9
y10
y11
0
1000
2000
3000
4000
5000
6000
200
400
600
800
1000
1200
1400

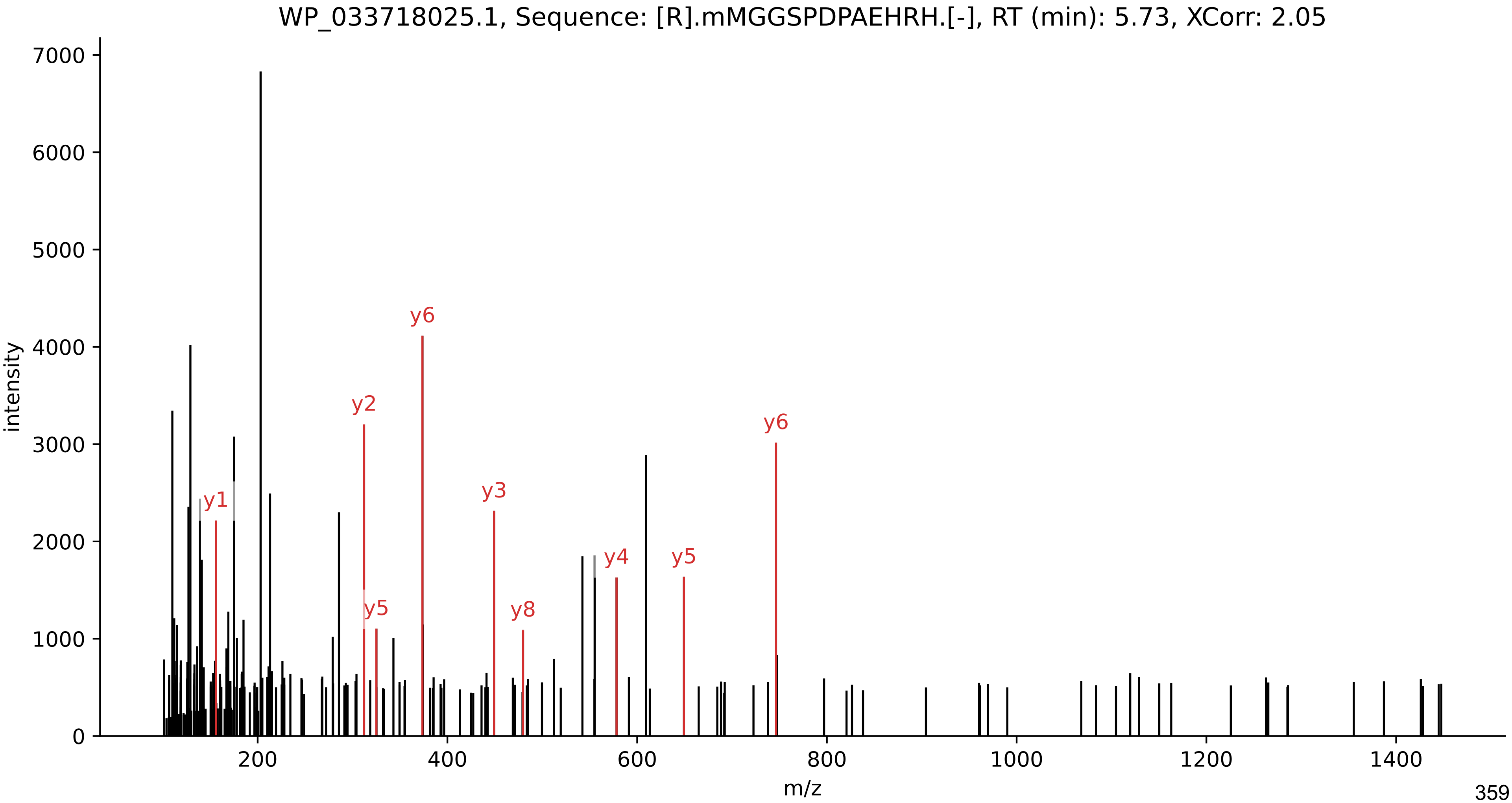

WP_033718025.1, Sequence: [R].mMGGSPDPAEHRH.[-], RT (min): 5.73, XCorr: 2.05
intensity
m/z
y1
y2
y5
y6
y3
y8
y4
y5
y6

WP_033718025.1, Sequence: MMGGSPDPAEHRH, RT (min): 6.11, XCorr: 4.03

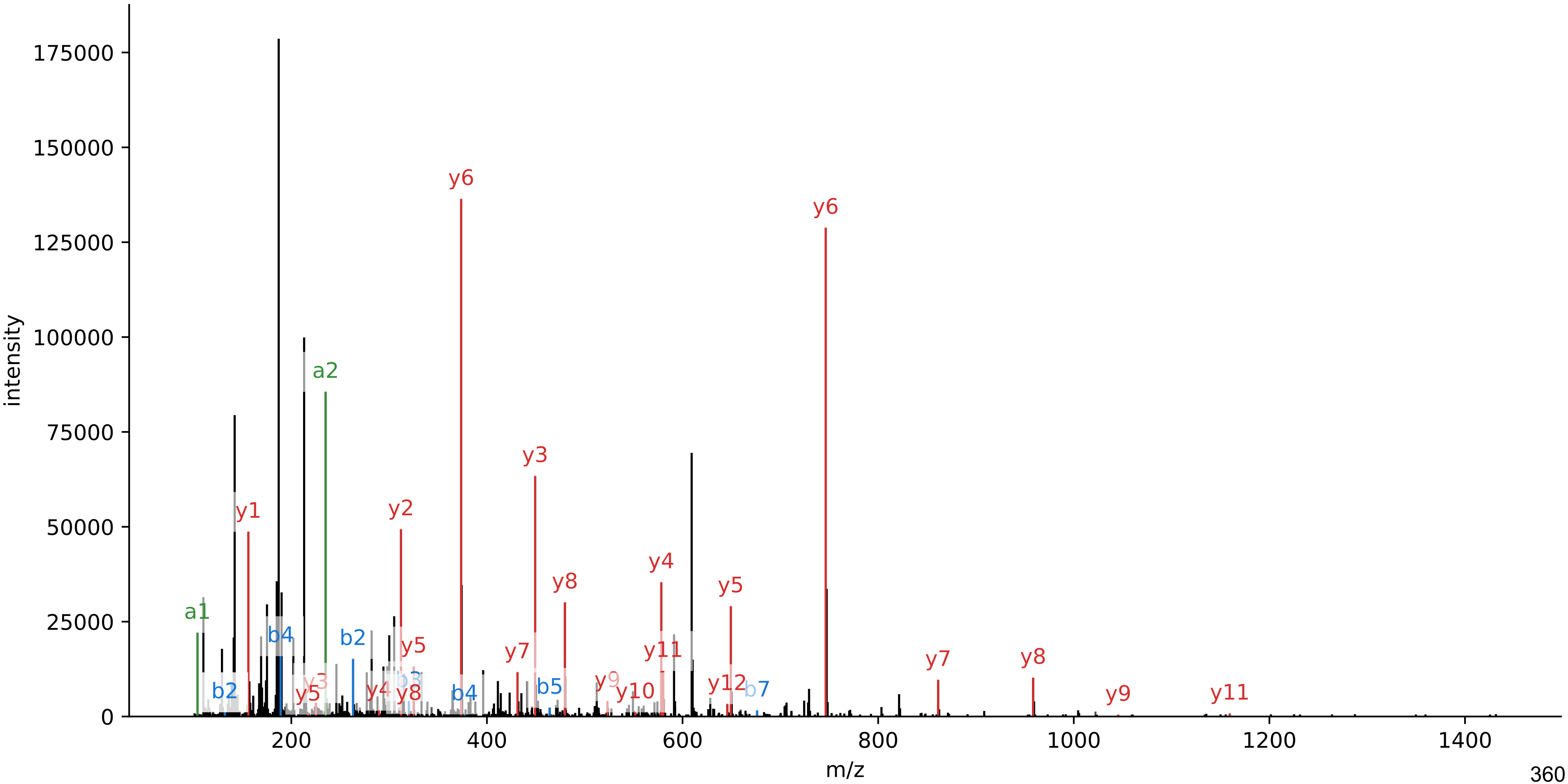

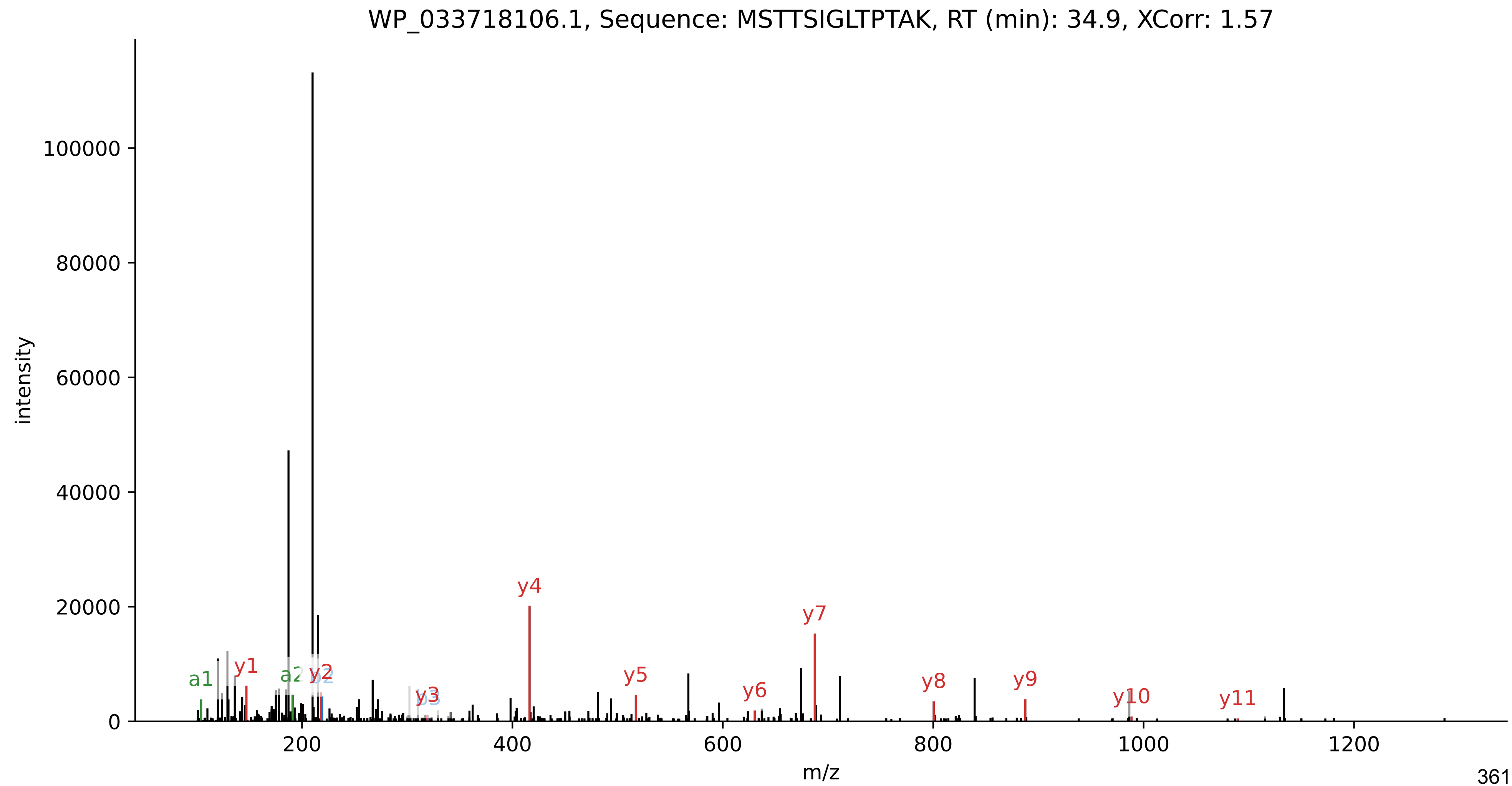
WP_033718106.1, Sequence: MSTTSIGLTPTAK, RT (min): 34.9, XCorr: 1.57
intensity
m/z
a1
y1
a2
y2
b2
y3
b3
y4
y5
y6
y7
y8
y9
y10
y11
0
20000
40000
60000
80000
100000
200
400
600
800
1000
1200

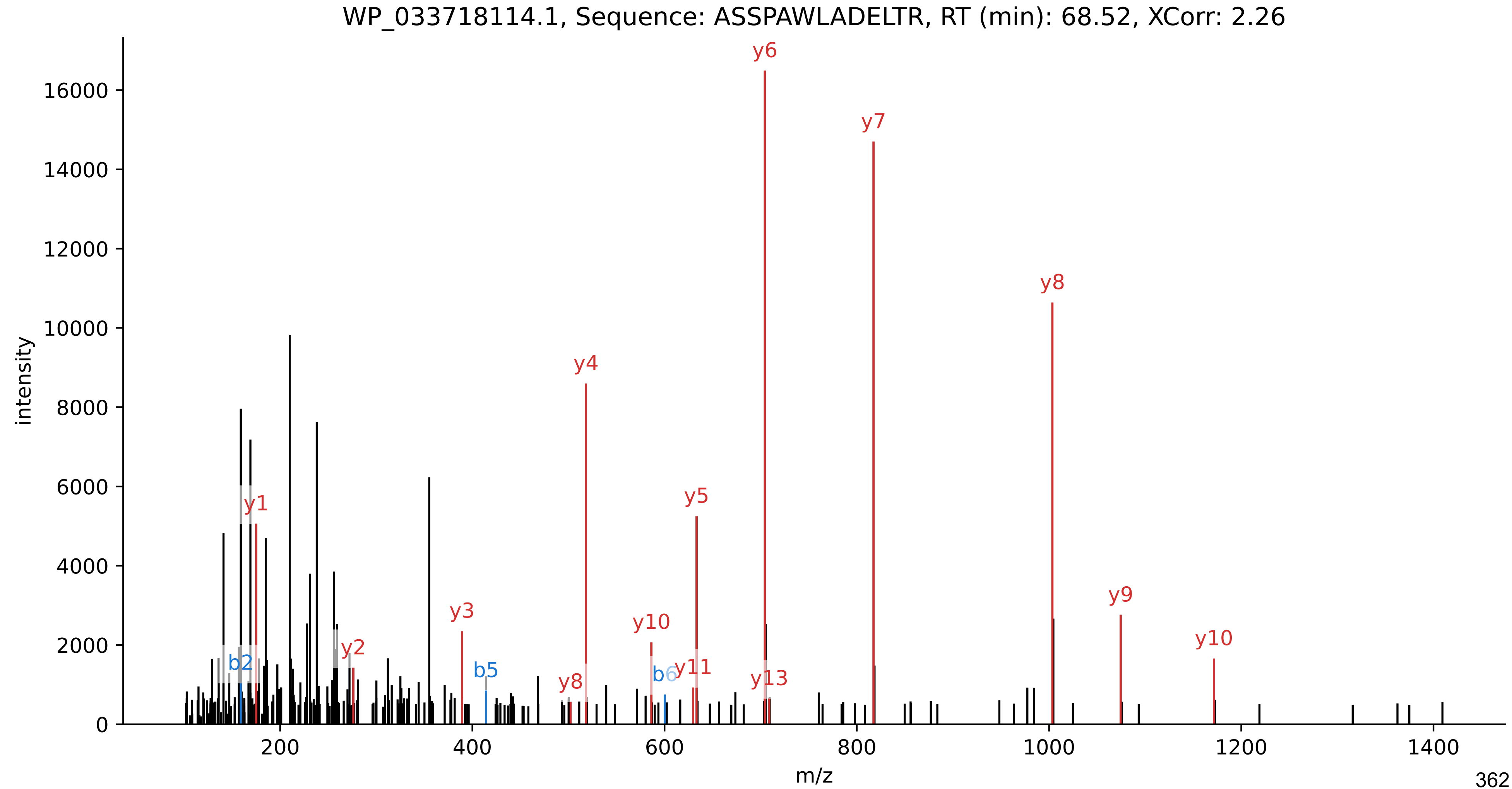

WP_033718114.1, Sequence: ASSPAWLADELTR, RT (min): 68.52, XCorr: 2.26
intensity
m/z
y6
y7
y8
y4
y5
y1
y3
y2
y9
y10
y10
b2
b5
b6
y8
y11
y13

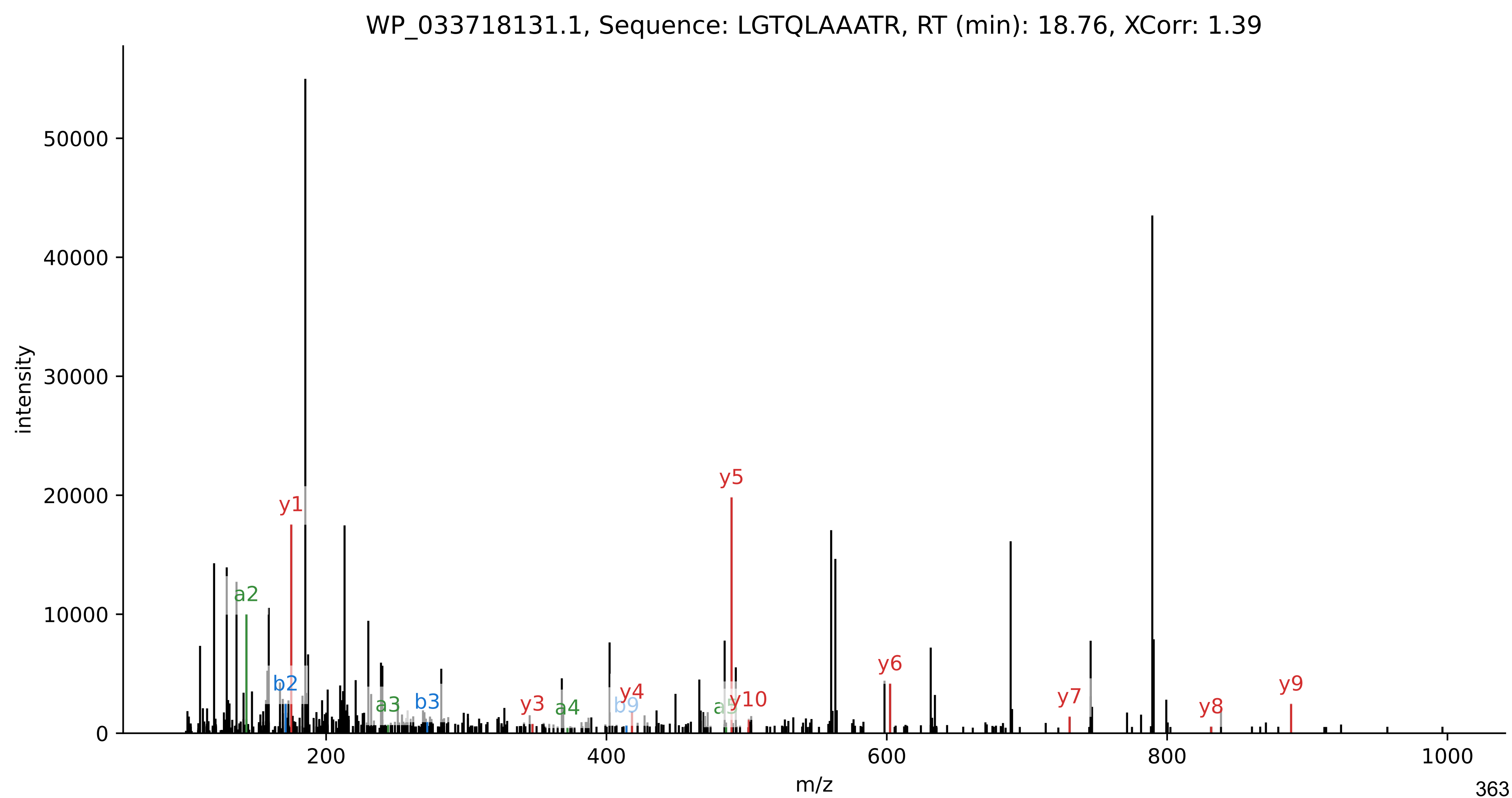

WP_033718131.1, Sequence: LGTQLAAATR, RT (min): 18.76, XCorr: 1.39
intensity
m/z
y1
y3
y4
y5
y6
y7
y8
y9
y10
a2
a3
a4
b2
b3
b9

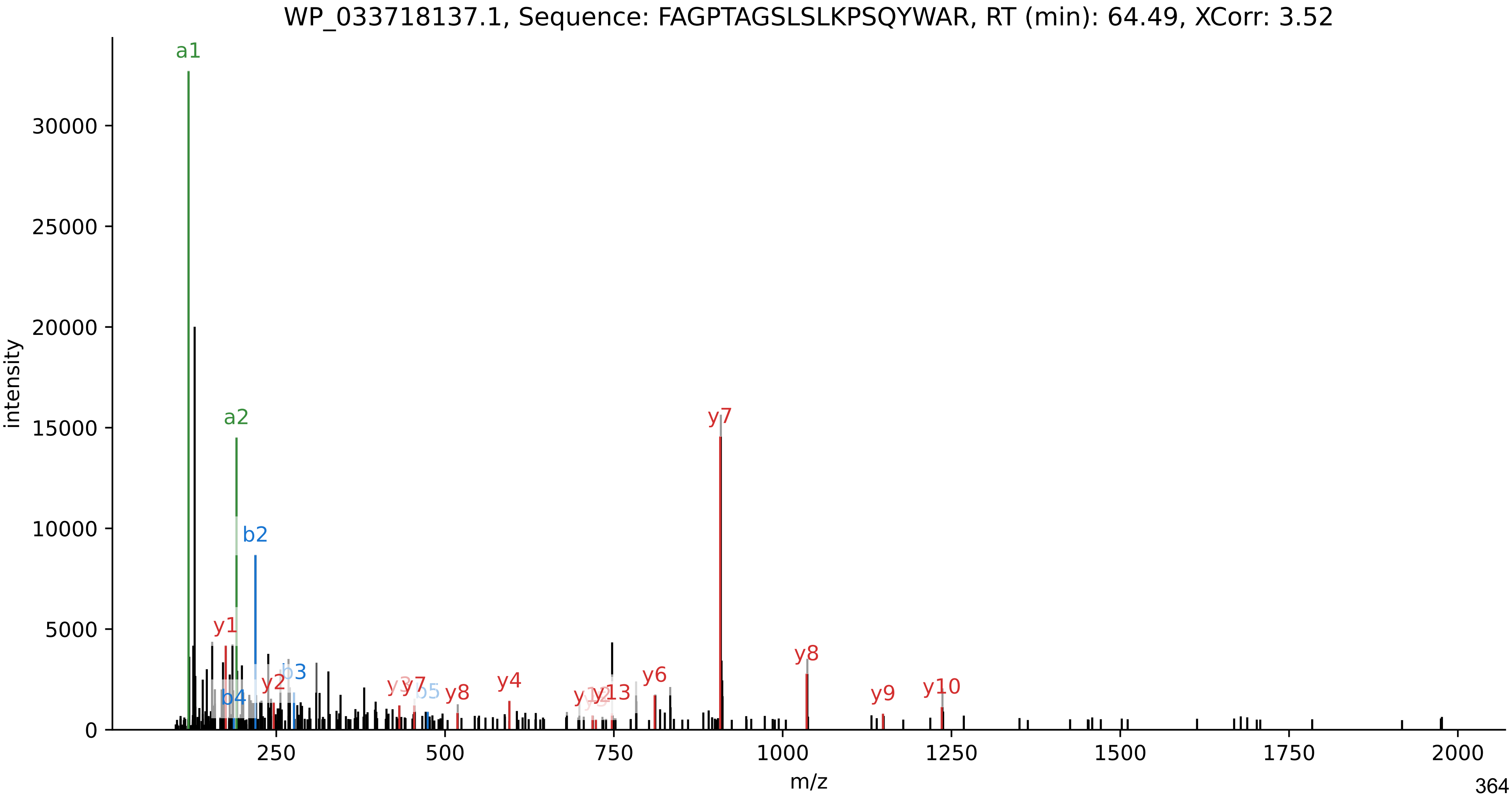
WP_033718137.1, Sequence: FAGPTAGSLSLKPSQYWAR, RT (min): 64.49, XCorr: 3.52
intensity
m/z
a1
a2
b2
y1
y2
b3
b4
b5
y7
y8
y4
y13
y6
y9
y10
0
5000
10000
15000
20000
25000
30000
250
500
750
1000
1250
1500
1750
2000

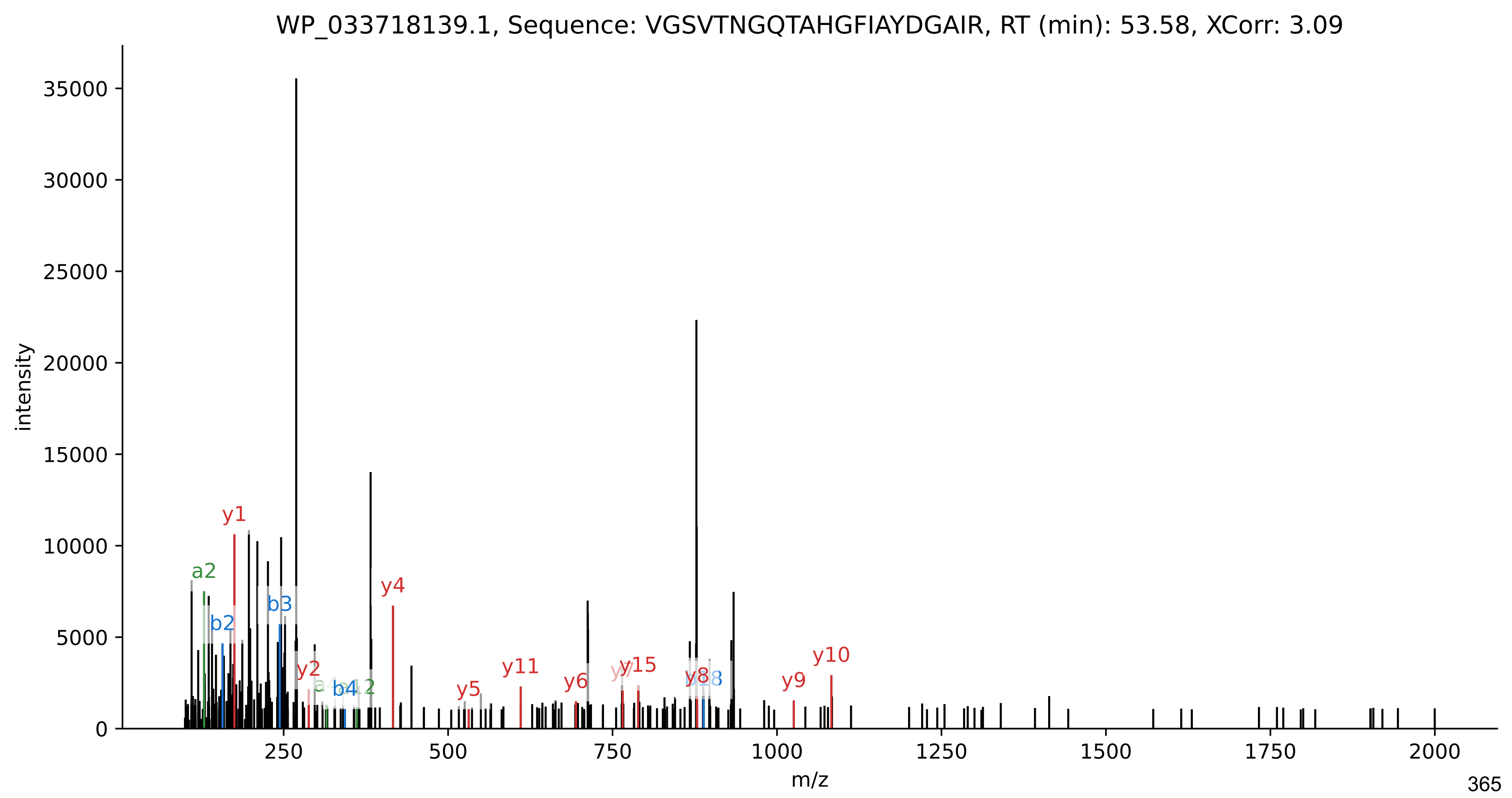
WP_033718139.1, Sequence: VGSVTNGQTAHGFIAYDGAIR, RT (min): 53.58, XCorr: 3.09
intensity
m/z
0
5000
10000
15000
20000
25000
30000
35000
250
500
750
1000
1250
1500
1750
2000
a2
b2
y1
b3
y2
b4
y4
y5
y11
y6
y15
y8
y9
y10

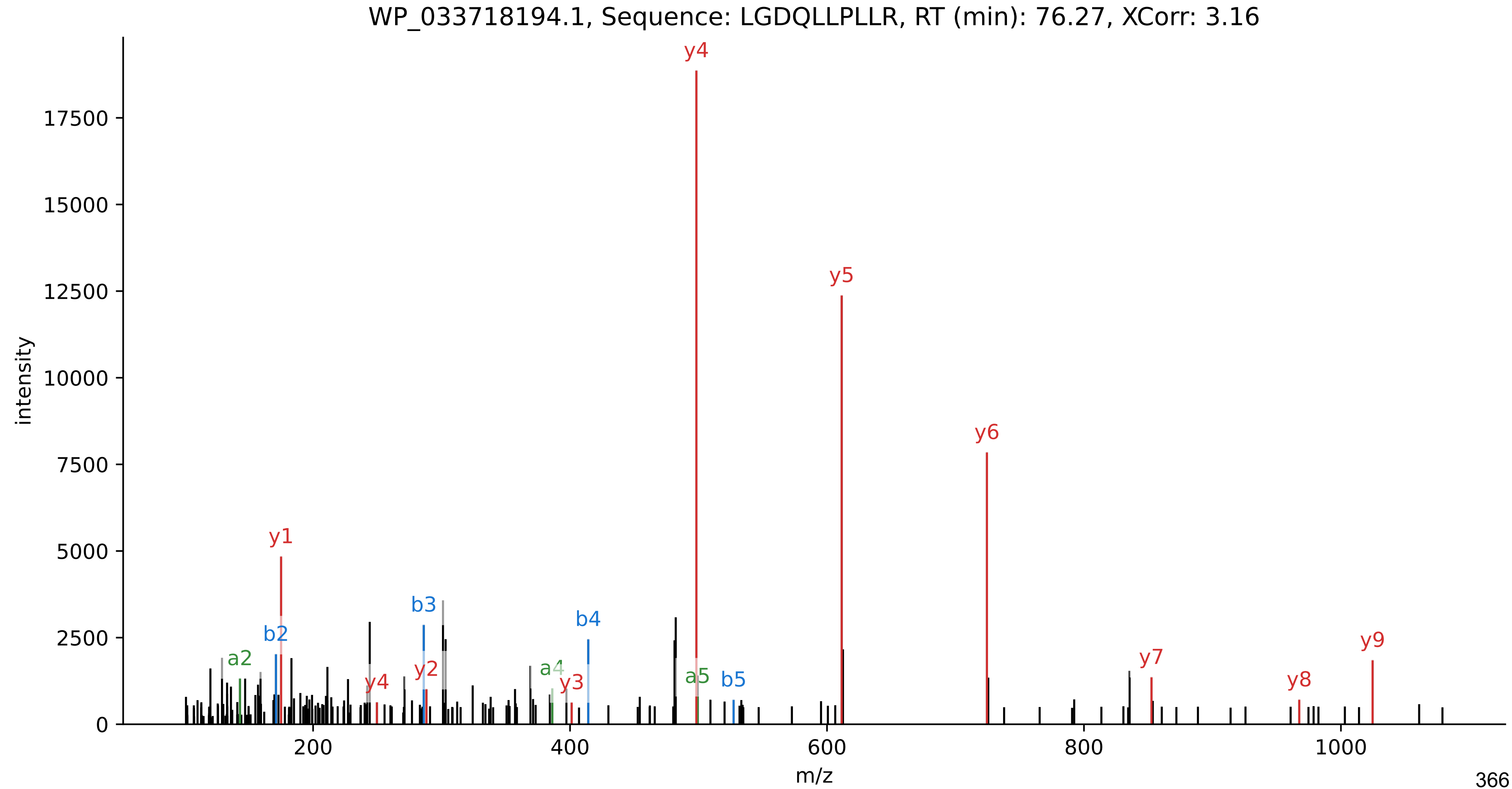

WP_033718194.1, Sequence: LGDQLLPLLR, RT (min): 76.27, XCorr: 3.16
intensity
m/z
0
2500
5000
7500
10000
12500
15000
17500
200
400
600
800
1000
y1
y2
y3
y4
y4
y5
y6
y7
y8
y9
b2
b3
b4
b5
a2
a4
a5

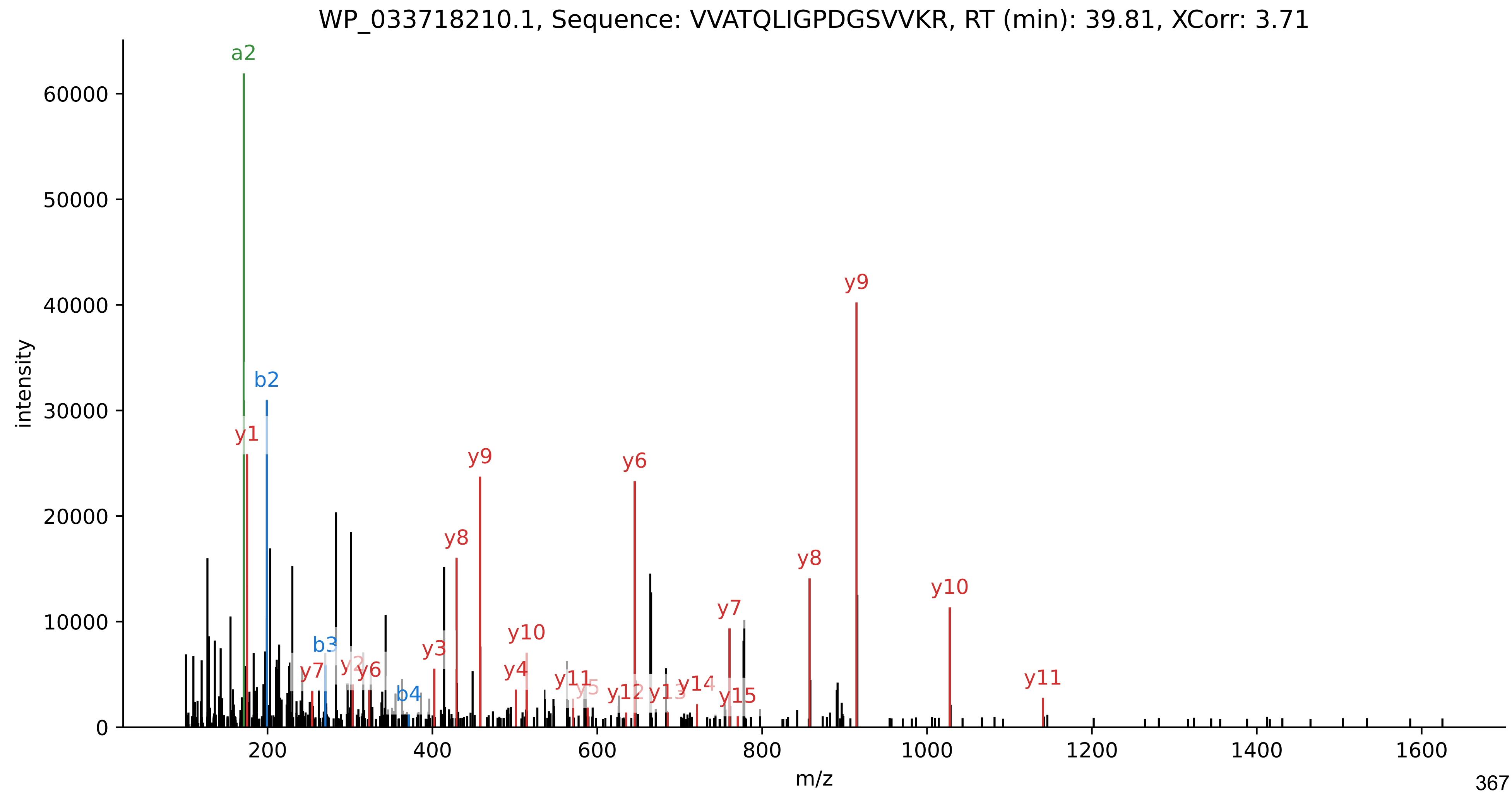

WP_033718210.1, Sequence: VVATQLIGPDGSVVKR, RT (min): 39.81, XCorr: 3.71
intensity
m/z
a2
b2
y1
y9
y6
y8
y9
y8
y10
y7
y11
b3
y7
y2
y6
b4
y3
y4
y10
y11
y5
y12
y13
y14
y15

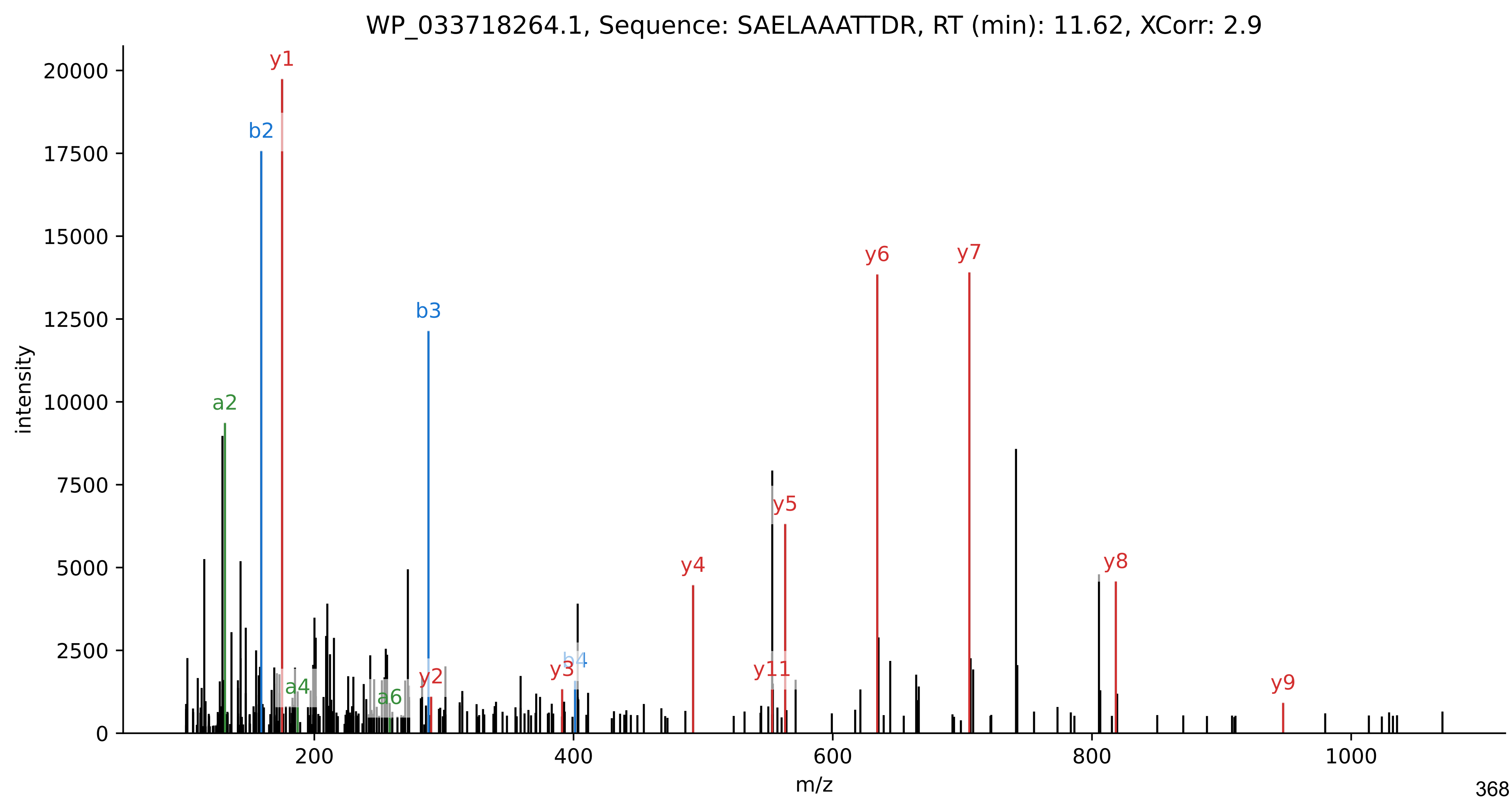

WP_033718264.1, Sequence: SAELAAATTDR, RT (min): 11.62, XCorr: 2.9
intensity
m/z
y1
b2
b3
a2
y6
y7
y5
y4
y8
y2
y3
b4
y11
a4
a6
y9

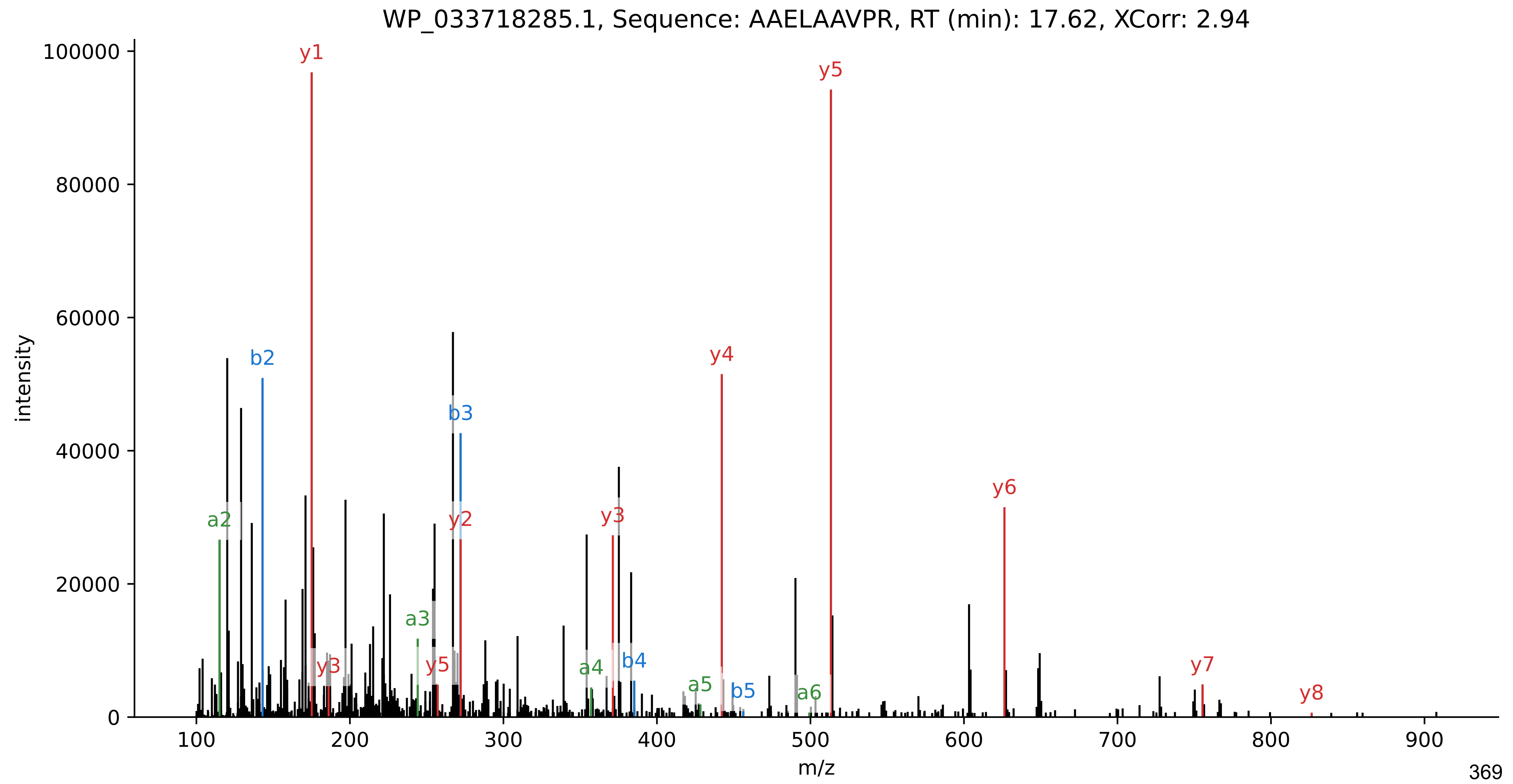

WP_033718285.1, Sequence: AAELAAVPR, RT (min): 17.62, XCorr: 2.94
intensity
m/z
y1
y5
y4
b2
b3
y2
a2
y3
y6
a3
y3
y5
a4
b4
a5
b5
a6
y7
y8

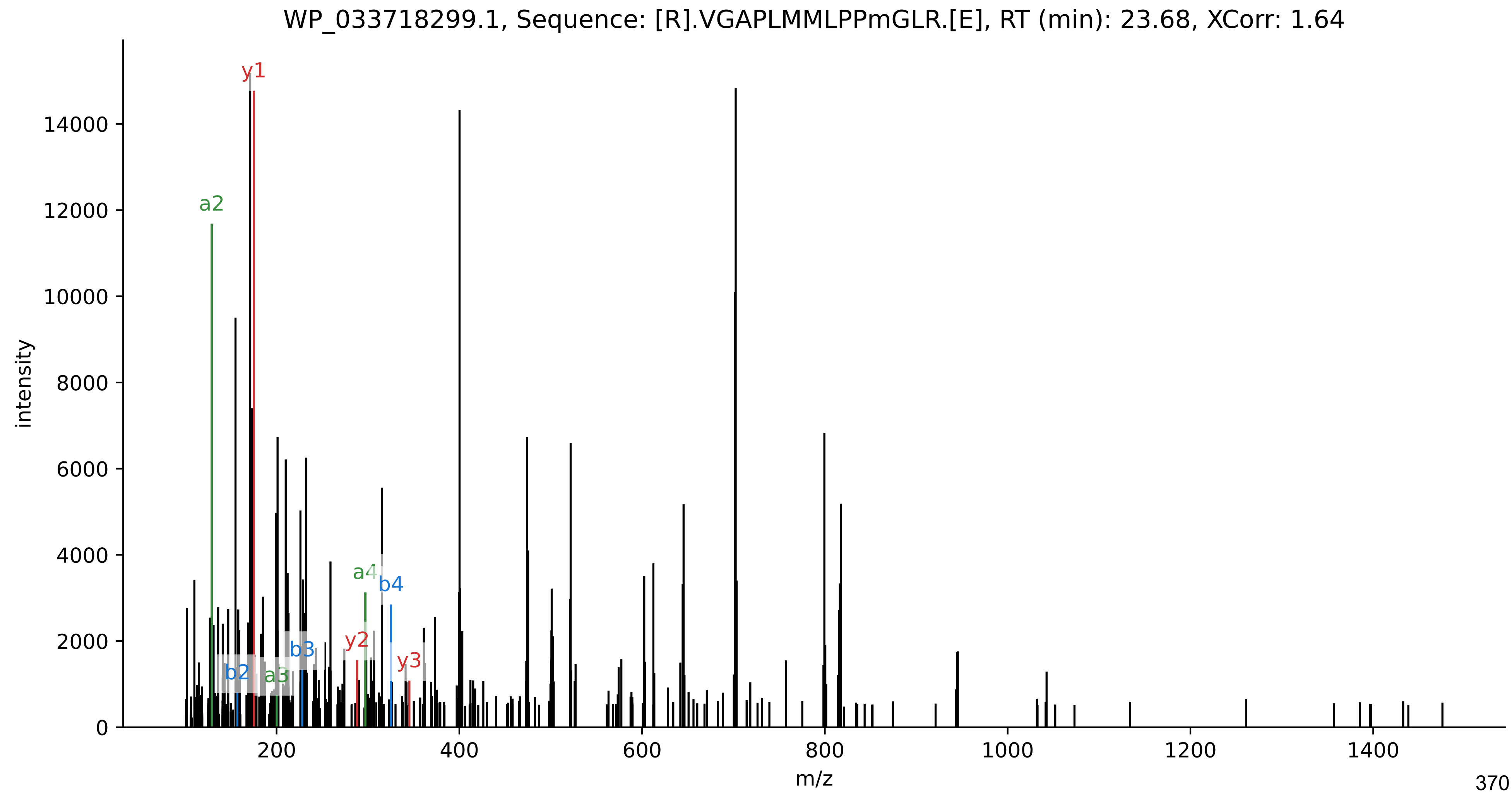
WP_033718299.1, Sequence: [R].VGAPLMMLPPmGLR.[E], RT (min): 23.68, XCorr: 1.64
intensity
m/z
y1
a2
a4
b4
y2
b3
y3
b2
a3
0
2000
4000
6000
8000
10000
12000
14000
200
400
600
800
1000
1200
1400

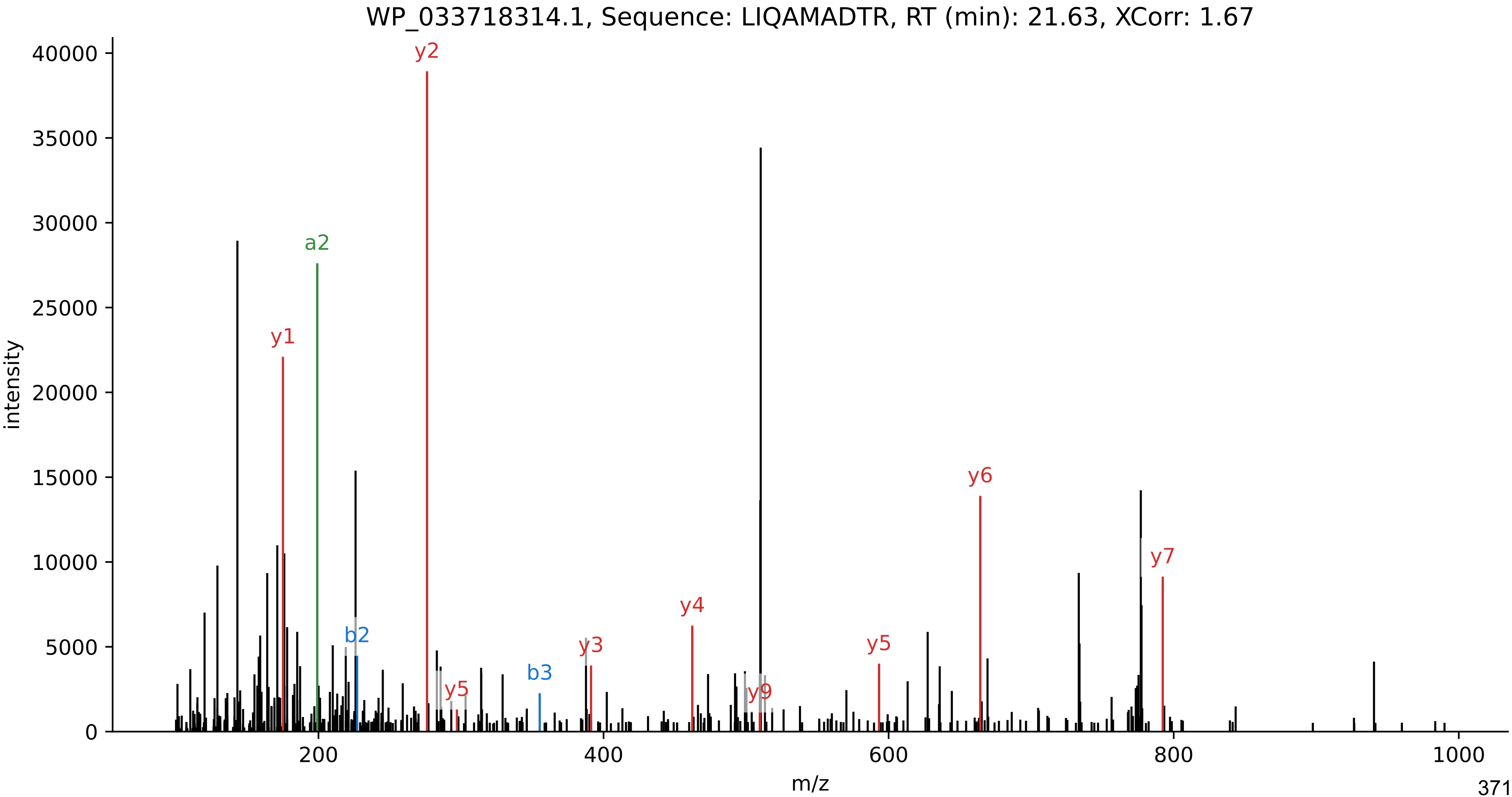

WP_033718314.1, Sequence: LIQAMADTR, RT (min): 21.63, XCorr: 1.67
intensity
m/z
y2
a2
y1
b2
b3
y5
y3
y4
y9
y5
y6
y7
0
5000
10000
15000
20000
25000
30000
35000
40000
200
400
600
800
1000

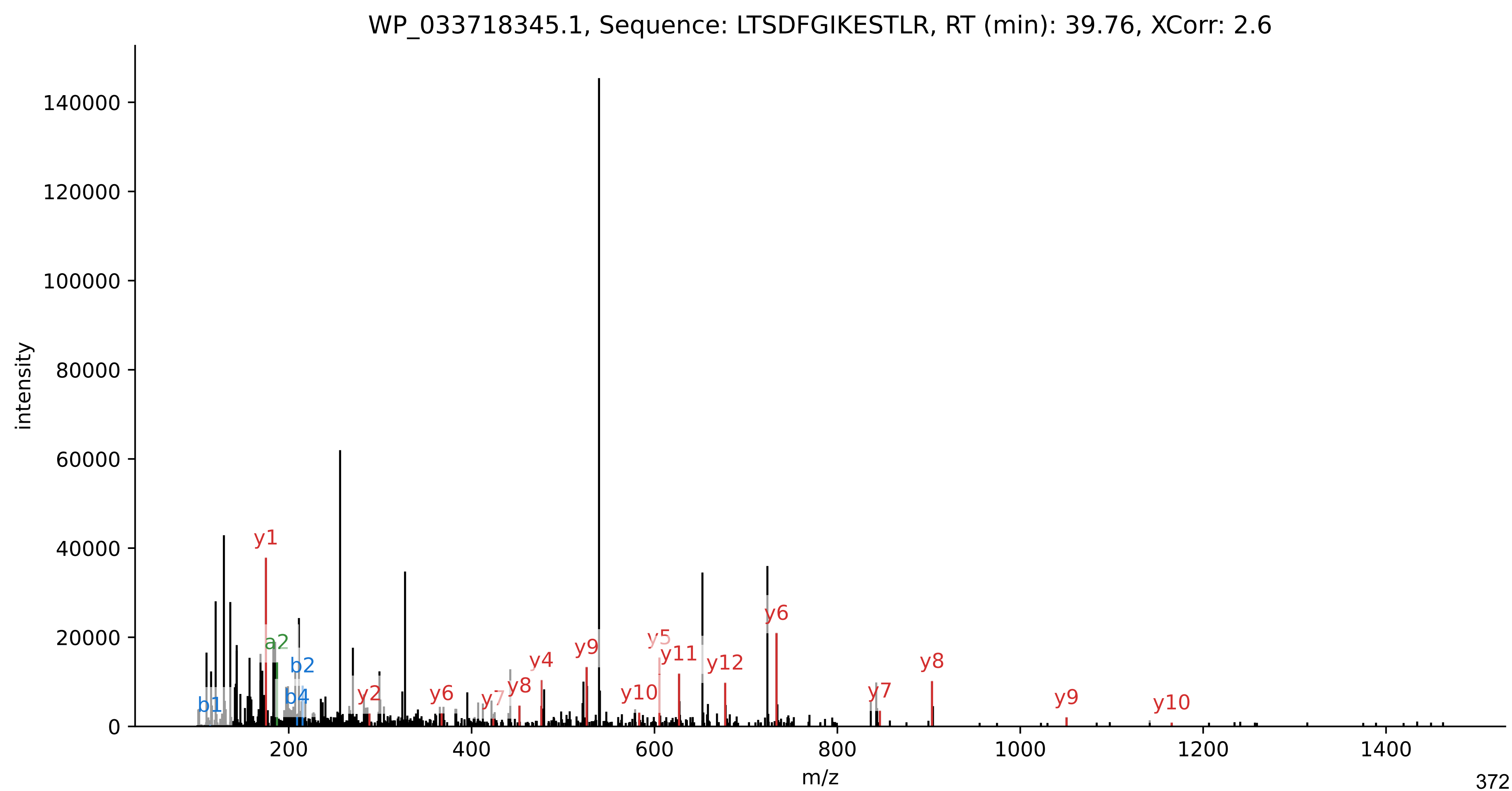
WP_033718345.1, Sequence: LTSDFGIKESTLR, RT (min): 39.76, XCorr: 2.6
intensity
m/z
0
20000
40000
60000
80000
100000
120000
140000
200
400
600
800
1000
1200
1400
y1
a2
b2
b1
b4
y2
y6
y7
y8
y4
y9
y10
y5
y11
y12
y6
y7
y8
y9
y10

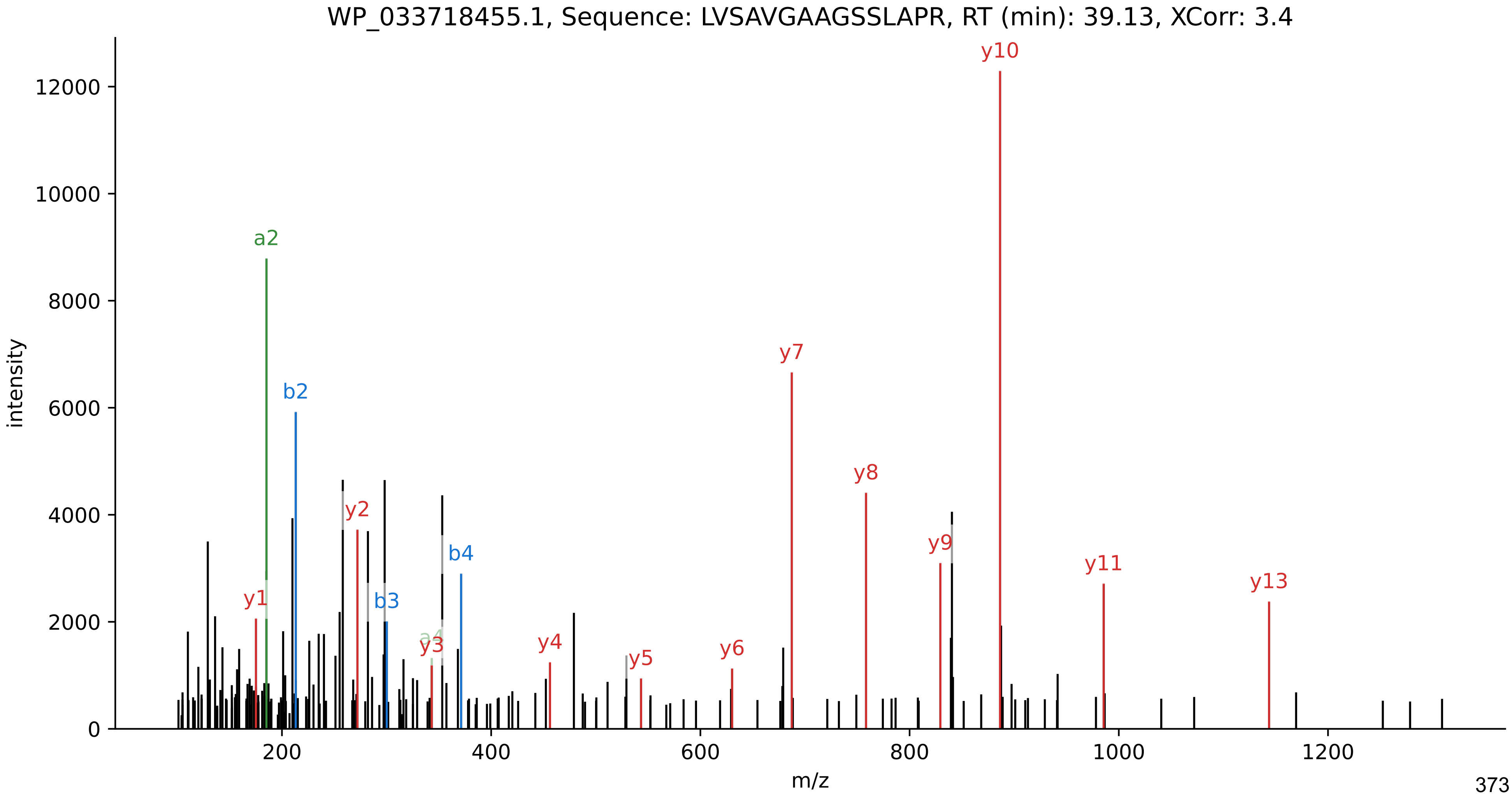
WP_033718455.1, Sequence: LVSAVGAAGSSLAPR, RT (min): 39.13, XCorr: 3.4
intensity
m/z
a2
b2
b3
b4
a4
y1
y2
y3
y4
y5
y6
y7
y8
y9
y10
y11
y13
0
2000
4000
6000
8000
10000
12000
200
400
600
800
1000
1200

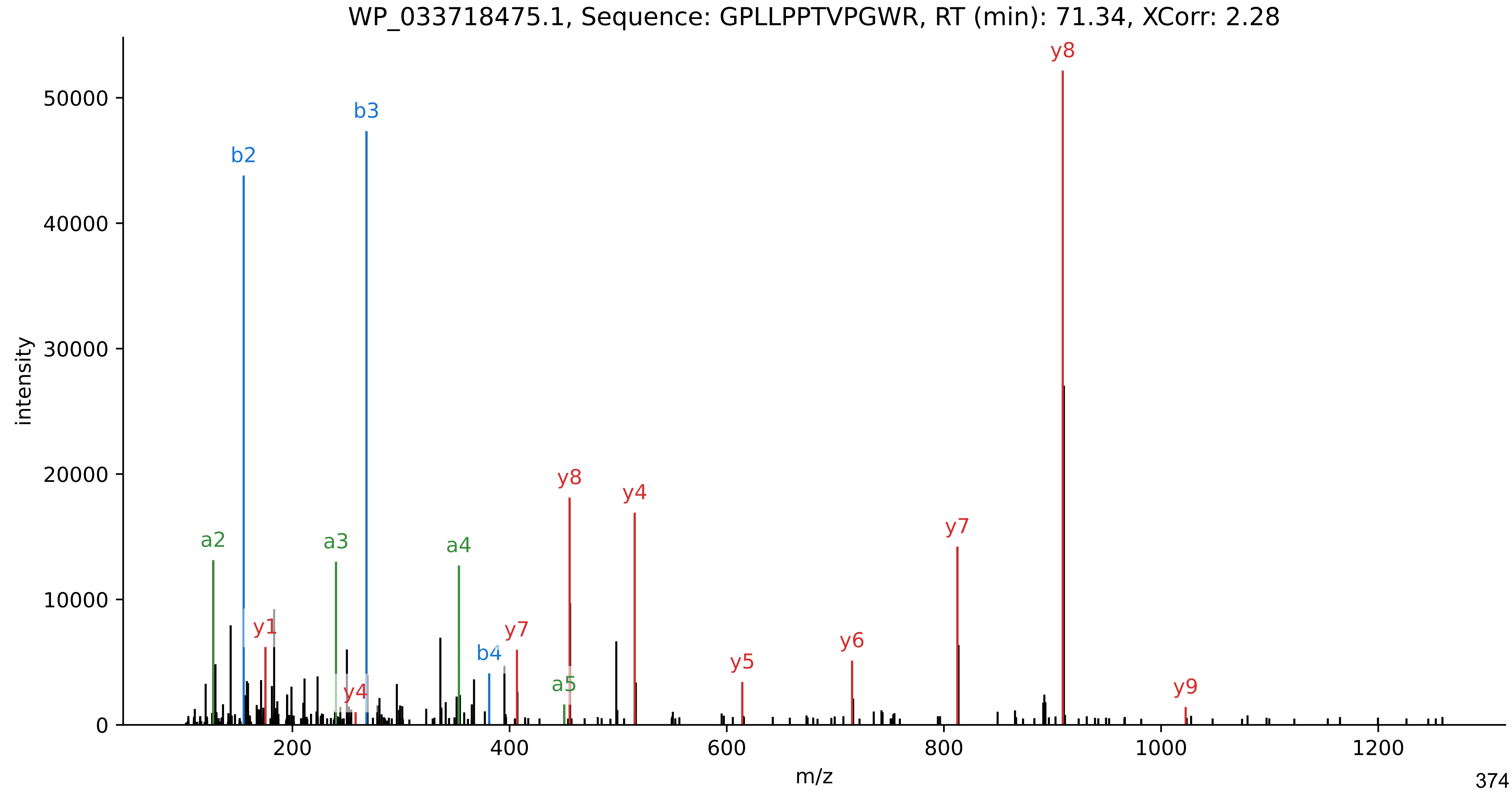
WP_033718475.1, Sequence: GPLLPPTVPGWR, RT (min): 71.34, XCorr: 2.28
intensity
m/z
0
10000
20000
30000
40000
50000
200
400
600
800
1000
1200
y8
b3
b2
y8
y4
y7
a2
a3
a4
y1
b4
y7
y6
y5
a5
y4
y9

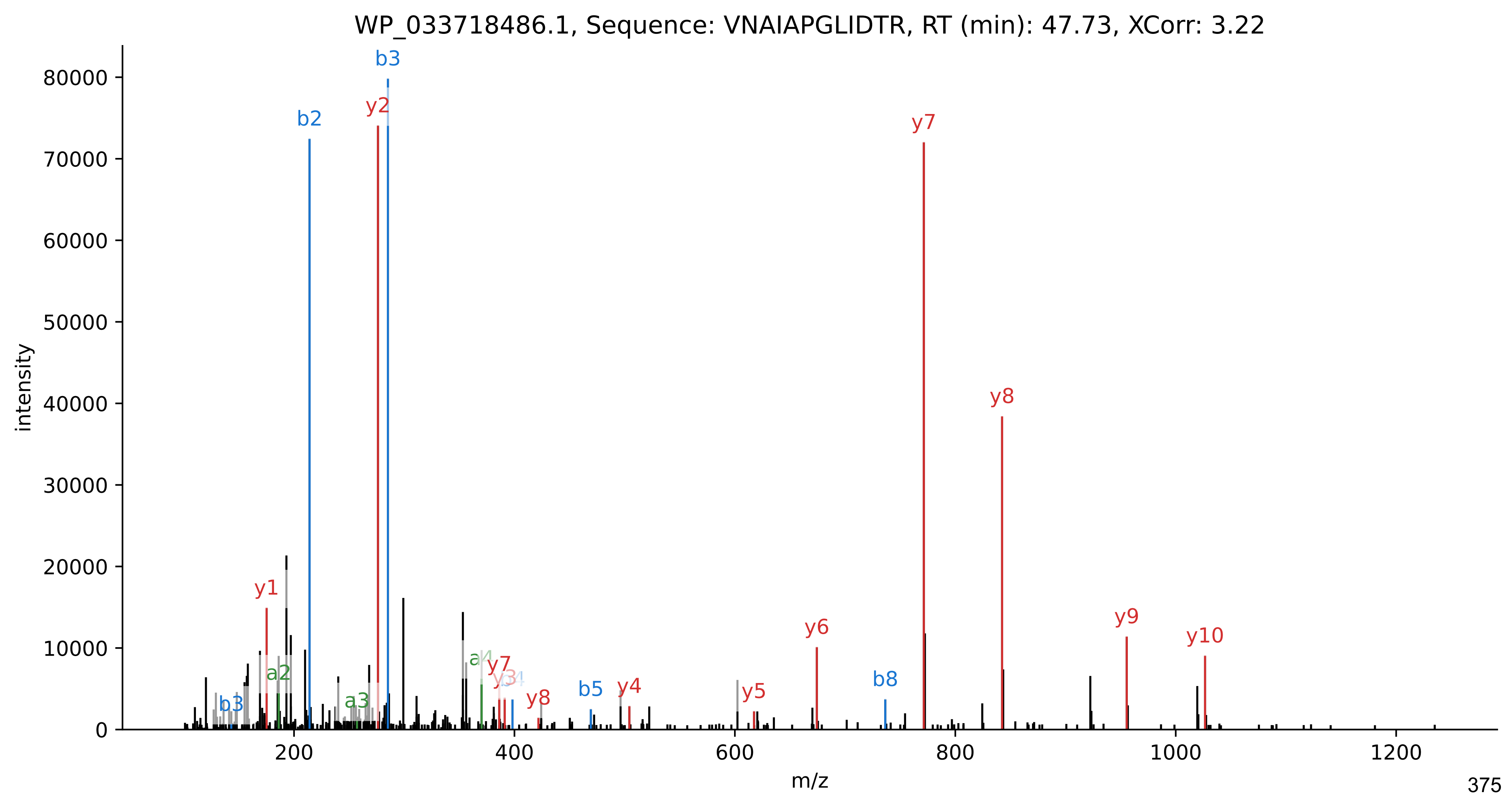
WP_033718486.1, Sequence: VNAIAPGLIDTR, RT (min): 47.73, XCorr: 3.22
intensity
m/z
b3
b2
y2
y7
y8
y1
a2
a3
a4
y7
y3
b4
y8
b5
y4
y5
y6
b8
y9
y10
b3
0
10000
20000
30000
40000
50000
60000
70000
80000
200
400
600
800
1000
1200

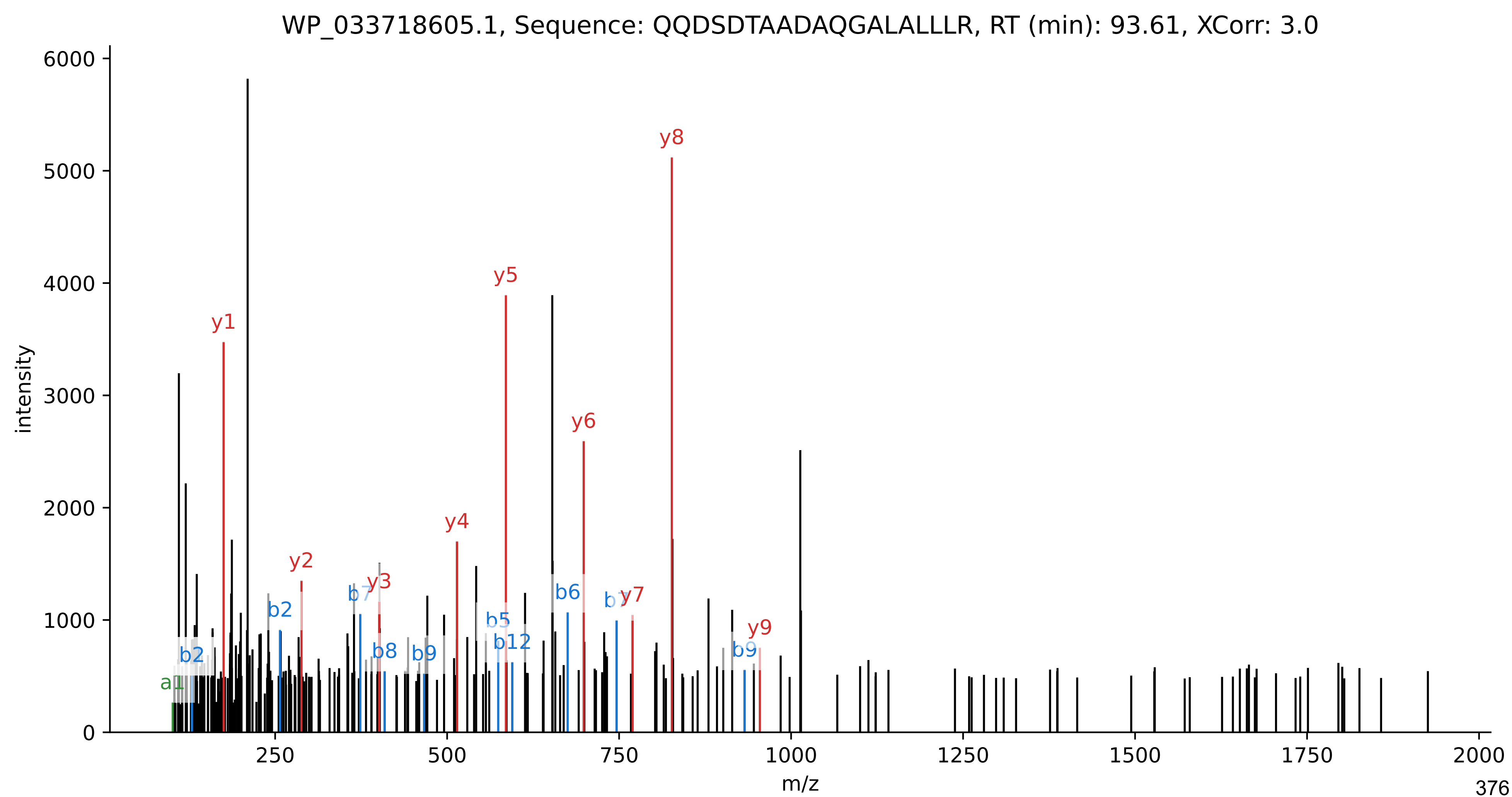

WP_033718605.1, Sequence: QQDSDTAADAQGALALLLR, RT (min): 93.61, XCorr: 3.0
intensity
m/z
y1
y2
y3
y4
y5
y6
y7
y8
y9
a1
b2
b2
b5
b6
b7
b8
b9
b9
b12

WP_033718823.1, Sequence: DAWQFFQR, RT (min): 68.87, XCorr: 2.1

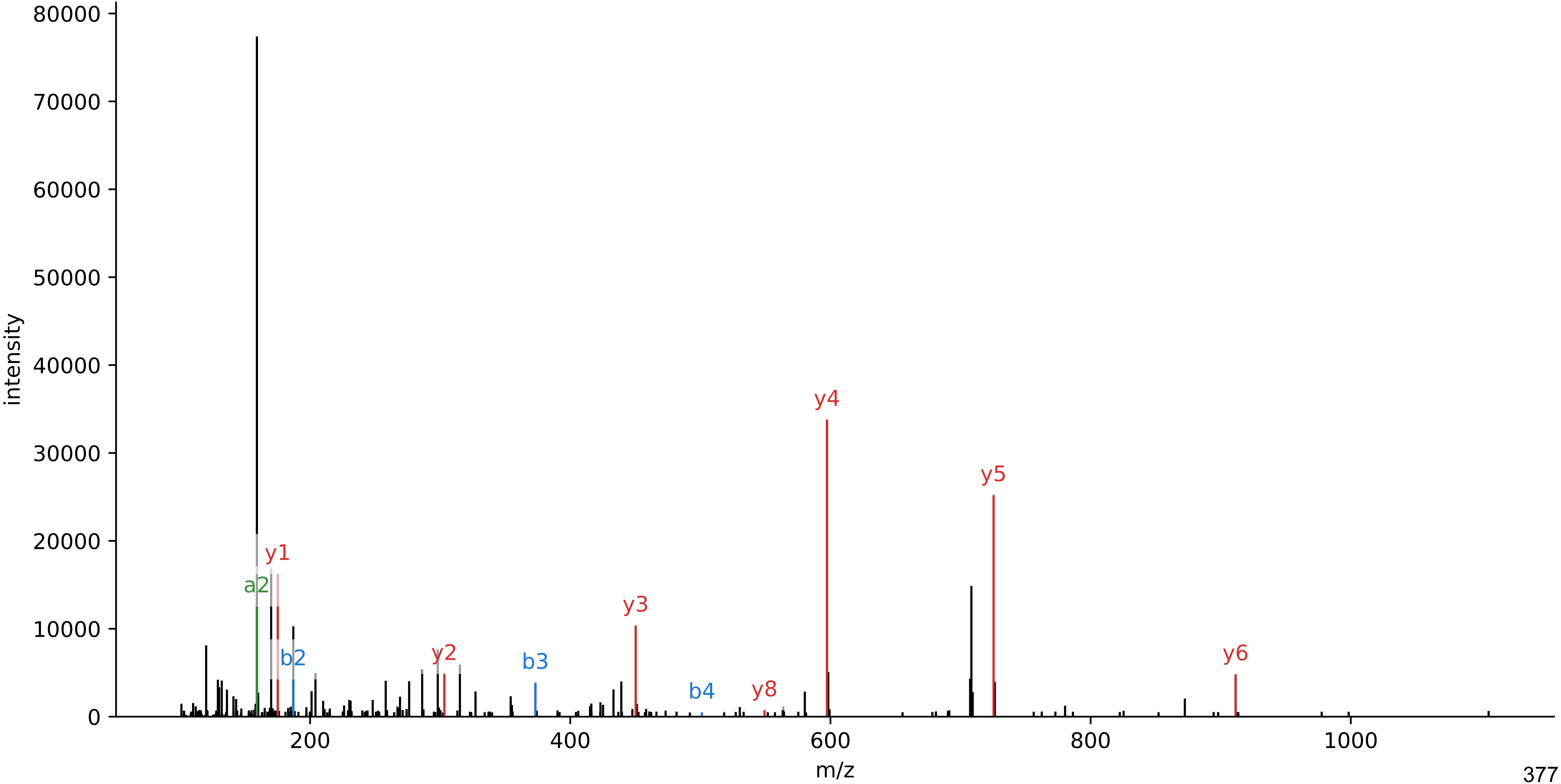

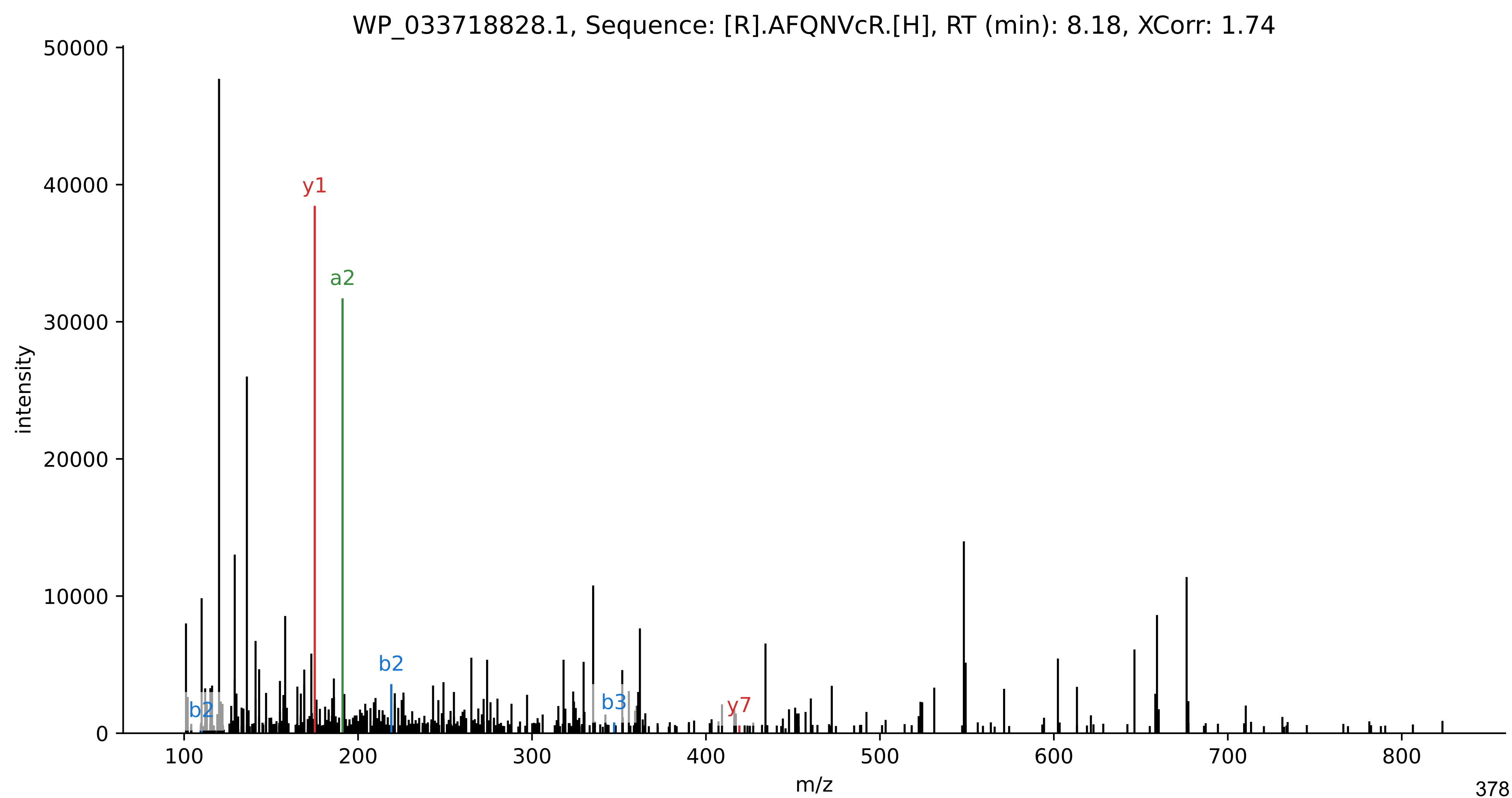
WP_033718828.1, Sequence: [R].AFQNVcR.[H], RT (min): 8.18, XCorr: 1.74
intensity
m/z
y1
a2
b2
b2
b3
y7
0
10000
20000
30000
40000
50000
100
200
300
400
500
600
700
800

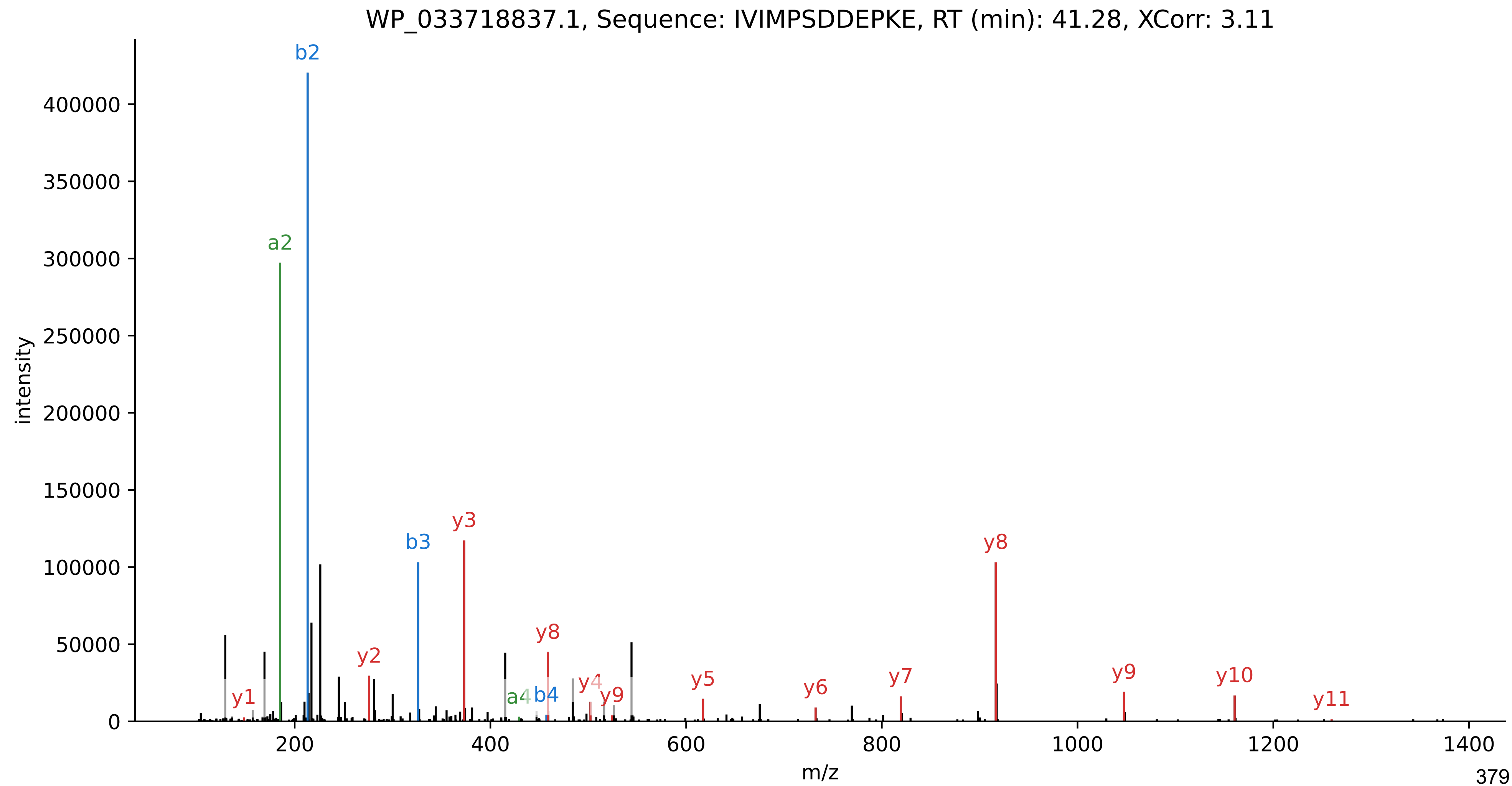
WP_033718837.1, Sequence: IVIMPSDDEPKE, RT (min): 41.28, XCorr: 3.11
intensity
m/z
b2
a2
b3
y3
y8
y2
y1
a4
b4
y4
y9
y5
y6
y7
y8
y9
y10
y11
0
50000
100000
150000
200000
250000
300000
350000
400000
200
400
600
800
1000
1200
1400

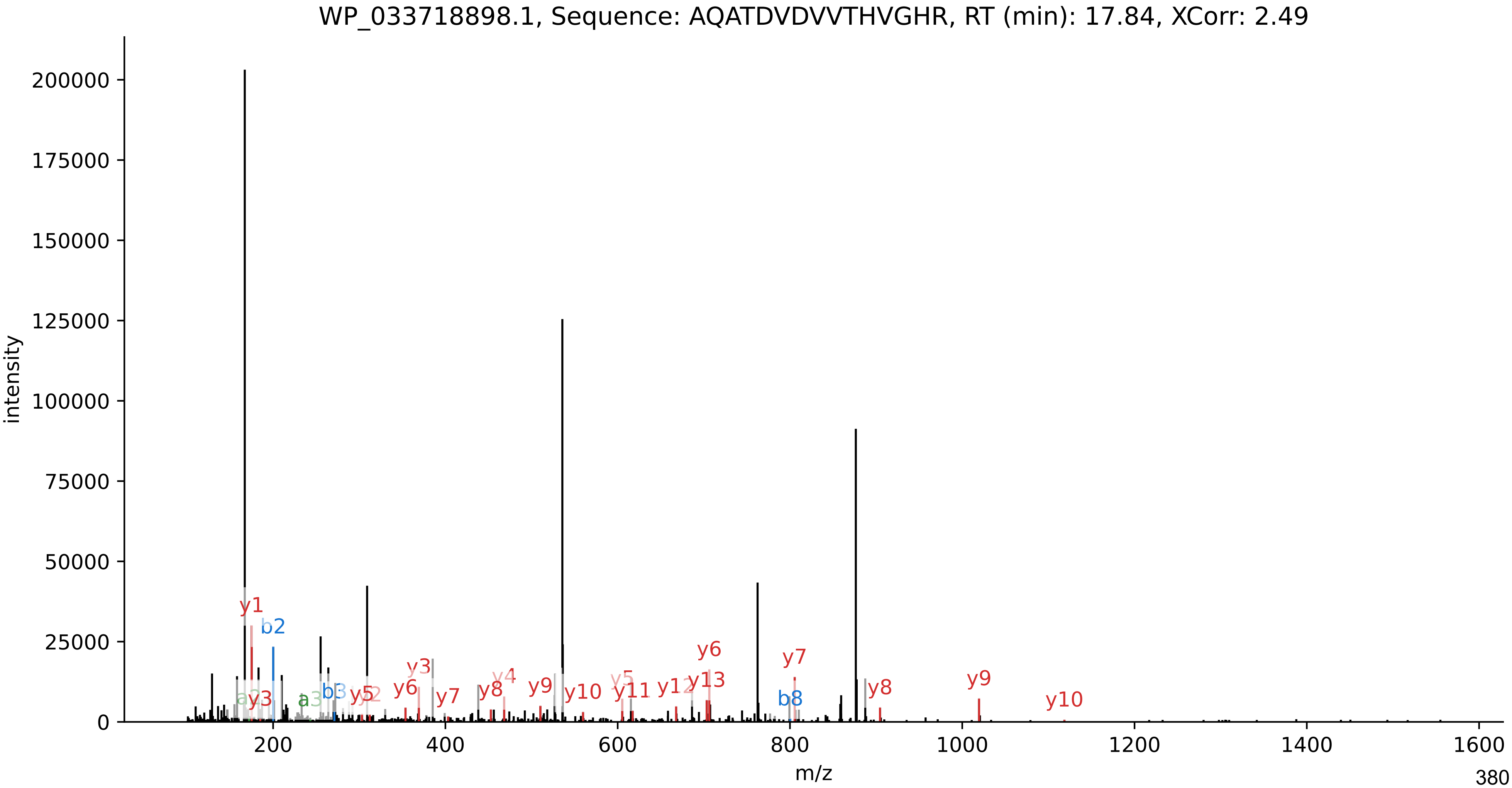
WP_033718898.1, Sequence: AQATDVDVVTHVGHR, RT (min): 17.84, XCorr: 2.49
intensity
m/z
y1
b2
y3
a3
b3
y5
y2
y6
y3
y7
y8
y4
y9
y10
y5
y11
y12
y13
y6
y7
b8
y8
y9
y10

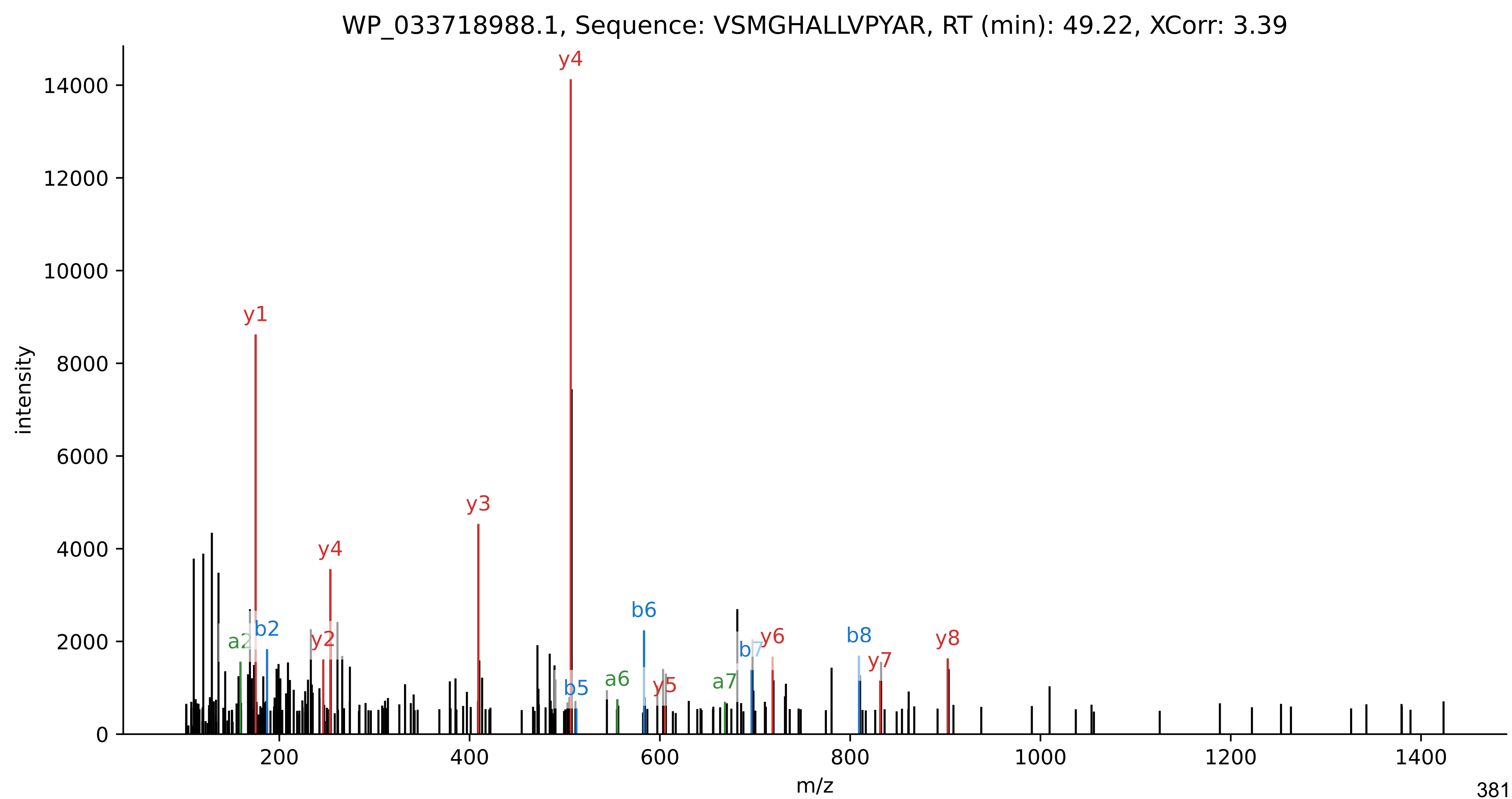

WP_033718988.1, Sequence: VSMGHALLVPYAR, RT (min): 49.22, XCorr: 3.39
intensity
m/z
y4
y1
y3
y4
b2
a2
y2
b6
b5
a6
y5
a7
b7
y6
b8
y7
y8

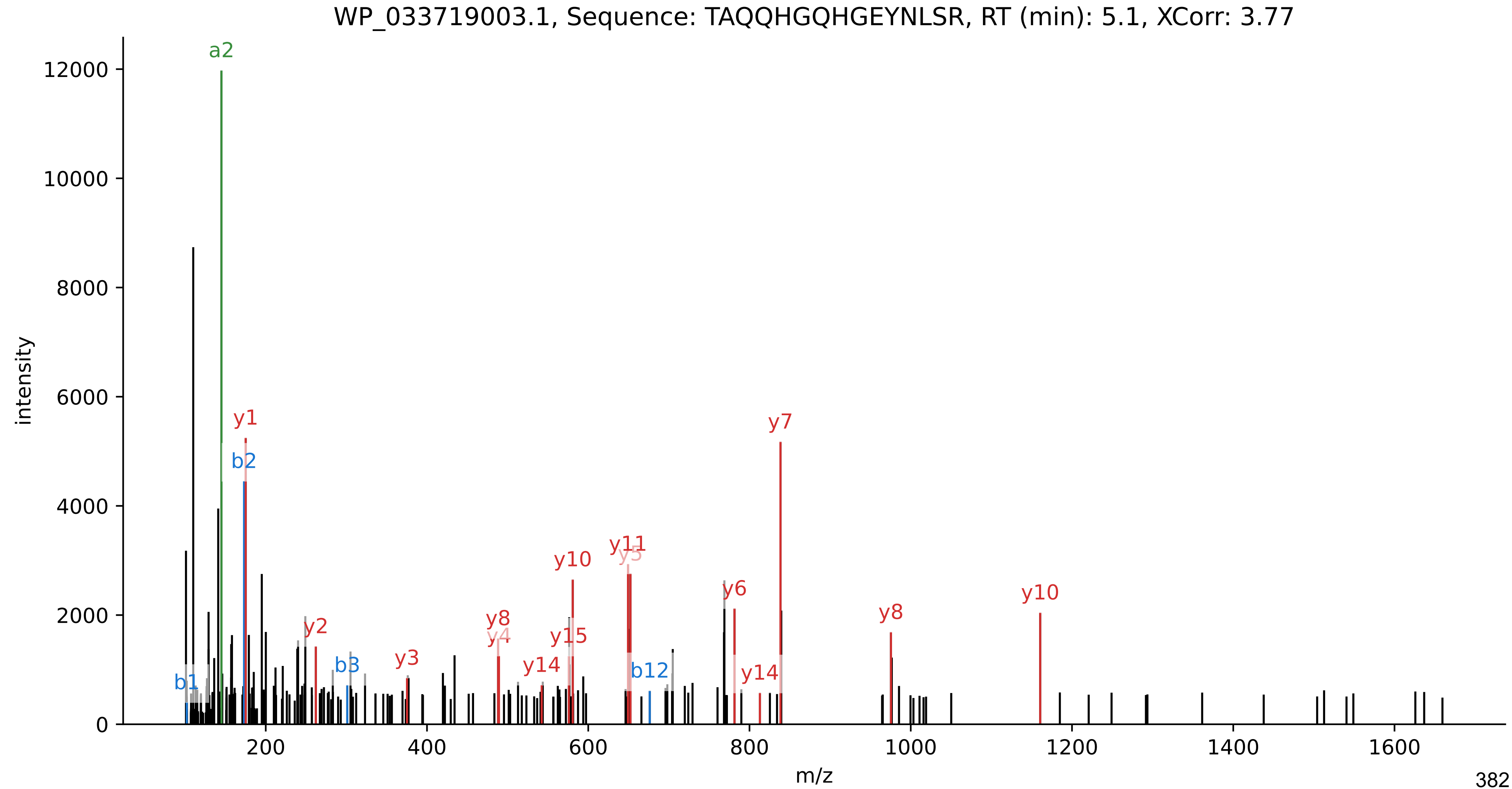
WP_033719003.1, Sequence: TAQQHGQHGEYNLSR, RT (min): 5.1, XCorr: 3.77
a2
y1
b2
y7
y11
y5
y10
y6
y10
y8
y2
y8
y4
y15
b3
y3
y14
b12
y14
b1
intensity
m/z
0
2000
4000
6000
8000
10000
12000
200
400
600
800
1000
1200
1400
1600

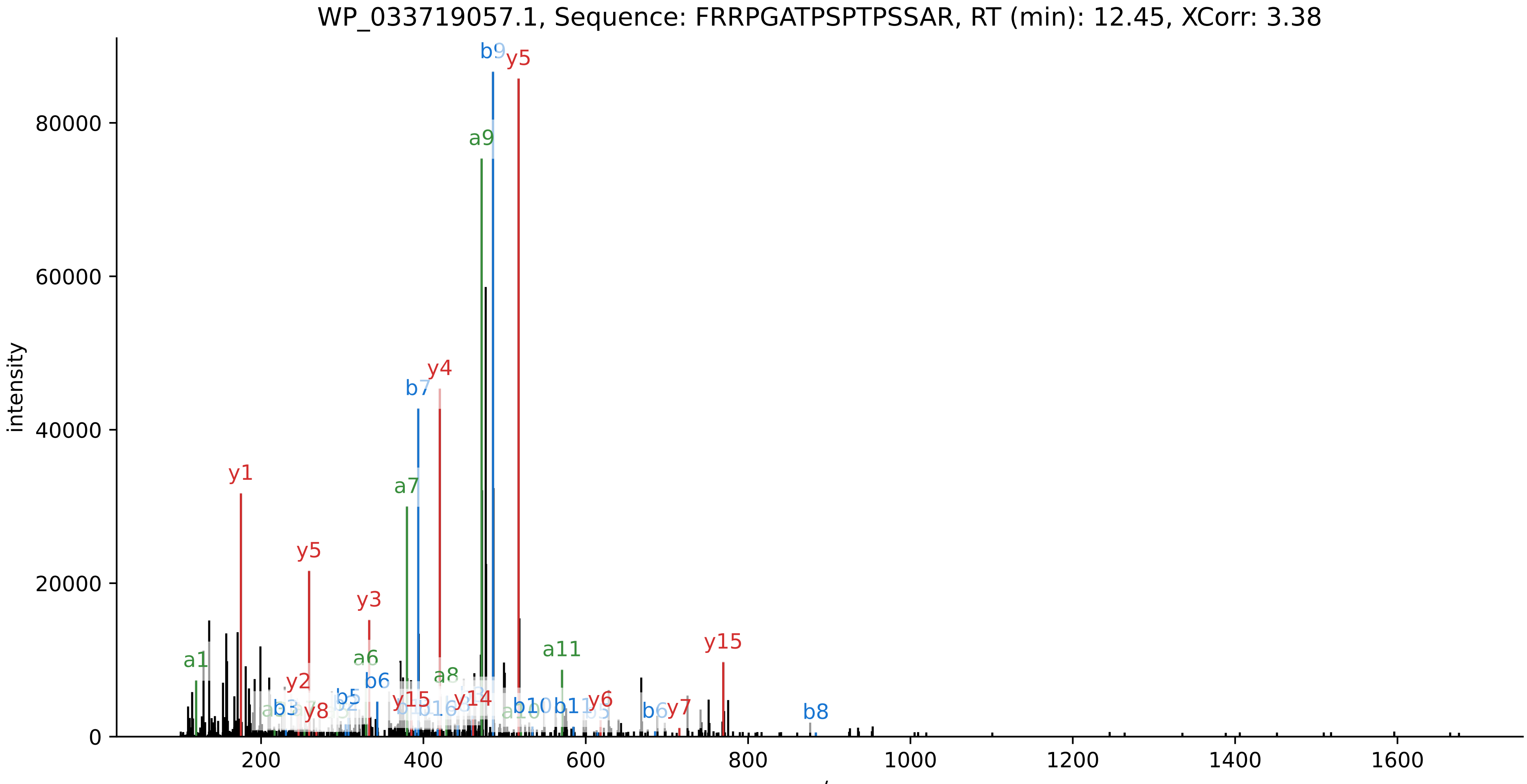
WP_033719057.1, Sequence: FRRPGATPSPTPSSAR, RT (min): 12.45, XCorr: 3.38
intensity
m/z
0
20000
40000
60000
80000
200
400
600
800
1000
1200
1400
1600
b9
y5
a9
y4
b7
a7
y1
y5
y3
a11
y15
a1
a6
a8
y2
b6
b5
y15
y14
y6
b3
y8
b10
b11
b6
y7
b8

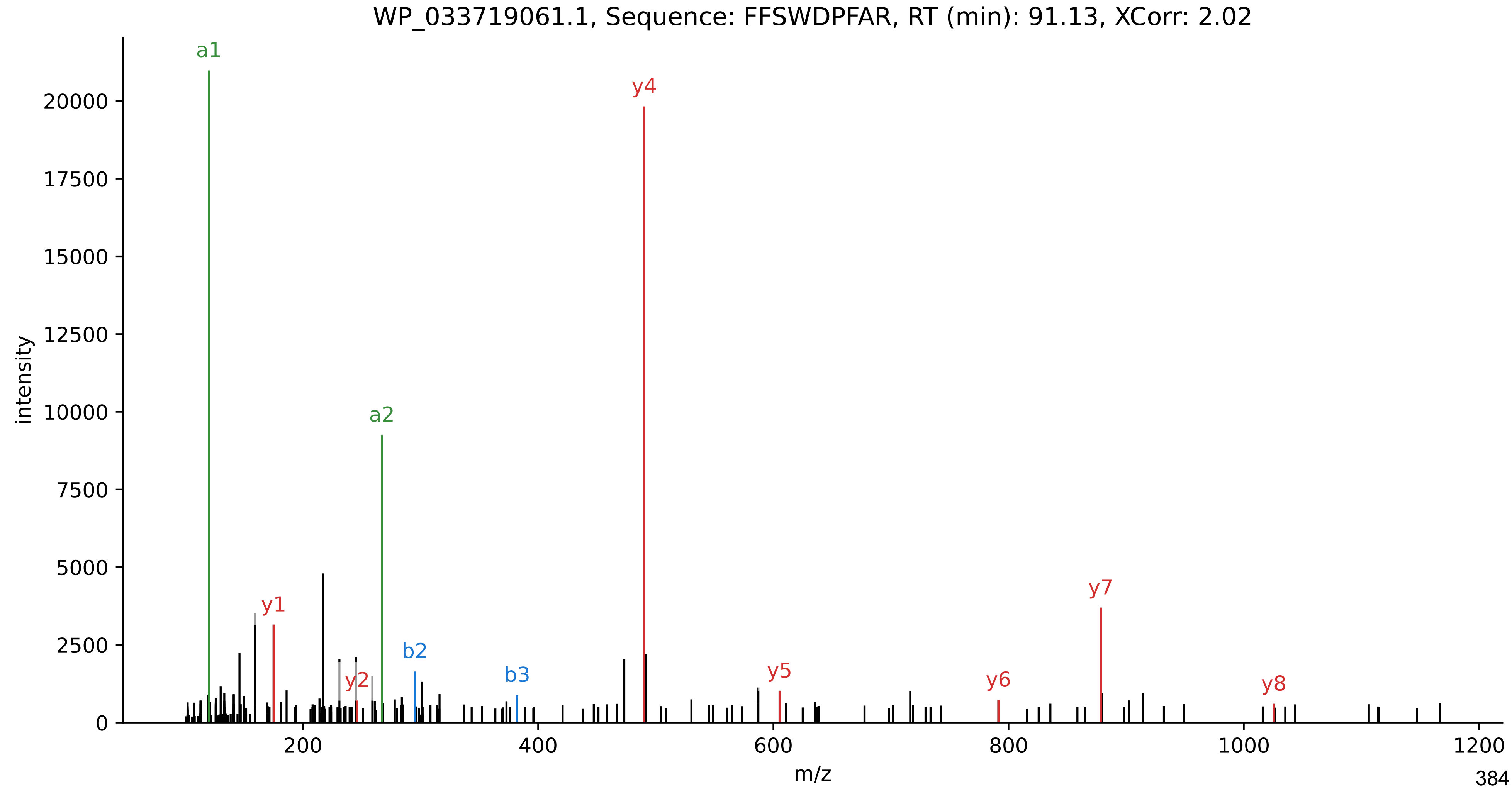
WP_033719061.1, Sequence: FFSWDPFAR, RT (min): 91.13, XCorr: 2.02
intensity
m/z
a1
a2
y1
y2
b2
b3
y4
y5
y6
y7
y8
0
2500
5000
7500
10000
12500
15000
17500
20000
200
400
600
800
1000
1200

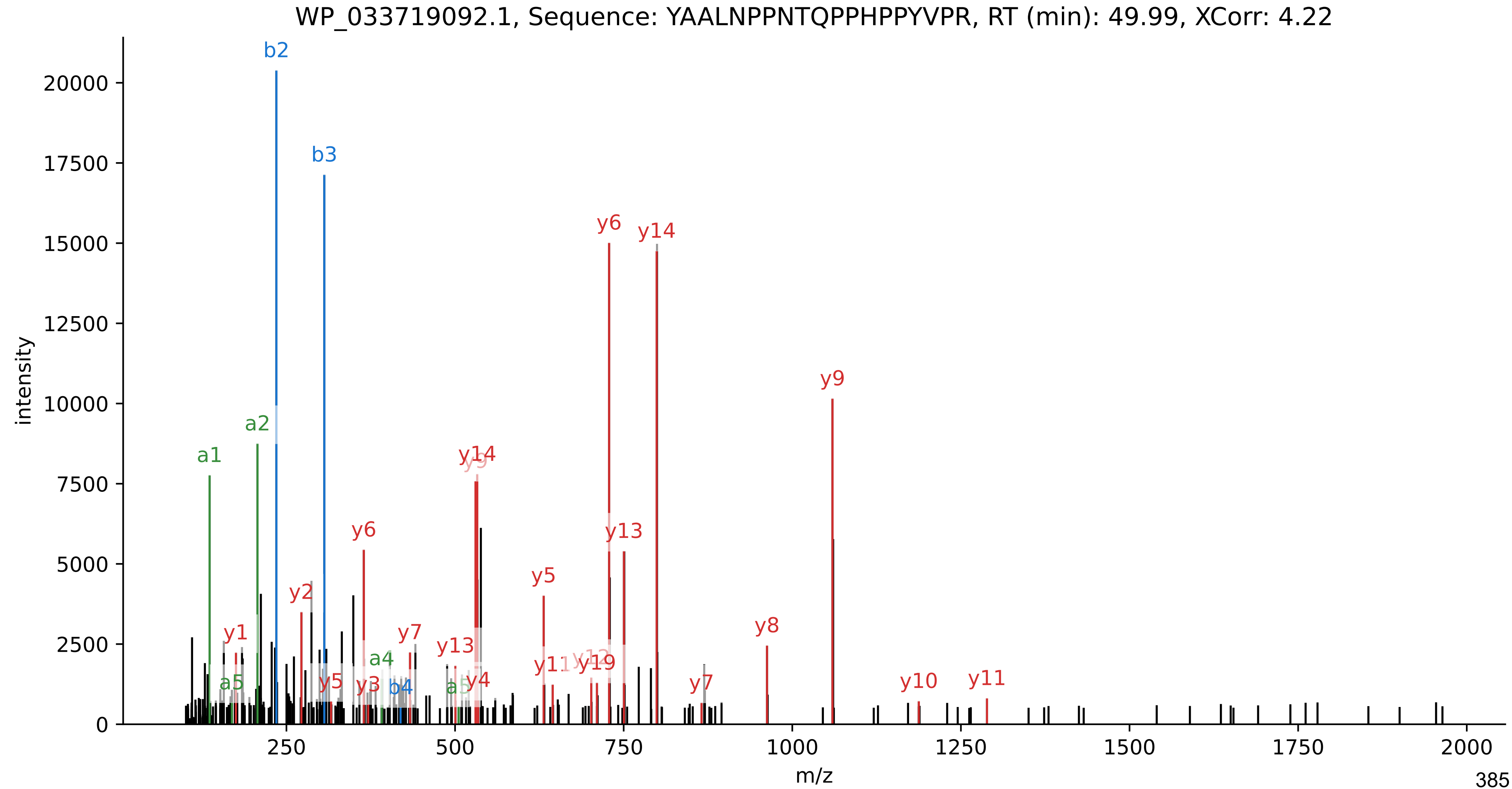
WP_033719092.1, Sequence: YAALNPPNTQPPHPPYVPR, RT (min): 49.99, XCorr: 4.22
intensity
m/z
b2
b3
y6
y14
y9
a2
a1
y14
y6
y13
y5
y2
y1
y7
a4
y13
y8
a5
y5
y3
b4
y4
y19
y7
y10
y11

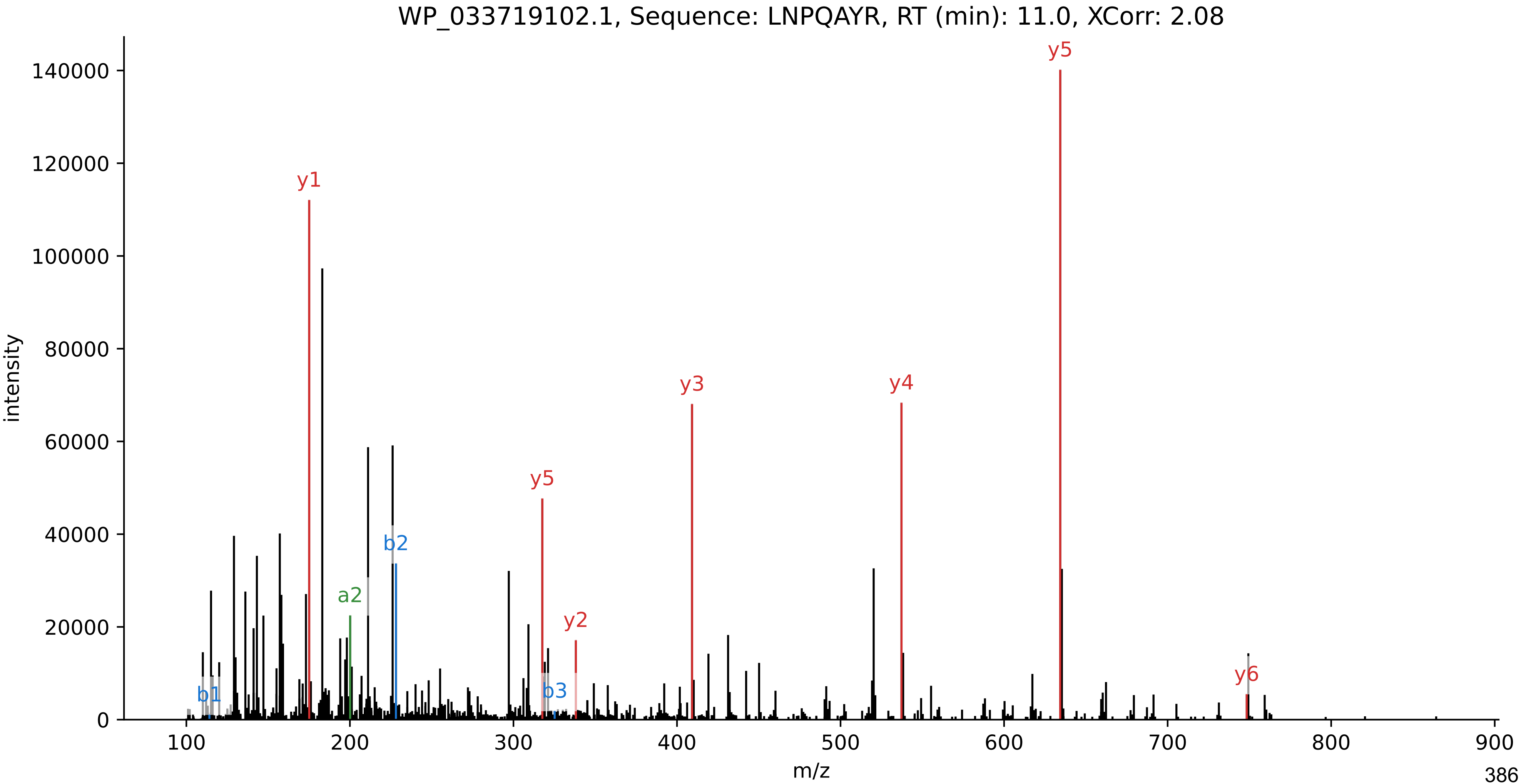
WP_033719102.1, Sequence: LNPQAYR, RT (min): 11.0, XCorr: 2.08
intensity
m/z
y5
y1
y3
y4
y5
b2
a2
y2
b1
b3
y6
0
20000
40000
60000
80000
100000
120000
140000
100
200
300
400
500
600
700
800
900

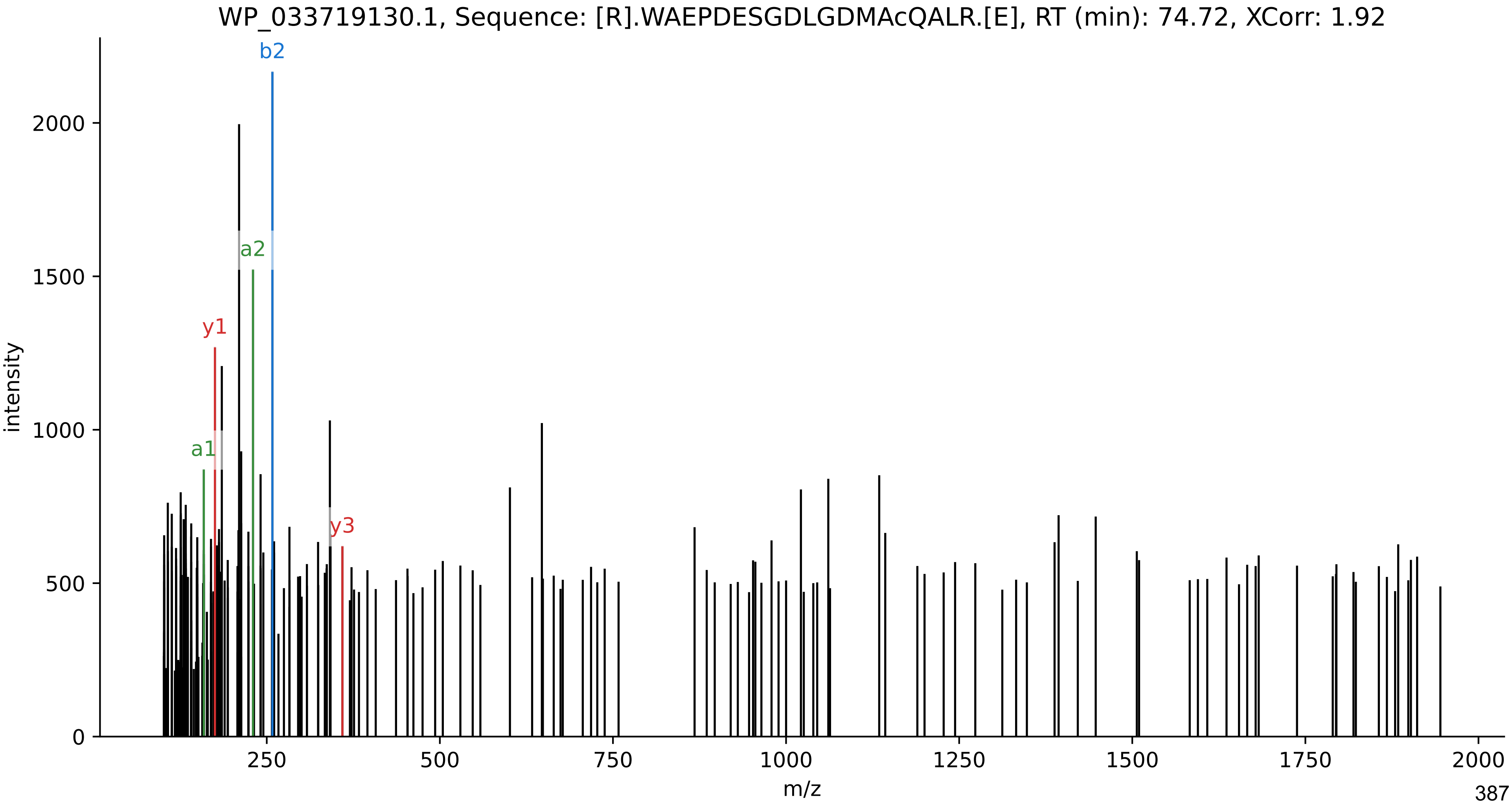
WP_033719130.1, Sequence: [R].WAEPDESGDLGDMAcQALR.[E], RT (min): 74.72, XCorr: 1.92
b2
a2
y1
a1
y3
intensity
m/z
0
500
1000
1500
2000
250
500
750
1000
1250
1500
1750
2000

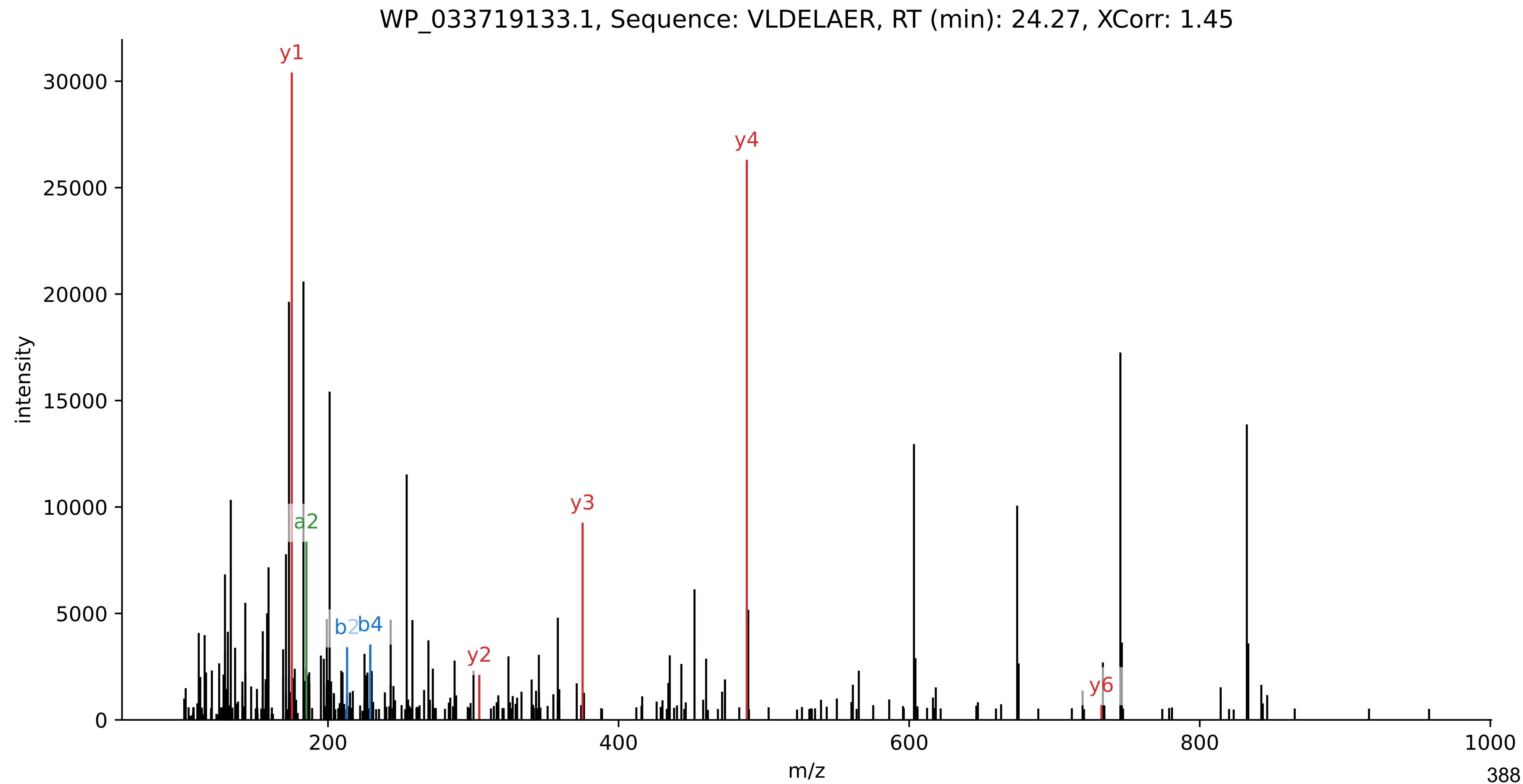

WP_033719133.1, Sequence: VLDELAER, RT (min): 24.27, XCorr: 1.45
intensity
m/z
y1
y4
a2
y3
b2
b4
y2
y6
0
5000
10000
15000
20000
25000
30000
200
400
600
800
1000

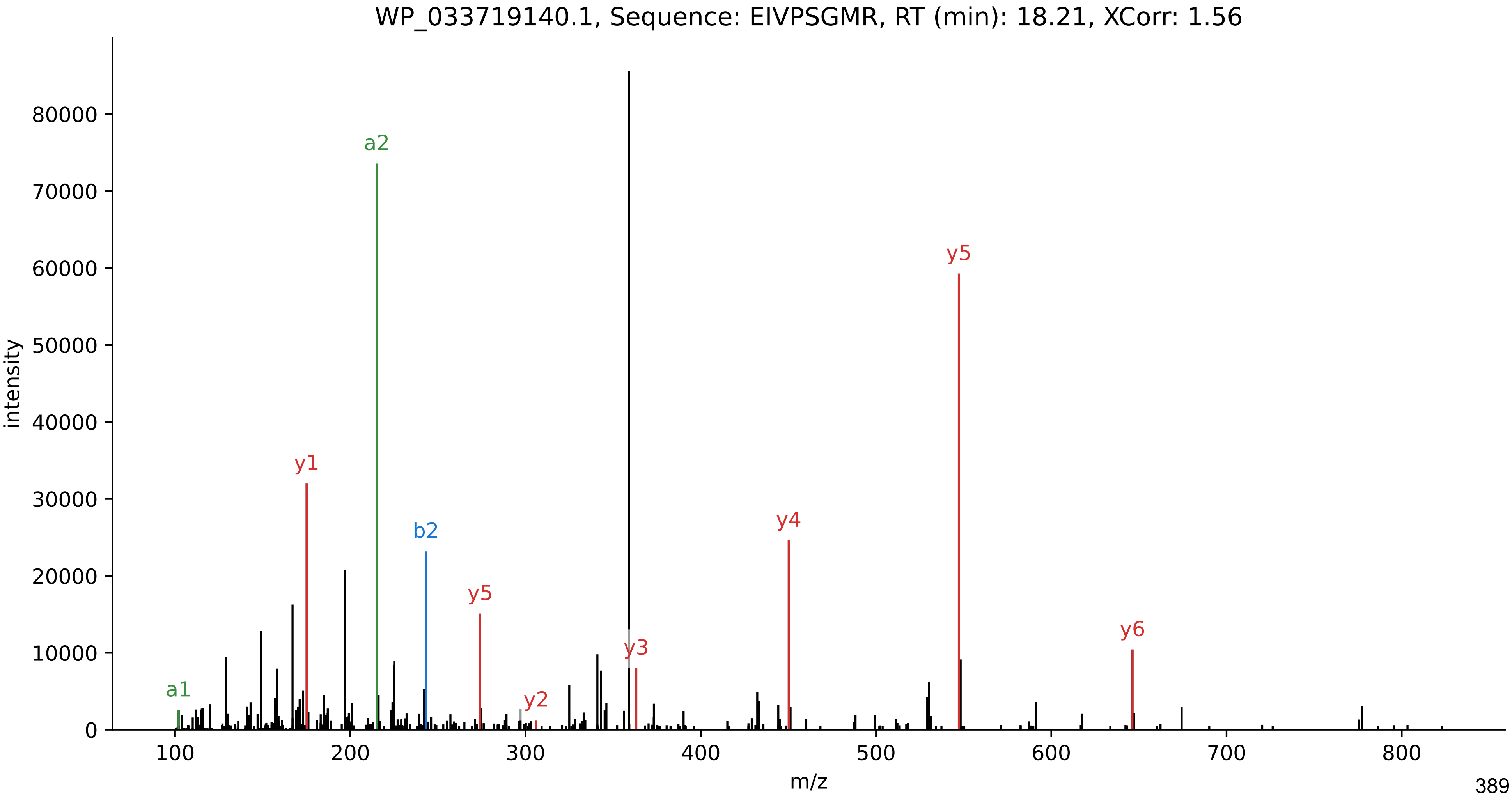

WP_033719140.1, Sequence: EIVPSGMR, RT (min): 18.21, XCorr: 1.56
intensity
m/z
a1
a2
b2
y1
y2
y3
y4
y5
y5
y6

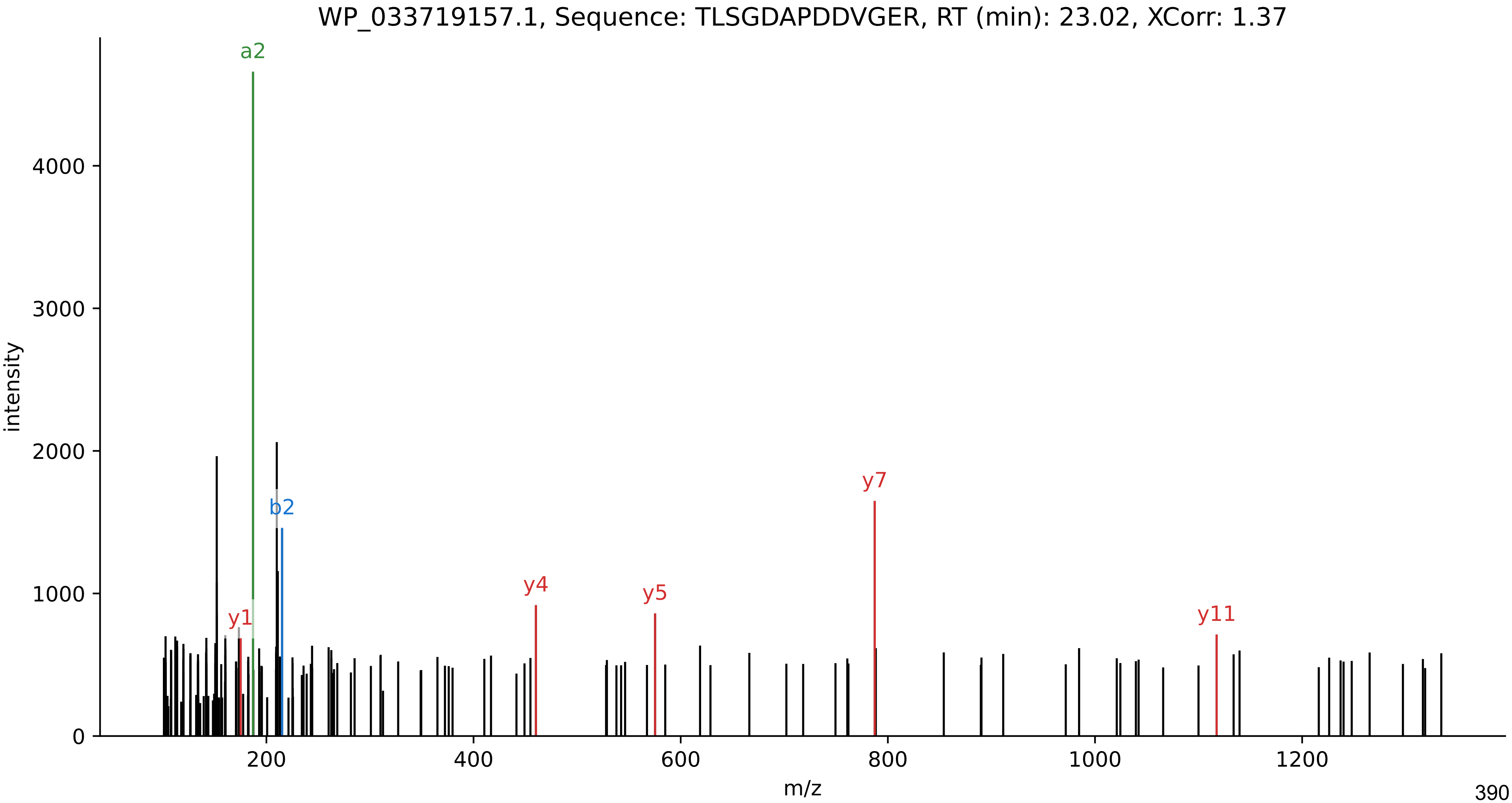

WP_033719157.1, Sequence: TLSGDAPDDVGER, RT (min): 23.02, XCorr: 1.37
a2
b2
y1
y4
y5
y7
y11
intensity
m/z
0
1000
2000
3000
4000
200
400
600
800
1000
1200

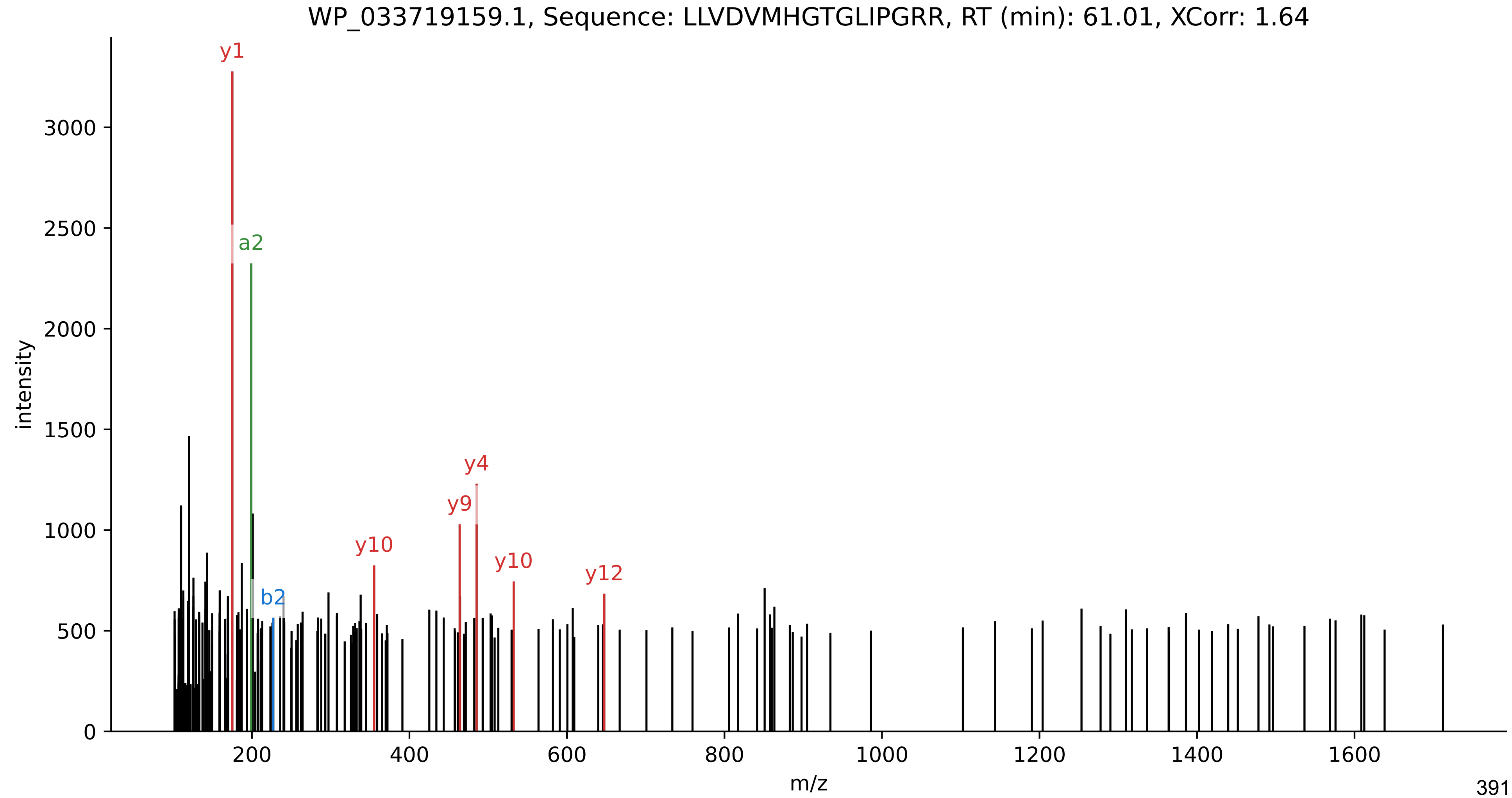
WP_033719159.1, Sequence: LLVDVMHGTGLIPGRR, RT (min): 61.01, XCorr: 1.64
intensity
m/z
y1
a2
b2
y10
y9
y4
y10
y12
0
500
1000
1500
2000
2500
3000
200
400
600
800
1000
1200
1400
1600

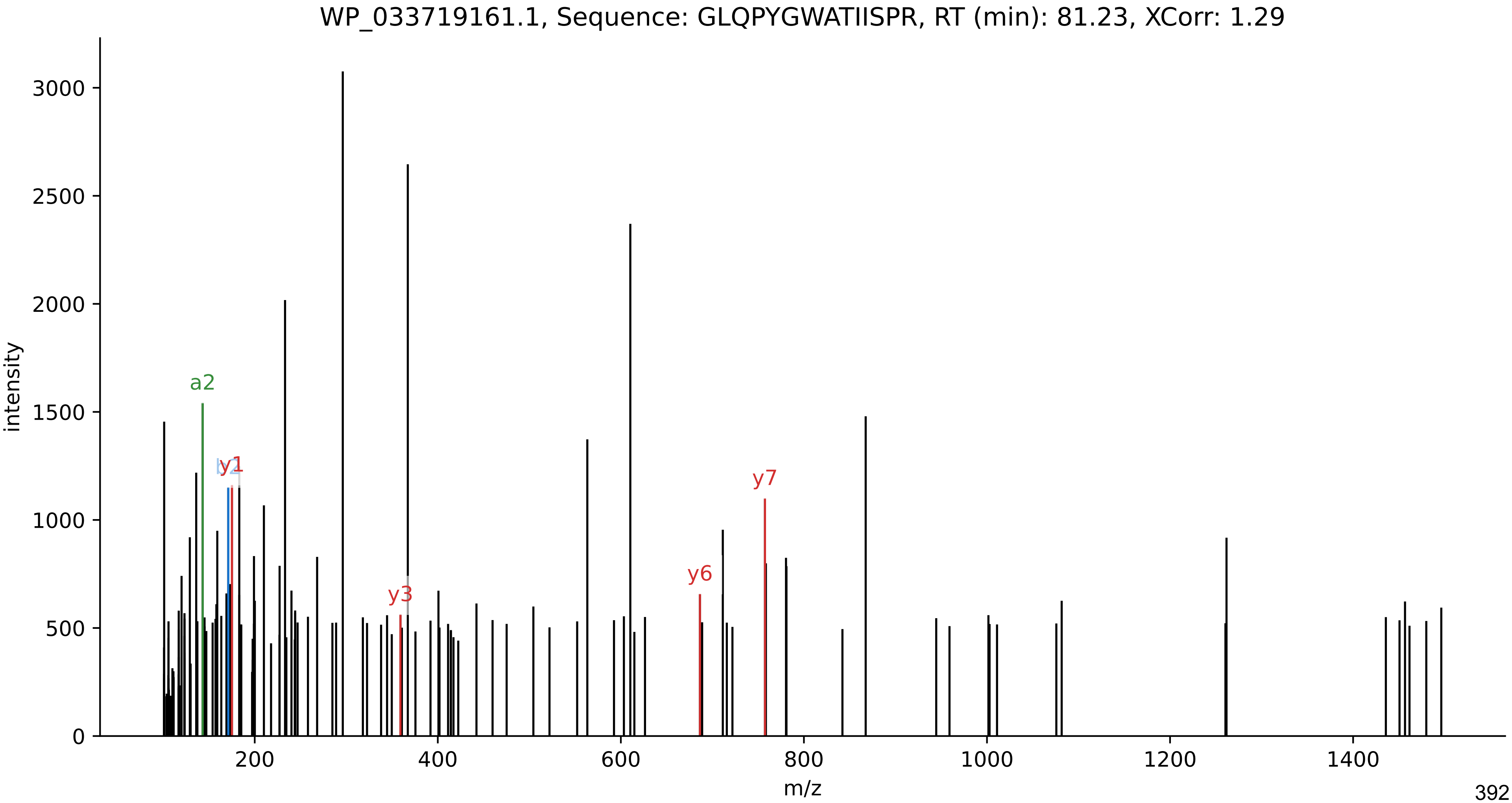

WP_033719161.1, Sequence: GLQPYGWATIISPR, RT (min): 81.23, XCorr: 1.29
intensity
m/z
a2
b2
y1
y3
y6
y7
0
500
1000
1500
2000
2500
3000
200
400
600
800
1000
1200
1400

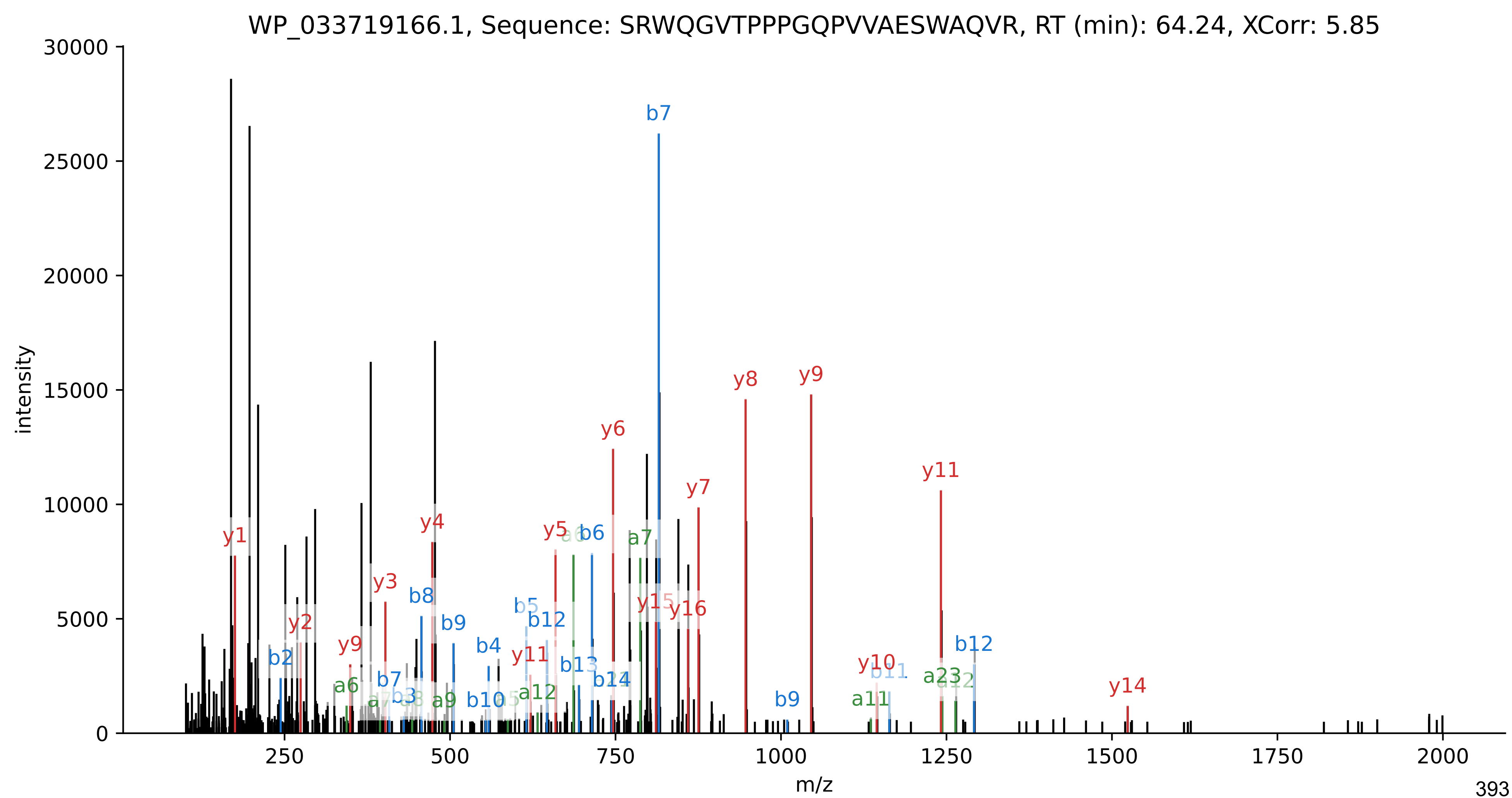
WP_033719166.1, Sequence: SRWQGVTPPPGQPVVAESWAQVR, RT (min): 64.24, XCorr: 5.85
intensity
m/z
0
5000
10000
15000
20000
25000
30000
250
500
750
1000
1250
1500
1750
2000
b7
y6
y8
y9
y11
y7
y1
y4
y5
b6
a7
y3
b8
y2
b9
b5
b12
y15
y16
y9
b4
y11
b13
b2
b14
a6
b7
b3
a9
b10
a12
y10
b11
a23
b12
b9
a11
y14

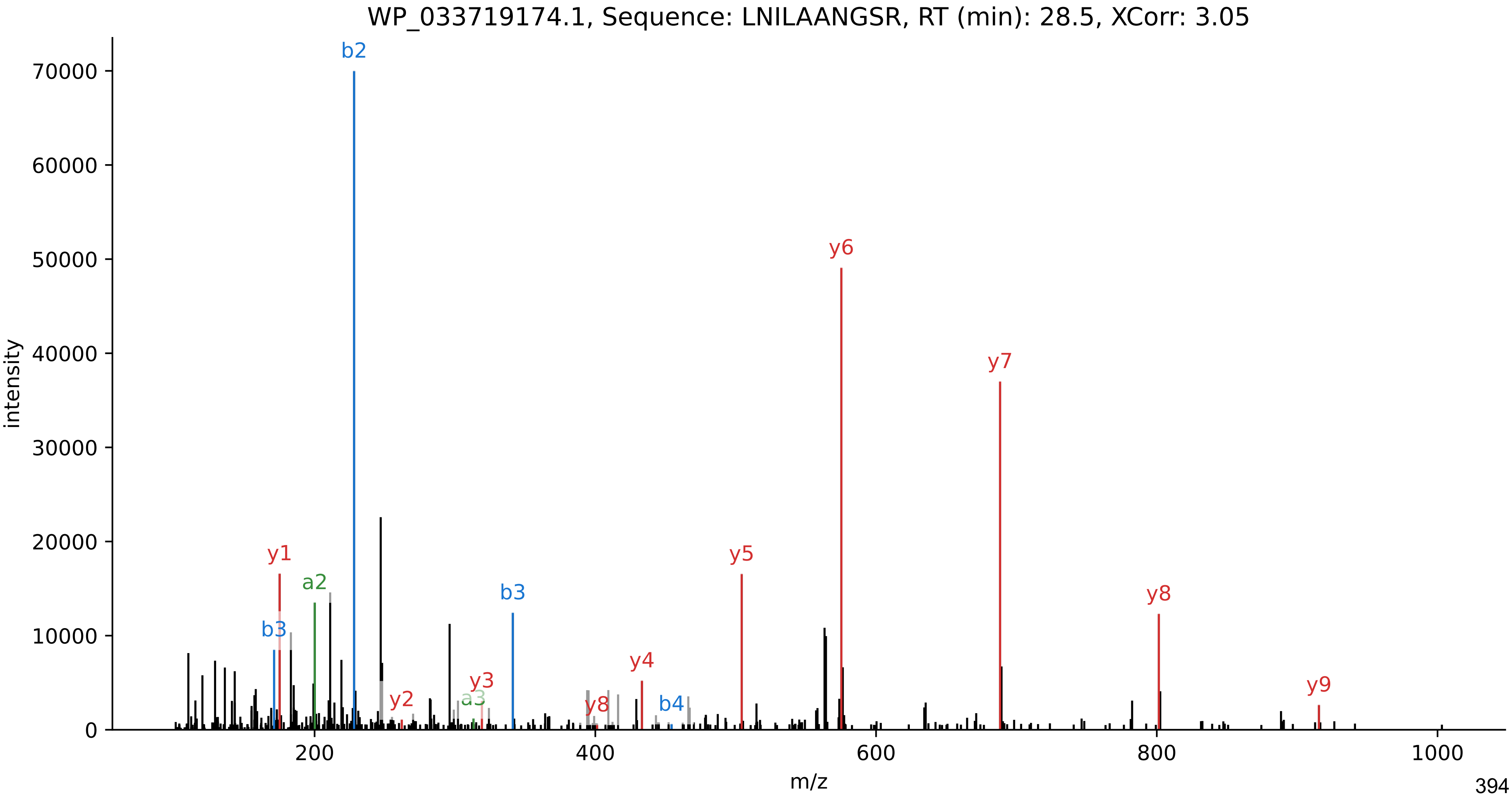
WP_033719174.1, Sequence: LNILAANGSR, RT (min): 28.5, XCorr: 3.05
intensity
m/z
b2
y6
y7
y1
a2
b3
b3
y8
y5
y4
y3
a3
y2
y8
b4
y9
70000
60000
50000
40000
30000
20000
10000
0
200
400
600
800
1000

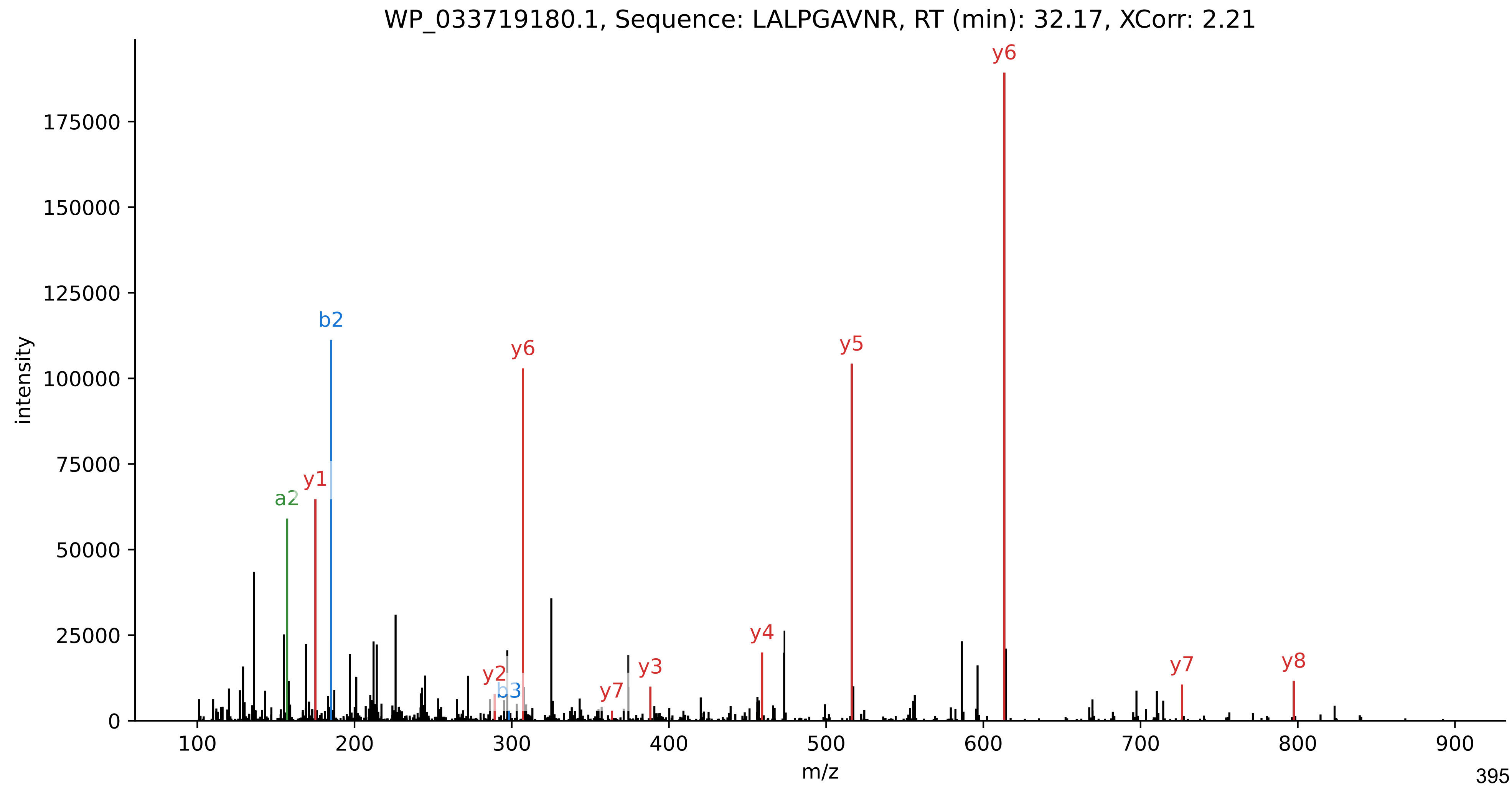
WP_033719180.1, Sequence: LALPGAVNR, RT (min): 32.17, XCorr: 2.21
intensity
m/z
y6
b2
y6
y5
y1
a2
y4
y2
b3
y7
y3
y7
y8
0
25000
50000
75000
100000
125000
150000
175000
100
200
300
400
500
600
700
800
900

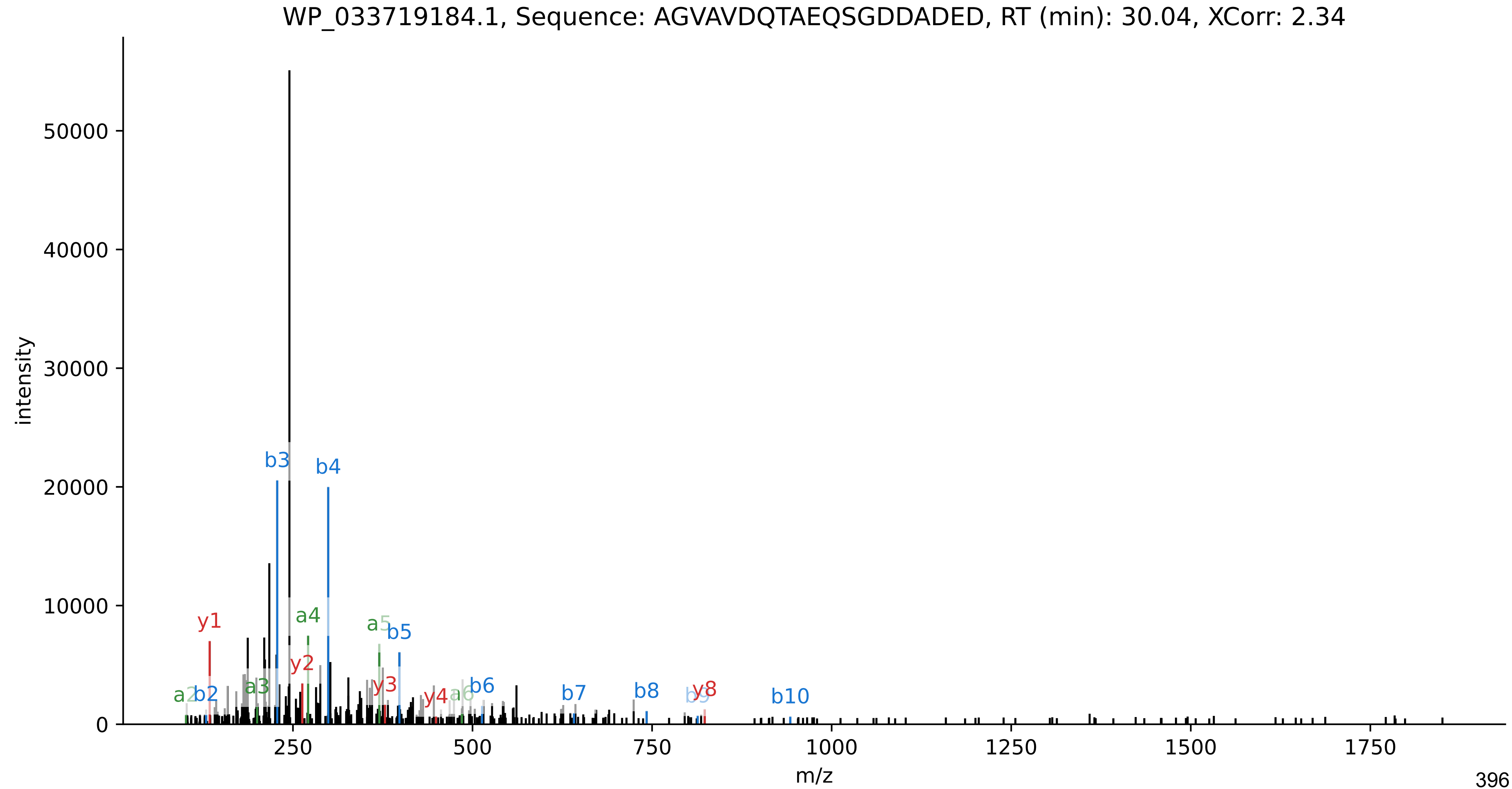

WP_033719184.1, Sequence: AGVAVDQTAEQSGDDADED, RT (min): 30.04, XCorr: 2.34
intensity
m/z
b3
b4
y1
a4
a5
b5
y2
y3
y4
a6
b6
a2
b2
a3
b7
b8
b9
y8
b10

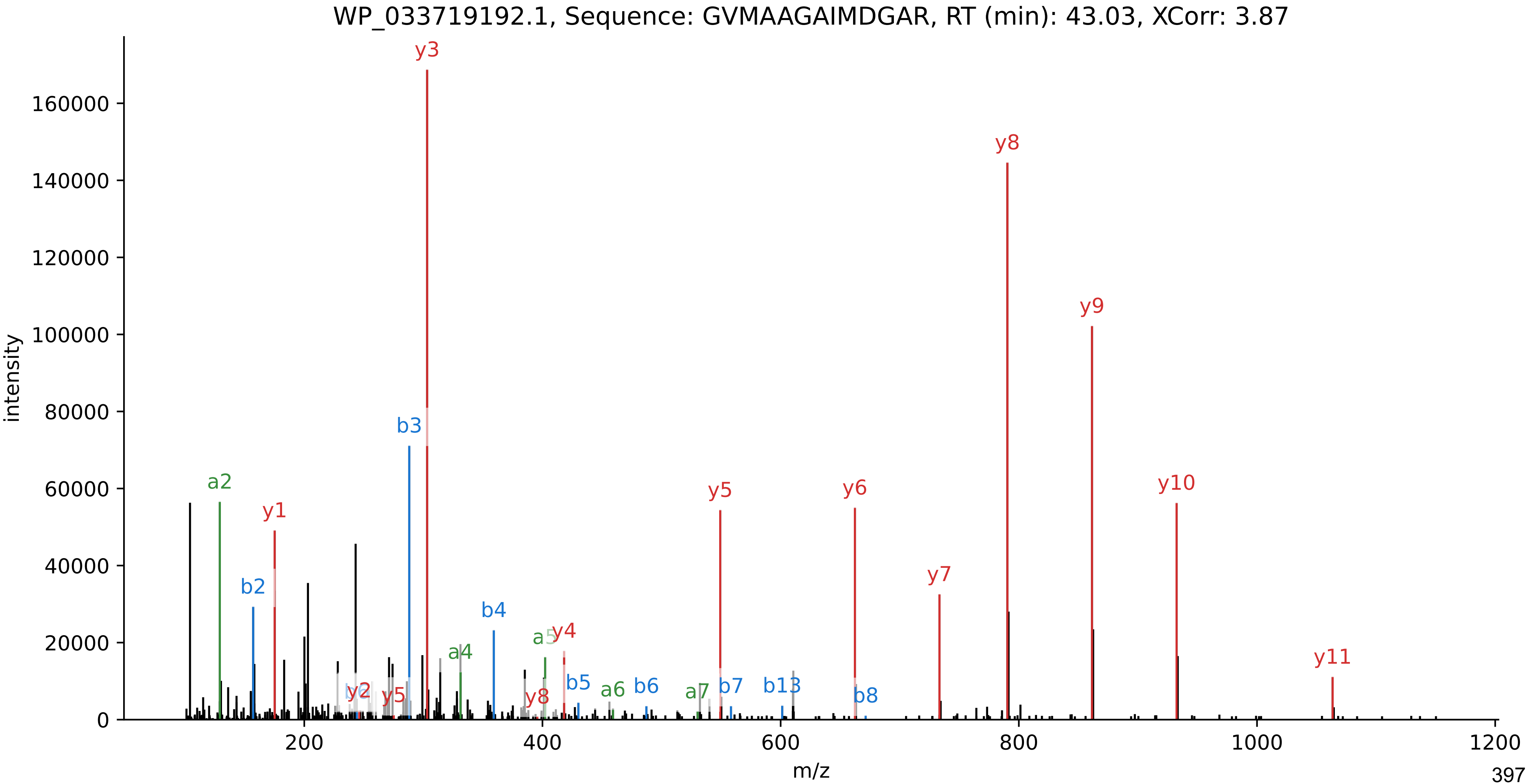
WP_033719192.1, Sequence: GVMAAGAIMDGAR, RT (min): 43.03, XCorr: 3.87
intensity
m/z
y3
y8
y9
b3
a2
y1
y5
y6
y10
y7
b2
b4
a4
a5
y4
b5
a6
b6
a7
b7
b13
b8
y2
y5
y8
y11
0
20000
40000
60000
80000
100000
120000
140000
160000
200
400
600
800
1000
1200

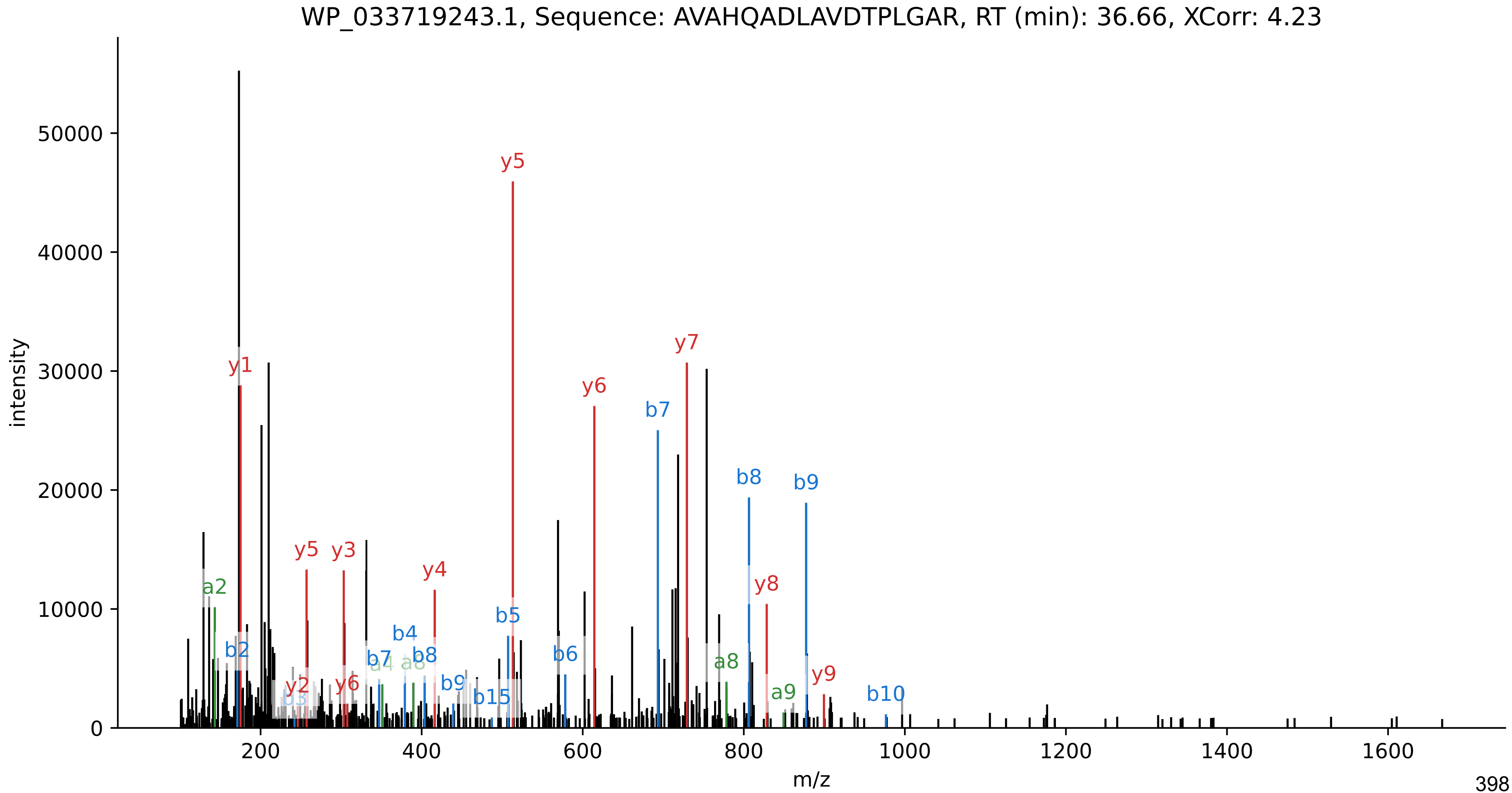
WP_033719243.1, Sequence: AVAHQADLAVDTPLGAR, RT (min): 36.66, XCorr: 4.23
intensity
m/z
0
10000
20000
30000
40000
50000
200
400
600
800
1000
1200
1400
1600
y5
y7
y6
b7
y1
b8
b9
y5
y3
y4
a2
y8
b5
b4
b2
b7
b8
b6
a8
y9
y2
y6
b9
a9
b10
b15

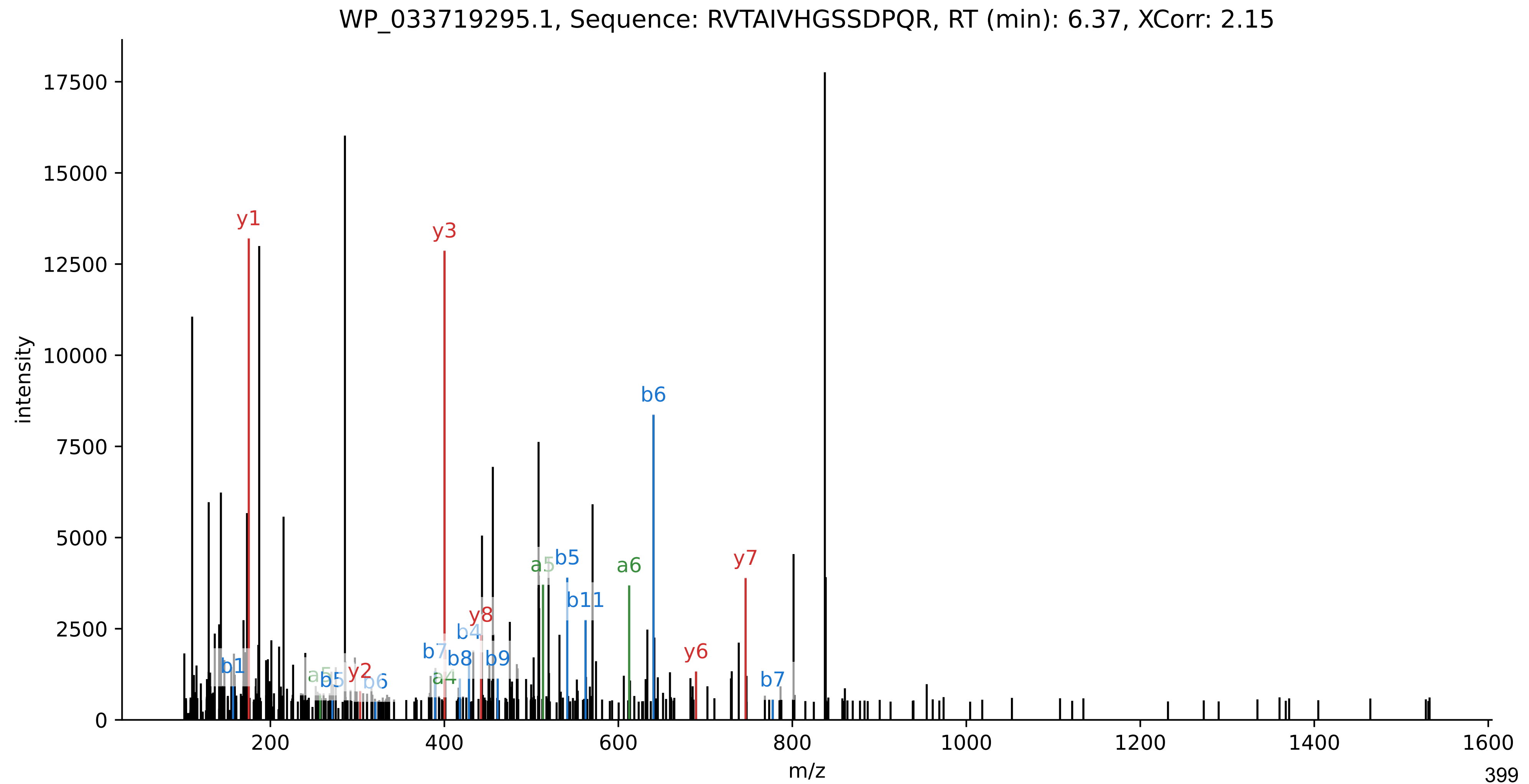
WP_033719295.1, Sequence: RVTAIVHGSSDPQR, RT (min): 6.37, XCorr: 2.15
intensity
m/z
y1
y3
b6
a5
b5
a6
y7
b11
y8
b4
b7
b8
b9
y6
b1
y2
b5
b6
a4
b7

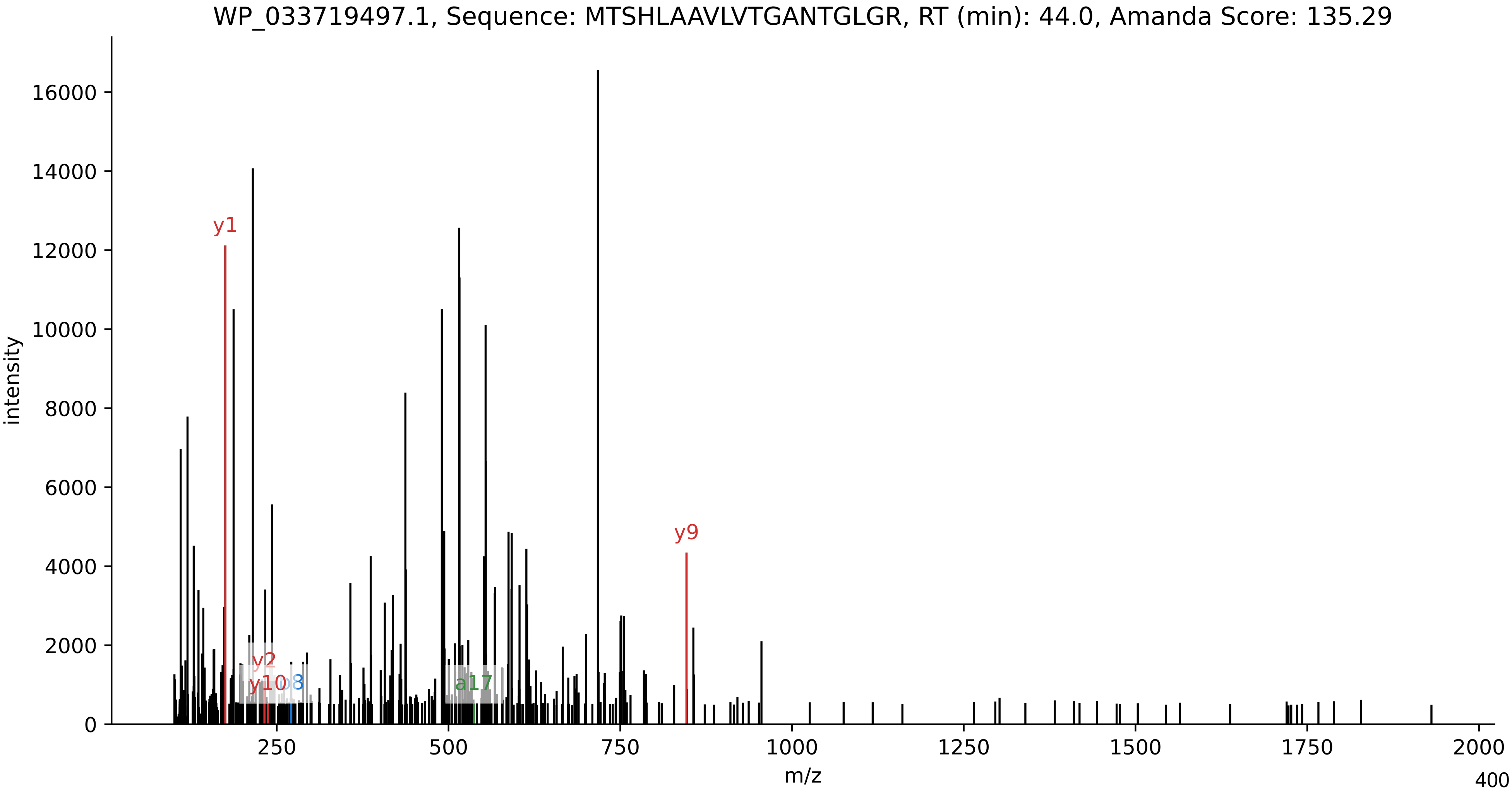

WP_033719497.1, Sequence: MTSHLAAVLVTGANTGLGR, RT (min): 44.0, Amanda Score: 135.29
intensity
m/z
y1
y9
y2
y10
a17

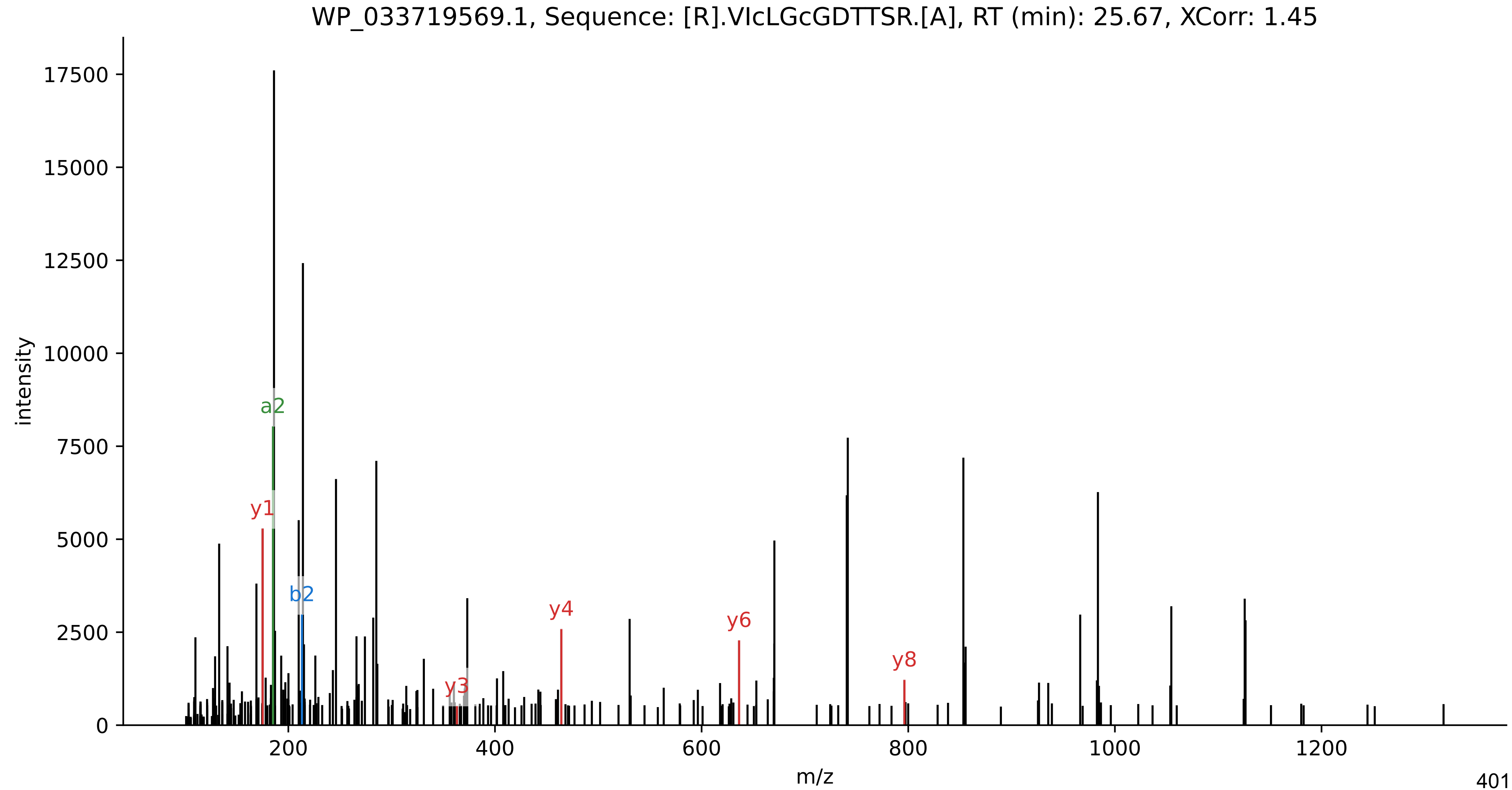
WP_033719569.1, Sequence: [R].VIcLGcGDTTSR.[A], RT (min): 25.67, XCorr: 1.45
intensity
m/z
a2
y1
b2
y3
y4
y6
y8
0
2500
5000
7500
10000
12500
15000
17500
200
400
600
800
1000
1200

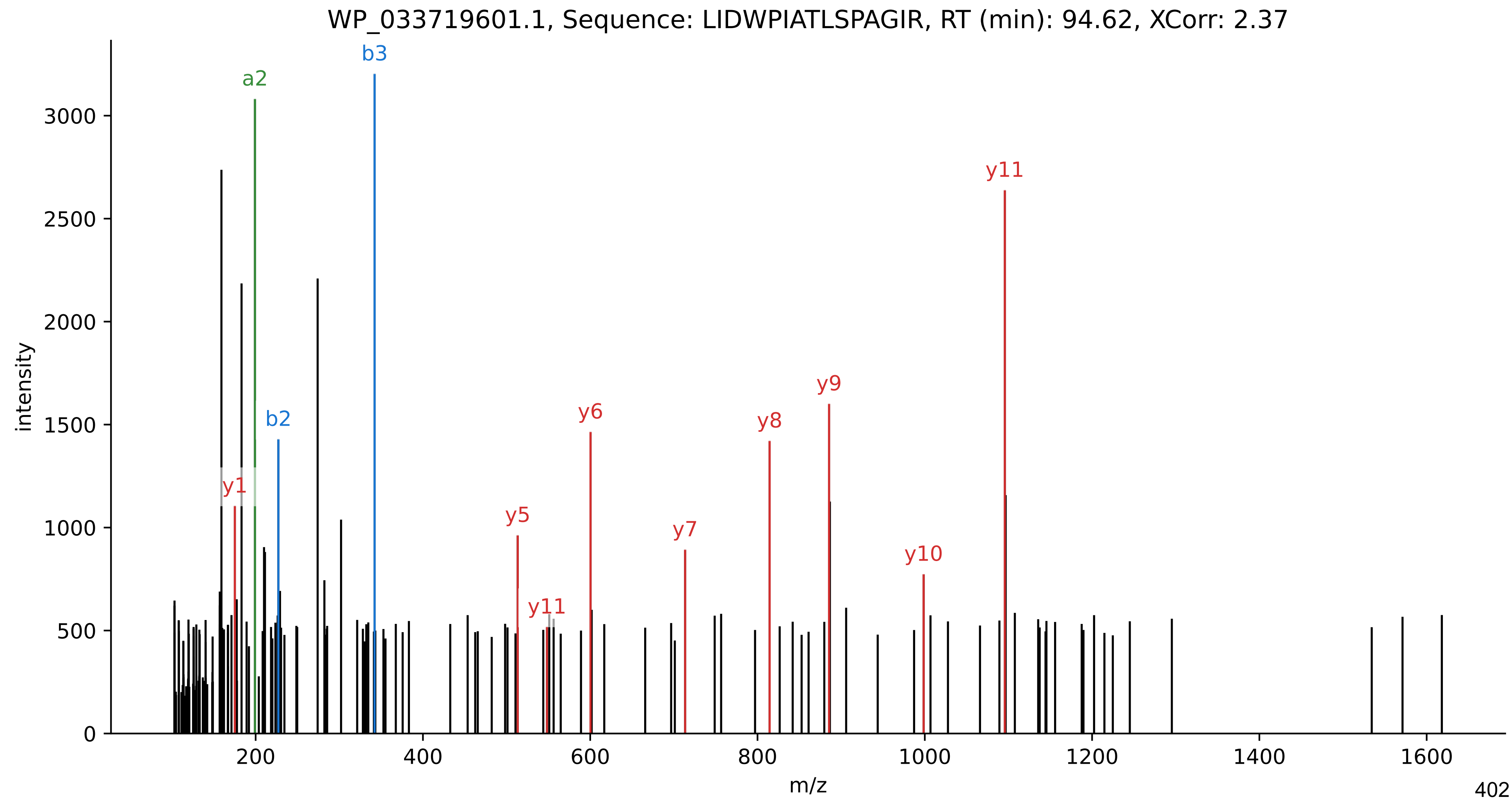
WP_033719601.1, Sequence: LIDWPIATLSPAGIR, RT (min): 94.62, XCorr: 2.37
intensity
m/z
a2
b3
b2
y1
y5
y11
y6
y7
y8
y9
y10
y11
0
500
1000
1500
2000
2500
3000
200
400
600
800
1000
1200
1400
1600

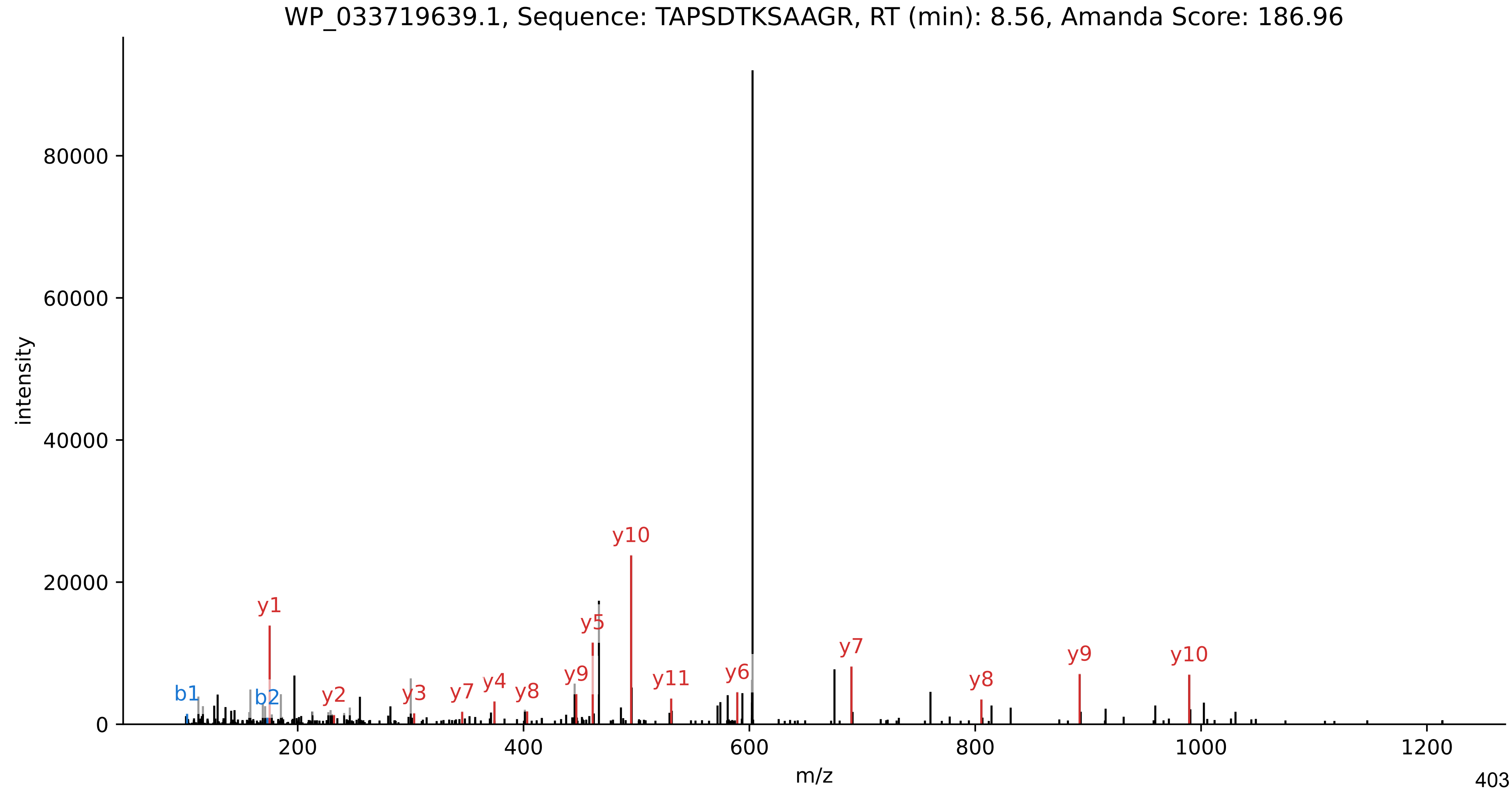
WP_033719639.1, Sequence: TAPSDTKSAAGR, RT (min): 8.56, Amanda Score: 186.96
intensity
m/z
80000
60000
40000
20000
0
200
400
600
800
1000
1200
b1
b2
y1
y2
y3
y7
y4
y8
y9
y5
y10
y11
y6
y7
y8
y9
y10

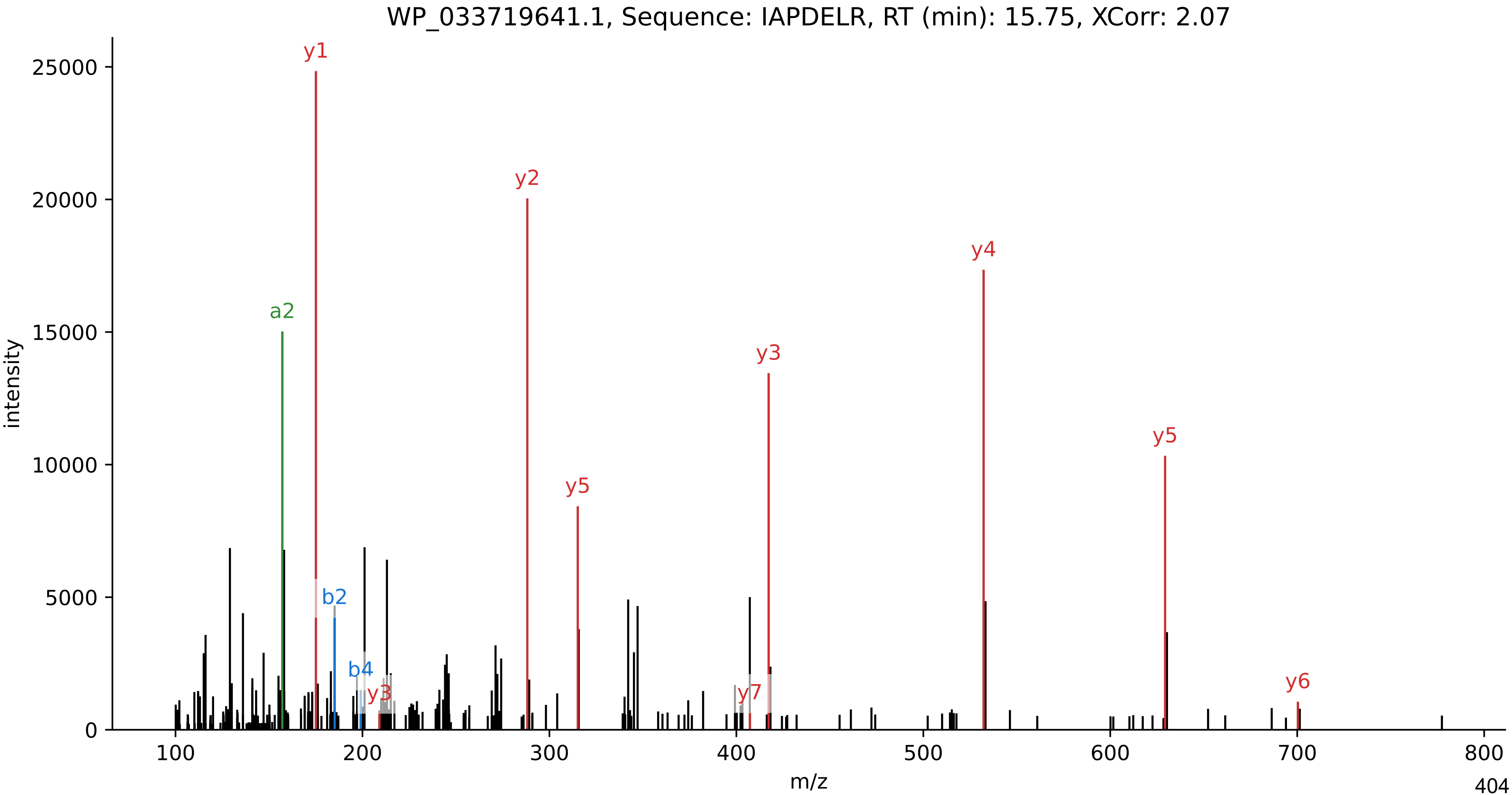

WP_033719641.1, Sequence: IAPDELR, RT (min): 15.75, XCorr: 2.07
y1
y2
a2
y4
y3
y5
y5
b2
b4
y3
y7
y6
intensity
m/z
0
5000
10000
15000
20000
25000
100
200
300
400
500
600
700
800

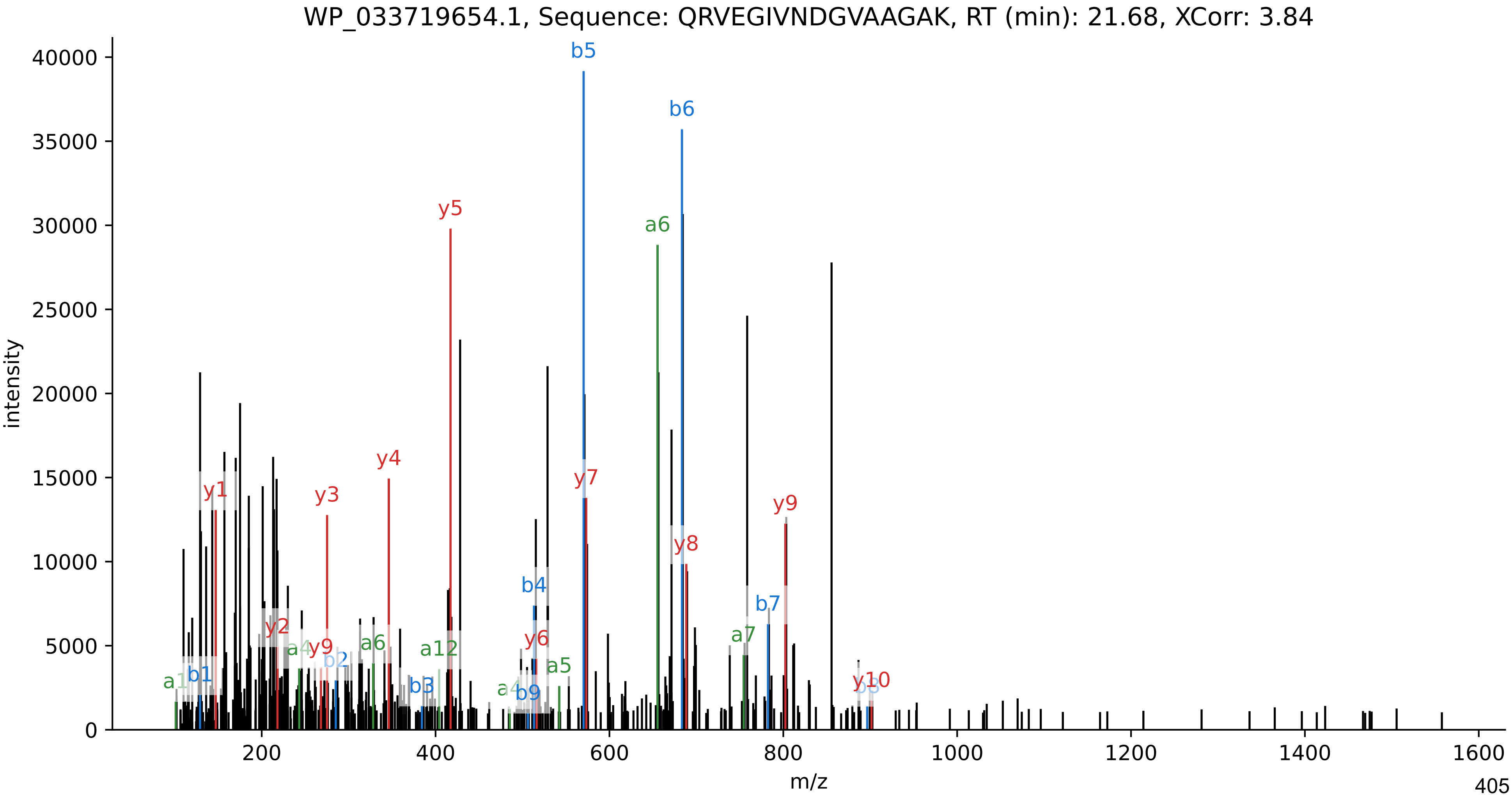
WP_033719654.1, Sequence: QRVEGIVNDGVAAGAK, RT (min): 21.68, XCorr: 3.84
intensity
m/z
0
5000
10000
15000
20000
25000
30000
35000
40000
200
400
600
800
1000
1200
1400
1600
b5
b6
y5
a6
y4
y7
y1
y3
y9
y8
b4
b7
y2
a4
y9
a6
a12
y6
a7
a5
b2
b1
a1
b3
a4
b9
y10

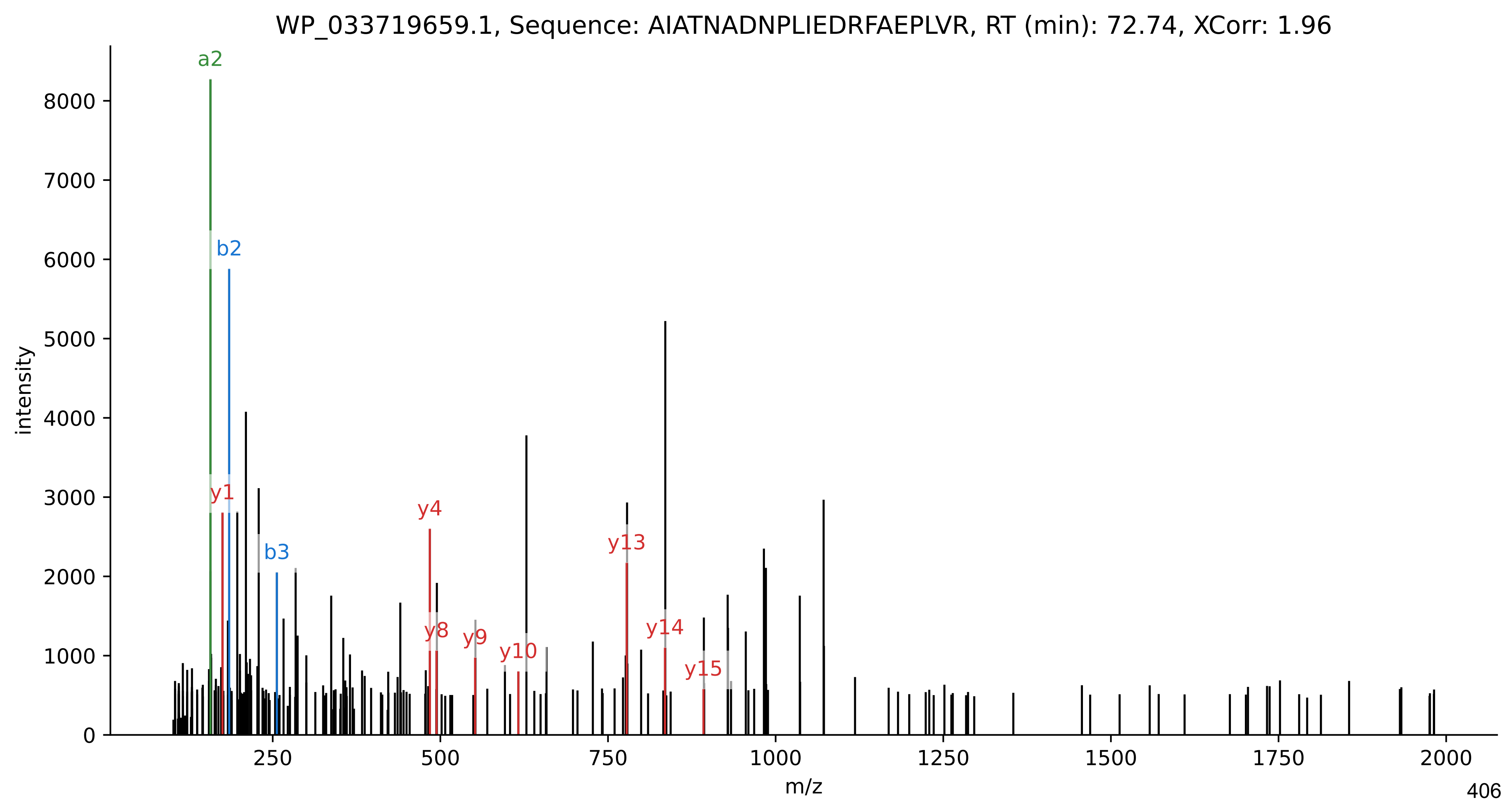

WP_033719659.1, Sequence: AIATNADNPLIEDRFAEPLVR, RT (min): 72.74, XCorr: 1.96
intensity
m/z
a2
b2
b3
y1
y4
y8
y9
y10
y13
y14
y15

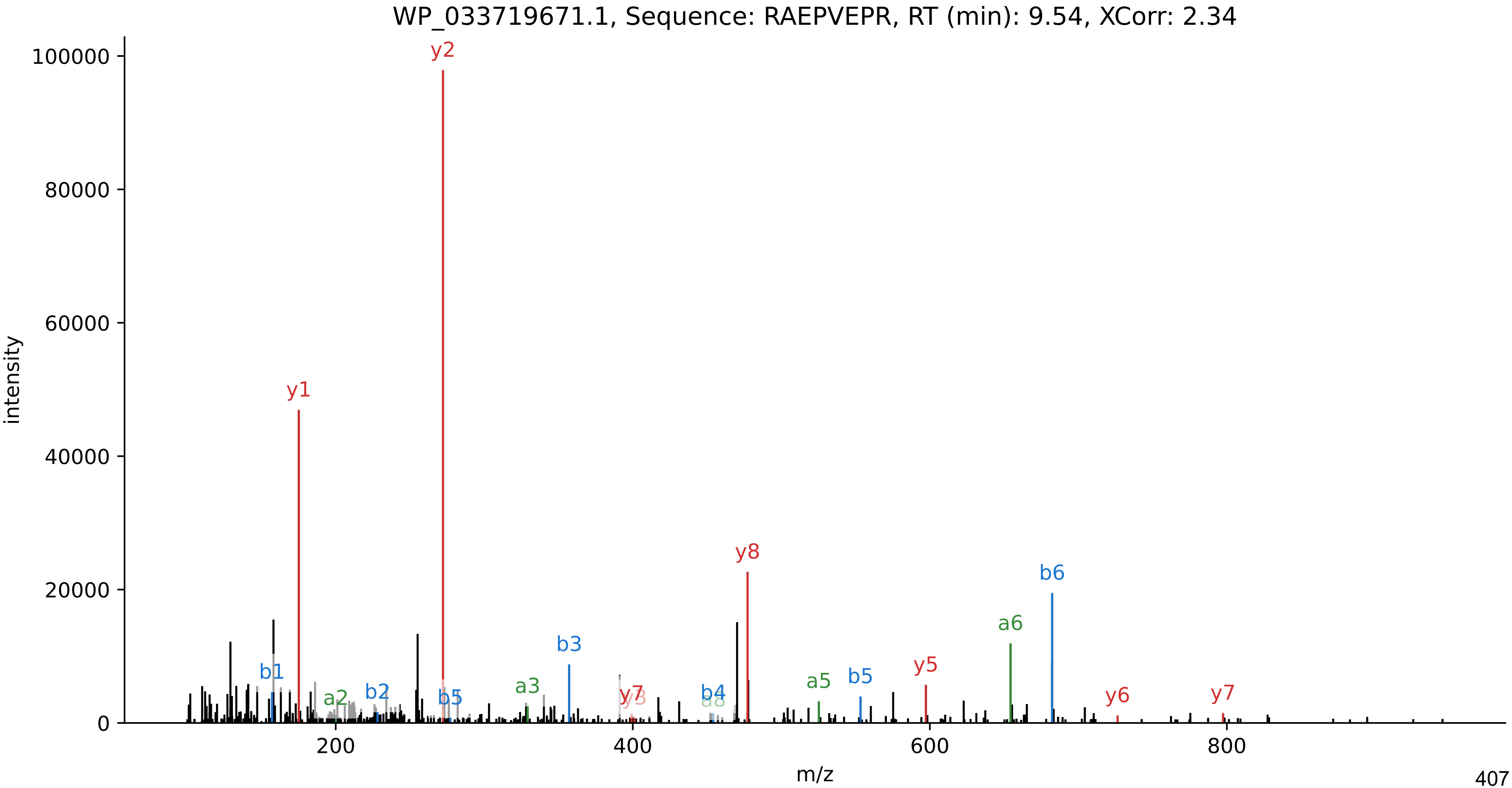

WP_033719671.1, Sequence: RAEPVEPR, RT (min): 9.54, XCorr: 2.34
intensity
m/z
100000
80000
60000
40000
20000
0
200
400
600
800
y2
y1
y8
b6
a6
b3
b1
a2
b2
b5
a3
y7
b4
a5
b5
y5
y6
y7

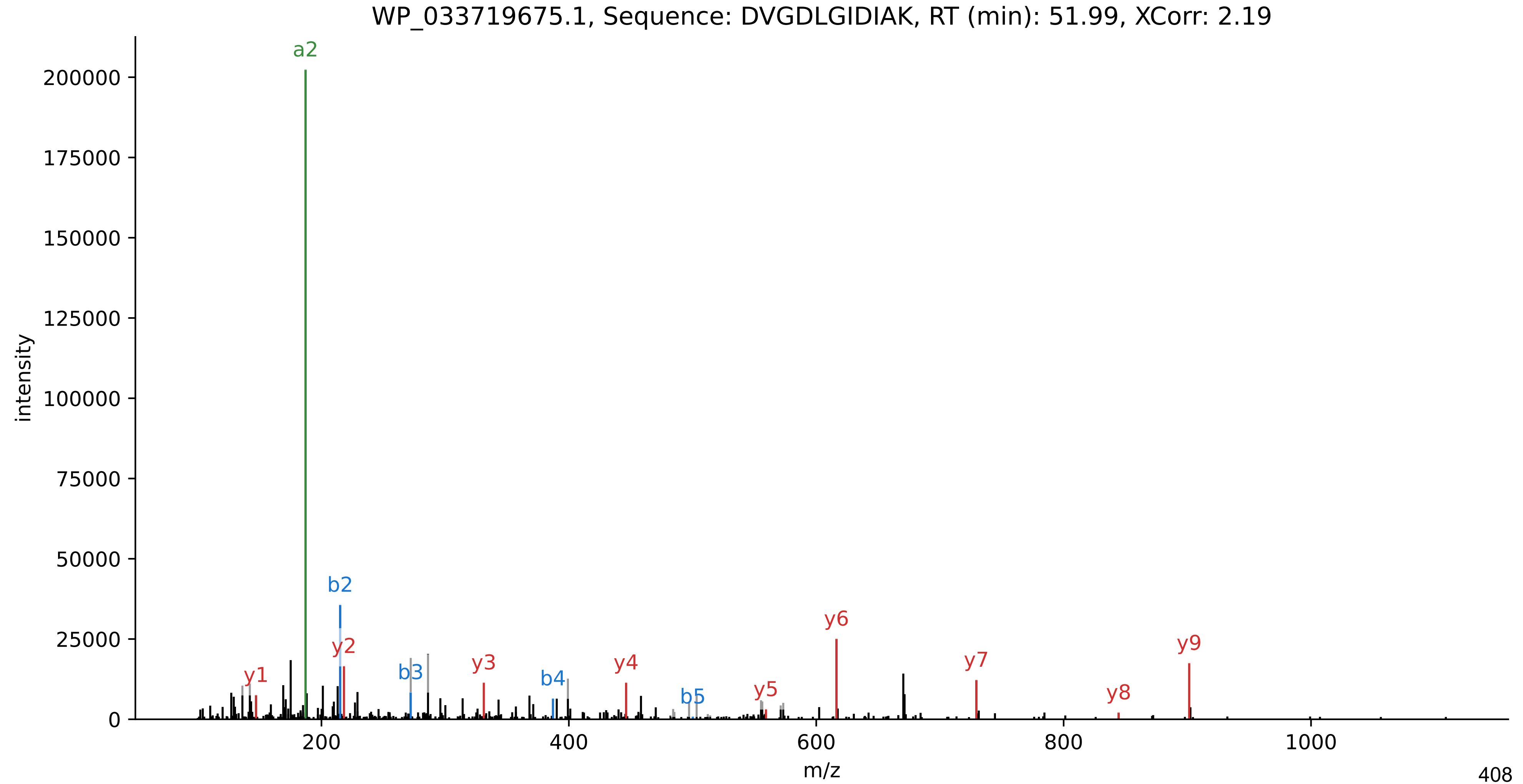

WP_033719675.1, Sequence: DVGDLGIDIAK, RT (min): 51.99, XCorr: 2.19
a2
b2
y2
b3
y1
y3
b4
y4
b5
y5
y6
y7
y8
y9
intensity
m/z
0
25000
50000
75000
100000
125000
150000
175000
200000
200
400
600
800
1000

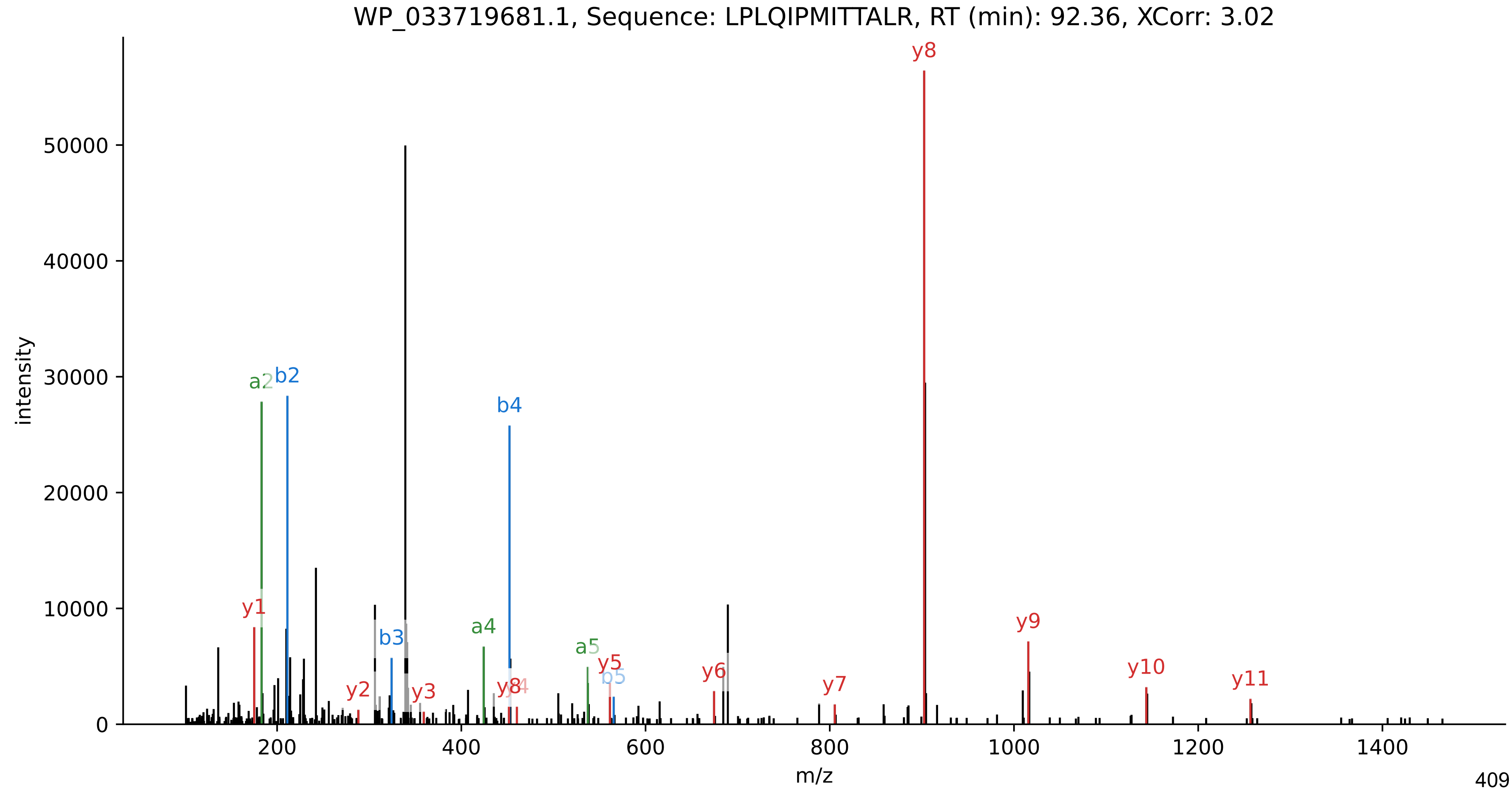
WP_033719681.1, Sequence: LPLQIPMITTALR, RT (min): 92.36, XCorr: 3.02
intensity
m/z
y8
a2
b2
b4
y1
b3
a4
a5
y5
b5
y6
y9
y10
y11
y7
y2
y3
y8
y4

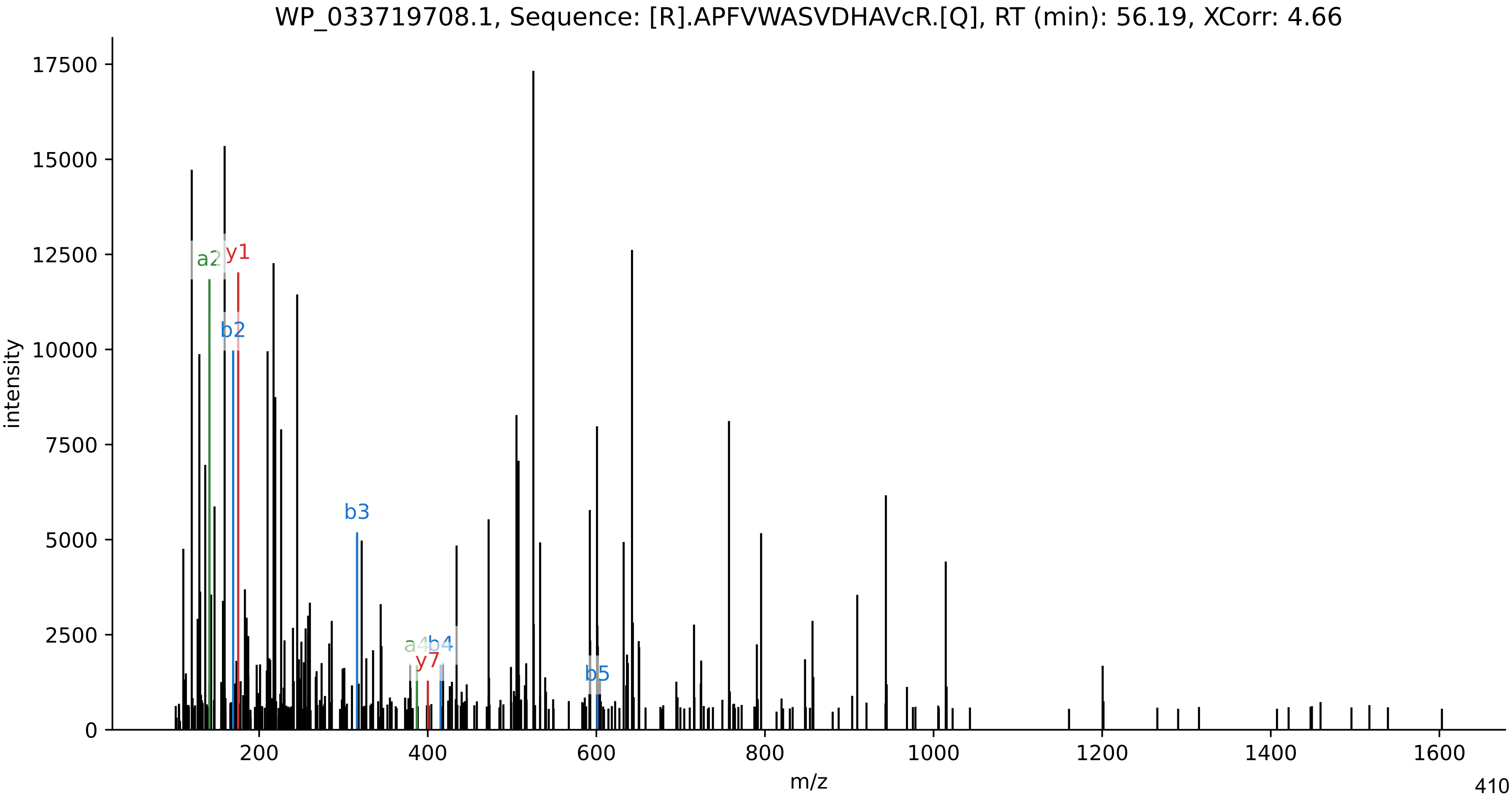

WP_033719708.1, Sequence: [R].APFVWASVDHAVcR.[Q], RT (min): 56.19, XCorr: 4.66
intensity
m/z
a2
y1
b2
b3
a4
b4
y7
b5

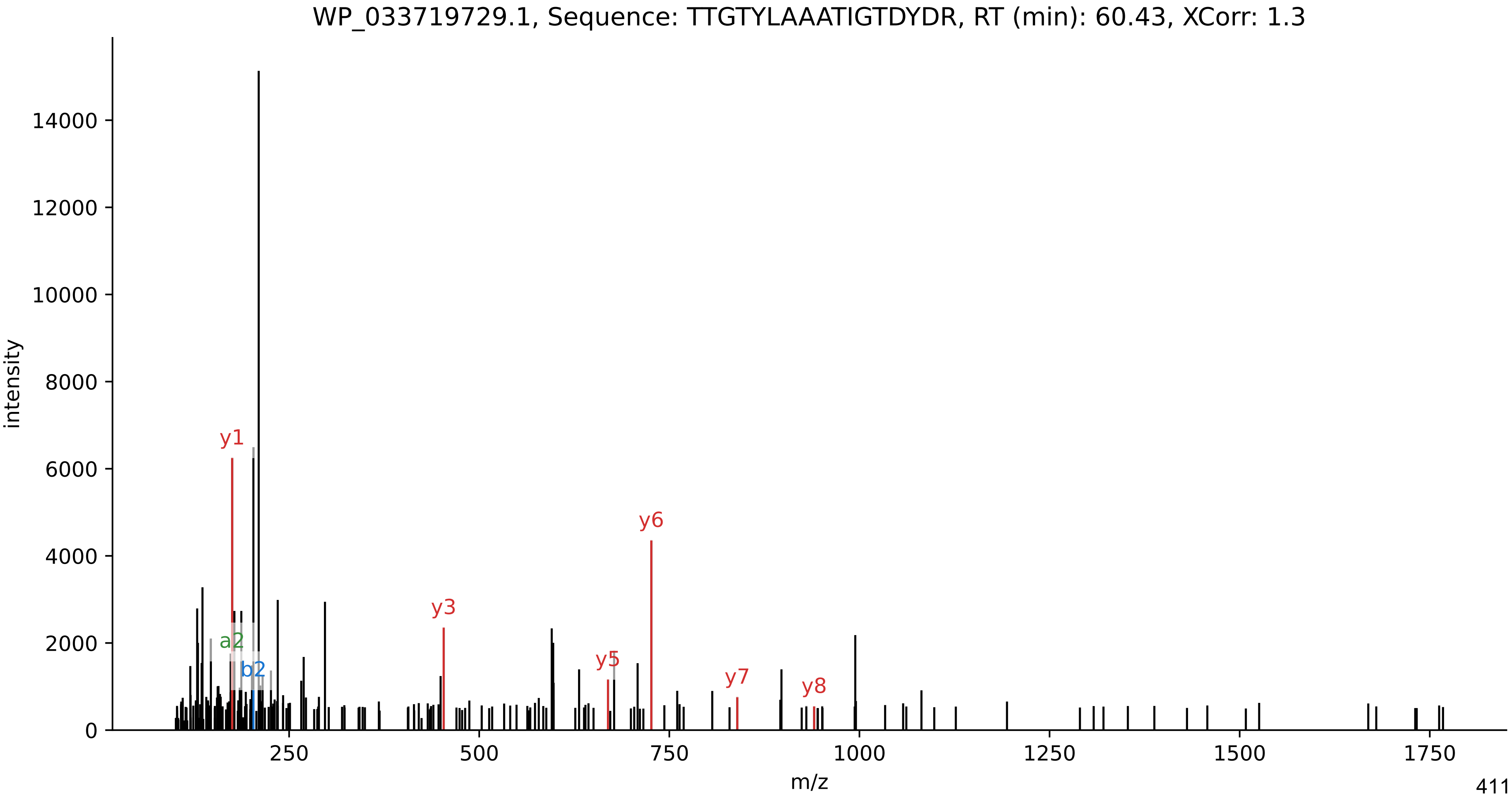
WP_033719729.1, Sequence: TTGTYLAAATIGTDYDR, RT (min): 60.43, XCorr: 1.3
intensity
m/z
y1
a2
b2
y3
y5
y6
y7
y8
0
2000
4000
6000
8000
10000
12000
14000
250
500
750
1000
1250
1500
1750

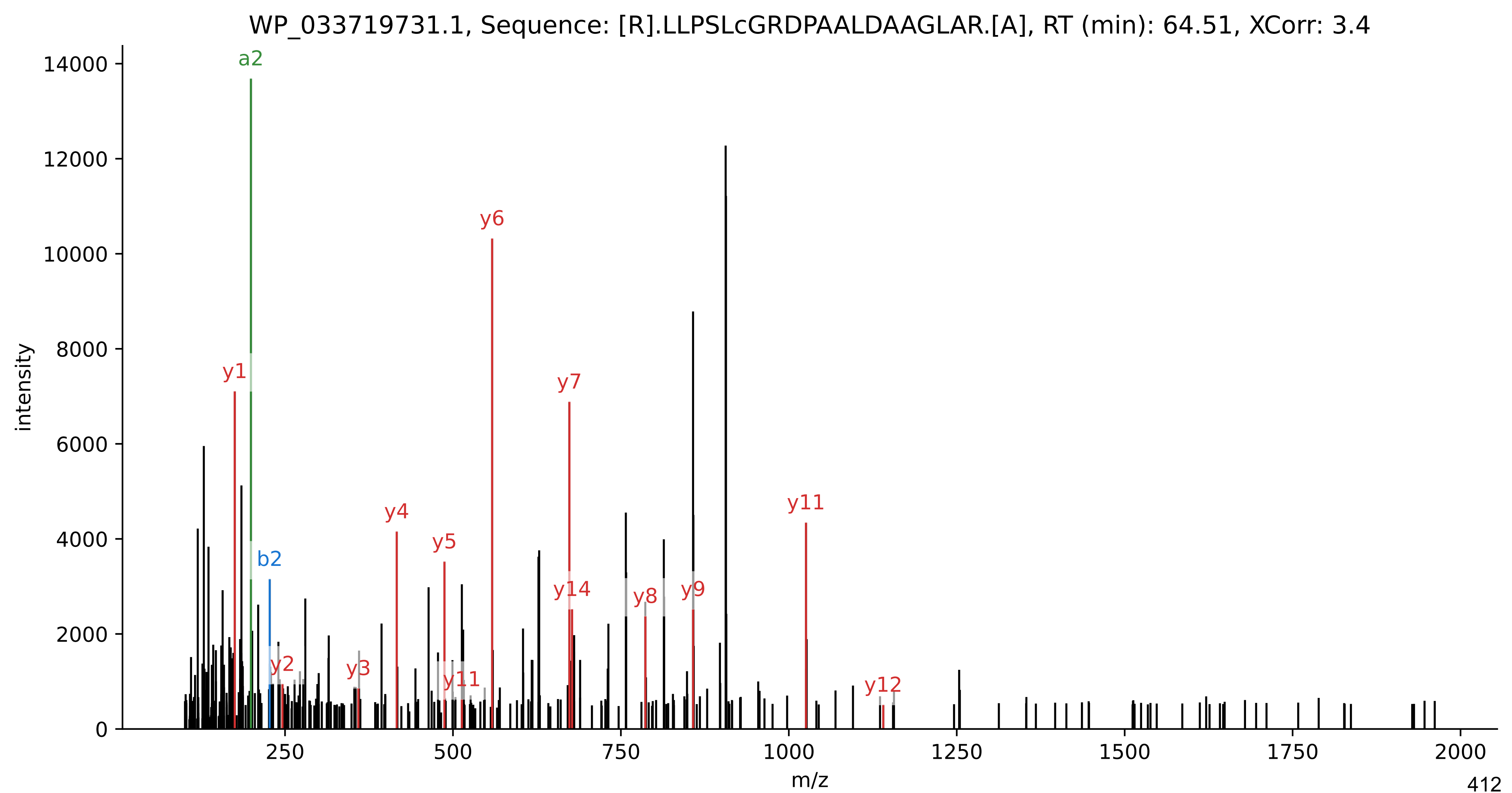

WP_033719731.1, Sequence: [R].LLPSLcGRDPAALDAAGLAR.[A], RT (min): 64.51, XCorr: 3.4
intensity
m/z
a2
b2
y1
y2
y3
y4
y5
y6
y7
y8
y9
y11
y11
y12
y14

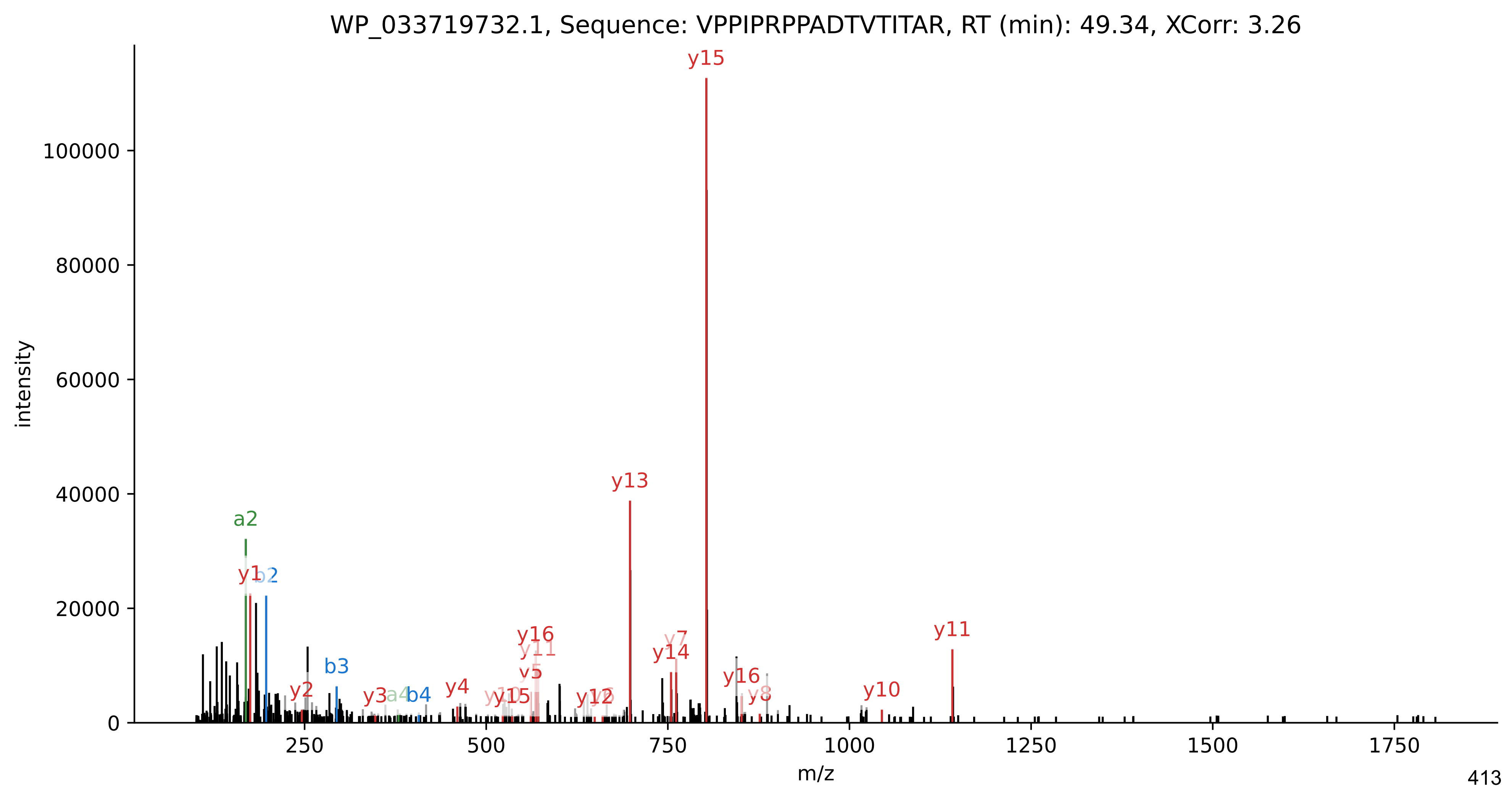
WP_033719732.1, Sequence: VPPIPRPPADTVTITAR, RT (min): 49.34, XCorr: 3.26
intensity
m/z
y15
y13
a2
y1
b2
b3
y2
y3
a4
b4
y4
y16
y11
y5
y15
y12
y7
y14
y16
y8
y10
y11
0
20000
40000
60000
80000
100000
250
500
750
1000
1250
1500
1750

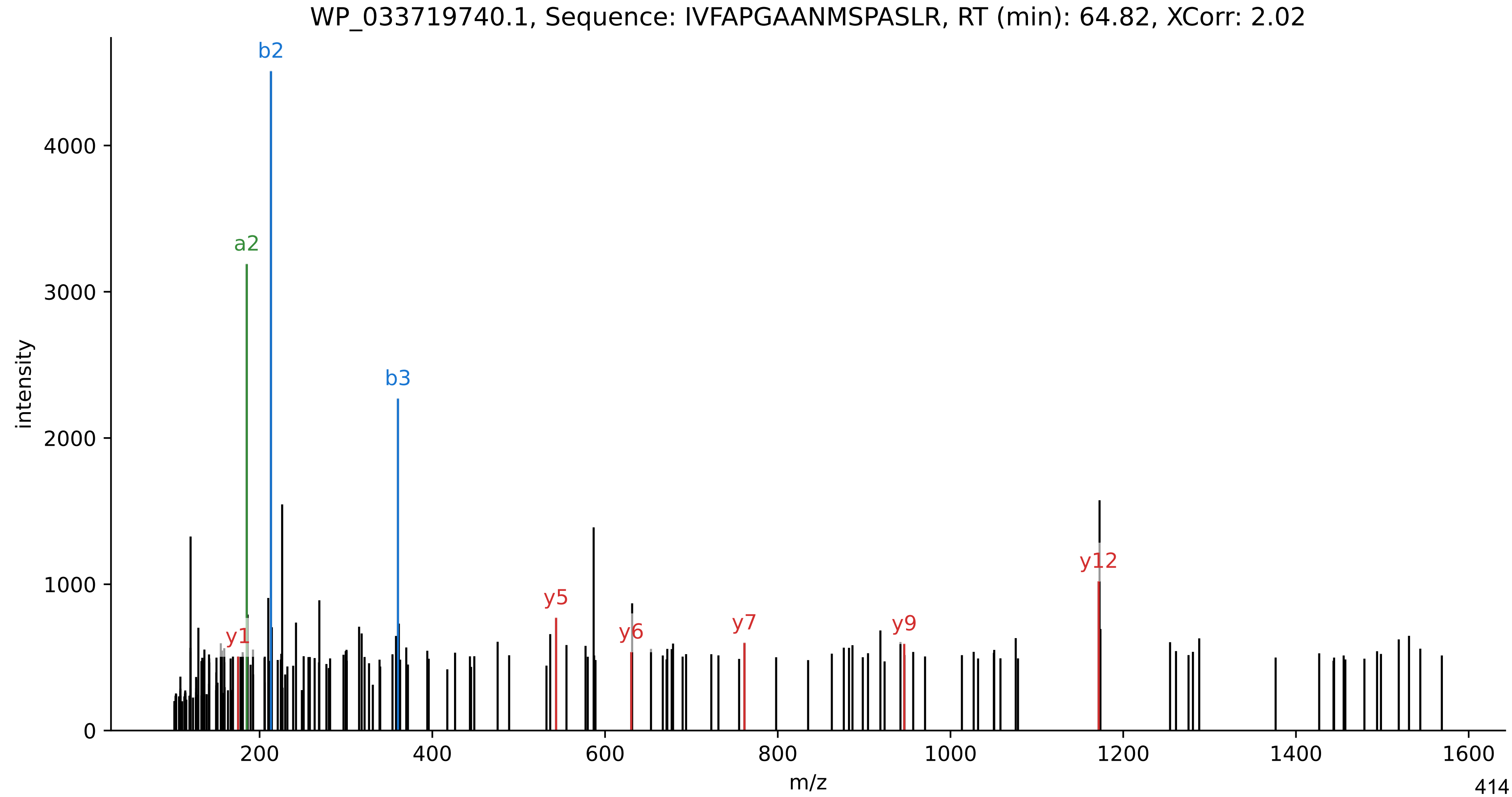

WP_033719740.1, Sequence: IVFAPGAANMSPASLR, RT (min): 64.82, XCorr: 2.02
intensity
m/z
b2
a2
b3
y1
y5
y6
y7
y9
y12
0
1000
2000
3000
4000
200
400
600
800
1000
1200
1400
1600

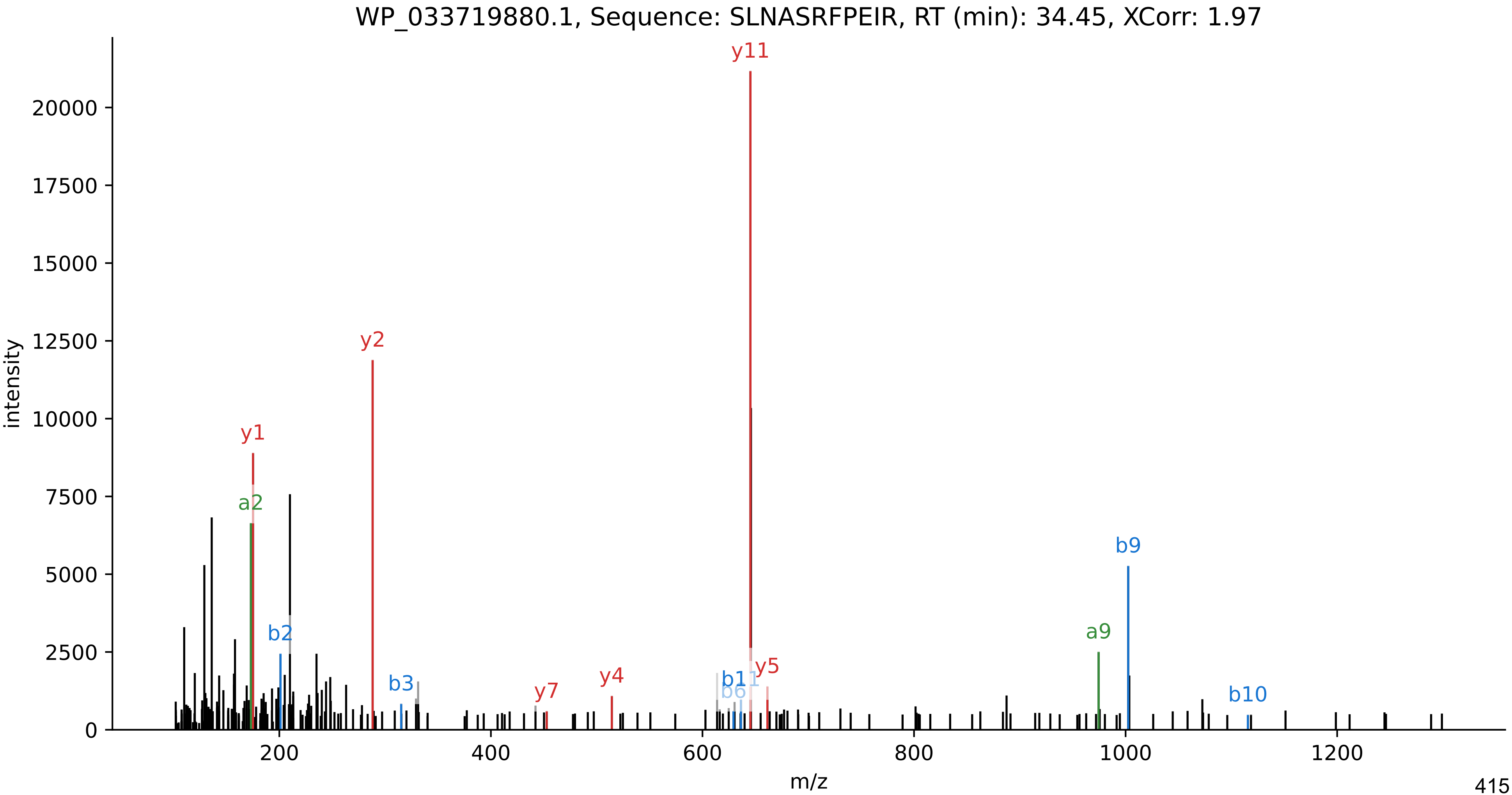
WP_033719880.1, Sequence: SLNASRFPEIR, RT (min): 34.45, XCorr: 1.97
intensity
m/z
y11
y2
y1
a2
b2
b3
y7
y4
b11
b6
y5
a9
b9
b10
0
2500
5000
7500
10000
12500
15000
17500
20000
200
400
600
800
1000
1200

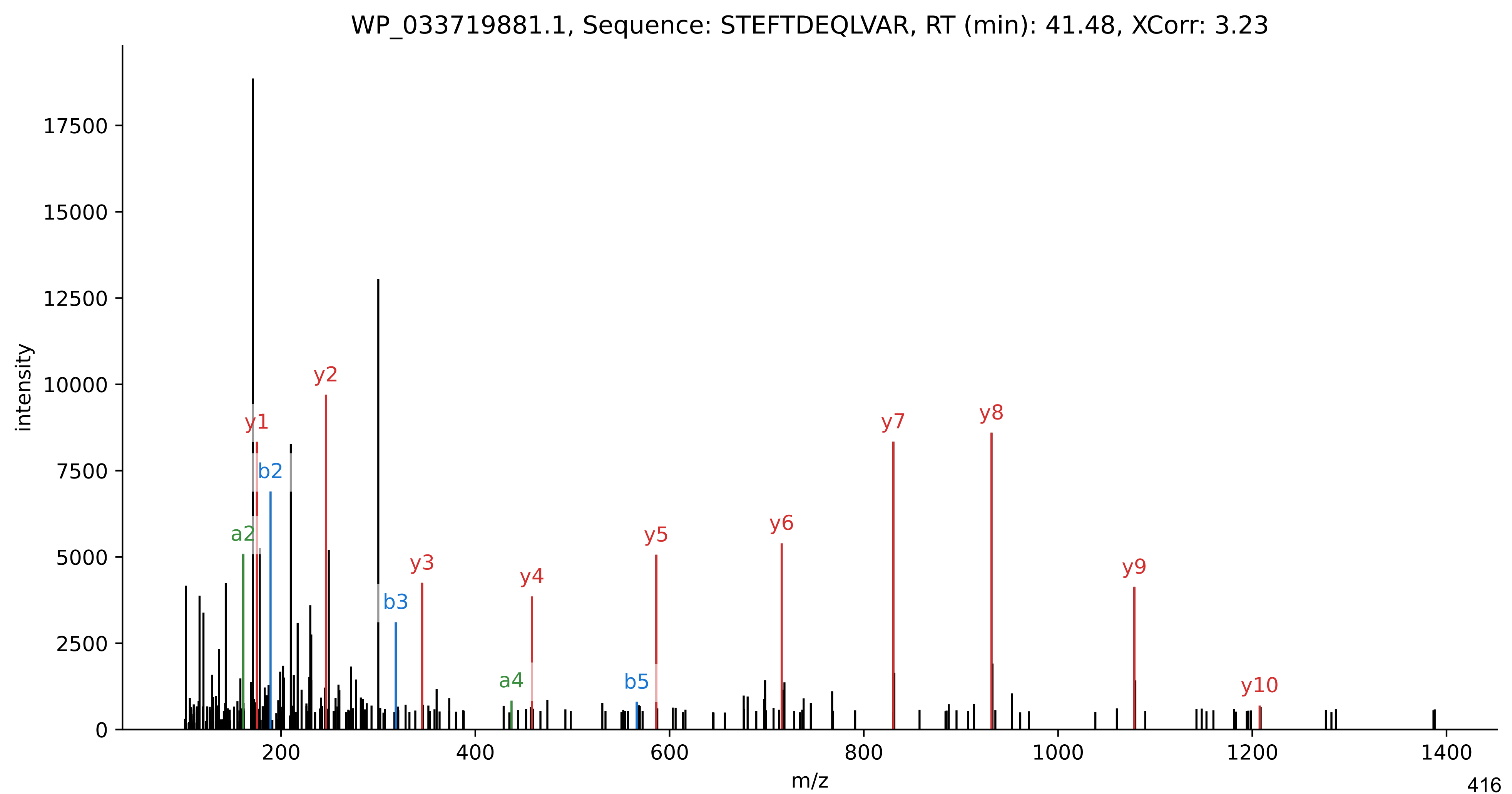

WP_033719881.1, Sequence: STEFTDEQLVAR, RT (min): 41.48, XCorr: 3.23
intensity
m/z
y1
y2
y3
y4
y5
y6
y7
y8
y9
y10
b2
b3
b5
a2
a4

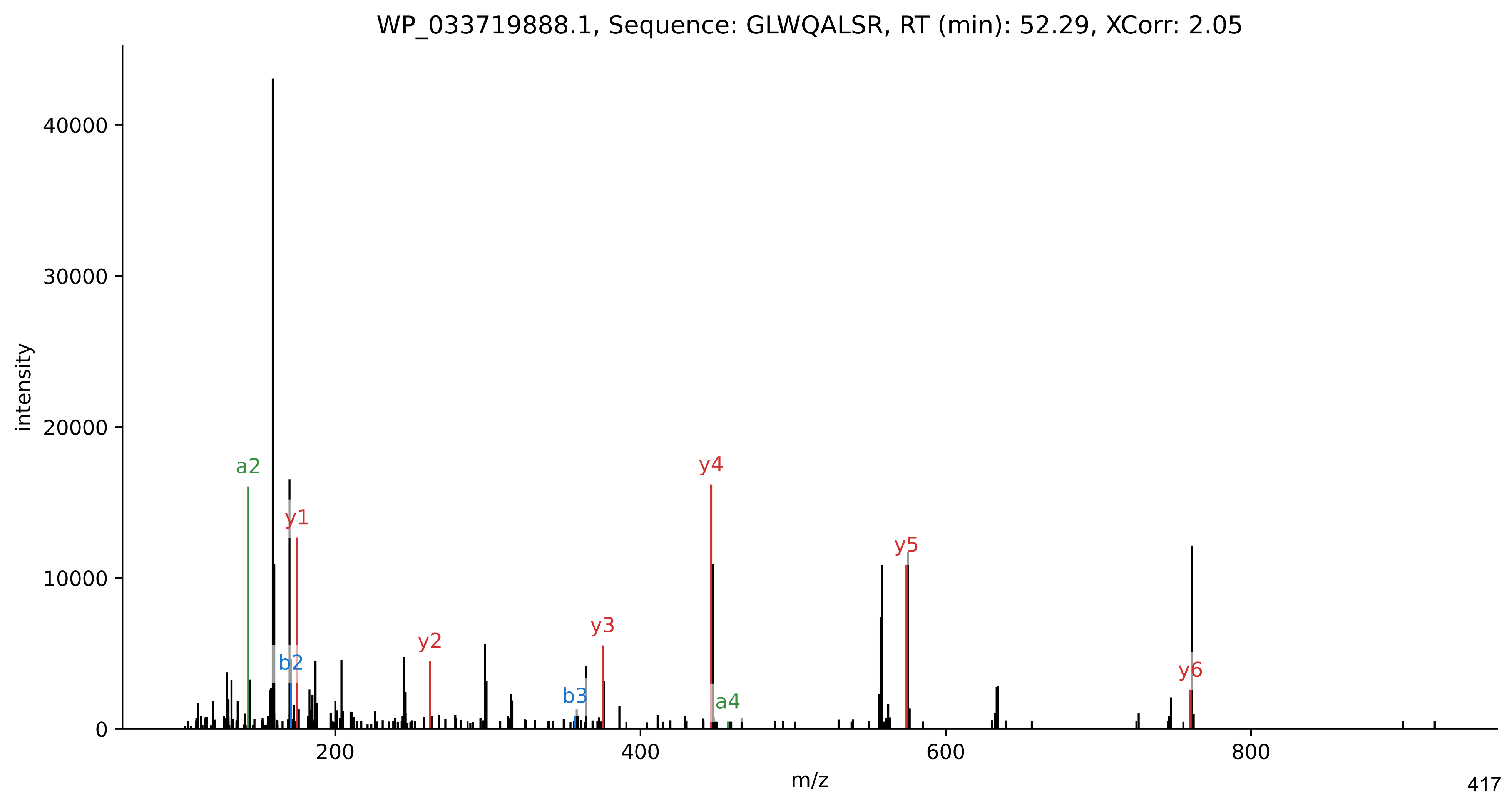
WP_033719888.1, Sequence: GLWQALSR, RT (min): 52.29, XCorr: 2.05
intensity
m/z
40000
30000
20000
10000
0
200
400
600
800
a2
y1
b2
y2
b3
y3
y4
a4
y5
y6

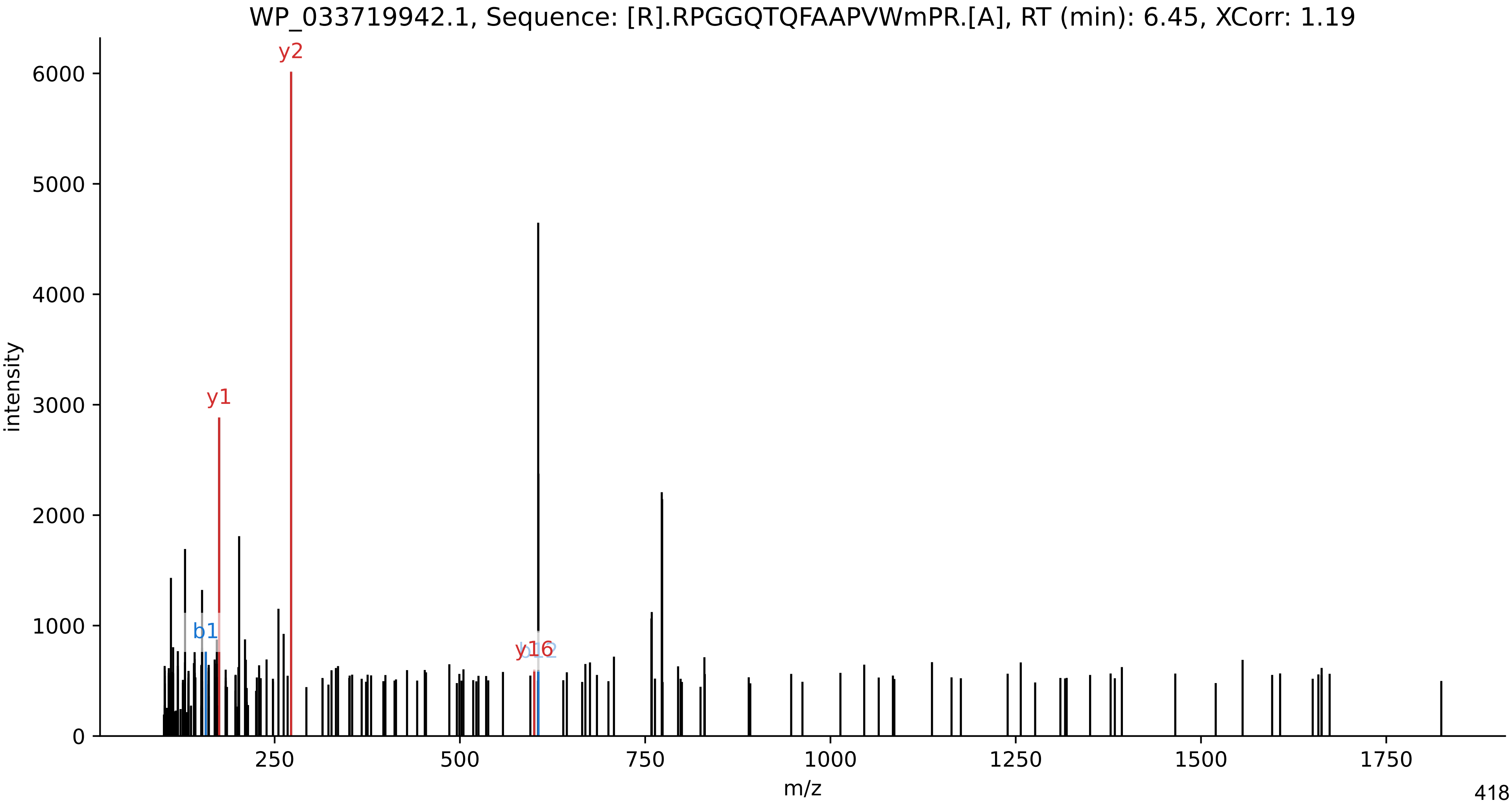
WP_033719942.1, Sequence: [R].RPGGQTQFAAPVWmPR.[A], RT (min): 6.45, XCorr: 1.19
y2
y1
b1
y16
intensity
m/z
0
1000
2000
3000
4000
5000
6000
250
500
750
1000
1250
1500
1750

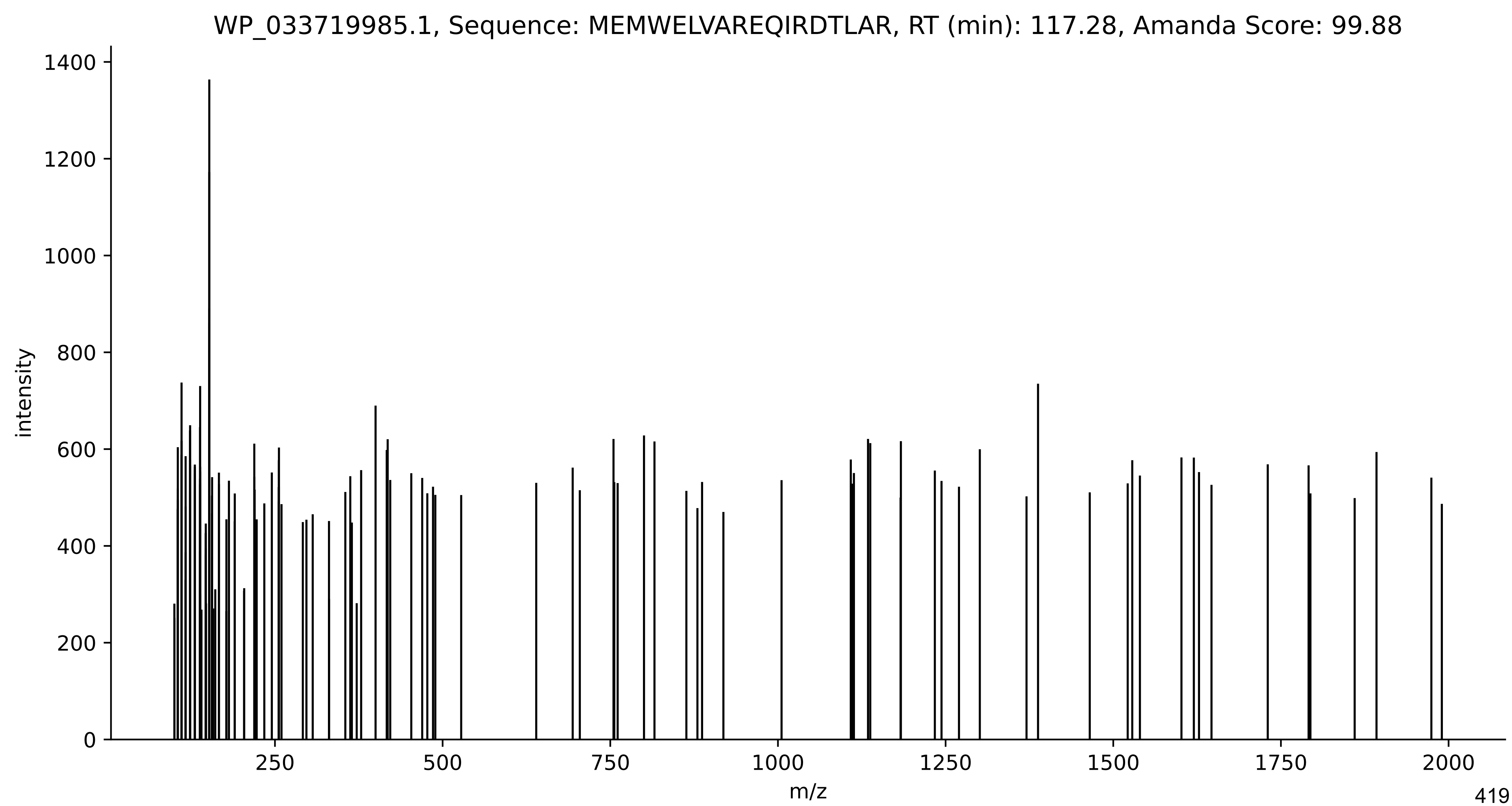
WP_033719985.1, Sequence: MEMWELVAREQIRDTLAR, RT (min): 117.28, Amanda Score: 99.88
intensity
m/z
0
200
400
600
800
1000
1200
1400
250
500
750
1000
1250
1500
1750
2000

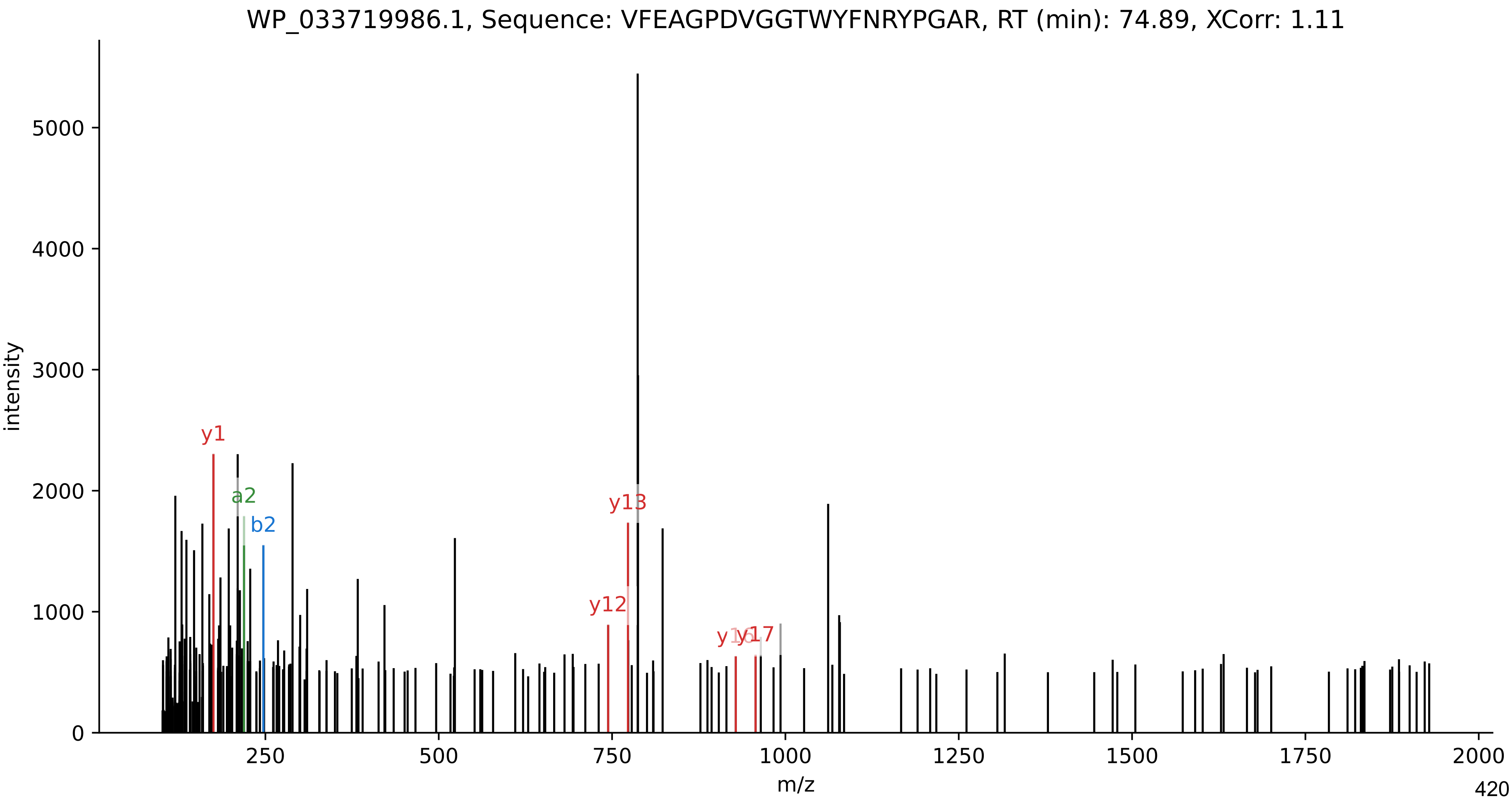
WP_033719986.1, Sequence: VFEAGPDVGGTWYFNRYPGAR, RT (min): 74.89, XCorr: 1.11
intensity
m/z
y1
a2
b2
y12
y13
y17
0
1000
2000
3000
4000
5000
250
500
750
1000
1250
1500
1750
2000

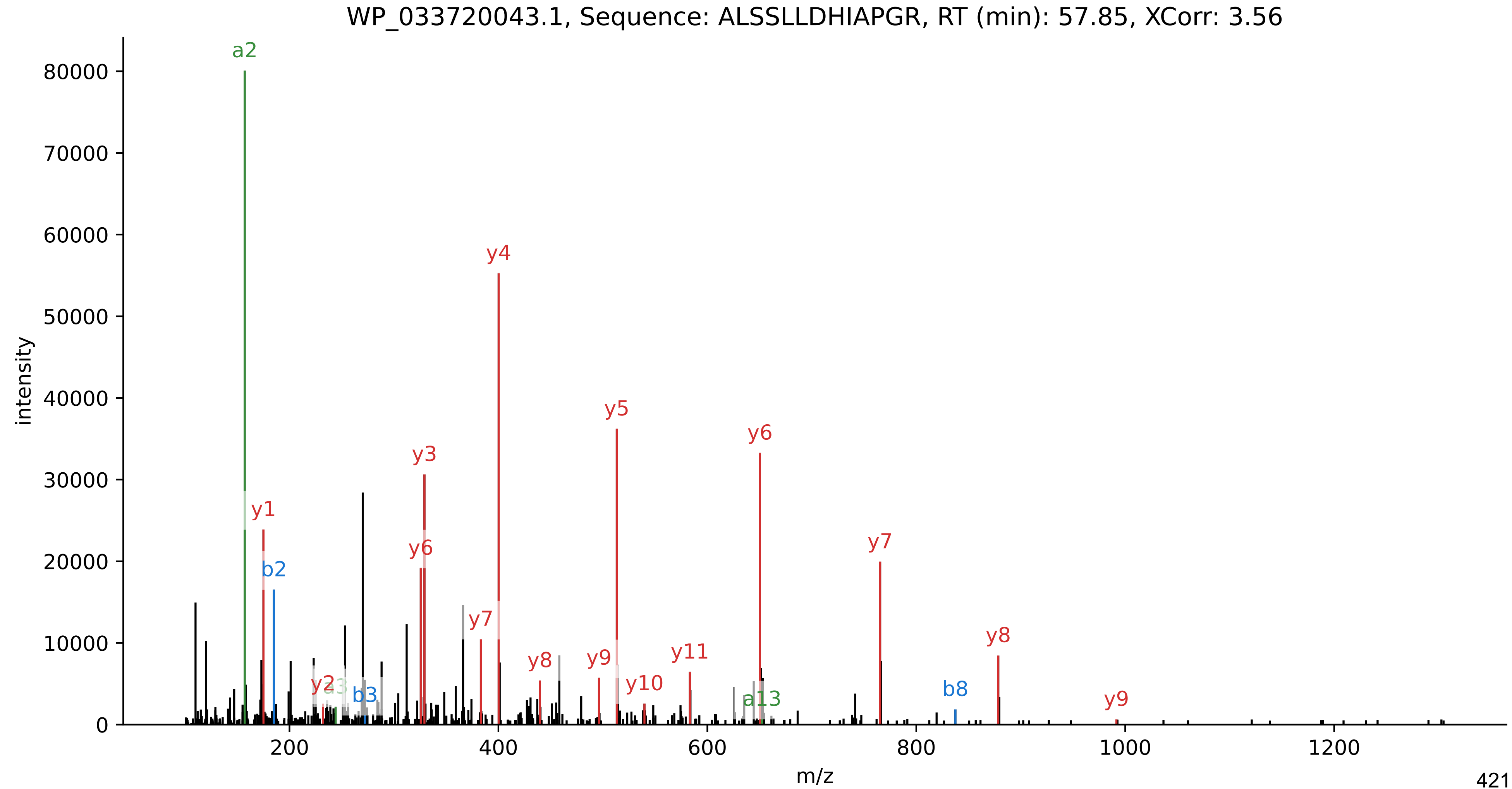

WP_033720043.1, Sequence: ALSSLLDHIAPGR, RT (min): 57.85, XCorr: 3.56
intensity
m/z
a2
y1
b2
y2
b3
y3
y6
y7
y4
y8
y9
y5
y10
y11
y6
a13
y7
b8
y8
y9
80000
70000
60000
50000
40000
30000
20000
10000
0
200
400
600
800
1000
1200

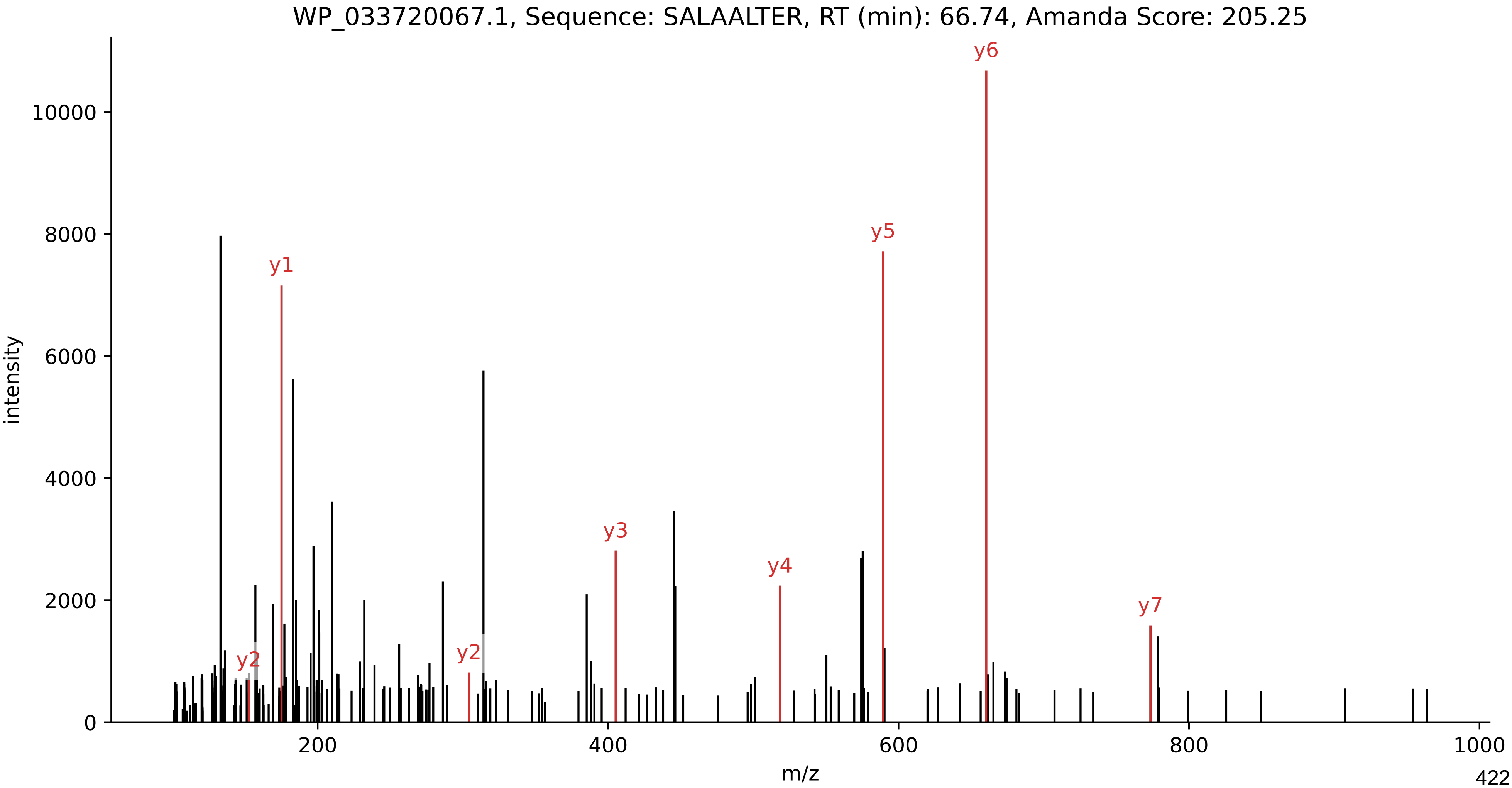

WP_033720067.1, Sequence: SALAALTER, RT (min): 66.74, Amanda Score: 205.25
intensity
m/z
y1
y2
y2
y3
y4
y5
y6
y7
0
2000
4000
6000
8000
10000
200
400
600
800
1000

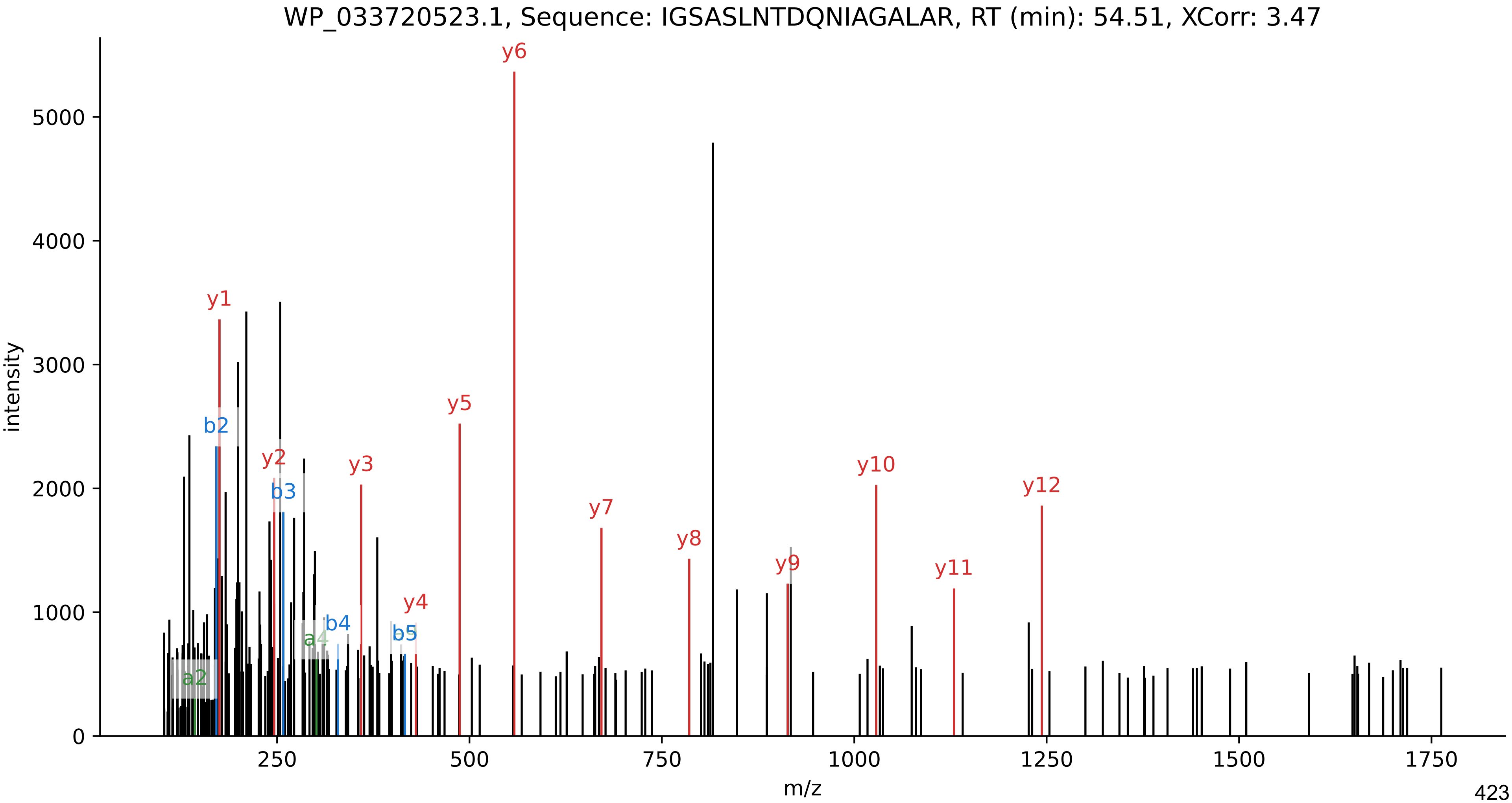
WP_033720523.1, Sequence: IGSASLNTDQNIAGALAR, RT (min): 54.51, XCorr: 3.47
intensity
m/z
y1
y2
y3
y4
y5
y6
y7
y8
y9
y10
y11
y12
b2
b3
b4
b5
a2
a4
0
1000
2000
3000
4000
5000
250
500
750
1000
1250
1500
1750

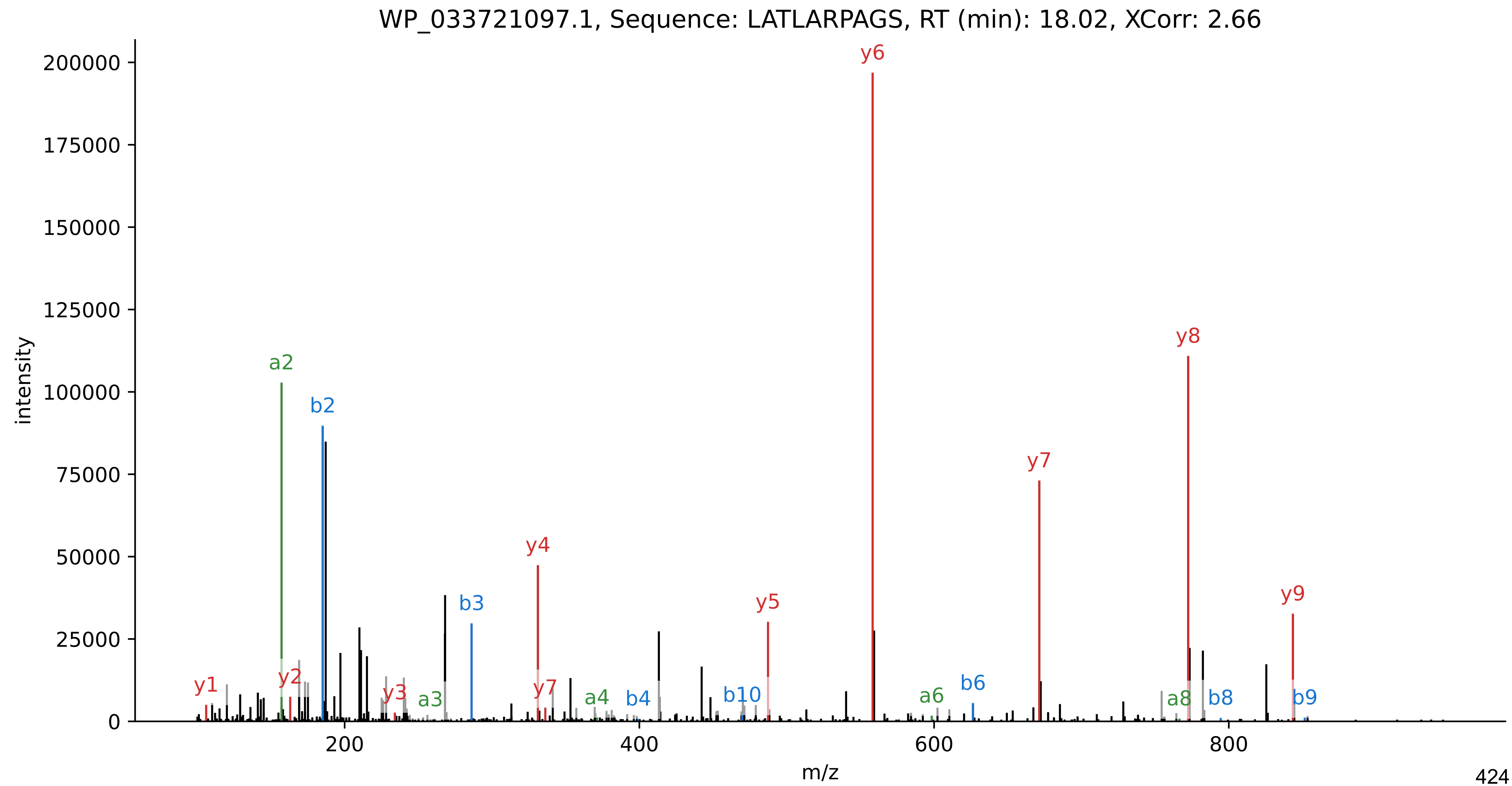
WP_033721097.1, Sequence: LATLARPAGS, RT (min): 18.02, XCorr: 2.66
intensity
m/z
y1
y2
y3
y4
y5
y6
y7
y7
y8
y9
a2
a3
a4
a6
a8
b2
b3
b4
b6
b8
b9
b10
200000
175000
150000
125000
100000
75000
50000
25000
0
200
400
600
800

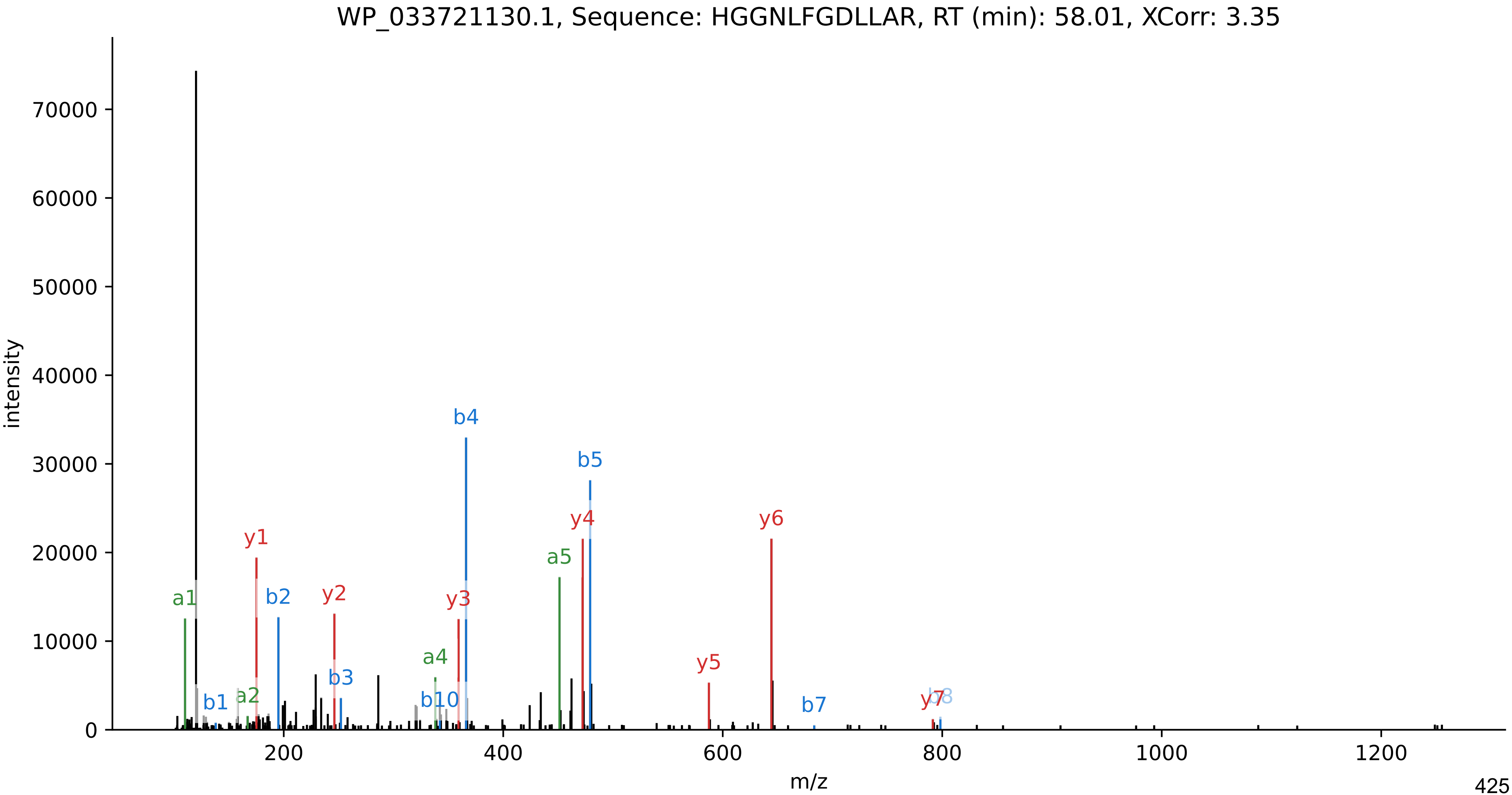
WP_033721130.1, Sequence: HGGNLFGDLLAR, RT (min): 58.01, XCorr: 3.35
intensity
m/z
a1
b1
a2
y1
b2
y2
b3
a4
b10
y3
b4
a5
y4
b5
y5
y6
b7
y7
b8
0
10000
20000
30000
40000
50000
60000
70000
200
400
600
800
1000
1200

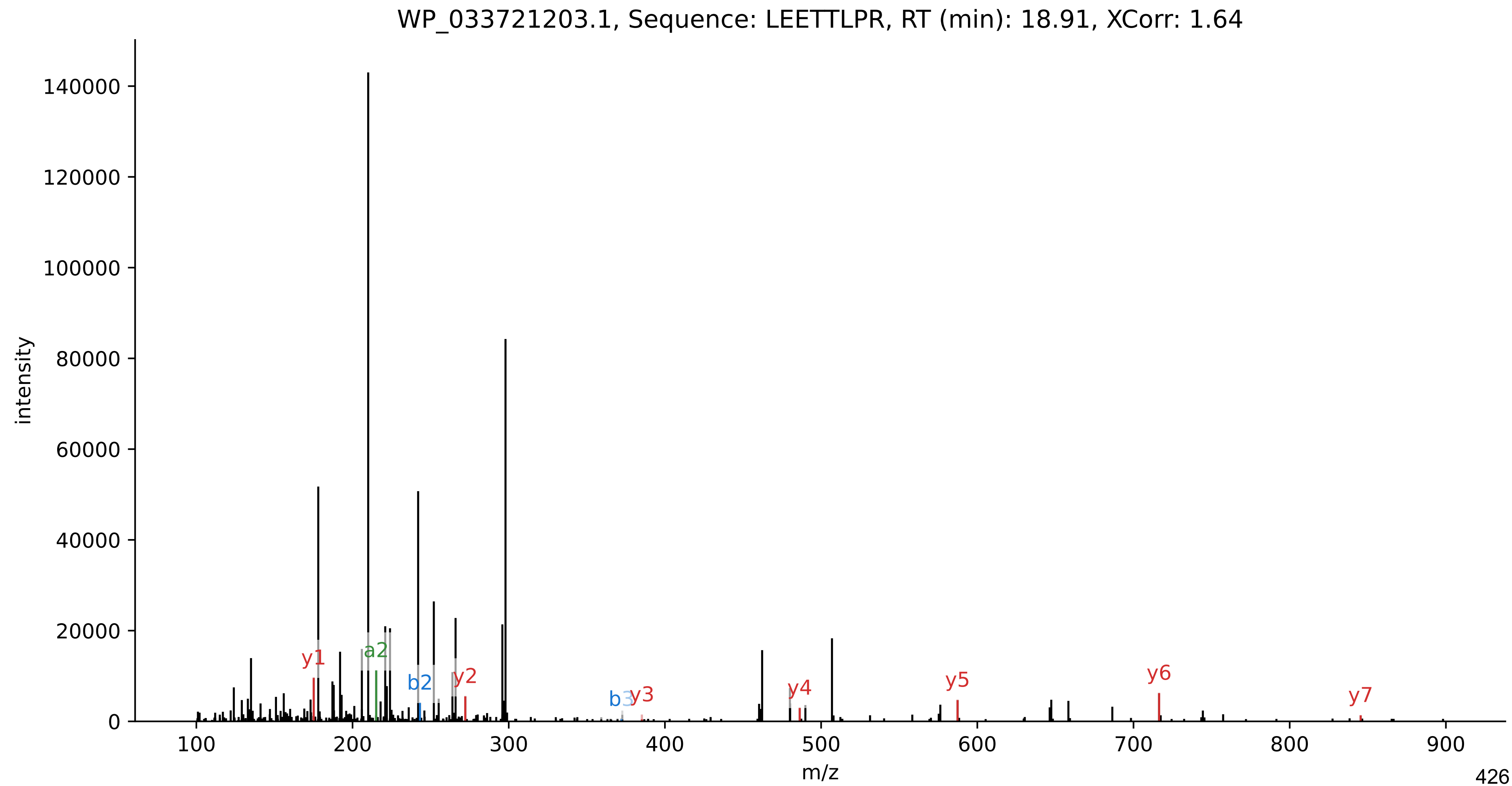

WP_033721203.1, Sequence: LEETTLPR, RT (min): 18.91, XCorr: 1.64
intensity
m/z
y1
a2
b2
y2
b3
y3
y4
y5
y6
y7

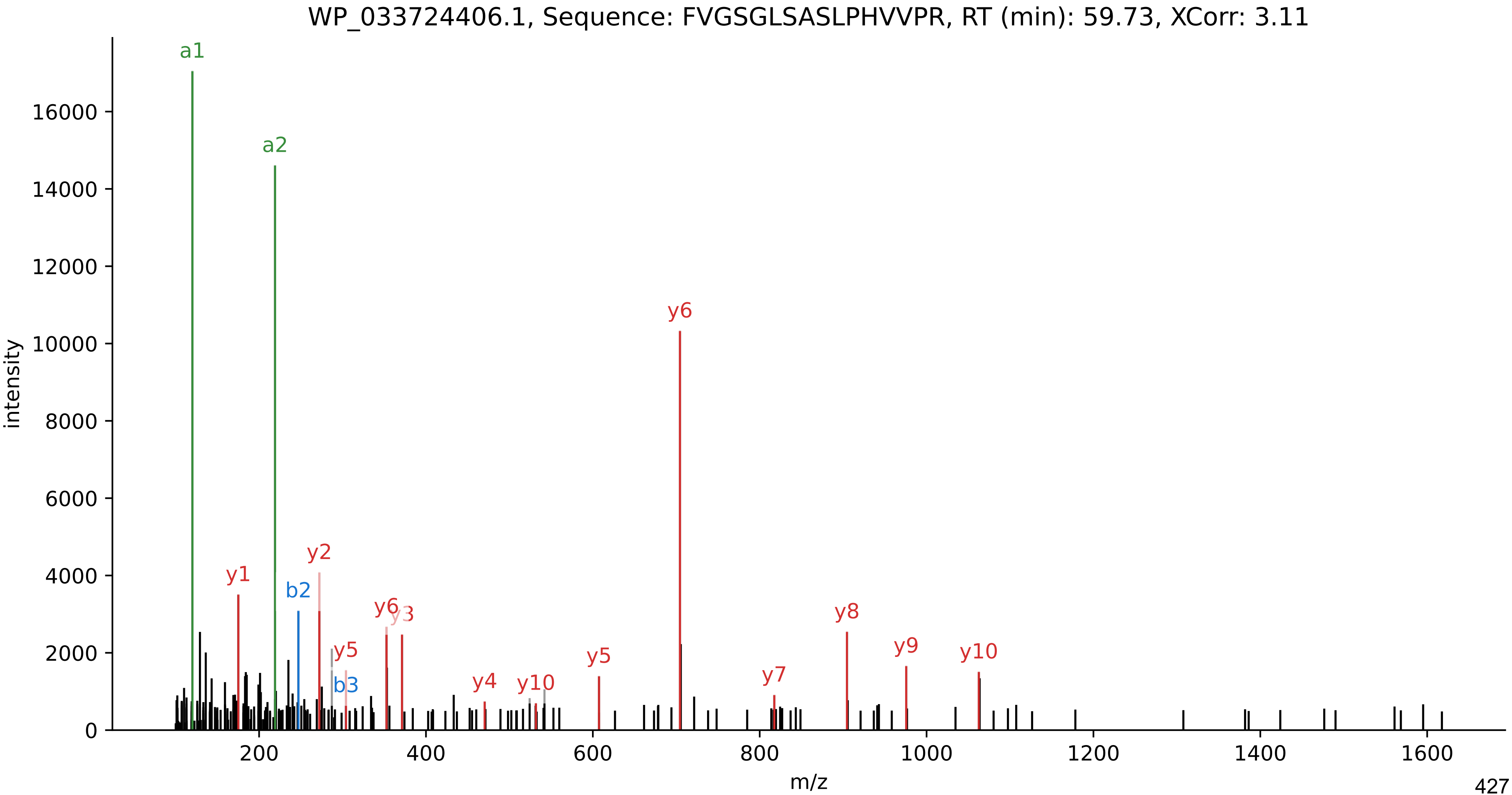

WP_033724406.1, Sequence: FVGSGLSASLPHVVPR, RT (min): 59.73, XCorr: 3.11
intensity
m/z
a1
a2
y6
y1
y2
b2
y6
y3
y5
b3
y8
y9
y10
y5
y7
y4
y10

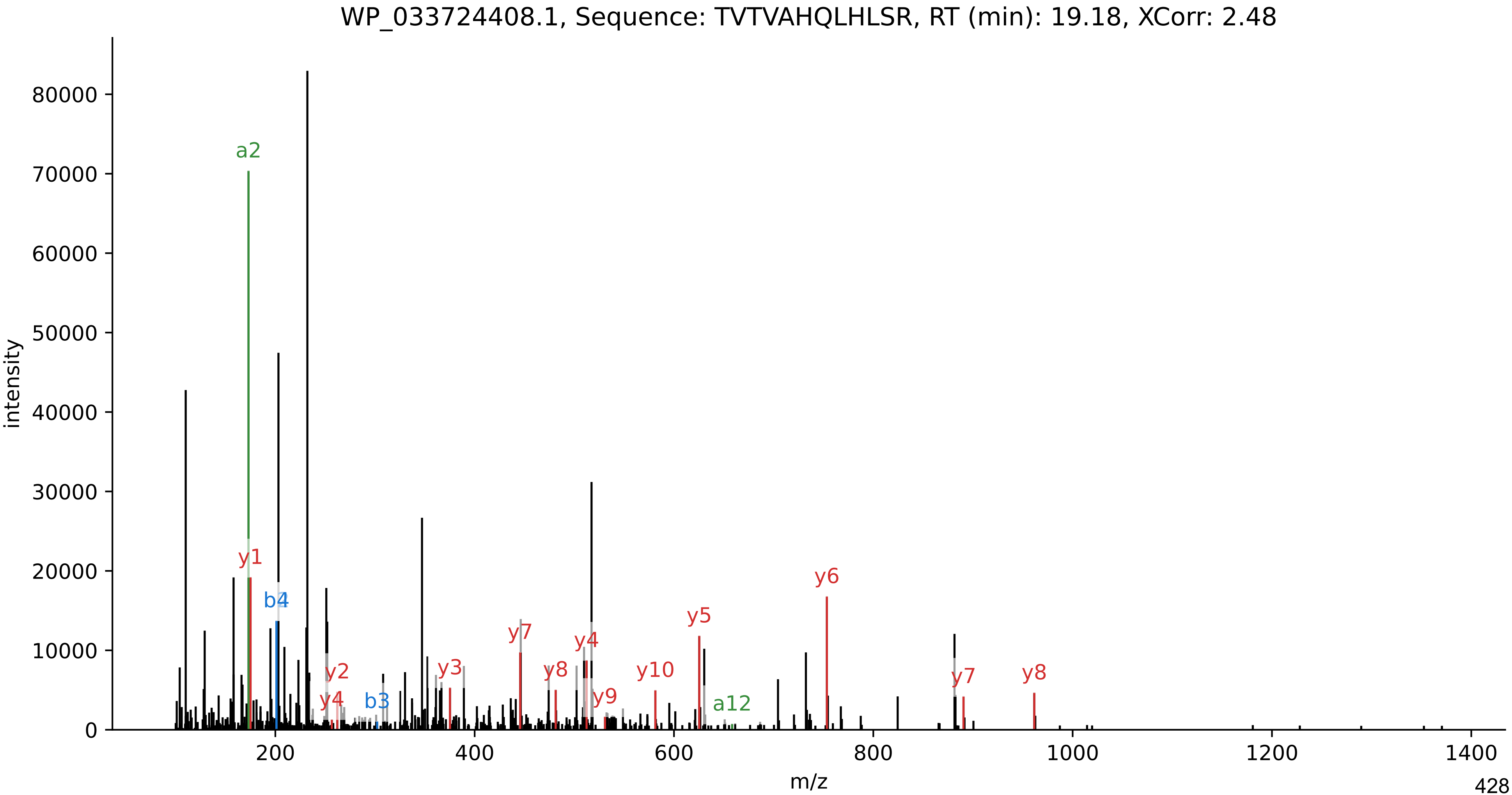
WP_033724408.1, Sequence: TVTVAHQLHLSR, RT (min): 19.18, XCorr: 2.48
intensity
m/z
a2
y1
b4
y2
y4
b3
y3
y7
y8
y4
y9
y10
y5
a12
y6
y7
y8
0
10000
20000
30000
40000
50000
60000
70000
80000
200
400
600
800
1000
1200
1400

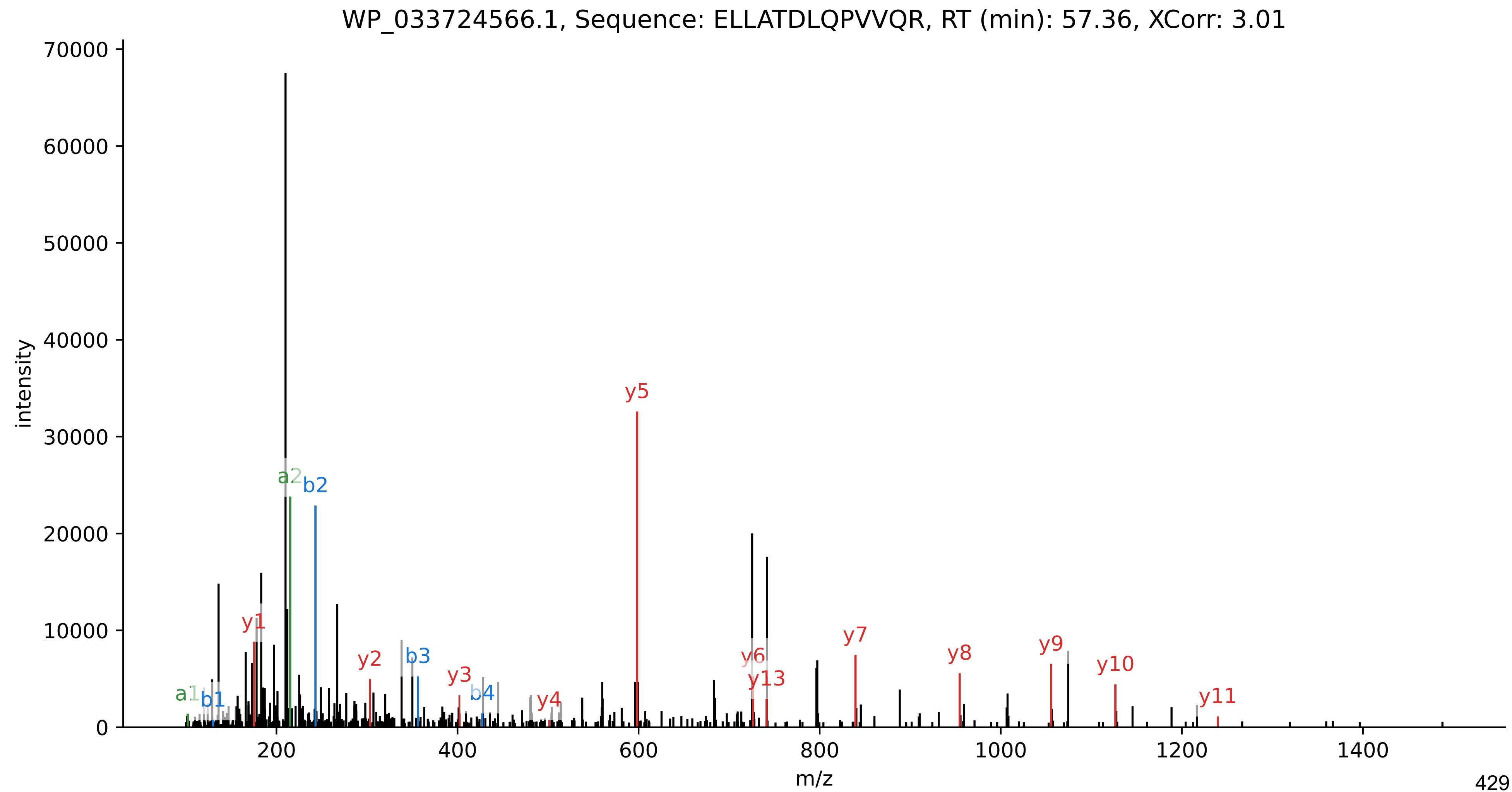

WP_033724566.1, Sequence: ELLATDLQPVVQR, RT (min): 57.36, XCorr: 3.01
intensity
m/z
y5
a2
b2
y1
y2
b3
y3
b4
y4
a1
b1
y6
y13
y7
y8
y9
y10
y11

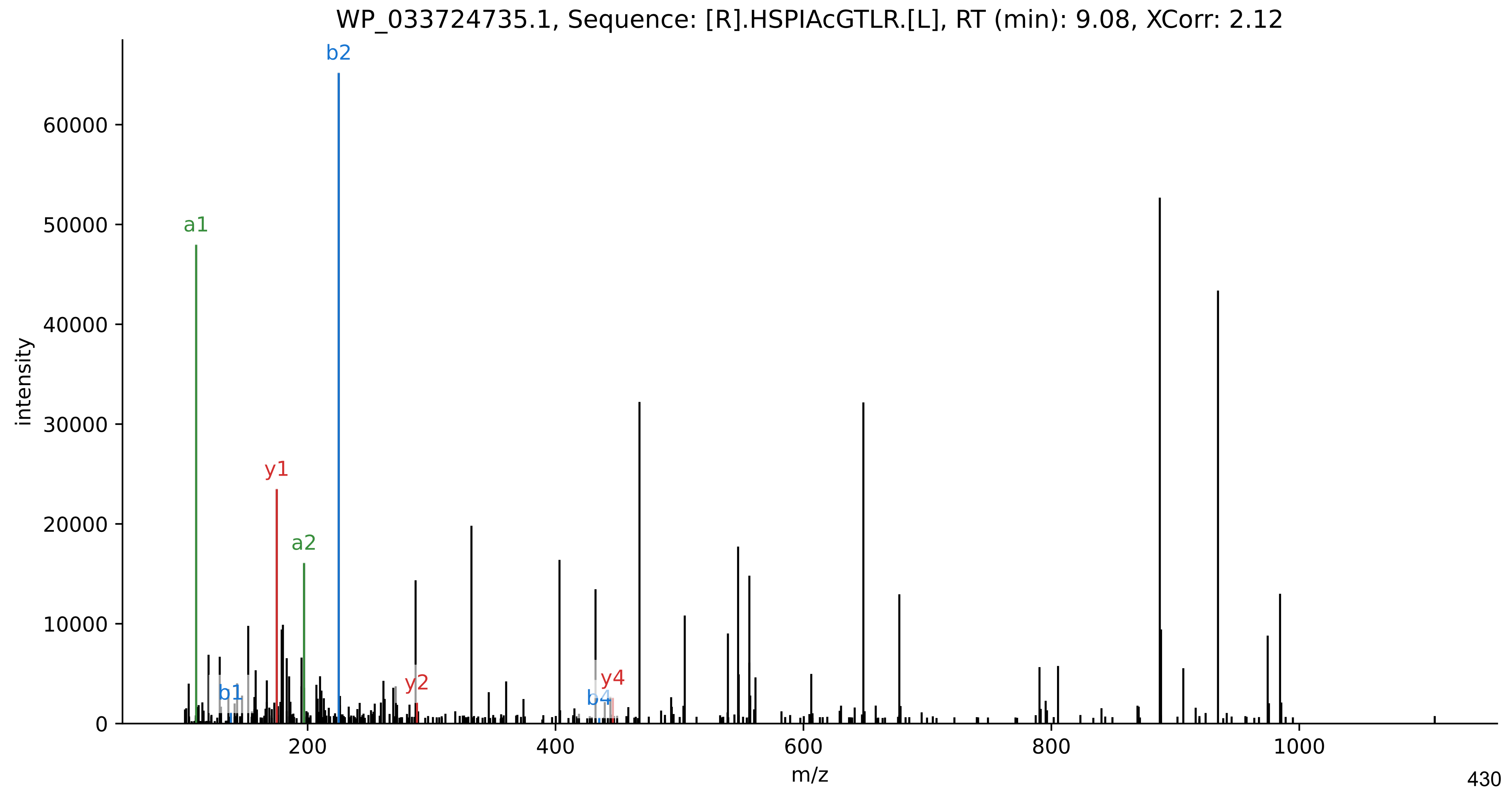
WP_033724735.1, Sequence: [R].HSPIAcGTLR.[L], RT (min): 9.08, XCorr: 2.12
b2
a1
y1
a2
y2
b1
b4
y4
intensity
m/z
0
10000
20000
30000
40000
50000
60000
200
400
600
800
1000

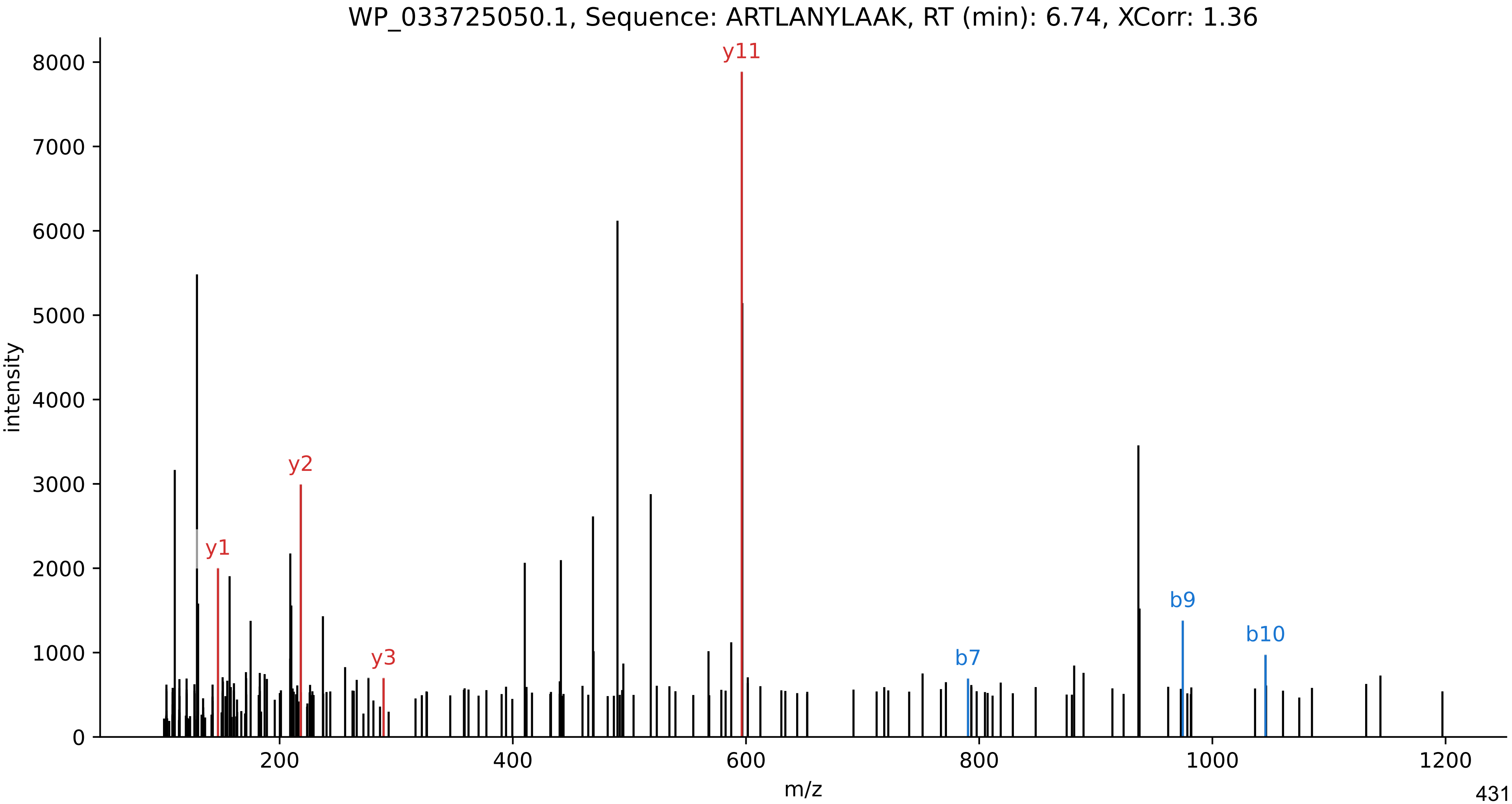

WP_033725050.1, Sequence: ARTLANYLAAK, RT (min): 6.74, XCorr: 1.36
y11
y2
y1
y3
b7
b9
b10
intensity
m/z
0
1000
2000
3000
4000
5000
6000
7000
8000
200
400
600
800
1000
1200

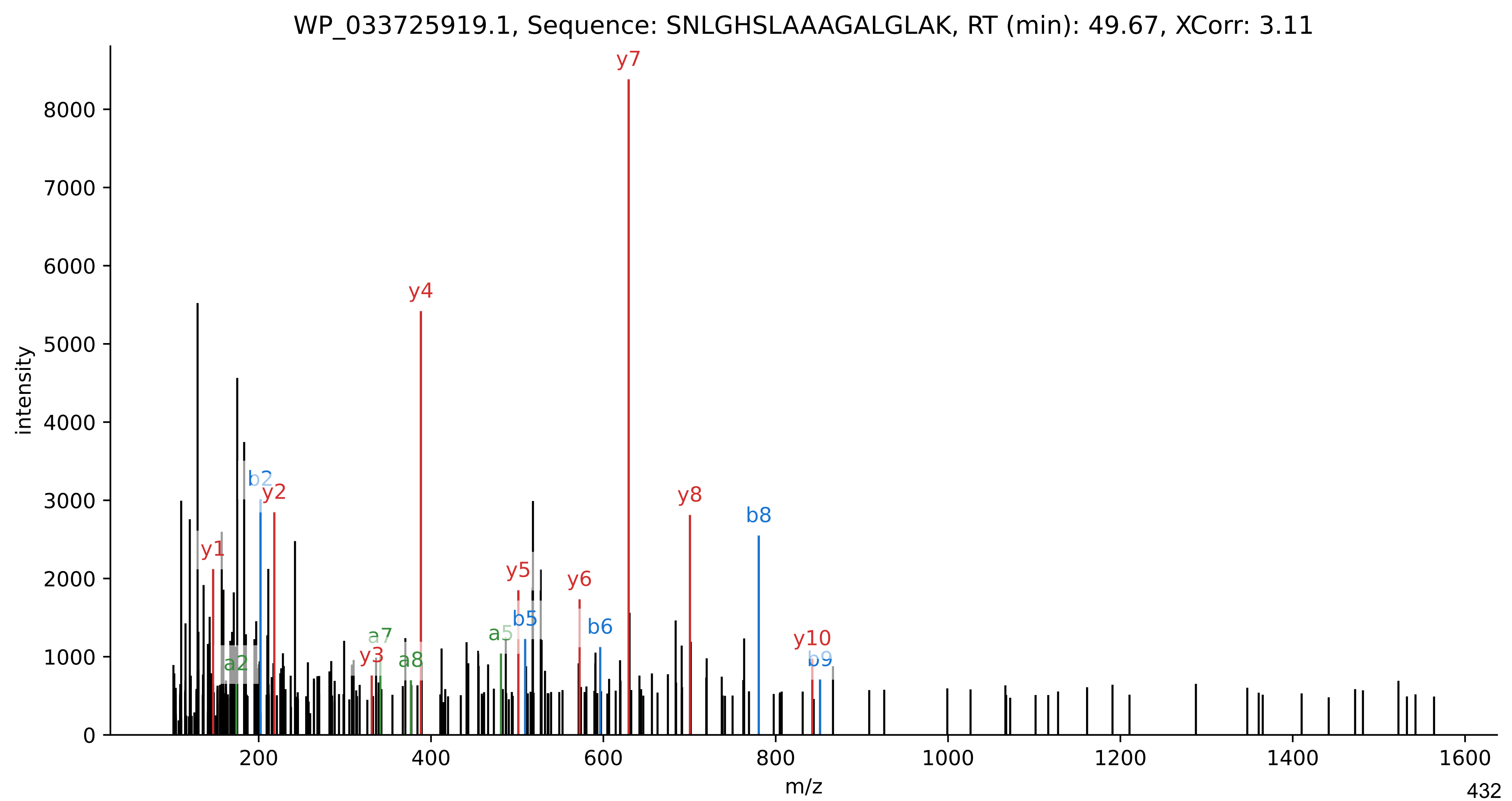
WP_033725919.1, Sequence: SNLGHSLAAAGALGLAK, RT (min): 49.67, XCorr: 3.11
intensity
m/z
y1
y2
b2
a2
y3
a7
a8
y4
a5
y5
b5
y6
b6
y7
y8
b8
y10
b9
0
1000
2000
3000
4000
5000
6000
7000
8000
200
400
600
800
1000
1200
1400
1600

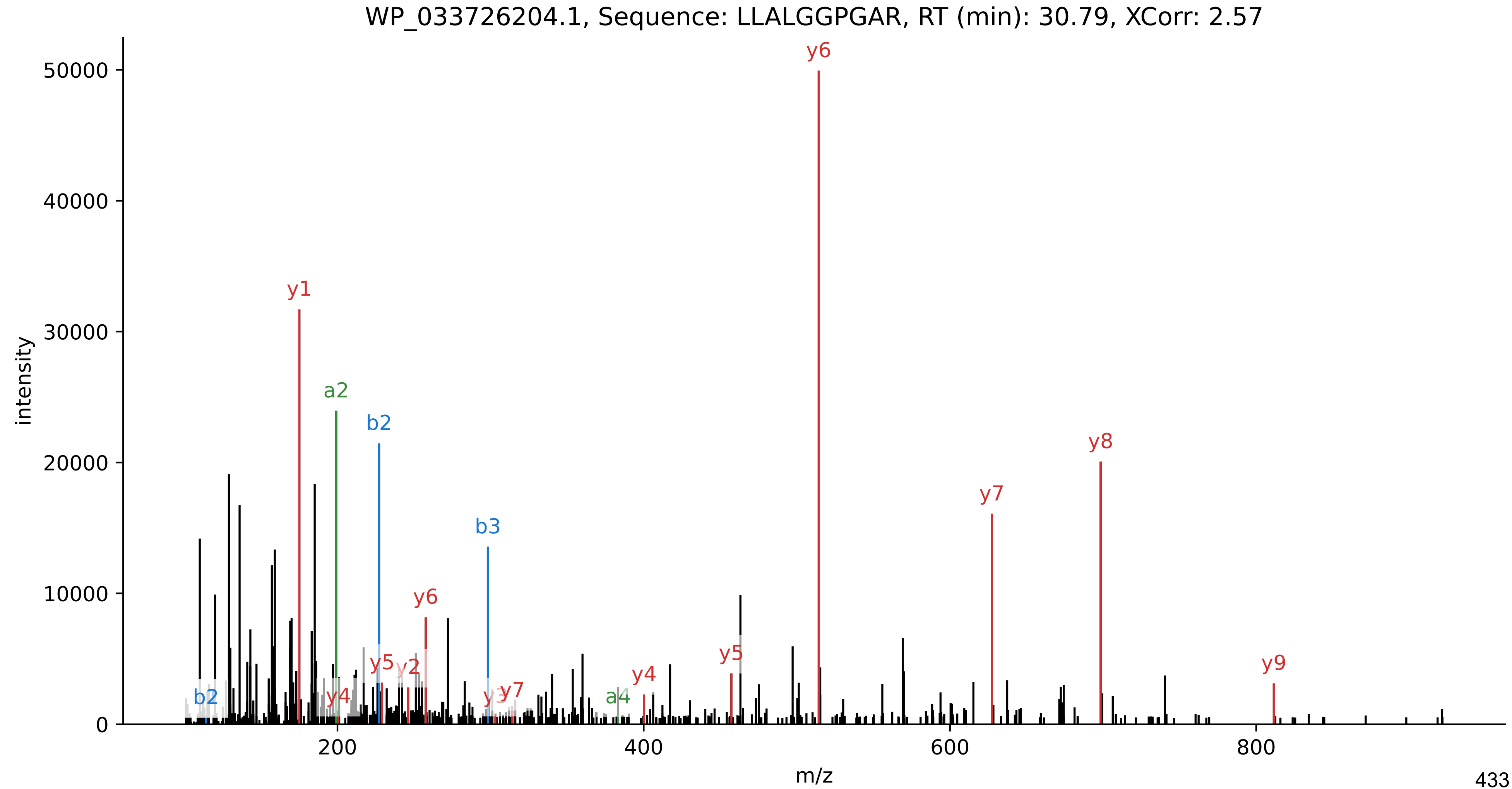
WP_033726204.1, Sequence: LLALGGPGAR, RT (min): 30.79, XCorr: 2.57
intensity
m/z
50000
40000
30000
20000
10000
0
200
400
600
800
y6
y1
a2
b2
y8
y7
b3
y6
y5
y9
y4
y5
y2
b2
y4
y7
a4

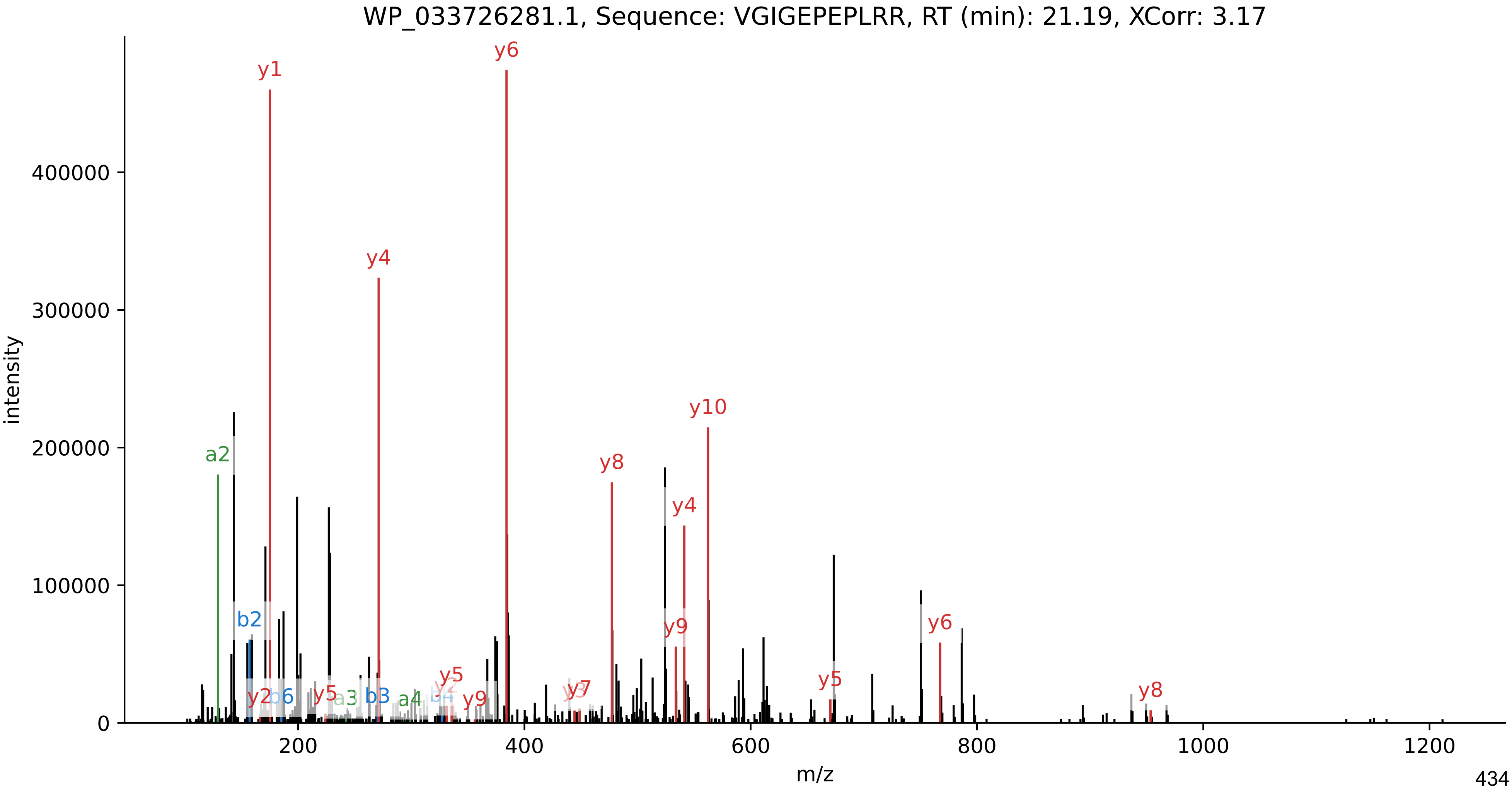
WP_033726281.1, Sequence: VGIGEPEPLRR, RT (min): 21.19, XCorr: 3.17
intensity
m/z
0
100000
200000
300000
400000
200
400
600
800
1000
1200
y1
y6
y4
y10
y8
y4
y9
y6
y5
y8
a2
b2
y2
b6
y5
a3
b3
a4
b4
y2
y5
y9
y3
y7

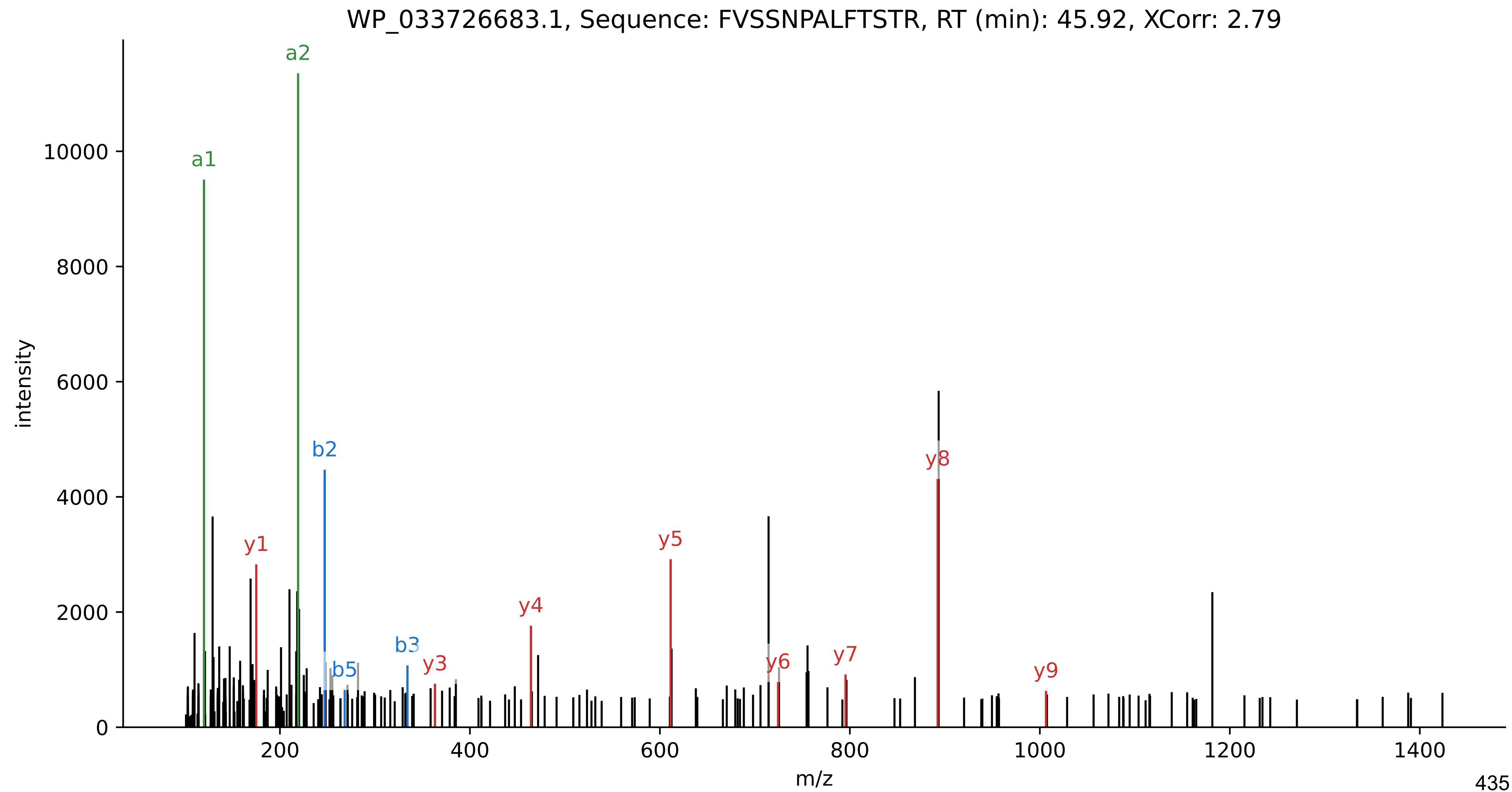
WP_033726683.1, Sequence: FVSSNPALFTSTR, RT (min): 45.92, XCorr: 2.79
intensity
m/z
a1
a2
b2
b3
b5
y1
y3
y4
y5
y6
y7
y8
y9
0
2000
4000
6000
8000
10000
200
400
600
800
1000
1200
1400

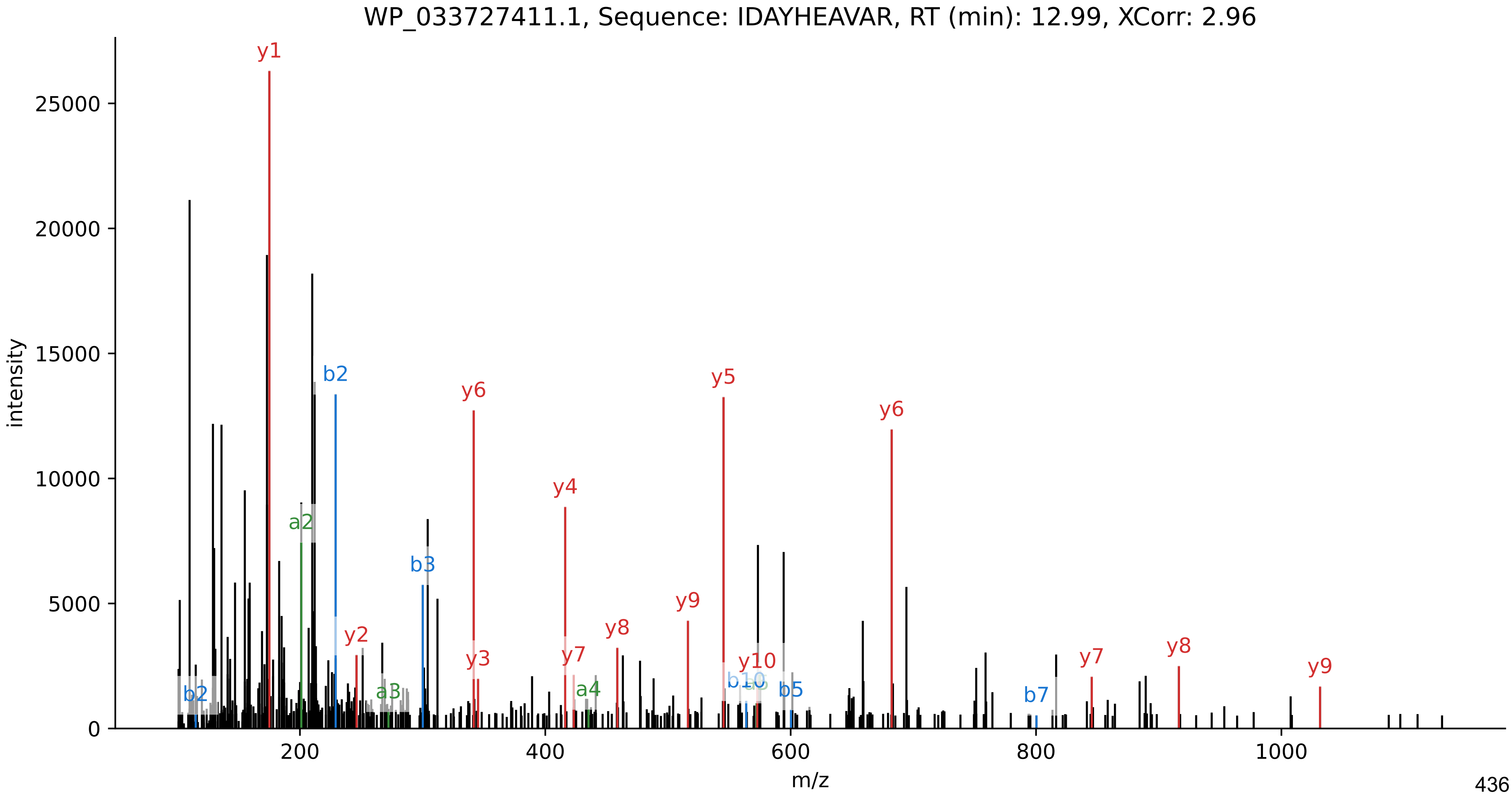
WP_033727411.1, Sequence: IDAYHEAVAR, RT (min): 12.99, XCorr: 2.96
intensity
m/z
0
5000
10000
15000
20000
25000
200
400
600
800
1000
y1
b2
y6
y5
y6
y4
a2
b3
y9
y8
y2
y3
y7
y10
b10
b2
a3
a4
b5
b7
y7
y8
y9

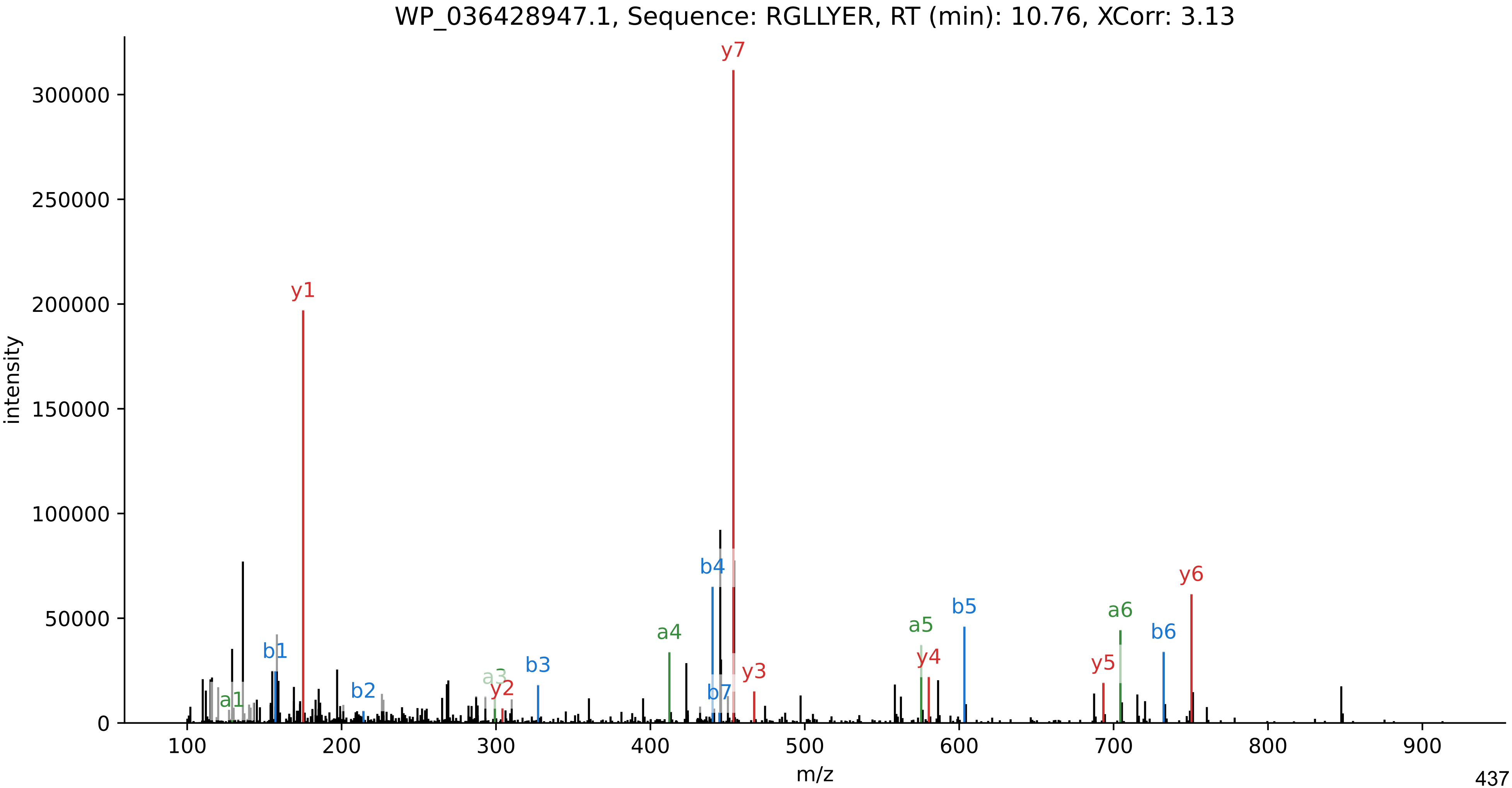

WP_036428947.1, Sequence: RGLLYER, RT (min): 10.76, XCorr: 3.13
intensity
m/z
y7
y1
y6
b4
b5
a6
a5
a4
b6
b1
y4
y5
b3
y3
a3
y2
b2
b7
a1
0
50000
100000
150000
200000
250000
300000
100
200
300
400
500
600
700
800
900

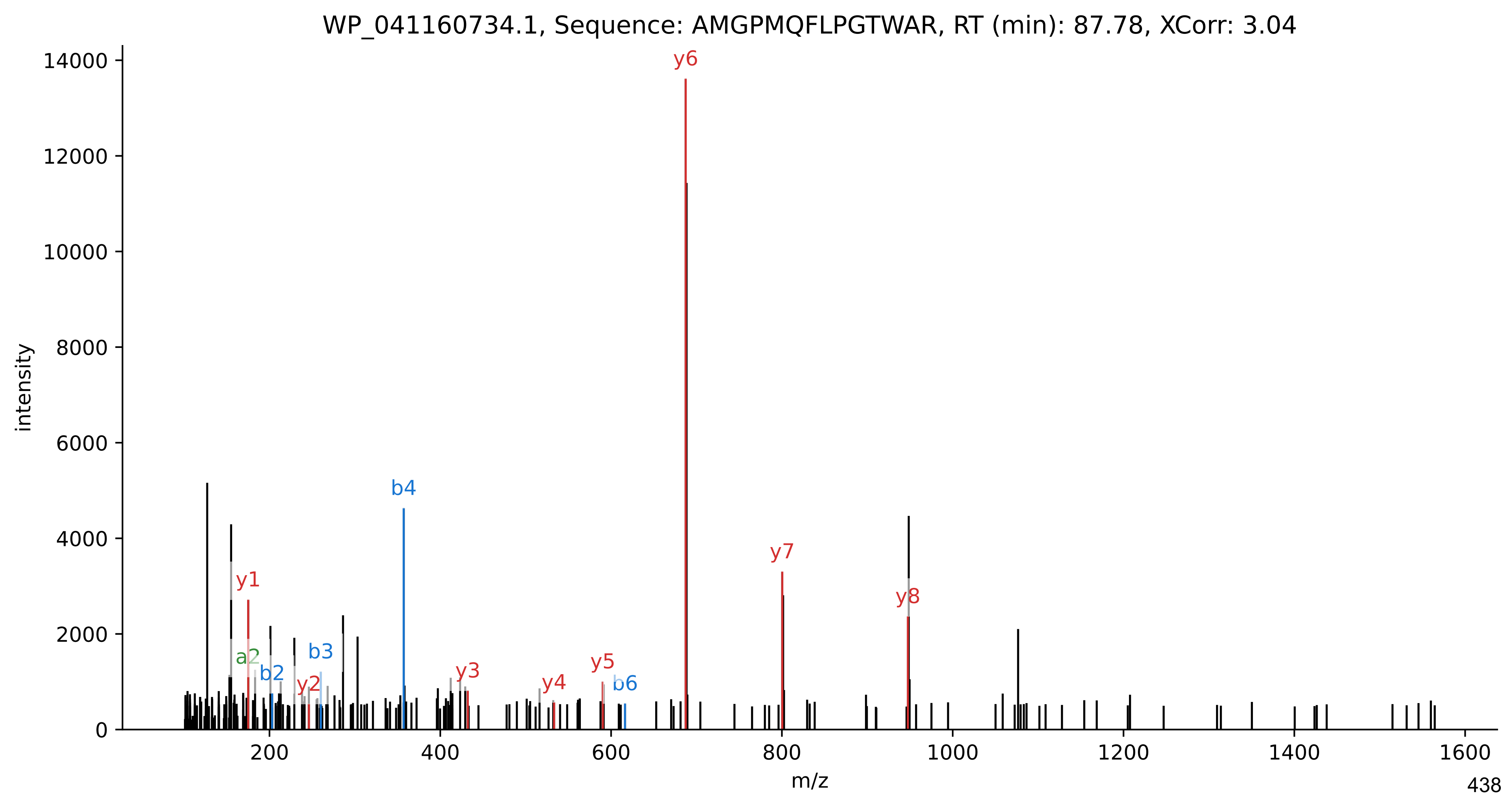

WP_041160734.1, Sequence: AMGPMQFLPGTWAR, RT (min): 87.78, XCorr: 3.04
intensity
m/z
y6
b4
y7
y8
y1
a2
b2
b3
y2
y3
y4
y5
b6

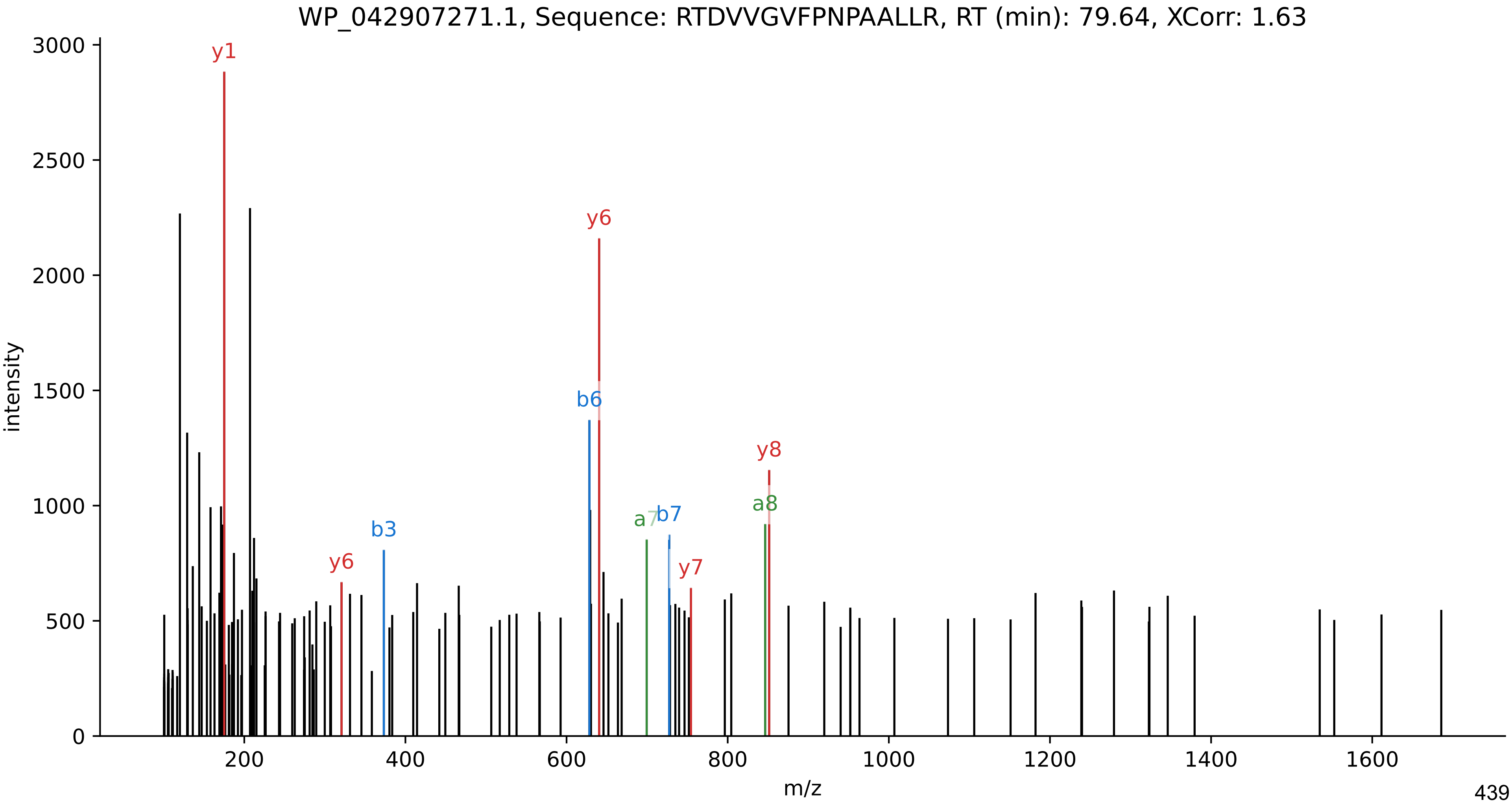
WP_042907271.1, Sequence: RTDVVGVFPNPAALLR, RT (min): 79.64, XCorr: 1.63
intensity
m/z
y1
y6
b6
y8
a8
a7
b7
b3
y6
y7
0
500
1000
1500
2000
2500
3000
200
400
600
800
1000
1200
1400
1600

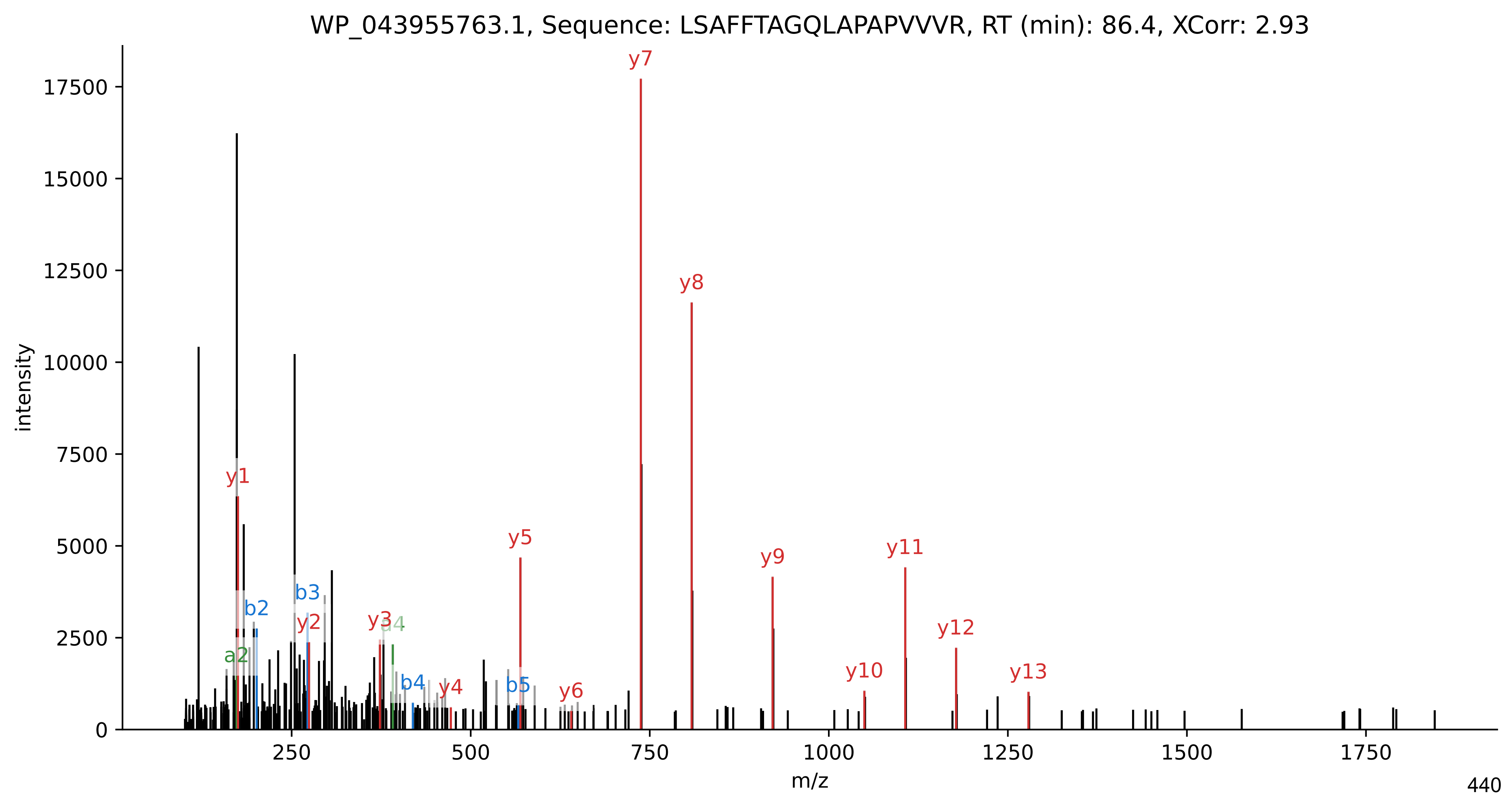

WP_043955763.1, Sequence: LSAFFTAGQLAPAPVVVR, RT (min): 86.4, XCorr: 2.93
intensity
m/z
y1
y2
y3
y4
y5
y6
y7
y8
y9
y10
y11
y12
y13
b2
b3
b4
b5
a2
a4

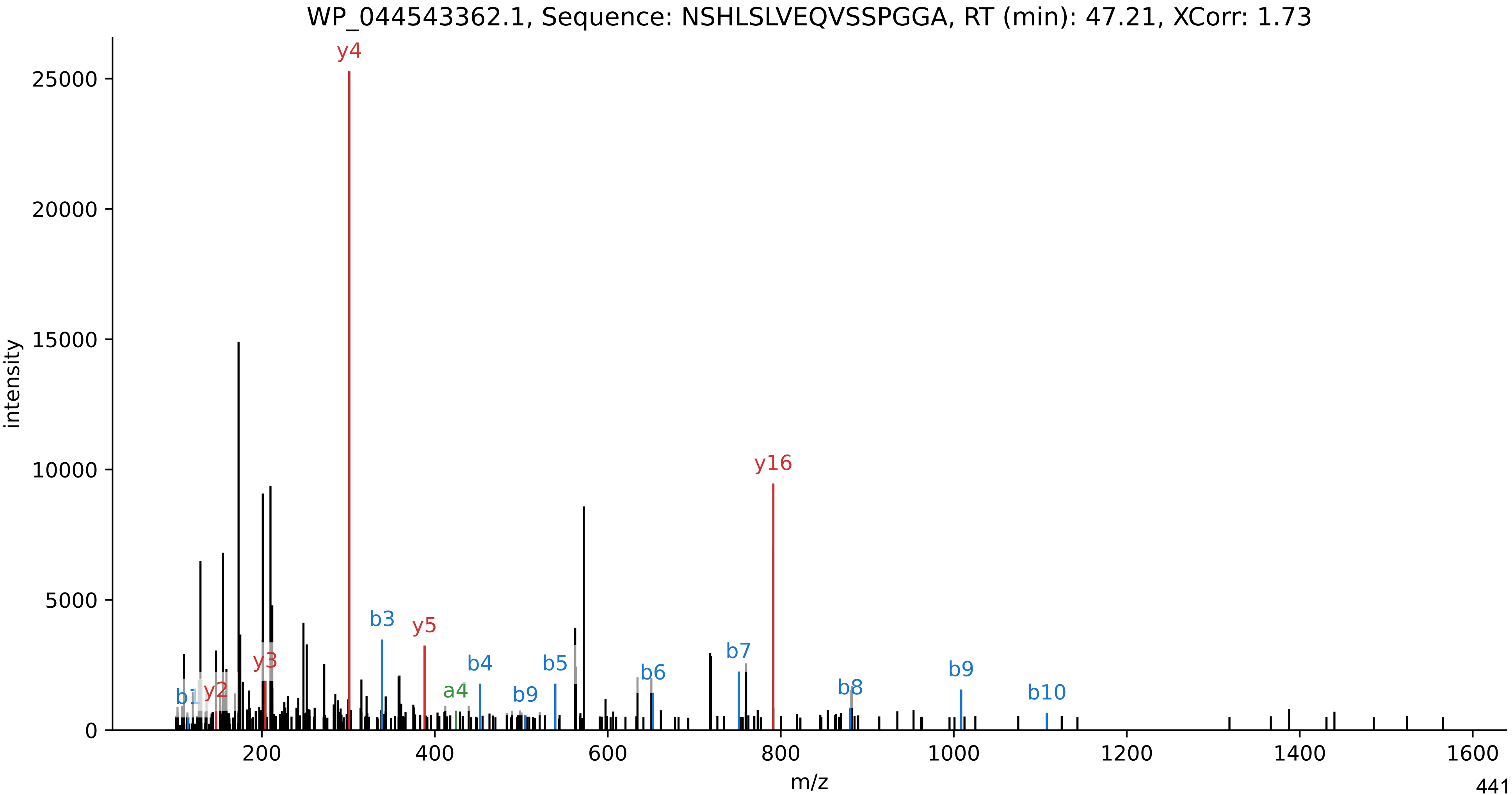
WP_044543362.1, Sequence: NSHLSLVEQVSSPGGA, RT (min): 47.21, XCorr: 1.73
y4
y16
b3
y5
b7
b4
b5
b6
b9
y3
a4
b8
b9
b10
b1
y2
intensity
m/z
25000
20000
15000
10000
5000
0
200
400
600
800
1000
1200
1400
1600

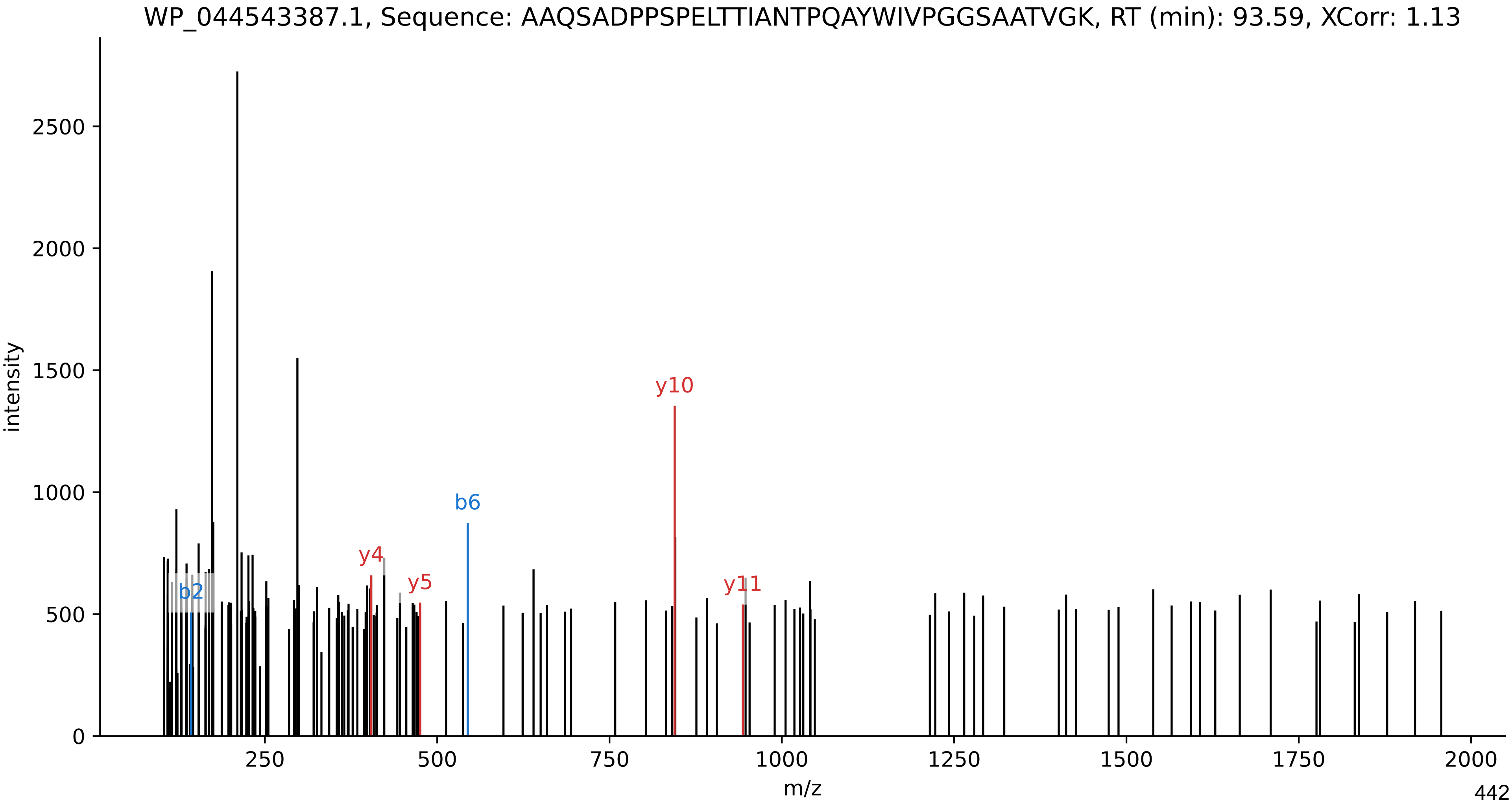
WP_044543387.1, Sequence: AAQSADPPSPELTTIANTPQAYWIVPGGSAATVGK, RT (min): 93.59, XCorr: 1.13
intensity
m/z
b2
b6
y4
y5
y10
y11
0
500
1000
1500
2000
2500
250
500
750
1000
1250
1500
1750
2000

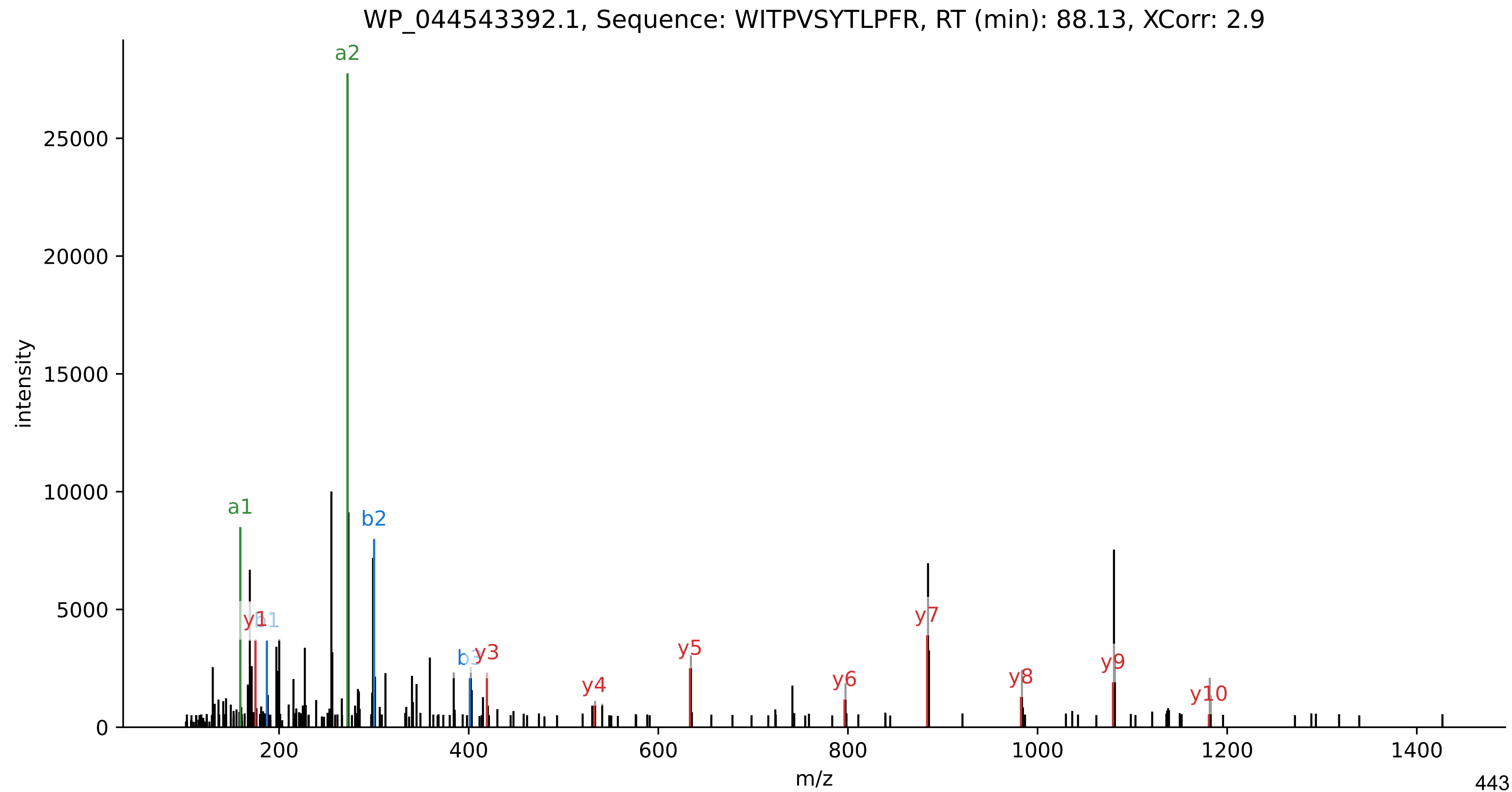
WP_044543392.1, Sequence: WITPVSYTLPFR, RT (min): 88.13, XCorr: 2.9
intensity
m/z
a2
a1
b2
y1
b1
b3
y3
y4
y5
y6
y7
y8
y9
y10
25000
20000
15000
10000
5000
0
200
400
600
800
1000
1200
1400

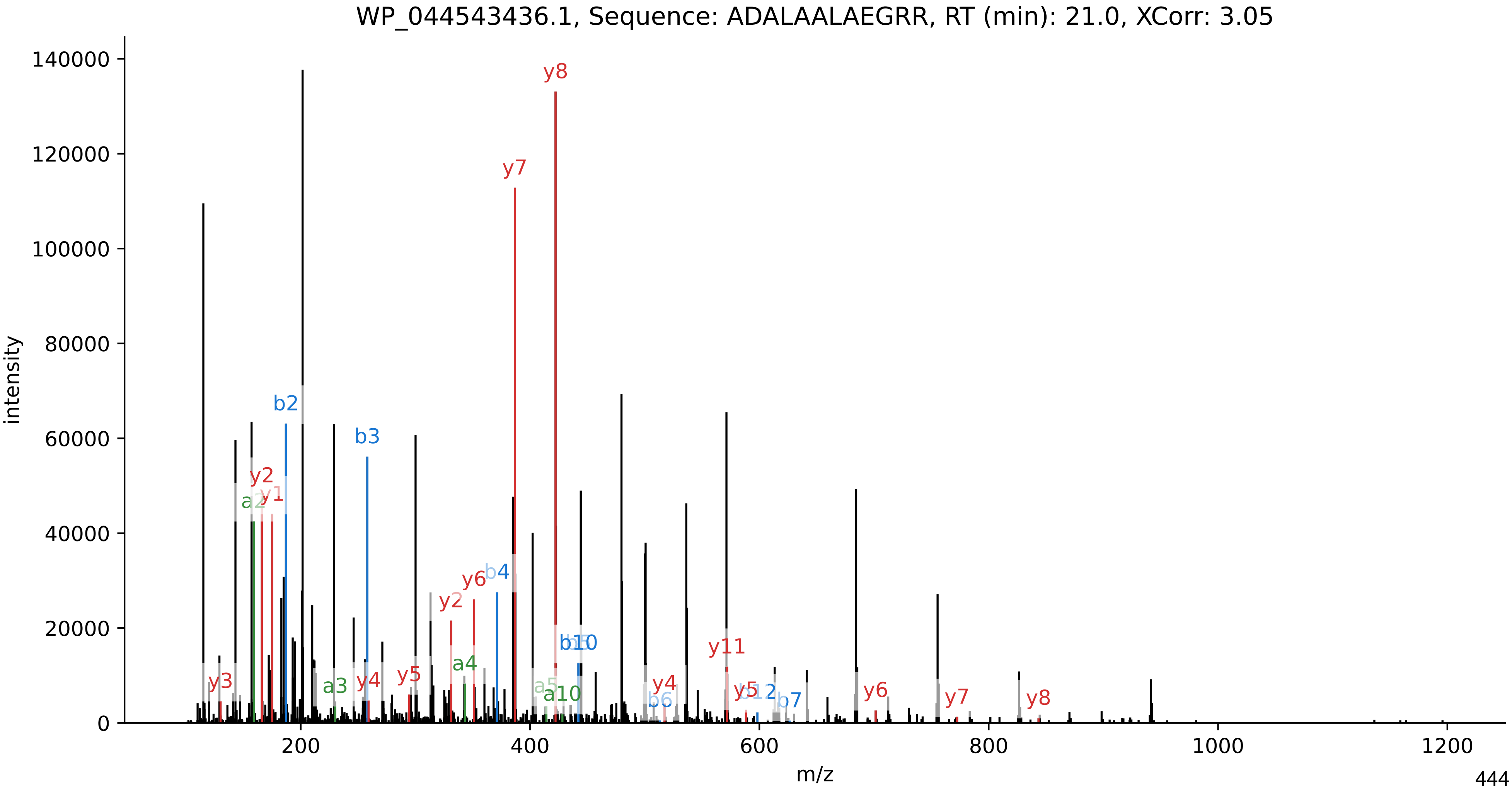

WP_044543436.1, Sequence: ADALAALAEGRR, RT (min): 21.0, XCorr: 3.05
intensity
m/z
y8
y7
b2
b3
y2
y1
a2
y6
b4
y2
b10
y11
a4
y3
a3
y4
y5
a5
a10
y4
b6
y5
b12
b7
y6
y7
y8

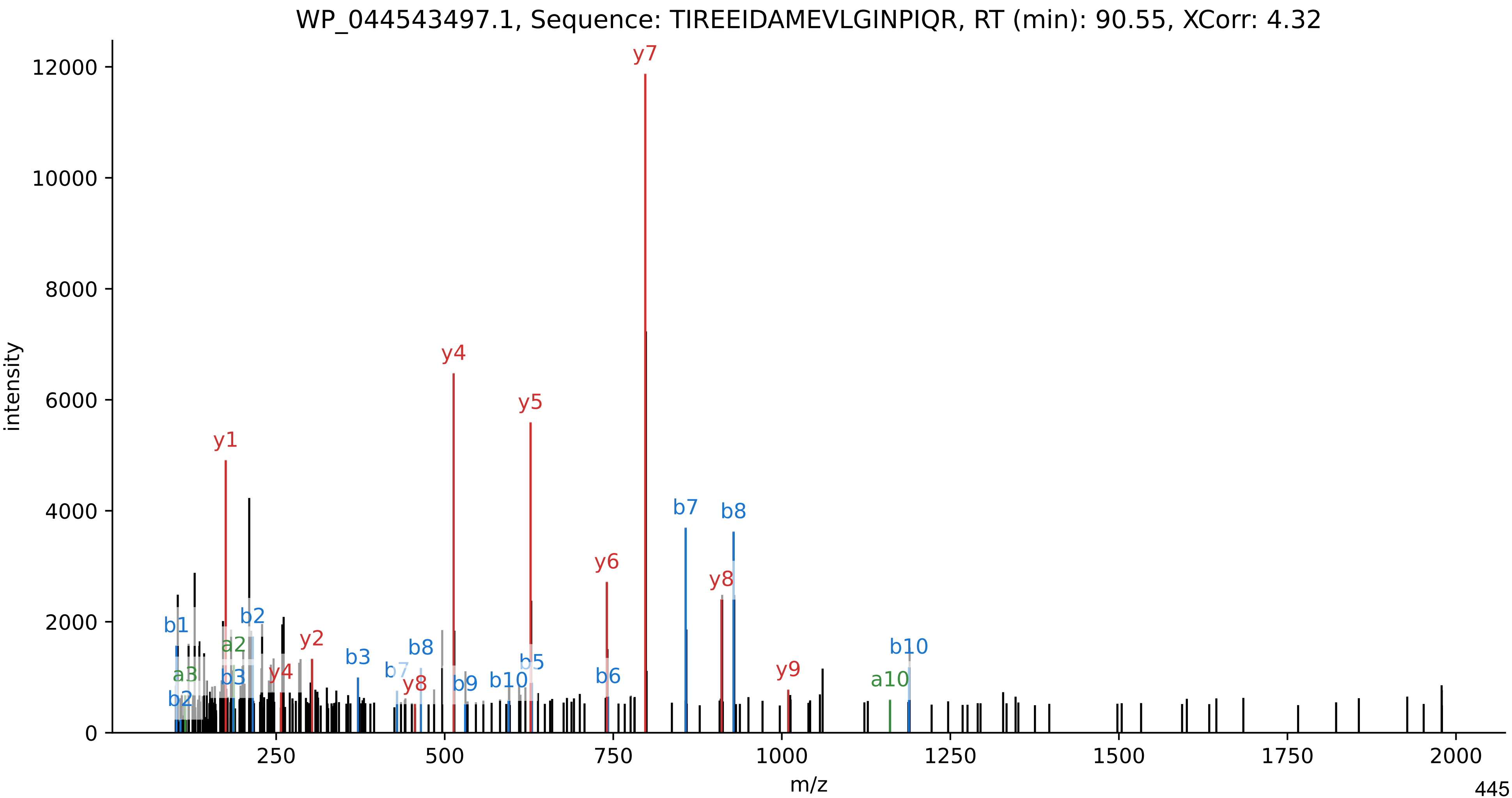

WP_044543497.1, Sequence: TIREEIDAMEVLGINPIQR, RT (min): 90.55, XCorr: 4.32
intensity
m/z
y7
y4
y5
y1
y6
b7
b8
y8
b2
b1
a2
y2
b3
b8
b5
b10
a3
b3
y4
b7
y8
b9
b10
b6
y9
a10
b2
0
2000
4000
6000
8000
10000
12000
250
500
750
1000
1250
1500
1750
2000

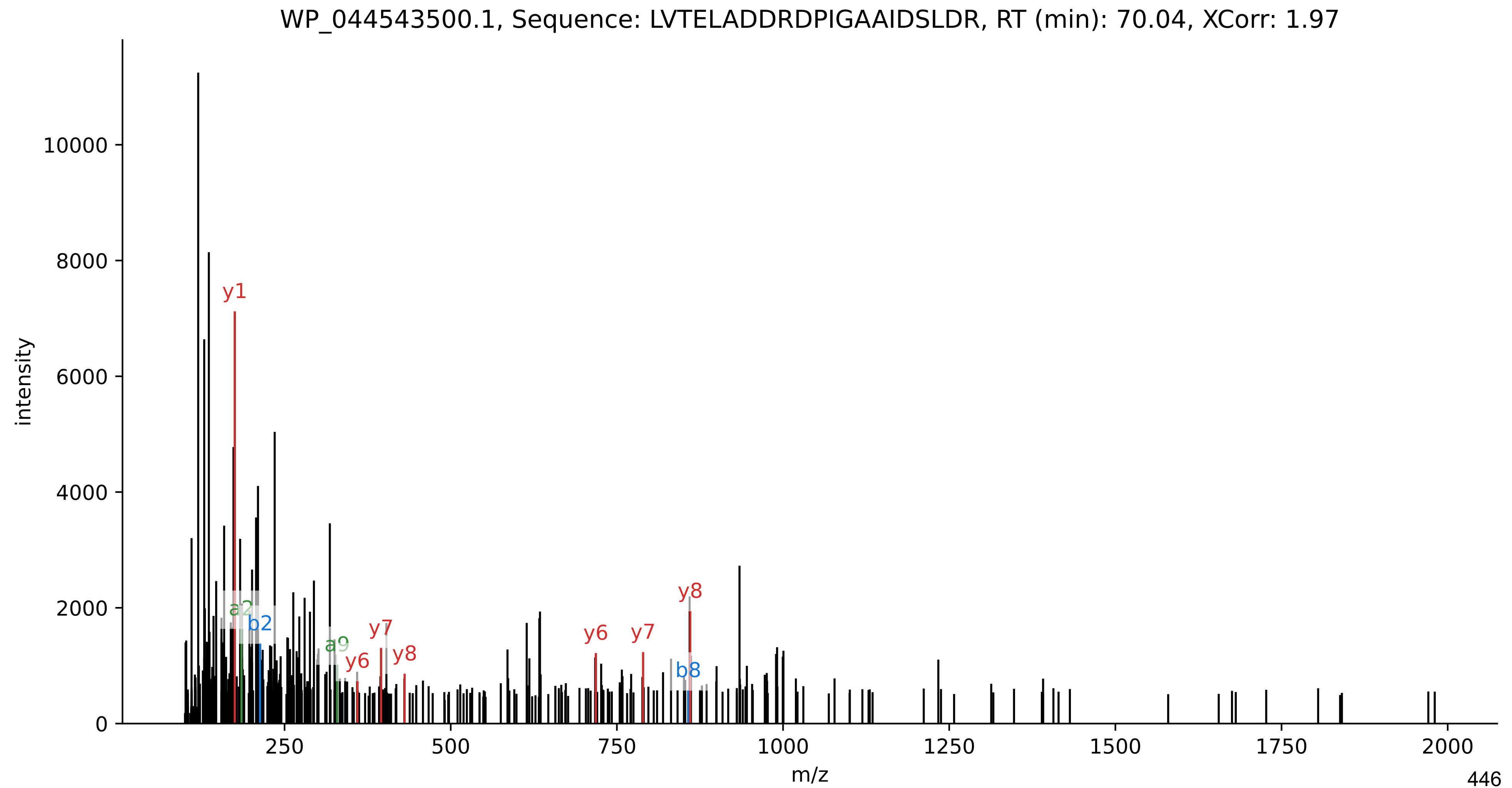

WP_044543500.1, Sequence: LVTELADDRDPIGAAIDSLDR, RT (min): 70.04, XCorr: 1.97
intensity
m/z
y1
a2
b2
a9
y6
y7
y8
y6
y7
y8
b8

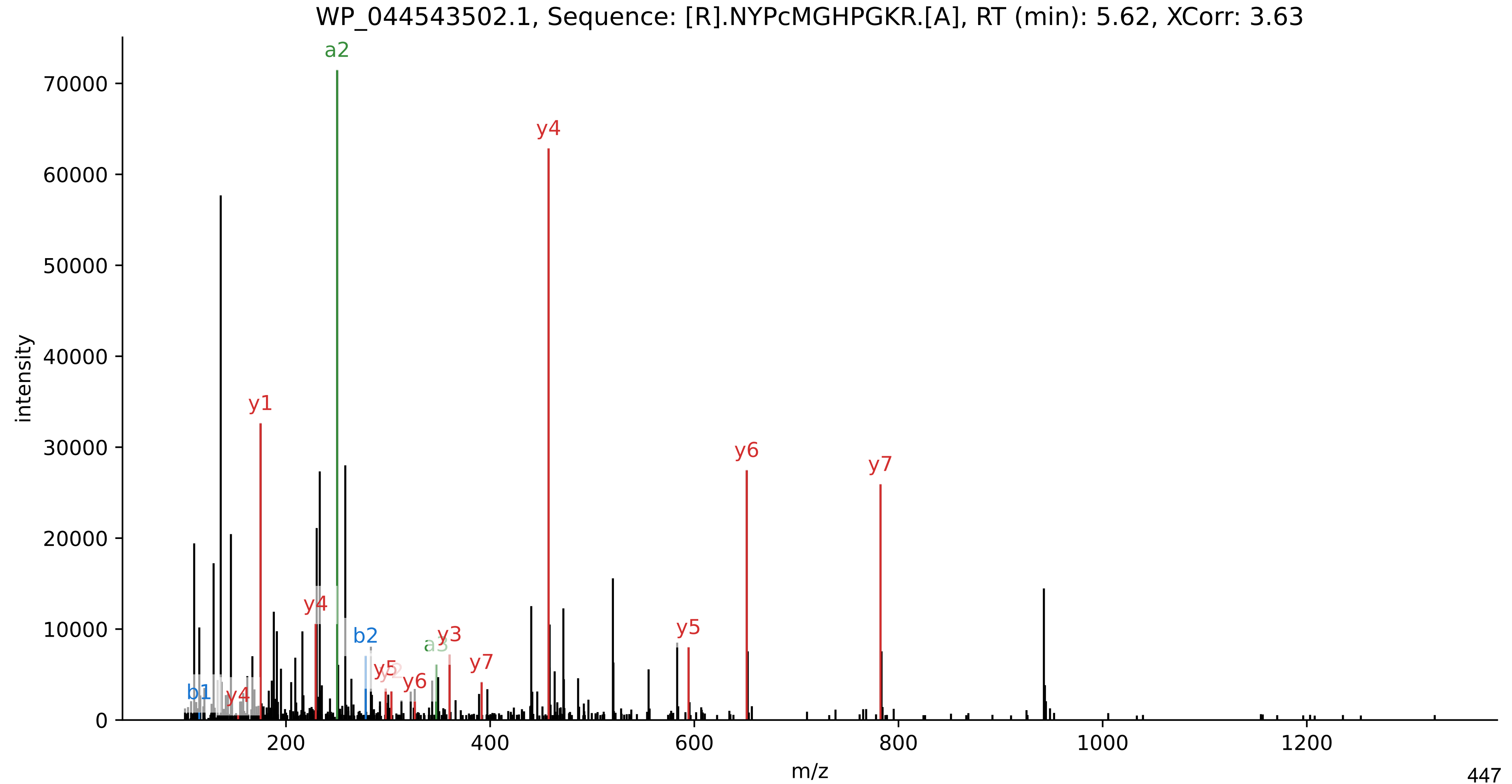

WP_044543502.1, Sequence: [R].NYPcMGHPGKR.[A], RT (min): 5.62, XCorr: 3.63
intensity
m/z
a2
y4
y1
y6
y7
y4
b2
a3
y3
y7
y5
y5
y6
b1
y4

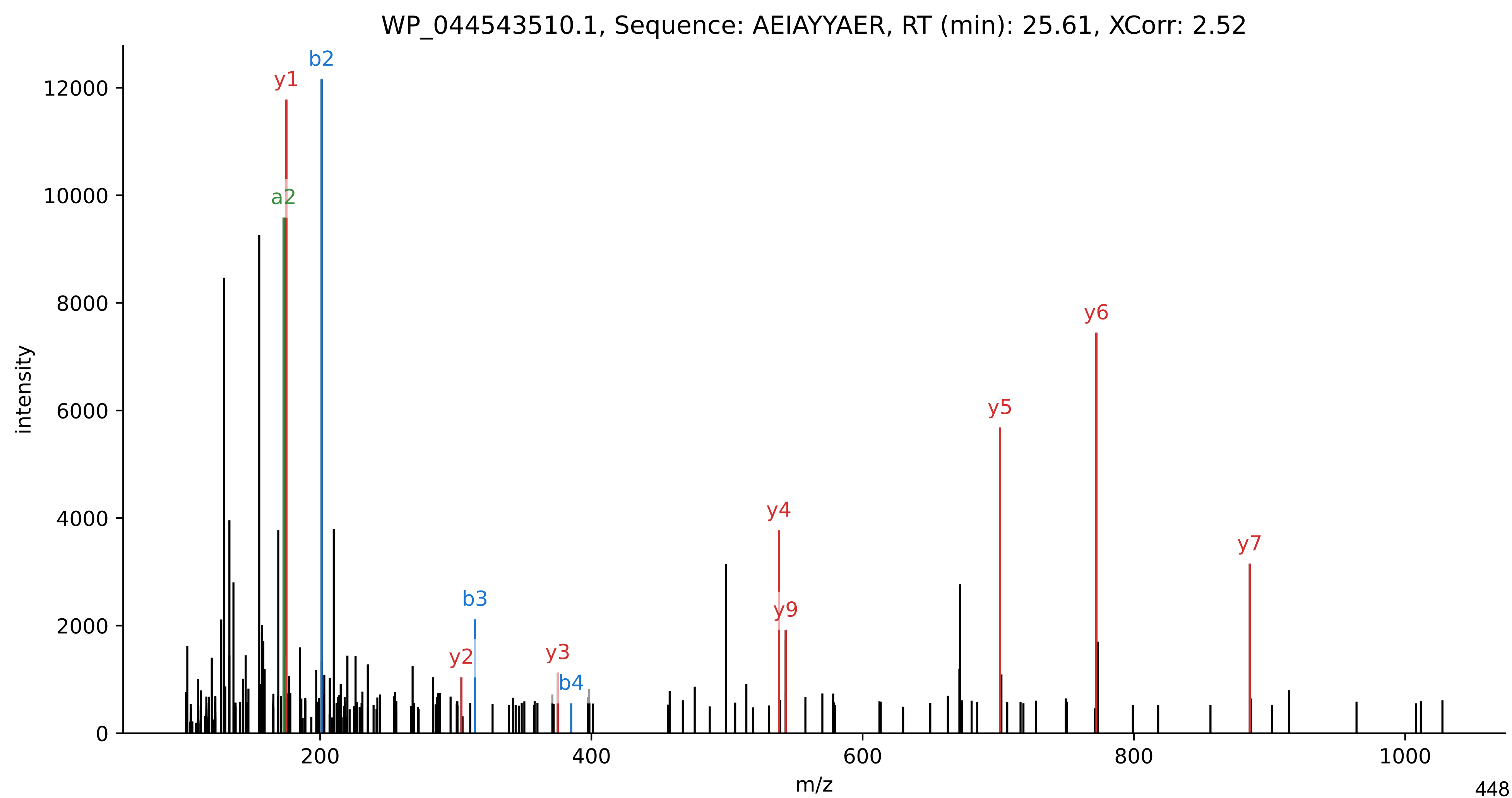

WP_044543510.1, Sequence: AEIAYYAER, RT (min): 25.61, XCorr: 2.52
intensity
m/z
b2
y1
a2
b3
y2
y3
b4
y4
y9
y5
y6
y7
0
2000
4000
6000
8000
10000
12000
200
400
600
800
1000

WP_044543564.1, Sequence: LTALLAEERR, RT (min): 23.87, XCorr: 1.48

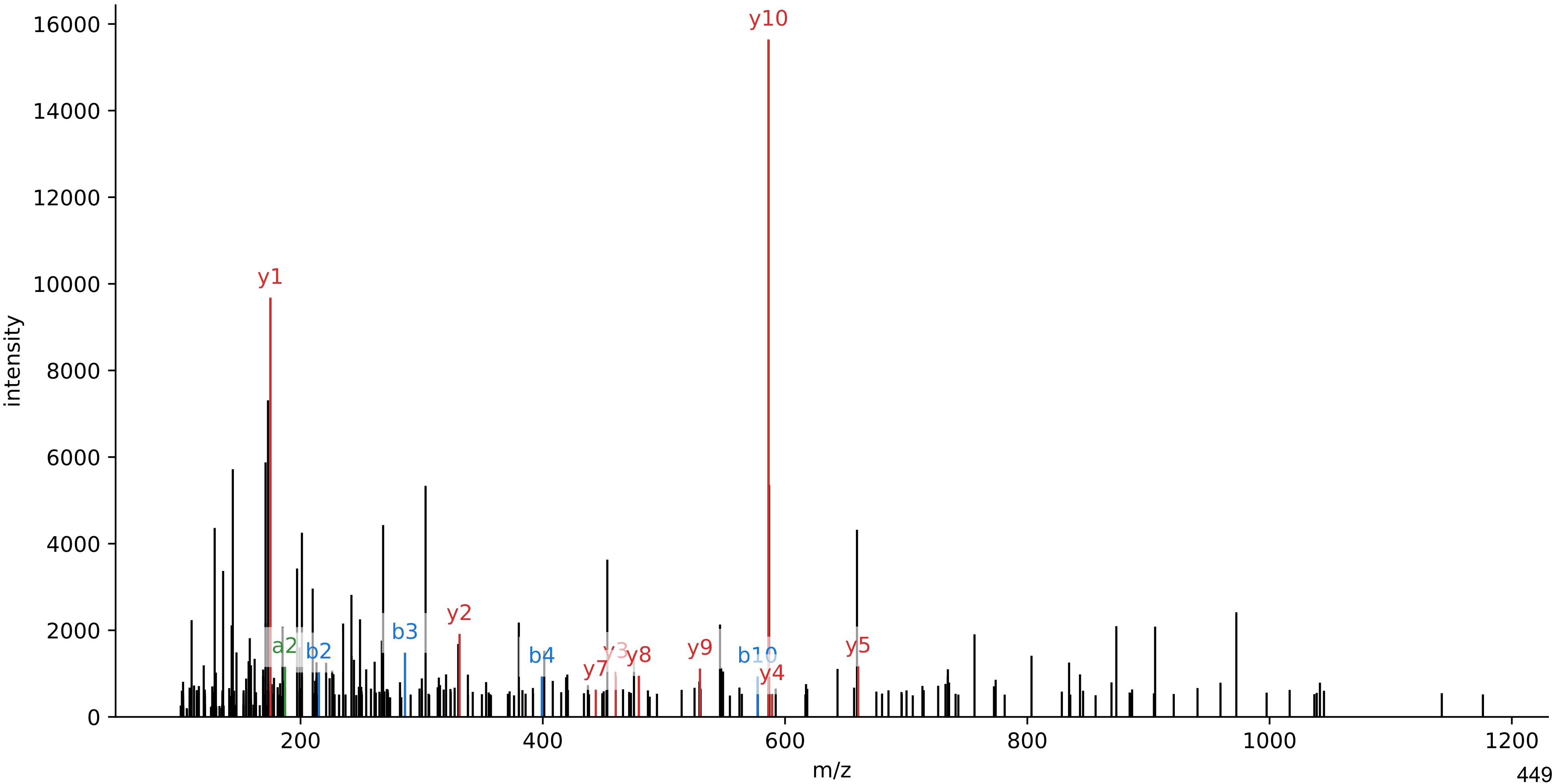

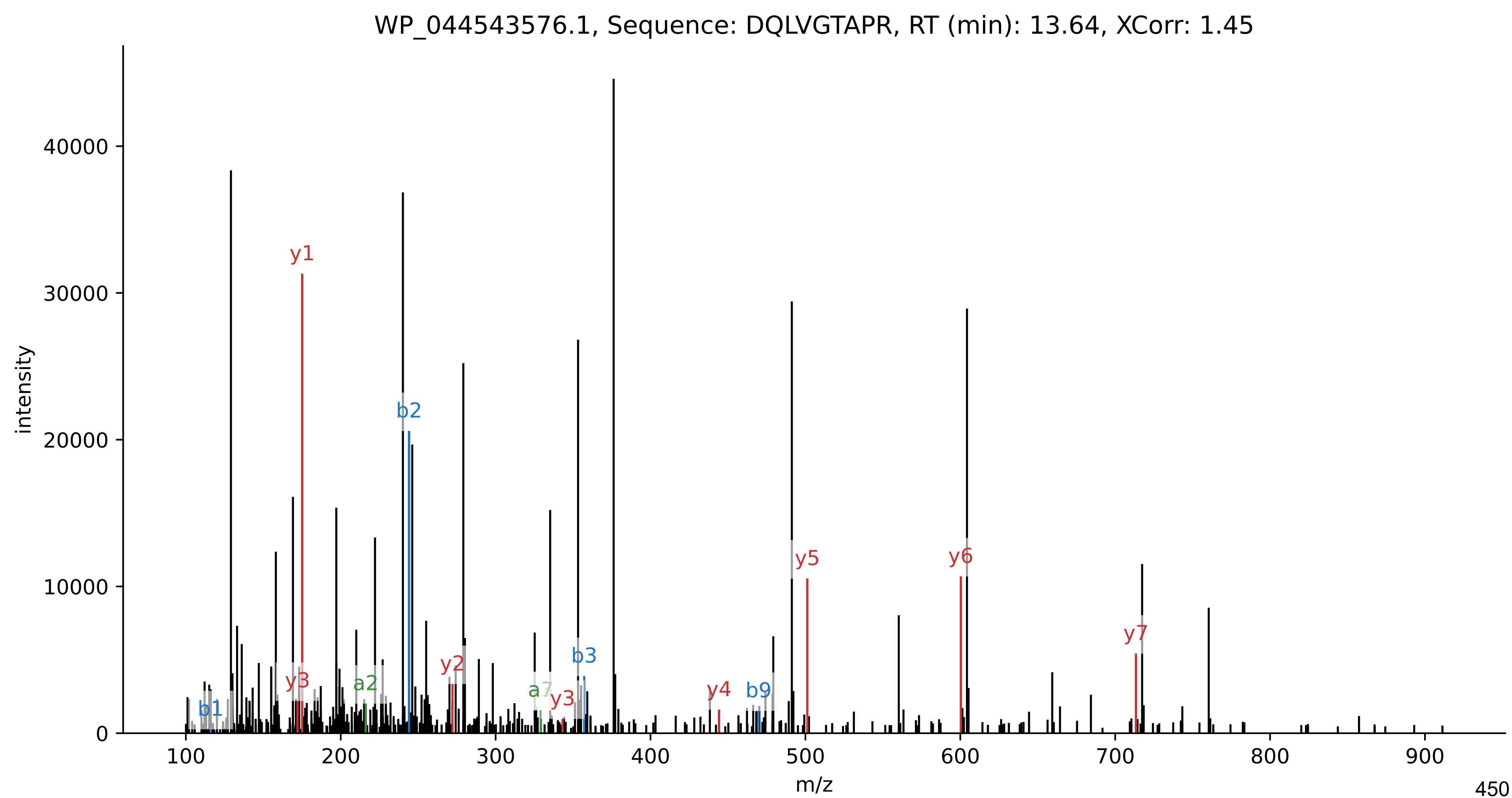
WP_044543576.1, Sequence: DQLVGTAPR, RT (min): 13.64, XCorr: 1.45
intensity
m/z
0
10000
20000
30000
40000
100
200
300
400
500
600
700
800
900
y1
b2
y2
b3
y5
y6
y7
y3
a2
a7
y3
b1
y4
b9

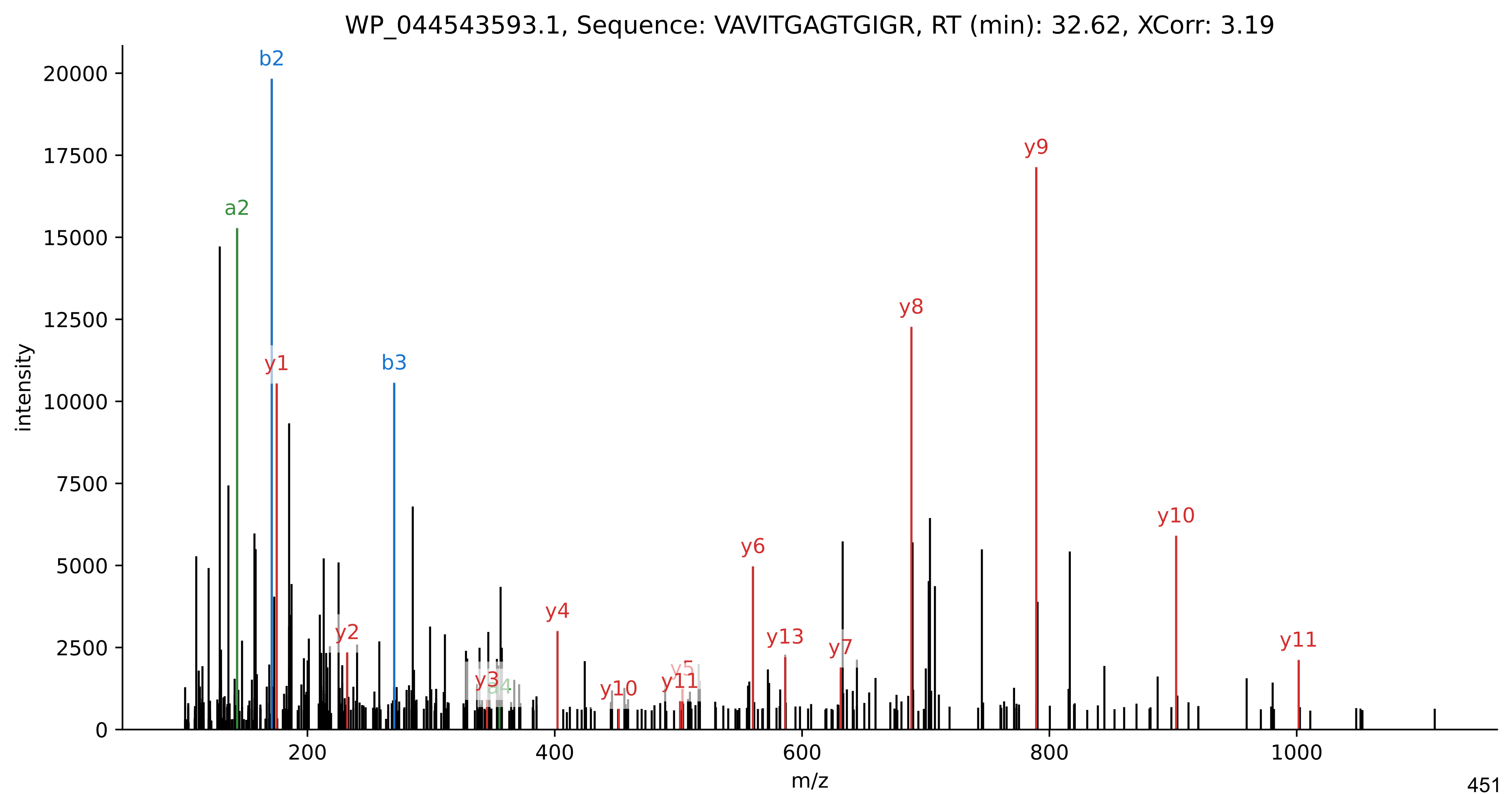

WP_044543593.1, Sequence: VAVITGAGTGIGR, RT (min): 32.62, XCorr: 3.19
b2
a2
y9
y8
y1
b3
y10
y6
y4
y2
y13
y7
y11
y3
y10
y11
intensity
m/z

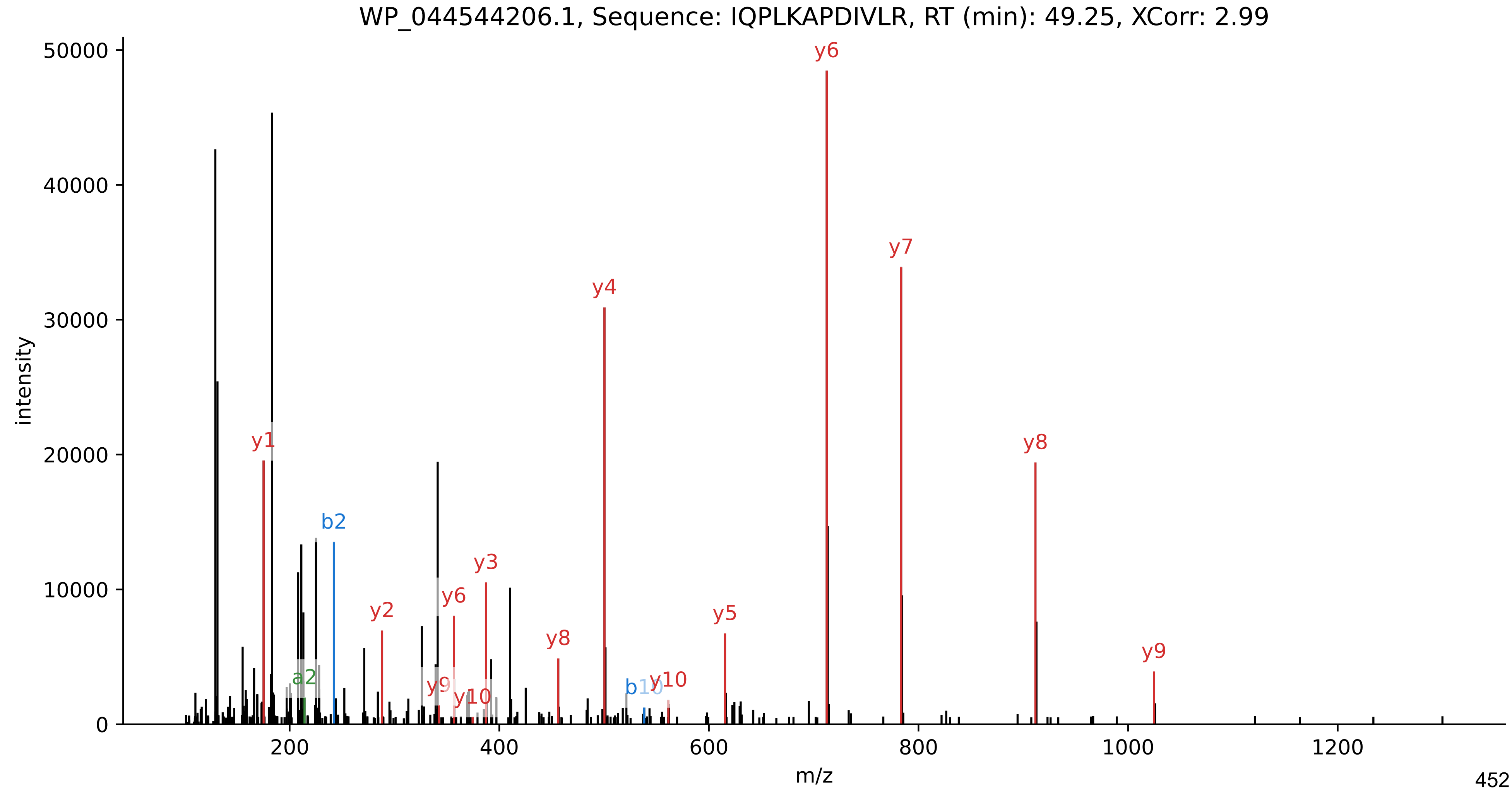

WP_044544206.1, Sequence: IQPLKAPDIVLR, RT (min): 49.25, XCorr: 2.99
intensity
m/z
y1
b2
a2
y2
y9
y6
y10
y3
y8
y4
b10
y10
y5
y6
y7
y8
y9
0
10000
20000
30000
40000
50000
200
400
600
800
1000
1200

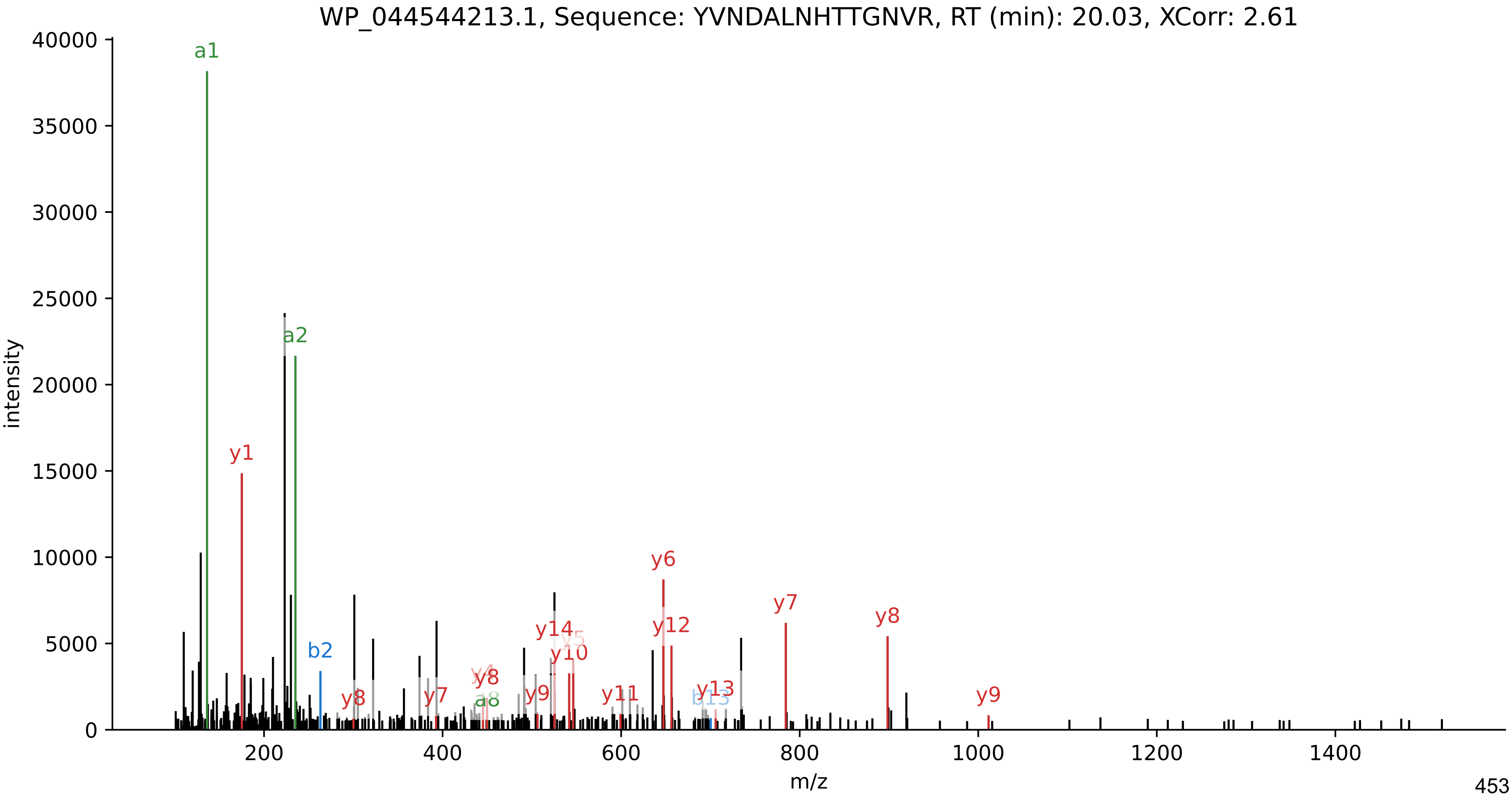
WP_044544213.1, Sequence: YVNDALNHTTGNVR, RT (min): 20.03, XCorr: 2.61
intensity
m/z
0
5000
10000
15000
20000
25000
30000
35000
40000
200
400
600
800
1000
1200
1400
a1
a2
y1
b2
y8
y7
y4
y8
a8
y9
y14
y5
y10
y11
y6
y12
b13
y13
y7
y8
y9

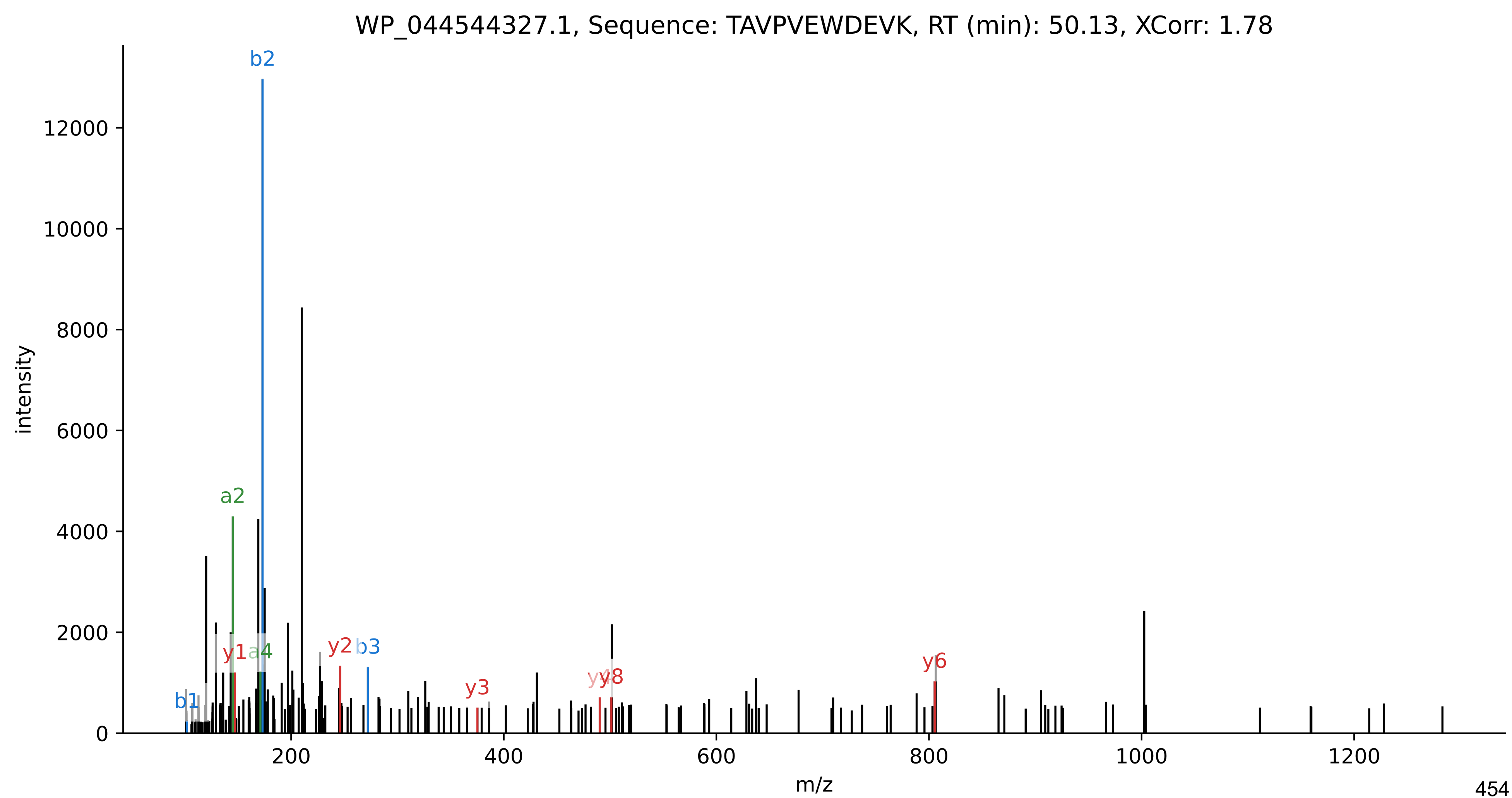
WP_044544327.1, Sequence: TAVPVEWDEVK, RT (min): 50.13, XCorr: 1.78
intensity
m/z
b1
b2
b3
a2
a4
y1
y2
y3
y4
y8
y6
0
2000
4000
6000
8000
10000
12000
200
400
600
800
1000
1200

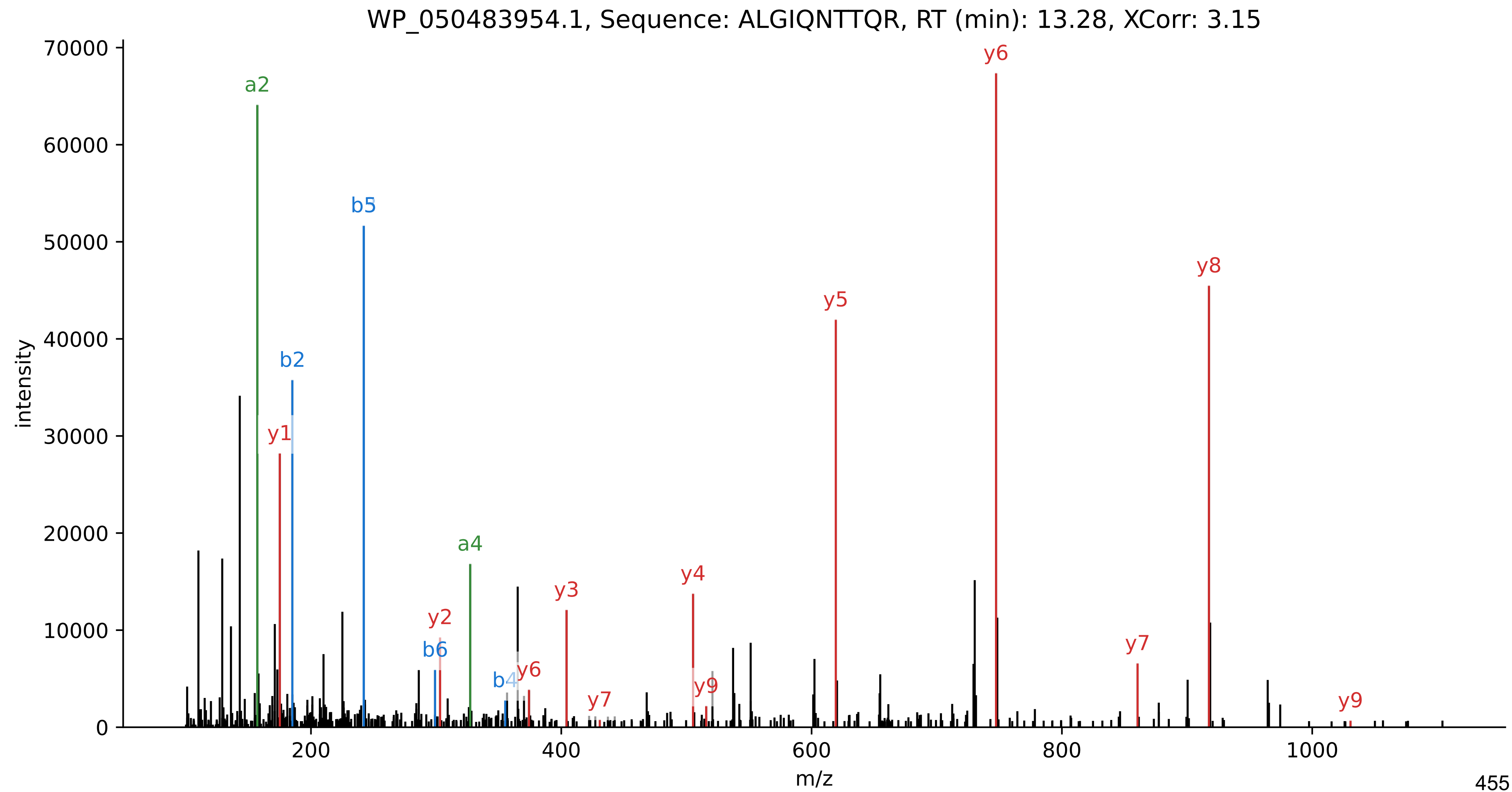

WP_050483954.1, Sequence: ALGIQNTTQR, RT (min): 13.28, XCorr: 3.15
intensity
m/z
a2
b5
b2
y1
a4
y2
b6
b4
y6
y3
y7
y4
y9
y5
y6
y8
y7
y9

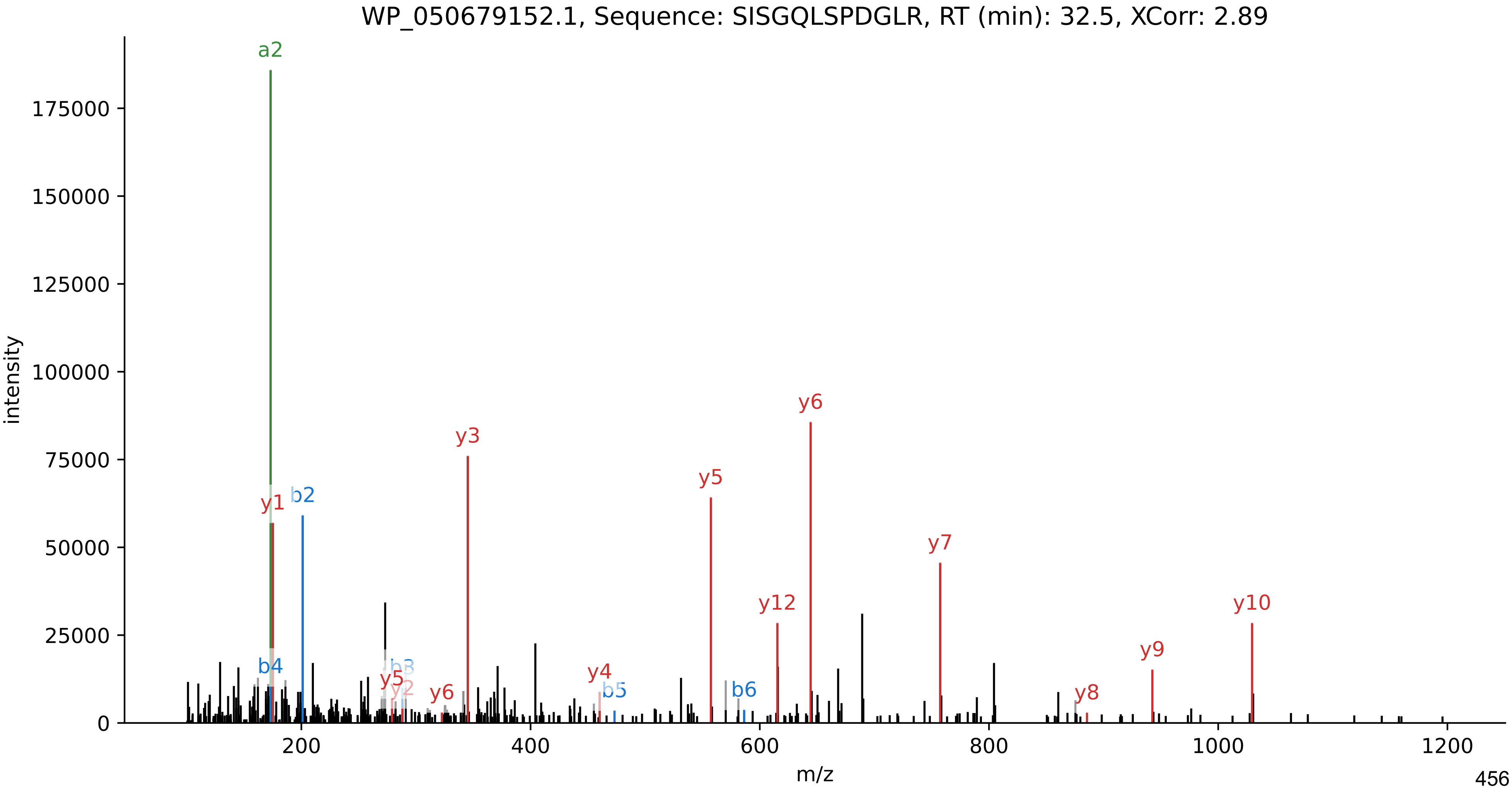
WP_050679152.1, Sequence: SISGQLSPDGLR, RT (min): 32.5, XCorr: 2.89
intensity
m/z
a2
y1
b2
y3
y6
y5
y7
y12
y10
y9
y8
y4
b5
b6
b4
b3
y2

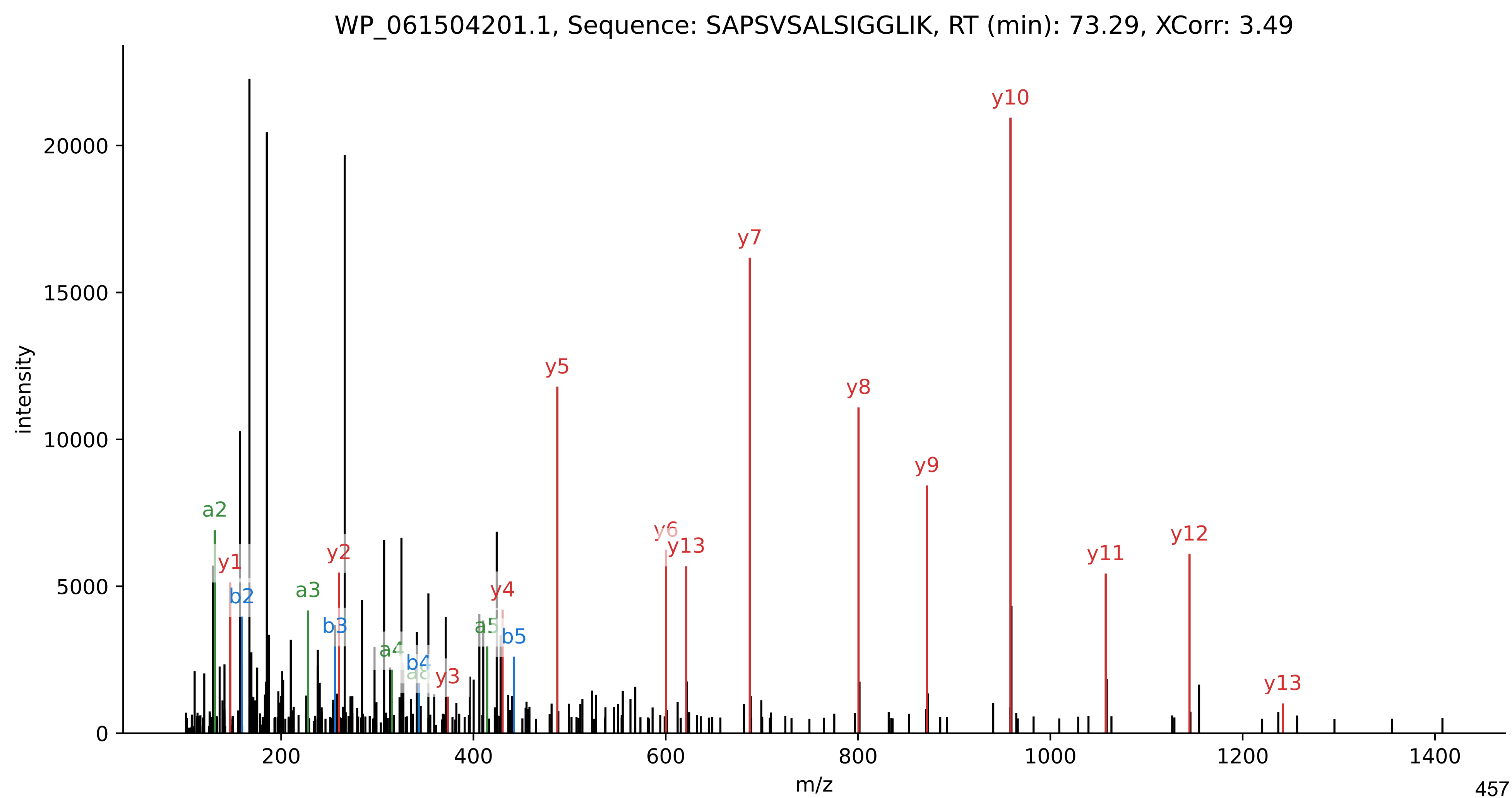

WP_061504201.1, Sequence: SAPSVSALSIGGLIK, RT (min): 73.29, XCorr: 3.49
intensity
m/z
y10
y7
y5
y8
y9
a2
y1
b2
y2
a3
b3
a4
b4
y3
a5
b5
y4
y6
y13
y11
y12
y13
0
5000
10000
15000
20000
200
400
600
800
1000
1200
1400

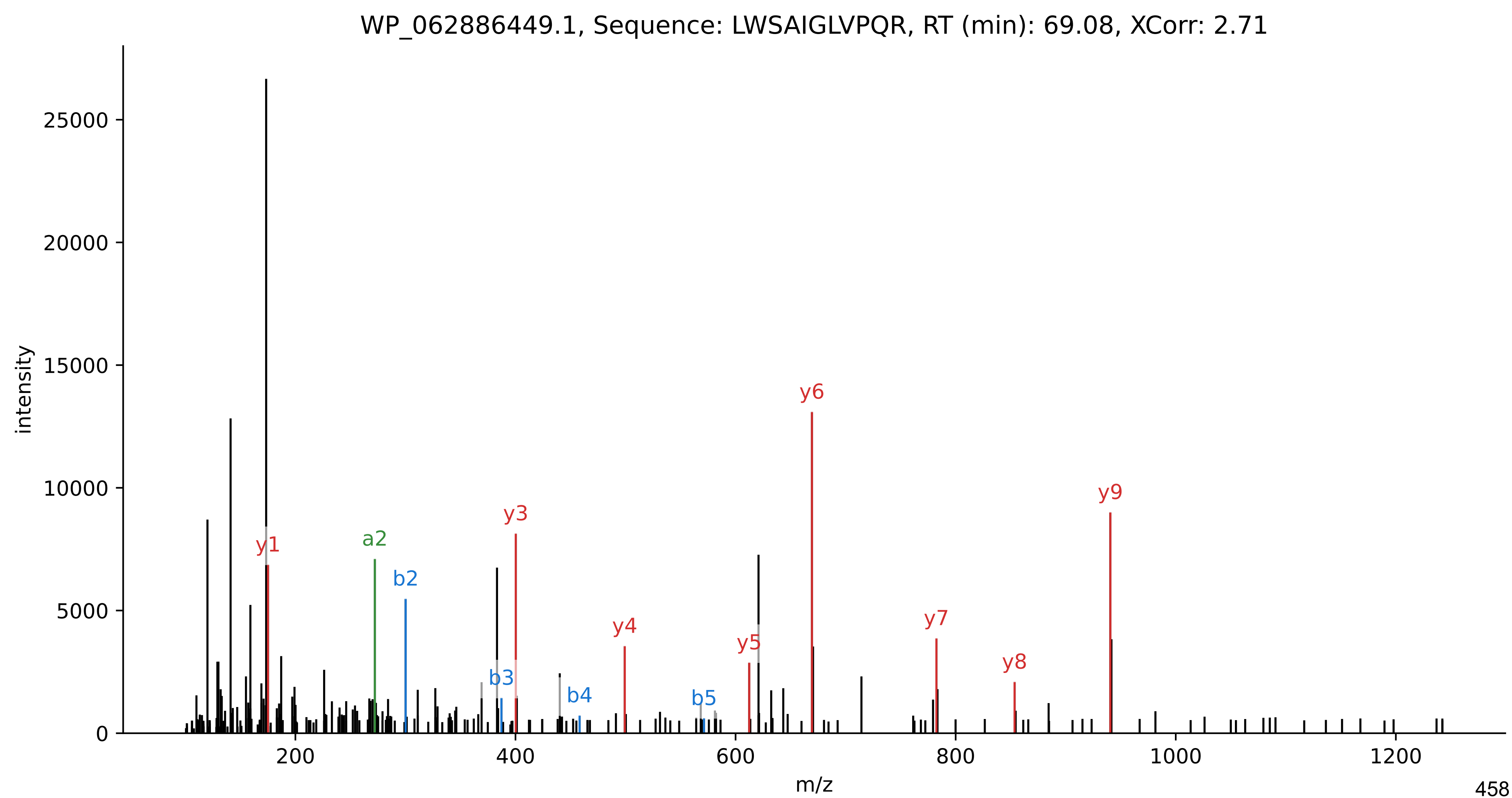

WP_062886449.1, Sequence: LWSAIGLVPQR, RT (min): 69.08, XCorr: 2.71
intensity
m/z
y1
a2
b2
b3
y3
b4
y4
b5
y5
y6
y7
y8
y9

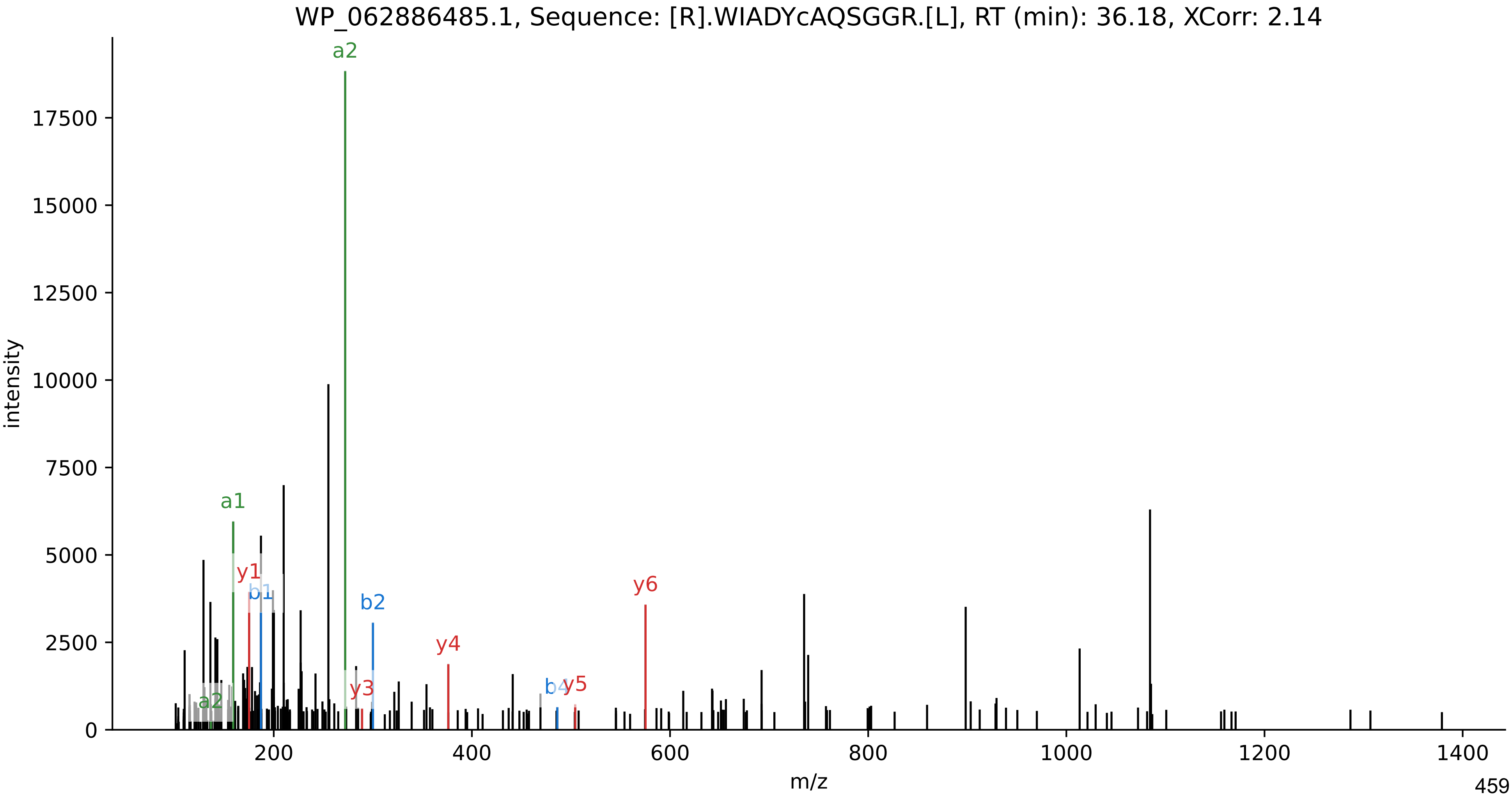
WP_062886485.1, Sequence: [R].WIADYcAQSGGR.[L], RT (min): 36.18, XCorr: 2.14
intensity
m/z
a2
a1
y1
b1
b2
y6
y4
y3
b4
y5
0
2500
5000
7500
10000
12500
15000
17500
200
400
600
800
1000
1200
1400

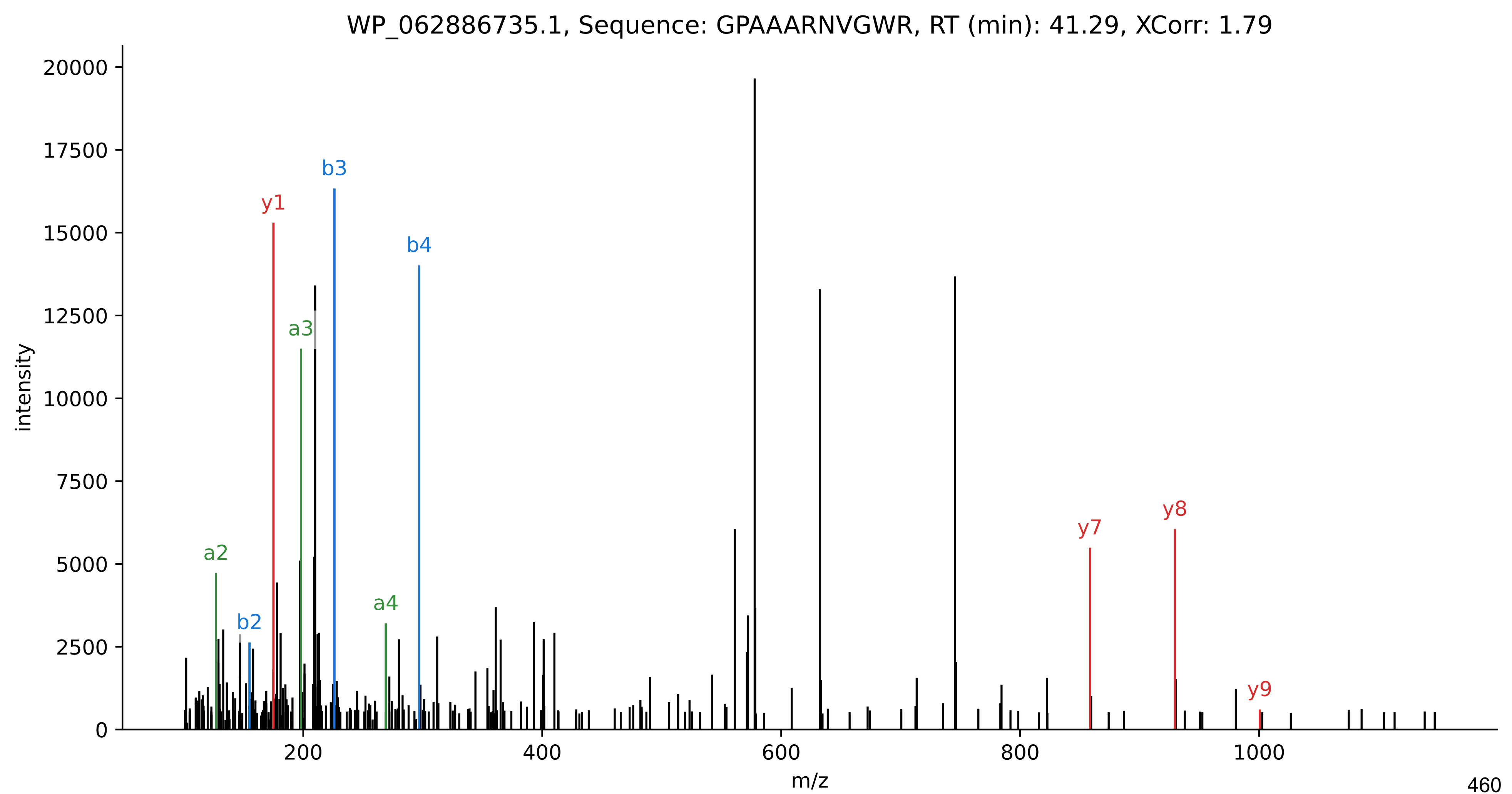
WP_062886735.1, Sequence: GPAAARNVGWR, RT (min): 41.29, XCorr: 1.79
intensity
m/z
0
2500
5000
7500
10000
12500
15000
17500
20000
200
400
600
800
1000
a2
b2
y1
a3
b3
a4
b4
y7
y8
y9

WP_062886737.1, Sequence: AMFANTLAASGK, RT (min): 35.18, XCorr: 2.96

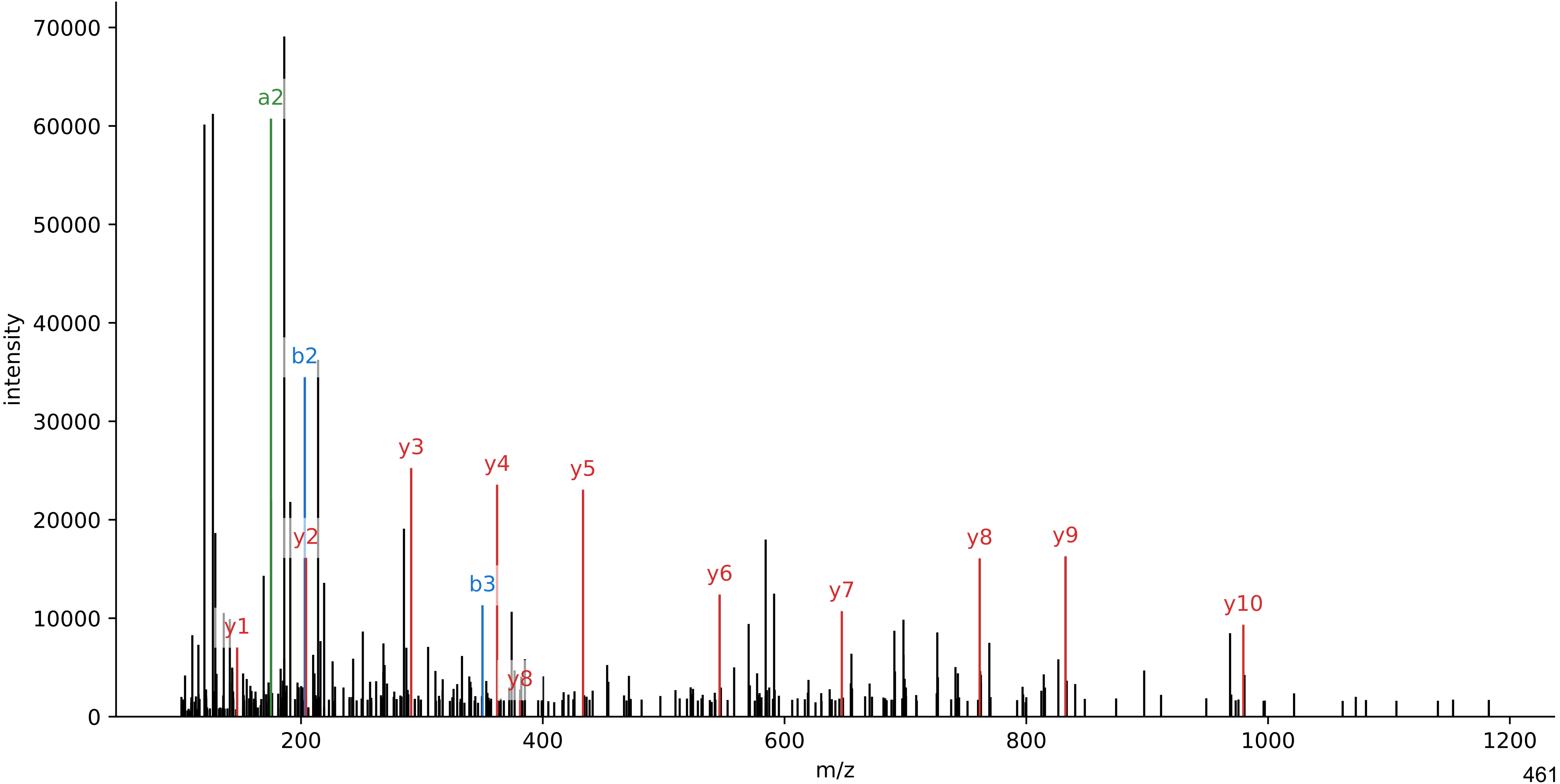

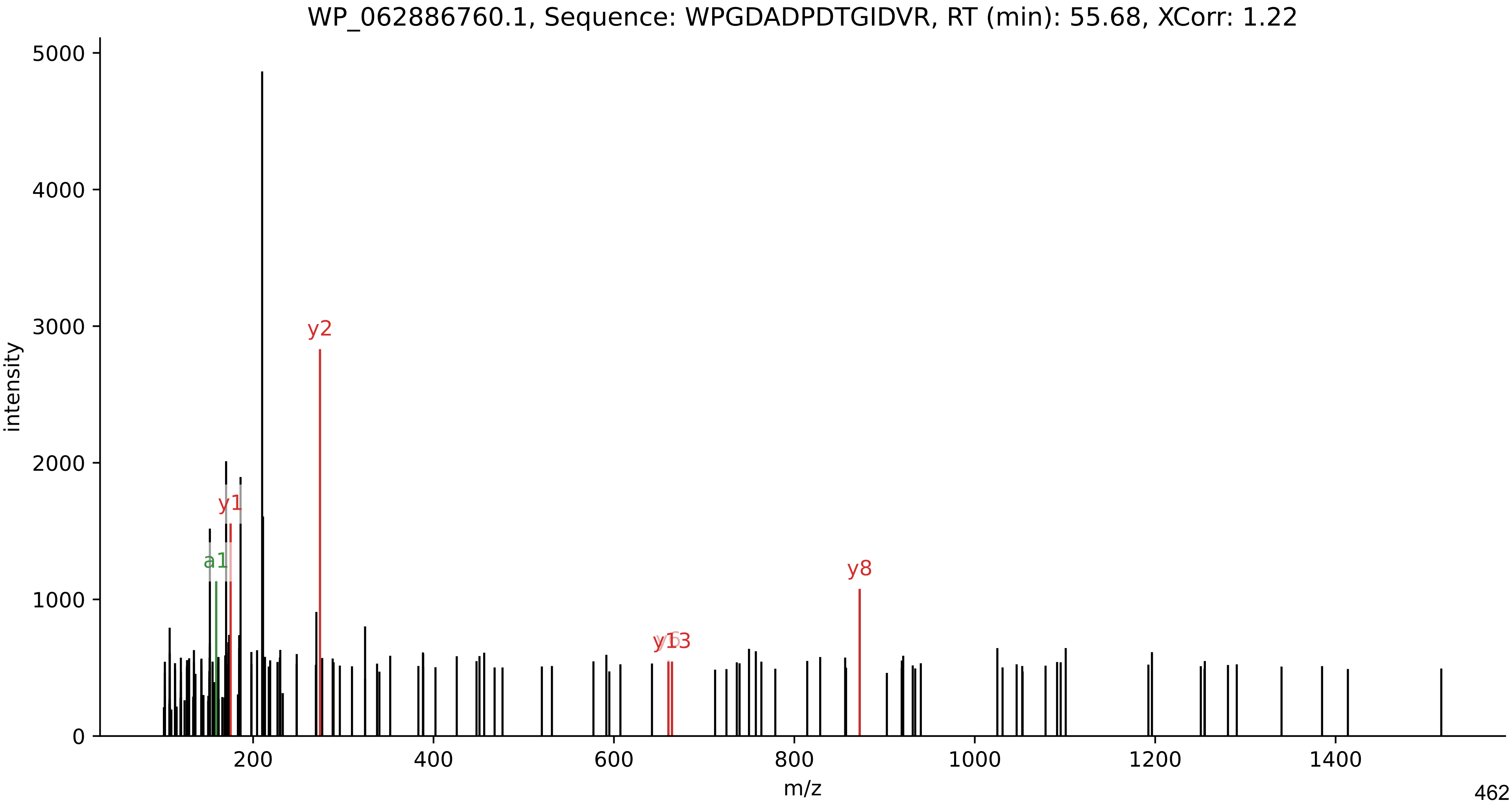
WP_062886760.1, Sequence: WPGDADPDTGIDVR, RT (min): 55.68, XCorr: 1.22
intensity
m/z
y2
y1
a1
y8
y13
0
1000
2000
3000
4000
5000
200
400
600
800
1000
1200
1400

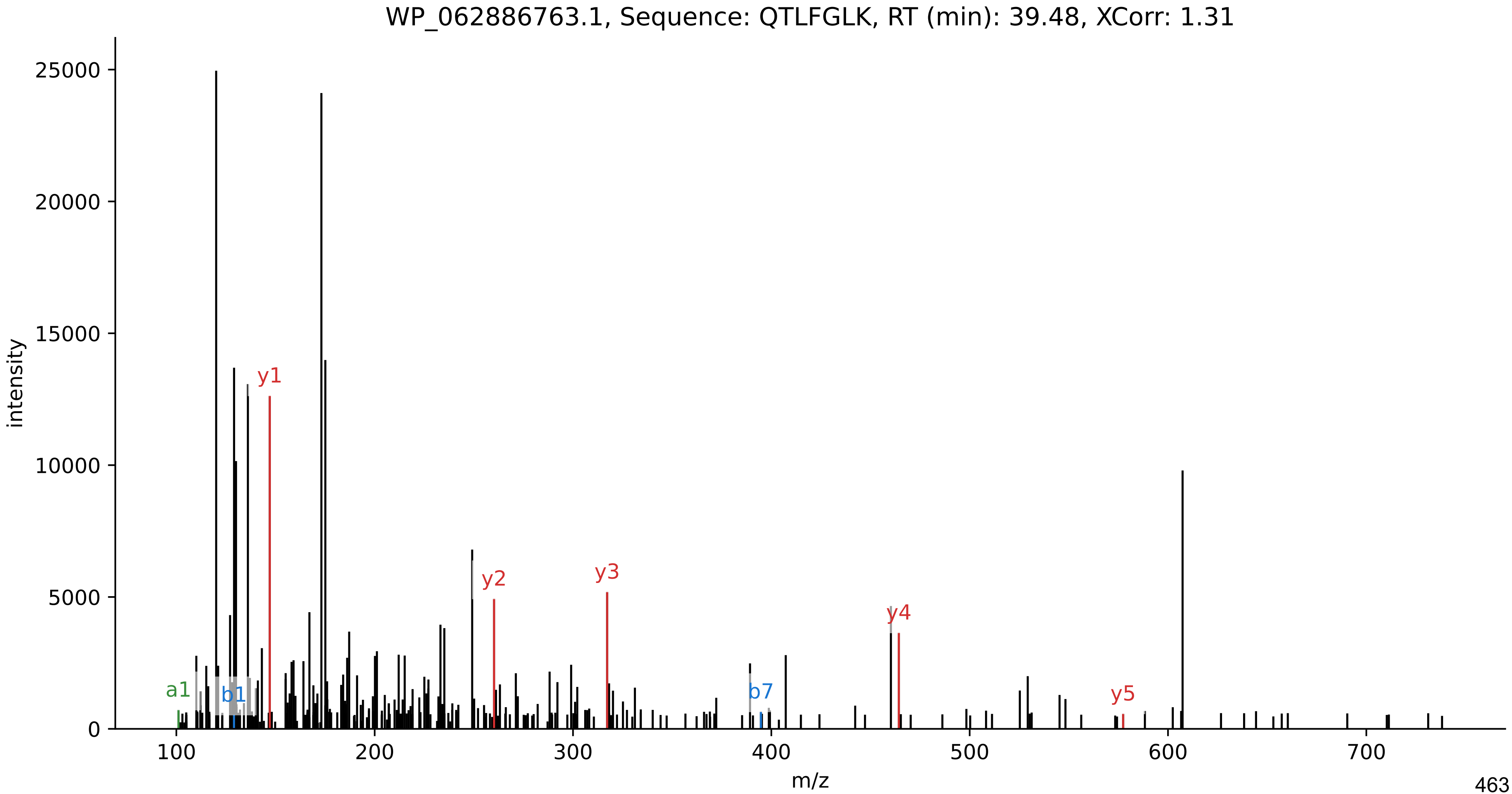

WP_062886763.1, Sequence: QTLFGLK, RT (min): 39.48, XCorr: 1.31
intensity
m/z
y1
y2
y3
y4
y5
a1
b1
b7

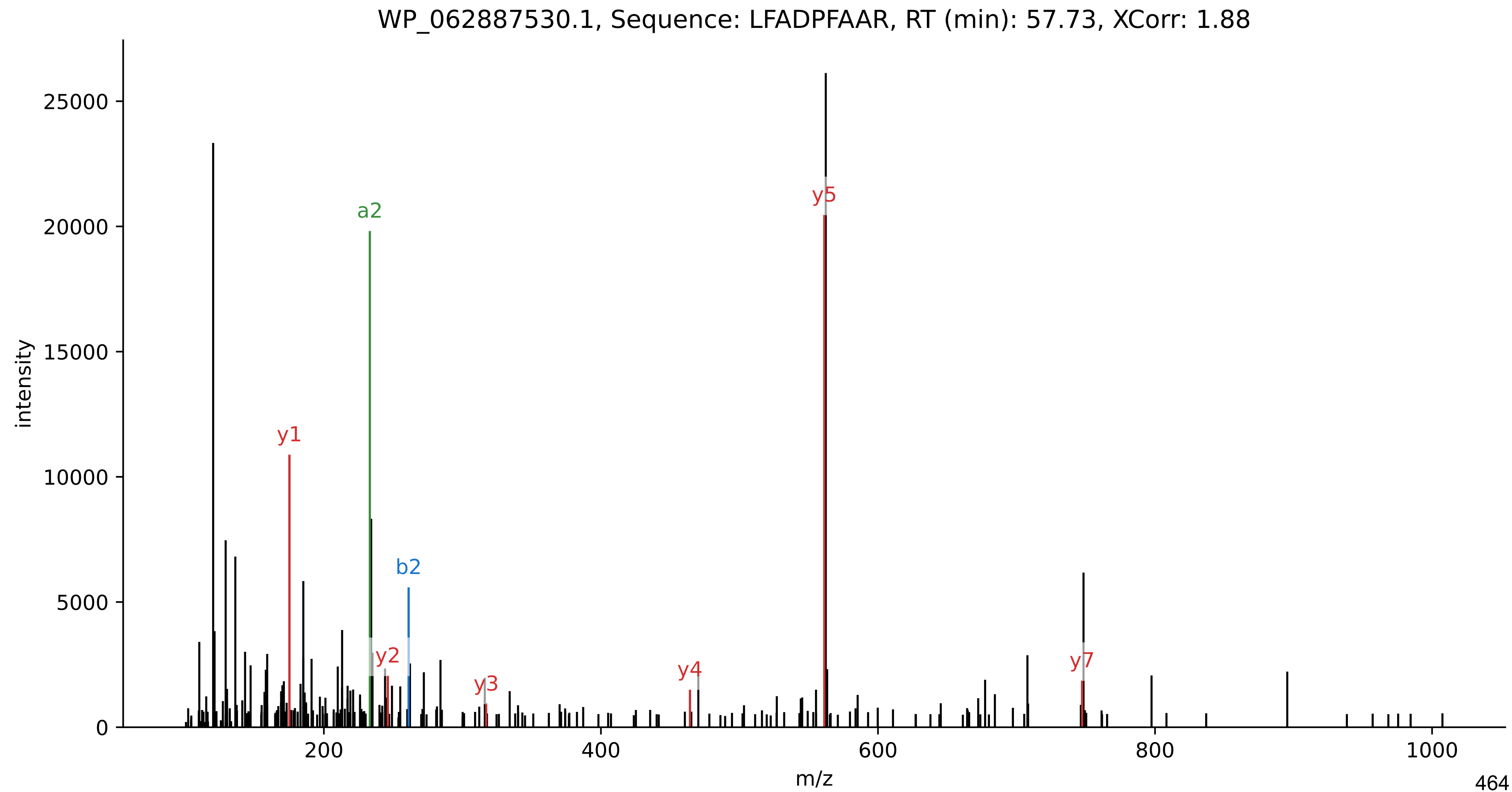

WP_062887530.1, Sequence: LFADPFAAR, RT (min): 57.73, XCorr: 1.88
intensity
m/z
a2
y1
b2
y2
y3
y4
y5
y7

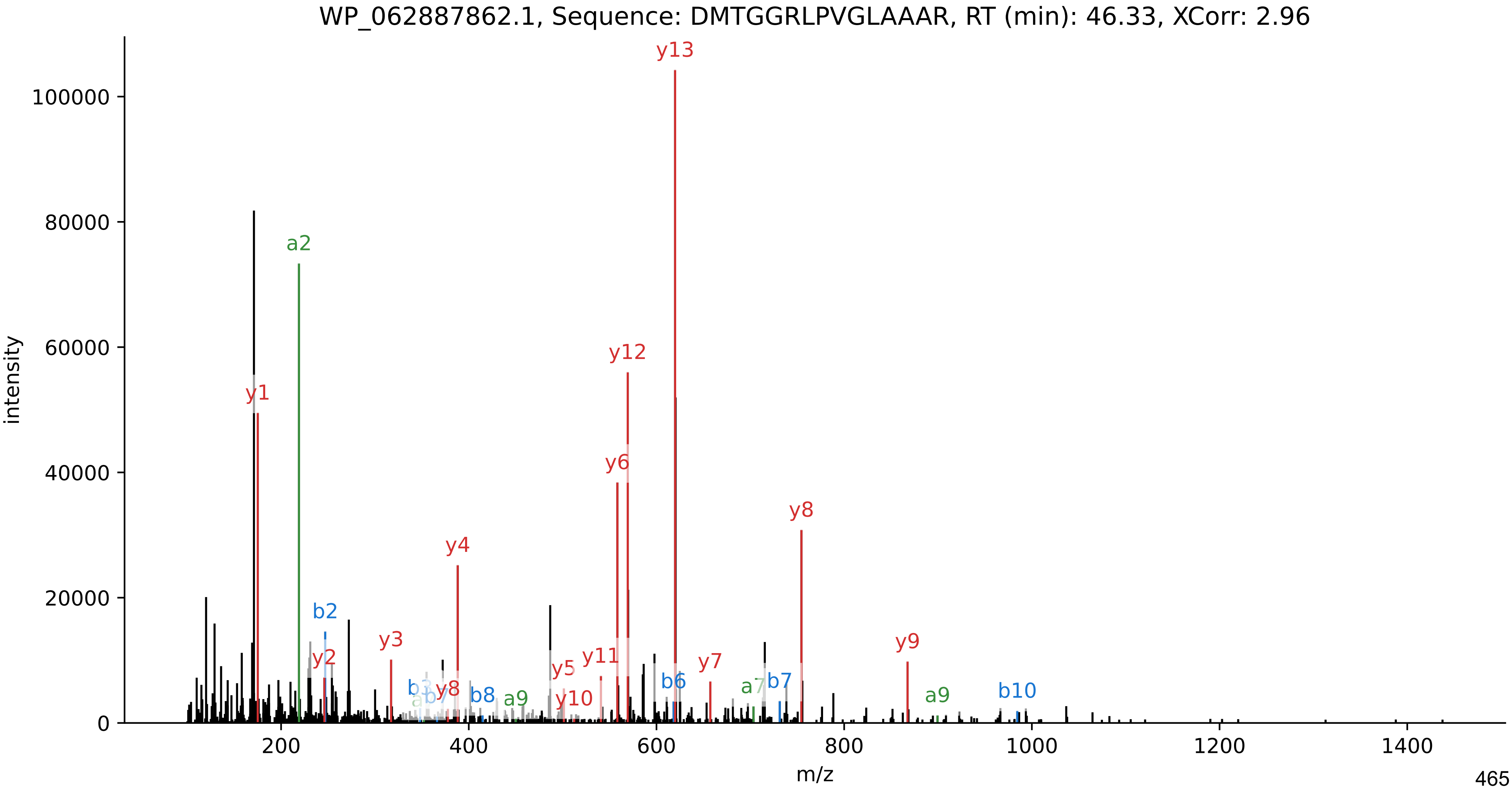

WP_062887862.1, Sequence: DMTGGRLPVGLAAAR, RT (min): 46.33, XCorr: 2.96
intensity
m/z
y13
y12
y1
a2
y6
y8
y4
b2
y3
y2
y9
y11
y7
y5
y10
b6
b7
a7
b8
a9
y8
a9
b10
0
20000
40000
60000
80000
100000
200
400
600
800
1000
1200
1400

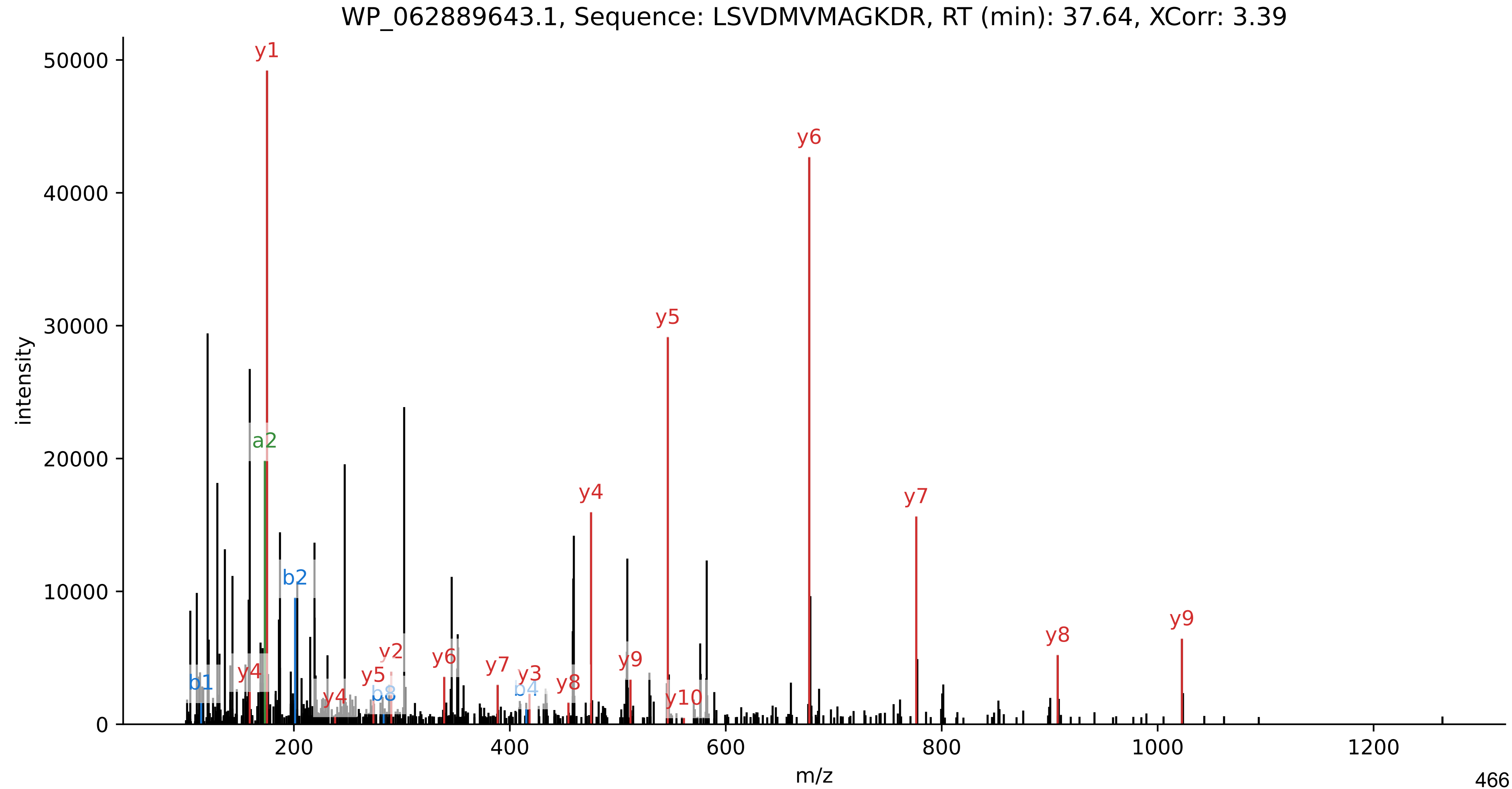

WP_062889643.1, Sequence: LSVDMVMAGKDR, RT (min): 37.64, XCorr: 3.39
intensity
m/z
y1
y6
y5
a2
y4
y7
b2
y9
y8
b1
y4
y2
y6
y7
y3
y9
y5
b8
b4
y8
y4
y10

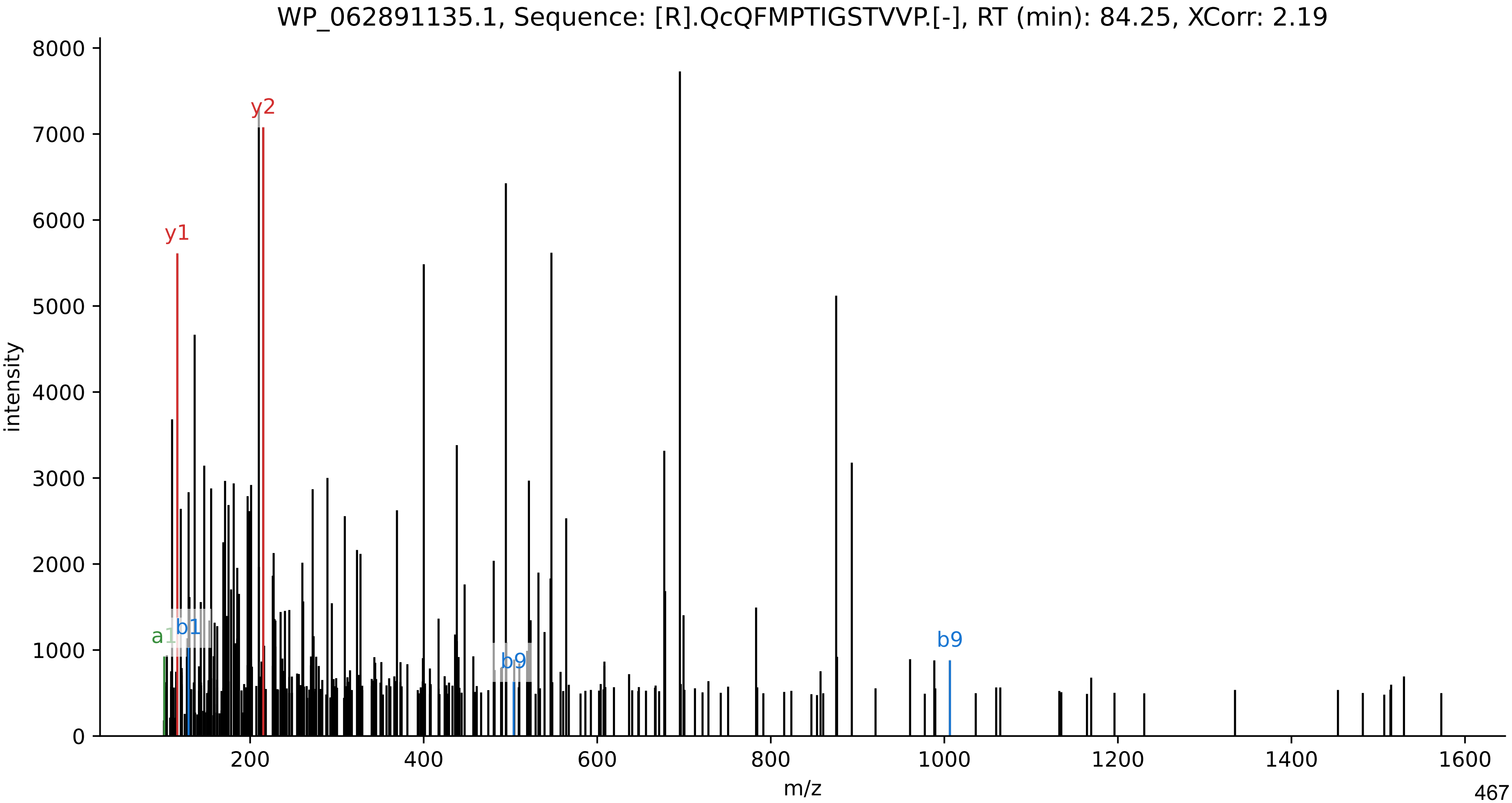
WP_062891135.1, Sequence: [R].QcQFMPTIGSTVVP.[-], RT (min): 84.25, XCorr: 2.19
intensity
m/z
y1
y2
a1
b1
b9
b9

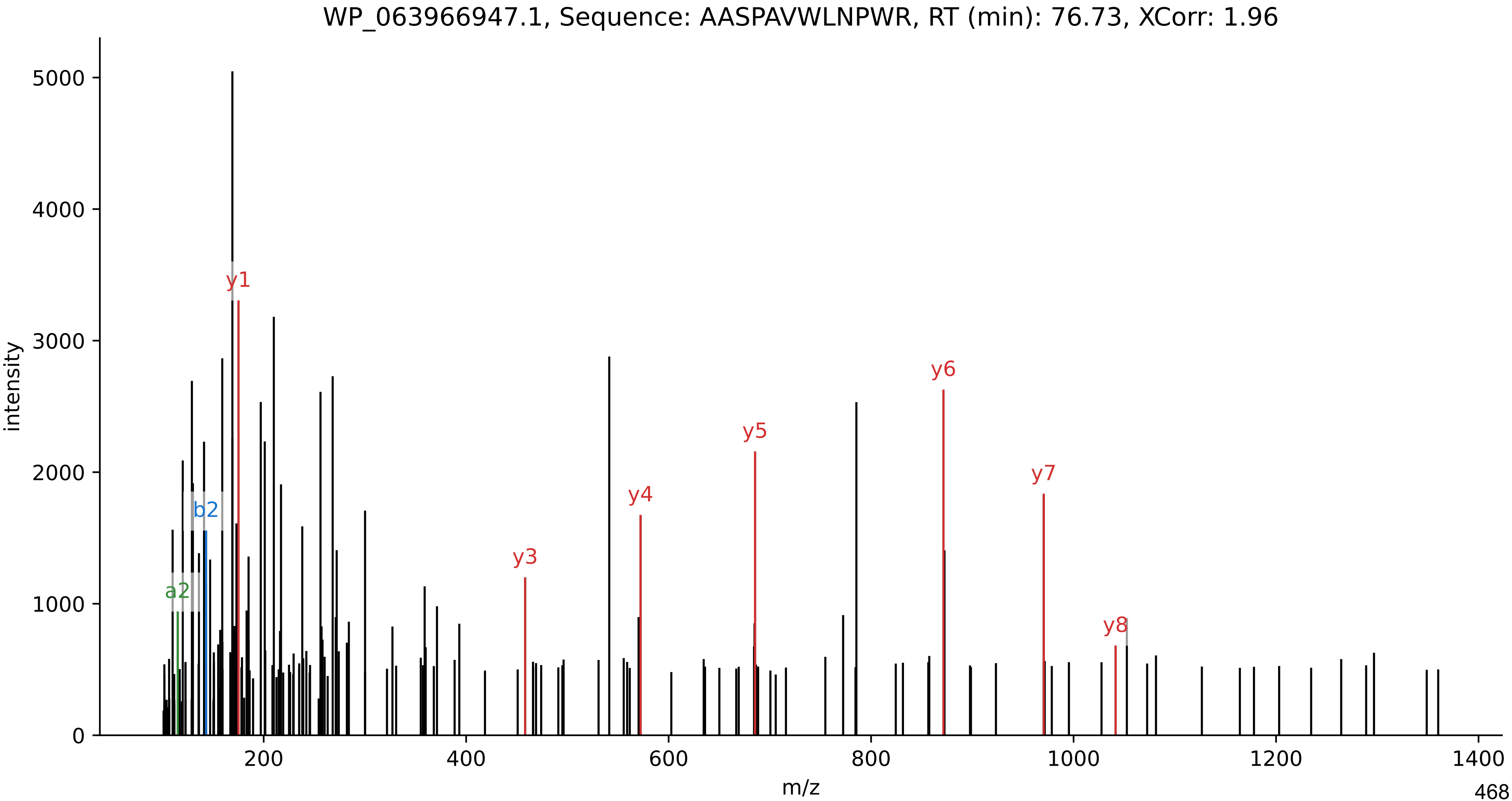
WP_063966947.1, Sequence: AASPAVWLNPWR, RT (min): 76.73, XCorr: 1.96
intensity
m/z
y1
b2
a2
y3
y4
y5
y6
y7
y8

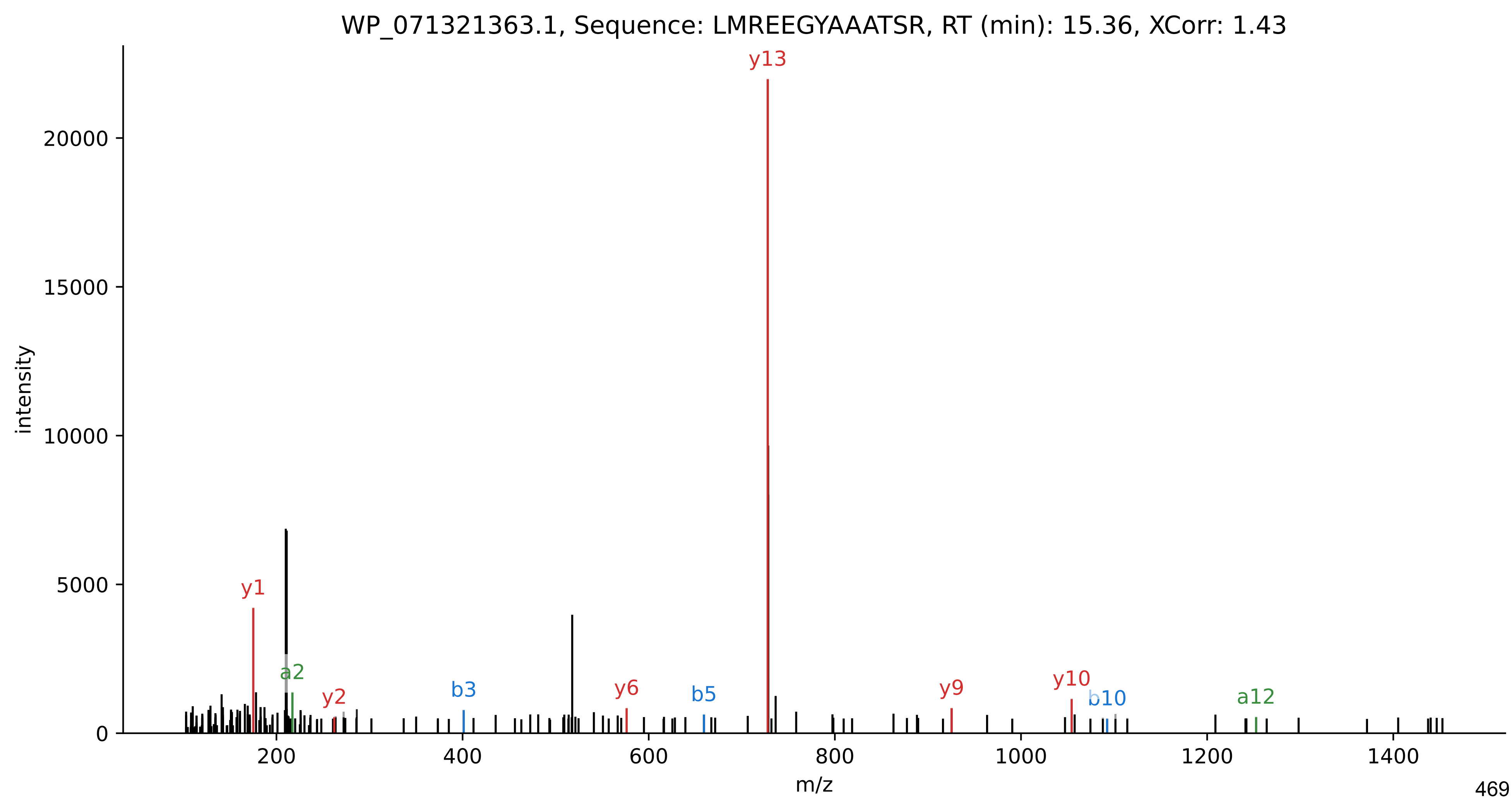

WP_071321363.1, Sequence: LMREEGYAAATSR, RT (min): 15.36, XCorr: 1.43
y13
y1
a2
y2
b3
y6
b5
y9
y10
b10
a12
intensity
m/z
0
5000
10000
15000
20000
200
400
600
800
1000
1200
1400

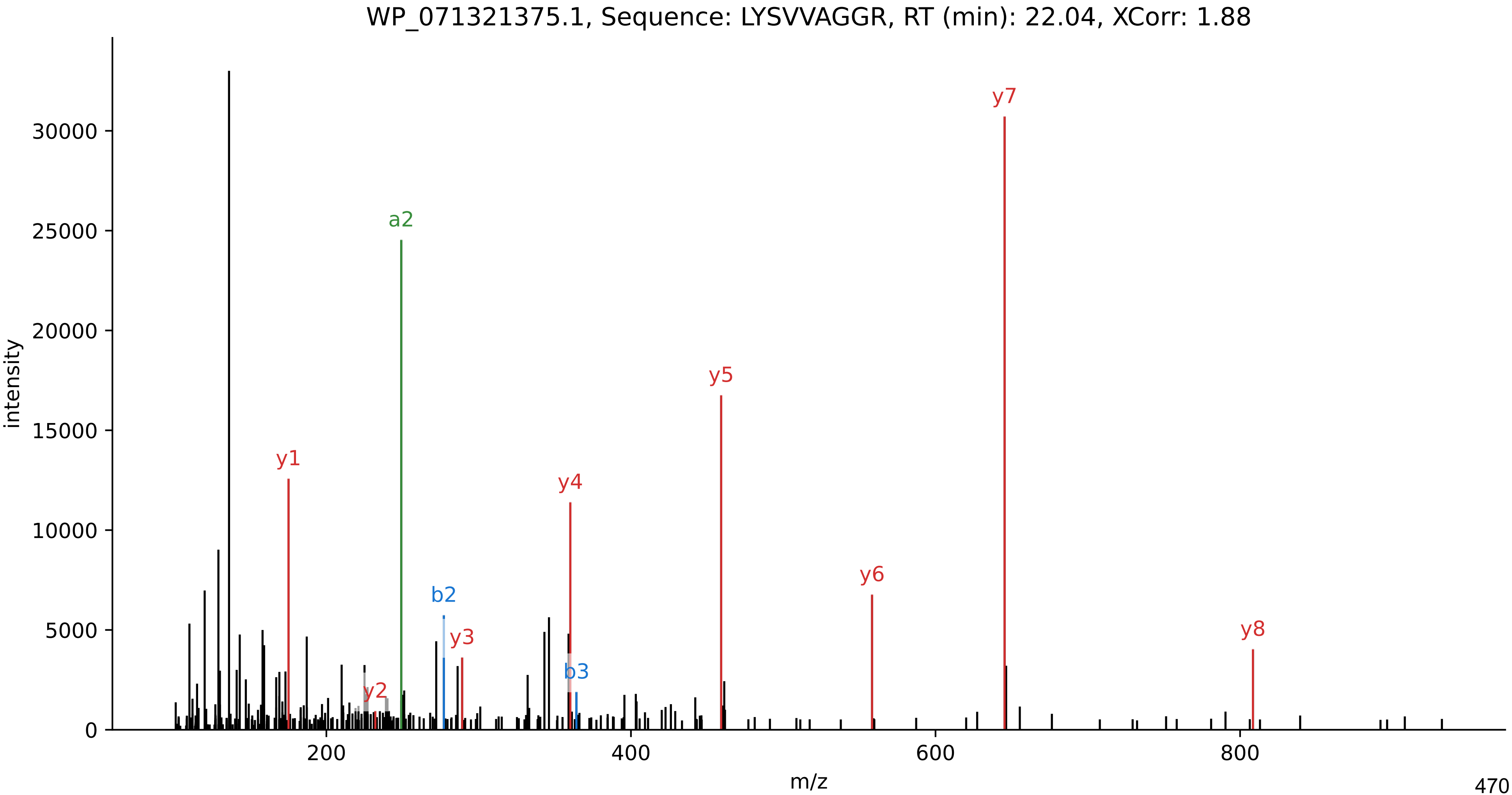
WP_071321375.1, Sequence: LYSVVAGGR, RT (min): 22.04, XCorr: 1.88
intensity
m/z
a2
y1
y2
y3
y4
y5
y6
y7
y8
b2
b3

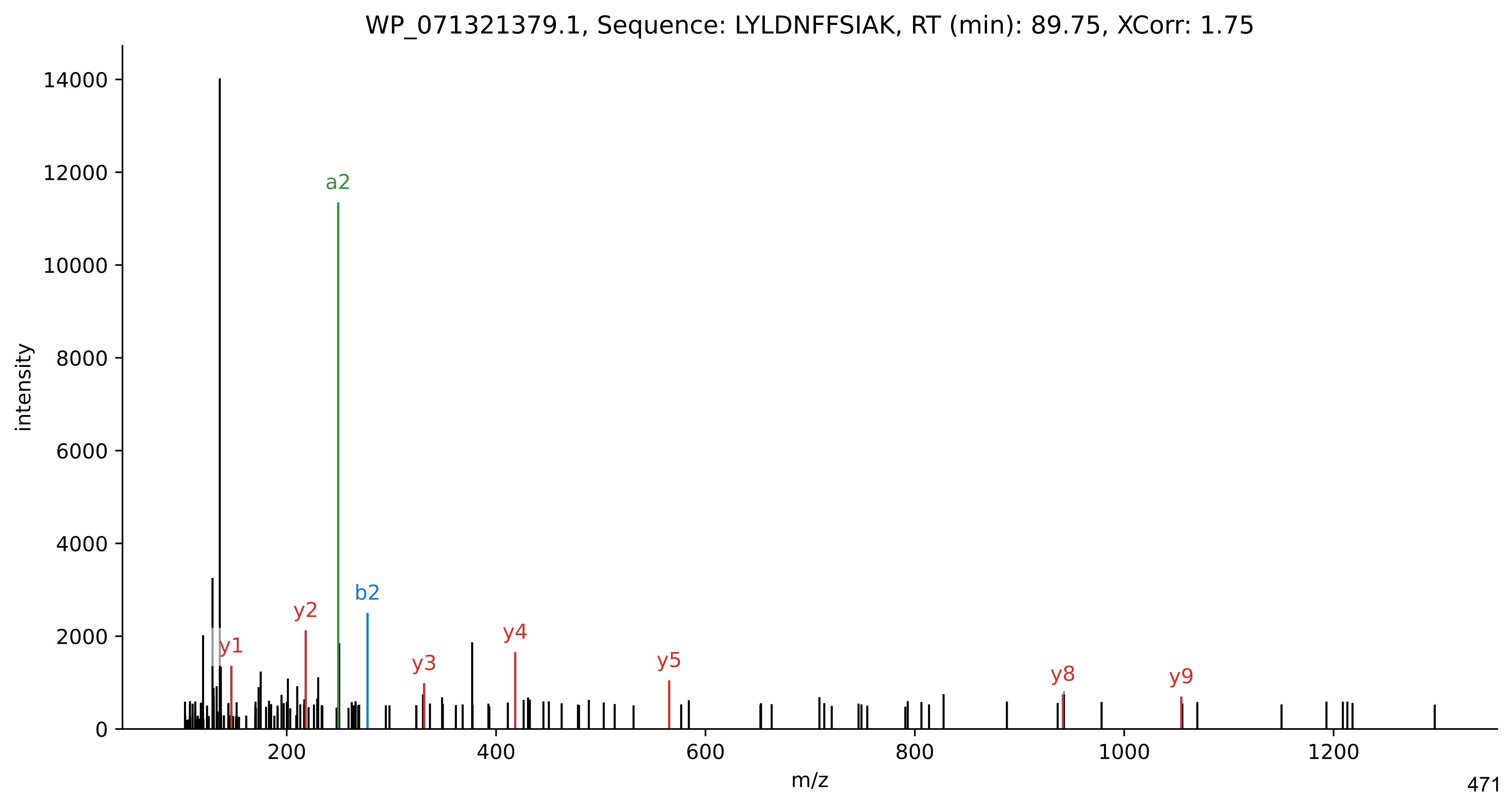
WP_071321379.1, Sequence: LYLDNFFSIAK, RT (min): 89.75, XCorr: 1.75
intensity
m/z
a2
b2
y1
y2
y3
y4
y5
y8
y9
0
2000
4000
6000
8000
10000
12000
14000
200
400
600
800
1000
1200

WP_071321387.1, Sequence: SASAHQVGDRLR, RT (min): 4.56, XCorr: 3.86

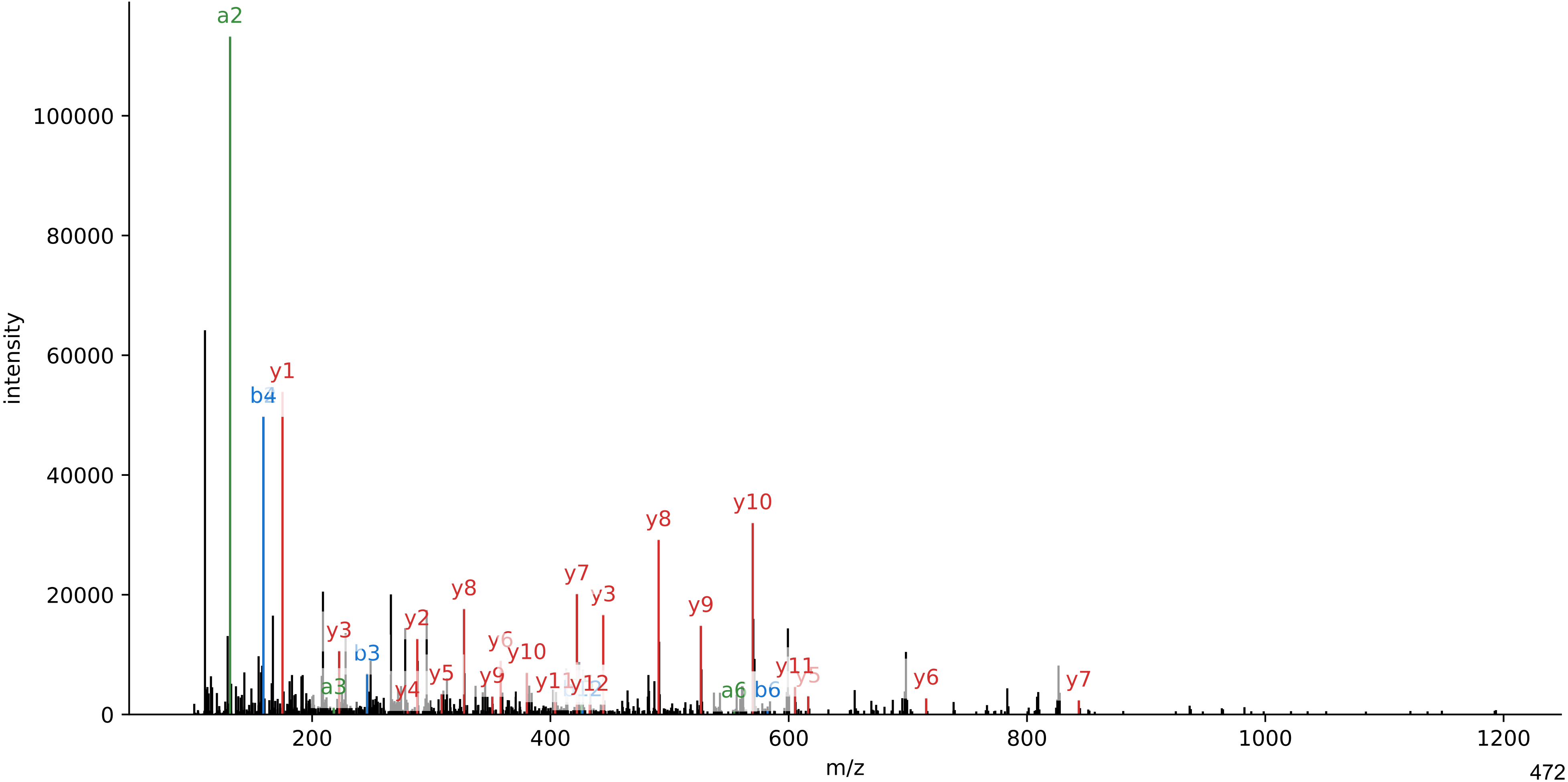

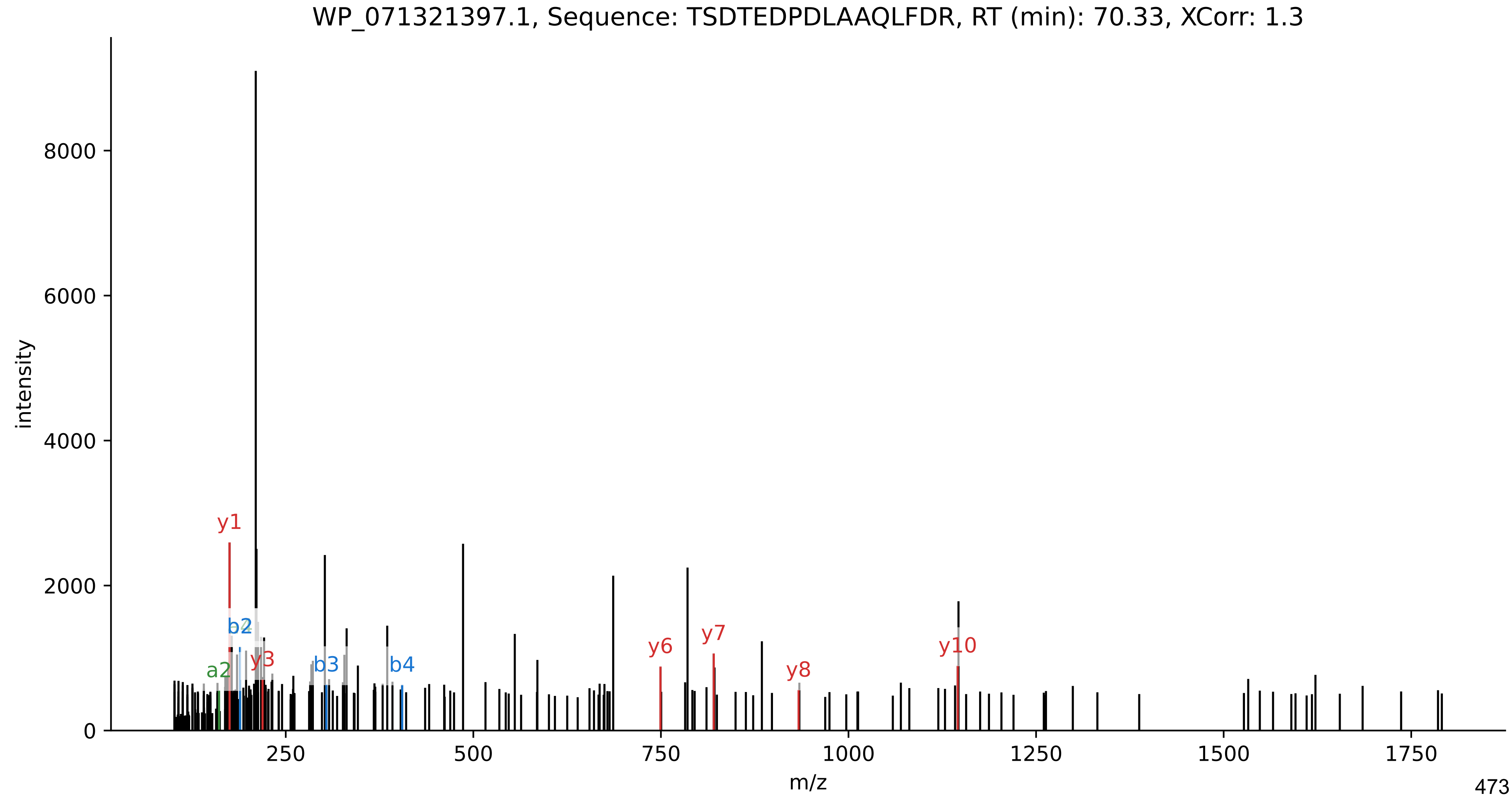
WP_071321397.1, Sequence: TSDTEDPDLAAQLFDR, RT (min): 70.33, XCorr: 1.3
intensity
m/z
8000
6000
4000
2000
0
250
500
750
1000
1250
1500
1750
y1
b2
a2
y3
b3
b4
y6
y7
y8
y10

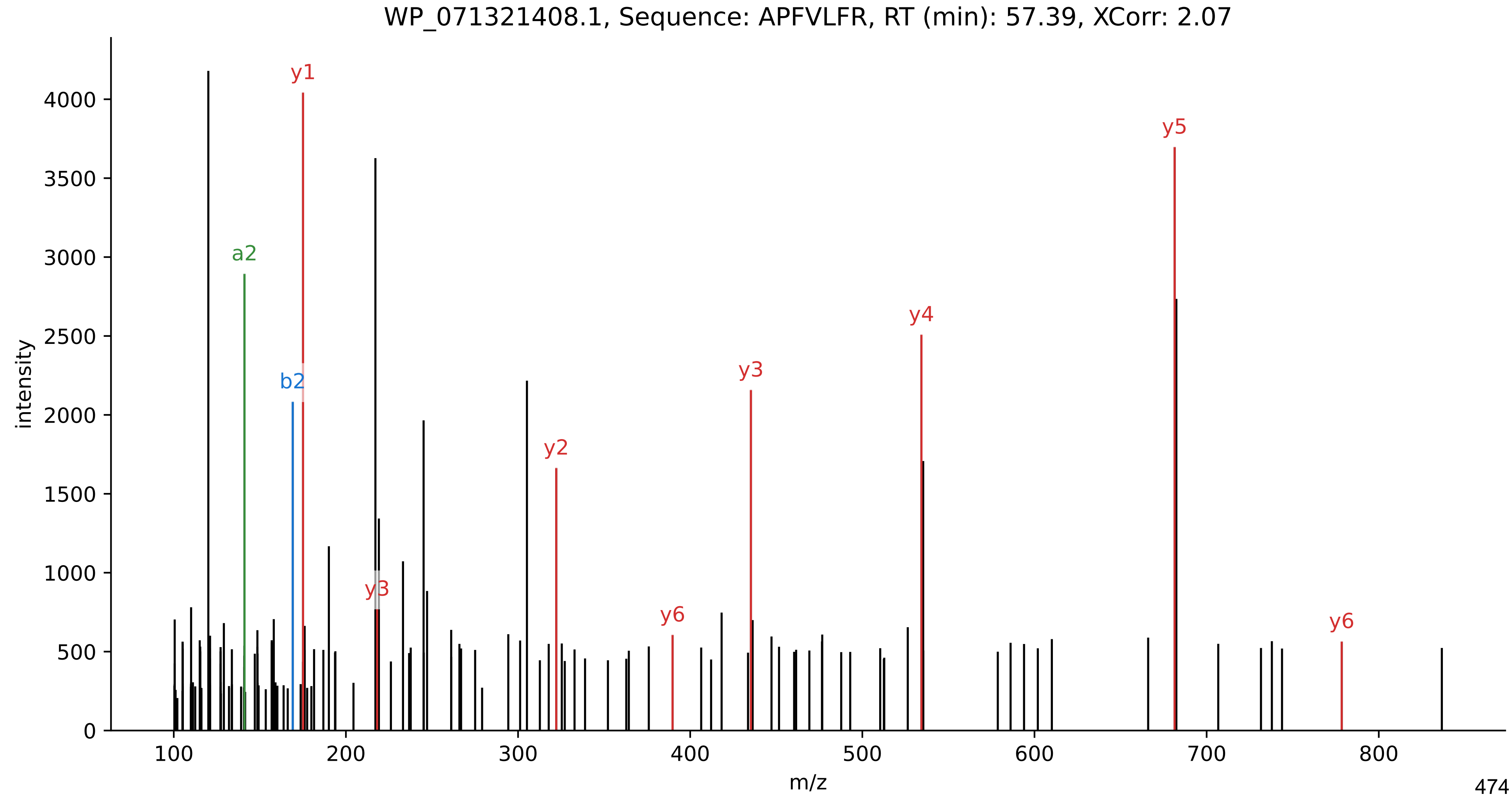

WP_071321408.1, Sequence: APFVLFR, RT (min): 57.39, XCorr: 2.07
intensity
m/z
y1
y5
a2
y4
b2
y3
y2
y3
y6
y6

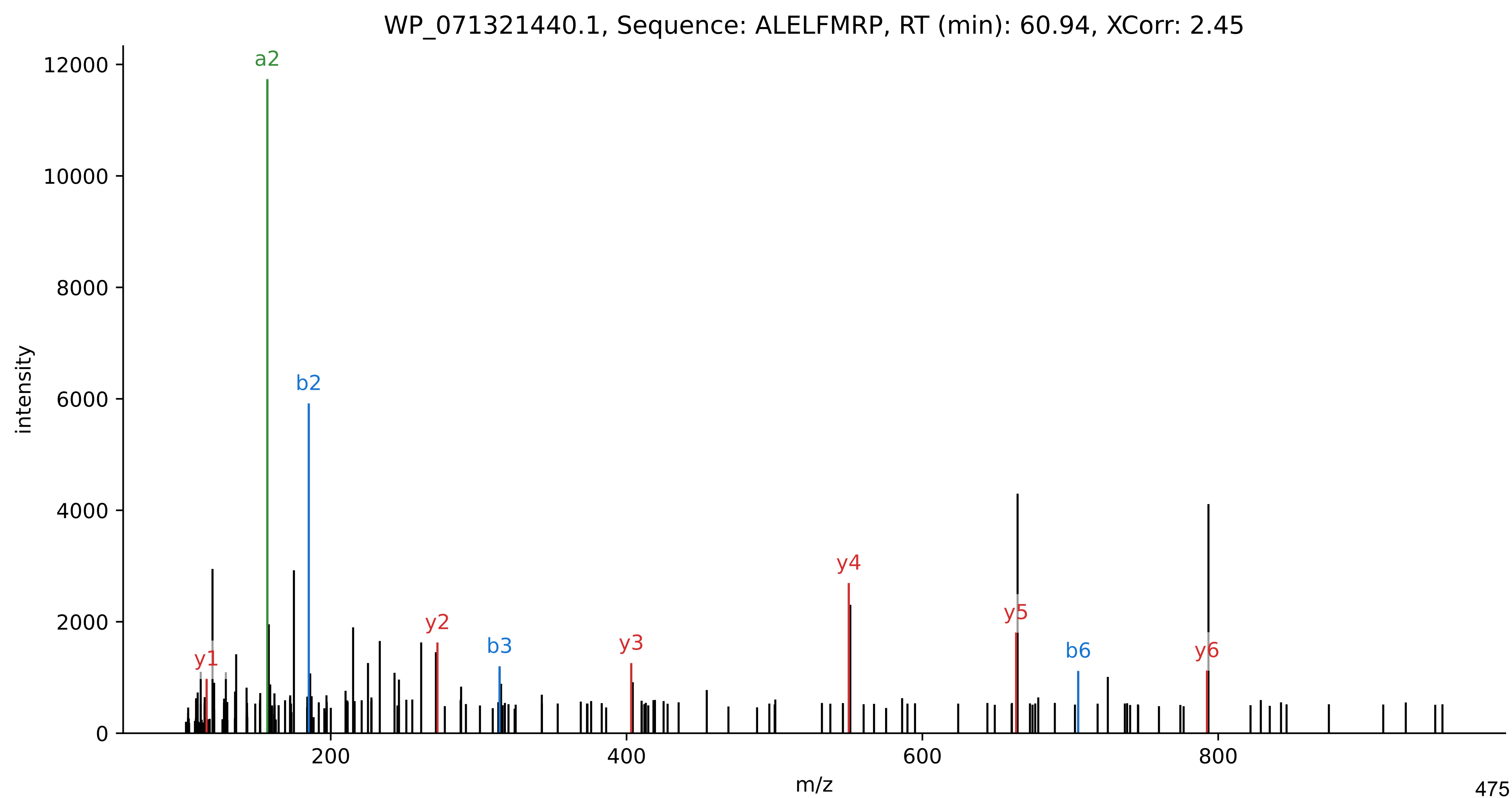
WP_071321440.1, Sequence: ALELFMRP, RT (min): 60.94, XCorr: 2.45
intensity
m/z
a2
b2
b3
b6
y1
y2
y3
y4
y5
y6
0
2000
4000
6000
8000
10000
12000
200
400
600
800

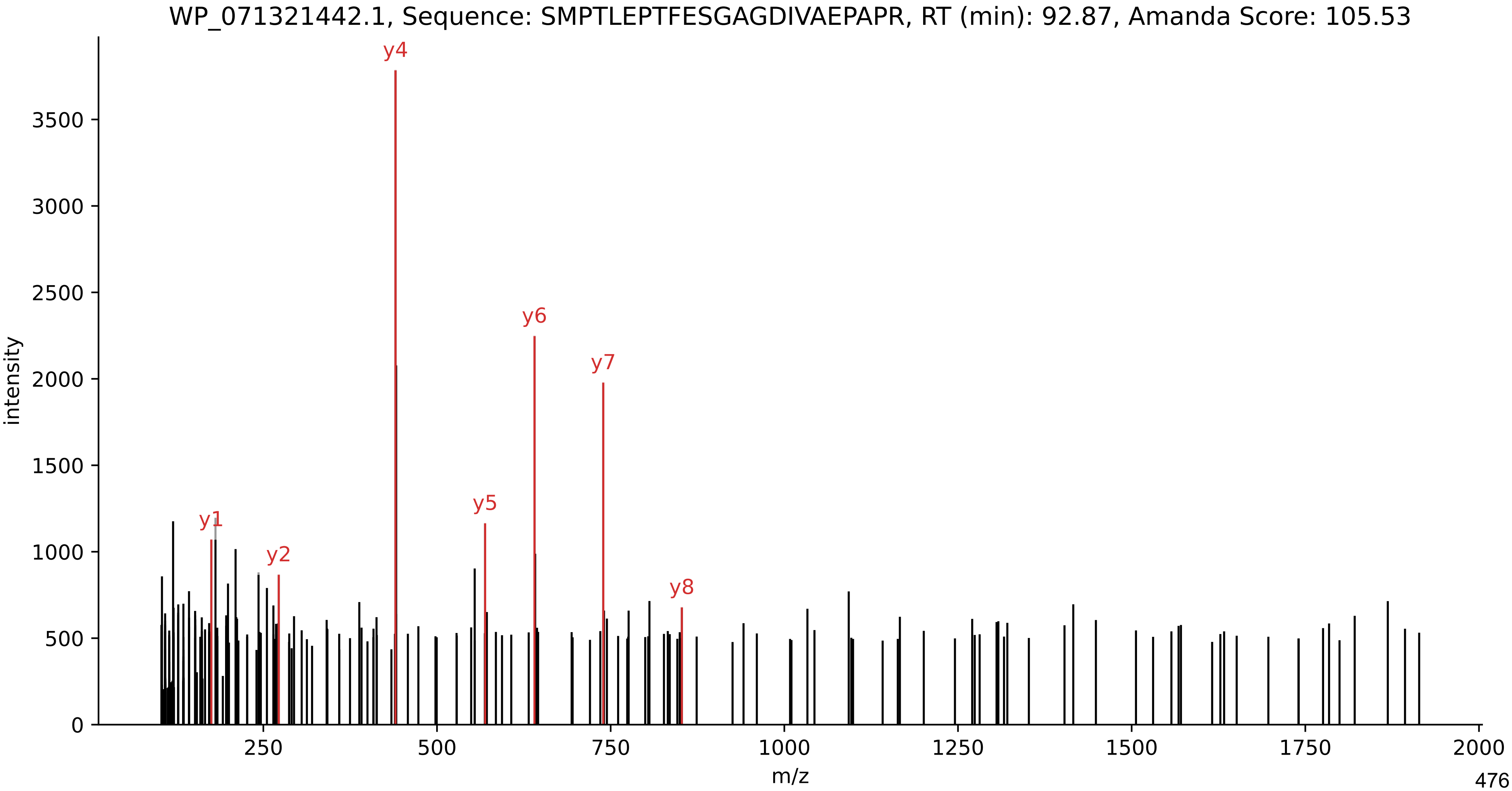

WP_071321442.1, Sequence: SMPTLEPTFESGAGDIVAEPAPR, RT (min): 92.87, Amanda Score: 105.53
intensity
m/z
y1
y2
y4
y5
y6
y7
y8

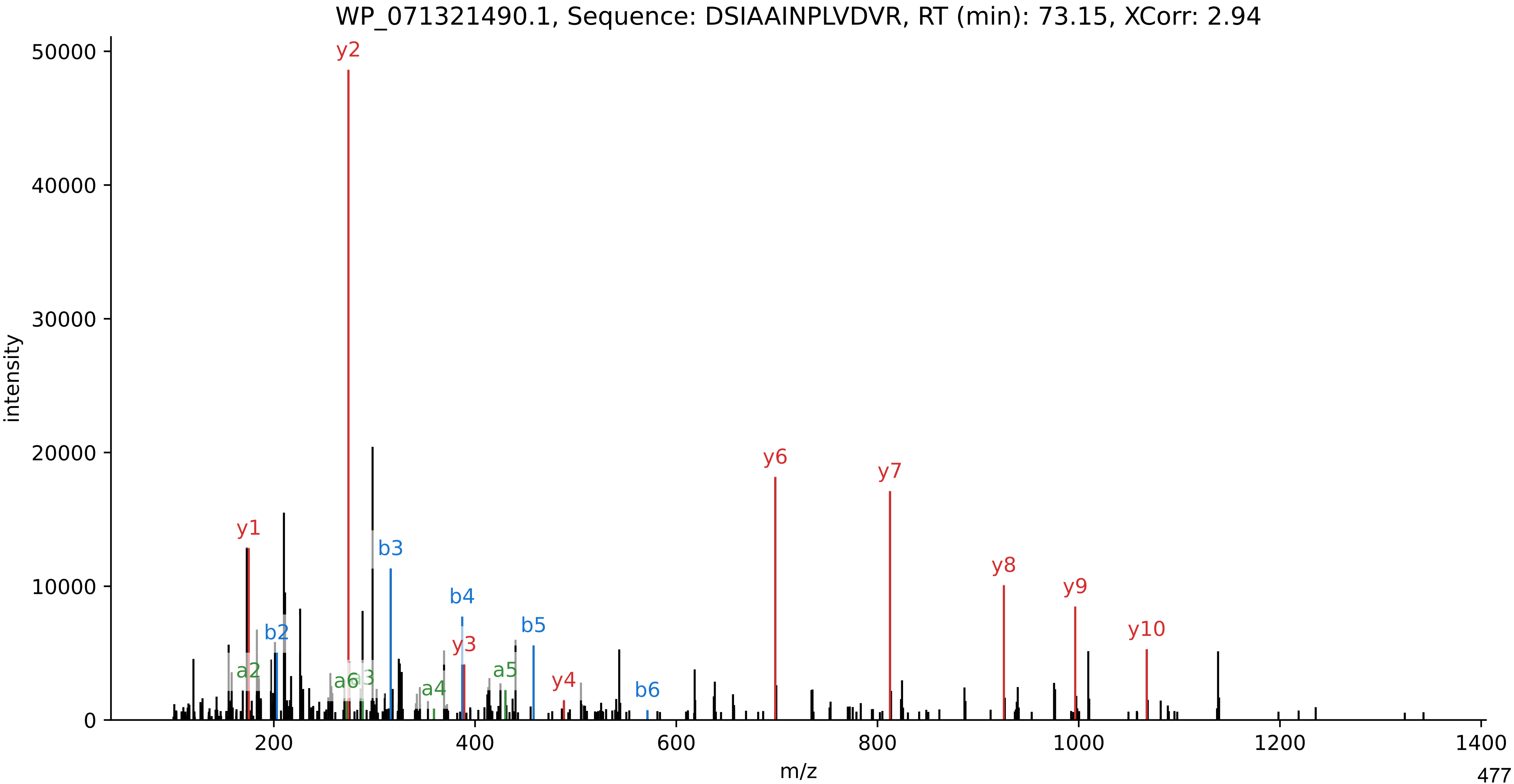

WP_071321490.1, Sequence: DSIAAINPLVDVR, RT (min): 73.15, XCorr: 2.94
intensity
m/z
y1
y2
y3
y4
y6
y7
y8
y9
y10
b2
b3
b4
b5
b6
a2
a4
a5
a6
0
10000
20000
30000
40000
50000
200
400
600
800
1000
1200
1400

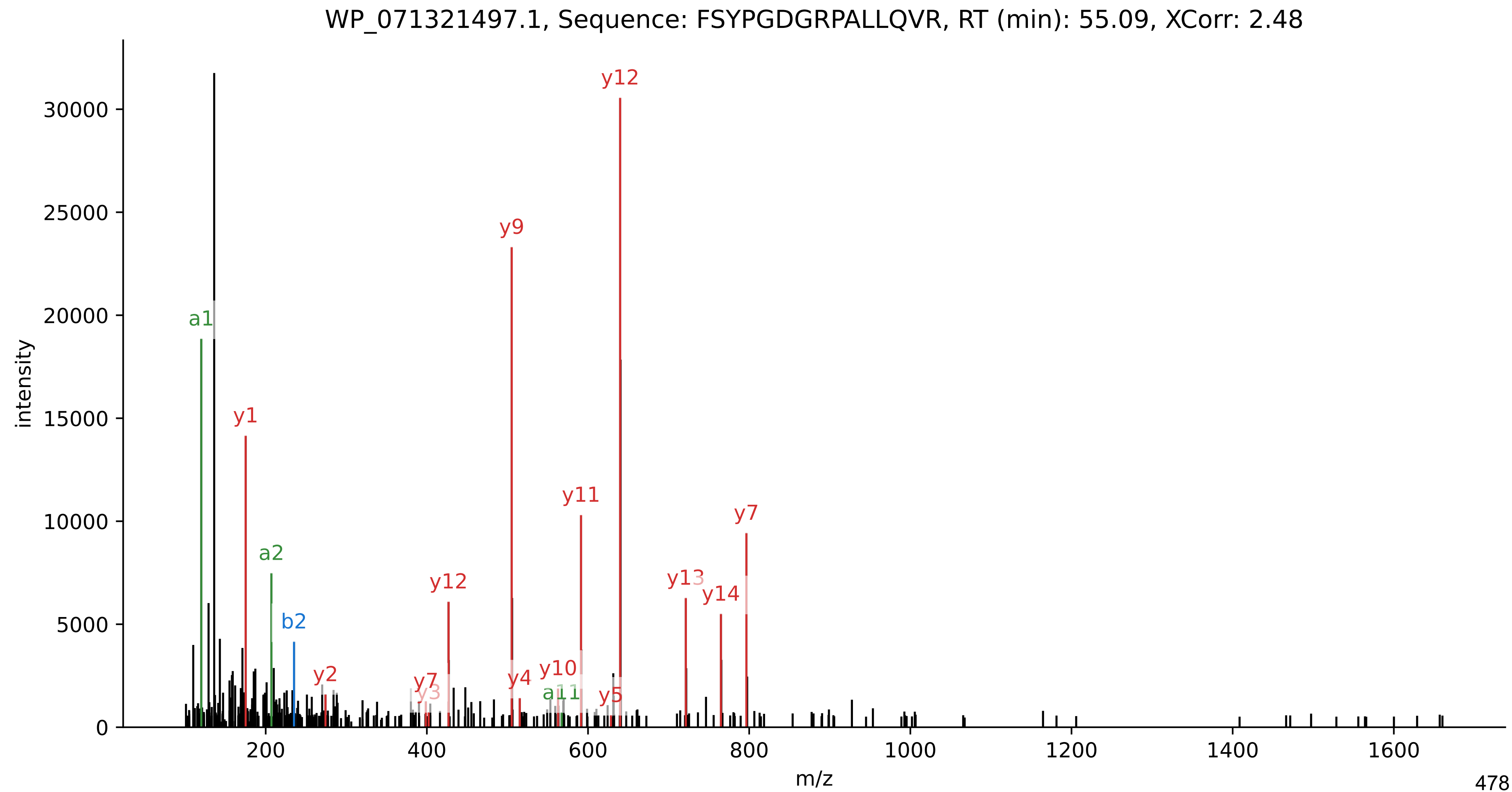

WP_071321497.1, Sequence: FSYPGDGRPALLQVR, RT (min): 55.09, XCorr: 2.48
intensity
m/z
a1
a2
b2
y1
y2
y7
y3
y12
y9
y4
y10
a11
y5
y11
y12
y13
y14
y7

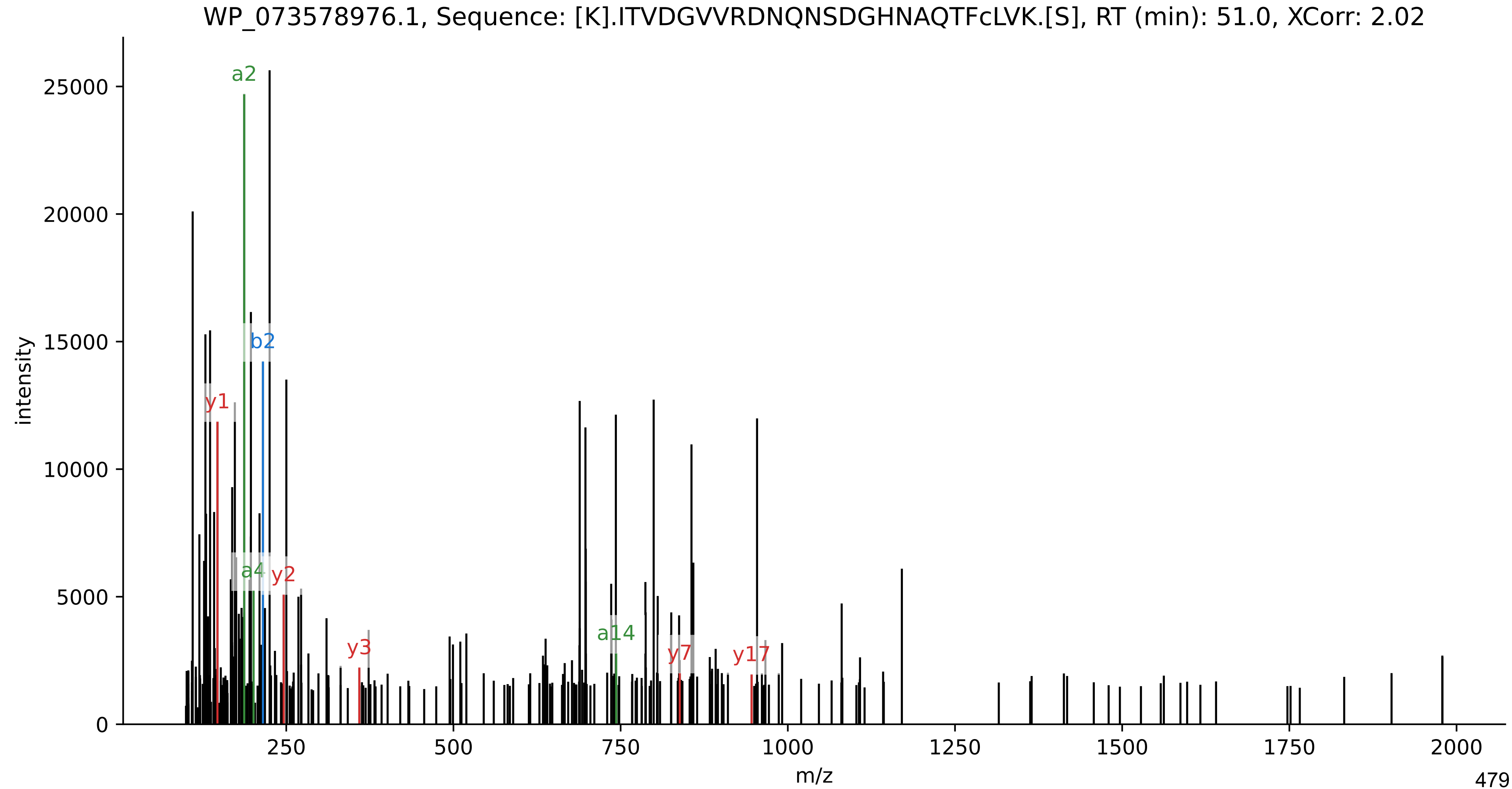
WP_073578976.1, Sequence: [K].ITVDGVVRDNQNSDGHNAQTFcLVK.[S], RT (min): 51.0, XCorr: 2.02
intensity
m/z
a2
b2
y1
a4
y2
y3
a14
y7
y17

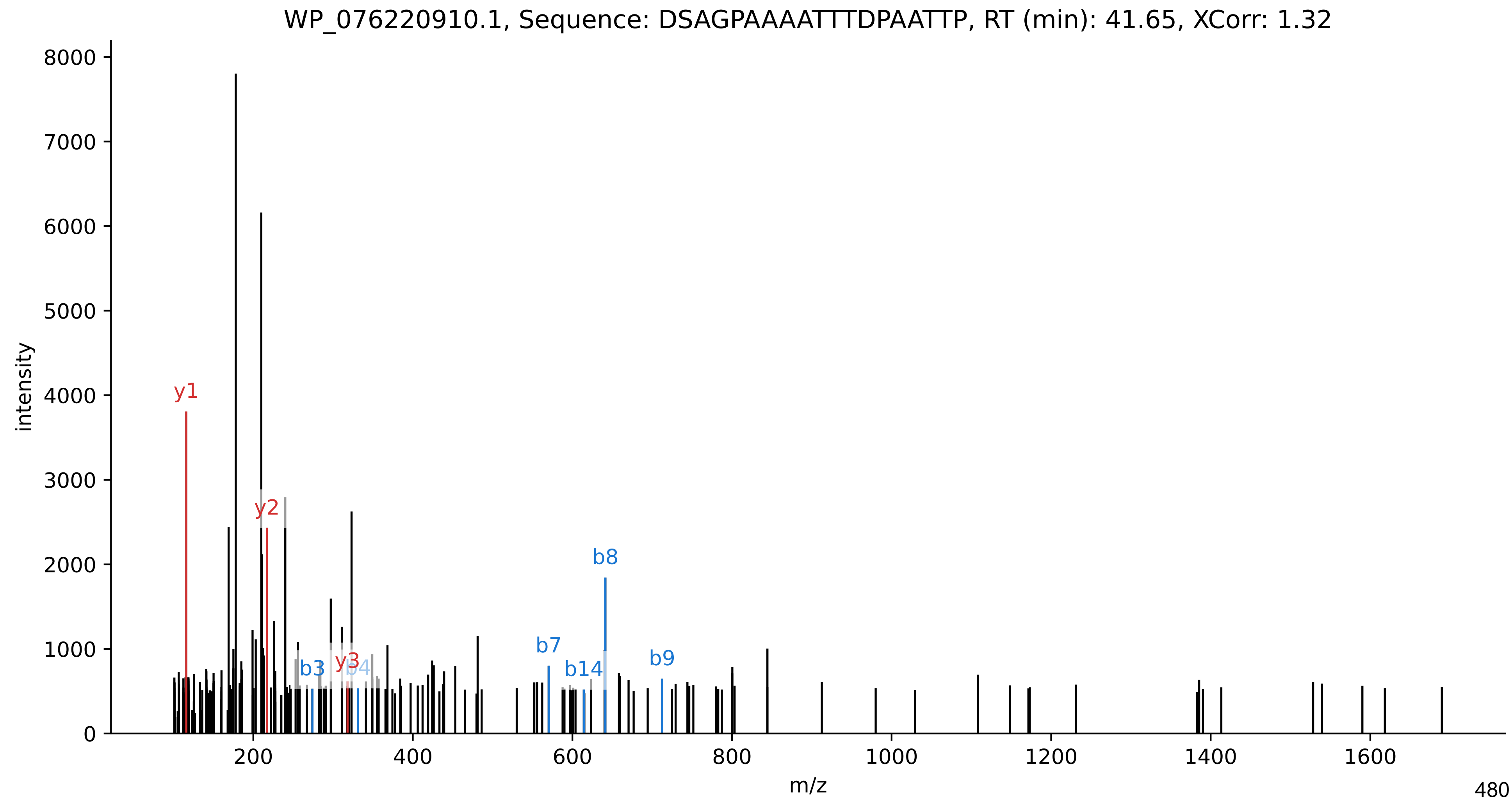
WP_076220910.1, Sequence: DSAGPAAAATTTDPAATTP, RT (min): 41.65, XCorr: 1.32
intensity
m/z
y1
y2
y3
b3
b4
b7
b14
b8
b9
0
1000
2000
3000
4000
5000
6000
7000
8000
200
400
600
800
1000
1200
1400
1600

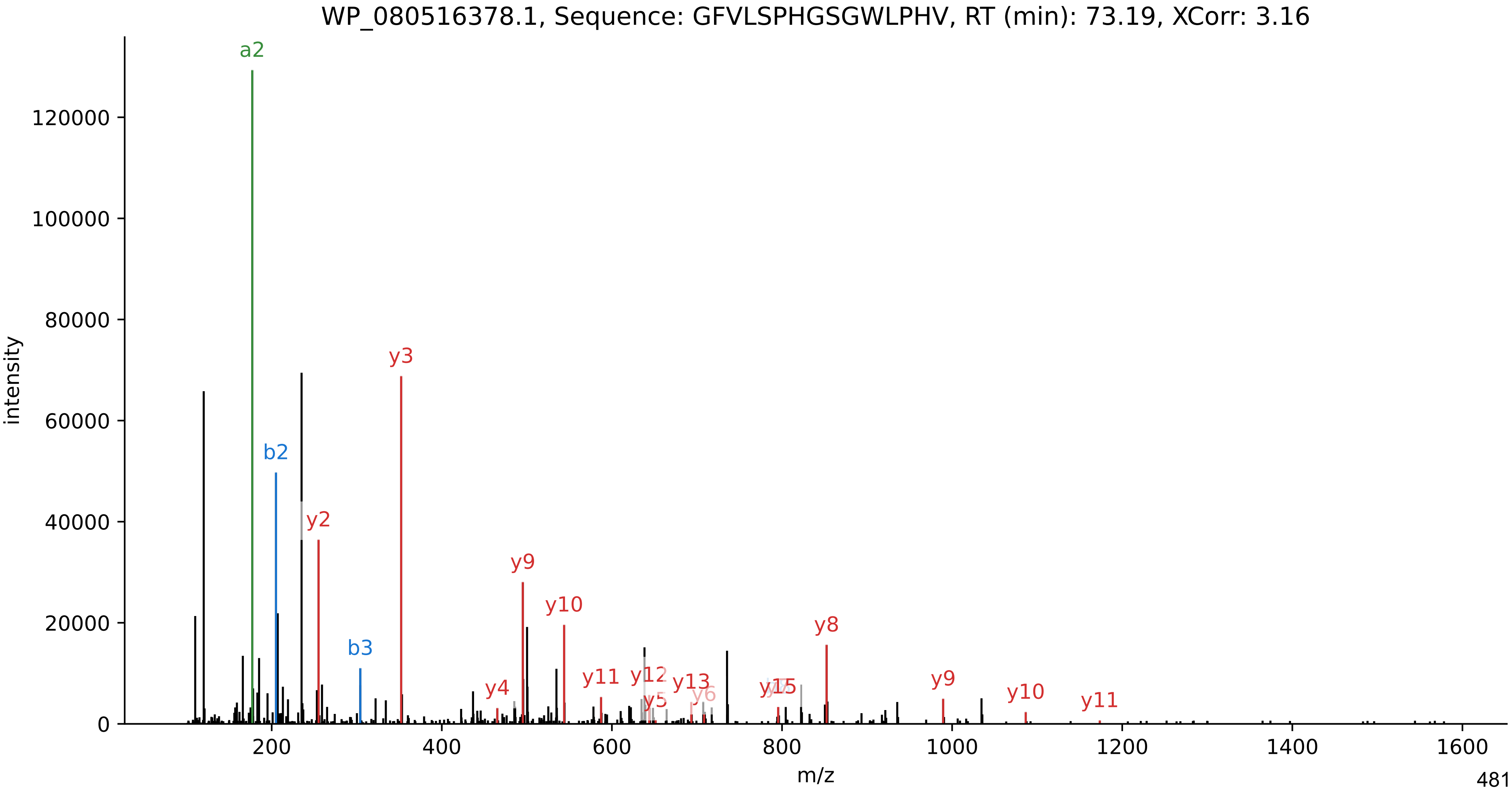
WP_080516378.1, Sequence: GFVLSPHGSGWLPHV, RT (min): 73.19, XCorr: 3.16
a2
b2
b3
y2
y3
y4
y9
y10
y11
y12
y5
y13
y6
y15
y8
y9
y10
y11
intensity
m/z
0
20000
40000
60000
80000
100000
120000
200
400
600
800
1000
1200
1400
1600

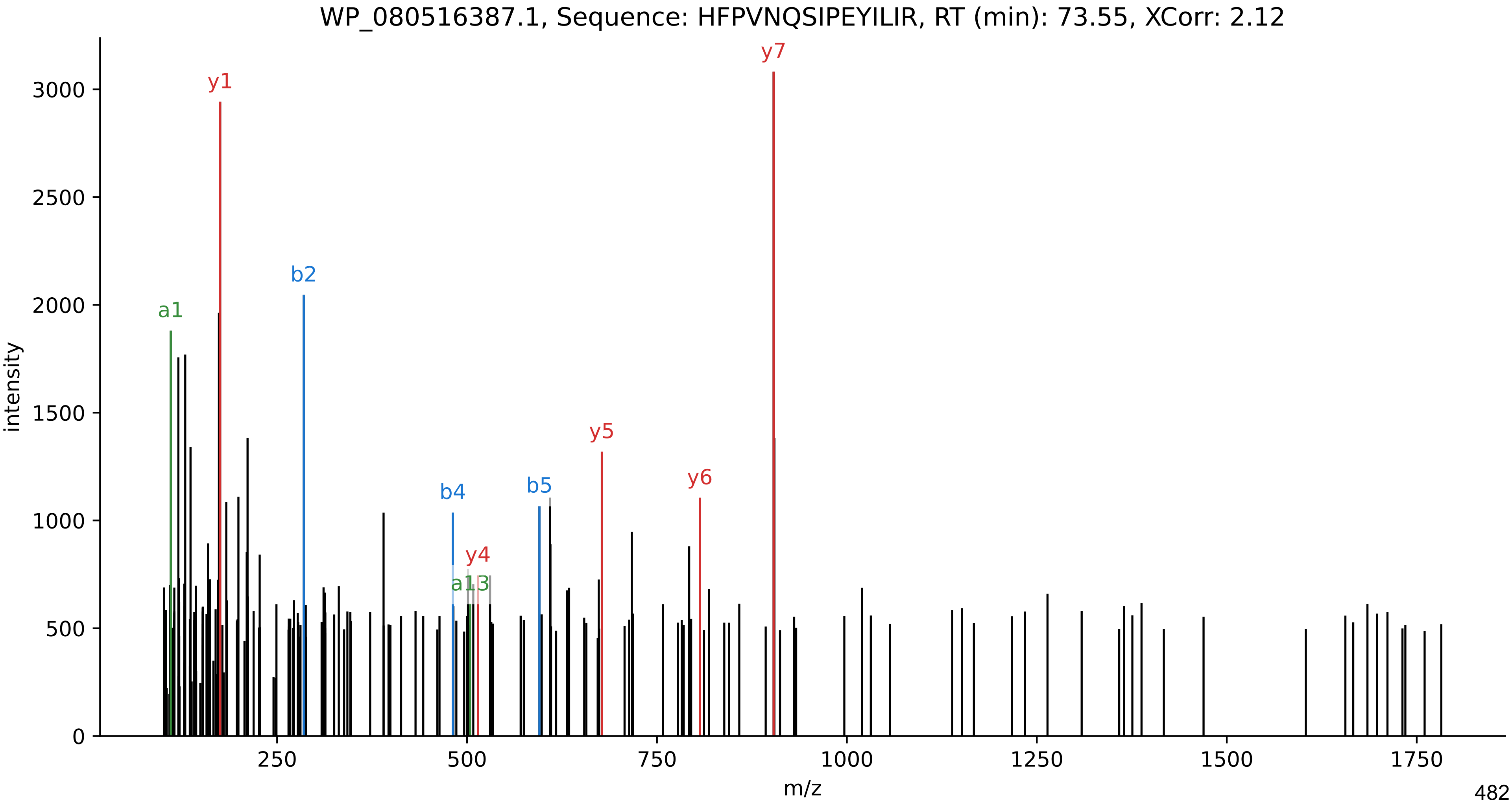

WP_080516387.1, Sequence: HFPVNQSIPEYILIR, RT (min): 73.55, XCorr: 2.12
intensity
m/z
y1
y7
b2
a1
b4
b5
y5
y6
y4
a13
3000
2500
2000
1500
1000
500
0
250
500
750
1000
1250
1500
1750

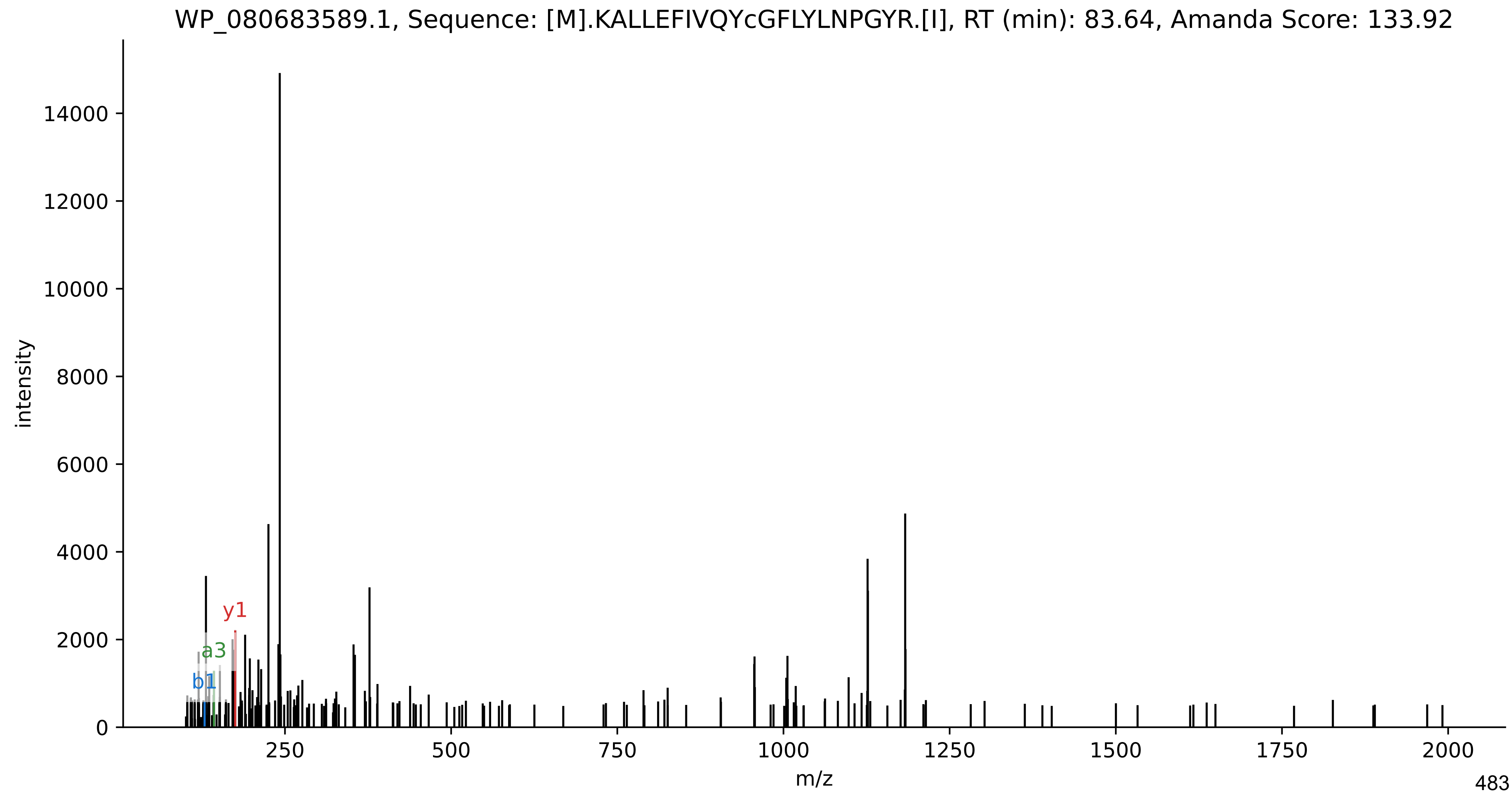

WP_080683589.1, Sequence: [M].KALLEFIVQYcGFLYLNPGYR.[I], RT (min): 83.64, Amanda Score: 133.92
intensity
m/z
y1
a3
b1
0
2000
4000
6000
8000
10000
12000
14000
250
500
750
1000
1250
1500
1750
2000

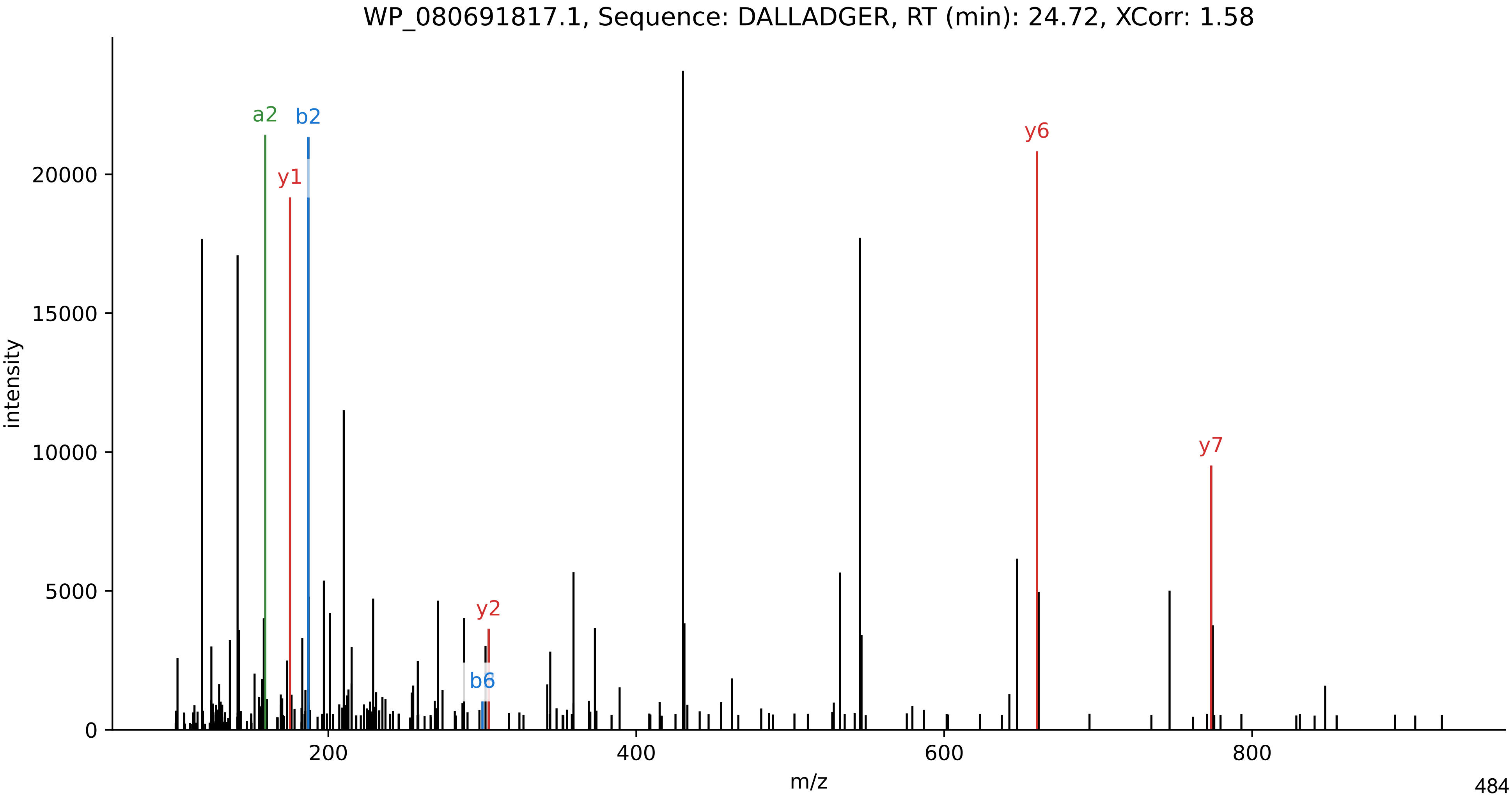

WP_080691817.1, Sequence: DALLADGER, RT (min): 24.72, XCorr: 1.58
a2
b2
y1
y6
y7
y2
b6
intensity
m/z
0
5000
10000
15000
20000
200
400
600
800

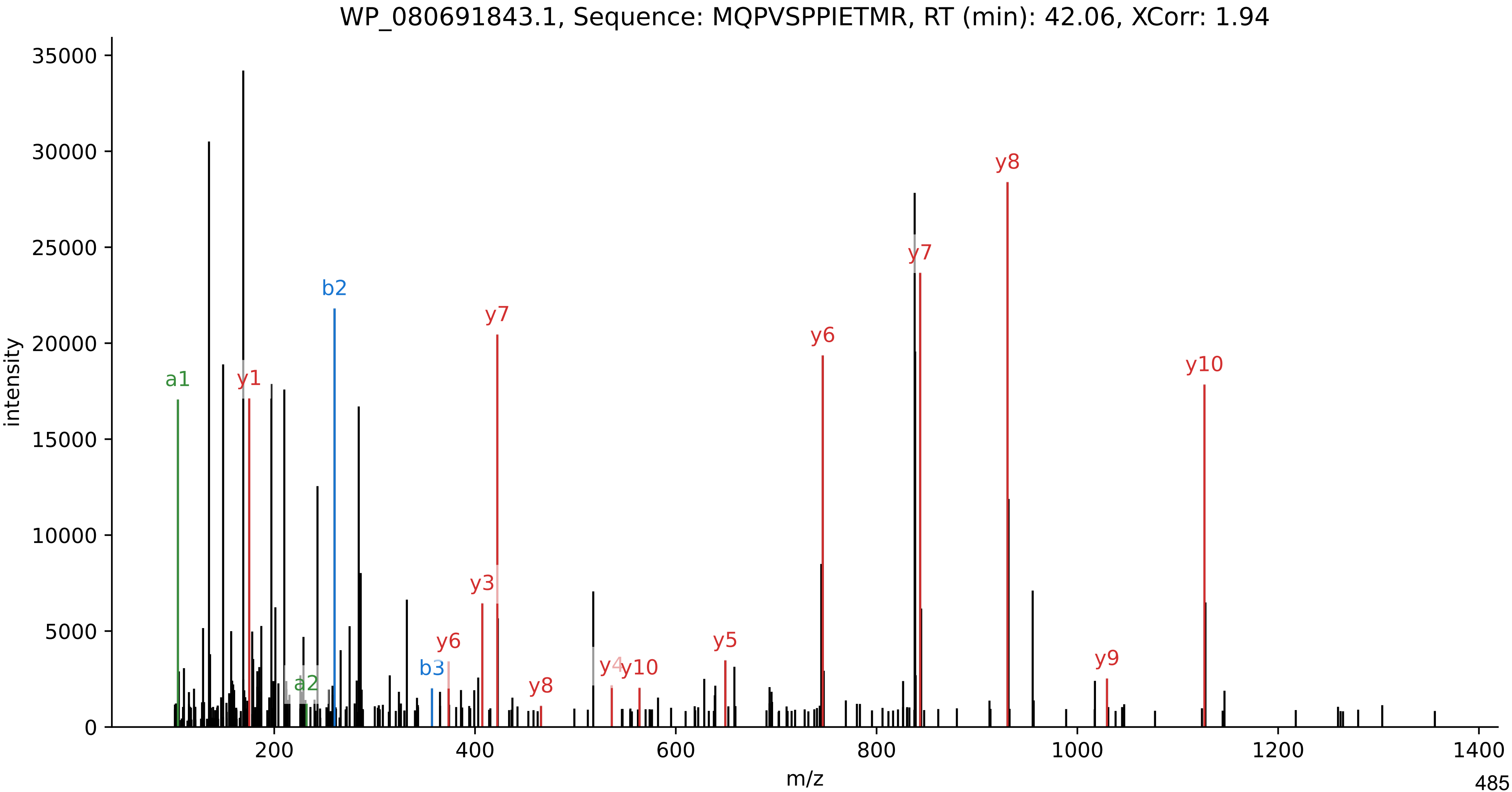

WP_080691843.1, Sequence: MQPVSPPIETMR, RT (min): 42.06, XCorr: 1.94
intensity
m/z
a1
y1
b2
y7
y6
y8
y7
y10
y3
y6
b3
a2
y8
y4
y10
y5
y9

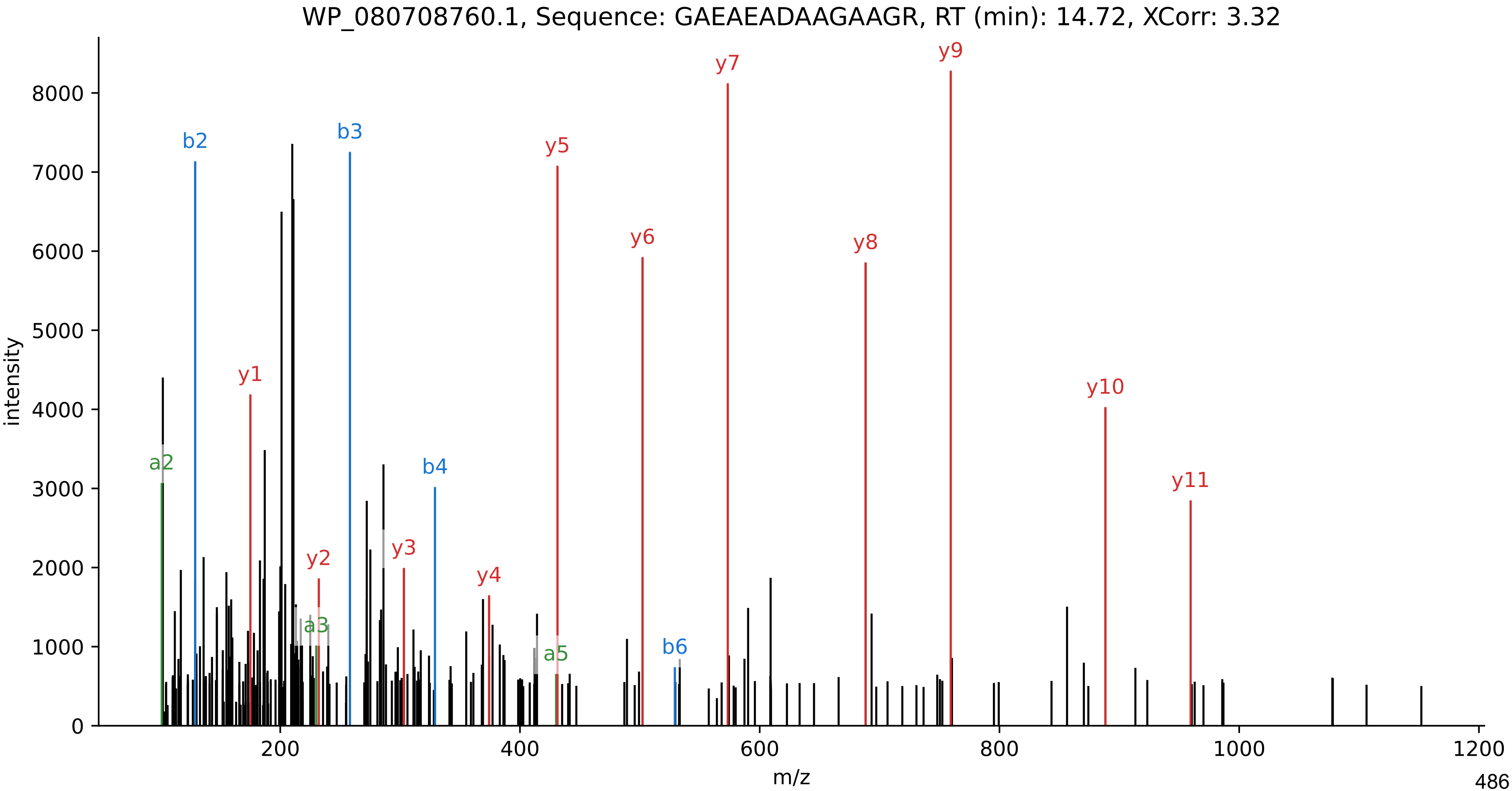
WP_080708760.1, Sequence: GAEAEADAAGAAGR, RT (min): 14.72, XCorr: 3.32
intensity
m/z
b2
b3
b4
b6
a2
a3
a5
y1
y2
y3
y4
y5
y6
y7
y8
y9
y10
y11
0
1000
2000
3000
4000
5000
6000
7000
8000
200
400
600
800
1000
1200

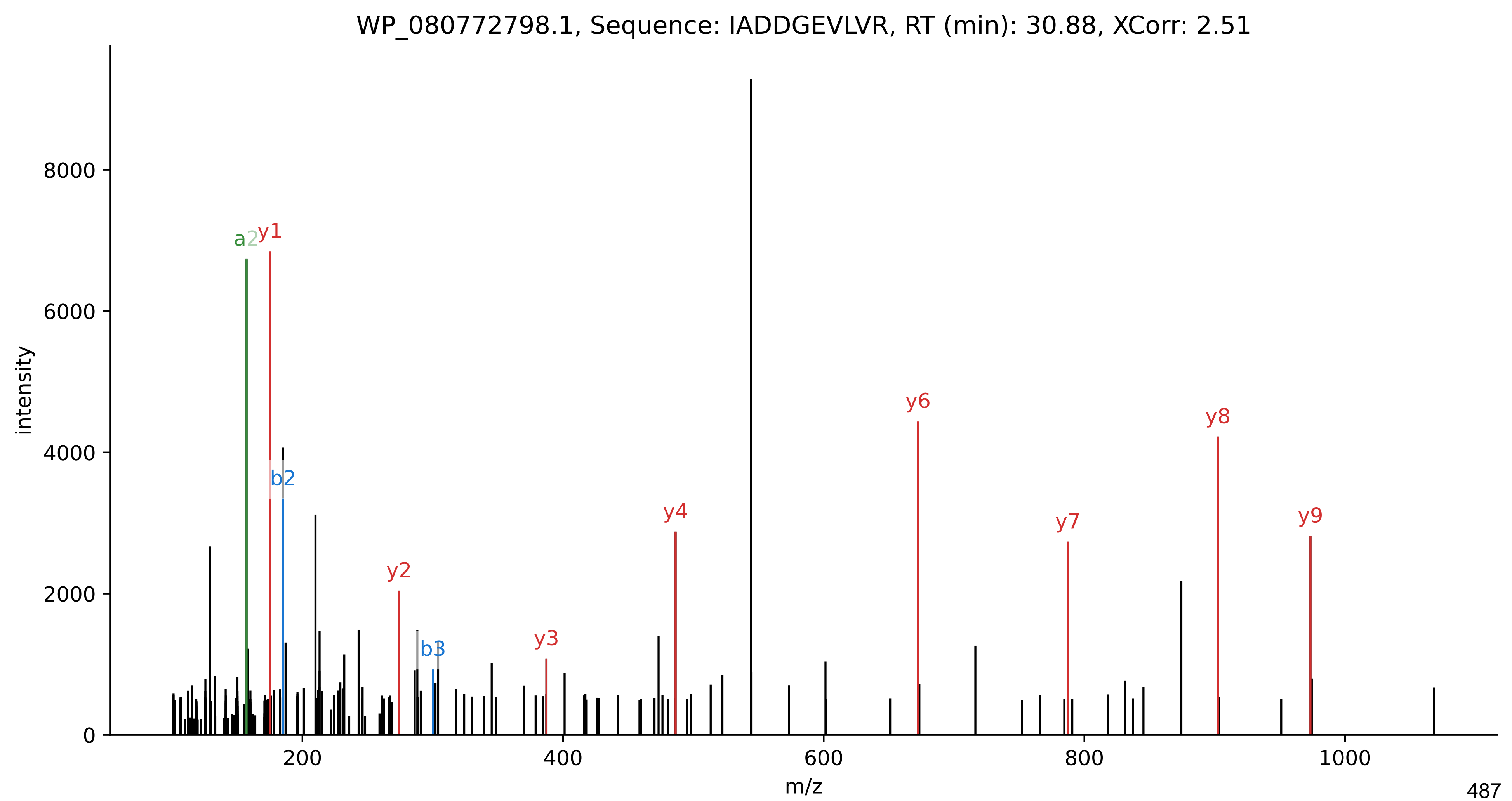
WP_080772798.1, Sequence: IADDGEVLVR, RT (min): 30.88, XCorr: 2.51
intensity
m/z
a2
y1
b2
y2
b3
y3
y4
y6
y7
y8
y9
0
2000
4000
6000
8000
200
400
600
800
1000

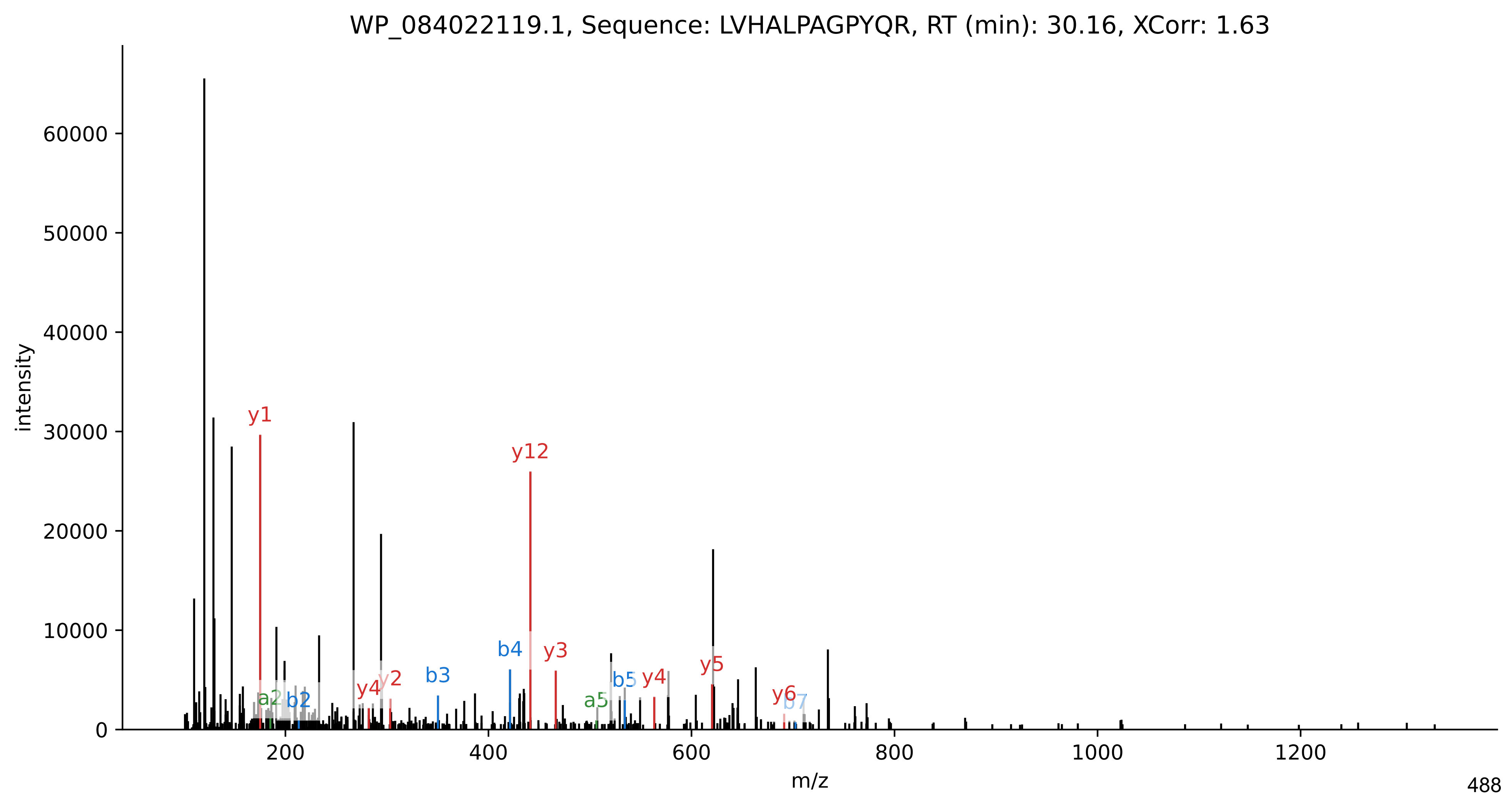

WP_084022119.1, Sequence: LVHALPAGPYQR, RT (min): 30.16, XCorr: 1.63
intensity
m/z
y1
y12
b4
y3
b3
y4
y2
a2
b2
a5
b5
y4
y5
y6
b7

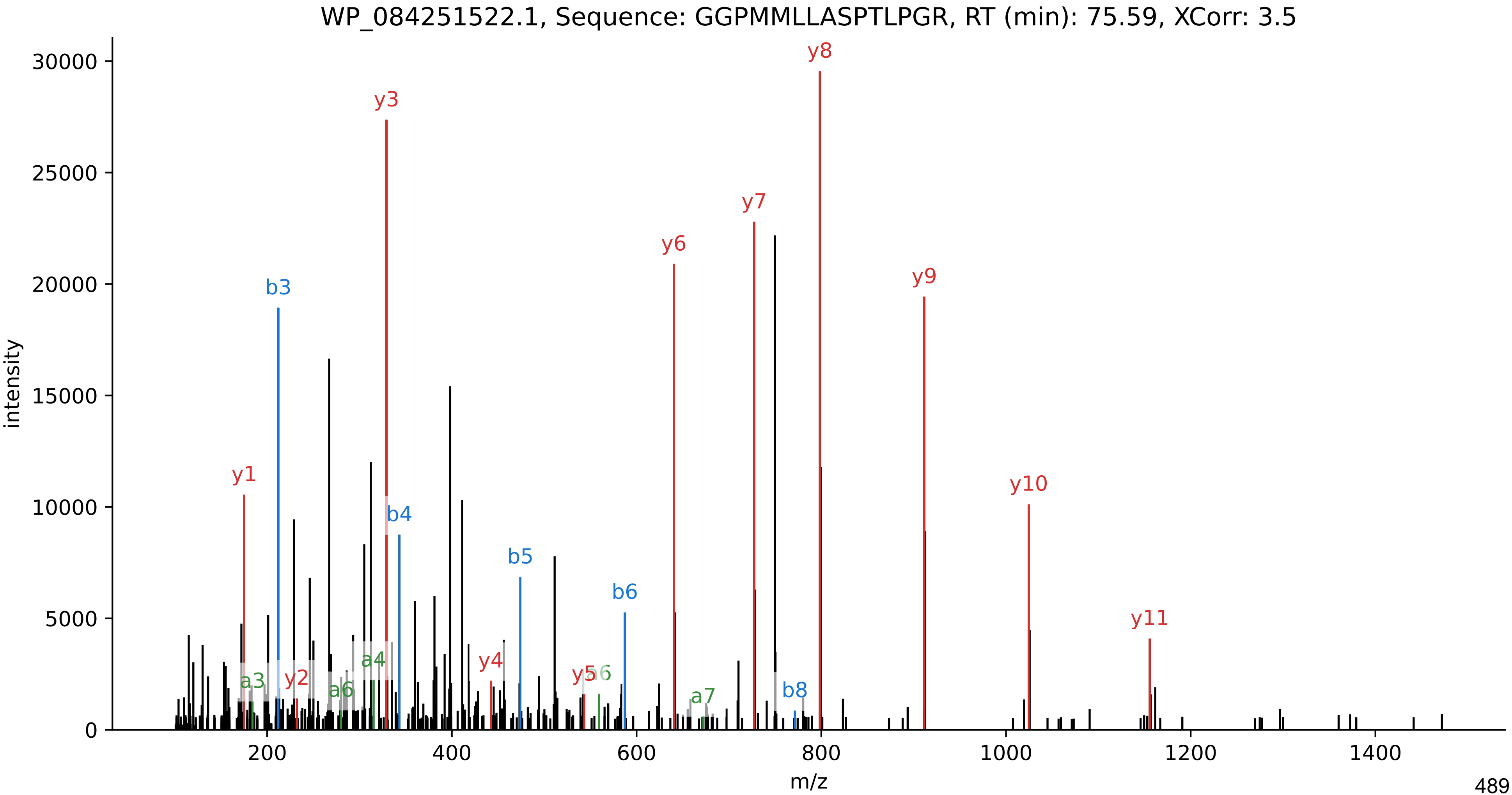
WP_084251522.1, Sequence: GGPMMLLASPTLPGR, RT (min): 75.59, XCorr: 3.5
intensity
m/z
y1
y2
y3
y4
y5
y6
y7
y8
y9
y10
y11
b3
b4
b5
b6
b8
a3
a4
a6
a7
0
5000
10000
15000
20000
25000
30000
200
400
600
800
1000
1200
1400

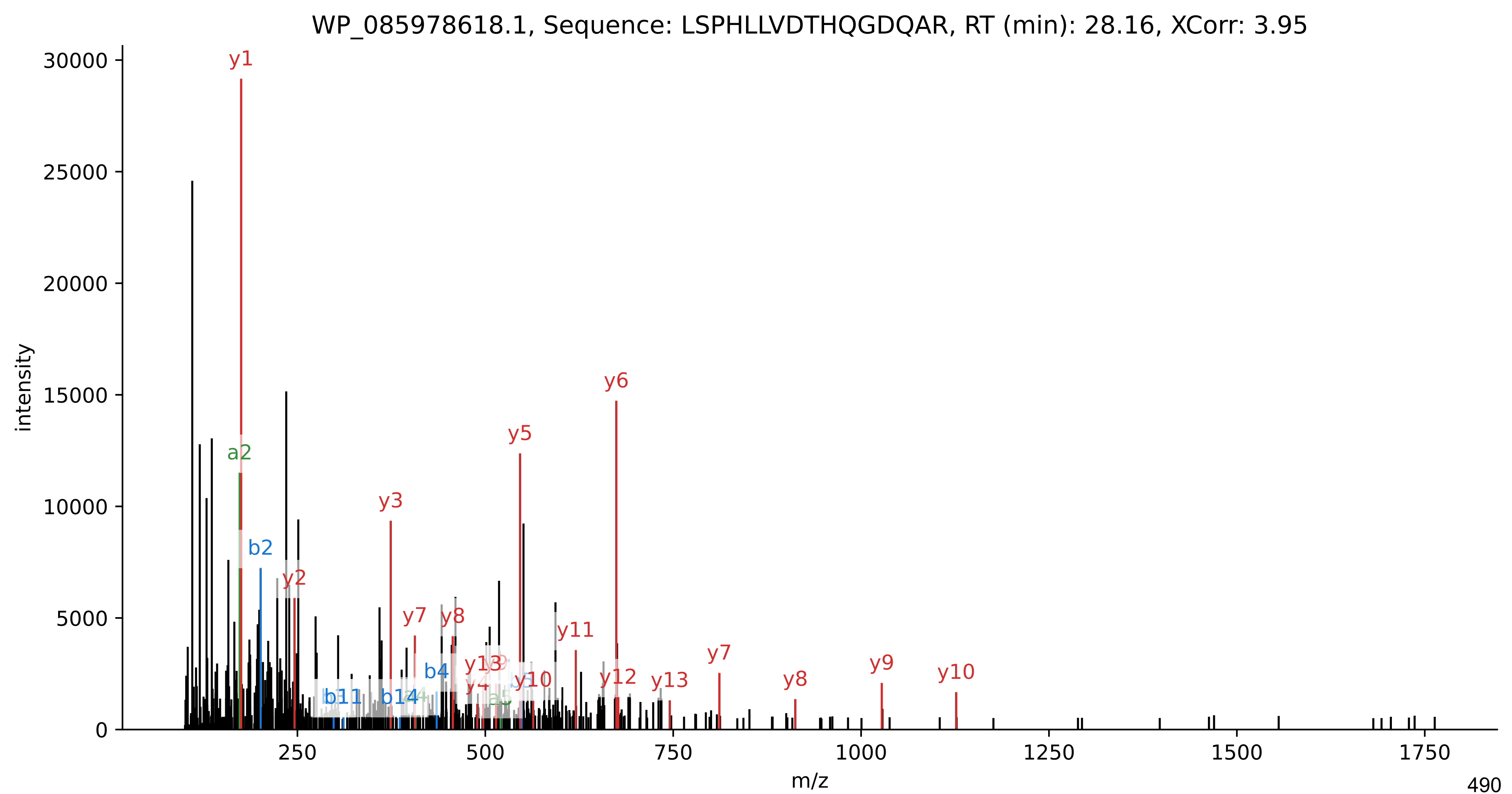
WP_085978618.1, Sequence: LSPHLLVDTHQGDQAR, RT (min): 28.16, XCorr: 3.95
intensity
m/z
0
5000
10000
15000
20000
25000
30000
250
500
750
1000
1250
1500
1750
y1
a2
b2
y2
y3
y7
y8
b4
b11
b14
y13
y4
y5
y10
y6
y11
y12
y13
y7
y8
y9
y10

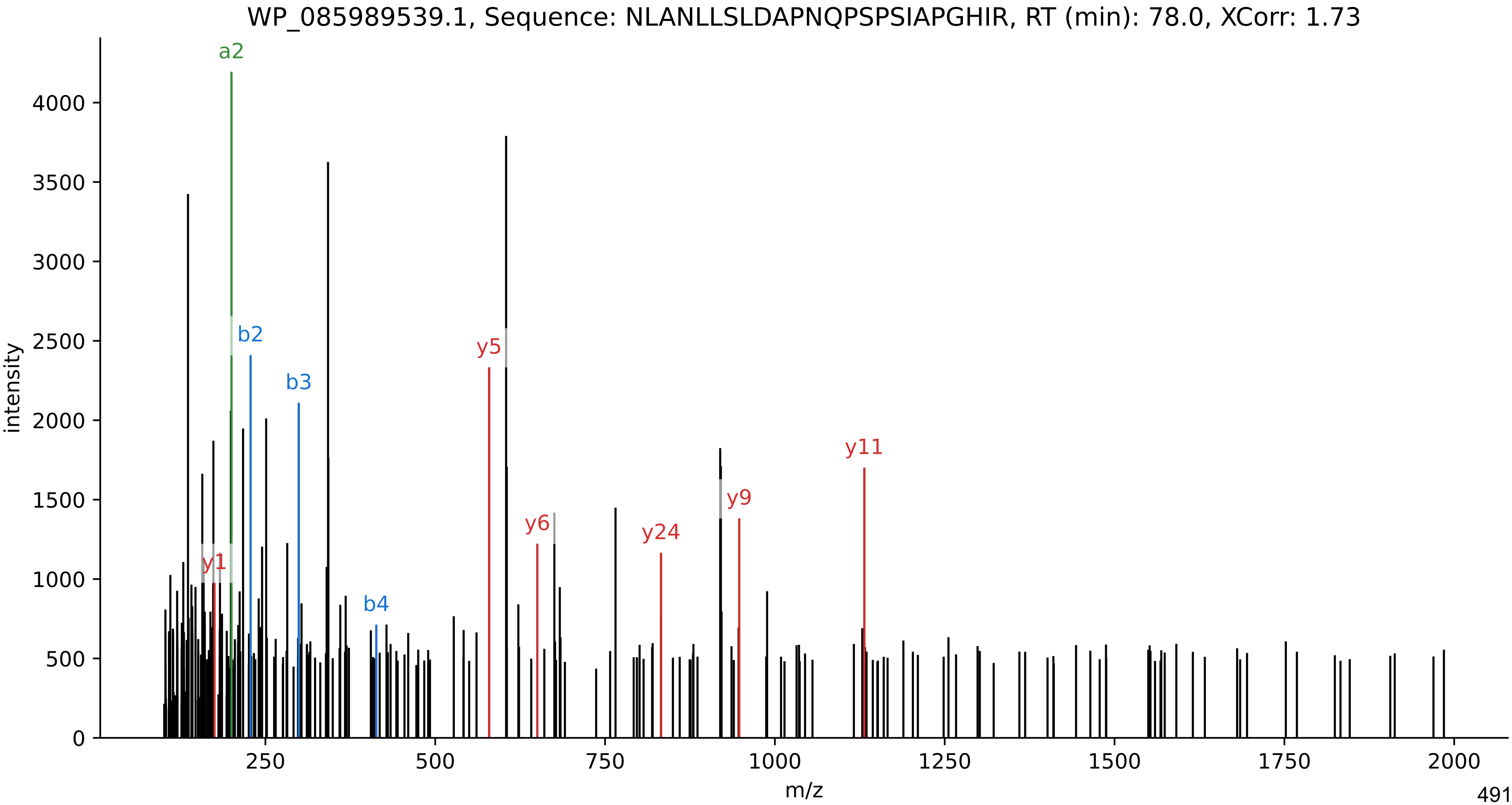

WP_085989539.1, Sequence: NLANLLSLDAPNQPSPSIAPGHIR, RT (min): 78.0, XCorr: 1.73
a2
b2
b3
b4
y1
y5
y6
y24
y9
y11
intensity
m/z
0
500
1000
1500
2000
2500
3000
3500
4000
250
500
750
1000
1250
1500
1750
2000

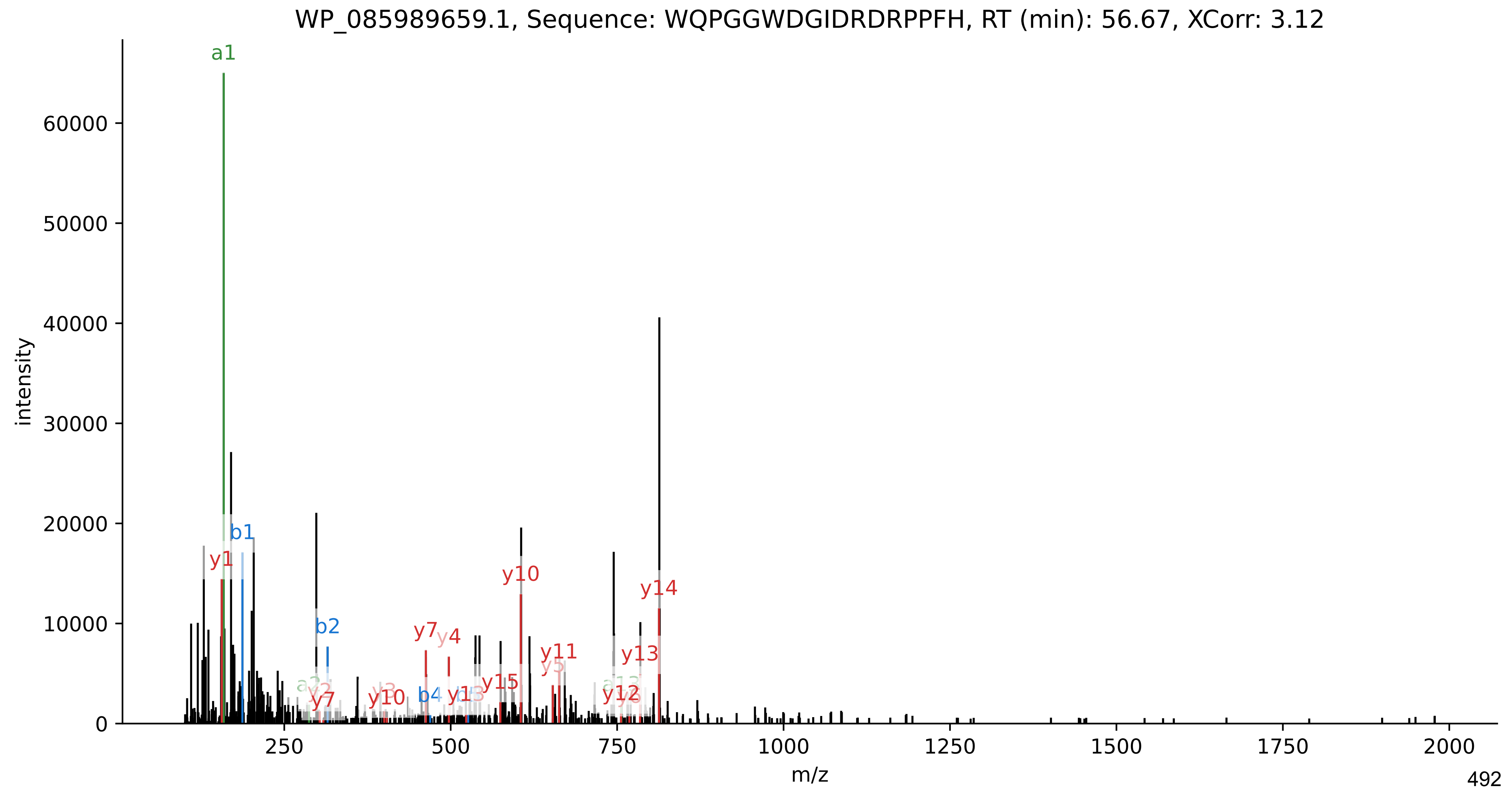

WP_085989659.1, Sequence: WQPGGWDGIDRDRPPFH, RT (min): 56.67, XCorr: 3.12
intensity
m/z
a1
b1
y1
b2
y7
y4
y10
y14
y11
y13
y15
y5
a2
y2
y7
y3
y10
b4
y13
a13
y12
0
10000
20000
30000
40000
50000
60000
250
500
750
1000
1250
1500
1750
2000

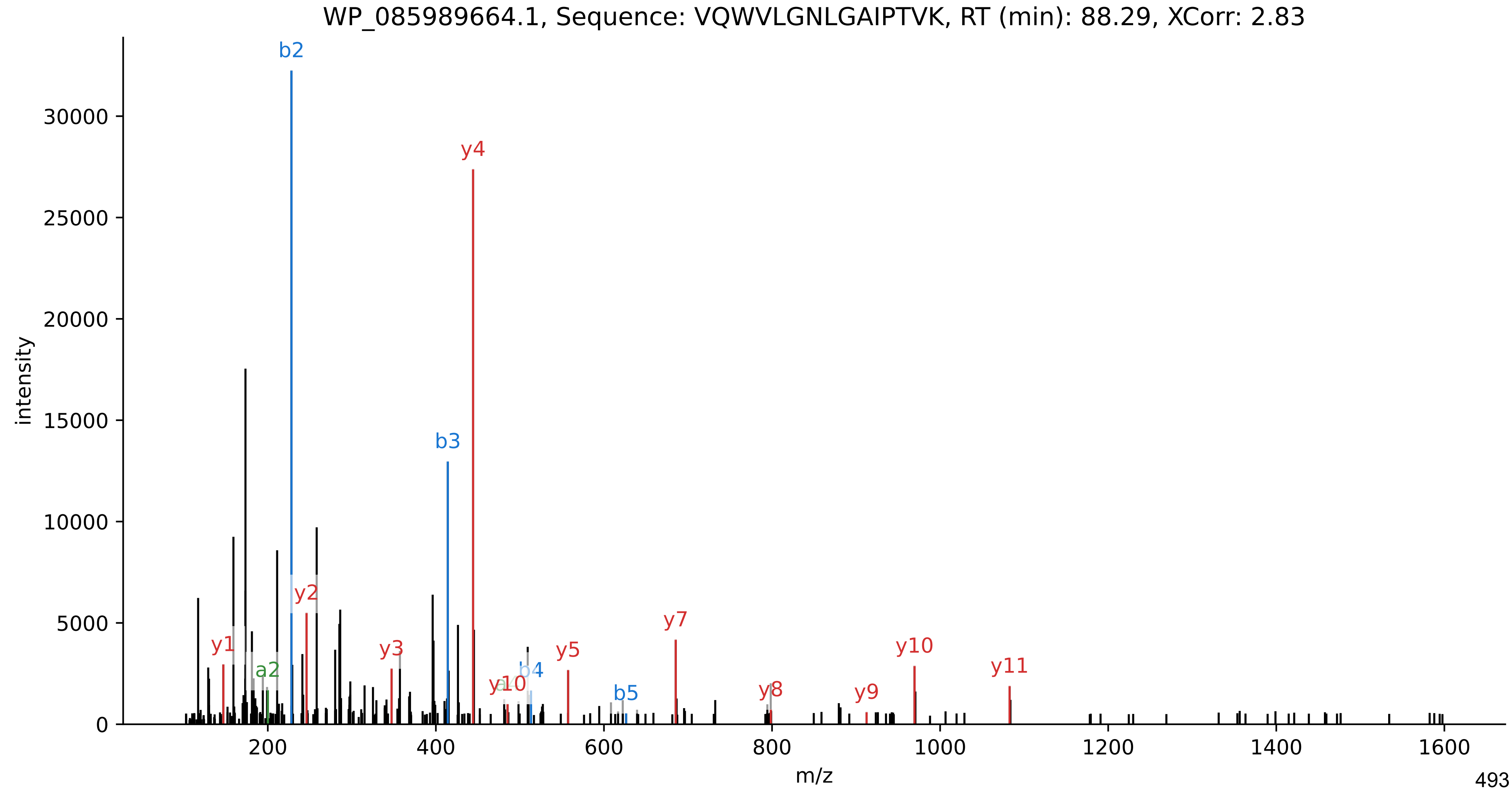
WP_085989664.1, Sequence: VQWVLGNLGAIPTVK, RT (min): 88.29, XCorr: 2.83
intensity
m/z
b2
y4
b3
y2
y7
y1
y3
y5
a2
b4
y10
b5
y8
y9
y10
y11
30000
25000
20000
15000
10000
5000
0
200
400
600
800
1000
1200
1400
1600

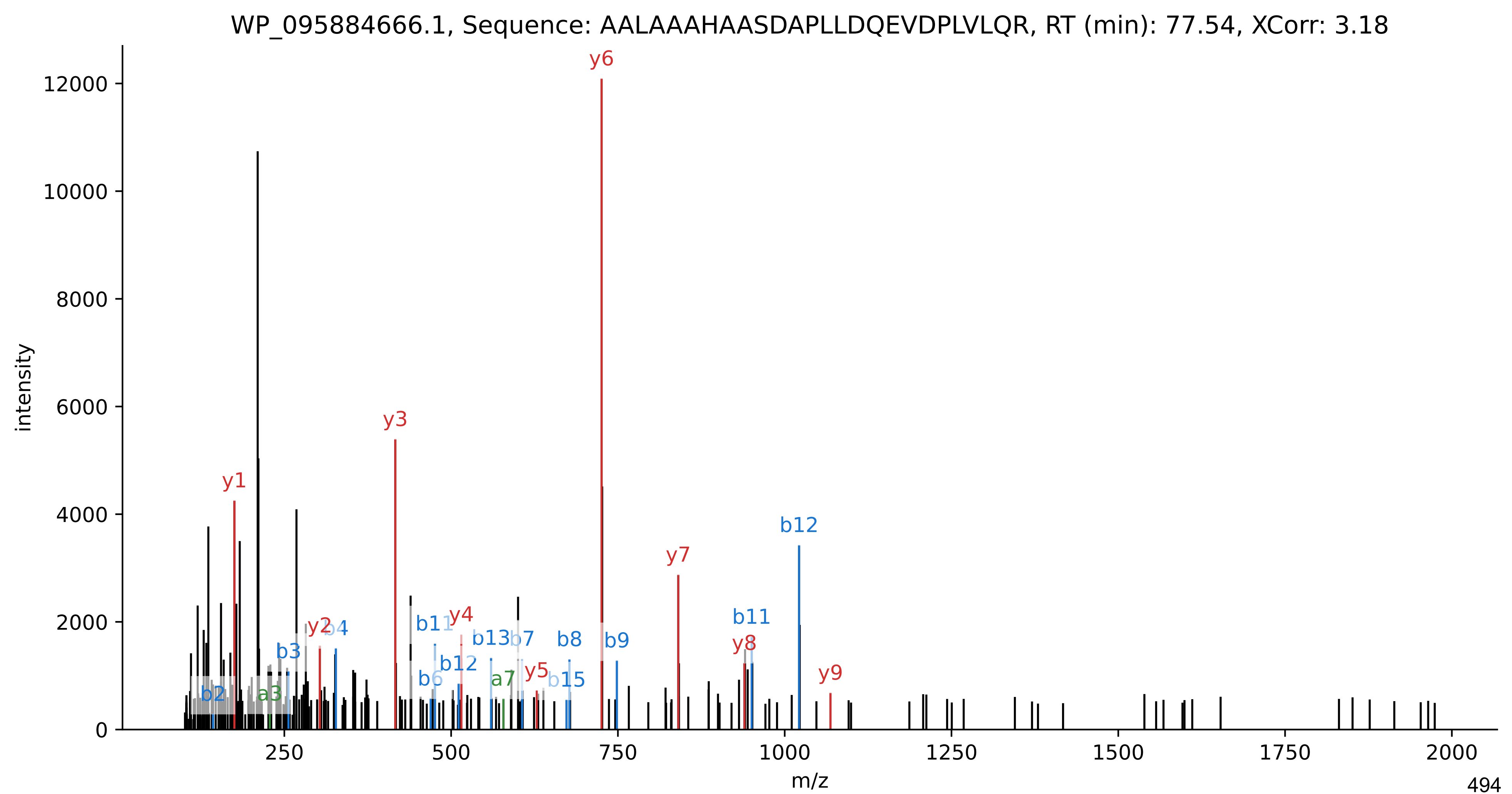

WP_095884666.1, Sequence: AALAAAHAASDAPLLDQEVDPLVLQR, RT (min): 77.54, XCorr: 3.18
intensity
m/z
y6
y3
y1
y7
b12
y2
b4
b3
b2
a3
b11
y4
b6
b12
b13
b7
a7
y5
b15
b8
b9
b11
y8
y9

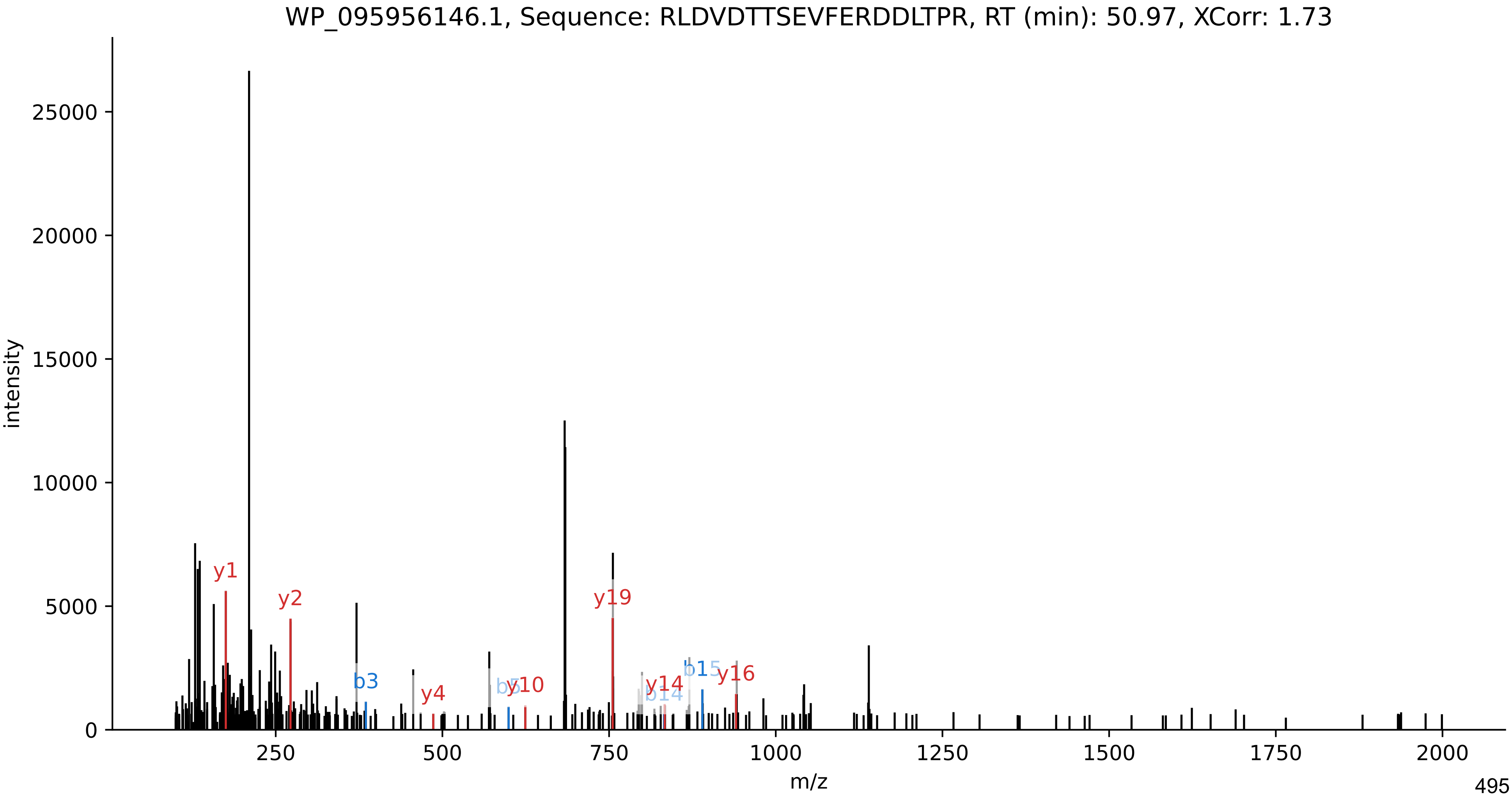

WP_095956146.1, Sequence: RLDVDTTSEVFERDDLTPR, RT (min): 50.97, XCorr: 1.73
intensity
m/z
y1
y2
b3
y4
b5
y10
y19
y14
b14
b15
y16

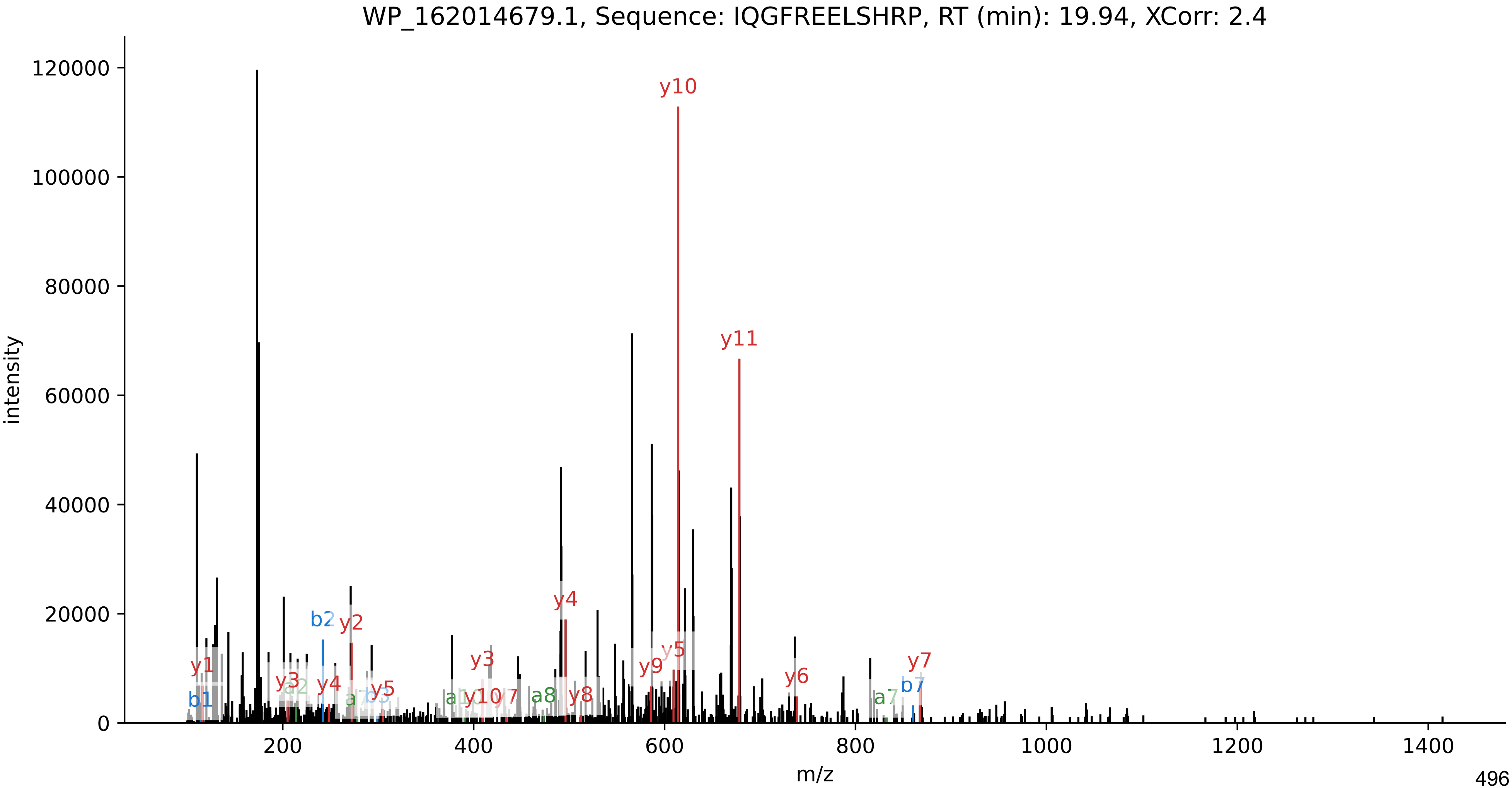
WP_162014679.1, Sequence: IQGFREELSHRP, RT (min): 19.94, XCorr: 2.4
intensity
m/z
0
20000
40000
60000
80000
100000
120000
200
400
600
800
1000
1200
1400
y10
y11
y4
b2
y2
y1
b1
y3
y4
y5
y3
y10
a8
y8
y9
y5
y6
y7
b7

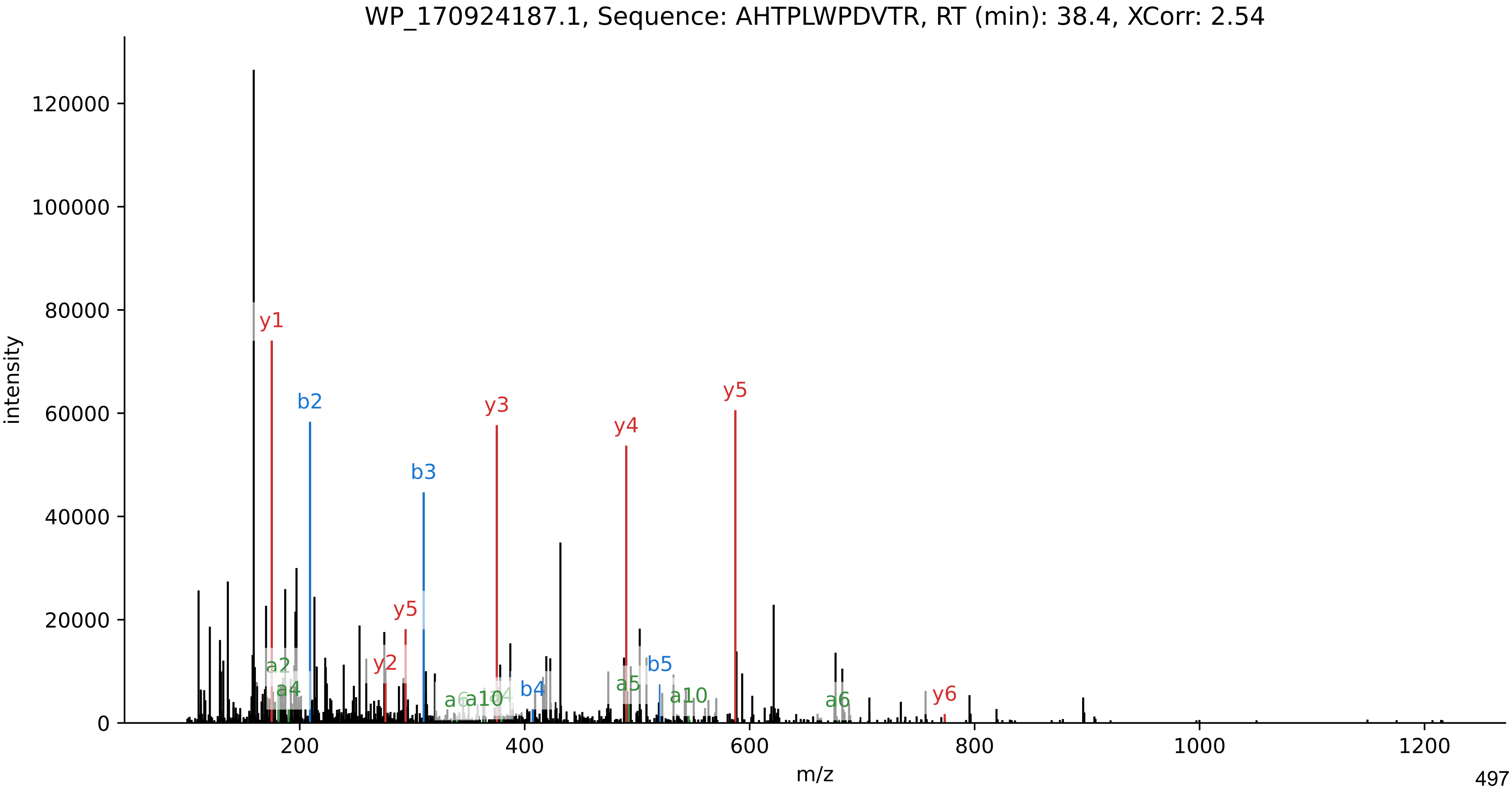
WP_170924187.1, Sequence: AHTPLWPDVTR, RT (min): 38.4, XCorr: 2.54
intensity
m/z
y1
b2
y3
y4
y5
b3
y5
y2
a2
a4
b4
a5
b5
a10
a6
y6
a10

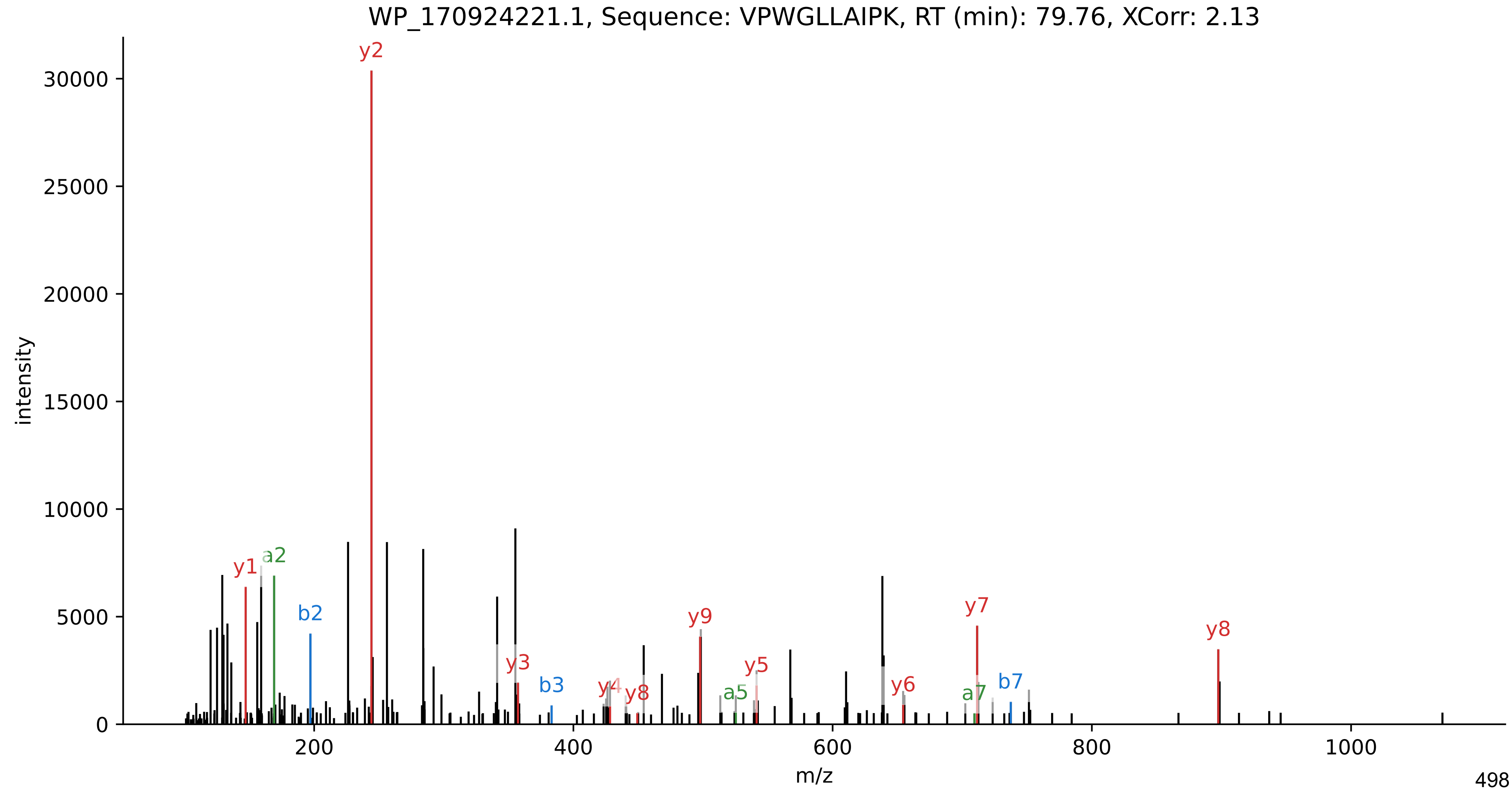

WP_170924221.1, Sequence: VPWGLLAIPK, RT (min): 79.76, XCorr: 2.13
intensity
m/z
y2
y1
a2
b2
y3
b3
y4
y8
y9
a5
y5
y6
y7
a7
b7
y8
30000
25000
20000
15000
10000
5000
0
200
400
600
800
1000

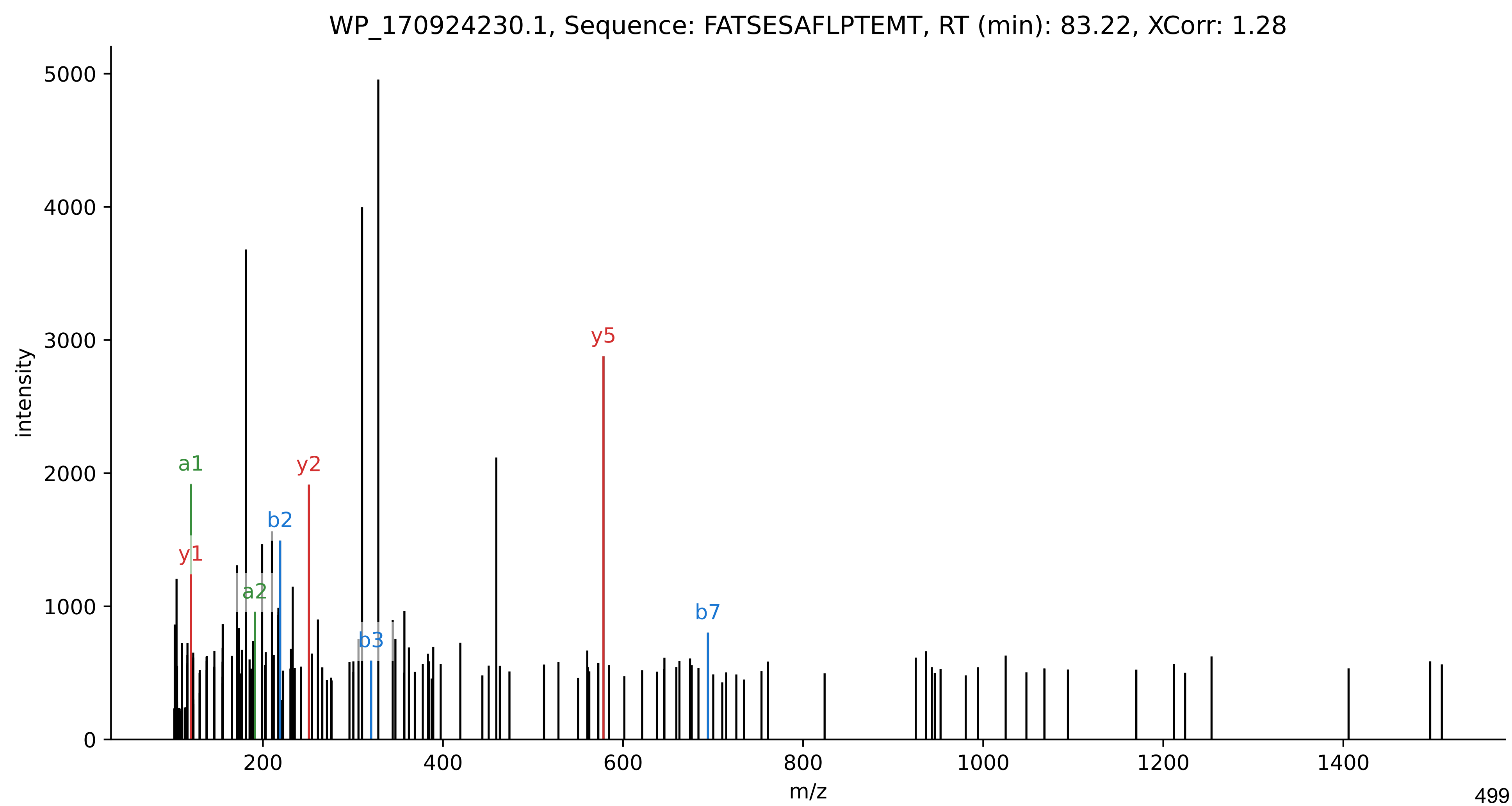
WP_170924230.1, Sequence: FATSESAFLPTEMT, RT (min): 83.22, XCorr: 1.28
intensity
m/z
0
1000
2000
3000
4000
5000
200
400
600
800
1000
1200
1400
a1
y1
a2
b2
y2
b3
y5
b7

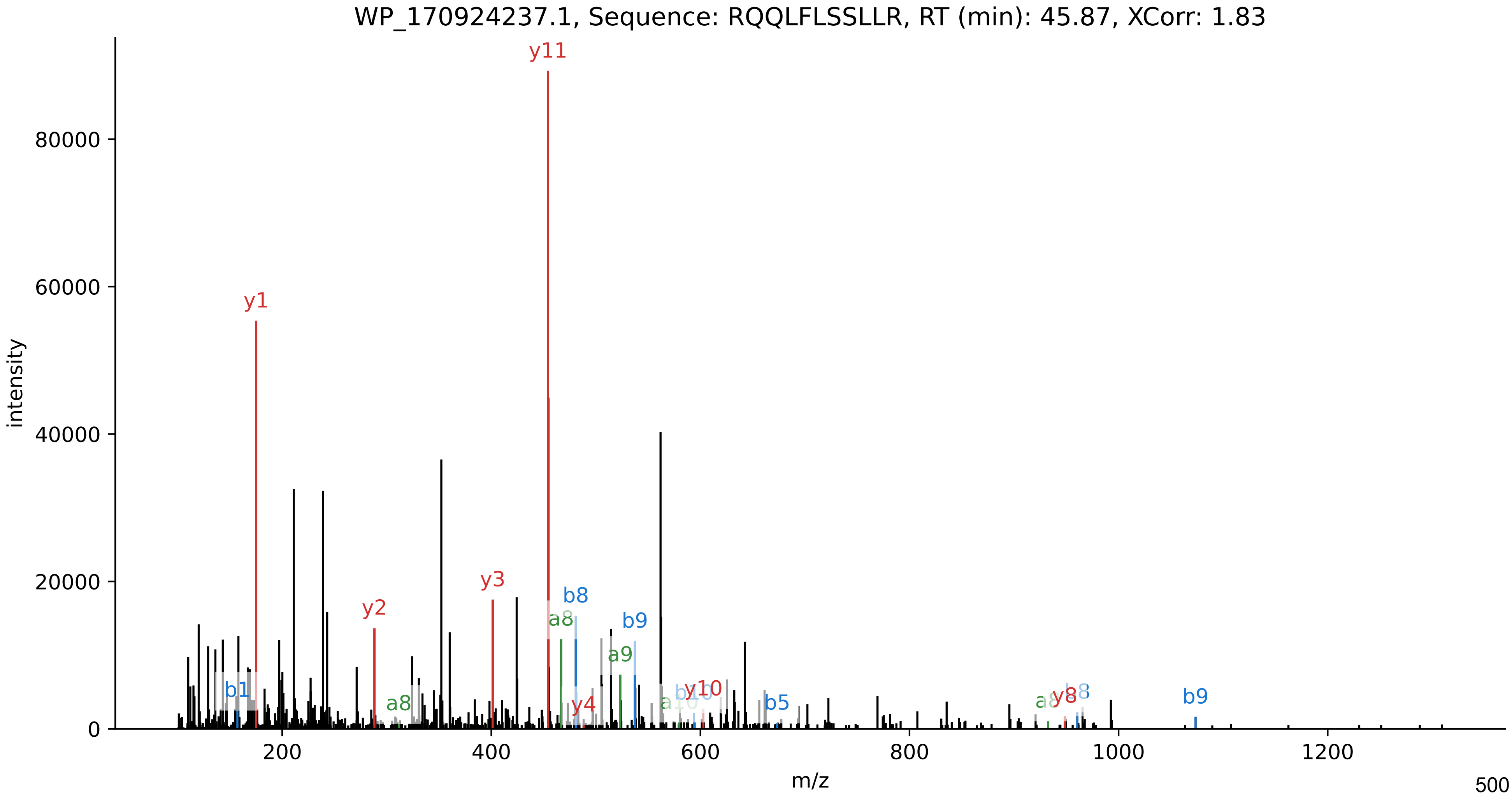
WP_170924237.1, Sequence: RQQLFLSSLLR, RT (min): 45.87, XCorr: 1.83
intensity
m/z
y11
y1
y3
y2
b8
a8
b9
a9
b1
a8
y4
y10
b5
a8
y8
b9
0
20000
40000
60000
80000
200
400
600
800
1000
1200

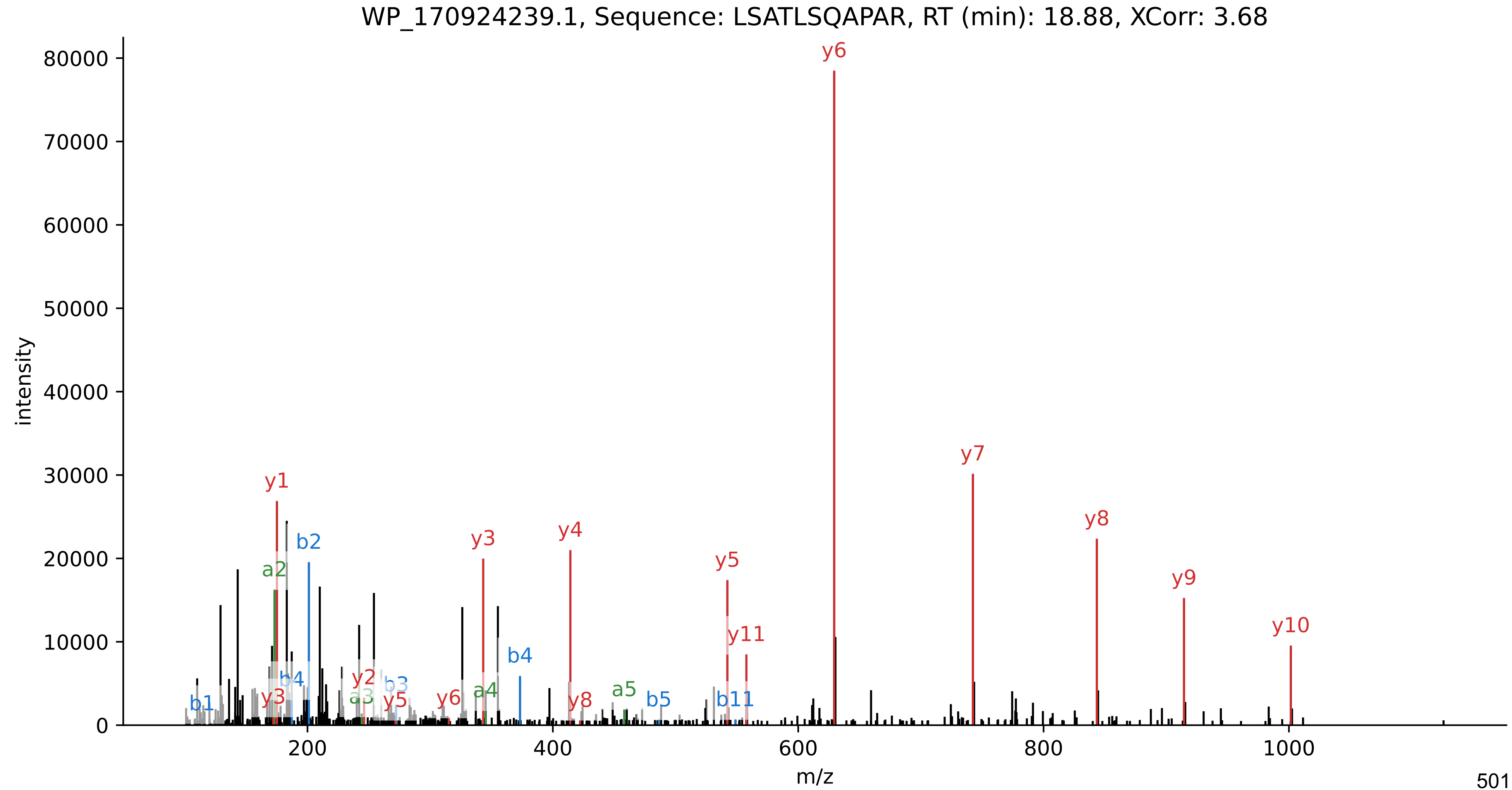
WP_170924239.1, Sequence: LSATLSQAPAR, RT (min): 18.88, XCorr: 3.68
intensity
m/z
y1
y2
y3
y4
y5
y6
y7
y8
y9
y10
y11
b1
b2
b3
b4
b5
b11
a2
a3
a4
a5

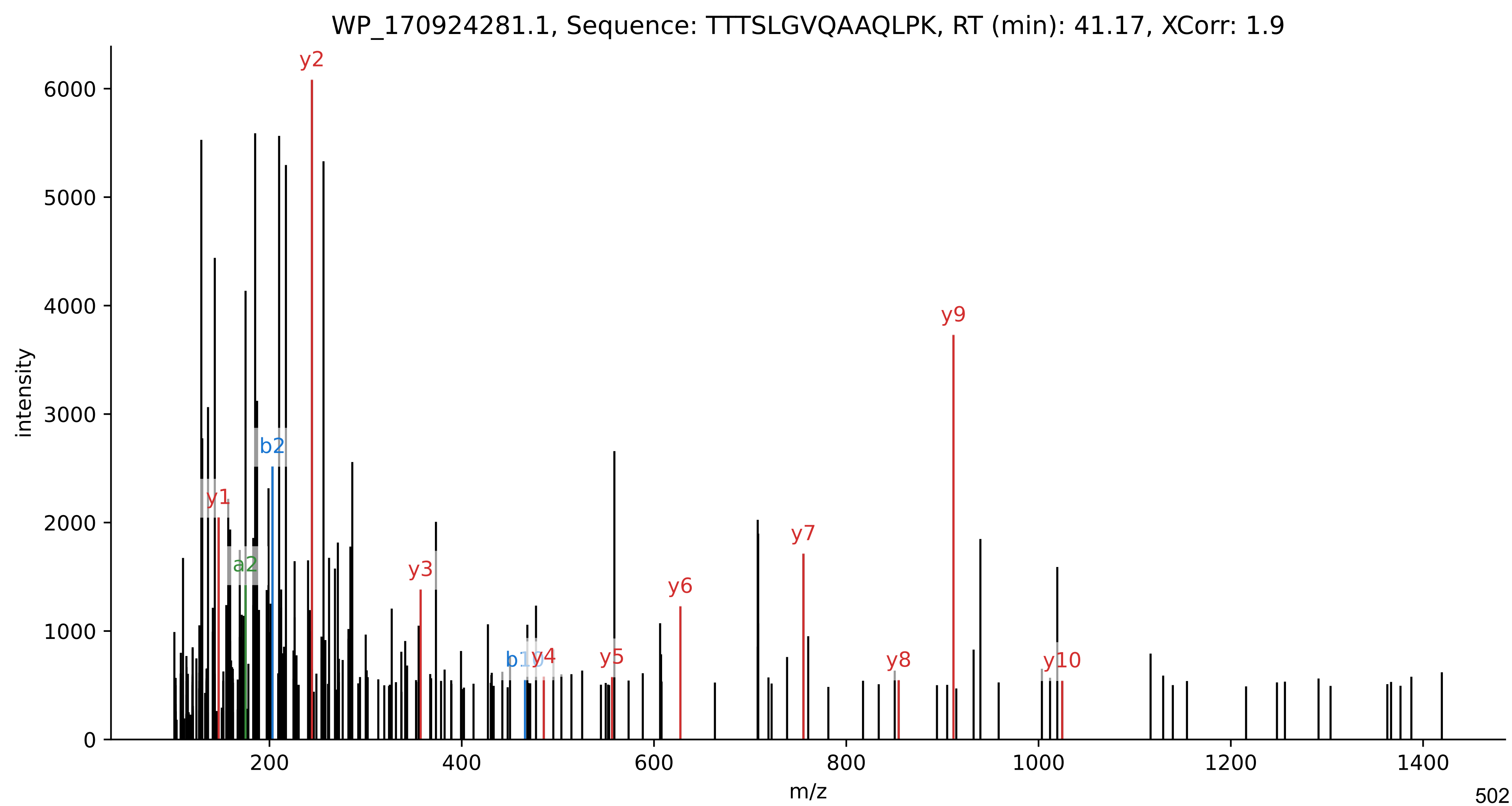
WP_170924281.1, Sequence: TTTSLGVQAAQLPK, RT (min): 41.17, XCorr: 1.9
intensity
m/z
y1
y2
y3
y4
y5
y6
y7
y8
y9
y10
b2
b10
a2

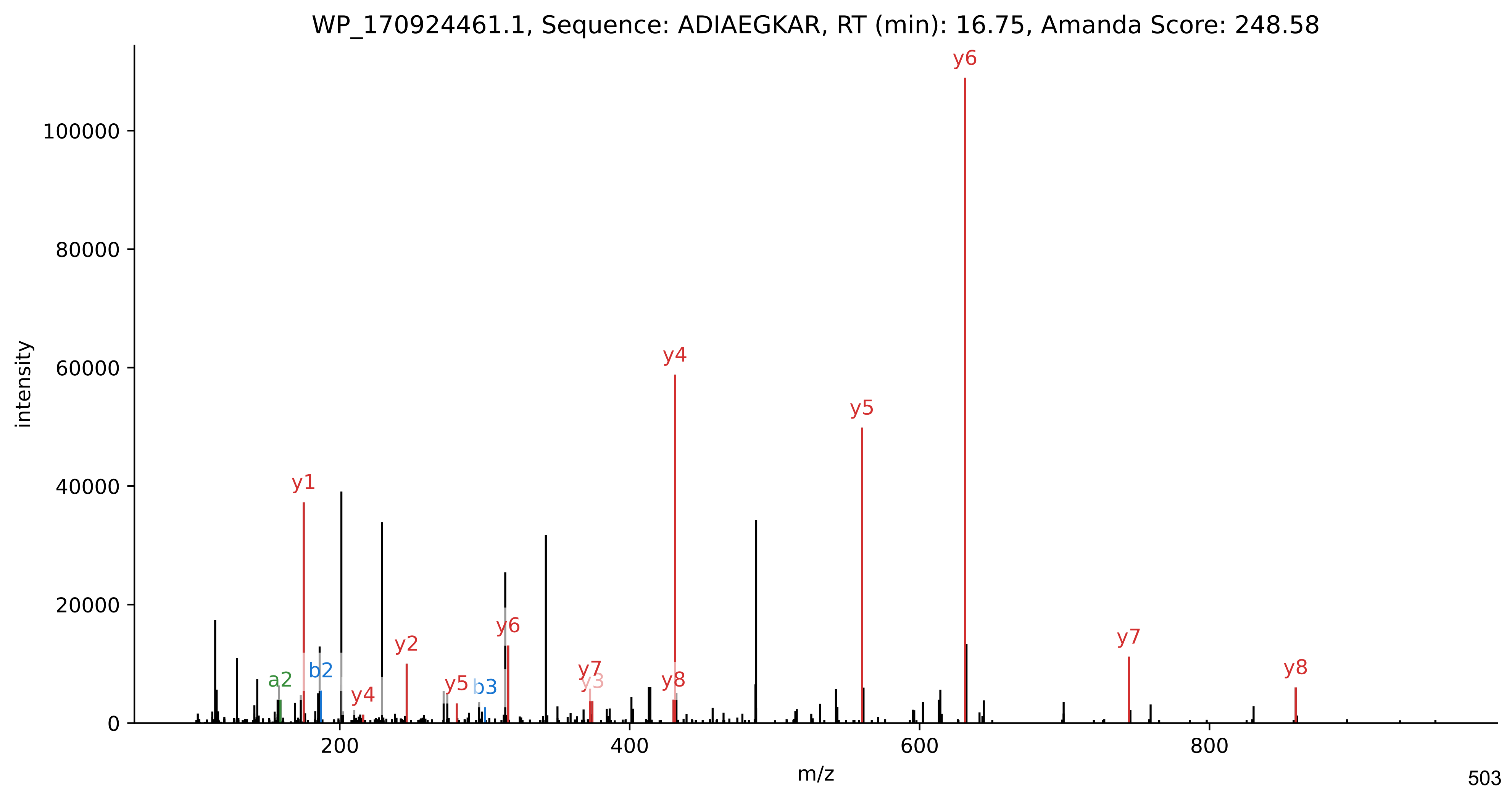
WP_170924461.1, Sequence: ADIAEGKAR, RT (min): 16.75, Amanda Score: 248.58
intensity
m/z
y6
y4
y5
y1
y6
y2
a2
b2
y4
y5
b3
y7
y3
y8
y7
y8
0
20000
40000
60000
80000
100000
200
400
600
800

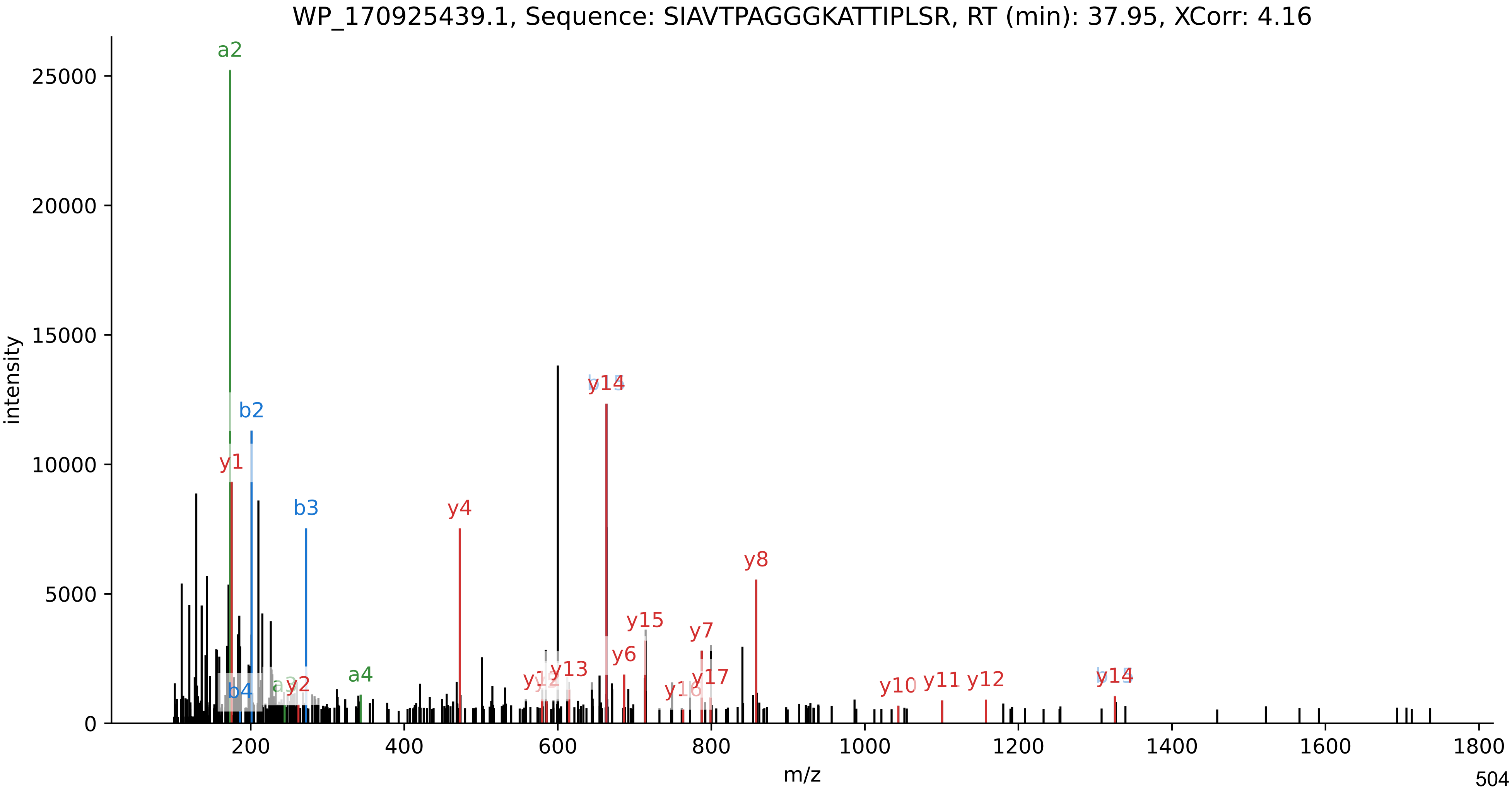
WP_170925439.1, Sequence: SIAVTPAGGGKATTIPLSR, RT (min): 37.95, XCorr: 4.16
intensity
m/z
a2
b2
y1
b3
y4
y14
y8
y15
y7
y6
y13
y17
a4
y2
b4
y10
y11
y12
y14
0
5000
10000
15000
20000
25000
200
400
600
800
1000
1200
1400
1600
1800

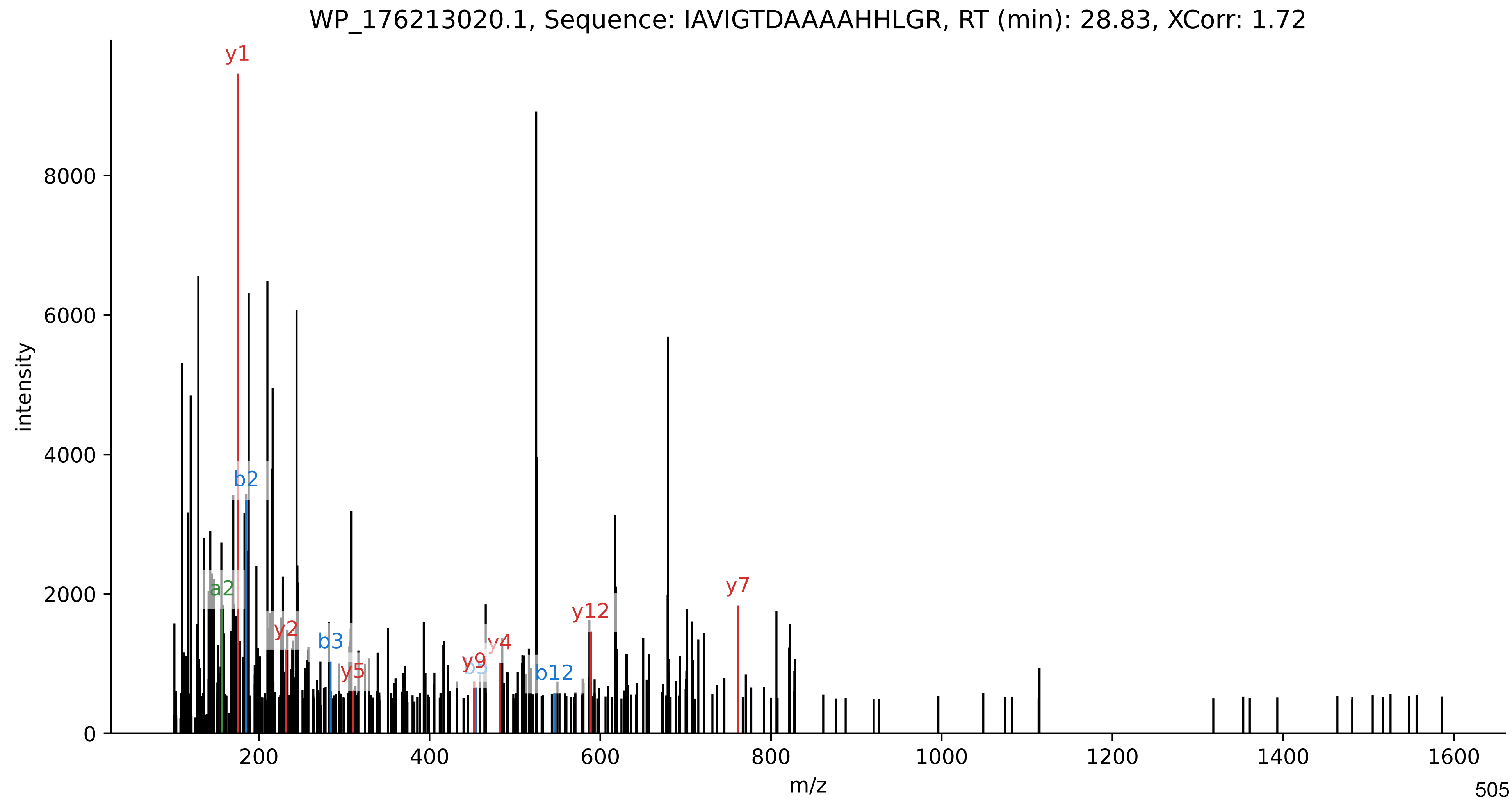
WP_176213020.1, Sequence: IAVIGTDAAAAHHLGR, RT (min): 28.83, XCorr: 1.72
intensity
m/z
y1
b2
a2
y2
b3
y5
y9
y4
b12
y12
y7

WP_176213036.1, Sequence: AAEAAVTAGGGESFGR, RT (min): 26.41, XCorr: 2.76

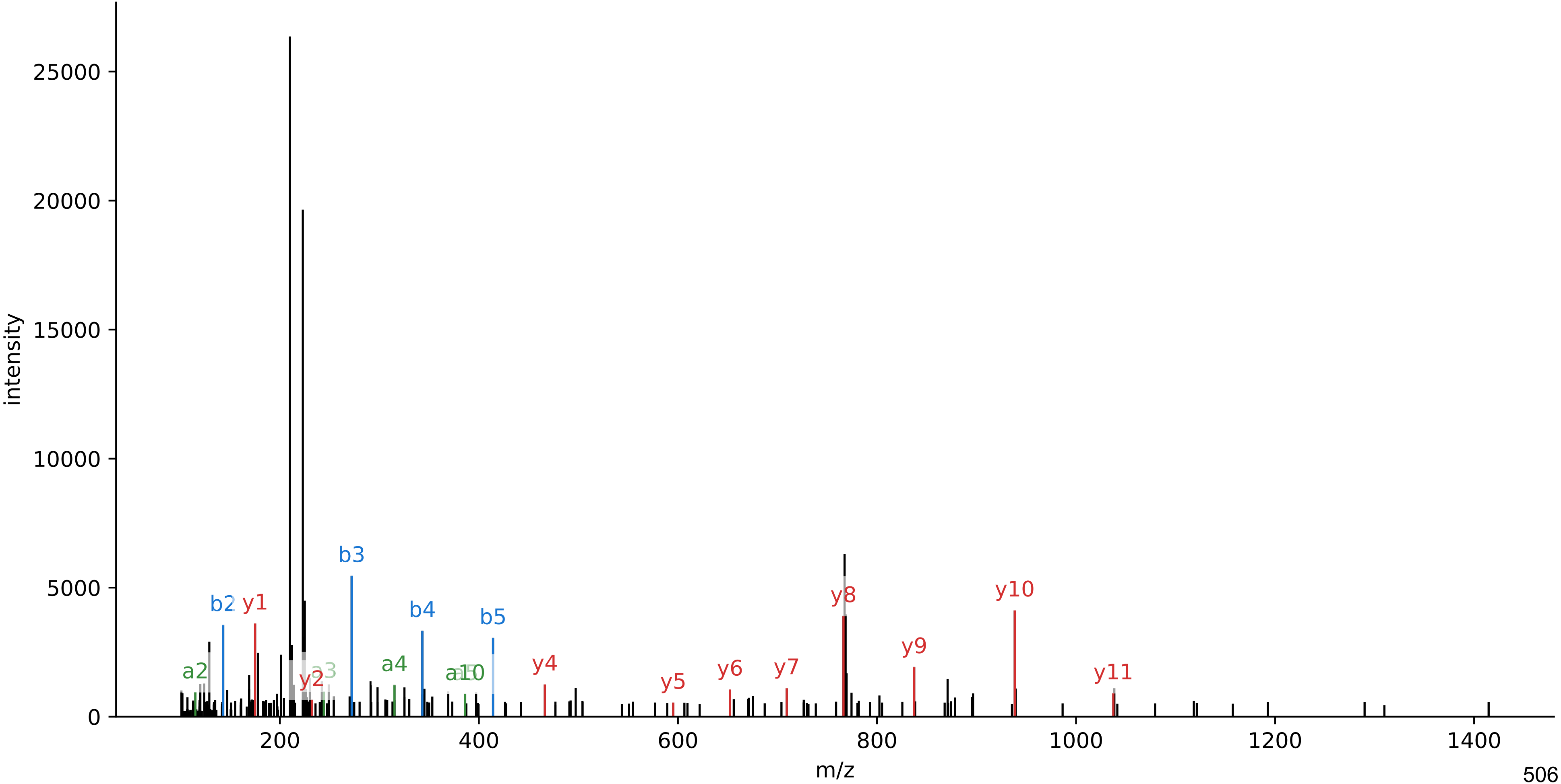

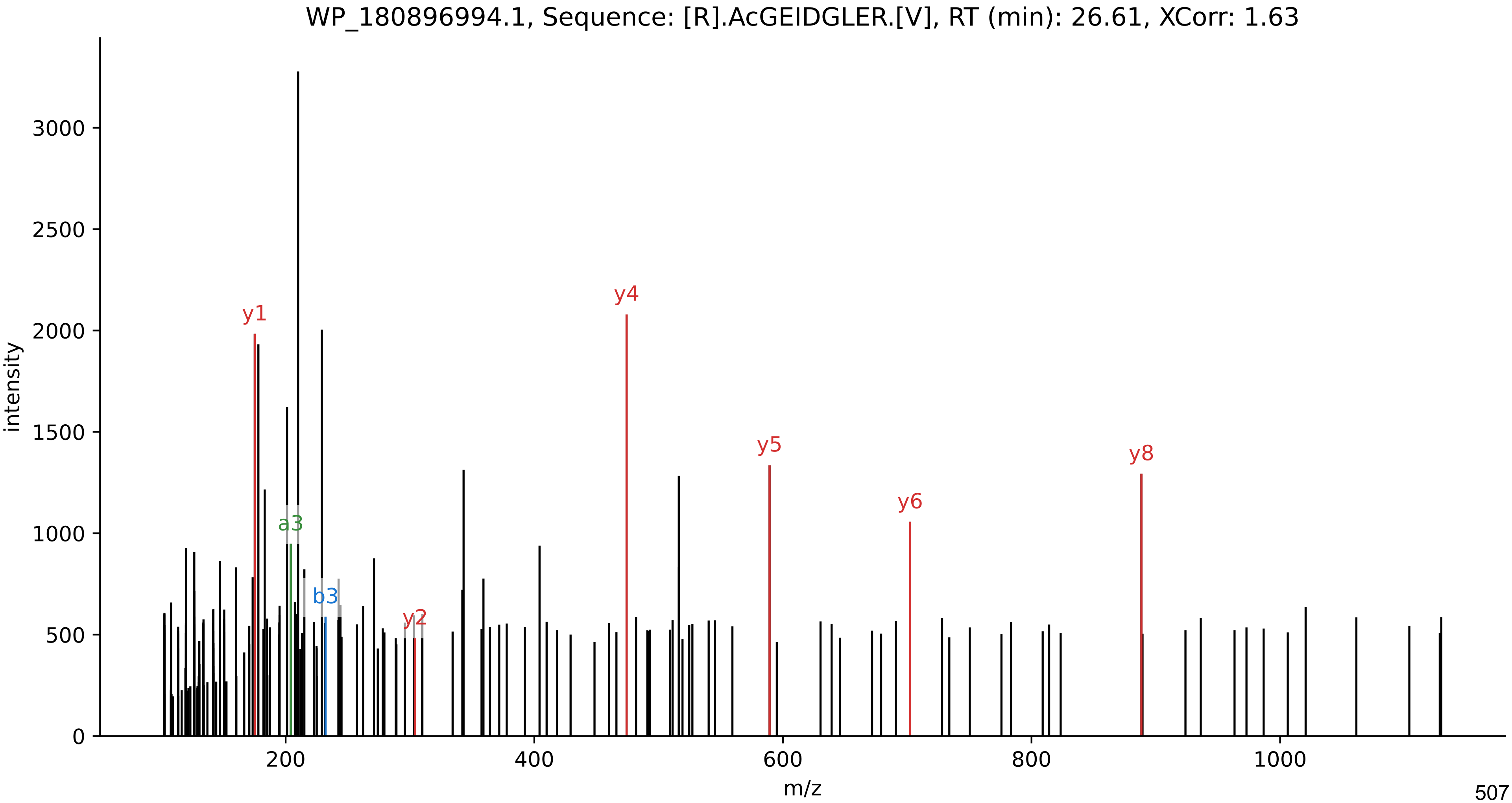
WP_180896994.1, Sequence: [R].AcGEIDGLER.[V], RT (min): 26.61, XCorr: 1.63
intensity
m/z
y1
a3
b3
y2
y4
y5
y6
y8
0
500
1000
1500
2000
2500
3000
200
400
600
800
1000

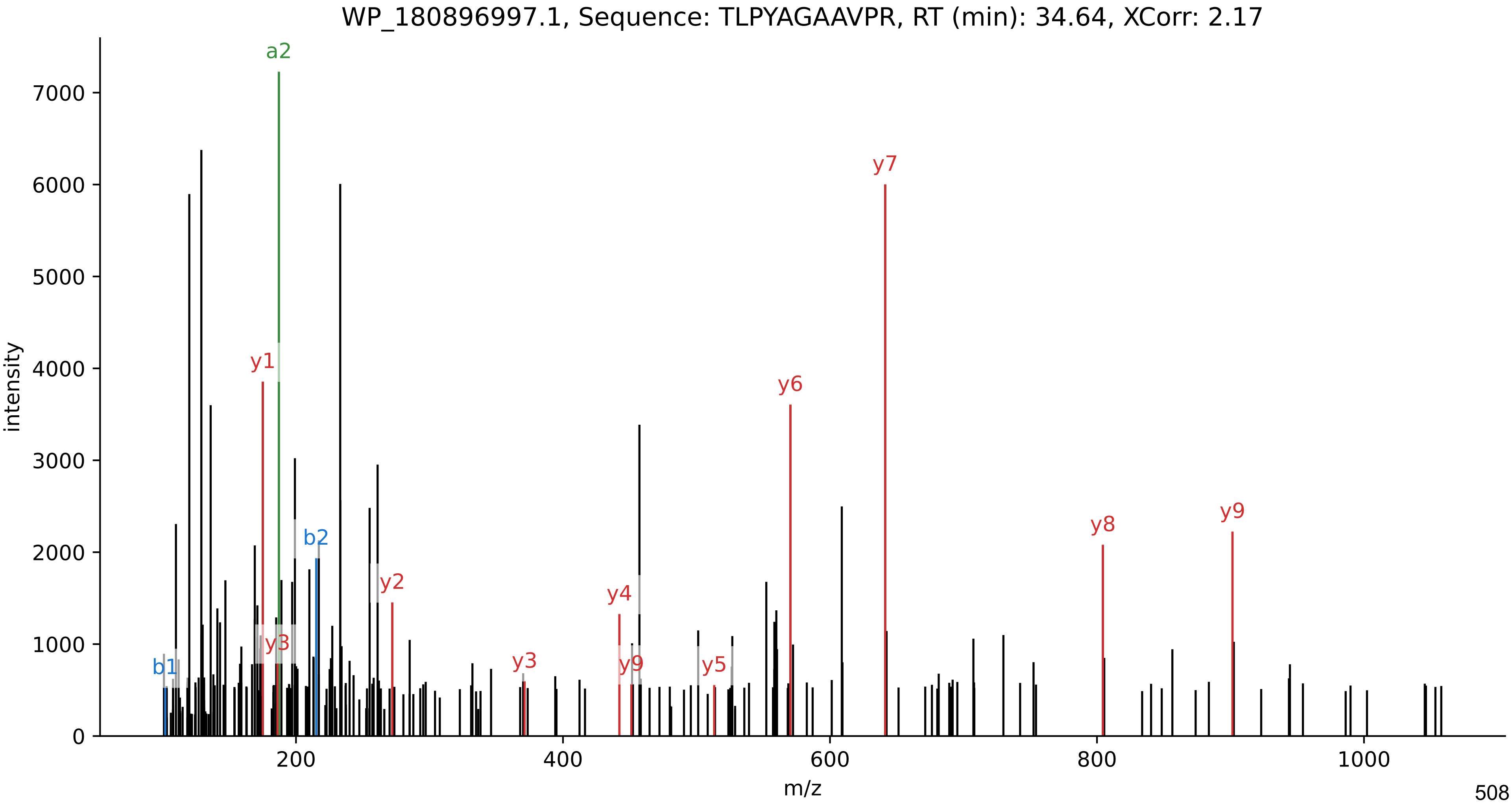
WP_180896997.1, Sequence: TLPYAGAAVPR, RT (min): 34.64, XCorr: 2.17
a2
y7
y1
y6
y8
y9
b2
y2
y4
y3
b1
y3
y9
y5
intensity
m/z
0
1000
2000
3000
4000
5000
6000
7000
200
400
600
800
1000

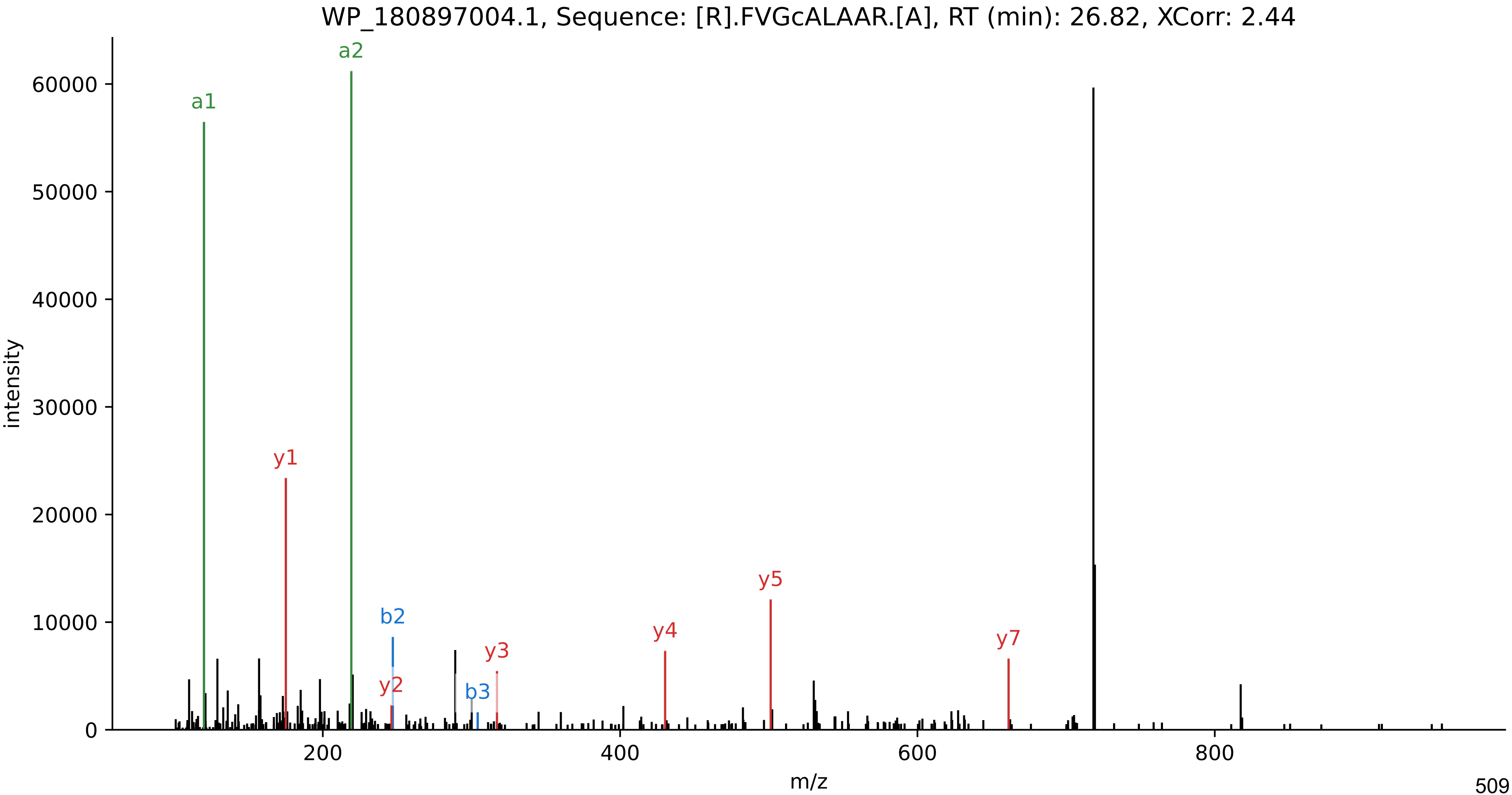
WP_180897004.1, Sequence: [R].FVGcALAAR.[A], RT (min): 26.82, XCorr: 2.44
a1
a2
y1
b2
y2
b3
y3
y4
y5
y7
intensity
0
10000
20000
30000
40000
50000
60000
200
400
600
800
m/z
