# Supplementary material for: Development of a Spectral Library for the Discovery of Altered Genomic Events in Mycobacterium avium Associated With Virulence Using Mass Spectrometry–Based Proteogenomic Analysis
Source: Mol Cell Proteomics. 2023 Mar 21;22(5):100533. doi: 10.1016/j.mcpro.2023.100533 (PMC10149365; doi:10.1016/j.mcpro.2023.100533)
Supplement: Supplemental Data 4 [file mmc10.pdf]

Sequence: QIVISSDRPPK, RT (min): 12.95, XCorr: 3.37

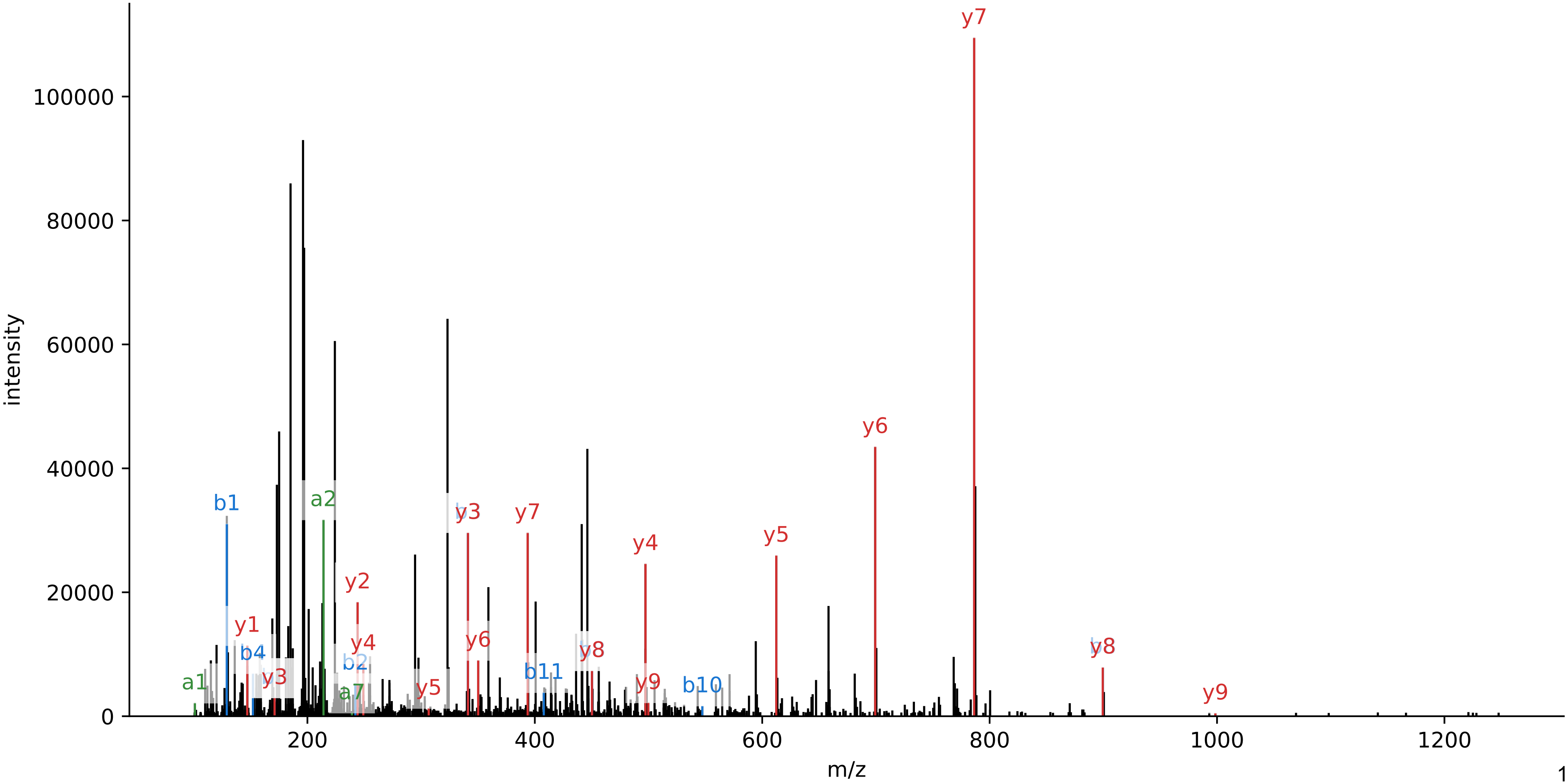

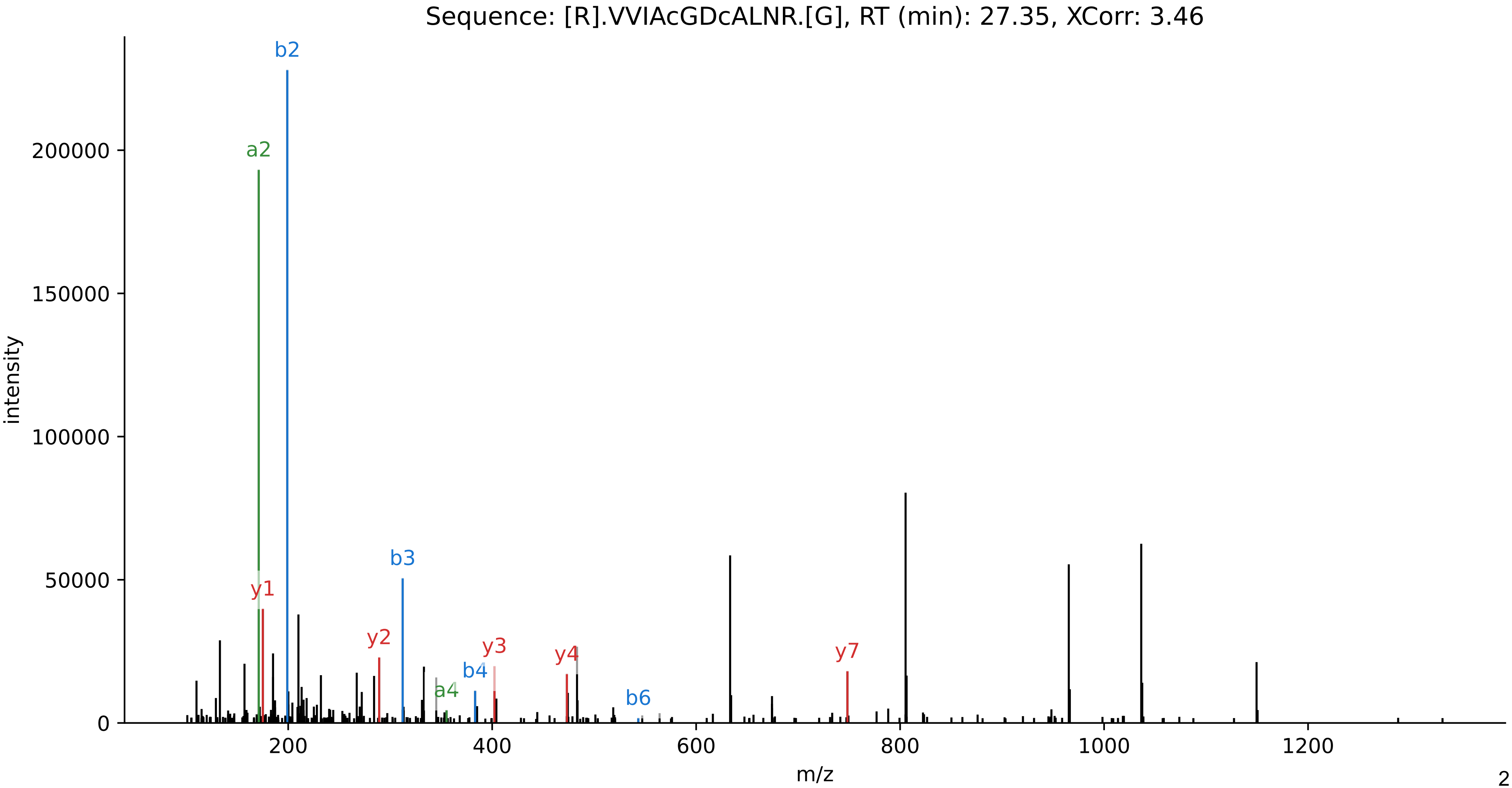

Sequence: [R].VVIAcGDcALNR.[G], RT (min): 27.35, XCorr: 3.46
intensity
m/z
b2
a2
b3
y1
y2
y3
y4
b4
a4
b6
y7
200000
150000
100000
50000
0
200
400
600
800
1000
1200

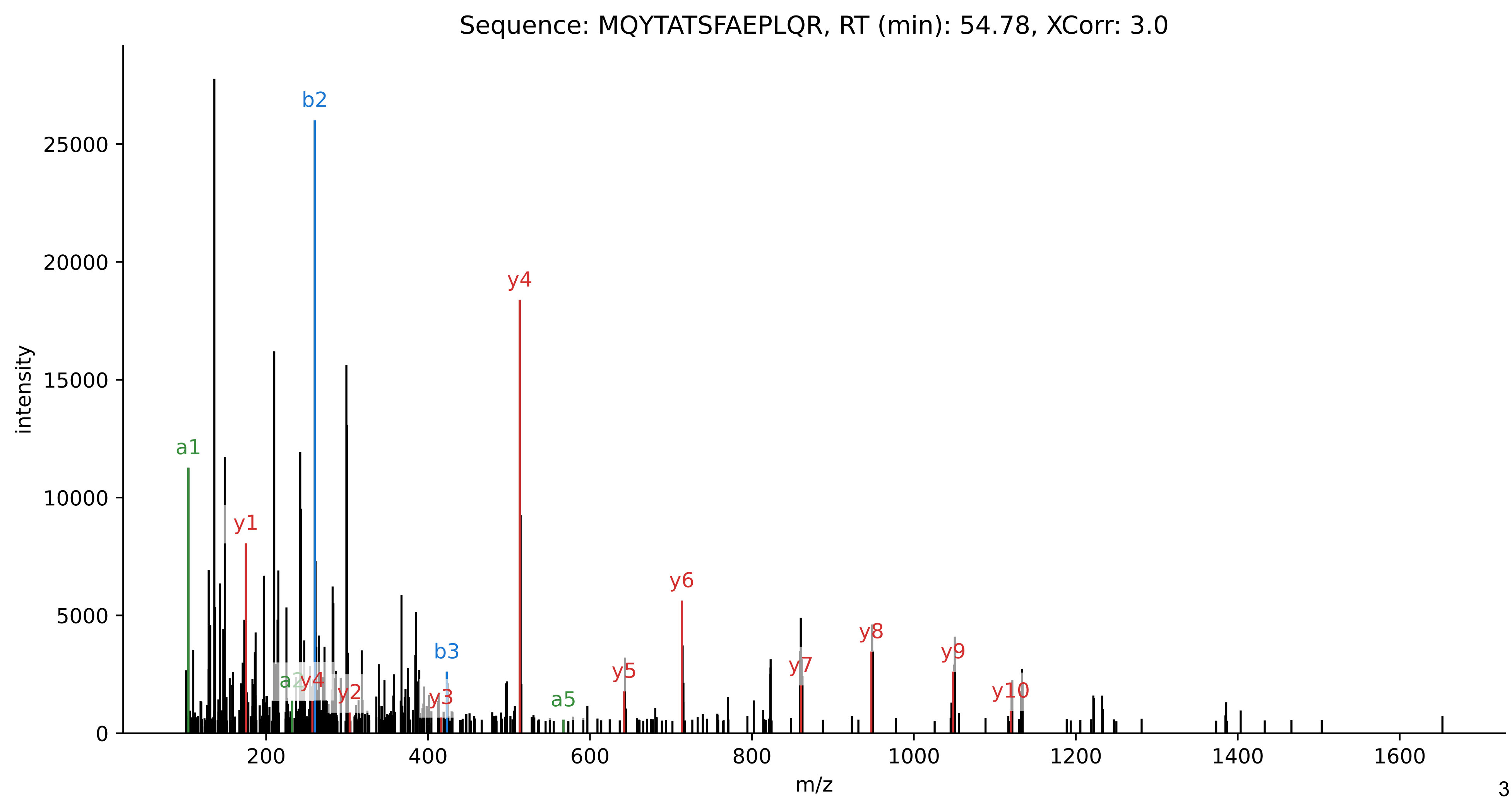
Sequence: MQYTATSFAEPLQR, RT (min): 54.78, XCorr: 3.0
intensity
m/z
a1
b2
y1
y4
a2
y4
y2
b3
y3
a5
y5
y6
y7
y8
y9
y10
0
5000
10000
15000
20000
25000
200
400
600
800
1000
1200
1400
1600

Sequence: AIARQLGLPADAR, RT (min): 29.2, XCorr: 3.09

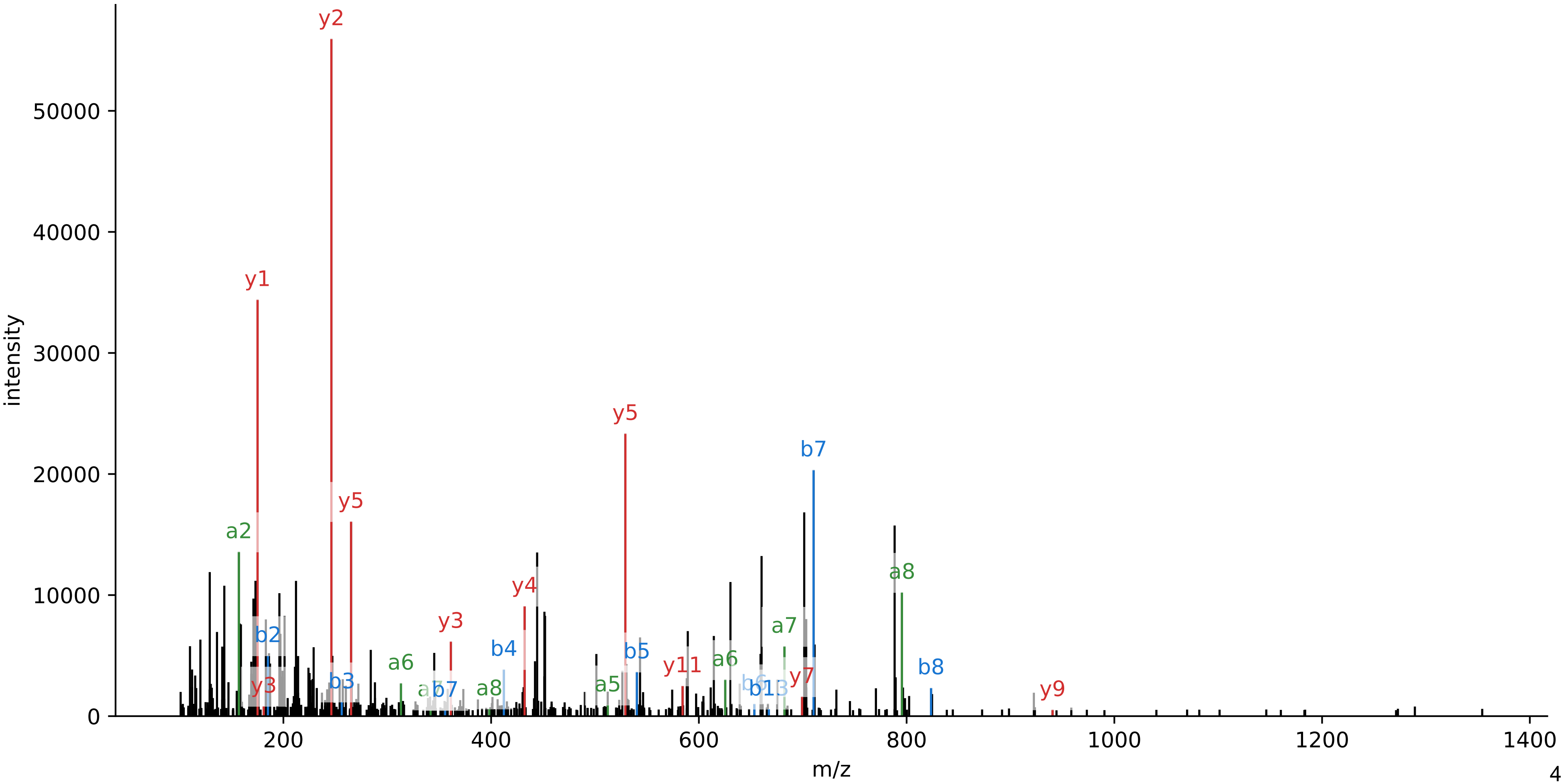

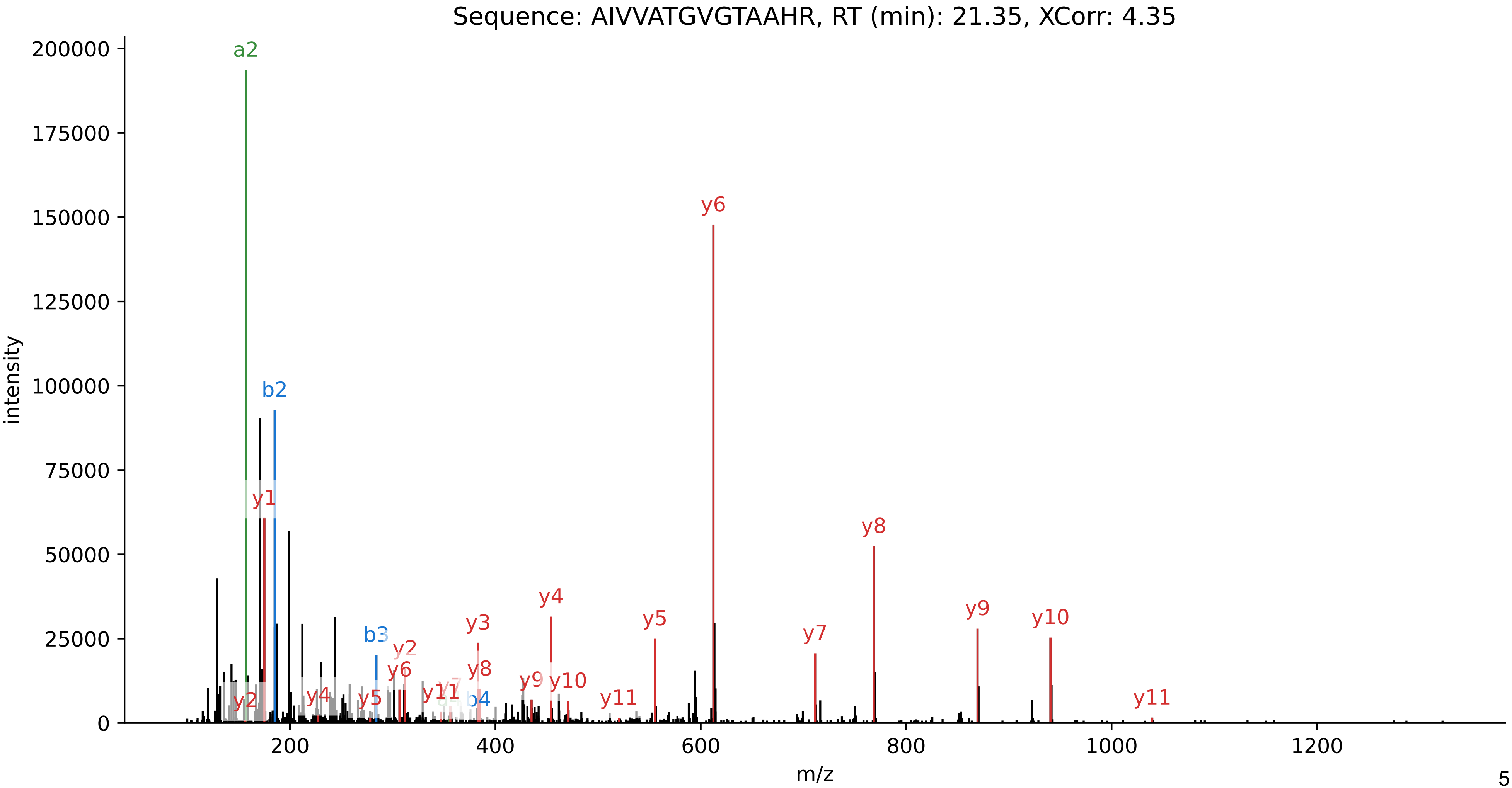
Sequence: AIVVATGVGTAAHR, RT (min): 21.35, XCorr: 4.35
intensity
m/z
a2
b2
y1
y6
y8
y4
y3
b3
y2
y6
y8
y9
y10
y5
y7
y9
y10
y11
y11
y2
y4
y5
b4
0
25000
50000
75000
100000
125000
150000
175000
200000
200
400
600
800
1000
1200

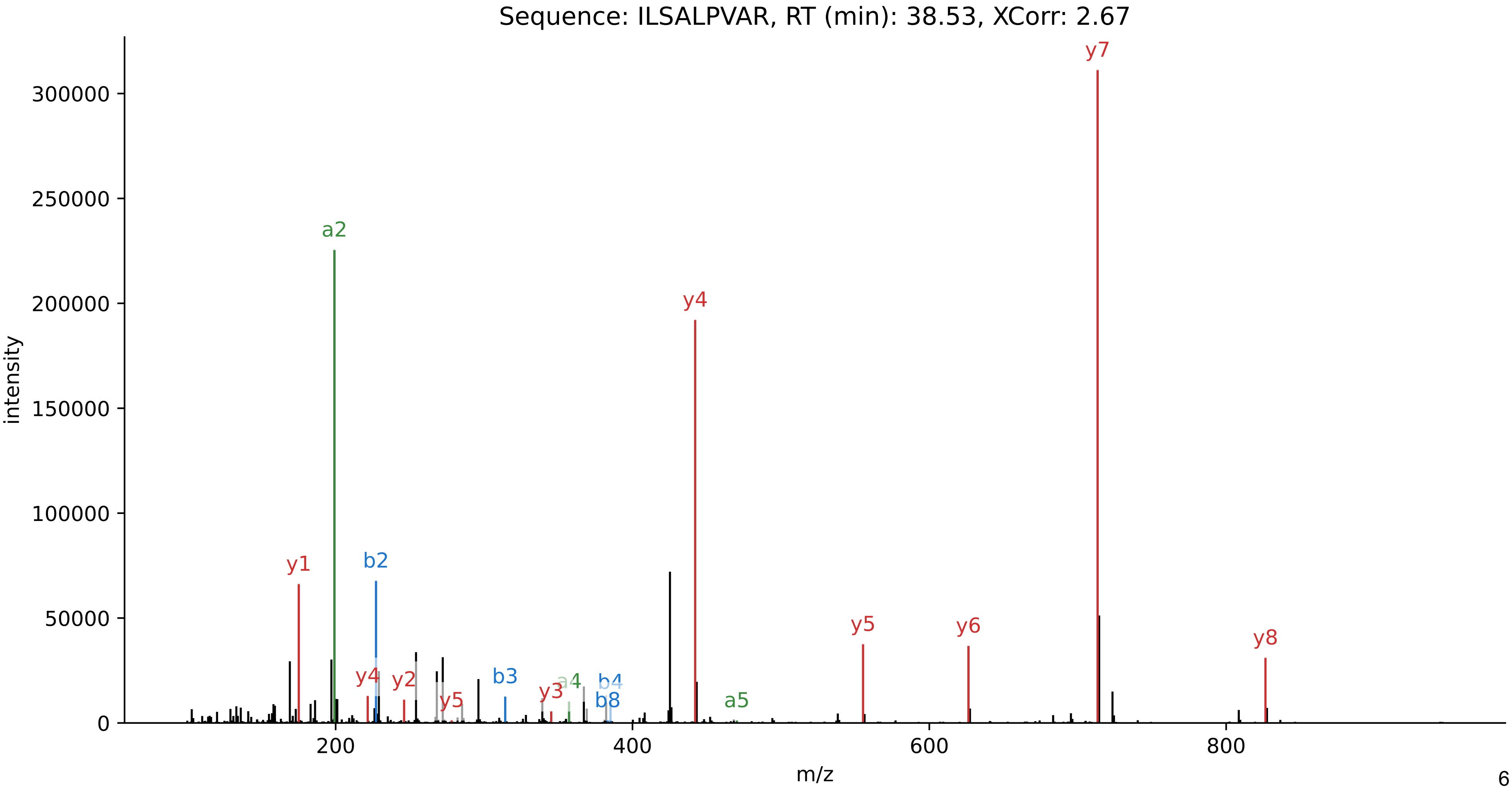

Sequence: ILSALPVAR, RT (min): 38.53, XCorr: 2.67
intensity
m/z
0
50000
100000
150000
200000
250000
300000
200
400
600
800
y7
a2
y4
y1
b2
y5
y6
y8
y4
y2
y5
b3
y3
a4
b4
b8
a5

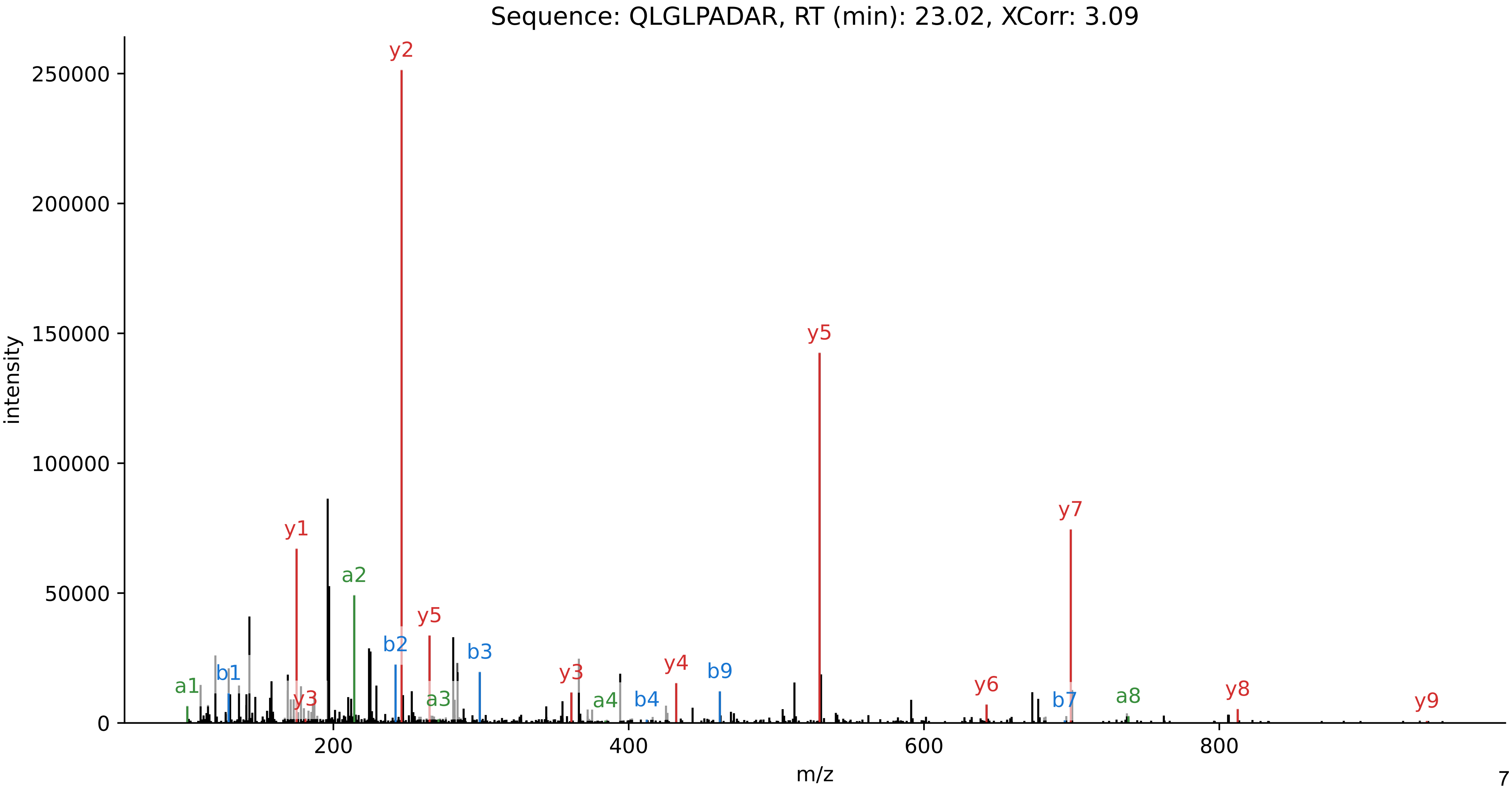

Sequence: QLGLPADAR, RT (min): 23.02, XCorr: 3.09
intensity
m/z
0
50000
100000
150000
200000
250000
200
400
600
800
y2
y5
y7
y1
a2
y5
b2
b3
a1
b1
y3
a3
y3
a4
b4
y4
b9
y6
b7
a8
y8
y9

Sequence: RVVVPAVATTRR, RT (min): 14.1, XCorr: 5.26

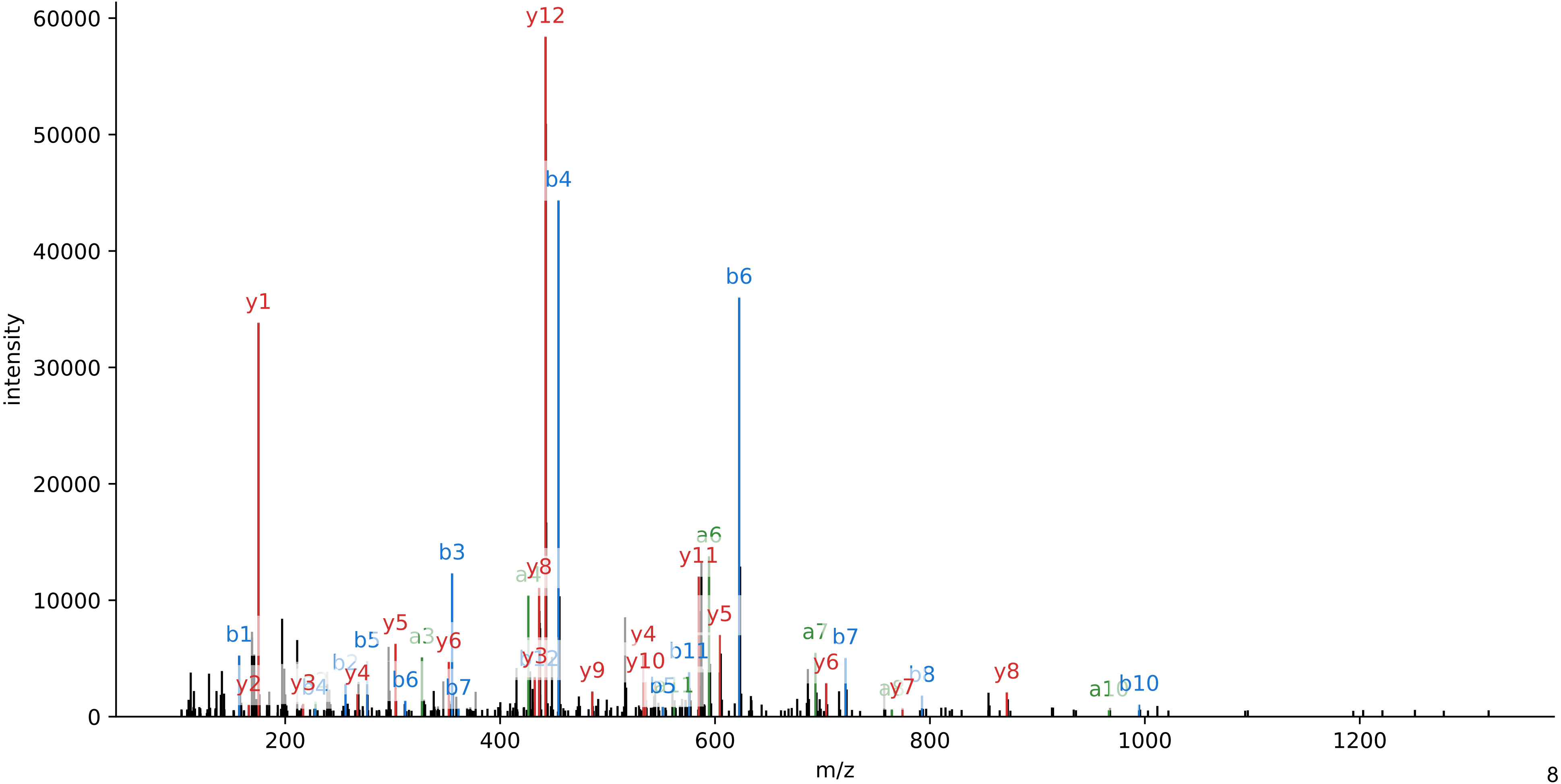

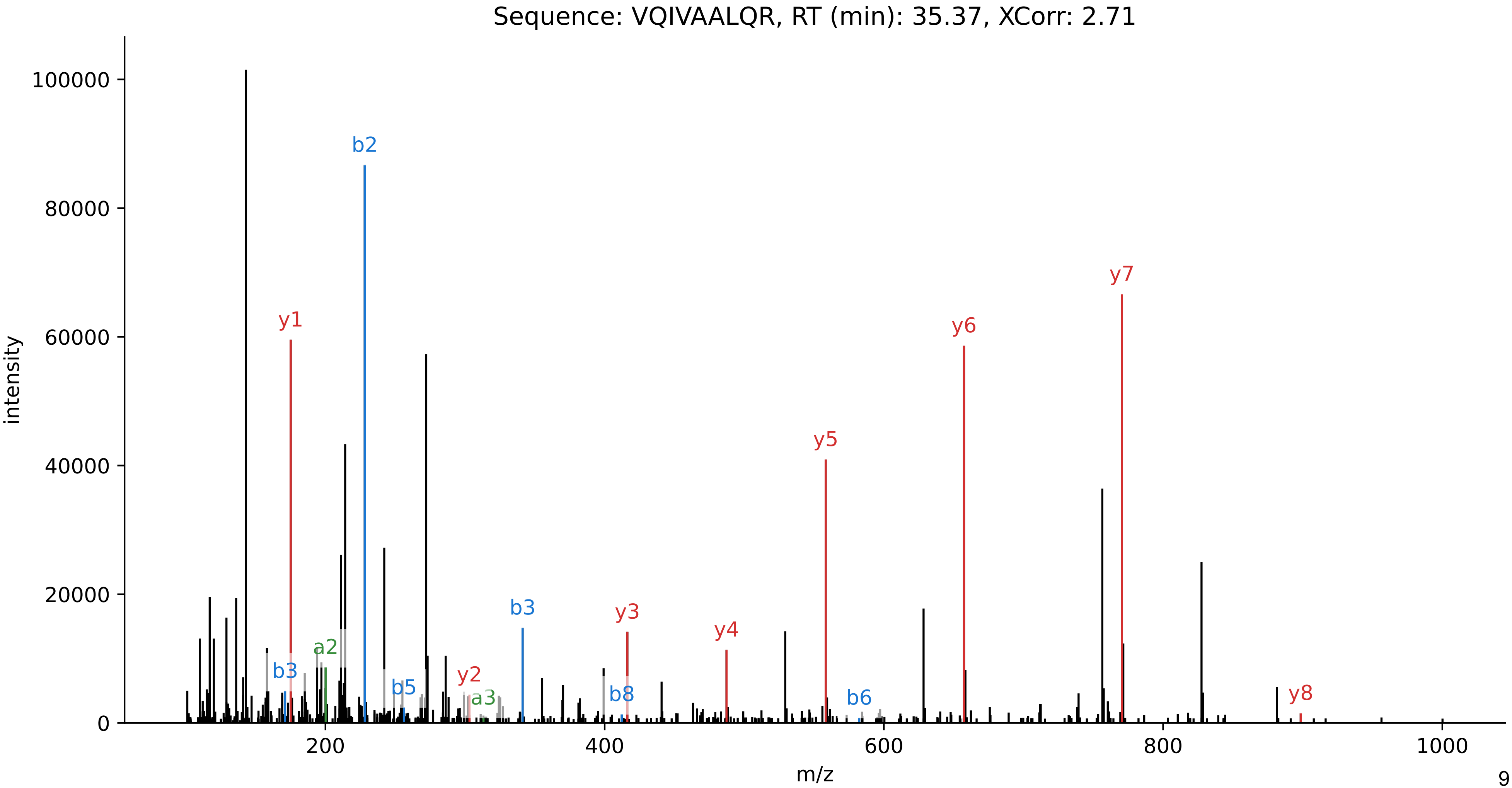
Sequence: VQIVAALQR, RT (min): 35.37, XCorr: 2.71
intensity
m/z
0
20000
40000
60000
80000
100000
200
400
600
800
1000
b2
y1
y7
y6
y5
b3
y3
y4
a2
b3
y2
b5
a3
b8
b6
y8

Sequence: VSPEQKVQIVAALQR, RT (min): 45.08, XCorr: 4.1

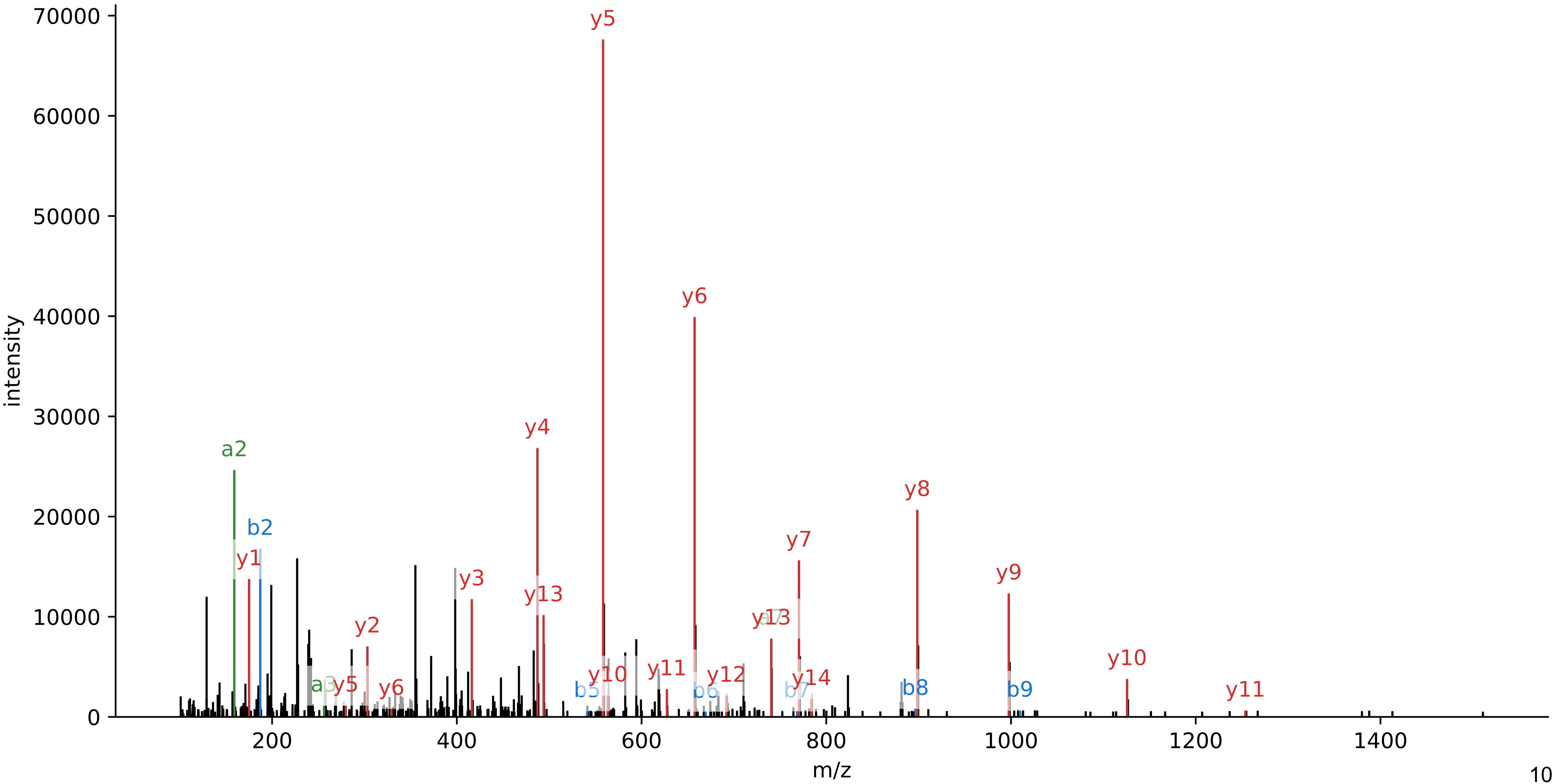

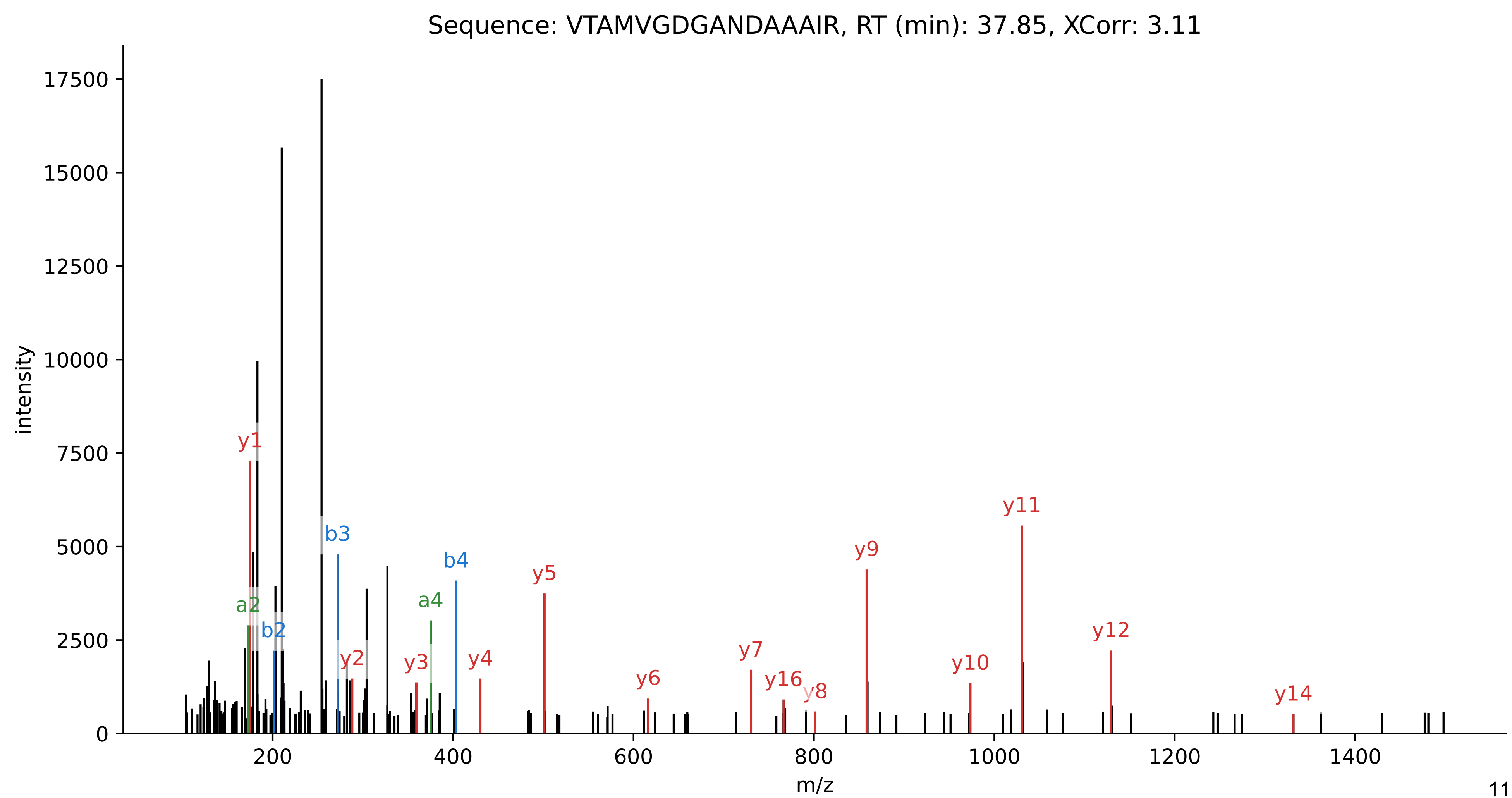
Sequence: VTAMVGDGANDAAAIR, RT (min): 37.85, XCorr: 3.11
intensity
m/z
y1
a2
b2
b3
y2
y3
a4
b4
y4
y5
y6
y7
y16
y8
y9
y10
y11
y12
y14
0
2500
5000
7500
10000
12500
15000
17500
200
400
600
800
1000
1200
1400

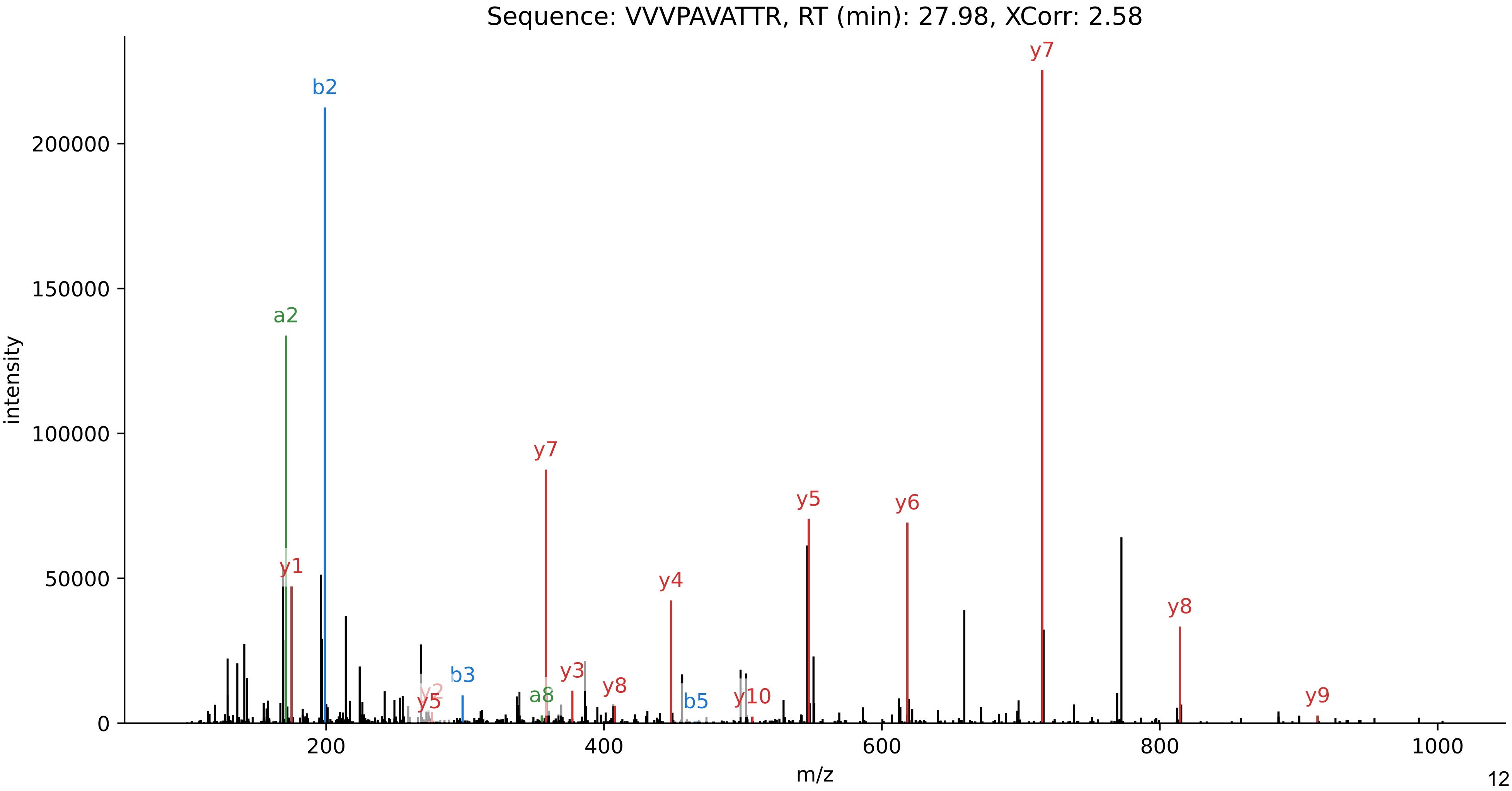
Sequence: VVVPAVATTR, RT (min): 27.98, XCorr: 2.58
intensity
m/z
0
50000
100000
150000
200000
200
400
600
800
1000
y7
b2
a2
y1
y7
y5
y6
y4
y8
b3
y2
y5
a8
y3
y8
b5
y10
y9

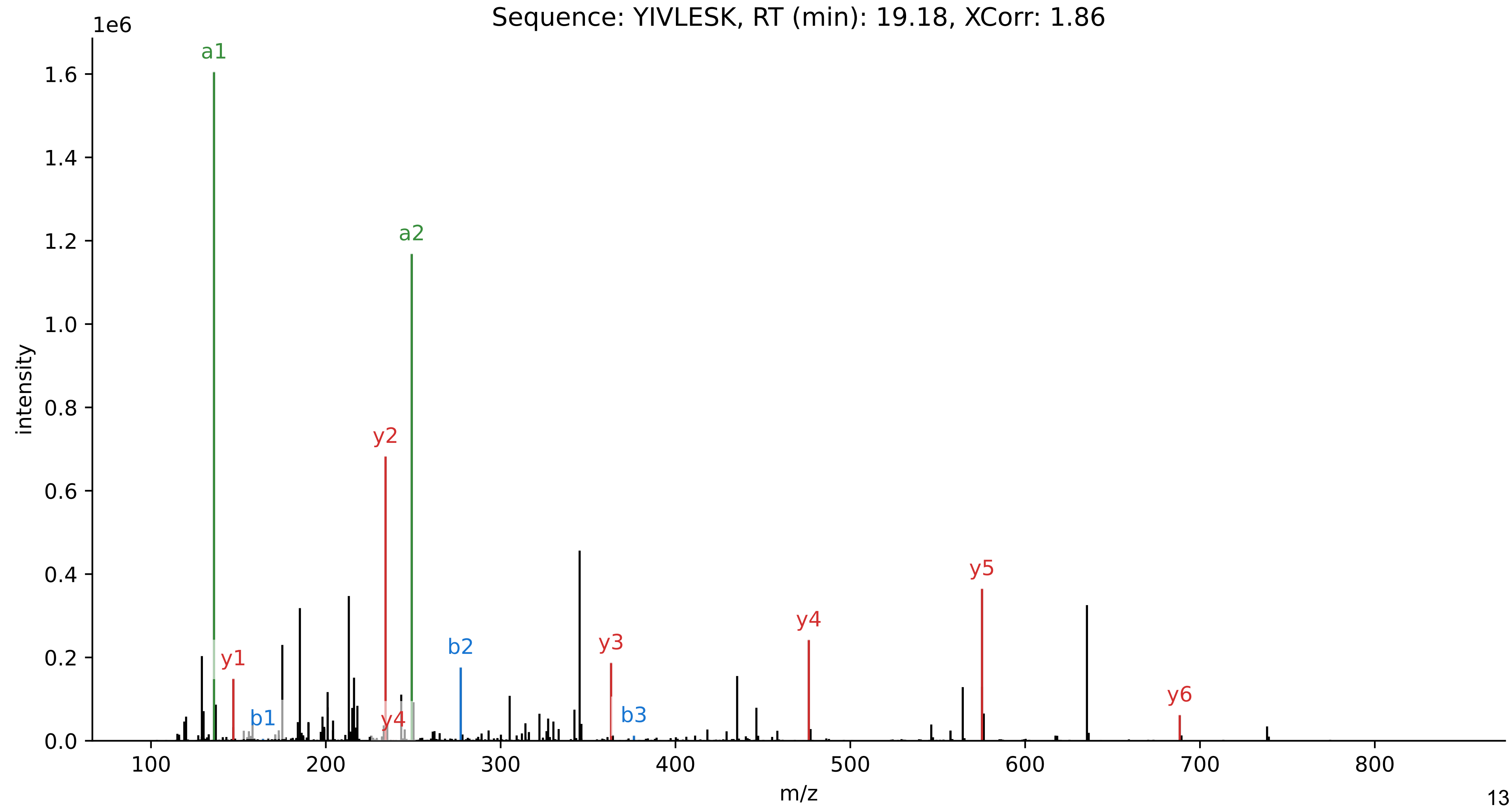
Sequence: YIVLESK, RT (min): 19.18, XCorr: 1.86
1e6
intensity
m/z
a1
a2
y1
y2
y3
y4
y4
y5
y6
b1
b2
b3
0.0
0.2
0.4
0.6
0.8
1.0
1.2
1.4
1.6
100
200
300
400
500
600
700
800

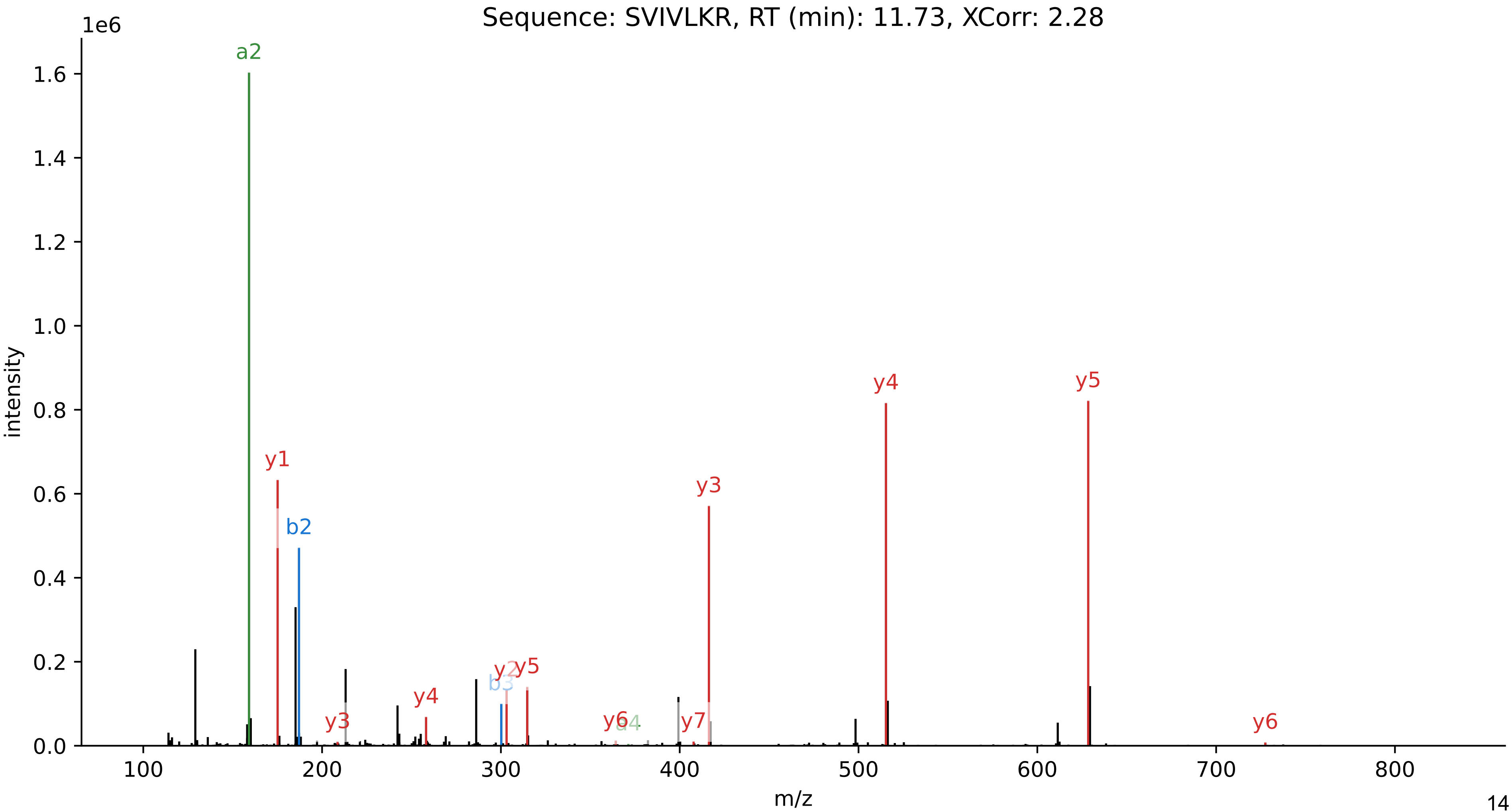
Sequence: SVIVLKR, RT (min): 11.73, XCorr: 2.28
1e6
intensity
m/z
a2
y1
b2
y3
y4
y5
y2
b3
y6
a4
y7
0.0
0.2
0.4
0.6
0.8
1.0
1.2
1.4
1.6
100
200
300
400
500
600
700
800

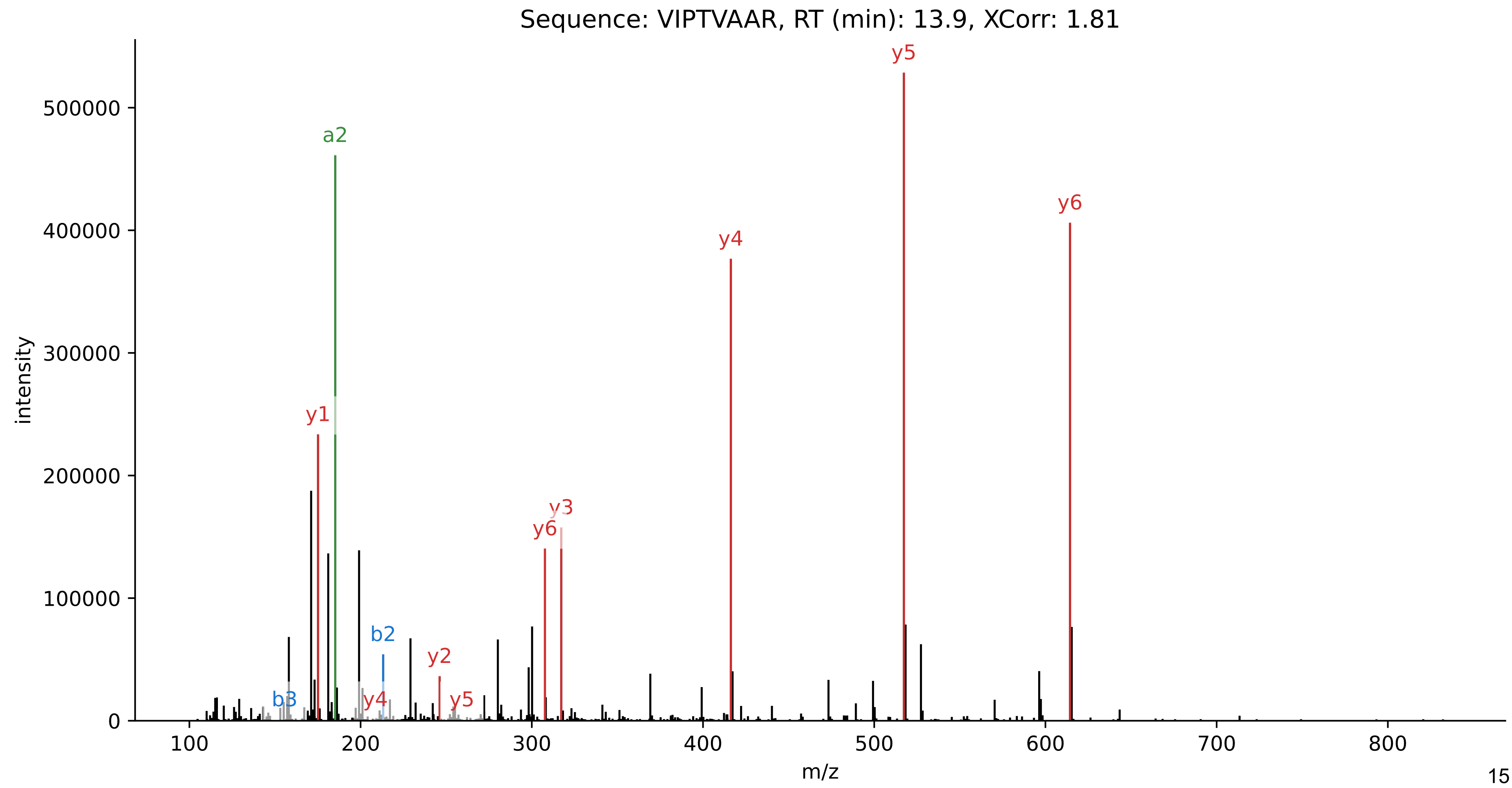
Sequence: VIPTVAAR, RT (min): 13.9, XCorr: 1.81
intensity
m/z
0
100000
200000
300000
400000
500000
100
200
300
400
500
600
700
800
a2
y1
b3
b2
y4
y2
y5
y6
y3
y4
y5
y6

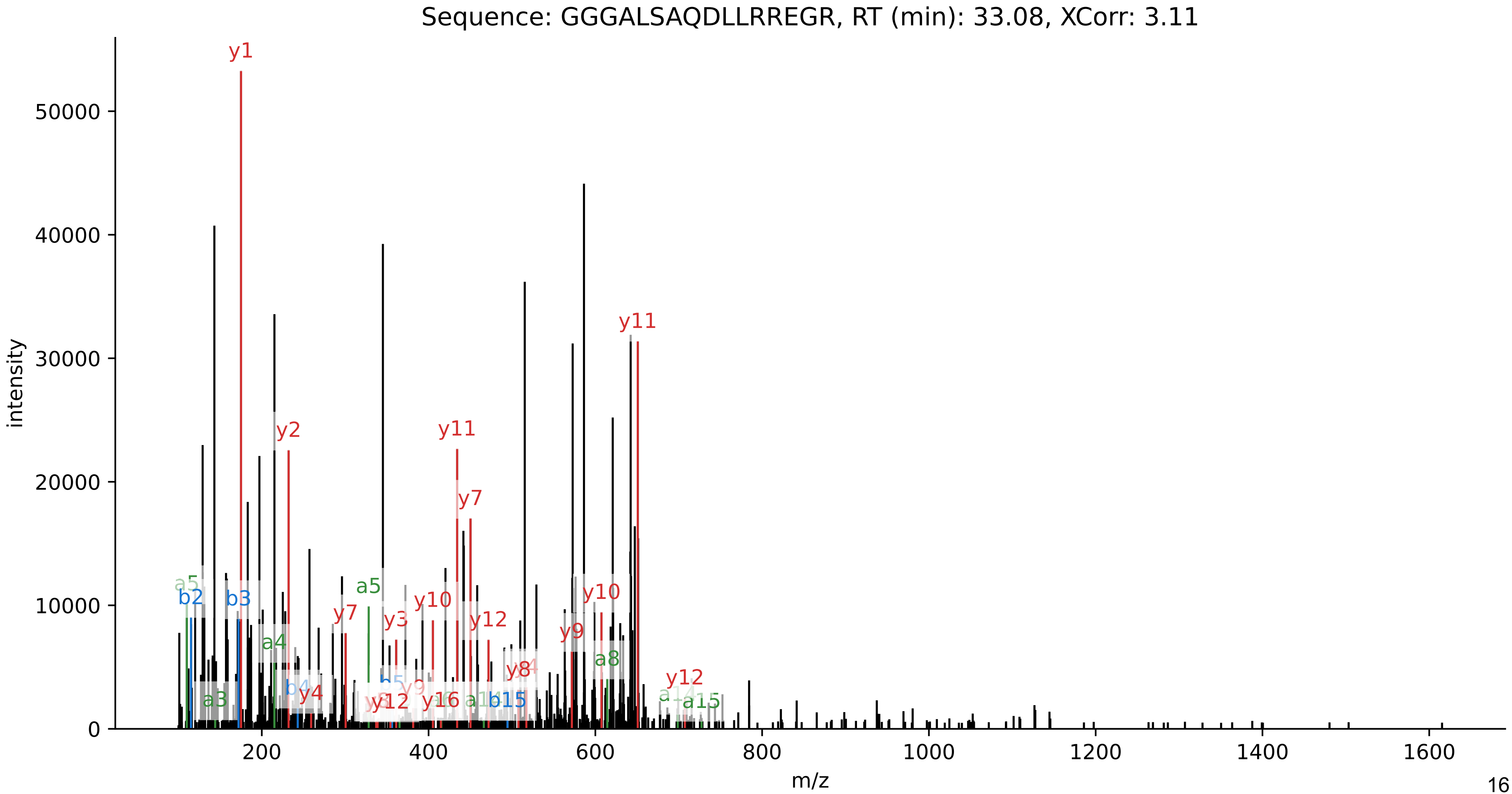

Sequence: GGGALSAQDLLRREGR, RT (min): 33.08, XCorr: 3.11
intensity
m/z
0
10000
20000
30000
40000
50000
200
400
600
800
1000
1200
1400
1600
y1
y2
y11
y7
y10
y12
y9
y11
y10
y12
a5
b2
b3
a4
a3
b4
y4
y7
a5
y3
y12
y16
b15
y8
a8
a15

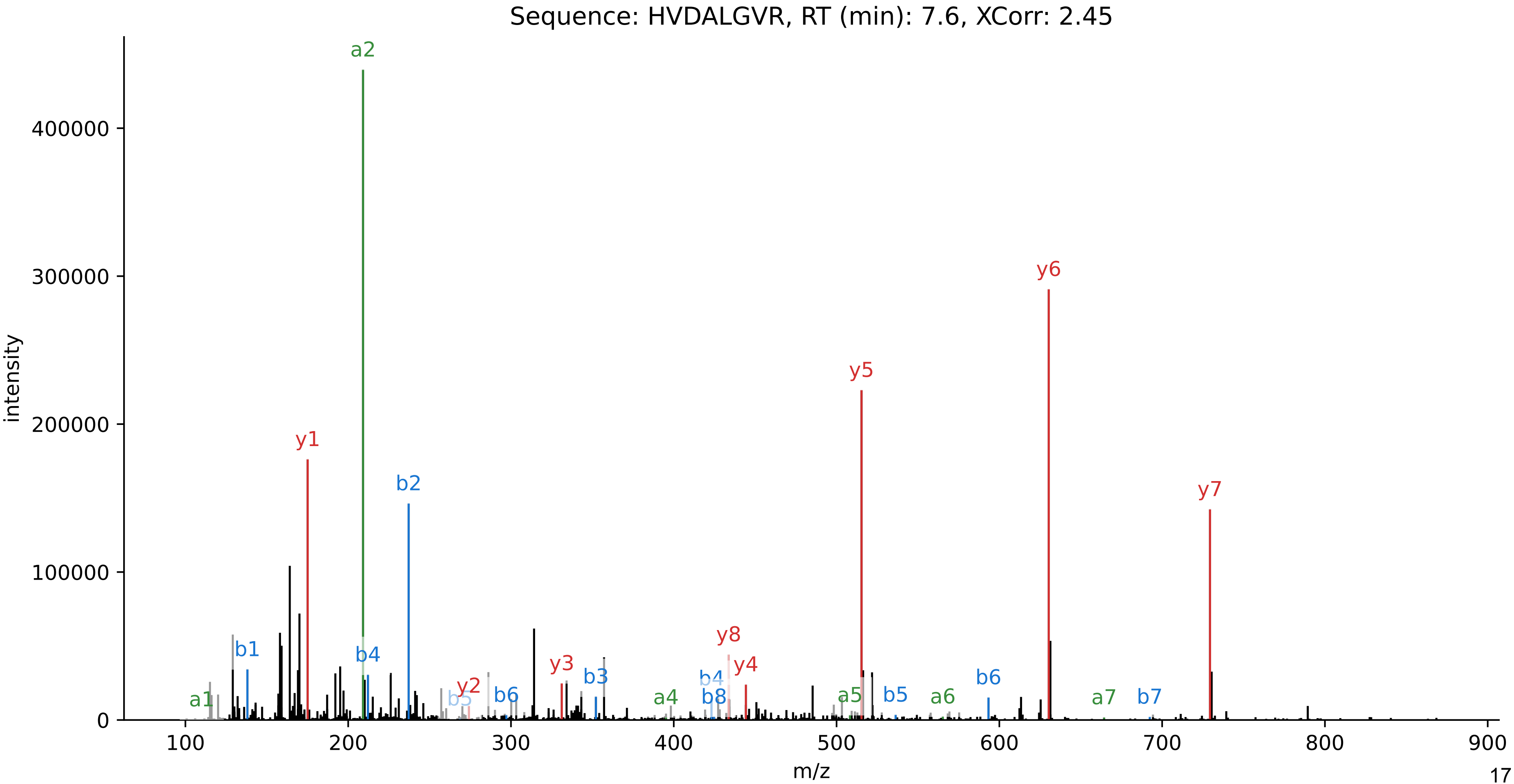
Sequence: HVDALGVR, RT (min): 7.6, XCorr: 2.45
a2
y6
y5
y1
b2
y7
b1
b4
y8
y3
y4
b3
b4
b8
y2
b5
b6
a1
a4
a5
b5
a6
b6
a7
b7
intensity
m/z
0
100000
200000
300000
400000
100
200
300
400
500
600
700
800
900

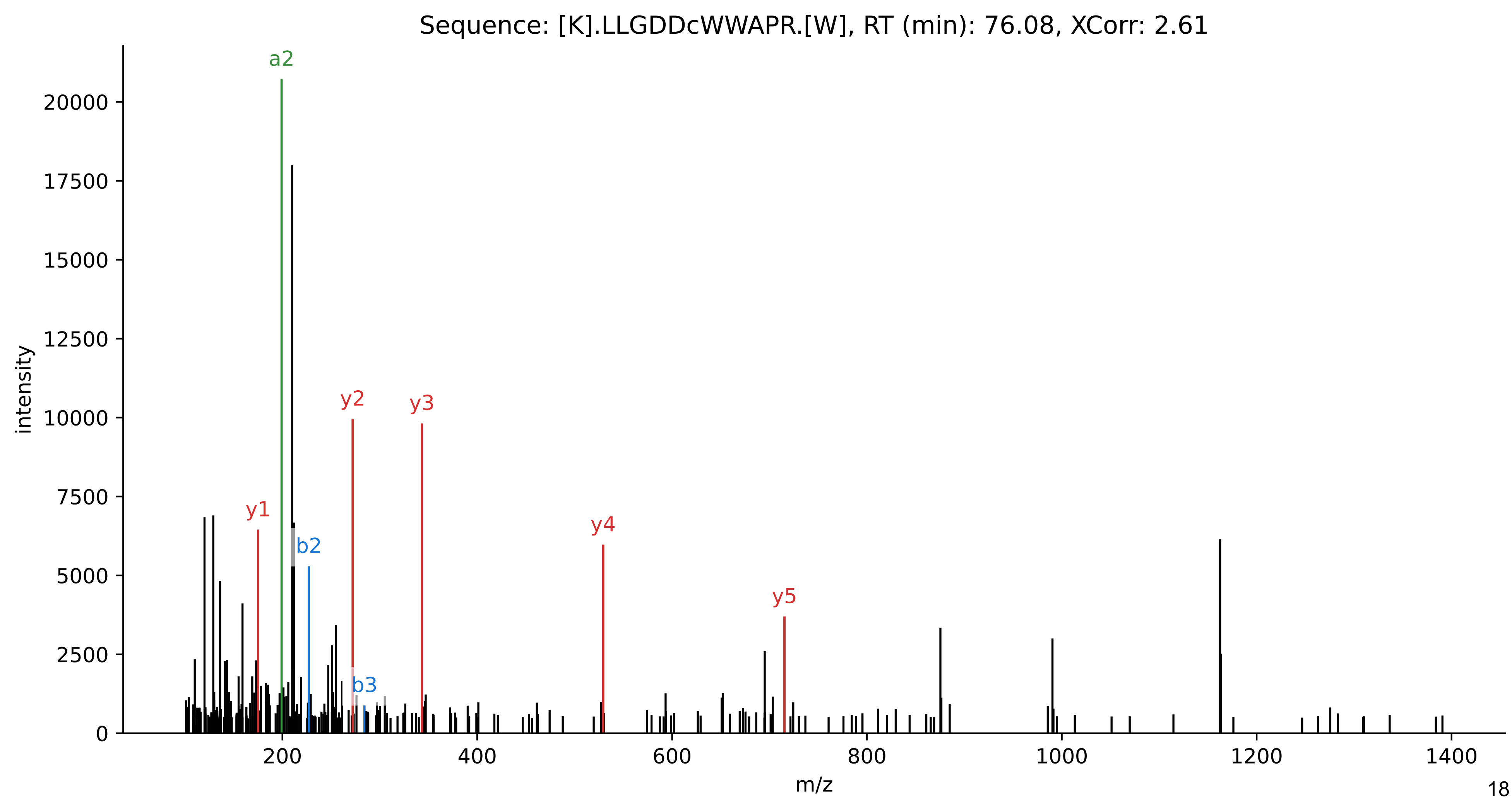

Sequence: [K].LLGDDcWWAPR.[W], RT (min): 76.08, XCorr: 2.61
a2
y1
b2
y2
b3
y3
y4
y5
intensity
m/z
0
2500
5000
7500
10000
12500
15000
17500
20000
200
400
600
800
1000
1200
1400

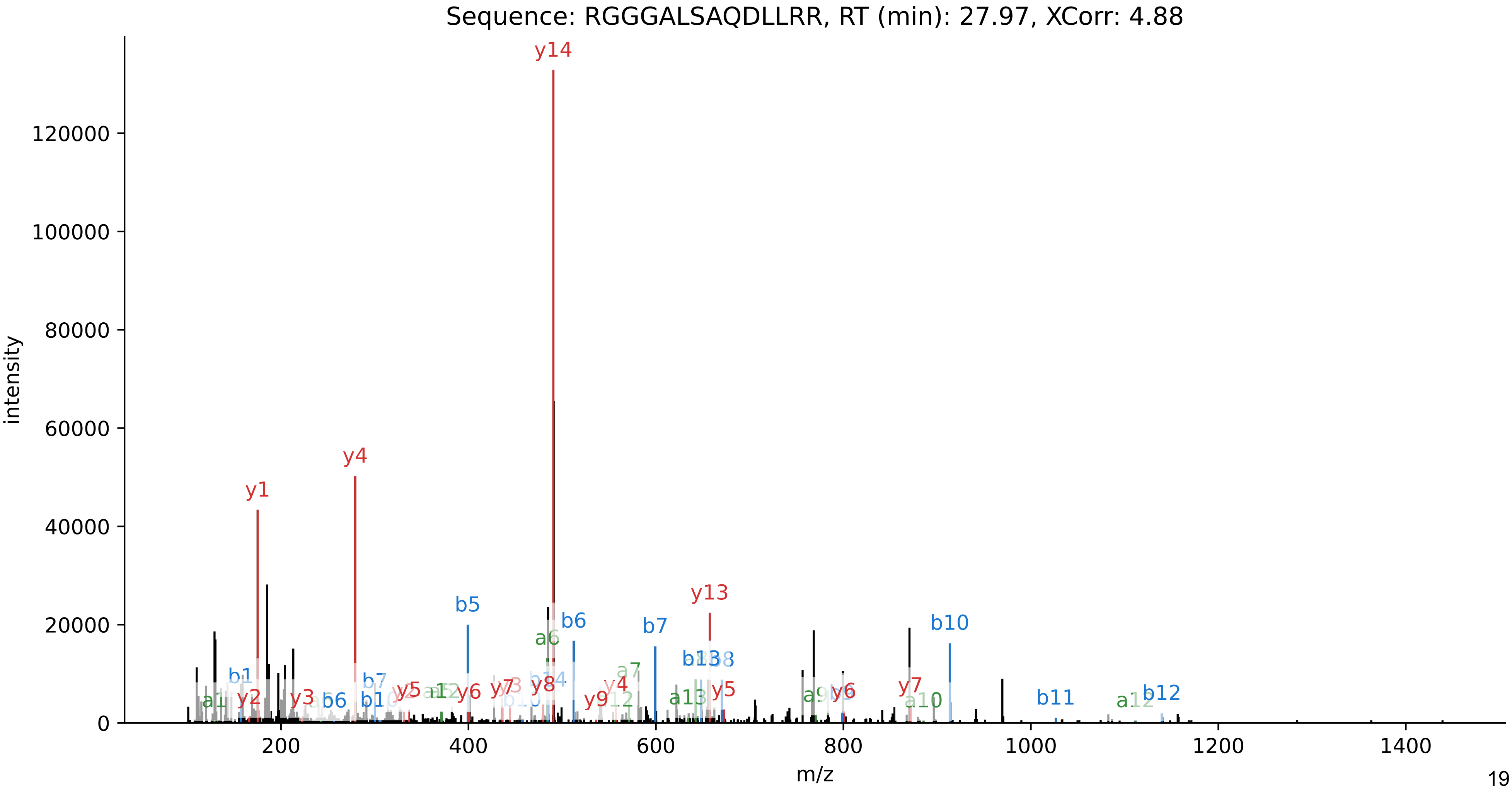

Sequence: RGGGALSAQDLLRR, RT (min): 27.97, XCorr: 4.88
intensity
m/z
y14
y4
y1
y13
b5
b6
b7
b10
a6
b11
b12

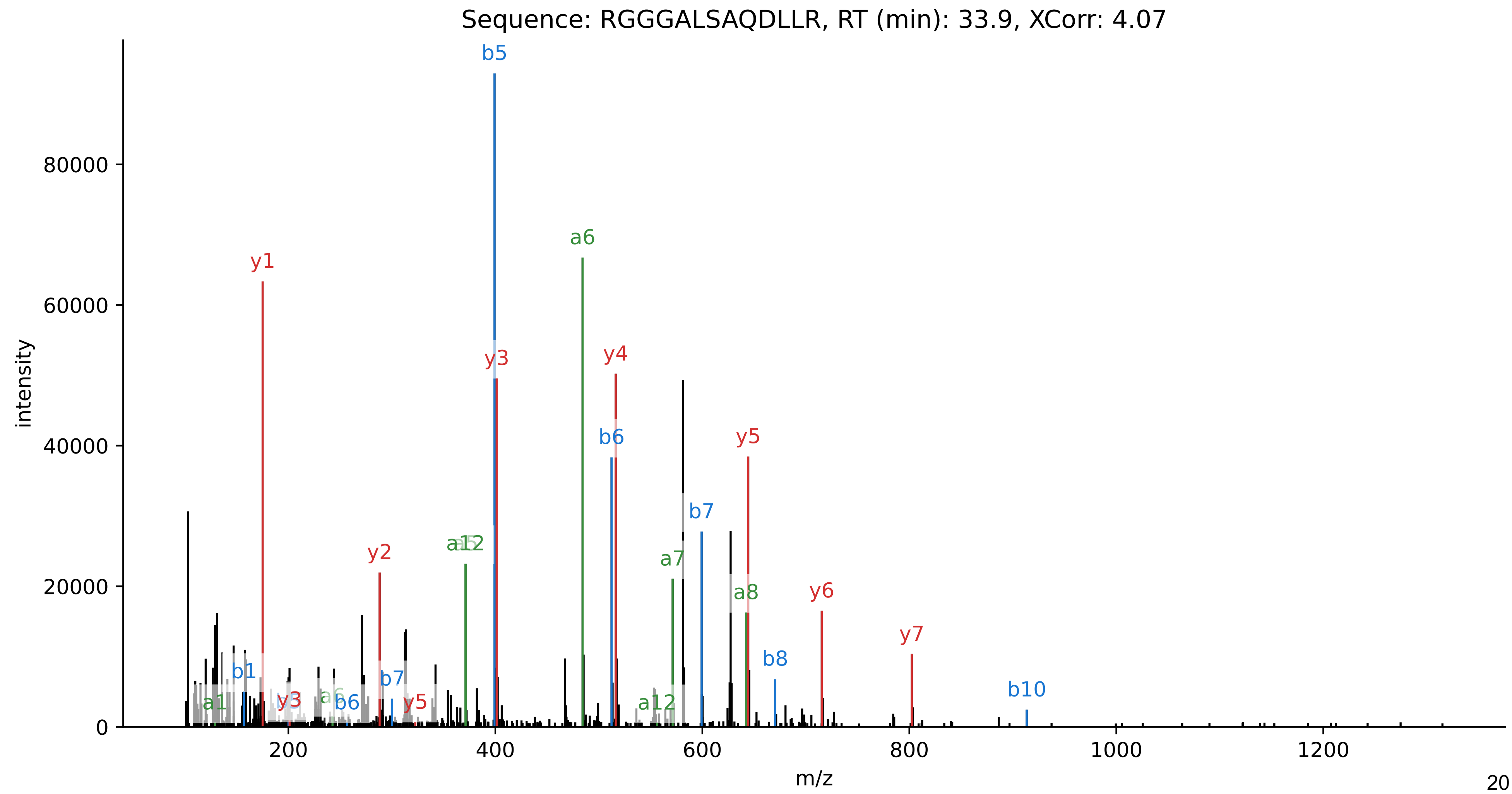
Sequence: RGGGALSAQDLLR, RT (min): 33.9, XCorr: 4.07
intensity
m/z
b5
a6
y1
y3
y4
b6
y5
b7
a12
a7
y2
a8
y6
y7
b8
b1
b7
b10
a1
y3
b6
y5
a12
80000
60000
40000
20000
0
200
400
600
800
1000
1200

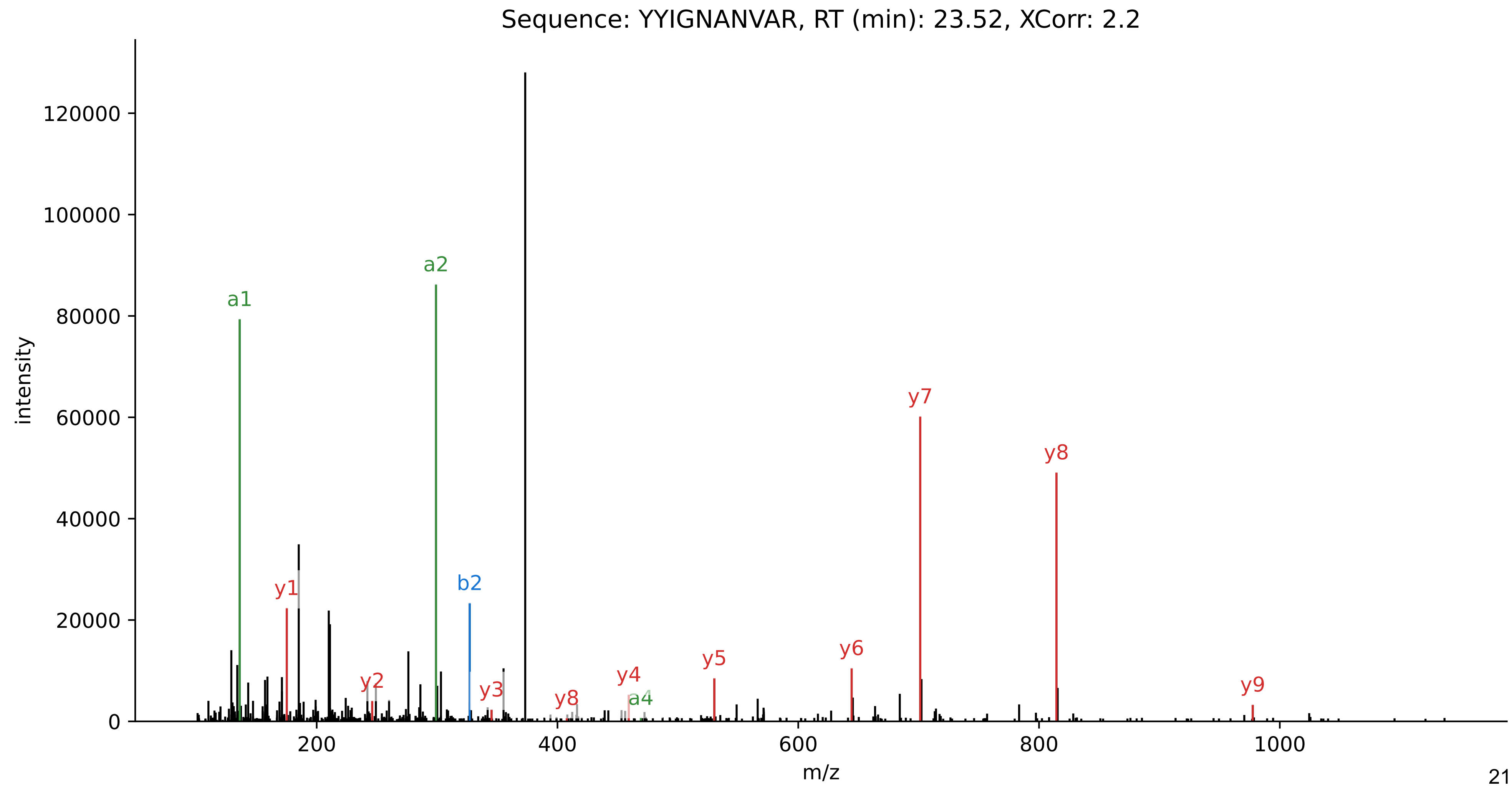
Sequence: YYIGNANVAR, RT (min): 23.52, XCorr: 2.2
intensity
m/z
0
20000
40000
60000
80000
100000
120000
200
400
600
800
1000
a1
a2
b2
y1
y2
y3
y8
y4
a4
y5
y6
y7
y8
y9

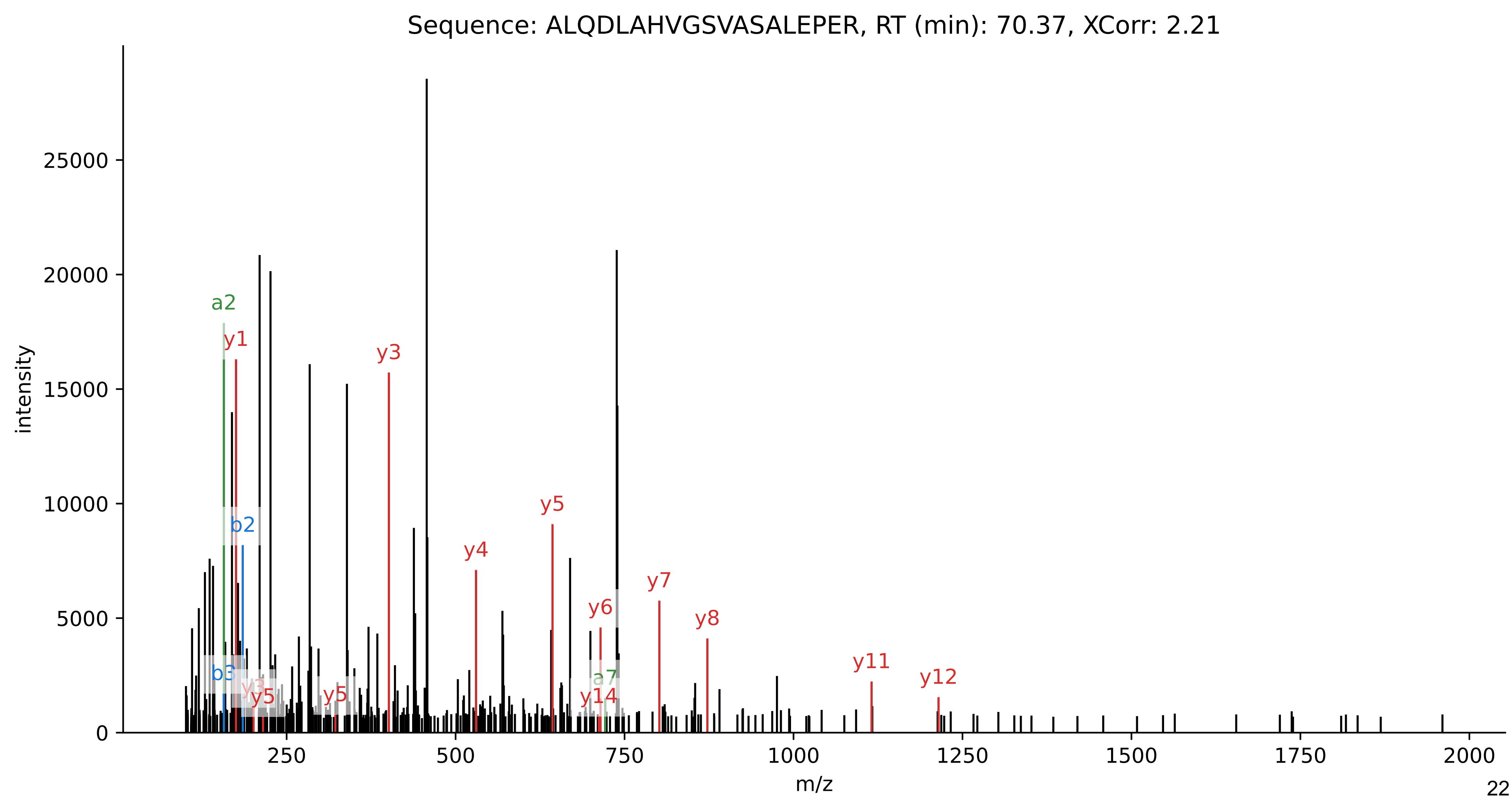

Sequence: ALQDLAHVGSVASALEPER, RT (min): 70.37, XCorr: 2.21
intensity
m/z
25000
20000
15000
10000
5000
0
250
500
750
1000
1250
1500
1750
2000
a2
y1
b2
b3
y3
y5
y5
y3
y4
y5
y6
a7
y14
y7
y8
y11
y12

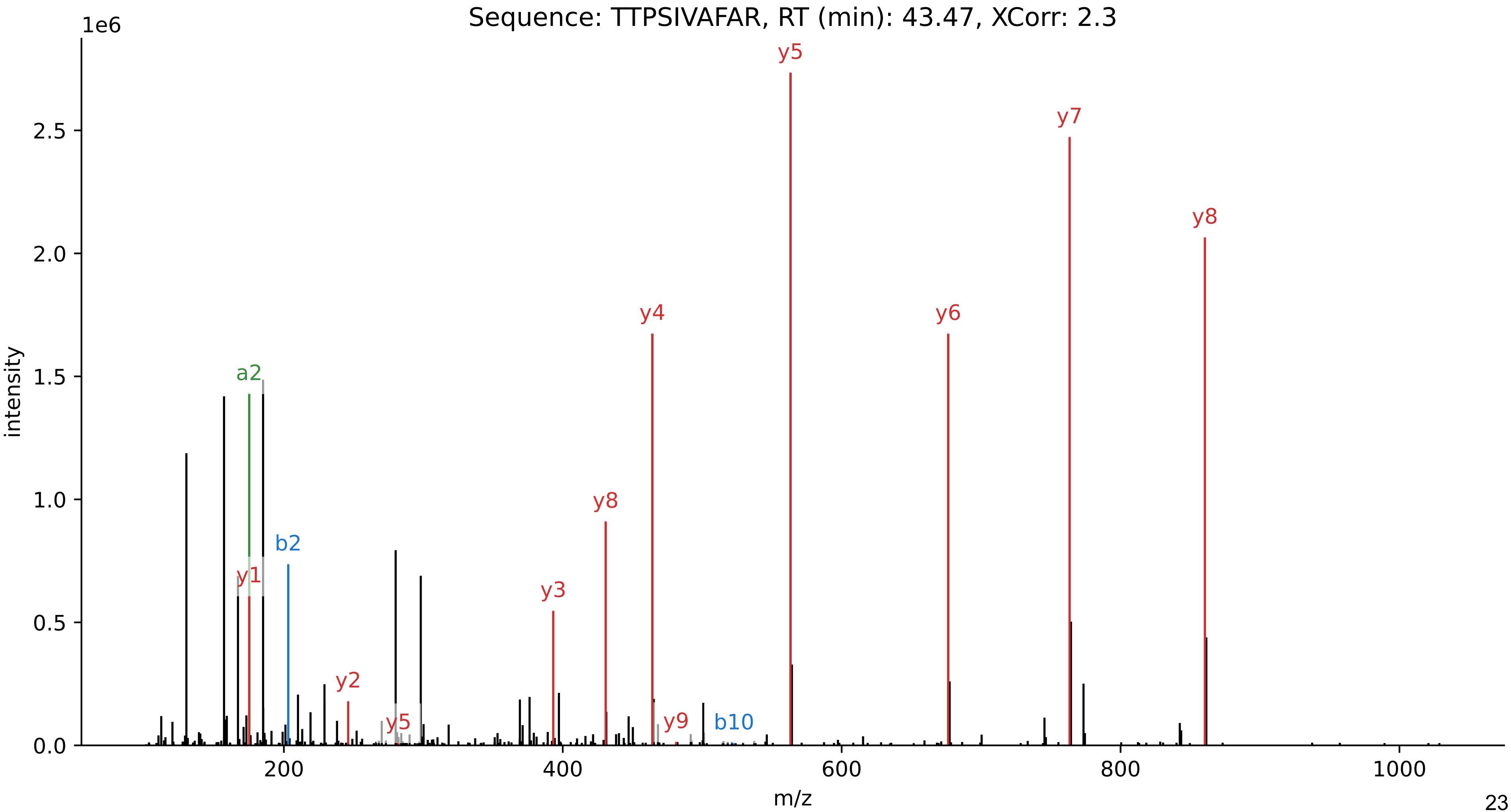

Sequence: TTPSIVAFAR, RT (min): 43.47, XCorr: 2.3
1e6
intensity
m/z
0.0
0.5
1.0
1.5
2.0
2.5
200
400
600
800
1000
a2
b2
y1
y2
y5
y3
y8
y4
y9
b10
y5
y6
y7
y8

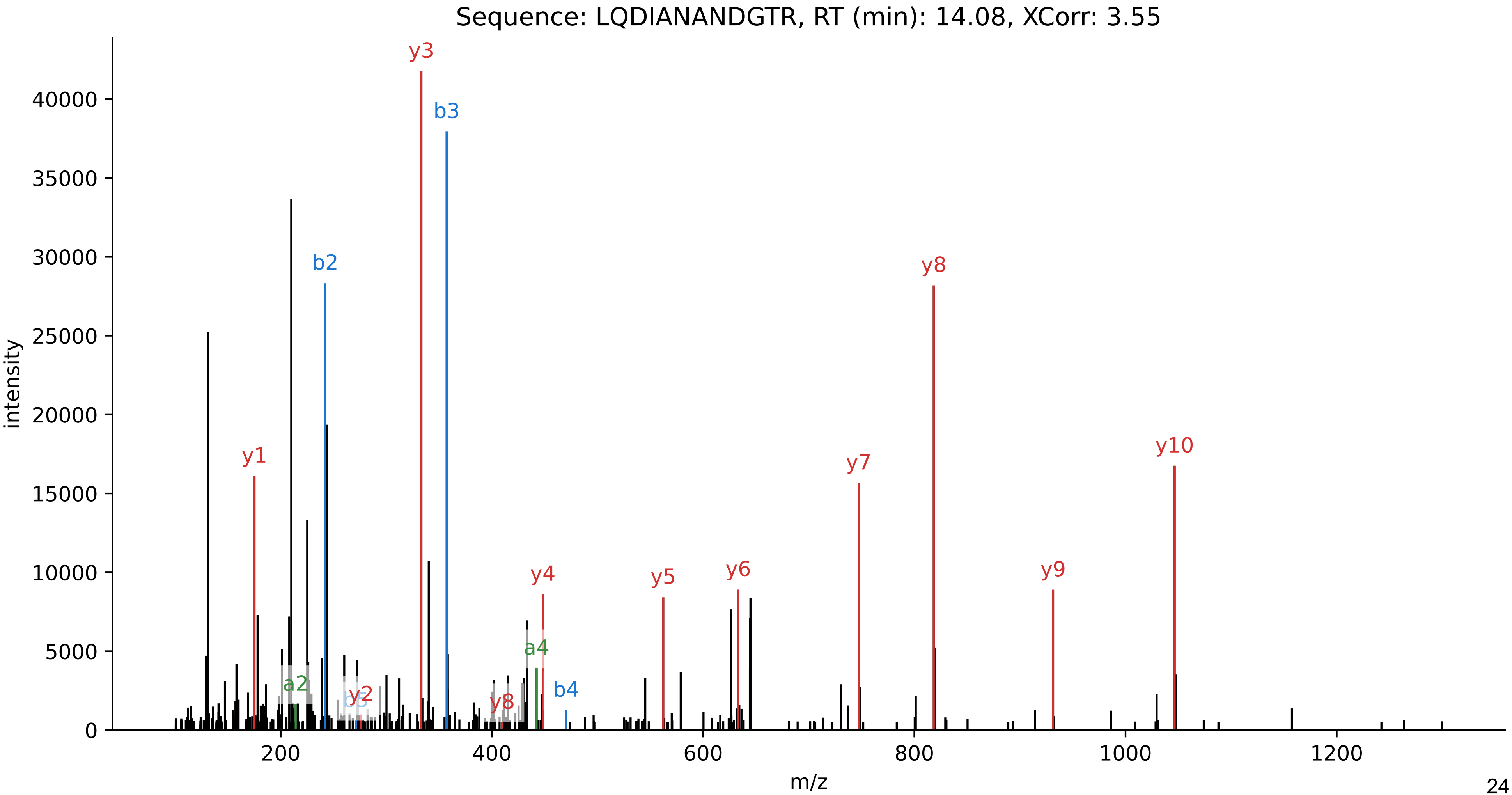
Sequence: LQDIANANDGTR, RT (min): 14.08, XCorr: 3.55
intensity
m/z
y1
y2
y3
y4
y5
y6
y7
y8
y9
y10
b2
b3
b4
a2
a4
y8
0
5000
10000
15000
20000
25000
30000
35000
40000
200
400
600
800
1000
1200

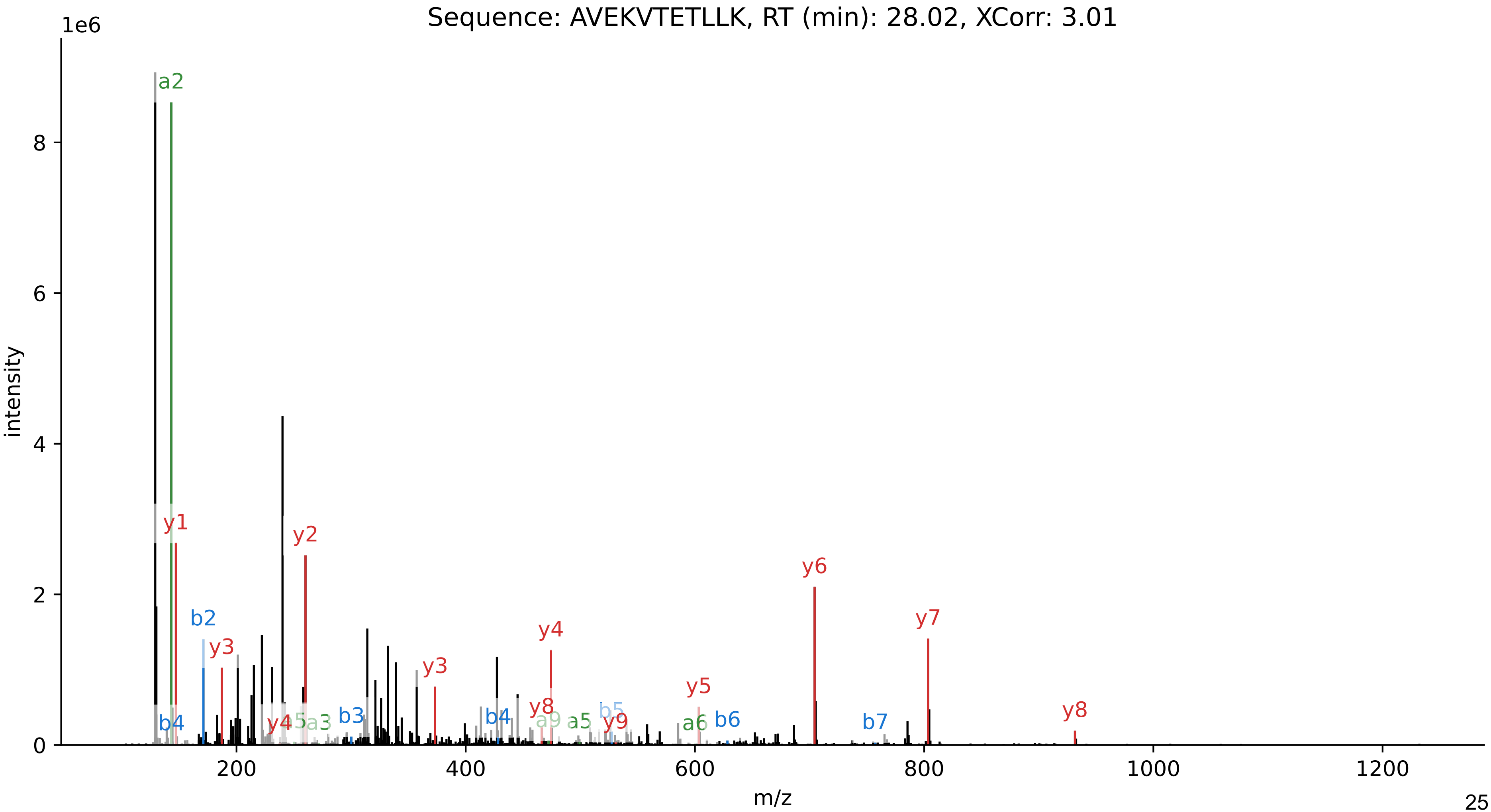

Sequence: AVEKVTETLLK, RT (min): 28.02, XCorr: 3.01
1e6
intensity
m/z
a2
y1
y2
b2
y3
y3
y4
y6
y7
y5
y8
y8
b4
y4
a5
a3
b3
b4
a9
a5
b5
y9
a6
b6
b7

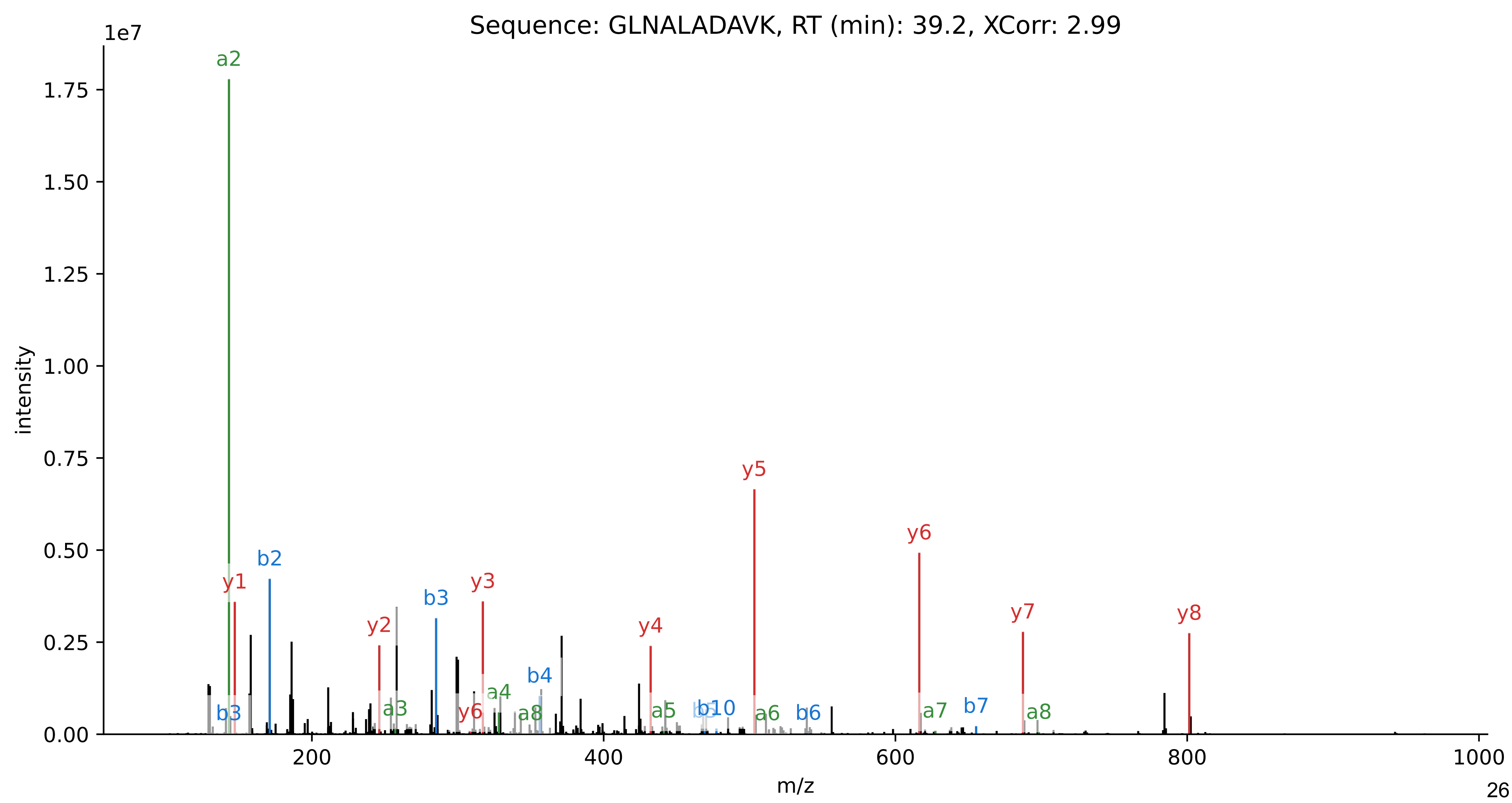

Sequence: GLNALADAVK, RT (min): 39.2, XCorr: 2.99
1e7
intensity
m/z
a2
b2
y1
b3
y2
a3
b3
y6
y3
a4
a8
b4
y4
a5
b10
a6
b6
y5
y6
a7
b7
y7
a8
y8

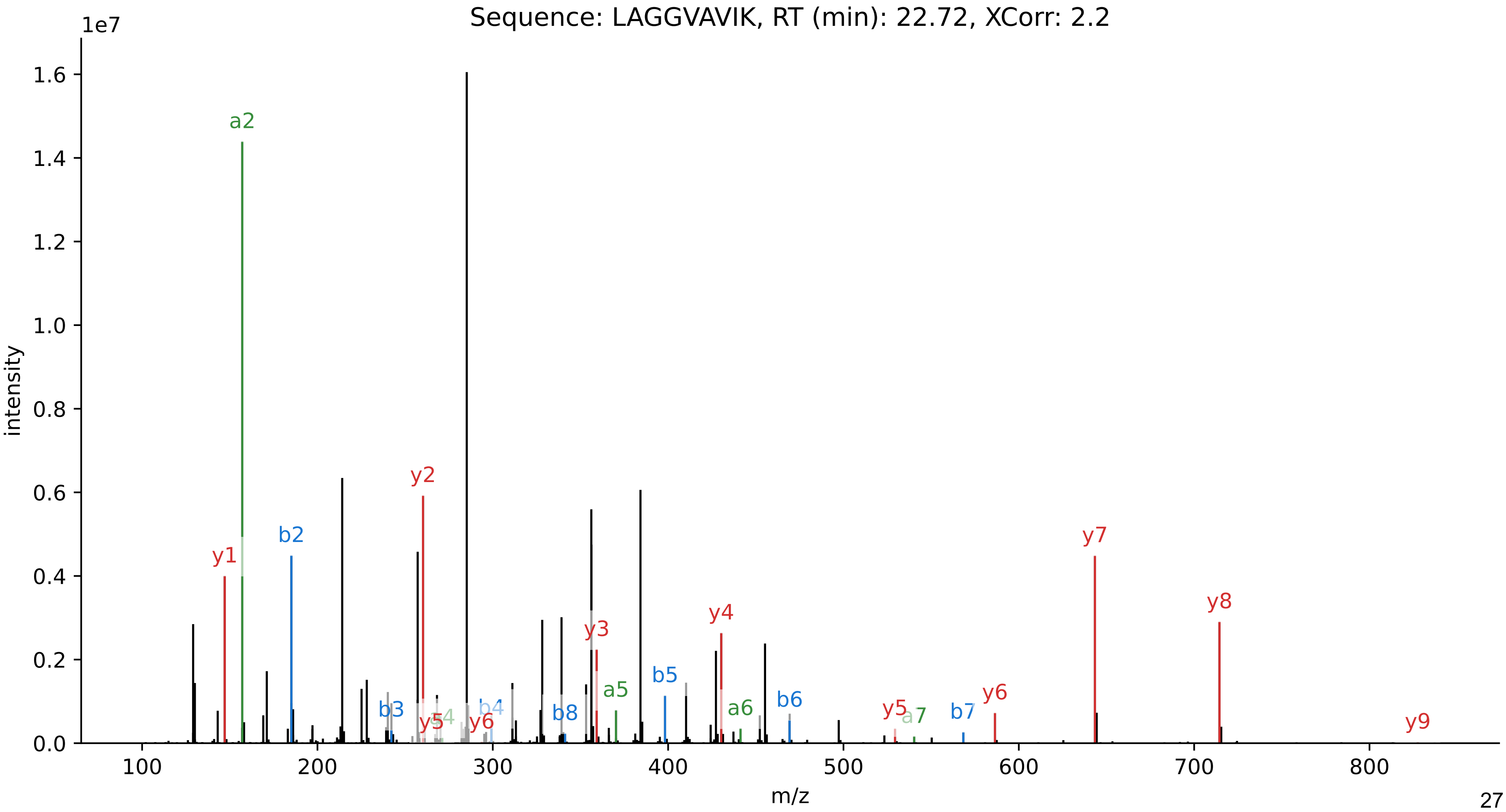

Sequence: LAGGVAVIK, RT (min): 22.72, XCorr: 2.2
1e7
intensity
m/z
a2
y1
b2
y2
y3
y4
a5
b5
a6
b6
y5
a7
b7
y6
y7
y8
y9
b3
b4
y6
b8

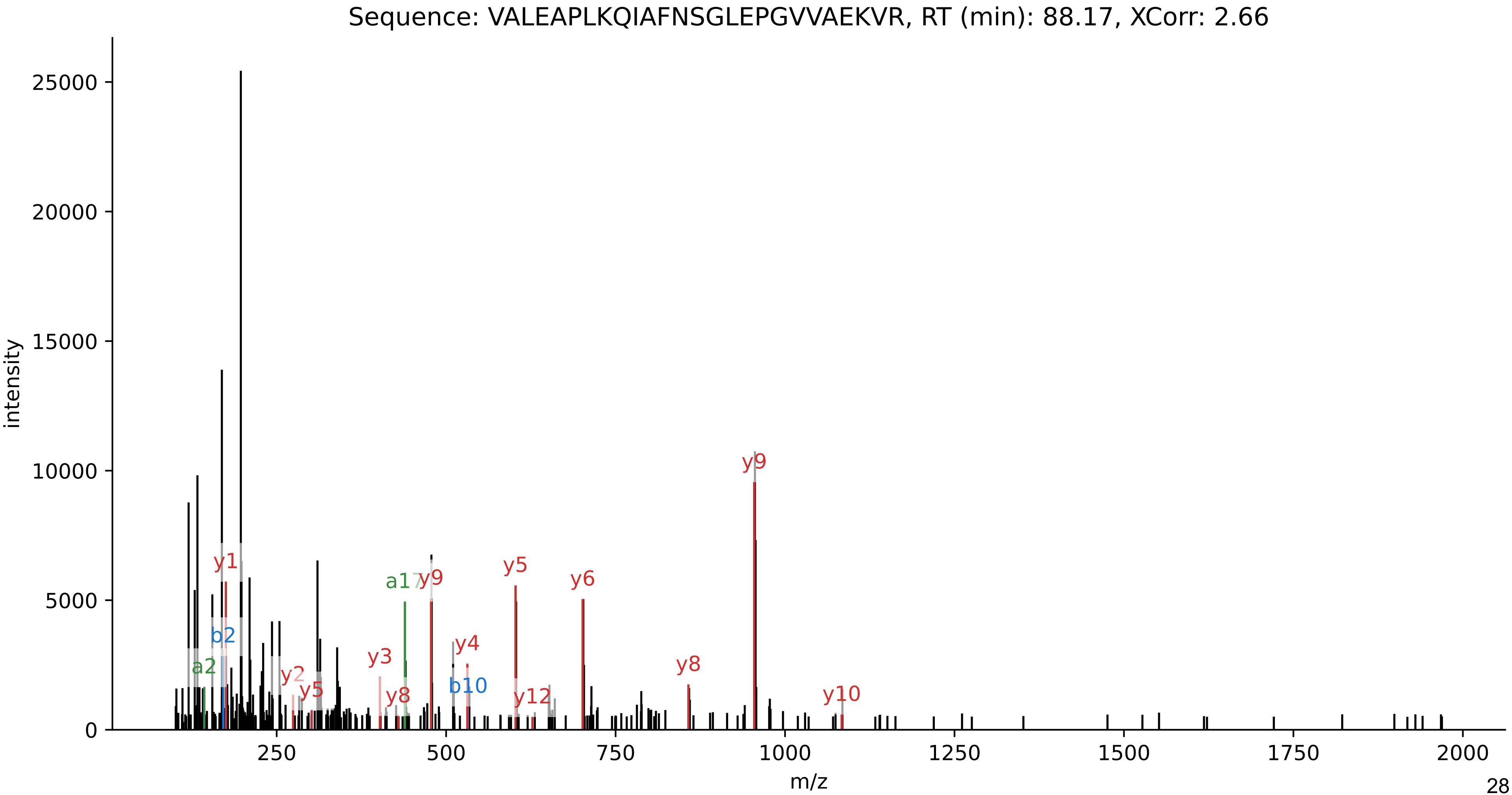
Sequence: VALEAPLKQIAFNSGLEPGVVAEKVR, RT (min): 88.17, XCorr: 2.66
intensity
m/z
y1
b2
a2
y2
y5
y3
y8
a17
y9
y4
b10
y5
y12
y6
y8
y9
y10
25000
20000
15000
10000
5000
0
250
500
750
1000
1250
1500
1750
2000

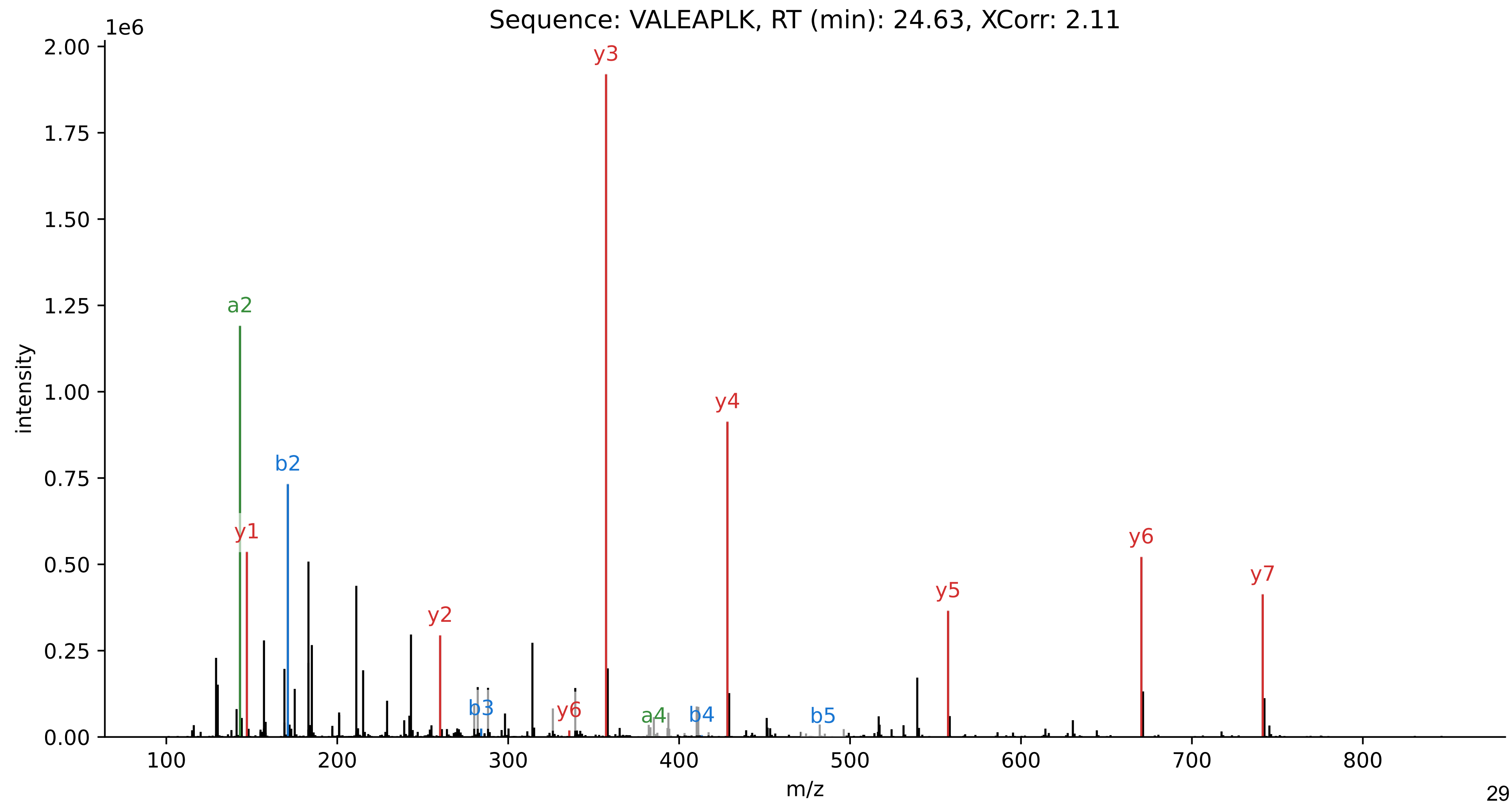

Sequence: VALEAPLK, RT (min): 24.63, XCorr: 2.11
1e6
2.00
1.75
1.50
1.25
1.00
0.75
0.50
0.25
0.00
intensity
y3
a2
y4
b2
y1
y6
y7
y5
y2
b3
y6
a4
b4
b5
100
200
300
400
500
600
700
800
m/z

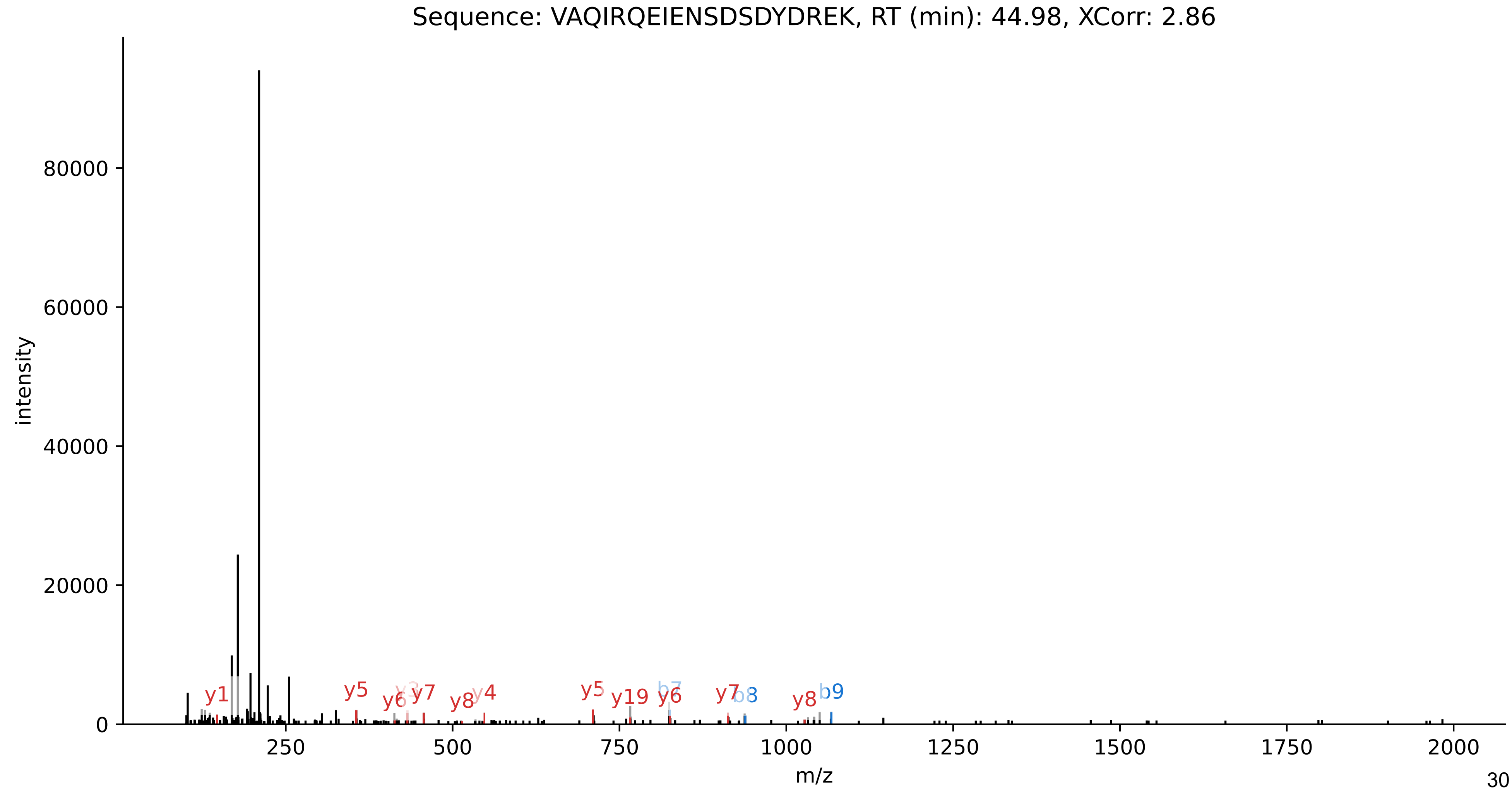

Sequence: VAQIRQEIENSDSDYDREK, RT (min): 44.98, XCorr: 2.86
intensity
80000
60000
40000
20000
0
y1
y5
y6
y3
y7
y8
y4
y5
y19
b7
y6
y7
b8
y8
b9
250
500
750
1000
1250
1500
1750
2000
m/z

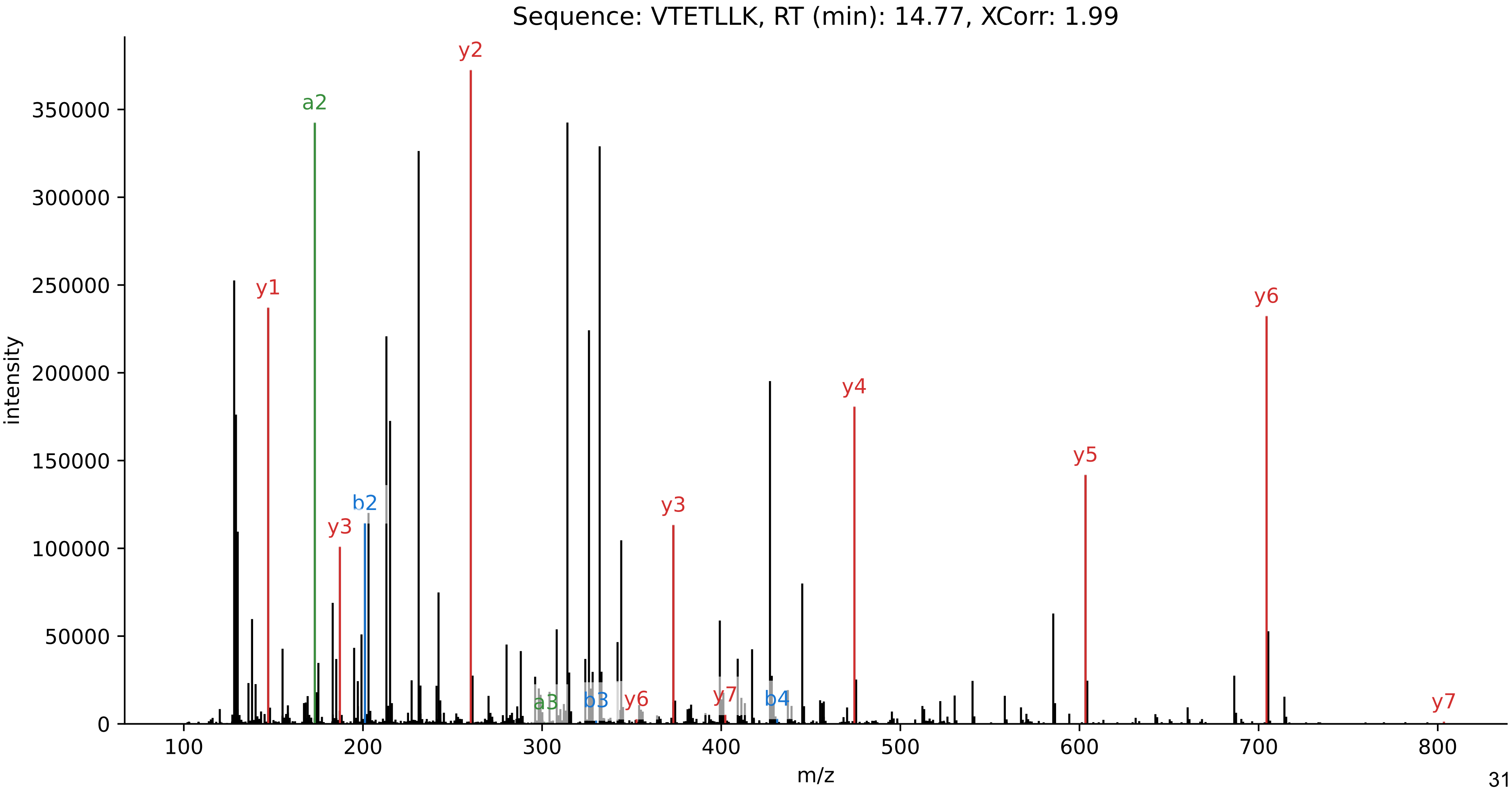

Sequence: VTETLLK, RT (min): 14.77, XCorr: 1.99
intensity
m/z
y1
y2
y3
y4
y5
y6
y7
a2
a3
b2
b3
b4

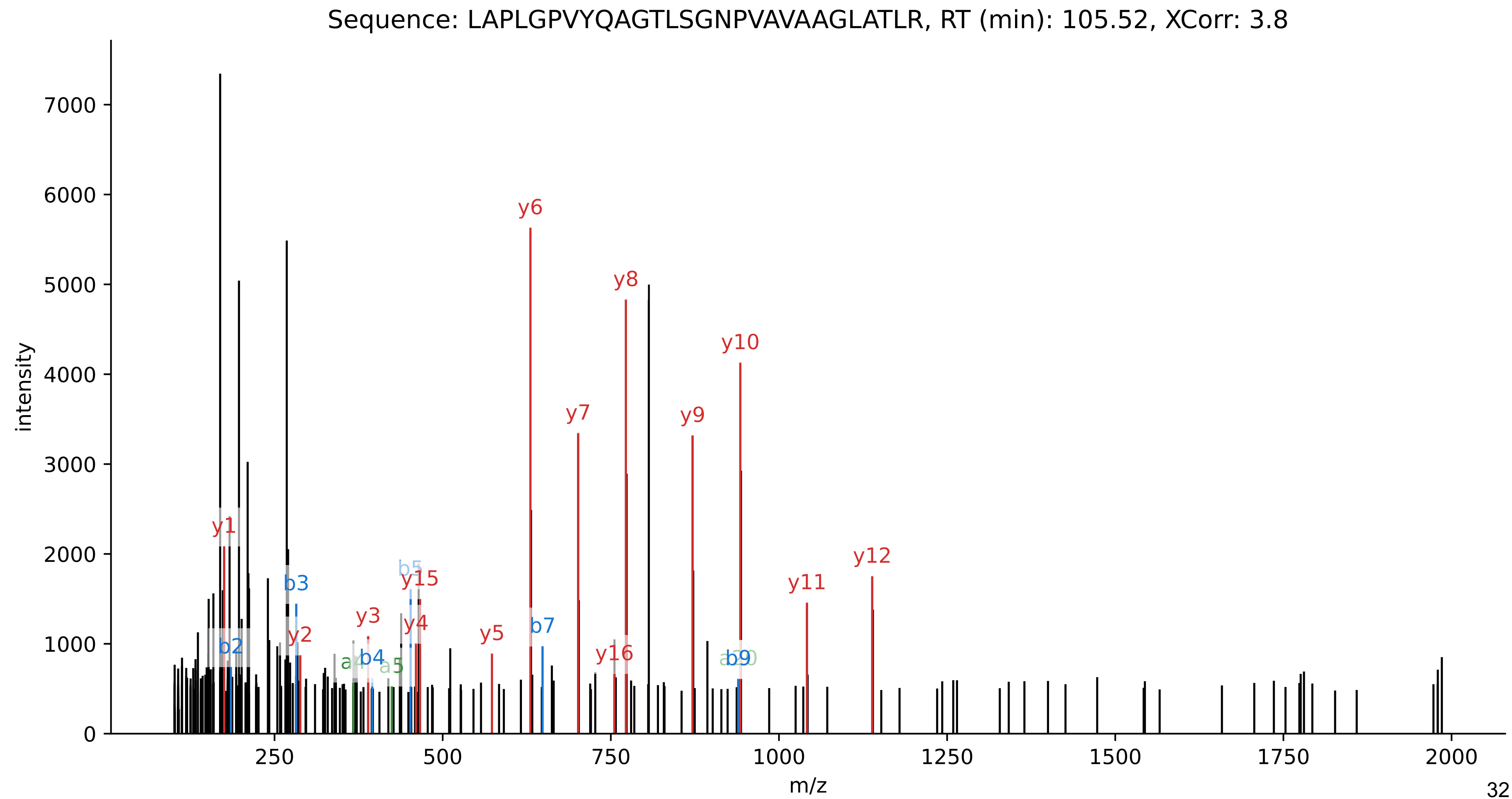
Sequence: LAPLGPVYQAGTLSGNPVAVAAGLATLR, RT (min): 105.52, XCorr: 3.8
intensity
m/z
y1
b2
b3
y2
y3
a4
b4
a5
b5
y15
y4
y5
b7
y6
y7
y16
y8
y9
b9
y10
y11
y12

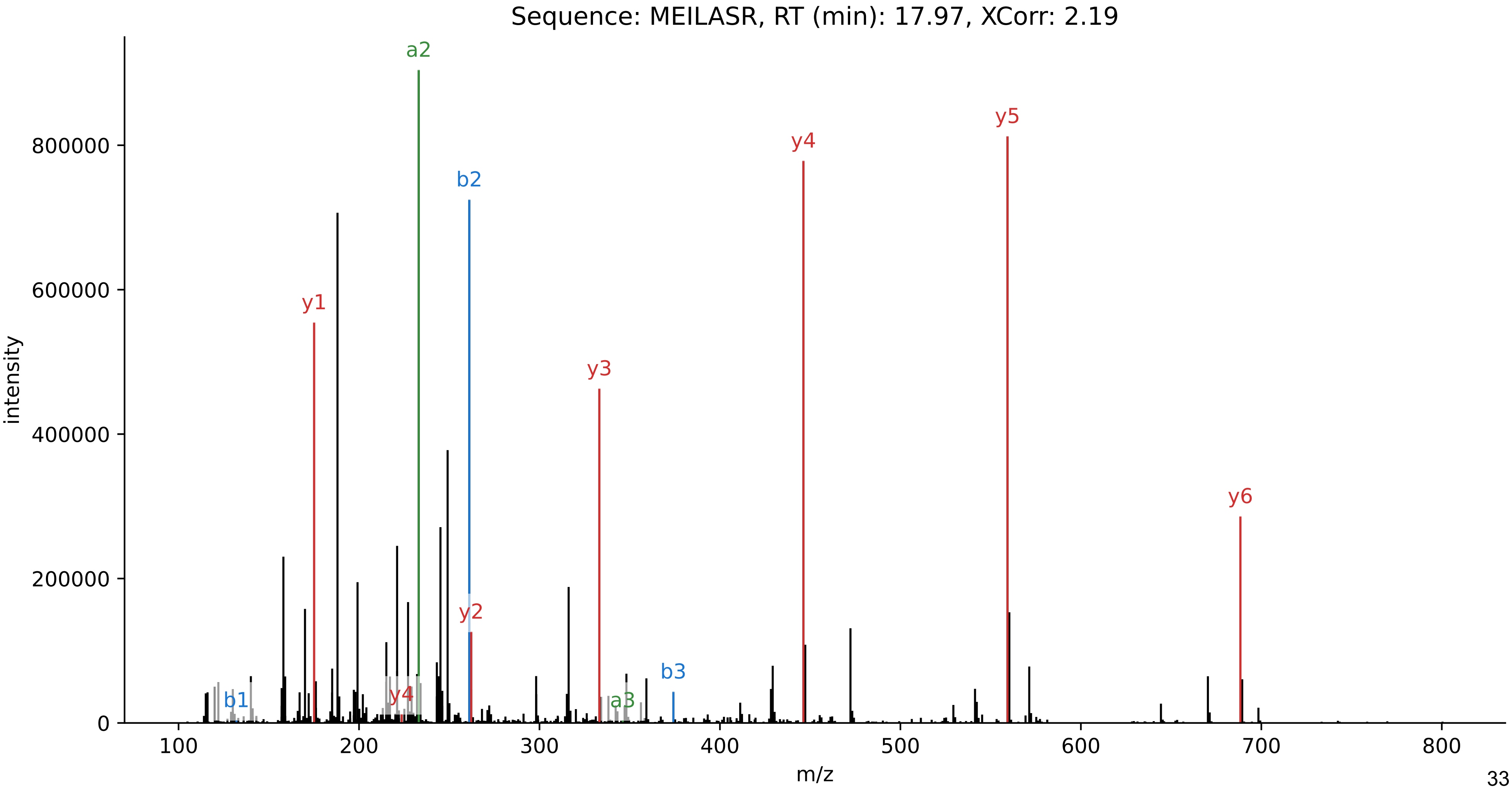
Sequence: MEILASR, RT (min): 17.97, XCorr: 2.19
intensity
m/z
a2
b2
y1
y3
y4
y5
y6
y2
b3
b1
y4
a3
0
200000
400000
600000
800000
100
200
300
400
500
600
700
800

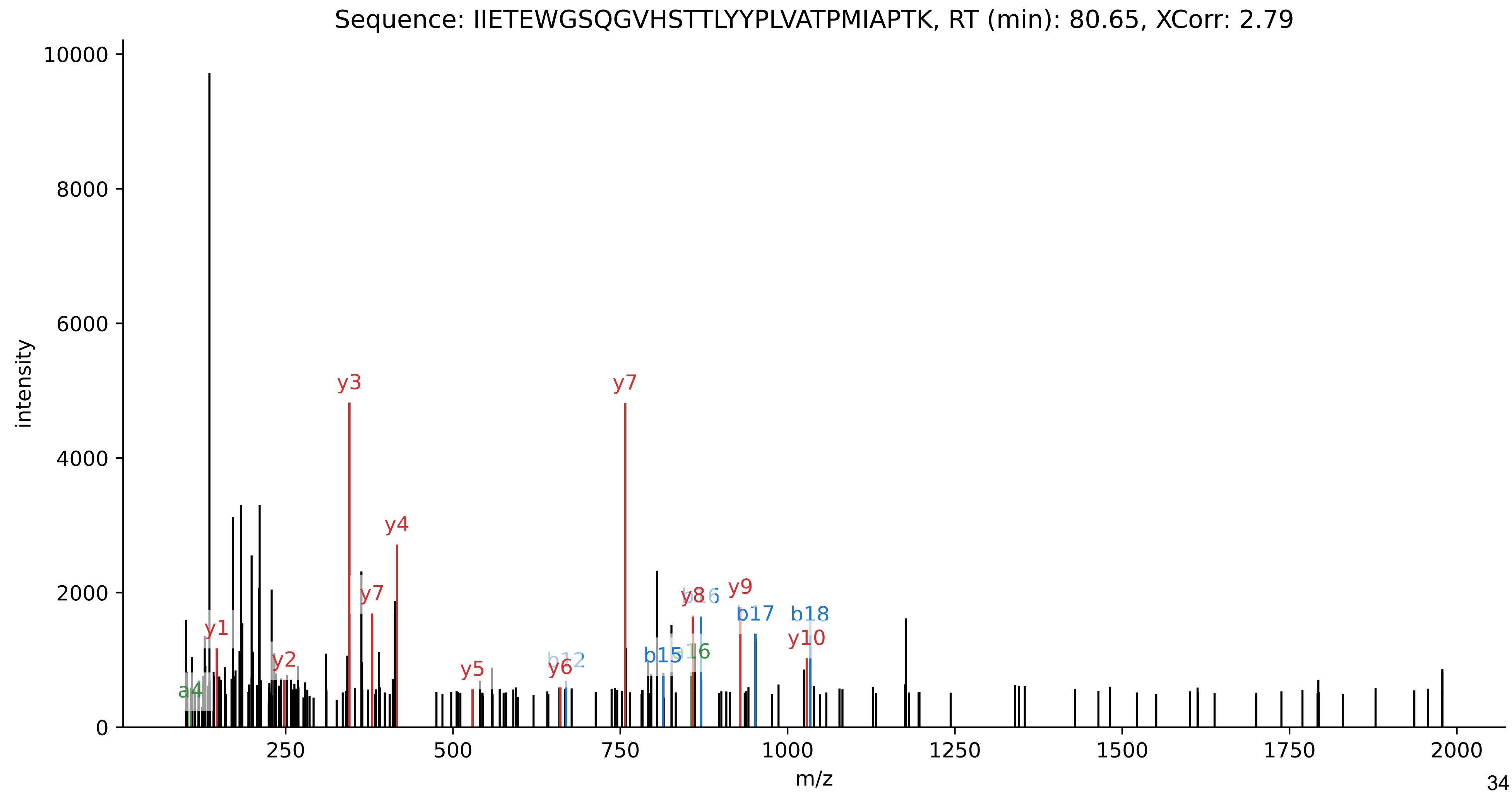
Sequence: IIETEWGSQGVHSTTLYYPLVATPMIAPTK, RT (min): 80.65, XCorr: 2.79
intensity
m/z
0
2000
4000
6000
8000
10000
250
500
750
1000
1250
1500
1750
2000
a4
y1
y2
y3
y7
y4
y5
y6
b12
y7
b15
a16
y8
b16
y9
b17
b18
y10

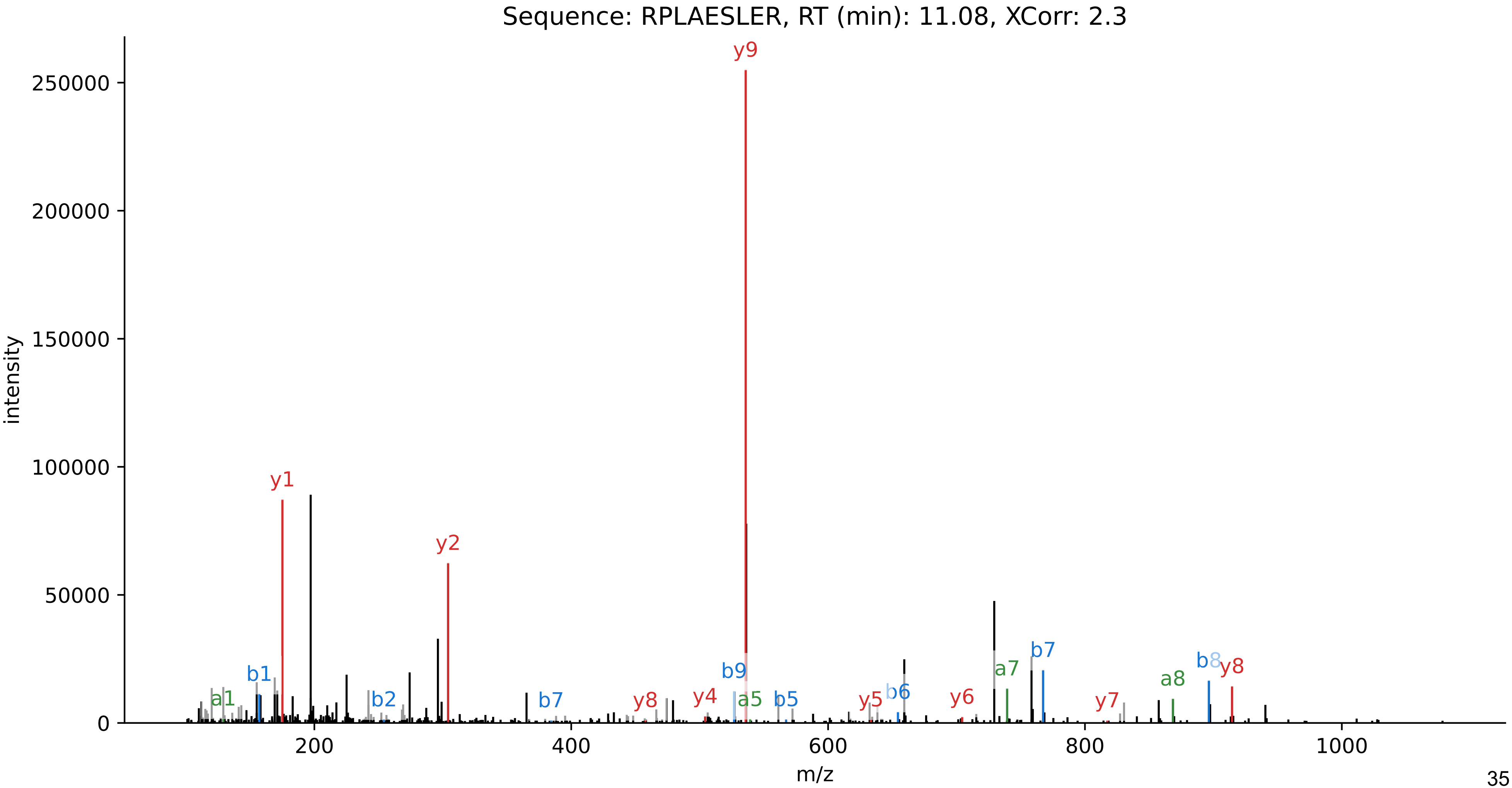
Sequence: RPLAESLER, RT (min): 11.08, XCorr: 2.3
intensity
m/z
250000
200000
150000
100000
50000
0
200
400
600
800
1000
y9
y1
y2
b1
a1
b2
b7
y8
y4
b9
a5
b5
y5
b6
y6
a7
b7
y7
a8
b8
y8

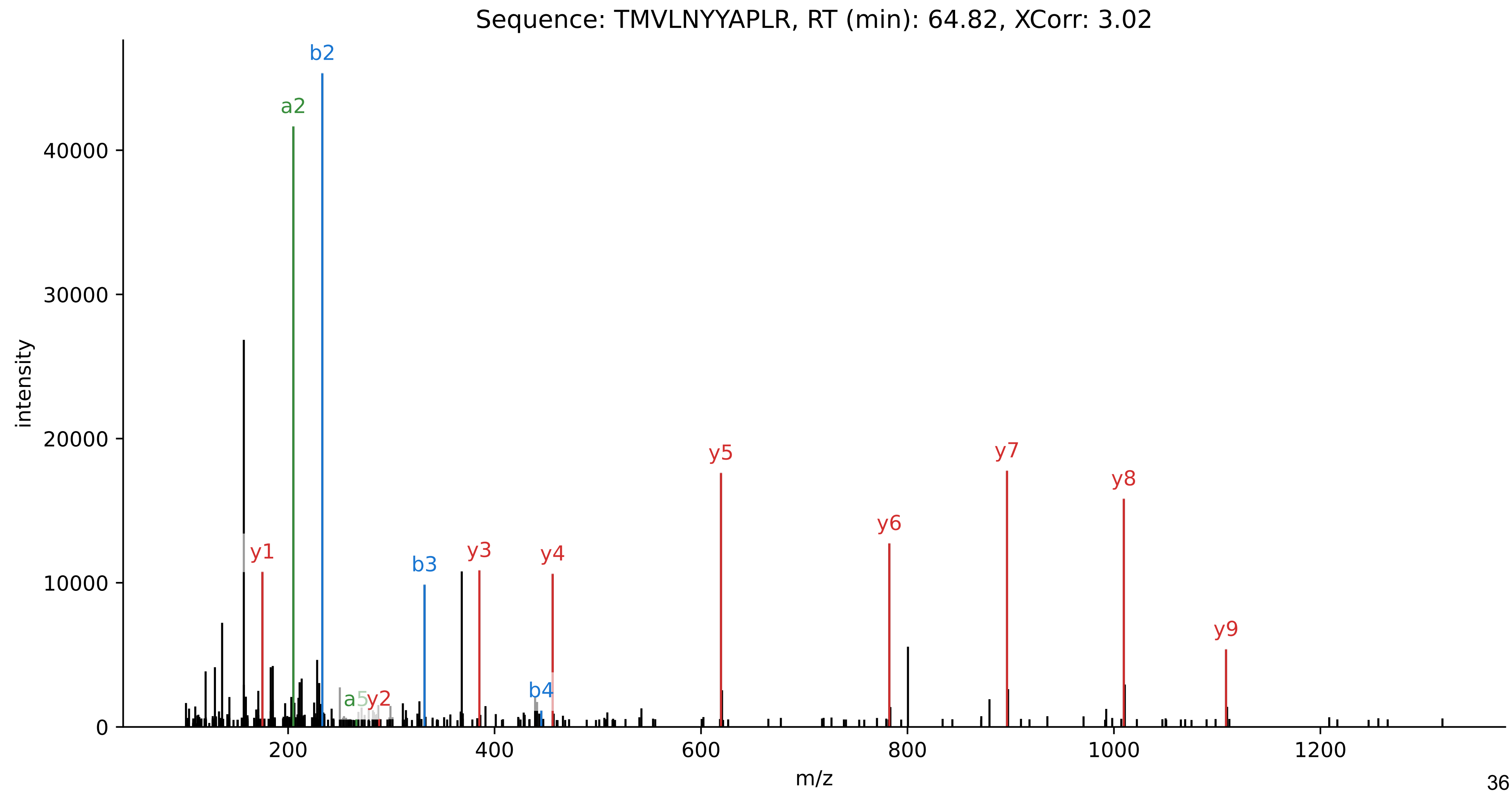

Sequence: TMVLNYYAPLR, RT (min): 64.82, XCorr: 3.02
intensity
m/z
b2
a2
y1
y2
a5
b3
y3
y4
b4
y5
y6
y7
y8
y9
0
10000
20000
30000
40000
200
400
600
800
1000
1200

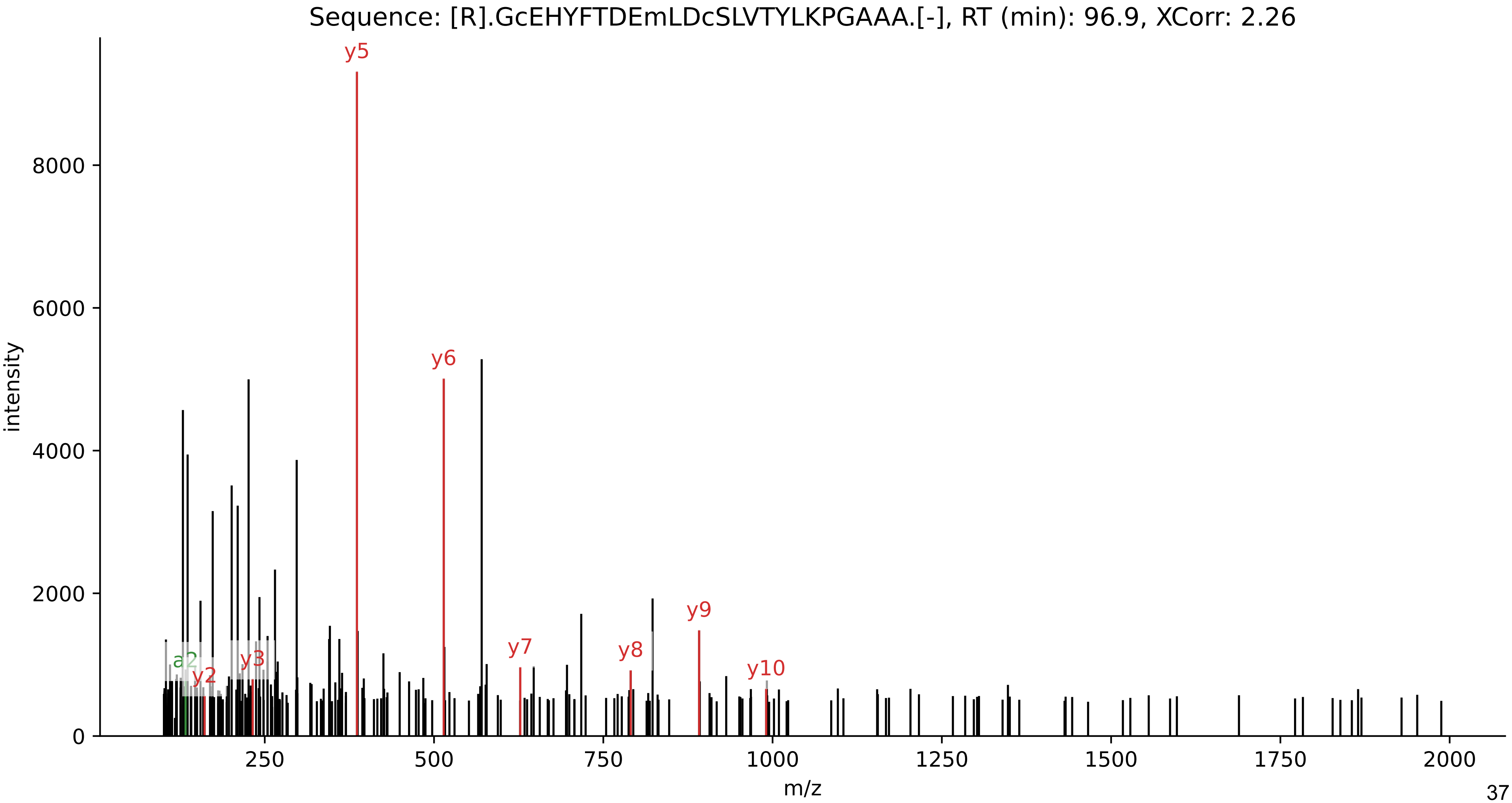

Sequence: [R].GcEHYFTDEmLDcSLVTYLKPGAAA.[-], RT (min): 96.9, XCorr: 2.26
y5
y6
y7
y8
y9
y10
a2
y2
y3
intensity
m/z
0
2000
4000
6000
8000
250
500
750
1000
1250
1500
1750
2000

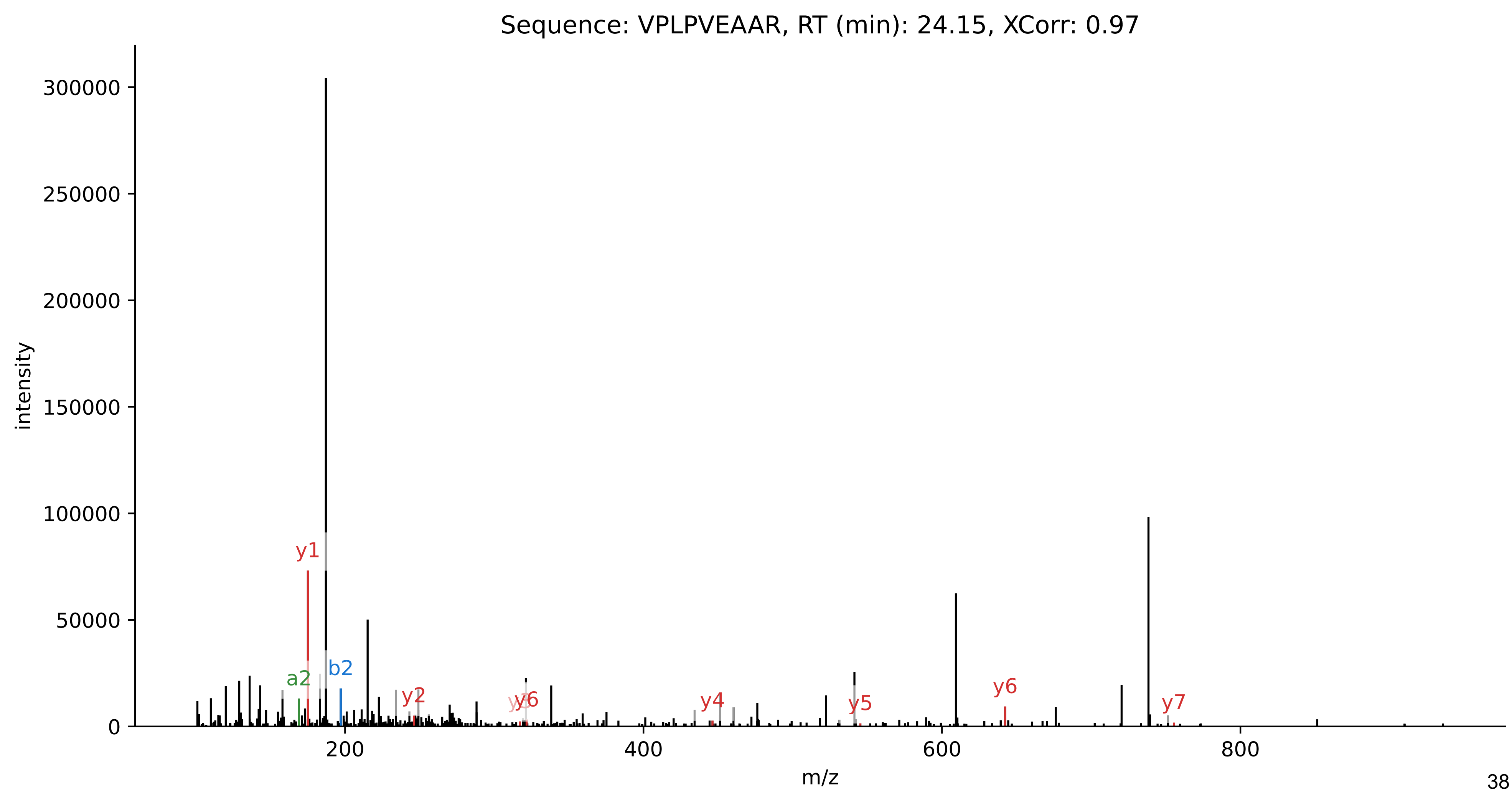
Sequence: VPLPVEAAR, RT (min): 24.15, XCorr: 0.97
intensity
m/z
300000
250000
200000
150000
100000
50000
0
200
400
600
800
y1
a2
b2
y2
y6
y4
y5
y6
y7

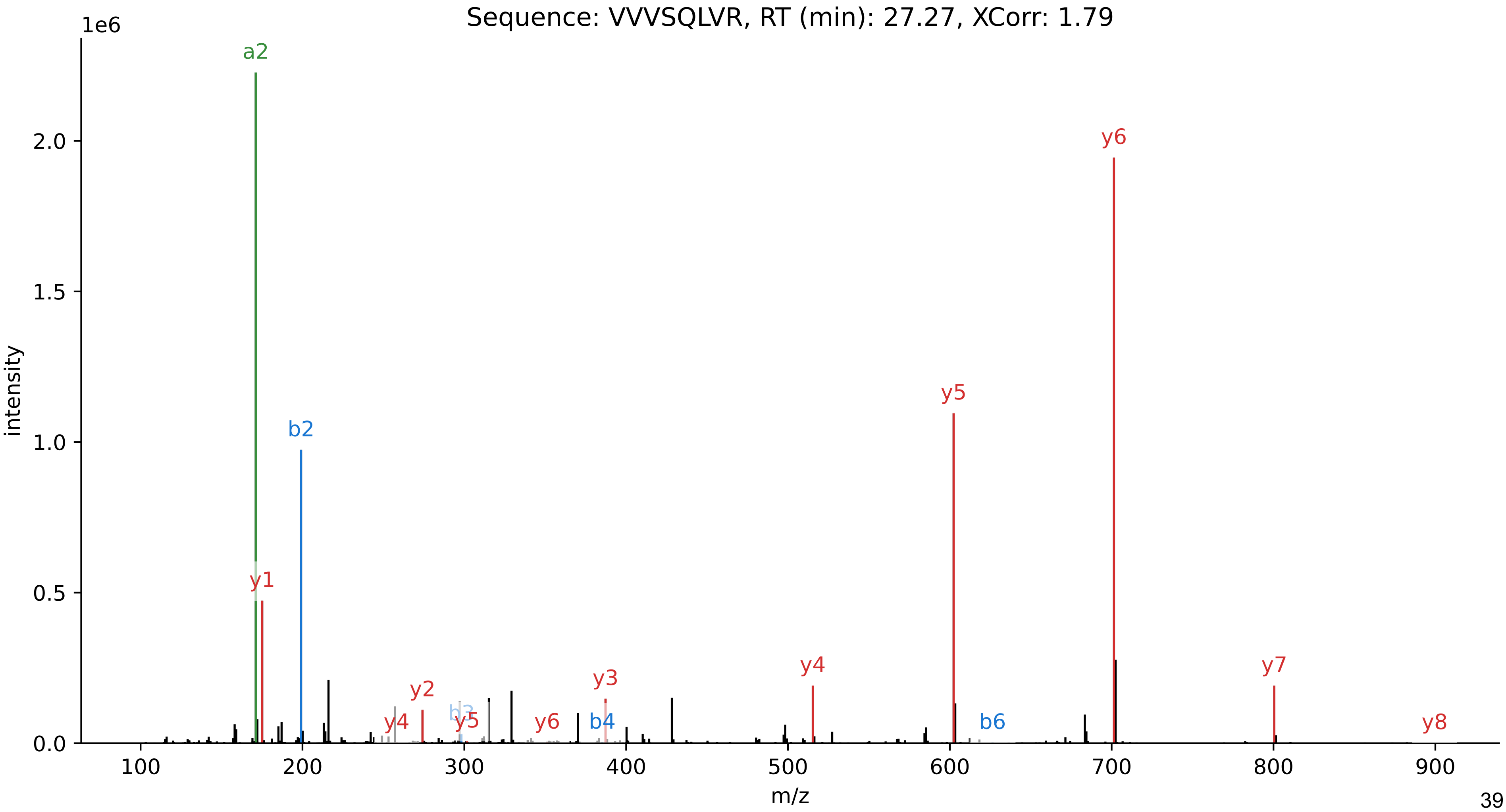

Sequence: VVVSQLVR, RT (min): 27.27, XCorr: 1.79
1e6
intensity
m/z
a2
y6
y5
b2
y1
y4
y7
y3
y2
y4
y5
y6
b4
b6
y8
0.0
0.5
1.0
1.5
2.0
100
200
300
400
500
600
700
800
900

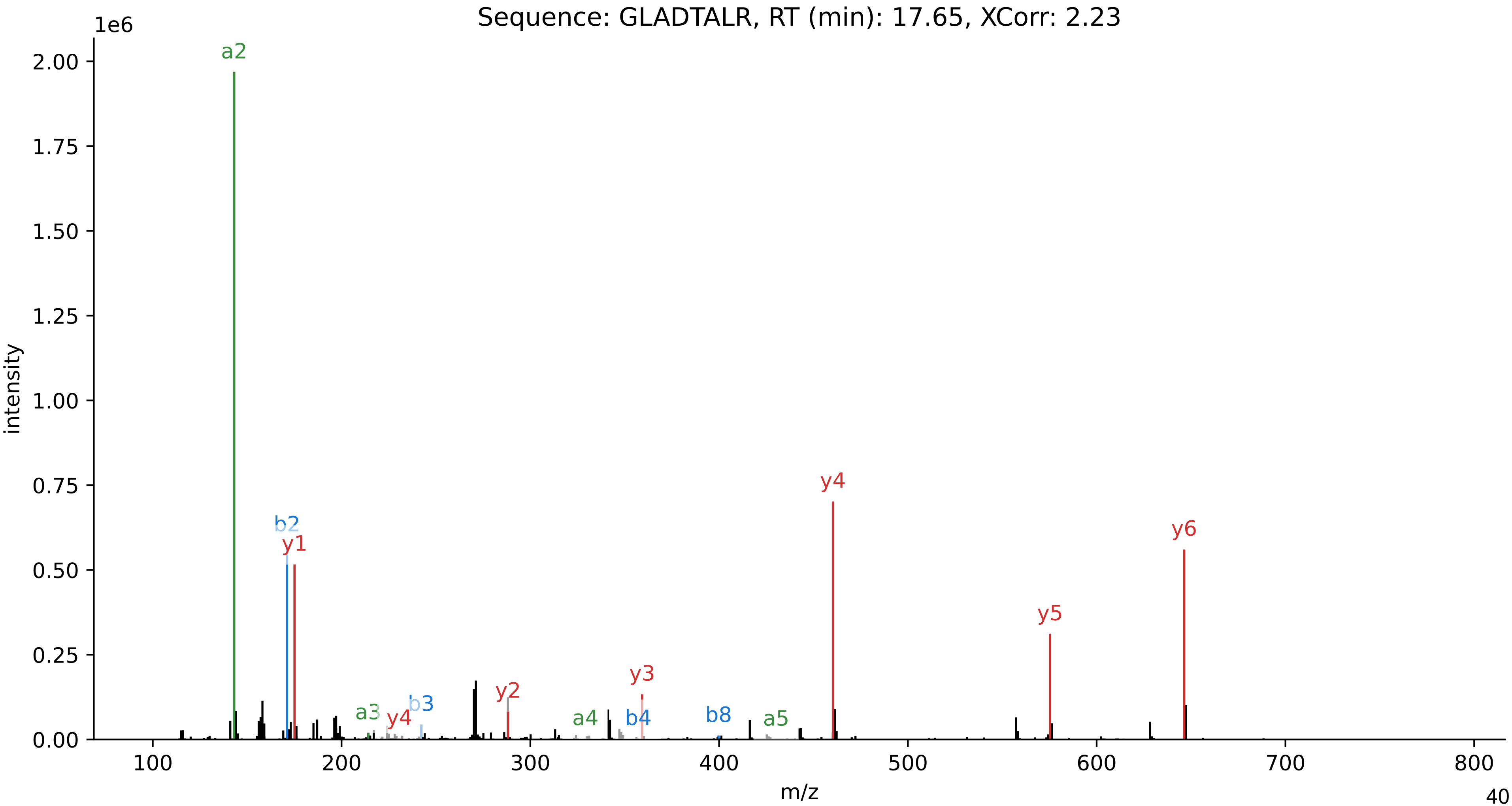
Sequence: GLADTALR, RT (min): 17.65, XCorr: 2.23
1e6
2.00
1.75
1.50
1.25
1.00
0.75
0.50
0.25
0.00
intensity
a2
b2
y1
a3
y4
b3
y2
a4
b4
y3
b8
a5
y4
y5
y6
100
200
300
400
500
600
700
800
m/z

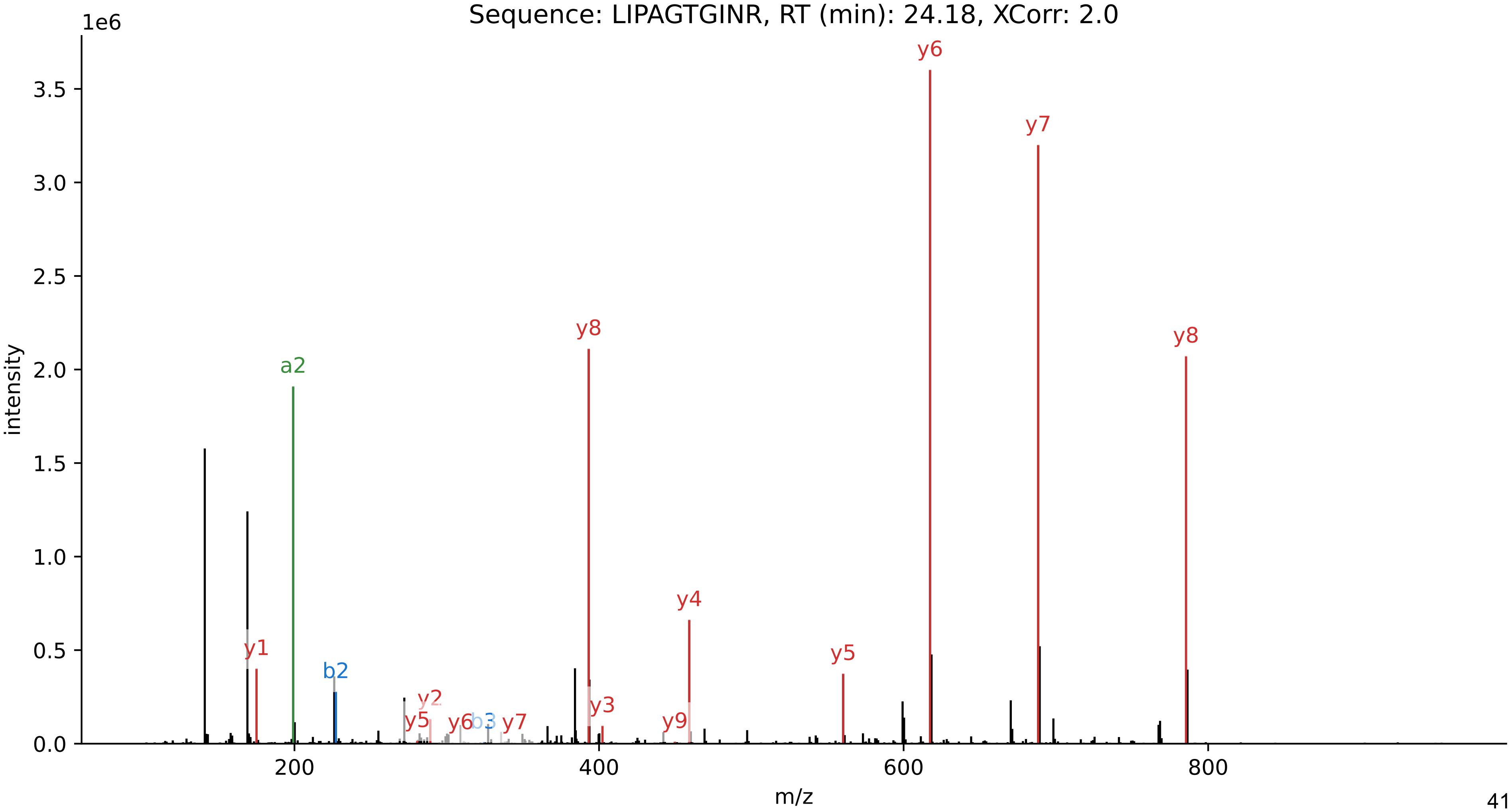

Sequence: LIPAGTGINR, RT (min): 24.18, XCorr: 2.0
1e6
intensity
m/z
y6
y7
y8
y8
a2
y4
y5
y1
b2
y2
y5
y6
b3
y7
y3
y9
3.5
3.0
2.5
2.0
1.5
1.0
0.5
0.0
200
400
600
800

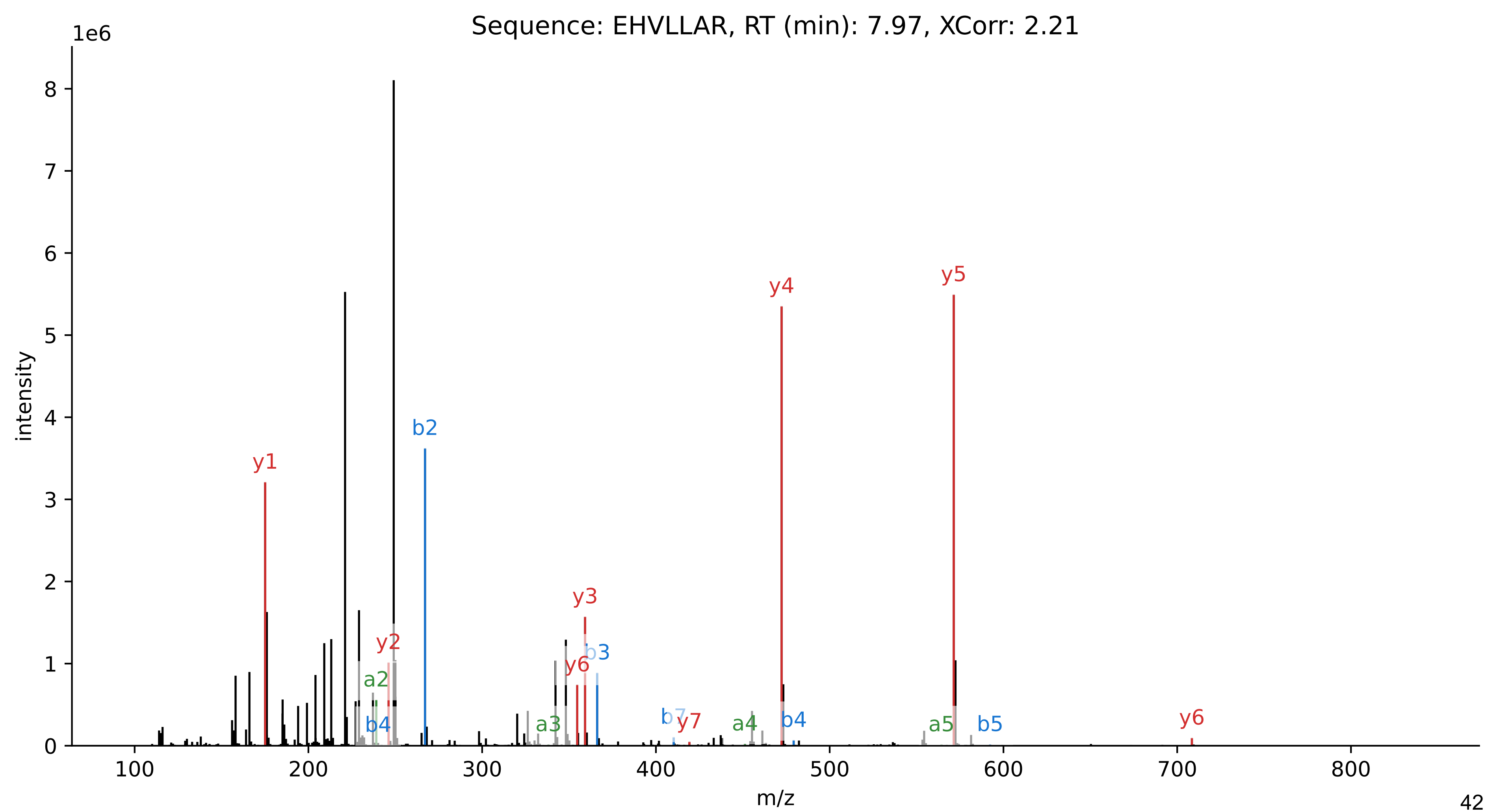
Sequence: EHVLLAR, RT (min): 7.97, XCorr: 2.21
1e6
intensity
m/z
0
1
2
3
4
5
6
7
8
100
200
300
400
500
600
700
800
y1
y2
a2
b4
b2
y3
b3
y6
a3
b7
y7
a4
y4
b4
y5
a5
b5
y6

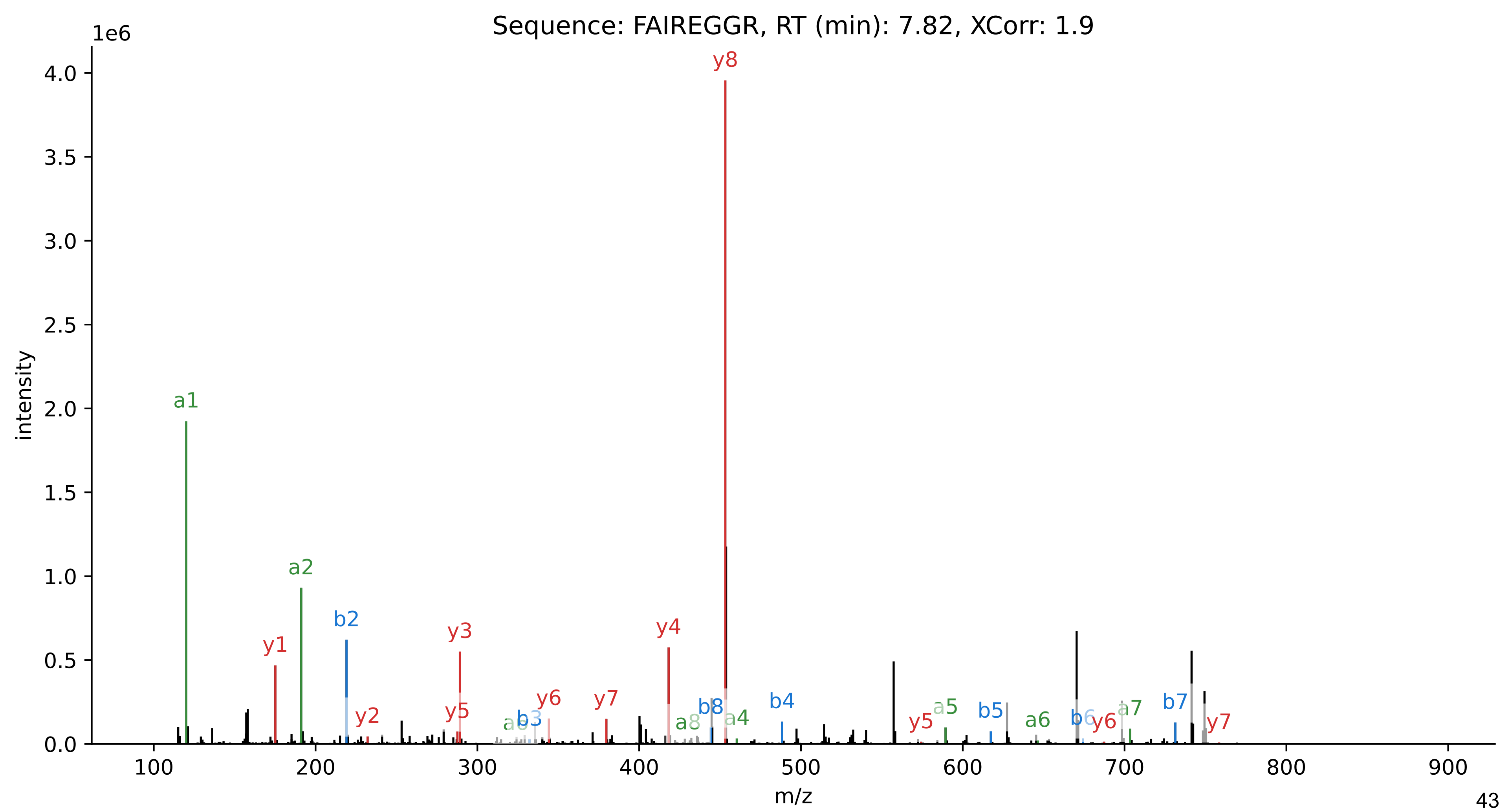

Sequence: FAIREGGR, RT (min): 7.82, XCorr: 1.9
1e6
intensity
m/z
0.0
0.5
1.0
1.5
2.0
2.5
3.0
3.5
4.0
100
200
300
400
500
600
700
800
900
y8
a1
a2
b2
y1
y3
y4
y2
y5
y6
y7
b3
b8
a8
a4
b4
y5
a5
b5
a6
b6
y6
a7
b7
y7

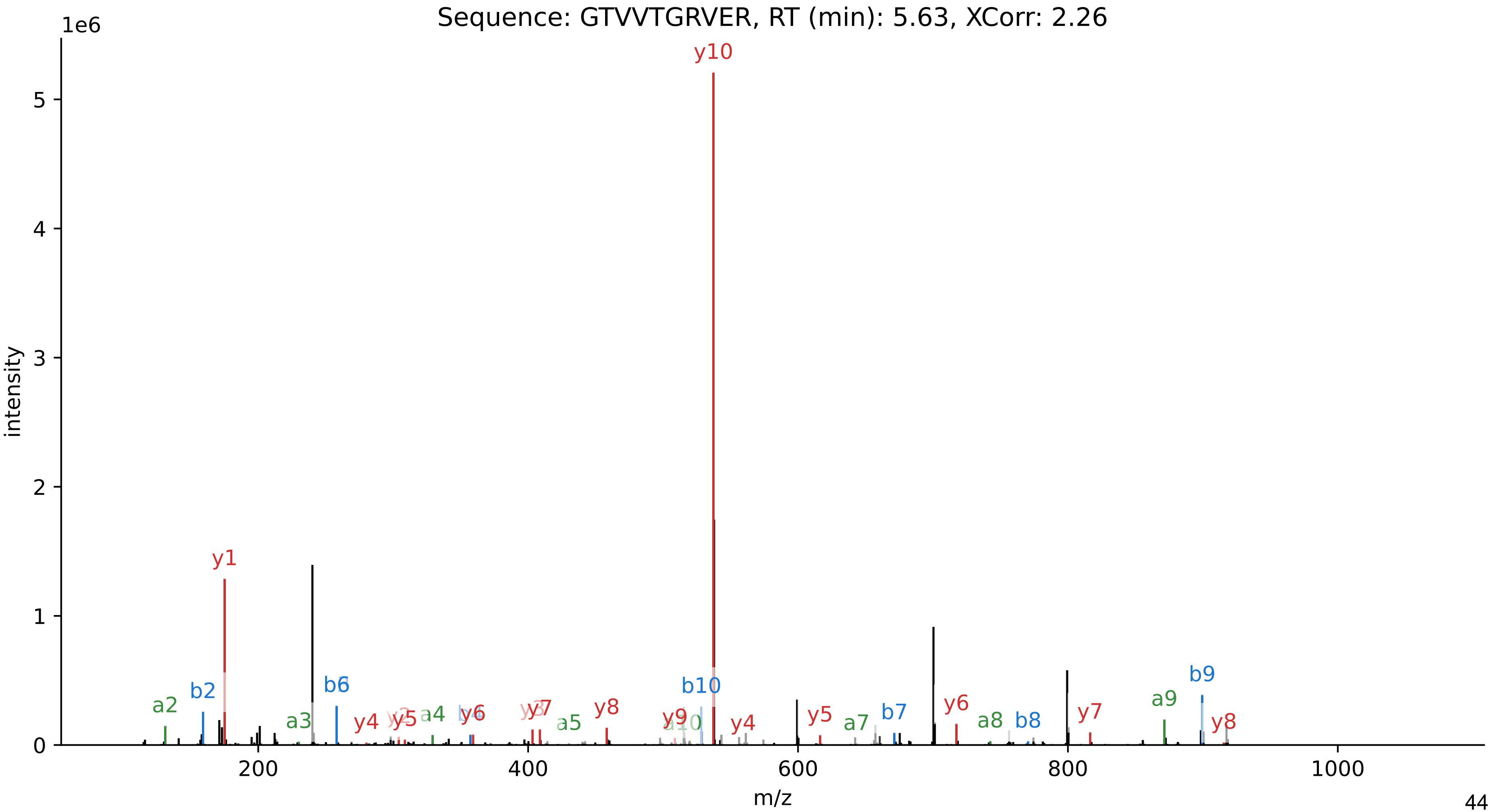

Sequence: GTVVTGRVER, RT (min): 5.63, XCorr: 2.26
1e6
intensity
m/z
0
1
2
3
4
5
200
400
600
800
1000
y10
y1
a2
b2
a3
b6
y4
y5
a4
y6
y7
a5
y8
y9
a10
b10
y4
y5
a7
b7
y6
a8
b8
y7
a9
b9
y8

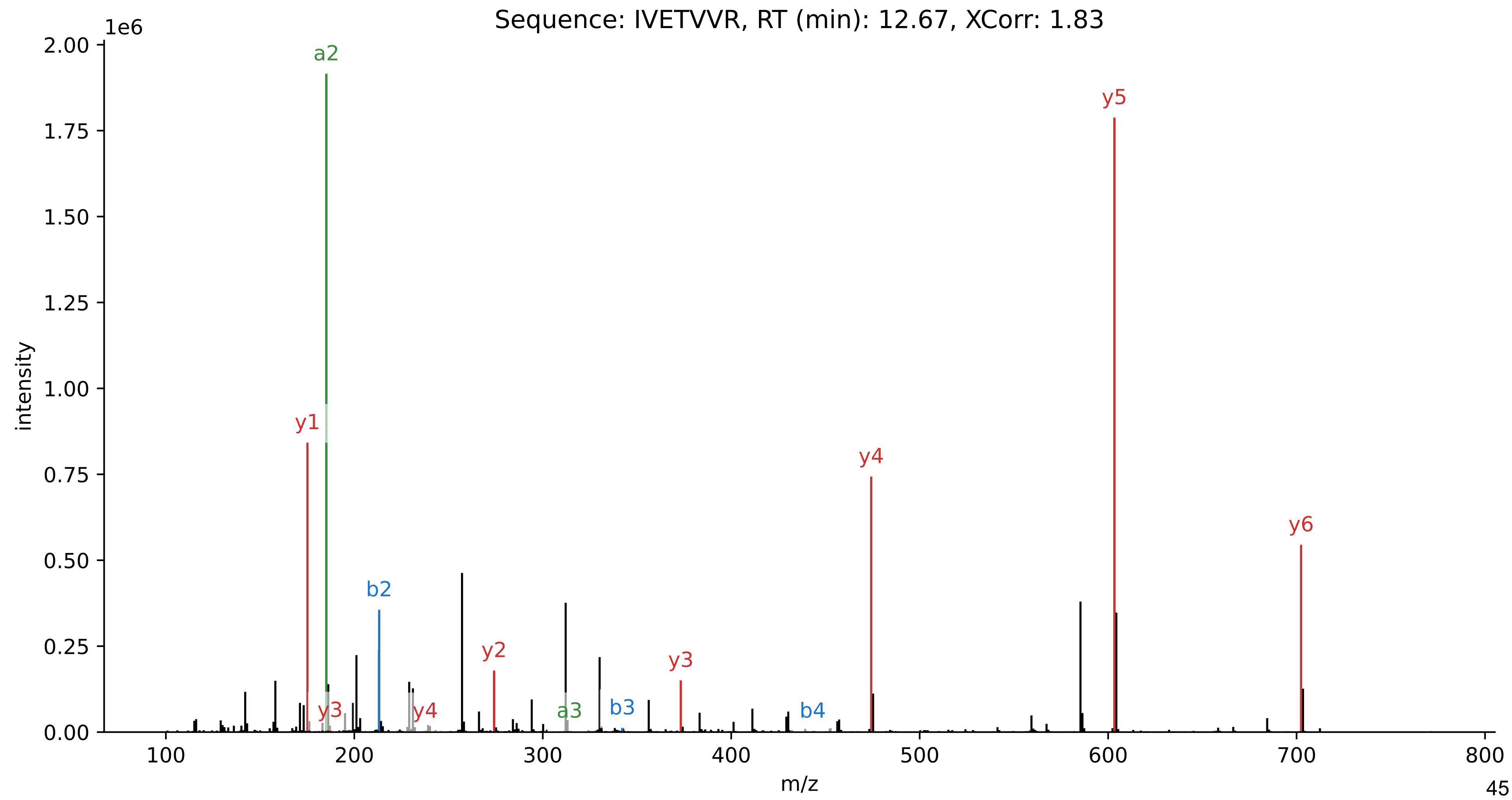
Sequence: IVETVVR, RT (min): 12.67, XCorr: 1.83
1e6
2.00
1.75
1.50
1.25
1.00
0.75
0.50
0.25
0.00
intensity
a2
y5
y1
y4
y6
b2
y2
y3
y3
y4
a3
b3
b4
100
200
300
400
500
600
700
800
m/z

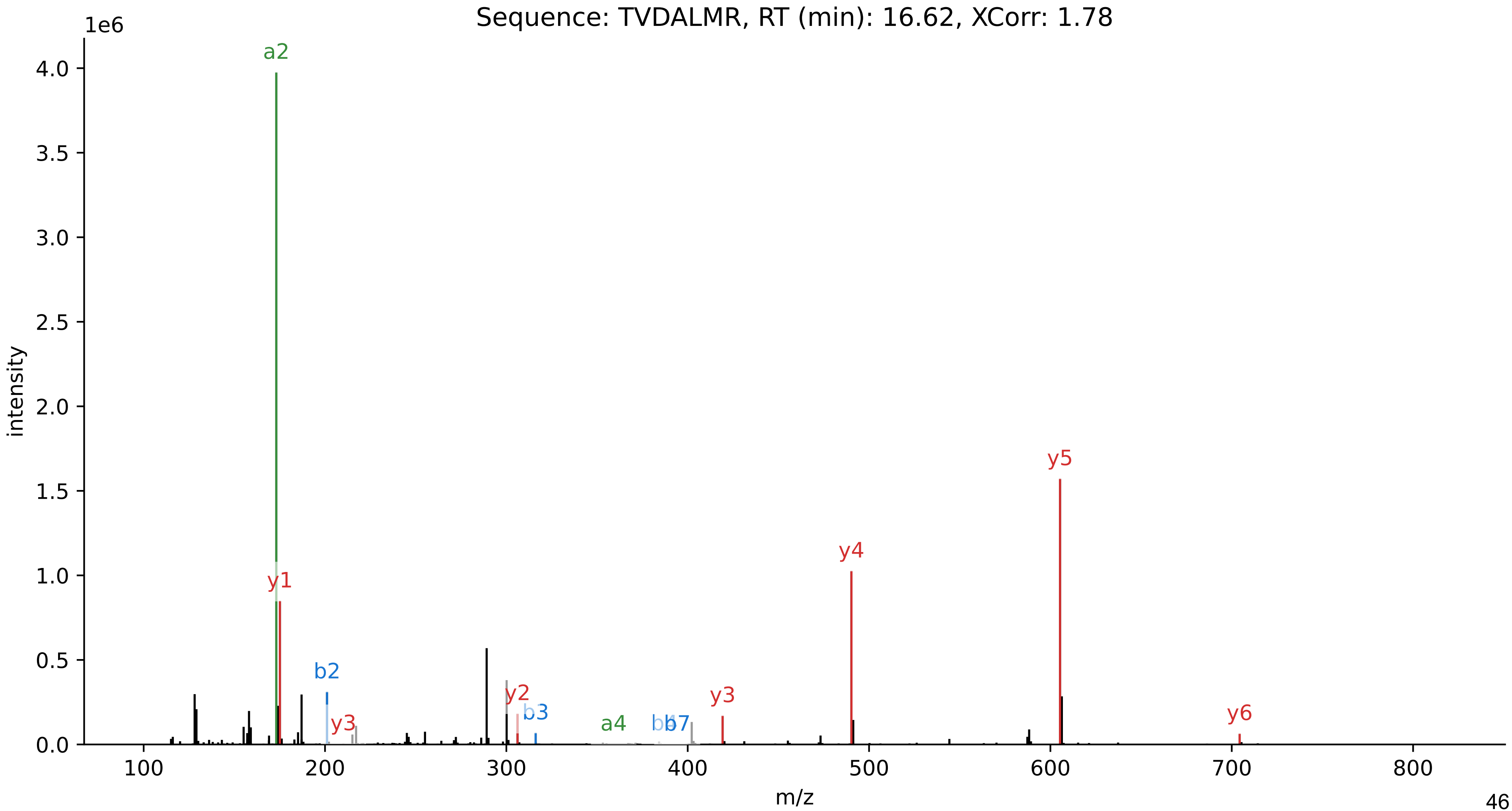
Sequence: TVDALMR, RT (min): 16.62, XCorr: 1.78
1e6
intensity
m/z
4.0
3.5
3.0
2.5
2.0
1.5
1.0
0.5
0.0
100
200
300
400
500
600
700
800
a2
y1
b2
y3
y2
b3
a4
b6
b7
y3
y4
y5
y6

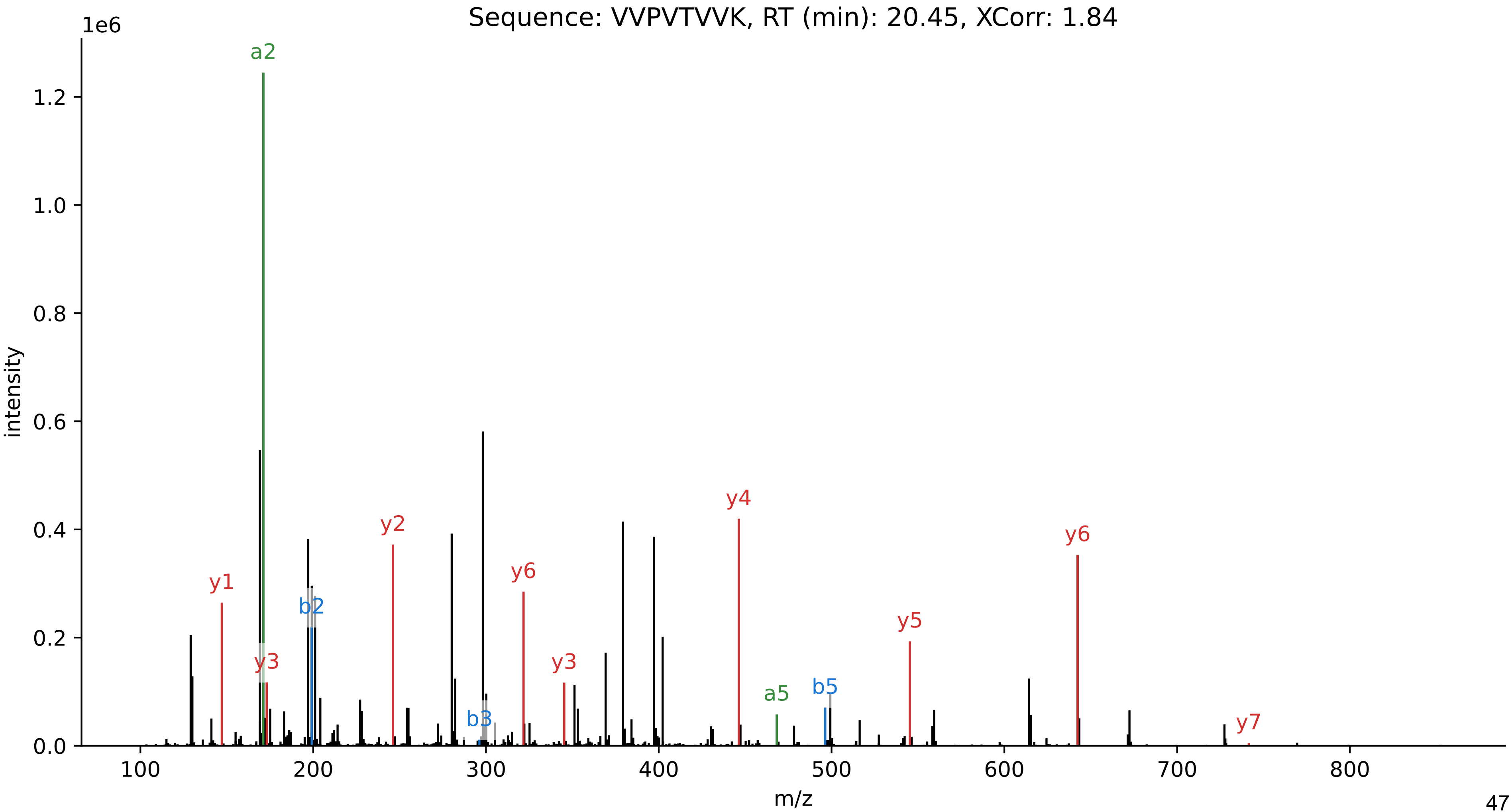
Sequence: VVPVTVVK, RT (min): 20.45, XCorr: 1.84
1e6
intensity
m/z
a2
y1
y3
b2
y2
y6
b3
y3
y4
a5
b5
y5
y6
y7
0.0
0.2
0.4
0.6
0.8
1.0
1.2
100
200
300
400
500
600
700
800

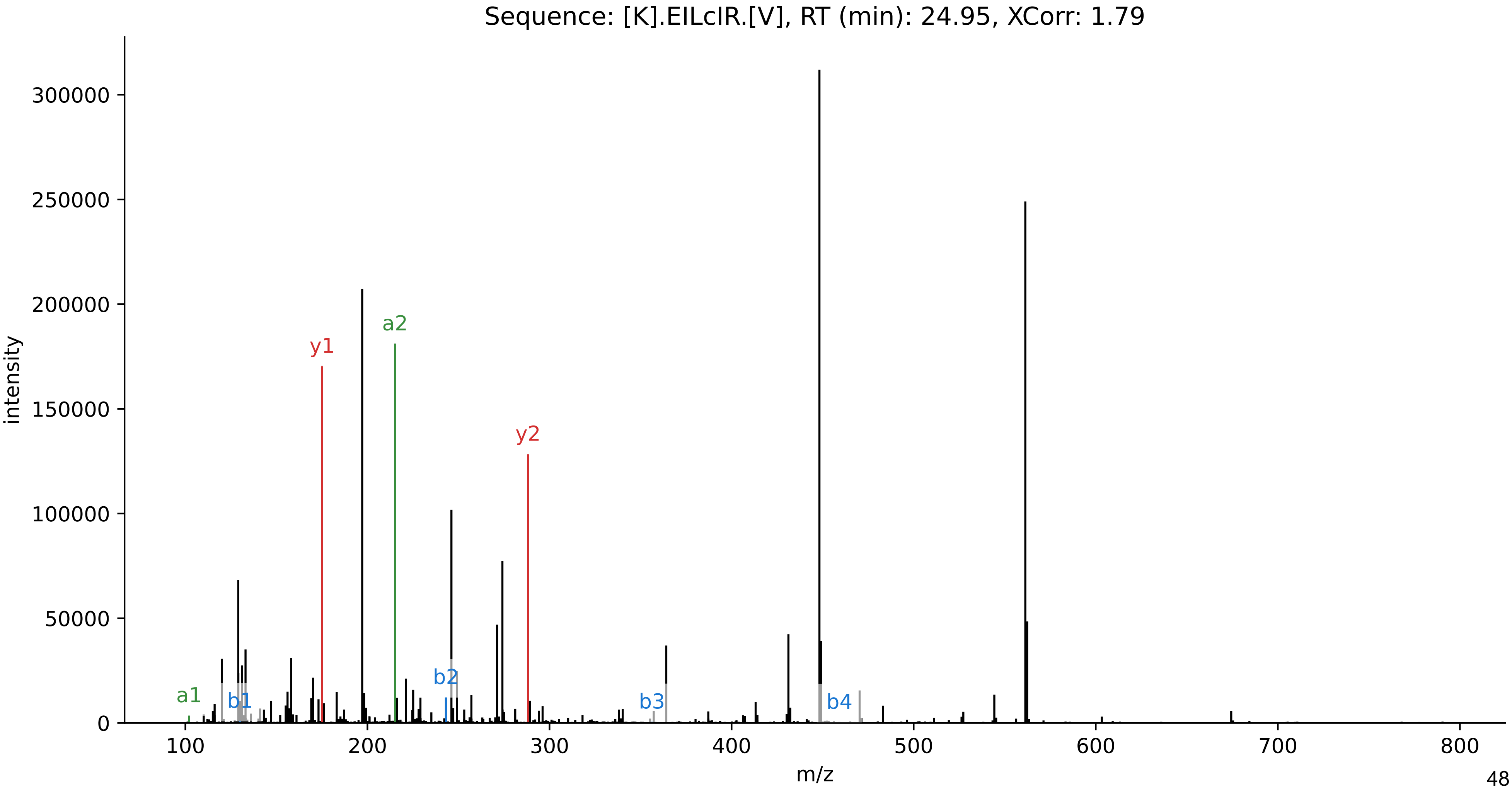

Sequence: [K].EILcIR.[V], RT (min): 24.95, XCorr: 1.79
intensity
m/z
y1
a2
y2
a1
b1
b2
b3
b4

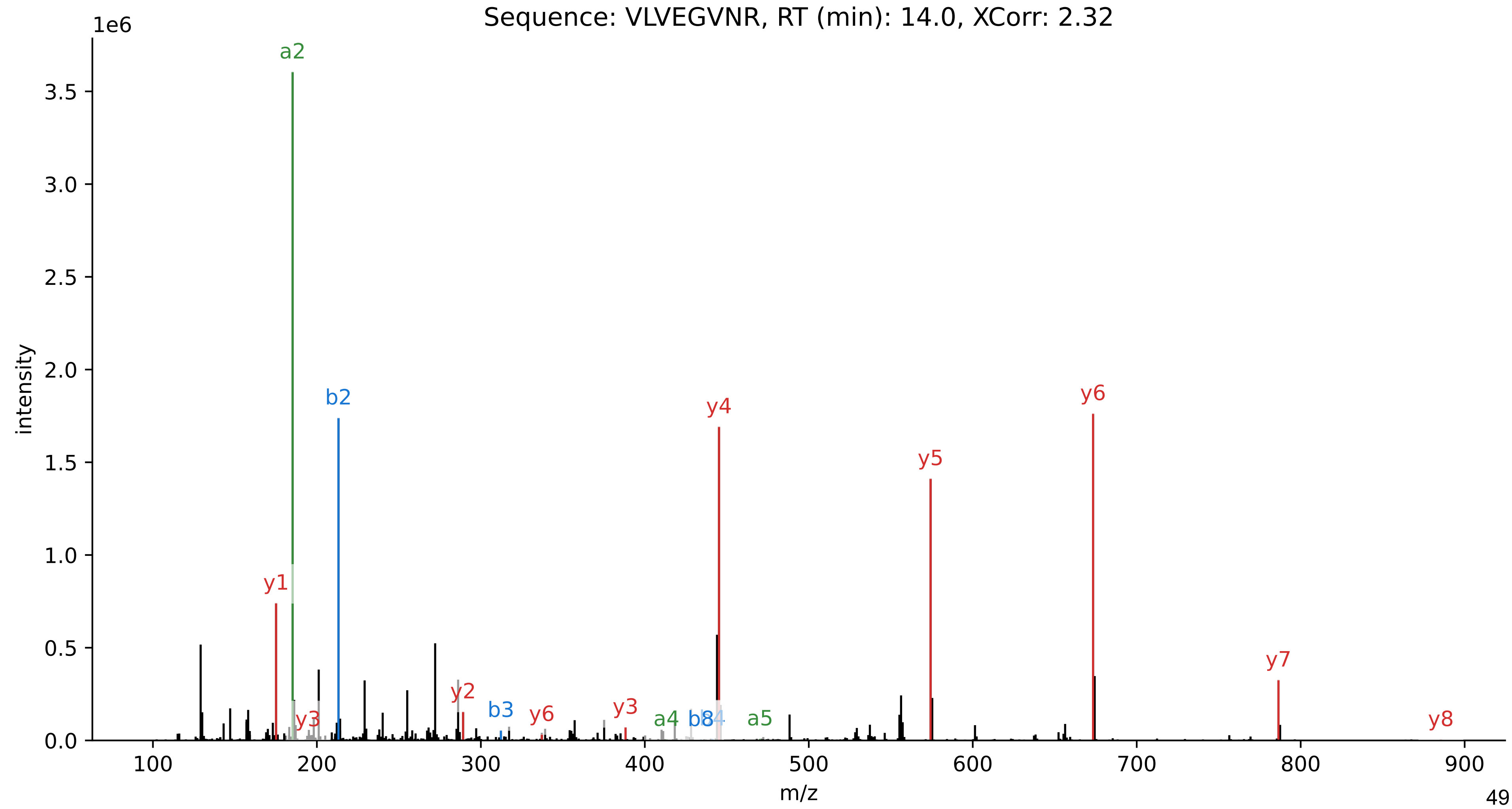
Sequence: VLVEGVNR, RT (min): 14.0, XCorr: 2.32
1e6
intensity
m/z
0.0
0.5
1.0
1.5
2.0
2.5
3.0
3.5
100
200
300
400
500
600
700
800
900
a2
b2
y1
y3
y2
b3
y6
y3
a4
b8
a5
y4
y5
y6
y7
y8

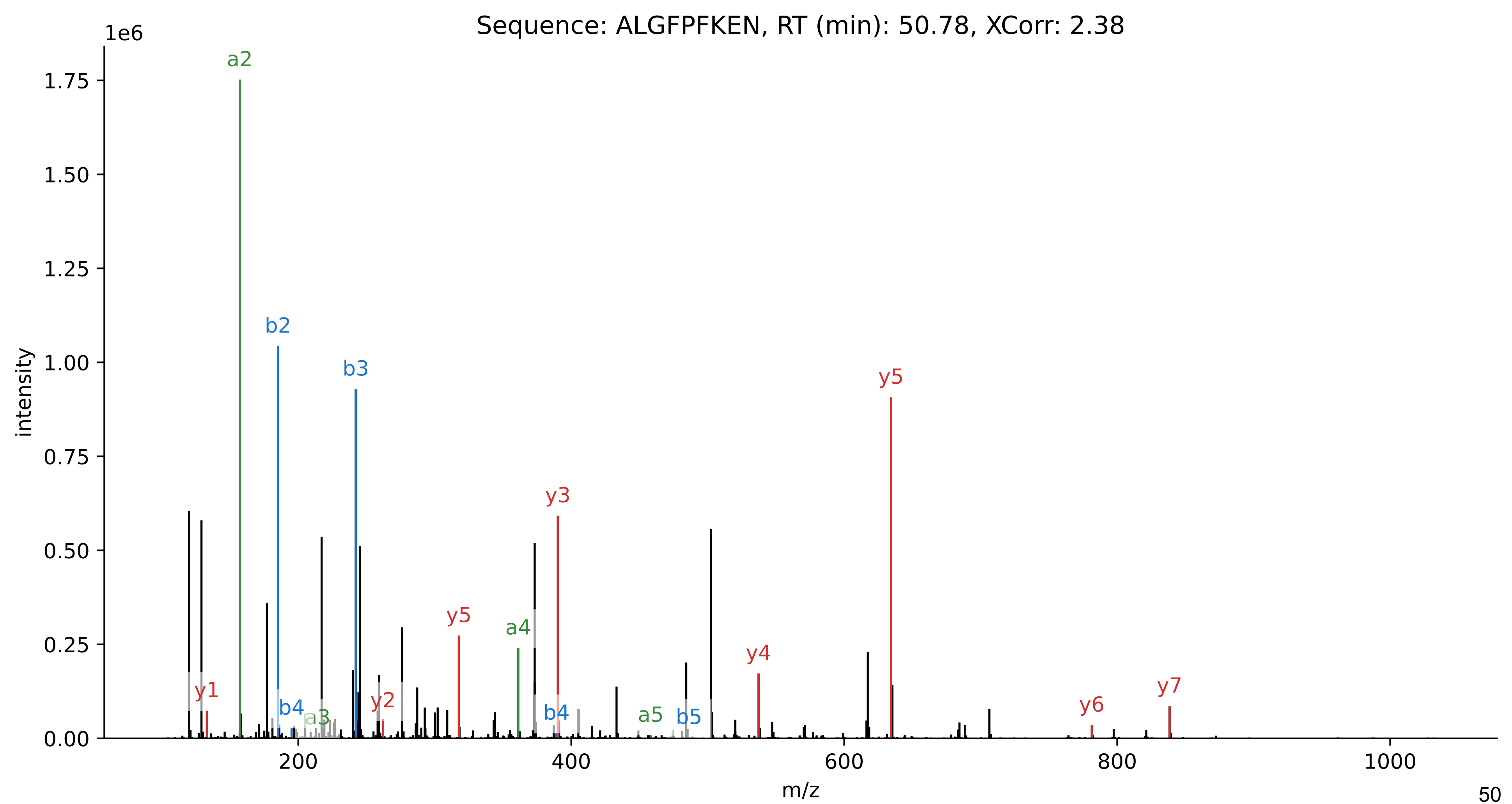

Sequence: ALGFPFKEN, RT (min): 50.78, XCorr: 2.38
1e6
1.75
1.50
1.25
1.00
0.75
0.50
0.25
0.00
intensity
a2
b2
b3
y5
y3
y5
a4
y4
y1
b4
a3
y2
b4
a5
b5
y6
y7
200
400
600
800
1000
m/z

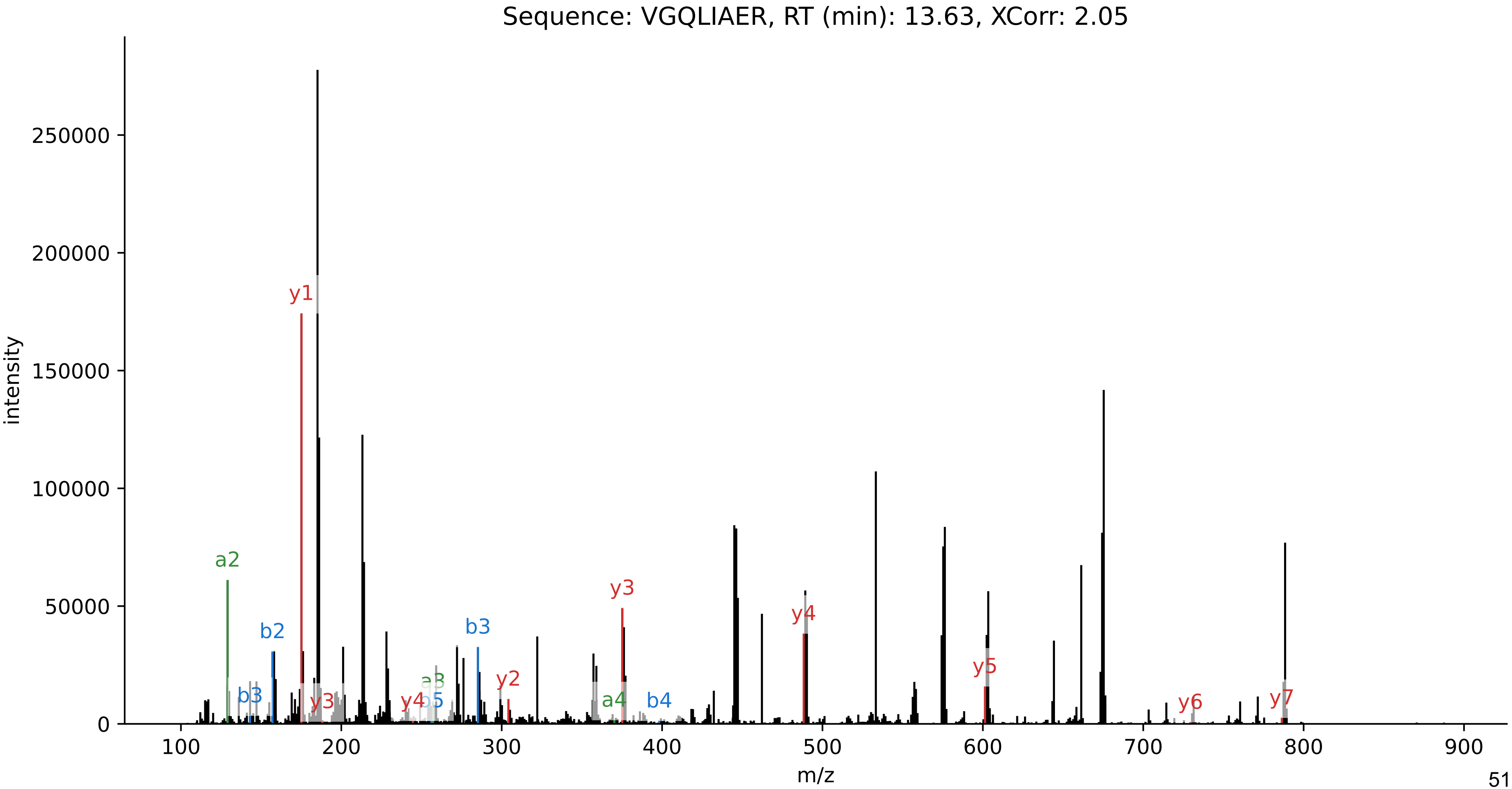

Sequence: VGQLIAER, RT (min): 13.63, XCorr: 2.05
intensity
m/z
0
50000
100000
150000
200000
250000
100
200
300
400
500
600
700
800
900
y1
a2
b2
b3
y3
y4
a3
b5
b3
y2
y3
a4
b4
y4
y5
y6
y7

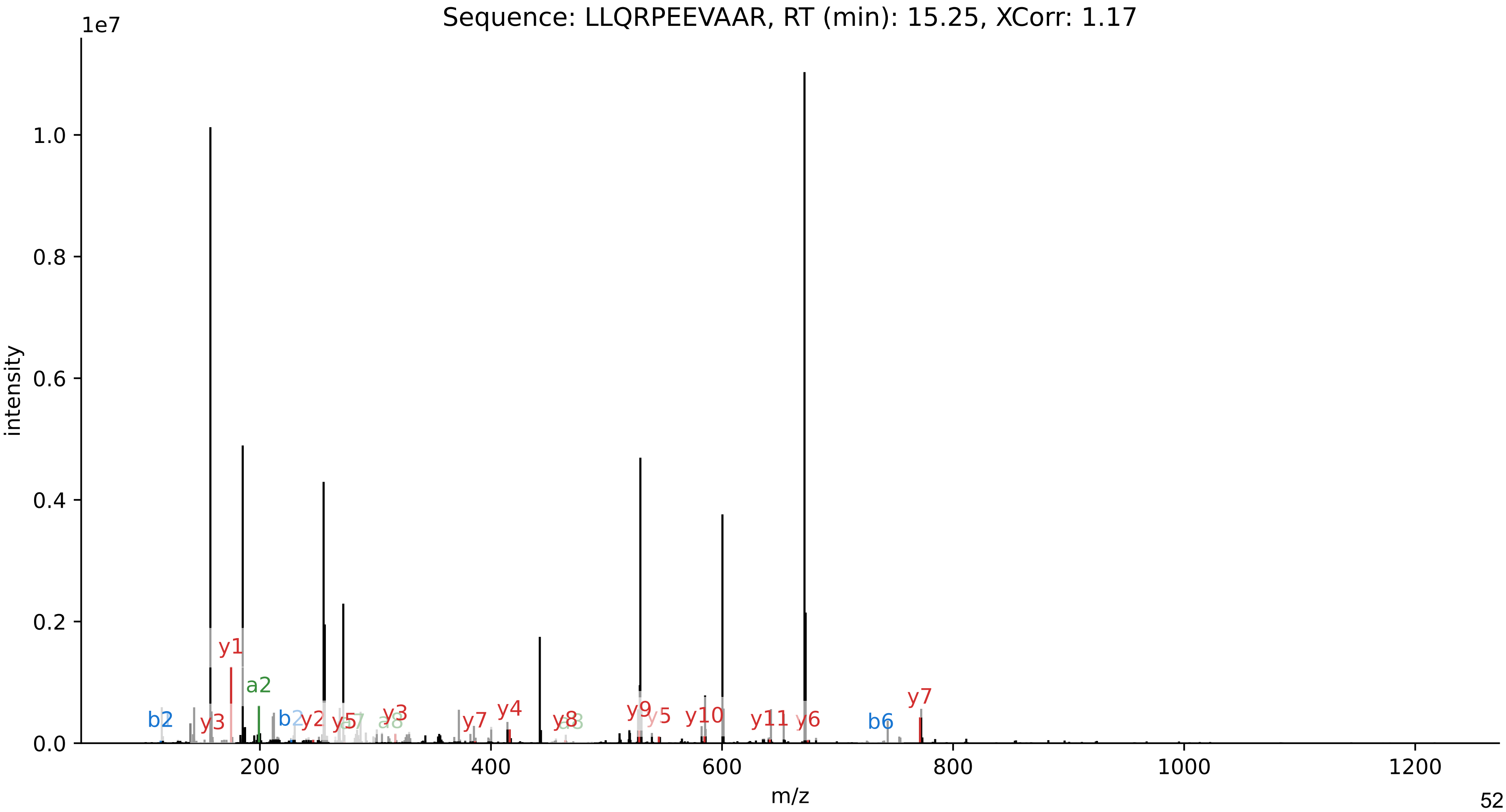
Sequence: LLQRPEEVAAR, RT (min): 15.25, XCorr: 1.17
1e7
intensity
m/z
y1
a2
b2
y3
b2
y2
y5
y3
y7
y4
y8
y9
y5
y10
y11
y6
b6
y7

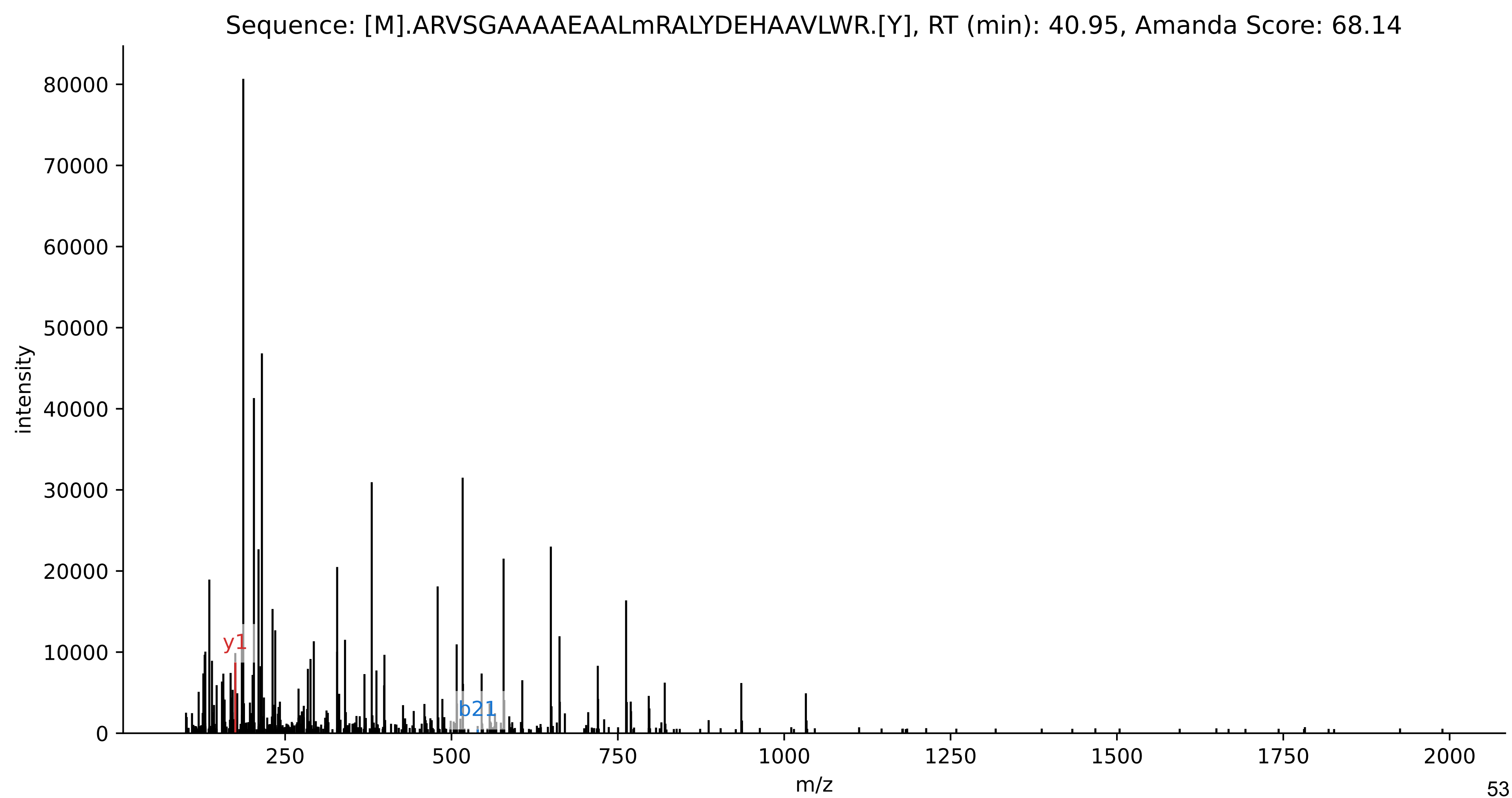

Sequence: [M].ARVSGAAAAEAALmRALYDEHAAVLWR.[Y], RT (min): 40.95, Amanda Score: 68.14
intensity
m/z
y1
b21

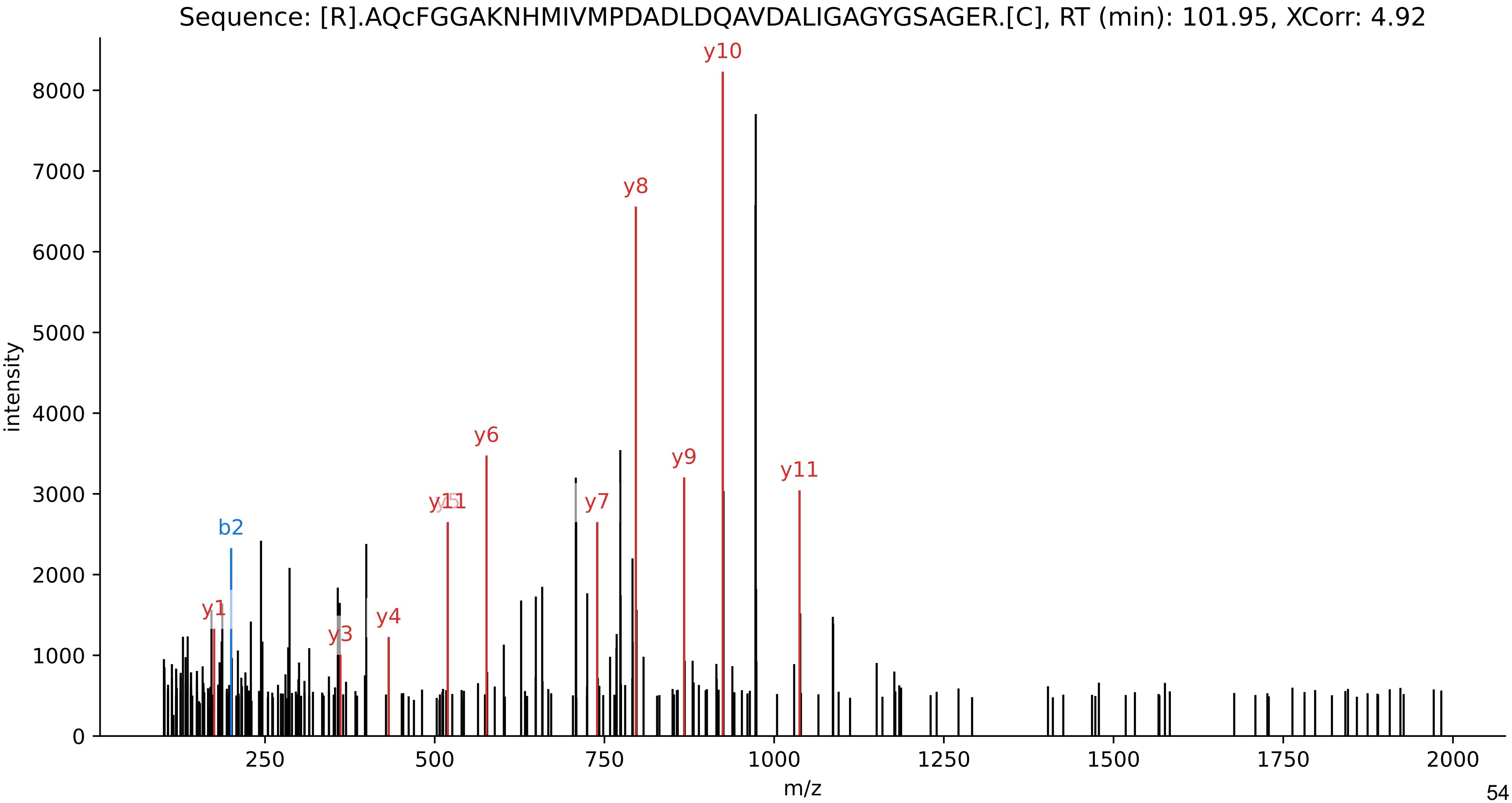
Sequence: [R].AQcFGGAKNHMIVMPDADLDQAVDALIGAGYGSAGER.[C], RT (min): 101.95, XCorr: 4.92
intensity
m/z
b2
y1
y3
y4
y5
y11
y6
y7
y8
y9
y10
y11

Sequence: LAFVEDR, RT (min): 23.83, XCorr: 2.12

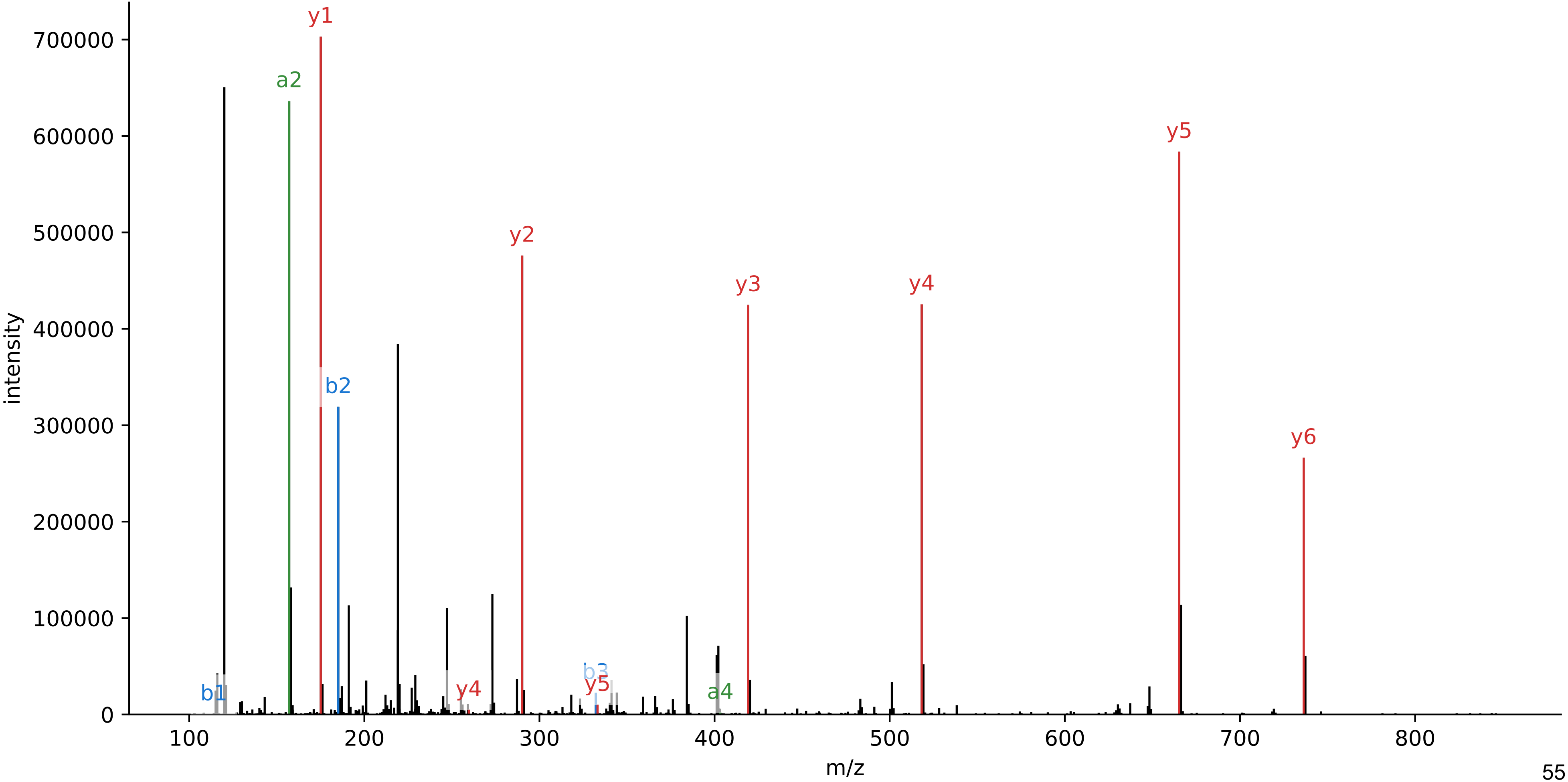

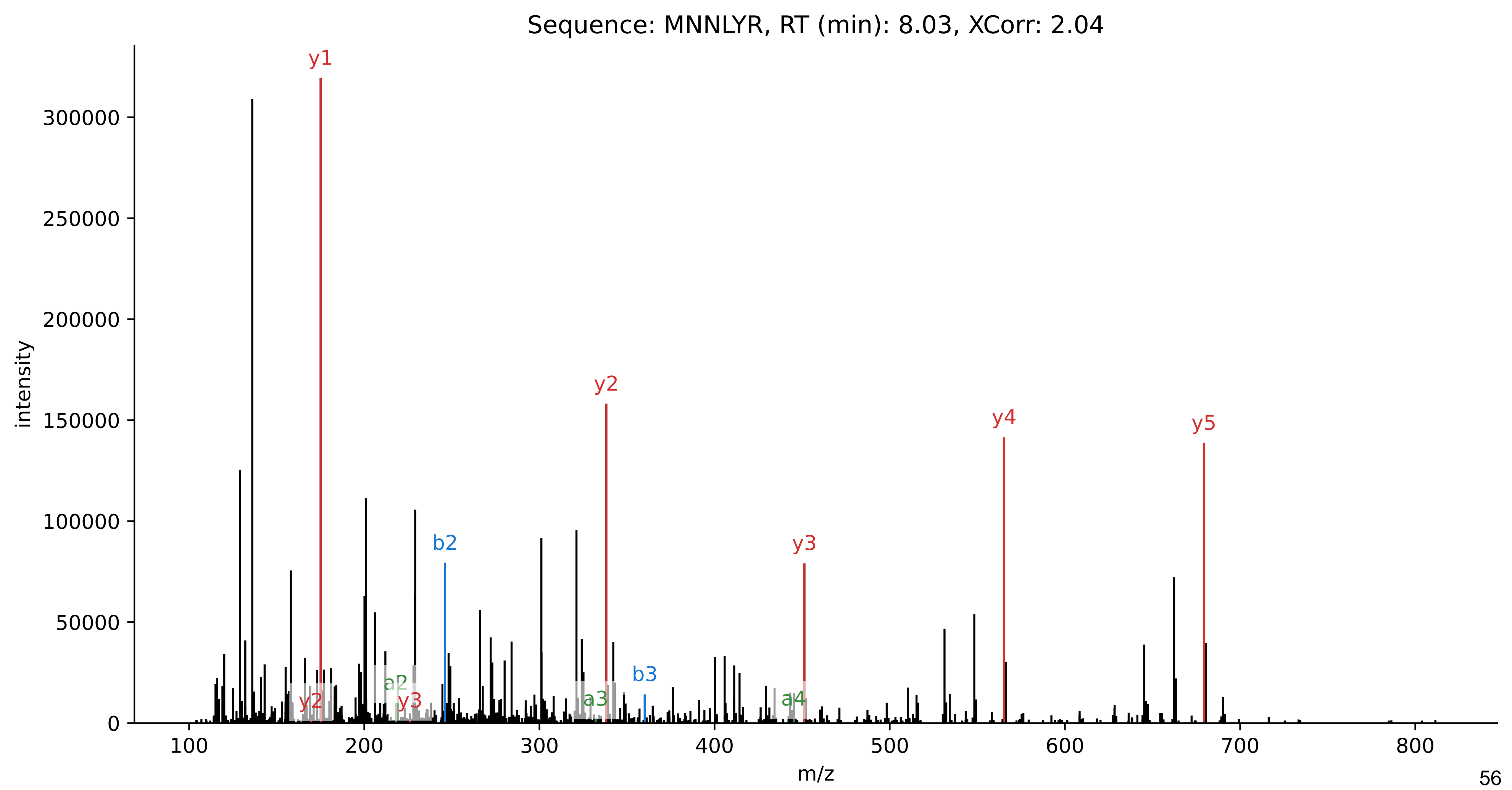

Sequence: MNNLYR, RT (min): 8.03, XCorr: 2.04
intensity
m/z
y1
y2
y4
y5
b2
y3
b3
a2
a3
a4
y2
y3
300000
250000
200000
150000
100000
50000
0
100
200
300
400
500
600
700
800

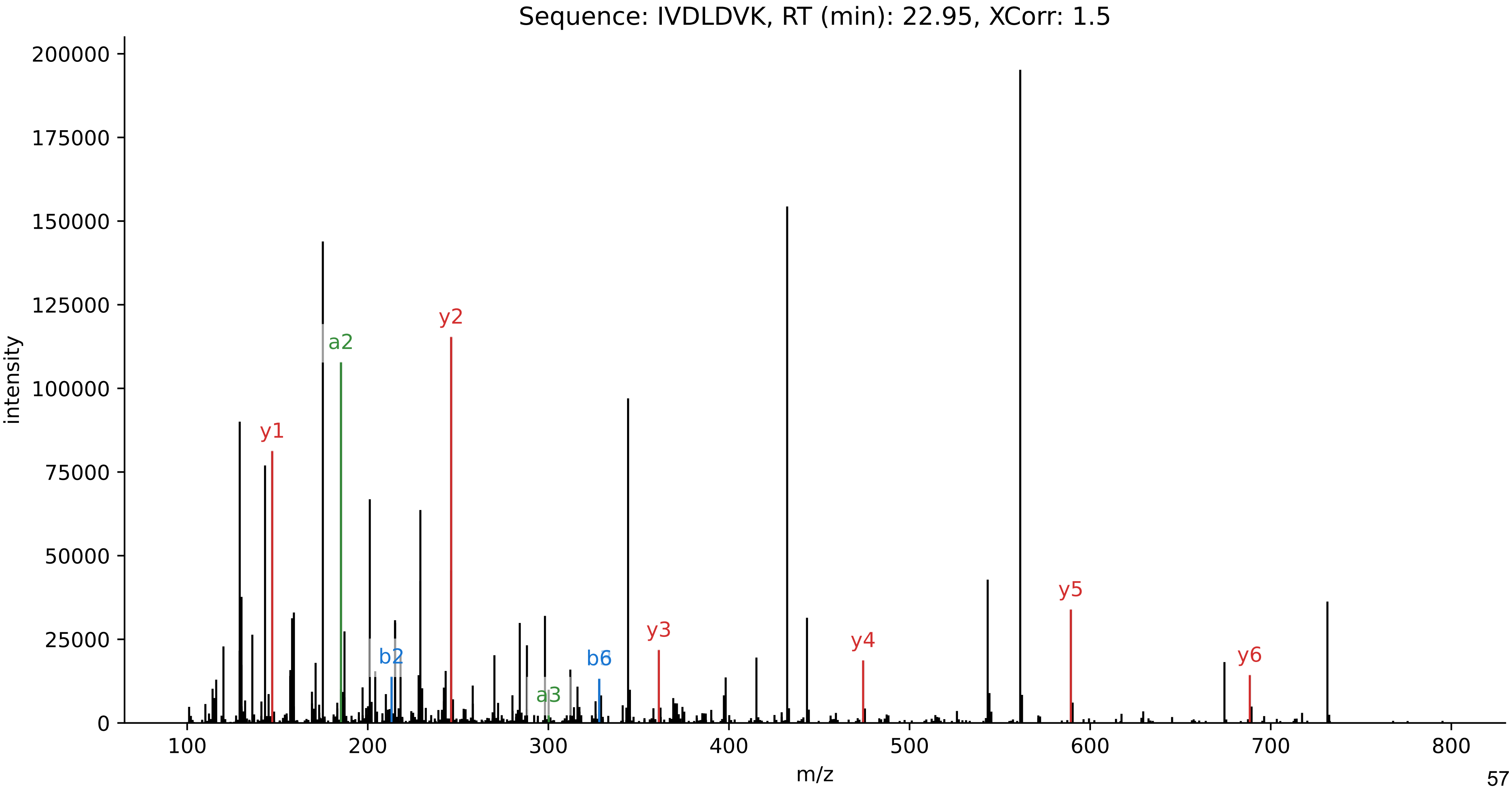
Sequence: IVDLDVK, RT (min): 22.95, XCorr: 1.5
intensity
m/z
y1
a2
y2
b2
a3
b6
y3
y4
y5
y6
0
25000
50000
75000
100000
125000
150000
175000
200000
100
200
300
400
500
600
700
800

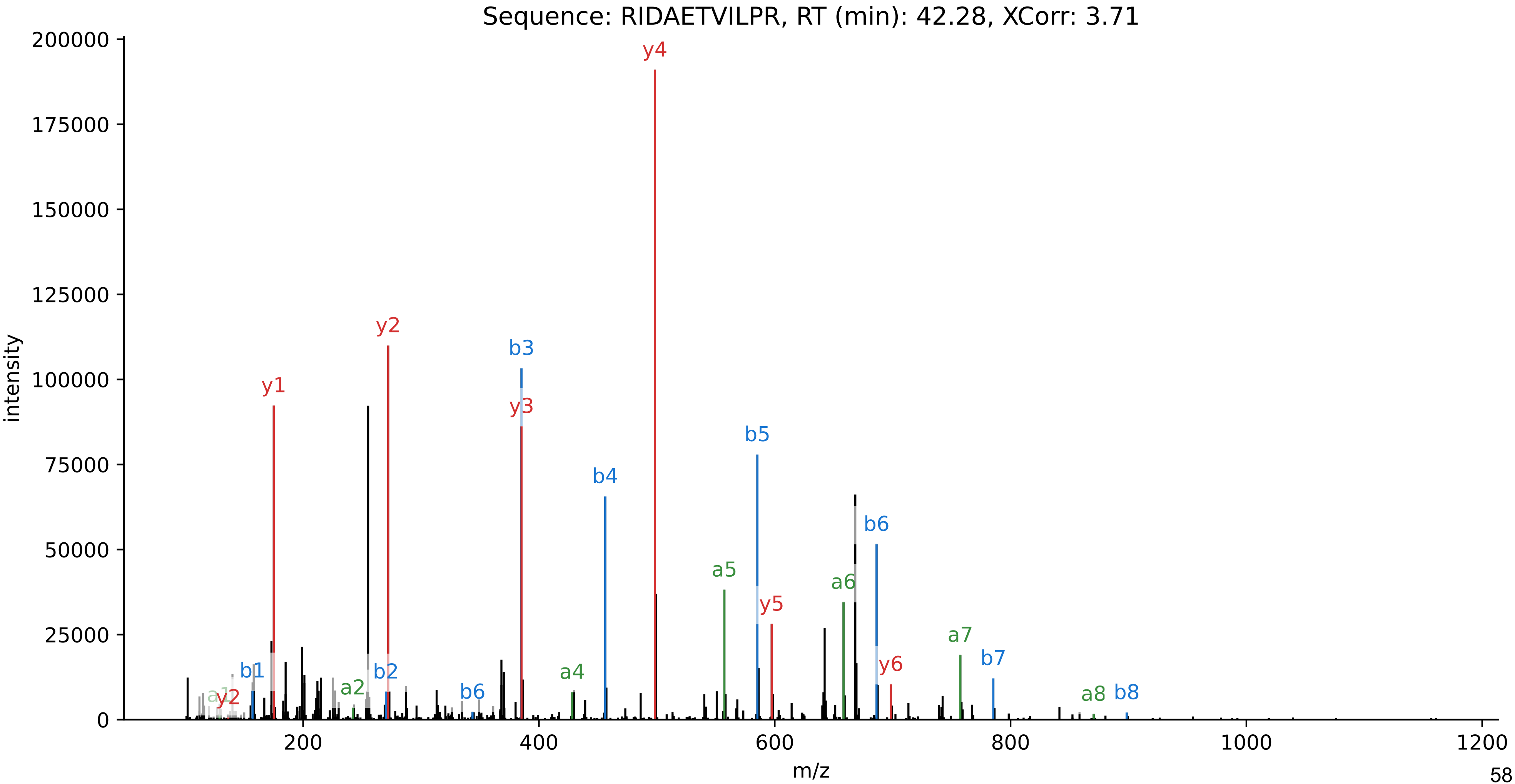

Sequence: RIDAETVILPR, RT (min): 42.28, XCorr: 3.71
intensity
m/z
y4
y2
b3
y3
y1
b4
b5
b6
a5
y5
a6
a7
b7
y6
b1
b2
a2
a4
b6
a8
b8
a1
y2
0
25000
50000
75000
100000
125000
150000
175000
200000
200
400
600
800
1000
1200

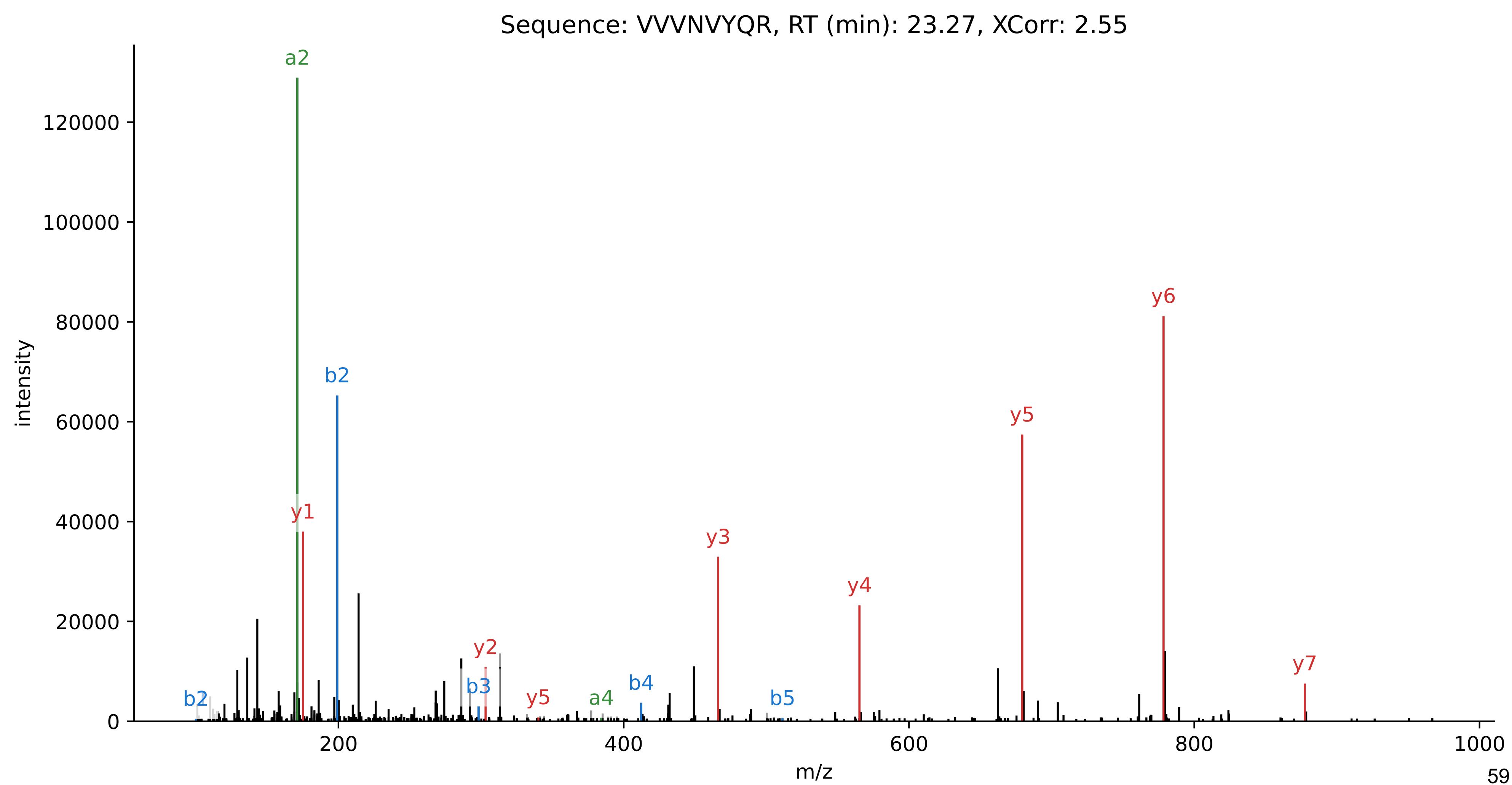
Sequence: VVVNVYQR, RT (min): 23.27, XCorr: 2.55
intensity
m/z
a2
b2
y1
y2
b3
y5
a4
b4
y3
b5
y4
y5
y6
y7
0
20000
40000
60000
80000
100000
120000
200
400
600
800
1000

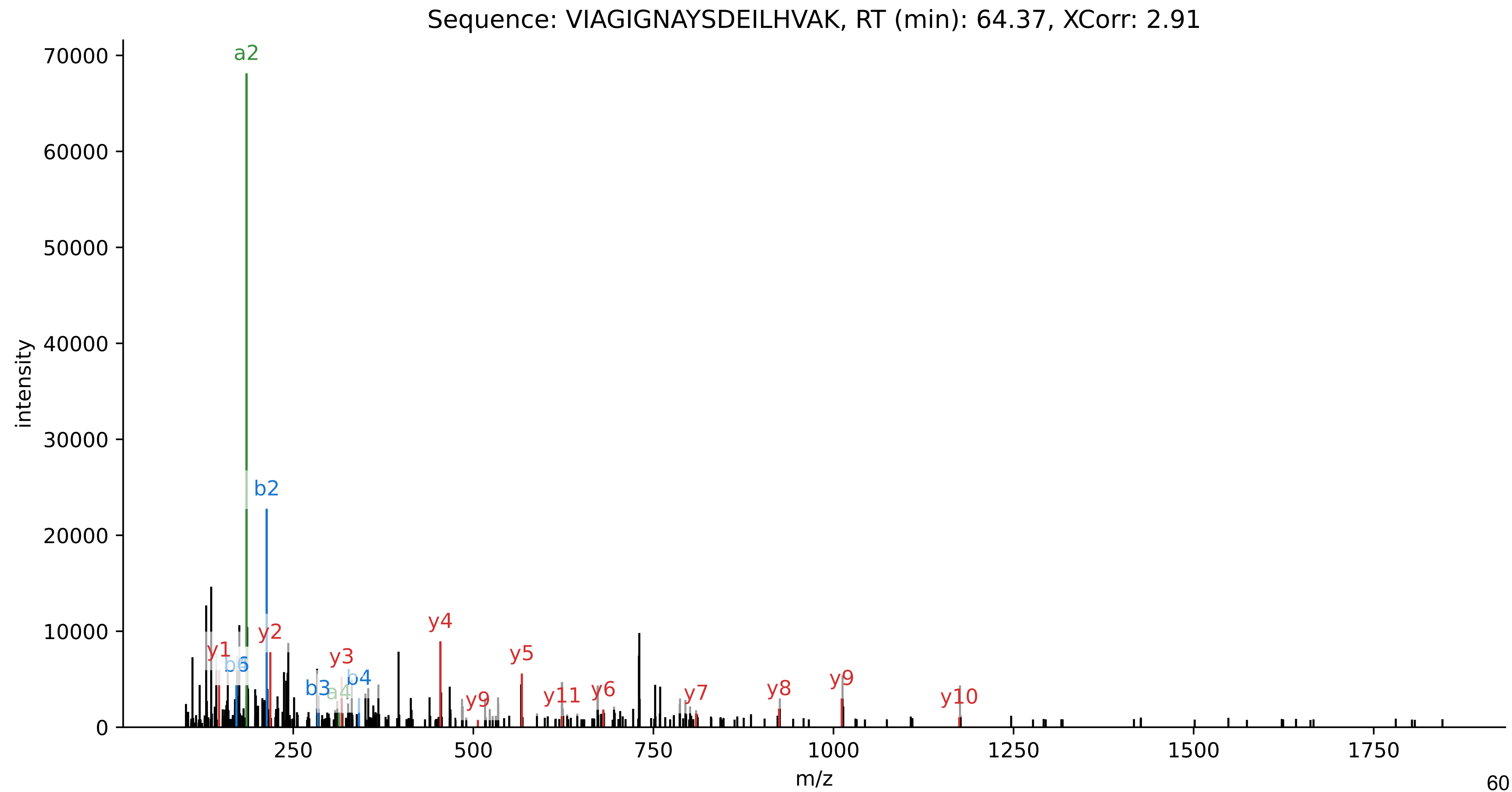

Sequence: VIAGIGNAYSDEILHVAK, RT (min): 64.37, XCorr: 2.91
intensity
m/z
a2
b2
y1
b6
y2
y3
b3
a4
b4
y4
y5
y9
y11
y6
y7
y8
y9
y10

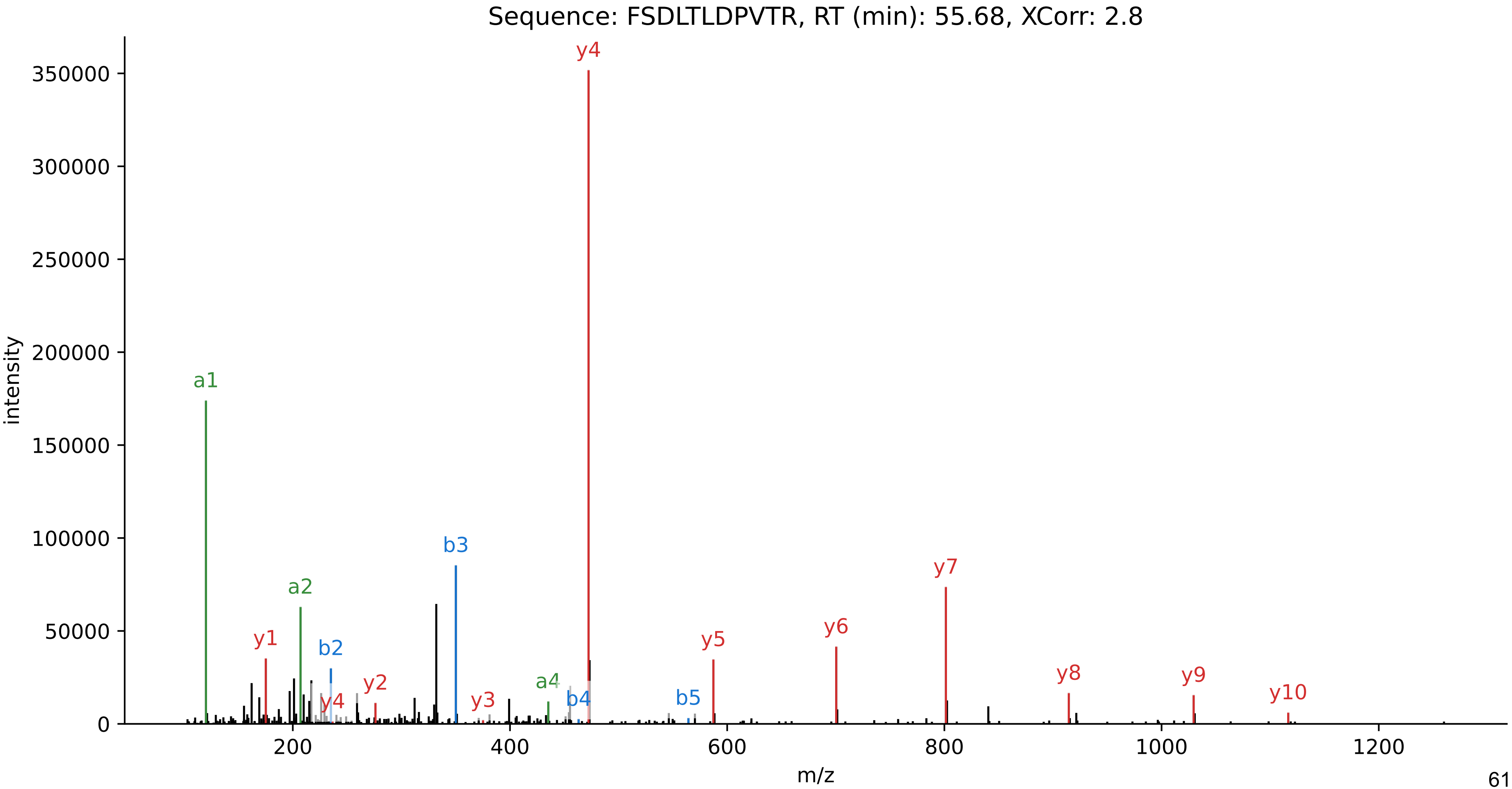

Sequence: FSDLTLDPVTR, RT (min): 55.68, XCorr: 2.8
intensity
m/z
y4
a1
b3
y7
a2
y6
y5
y1
b2
y2
y4
y3
a4
b4
b5
y8
y9
y10

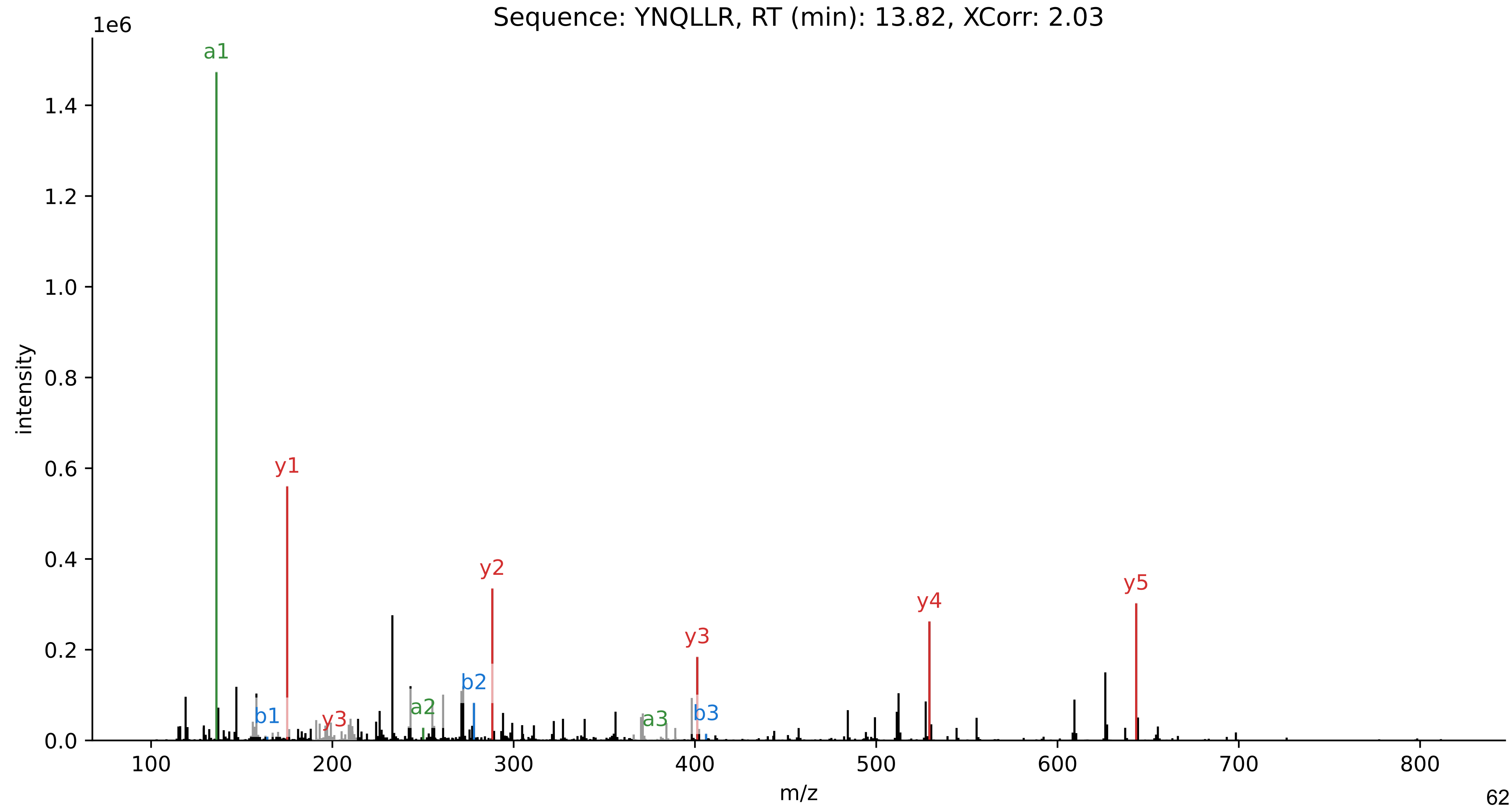

Sequence: YNQLLR, RT (min): 13.82, XCorr: 2.03
1e6
intensity
m/z
a1
y1
y2
y3
y4
y5
b1
b2
b3
a2
a3
y3

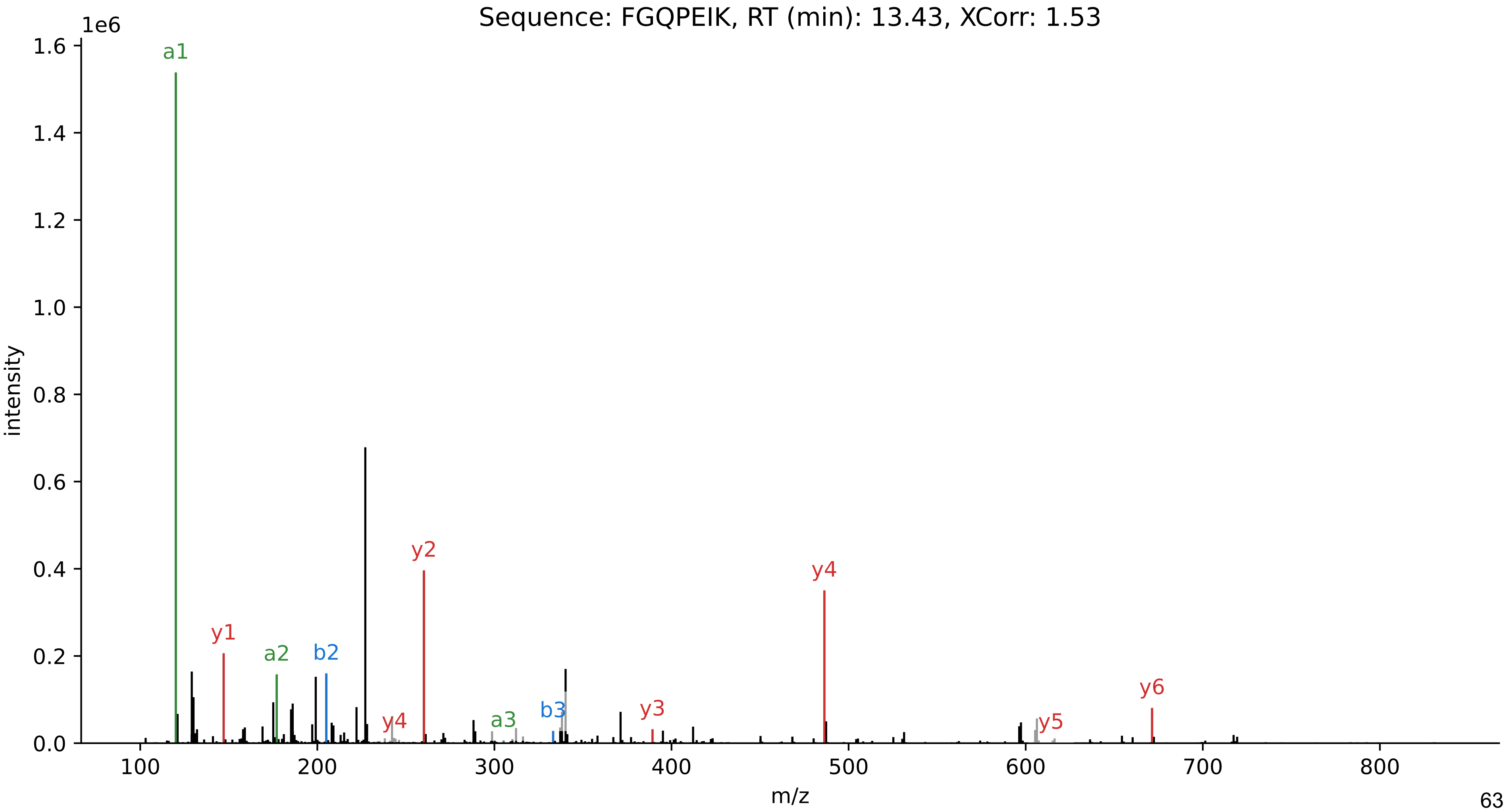

Sequence: FGQPEIK, RT (min): 13.43, XCorr: 1.53
1e6
1.6
1.4
1.2
1.0
0.8
0.6
0.4
0.2
0.0
intensity
a1
y1
a2
b2
y4
y2
a3
b3
y3
y4
y5
y6
100
200
300
400
500
600
700
800
m/z

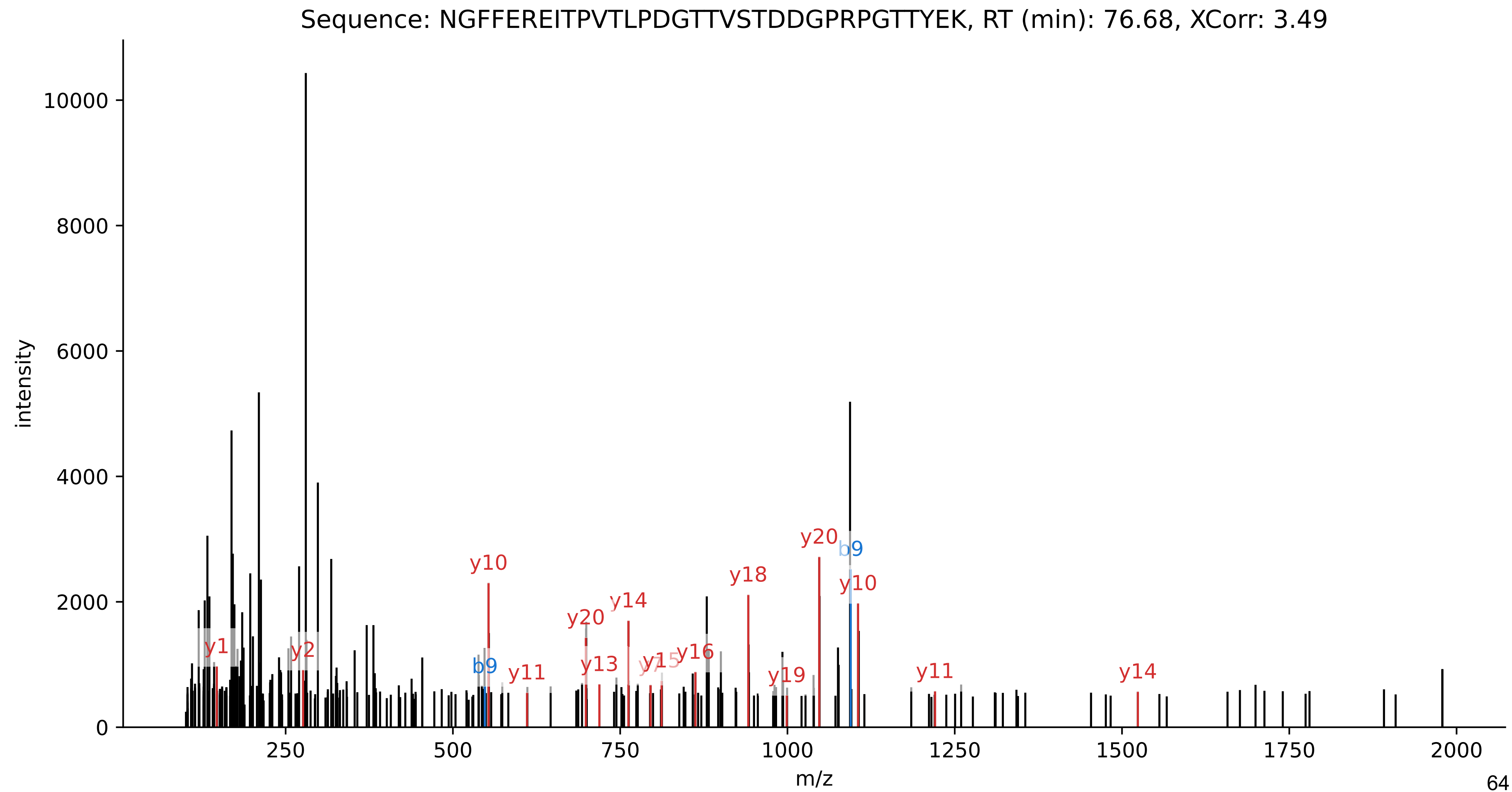

Sequence: NGFFEREITPVTLPDGTTVSTDDGPRPGTTYEK, RT (min): 76.68, XCorr: 3.49
intensity
m/z
0
2000
4000
6000
8000
10000
250
500
750
1000
1250
1500
1750
2000
y1
y2
b9
y10
y11
y20
y13
y14
y16
y18
y19
y20
b9
y10
y11
y14

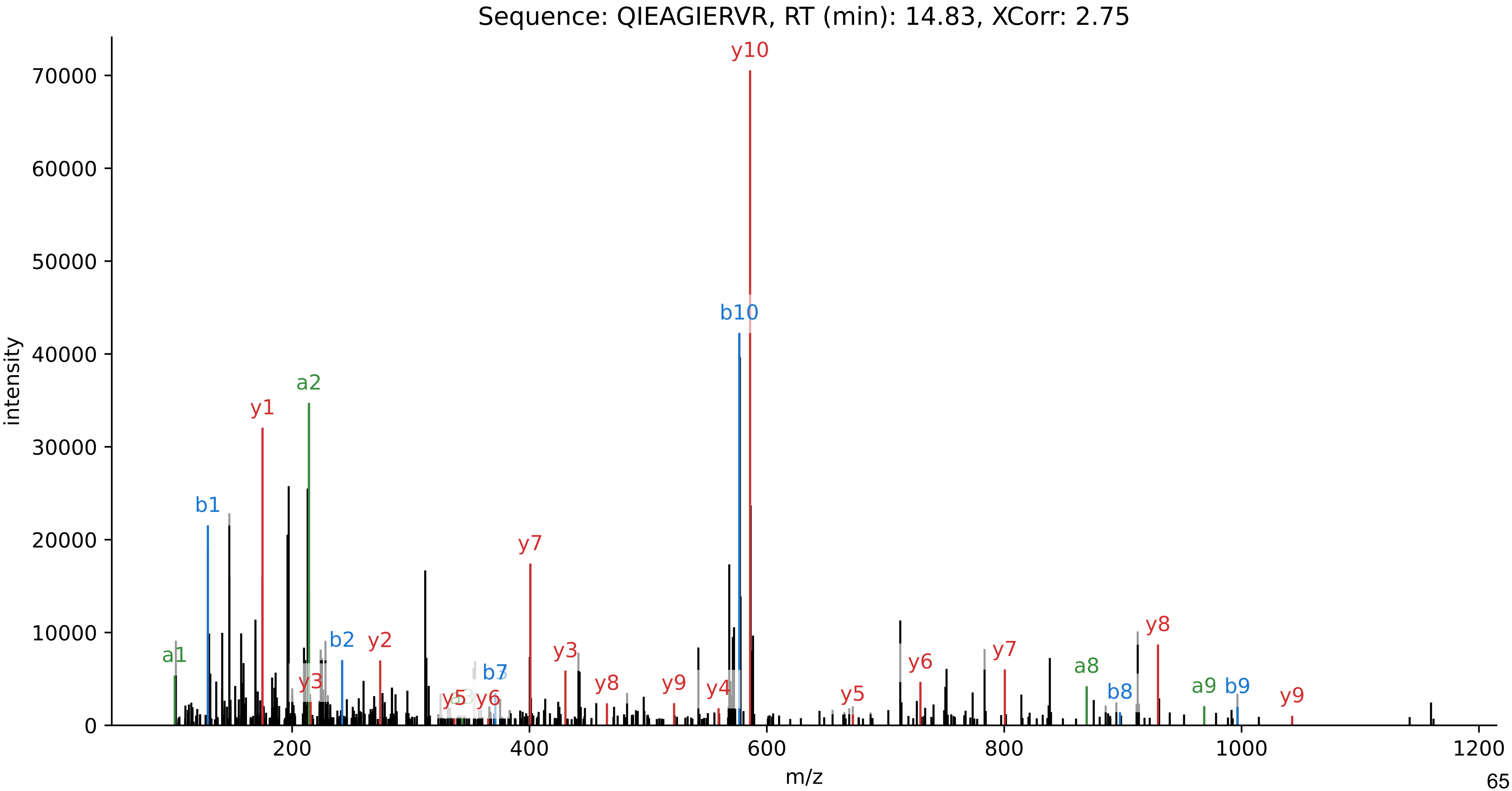
Sequence: QIEAGIERVR, RT (min): 14.83, XCorr: 2.75
intensity
m/z
y10
b10
a2
y1
b1
y7
a1
b2
y2
y3
y8
y9
y4
y5
y6
y7
a8
y8
b8
a9
b9
y9
0
10000
20000
30000
40000
50000
60000
70000
200
400
600
800
1000
1200

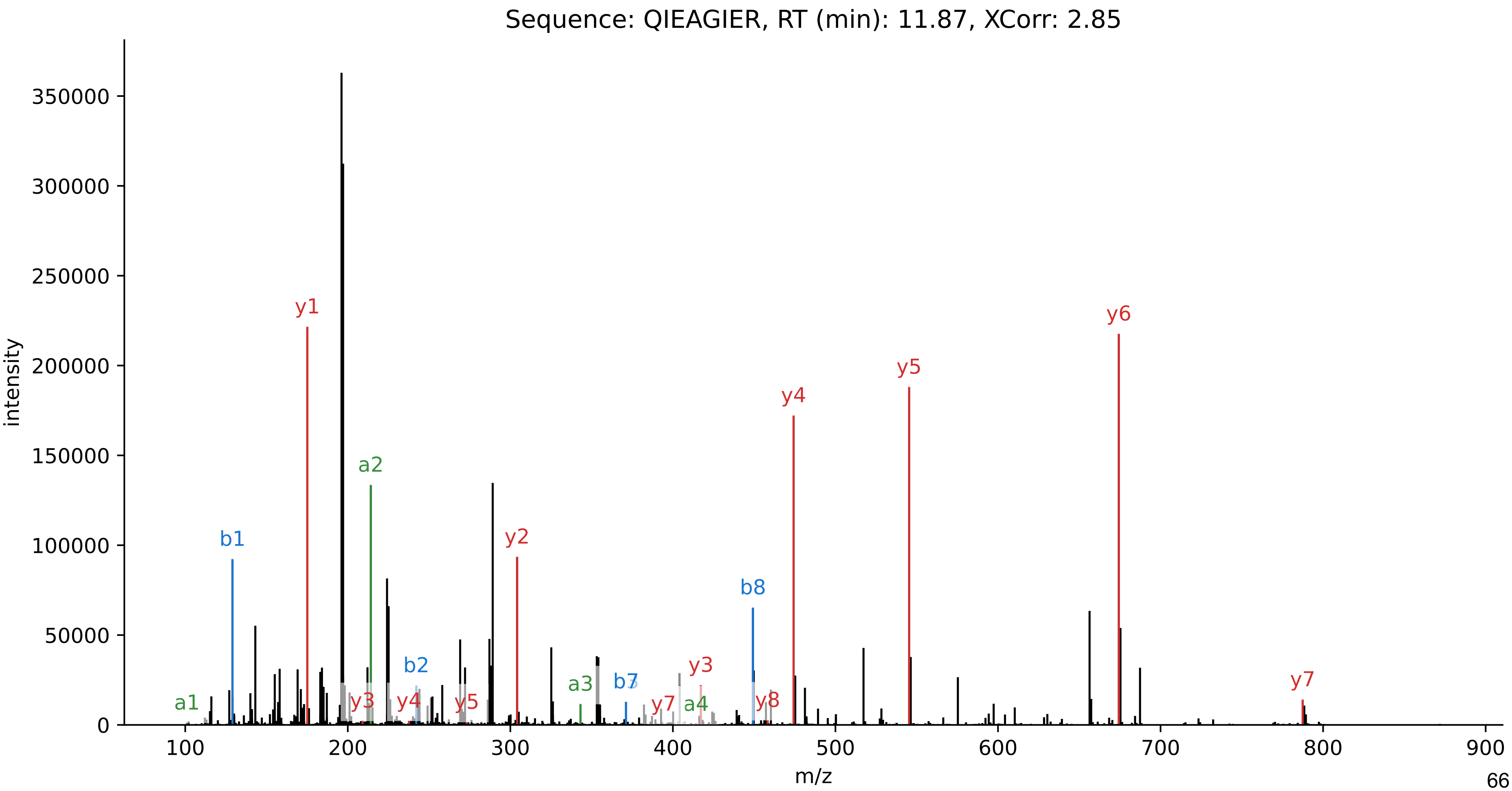

Sequence: QIEAGIER, RT (min): 11.87, XCorr: 2.85
intensity
m/z
350000
300000
250000
200000
150000
100000
50000
0
100
200
300
400
500
600
700
800
900
y1
y2
y3
y4
y5
y6
y7
y8
b1
b2
b7
b8
a1
a2
a3
a4

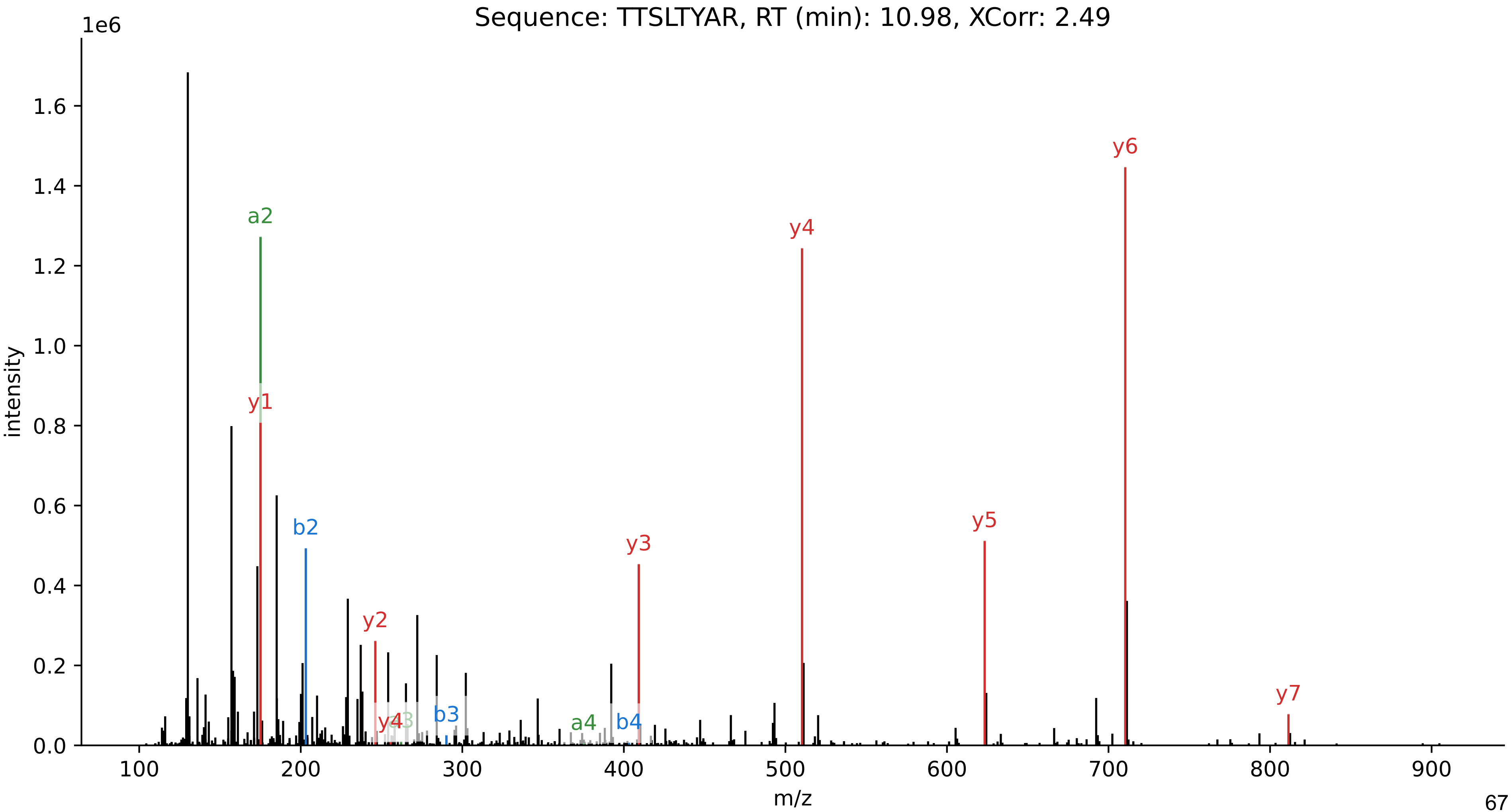
Sequence: TTSLTYAR, RT (min): 10.98, XCorr: 2.49
1e6
intensity
m/z
a2
y1
b2
y2
y4
b3
a4
b4
y3
y4
y5
y6
y7
0.0
0.2
0.4
0.6
0.8
1.0
1.2
1.4
1.6
100
200
300
400
500
600
700
800
900

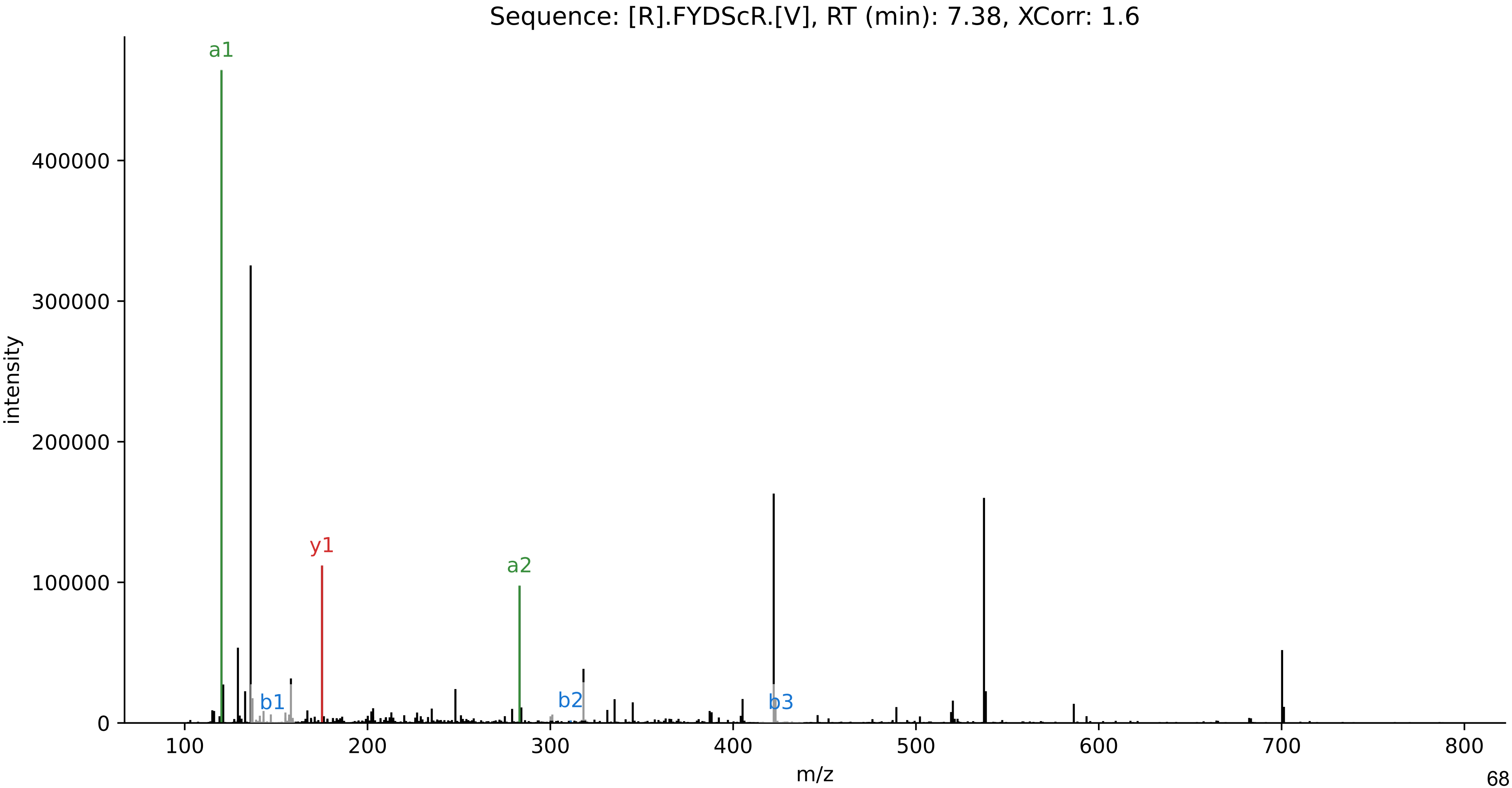
Sequence: [R].FYDScR.[V], RT (min): 7.38, XCorr: 1.6
a1
y1
a2
b1
b2
b3
intensity
m/z
0
100000
200000
300000
400000
100
200
300
400
500
600
700
800

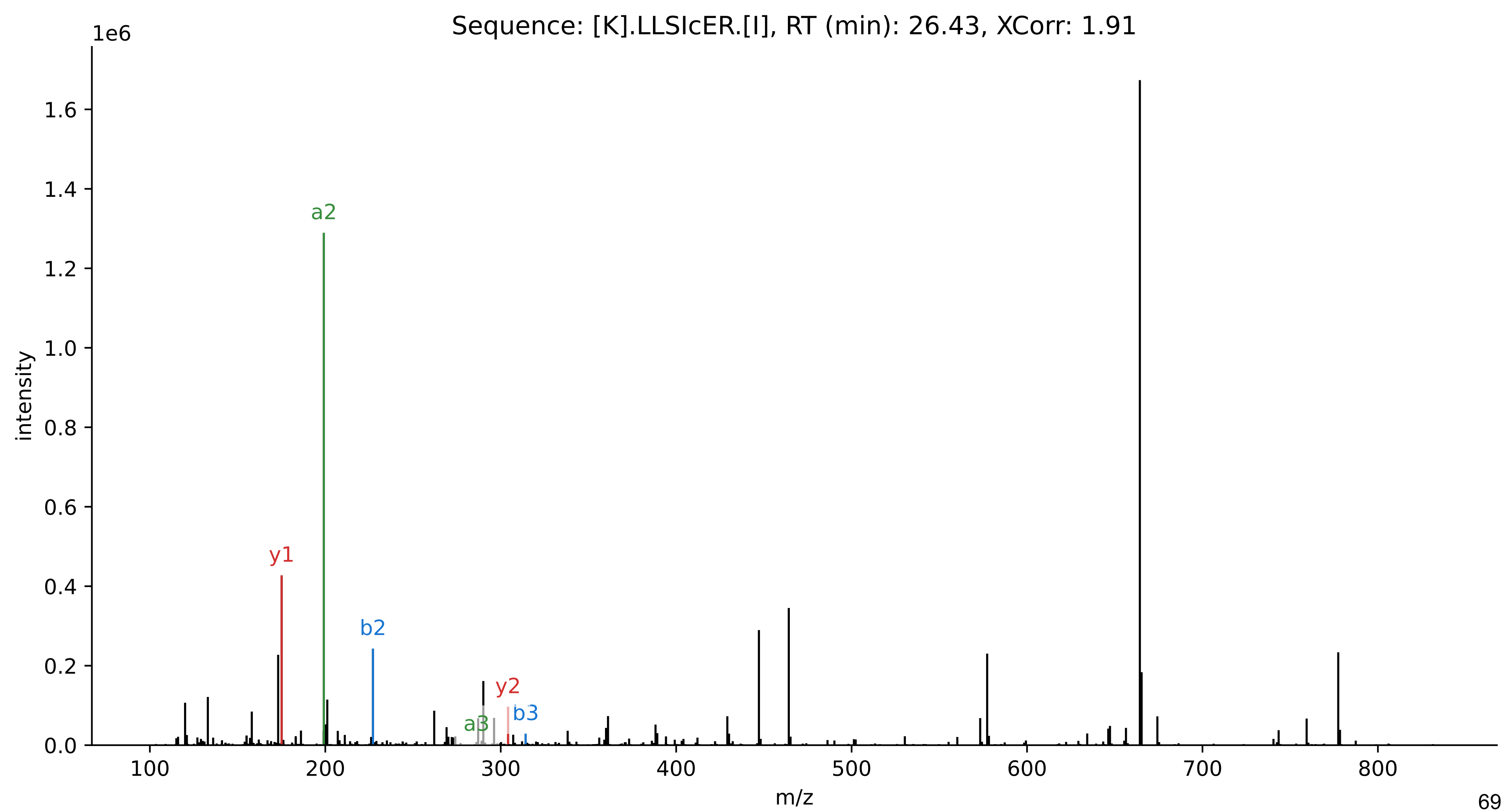
Sequence: [K].LLSIcER.[I], RT (min): 26.43, XCorr: 1.91
1e6
intensity
m/z
a2
y1
b2
y2
a3
b3
0.0
0.2
0.4
0.6
0.8
1.0
1.2
1.4
1.6
100
200
300
400
500
600
700
800

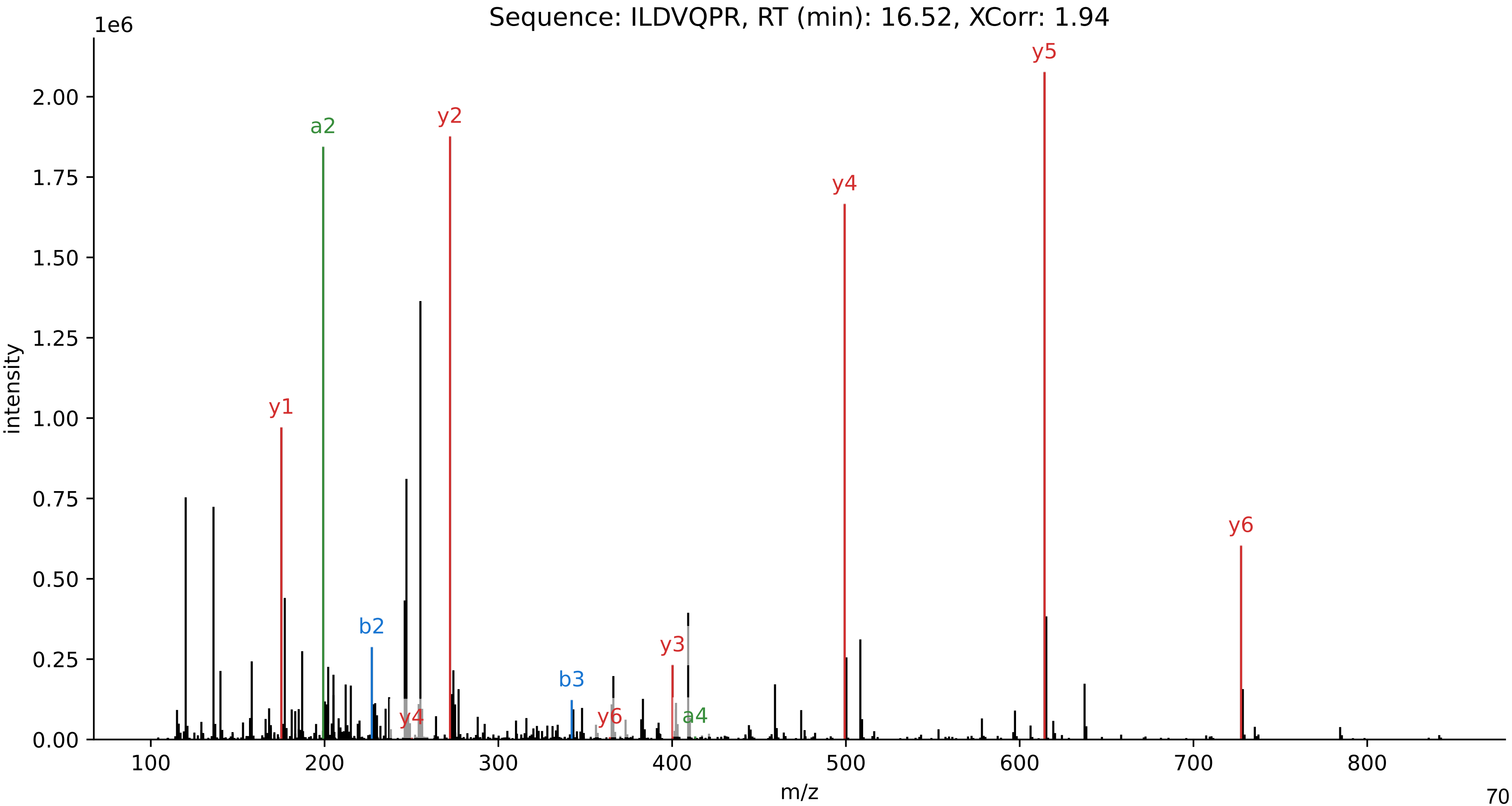

Sequence: ILDVQPR, RT (min): 16.52, XCorr: 1.94
1e6
intensity
m/z
0.00
0.25
0.50
0.75
1.00
1.25
1.50
1.75
2.00
100
200
300
400
500
600
700
800
y1
a2
b2
y4
y2
b3
y6
y3
a4
y4
y5
y6

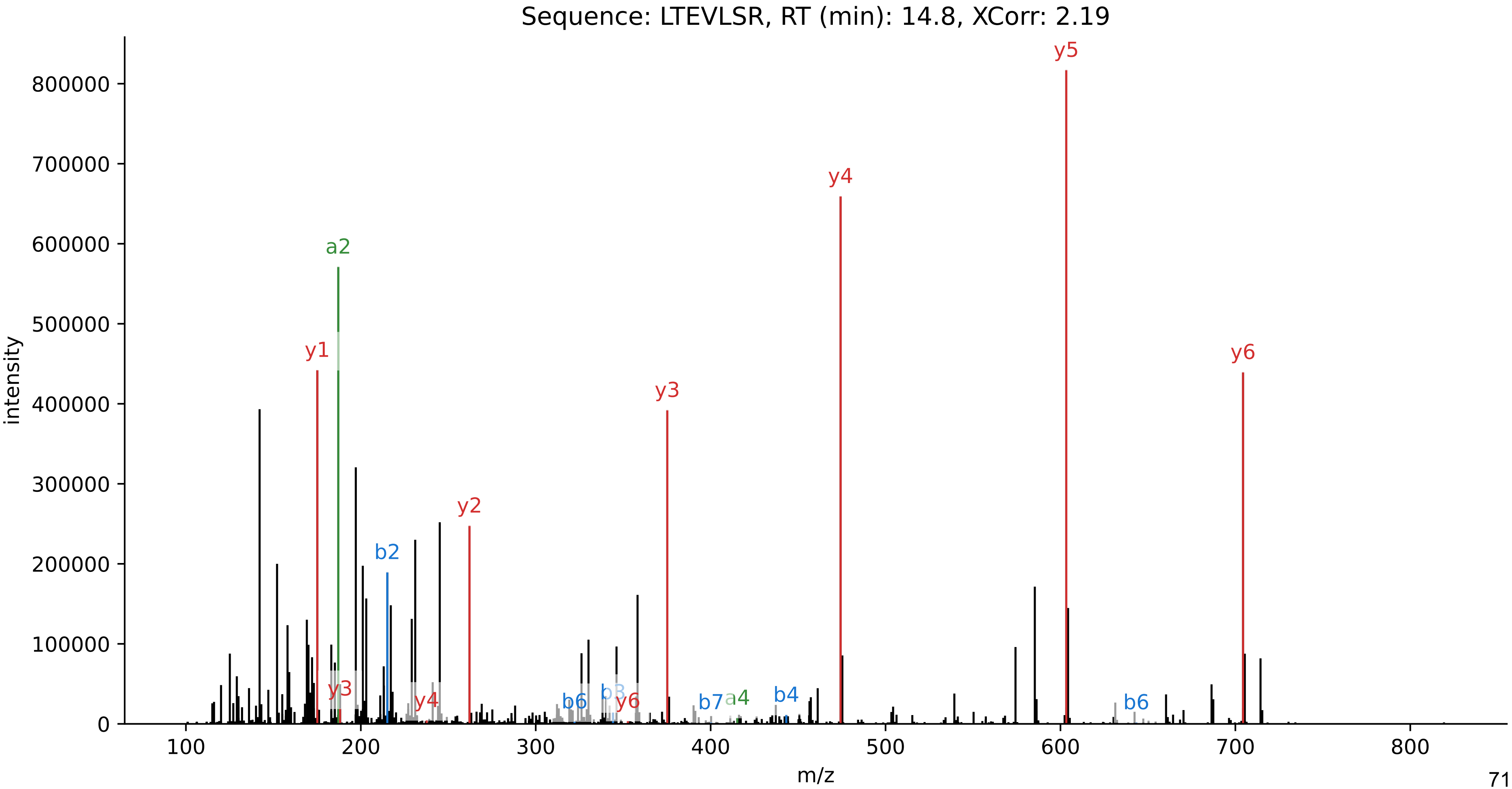

Sequence: LTEVLSR, RT (min): 14.8, XCorr: 2.19
intensity
m/z
y5
y4
a2
y1
y6
y3
y2
b2
y3
y4
b6
b3
y6
b7
a4
b4
b6
800000
700000
600000
500000
400000
300000
200000
100000
0
100
200
300
400
500
600
700
800

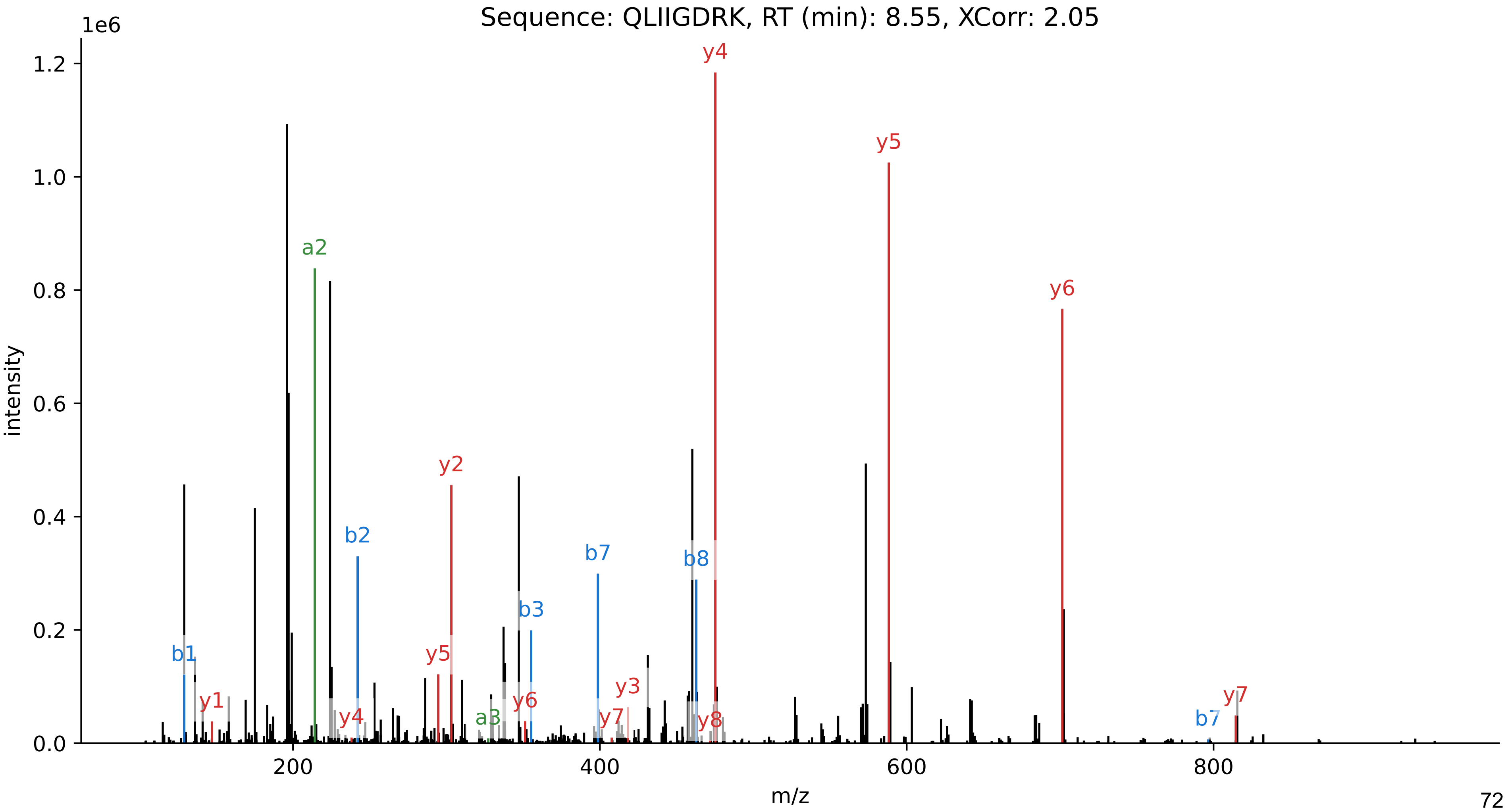

Sequence: QLIIGDRK, RT (min): 8.55, XCorr: 2.05
1e6
intensity
m/z
y4
y5
y6
a2
y2
b2
b7
b8
b3
b1
y5
y1
y4
a3
y6
y7
y3
y8
y7
b7

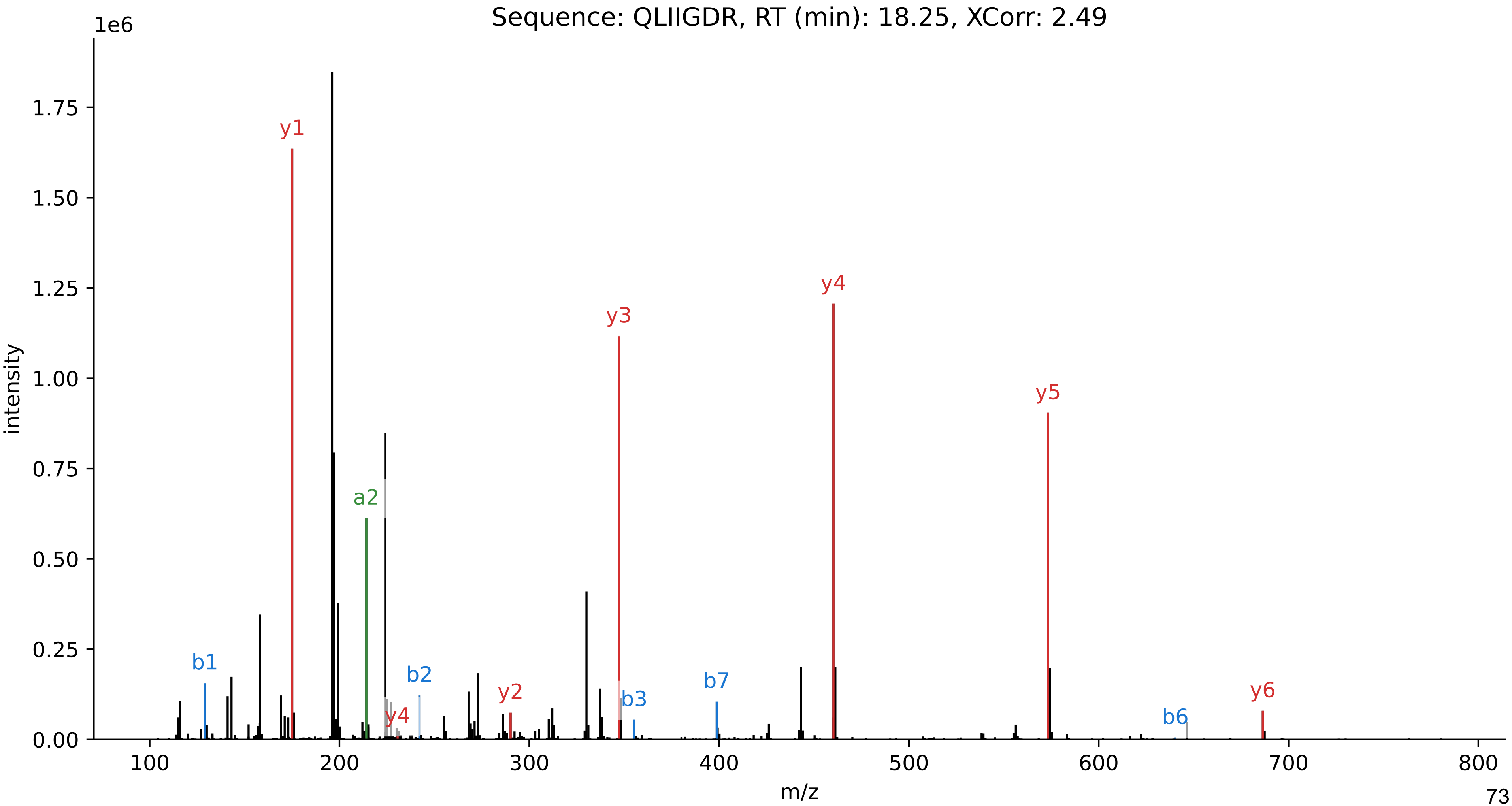
Sequence: QLIIGDR, RT (min): 18.25, XCorr: 2.49
1e6
intensity
m/z
y1
y3
y4
y5
a2
b1
b2
y2
y4
b3
b7
b6
y6
0.00
0.25
0.50
0.75
1.00
1.25
1.50
1.75
100
200
300
400
500
600
700
800

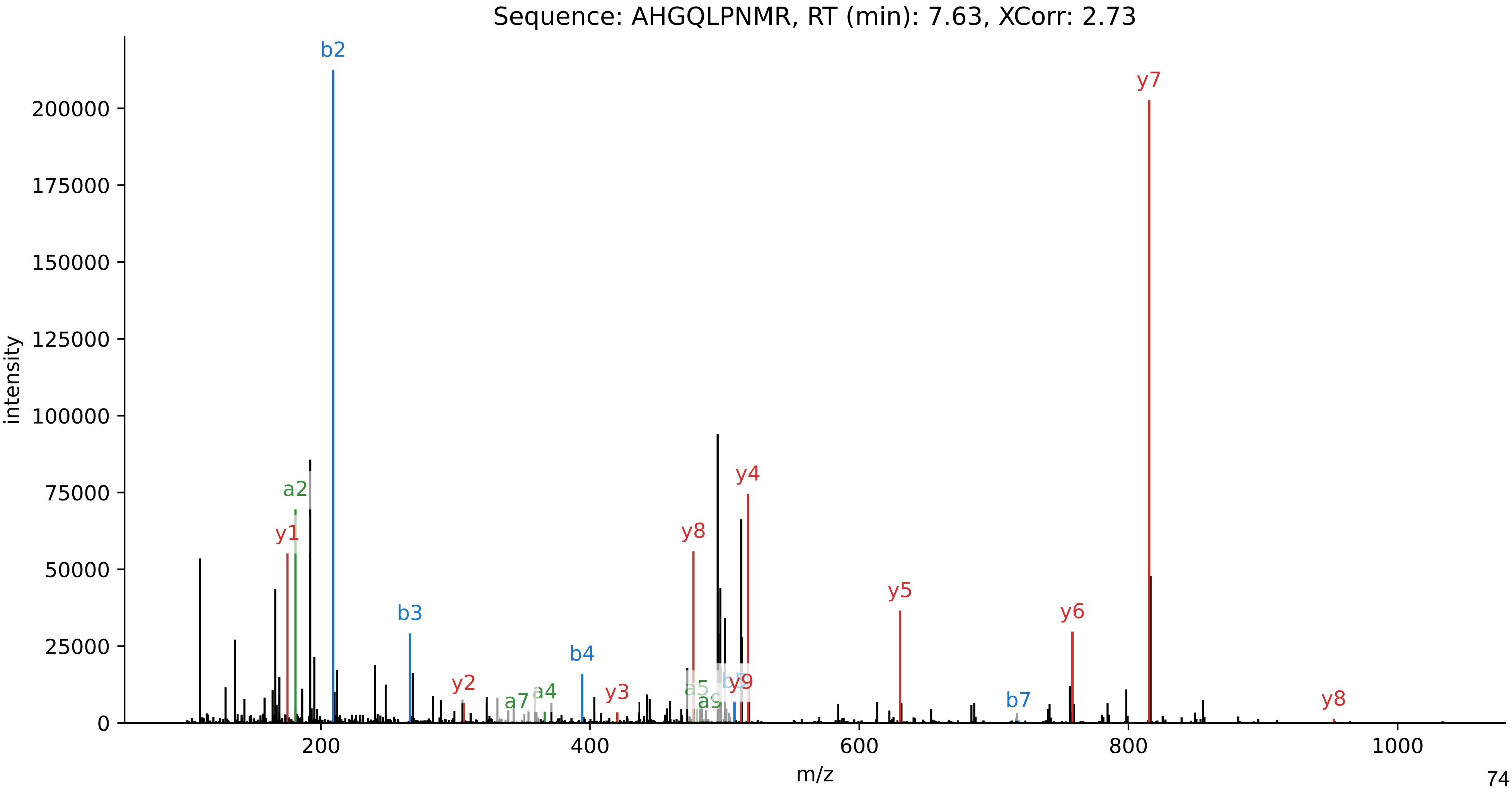

Sequence: AHGQLPNMR, RT (min): 7.63, XCorr: 2.73
intensity
m/z
b2
y7
y4
a2
y1
y8
y5
y6
b3
b4
y2
y3
a7
a4
a5
a9
y9
b7
y8
0
25000
50000
75000
100000
125000
150000
175000
200000
200
400
600
800
1000

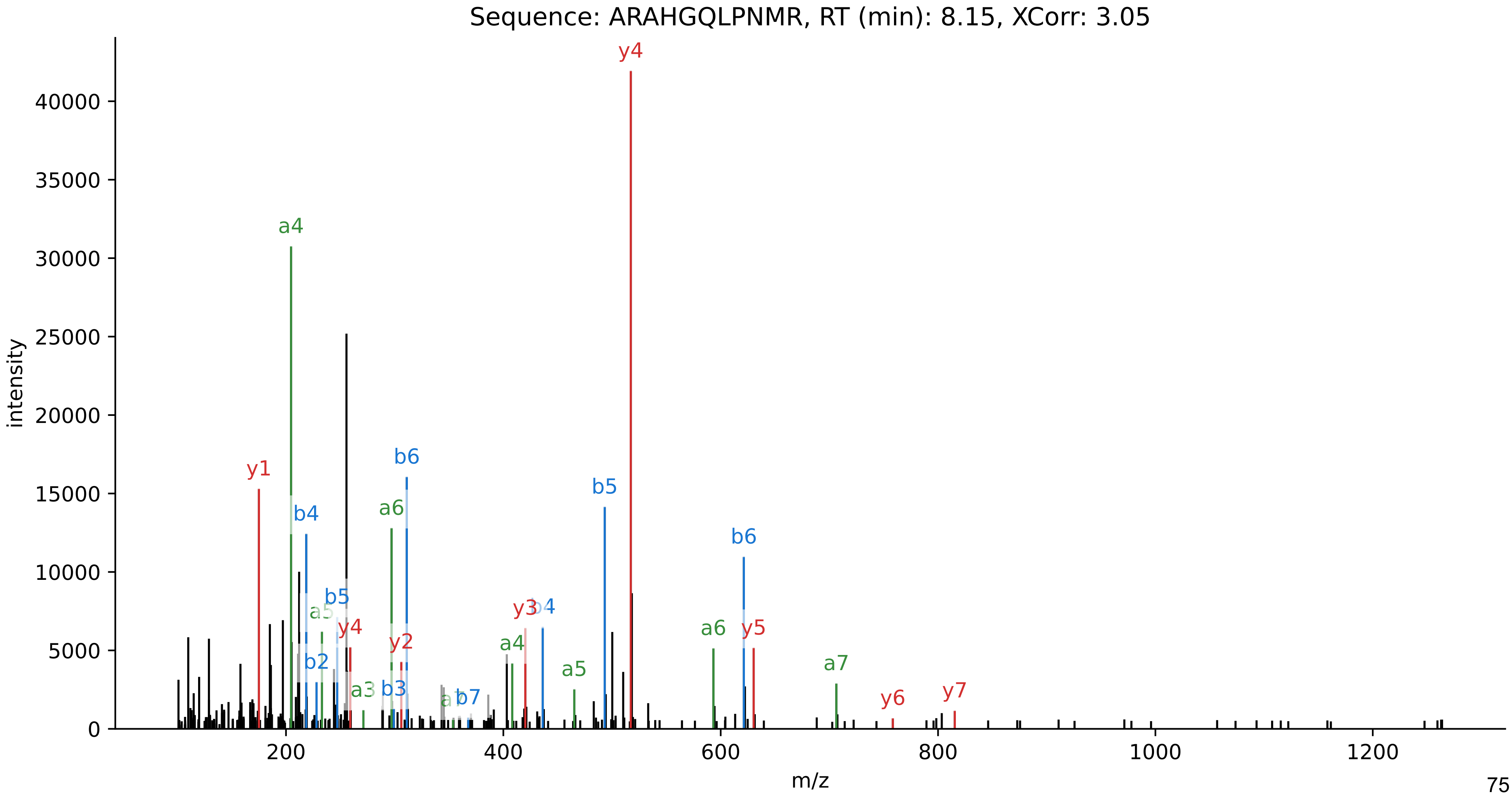
Sequence: ARAHGQLPNMR, RT (min): 8.15, XCorr: 3.05
intensity
m/z
y4
a4
y1
b4
b6
a6
b5
b6
b5
a5
y4
y3
b4
a6
y5
y2
b2
a4
a5
a7
a3
b3
b7
y6
y7
0
5000
10000
15000
20000
25000
30000
35000
40000
200
400
600
800
1000
1200

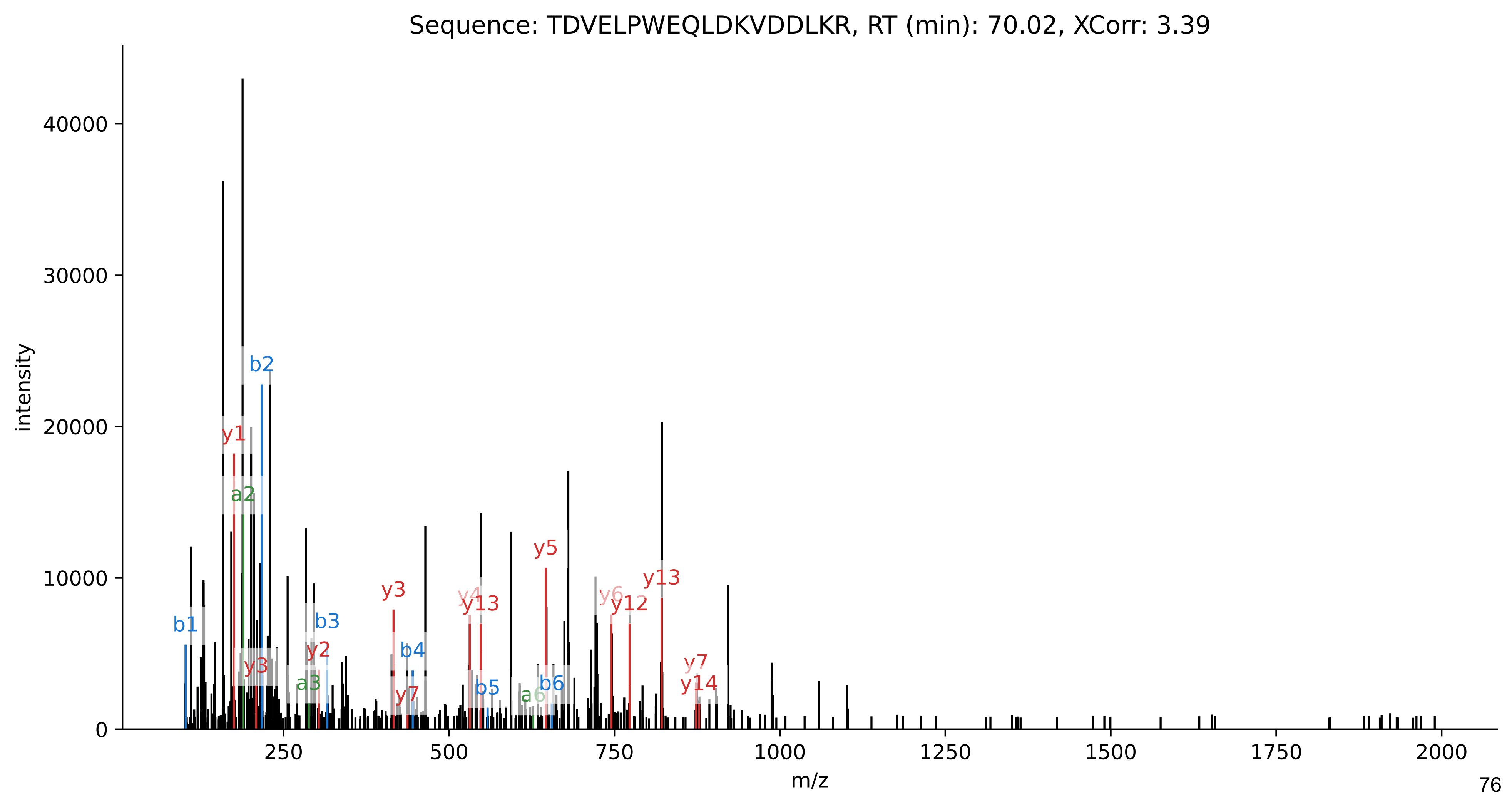
Sequence: TDVELPWEQLDKVDDLKR, RT (min): 70.02, XCorr: 3.39
intensity
m/z
0
10000
20000
30000
40000
250
500
750
1000
1250
1500
1750
2000
b1
b2
b3
b4
b5
b6
y1
y2
y3
y3
y4
y5
y6
y7
y7
y12
y13
y13
y14
a2
a3
a6

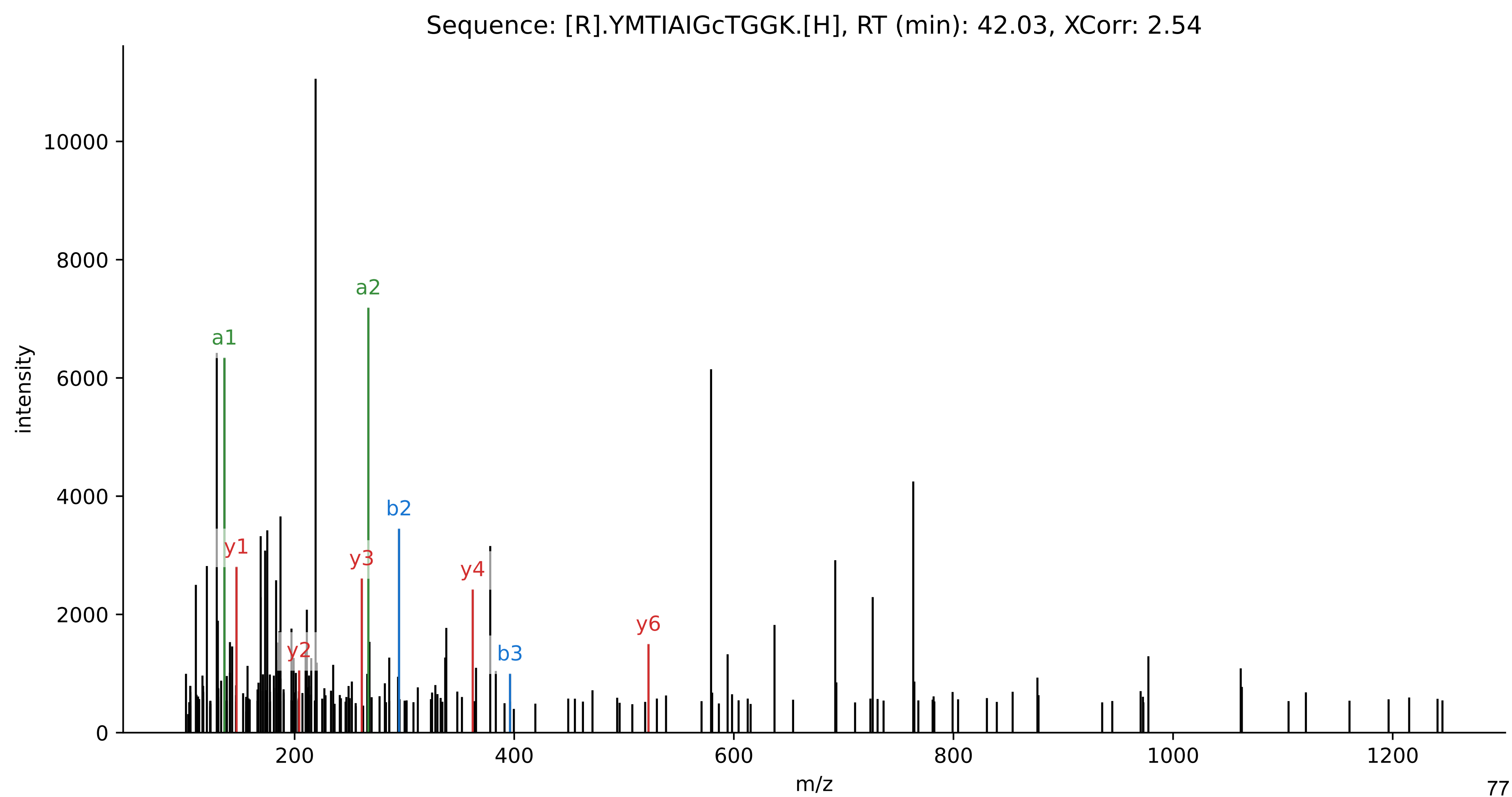
Sequence: [R].YMTIAIGcTGGK.[H], RT (min): 42.03, XCorr: 2.54
intensity
m/z
0
2000
4000
6000
8000
10000
200
400
600
800
1000
1200
a1
a2
b2
b3
y1
y2
y3
y4
y6

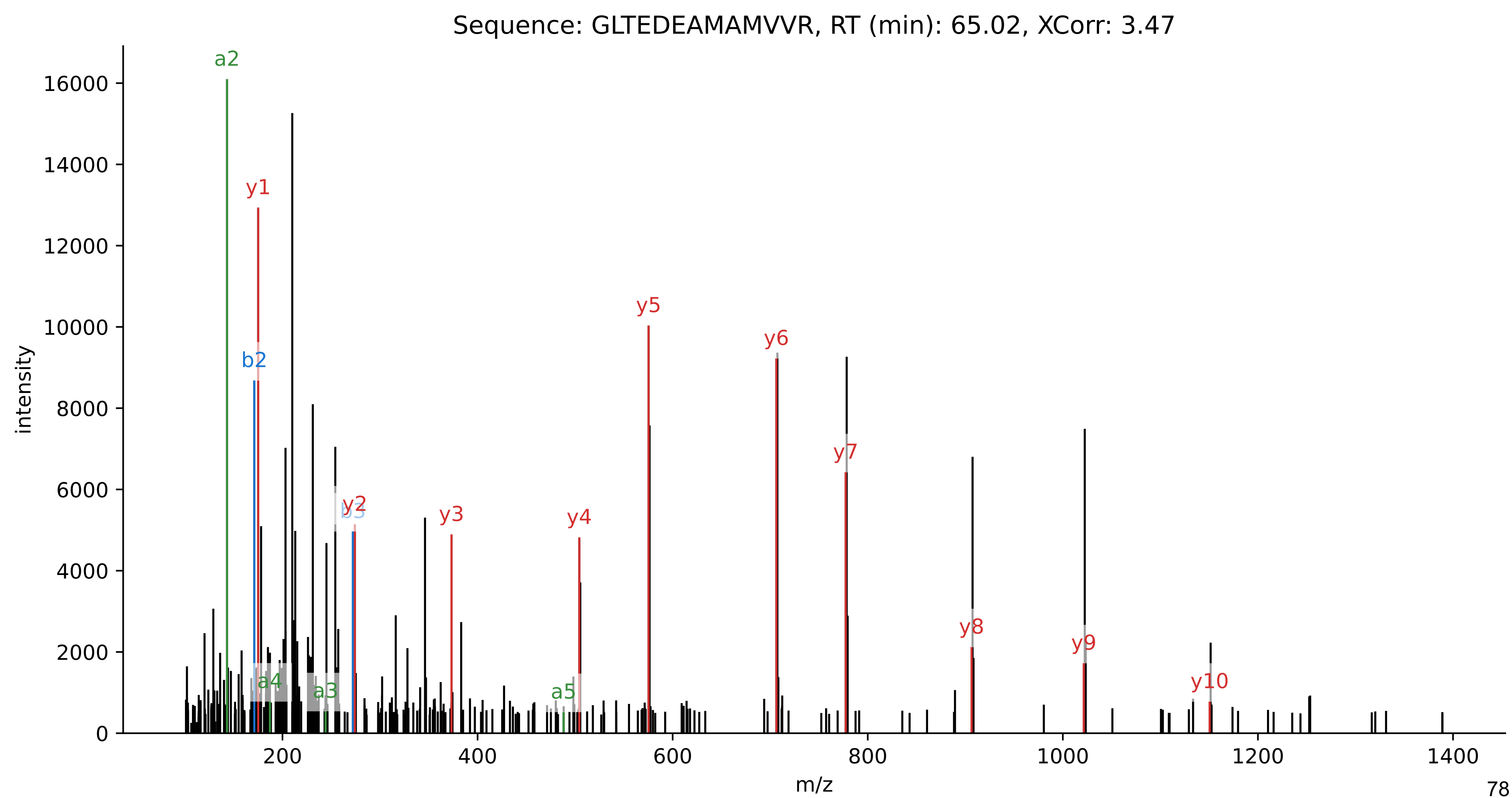
Sequence: GLTEDEAMAMVVR, RT (min): 65.02, XCorr: 3.47
intensity
m/z
a2
y1
b2
y2
b3
y3
y4
y5
y6
y7
y8
y9
y10
a3
a4
a5
0
2000
4000
6000
8000
10000
12000
14000
16000
200
400
600
800
1000
1200
1400

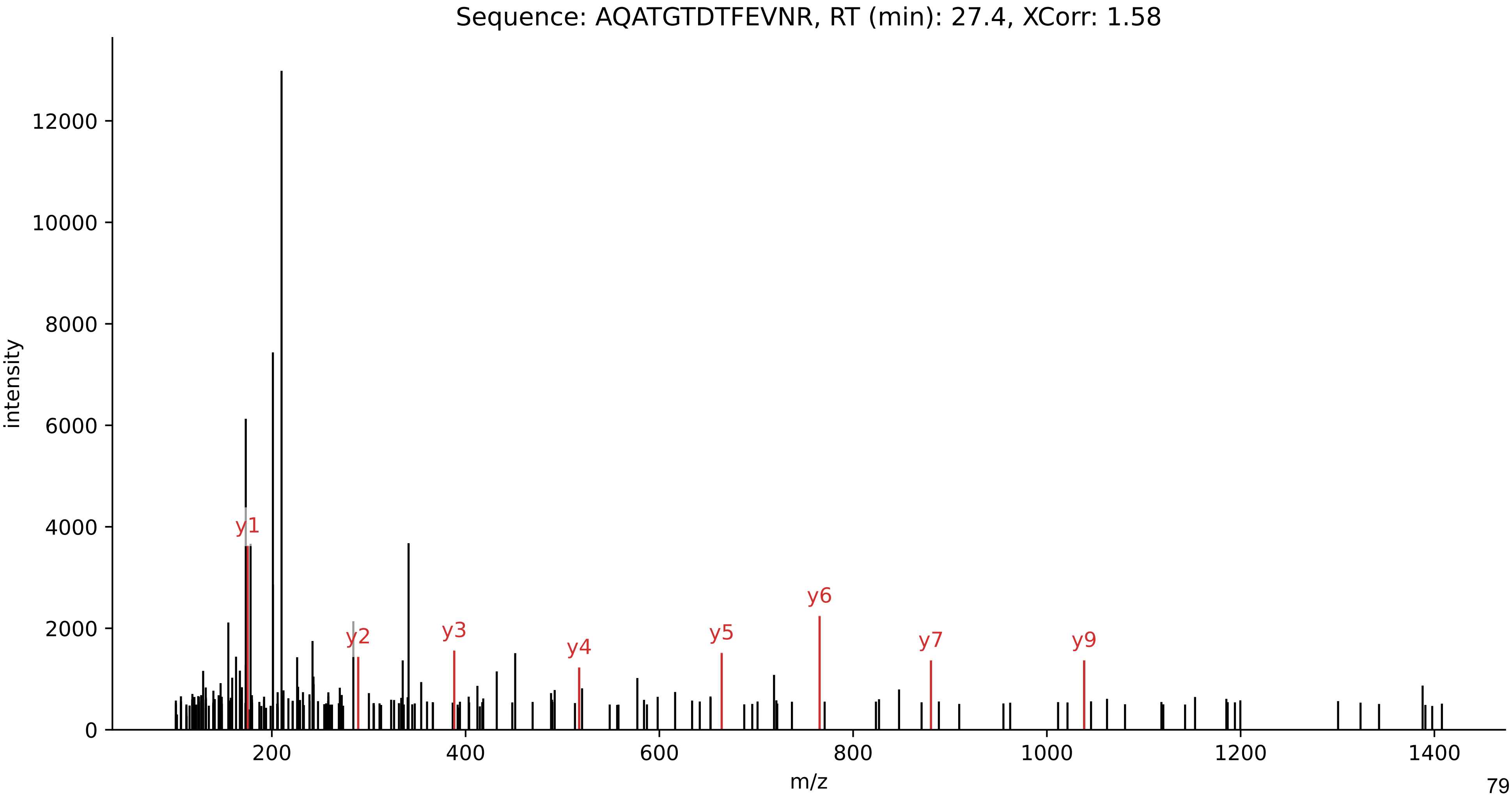
Sequence: AQATGTDTFEVNR, RT (min): 27.4, XCorr: 1.58
intensity
m/z
y1
y2
y3
y4
y5
y6
y7
y9
0
2000
4000
6000
8000
10000
12000
200
400
600
800
1000
1200
1400

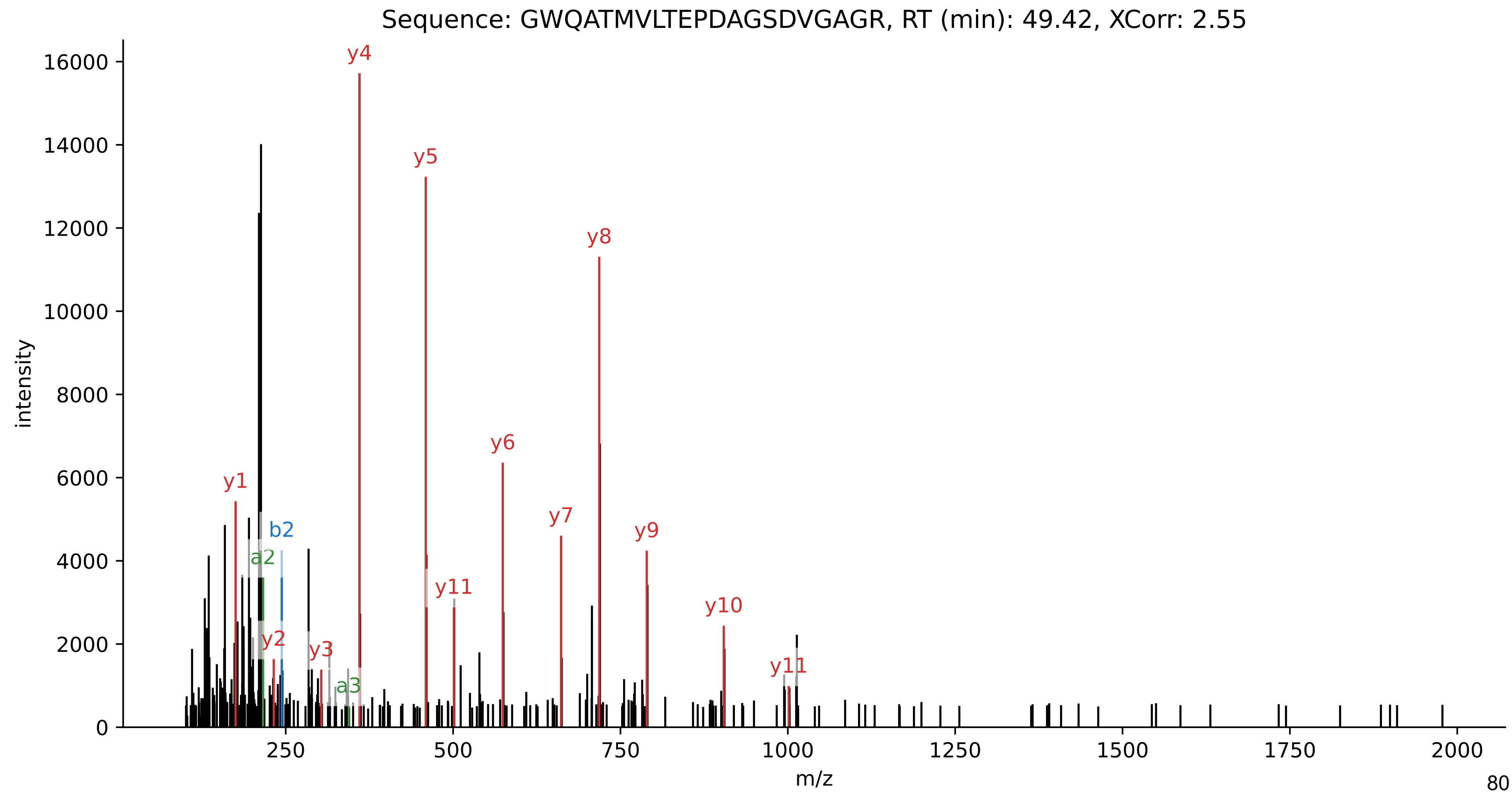
Sequence: GWQATMVLTEPDAGSDVGAGR, RT (min): 49.42, XCorr: 2.55
intensity
m/z
0
2000
4000
6000
8000
10000
12000
14000
16000
250
500
750
1000
1250
1500
1750
2000
y1
y2
y3
y4
y5
y6
y7
y8
y9
y10
y11
y11
a2
a3
b2

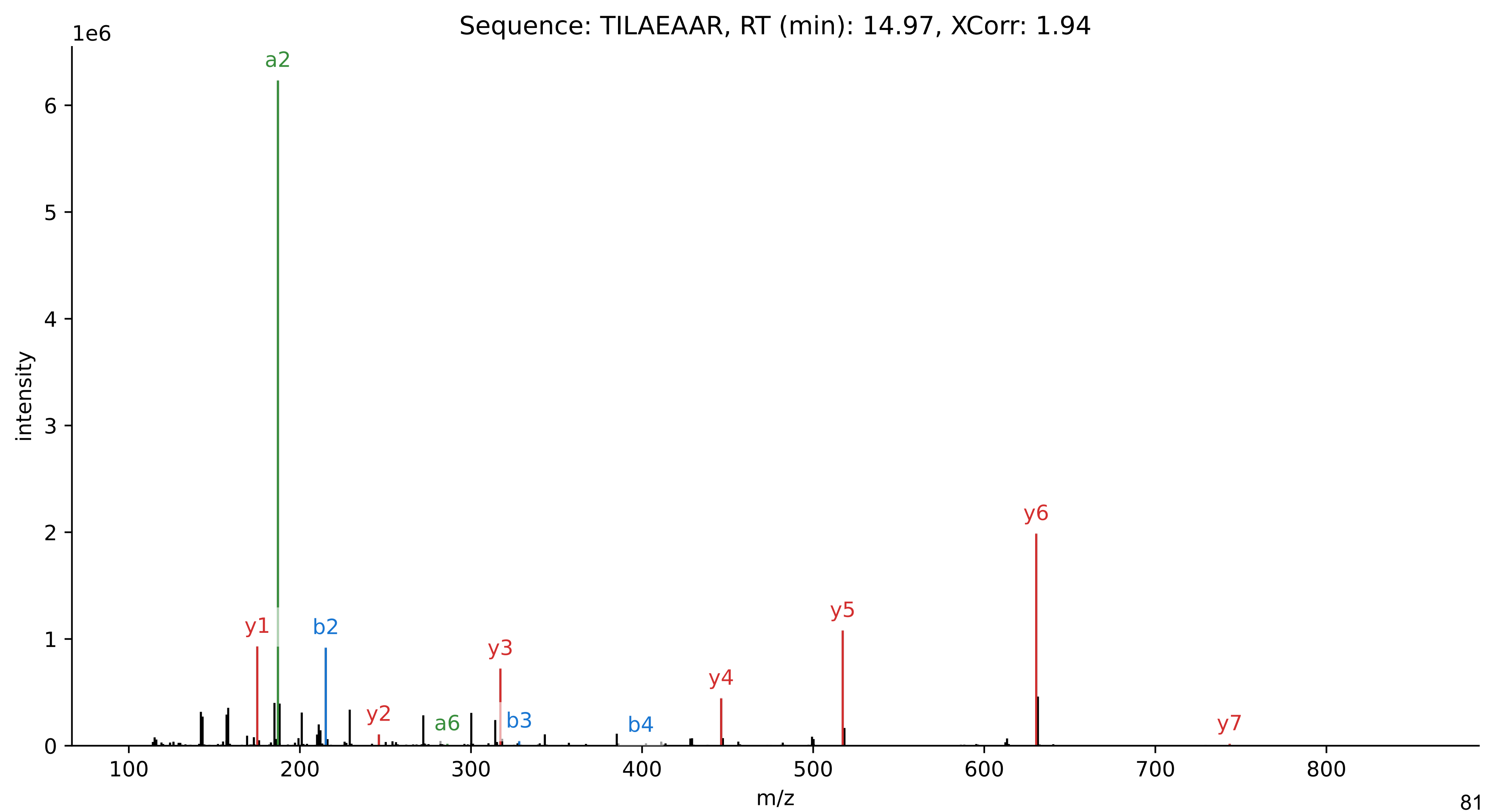

Sequence: TILAEAAR, RT (min): 14.97, XCorr: 1.94
1e6
intensity
m/z
a2
y1
b2
y2
y3
a6
b3
b4
y4
y5
y6
y7
0
1
2
3
4
5
6
100
200
300
400
500
600
700
800

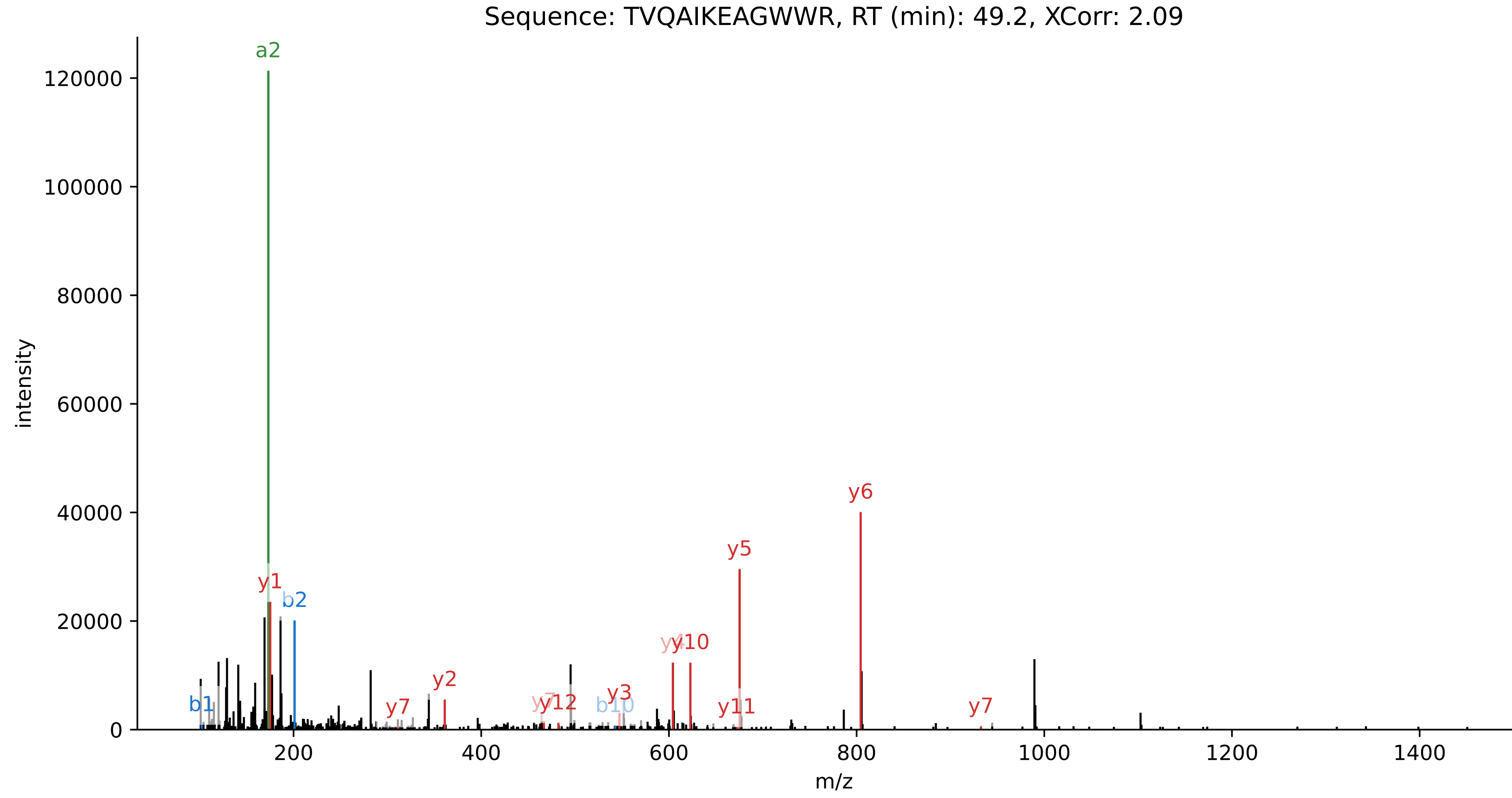
Sequence: TVQAIKEAGWWR, RT (min): 49.2, XCorr: 2.09
intensity
m/z
a2
y1
b2
b1
y7
y2
y12
y3
b10
y10
y5
y6
y11
y7
0
20000
40000
60000
80000
100000
120000
200
400
600
800
1000
1200
1400

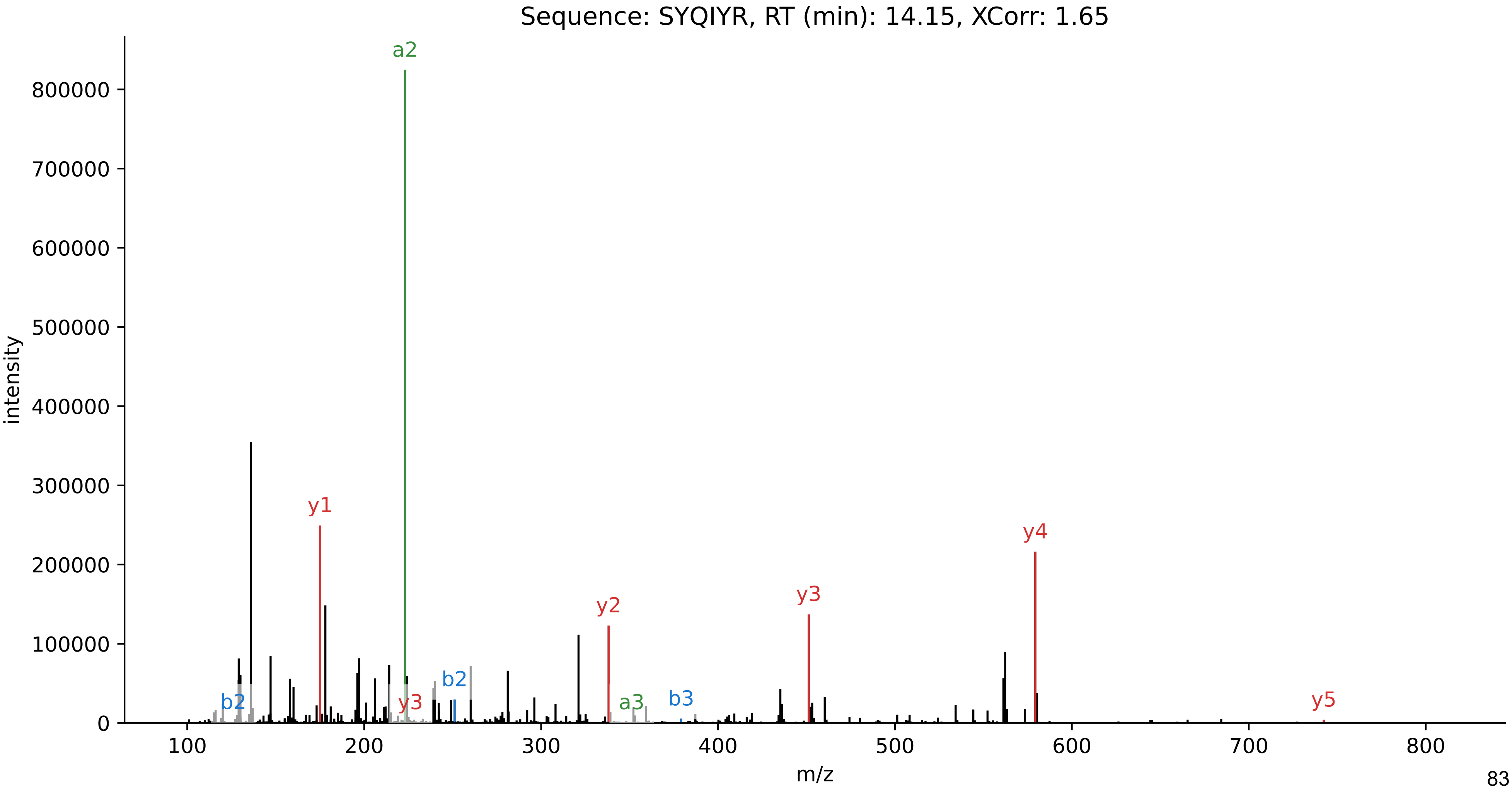
Sequence: SYQIYR, RT (min): 14.15, XCorr: 1.65
a2
y1
y4
y2
y3
b2
a3
b3
y3
b2
y5
intensity
m/z

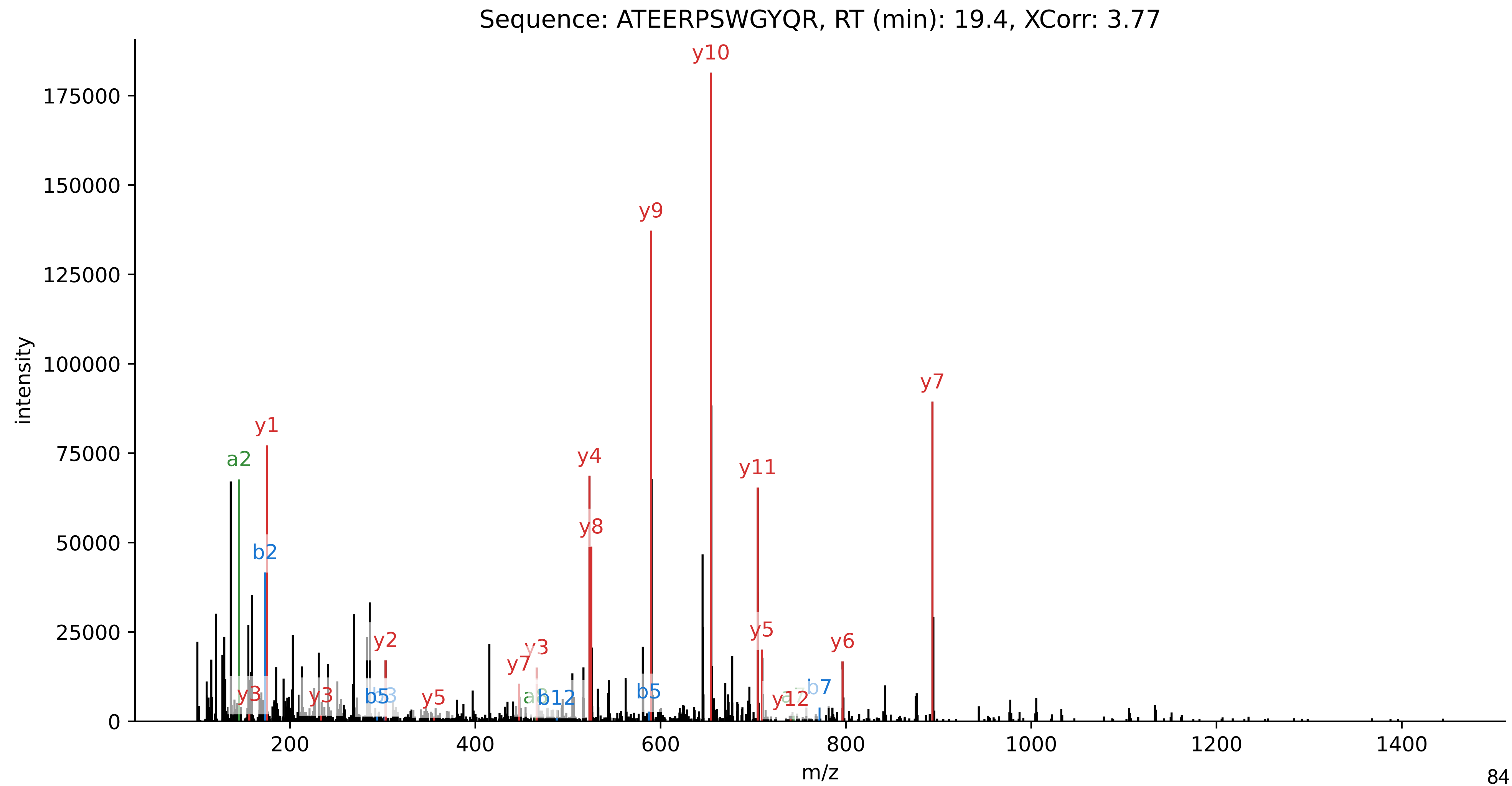

Sequence: ATEERPSWGYQR, RT (min): 19.4, XCorr: 3.77
intensity
m/z
y10
y9
y7
y1
a2
y4
y11
y8
b2
y5
y6
y2
y3
y7
b5
b7
y12
b12
y3
y3
b5
y5
0
25000
50000
75000
100000
125000
150000
175000
200
400
600
800
1000
1200
1400

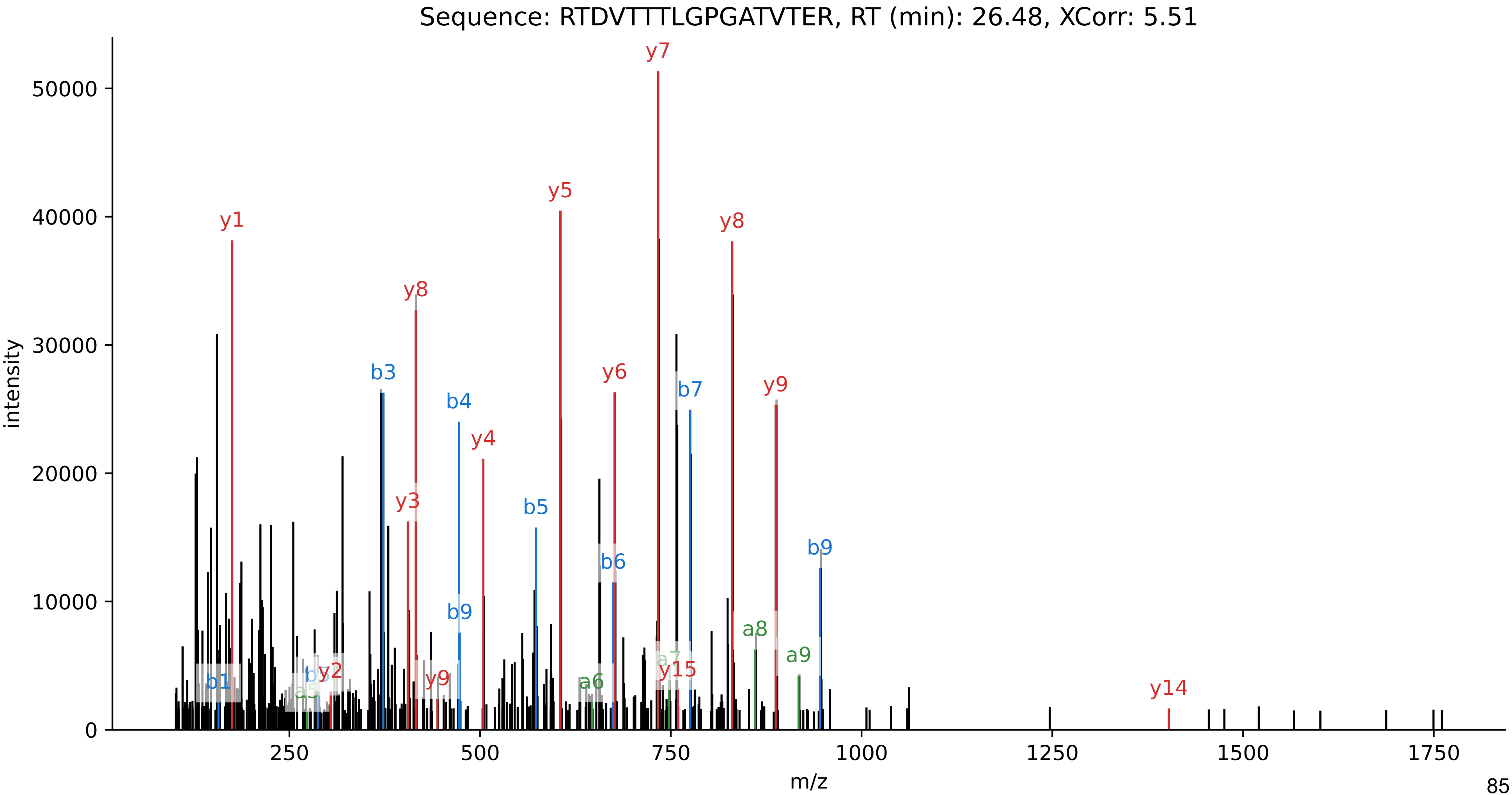

Sequence: RTDVTTTLGPGATVTER, RT (min): 26.48, XCorr: 5.51
intensity
m/z
y7
y5
y1
y8
y8
y9
b3
y6
b7
b4
y4
y3
b5
b9
b6
b9
a8
a9
a7
y15
b1
y2
y9
a6
y14
a5

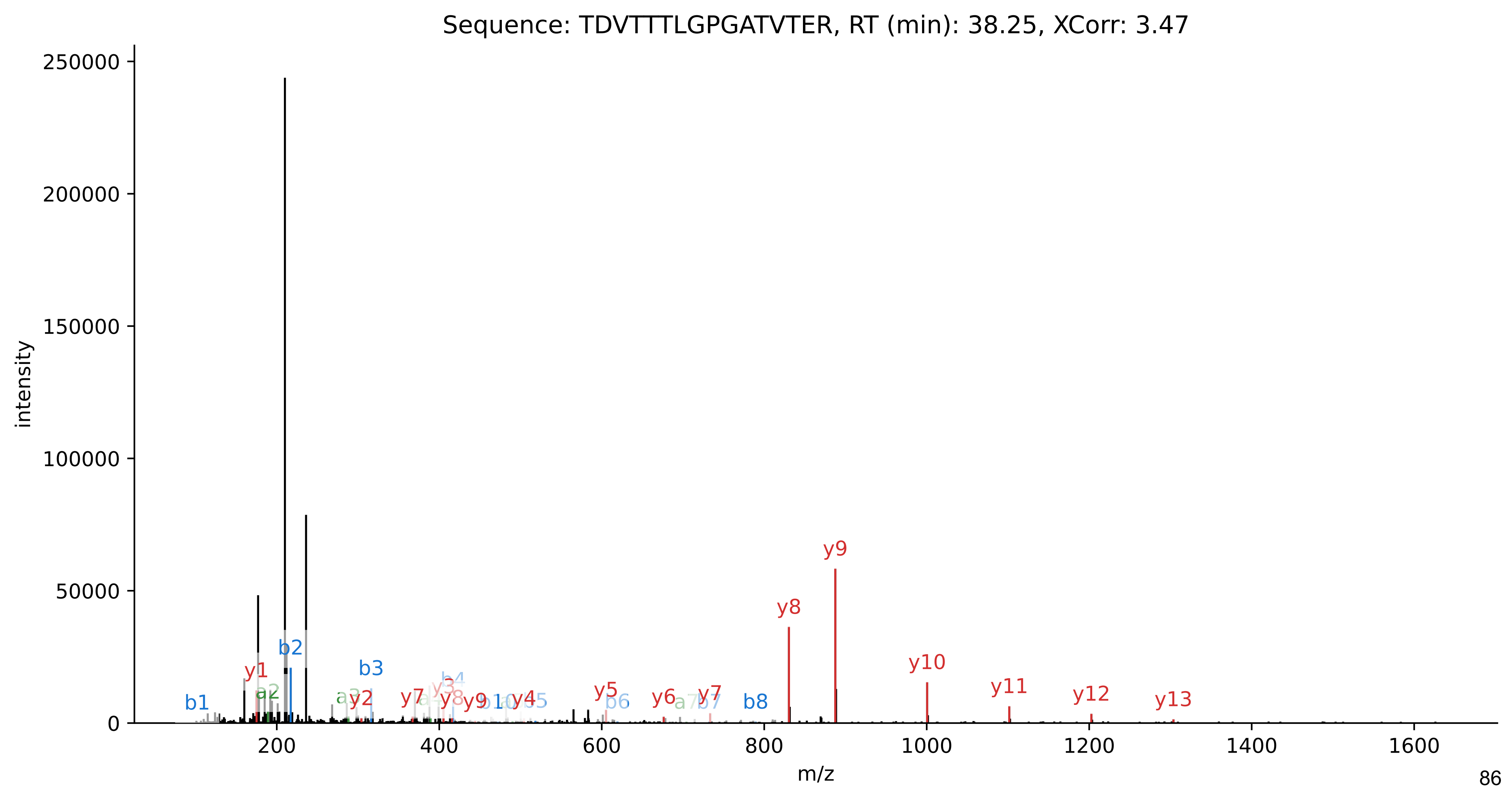

Sequence: TDVTTTLGPGATVTER, RT (min): 38.25, XCorr: 3.47
intensity
m/z
y9
y8
y10
y11
y12
y13
b2
b3
y1
b1
y5
b6
y6
y7
b8

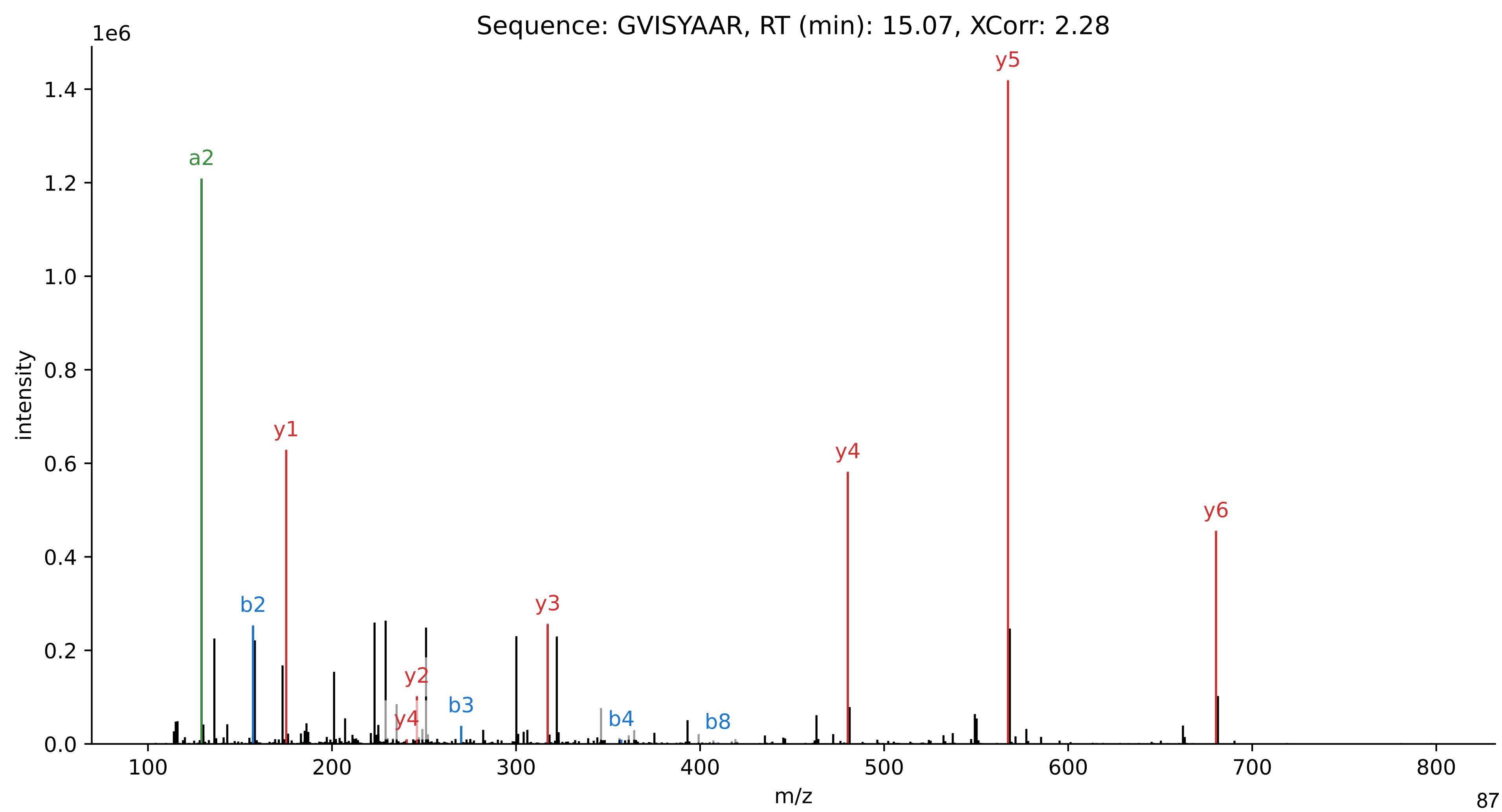
Sequence: GVISYAAR, RT (min): 15.07, XCorr: 2.28
1e6
intensity
m/z
a2
y5
y1
y4
y6
b2
y3
y2
y4
b3
b4
b8
0.0
0.2
0.4
0.6
0.8
1.0
1.2
1.4
100
200
300
400
500
600
700
800

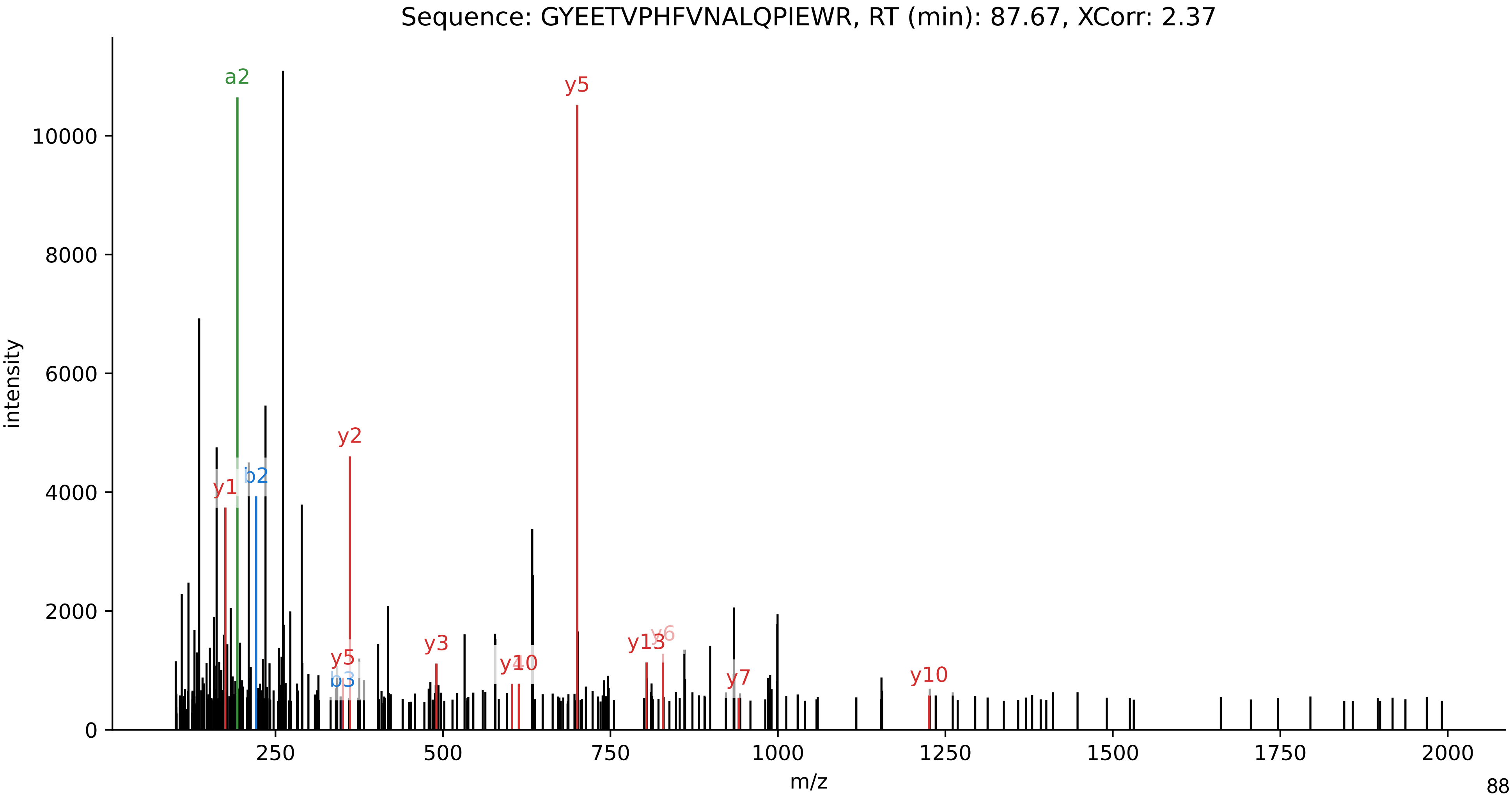

Sequence: GYEETVPHFVNALQPIEWR, RT (min): 87.67, XCorr: 2.37
intensity
m/z
a2
y5
y2
b2
y1
y5
b3
y3
y10
y13
y6
y7
y10

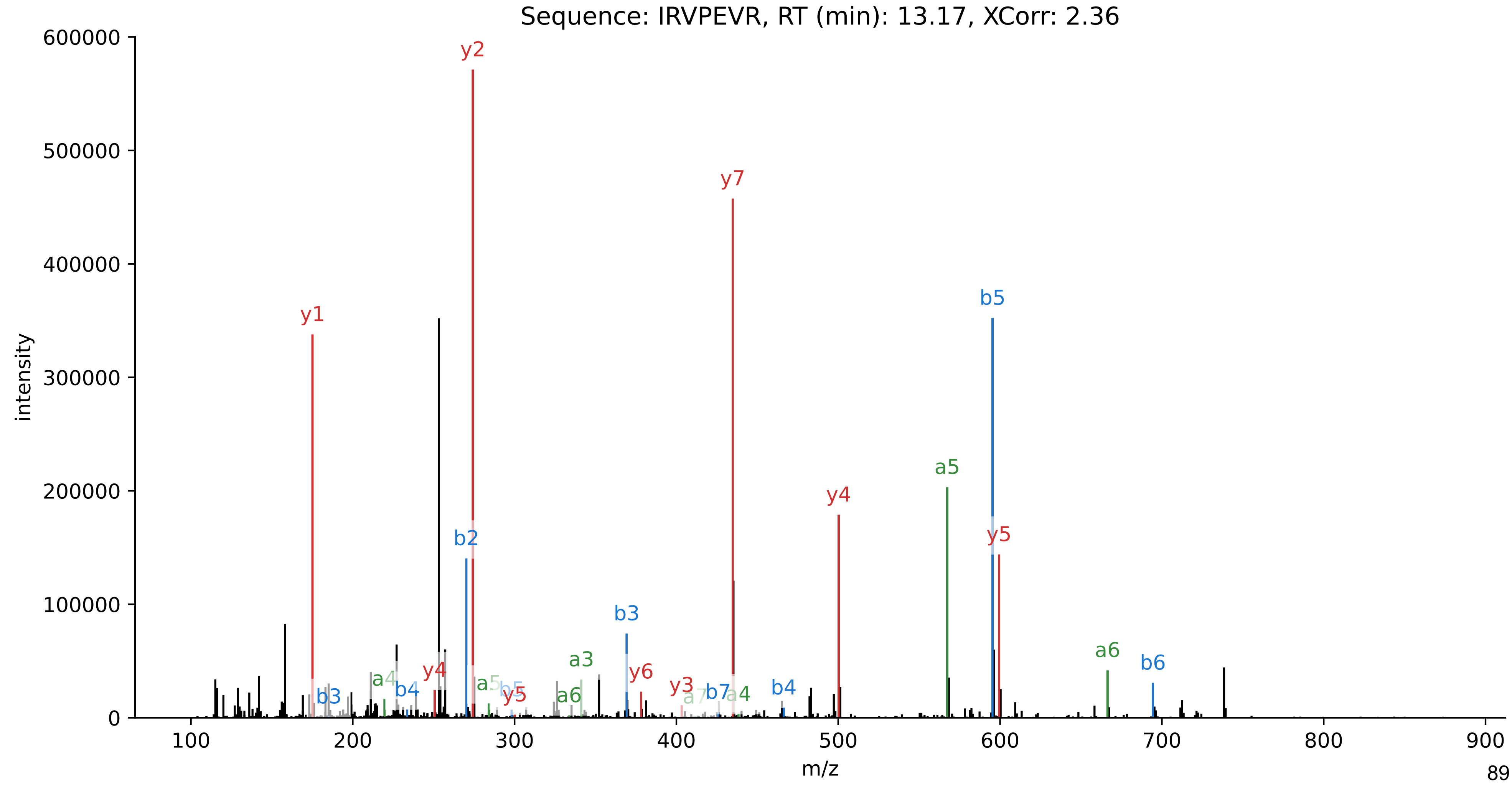
Sequence: IRVPEVR, RT (min): 13.17, XCorr: 2.36
intensity
m/z
0
100000
200000
300000
400000
500000
600000
100
200
300
400
500
600
700
800
900
y1
y2
y7
b5
a5
y4
y5
b2
b3
a6
b6
a3
y6
y3
b7
a4
b4
y4
a4
b3
b4
a5
b5
y5
a6
a7

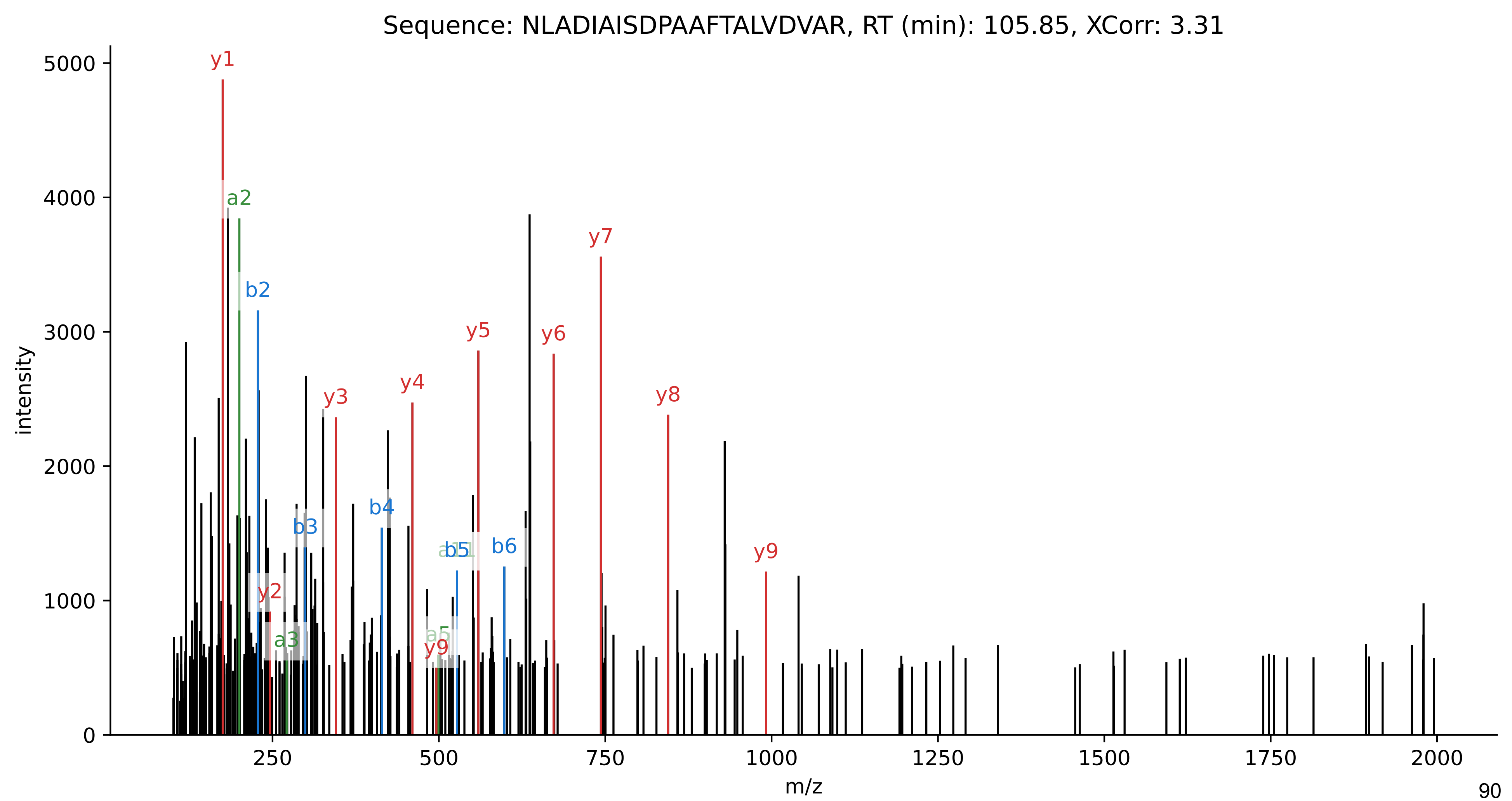

Sequence: NLADIAISDPAAFTALVDVAR, RT (min): 105.85, XCorr: 3.31
intensity
m/z
y1
a2
b2
y7
y5
y6
y4
y3
y8
b4
b3
b5
b6
y9
y2
a3
y9

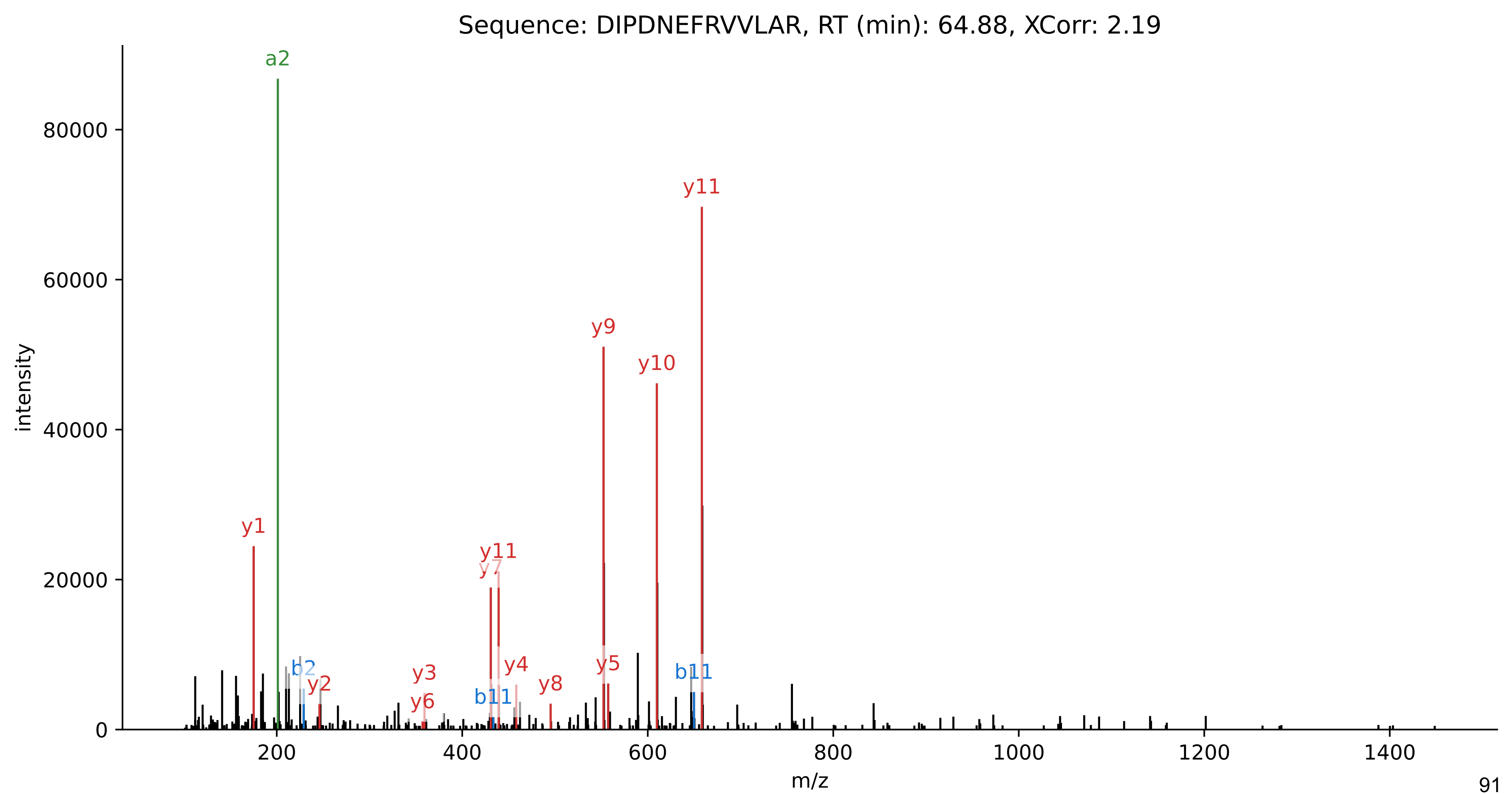

Sequence: DIPDNEFRVVLAR, RT (min): 64.88, XCorr: 2.19
a2
y11
y9
y10
y1
y11
y7
b2
y2
y3
y6
b11
y4
y8
y5
b11
intensity
m/z
0
20000
40000
60000
80000
200
400
600
800
1000
1200
1400

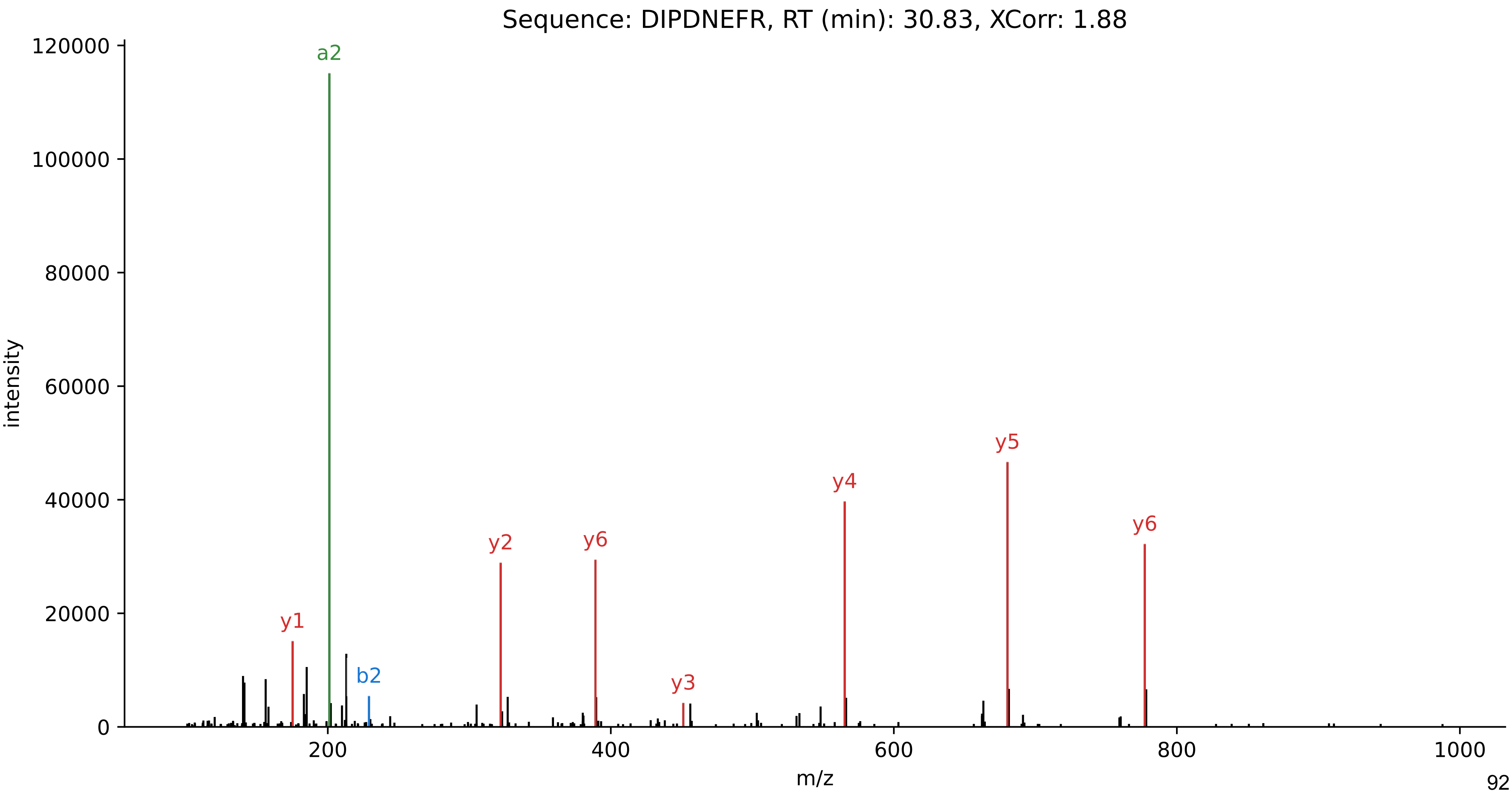

Sequence: DIPDNEFR, RT (min): 30.83, XCorr: 1.88
a2
y1
b2
y2
y6
y3
y4
y5
y6
intensity
m/z
0
20000
40000
60000
80000
100000
120000
200
400
600
800
1000

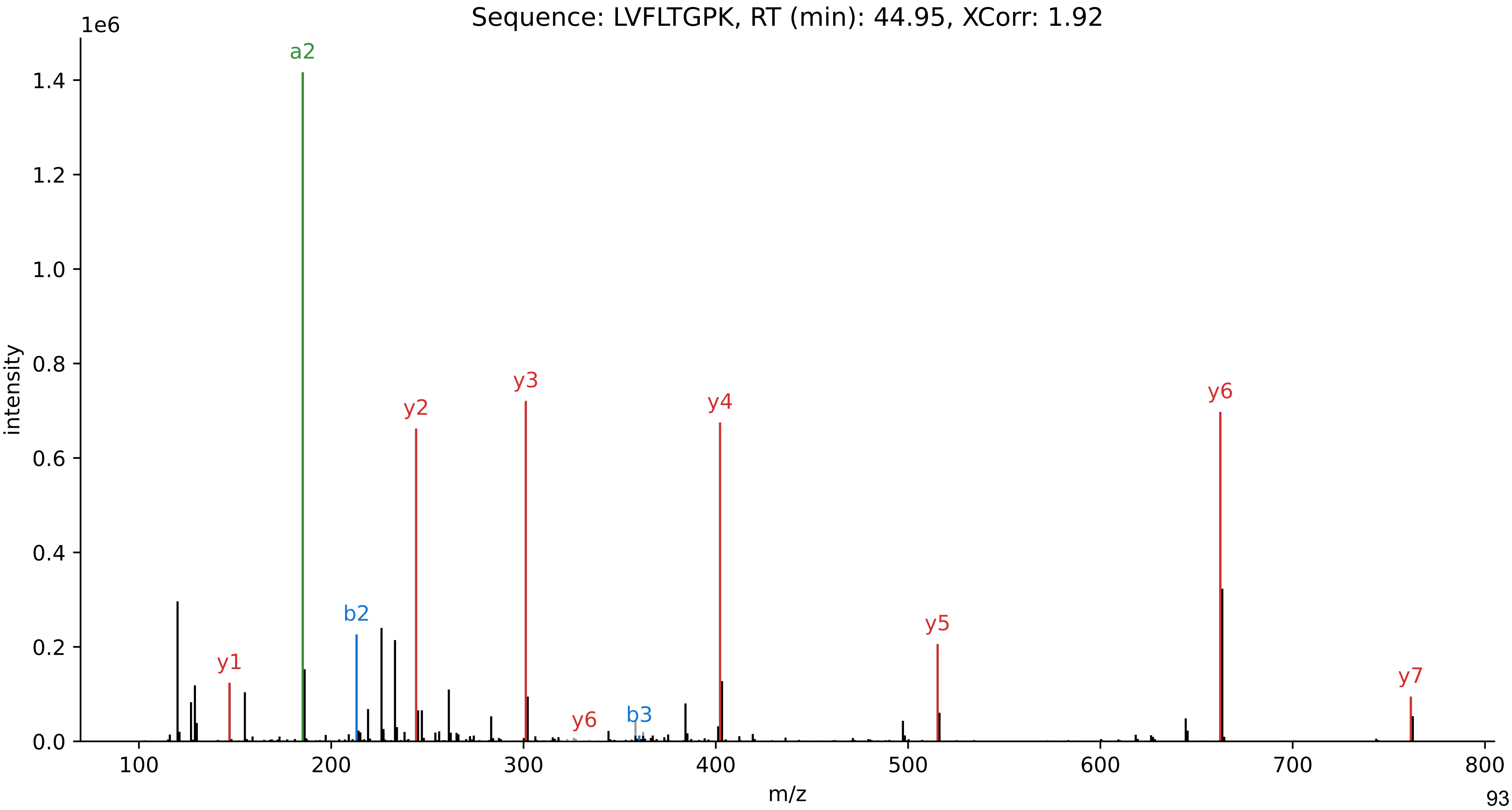

Sequence: LVFLTGPK, RT (min): 44.95, XCorr: 1.92
1e6
1.4
1.2
1.0
0.8
0.6
0.4
0.2
0.0
intensity
a2
b2
b3
y1
y2
y3
y4
y5
y6
y6
y7
100
200
300
400
500
600
700
800
m/z

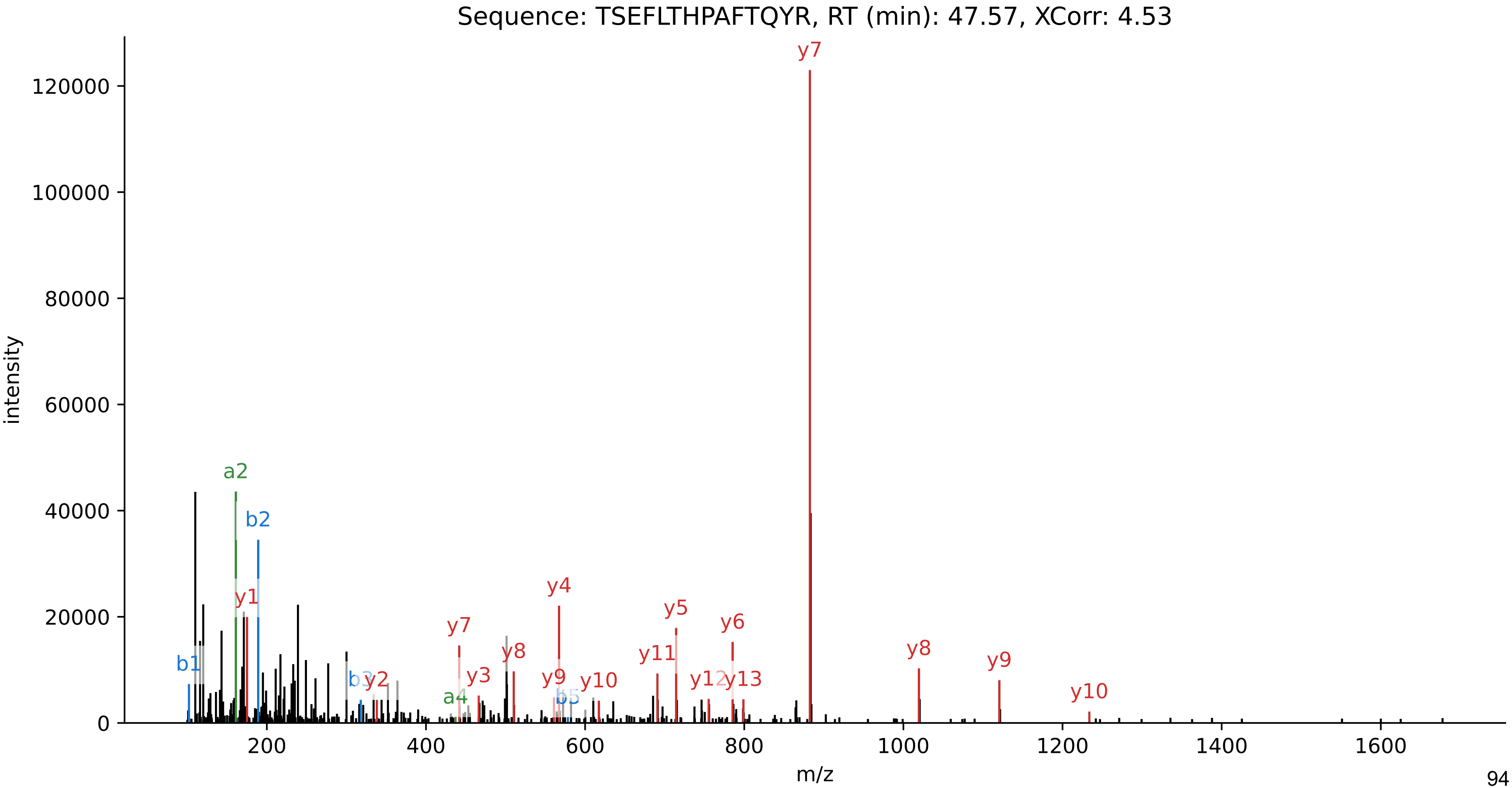
Sequence: TSEFLTHPAFTQYR, RT (min): 47.57, XCorr: 4.53
intensity
m/z
0
20000
40000
60000
80000
100000
120000
200
400
600
800
1000
1200
1400
1600
y7
a2
b2
y1
b1
y4
y5
y6
y7
y8
y3
y9
y10
y11
y12
y13
y2
a4
b5
y8
y9
y10

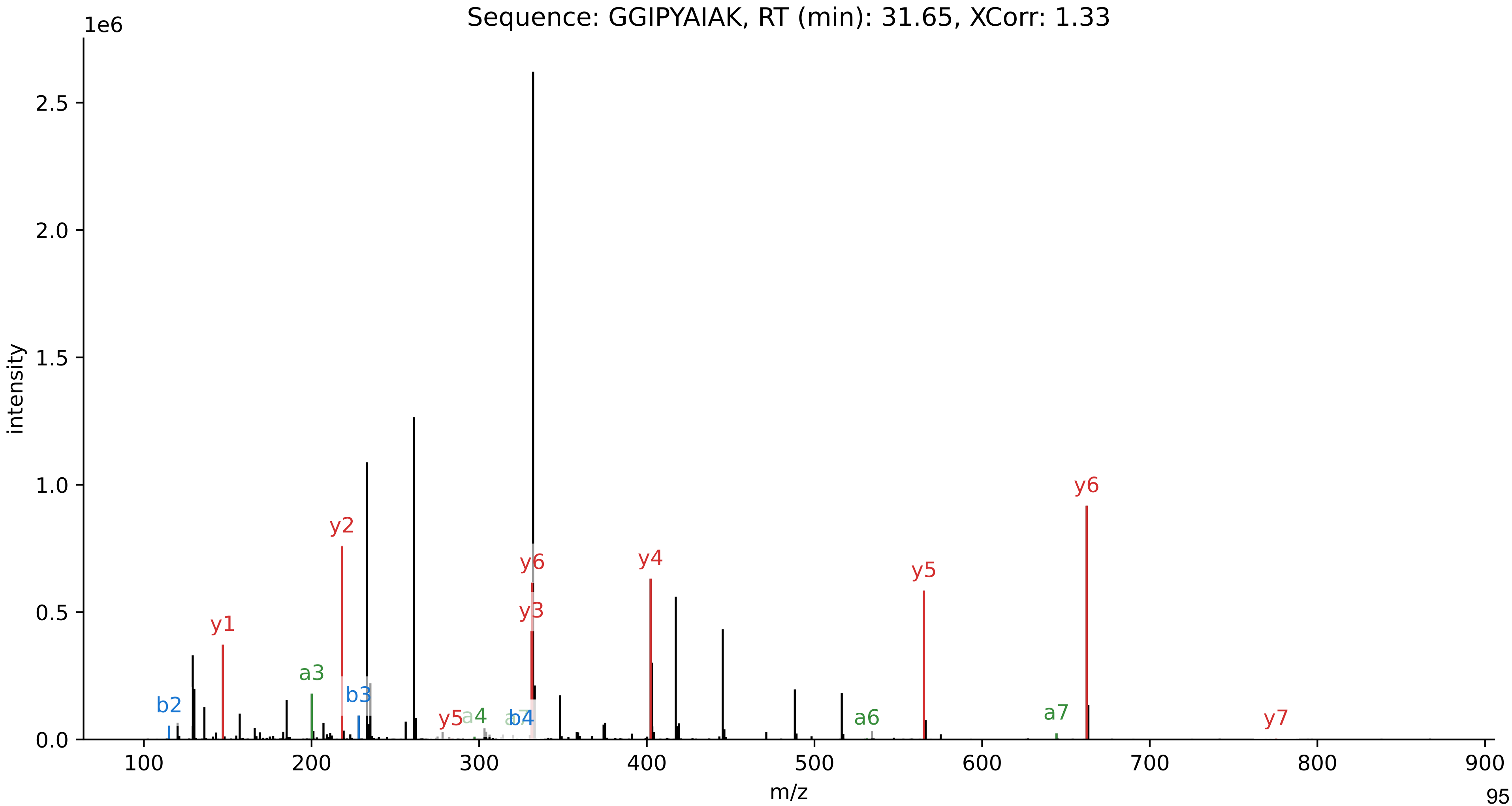
Sequence: GGIPYAIAK, RT (min): 31.65, XCorr: 1.33
1e6
intensity
m/z
0.0
0.5
1.0
1.5
2.0
2.5
100
200
300
400
500
600
700
800
900
b2
y1
a3
y2
b3
y5
a4
b4
y6
y3
y4
a6
y5
a7
y6
y7

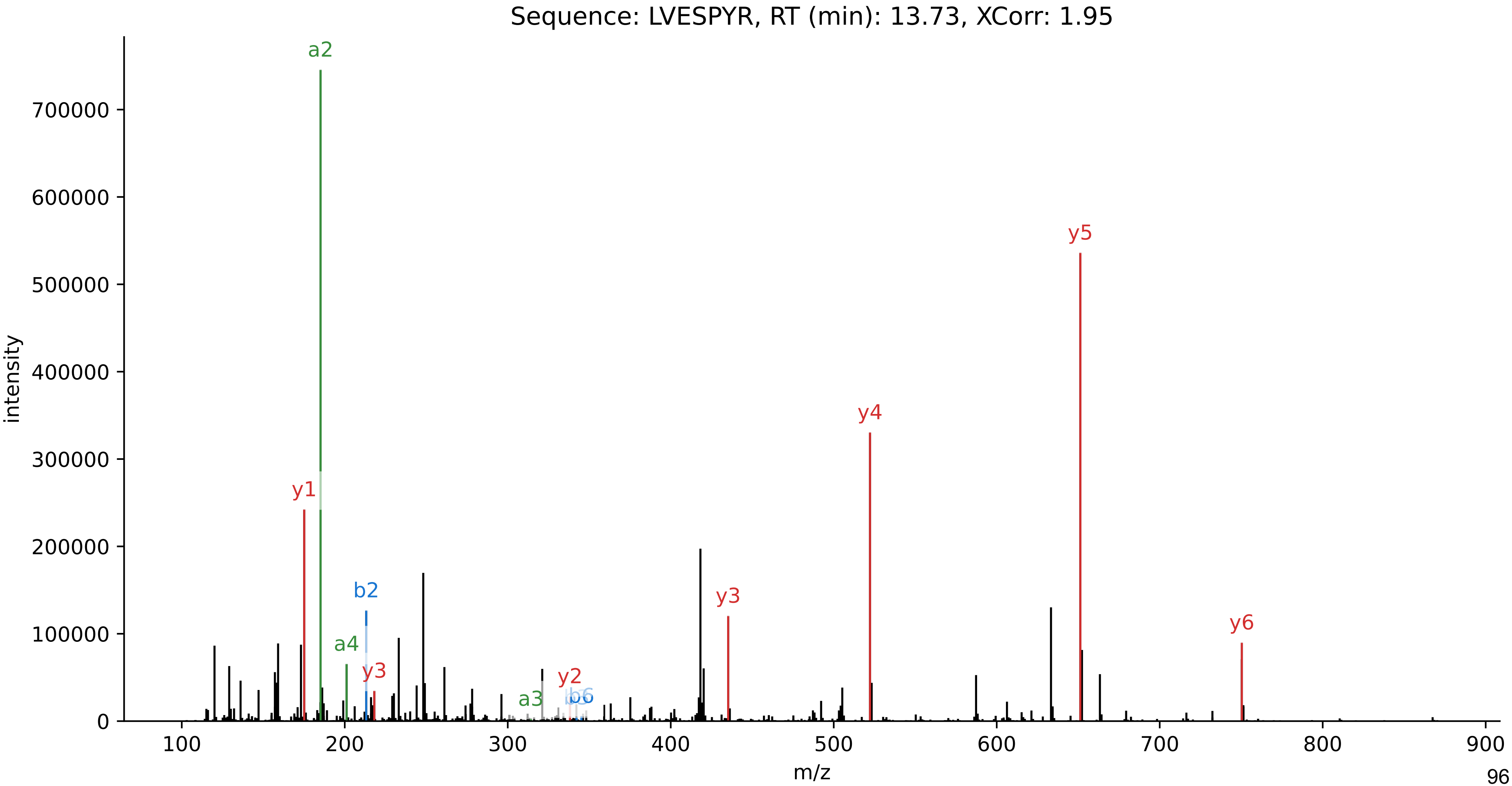

Sequence: LVESPYR, RT (min): 13.73, XCorr: 1.95
a2
y5
y4
y1
b2
y3
a4
y3
y2
a3
b6
y6
intensity
m/z
0
100000
200000
300000
400000
500000
600000
700000
100
200
300
400
500
600
700
800
900

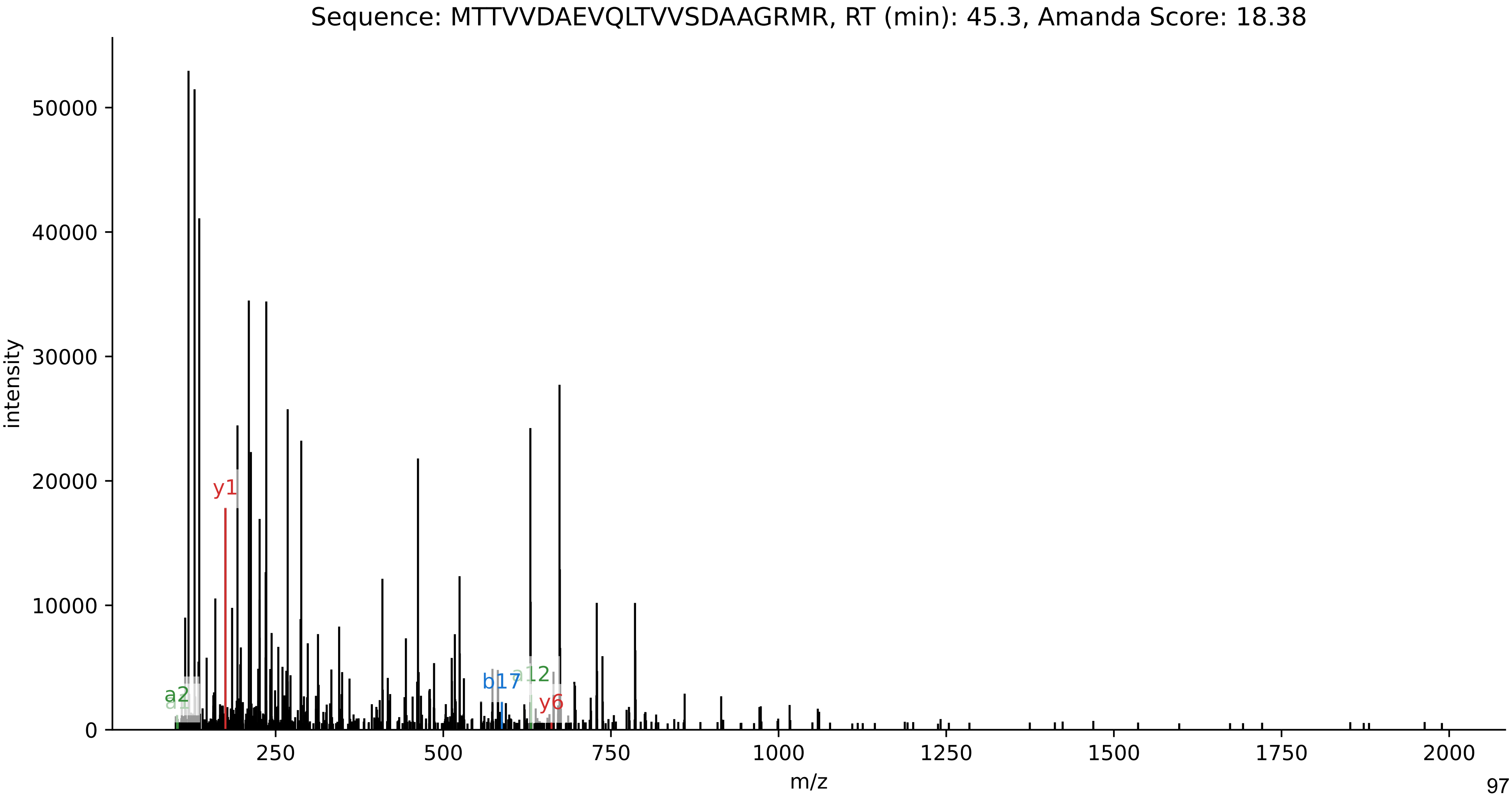

Sequence: MTTVVDAEVQLTVVSDAAGRMR, RT (min): 45.3, Amanda Score: 18.38
intensity
m/z
0
10000
20000
30000
40000
50000
250
500
750
1000
1250
1500
1750
2000
y1
a2
b17
a12
y6

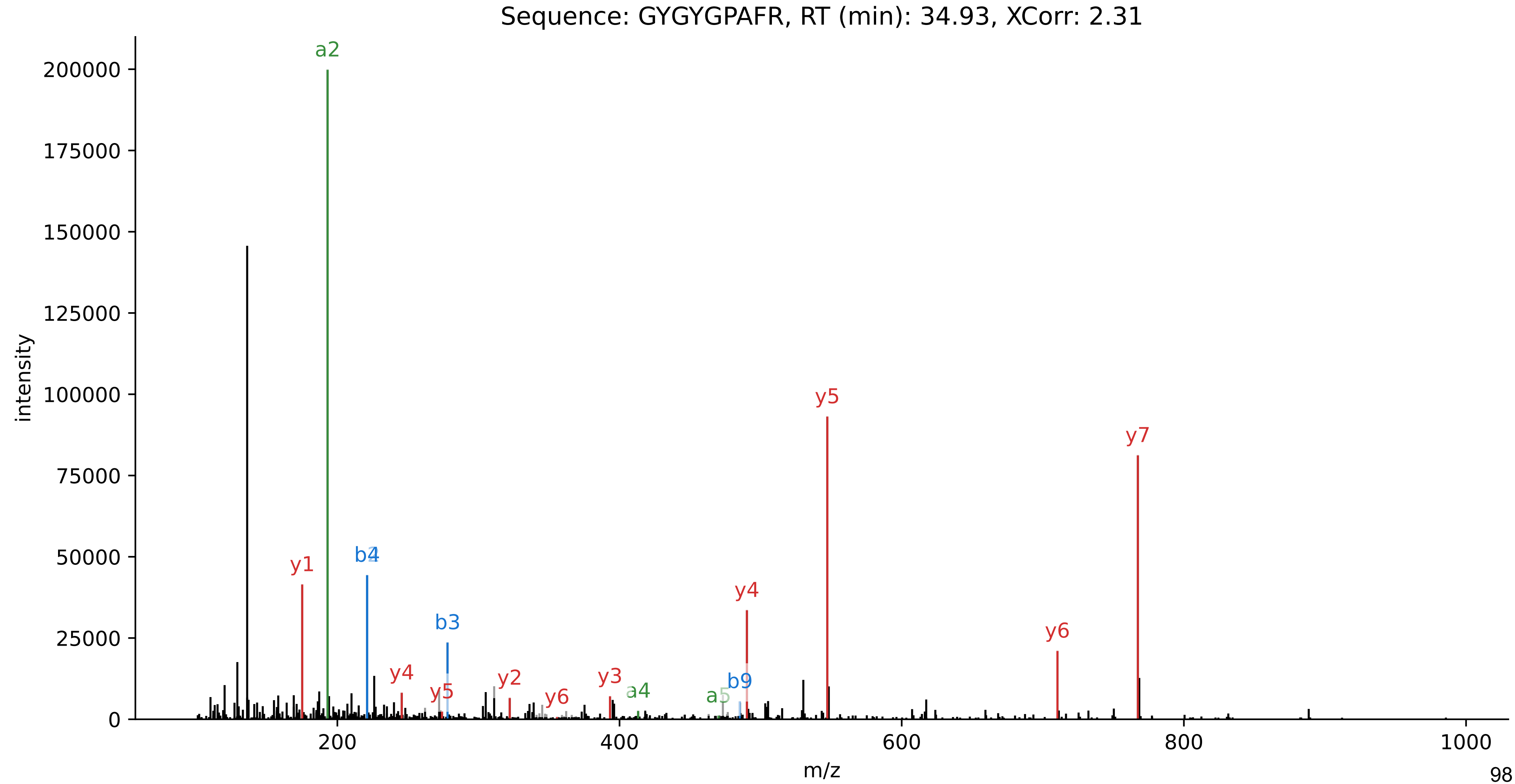

Sequence: GYGYGPAFR, RT (min): 34.93, XCorr: 2.31
a2
y5
y7
y1
b4
b3
y4
y6
y4
y2
y3
y5
y6
a4
a5
b9
intensity
200000
175000
150000
125000
100000
75000
50000
25000
0
200
400
600
800
1000
m/z

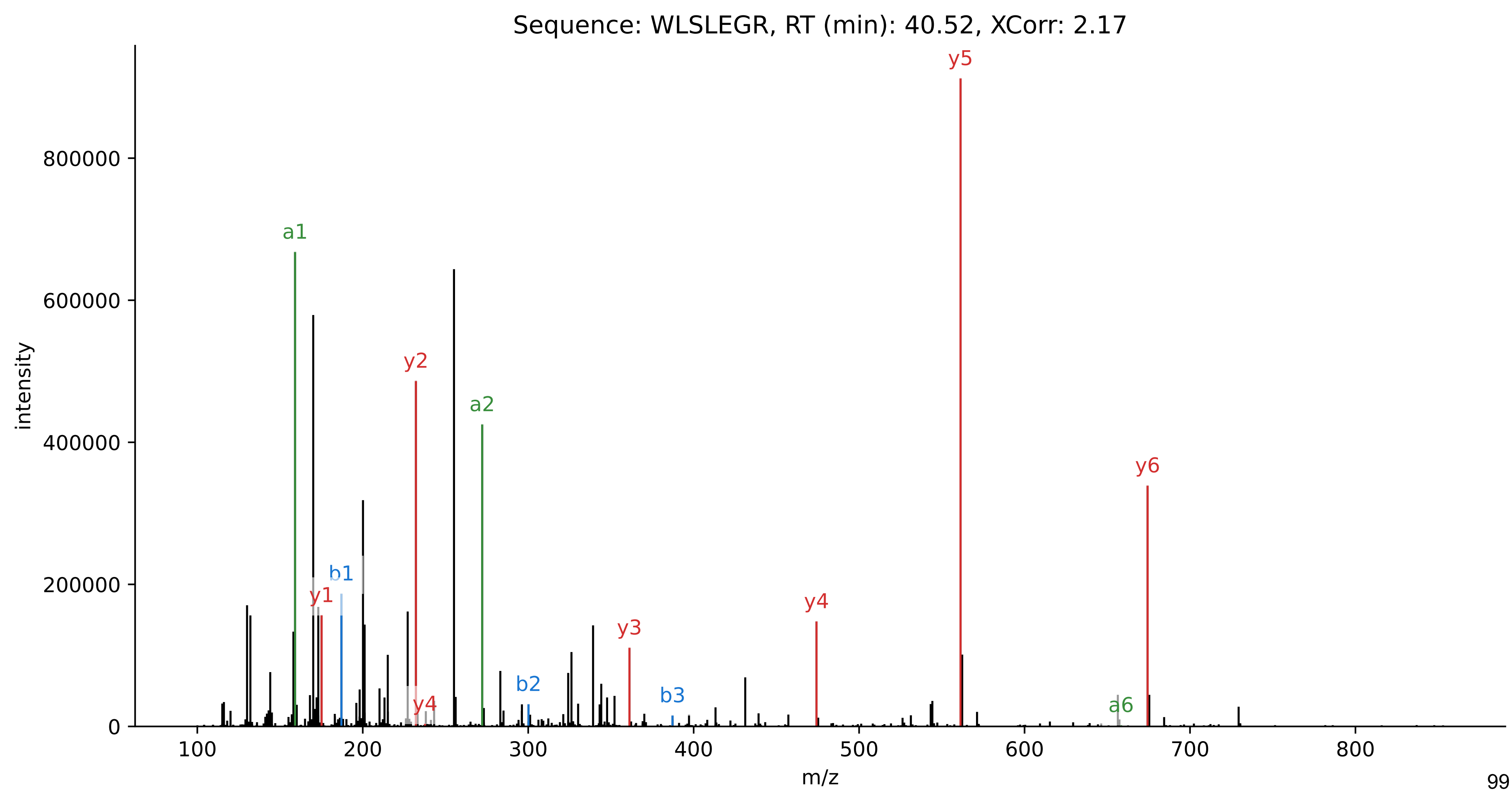

Sequence: WLSLEGR, RT (min): 40.52, XCorr: 2.17
intensity
m/z
800000
600000
400000
200000
0
100
200
300
400
500
600
700
800
y5
a1
y2
a2
y6
b1
y1
y4
y3
b2
b3
y4
a6

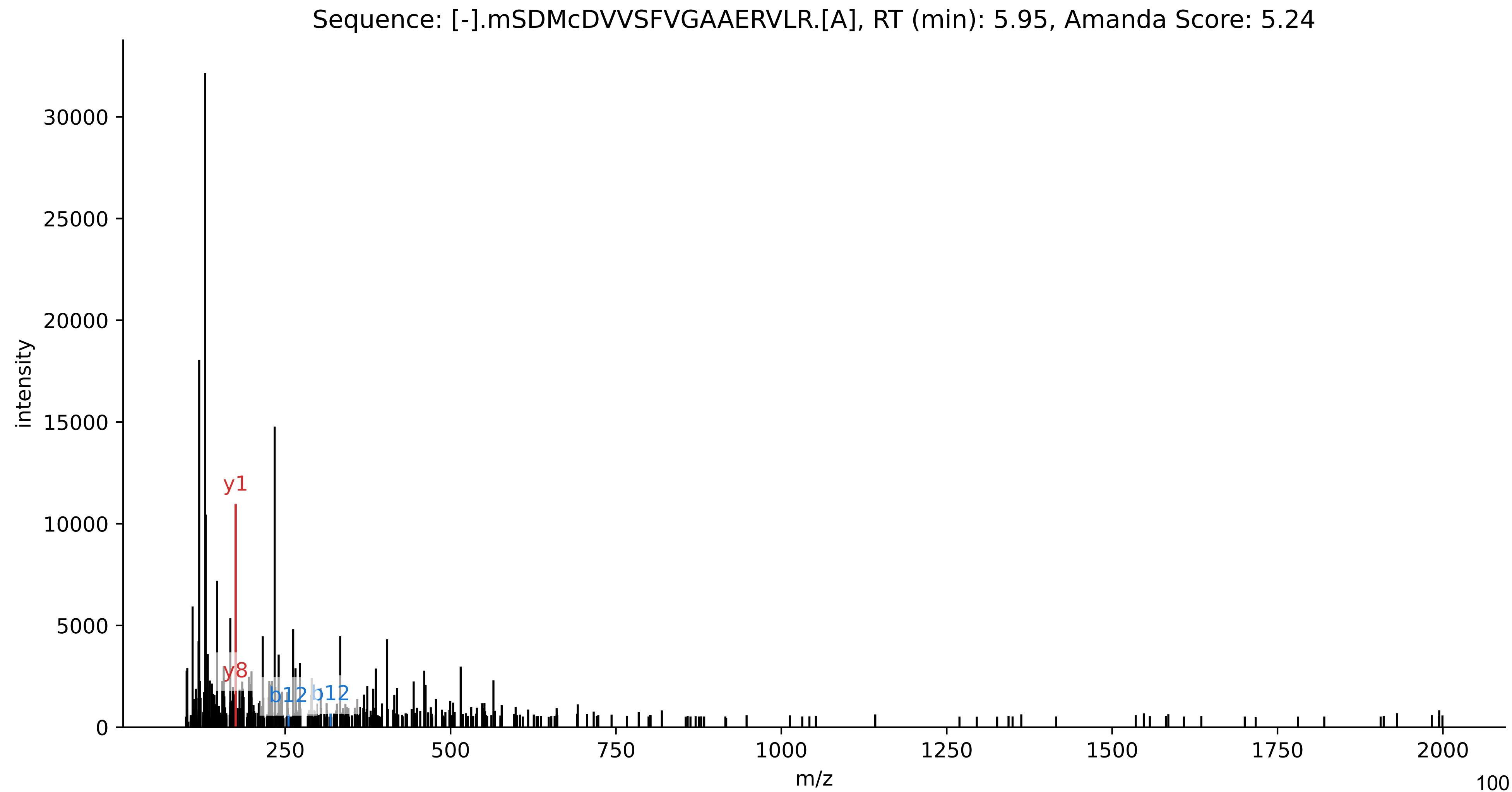

Sequence: [-].mSDMcDVVSFVGAAERVLR.[A], RT (min): 5.95, Amanda Score: 5.24
intensity
m/z
y1
y8
b12
b12
0
5000
10000
15000
20000
25000
30000
250
500
750
1000
1250
1500
1750
2000

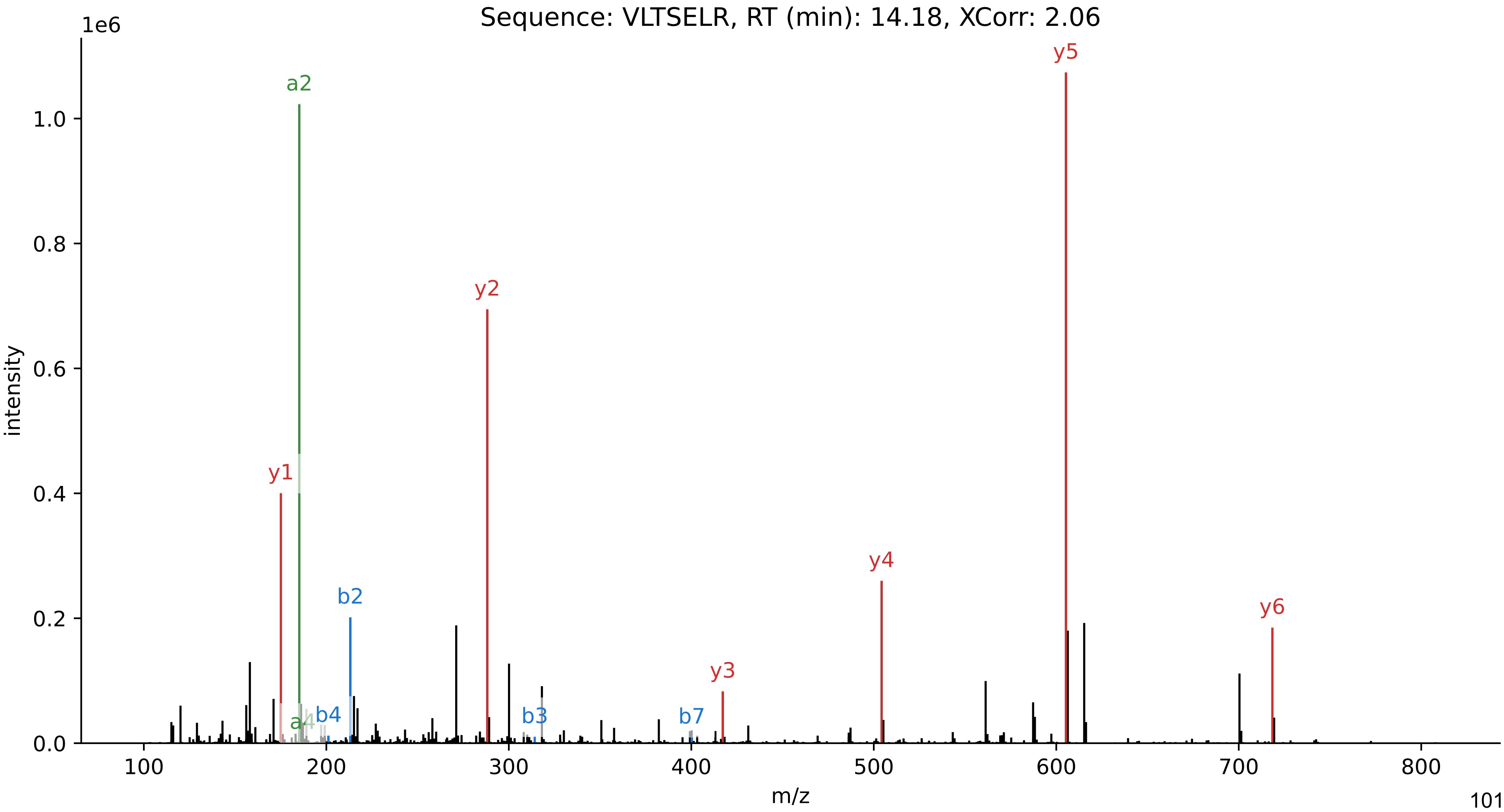
Sequence: VLTSELR, RT (min): 14.18, XCorr: 2.06
1e6
intensity
m/z
a2
y5
y2
y1
b2
y4
y6
y3
a4
b4
b3
b7
1.0
0.8
0.6
0.4
0.2
0.0
100
200
300
400
500
600
700
800

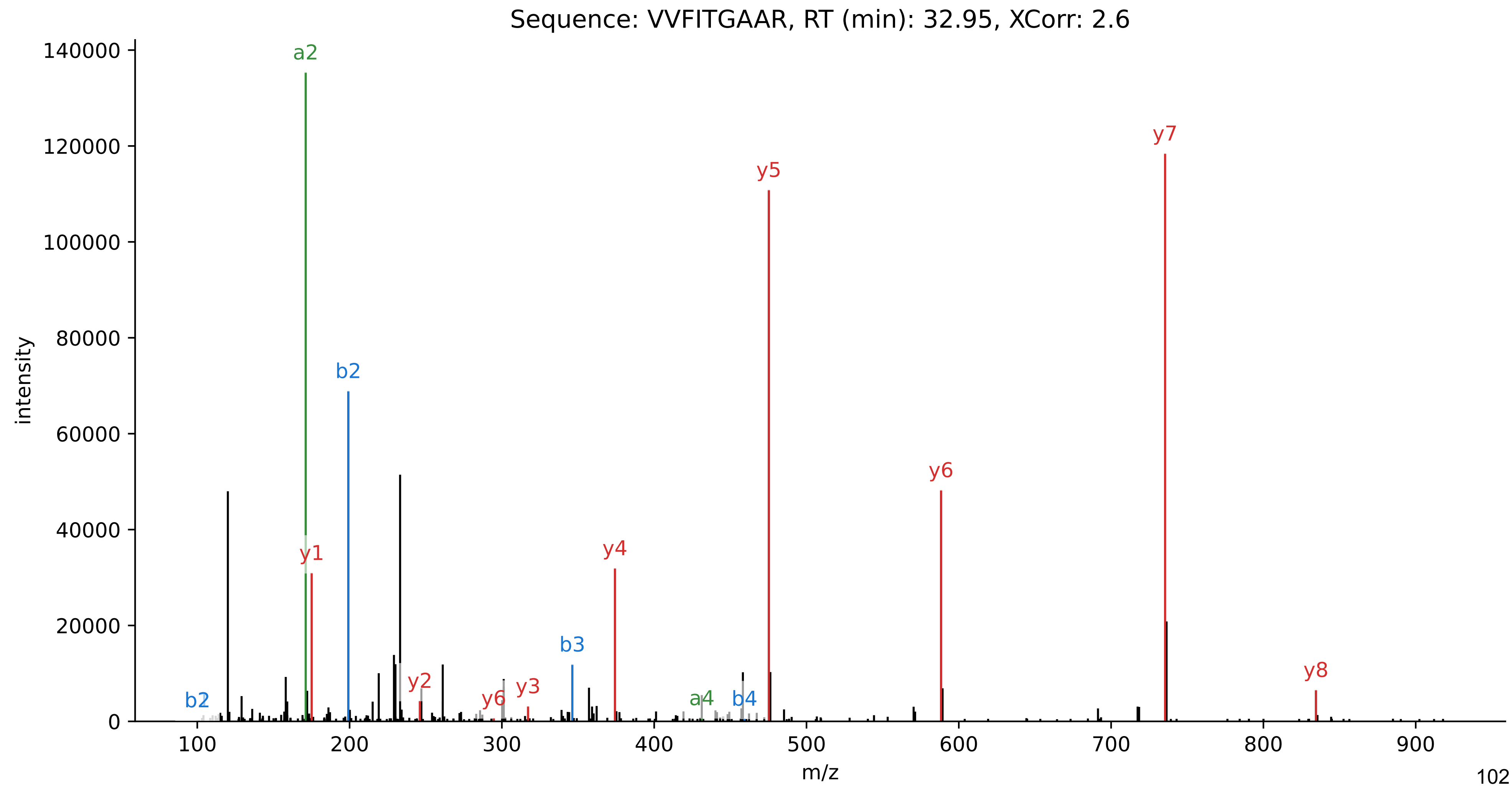

Sequence: VVFITGAAR, RT (min): 32.95, XCorr: 2.6
intensity
m/z
a2
b2
y1
y2
y3
y4
y5
y6
y7
y8
b3
b4
a4
0
20000
40000
60000
80000
100000
120000
140000
100
200
300
400
500
600
700
800
900

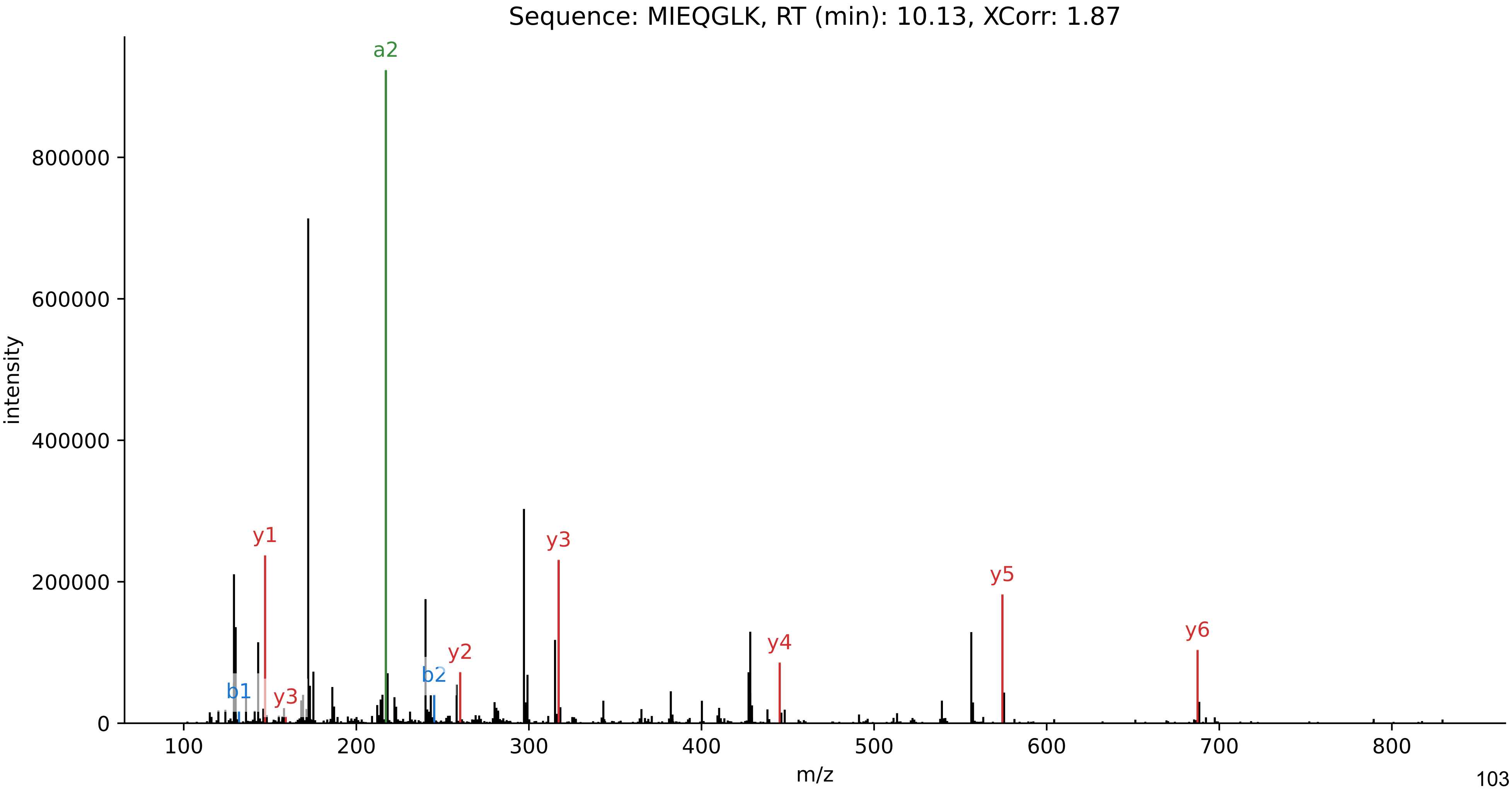
Sequence: MIEQGLK, RT (min): 10.13, XCorr: 1.87
a2
y1
b1
y3
b2
y2
y3
y4
y5
y6
intensity
0
200000
400000
600000
800000
m/z
100
200
300
400
500
600
700
800

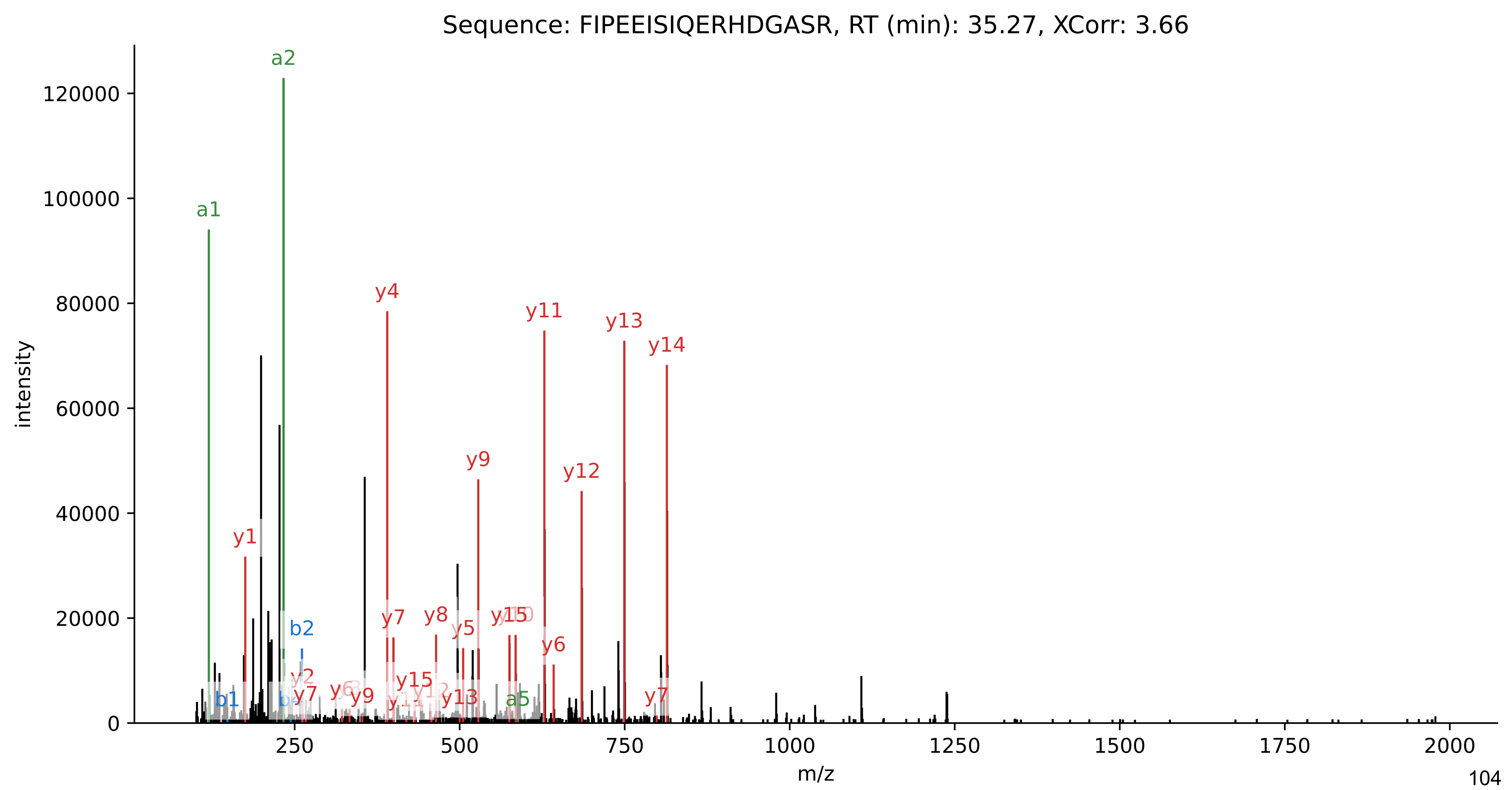
Sequence: FIPEEISIQERHDGASR, RT (min): 35.27, XCorr: 3.66
intensity
m/z
a1
a2
y1
y4
y11
y13
y14
y9
y12
y7
y8
y5
y15
y6
b2
b1
y2
a5
y13
y9
y15

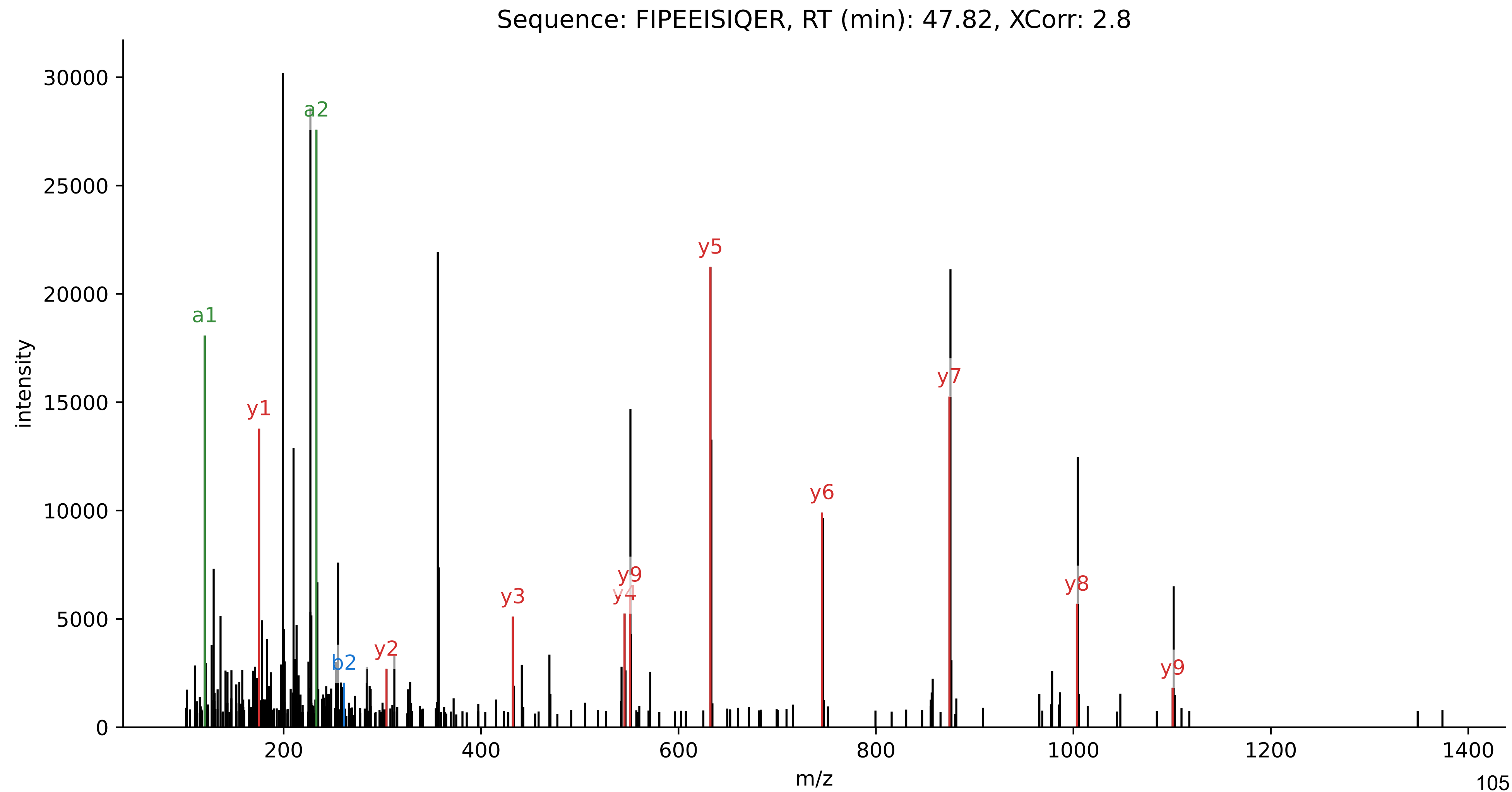

Sequence: FIPEEISIQER, RT (min): 47.82, XCorr: 2.8
intensity
m/z
a1
a2
y1
y2
b2
y3
y4
y9
y5
y6
y7
y8
y9

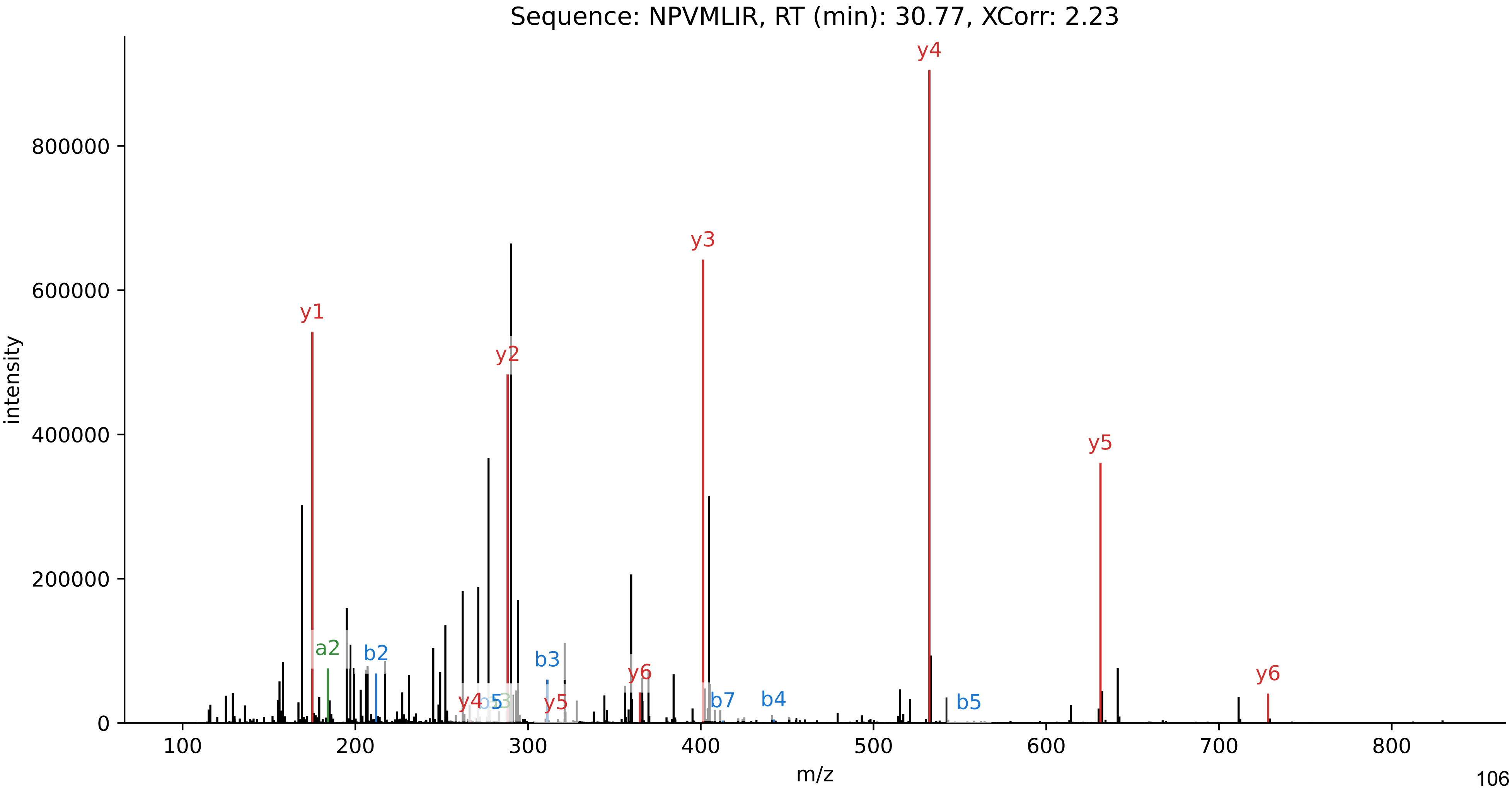

Sequence: NPVMLIR, RT (min): 30.77, XCorr: 2.23
intensity
m/z
0
200000
400000
600000
800000
100
200
300
400
500
600
700
800
y1
y2
y3
y4
y5
y6
a2
b2
b3
b4
b5
b7

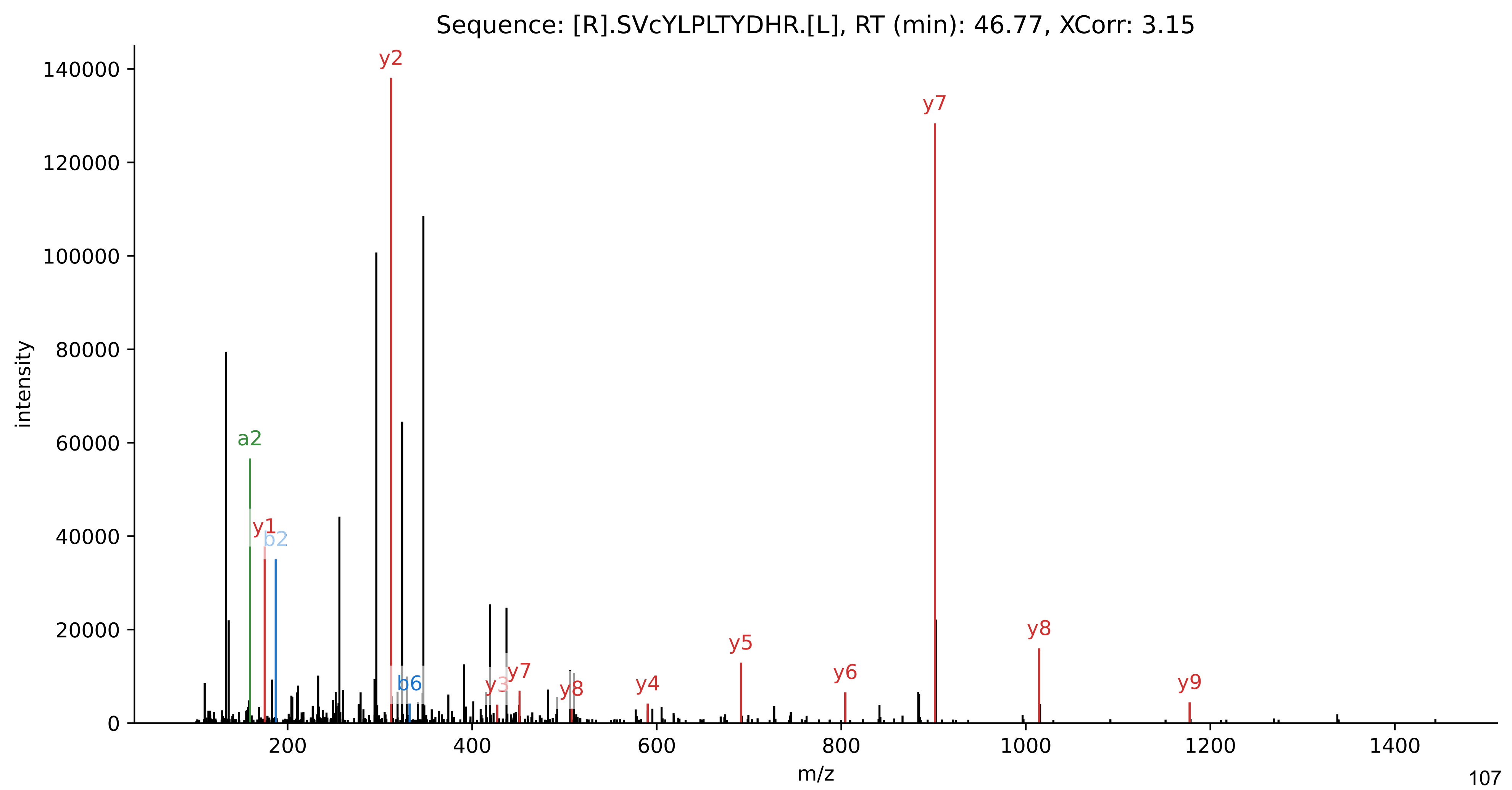
Sequence: [R].SVcYLPLTYDHR.[L], RT (min): 46.77, XCorr: 3.15
intensity
m/z
y2
y7
a2
y1
b2
b6
y3
y7
y8
y4
y5
y6
y8
y9
0
20000
40000
60000
80000
100000
120000
140000
200
400
600
800
1000
1200
1400

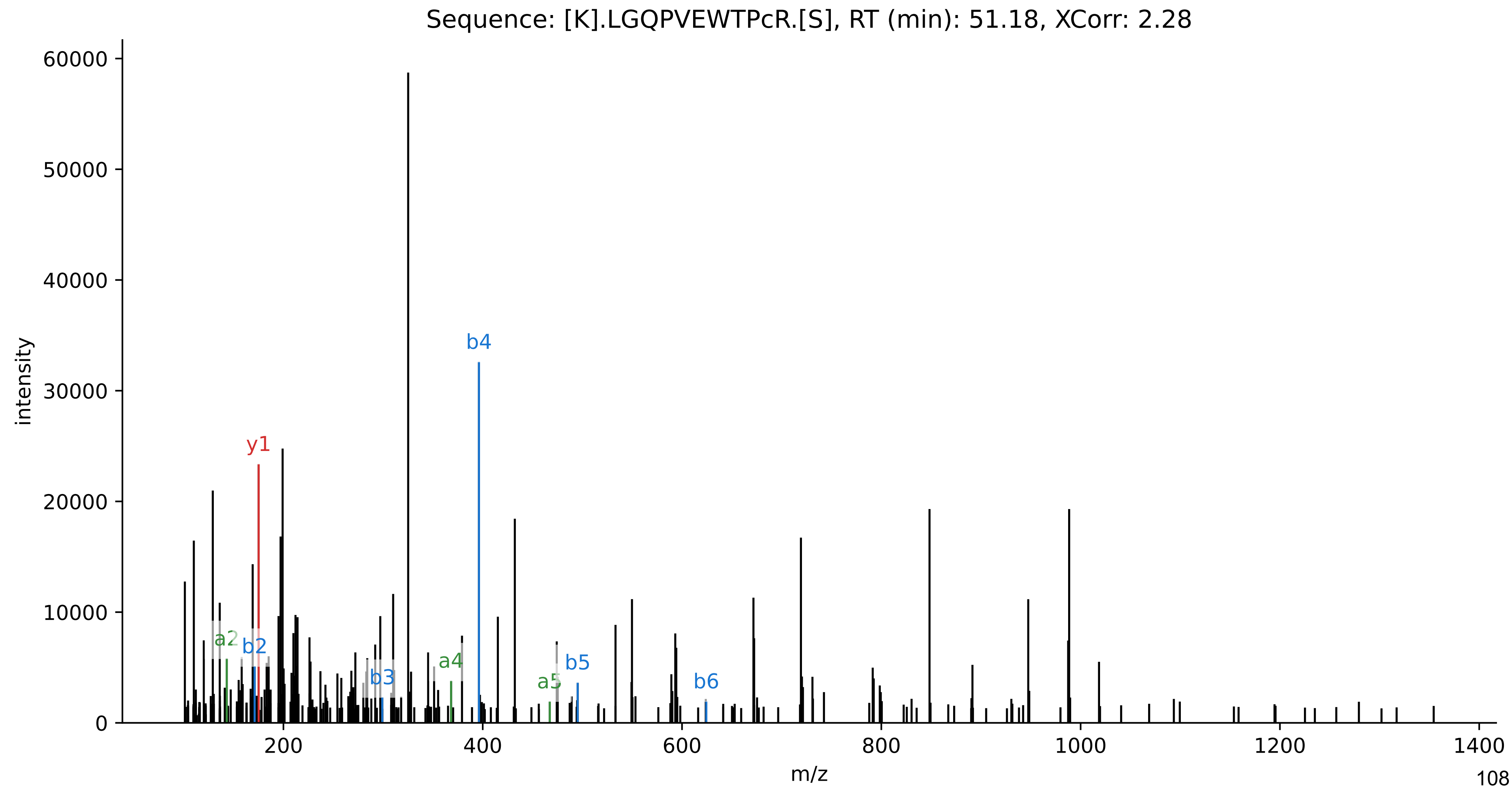

Sequence: [K].LGQPVEWTPcR.[S], RT (min): 51.18, XCorr: 2.28
intensity
m/z
y1
a2
b2
b3
a4
b4
a5
b5
b6

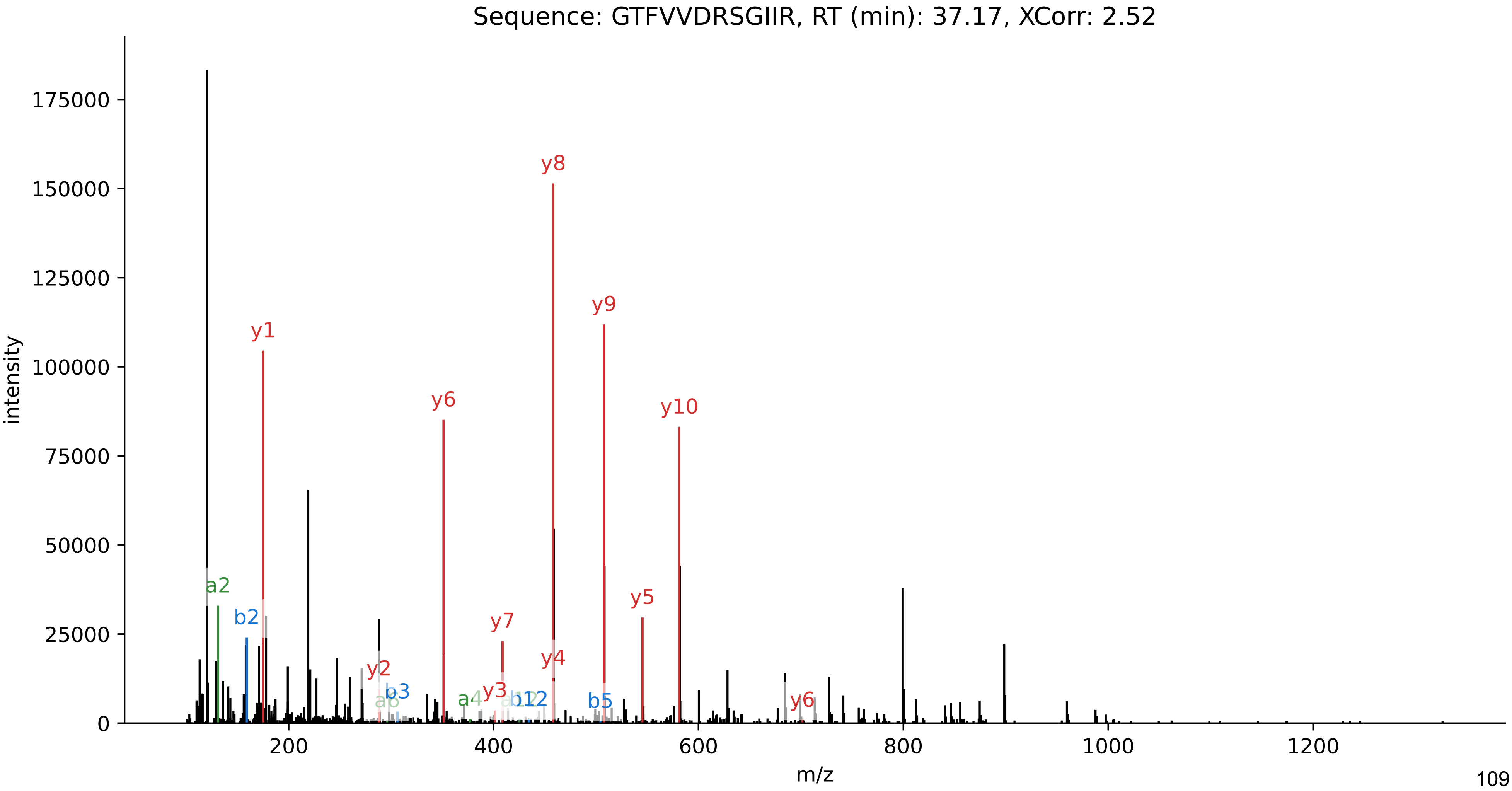

Sequence: GTFVVDRSGIIR, RT (min): 37.17, XCorr: 2.52
intensity
m/z
175000
150000
125000
100000
75000
50000
25000
0
200
400
600
800
1000
1200
y8
y9
y1
y6
y10
a2
b2
y5
y7
y2
y4
b3
y3
a4
b5
y6

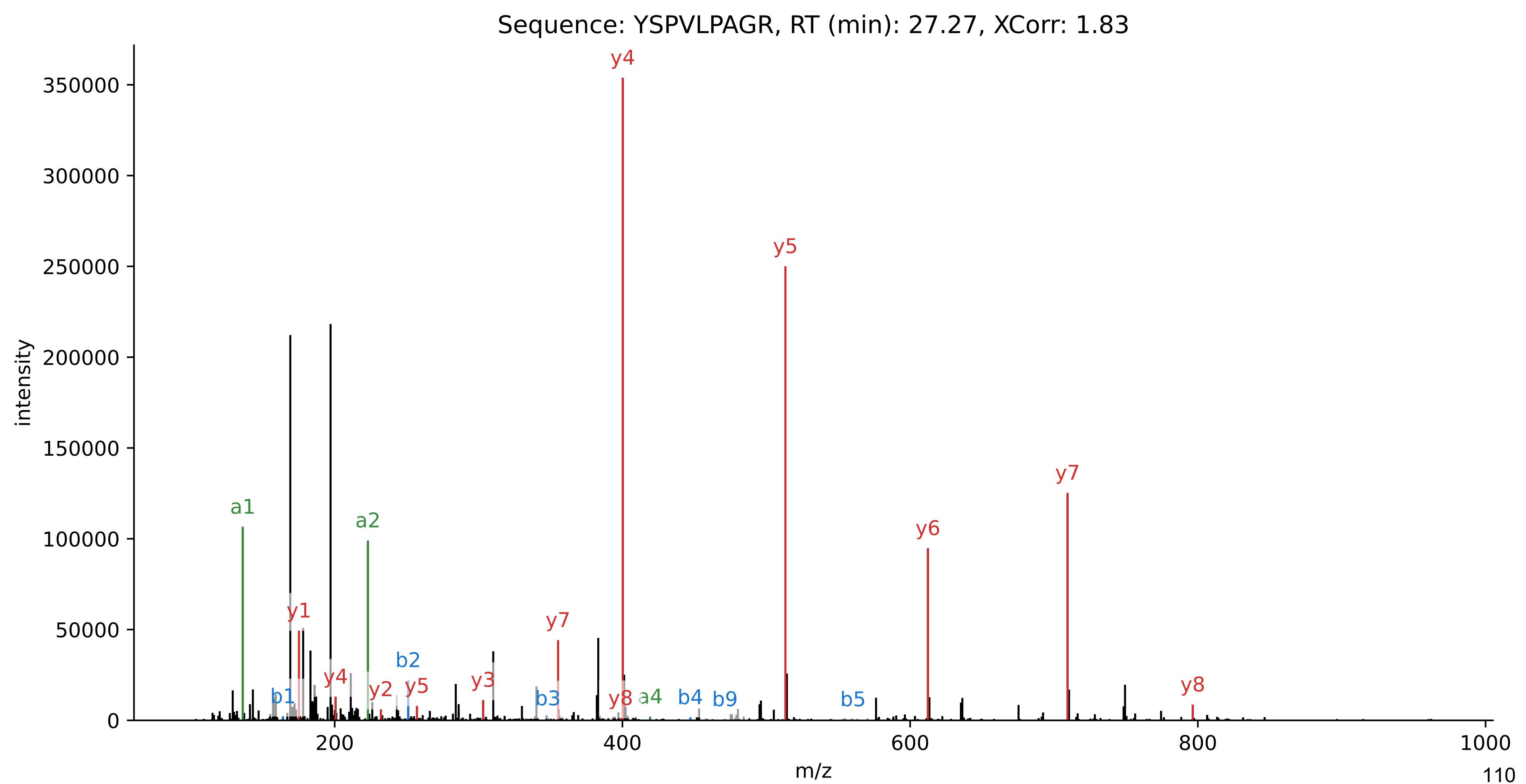

Sequence: YSPVLPAGR, RT (min): 27.27, XCorr: 1.83
intensity
m/z
y4
y5
y7
y6
a1
a2
y1
y7
b2
y4
y2
y5
y3
b1
b3
y8
a4
b4
b9
b5
y8

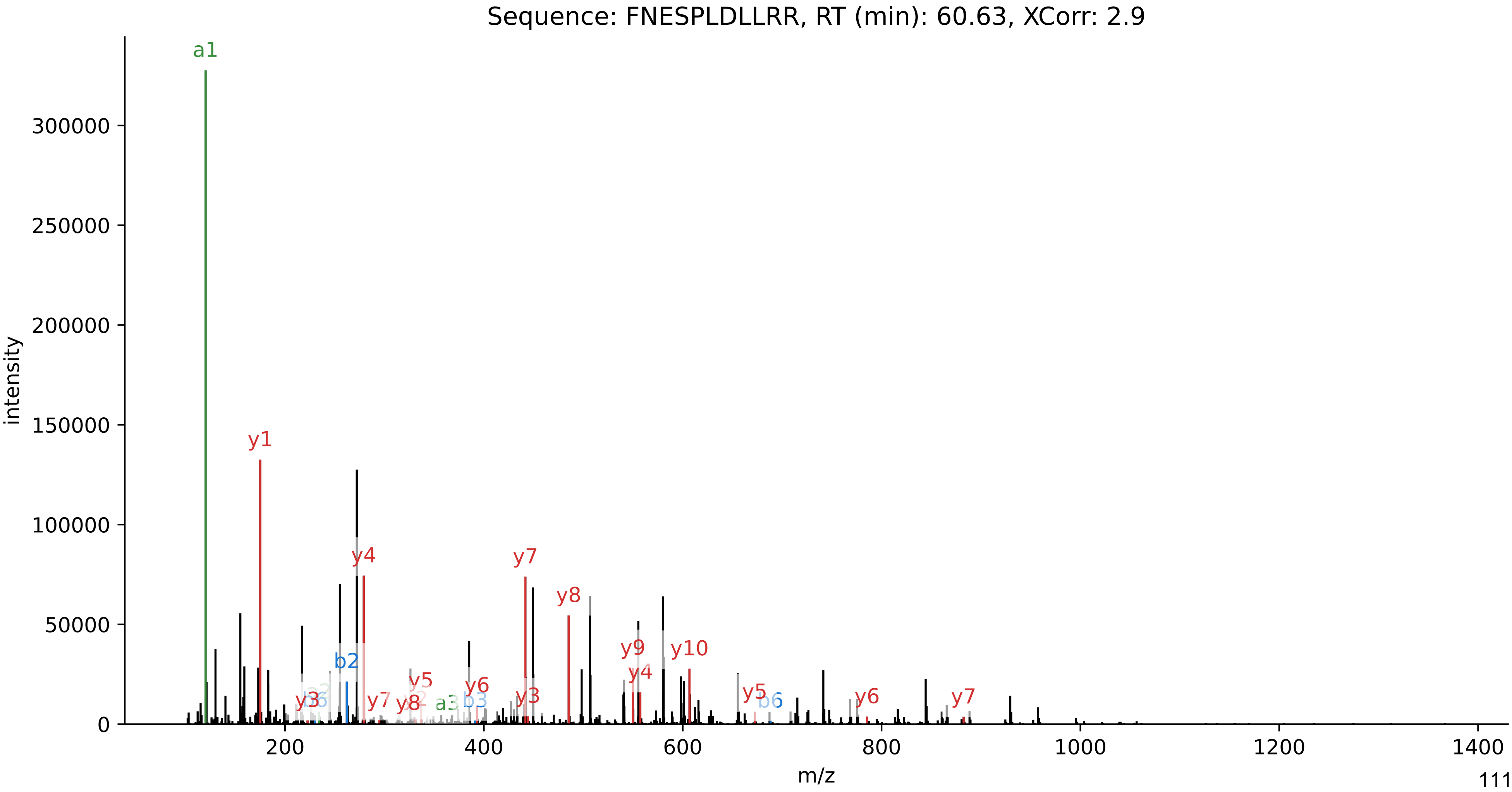
Sequence: FNESPLDLLRR, RT (min): 60.63, XCorr: 2.9
intensity
m/z
a1
y1
y4
y7
y8
b2
y9
y4
y10
y5
y6
y3
y7
y8
a3
b3
y3
y5
b6
y6
y7
0
50000
100000
150000
200000
250000
300000
200
400
600
800
1000
1200
1400

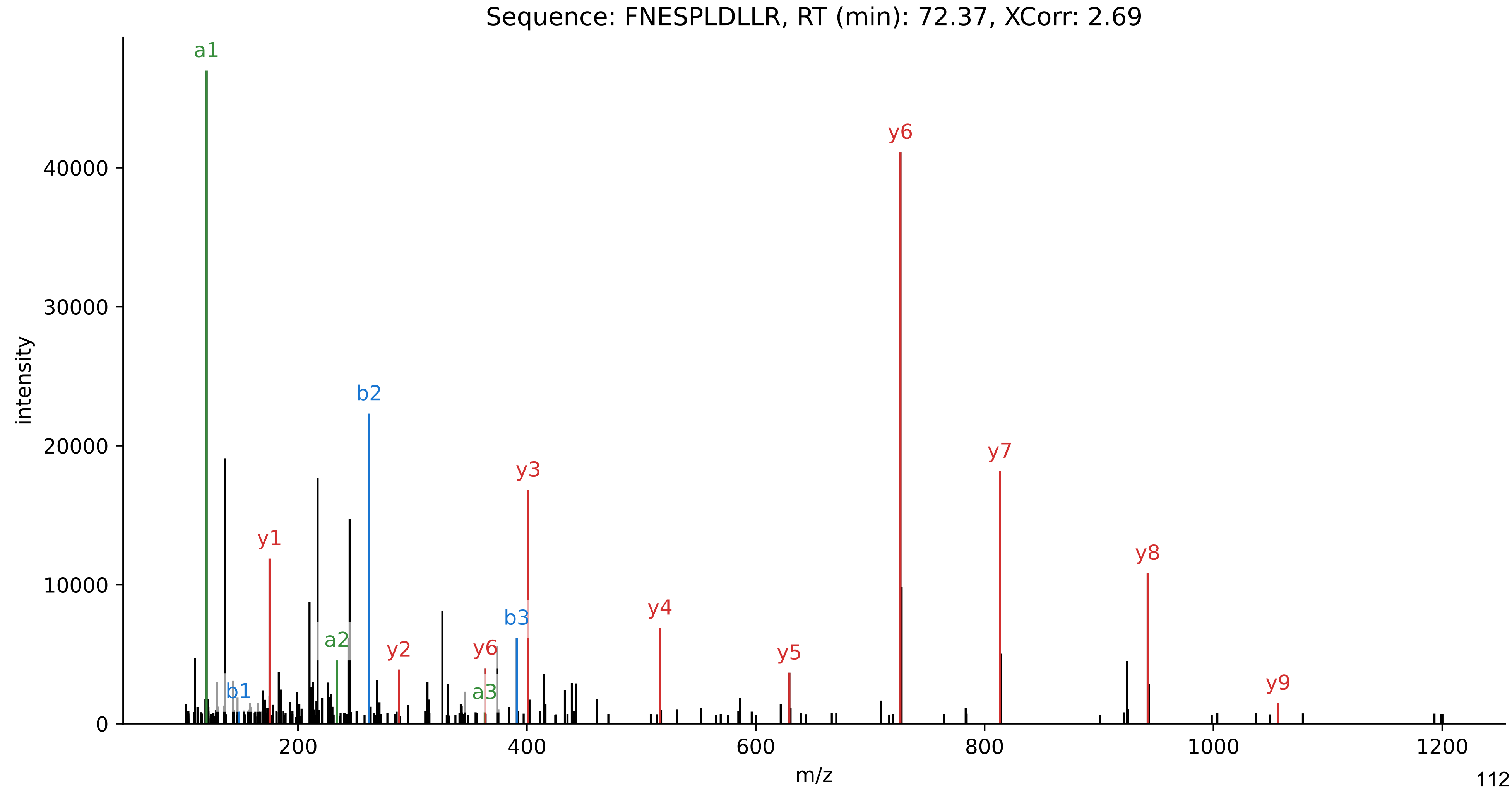

Sequence: FNESPLDLLR, RT (min): 72.37, XCorr: 2.69
intensity
m/z
a1
b1
y1
a2
b2
y2
y6
a3
b3
y3
y4
y5
y6
y7
y8
y9
0
10000
20000
30000
40000
200
400
600
800
1000
1200

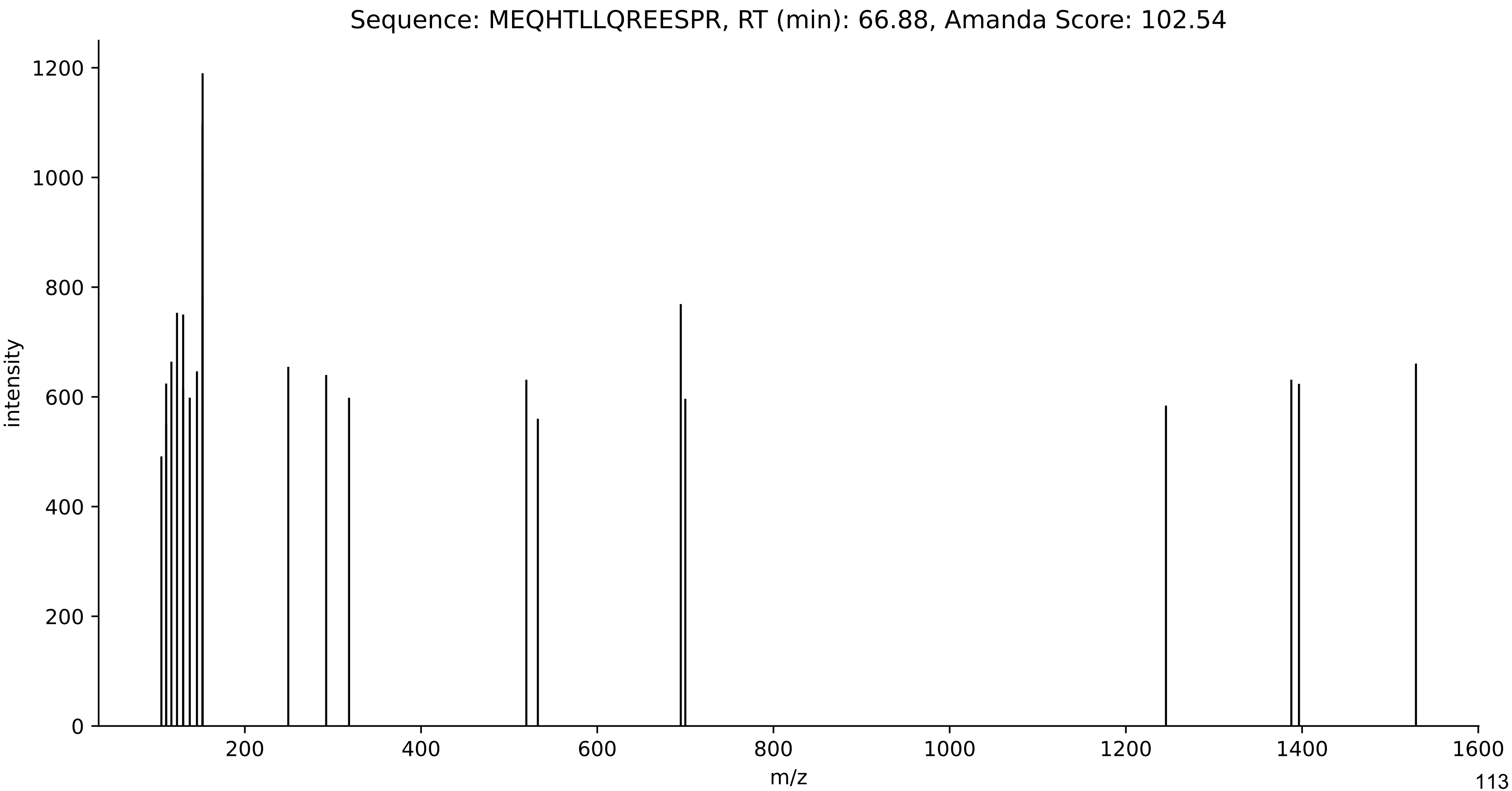
Sequence: MEQHTLLQREESPR, RT (min): 66.88, Amanda Score: 102.54
intensity
m/z
0
200
400
600
800
1000
1200
1400
1600

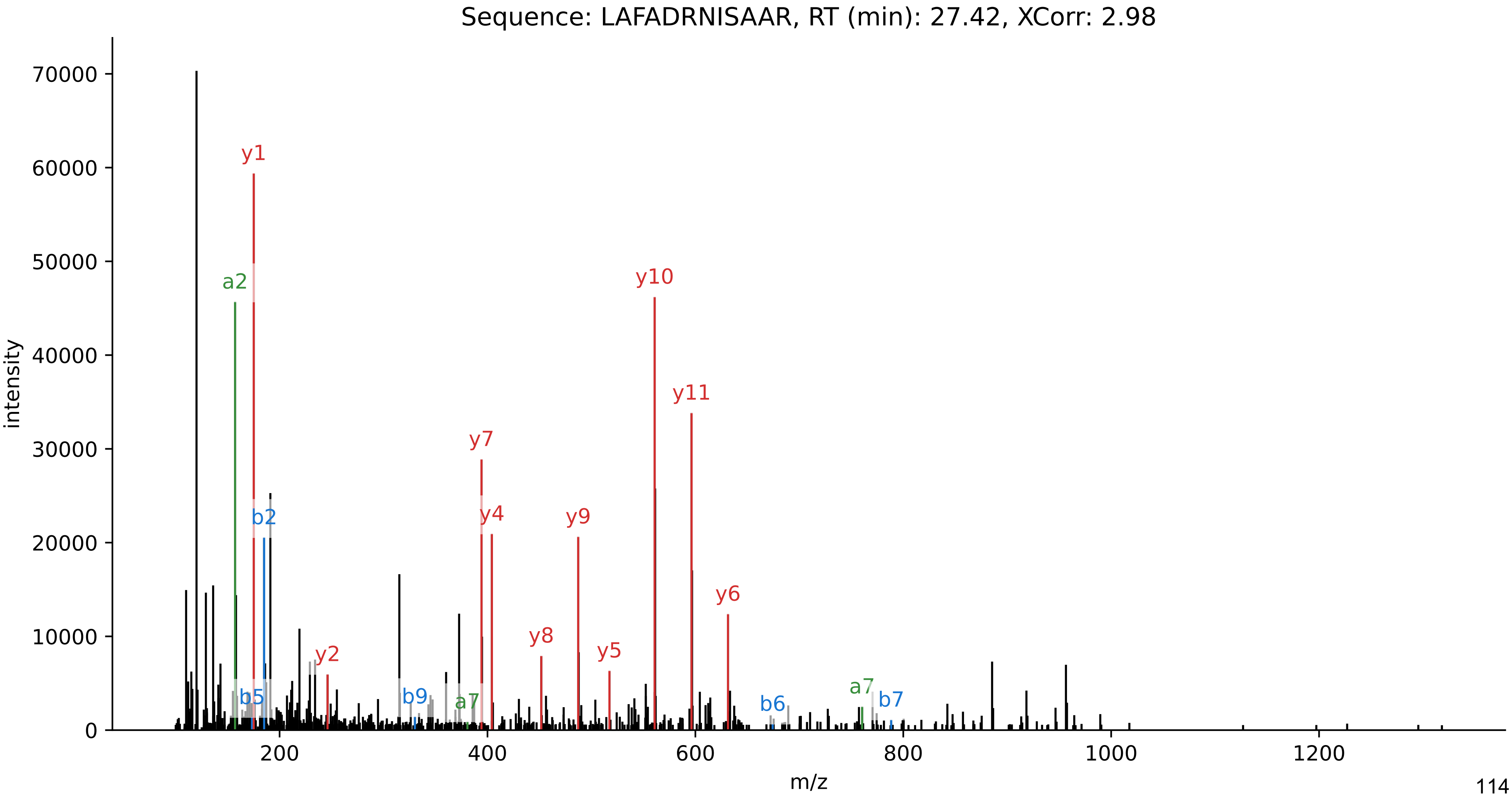

Sequence: LAFADRNISAAR, RT (min): 27.42, XCorr: 2.98
intensity
m/z
y1
a2
y10
y11
y7
y4
b2
y9
y6
y8
y5
y2
b5
b9
a7
b6
a7
b7
0
10000
20000
30000
40000
50000
60000
70000
200
400
600
800
1000
1200

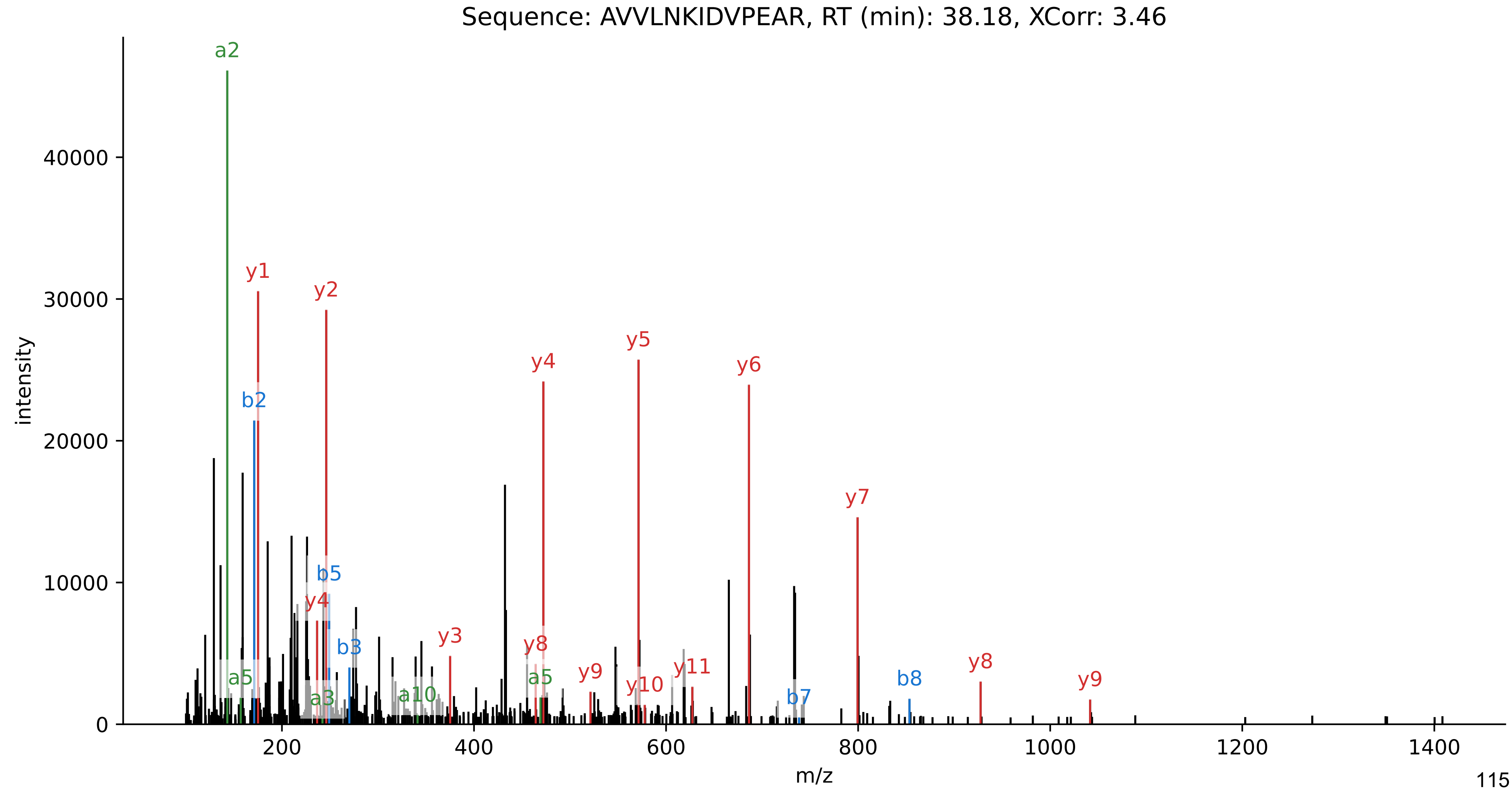

Sequence: AVVLNKIDVPEAR, RT (min): 38.18, XCorr: 3.46
intensity
m/z
a2
y1
y2
b2
y5
y4
y6
y7
b5
y4
y3
b3
y8
a5
y9
y11
y10
a3
a10
a5
b7
b8
y8
y9

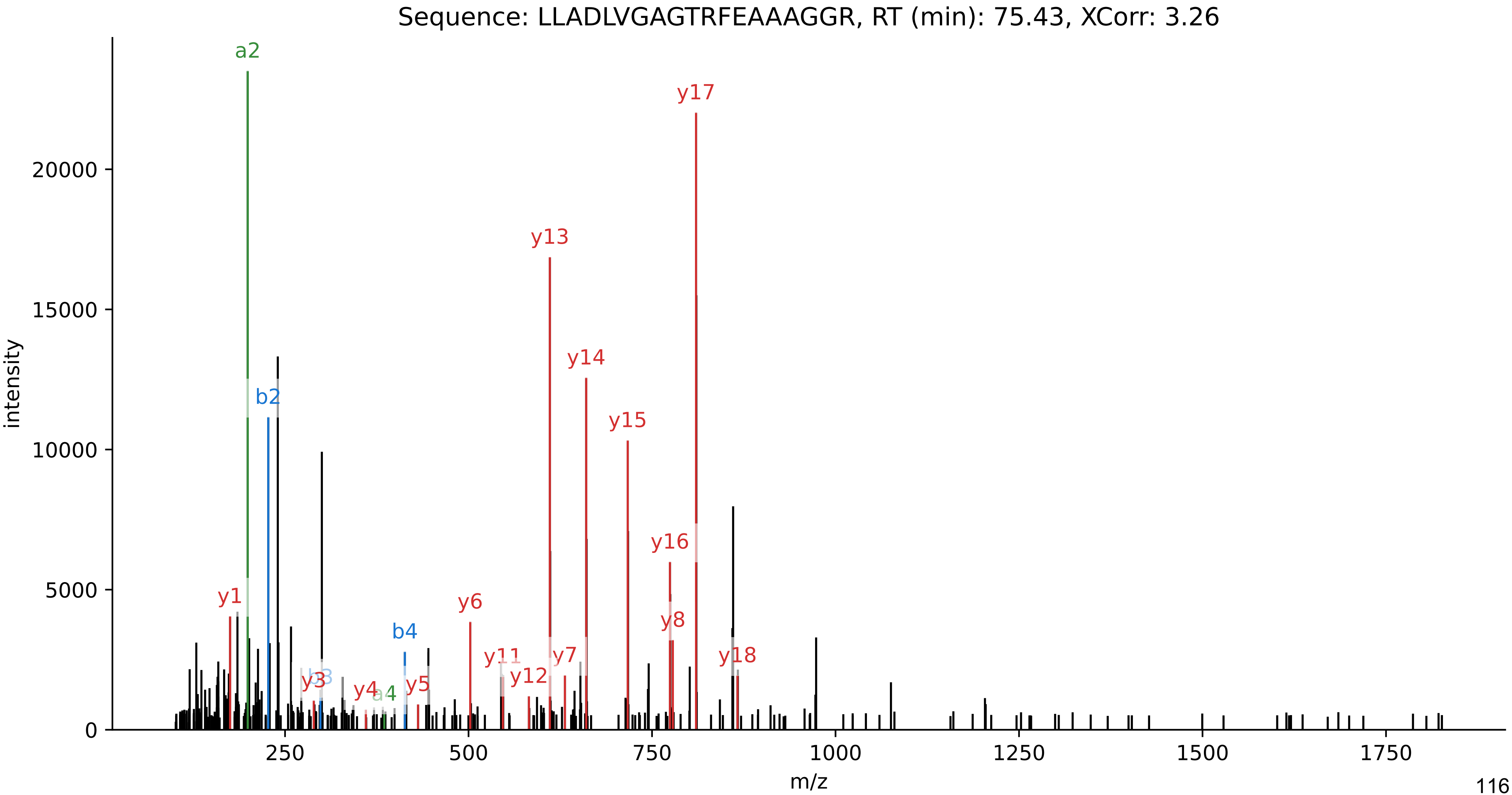

Sequence: LLADLVGAGTRFEAAAGGR, RT (min): 75.43, XCorr: 3.26
a2
y17
y13
y14
b2
y15
y16
y1
y6
y8
b4
y11
y7
y18
y12
b3
y3
y4
a4
y5
intensity
m/z
0
5000
10000
15000
20000
250
500
750
1000
1250
1500
1750

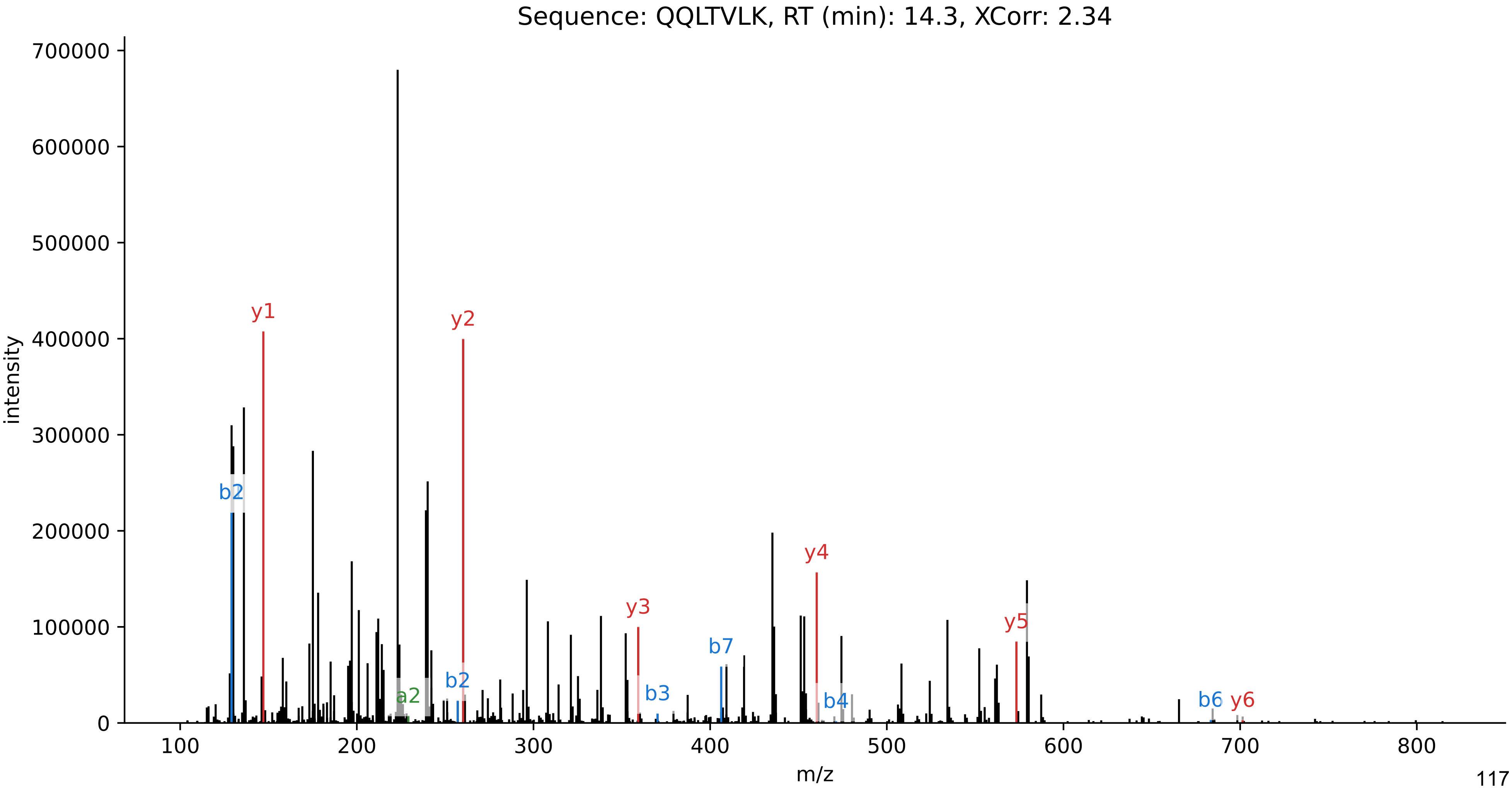
Sequence: QQLTVLK, RT (min): 14.3, XCorr: 2.34
intensity
m/z
y1
y2
b2
b2
a2
y3
b3
b7
y4
b4
y5
b6
y6
0
100000
200000
300000
400000
500000
600000
700000
100
200
300
400
500
600
700
800

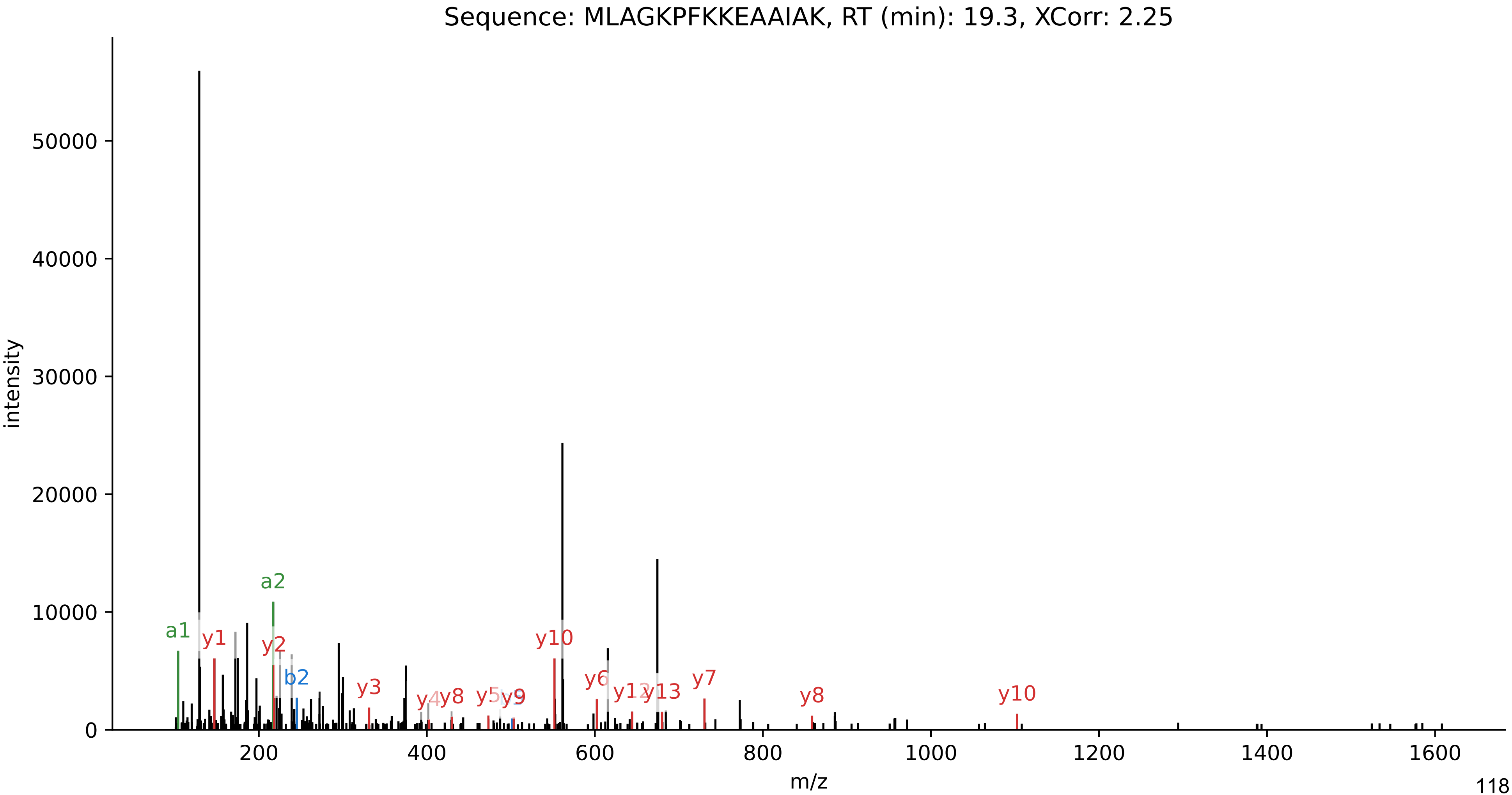

Sequence: MLAGKPFKKEAAIAK, RT (min): 19.3, XCorr: 2.25
intensity
m/z
a1
a2
y1
y2
b2
y3
y4
y8
y5
y9
y10
y6
y12
y13
y7
y8
y10

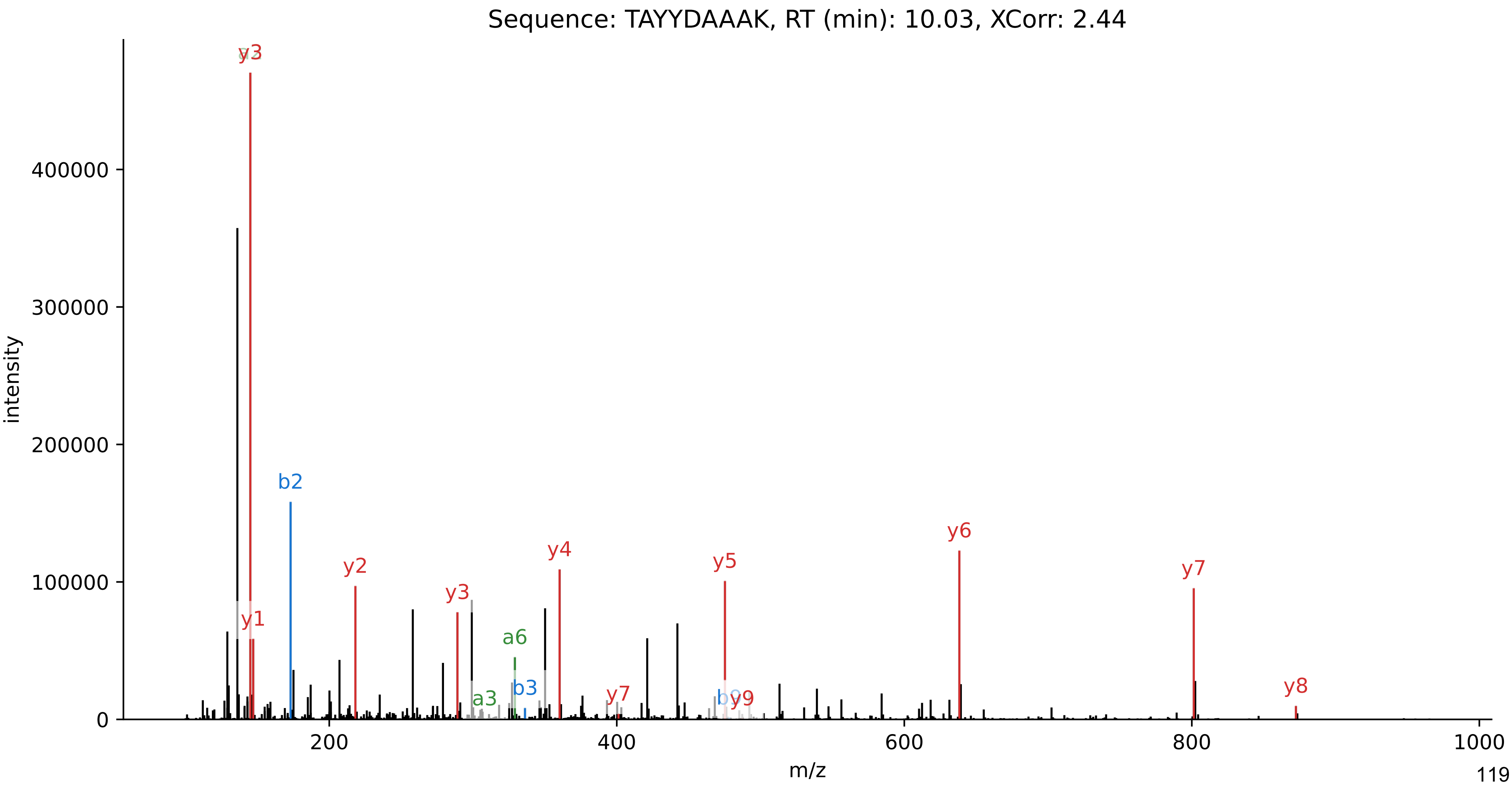

Sequence: TAYYDAAAK, RT (min): 10.03, XCorr: 2.44
y3
b2
y2
y3
y1
a6
a3
b3
y4
y7
y5
y9
y6
y7
y8
intensity
m/z
0
100000
200000
300000
400000
200
400
600
800
1000

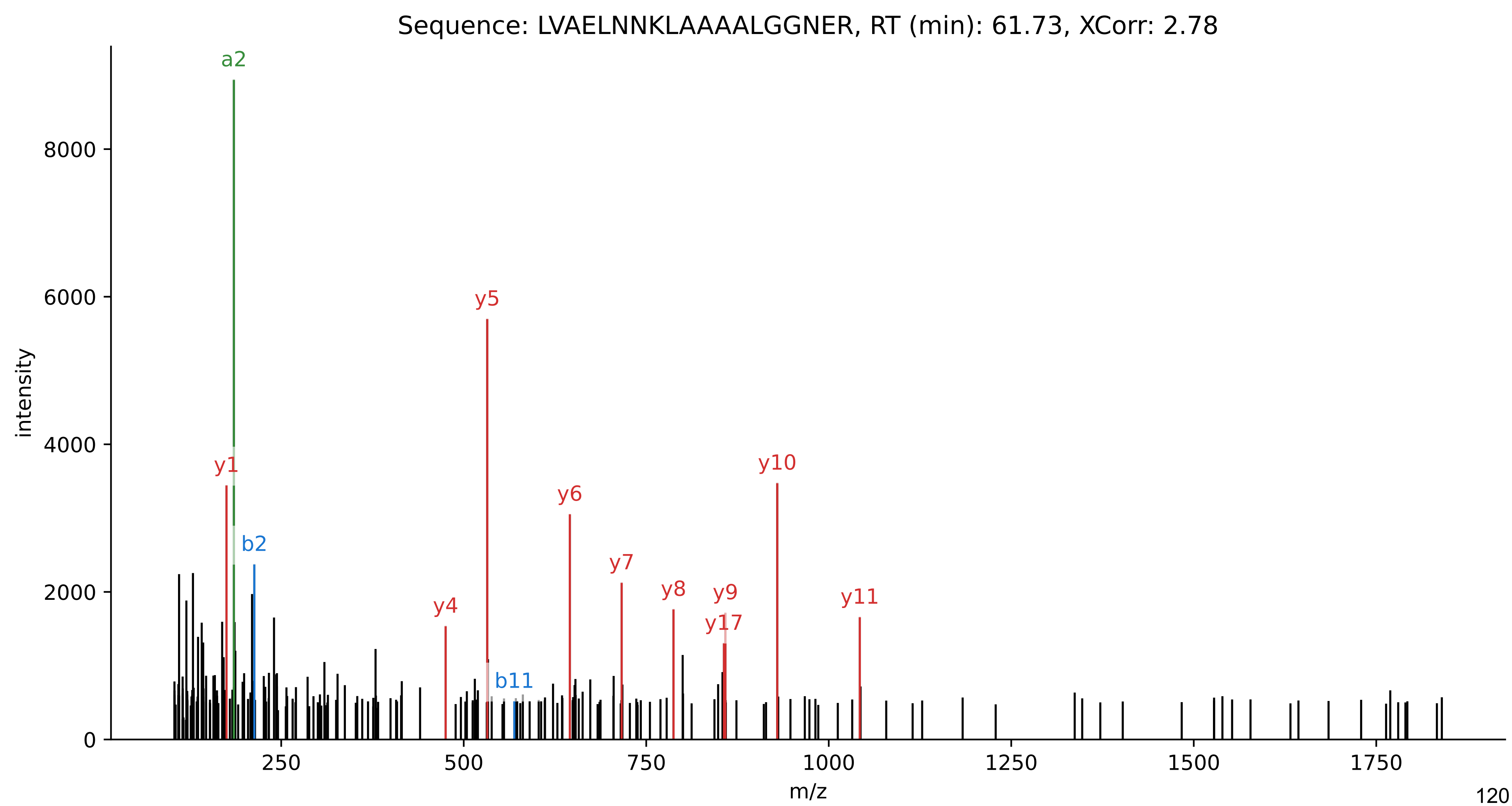
Sequence: LVAELNNKLAAAALGGNER, RT (min): 61.73, XCorr: 2.78
a2
y5
y1
y10
y6
b2
y7
y8
y9
y11
y4
y17
b11
intensity
0
2000
4000
6000
8000
250
500
750
1000
1250
1500
1750
m/z

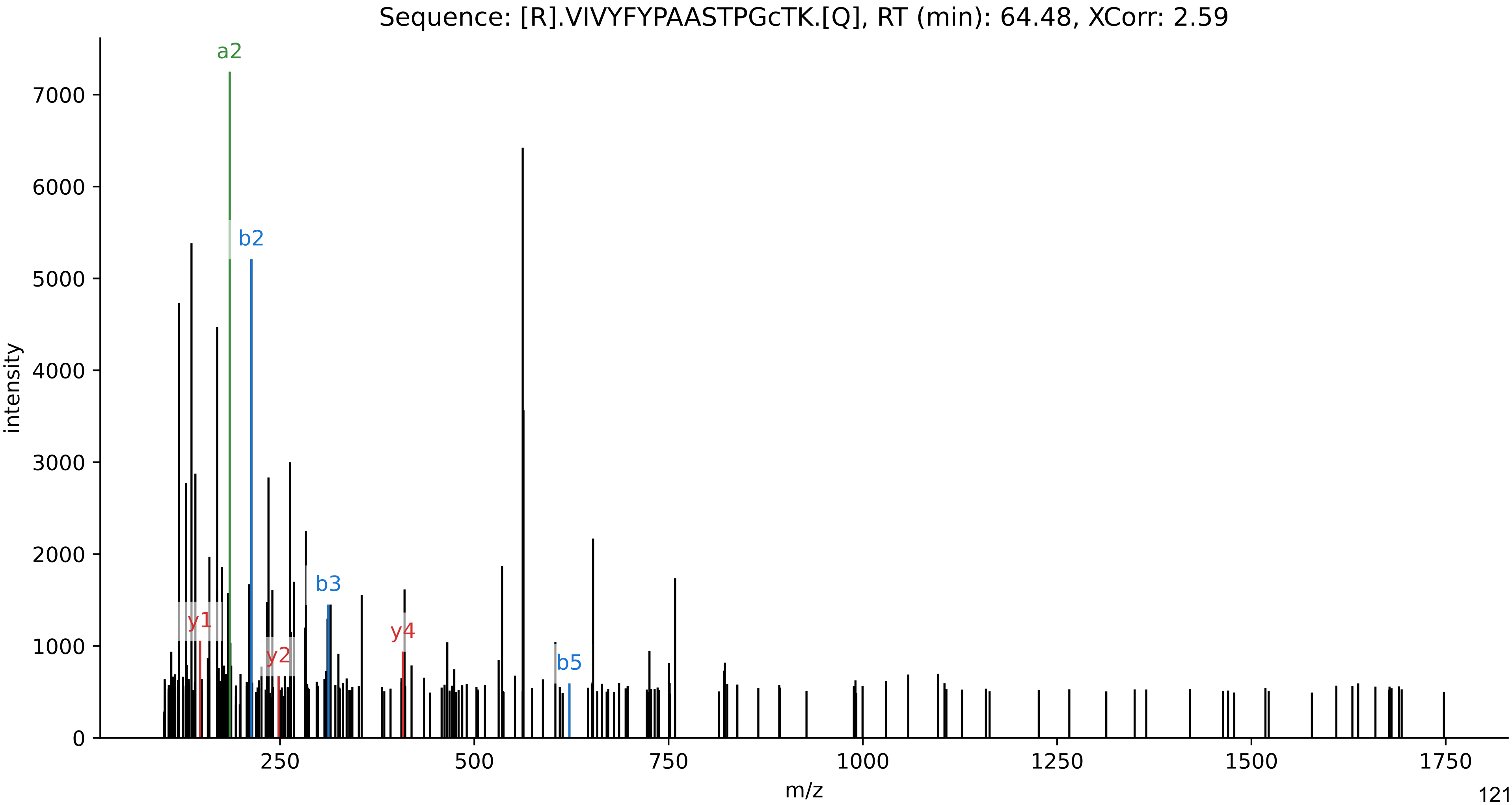

Sequence: [R].VIVYFYPAASTPGcTK.[Q], RT (min): 64.48, XCorr: 2.59
a2
b2
b3
b5
y1
y2
y4
intensity
m/z
0
1000
2000
3000
4000
5000
6000
7000
250
500
750
1000
1250
1500
1750

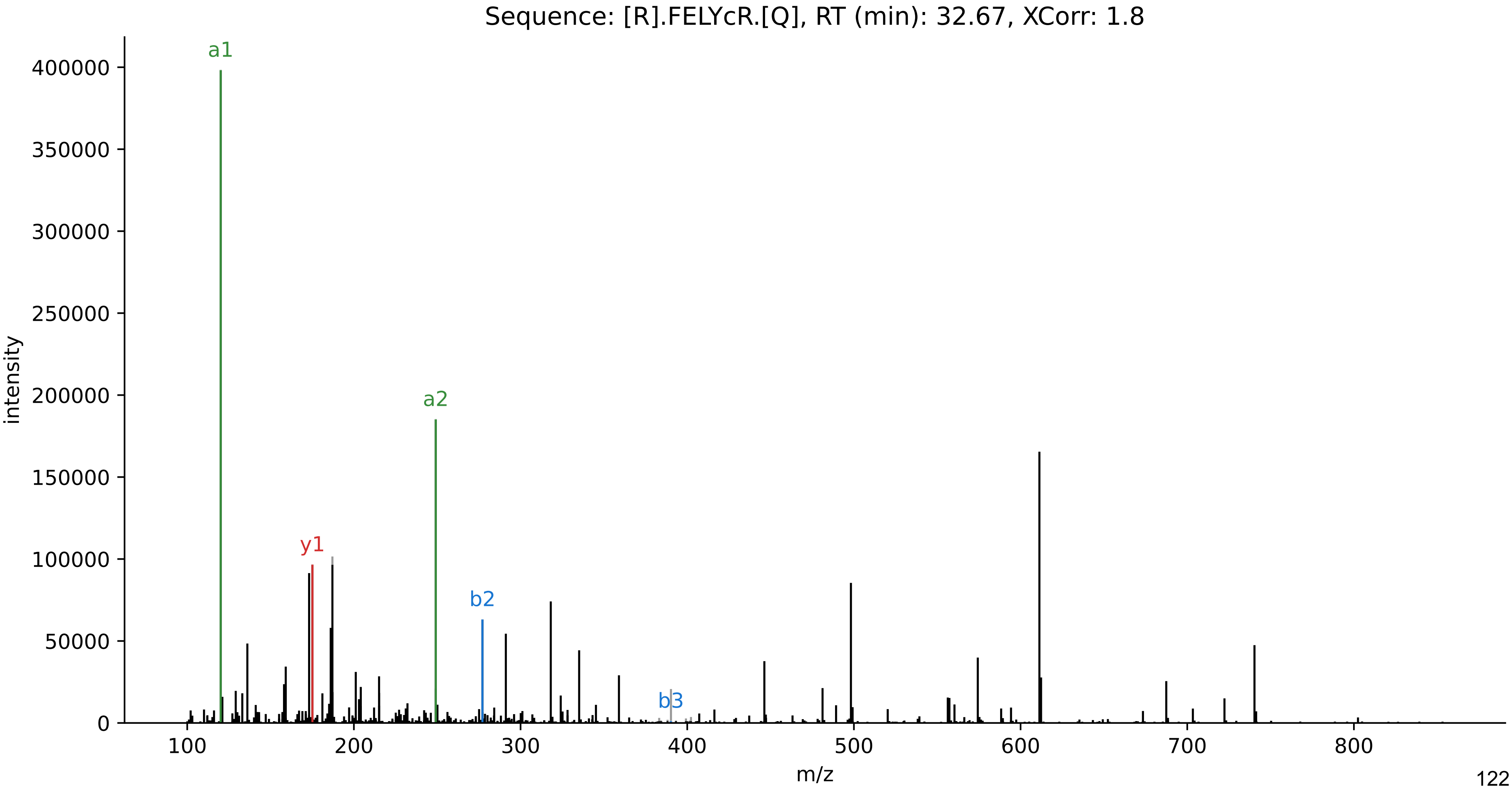

Sequence: [R].FELYcR.[Q], RT (min): 32.67, XCorr: 1.8
a1
a2
y1
b2
b3
intensity
m/z
0
50000
100000
150000
200000
250000
300000
350000
400000
100
200
300
400
500
600
700
800

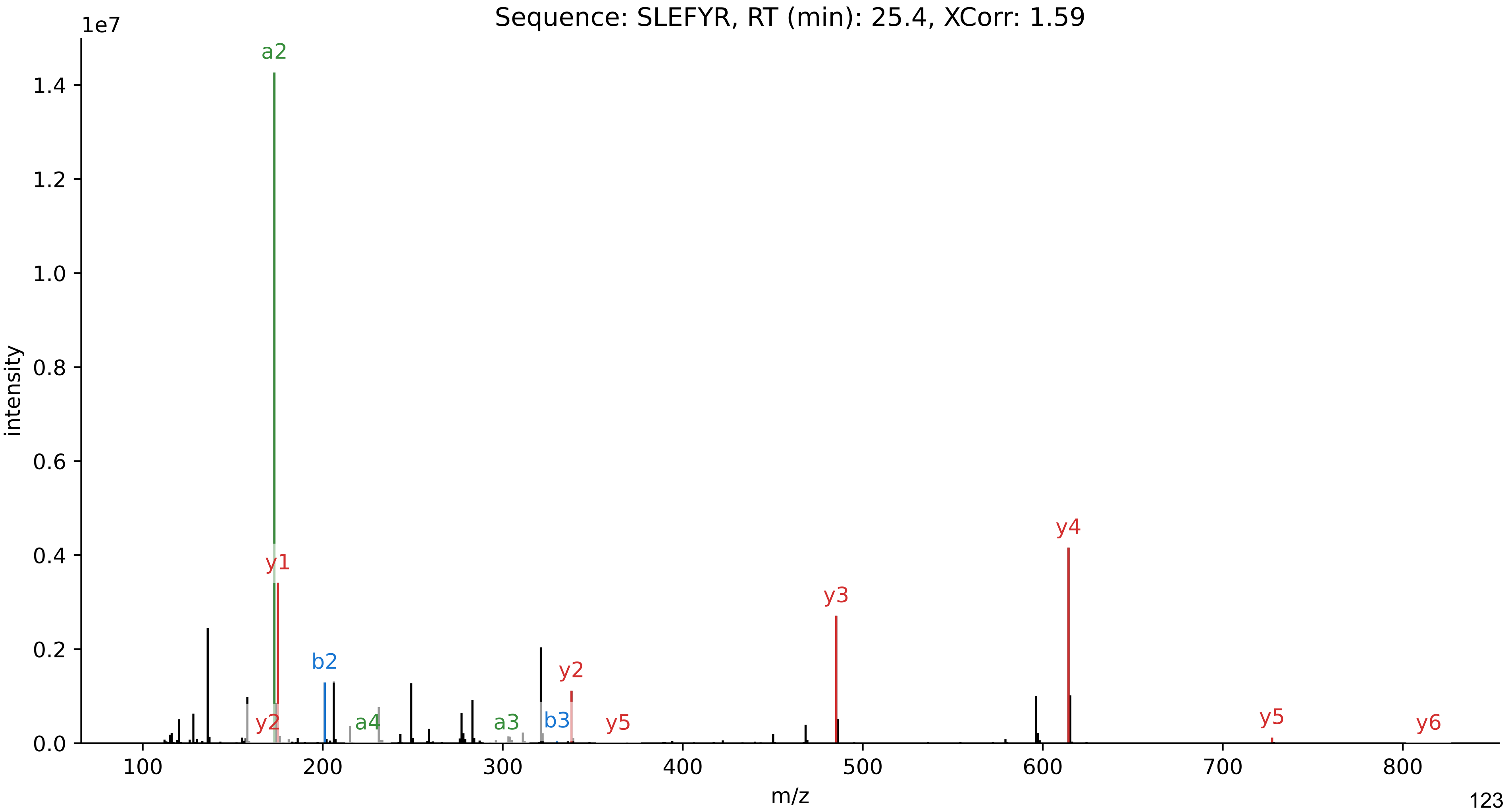

Sequence: SLEFYR, RT (min): 25.4, XCorr: 1.59
1e7
intensity
m/z
a2
y1
b2
y2
a4
a3
b3
y2
y5
y3
y4
y5
y6
0.0
0.2
0.4
0.6
0.8
1.0
1.2
1.4
100
200
300
400
500
600
700
800

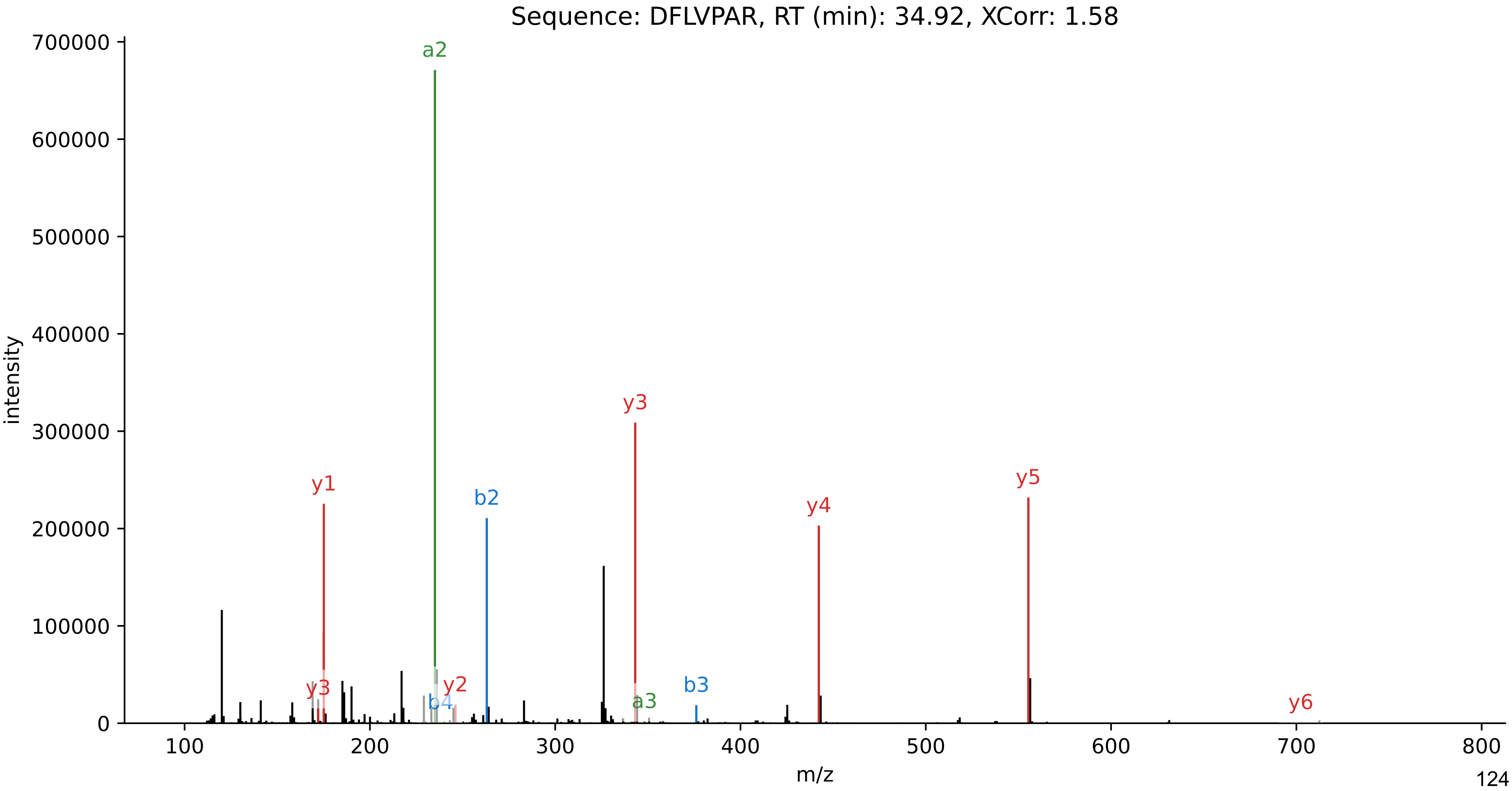

Sequence: DFLVPAR, RT (min): 34.92, XCorr: 1.58
intensity
m/z
a2
y3
y1
b2
y4
y5
y3
y2
b4
b3
a3
y6

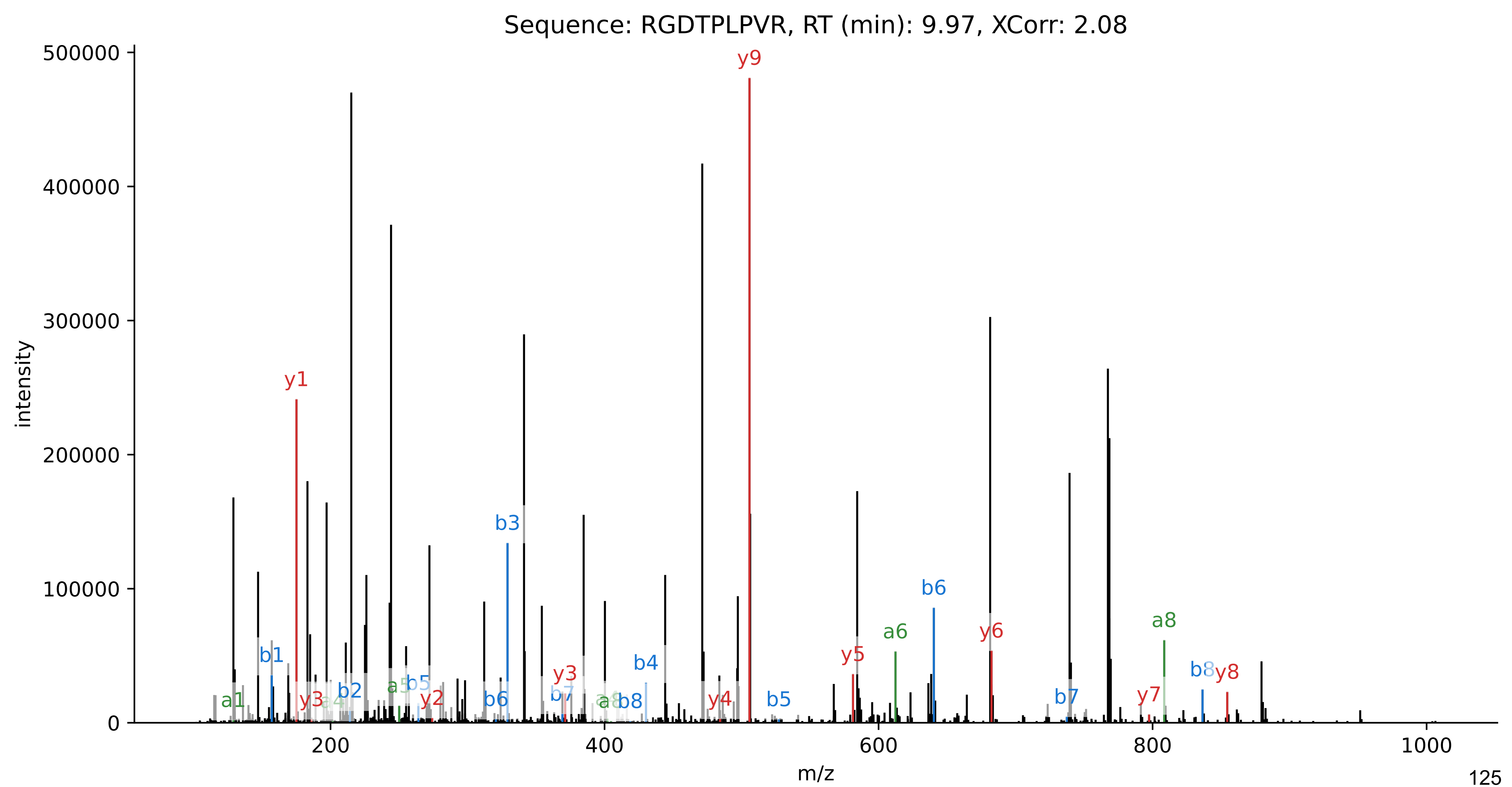

Sequence: RGDTPLPVR, RT (min): 9.97, XCorr: 2.08
intensity
m/z
y9
y1
b3
b6
y6
a6
a8
y5
b1
b4
y3
b8
y8
b7
y7
b5
y2
b2
y4
a1

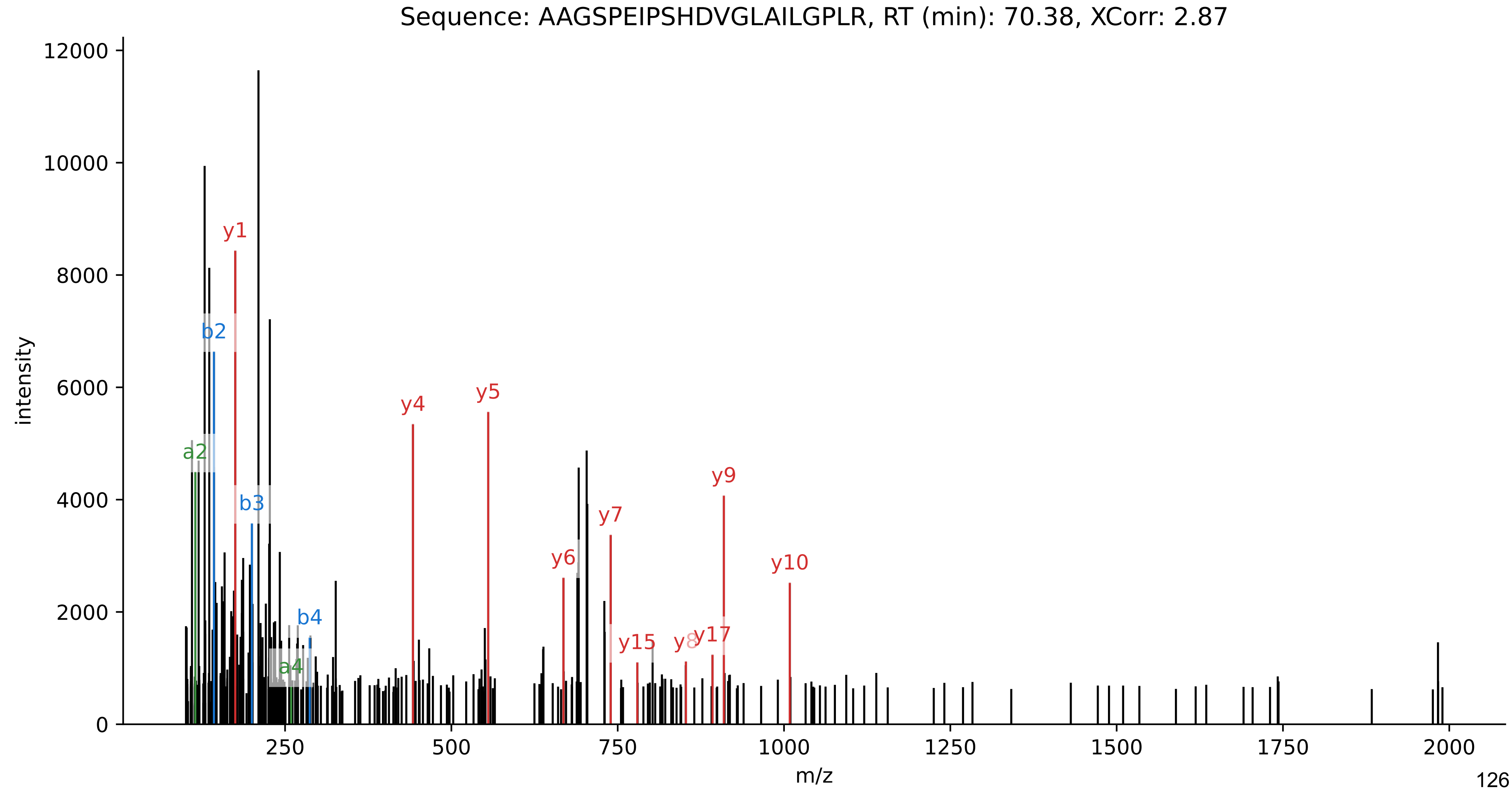

Sequence: AAGSPEIPSHDVGLAILGPLR, RT (min): 70.38, XCorr: 2.87
intensity
m/z
y1
b2
a2
b3
b4
a4
y4
y5
y6
y7
y15
y8
y17
y9
y10
0
2000
4000
6000
8000
10000
12000
250
500
750
1000
1250
1500
1750
2000

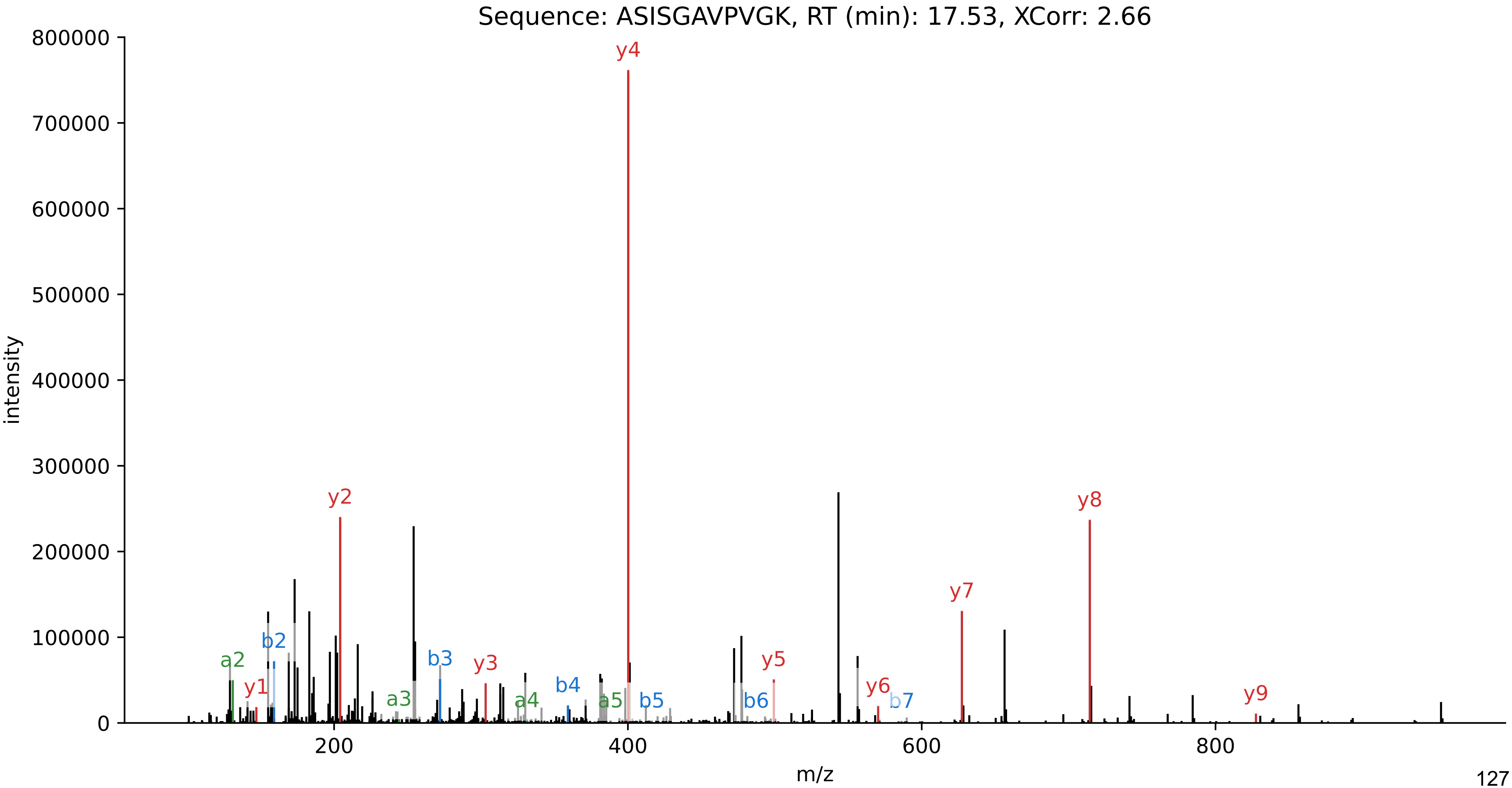

Sequence: ASISGAVPVGK, RT (min): 17.53, XCorr: 2.66
intensity
m/z
800000
700000
600000
500000
400000
300000
200000
100000
0
200
400
600
800
y4
y2
y8
y7
a2
b2
y1
b3
y3
a3
a4
b4
a5
b5
y5
b6
y6
b7
y9

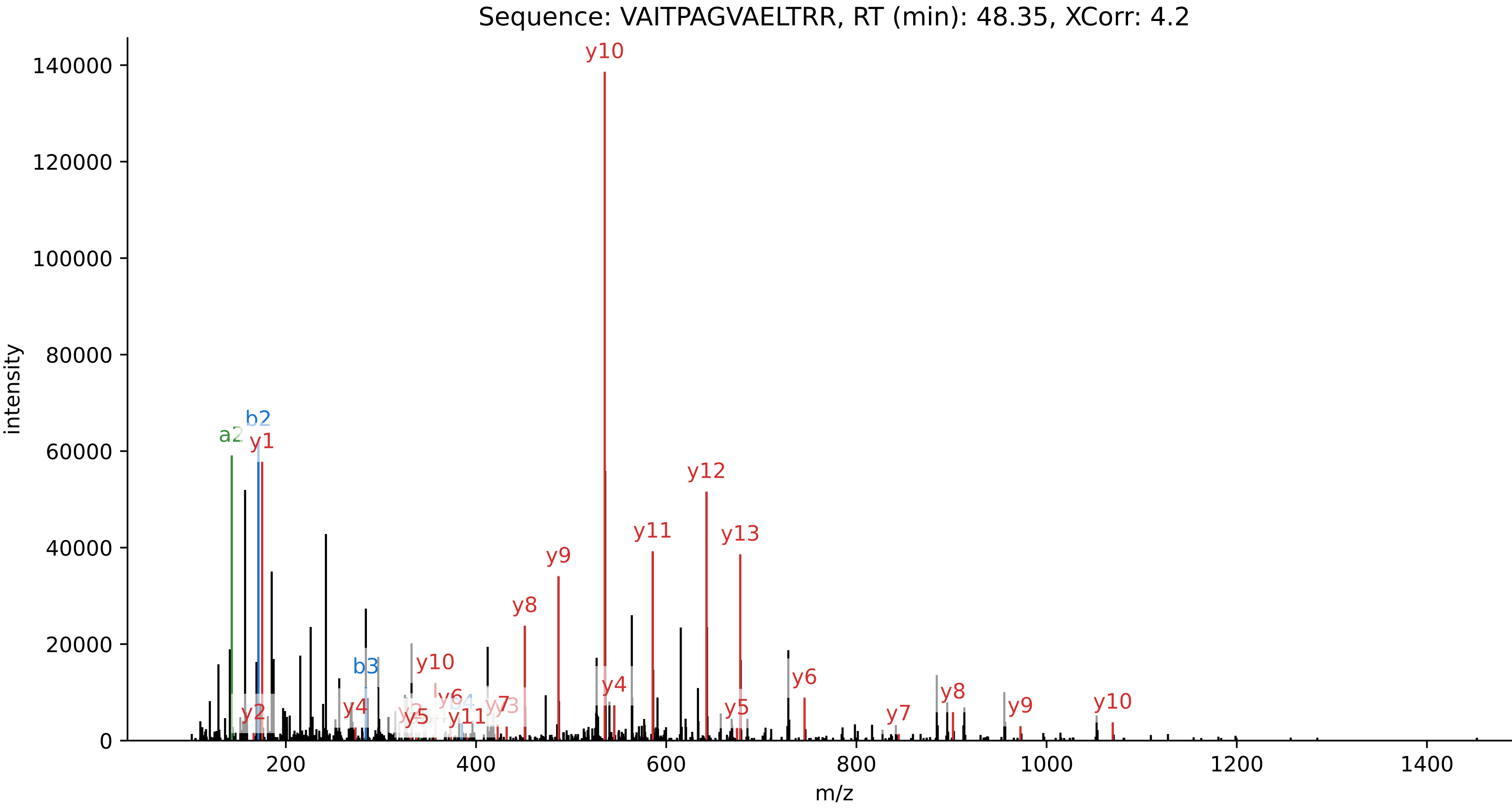
Sequence: VAITPAGVAELTRR, RT (min): 48.35, XCorr: 4.2
intensity
m/z
0
20000
40000
60000
80000
100000
120000
140000
200
400
600
800
1000
1200
1400
y10
a2
b2
y1
y12
y11
y13
y9
y8
b3
y10
y4
y2
y5
y6
y11
y4
y5
y6
y7
y8
y9
y10

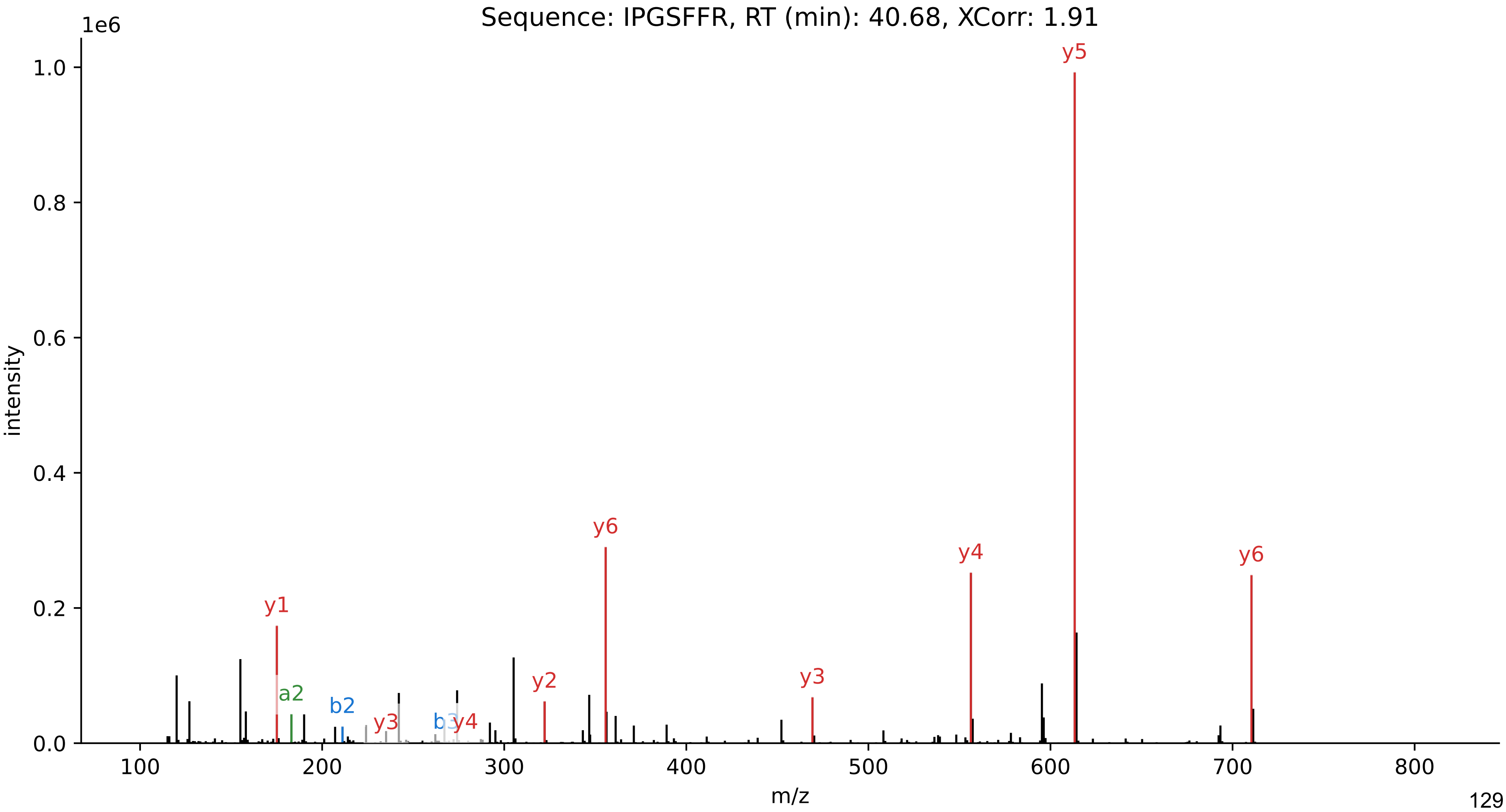

Sequence: IPGSFFR, RT (min): 40.68, XCorr: 1.91
1e6
1.0
0.8
0.6
0.4
0.2
0.0
intensity
y5
y6
y4
y6
y1
a2
b2
y3
b3
y4
y2
y3
100
200
300
400
500
600
700
800
m/z

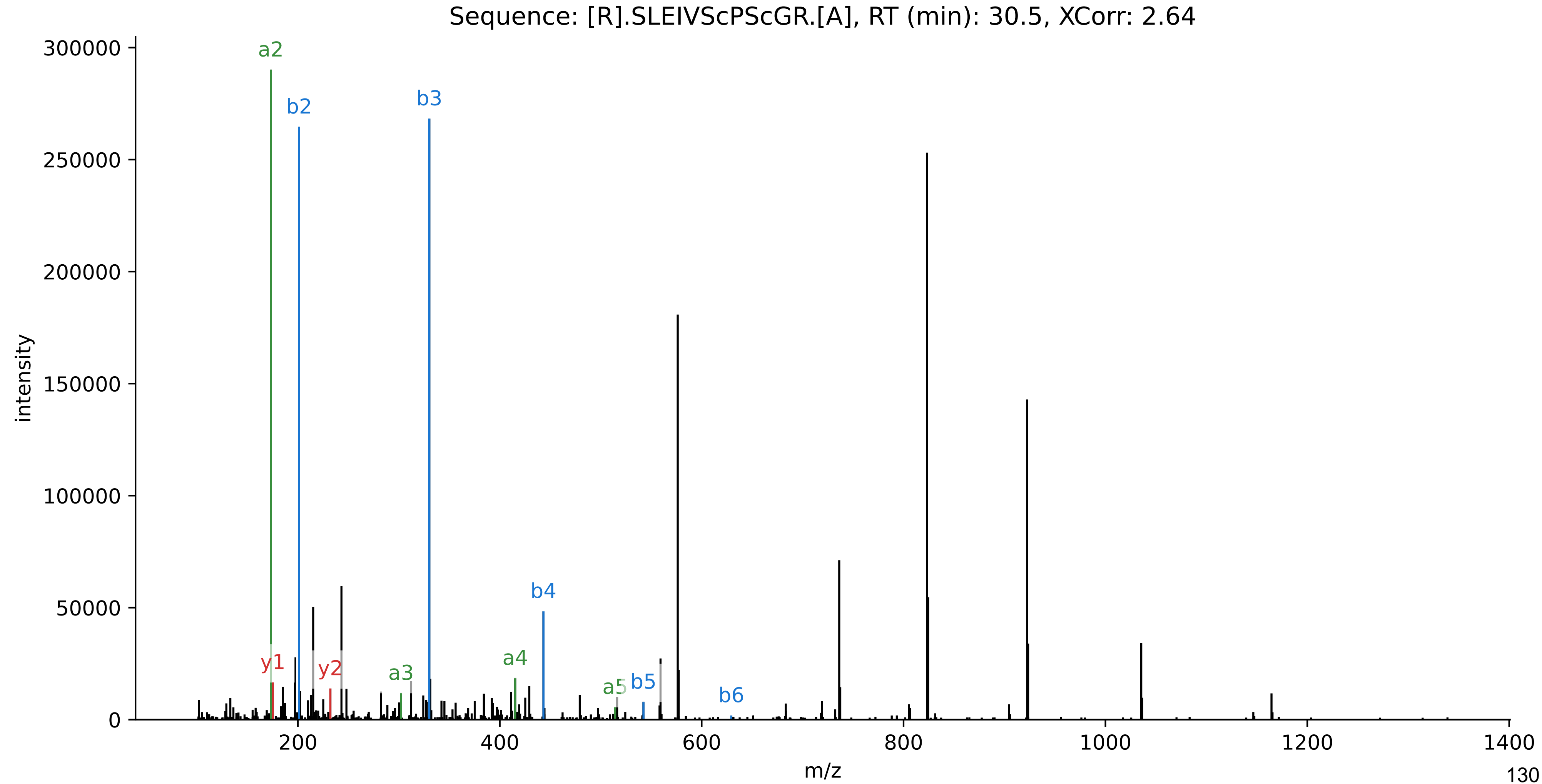
Sequence: [R].SLEIVScPScGR.[A], RT (min): 30.5, XCorr: 2.64
a2
b2
b3
y1
y2
a3
a4
b4
a5
b5
b6
intensity
m/z
300000
250000
200000
150000
100000
50000
0
200
400
600
800
1000
1200
1400

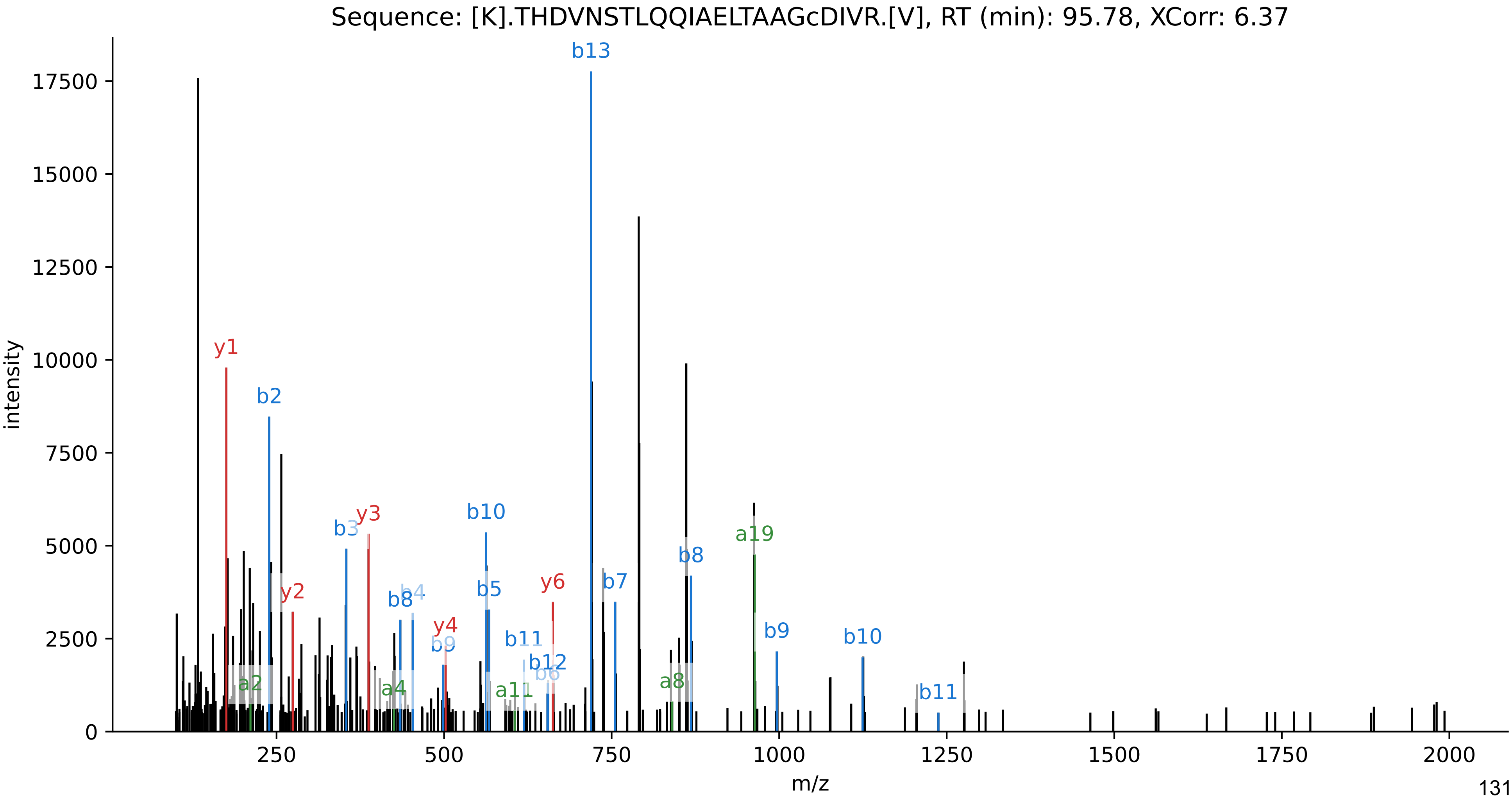
Sequence: [K].THDVNSTLQQIAELTAAGcDIVR.[V], RT (min): 95.78, XCorr: 6.37
intensity
m/z
y1
b2
y2
b3
y3
b8
b4
y4
b9
b10
b5
b11
b12
b6
y6
b13
b7
b8
a19
b9
b10
b11
a2
a4
a11
a8

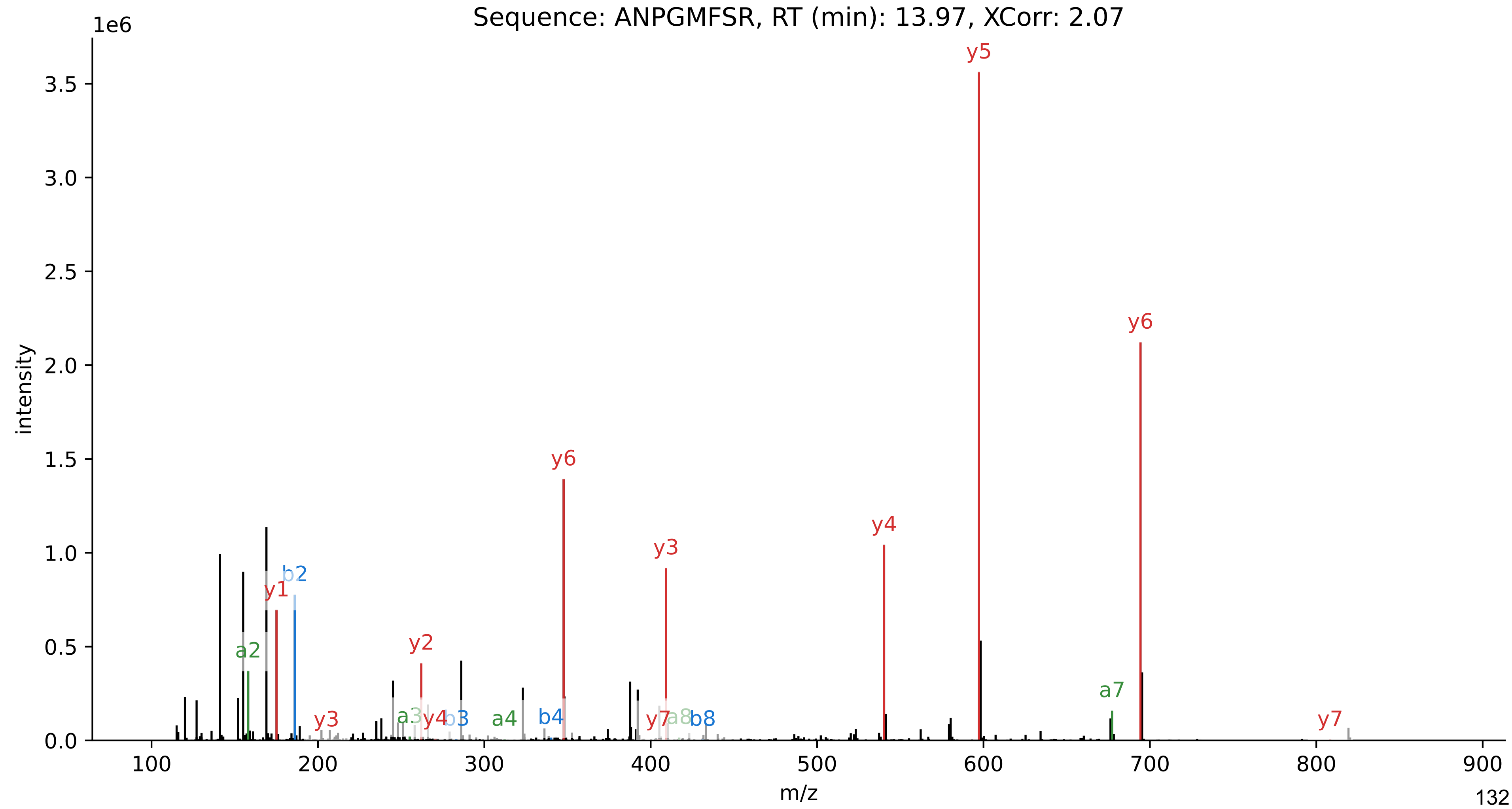

Sequence: ANPGMFSR, RT (min): 13.97, XCorr: 2.07
1e6
intensity
m/z
y5
y6
y6
y4
y3
b2
y1
y2
a2
y3
a3
y4
b3
a4
b4
y7
a8
b8
a7
y7

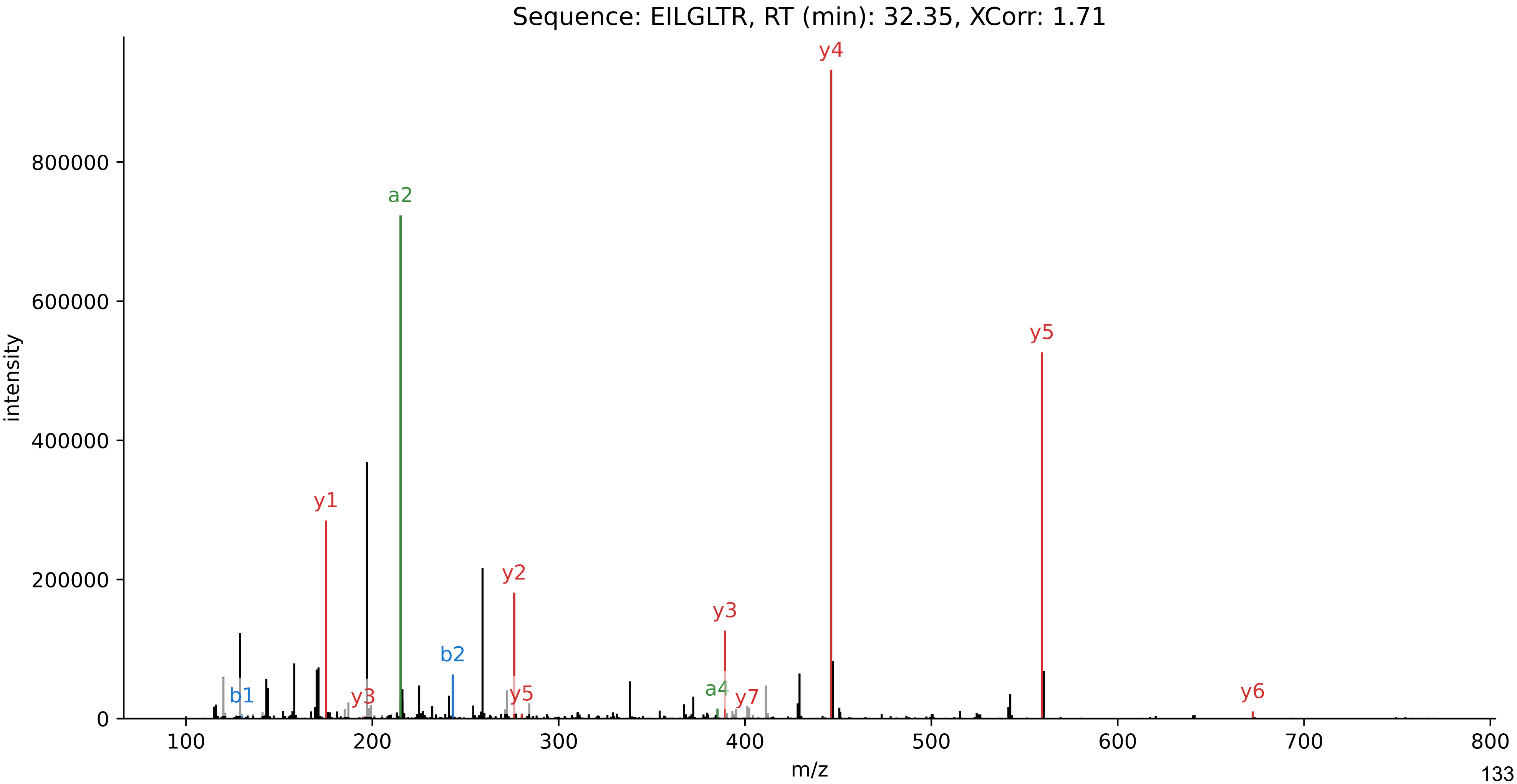

Sequence: EILGLTR, RT (min): 32.35, XCorr: 1.71
intensity
m/z
y4
a2
y5
y1
y2
y3
b2
b1
y3
y5
a4
y7
y6
800000
600000
400000
200000
0
100
200
300
400
500
600
700
800

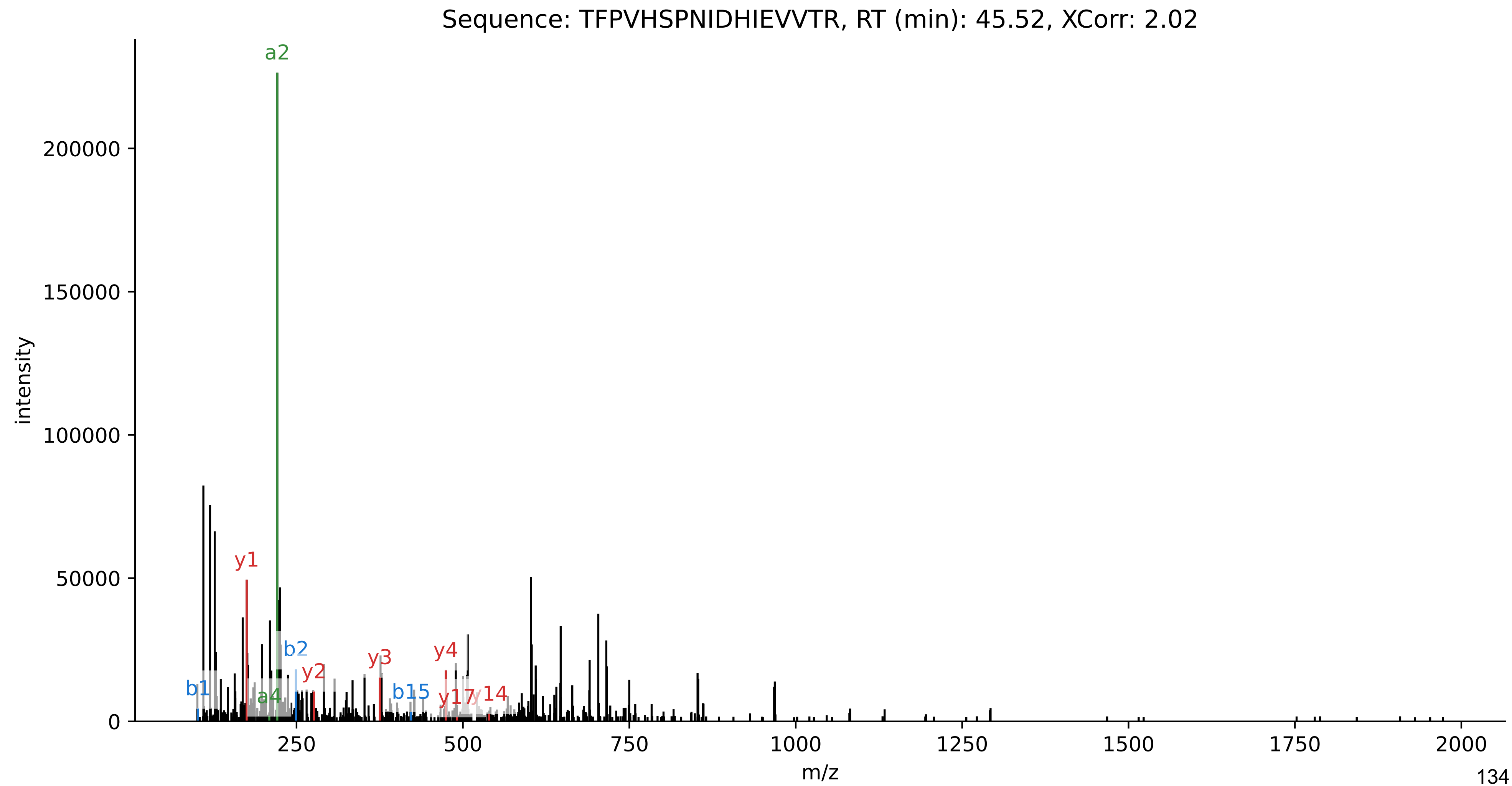
Sequence: TFPVHSPNIDHIEVVTR, RT (min): 45.52, XCorr: 2.02
a2
y1
b2
y2
y3
y4
b1
a4
b15
y17
y14
intensity
m/z
0
50000
100000
150000
200000
250
500
750
1000
1250
1500
1750
2000

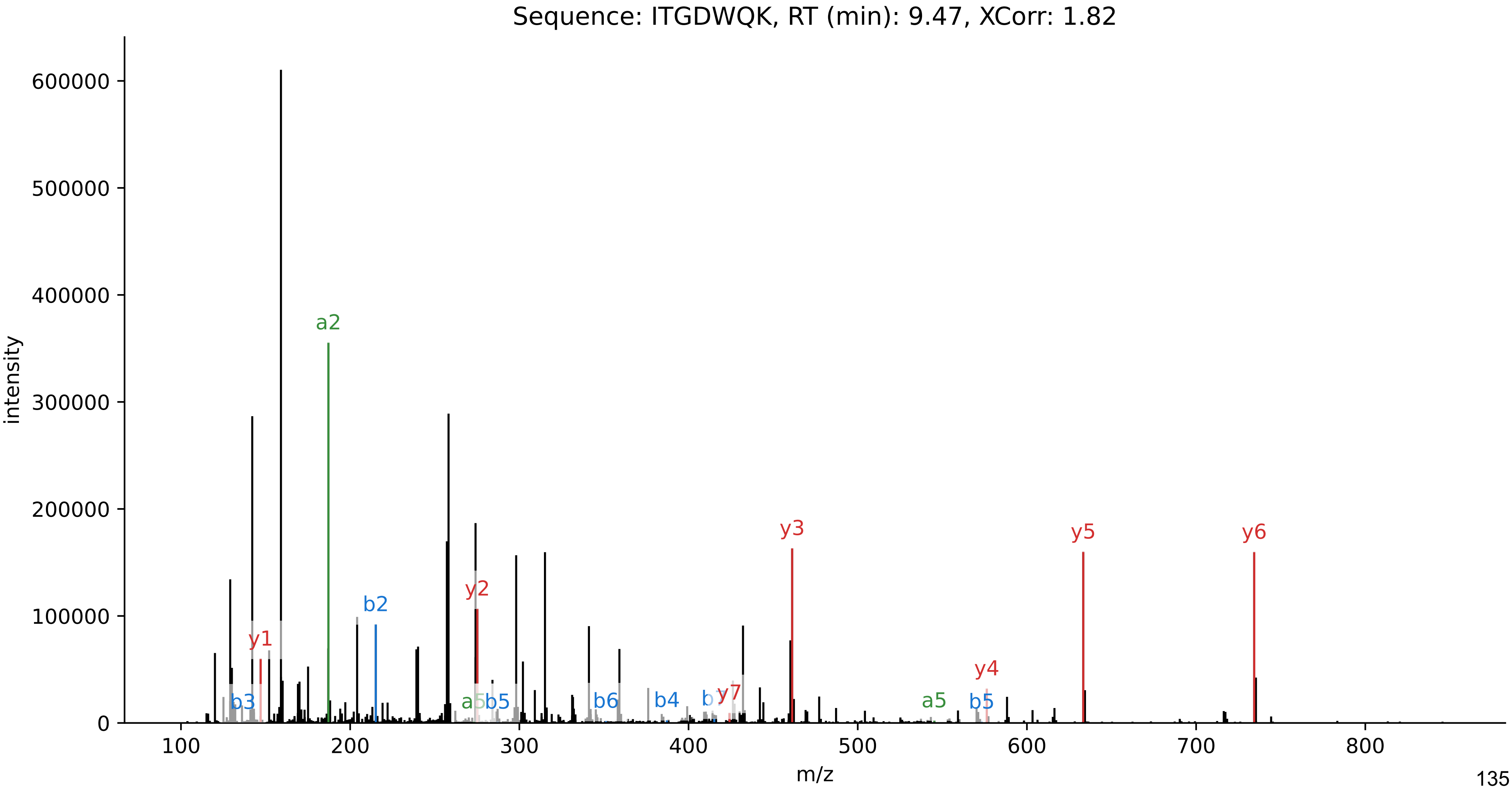
Sequence: ITGDWQK, RT (min): 9.47, XCorr: 1.82
intensity
m/z
a2
b2
y1
y2
y3
y5
y6
y4
a5
b3
b5
b6
b4
y7
0
100000
200000
300000
400000
500000
600000
100
200
300
400
500
600
700
800

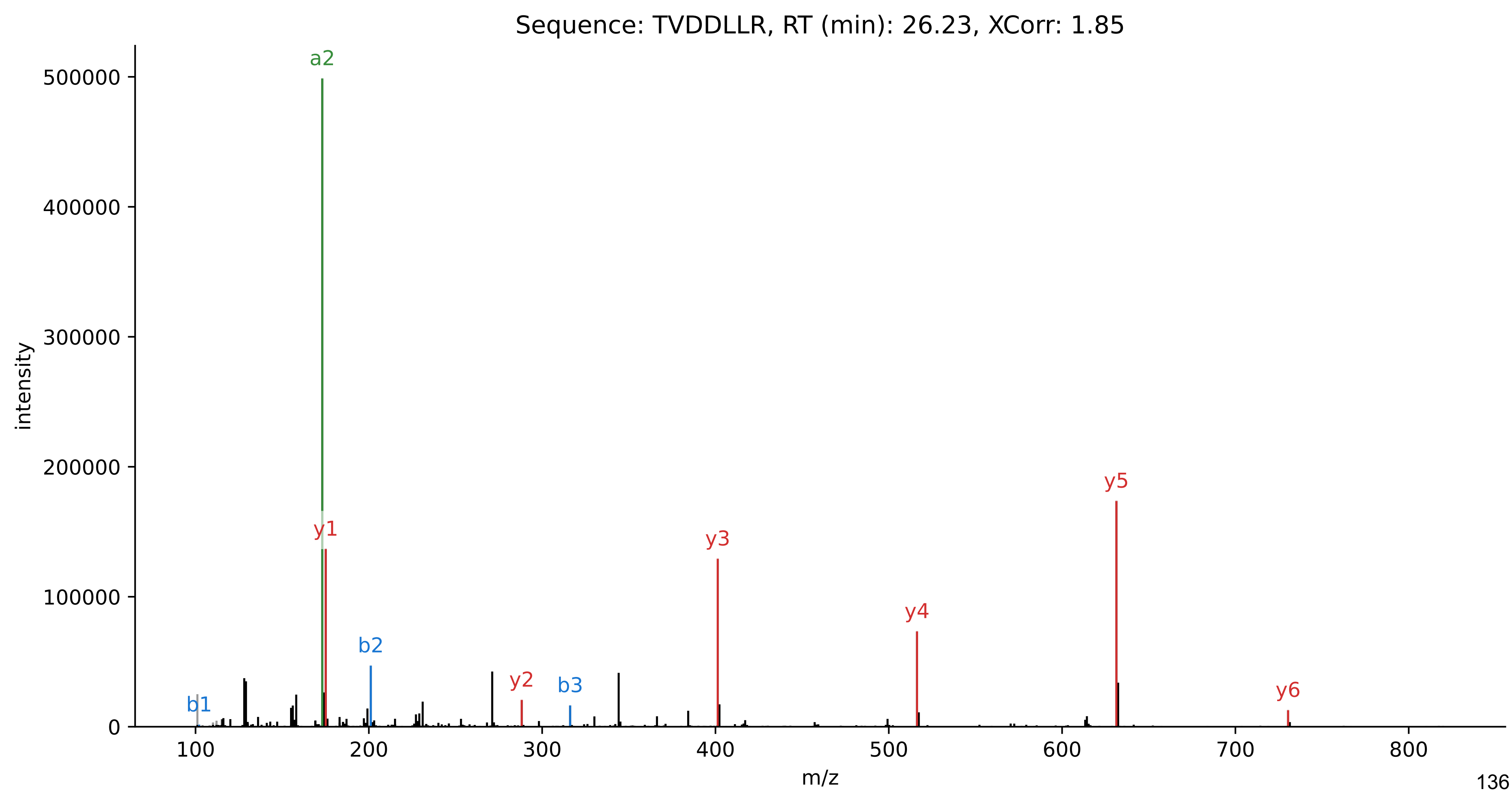

Sequence: TVDDLLR, RT (min): 26.23, XCorr: 1.85
intensity
m/z
a2
y1
b1
b2
y2
b3
y3
y4
y5
y6
0
100000
200000
300000
400000
500000
100
200
300
400
500
600
700
800

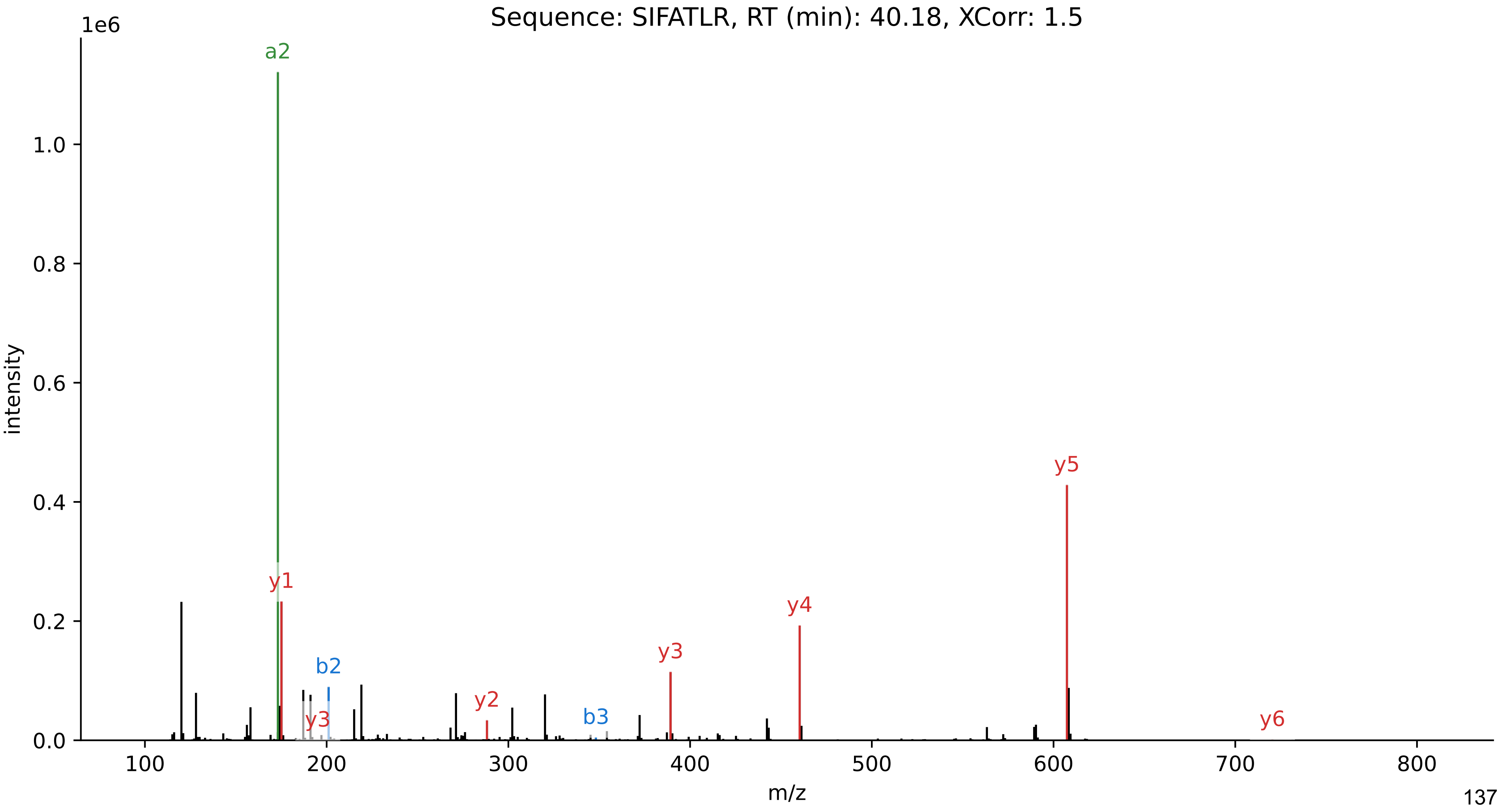
Sequence: SIFATLR, RT (min): 40.18, XCorr: 1.5
1e6
a2
y5
y1
y4
y3
b2
y2
b3
y3
y6
intensity
m/z
0.0
0.2
0.4
0.6
0.8
1.0
100
200
300
400
500
600
700
800

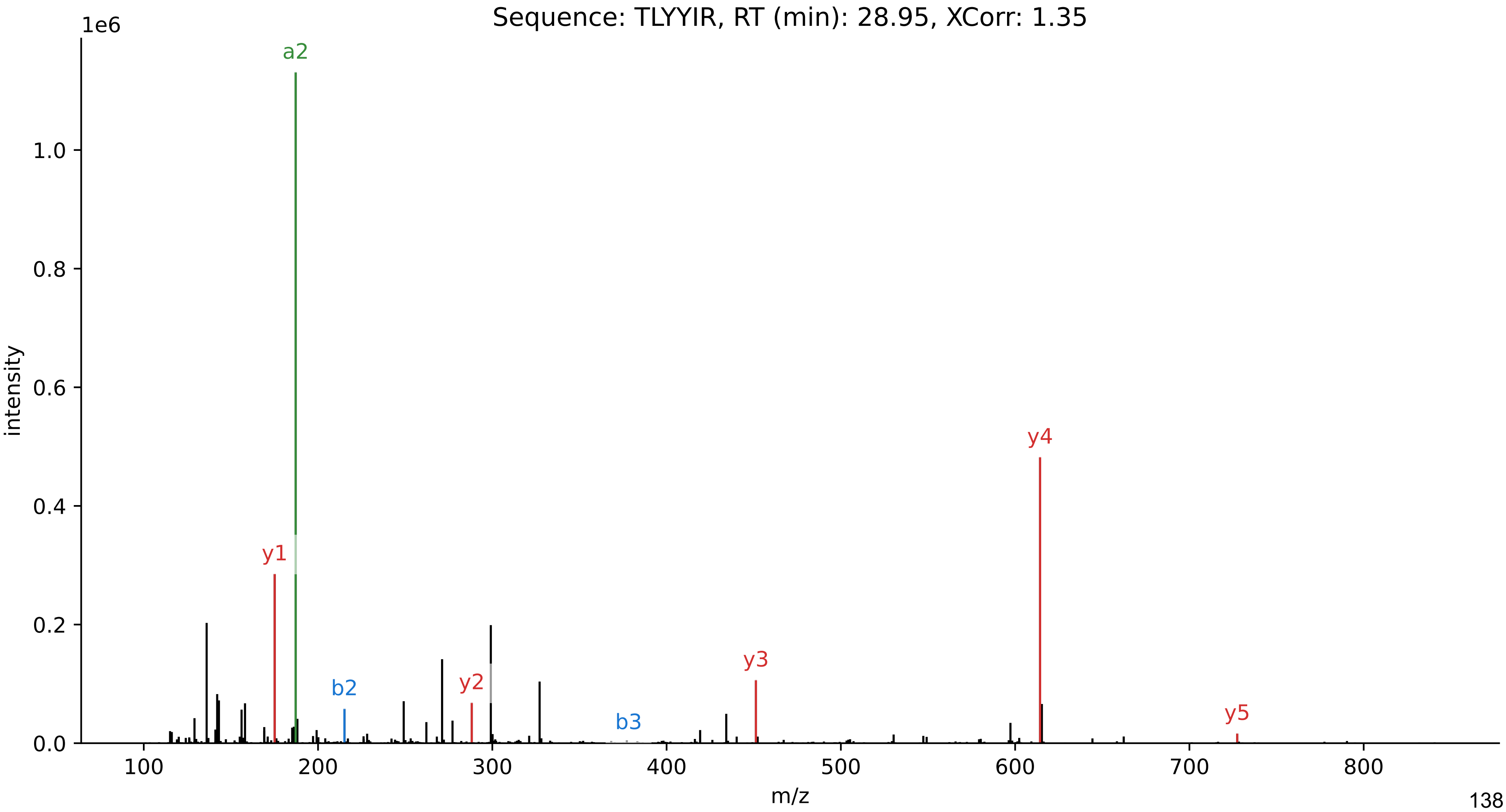

Sequence: TLYYIR, RT (min): 28.95, XCorr: 1.35
1e6
a2
y1
b2
y2
b3
y3
y4
y5
intensity
m/z
0.0
0.2
0.4
0.6
0.8
1.0
100
200
300
400
500
600
700
800

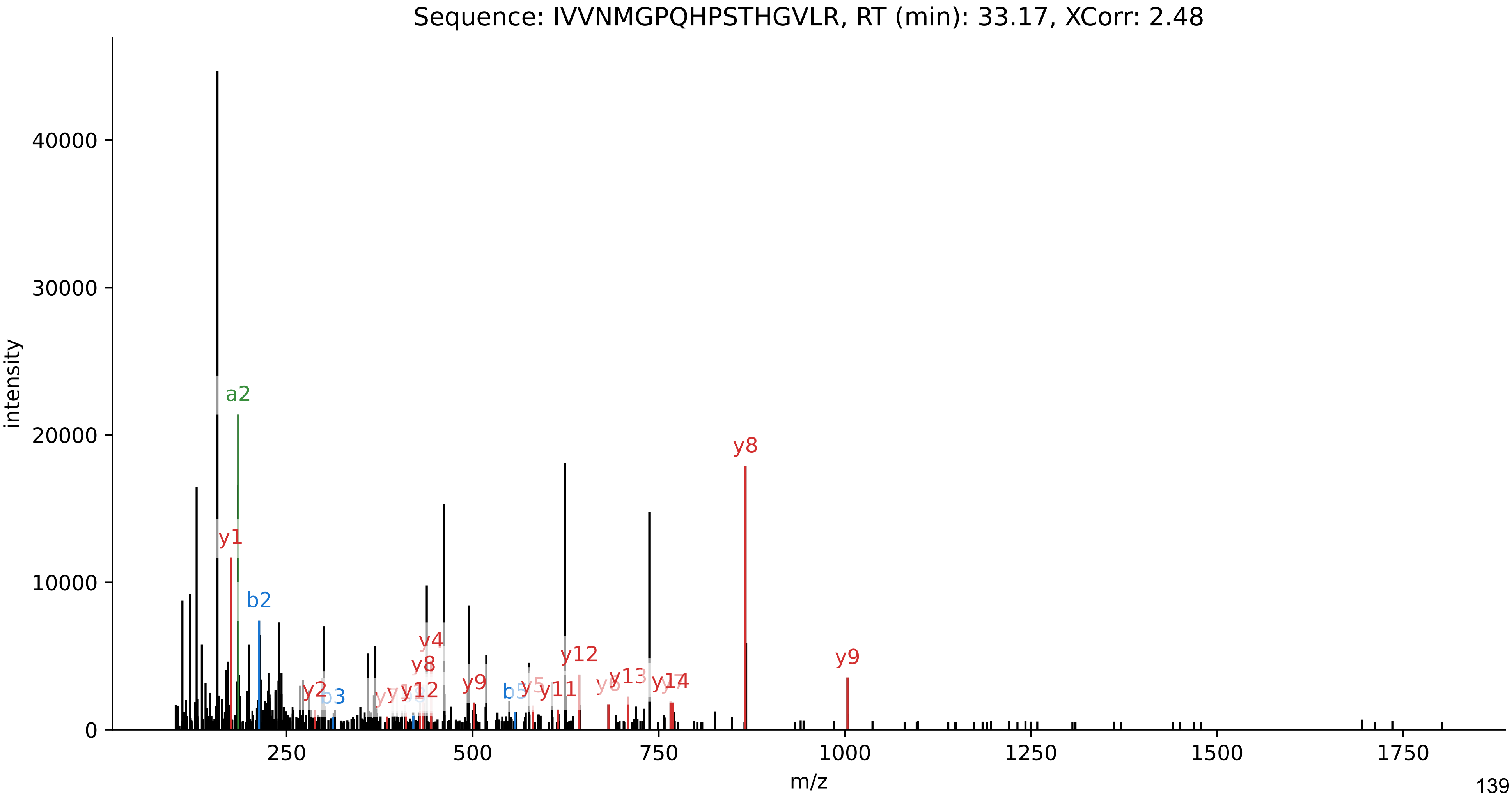

Sequence: IVVNMGPQHPSTHGVLR, RT (min): 33.17, XCorr: 2.48
intensity
m/z
0
10000
20000
30000
40000
250
500
750
1000
1250
1500
1750
a2
y1
b2
y2
b3
y4
y8
y12
y9
b5
y5
y11
y12
y13
y14
y8
y9

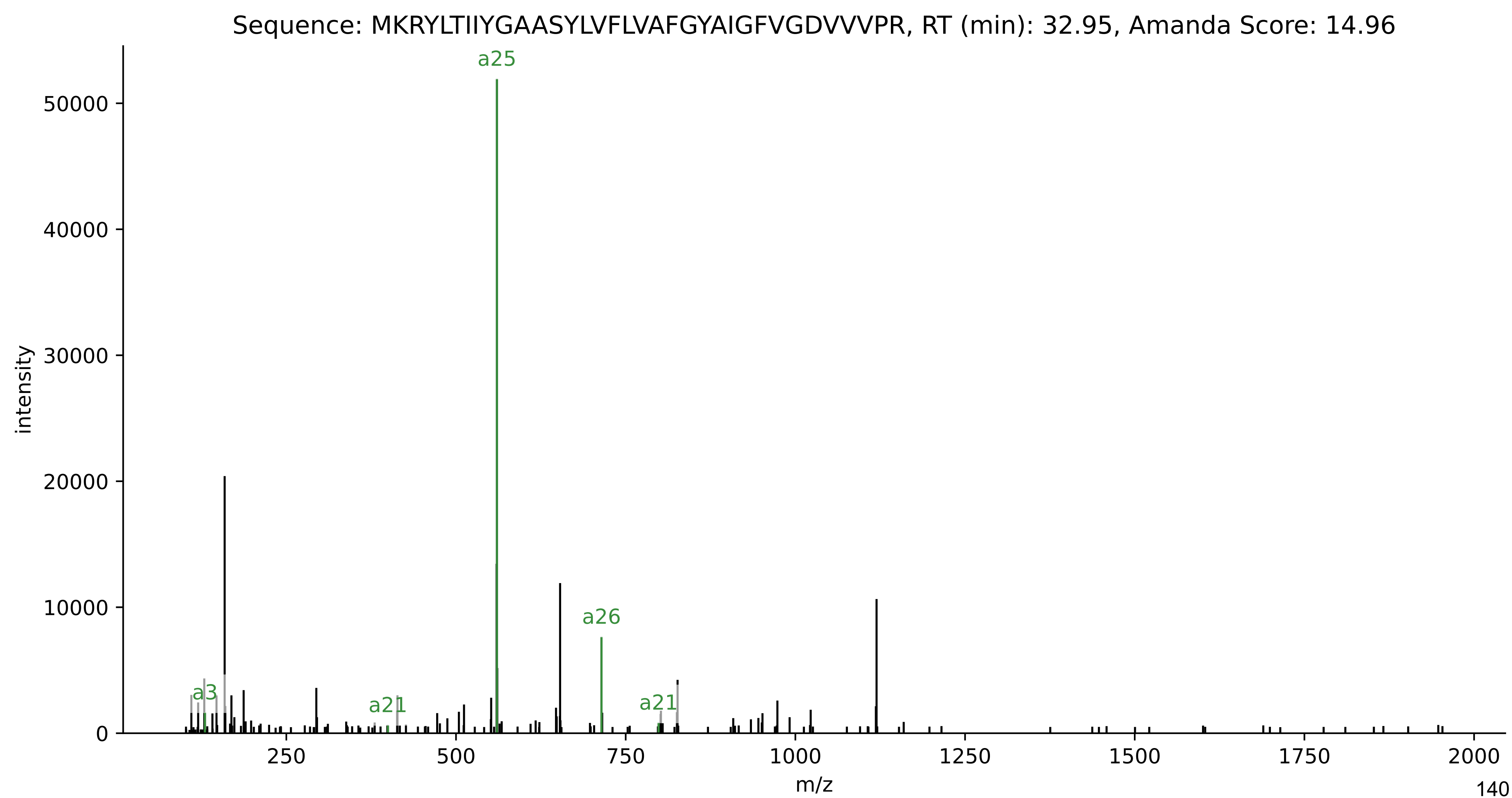
Sequence: MKRYLTIIYGAASYLVFLVAFGYAIGFVGDVVVPR, RT (min): 32.95, Amanda Score: 14.96
a25
a26
a3
a21
a21
intensity
m/z
0
10000
20000
30000
40000
50000
250
500
750
1000
1250
1500
1750
2000

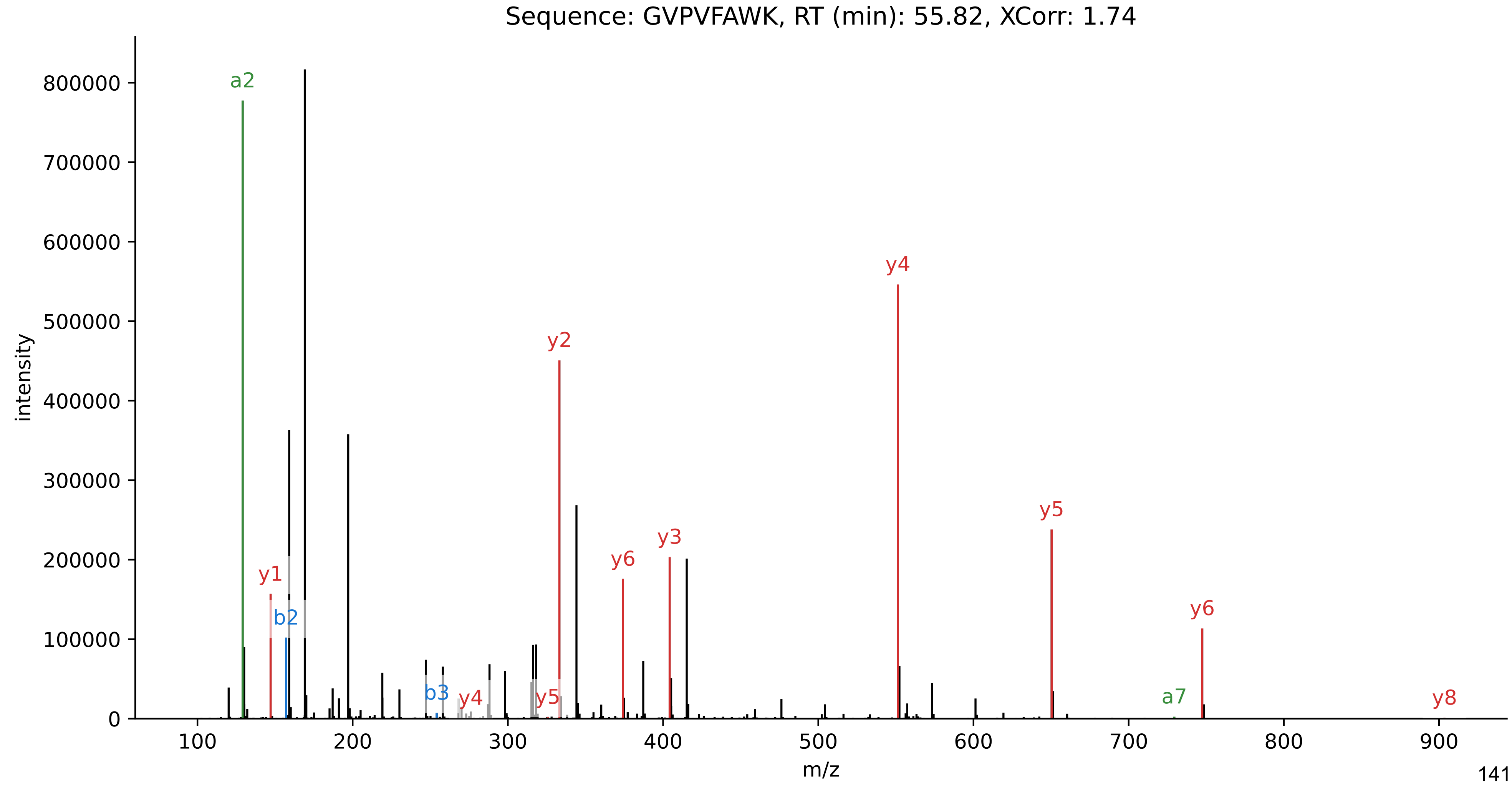

Sequence: GVPVFAWK, RT (min): 55.82, XCorr: 1.74
intensity
m/z
a2
y1
b2
b3
y4
y5
y2
y6
y3
y4
y5
a7
y6
y8
0
100000
200000
300000
400000
500000
600000
700000
800000
100
200
300
400
500
600
700
800
900

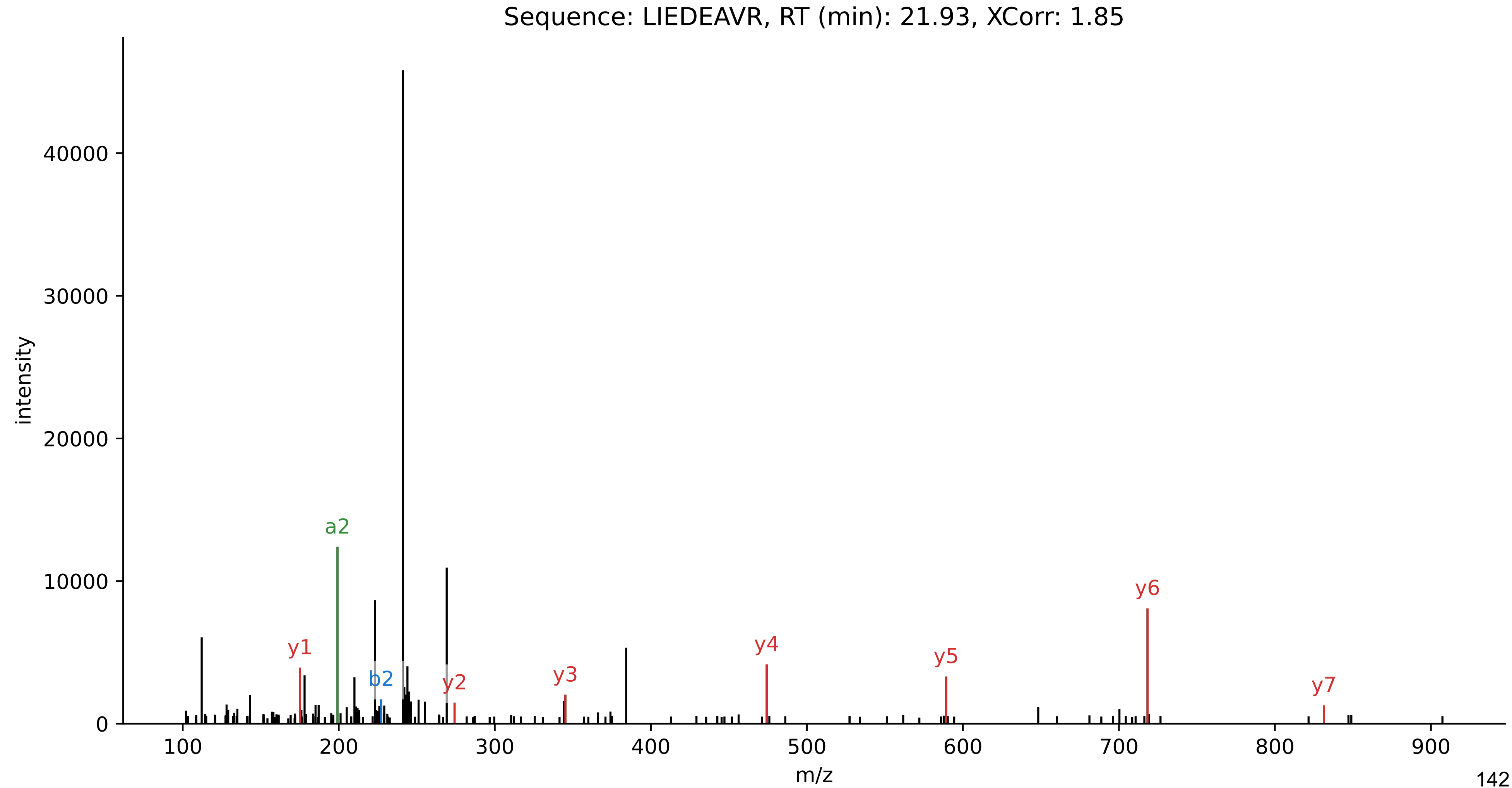

Sequence: LIEDEAVR, RT (min): 21.93, XCorr: 1.85
intensity
m/z
a2
b2
y1
y2
y3
y4
y5
y6
y7
0
10000
20000
30000
40000
100
200
300
400
500
600
700
800
900

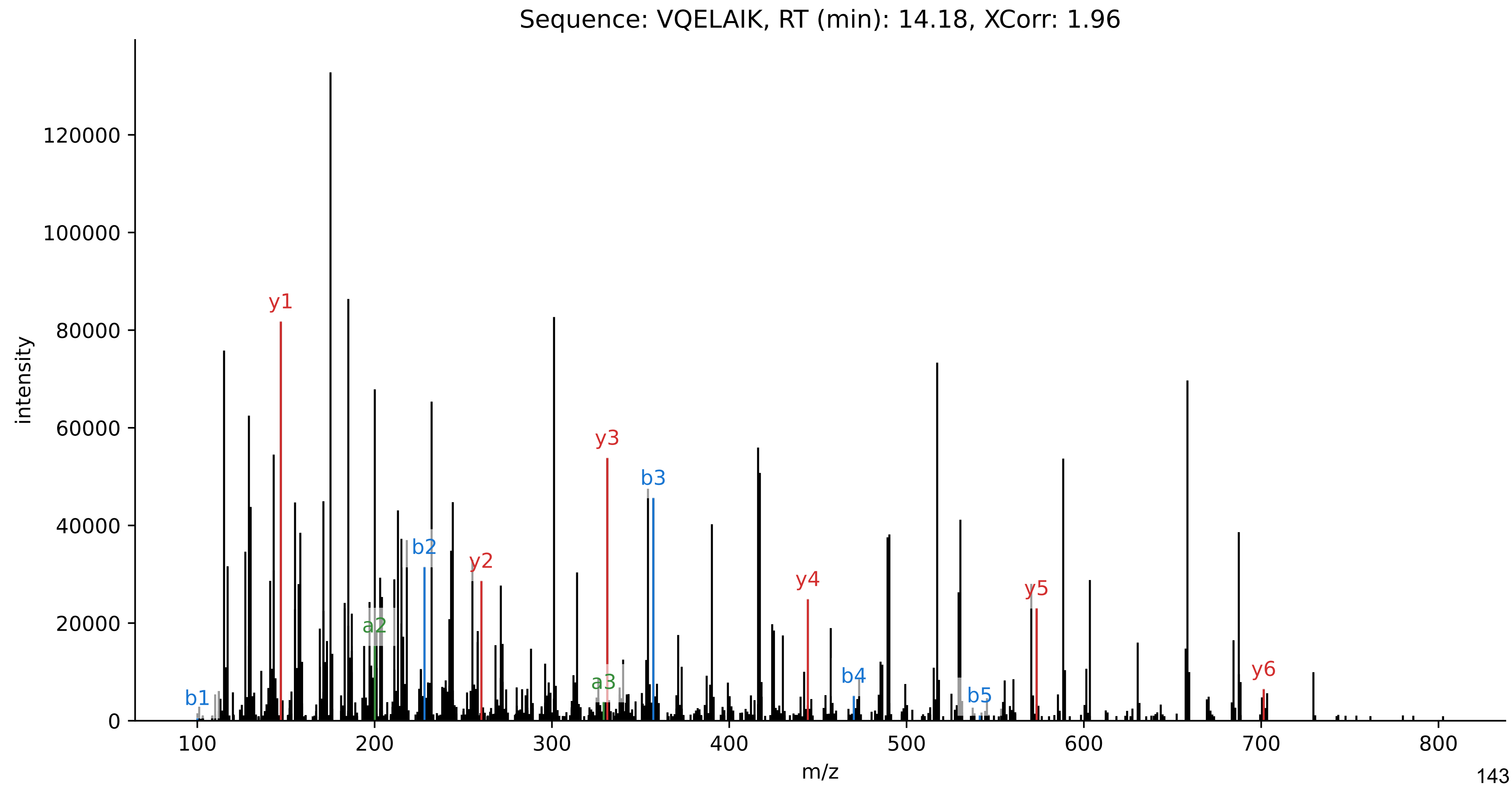
Sequence: VQELAIK, RT (min): 14.18, XCorr: 1.96
intensity
m/z
y1
y2
y3
y4
y5
y6
b1
b2
b3
b4
b5
a2
a3
0
20000
40000
60000
80000
100000
120000
100
200
300
400
500
600
700
800

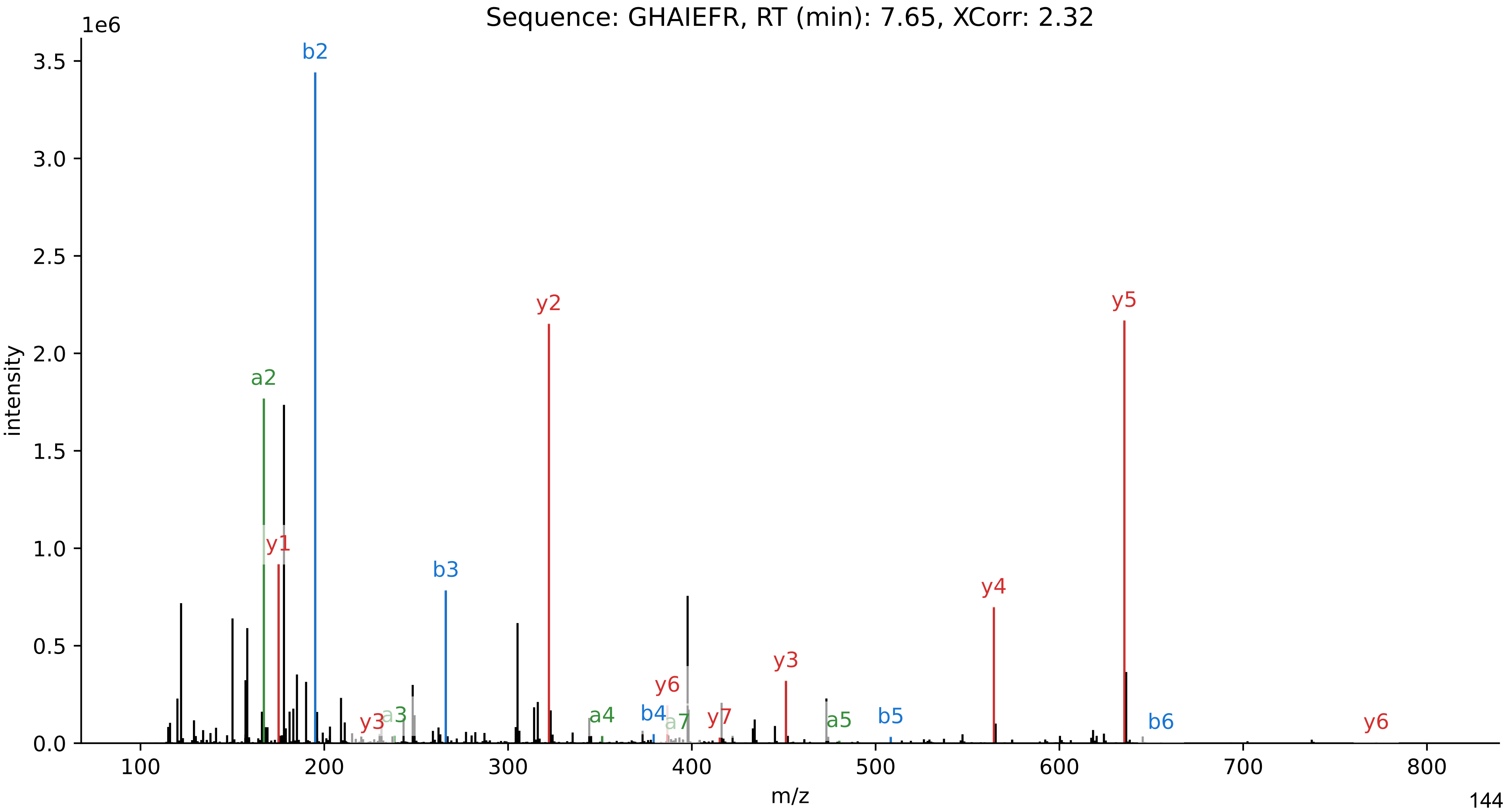

Sequence: GHAIEFR, RT (min): 7.65, XCorr: 2.32
1e6
3.5
3.0
2.5
2.0
1.5
1.0
0.5
0.0
intensity
100
200
300
400
500
600
700
800
m/z
b2
y2
y5
a2
y1
b3
y4
y3
y6
a3
a4
b4
a7
y7
a5
b5
b6

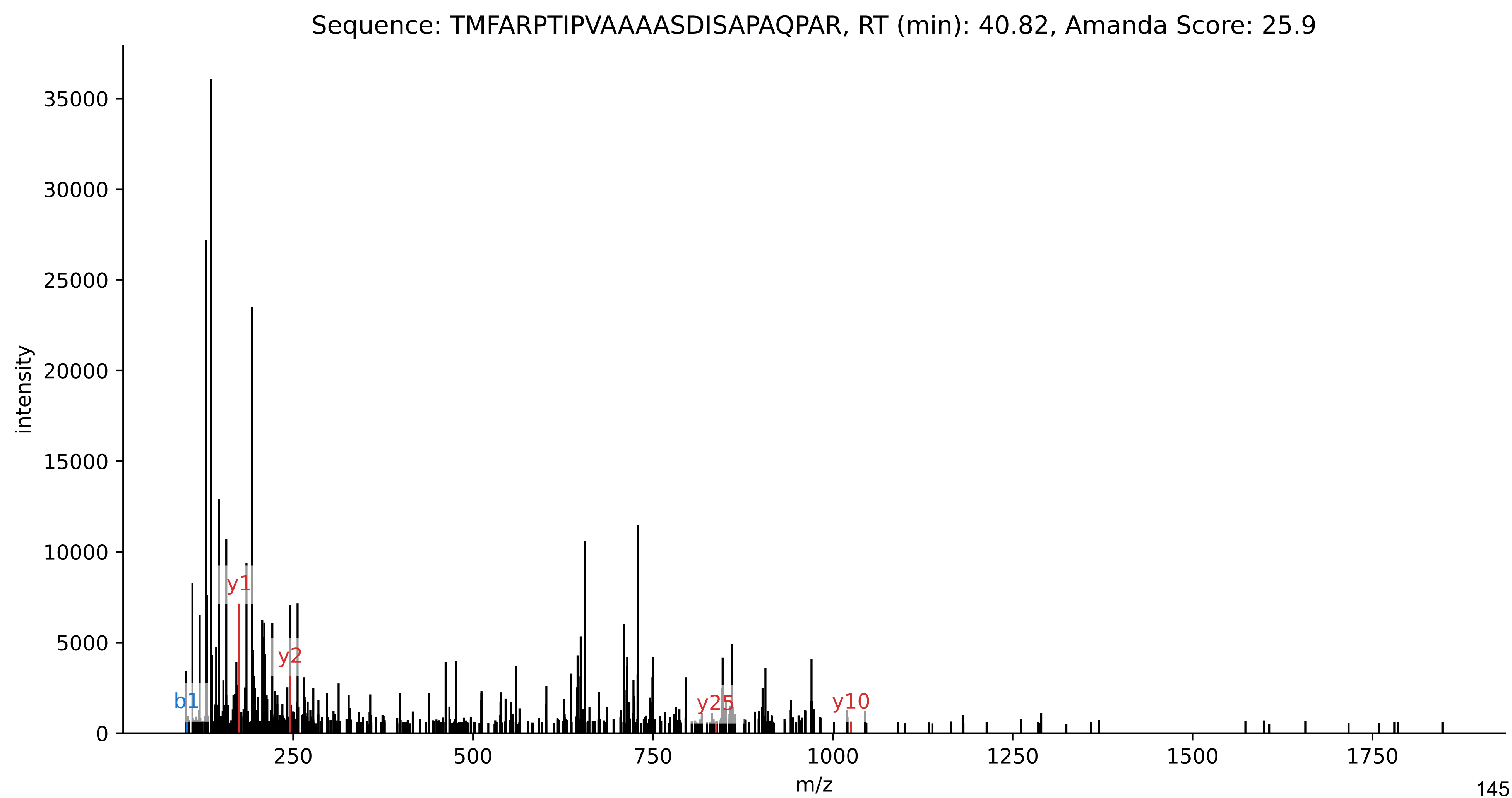

Sequence: TMFARPTIPVAAAASDISAPAQPAR, RT (min): 40.82, Amanda Score: 25.9
intensity
m/z
0
5000
10000
15000
20000
25000
30000
35000
250
500
750
1000
1250
1500
1750
b1
y1
y2
y25
y10

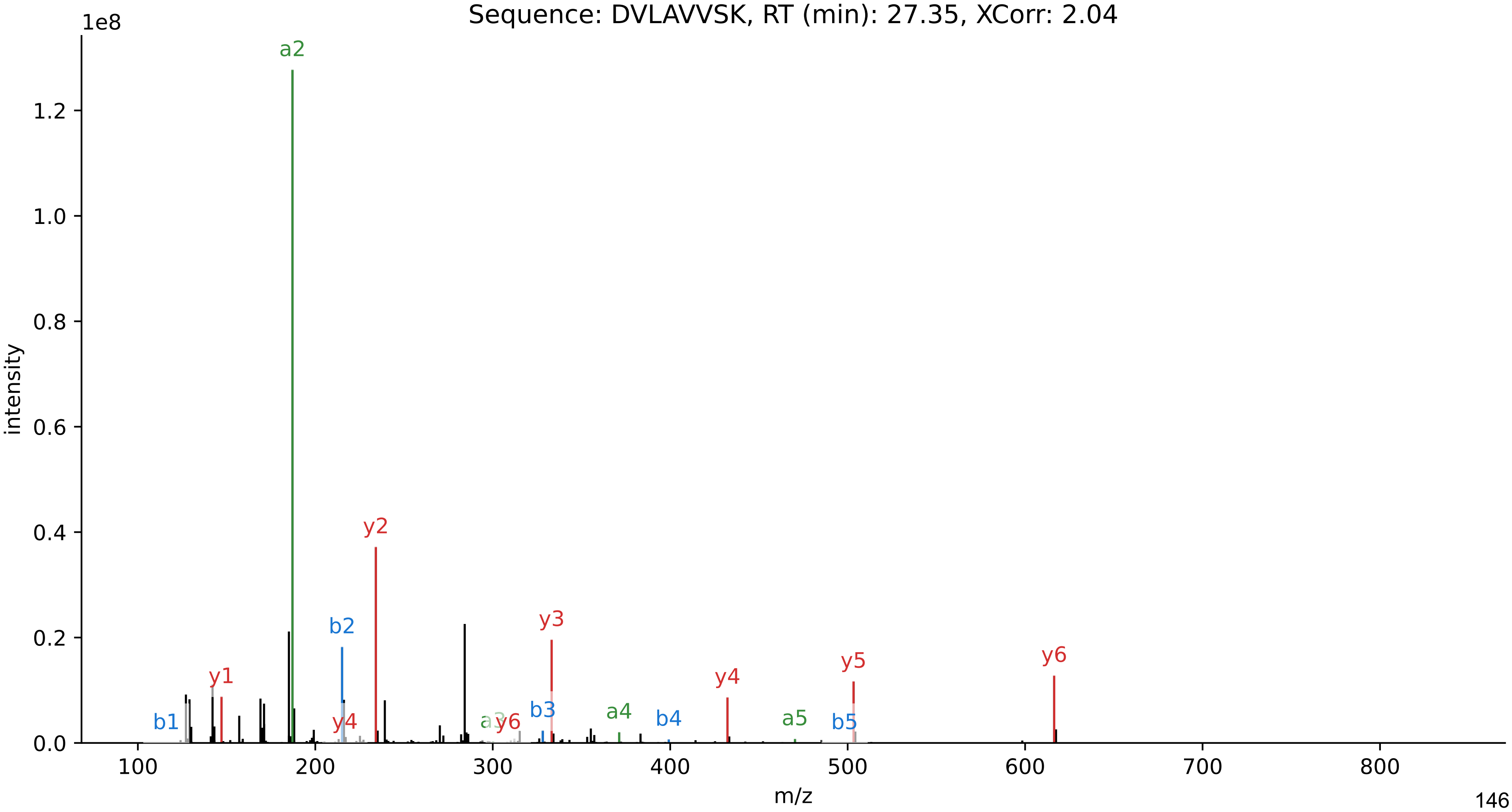

Sequence: DVLAVVSK, RT (min): 27.35, XCorr: 2.04
1e8
intensity
m/z
a2
y2
b2
y3
y1
y5
y6
y4
b1
y4
y6
b3
a4
b4
a5
b5

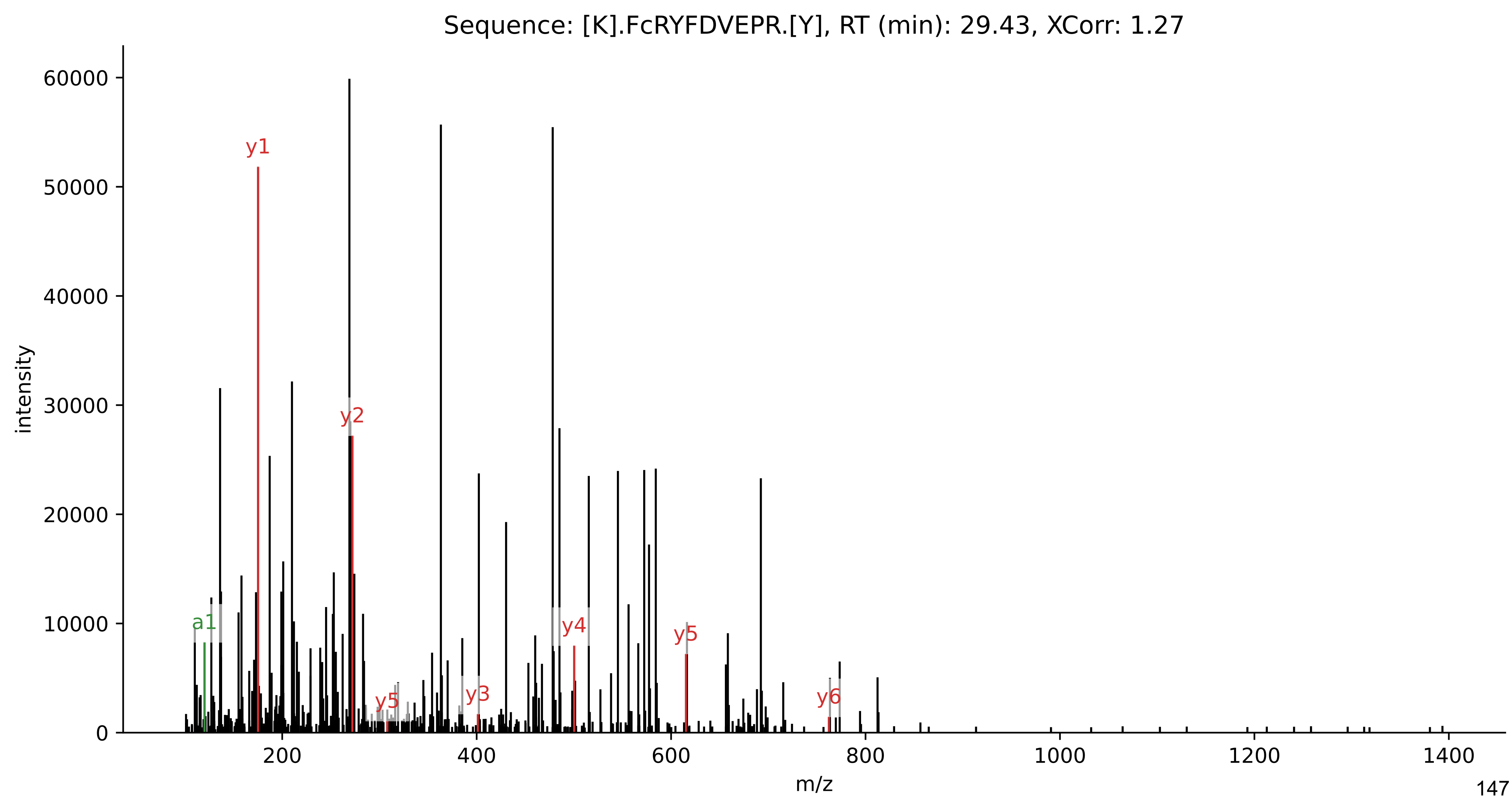
Sequence: [K].FcRYFDVEPR.[Y], RT (min): 29.43, XCorr: 1.27
intensity
m/z
y1
y2
a1
y4
y5
y3
y5
y6
0
10000
20000
30000
40000
50000
60000
200
400
600
800
1000
1200
1400

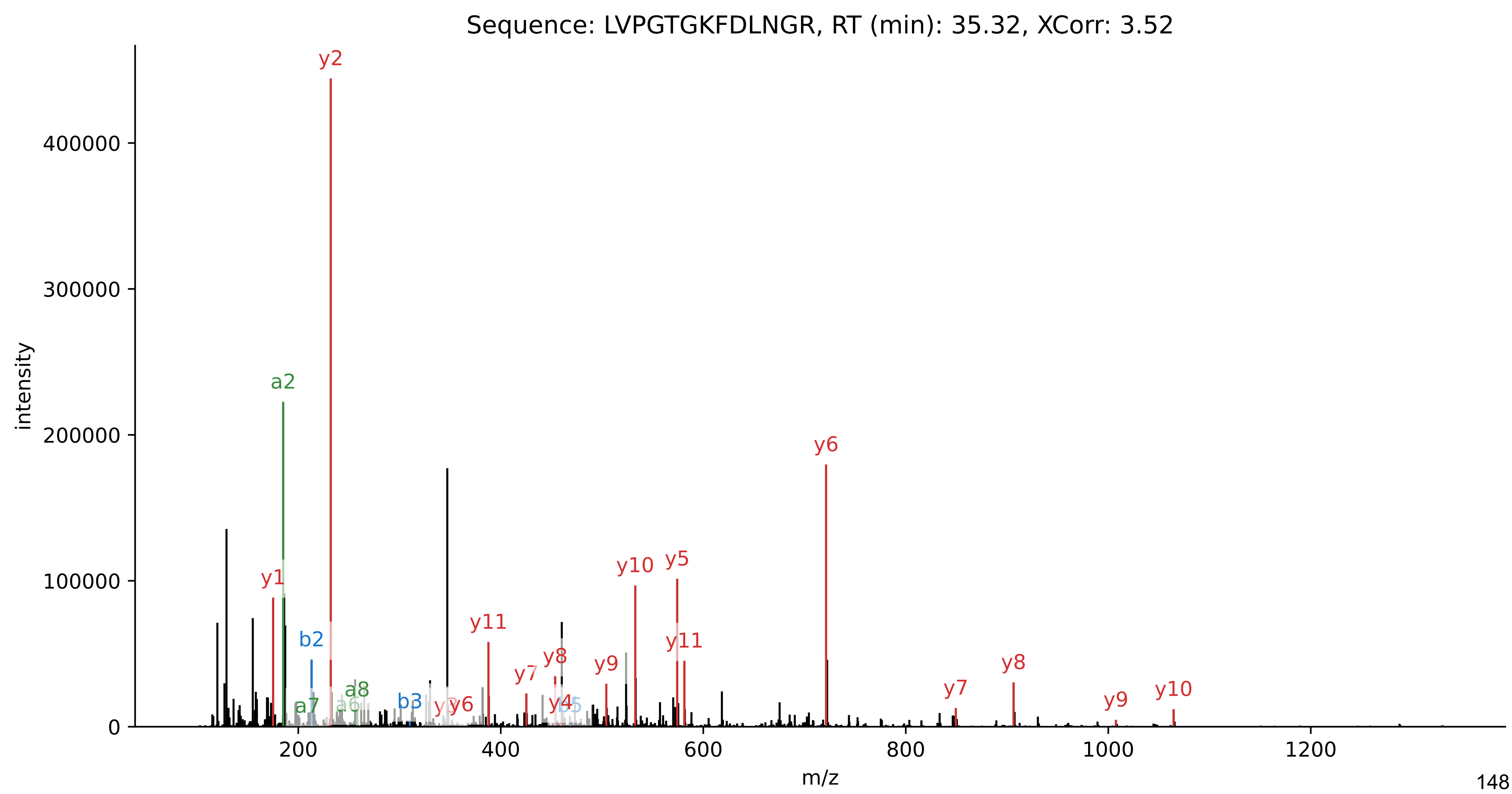

Sequence: LVPGTGKFDLNGR, RT (min): 35.32, XCorr: 3.52
intensity
m/z
0
100000
200000
300000
400000
200
400
600
800
1000
1200
y2
a2
y6
y1
y10
y5
y11
b2
y11
y8
y9
y7
y8
a8
b3
a7
y6
y4
y7
y9
y10

Sequence: VRLVPGTGKFDLNGR, RT (min): 41.8, XCorr: 4.19

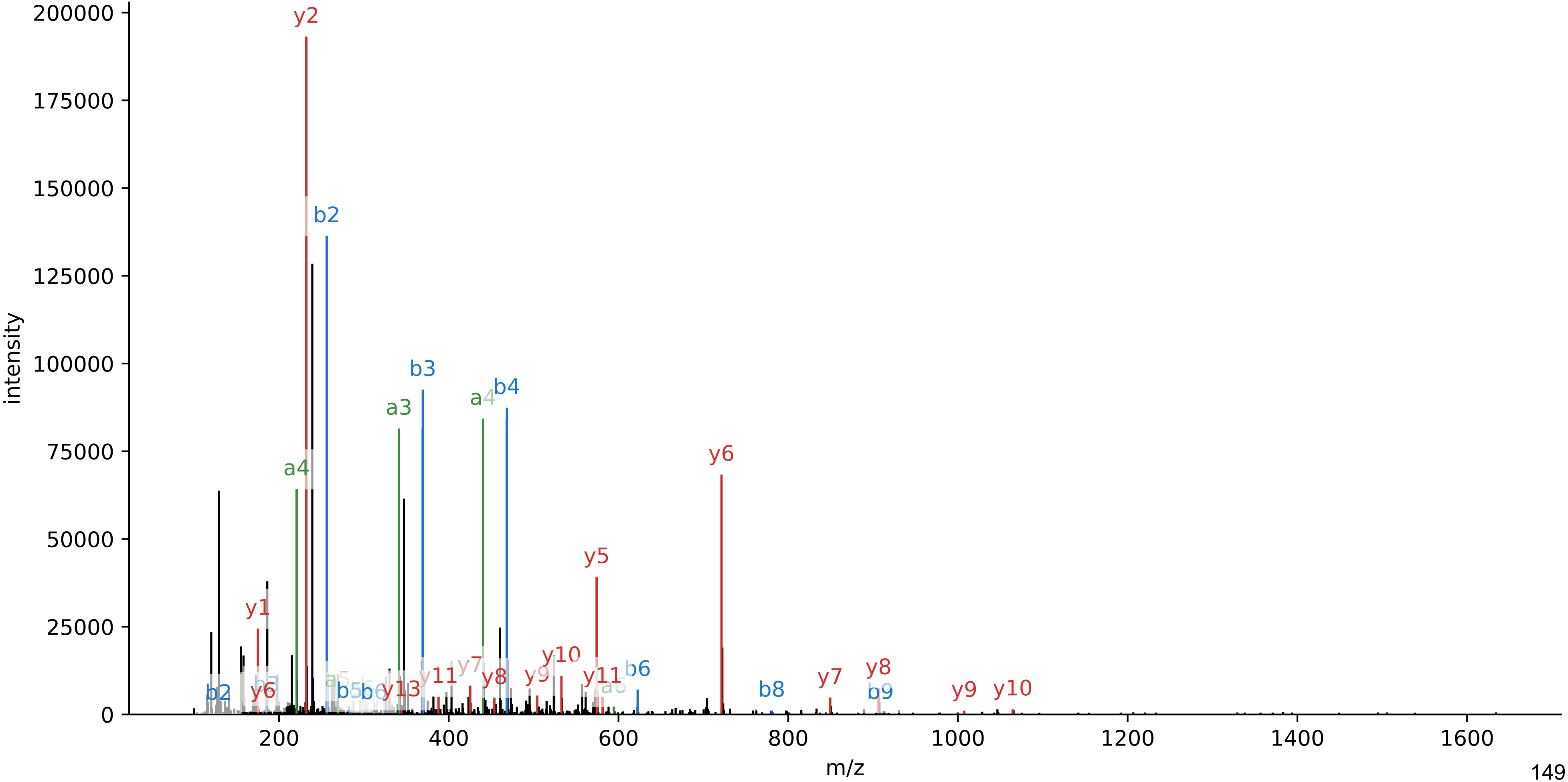

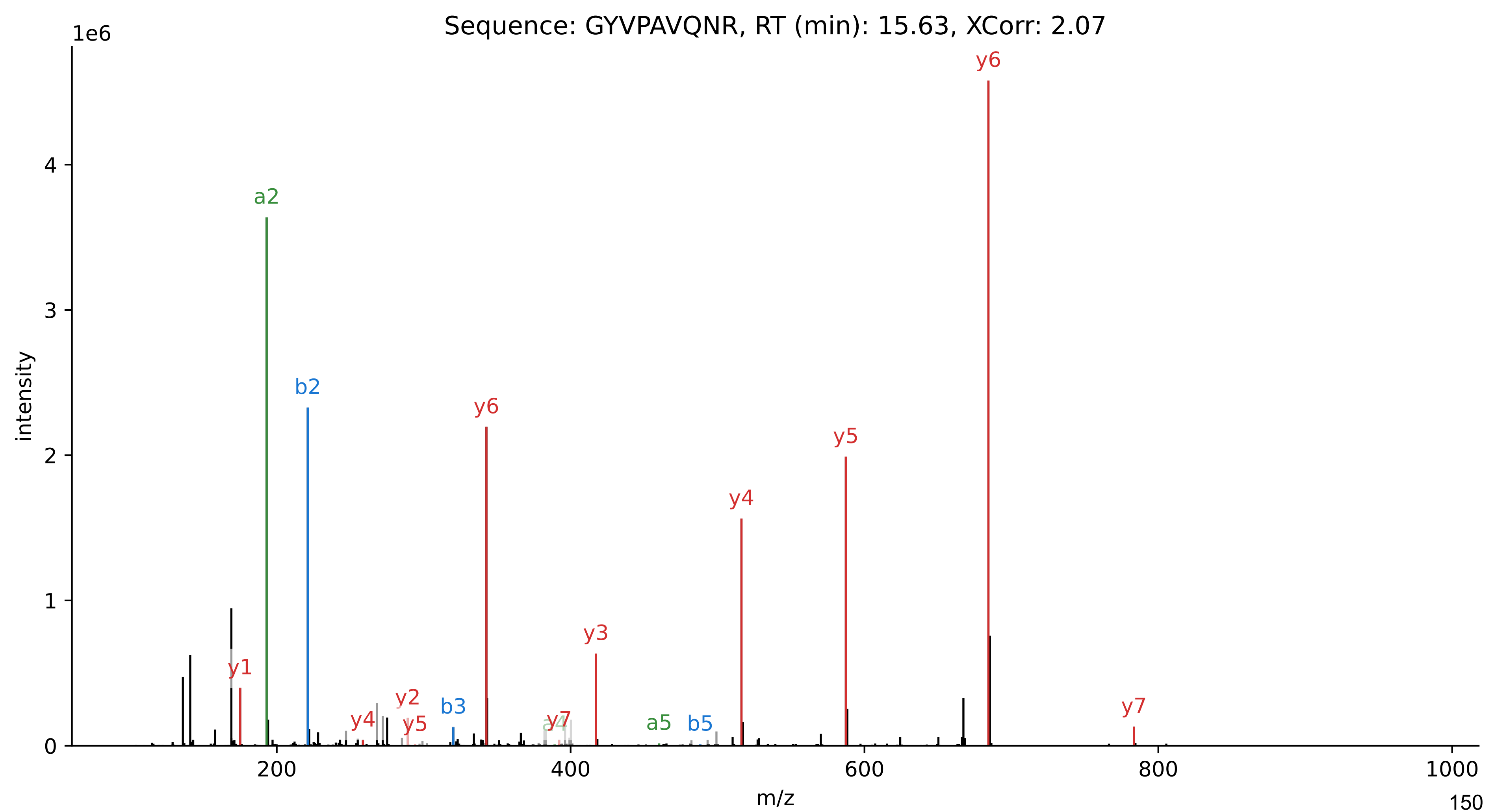

Sequence: GYVPAVQNR, RT (min): 15.63, XCorr: 2.07
1e6
intensity
m/z
y6
a2
b2
y6
y5
y4
y3
y1
y2
y4
y5
b3
y7
a5
b5
y7
0
1
2
3
4
200
400
600
800
1000

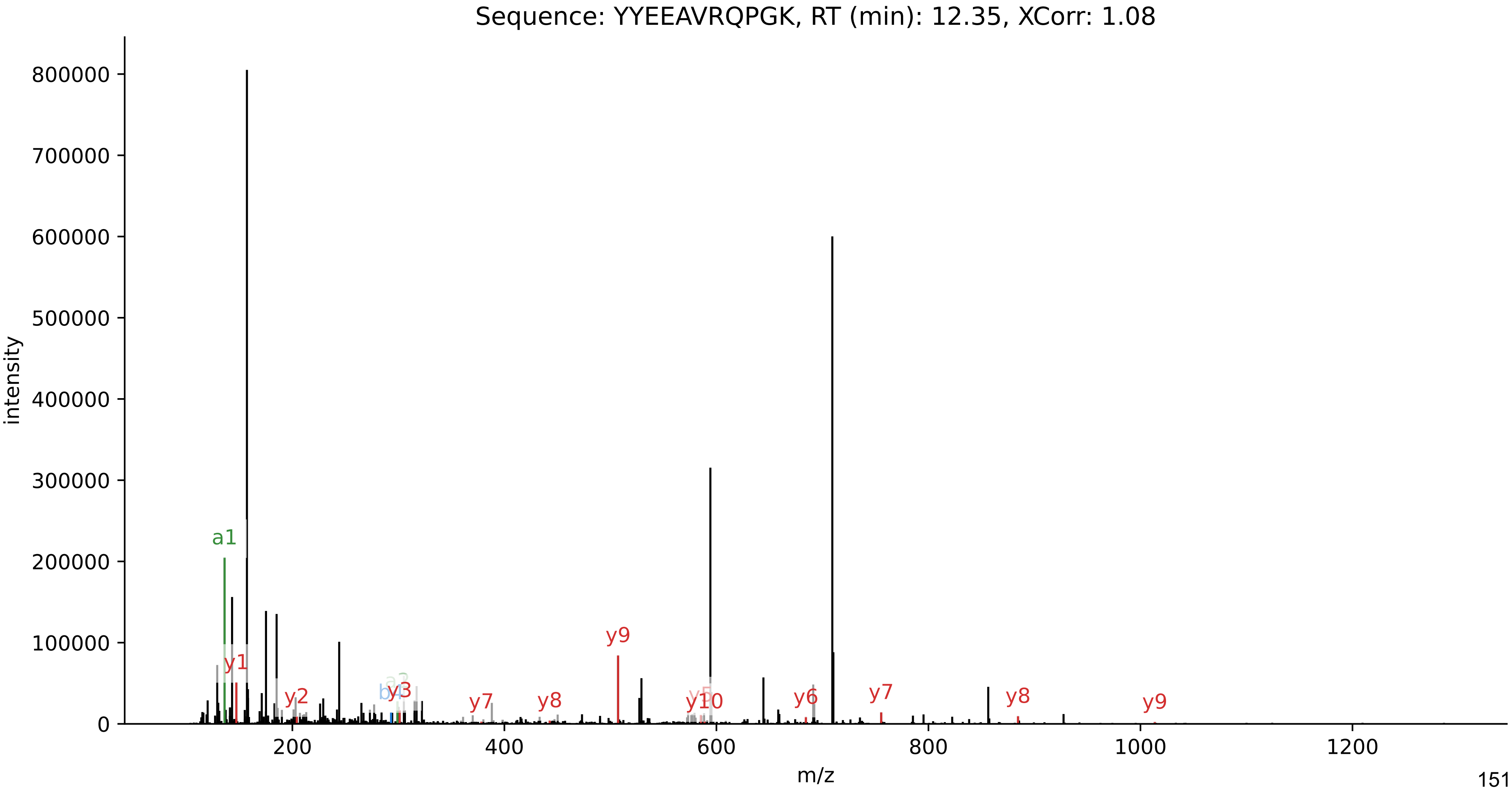
Sequence: YYEEAVRQPGK, RT (min): 12.35, XCorr: 1.08
intensity
m/z
a1
y1
y2
b4
y3
y7
y8
y9
y10
y6
y7
y8
y9

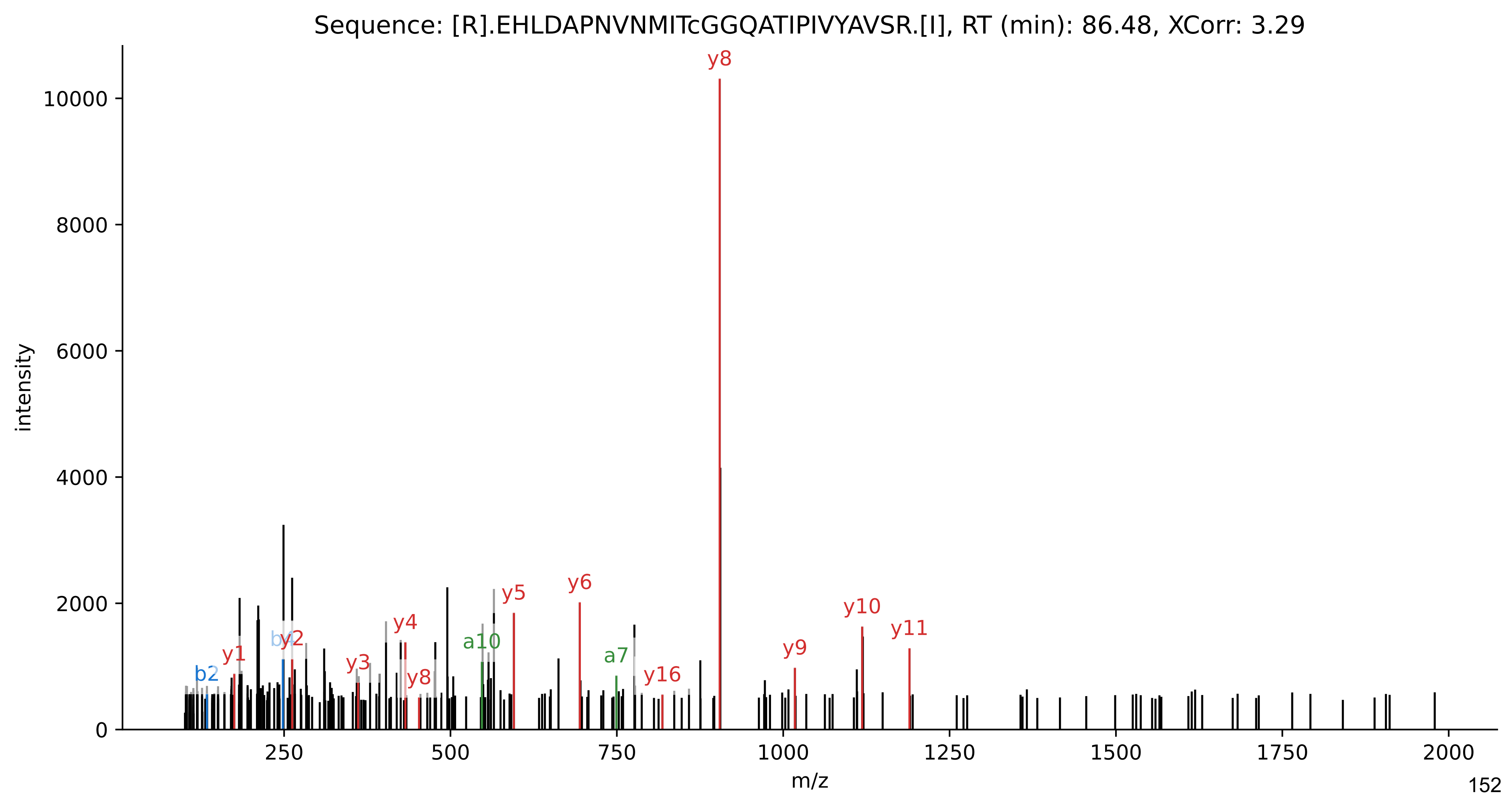

Sequence: [R].EHLDAPNVNMITcGGQATIPIVYAVSR.[I], RT (min): 86.48, XCorr: 3.29
intensity
m/z
y8
y5
y6
y4
b4
y2
y1
b2
y3
y8
a10
a7
y16
y9
y10
y11
0
2000
4000
6000
8000
10000
250
500
750
1000
1250
1500
1750
2000

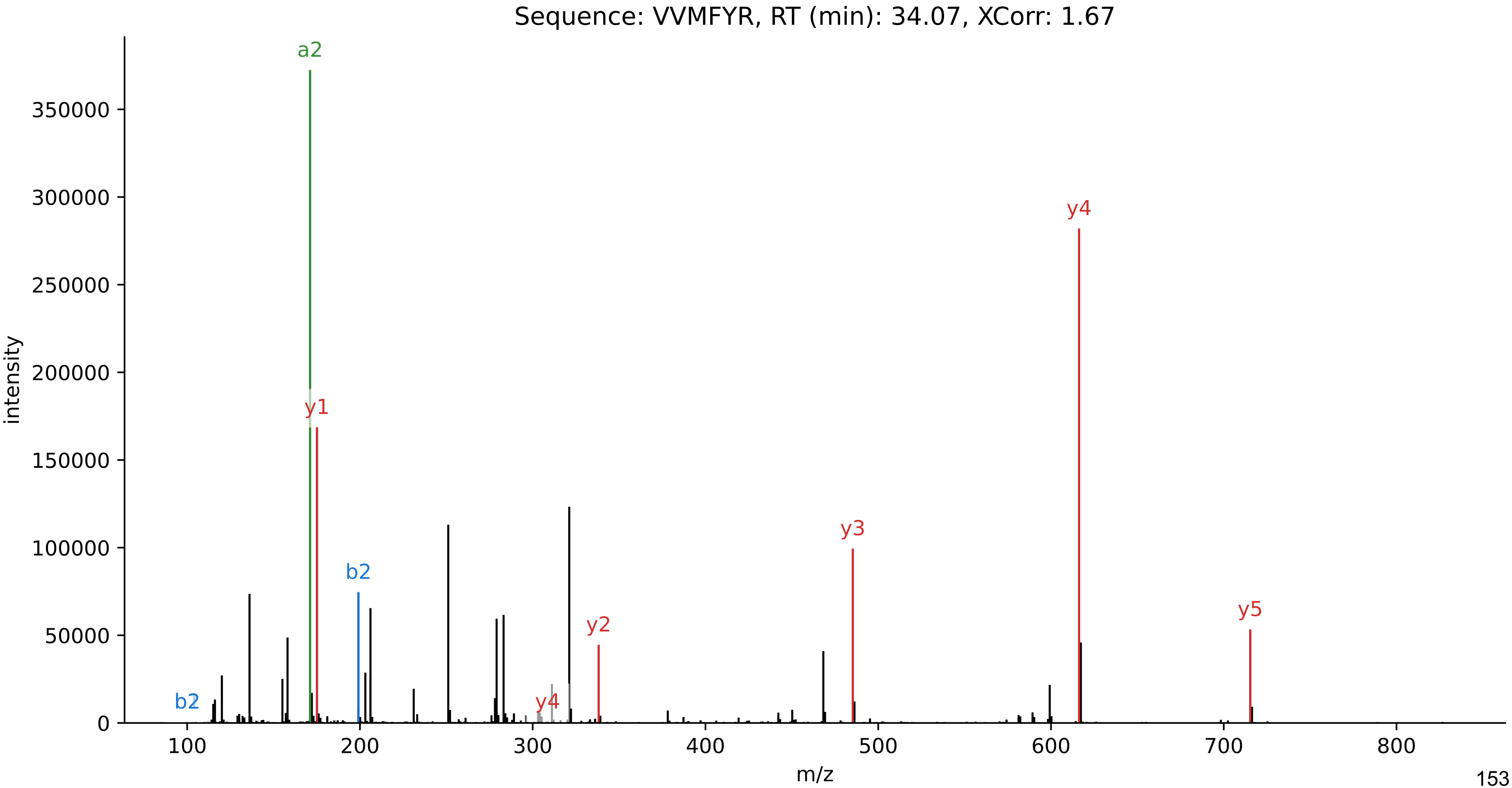
Sequence: VVMFYR, RT (min): 34.07, XCorr: 1.67
a2
y4
y1
y3
b2
y5
y2
b2
y4
intensity
m/z
0
50000
100000
150000
200000
250000
300000
350000
100
200
300
400
500
600
700
800

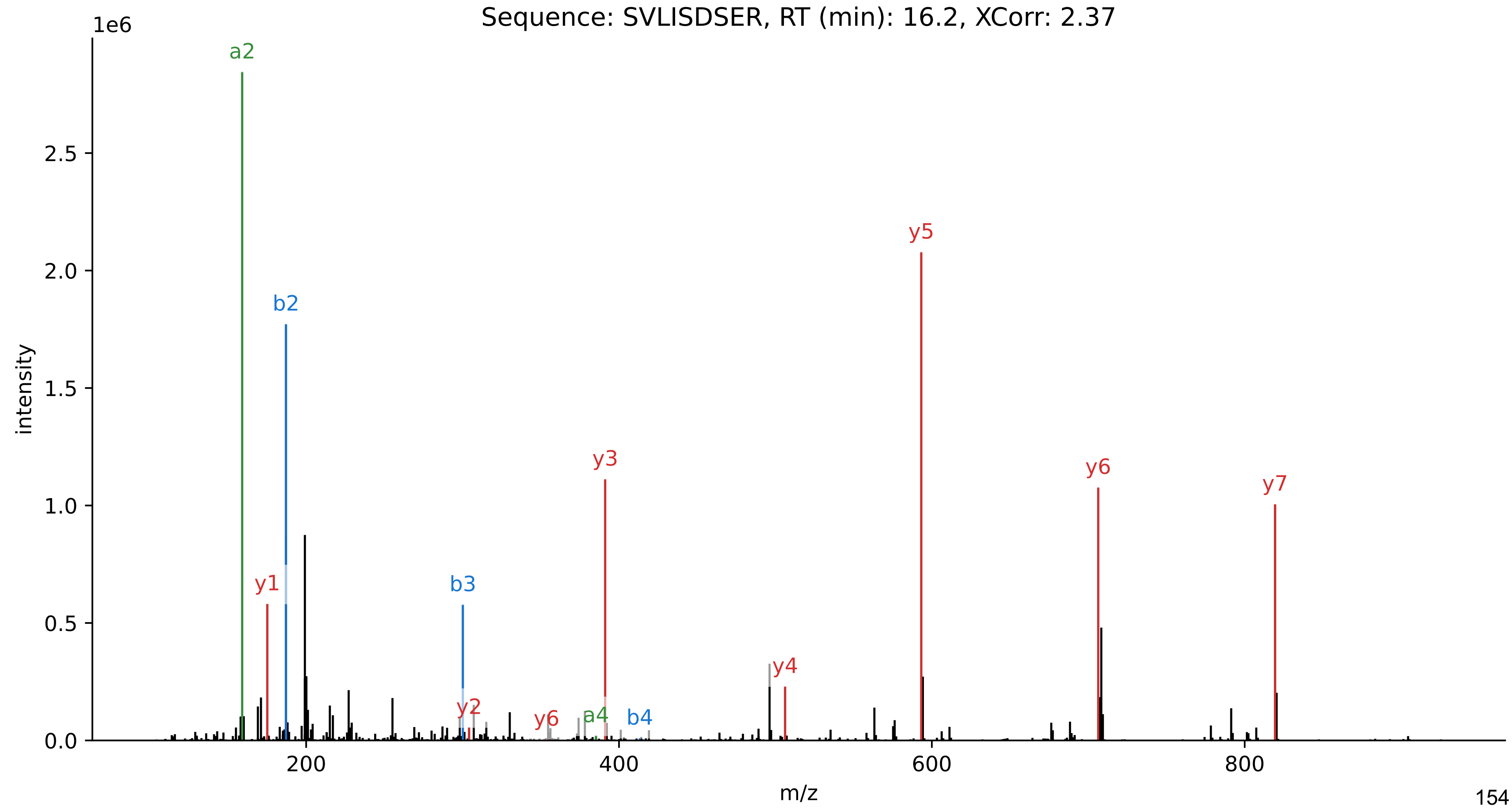

Sequence: SVLISDSER, RT (min): 16.2, XCorr: 2.37
1e6
a2
b2
y5
y3
y6
y7
y1
b3
y4
y2
y6
a4
b4
intensity
m/z
0.0
0.5
1.0
1.5
2.0
2.5
200
400
600
800

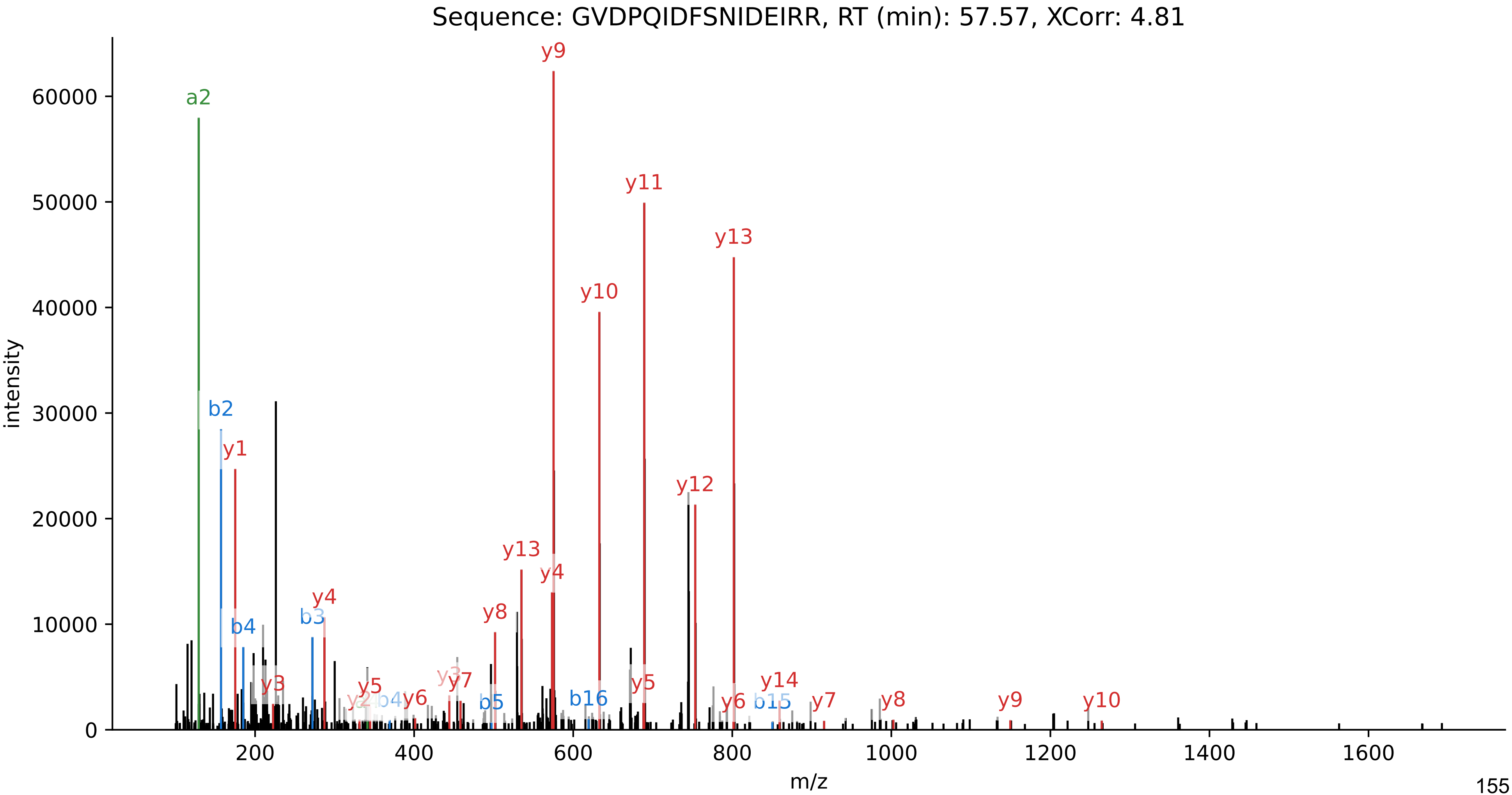
Sequence: GVDPQIDFSNIDEIRR, RT (min): 57.57, XCorr: 4.81
intensity
m/z
0
10000
20000
30000
40000
50000
60000
200
400
600
800
1000
1200
1400
1600
a2
b2
y1
b4
b3
y4
y3
y5
y6
y7
y8
y13
y4
b5
y9
y10
b16
y11
y5
y12
y13
y6
b15
y14
y7
y8
y9
y10

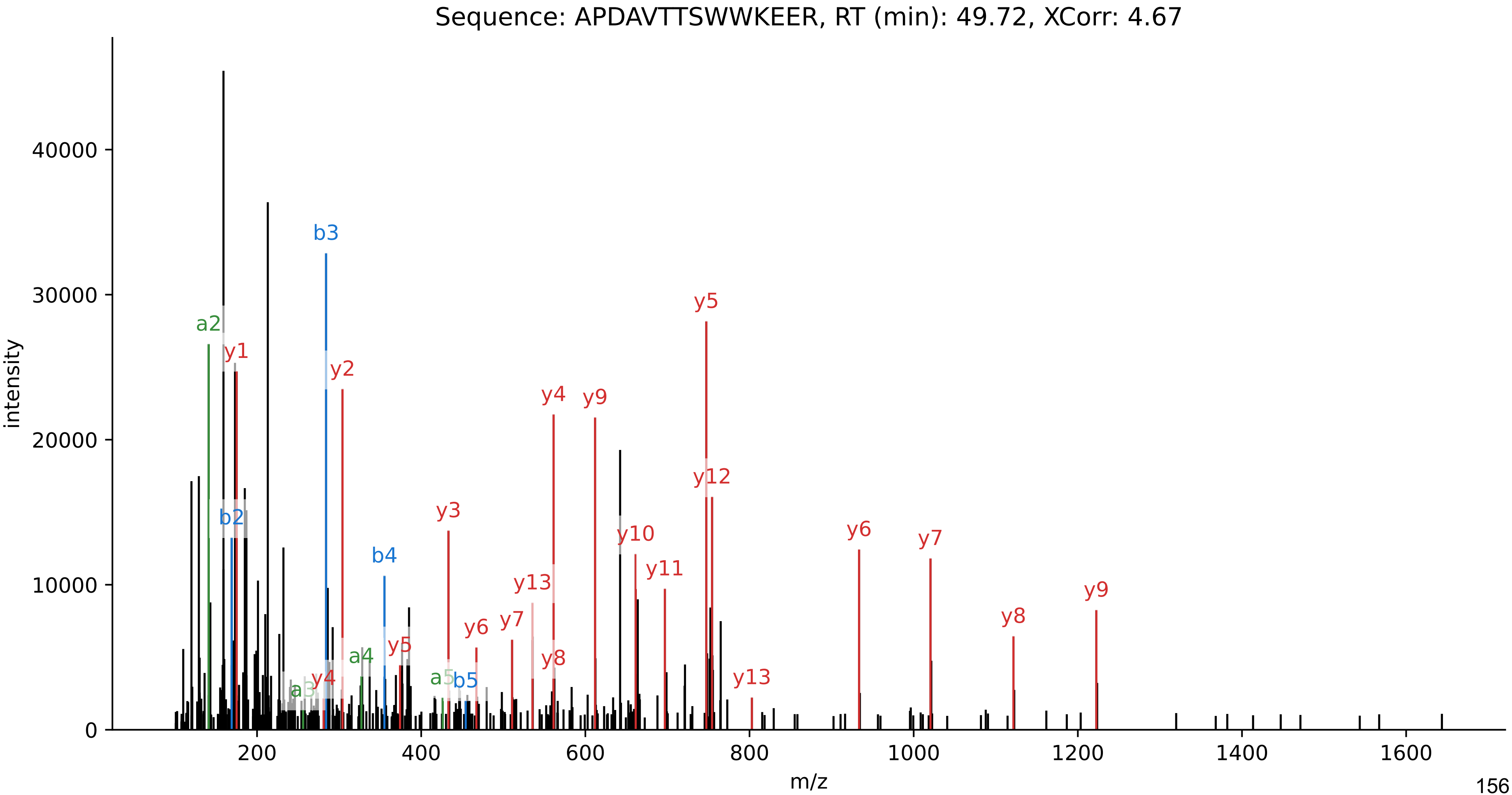
Sequence: APDAVTTSWWKEER, RT (min): 49.72, XCorr: 4.67
intensity
m/z
0
10000
20000
30000
40000
200
400
600
800
1000
1200
1400
1600
a2
y1
b2
b3
y2
b4
a3
y4
a4
y5
a5
b5
y3
y6
y7
y13
y8
y4
y9
y10
y11
y5
y12
y13
y6
y7
y8
y9

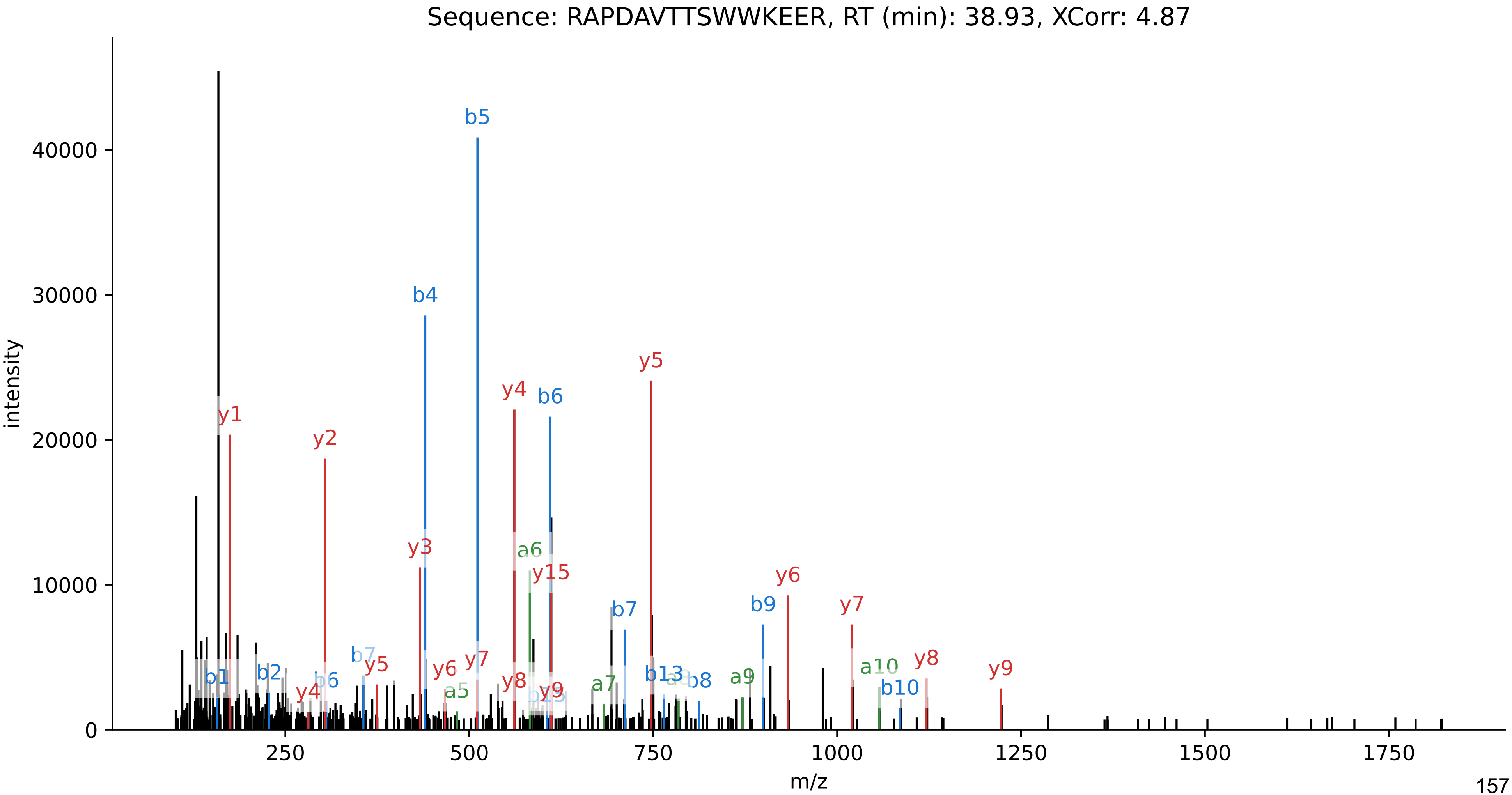
Sequence: RAPDAVTTSWWKEER, RT (min): 38.93, XCorr: 4.87
intensity
m/z
b5
b4
y5
y4
b6
y1
y2
y3
a6
y15
b7
b9
y6
y7
y8
y9
a10
b10
b13
b8
a9
a7
a5
b1
b2
b6
b7
y5
y4
y6
y7
y8
y9

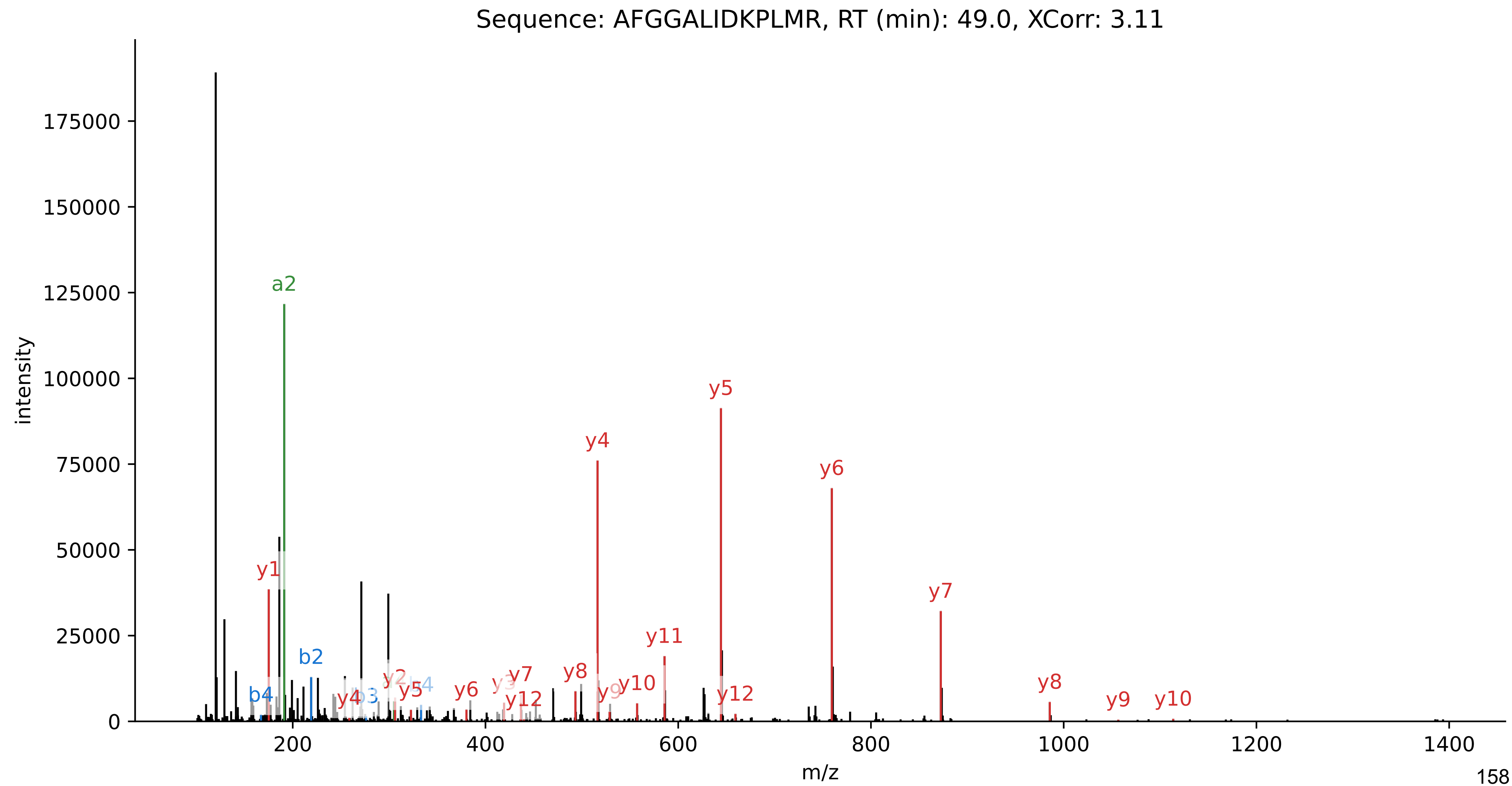

Sequence: AFGGALIDKPLMR, RT (min): 49.0, XCorr: 3.11
intensity
m/z
0
25000
50000
75000
100000
125000
150000
175000
200
400
600
800
1000
1200
1400
a2
y1
b2
b4
y2
y4
y5
y6
y7
y8
y9
y10
y11
y12

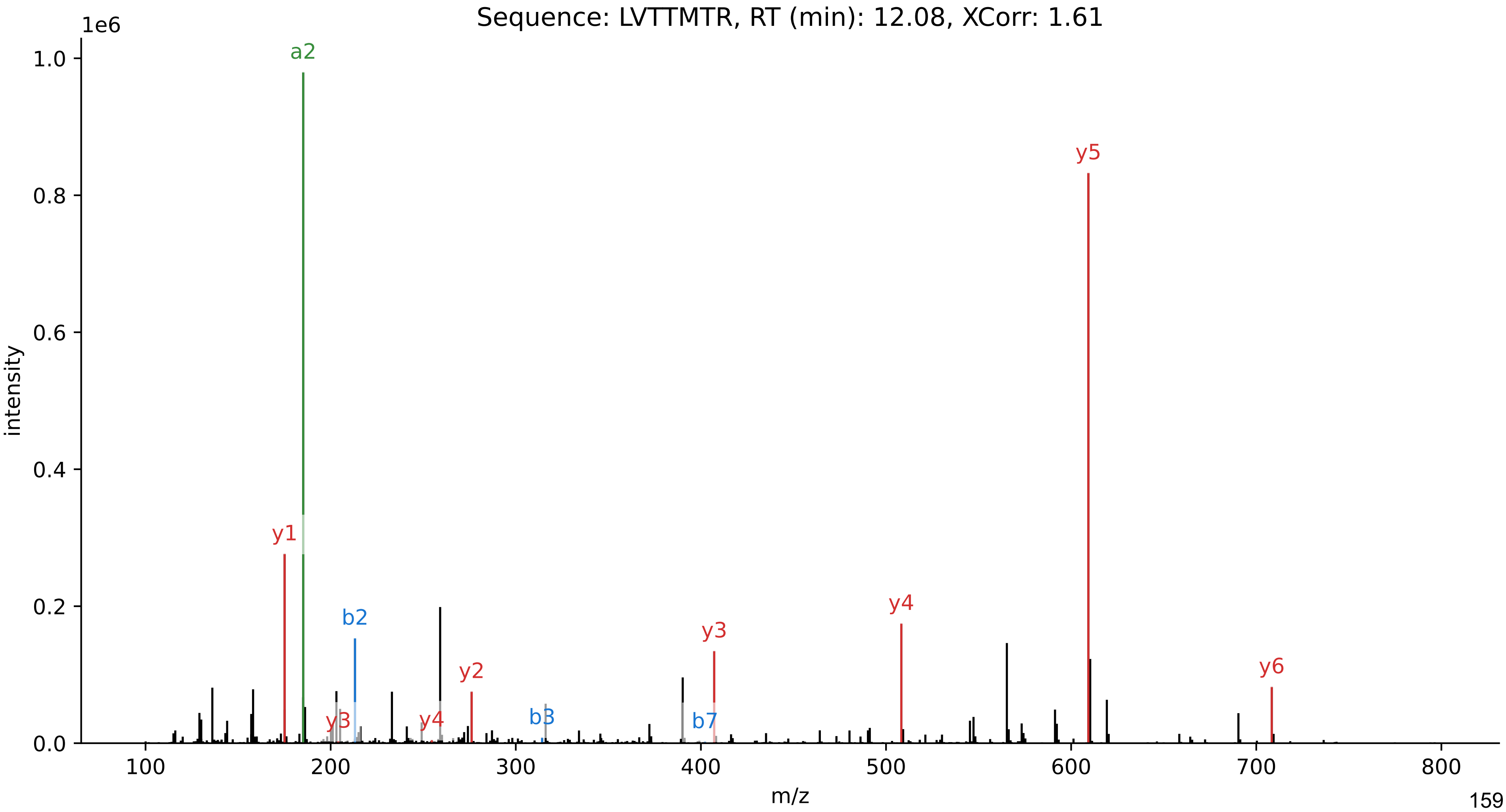

Sequence: LVTTMTR, RT (min): 12.08, XCorr: 1.61
1e6
1.0
0.8
0.6
0.4
0.2
0.0
intensity
a2
y1
b2
y3
y4
y2
b3
b7
y3
y4
y5
y6
100
200
300
400
500
600
700
800
m/z

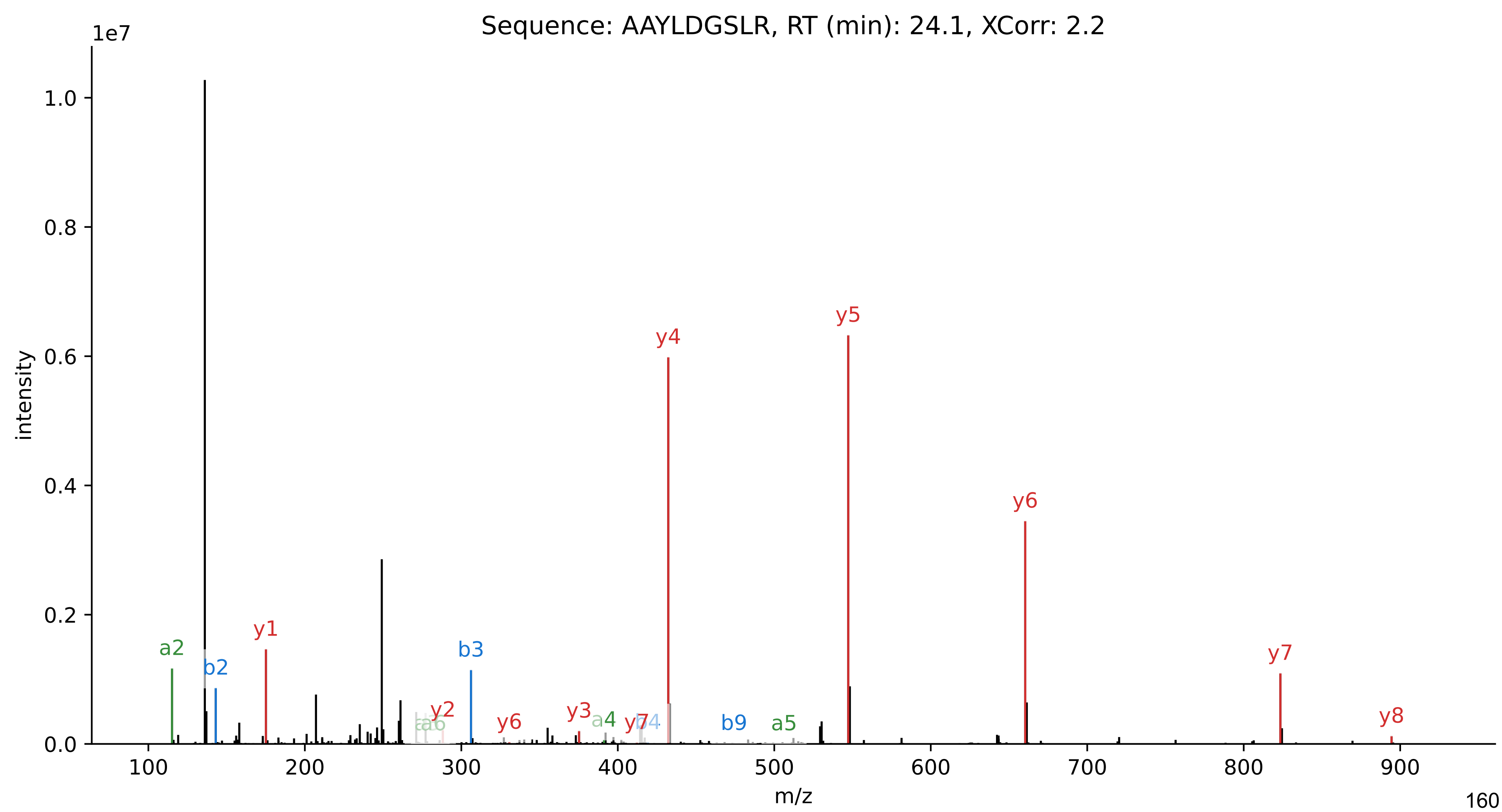
Sequence: AAYLDGSLR, RT (min): 24.1, XCorr: 2.2
1e7
1.0
0.8
0.6
0.4
0.2
0.0
intensity
100
200
300
400
500
600
700
800
900
m/z
a2
b2
y1
b3
y2
y6
y3
a4
y4
y5
b9
a5
y6
y7
y8

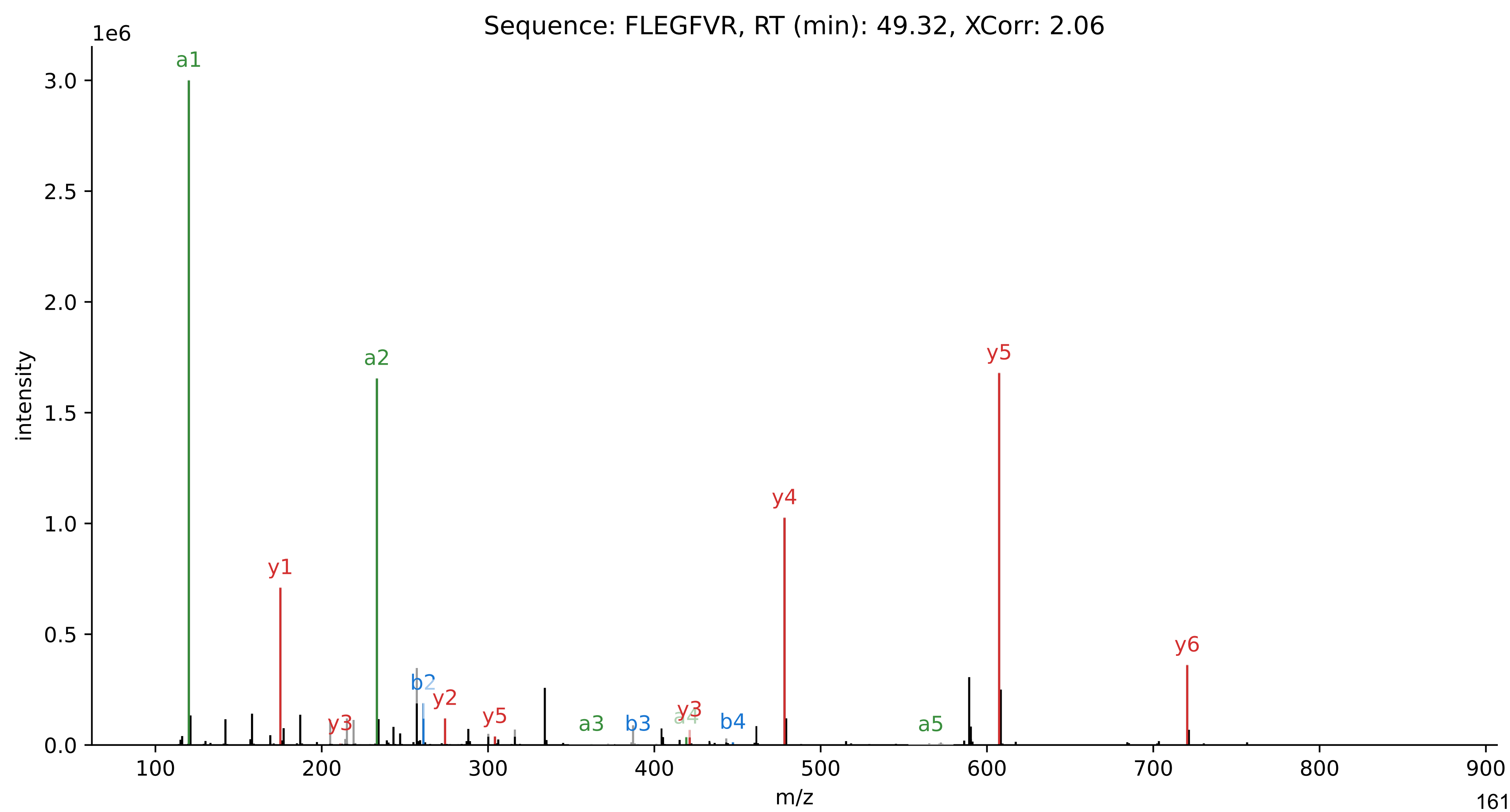
Sequence: FLEGFVR, RT (min): 49.32, XCorr: 2.06
1e6
3.0
2.5
2.0
1.5
1.0
0.5
0.0
intensity
a1
a2
y1
y4
y5
y6
b2
y2
y3
y5
a3
b3
a4
y3
b4
a5
100
200
300
400
500
600
700
800
900
m/z

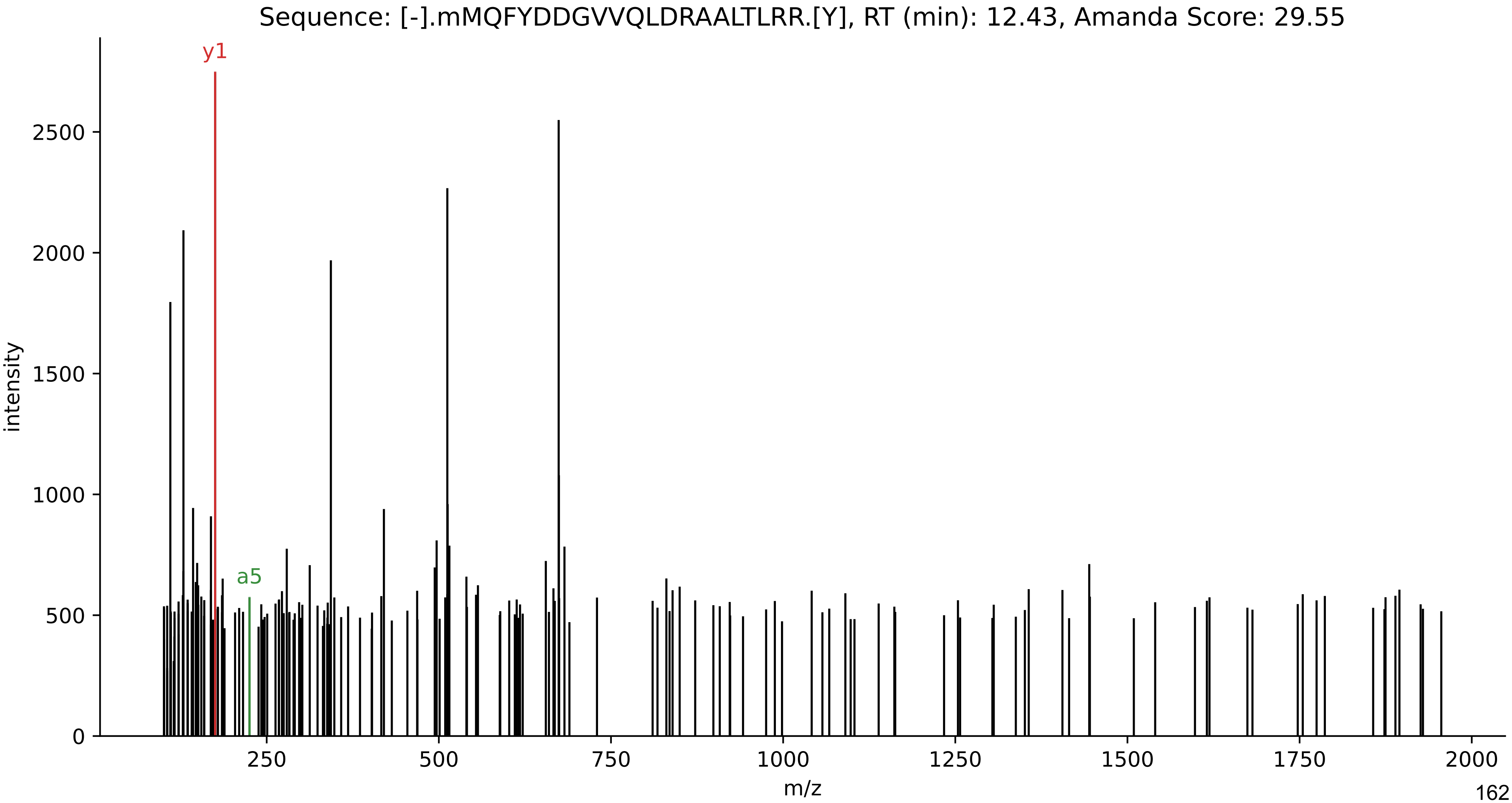

Sequence: [-].mMQFYDDGVVQLDRAALTLRR.[Y], RT (min): 12.43, Amanda Score: 29.55
y1
a5
intensity
m/z
0
500
1000
1500
2000
2500
250
500
750
1000
1250
1500
1750
2000

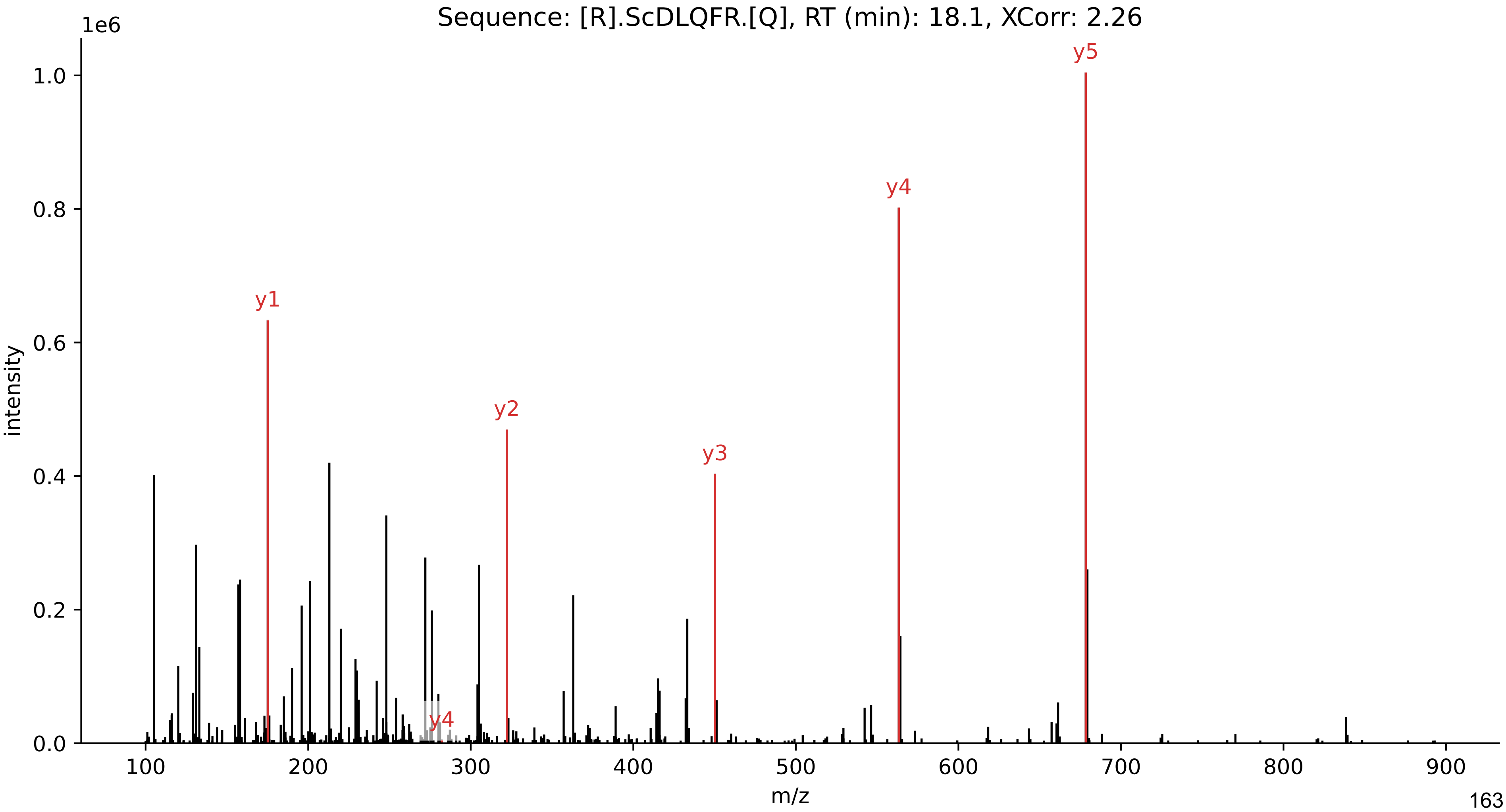

Sequence: [R].ScDLQFR.[Q], RT (min): 18.1, XCorr: 2.26
1e6
intensity
m/z
y1
y2
y3
y4
y5
y4

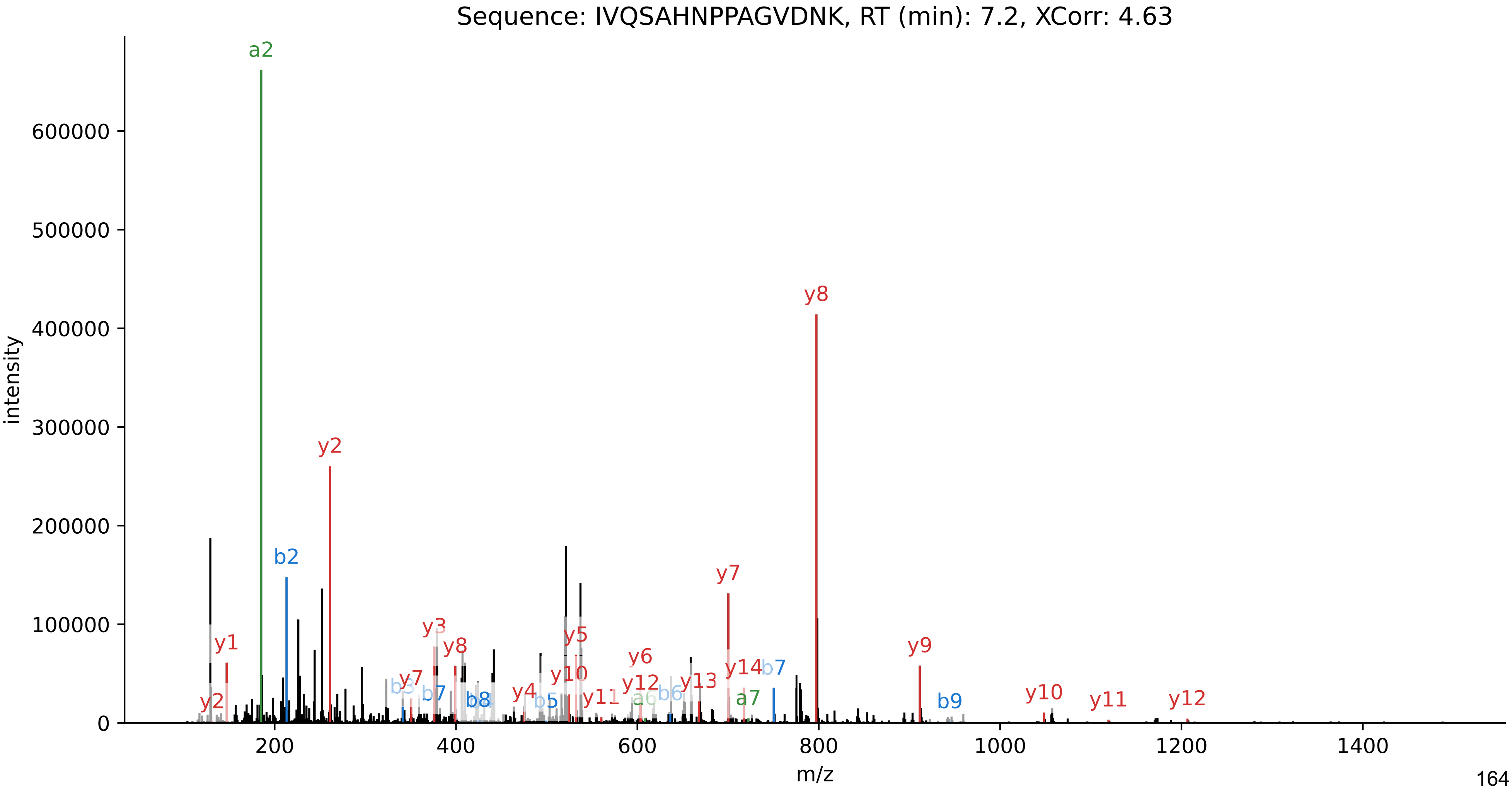

Sequence: IVQSAHNPPAGVDNK, RT (min): 7.2, XCorr: 4.63
intensity
m/z
a2
y8
y2
b2
y7
y1
y2
y3
y8
y7
b3
b7
b8
y4
b5
y10
y5
y11
y6
y12
a6
b6
y13
y14
b7
a7
y9
b9
y10
y11
y12

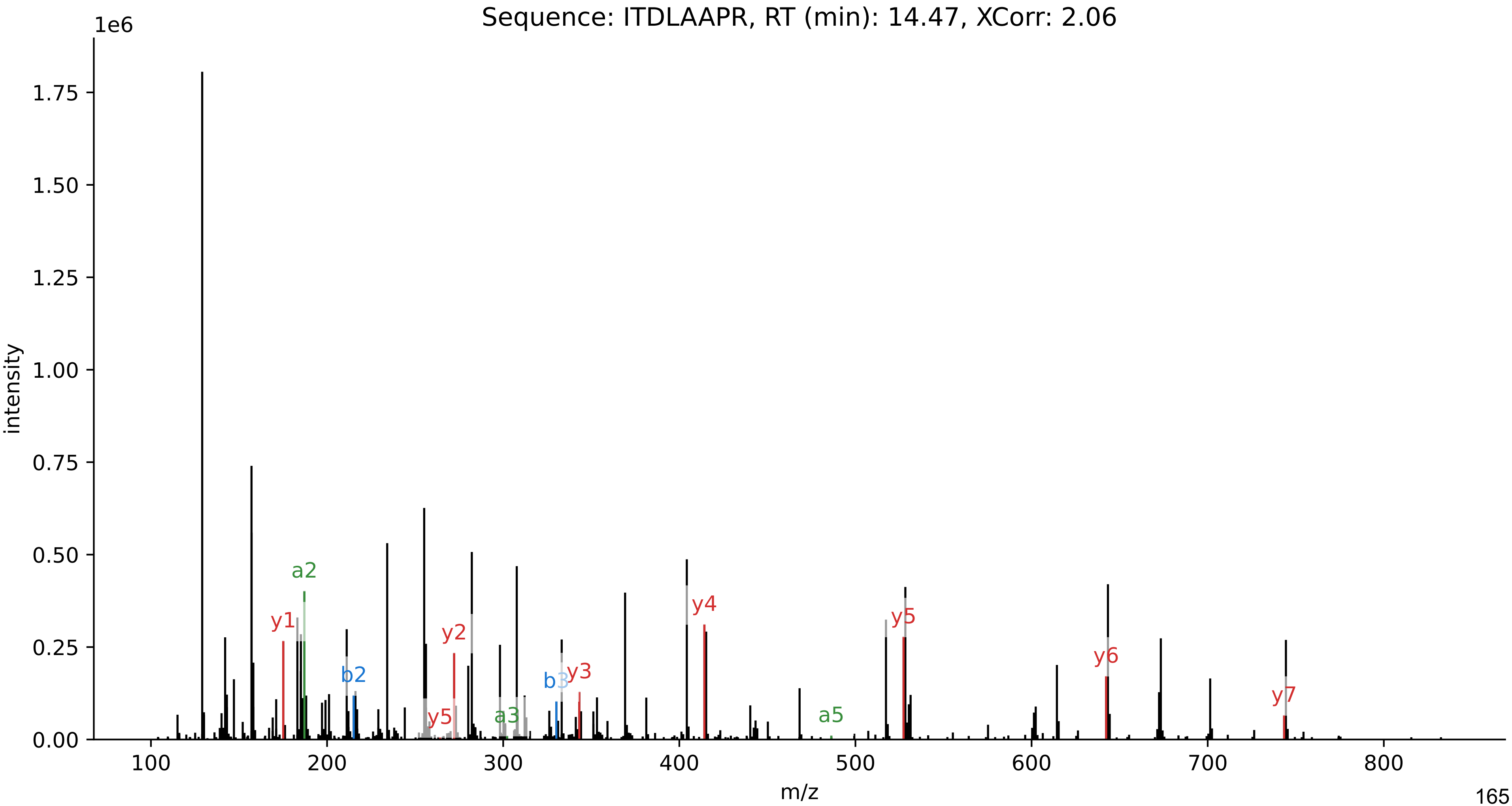
Sequence: ITDLAAPR, RT (min): 14.47, XCorr: 2.06
1e6
intensity
m/z
y1
a2
b2
y2
y5
a3
b3
y3
y4
a5
y5
y6
y7

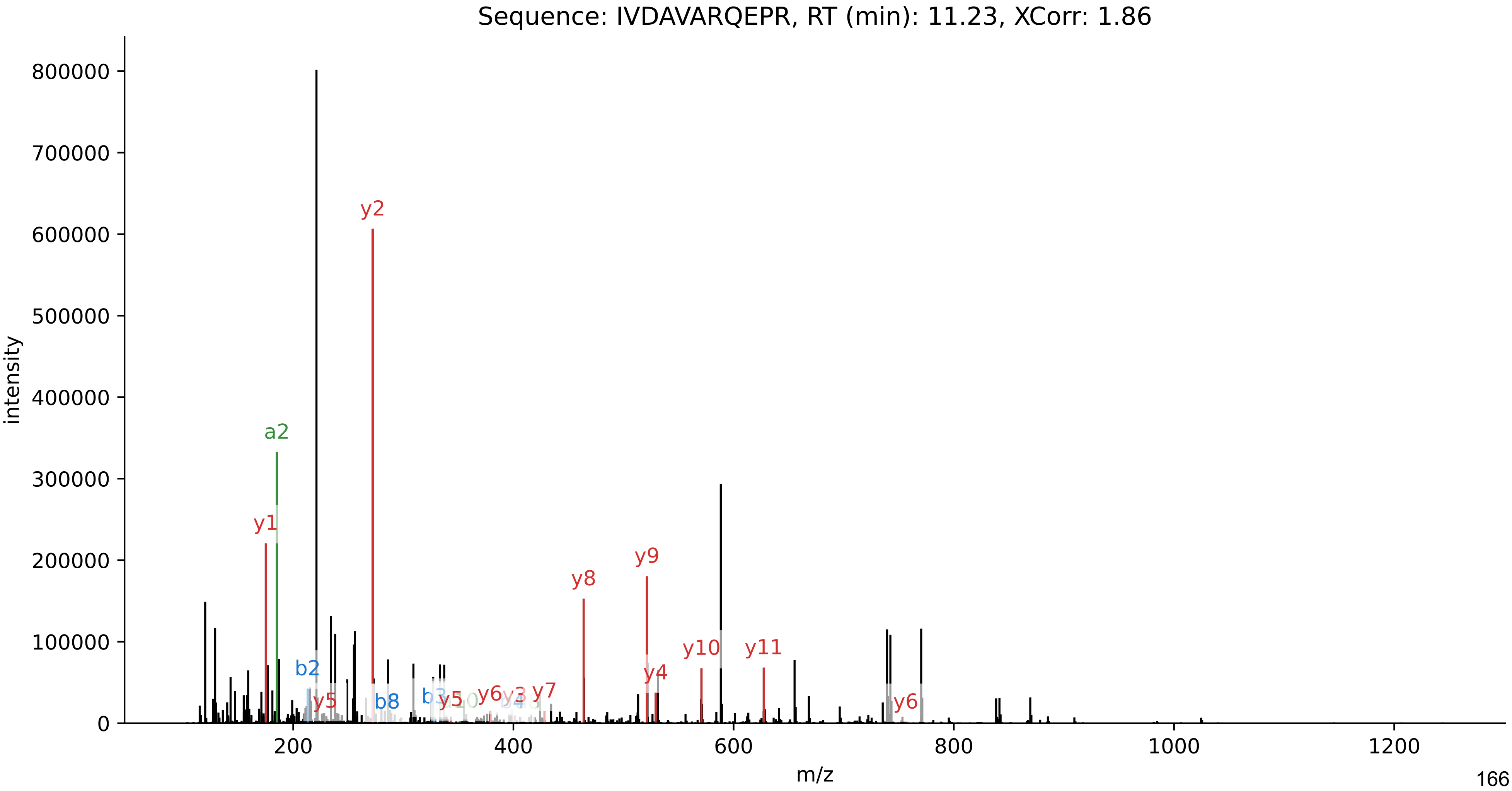

Sequence: IVDAVARQEPR, RT (min): 11.23, XCorr: 1.86
intensity
m/z
0
100000
200000
300000
400000
500000
600000
700000
800000
200
400
600
800
1000
1200
a2
y1
y2
b2
y5
b8
b3
y5
y6
y3
y7
y8
y9
y4
y10
y11
y6

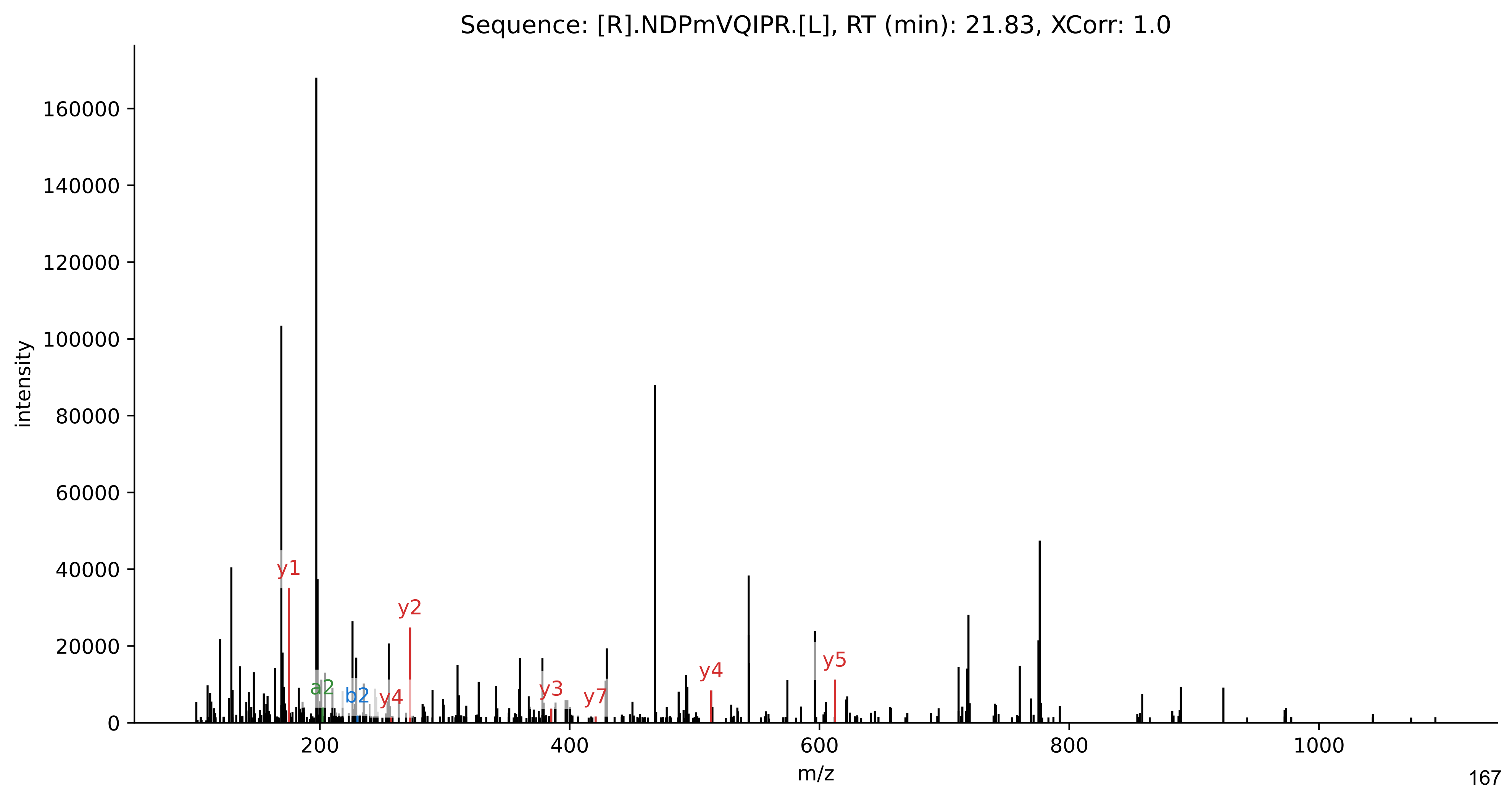

Sequence: [R].NDPmVQIPR.[L], RT (min): 21.83, XCorr: 1.0
intensity
m/z
y1
y2
a2
b2
y4
y3
y7
y4
y5
0
20000
40000
60000
80000
100000
120000
140000
160000
200
400
600
800
1000

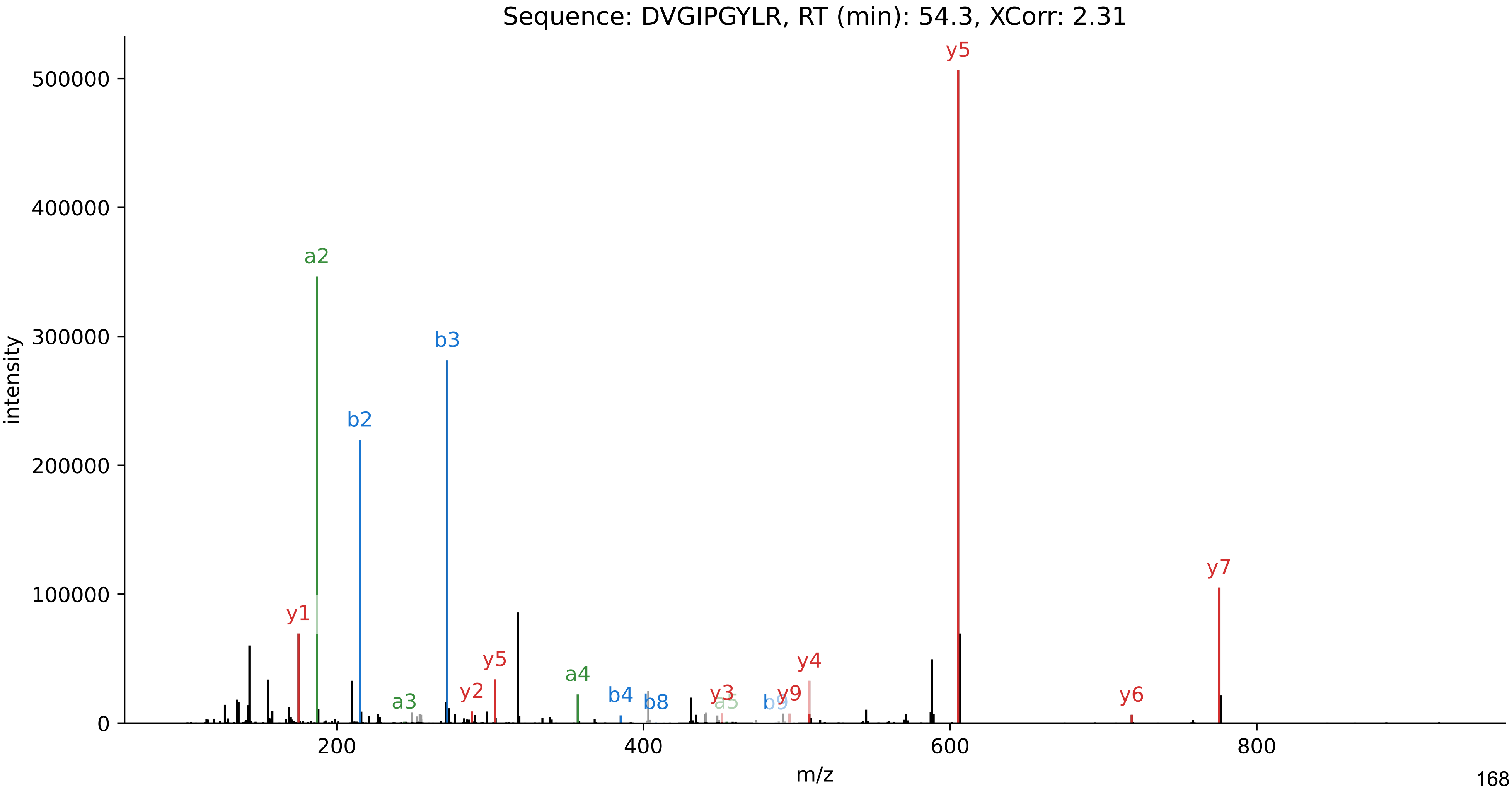
Sequence: DVGIPGYLR, RT (min): 54.3, XCorr: 2.31
intensity
m/z
500000
400000
300000
200000
100000
0
200
400
600
800
y5
a2
b3
b2
y7
y1
y5
a4
y4
b4
b8
y3
y9
y2
a3
y6
a5
b9

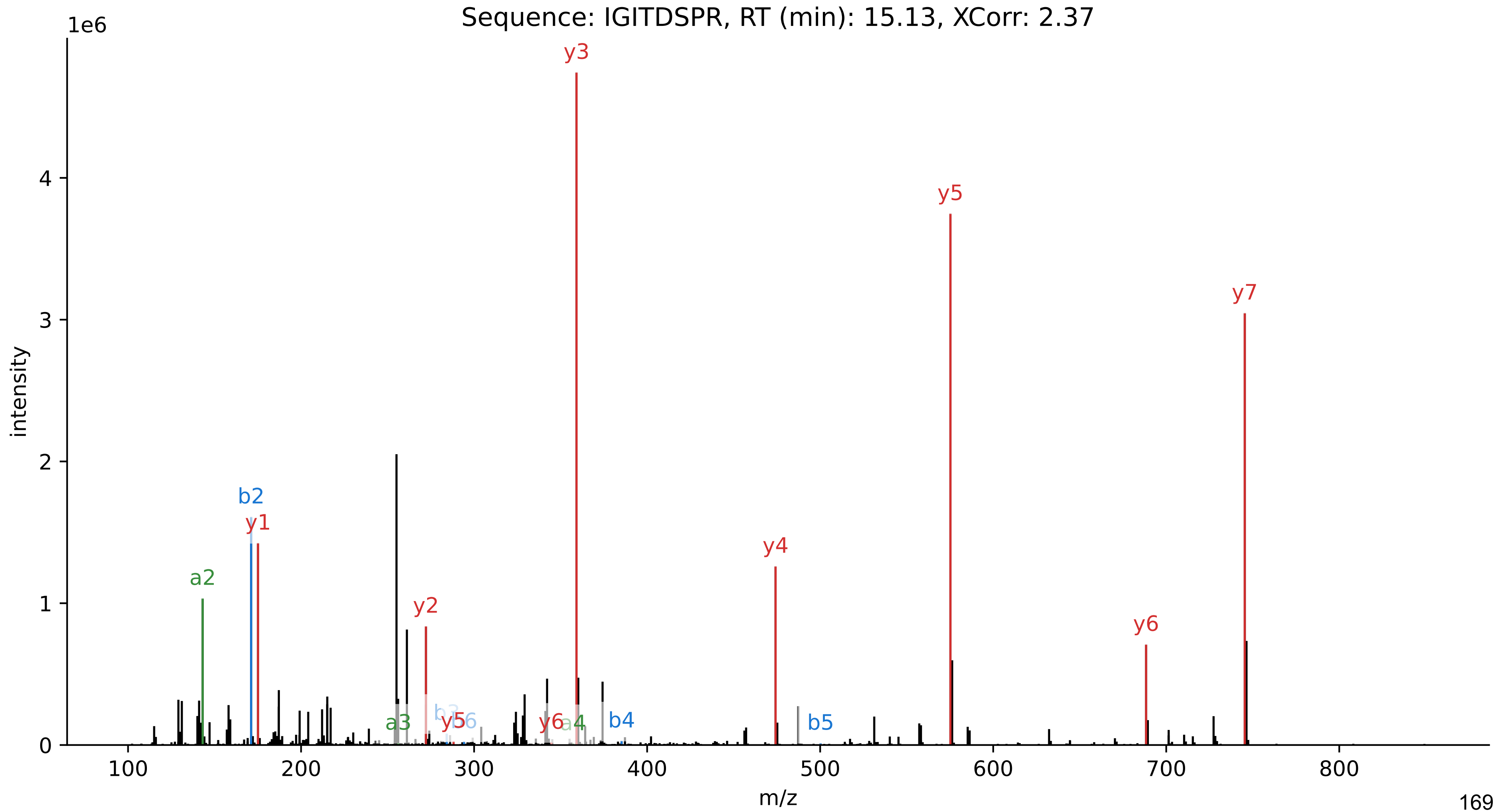
Sequence: IGITDSPR, RT (min): 15.13, XCorr: 2.37
1e6
intensity
m/z
0
1
2
3
4
100
200
300
400
500
600
700
800
y3
y5
y7
b2
y1
a2
y2
y4
y6
a3
y5
y6
a4
b4
b5

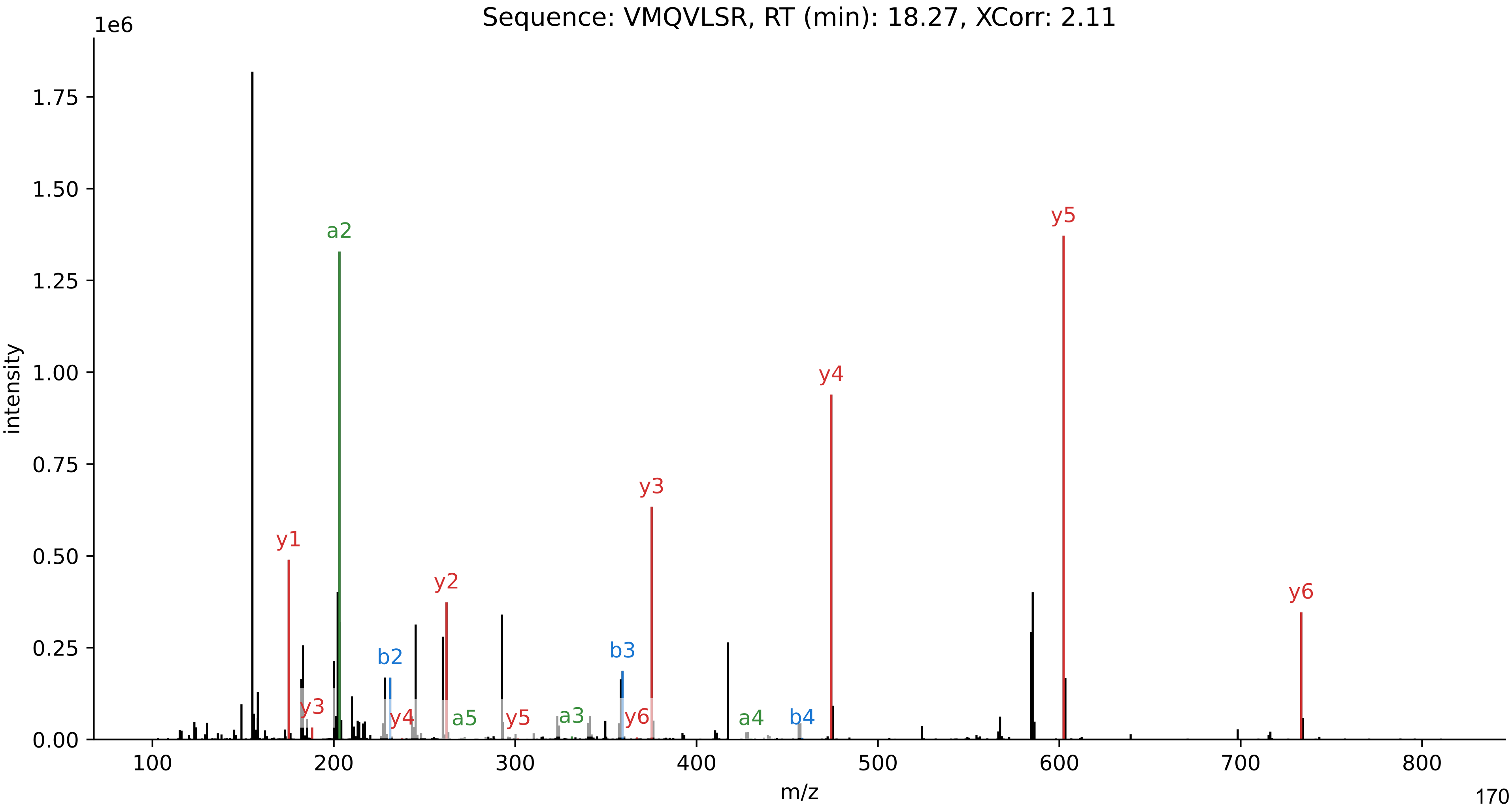

Sequence: VMQVLSR, RT (min): 18.27, XCorr: 2.11
1e6
1.75
1.50
1.25
1.00
0.75
0.50
0.25
0.00
intensity
100
200
300
400
500
600
700
800
m/z
a2
y5
y4
y3
y1
y2
y6
b2
b3
y3
y4
a5
y5
a3
y6
a4
b4
