# Supplementary material for: Development of a Spectral Library for the Discovery of Altered Genomic Events in Mycobacterium avium Associated With Virulence Using Mass Spectrometry–Based Proteogenomic Analysis
Source: Mol Cell Proteomics. 2023 Mar 21;22(5):100533. doi: 10.1016/j.mcpro.2023.100533 (PMC10149365; doi:10.1016/j.mcpro.2023.100533)
Supplement: Supplemental Data 6 [file mmc12.pdf]

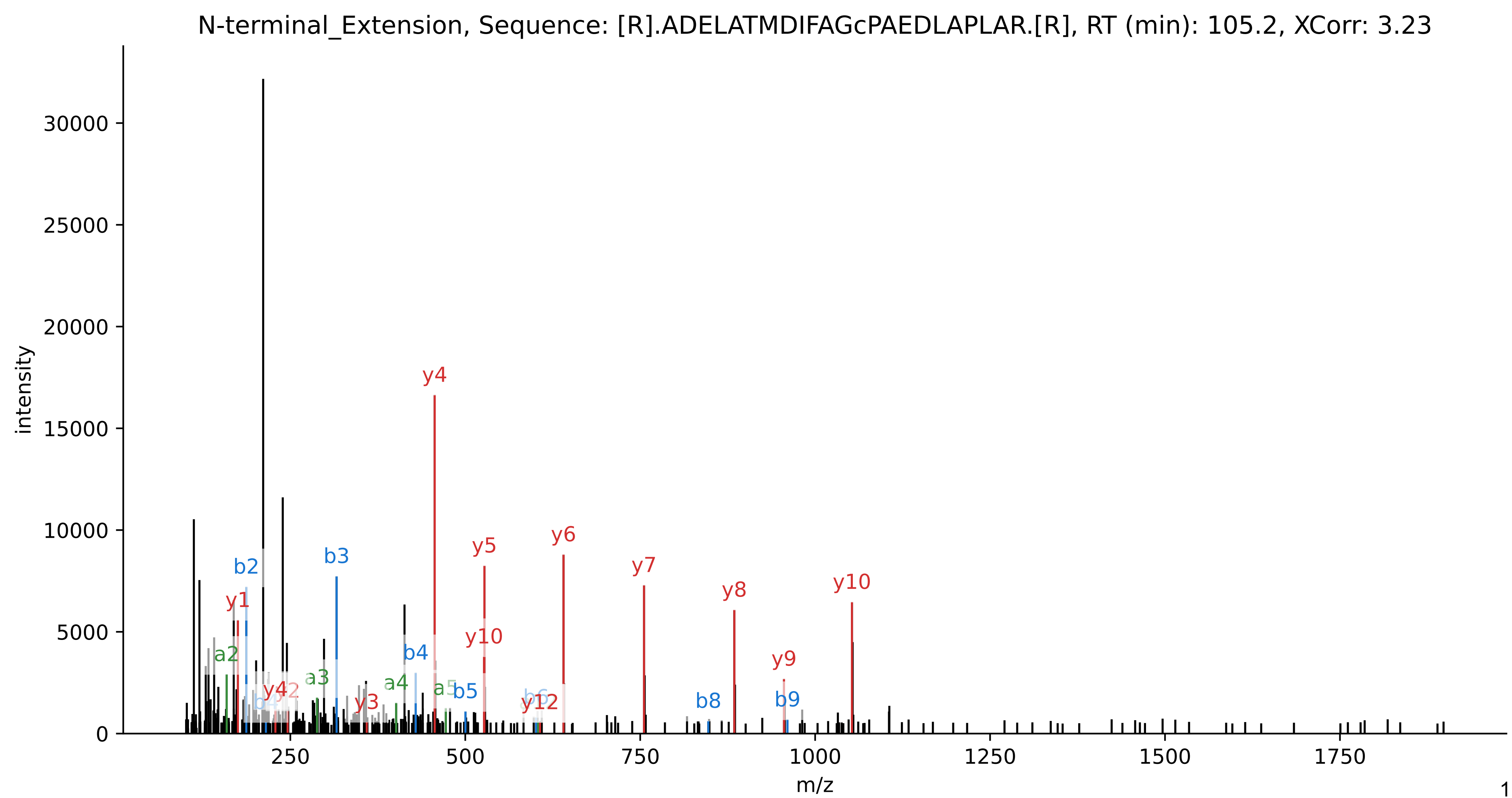
N-terminal_Extension, Sequence: [R].ADELATMDIFAGcPAEDLAPLAR.[R], RT (min): 105.2, XCorr: 3.23
intensity
m/z
y4
y5
y6
y7
y8
y9
y10
y1
y3
y12
b2
b3
b4
b5
b8
b9
a2
a3
a4

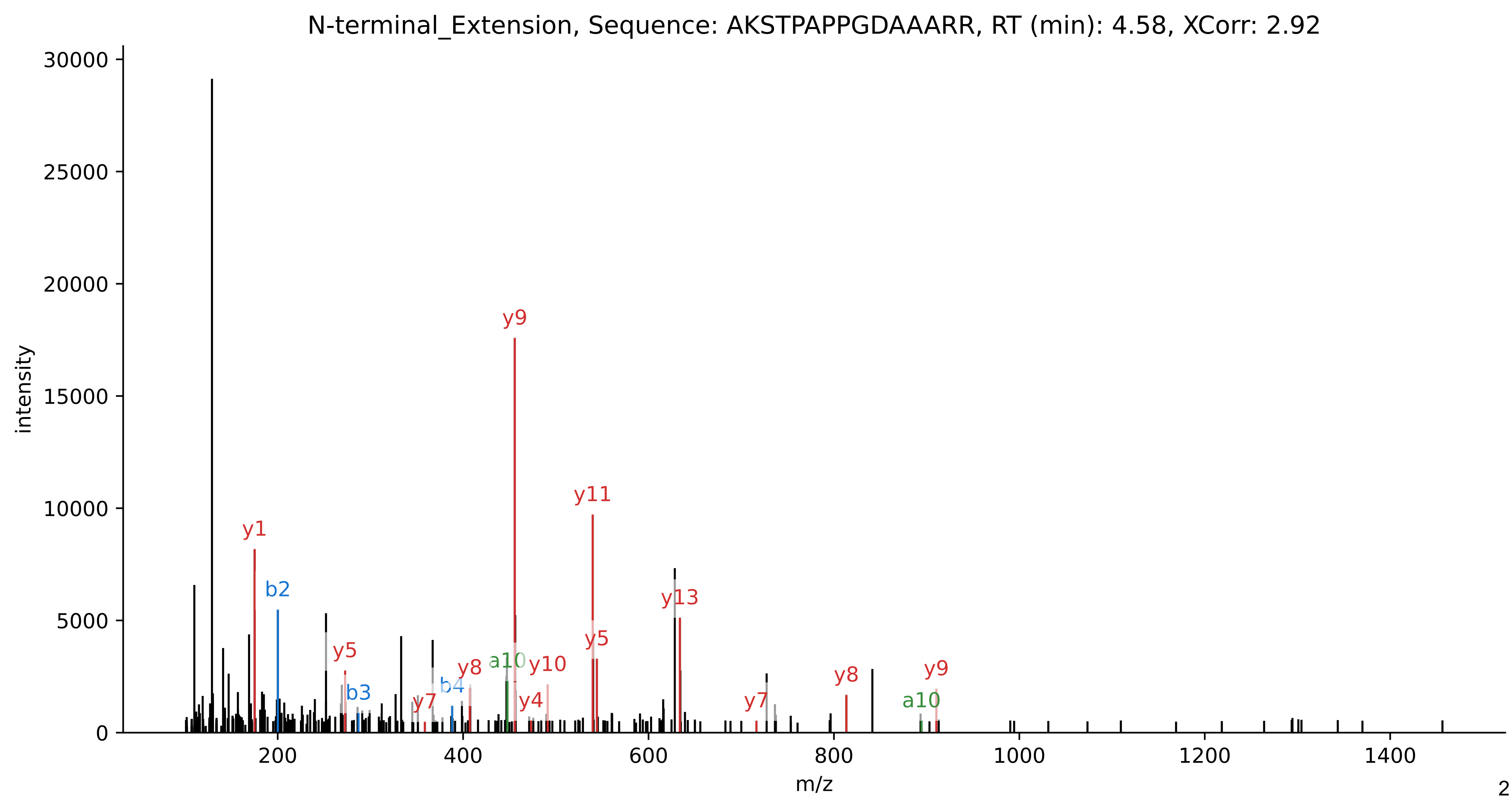

N-terminal_Extension, Sequence: AKSTPAPPGDAAARR, RT (min): 4.58, XCorr: 2.92
intensity
m/z
y1
b2
y5
b3
y7
b4
y8
a10
y9
y10
y4
y11
y5
y13
y7
y8
a10
y9

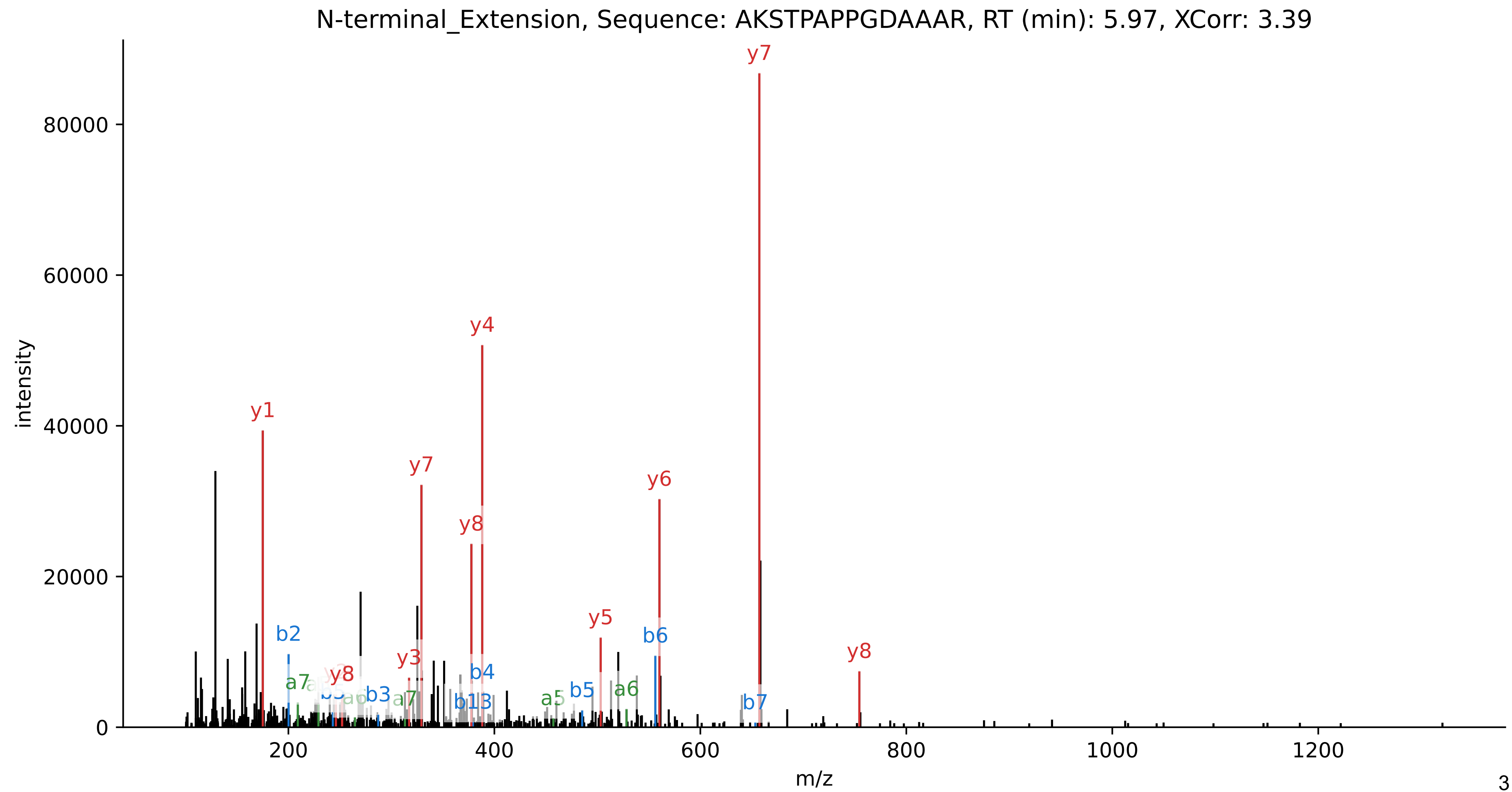
N-terminal_Extension, Sequence: AKSTPAPPGDAAAR, RT (min): 5.97, XCorr: 3.39
intensity
m/z
y7
y4
y1
y7
y8
y6
y5
b6
y8
b2
y3
b4
y8
a7
b3
a7
b13
a5
b5
a6
b7

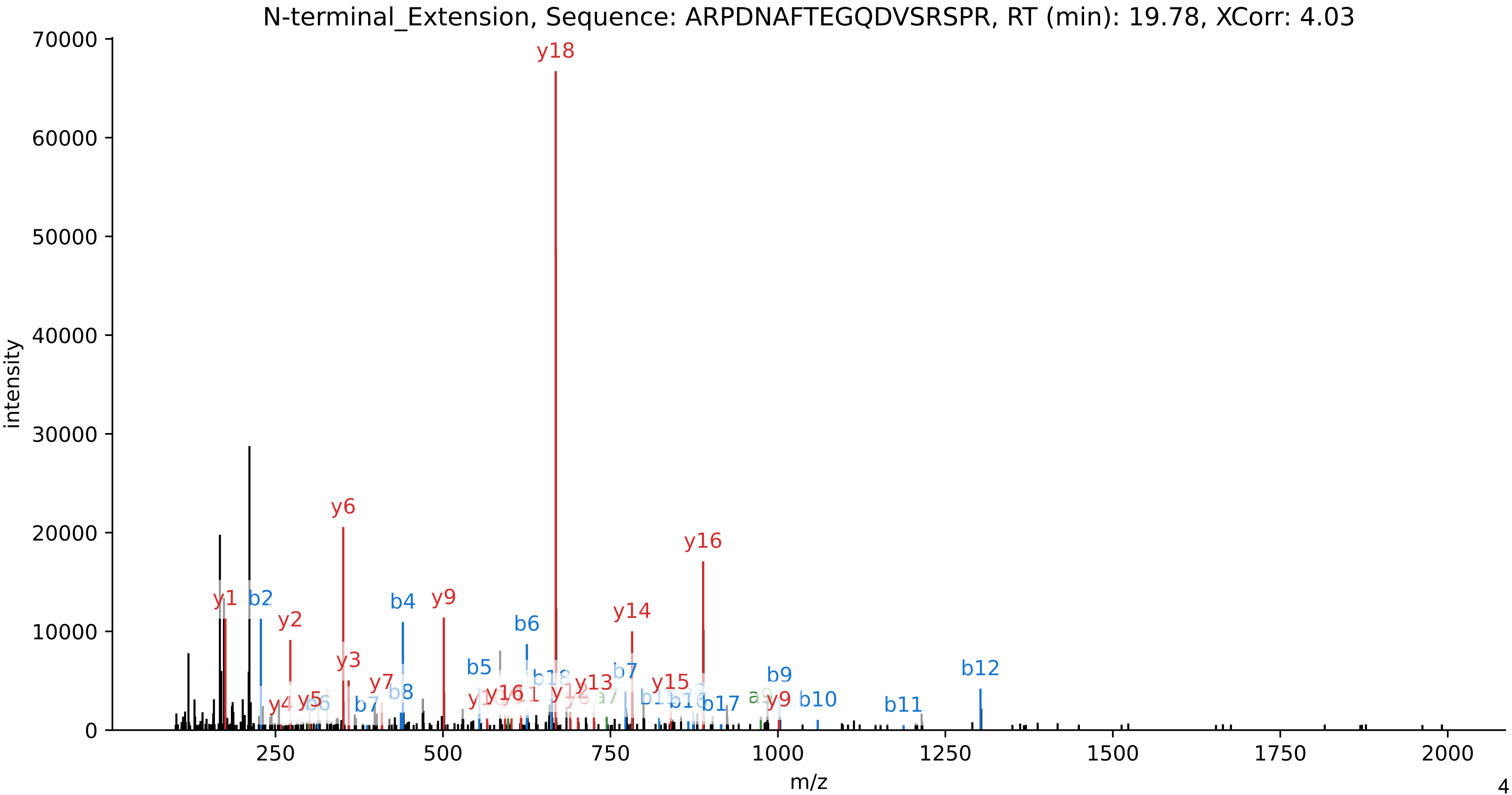
N-terminal_Extension, Sequence: ARPDNAFTEGQDVSRSPR, RT (min): 19.78, XCorr: 4.03
intensity
m/z
y18
y6
y16
y1
b2
y2
b4
y9
b6
y14
b12
b5
b9
y3
y7
b8
y13
y15
b7
b10
b11
y4
y5
b17
y9

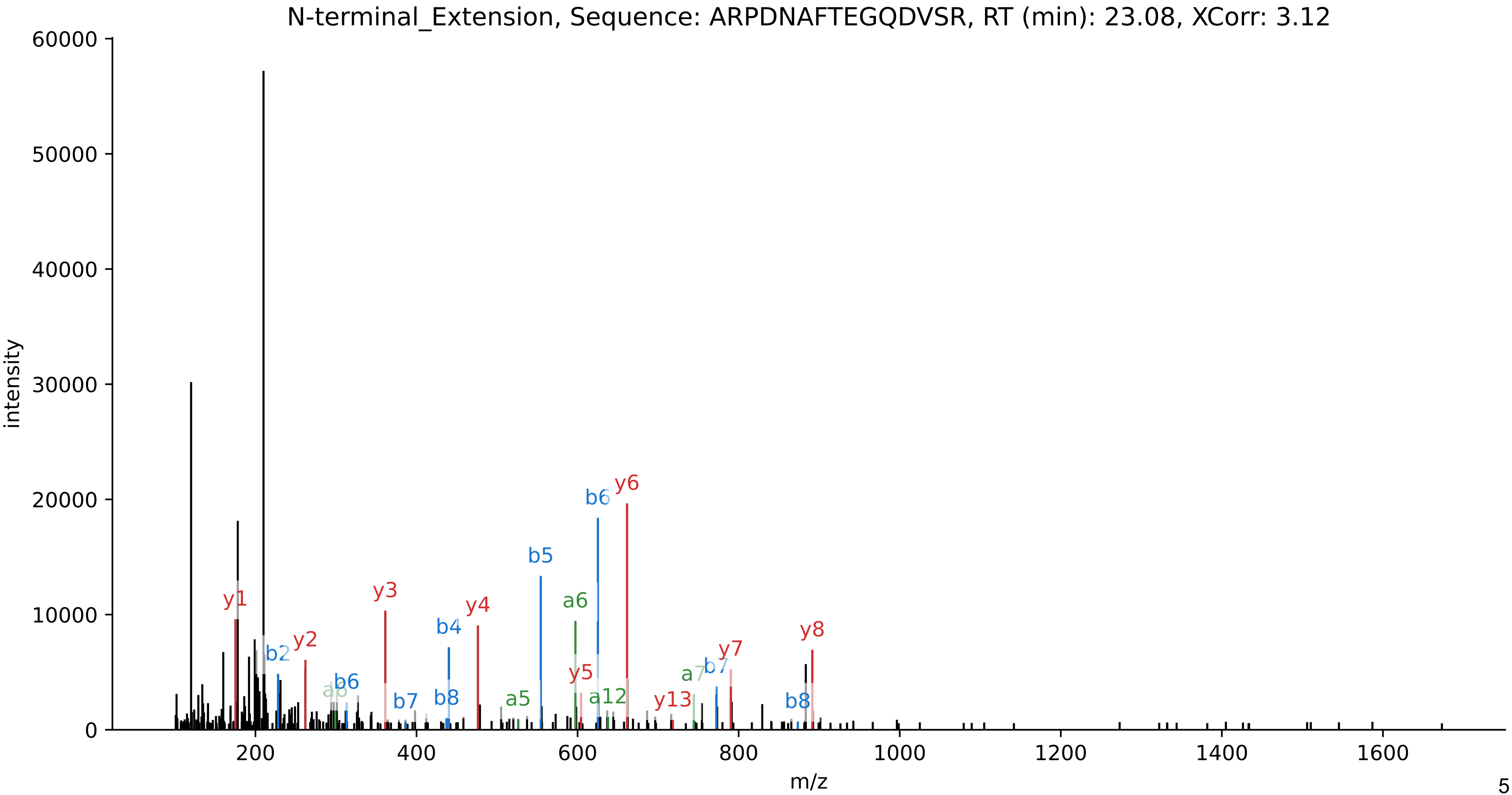

N-terminal_Extension, Sequence: ARPDNAFTEGQDVSR, RT (min): 23.08, XCorr: 3.12
intensity
m/z
y1
b2
y2
b6
y3
b7
b8
b4
y4
b5
a5
a6
y5
b6
y6
a12
y13
a7
b7
y7
b8
y8

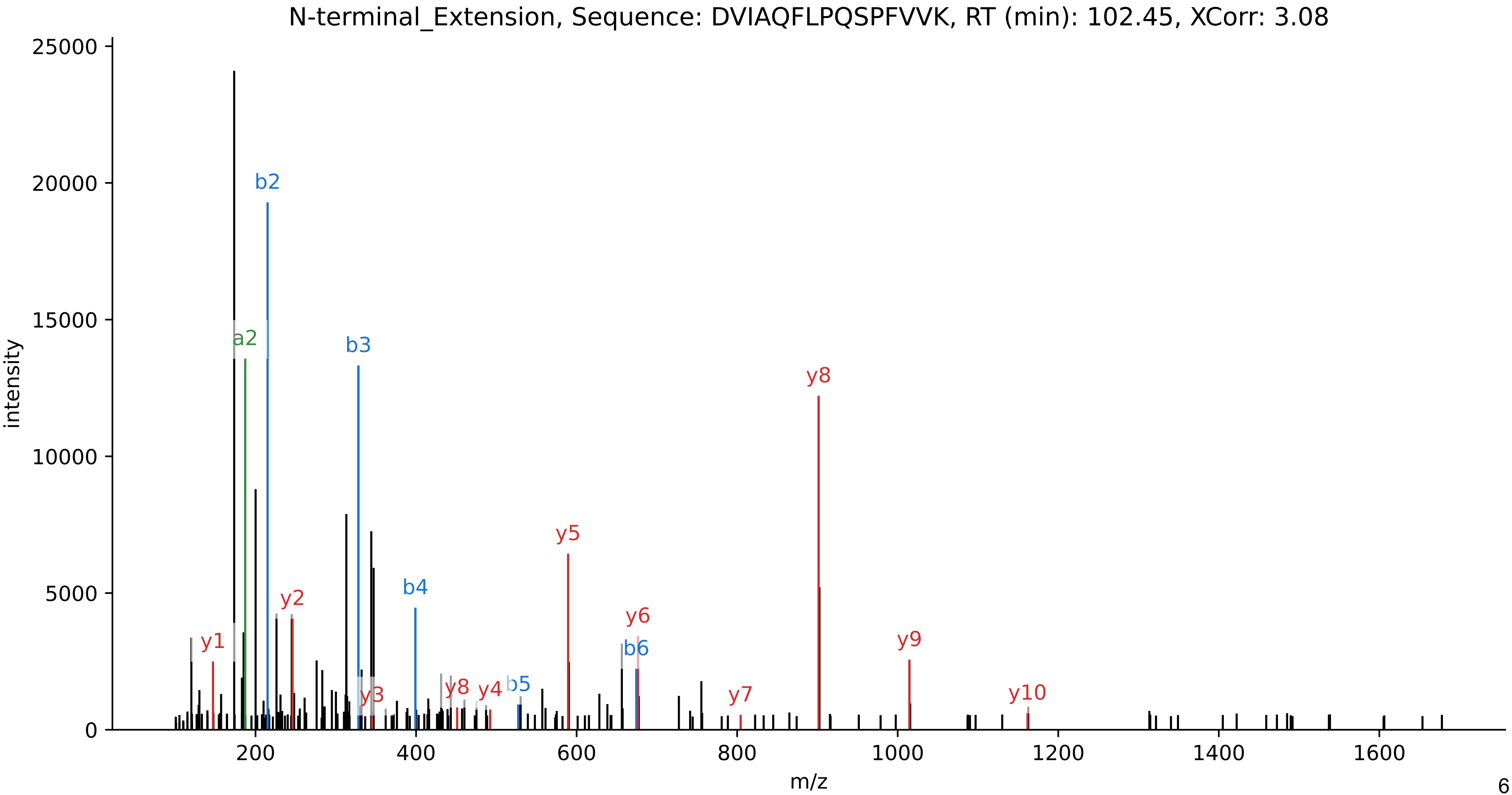
N-terminal_Extension, Sequence: DVIAQFLPQSPFVVK, RT (min): 102.45, XCorr: 3.08
intensity
m/z
b2
a2
b3
y8
y5
b4
y2
y6
b6
y1
y9
y8
y4
b5
y3
y7
y10
25000
20000
15000
10000
5000
0
200
400
600
800
1000
1200
1400
1600

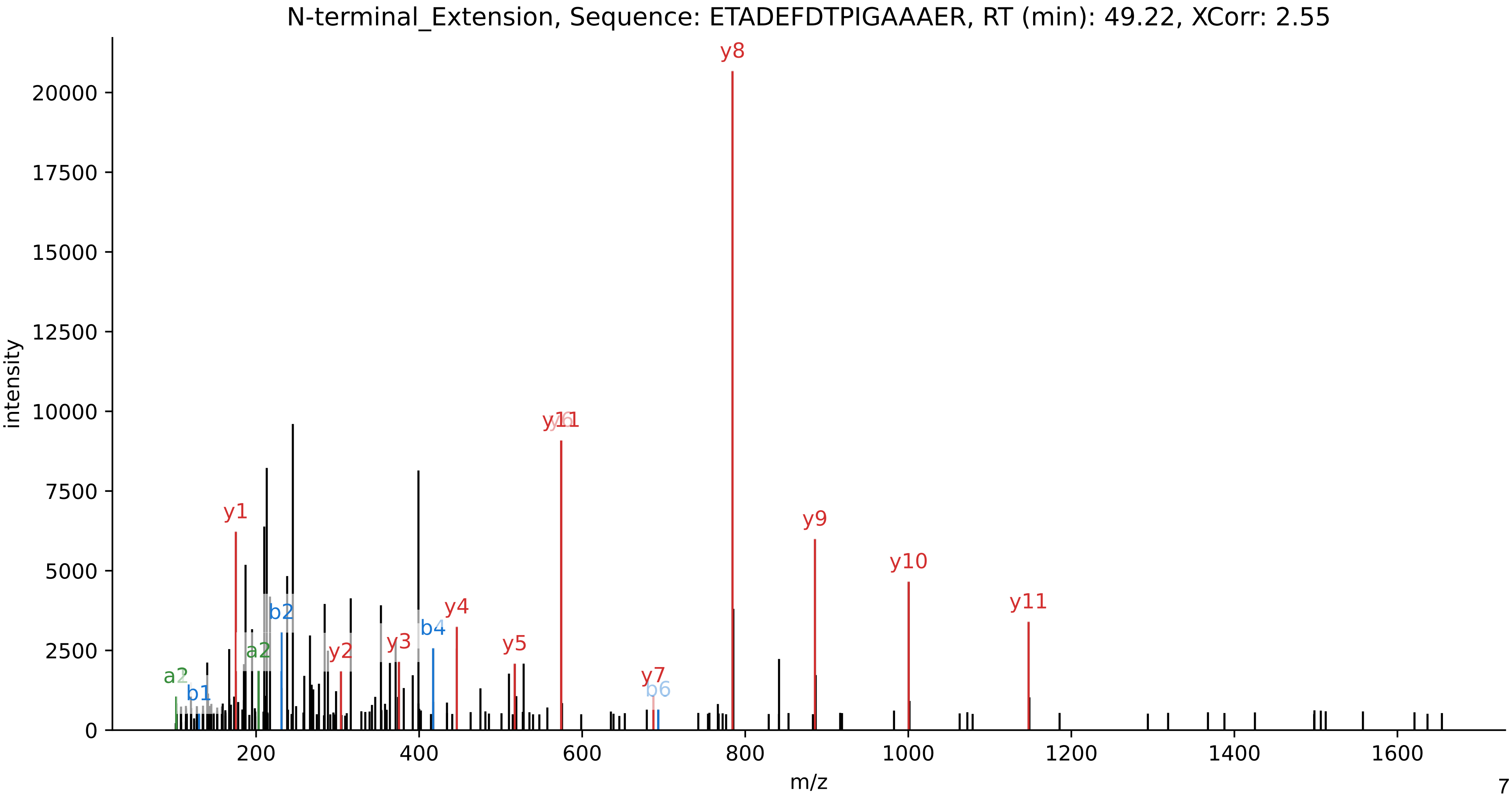
N-terminal_Extension, Sequence: ETADEFDTPIGAAAER, RT (min): 49.22, XCorr: 2.55
intensity
m/z
y8
y11
y6
y1
y9
y10
y11
y4
b2
b4
y3
y5
a2
y2
a2
b1
y7
b6

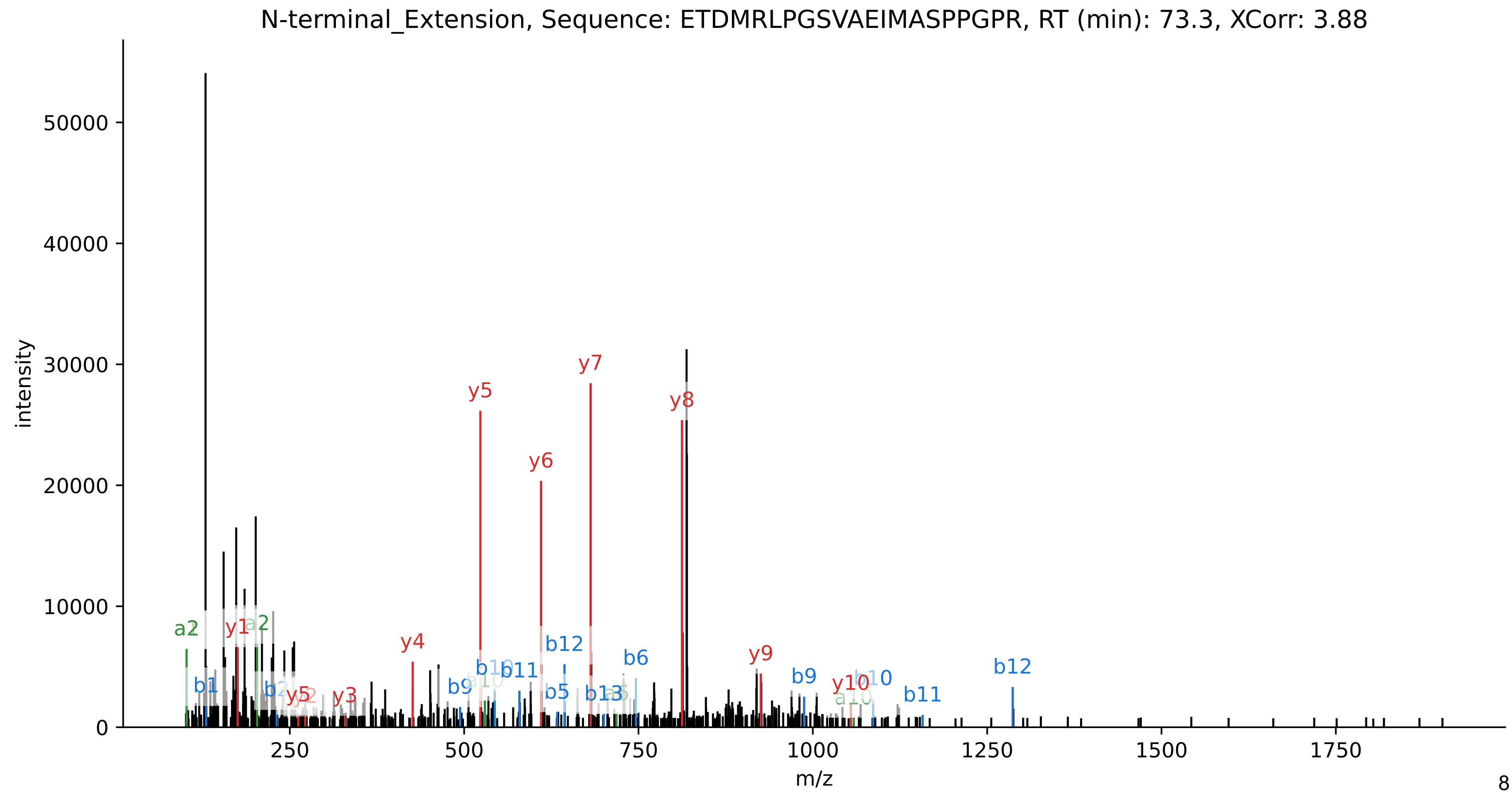

N-terminal_Extension, Sequence: ETDMRLPGSVAEIMASPPGPR, RT (min): 73.3, XCorr: 3.88
intensity
m/z
y5
y7
y8
y6
y4
y9
y1
a2
b12
b6
b9
y10
b10
b11
b1
b2
y3
b5
b13

N-terminal_Extension, Sequence: EVMNVAVLAESELGSEAQR, RT (min): 78.93, XCorr: 2.43

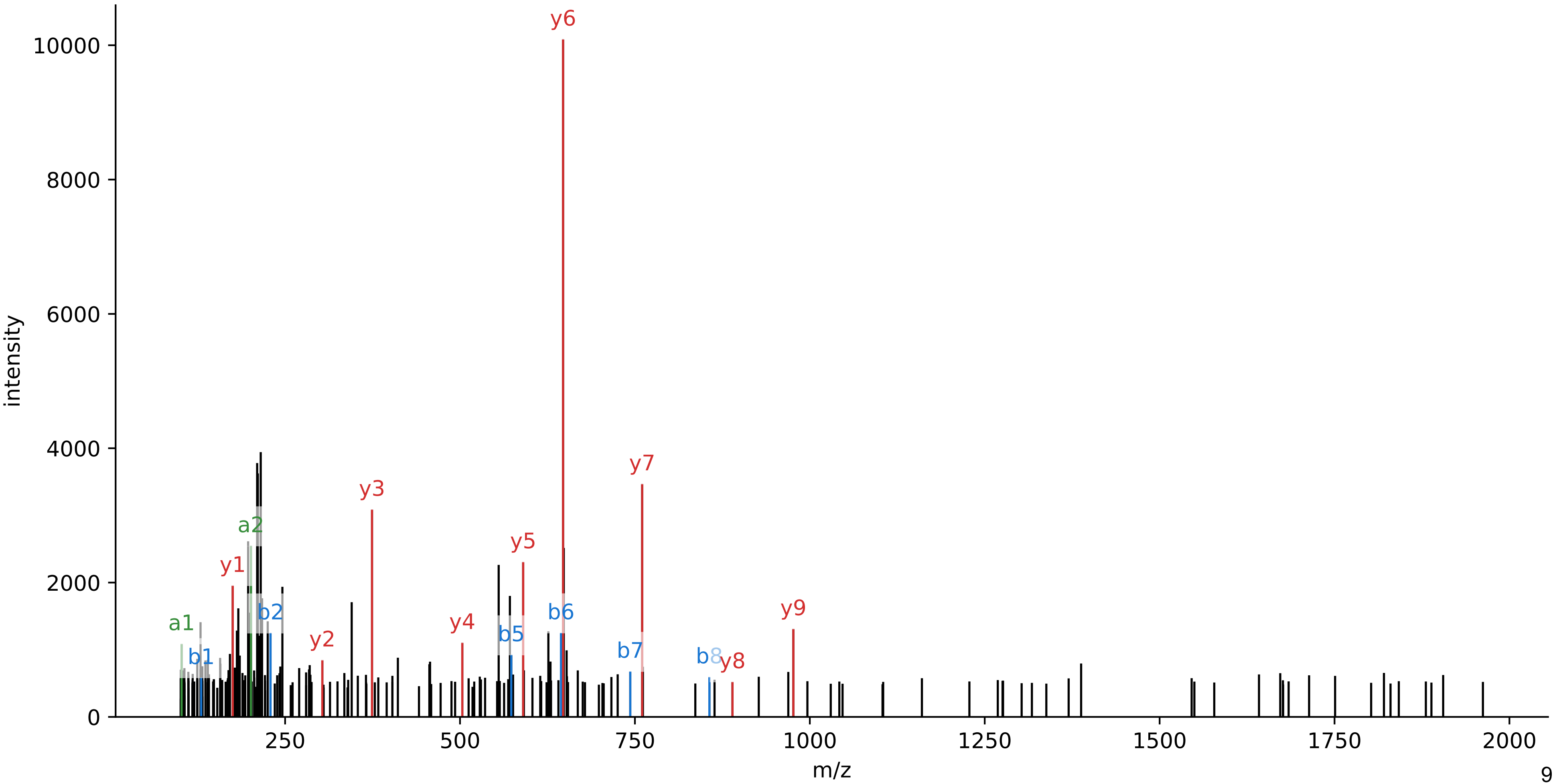

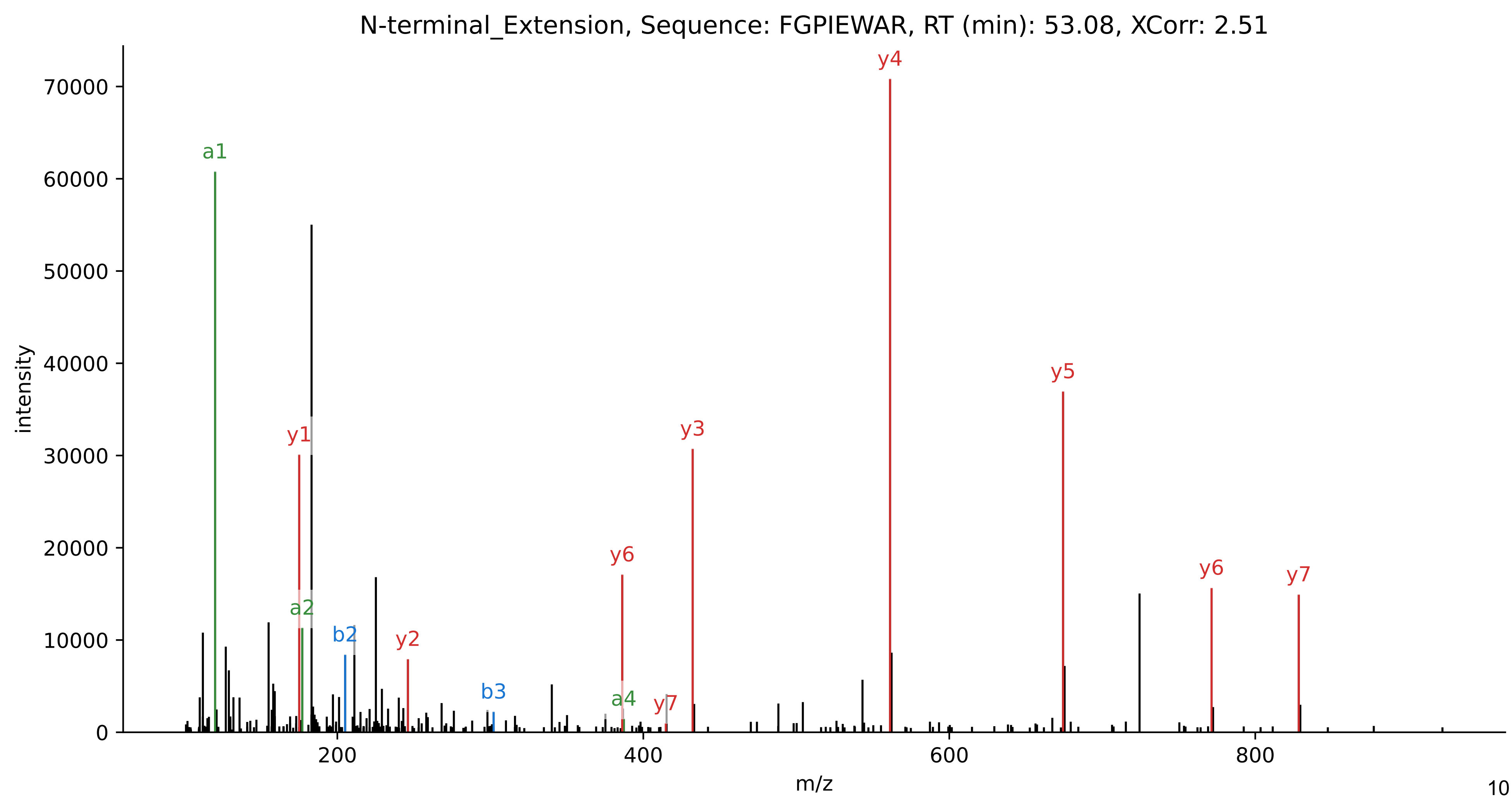

N-terminal_Extension, Sequence: FGPIEWAR, RT (min): 53.08, XCorr: 2.51
intensity
m/z
a1
y1
a2
b2
y2
b3
y6
a4
y7
y3
y4
y5
y6
y7
70000
60000
50000
40000
30000
20000
10000
0
200
400
600
800

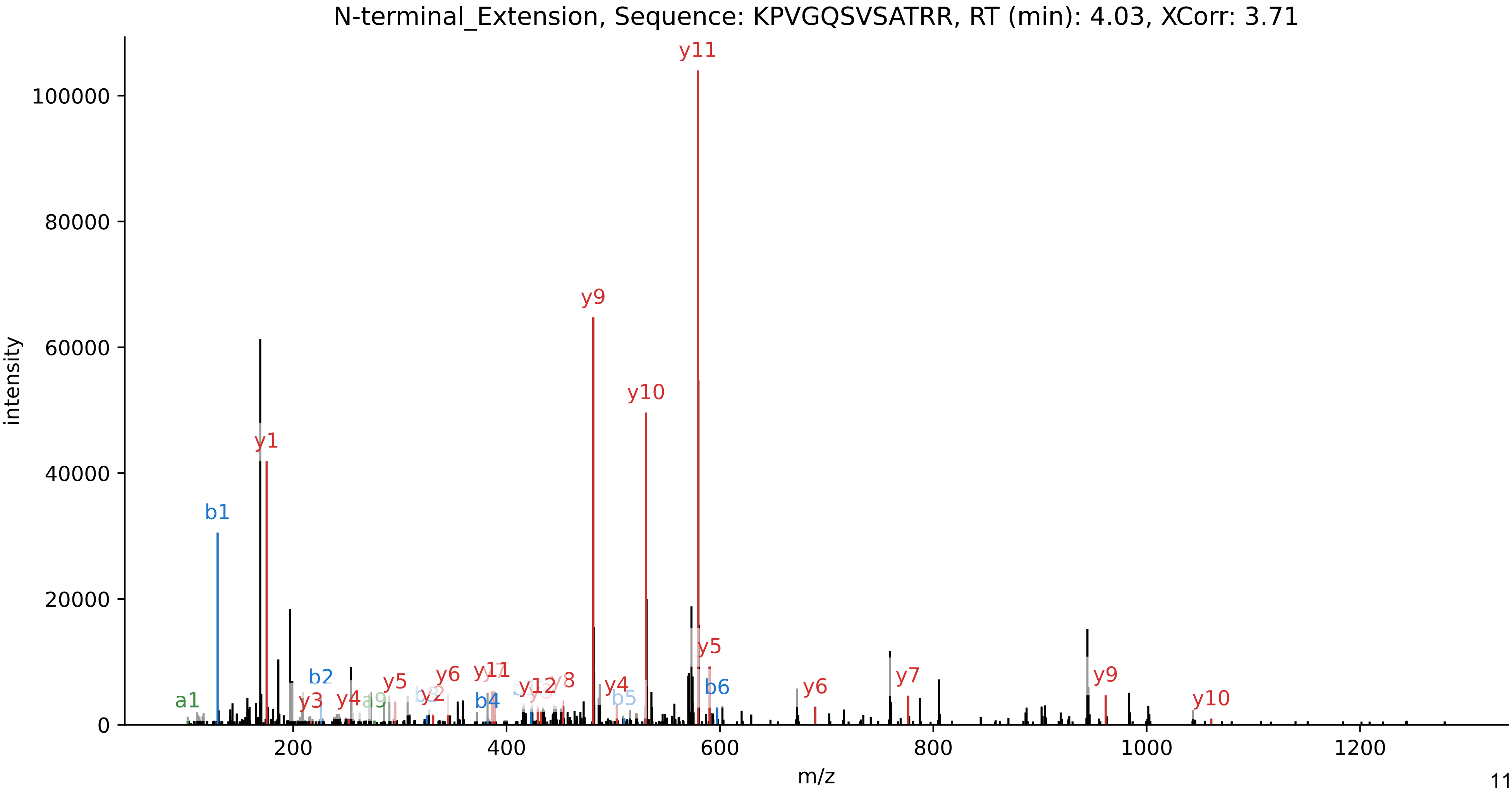
N-terminal_Extension, Sequence: KPVGQSVSATRR, RT (min): 4.03, XCorr: 3.71
intensity
m/z
0
20000
40000
60000
80000
100000
200
400
600
800
1000
1200
y11
y9
y10
y1
b1
b2
a1
y3
y4
y5
y6
b4
y4
b5
y5
b6
y6
y7
y9
y10

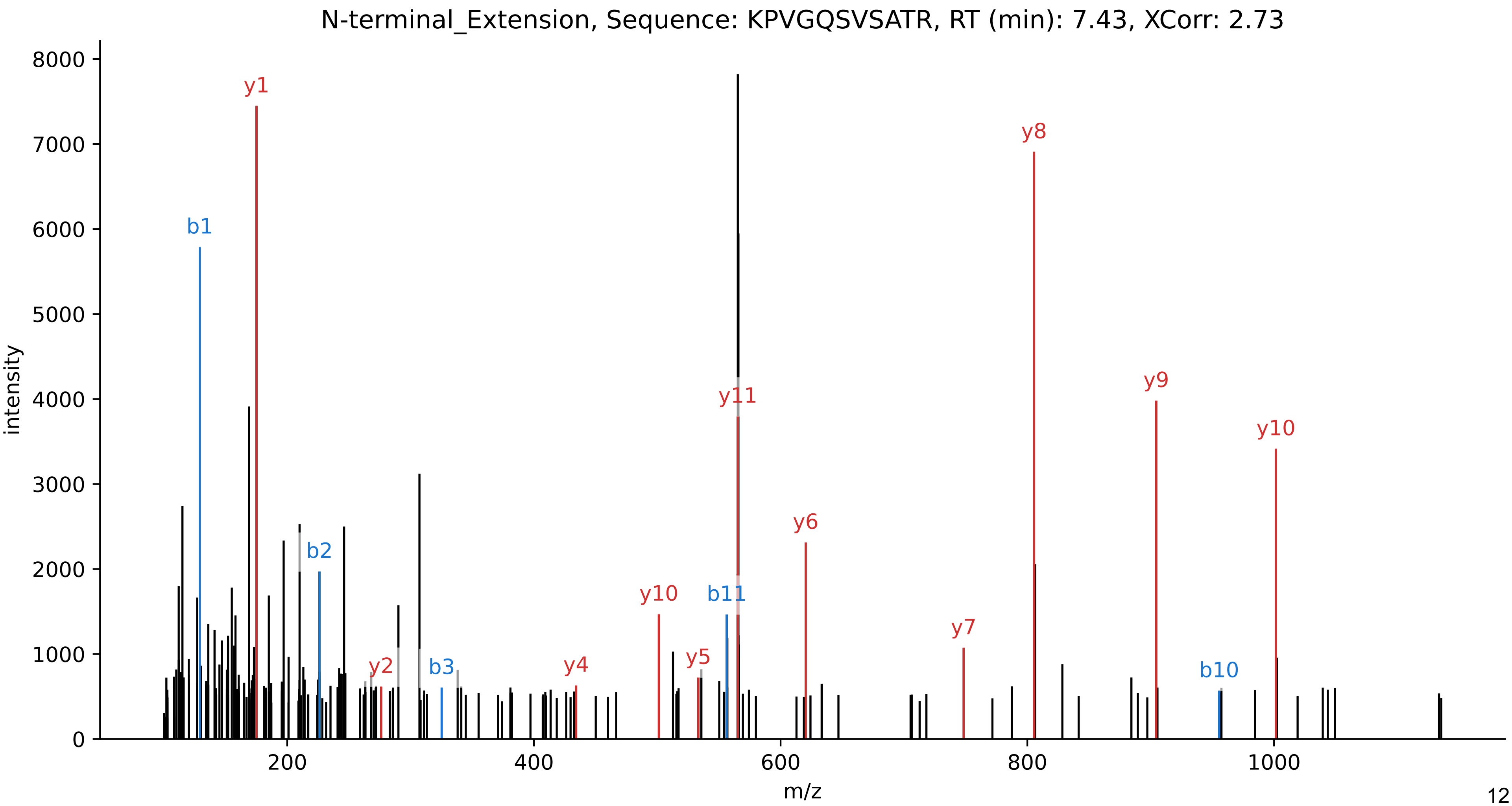

N-terminal_Extension, Sequence: KPVGQSVSATR, RT (min): 7.43, XCorr: 2.73
intensity
m/z
0
1000
2000
3000
4000
5000
6000
7000
8000
200
400
600
800
1000
y1
b1
b2
y2
b3
y4
y10
y5
b11
y11
y6
y7
y8
y9
b10
y10

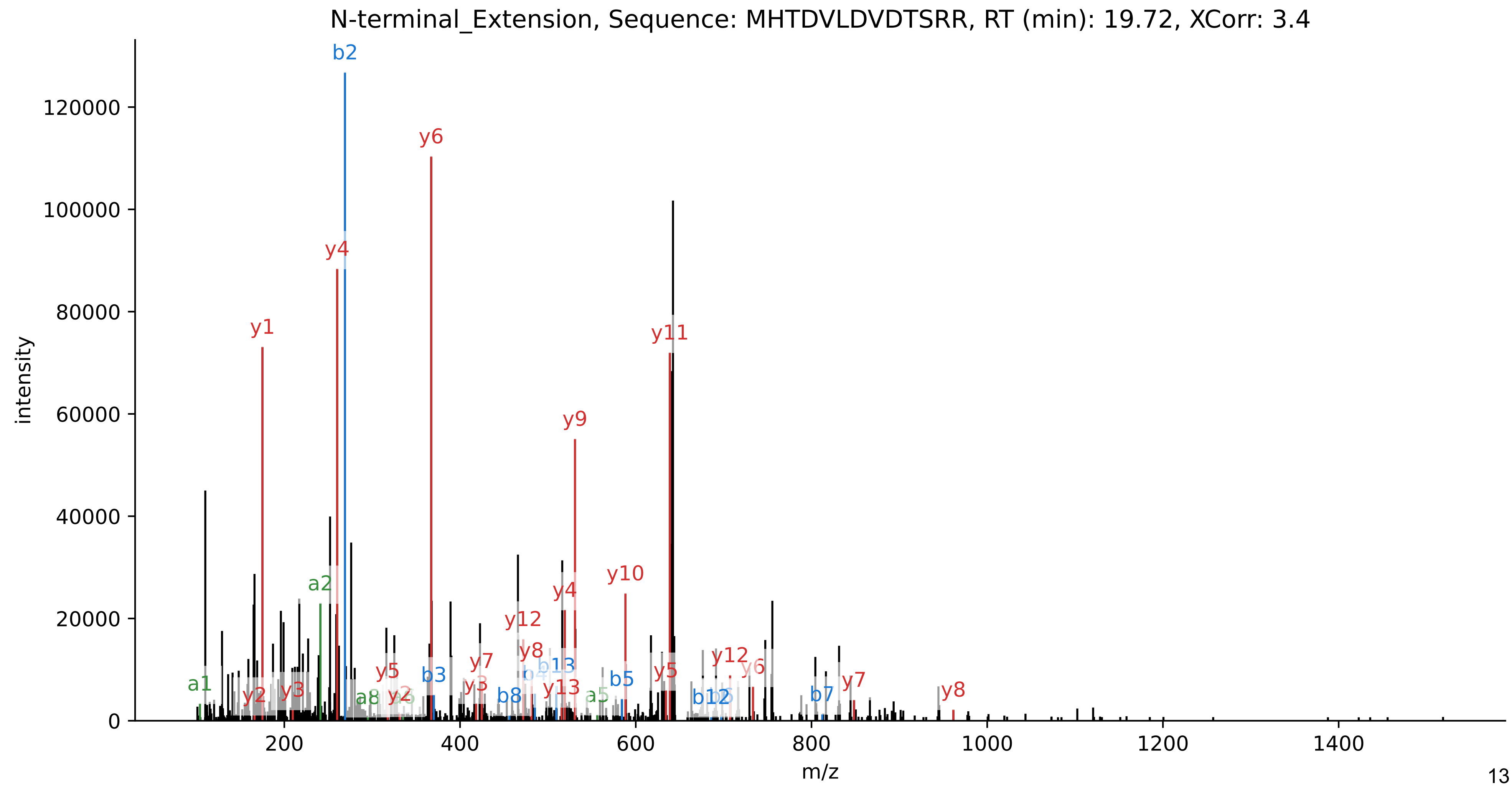
N-terminal_Extension, Sequence: MHTDVLDVDTSRR, RT (min): 19.72, XCorr: 3.4
intensity
m/z
b2
y6
y4
y1
y11
y9
y10
a2
y12
y8
b3
b5
y5
y7
y13
b8
b7
b12
a1
y2
y3

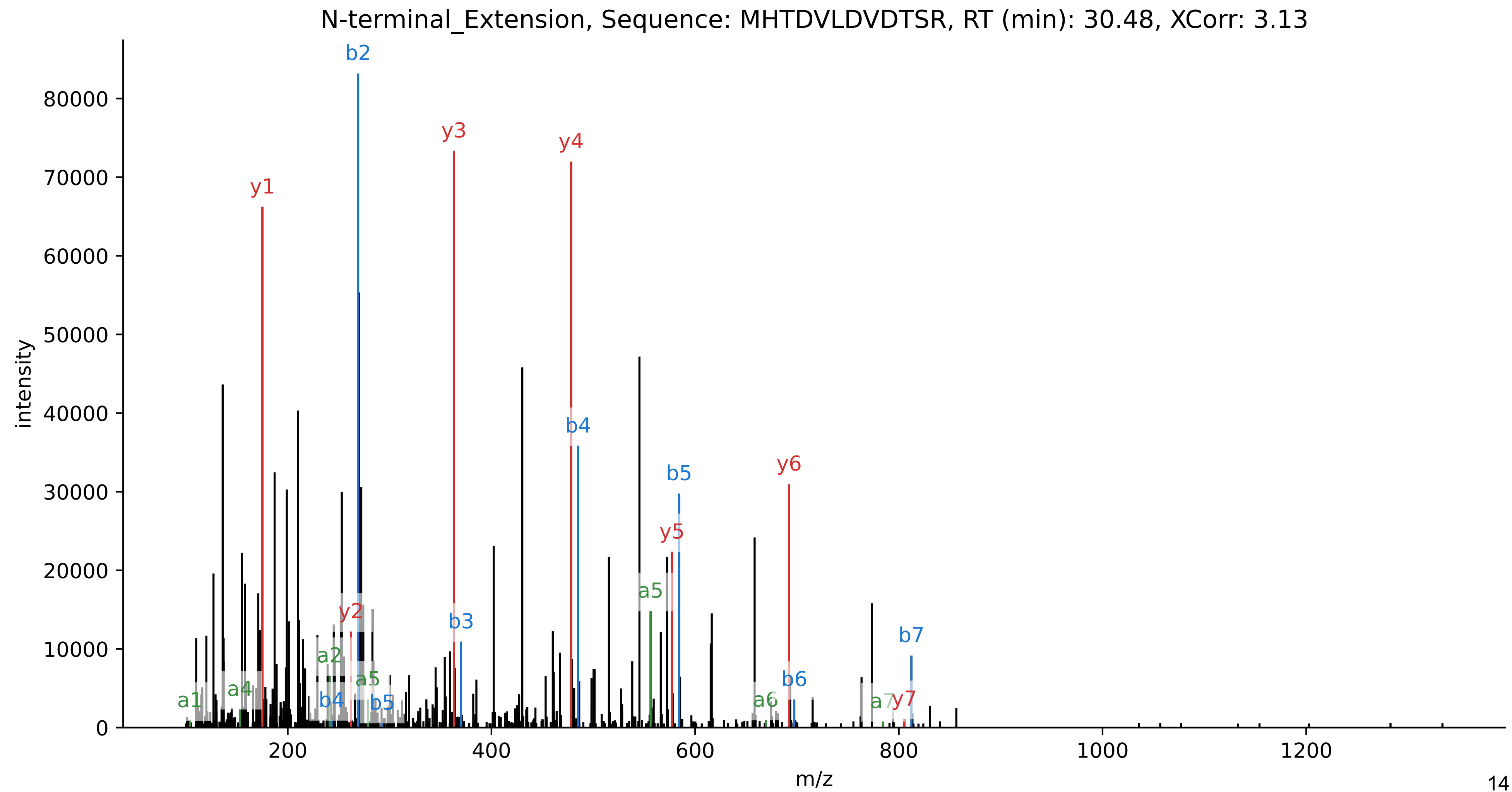

N-terminal_Extension, Sequence: MHTDVLDVDTSR, RT (min): 30.48, XCorr: 3.13
intensity
m/z
b2
y3
y4
y1
b4
b5
y6
y5
a5
y2
b3
b7
a2
a5
b6
a4
a1
b4
b5
a6
a7
y7

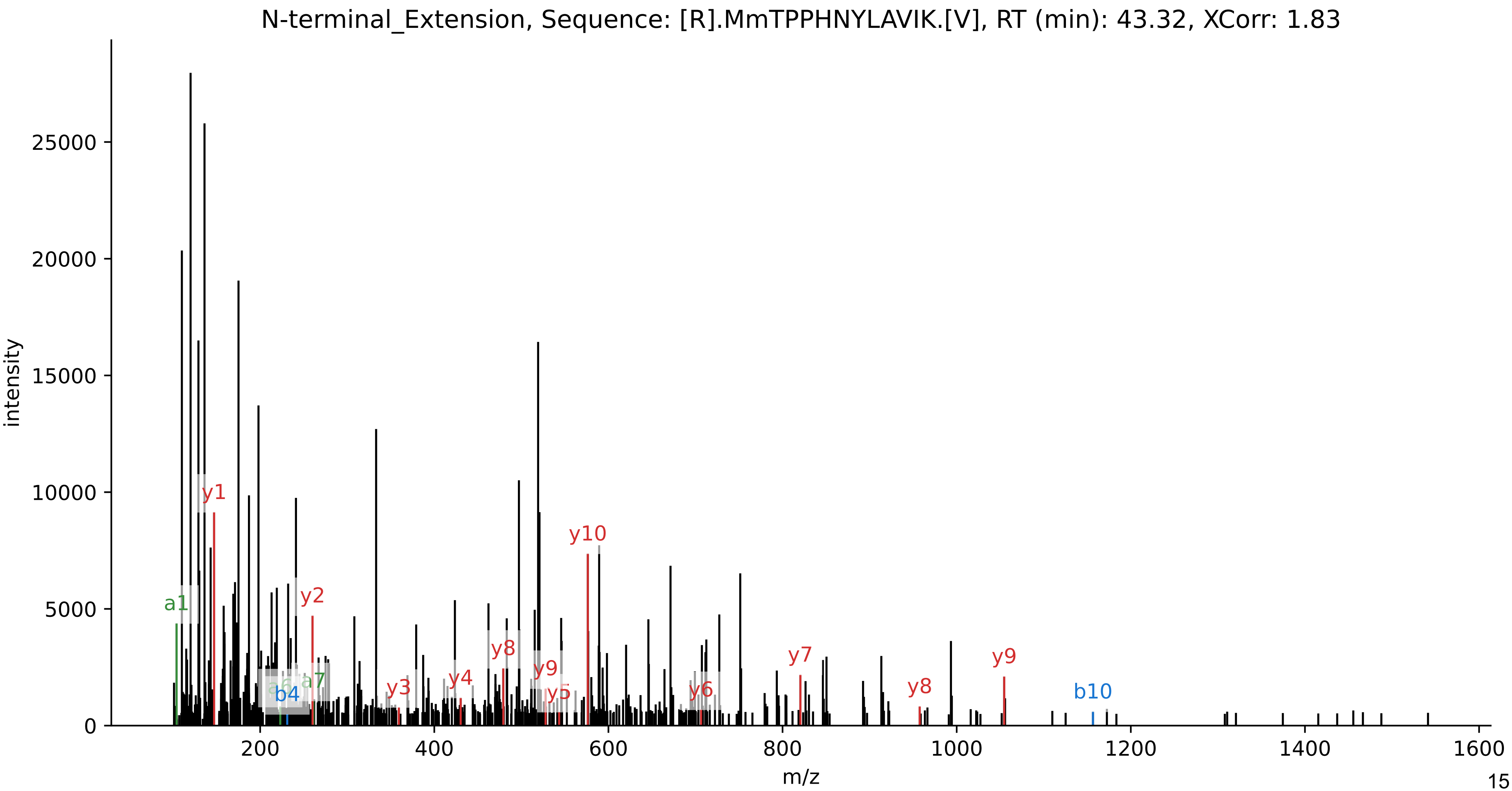
N-terminal_Extension, Sequence: [R].MmTPPHNYLAVIK.[V], RT (min): 43.32, XCorr: 1.83
intensity
m/z
0
5000
10000
15000
20000
25000
200
400
600
800
1000
1200
1400
1600
a1
y1
y2
b4
a7
y3
y4
y8
y9
y5
y10
y6
y7
y8
y9
b10

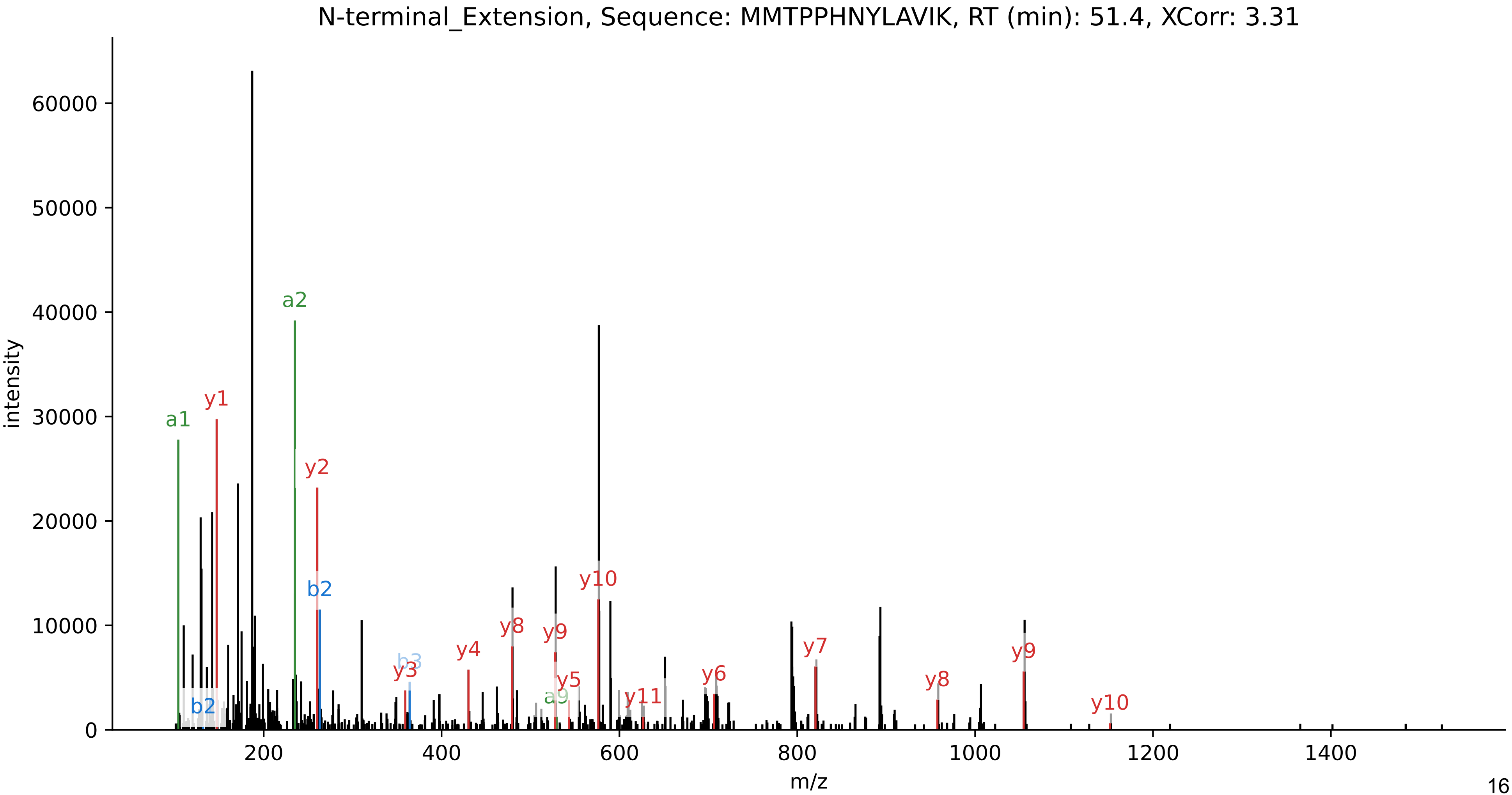

N-terminal_Extension, Sequence: MMTPPHNYLAVIK, RT (min): 51.4, XCorr: 3.31
intensity
m/z
a1
y1
a2
y2
b2
b2
b3
y3
y4
y8
y9
a9
y5
y10
y11
y6
y7
y8
y9
y10

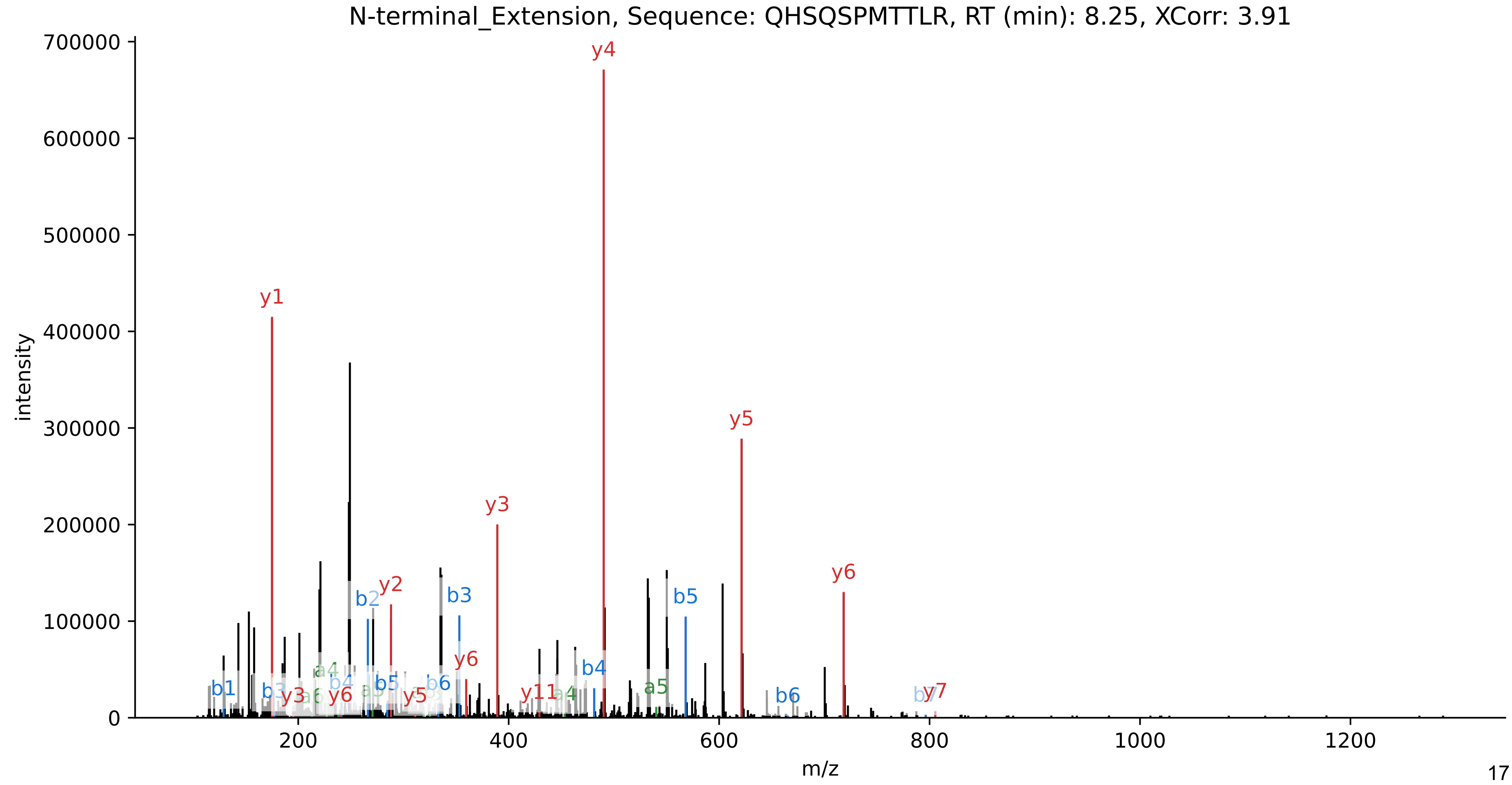
N-terminal_Extension, Sequence: QHSQSPMTTLR, RT (min): 8.25, XCorr: 3.91
intensity
m/z
y4
y1
y5
y3
y6
y2
b2
b3
b5
b4
y6
b1
b3
y3
y6
b4
b5
b6
y5
y11
a4
a5
b6
y7
a4

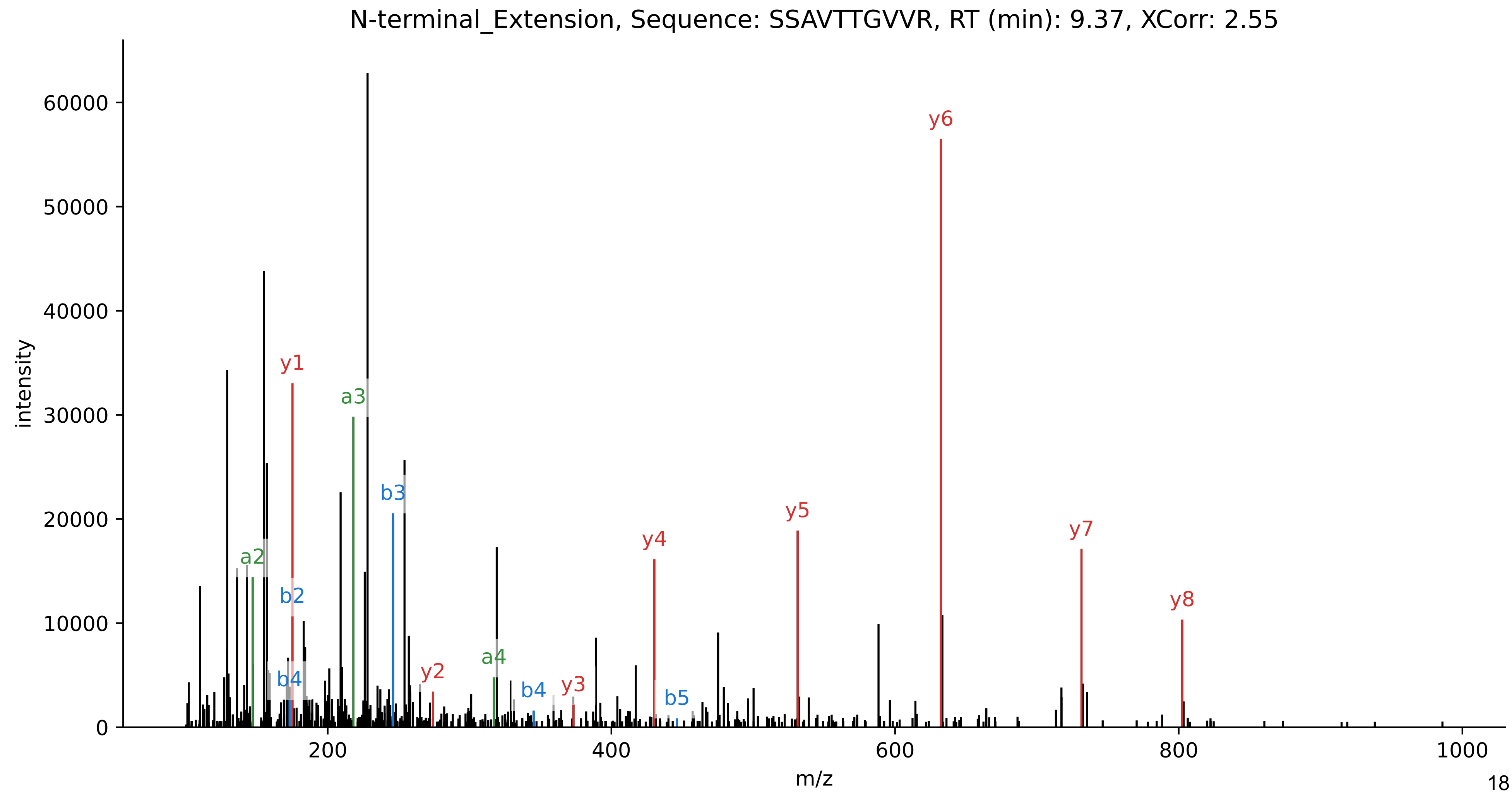
N-terminal_Extension, Sequence: SSAVTTGVVR, RT (min): 9.37, XCorr: 2.55
intensity
m/z
0
10000
20000
30000
40000
50000
60000
200
400
600
800
1000
y1
y2
y3
y4
y5
y6
y7
y8
a2
a3
a4
b2
b3
b4
b4
b5

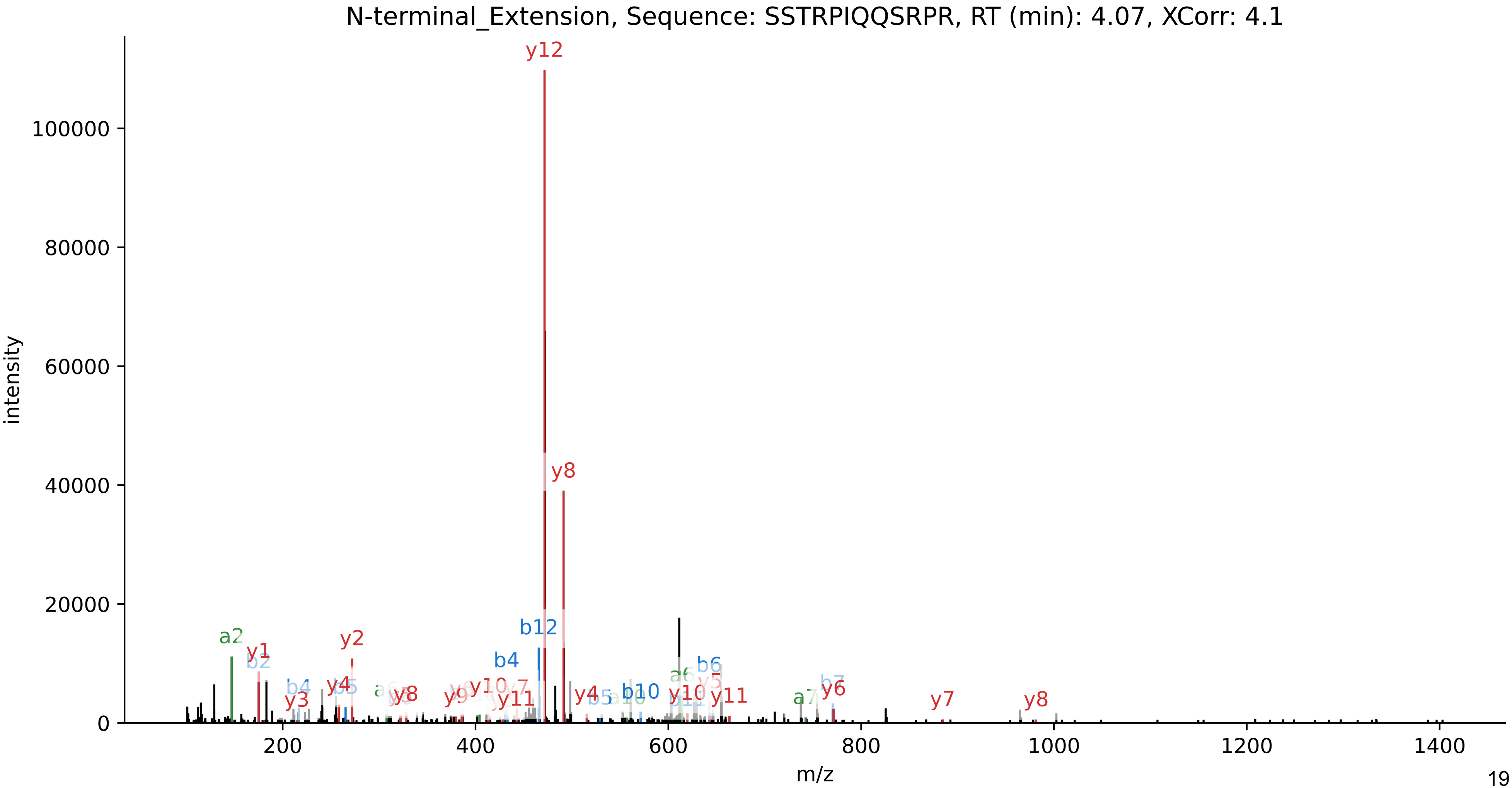
N-terminal_Extension, Sequence: SSTRPIQQSRPR, RT (min): 4.07, XCorr: 4.1
intensity
m/z
y12
y8
b12
b4
a2
y1
b2
y2
y3
y4
y8
y9
y10
y11
y4
b10
y10
y11
a6
b6
y5
a7
b7
y6
y7
y8

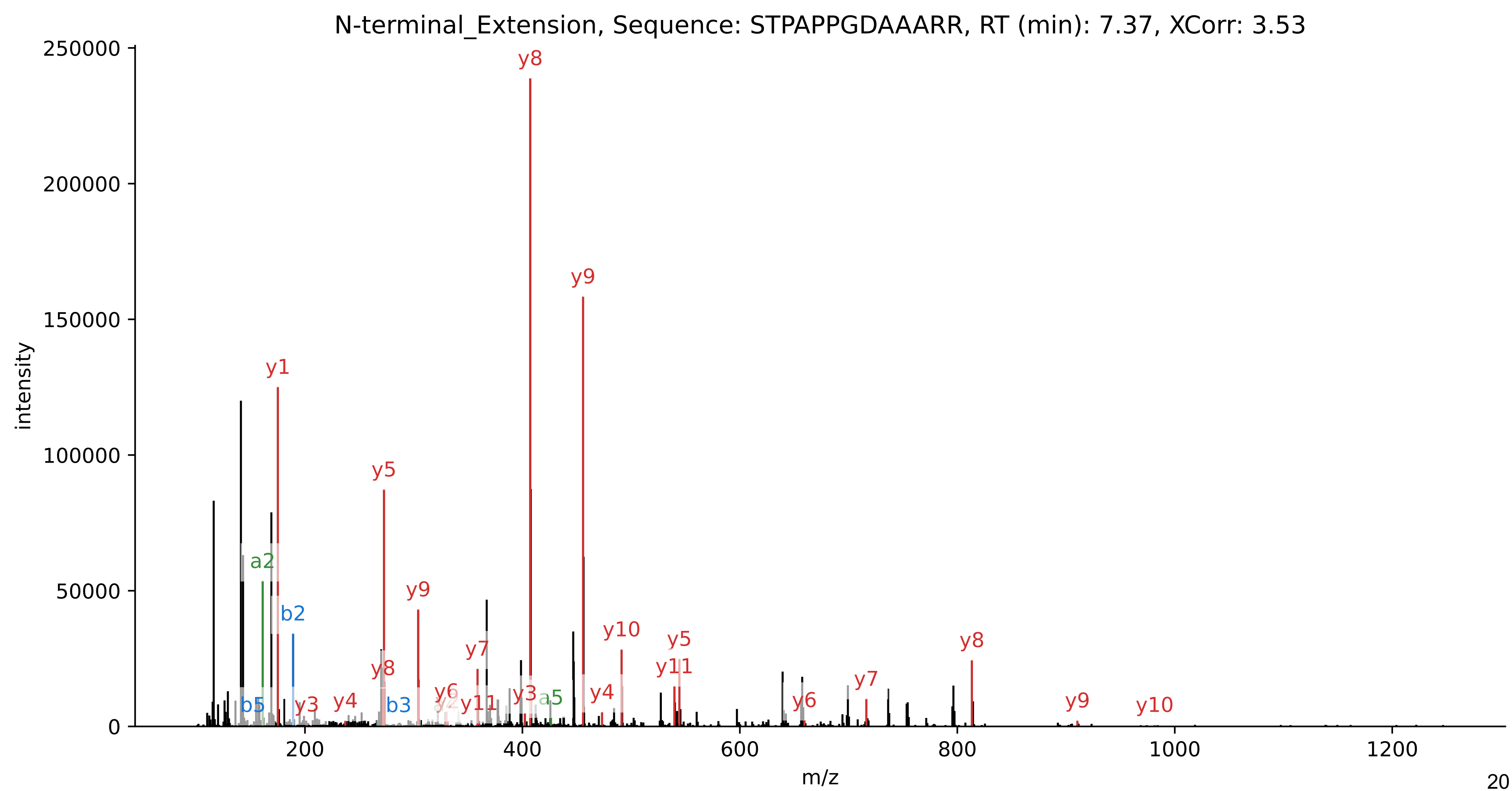

N-terminal_Extension, Sequence: STPAPPGDAAARR, RT (min): 7.37, XCorr: 3.53
intensity
m/z
y8
y9
y1
y5
a2
b2
y9
y10
y5
y8
y11
y7
y8
y7
y4
y3
y4
b5
b3
y6
y11
y3
a5
y6
y9
y10

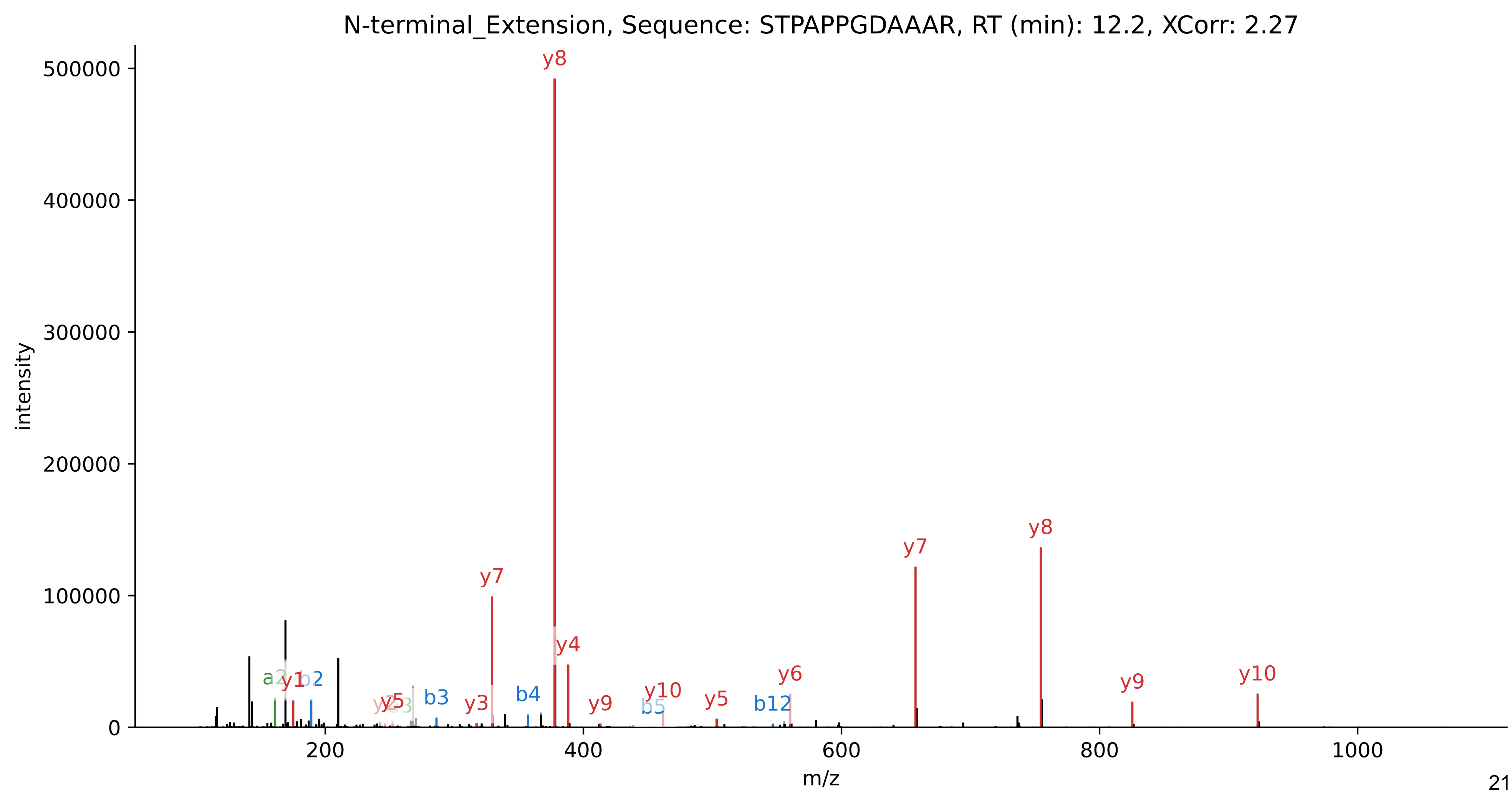

N-terminal_Extension, Sequence: STPAPPGDAAAR, RT (min): 12.2, XCorr: 2.27
intensity
m/z
y8
y7
y4
y1
b2
a2
b3
y3
b4
y9
y10
b5
y5
y6
b12
y7
y8
y9
y10

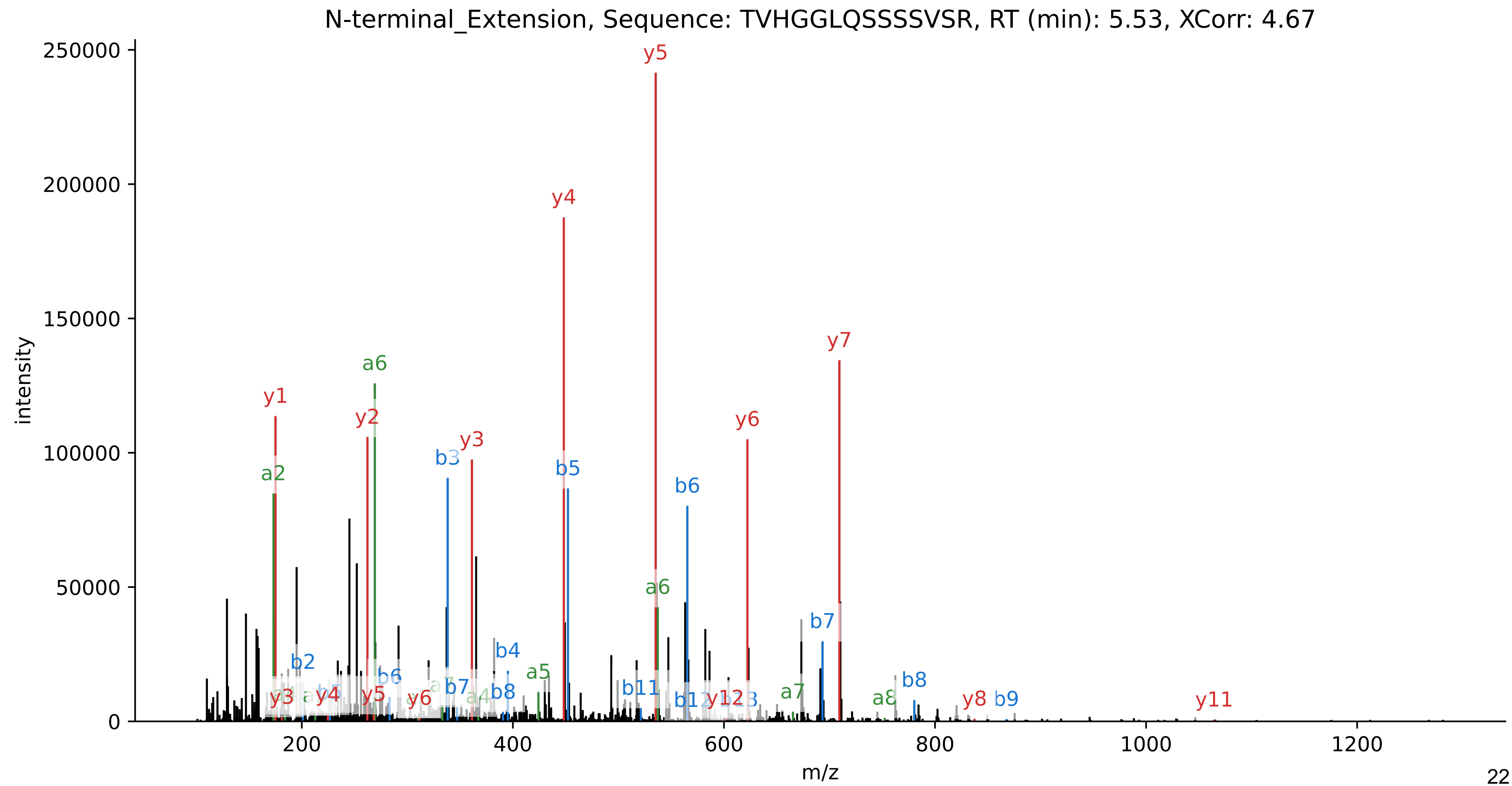
N-terminal_Extension, Sequence: TVHGGLQSSSSVSR, RT (min): 5.53, XCorr: 4.67
intensity
m/z
250000
200000
150000
100000
50000
0
200
400
600
800
1000
1200
y5
y4
y7
a6
y1
y2
y6
y3
b3
b5
b6
a2
a6
b7
b2
b4
a5
b8
b11
a7
a8
y8
b9
y11
y3
y4
y5
y6
b6
b7
b8
y12

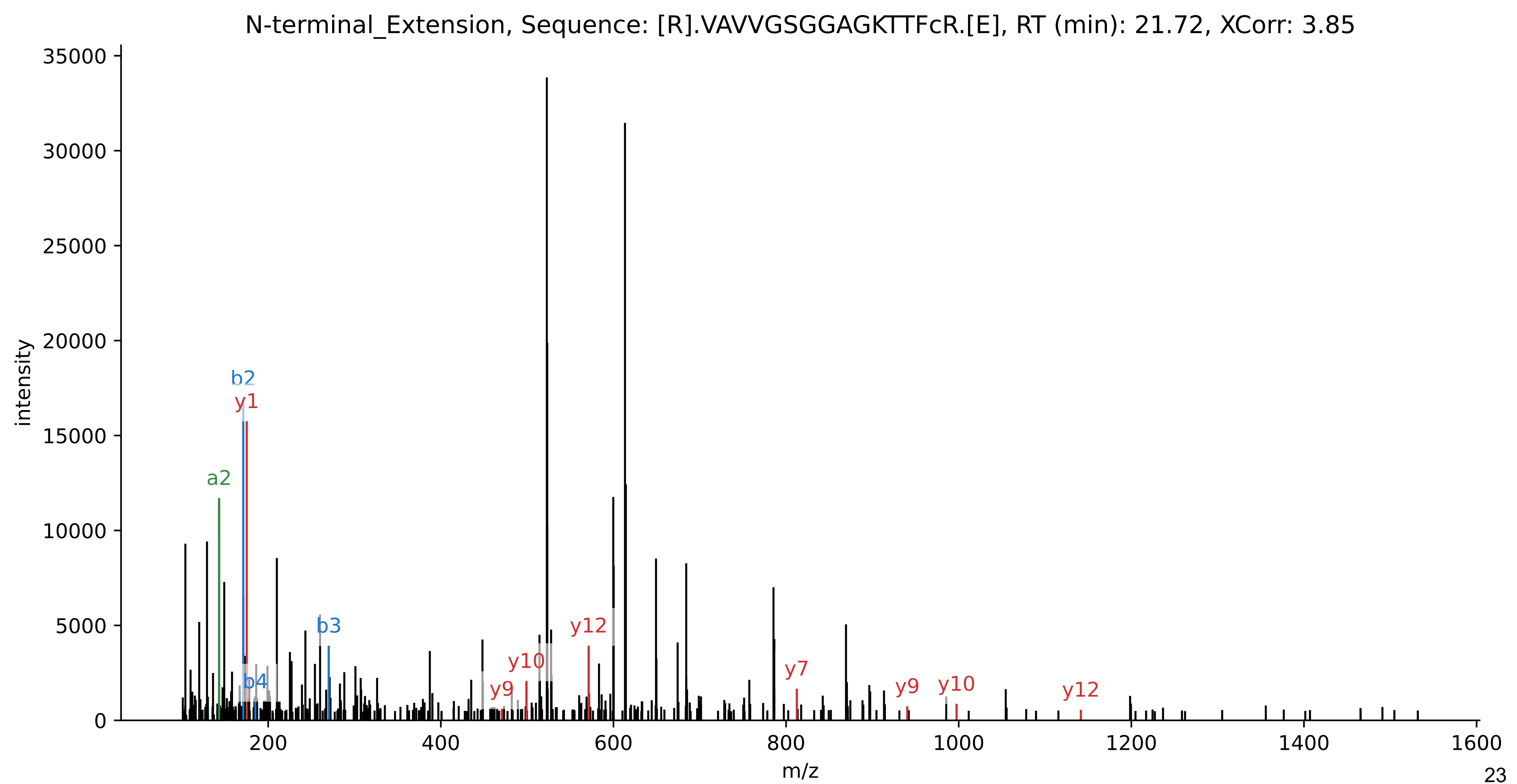

N-terminal_Extension, Sequence: [R].VAVVGSGGAGKTTFcR.[E], RT (min): 21.72, XCorr: 3.85
intensity
m/z
b2
y1
a2
b3
b4
y9
y10
y12
y7
y9
y10
y12
