# Supplementary material for: Development of a Spectral Library for the Discovery of Altered Genomic Events in Mycobacterium avium Associated With Virulence Using Mass Spectrometry–Based Proteogenomic Analysis
Source: Mol Cell Proteomics. 2023 Mar 21;22(5):100533. doi: 10.1016/j.mcpro.2023.100533 (PMC10149365; doi:10.1016/j.mcpro.2023.100533)
Supplement: Supplemental Material Legends [file mmc14.docx]

# **Supplementary Material Legends**

**Supplementary Table 1:** A) *Mycobacterium avium* (*M. avium*) proteome annotated genome variants. B) *M. avium* genome alignment output with percentage (%) mapped and paired post-BWA analysis

**Supplementary Table 2:** Proteins and peptides identified from proteome database searches

**Supplementary Table 3:** Genome search-specific peptides (GSSPs) identified from the *Mycobacterium avium* (*M. avium*) six-frame translated genome database search with manual inspection details

**Supplementary Table 4:** A) List of single nucleotide variants (SNVs) identified at the proteome level from the variant protein database search. B) Output from the virulence factor database search of variant proteins

**Supplementary Table 5:** List of peptides in the spectral library after the data-independent acquisition (DIA) raw data search with q-value

**Supplementary Table 6:** Peptide spectrum matches (PSMs) corresponding to variant peptides from *Mycobacterium avium* (*M. avium*) variant protein database search with their predicted retention time (RT), absolute RT error, class-FDR and class-FRD error

**Supplementary Document 1:** Annotated peptide spectrum matches for i) Single unique peptides responsible for protein identifications against *Mycobacterium avium* (*M. avium*) protein database search; ii) Peptides identified from the search against *Mycobacterium tuberculosis* (*Mtb*) protein database; and iii) Genome search-specific peptides (GSSPs) and variant peptides identified from searches against six-frame translated genome database and a variant proteins database, respectively.

**Supplementary Document 2:** Least square regression curve from proportion of variant decoy peptide among decoy peptides at different Xcorr thresholds. Except the tail generated in the high-score region, Coefficient’s correlation (r) and slope was calculated using the data point within 0.4 > Xcorr < 2.0. Using the coefficients of slope, class-FDR was calculated for all variant peptide hits from Sequest HT search engine based on transferred FDR strategy as reported by Y. Fu et. *al.* (2014).

**Supplementary Figure 1:** Summary of single nucleotide variants (SNVs) detected from the genome variant analysis and proteomic search has been depicted using bar plots. A bar plot shows the number of annotated non-synonymous variants detected in 24 variant categories.

**Supplementary Figure 2:** Retention time prediction output of peptides identified from the unassigned spectra searches. **a)** A violin plot shows the interquartile range (IQR) and median absolute retention time error (MAE) from the absolute retention time (RT) errors of peptides from *Mycobacterium tuberculosis* (*Mtb*) H37Rv and H37Ra protein database, *Mycobacterium avium* (*M. avium*) six-frame translated genome and variant proteins database searches, respectively. On top of each violin graph, MAE values and the number of peptides to which the RTs were predicted have been mentioned. The difference between predicted and observed RT of peptides from **b)** *Mtb* H37Rv and H37Ra protein database (r^2^ = 0.95), **c)** *M. avium* six-frame translated genome database (r^2^ = 78), **d)** *M. avium* variant protein (r^2^ = 0.93) database have been visualized with regression plot. Where, the observed and predicted RTs of peptides are shown in red and blue color, respectively. The regression coefficient values for each dataset show that the difference between predicted and observed RTs for most of the peptides are minimal or within the acceptable limits

**Supplementary Figure 3:** Protein-coding evidence for pseudogene 25 (GeneID: 61329414, HAD-1C family P-type ATPase) falling between the genome region of NZ_CP009360.4: 5,070,791-5,075,626 was observed with the identification of 21 genome search-specific peptides (GSSPs). **a)** Schematic representation of the revised pseudogene coding region with GSSPs supported in were depicted. **b)** Peptide spectrum match and spectral library match evidence for the GSSP: SLRPGDVIDLAAPEVVPADAR from data-dependent acquisition (DDA) and data-independent acquisition (DIA) experiment were depicted.

**Supplementary Figure 4:** *Mycobacterium avium* subsp. *hominissuis* 11 (MAH11) proteins known to be involved in the bacterial infection in mice from Dragset et. *al.* (2019) were compared with *Mycobacterium avium* (*M. avium*) variant proteins identified from the database search in the current study. There were 25 proteins known to be involved in the infection of *M. avium* in mice that have been identified with single nucleotide variants (SNVs) from our variant proteins database search.
