# Supplementary material for: Development of a Spectral Library for the Discovery of Altered Genomic Events in Mycobacterium avium Associated With Virulence Using Mass Spectrometry–Based Proteogenomic Analysis
Source: Mol Cell Proteomics. 2023 Mar 21;22(5):100533. doi: 10.1016/j.mcpro.2023.100533 (PMC10149365; doi:10.1016/j.mcpro.2023.100533)

WP_003872292.1_S57P, Sequence: [R].SGNRTNPPSATmAALANFFR.[I], RT (min): 88.5, XCorr: 2.97

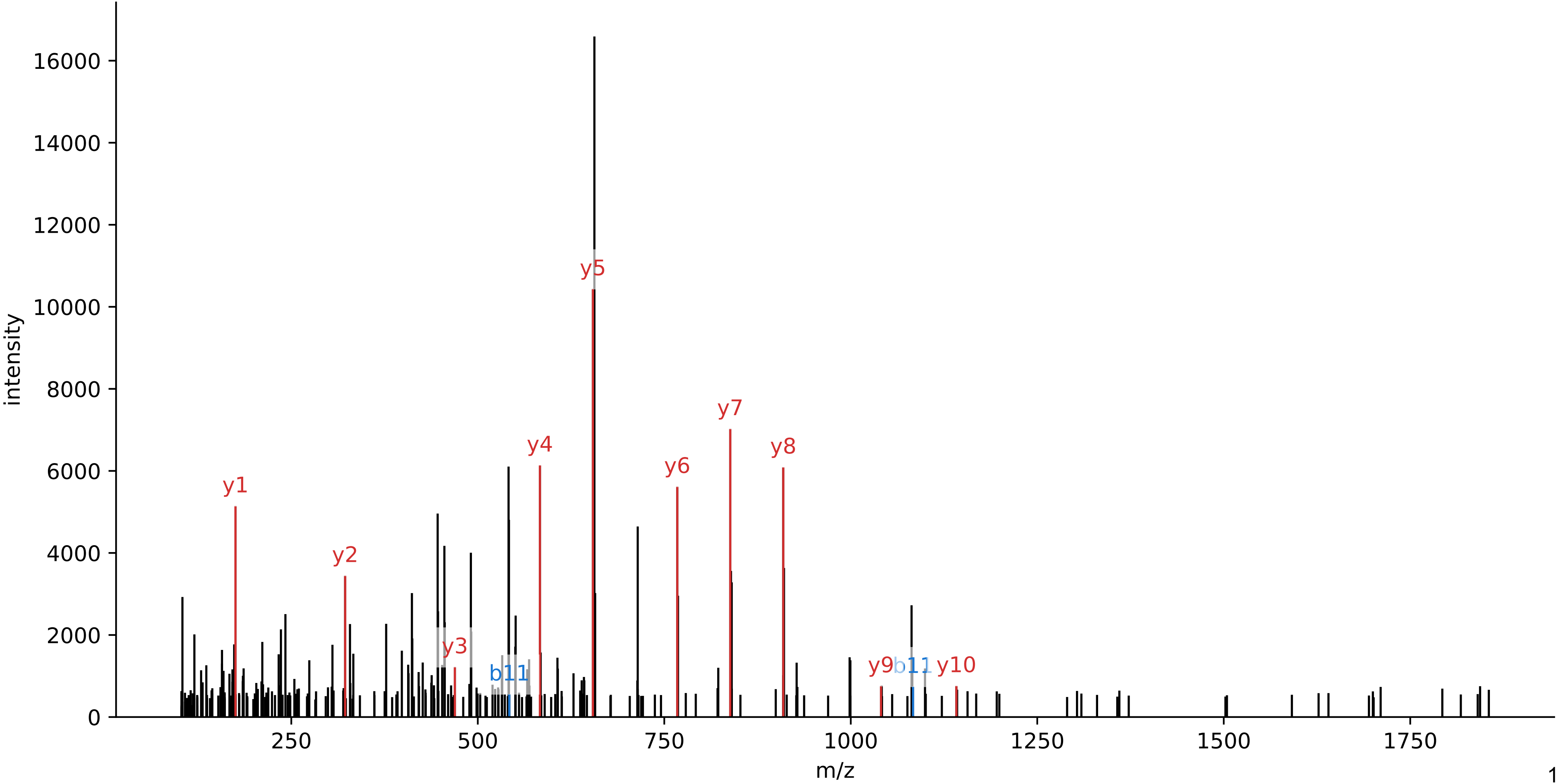

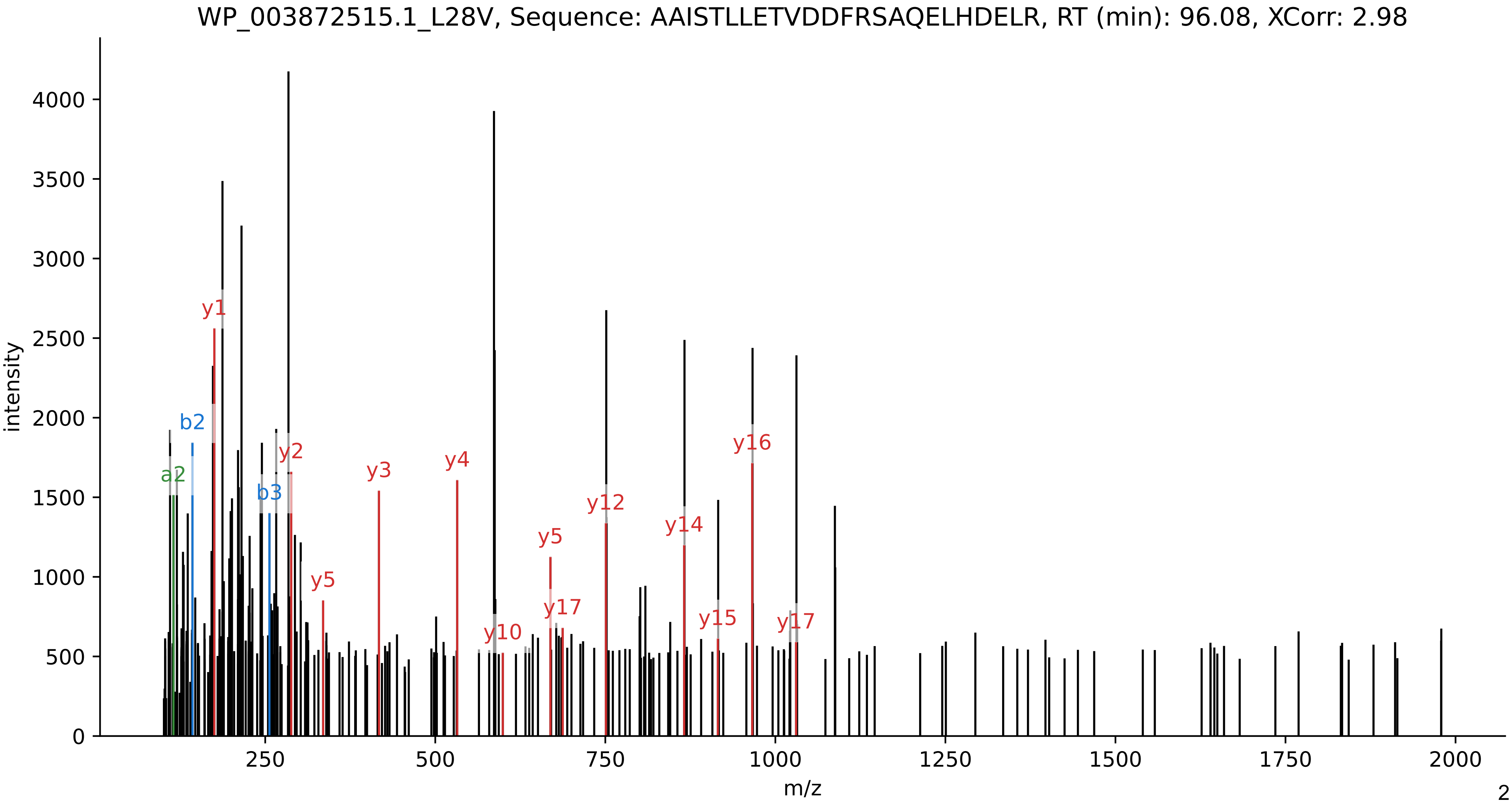
WP_003872515.1_L28V, Sequence: AAISTLLETVDDFRSAQELHDELR, RT (min): 96.08, XCorr: 2.98
intensity
m/z
0
500
1000
1500
2000
2500
3000
3500
4000
250
500
750
1000
1250
1500
1750
2000
a2
b2
b3
y1
y2
y5
y3
y4
y10
y5
y17
y12
y14
y15
y16
y17

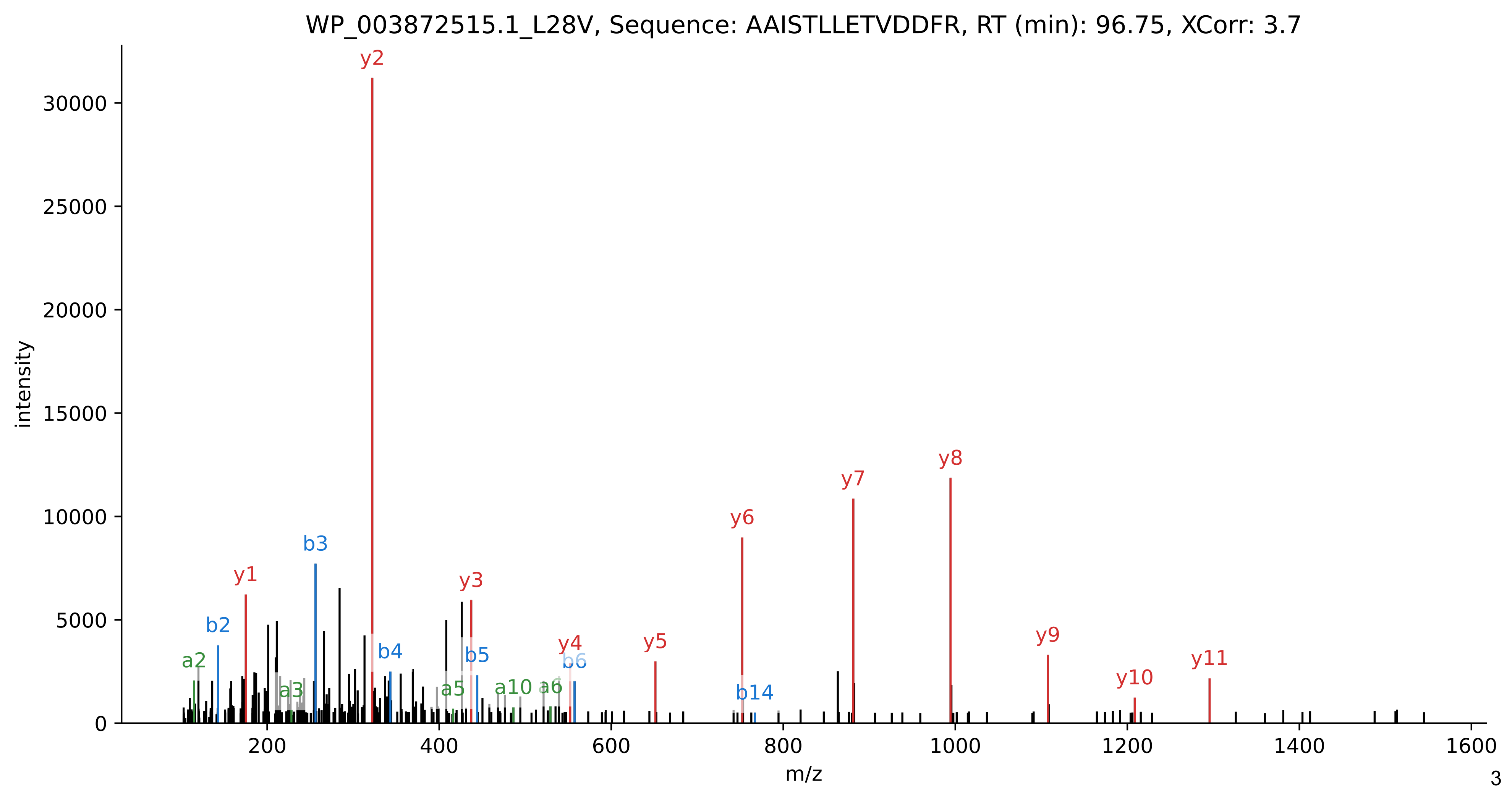
WP_003872515.1_L28V, Sequence: AAISTLLETVDDFR, RT (min): 96.75, XCorr: 3.7
intensity
m/z
y2
y8
y7
y6
b3
y1
y3
b2
y4
y5
y9
y11
y10
a2
b4
b5
b6
a3
a5
a10
a6
b14

WP_003872516.1_E31Q, Sequence: EVLPSQEESDGALTVHHQGTIASLR, RT (min): 45.42, XCorr: 5.58

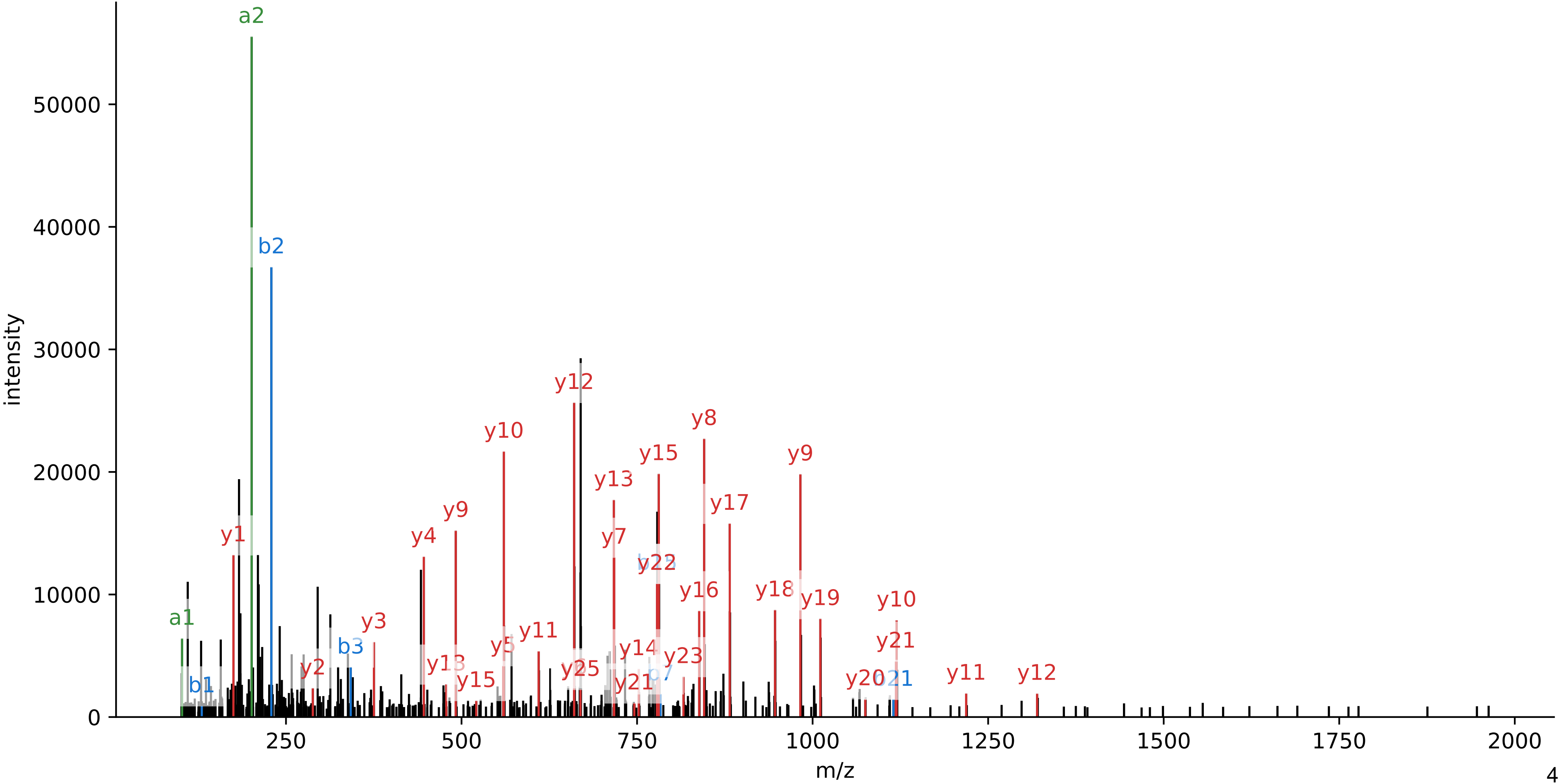

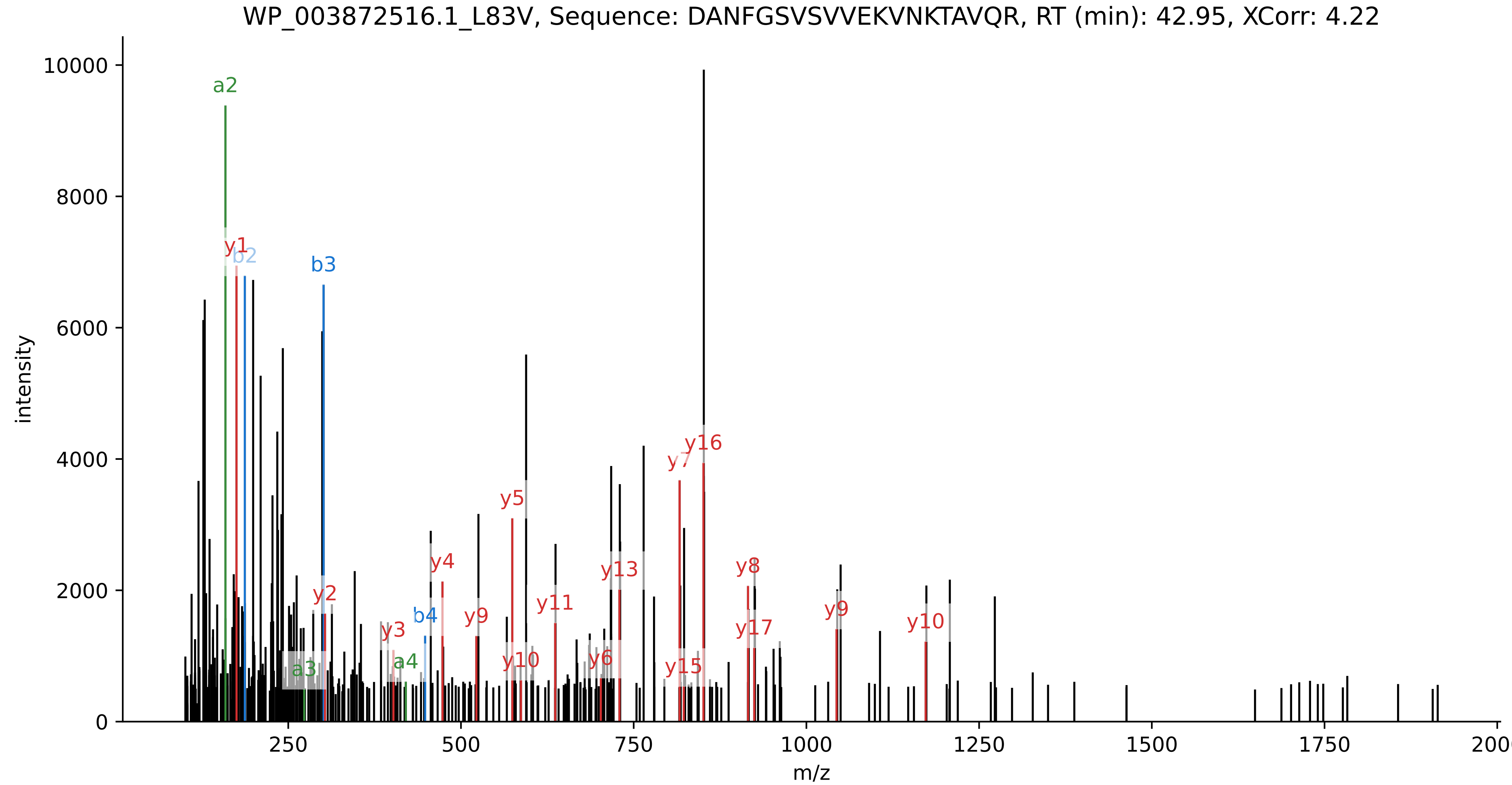
WP_003872516.1_L83V, Sequence: DANFGSVSVVEKVNKTAVQR, RT (min): 42.95, XCorr: 4.22
intensity
m/z
a2
y1
b2
b3
y2
y3
a3
a4
b4
y4
y9
y5
y10
y11
y6
y13
y7
y16
y15
y8
y17
y9
y10
0
2000
4000
6000
8000
10000
250
500
750
1000
1250
1500
1750
2000

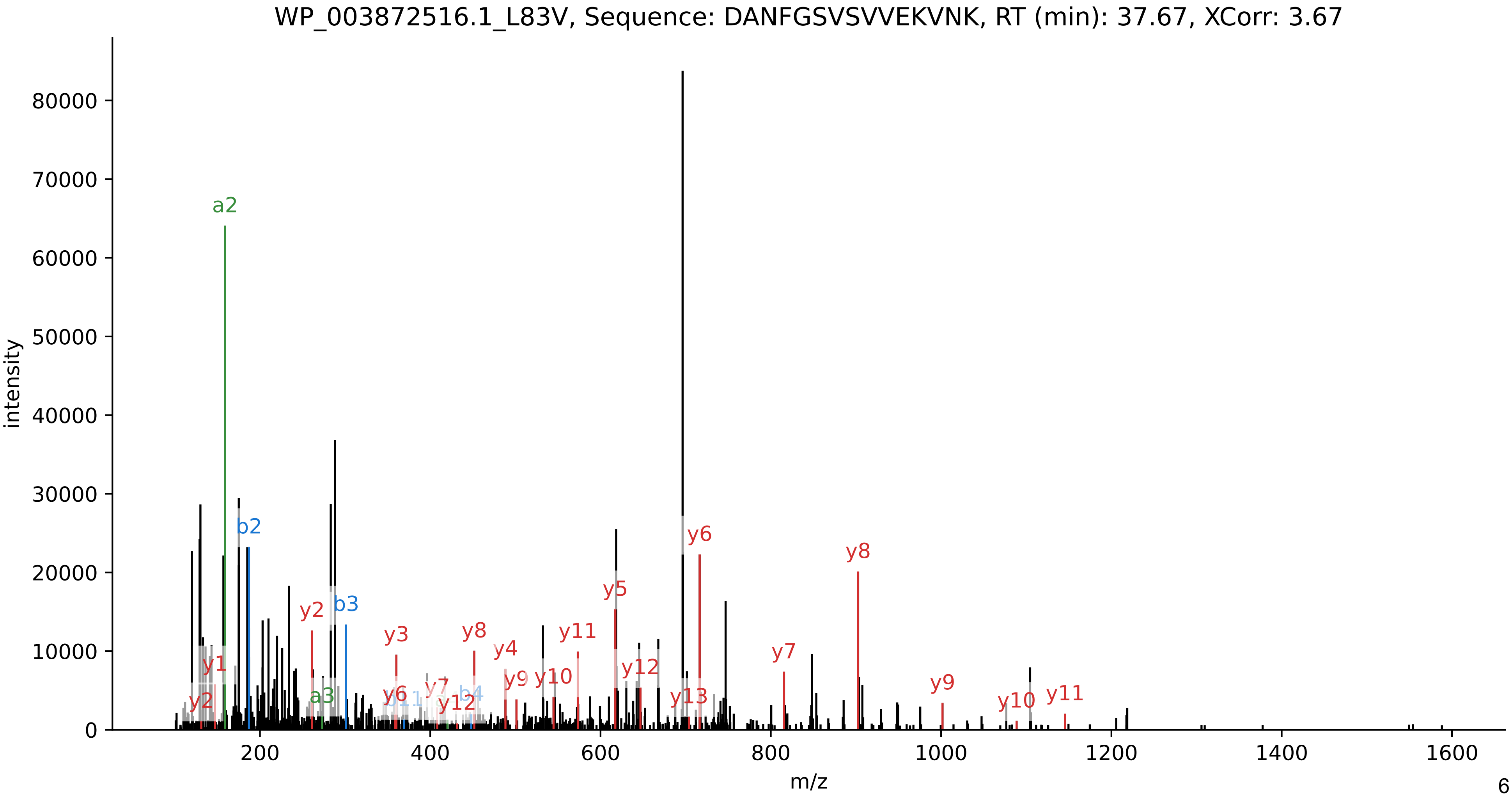
WP_003872516.1_L83V, Sequence: DANFGSVSVVEKVNK, RT (min): 37.67, XCorr: 3.67
intensity
m/z
80000
70000
60000
50000
40000
30000
20000
10000
0
200
400
600
800
1000
1200
1400
1600
a2
b2
b3
a3
b4
y1
y2
y2
y3
y4
y5
y6
y6
y7
y7
y8
y8
y9
y9
y10
y10
y11
y11
y12
y12
y13

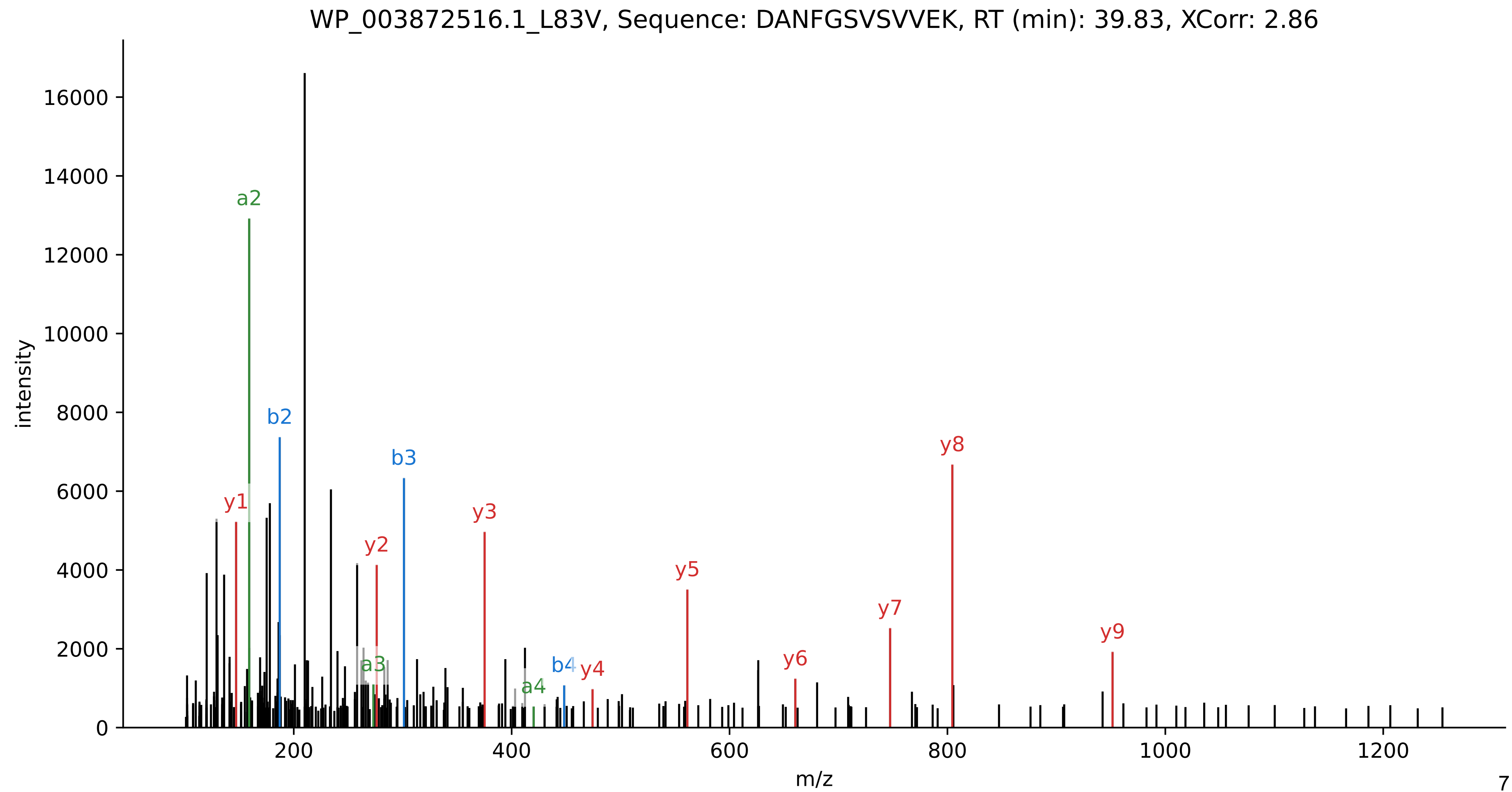

WP_003872516.1_L83V, Sequence: DANFGSVSVVEK, RT (min): 39.83, XCorr: 2.86
intensity
m/z
0
2000
4000
6000
8000
10000
12000
14000
16000
200
400
600
800
1000
1200
a2
b2
b3
y1
y2
y3
a3
a4
b4
y4
y5
y6
y7
y8
y9

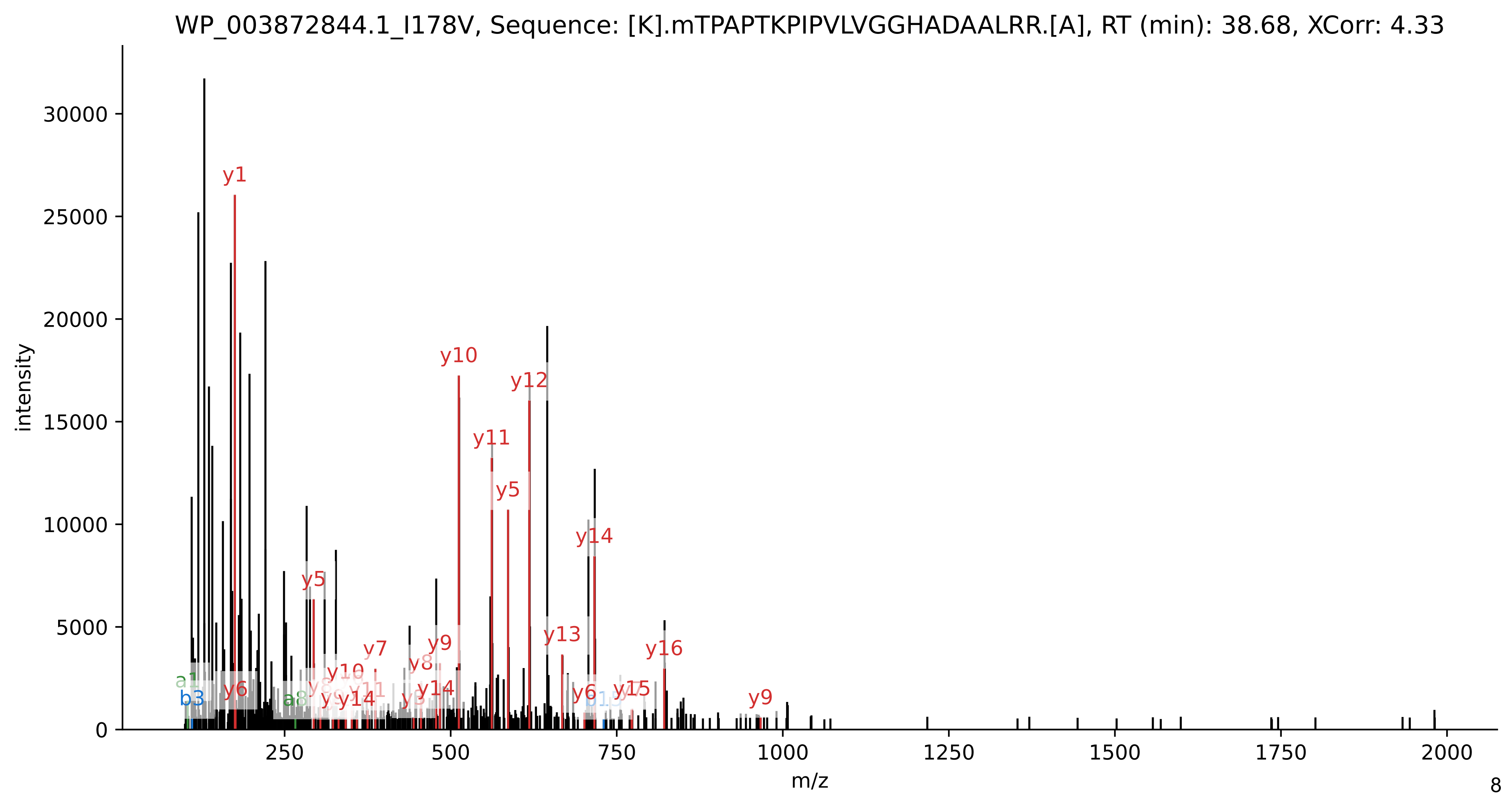
WP_003872844.1_I178V, Sequence: [K].mTPAPTKPIPVLVGGHADAALRR.[A], RT (min): 38.68, XCorr: 4.33
intensity
m/z
y1
y10
y12
y11
y5
y14
y5
y13
y16
y9
y7
y8
y9
y6
y14
y15
a1
b3
a8

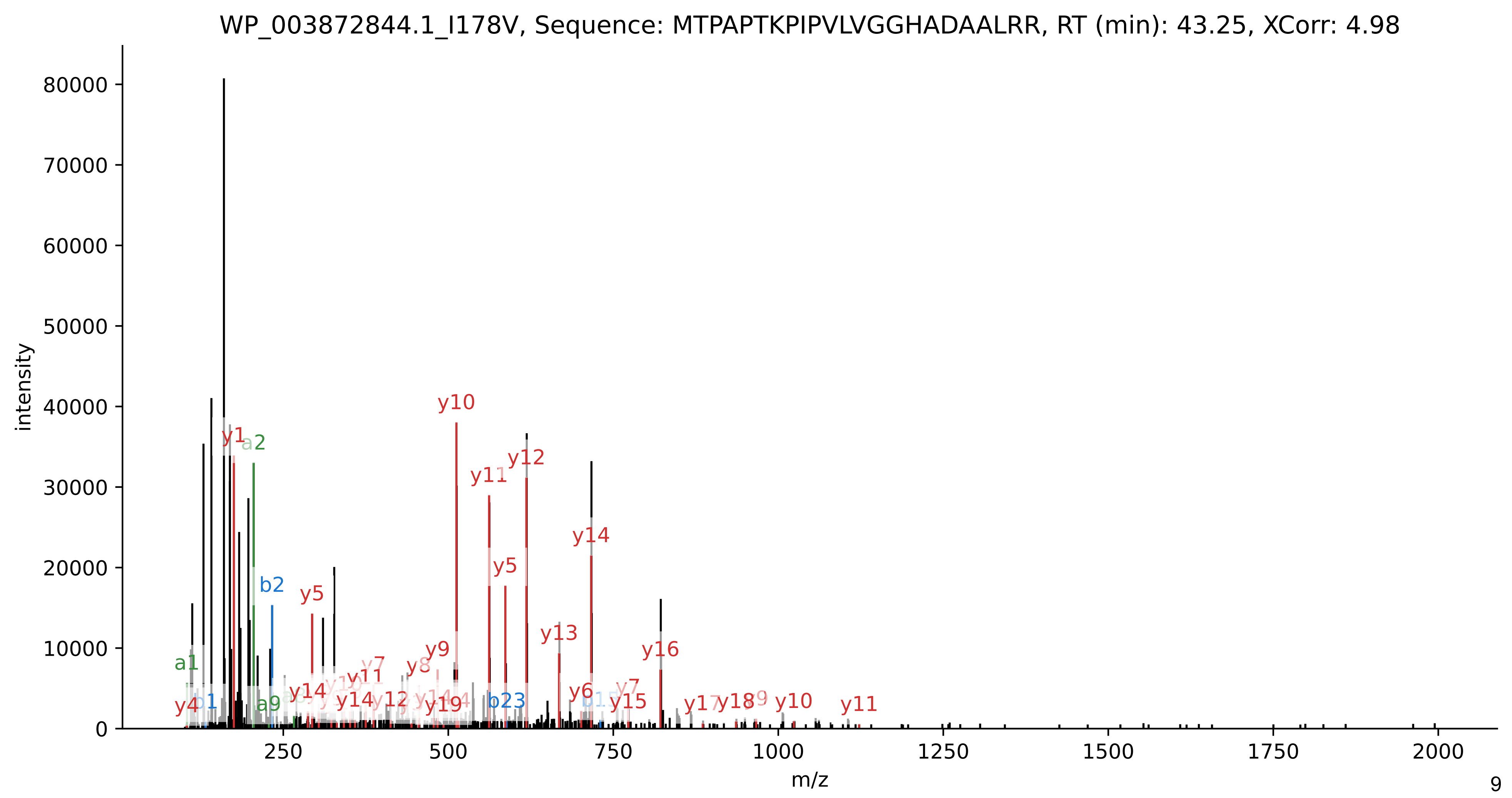

WP_003872844.1_I178V, Sequence: MTPAPTKPIPVLVGGHADAALRR, RT (min): 43.25, XCorr: 4.98
intensity
m/z
y10
y1
a2
y12
y11
y14
y5
b2
y5
y13
y16
a1
y9
y8
y6
y4
b1
a9
y14
y14
y12
y19
b23
y15
y18
y10
y11

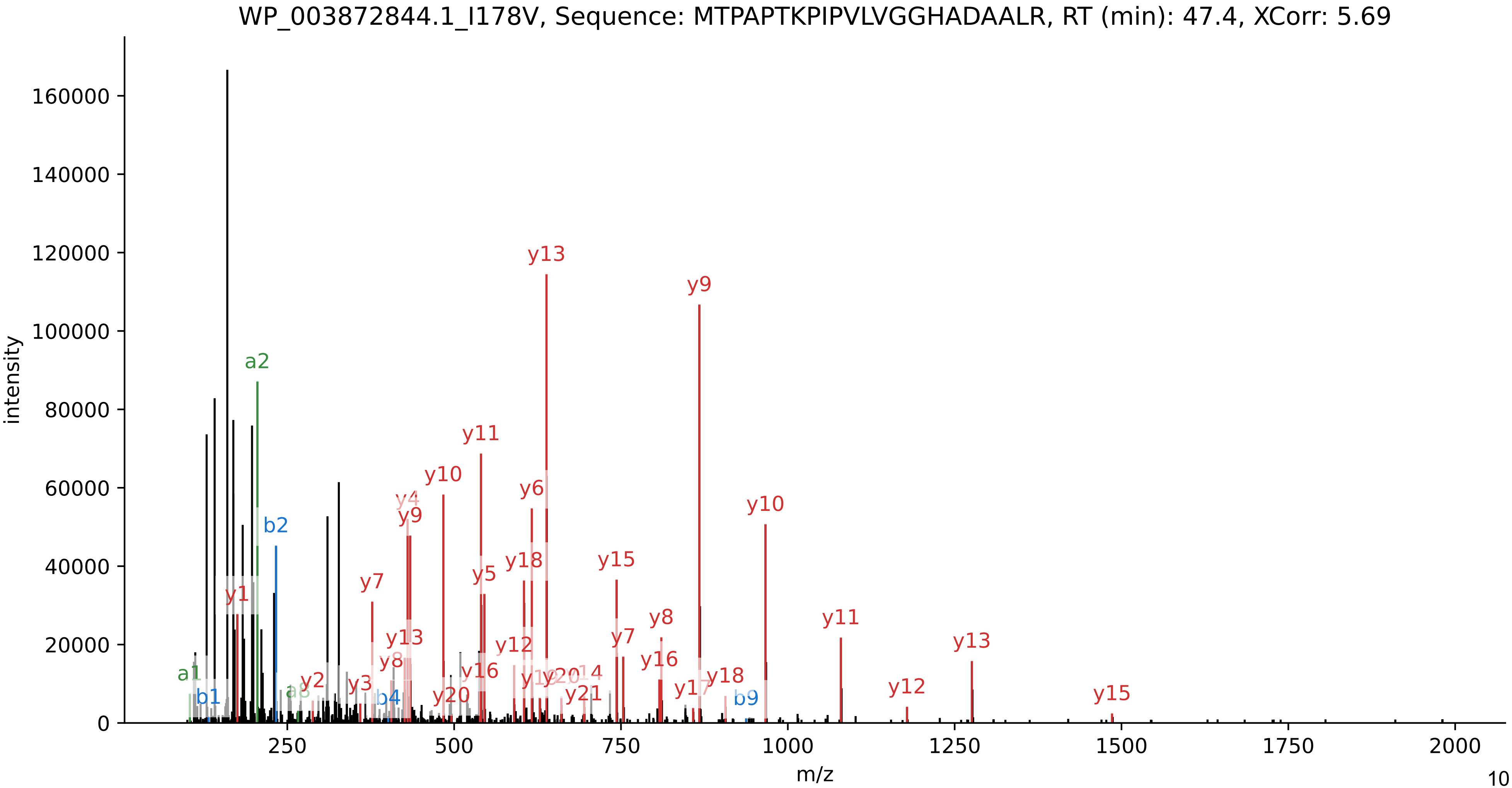
WP_003872844.1_I178V, Sequence: MTPAPTKPIPVLVGGHADAALR, RT (min): 47.4, XCorr: 5.69
intensity
m/z
0
20000
40000
60000
80000
100000
120000
140000
160000
250
500
750
1000
1250
1500
1750
2000
a1
b1
a2
b2
y1
y2
y3
y7
b4
y8
y13
y4
y9
y10
y20
y16
y11
y5
y12
y18
y6
y13
y21
y15
y7
y8
y16
y17
y9
y18
b9
y10
y11
y12
y13
y15

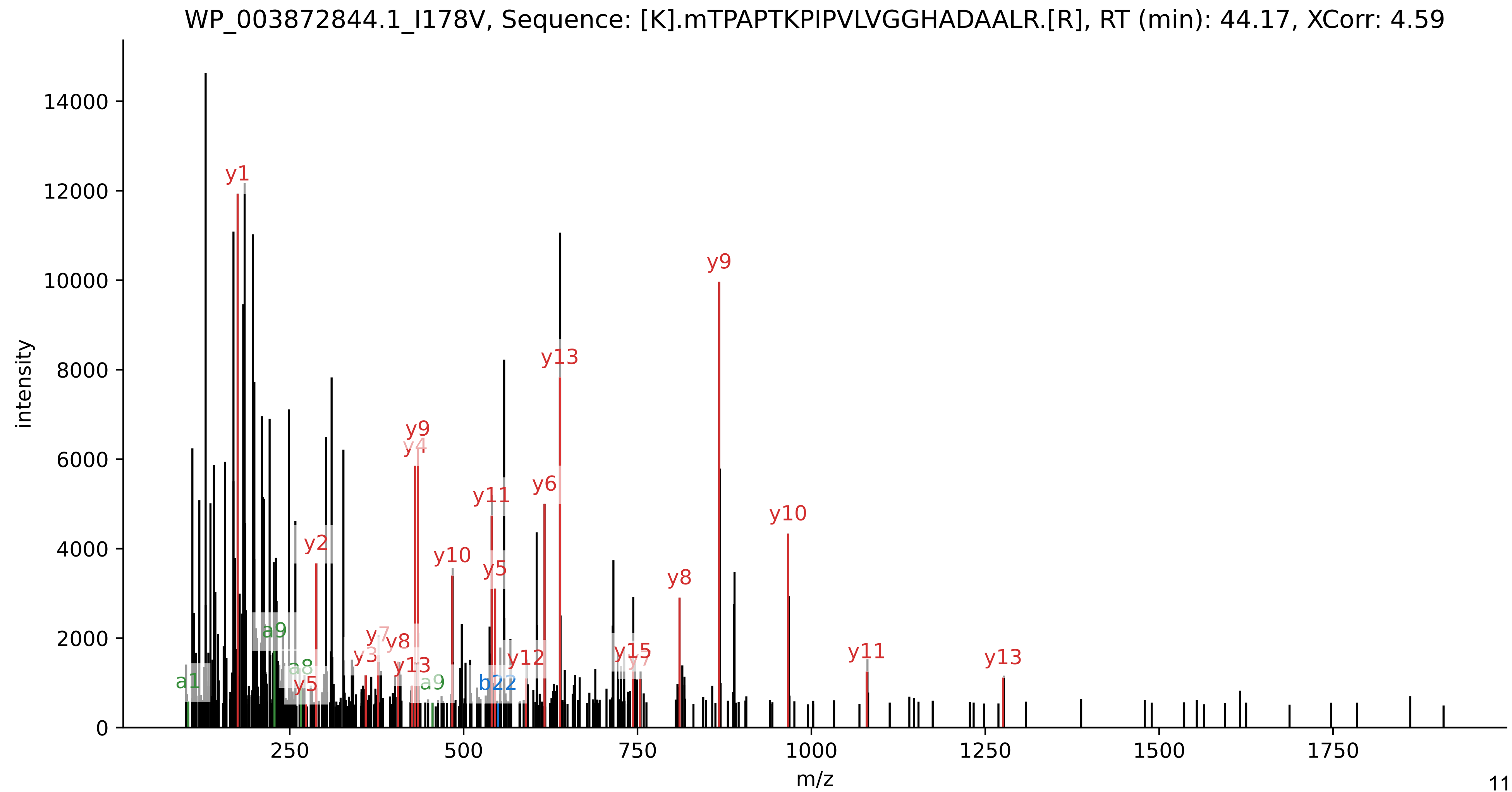

WP_003872844.1_I178V, Sequence: [K].mTPAPTKPIPVLVGGHADAALR.[R], RT (min): 44.17, XCorr: 4.59
intensity
m/z
y1
y9
y13
y9
y4
y6
y11
y10
y2
y10
y5
y8
y8
y7
y3
y13
y12
y15
y7
y11
y13
a9
a8
a1
y5
a9
b22

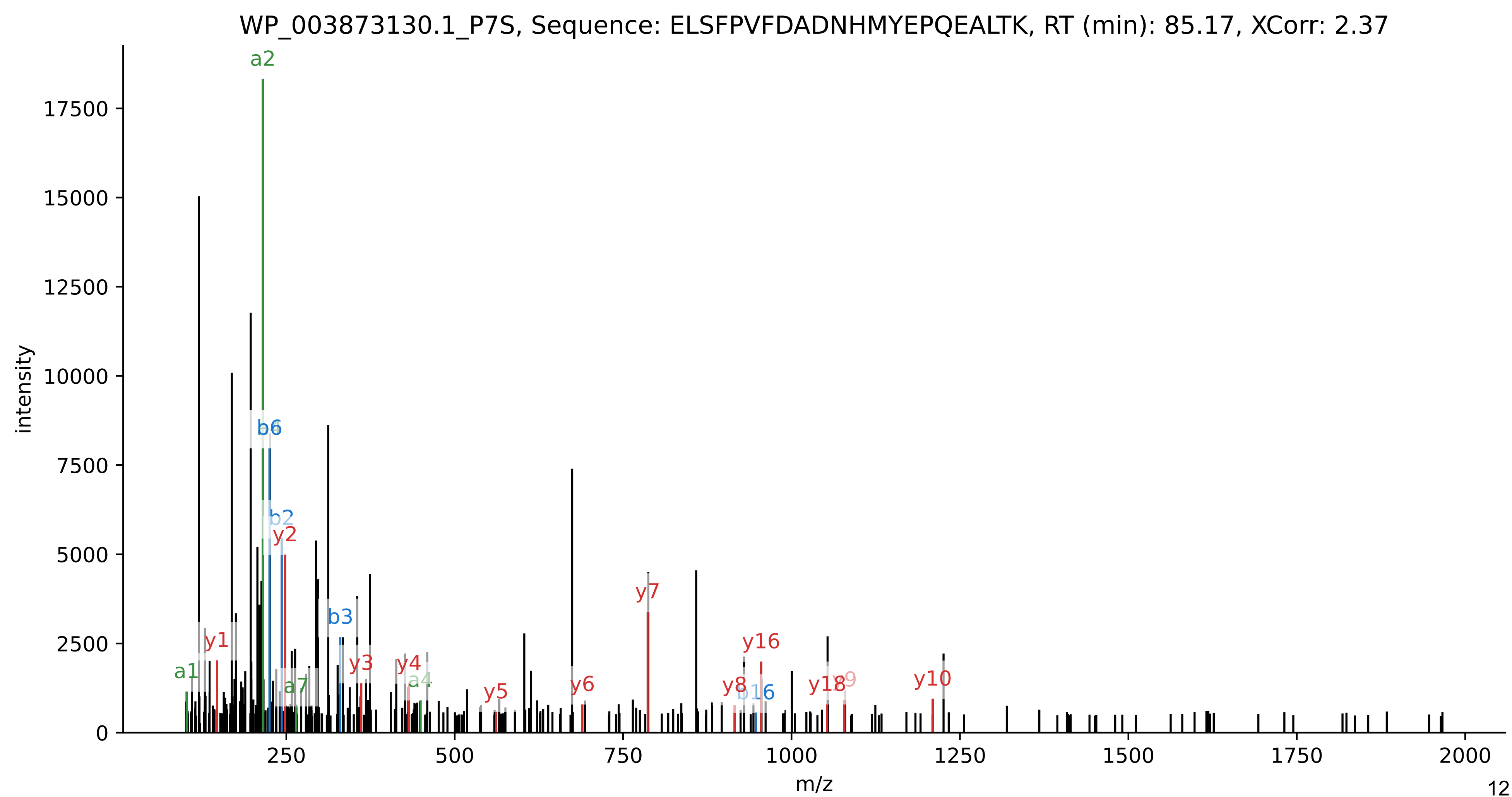
WP_003873130.1_P7S, Sequence: ELSFPVFDADNHMYEPQEALTK, RT (min): 85.17, XCorr: 2.37
intensity
m/z
a1
a2
a4
a7
b2
b3
b6
b16
y1
y2
y3
y4
y5
y6
y7
y8
y10
y16
y18
0
2500
5000
7500
10000
12500
15000
17500
250
500
750
1000
1250
1500
1750
2000

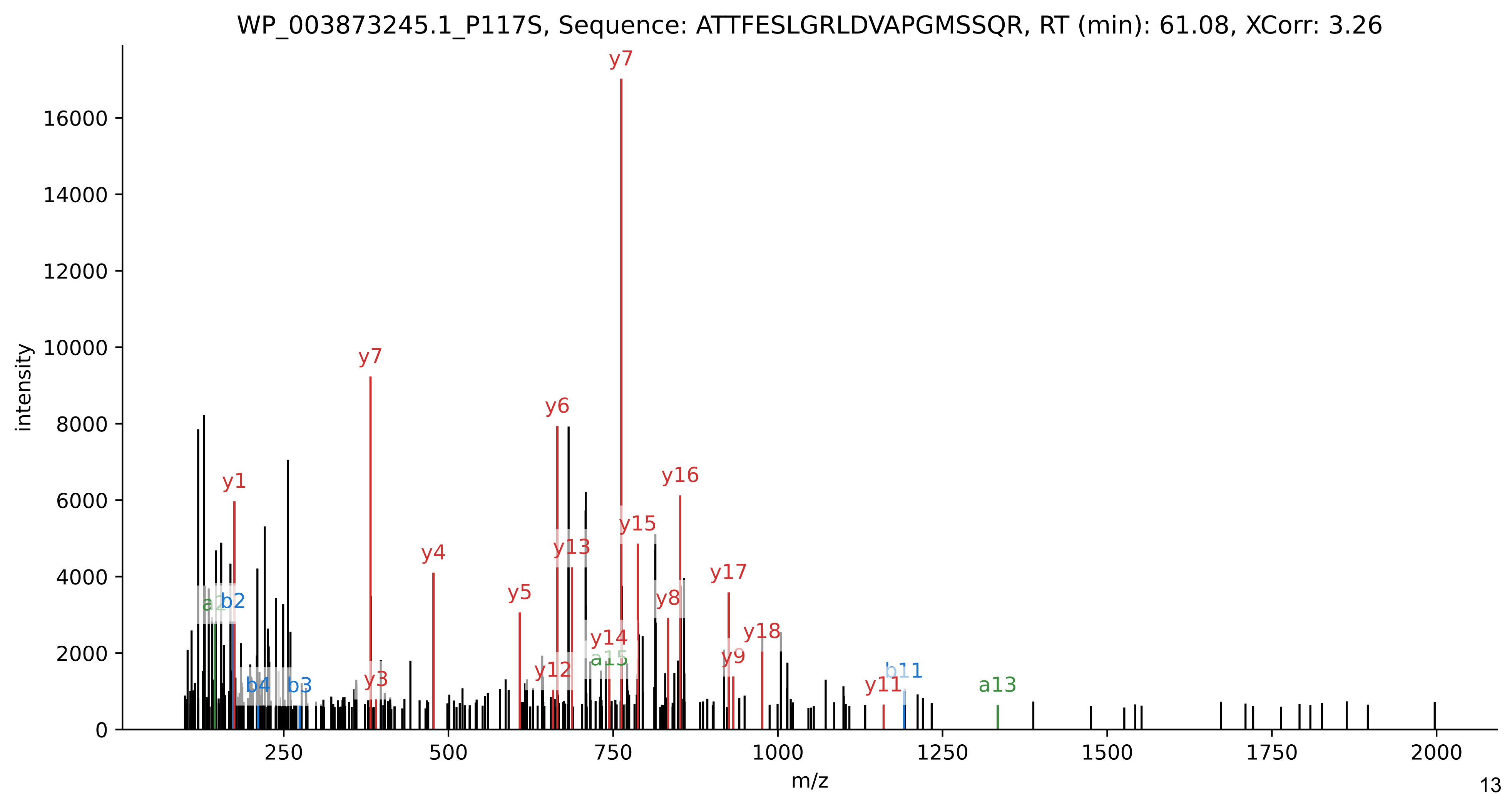
WP_003873245.1_P117S, Sequence: ATTFESLGRLDVAPGMSSQR, RT (min): 61.08, XCorr: 3.26
intensity
m/z
y7
y7
y6
y1
y16
y15
y13
y4
y17
y5
y8
a2
b2
y18
y14
a15
y9
y12
b11
b4
b3
y3
y11
a13
0
2000
4000
6000
8000
10000
12000
14000
16000
250
500
750
1000
1250
1500
1750
2000

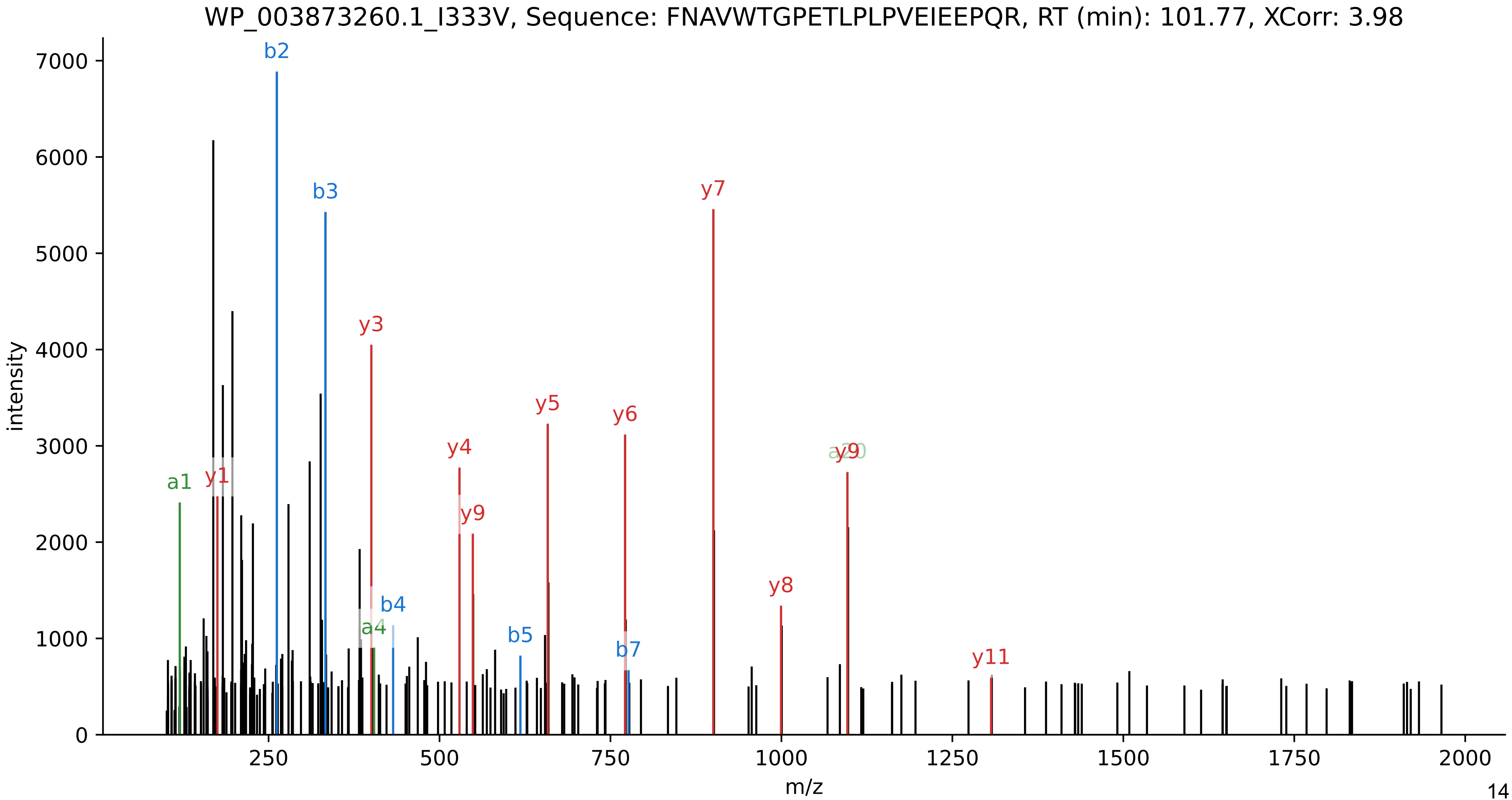
WP_003873260.1_I333V, Sequence: FNAVWTGPETLPLPVEIEEPQR, RT (min): 101.77, XCorr: 3.98
intensity
m/z
b2
b3
y7
y3
y5
y6
y4
y9
y9
y1
a1
y8
b4
a4
b5
b7
y11
0
1000
2000
3000
4000
5000
6000
7000
250
500
750
1000
1250
1500
1750
2000

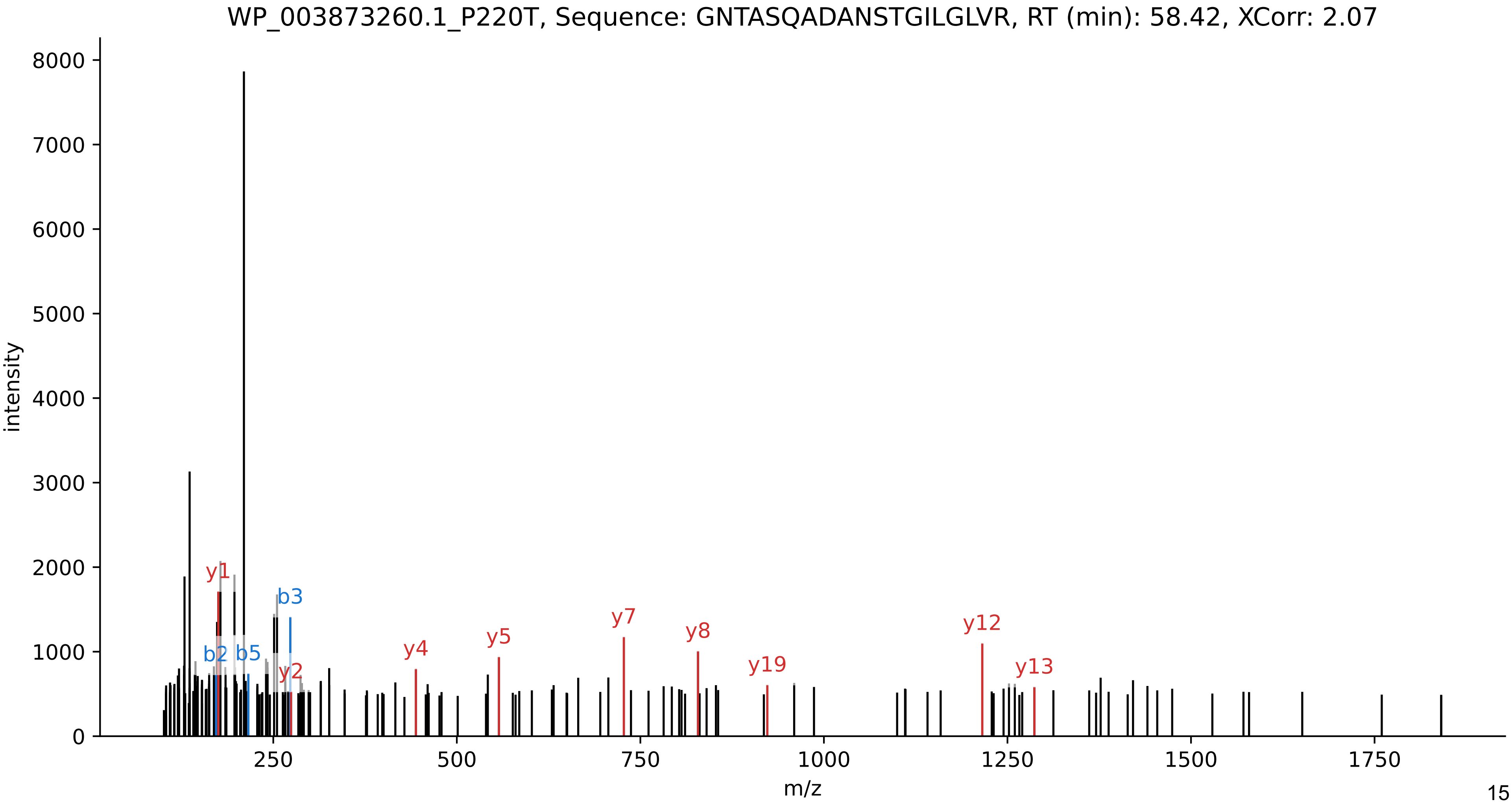
WP_003873260.1_P220T, Sequence: GNTASQADANSTGILGLVR, RT (min): 58.42, XCorr: 2.07
intensity
m/z
y1
b3
b2
b5
y2
y4
y5
y7
y8
y19
y12
y13
0
1000
2000
3000
4000
5000
6000
7000
8000
250
500
750
1000
1250
1500
1750

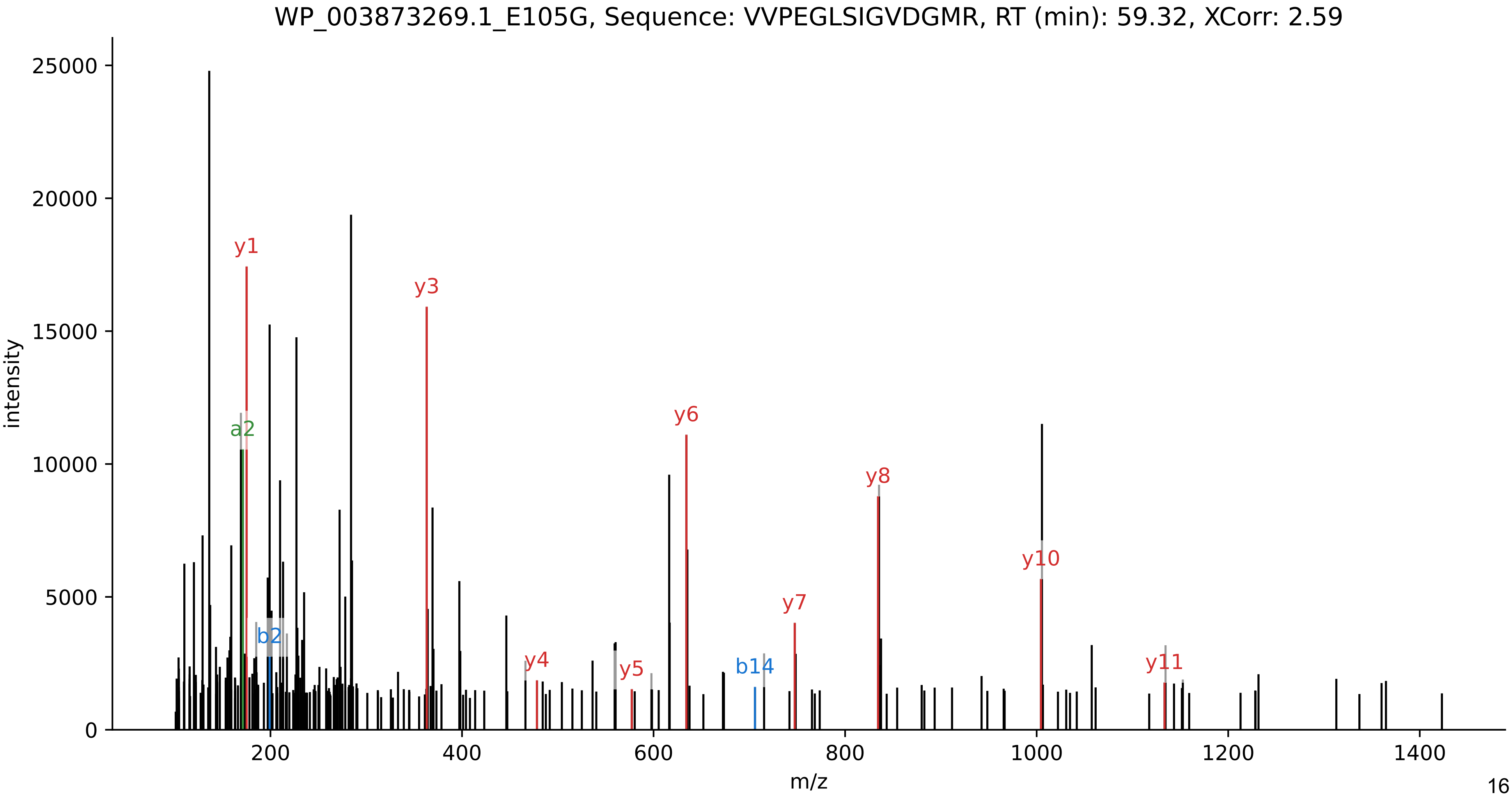

WP_003873269.1_E105G, Sequence: VVPEGLSIGVDGMR, RT (min): 59.32, XCorr: 2.59
intensity
m/z
y1
y3
a2
b2
y4
y5
b14
y6
y7
y8
y10
y11

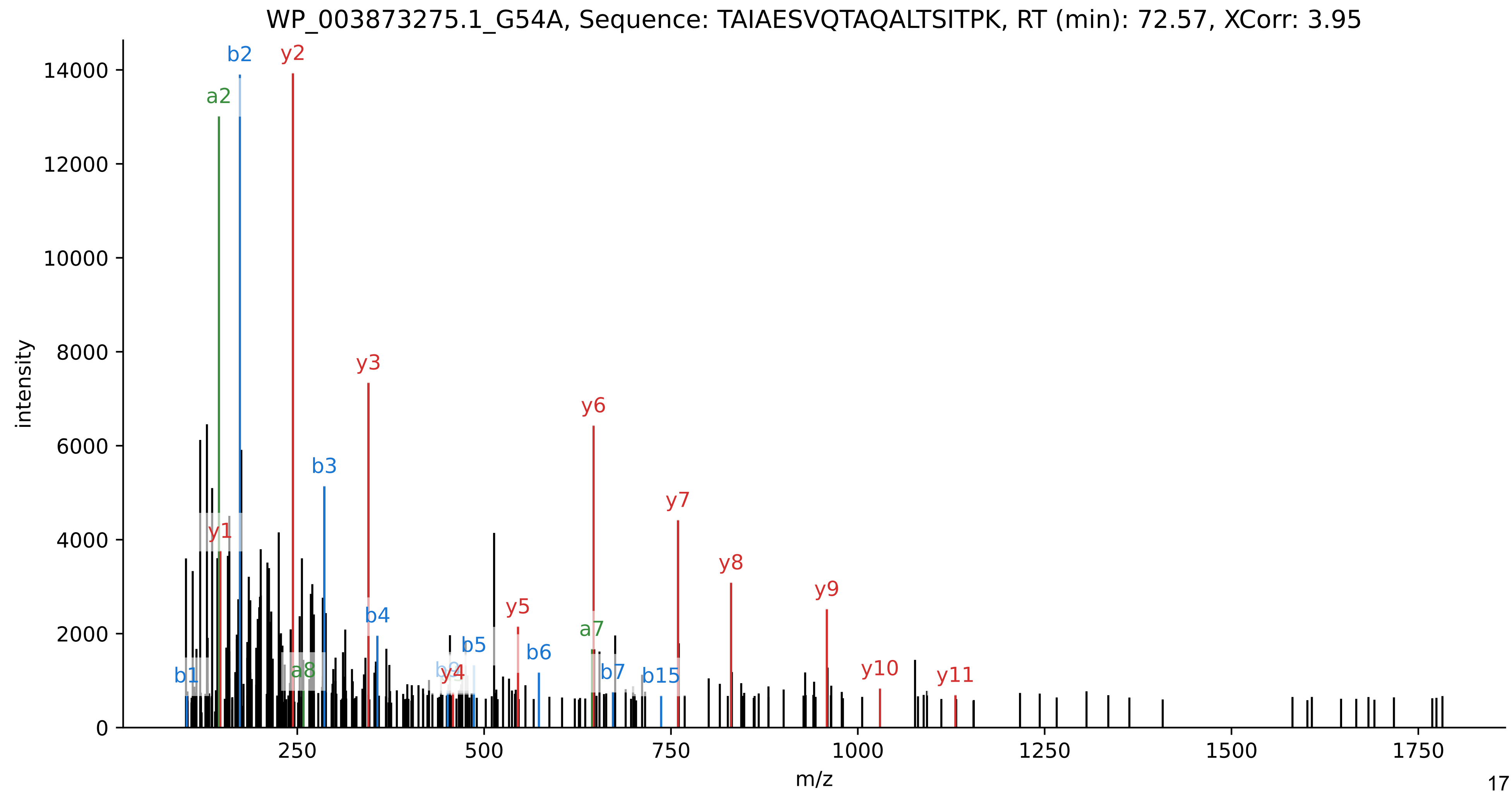
WP_003873275.1_G54A, Sequence: TAIAESVQTAQALTSITPK, RT (min): 72.57, XCorr: 3.95
intensity
m/z
b2
y2
a2
y3
b3
y6
y7
y8
y9
y1
y5
a7
b4
b5
b6
b7
b15
y10
y11
b1
a8
y4
0
2000
4000
6000
8000
10000
12000
14000
250
500
750
1000
1250
1500
1750

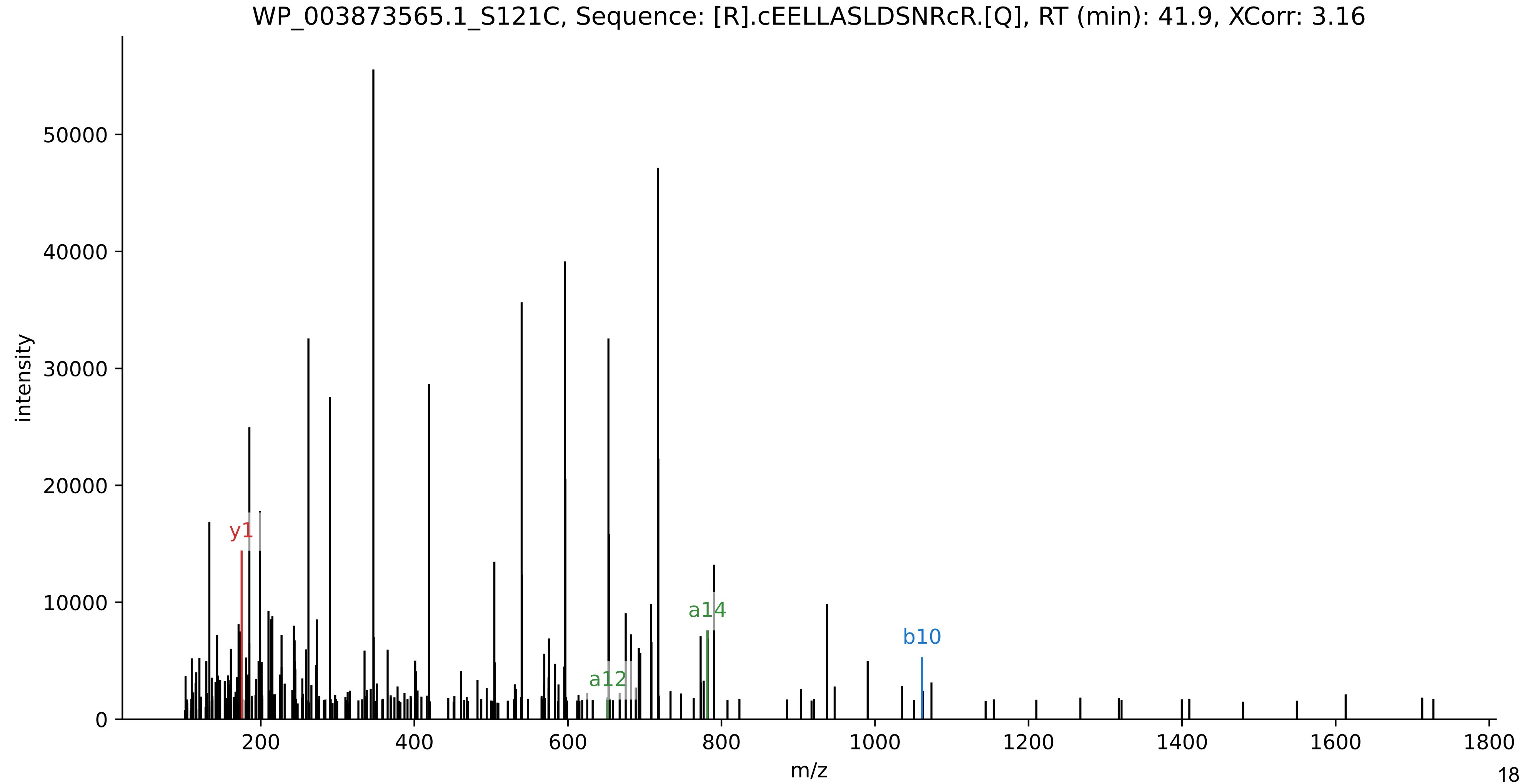
WP_003873565.1_S121C, Sequence: [R].cEELLASLDSNRcR.[Q], RT (min): 41.9, XCorr: 3.16
intensity
m/z
0
10000
20000
30000
40000
50000
200
400
600
800
1000
1200
1400
1600
1800
y1
a12
a14
b10

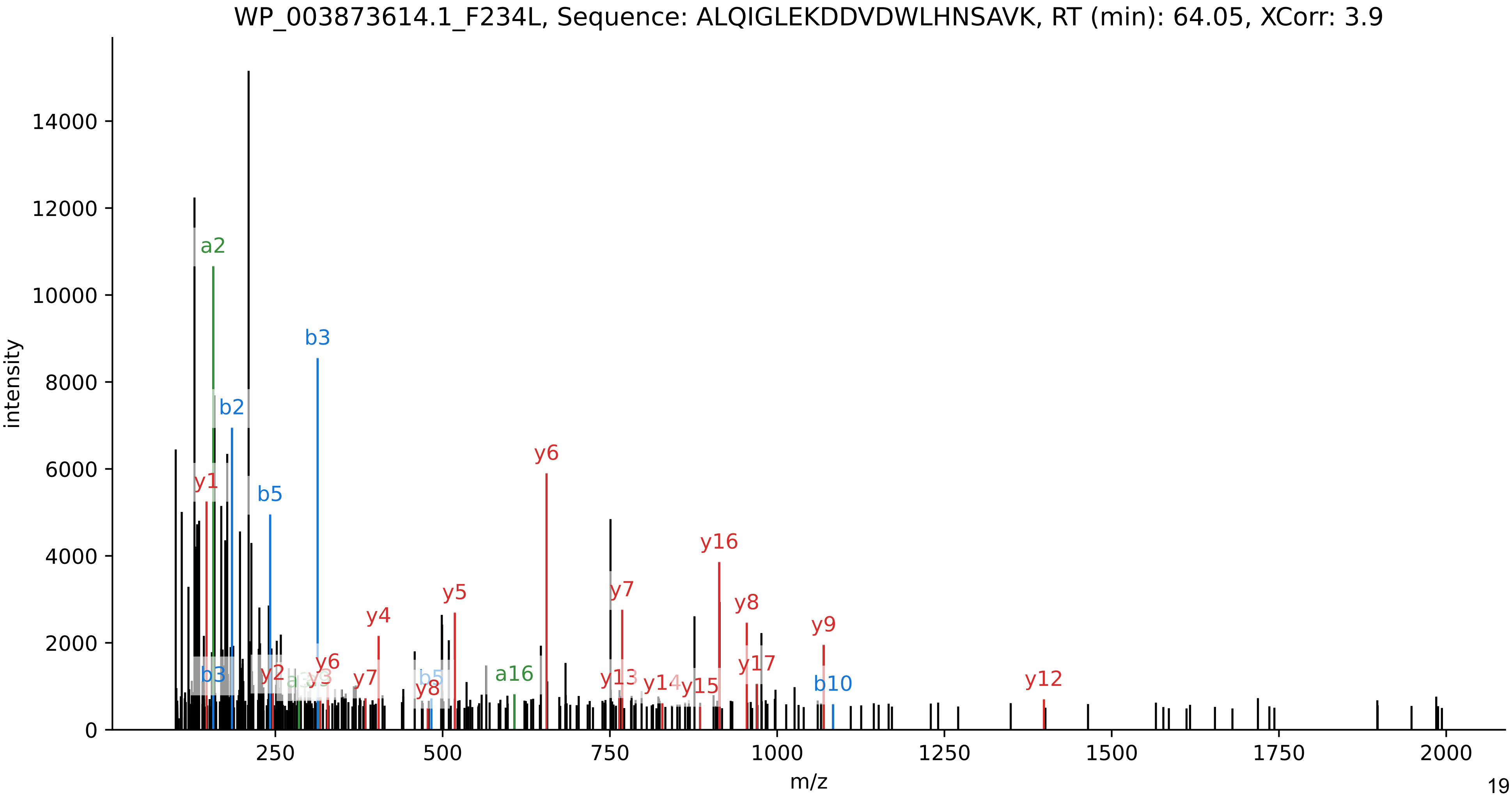
WP_003873614.1_F234L, Sequence: ALQIGLEKDDVDWLHNSAVK, RT (min): 64.05, XCorr: 3.9
intensity
m/z
0
2000
4000
6000
8000
10000
12000
14000
250
500
750
1000
1250
1500
1750
2000
a2
b2
b3
y1
b5
y6
y16
y7
y5
y4
y8
y9
y17
b10
y12
a16
y13
y14
y15
b3
y2
y6
y3
y7
y8
b5

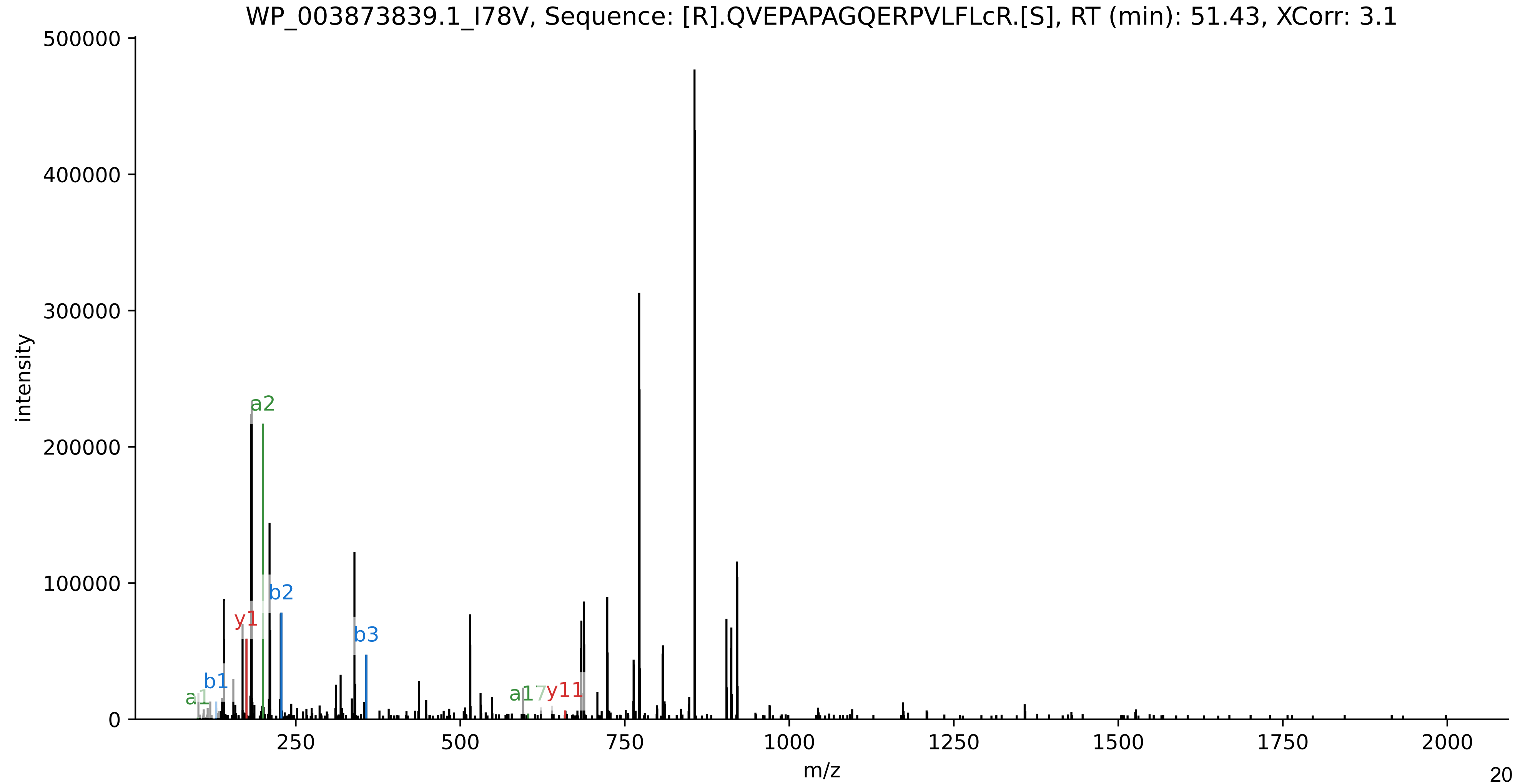

WP_003873839.1_I78V, Sequence: [R].QVEPAPAGQERPVLFLcR.[S], RT (min): 51.43, XCorr: 3.1
intensity
m/z
a2
b2
y1
b3
b1
a1
y11

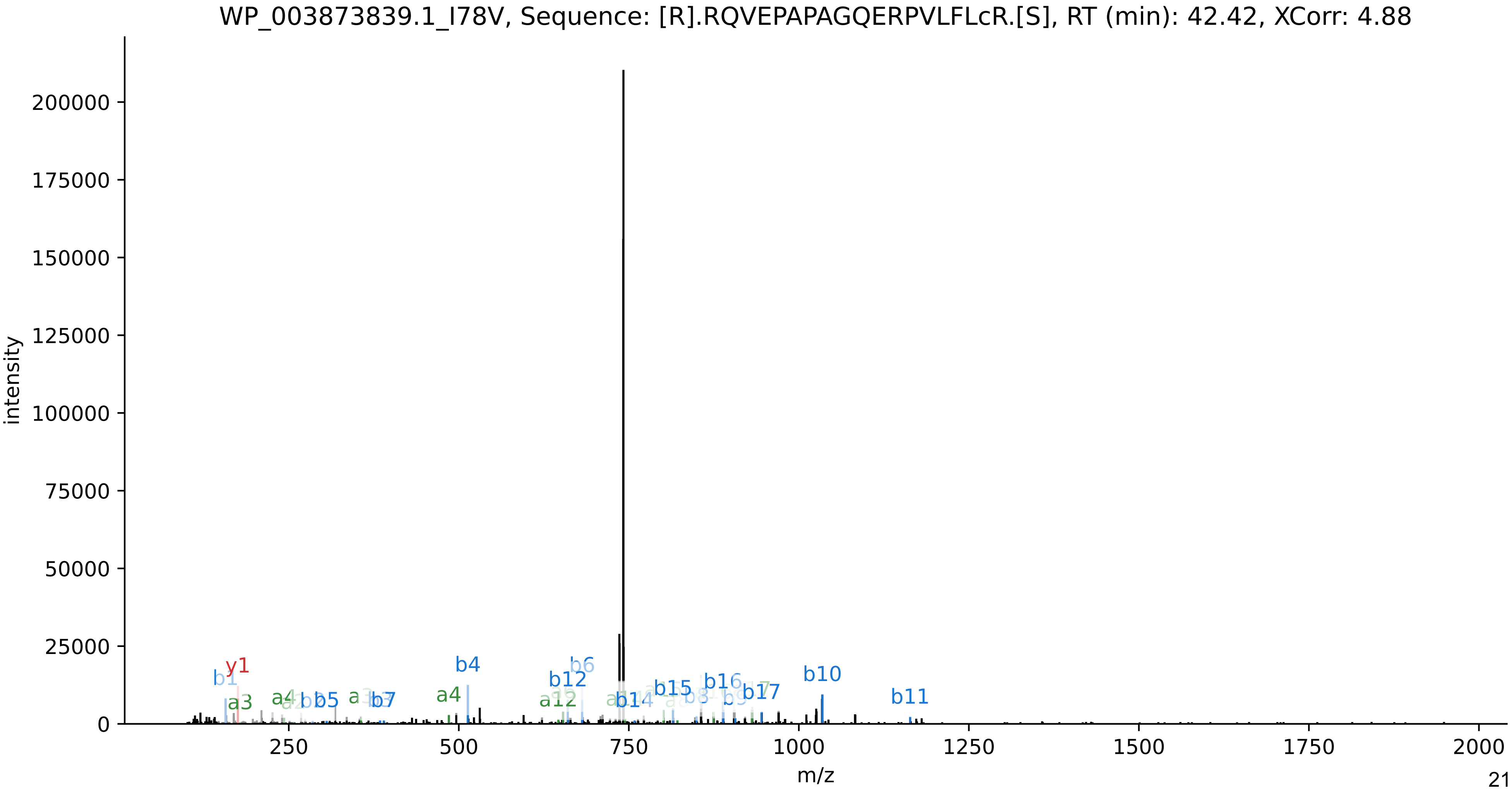
WP_003873839.1_I78V, Sequence: [R].RQVEPAPAGQERPVLFLcR.[S], RT (min): 42.42, XCorr: 4.88
intensity
m/z
0
25000
50000
75000
100000
125000
150000
175000
200000
250
500
750
1000
1250
1500
1750
2000
y1
b1
a3
a4
b5
b7
b4
a4
b6
b12
a12
b14
b15
b16
b17
b10
b11

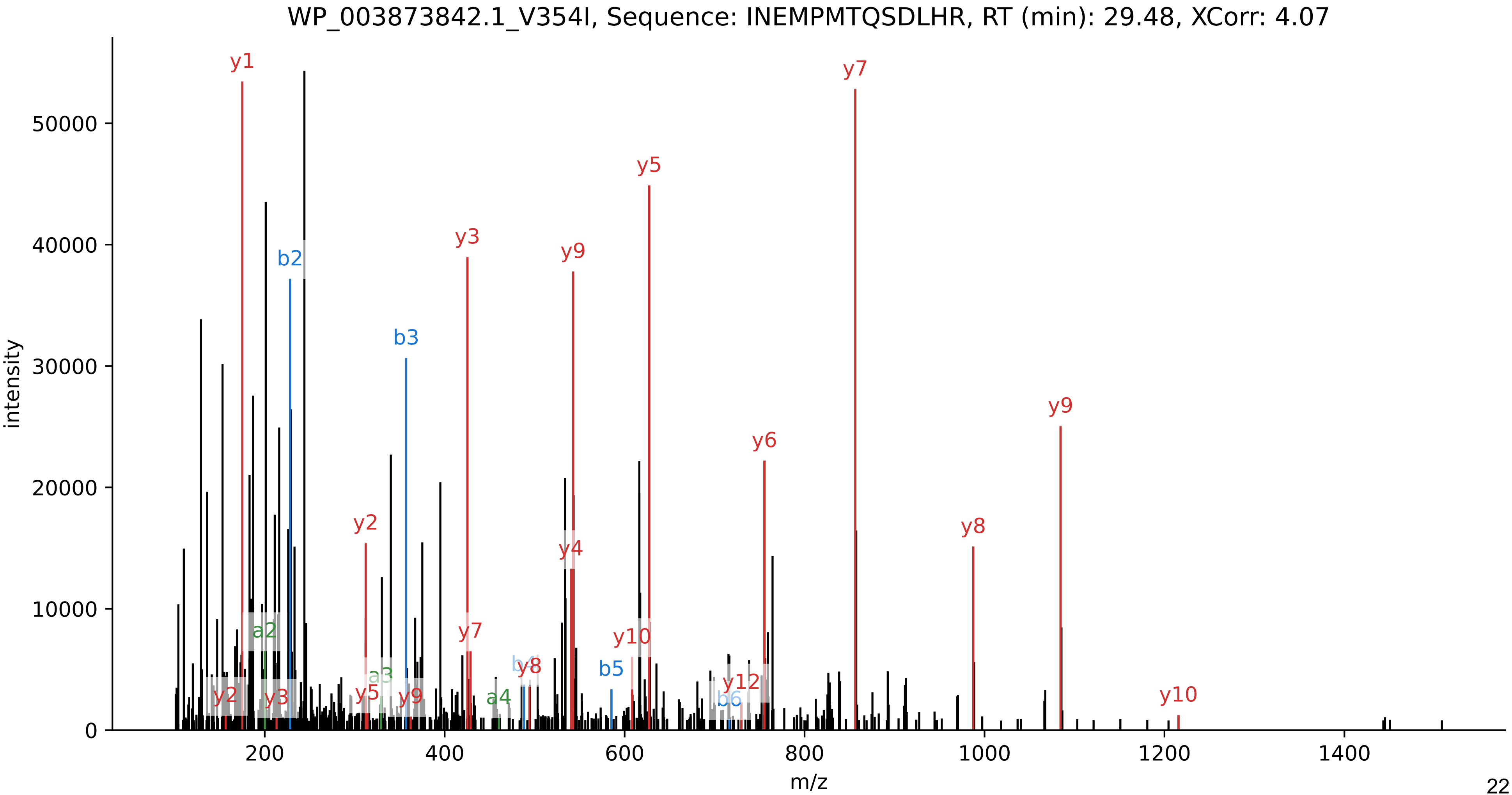

WP_003873842.1_V354I, Sequence: INEMPMTQSDLHR, RT (min): 29.48, XCorr: 4.07
intensity
m/z
y1
y7
y5
y3
b2
y9
b3
y6
y9
y2
y8
y4
y7
y10
a2
b5
y12
y10
y2
y3
y5
y9
a3
a4
y8
b6

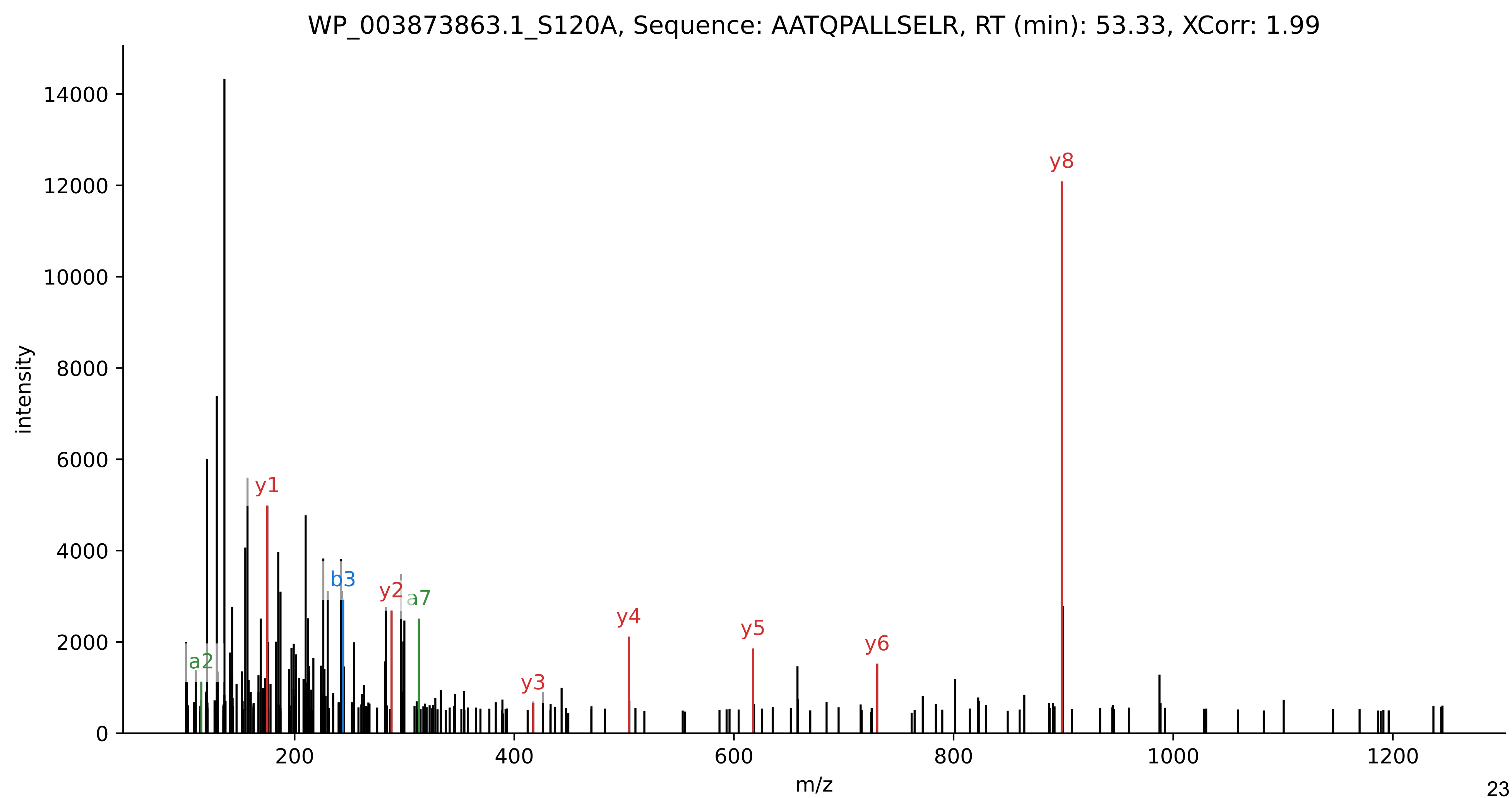
WP_003873863.1_S120A, Sequence: AATQPALLSELR, RT (min): 53.33, XCorr: 1.99
intensity
m/z
y8
y1
b3
y2
a7
y4
y5
y6
y3
a2
14000
12000
10000
8000
6000
4000
2000
0
200
400
600
800
1000
1200

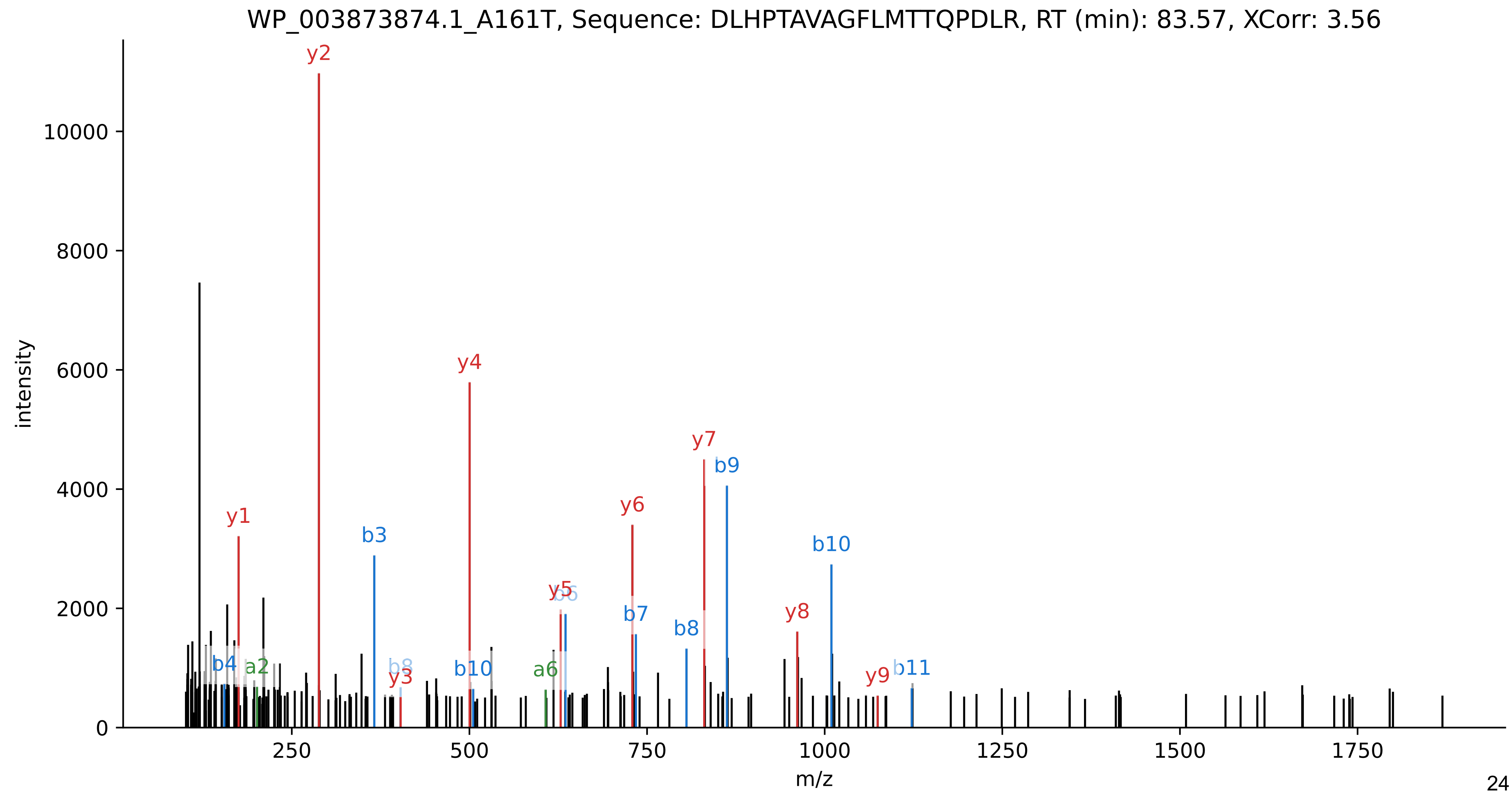

WP_003873874.1_A161T, Sequence: DLHPTAVAGFLMTTQPDLR, RT (min): 83.57, XCorr: 3.56
intensity
m/z
y2
y4
y7
b9
y6
y1
b3
b10
y5
b6
b7
b8
y8
b4
a2
b8
y3
b10
a6
y9
b11
0
2000
4000
6000
8000
10000
250
500
750
1000
1250
1500
1750

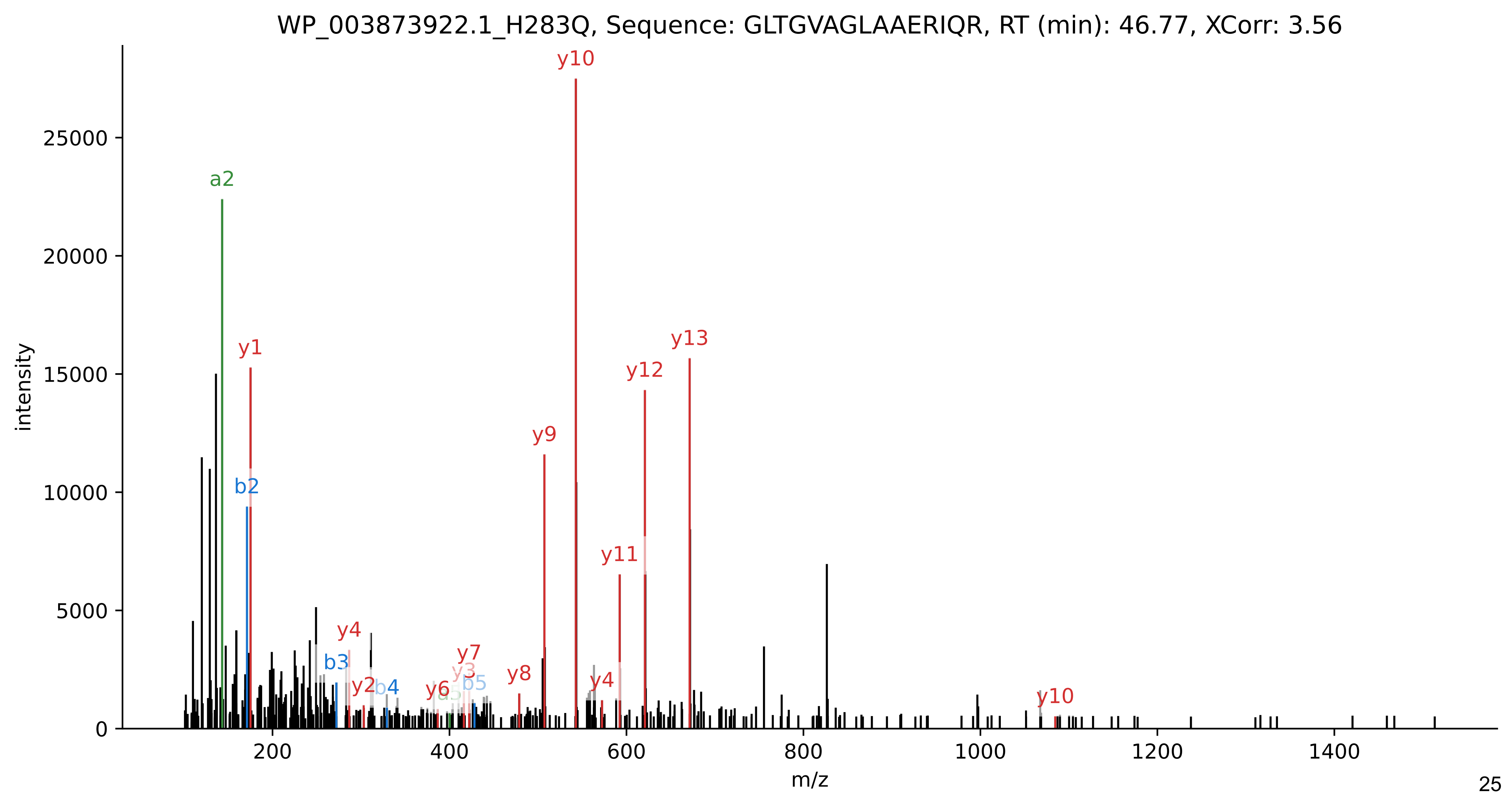
WP_003873922.1_H283Q, Sequence: GLTGVAGLAAERIQR, RT (min): 46.77, XCorr: 3.56
intensity
m/z
y10
a2
y1
b2
y9
y13
y12
y11
y4
b3
y2
b4
y6
y7
y3
b5
y8
y4
y10

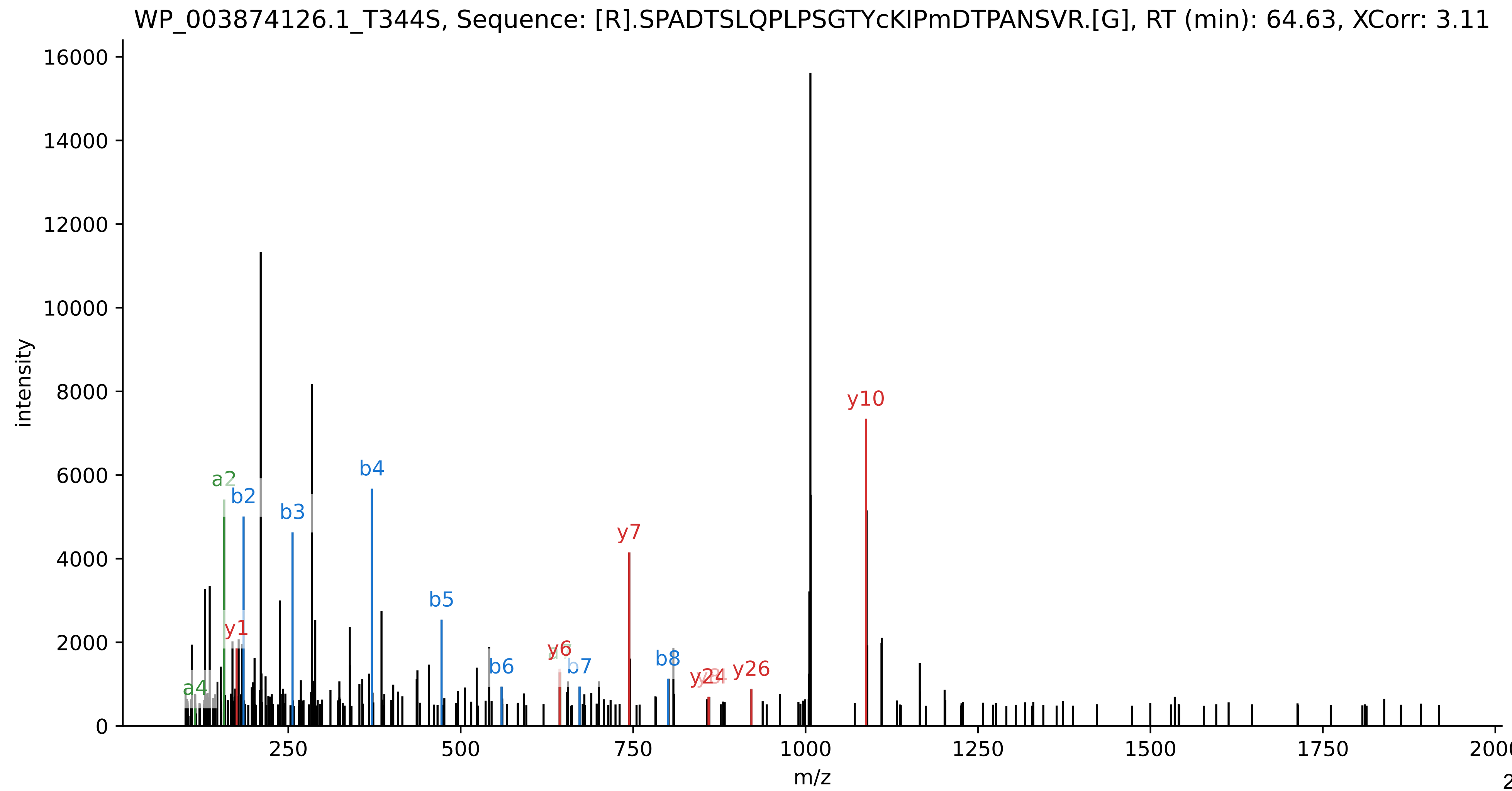

WP_003874126.1_T344S, Sequence: [R].SPADTSLQPLPSGTYcKIPmDTPANSVR.[G], RT (min): 64.63, XCorr: 3.11
intensity
m/z
a2
a4
b2
b3
b4
b5
b6
b7
b8
y1
y6
y7
y10
y24
y26

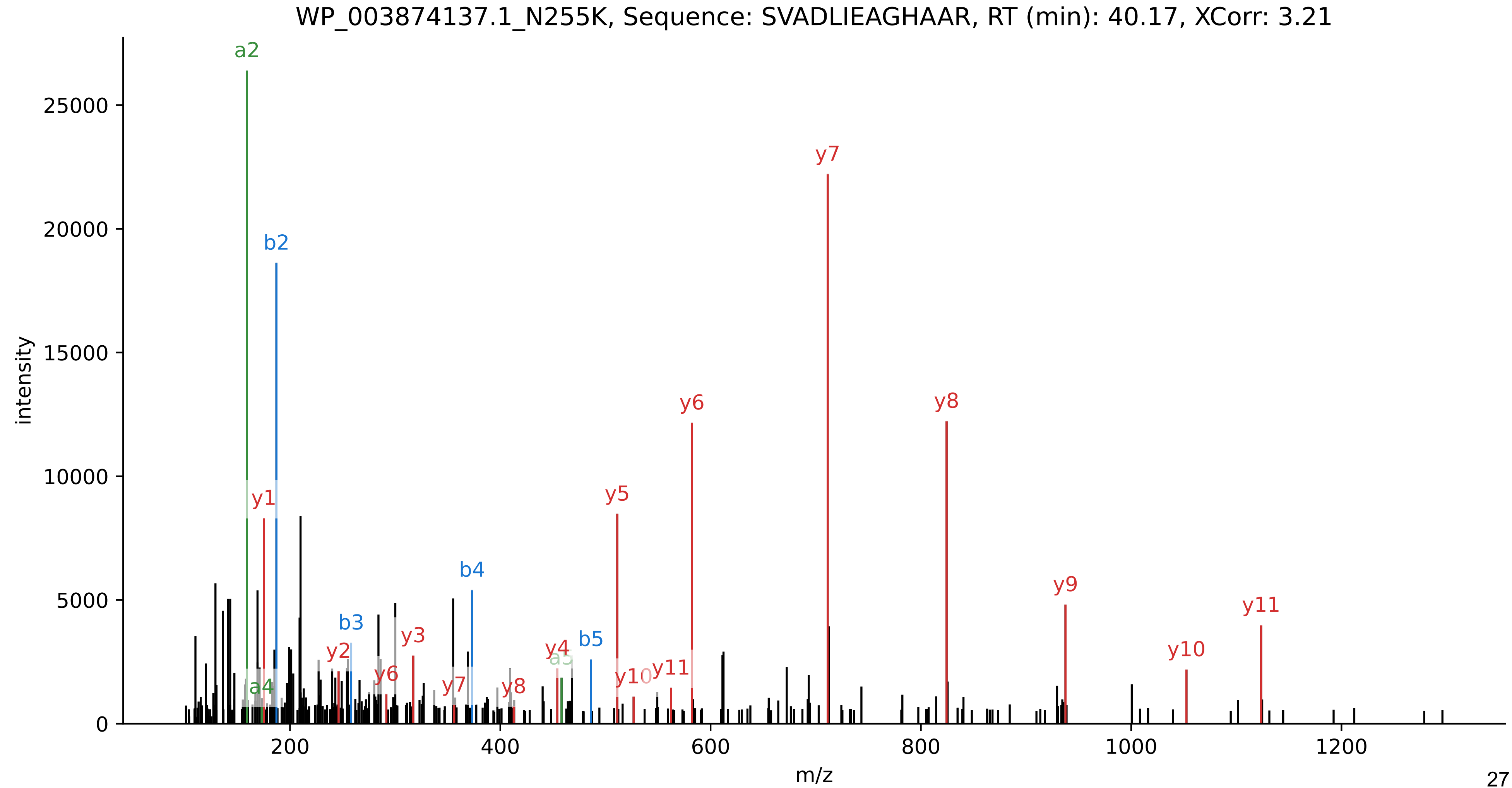
WP_003874137.1_N255K, Sequence: SVADLIEAGHAAR, RT (min): 40.17, XCorr: 3.21
intensity
m/z
a2
b2
y1
y7
y6
y8
y5
b4
b3
y3
y2
y6
y7
y8
a4
y4
a3
b5
y10
y11
y9
y11
y10

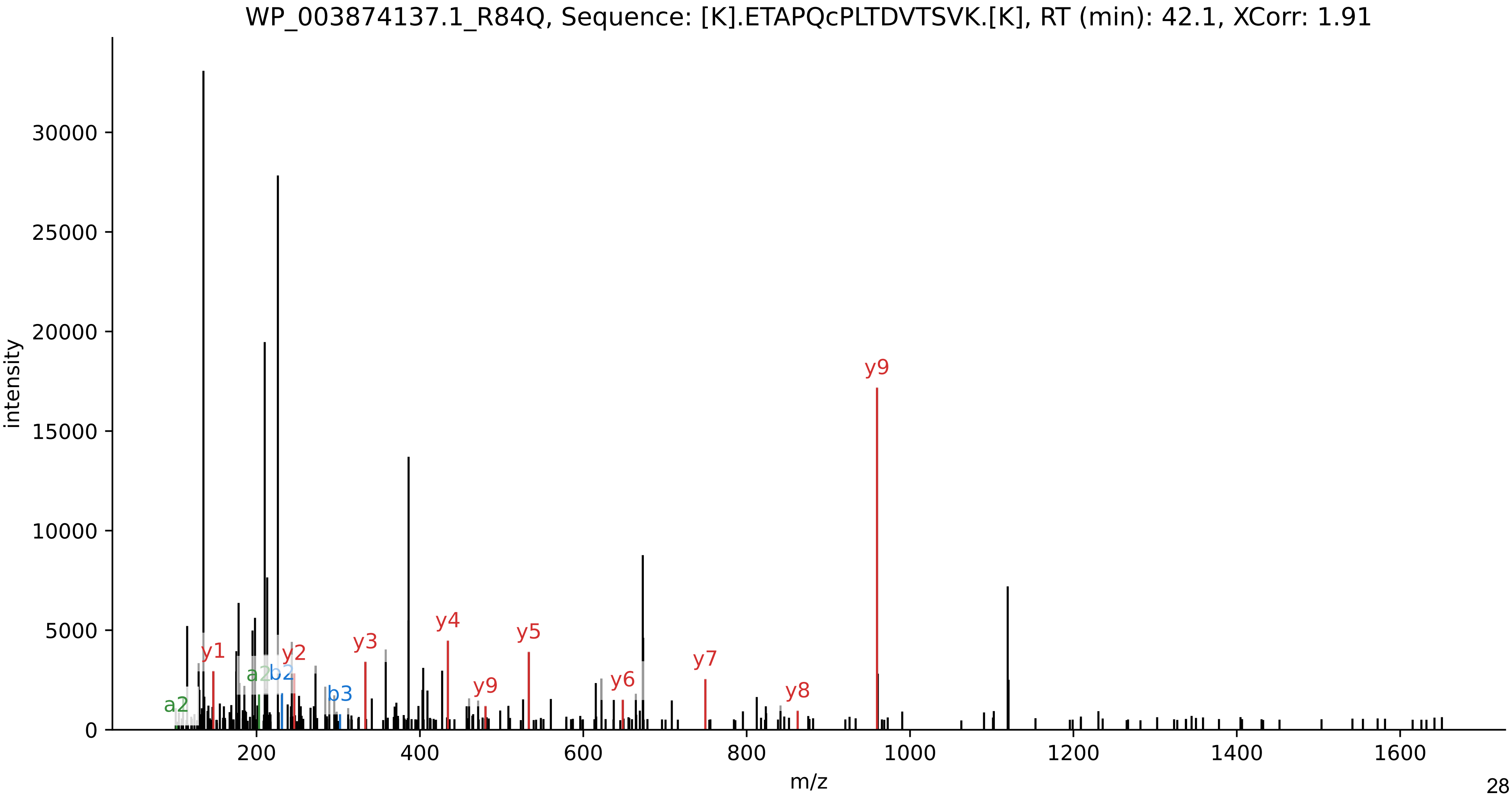
WP_003874137.1_R84Q, Sequence: [K].ETAPQcPLTDVTSVK.[K], RT (min): 42.1, XCorr: 1.91
intensity
m/z
y1
y2
y3
y4
y5
y6
y7
y8
y9
y9
a2
a2
b2
b3
0
5000
10000
15000
20000
25000
30000
200
400
600
800
1000
1200
1400
1600

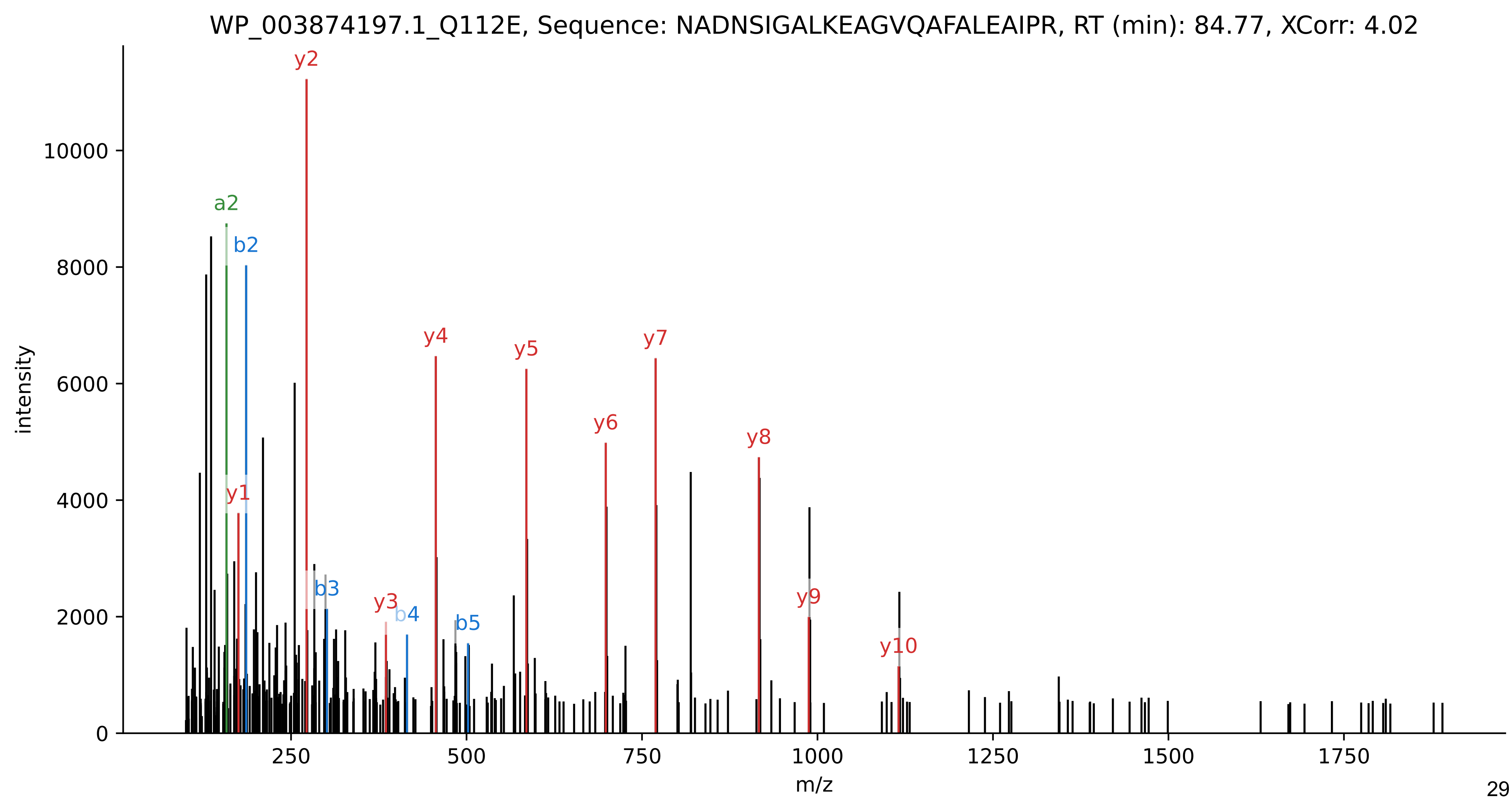

WP_003874197.1_Q112E, Sequence: NADNSIGALKEAGVQAFALEAIPR, RT (min): 84.77, XCorr: 4.02
intensity
m/z
0
2000
4000
6000
8000
10000
250
500
750
1000
1250
1500
1750
a2
b2
b3
b4
b5
y1
y2
y3
y4
y5
y6
y7
y8
y9
y10

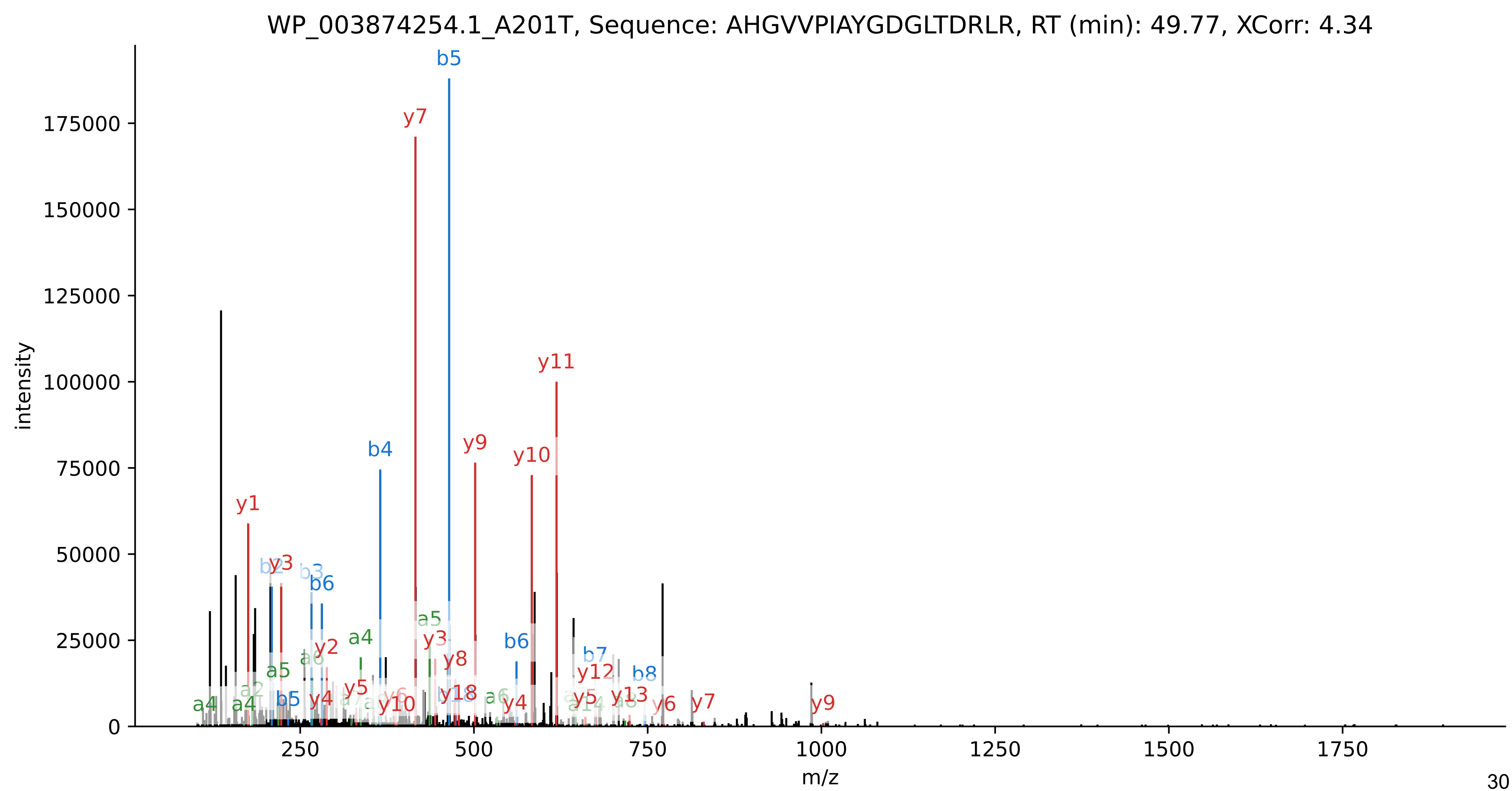
WP_003874254.1_A201T, Sequence: AHGVVPIAYGDGLTDRLR, RT (min): 49.77, XCorr: 4.34
intensity
m/z

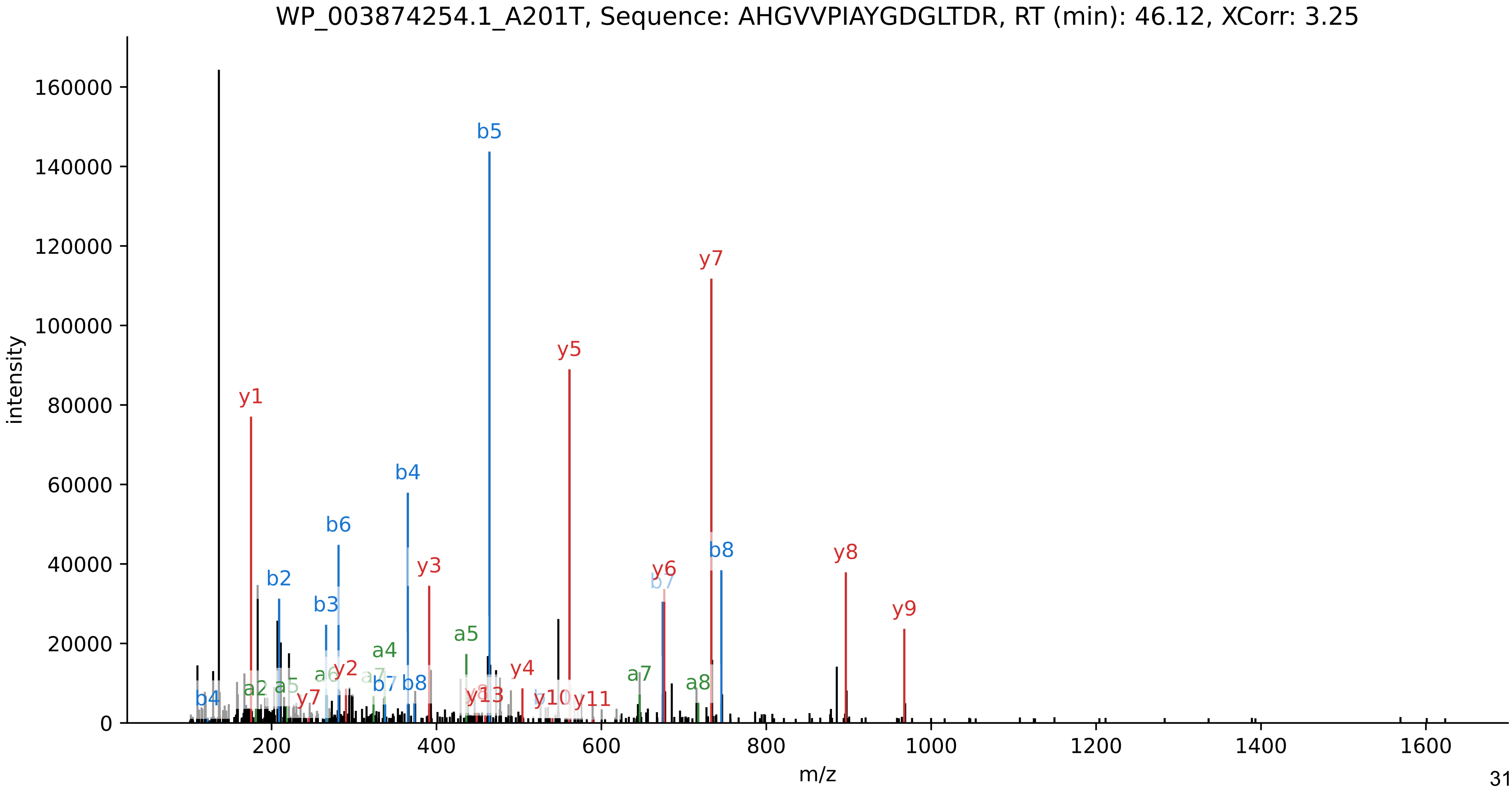
WP_003874254.1_A201T, Sequence: AHGVVPIAYGDGLTDR, RT (min): 46.12, XCorr: 3.25
intensity
m/z
0
20000
40000
60000
80000
100000
120000
140000
160000
200
400
600
800
1000
1200
1400
1600
b5
y7
y5
y1
b4
b6
y3
b8
y8
y6
b2
b3
y9
a5
a4
y4
a7
a8
y2
b7
b8
a2
y13
y10
y11
y7
b4

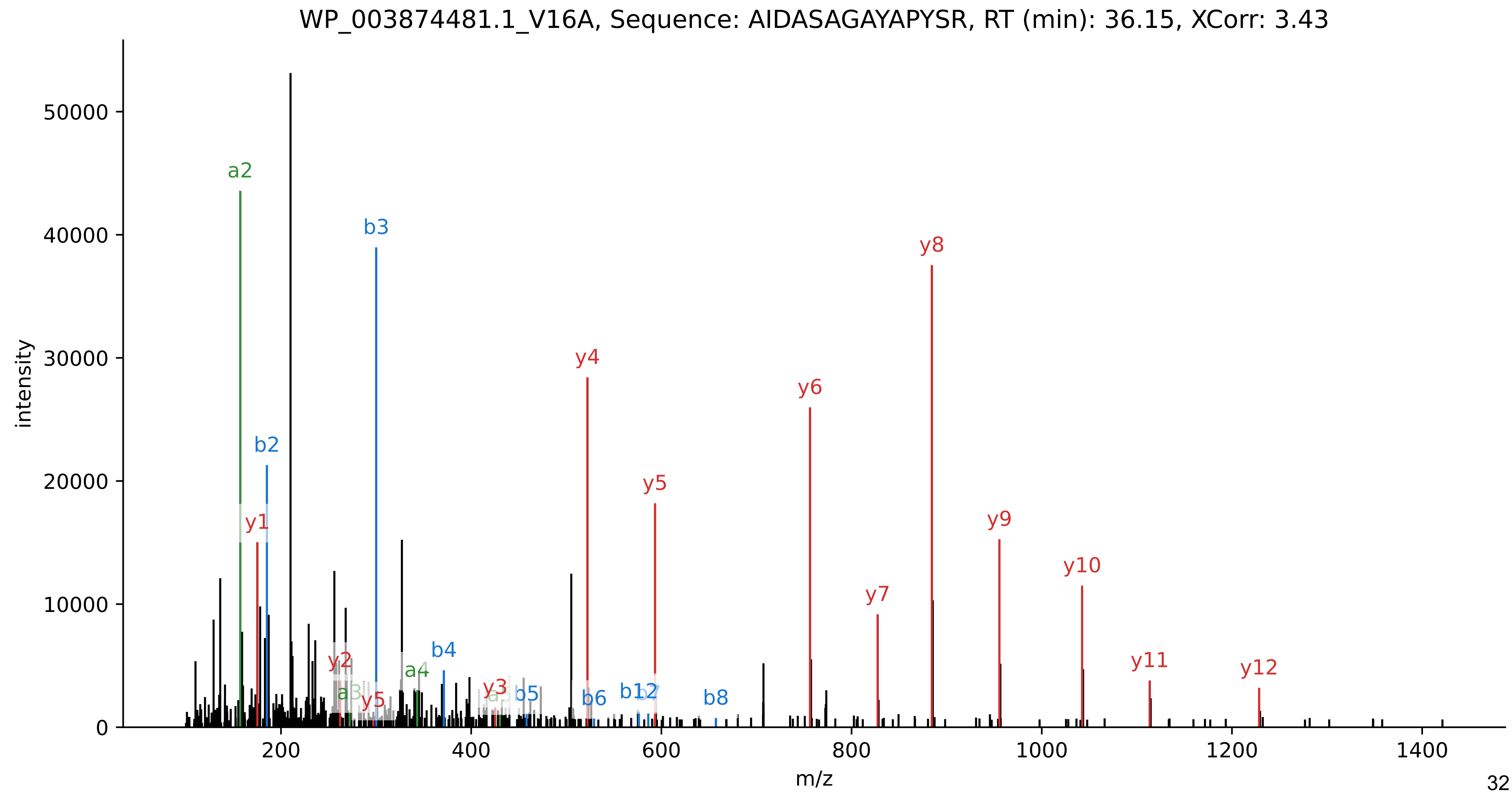
WP_003874481.1_V16A, Sequence: AIDASAGAYAPYSR, RT (min): 36.15, XCorr: 3.43
intensity
m/z
0
10000
20000
30000
40000
50000
200
400
600
800
1000
1200
1400
a2
b2
y1
b3
y2
a3
y5
a4
b4
y3
b5
y4
b6
y5
b12
b8
y6
y7
y8
y9
y10
y11
y12

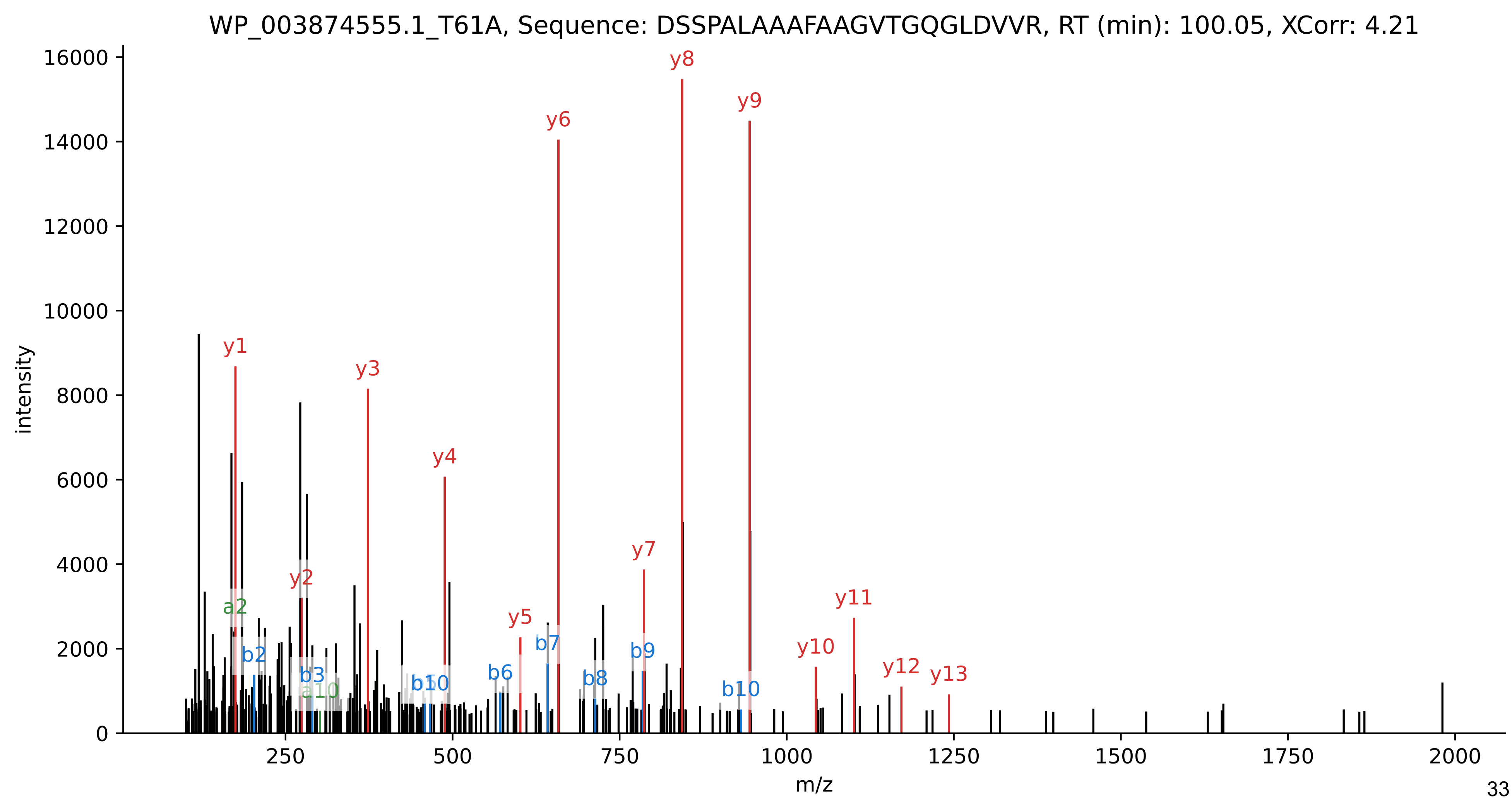

WP_003874555.1_T61A, Sequence: DSSPALAAAFAAGVTGQGLDVVR, RT (min): 100.05, XCorr: 4.21
intensity
m/z
y1
y2
y3
y4
y5
y6
y7
y8
y9
y10
y11
y12
y13
a2
b2
b3
b6
b7
b8
b9
b10

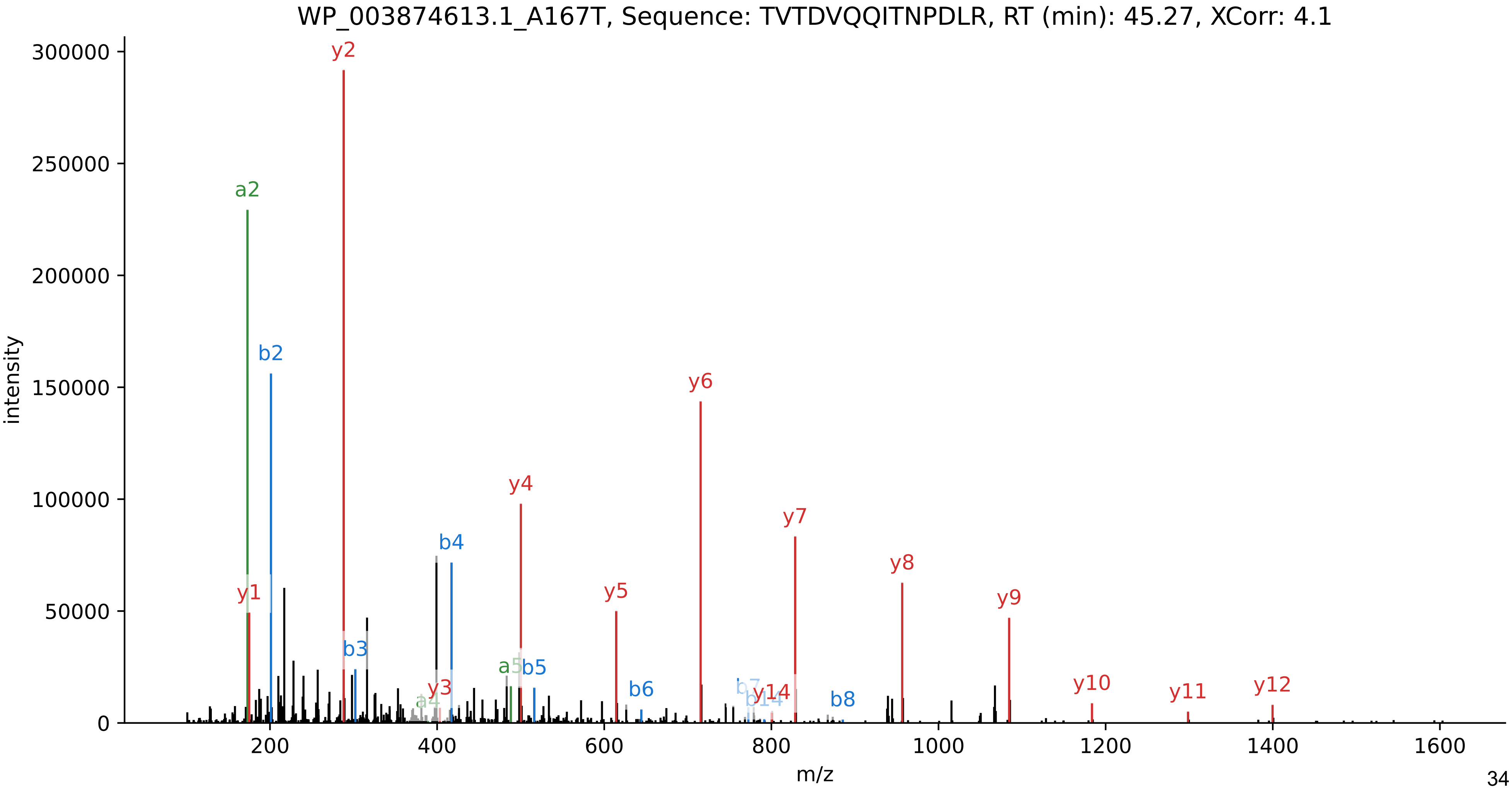

WP_003874613.1_A167T, Sequence: TVTDVQQITNPDLR, RT (min): 45.27, XCorr: 4.1
intensity
m/z
y2
a2
b2
y6
y4
b4
y7
y8
y9
y5
y1
b3
a5
b5
y3
a4
b6
b7
y14
b14
b8
y10
y11
y12
300000
250000
200000
150000
100000
50000
0
200
400
600
800
1000
1200
1400
1600

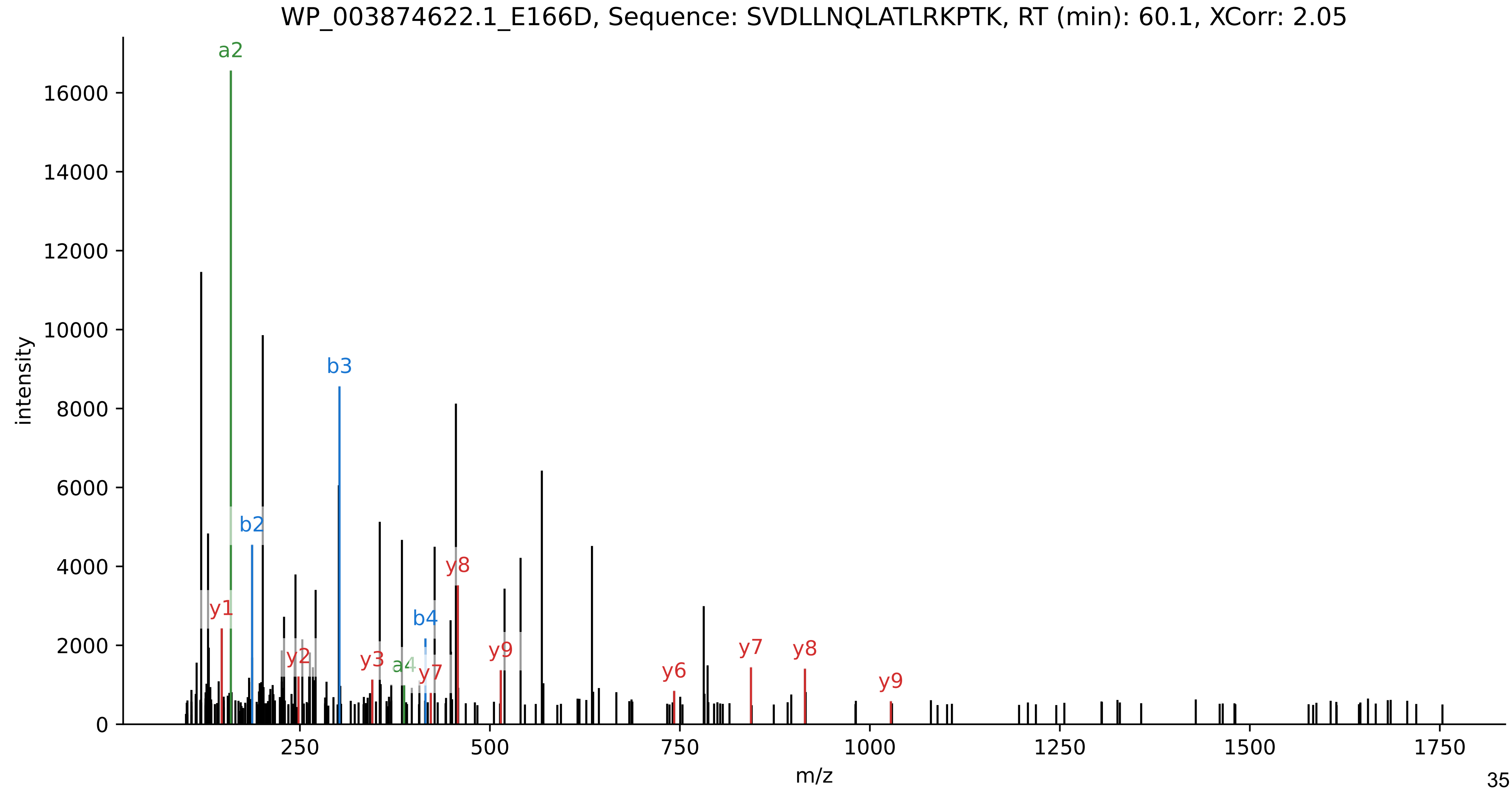

WP_003874622.1_E166D, Sequence: SVDLLNQLATLRKPTK, RT (min): 60.1, XCorr: 2.05
intensity
m/z
a2
b2
b3
b4
a4
y1
y2
y3
y7
y8
y9
y6
y7
y8
y9

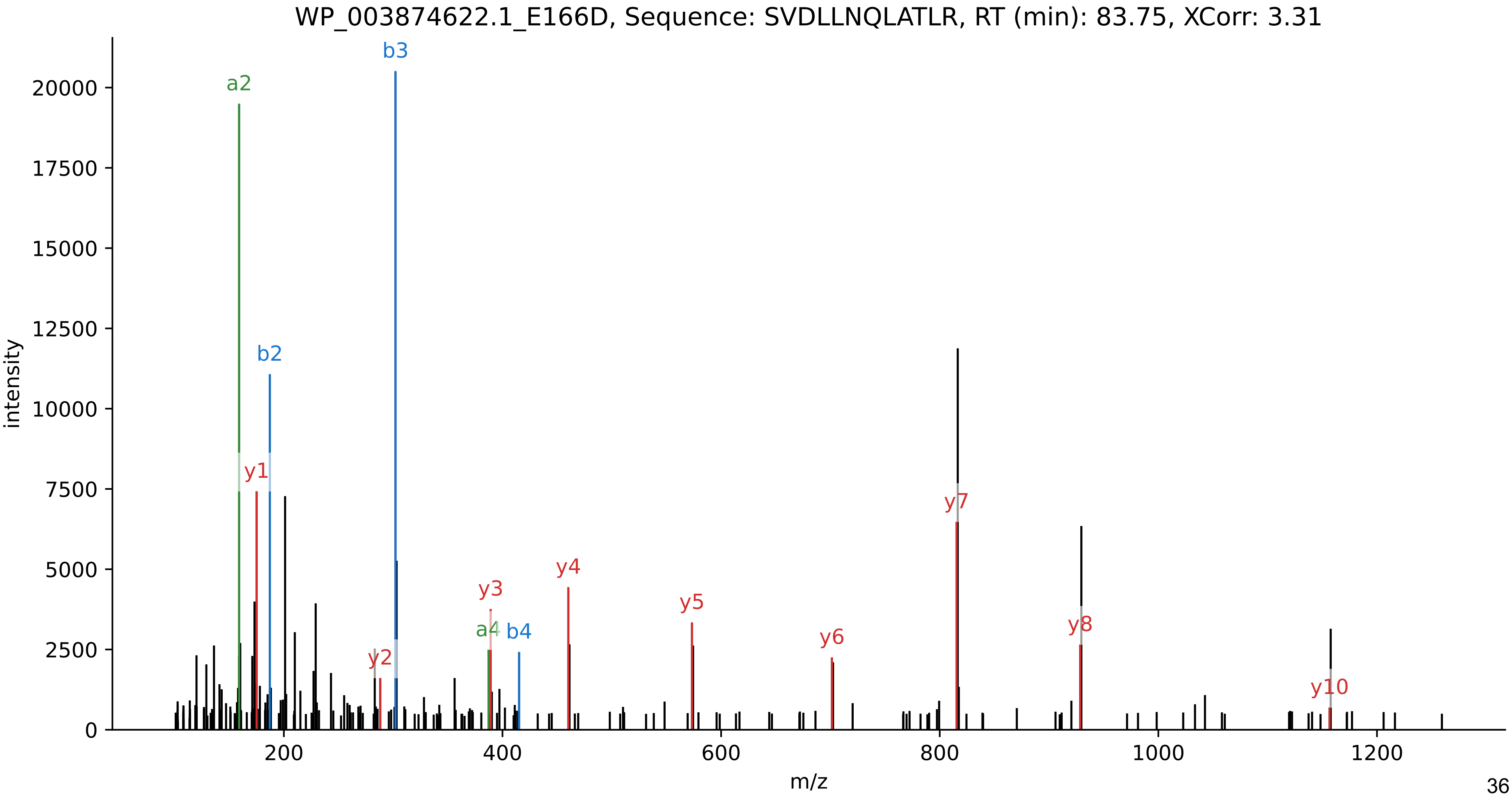
WP_003874622.1_E166D, Sequence: SVDLLNQLATLR, RT (min): 83.75, XCorr: 3.31
intensity
m/z
a2
b3
b2
y1
y7
y8
y2
y3
a4
b4
y4
y5
y6
y10

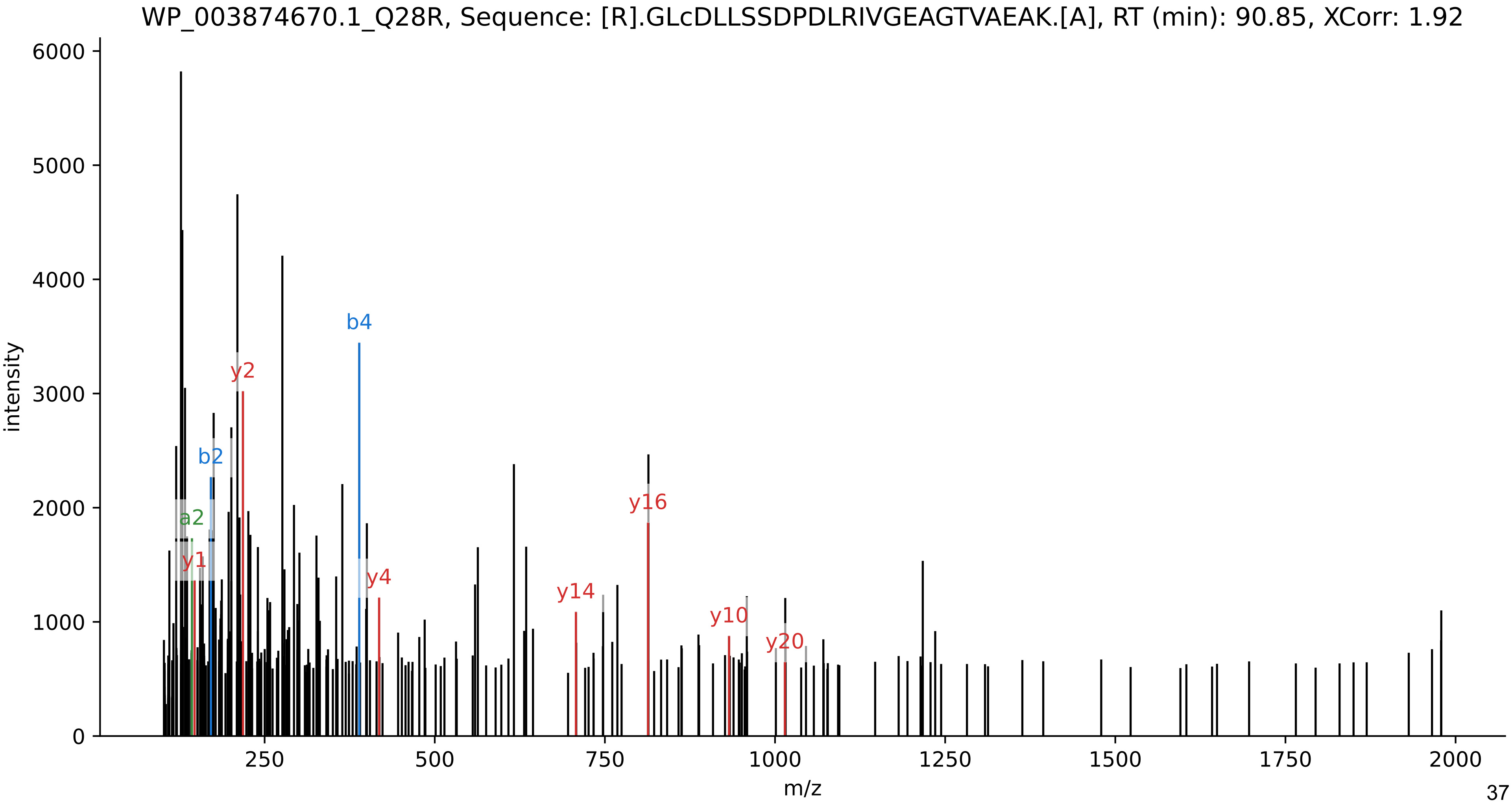

WP_003874670.1_Q28R, Sequence: [R].GLcDLLSSDPDLRIVGEAGTVAEAK.[A], RT (min): 90.85, XCorr: 1.92
intensity
m/z
b4
y2
b2
a2
y1
y4
y16
y14
y10
y20

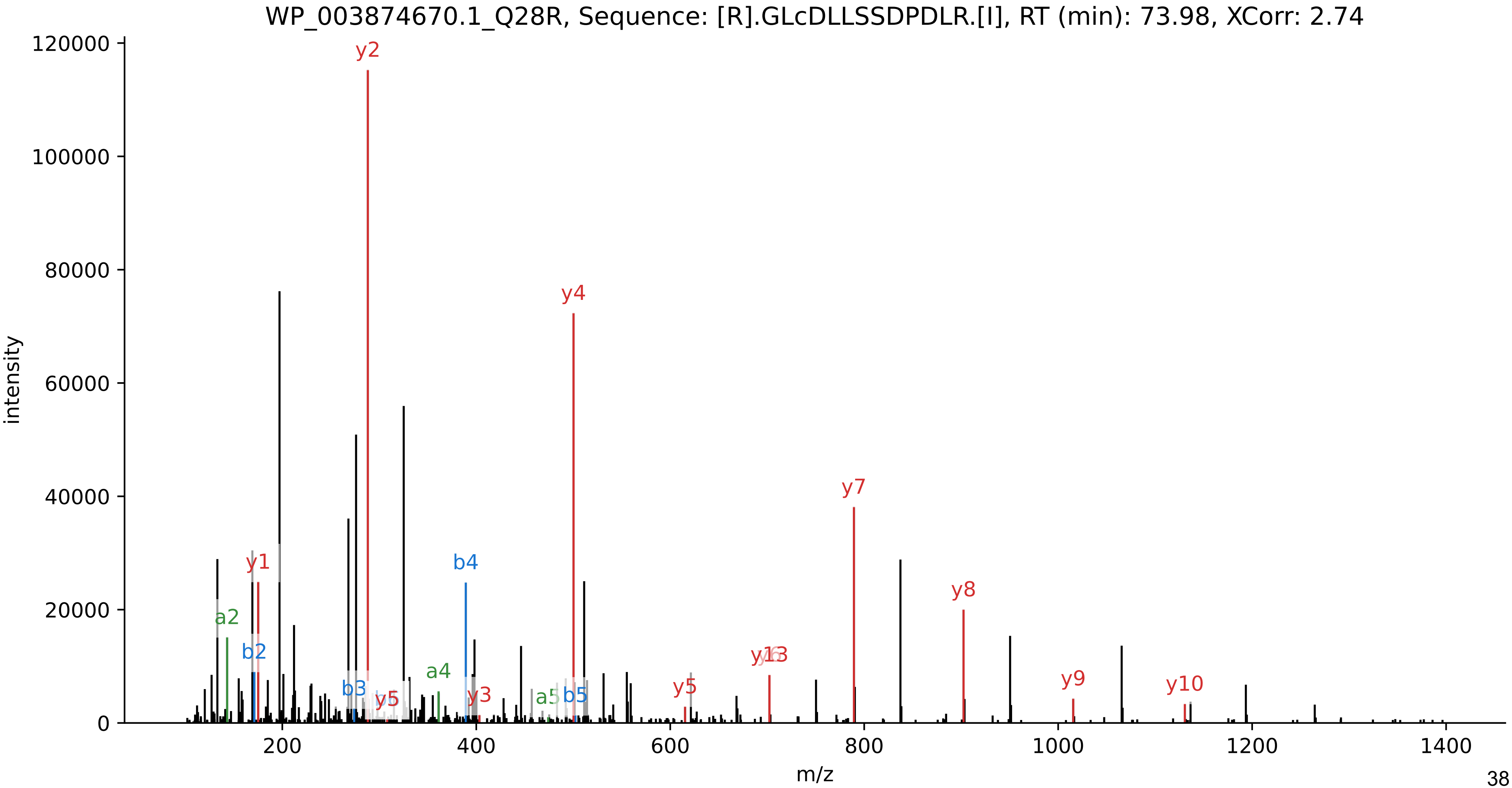

WP_003874670.1_Q28R, Sequence: [R].GLcDLLSSDPDLR.[I], RT (min): 73.98, XCorr: 2.74
intensity
m/z
0
20000
40000
60000
80000
100000
120000
200
400
600
800
1000
1200
1400
y2
y4
y7
y8
y1
b4
a2
b2
a4
b3
y5
y3
a5
b5
y5
y13
y9
y10

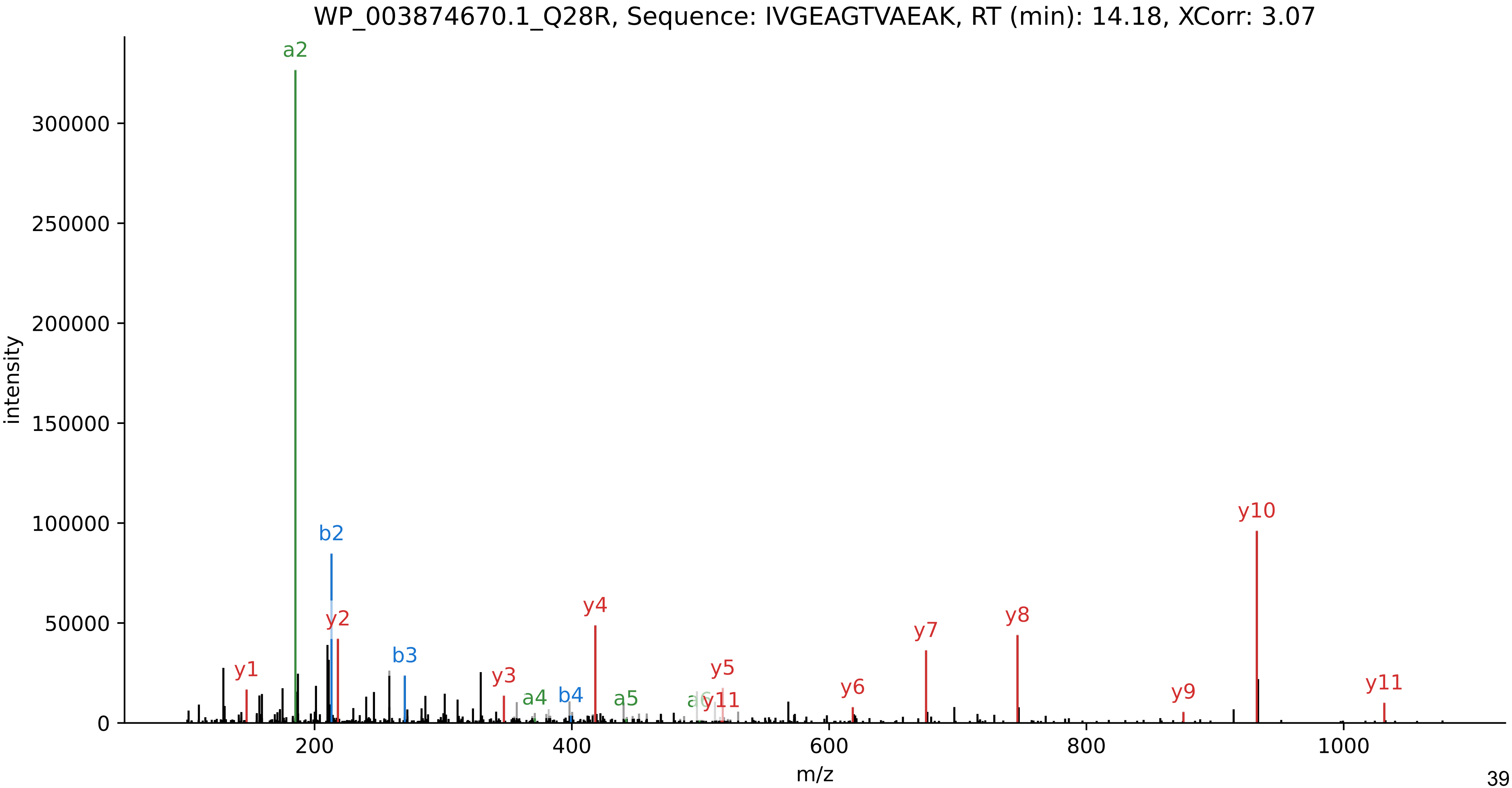

WP_003874670.1_Q28R, Sequence: IVGEAGTVAEAK, RT (min): 14.18, XCorr: 3.07
intensity
m/z
a2
b2
y2
b3
y1
y3
a4
b4
y4
a5
y5
y11
y6
y7
y8
y9
y10
y11

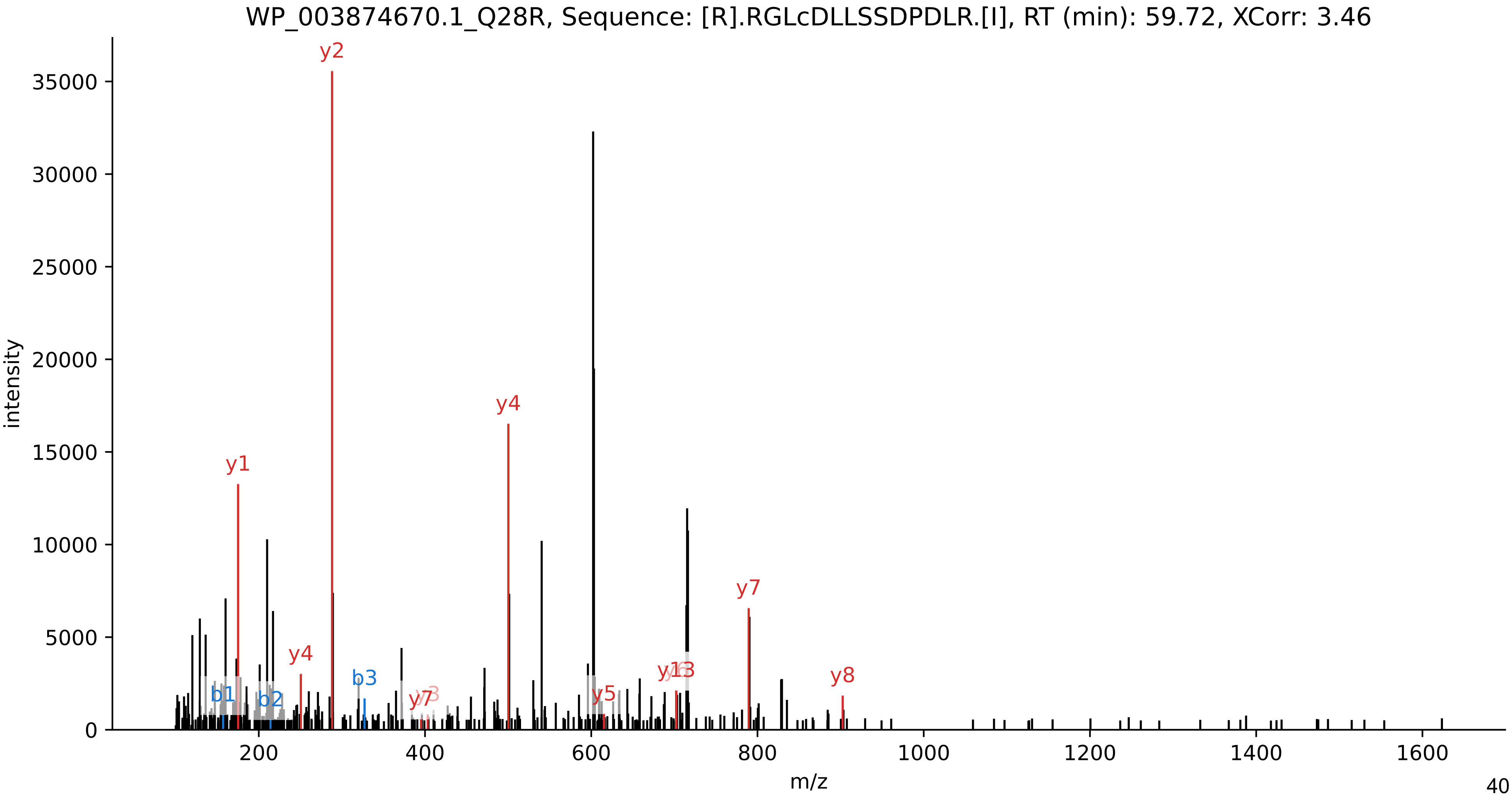
WP_003874670.1_Q28R, Sequence: [R].RGLcDLLSSDPDLR.[I], RT (min): 59.72, XCorr: 3.46
intensity
m/z
0
5000
10000
15000
20000
25000
30000
35000
200
400
600
800
1000
1200
1400
1600
y2
y4
y1
y4
b3
b1
b2
y7
y7
y5
y13
y8

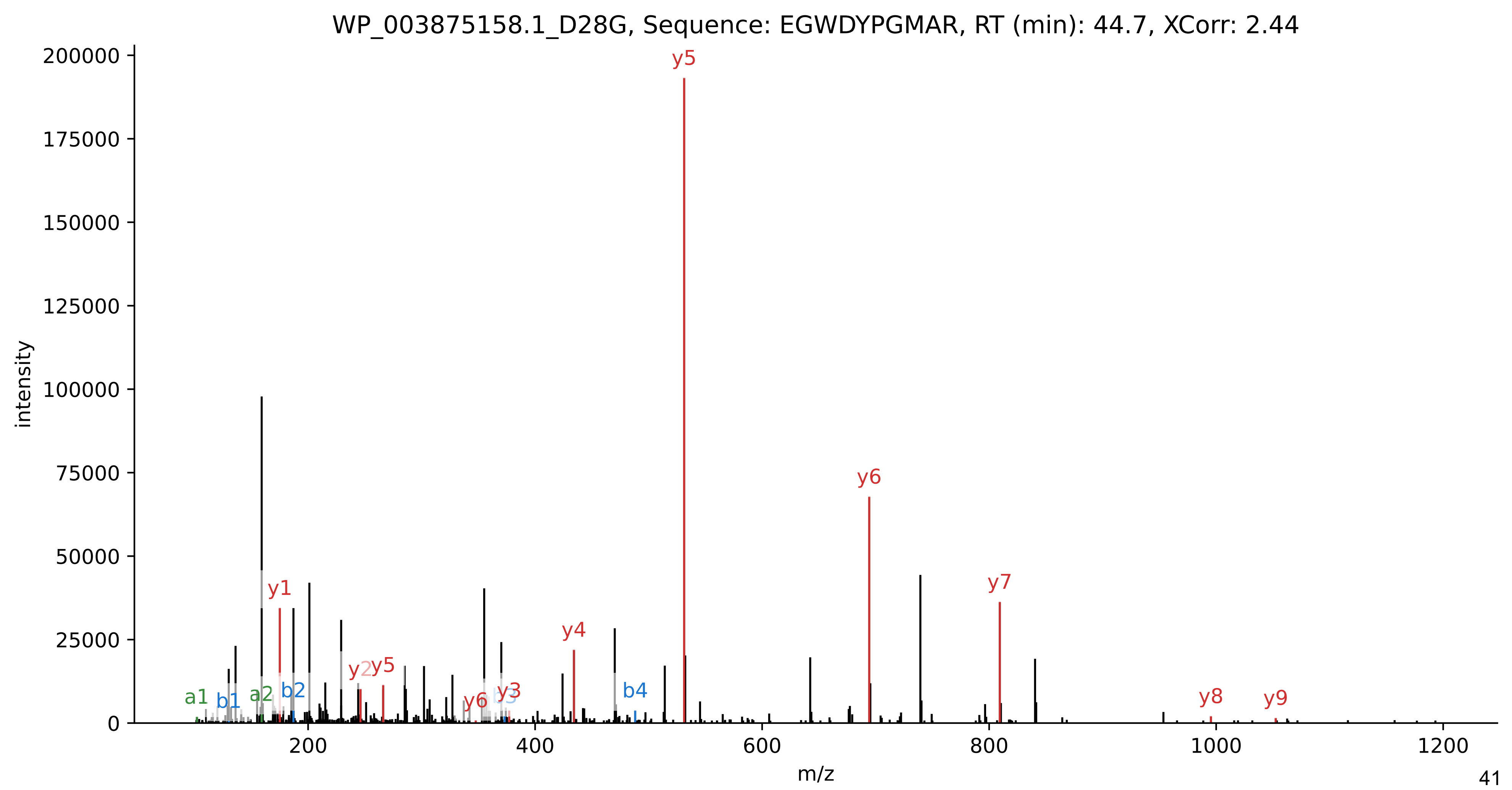
WP_003875158.1_D28G, Sequence: EGWDYPGMAR, RT (min): 44.7, XCorr: 2.44
intensity
m/z
y5
y6
y7
y1
y4
y2
y5
y6
y3
b3
a1
b1
a2
b2
b4
y8
y9
0
25000
50000
75000
100000
125000
150000
175000
200000
200
400
600
800
1000
1200

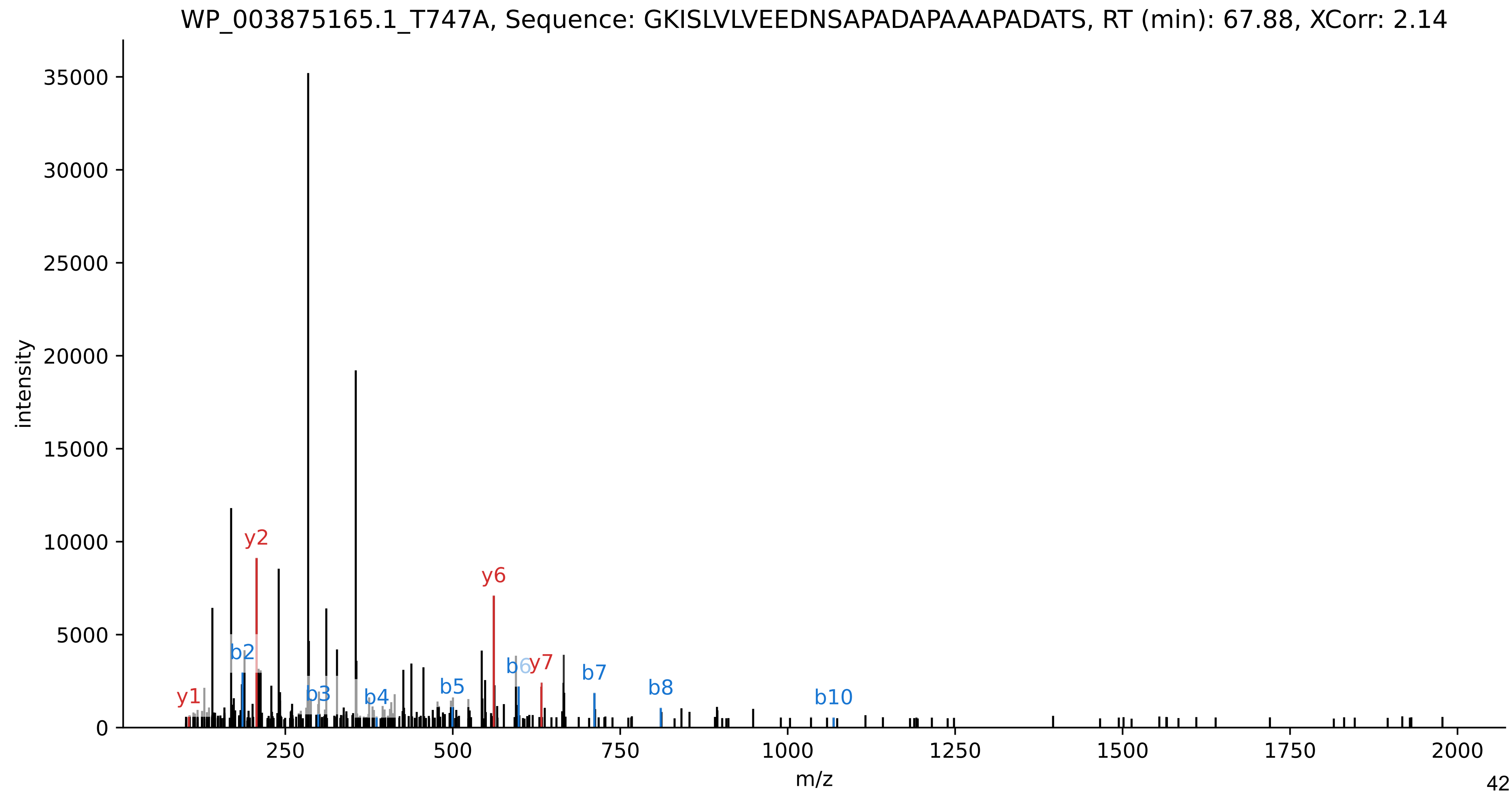
WP_003875165.1_T747A, Sequence: GKISLVLVEEDNSAPADAPAAAPADATS, RT (min): 67.88, XCorr: 2.14
intensity
m/z
y1
y2
b2
b3
b4
b5
y6
b6
y7
b7
b8
b10

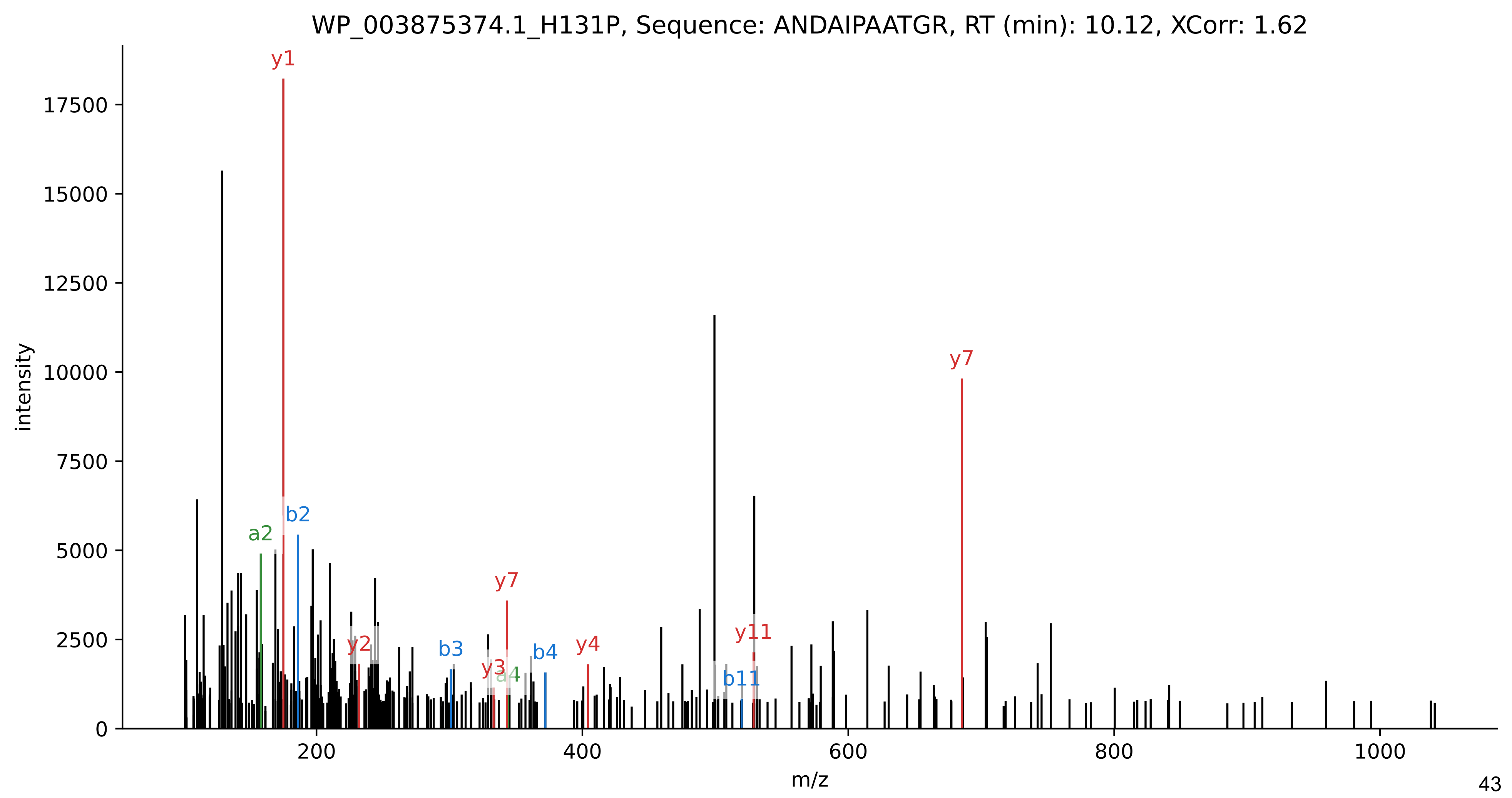

WP_003875374.1_H131P, Sequence: ANDAIPAATGR, RT (min): 10.12, XCorr: 1.62
intensity
m/z
y1
b2
a2
y7
y2
b3
y3
a4
b4
y4
y11
b11
y7
17500
15000
12500
10000
7500
5000
2500
0
200
400
600
800
1000

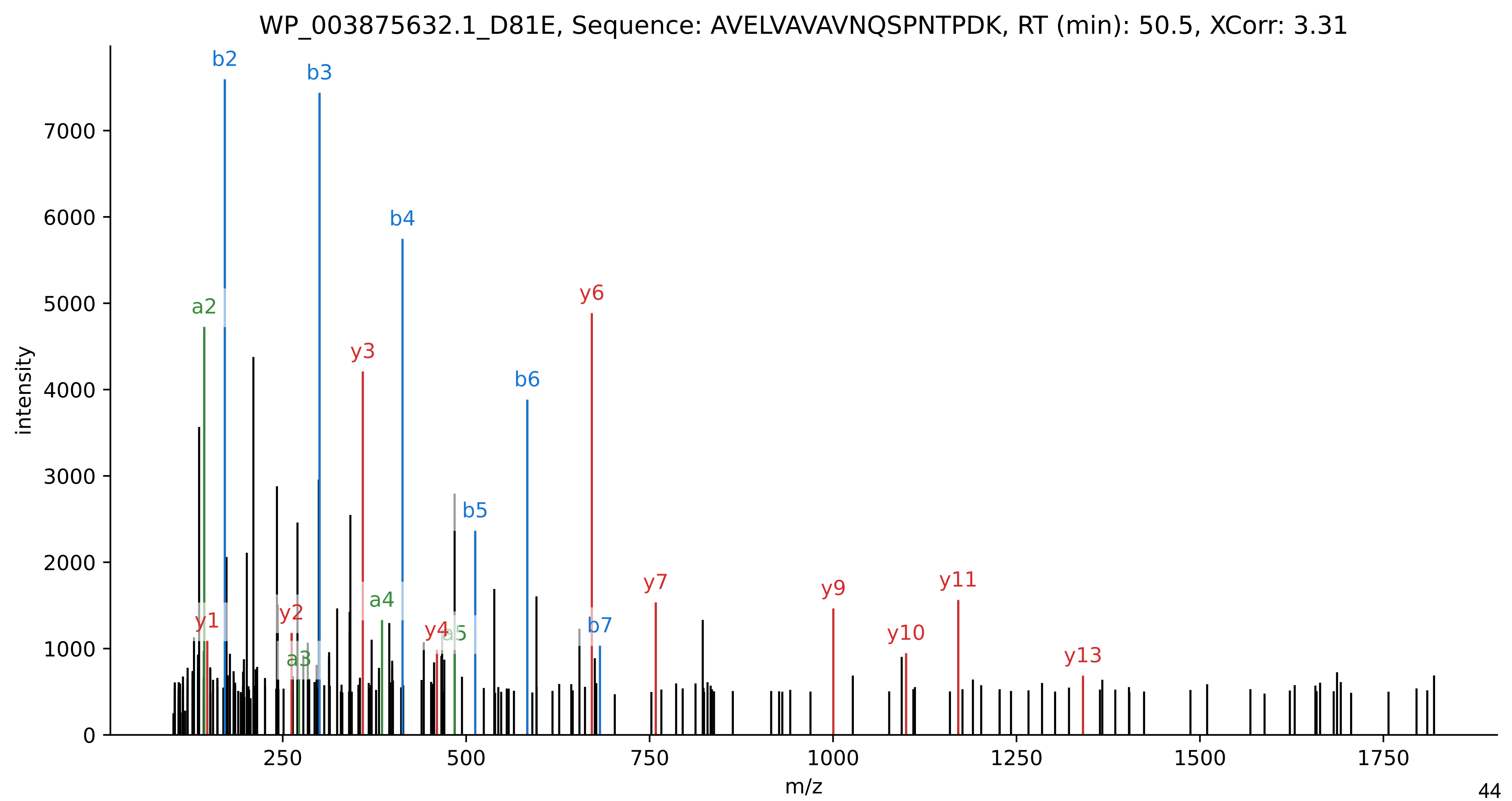
WP_003875632.1_D81E, Sequence: AVELVAVAVNQSPNTPDK, RT (min): 50.5, XCorr: 3.31
intensity
m/z
0
1000
2000
3000
4000
5000
6000
7000
250
500
750
1000
1250
1500
1750
b2
b3
b4
b5
b6
b7
a2
a3
a4
a5
y1
y2
y3
y4
y6
y7
y9
y10
y11
y13

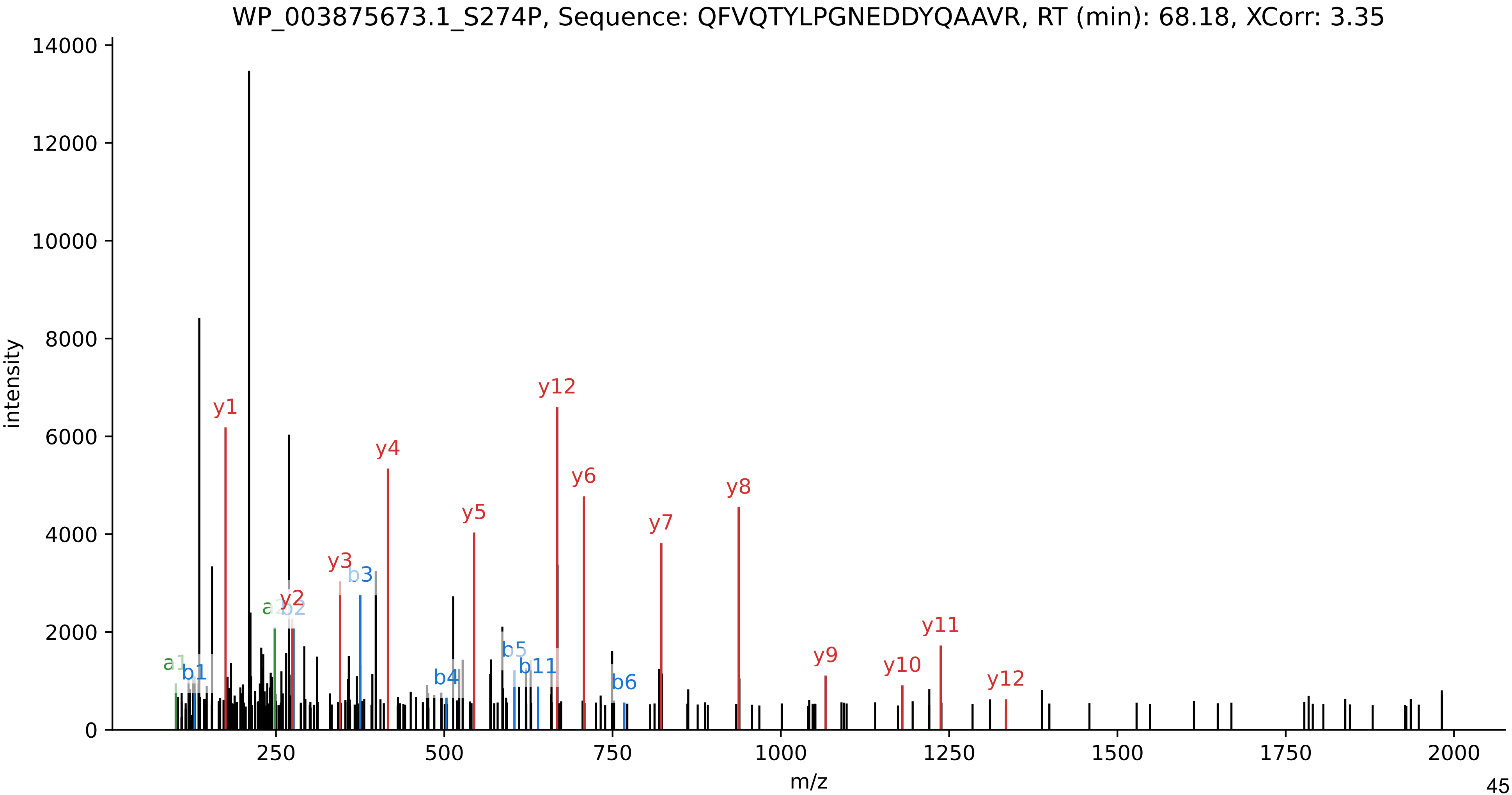

WP_003875673.1_S274P, Sequence: QFVQTYLPGNEDDYQAAVR, RT (min): 68.18, XCorr: 3.35
intensity
m/z
y1
y2
y3
y4
y5
y6
y7
y8
y9
y10
y11
y12
y12
a1
a2
b1
b2
b3
b4
b5
b6
b11

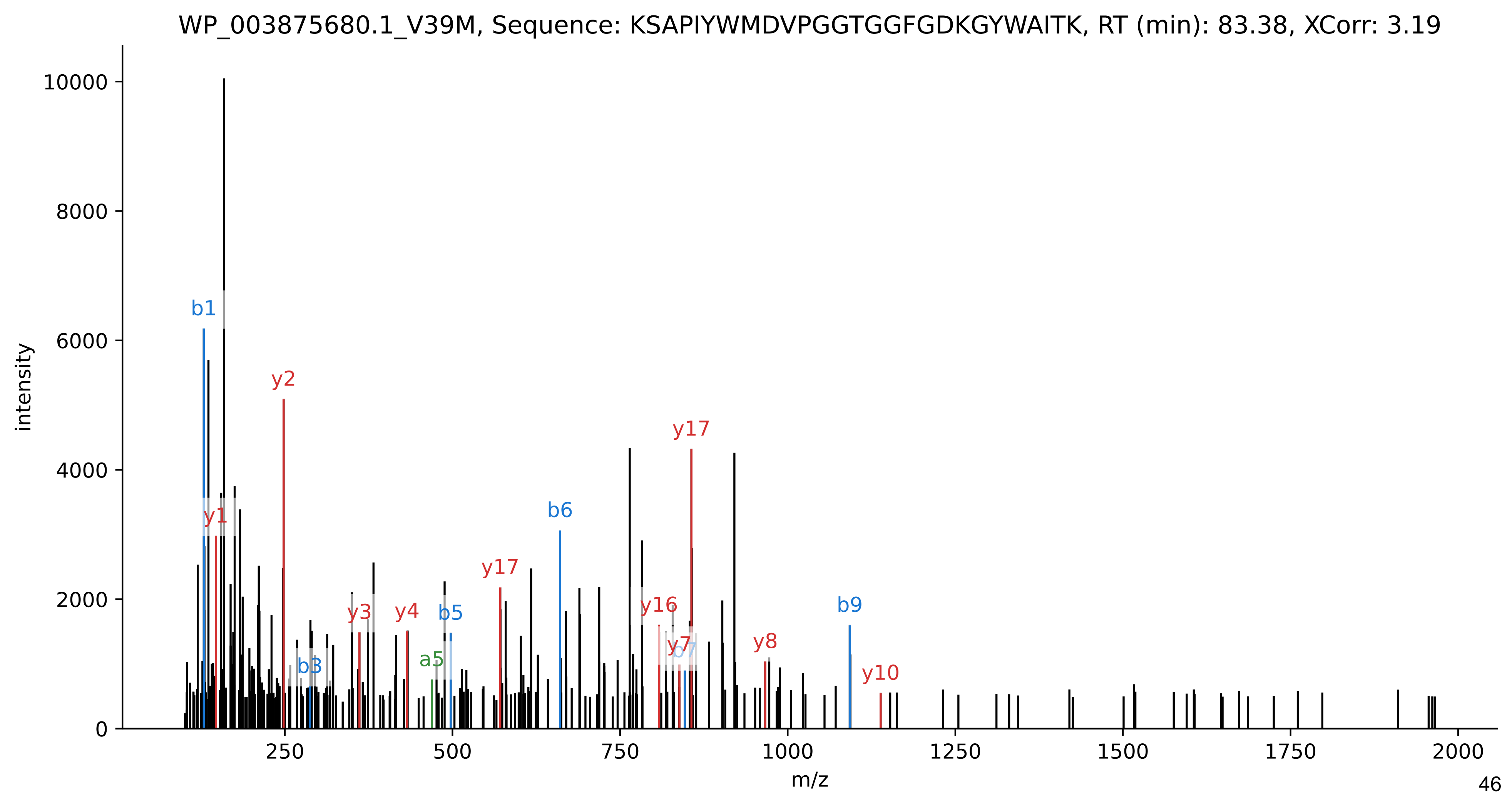
WP_003875680.1_V39M, Sequence: KSAPIYWMDVPGGTGGFGDKGYWAITK, RT (min): 83.38, XCorr: 3.19
intensity
m/z
b1
y1
y2
b3
y3
y4
a5
b5
y17
b6
y16
y17
y7
b7
y8
b9
y10
0
2000
4000
6000
8000
10000
250
500
750
1000
1250
1500
1750
2000

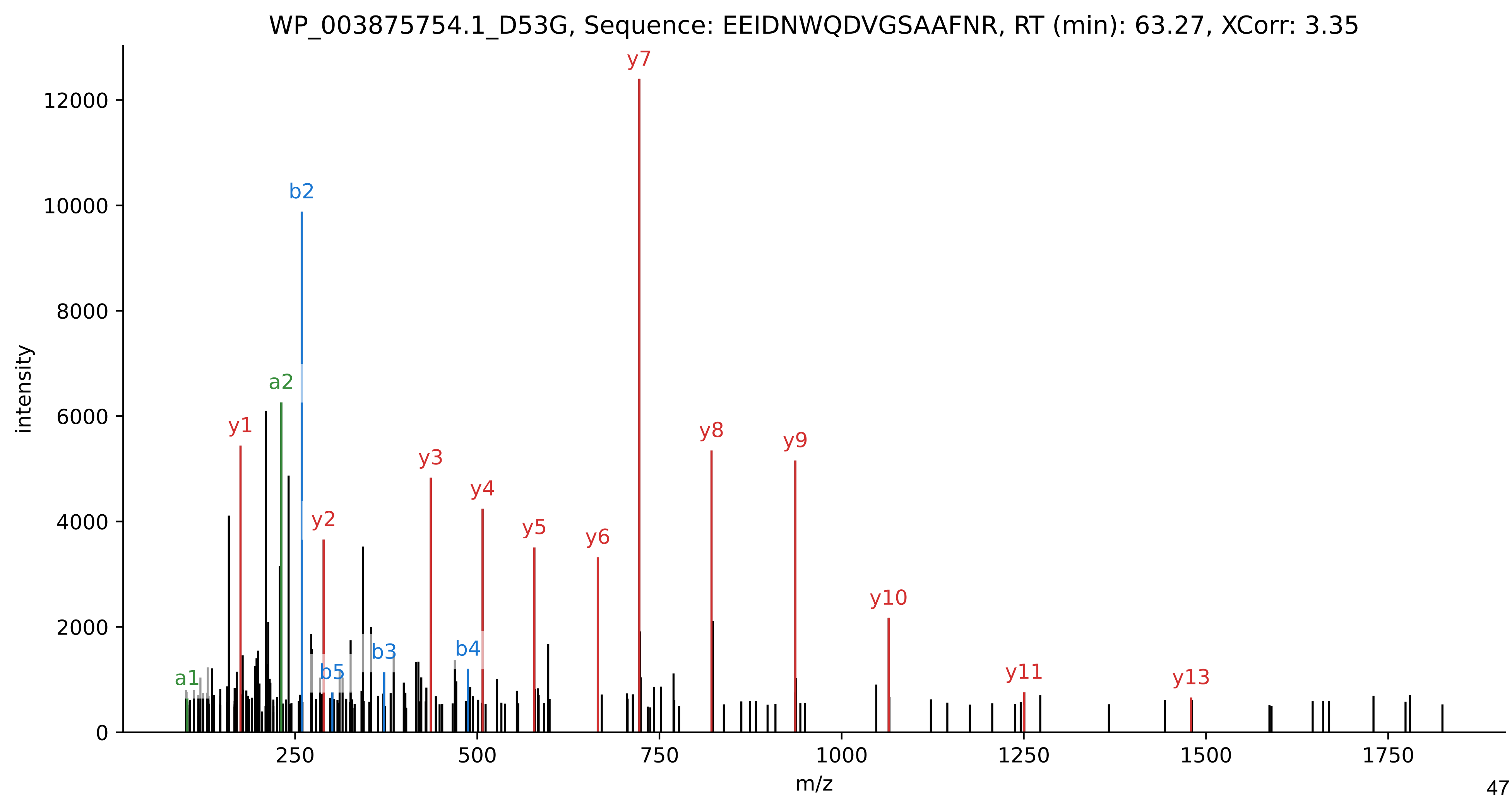
WP_003875754.1_D53G, Sequence: EEIDNWQDVGSAAFNR, RT (min): 63.27, XCorr: 3.35
intensity
m/z
y7
b2
a2
y1
y8
y9
y3
y4
y2
y5
y6
y10
b3
b4
b5
a1
y11
y13

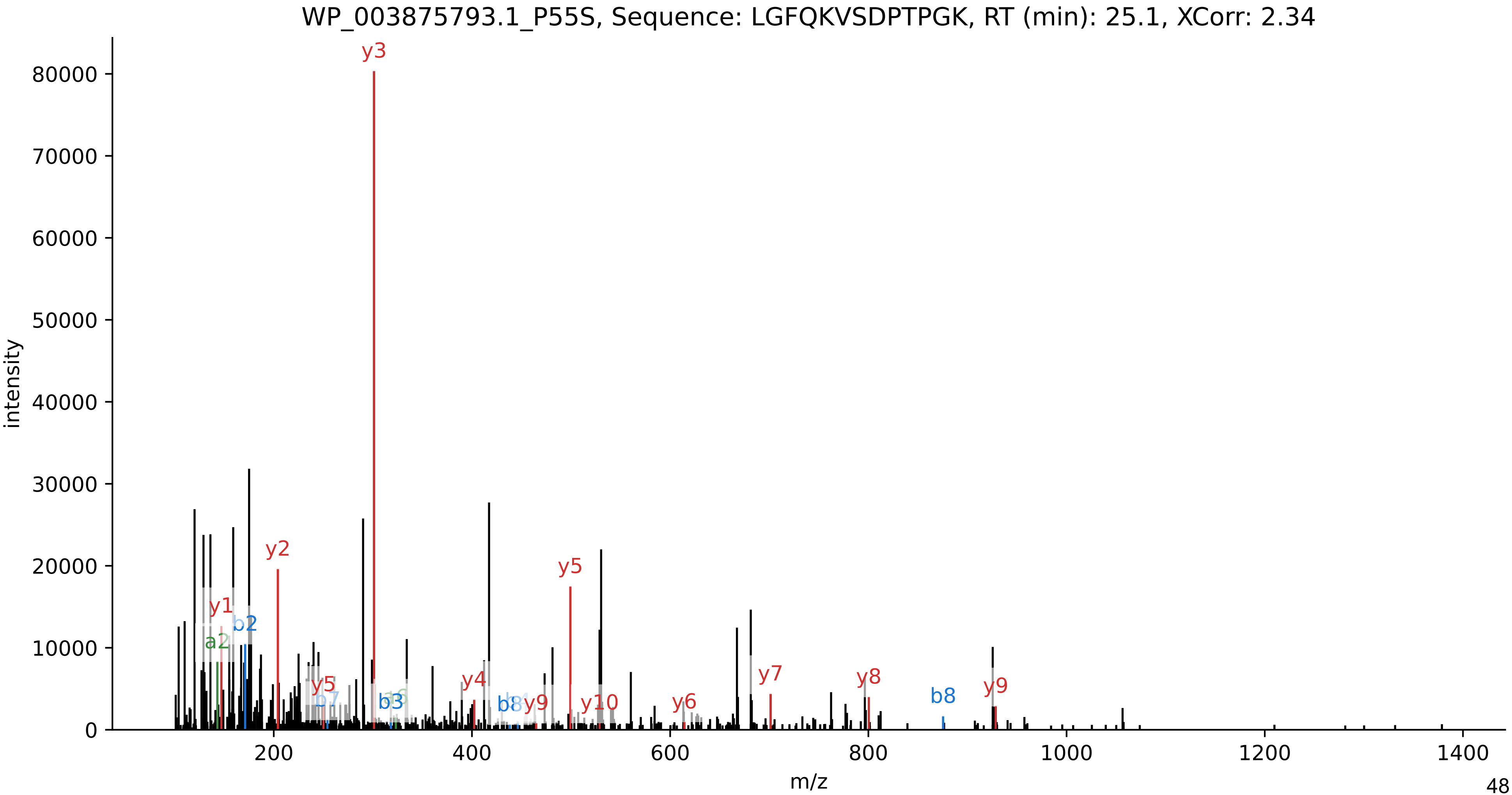
WP_003875793.1_P55S, Sequence: LGFQKVSDPTPGK, RT (min): 25.1, XCorr: 2.34
intensity
m/z
y3
y2
y5
y1
b2
a2
y5
b3
y4
b8
y9
y10
y6
y7
y8
b8
y9
0
10000
20000
30000
40000
50000
60000
70000
80000
200
400
600
800
1000
1200
1400

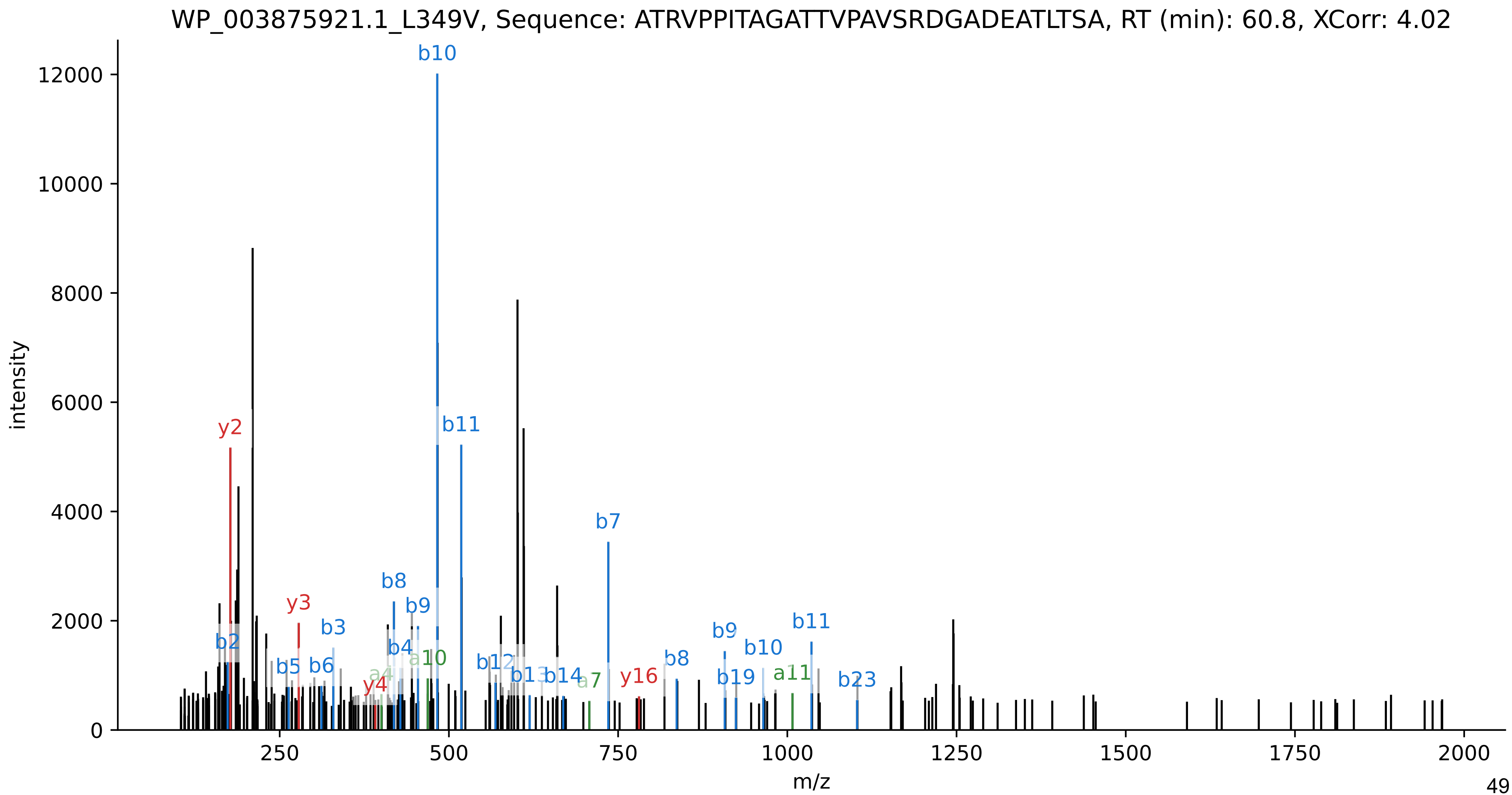
WP_003875921.1_L349V, Sequence: ATRVPPITAGATTVPAVSRDGADEATLTSA, RT (min): 60.8, XCorr: 4.02
intensity
m/z
0
2000
4000
6000
8000
10000
12000
250
500
750
1000
1250
1500
1750
2000
b10
b11
y2
b7
b8
b9
y3
b3
b2
b4
a10
b5
b6
a4
y4
b12
b13
b14
a7
y16
b8
b9
b10
b19
a11
b11
b23

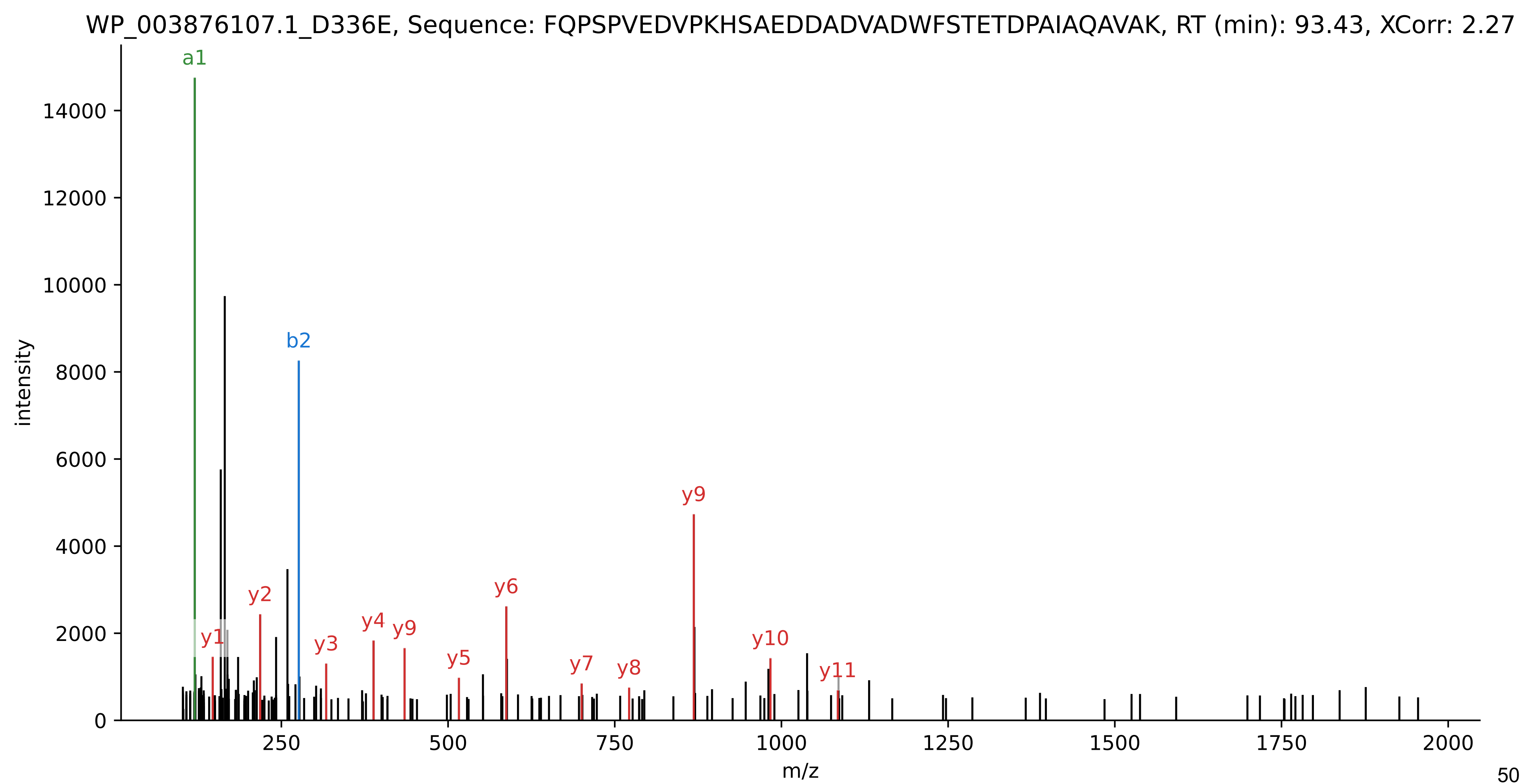
WP_003876107.1_D336E, Sequence: FQPSPVEDVPKHSAEDDADVADWFSTETDPAIAQAVAK, RT (min): 93.43, XCorr: 2.27
a1
b2
y1
y2
y3
y4
y9
y5
y6
y7
y8
y9
y10
y11
intensity
m/z
0
2000
4000
6000
8000
10000
12000
14000
250
500
750
1000
1250
1500
1750
2000

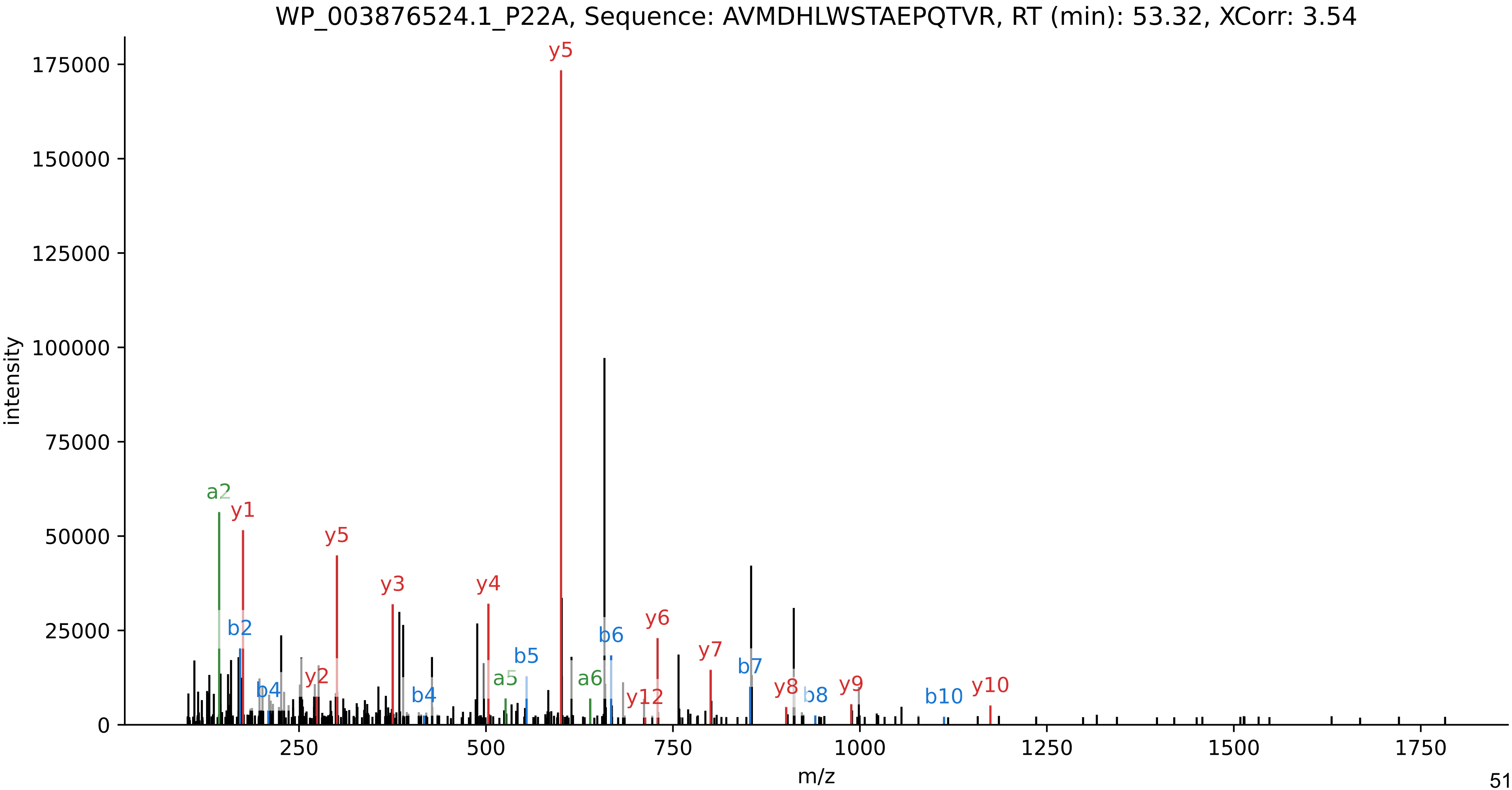
WP_003876524.1_P22A, Sequence: AVMDHLWSTAEPQTVR, RT (min): 53.32, XCorr: 3.54
intensity
m/z
y5
a2
y1
y5
y3
y4
b2
b4
y2
b4
b5
a5
a6
b6
y6
y7
b7
y12
y8
b8
y9
b10
y10
0
25000
50000
75000
100000
125000
150000
175000
250
500
750
1000
1250
1500
1750

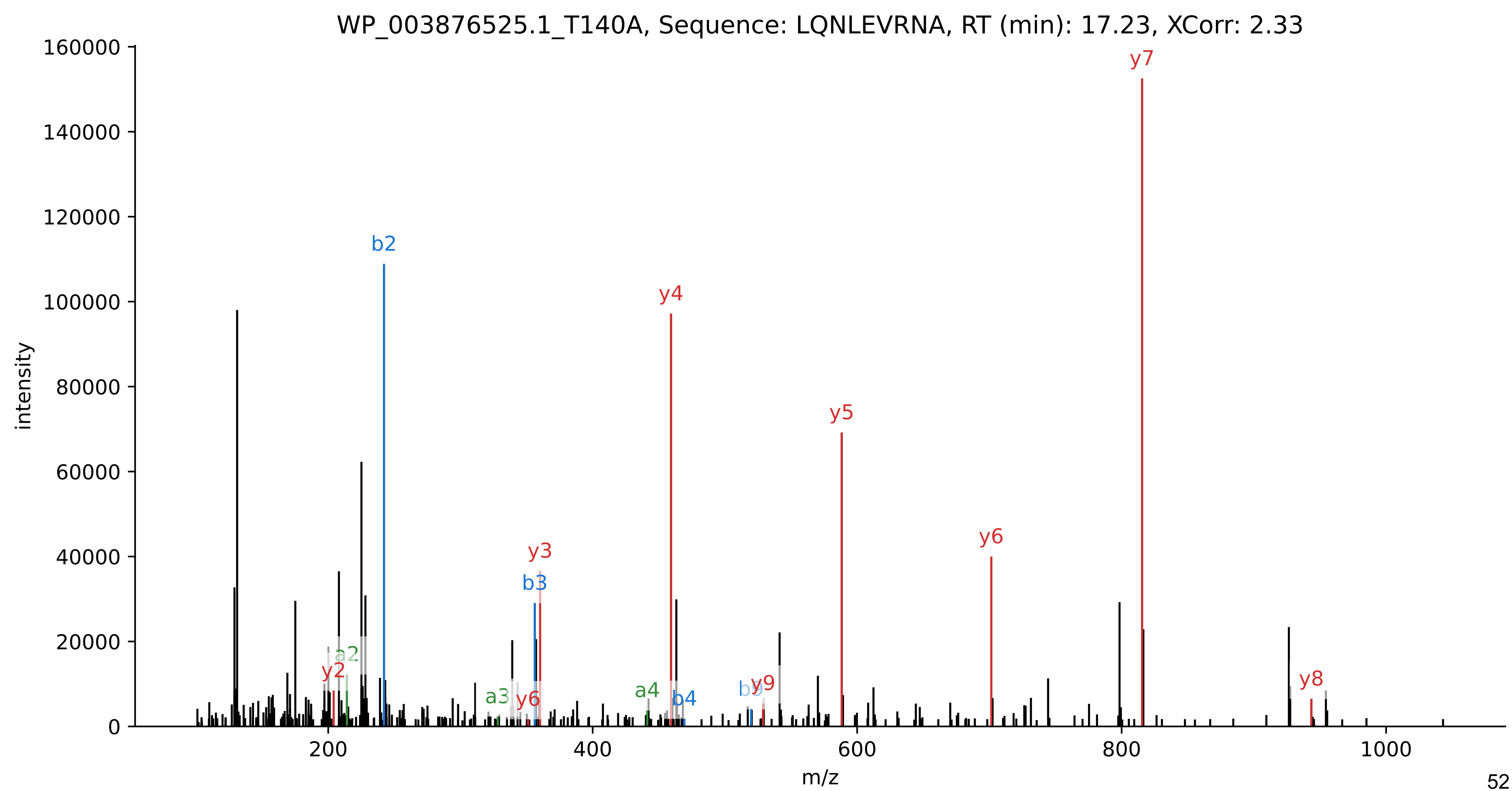

WP_003876525.1_T140A, Sequence: LQNLEVRNA, RT (min): 17.23, XCorr: 2.33
intensity
m/z
0
20000
40000
60000
80000
100000
120000
140000
160000
200
400
600
800
1000
y7
b2
y4
y5
y6
y3
b3
a2
y2
a3
y6
a4
b4
b5
y9
y8

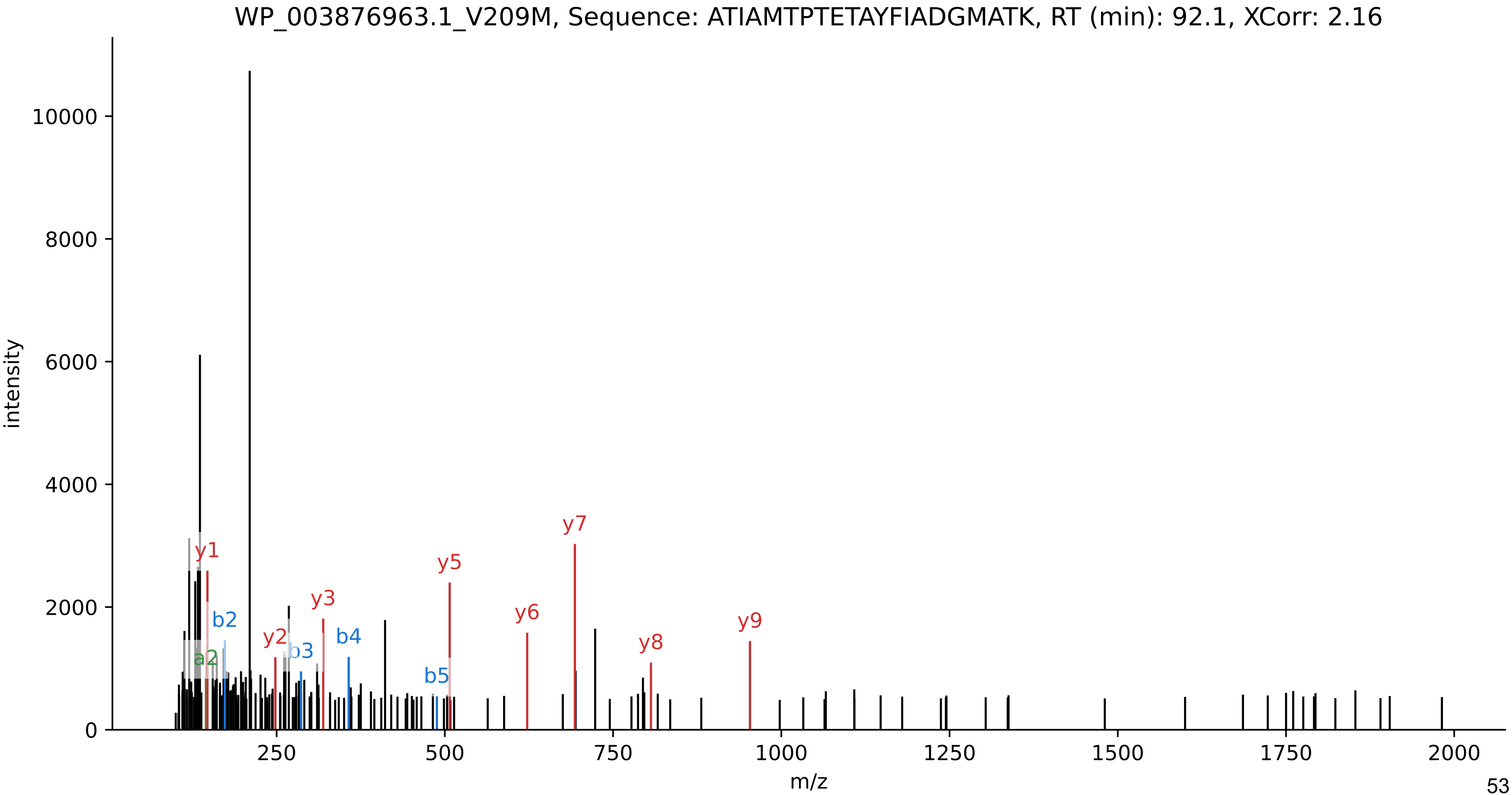
WP_003876963.1_V209M, Sequence: ATIAMTPTETAYFIADGMATK, RT (min): 92.1, XCorr: 2.16
intensity
m/z
y1
a2
b2
y2
b3
y3
b4
b5
y5
y6
y7
y8
y9
0
2000
4000
6000
8000
10000
250
500
750
1000
1250
1500
1750
2000

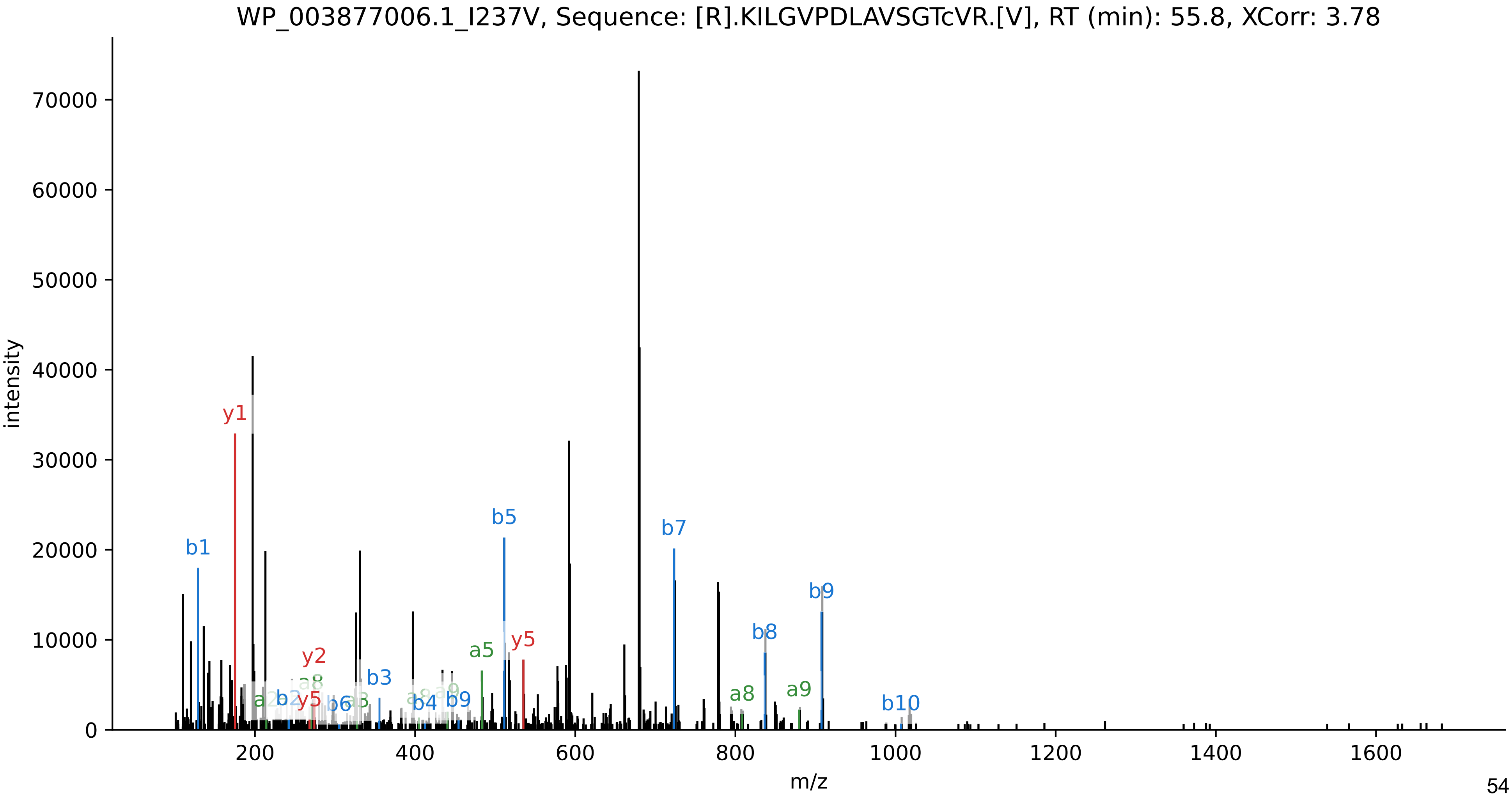

WP_003877006.1_I237V, Sequence: [R].KILGVPDLAVSGTcVR.[V], RT (min): 55.8, XCorr: 3.78
intensity
m/z
y1
b1
b5
b7
b9
b8
y5
a5
y2
b3
a8
a9
b10
b4
b9
b6
b2
a2

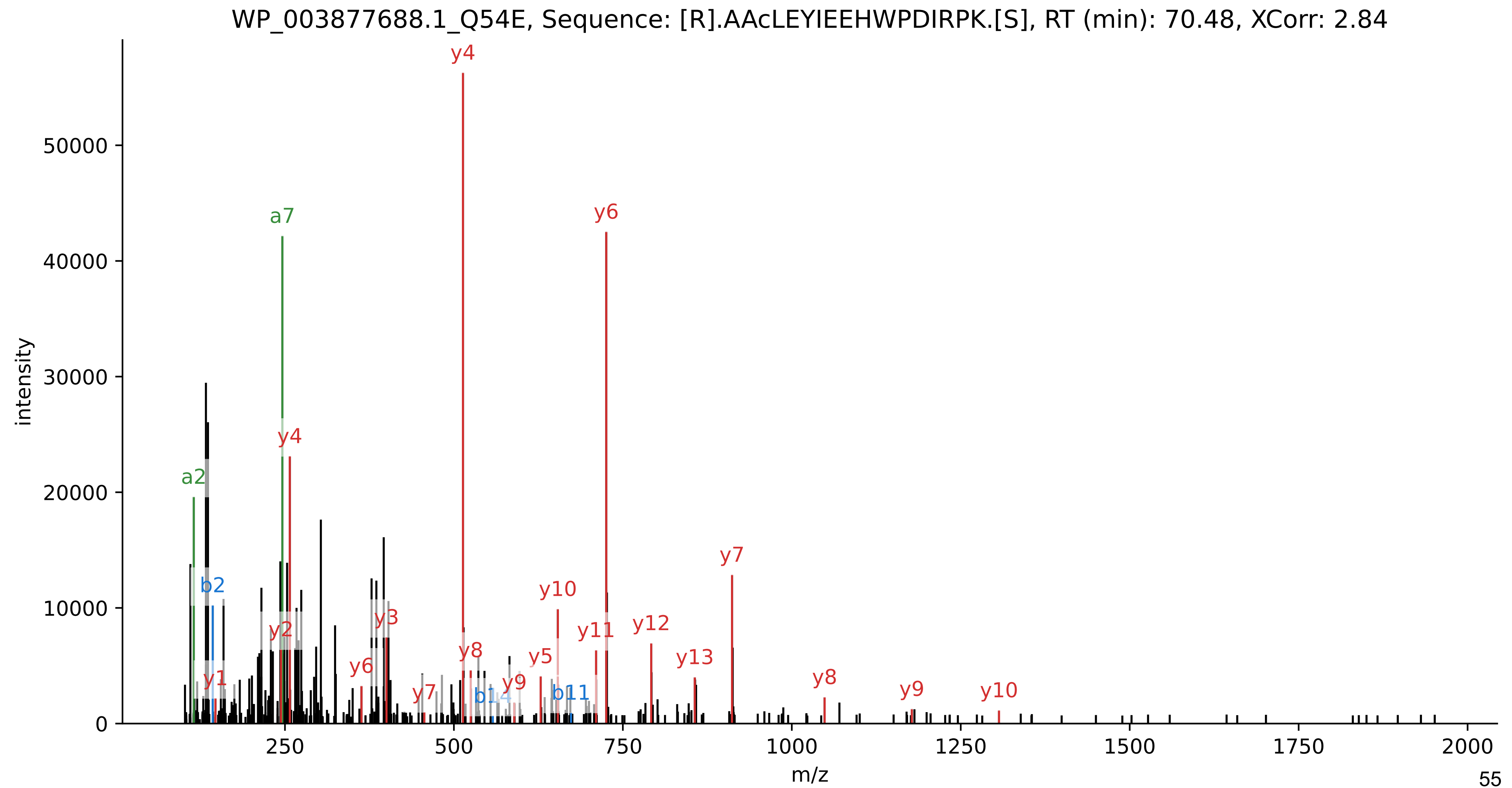

WP_003877688.1_Q54E, Sequence: [R].AAcLEYIEEHWPDIRPK.[S], RT (min): 70.48, XCorr: 2.84
intensity
m/z
y4
a7
y6
y4
a2
b2
y1
y2
y3
y6
y7
y8
b14
y9
y5
y10
b11
y11
y12
y13
y7
y8
y9
y10

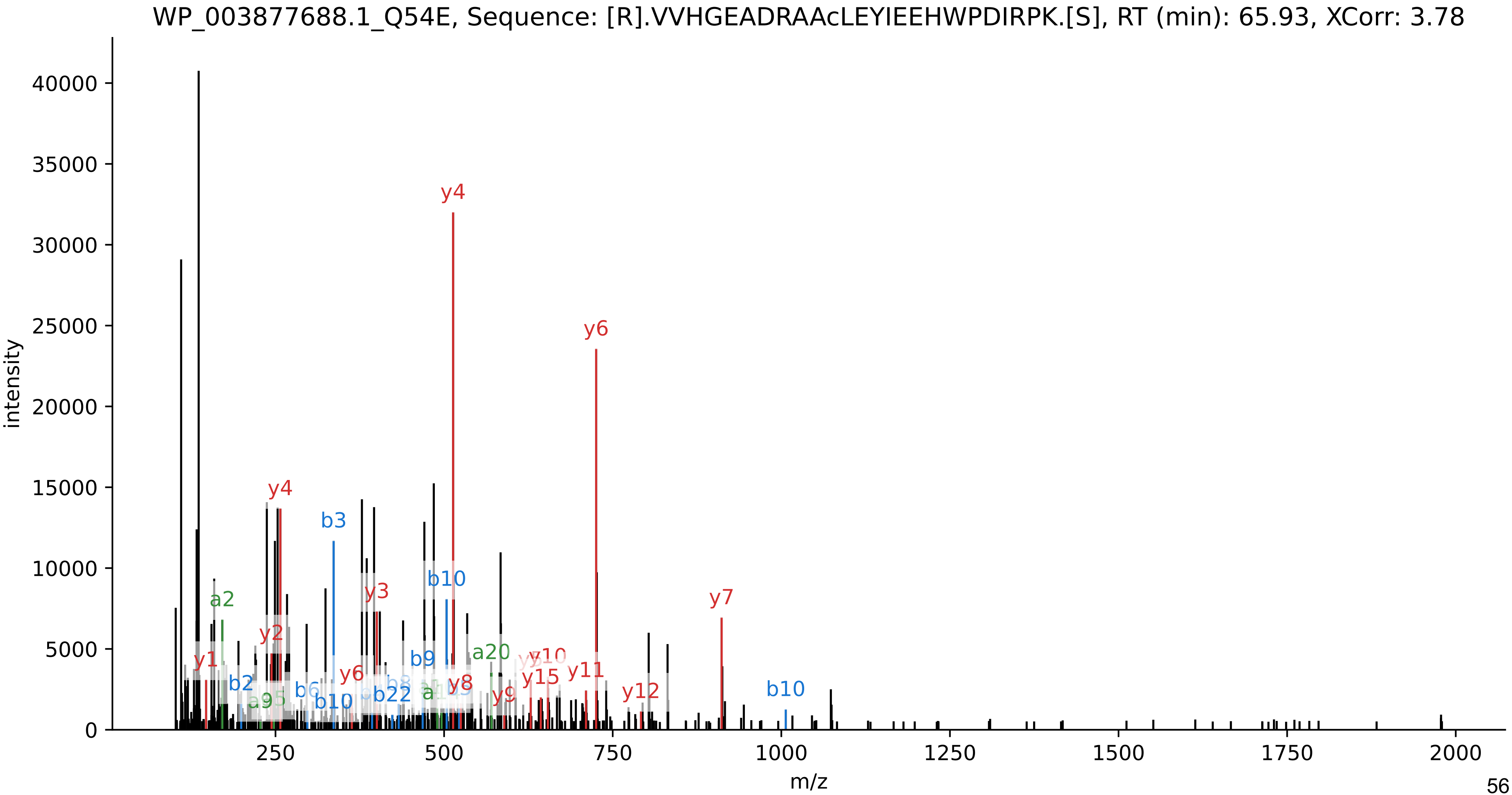
WP_003877688.1_Q54E, Sequence: [R].VVHGEADRAAcLEYIEEHWPDIRPK.[S], RT (min): 65.93, XCorr: 3.78
intensity
m/z
y4
y6
y4
b3
b10
y3
a2
y7
y2
a20
y1
b9
y10
y11
y15
b2
y6
y8
b10
y12
b6
b22
y9
b10
a9
0
5000
10000
15000
20000
25000
30000
35000
40000
250
500
750
1000
1250
1500
1750
2000

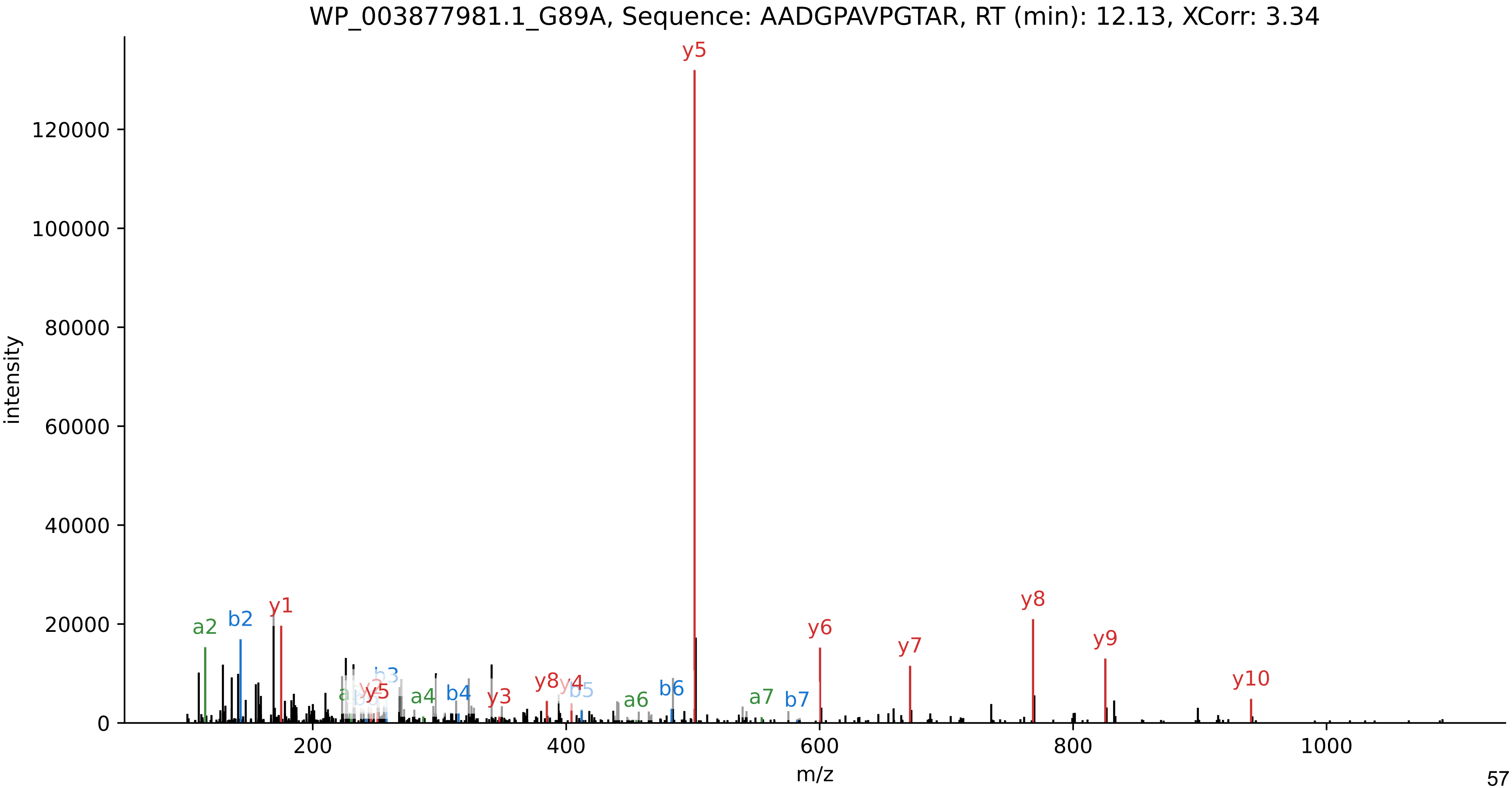
WP_003877981.1_G89A, Sequence: AADGPAVPGTAR, RT (min): 12.13, XCorr: 3.34
intensity
m/z
y5
y1
a2
b2
y6
y7
y8
y9
y10
b3
a4
b4
y3
y8
y4
b5
a6
b6
a7
b7
0
20000
40000
60000
80000
100000
120000
200
400
600
800
1000

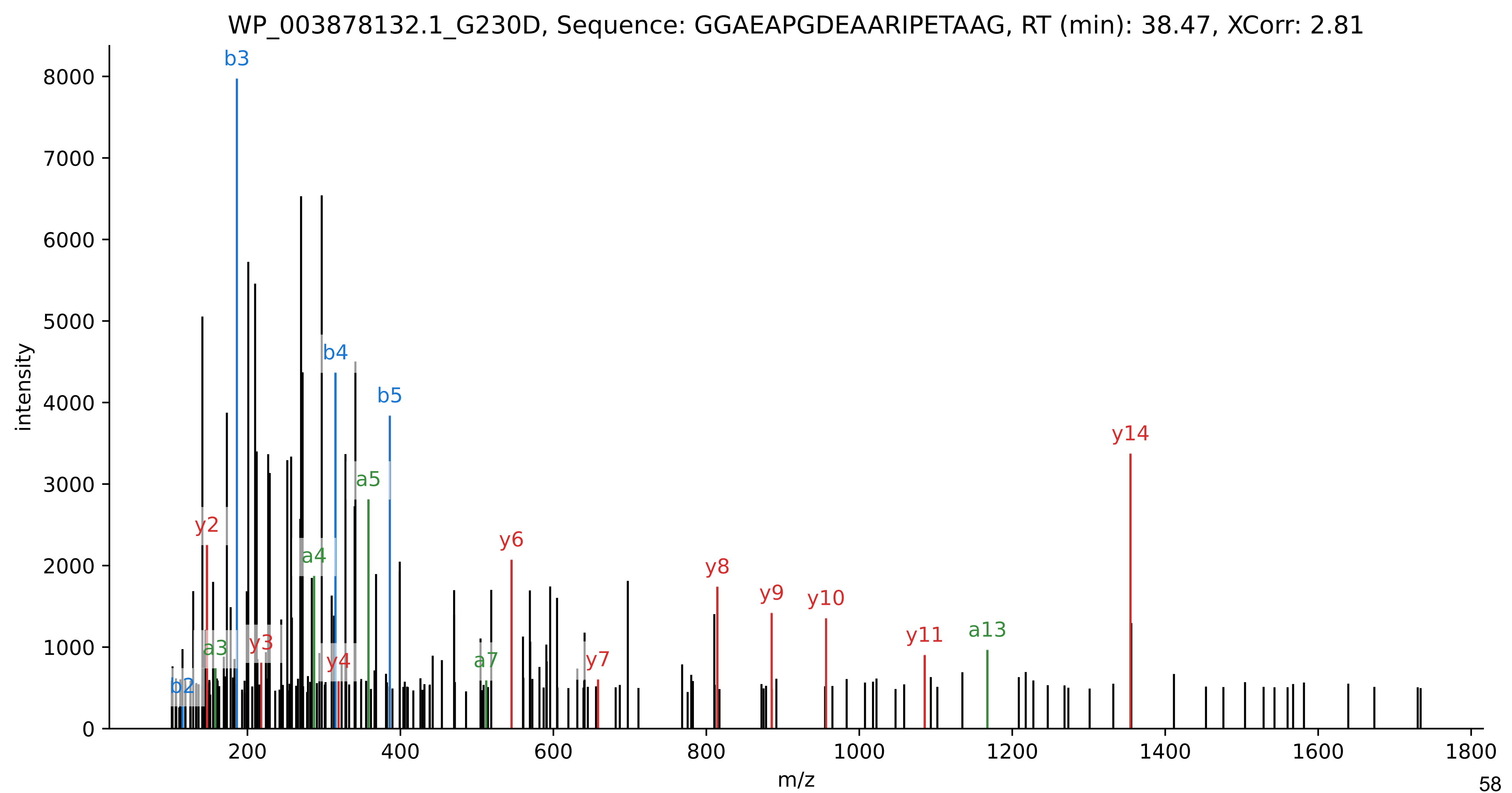
WP_003878132.1_G230D, Sequence: GGAEAPGDEAARIPETAAG, RT (min): 38.47, XCorr: 2.81
intensity
m/z
b2
b3
b4
b5
a3
a4
a5
a7
a13
y2
y3
y4
y6
y7
y8
y9
y10
y11
y14

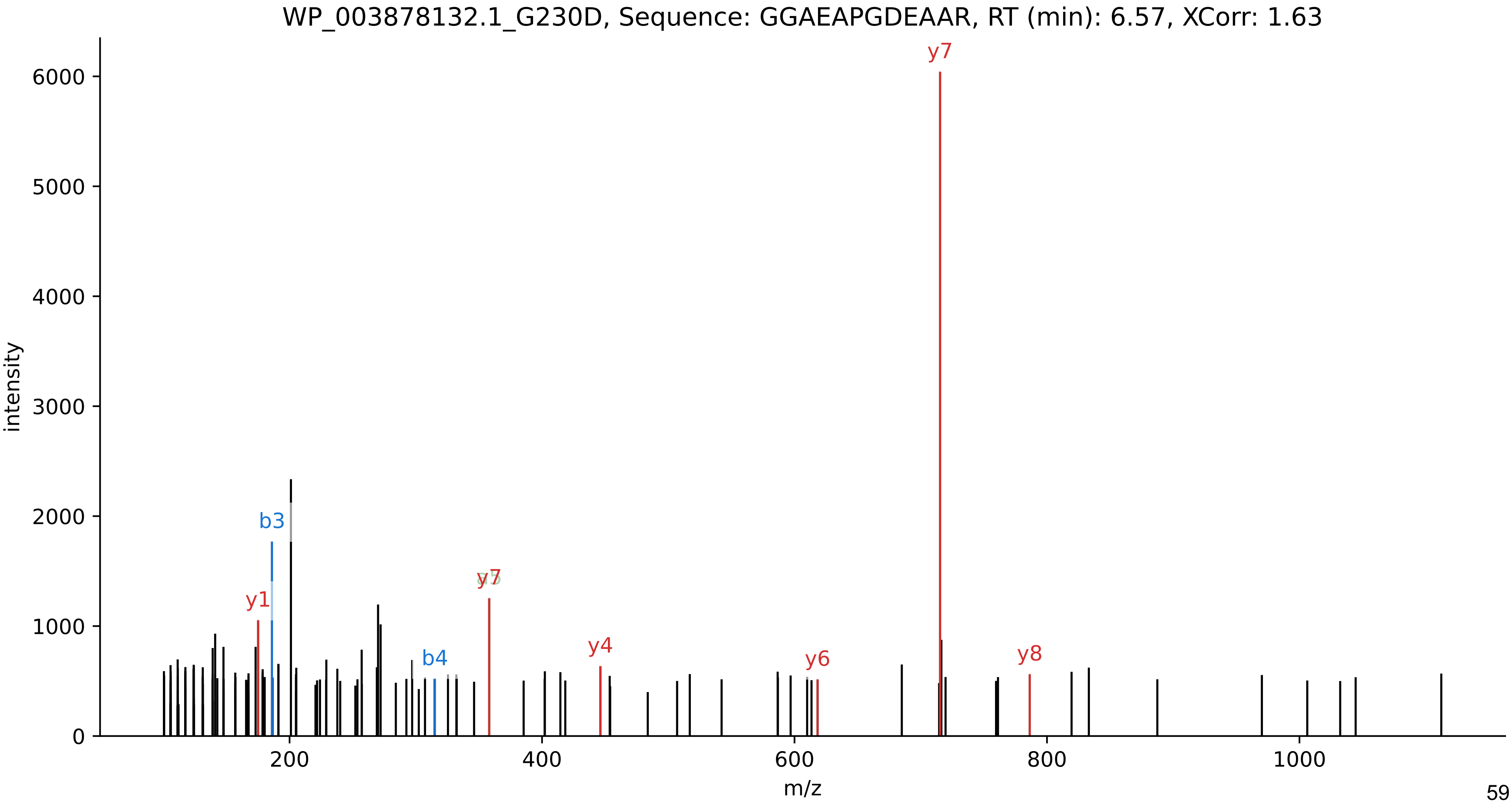
WP_003878132.1_G230D, Sequence: GGAEAPGDEAAR, RT (min): 6.57, XCorr: 1.63
y7
b3
y1
y7
b4
y4
y6
y8
intensity
m/z
0
1000
2000
3000
4000
5000
6000
200
400
600
800
1000

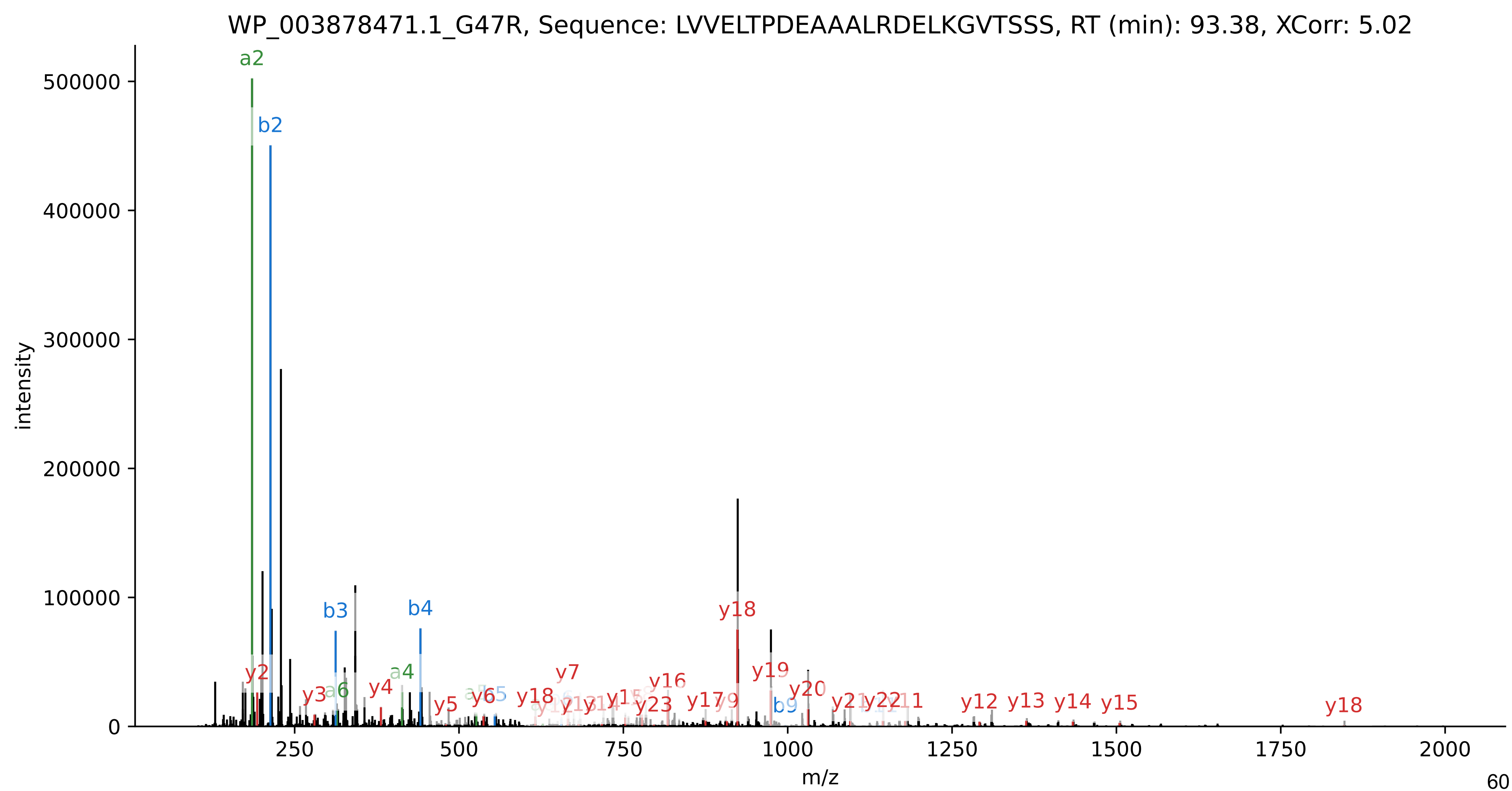
WP_003878471.1_G47R, Sequence: LVVELTPDEAAALRDELKGVTSSS, RT (min): 93.38, XCorr: 5.02
intensity
m/z
a2
b2
b3
b4
a4
a6
y2
y3
y4
y5
y6
y7
y16
y18
y19
y20
y23
y17
b9
y12
y13
y14
y15
y18

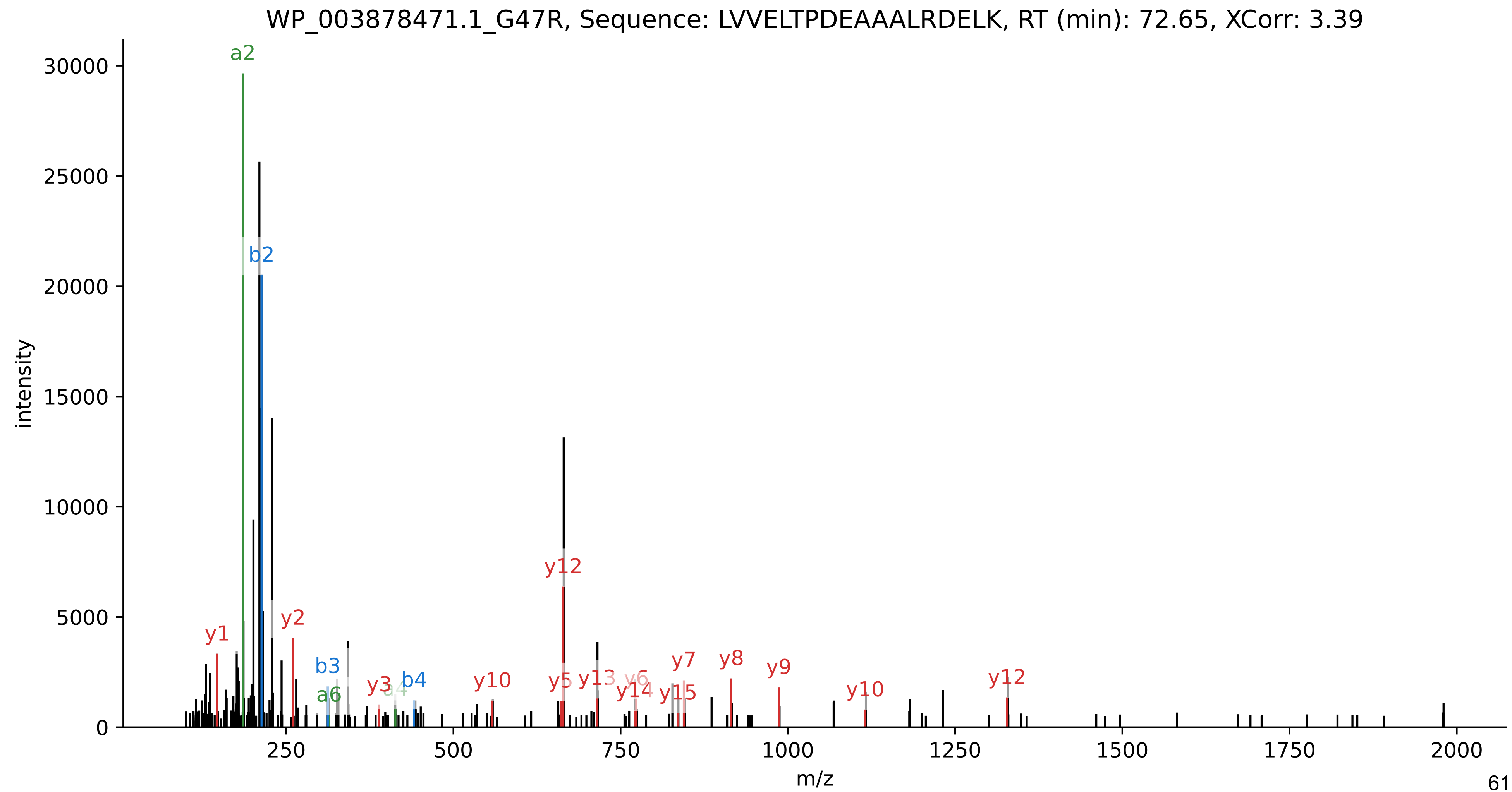
WP_003878471.1_G47R, Sequence: LVVELTPDEAAALRDELK, RT (min): 72.65, XCorr: 3.39
intensity
m/z
a2
b2
y1
y2
b3
a6
y3
a4
b4
y10
y12
y5
y13
y6
y14
y7
y15
y8
y9
y10
y12
0
5000
10000
15000
20000
25000
30000
250
500
750
1000
1250
1500
1750
2000

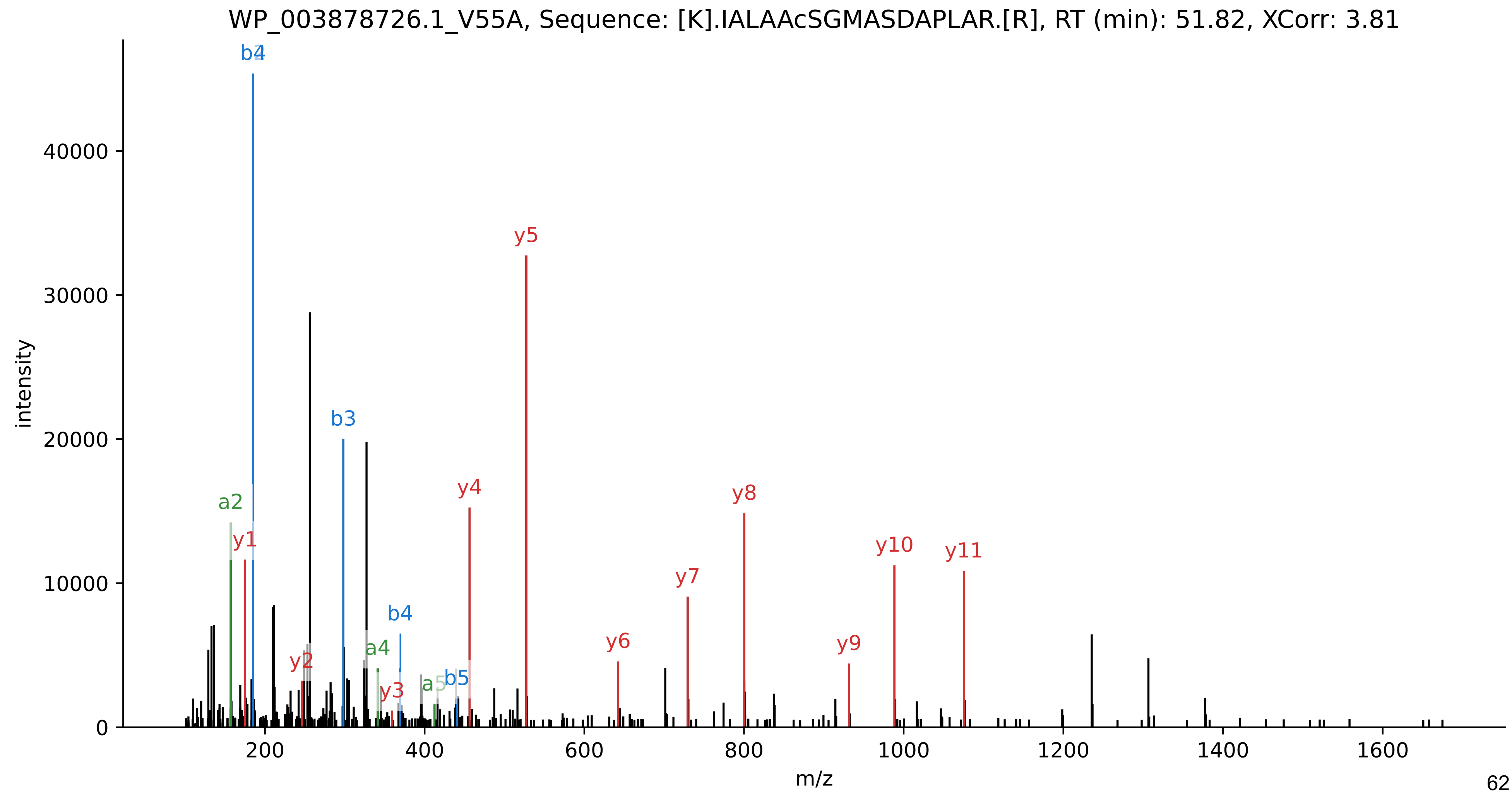

WP_003878726.1_V55A, Sequence: [K].IALAAcSGMASDAPLAR.[R], RT (min): 51.82, XCorr: 3.81
intensity
m/z
b4
y5
b3
y4
y8
a2
y1
y10
y11
y7
b4
y6
y9
a4
y2
b5
a5
y3

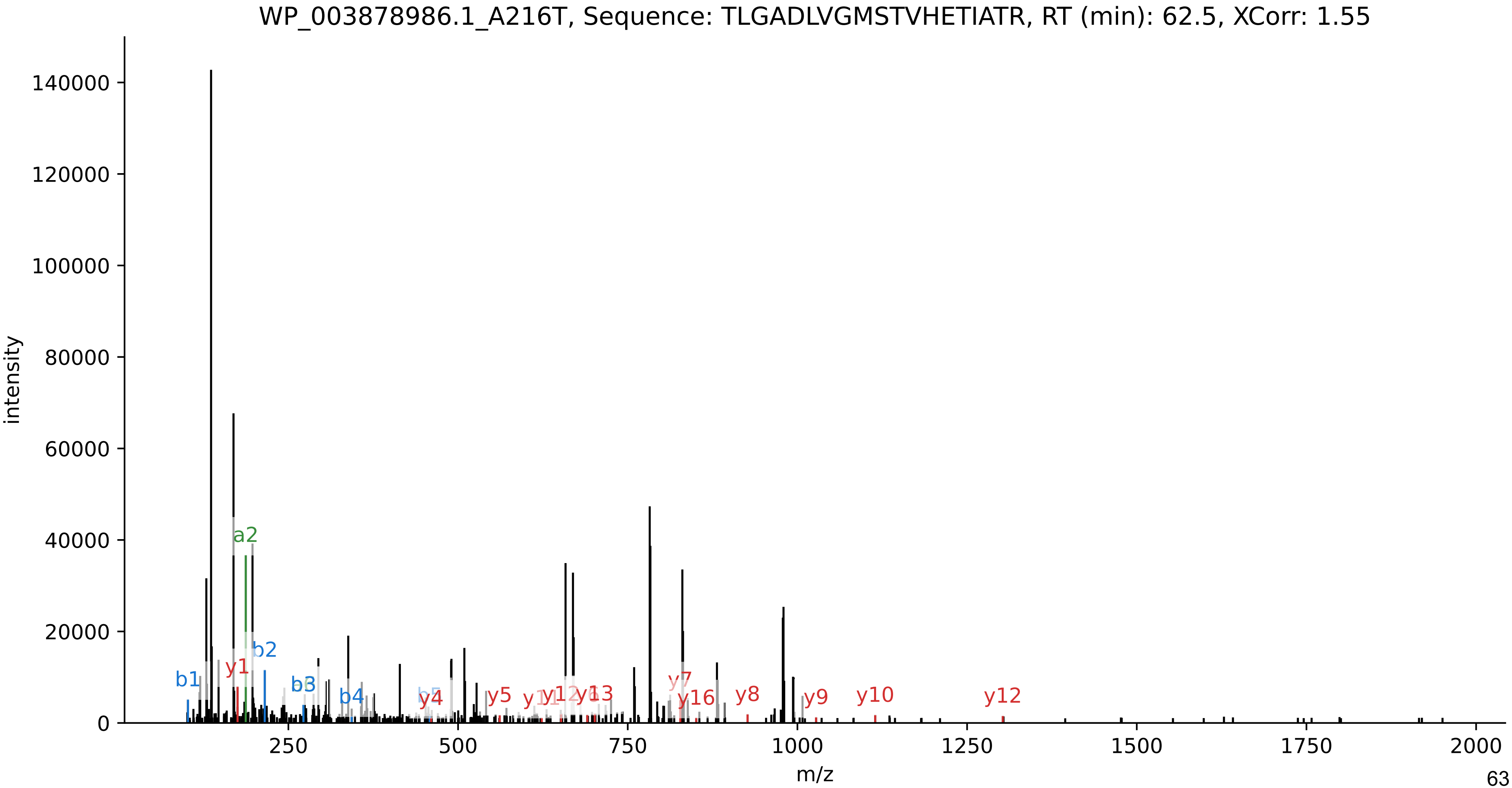
WP_003878986.1_A216T, Sequence: TLGADLVGMSTVHETIATR, RT (min): 62.5, XCorr: 1.55
intensity
m/z
0
20000
40000
60000
80000
100000
120000
140000
250
500
750
1000
1250
1500
1750
2000
b1
y1
a2
b2
b3
b4
y4
y5
y7
y16
y8
y9
y10
y12

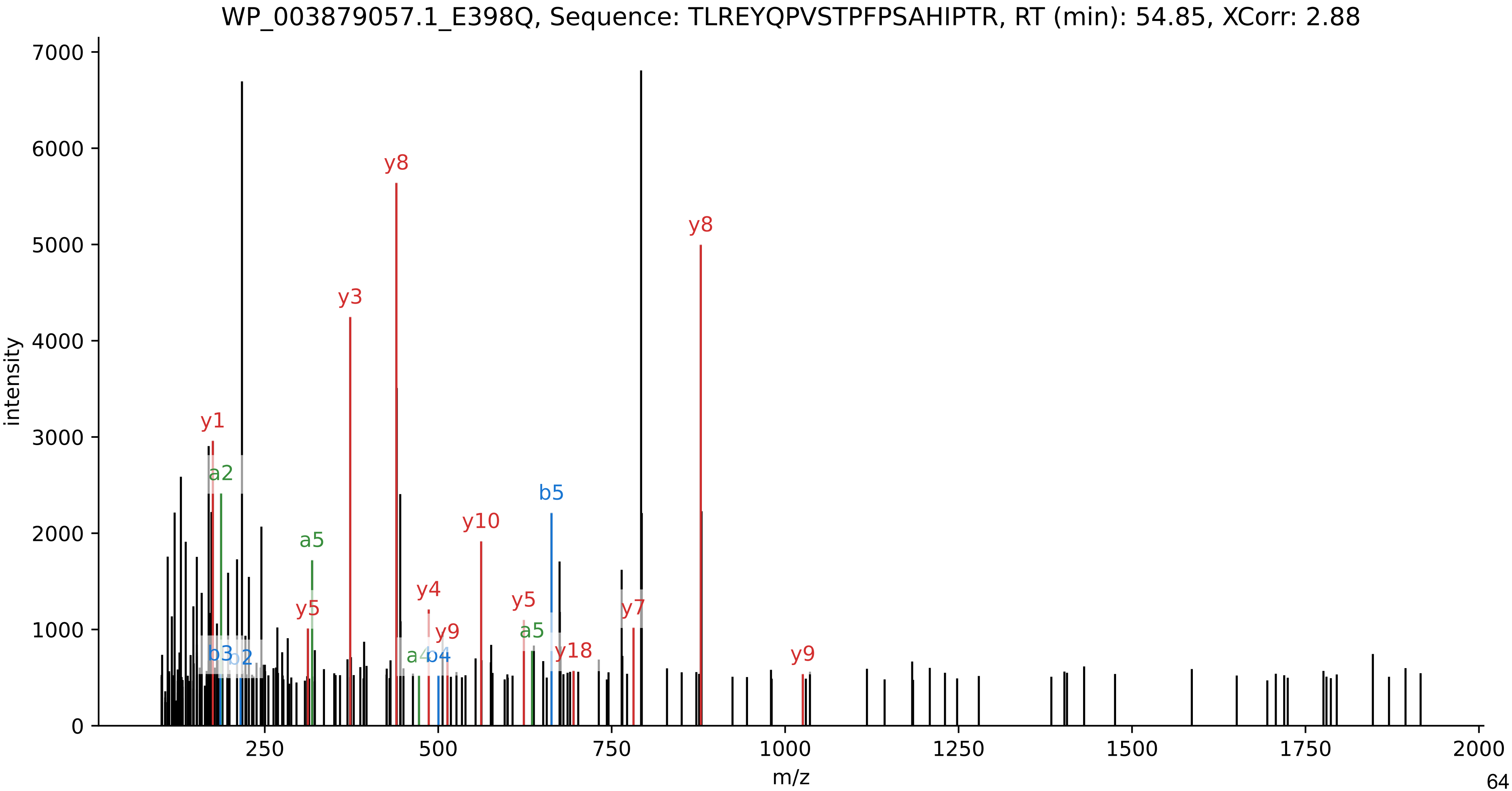
WP_003879057.1_E398Q, Sequence: TLREYQPVSTPFPSAHIPTR, RT (min): 54.85, XCorr: 2.88
intensity
m/z
0
1000
2000
3000
4000
5000
6000
7000
250
500
750
1000
1250
1500
1750
2000
y1
a2
b3
b2
a5
y5
y3
y8
y4
y9
a4
b4
y10
b5
y5
a5
y18
y7
y8
y9

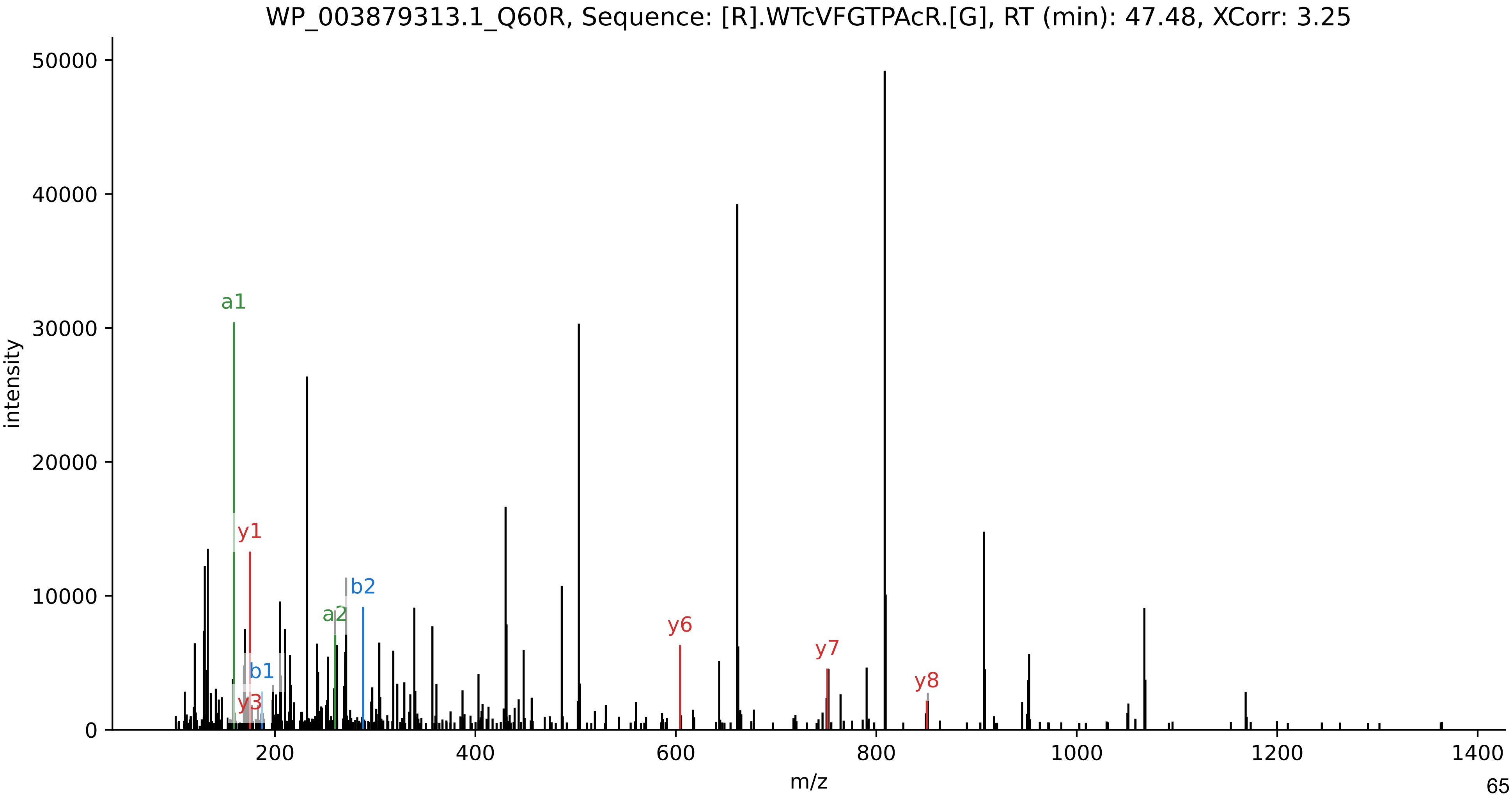

WP_003879313.1_Q60R, Sequence: [R].WTcVFGTPAcR.[G], RT (min): 47.48, XCorr: 3.25
intensity
m/z
a1
y1
b1
y3
a2
b2
y6
y7
y8
0
10000
20000
30000
40000
50000
200
400
600
800
1000
1200
1400

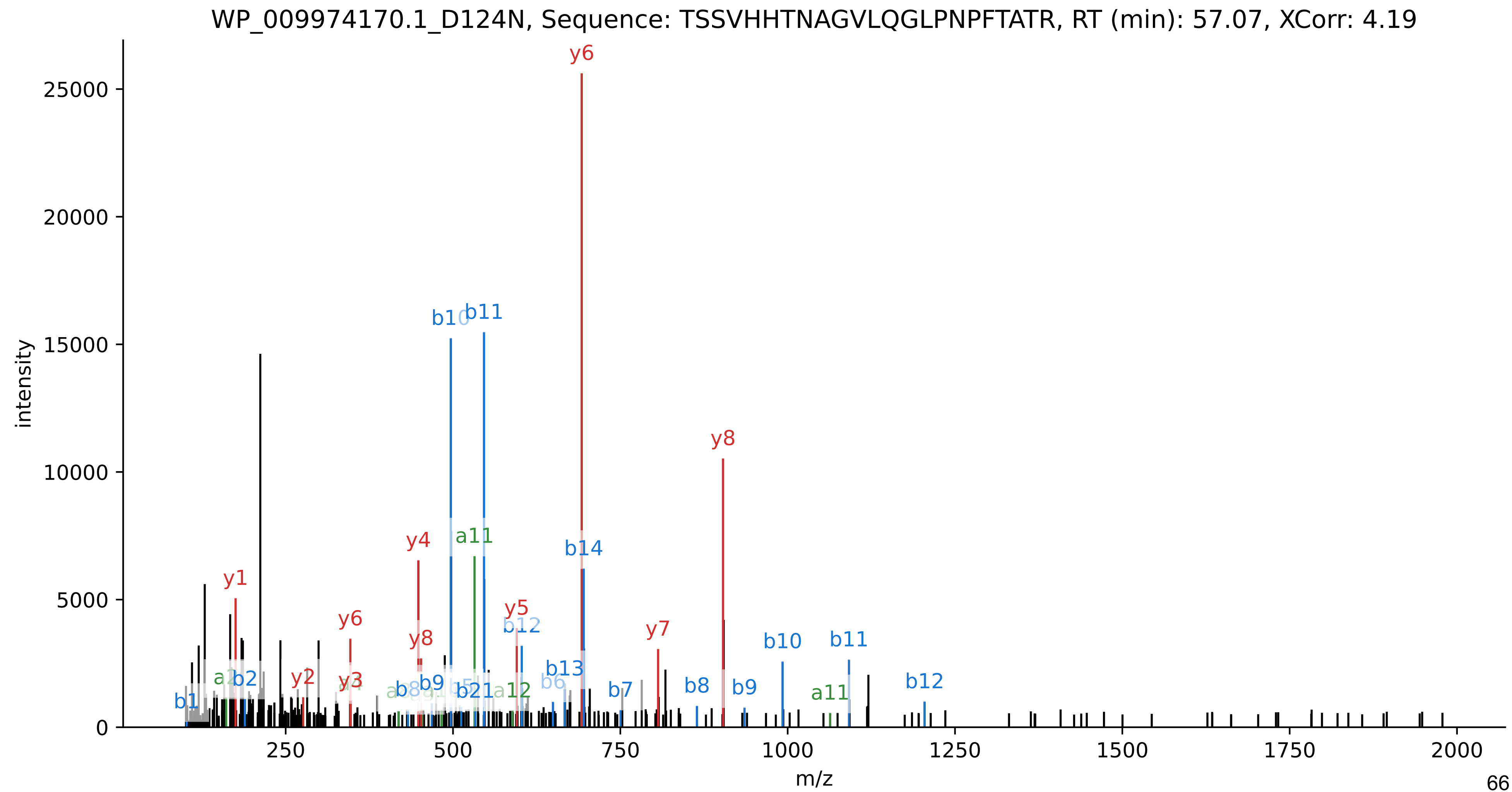

WP_009974170.1_D124N, Sequence: TSSVHHTNAGVLQGLPNPFTATR, RT (min): 57.07, XCorr: 4.19
intensity
m/z
y6
b10
b11
y8
y4
a11
b14
y1
y5
b12
y6
y8
y7
b10
b11
y2
y3
b13
a1
b2
b1
b9
b8
b21
a12
b6
b7
b8
b9
a11
b12

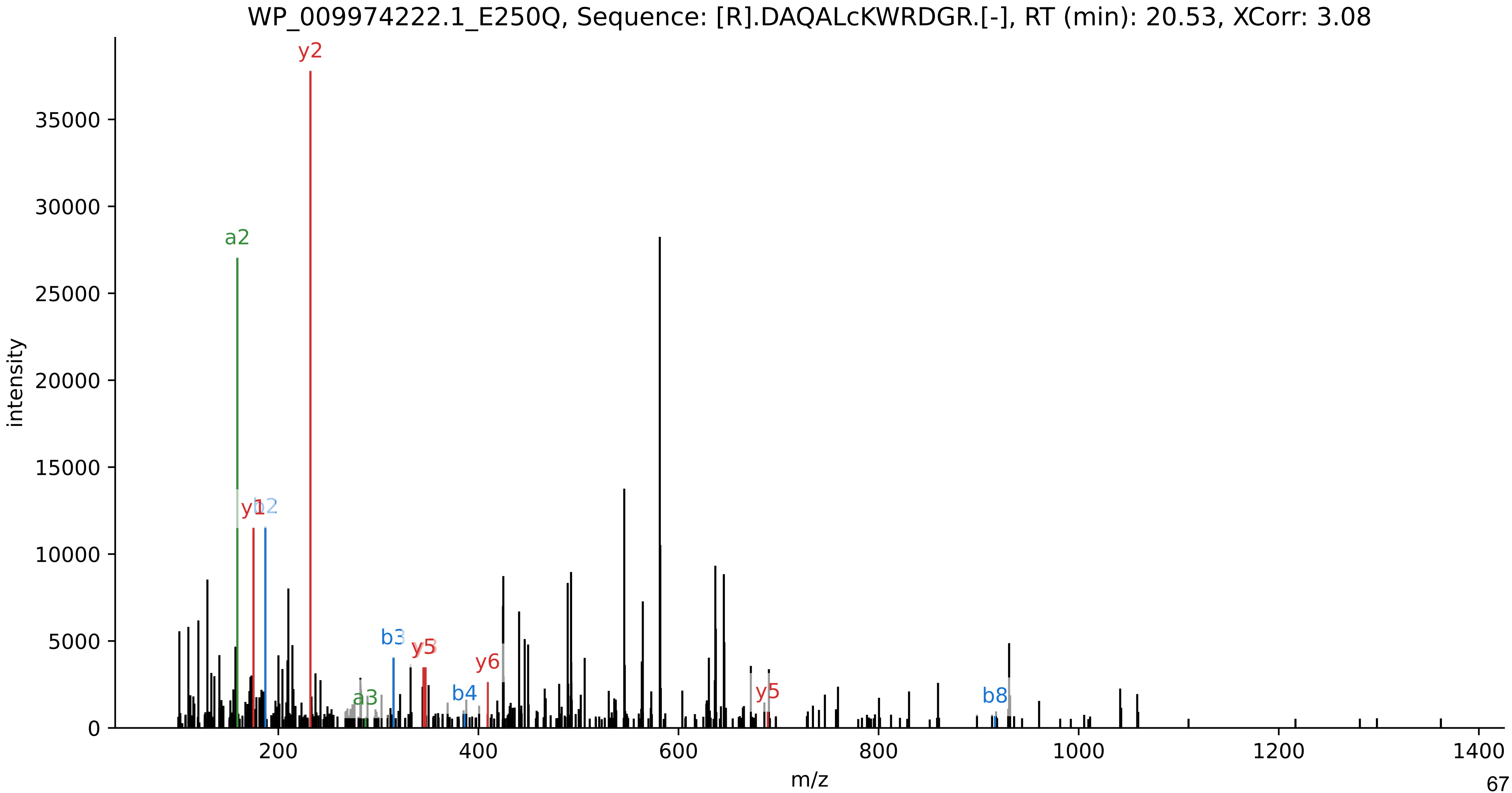
WP_009974222.1_E250Q, Sequence: [R].DAQALcKWRDGR.[-], RT (min): 20.53, XCorr: 3.08
intensity
m/z
y2
a2
y1
b2
b3
y5
y6
b4
a3
y5
b8
0
5000
10000
15000
20000
25000
30000
35000
200
400
600
800
1000
1200
1400

WP_009974331.1_R112P, Sequence: NQQAHVQRPLDNHPSPR, RT (min): 6.53, XCorr: 4.7

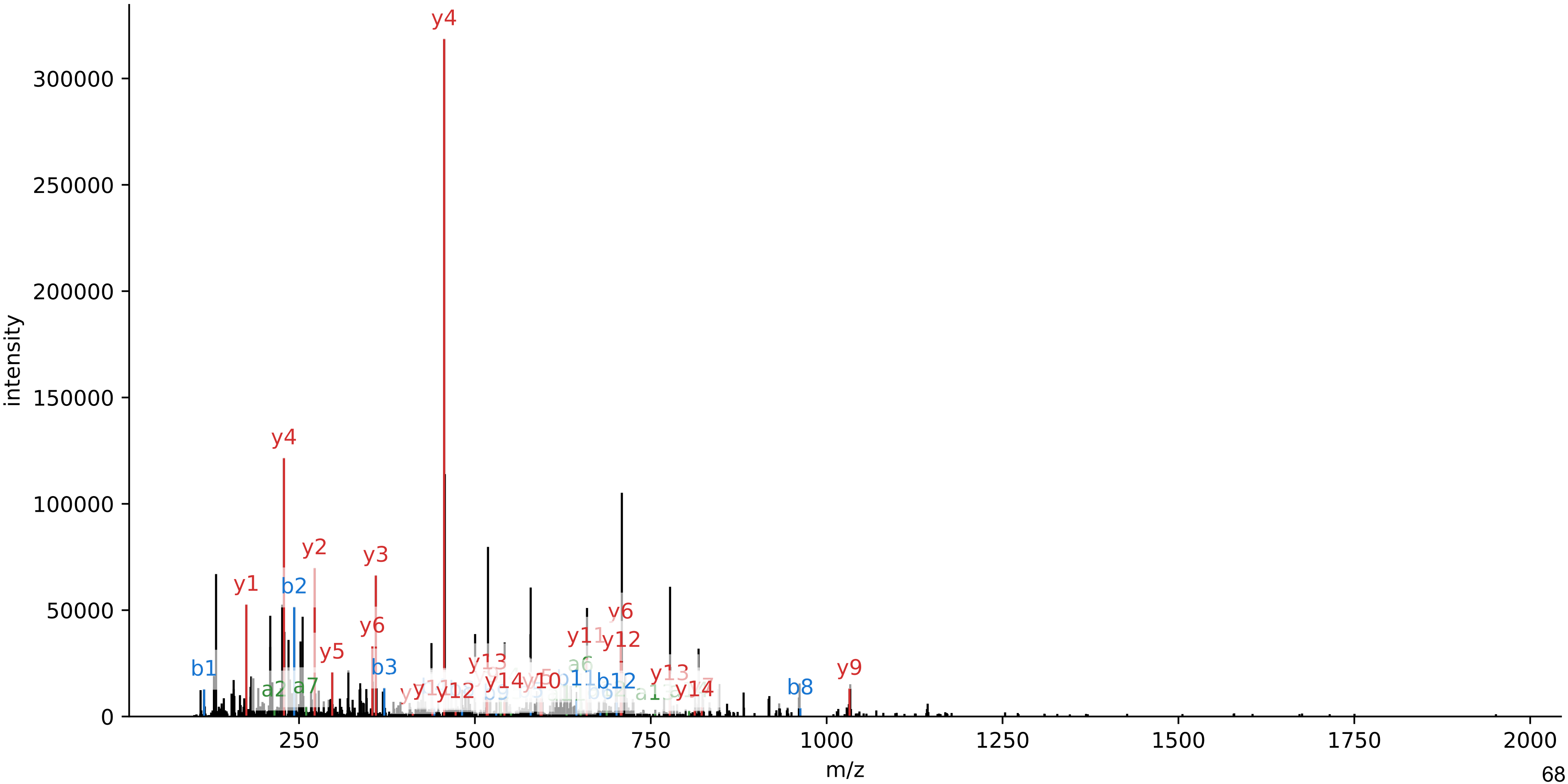

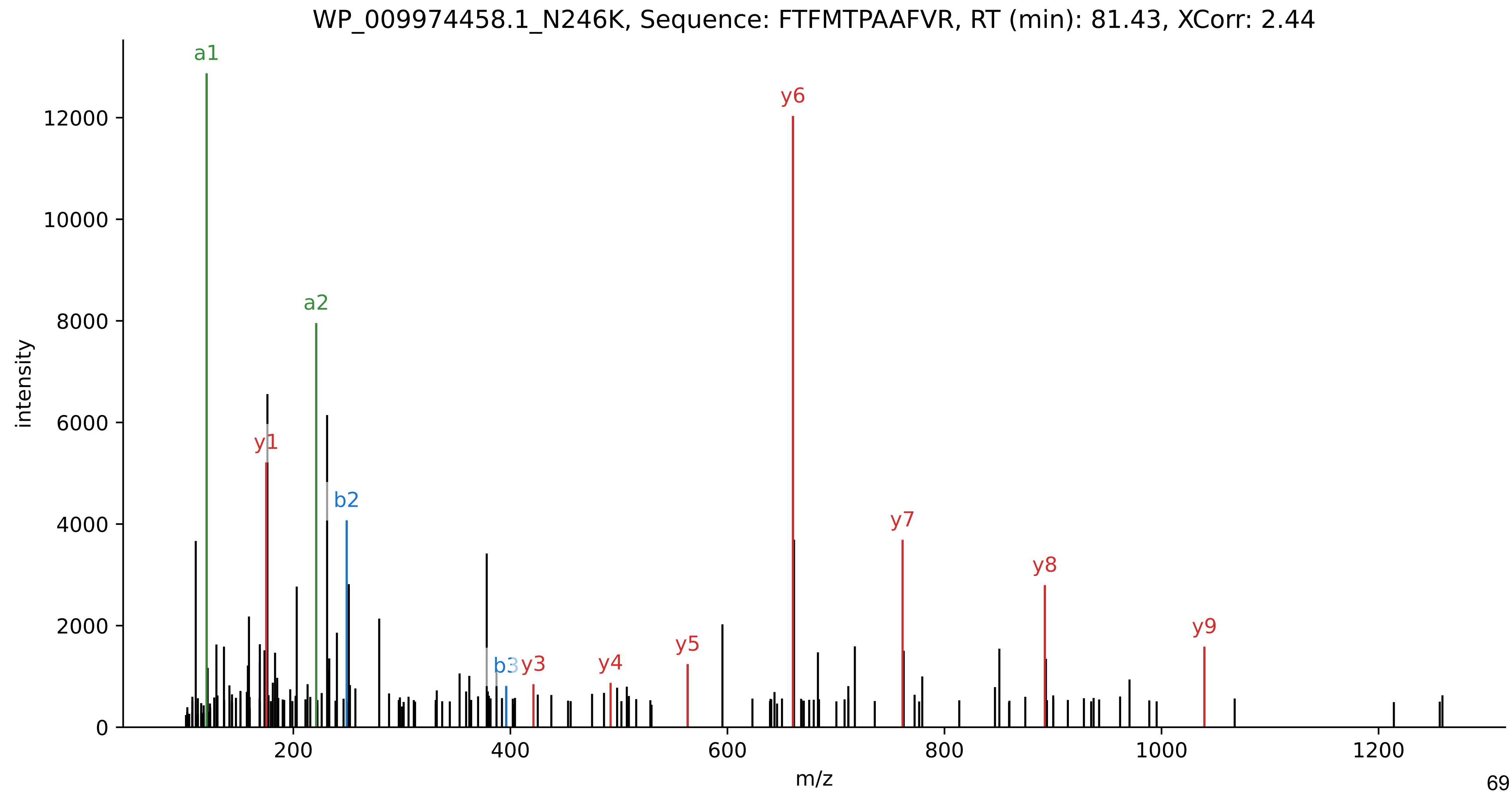

WP_009974458.1_N246K, Sequence: FTFMTPAAFVR, RT (min): 81.43, XCorr: 2.44
intensity
m/z
a1
a2
y1
b2
b3
y3
y4
y5
y6
y7
y8
y9
0
2000
4000
6000
8000
10000
12000
200
400
600
800
1000
1200

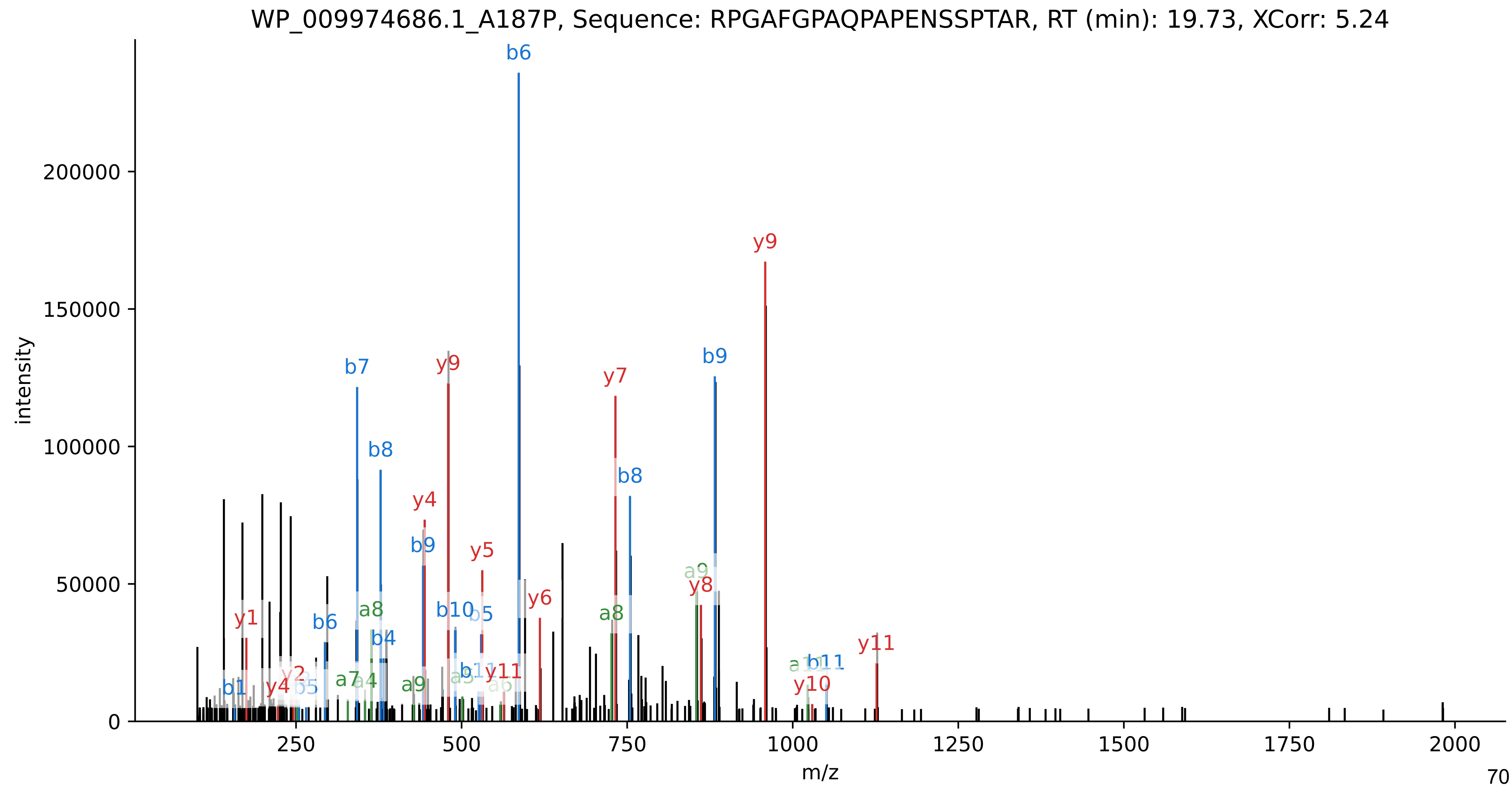
WP_009974686.1_A187P, Sequence: RPGAFGPAQPAPENSSPTAR, RT (min): 19.73, XCorr: 5.24
intensity
m/z

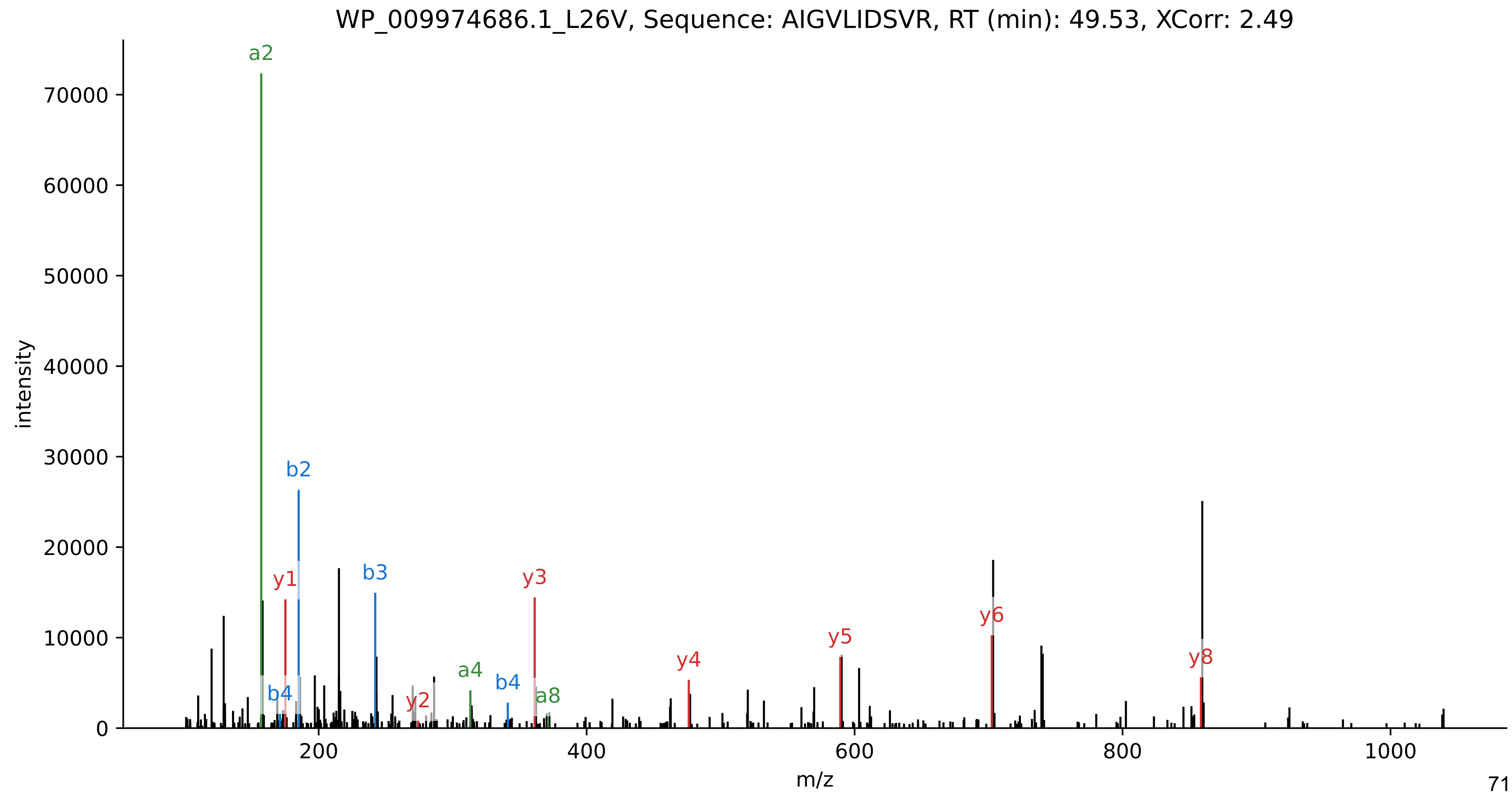
WP_009974686.1_L26V, Sequence: AIGVLIDSVR, RT (min): 49.53, XCorr: 2.49
intensity
m/z
a2
b2
b3
y1
y3
b4
y2
a4
b4
a8
y4
y5
y6
y8
0
10000
20000
30000
40000
50000
60000
70000
200
400
600
800
1000

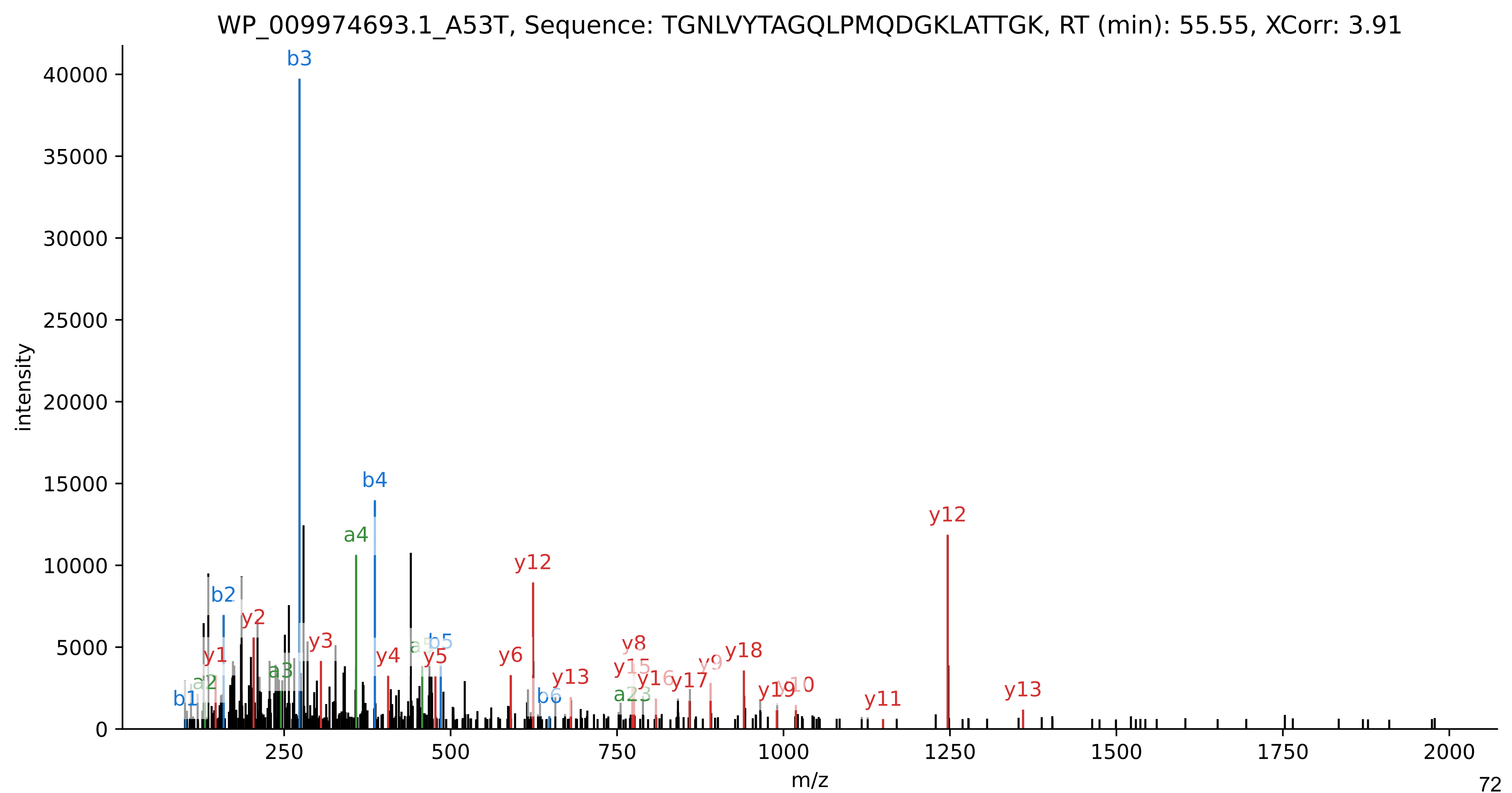
WP_009974693.1_A53T, Sequence: TGNLVYTAGQLPMQDGKLATTGK, RT (min): 55.55, XCorr: 3.91
intensity
m/z
b3
b4
y12
a4
y12
b2
y2
y1
y3
y4
y5
b5
y6
y13
y8
y15
y16
y17
y9
y18
y19
y10
y11
y13
a2
a3
b1
b6
a23

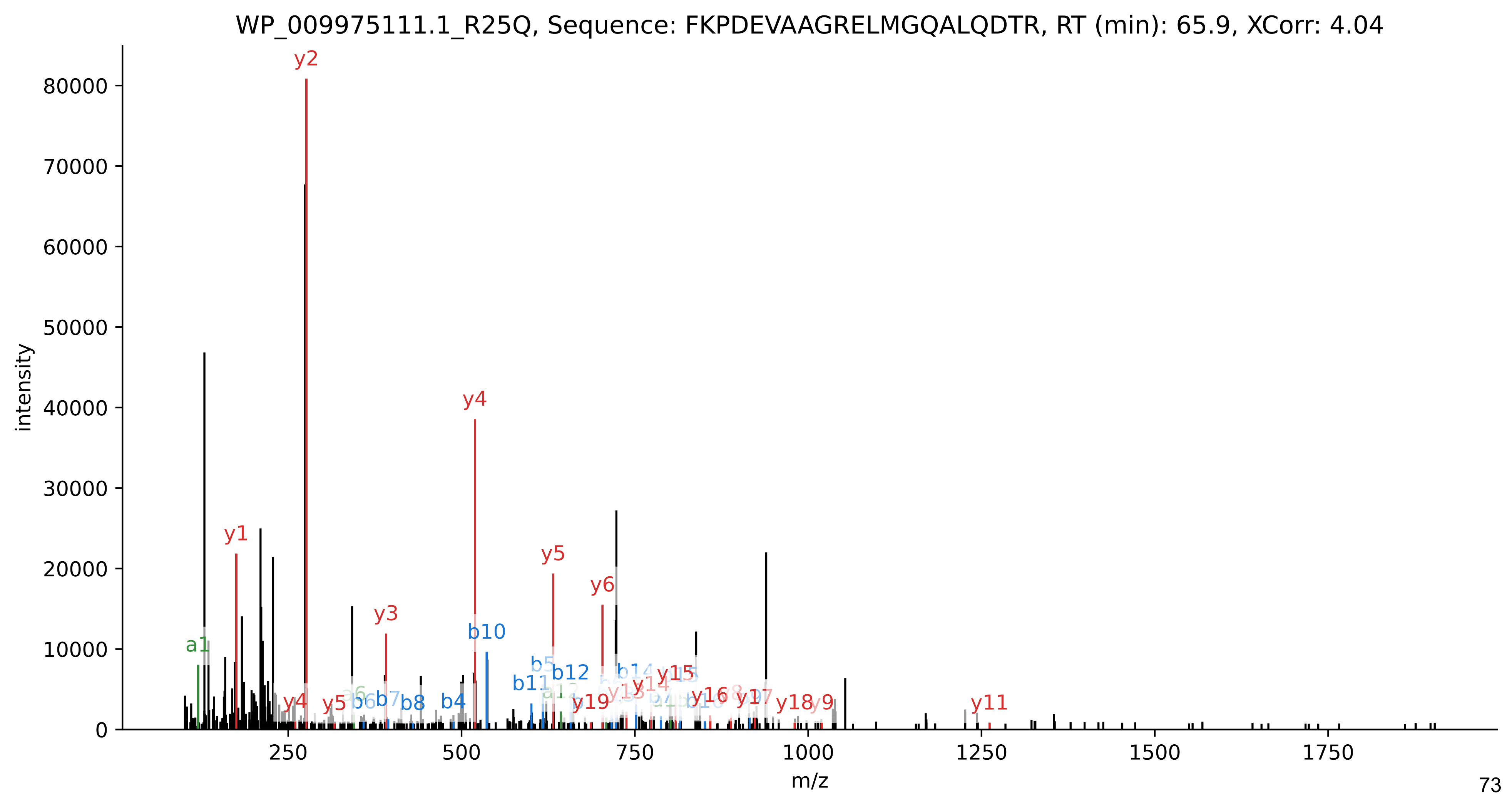
WP_009975111.1_R25Q, Sequence: FKPDEVAAGRELMGQALQDTR, RT (min): 65.9, XCorr: 4.04
intensity
m/z
y2
y4
y1
y5
y6
y3
b10
a1
b5
b12
b11
b14
y15
y14
y19
y16
y17
y18
y9
y11
y4
y5
b6
b7
b8
b4

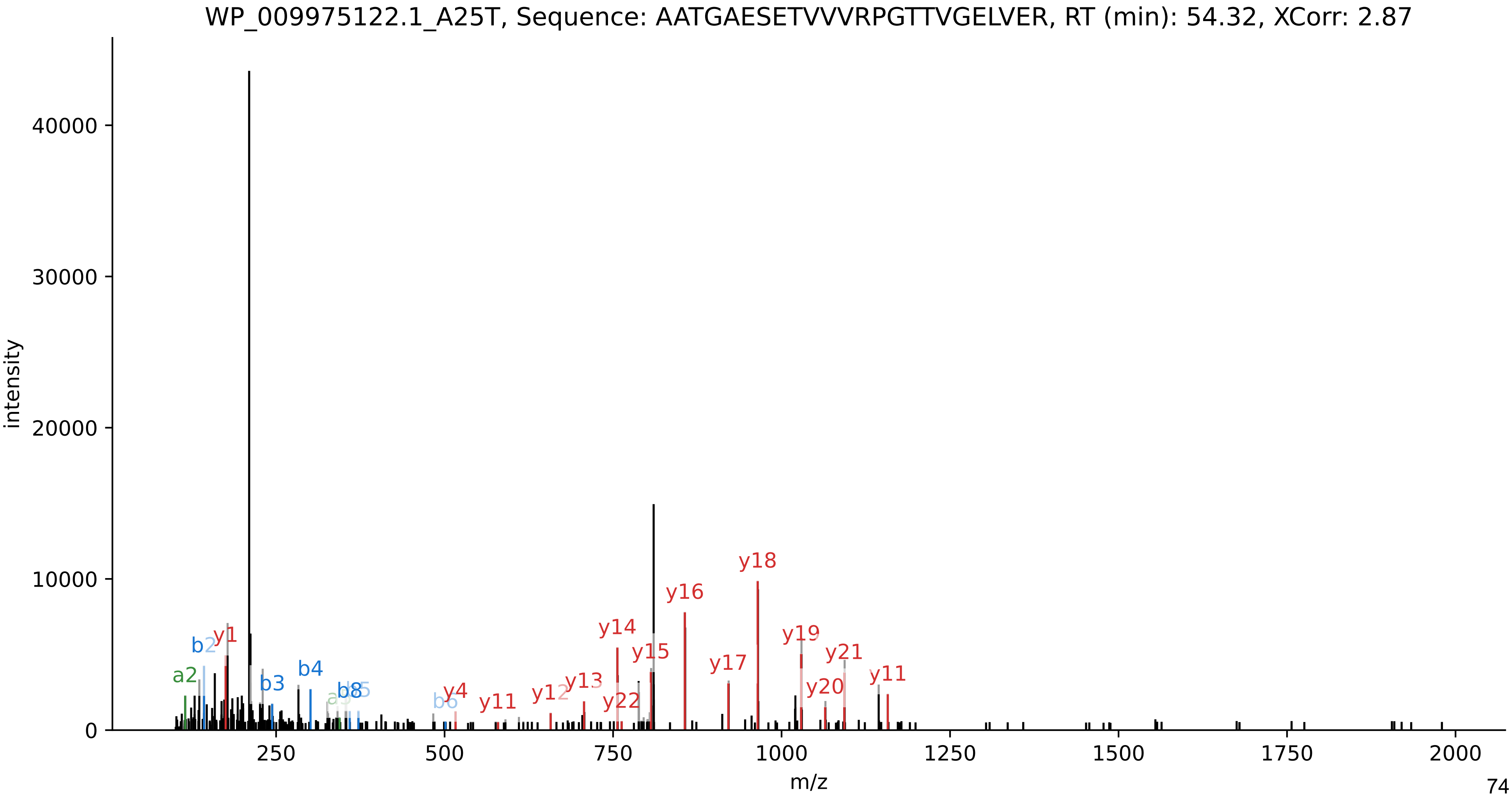
WP_009975122.1_A25T, Sequence: AATGAESETVVVRPGTTVGELVER, RT (min): 54.32, XCorr: 2.87
intensity
m/z
0
10000
20000
30000
40000
250
500
750
1000
1250
1500
1750
2000
a2
b2
y1
b3
b4
b8
b6
y4
y11
y12
y13
y14
y22
y15
y16
y17
y18
y19
y20
y21
y11

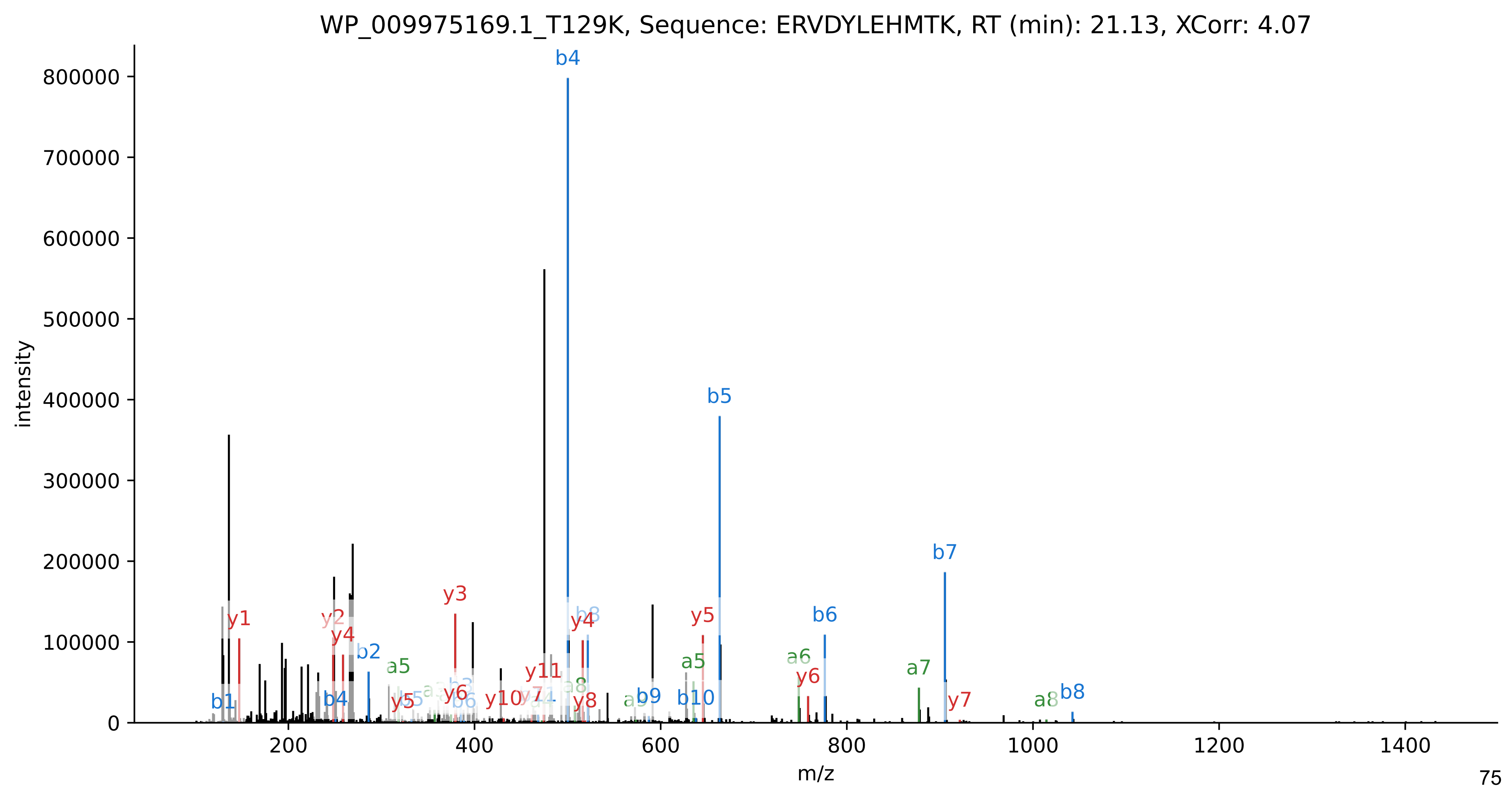
WP_009975169.1_T129K, Sequence: ERVDYLEHMTK, RT (min): 21.13, XCorr: 4.07
intensity
m/z
b4
b5
b7
b6
y3
y1
y2
y4
y5
b2
a5
a6
y6
a7
y7
b8
a8
b1
b9
b10
y8
y10
y11

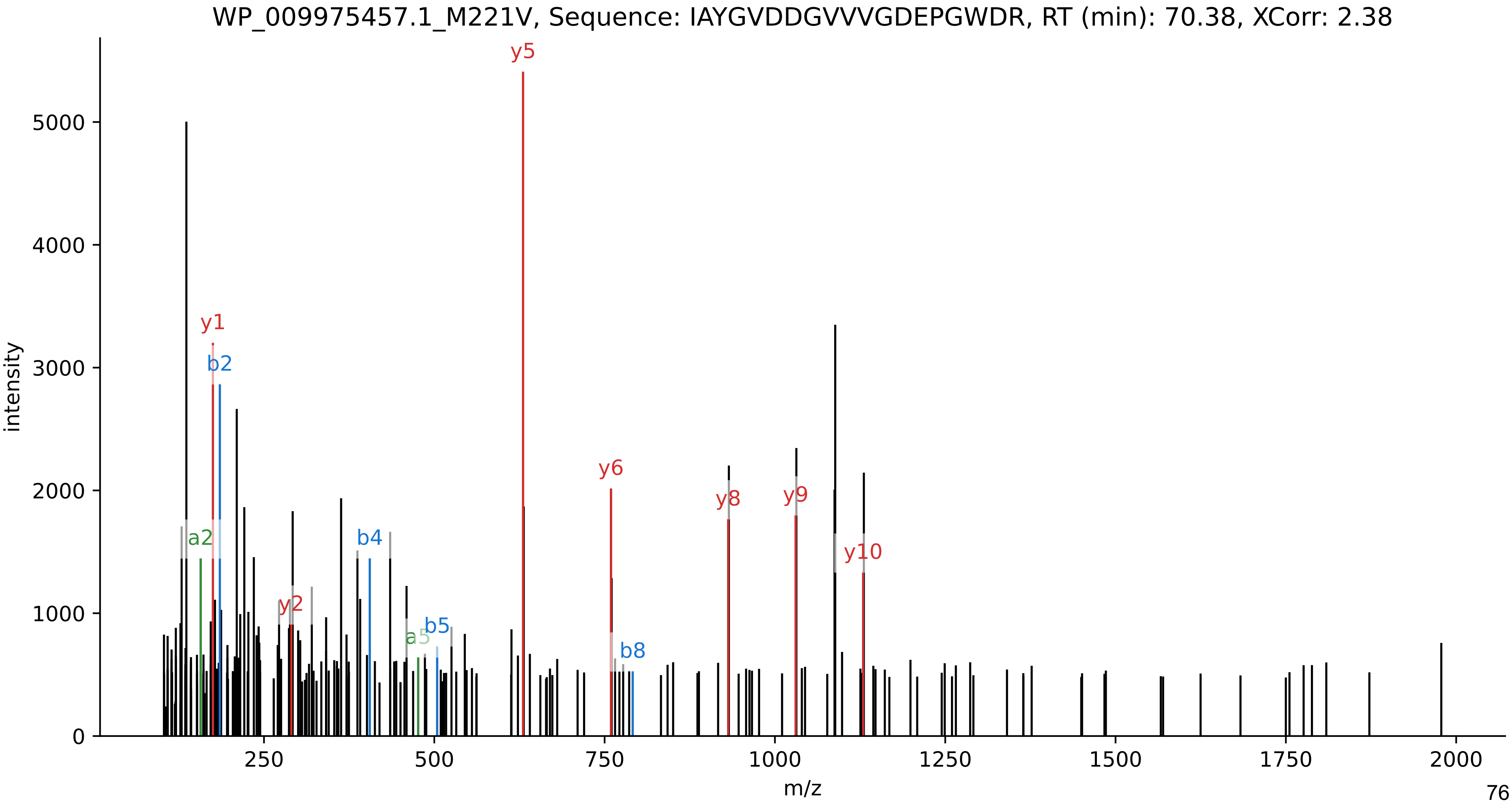

WP_009975457.1_M221V, Sequence: IAYGVDDGVVVGDEPGWDR, RT (min): 70.38, XCorr: 2.38
intensity
m/z
y5
y1
b2
a2
y2
b4
a5
b5
y6
b8
y8
y9
y10

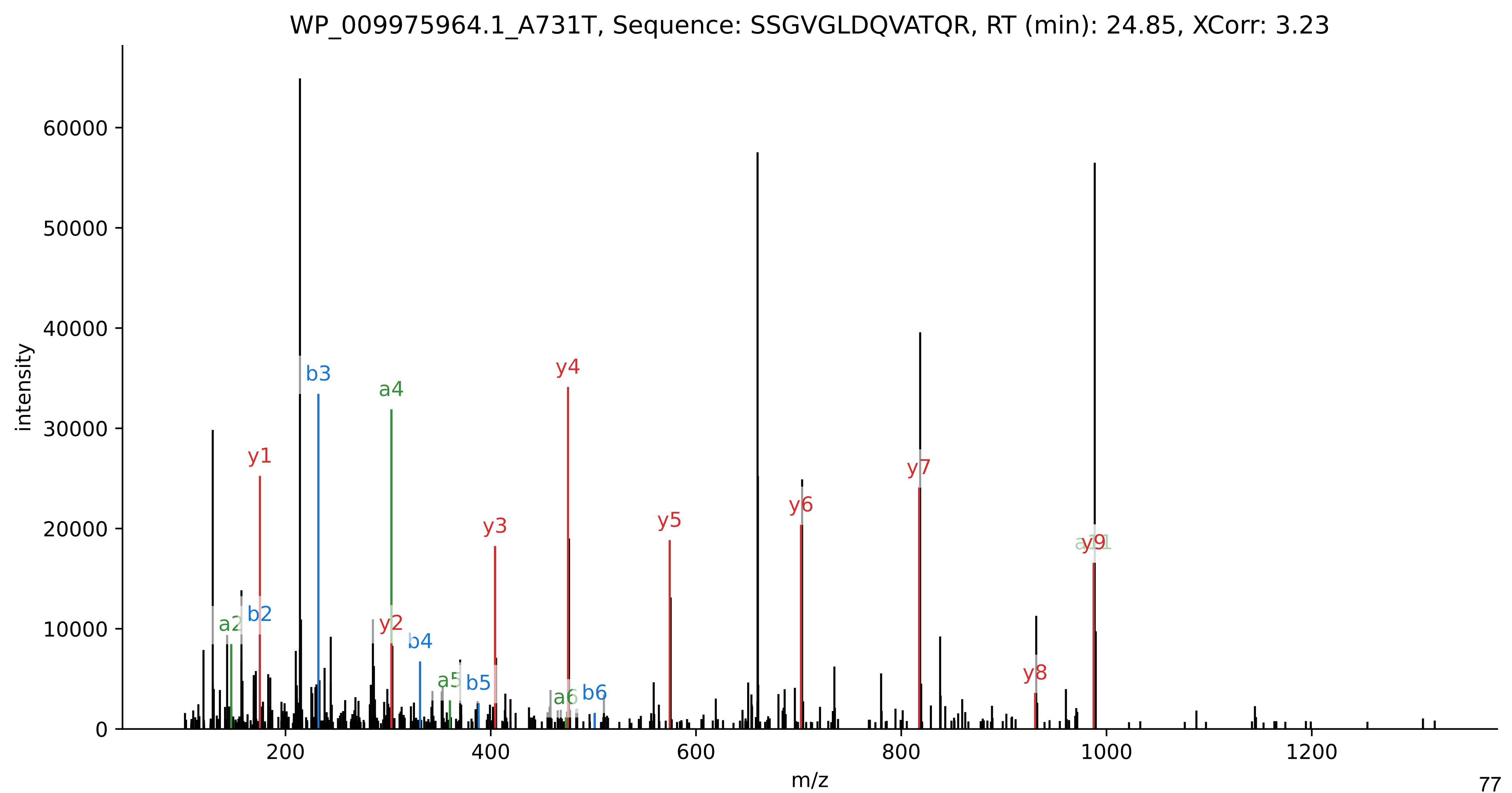

WP_009975964.1_A731T, Sequence: SSGVGLDQVATQR, RT (min): 24.85, XCorr: 3.23
intensity
m/z
y1
y2
y3
y4
y5
y6
y7
y8
y9
b2
b3
b4
b5
b6
a2
a4
a5
a6

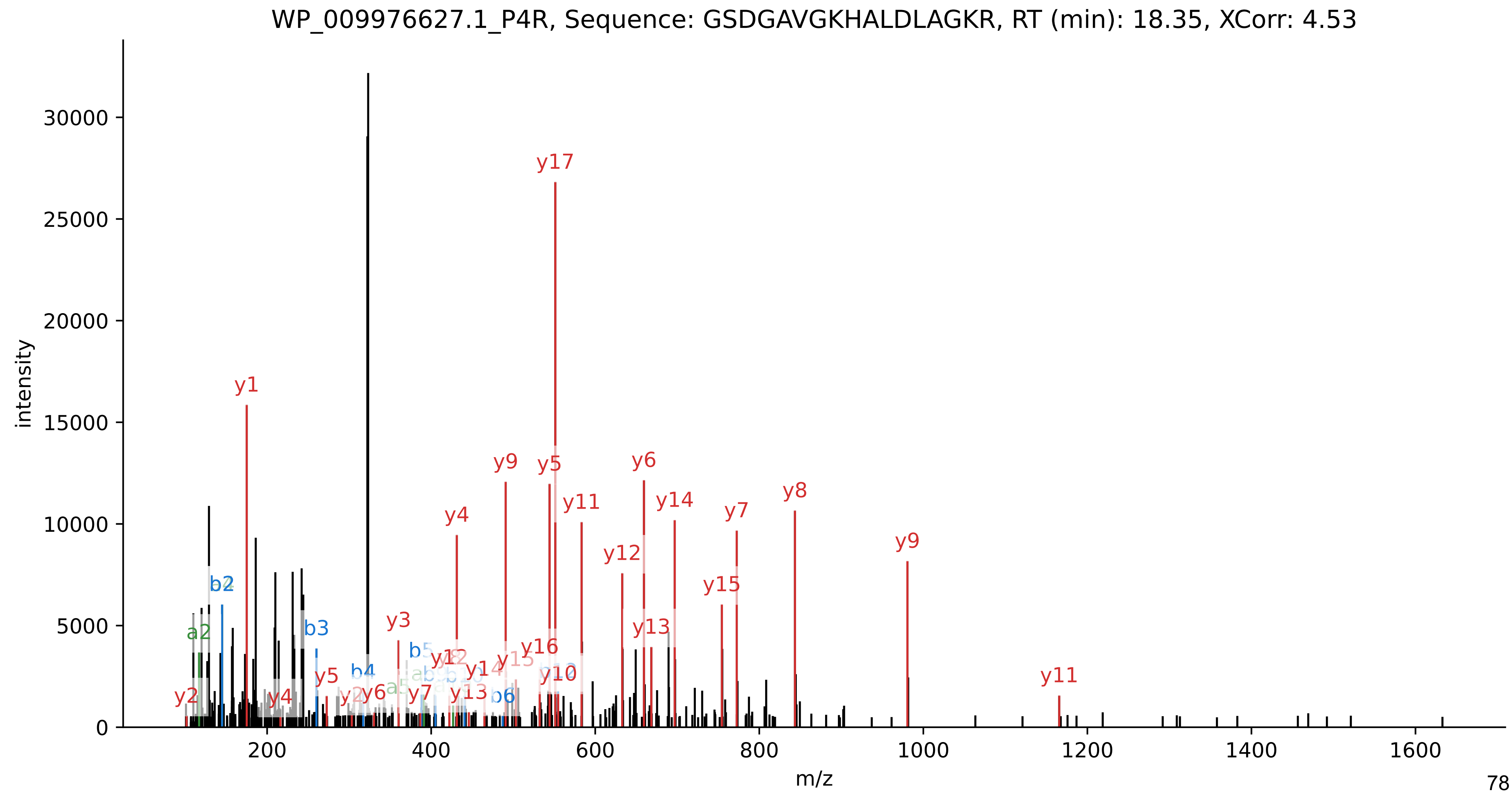

WP_009976627.1_P4R, Sequence: GSDGAVGKHALDLAGKR, RT (min): 18.35, XCorr: 4.53
intensity
m/z
y17
y1
y9
y5
y6
y11
y14
y4
y7
y8
y12
y9
y15
y13
y3
y16
y10
y11
b2
a2
b3
b4
b5
b6
y5
y2
y4
y6
y7
y13

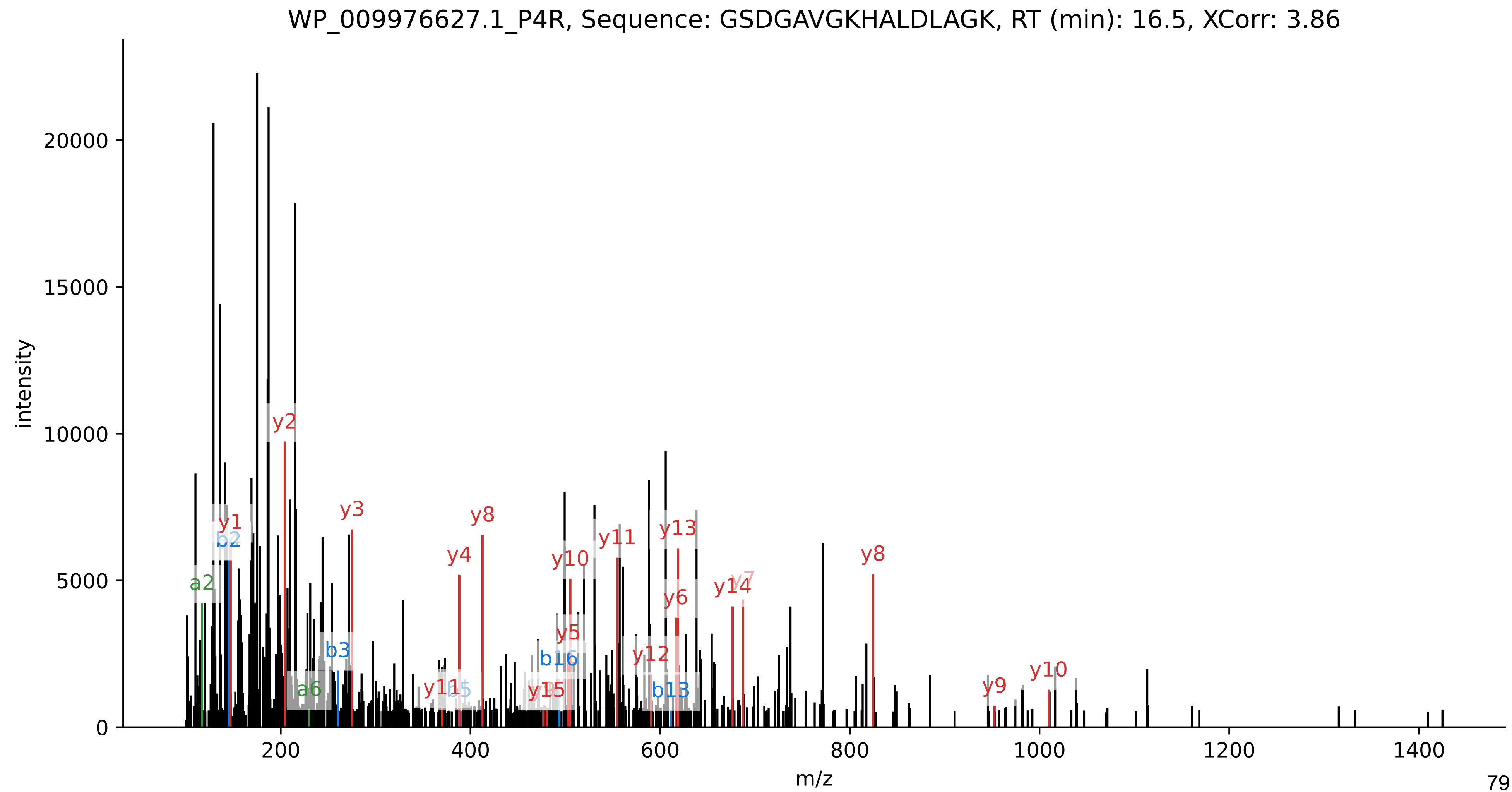
WP_009976627.1_P4R, Sequence: GSDGAVGKHALDLAGK, RT (min): 16.5, XCorr: 3.86
intensity
m/z
y2
y3
y8
y1
b2
y4
y10
y11
y13
y8
a2
y14
y6
y5
b3
y12
y10
y9
y11
a6
b13

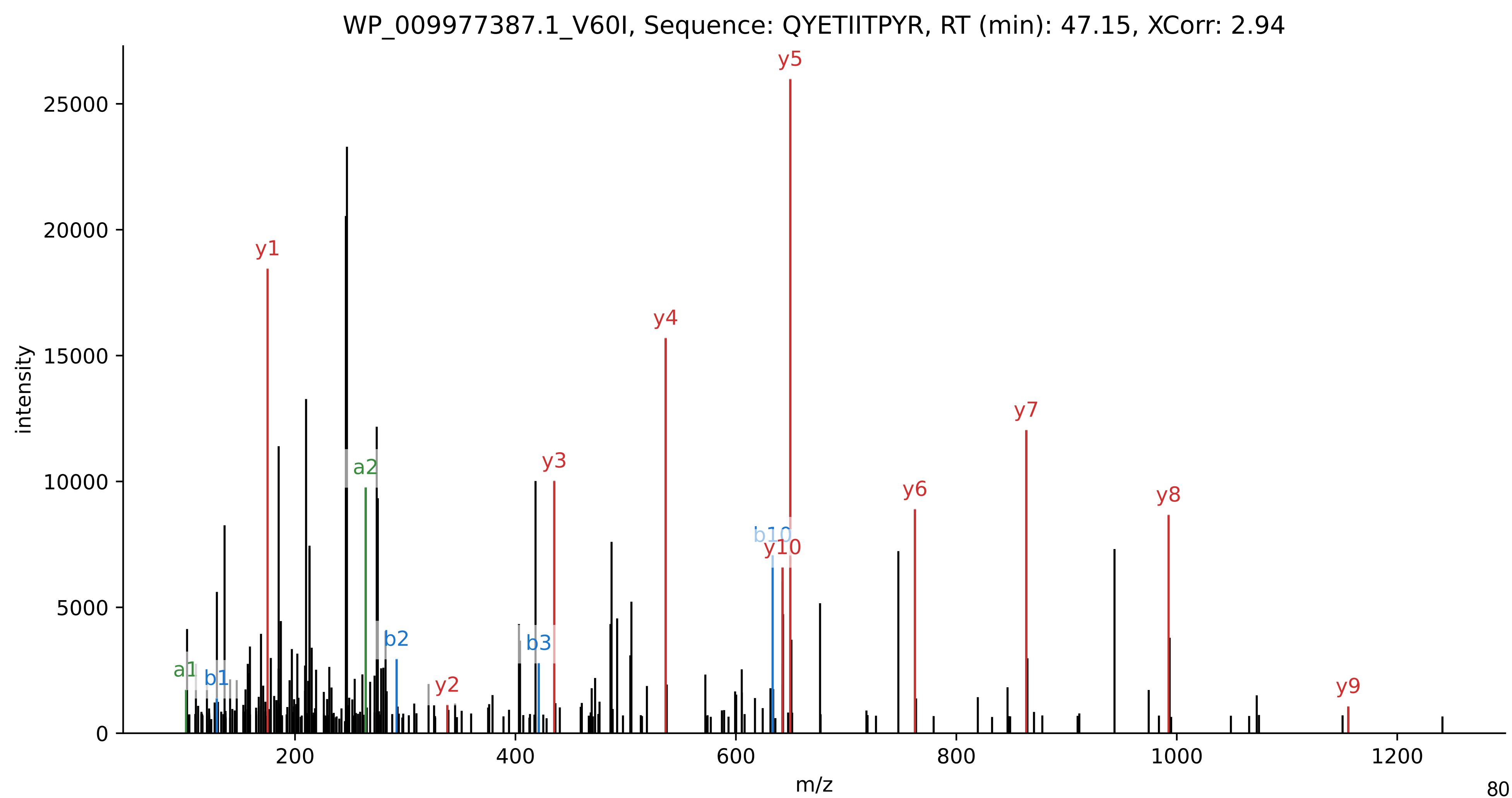
WP_009977387.1_V60I, Sequence: QYETIITPYR, RT (min): 47.15, XCorr: 2.94
intensity
m/z
y5
y1
y4
y7
y3
a2
y6
y8
b10
y10
b2
b3
a1
b1
y2
y9
25000
20000
15000
10000
5000
0
200
400
600
800
1000
1200

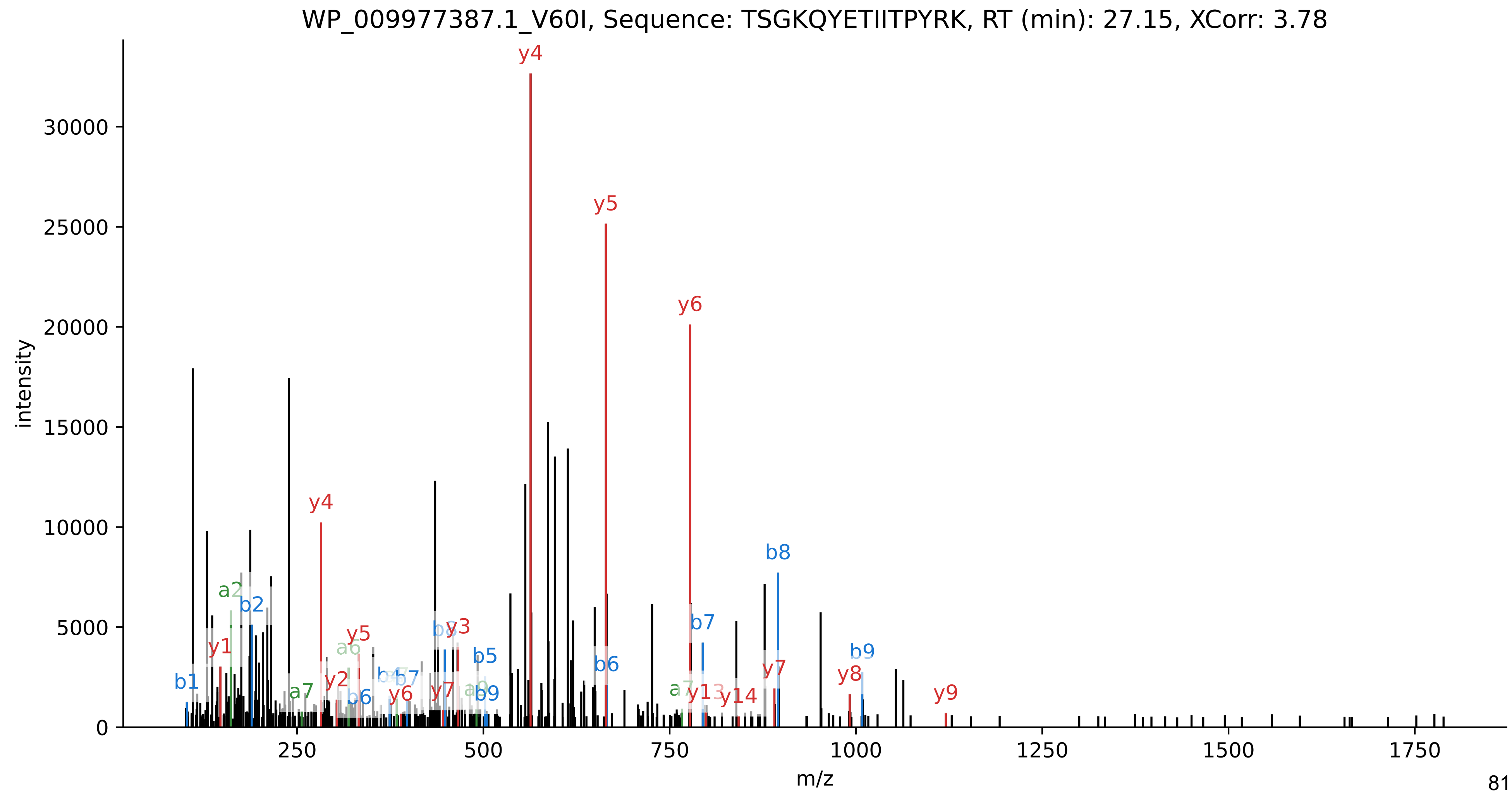
WP_009977387.1_V60I, Sequence: TSGKQYETIITPYRK, RT (min): 27.15, XCorr: 3.78
intensity
m/z
30000
25000
20000
15000
10000
5000
0
250
500
750
1000
1250
1500
1750
y4
y5
y6
y4
b8
b7
a2
b2
y1
b1
y5
y3
b5
b6
b9
y7
y8
y9
y2
a7
b6
y6
y7
b7
b9
y14

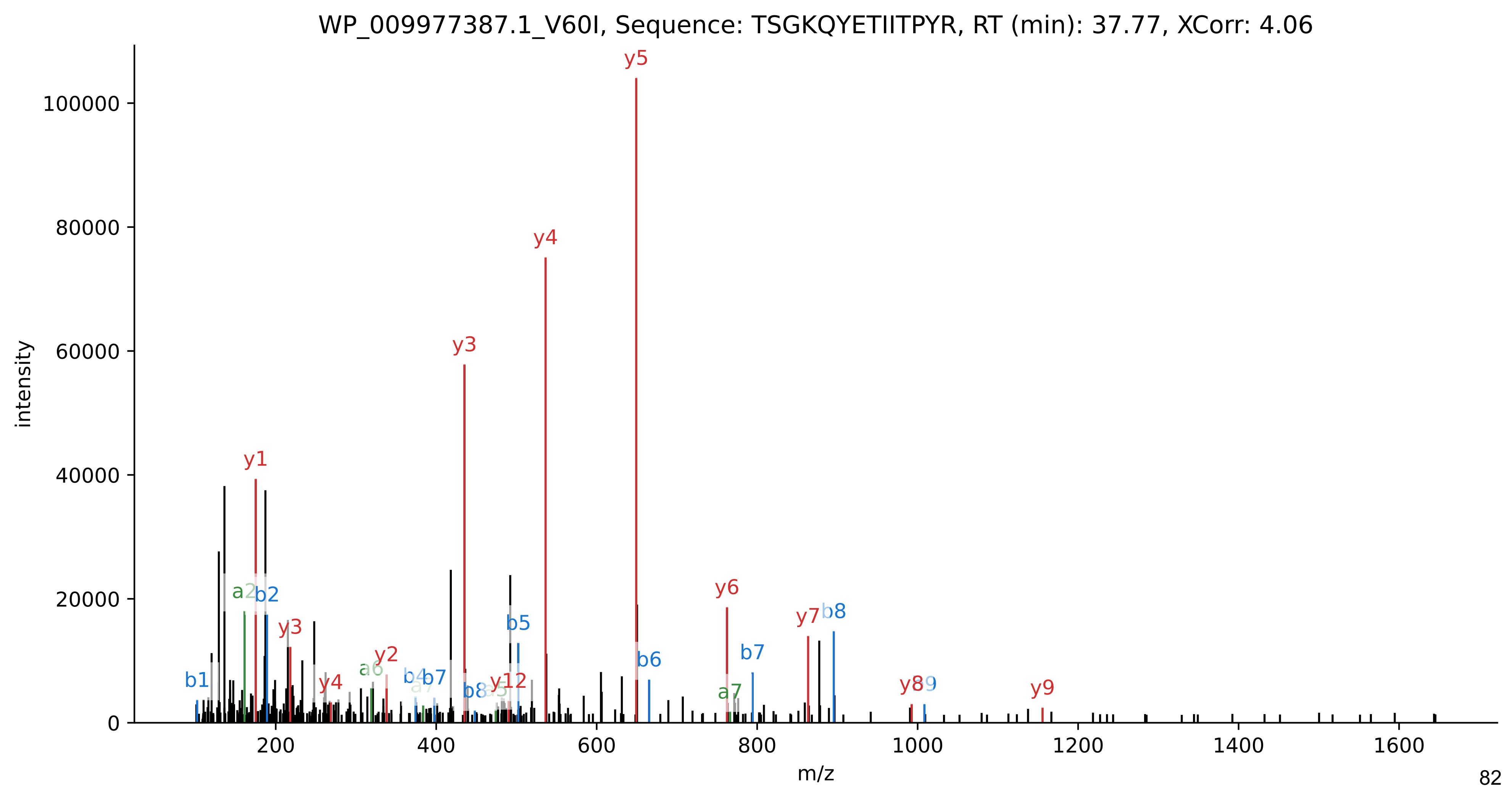

WP_009977387.1_V60I, Sequence: TSGKQYETIITPYR, RT (min): 37.77, XCorr: 4.06
intensity
m/z
y5
y4
y3
y1
a2
b2
y3
y4
a6
y2
b4
b7
b8
y12
b5
b6
y6
a7
b7
y7
b8
y8
y9

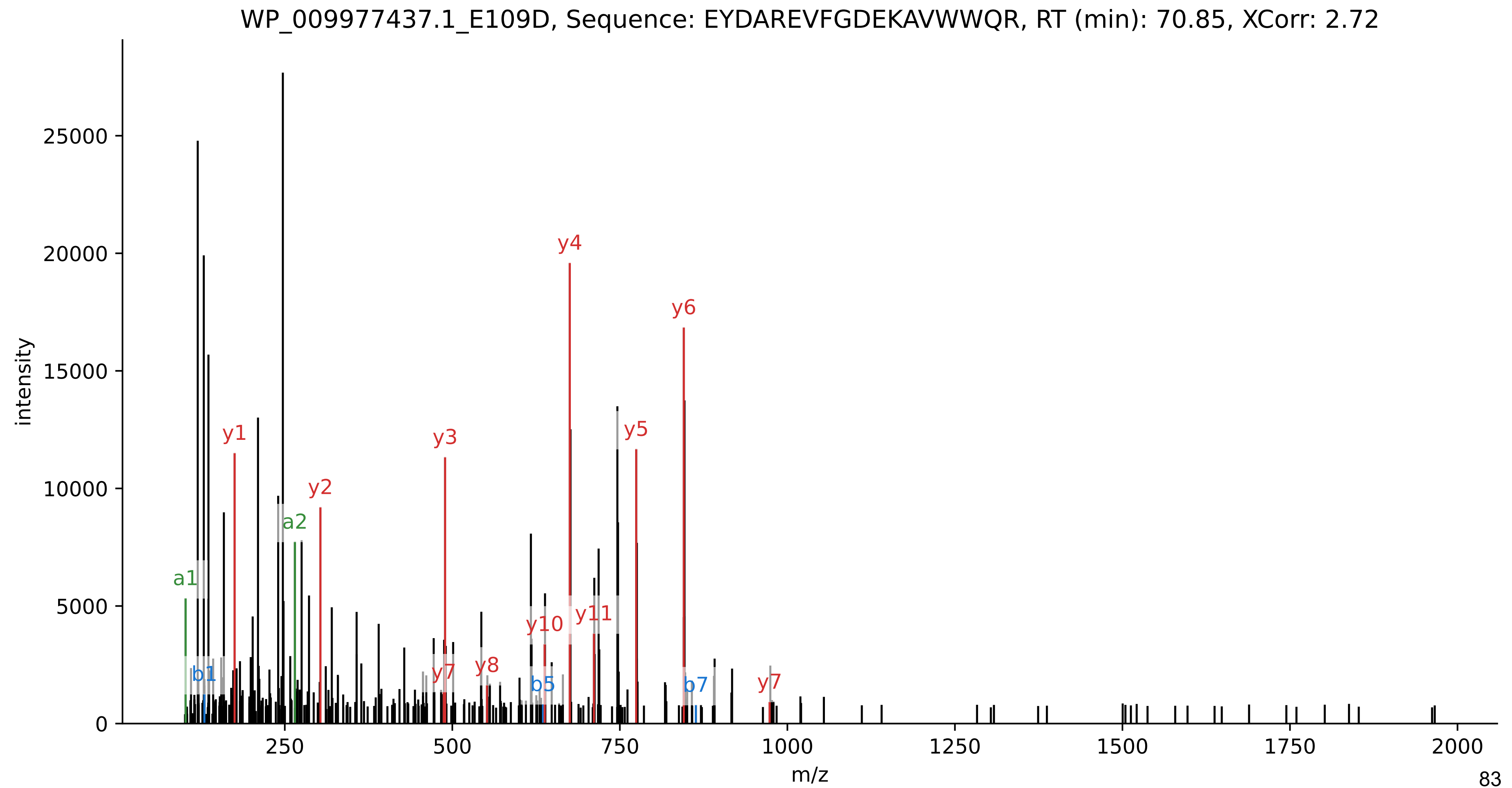
WP_009977437.1_E109D, Sequence: EYDAREVFGDEKAVWWQR, RT (min): 70.85, XCorr: 2.72
intensity
m/z
0
5000
10000
15000
20000
25000
250
500
750
1000
1250
1500
1750
2000
a1
b1
y1
a2
y2
y3
y7
y8
y10
b5
y4
y11
y5
y6
b7
y7

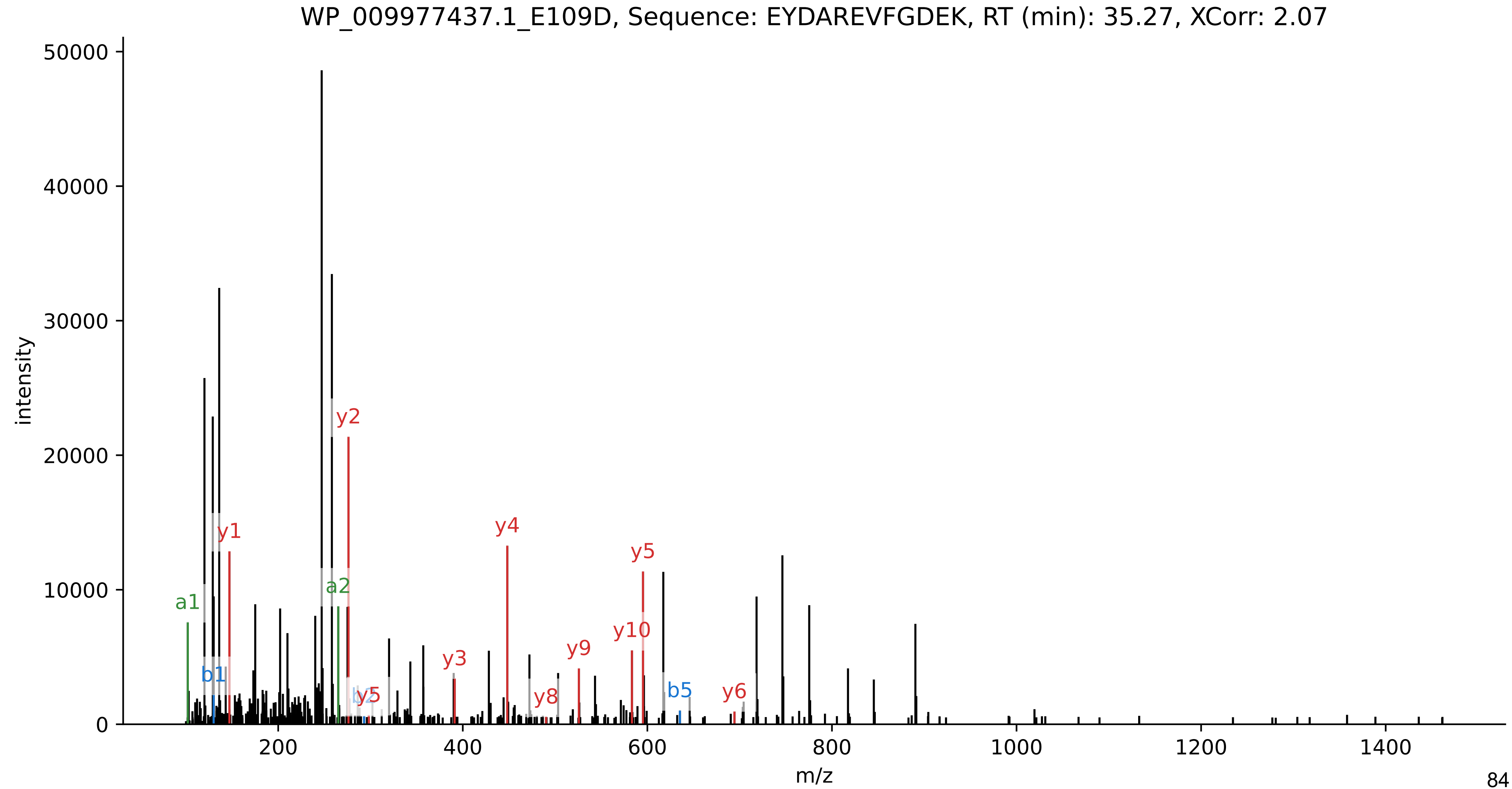

WP_009977437.1_E109D, Sequence: EYDAREVFGDEK, RT (min): 35.27, XCorr: 2.07
intensity
m/z
a1
b1
y1
a2
y2
y3
y4
y5
y8
y9
y10
b5
y6

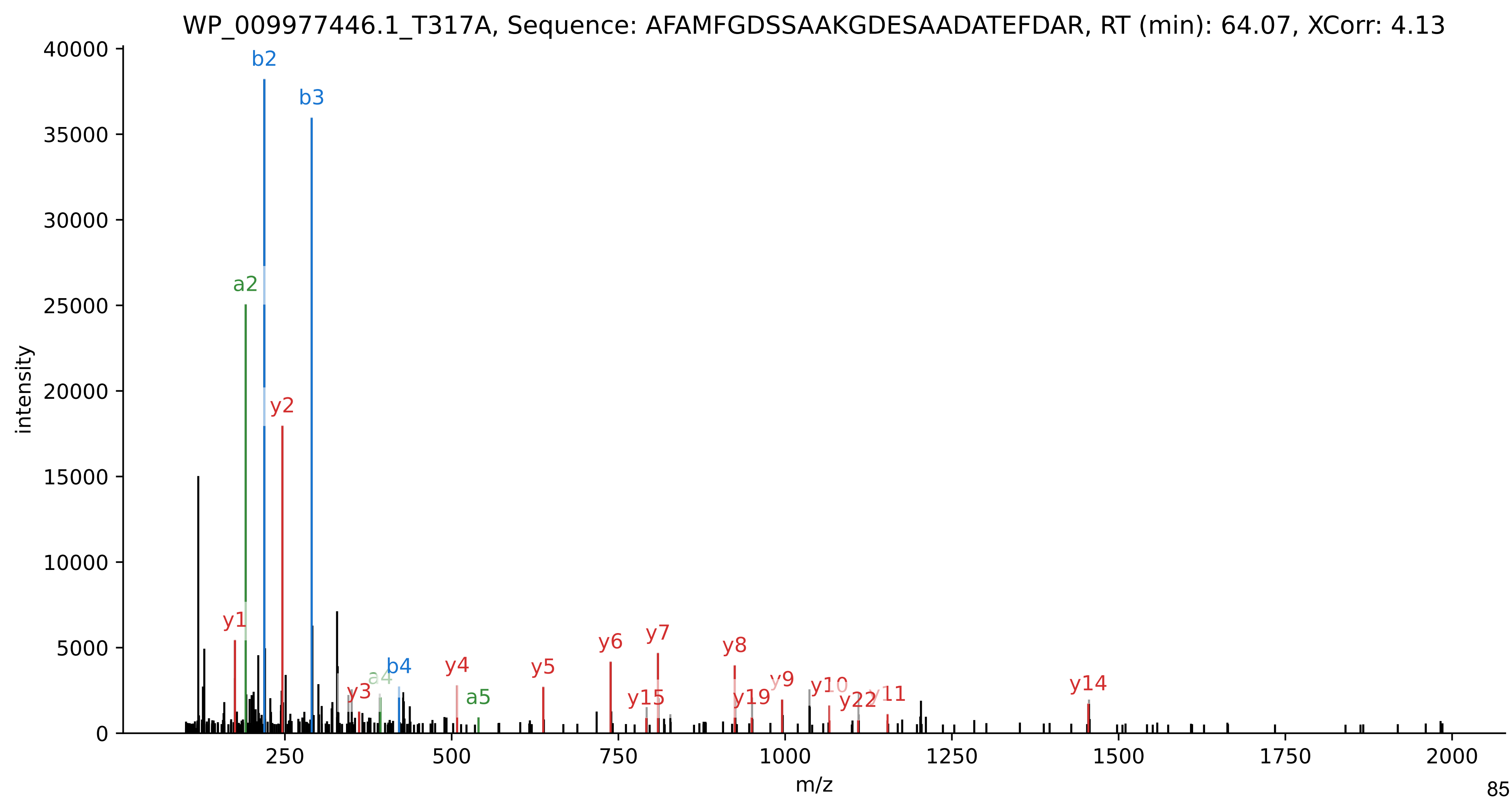

WP_009977446.1_T317A, Sequence: AFAMFGDSSAAKGDESAADATEFDAR, RT (min): 64.07, XCorr: 4.13
intensity
m/z
0
5000
10000
15000
20000
25000
30000
35000
40000
250
500
750
1000
1250
1500
1750
2000
b2
b3
a2
y2
y1
a4
b4
y3
y4
a5
y5
y6
y7
y15
y8
y19
y9
y10
y22
y11
y14

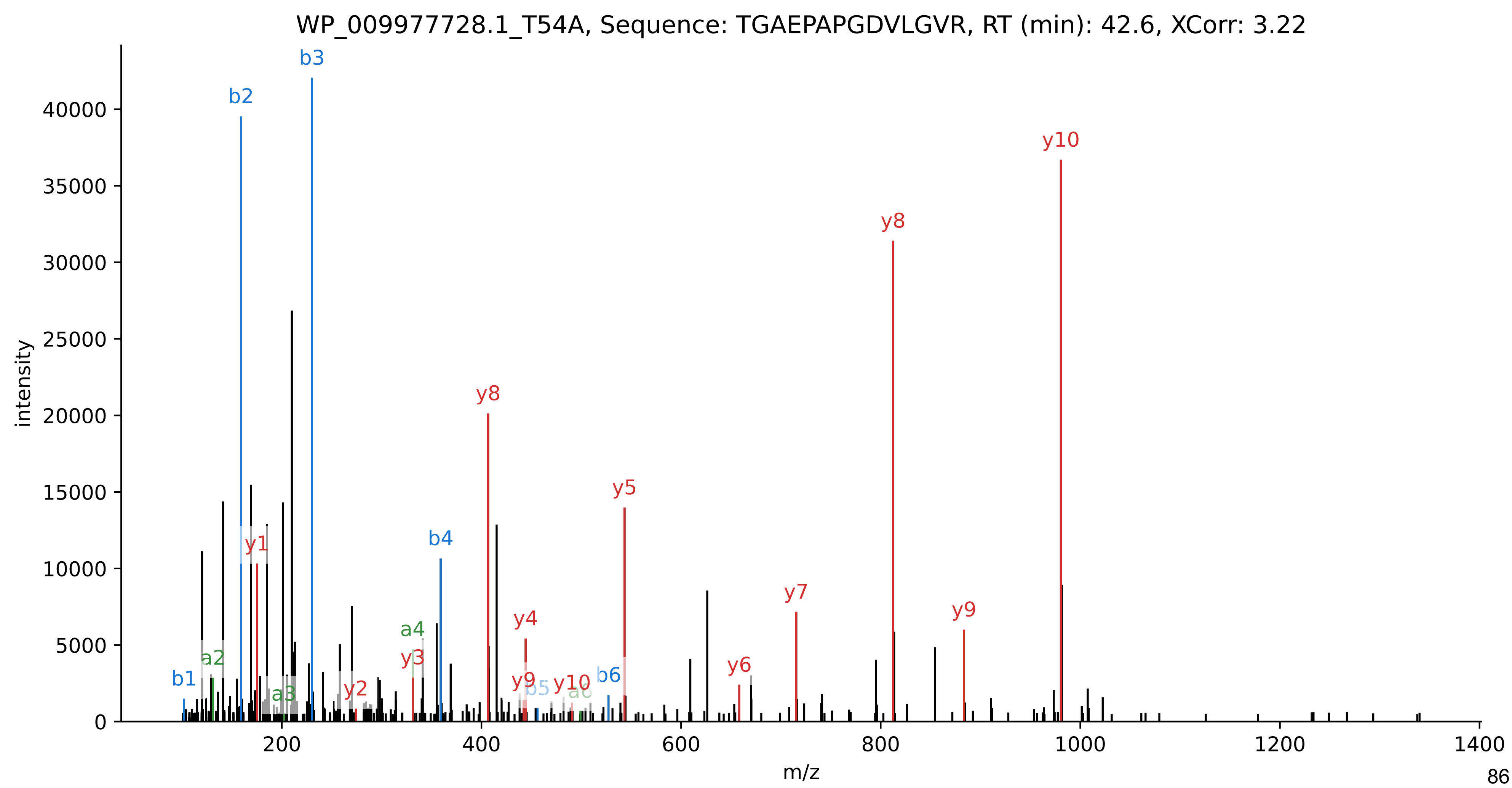

WP_009977728.1_T54A, Sequence: TGAEPAPGDVLGVR, RT (min): 42.6, XCorr: 3.22
intensity
m/z
b1
a2
b2
y1
a3
b3
y2
a4
y3
b4
y8
y4
y9
b5
y10
a6
b6
y5
y6
y7
y8
y9
y10
0
5000
10000
15000
20000
25000
30000
35000
40000
200
400
600
800
1000
1200
1400

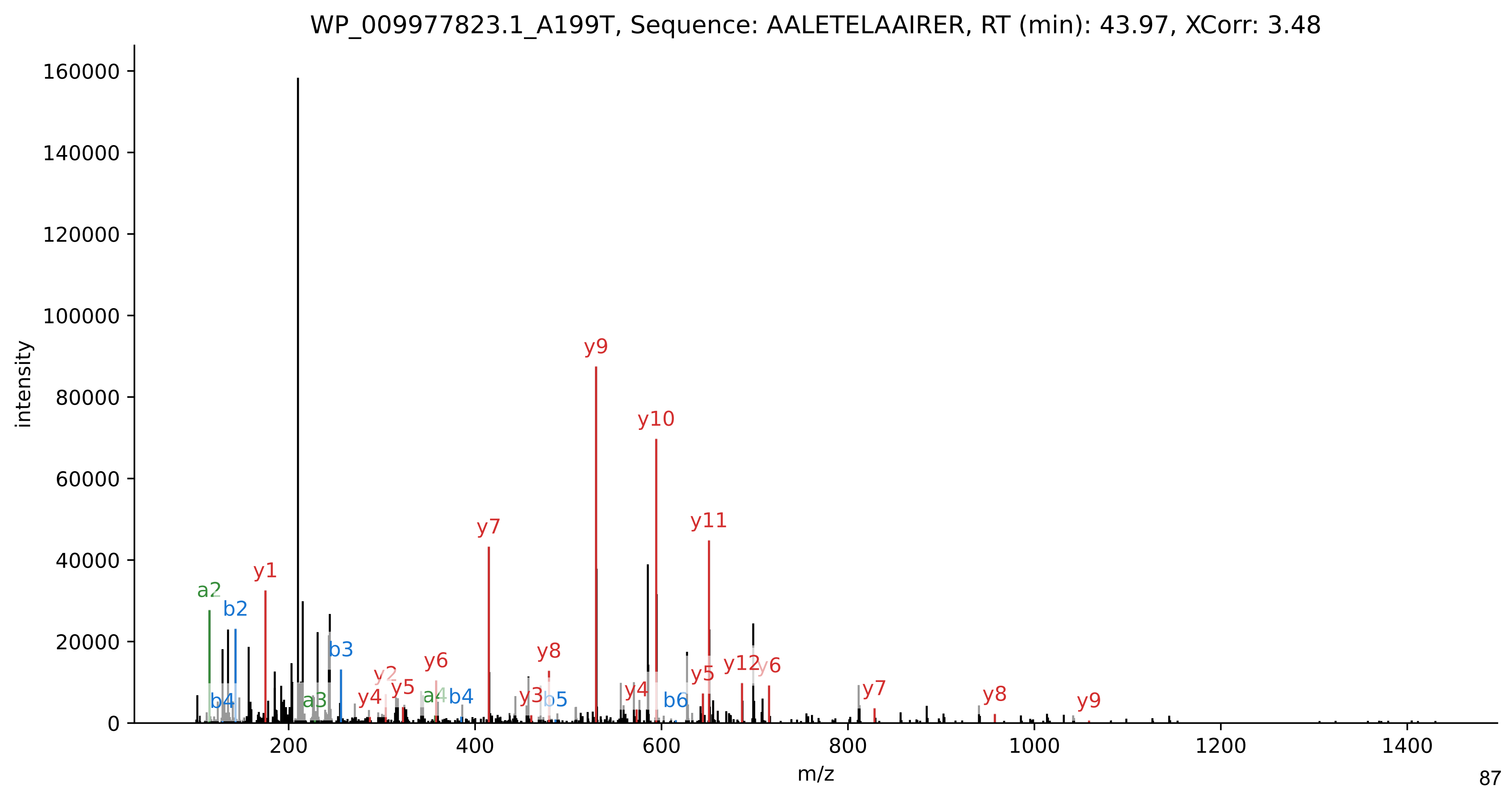
WP_009977823.1_A199T, Sequence: AALETELAAIRER, RT (min): 43.97, XCorr: 3.48
intensity
m/z
y9
y10
y11
y7
y1
a2
b2
b3
y8
y2
y6
y5
y4
a4
b4
a3
y3
b5
b6
y12
y7
y8
y9

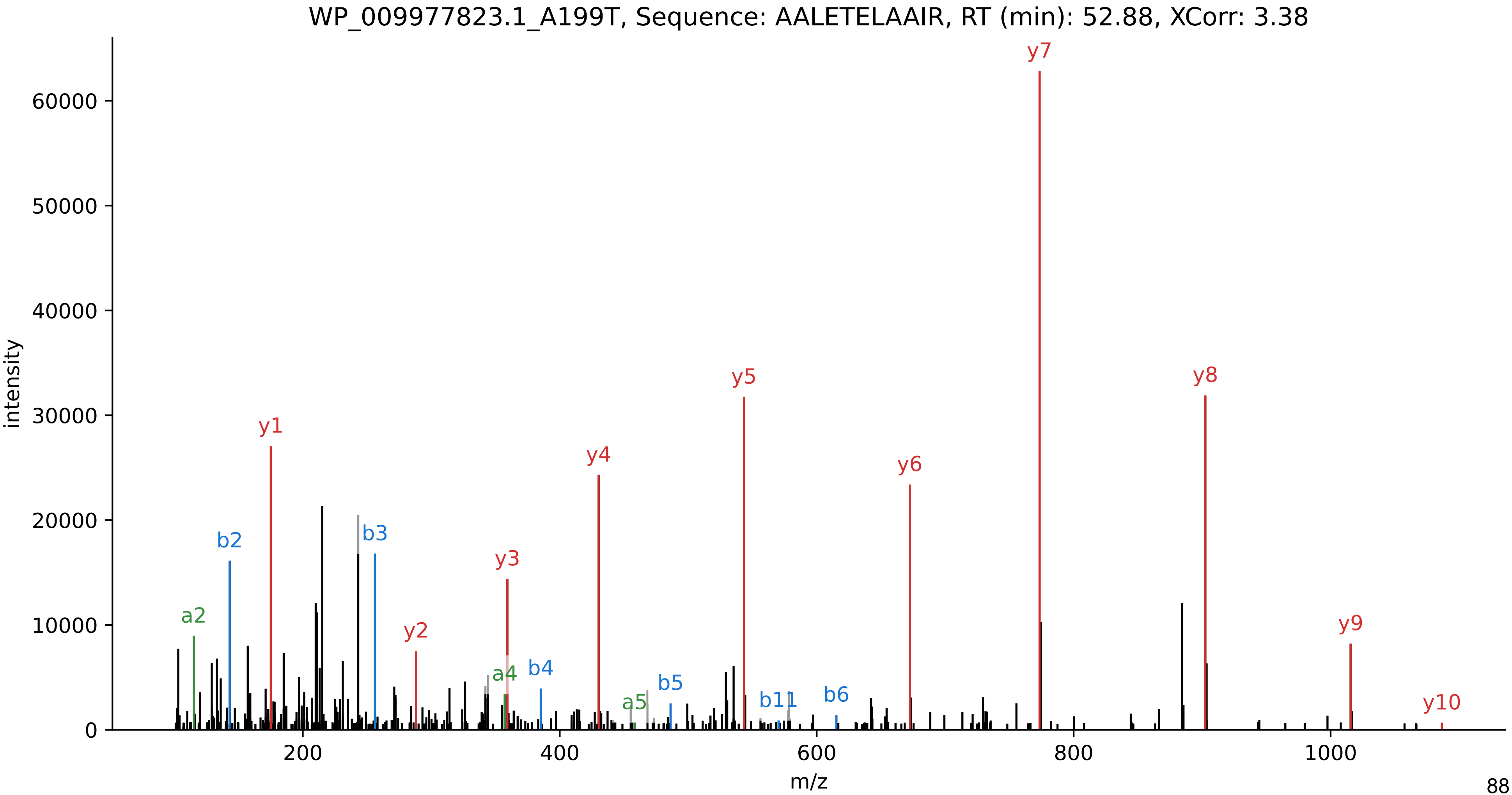

WP_009977823.1_A199T, Sequence: AALETELAAIR, RT (min): 52.88, XCorr: 3.38
intensity
m/z
60000
50000
40000
30000
20000
10000
0
200
400
600
800
1000
y7
y5
y8
y1
y4
y6
b2
b3
y3
a2
y2
y9
a4
b4
a5
b5
b11
b6
y10

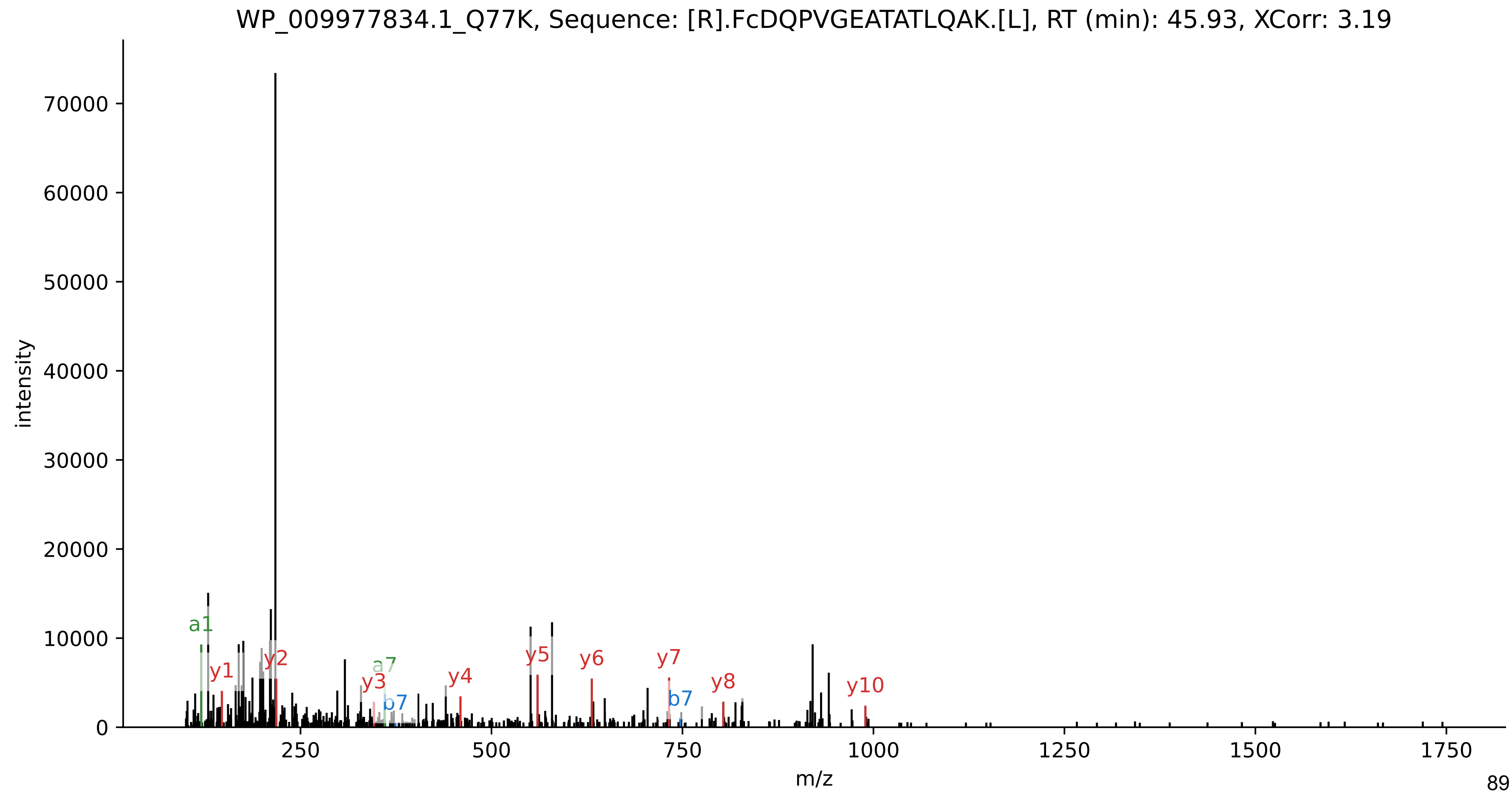
WP_009977834.1_Q77K, Sequence: [R].FcDQPVGEATATLQAK.[L], RT (min): 45.93, XCorr: 3.19
intensity
m/z
a1
y1
y2
y3
a7
b7
y4
y5
y6
y7
b7
y8
y10
0
10000
20000
30000
40000
50000
60000
70000
250
500
750
1000
1250
1500
1750

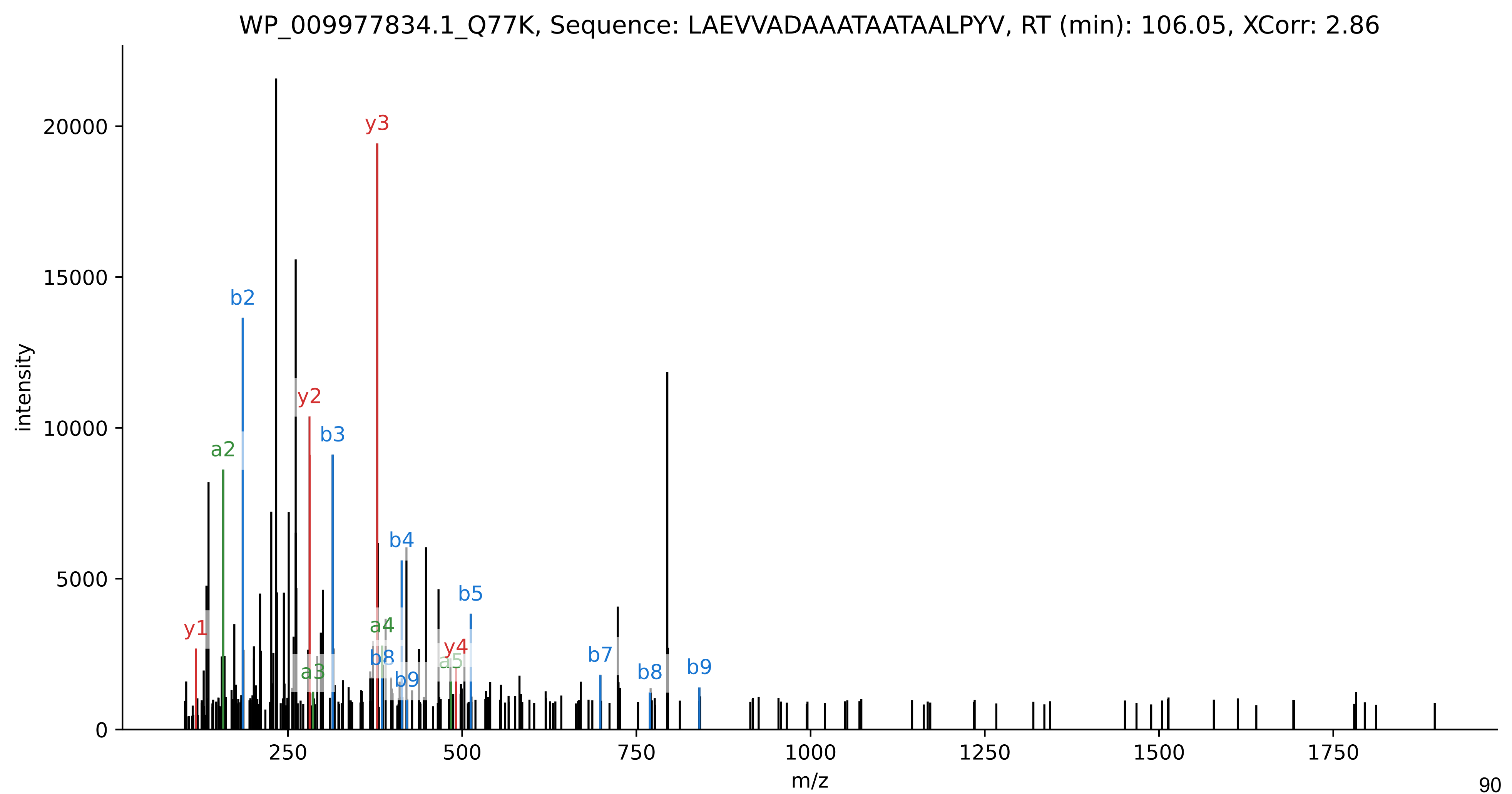
WP_009977834.1_Q77K, Sequence: LAEVVADAAATAATAALPYV, RT (min): 106.05, XCorr: 2.86
intensity
m/z
y1
a2
b2
y2
b3
y3
a3
b4
a4
b8
b9
a5
y4
b5
b7
b8
b9
0
5000
10000
15000
20000
250
500
750
1000
1250
1500
1750

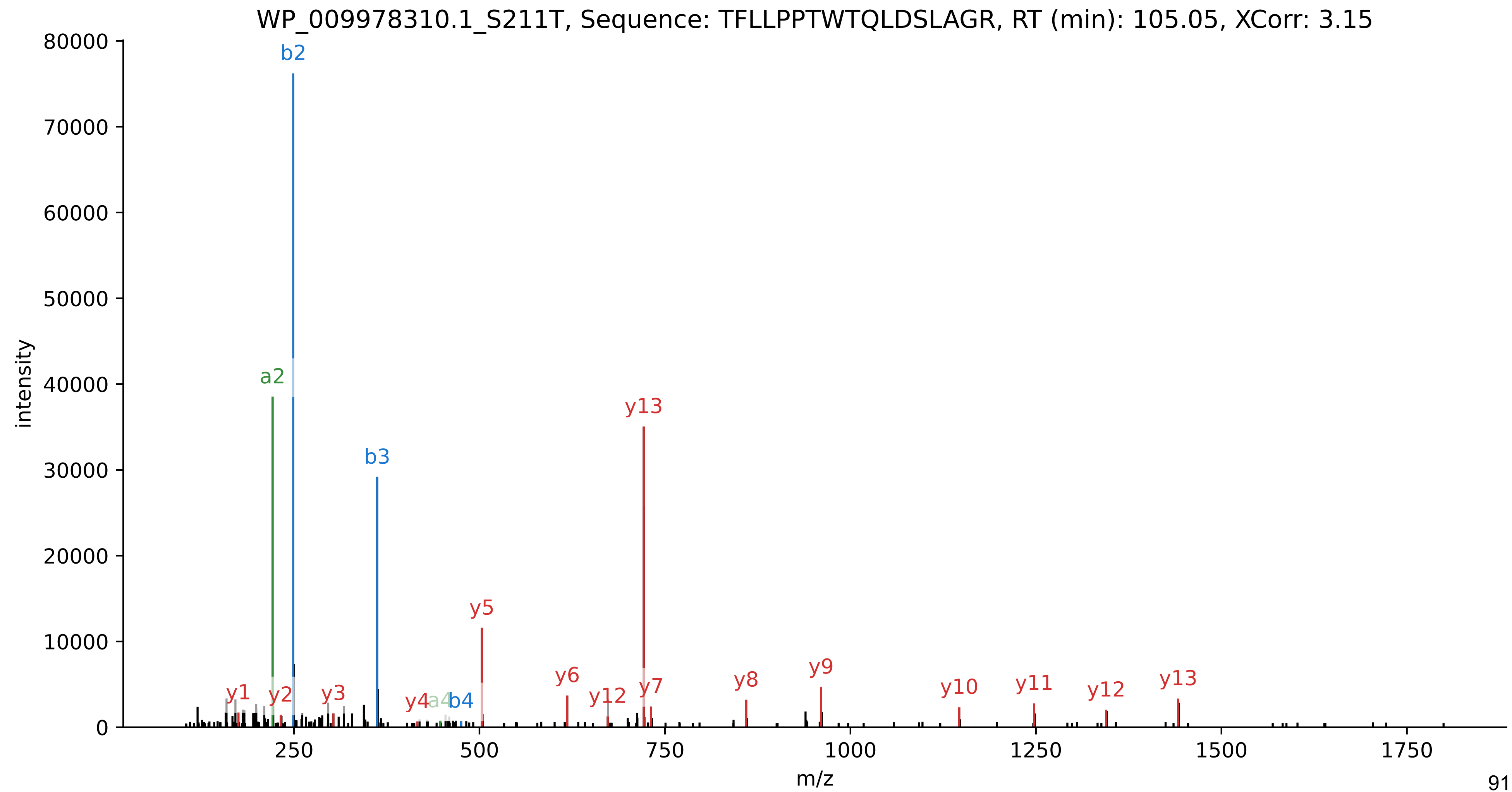
WP_009978310.1_S211T, Sequence: TFLLPPTWTQLDSLAGR, RT (min): 105.05, XCorr: 3.15
intensity
m/z
b2
a2
b3
y13
y5
y1
y2
y3
y4
a4
b4
y6
y12
y7
y8
y9
y10
y11
y12
y13

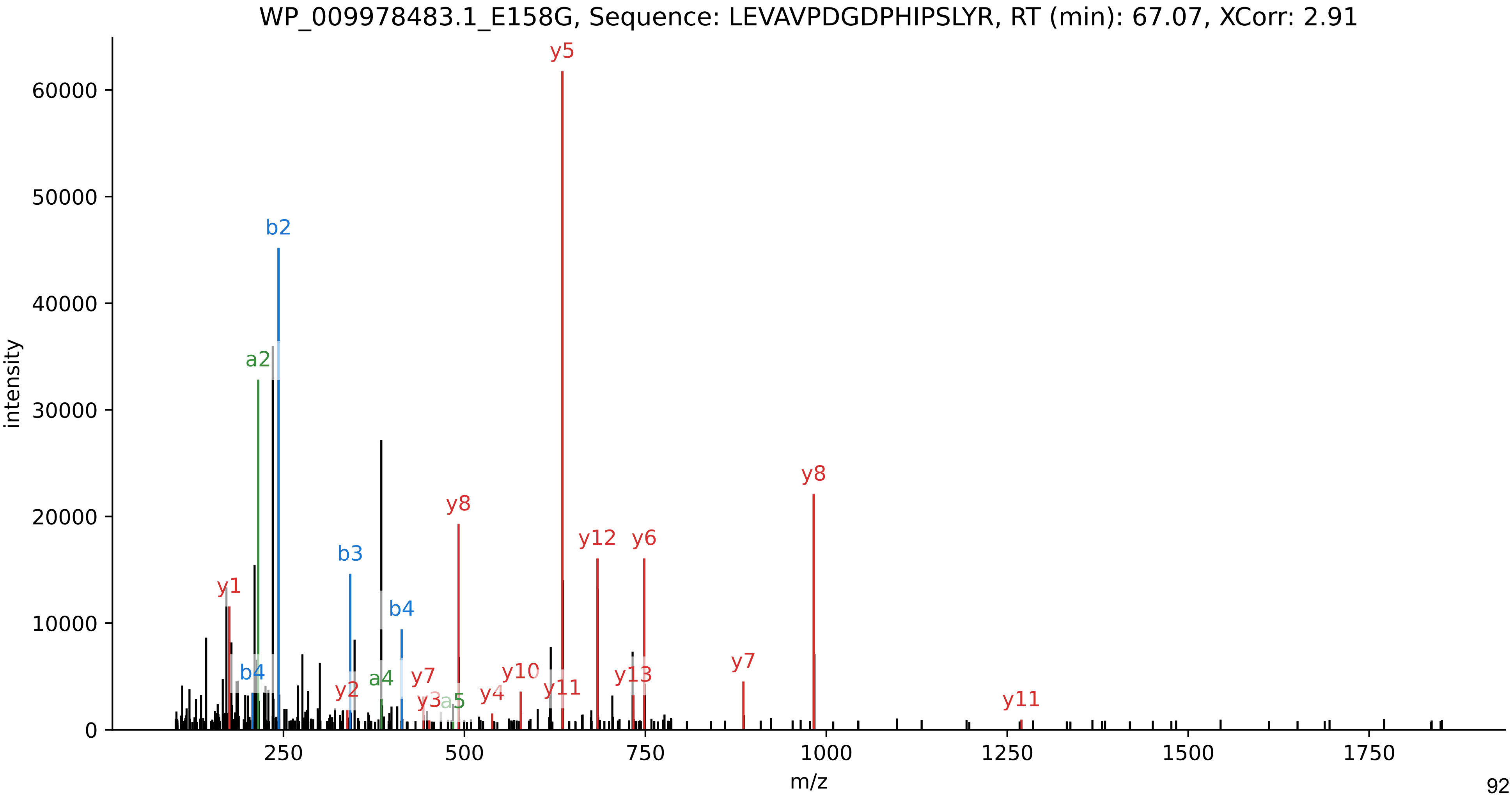
WP_009978483.1_E158G, Sequence: LEVAVPDGDPHIPSLYR, RT (min): 67.07, XCorr: 2.91
intensity
m/z
y5
b2
a2
y8
y8
y12
y6
b3
y1
b4
b4
a4
y7
y2
y3
a5
y4
y10
y11
y13
y7
y11
0
10000
20000
30000
40000
50000
60000
250
500
750
1000
1250
1500
1750

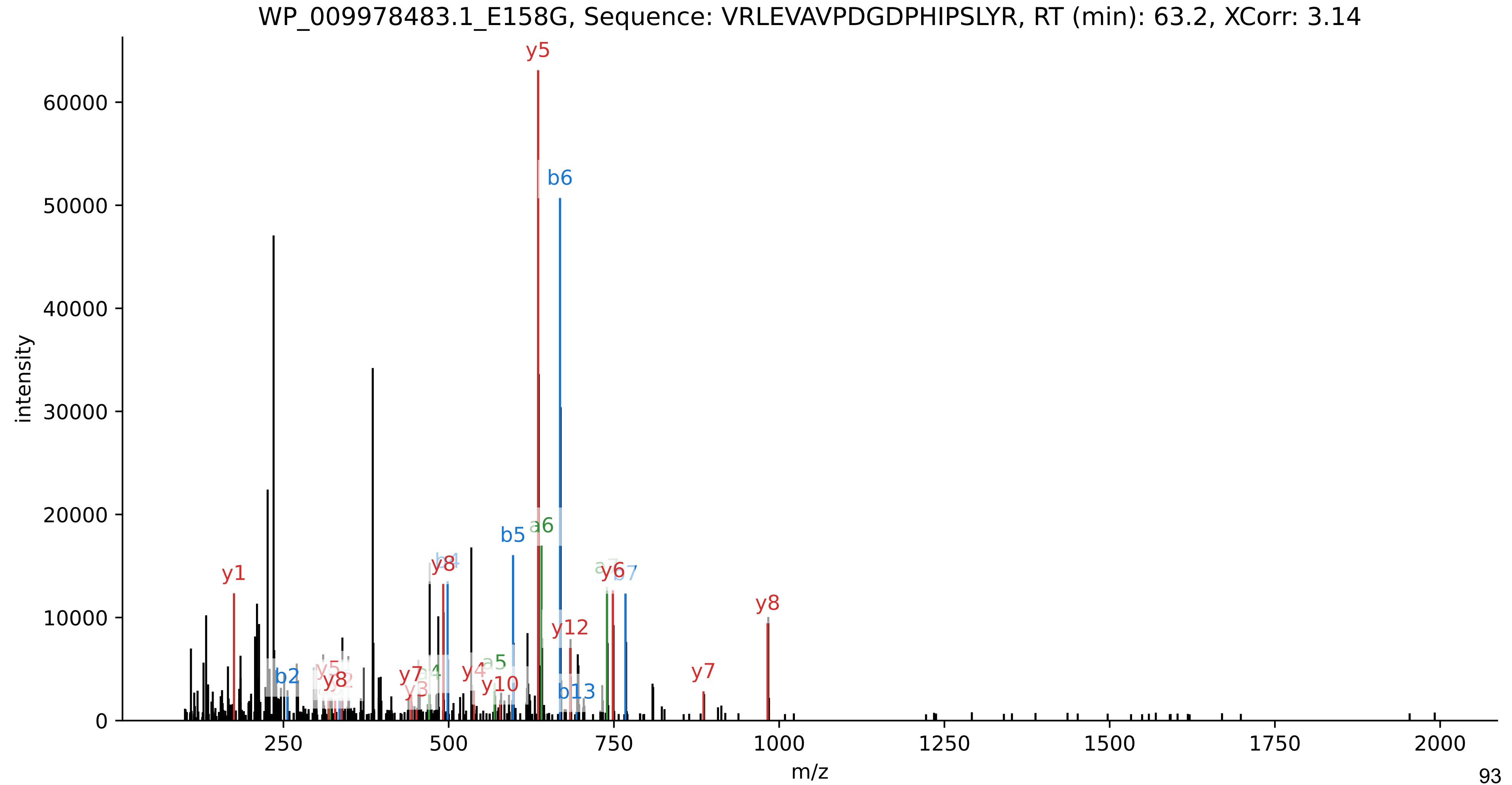

WP_009978483.1_E158G, Sequence: VRLEVAVPDGDPHIPSLYR, RT (min): 63.2, XCorr: 3.14
intensity
m/z
y5
b6
b5
a6
y1
y8
y6
y8
y12
y7
y7
y4
a5
y10
b13
b2
y8
y3

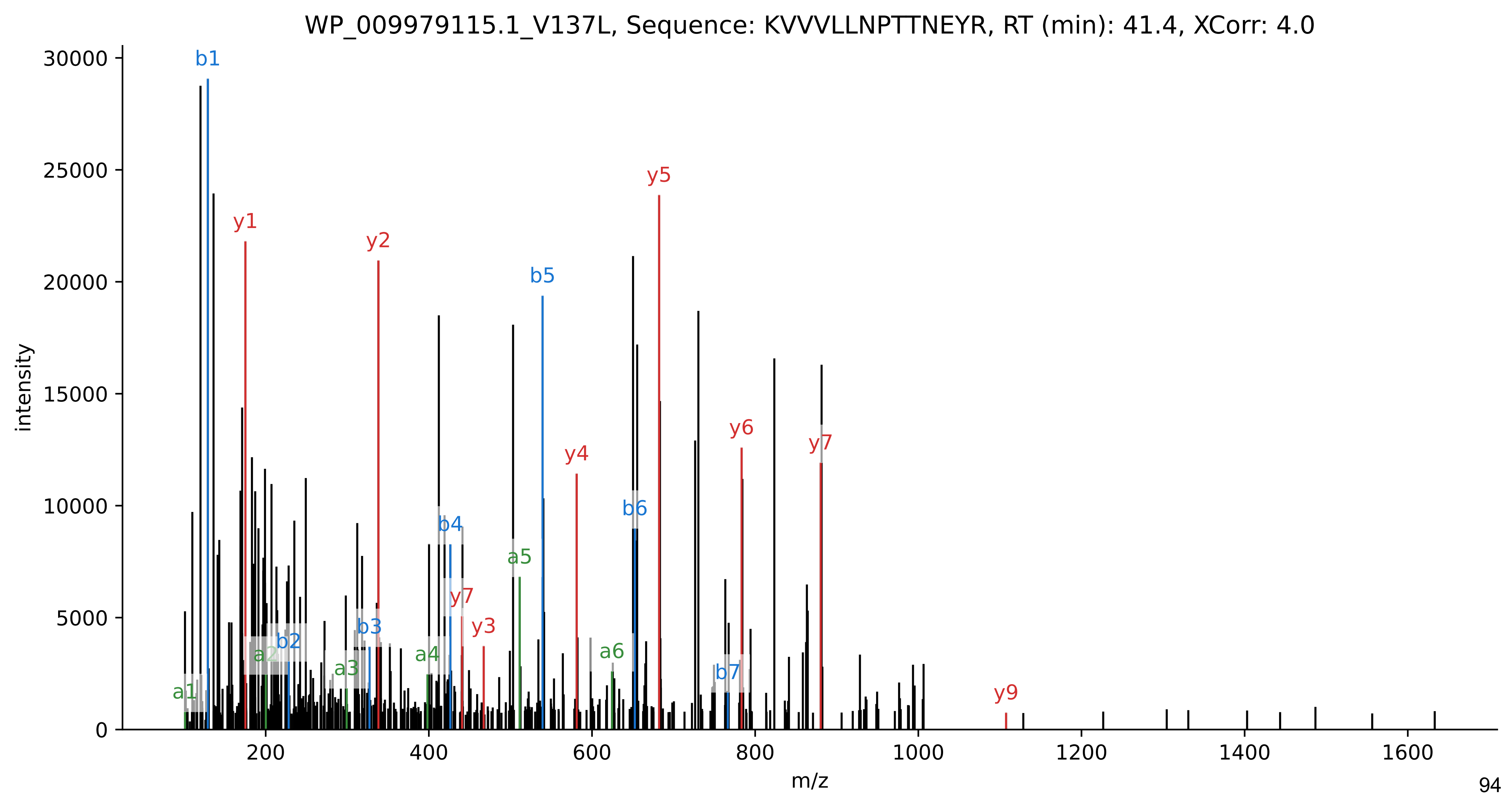

WP_009979115.1_V137L, Sequence: KVVVLLNPTTNEYR, RT (min): 41.4, XCorr: 4.0
intensity
m/z
b1
y1
y2
b5
y5
y6
y7
y4
b6
b4
a5
y7
y3
b3
b2
a2
a4
a3
a6
b7
a1
y9

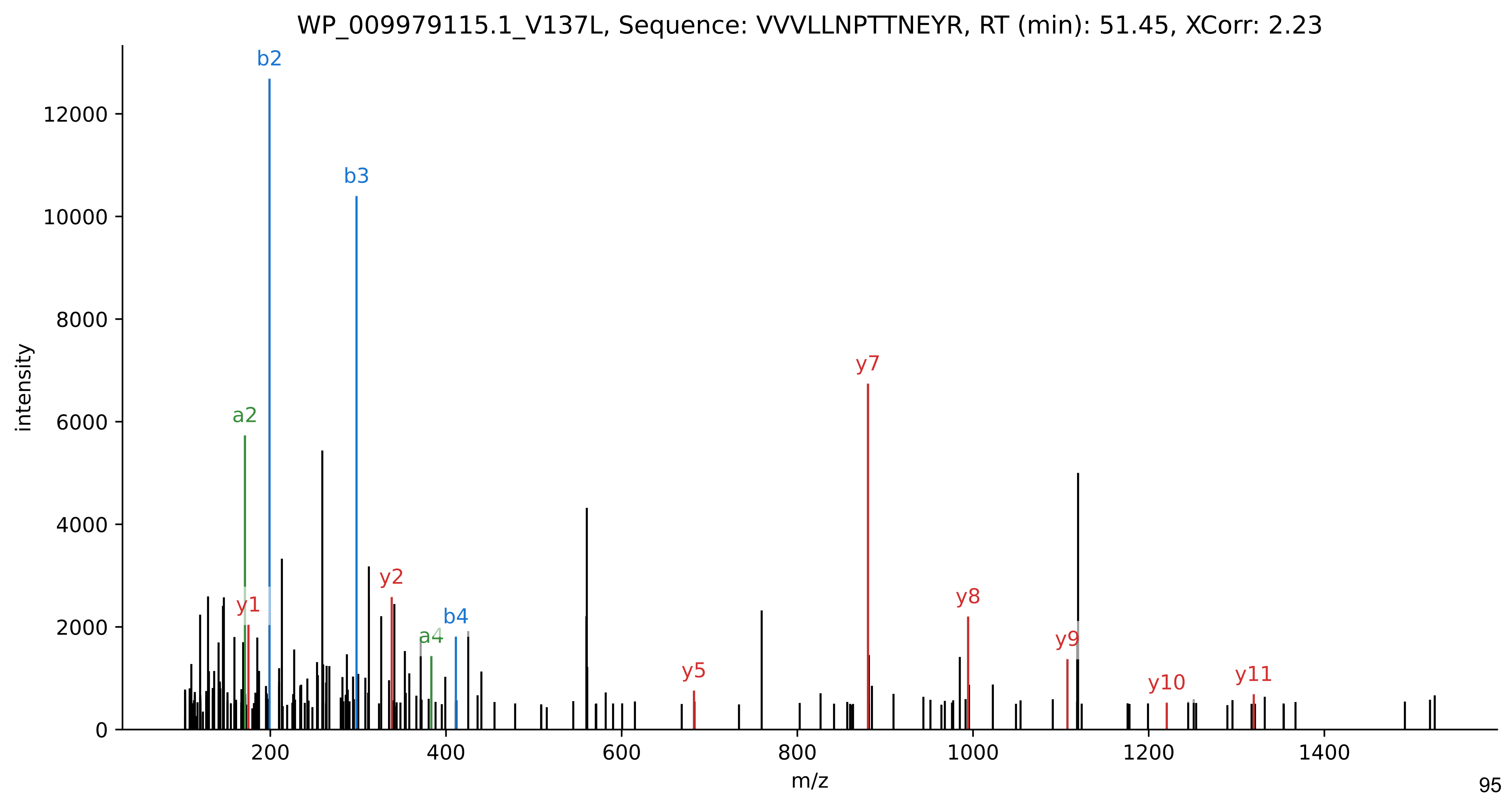
WP_009979115.1_V137L, Sequence: VVVLLNPTTNEYR, RT (min): 51.45, XCorr: 2.23
intensity
m/z
b2
b3
a2
y7
y2
y1
b4
a4
y8
y9
y5
y10
y11
0
2000
4000
6000
8000
10000
12000
200
400
600
800
1000
1200
1400

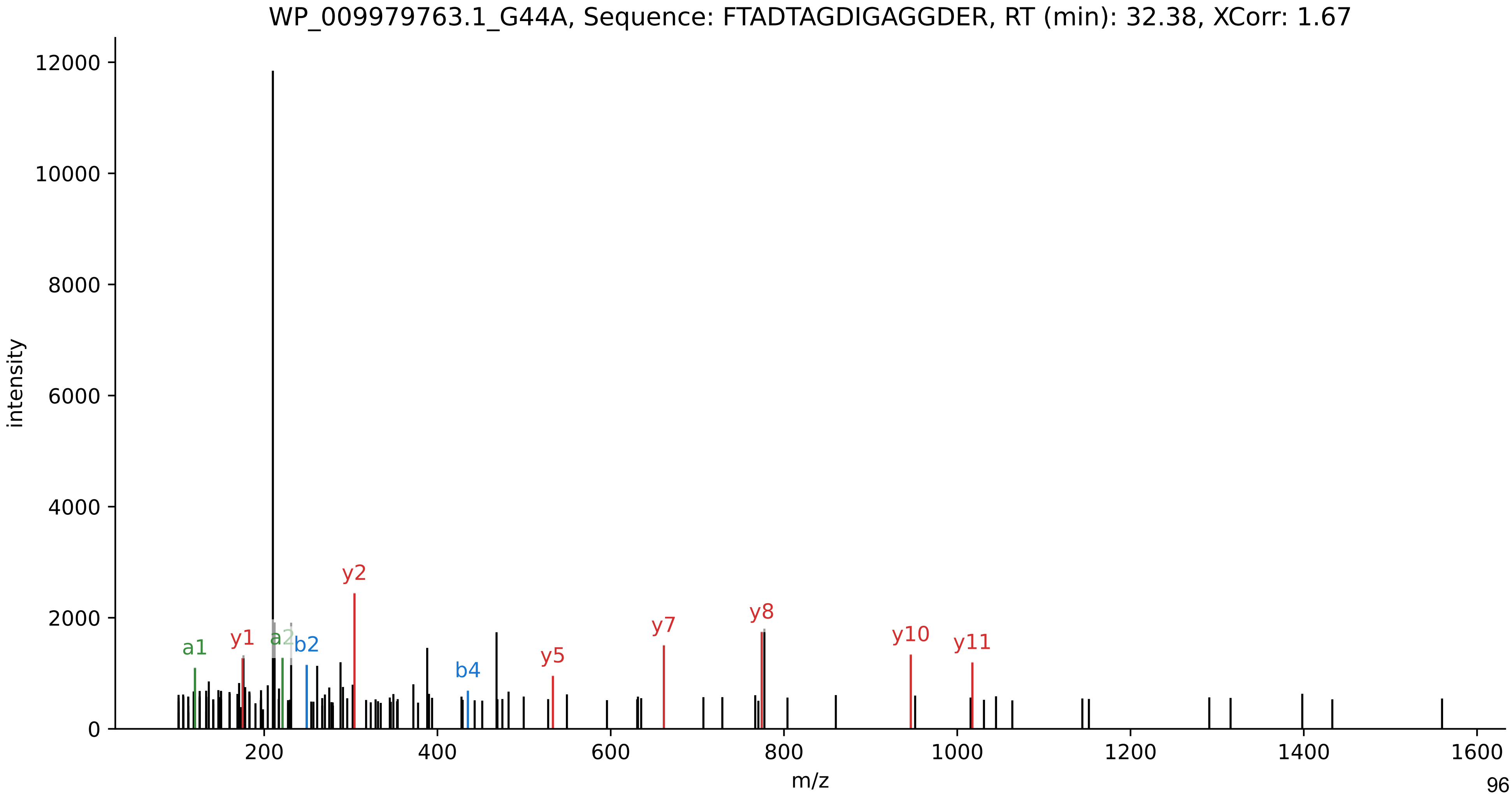
WP_009979763.1_G44A, Sequence: FTADTAGDIGAGGDER, RT (min): 32.38, XCorr: 1.67
intensity
m/z
0
2000
4000
6000
8000
10000
12000
200
400
600
800
1000
1200
1400
1600
a1
y1
a2
b2
y2
b4
y5
y7
y8
y10
y11

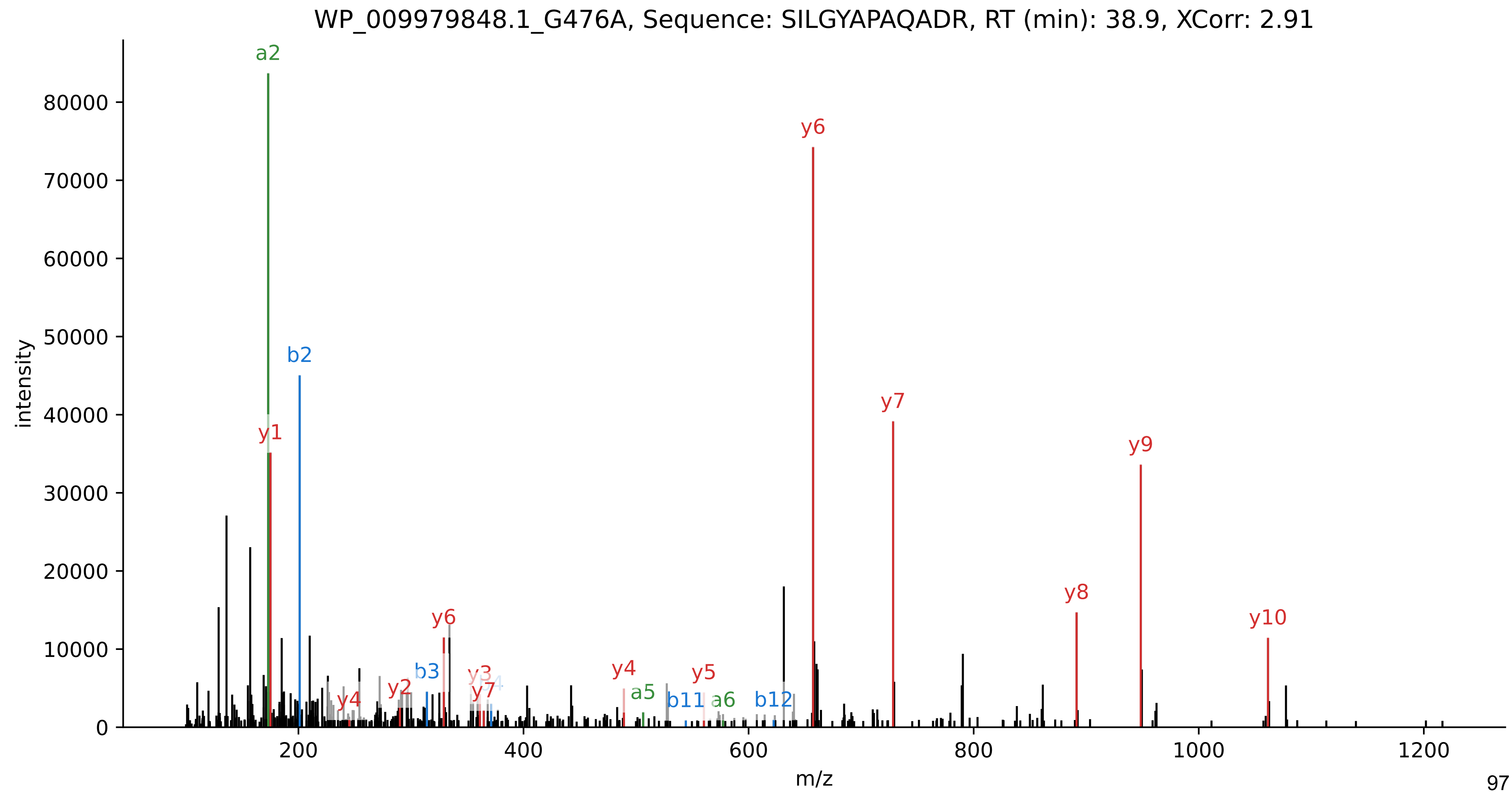

WP_009979848.1_G476A, Sequence: SILGYAPAQADR, RT (min): 38.9, XCorr: 2.91
intensity
m/z
a2
b2
y1
y6
y7
y9
y8
y10
b3
y3
y7
y4
y2
y4
a5
y5
b11
a6
b12

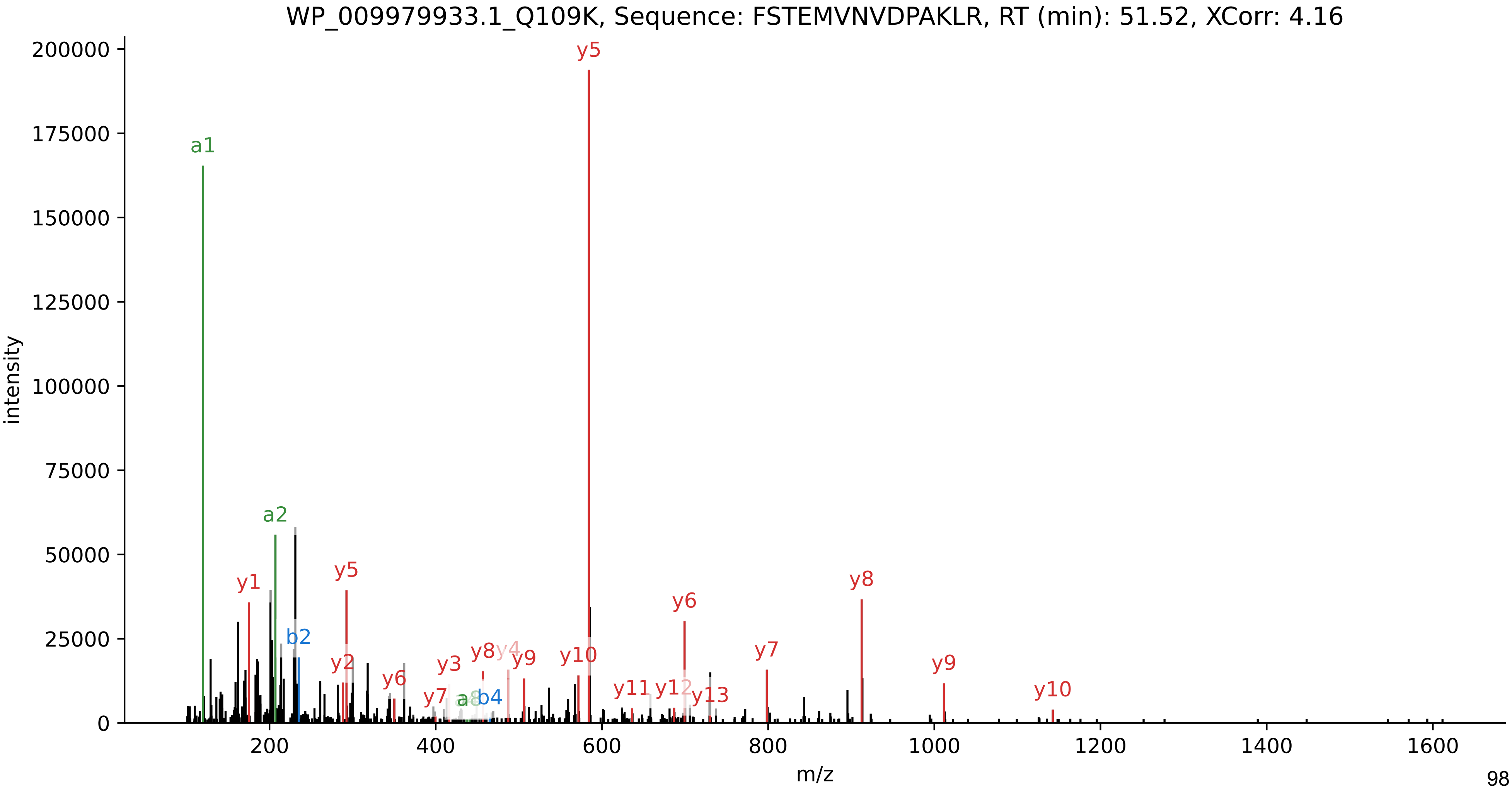

WP_009979933.1_Q109K, Sequence: FSTEMVNVDPAKLR, RT (min): 51.52, XCorr: 4.16
intensity
m/z
y5
a1
a2
y1
y5
b2
y2
y6
y3
y8
y4
y9
y10
y7
b4
y6
y11
y12
y13
y7
y8
y9
y10

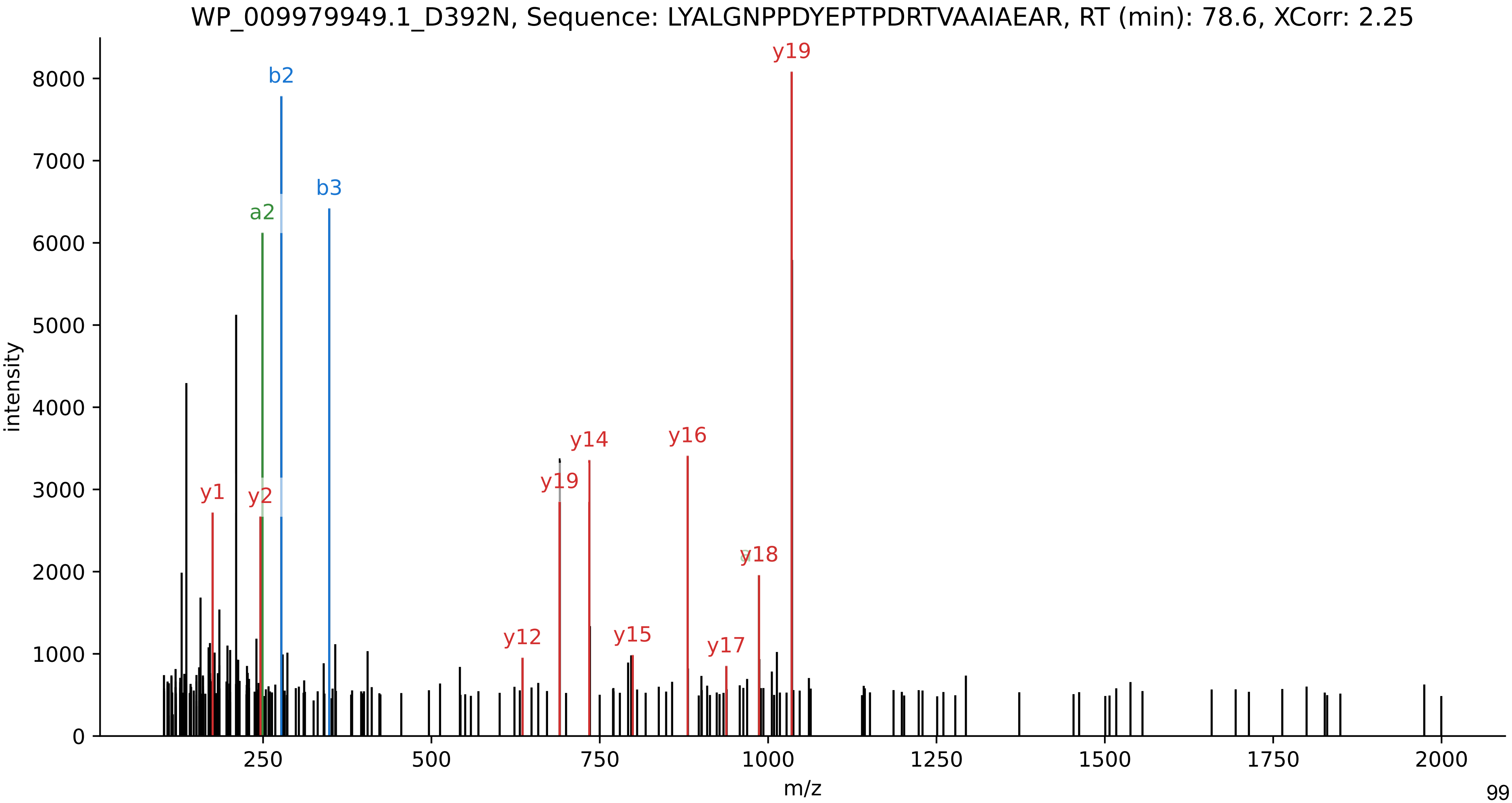
WP_009979949.1_D392N, Sequence: LYALGNPPDYEPTPDRTVAAIAEAR, RT (min): 78.6, XCorr: 2.25
intensity
m/z
b2
b3
a2
y19
y1
y2
y19
y14
y16
y18
y12
y15
y17
0
1000
2000
3000
4000
5000
6000
7000
8000
250
500
750
1000
1250
1500
1750
2000

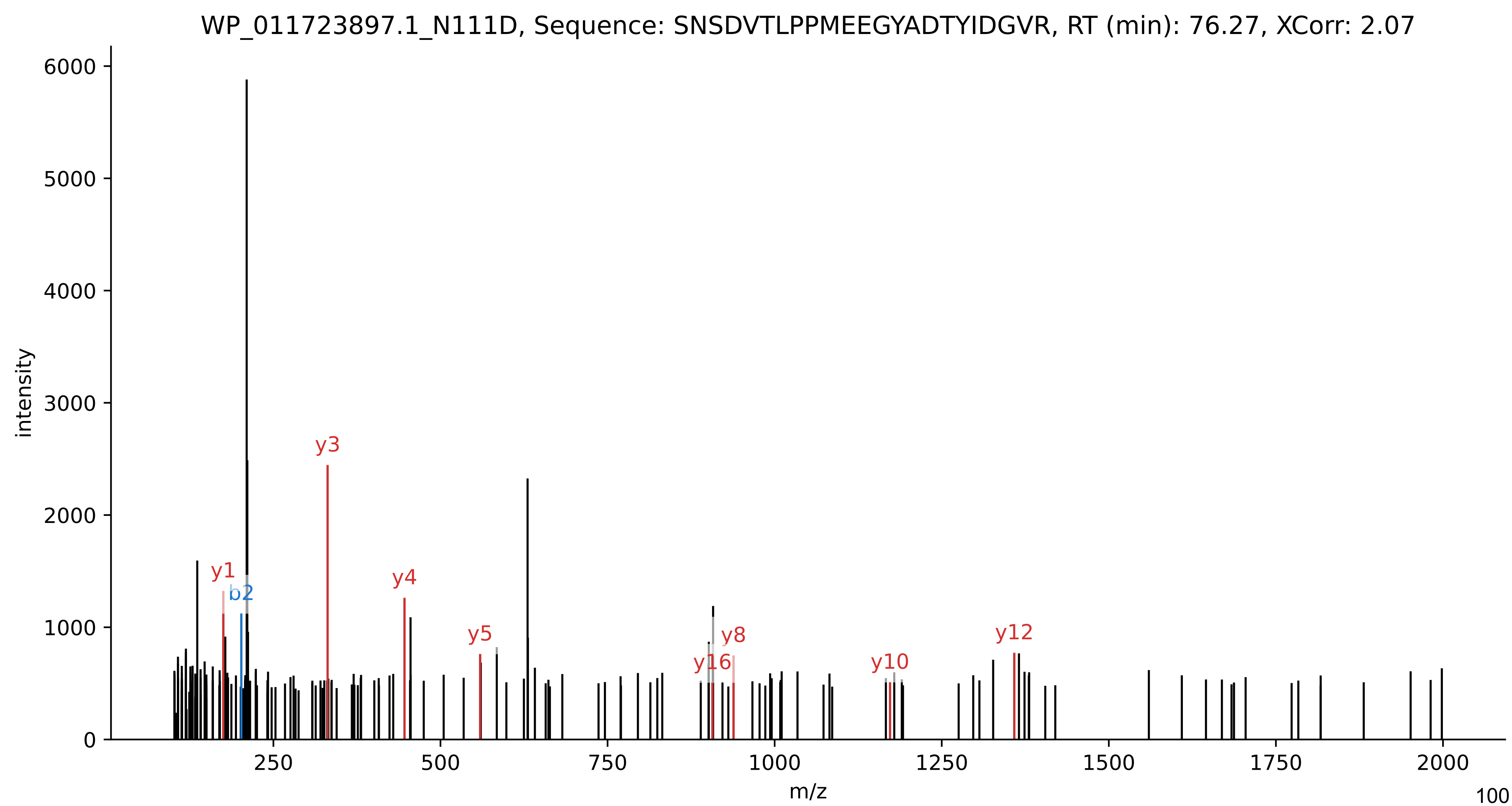
WP_011723897.1_N111D, Sequence: SNSDVTLPPMEEGYADTYIDGVR, RT (min): 76.27, XCorr: 2.07
intensity
m/z
y1
b2
y3
y4
y5
y16
y8
y10
y12
0
1000
2000
3000
4000
5000
6000
250
500
750
1000
1250
1500
1750
2000

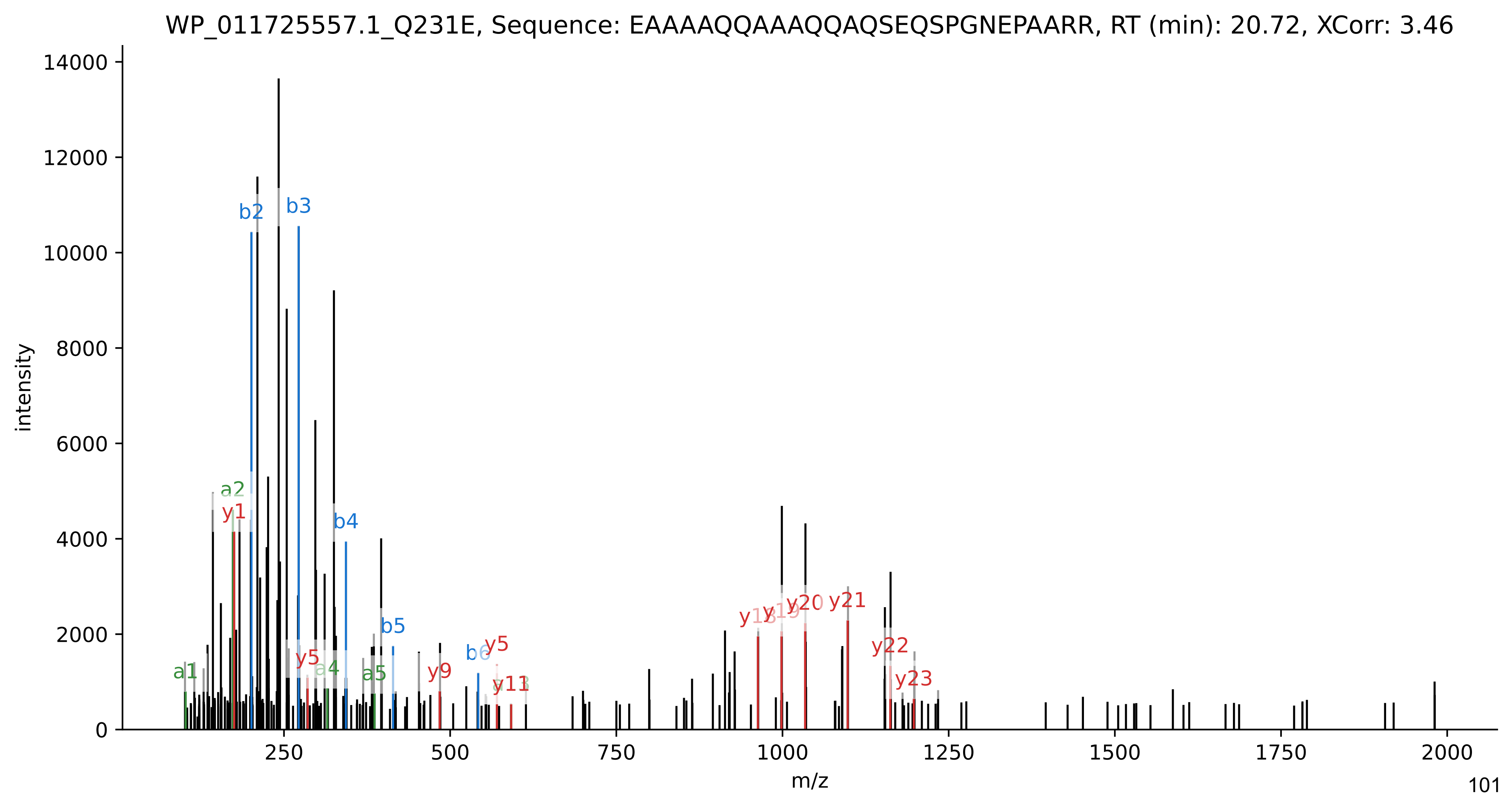

WP_011725557.1_Q231E, Sequence: EAAAAQQAAAQQAQSEQSPGNEPAARR, RT (min): 20.72, XCorr: 3.46
intensity
m/z
b2
b3
b4
b5
b6
a1
a2
a4
a5
y1
y5
y9
y11
y20
y21
y22
y23

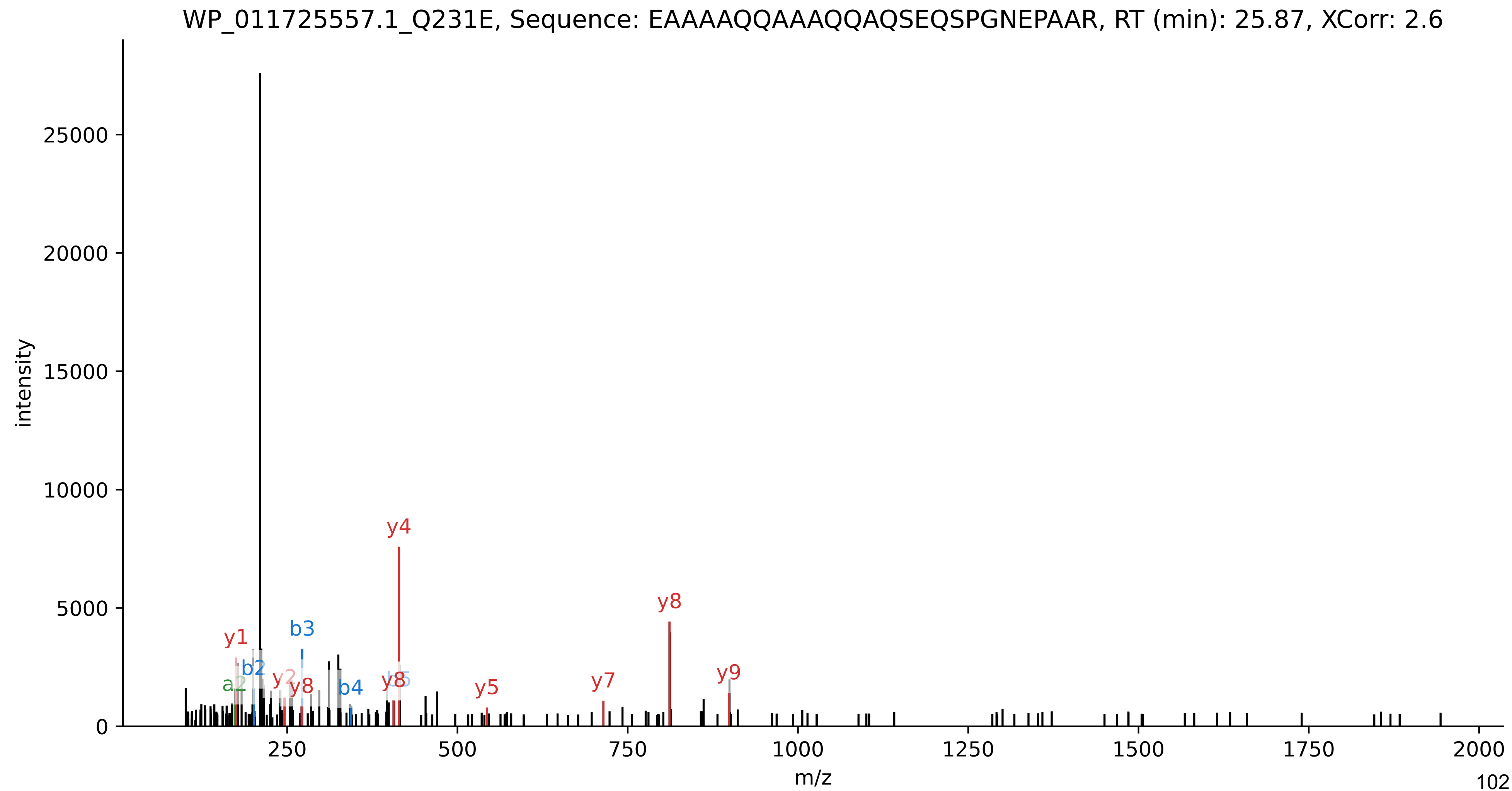
WP_011725557.1_Q231E, Sequence: EAAAAQQAAAQQAQSEQSPGNEPAAR, RT (min): 25.87, XCorr: 2.6
intensity
m/z
0
5000
10000
15000
20000
25000
250
500
750
1000
1250
1500
1750
2000
y1
a2
b2
y2
y8
b3
b4
y8
y4
y5
y7
y8
y9

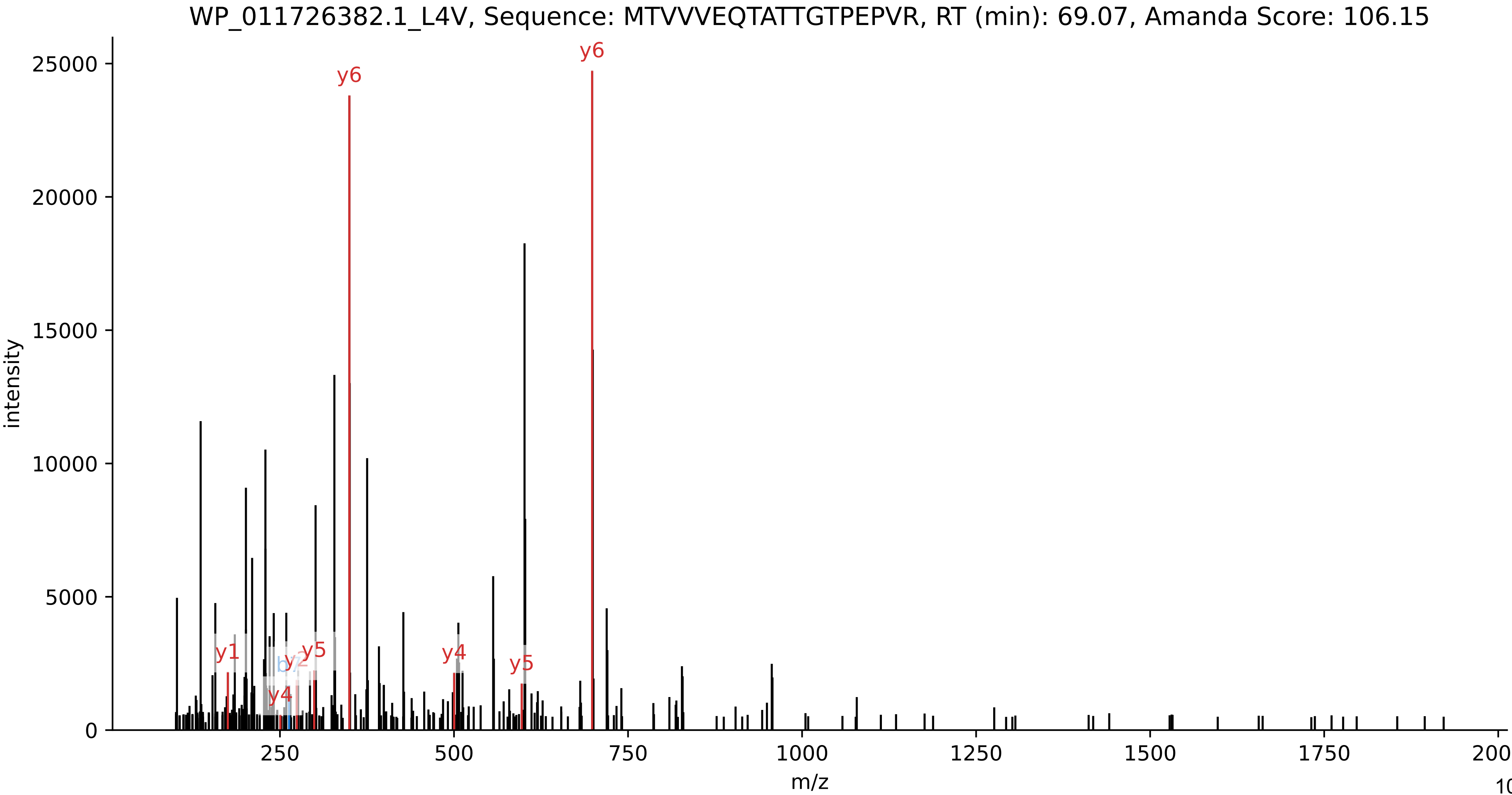
WP_011726382.1_L4V, Sequence: MTVVVEQTATTGTPEPVR, RT (min): 69.07, Amanda Score: 106.15
intensity
m/z
y6
y6
y1
y5
y4
y4
y5
25000
20000
15000
10000
5000
0
250
500
750
1000
1250
1500
1750
2000

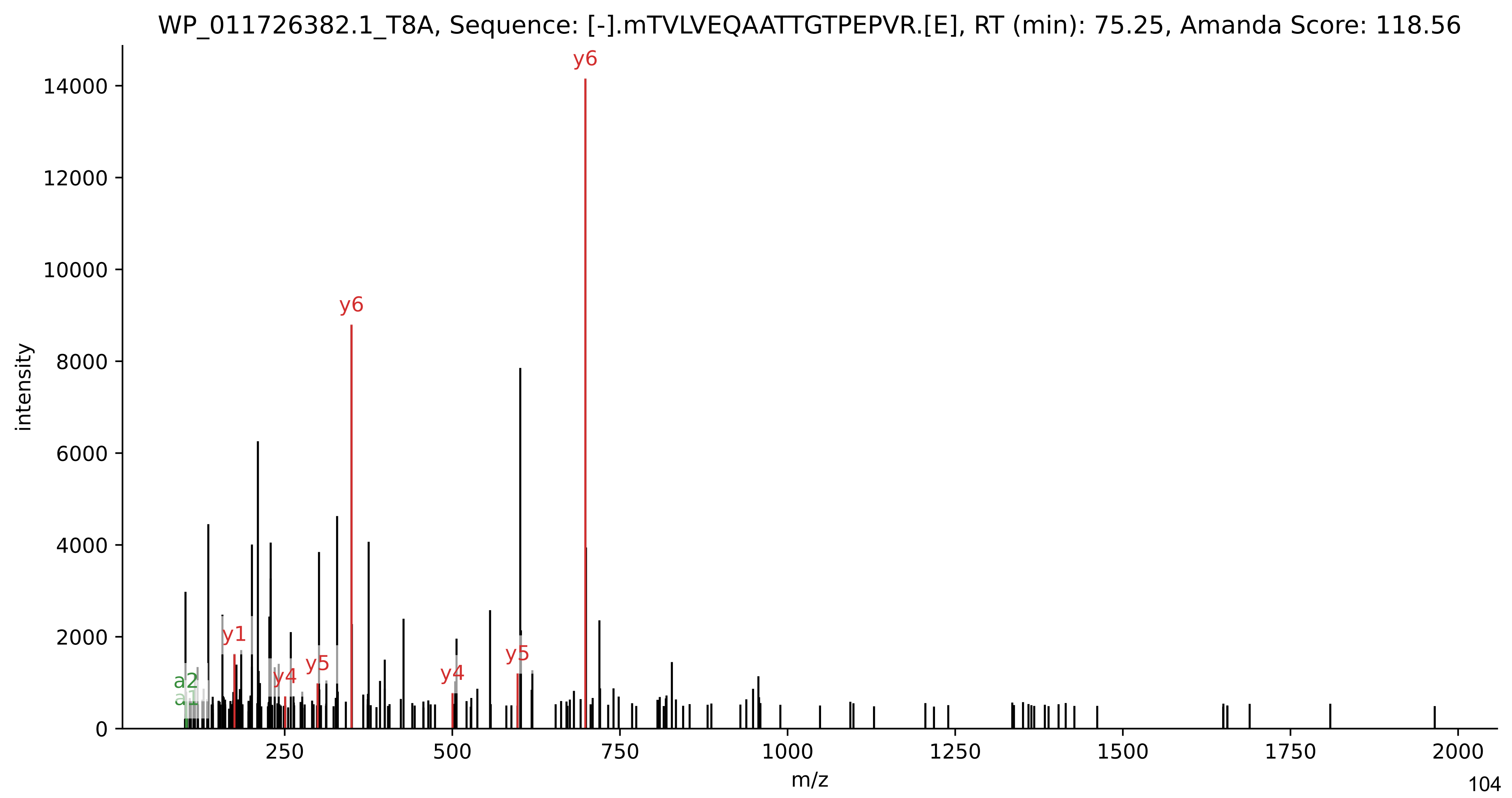
WP_011726382.1_T8A, Sequence: [-].mTVLVEQAATTGTPEPVR.[E], RT (min): 75.25, Amanda Score: 118.56
intensity
m/z
y6
y6
y1
y4
y5
y4
y5
a2
a1
0
2000
4000
6000
8000
10000
12000
14000
250
500
750
1000
1250
1500
1750
2000

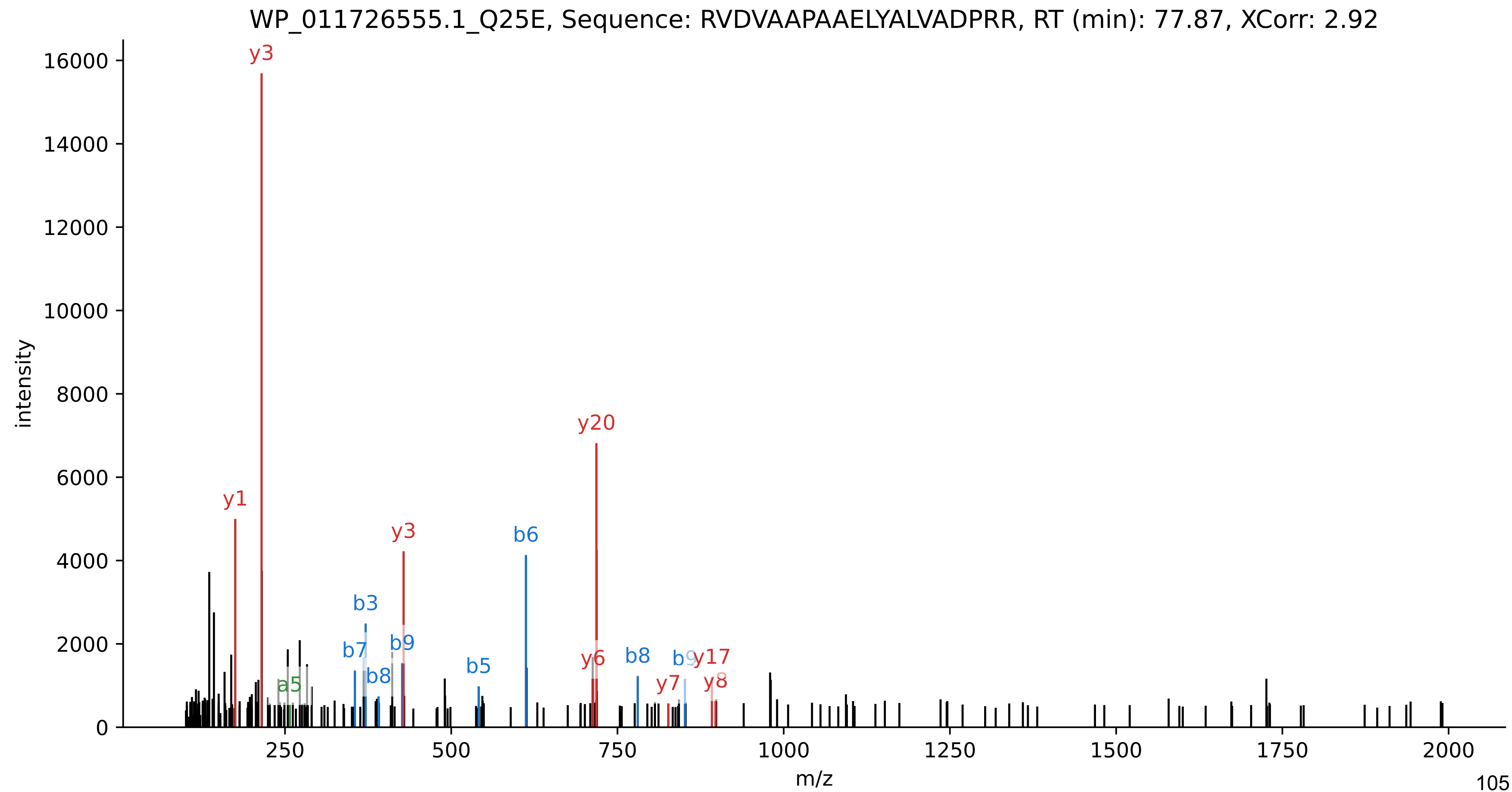

WP_011726555.1_Q25E, Sequence: RVDVAAPAAELYALVADPRR, RT (min): 77.87, XCorr: 2.92
intensity
m/z
y3
y20
y1
y3
b6
b3
b7
b9
b8
b5
y6
b8
b9
y17
y7
y8
a5

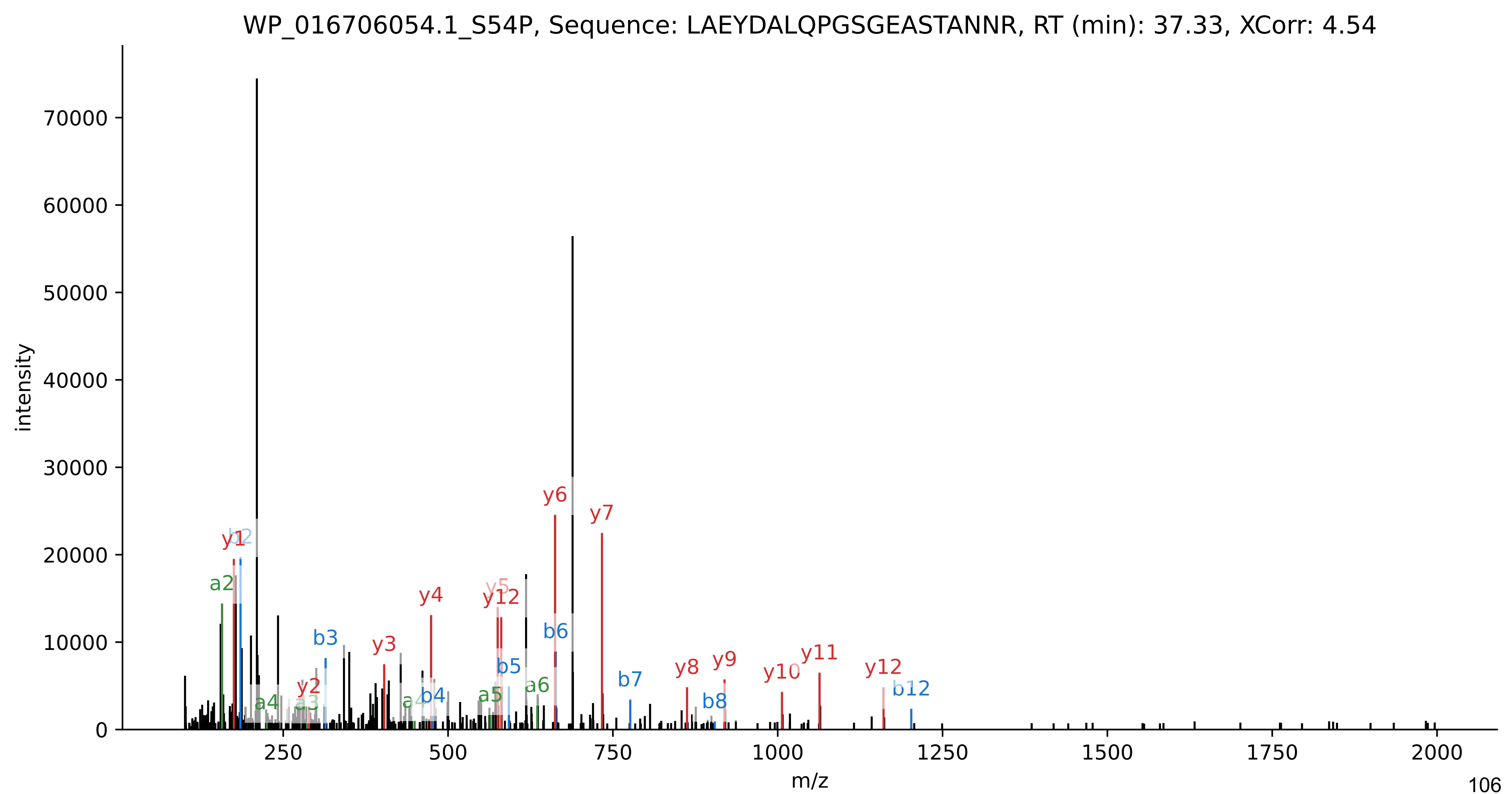
WP_016706054.1_S54P, Sequence: LAEYDALQPGSGEASTANNR, RT (min): 37.33, XCorr: 4.54
intensity
m/z
70000
60000
50000
40000
30000
20000
10000
0
250
500
750
1000
1250
1500
1750
2000
y1
b2
a2
a4
y2
a3
b3
y3
a4
b4
y4
a5
y5
y12
b5
a6
y6
b6
y7
b7
y8
b8
y9
y10
y11
y12
b12

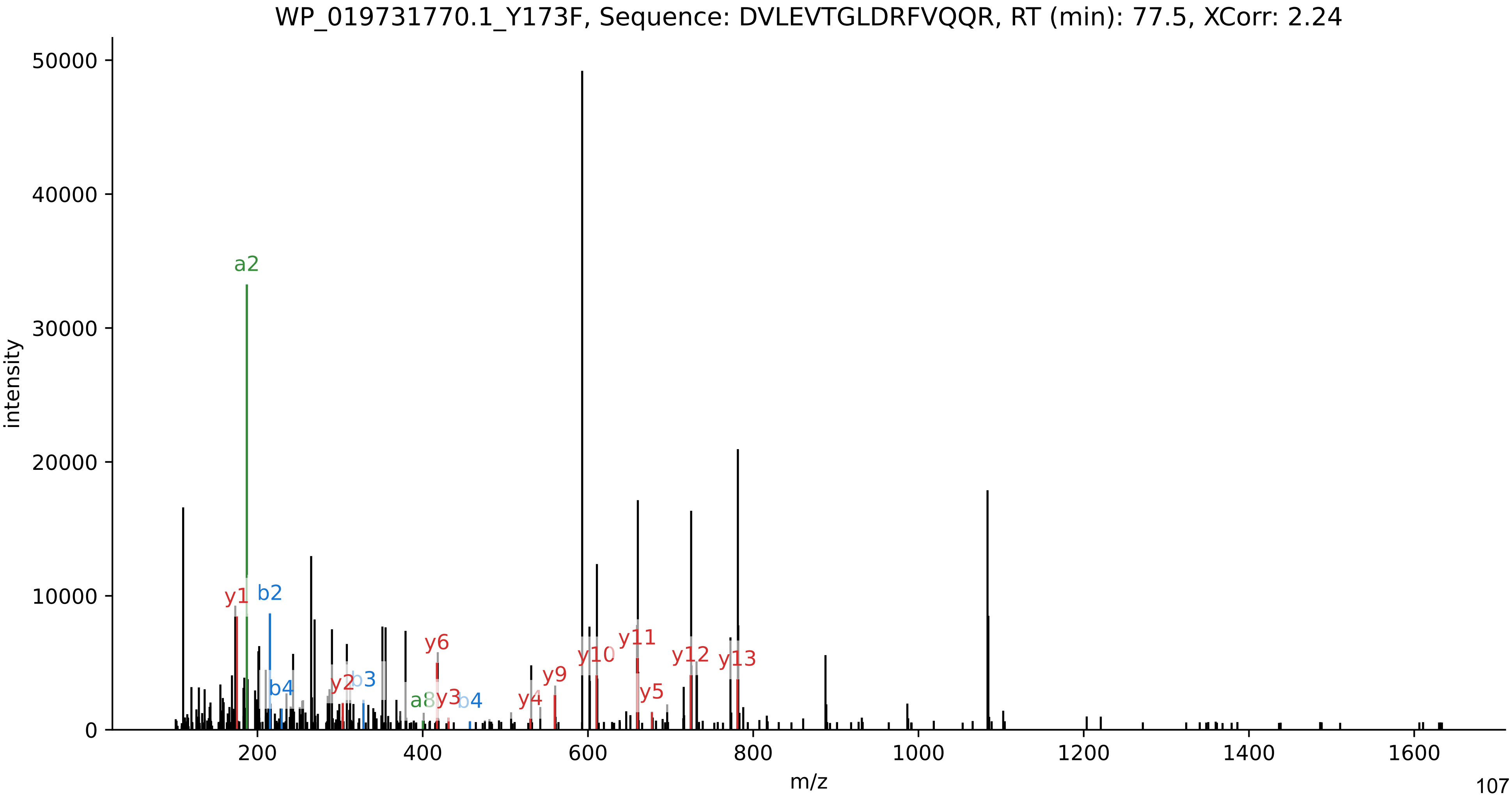

WP_019731770.1_Y173F, Sequence: DVLEVTGLDRFVQQR, RT (min): 77.5, XCorr: 2.24
intensity
m/z
a2
y1
b2
b4
y2
b3
a8
y3
b4
y6
y4
y9
y10
y11
y5
y12
y13

WP_019731959.1_K143E, Sequence: ITGETTLDRAR, RT (min): 11.17, XCorr: 2.61

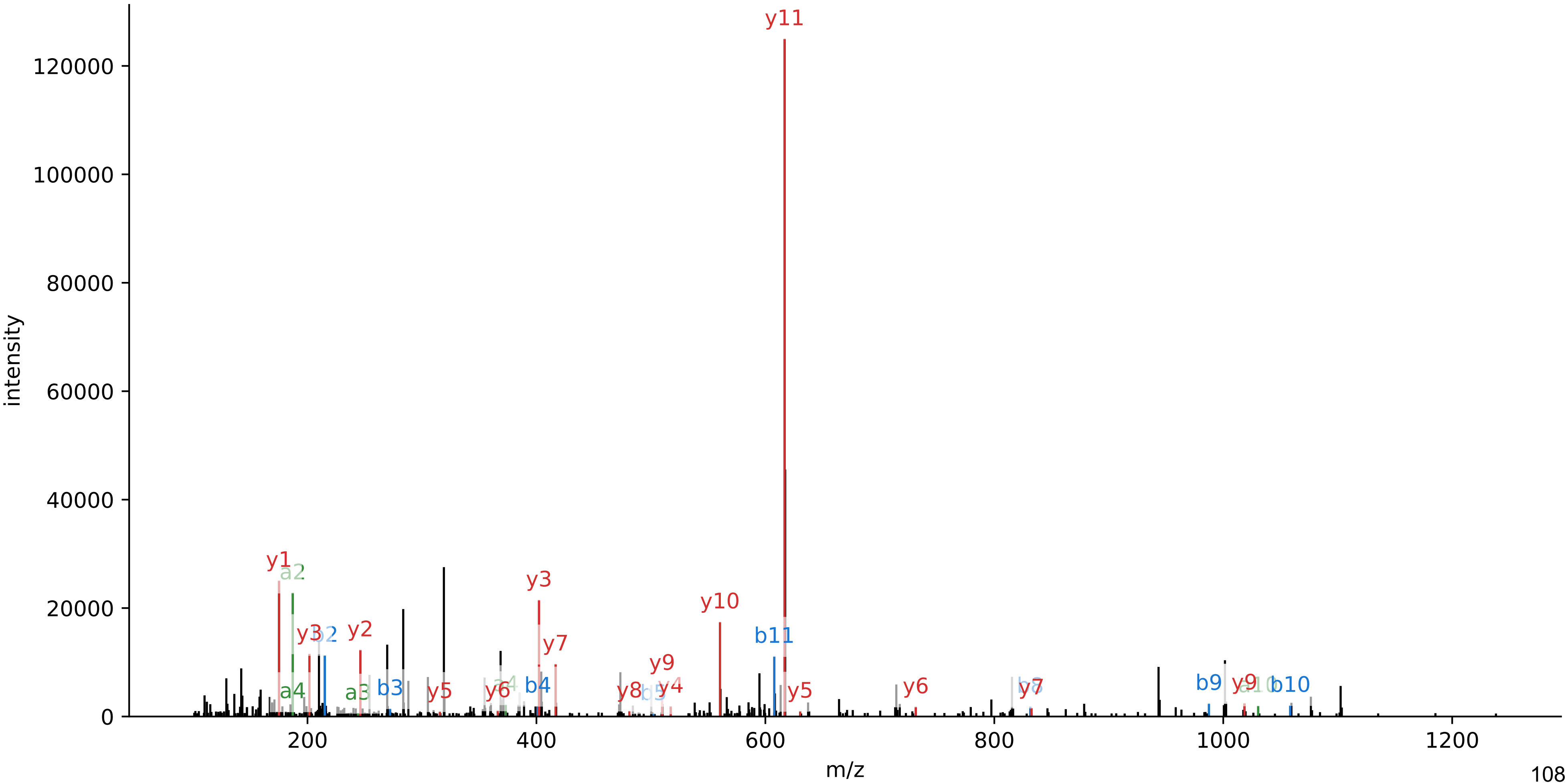

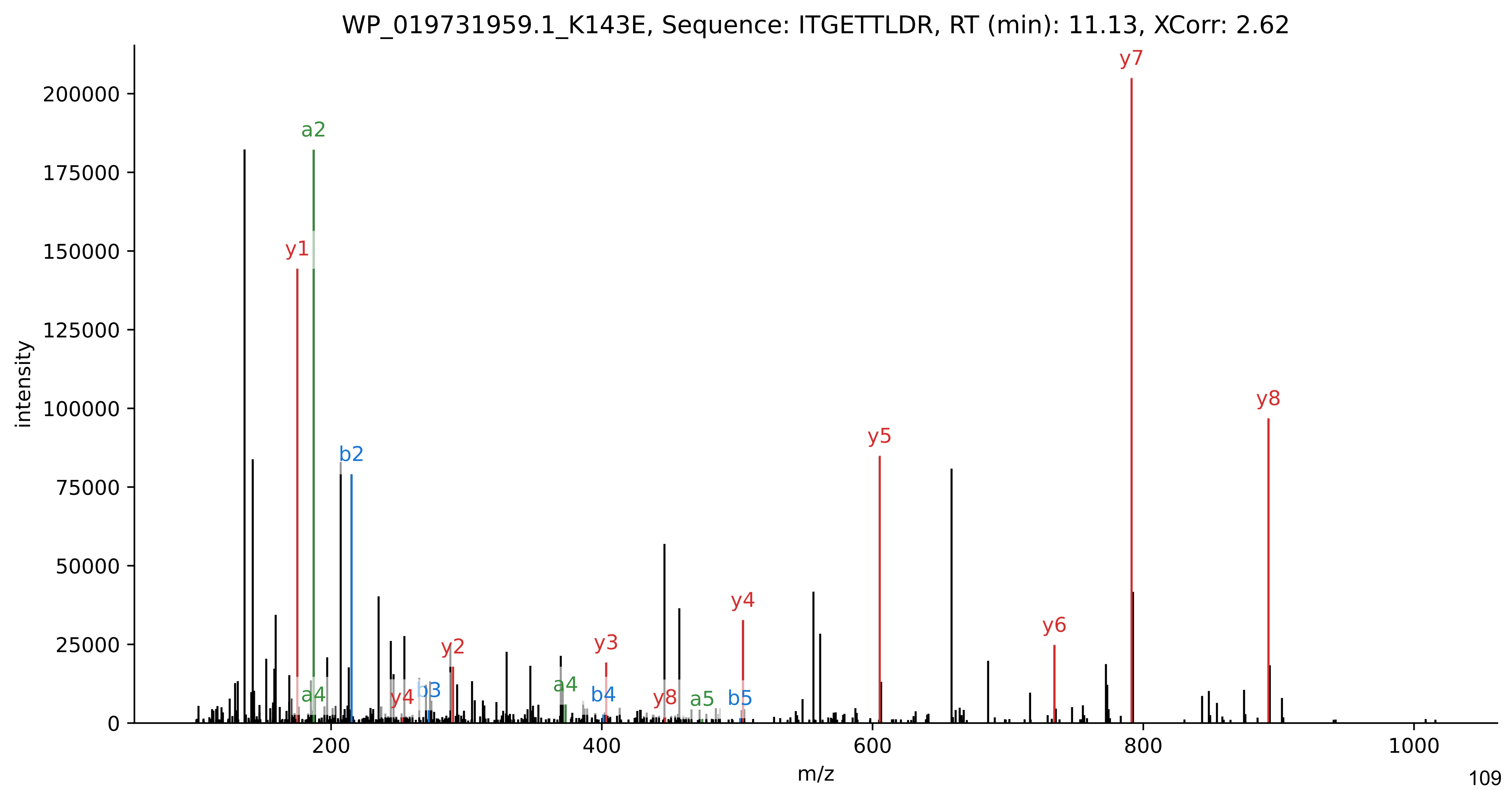
WP_019731959.1_K143E, Sequence: ITGETTLDR, RT (min): 11.13, XCorr: 2.62
intensity
m/z
y7
a2
y1
b2
y8
y5
y4
y6
y3
y2
a4
y4
b3
a4
b4
y8
a5
b5

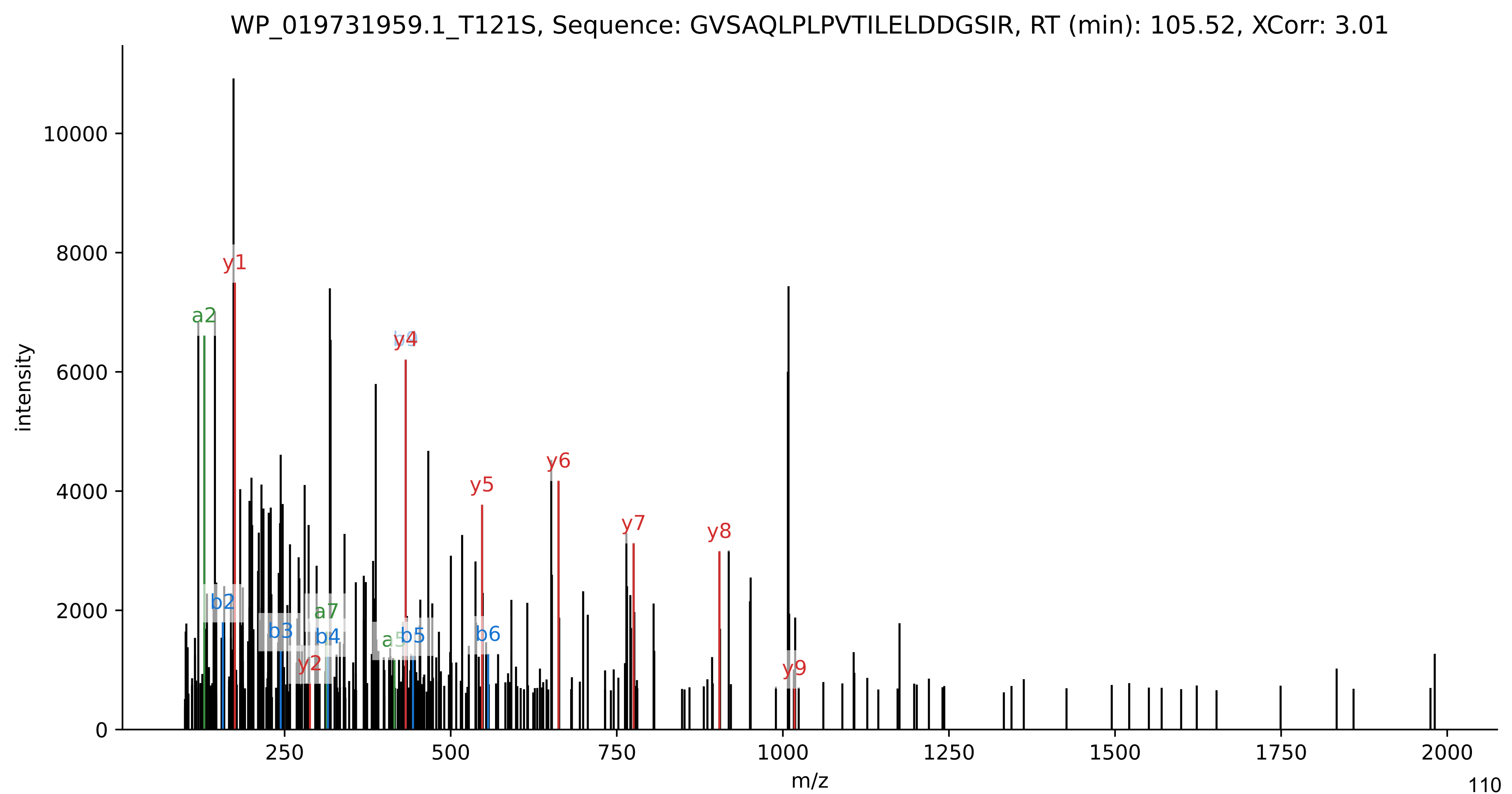

WP_019731959.1_T121S, Sequence: GVSAQLPLPVTILELDDGSIR, RT (min): 105.52, XCorr: 3.01
intensity
m/z
y1
a2
y4
y5
y6
y7
y8
y9
y2
b2
b3
b4
b5
b6
a7

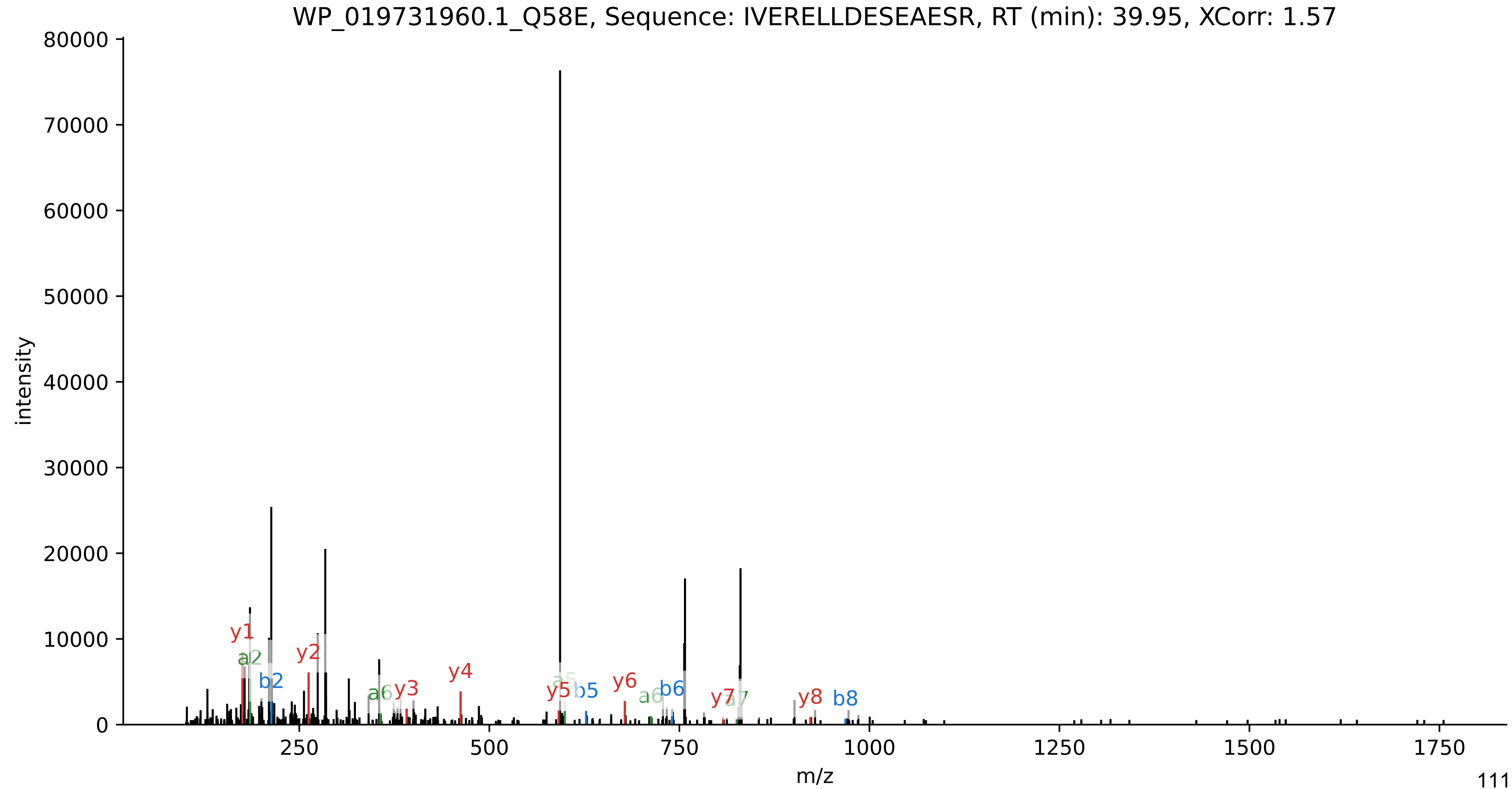

WP_019731960.1_Q58E, Sequence: IVERELLDESEAESR, RT (min): 39.95, XCorr: 1.57
intensity
m/z
y1
a2
b2
y2
a6
y3
y4
y5
b5
y6
a6
b6
y7
a7
y8
b8

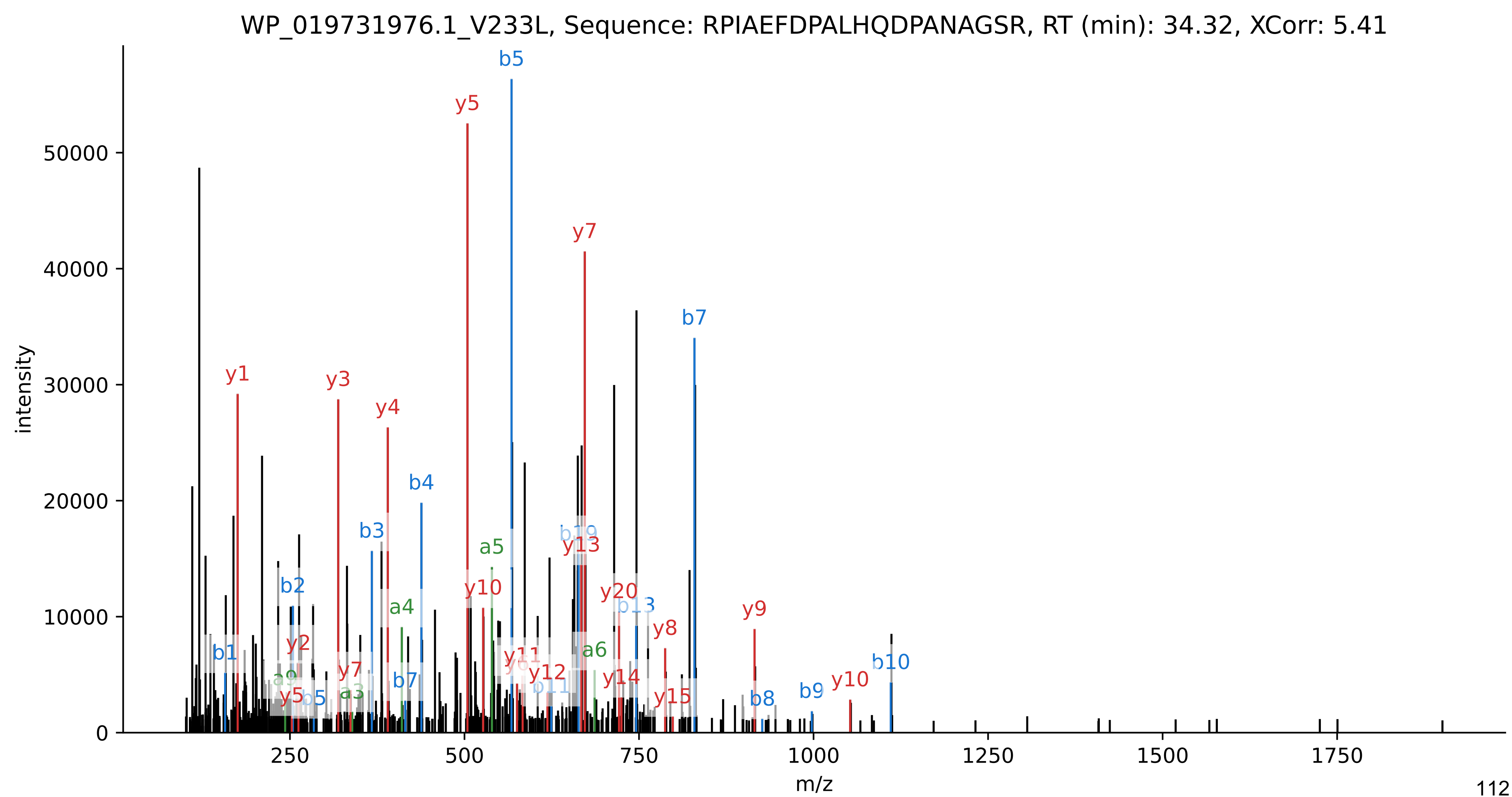
WP_019731976.1_V233L, Sequence: RPIAEFDPALHQDPANAGSR, RT (min): 34.32, XCorr: 5.41
intensity
m/z
b5
y5
y7
b7
y1
y3
y4
b4
b3
a5
y13
b2
y10
y20
a4
b13
y9
y8
b1
y2
a6
y11
y12
b10
y7
y14
y10
b7
b11
a3
y5
b5
y15
b8
b9
0
10000
20000
30000
40000
50000
250
500
750
1000
1250
1500
1750

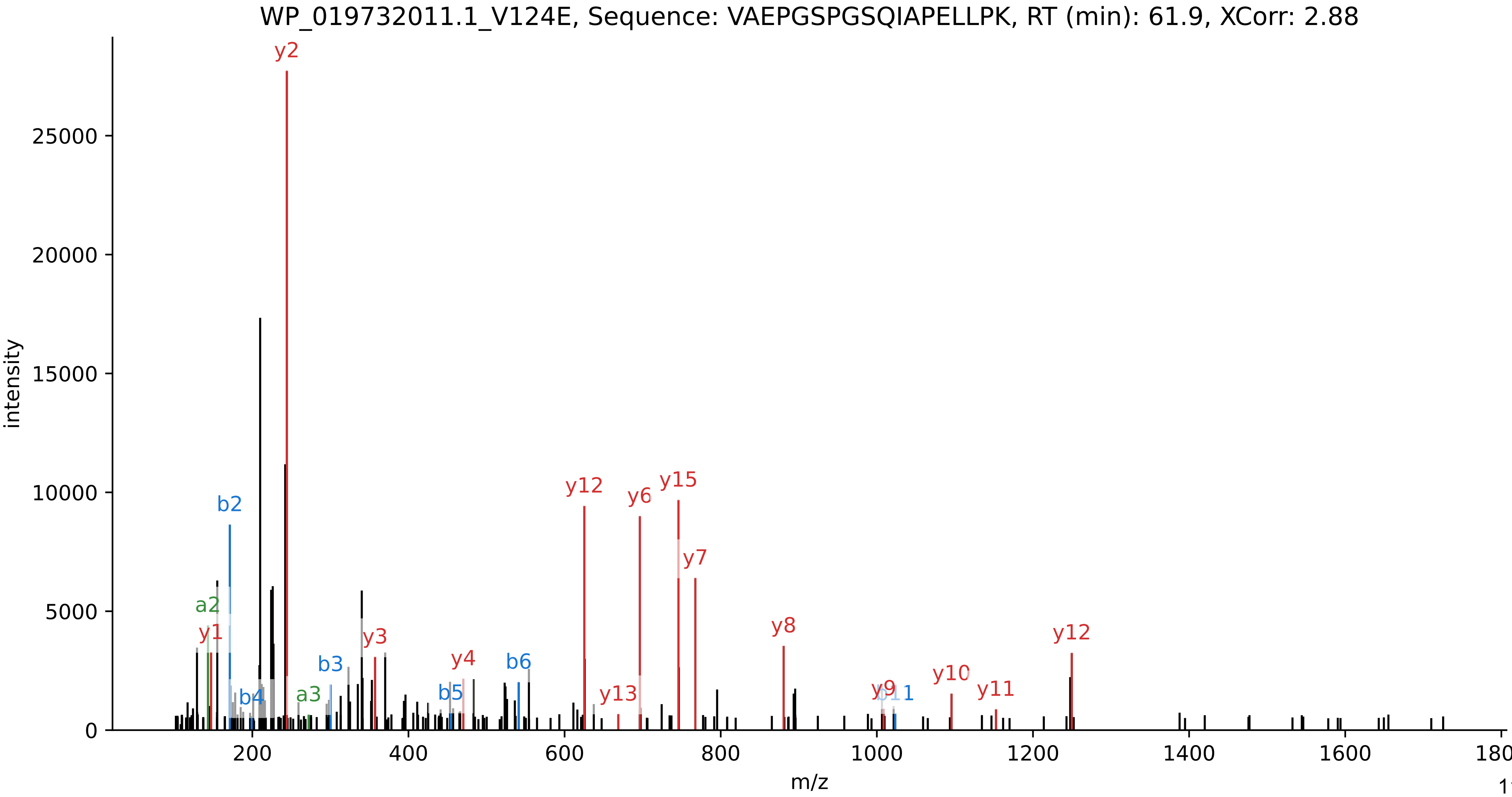
WP_019732011.1_V124E, Sequence: VAEPGSPGSQIAPELLPK, RT (min): 61.9, XCorr: 2.88
intensity
m/z
y2
b2
a2
y1
b4
a3
b3
y3
y4
b5
b6
y12
y13
y6
y15
y7
y8
y9
b11
y10
y11
y12
0
5000
10000
15000
20000
25000
200
400
600
800
1000
1200
1400
1600
1800

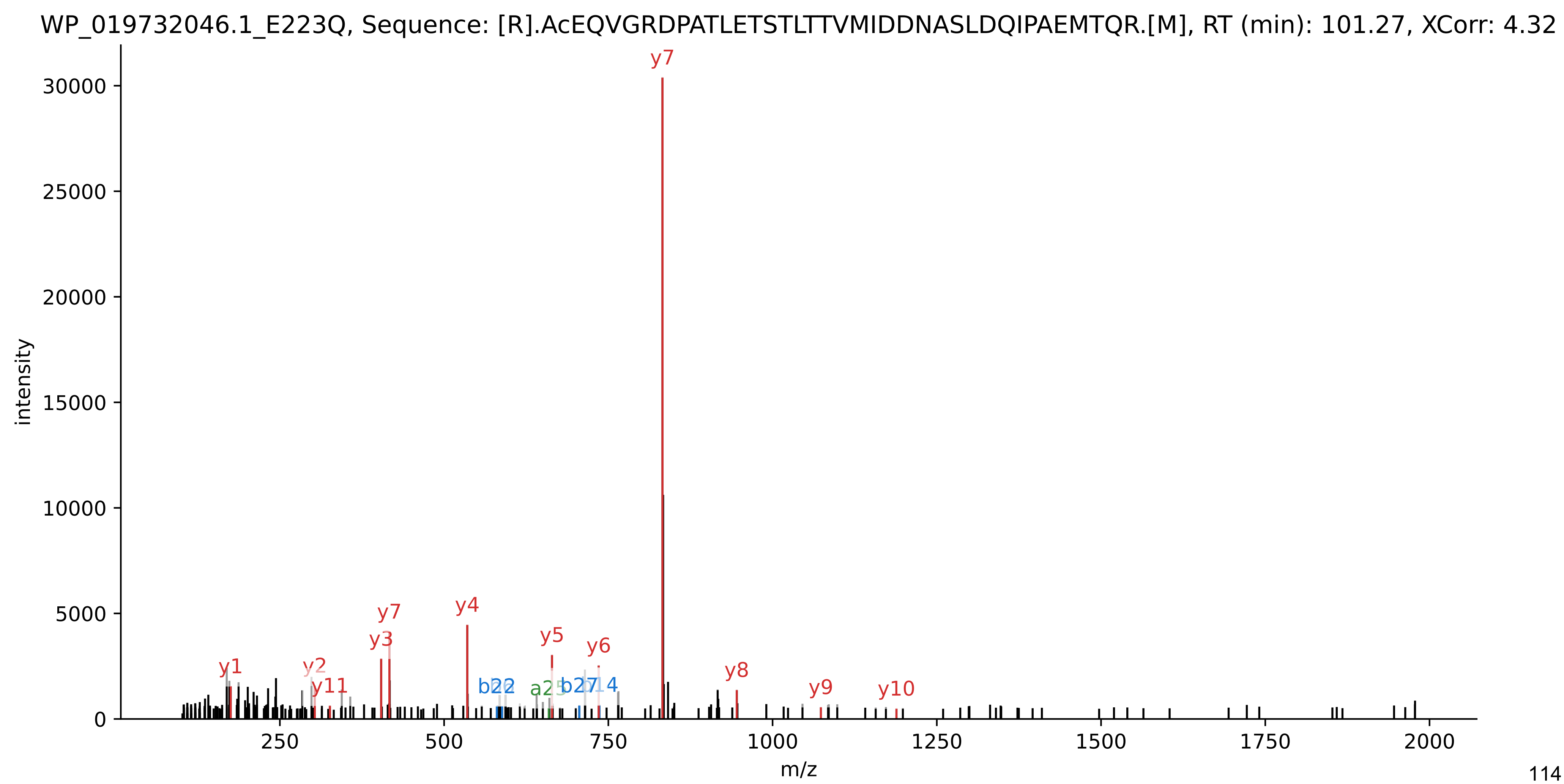
WP_019732046.1_E223Q, Sequence: [R].AcEQVGRDPATLETSTLTTVMIDDNASLDQIPAEMTQR.[M], RT (min): 101.27, XCorr: 4.32
intensity
m/z
y7
y1
y2
y11
y3
y7
y4
b22
a25
b27
y5
y6
y8
y9
y10

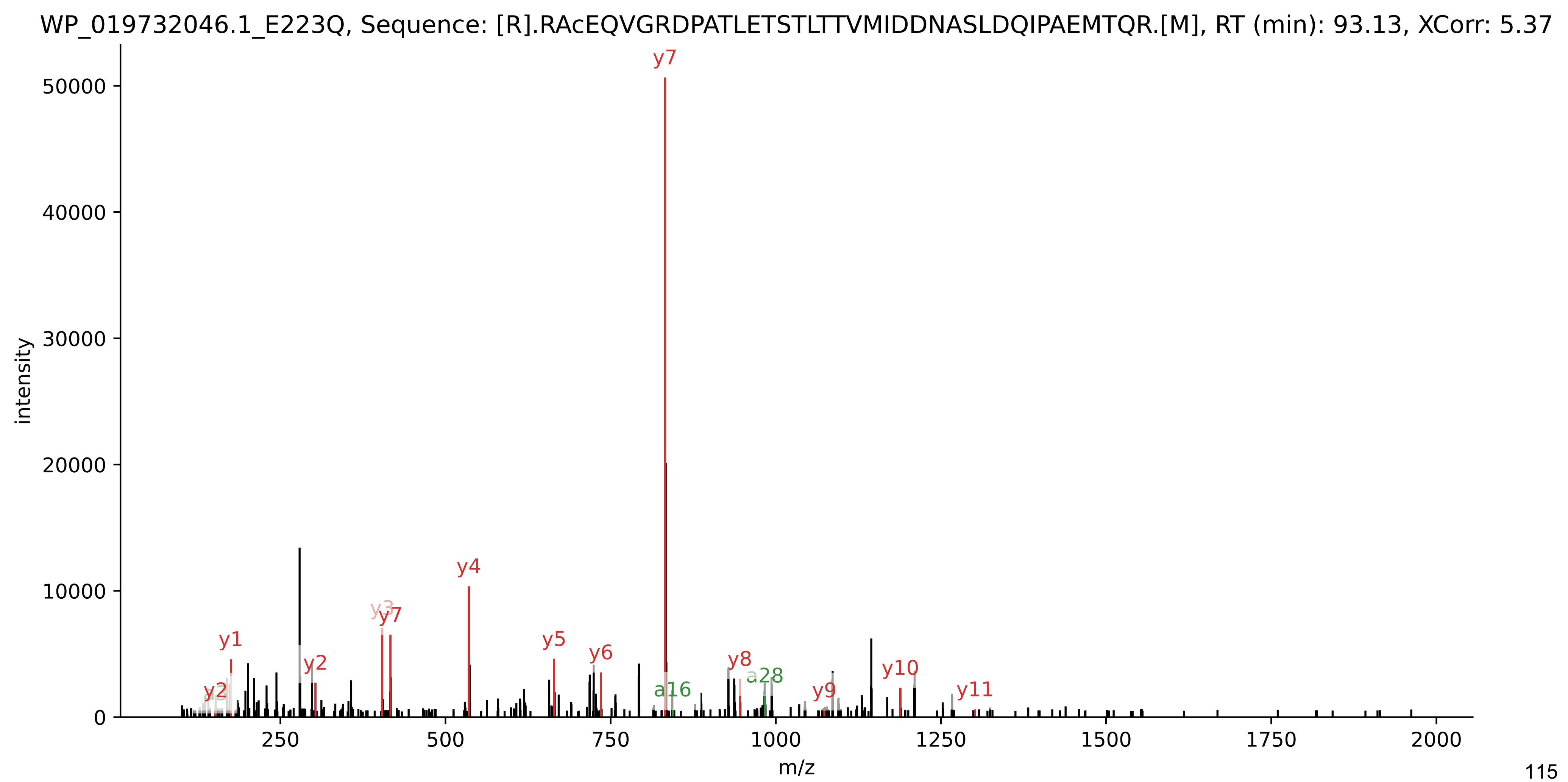
WP_019732046.1_E223Q, Sequence: [R].RAcEQVGRDPATLETSTLTTVMIDDNASLDQIPAEMTQR.[M], RT (min): 93.13, XCorr: 5.37
intensity
m/z
y7
y4
y3
y7
y1
y2
y5
y6
y8
a28
y10
a16
y2
y9
y11
50000
40000
30000
20000
10000
0
250
500
750
1000
1250
1500
1750
2000

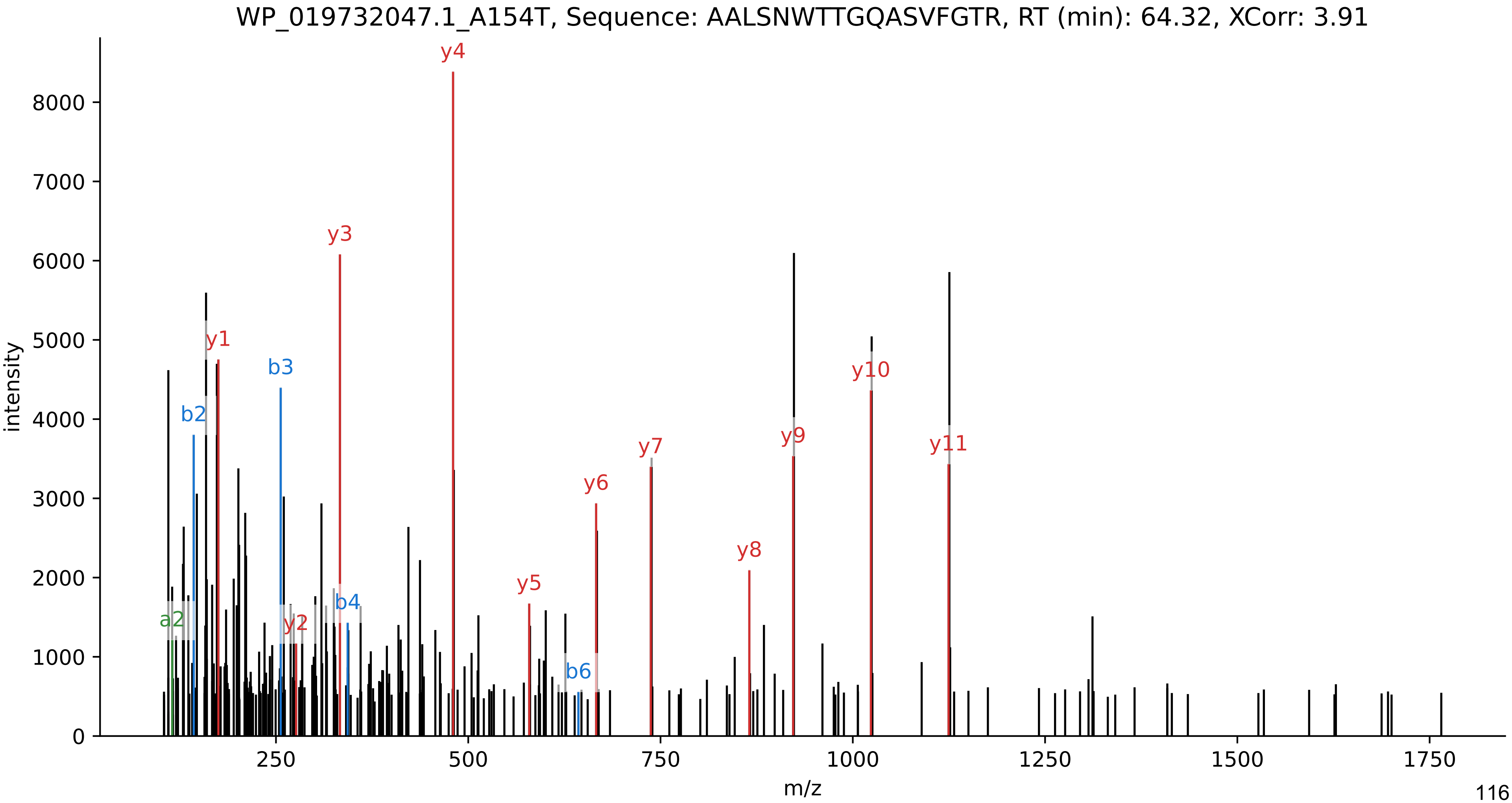

WP_019732047.1_A154T, Sequence: AALSNWTTGQASVFGTR, RT (min): 64.32, XCorr: 3.91
intensity
m/z
0
1000
2000
3000
4000
5000
6000
7000
8000
250
500
750
1000
1250
1500
1750
y4
y3
y1
b3
b2
y10
y9
y7
y11
y6
y8
y5
b4
a2
y2
b6

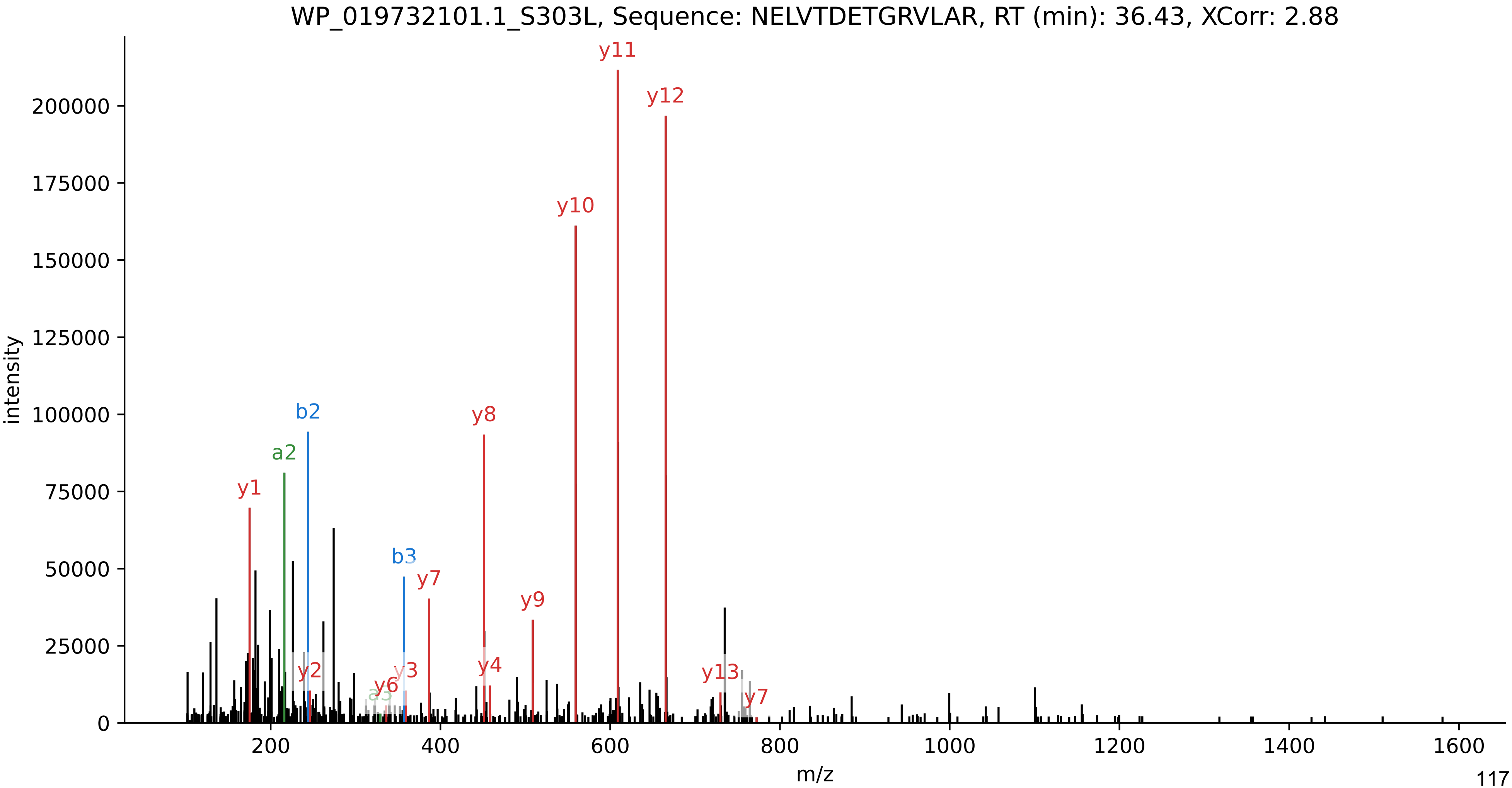
WP_019732101.1_S303L, Sequence: NELVTDETGRVLAR, RT (min): 36.43, XCorr: 2.88
intensity
m/z
y11
y12
y10
b2
y8
a2
y1
b3
y7
y9
y2
y3
y6
a3
y4
y13
y7
0
25000
50000
75000
100000
125000
150000
175000
200000
200
400
600
800
1000
1200
1400
1600

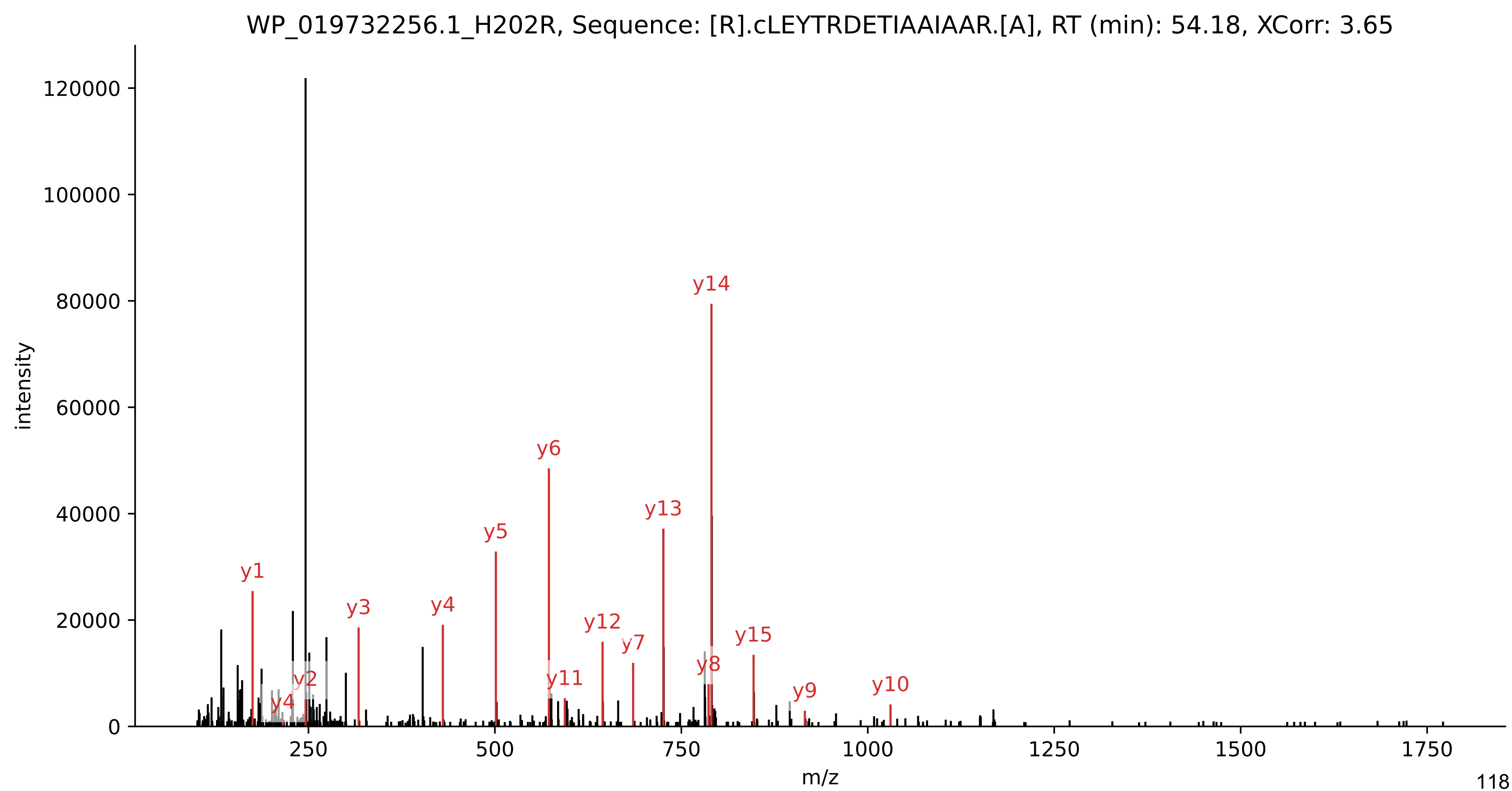

WP_019732256.1_H202R, Sequence: [R].cLEYTRDETIAAIAAR.[A], RT (min): 54.18, XCorr: 3.65
intensity
m/z
0
20000
40000
60000
80000
100000
120000
250
500
750
1000
1250
1500
1750
y1
y2
y3
y4
y4
y5
y6
y7
y8
y9
y10
y11
y12
y13
y14
y15

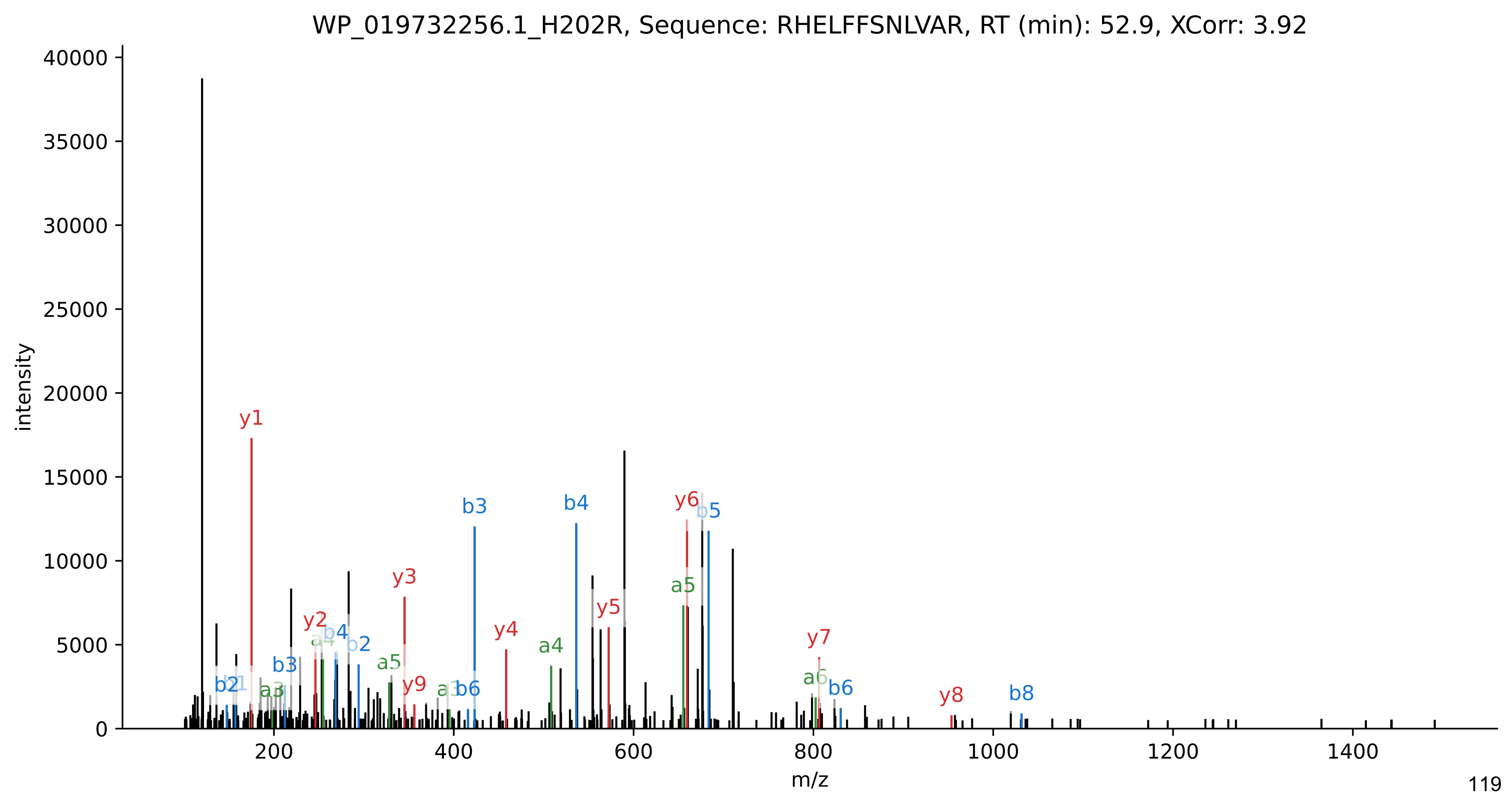
WP_019732256.1_H202R, Sequence: RHELFFSNLVAR, RT (min): 52.9, XCorr: 3.92
intensity
m/z
y1
y2
y3
y4
y5
y6
y7
y8
y9
b2
b3
b4
b5
b6
b8
a3
a4
a5
a6

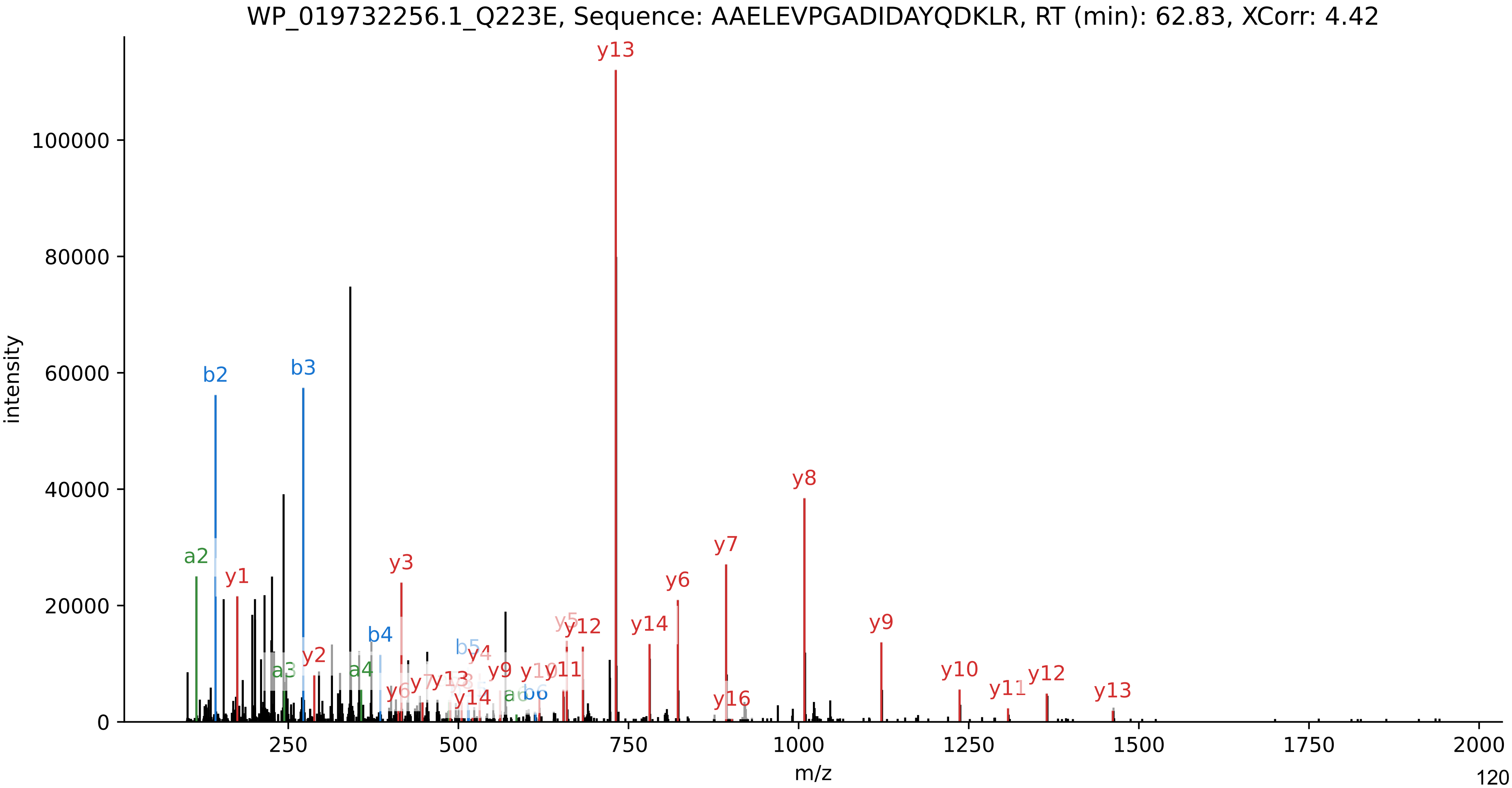
WP_019732256.1_Q223E, Sequence: AAELEVPGADIDAYQDKLR, RT (min): 62.83, XCorr: 4.42
intensity
m/z
y13
y8
y7
y6
y3
y1
y2
a2
b2
b3
b4
b5
a3
a4
y4
y5
y12
y14
y9
y10
y11
y16
y6
y7
y14
y11
y10
y12
y13
b6
0
20000
40000
60000
80000
100000
250
500
750
1000
1250
1500
1750
2000

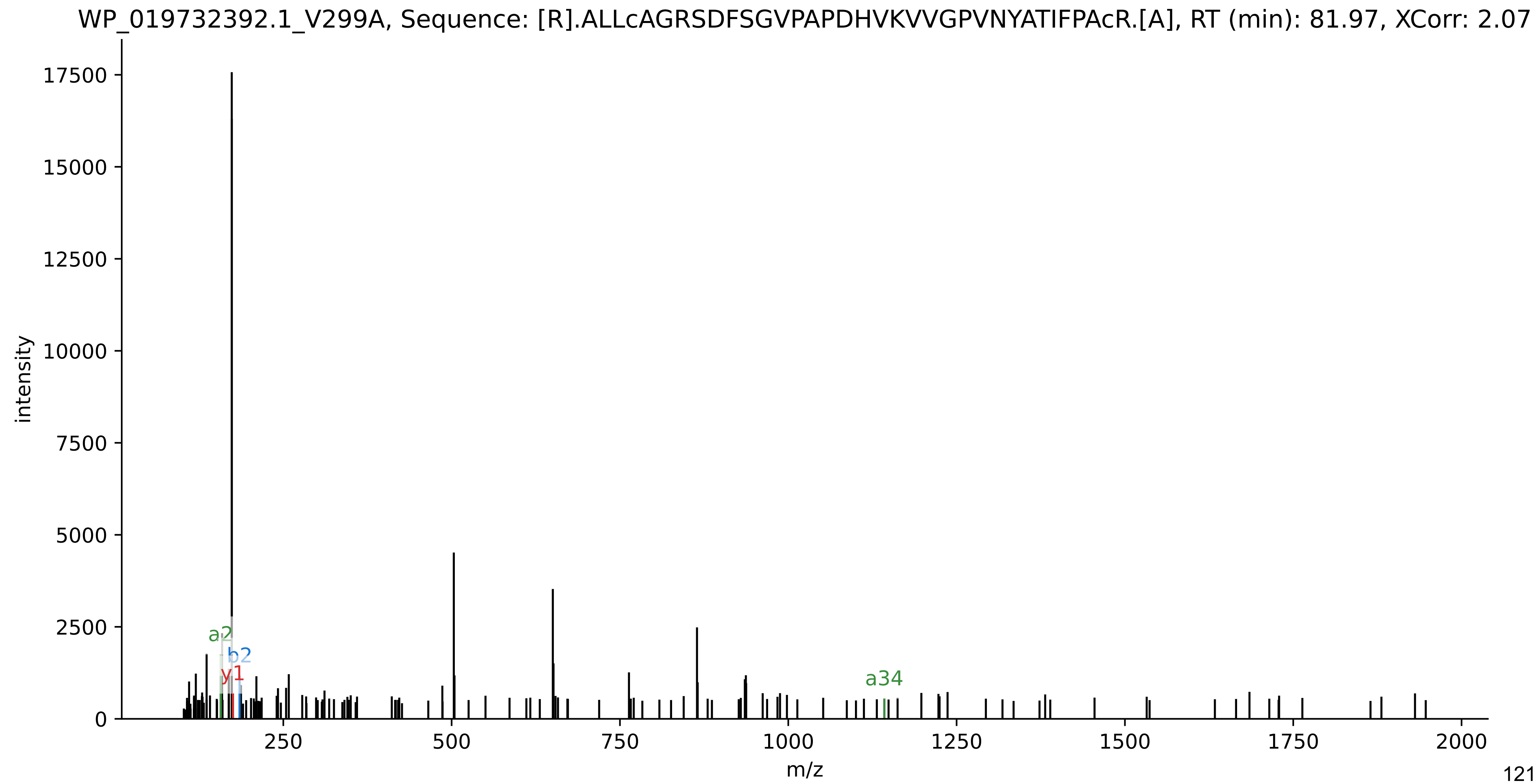
WP_019732392.1_V299A, Sequence: [R].ALLcAGRSDFSGVPAPDHVKVVGPVNYATIFPAcR.[A], RT (min): 81.97, XCorr: 2.07
intensity
m/z
a2
b2
y1
a34
0
2500
5000
7500
10000
12500
15000
17500
250
500
750
1000
1250
1500
1750
2000

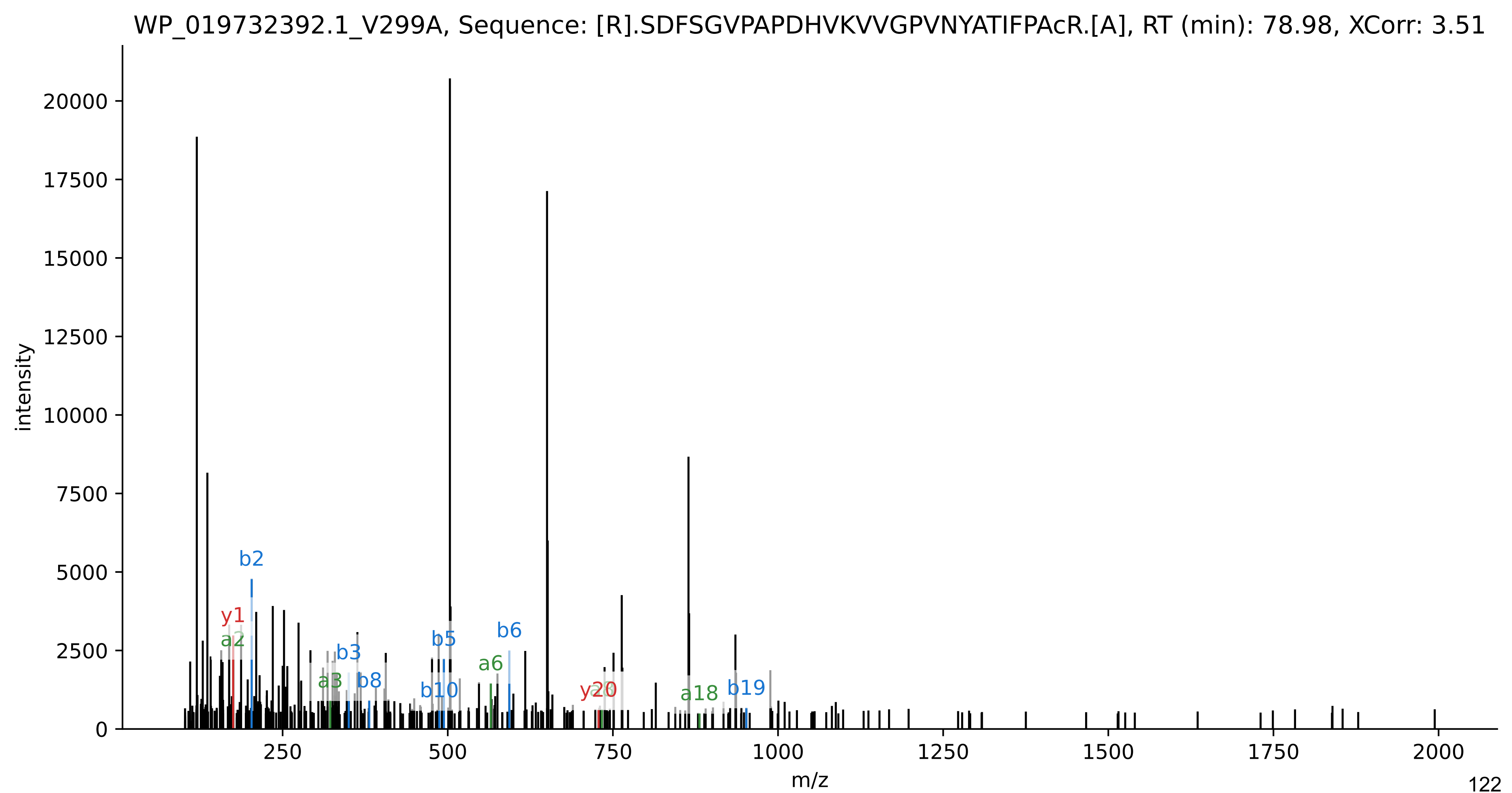
WP_019732392.1_V299A, Sequence: [R].SDFSGVPAPDHVKVVGPVNYATIFPAcR.[A], RT (min): 78.98, XCorr: 3.51
intensity
m/z
0
2500
5000
7500
10000
12500
15000
17500
20000
250
500
750
1000
1250
1500
1750
2000
b2
y1
a2
b3
a3
b8
b5
b10
b6
a6
y20
a18
b19

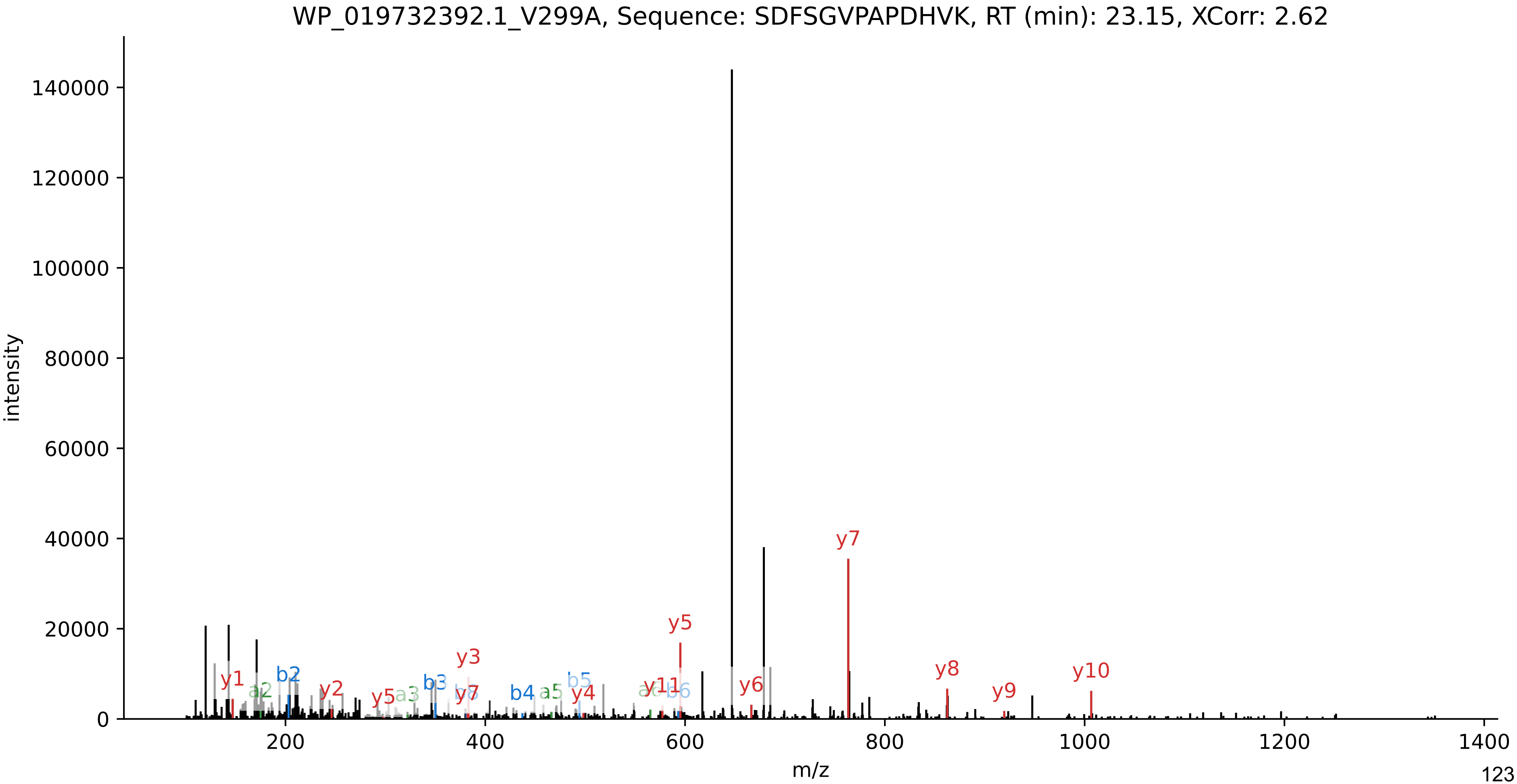

WP_019732392.1_V299A, Sequence: SDFSGVPAPDHVK, RT (min): 23.15, XCorr: 2.62
intensity
m/z
140000
120000
100000
80000
60000
40000
20000
0
200
400
600
800
1000
1200
1400
y1
a2
b2
y2
y5
a3
b3
y3
b8
y7
b4
a5
b5
y4
a6
y11
b6
y5
y6
y7
y8
y9
y10

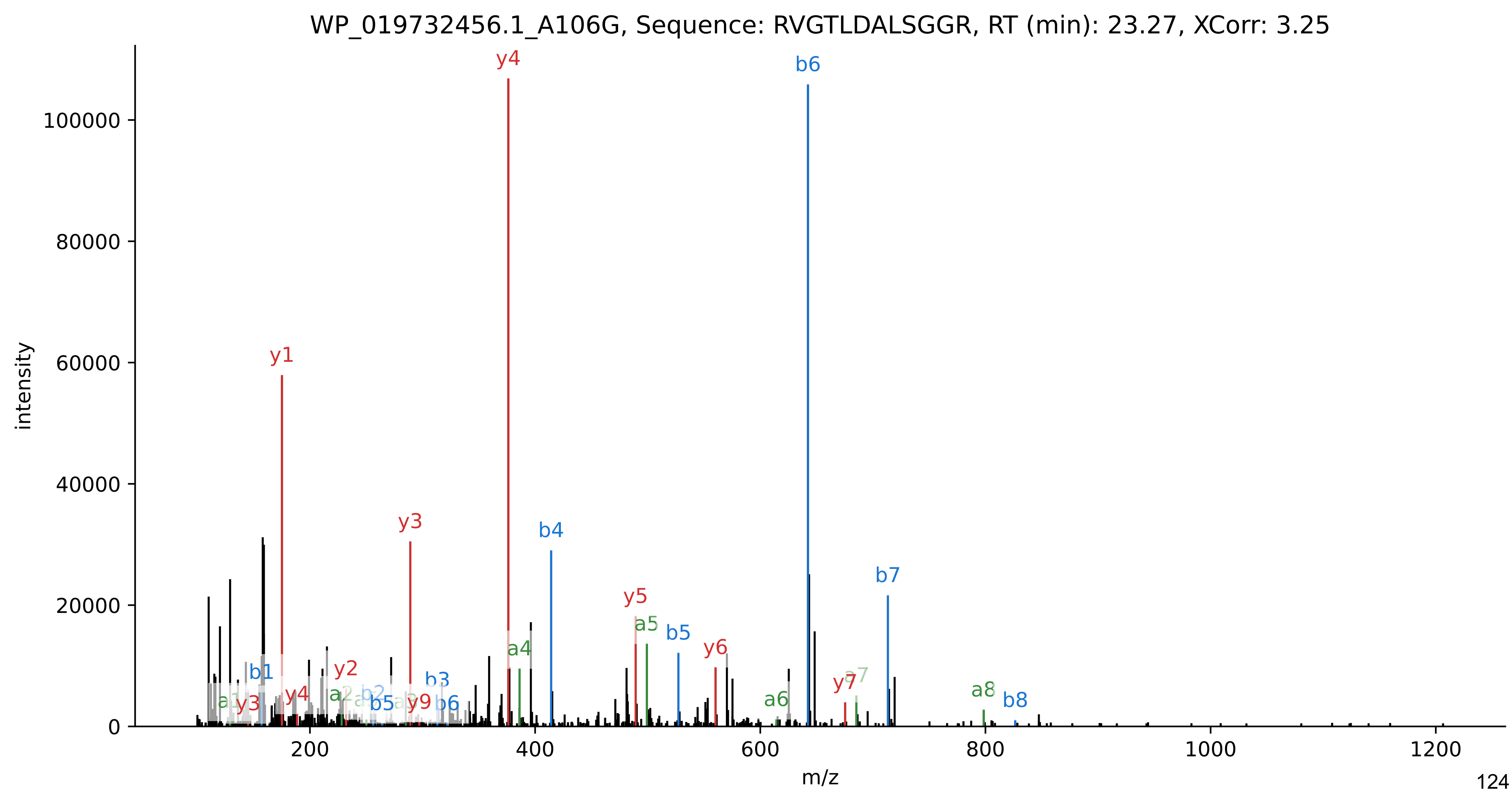

WP_019732456.1_A106G, Sequence: RVGTLDALSGGR, RT (min): 23.27, XCorr: 3.25
intensity
m/z
y4
b6
y1
y3
b4
b7
y5
a5
b5
y6
a4
y2
b1
b3
y7
a8
b8
a6

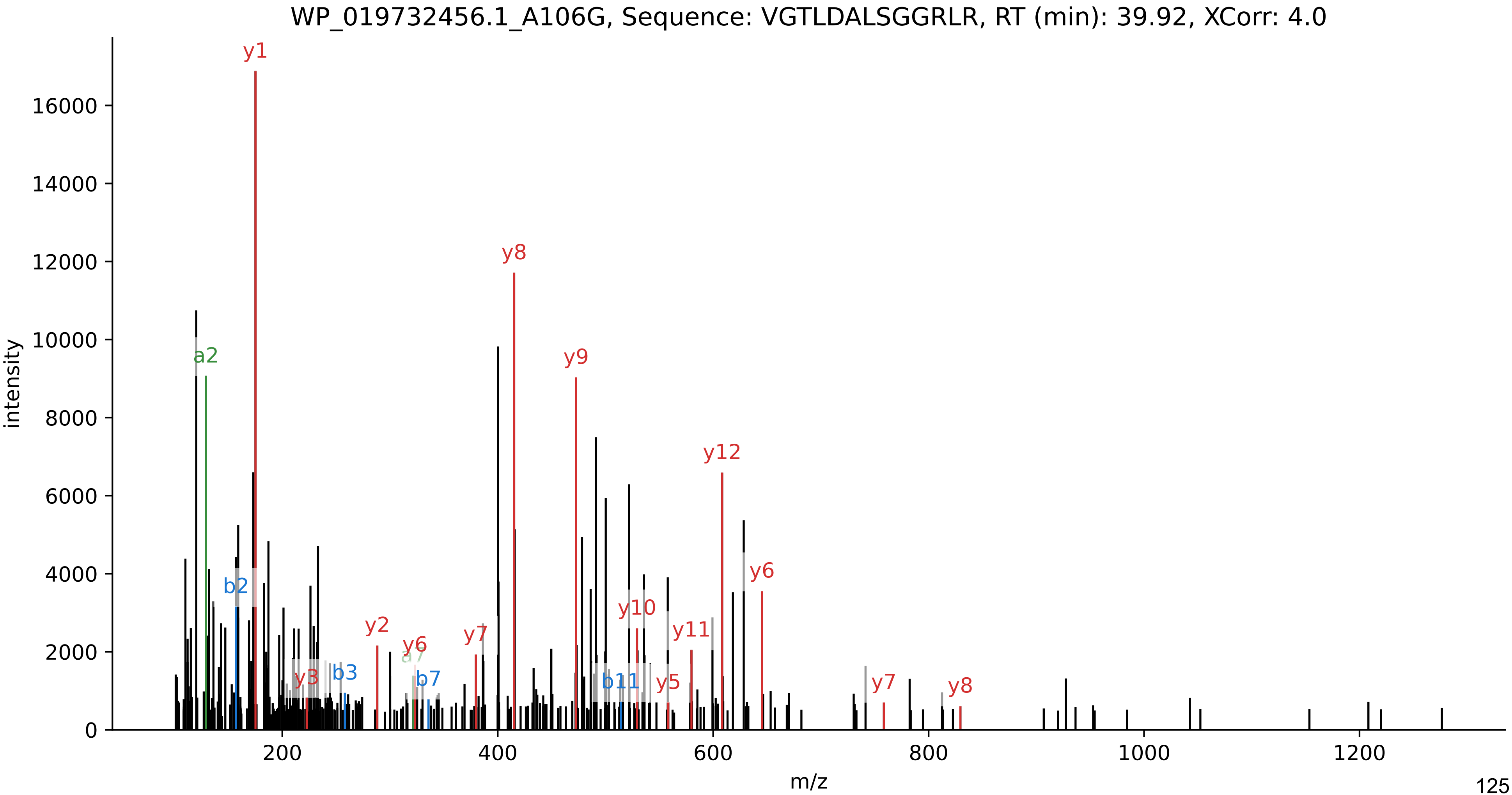

WP_019732456.1_A106G, Sequence: VGTLDALSGGRLR, RT (min): 39.92, XCorr: 4.0
intensity
m/z
0
2000
4000
6000
8000
10000
12000
14000
16000
200
400
600
800
1000
1200
y1
y8
y9
y12
a2
b2
y6
y10
y11
y2
y7
y6
y3
b3
b7
b11
y5
y7
y8

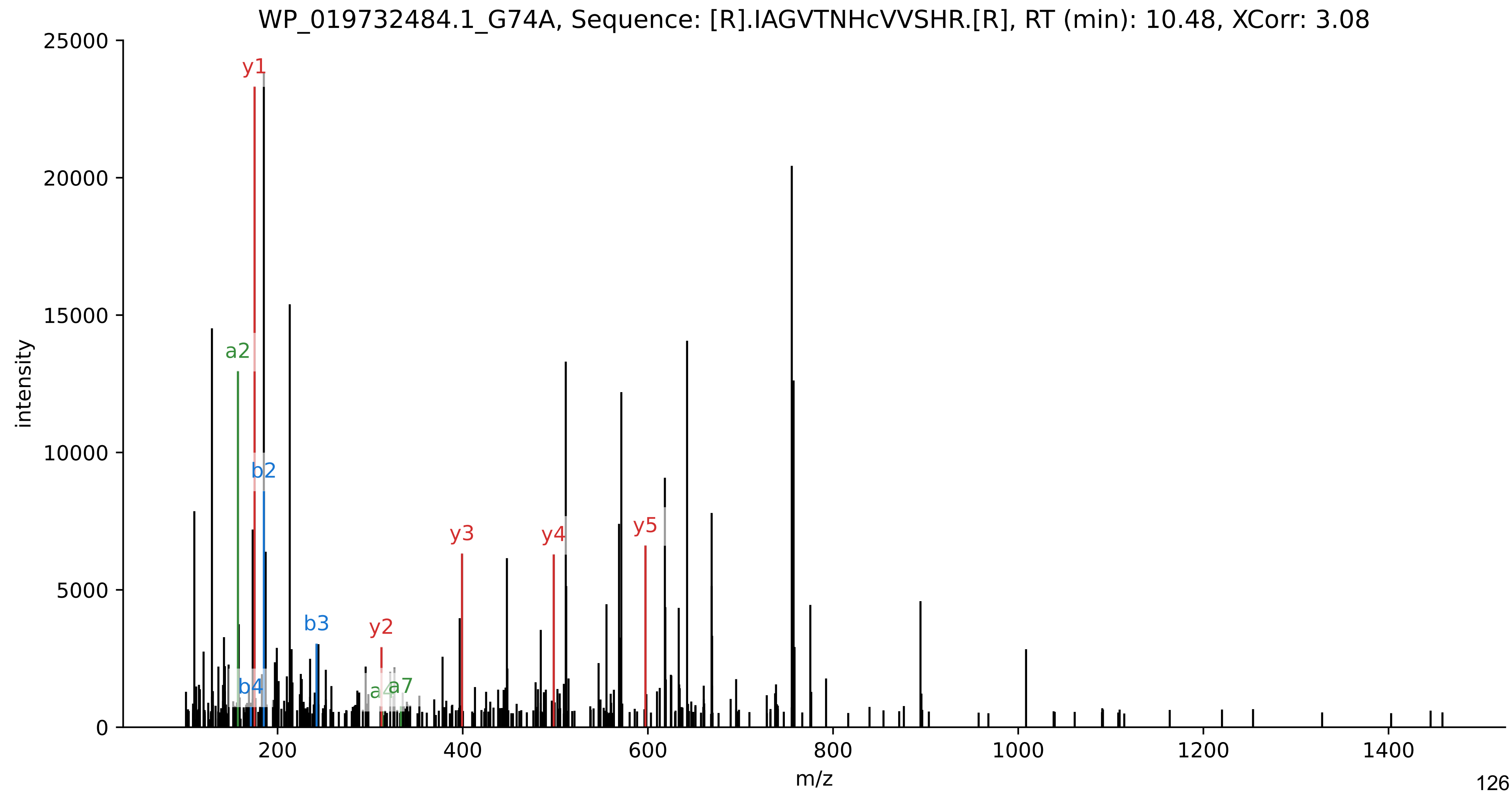

WP_019732484.1_G74A, Sequence: [R].IAGVTNHcVVSHR.[R], RT (min): 10.48, XCorr: 3.08
intensity
m/z
y1
a2
b2
b3
b4
y2
a7
y3
y4
y5
25000
20000
15000
10000
5000
0
200
400
600
800
1000
1200
1400

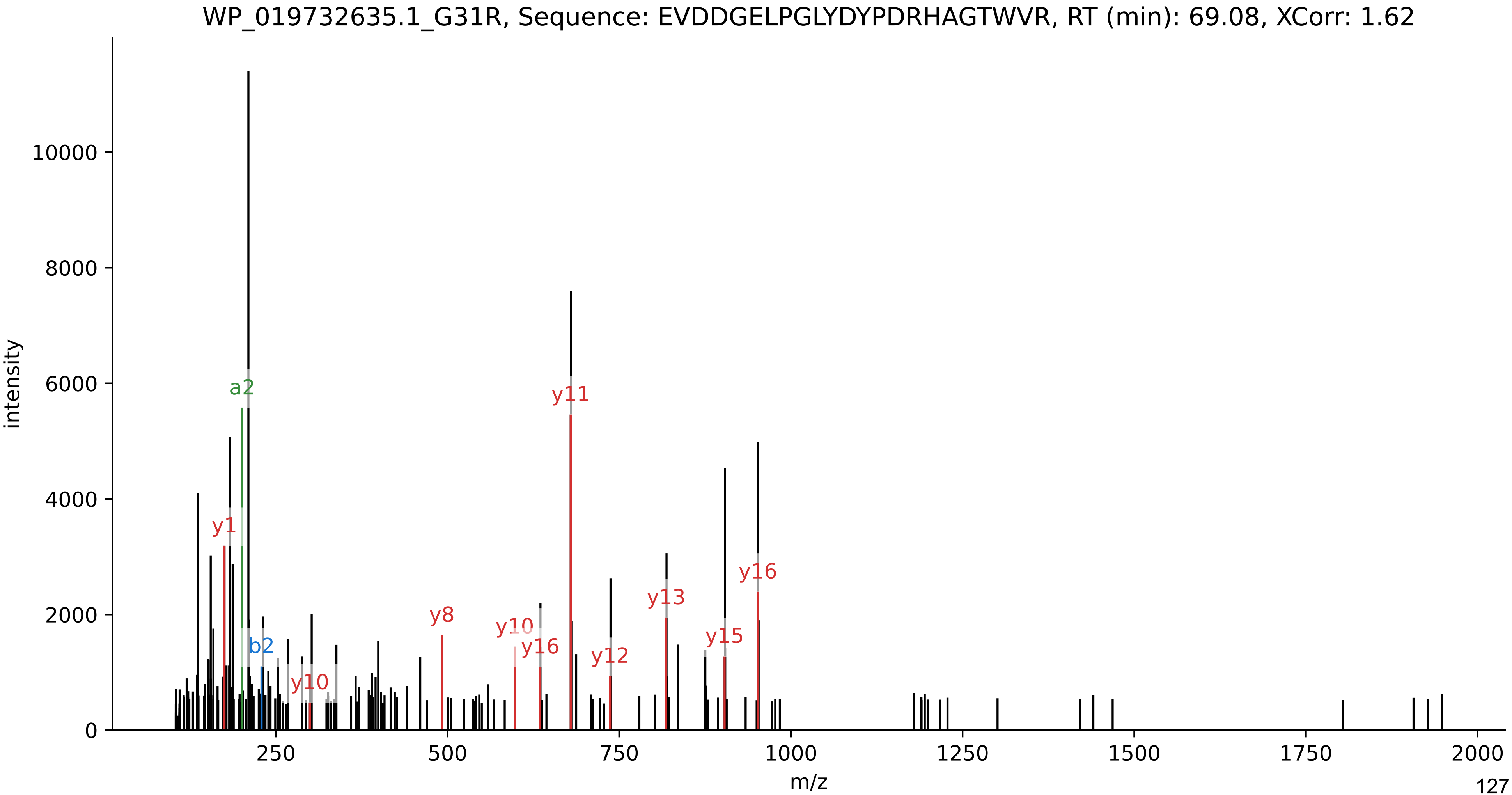

WP_019732635.1_G31R, Sequence: EVDDGELPGLYDYPDRHAGTWVR, RT (min): 69.08, XCorr: 1.62
intensity
m/z
a2
y1
b2
y10
y8
y10
y16
y11
y12
y13
y15
y16
0
2000
4000
6000
8000
10000
250
500
750
1000
1250
1500
1750
2000

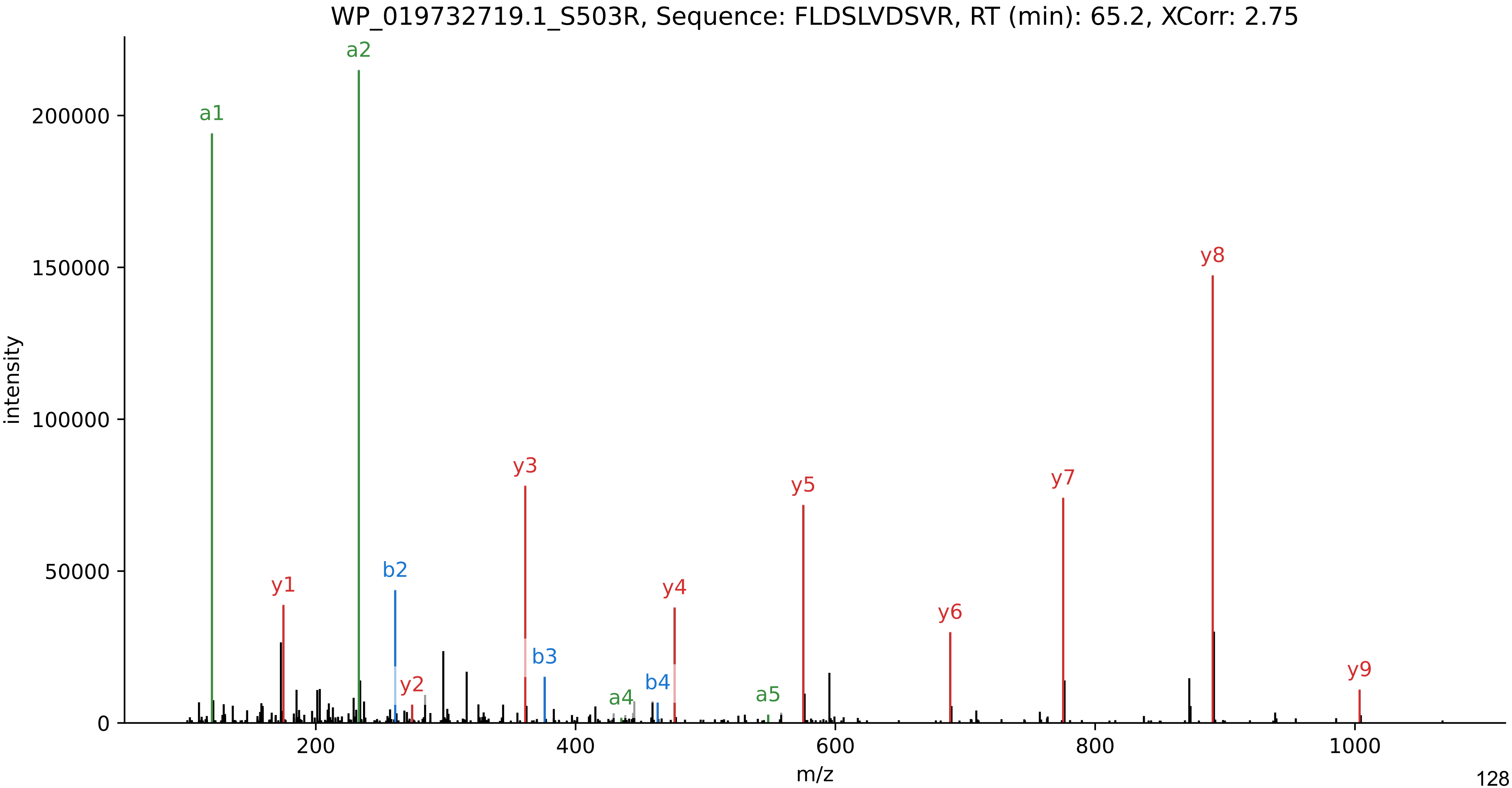

WP_019732719.1_S503R, Sequence: FLDSLVDSVR, RT (min): 65.2, XCorr: 2.75
intensity
m/z
0
50000
100000
150000
200000
200
400
600
800
1000
a1
a2
a4
a5
b2
b3
b4
y1
y2
y3
y4
y5
y6
y7
y8
y9

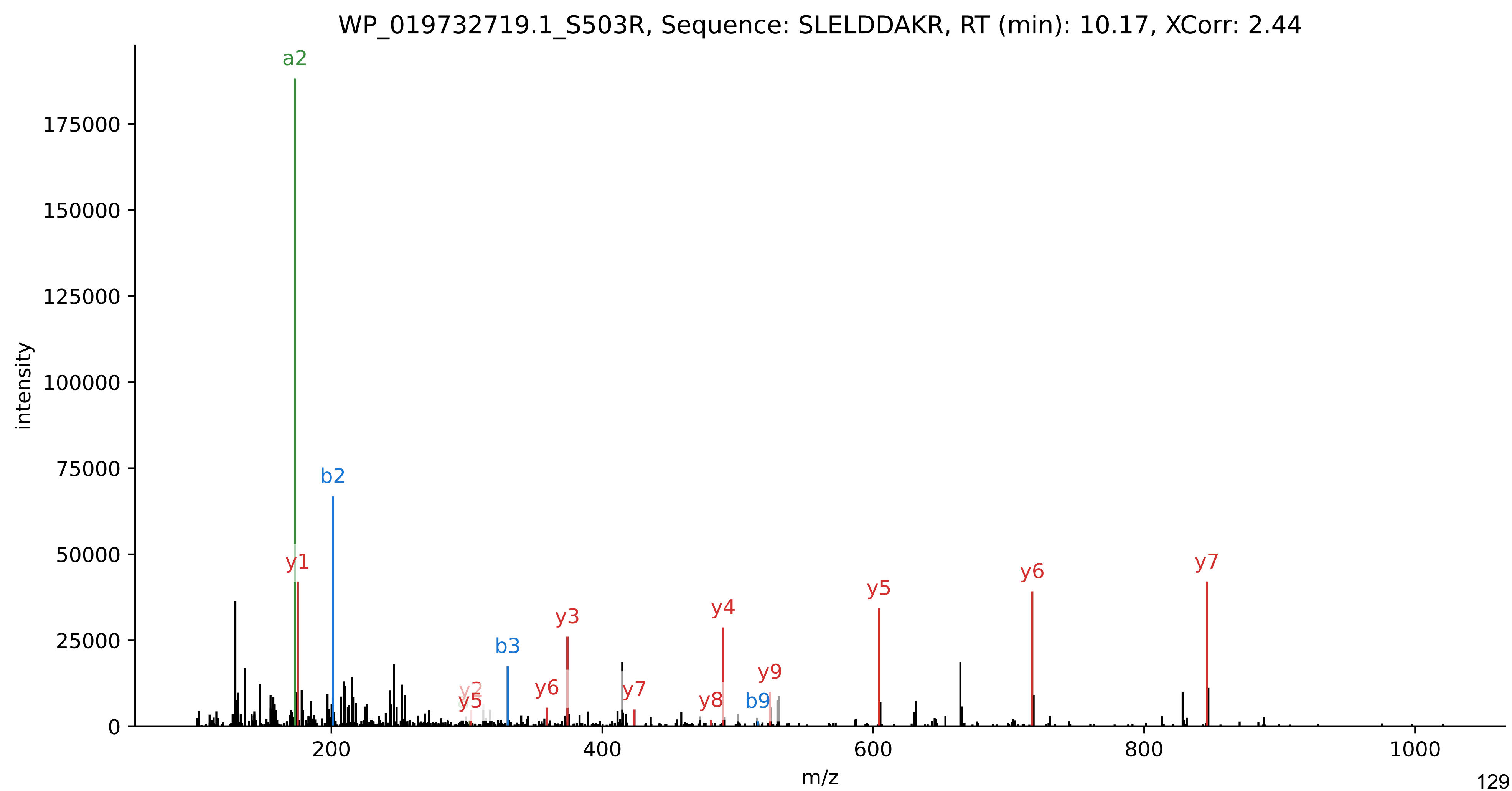
WP_019732719.1_S503R, Sequence: SLELDDAKR, RT (min): 10.17, XCorr: 2.44
intensity
m/z
a2
b2
y1
y7
y6
y5
y4
y3
b3
y9
y6
y2
y5
y7
y8
b9
175000
150000
125000
100000
75000
50000
25000
0
200
400
600
800
1000

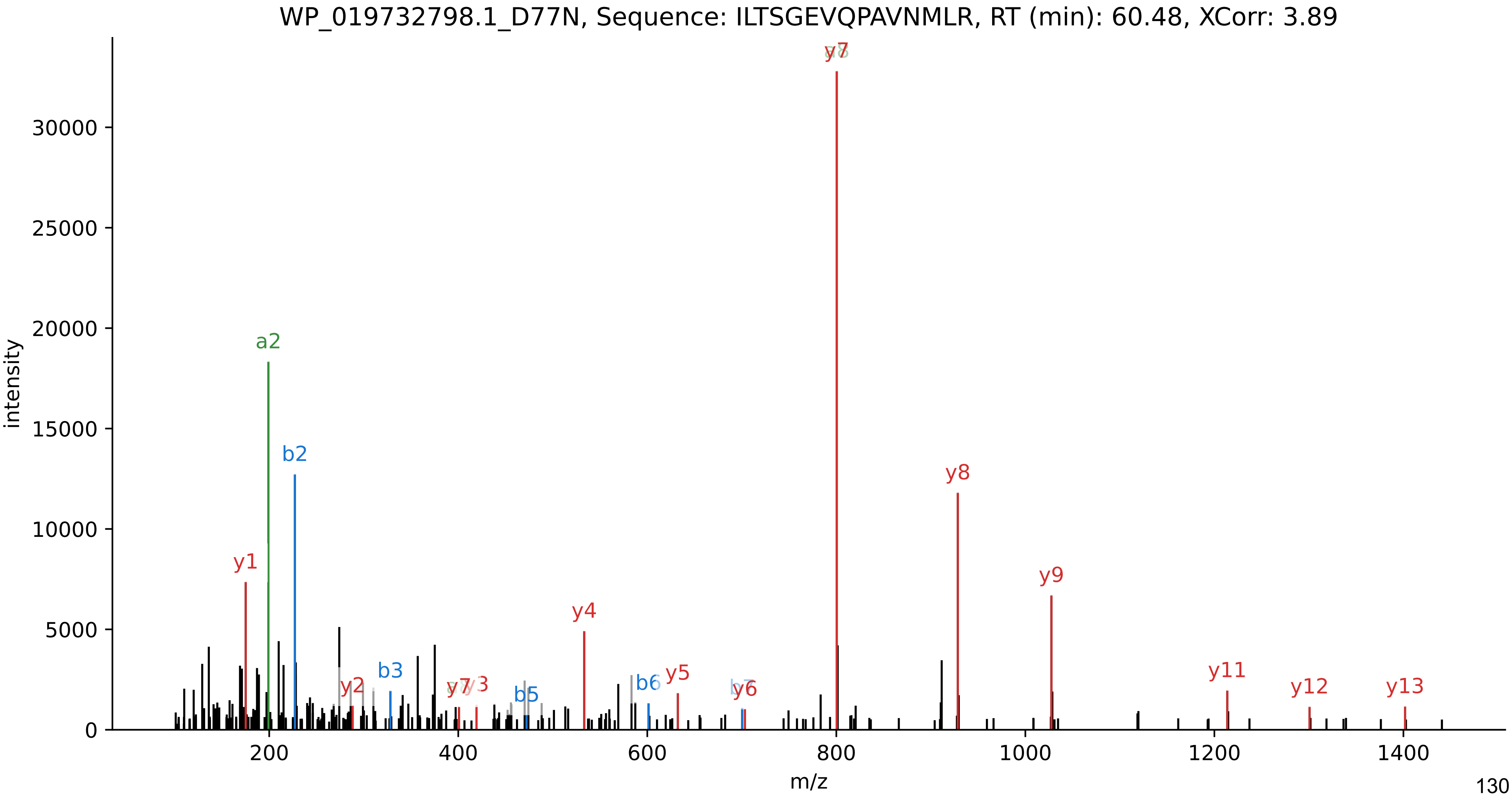
WP_019732798.1_D77N, Sequence: ILTSGEVQPAVNMLR, RT (min): 60.48, XCorr: 3.89
intensity
m/z
a2
b2
y1
y2
b3
y7
y3
b5
y4
b6
y5
y6
y7
y8
y9
y11
y12
y13
0
5000
10000
15000
20000
25000
30000
200
400
600
800
1000
1200
1400

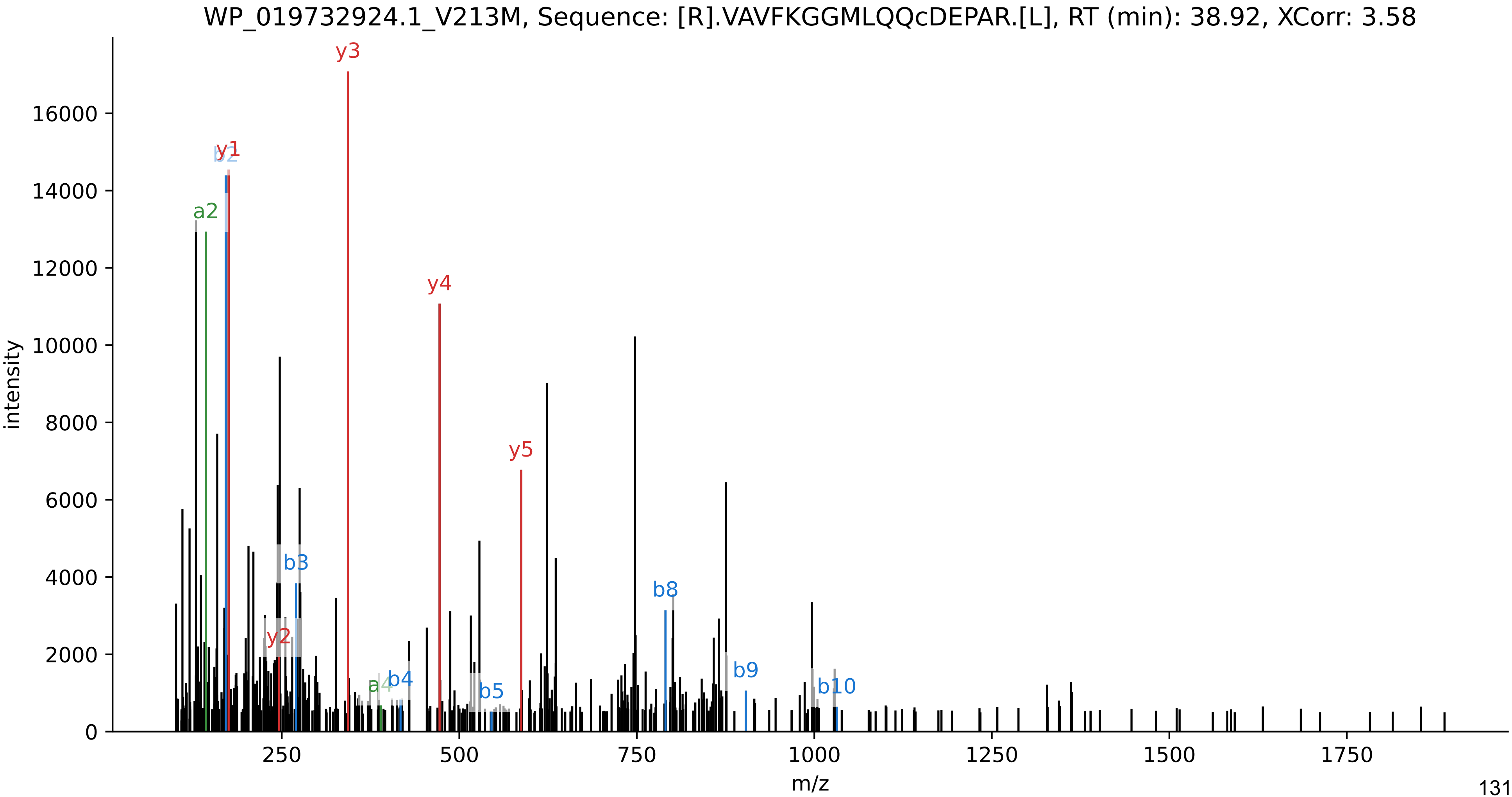
WP_019732924.1_V213M, Sequence: [R].VAVFKGGMLQQcDEPAR.[L], RT (min): 38.92, XCorr: 3.58
intensity
m/z
y3
y1
a2
y4
y5
b3
y2
b4
b5
b8
b9
b10

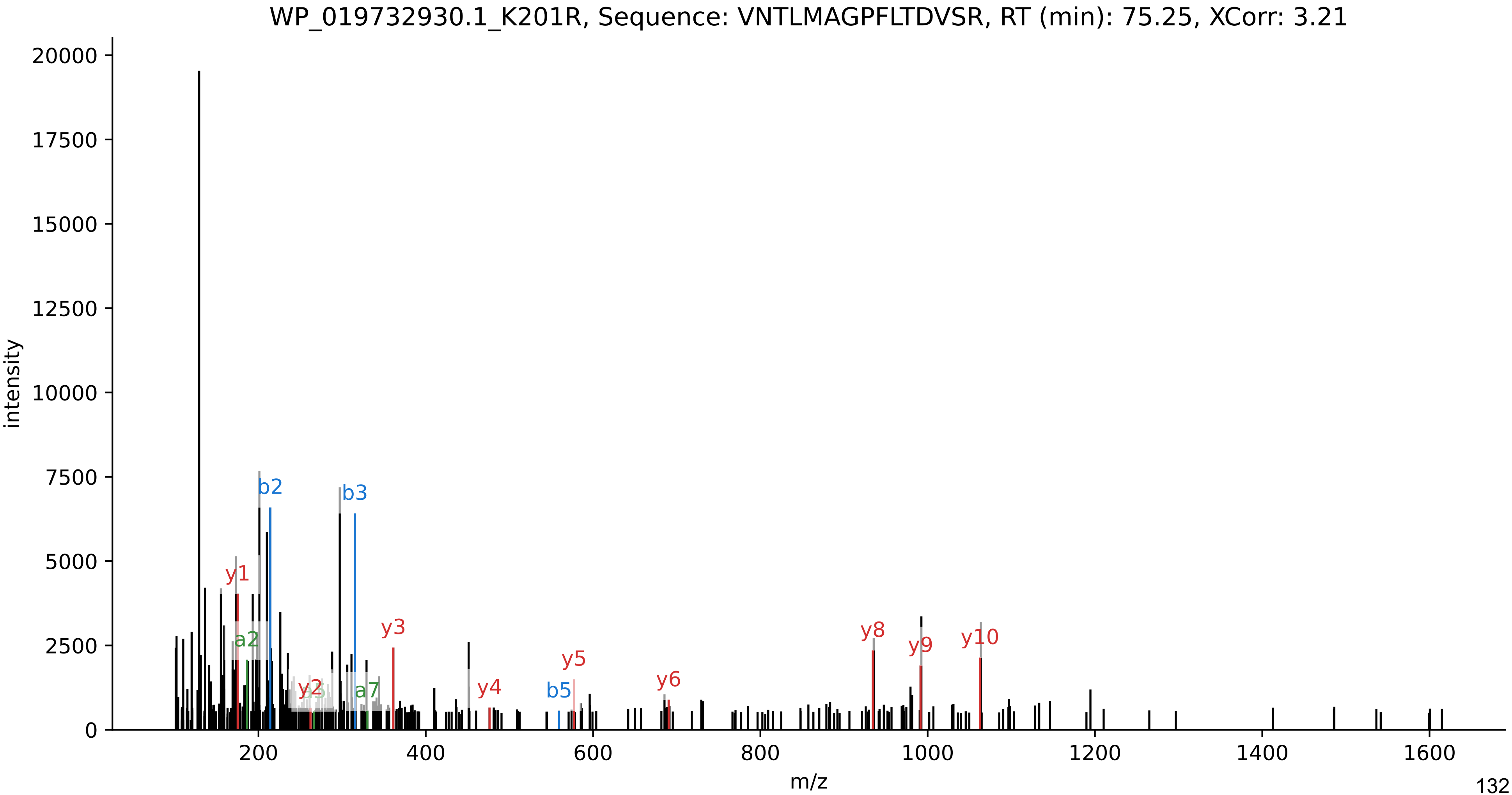
WP_019732930.1_K201R, Sequence: VNTLMAGPFLTDVSR, RT (min): 75.25, XCorr: 3.21
intensity
m/z
b2
b3
b5
y1
y2
y3
y4
y5
y6
y8
y9
y10
a2
a7
0
2500
5000
7500
10000
12500
15000
17500
20000
200
400
600
800
1000
1200
1400
1600

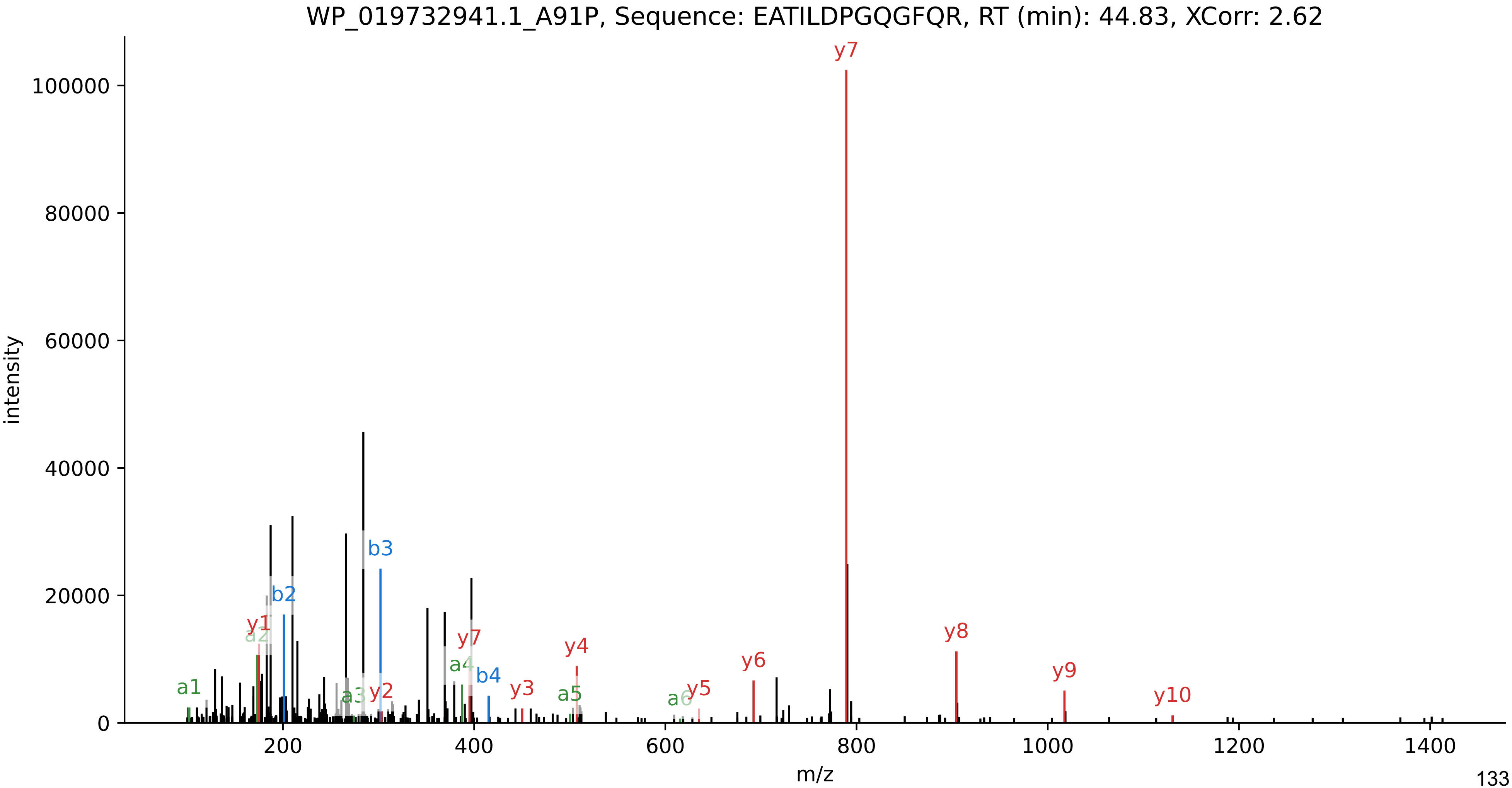
WP_019732941.1_A91P, Sequence: EATILDPGQGFQR, RT (min): 44.83, XCorr: 2.62
intensity
m/z
y7
b3
b2
y1
a2
y7
a4
b4
y4
y3
a5
y2
a3
a1
y6
y5
a6
y8
y9
y10
100000
80000
60000
40000
20000
0
200
400
600
800
1000
1200
1400

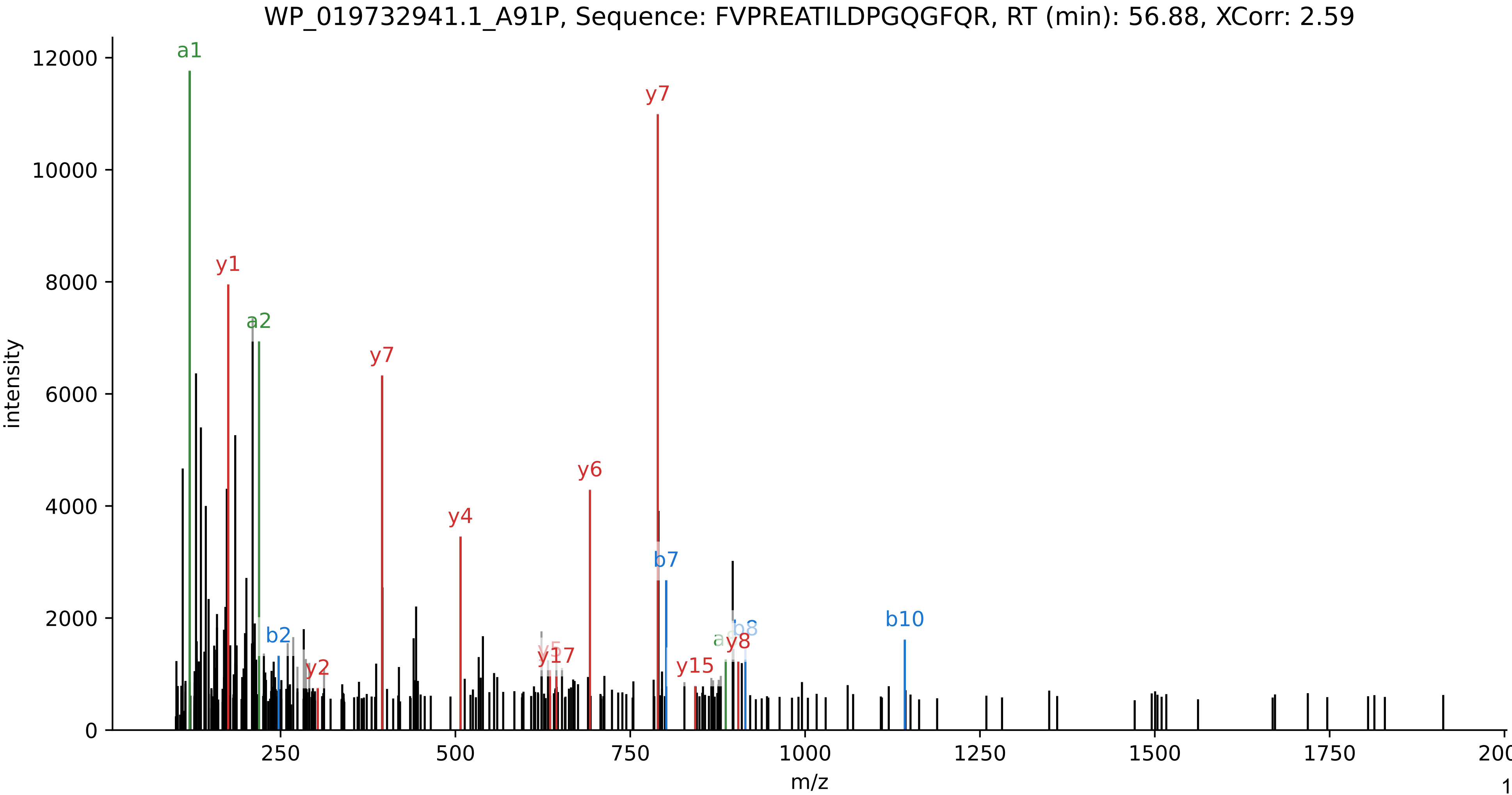

WP_019732941.1_A91P, Sequence: FVPREATILDPGQGFQR, RT (min): 56.88, XCorr: 2.59
a1
y7
y1
a2
y7
y6
y4
b7
b10
b2
y2
y17
y15
y8
b8
intensity
m/z
0
2000
4000
6000
8000
10000
12000
250
500
750
1000
1250
1500
1750
2000

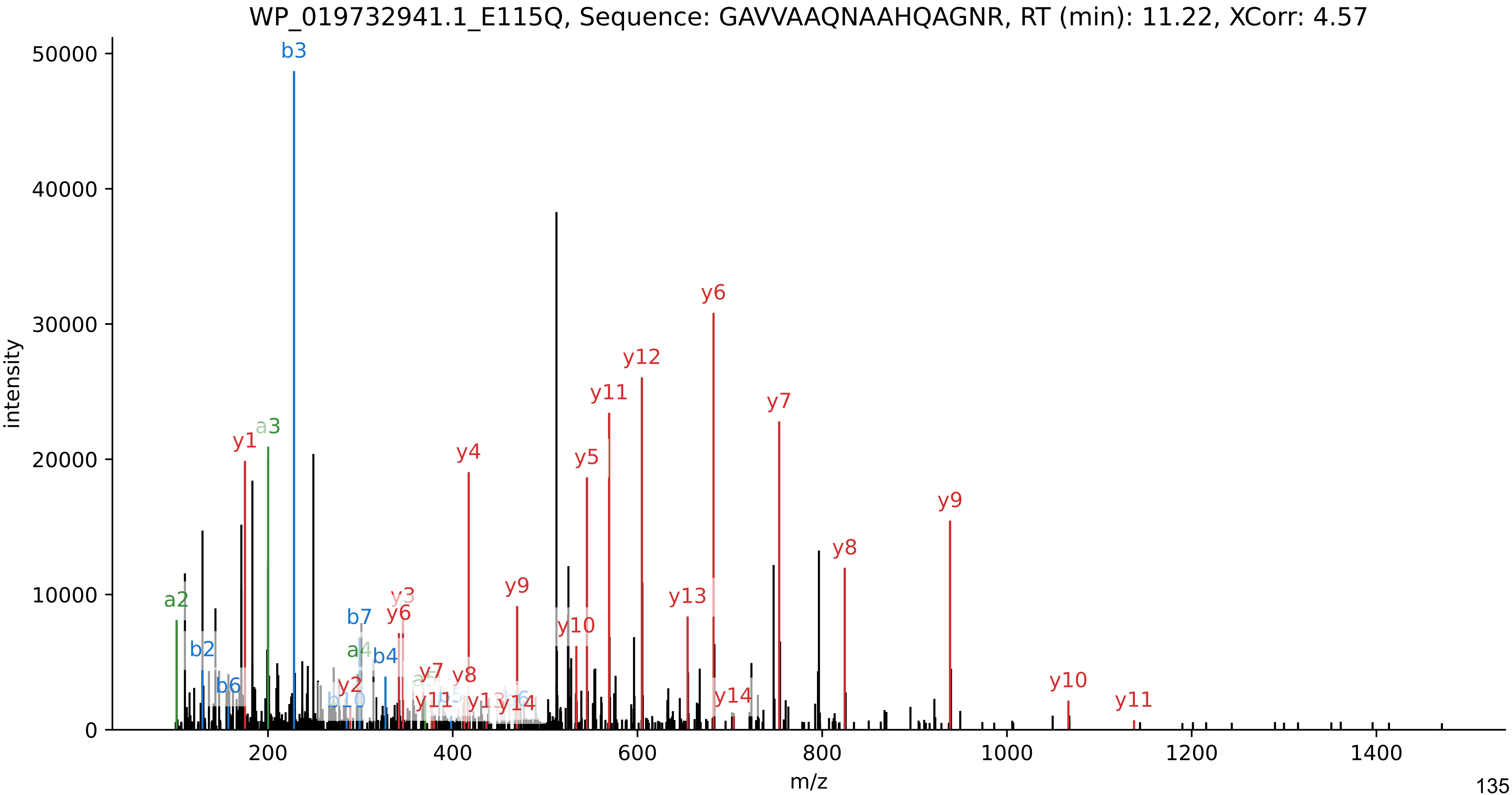
WP_019732941.1_E115Q, Sequence: GAVVAAQNAAHQAGNR, RT (min): 11.22, XCorr: 4.57
intensity
m/z
b3
y6
y12
y11
y7
a3
y1
y4
y5
y9
y8
y13
y9
y10
a2
b7
y6
y3
b2
a4
b4
b6
y2
y7
y8
b10
y11
y14
y10
y11

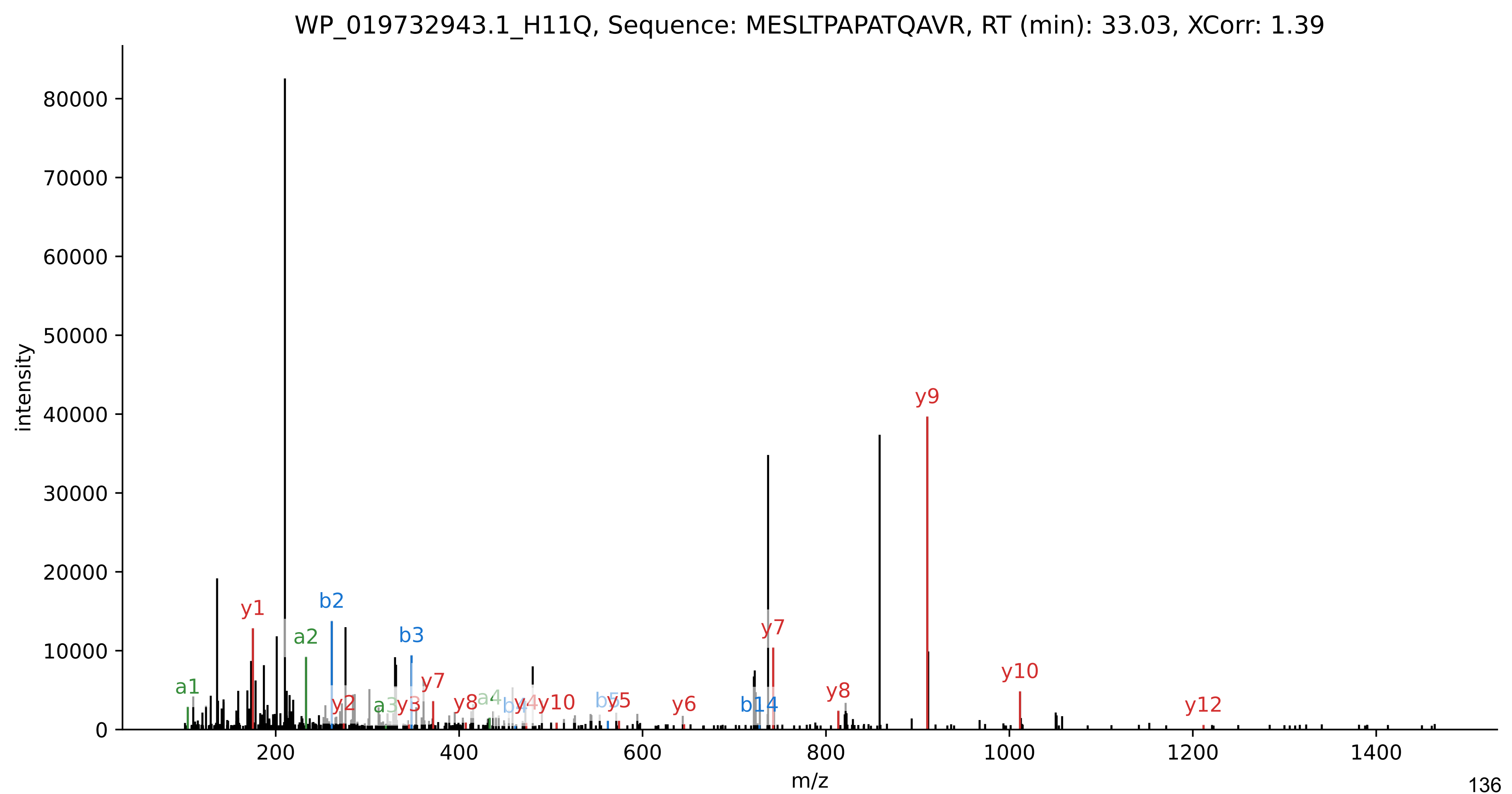
WP_019732943.1_H11Q, Sequence: MESLTPAPATQAVR, RT (min): 33.03, XCorr: 1.39
intensity
m/z
80000
70000
60000
50000
40000
30000
20000
10000
0
200
400
600
800
1000
1200
1400
y9
y1
b2
a2
b3
y7
a1
y2
a3
y3
y8
a4
y4
y10
y5
y6
b14
y8
y10
y12

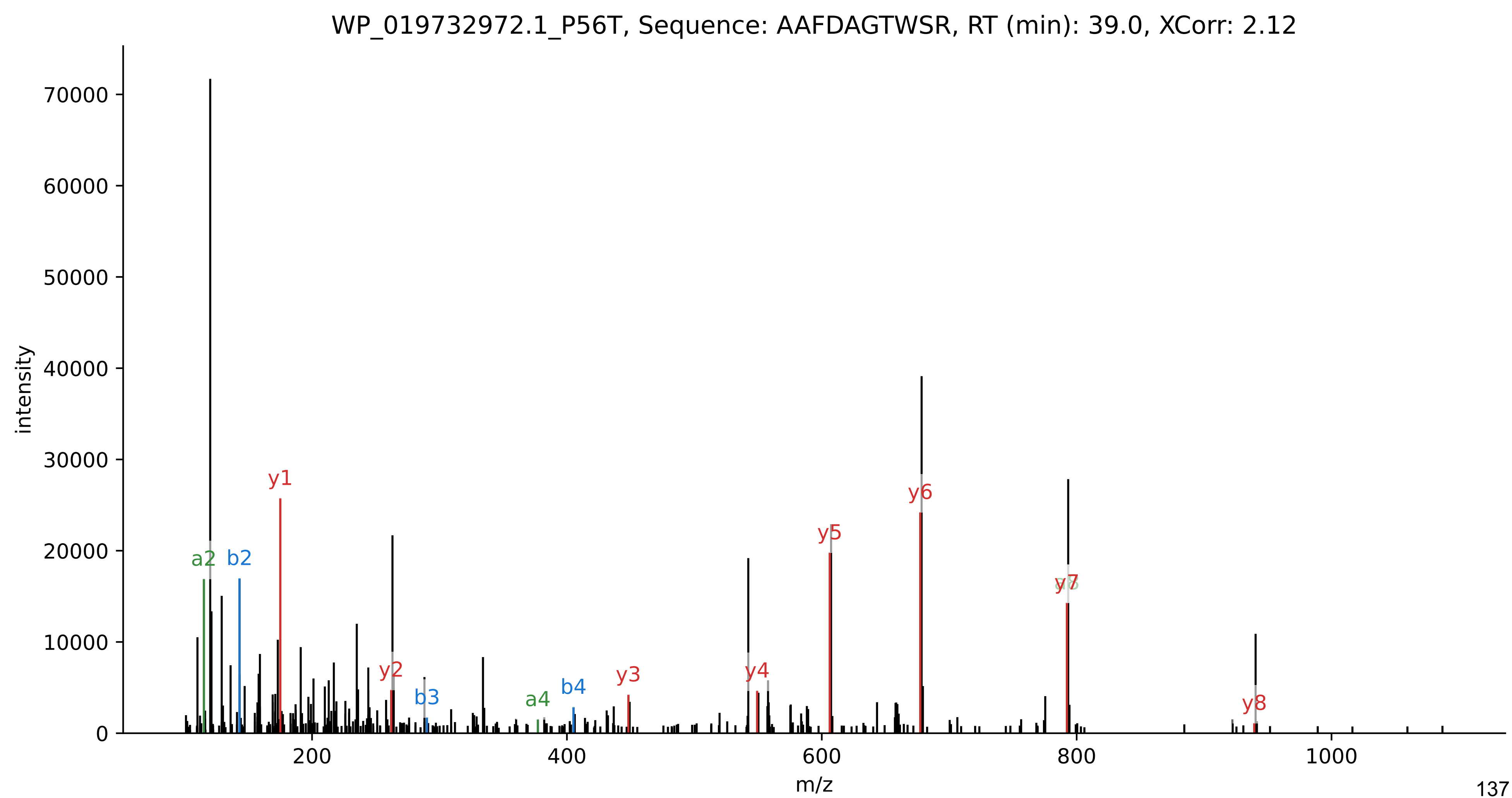
WP_019732972.1_P56T, Sequence: AAFDAGTWSR, RT (min): 39.0, XCorr: 2.12
intensity
m/z
a2
b2
y1
y2
b3
a4
b4
y3
y4
y5
y6
y7
y8

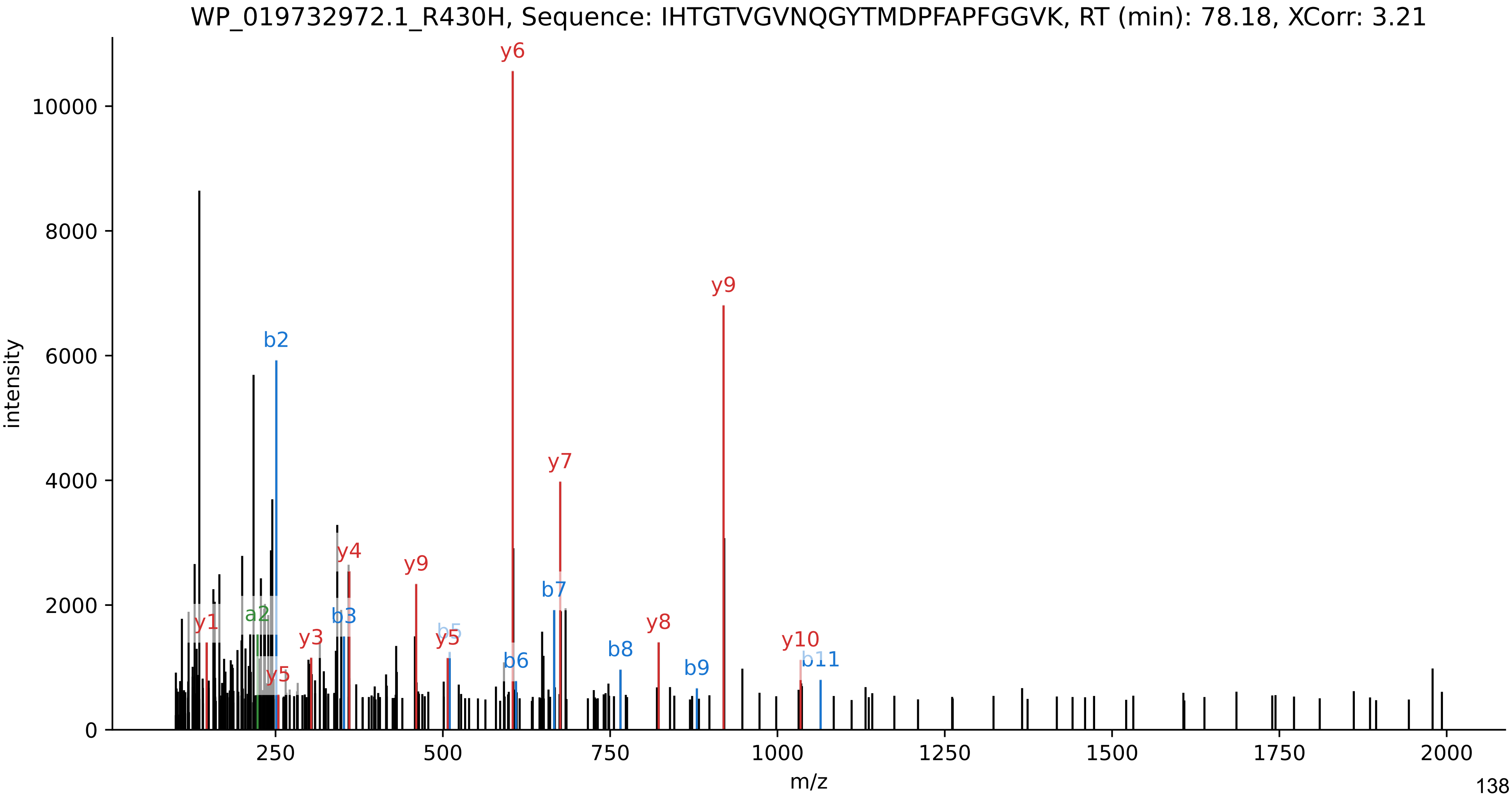

WP_019732972.1_R430H, Sequence: IHTGTVGVNQGYTMDPFAPFGGVK, RT (min): 78.18, XCorr: 3.21
intensity
m/z
y6
y9
b2
y7
y4
y9
b7
a2
b3
y1
y8
y3
y5
b5
y10
b8
b11
b6
b9
y5
0
2000
4000
6000
8000
10000
250
500
750
1000
1250
1500
1750
2000

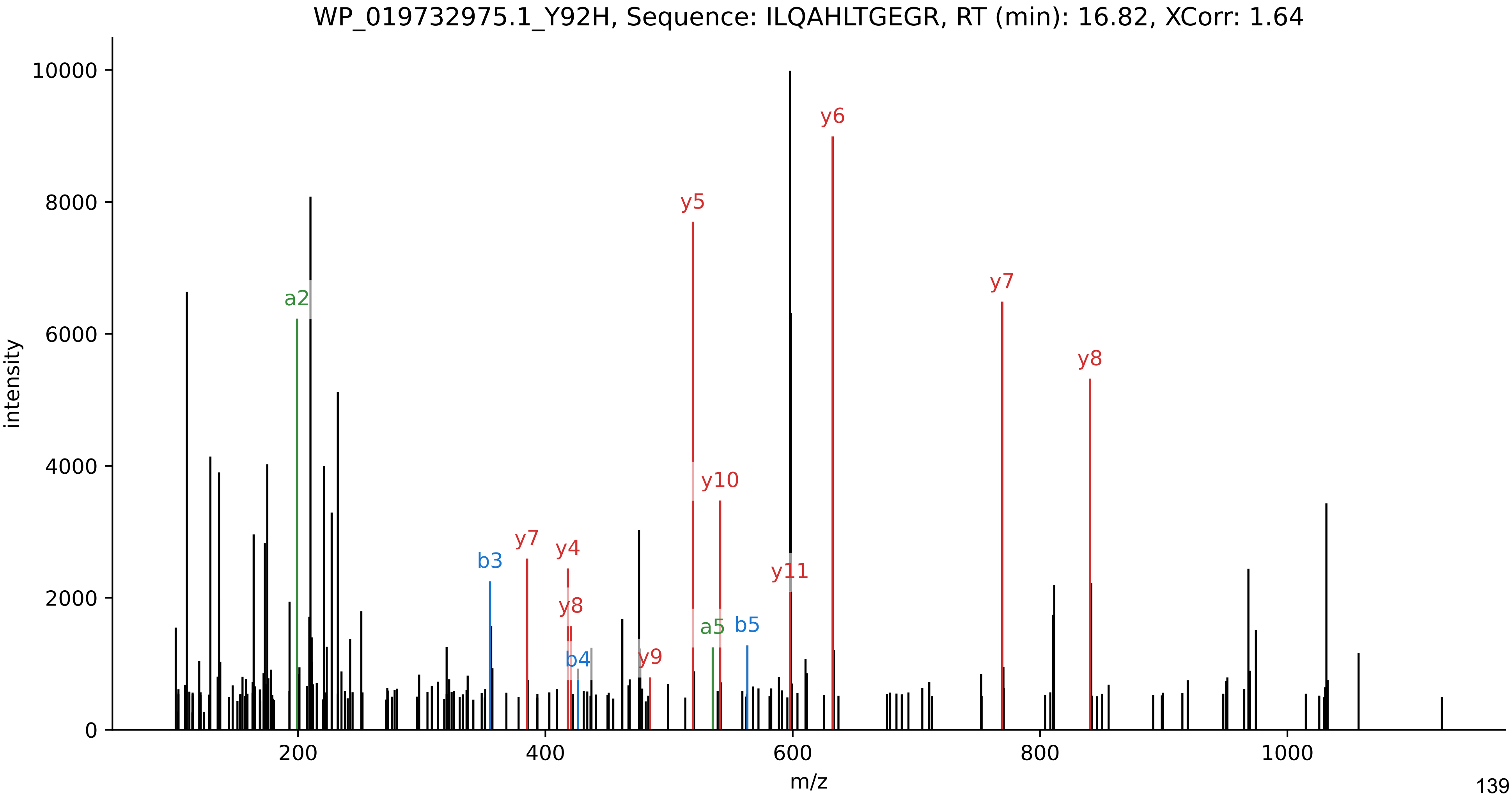

WP_019732975.1_Y92H, Sequence: ILQAHLTGEGR, RT (min): 16.82, XCorr: 1.64
intensity
m/z
a2
b3
y7
y4
y8
b4
y9
y5
y10
a5
b5
y11
y6
y7
y8

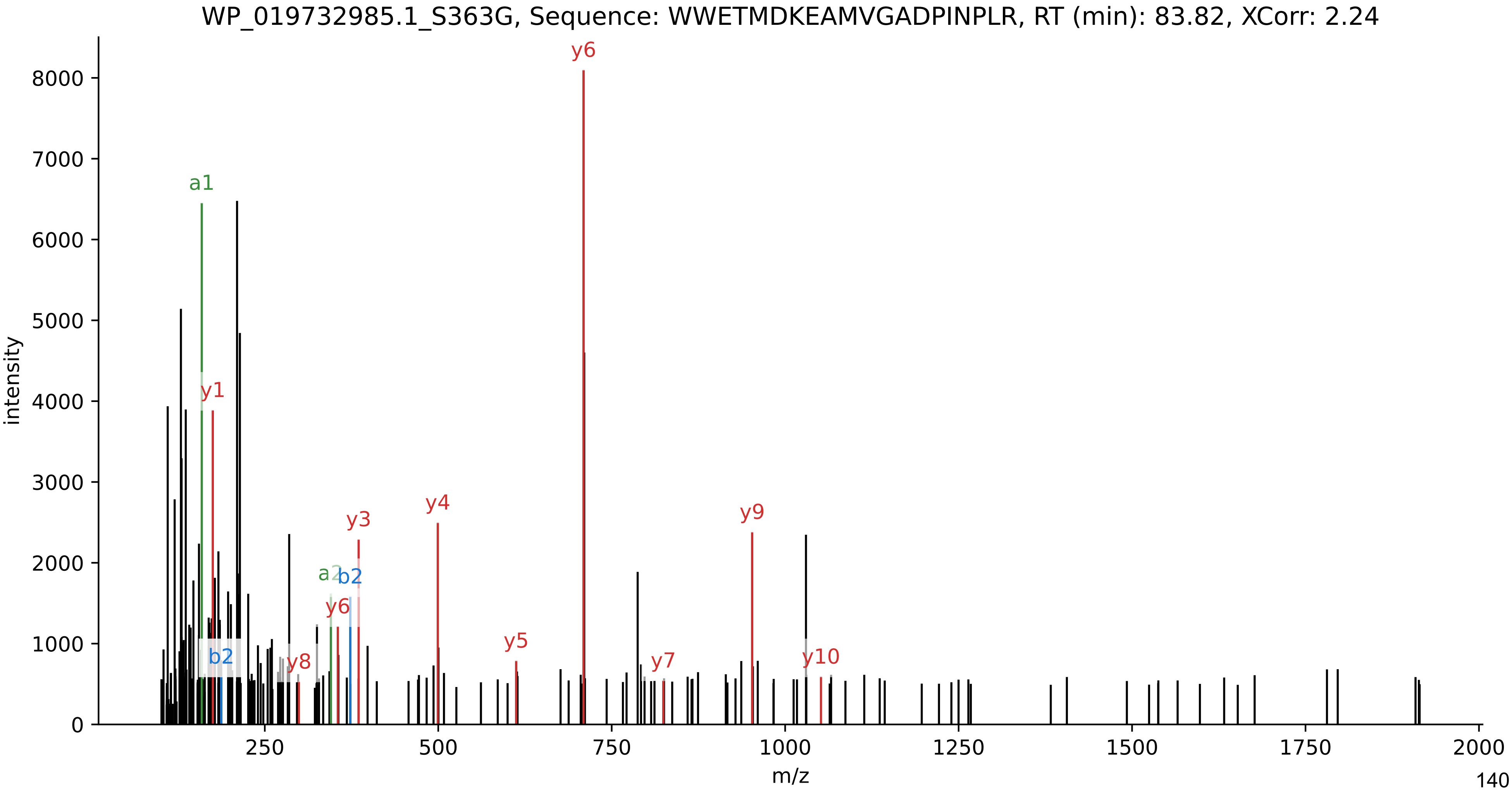
WP_019732985.1_S363G, Sequence: WWETMDKEAMVGADPINPLR, RT (min): 83.82, XCorr: 2.24
intensity
m/z
y6
a1
y1
b2
y8
a2
b2
y6
y3
y4
y5
y7
y9
y10
0
1000
2000
3000
4000
5000
6000
7000
8000
250
500
750
1000
1250
1500
1750
2000

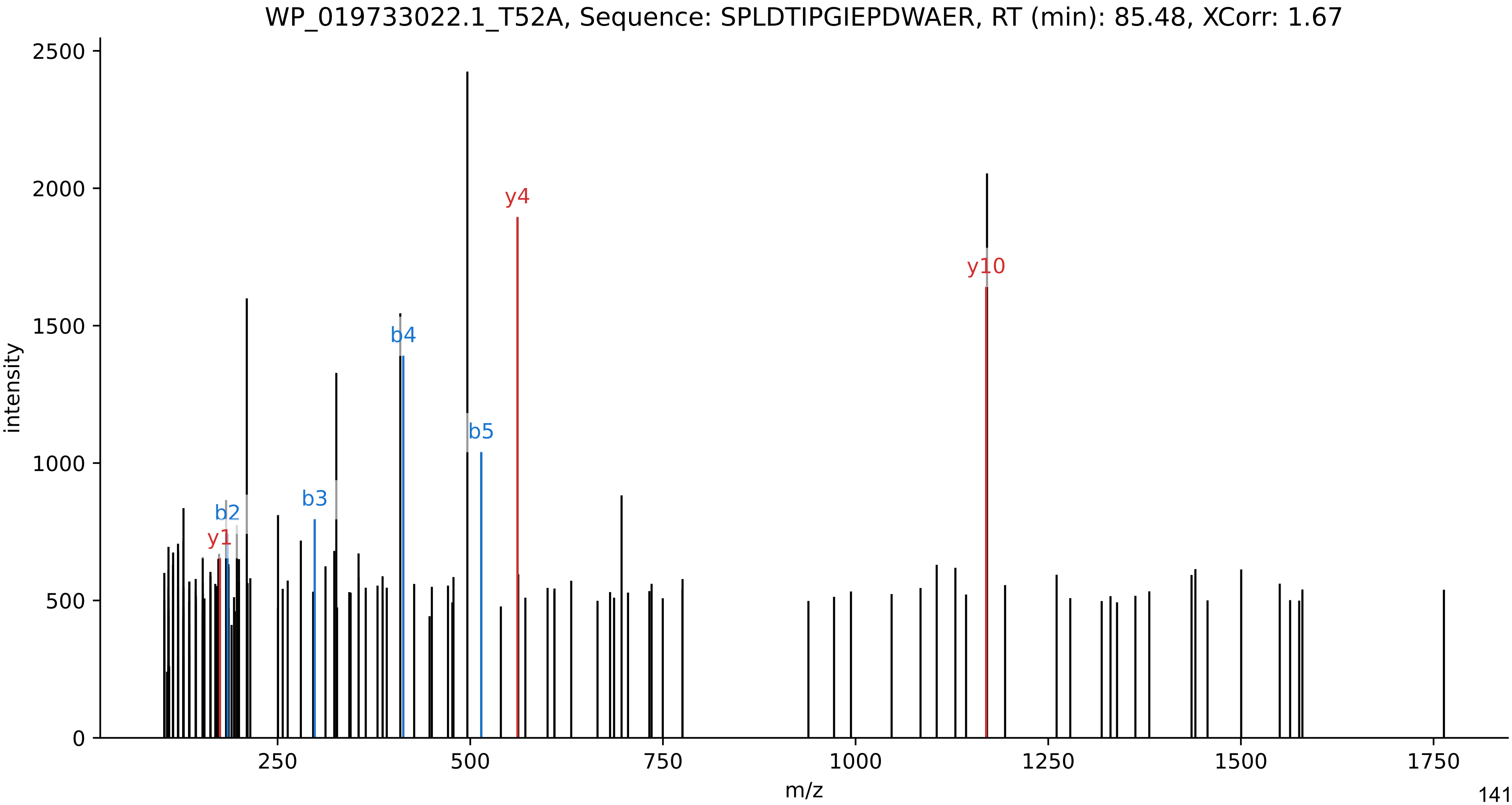

WP_019733022.1_T52A, Sequence: SPLDTIPGIEPDWAER, RT (min): 85.48, XCorr: 1.67
intensity
m/z
y1
b2
b3
b4
b5
y4
y10
0
500
1000
1500
2000
2500
250
500
750
1000
1250
1500
1750

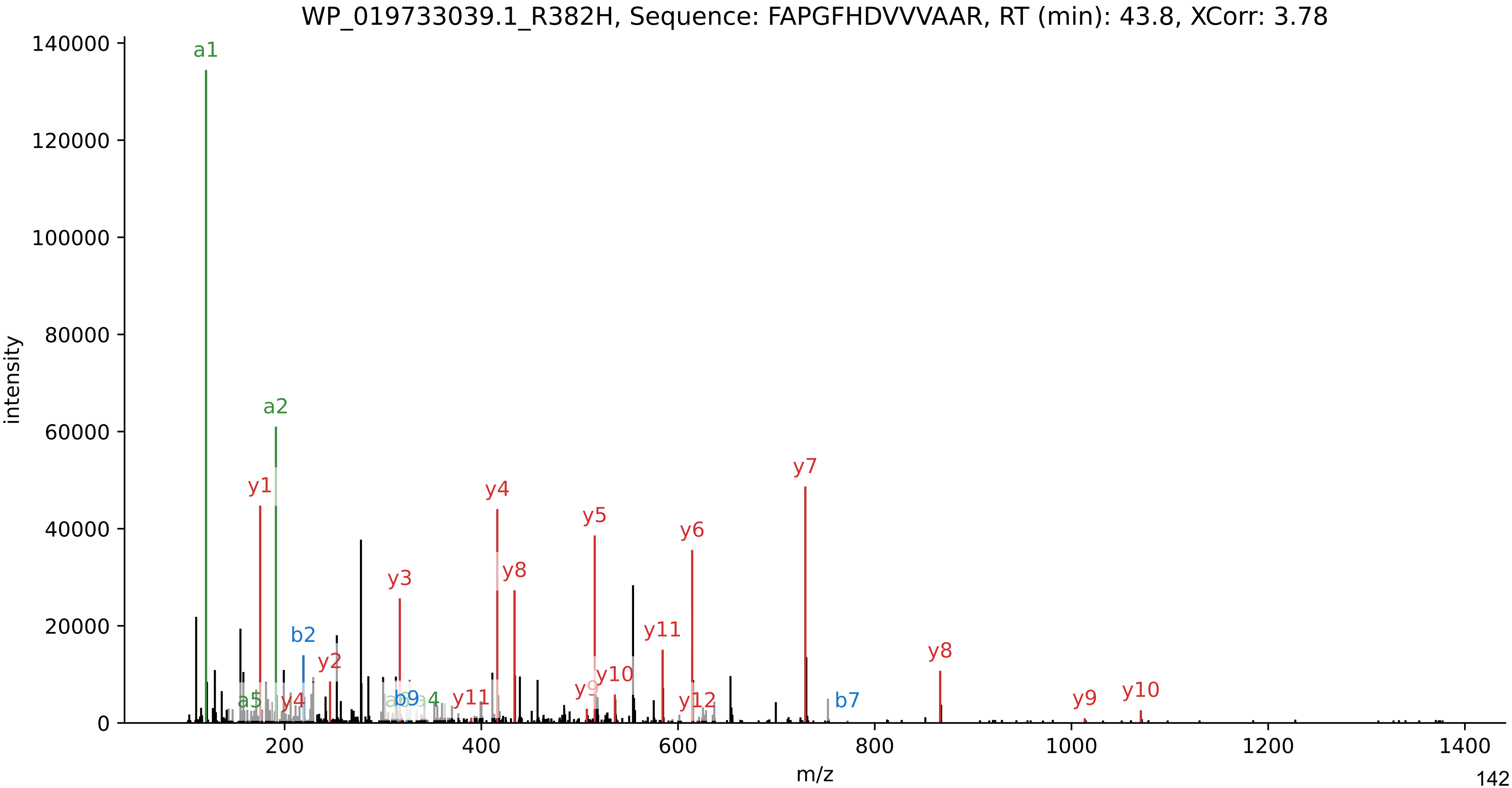
WP_019733039.1_R382H, Sequence: FAPGFHDVVVAAR, RT (min): 43.8, XCorr: 3.78
intensity
m/z
a1
a2
y1
y4
y5
y6
y7
y3
y8
b2
y2
y11
y10
y9
y12
b7
a5
y4
b9
a4
y11
y8
y9
y10

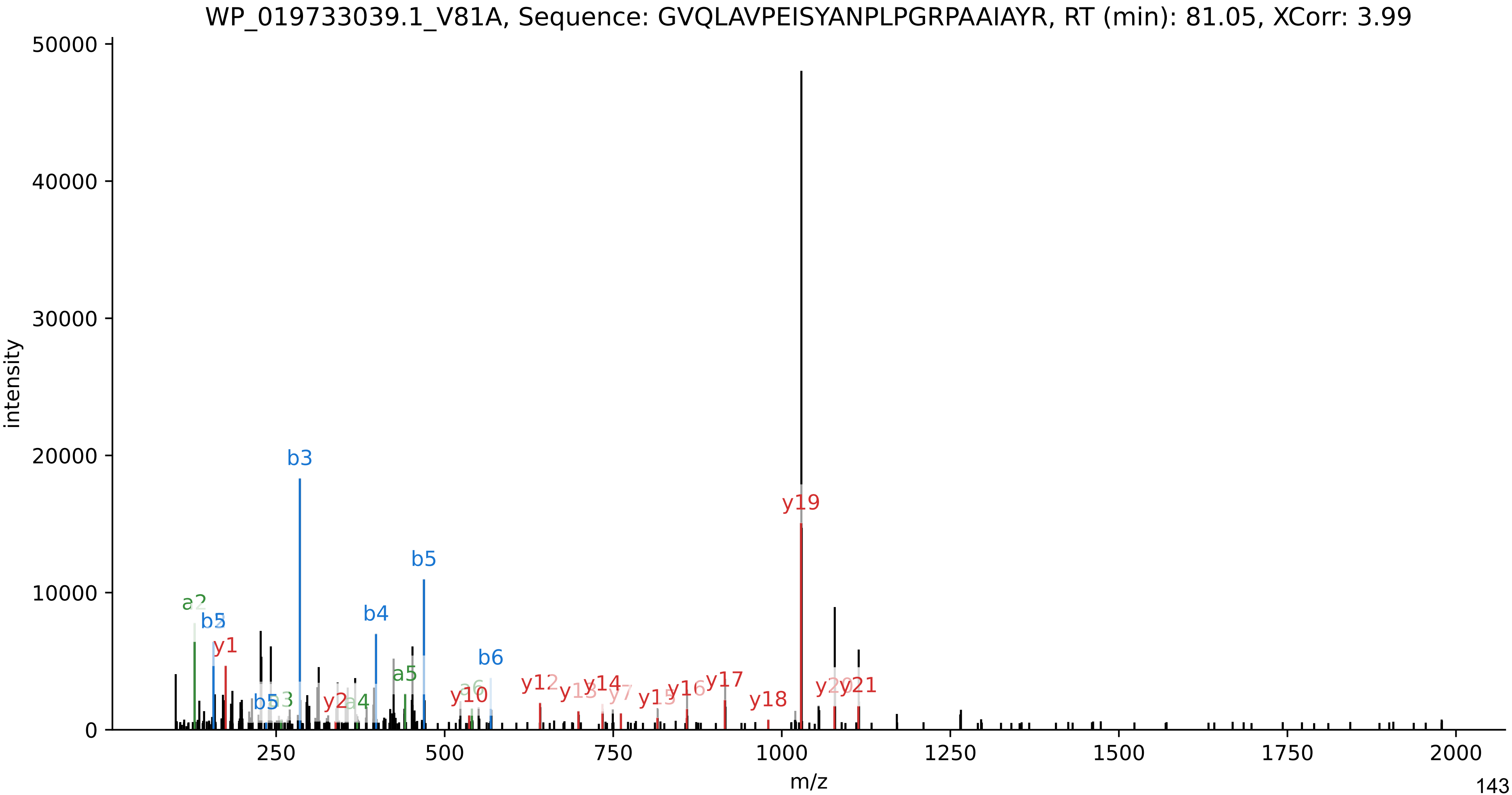

WP_019733039.1_V81A, Sequence: GVQLAVPEISYANPLPGRPAAIAYR, RT (min): 81.05, XCorr: 3.99
intensity
m/z
b3
y19
b5
b4
a2
y1
b6
a5
y12
y14
y17
y10
y16
y15
y18
y21
b5
y2
a4

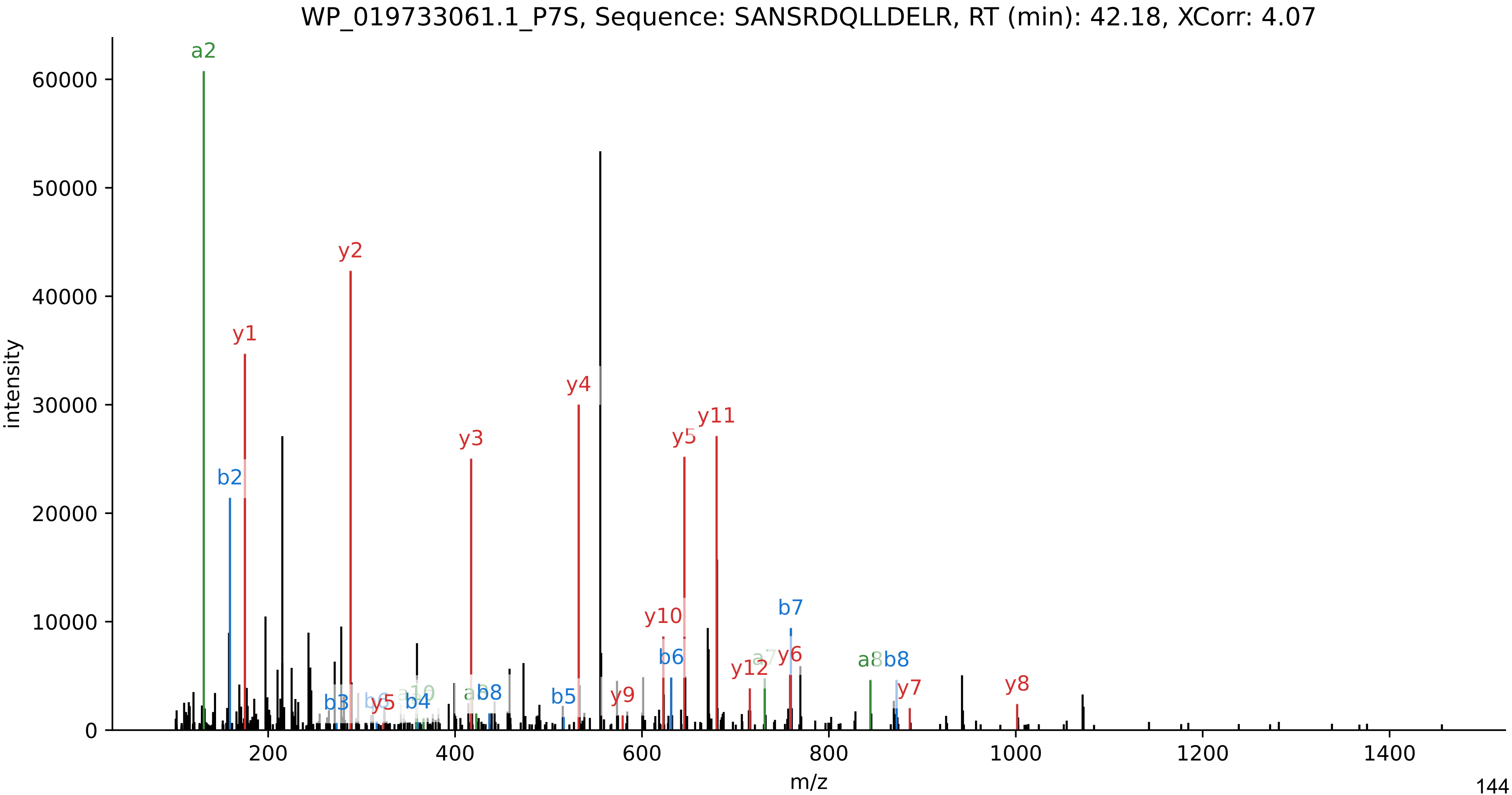

WP_019733061.1_P7S, Sequence: SANSRDQLLDELR, RT (min): 42.18, XCorr: 4.07
intensity
m/z
a2
y2
y1
y4
y3
y11
y5
b2
y10
b7
b6
y12
y6
a8
b8
y7
y8
y9
b5
b3
y5
b4
b8
0
10000
20000
30000
40000
50000
60000
200
400
600
800
1000
1200
1400

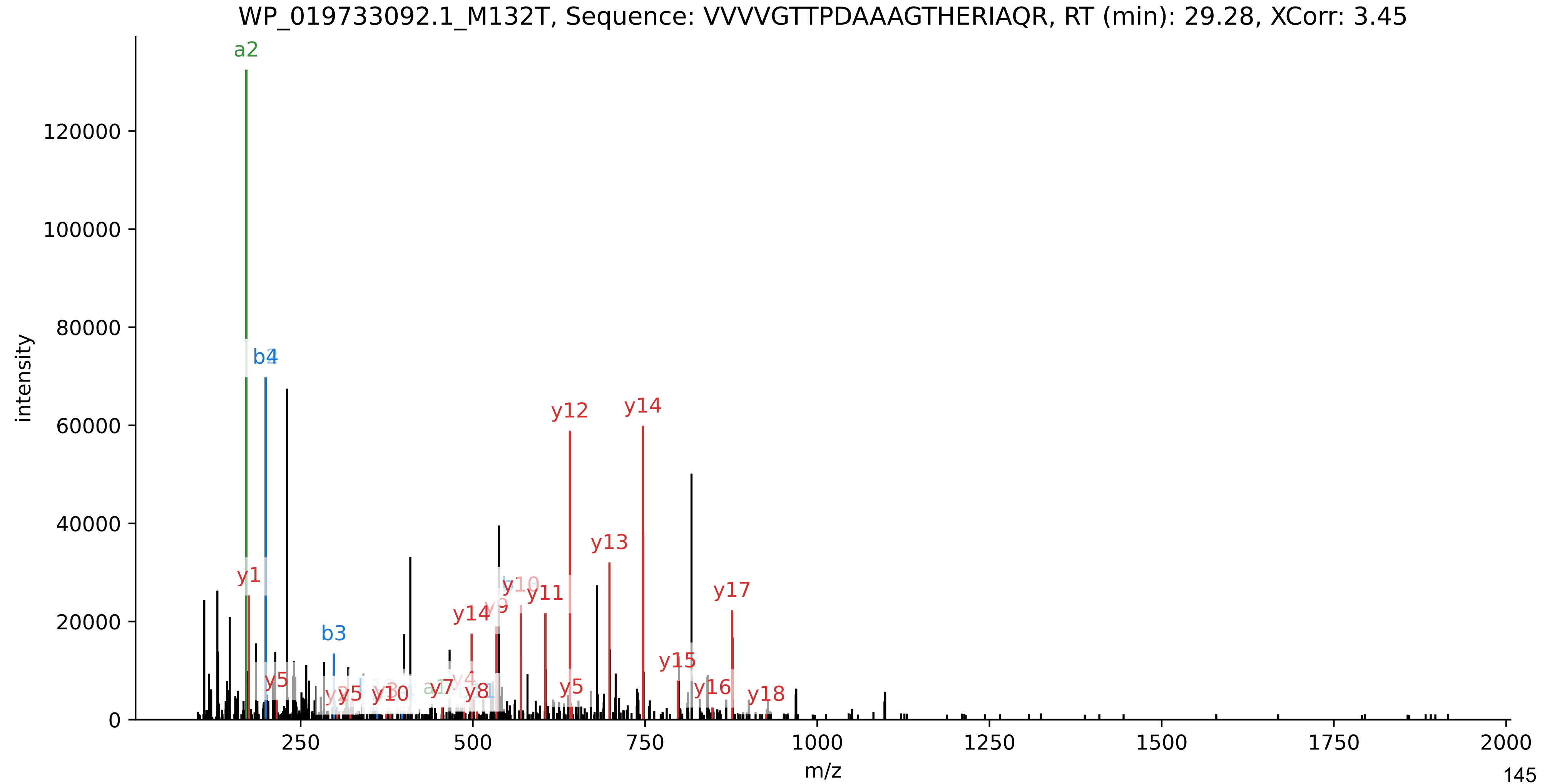
WP_019733092.1_M132T, Sequence: VVVVGTTPDAAAGTHERIAQR, RT (min): 29.28, XCorr: 3.45
intensity
m/z
a2
b4
y1
b3
y5
y10
y7
y8
y14
y9
y11
y12
y14
y13
y5
y15
y16
y17
y18
0
20000
40000
60000
80000
100000
120000
250
500
750
1000
1250
1500
1750
2000

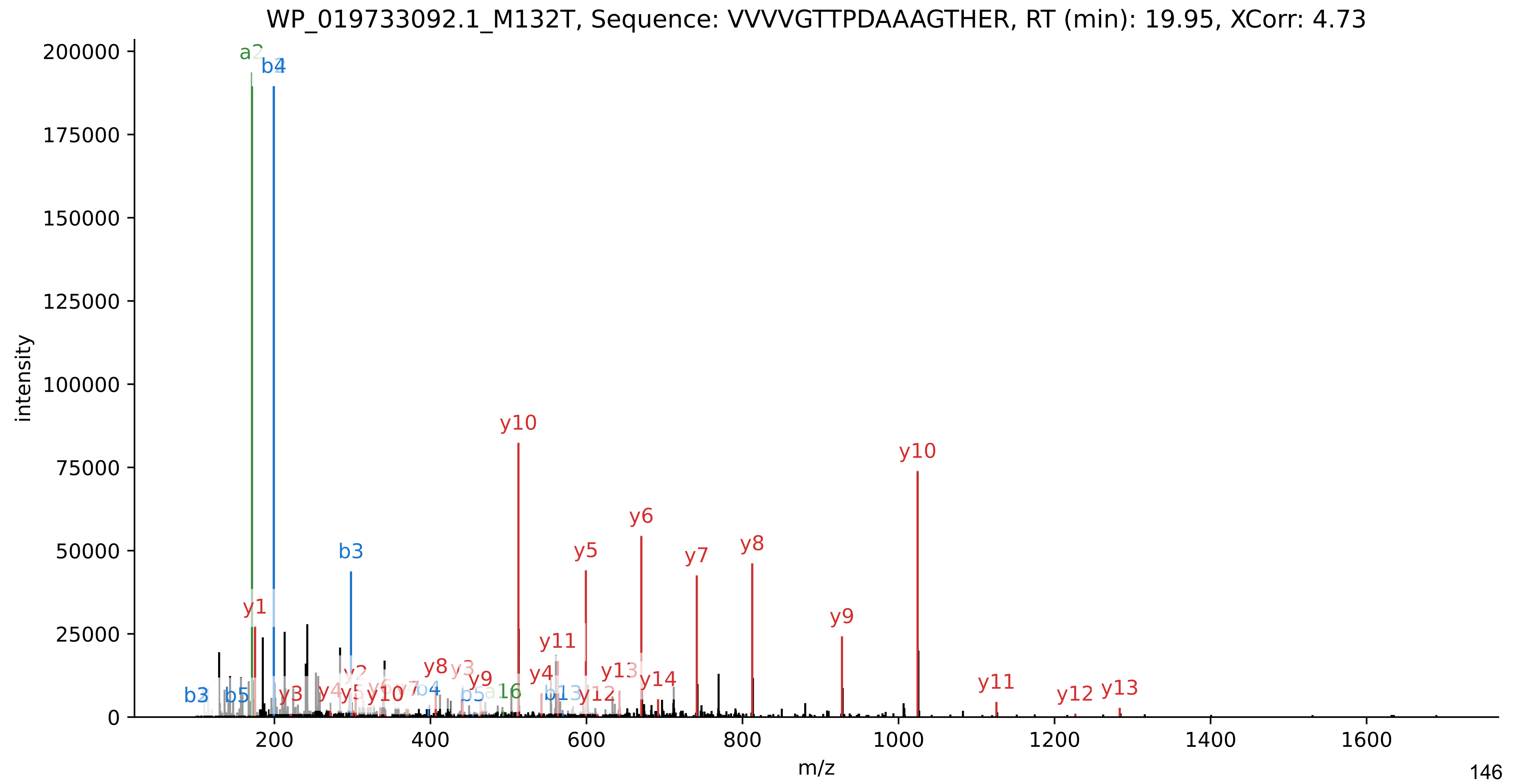

WP_019733092.1_M132T, Sequence: VVVVGTTPDAAAGTHER, RT (min): 19.95, XCorr: 4.73
intensity
m/z
a2
b4
b3
y1
y10
y10
y6
y5
y7
y8
y9
y11
y4
y13
y14
y11
y12
y13
y12
b3
b5
y3
y8
y3
y9
b13

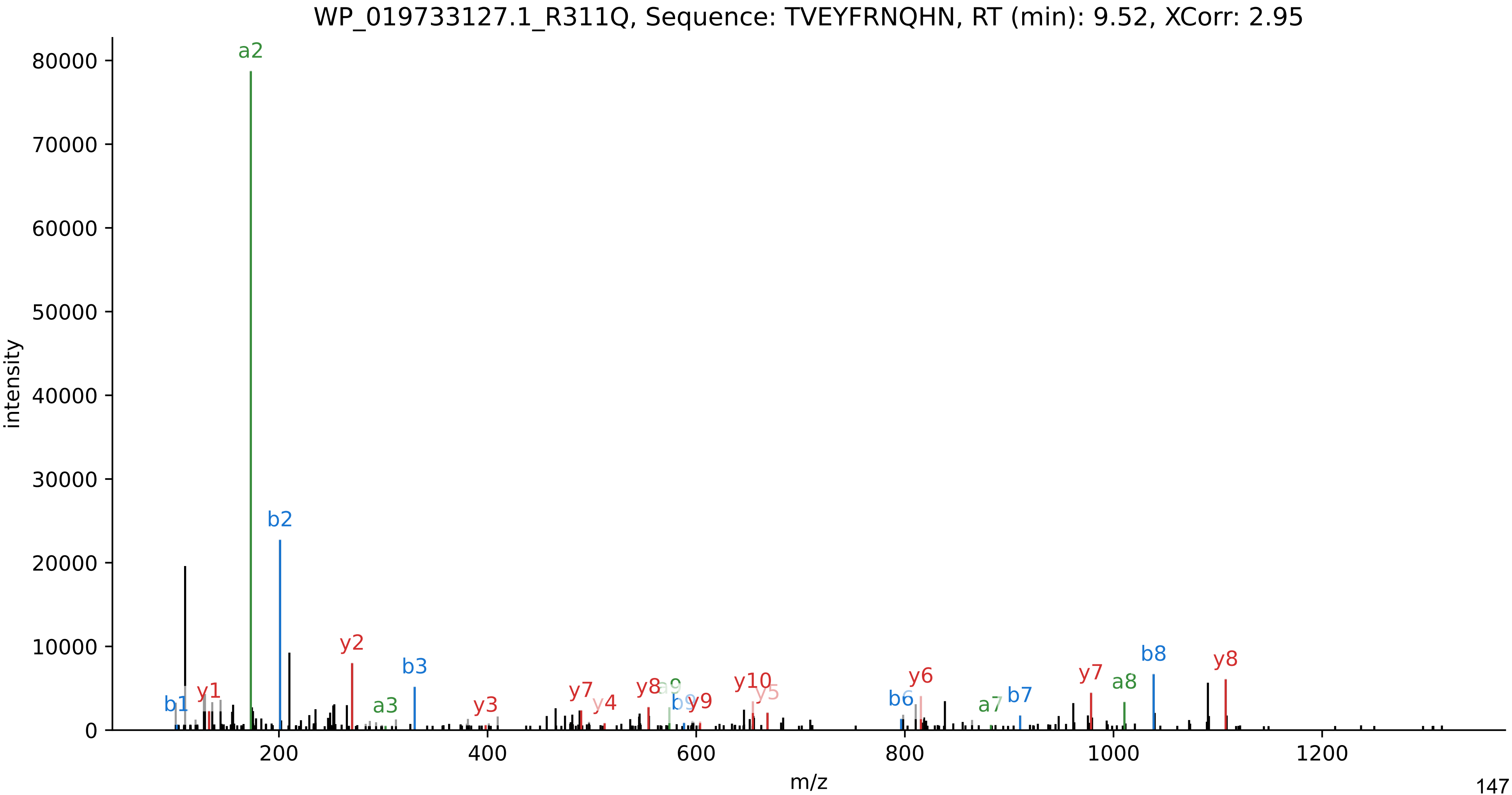
WP_019733127.1_R311Q, Sequence: TVEYFRNQHN, RT (min): 9.52, XCorr: 2.95
intensity
m/z
0
10000
20000
30000
40000
50000
60000
70000
80000
200
400
600
800
1000
1200
a2
b2
y2
b3
b1
y1
a3
y3
y7
y4
y8
y9
y10
y5
b6
y6
a7
b7
y7
a8
b8
y8

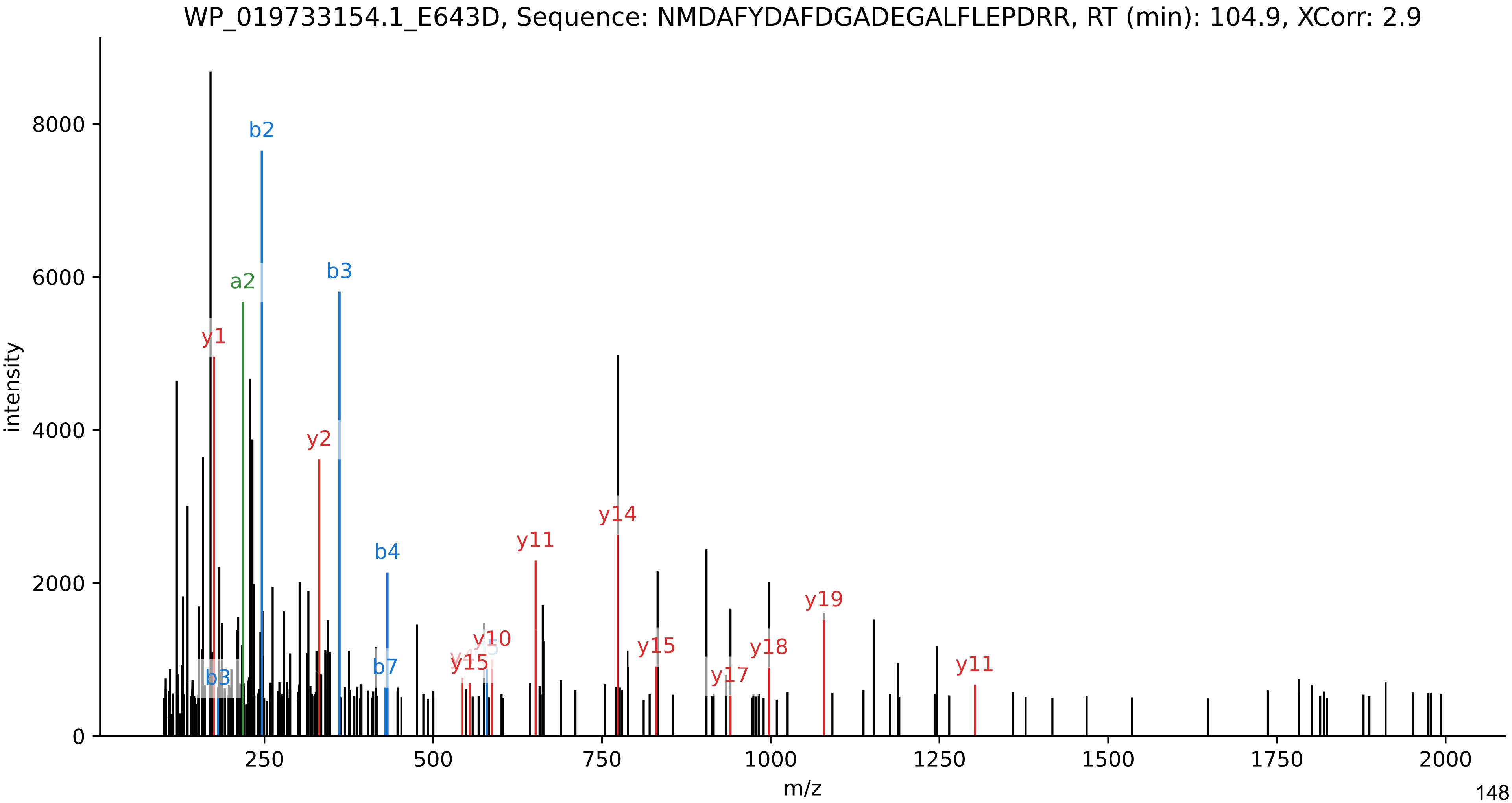
WP_019733154.1_E643D, Sequence: NMDAFYDAFDGADEGALFLEPDRR, RT (min): 104.9, XCorr: 2.9
intensity
m/z
b2
a2
b3
y1
y2
y14
y11
b4
y19
y10
y15
y18
y11
y17
y15
b7
b3
0
2000
4000
6000
8000
250
500
750
1000
1250
1500
1750
2000

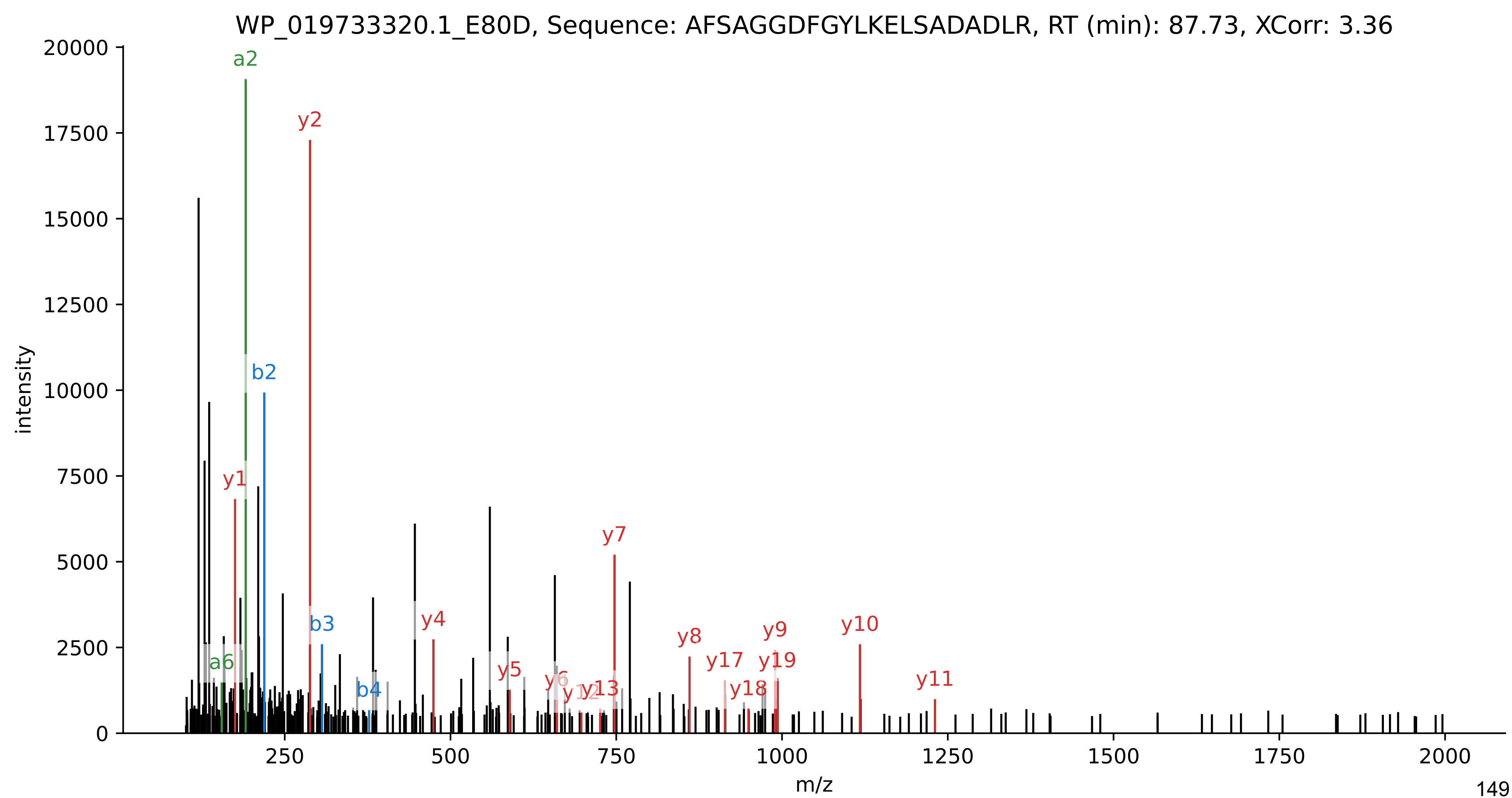

WP_019733320.1_E80D, Sequence: AFSAGGDFGYLKELSADADLR, RT (min): 87.73, XCorr: 3.36
intensity
m/z
a2
y2
b2
y1
a6
b3
b4
y4
y5
y6
y12
y13
y7
y8
y17
y18
y9
y19
y10
y11

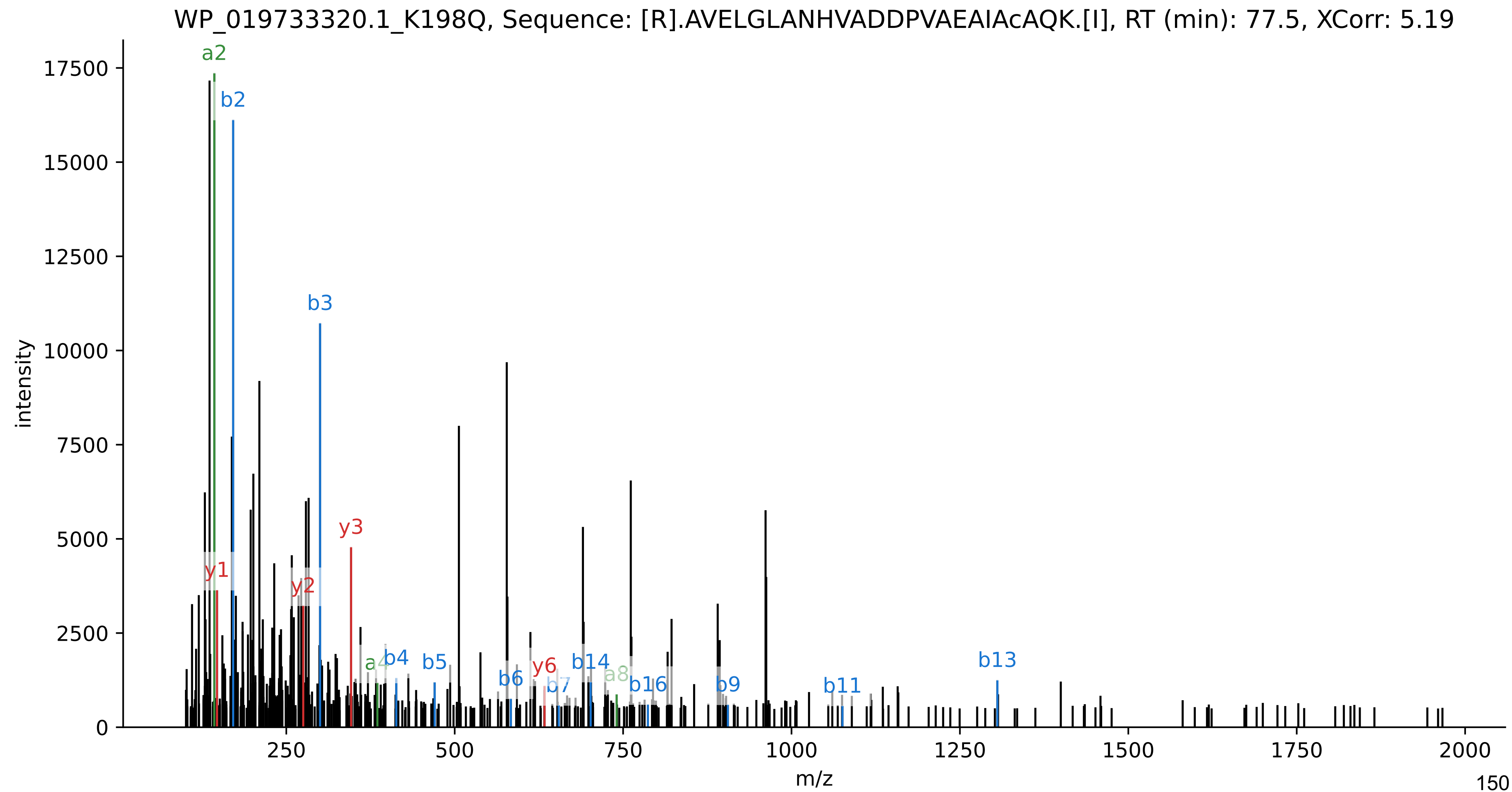

WP_019733320.1_K198Q, Sequence: [R].AVELGLANHVADDPVAEAIAcAQK.[I], RT (min): 77.5, XCorr: 5.19
intensity
m/z
a2
b2
b3
y3
y1
y2
a4
b4
b5
b6
y6
b7
b14
a8
b16
b9
b11
b13

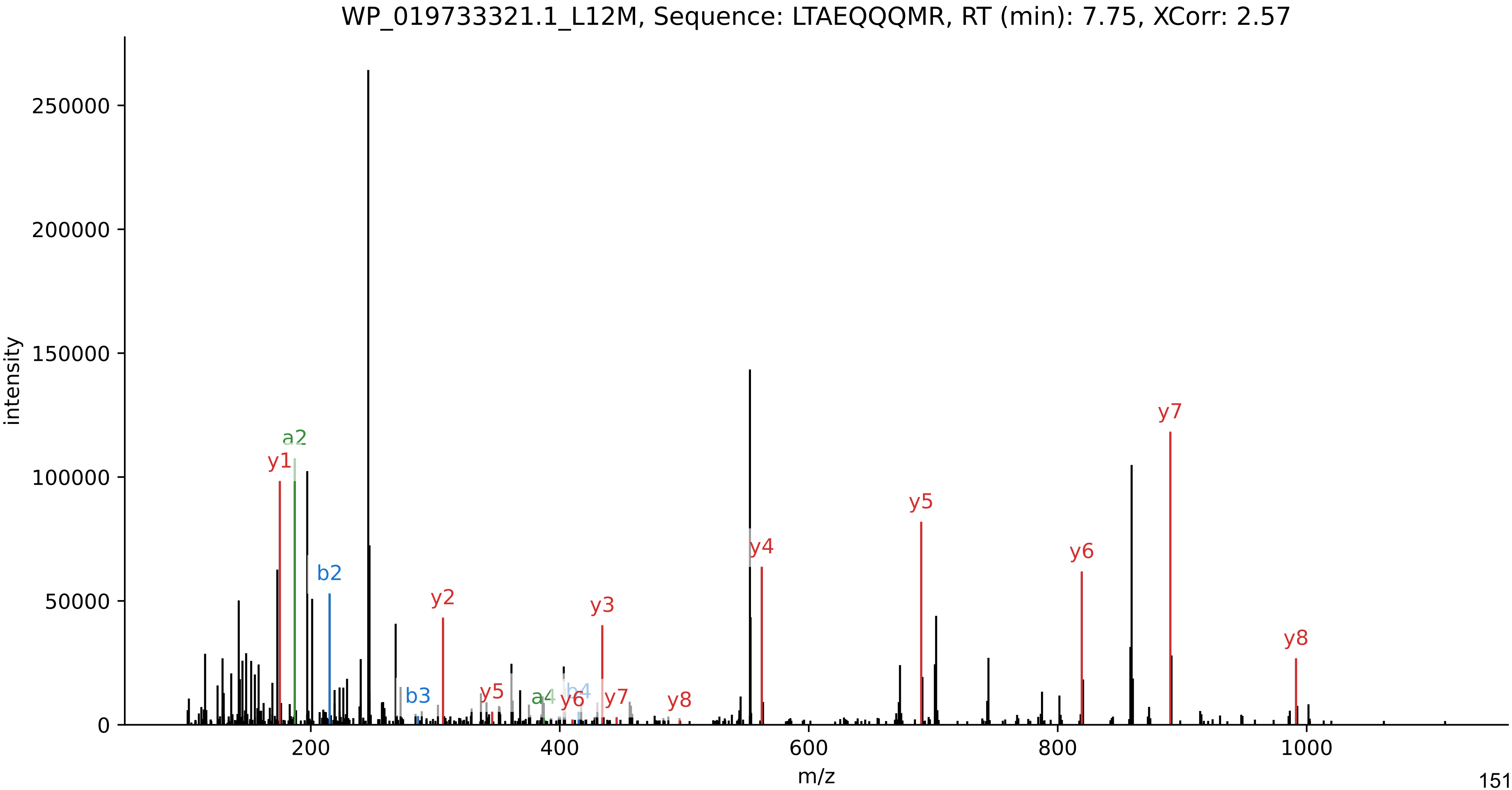

WP_019733321.1_L12M, Sequence: LTAEQQQMR, RT (min): 7.75, XCorr: 2.57
intensity
m/z
250000
200000
150000
100000
50000
0
200
400
600
800
1000
a2
y1
b2
y2
b3
y5
a4
y6
b4
y7
y3
y8
y4
y5
y6
y7
y8

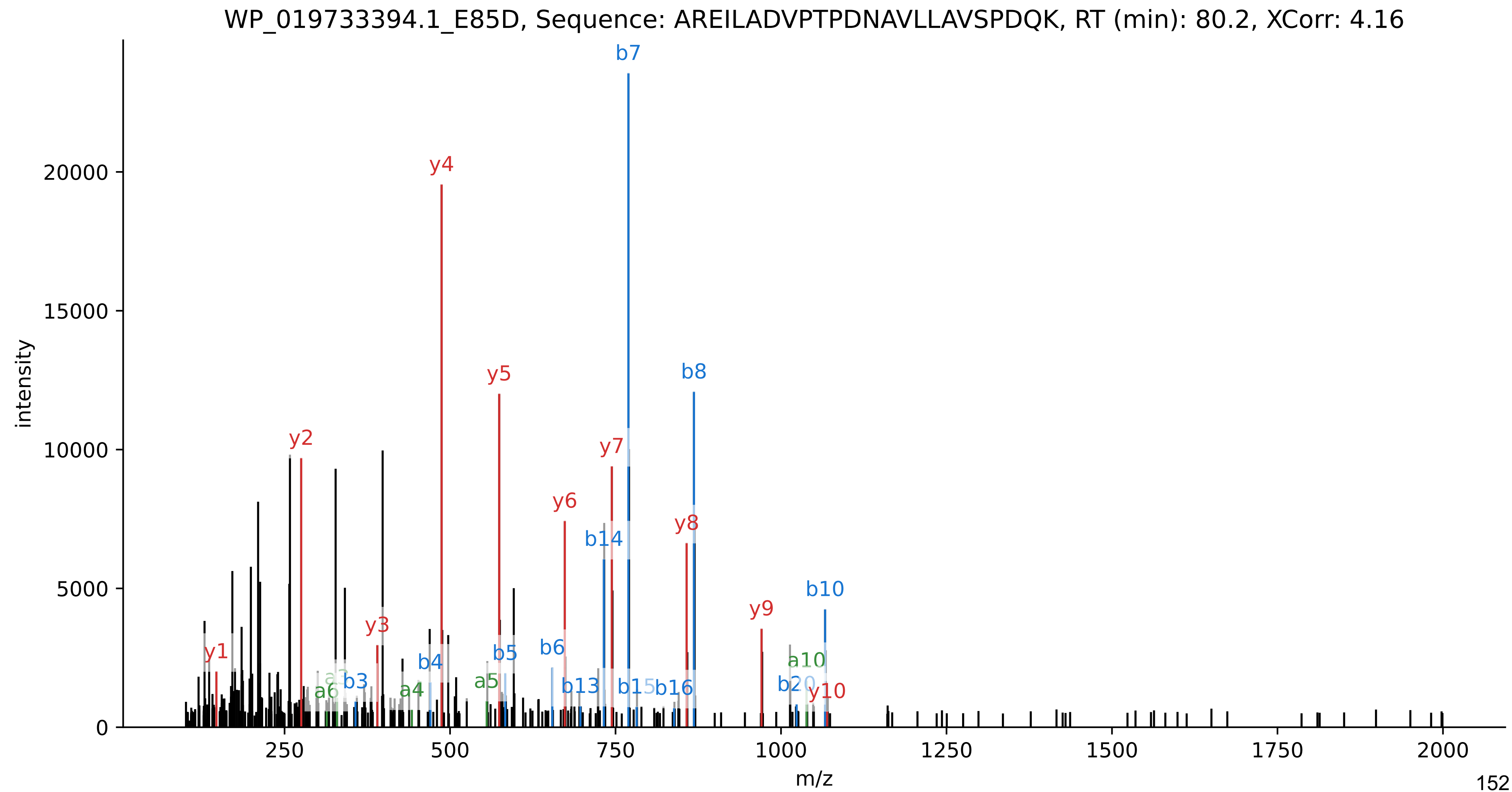
WP_019733394.1_E85D, Sequence: AREILADVPTPDNAVLLAVSPDQK, RT (min): 80.2, XCorr: 4.16
intensity
m/z
0
5000
10000
15000
20000
250
500
750
1000
1250
1500
1750
2000
b7
y4
y5
b8
y2
y7
y6
b14
y8
b10
y9
y3
y1
b6
b5
b4
a10
a5
b3
a6
a4
b13
b16
b20
y10

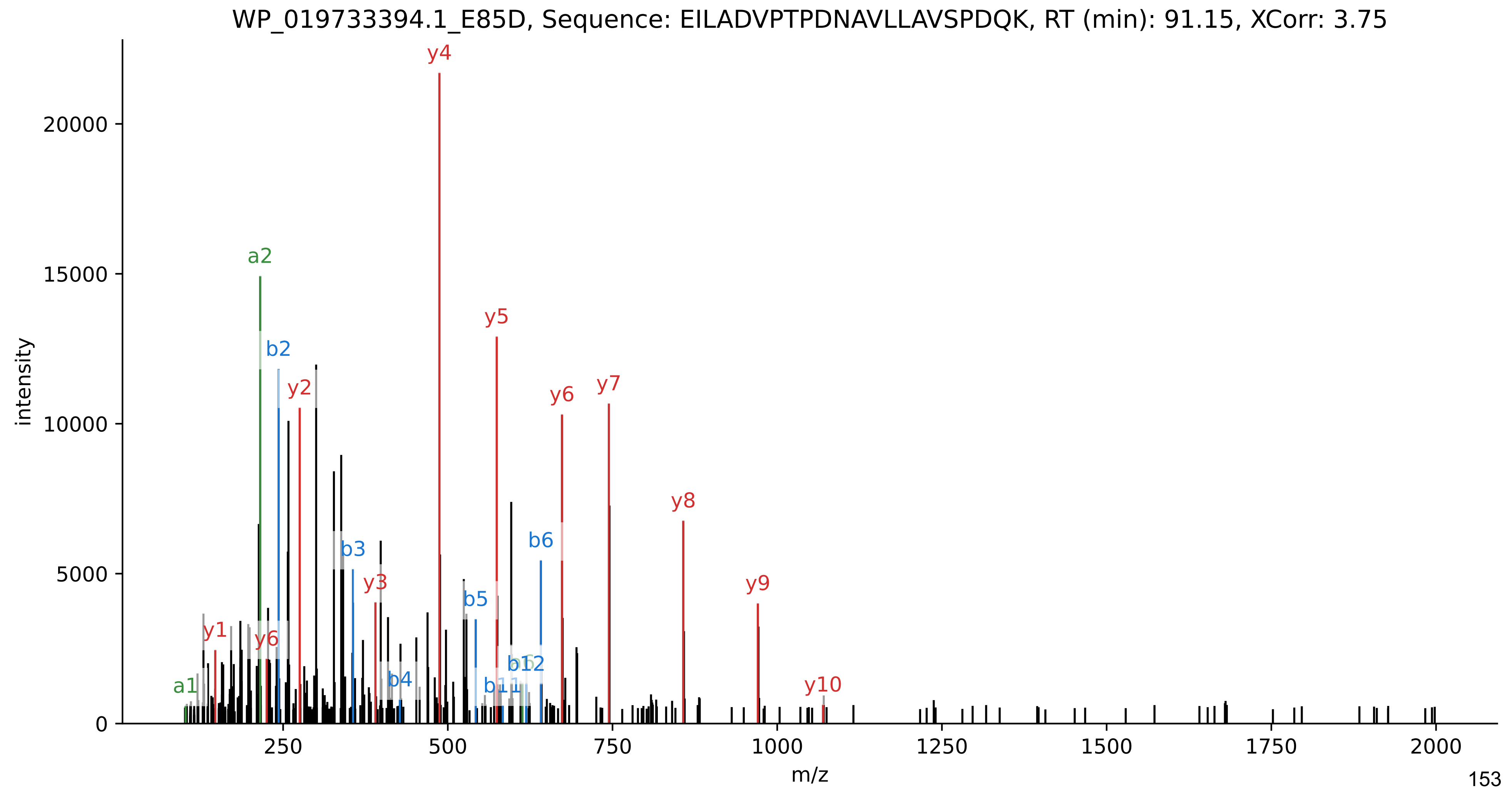
WP_019733394.1_E85D, Sequence: EILADVPTPDNAVLLAVSPDQK, RT (min): 91.15, XCorr: 3.75
intensity
m/z
y4
a2
b2
y2
y5
y6
y7
y8
b6
b3
y3
b5
y9
y1
y6
b12
b4
b11
a1
y10

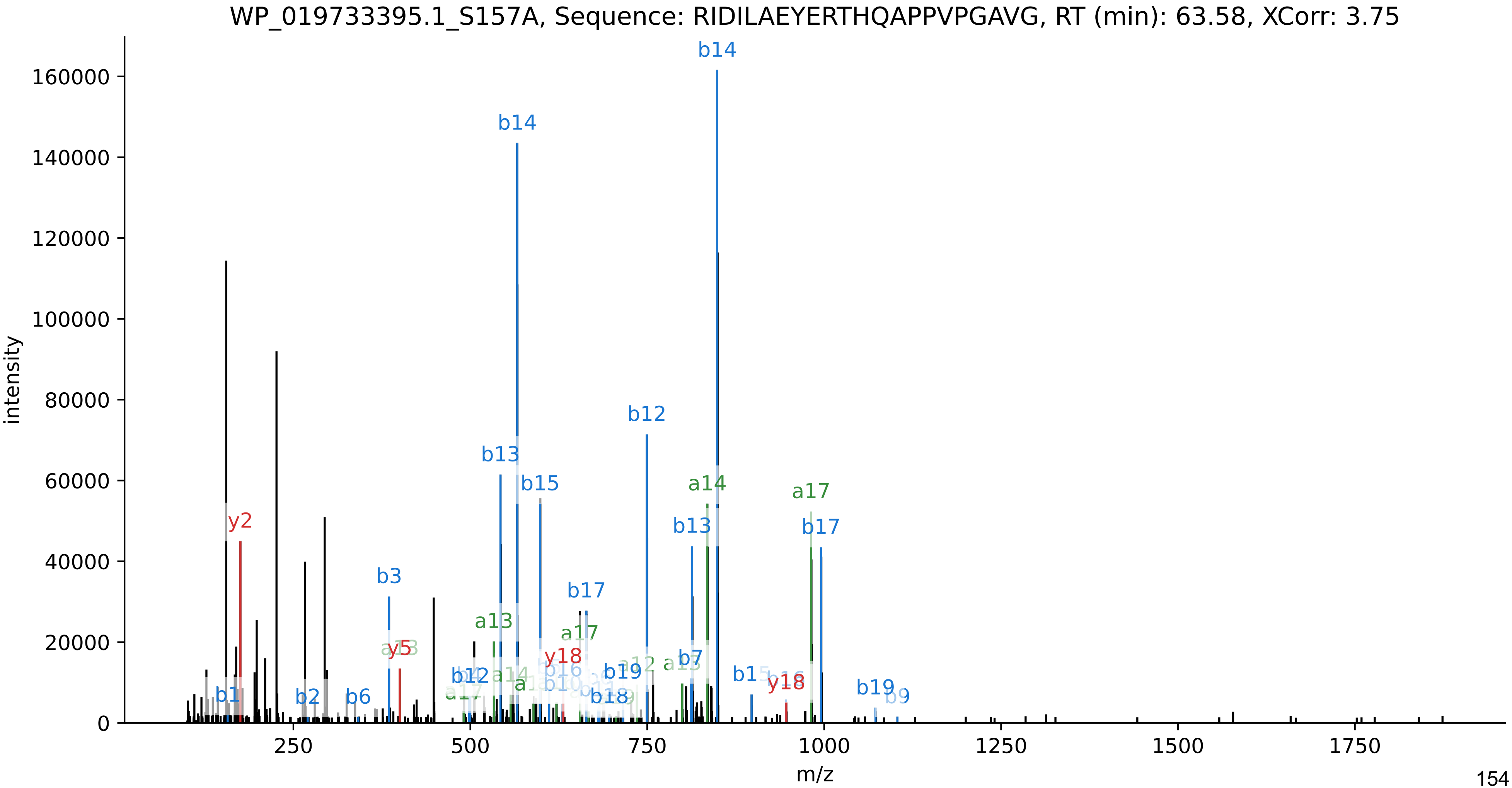
WP_019733395.1_S157A, Sequence: RIDILAEYERTHQAPPVPGAVG, RT (min): 63.58, XCorr: 3.75
intensity
m/z
b14
b14
b12
b13
b15
a14
a17
b13
b17
y2
b3
b17
a13
a17
y5
y18
b1
b2
b6
b12
b19
b7
b15
y18
b19
b18
0
20000
40000
60000
80000
100000
120000
140000
160000
250
500
750
1000
1250
1500
1750

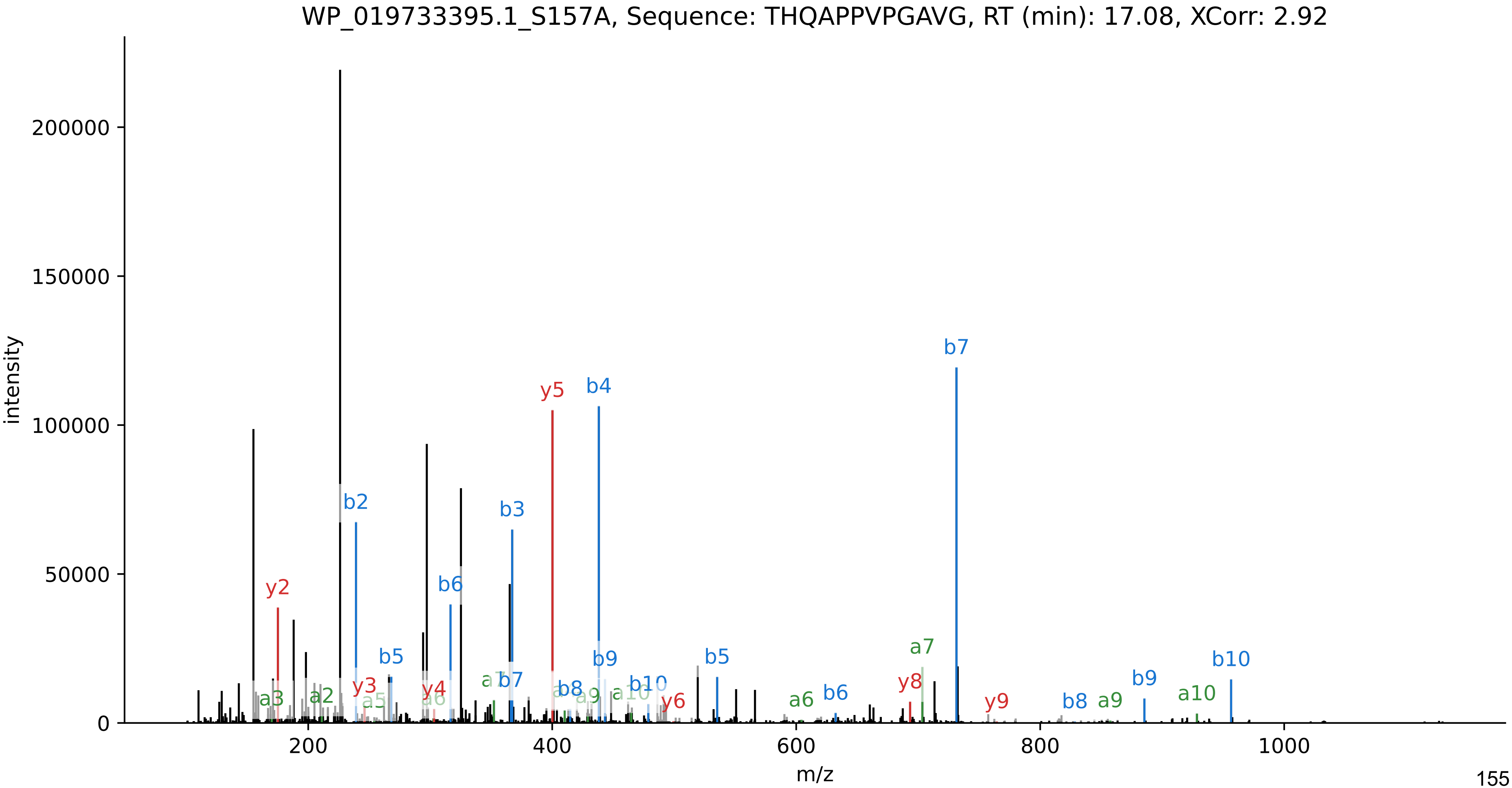
WP_019733395.1_S157A, Sequence: THQAPPVPGAVG, RT (min): 17.08, XCorr: 2.92
intensity
m/z
0
50000
100000
150000
200000
200
400
600
800
1000
y2
y3
y4
y5
y6
y8
y9
b2
b3
b4
b5
b6
b7
b8
b9
b10
a2
a3
a5
a6
a7
a9
a10

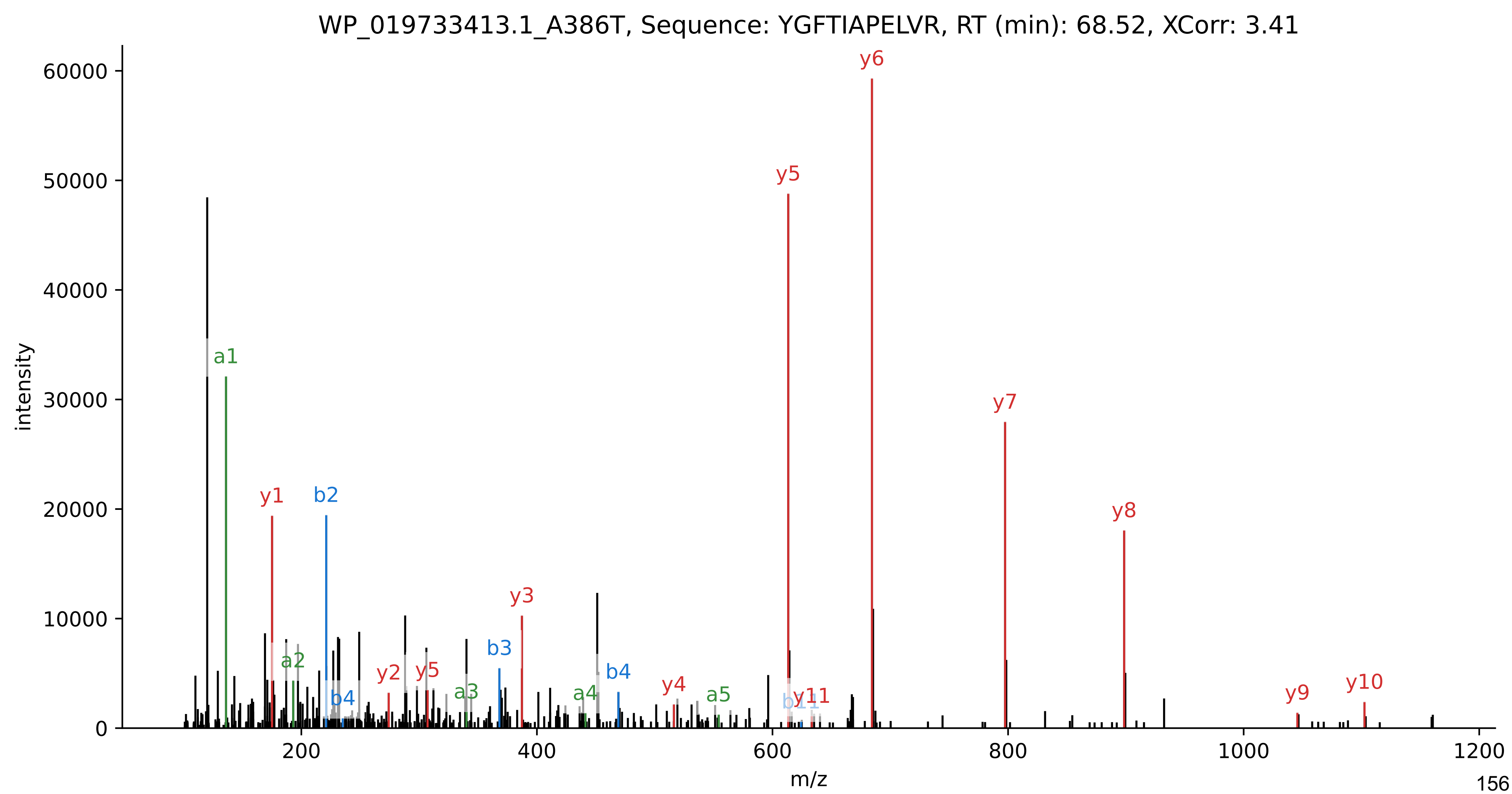
WP_019733413.1_A386T, Sequence: YGFTIAPELVR, RT (min): 68.52, XCorr: 3.41
intensity
m/z
a1
y1
b2
a2
y2
y5
b4
a3
b3
y3
a4
b4
y4
a5
b11
y11
y5
y6
y7
y8
y9
y10

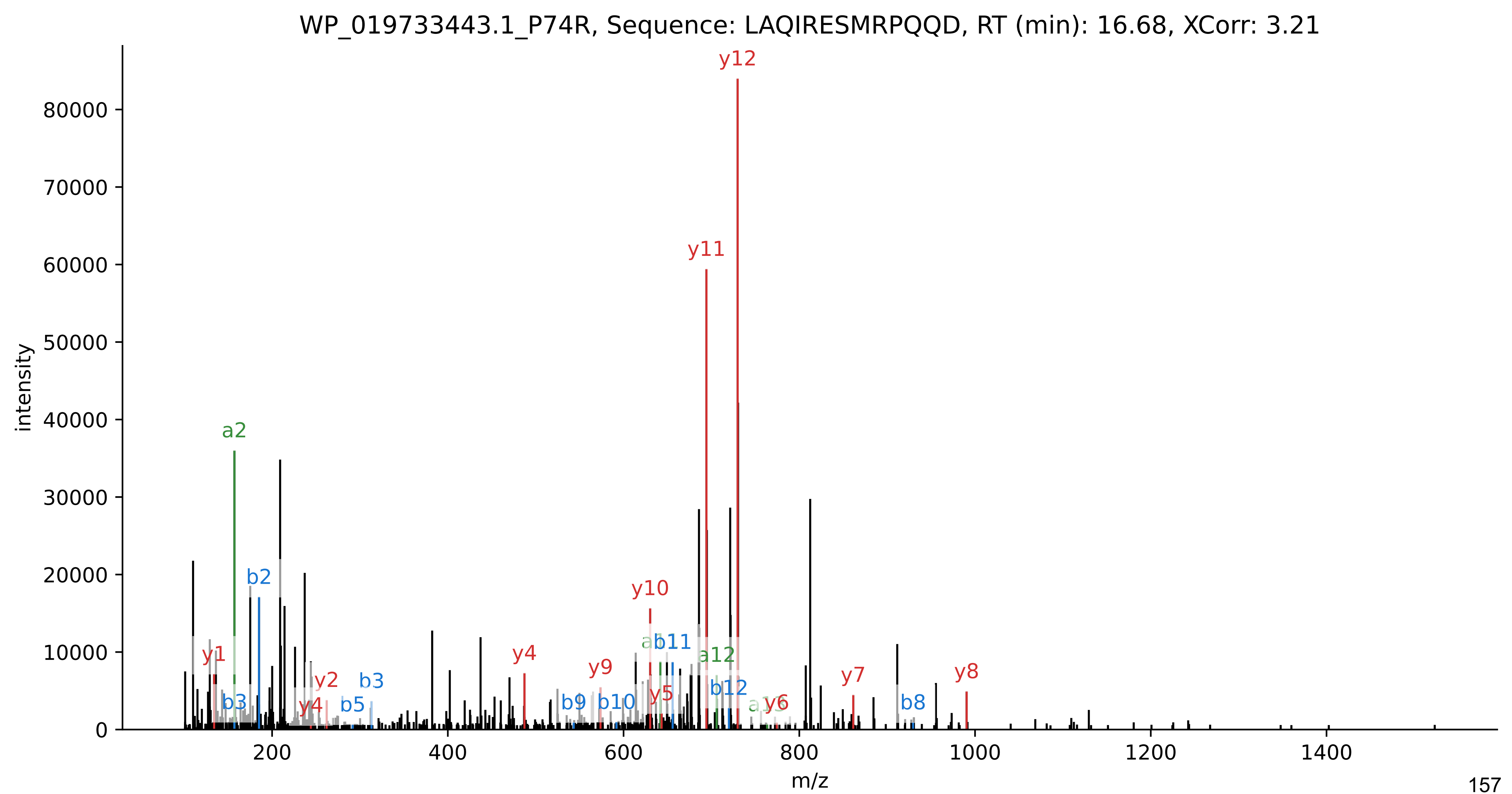

WP_019733443.1_P74R, Sequence: LAQIRESMRPQQD, RT (min): 16.68, XCorr: 3.21
intensity
m/z
y12
y11
y10
y1
y2
y4
y5
y6
y7
y8
y9
a2
a12
b2
b3
b5
b8
b9
b10
b11
b12

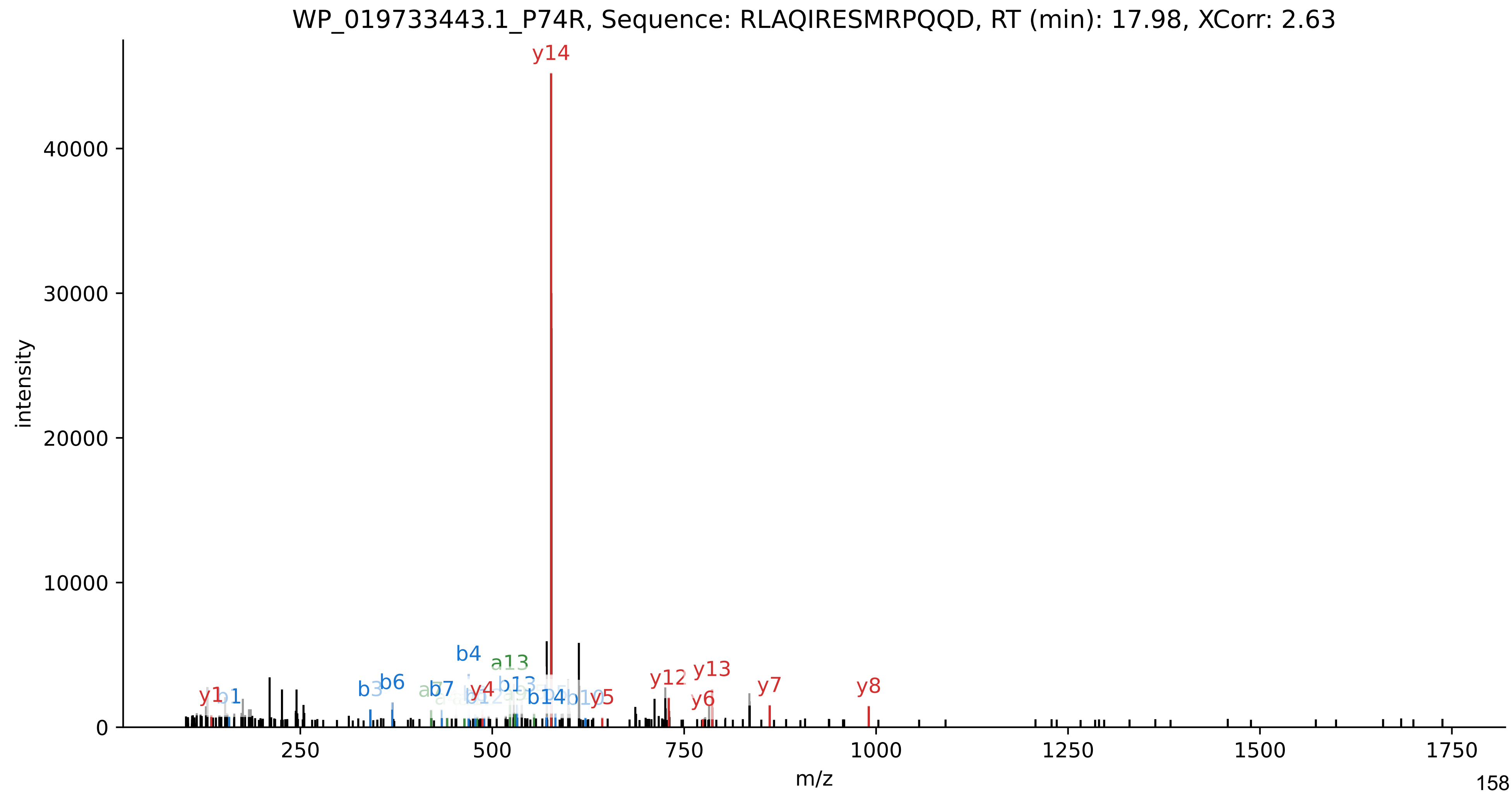

WP_019733443.1_P74R, Sequence: RLAQIRESMRPQQD, RT (min): 17.98, XCorr: 2.63
y14
b4
a13
y12
y13
b3
b6
b7
y4
b13
b14
y5
y6
y7
y8
y1
b1
intensity
m/z
0
10000
20000
30000
40000
250
500
750
1000
1250
1500
1750

WP_019733460.1_R456G, Sequence: GAGRPGTATARPSP, RT (min): 5.93, XCorr: 5.53

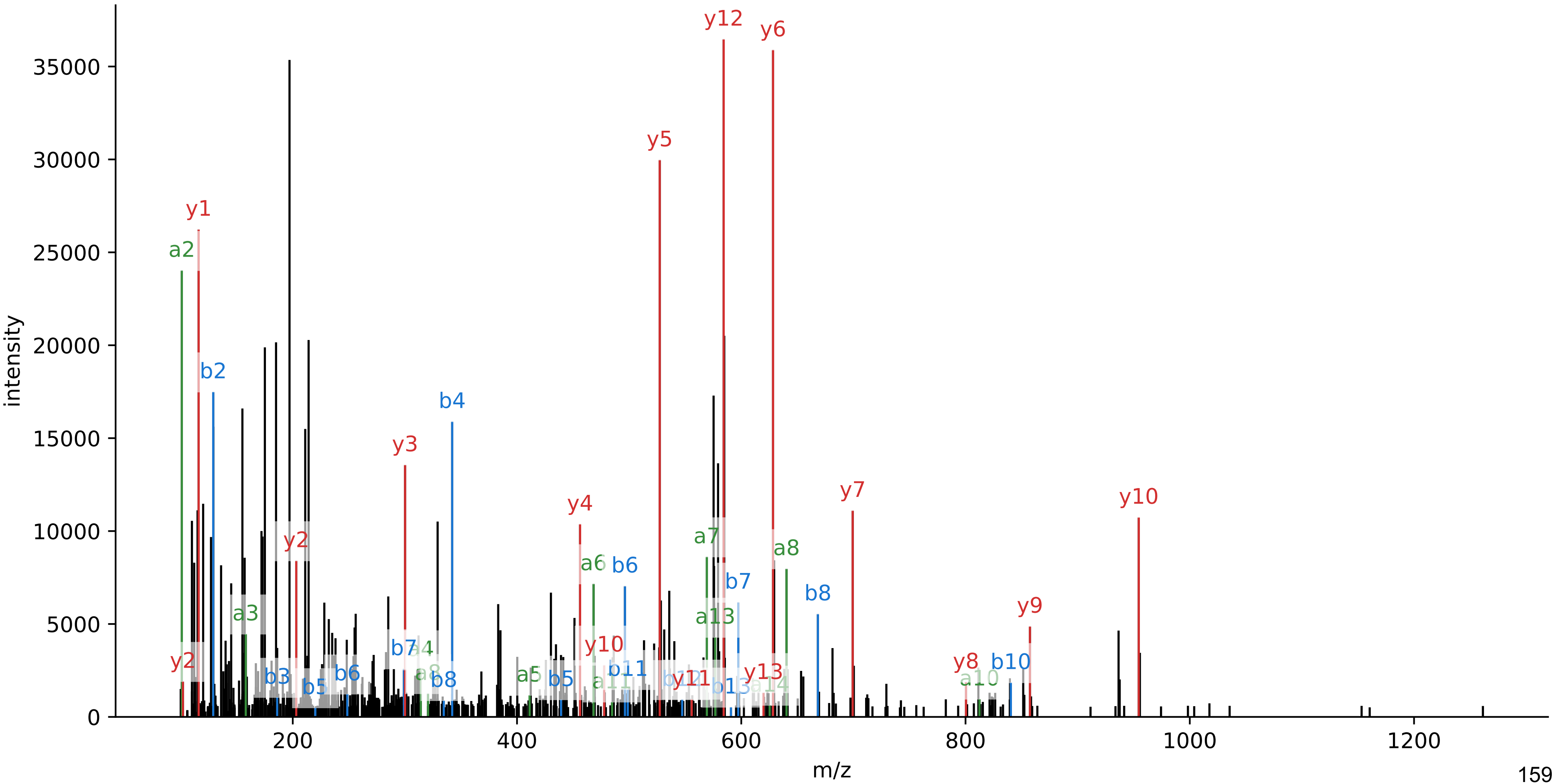

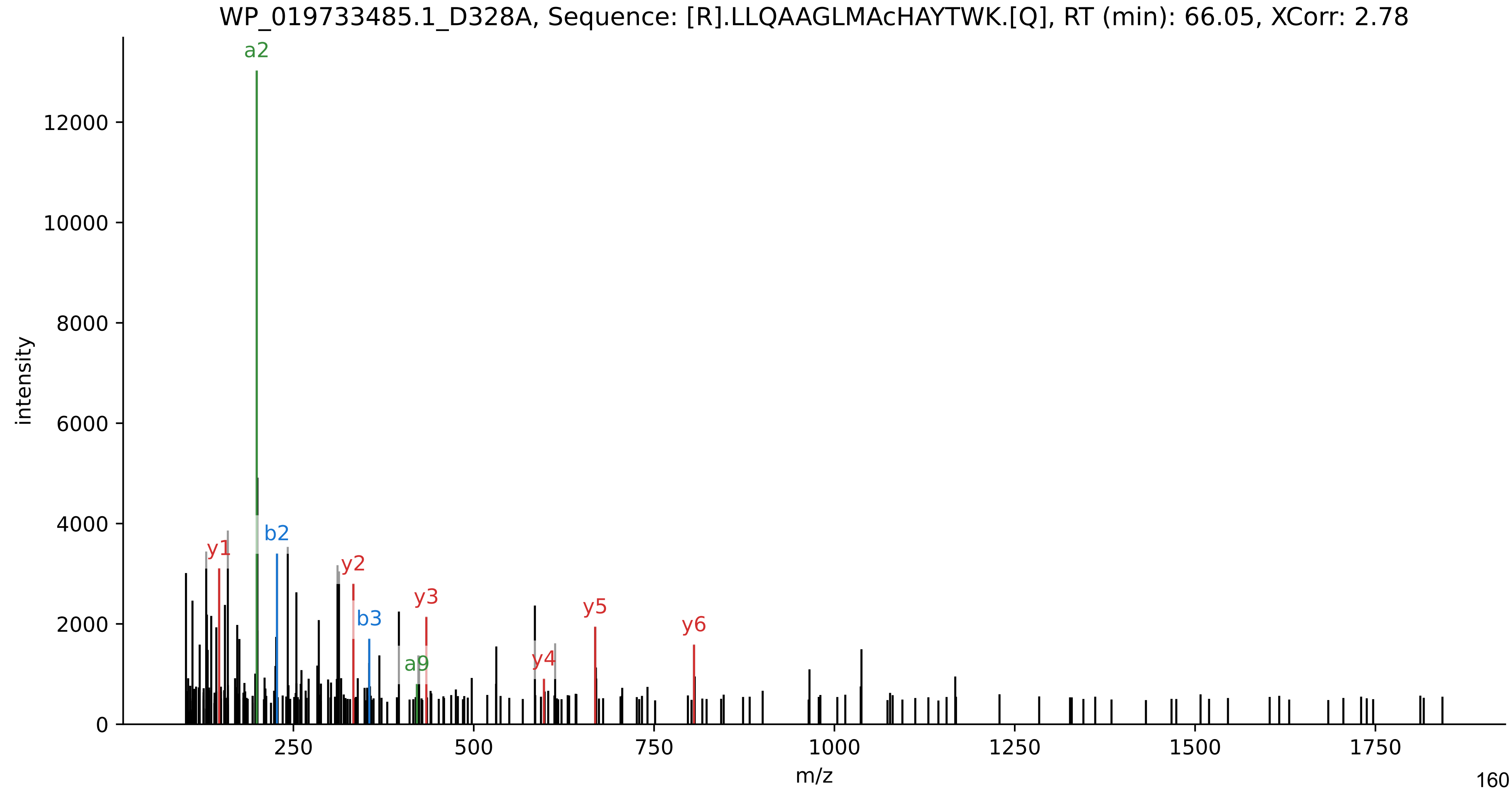
WP_019733485.1_D328A, Sequence: [R].LLQAAGLMAcHAYTWK.[Q], RT (min): 66.05, XCorr: 2.78
a2
b2
y1
y2
b3
y3
a9
y4
y5
y6
intensity
m/z
0
2000
4000
6000
8000
10000
12000
250
500
750
1000
1250
1500
1750

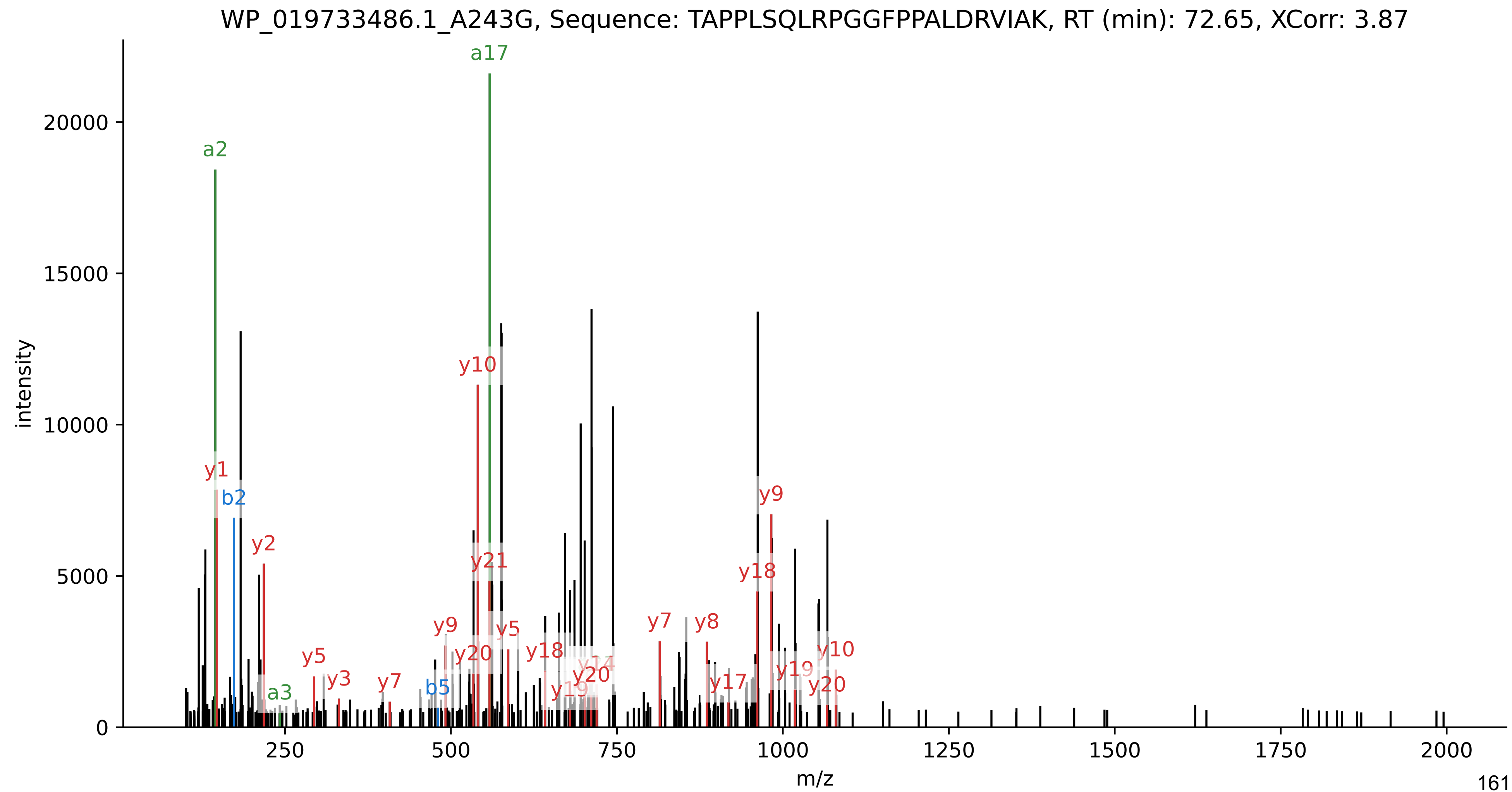

WP_019733486.1_A243G, Sequence: TAPPLSQLRPGGFPPALDRVIAK, RT (min): 72.65, XCorr: 3.87
intensity
m/z
a17
a2
y10
y1
b2
y2
y21
y9
y18
y5
y3
y7
b5
a3
y20
y19
y14
y8
y17
y10

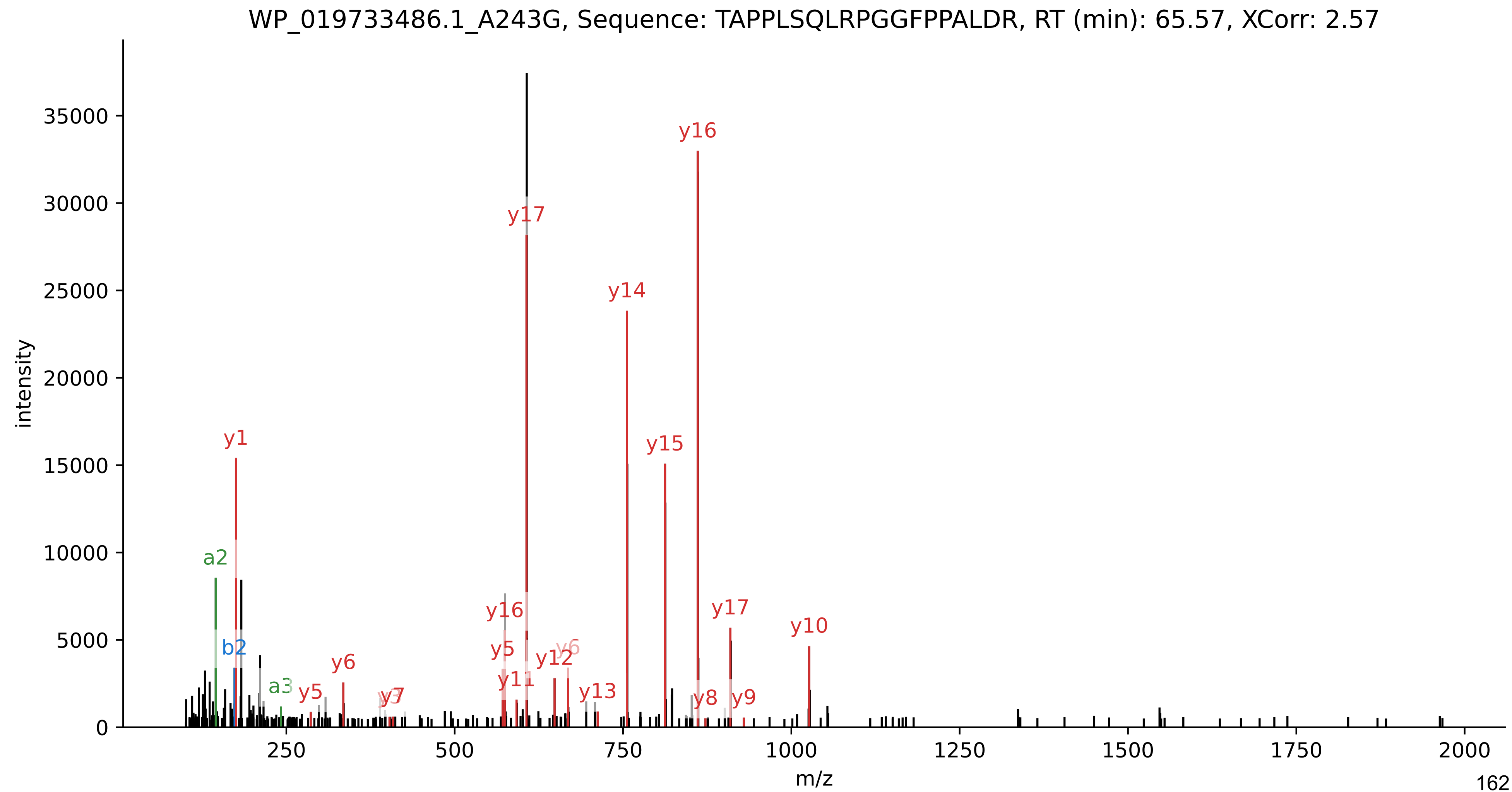

WP_019733486.1_A243G, Sequence: TAPPLSQLRPGGFPPALDR, RT (min): 65.57, XCorr: 2.57
intensity
m/z
y16
y17
y14
y15
y1
a2
b2
a3
y5
y6
y7
y16
y5
y11
y12
y6
y13
y8
y9
y17
y10

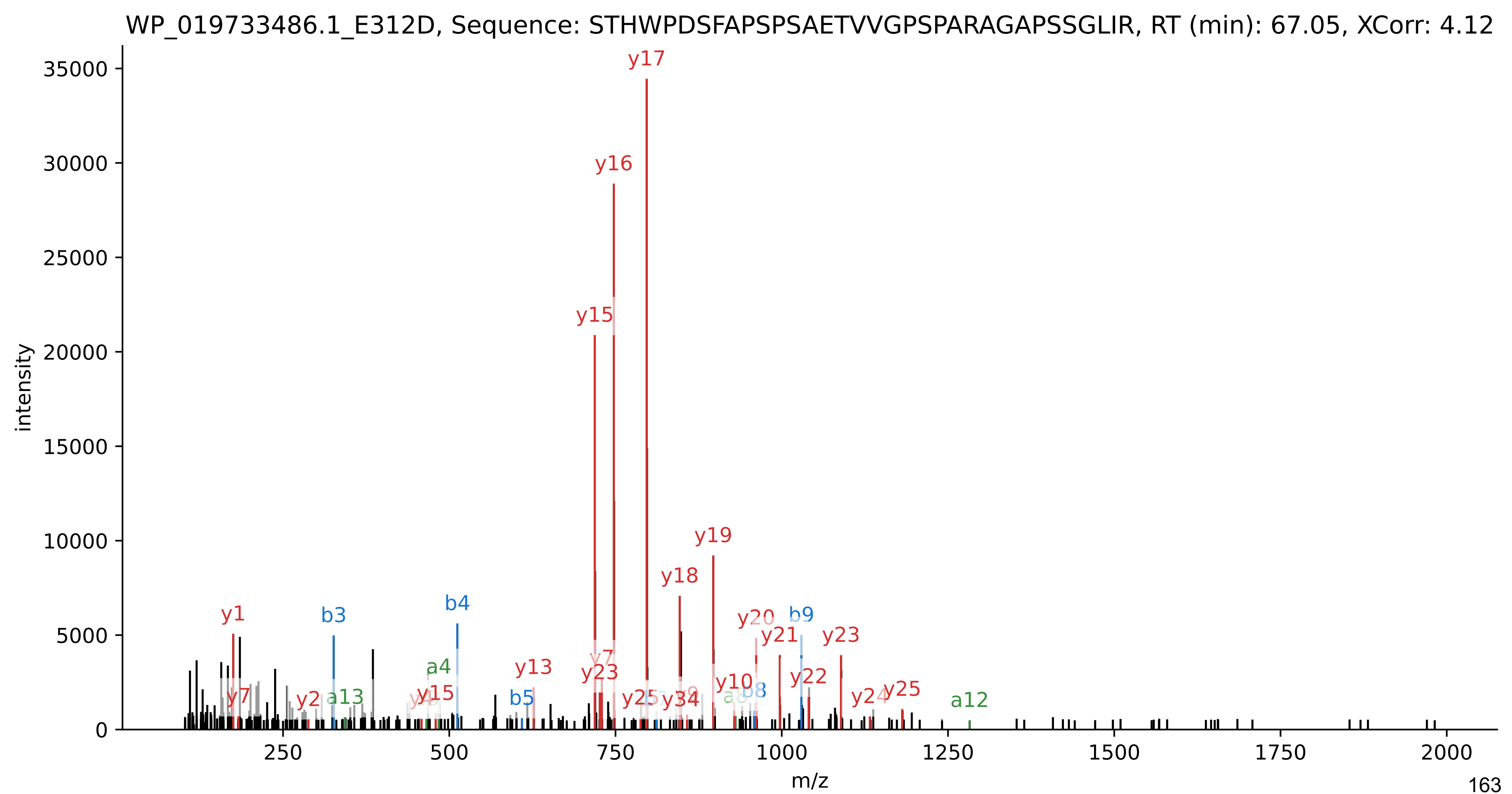

WP_019733486.1_E312D, Sequence: STHWPDSFAPSPSAETVVGPSPARAGAPSSGLIR, RT (min): 67.05, XCorr: 4.12
intensity
m/z
y17
y16
y15
y19
y18
y1
b3
b4
y20
b9
y21
y23
y13
a4
y23
y22
y10
y15
y7
y2
a13
b5
y25
y34
b8
y24
y25
a12

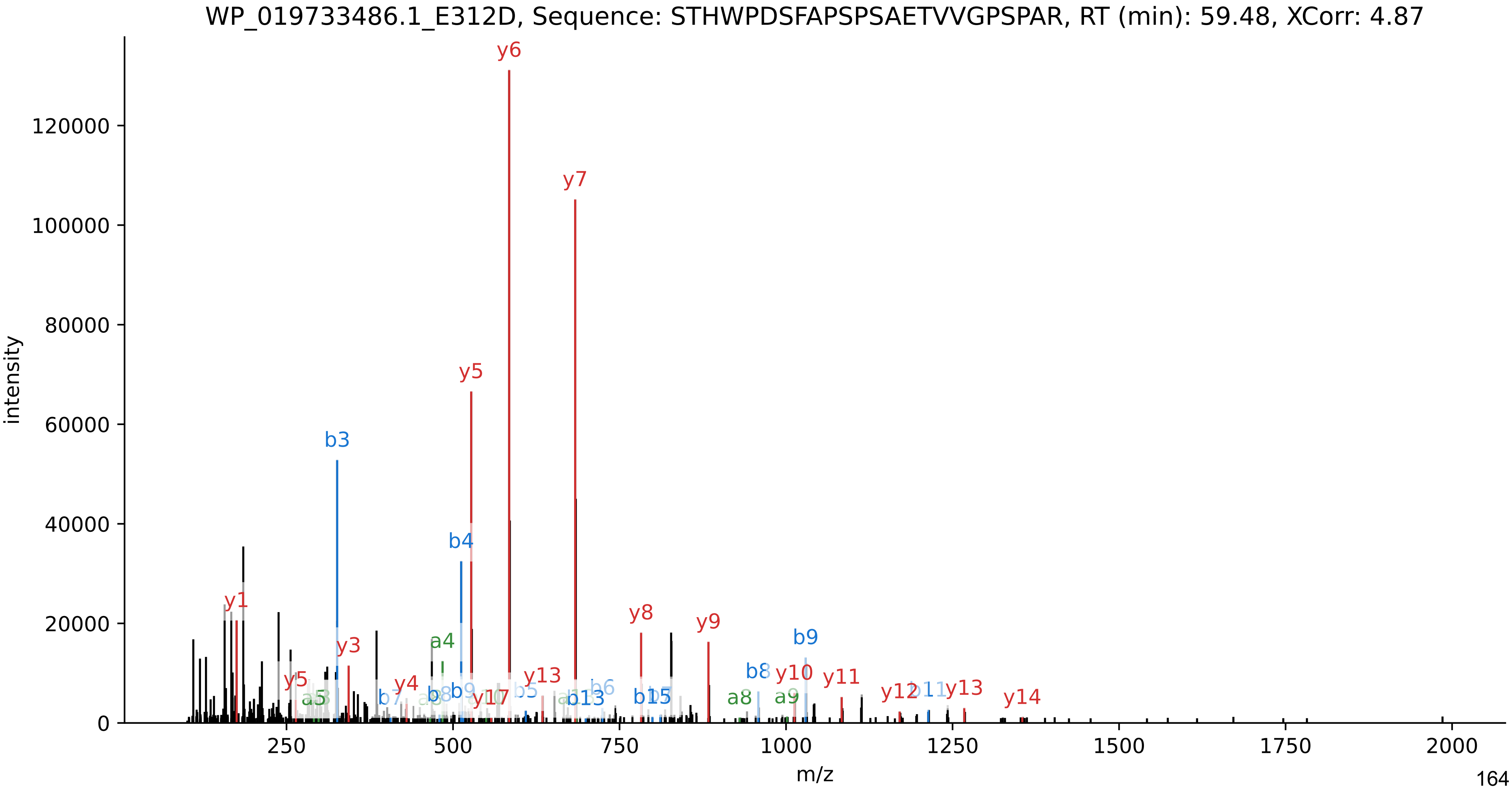

WP_019733486.1_E312D, Sequence: STHWPDSFAPSPSAETVVGPSPAR, RT (min): 59.48, XCorr: 4.87
intensity
m/z
y6
y7
y5
b3
b4
y1
y8
y9
b9
a4
y3
b8
y10
y11
y13
y12
y13
y14
a8
a9
b15
b13
y4
y17
b5
b6
b7
b9

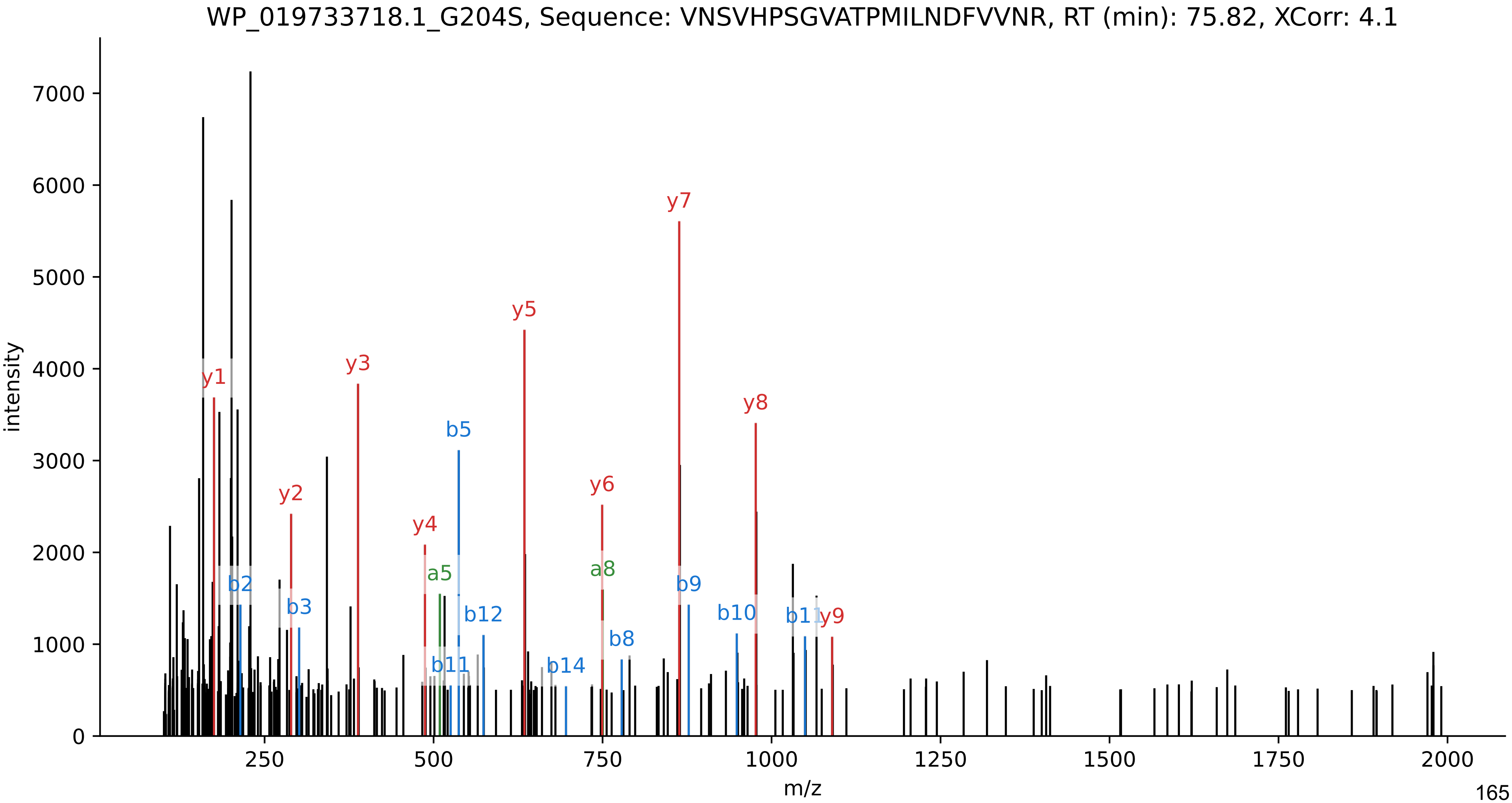

WP_019733718.1_G204S, Sequence: VNSVHPSGVATPMILNDFVVNR, RT (min): 75.82, XCorr: 4.1
intensity
m/z
y1
y2
y3
y4
y5
y6
y7
y8
y9
a5
a8
b2
b3
b5
b8
b9
b10
b11
b12
b14

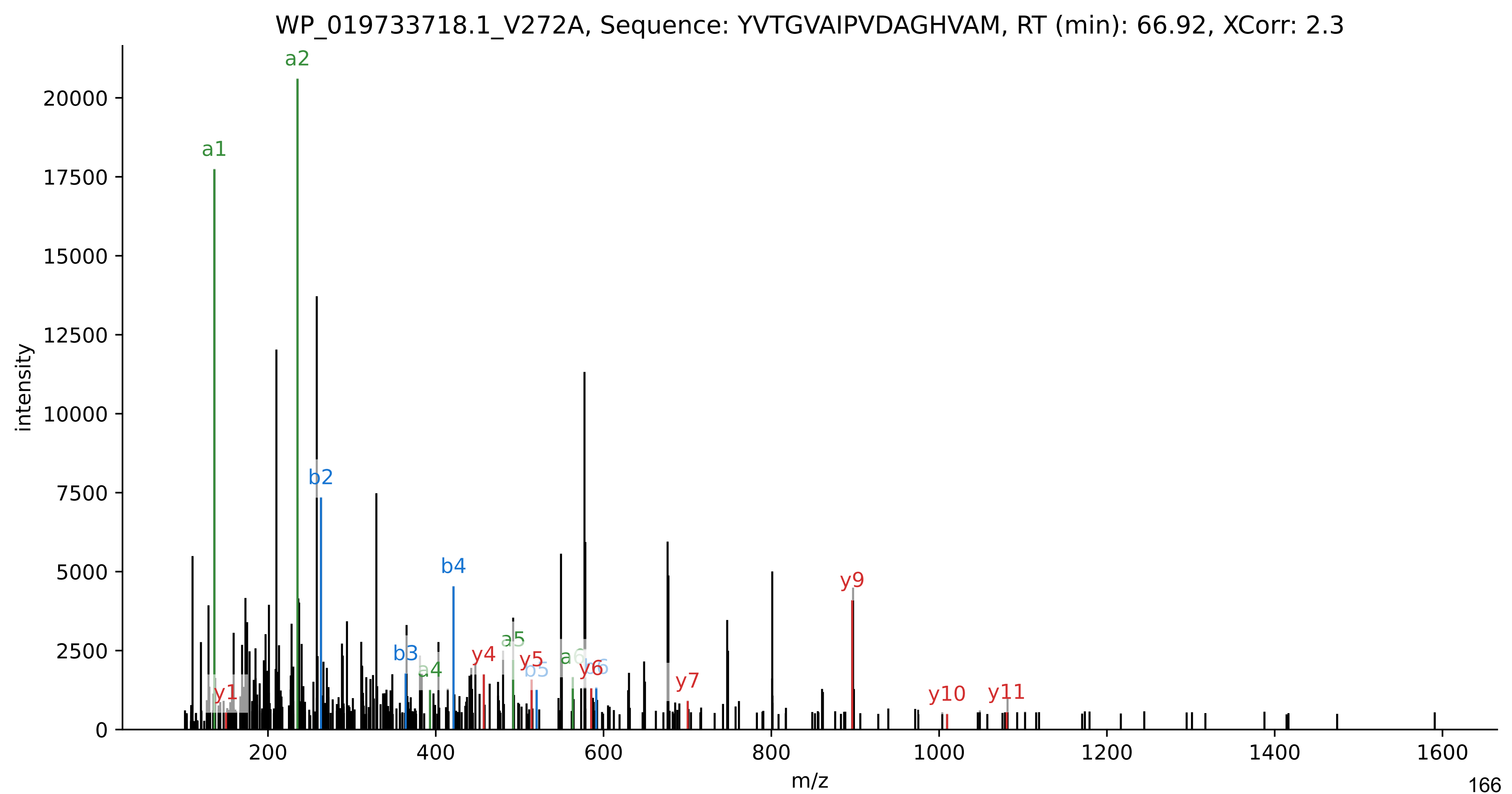

WP_019733718.1_V272A, Sequence: YVTGVAIPVDAGHVAM, RT (min): 66.92, XCorr: 2.3
intensity
m/z
a1
a2
b2
b4
b3
a4
y4
a5
y5
b5
a6
y6
y1
y7
y9
y10
y11

WP_019733719.1_L76V, Sequence: AAQMGGSTAVEKLDLADLSSVR, RT (min): 65.05, XCorr: 4.59

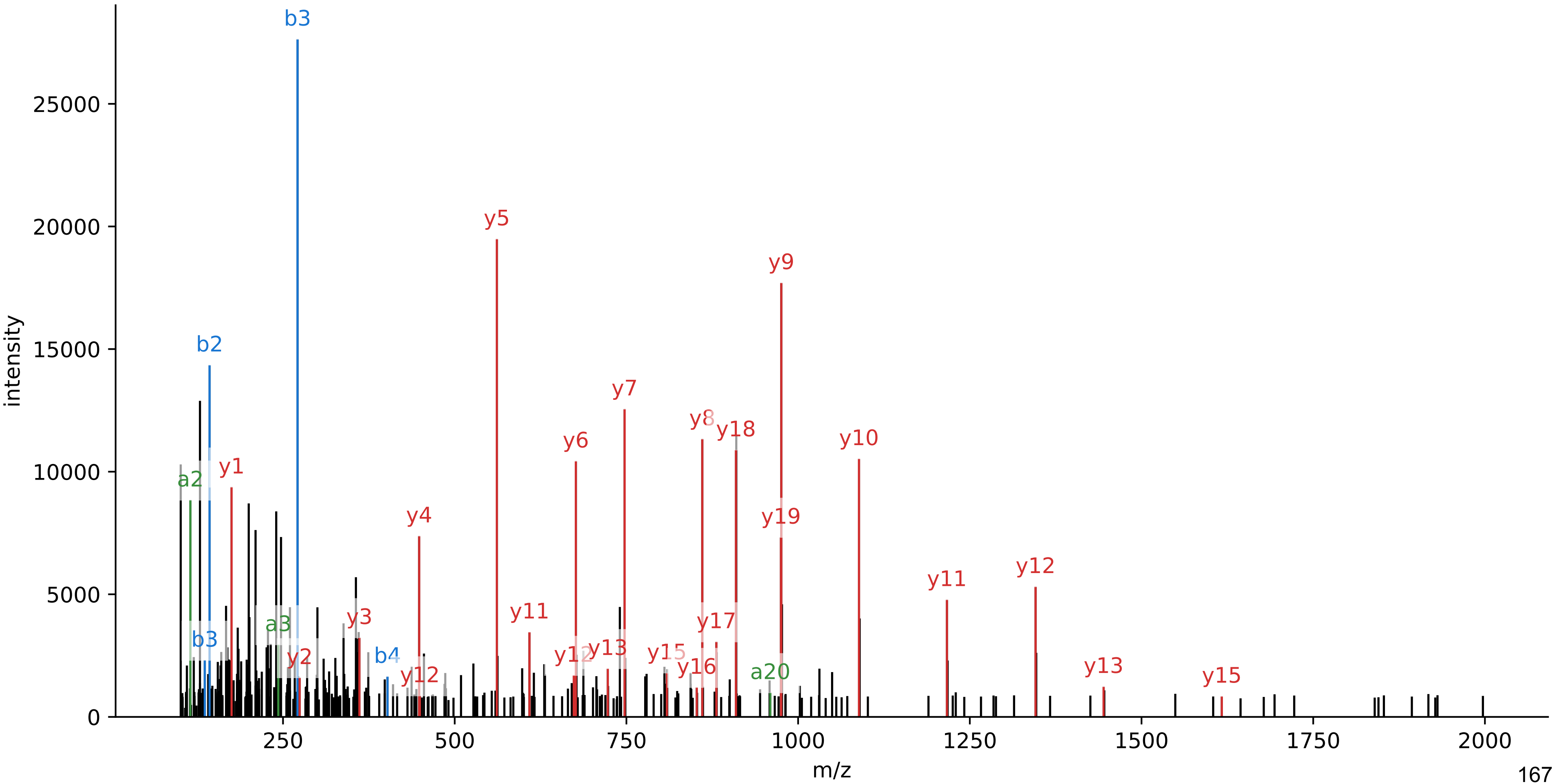

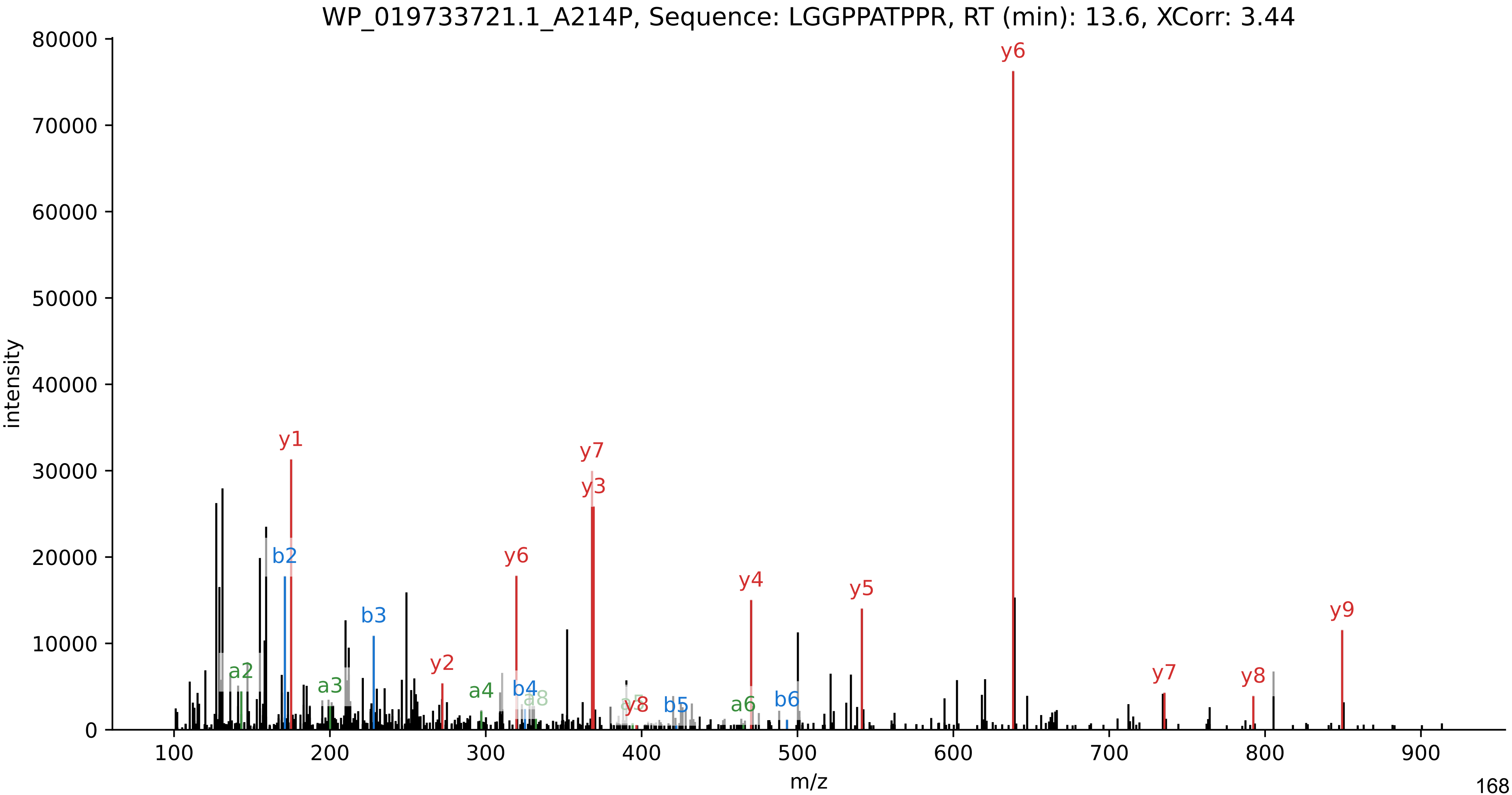

WP_019733721.1_A214P, Sequence: LGGPPATPPR, RT (min): 13.6, XCorr: 3.44
intensity
m/z
0
10000
20000
30000
40000
50000
60000
70000
80000
100
200
300
400
500
600
700
800
900
y1
y2
y3
y4
y5
y6
y6
y7
y7
y8
y8
y9
b2
b3
b4
b5
b6
a2
a3
a4
a6
a8

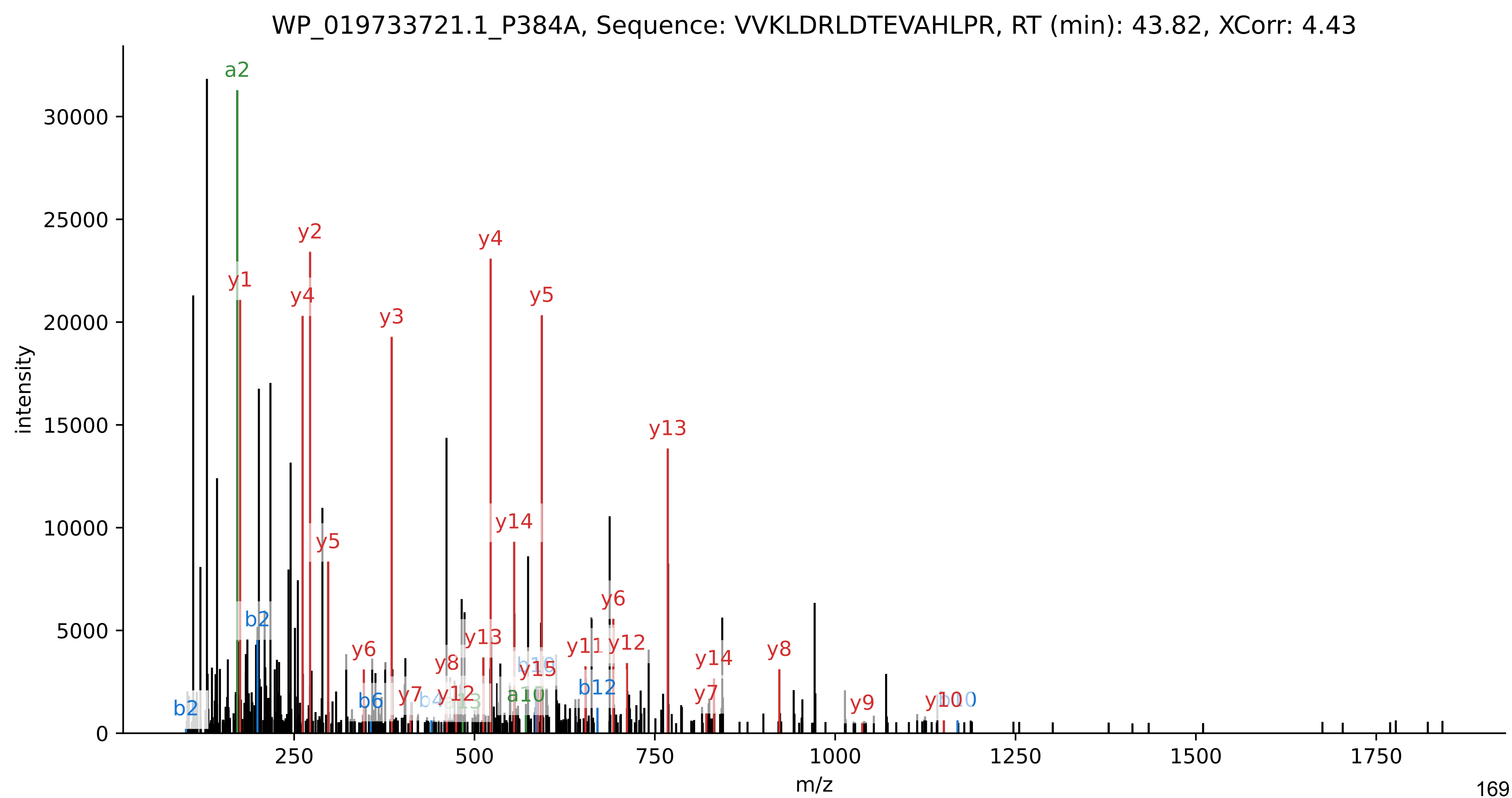
WP_019733721.1_P384A, Sequence: VVKLDRLDTEVAHLPR, RT (min): 43.82, XCorr: 4.43
intensity
m/z
a2
y1
y2
y4
y4
y3
y5
y5
y14
y13
y6
y6
b2
b2
b6
y7
y8
y12
y13
y15
a10
b12
y11
y12
y14
y7
y8
y9
y10

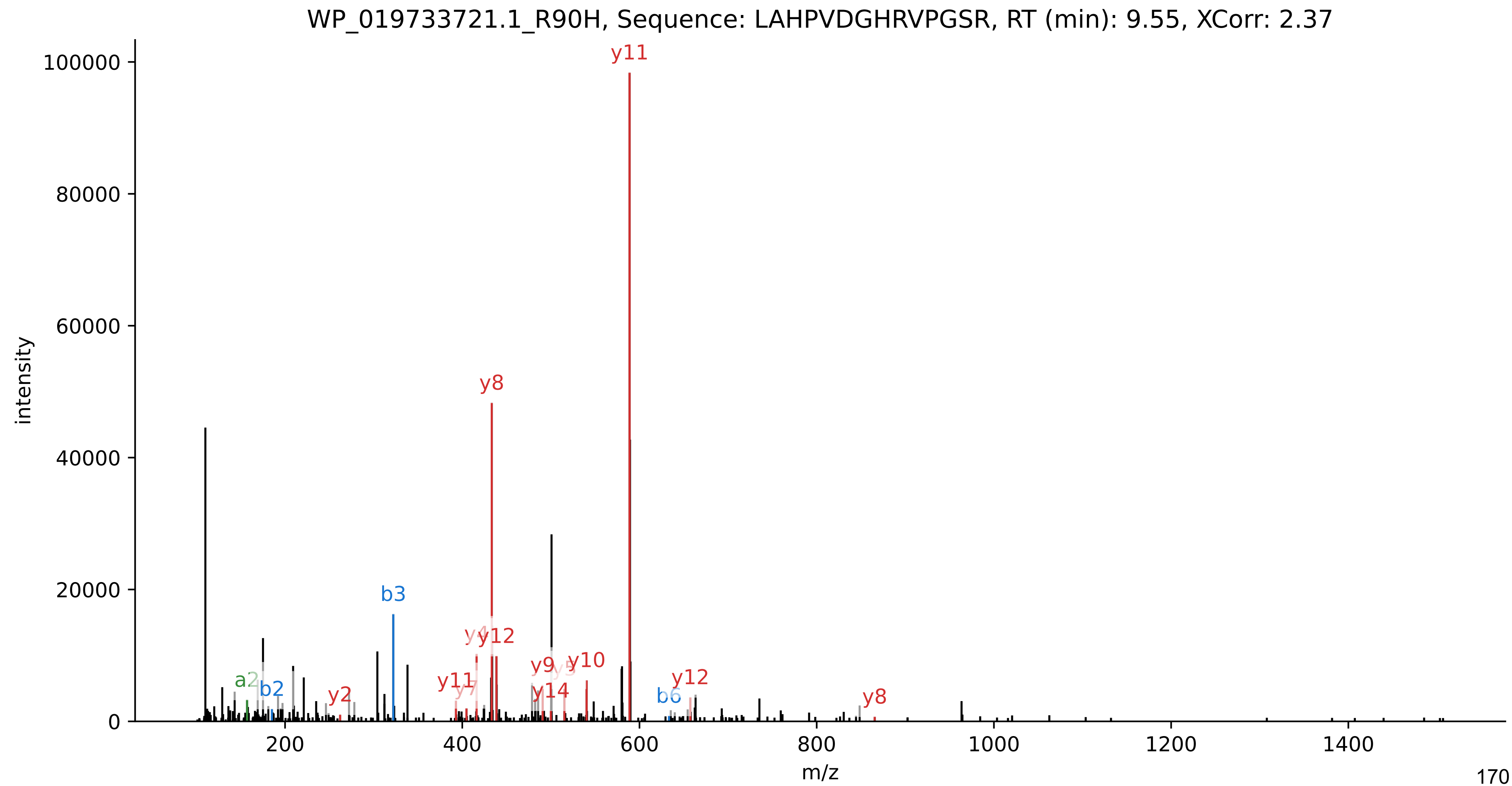

WP_019733721.1_R90H, Sequence: LAHPVDGHRVPGSR, RT (min): 9.55, XCorr: 2.37
intensity
m/z
y11
y8
b3
y12
y10
y9
y14
y11
a2
b2
y2
b6
y12
y8

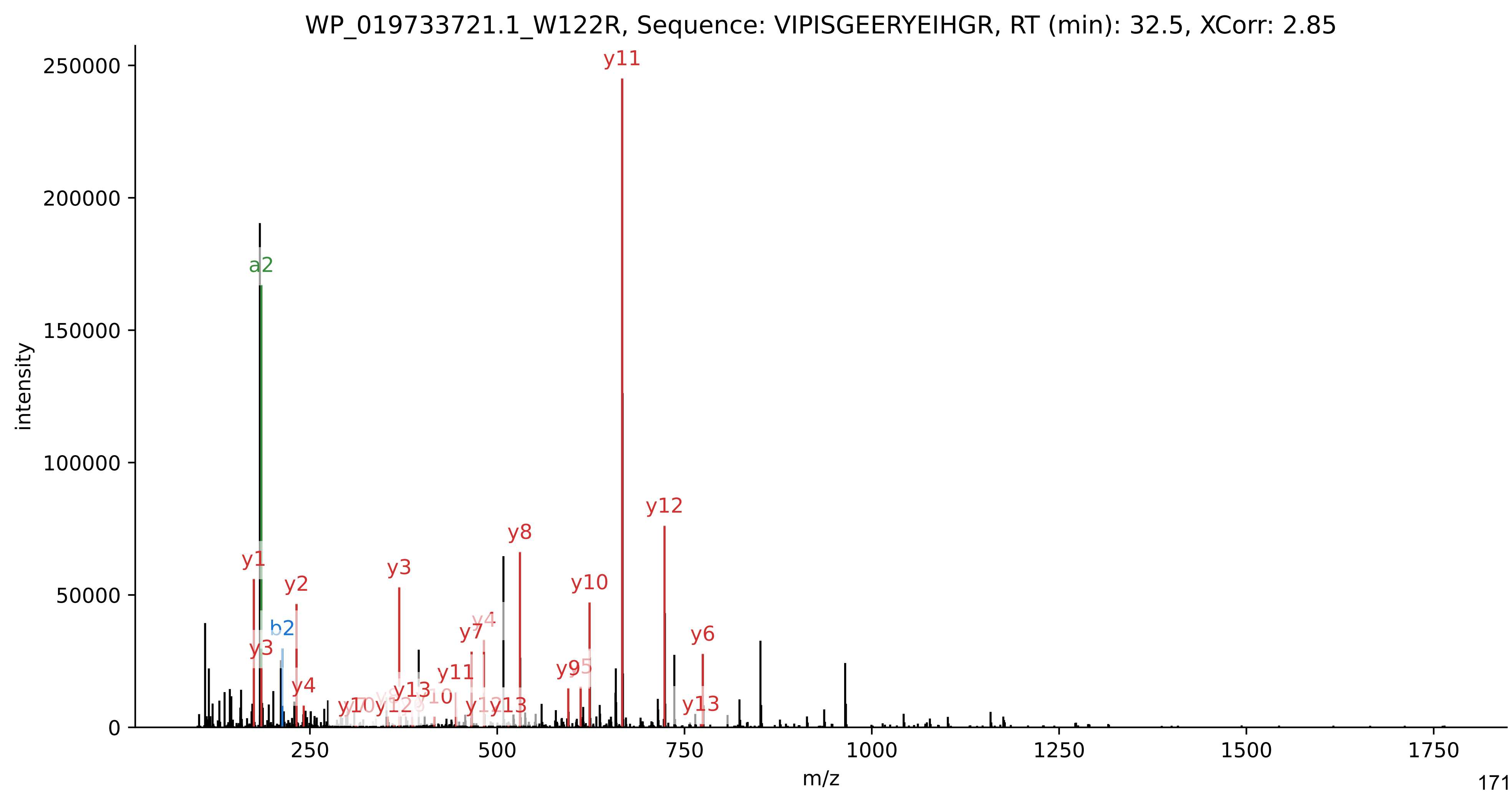
WP_019733721.1_W122R, Sequence: VIPISGEERYEIHGR, RT (min): 32.5, XCorr: 2.85
intensity
m/z
y11
a2
y12
y8
y1
y3
y2
y10
b2
y4
y7
y6
y3
y9
y11
y4
y13
y10
y12
y13
y13

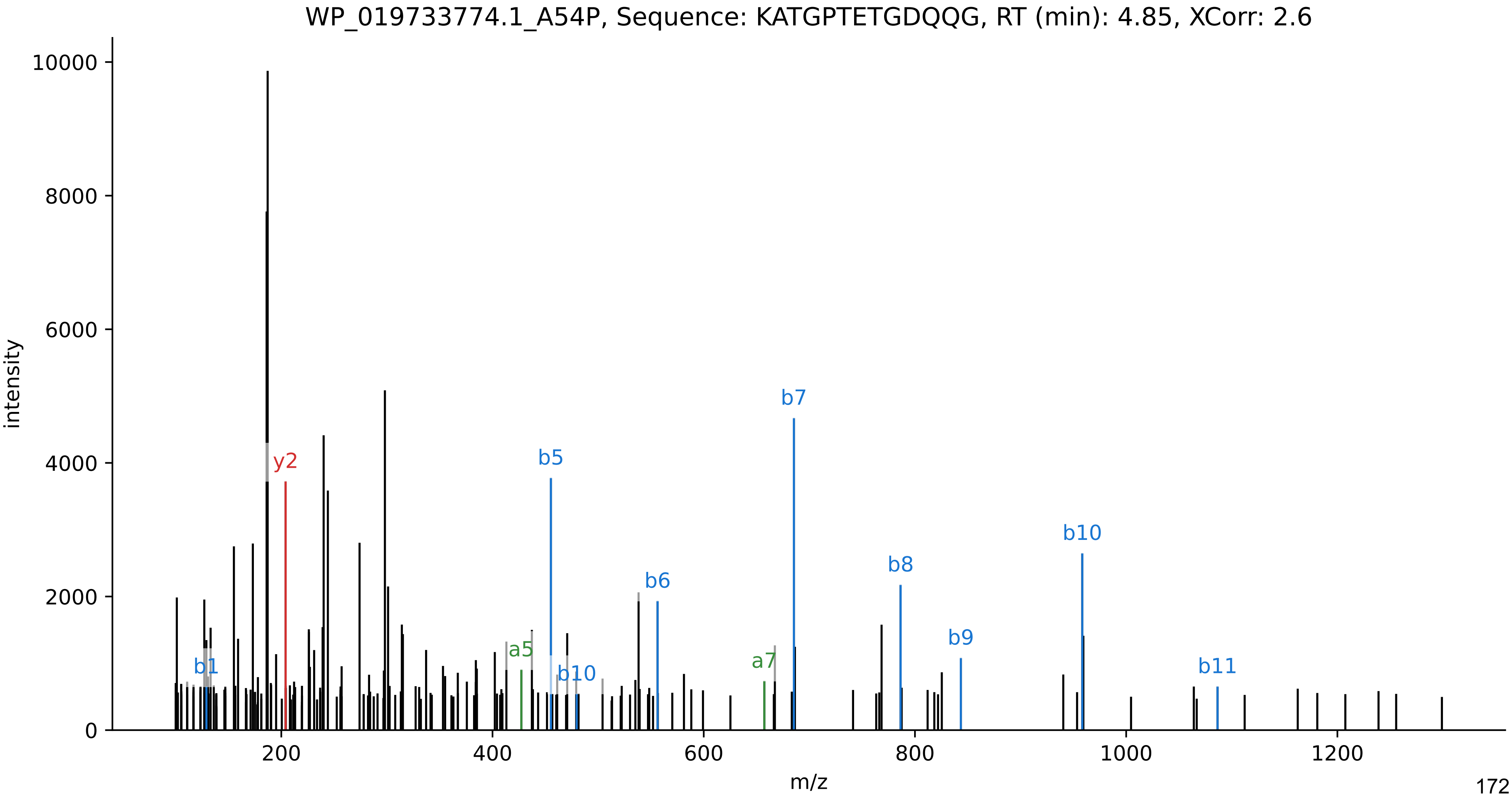
WP_019733774.1_A54P, Sequence: KATGPTETGDQQG, RT (min): 4.85, XCorr: 2.6
intensity
10000
8000
6000
4000
2000
0
200
400
600
800
1000
1200
m/z
b1
y2
a5
b5
b10
b6
a7
b7
b8
b9
b10
b11

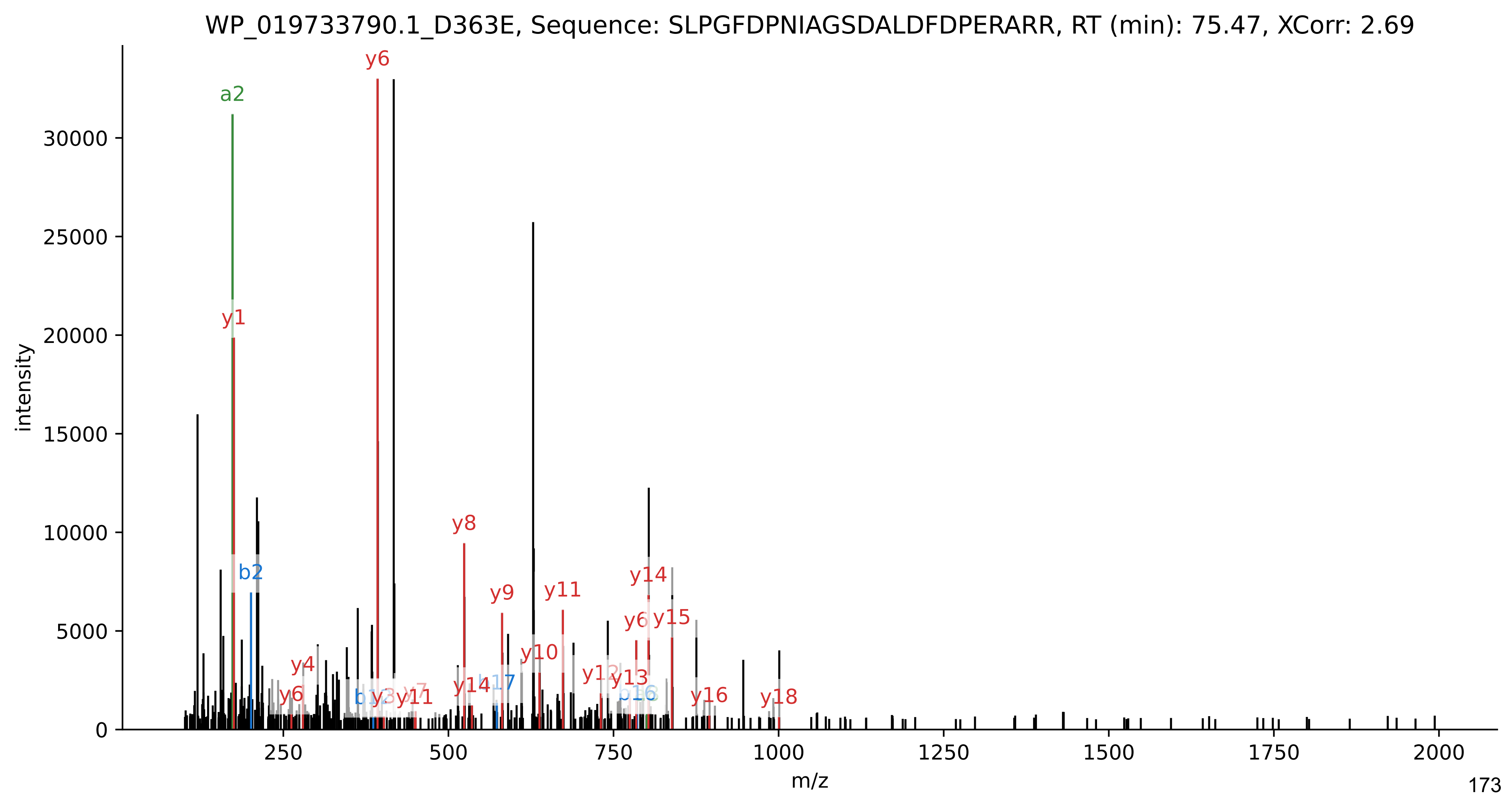
WP_019733790.1_D363E, Sequence: SLPGFDPNIAGSDALDFDPERARR, RT (min): 75.47, XCorr: 2.69
intensity
m/z
a2
y6
y1
b2
y8
y9
y11
y14
y6
y15
y10
y4
y6
y11
y14
b17
y12
y13
b16
y16
y18

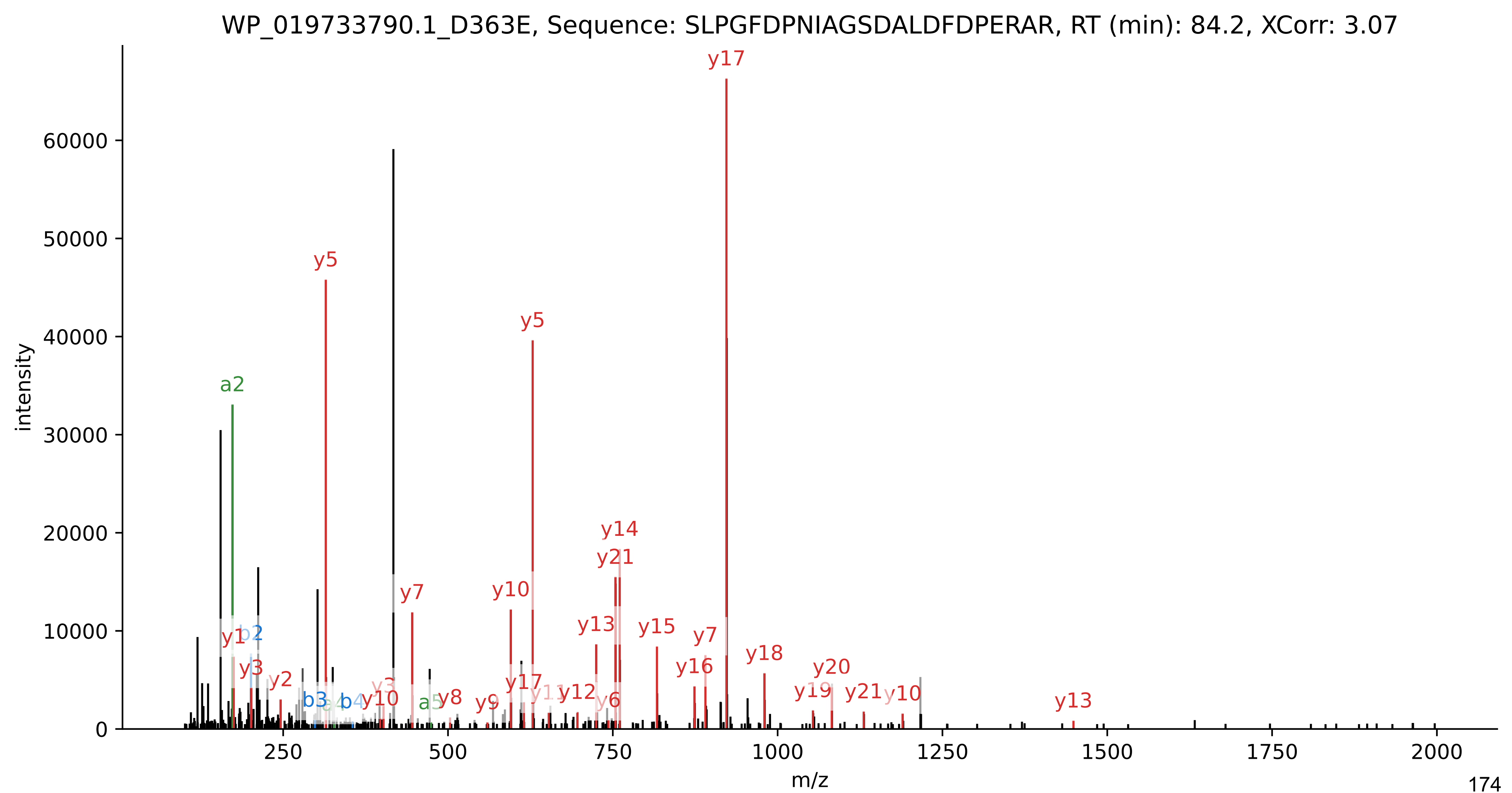
WP_019733790.1_D363E, Sequence: SLPGFDPNIAGSDALDFDPERAR, RT (min): 84.2, XCorr: 3.07
intensity
m/z
y17
y5
y5
a2
y14
y21
y10
y7
y13
y15
y7
y18
y16
y20
y19
y21
y10
y13
y12
y6
y1
b2
y3
y2
b3
b4
y10
a5
y8
y9
y17

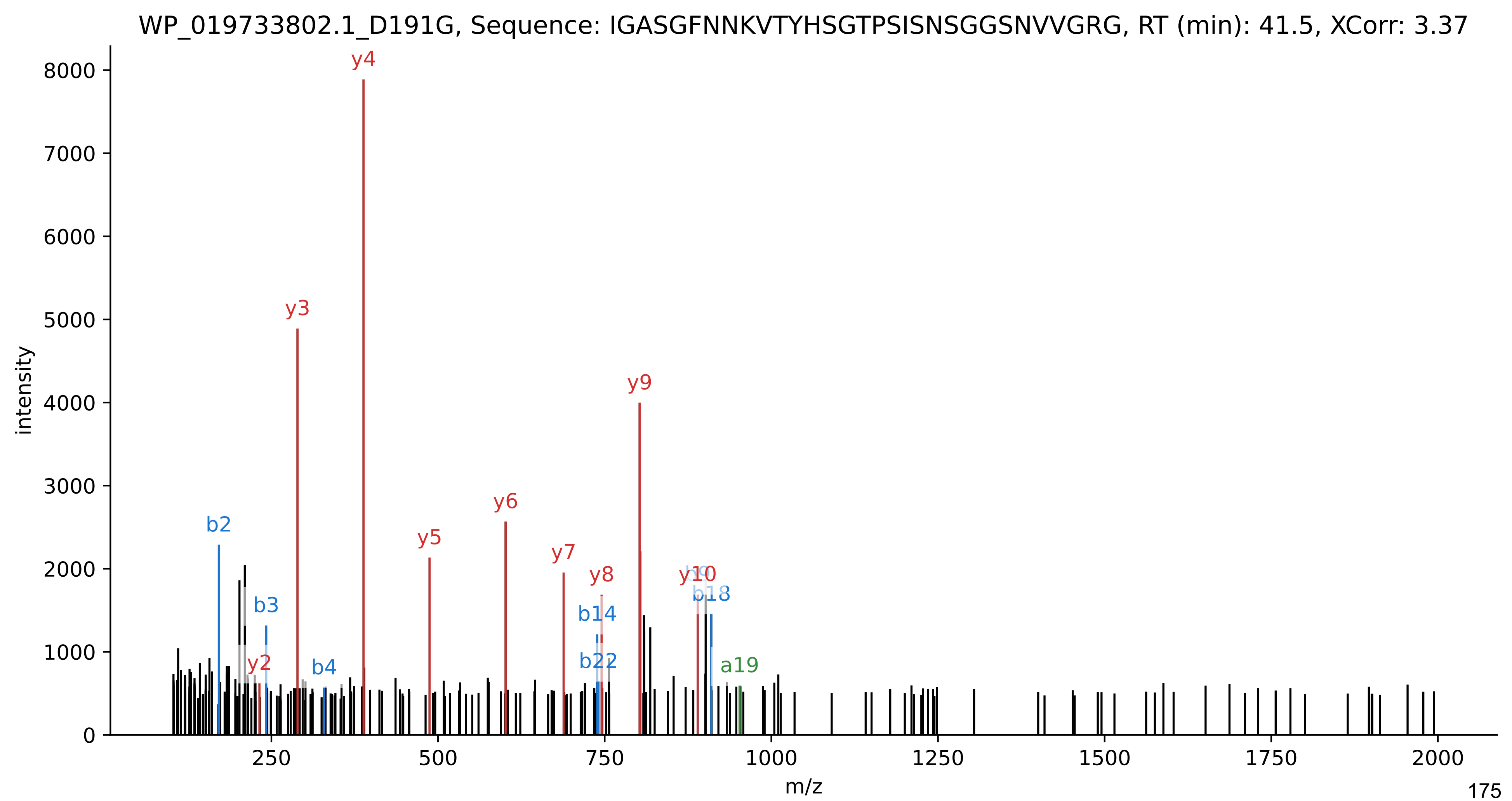

WP_019733802.1_D191G, Sequence: IGASGFNNKVTYHSGTPSISNSGGSNVVGRG, RT (min): 41.5, XCorr: 3.37
intensity
m/z
y4
y3
y9
y6
b2
y5
y7
y8
y10
b18
b3
b14
y2
b4
b22
a19

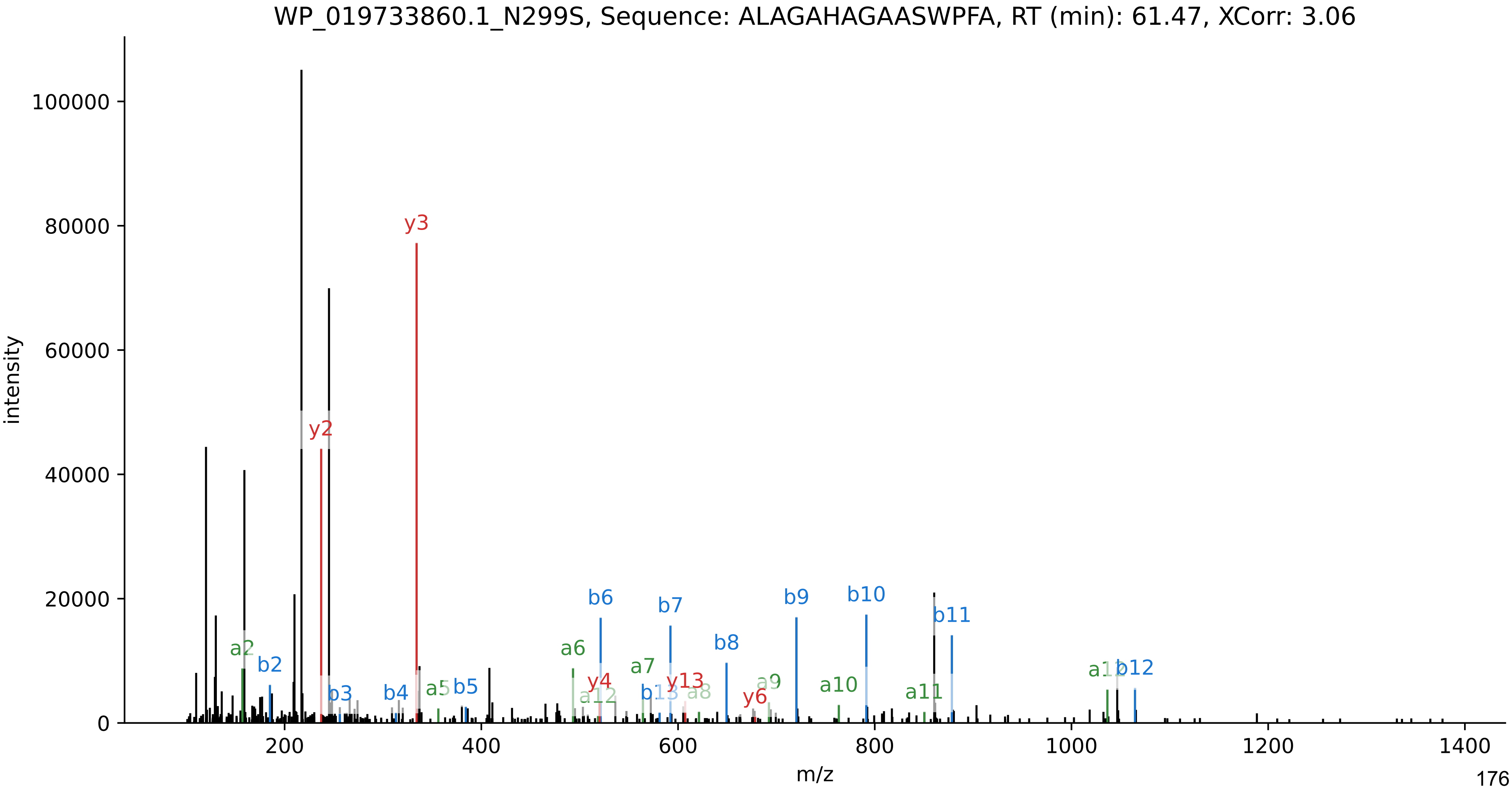
WP_019733860.1_N299S, Sequence: ALAGAHAGAASWPFA, RT (min): 61.47, XCorr: 3.06
intensity
m/z
y3
y2
a2
b2
b3
b4
a5
b5
a6
y4
a12
b6
a7
b13
b7
y13
a8
b8
y6
a9
b9
a10
b10
a11
b11
b12
0
20000
40000
60000
80000
100000
200
400
600
800
1000
1200
1400

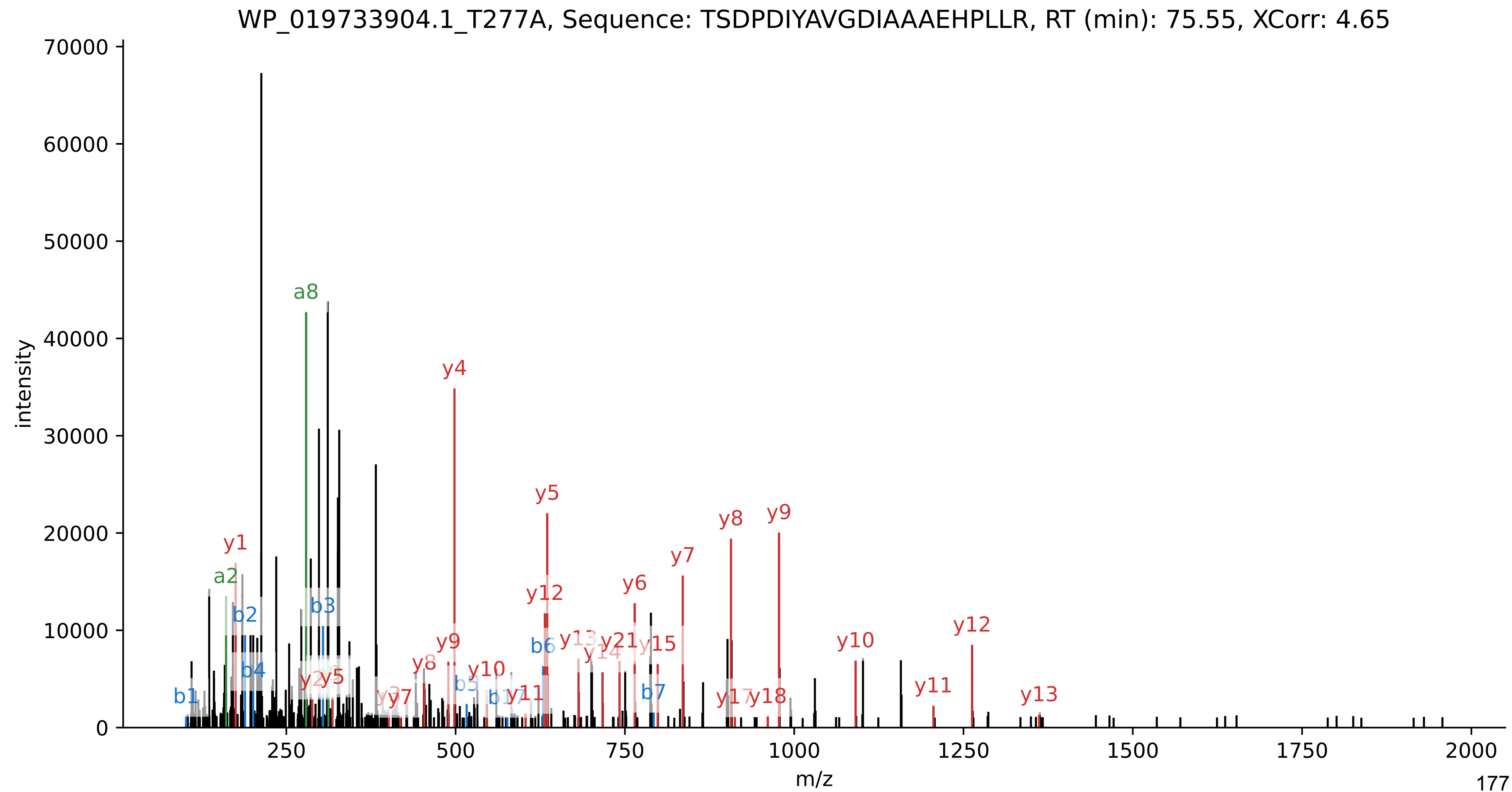
WP_019733904.1_T277A, Sequence: TSDPDIYAVGDIAAAEHPLLR, RT (min): 75.55, XCorr: 4.65
intensity
m/z
0
10000
20000
30000
40000
50000
60000
70000
250
500
750
1000
1250
1500
1750
2000
a8
a2
y1
b1
b2
b3
b4
b5
b6
b7
y4
y5
y12
y6
y7
y8
y9
y10
y11
y12
y13
y15
y18
y21

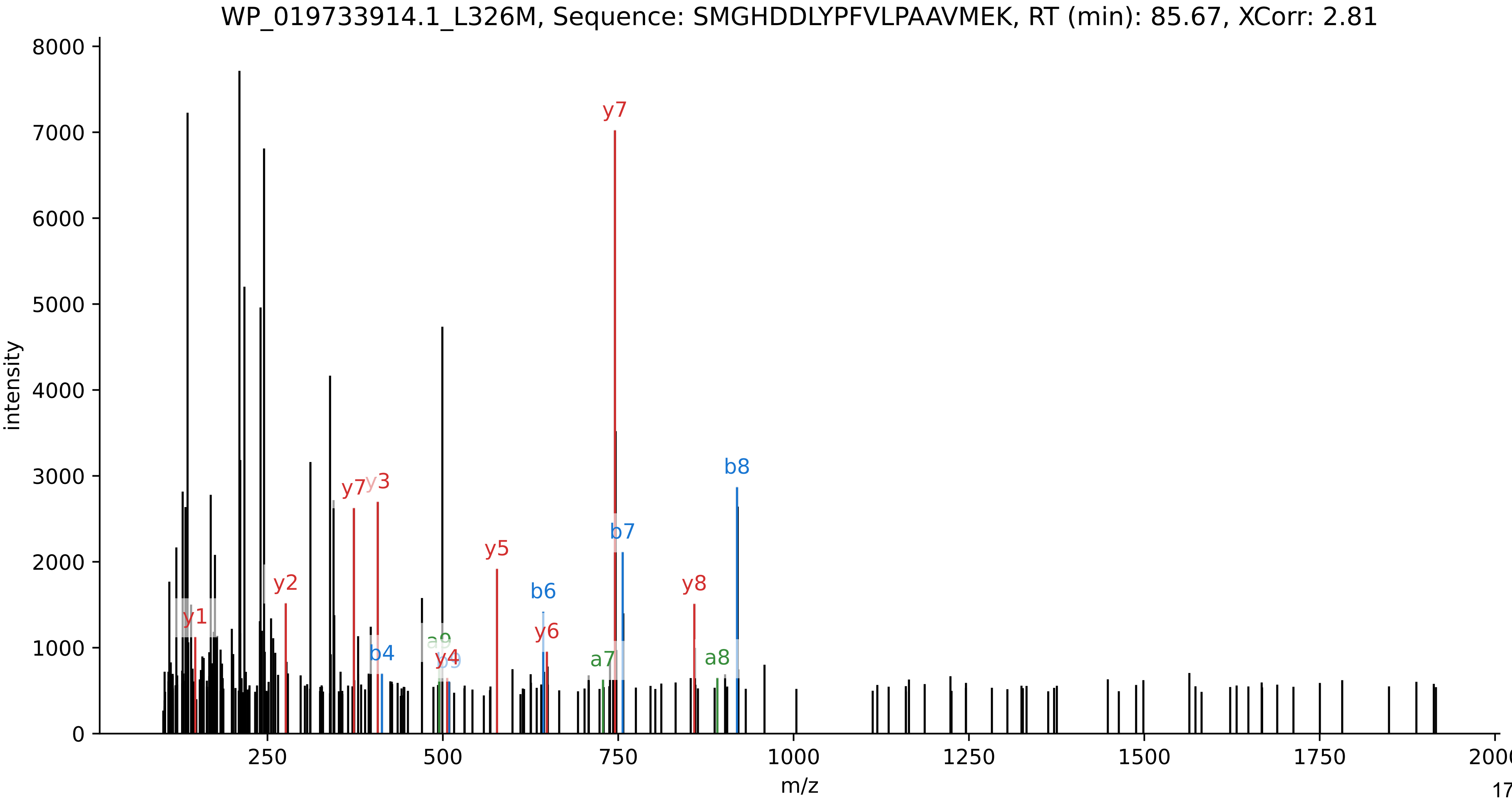

WP_019733914.1_L326M, Sequence: SMGHDDLYPFVLPAAVMEK, RT (min): 85.67, XCorr: 2.81
intensity
m/z
0
1000
2000
3000
4000
5000
6000
7000
8000
250
500
750
1000
1250
1500
1750
2000
y1
y2
y7
y3
y4
y5
y6
y7
y8
b4
b6
b7
b8
a7
a8

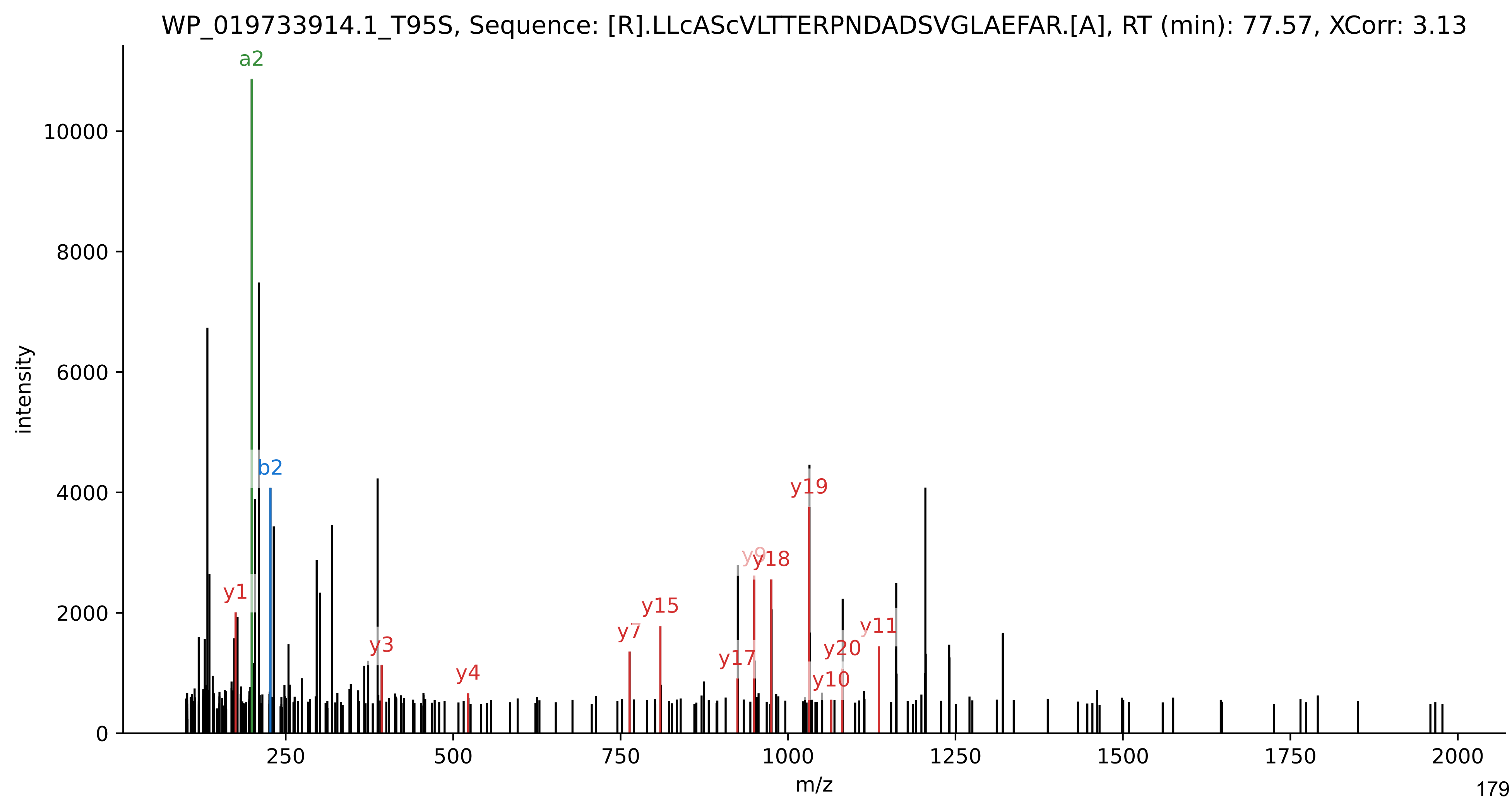
WP_019733914.1_T95S, Sequence: [R].LLcAScVLTTERPNDADSVGLAEFAR.[A], RT (min): 77.57, XCorr: 3.13
a2
b2
y1
y3
y4
y7
y15
y17
y9
y18
y19
y10
y20
y11
intensity
m/z
0
2000
4000
6000
8000
10000
250
500
750
1000
1250
1500
1750
2000

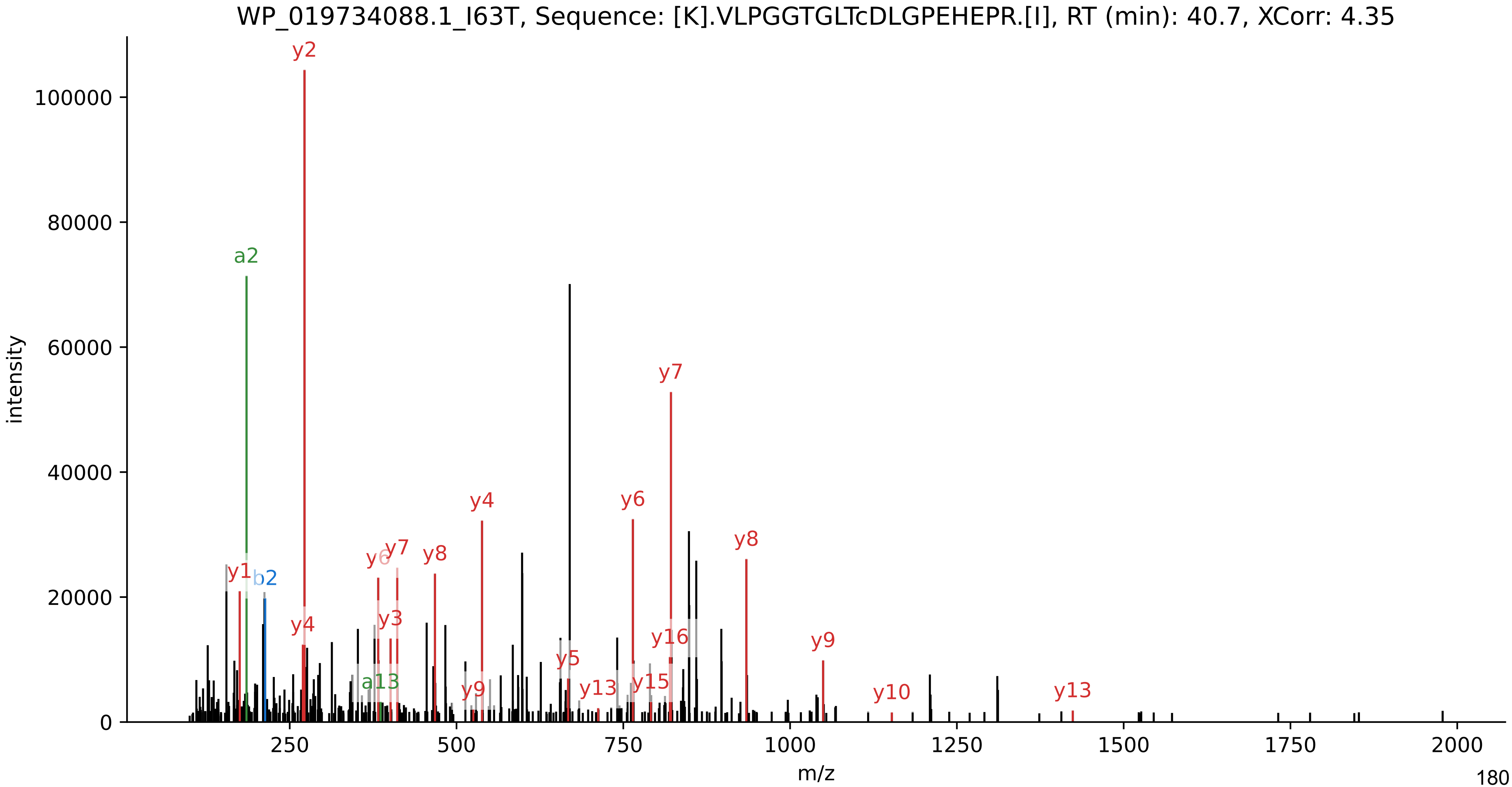

WP_019734088.1_I63T, Sequence: [K].VLPGGTGLTcDLGPEHEPR.[I], RT (min): 40.7, XCorr: 4.35
intensity
m/z
y2
a2
y7
y4
y6
y8
y1
b2
y6
y7
y8
y4
y3
y16
y9
y5
a13
y9
y13
y15
y10
y13
0
20000
40000
60000
80000
100000
250
500
750
1000
1250
1500
1750
2000

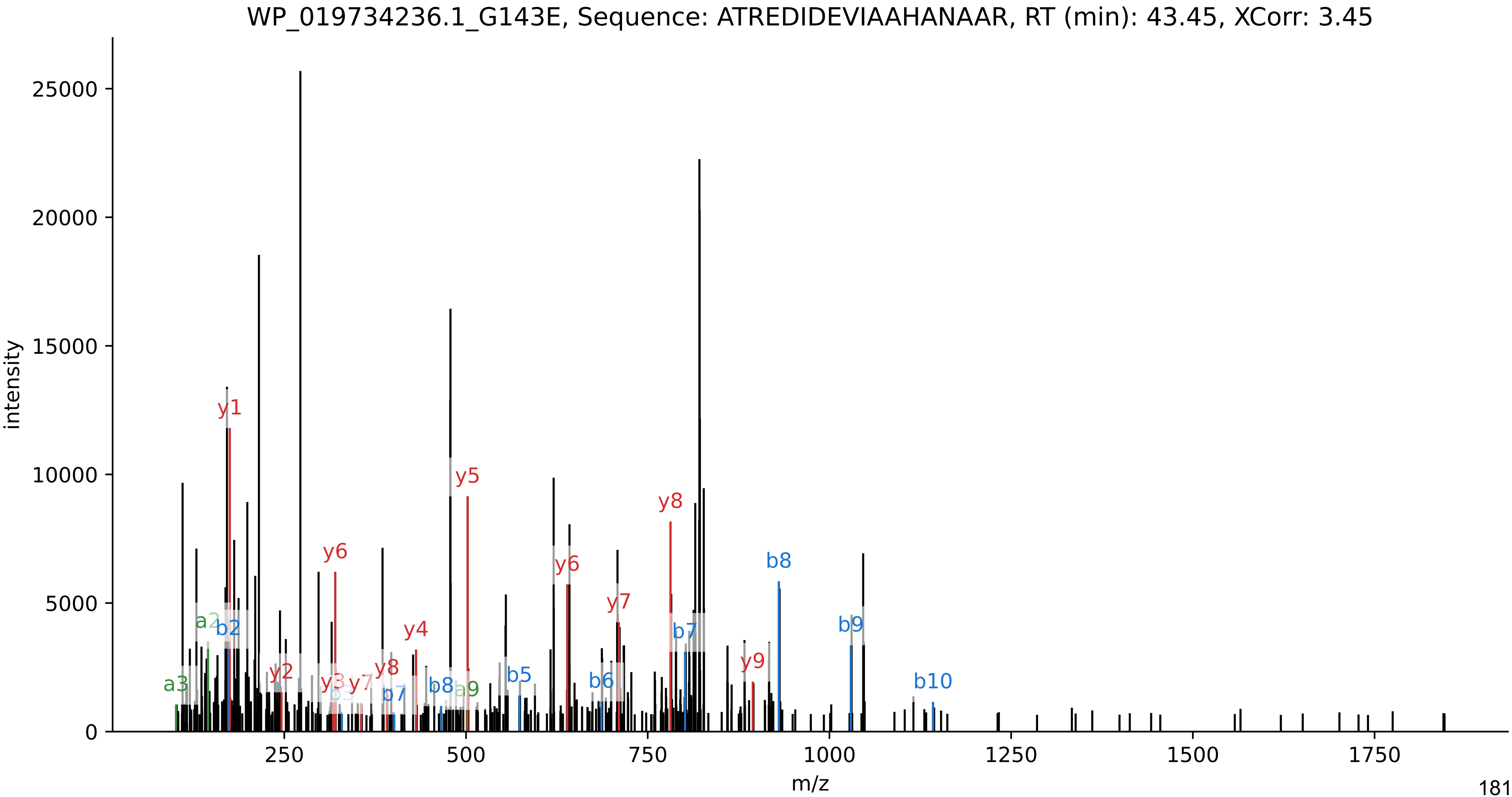
WP_019734236.1_G143E, Sequence: ATREDIDEVIAAHANAAR, RT (min): 43.45, XCorr: 3.45
intensity
m/z
25000
20000
15000
10000
5000
0
250
500
750
1000
1250
1500
1750
y1
y5
y8
y6
y6
y7
b8
b7
b9
y9
b10
a2
b2
a3
y2
y3
y7
y8
y4
b8
a9
b5
b6
b7

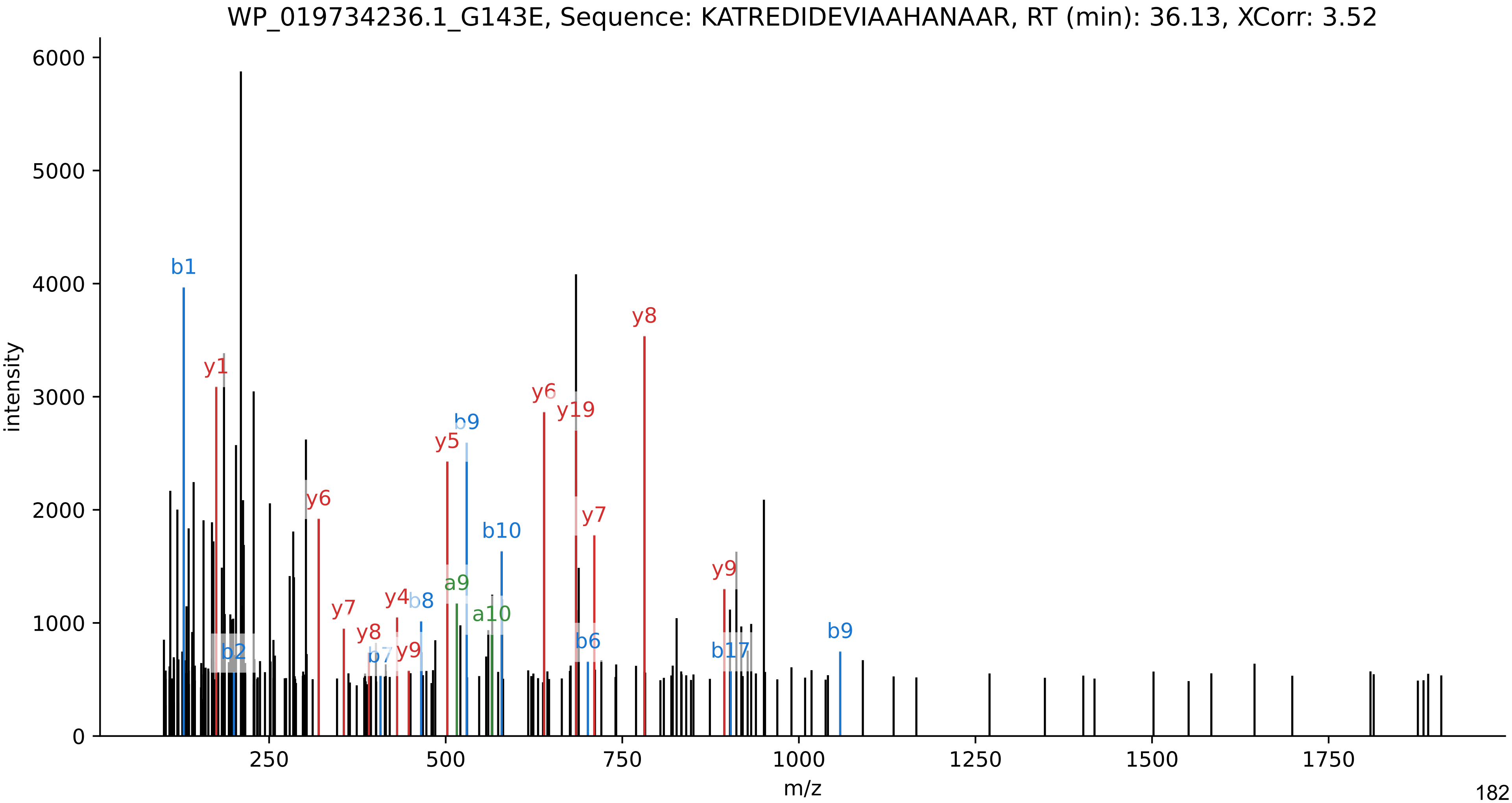
WP_019734236.1_G143E, Sequence: KATREDIDEVIAAHANAAR, RT (min): 36.13, XCorr: 3.52
intensity
m/z
b1
y1
b2
y6
y7
y8
b7
y9
y4
b8
y5
b9
a9
a10
b10
y6
y19
y7
b6
y8
y9
b17
b9

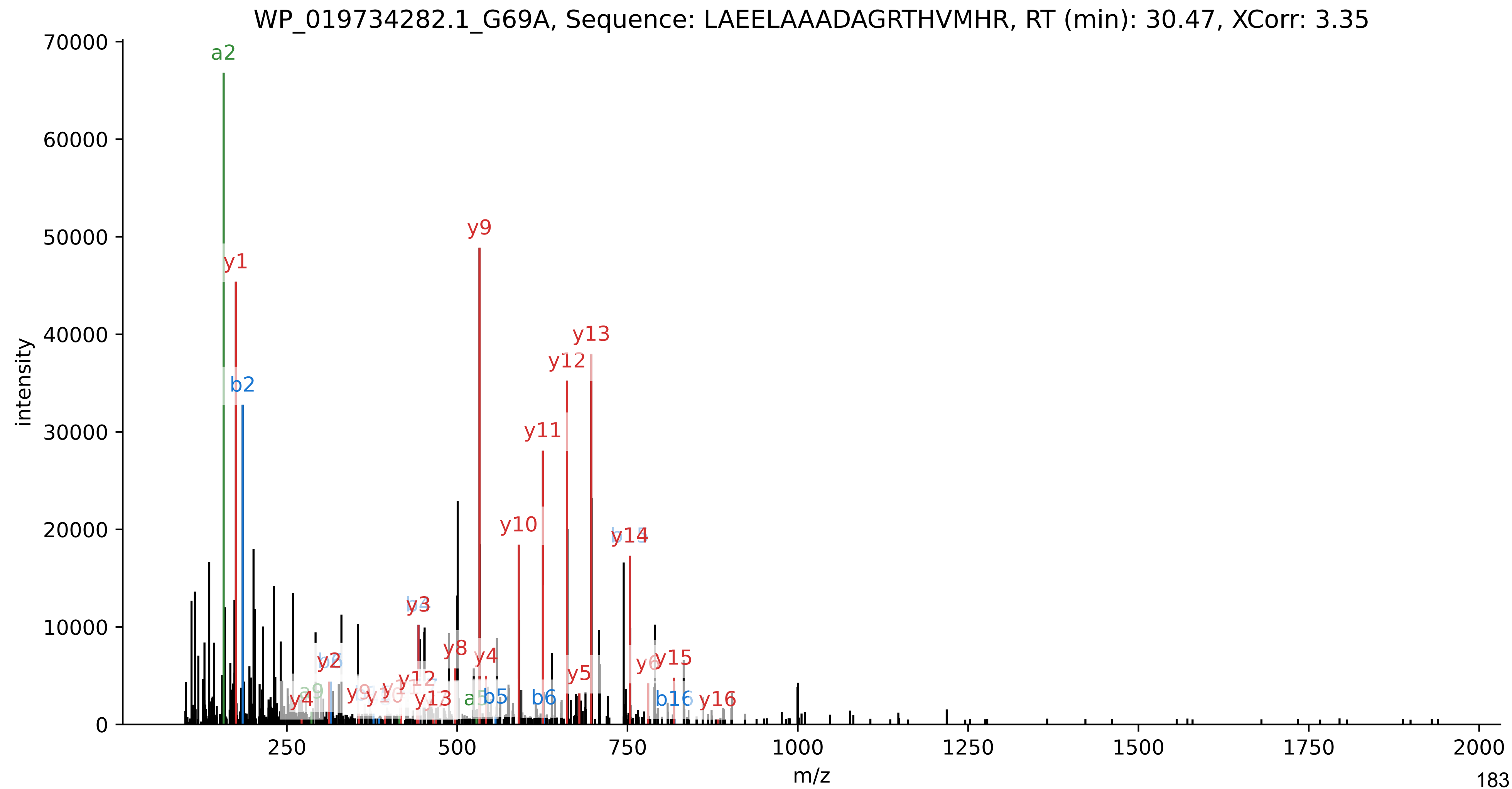

WP_019734282.1_G69A, Sequence: LAEELAAADAGRTHVMHR, RT (min): 30.47, XCorr: 3.35
intensity
m/z
a2
y1
b2
y9
y13
y12
y11
y10
y14
y3
y8
y4
y2
y5
y6
y15
y16
b16
b6
b5
y4

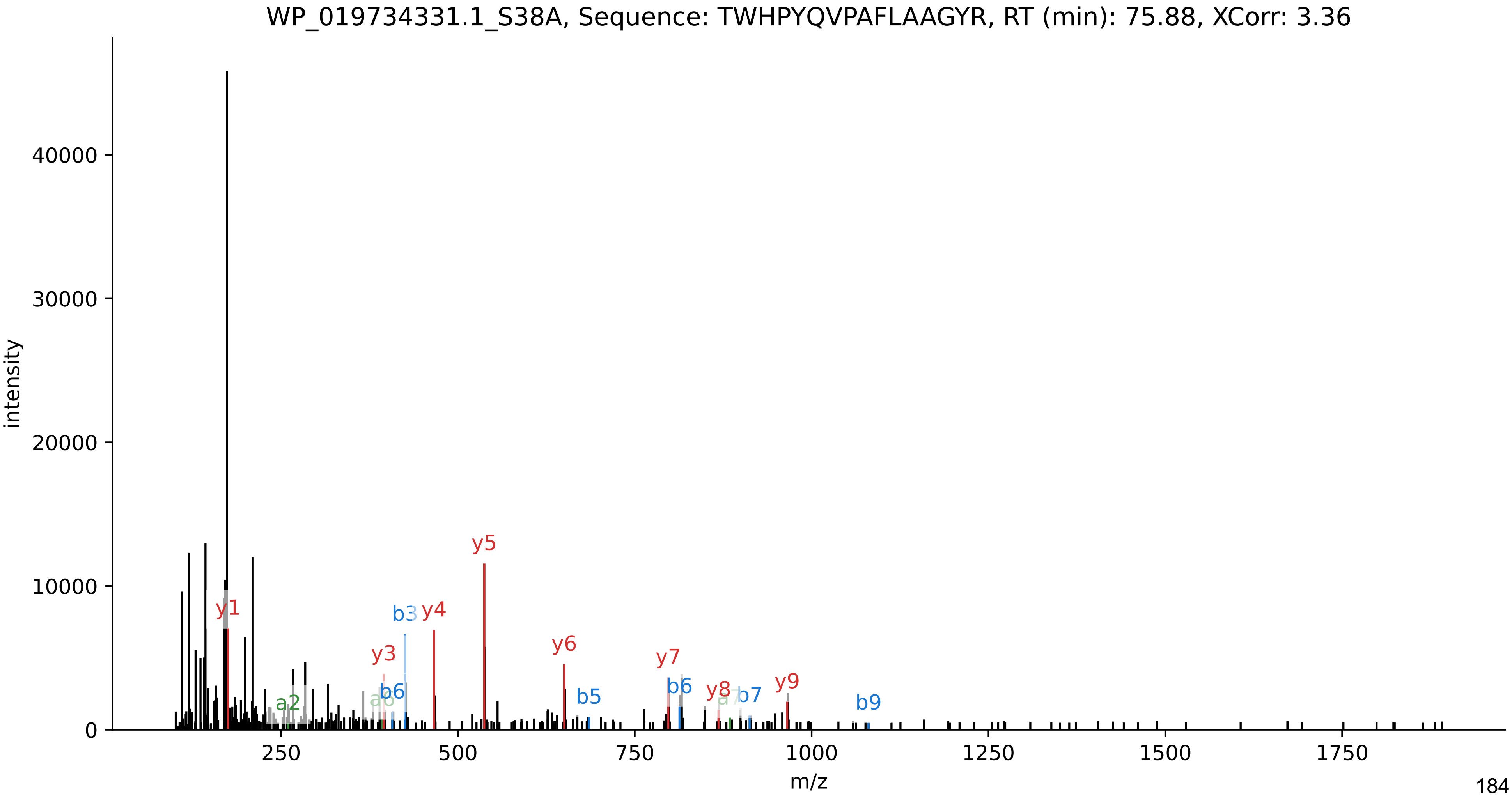

WP_019734331.1_S38A, Sequence: TWHPYQVPAFLAAGYR, RT (min): 75.88, XCorr: 3.36
intensity
m/z
40000
30000
20000
10000
0
250
500
750
1000
1250
1500
1750
y1
a2
y3
b6
b3
y4
y5
y6
b5
y7
b6
y8
b7
y9
b9

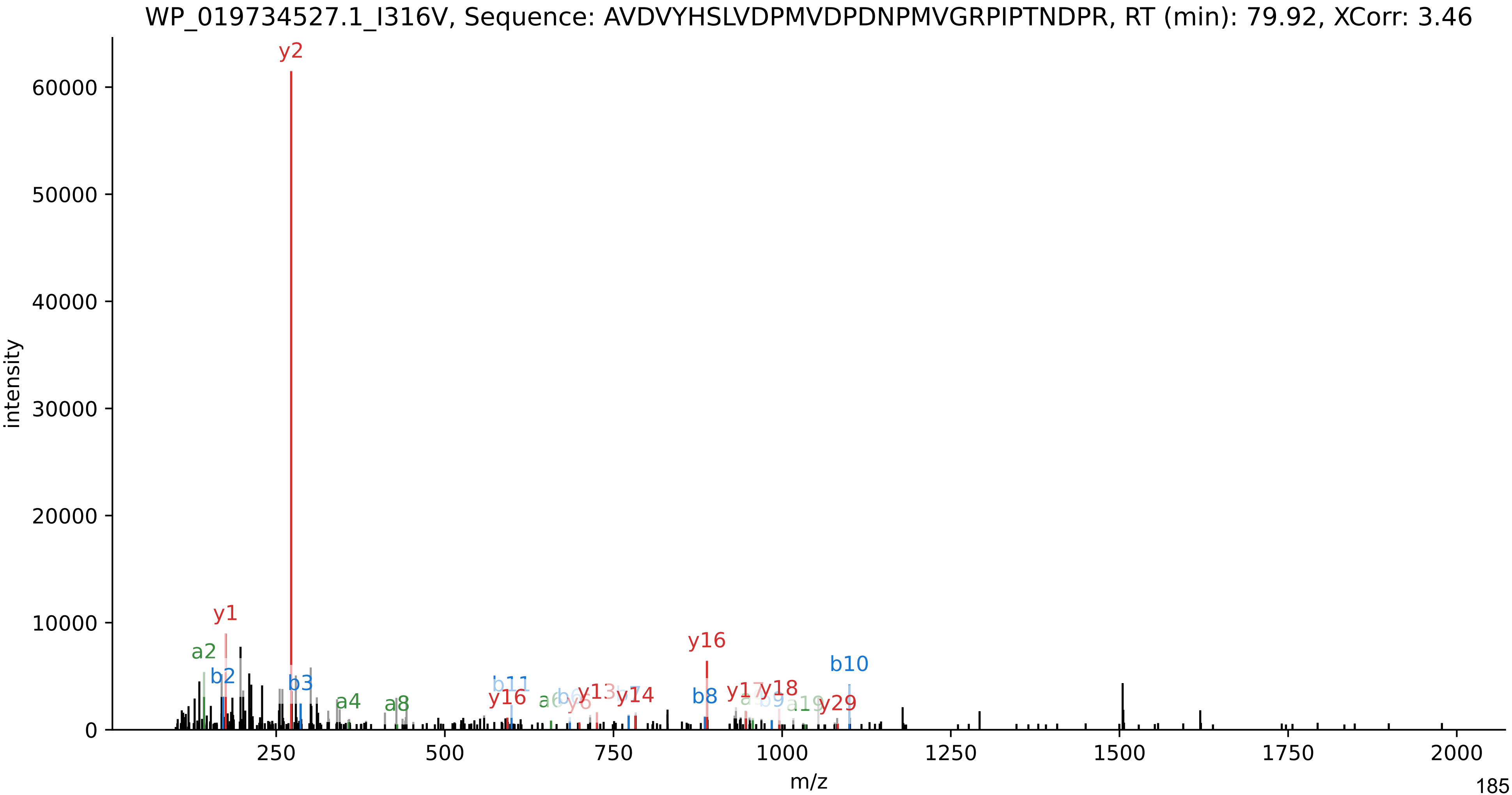
WP_019734527.1_I316V, Sequence: AVDVYHSLVDPMVDPDNPMVGRPIPTNDPR, RT (min): 79.92, XCorr: 3.46
intensity
m/z
y2
y1
a2
b2
b3
a4
a8
b11
y16
y13
y14
b8
y16
y17
y18
b10
y29

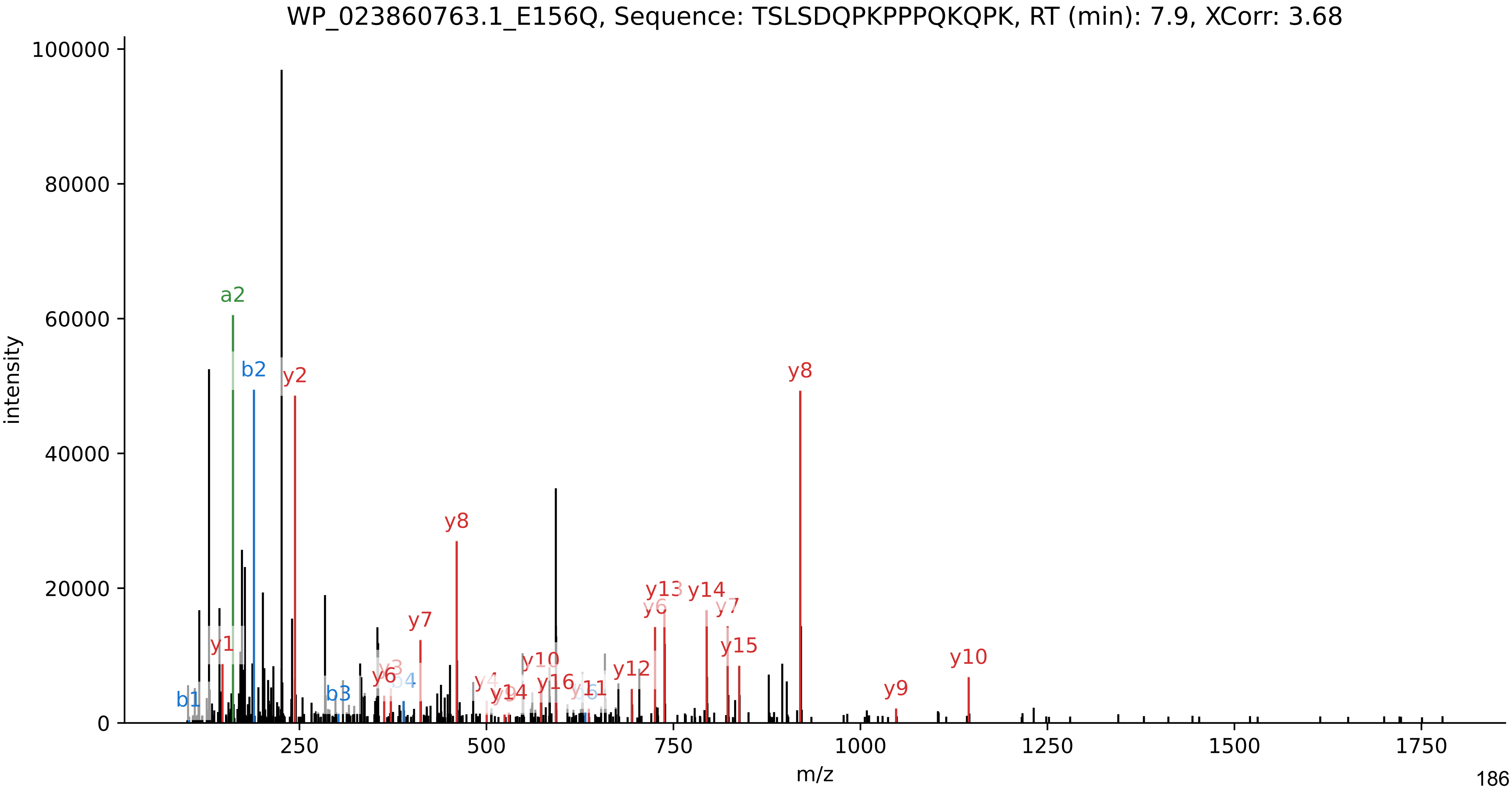
WP_023860763.1_E156Q, Sequence: TSLSDQPKPPPQKQPK, RT (min): 7.9, XCorr: 3.68
intensity
m/z
a2
b2
y2
y8
y8
y7
y1
b1
b3
y6
y3
y10
y16
y14
y12
y13
y6
y14
y7
y15
y9
y10

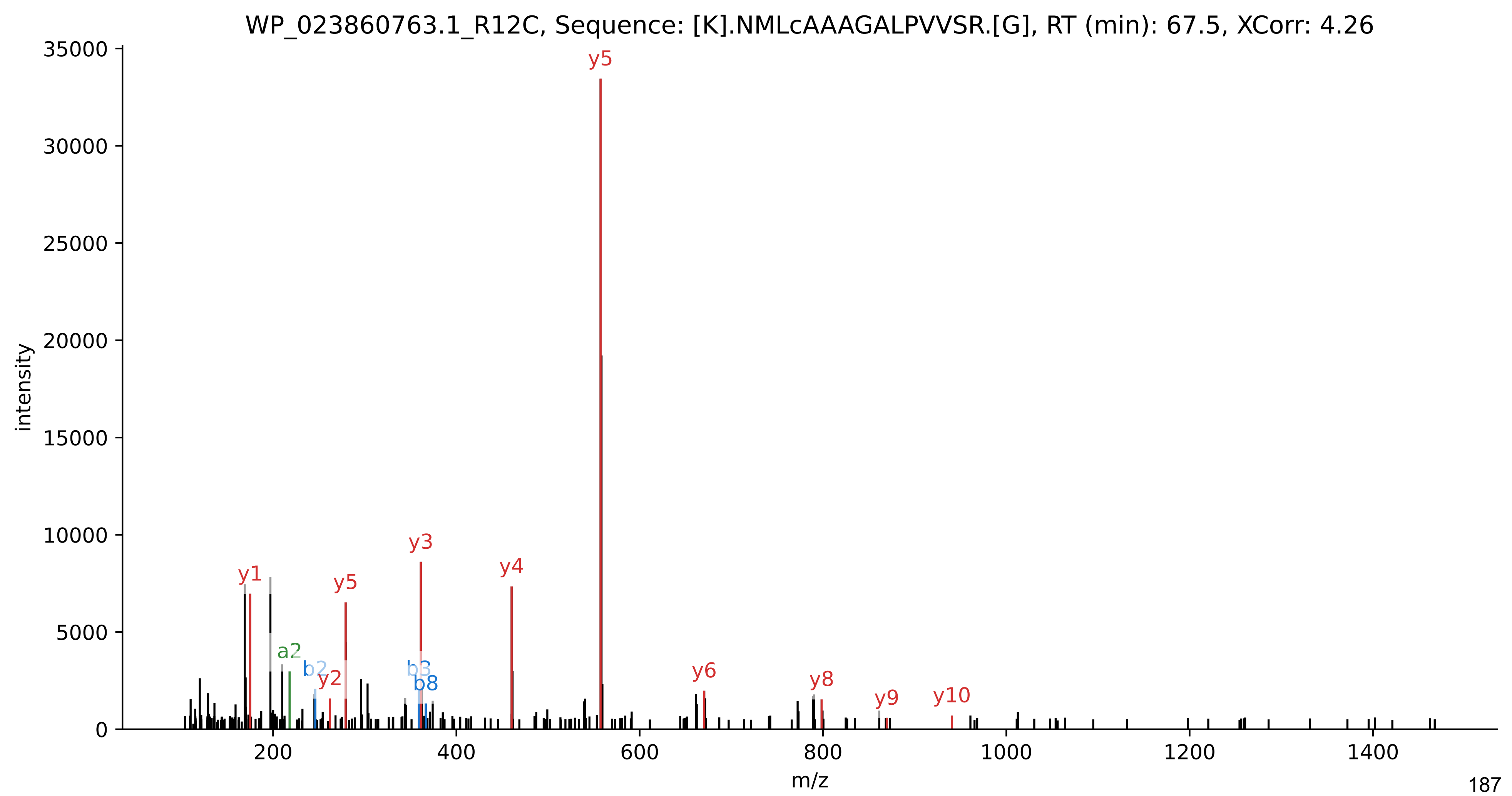
WP_023860763.1_R12C, Sequence: [K].NMLcAAAGALPVVSR.[G], RT (min): 67.5, XCorr: 4.26
intensity
m/z
y5
y1
y5
y3
y4
a2
b2
y2
b3
b8
y6
y8
y9
y10
0
5000
10000
15000
20000
25000
30000
35000
200
400
600
800
1000
1200
1400

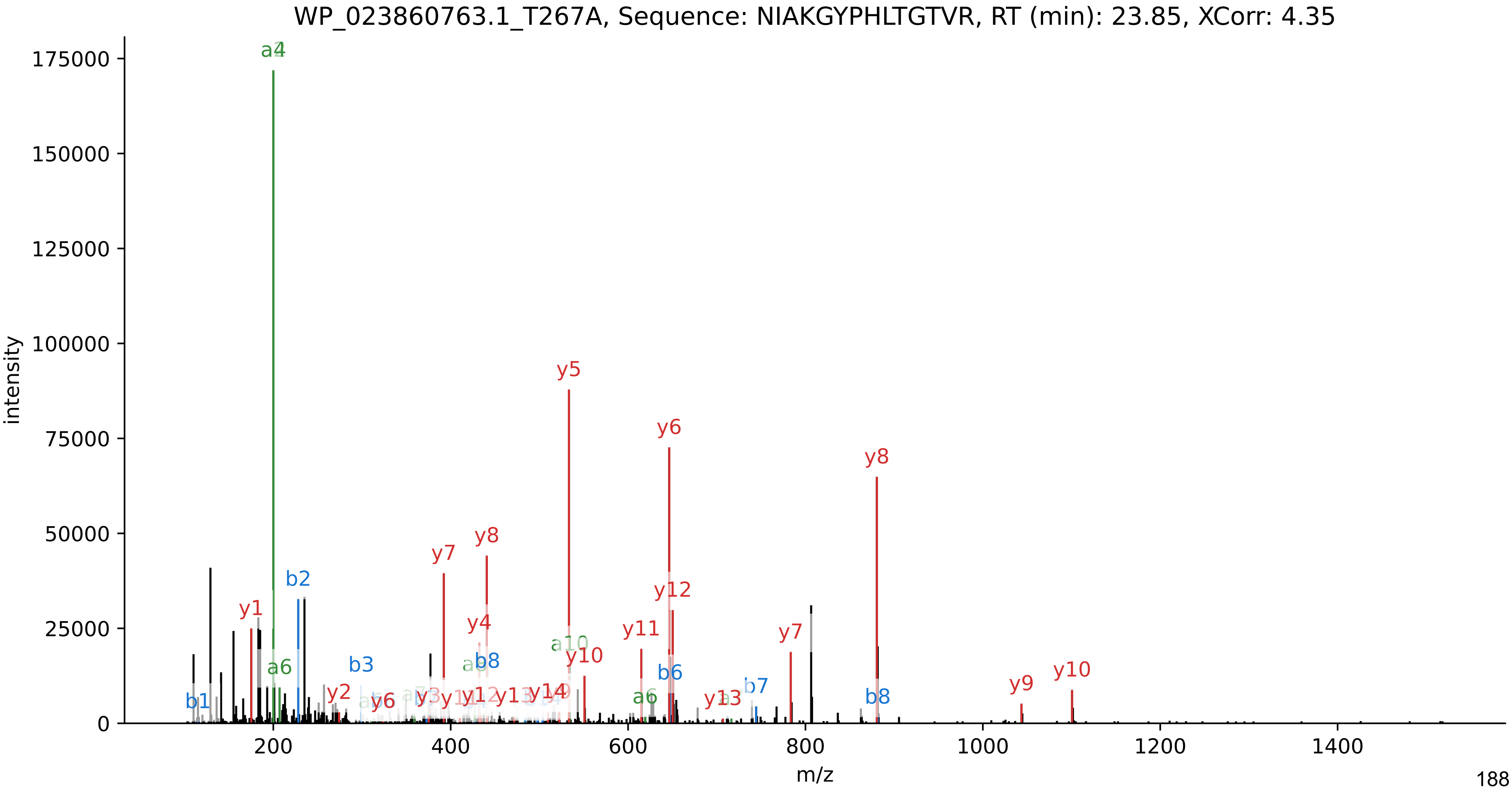
WP_023860763.1_T267A, Sequence: NIAKGYPHLTGTVR, RT (min): 23.85, XCorr: 4.35
intensity
m/z
175000
150000
125000
100000
75000
50000
25000
0
200
400
600
800
1000
1200
1400
a4
y5
y6
y8
y8
y7
b2
y12
y1
y4
y11
a10
y10
y7
b8
b3
a6
b6
y10
y9
b7
y2
b1
y6
a6
y13
b8

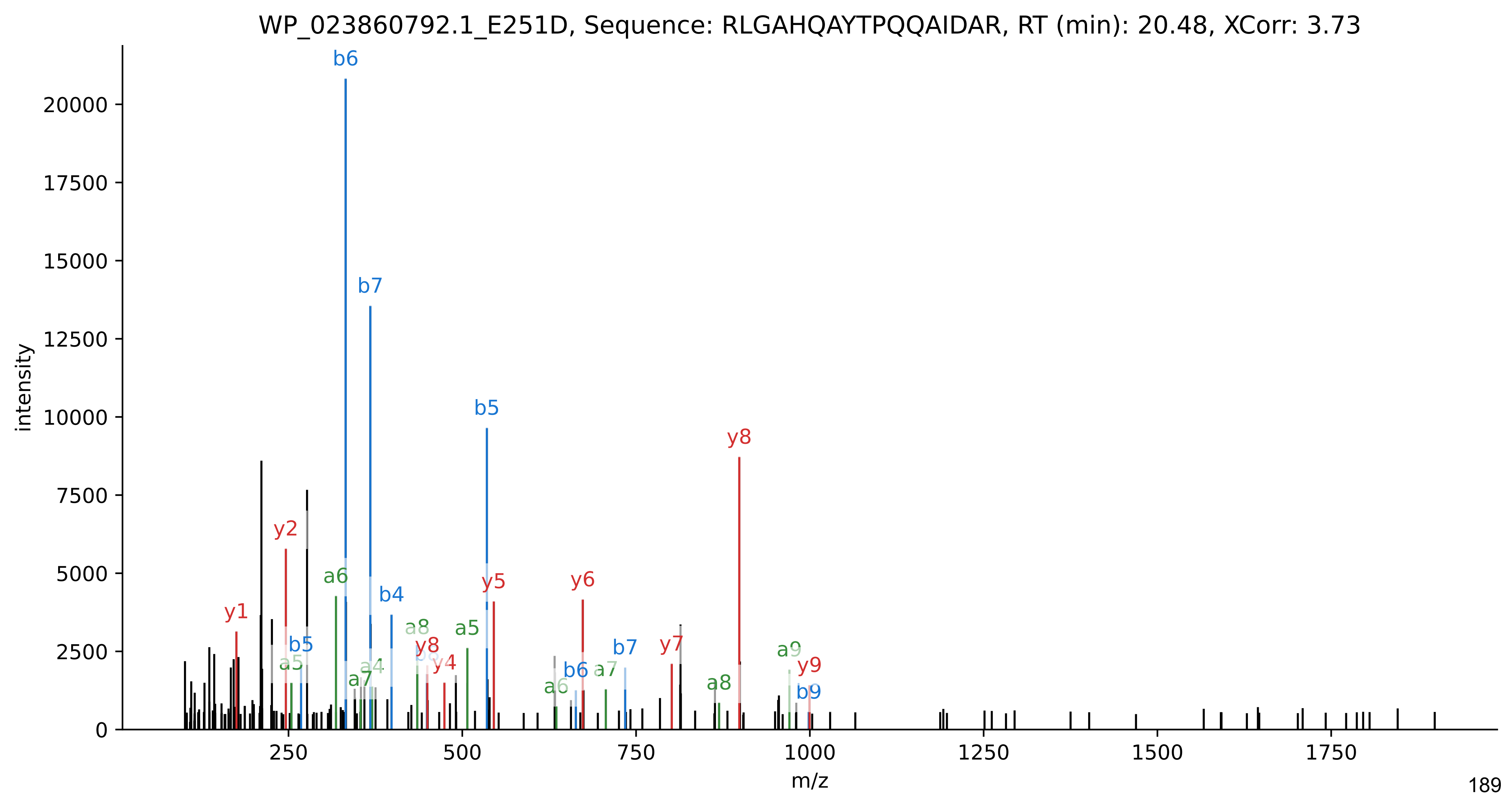
WP_023860792.1_E251D, Sequence: RLGAHQAYTPQQAIDAR, RT (min): 20.48, XCorr: 3.73
intensity
m/z
b6
b7
b5
y8
y2
a6
b4
y5
y6
y1
a8
a5
b5
y8
y4
a5
a4
a7
b7
y7
a6
b6
a7
a8
a9
y9
b9

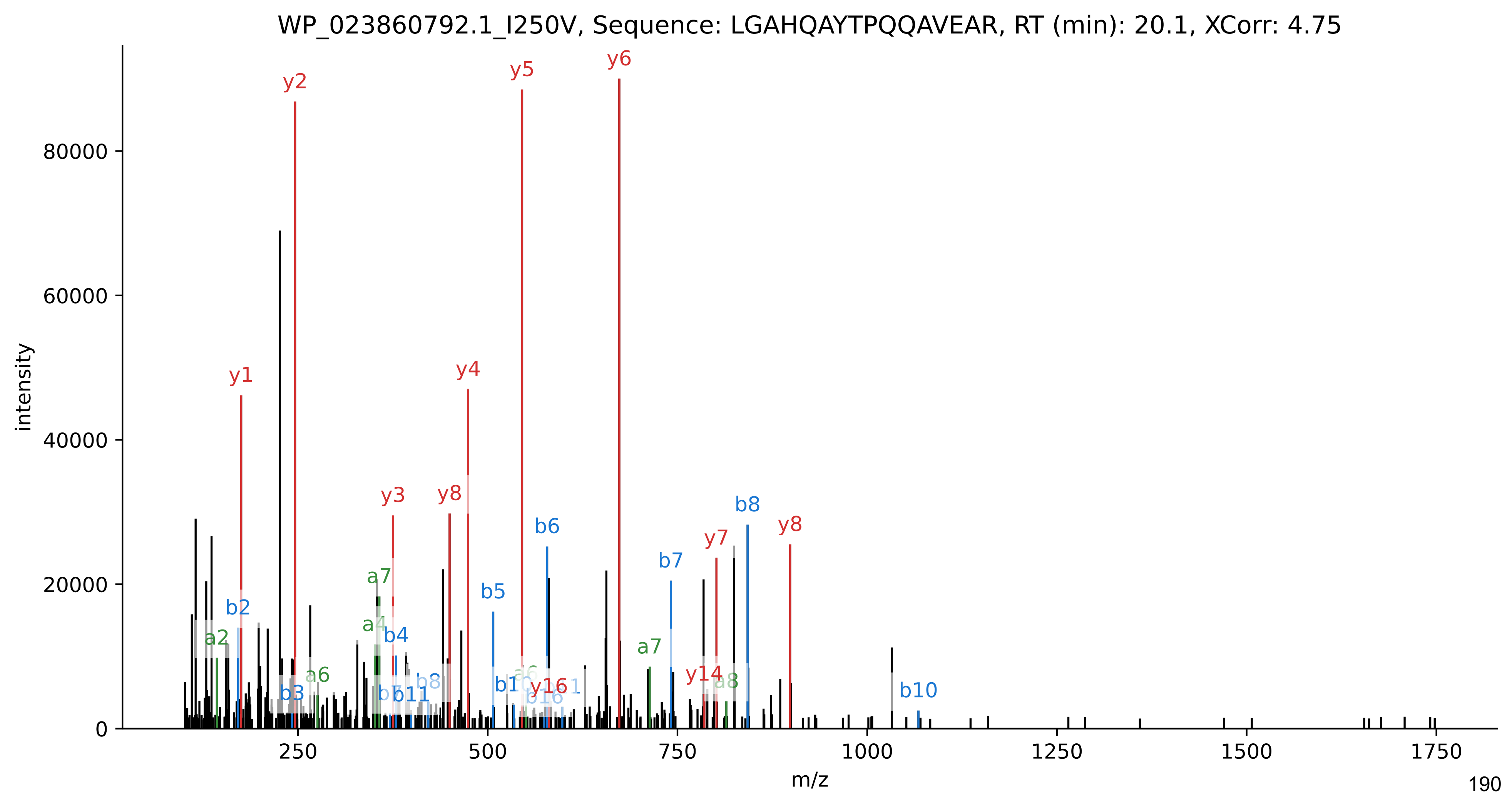
WP_023860792.1_I250V, Sequence: LGAHQAYTPQQAVEAR, RT (min): 20.1, XCorr: 4.75
intensity
m/z
y1
y2
y3
y4
y5
y6
y7
y8
y14
y16
b2
b3
b4
b5
b6
b7
b8
b10
b11
a2
a4
a6
a7
a8

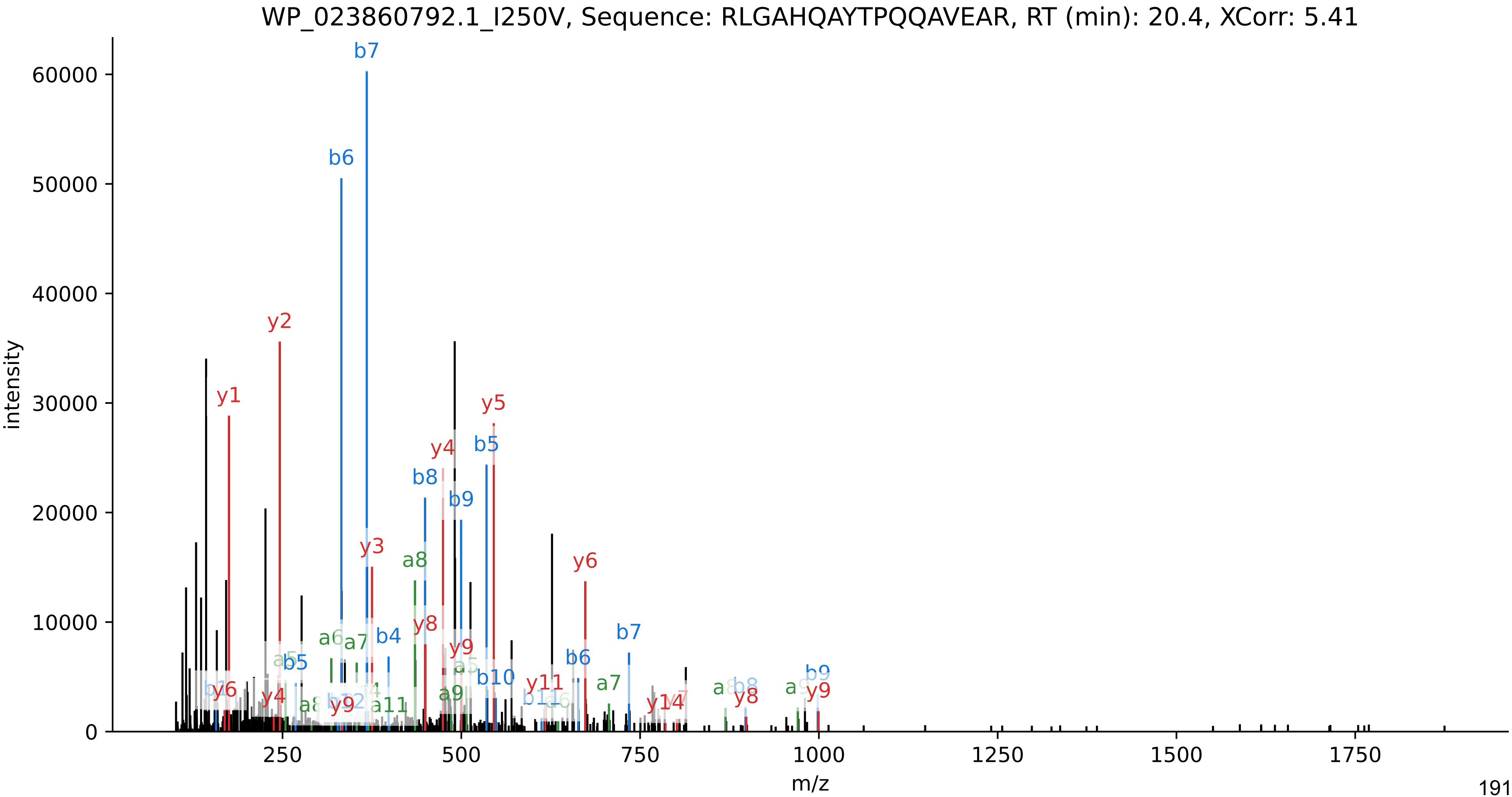

WP_023860792.1_I250V, Sequence: RLGAHQAYTPQQAVEAR, RT (min): 20.4, XCorr: 5.41
intensity
m/z

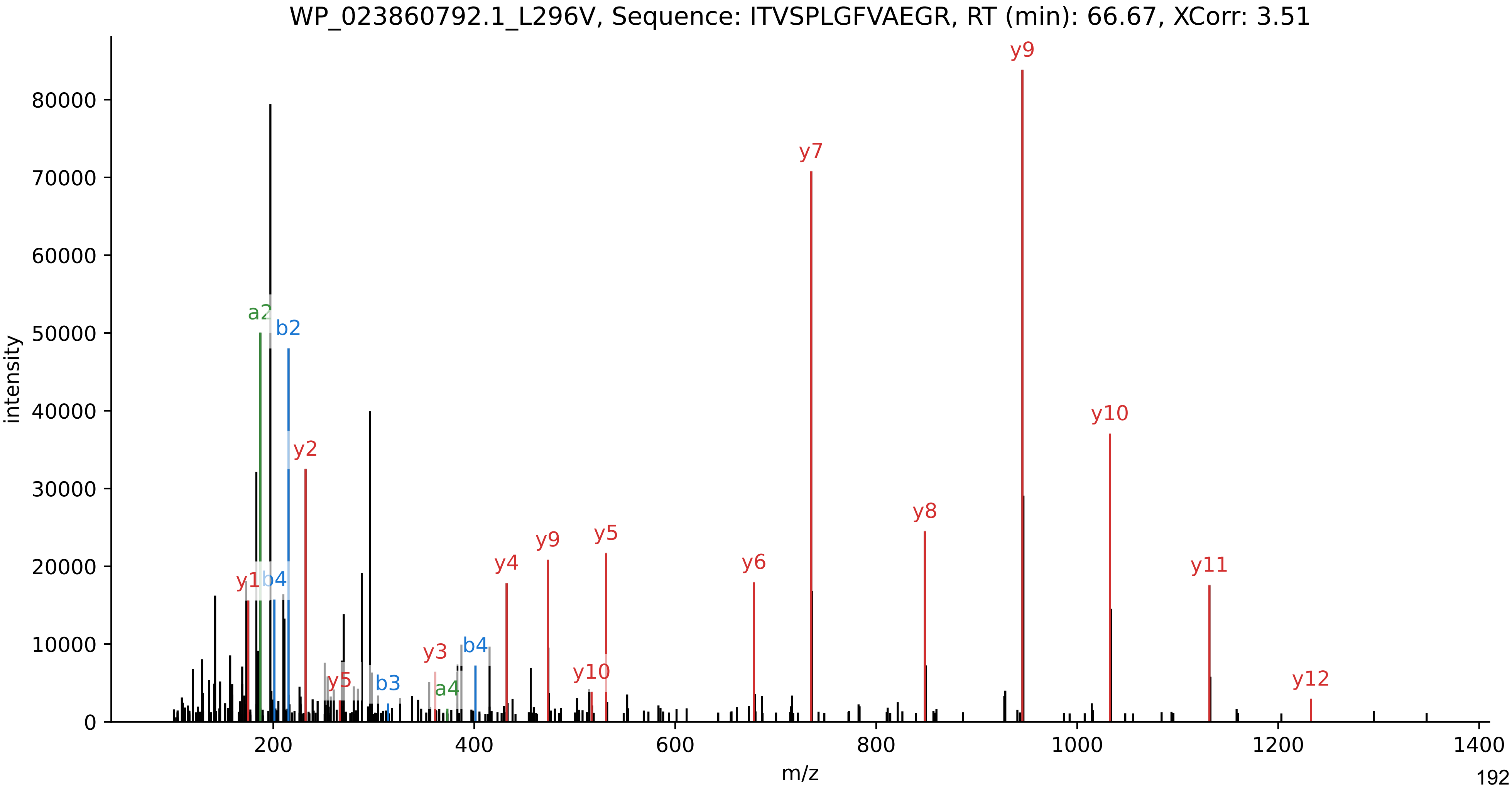

WP_023860792.1_L296V, Sequence: ITVSPLGFVAEGR, RT (min): 66.67, XCorr: 3.51
intensity
m/z
y9
y7
a2
b2
y2
y10
y8
y1
b4
y4
y9
y5
y6
y11
y3
b4
y10
y5
b3
a4
y12

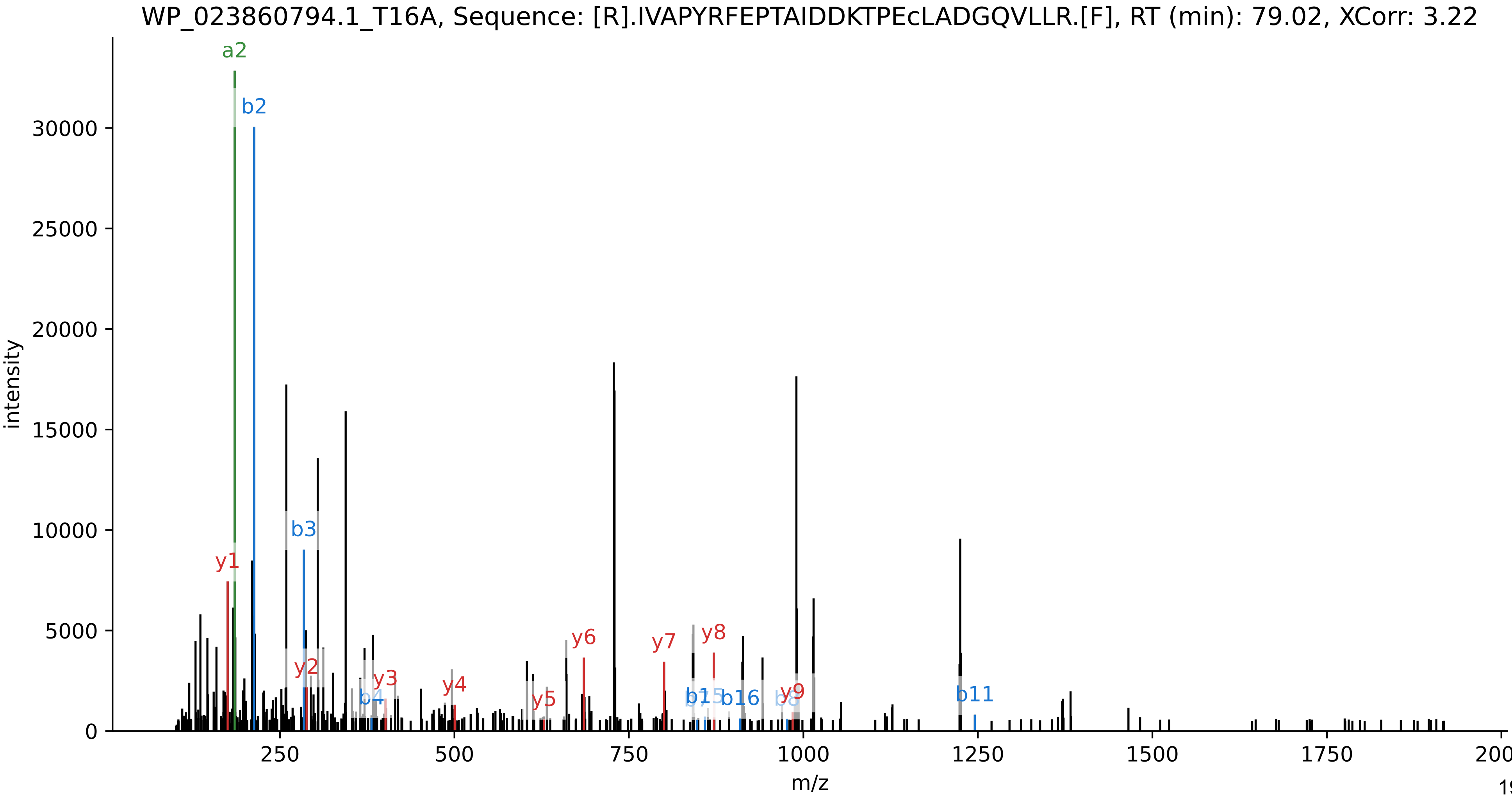

WP_023860794.1_T16A, Sequence: [R].IVAPYRFEPTAIDDKTPEcLADGQVLLR.[F], RT (min): 79.02, XCorr: 3.22
intensity
m/z
a2
b2
b3
y1
y2
y3
b4
y4
y5
y6
y7
y8
b16
y9
b11

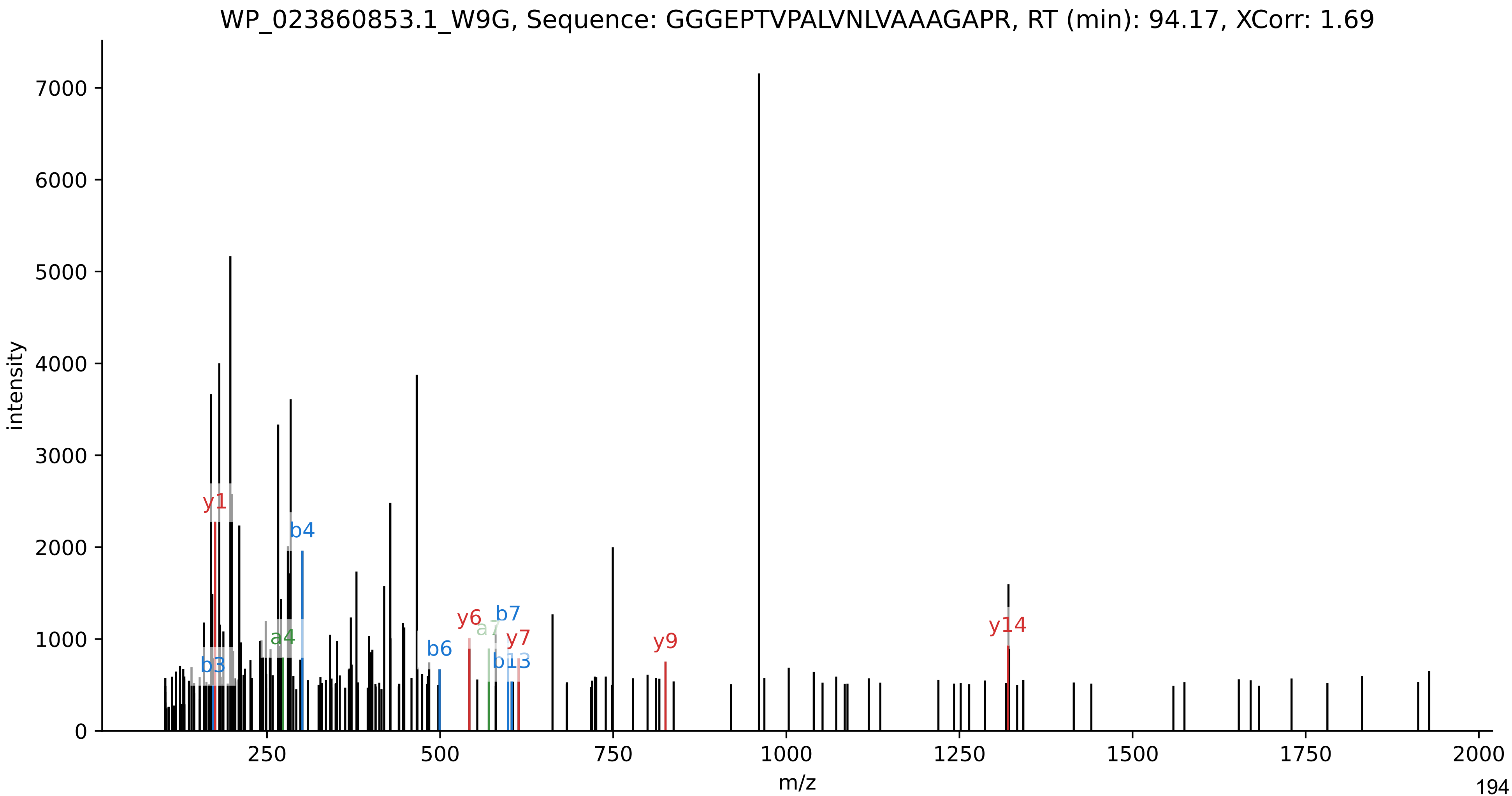

WP_023860853.1_W9G, Sequence: GGGEPTVPALVNLVAAAGAPR, RT (min): 94.17, XCorr: 1.69
intensity
m/z
0
1000
2000
3000
4000
5000
6000
7000
250
500
750
1000
1250
1500
1750
2000
y1
b4
a4
b3
b6
y6
b7
a7
y7
b13
y9
y14

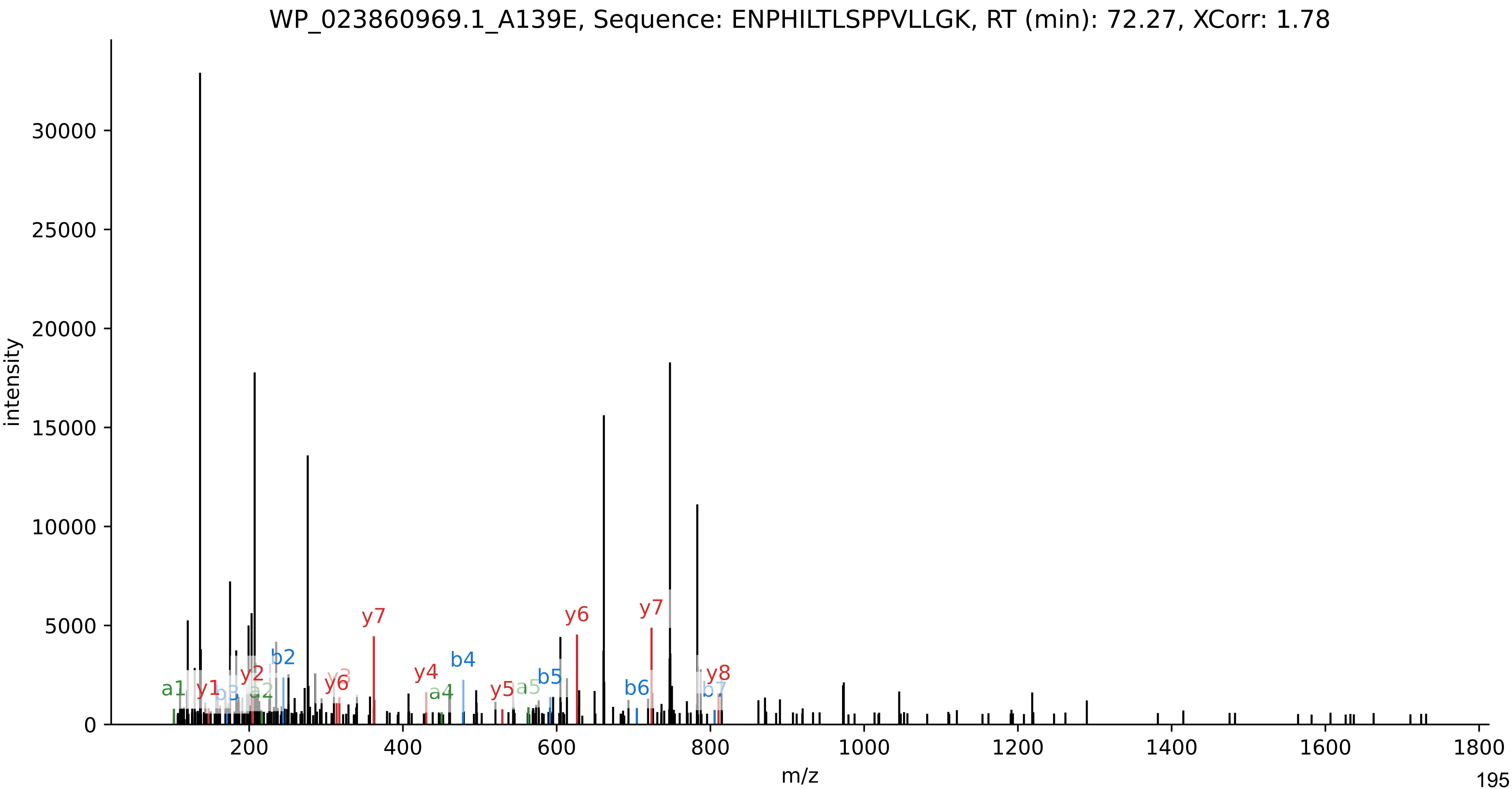
WP_023860969.1_A139E, Sequence: ENPHILTLSPPVLLGK, RT (min): 72.27, XCorr: 1.78
intensity
m/z
a1
y1
b3
y2
a2
b2
y3
y6
y7
y4
a4
b4
y5
a5
b5
y6
b6
y7
y8
b7

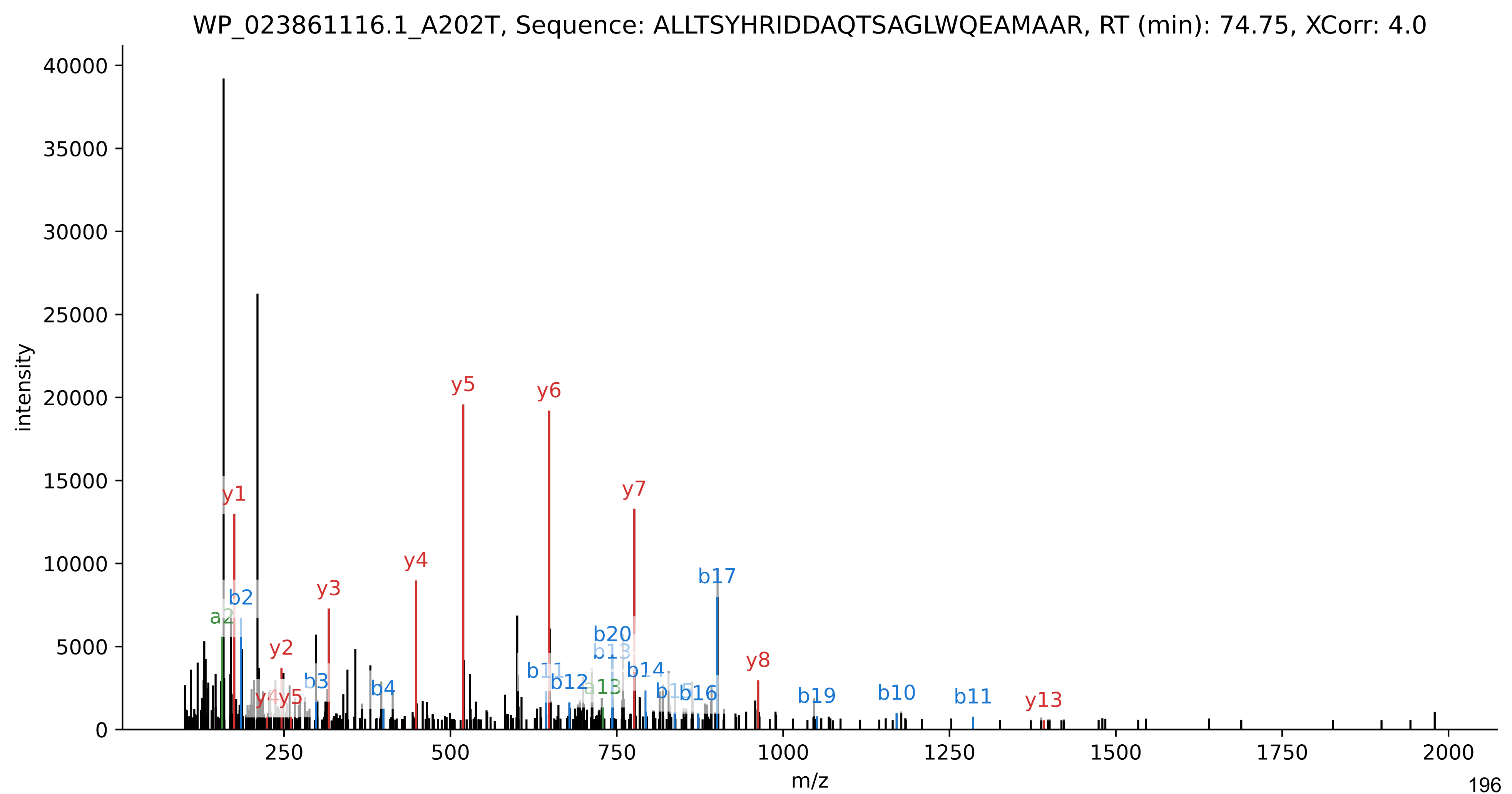

WP_023861116.1_A202T, Sequence: ALLTSYHRIDDAQTSAGLWQEAMAAR, RT (min): 74.75, XCorr: 4.0
intensity
m/z
y1
y2
y3
y4
y5
y6
y7
y8
y13
b2
b3
b4
b10
b11
b12
b14
b16
b17
b19
b20
a2
a13

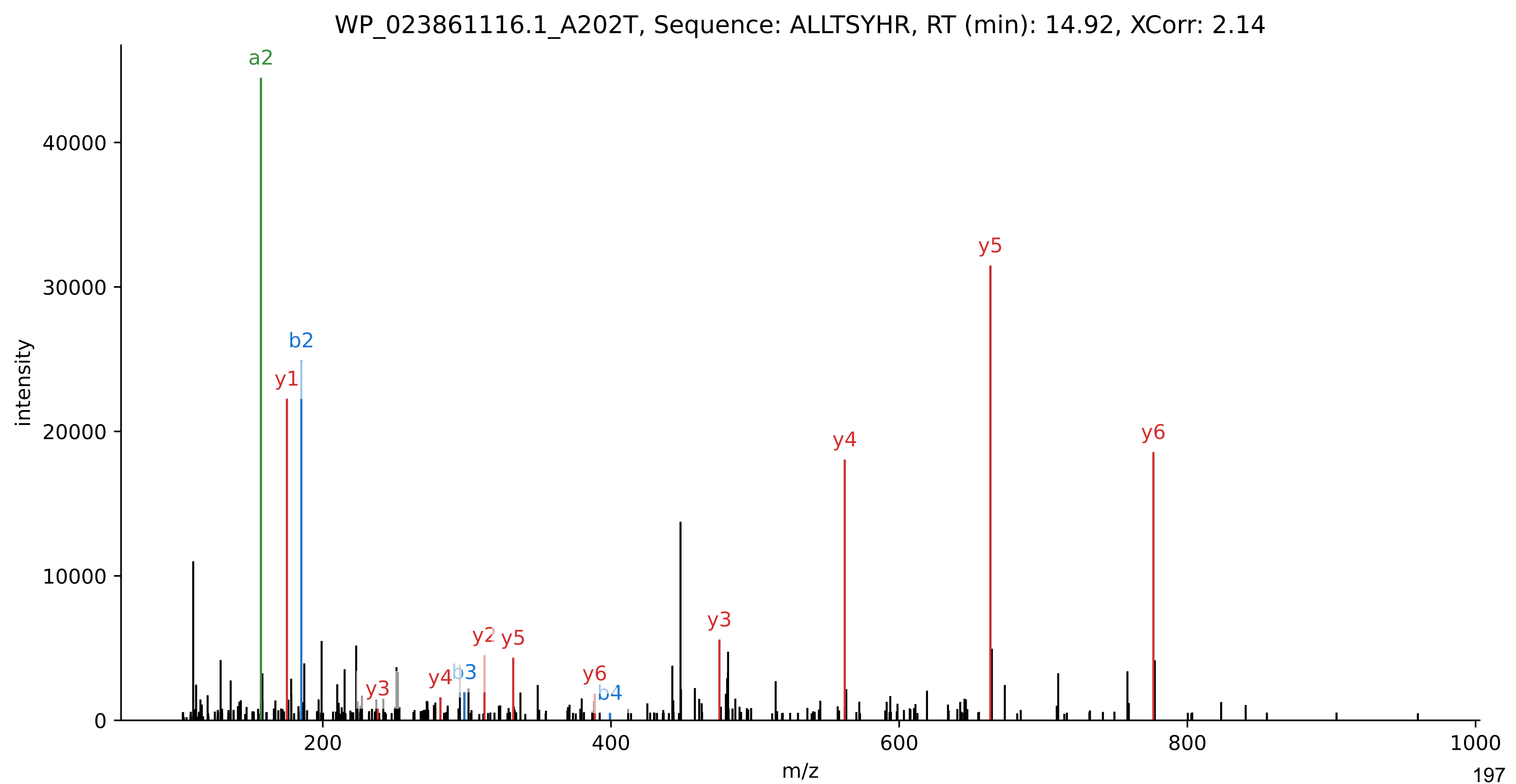

WP_023861116.1_A202T, Sequence: ALLTSYHR, RT (min): 14.92, XCorr: 2.14
a2
b2
y1
y5
y4
y6
y3
y2
y5
y3
y4
b3
y6
b4
intensity
m/z
40000
30000
20000
10000
0
200
400
600
800
1000

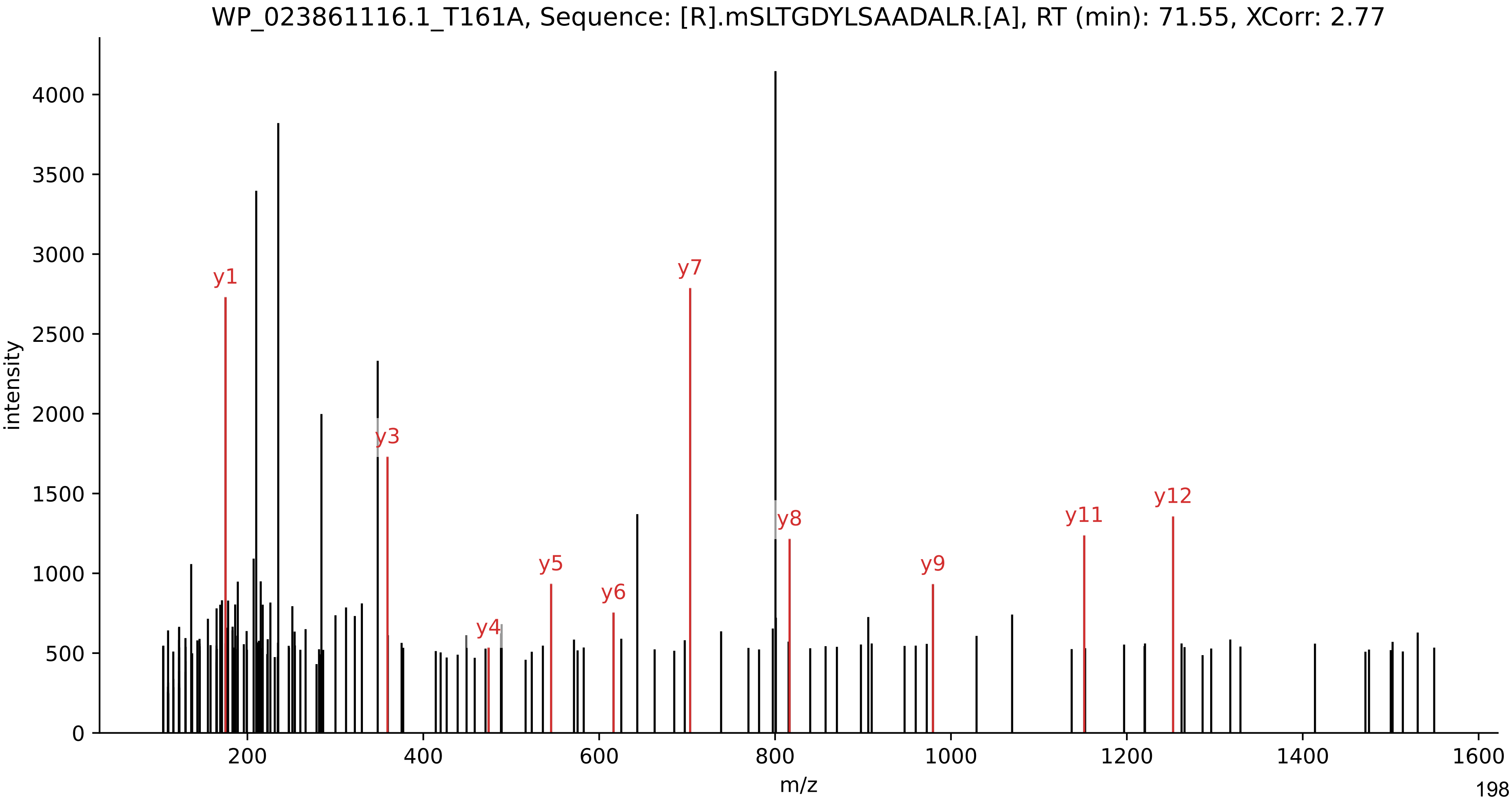

WP_023861116.1_T161A, Sequence: [R].mSLTGDYLSAADALR.[A], RT (min): 71.55, XCorr: 2.77
intensity
m/z
y1
y3
y4
y5
y6
y7
y8
y9
y11
y12

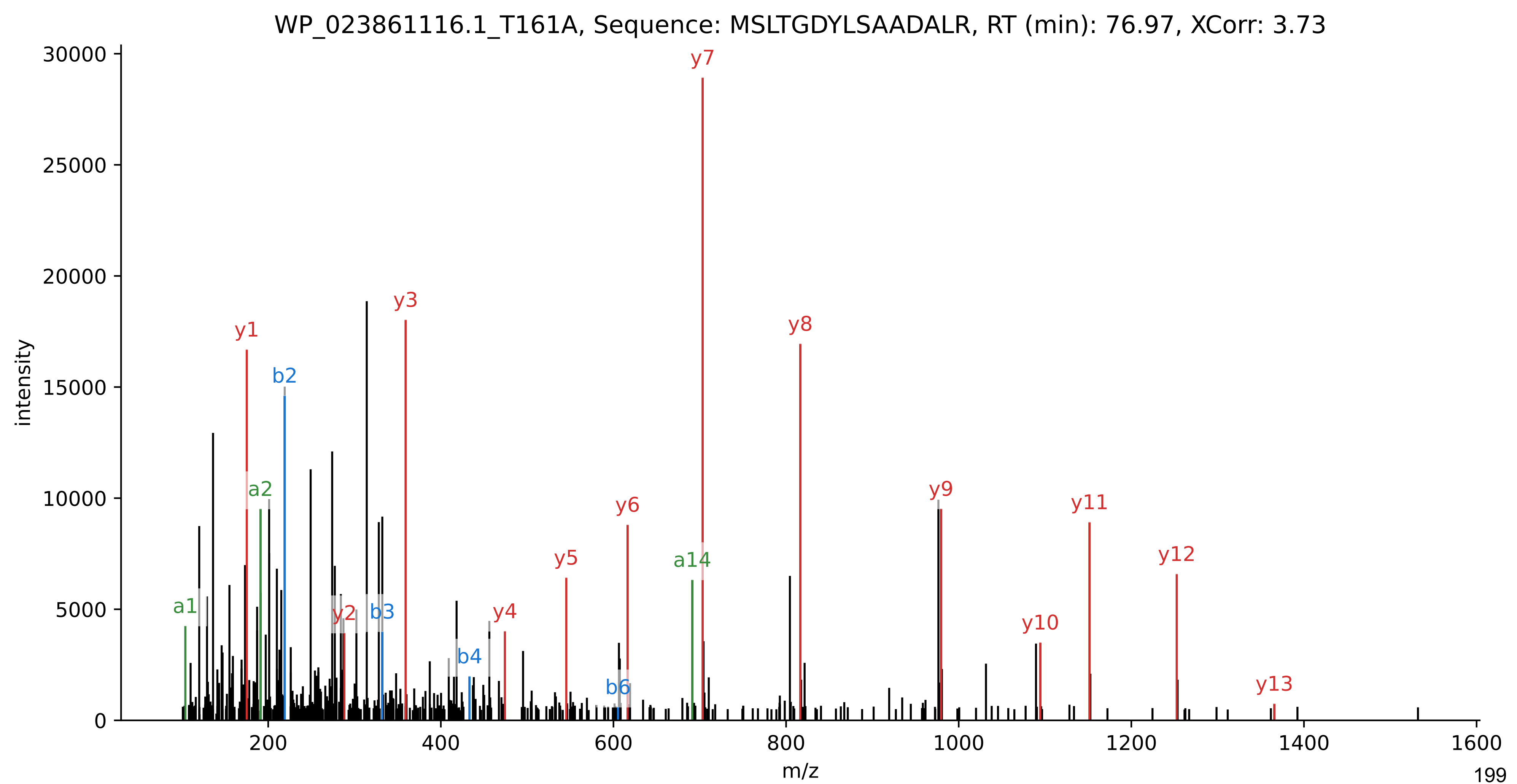
WP_023861116.1_T161A, Sequence: MSLTGDYLSAADALR, RT (min): 76.97, XCorr: 3.73
intensity
m/z
a1
y1
a2
b2
y2
b3
y3
b4
y4
y5
b6
y6
a14
y7
y8
y9
y10
y11
y12
y13

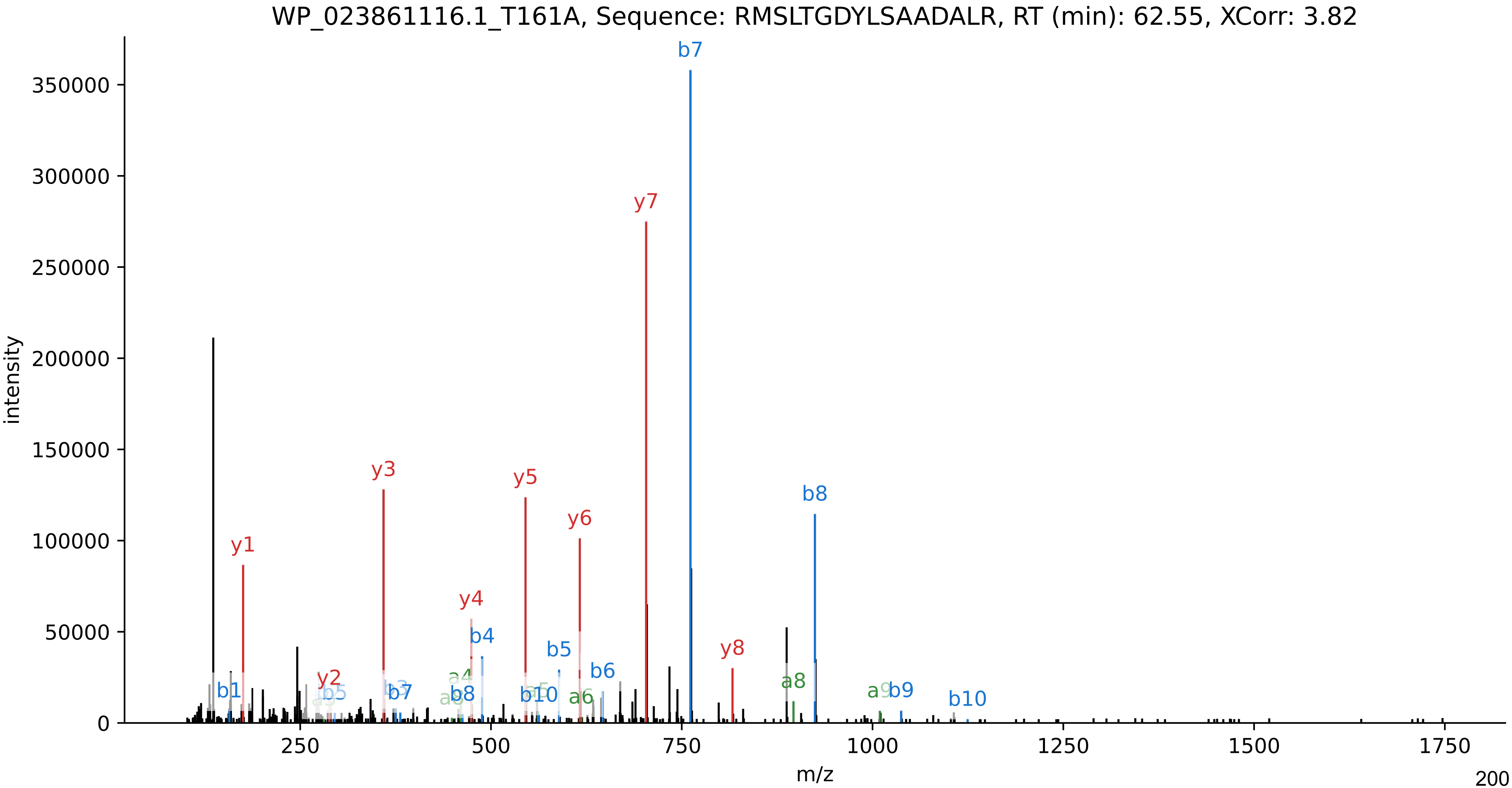
WP_023861116.1_T161A, Sequence: RMSLTGDYLSAADALR, RT (min): 62.55, XCorr: 3.82
intensity
m/z
b7
y7
y3
y5
y6
b8
y1
y4
b4
b5
y8
b6
a8
y2
b1
b5
b3
b7
a4
b8
a5
b10
a6
a9
b9
b10
0
50000
100000
150000
200000
250000
300000
350000
250
500
750
1000
1250
1500
1750

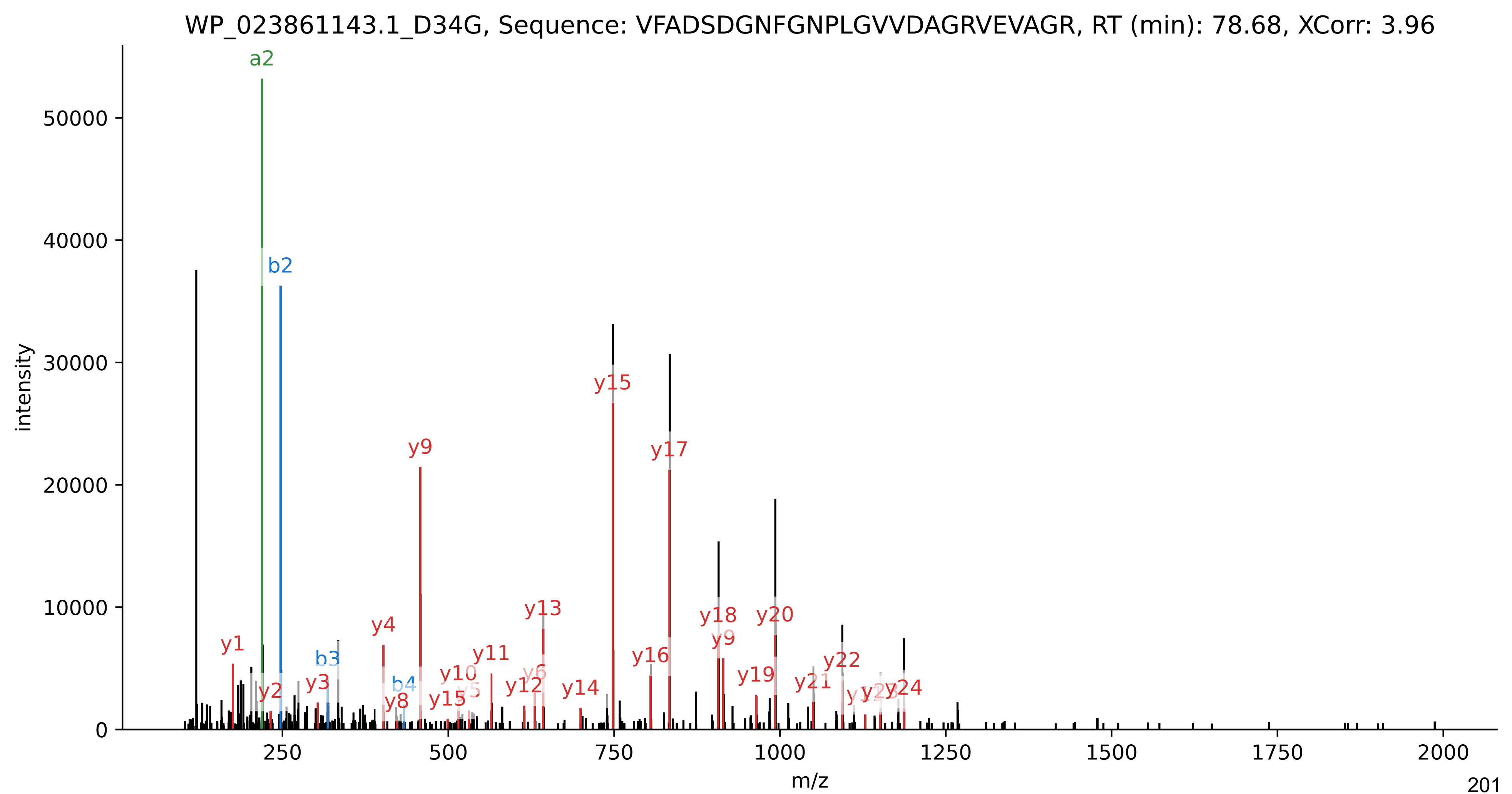
WP_023861143.1_D34G, Sequence: VFADSDGNFGNPLGVVDAGRVEVAGR, RT (min): 78.68, XCorr: 3.96
intensity
m/z
a2
b2
b3
b4
y1
y2
y3
y4
y8
y9
y10
y11
y12
y13
y14
y15
y16
y17
y18
y19
y20
y21
y22
y24

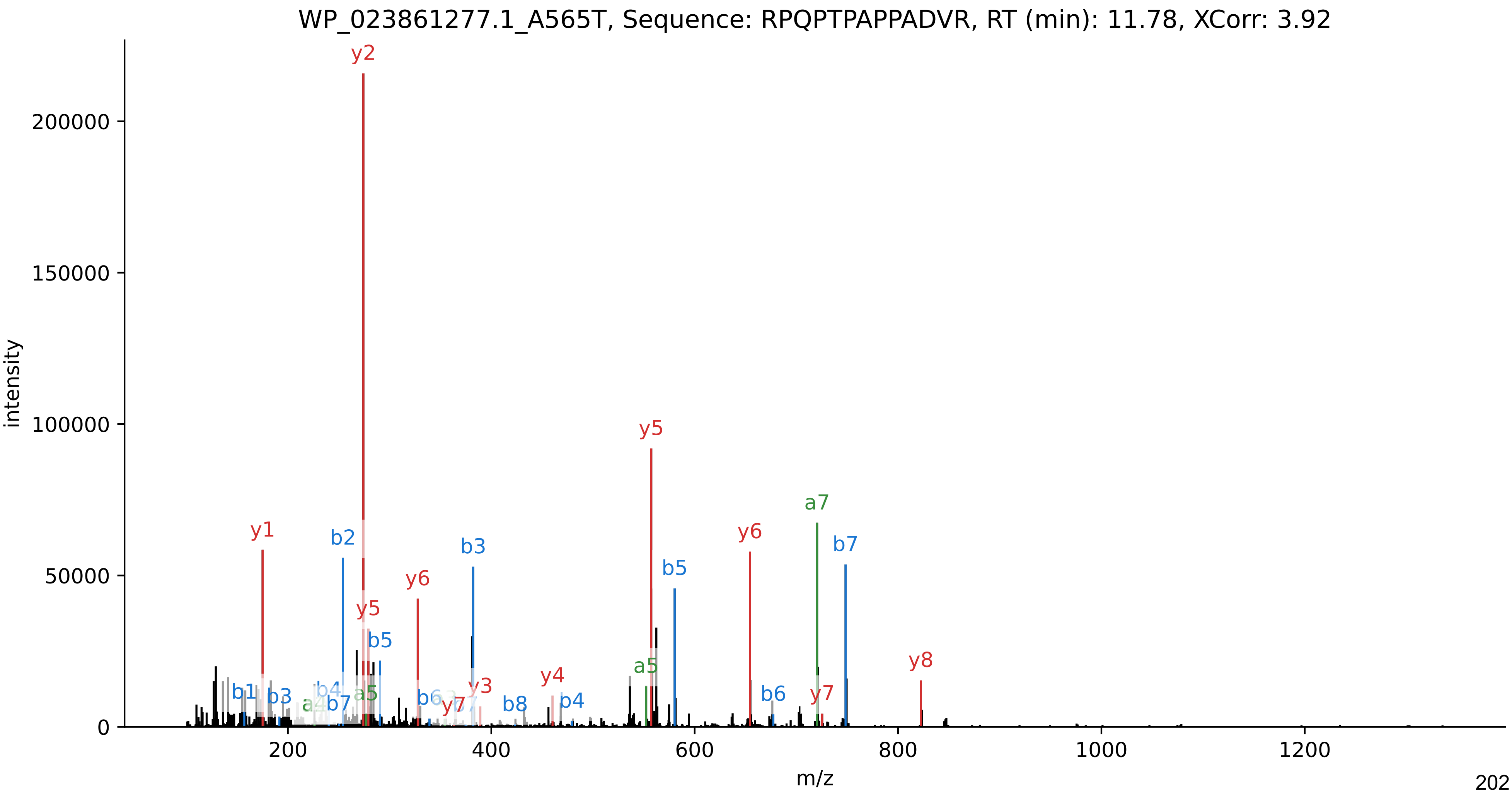
WP_023861277.1_A565T, Sequence: RPQPTPAPPADVR, RT (min): 11.78, XCorr: 3.92
intensity
m/z
y2
y5
a7
y1
b2
b3
y6
b7
y6
b5
y5
b5
y8
a5
y4
y3
b1
b3
b4
a5
a4
b7
b6
y7
b8
b4
b6
y7
200000
150000
100000
50000
0
200
400
600
800
1000
1200

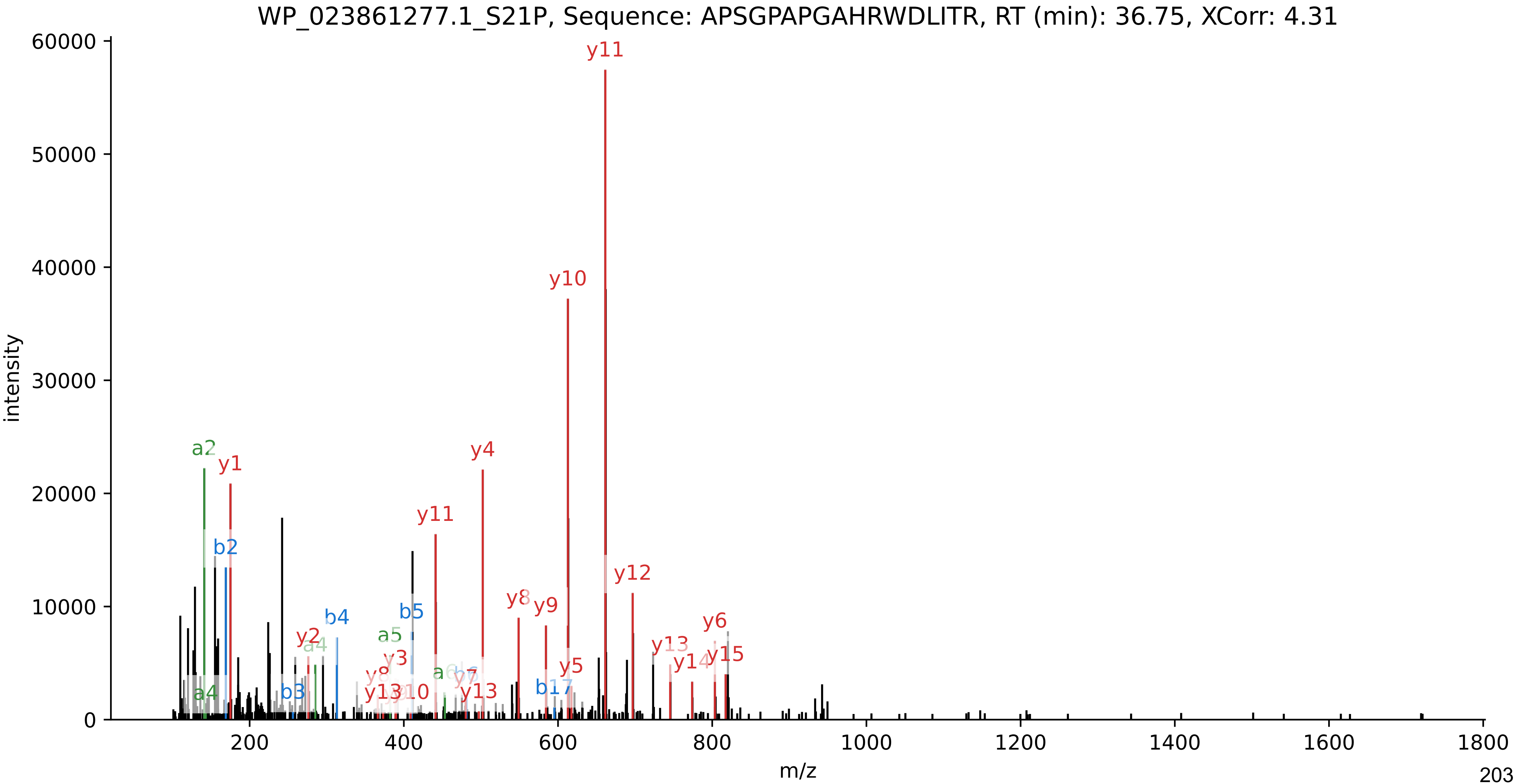

WP_023861277.1_S21P, Sequence: APSGPAPGAHRWDLITR, RT (min): 36.75, XCorr: 4.31
intensity
m/z
y11
y10
y4
a2
y1
b2
y11
y12
y8
y9
b4
b5
y2
a5
a4
y6
y13
y14
y15
y3
y5
a4
b3
b17
y13

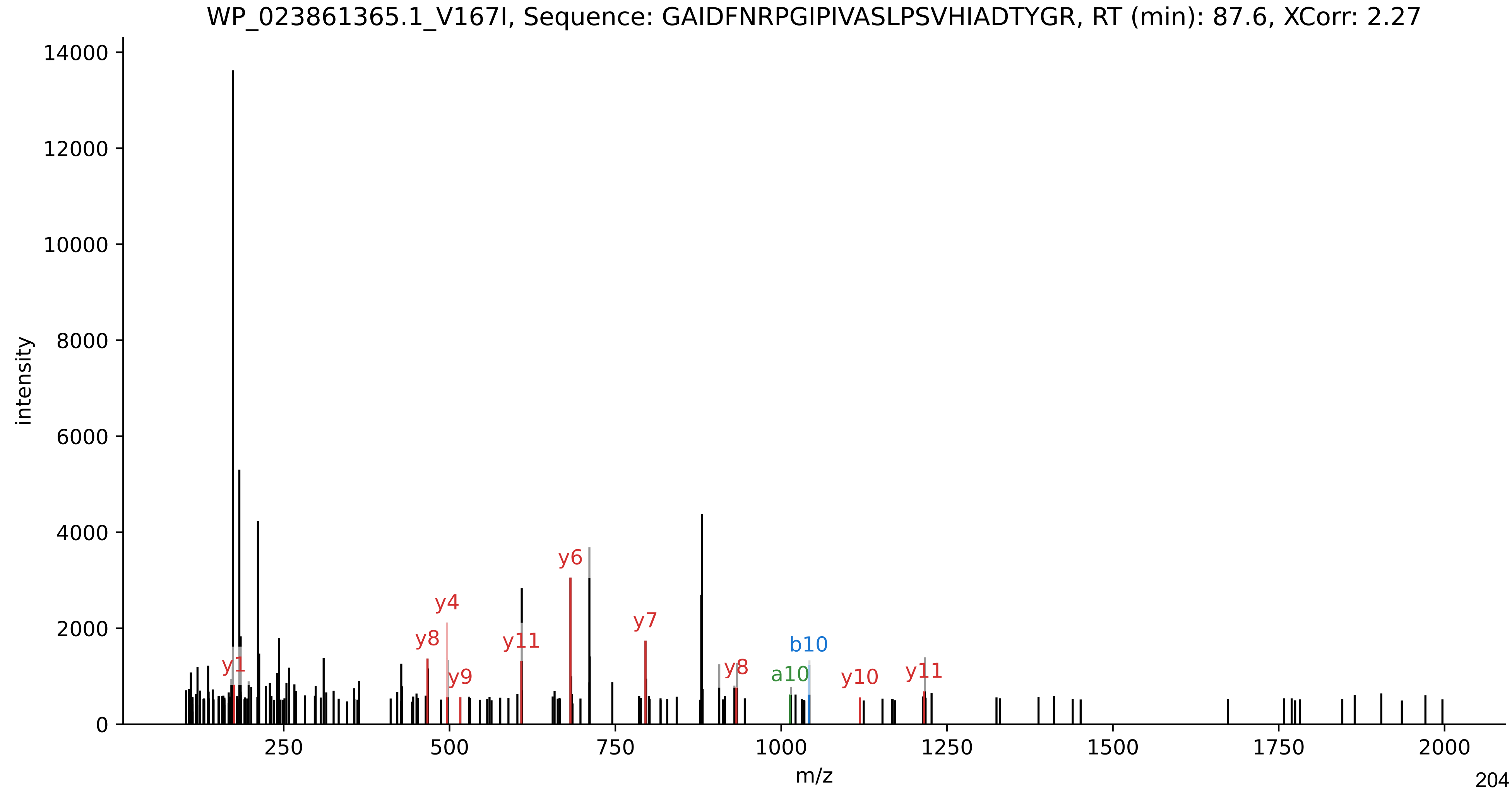

WP_023861365.1_V167I, Sequence: GAIDFNRPGIPIVASLPSVHIADTYGR, RT (min): 87.6, XCorr: 2.27
intensity
m/z
y1
y8
y4
y9
y11
y6
y7
y8
a10
b10
y10
y11

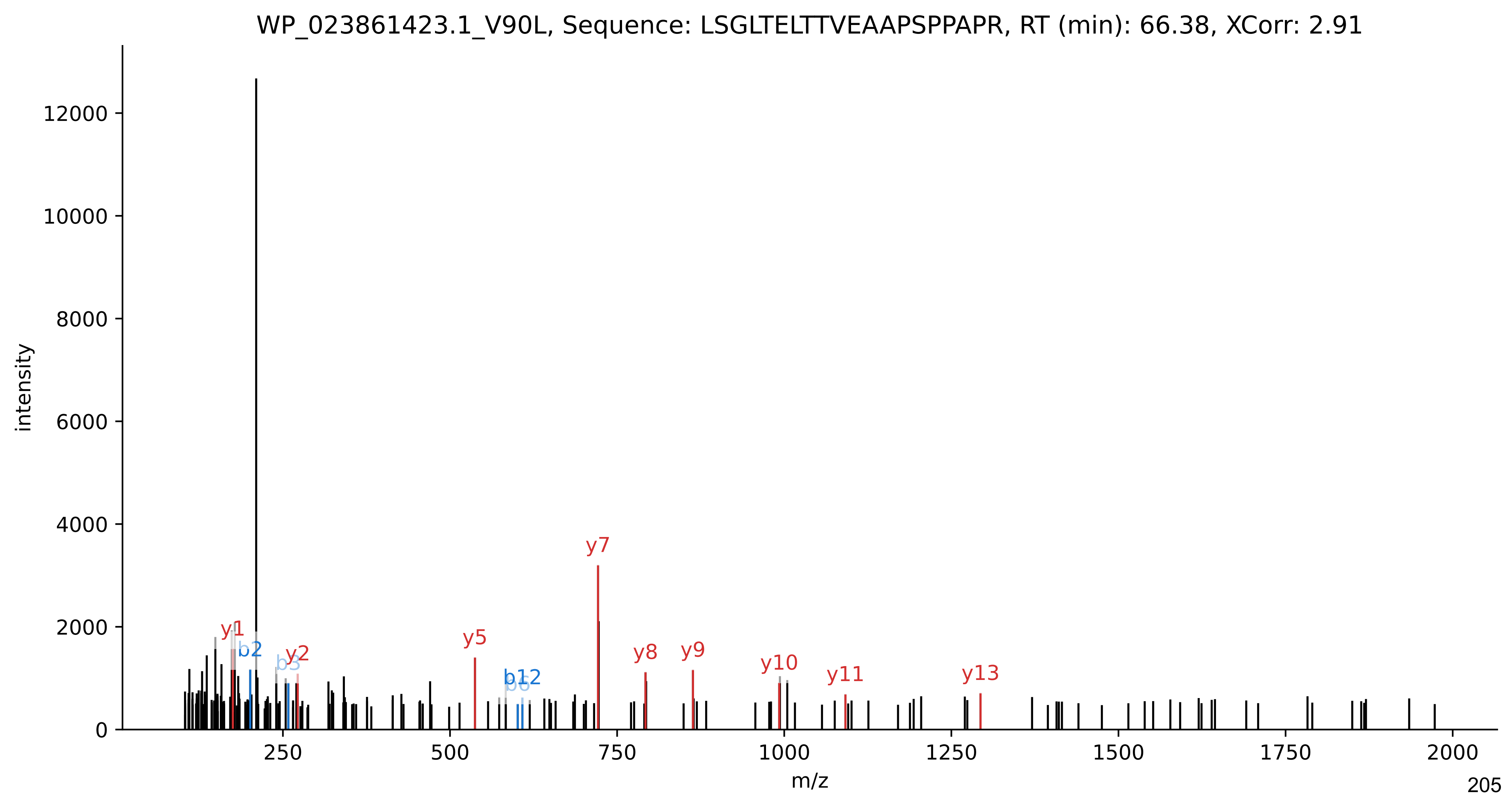
WP_023861423.1_V90L, Sequence: LSGLTELTTVEAAPSPPAPR, RT (min): 66.38, XCorr: 2.91
intensity
m/z
y1
b2
b3
y2
y5
b12
b6
y7
y8
y9
y10
y11
y13

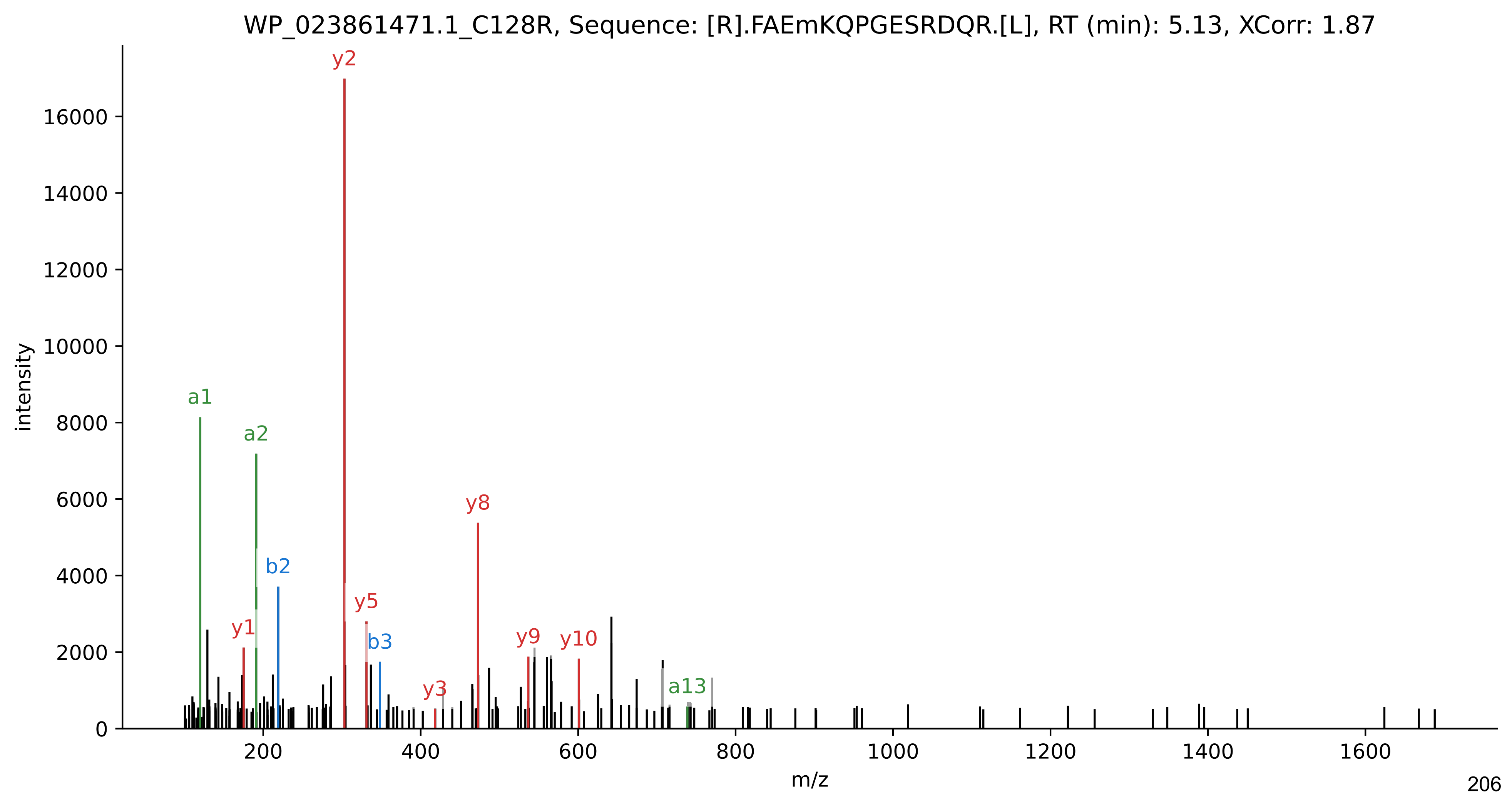
WP_023861471.1_C128R, Sequence: [R].FAEmKQPGESRDQR.[L], RT (min): 5.13, XCorr: 1.87
intensity
m/z
y2
a1
a2
y8
b2
y5
y1
b3
y9
y10
y3
a13
0
2000
4000
6000
8000
10000
12000
14000
16000
200
400
600
800
1000
1200
1400
1600

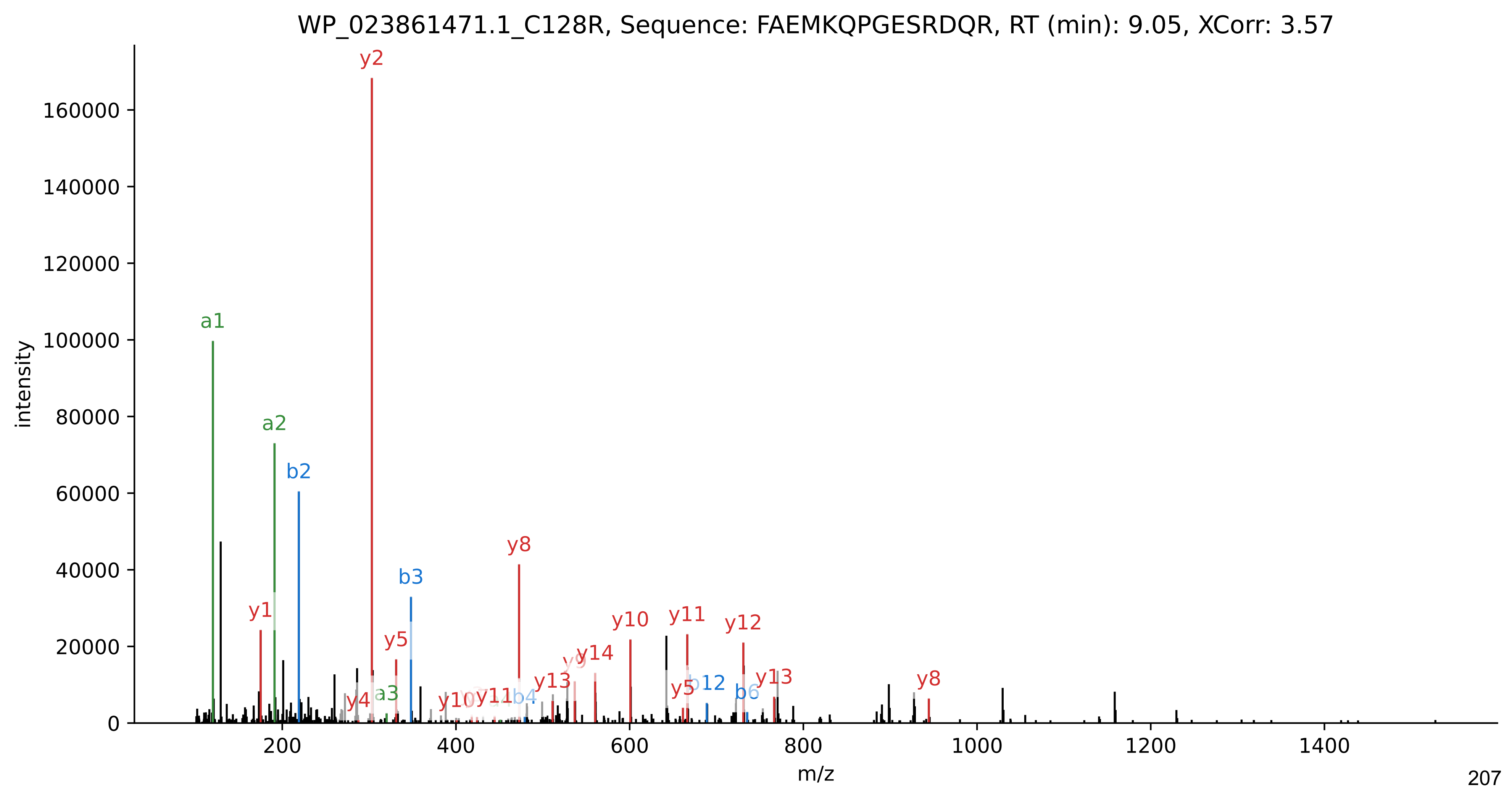
WP_023861471.1_C128R, Sequence: FAEMKQPGESRDQR, RT (min): 9.05, XCorr: 3.57
intensity
m/z
y2
a1
a2
b2
y8
b3
y1
y5
y10
y11
y12
y14
y13
y5
b12
b6
y13
y8
y4
a3
y10
y11
b4
0
20000
40000
60000
80000
100000
120000
140000
160000
200
400
600
800
1000
1200
1400

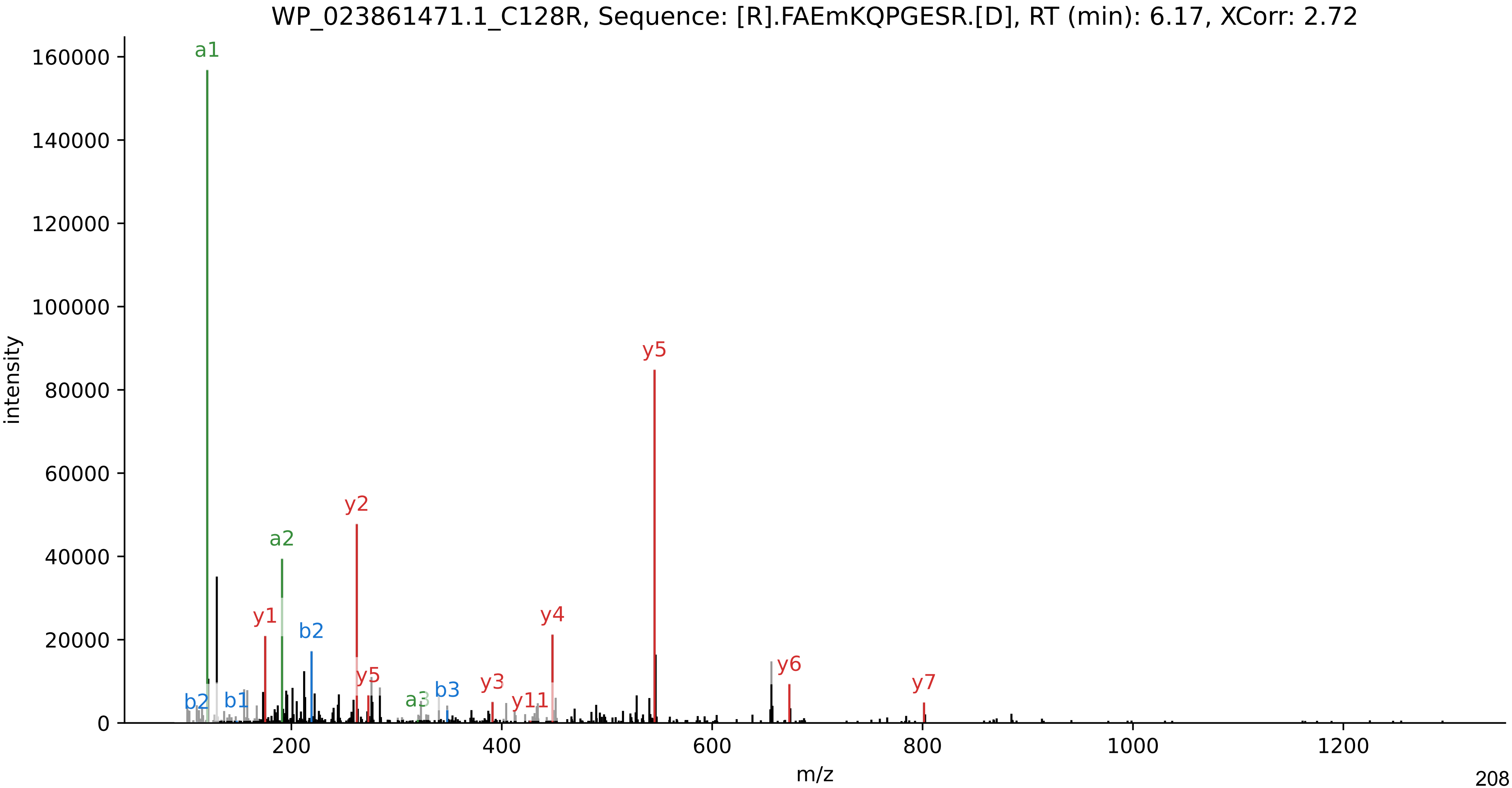
WP_023861471.1_C128R, Sequence: [R].FAEmKQPGESR.[D], RT (min): 6.17, XCorr: 2.72
intensity
m/z
0
20000
40000
60000
80000
100000
120000
140000
160000
200
400
600
800
1000
1200
a1
a2
a3
b1
b2
b2
b3
y1
y2
y5
y3
y11
y4
y5
y6
y7

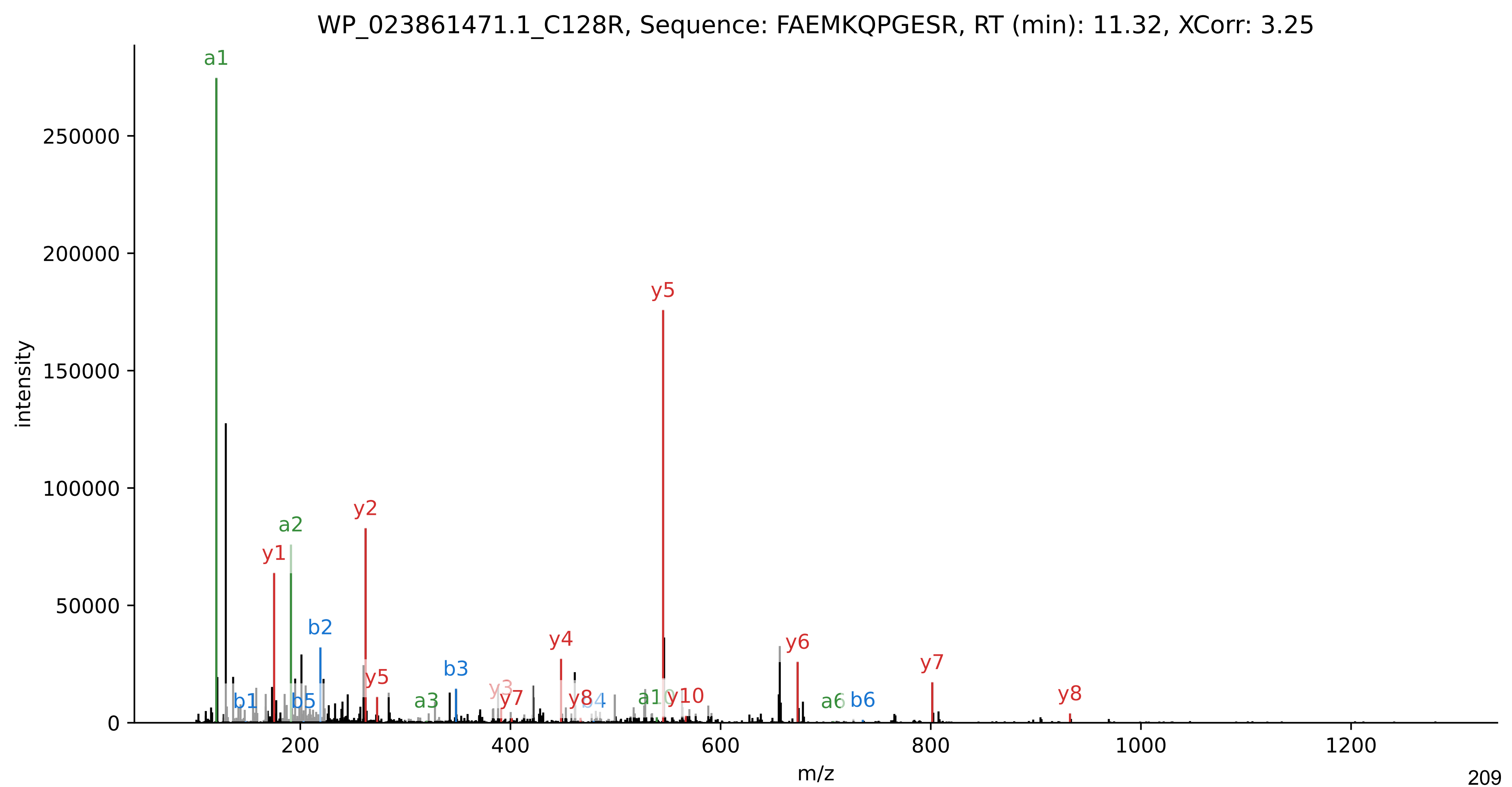

WP_023861471.1_C128R, Sequence: FAEMKQPGESR, RT (min): 11.32, XCorr: 3.25
intensity
m/z
a1
y5
y2
a2
y1
b2
y4
y6
y7
b3
y5
y3
y7
y8
a3
b1
b5
y10
a6
b6
y8

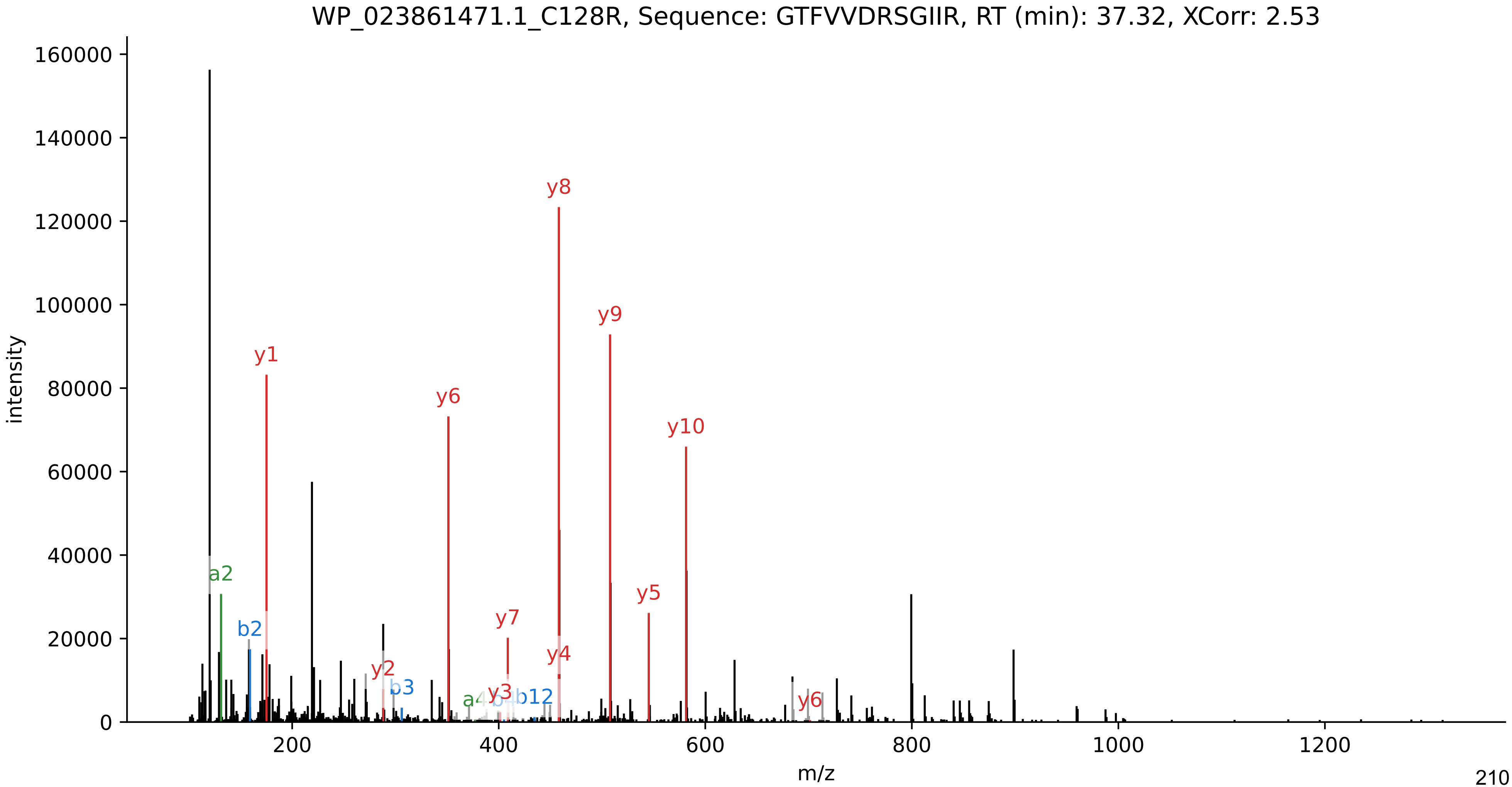
WP_023861471.1_C128R, Sequence: GTFVVDRSGIIR, RT (min): 37.32, XCorr: 2.53
intensity
m/z
0
20000
40000
60000
80000
100000
120000
140000
160000
200
400
600
800
1000
1200
a2
b2
y1
y2
b3
y6
a4
y3
b4
b12
y7
y8
y4
y9
y5
y10
y6

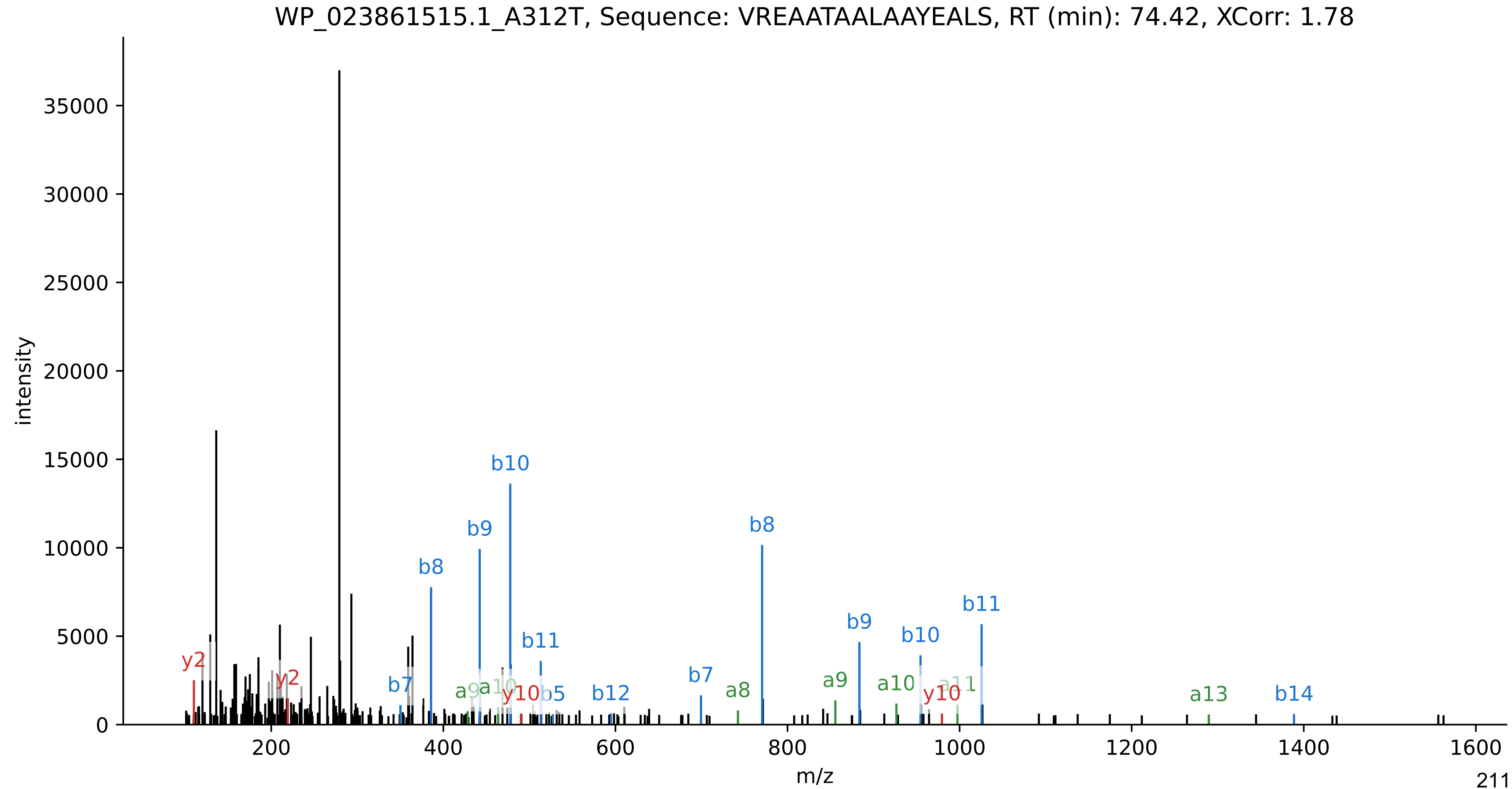
WP_023861515.1_A312T, Sequence: VREAATAALAAYEALS, RT (min): 74.42, XCorr: 1.78
intensity
m/z
0
5000
10000
15000
20000
25000
30000
35000
200
400
600
800
1000
1200
1400
1600
y2
y2
b7
b8
b9
a9
a10
b10
y10
b11
b5
b12
b7
a8
b8
a9
b9
a10
b10
y10
a11
b11
a13
b14

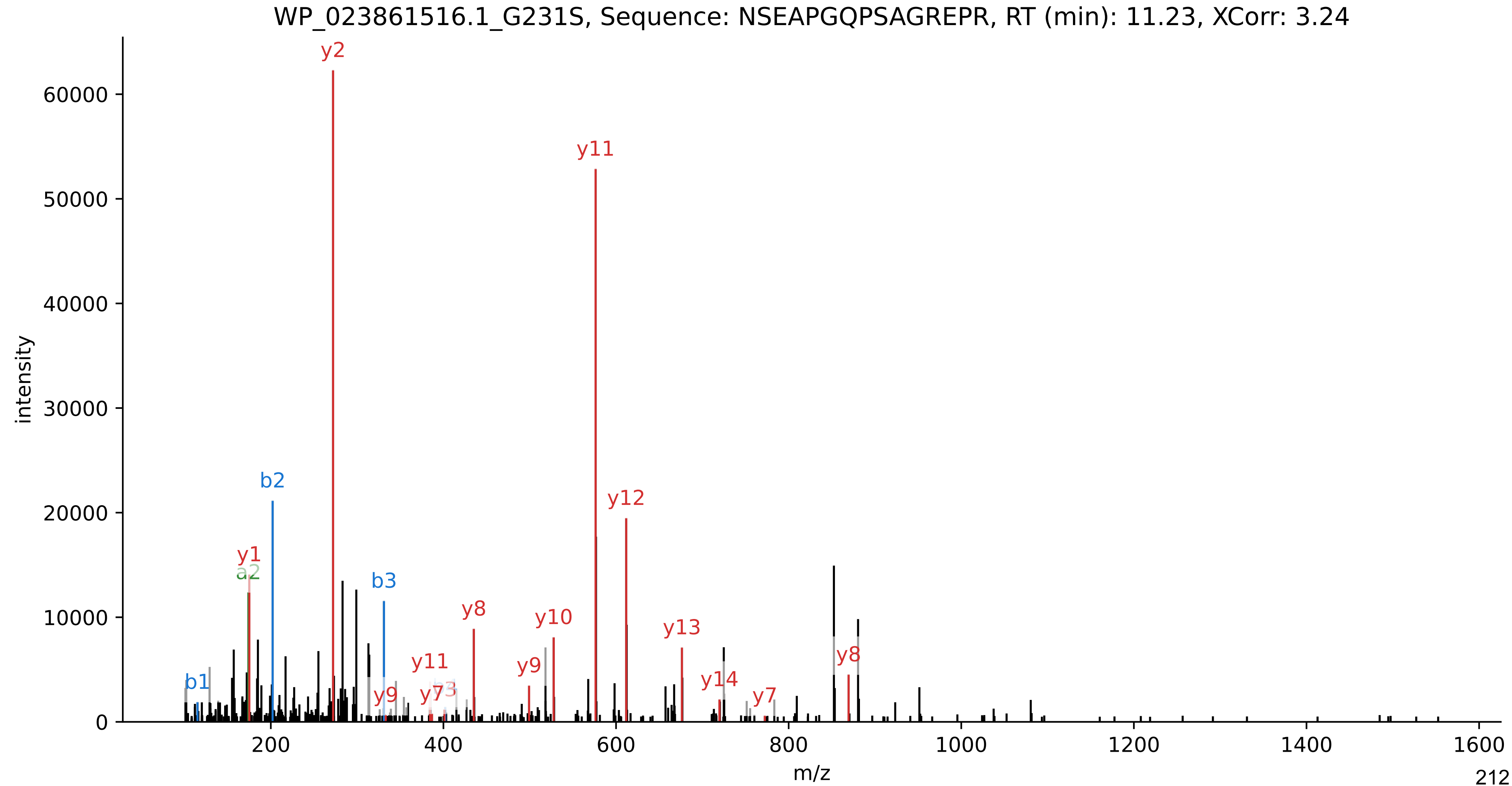

WP_023861516.1_G231S, Sequence: NSEAPGQPSAGREPR, RT (min): 11.23, XCorr: 3.24
intensity
m/z
y2
y11
y12
b2
y1
a2
b3
y8
y10
y13
y11
y9
y9
y7
y14
y7
y8
b1
0
10000
20000
30000
40000
50000
60000
200
400
600
800
1000
1200
1400
1600

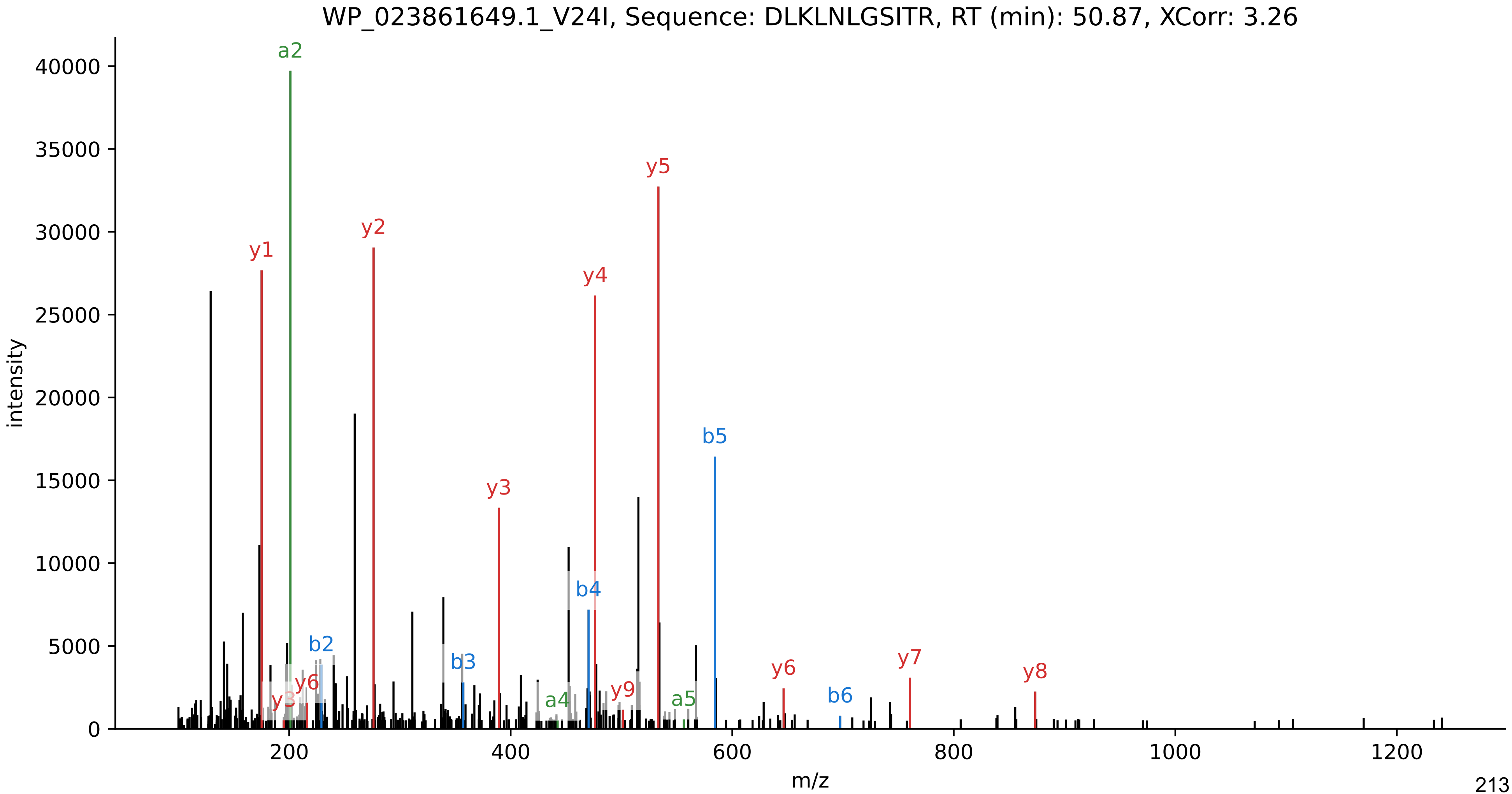

WP_023861649.1_V24I, Sequence: DLKLNLGSITR, RT (min): 50.87, XCorr: 3.26
intensity
m/z
a2
y5
y2
y1
y4
b5
y3
b4
b2
b3
y6
y3
y9
a4
a5
y6
b6
y7
y8
0
5000
10000
15000
20000
25000
30000
35000
40000
200
400
600
800
1000
1200

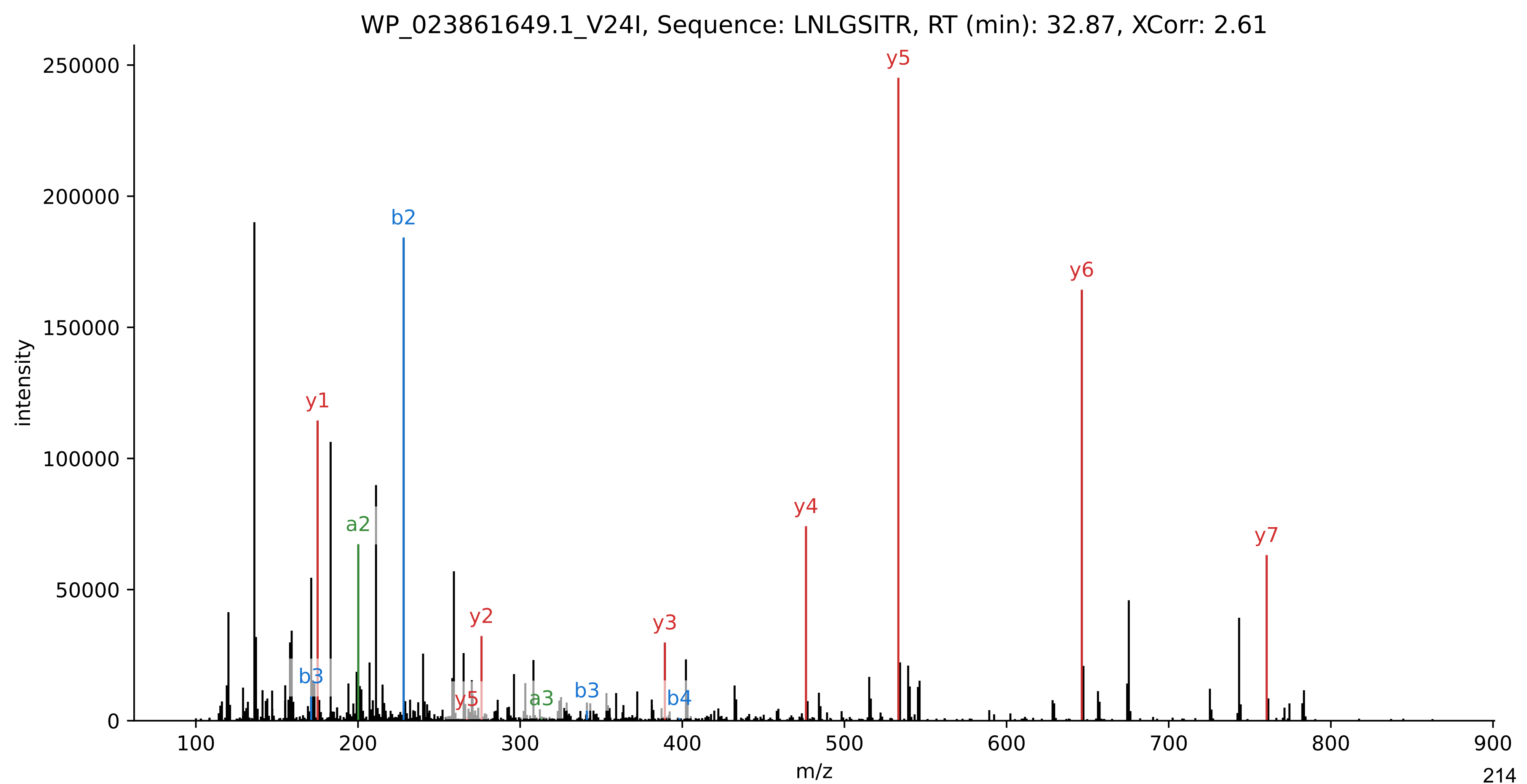
WP_023861649.1_V24I, Sequence: LNLGSITR, RT (min): 32.87, XCorr: 2.61
intensity
m/z
y5
b2
y6
y1
a2
y4
y7
y2
y3
b3
y5
a3
b3
b4

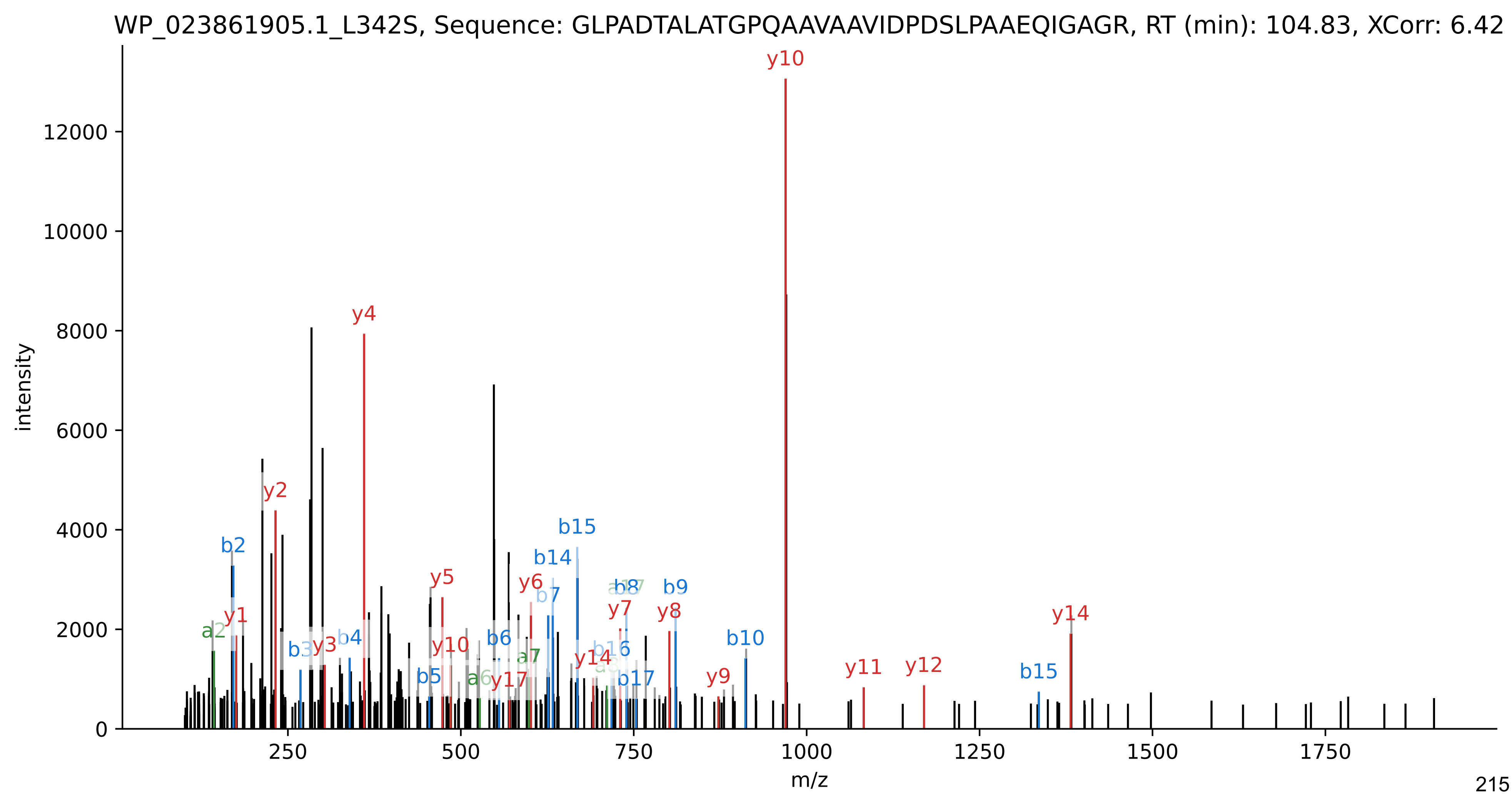
WP_023861905.1_L342S, Sequence: GLPADTALATGPQAAVAAVIDPDSLPAAEQIGAGR, RT (min): 104.83, XCorr: 6.42
intensity
m/z
0
2000
4000
6000
8000
10000
12000
250
500
750
1000
1250
1500
1750
y10
y4
y2
b2
a2
y1
b3
y3
b4
y5
y10
b5
a6
y17
b6
a7
y6
b7
b14
b15
y14
b16
b17
b8
y7
y8
b9
b10
y9
y11
y12
b15
y14

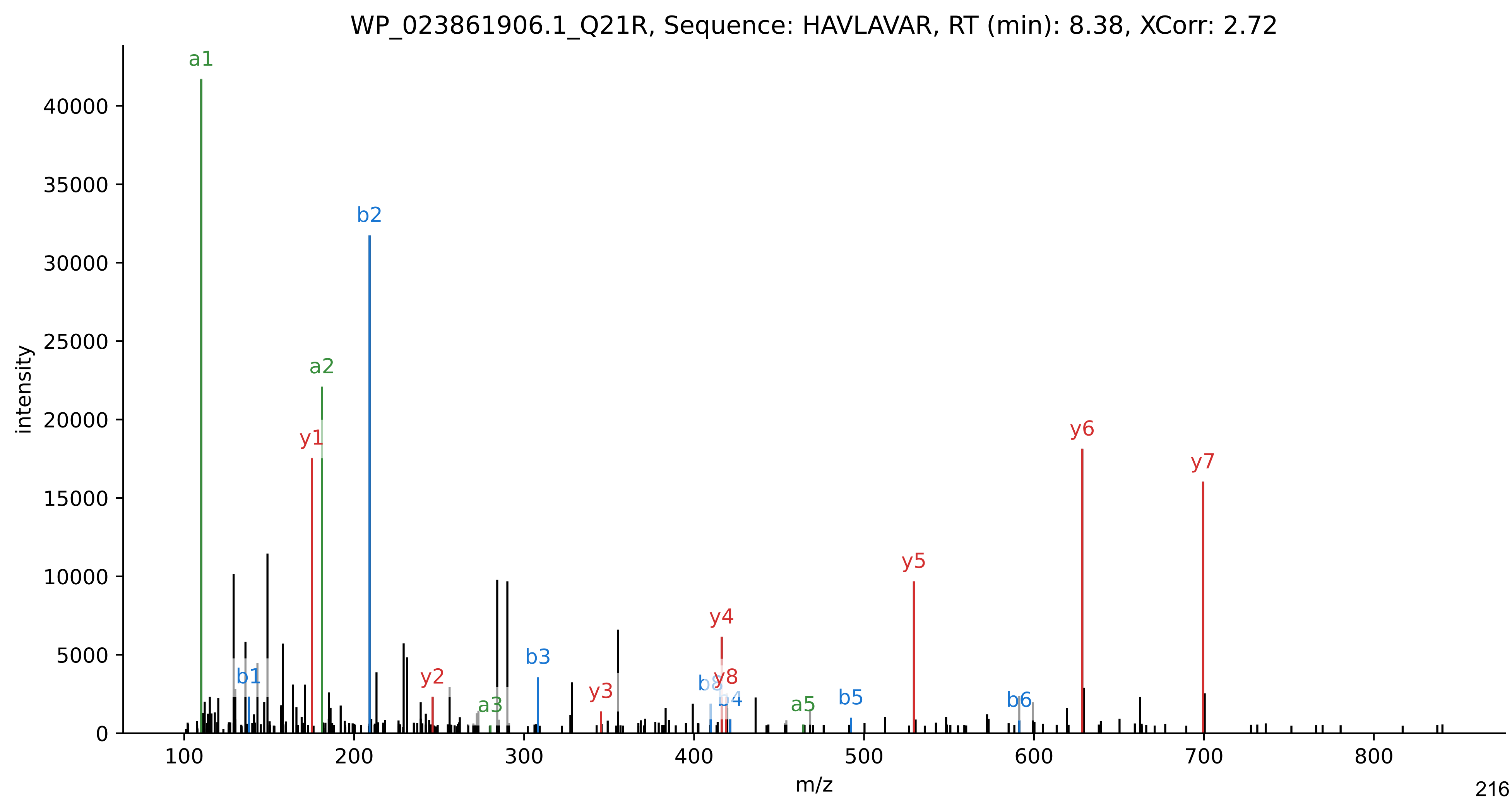

WP_023861906.1_Q21R, Sequence: HAVLAVAR, RT (min): 8.38, XCorr: 2.72
intensity
m/z
a1
a2
a3
a5
b1
b2
b3
b4
b5
b6
b8
y1
y2
y3
y4
y5
y6
y7
y8
0
5000
10000
15000
20000
25000
30000
35000
40000
100
200
300
400
500
600
700
800

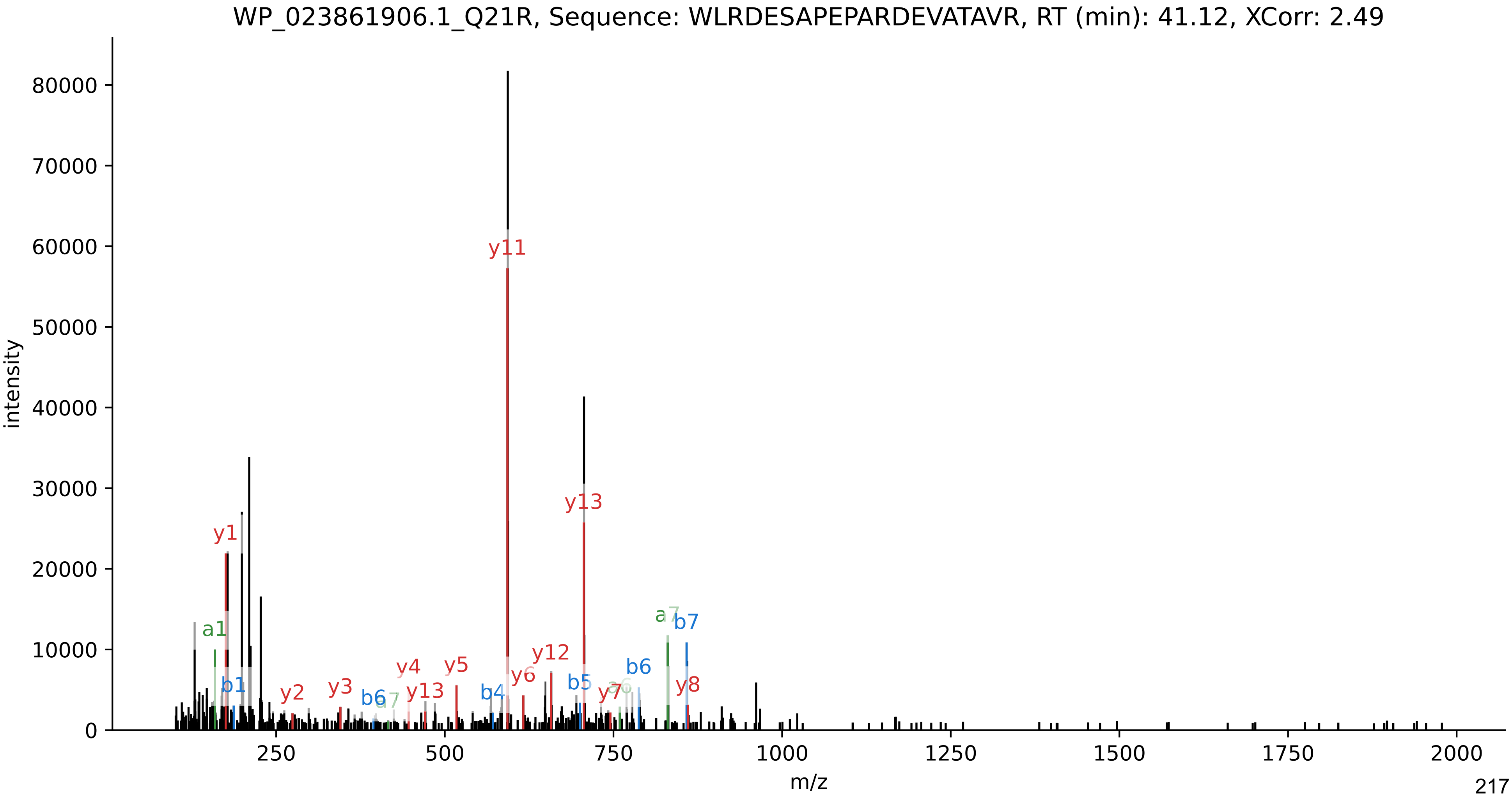

WP_023861906.1_Q21R, Sequence: WLRDESAPEPARDEVATAVR, RT (min): 41.12, XCorr: 2.49
intensity
m/z
y11
y13
y1
a1
b1
y2
y3
b6
a7
y4
y13
y5
b4
y6
y12
b5
y7
a6
b6
a7
b7
y8

WP_023861922.1_T288K, Sequence: LAYEGLAAGLGVPHKEKEA, RT (min): 44.53, XCorr: 4.8

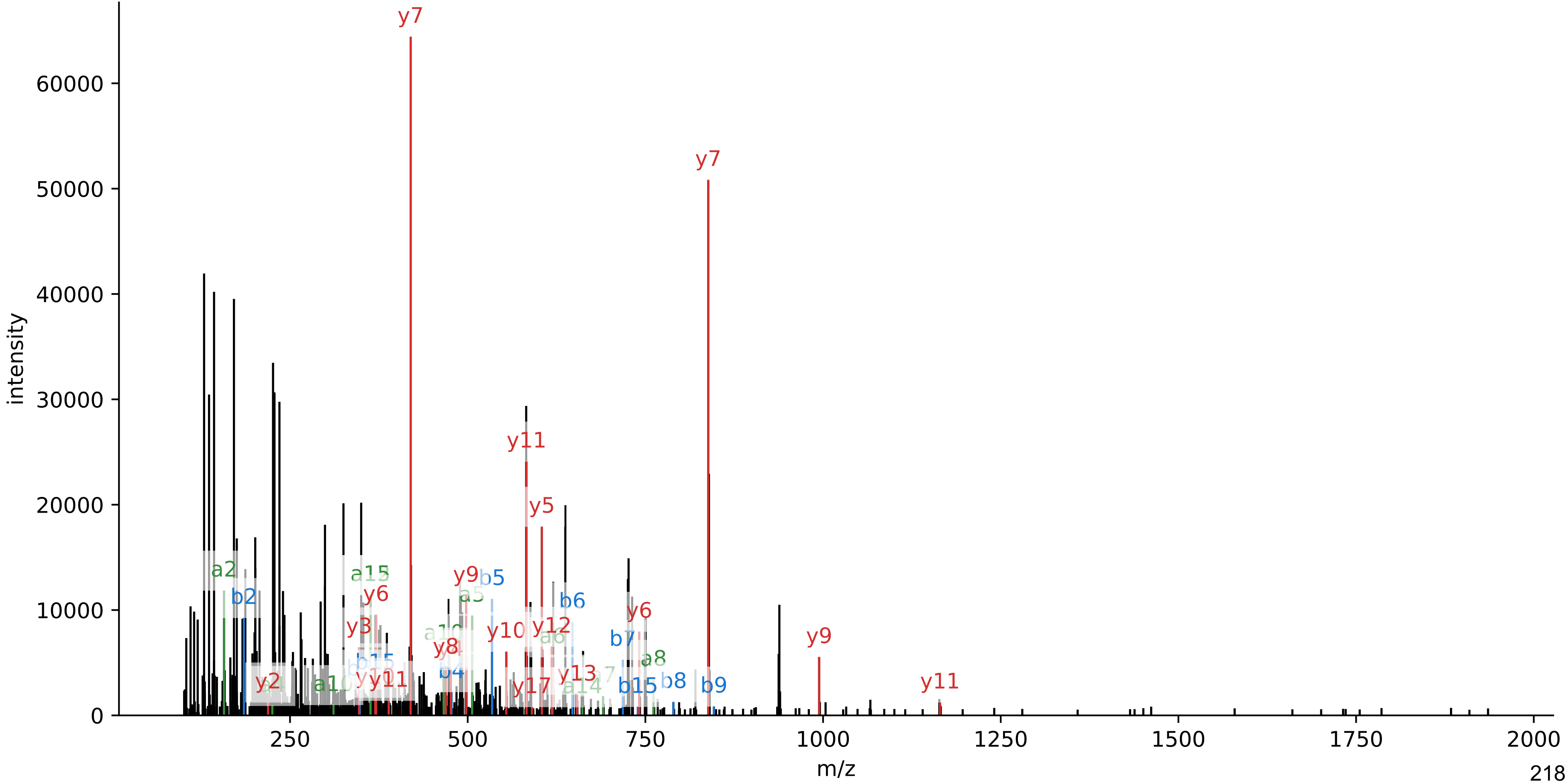

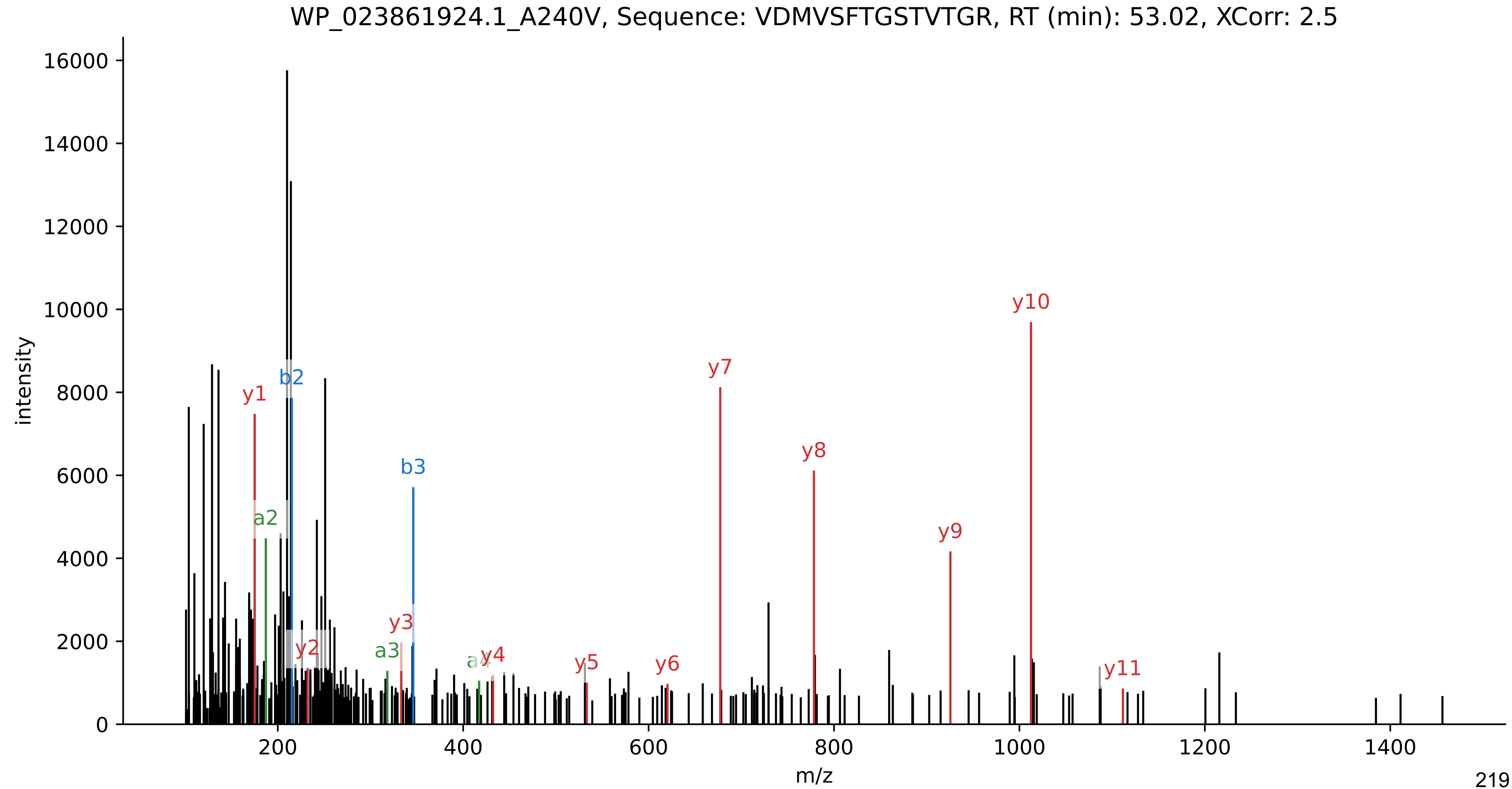

WP_023861924.1_A240V, Sequence: VDMVSFTGSTVTGR, RT (min): 53.02, XCorr: 2.5
intensity
m/z
y1
b2
a2
b3
y3
a3
y2
a4
y4
y5
y6
y7
y8
y9
y10
y11

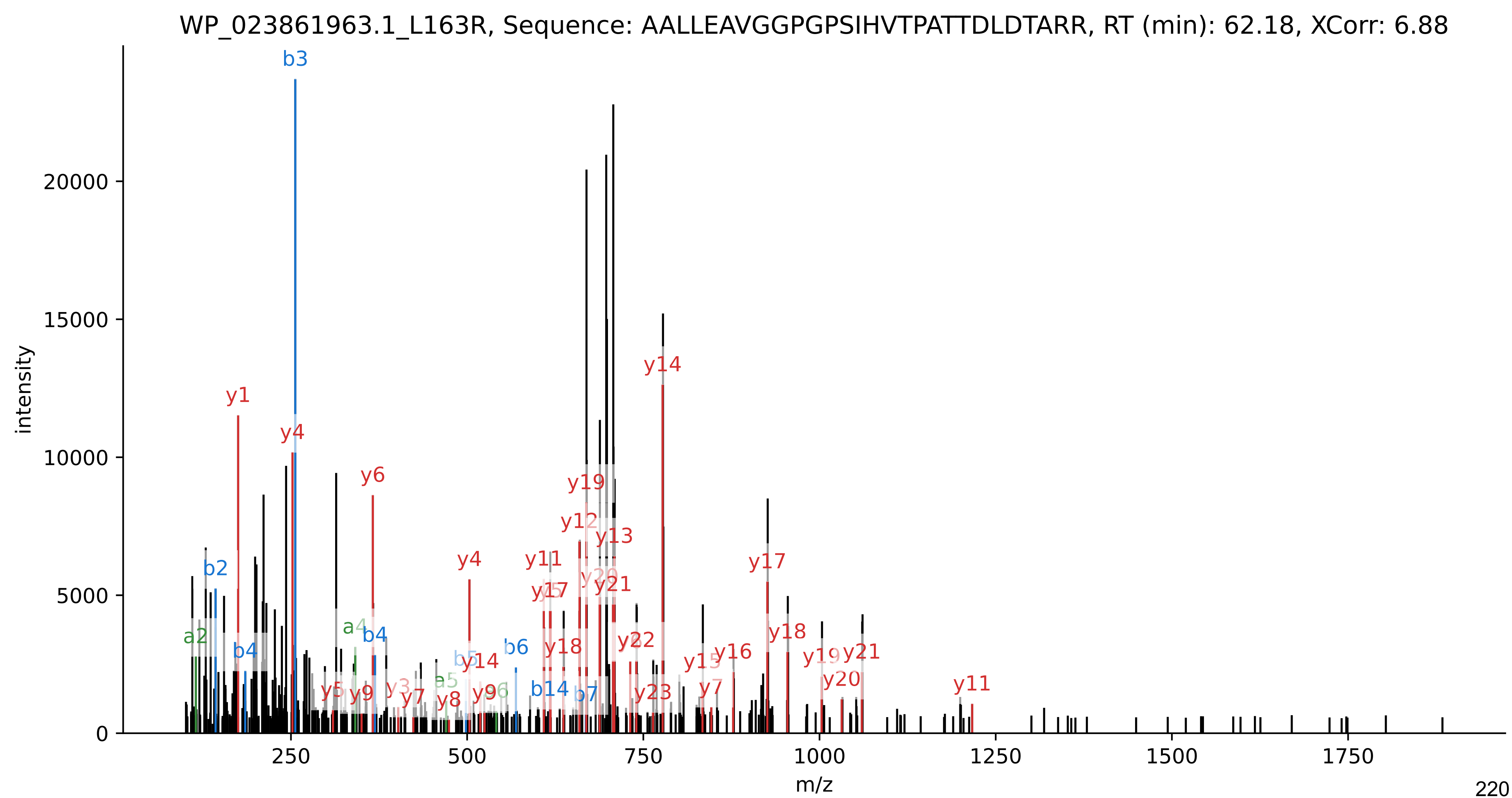
WP_023861963.1_L163R, Sequence: AALLEAVGGPGPSIHVTPATTDLDTARR, RT (min): 62.18, XCorr: 6.88
intensity
m/z
0
5000
10000
15000
20000
250
500
750
1000
1250
1500
1750
b3
y1
y4
y6
b2
a2
b4
a4
b4
y4
y14
b6
y11
y17
y18
y19
y12
y13
y21
y14
y22
y23
y17
y18
y16
y19
y21
y20
y11
b14
b7
y9
y7
y8
y9

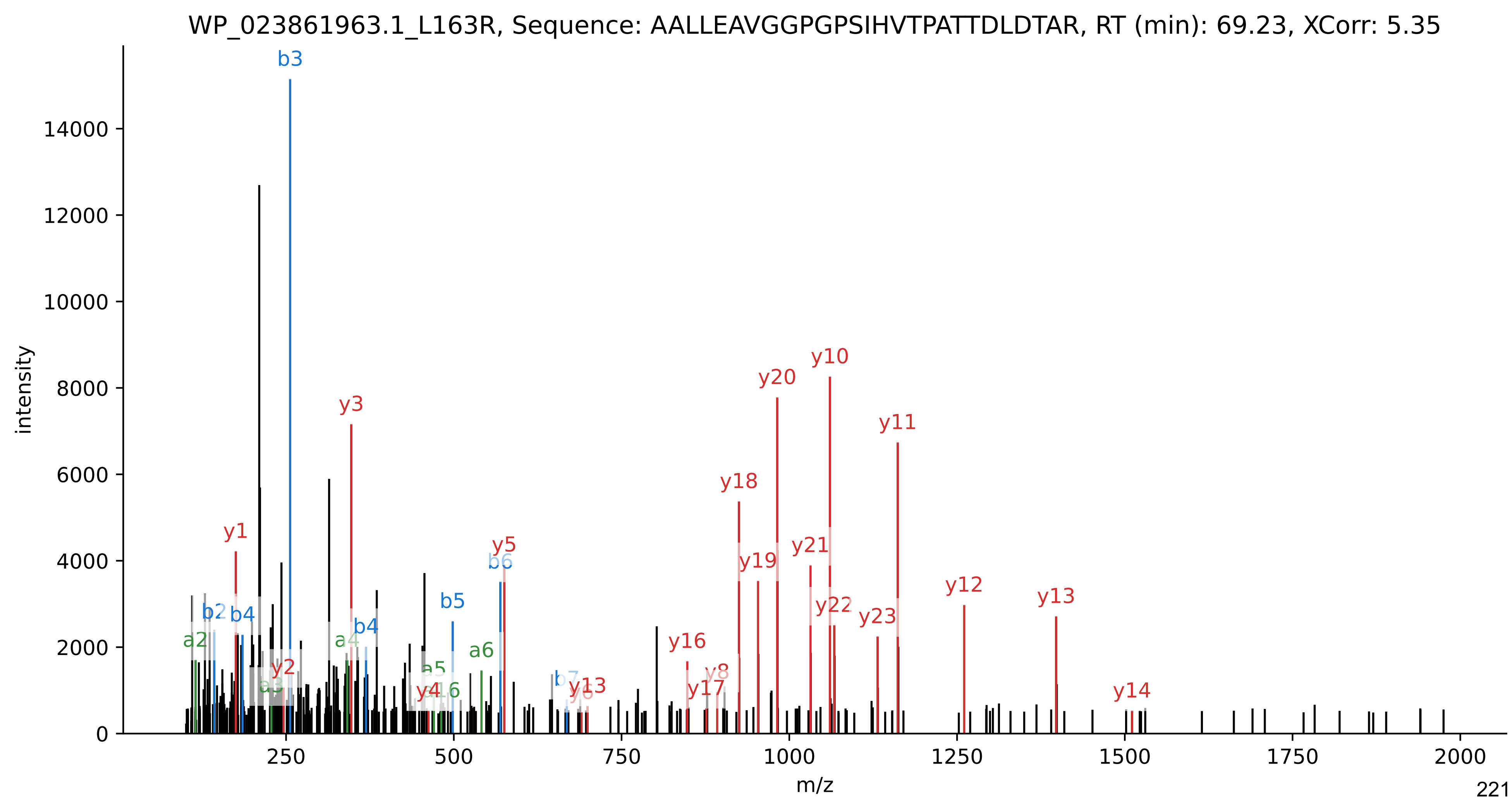

WP_023861963.1_L163R, Sequence: AALLEAVGGPGPSIHVTPATTDLDTAR, RT (min): 69.23, XCorr: 5.35
intensity
m/z
b3
y1
y3
y5
b6
b5
y10
y20
y11
y18
y19
y21
y22
y23
y12
y13
y14
y16
y17
a2
a4
b4
a6
y2
b7

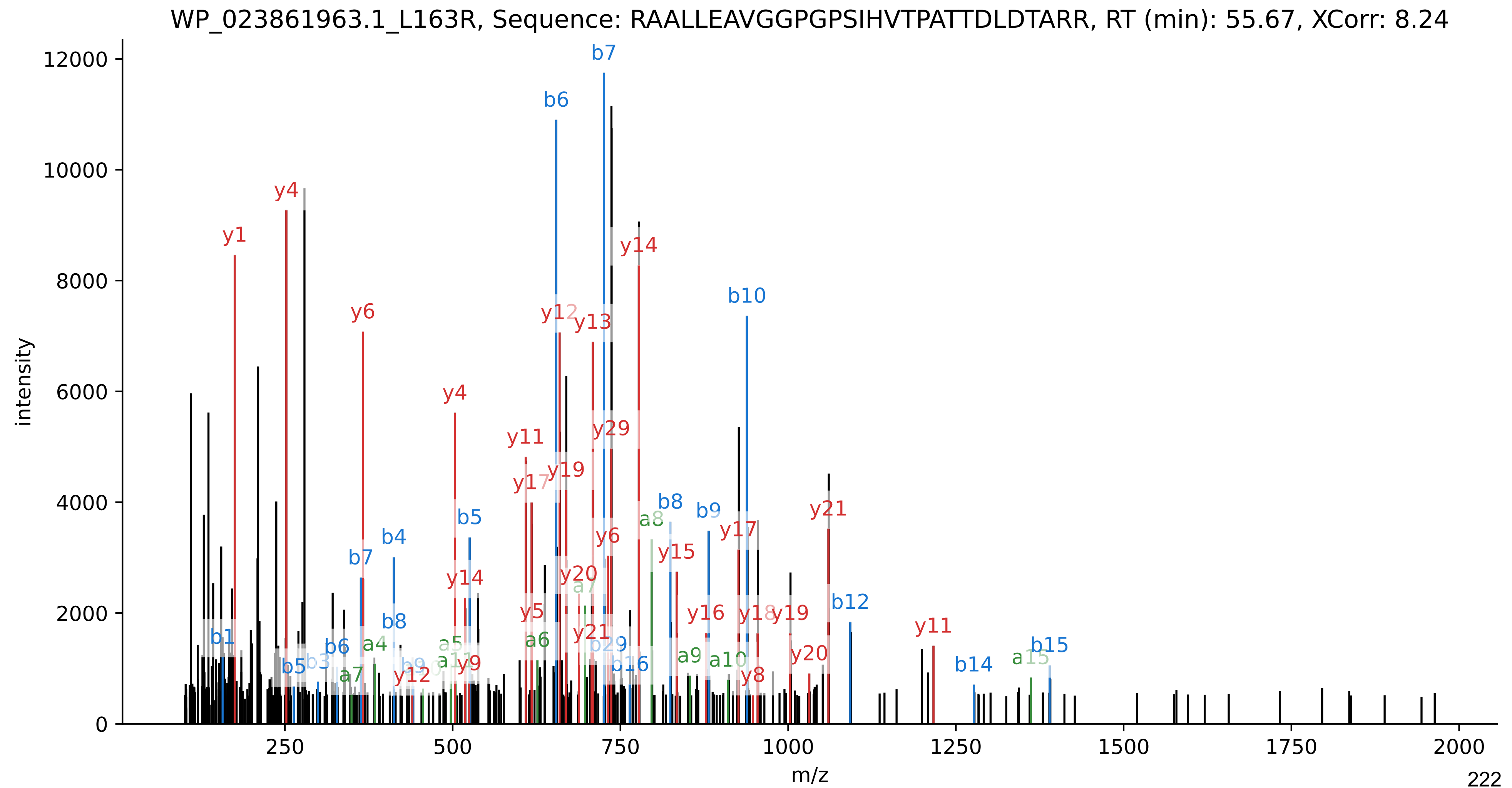

WP_023861963.1_L163R, Sequence: RAALLEAVGGPGPSIHVTPATTDLDTARR, RT (min): 55.67, XCorr: 8.24
intensity
m/z

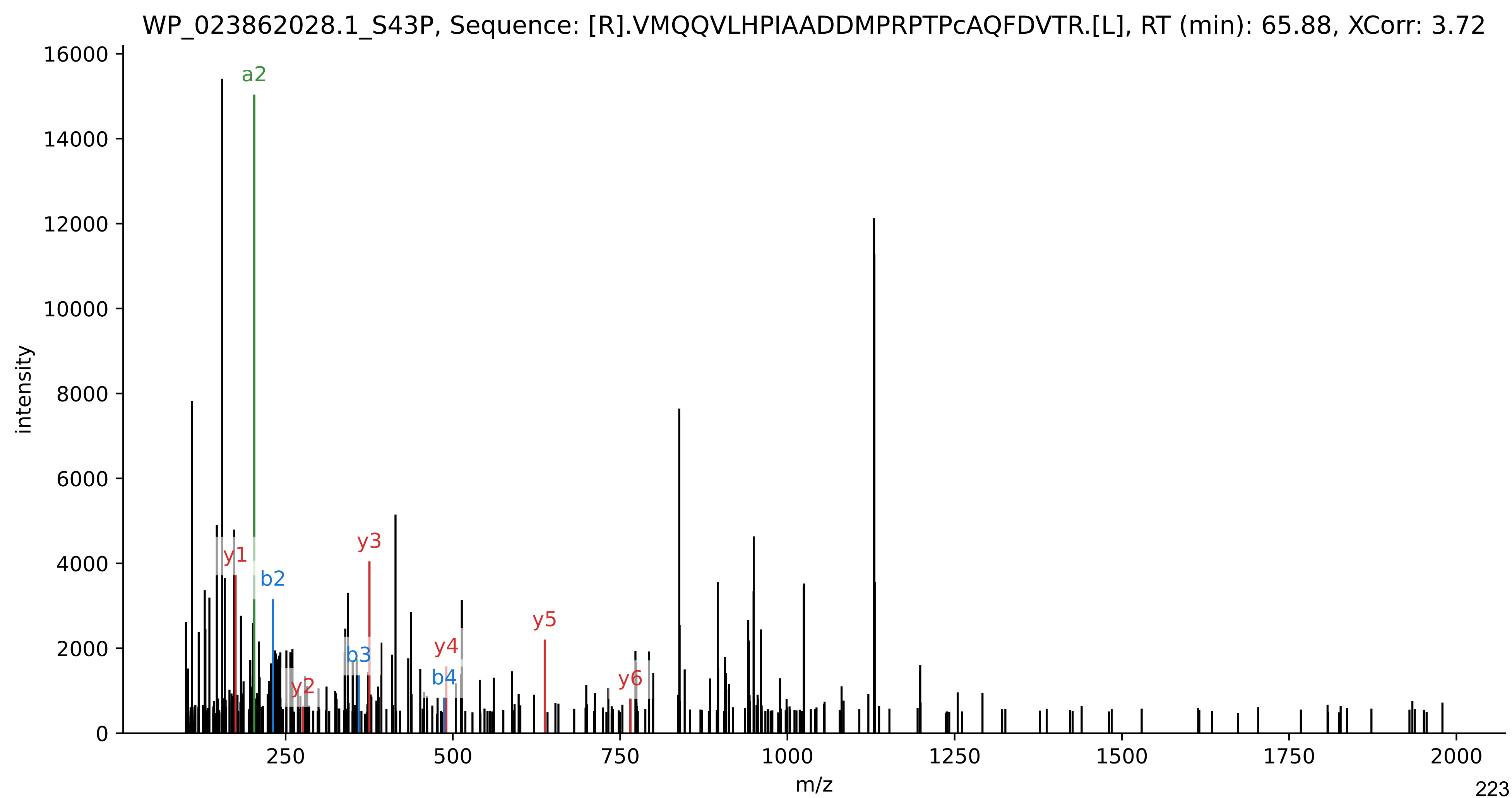

WP_023862028.1_S43P, Sequence: [R].VMQQVLHPIAADDMPRPTPcAQFDVTR.[L], RT (min): 65.88, XCorr: 3.72
intensity
m/z
a2
y1
b2
y3
y2
b3
y4
b4
y5
y6

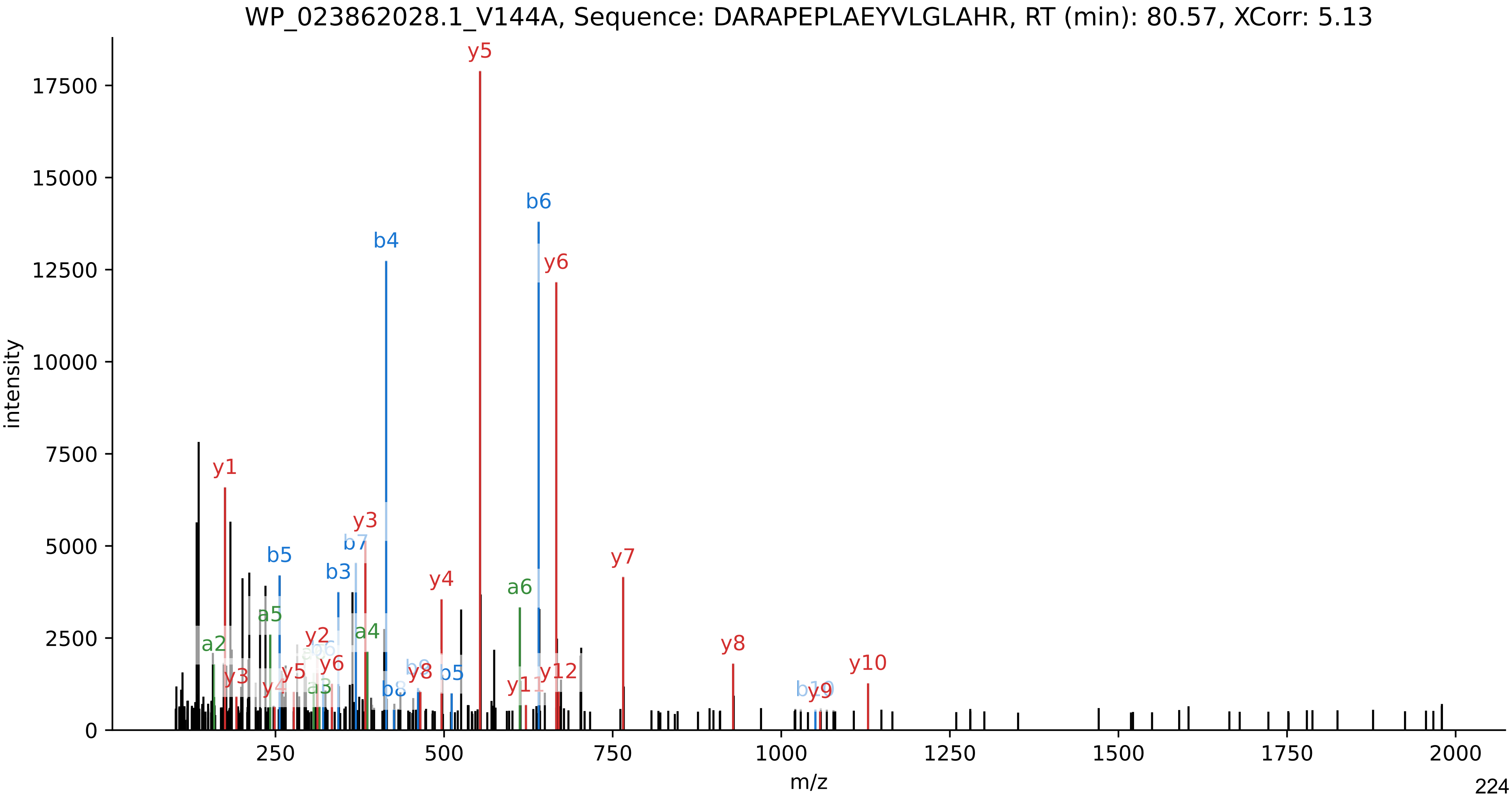

WP_023862028.1_V144A, Sequence: DARAPEPLAEYVLGLAHR, RT (min): 80.57, XCorr: 5.13
intensity
m/z
y5
b6
b4
y6
y1
y3
b7
b5
b3
y4
y7
a6
a5
a4
a2
y2
y6
y3
y5
y4
a3
y8
b5
b8
y11
y12
y8
y9
y10

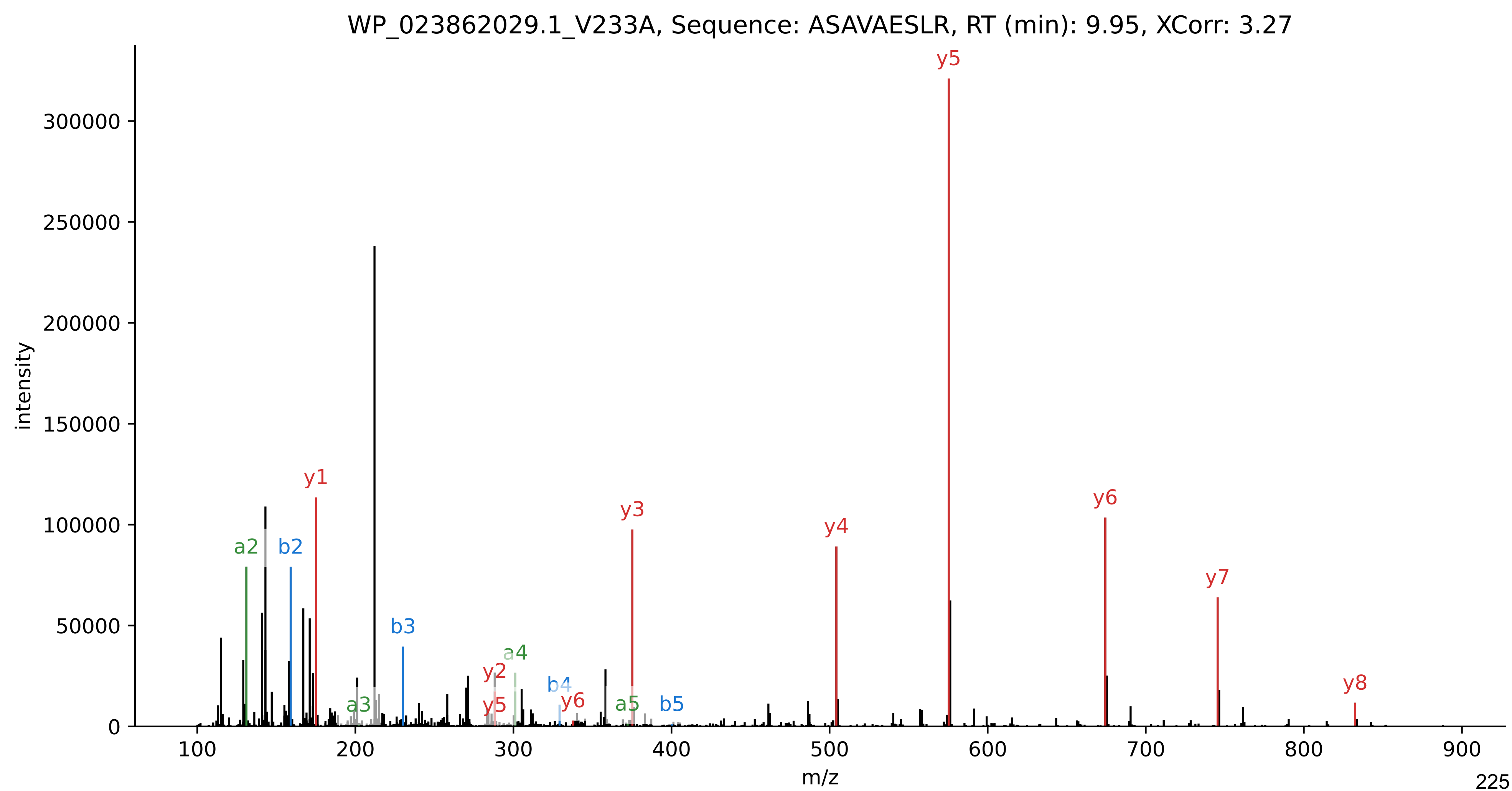

WP_023862029.1_V233A, Sequence: ASAVAESLR, RT (min): 9.95, XCorr: 3.27
intensity
m/z
y5
y1
y3
y4
y6
y7
y8
a2
b2
b3
a4
y2
b4
y6
a3
y5
a5
b5

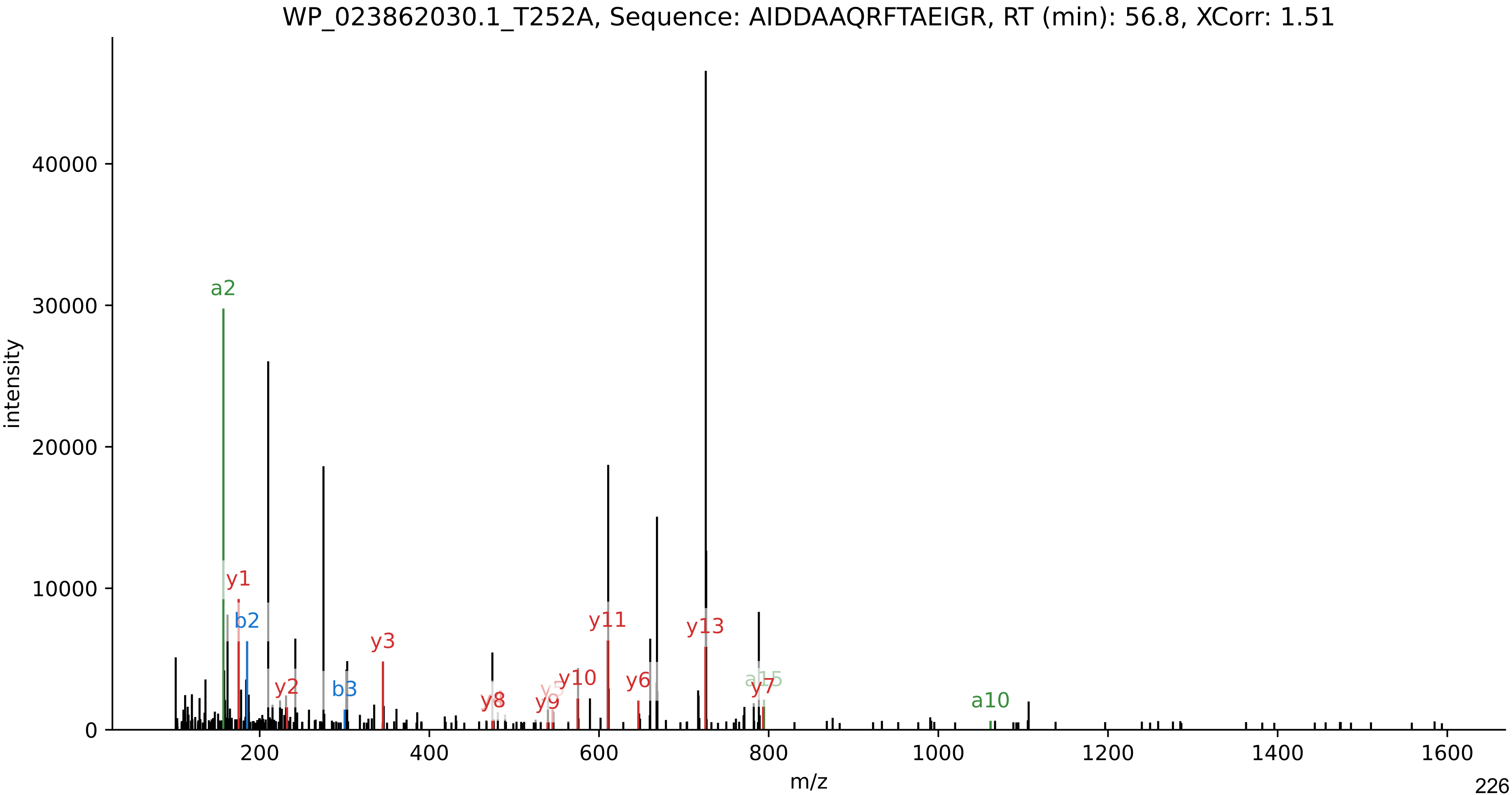
WP_023862030.1_T252A, Sequence: AIDDAAQRFTAEIGR, RT (min): 56.8, XCorr: 1.51
intensity
m/z
a2
y1
b2
y2
b3
y3
y8
y9
y5
y10
y11
y6
y13
a15
y7
a10
0
10000
20000
30000
40000
200
400
600
800
1000
1200
1400
1600

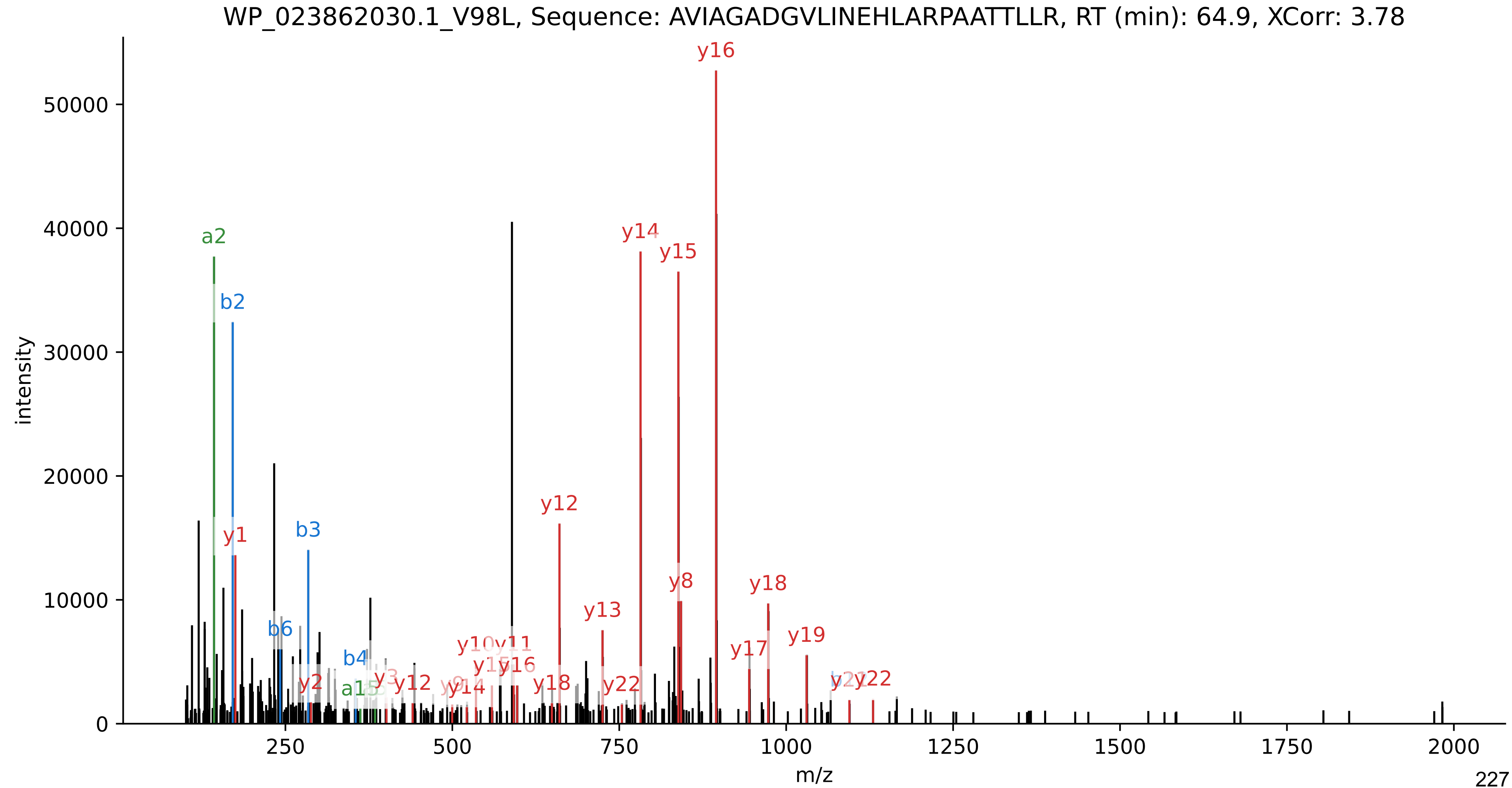

WP_023862030.1_V98L, Sequence: AVIAGADGVLINEHLARPAATTLLR, RT (min): 64.9, XCorr: 3.78
intensity
m/z

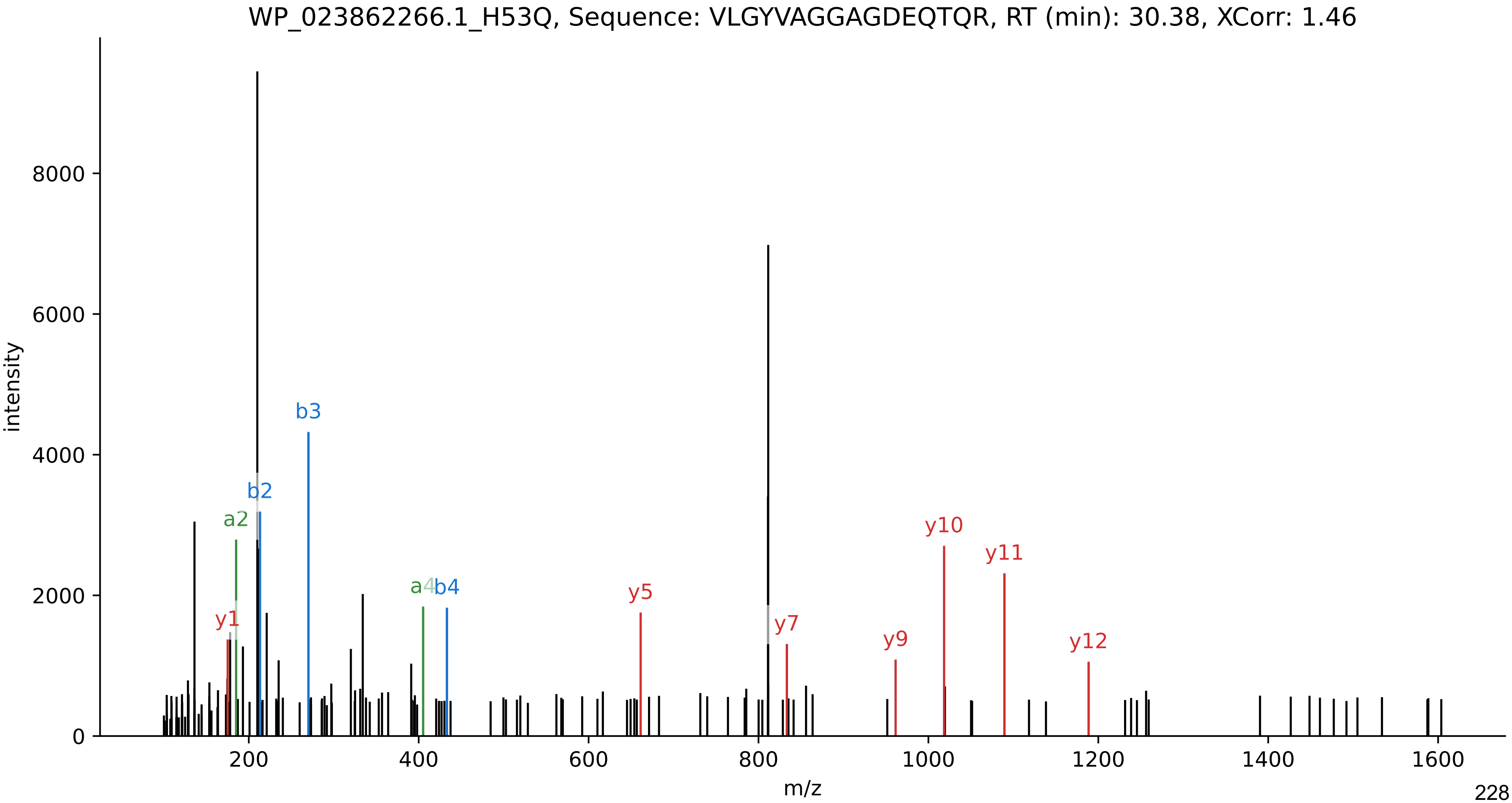
WP_023862266.1_H53Q, Sequence: VLGYVAGGAGDEQTQR, RT (min): 30.38, XCorr: 1.46
intensity
m/z
0
2000
4000
6000
8000
200
400
600
800
1000
1200
1400
1600
y1
a2
b2
b3
a4
b4
y5
y7
y9
y10
y11
y12

WP_023863794.1_A276V, Sequence: RPNTDDLDLAVAGVR, RT (min): 44.83, XCorr: 4.37

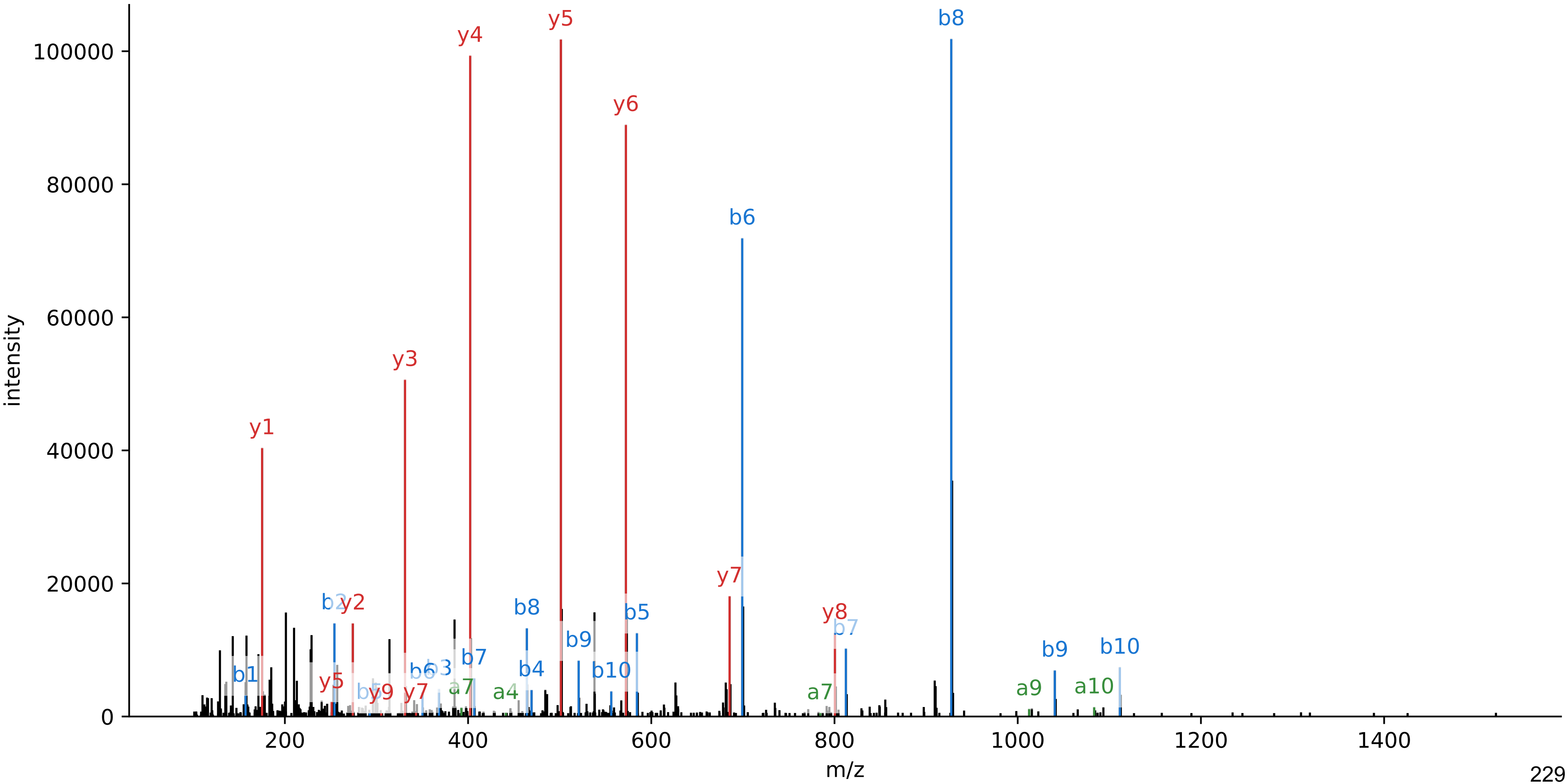

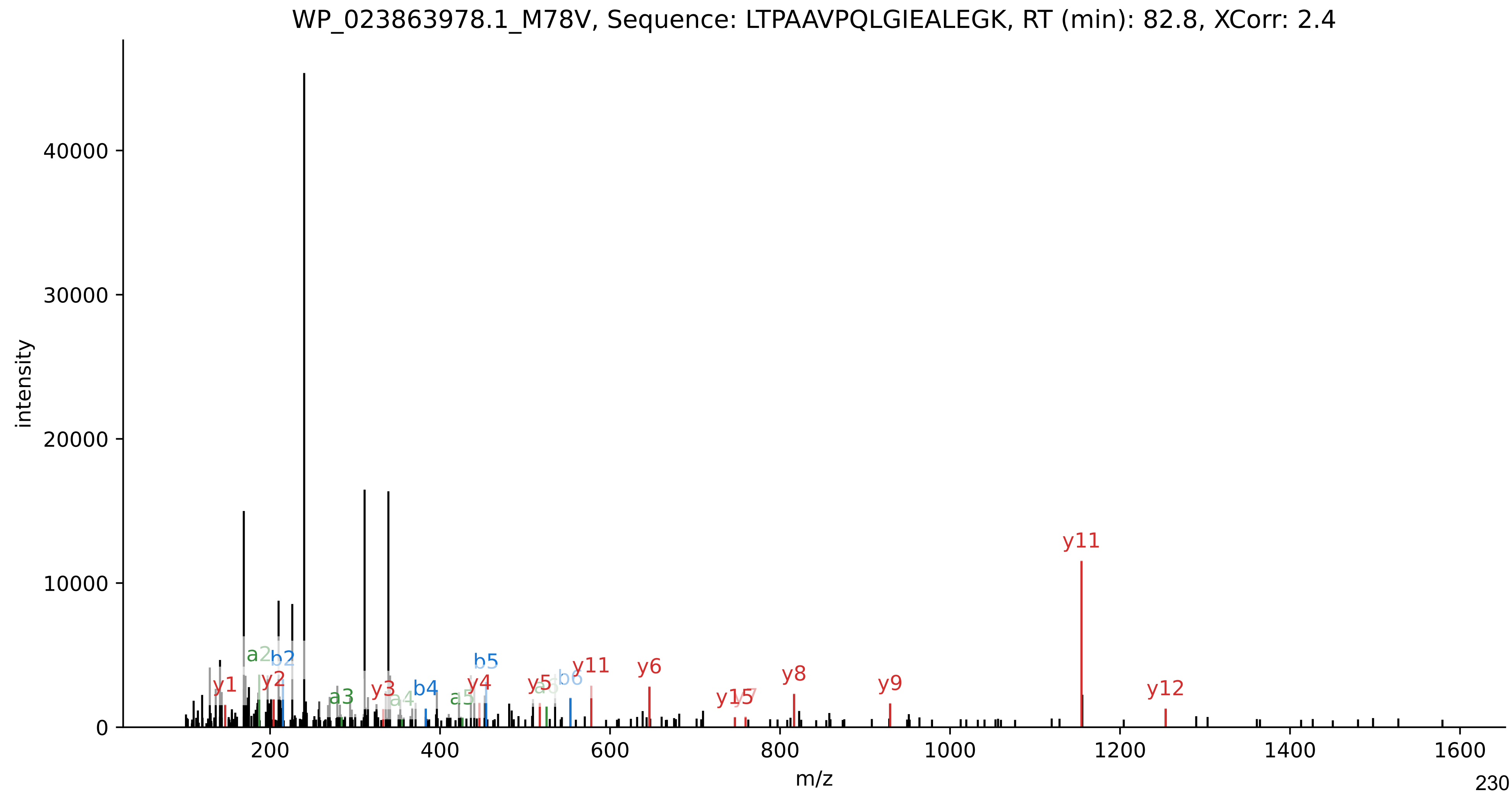
WP_023863978.1_M78V, Sequence: LTPAAVPQLGIEALEGK, RT (min): 82.8, XCorr: 2.4
intensity
m/z
0
10000
20000
30000
40000
200
400
600
800
1000
1200
1400
1600
y1
a2
b2
y2
a3
y3
a4
b4
a5
y4
b5
y5
b6
y11
y6
y15
y8
y9
y11
y12

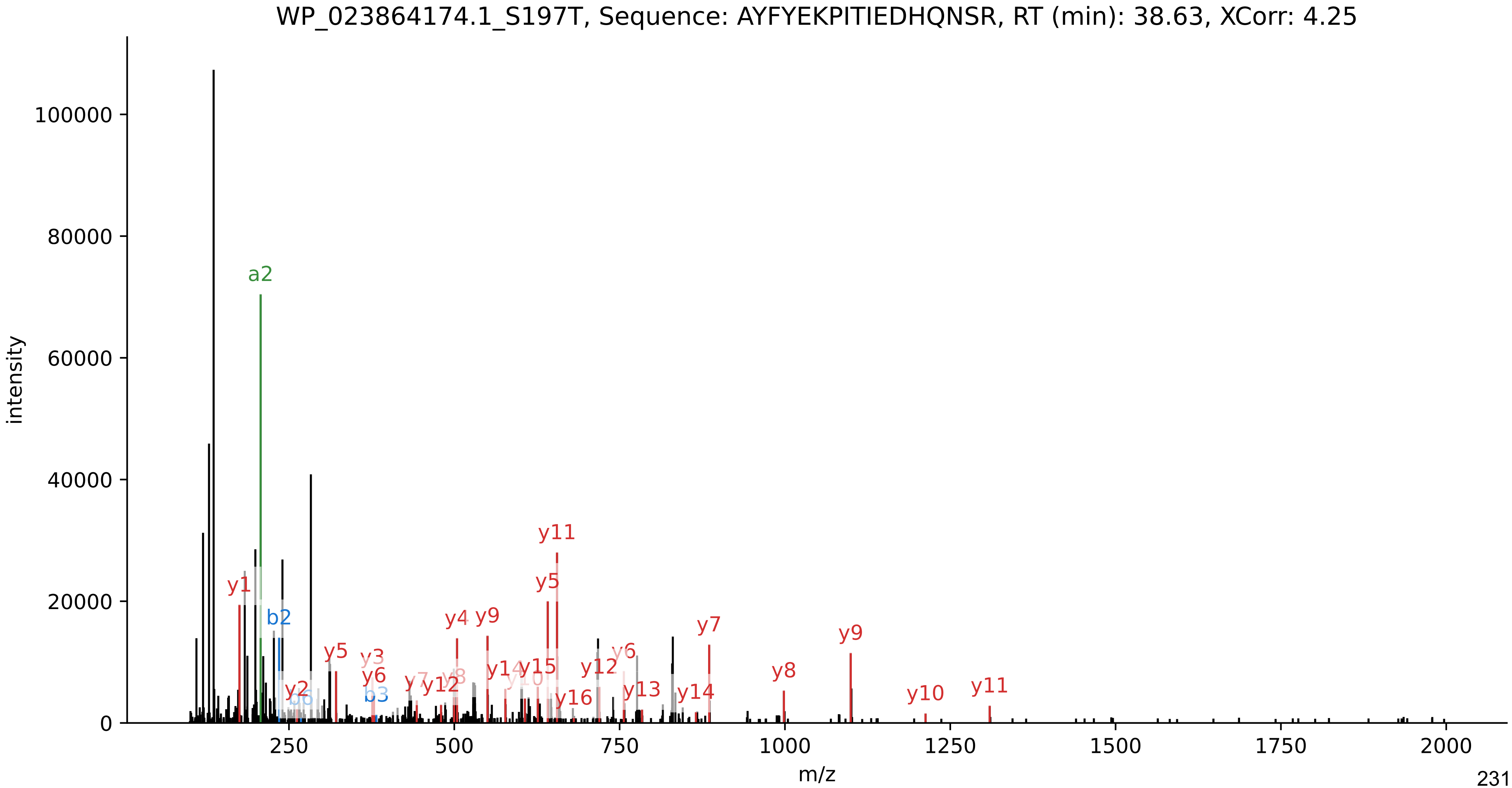
WP_023864174.1_S197T, Sequence: AYFYEKPITIEDHQNSR, RT (min): 38.63, XCorr: 4.25
intensity
m/z
0
20000
40000
60000
80000
100000
250
500
750
1000
1250
1500
1750
2000
a2
y1
b2
y5
y3
y6
b3
y2
y7
y12
y8
y4
y9
y14
y10
y15
y5
y11
y16
y12
y6
y13
y14
y7
y8
y9
y10
y11

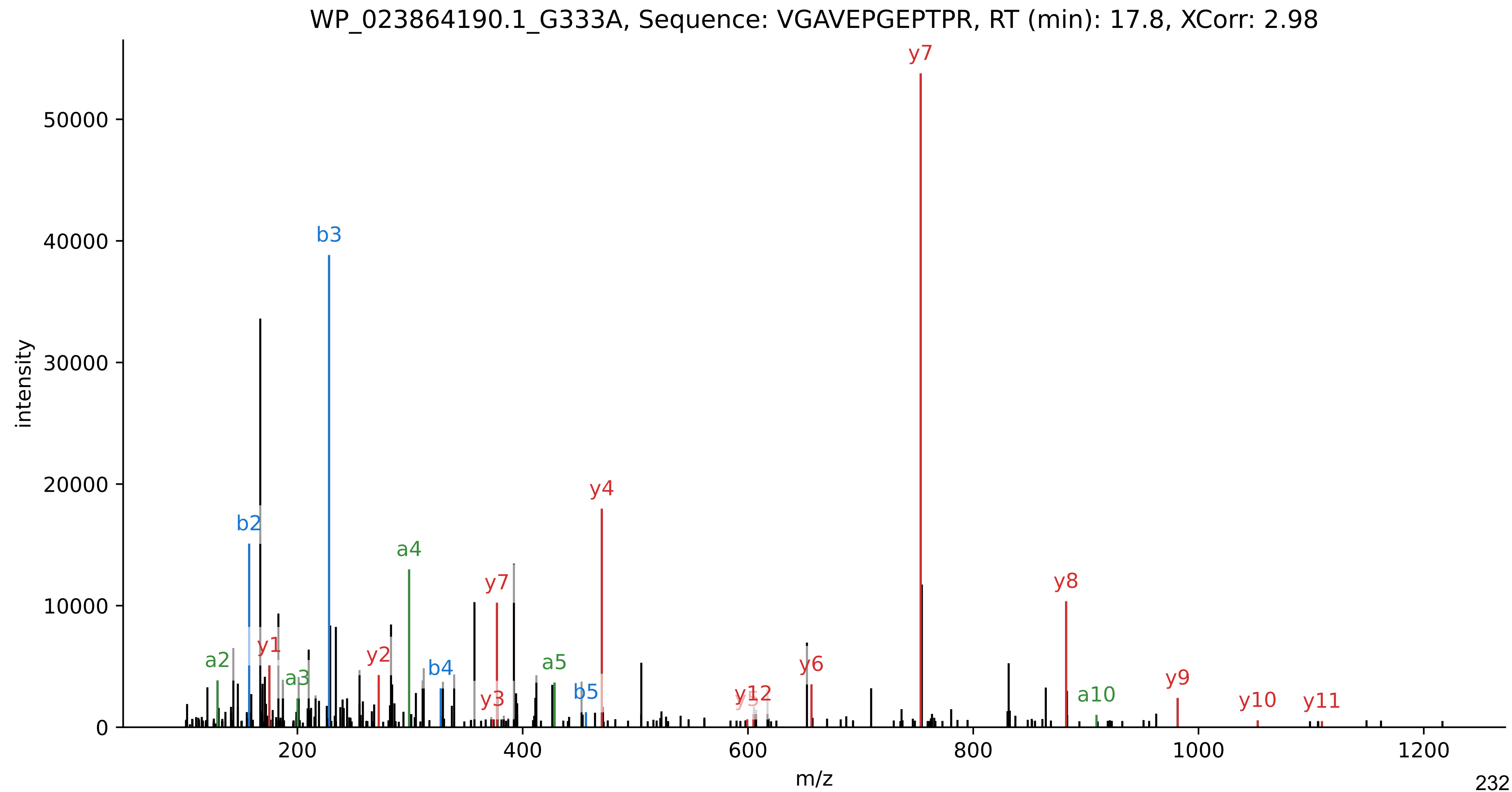

WP_023864190.1_G333A, Sequence: VGAVEPGEPTPR, RT (min): 17.8, XCorr: 2.98
intensity
m/z
y7
b3
y4
b2
a4
y7
y8
a2
y1
y2
b4
a3
a5
b5
y3
y12
y5
y6
a10
y9
y10
y11
0
10000
20000
30000
40000
50000
200
400
600
800
1000
1200

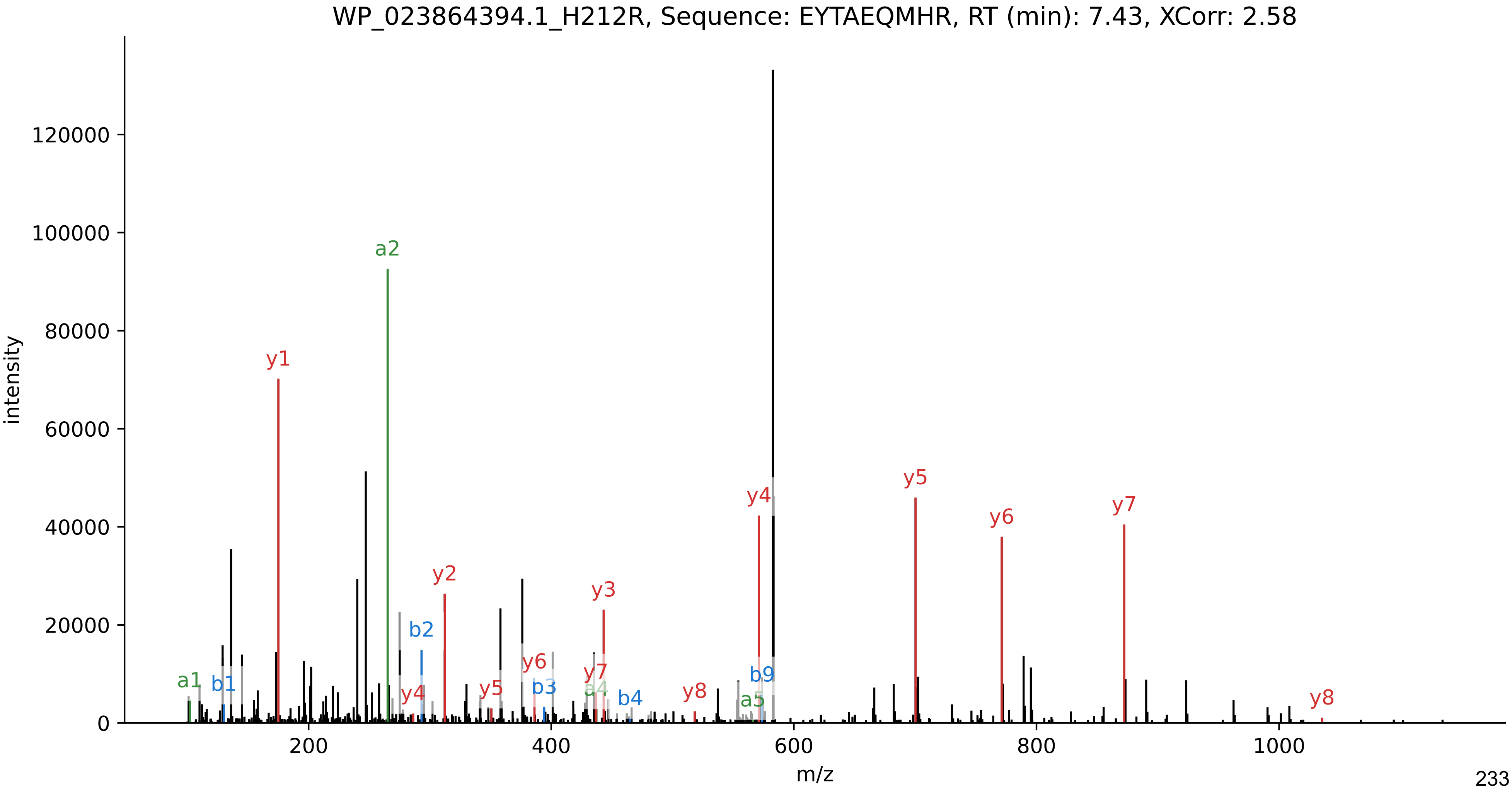

WP_023864394.1_H212R, Sequence: EYTAEQMHR, RT (min): 7.43, XCorr: 2.58
intensity
m/z
0
20000
40000
60000
80000
100000
120000
200
400
600
800
1000
a1
b1
y1
a2
y4
b2
y2
y5
y6
b3
y7
a4
y3
b4
y8
a5
b9
y4
y5
y6
y7
y8

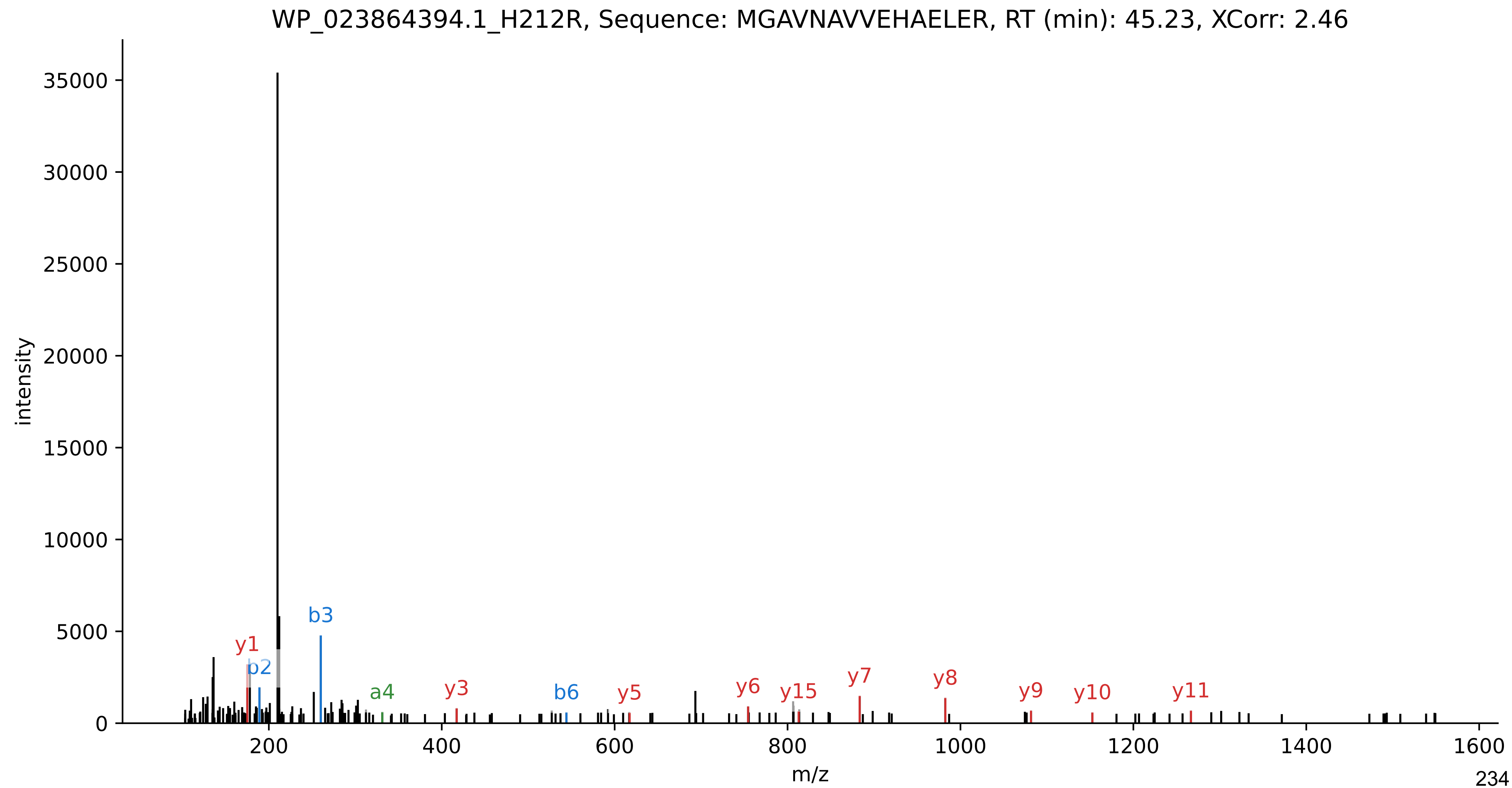
WP_023864394.1_H212R, Sequence: MGAVNAVVEHAELER, RT (min): 45.23, XCorr: 2.46
intensity
m/z
y1
b2
b3
a4
y3
b6
y5
y6
y15
y7
y8
y9
y10
y11

WP_023864936.1_D79G, Sequence: AGPGGAPETKGPMLINAR, RT (min): 30.42, XCorr: 4.3

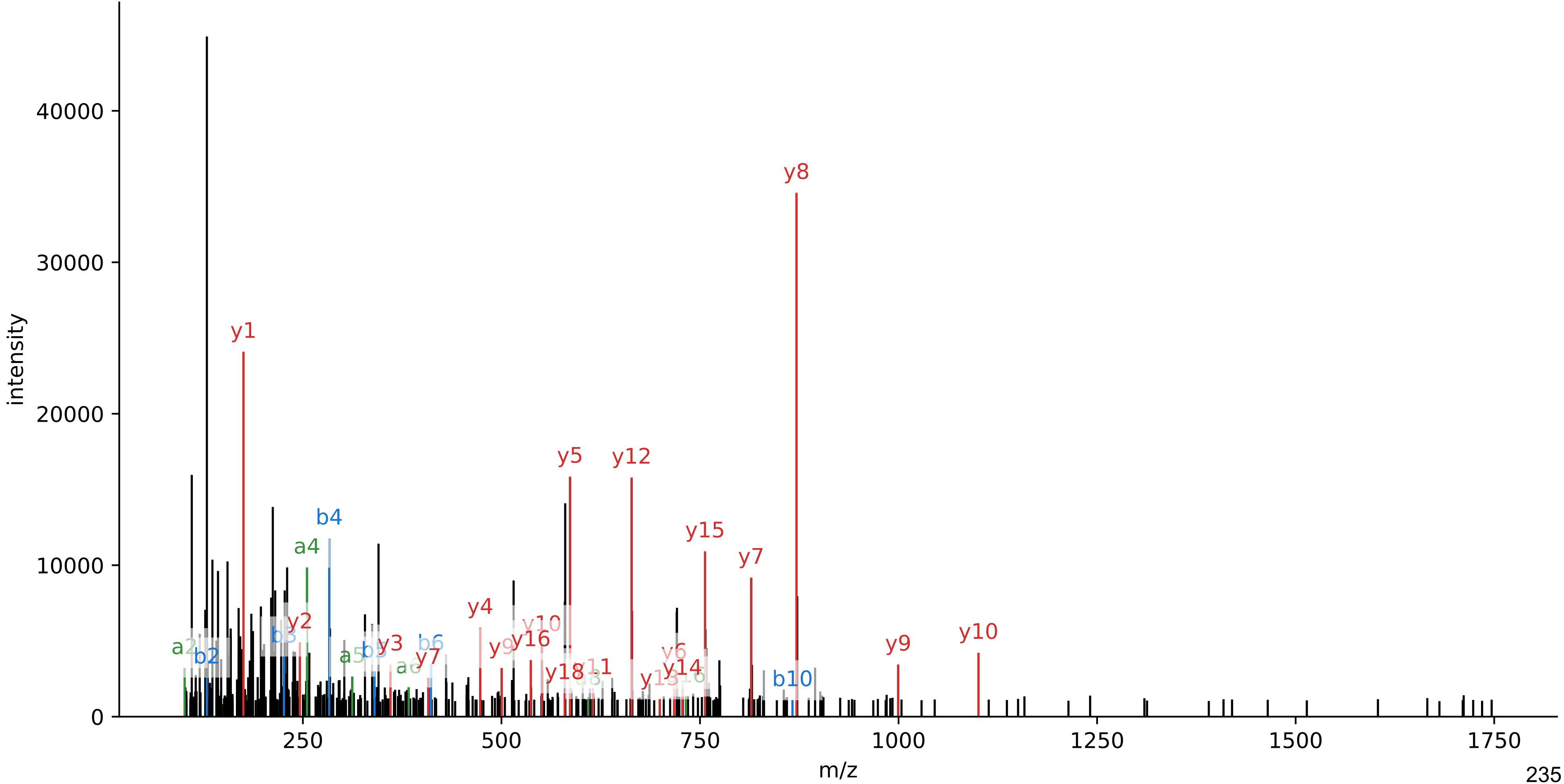

WP_023865474.1_V25A, Sequence: RVEAGPDGHDYEVRPIAAAR, RT (min): 23.58, XCorr: 5.34

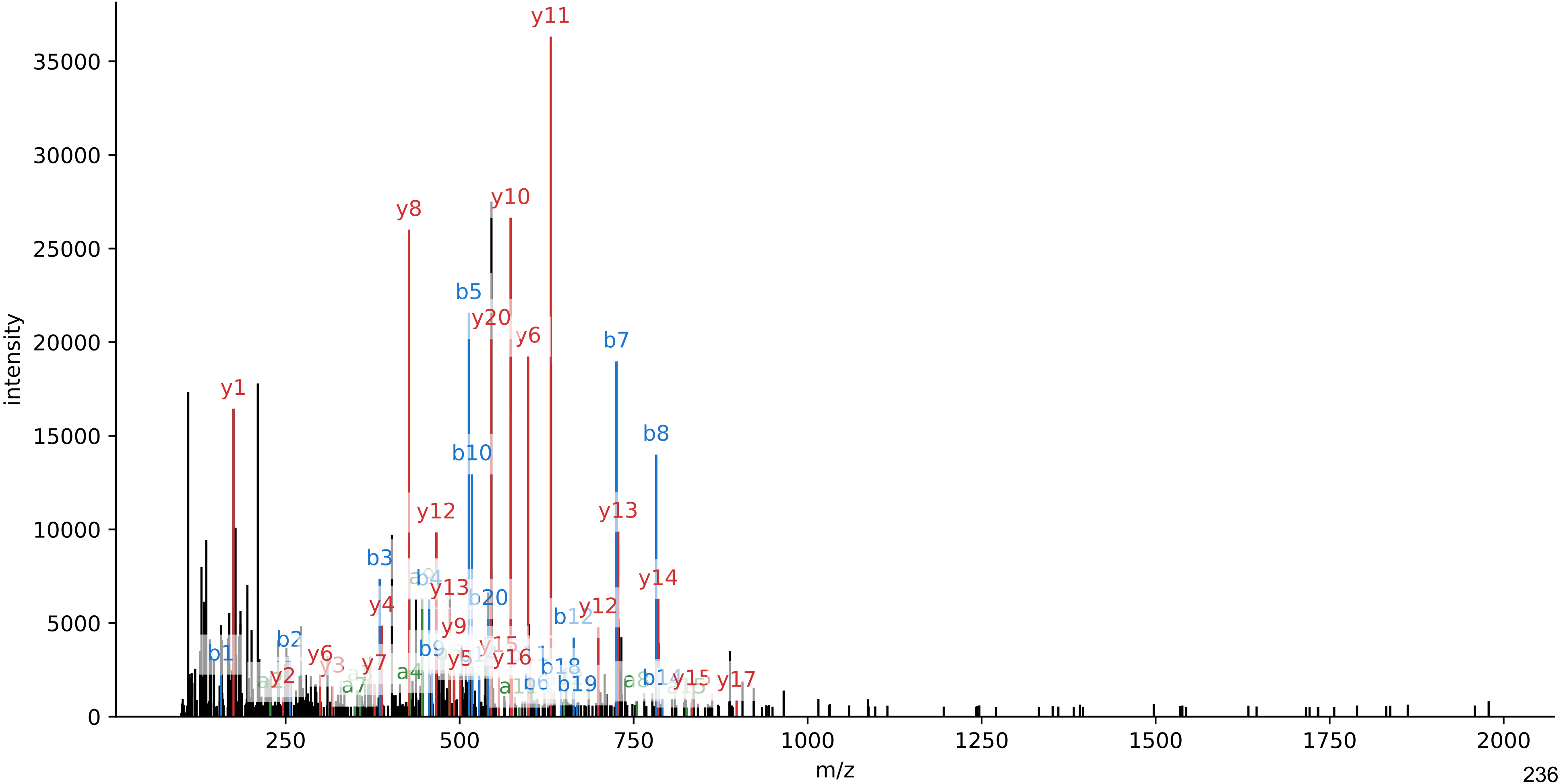

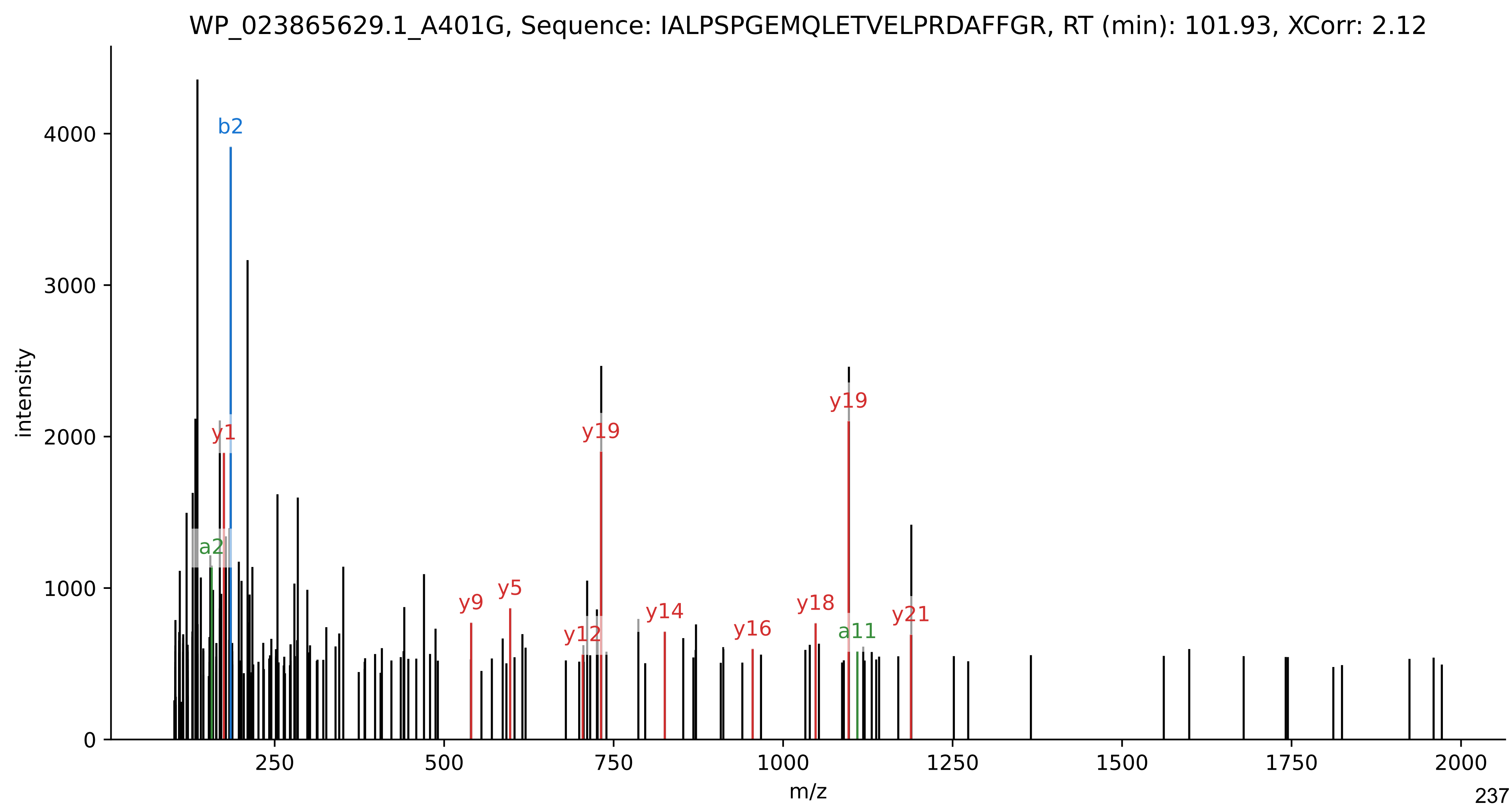

WP_023865629.1_A401G, Sequence: IALPSPGEMQLETVELPRDAFFGR, RT (min): 101.93, XCorr: 2.12
intensity
m/z
b2
y1
a2
y19
y19
y9
y5
y12
y14
y16
y18
a11
y21
0
1000
2000
3000
4000
250
500
750
1000
1250
1500
1750
2000

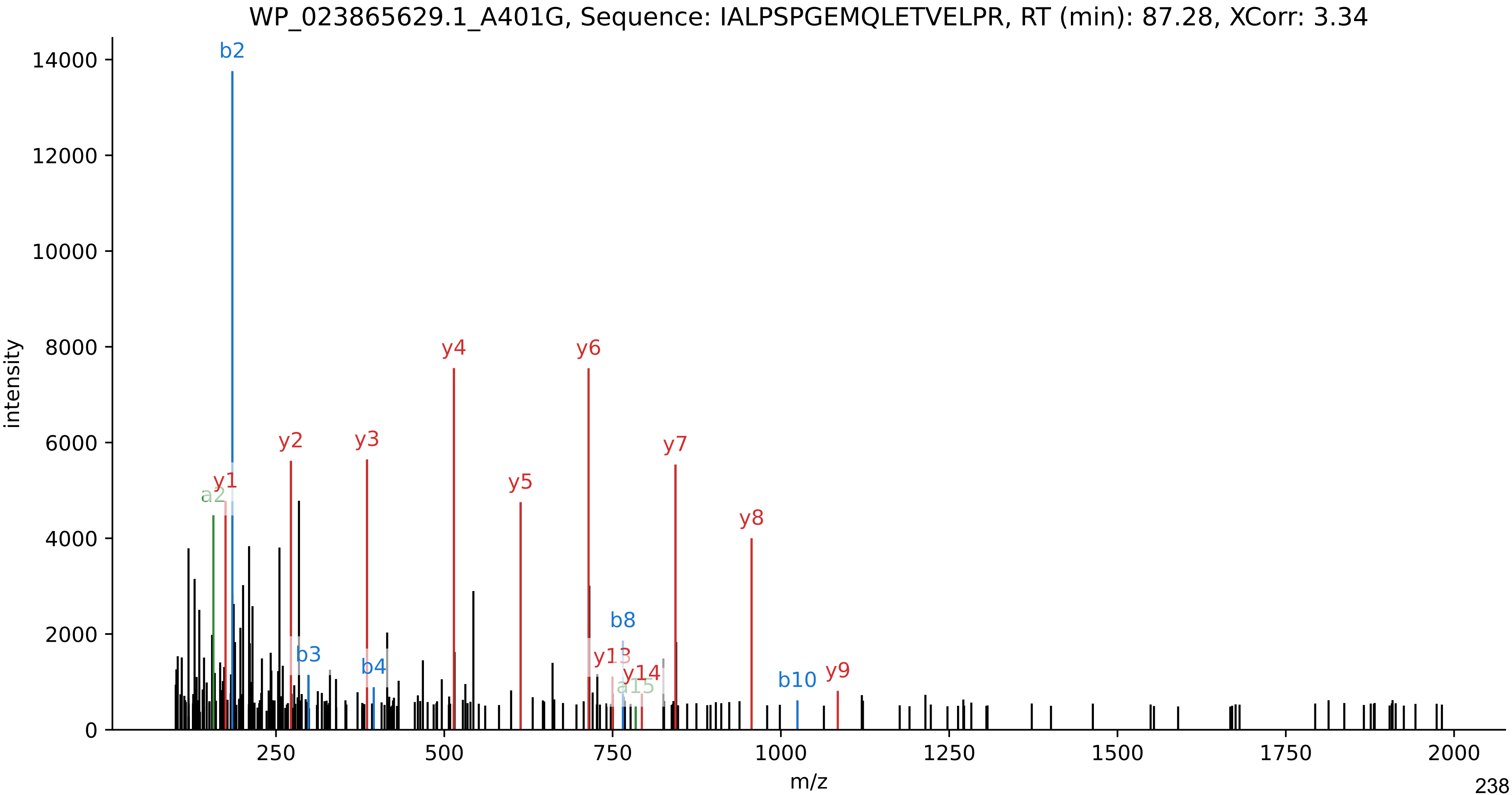

WP_023865629.1_A401G, Sequence: IALPSPGEMQLETVELPR, RT (min): 87.28, XCorr: 3.34
intensity
m/z
b2
a2
y1
y2
y3
y4
y5
y6
y7
y8
y9
b3
b4
b8
b10
y13
y14
a15

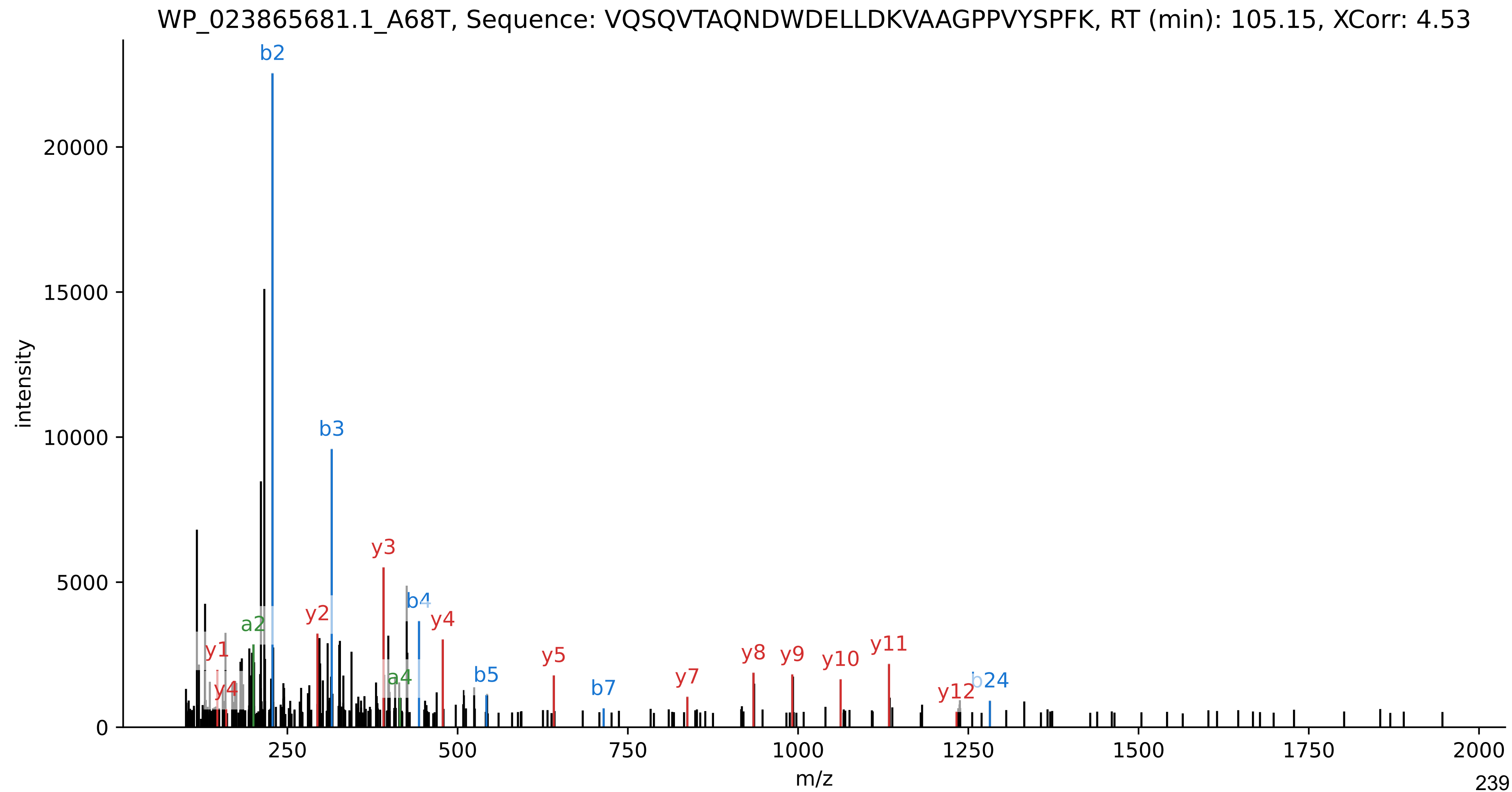

WP_023865681.1_A68T, Sequence: VQSQVTAQNDWDELLDKVAAGPPVYSPFK, RT (min): 105.15, XCorr: 4.53
intensity
m/z
0
5000
10000
15000
20000
250
500
750
1000
1250
1500
1750
2000
b2
b3
b4
b5
b7
b24
a2
a4
y1
y2
y3
y4
y4
y5
y7
y8
y9
y10
y11
y12

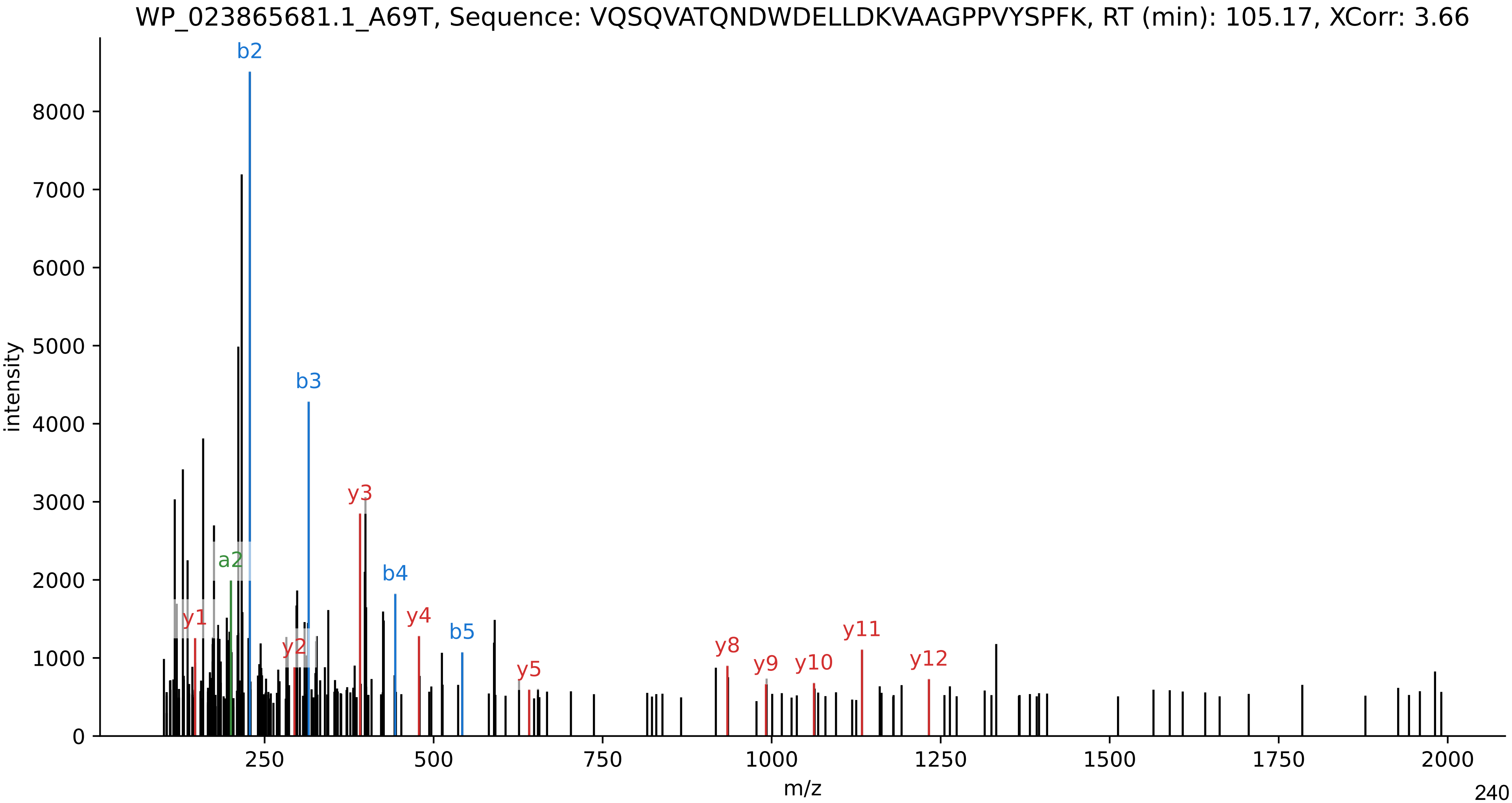
WP_023865681.1_A69T, Sequence: VQSQVATQNDWDELLDKVAAGPPVYSPFK, RT (min): 105.17, XCorr: 3.66
intensity
m/z
b2
b3
b4
b5
a2
y1
y2
y3
y4
y5
y8
y9
y10
y11
y12
0
1000
2000
3000
4000
5000
6000
7000
8000
250
500
750
1000
1250
1500
1750
2000

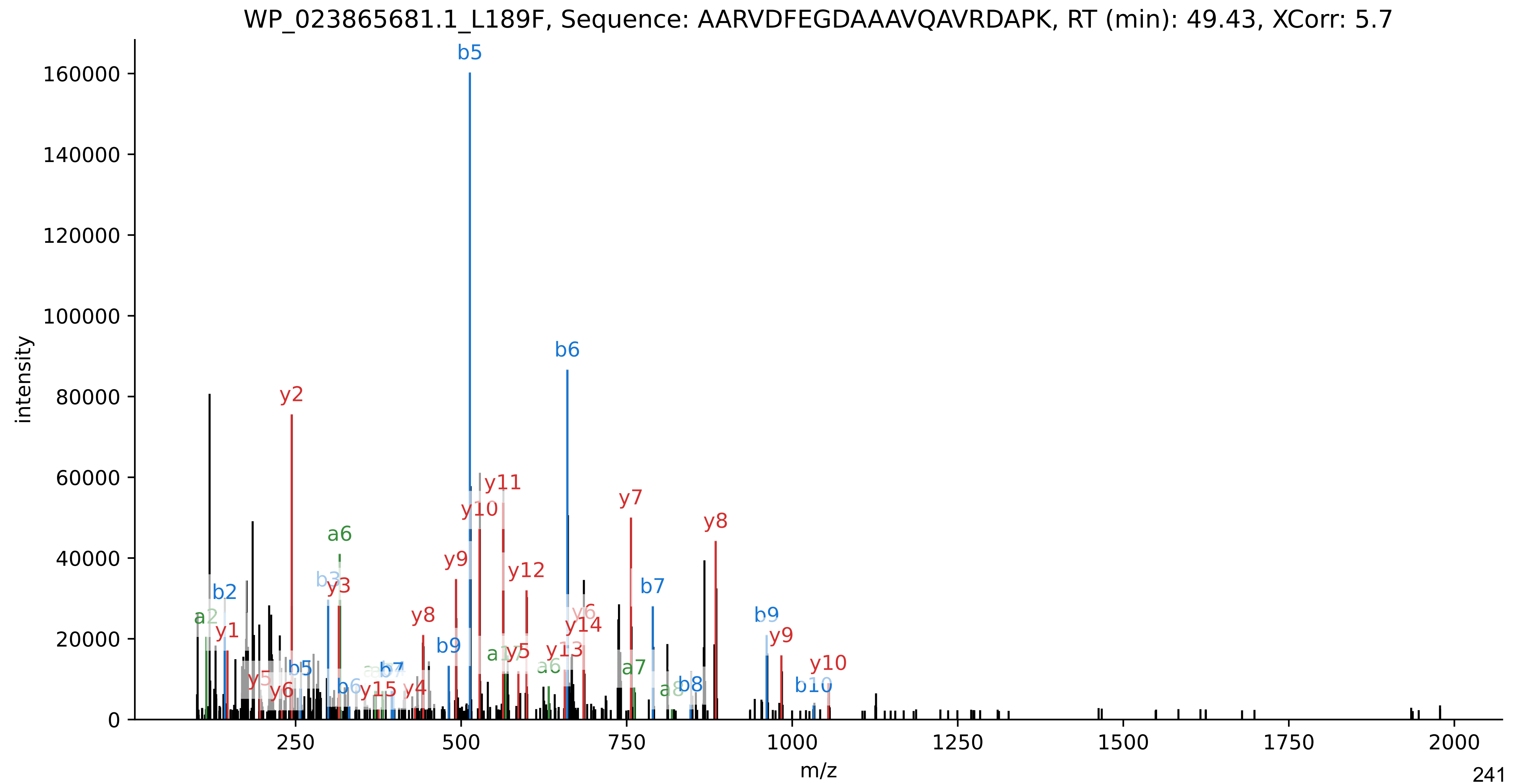
WP_023865681.1_L189F, Sequence: AARVDFEGDAAAVQAVRDAPK, RT (min): 49.43, XCorr: 5.7
intensity
m/z
b5
b6
y2
y11
y10
y7
y8
a6
y9
y12
b3
y3
b2
b7
a2
y1
y8
y6
y14
b9
y9
b9
y5
y13
y10
a6
a7
b5
b7
y5
y6
b6
y15
y4
b8
a8
b10
0
20000
40000
60000
80000
100000
120000
140000
160000
250
500
750
1000
1250
1500
1750
2000

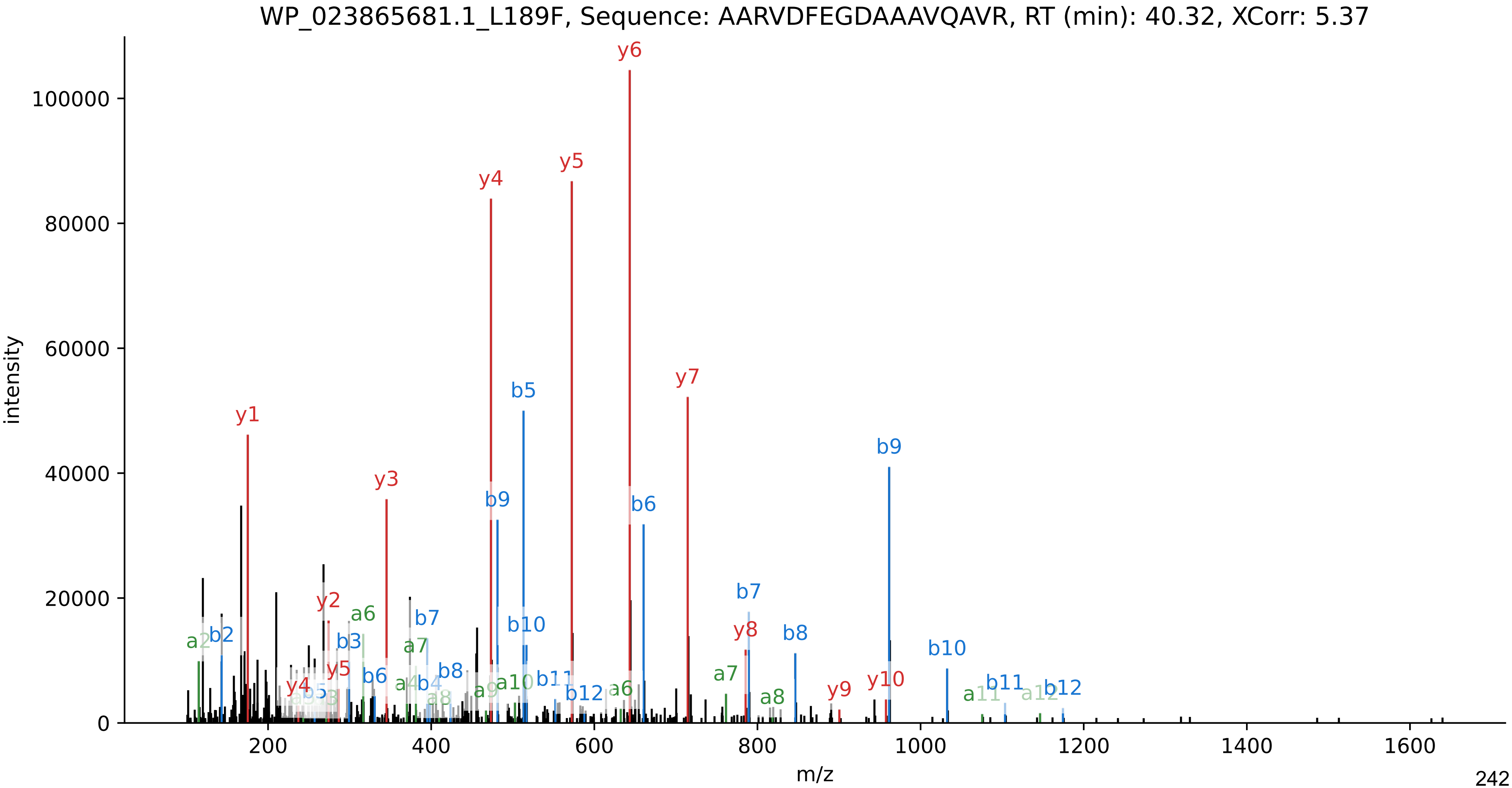

WP_023865681.1_L189F, Sequence: AARVDFEGDAAAVQAVR, RT (min): 40.32, XCorr: 5.37
intensity
m/z
0
20000
40000
60000
80000
100000
200
400
600
800
1000
1200
1400
1600
y1
y2
y3
y4
y5
y6
y7
y8
y9
y10
b2
b3
b5
b6
b7
b8
b9
b10
b11
b12
a2
a6
a7
a8
a11
a12

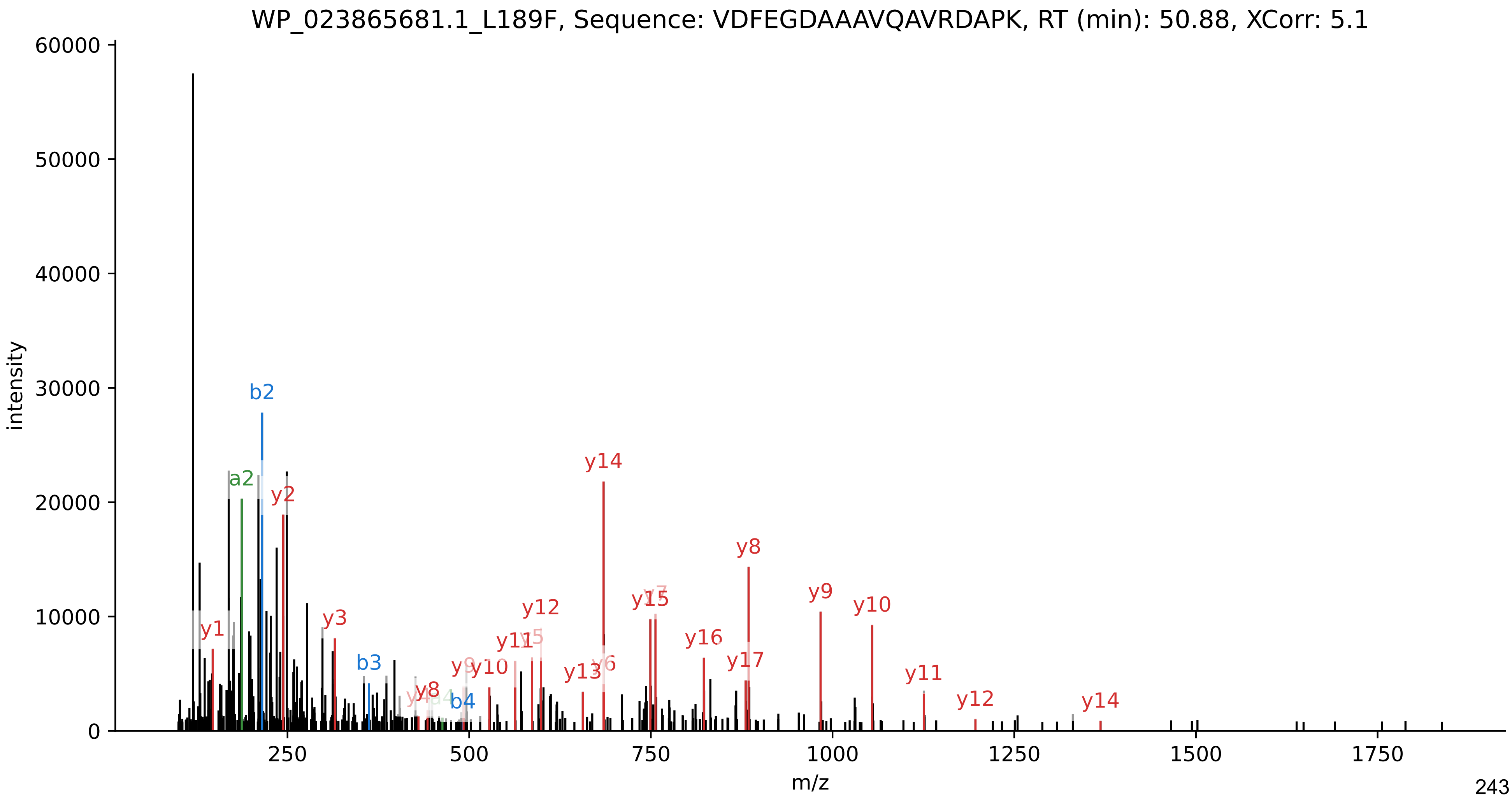
WP_023865681.1_L189F, Sequence: VDFEGDAAAVQAVRDAPK, RT (min): 50.88, XCorr: 5.1
intensity
m/z
b2
a2
y2
y1
y3
b3
b4
y8
y9
y10
y11
y12
y13
y14
y15
y16
y17
y8
y9
y10
y11
y12
y14

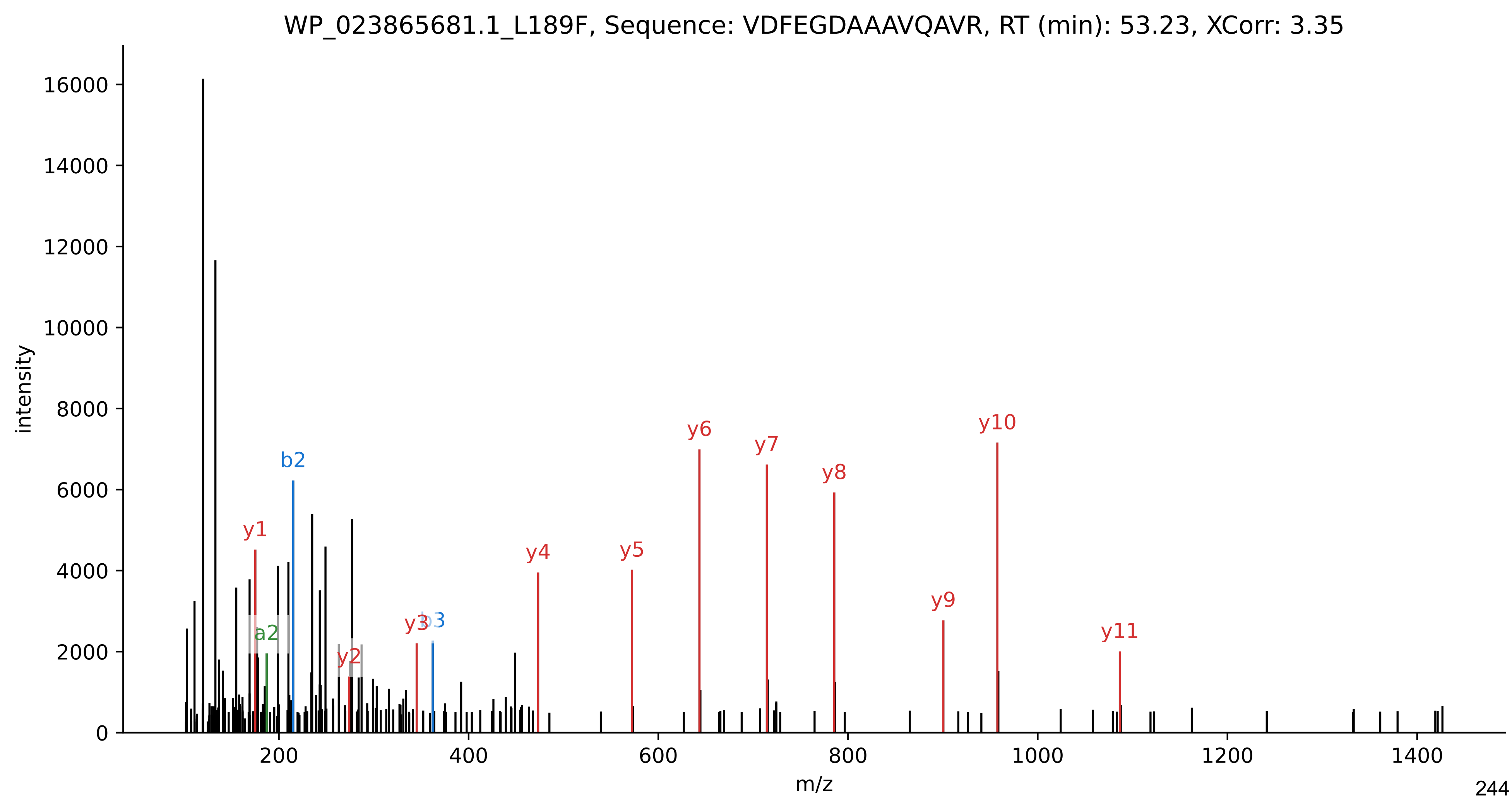

WP_023865681.1_L189F, Sequence: VDFEGDAAAVQAVR, RT (min): 53.23, XCorr: 3.35
intensity
m/z
0
2000
4000
6000
8000
10000
12000
14000
16000
200
400
600
800
1000
1200
1400
y1
a2
b2
y2
y3
b3
y4
y5
y6
y7
y8
y9
y10
y11

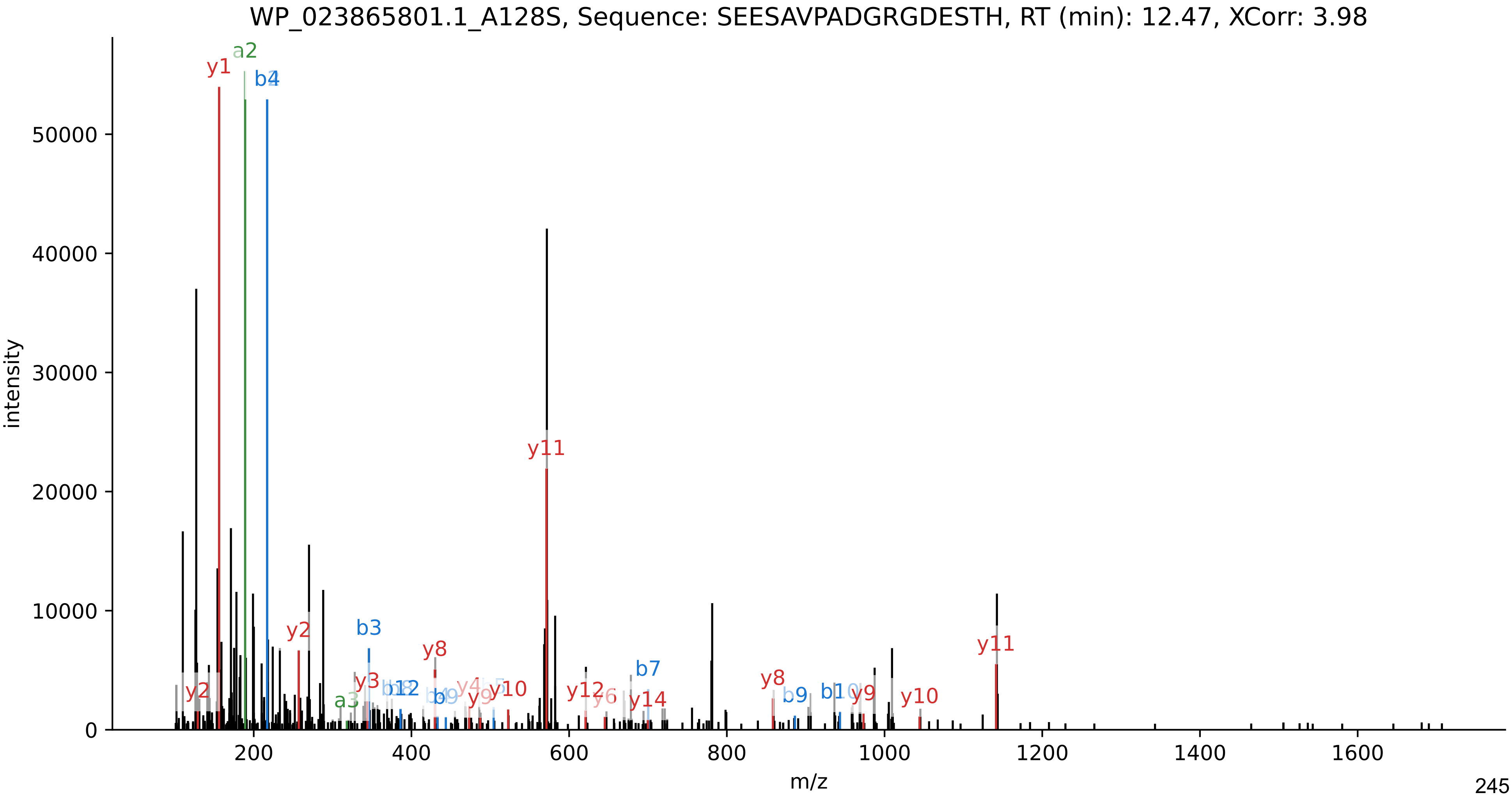
WP_023865801.1_A128S, Sequence: SEESAVPADGRGDESTH, RT (min): 12.47, XCorr: 3.98
intensity
m/z
y1
a2
b4
y11
y2
b3
y8
y3
a3
b12
y4
y9
y10
y12
y6
y14
b7
b9
b10

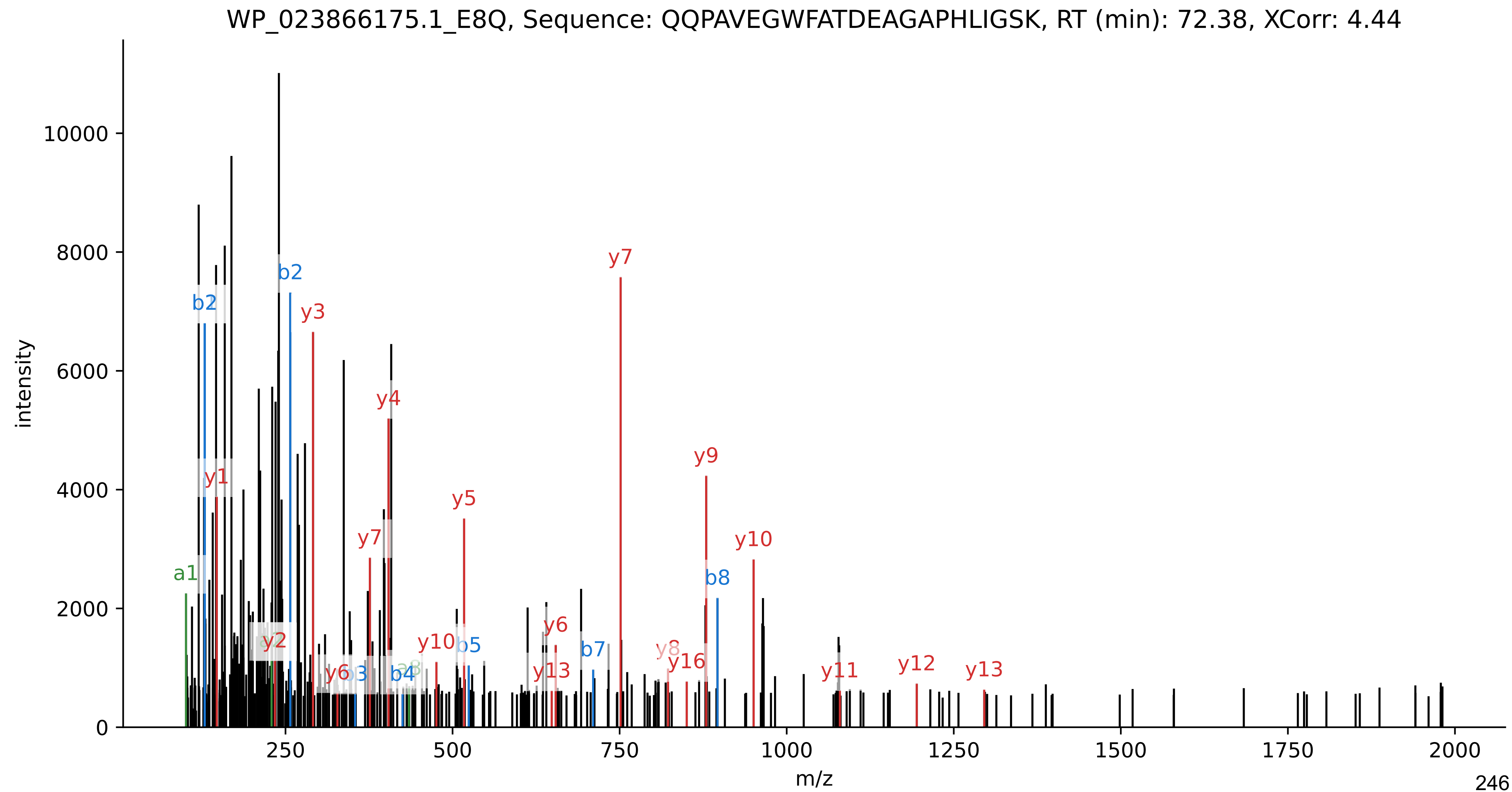

WP_023866175.1_E8Q, Sequence: QQPAVEGWFATDEAGAPHLIGSK, RT (min): 72.38, XCorr: 4.44
intensity
m/z
0
2000
4000
6000
8000
10000
250
500
750
1000
1250
1500
1750
2000
a1
b2
b2
y1
y2
y3
b3
y6
b4
y7
y4
y10
b5
y5
y13
y6
b7
y7
y8
y16
y9
b8
y10
y11
y12
y13

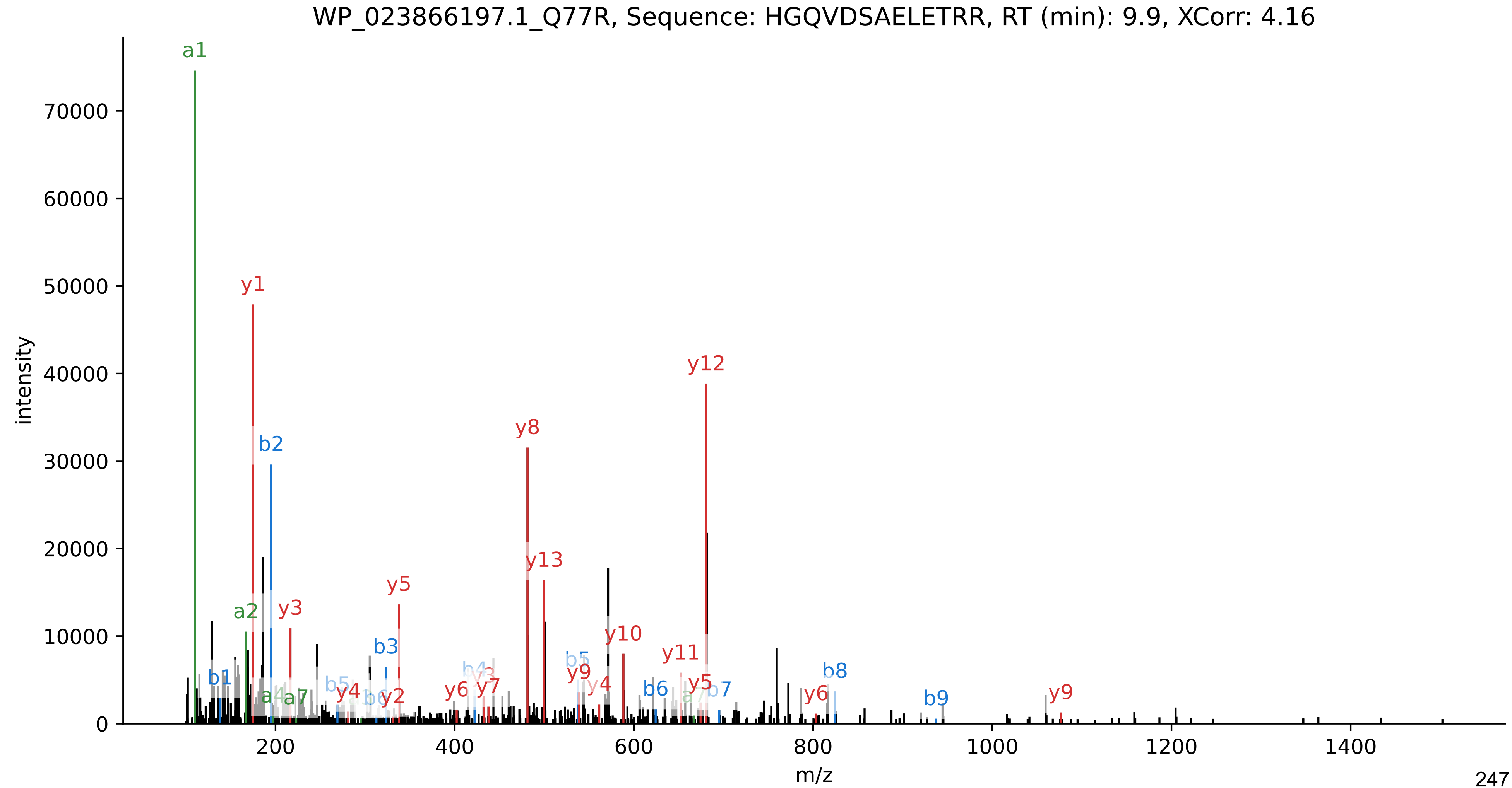

WP_023866197.1_Q77R, Sequence: HGQVDSAELETRR, RT (min): 9.9, XCorr: 4.16
intensity
m/z
a1
y1
b2
y12
y8
y13
y5
a2
y3
y10
b3
y11
b8
b5
y9
b1
y5
b7
b6
y6
y7
y4
y2
y6
b9
y9

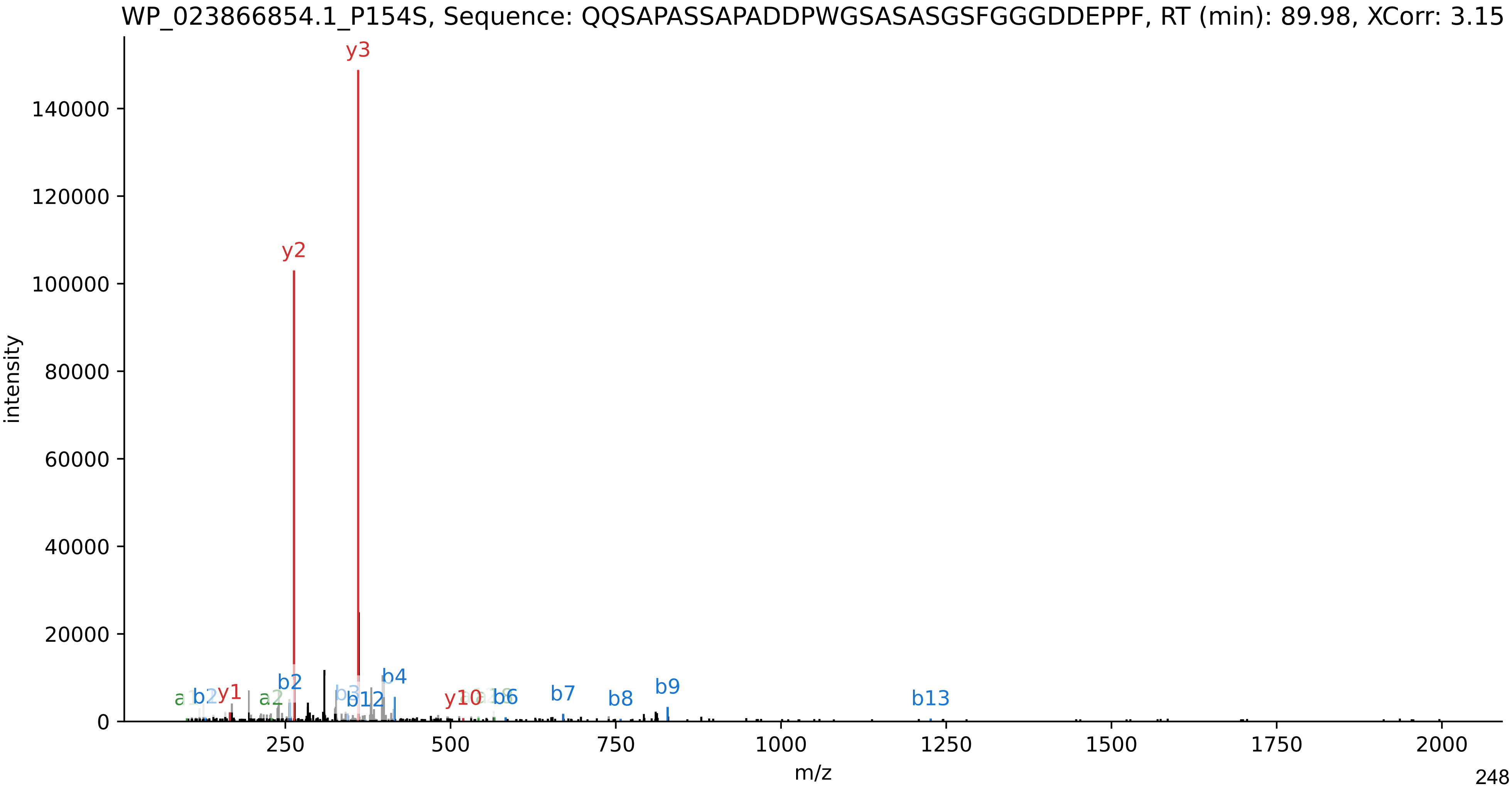
WP_023866854.1_P154S, Sequence: QQSAPASSAPADDPWGSASASGSFGGGDDEPPF, RT (min): 89.98, XCorr: 3.15
intensity
m/z
y3
y2
b4
b2
y1
a2
b12
y10
b6
b7
b8
b9
b13
0
20000
40000
60000
80000
100000
120000
140000
250
500
750
1000
1250
1500
1750
2000

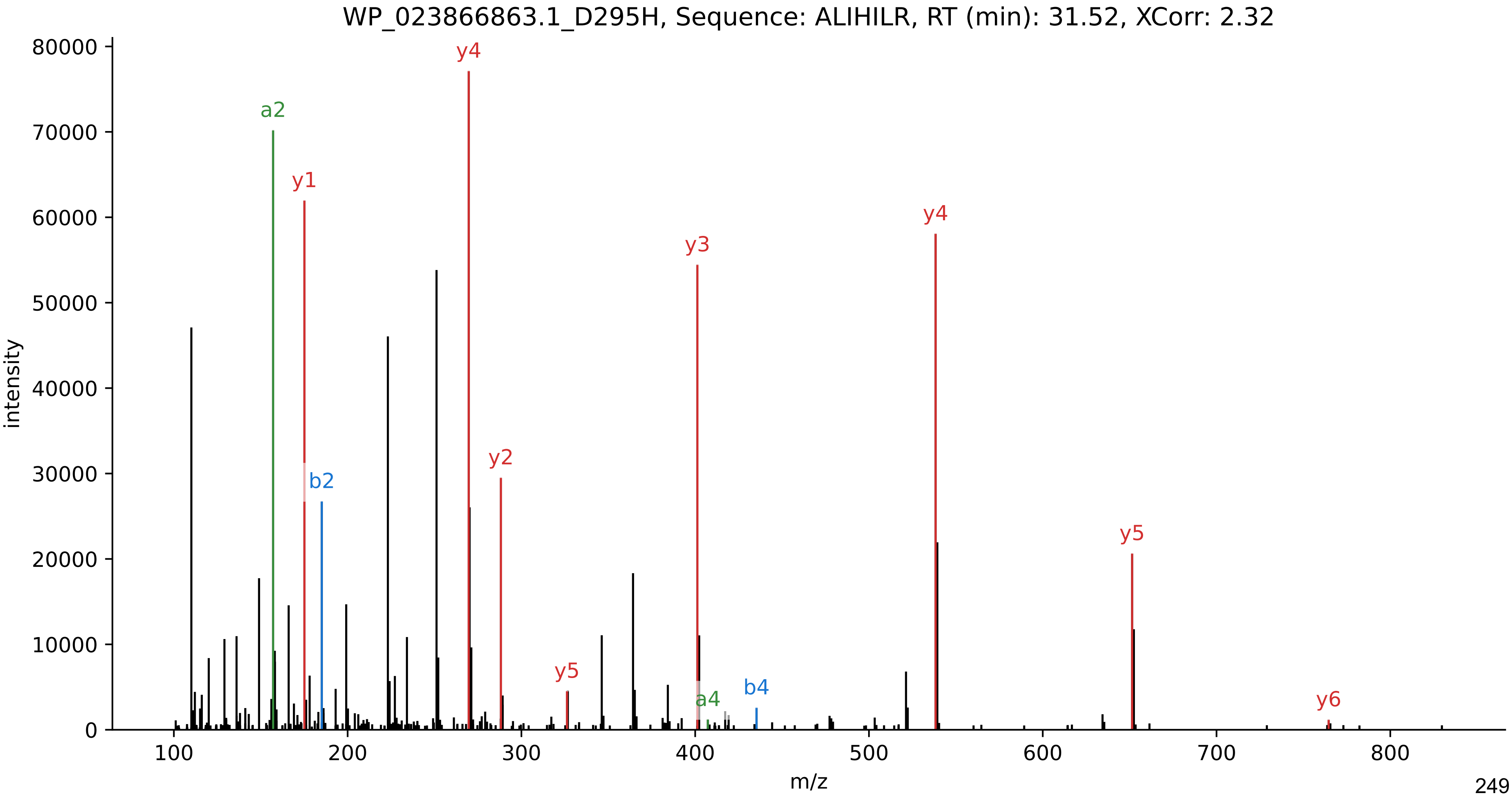

WP_023866863.1_D295H, Sequence: ALIHILR, RT (min): 31.52, XCorr: 2.32
intensity
m/z
a2
y1
b2
y4
y2
y5
y3
a4
b4
y4
y5
y6

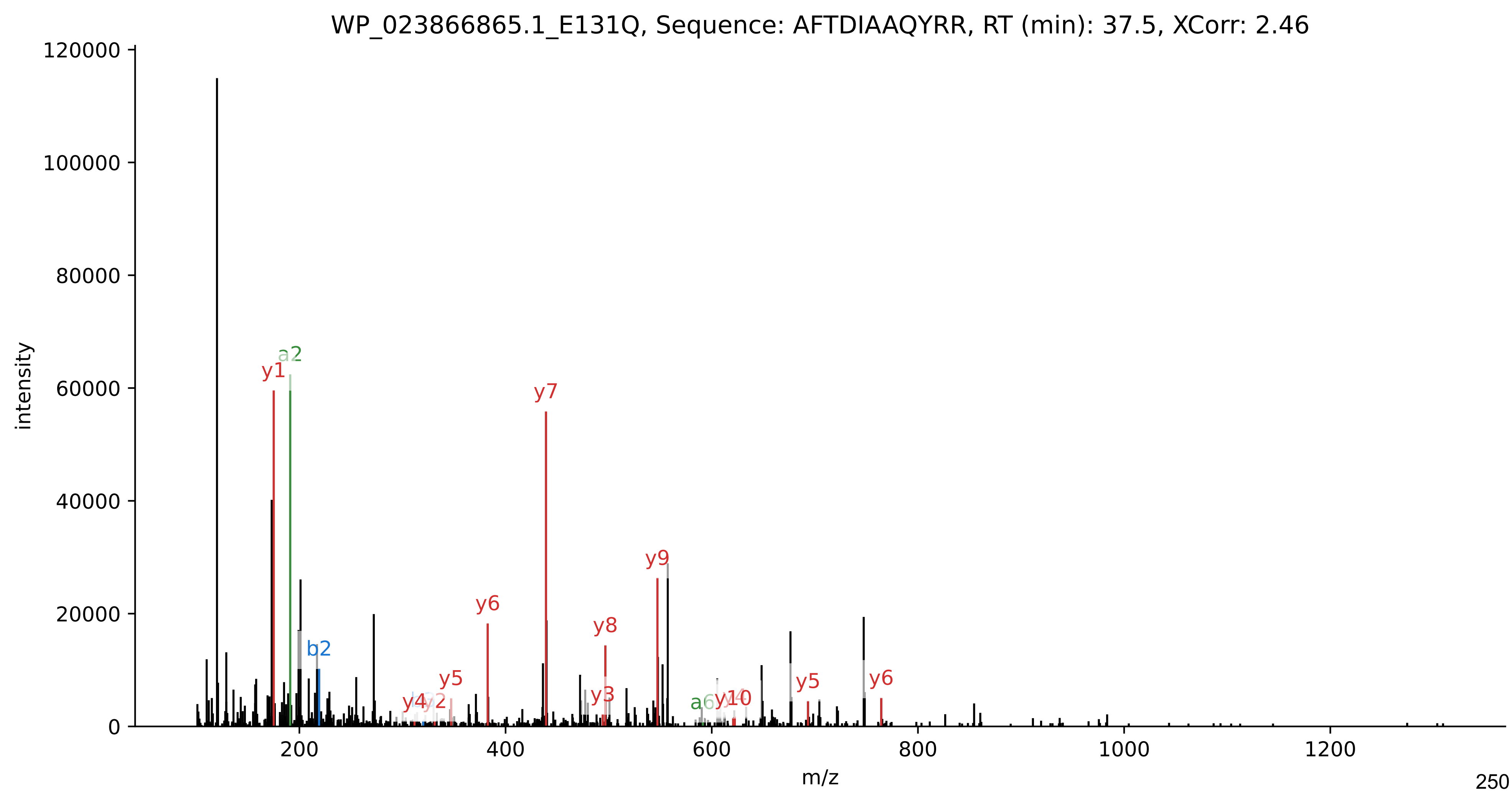
WP_023866865.1_E131Q, Sequence: AFTDIAAQYRR, RT (min): 37.5, XCorr: 2.46
intensity
m/z
y1
a2
y7
b2
y6
y9
y8
y5
y4
y2
y3
a6
y10
y5
y6

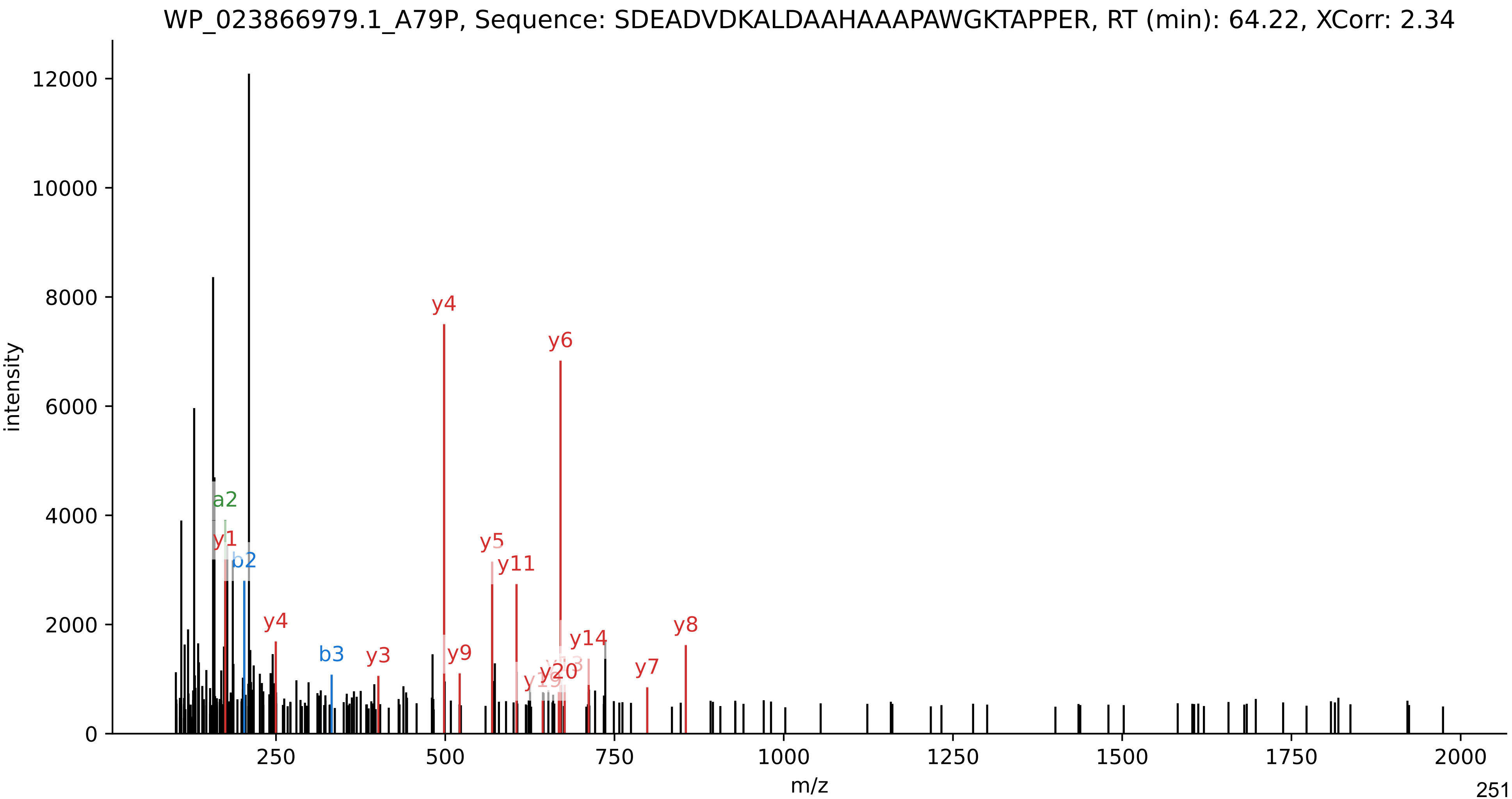

WP_023866979.1_A79P, Sequence: SDEADVDKALDAAHAAAPAWGKTAPPER, RT (min): 64.22, XCorr: 2.34
intensity
m/z
0
2000
4000
6000
8000
10000
12000
250
500
750
1000
1250
1500
1750
2000
a2
y1
b2
y4
b3
y3
y4
y9
y5
y11
y6
y14
y20
y7
y8

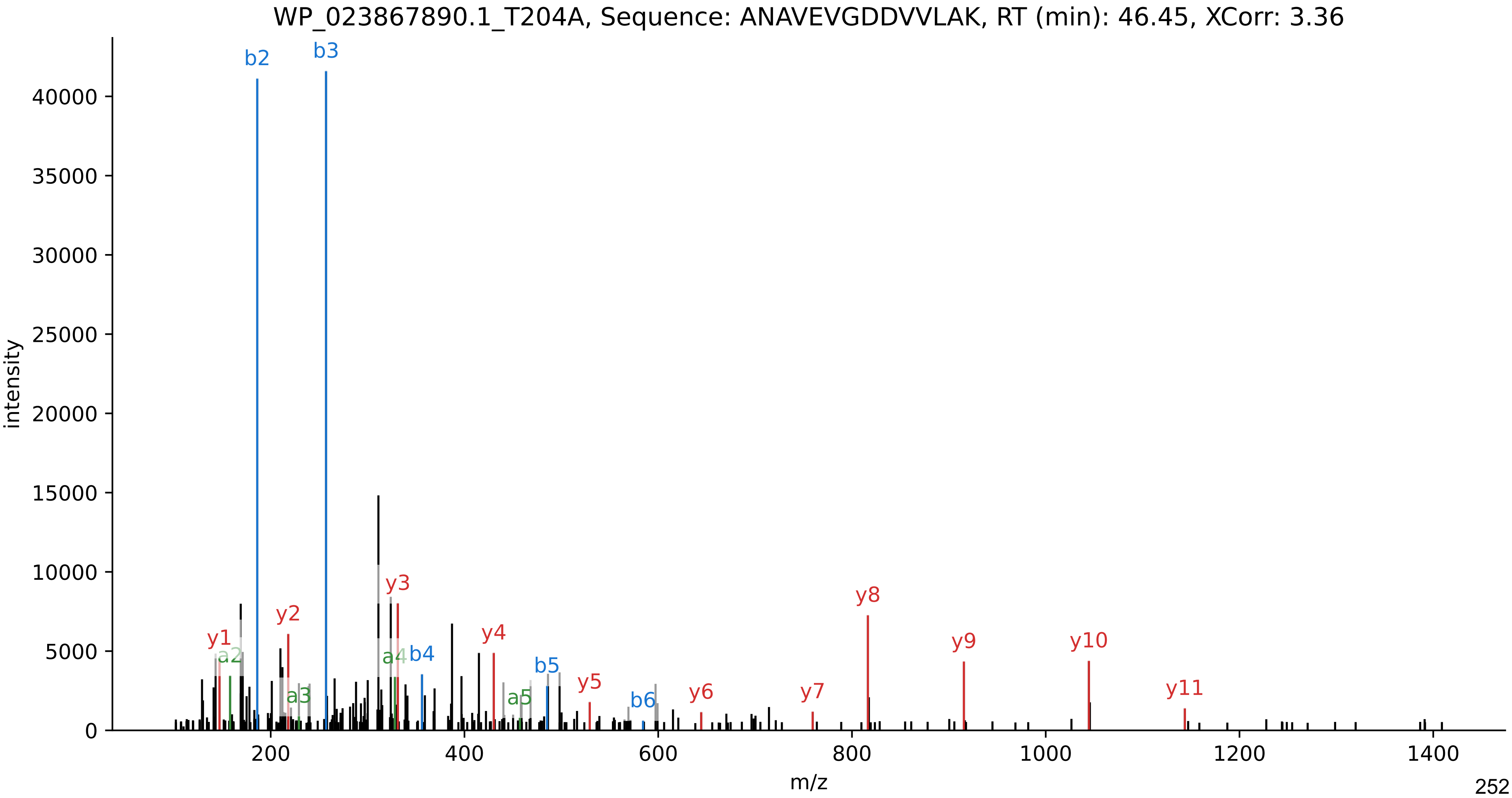

WP_023867890.1_T204A, Sequence: ANAVEVGDDVVLAK, RT (min): 46.45, XCorr: 3.36
b2
b3
y1
a2
y2
a3
y3
a4
b4
y4
a5
b5
y5
b6
y6
y7
y8
y9
y10
y11
intensity
m/z
0
5000
10000
15000
20000
25000
30000
35000
40000
200
400
600
800
1000
1200
1400

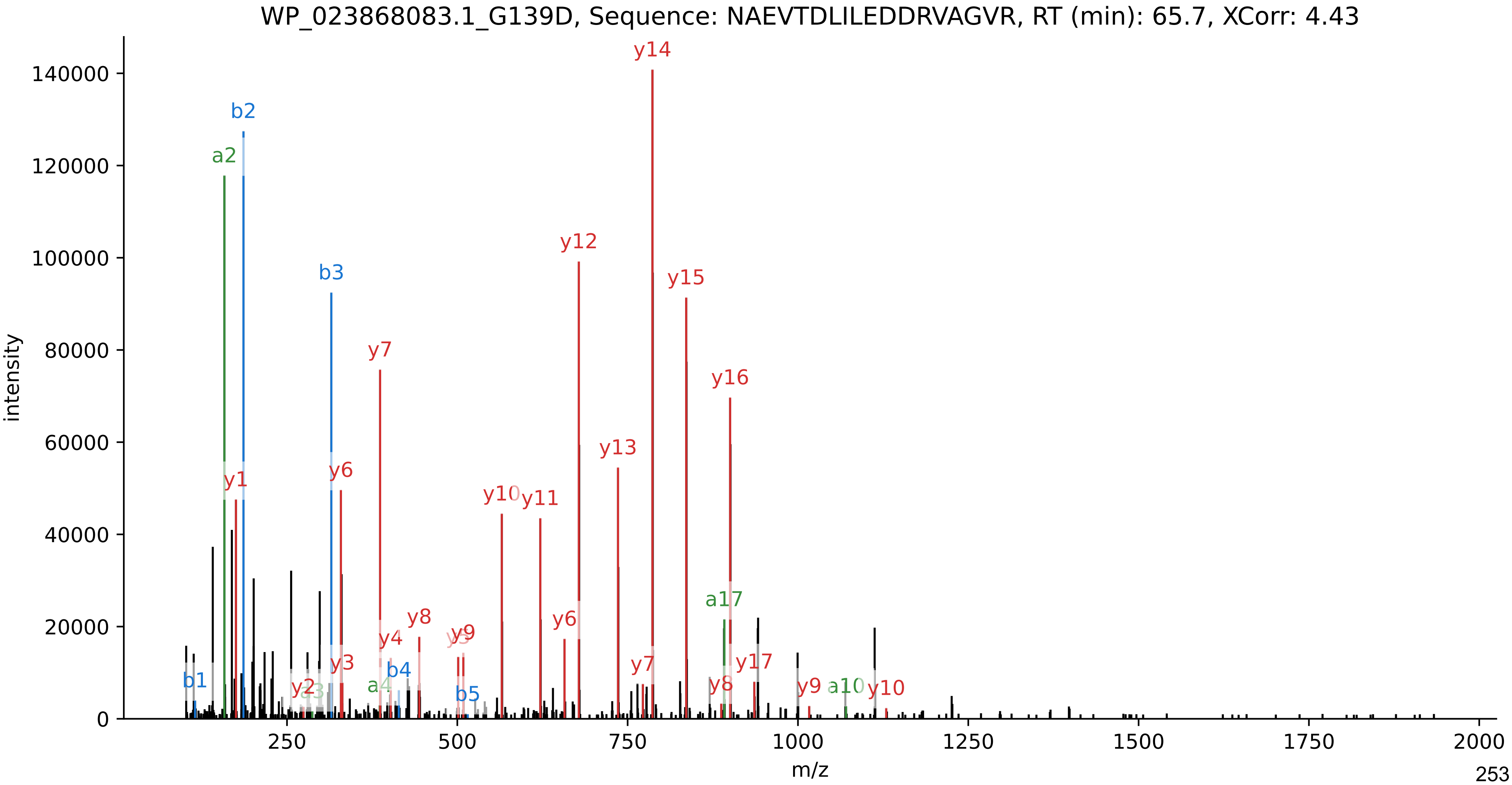

WP_023868083.1_G139D, Sequence: NAEVTDLILEDDRVAGVR, RT (min): 65.7, XCorr: 4.43
intensity
m/z
y14
b2
a2
y12
b3
y15
y7
y16
y13
y6
y1
y10
y11
a17
y8
y6
y4
y9
y3
b4
y7
y17
b1
y2
a4
y8
y9
a10
y10
b5

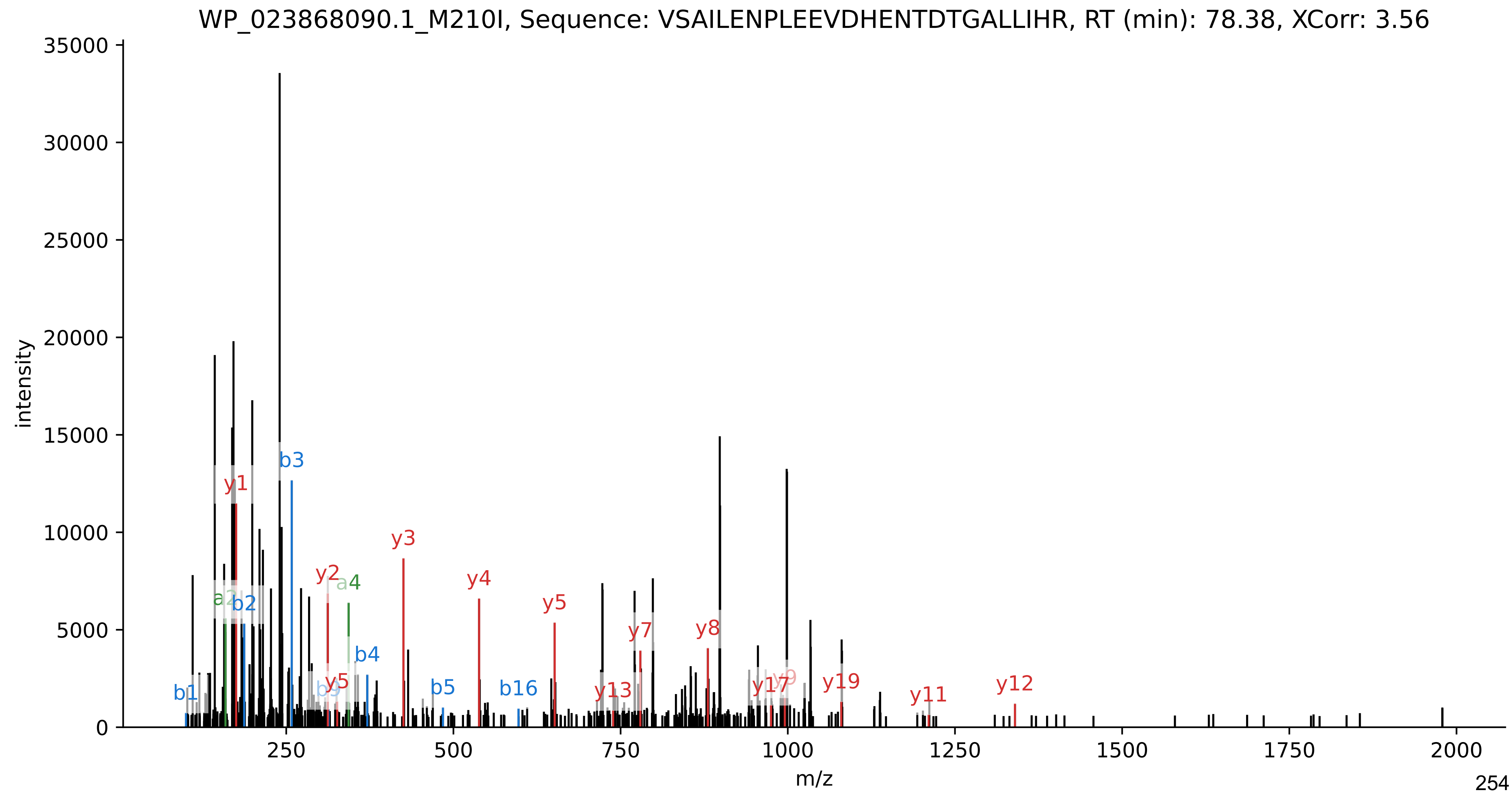

WP_023868090.1_M210I, Sequence: VSAILENPLEEVDHENTDTGALLIHR, RT (min): 78.38, XCorr: 3.56
intensity
m/z
b1
a2
b2
y1
b3
y2
a4
b4
y5
y3
y4
b5
b16
y5
y7
y8
y13
y17
y19
y11
y12

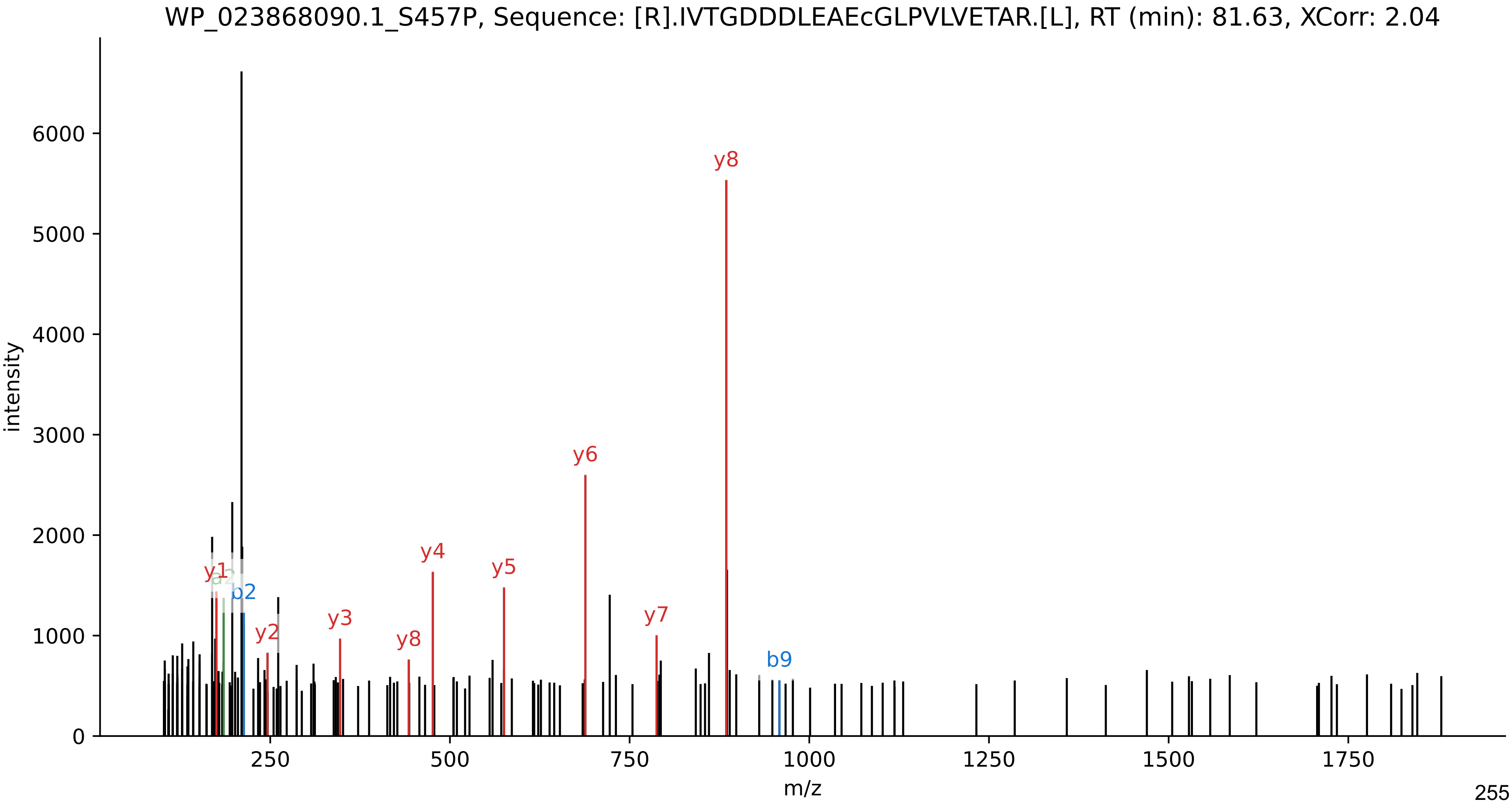
WP_023868090.1_S457P, Sequence: [R].IVTGDDDLEAEcGLPVLVETAR.[L], RT (min): 81.63, XCorr: 2.04
intensity
m/z
y1
a2
b2
y2
y3
y8
y4
y5
y6
y7
y8
b9
0
1000
2000
3000
4000
5000
6000
250
500
750
1000
1250
1500
1750

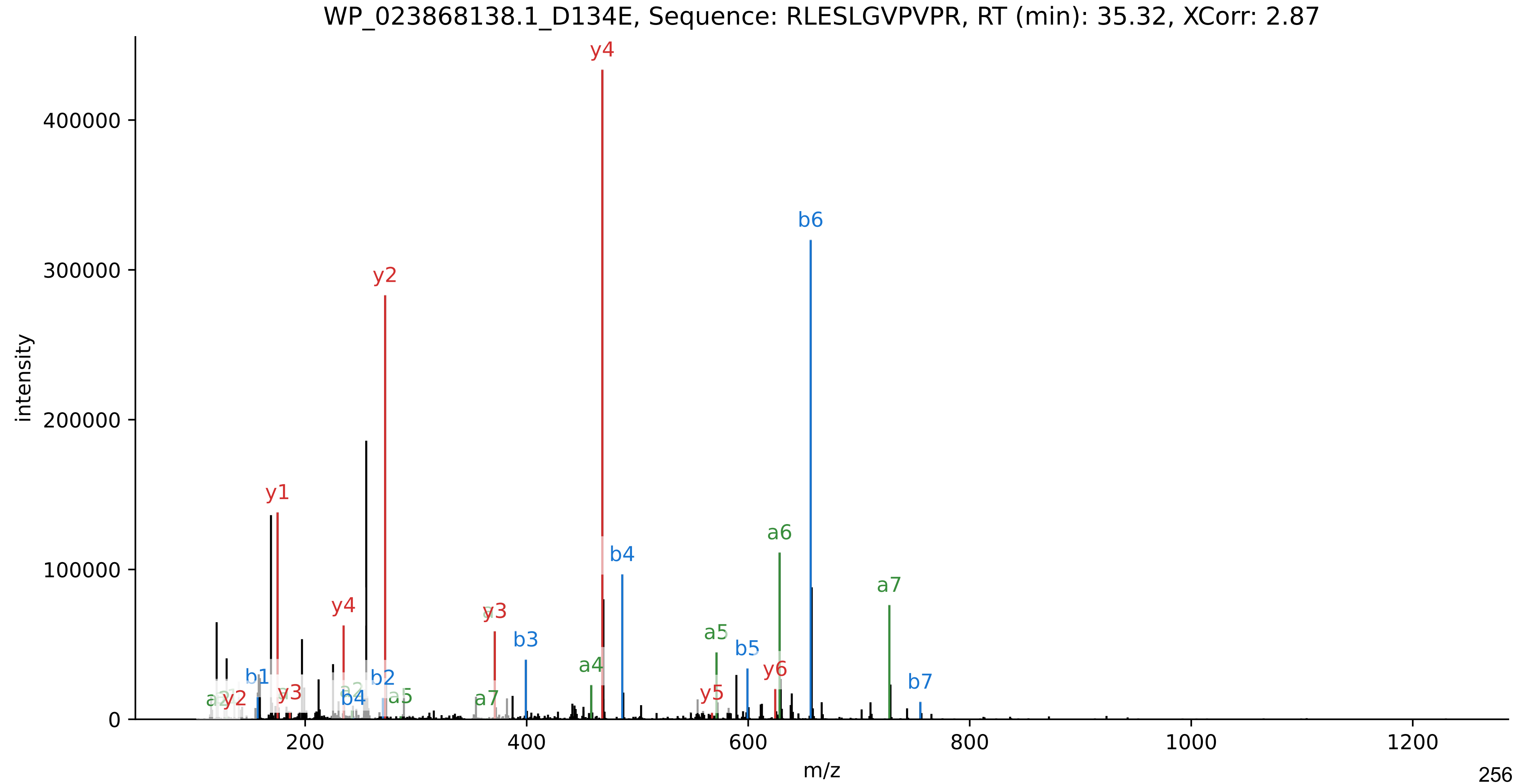
WP_023868138.1_D134E, Sequence: RLESLGVPVPR, RT (min): 35.32, XCorr: 2.87
intensity
m/z
0
100000
200000
300000
400000
200
400
600
800
1000
1200
y4
b6
y2
y1
b4
a6
a7
y4
y3
b3
a5
b5
a4
y6
b7
y5
b1
b2
y3
y2
b4
a5
a7

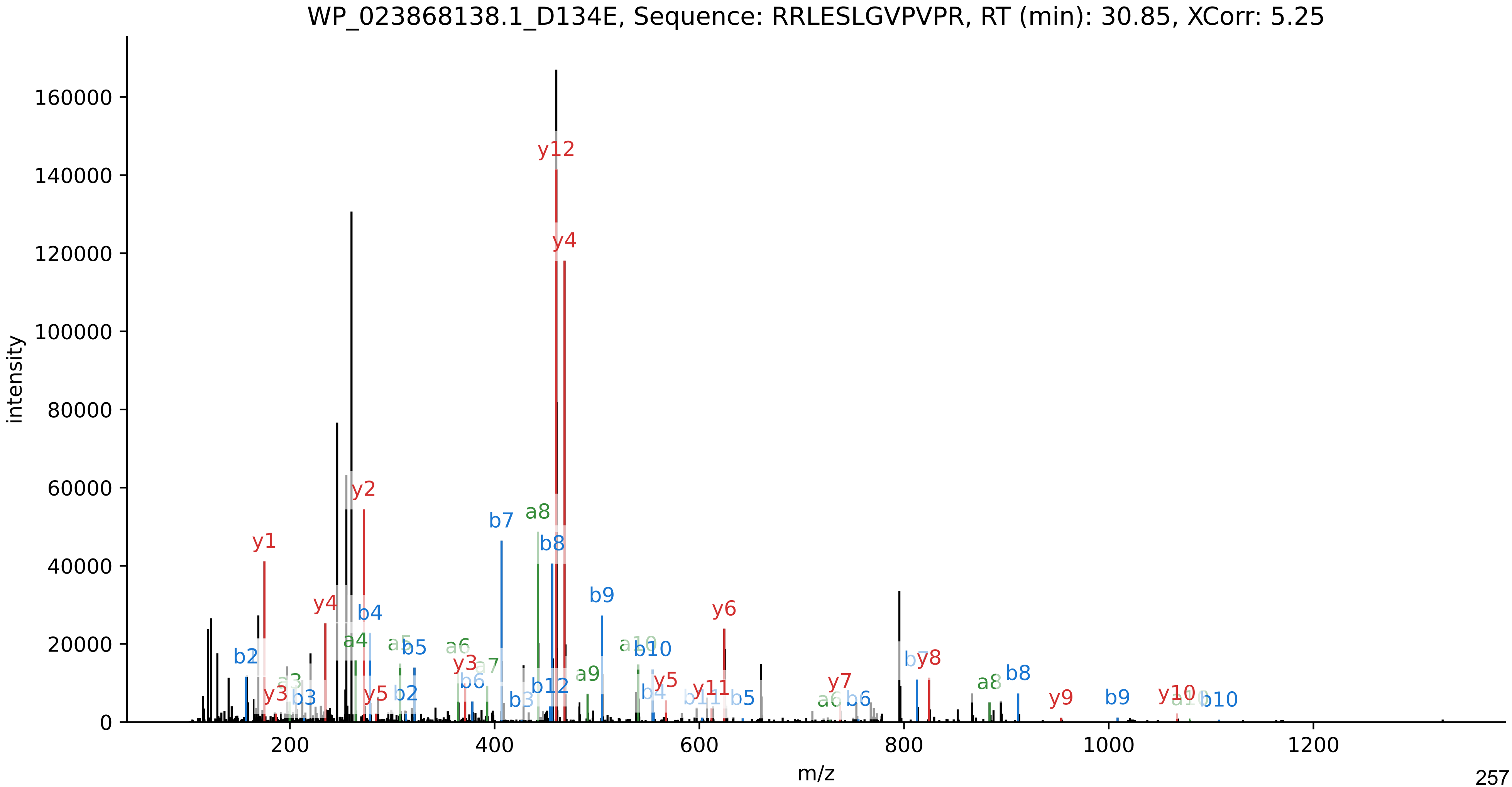
WP_023868138.1_D134E, Sequence: RRLESLGVPVPR, RT (min): 30.85, XCorr: 5.25
intensity
m/z
0
20000
40000
60000
80000
100000
120000
140000
160000
200
400
600
800
1000
1200
y12
y4
y2
y1
y4
b4
b7
a8
b8
b9
y6
a4
b5
a6
b2
y3
a7
b6
b12
a9
b10
y5
y11
y7
a6
b6
b7
y8
a8
b8
y9
b9
y10
b10
y3
b3
y5
b2
b3
b4
b5

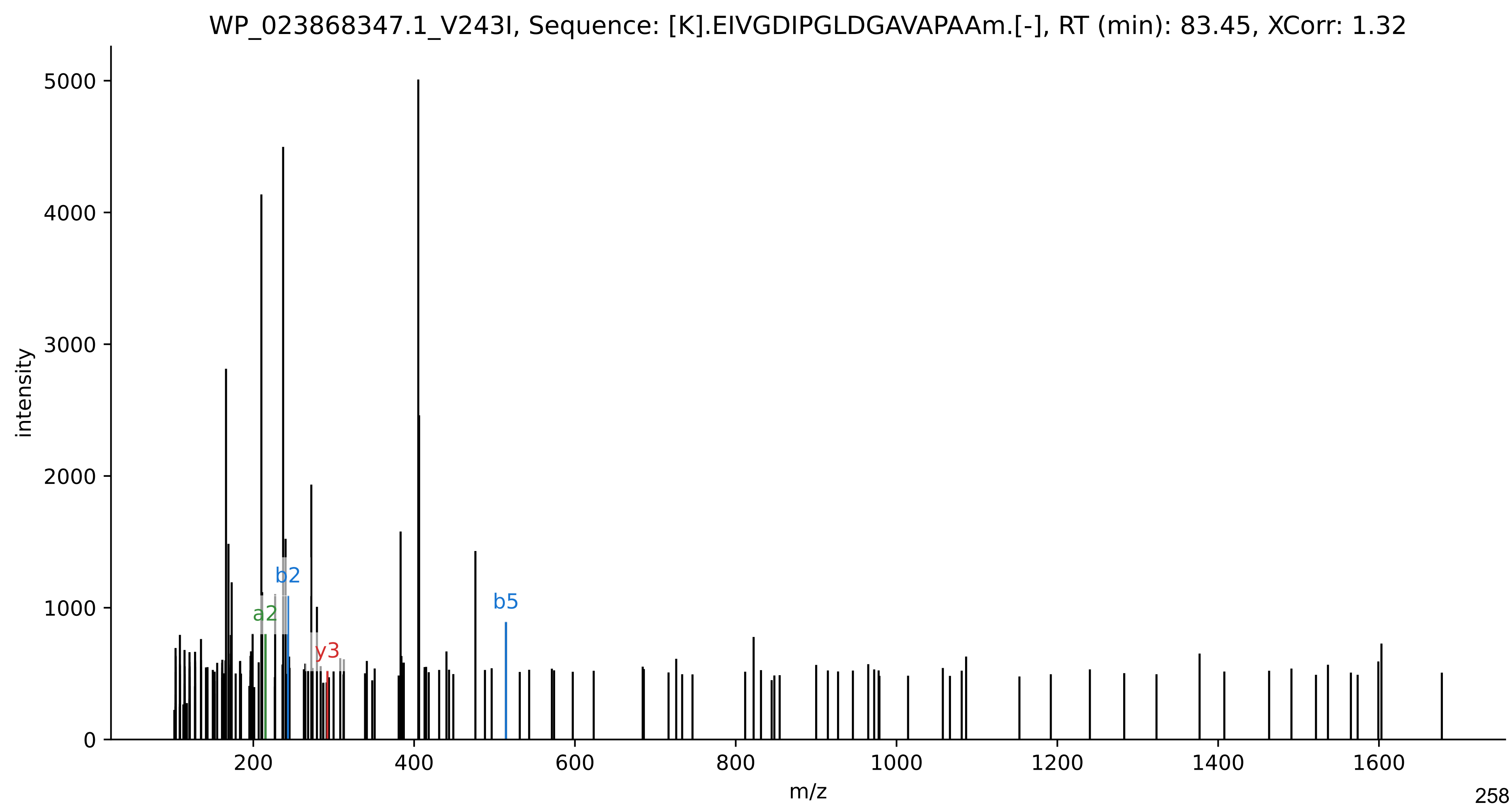

WP_023868347.1_V243I, Sequence: [K].EIVGDIPGLDGAVAPAAm.[-], RT (min): 83.45, XCorr: 1.32
intensity
0
1000
2000
3000
4000
5000
200
400
600
800
1000
1200
1400
1600
m/z
a2
b2
y3
b5

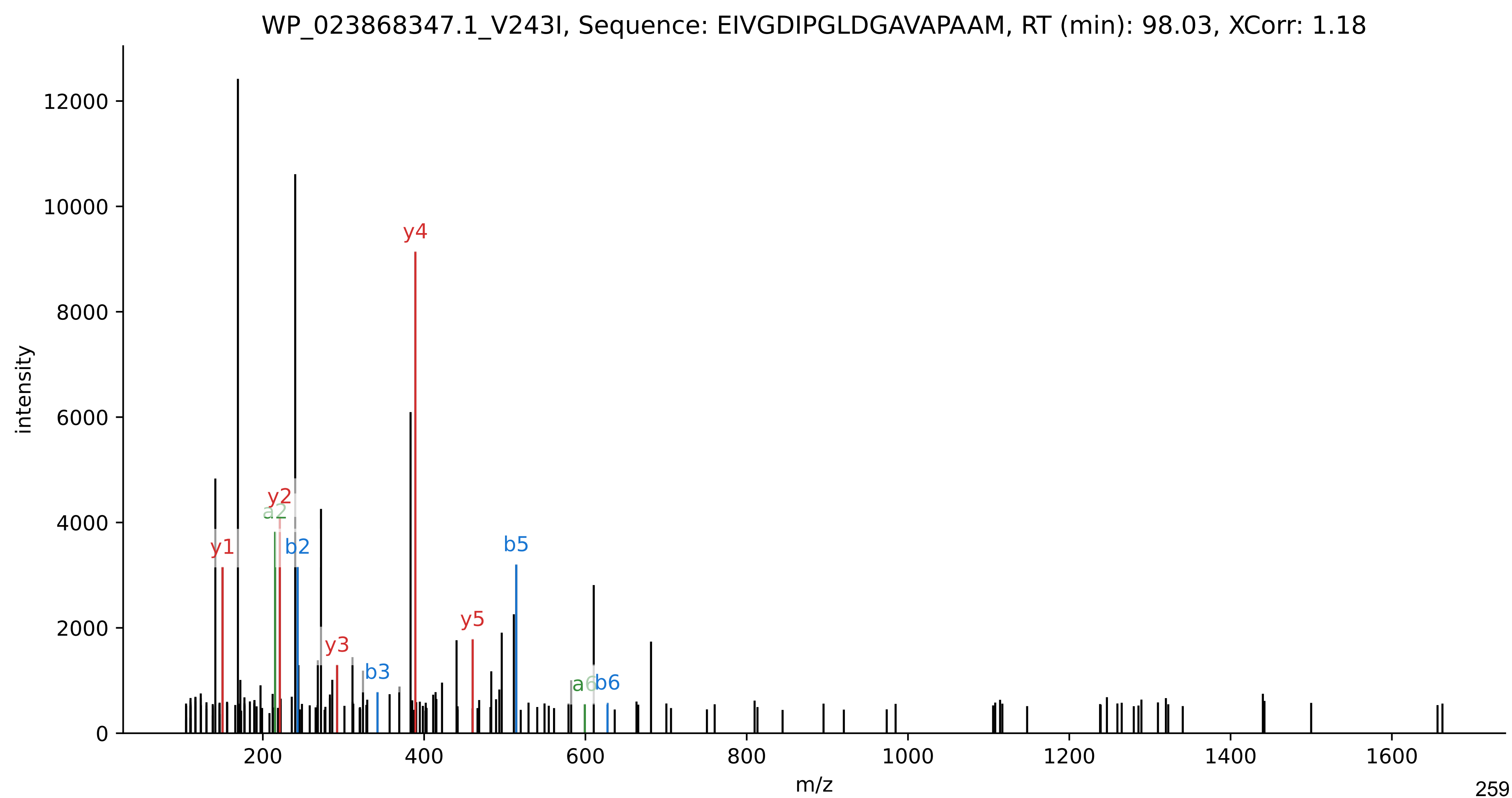

WP_023868347.1_V243I, Sequence: EIVGDIPGLDGAVAPAAM, RT (min): 98.03, XCorr: 1.18
intensity
m/z
y1
y2
a2
b2
y3
b3
y4
y5
b5
a6
b6

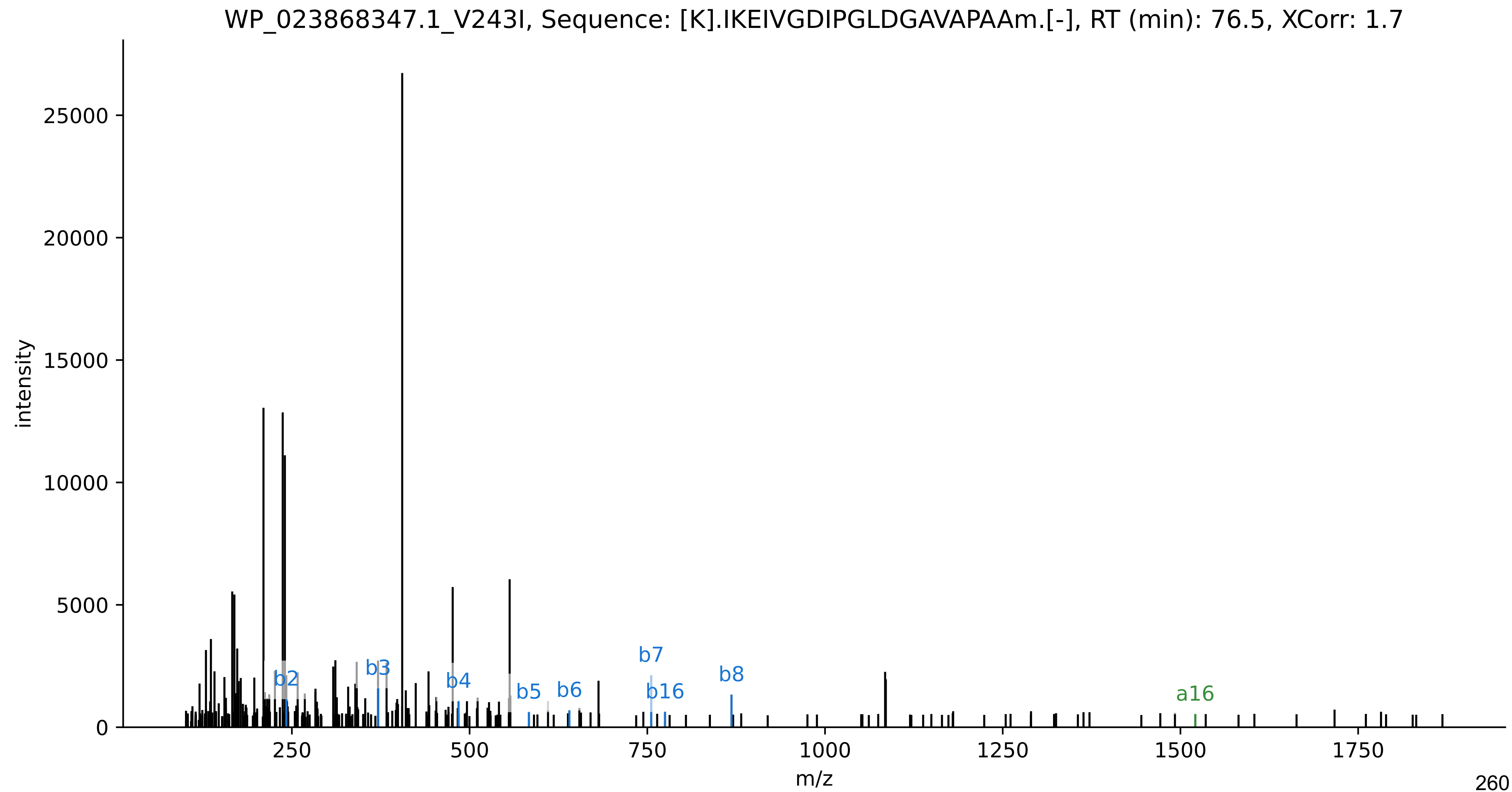
WP_023868347.1_V243I, Sequence: [K].IKEIVGDIPGLDGAVAPAAm.[-], RT (min): 76.5, XCorr: 1.7
intensity
m/z
b2
b3
b4
b5
b6
b7
b16
b8
a16
0
5000
10000
15000
20000
25000
250
500
750
1000
1250
1500
1750

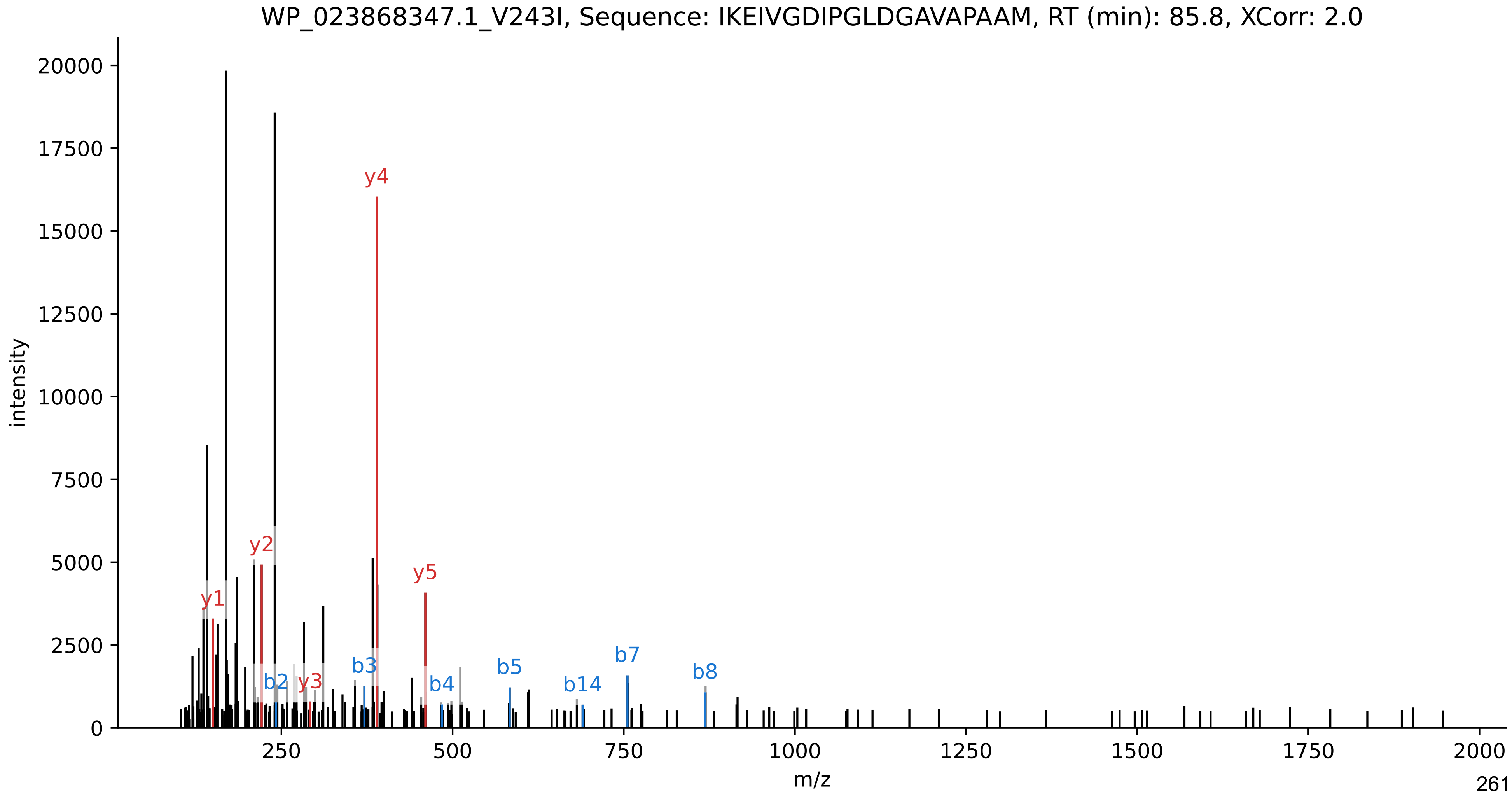

WP_023868347.1_V243I, Sequence: IKEIVGDIPGLDGAVAPAAM, RT (min): 85.8, XCorr: 2.0
intensity
m/z
y1
y2
y3
y4
y5
b2
b3
b4
b5
b14
b7
b8
0
2500
5000
7500
10000
12500
15000
17500
20000
250
500
750
1000
1250
1500
1750
2000

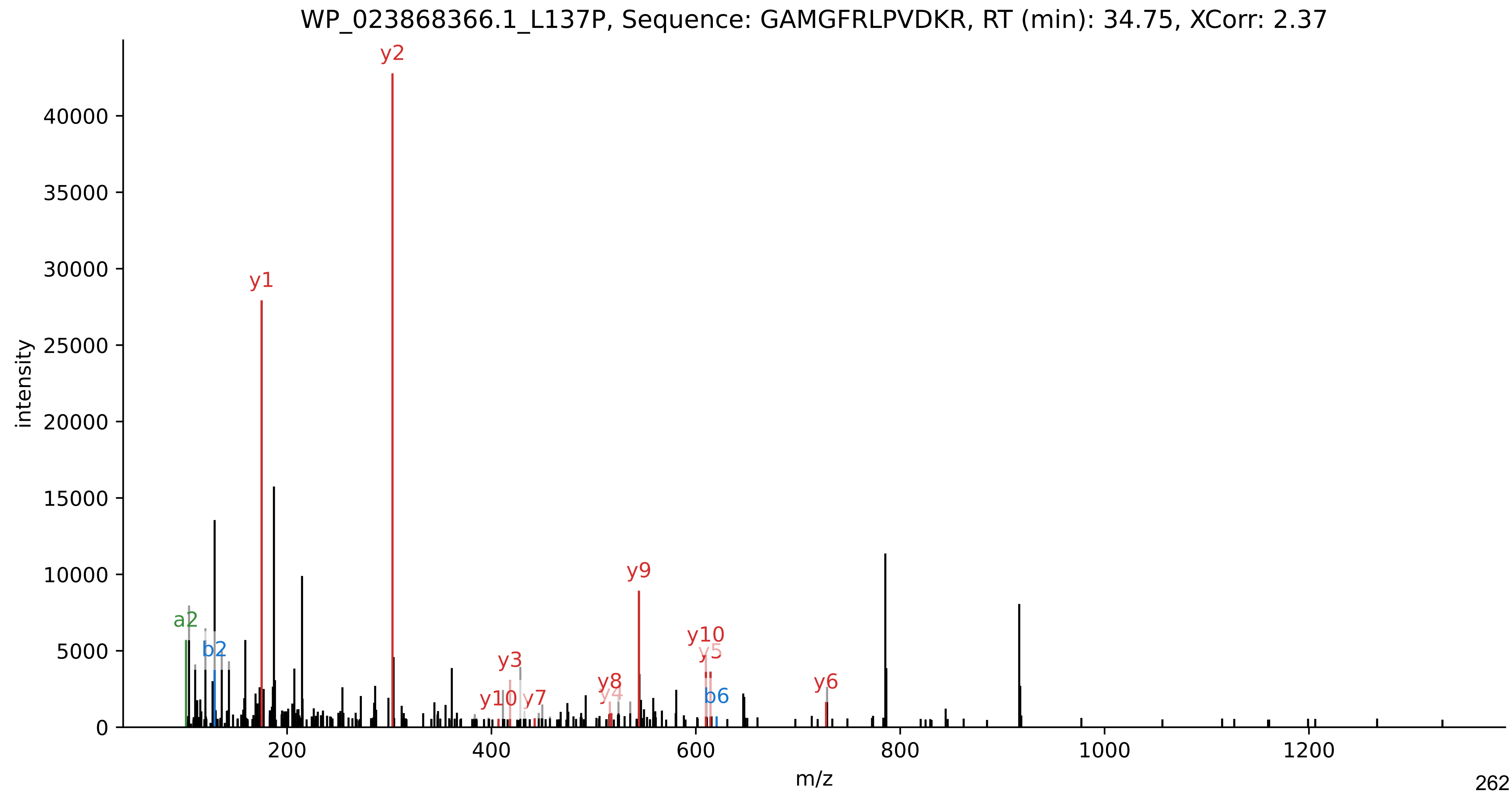

WP_023868366.1_L137P, Sequence: GAMGFRLPVDKR, RT (min): 34.75, XCorr: 2.37
intensity
m/z
y2
y1
a2
b2
y3
y10
y7
y8
y4
y9
y10
y5
b6
y6

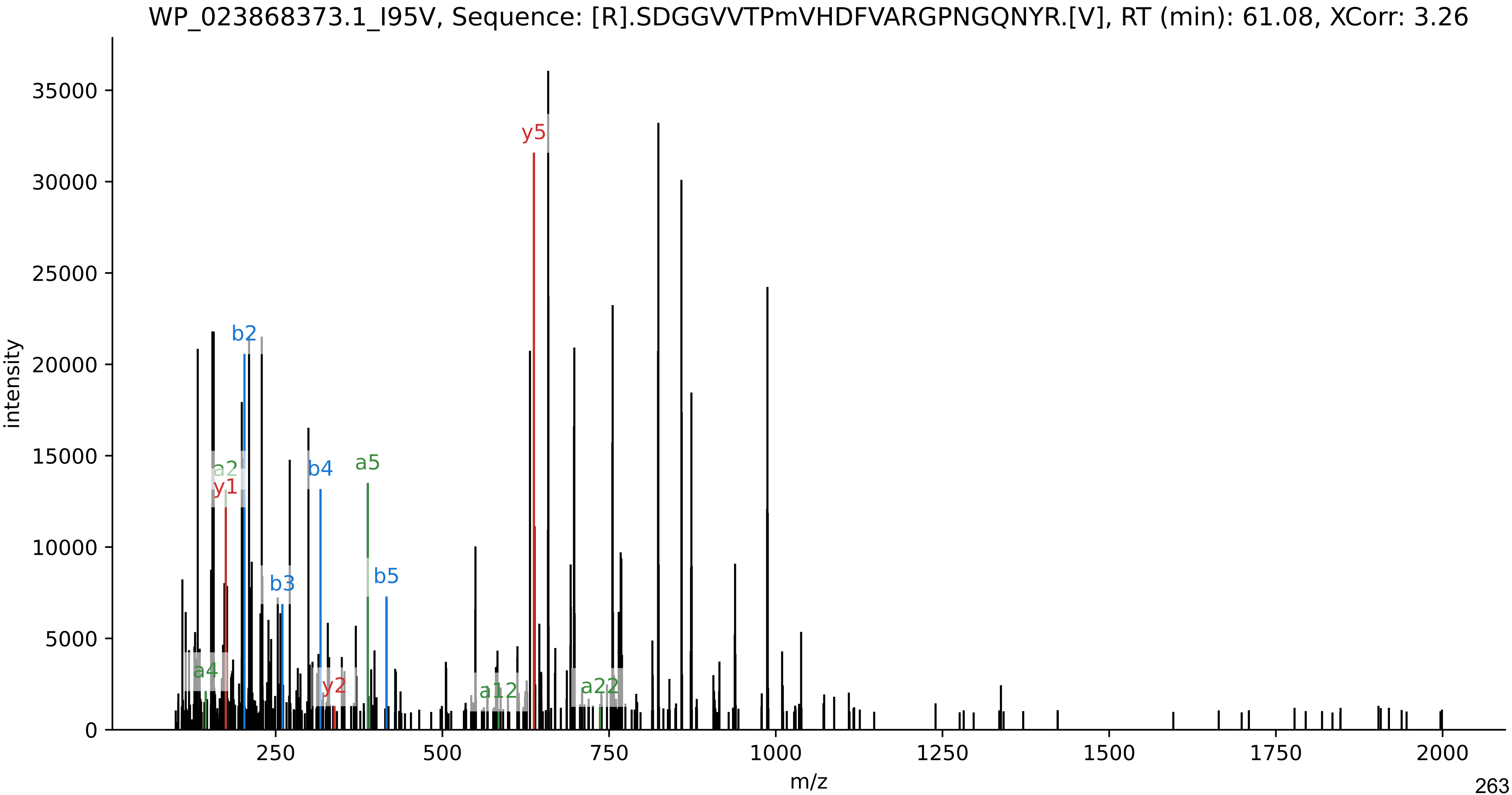
WP_023868373.1_I95V, Sequence: [R].SDGGVVTPmVHDFVARGPNGQNYR.[V], RT (min): 61.08, XCorr: 3.26
intensity
m/z
y5
b2
a2
y1
b4
a5
b3
b5
a4
y2
a12
a22
35000
30000
25000
20000
15000
10000
5000
0
250
500
750
1000
1250
1500
1750
2000

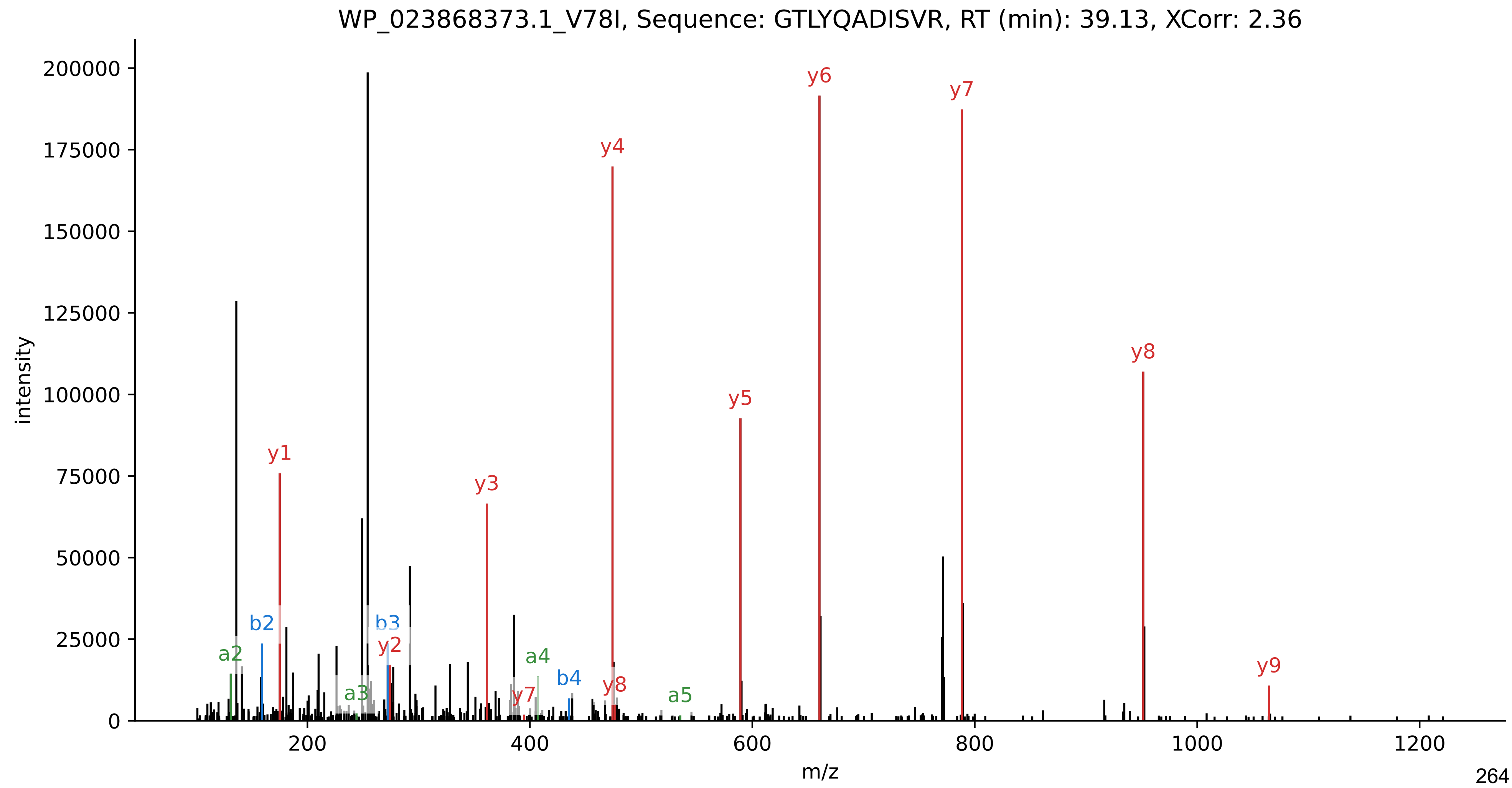
WP_023868373.1_V78I, Sequence: GTLYQADISVR, RT (min): 39.13, XCorr: 2.36
intensity
m/z
y1
y2
y3
y4
y5
y6
y7
y7
y8
y8
y9
b2
b3
b4
a2
a3
a4
a5
0
25000
50000
75000
100000
125000
150000
175000
200000
200
400
600
800
1000
1200

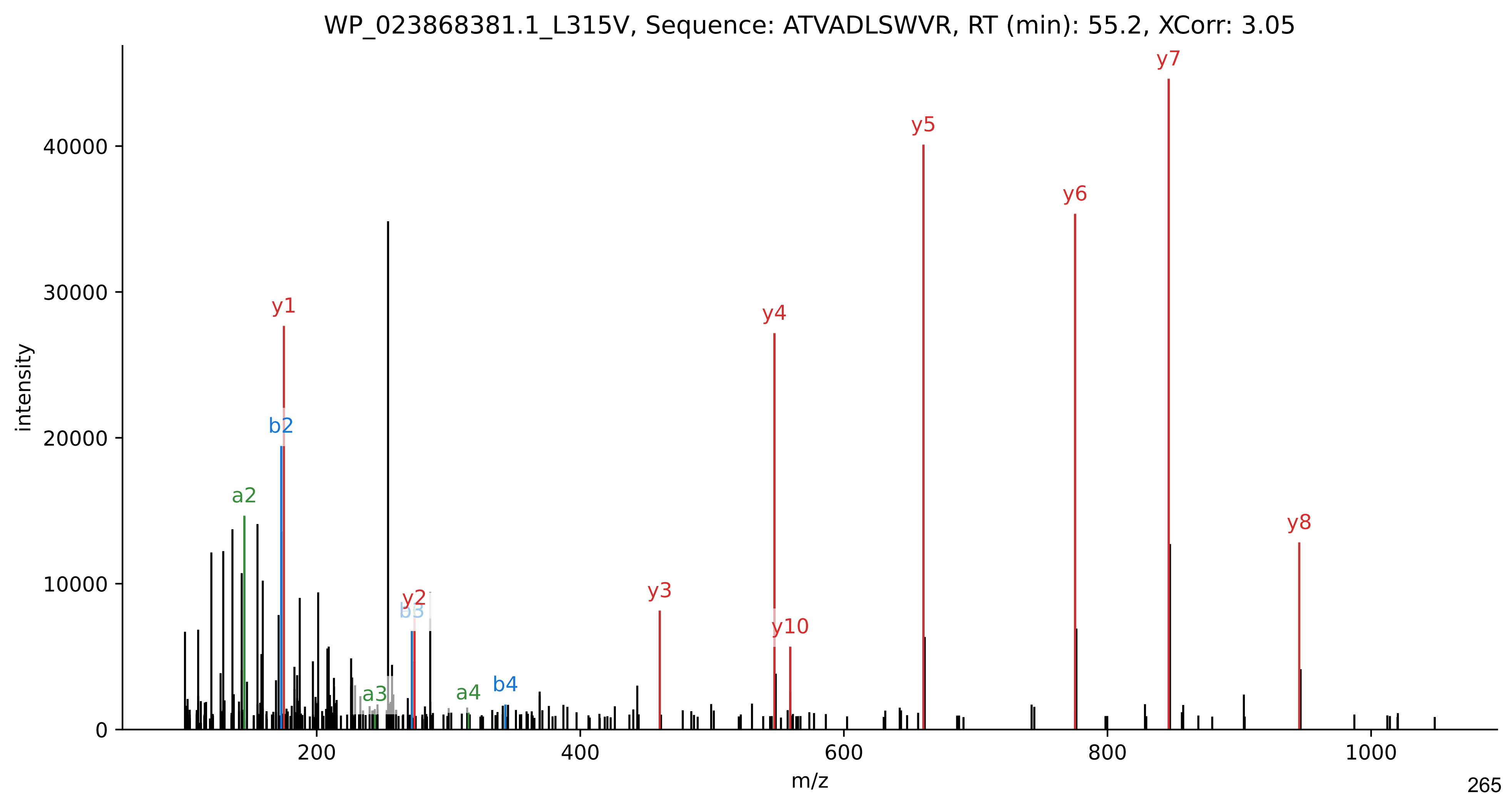
WP_023868381.1_L315V, Sequence: ATVADLSWVR, RT (min): 55.2, XCorr: 3.05
intensity
m/z
y1
b2
a2
y2
b3
a3
a4
b4
y3
y4
y10
y5
y6
y7
y8
0
10000
20000
30000
40000
200
400
600
800
1000

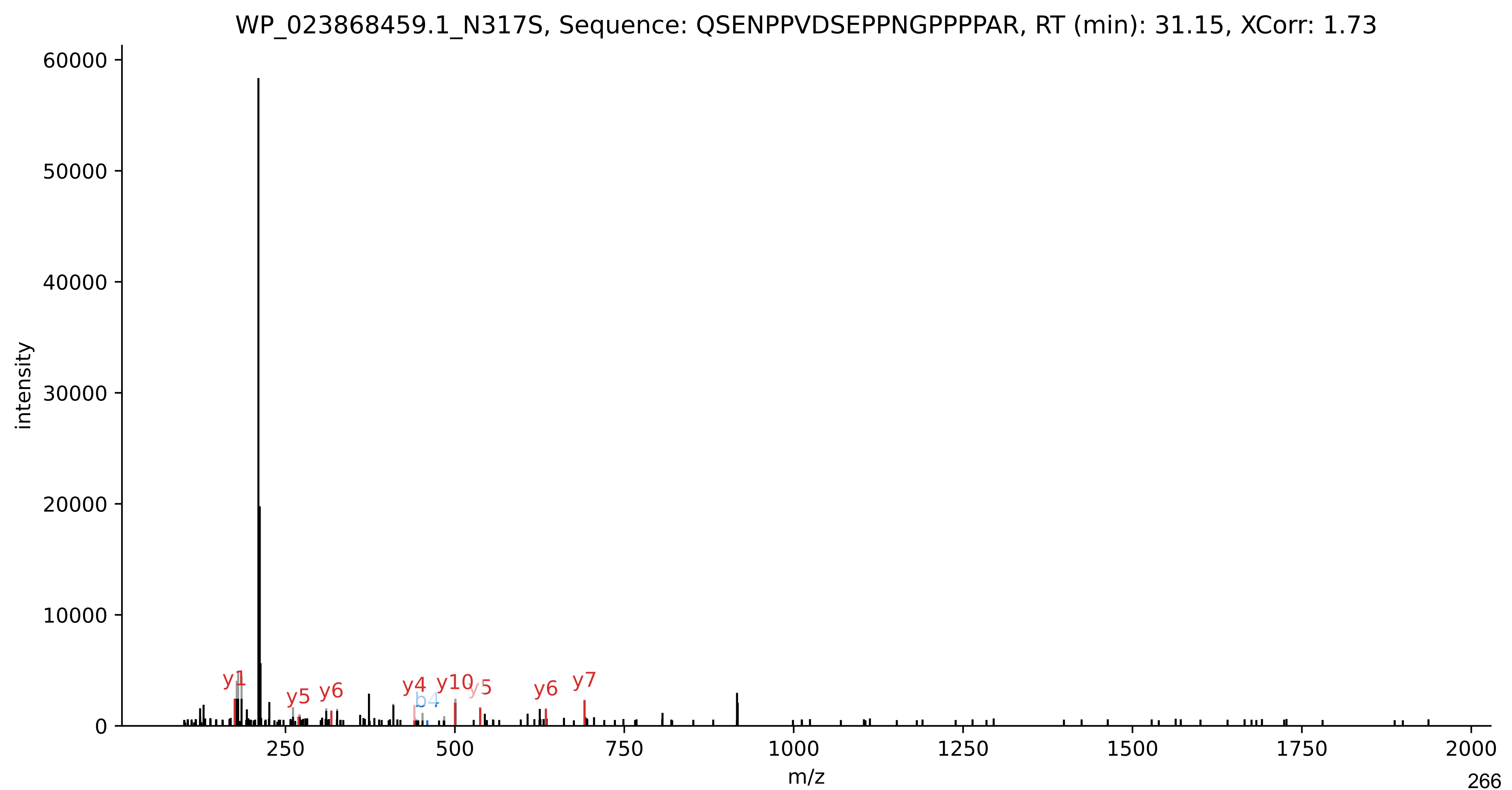
WP_023868459.1_N317S, Sequence: QSENPPVDSEPPNGPPPPAR, RT (min): 31.15, XCorr: 1.73
intensity
m/z
y1
y5
y6
y4
b4
y10
y5
y6
y7

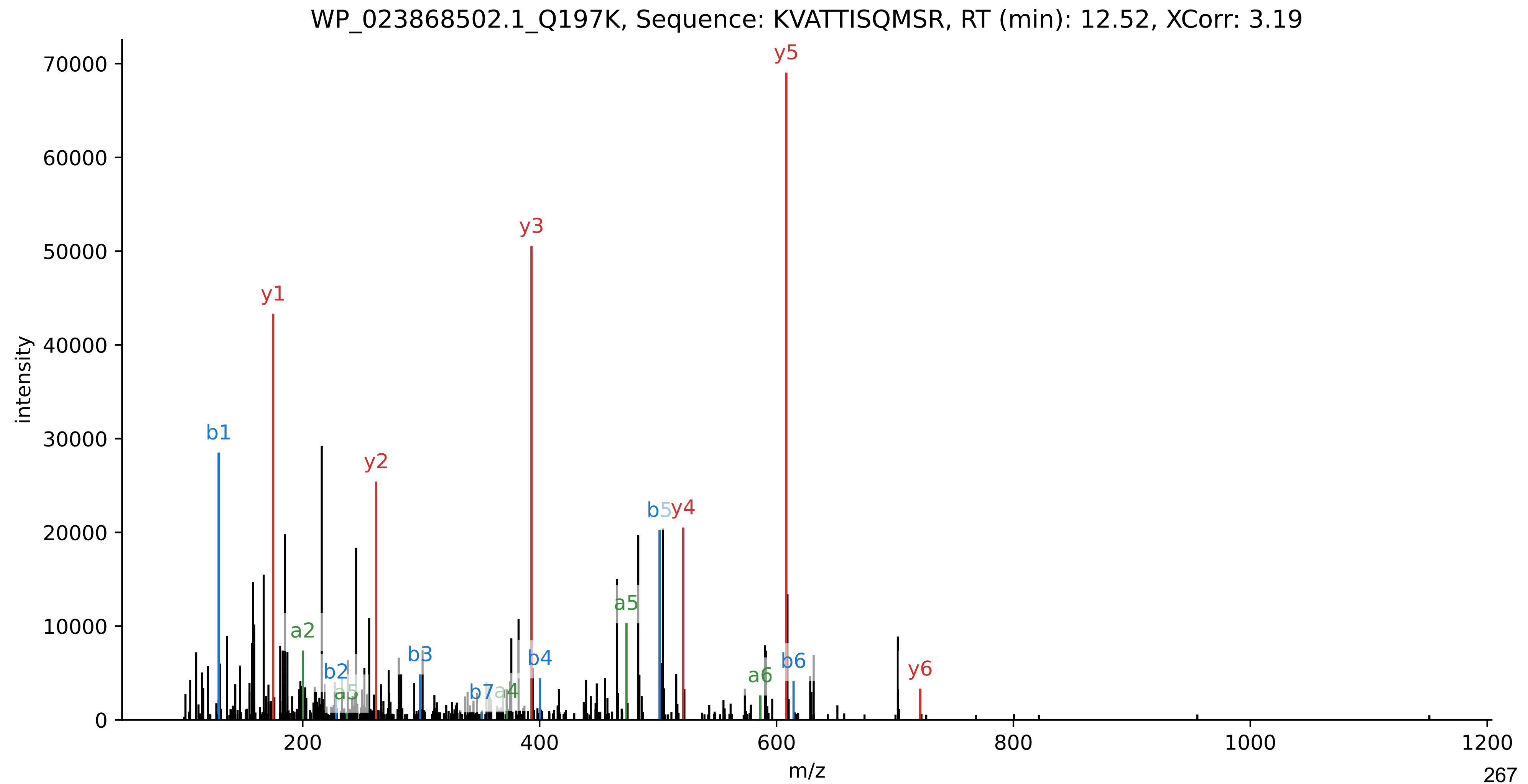

WP_023868502.1_Q197K, Sequence: KVATTISQMSR, RT (min): 12.52, XCorr: 3.19
intensity
m/z
y5
y3
y1
b1
y2
b5
y4
a5
a2
b3
b4
b2
a6
b6
y6
b7
a4
a3
0
10000
20000
30000
40000
50000
60000
70000
200
400
600
800
1000
1200

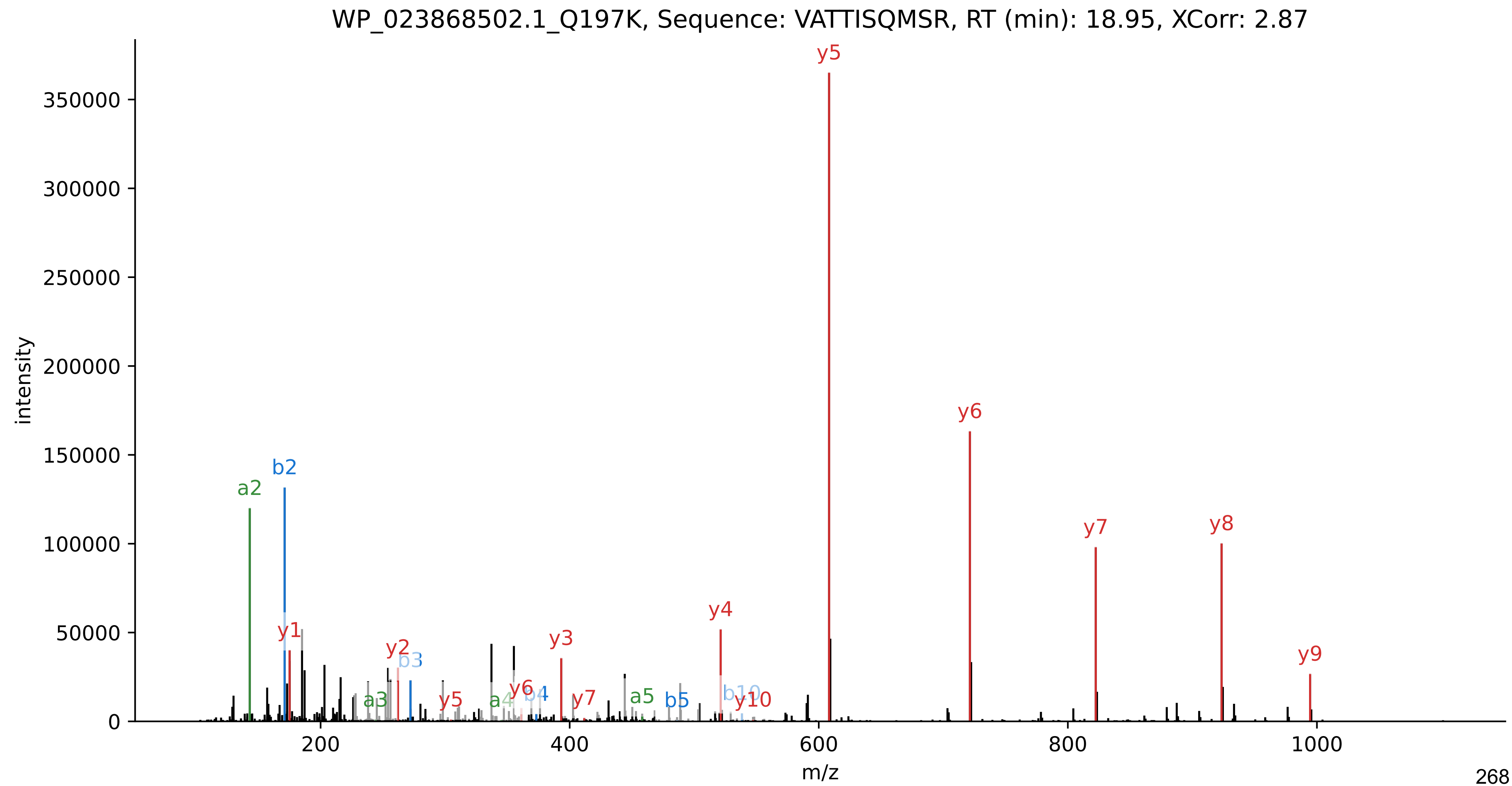
WP_023868502.1_Q197K, Sequence: VATTISQMSR, RT (min): 18.95, XCorr: 2.87
intensity
m/z
y5
y6
y7
y8
y9
y4
y3
y2
y1
b2
a2
b3
a3
y5
a4
y6
b4
y7
a5
b5
b10
y10
0
50000
100000
150000
200000
250000
300000
350000
200
400
600
800
1000

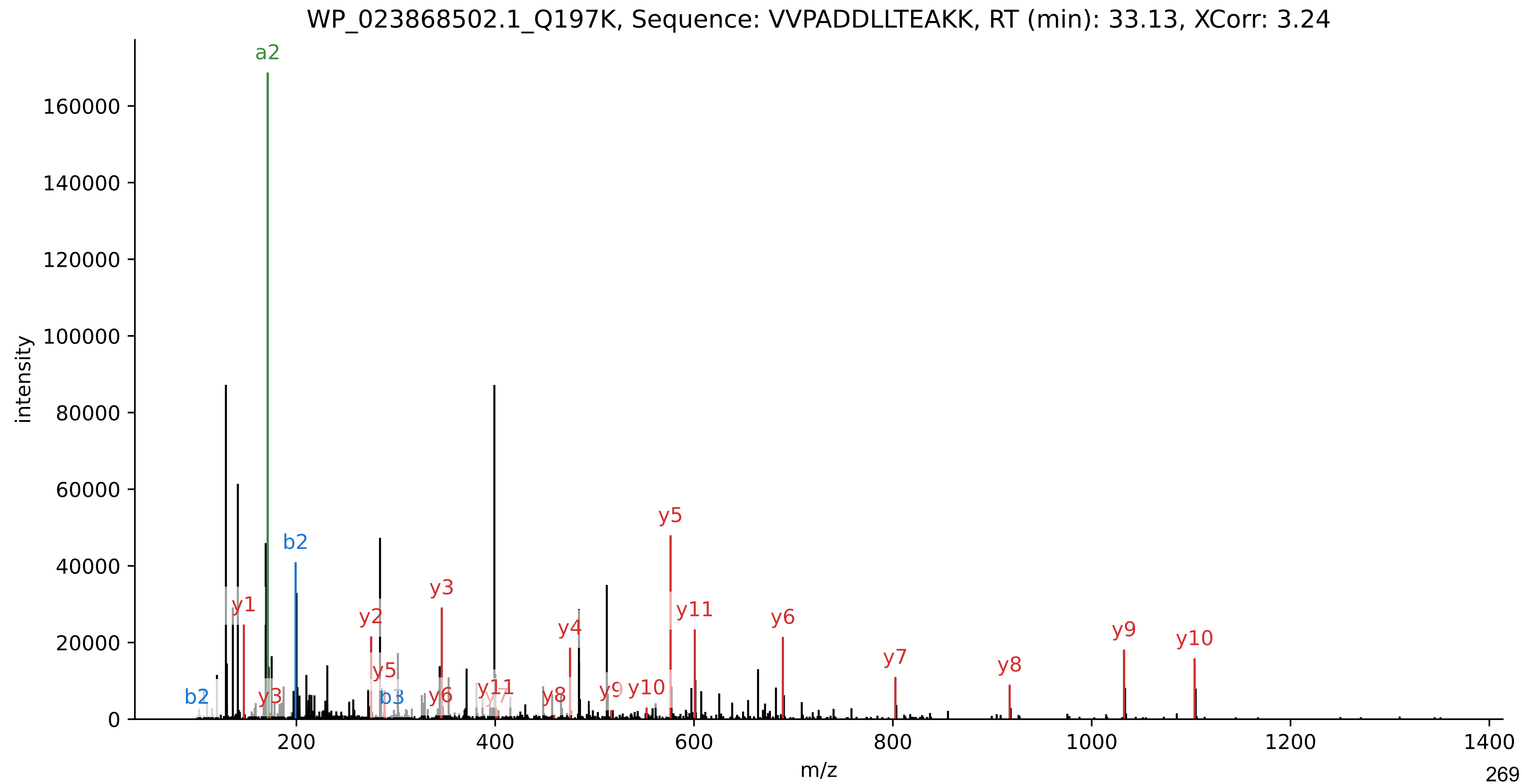

WP_023868502.1_Q197K, Sequence: VVPADDLLTEAKK, RT (min): 33.13, XCorr: 3.24
intensity
m/z
a2
b2
b3
y1
y2
y3
y4
y5
y6
y7
y8
y9
y10
y11

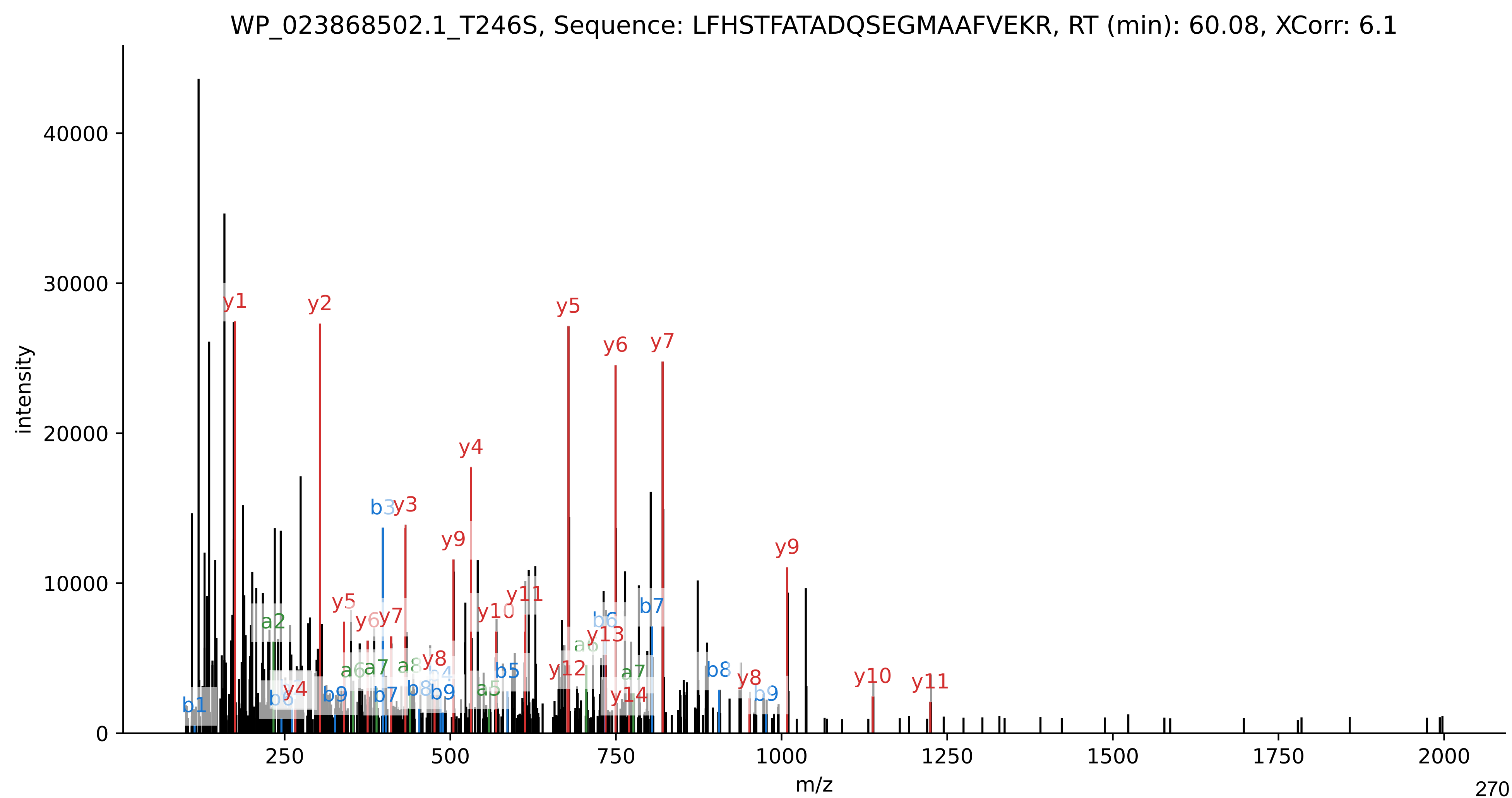

WP_023868502.1_T246S, Sequence: LFHSTFATADQSEGMAAFVEKR, RT (min): 60.08, XCorr: 6.1
intensity
m/z

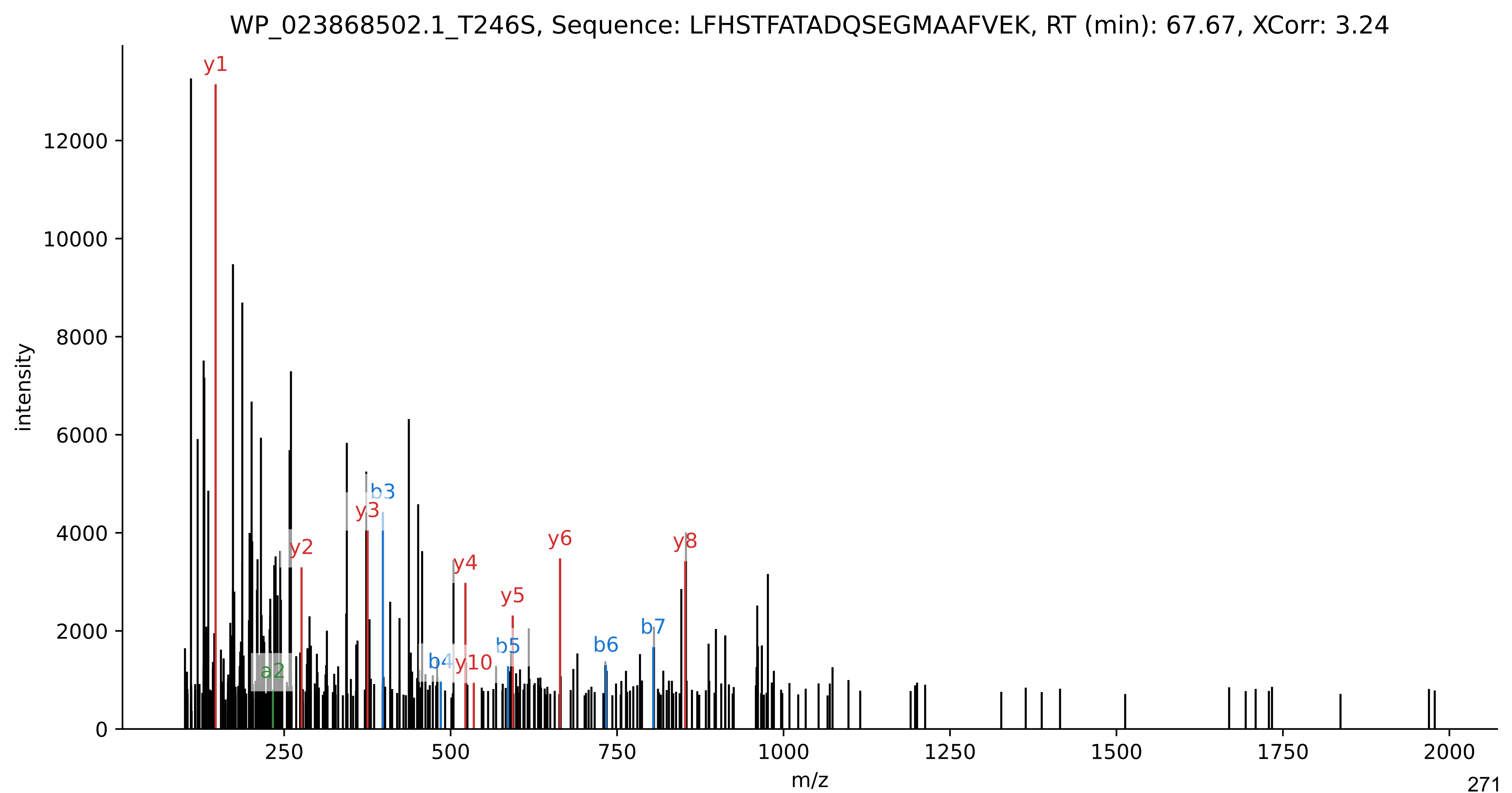

WP_023868502.1_T246S, Sequence: LFHSTFATADQSEGMAAFVEK, RT (min): 67.67, XCorr: 3.24
intensity
m/z
y1
y2
y3
b3
y4
y5
y6
y8
b4
y10
b5
b6
b7
a2
0
2000
4000
6000
8000
10000
12000
250
500
750
1000
1250
1500
1750
2000

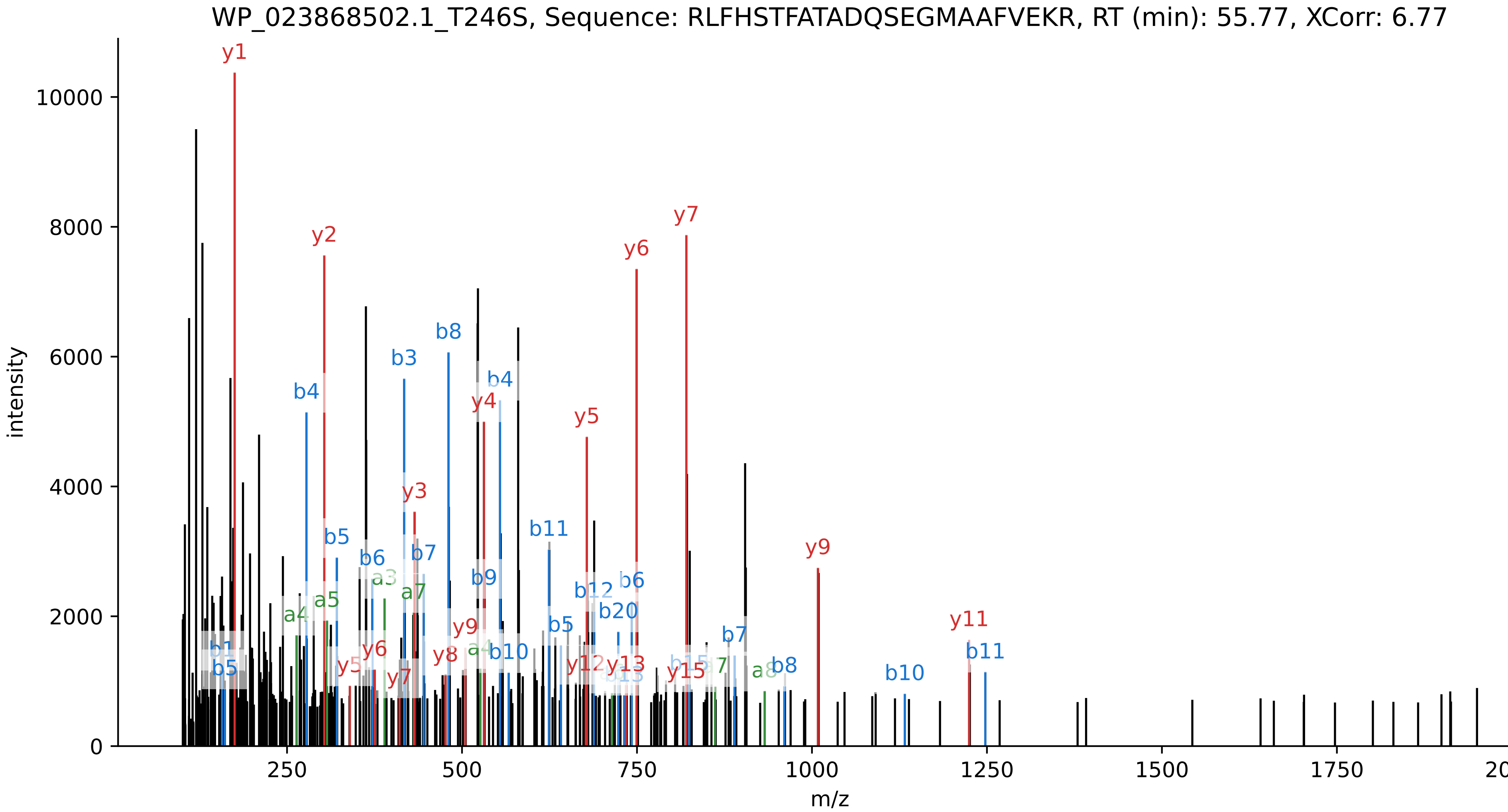
WP_023868502.1_T246S, Sequence: RLFHSTFATADQSEGMAAFVEKR, RT (min): 55.77, XCorr: 6.77
intensity
m/z
0
2000
4000
6000
8000
10000
250
500
750
1000
1250
1500
1750
2000
y1
y2
y3
y4
y5
y6
y7
y9
y11
b3
b4
b5
b6
b7
b8
b9
b10
b11
b12
b20
a4
a5
a7
y8
y12
y13
y15

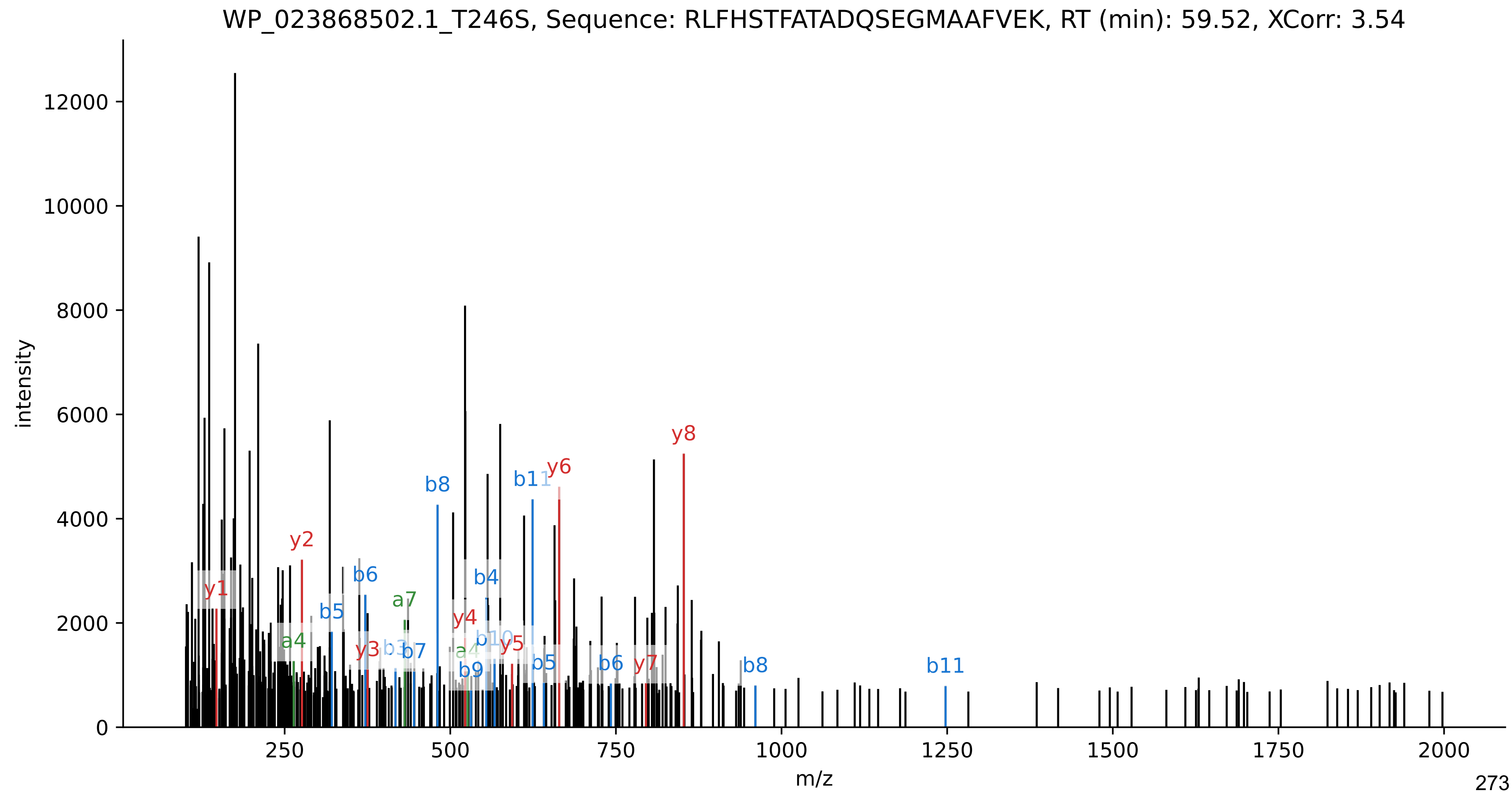
WP_023868502.1_T246S, Sequence: RLFHSTFATADQSEGMAAFVEK, RT (min): 59.52, XCorr: 3.54
intensity
m/z
y1
y2
b5
b6
a4
a7
y3
b3
b7
b8
y4
a4
b9
b10
b4
y5
b11
y6
b5
b6
y7
y8
b8
b11

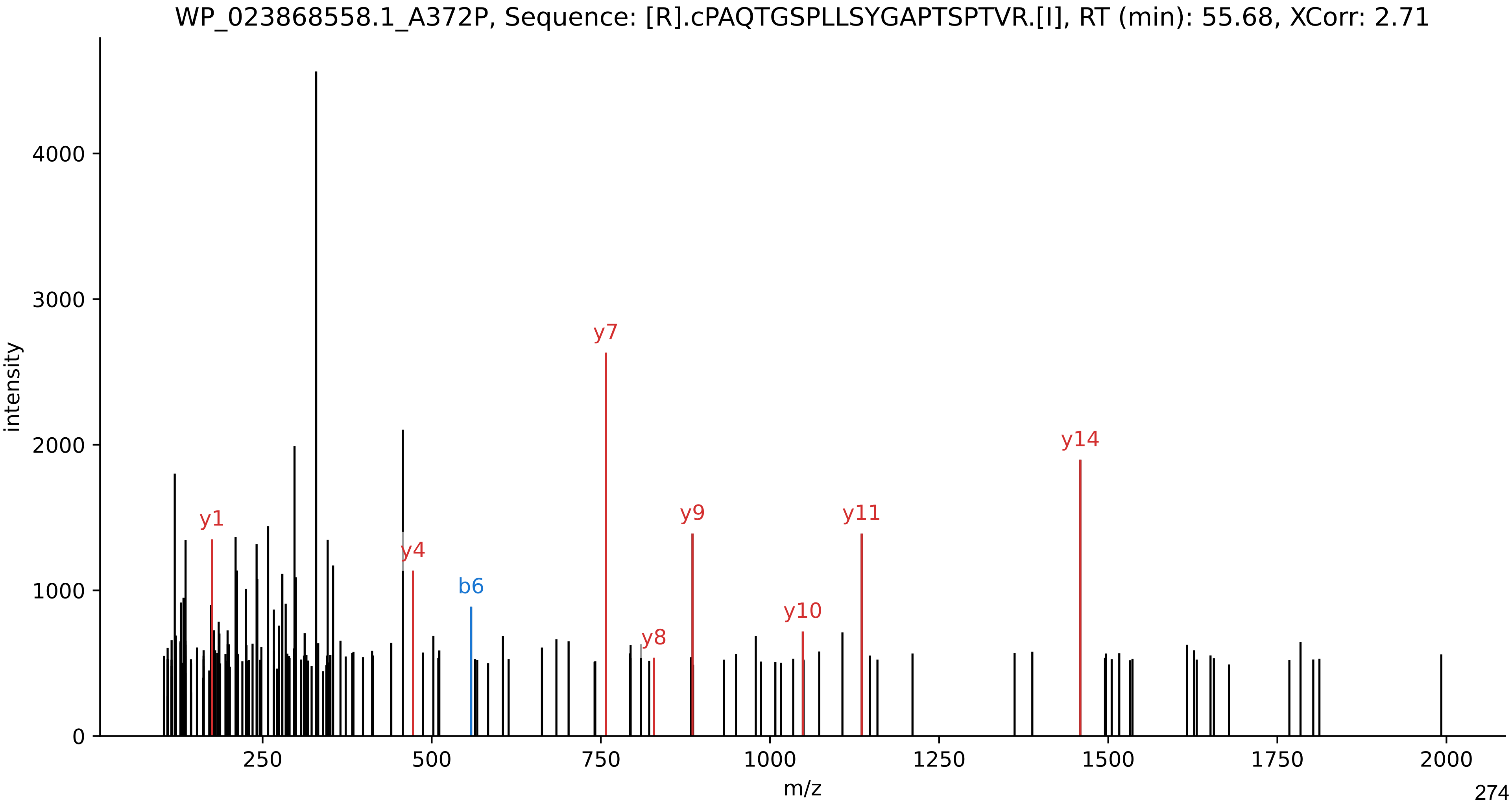
WP_023868558.1_A372P, Sequence: [R].cPAQTGSPLLSYGAPTSPTVR.[I], RT (min): 55.68, XCorr: 2.71
intensity
m/z
0
1000
2000
3000
4000
250
500
750
1000
1250
1500
1750
2000
y1
y4
b6
y7
y8
y9
y10
y11
y14

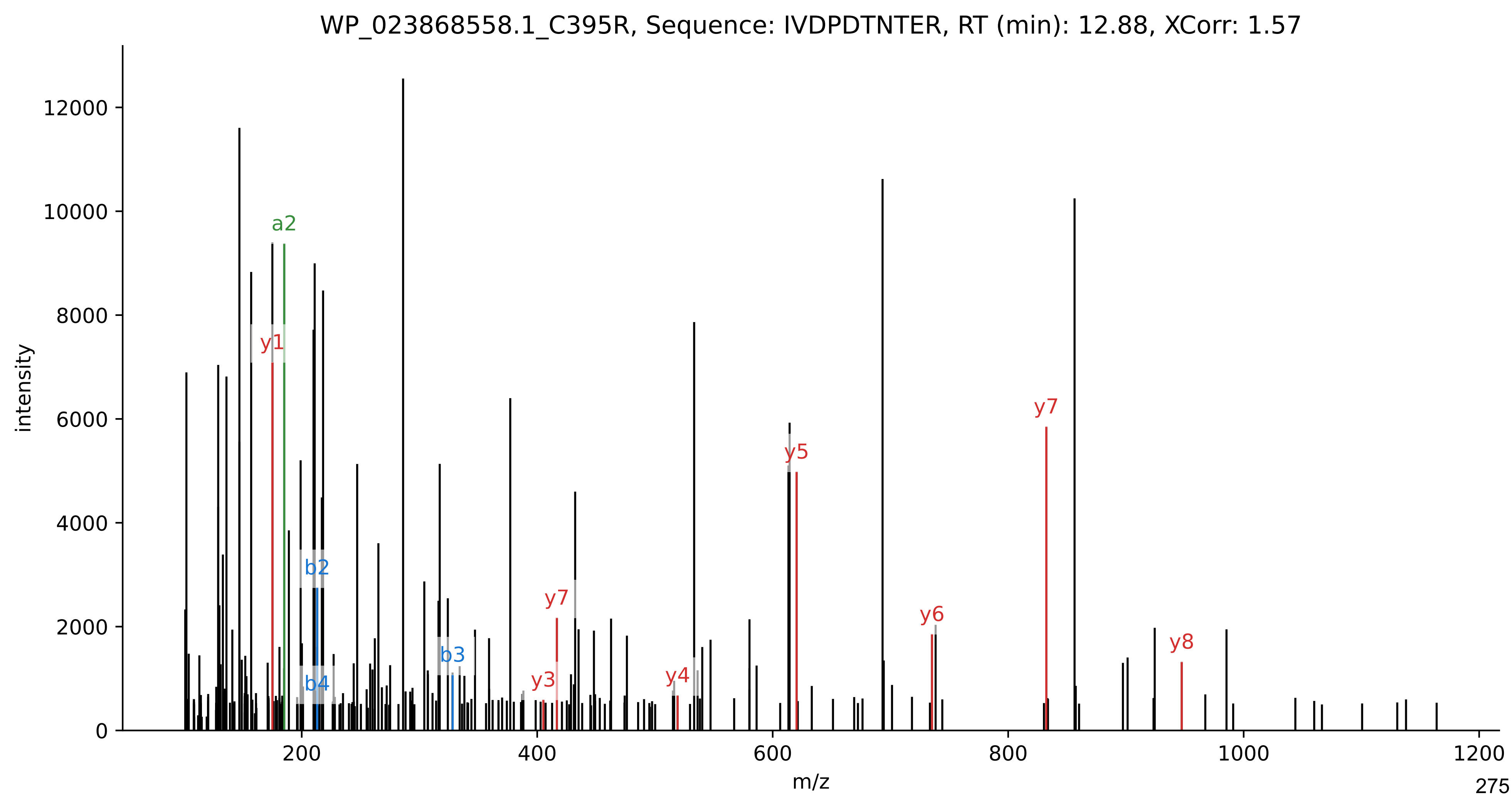

WP_023868558.1_C395R, Sequence: IVDPDTNTER, RT (min): 12.88, XCorr: 1.57
intensity
m/z
a2
y1
b2
b3
b4
y7
y3
y4
y5
y6
y7
y8
0
2000
4000
6000
8000
10000
12000
200
400
600
800
1000
1200

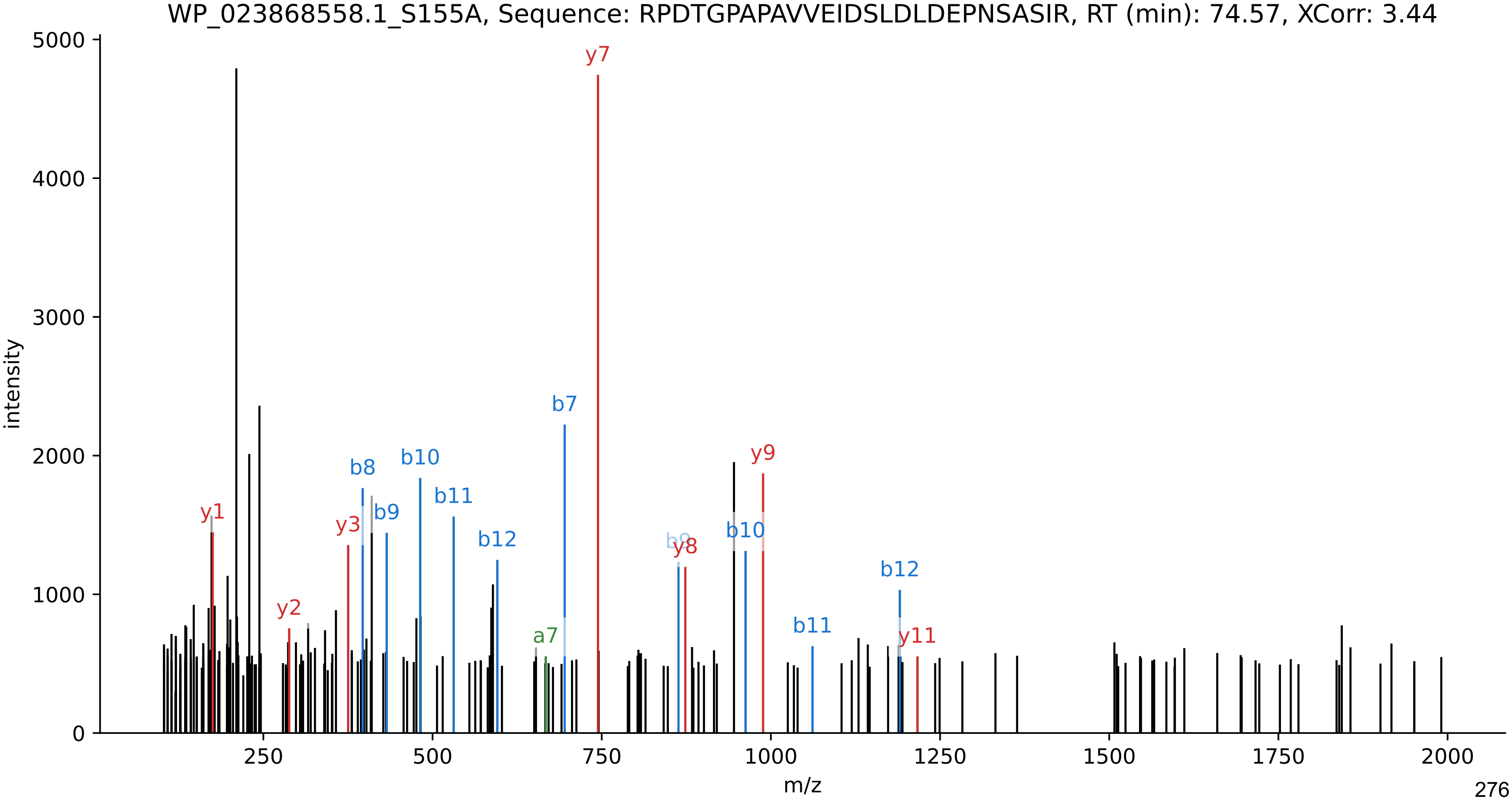

WP_023868558.1_S155A, Sequence: RPDTGPAPAVVEIDSLDLDEPNSASIR, RT (min): 74.57, XCorr: 3.44
intensity
m/z
y7
b7
y9
b10
b8
b11
y1
b9
y3
b12
b10
y8
b12
y2
b11
a7
y11
0
1000
2000
3000
4000
5000
250
500
750
1000
1250
1500
1750
2000

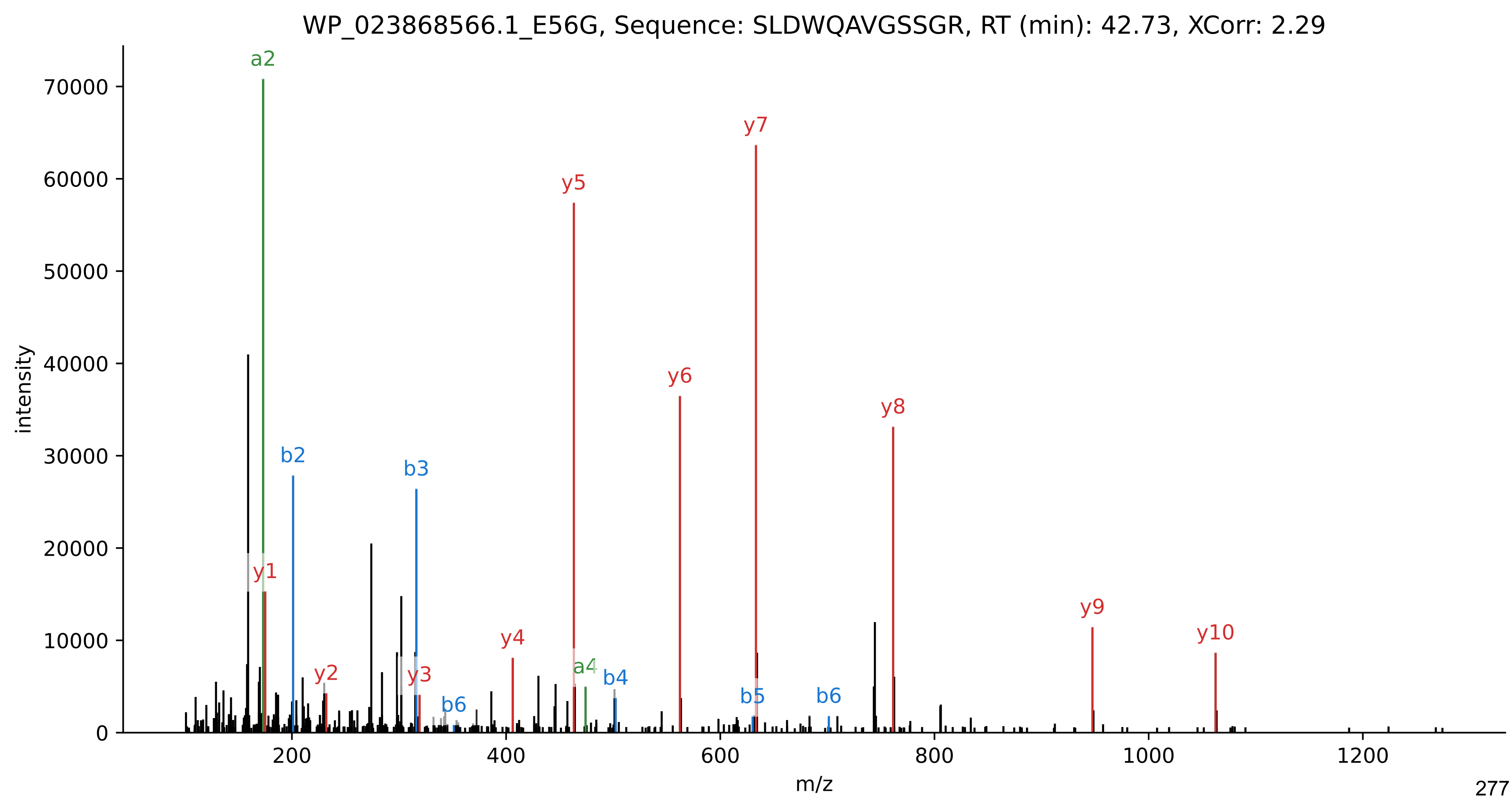

WP_023868566.1_E56G, Sequence: SLDWQAVGSSGR, RT (min): 42.73, XCorr: 2.29
a2
y7
y5
y6
y8
b2
b3
y1
y9
y10
y4
y2
y3
a4
b4
b6
b5
b6
intensity
m/z
0
10000
20000
30000
40000
50000
60000
70000
200
400
600
800
1000
1200

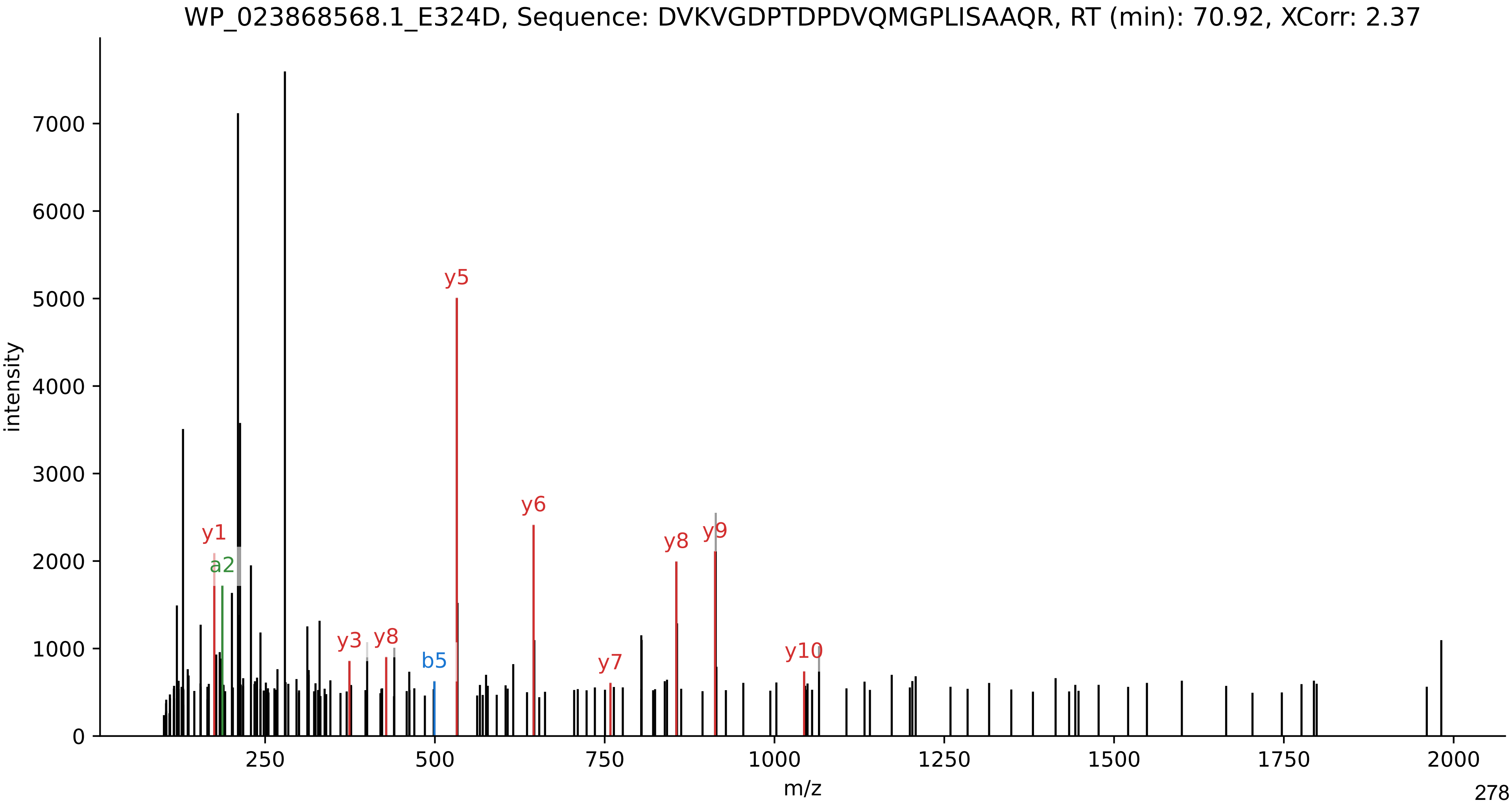

WP_023868568.1_E324D, Sequence: DVKVGDPTDPDVQMGPLISAAQR, RT (min): 70.92, XCorr: 2.37
intensity
m/z
y1
a2
y3
y8
b5
y5
y6
y7
y8
y9
y10
0
1000
2000
3000
4000
5000
6000
7000
250
500
750
1000
1250
1500
1750
2000

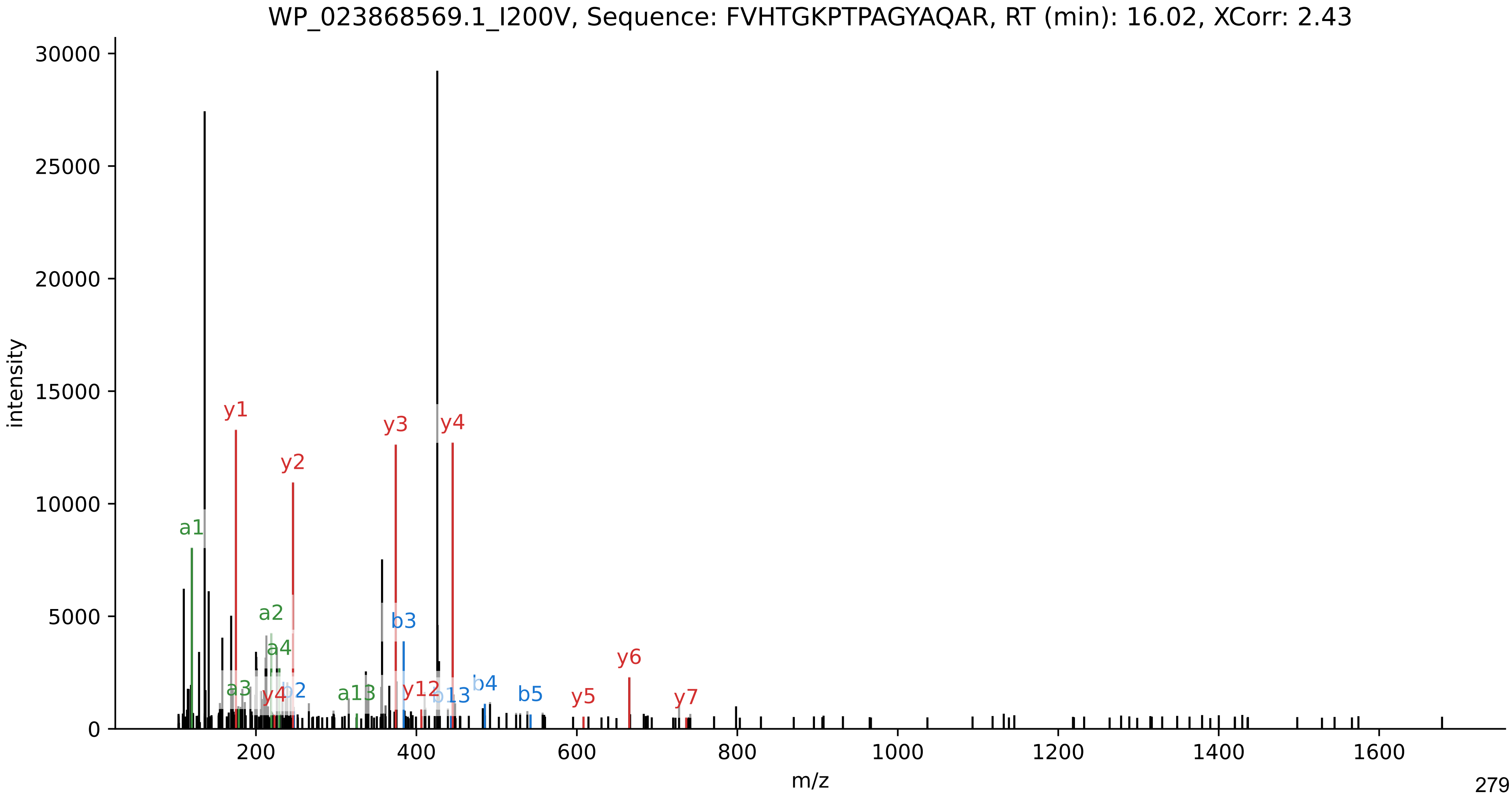

WP_023868569.1_I200V, Sequence: FVHTGKPTPAGYAQAR, RT (min): 16.02, XCorr: 2.43
intensity
m/z
0
5000
10000
15000
20000
25000
30000
200
400
600
800
1000
1200
1400
1600
a1
y1
y2
y3
y4
a2
b3
a4
a3
y4
b2
a13
y12
b13
b4
b5
y5
y6
y7

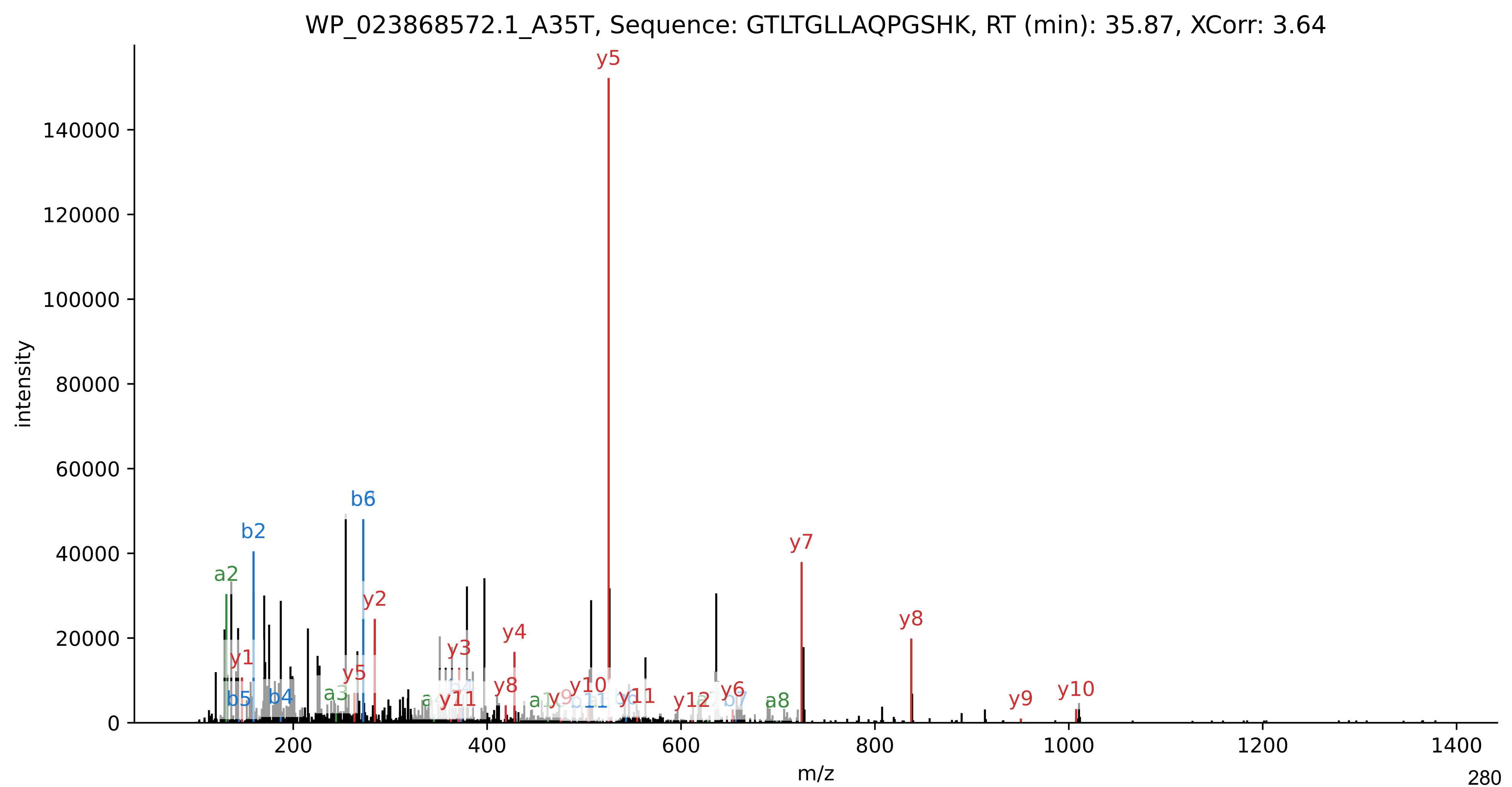
WP_023868572.1_A35T, Sequence: GTLTGLLAQPGSHK, RT (min): 35.87, XCorr: 3.64
intensity
m/z
y5
b6
b2
a2
y7
y2
y8
y4
y3
y1
y5
y8
y10
y11
y12
y6
y9
y10
b5
b4
a3
a8
0
20000
40000
60000
80000
100000
120000
140000
200
400
600
800
1000
1200
1400

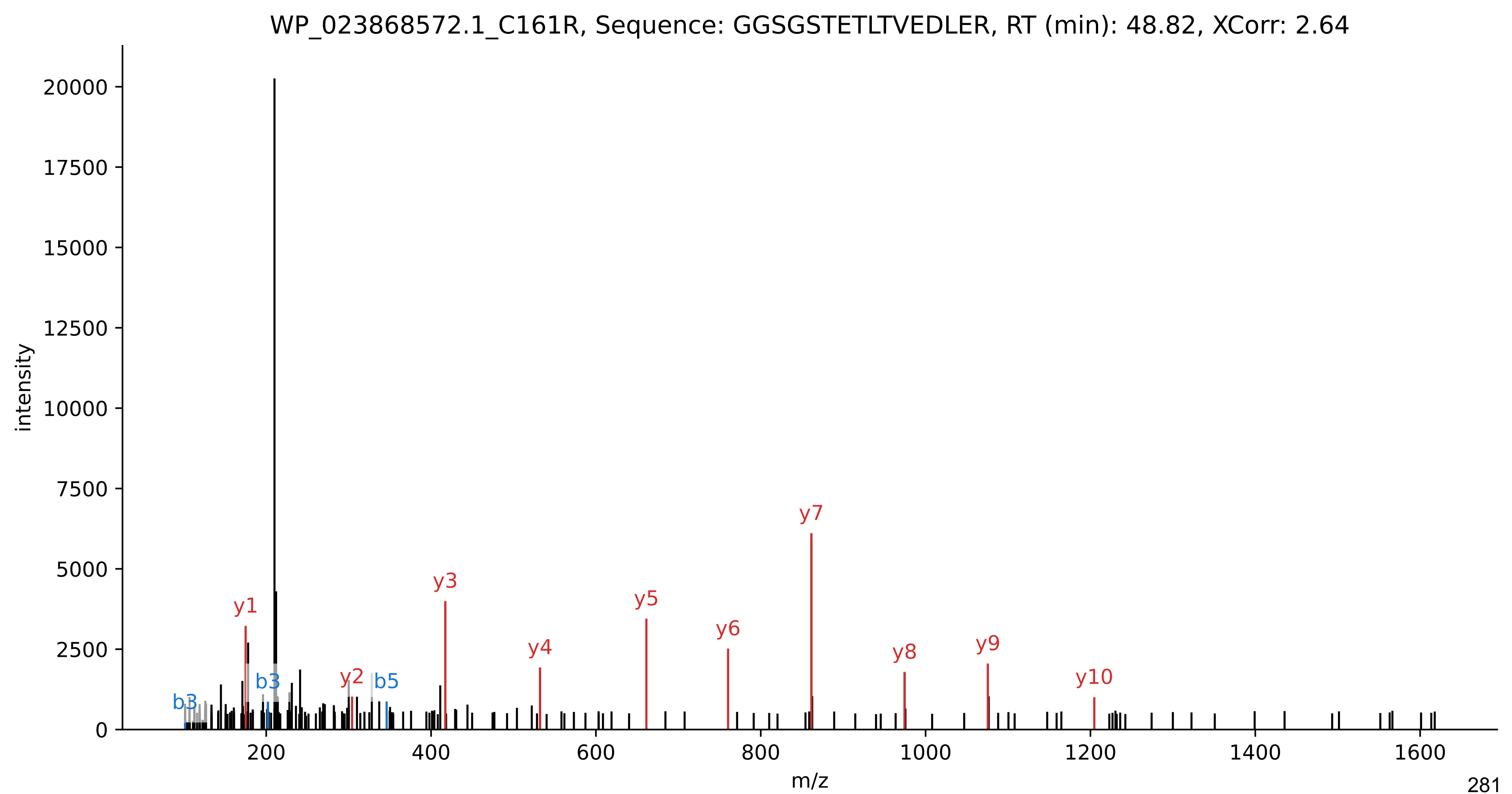

WP_023868572.1_C161R, Sequence: GGSGSTETLTVEDLER, RT (min): 48.82, XCorr: 2.64
intensity
m/z
y1
y2
y3
y4
y5
y6
y7
y8
y9
y10
b3
b3
b5

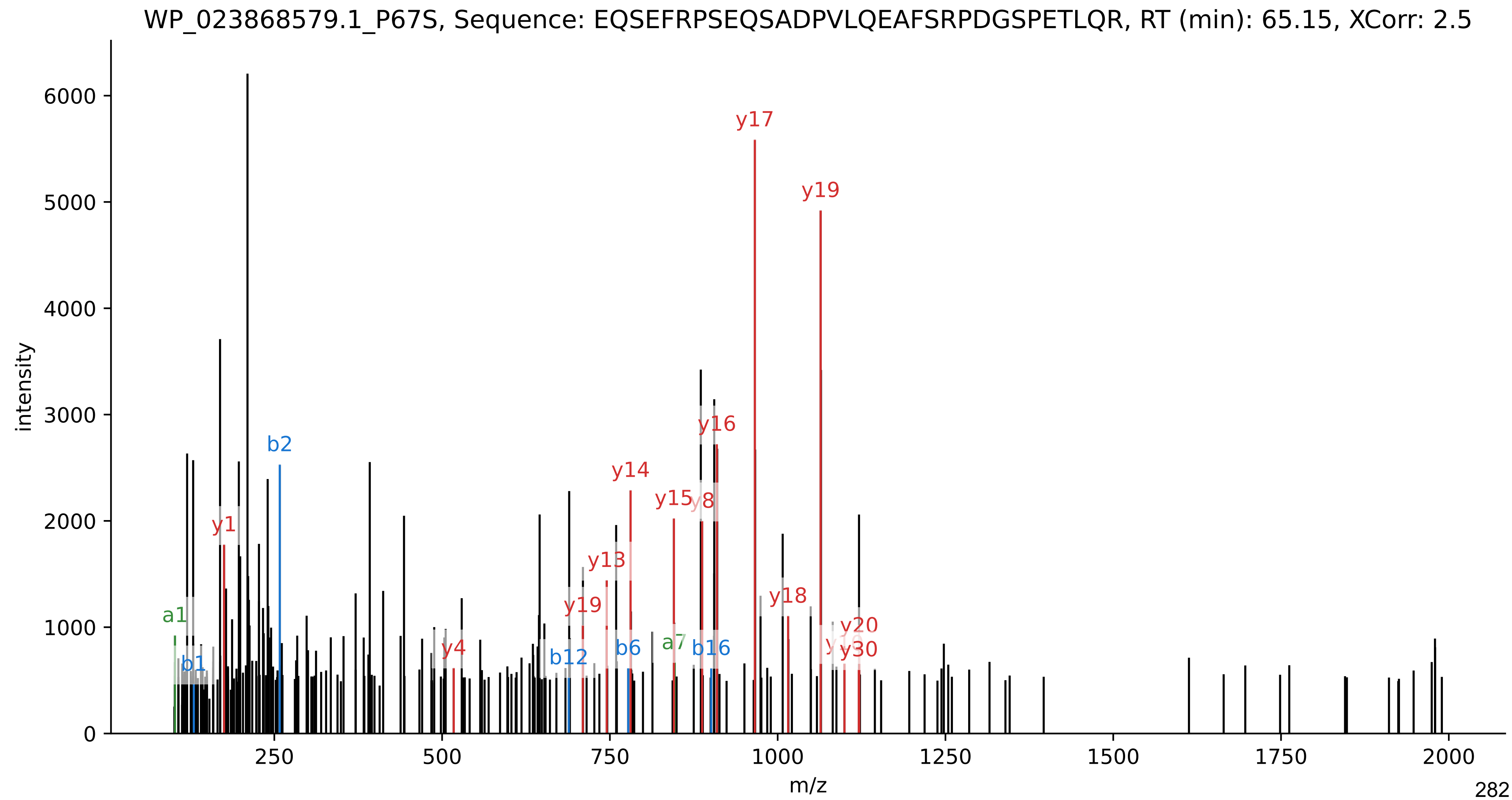

WP_023868579.1_P67S, Sequence: EQSEFRPSEQSADPVLQEAFSRPDGSPETLQR, RT (min): 65.15, XCorr: 2.5
intensity
m/z
y17
y19
y16
b2
y14
y15
y8
y1
y13
y18
y19
a1
y20
y4
a7
b6
b16
b12
y30
b1

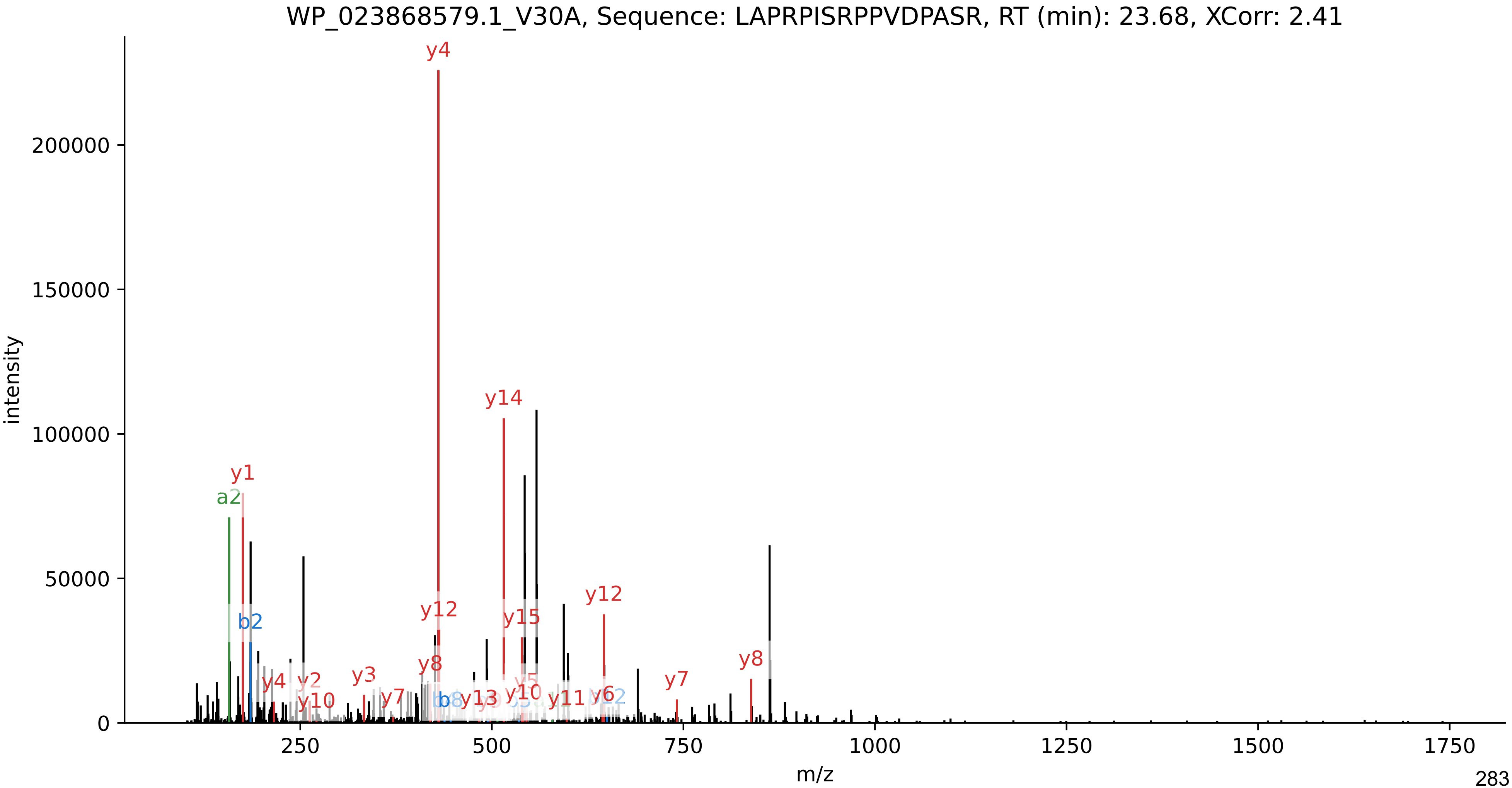

WP_023868579.1_V30A, Sequence: LAPRPISRPPVDPASR, RT (min): 23.68, XCorr: 2.41
intensity
m/z
y4
y14
y1
a2
b2
y12
y15
y12
y8
y8
y7
y3
y4
y2
y10
y7
y13
y10
y11
y6
0
50000
100000
150000
200000
250
500
750
1000
1250
1500
1750

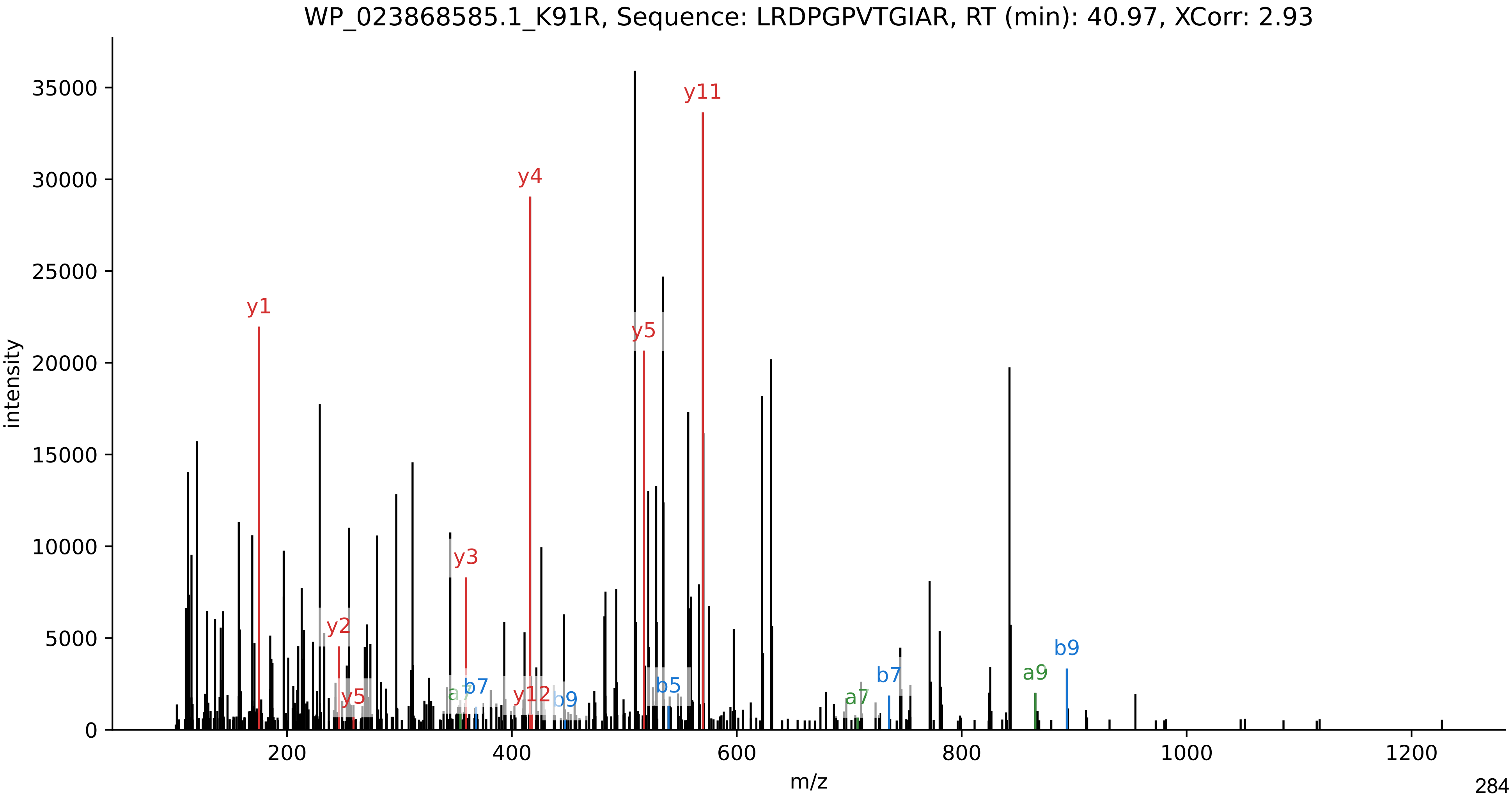

WP_023868585.1_K91R, Sequence: LRDPGPVTGIAR, RT (min): 40.97, XCorr: 2.93
intensity
m/z
y1
y2
y3
y4
y5
y11
y12
b5
b7
b9
a7
a9

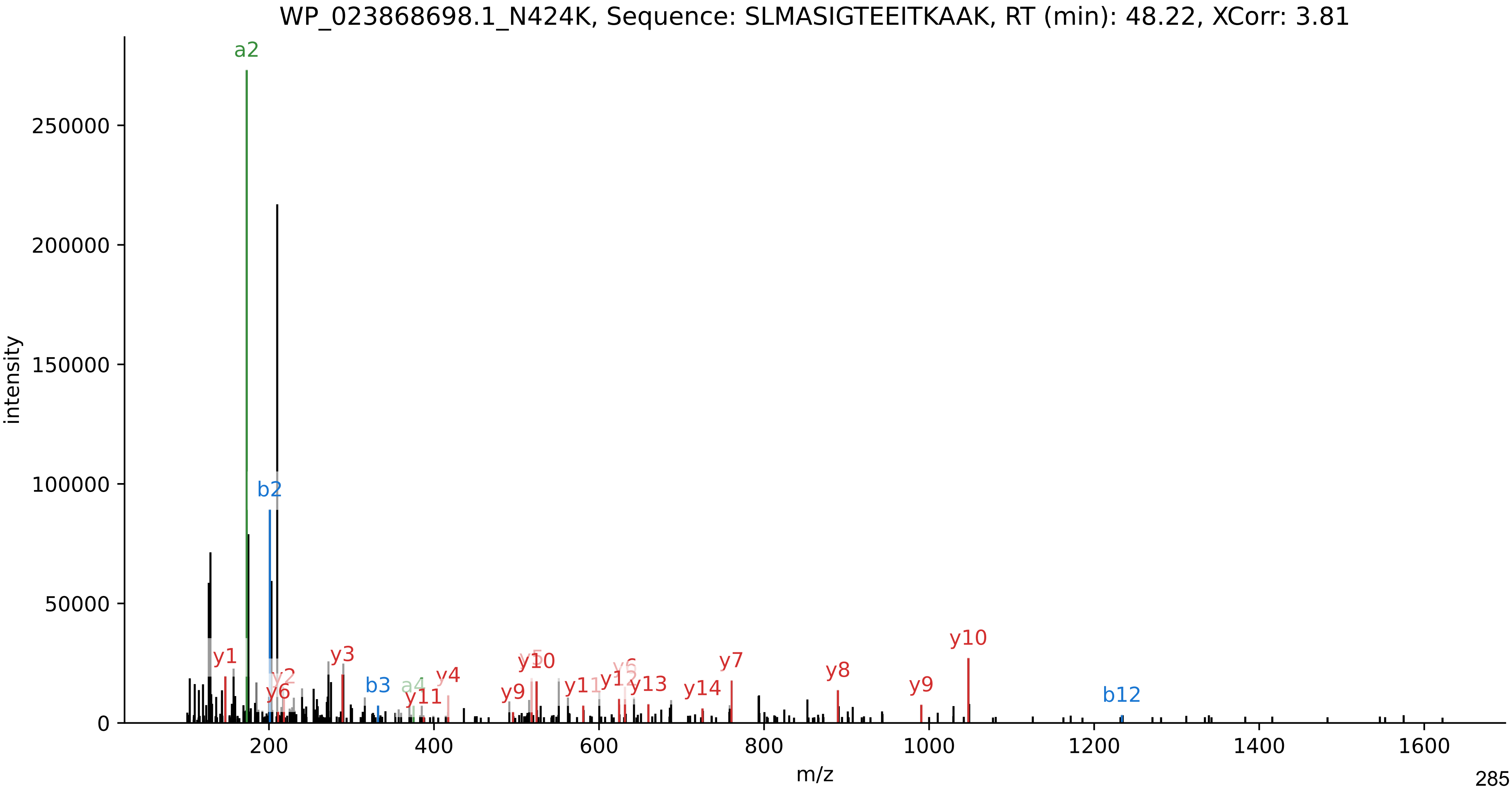
WP_023868698.1_N424K, Sequence: SLMASIGTEEITKAAK, RT (min): 48.22, XCorr: 3.81
intensity
m/z
a2
b2
y1
y2
y6
y3
b3
a4
y11
y4
y9
y5
y10
y11
y12
y6
y13
y14
y7
y8
y9
y10
b12

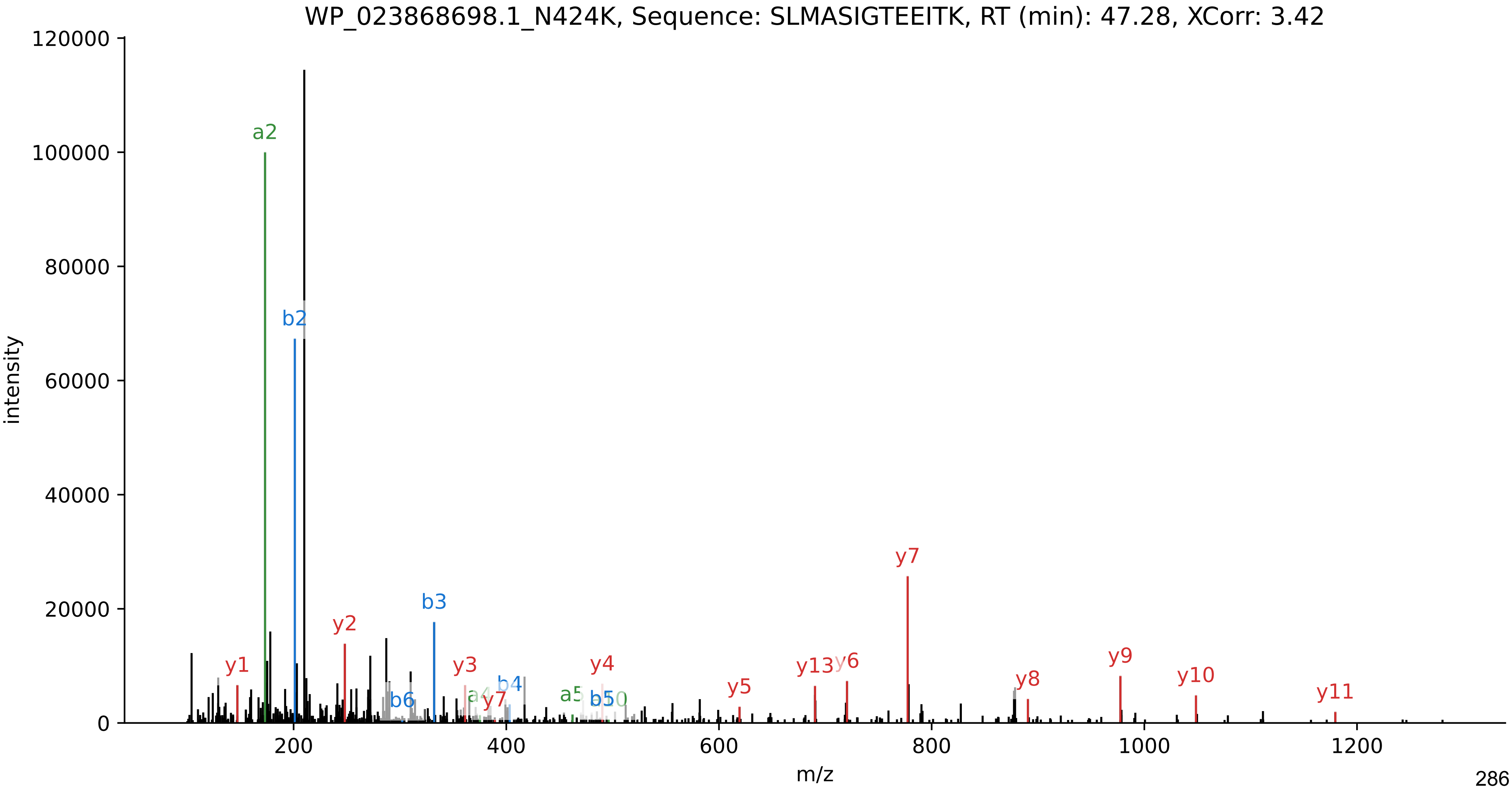

WP_023868698.1_N424K, Sequence: SLMASIGTEEITK, RT (min): 47.28, XCorr: 3.42
intensity
m/z
a2
b2
b3
y2
y1
y3
y4
y5
y13
y6
y7
y8
y9
y10
y11
b4
b6
a5
b5

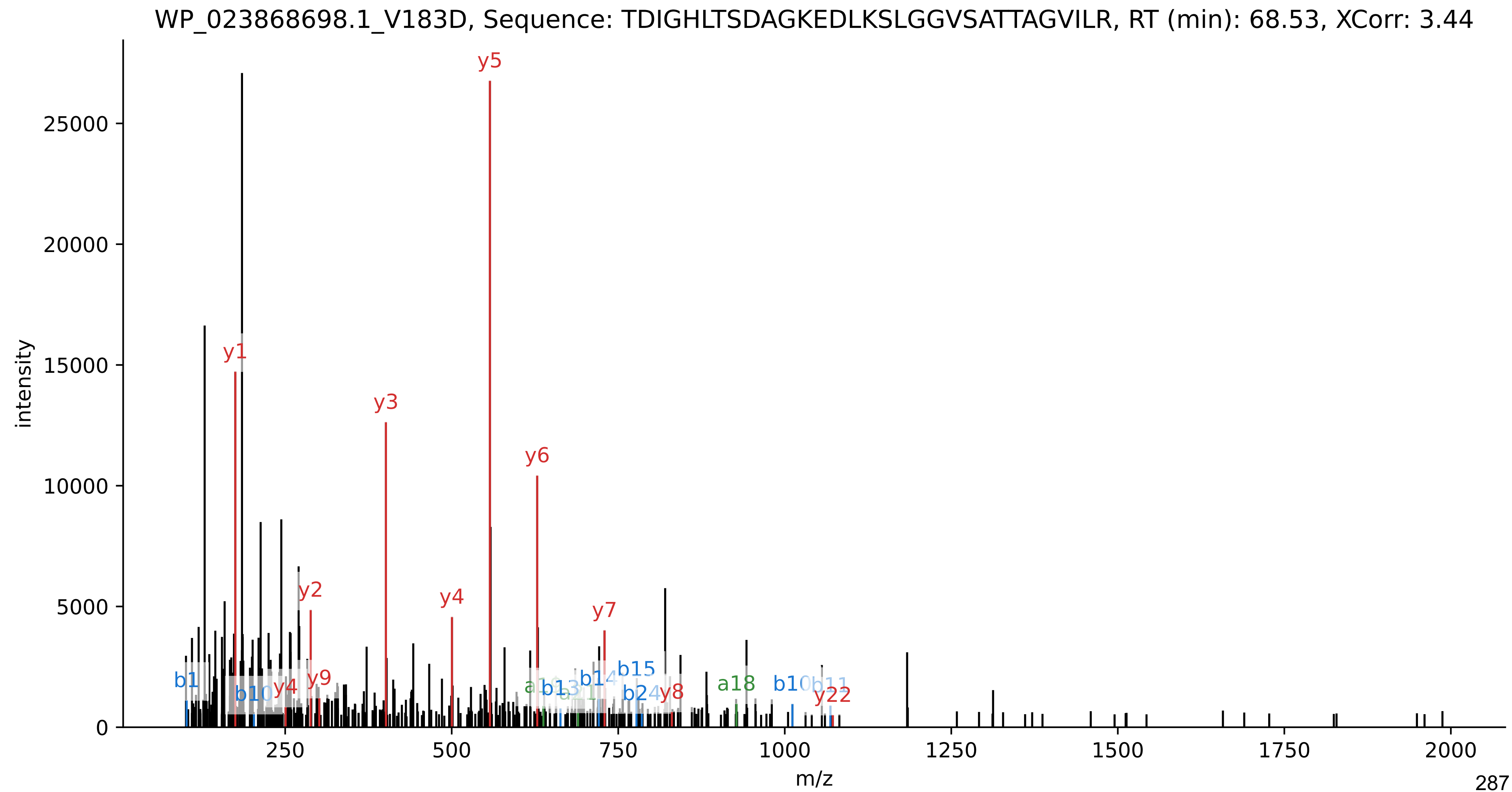
WP_023868698.1_V183D, Sequence: TDIGHLTSDAGKEDLKSLGGVSATTAGVILR, RT (min): 68.53, XCorr: 3.44
intensity
m/z
y5
y1
y3
y6
y2
y4
y7
y9
y8
y22
b1
b15
a18
b10
b11

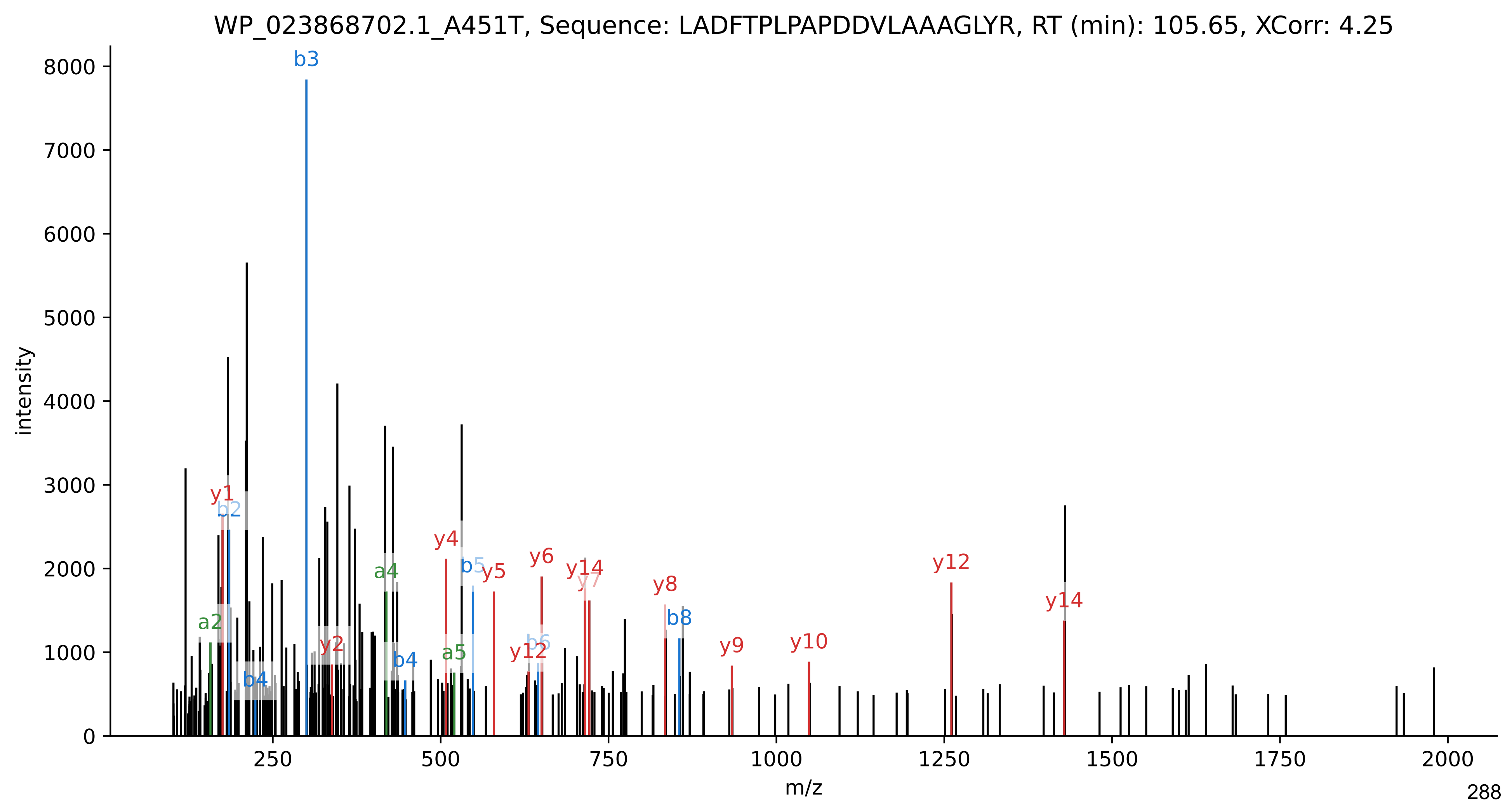
WP_023868702.1_A451T, Sequence: LADFTPLPAPDDVLAAAGLYR, RT (min): 105.65, XCorr: 4.25
intensity
m/z
b3
y1
b2
a2
b4
y2
b4
a4
y4
b5
y5
a5
y12
b6
y6
y14
y7
y8
b8
y9
y10
y12
y14
0
1000
2000
3000
4000
5000
6000
7000
8000
250
500
750
1000
1250
1500
1750
2000

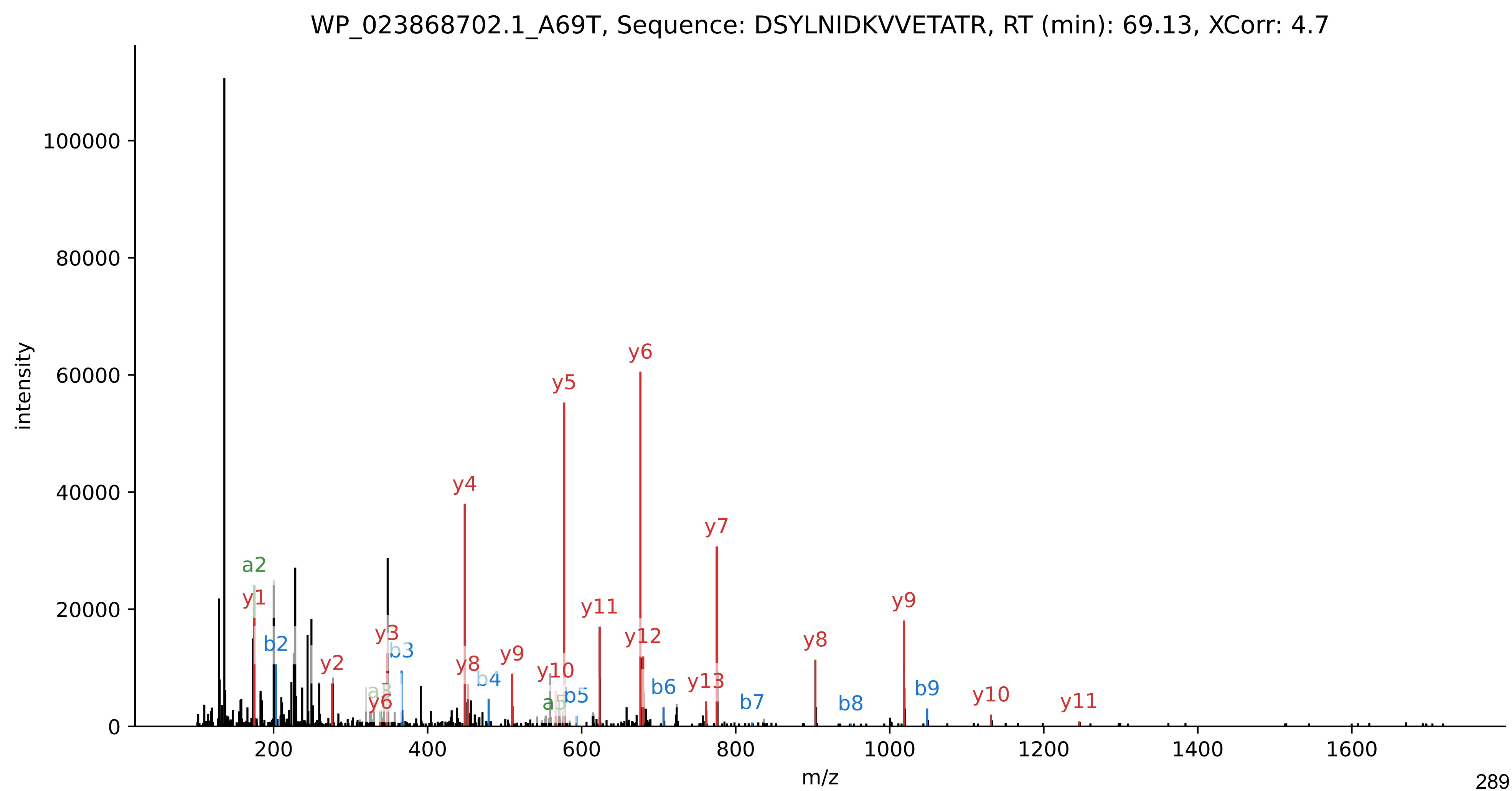

WP_023868702.1_A69T, Sequence: DSYLNIDKVVETATR, RT (min): 69.13, XCorr: 4.7
intensity
m/z
y6
y5
y4
y7
y11
y12
y9
y8
y13
y10
y1
y2
y3
a2
b2
b3
b4
b5
b6
b7
b8
b9
a5

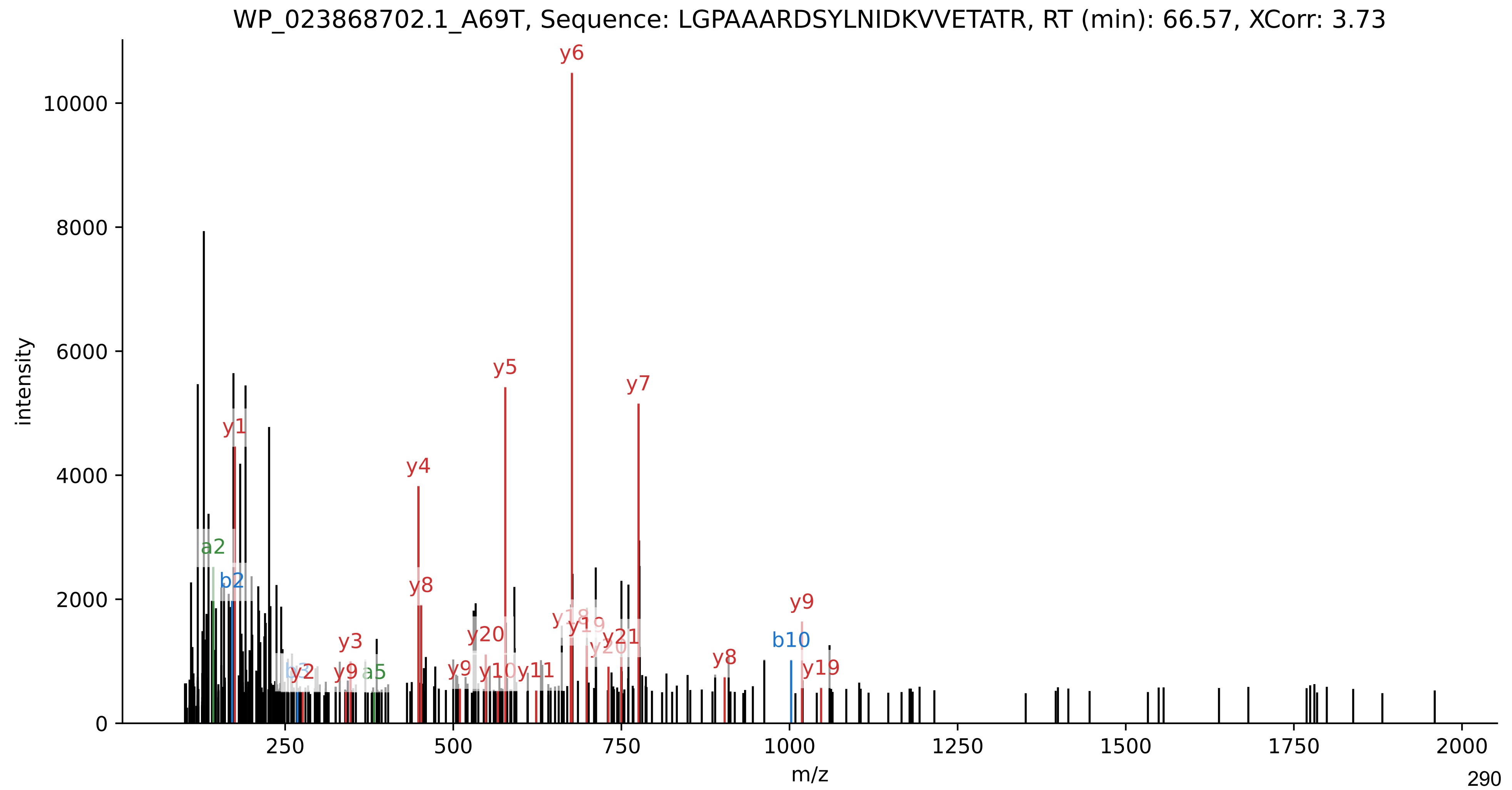
WP_023868702.1_A69T, Sequence: LGPAAARDSYLNIDKVVETATR, RT (min): 66.57, XCorr: 3.73
intensity
m/z
10000
8000
6000
4000
2000
0
250
500
750
1000
1250
1500
1750
2000
y6
y5
y7
y1
y4
a2
b2
y8
y9
b10
y20
y18
y19
y21
y20
y3
y8
y19
y2
y9
a5
y9
y10
y11

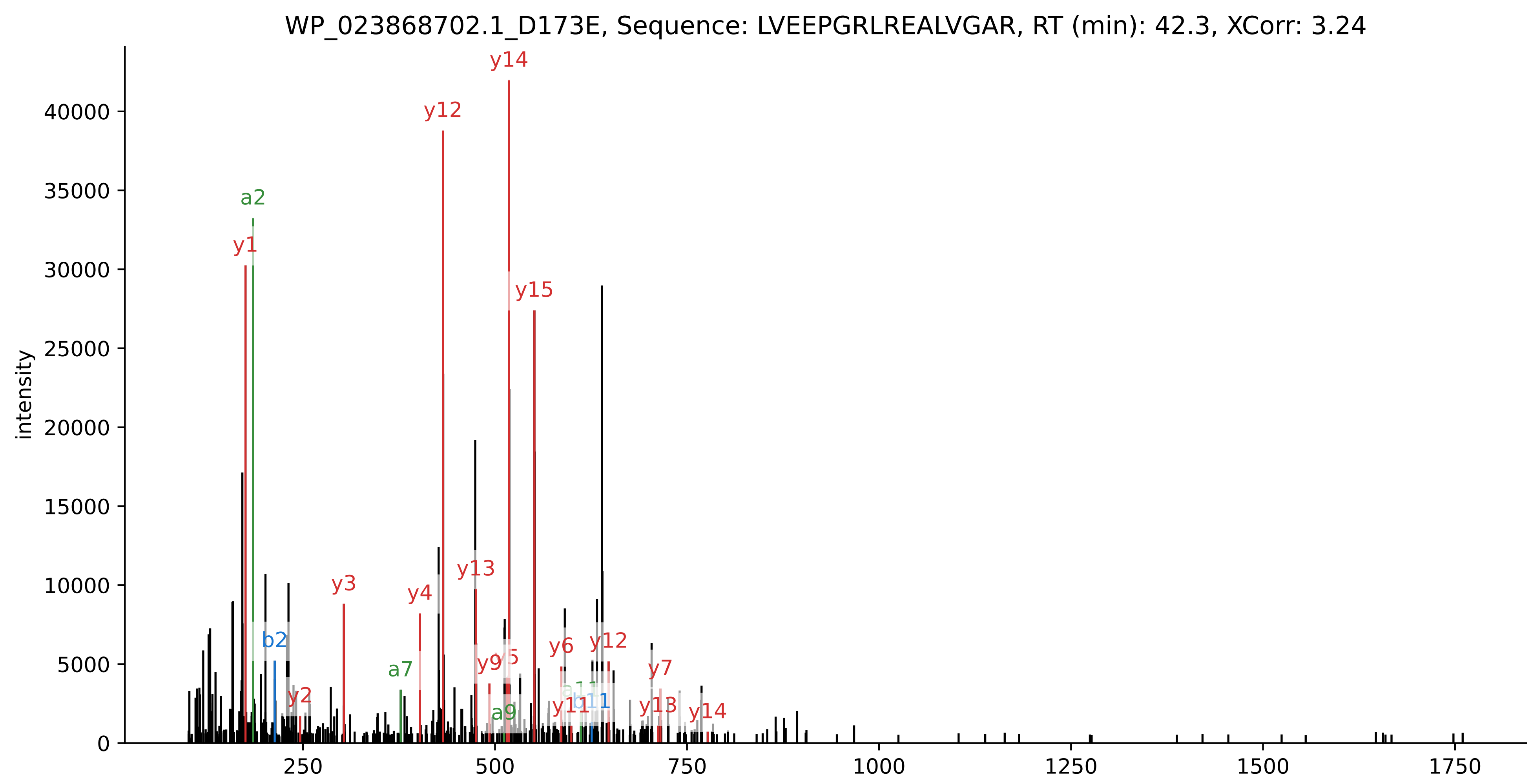
WP_023868702.1_D173E, Sequence: LVEEPGRLREALVGAR, RT (min): 42.3, XCorr: 3.24
intensity
m/z
0
5000
10000
15000
20000
25000
30000
35000
40000
250
500
750
1000
1250
1500
1750
y14
y12
a2
y1
y15
y13
y3
y4
b2
y2
a7
y9
y6
y12
y7
y13
y14
a9
y11
b11

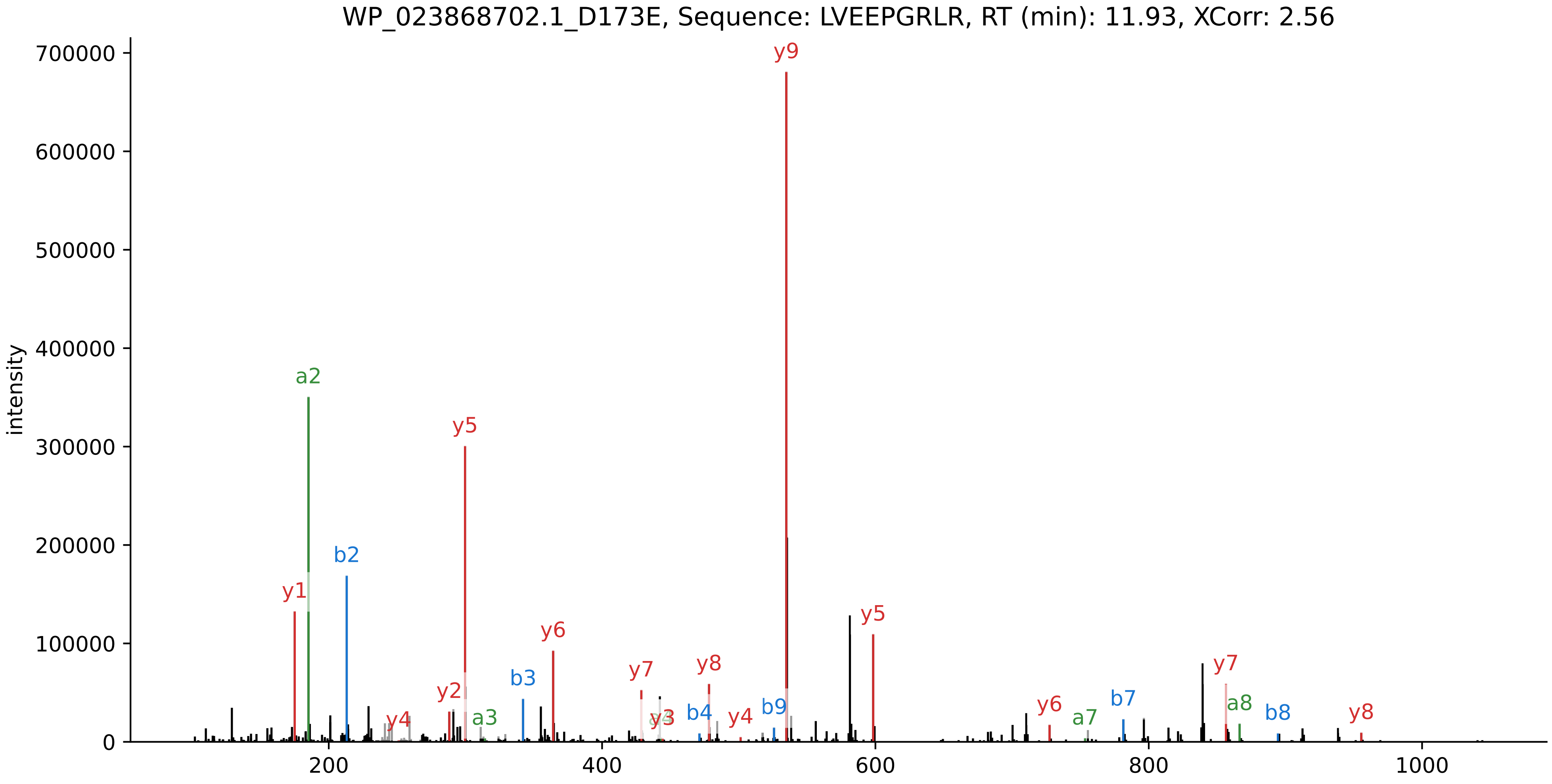
WP_023868702.1_D173E, Sequence: LVEEPGRLR, RT (min): 11.93, XCorr: 2.56
intensity
m/z
0
100000
200000
300000
400000
500000
600000
700000
200
400
600
800
1000
y9
a2
y5
b2
y1
y6
y5
y7
y8
b3
y2
y4
a3
y3
b4
y4
b9
y6
a7
b7
y7
a8
b8
y8

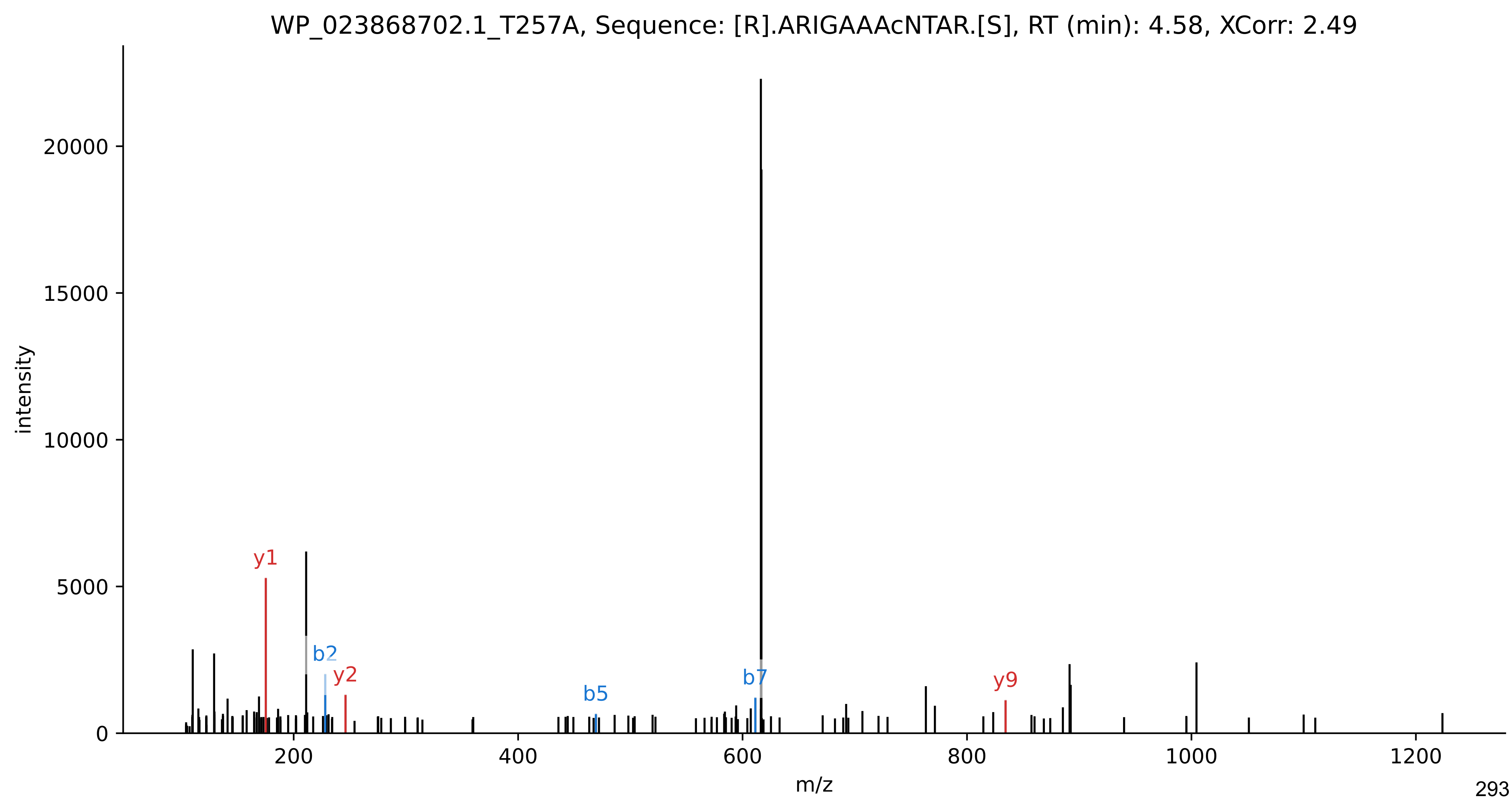
WP_023868702.1_T257A, Sequence: [R].ARIGAAAcNTAR.[S], RT (min): 4.58, XCorr: 2.49
intensity
m/z
y1
b2
y2
b5
b7
y9
0
5000
10000
15000
20000
200
400
600
800
1000
1200

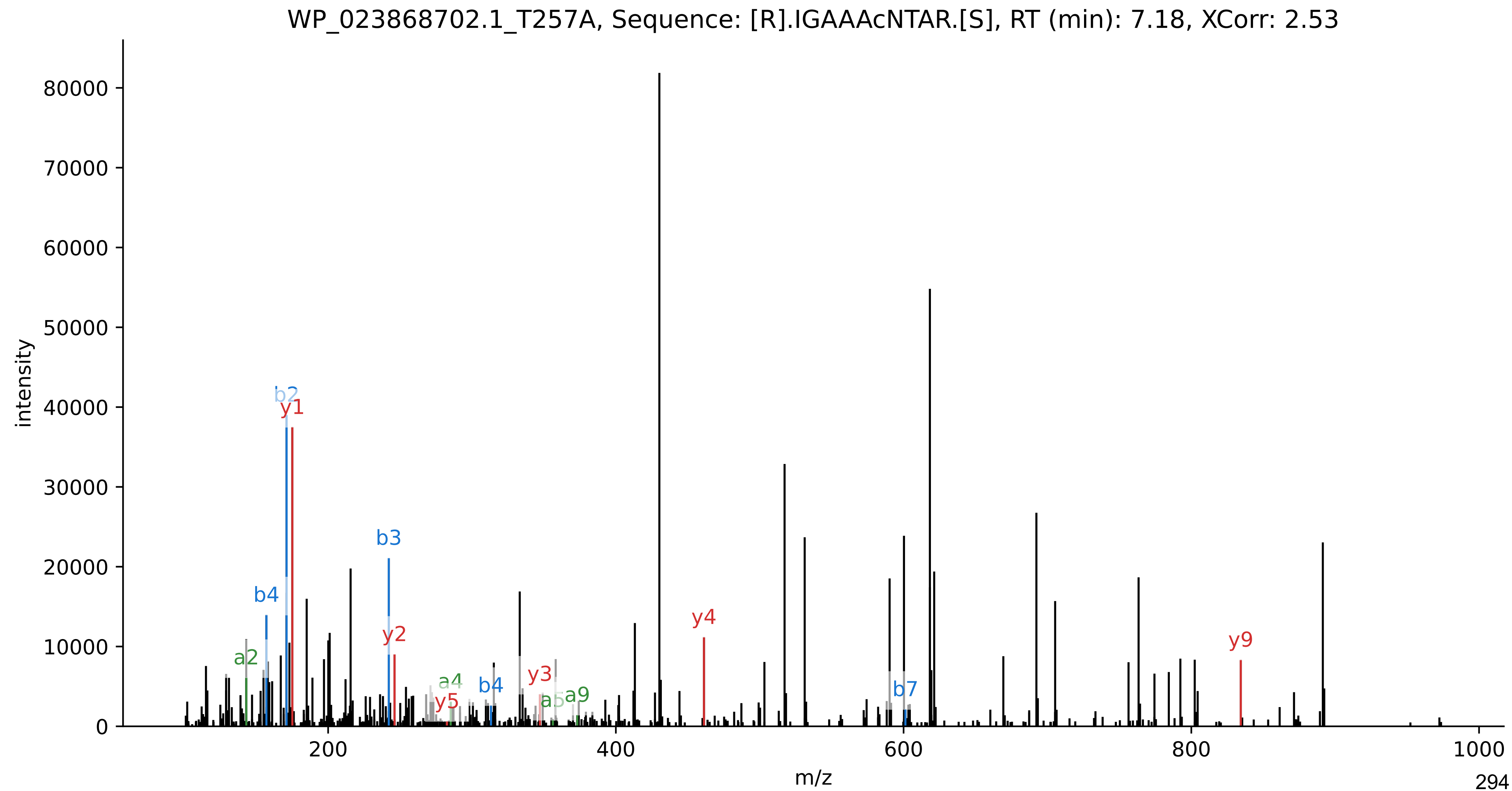

WP_023868702.1_T257A, Sequence: [R].IGAAAcNTAR.[S], RT (min): 7.18, XCorr: 2.53
intensity
m/z
b2
y1
b3
b4
y2
a2
a4
y5
b4
y3
a5
a9
y4
b7
y9

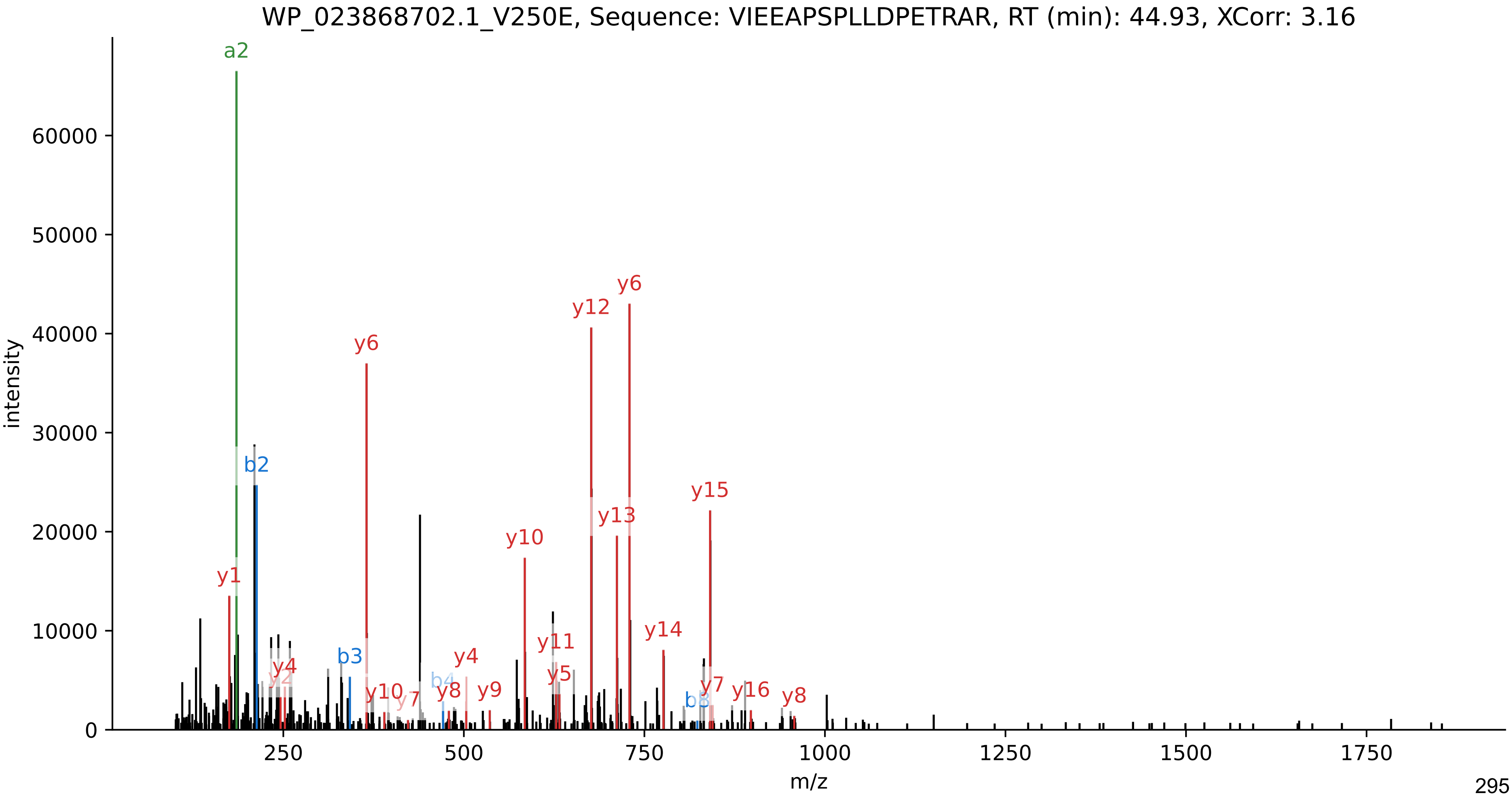
WP_023868702.1_V250E, Sequence: VIEEAPSPLLDPETRAR, RT (min): 44.93, XCorr: 3.16
intensity
m/z
a2
b2
b3
b4
b8
y1
y2
y4
y6
y7
y8
y9
y10
y11
y12
y13
y14
y15
y16
y5
0
10000
20000
30000
40000
50000
60000
250
500
750
1000
1250
1500
1750

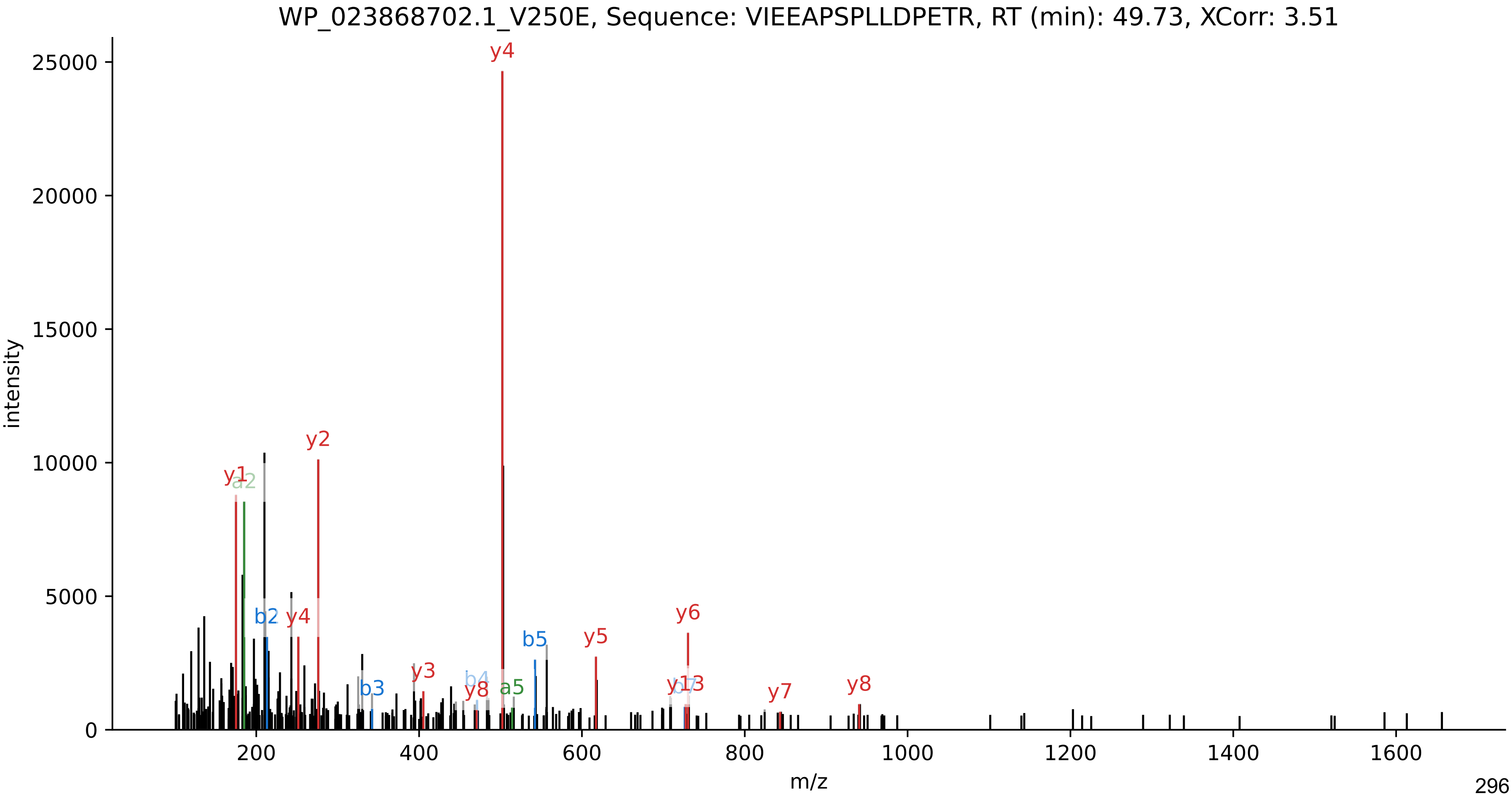

WP_023868702.1_V250E, Sequence: VIEEAPSPLLDPETR, RT (min): 49.73, XCorr: 3.51
intensity
m/z
y4
y2
y1
a2
b2
y4
b3
y3
b4
y8
a5
b5
y5
y6
y13
b7
y7
y8

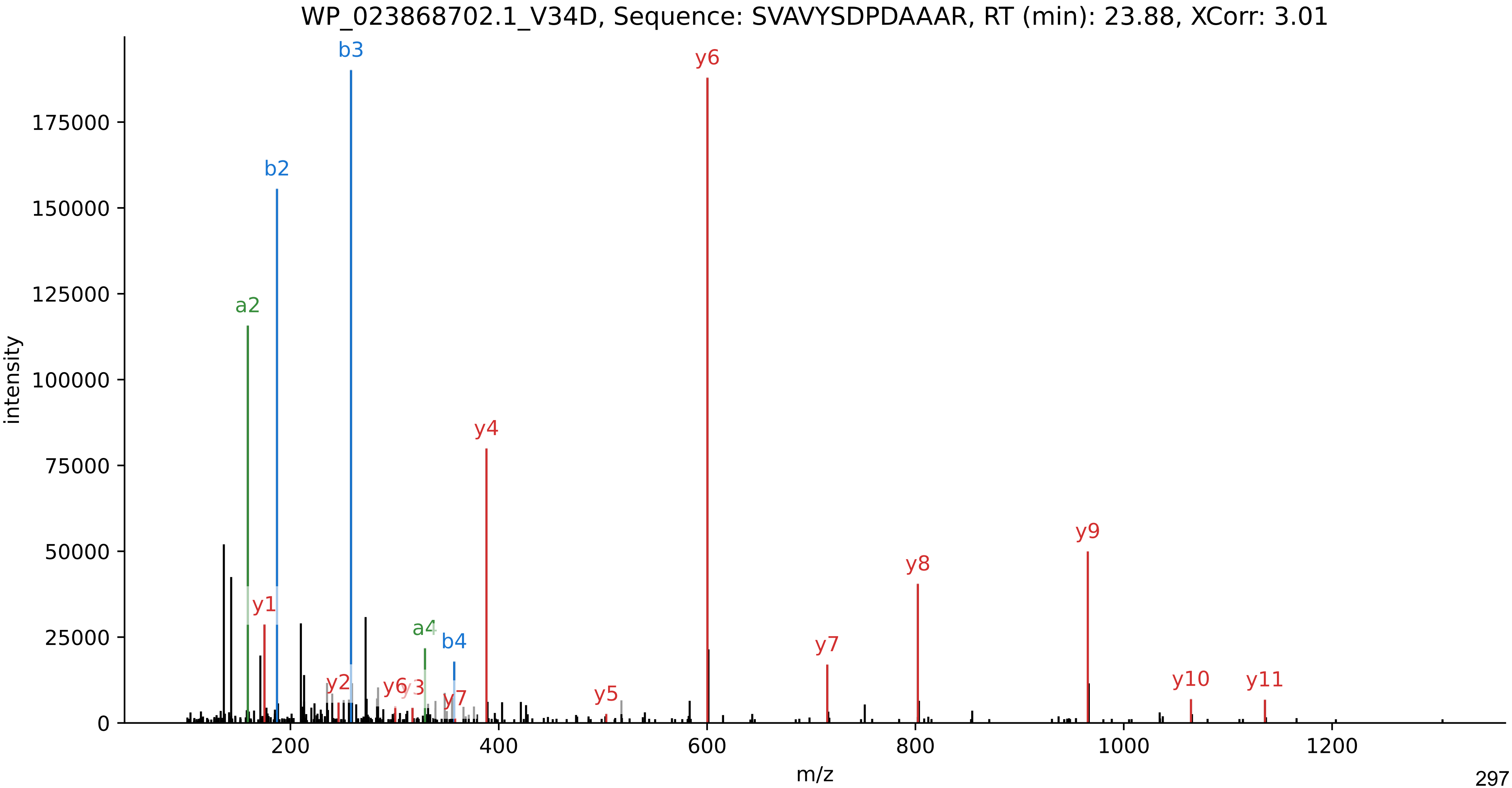
WP_023868702.1_V34D, Sequence: SVAVYSDPDAAAR, RT (min): 23.88, XCorr: 3.01
intensity
m/z
b3
y6
b2
a2
y4
y9
y8
y1
a4
b4
y7
y2
y6
y3
y7
y5
y10
y11
0
25000
50000
75000
100000
125000
150000
175000
200
400
600
800
1000
1200

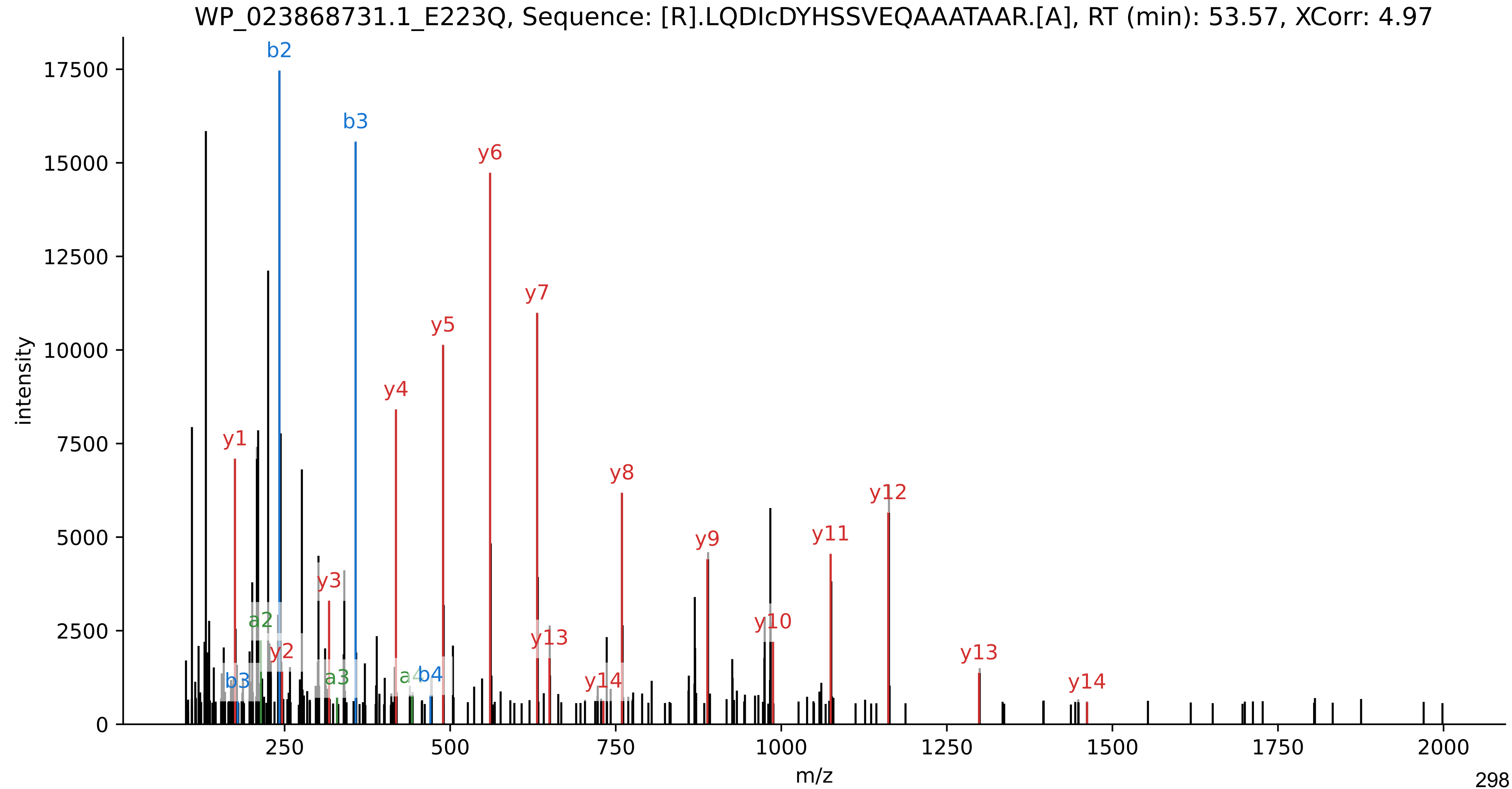
WP_023868731.1_E223Q, Sequence: [R].LQDIcDYHSSVEQAAATAAR.[A], RT (min): 53.57, XCorr: 4.97
intensity
m/z
b2
b3
y6
y7
y5
y4
y1
y8
y12
y11
y9
y3
a2
y2
y10
y13
b3
a3
b4
y14
y13
y14
17500
15000
12500
10000
7500
5000
2500
0
250
500
750
1000
1250
1500
1750
2000

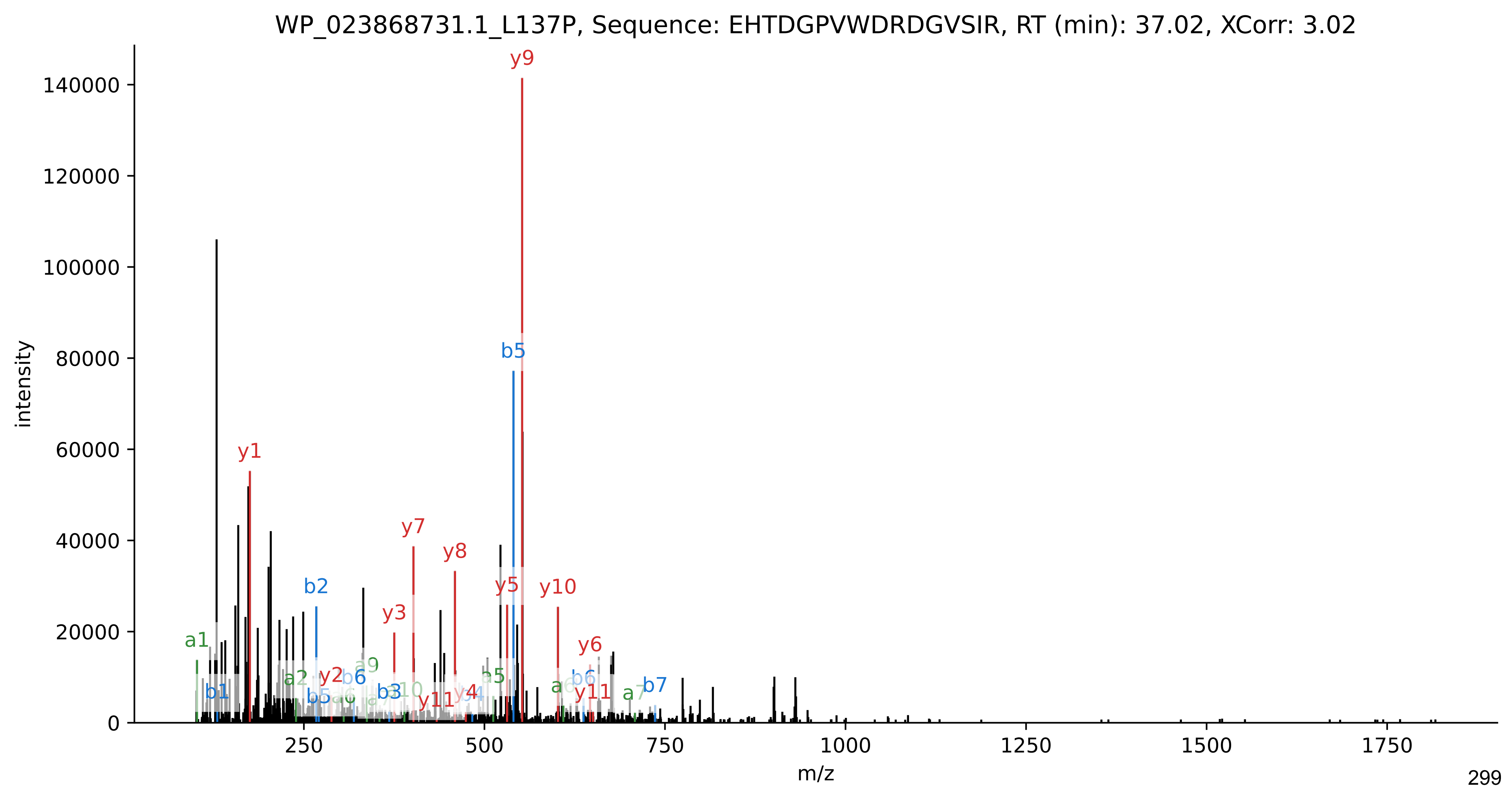
WP_023868731.1_L137P, Sequence: EHTDGPVWDRDGVSIR, RT (min): 37.02, XCorr: 3.02
intensity
m/z
y9
b5
y1
y7
y8
y5
y10
b2
y3
y6
a1
a2
y2
b6
a9
b1
b5
b3
y11
y4
a5
a6
b6
y11
a7
b7
0
20000
40000
60000
80000
100000
120000
140000
250
500
750
1000
1250
1500
1750

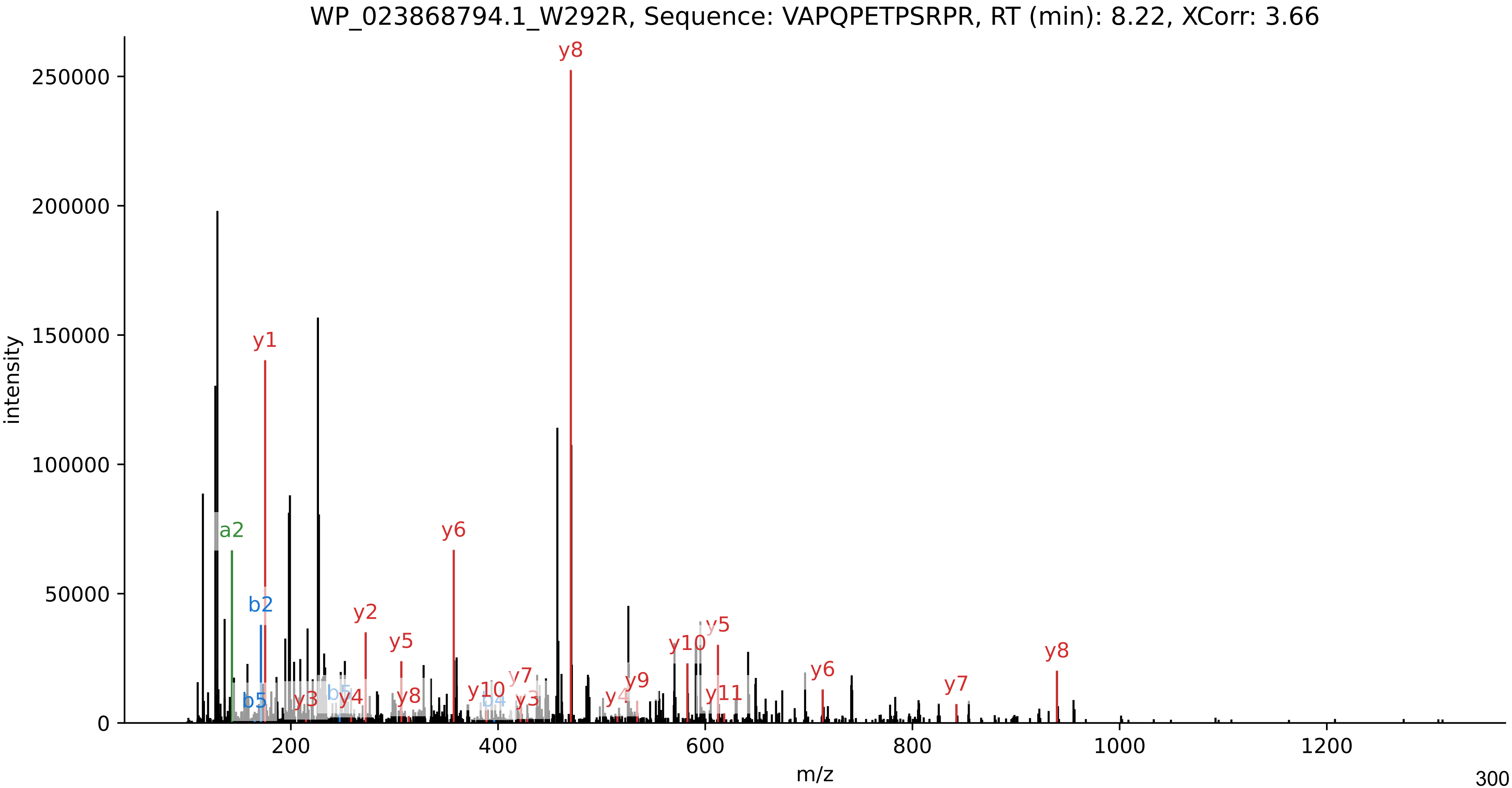
WP_023868794.1_W292R, Sequence: VAPQPETPSRPR, RT (min): 8.22, XCorr: 3.66
intensity
m/z
y8
y1
a2
b2
y2
y5
y6
y10
y5
y8
y6
y7
y9
y4
y11
y3
y4
y8
y10
b4
y7
y3
b5
y4

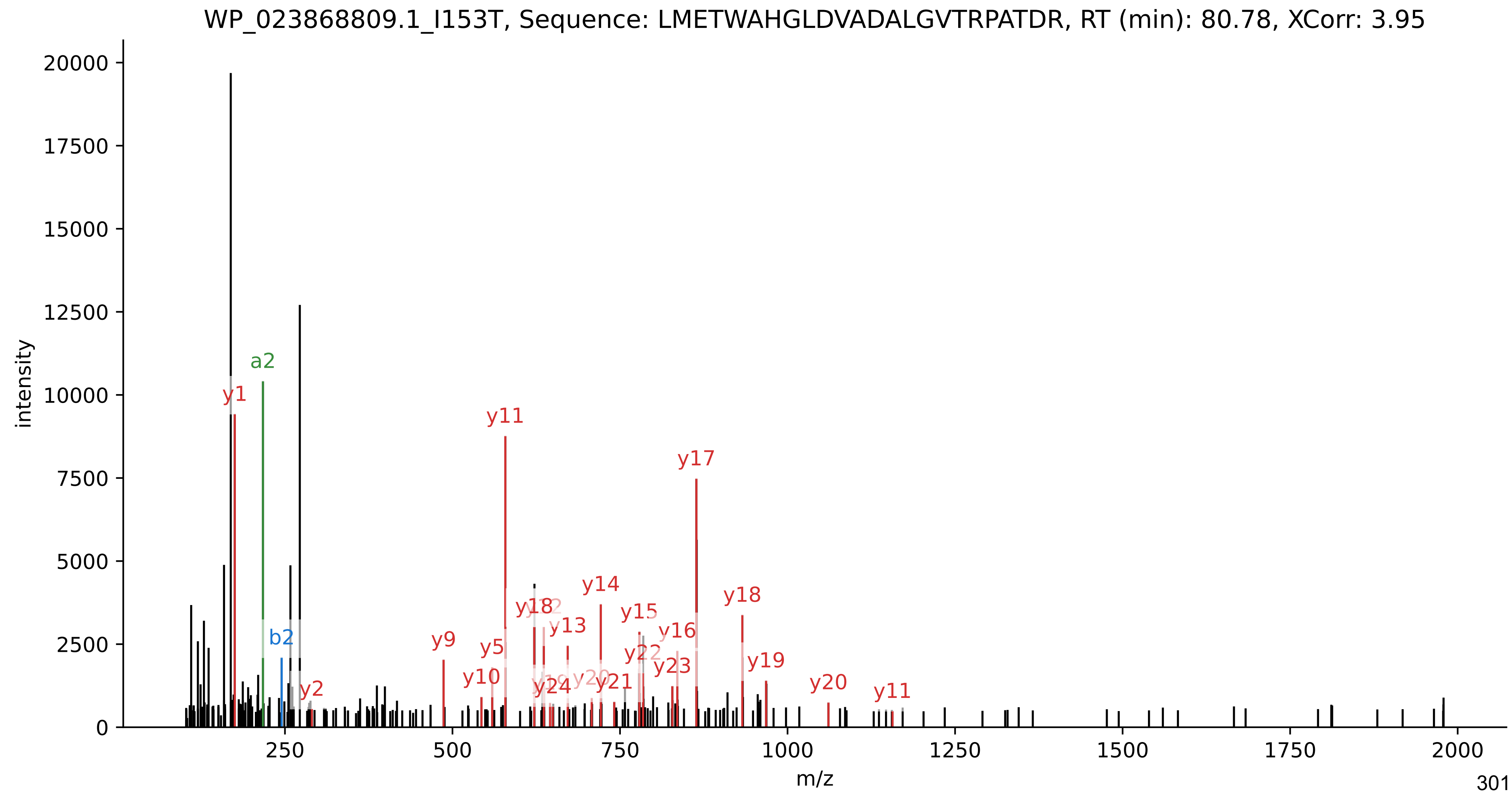
WP_023868809.1_I153T, Sequence: LMETWAHGLDVADALGVTRPATDR, RT (min): 80.78, XCorr: 3.95
intensity
m/z
a2
y1
b2
y2
y9
y10
y5
y11
y18
y13
y14
y24
y21
y15
y22
y16
y23
y17
y18
y19
y20
y11

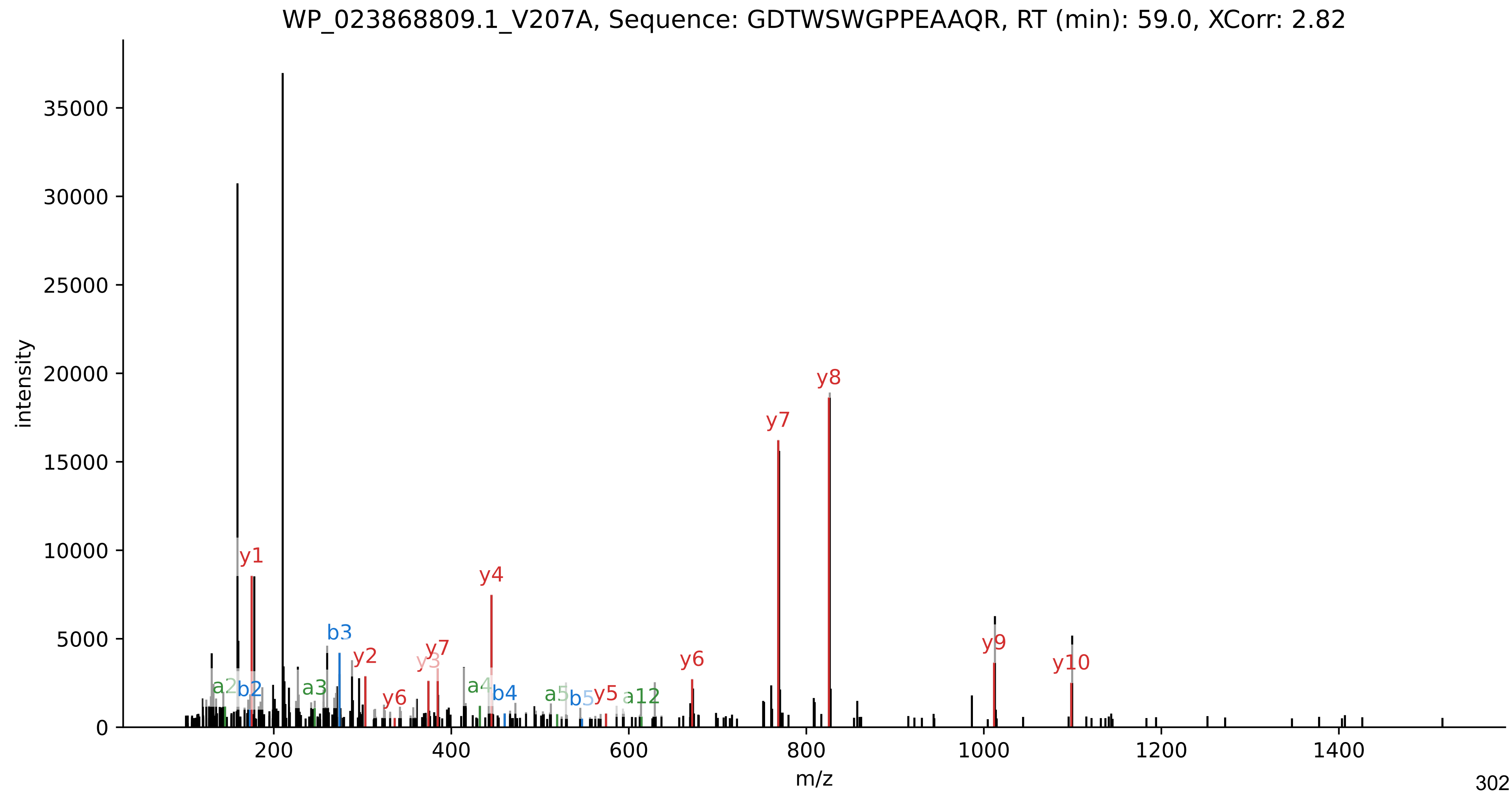
WP_023868809.1_V207A, Sequence: GDTWSWGPPEAAQR, RT (min): 59.0, XCorr: 2.82
intensity
m/z
y1
y2
y3
y4
y5
y6
y7
y8
y9
y10
b2
b3
b4
b5
a2
a3
a4
a5
a12

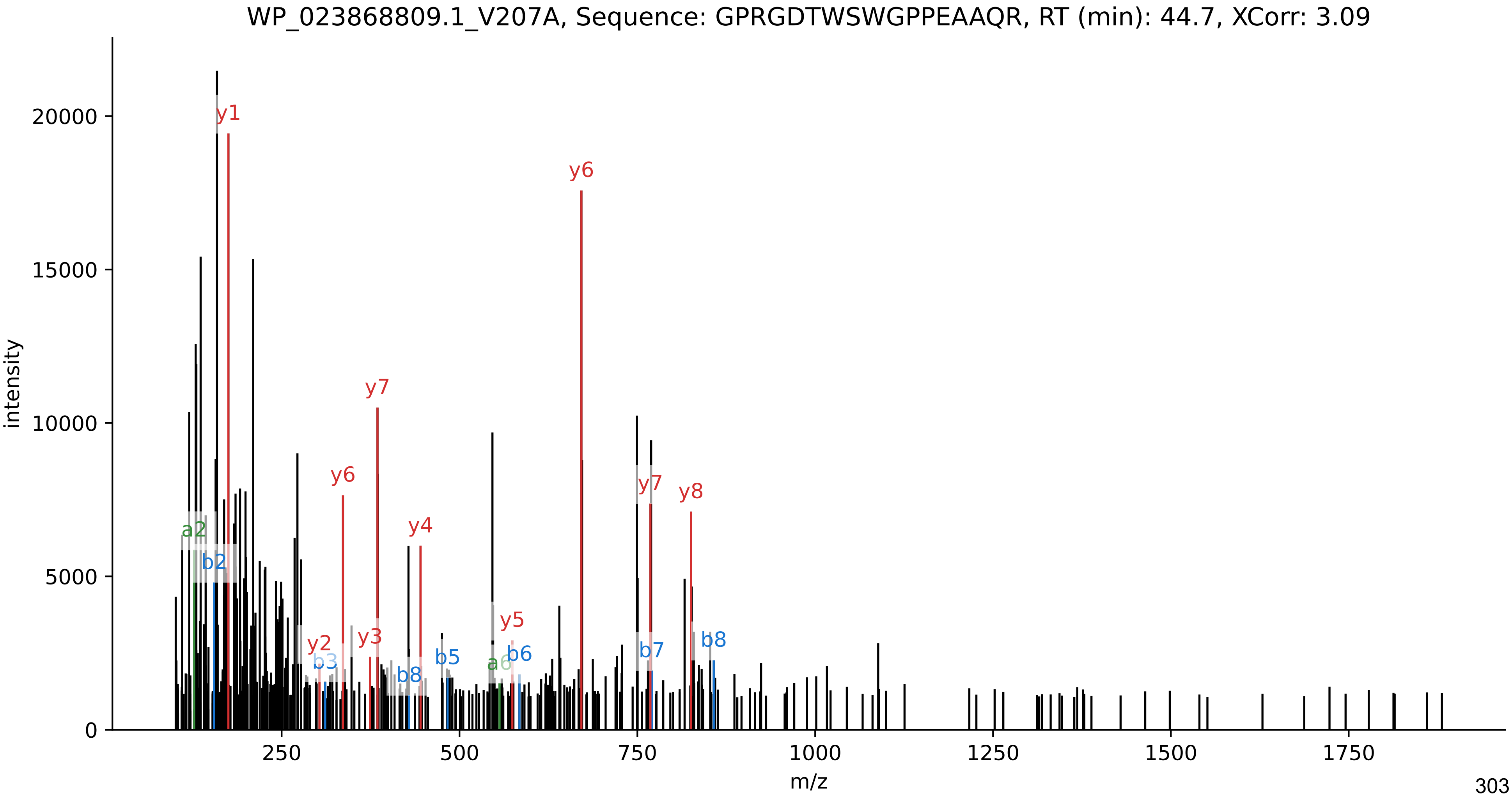

WP_023868809.1_V207A, Sequence: GPRGDTWSWGPPEAAQR, RT (min): 44.7, XCorr: 3.09
intensity
m/z
y1
y6
y7
y6
y7
y8
y4
a2
b2
y5
y2
y3
b3
b8
b5
a6
b6
b7
b8
0
5000
10000
15000
20000
250
500
750
1000
1250
1500
1750

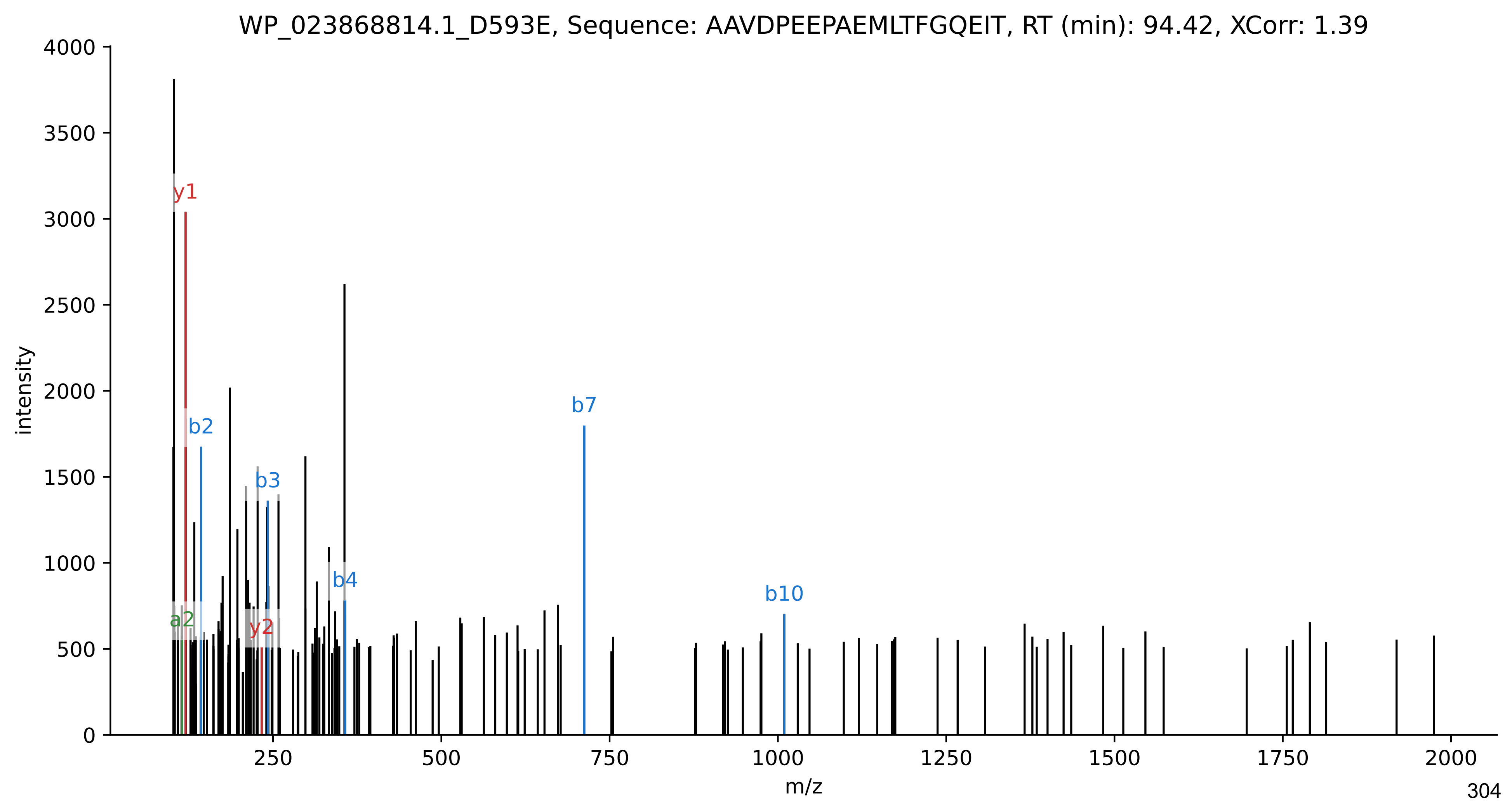

WP_023868814.1_D593E, Sequence: AAVDPEEPAEMLTFGQEIT, RT (min): 94.42, XCorr: 1.39
4000
3500
3000
2500
2000
1500
1000
500
0
intensity
y1
b2
b3
b4
a2
y2
b7
b10
250
500
750
1000
1250
1500
1750
2000
m/z

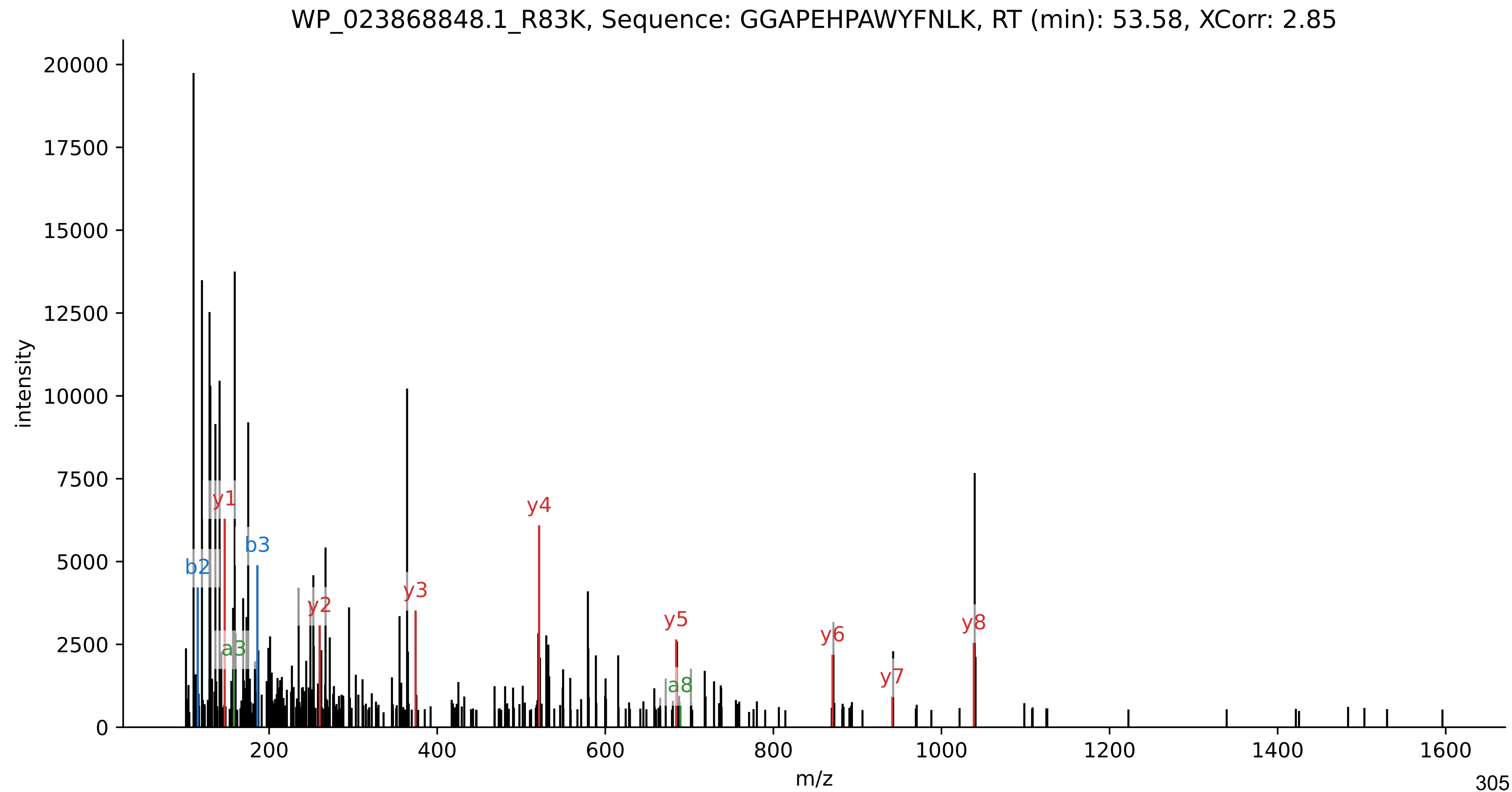

WP_023868848.1_R83K, Sequence: GGAPEHPAWYFNLK, RT (min): 53.58, XCorr: 2.85
intensity
m/z
b2
b3
y1
a3
y2
y3
y4
y5
a8
y6
y7
y8

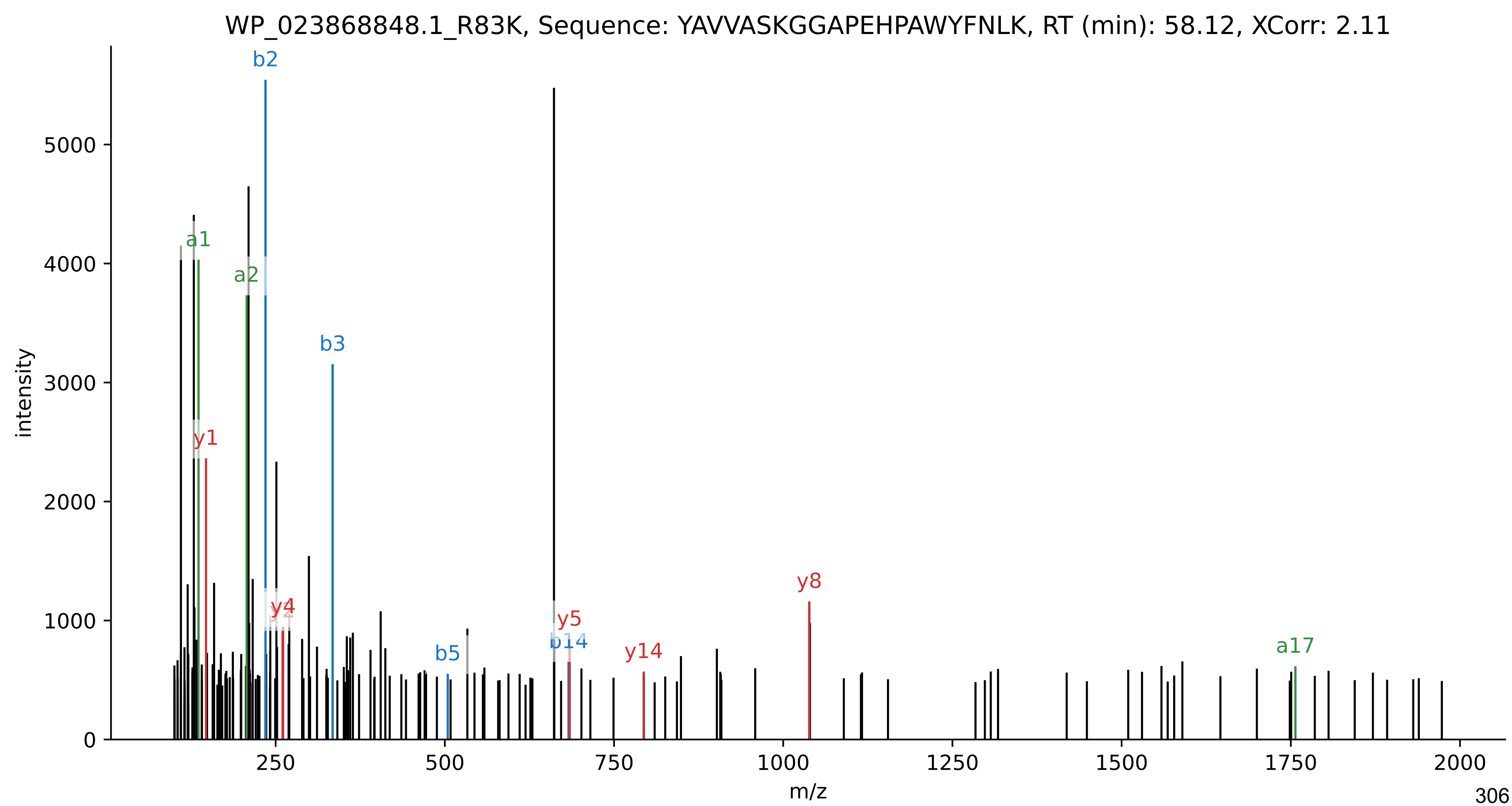

WP_023868848.1_R83K, Sequence: YAVVASKGGAPEHPAWYFNLK, RT (min): 58.12, XCorr: 2.11
intensity
m/z
b2
a1
a2
b3
y1
y4
y5
b14
b5
y14
y8
a17
0
1000
2000
3000
4000
5000
250
500
750
1000
1250
1500
1750
2000

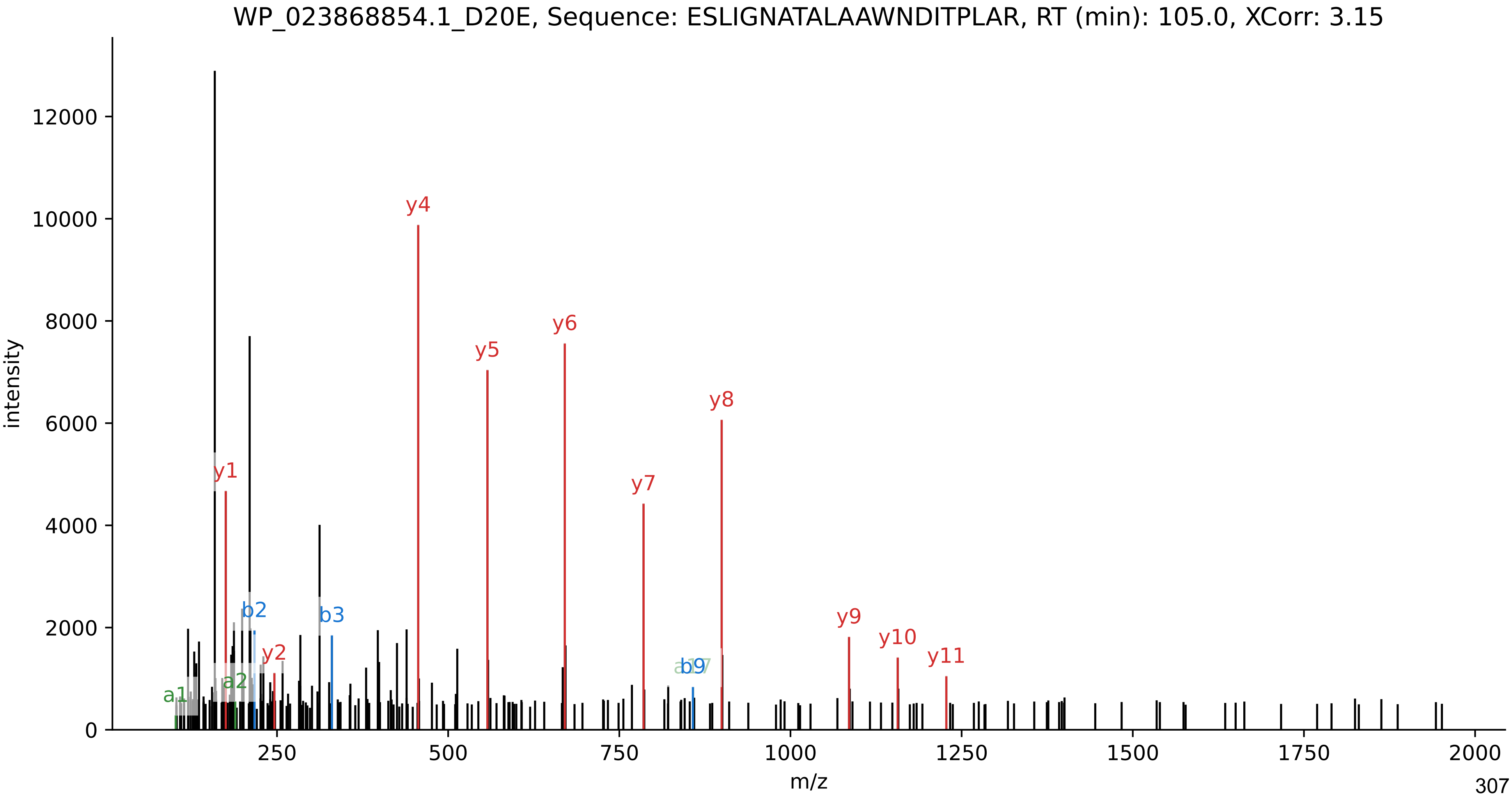

WP_023868854.1_D20E, Sequence: ESLIGNATALAAWNDITPLAR, RT (min): 105.0, XCorr: 3.15
intensity
m/z
y1
y2
y4
y5
y6
y7
y8
y9
y10
y11
b2
b3
b9
a1
a2

WP_023868988.1_M49L, Sequence: YELIPQLIVHDGEAAVR, RT (min): 77.78, XCorr: 3.24

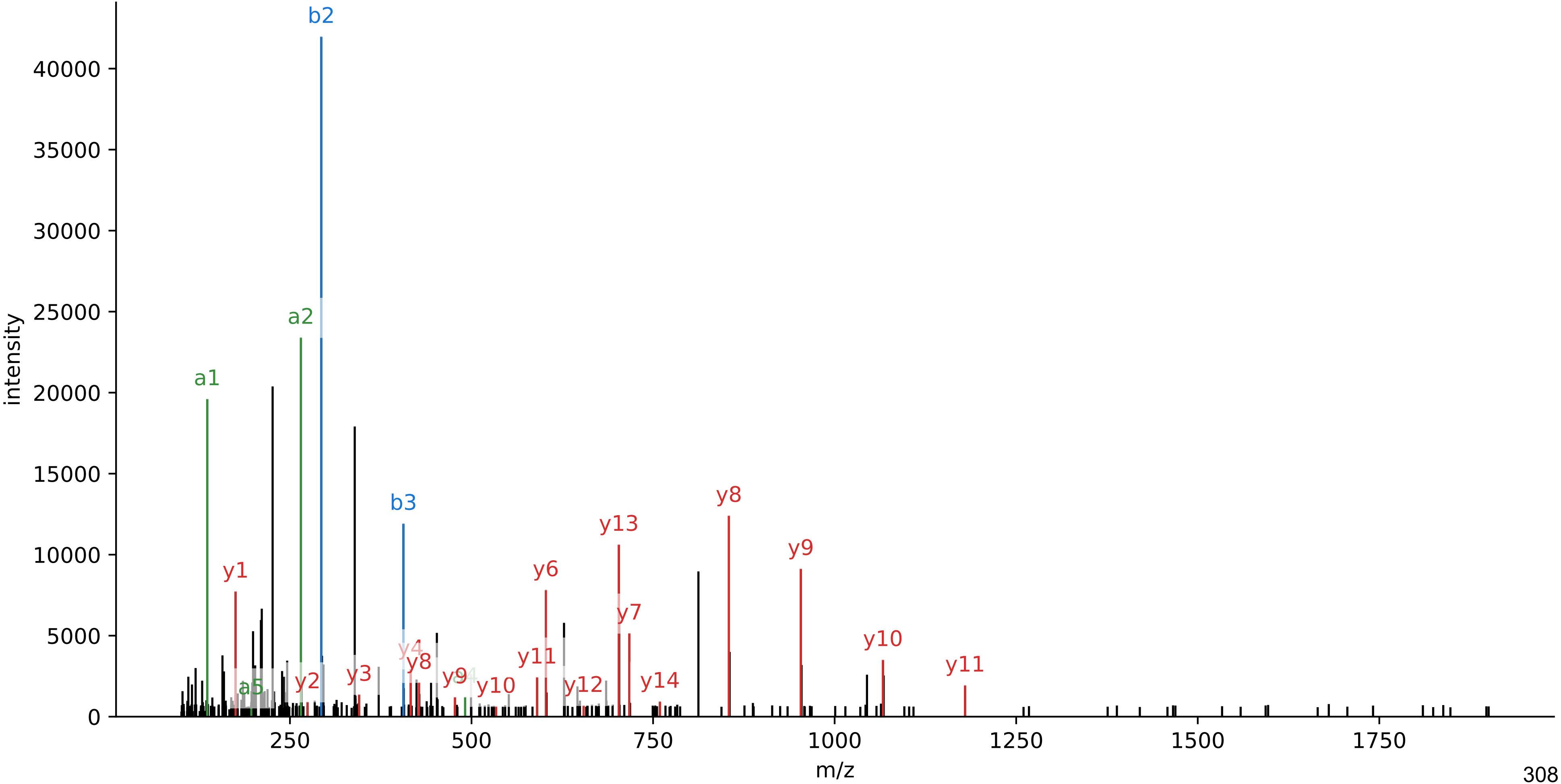

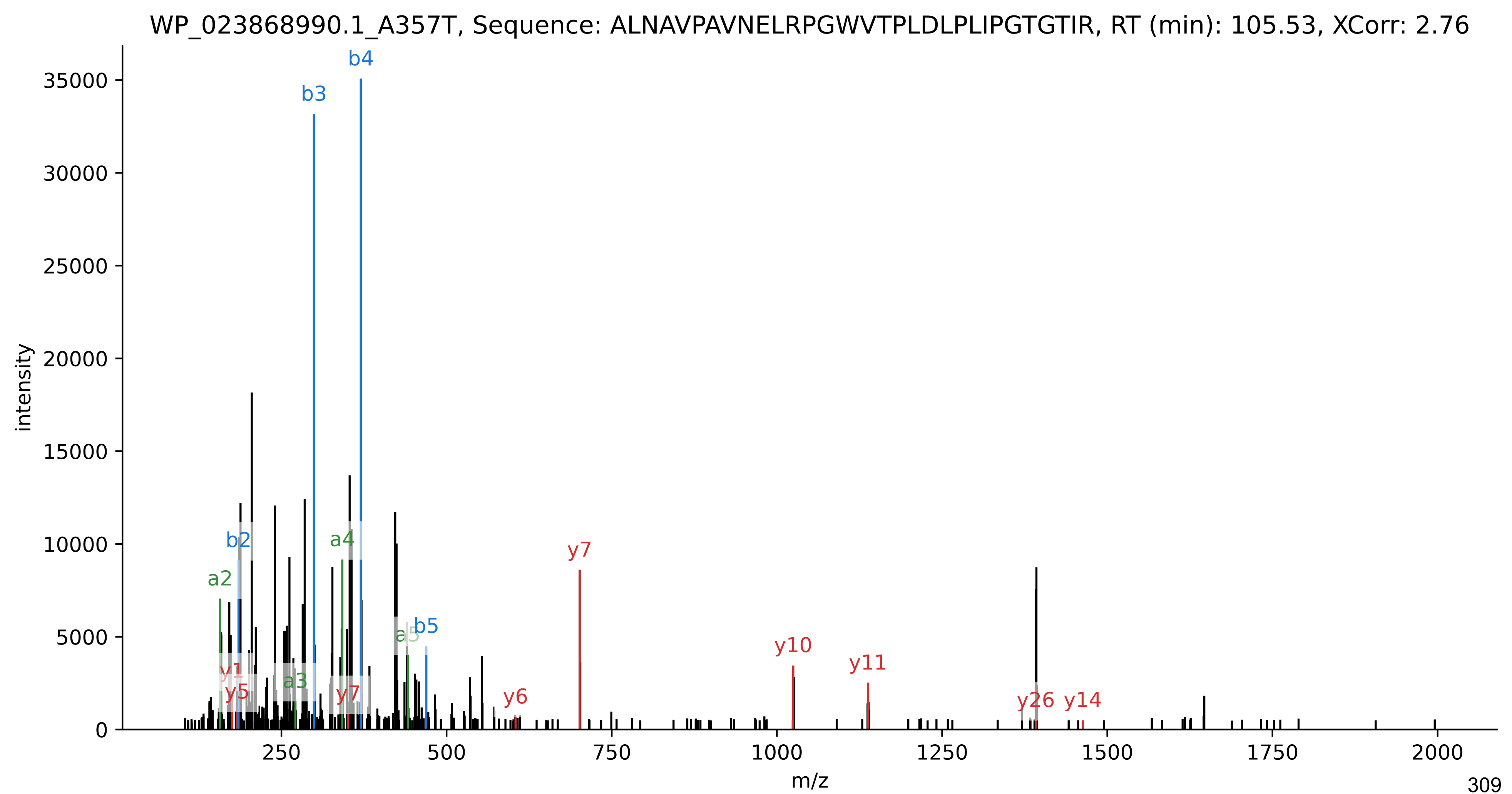

WP_023868990.1_A357T, Sequence: ALNAVPAVNELRPGWVTPLDLPLIPGTGTIR, RT (min): 105.53, XCorr: 2.76
intensity
m/z
b4
b3
b2
a4
a2
y7
b5
y10
y11
y1
y5
a3
y7
y6
y26
y14

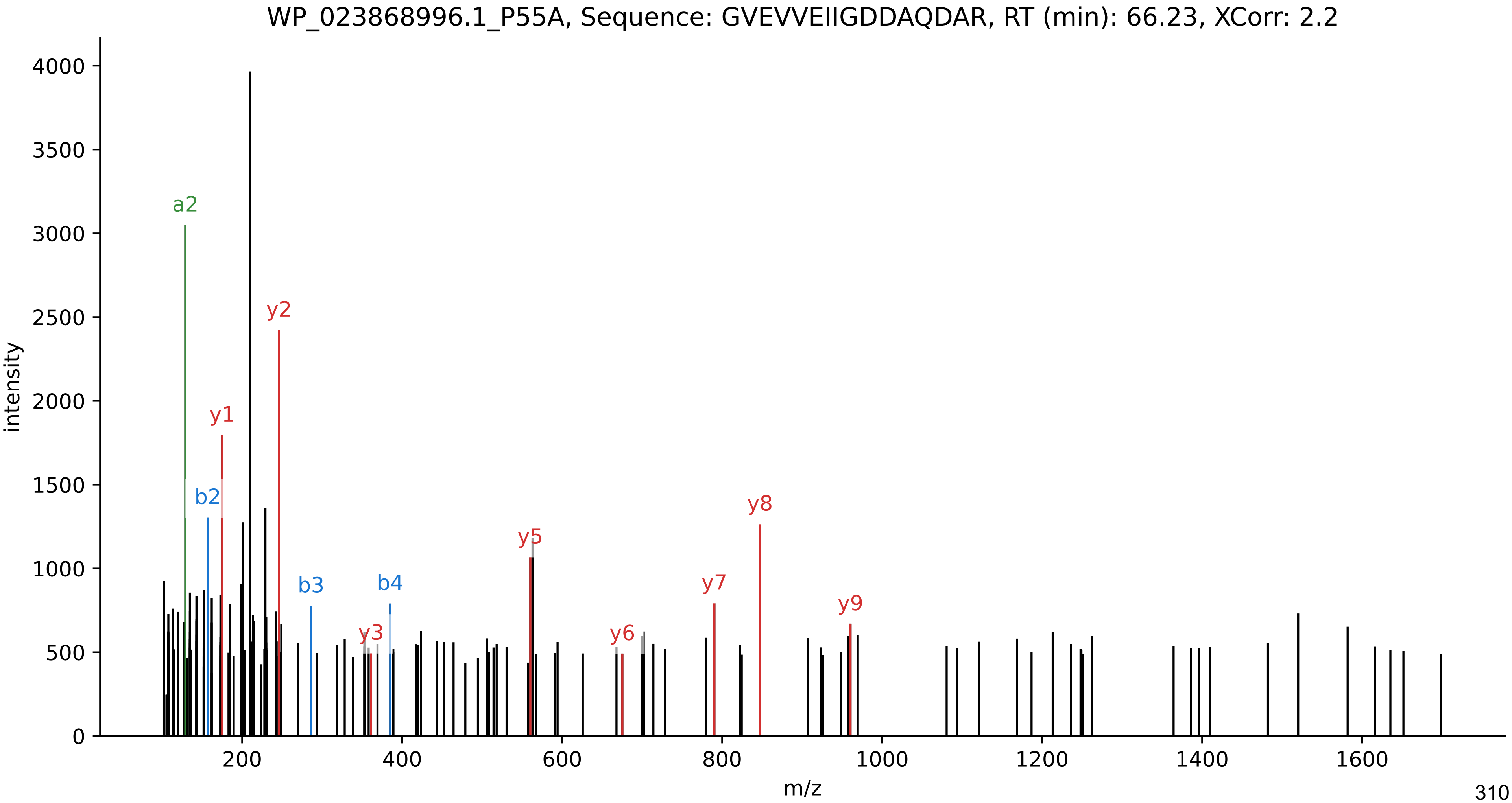

WP_023868996.1_P55A, Sequence: GVEVVEIIGDDAQDAR, RT (min): 66.23, XCorr: 2.2
intensity
m/z
a2
y2
y1
b2
b3
b4
y3
y5
y6
y7
y8
y9

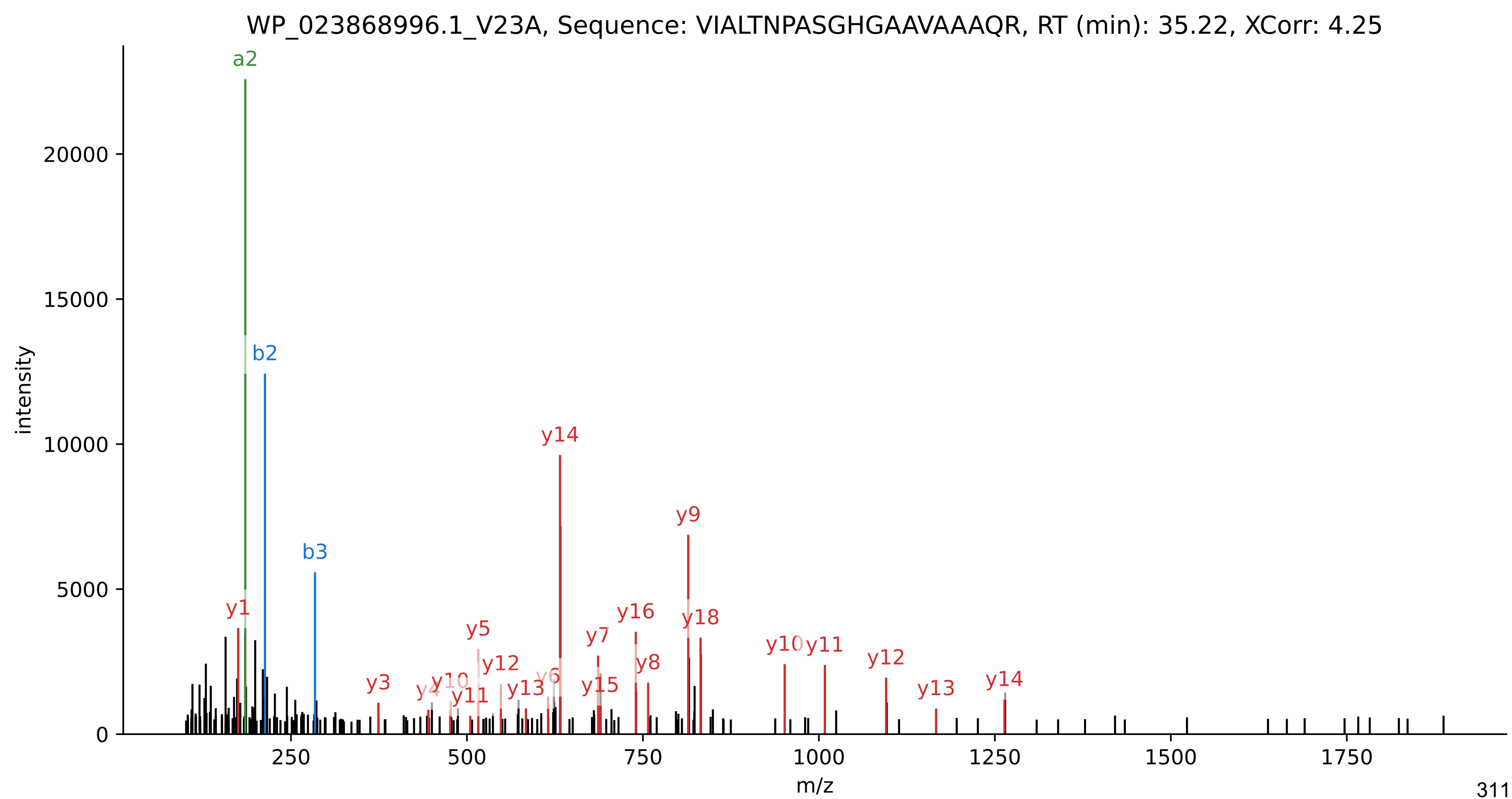
WP_023868996.1_V23A, Sequence: VIALTNPASGHGAAVAAAQR, RT (min): 35.22, XCorr: 4.25
intensity
m/z
a2
b2
b3
y1
y3
y4
y10
y11
y5
y12
y13
y6
y14
y7
y15
y16
y8
y9
y18
y10
y11
y12
y13
y14
0
5000
10000
15000
20000
250
500
750
1000
1250
1500
1750

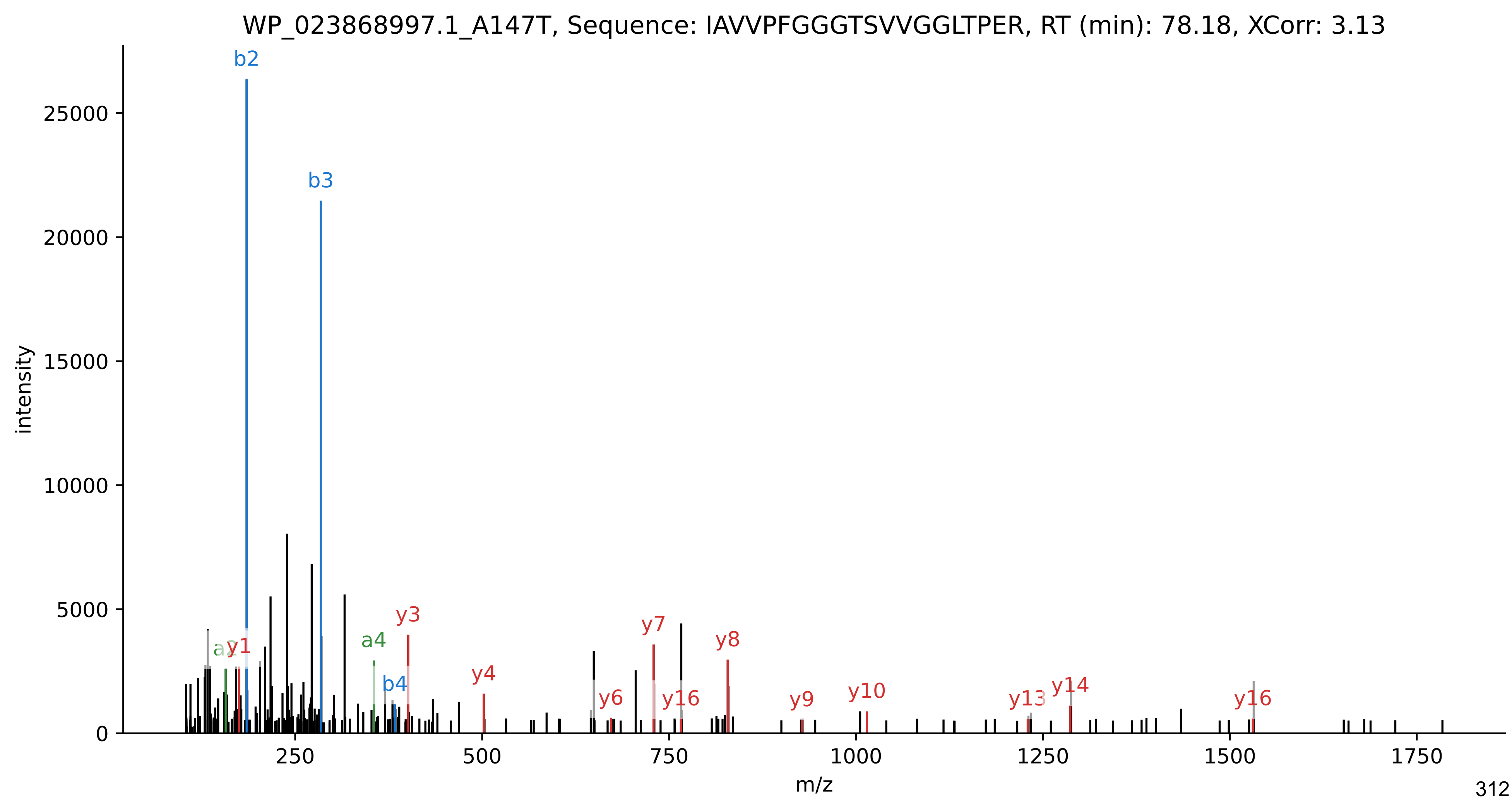

WP_023868997.1_A147T, Sequence: IAVVPFGGGTSVVGGLTPER, RT (min): 78.18, XCorr: 3.13
intensity
m/z
b2
b3
b4
a2
a4
y1
y3
y4
y6
y7
y16
y8
y9
y10
y13
y14
y16
0
5000
10000
15000
20000
25000
250
500
750
1000
1250
1500
1750

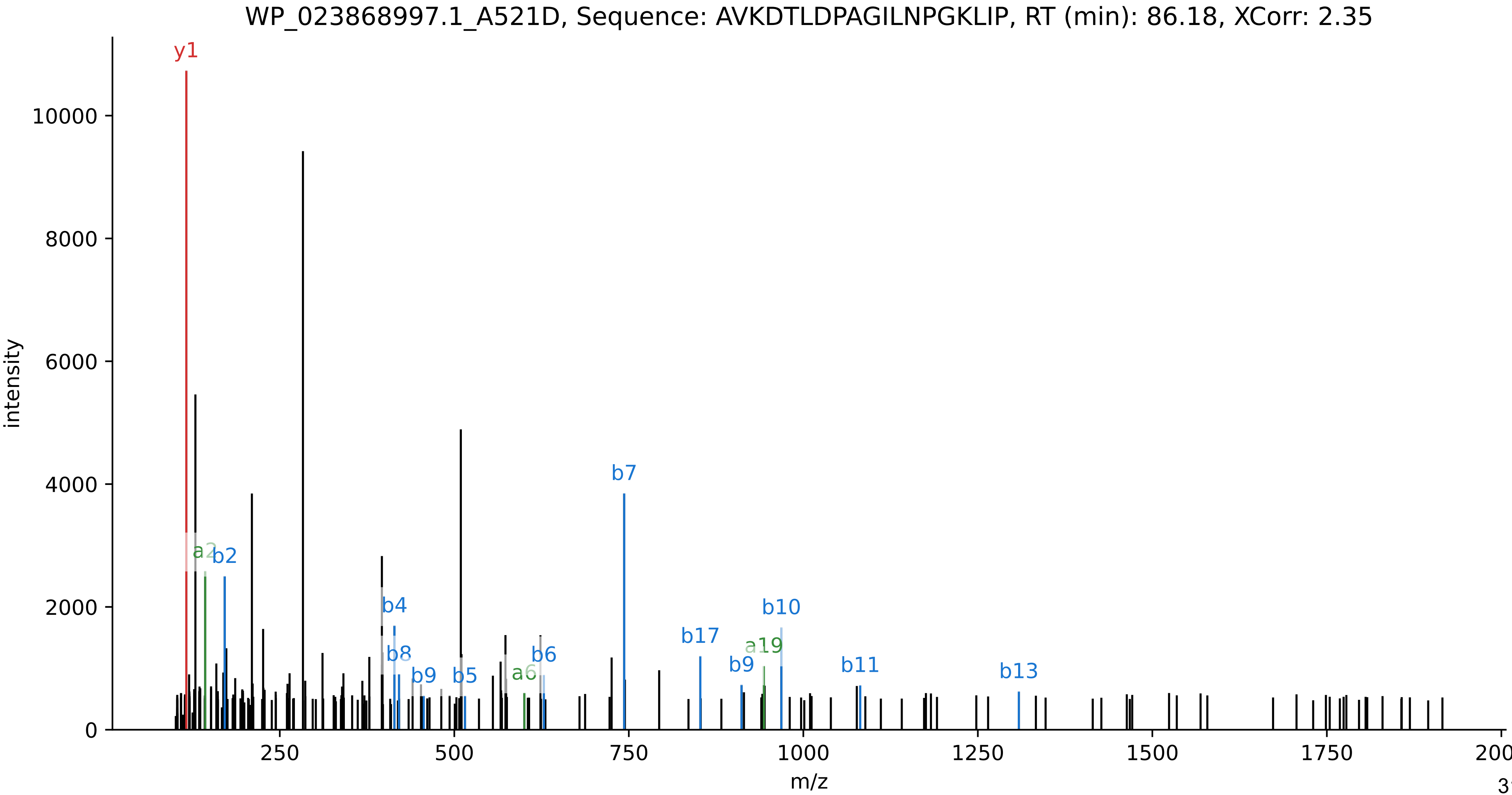

WP_023868997.1_A521D, Sequence: AVKDTLDPAGILNPGKLIP, RT (min): 86.18, XCorr: 2.35
y1
a2
b2
b4
b8
b9
b5
a6
b6
b7
b17
b9
a19
b10
b11
b13
intensity
m/z

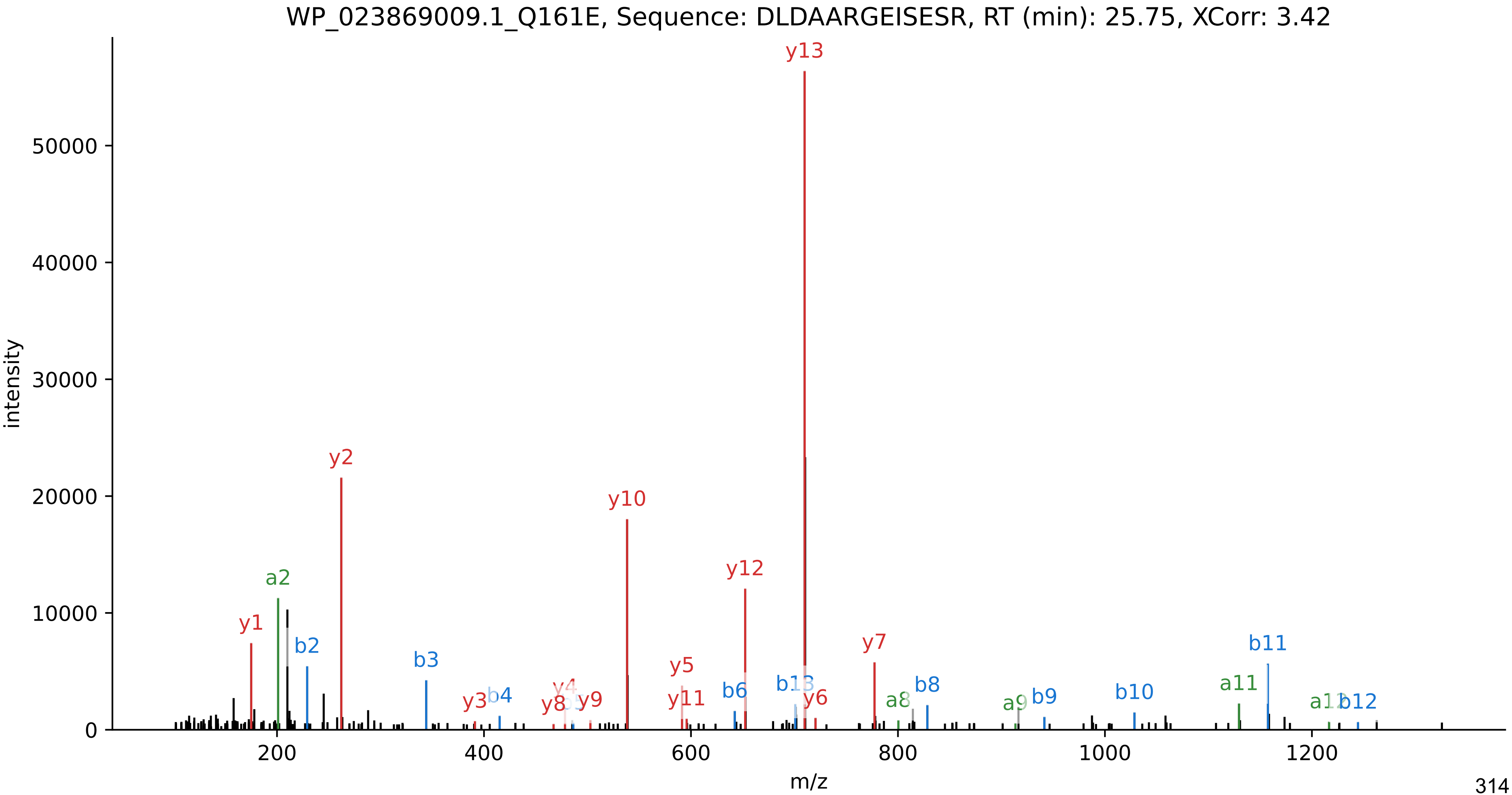
WP_023869009.1_Q161E, Sequence: DLDAARGEISESR, RT (min): 25.75, XCorr: 3.42
intensity
m/z
y13
y2
y10
y12
a2
y1
b2
b3
y7
b11
y5
b13
b8
a11
b6
y4
y3
b4
y8
y9
y11
y6
a8
a9
b9
b10
a12
b12
50000
40000
30000
20000
10000
0
200
400
600
800
1000
1200

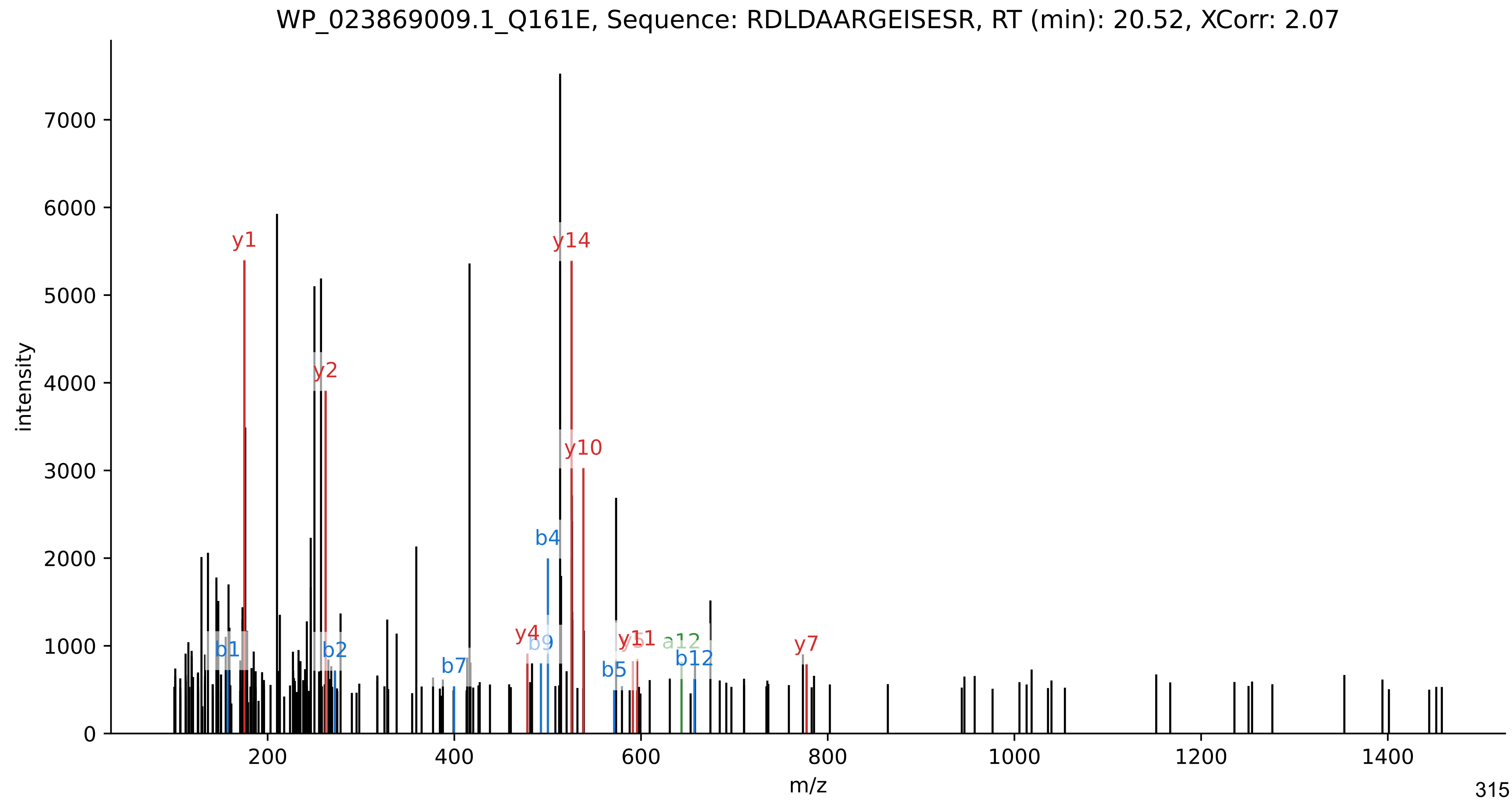

WP_023869009.1_Q161E, Sequence: RDLDAARGEISESR, RT (min): 20.52, XCorr: 2.07
intensity
m/z
y1
y2
y14
y10
b4
y4
b9
y11
a12
b12
b5
y7
b1
b2
b7

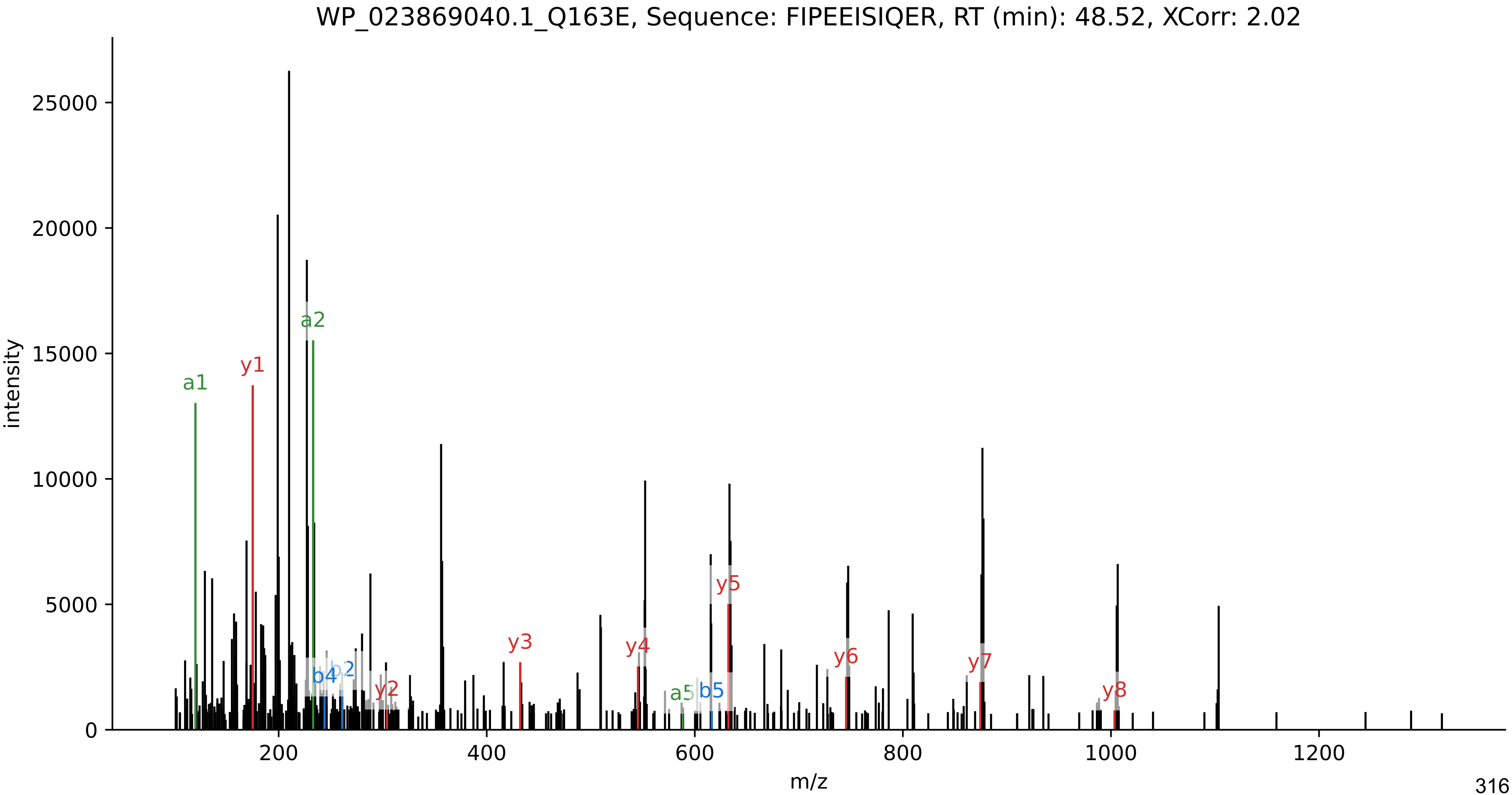

WP_023869040.1_Q163E, Sequence: FIPEEISIQER, RT (min): 48.52, XCorr: 2.02
intensity
m/z
a1
y1
a2
b4
b2
y2
y3
y4
a5
b5
y5
y6
y7
y8

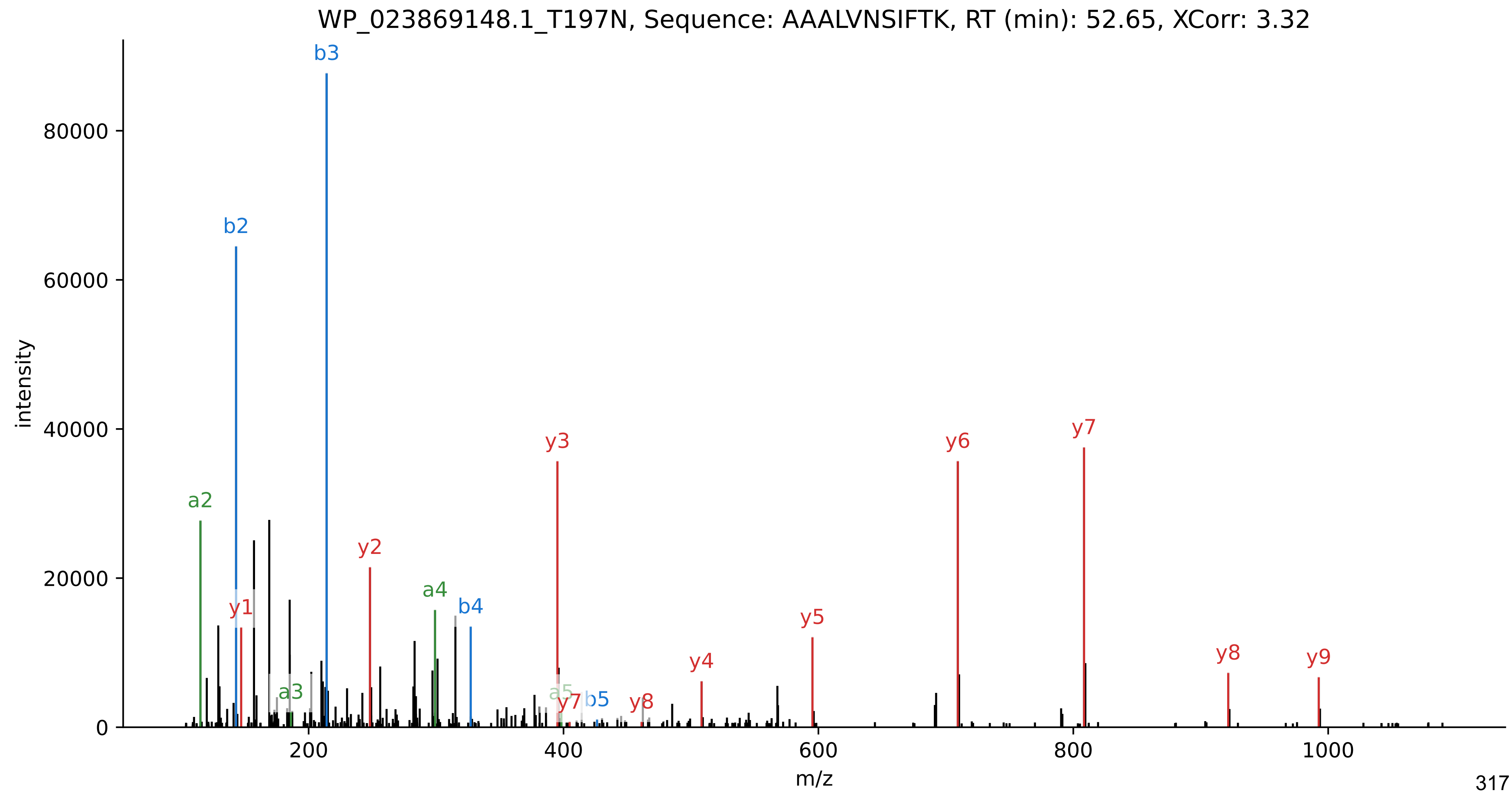
WP_023869148.1_T197N, Sequence: AAALVNSIFTK, RT (min): 52.65, XCorr: 3.32
intensity
m/z
b3
b2
a2
y1
a3
y2
a4
b4
y3
a5
y7
b5
y8
y4
y5
y6
y7
y8
y9
80000
60000
40000
20000
0
200
400
600
800
1000

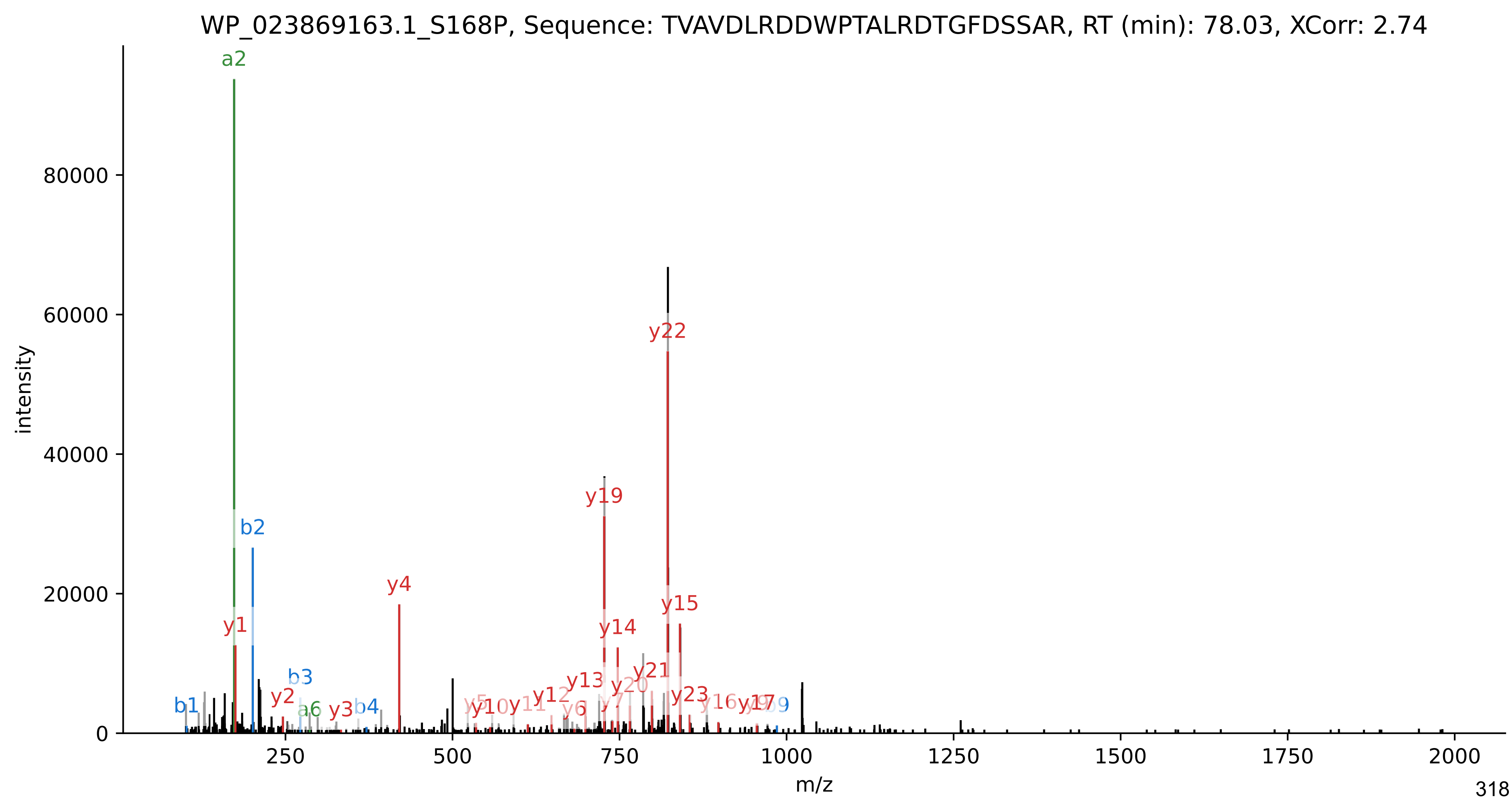

WP_023869163.1_S168P, Sequence: TVAVDLRDDWPTALRDTGFDSSAR, RT (min): 78.03, XCorr: 2.74
intensity
m/z
a2
b2
y1
y4
y19
y22
y14
y15
y13
y21
y20
y12
y23
b1
y2
b3
a6
y3
b4
y10
y11
y6
y16
y17

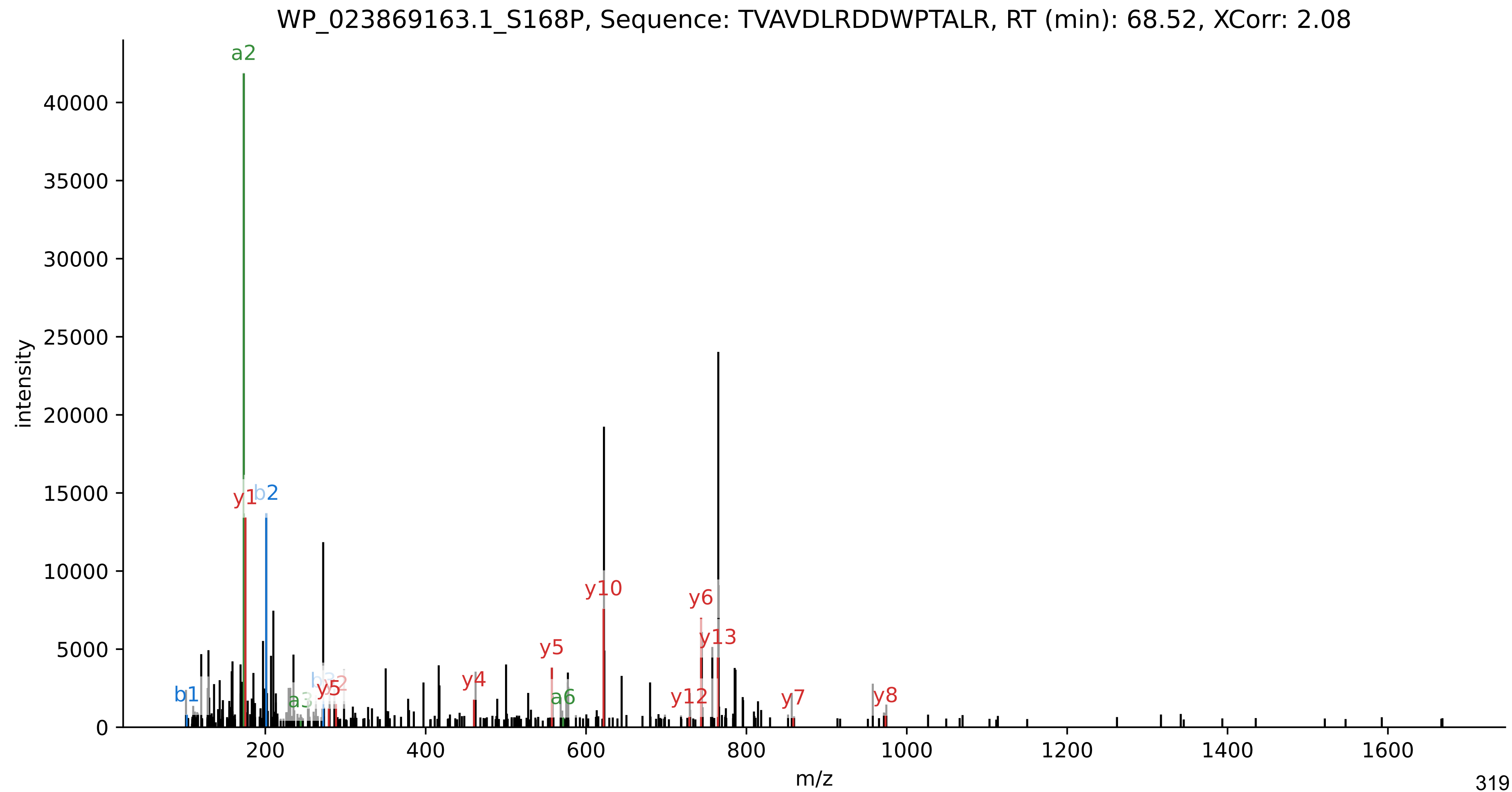
WP_023869163.1_S168P, Sequence: TVAVDLRDDWPTALR, RT (min): 68.52, XCorr: 2.08
intensity
m/z
a2
y1
b2
y10
y6
y13
y5
y4
b1
a3
y5
a6
y12
y7
y8
0
5000
10000
15000
20000
25000
30000
35000
40000
200
400
600
800
1000
1200
1400
1600

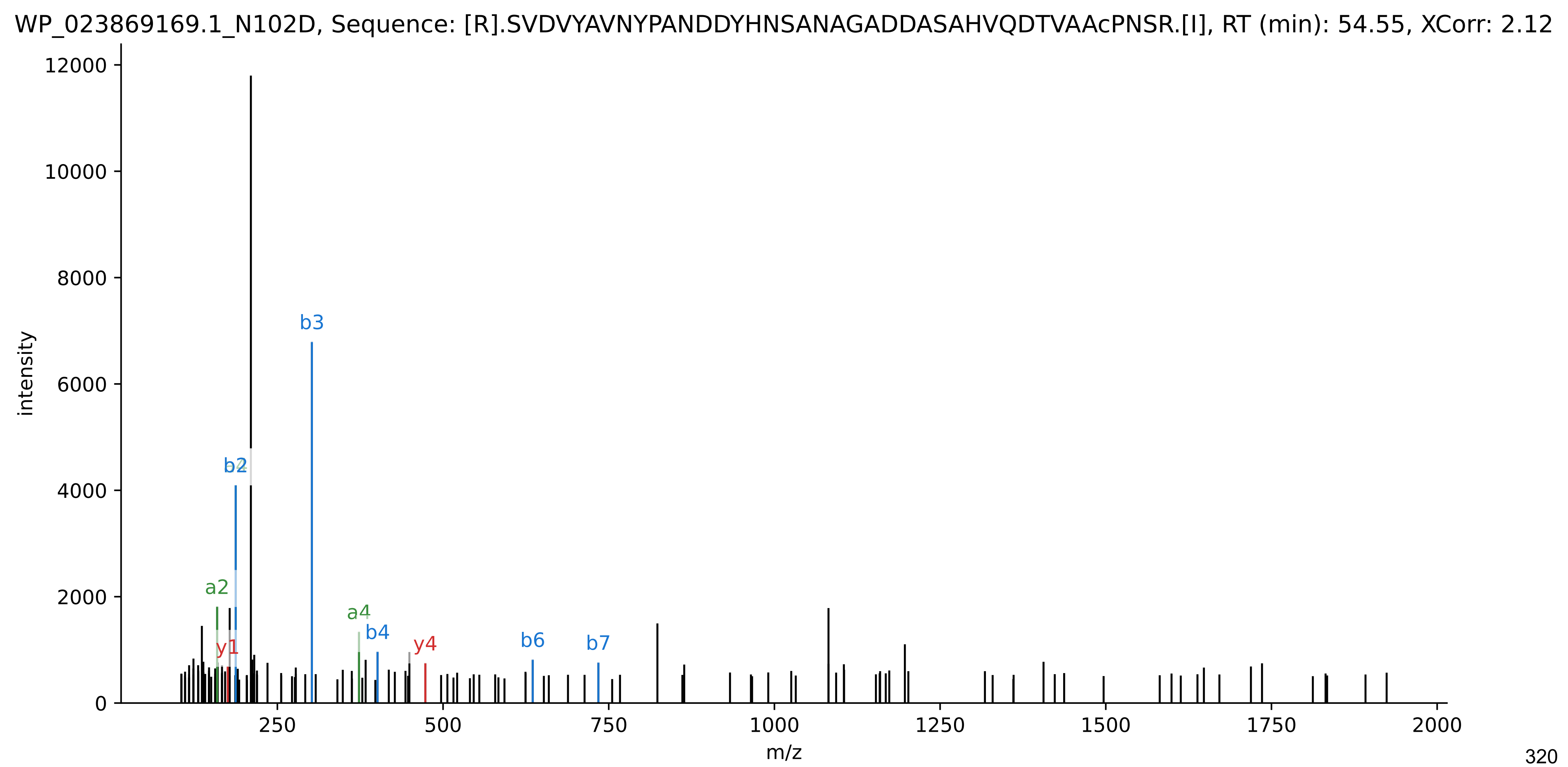
WP_023869169.1_N102D, Sequence: [R].SVDVYAVNYPANDDYHNSANAGADDASAHVQDTVAAcPNSR.[I], RT (min): 54.55, XCorr: 2.12
intensity
m/z
b3
b2
a2
a4
b4
y1
y4
b6
b7
0
2000
4000
6000
8000
10000
12000
250
500
750
1000
1250
1500
1750
2000

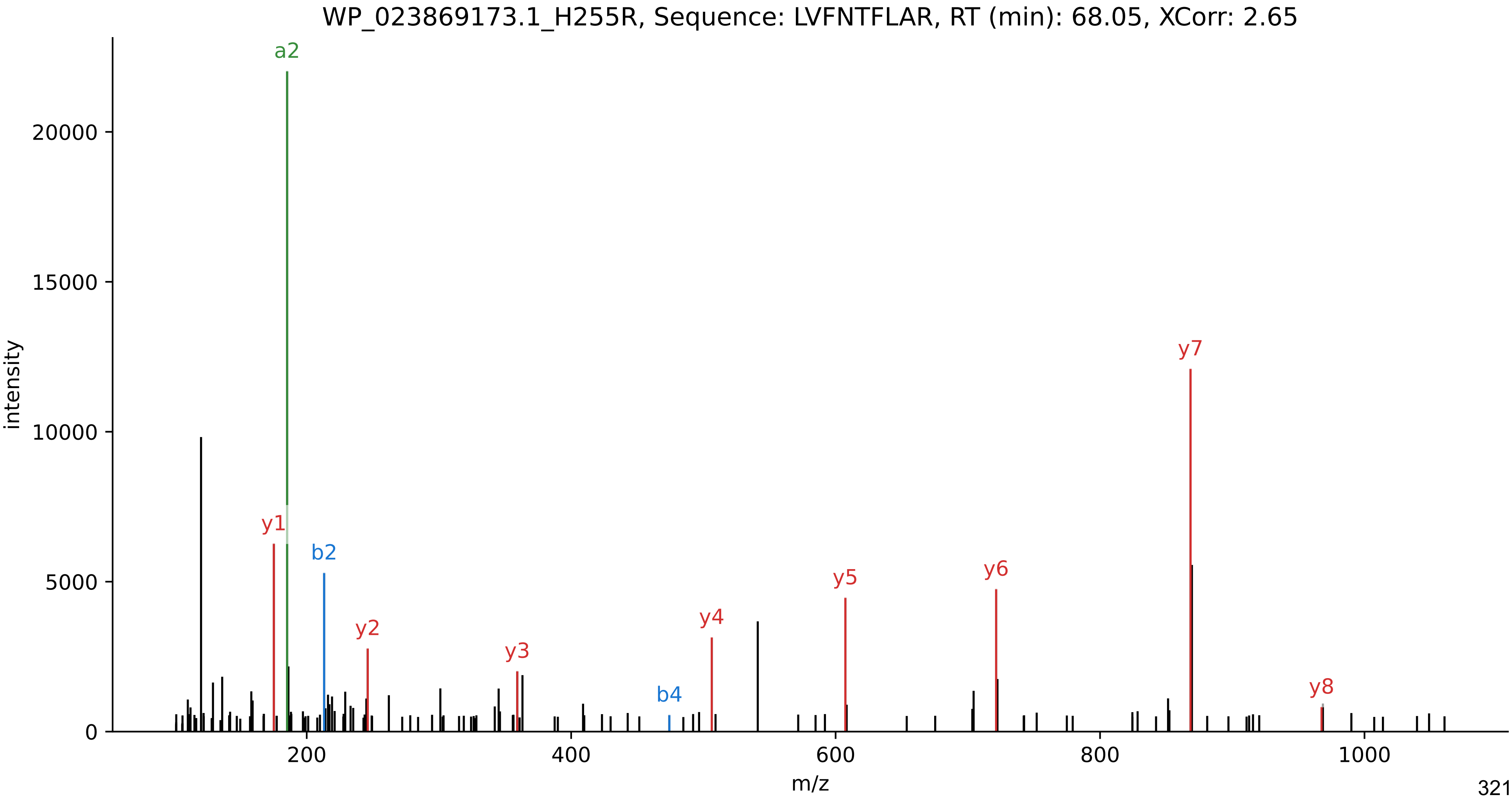

WP_023869173.1_H255R, Sequence: LVFNTFLAR, RT (min): 68.05, XCorr: 2.65
a2
y1
b2
y2
y3
b4
y4
y5
y6
y7
y8
intensity
0
5000
10000
15000
20000
200
400
600
800
1000
m/z

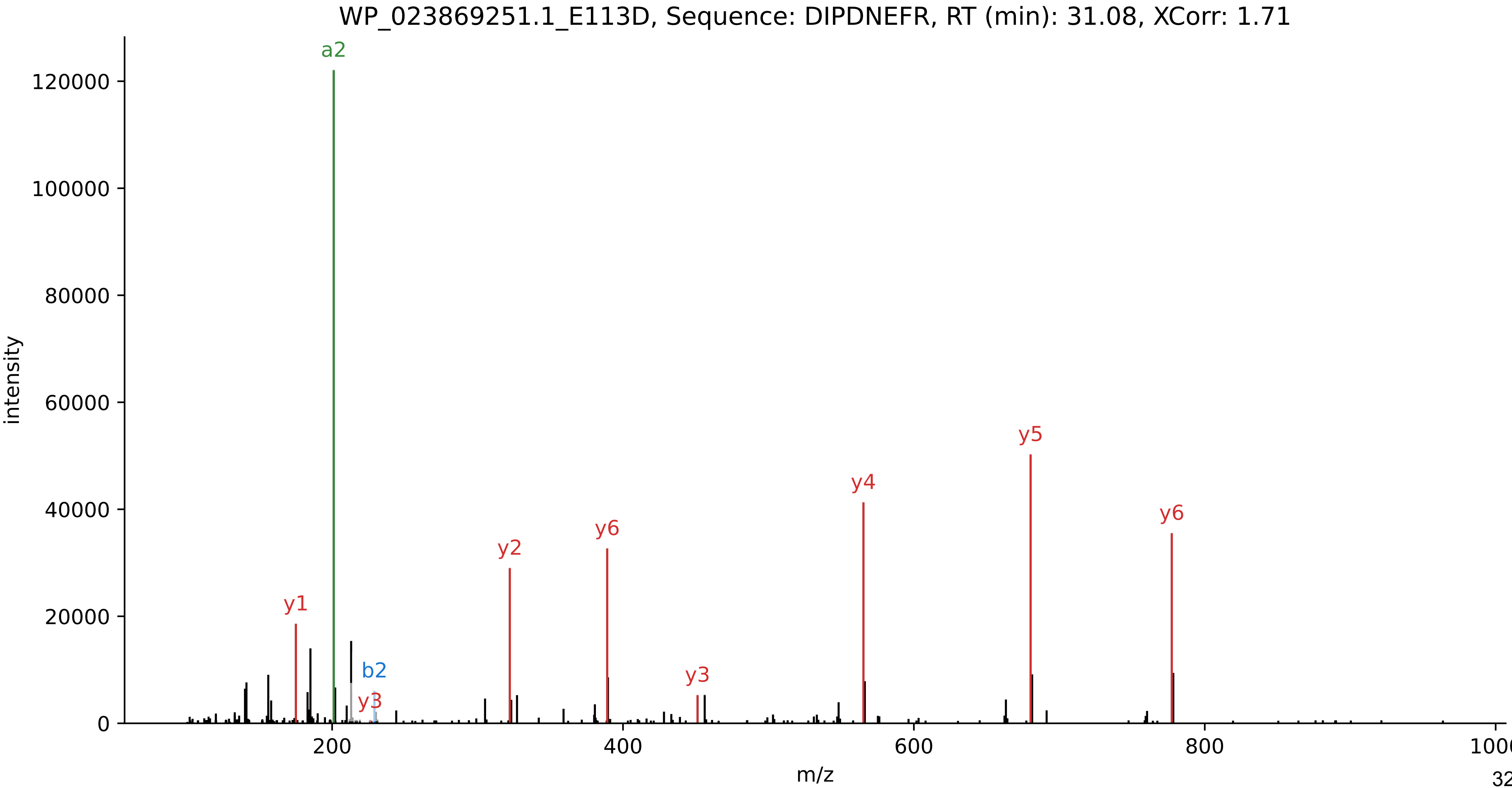

WP_023869251.1_E113D, Sequence: DIPDNEFR, RT (min): 31.08, XCorr: 1.71
intensity
m/z
a2
y1
b2
y3
y2
y6
y3
y4
y5
y6
0
20000
40000
60000
80000
100000
120000
200
400
600
800
1000

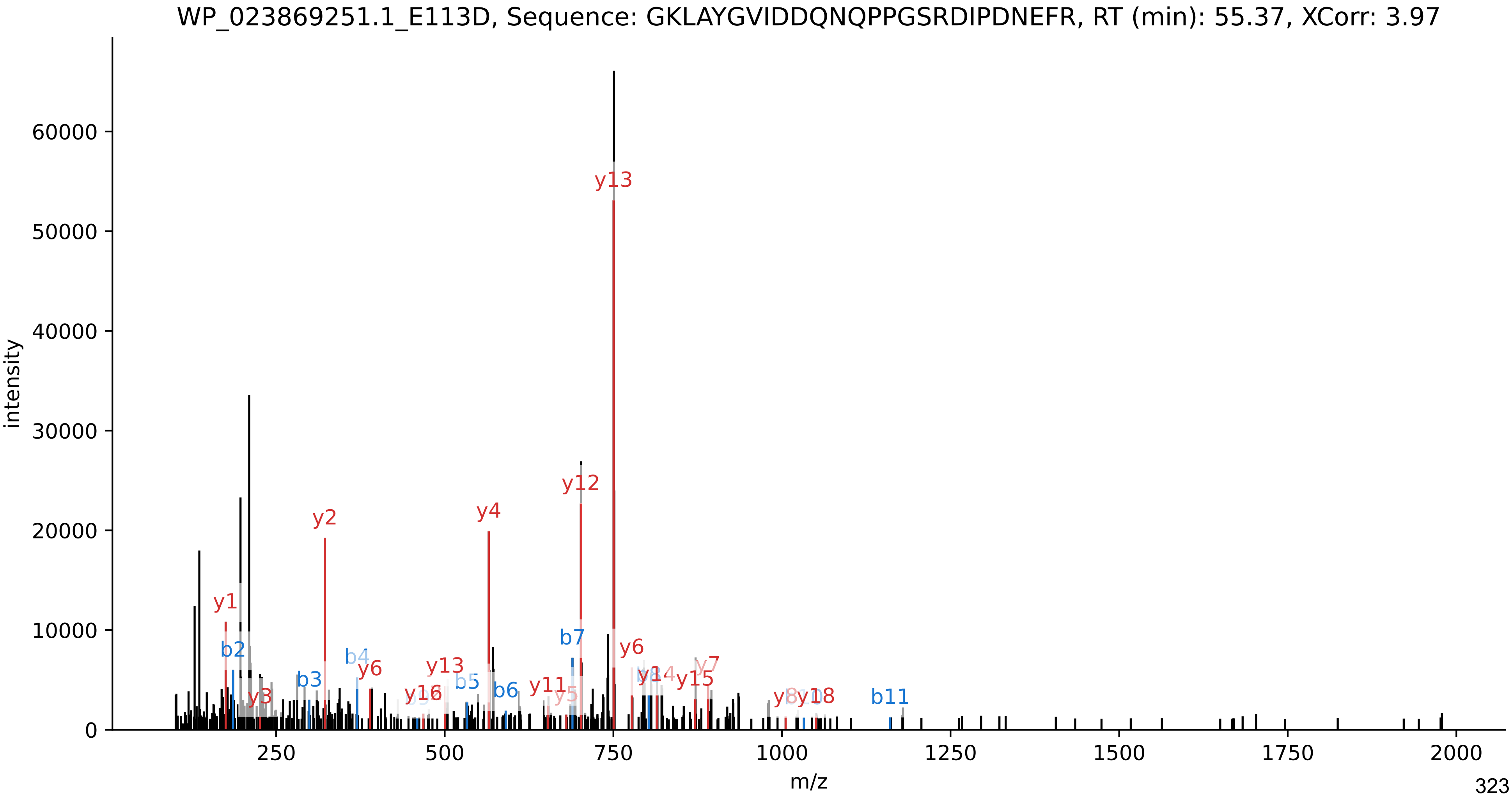
WP_023869251.1_E113D, Sequence: GKLAYGVIDDQNQPPGSRDIPDNEFR, RT (min): 55.37, XCorr: 3.97
intensity
m/z
y13
y12
y4
y2
y1
b2
b3
b4
y6
y13
y16
b5
b6
y11
b7
y6
y15
y7
y18
b11

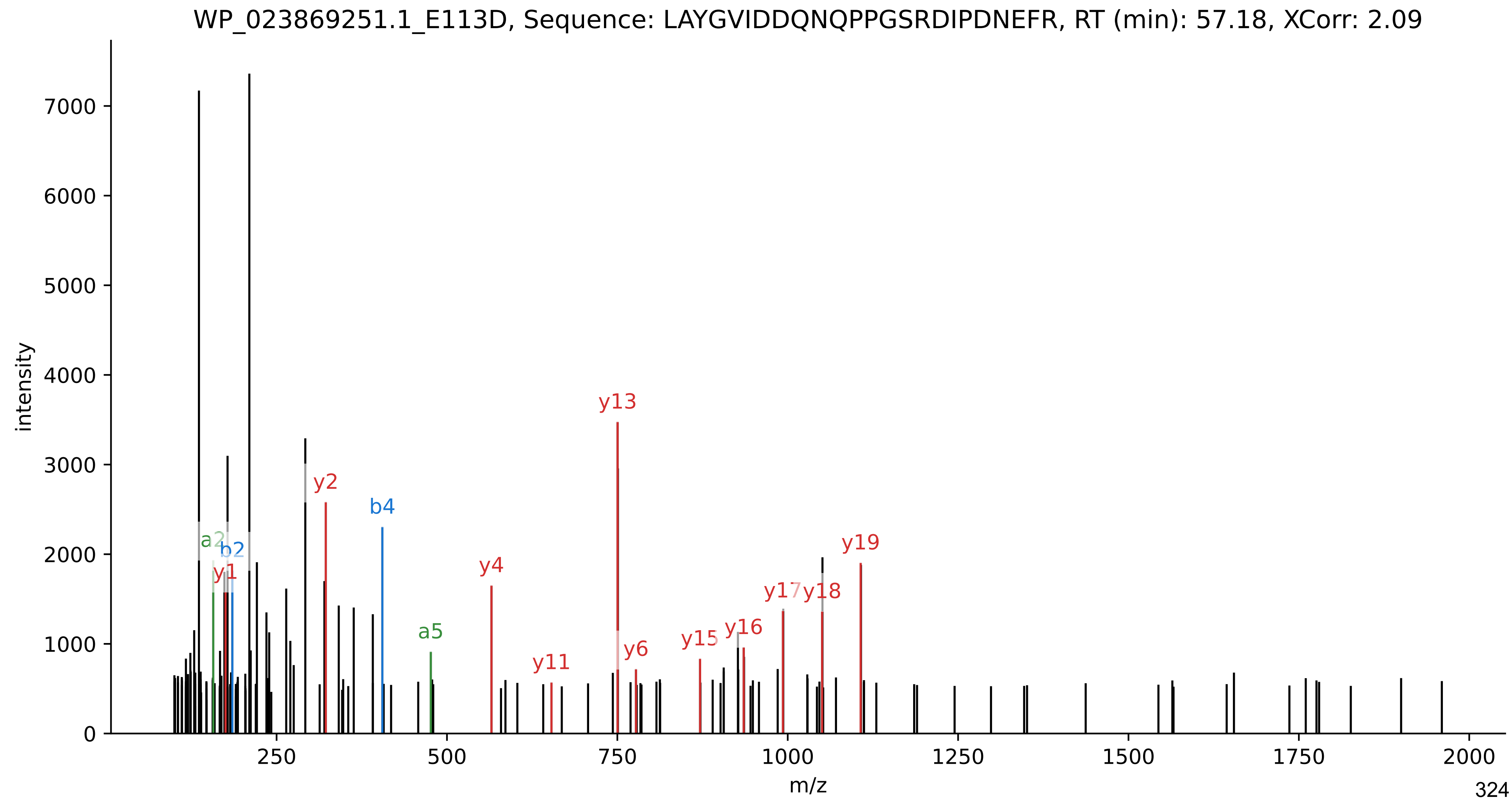
WP_023869251.1_E113D, Sequence: LAYGVIDDQNQPPGSRDIPDNEFR, RT (min): 57.18, XCorr: 2.09
intensity
m/z
a2
b2
y1
y2
b4
y4
a5
y13
y11
y6
y15
y16
y17
y18
y19
0
1000
2000
3000
4000
5000
6000
7000
250
500
750
1000
1250
1500
1750
2000

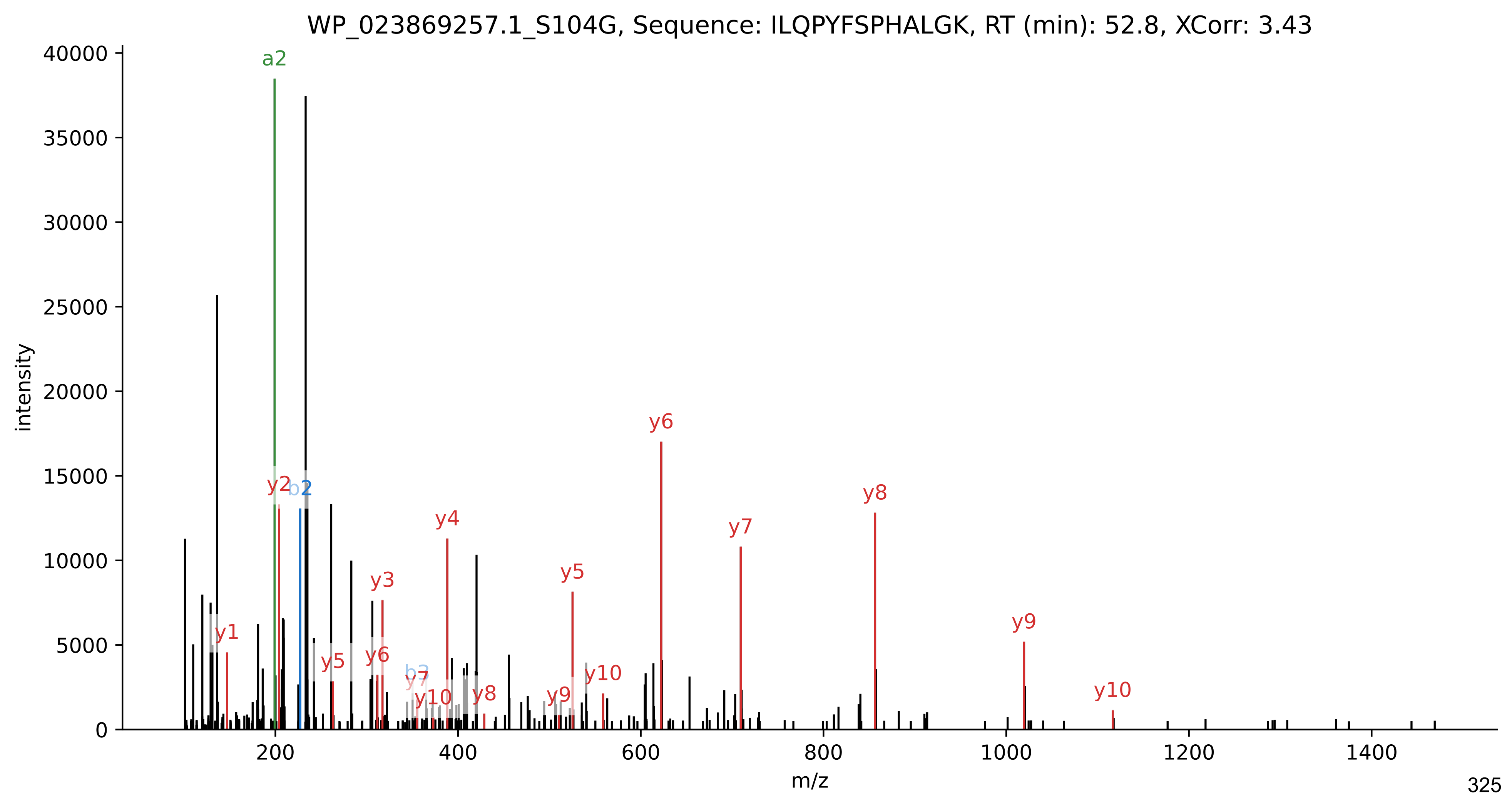
WP_023869257.1_S104G, Sequence: ILQPYFSPHALGK, RT (min): 52.8, XCorr: 3.43
intensity
m/z
a2
y1
y2
b2
y3
y4
y5
y6
y7
y8
y9
y10
b3
40000
35000
30000
25000
20000
15000
10000
5000
0
200
400
600
800
1000
1200
1400

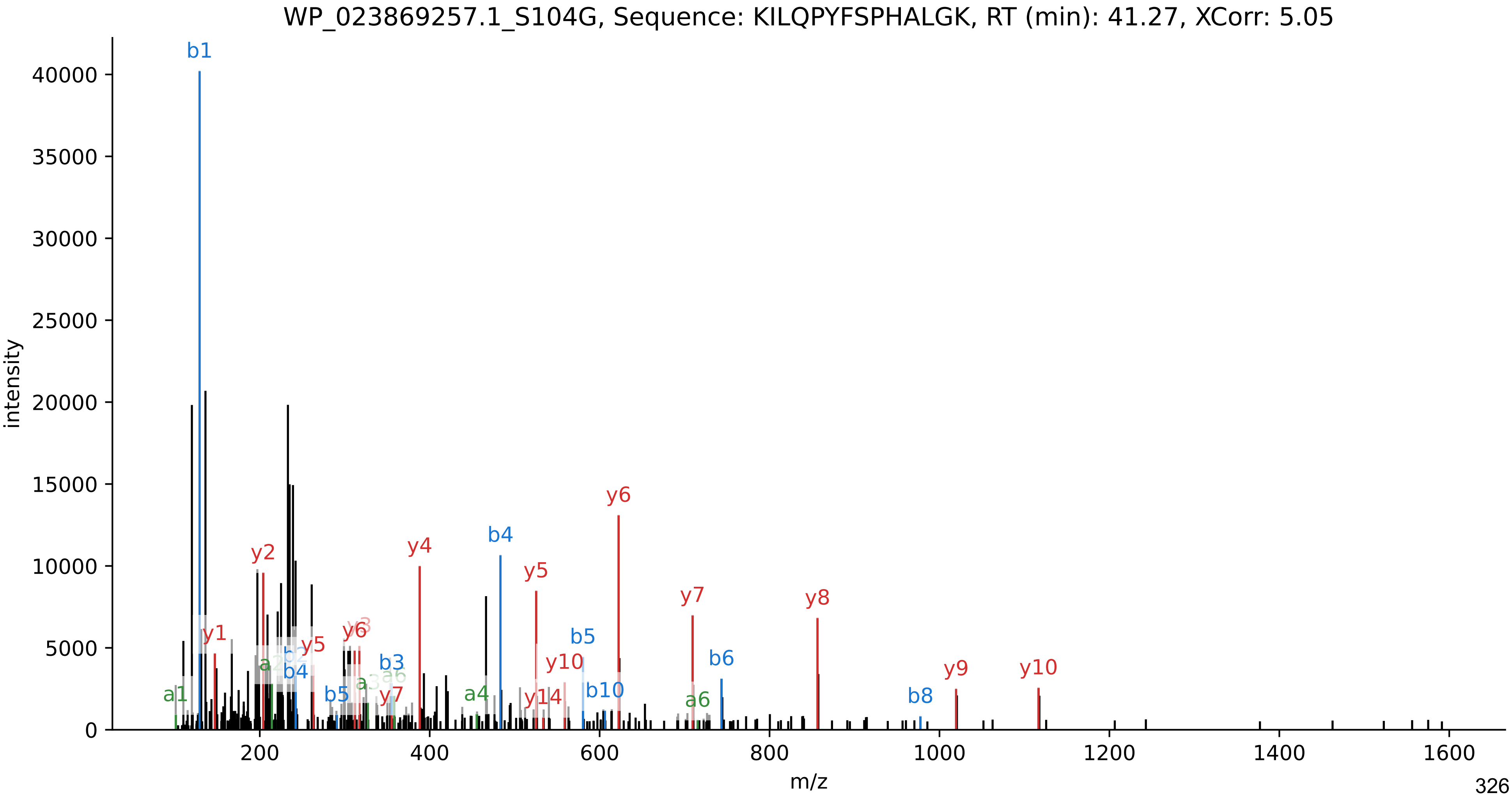

WP_023869257.1_S104G, Sequence: KILQPYFSPHALGK, RT (min): 41.27, XCorr: 5.05
intensity
m/z
b1
y1
y2
y4
b4
y5
y6
y7
y8
y9
y10
b5
b6
b8
b10
y10
y14
a1
a4
a6
b3
b5
b4
y5
y6
y7

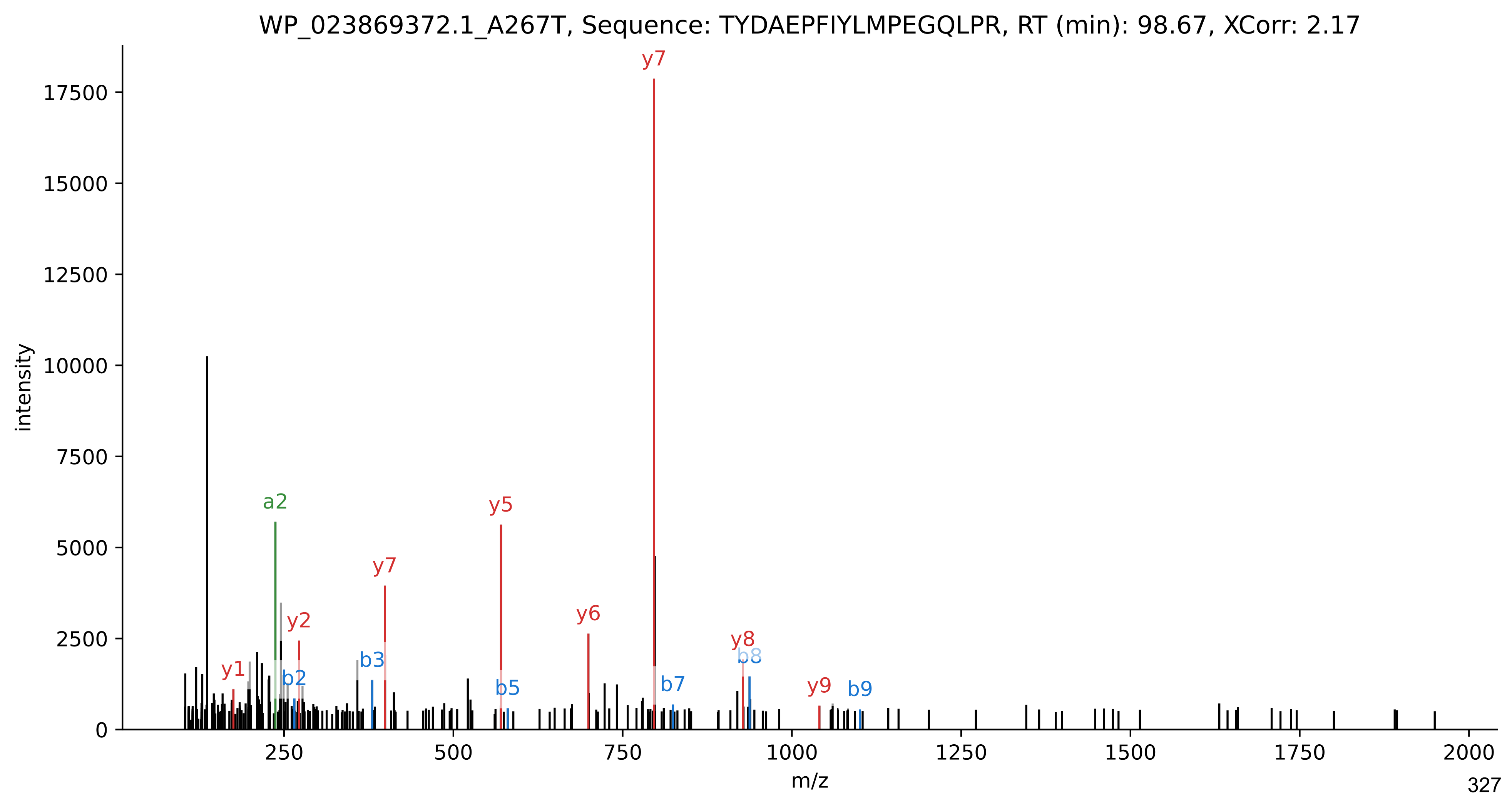
WP_023869372.1_A267T, Sequence: TYDAEPFIYLMPEGQLPR, RT (min): 98.67, XCorr: 2.17
intensity
m/z
y7
a2
y5
y7
y2
y6
y1
b2
b3
b5
b7
y8
b8
y9
b9
17500
15000
12500
10000
7500
5000
2500
0
250
500
750
1000
1250
1500
1750
2000

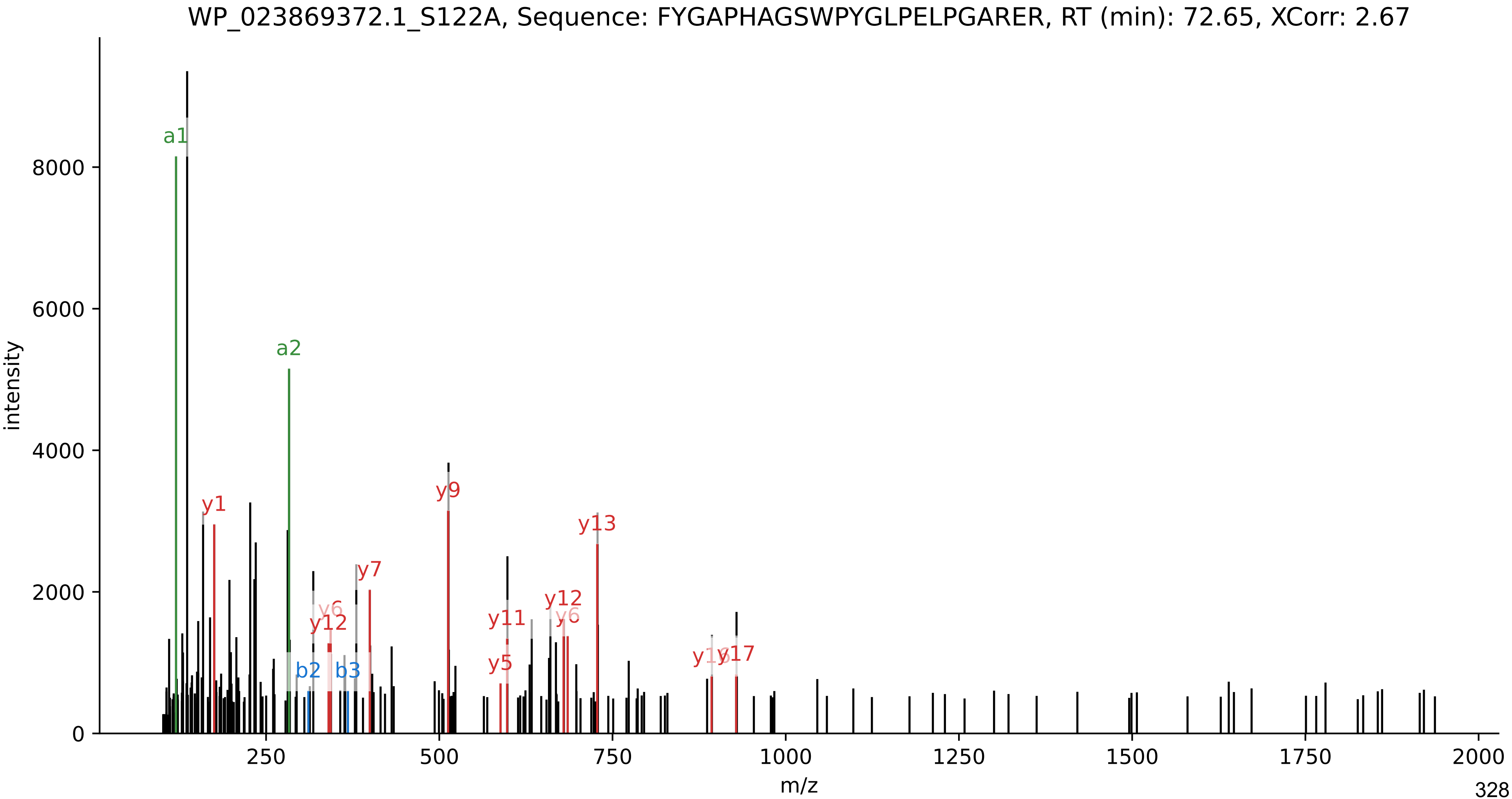
WP_023869372.1_S122A, Sequence: FYGAPHAGSWPYGLPELPGARER, RT (min): 72.65, XCorr: 2.67
intensity
m/z
0
2000
4000
6000
8000
250
500
750
1000
1250
1500
1750
2000
a1
a2
y1
y9
y13
y7
y6
y12
b2
b3
y11
y5
y12
y6
y17

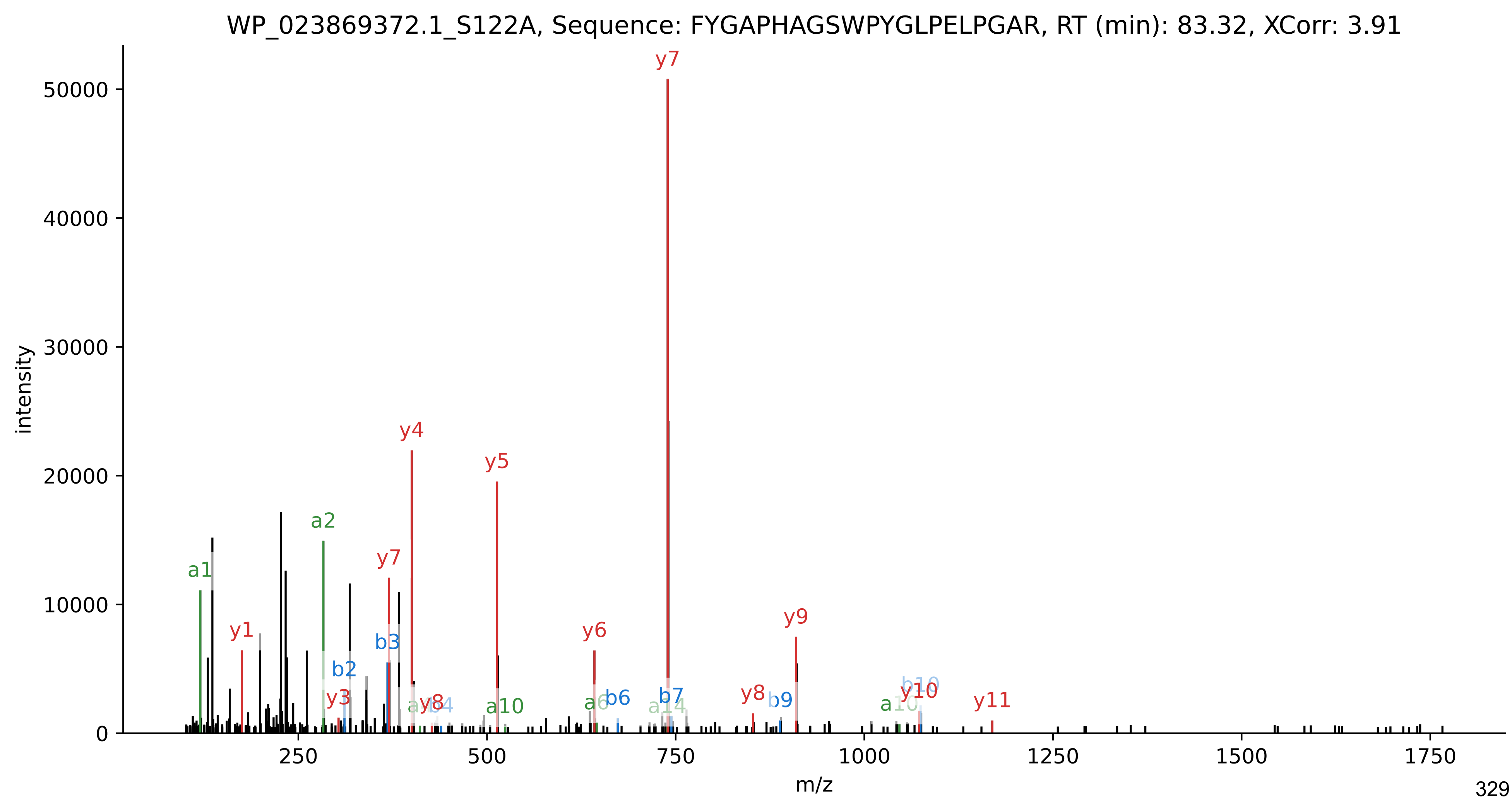

WP_023869372.1_S122A, Sequence: FYGAPHAGSWPYGLPELPGAR, RT (min): 83.32, XCorr: 3.91
intensity
m/z
y7
y4
y5
a2
a1
y7
y1
b3
b2
y3
y8
y6
b6
b7
y9
y8
b9
y10
y11
a10

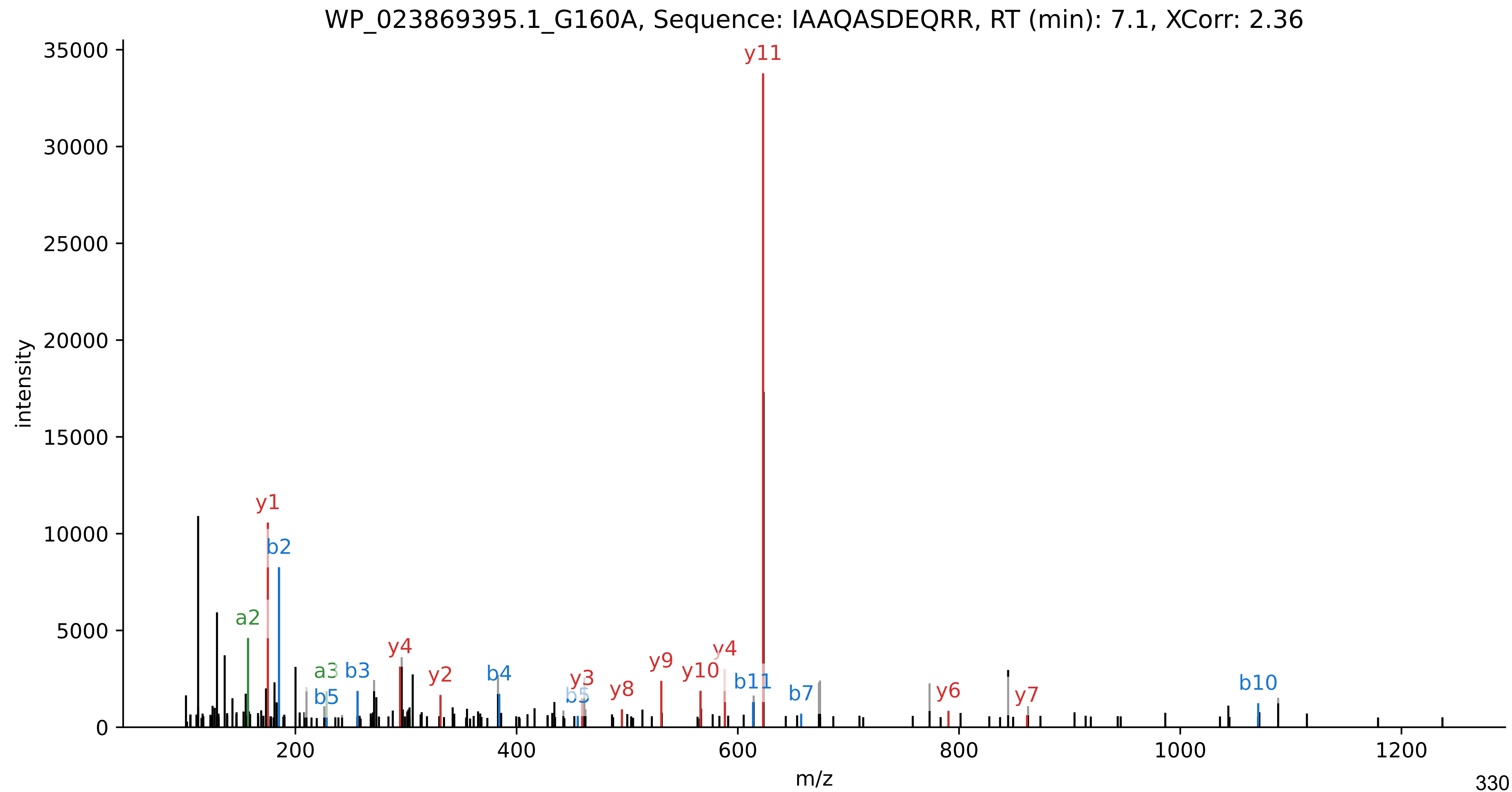

WP_023869395.1_G160A, Sequence: IAAQASDEQRR, RT (min): 7.1, XCorr: 2.36
intensity
m/z
y11
y1
b2
a2
a3
b3
b5
y4
y2
b4
b5
y3
y8
y9
y10
y4
b11
b7
y6
y7
b10
0
5000
10000
15000
20000
25000
30000
35000
200
400
600
800
1000
1200

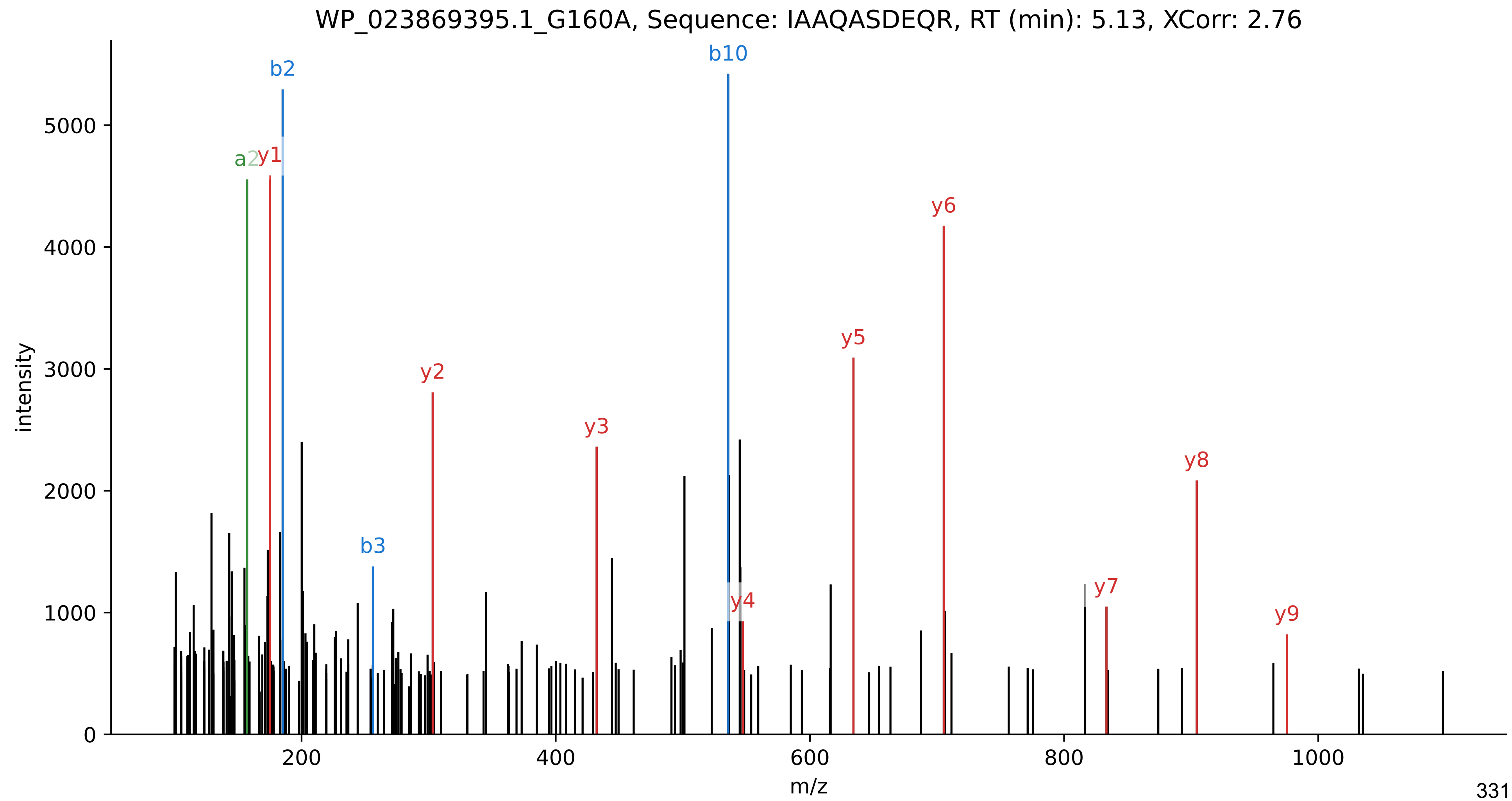
WP_023869395.1_G160A, Sequence: IAAQASDEQR, RT (min): 5.13, XCorr: 2.76
intensity
m/z
b2
b10
a2
y1
y6
y5
y2
y3
y8
b3
y7
y4
y9
0
1000
2000
3000
4000
5000
200
400
600
800
1000

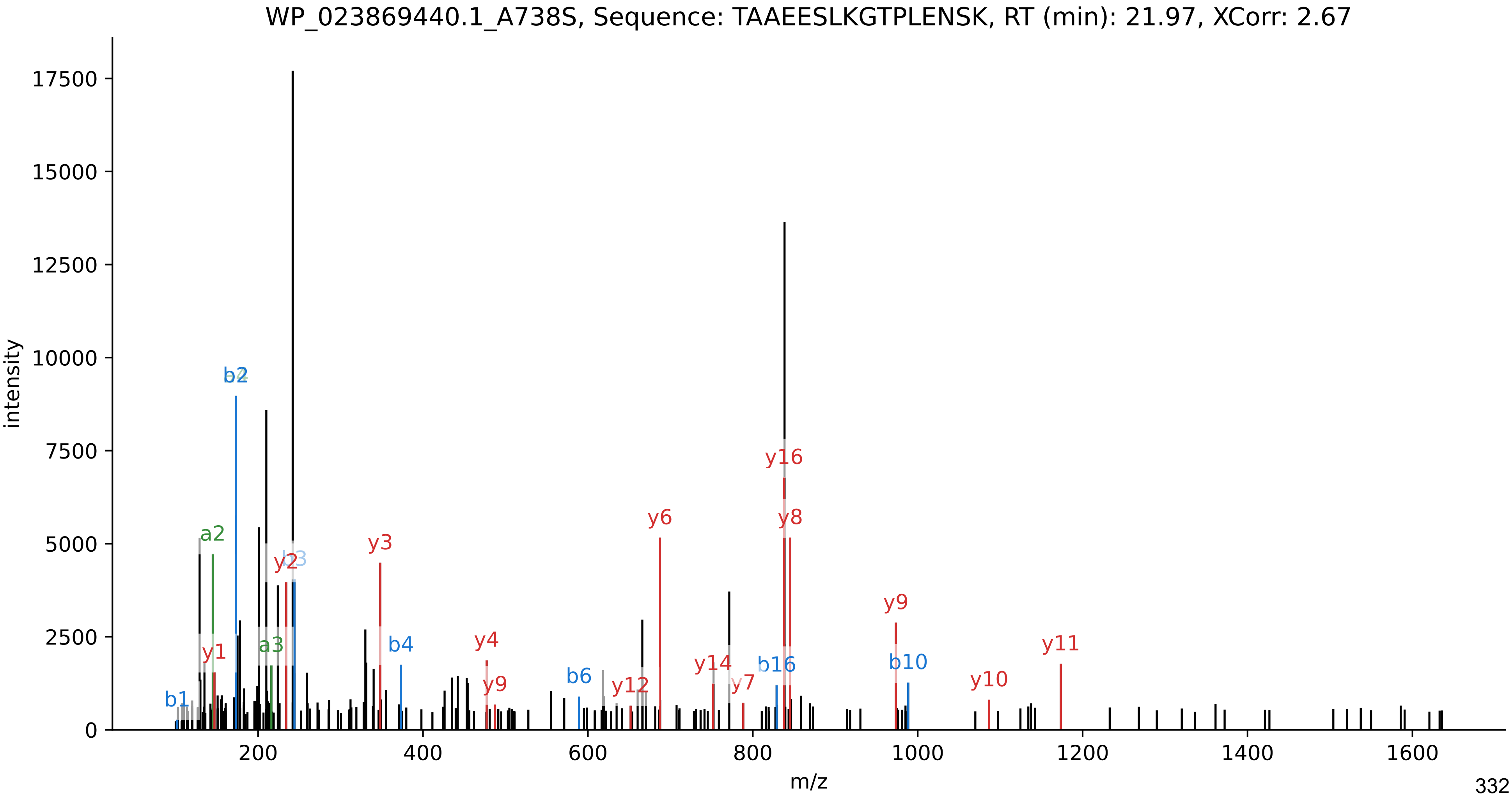

WP_023869440.1_A738S, Sequence: TAAEESLKGTPLENSK, RT (min): 21.97, XCorr: 2.67
intensity
m/z
17500
15000
12500
10000
7500
5000
2500
0
200
400
600
800
1000
1200
1400
1600
b1
a2
y1
b2
a3
y2
b3
y3
b4
y4
y9
b6
y12
y6
y14
y7
b16
y16
y8
y9
b10
y10
y11

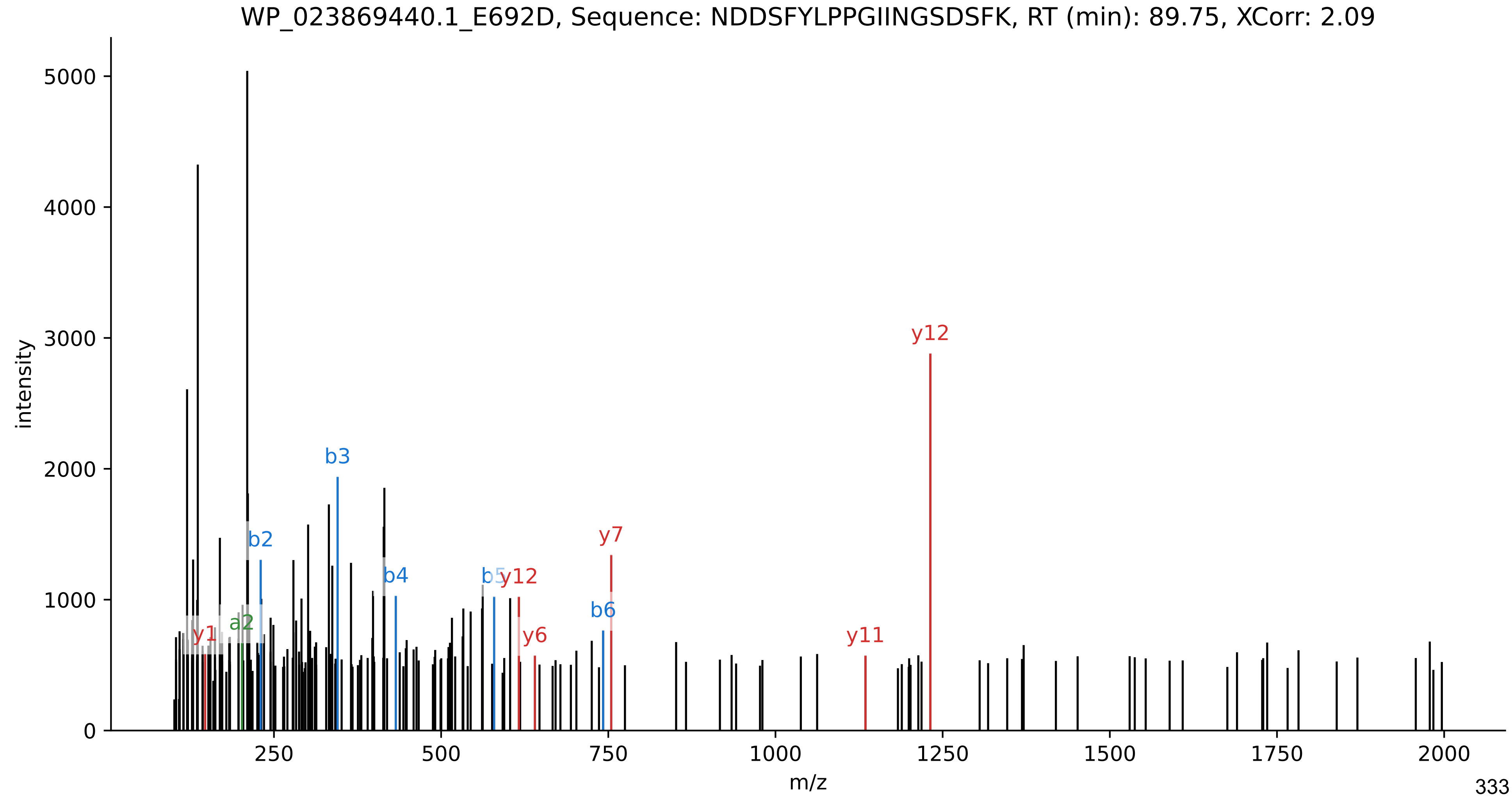

WP_023869440.1_E692D, Sequence: NDDSFYLPPGIINGSDSFK, RT (min): 89.75, XCorr: 2.09
intensity
m/z
y12
b3
b2
y7
b4
b5
y12
b6
a2
y1
y6
y11

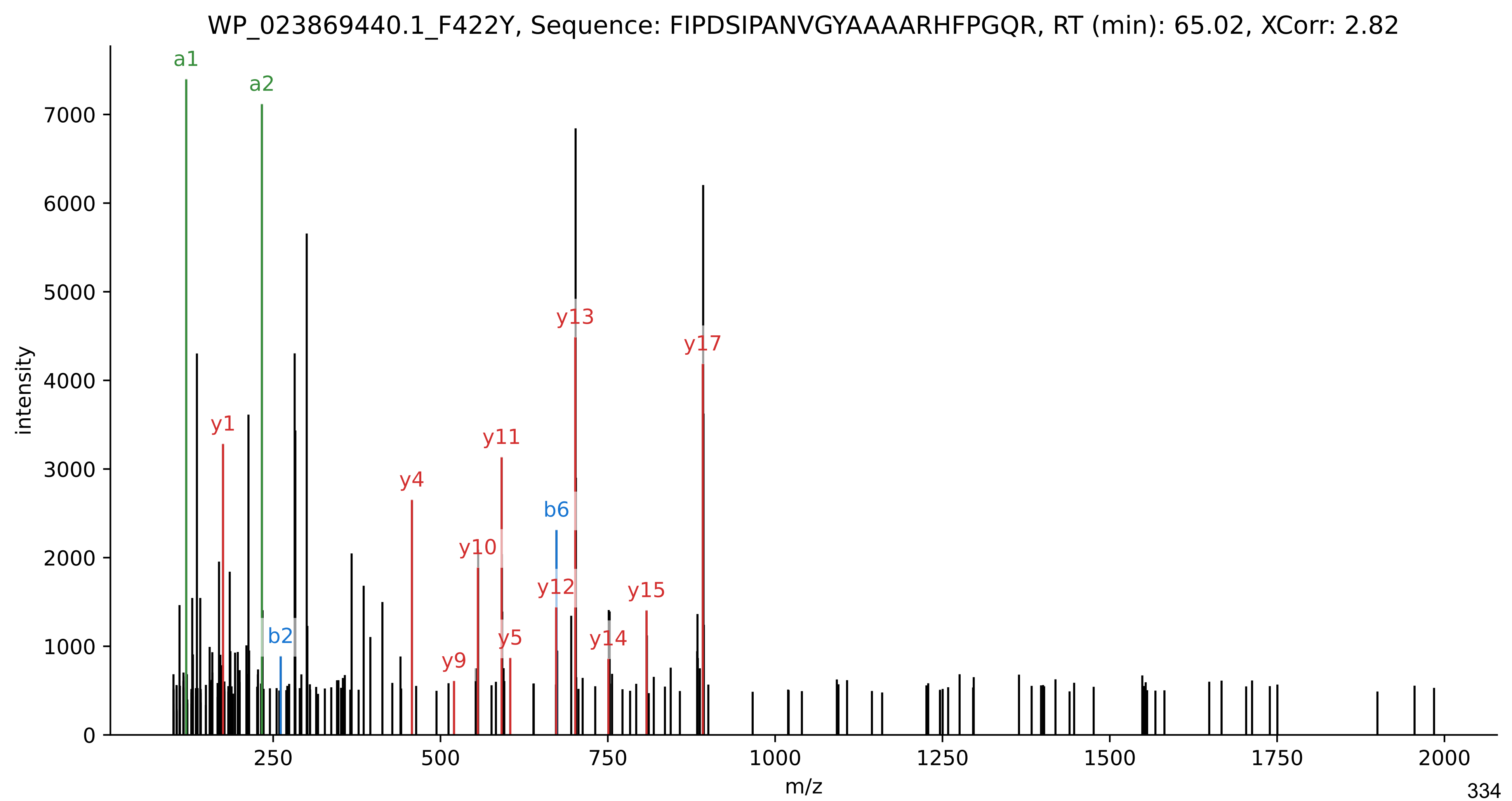

WP_023869440.1_F422Y, Sequence: FIPDSIPANVGYAAAARHFPGQR, RT (min): 65.02, XCorr: 2.82
a1
a2
y1
y4
y9
y10
y11
y5
b2
b6
y12
y13
y14
y15
y17
intensity
m/z
0
1000
2000
3000
4000
5000
6000
7000
250
500
750
1000
1250
1500
1750
2000

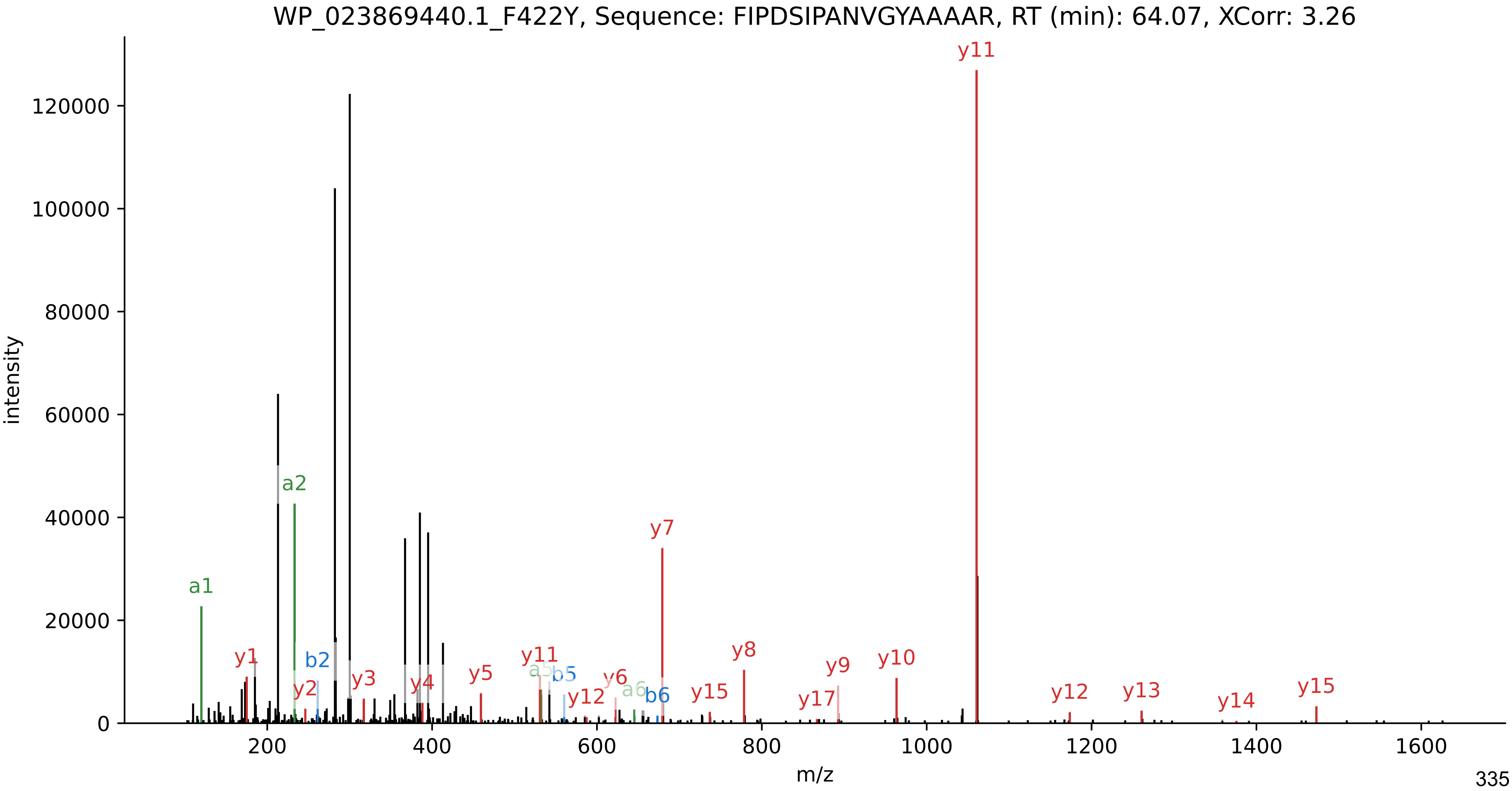

WP_023869440.1_F422Y, Sequence: FIPDSIPANVGYAAAAR, RT (min): 64.07, XCorr: 3.26
intensity
m/z
y11
a2
y7
a1
y1
b2
y2
y3
y4
y5
y11
b5
y12
y6
a6
b6
y15
y8
y17
y9
y10
y12
y13
y14
y15

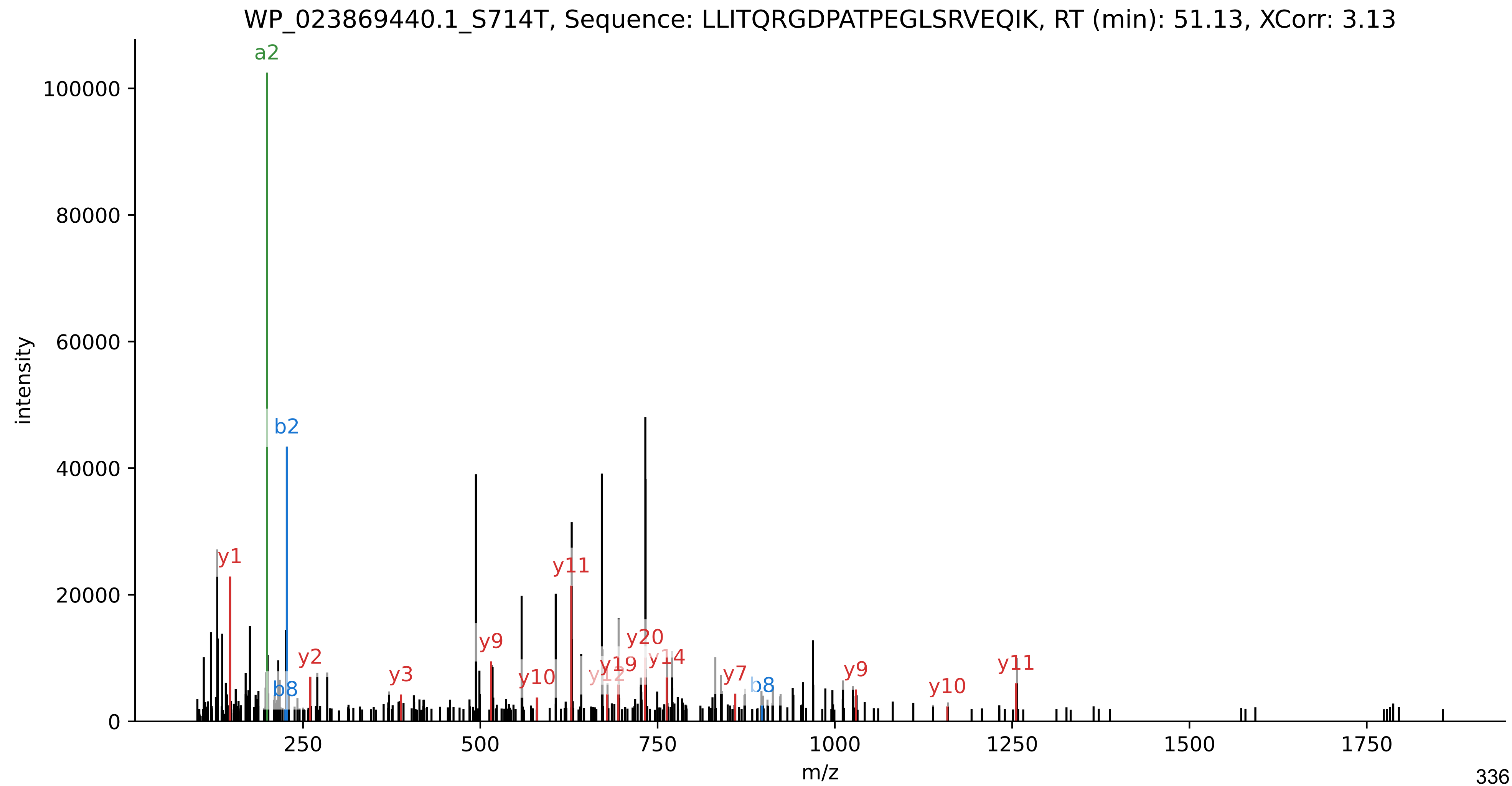

WP_023869440.1_S714T, Sequence: LLITQRGDPATPEGLSRVEQIK, RT (min): 51.13, XCorr: 3.13
intensity
m/z
a2
b2
y1
y11
y2
b8
y3
y9
y10
y19
y12
y20
y14
y7
b8
y9
y10
y11

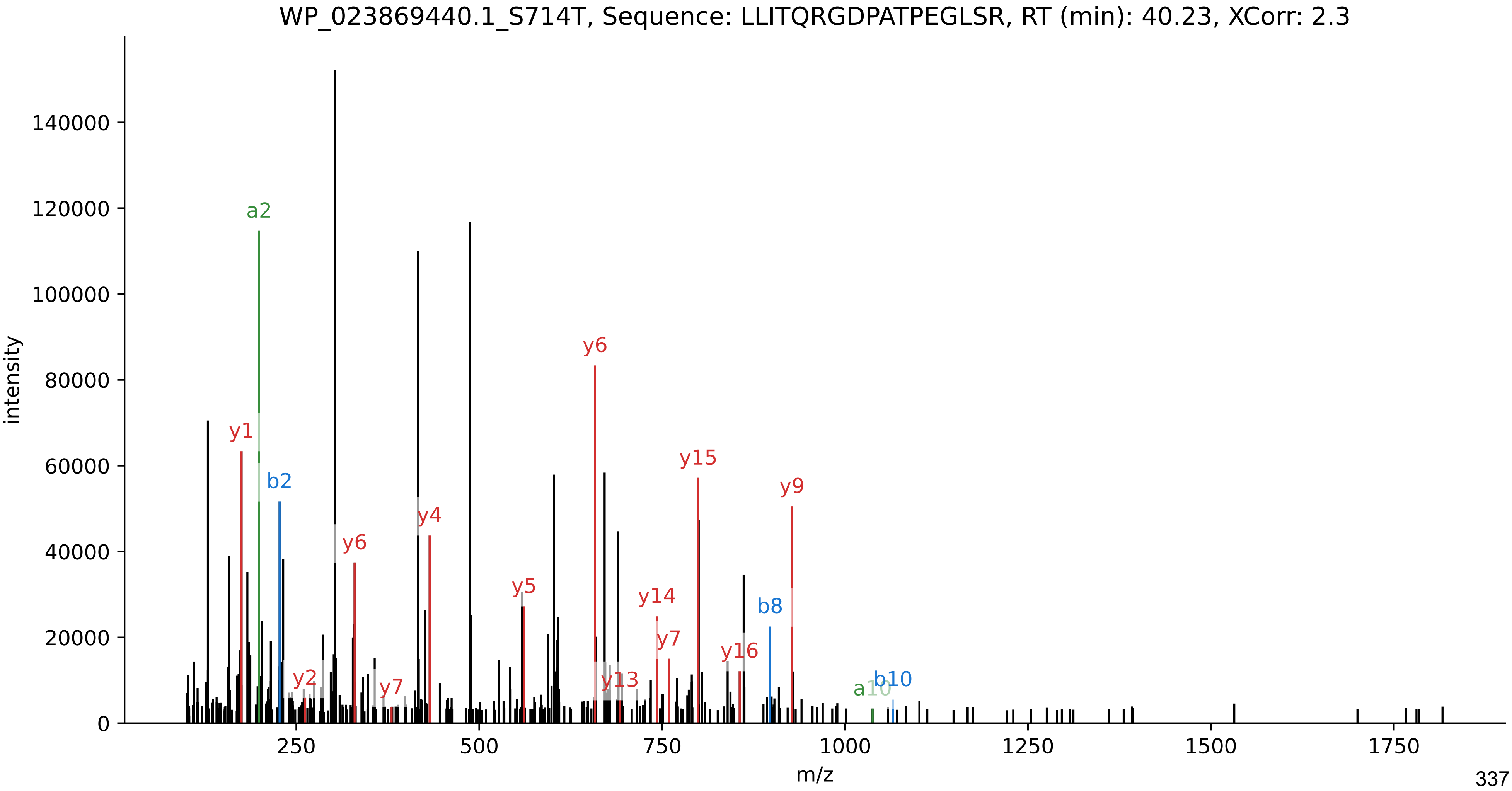

WP_023869440.1_S714T, Sequence: LLITQRGDPATPEGLSR, RT (min): 40.23, XCorr: 2.3
intensity
m/z
0
20000
40000
60000
80000
100000
120000
140000
250
500
750
1000
1250
1500
1750
a2
y1
b2
y2
y6
y7
y4
y5
y6
y13
y14
y7
y15
y16
b8
y9
a10
b10

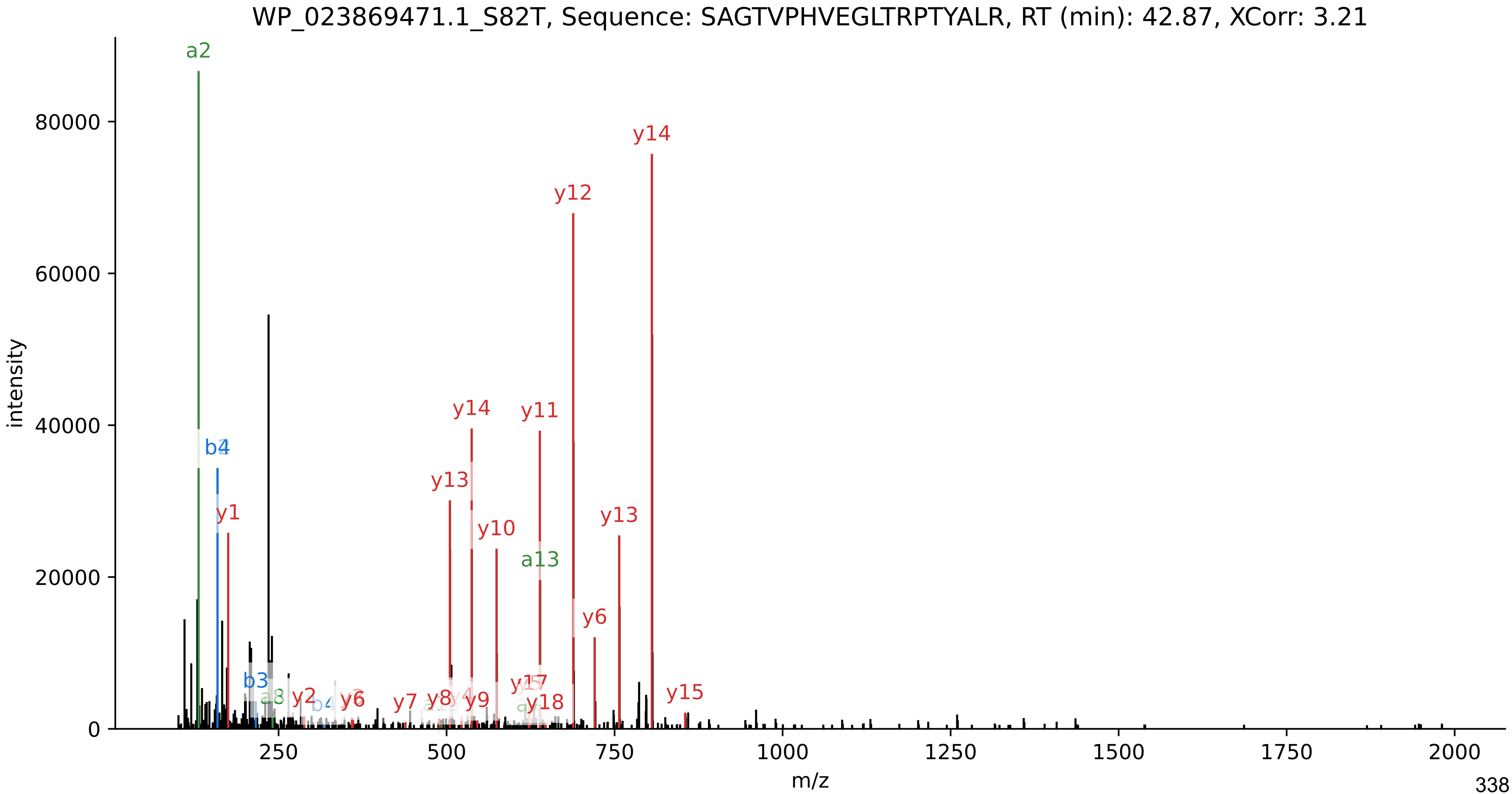
WP_023869471.1_S82T, Sequence: SAGTVPHVEGLTRPTYALR, RT (min): 42.87, XCorr: 3.21
intensity
m/z
a2
y14
y12
y14
y11
y13
y10
a13
y1
b4
y13
y6
b3
a8
y2
y6
y7
y8
y9
y17
y18
y15
0
20000
40000
60000
80000
250
500
750
1000
1250
1500
1750
2000

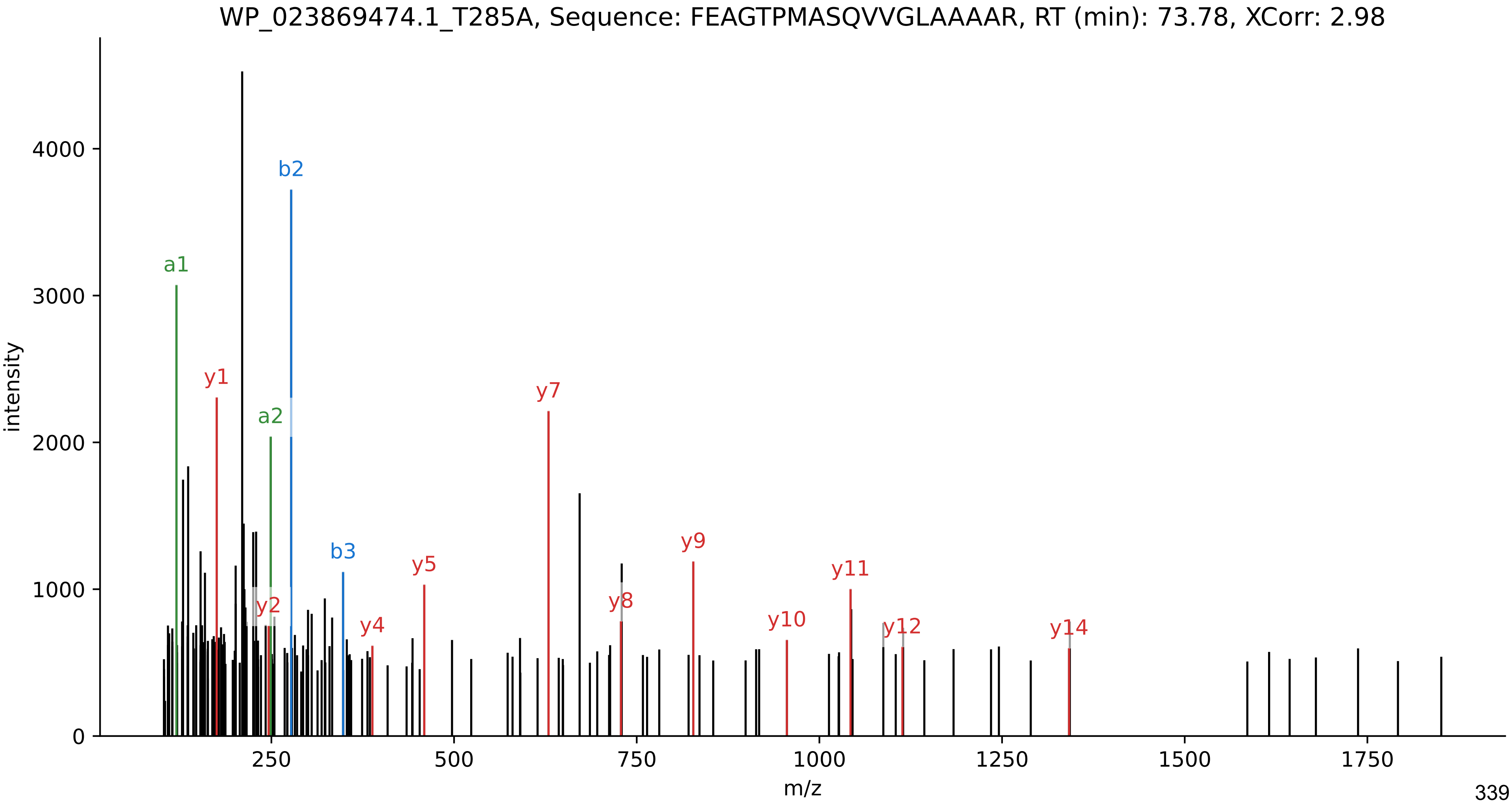

WP_023869474.1_T285A, Sequence: FEAGTPMASQVVGLAAAAR, RT (min): 73.78, XCorr: 2.98
intensity
m/z
a1
a2
b2
b3
y1
y2
y4
y5
y7
y8
y9
y10
y11
y12
y14
0
1000
2000
3000
4000
250
500
750
1000
1250
1500
1750

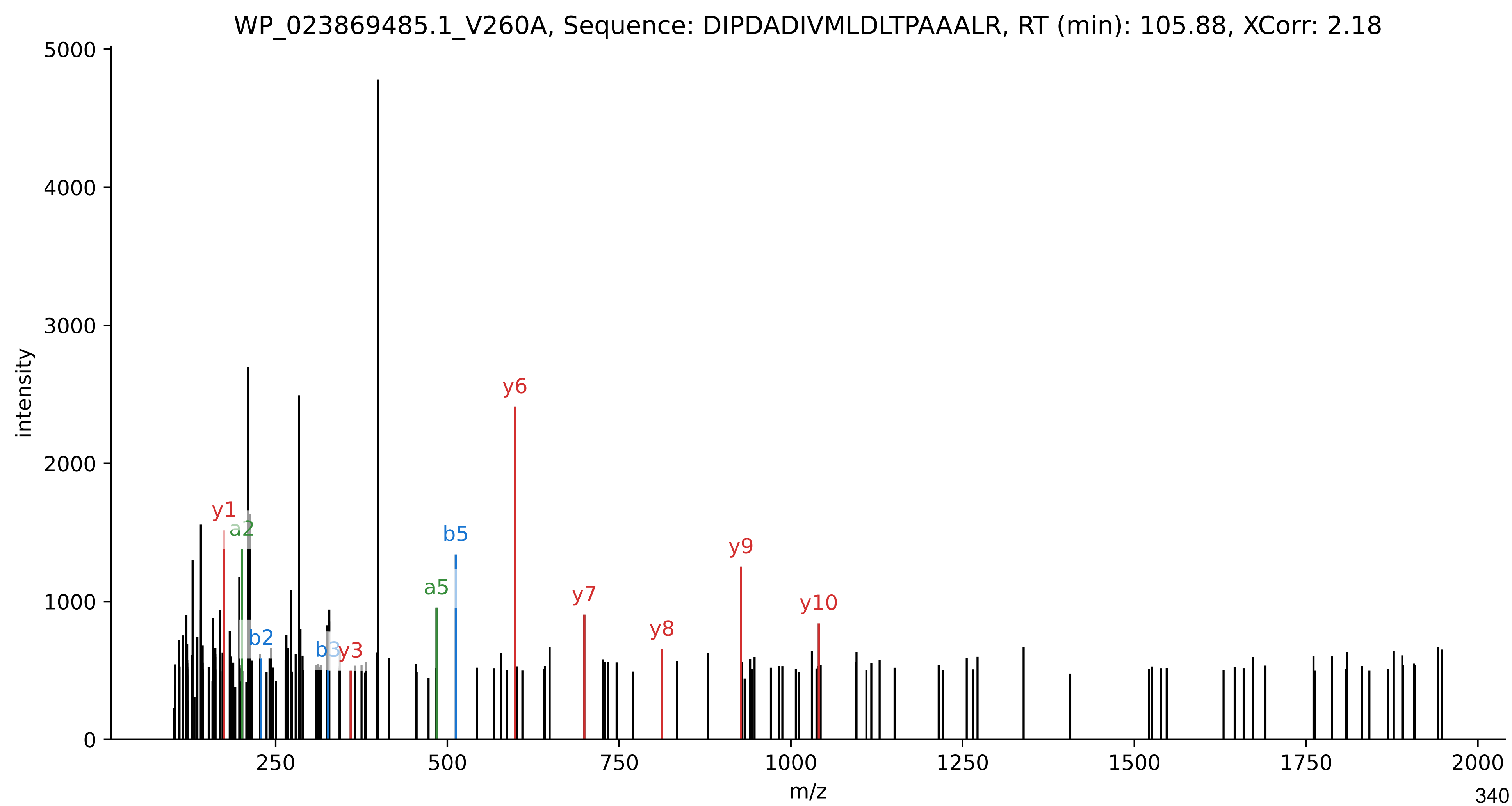

WP_023869485.1_V260A, Sequence: DIPDADIVMLDLTPAAALR, RT (min): 105.88, XCorr: 2.18
intensity
m/z
y1
a2
b2
b3
y3
a5
b5
y6
y7
y8
y9
y10

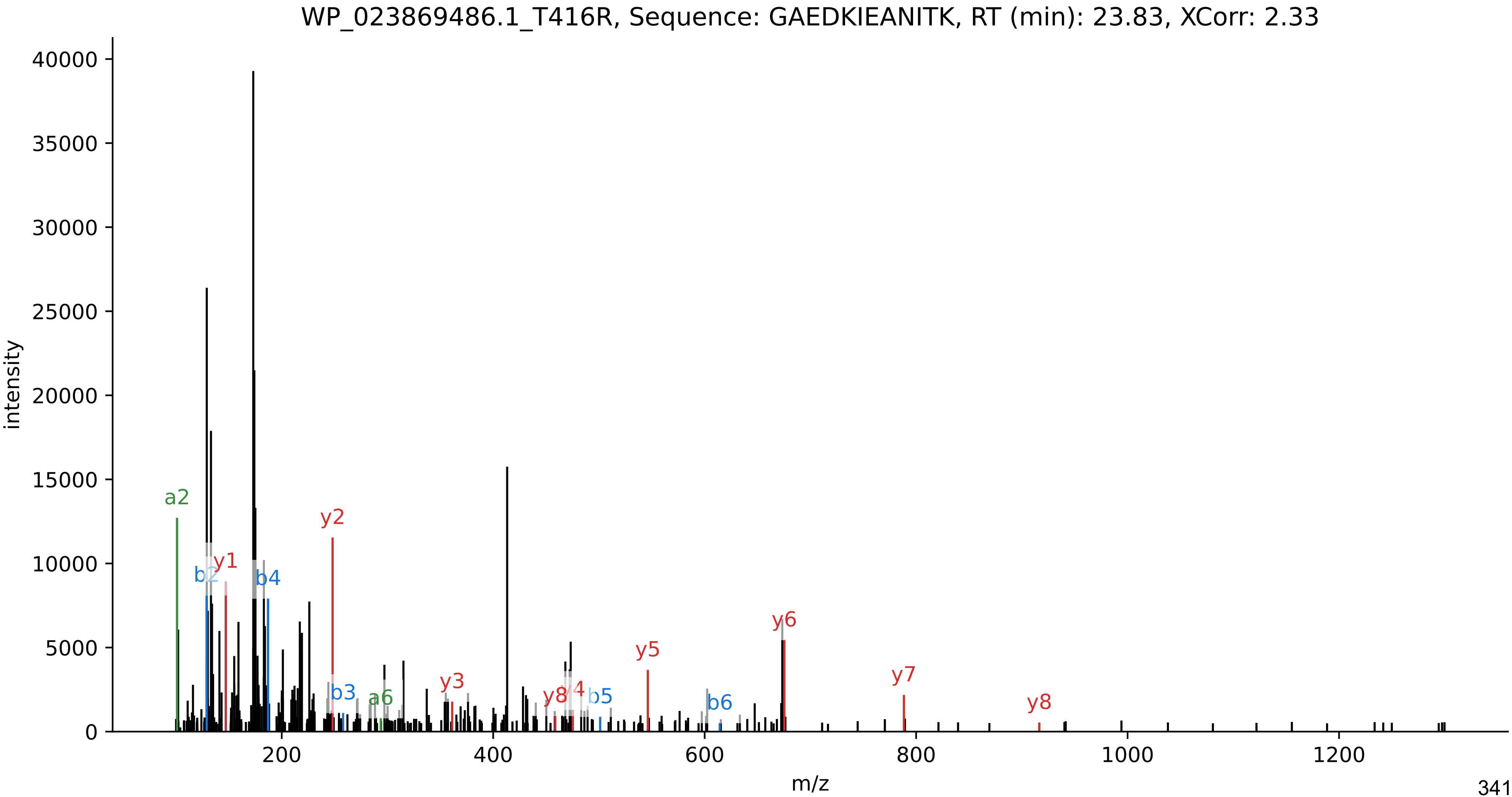
WP_023869486.1_T416R, Sequence: GAEDKIEANITK, RT (min): 23.83, XCorr: 2.33
intensity
m/z
a2
b2
y1
b4
y2
b3
a6
y3
y8
y4
b5
y5
b6
y6
y7
y8

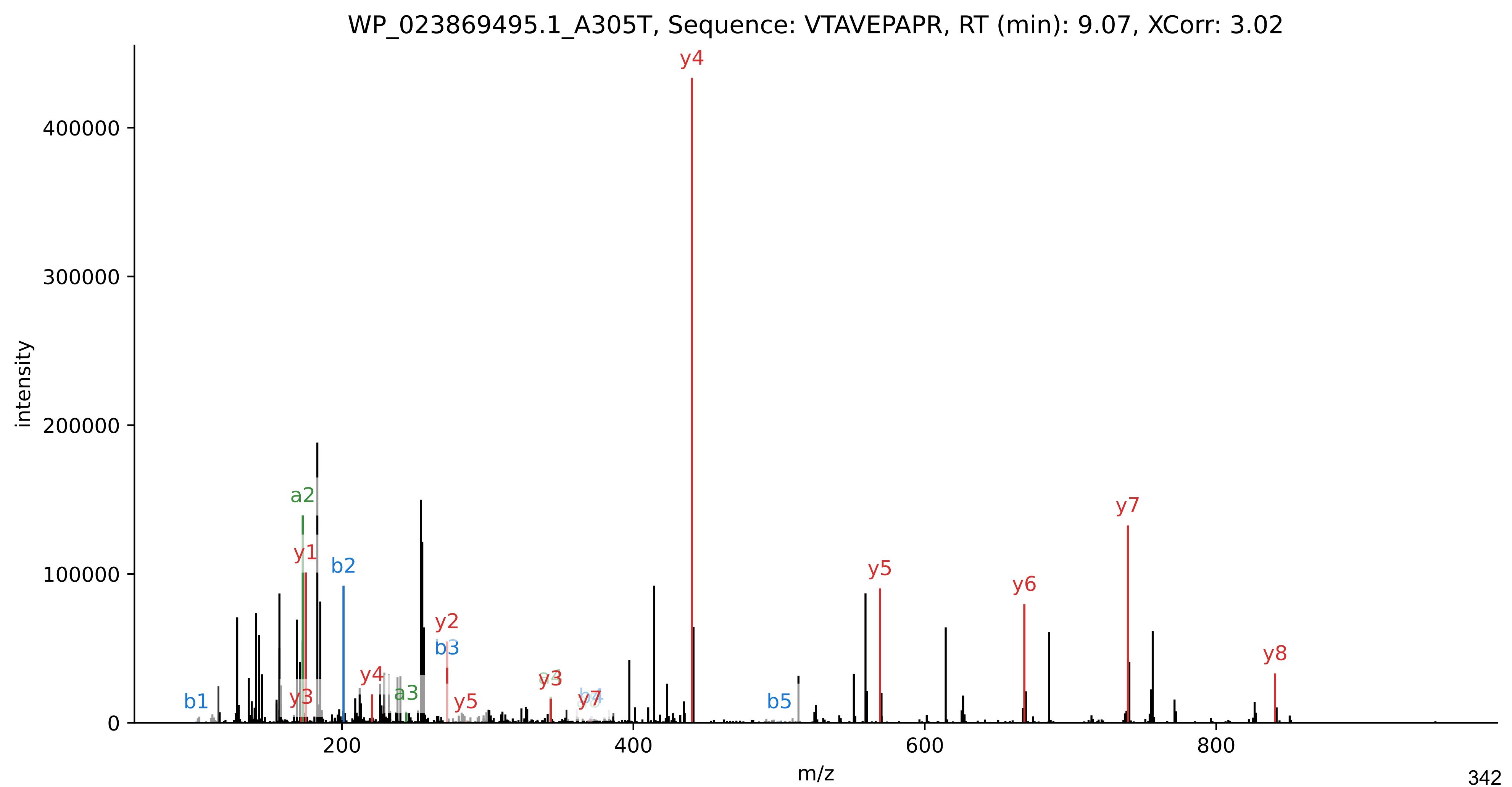
WP_023869495.1_A305T, Sequence: VTAVEPAPR, RT (min): 9.07, XCorr: 3.02
intensity
m/z
0
100000
200000
300000
400000
200
400
600
800
y4
a2
y1
b2
y2
b3
y4
a3
y3
y5
b1
y3
y7
b5
y5
y6
y7
y8

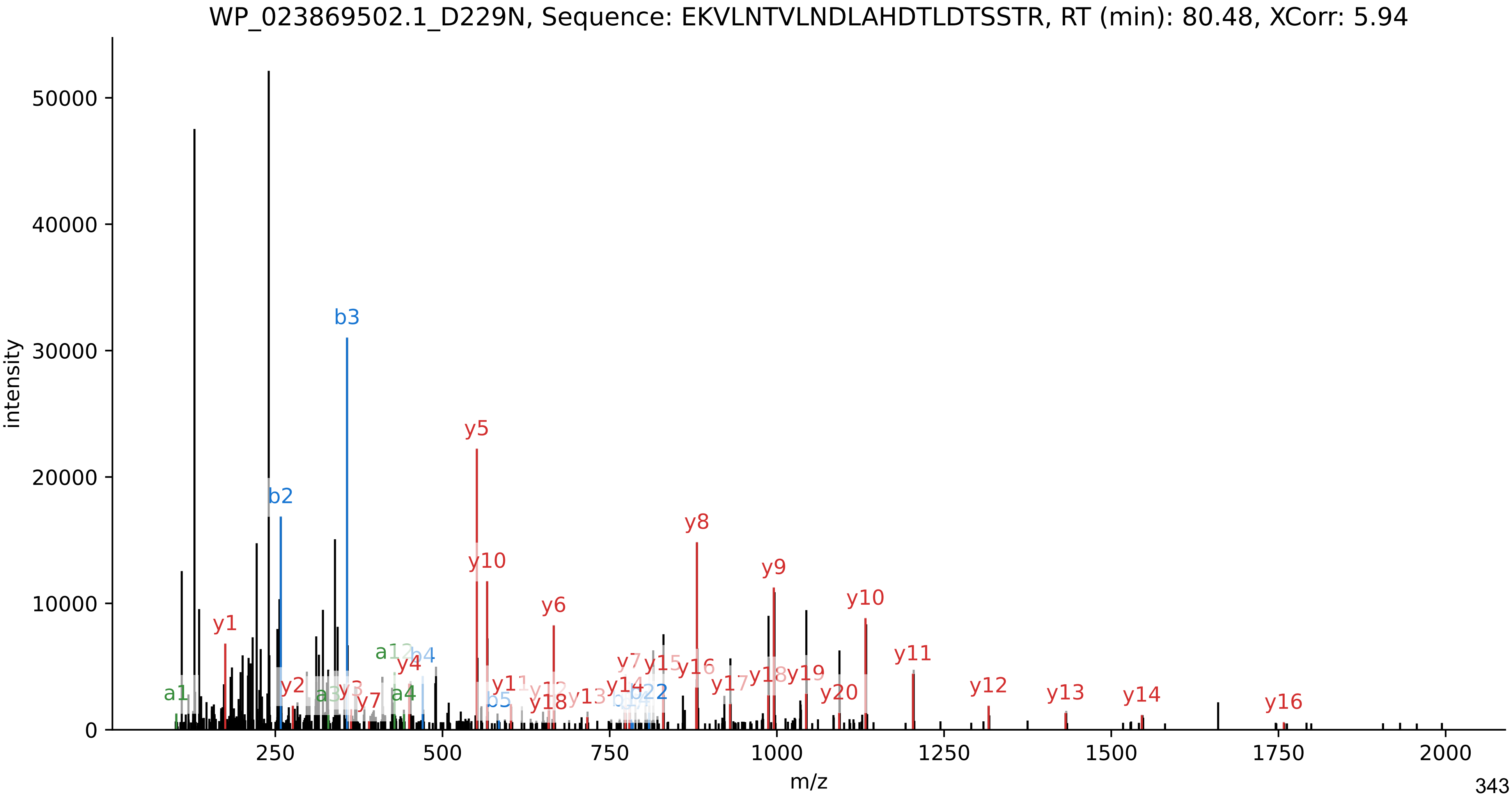
WP_023869502.1_D229N, Sequence: EKVLNTVLNDLAHDTLDTSSTR, RT (min): 80.48, XCorr: 5.94
intensity
m/z
b3
y5
b2
y8
y10
y9
y6
y10
y1
y11
y12
y13
y14
y16

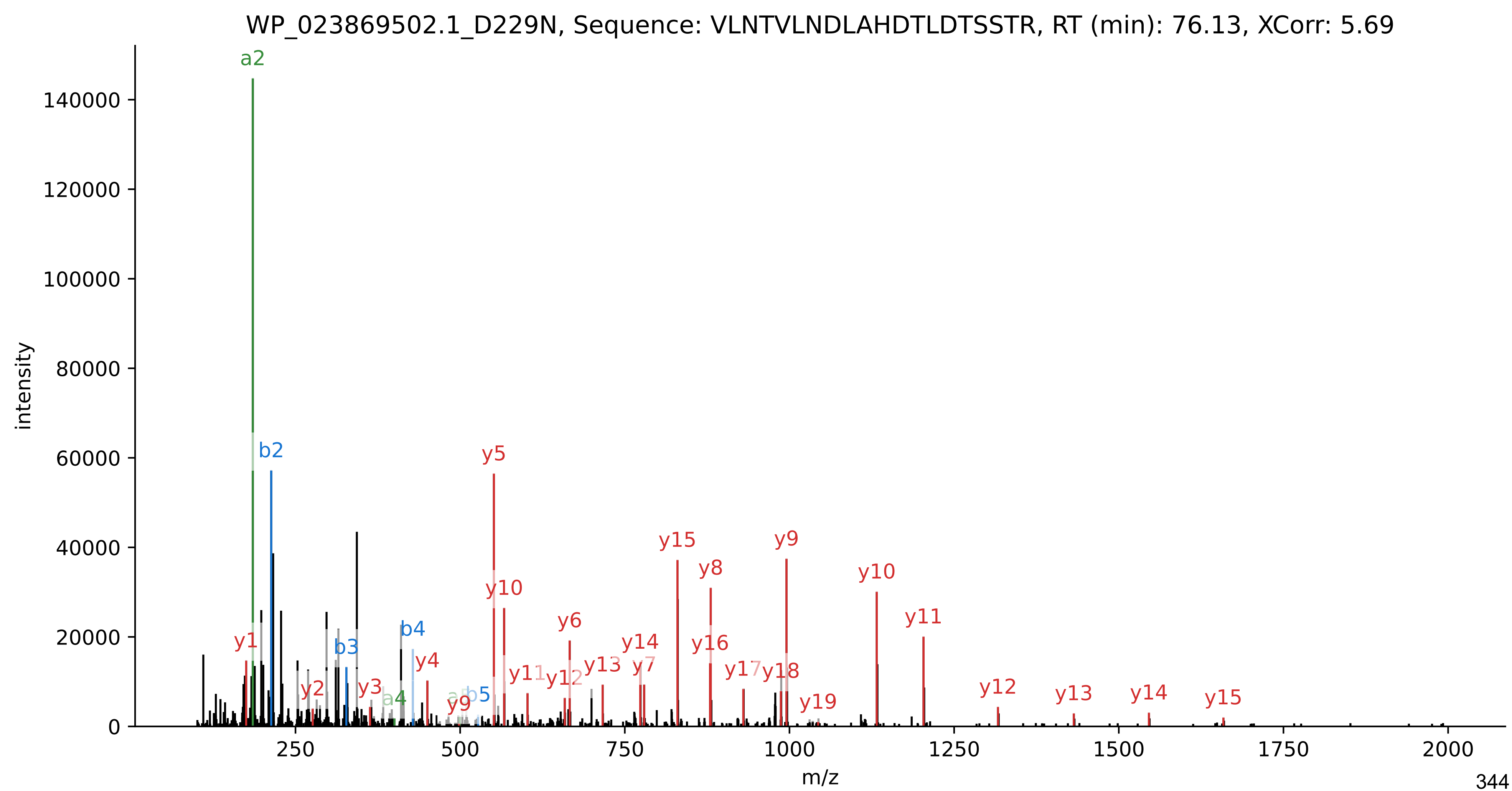
WP_023869502.1_D229N, Sequence: VLNTVLNDLAHDTLDTSSTR, RT (min): 76.13, XCorr: 5.69
intensity
m/z
a2
b2
y5
y15
y9
y8
y10
y10
y11
y6
y14
y16
y1
b4
b3
y4
y13
y7
y17
y18
y11
y12
y2
y3
a4
b5
y9
y19
y12
y13
y14
y15

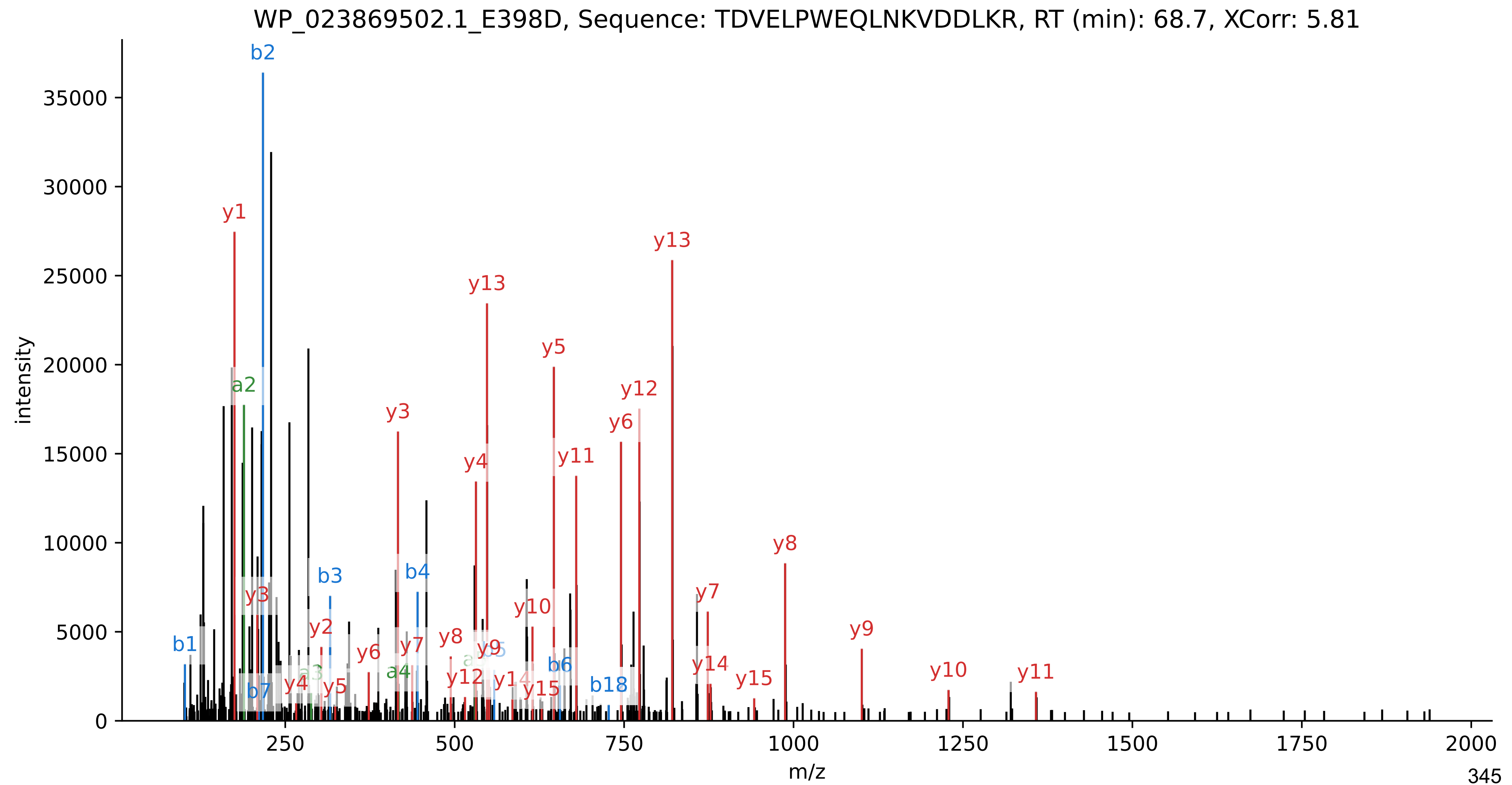
WP_023869502.1_E398D, Sequence: TDVELPWEQLNKVDDLKR, RT (min): 68.7, XCorr: 5.81
intensity
m/z
b1
b2
b3
b4
b6
b7
b18
a2
a4
y1
y2
y3
y4
y5
y6
y7
y8
y9
y10
y11
y12
y13
y14
y15

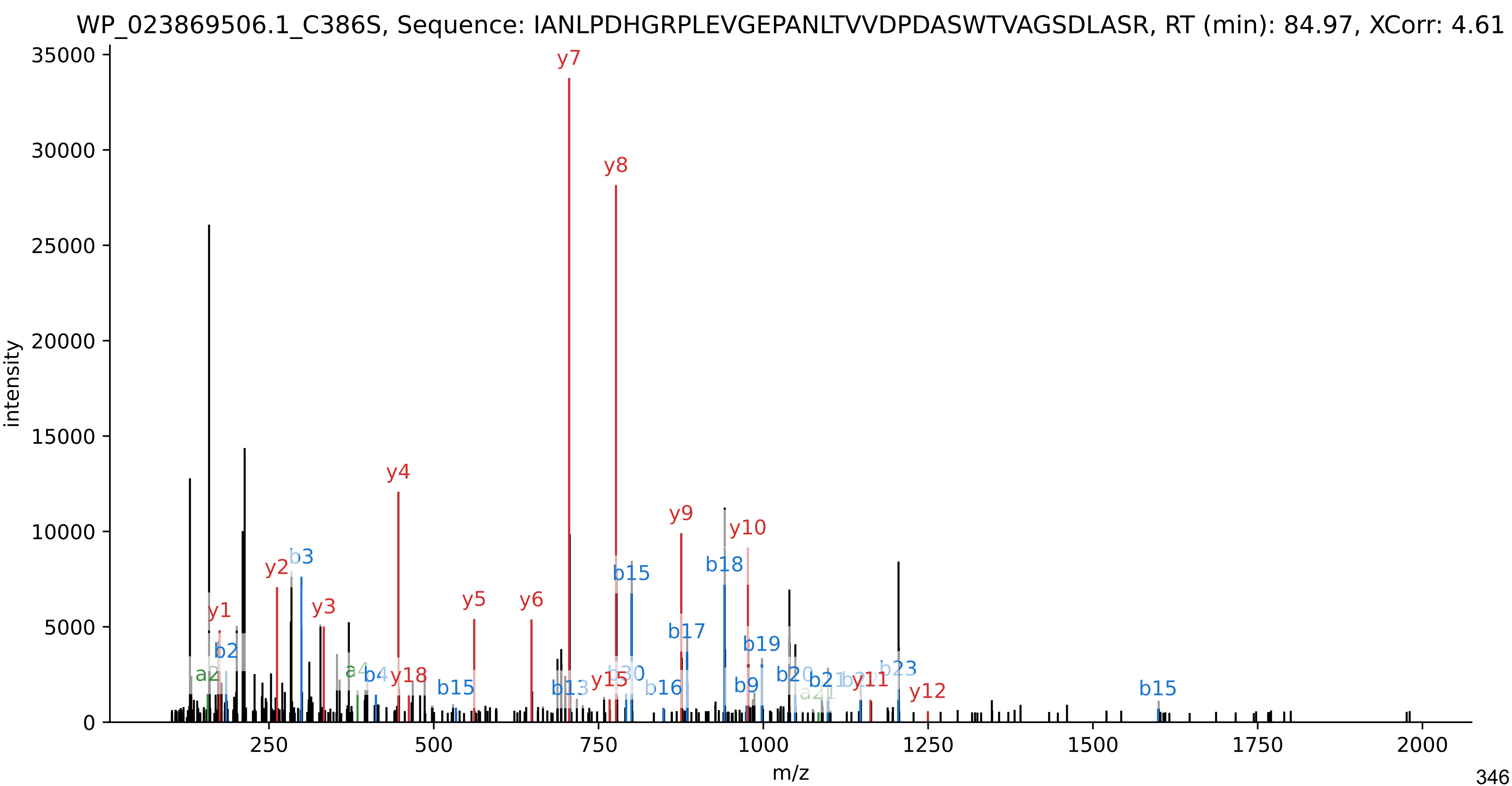
WP_023869506.1_C386S, Sequence: IANLPDHGRPLEVGEPANLTVVDPDASWTVAGSDLASR, RT (min): 84.97, XCorr: 4.61
intensity
m/z
0
5000
10000
15000
20000
25000
30000
35000
250
500
750
1000
1250
1500
1750
2000
y7
y8
y4
y9
y10
y2
b3
y1
y3
y5
y6
b15
b18
b17
b19
b2
a2
a4
b4
y18
b15
b13
y15
b30
b16
b9
b20
b21
y11
b23
y12
a21
b15

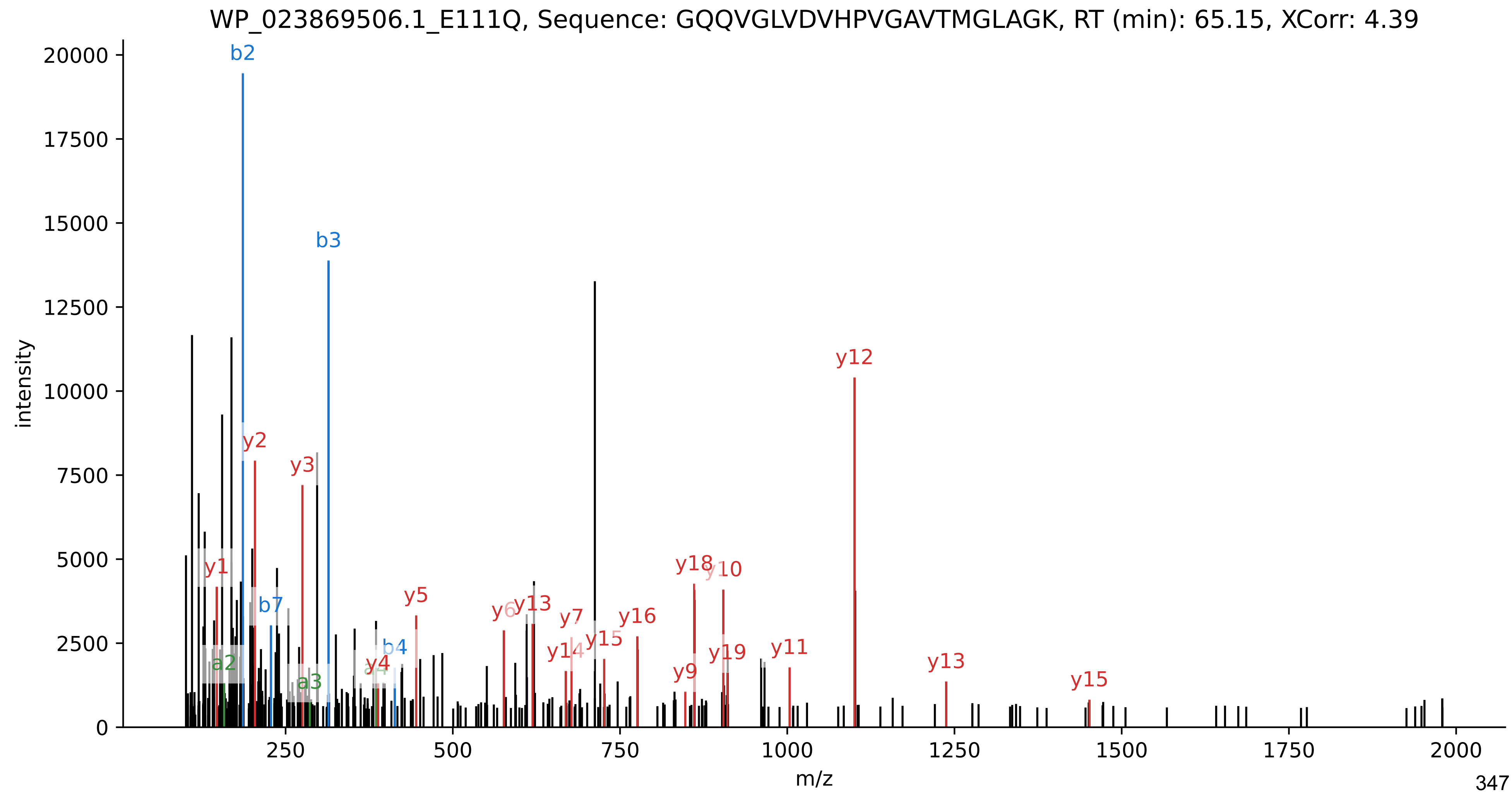

WP_023869506.1_E111Q, Sequence: GQQVGLVDVHPVGAVTMGLAGK, RT (min): 65.15, XCorr: 4.39
intensity
m/z
b2
b3
y12
y2
y3
y1
b7
y5
y6
y13
y7
y16
y18
y10
y15
y14
b4
y4
a2
a3
a4
y9
y19
y11
y13
y15

WP_023869589.1_C49R, Sequence: RGATVIMAVRDTR, RT (min): 18.75, XCorr: 3.4

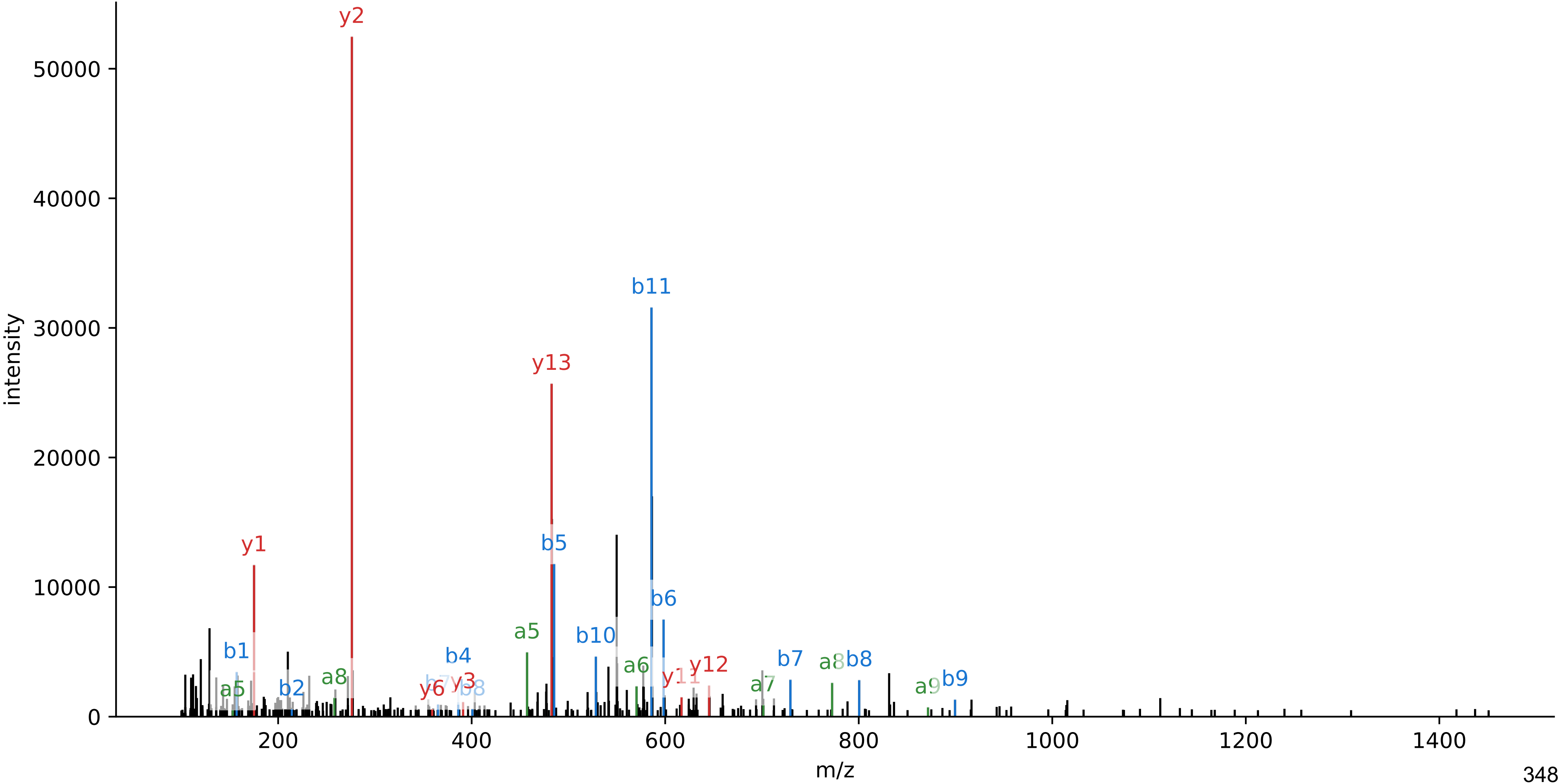

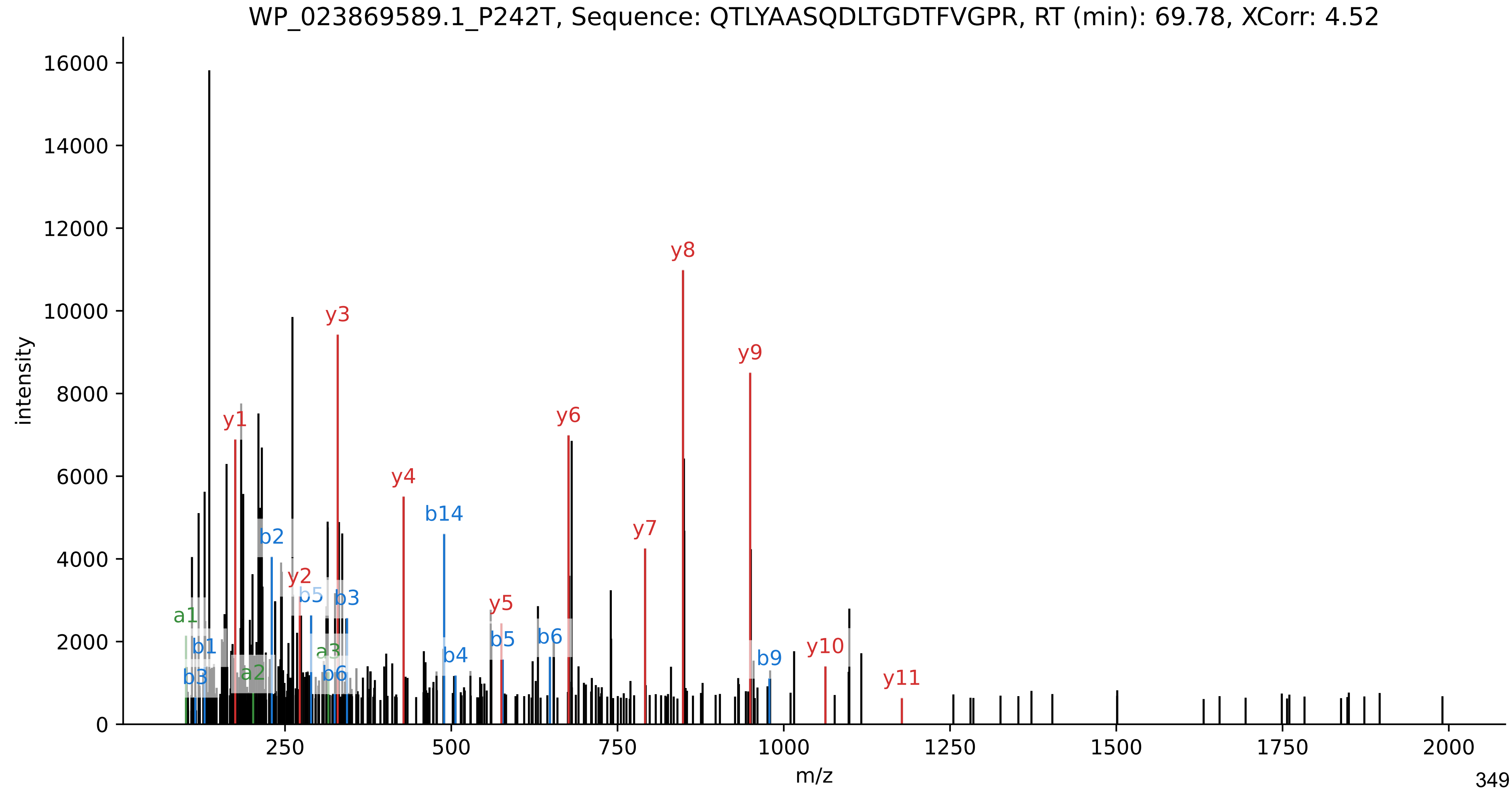
WP_023869589.1_P242T, Sequence: QTLYAASQDLTGDTFVGPR, RT (min): 69.78, XCorr: 4.52
intensity
m/z
16000
14000
12000
10000
8000
6000
4000
2000
0
250
500
750
1000
1250
1500
1750
2000
y1
y2
y3
y4
y5
y6
y7
y8
y9
y10
y11
b1
b2
b3
b4
b5
b6
b9
b14
a1
a2
a3

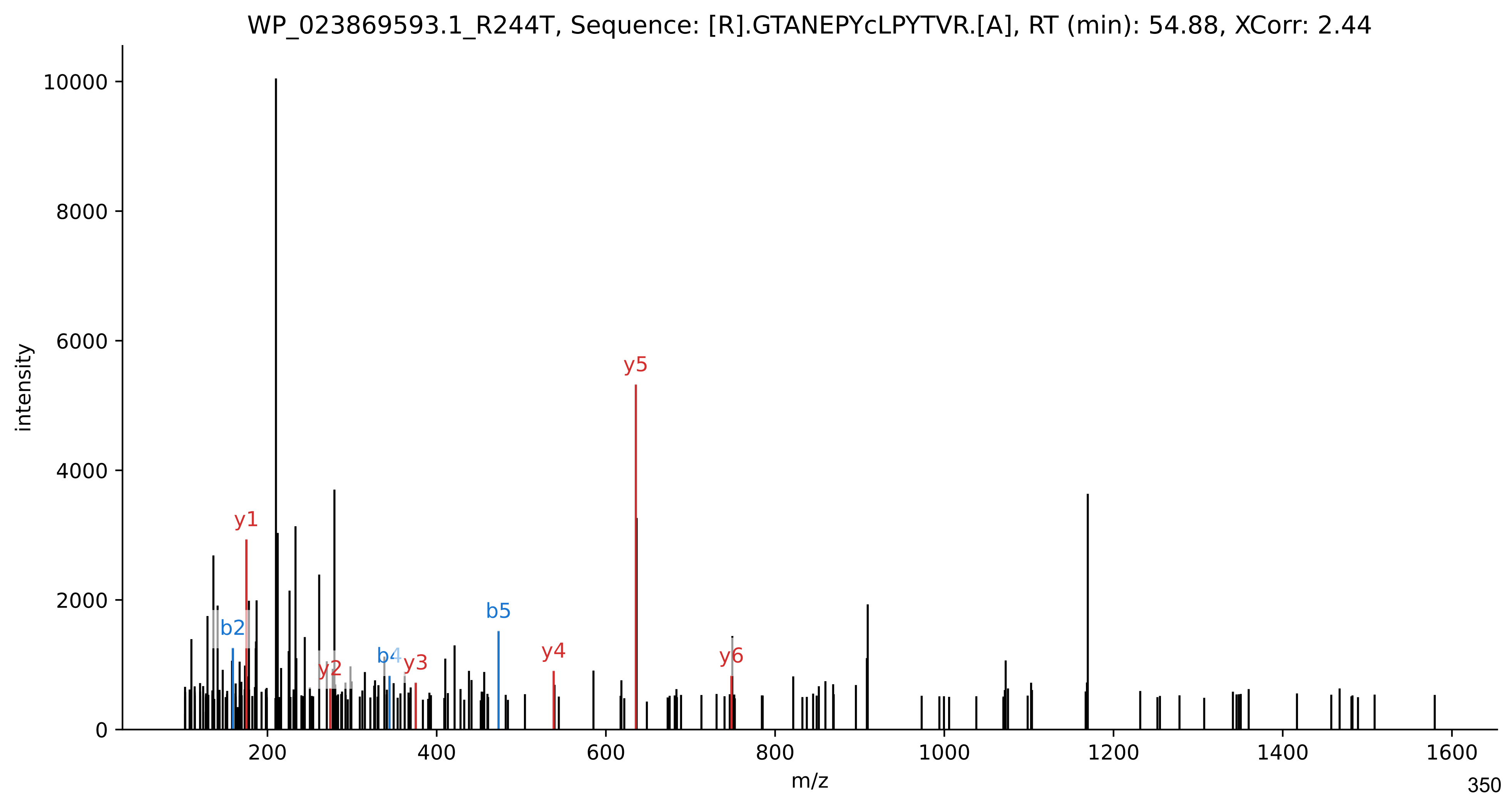
WP_023869593.1_R244T, Sequence: [R].GTANEPYcLPYTVR.[A], RT (min): 54.88, XCorr: 2.44
intensity
m/z
y1
y2
y3
y4
y5
y6
b2
b4
b5
0
2000
4000
6000
8000
10000
200
400
600
800
1000
1200
1400
1600

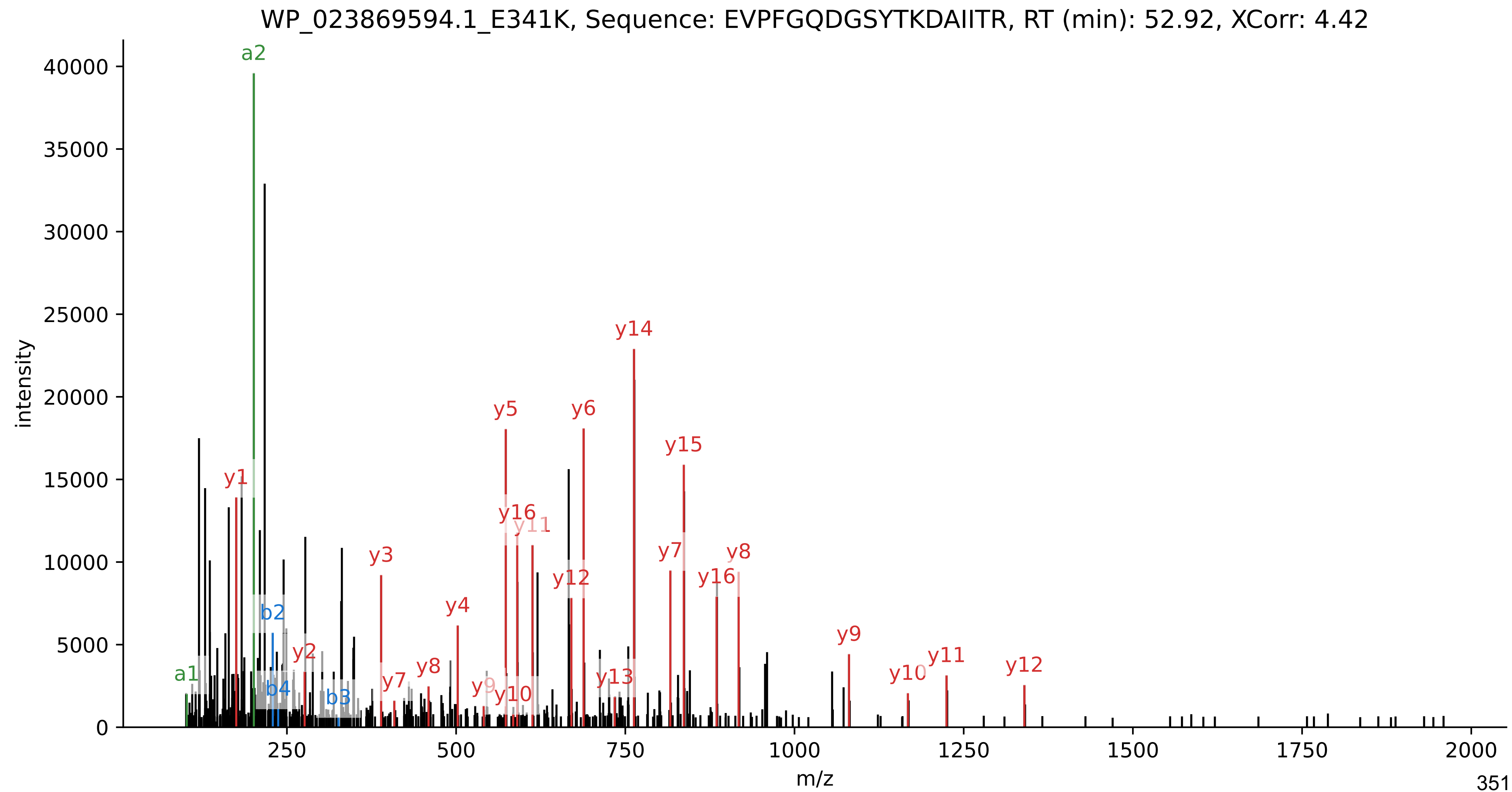

WP_023869594.1_E341K, Sequence: EVPFGQDGSYTKDAIITR, RT (min): 52.92, XCorr: 4.42
intensity
m/z
a1
a2
b2
b3
b4
y1
y2
y3
y4
y5
y6
y7
y8
y9
y10
y11
y12
y13
y14
y15
y16

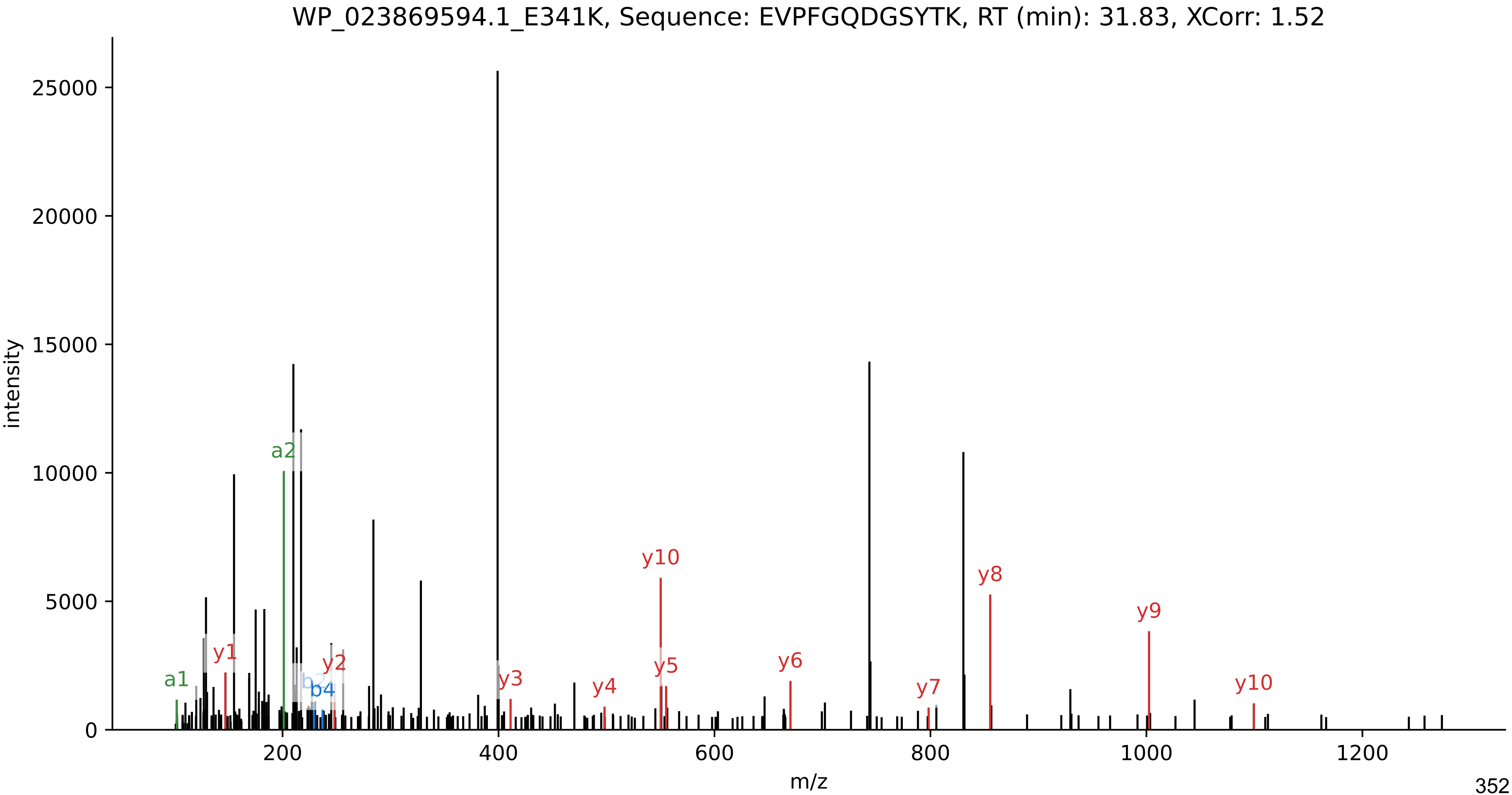
WP_023869594.1_E341K, Sequence: EVPFGQDGSYTK, RT (min): 31.83, XCorr: 1.52
intensity
m/z
0
5000
10000
15000
20000
25000
200
400
600
800
1000
1200
a1
a2
y1
y2
b2
b4
y3
y4
y10
y5
y6
y7
y8
y9
y10

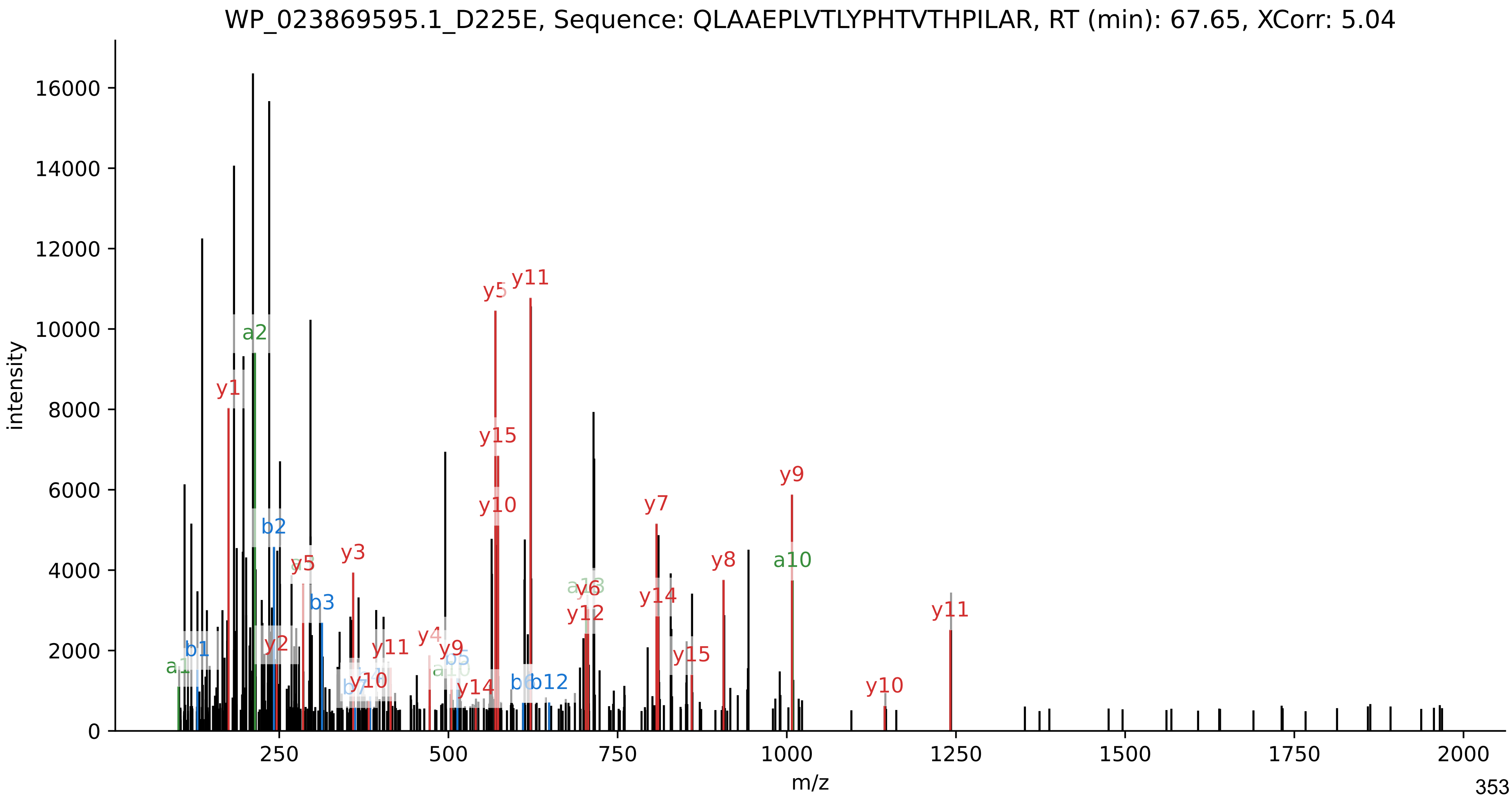
WP_023869595.1_D225E, Sequence: QLAAEPLVTLYPHTVTHPILAR, RT (min): 67.65, XCorr: 5.04
intensity
m/z
0
2000
4000
6000
8000
10000
12000
14000
16000
250
500
750
1000
1250
1500
1750
2000
a2
y1
b2
y5
y3
b3
y2
b1
y11
y4
y9
y10
y14
y5
y11
y15
y10
b12
y12
y7
y14
y15
y8
y9
a10
y10
y11

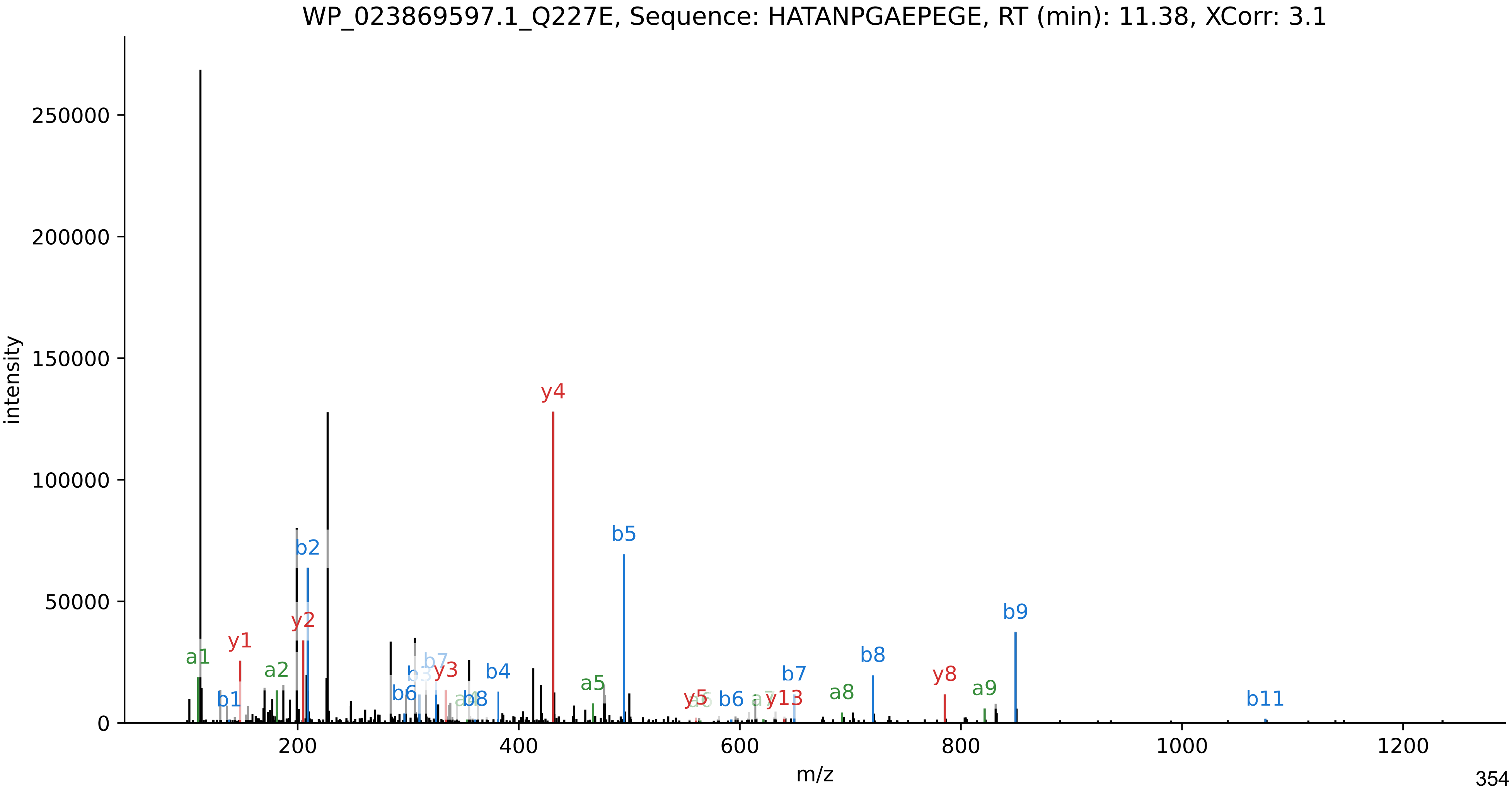
WP_023869597.1_Q227E, Sequence: HATANPGAEPEGE, RT (min): 11.38, XCorr: 3.1
intensity
m/z
250000
200000
150000
100000
50000
0
200
400
600
800
1000
1200
y4
b5
b2
y2
b9
y1
a1
a2
b1
b6
b7
y3
b4
b8
a5
y5
b6
y13
b7
a8
b8
y8
a9
b11

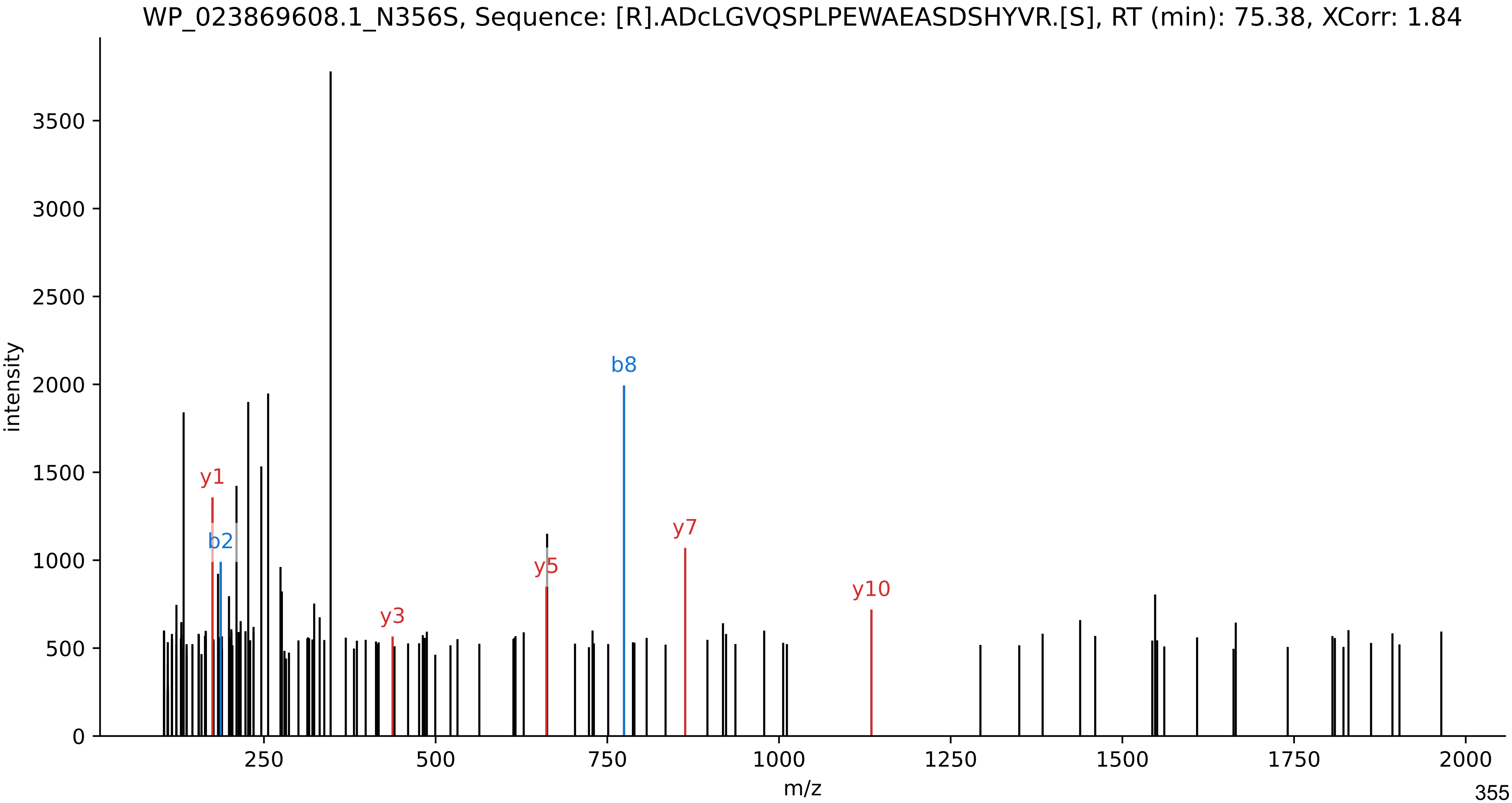

WP_023869608.1_N356S, Sequence: [R].ADcLGVQSPLPEWAEASDSHYVR.[S], RT (min): 75.38, XCorr: 1.84
intensity
m/z
y1
b2
y3
y5
b8
y7
y10

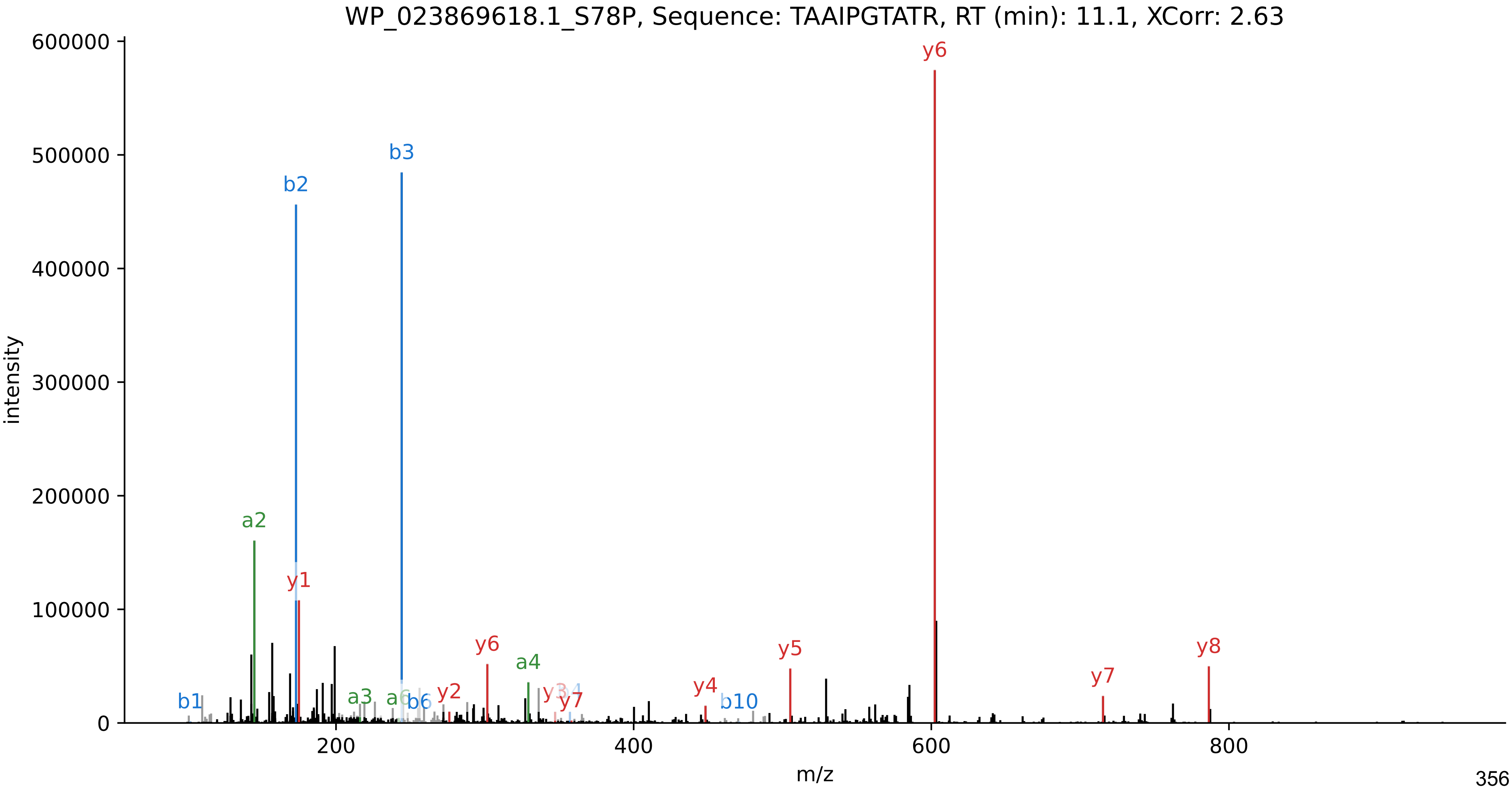
WP_023869618.1_S78P, Sequence: TAAIPGTATR, RT (min): 11.1, XCorr: 2.63
intensity
m/z
0
100000
200000
300000
400000
500000
600000
200
400
600
800
y6
b3
b2
a2
y1
y6
a4
y5
y8
y7
y4
b10
y2
a3
a6
b6
b1
y3
y7
b4

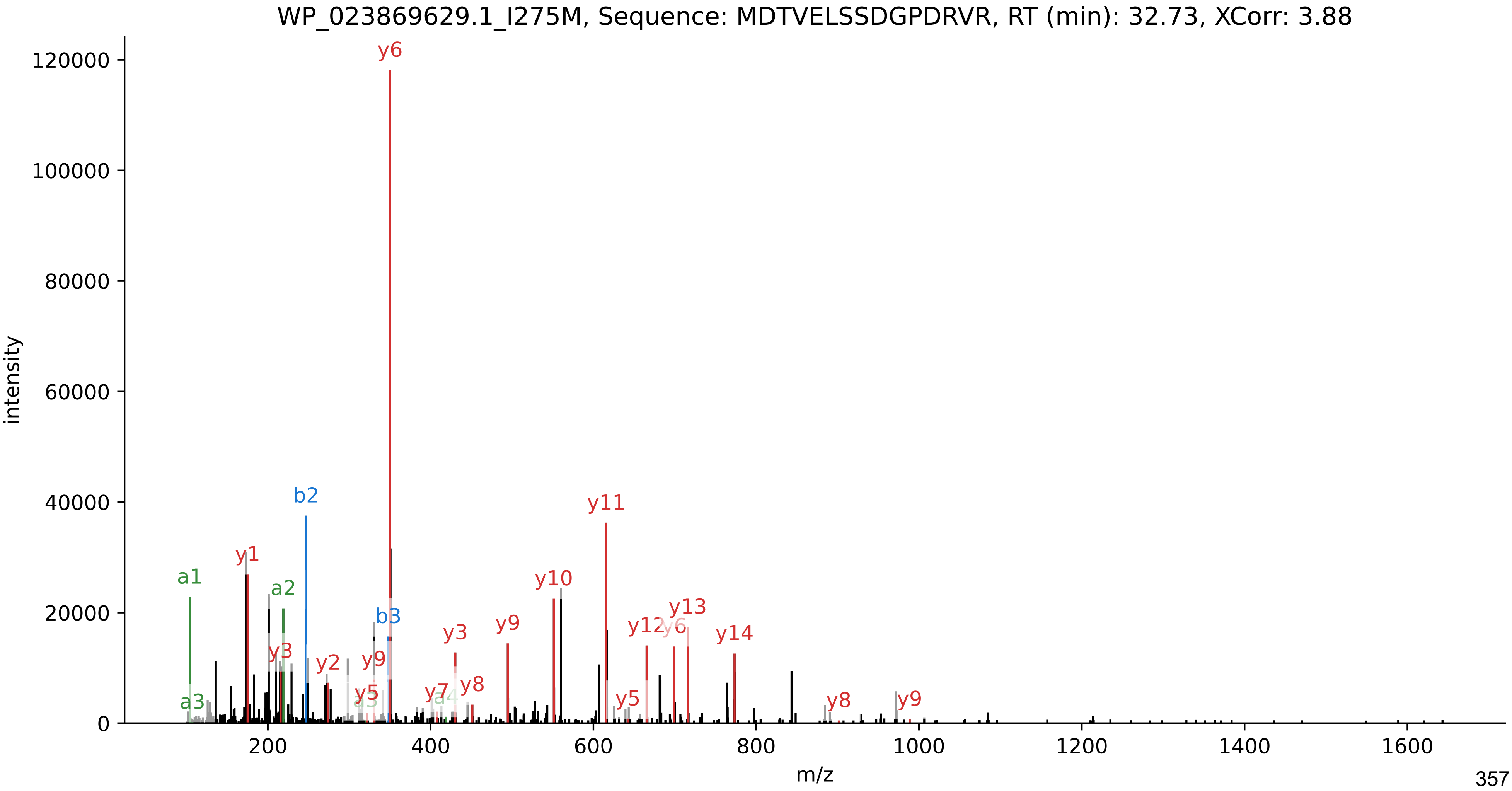

WP_023869629.1_I275M, Sequence: MDTVELSSDGPDRVR, RT (min): 32.73, XCorr: 3.88
intensity
m/z
0
20000
40000
60000
80000
100000
120000
200
400
600
800
1000
1200
1400
1600
y6
b2
y11
y1
a1
a2
y10
y13
b3
y12
y6
y14
y3
y9
y3
y2
y9
y8
y7
a4
y5
a3
y5
a3
y8
y9

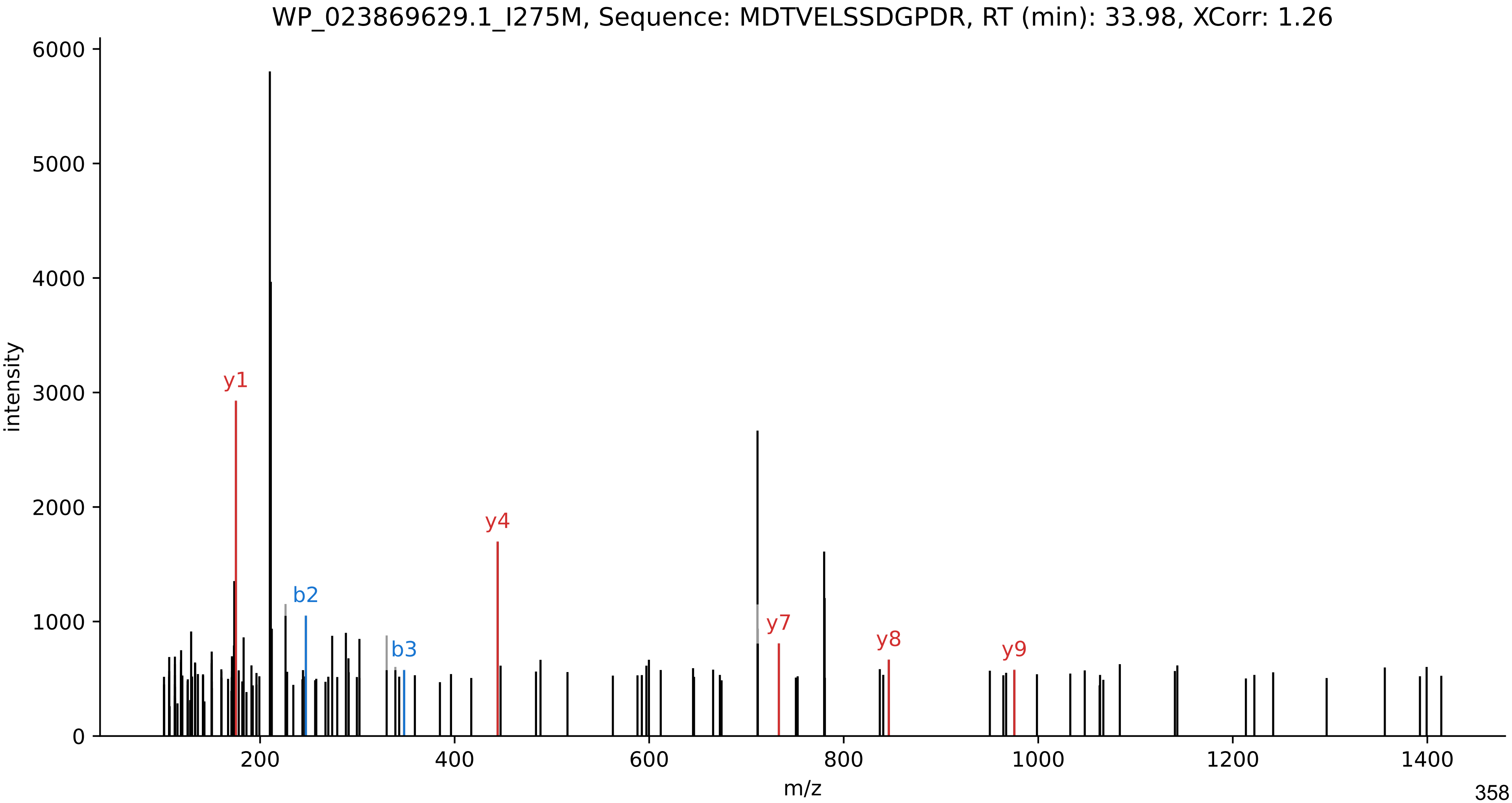

WP_023869629.1_I275M, Sequence: MDTVELSSDGPDR, RT (min): 33.98, XCorr: 1.26
intensity
m/z
y1
b2
b3
y4
y7
y8
y9
0
1000
2000
3000
4000
5000
6000
200
400
600
800
1000
1200
1400

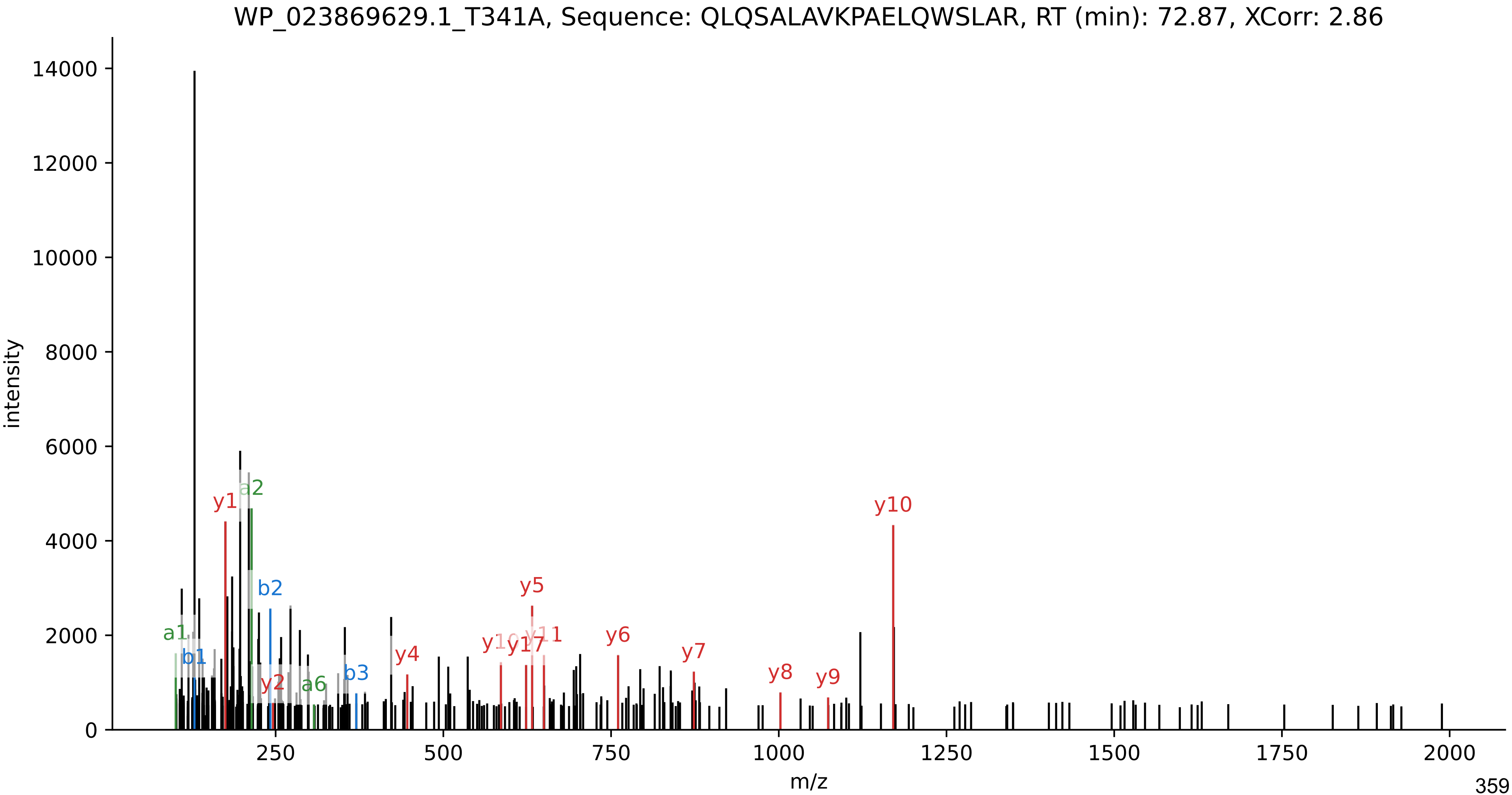

WP_023869629.1_T341A, Sequence: QLQSALAVKPAELQWSLAR, RT (min): 72.87, XCorr: 2.86
intensity
m/z
y10
y5
y1
a2
b2
a1
b1
y2
a6
b3
y4
y11
y17
y6
y7
y8
y9

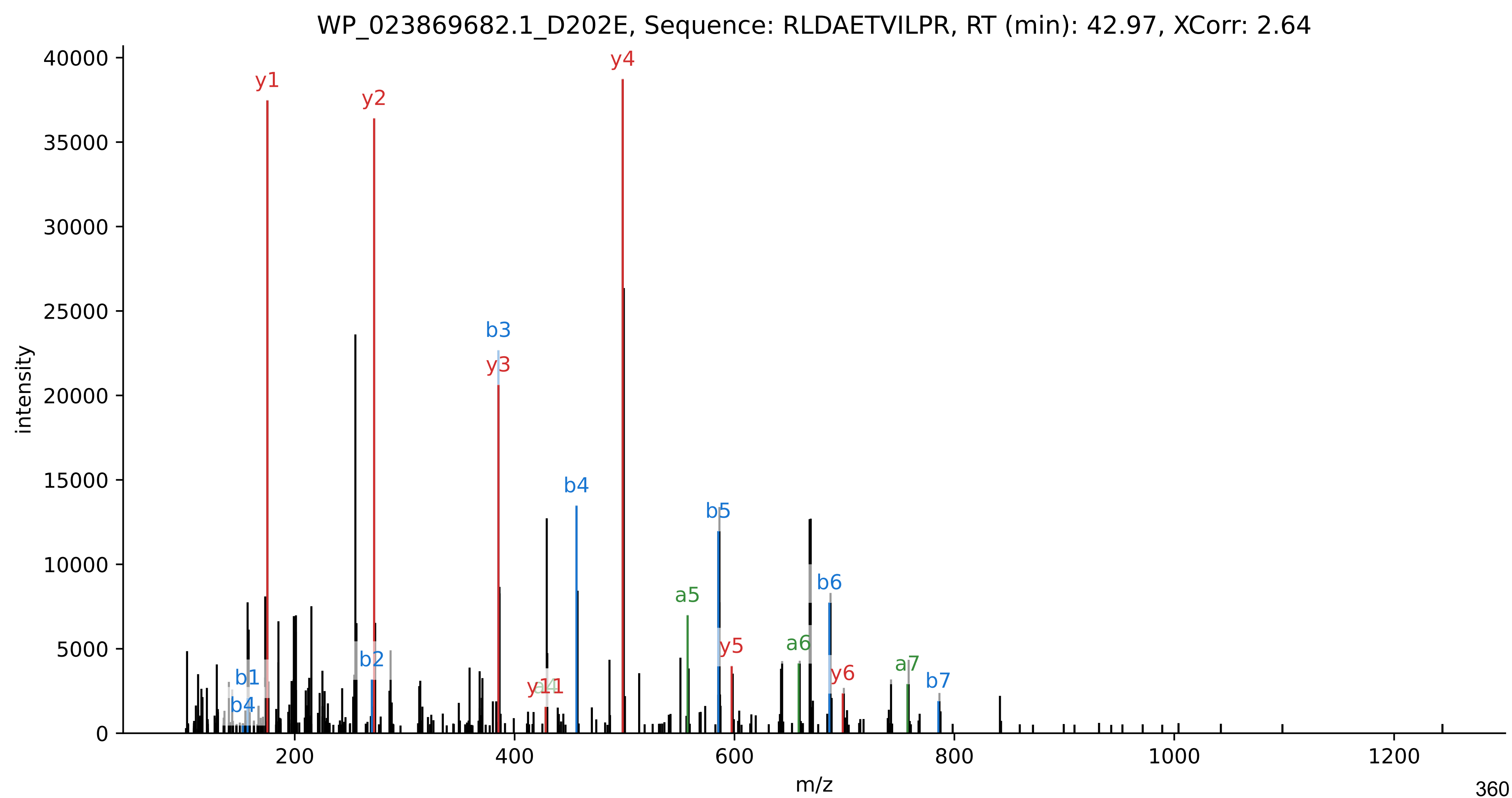

WP_023869682.1_D202E, Sequence: RLDAETVILPR, RT (min): 42.97, XCorr: 2.64
intensity
m/z
y1
y2
y3
y4
y5
y6
b1
b2
b3
b4
b5
b6
b7
a5
a6
a7

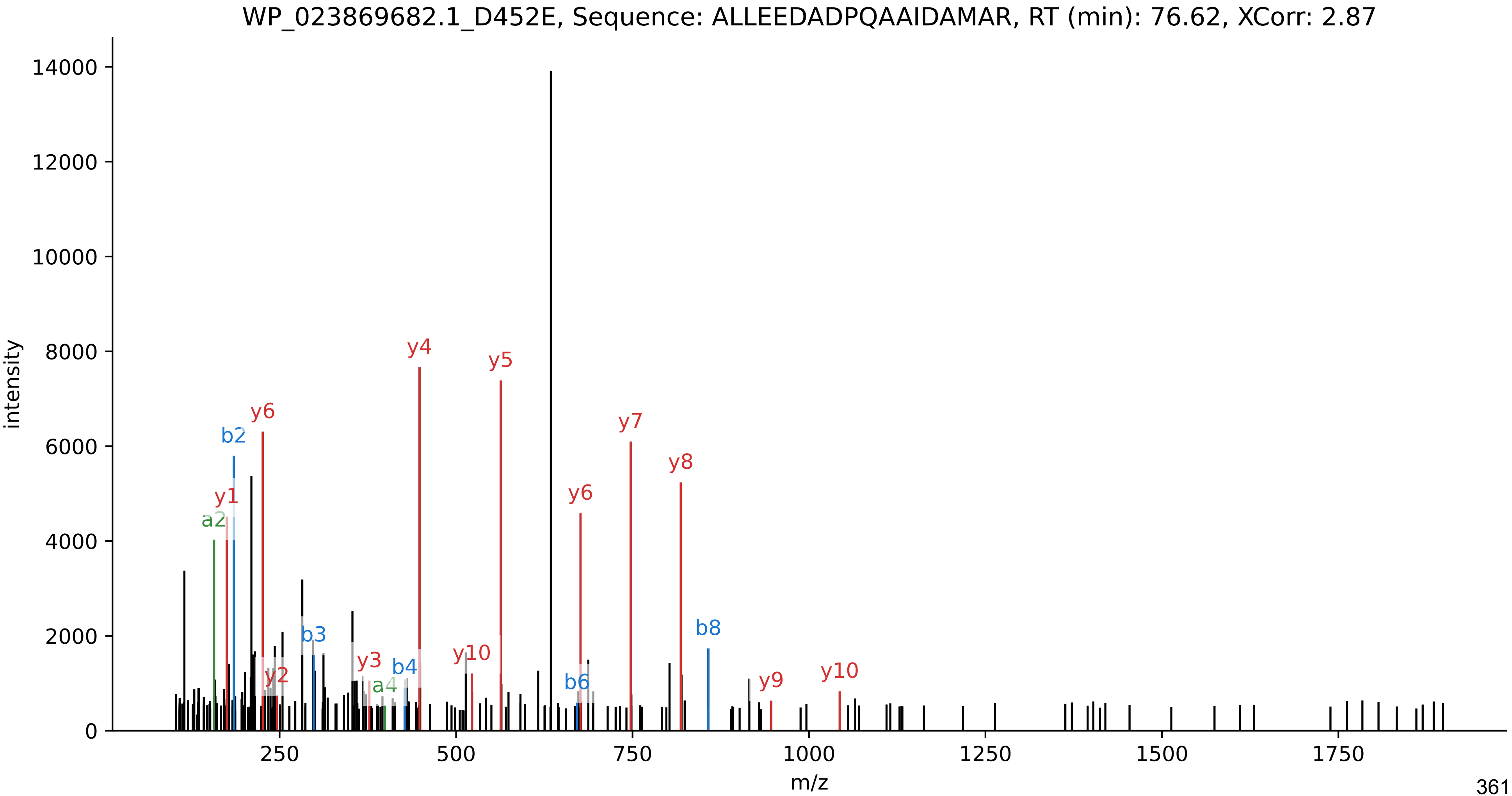

WP_023869682.1_D452E, Sequence: ALLEEDADPQAAIDAMAR, RT (min): 76.62, XCorr: 2.87
intensity
m/z
0
2000
4000
6000
8000
10000
12000
14000
250
500
750
1000
1250
1500
1750
a2
y1
b2
y6
y2
b3
y3
a4
b4
y4
y10
y5
b6
y6
y7
y8
b8
y9
y10

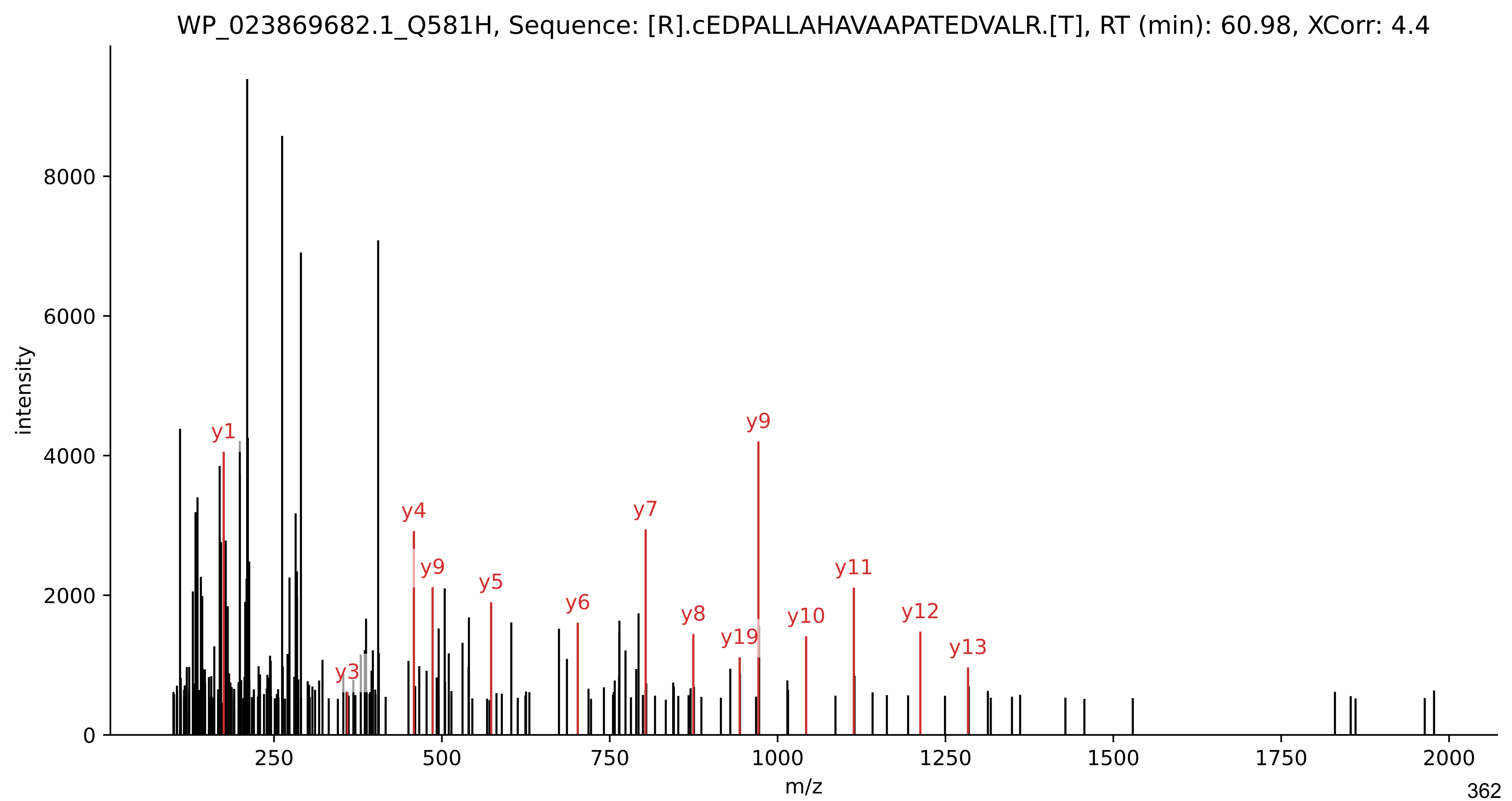

WP_023869682.1_Q581H, Sequence: [R].cEDPALLAHAVAAPATEDVALR.[T], RT (min): 60.98, XCorr: 4.4
intensity
m/z
0
2000
4000
6000
8000
250
500
750
1000
1250
1500
1750
2000
y1
y3
y4
y9
y5
y6
y7
y8
y19
y9
y10
y11
y12
y13

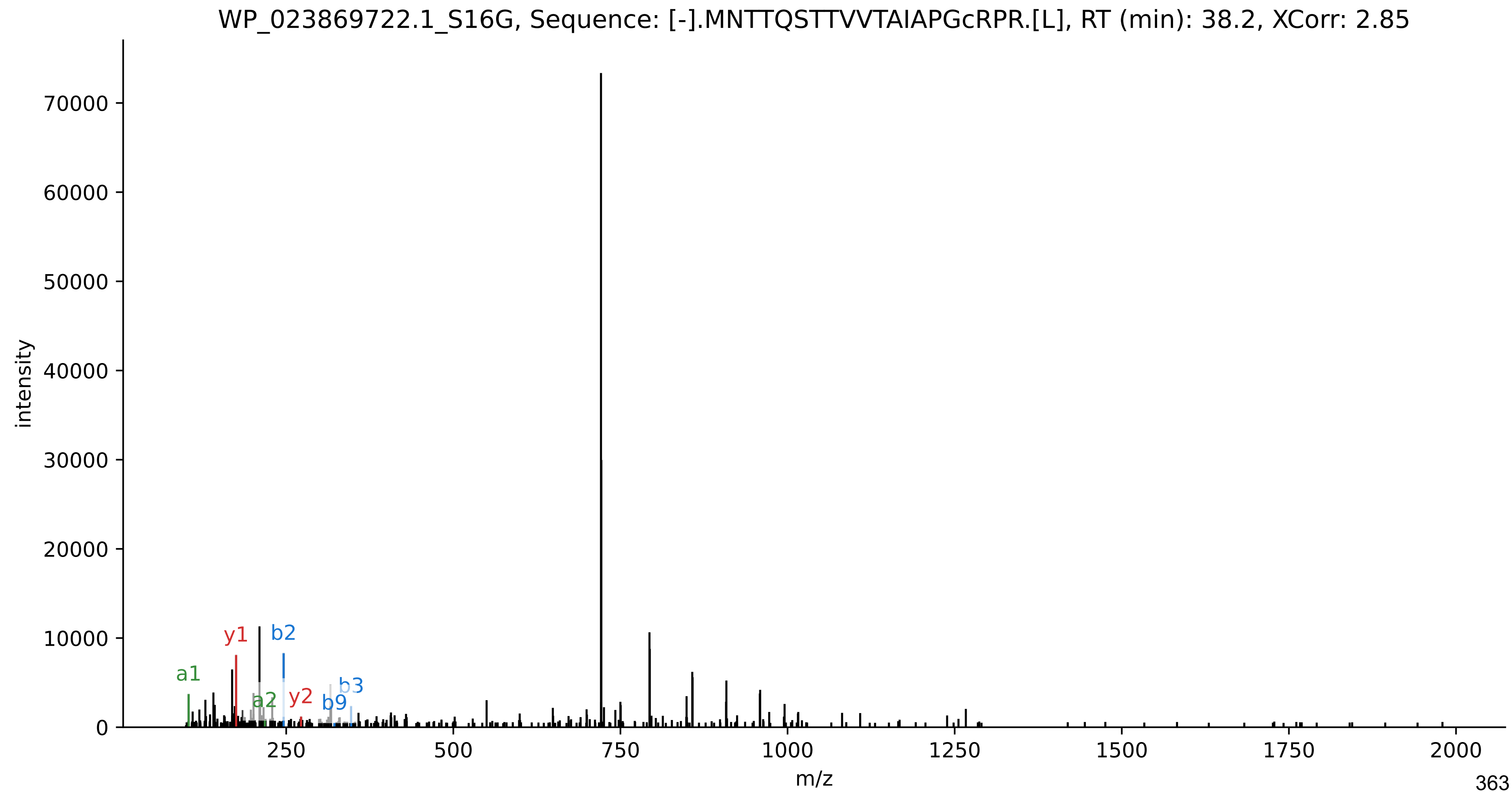
WP_023869722.1_S16G, Sequence: [-].MNTTQSTTVVTAIAPGcRPR.[L], RT (min): 38.2, XCorr: 2.85
intensity
m/z
a1
y1
b2
a2
y2
b9
b3
0
10000
20000
30000
40000
50000
60000
70000
250
500
750
1000
1250
1500
1750
2000

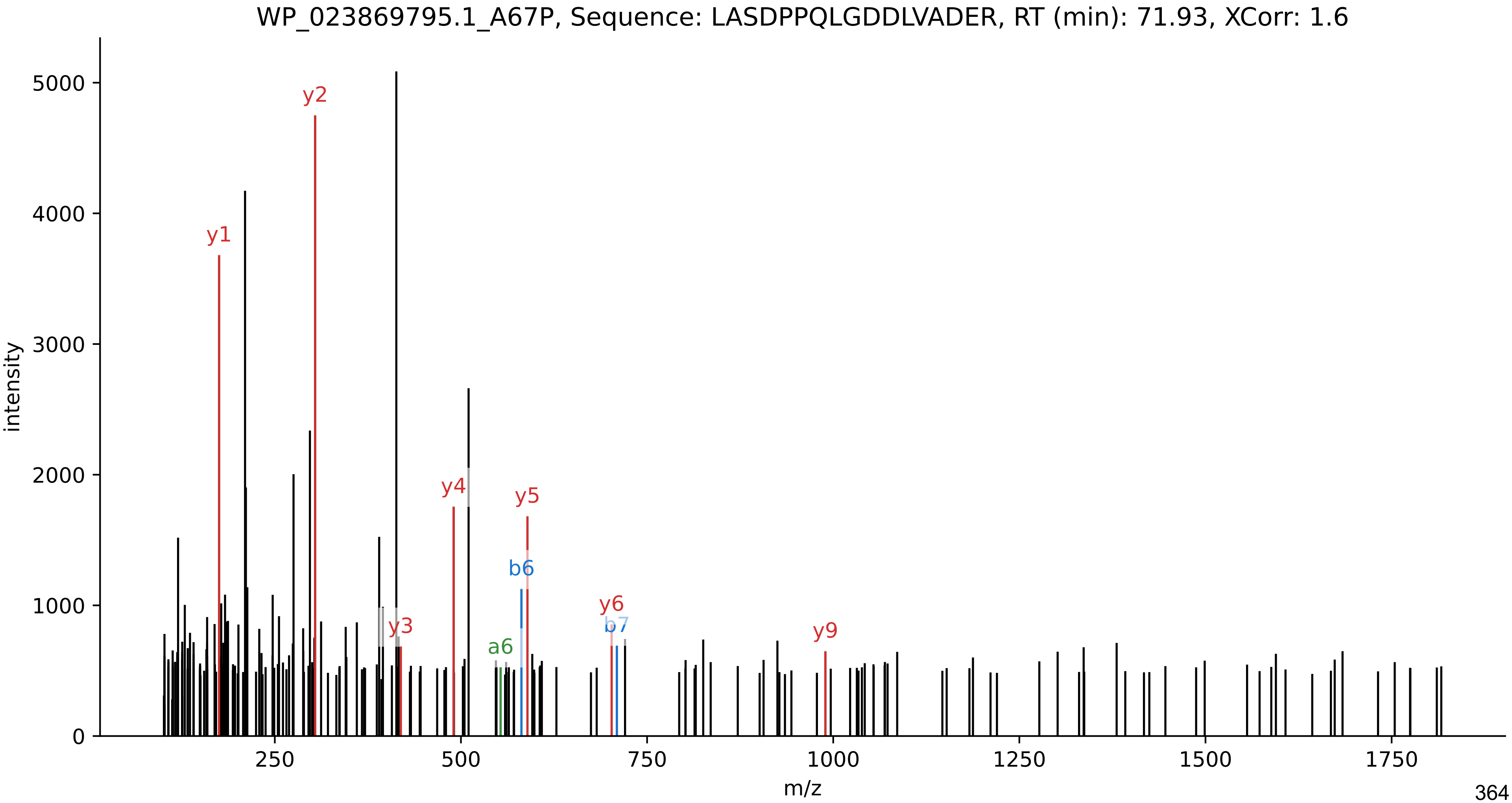

WP_023869795.1_A67P, Sequence: LASDPPQLGDDLVADER, RT (min): 71.93, XCorr: 1.6
intensity
m/z
y1
y2
y3
y4
y5
y6
y9
a6
b6
b7
0
1000
2000
3000
4000
5000
250
500
750
1000
1250
1500
1750

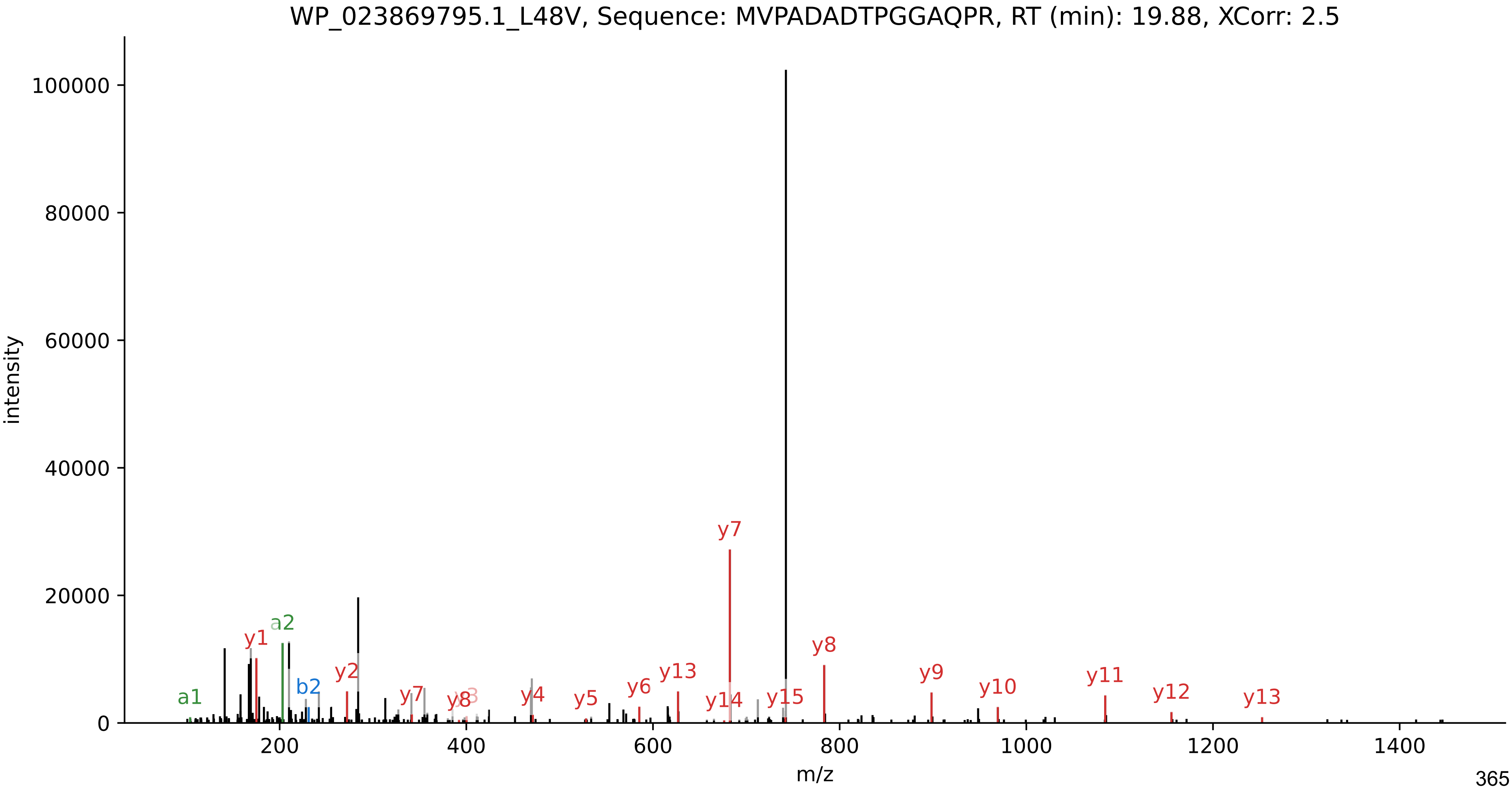
WP_023869795.1_L48V, Sequence: MVPADADTPGGAQPR, RT (min): 19.88, XCorr: 2.5
intensity
m/z
a1
y1
a2
b2
y2
y7
y8
y4
y5
y6
y13
y14
y15
y7
y8
y9
y10
y11
y12
y13

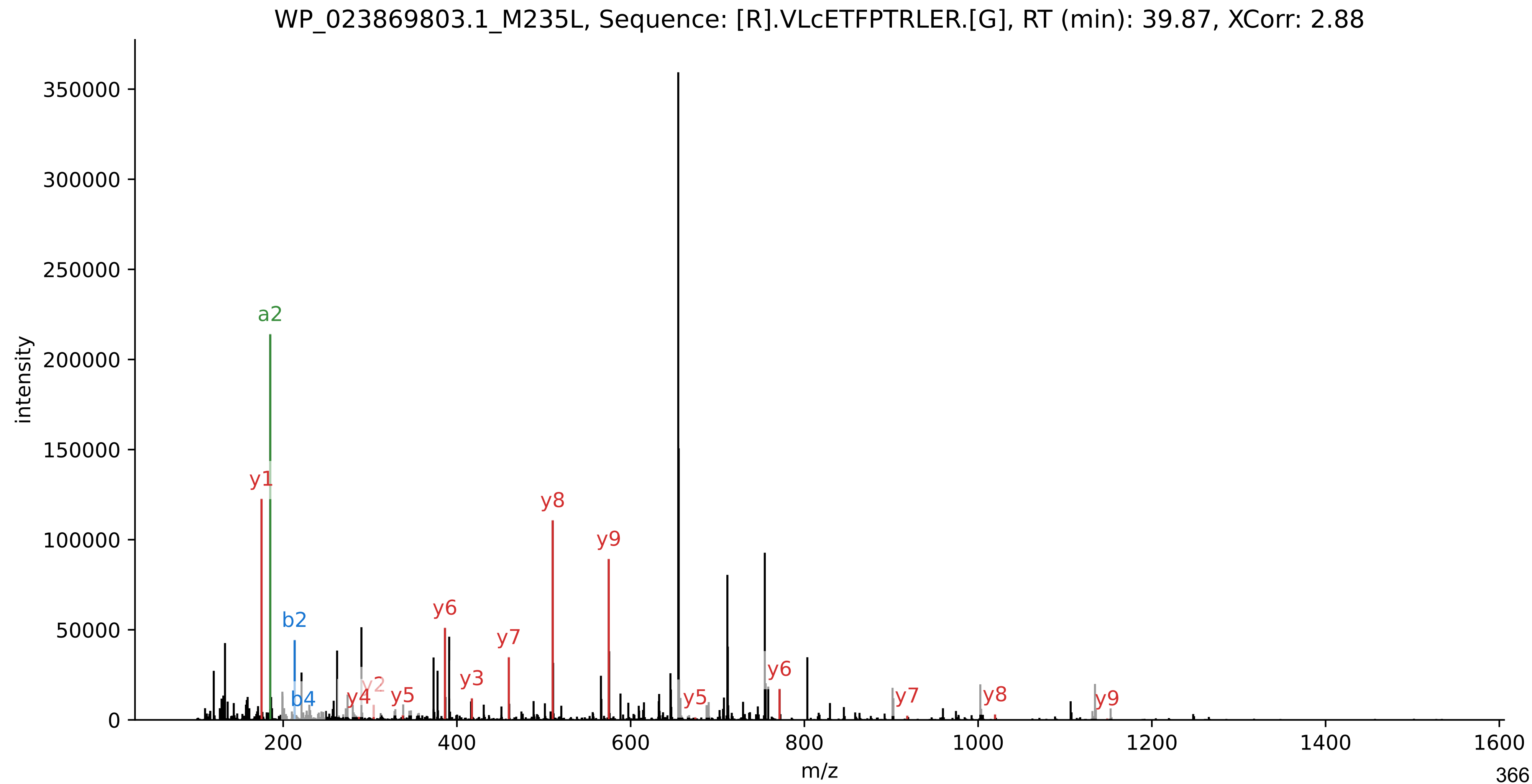
WP_023869803.1_M235L, Sequence: [R].VLcETFPTRLER.[G], RT (min): 39.87, XCorr: 2.88
intensity
m/z
a2
y1
b2
b4
y4
y2
y5
y6
y3
y7
y8
y9
y5
y6
y7
y8
y9

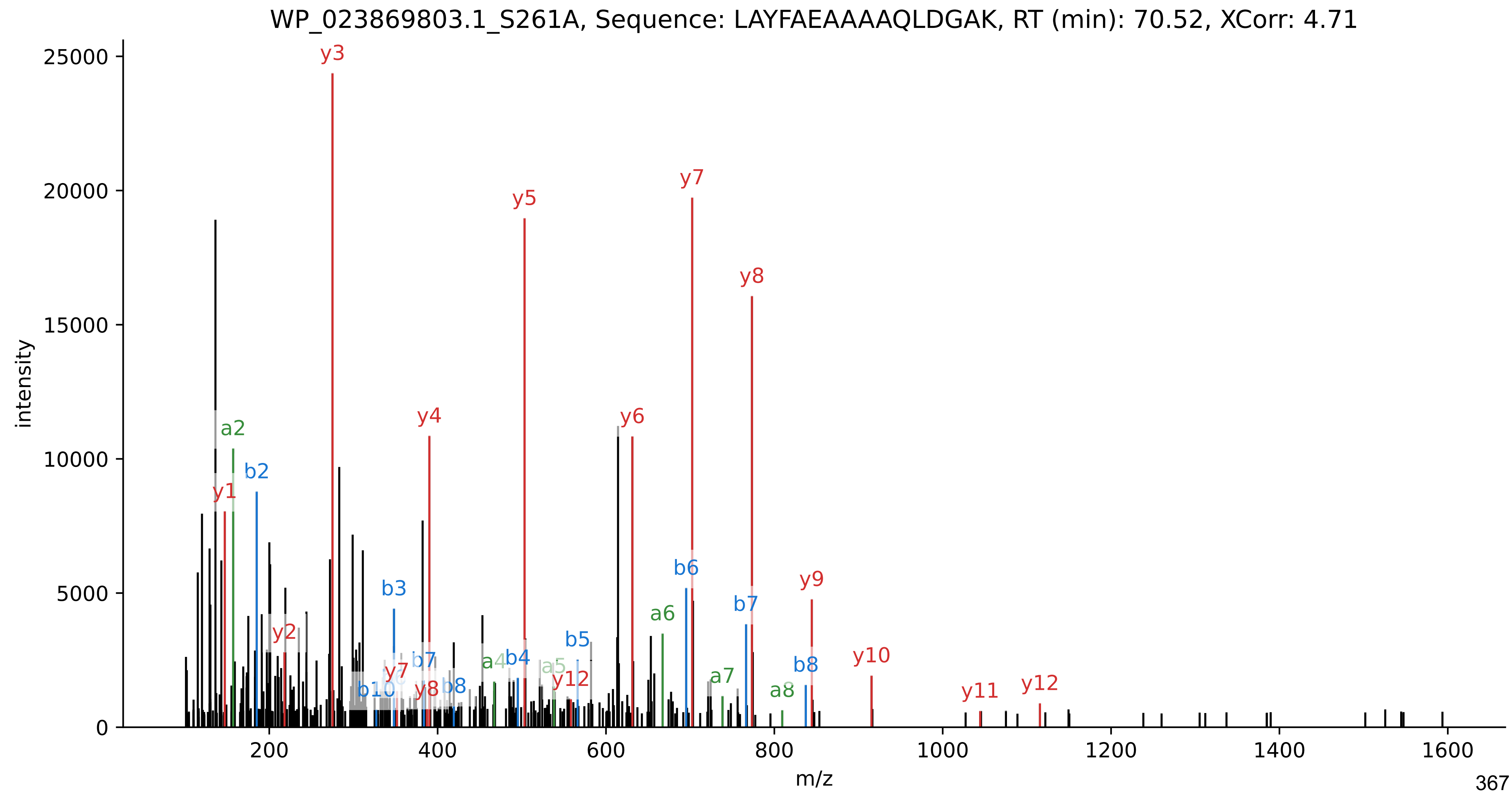

WP_023869803.1_S261A, Sequence: LAYFAEAAAAQLDGAK, RT (min): 70.52, XCorr: 4.71
intensity
m/z
y3
y5
y7
y8
y4
y6
a2
b2
y1
b6
y9
b3
a6
b7
y2
b5
y10
b8
a7
a8
y11
y12
b4
b7
y7
y12
b8
y8
b10

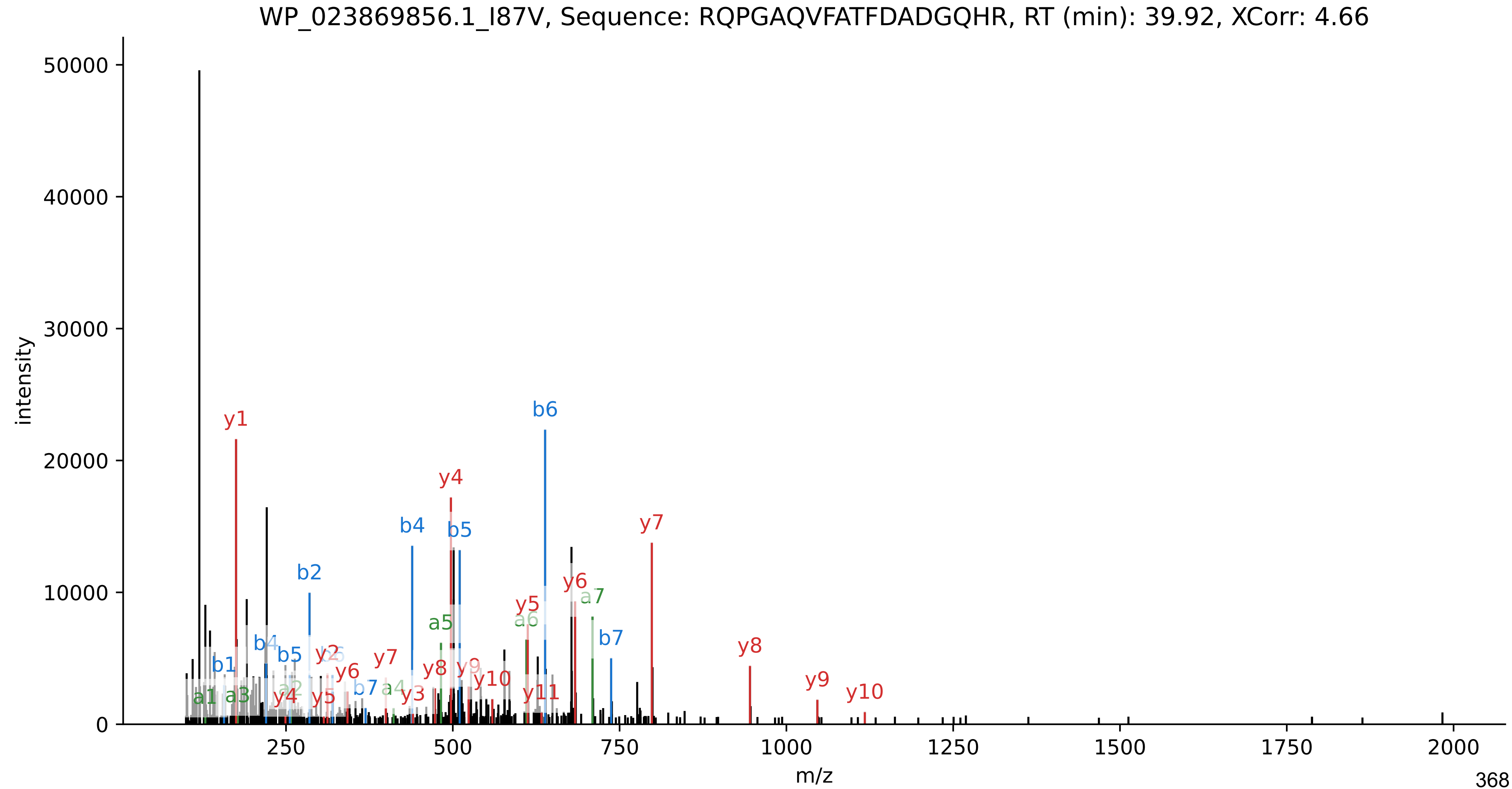
WP_023869856.1_I87V, Sequence: RQPGAQVFATFDADGQHR, RT (min): 39.92, XCorr: 4.66
intensity
m/z
0
10000
20000
30000
40000
50000
250
500
750
1000
1250
1500
1750
2000
y1
b6
y4
b4
b5
y7
b2
y6
a7
y5
a6
a5
b7
y8
b4
b5
y2
b1
y6
y7
y8
y9
y10
y9
y10
y11
y3
y4
y5
a1
a3
b7

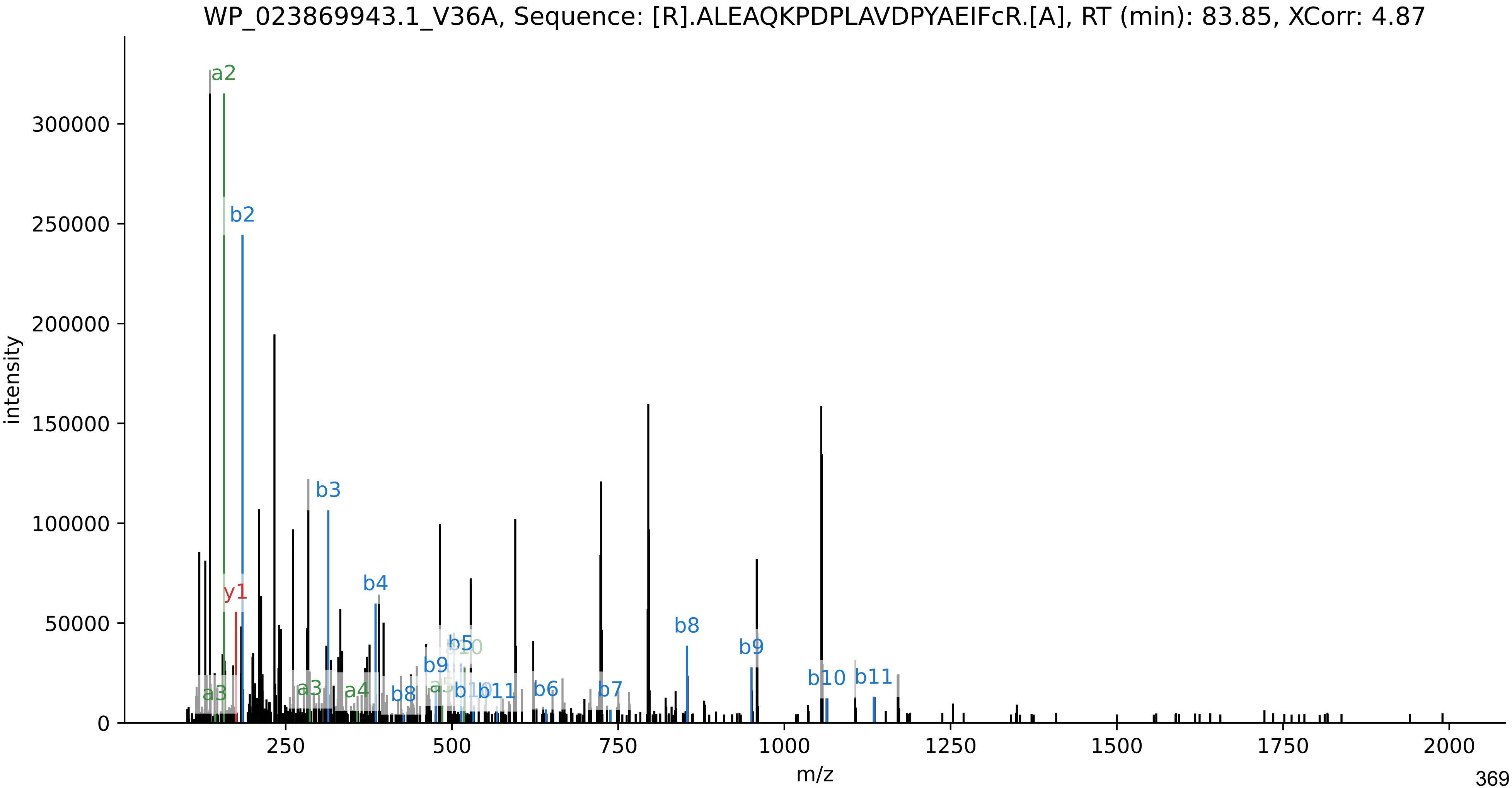
WP_023869943.1_V36A, Sequence: [R].ALEAQKPDPLAVDPYAEIFcR.[A], RT (min): 83.85, XCorr: 4.87
intensity
m/z
a2
b2
b3
b4
y1
a3
a4
b5
b6
b7
b8
b9
b10
b11

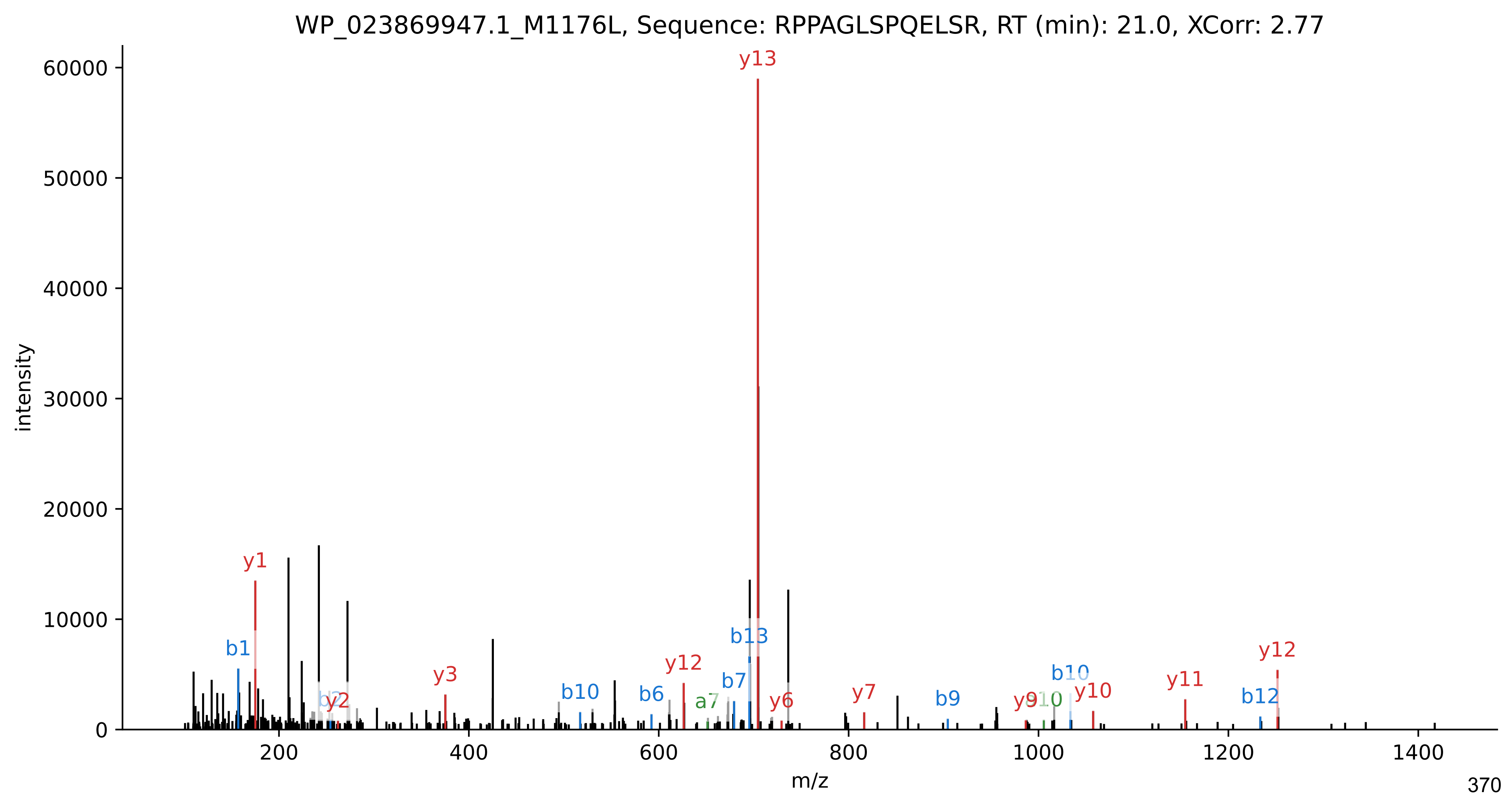

WP_023869947.1_M1176L, Sequence: RPPAGLSPQELSR, RT (min): 21.0, XCorr: 2.77
intensity
m/z
y13
y1
b1
y3
b10
b6
y12
a7
b7
b13
y6
y7
b9
y9
b10
y10
y11
b12
y12
y2

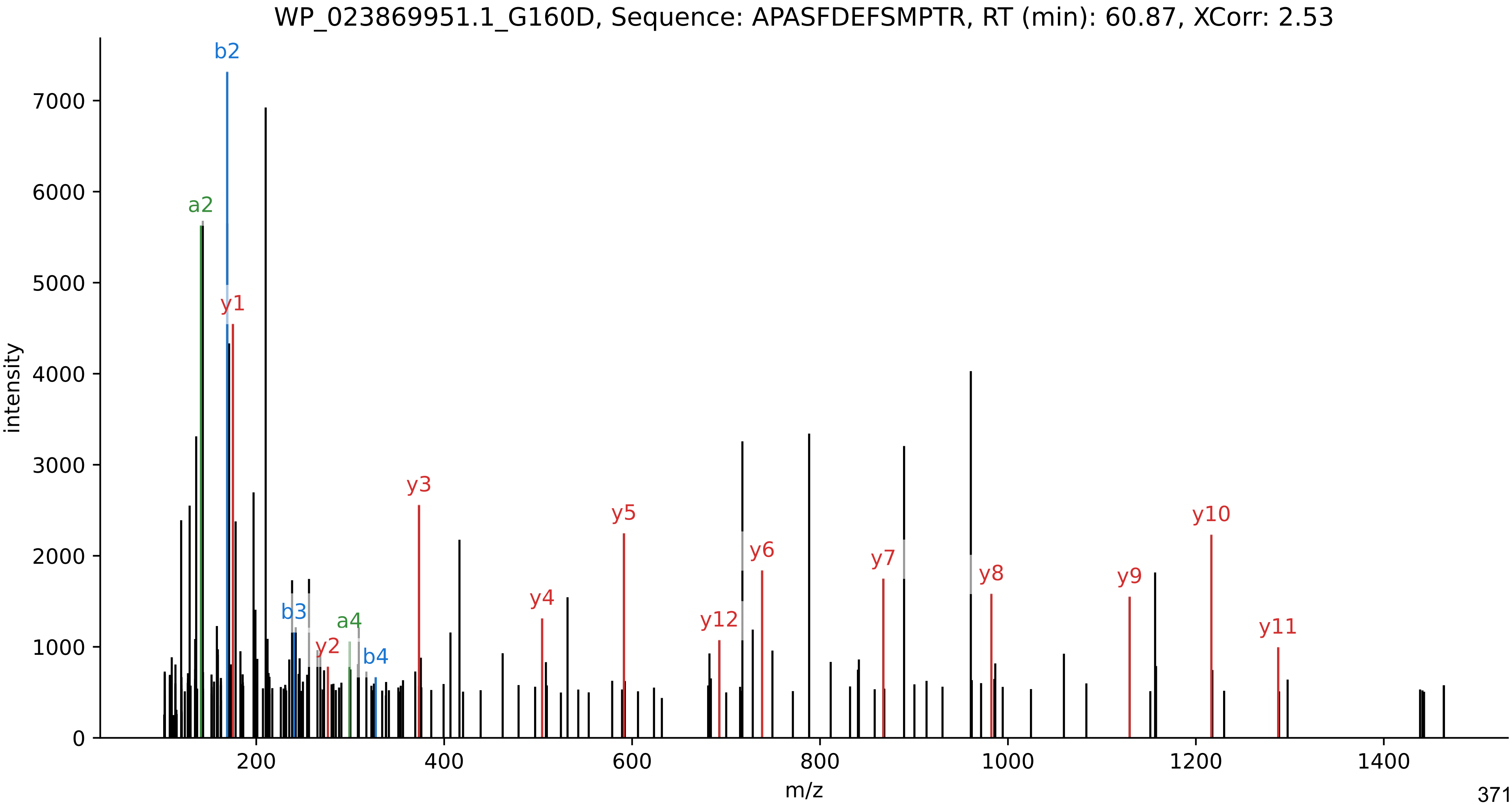

WP_023869951.1_G160D, Sequence: APASFDEFSMPTR, RT (min): 60.87, XCorr: 2.53
intensity
m/z
0
1000
2000
3000
4000
5000
6000
7000
200
400
600
800
1000
1200
1400
b2
a2
y1
b3
a4
y2
b4
y3
y4
y5
y12
y6
y7
y8
y9
y10
y11

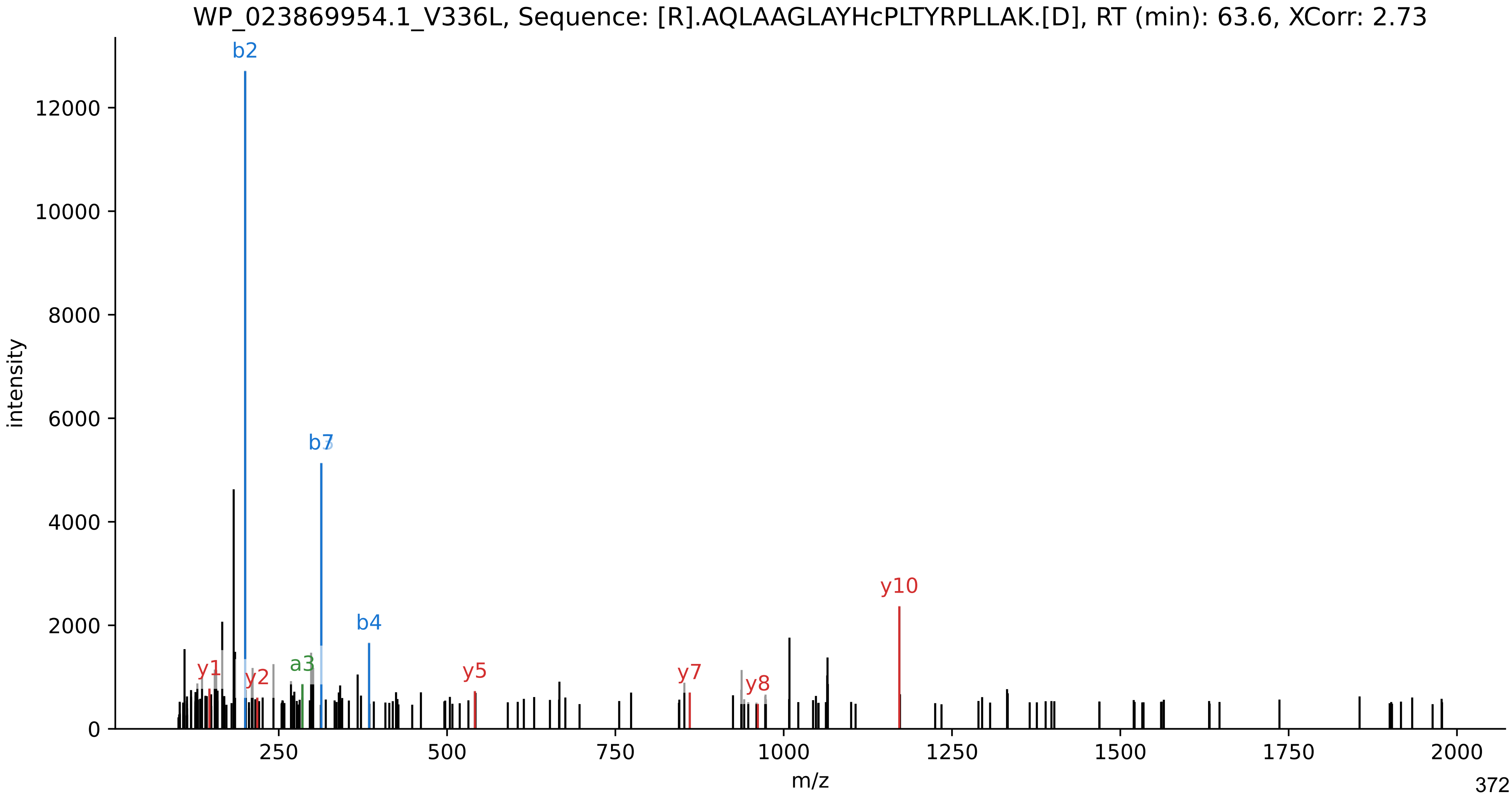
WP_023869954.1_V336L, Sequence: [R].AQLAAGLAYHcPLTYRPLLAK.[D], RT (min): 63.6, XCorr: 2.73
b2
b7
b4
y1
y2
a3
y5
y7
y8
y10
intensity
m/z
0
2000
4000
6000
8000
10000
12000
250
500
750
1000
1250
1500
1750
2000

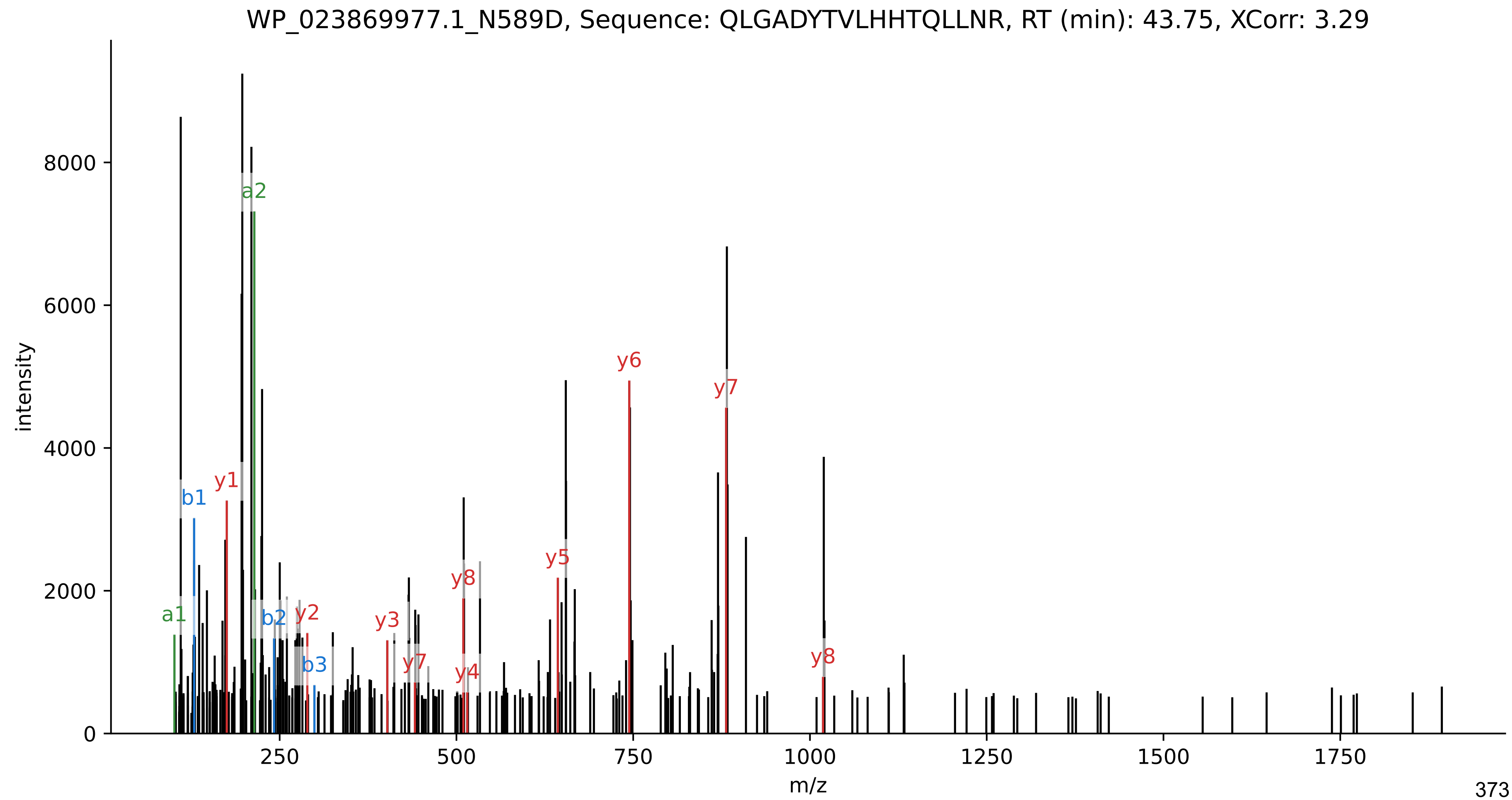
WP_023869977.1_N589D, Sequence: QLGADYTVLHHTQLLNR, RT (min): 43.75, XCorr: 3.29
intensity
m/z
0
2000
4000
6000
8000
250
500
750
1000
1250
1500
1750
a1
a2
b1
b2
b3
y1
y2
y3
y7
y8
y4
y5
y6
y7
y8

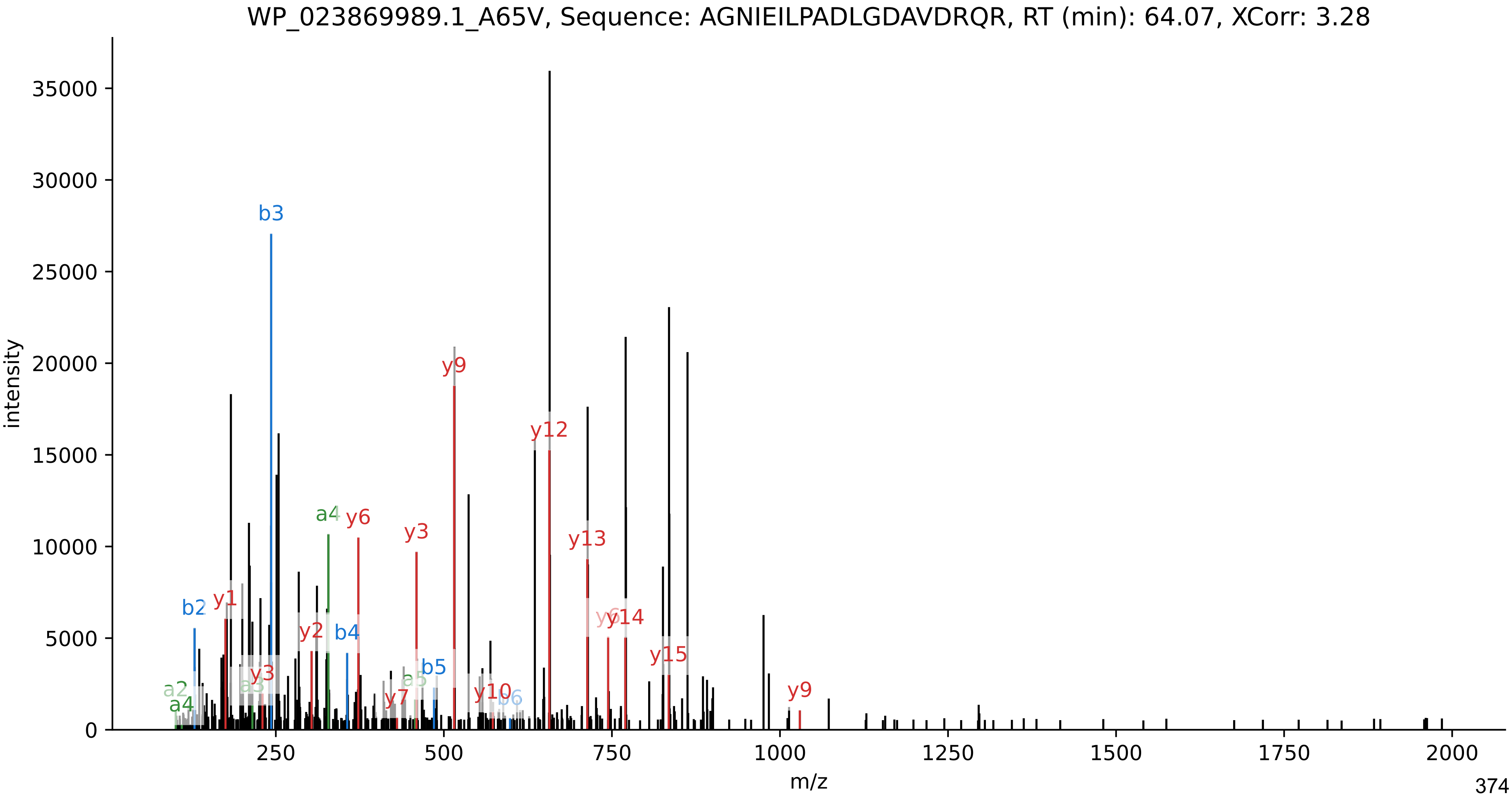
WP_023869989.1_A65V, Sequence: AGNIEILPADLGDAVDRQR, RT (min): 64.07, XCorr: 3.28
intensity
m/z
b3
y9
y12
a4
y6
y3
y13
b2
y1
y2
b4
y14
y15
y3
a2
a4
a3
b5
a5
y7
y10
b6
y9

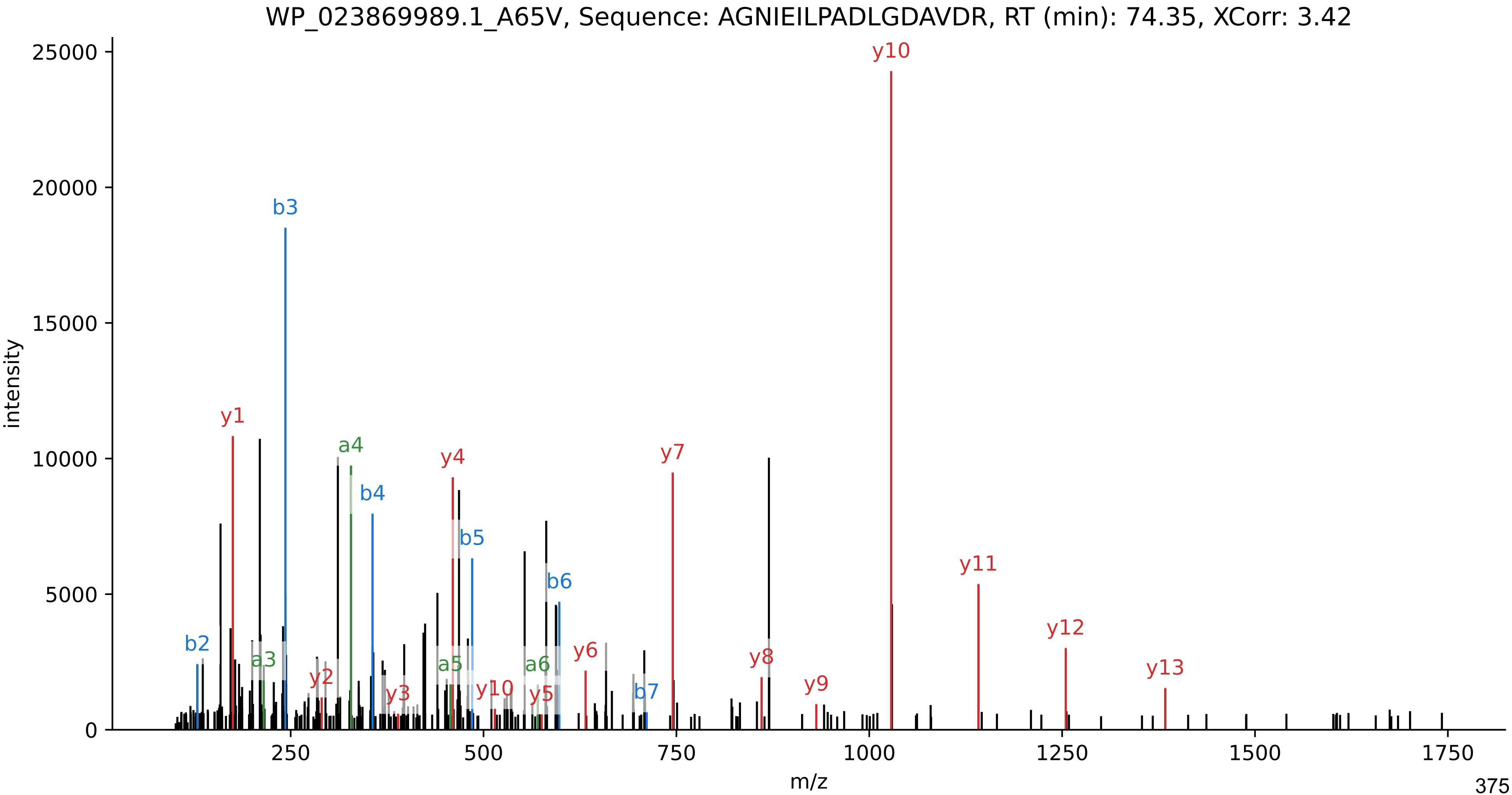
WP_023869989.1_A65V, Sequence: AGNIEILPADLGDAVDR, RT (min): 74.35, XCorr: 3.42
intensity
m/z
y10
b3
y1
a4
y4
y7
b4
b5
y11
b6
y12
b2
a3
y2
a5
a6
y6
y8
y13
y10
y3
y5
b7
y9

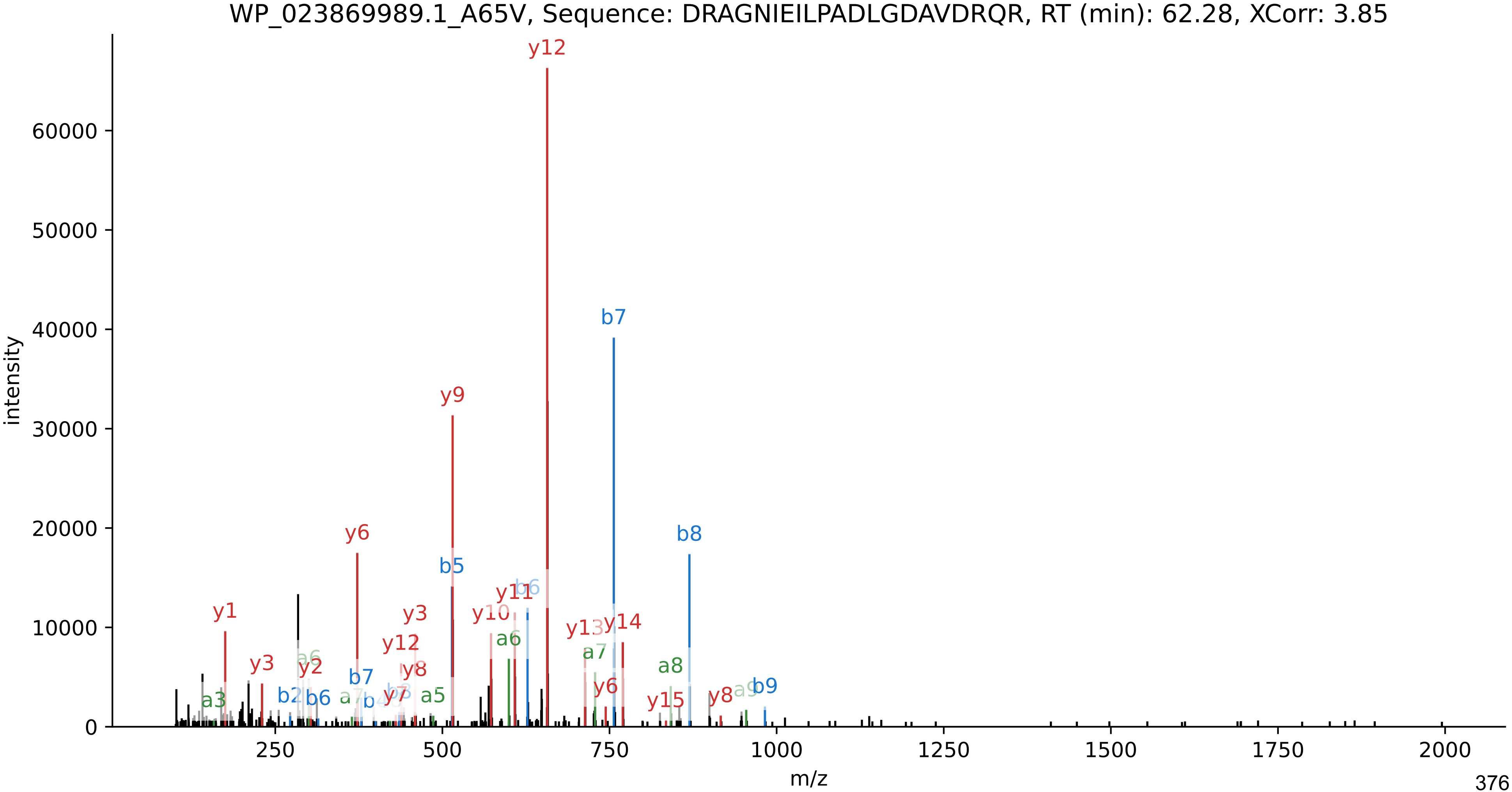

WP_023869989.1_A65V, Sequence: DRAGNIEILPADLGDAVDRQR, RT (min): 62.28, XCorr: 3.85
intensity
m/z
y12
b7
y9
y6
b8
b5
y11
b6
y10
y1
y3
y14
y13
a6
a7
y12
y8
y3
y2
a8
y6
b7
b2
b6
a3
a5
y7
y15
y8
b9

WP_023869989.1_A65V, Sequence: DRAGNIEILPADLGDAVDR, RT (min): 69.88, XCorr: 3.53

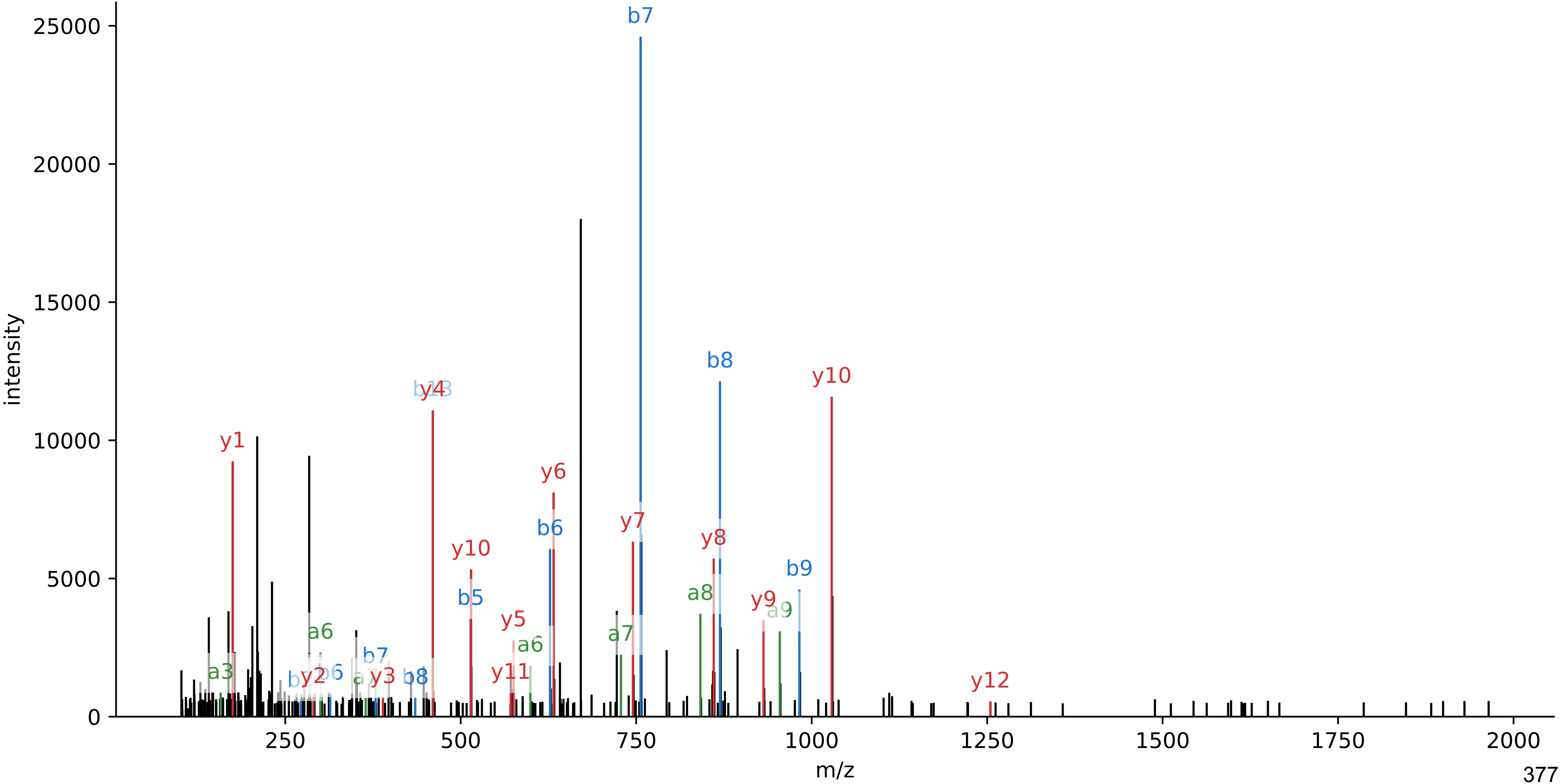

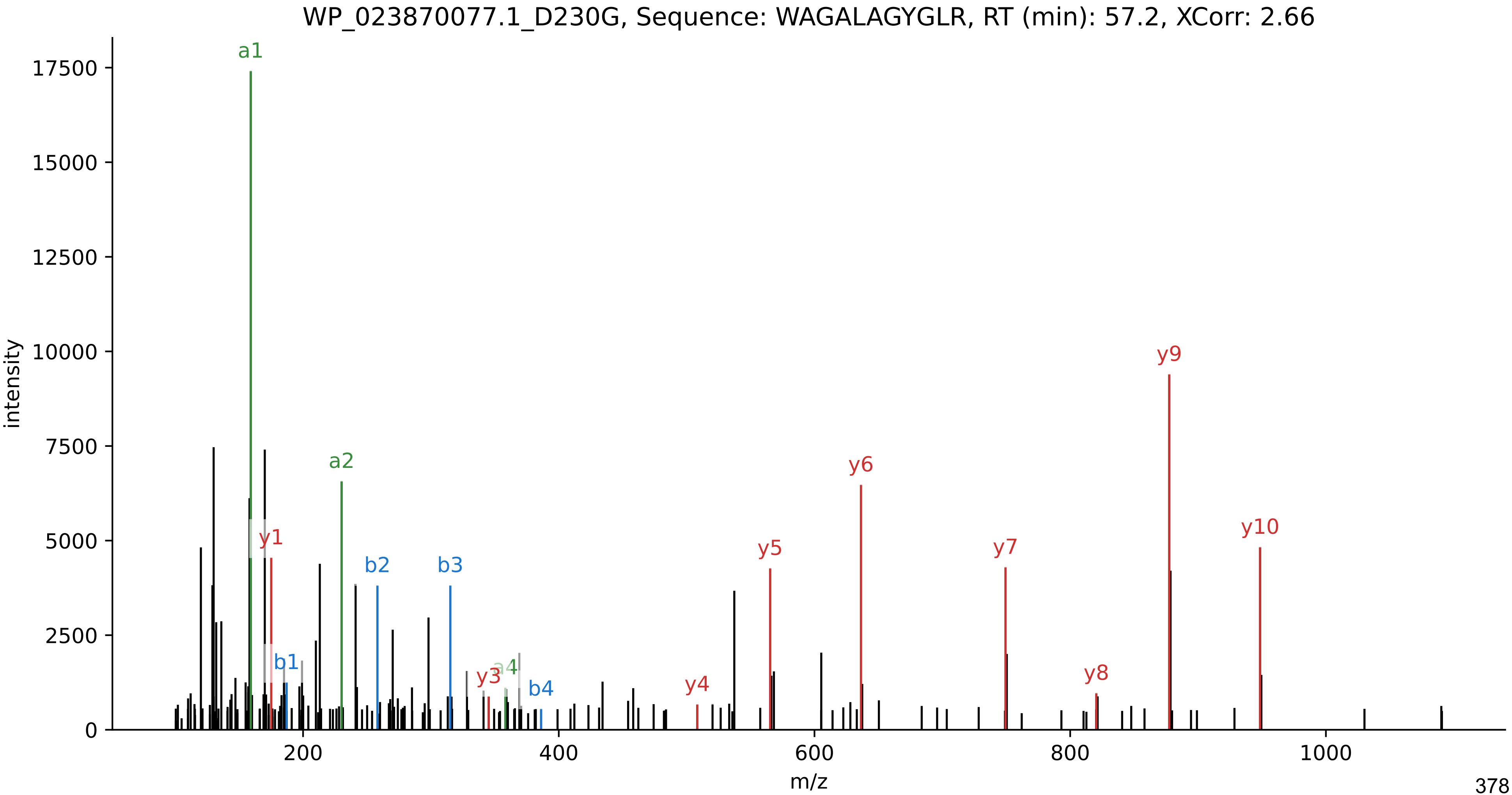
WP_023870077.1_D230G, Sequence: WAGALAGYGLR, RT (min): 57.2, XCorr: 2.66
intensity
m/z
a1
a2
a4
b1
b2
b3
b4
y1
y3
y4
y5
y6
y7
y8
y9
y10
0
2500
5000
7500
10000
12500
15000
17500
200
400
600
800
1000

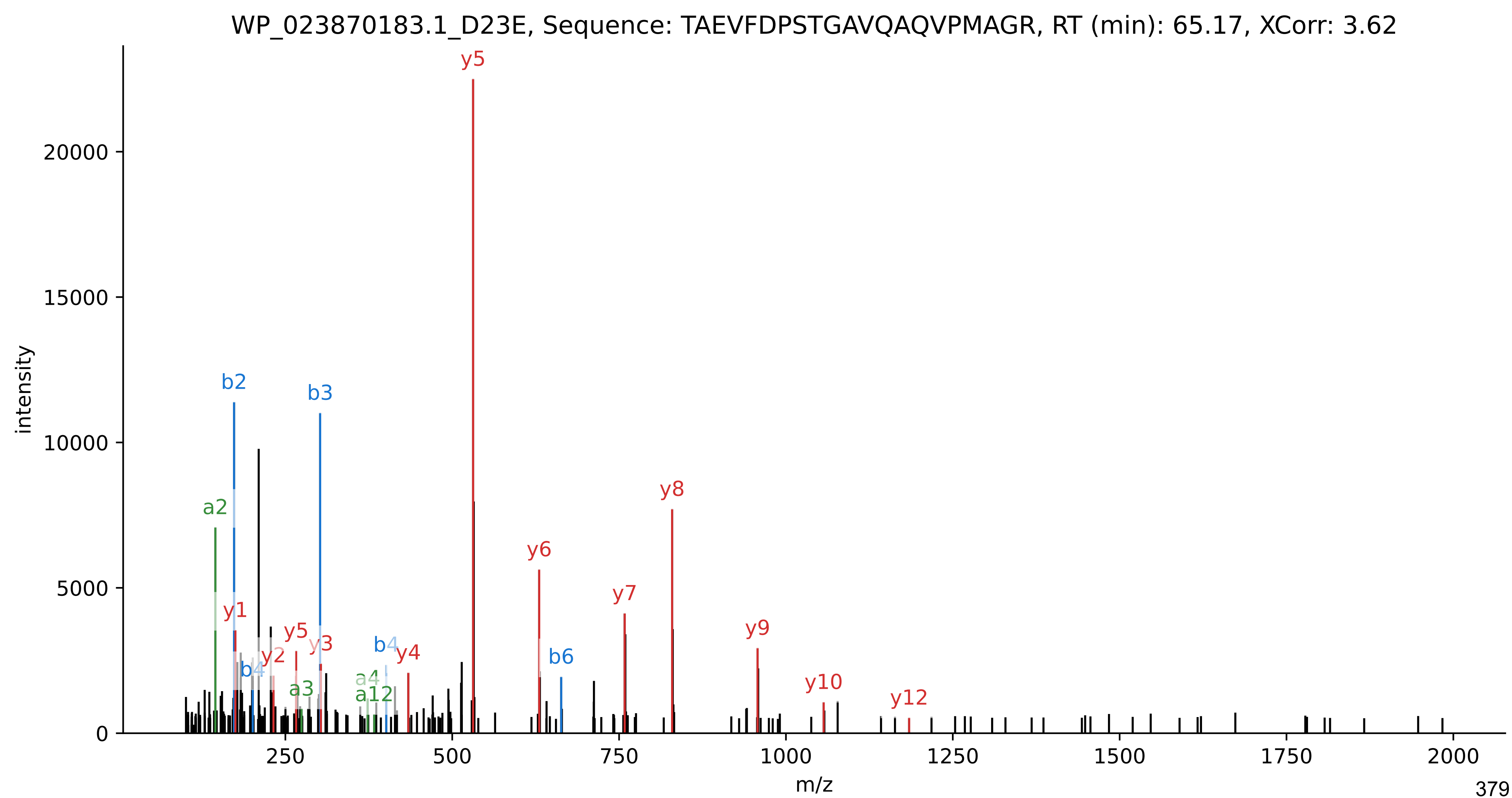
WP_023870183.1_D23E, Sequence: TAEVFDPSTGAVQAQVPMAGR, RT (min): 65.17, XCorr: 3.62
intensity
m/z
y5
b2
b3
a2
y1
b4
y2
y5
y3
a3
a4
a12
b4
y4
y6
b6
y7
y8
y9
y10
y12
0
5000
10000
15000
20000
250
500
750
1000
1250
1500
1750
2000

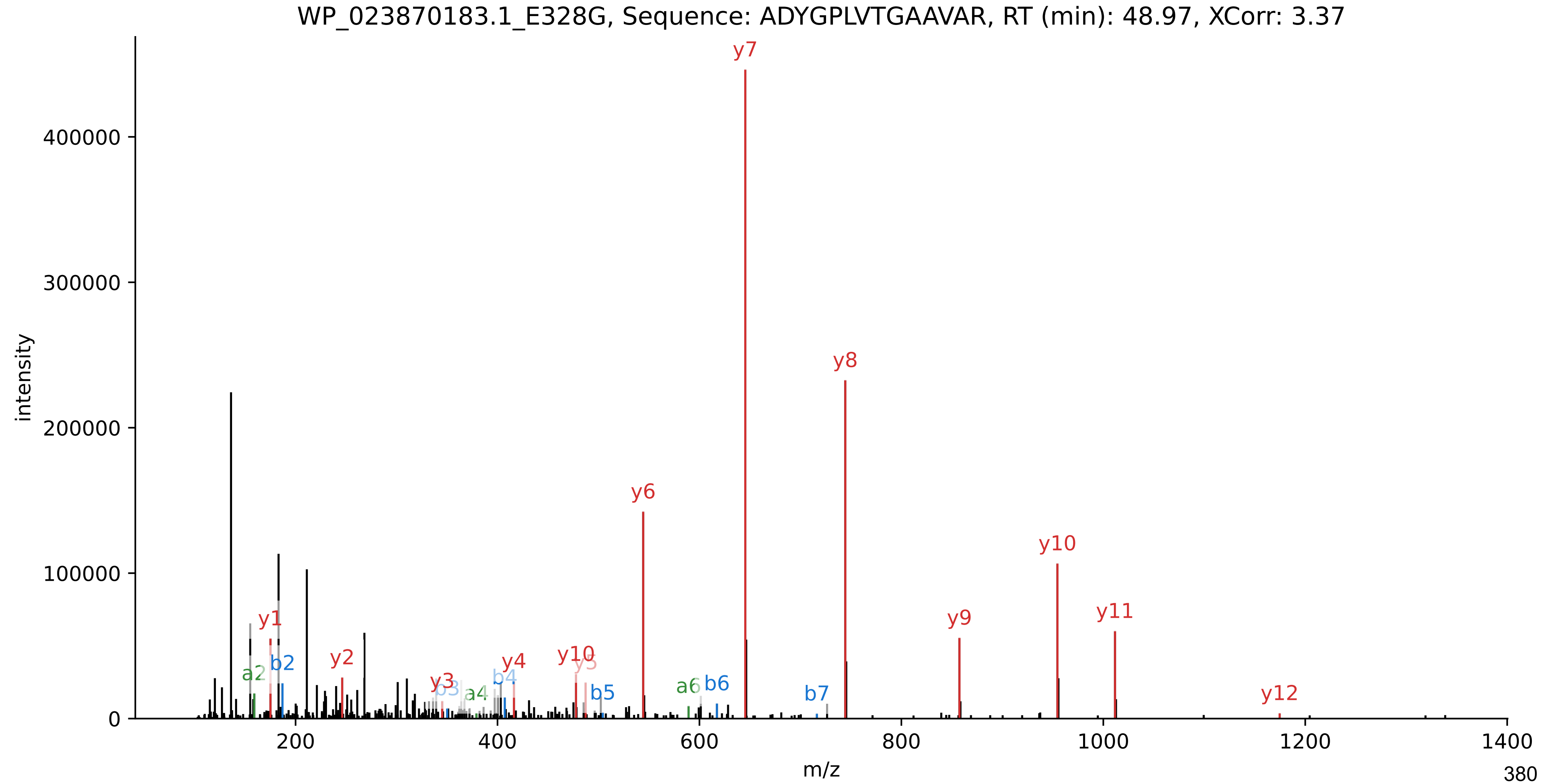
WP_023870183.1_E328G, Sequence: ADYGPLVTGAAVAR, RT (min): 48.97, XCorr: 3.37
intensity
m/z
y7
y8
y6
y10
y11
y9
y12
y1
b2
a2
y2
y3
b3
a4
b4
y4
y10
y5
b5
a6
b6
b7
0
100000
200000
300000
400000
200
400
600
800
1000
1200
1400

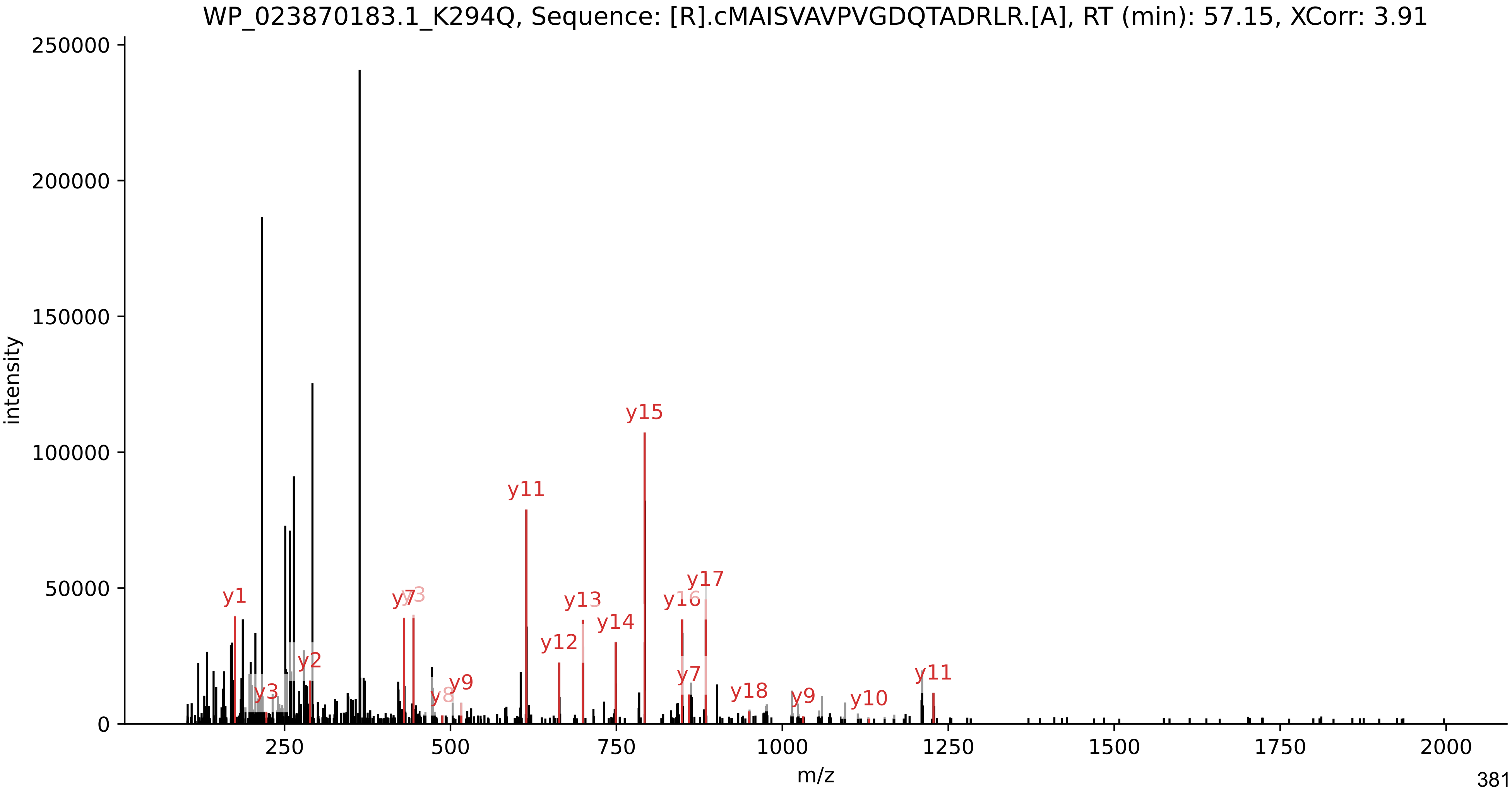
WP_023870183.1_K294Q, Sequence: [R].cMAISVAVPVGDQTADRLR.[A], RT (min): 57.15, XCorr: 3.91
intensity
m/z
y1
y2
y3
y7
y3
y8
y9
y11
y12
y13
y14
y15
y16
y17
y7
y18
y9
y10
y11

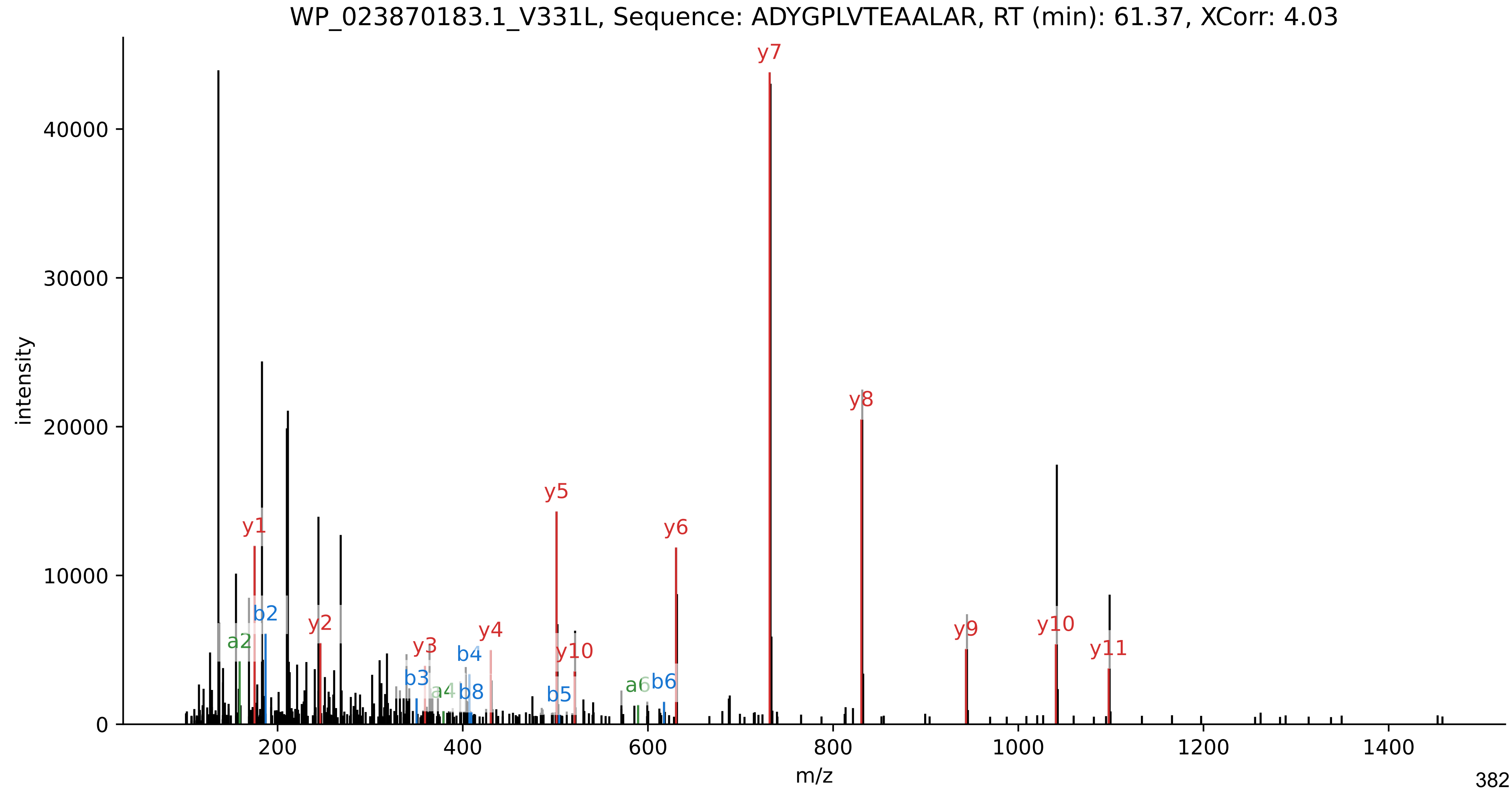

WP_023870183.1_V331L, Sequence: ADYGPLVTEAALAR, RT (min): 61.37, XCorr: 4.03
intensity
m/z
0
10000
20000
30000
40000
200
400
600
800
1000
1200
1400
y1
y2
y3
y4
y5
y6
y7
y8
y9
y10
y10
y11
a2
a4
a6
b2
b3
b4
b5
b6
b8

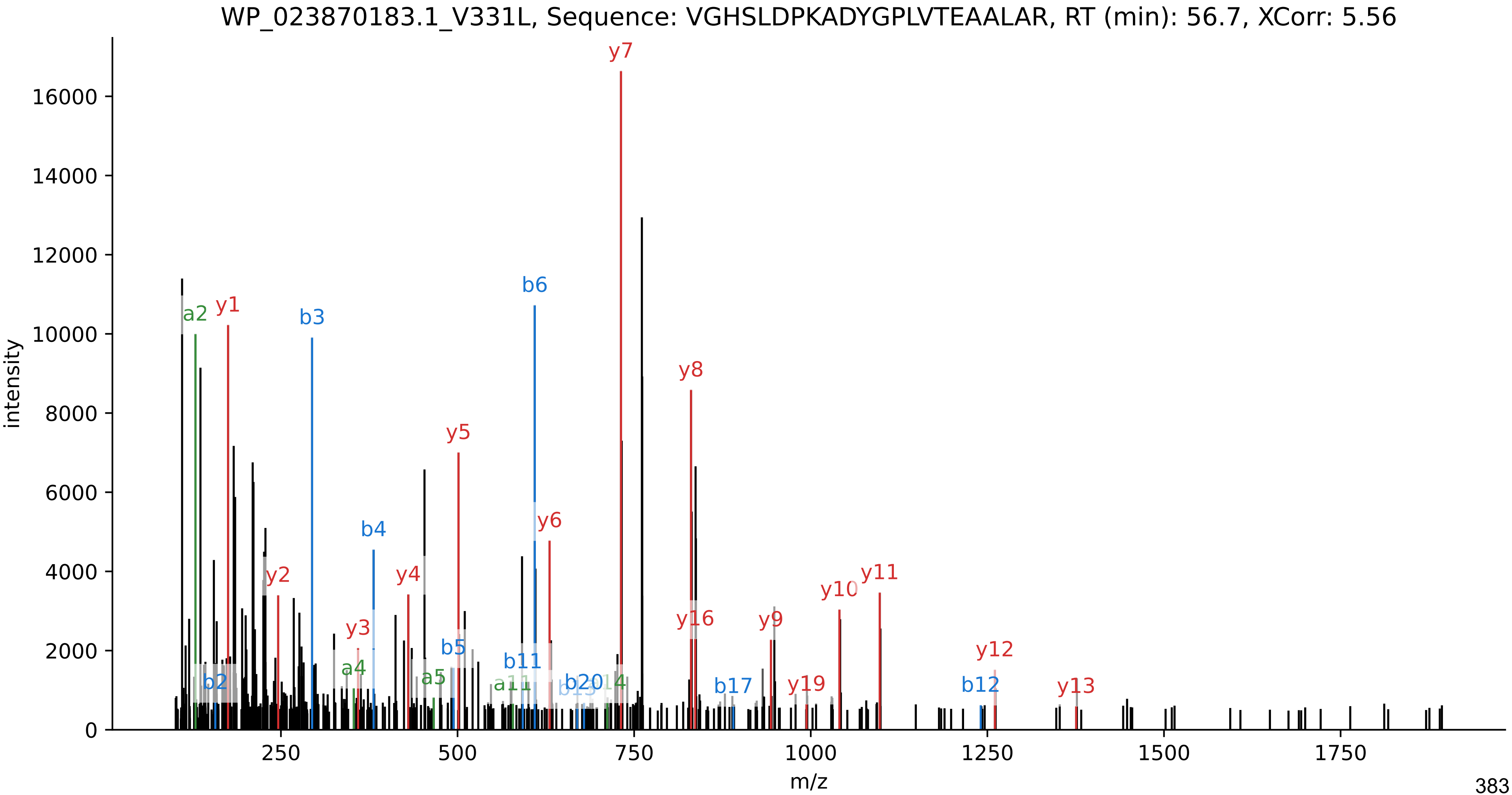

WP_023870183.1_V331L, Sequence: VGHSLDPKADYGPLVTEAALAR, RT (min): 56.7, XCorr: 5.56
intensity
m/z
a2
y1
b3
b6
y7
y8
y5
y6
b4
y2
y4
y3
a4
b2
a5
b5
b11
a11
b20
b17
y16
y9
y19
y10
y11
y12
b12
y13

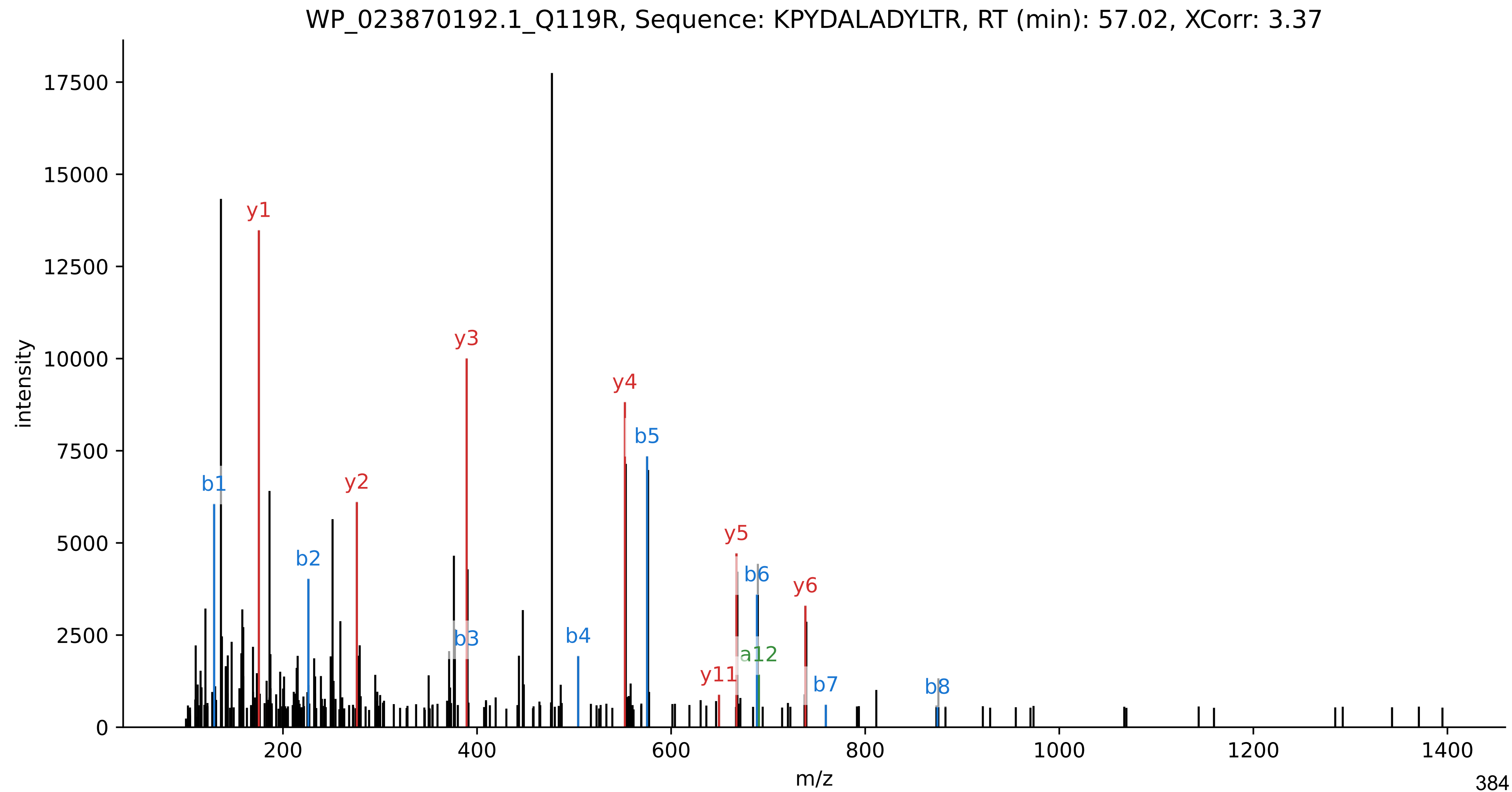

WP_023870192.1_Q119R, Sequence: KPYDALADYLTR, RT (min): 57.02, XCorr: 3.37
intensity
m/z
y1
y2
y3
y4
y5
y6
y11
b1
b2
b3
b4
b5
b6
b7
b8
a12

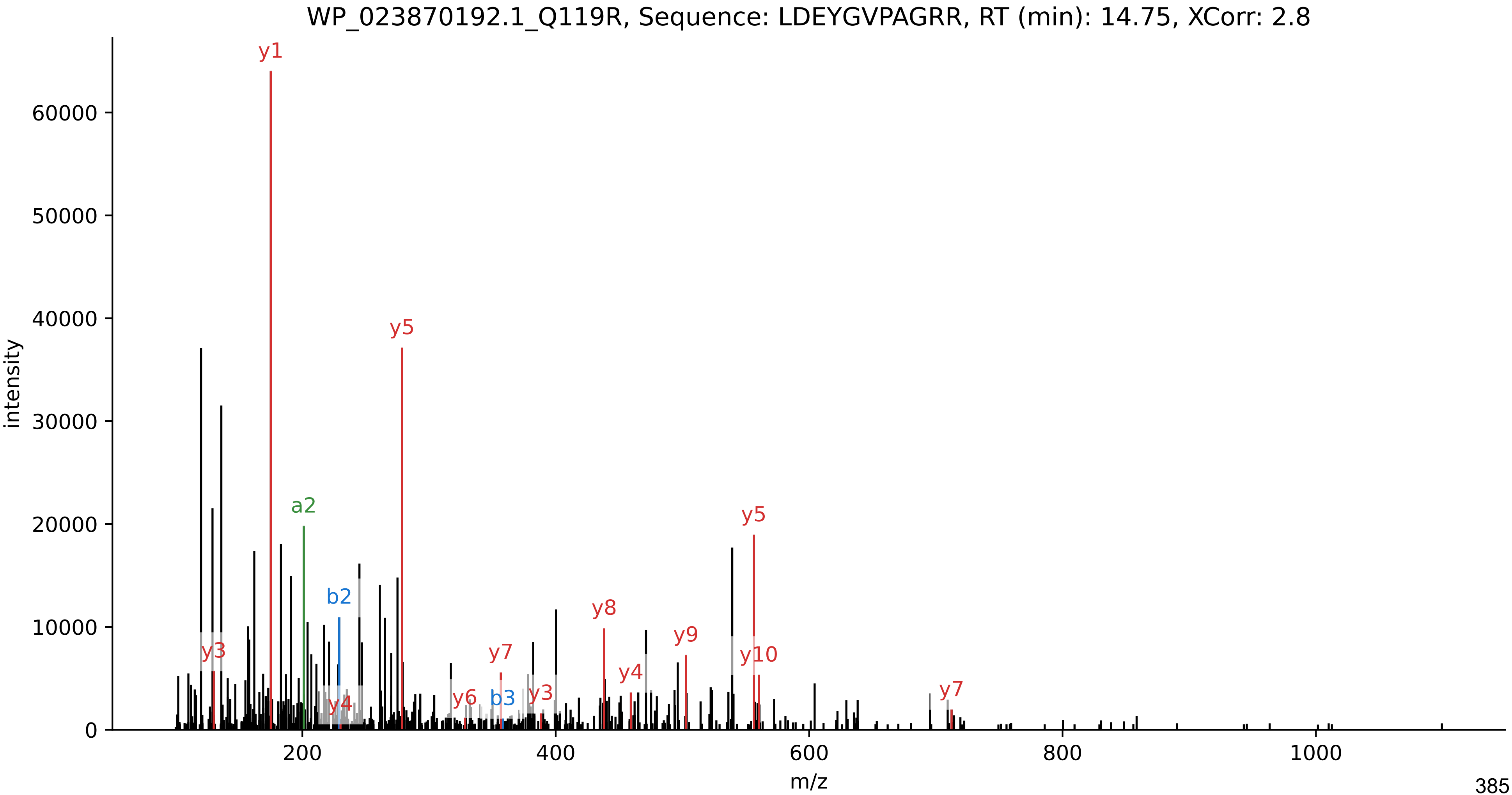
WP_023870192.1_Q119R, Sequence: LDEYGVPAGRR, RT (min): 14.75, XCorr: 2.8
intensity
m/z
0
10000
20000
30000
40000
50000
60000
200
400
600
800
1000
y1
y5
a2
b2
y3
y4
y6
y7
b3
y3
y8
y4
y9
y5
y10
y7

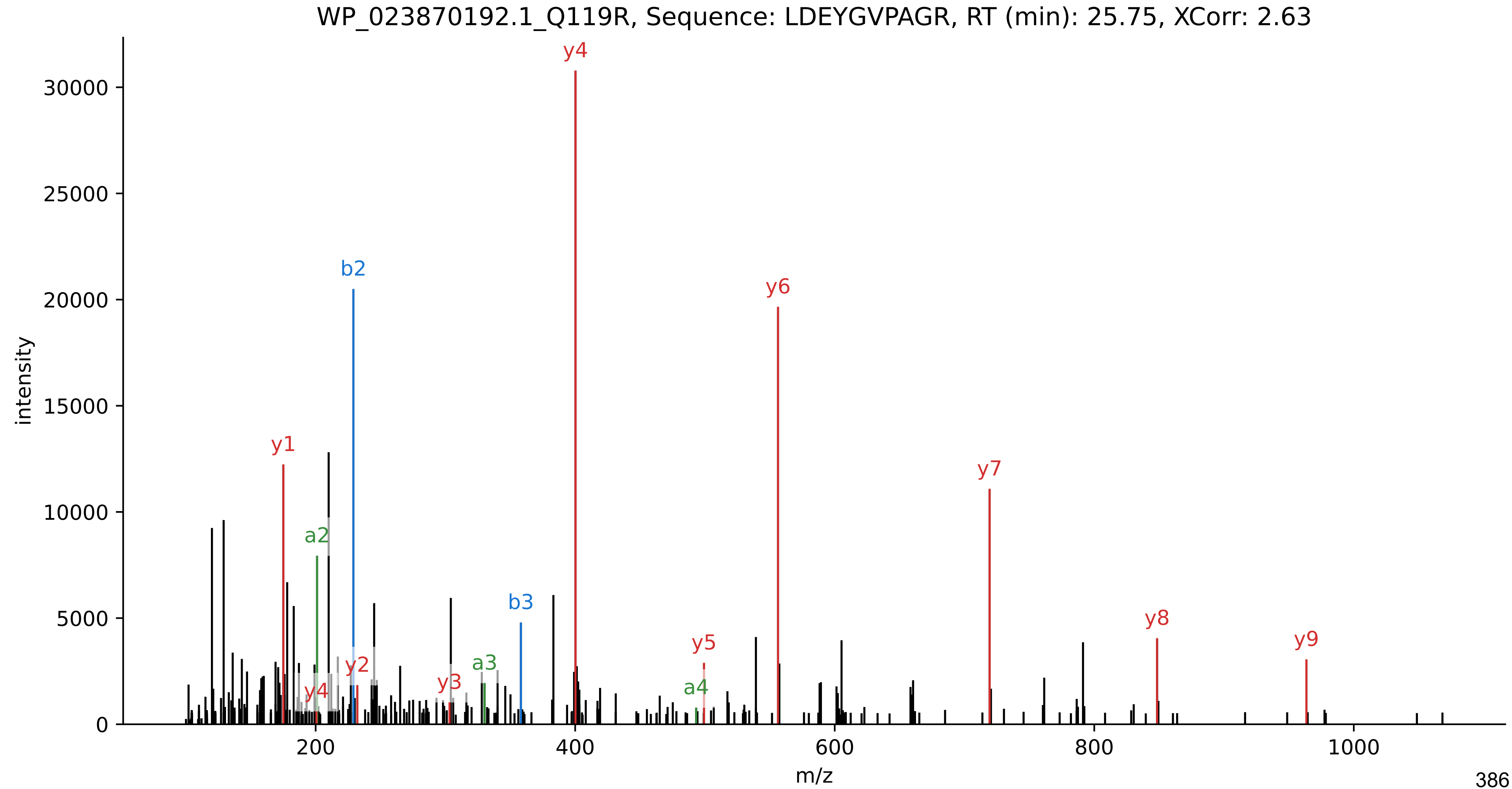

WP_023870192.1_Q119R, Sequence: LDEYGVPAGR, RT (min): 25.75, XCorr: 2.63
intensity
m/z
y1
y2
y3
y4
y4
y5
y6
y7
y8
y9
a2
a3
a4
b2
b3
0
5000
10000
15000
20000
25000
30000
200
400
600
800
1000

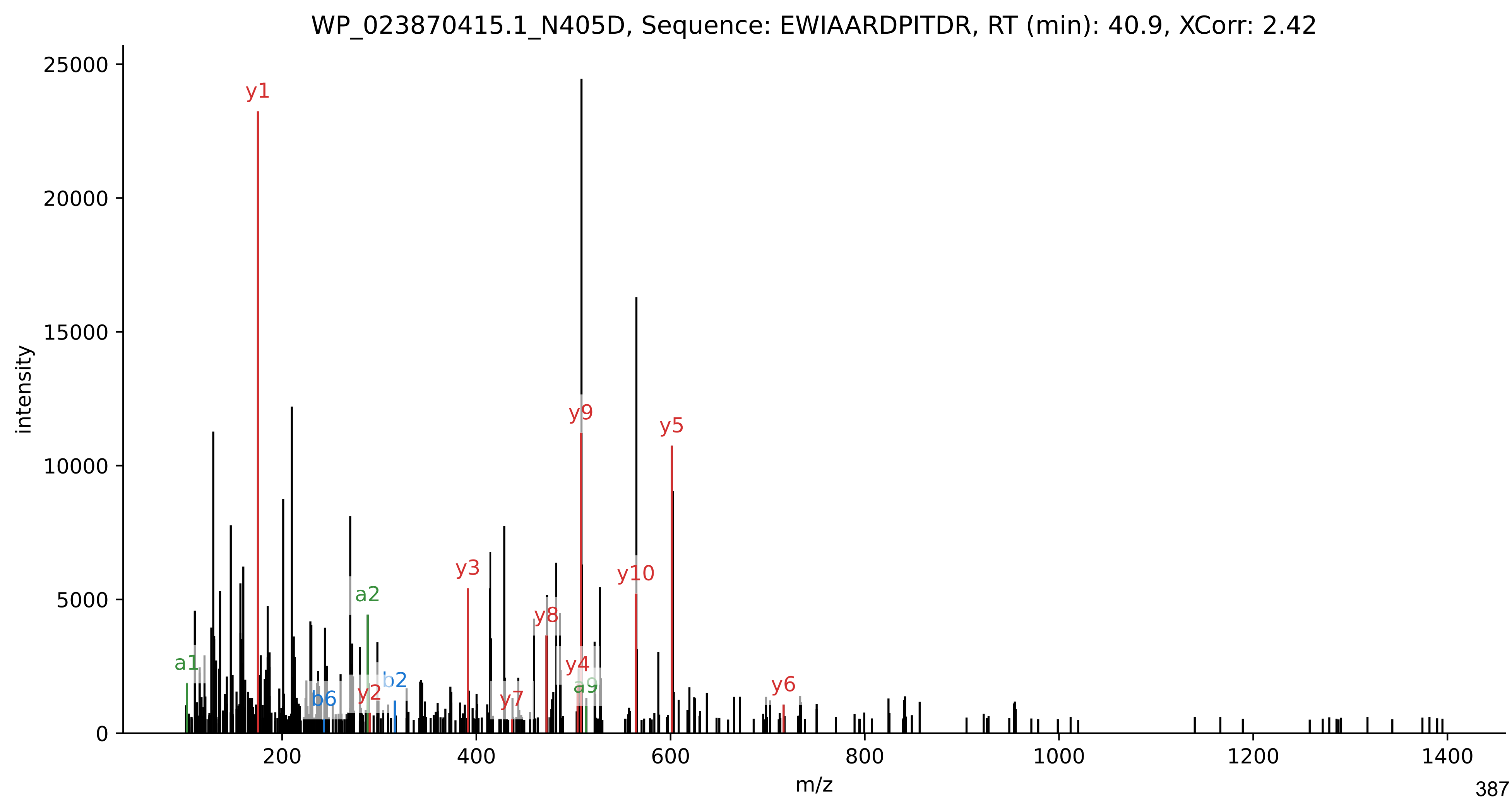

WP_023870415.1_N405D, Sequence: EWIAARDPITDR, RT (min): 40.9, XCorr: 2.42
intensity
m/z
y1
y9
y5
y3
y10
a2
y8
a1
y4
a9
b2
y6
b6
y2
y7

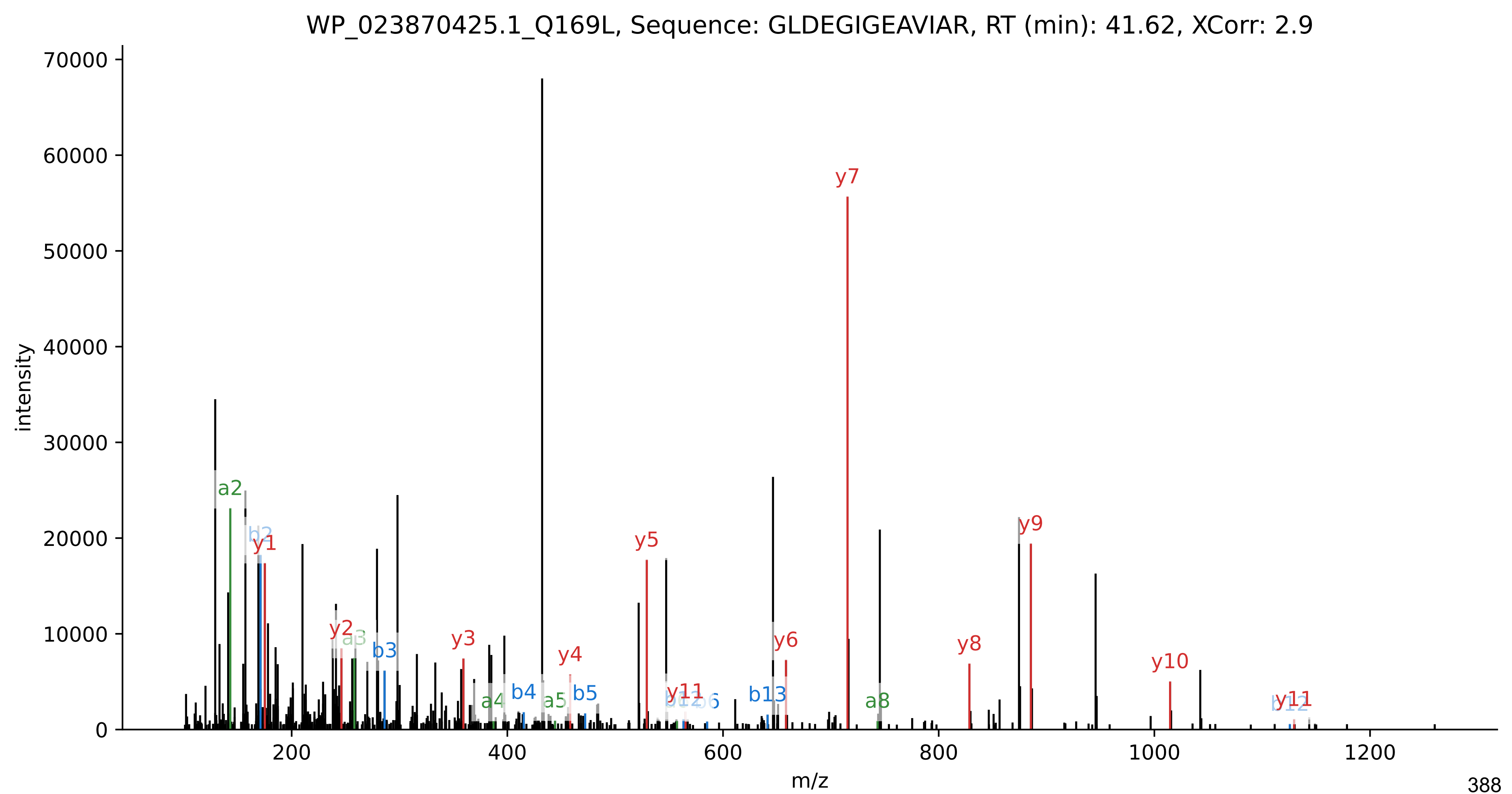
WP_023870425.1_Q169L, Sequence: GLDEGIGEAVIAR, RT (min): 41.62, XCorr: 2.9
intensity
m/z
0
10000
20000
30000
40000
50000
60000
70000
200
400
600
800
1000
1200
a2
b2
y1
y2
a3
b3
y3
a4
b4
a5
b5
y4
y5
y11
b13
y6
y7
a8
y8
y9
y10
y11
b12

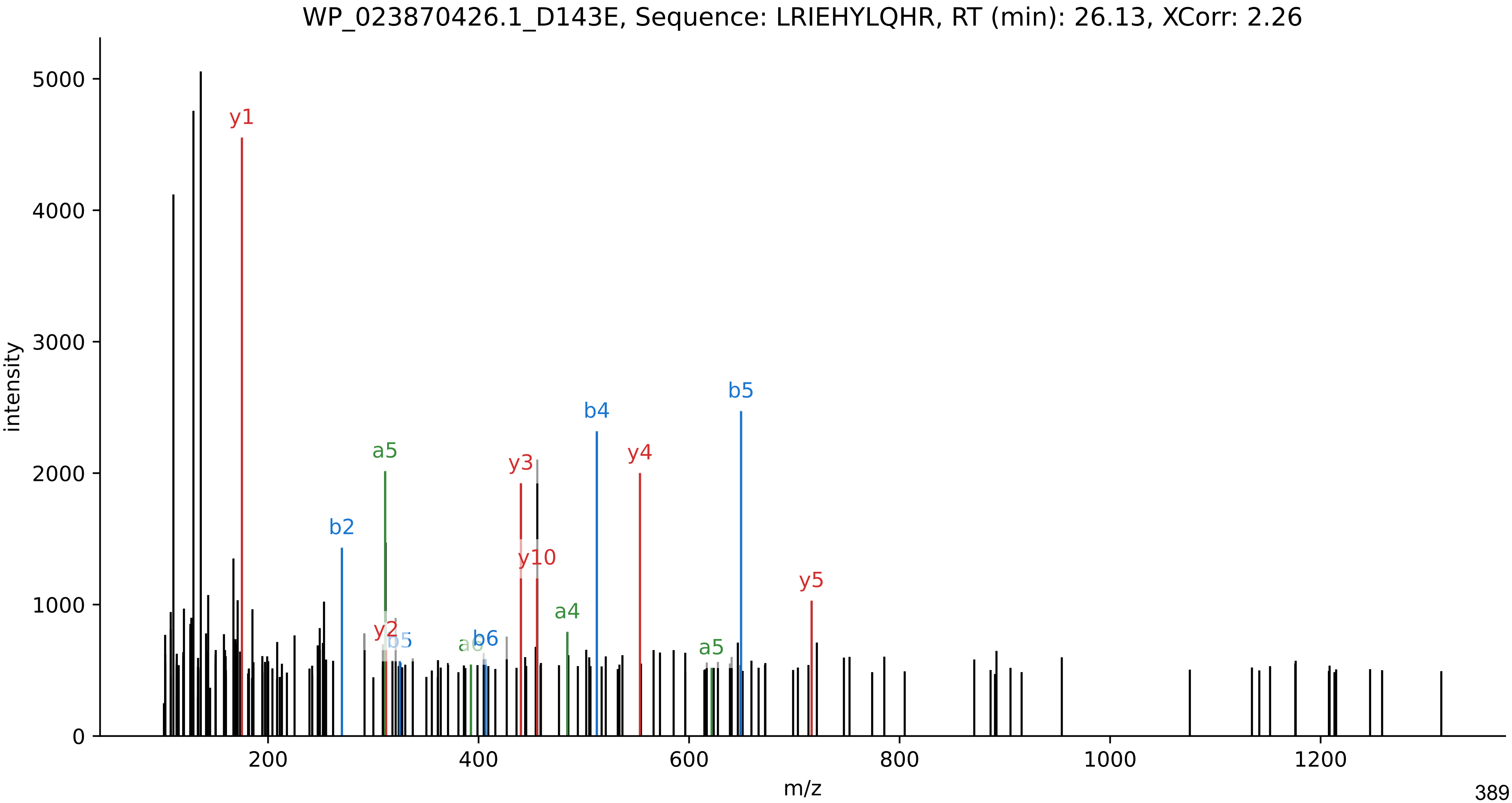

WP_023870426.1_D143E, Sequence: LRIEHYLQHR, RT (min): 26.13, XCorr: 2.26
intensity
m/z
y1
a5
b2
y2
b5
a6
b6
y3
y10
b4
y4
a4
a5
b5
y5

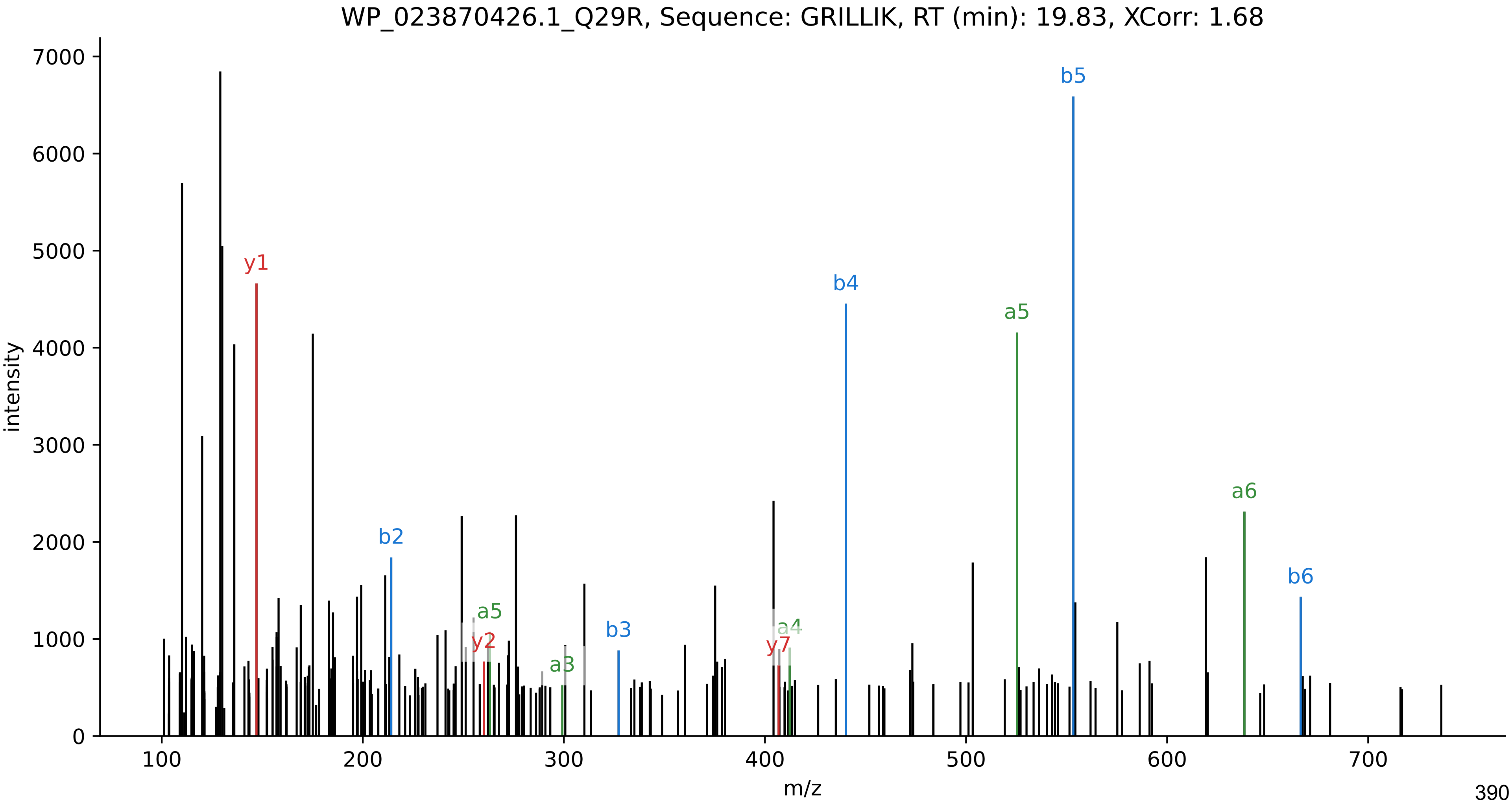
WP_023870426.1_Q29R, Sequence: GRILLIK, RT (min): 19.83, XCorr: 1.68
intensity
m/z
y1
b2
a5
y2
a3
b3
a4
y7
b4
a5
b5
a6
b6

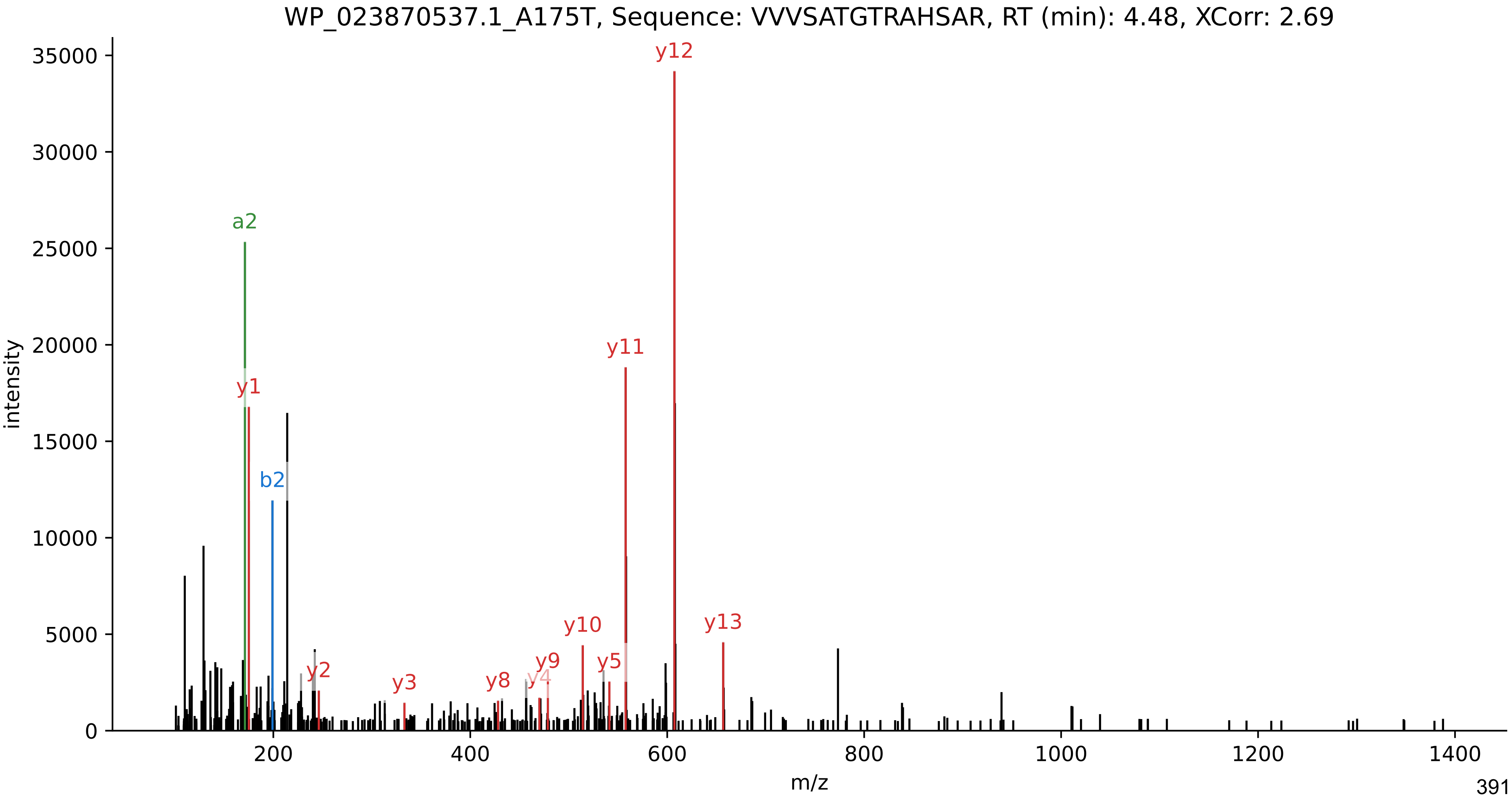
WP_023870537.1_A175T, Sequence: VVVSATGTRAHSAR, RT (min): 4.48, XCorr: 2.69
intensity
m/z
a2
y1
b2
y2
y3
y8
y4
y9
y10
y5
y11
y12
y13
0
5000
10000
15000
20000
25000
30000
35000
200
400
600
800
1000
1200
1400

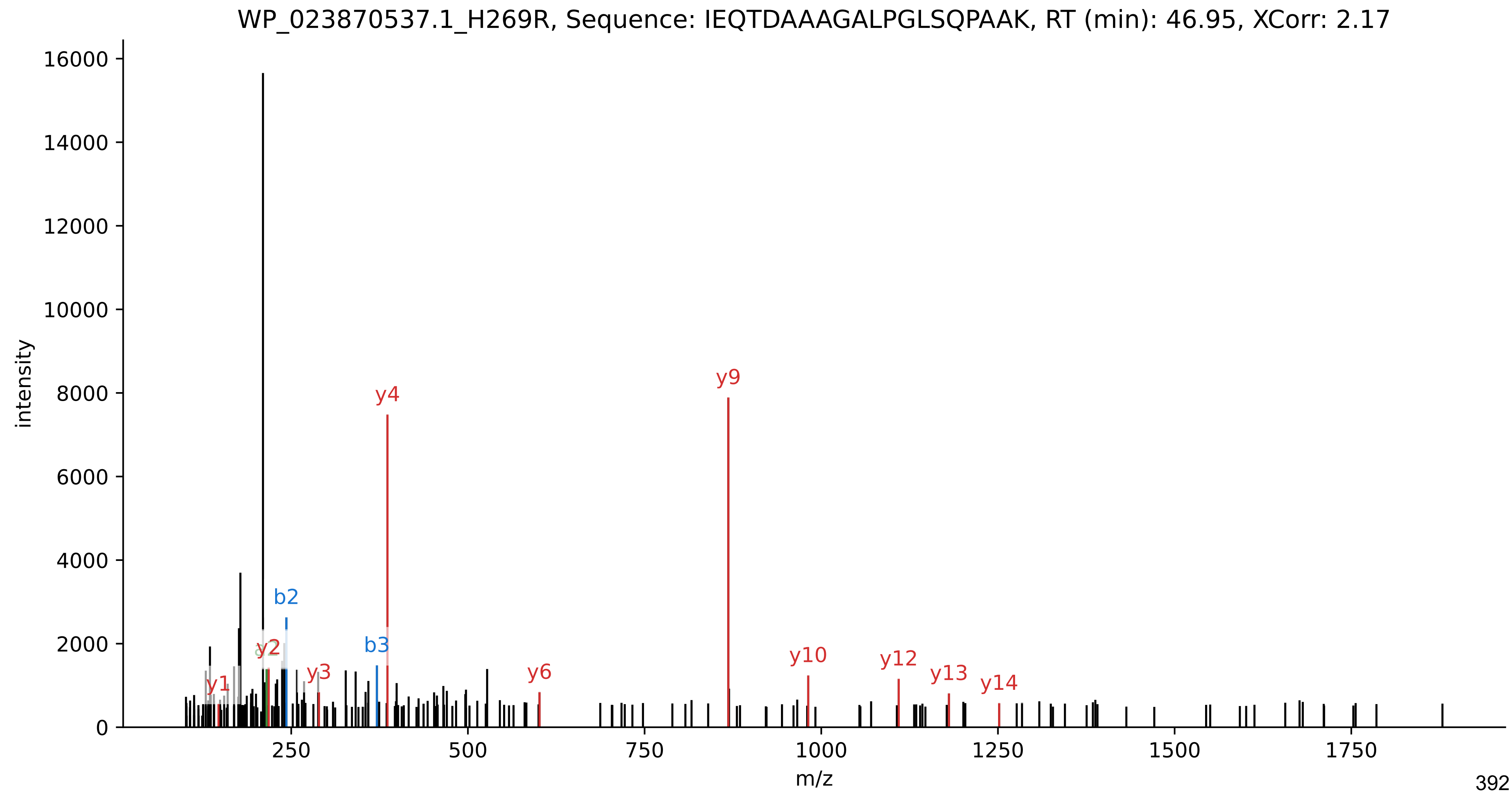

WP_023870537.1_H269R, Sequence: IEQTDAAAGALPGLSQPAAK, RT (min): 46.95, XCorr: 2.17
intensity
m/z
y1
y2
y3
b2
b3
y4
y6
y9
y10
y12
y13
y14

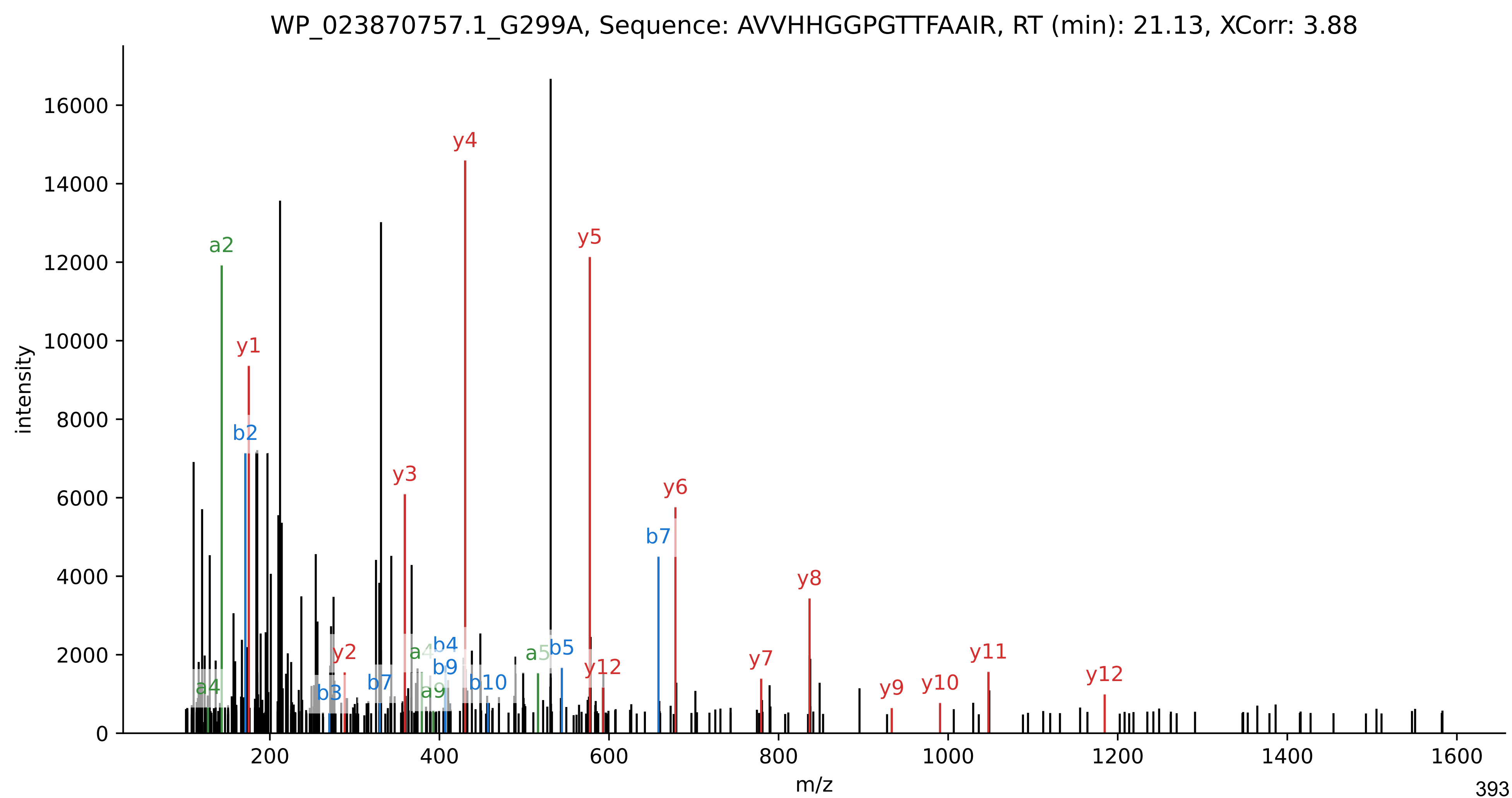
WP_023870757.1_G299A, Sequence: AVVHHGGPGTTFAAIR, RT (min): 21.13, XCorr: 3.88
intensity
m/z
a2
y1
b2
y3
y4
y5
y6
b7
y8
y2
a4
b3
b7
a4
b4
b9
a9
b10
a5
b5
y12
y7
y9
y10
y11
y12

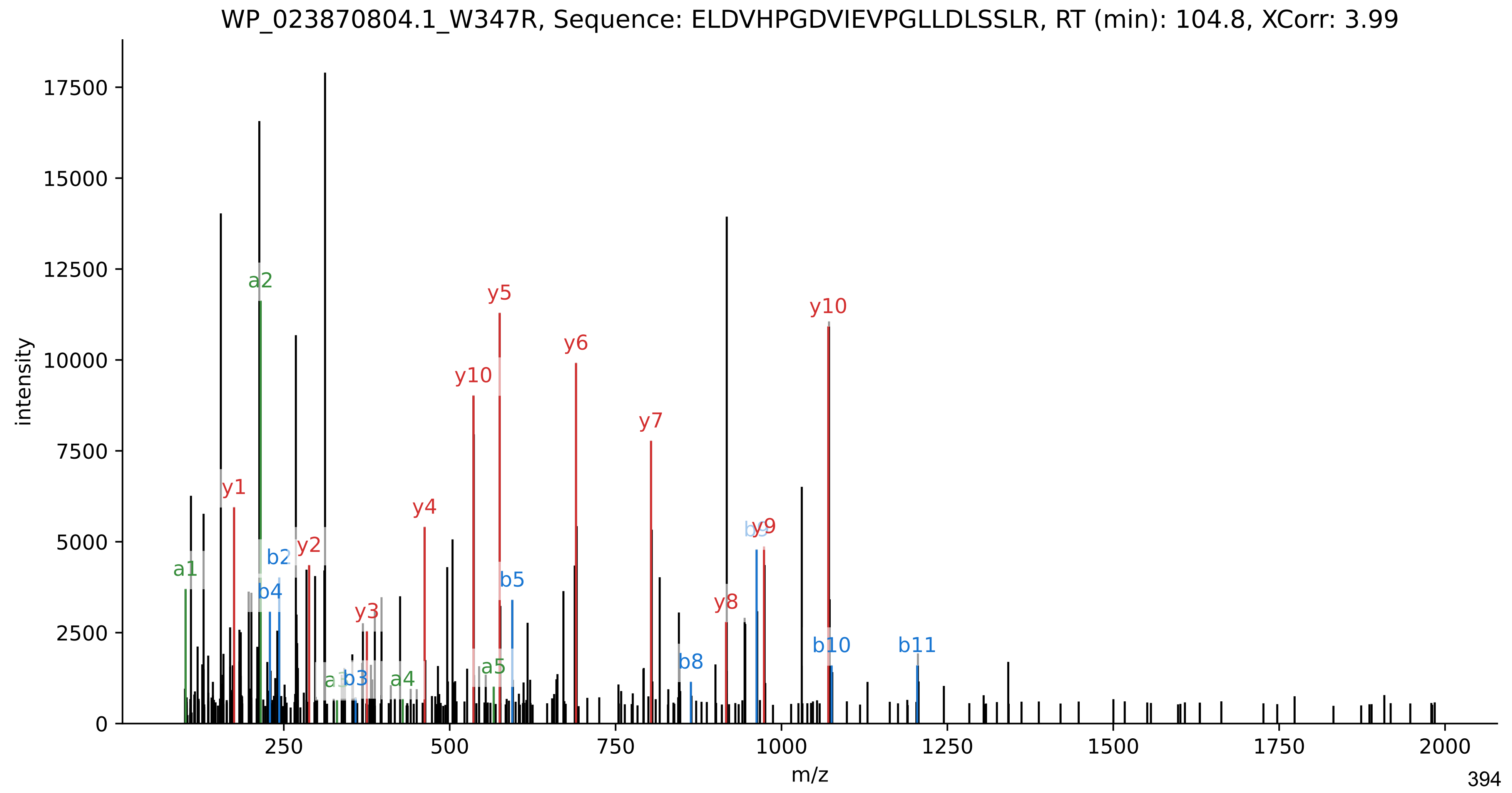

WP_023870804.1_W347R, Sequence: ELDVHPGDVIEVPGLLDLSSLR, RT (min): 104.8, XCorr: 3.99
intensity
m/z
a1
a2
a4
a5
b2
b3
b4
b5
b8
b10
b11
y1
y2
y3
y4
y5
y6
y7
y8
y9
y10

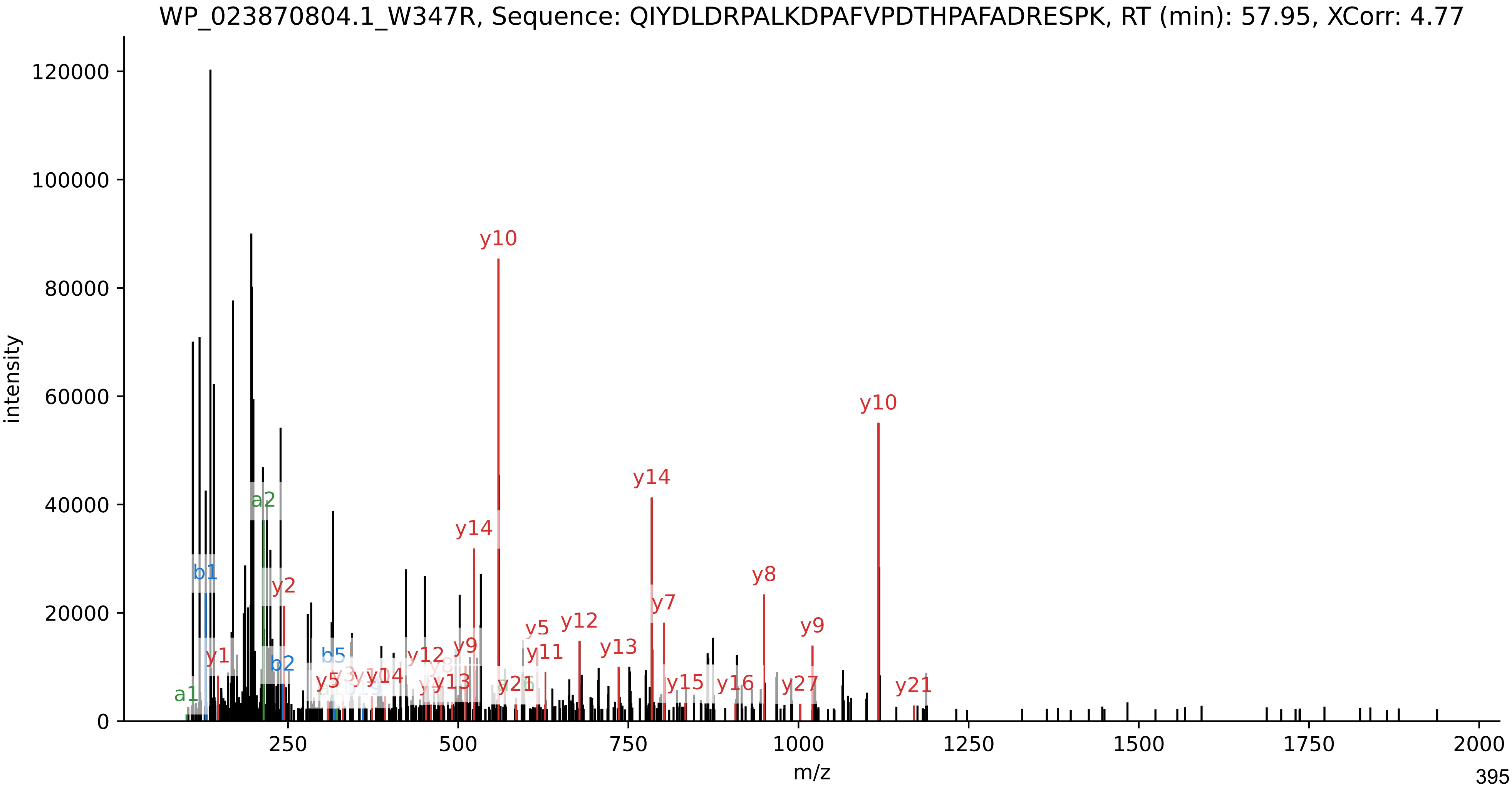
WP_023870804.1_W347R, Sequence: QIYDLDRPALKDPAFVPDTHPAFADRESPK, RT (min): 57.95, XCorr: 4.77
intensity
m/z
0
20000
40000
60000
80000
100000
120000
250
500
750
1000
1250
1500
1750
2000
y10
y10
y14
y14
a2
b1
y2
y7
y8
y12
y5
y9
y9
y11
y13
y1
b2
b5
a1
y5
y13
y21
y15
y16
y27
y21

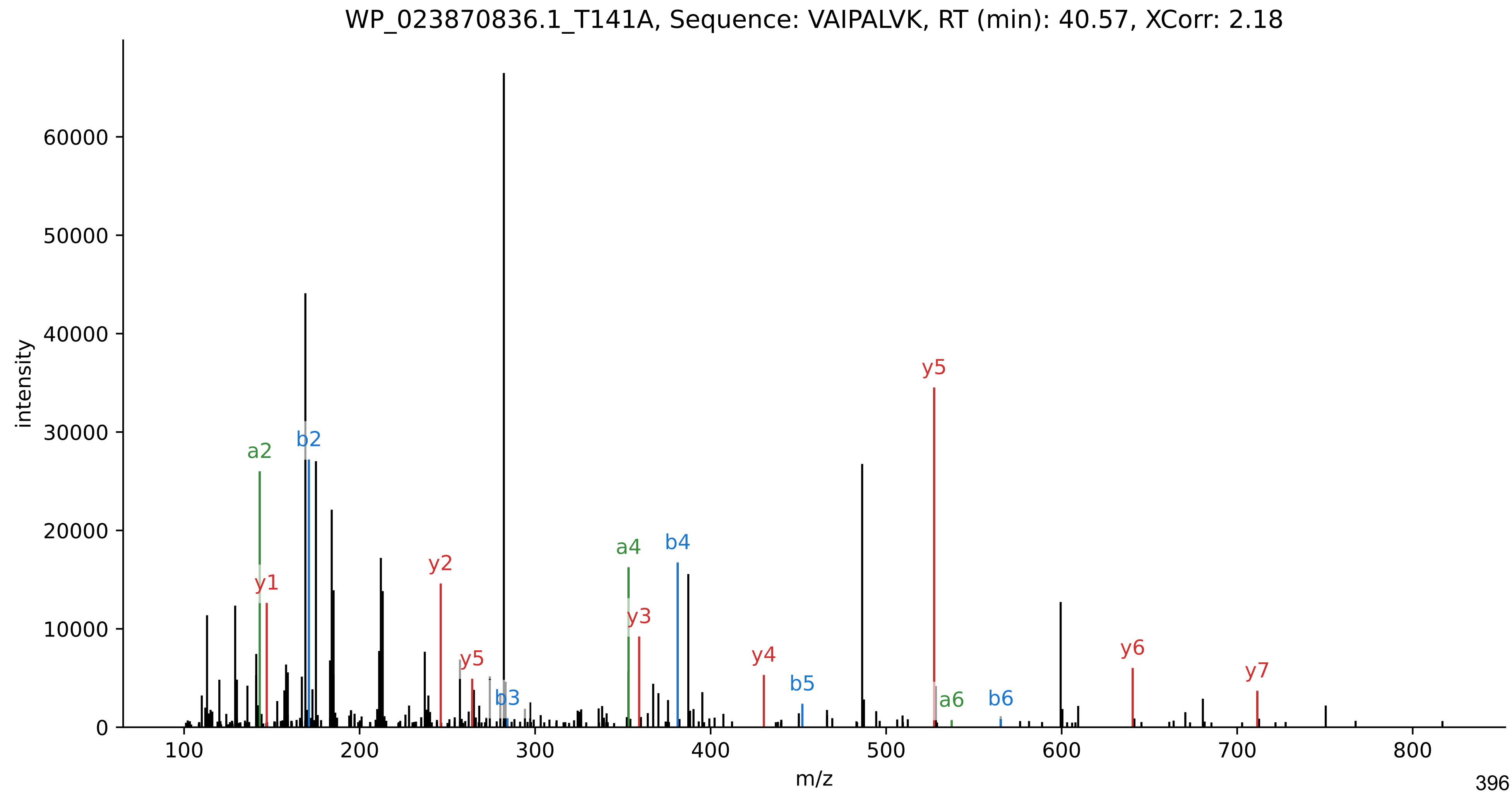

WP_023870836.1_T141A, Sequence: VAIPALVK, RT (min): 40.57, XCorr: 2.18
intensity
m/z
a2
b2
y1
y2
y5
b3
a4
b4
y3
y4
b5
y5
a6
b6
y6
y7

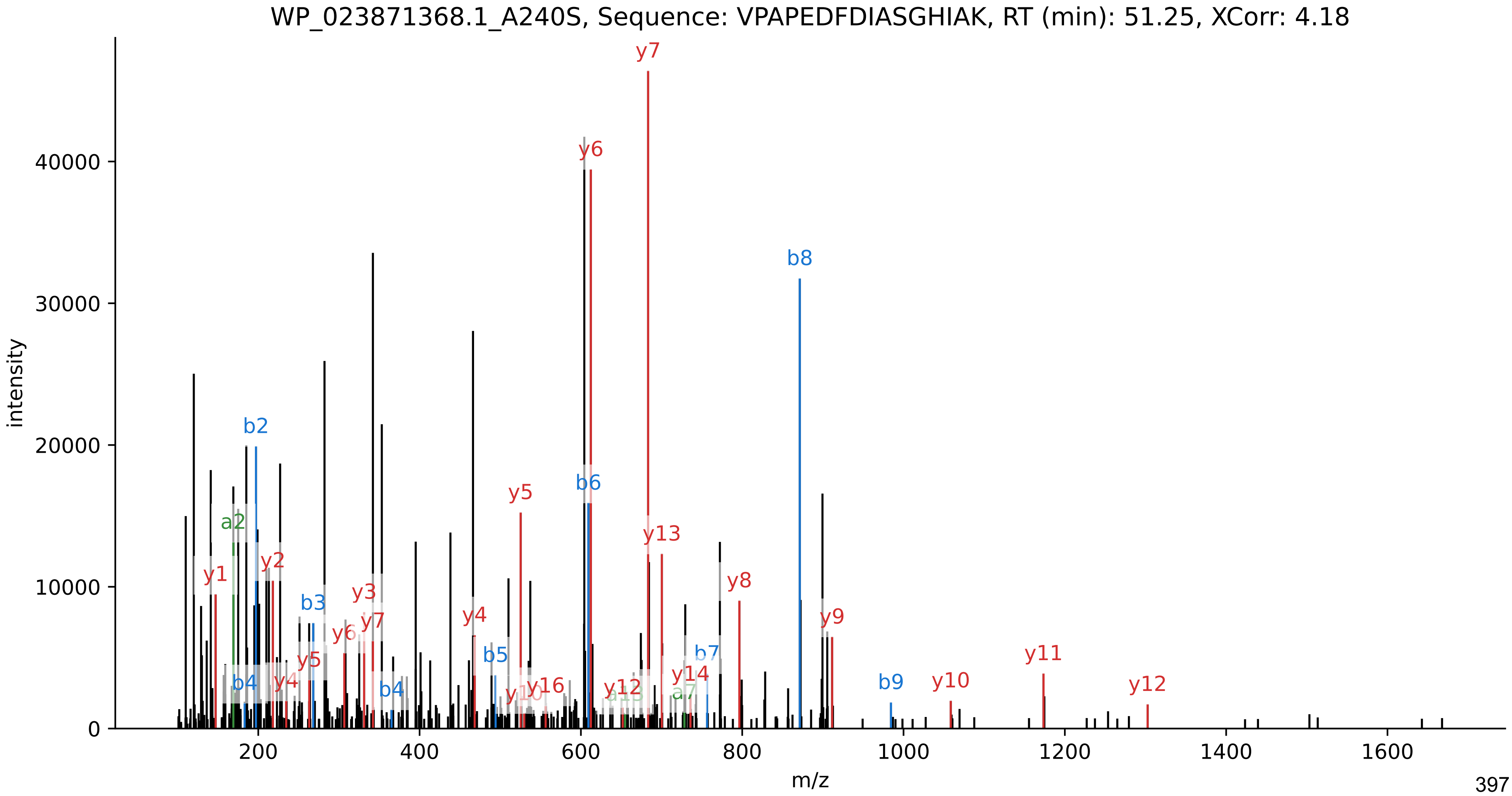
WP_023871368.1_A240S, Sequence: VPAPEDFDIASGHIAK, RT (min): 51.25, XCorr: 4.18
intensity
m/z
0
10000
20000
30000
40000
200
400
600
800
1000
1200
1400
1600
y7
y6
b8
b2
b6
y5
a2
y13
y2
y1
y8
b3
y3
y9
y4
y7
y6
b5
b7
y5
y11
y14
b4
y4
y16
y12
b9
y10
b4
a7
y12

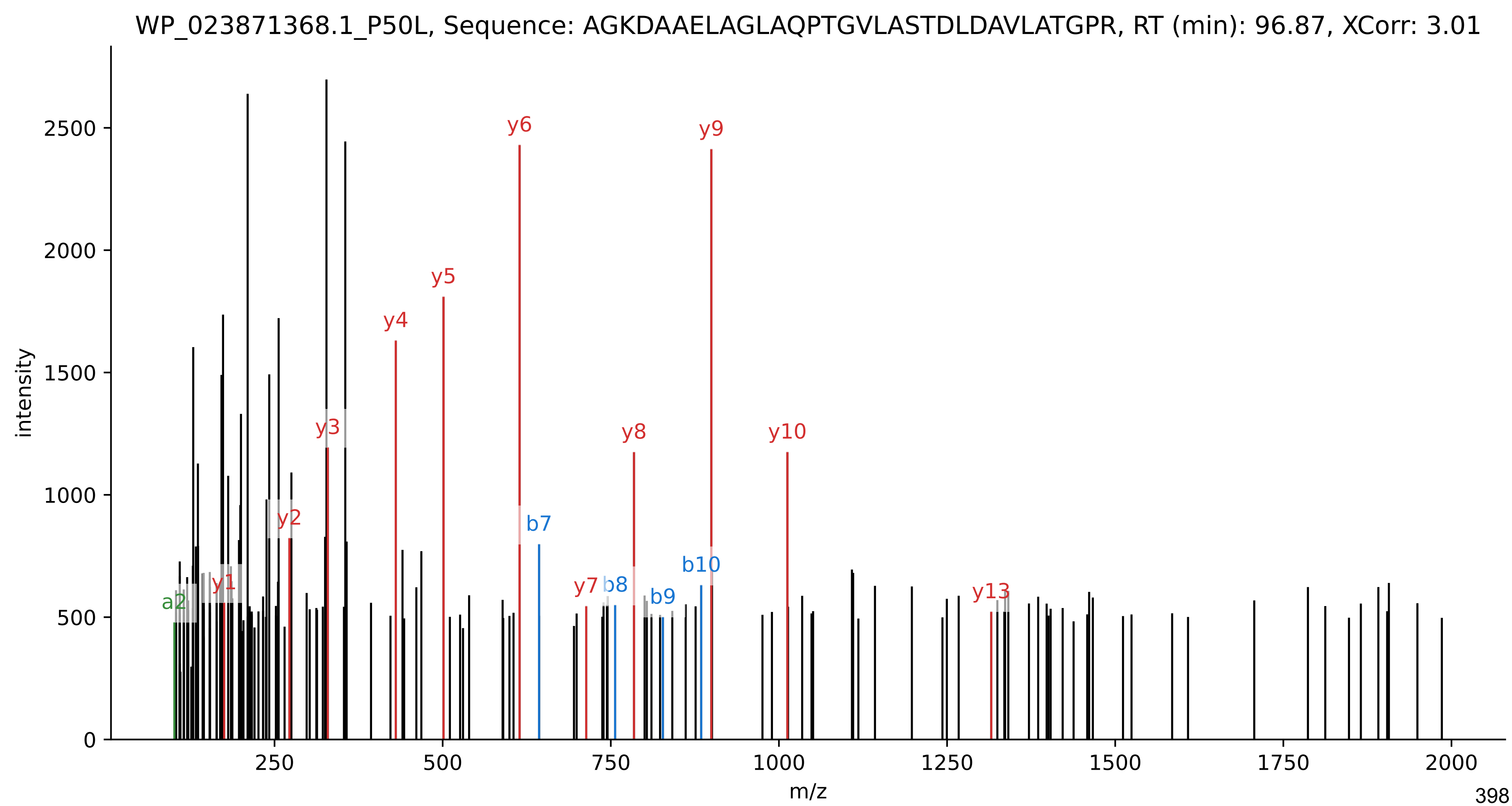

WP_023871368.1_P50L, Sequence: AGKDAAELAGLAQPTGVLASTDLDAVLATGPR, RT (min): 96.87, XCorr: 3.01
intensity
m/z
0
500
1000
1500
2000
2500
250
500
750
1000
1250
1500
1750
2000
a2
y1
y2
y3
y4
y5
y6
y7
y8
y9
y10
y13
b7
b8
b9
b10

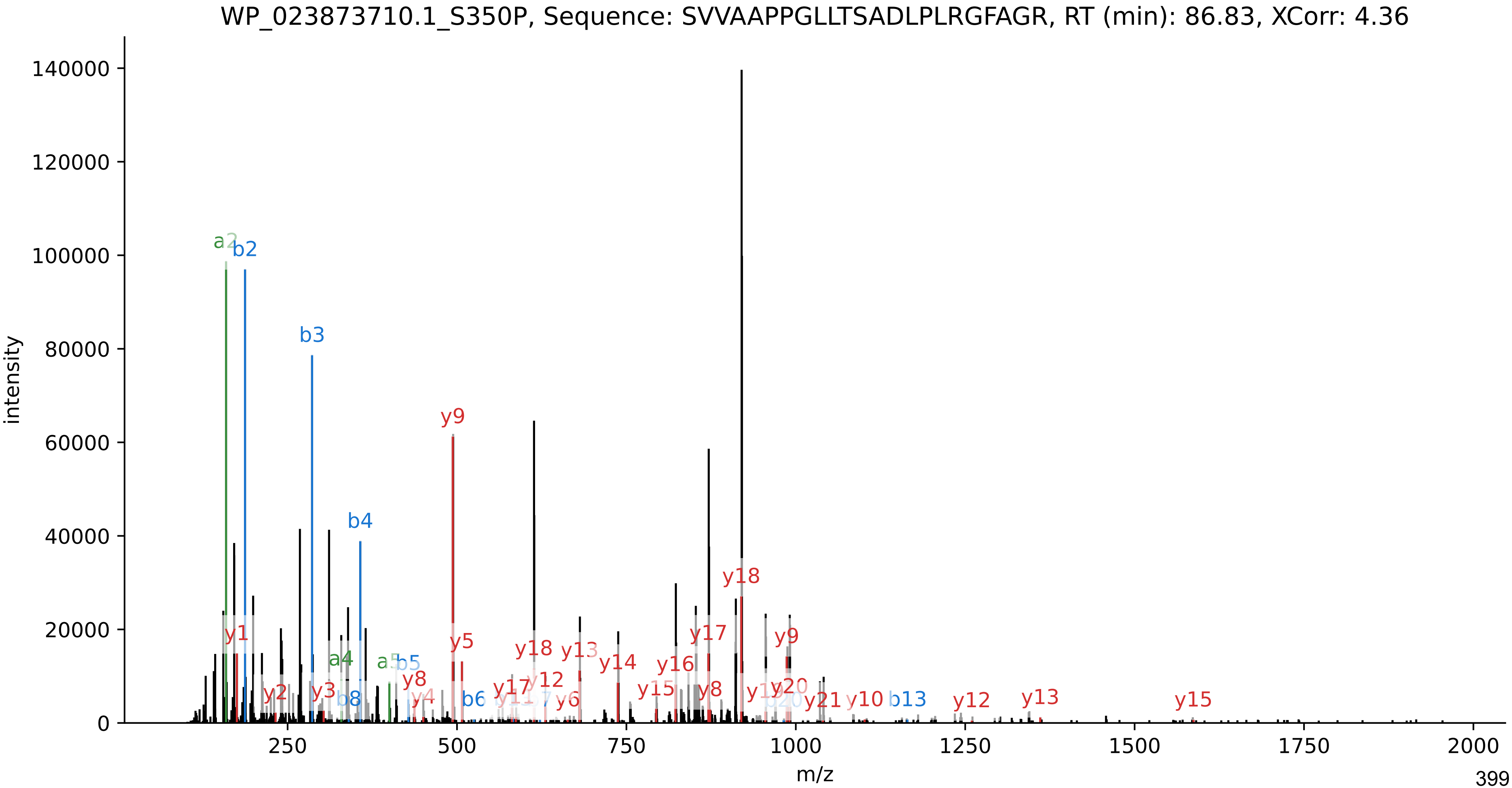

WP_023873710.1_S350P, Sequence: SVVAAPPGLLTSADLPLRGFAGR, RT (min): 86.83, XCorr: 4.36
intensity
m/z
a2
b2
b3
b4
y9
y18
y1
y5
y17
y9
a4
a5
b5
y18
y13
y14
y16
y2
y3
y8
y12
y15
y8
y20
b8
y4
y6
y21
y10
b13
y12
y13
y15

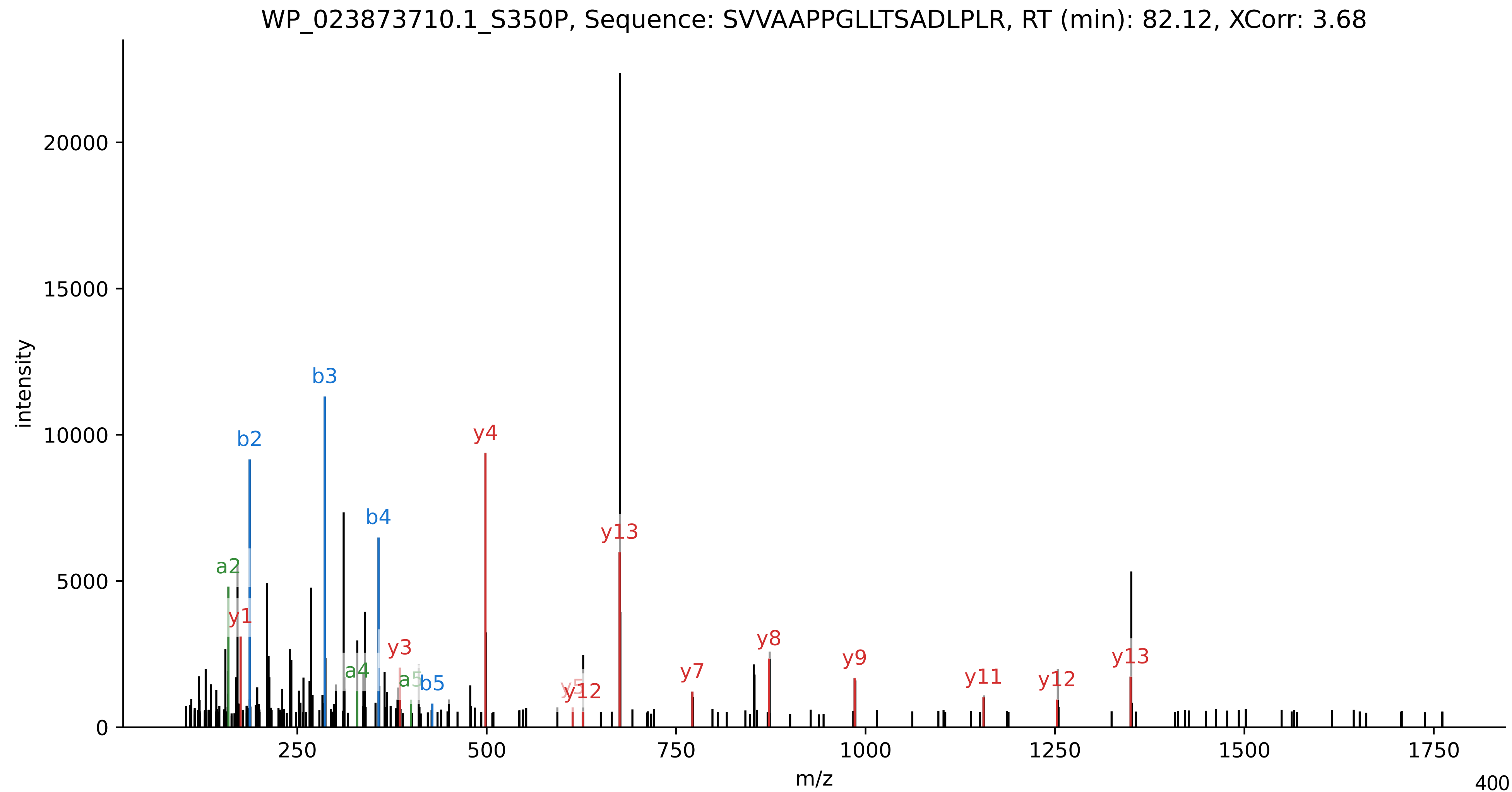

WP_023873710.1_S350P, Sequence: SVVAAPPGLLTSADLPLR, RT (min): 82.12, XCorr: 3.68
intensity
m/z
b2
b3
b4
b5
a2
a4
y1
y3
y4
y7
y8
y9
y11
y12
y13

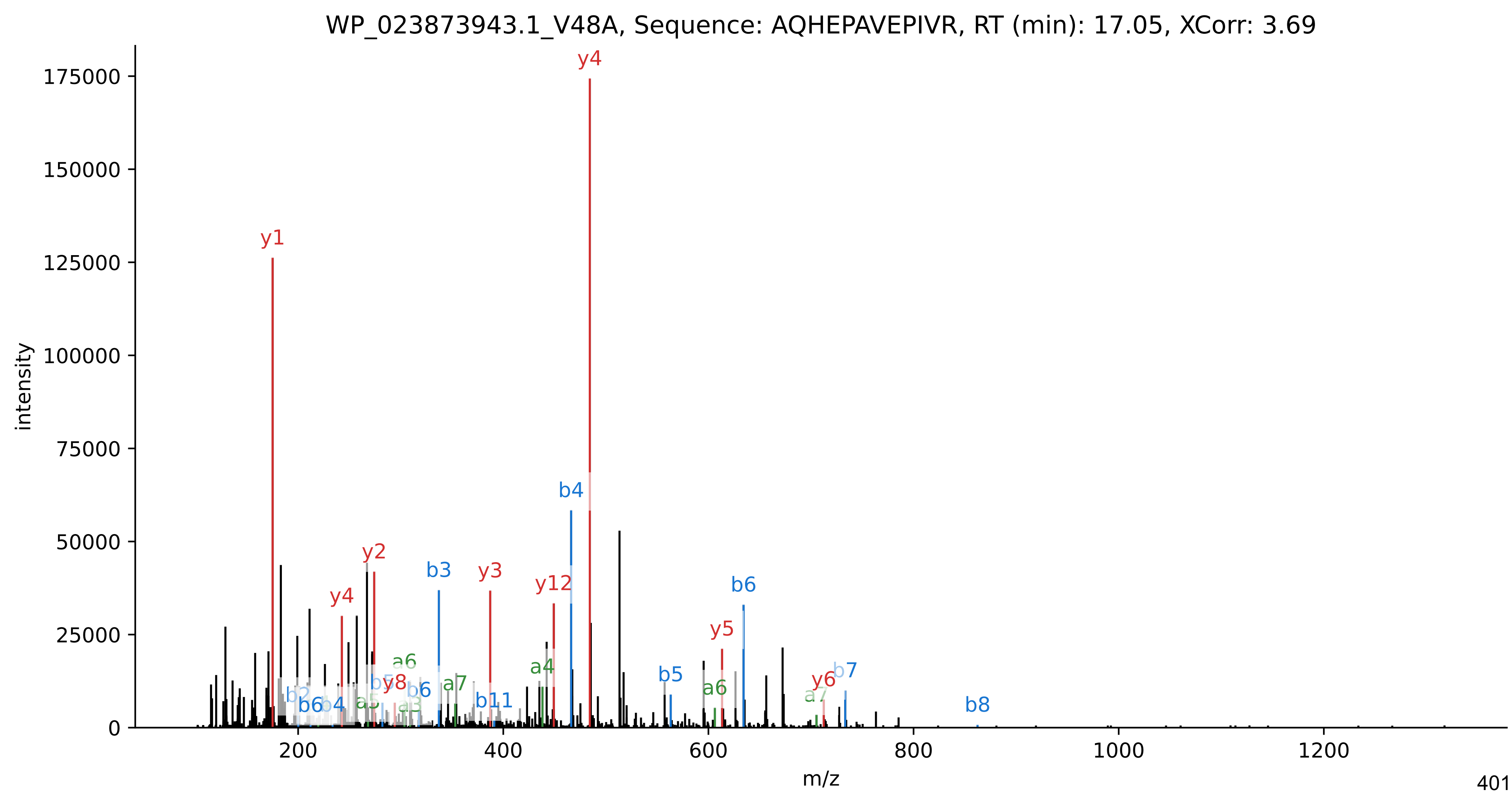

WP_023873943.1_V48A, Sequence: AQHEPAVEPIVR, RT (min): 17.05, XCorr: 3.69
intensity
m/z
y4
y1
b4
y2
y4
b3
y3
y12
b6
y5
b5
a6
a4
a7
y8
b11
a6
b7
y6
a7
b8
b6
b4
a5
b2
b6

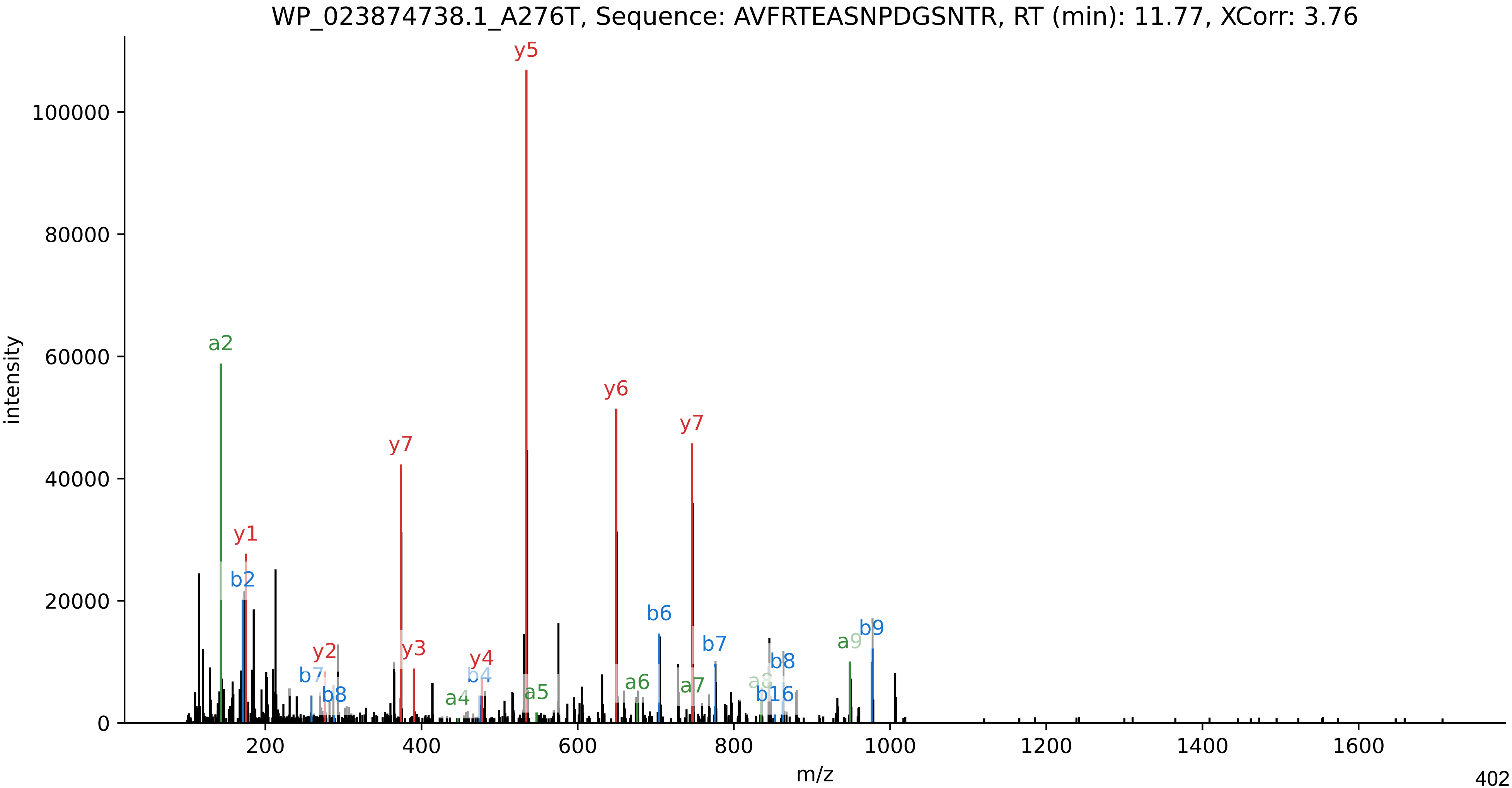
WP_023874738.1_A276T, Sequence: AVFRTEASNPDGSNTR, RT (min): 11.77, XCorr: 3.76
intensity
m/z
y5
a2
y6
y7
y7
y1
b2
b6
b9
a9
b7
y2
y3
y4
b8
b7
b4
b8
a6
a7
a8
b16
a5
a4
0
20000
40000
60000
80000
100000
200
400
600
800
1000
1200
1400
1600

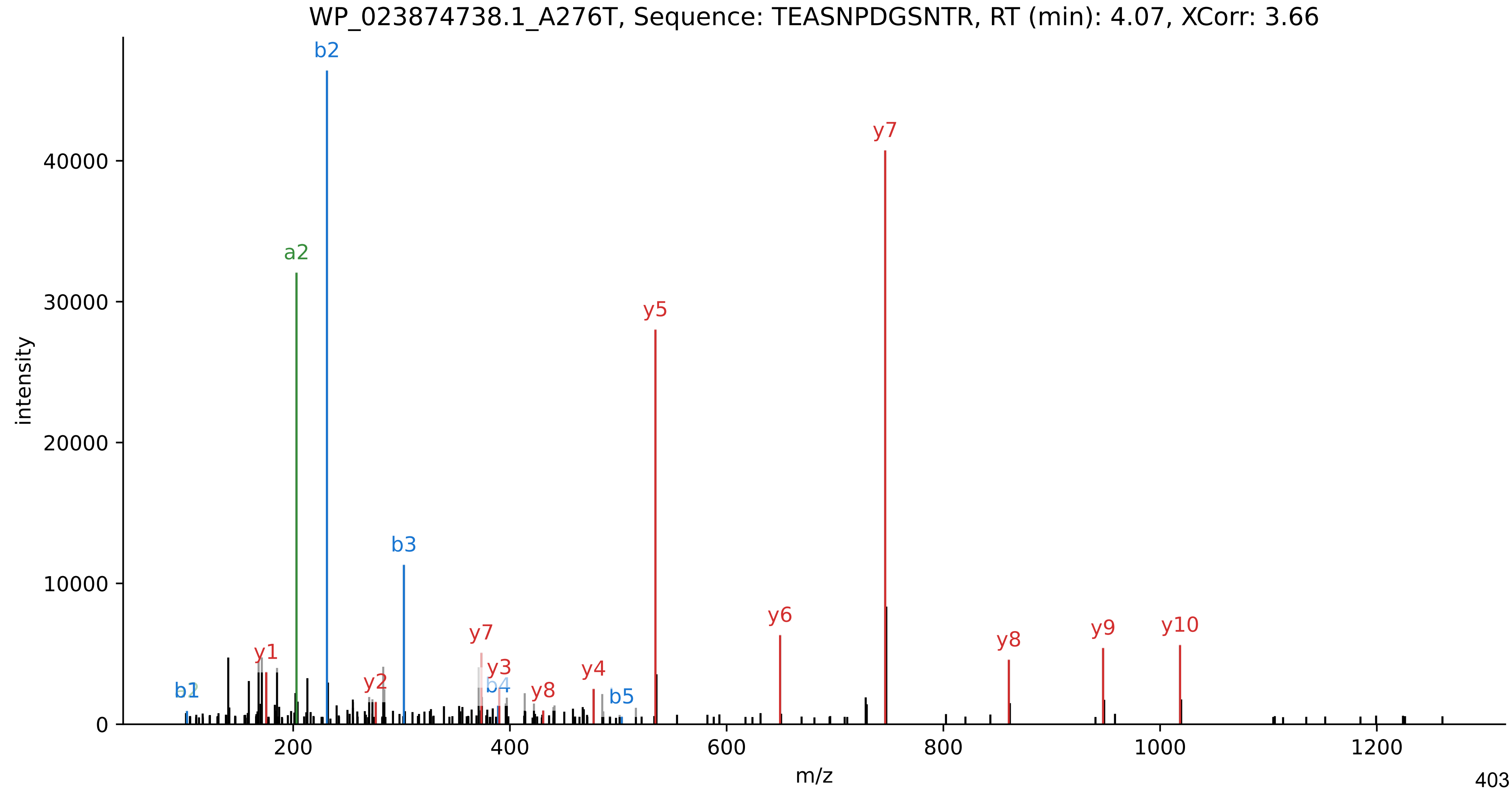
WP_023874738.1_A276T, Sequence: TEASNPDGSNTR, RT (min): 4.07, XCorr: 3.66
intensity
m/z
b2
a2
y7
y5
b3
y6
y10
y9
y8
y7
y1
y3
y4
y2
b4
y8
b5
b1
0
10000
20000
30000
40000
200
400
600
800
1000
1200

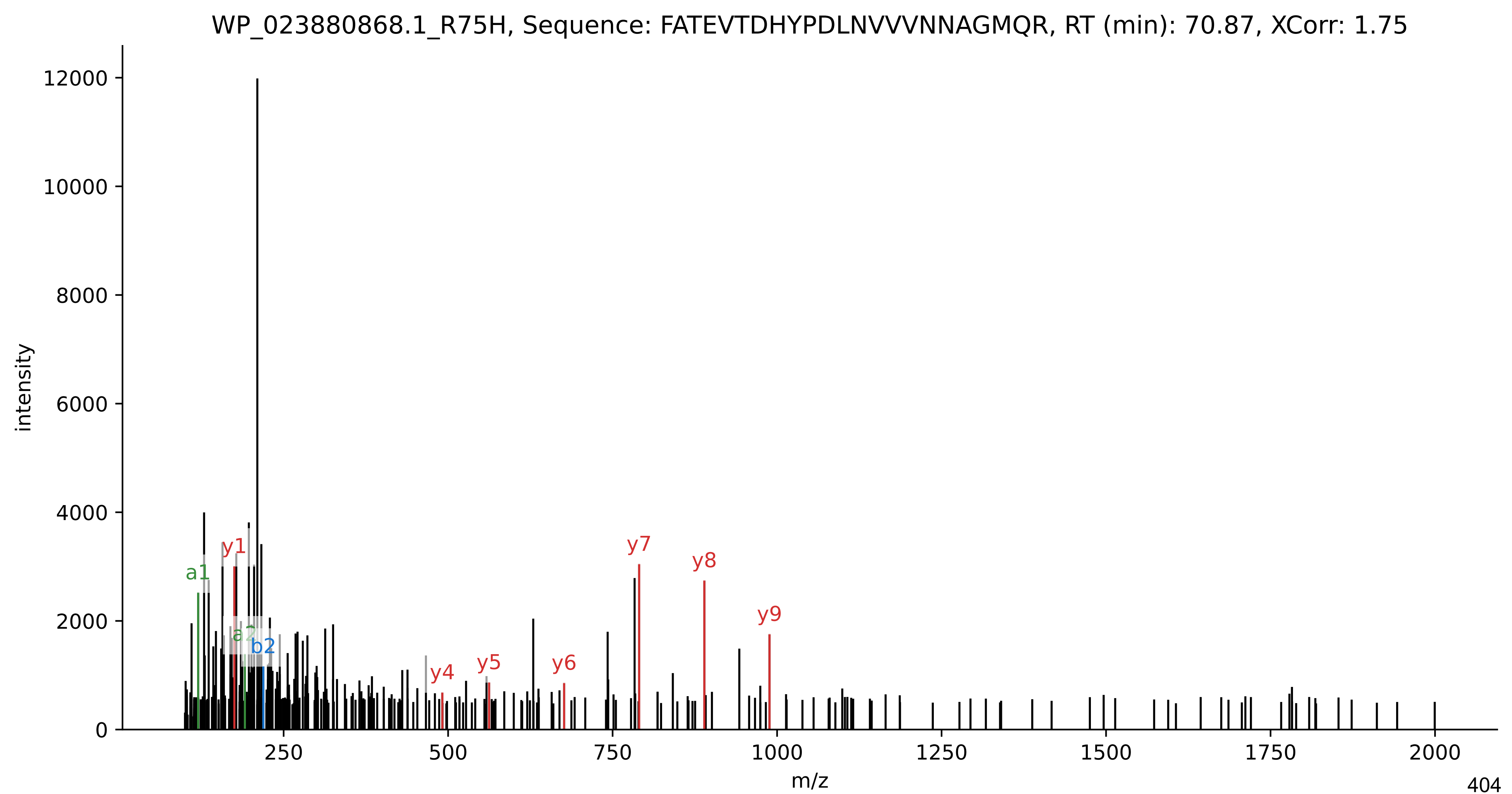

WP_023880868.1_R75H, Sequence: FATEVTDHYPDLNVVVNNAGMQR, RT (min): 70.87, XCorr: 1.75
intensity
m/z
0
2000
4000
6000
8000
10000
12000
250
500
750
1000
1250
1500
1750
2000
a1
a2
b2
y1
y4
y5
y6
y7
y8
y9

WP_023884191.1_V235D, Sequence: LASSPPDSEWTSGAYYAKR, RT (min): 46.78, XCorr: 2.94

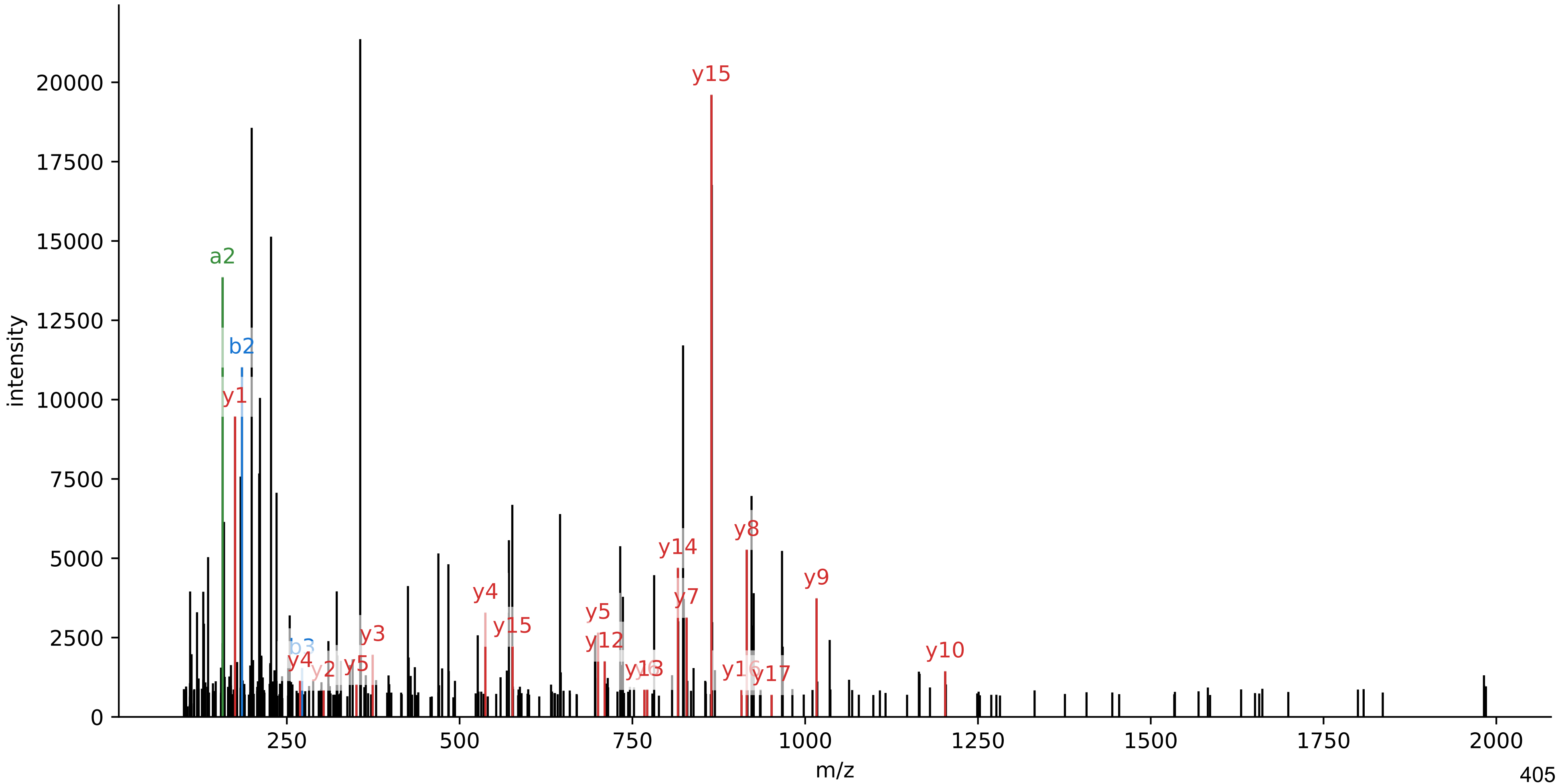

WP_023884191.1_V235D, Sequence: LASSPPDSEWTSGAYYAK, RT (min): 55.02, XCorr: 1.16

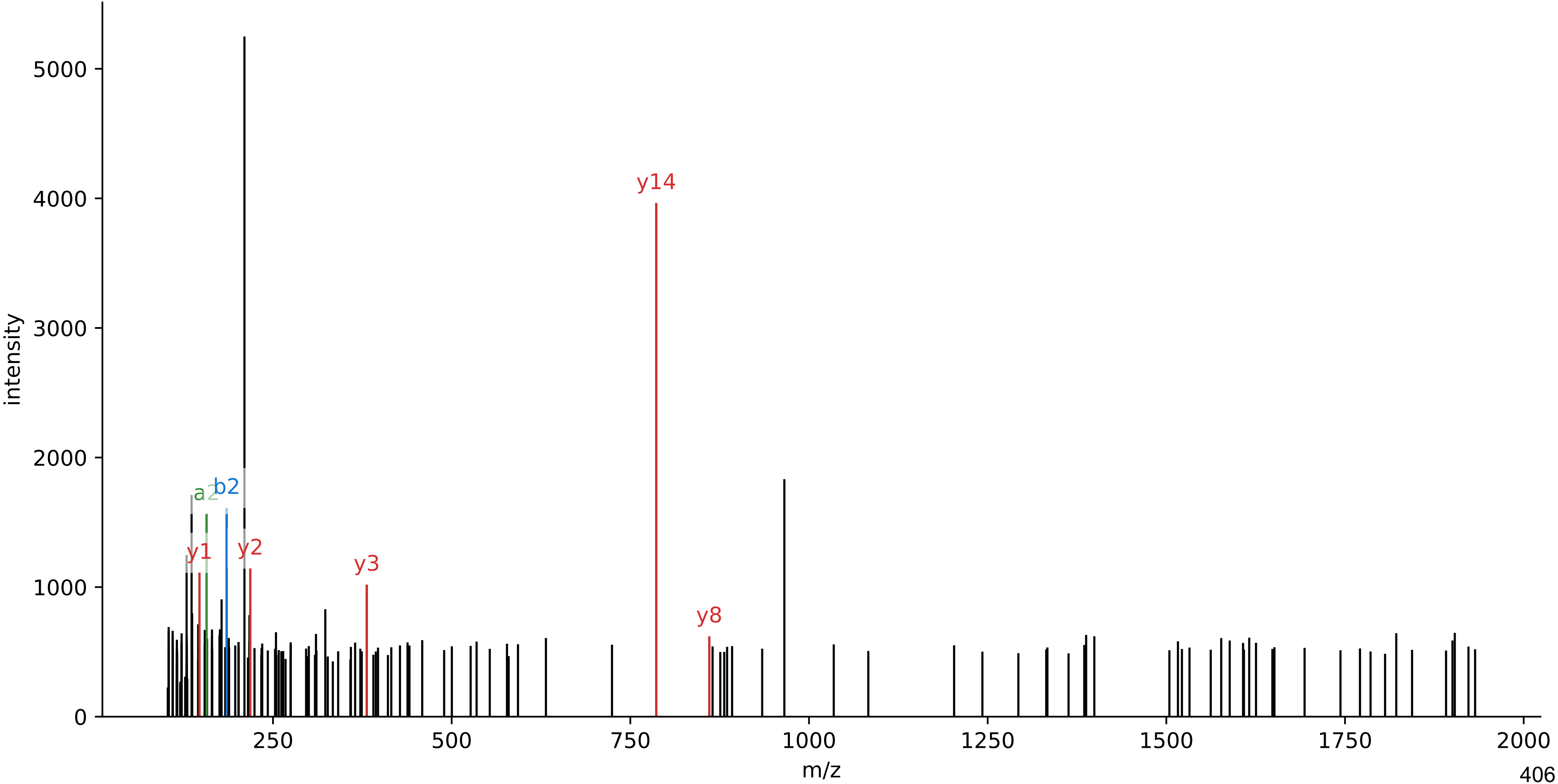

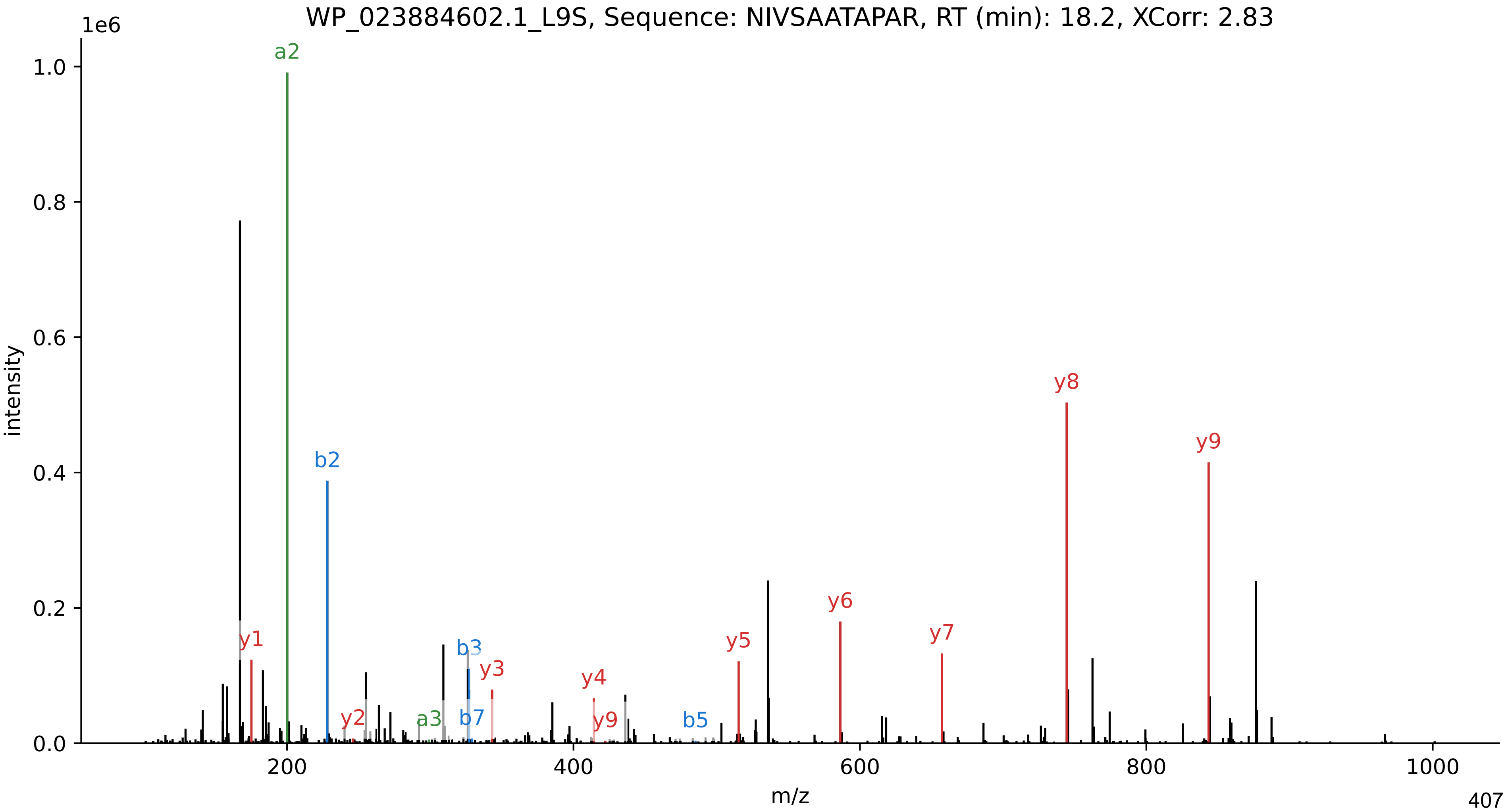
WP_023884602.1_L9S, Sequence: NIVSAATAPAR, RT (min): 18.2, XCorr: 2.83
1e6
intensity
m/z
a2
b2
y1
y2
a3
b3
b7
y3
y4
y9
b5
y5
y6
y7
y8
y9
0.0
0.2
0.4
0.6
0.8
1.0
200
400
600
800
1000

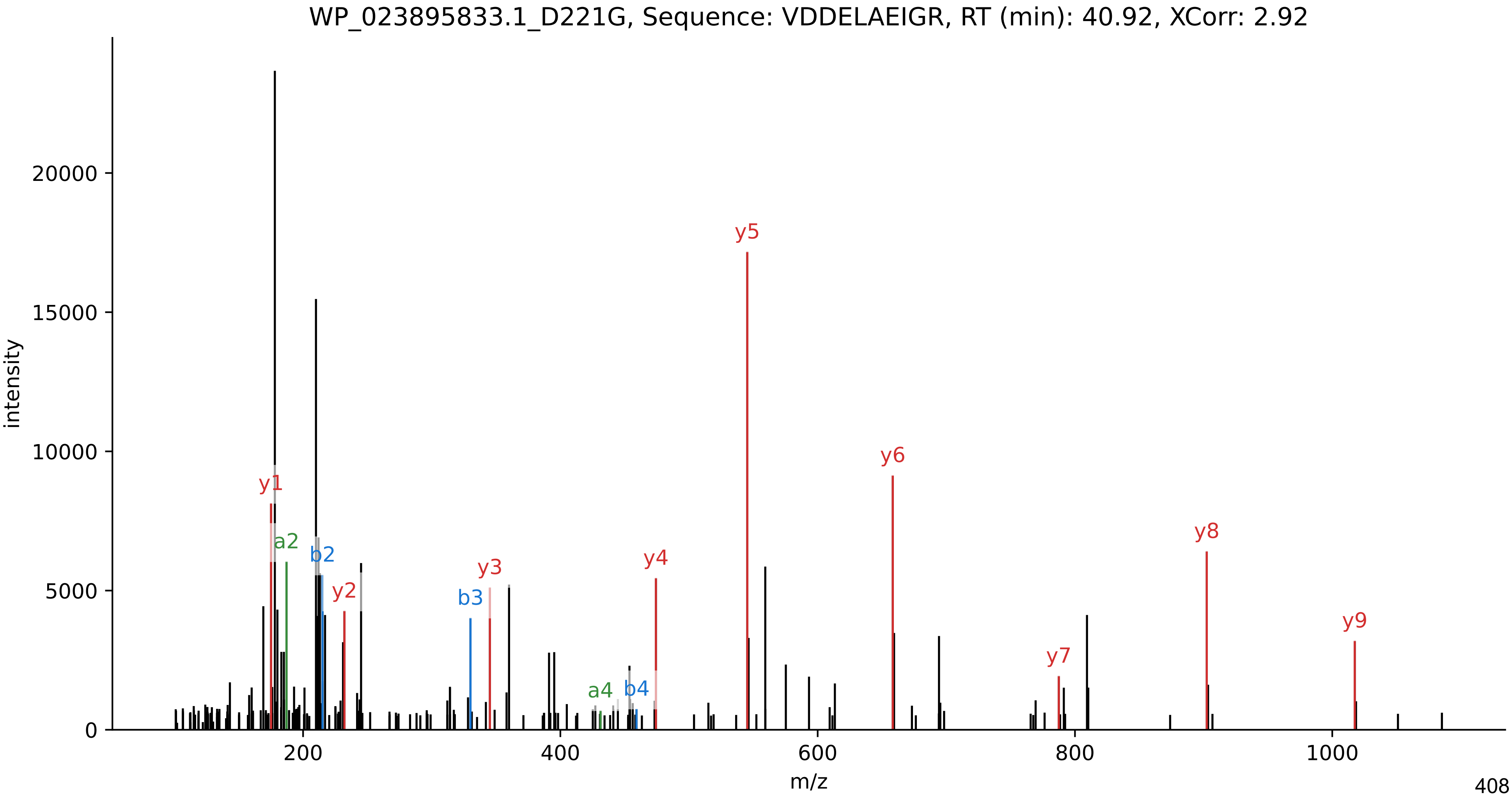

WP_023895833.1_D221G, Sequence: VDDELAEIGR, RT (min): 40.92, XCorr: 2.92
intensity
m/z
0
5000
10000
15000
20000
200
400
600
800
1000
y1
a2
b2
y2
b3
y3
a4
b4
y4
y5
y6
y7
y8
y9

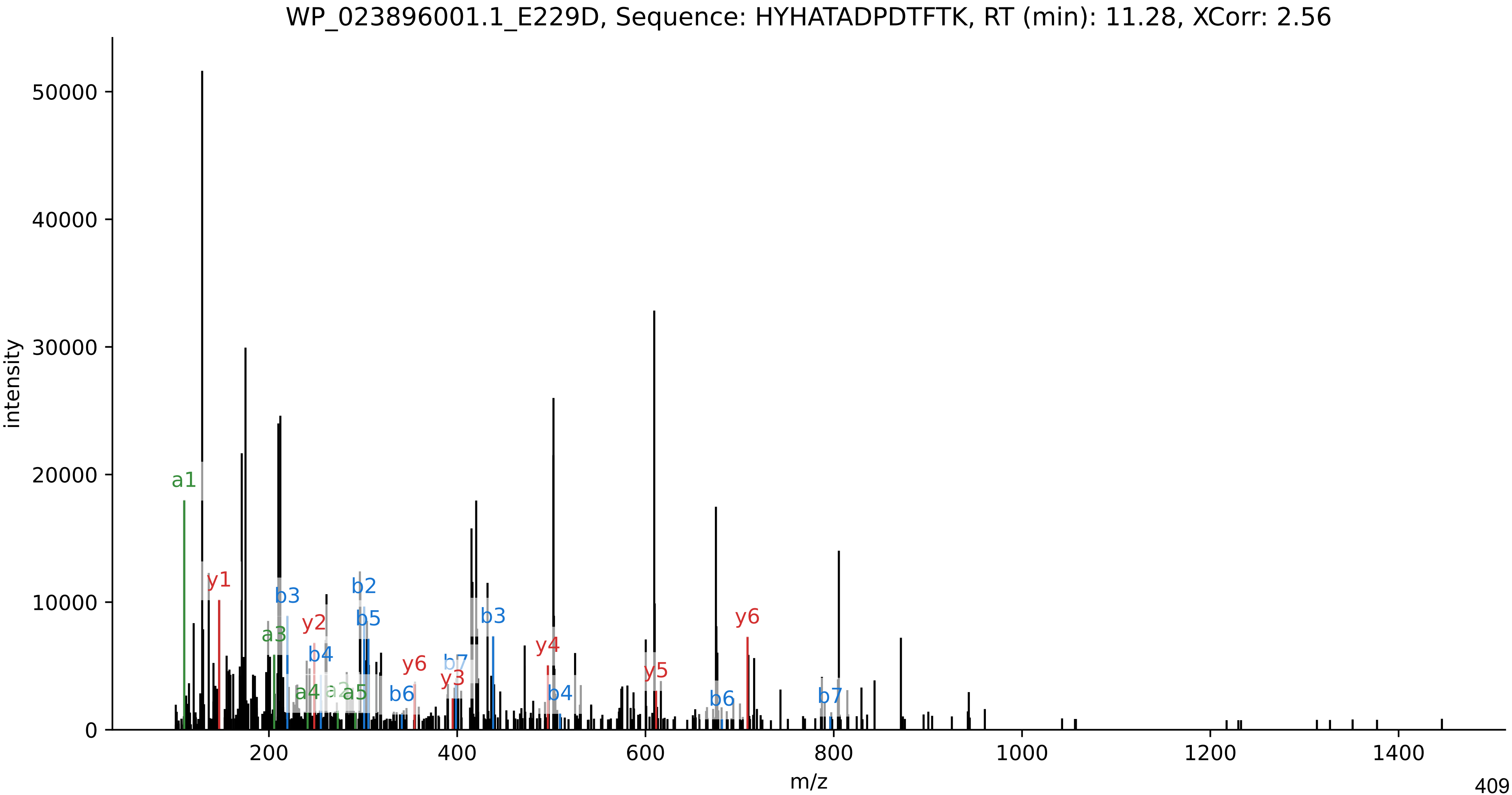
WP_023896001.1_E229D, Sequence: HYHATADPDTFTK, RT (min): 11.28, XCorr: 2.56
intensity
m/z
50000
40000
30000
20000
10000
0
200
400
600
800
1000
1200
1400
a1
y1
b3
b2
b5
a3
y2
b4
y6
b7
y3
b6
a4
a5
b3
y4
b4
y5
y6
b6
b7

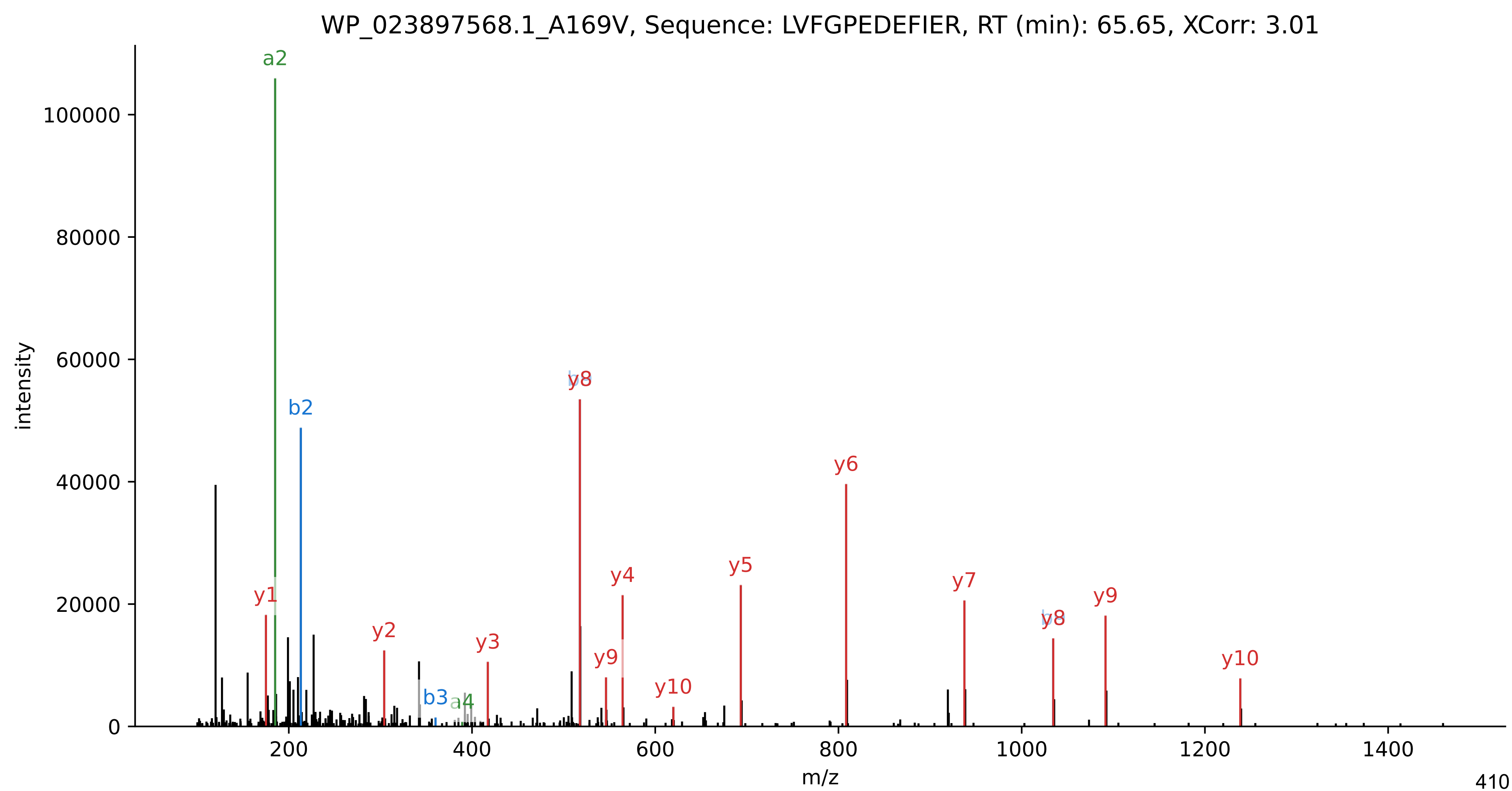
WP_023897568.1_A169V, Sequence: LVFGPEDEFIER, RT (min): 65.65, XCorr: 3.01
intensity
m/z
a2
b2
y8
y6
y5
y4
y7
y1
y8
y9
y2
y3
y9
y10
y10
b3
a4

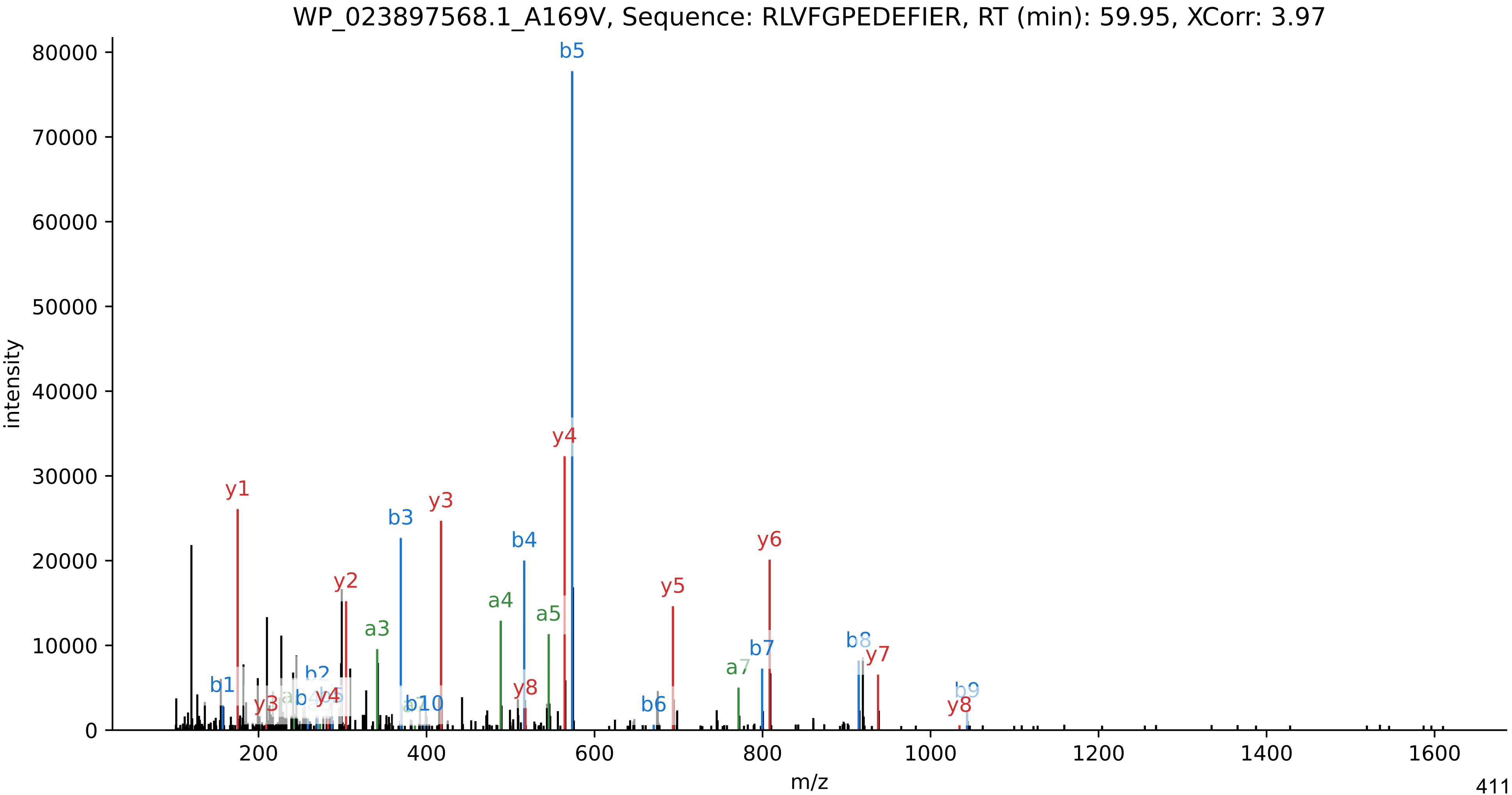
WP_023897568.1_A169V, Sequence: RLVFGPEDEFIER, RT (min): 59.95, XCorr: 3.97
intensity
m/z
0
10000
20000
30000
40000
50000
60000
70000
80000
200
400
600
800
1000
1200
1400
1600
b5
y4
y1
y3
b3
b4
y6
y2
y5
a4
a5
a3
b8
y7
b7
a7
b1
b2
y3
y4
y8
b10
b6
b9
y8

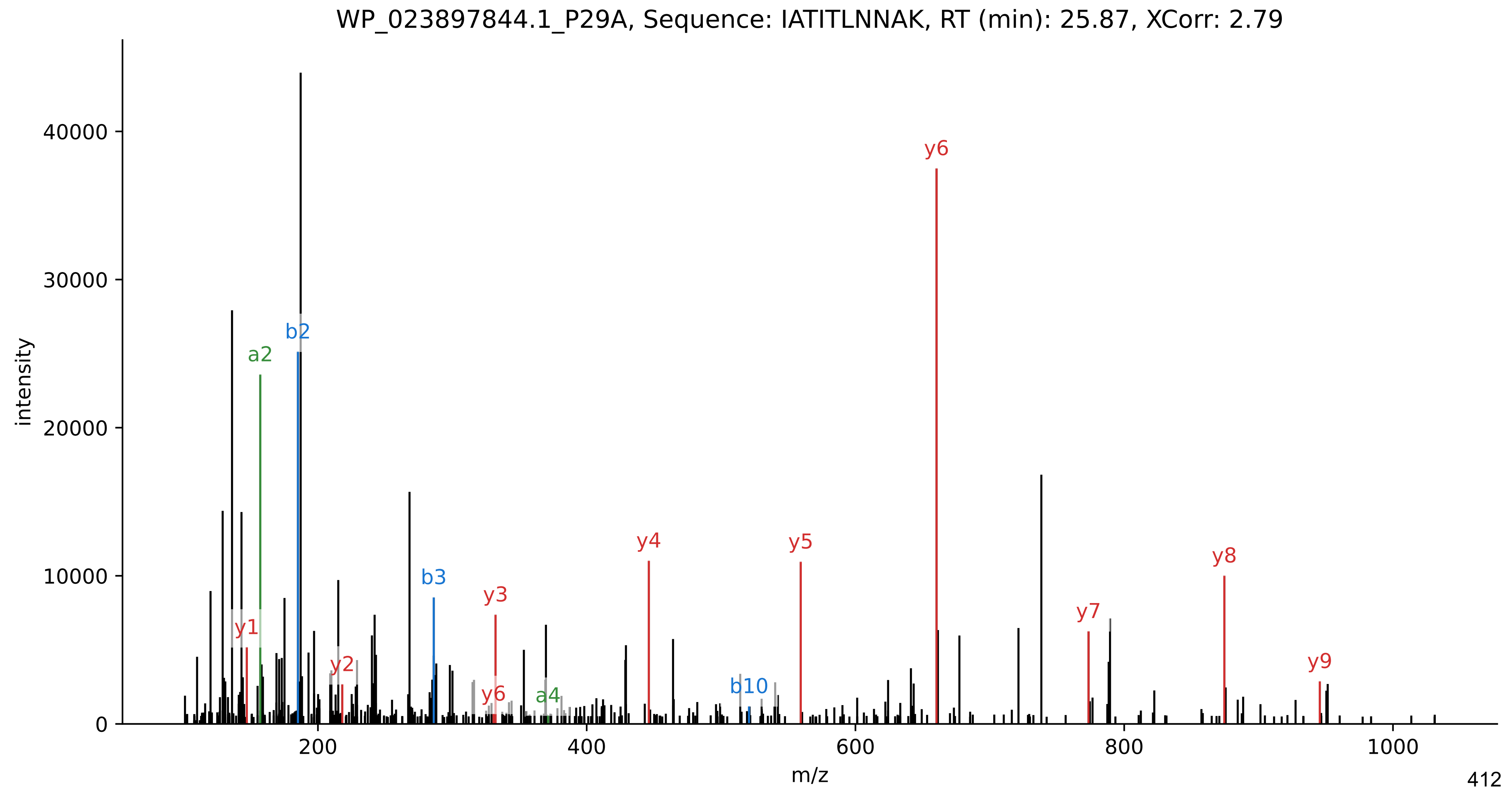
WP_023897844.1_P29A, Sequence: IATITLNNAK, RT (min): 25.87, XCorr: 2.79
intensity
m/z
y1
a2
b2
y2
y6
a4
b3
y3
y4
b10
y5
y6
y7
y8
y9

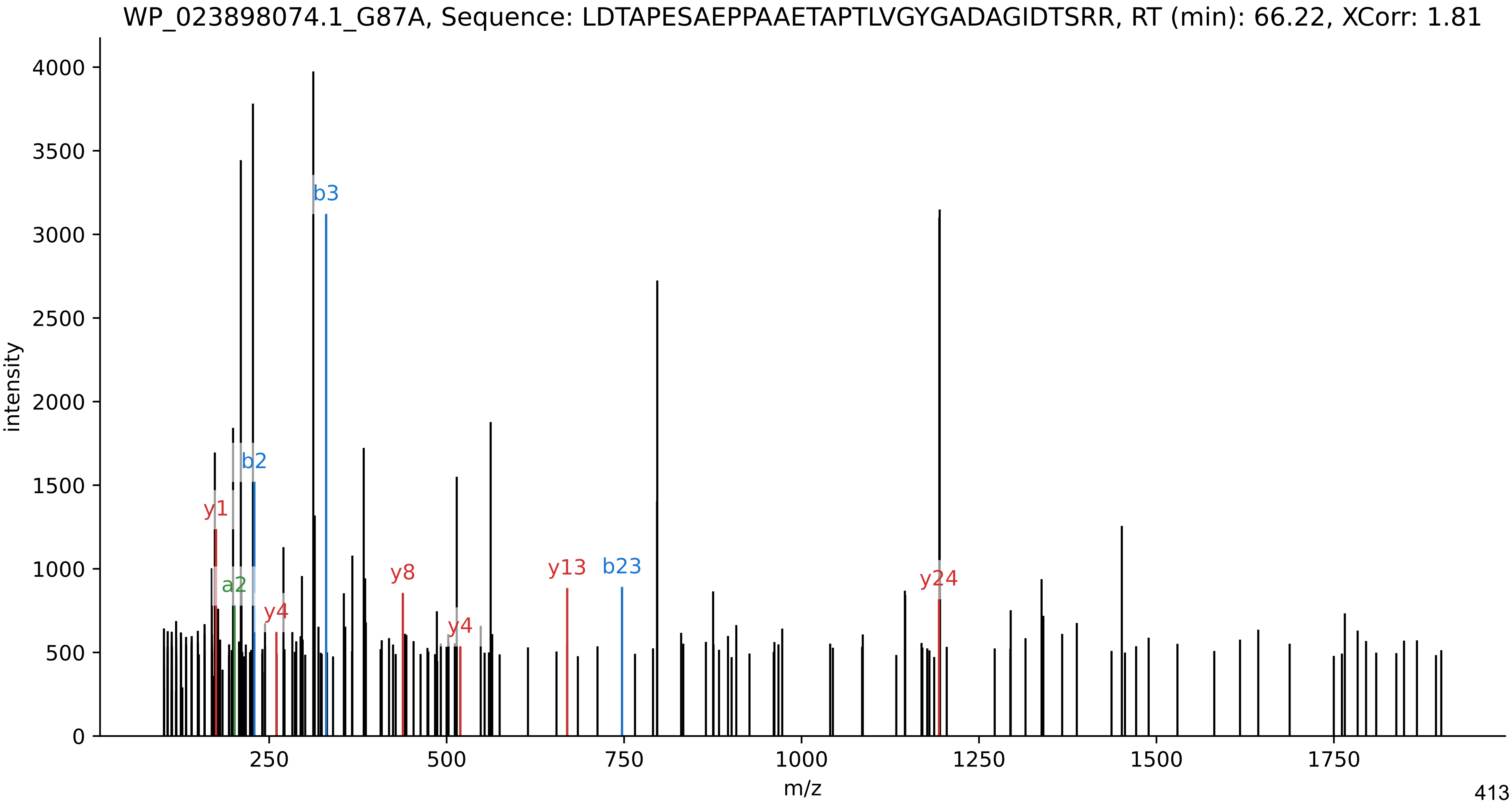

WP_023898074.1_G87A, Sequence: LDTAPESAEPPAAETAPTLVGYGADAGIDTSRR, RT (min): 66.22, XCorr: 1.81
intensity
m/z
y1
a2
b2
y4
b3
y8
y4
y13
b23
y24
0
500
1000
1500
2000
2500
3000
3500
4000
250
500
750
1000
1250
1500
1750

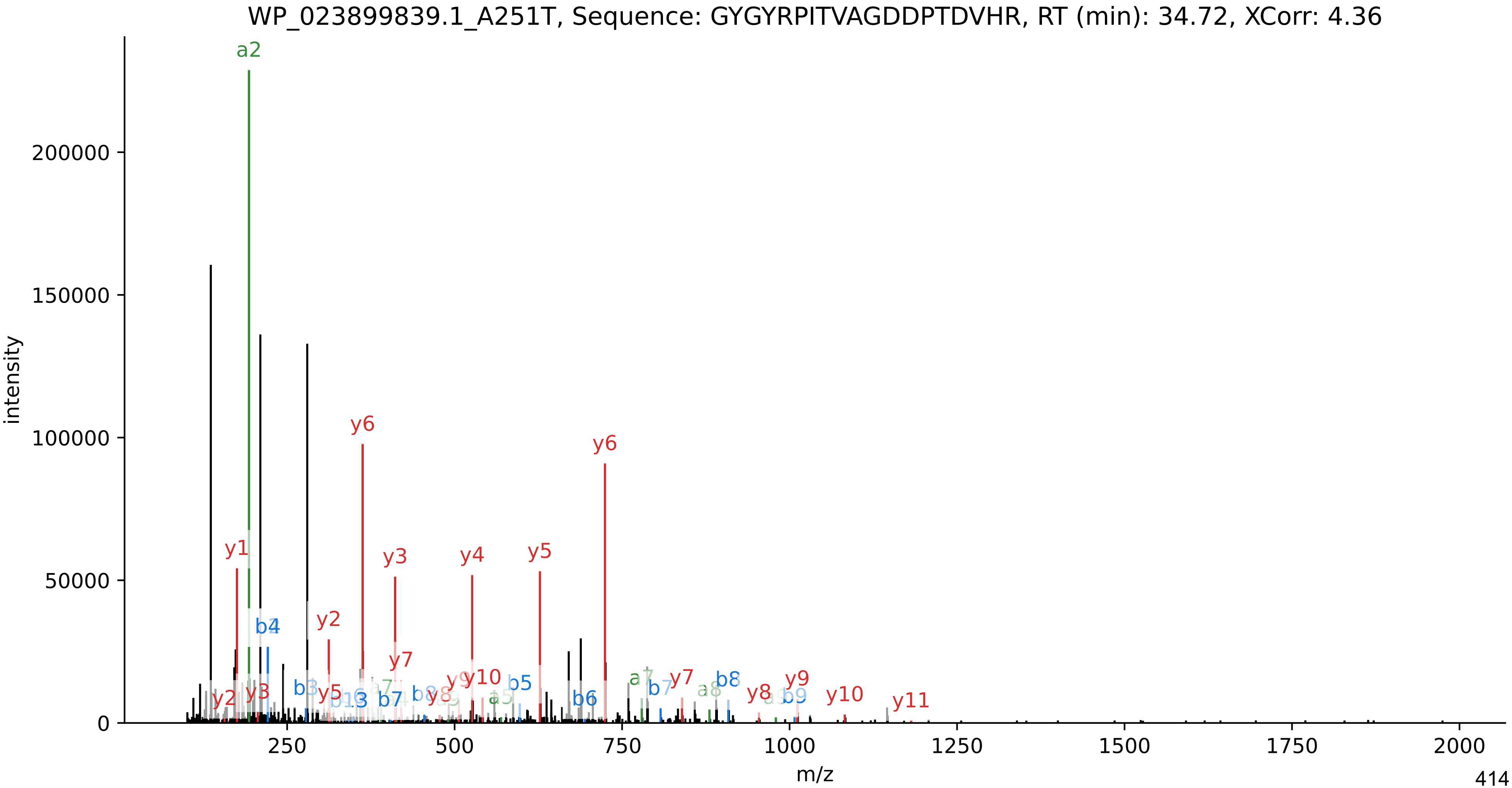

WP_023899839.1_A251T, Sequence: GYGYRPITVAGDDPTDVHR, RT (min): 34.72, XCorr: 4.36
intensity
m/z
a2
y6
y6
y1
y3
y4
y5
y2
b4
y7
y10
b5
a7
y7
b8
a8
b6
y8
y9
y10
y11
b7
b9

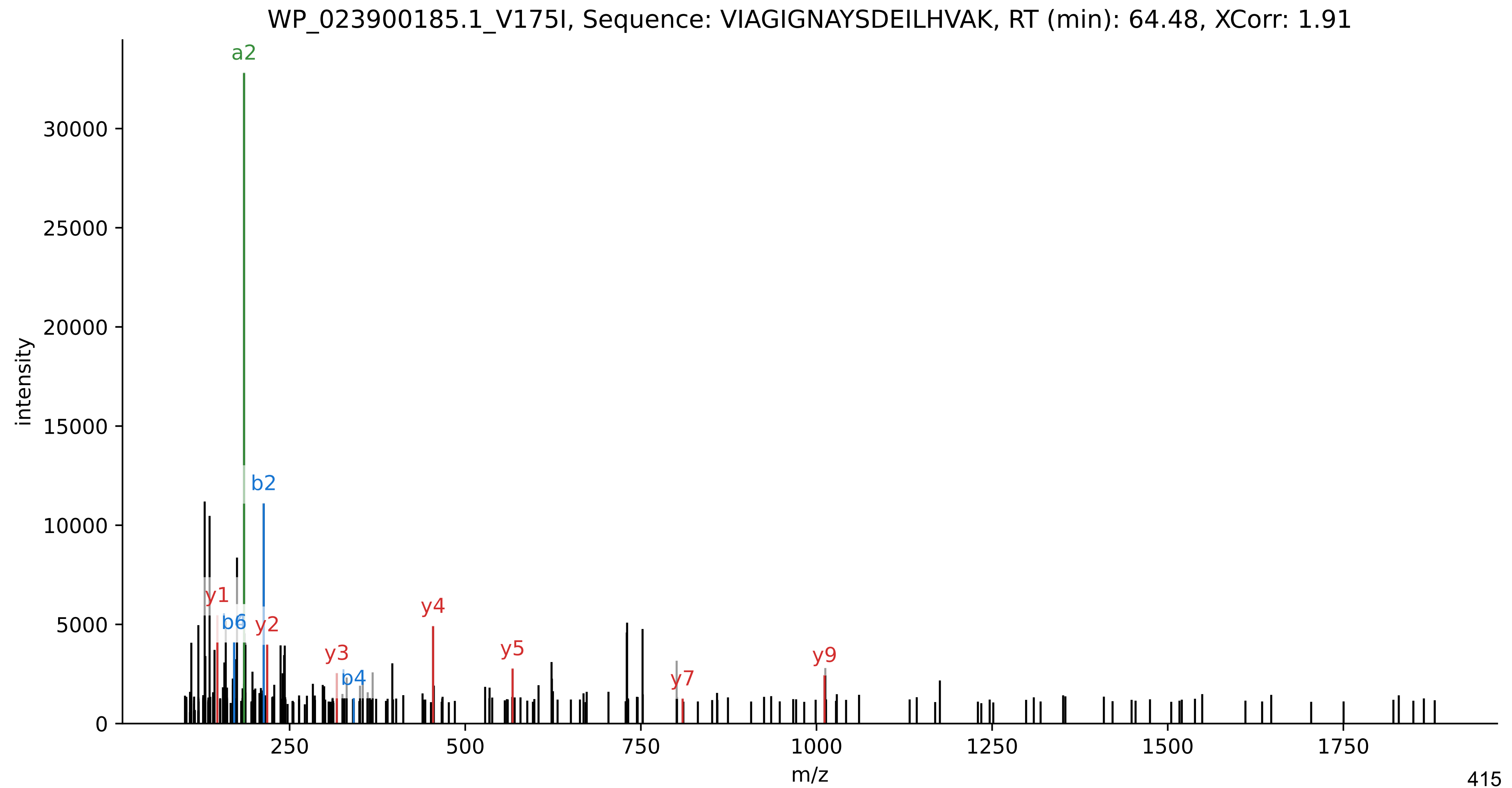

WP_023900185.1_V175I, Sequence: VIAGIGNAYSDEILHVAK, RT (min): 64.48, XCorr: 1.91
a2
b2
y1
b6
y2
y4
y3
b4
y5
y7
y9
intensity
m/z

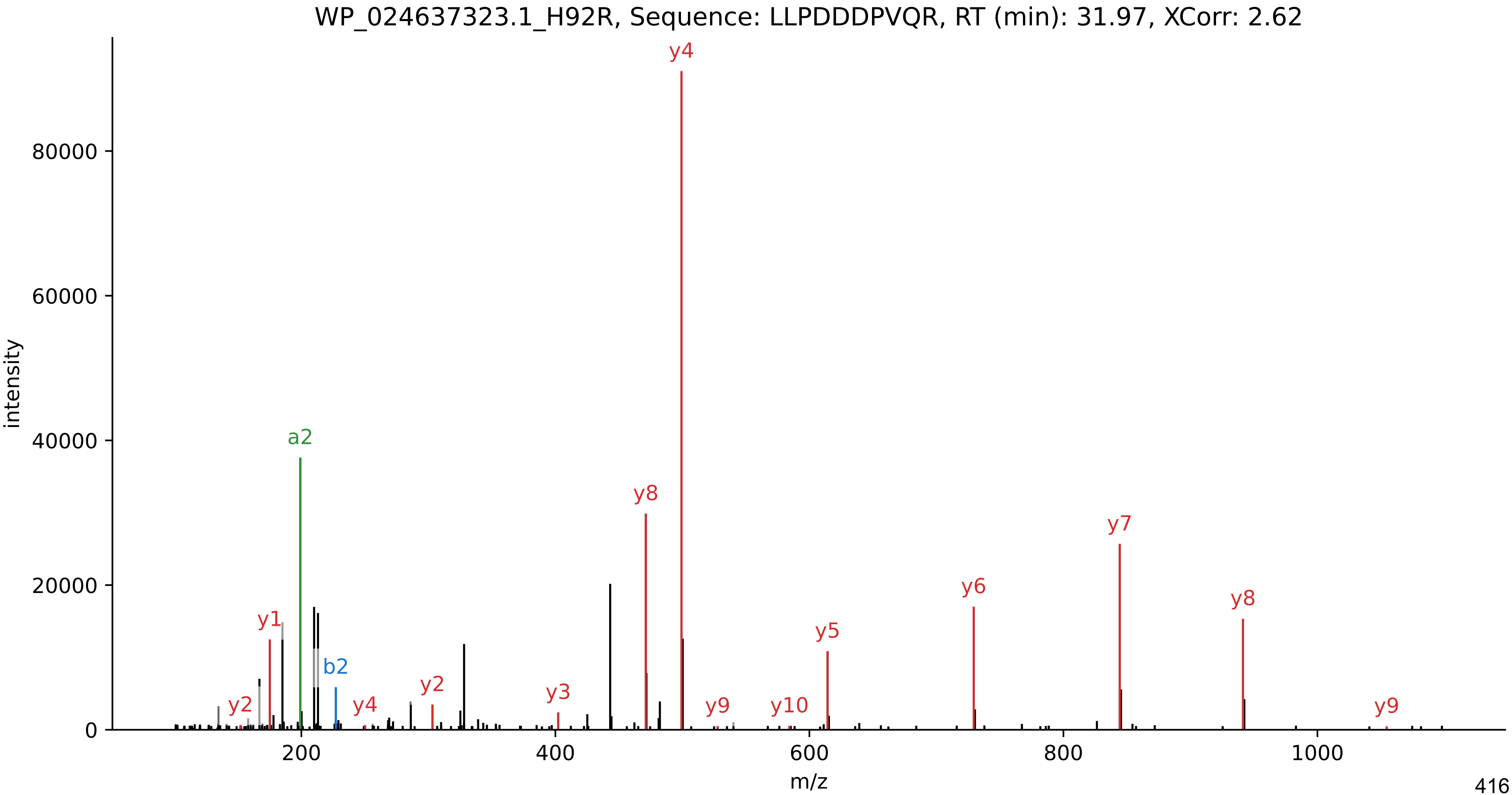
WP_024637323.1_H92R, Sequence: LLPDDDPVQR, RT (min): 31.97, XCorr: 2.62
intensity
m/z
0
20000
40000
60000
80000
200
400
600
800
1000
y4
a2
y8
y7
y6
y8
y1
b2
y5
y2
y2
y4
y3
y9
y10
y9

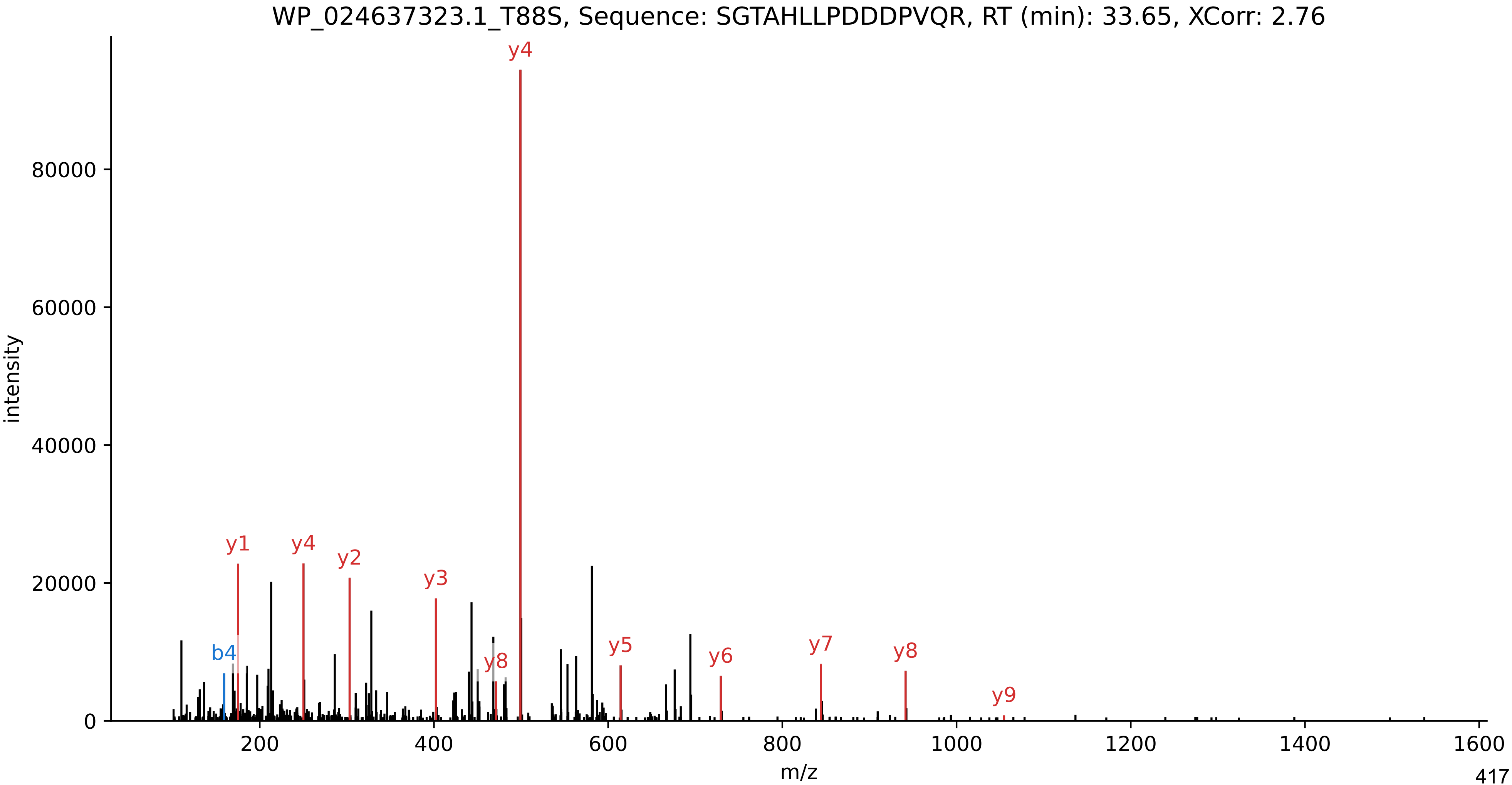

WP_024637323.1_T88S, Sequence: SGTAHLLPDDDPVQR, RT (min): 33.65, XCorr: 2.76
intensity
m/z
y4
y1
y4
y2
y3
b4
y8
y5
y6
y7
y8
y9
0
20000
40000
60000
80000
200
400
600
800
1000
1200
1400
1600

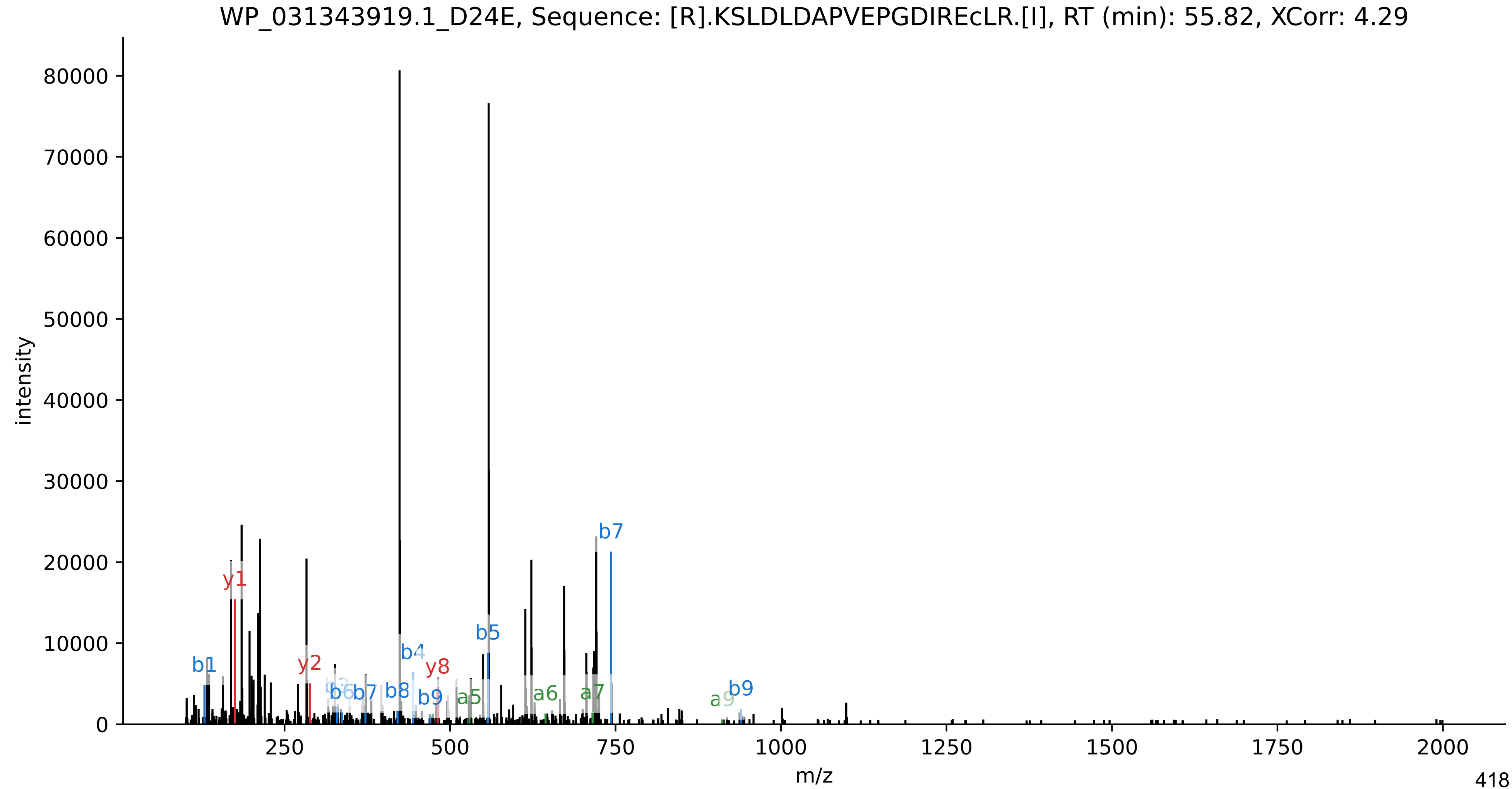

WP_031343919.1_D24E, Sequence: [R].KSLDLDAPVEPGDIREcLR.[I], RT (min): 55.82, XCorr: 4.29
intensity
m/z
b1
y1
y2
b6
b7
b8
b4
y8
b9
b5
a5
a6
a7
b7
a9
b9

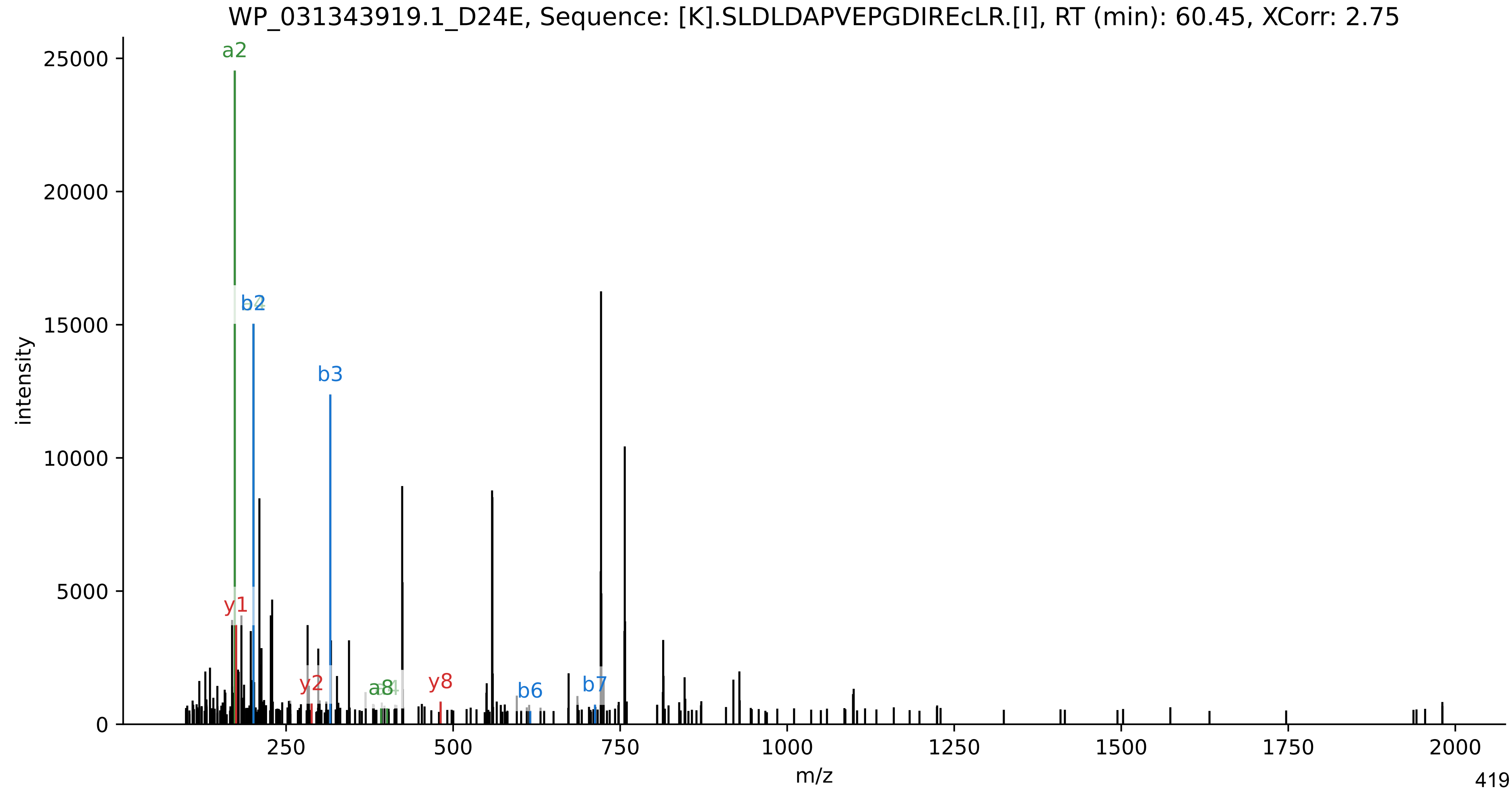

WP_031343919.1_D24E, Sequence: [K].SLDLDAPVEPGDIREcLR.[I], RT (min): 60.45, XCorr: 2.75
a2
b2
b3
y1
y2
a8
y8
b6
b7
intensity
m/z
0
5000
10000
15000
20000
25000
250
500
750
1000
1250
1500
1750
2000

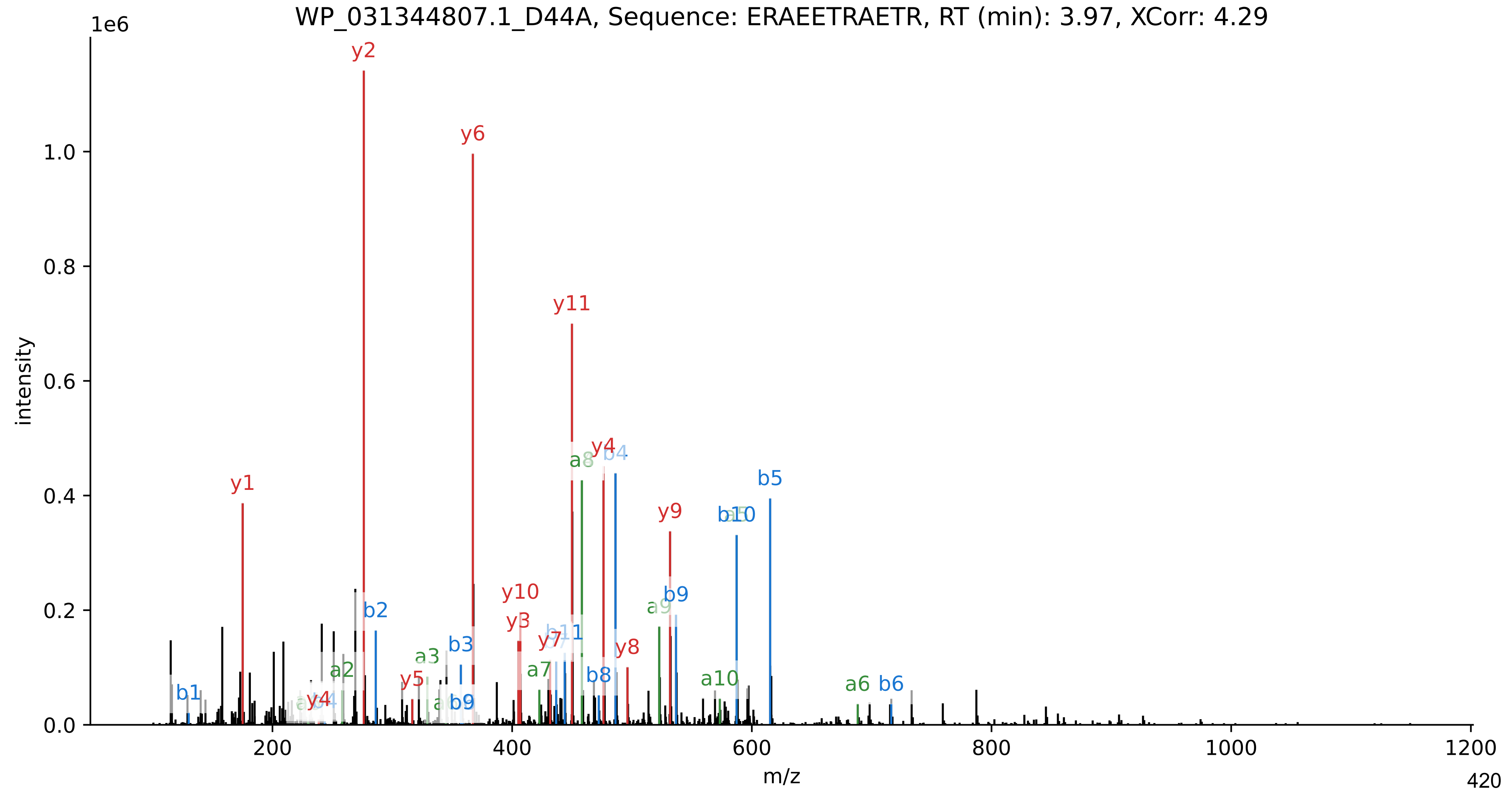

WP_031344807.1_D44A, Sequence: ERAEETRAETR, RT (min): 3.97, XCorr: 4.29
1e6
intensity
m/z
y2
y6
y11
y4
a8
b4
y1
b5
y9
b10
y10
b2
a9
b9
y3
b11
y7
y8
b3
a3
a2
a7
b8
a10
y5
a6
b6
b1
y4
b9

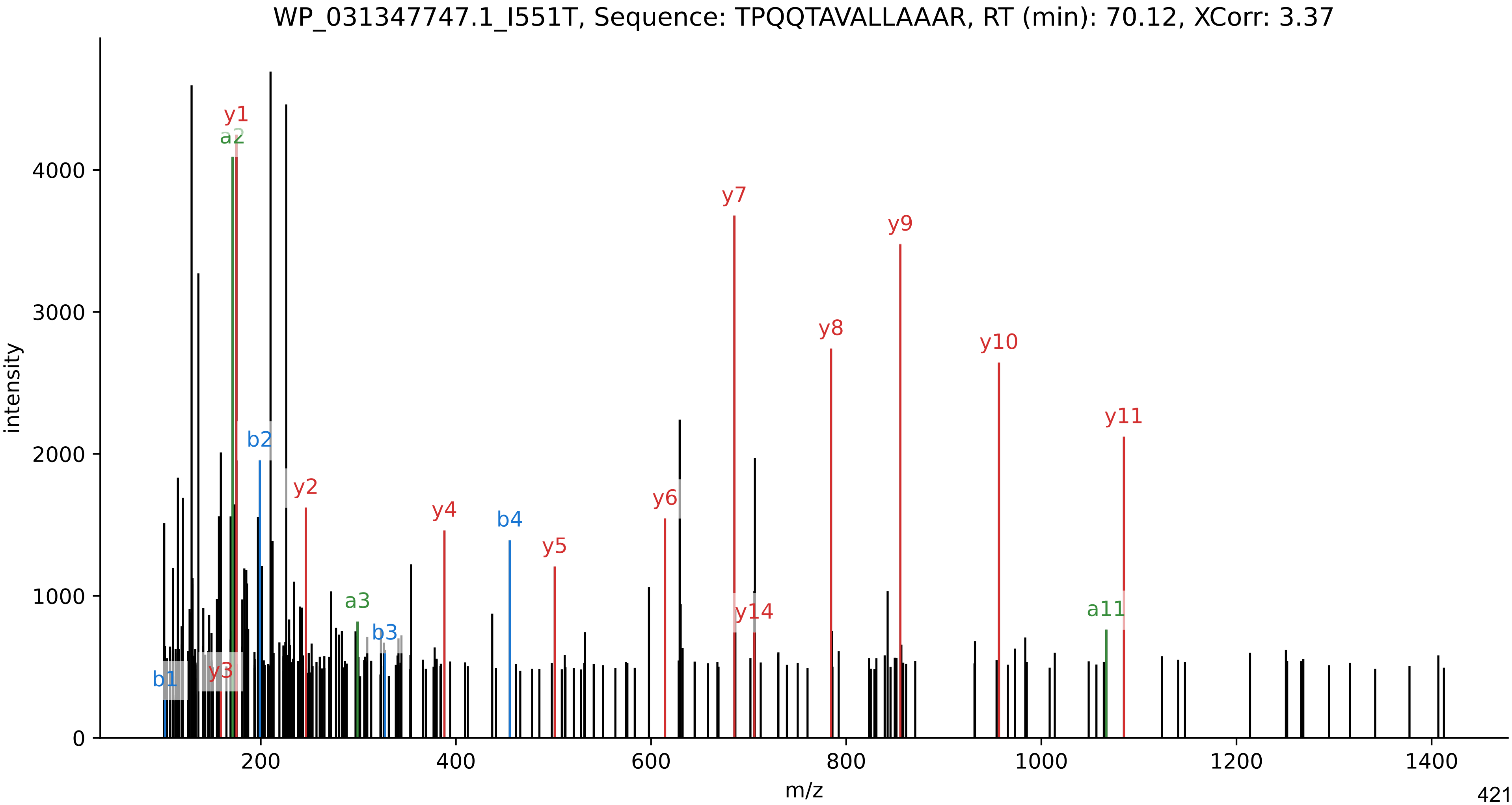

WP_031347747.1_I551T, Sequence: TPQQTAVALLAAAR, RT (min): 70.12, XCorr: 3.37
intensity
m/z
y1
a2
b2
y2
b1
y3
a3
b3
y4
b4
y5
y6
y7
y14
y8
y9
y10
a11
y11

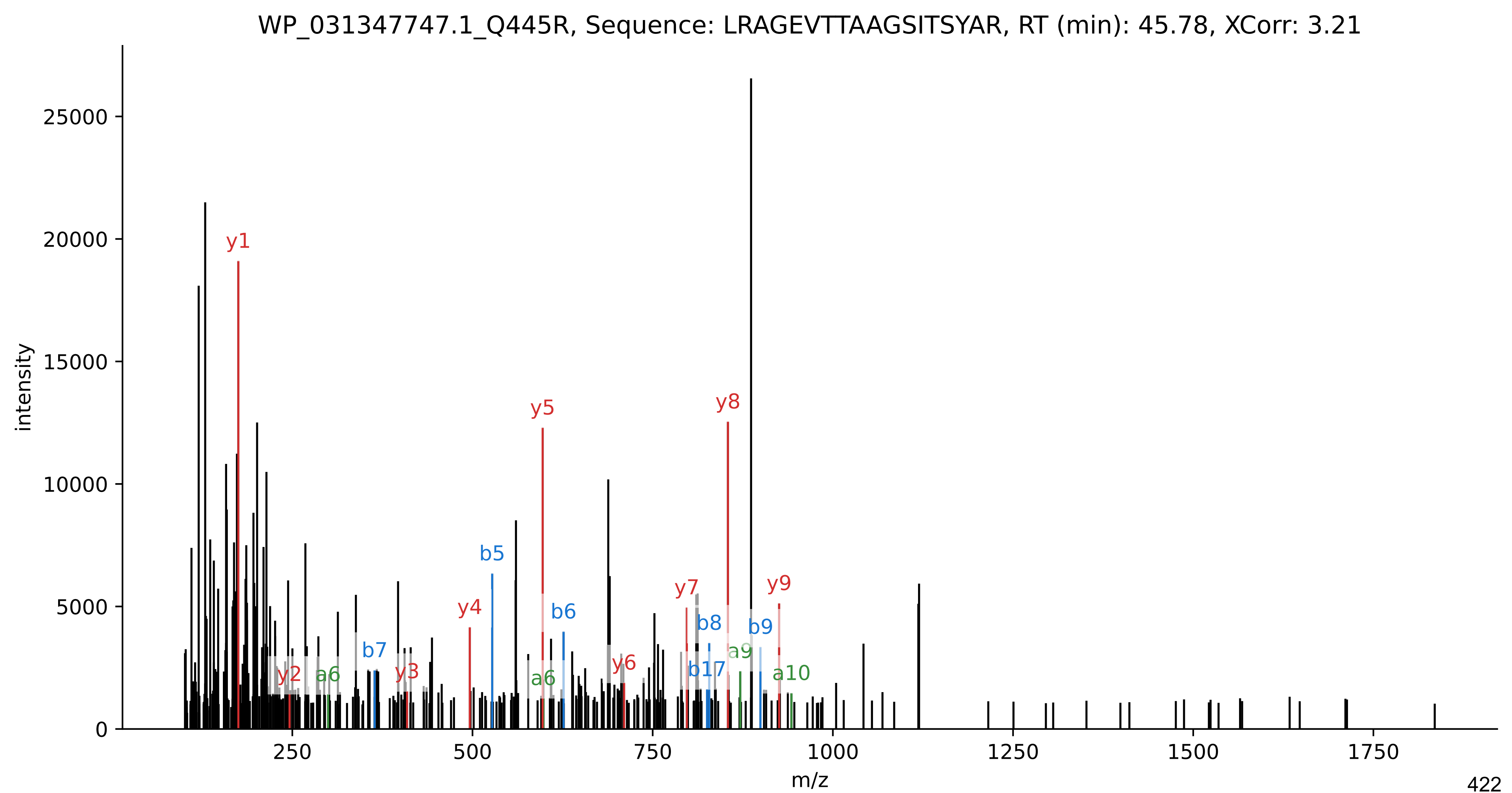
WP_031347747.1_Q445R, Sequence: LRAGEVTTAAGSITSYAR, RT (min): 45.78, XCorr: 3.21
intensity
m/z
y1
y2
a6
b7
y3
y4
b5
y5
a6
b6
y6
y7
b8
b17
y8
a9
b9
y9
a10
0
5000
10000
15000
20000
25000
250
500
750
1000
1250
1500
1750

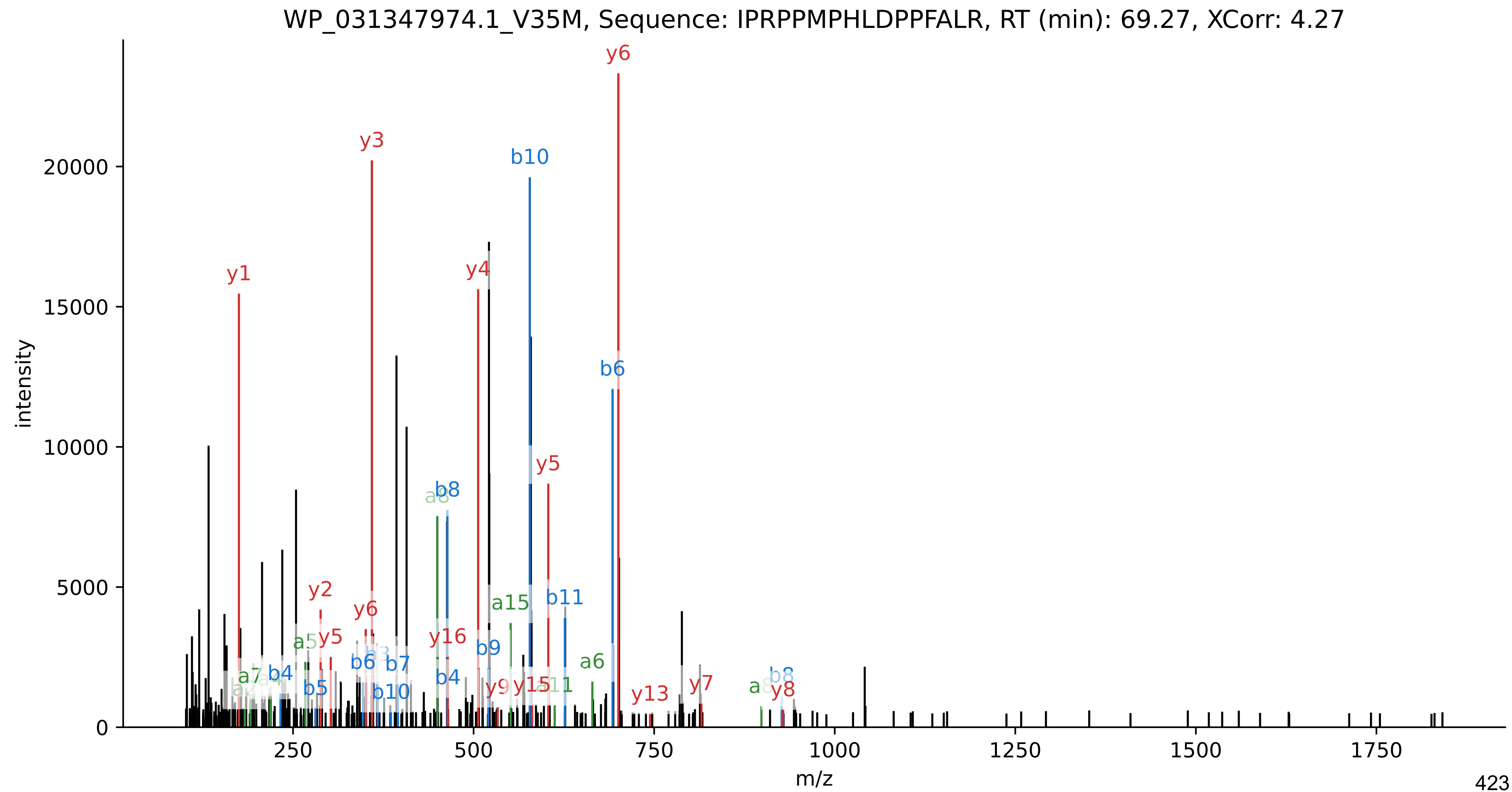
WP_031347974.1_V35M, Sequence: IPRPPMPHLDPPFALR, RT (min): 69.27, XCorr: 4.27
intensity
m/z
y6
y3
b10
y1
y4
b6
y5
b8
y2
y6
a15
b11
y16
b9
a6
y13
y7
y8
b4
b5
b7
b10
y9
y15
0
5000
10000
15000
20000
250
500
750
1000
1250
1500
1750

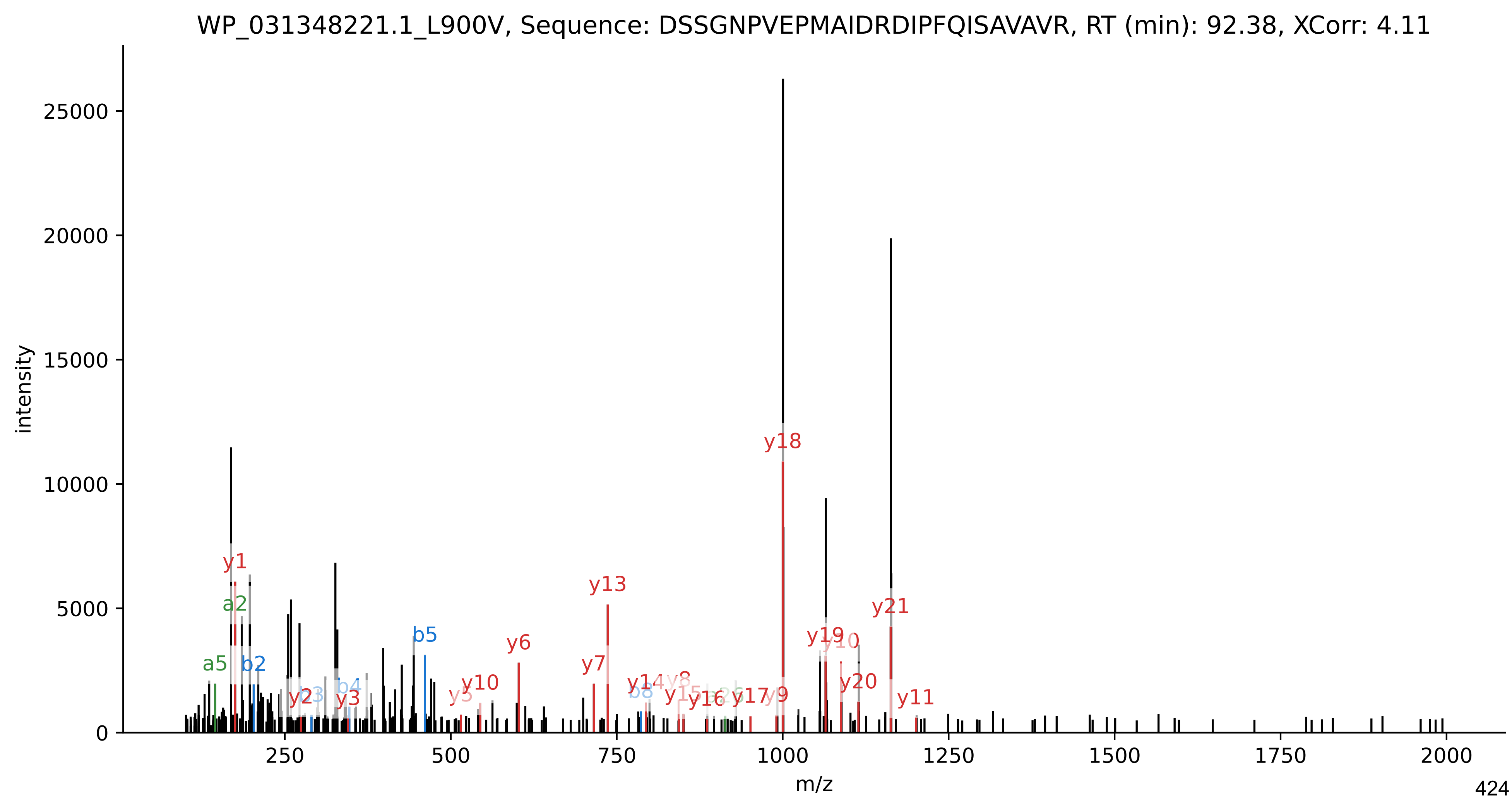

WP_031348221.1_L900V, Sequence: DSSGNPVEPMAIDRDIPFQISAVAVR, RT (min): 92.38, XCorr: 4.11
intensity
m/z
y18
y1
y13
y21
a2
b5
y6
y19
y20
a5
b2
y7
y10
y14
y8
y2
y3
b3
b4
y15
y16
y17
y9
y11
b8

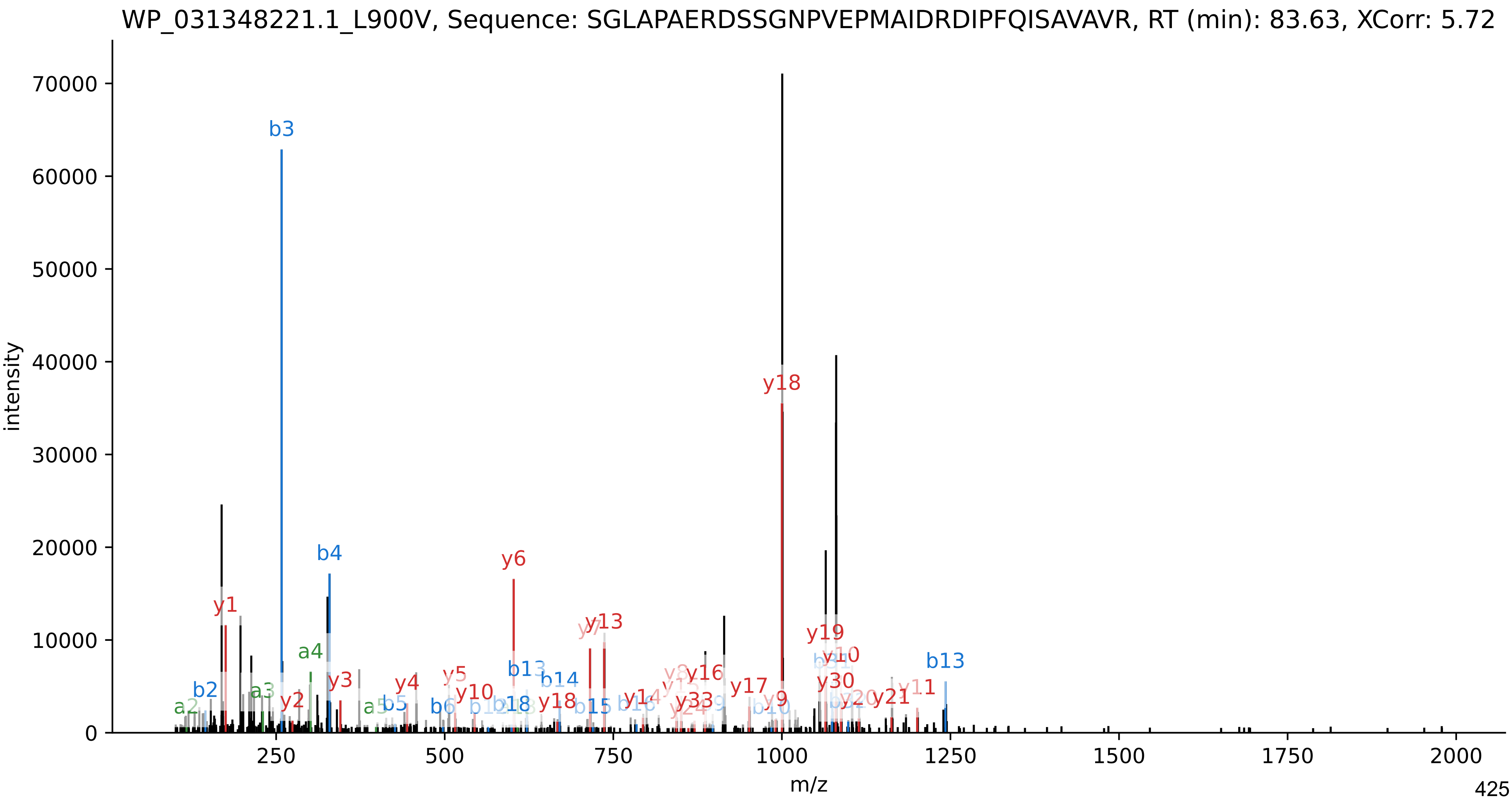
WP_031348221.1_L900V, Sequence: SGLAPAERDSSGNPVEPMAIDRDIPFQISAVAVR, RT (min): 83.63, XCorr: 5.72
intensity
m/z
0
10000
20000
30000
40000
50000
60000
70000
250
500
750
1000
1250
1500
1750
2000
b3
b4
y6
y1
y18
y19
y13
a4
b2
a3
a2
y2
y3
y4
y5
y10
b13
b14
b6
b5
y16
y17
y9
y30
y21
y11
b15
y33
b13

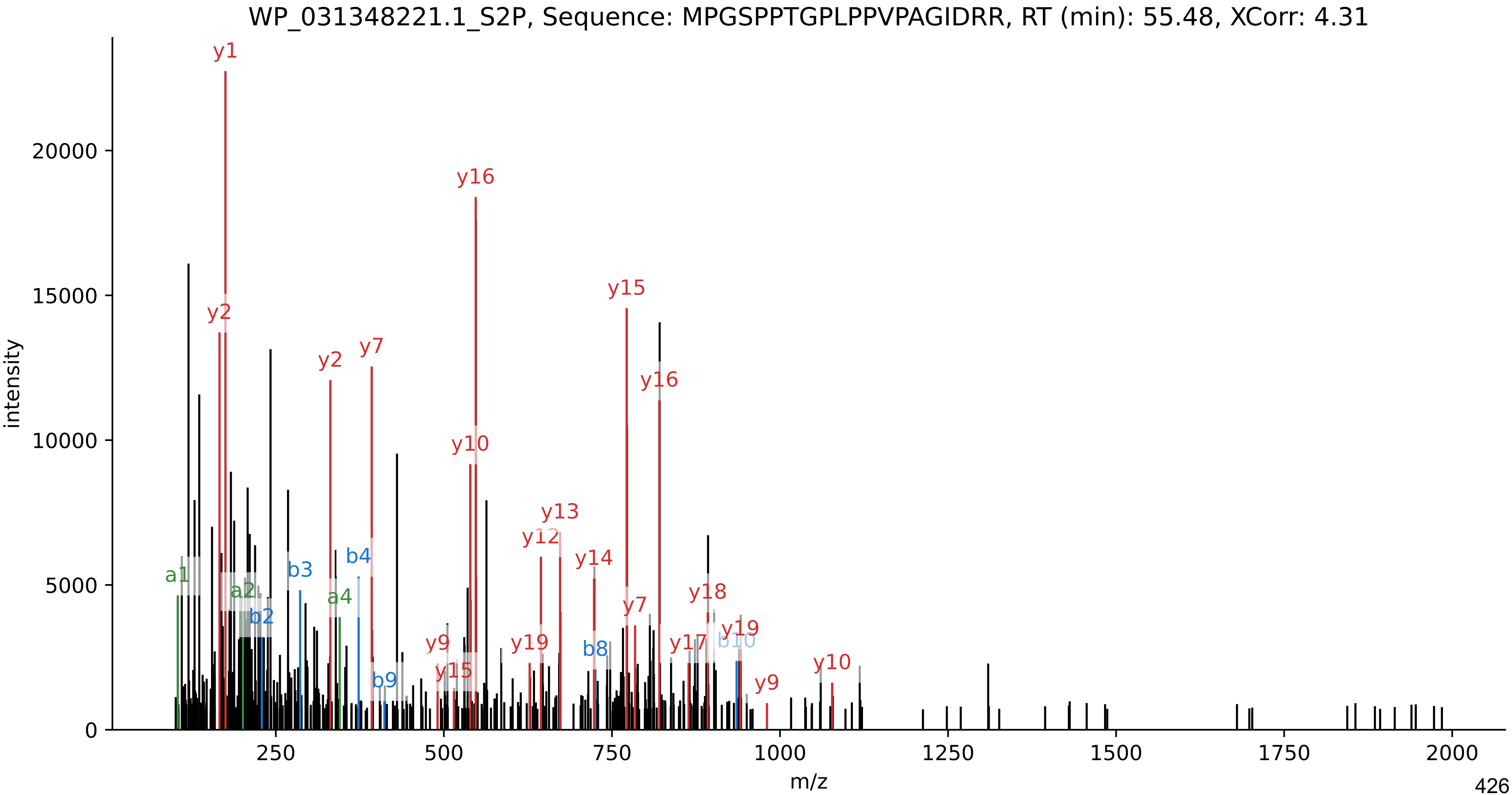

WP_031348221.1_S2P, Sequence: MPGSPPTGPLPPVPAGIDRR, RT (min): 55.48, XCorr: 4.31
intensity
m/z
20000
15000
10000
5000
0
250
500
750
1000
1250
1500
1750
2000
y1
y16
y2
y15
y2
y7
y16
y10
y13
y12
y14
a1
b4
b3
a2
a4
b2
y7
y18
y19
y9
y19
b10
y17
b8
y15
y10
b9
y9

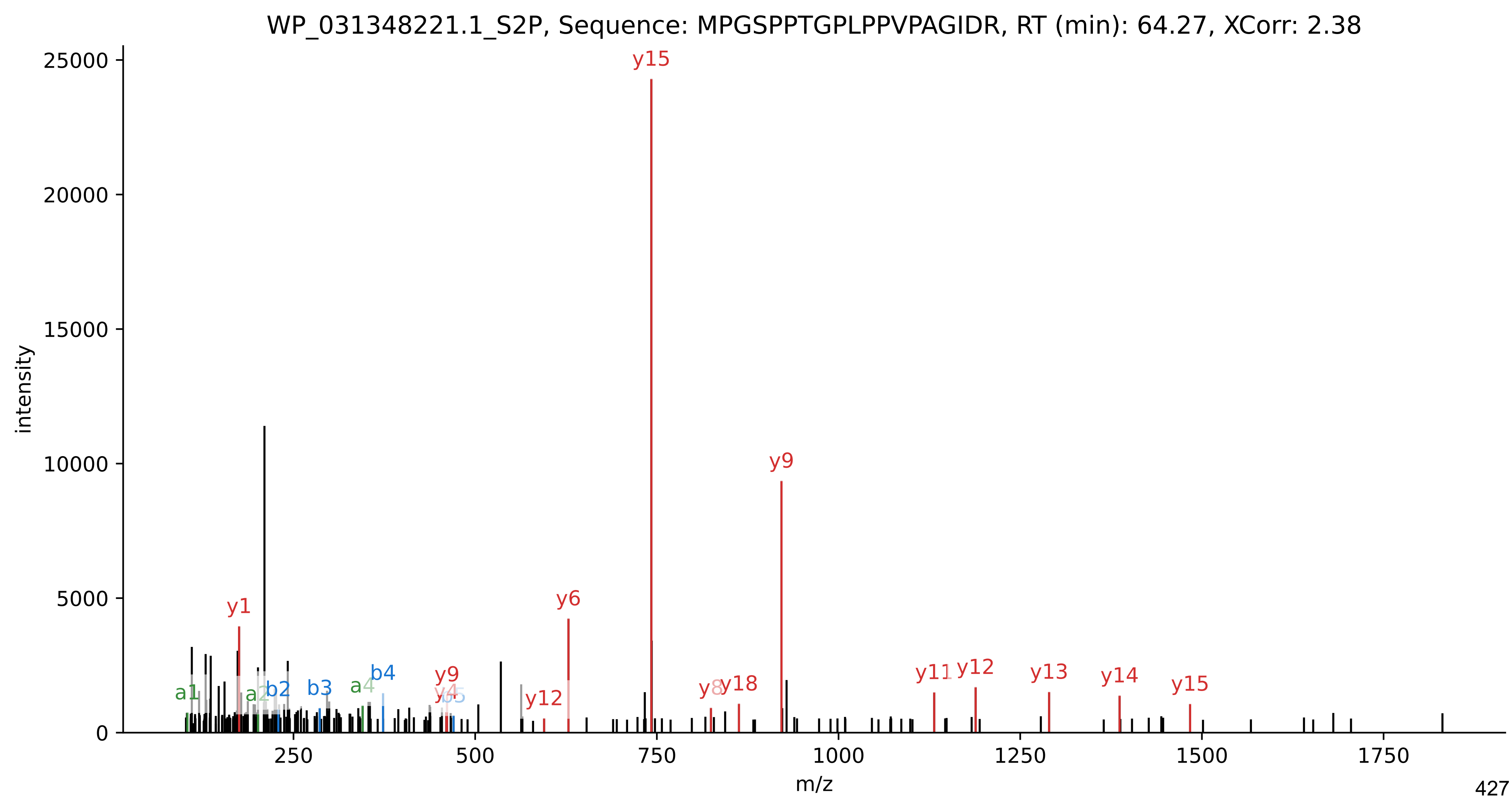
WP_031348221.1_S2P, Sequence: MPGSPPTGPLPPVPAGIDR, RT (min): 64.27, XCorr: 2.38
intensity
m/z
y15
y9
y1
y6
a1
a2
b2
b3
a4
b4
y9
y4
b5
y12
y8
y18
y11
y12
y13
y14
y15
25000
20000
15000
10000
5000
0
250
500
750
1000
1250
1500
1750

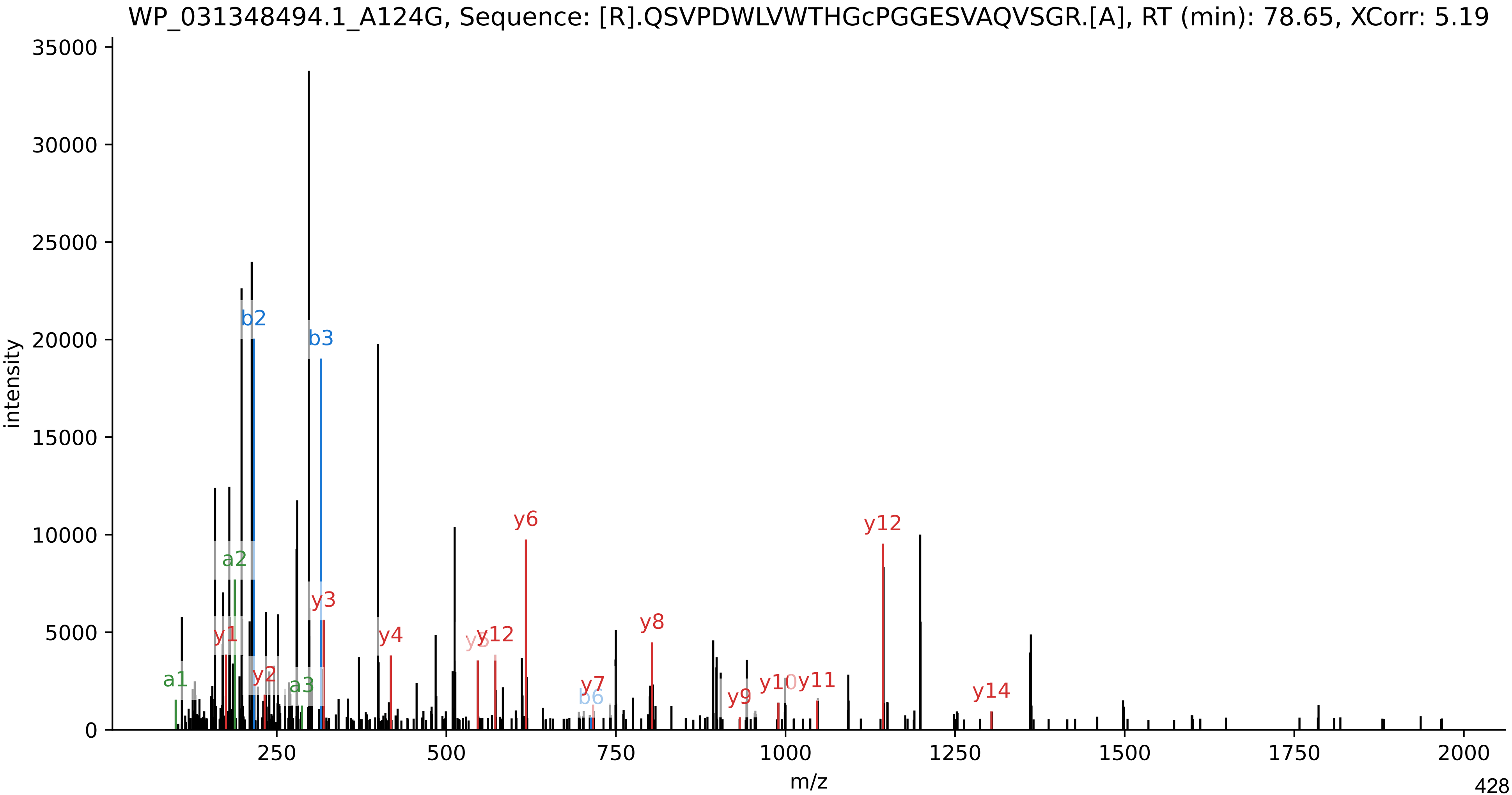
WP_031348494.1_A124G, Sequence: [R].QSVPDWLVWTHGcPGGESVAQVSGR.[A], RT (min): 78.65, XCorr: 5.19
intensity
m/z
0
5000
10000
15000
20000
25000
30000
35000
250
500
750
1000
1250
1500
1750
2000
b2
b3
a2
y3
y1
y4
y12
y6
a1
y2
a3
y7
b6
y8
y9
y11
y12
y14

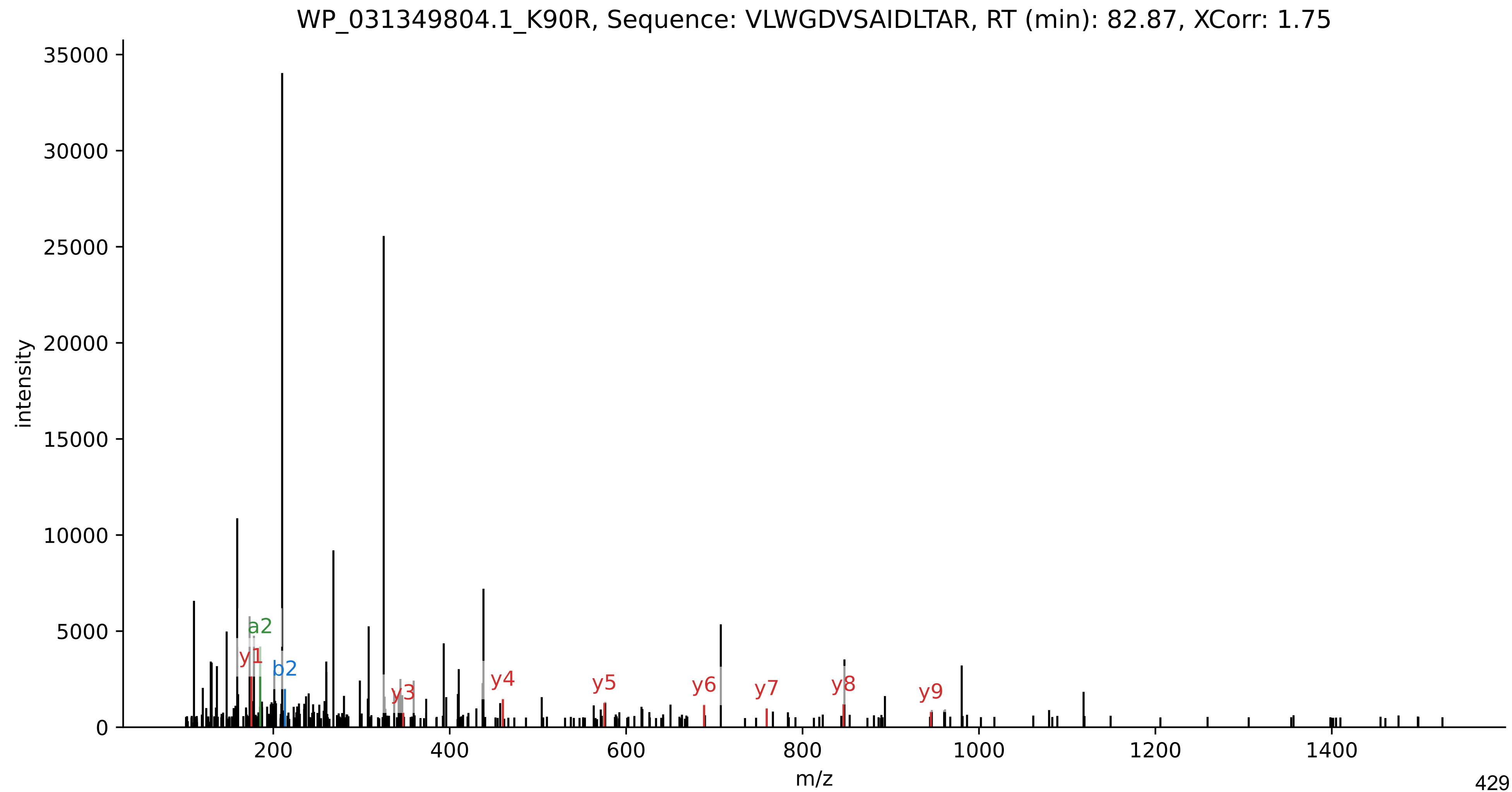

WP_031349804.1_K90R, Sequence: VLWGDVSAIDLTAR, RT (min): 82.87, XCorr: 1.75
intensity
m/z
35000
30000
25000
20000
15000
10000
5000
0
200
400
600
800
1000
1200
1400
a2
y1
b2
y3
y4
y5
y6
y7
y8
y9

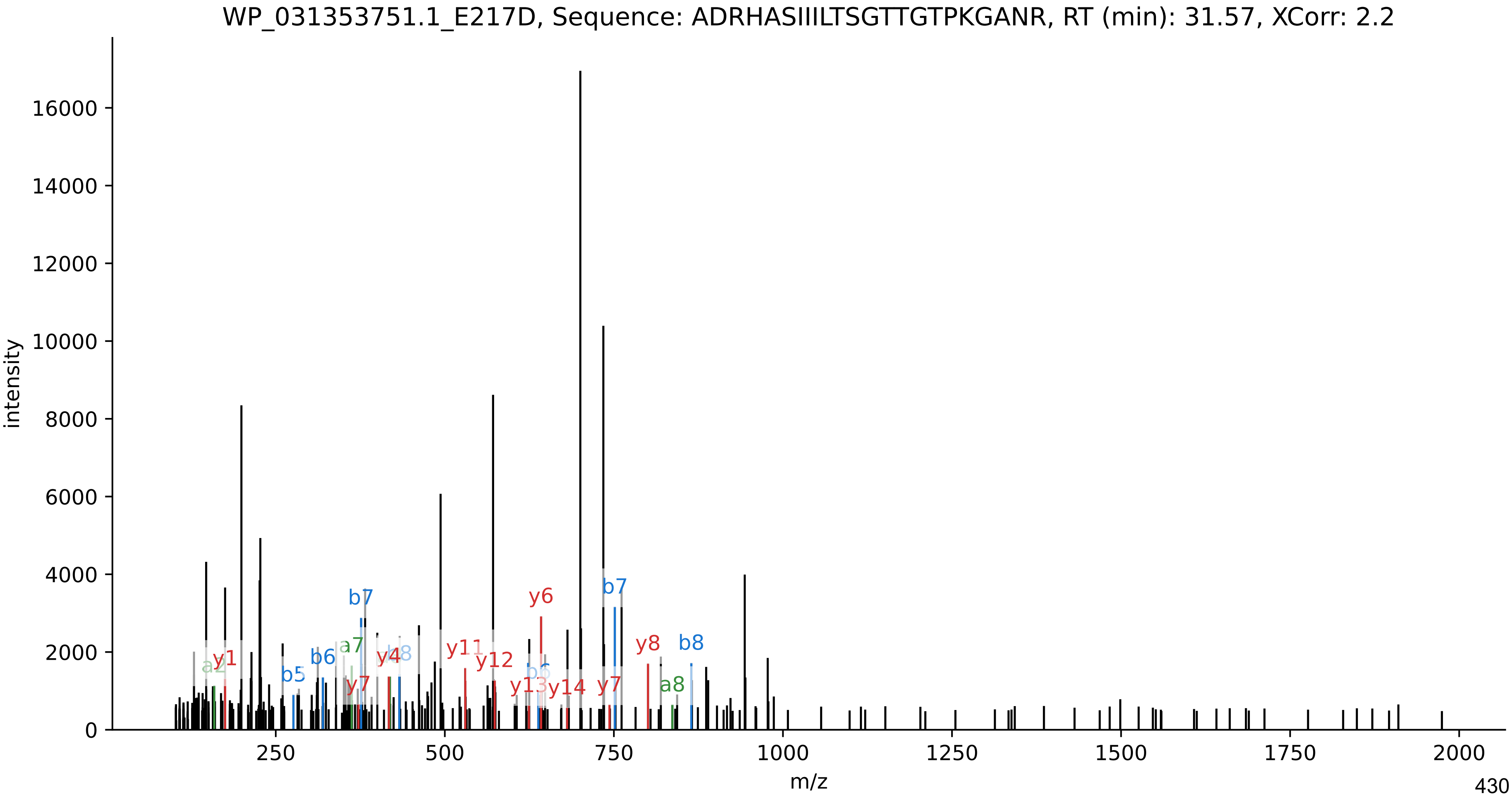
WP_031353751.1_E217D, Sequence: ADRHASIIILTSGTTGTPKGANR, RT (min): 31.57, XCorr: 2.2
intensity
m/z

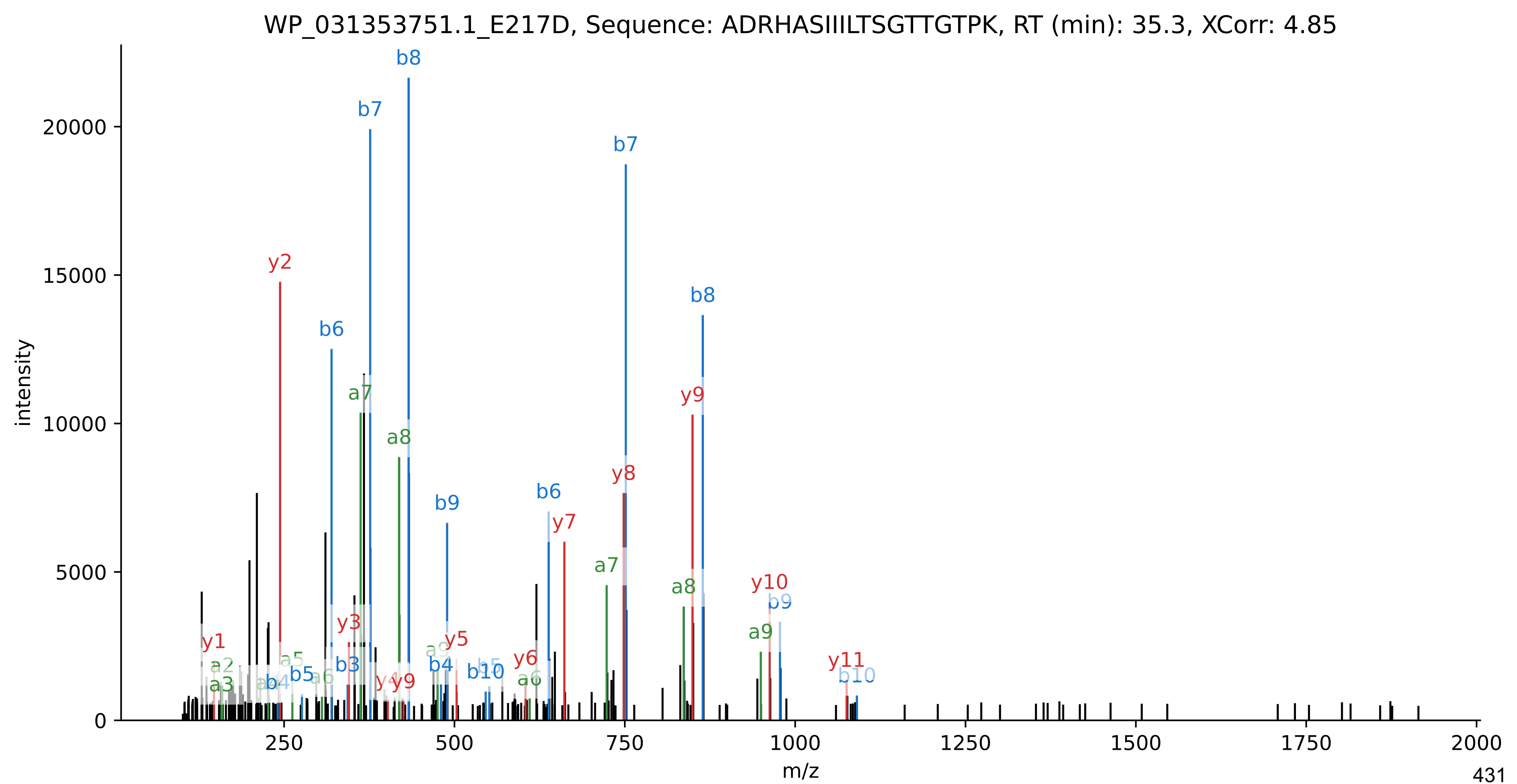

WP_031353751.1_E217D, Sequence: ADRHASIIILTSGTTGTPK, RT (min): 35.3, XCorr: 4.85
intensity
m/z

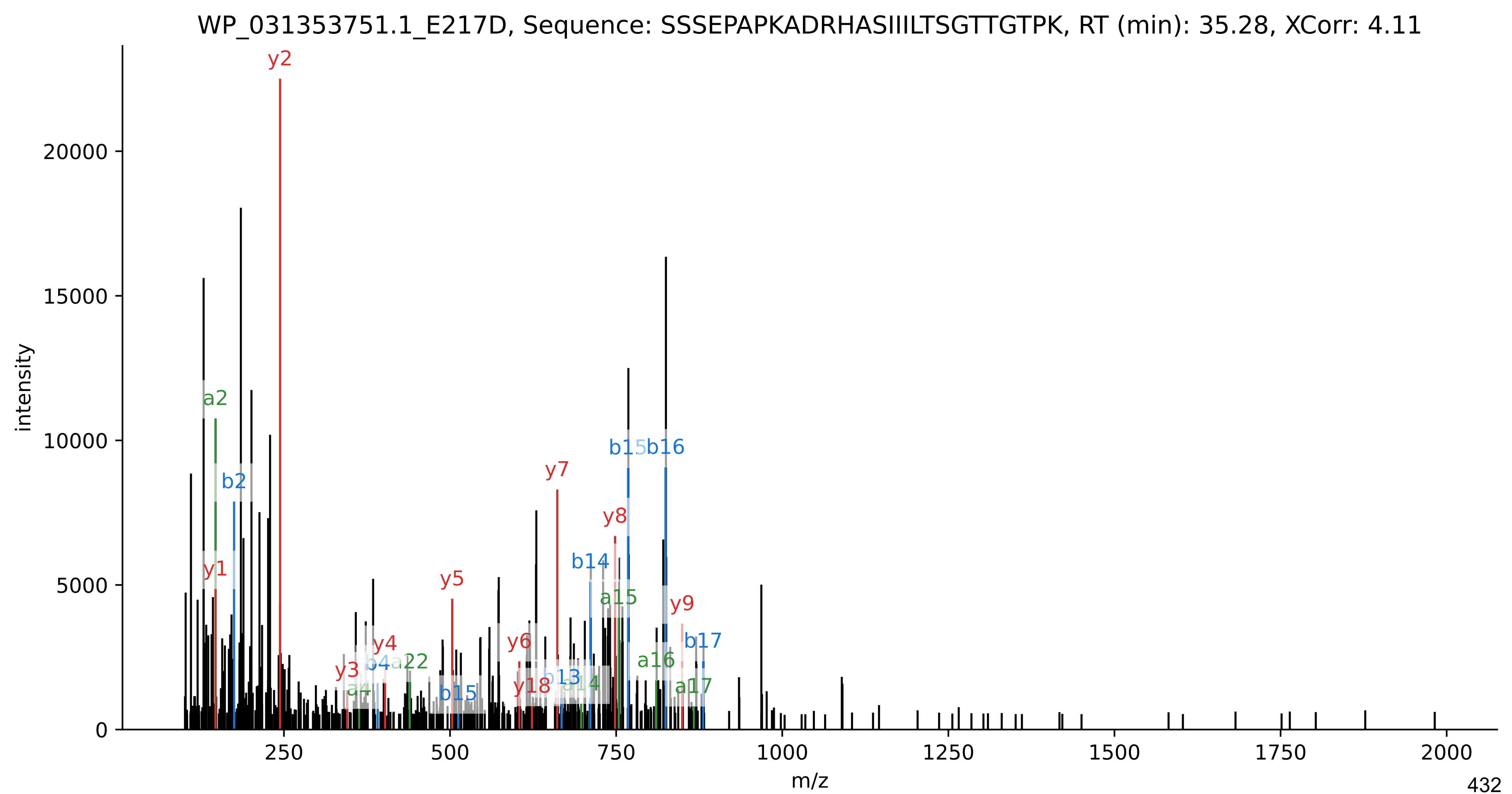
WP_031353751.1_E217D, Sequence: SSSEPAPKADRHASIIILTSGTTGTPK, RT (min): 35.28, XCorr: 4.11
intensity
m/z
y2
a2
b2
y1
y3
a4
b4
y4
a22
y5
b15
y6
y18
b13
a14
y7
b14
y8
a15
b15
b16
y9
b17
a16
a17
0
5000
10000
15000
20000
250
500
750
1000
1250
1500
1750
2000

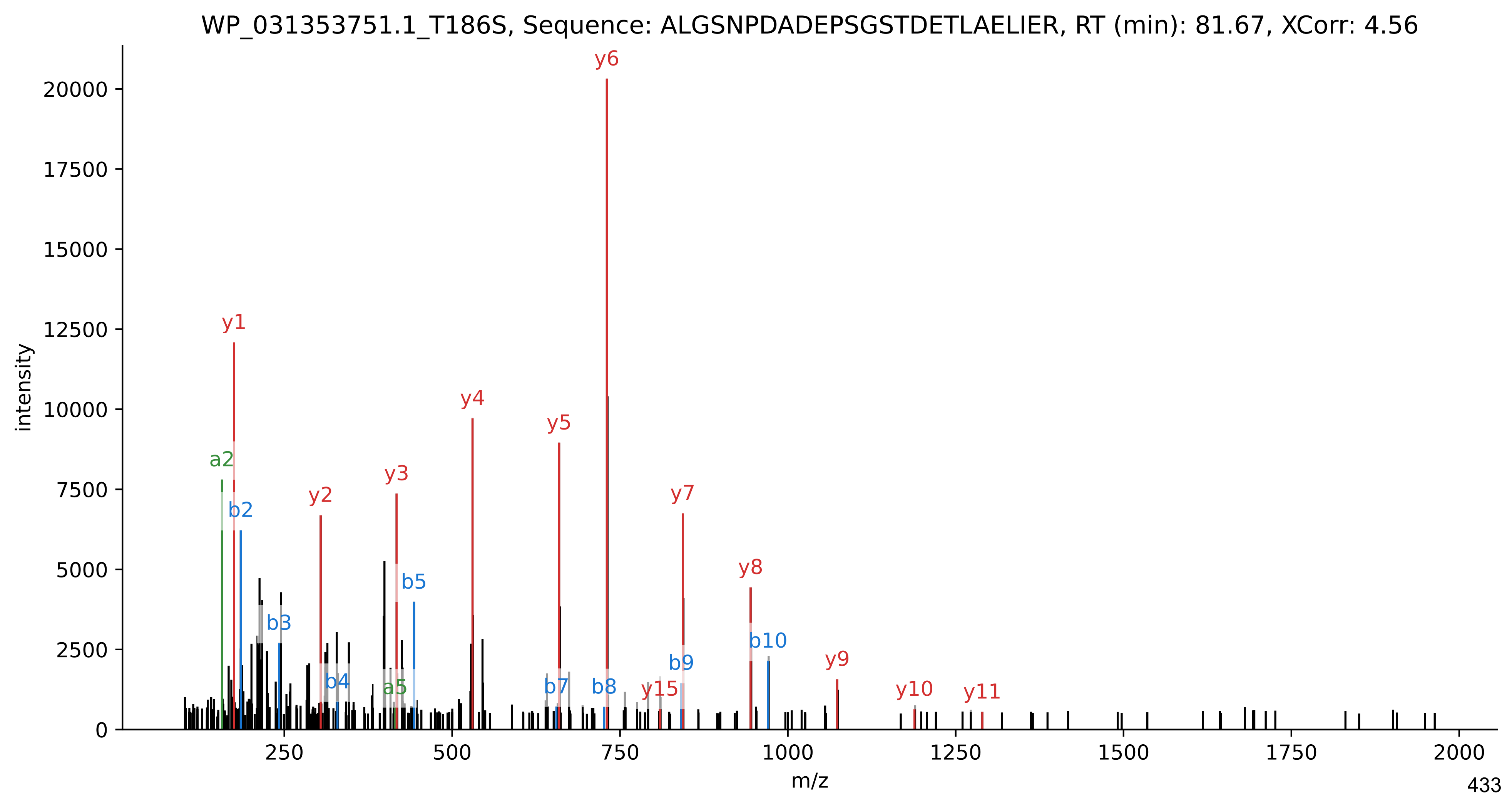

WP_031353751.1_T186S, Sequence: ALGSNPDADEPSGSTDETLAELIER, RT (min): 81.67, XCorr: 4.56
intensity
m/z
y6
y1
y4
y5
a2
y3
y2
y7
b2
y8
b5
b3
b10
b9
y9
b4
a5
b7
b8
y15
y10
y11
20000
17500
15000
12500
10000
7500
5000
2500
0
250
500
750
1000
1250
1500
1750
2000

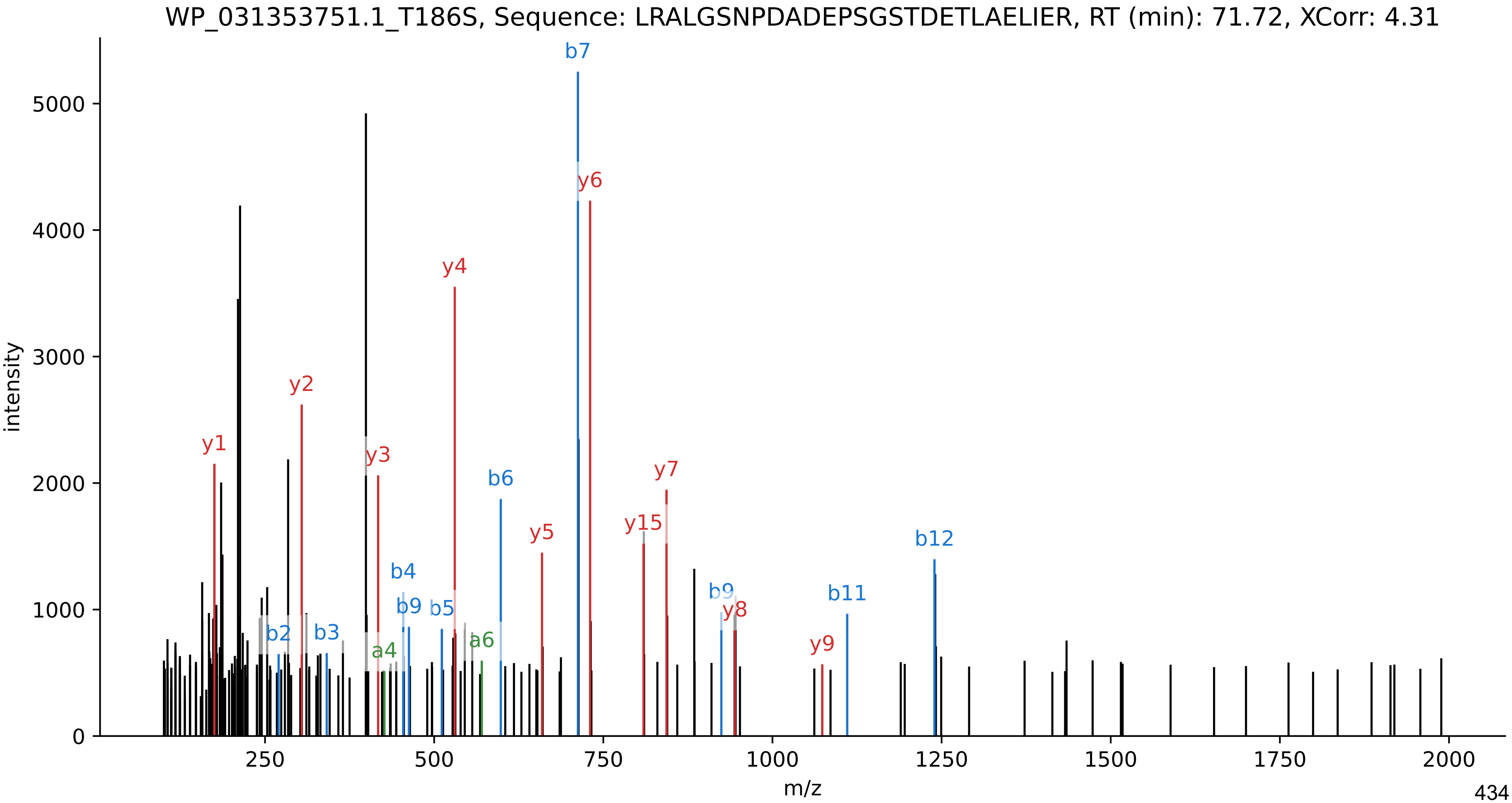
WP_031353751.1_T186S, Sequence: LRALGSNPDADEPSGSTDETLAELIER, RT (min): 71.72, XCorr: 4.31
intensity
m/z
0
1000
2000
3000
4000
5000
250
500
750
1000
1250
1500
1750
2000
y1
y2
y3
y4
y5
y6
y7
y8
y9
y15
b2
b3
b4
b5
b6
b7
b9
b9
b11
b12
a4
a6

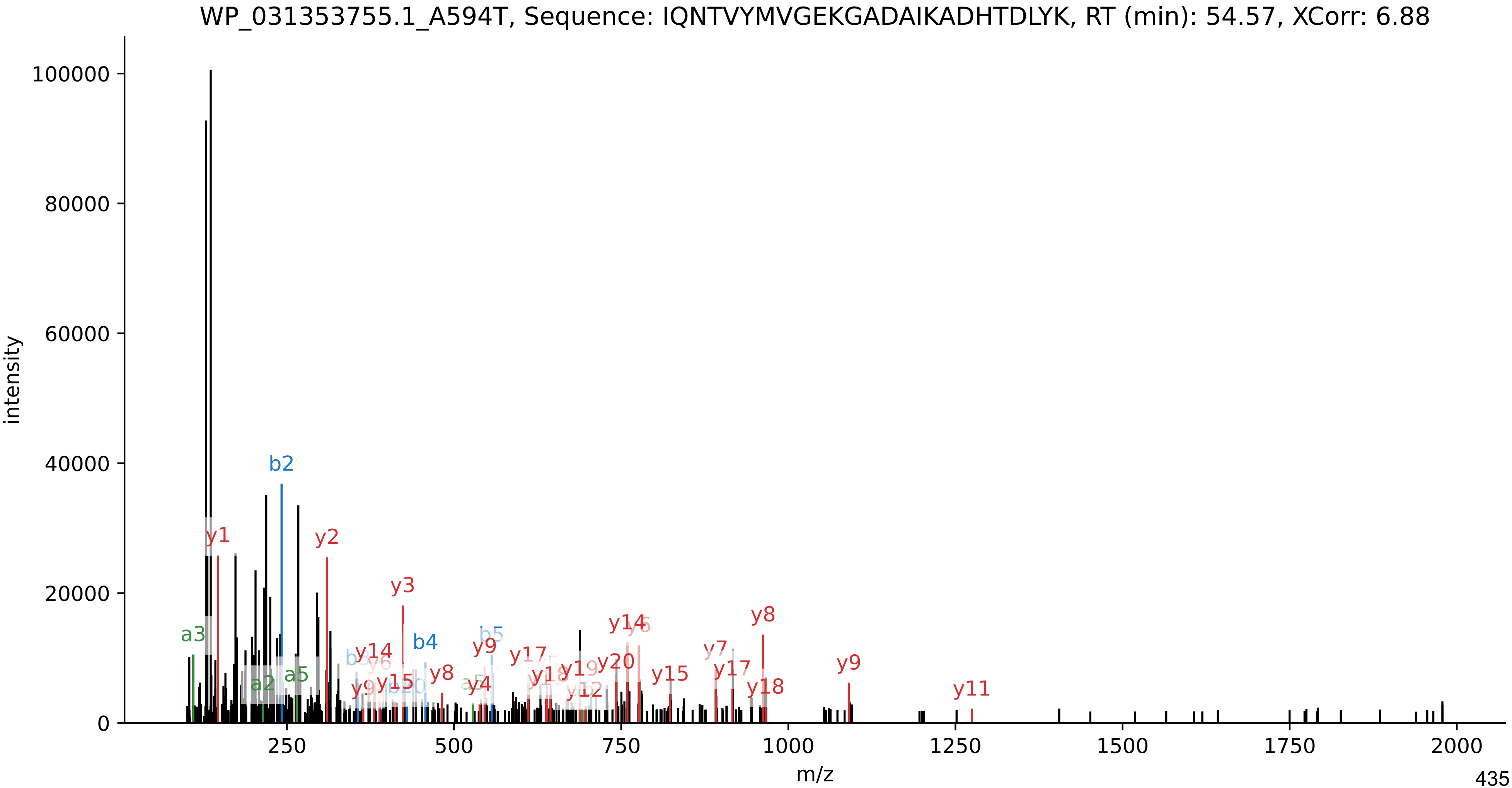

WP_031353755.1_A594T, Sequence: IQNTVYMVGEKGADAIKADHTDLYK, RT (min): 54.57, XCorr: 6.88
intensity
m/z
0
20000
40000
60000
80000
100000
250
500
750
1000
1250
1500
1750
2000
b2
y1
y2
y3
a3
y14
b4
y8
y9
y17
y14
y8
y7
y17
y9
y11
y15
y18
y20
y4
a2
a5
y15

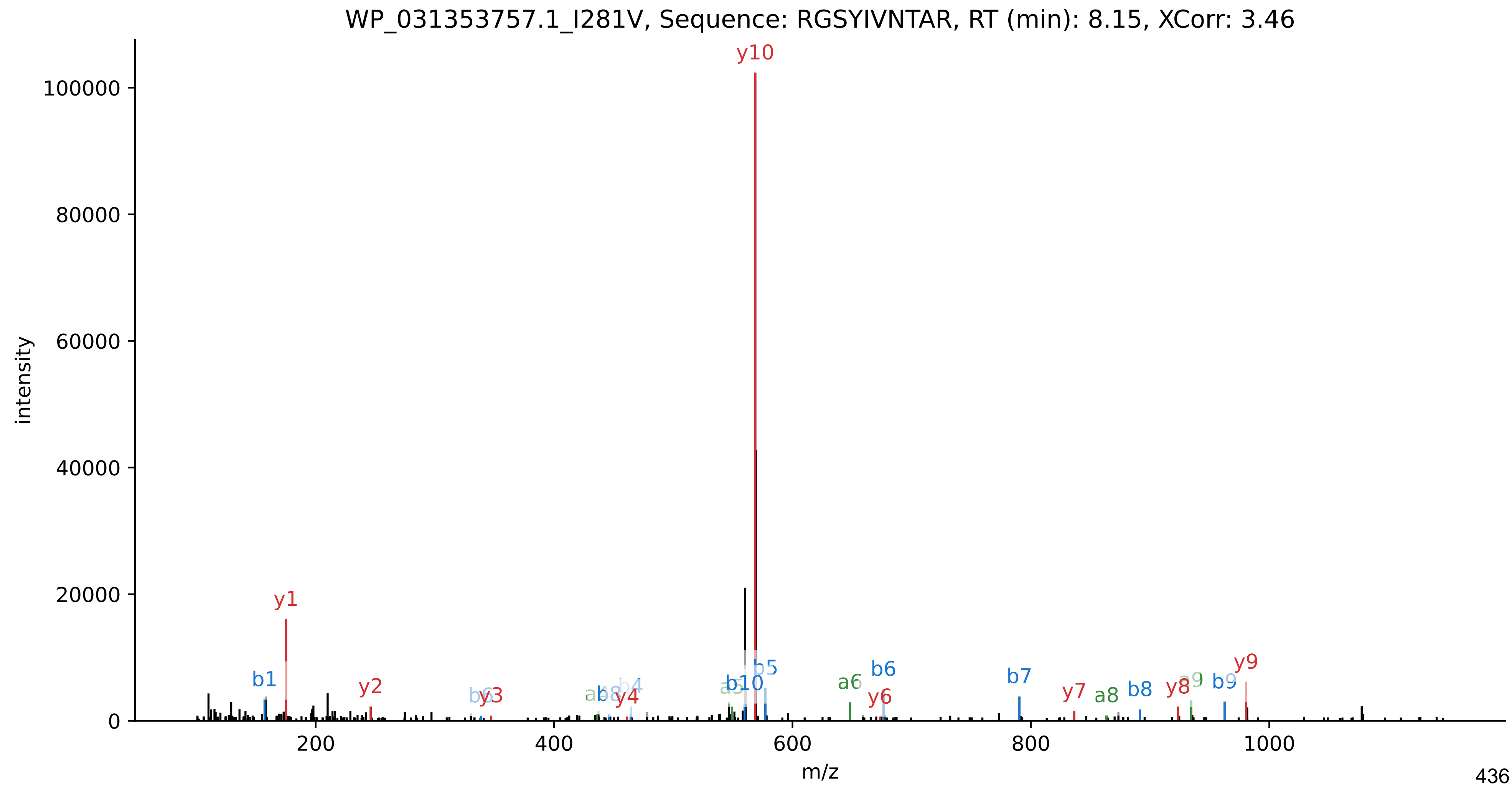
WP_031353757.1_I281V, Sequence: RGSYIVNTAR, RT (min): 8.15, XCorr: 3.46
intensity
m/z
0
20000
40000
60000
80000
100000
200
400
600
800
1000
y10
y1
b1
y2
y3
y4
b10
b5
a6
b6
y6
b7
y7
a8
b8
y8
a9
b9
y9

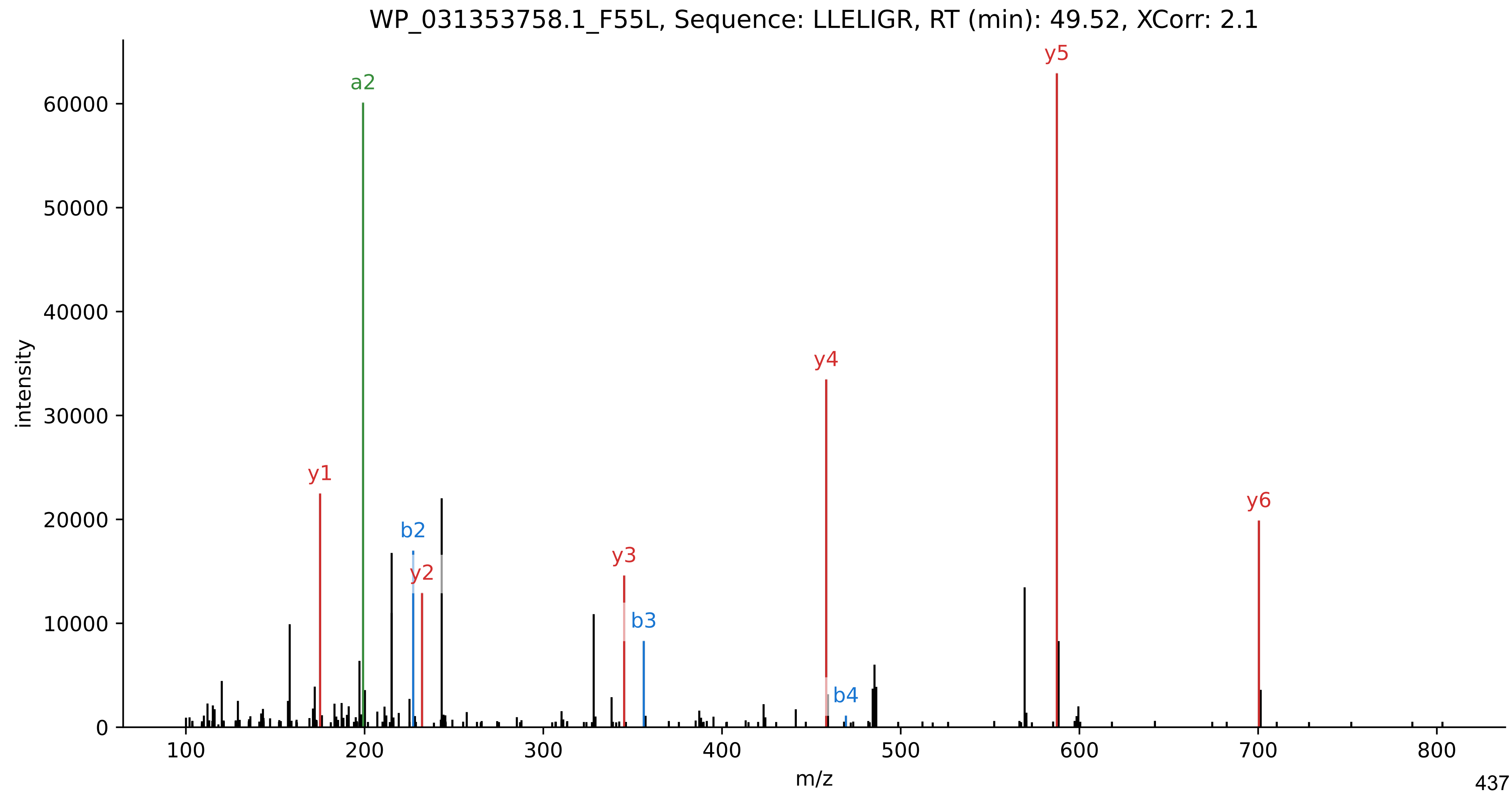

WP_031353758.1_F55L, Sequence: LLELIGR, RT (min): 49.52, XCorr: 2.1
intensity
m/z
a2
y5
y4
y1
y6
b2
y2
y3
b3
b4
0
10000
20000
30000
40000
50000
60000
100
200
300
400
500
600
700
800

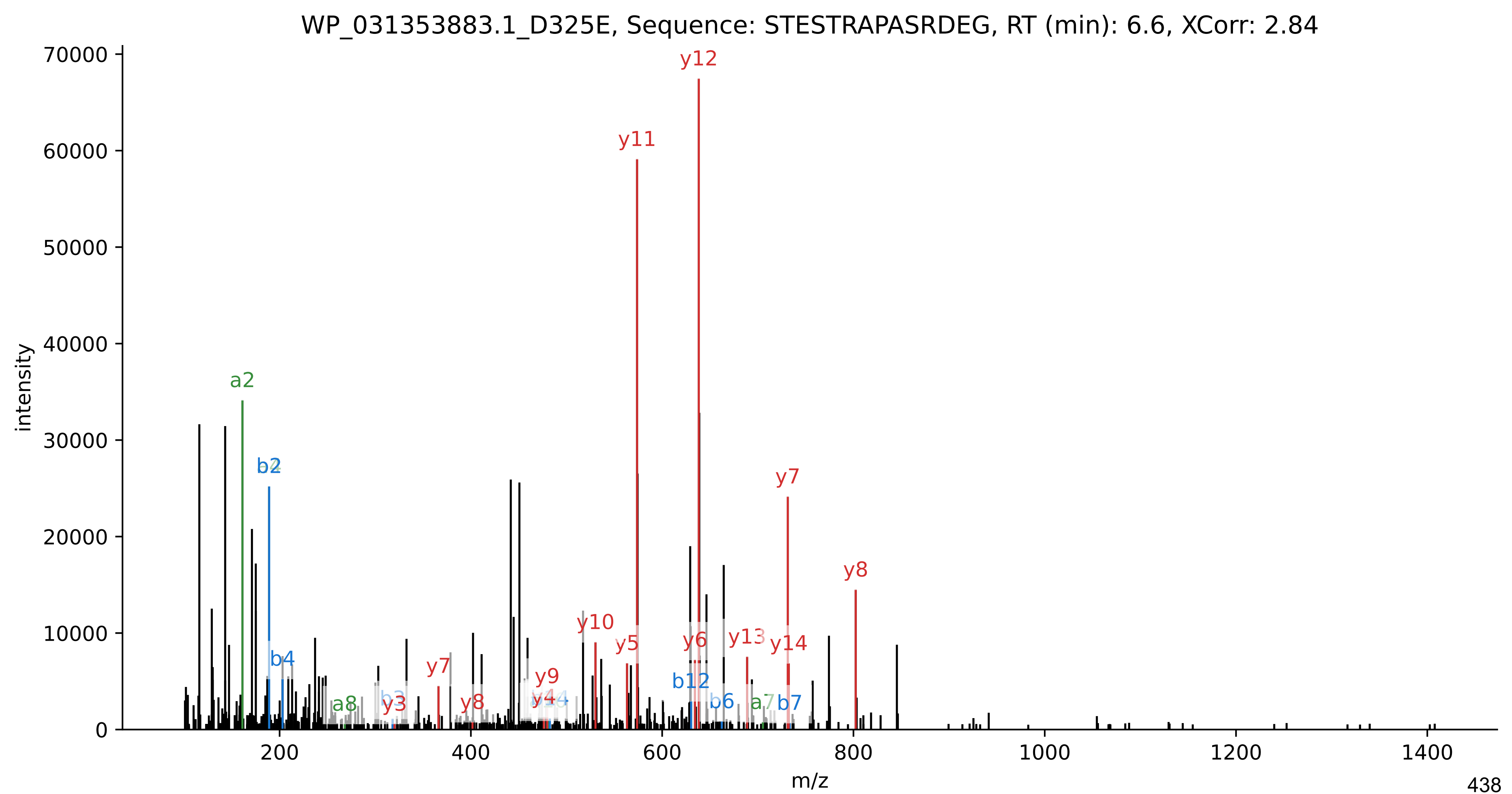

WP_031353883.1_D325E, Sequence: STESTRAPASRDEG, RT (min): 6.6, XCorr: 2.84
intensity
m/z
y12
y11
a2
b2
y7
y8
y10
y5
y6
y13
y14
b4
y7
y9
b12
b6
a8
b3
y3
y8
y4
a7
b7
0
10000
20000
30000
40000
50000
60000
70000
200
400
600
800
1000
1200
1400

WP_031353911.1_I285V, Sequence: AADLPTLKDVLVQDYRVSSASLR, RT (min): 84.68, XCorr: 3.98

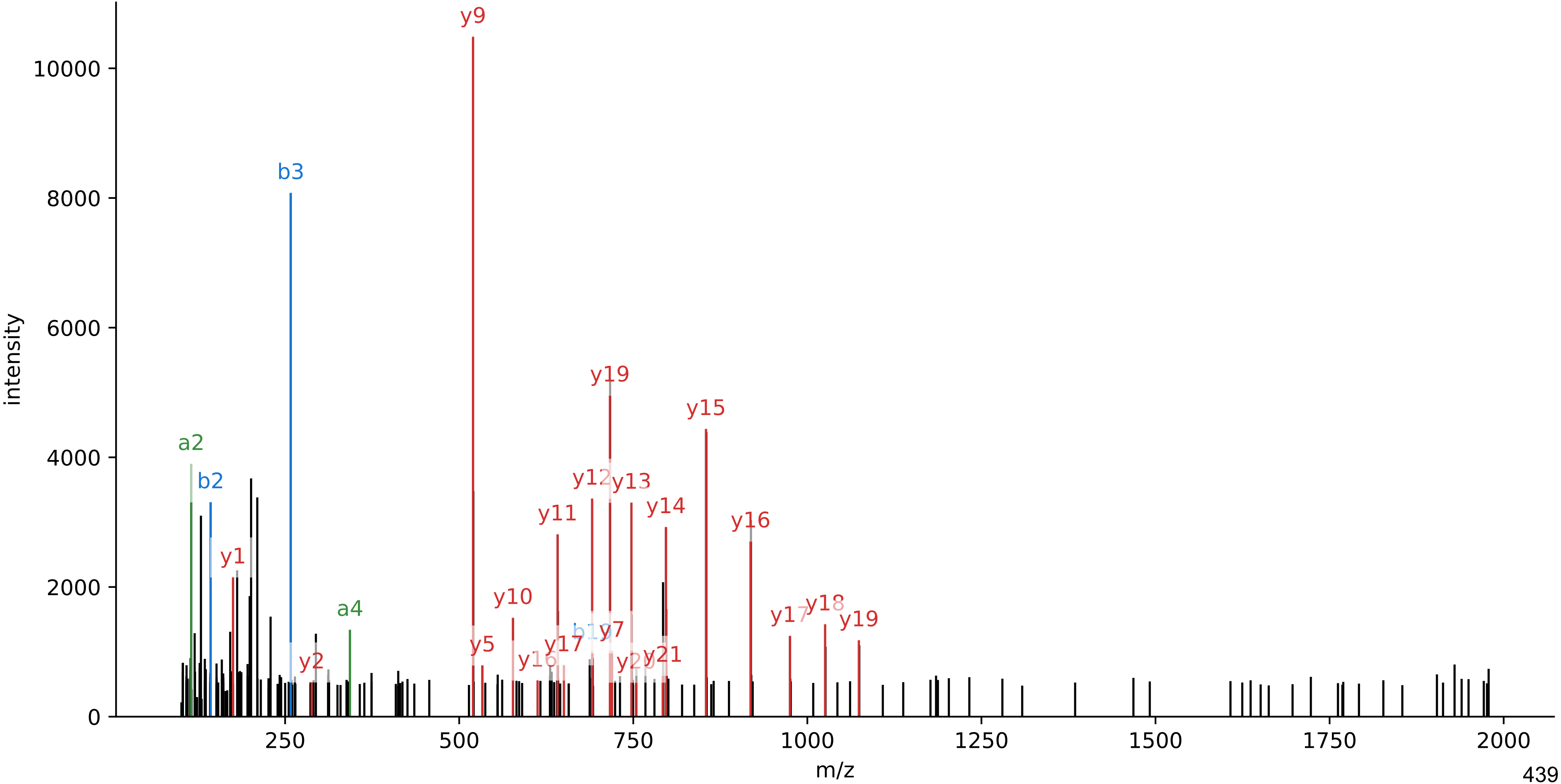

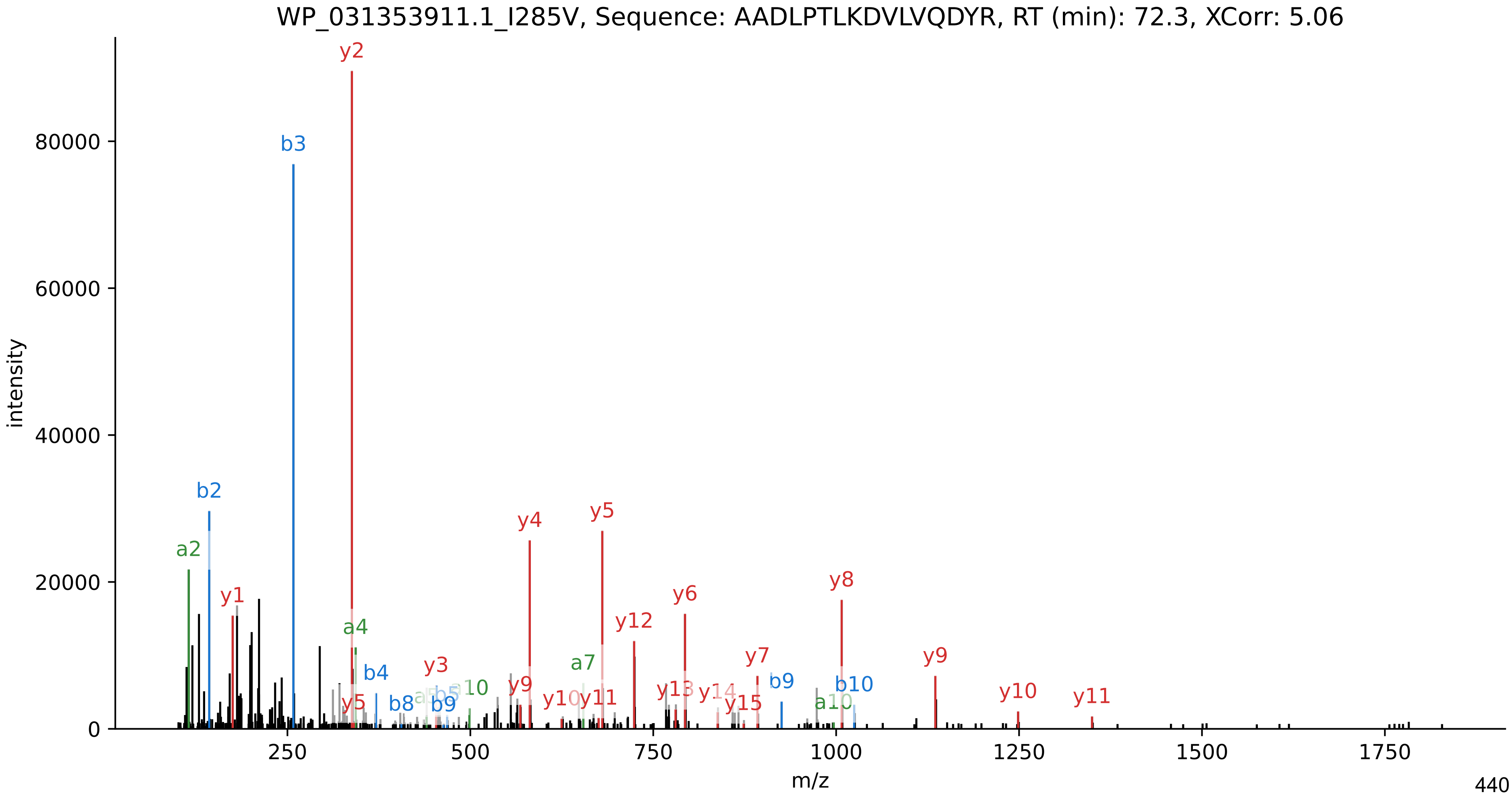
WP_031353911.1_I285V, Sequence: AADLPTLKDVLVQDYR, RT (min): 72.3, XCorr: 5.06
intensity
m/z
0
20000
40000
60000
80000
250
500
750
1000
1250
1500
1750
y2
b3
b2
a2
y1
a4
b4
y3
b8
b9
y5
y4
y9
y5
a7
y10
y11
y12
y6
y13
y14
y15
y7
b9
a10
b10
y8
y9
y10
y11

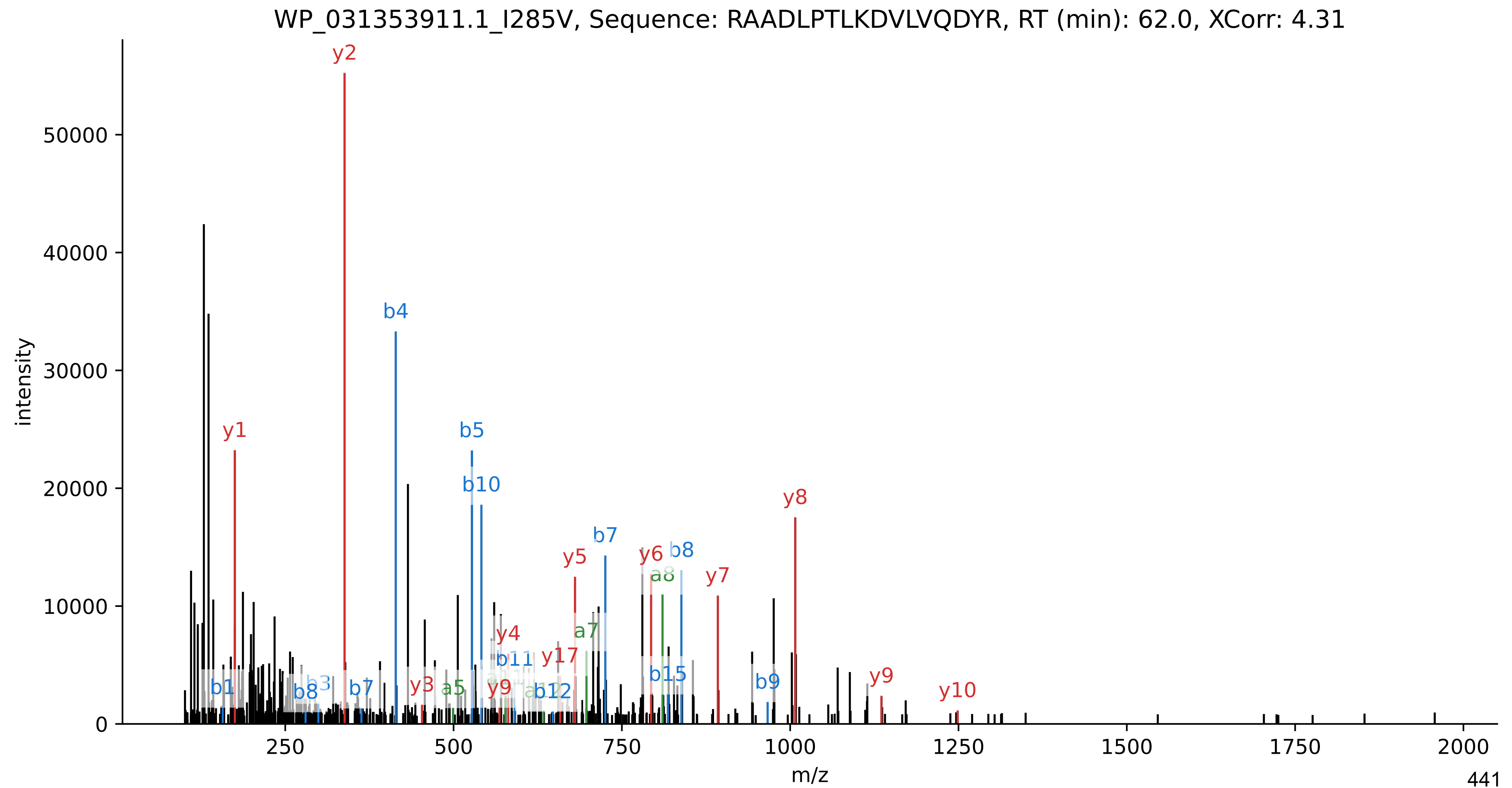

WP_031353911.1_I285V, Sequence: RAADLPTLKDVLVQDYR, RT (min): 62.0, XCorr: 4.31
intensity
m/z
y2
b4
y1
b5
b10
y8
b7
y5
y6
b8
a8
y7
a7
y4
b11
y17
b15
b1
b3
b8
b7
y3
a5
y9
b12
b9
y9
y10

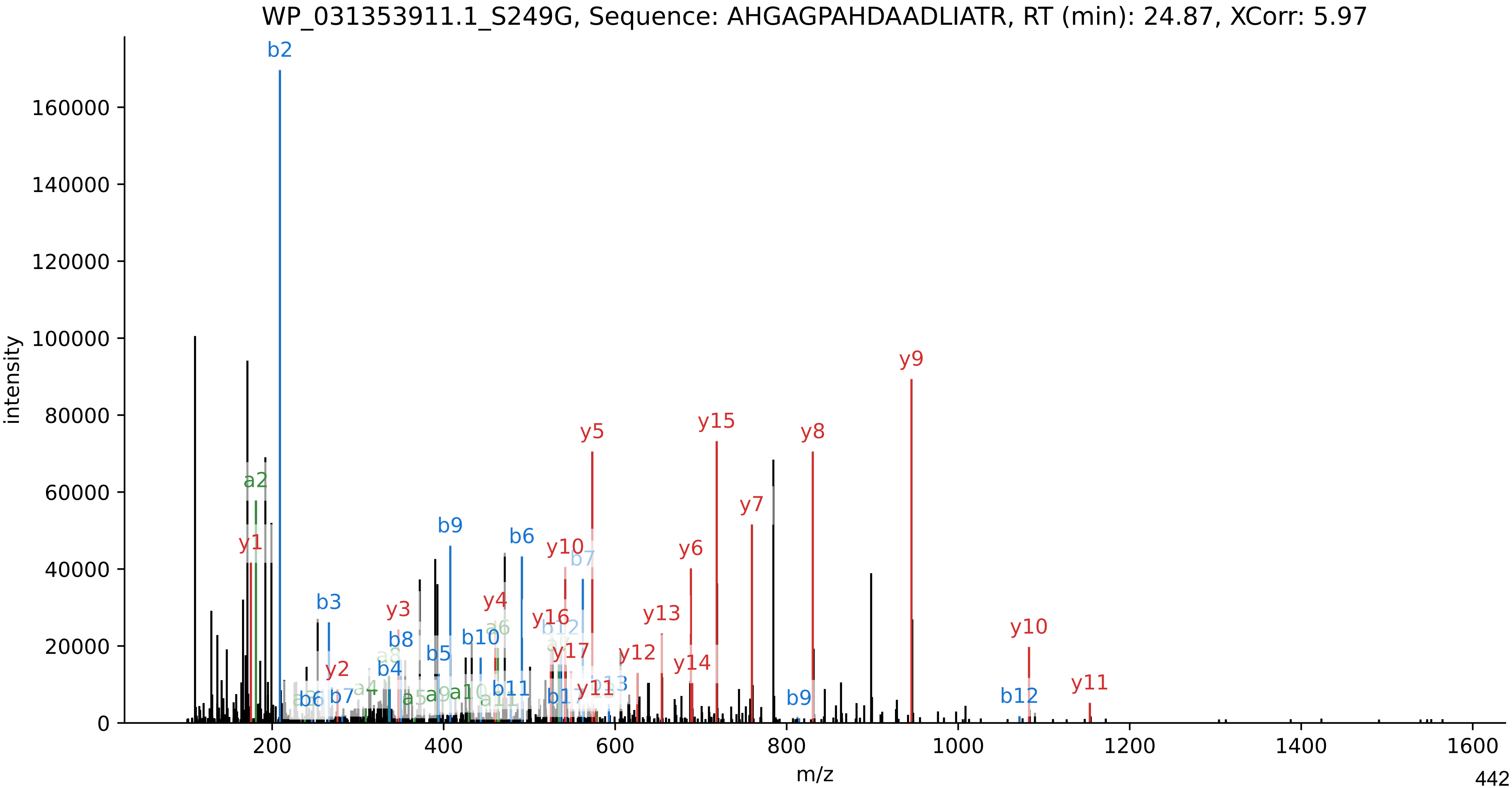
WP_031353911.1_S249G, Sequence: AHGAGPAHDAADLIATR, RT (min): 24.87, XCorr: 5.97
intensity
m/z
b2
y9
y5
y15
y8
y7
a2
y1
b9
b6
y10
y6
b3
y3
y4
y16
y13
y10
b8
b10
b5
y17
y12
b4
y2
y14
y11
b11
b9
b12
b6
b7

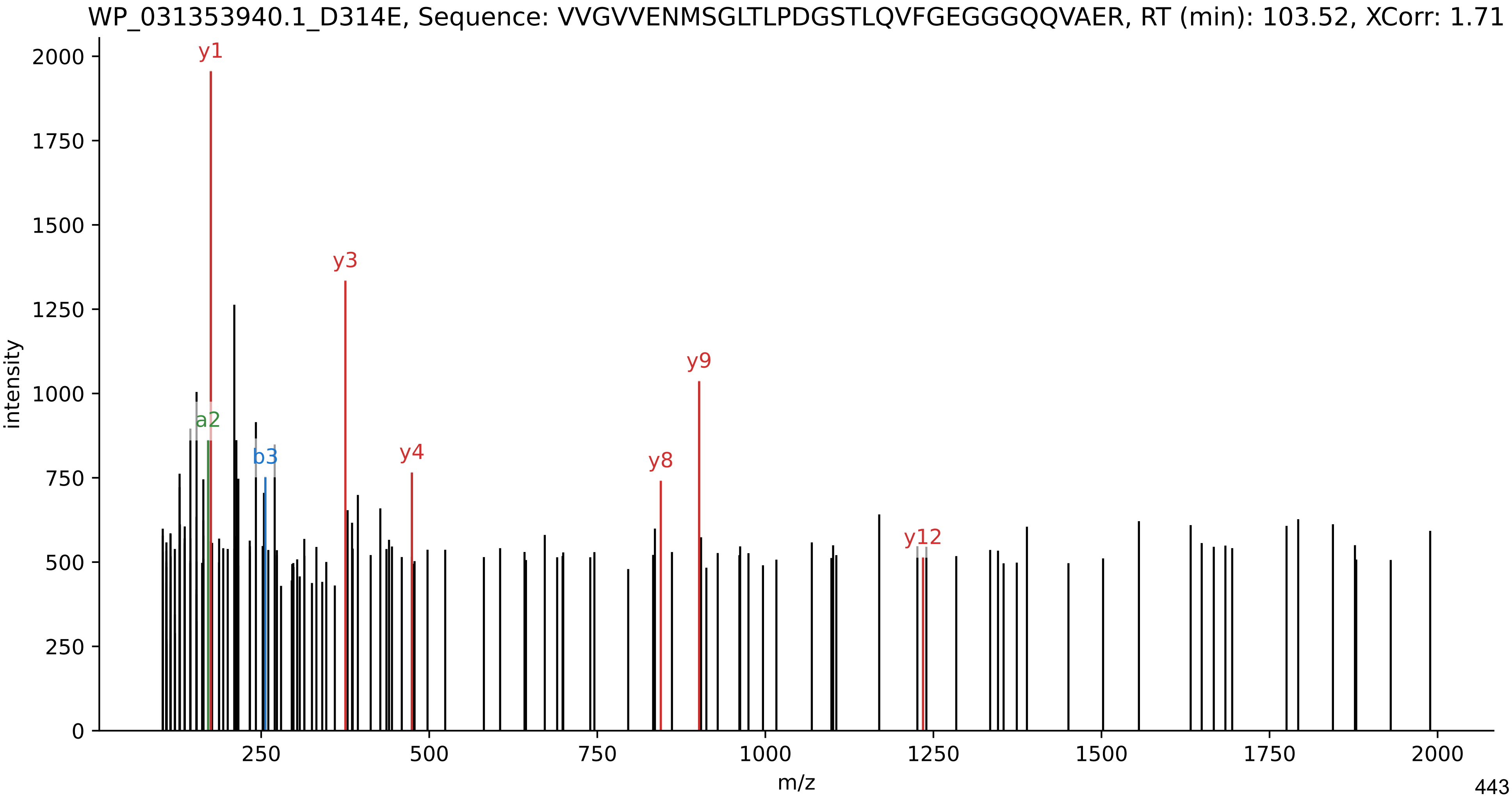

WP_031353940.1_D314E, Sequence: VVGVVENMSGLTLPDGSTLQVFGEGGGQQVAER, RT (min): 103.52, XCorr: 1.71
intensity
m/z
y1
y3
y9
y4
y8
y12
a2
b3
0
250
500
750
1000
1250
1500
1750
2000

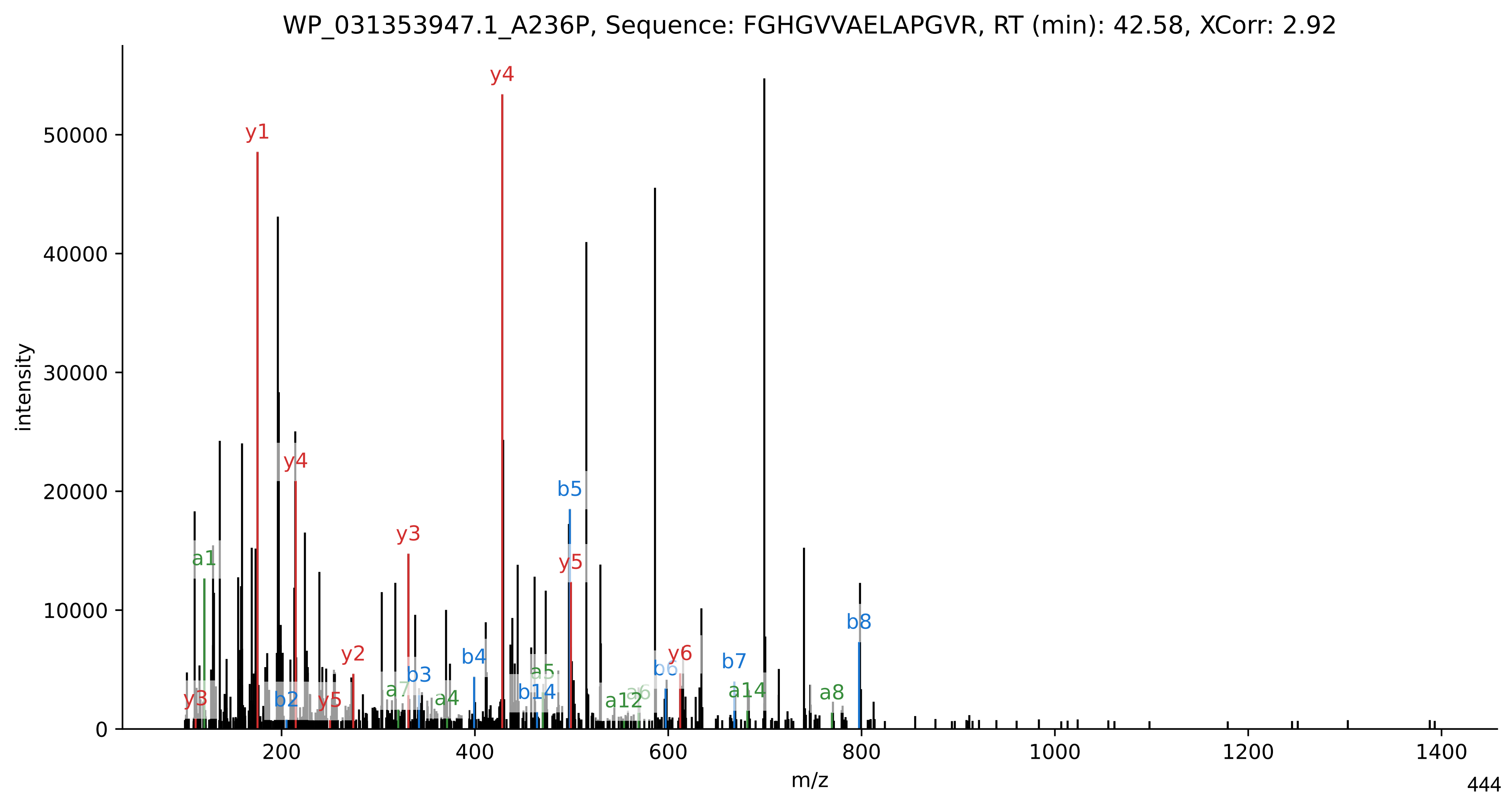

WP_031353947.1_A236P, Sequence: FGHGVVAELAPGVR, RT (min): 42.58, XCorr: 2.92
intensity
m/z
0
10000
20000
30000
40000
50000
200
400
600
800
1000
1200
1400
y1
y4
y4
y3
y2
y3
y5
y5
y6
b2
b3
b4
b5
b14
b6
b7
b8
a1
a7
a4
a5
a12
a6
a14
a8

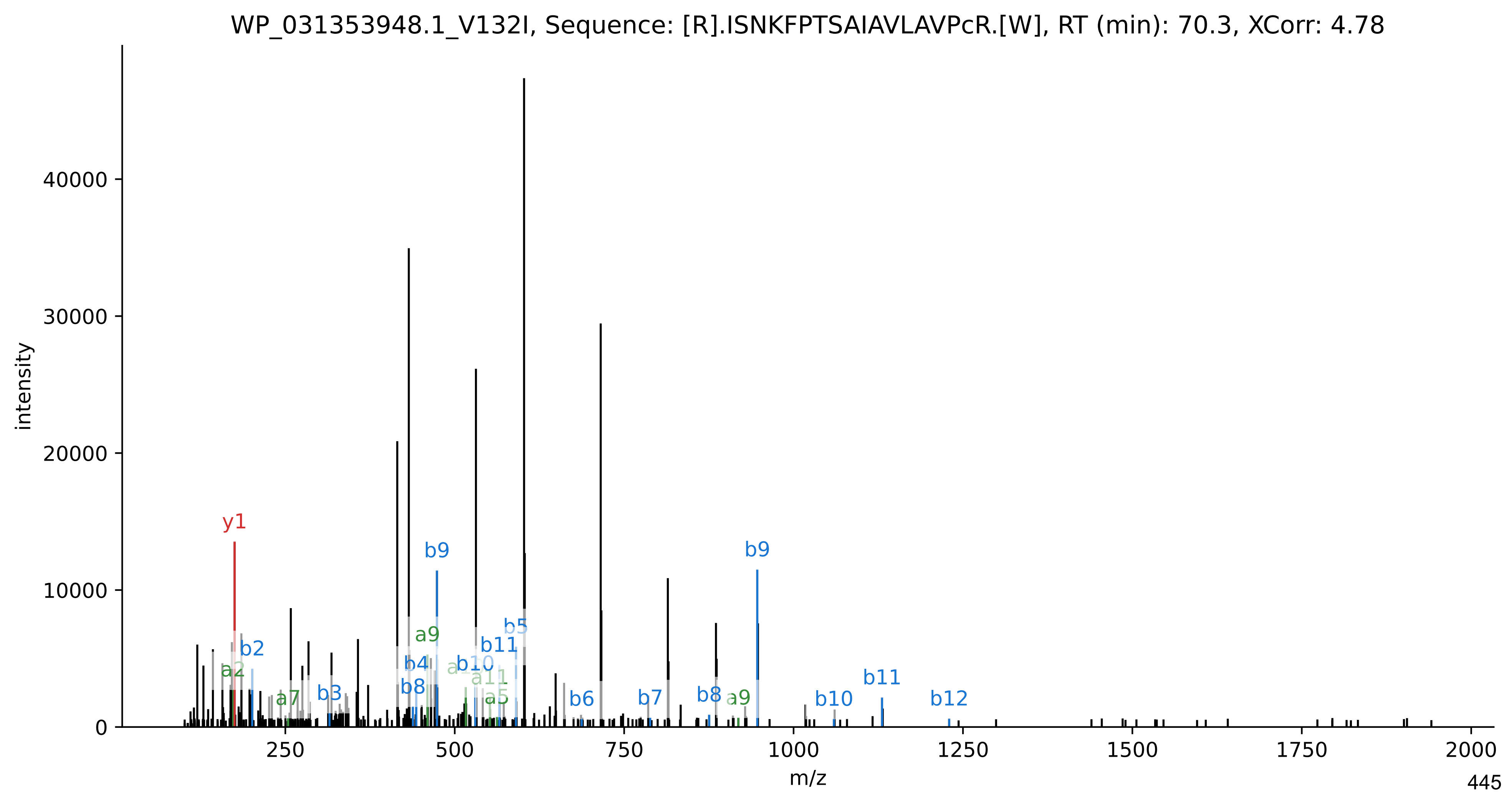

WP_031353948.1_V132I, Sequence: [R].ISNKFPTSAIAVLAVPcR.[W], RT (min): 70.3, XCorr: 4.78
intensity
m/z
y1
b2
a2
a7
b3
b9
a9
b4
b8
b10
b11
a11
a5
b5
b6
b7
b8
a9
b9
b10
b11
b12

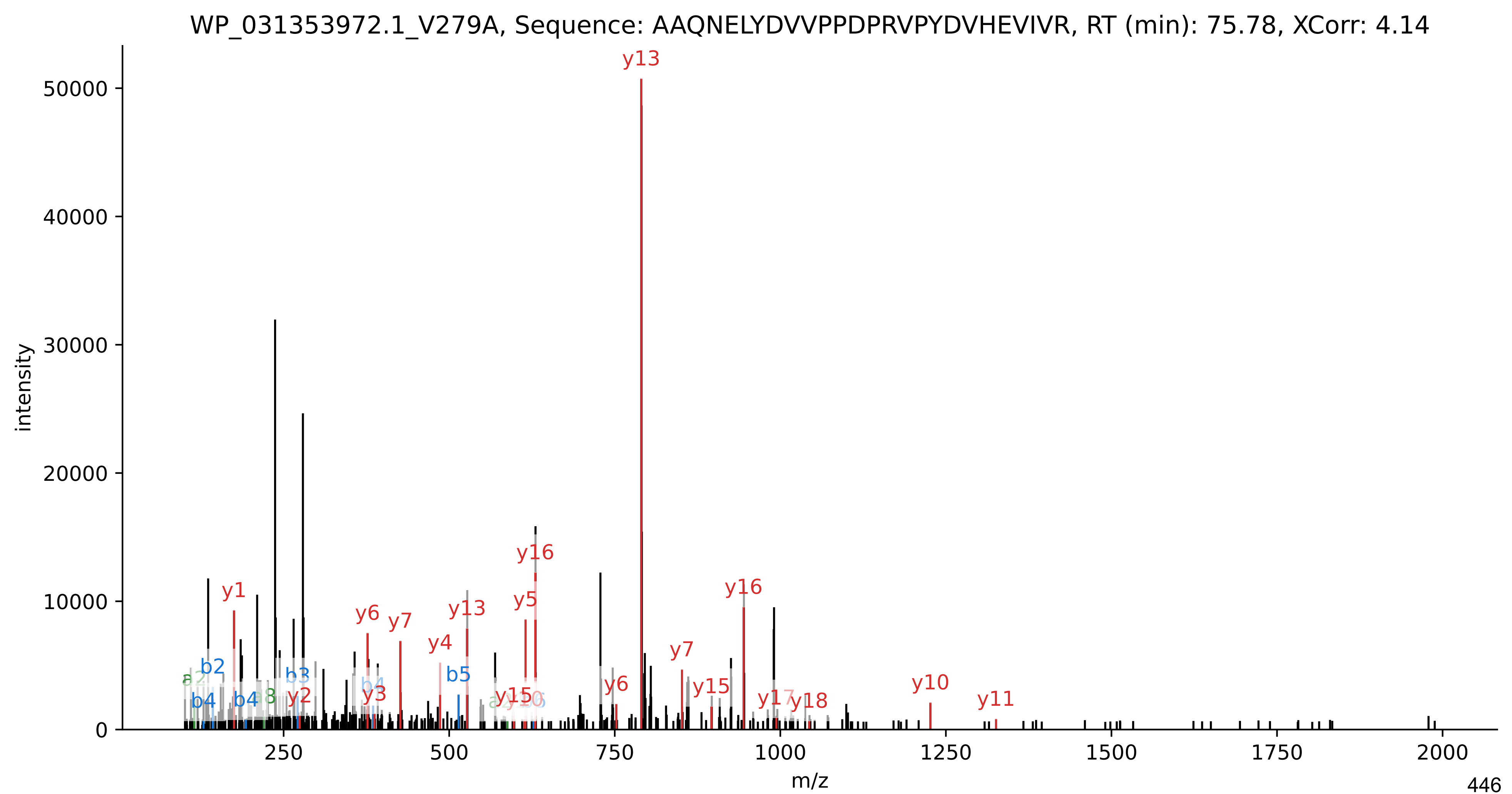
WP_031353972.1_V279A, Sequence: AAQNELYDVVPPDPRVPYDVHEVIVR, RT (min): 75.78, XCorr: 4.14
intensity
m/z
y13
y16
y1
y6
y7
y4
y13
y5
b2
b3
b5
b4
b4
a3
y2
y3
y15
y6
y7
y15
y16
y17
y18
y10
y11

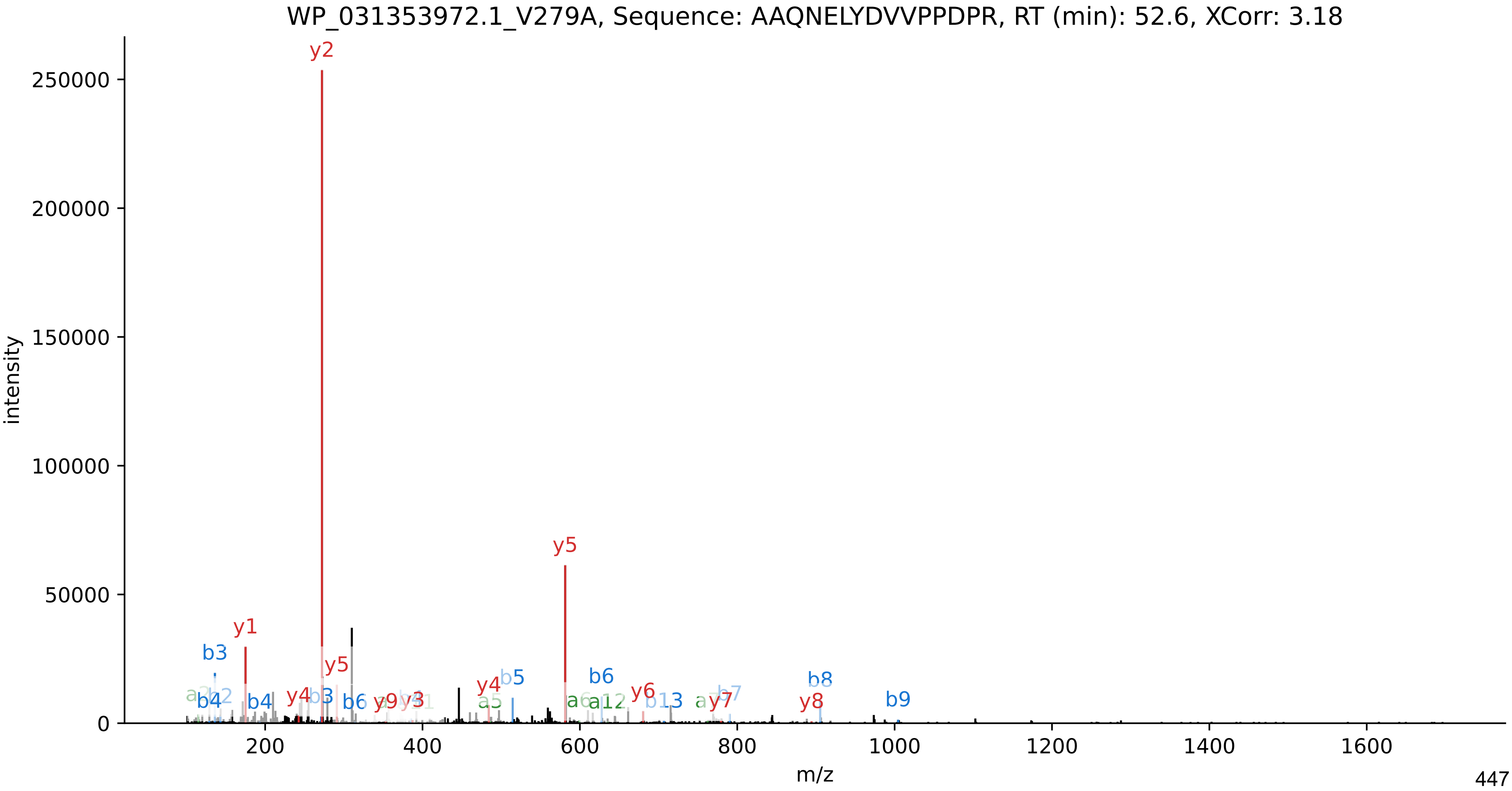
WP_031353972.1_V279A, Sequence: AAQNELYDVVPPDPR, RT (min): 52.6, XCorr: 3.18
y2
y5
y1
b3
y5
b6
b8
y6
y4
b5
b9
y8
y7
b13
a5
a12
b4
y9
intensity
m/z
0
50000
100000
150000
200000
250000
200
400
600
800
1000
1200
1400
1600

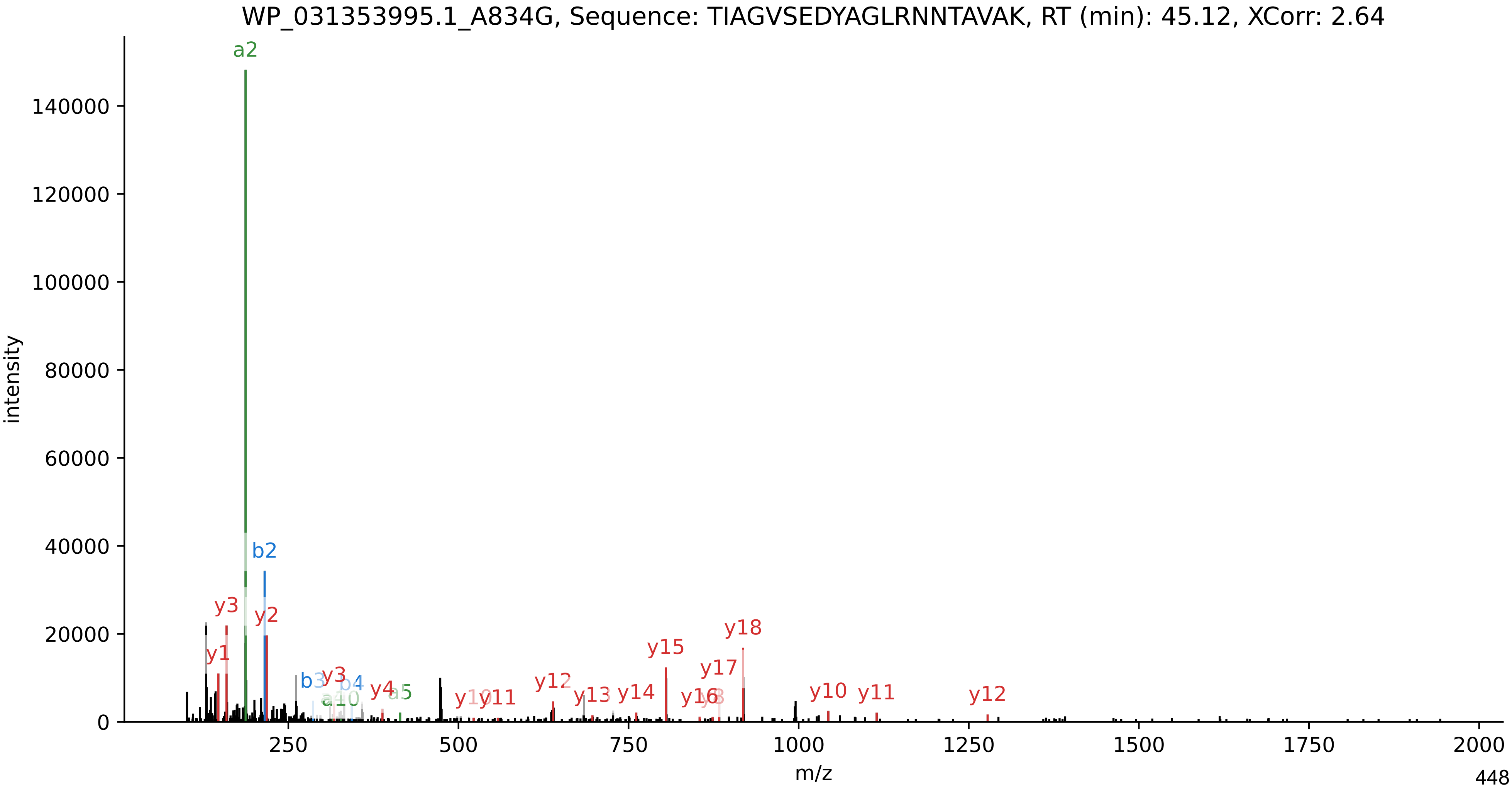

WP_031353995.1_A834G, Sequence: TIAGVSEDYAGLRNNTAVAK, RT (min): 45.12, XCorr: 2.64
intensity
m/z
a2
b2
y3
y2
y1
y18
y15
y17
y16
y12
y13
y14
y10
y11
y12
y4
a5
b3
y3
b4
a10

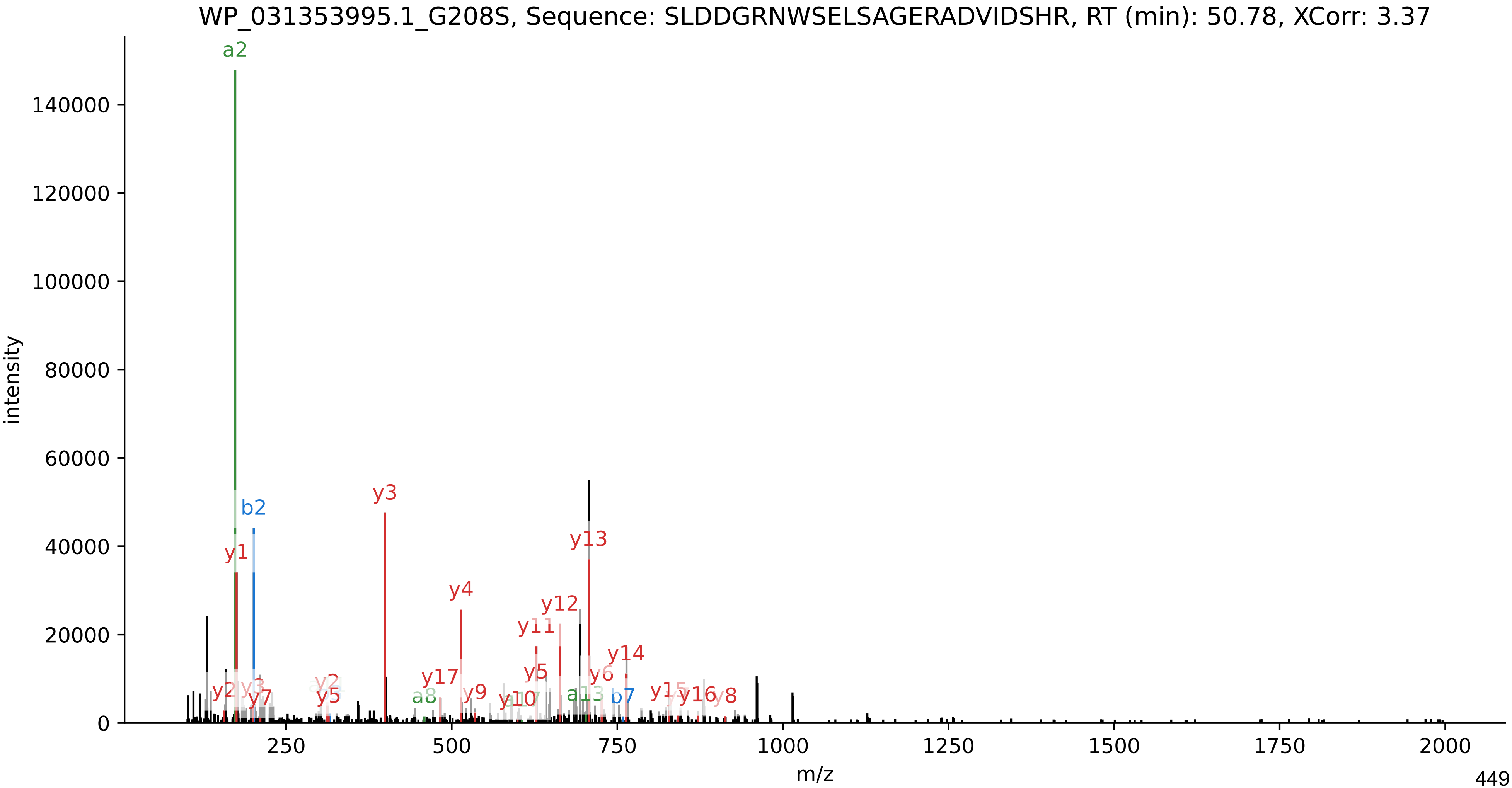
WP_031353995.1_G208S, Sequence: SLDDGRNWSELSAGERADVIDSHR, RT (min): 50.78, XCorr: 3.37
a2
b2
y1
y3
y4
y13
y12
y11
y14
y5
y6
y17
y9
y10
a8
a13
b7
y2
y3
y7
y5
y15
y16
y8
intensity
m/z
0
20000
40000
60000
80000
100000
120000
140000
250
500
750
1000
1250
1500
1750
2000

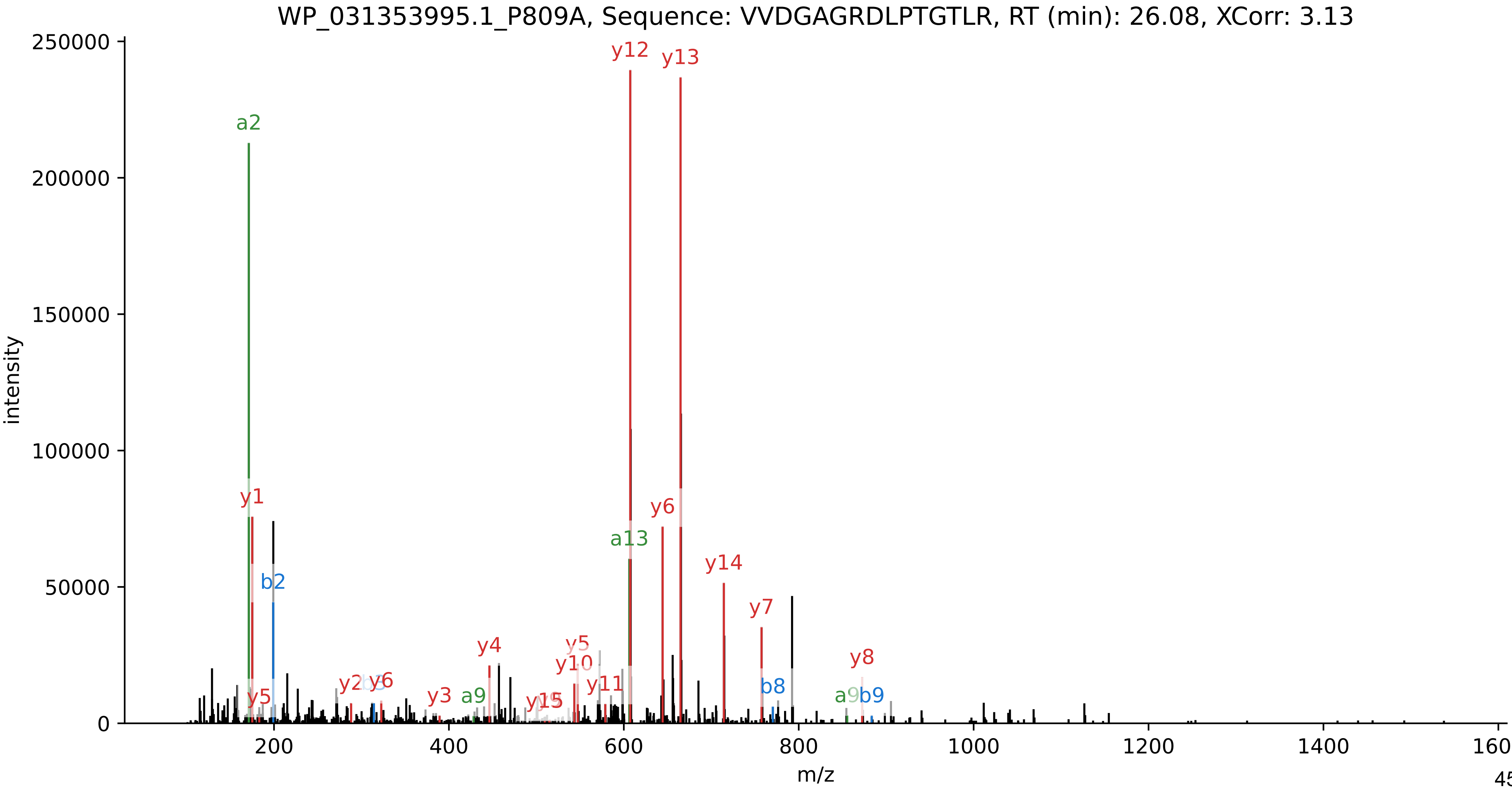

WP_031353995.1_P809A, Sequence: VVDGAGRDLPTGTLR, RT (min): 26.08, XCorr: 3.13
intensity
m/z
0
50000
100000
150000
200000
250000
200
400
600
800
1000
1200
1400
1600
a2
y1
b2
y5
y2
y6
y3
a9
y4
y15
y5
y10
y11
a13
y12
y13
y6
y14
y7
b8
y8
a9
b9

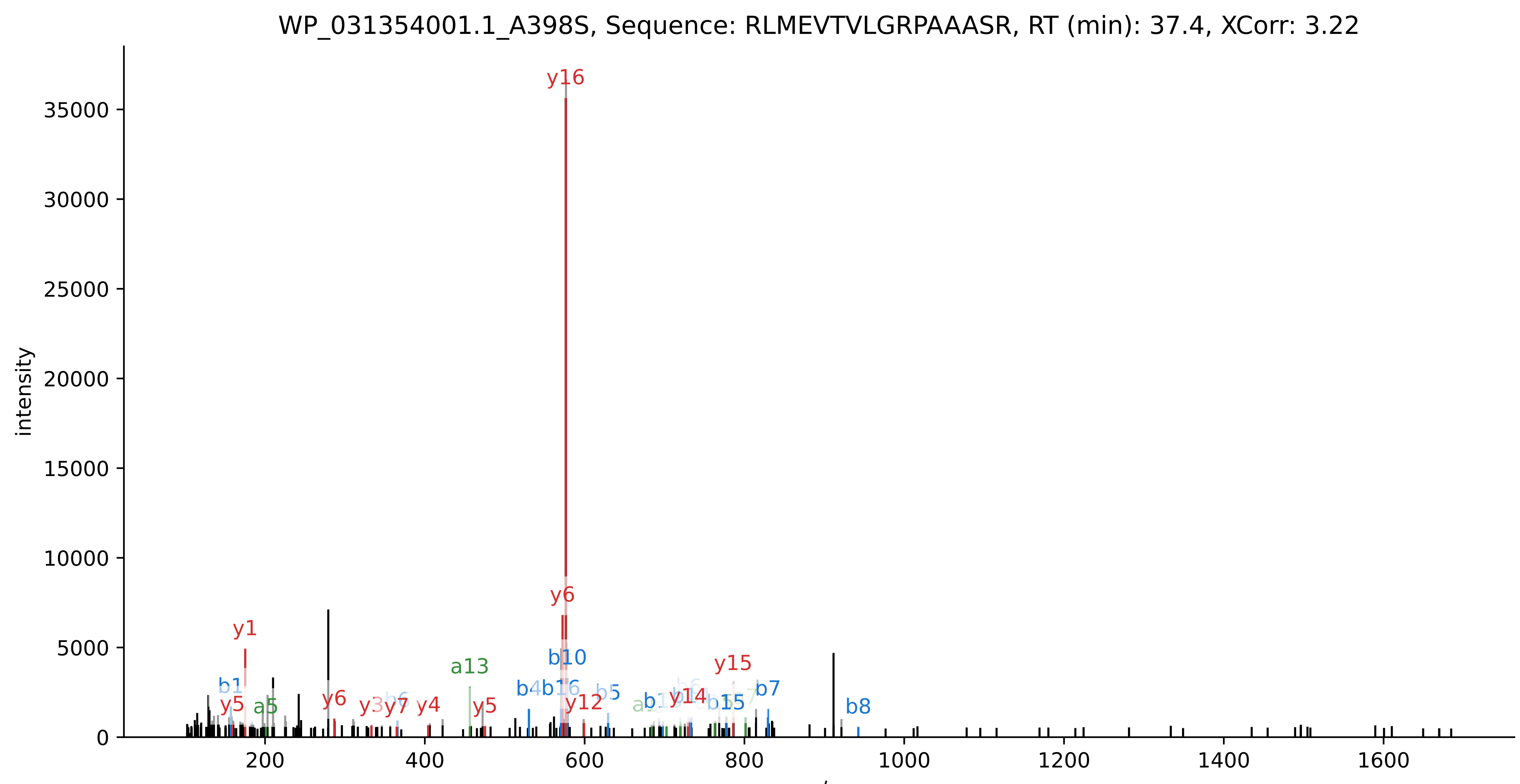
WP_031354001.1_A398S, Sequence: RLMEVTVLGRPAAASR, RT (min): 37.4, XCorr: 3.22
intensity
m/z
0
5000
10000
15000
20000
25000
30000
35000
200
400
600
800
1000
1200
1400
1600
y16
y6
y1
b10
a13
y15
b1
y5
a5
y6
y7
y4
y5
b4
y12
b5
y14
b15
b7
b8

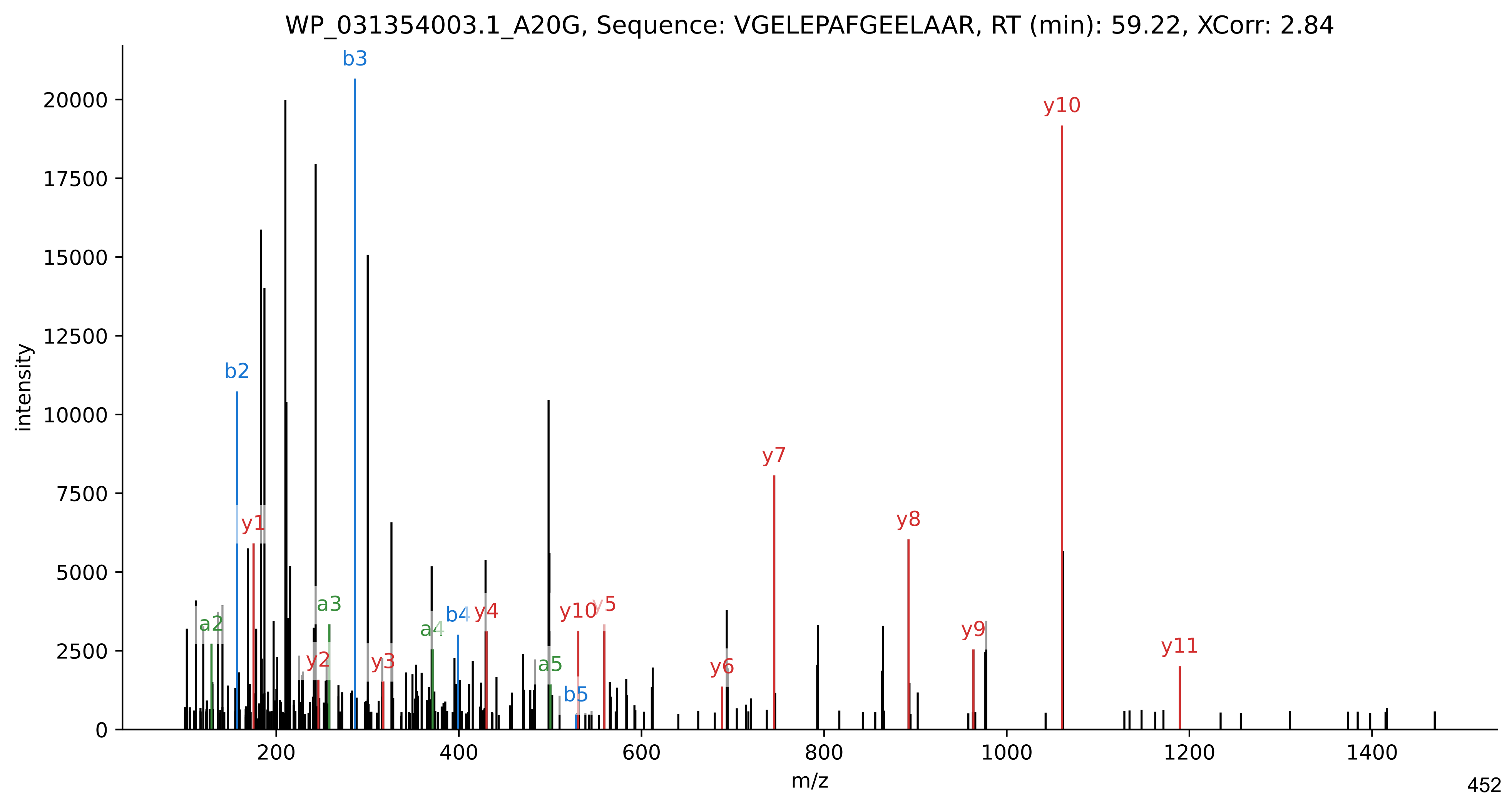
WP_031354003.1_A20G, Sequence: VGELEPAFGEELAAR, RT (min): 59.22, XCorr: 2.84
intensity
m/z
b2
b3
b4
b5
a2
a3
a4
a5
y1
y2
y3
y4
y5
y6
y7
y8
y9
y10
y10
y11

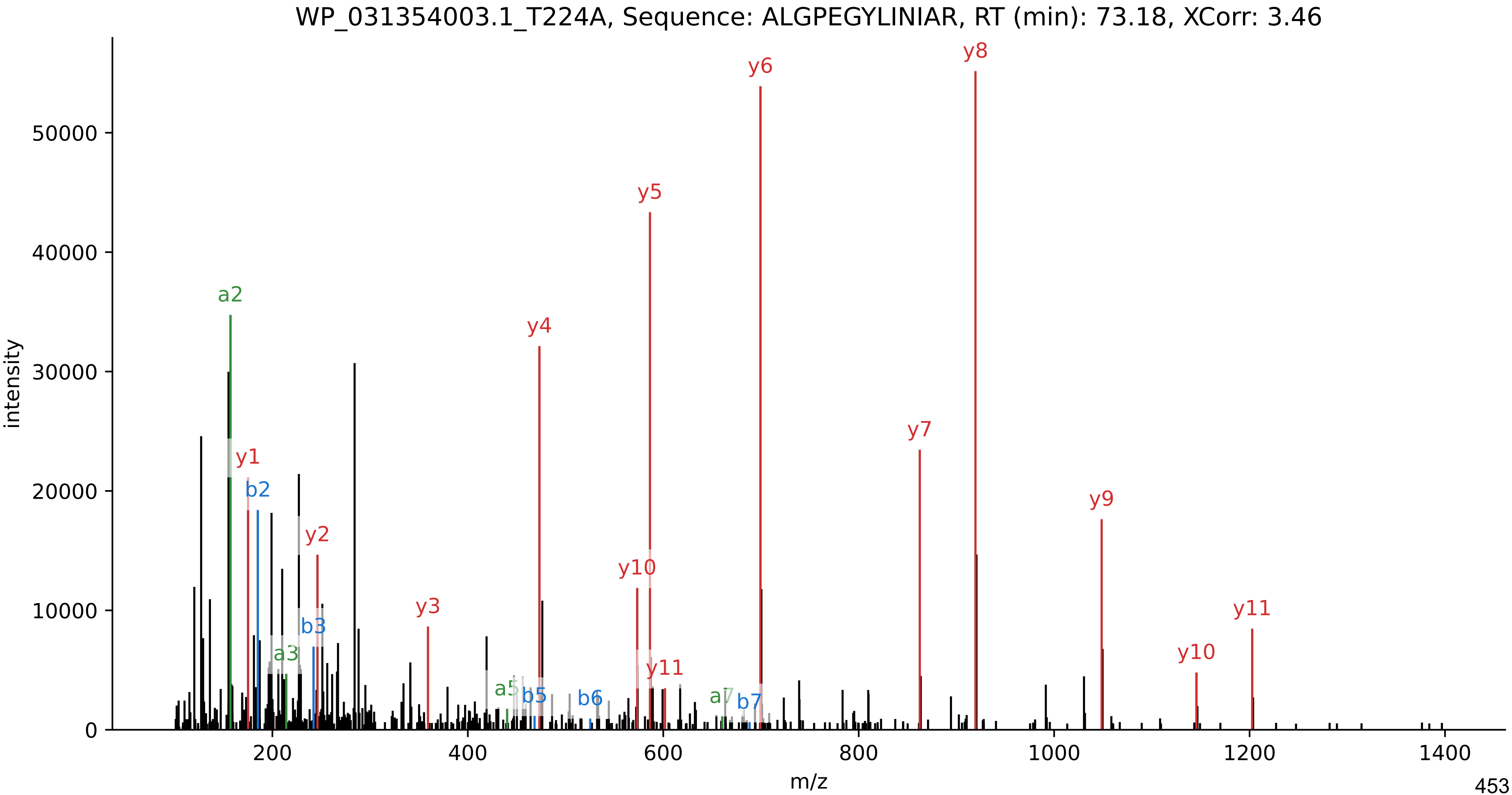
WP_031354003.1_T224A, Sequence: ALGPEGYLINIAR, RT (min): 73.18, XCorr: 3.46
intensity
m/z
a2
y1
b2
y2
a3
b3
y3
y4
a5
b5
b6
y10
y5
y11
a7
b7
y6
y7
y8
y9
y10
y11

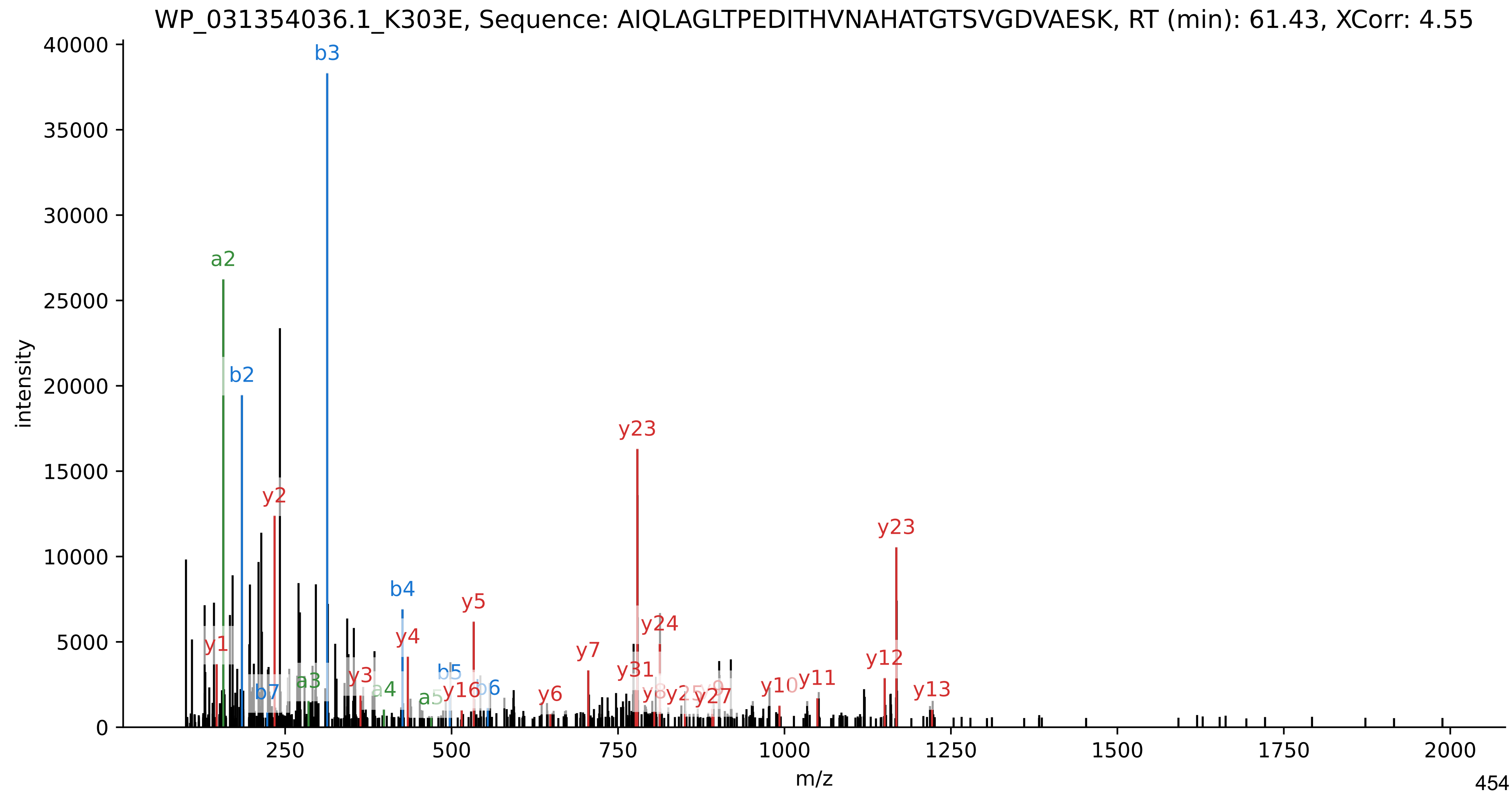
WP_031354036.1_K303E, Sequence: AIQLAGLTPEDITHVNAHATGTSVGDVAESK, RT (min): 61.43, XCorr: 4.55
intensity
m/z
b3
a2
b2
y2
y23
y23
b4
y5
y24
y4
y1
y7
y31
y12
b5
a3
y3
b6
y11
a4
y10
y13
b7
a5
y16
y6
y27
0
5000
10000
15000
20000
25000
30000
35000
40000
250
500
750
1000
1250
1500
1750
2000

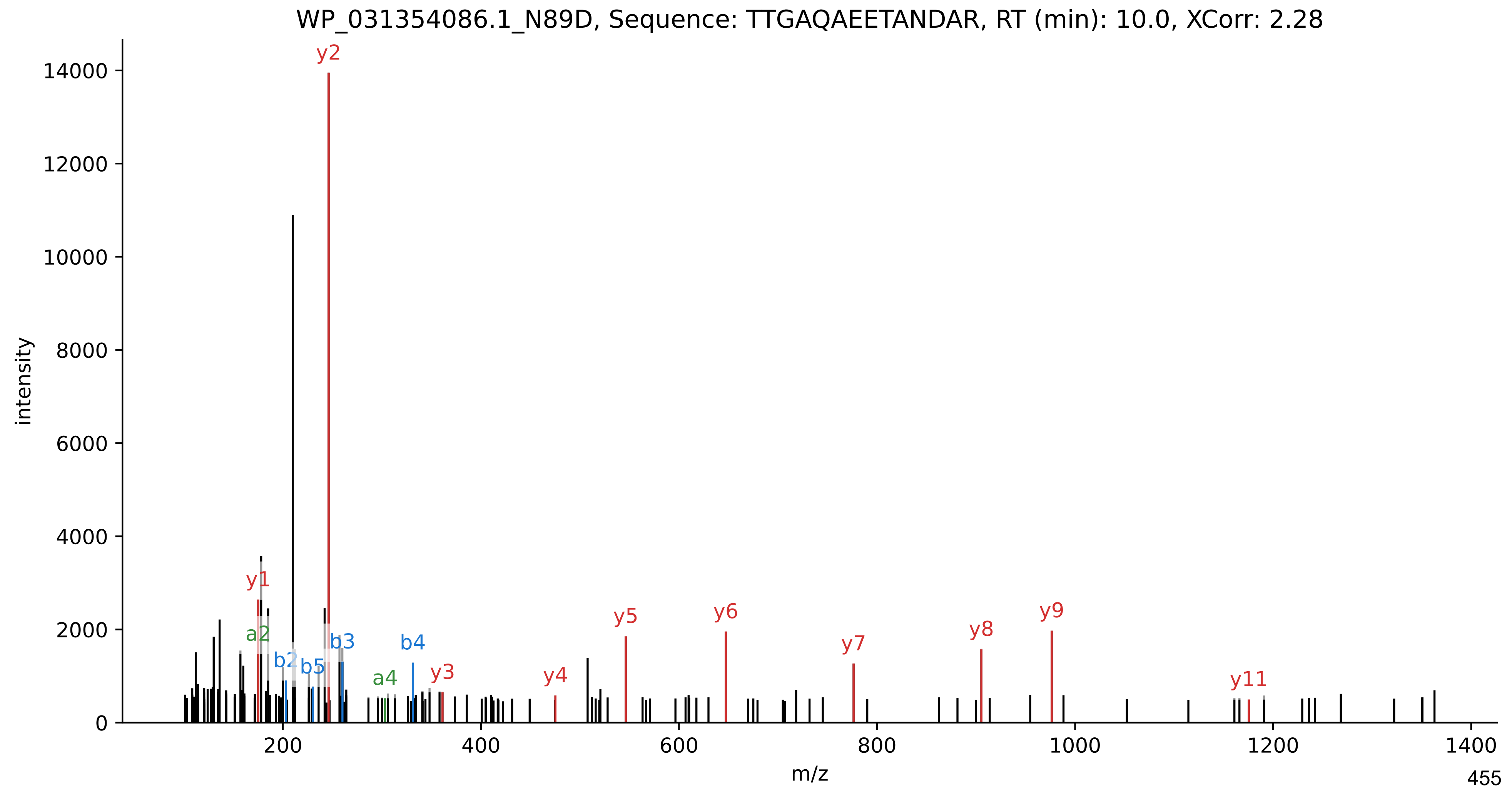

WP_031354086.1_N89D, Sequence: TTGAQAEETANDAR, RT (min): 10.0, XCorr: 2.28
intensity
m/z
y2
y1
a2
b2
b5
b3
a4
b4
y3
y4
y5
y6
y7
y8
y9
y11

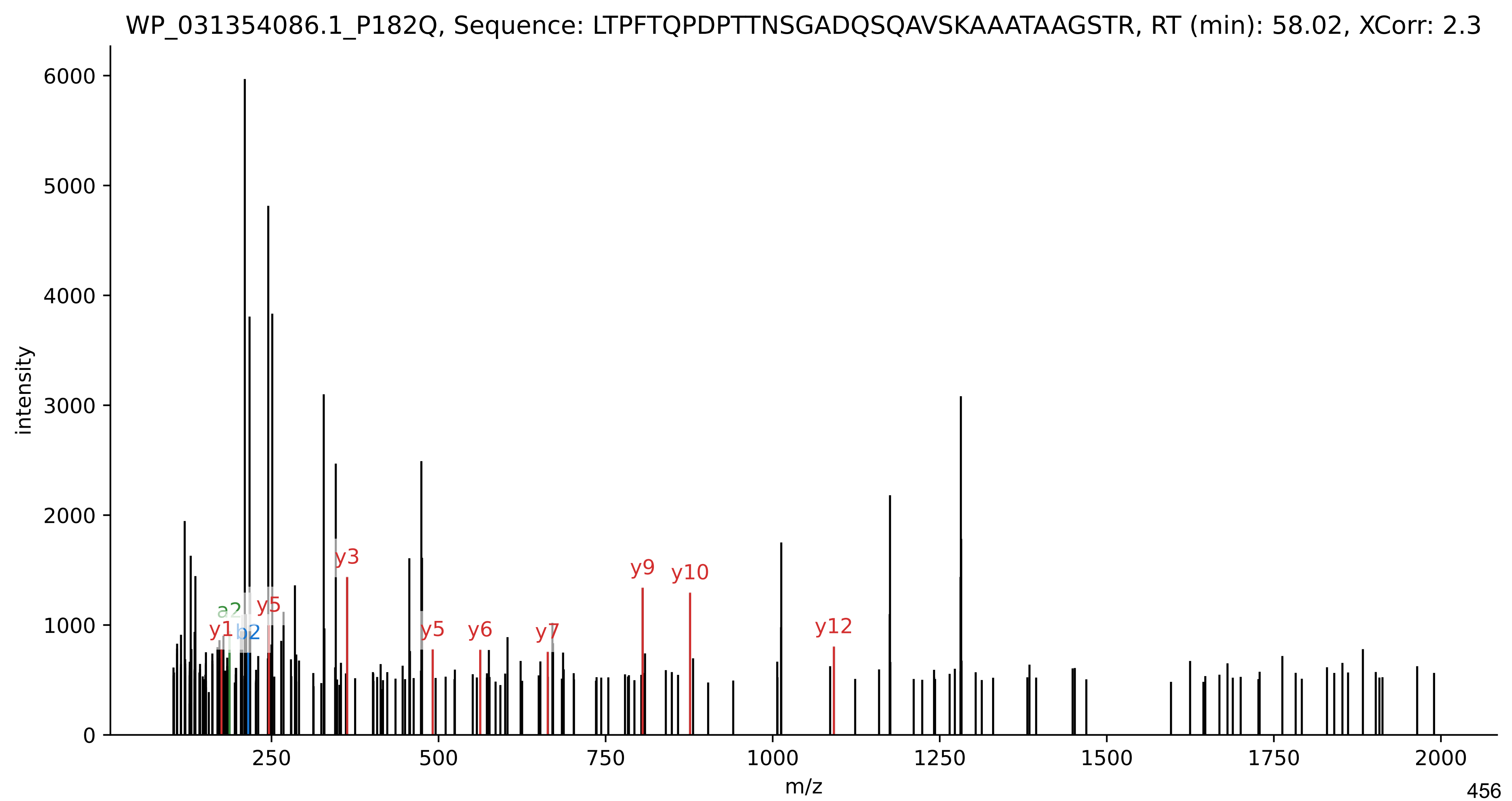

WP_031354086.1_P182Q, Sequence: LTPFTQPDPTTNSGADQSQAVSKAAATAAGSTR, RT (min): 58.02, XCorr: 2.3
intensity
m/z
y1
a2
b2
y5
y3
y5
y6
y7
y9
y10
y12

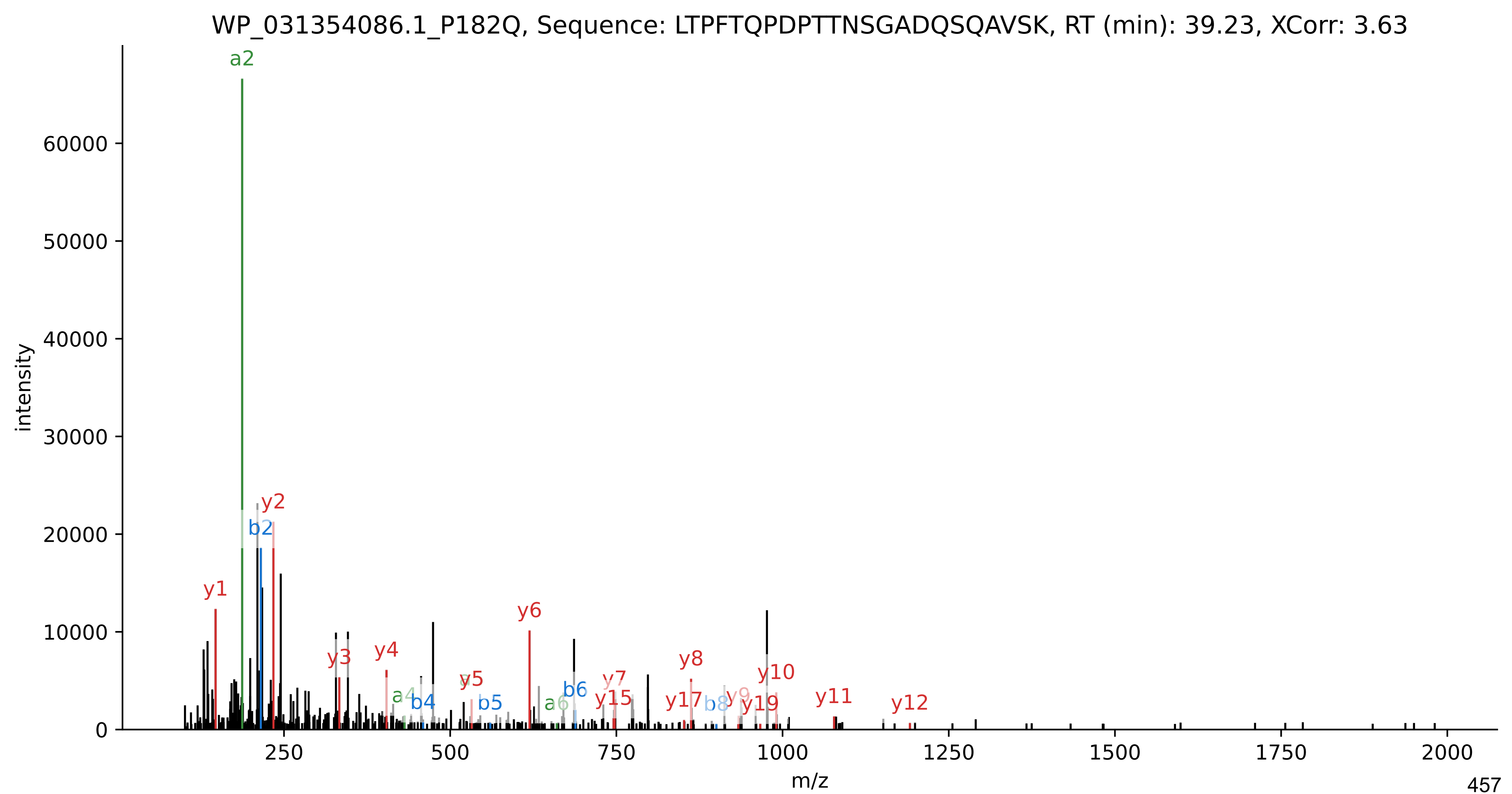
WP_031354086.1_P182Q, Sequence: LTPFTQPDPTTNSGADQSQAVSK, RT (min): 39.23, XCorr: 3.63
intensity
m/z
a2
y1
b2
y2
y3
y4
a4
b4
y5
b5
y6
a6
b6
y7
y15
y8
y17
b8
y9
y19
y10
y11
y12

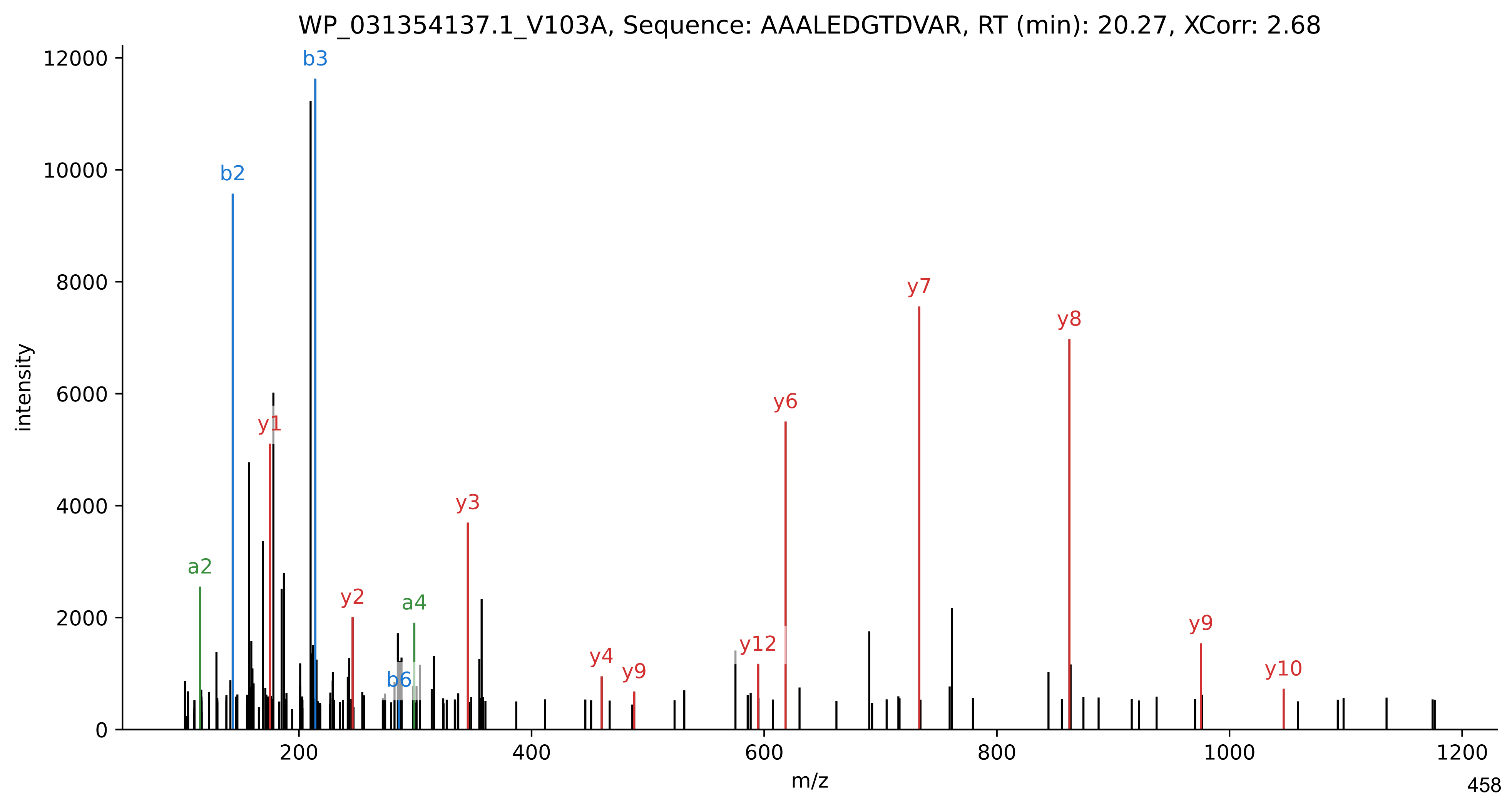
WP_031354137.1_V103A, Sequence: AAALEDGTDVAR, RT (min): 20.27, XCorr: 2.68
intensity
m/z
b2
b3
b6
a2
a4
y1
y2
y3
y4
y9
y12
y6
y7
y8
y9
y10
0
2000
4000
6000
8000
10000
12000
200
400
600
800
1000
1200

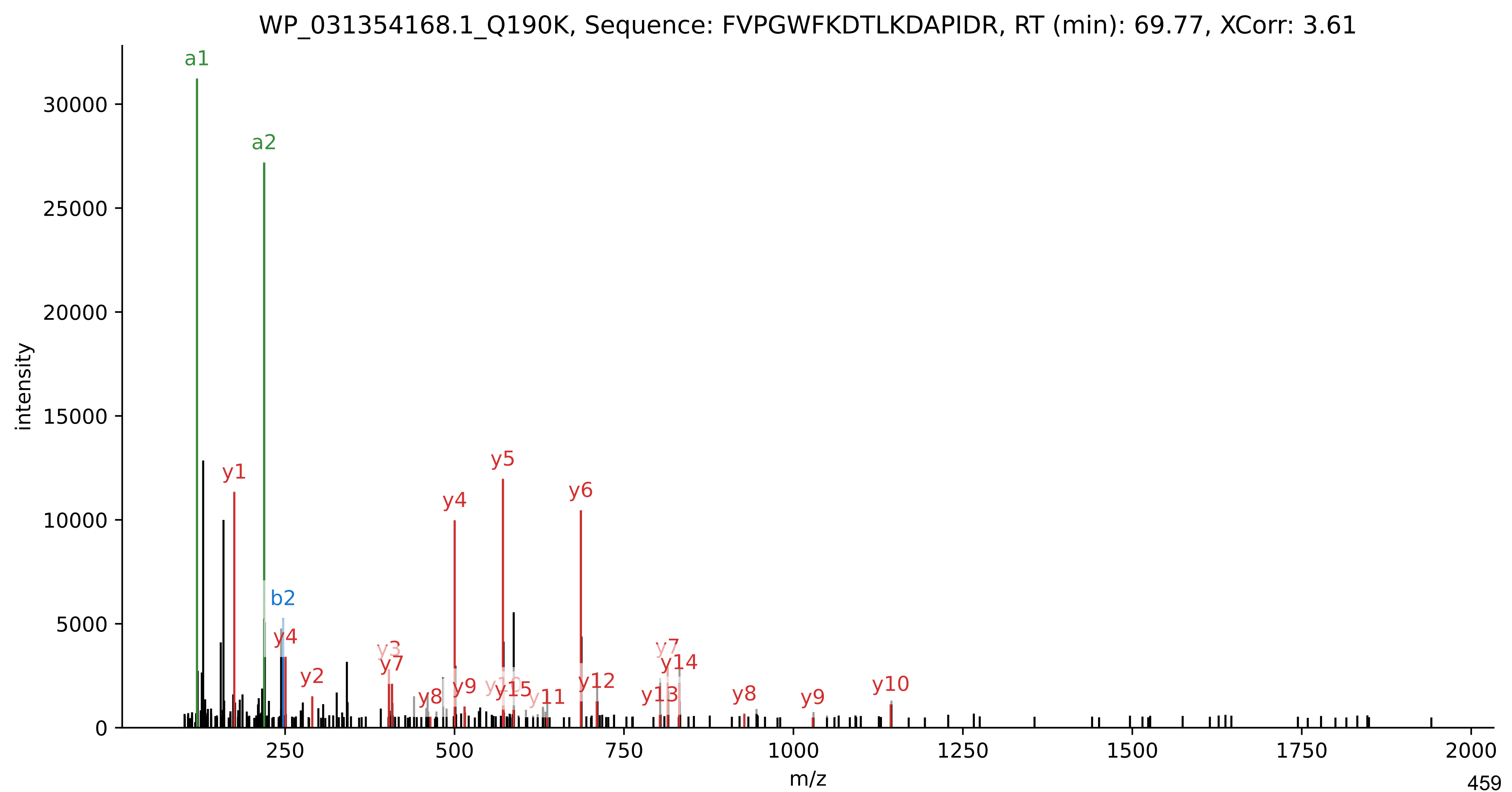
WP_031354168.1_Q190K, Sequence: FVPGWFKDTLKDAPIDR, RT (min): 69.77, XCorr: 3.61
intensity
m/z
a1
a2
b2
y1
y2
y3
y4
y5
y6
y7
y8
y9
y10
y11
y12
y13
y14
y15
y7
y8
y9
y10
0
5000
10000
15000
20000
25000
30000
250
500
750
1000
1250
1500
1750
2000

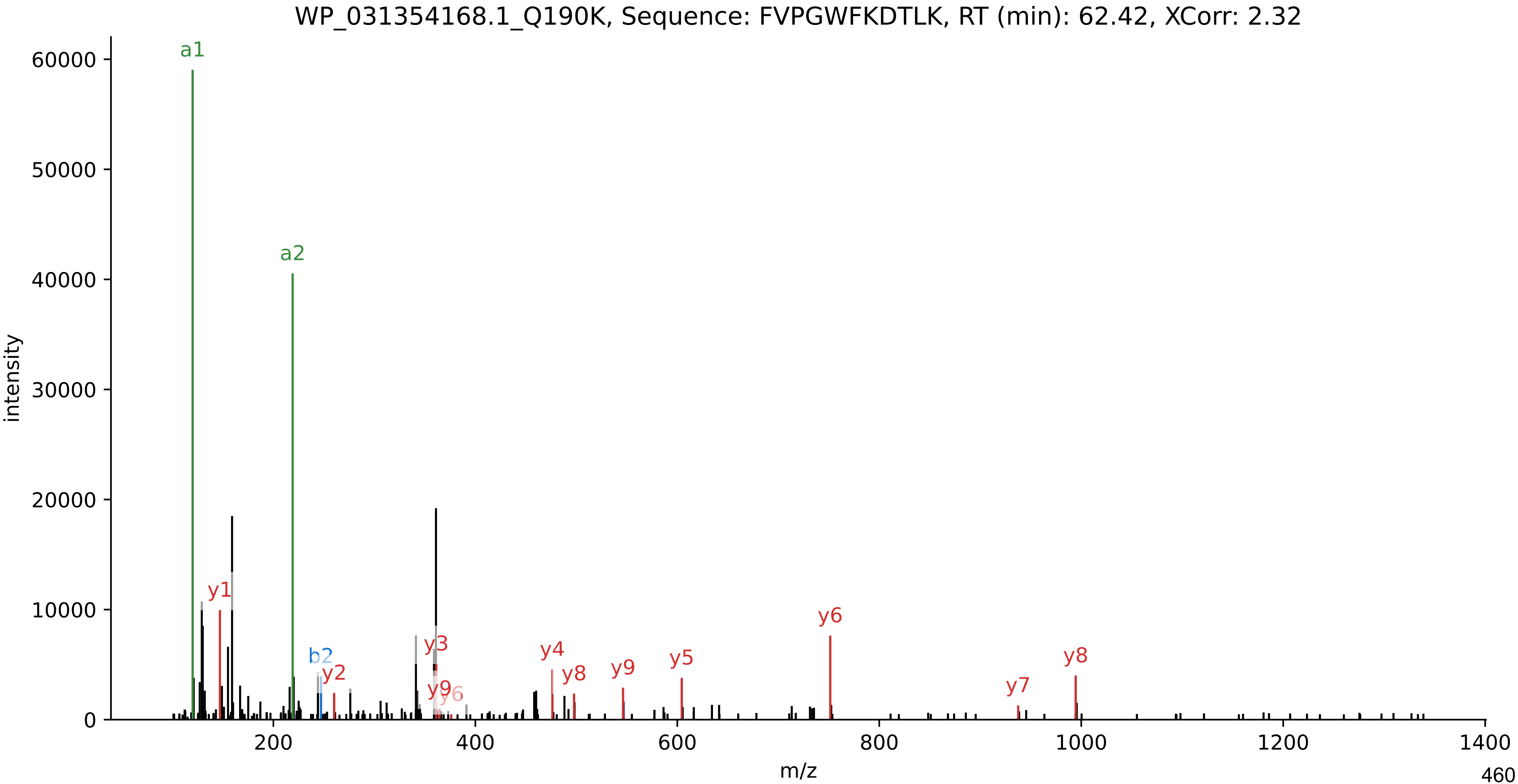

WP_031354168.1_Q190K, Sequence: FVPGWFKDTLK, RT (min): 62.42, XCorr: 2.32
a1
a2
y1
b2
y2
y3
y9
y6
y4
y8
y9
y5
y6
y7
y8
intensity
m/z

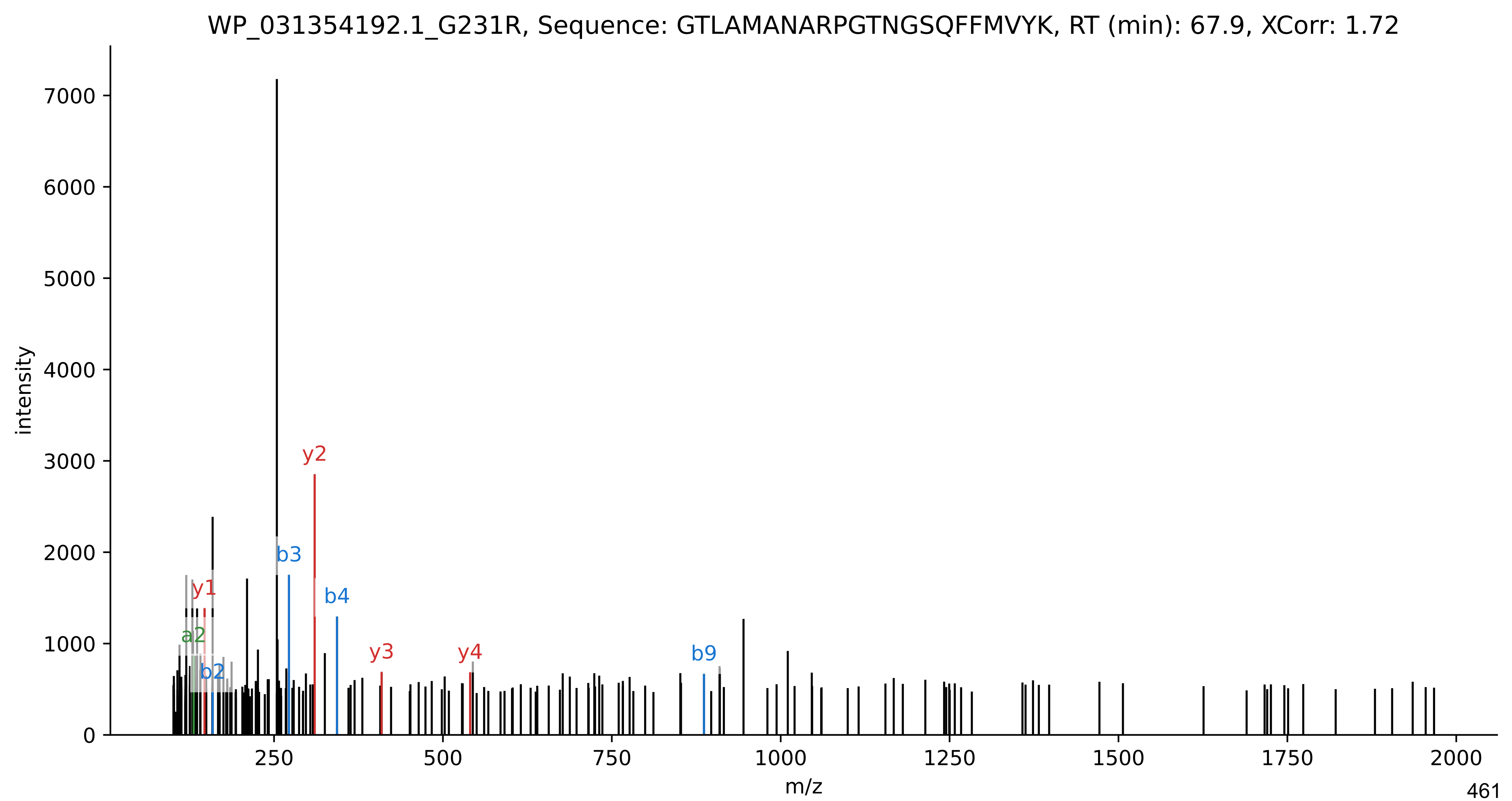
WP_031354192.1_G231R, Sequence: GTLAMANARPGTNGSQFFMVYK, RT (min): 67.9, XCorr: 1.72
intensity
m/z
y1
y2
y3
y4
a2
b2
b3
b4
b9
0
1000
2000
3000
4000
5000
6000
7000
250
500
750
1000
1250
1500
1750
2000

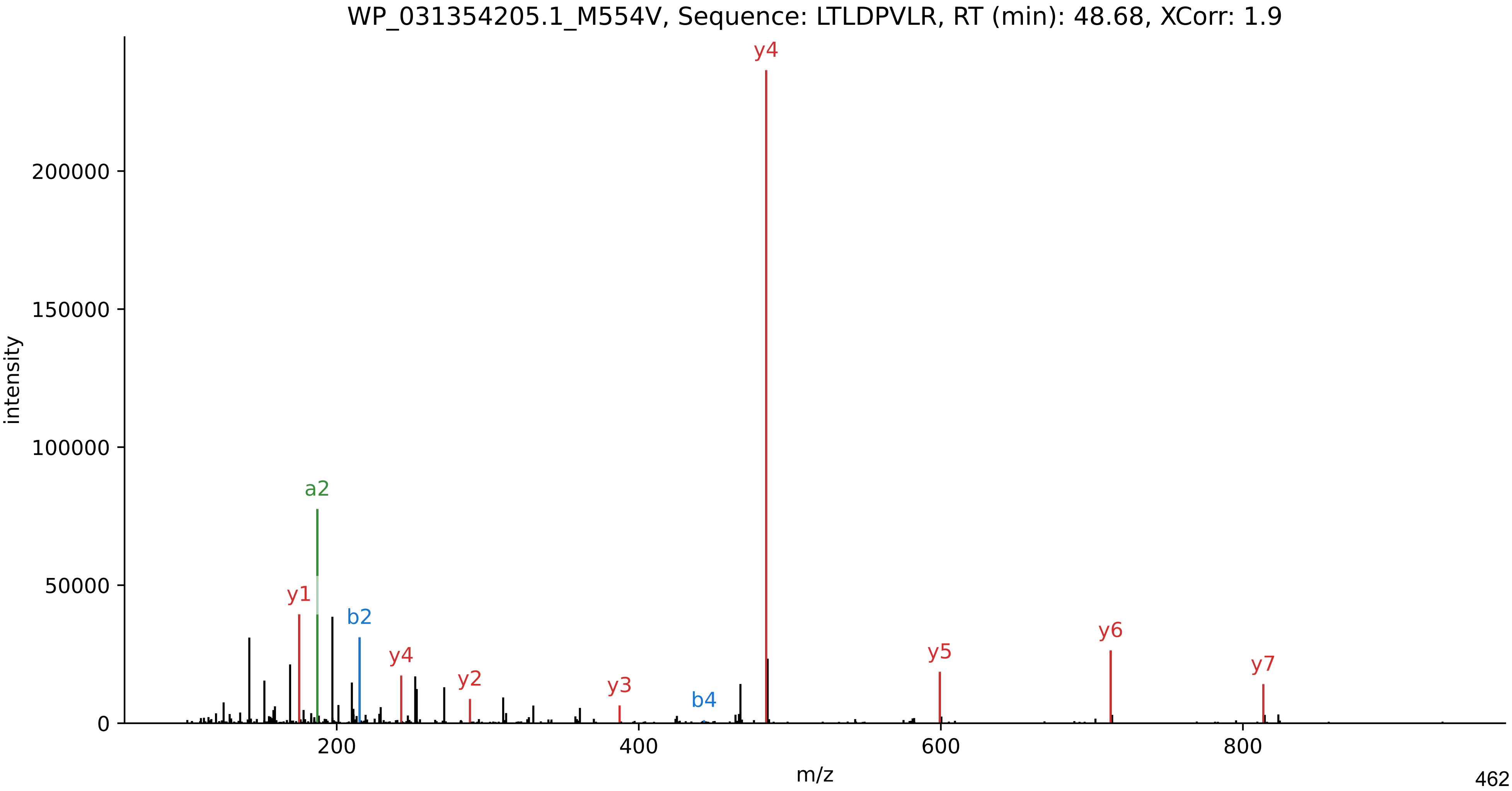

WP_031354205.1_M554V, Sequence: LTLDPVLR, RT (min): 48.68, XCorr: 1.9
intensity
m/z
y4
a2
y1
b2
y4
y2
y3
b4
y5
y6
y7
0
50000
100000
150000
200000
200
400
600
800

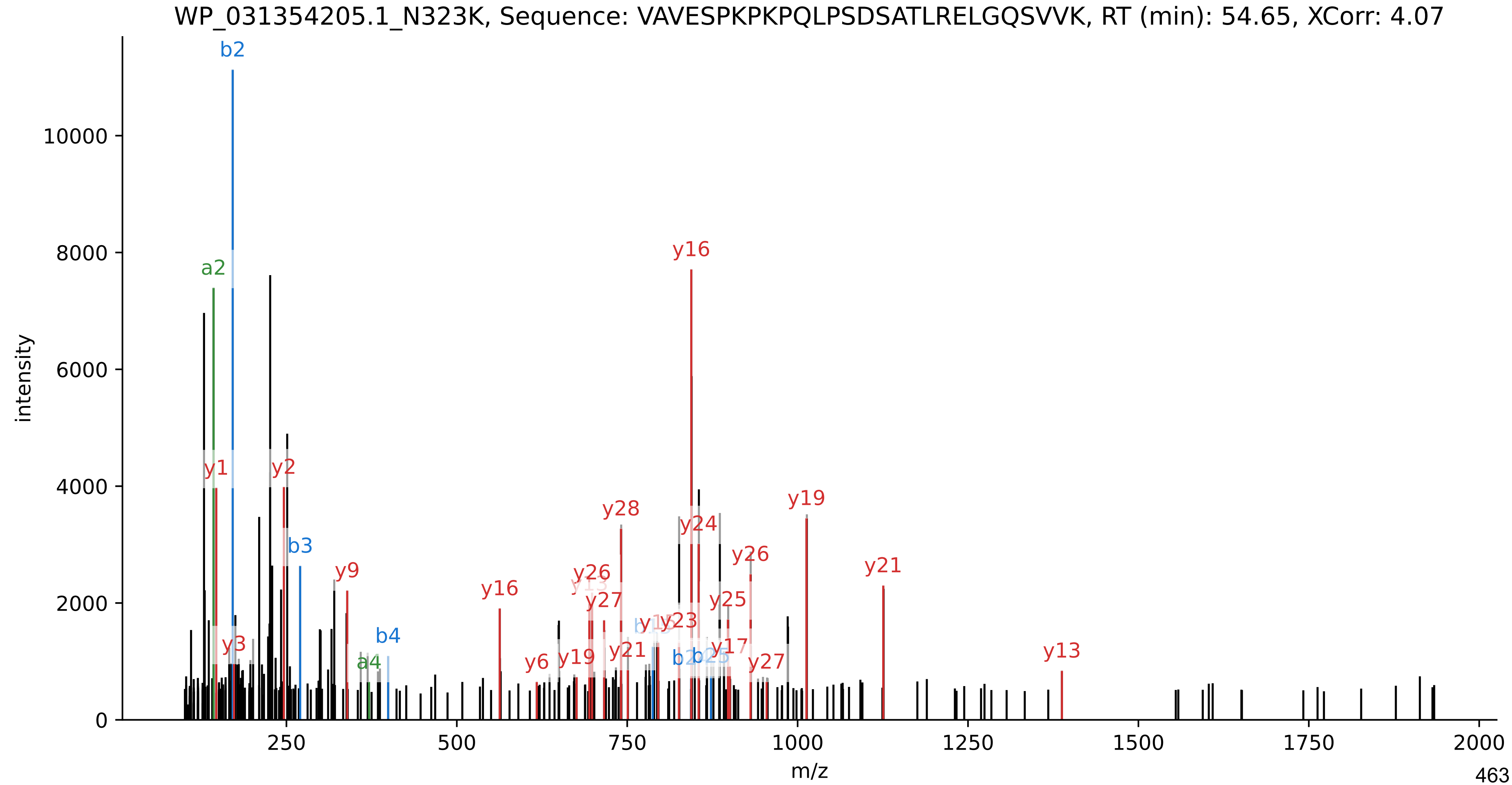

WP_031354205.1_N323K, Sequence: VAVESPKPKPQLPSDSATLRELGQSVVK, RT (min): 54.65, XCorr: 4.07
intensity
m/z
0
2000
4000
6000
8000
10000
250
500
750
1000
1250
1500
1750
2000
b2
a2
y1
y2
b3
y9
y3
a4
b4
y16
y6
y19
y26
y27
y21
y28
y23
y24
y25
y17
y26
y27
y19
y21
y13

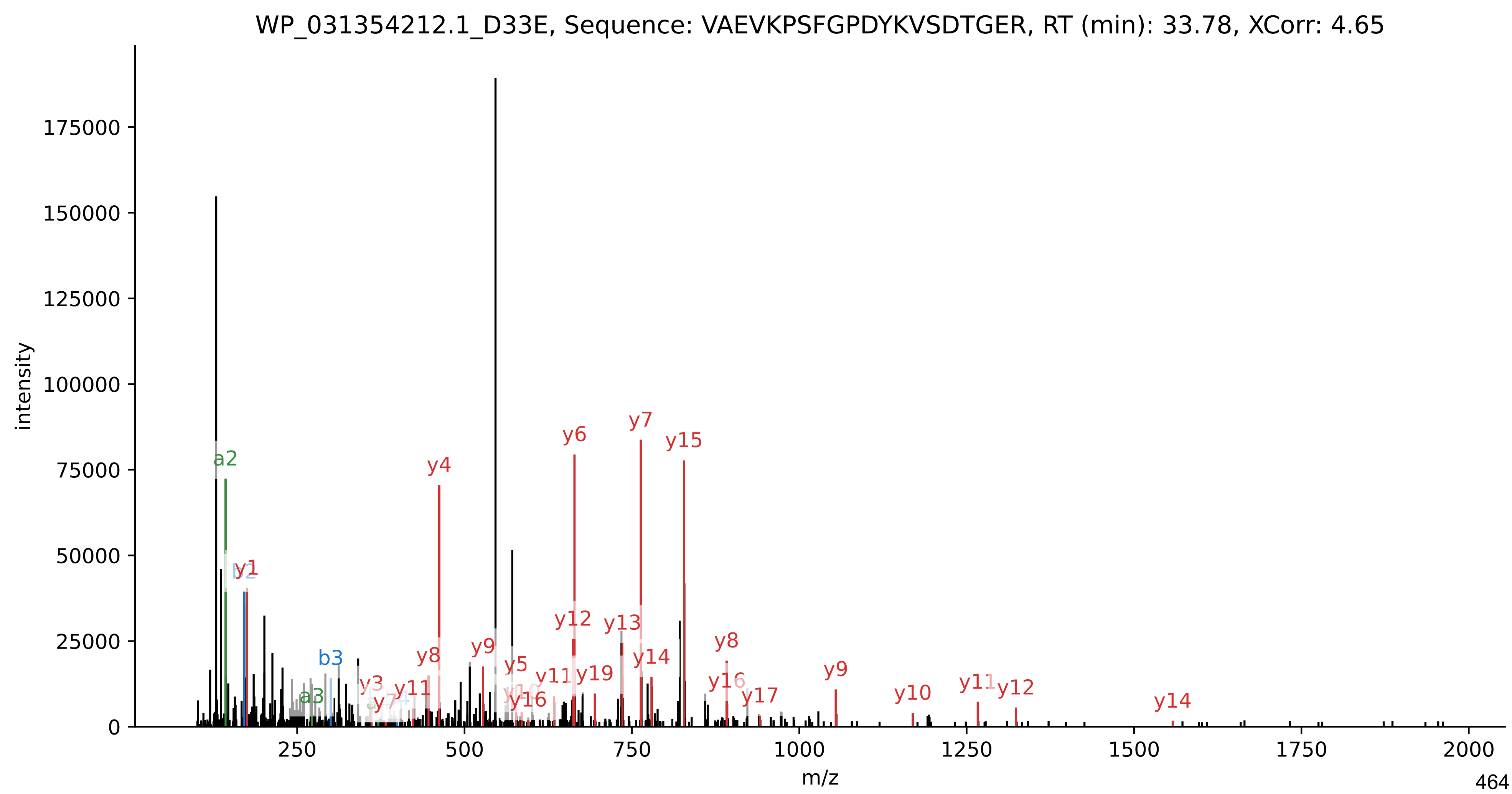

WP_031354212.1_D33E, Sequence: VAEVKPSFGPDYKVSDTGER, RT (min): 33.78, XCorr: 4.65
intensity
m/z
0
25000
50000
75000
100000
125000
150000
175000
250
500
750
1000
1250
1500
1750
2000
a2
y1
b3
a3
y3
y11
y8
y4
y9
y5
y16
y11
y19
y6
y12
y13
y7
y14
y15
y8
y16
y17
y9
y10
y11
y12
y14

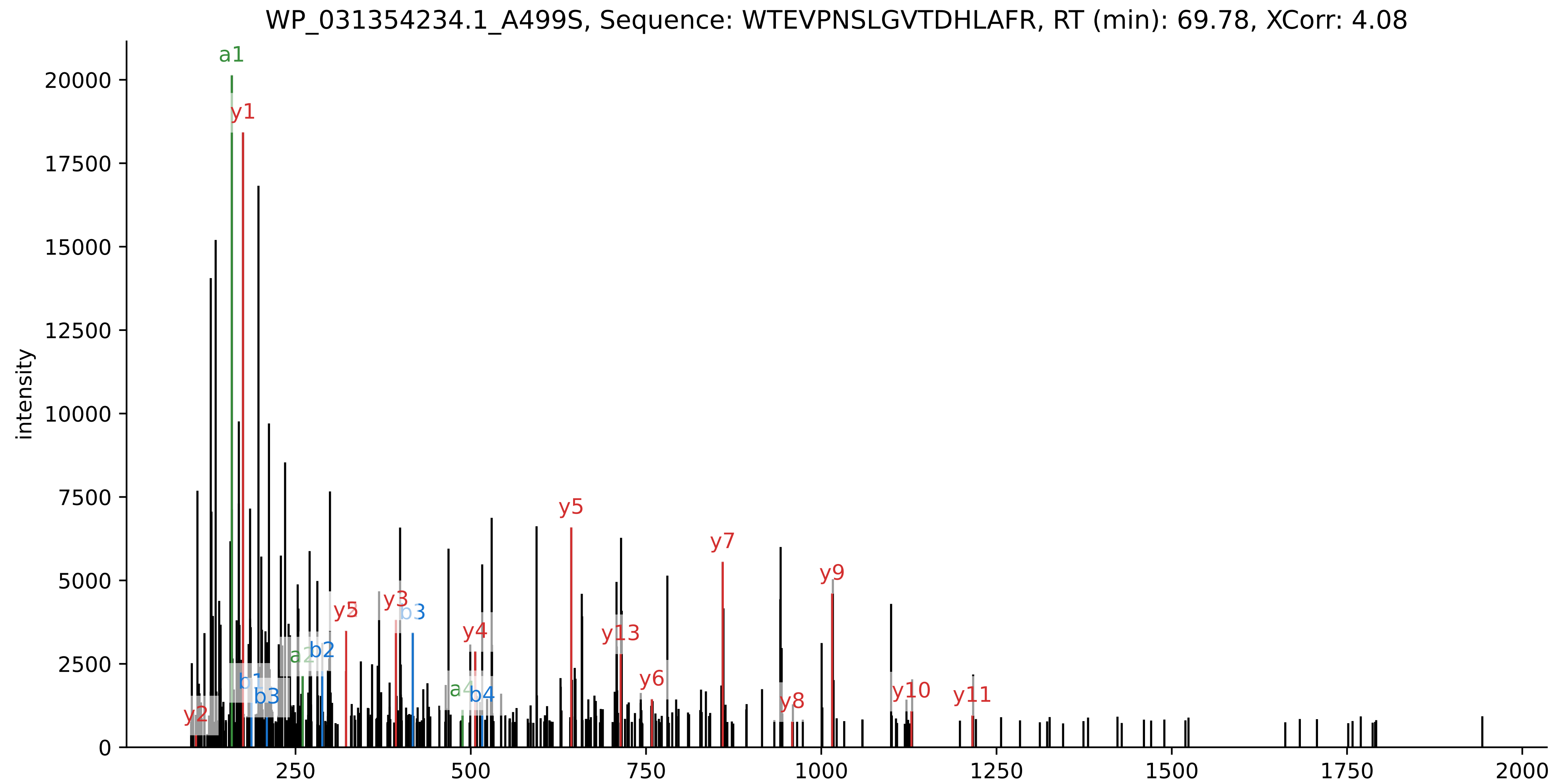

WP_031354234.1_A499S, Sequence: WTEVPNSLGVTDHLAFR, RT (min): 69.78, XCorr: 4.08
intensity
m/z
a1
y1
y2
b1
b3
a2
b2
y5
y3
b3
y4
a4
b4
y5
y13
y6
y7
y8
y9
y10
y11

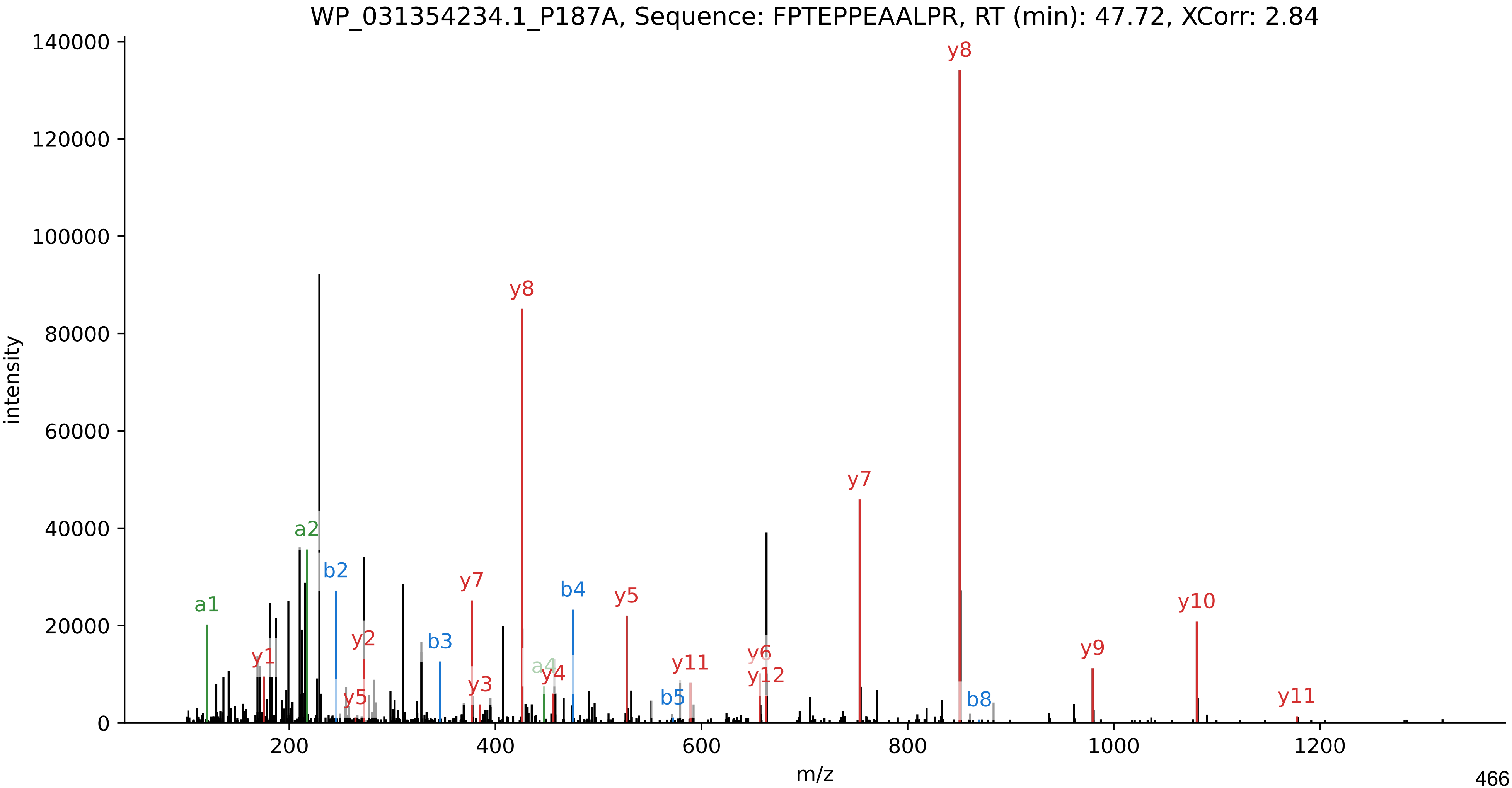

WP_031354234.1_P187A, Sequence: FPTEPPEAALPR, RT (min): 47.72, XCorr: 2.84
intensity
m/z
0
20000
40000
60000
80000
100000
120000
140000
200
400
600
800
1000
1200
a1
a2
b2
y1
y2
y5
y7
y3
b3
y8
a4
y4
b4
y5
b5
y11
y6
y12
y7
y8
b8
y9
y10
y11

WP_031354269.1_D424N, Sequence: AVHDLVDQVALPSSVLNRLPR, RT (min): 78.52, XCorr: 3.51

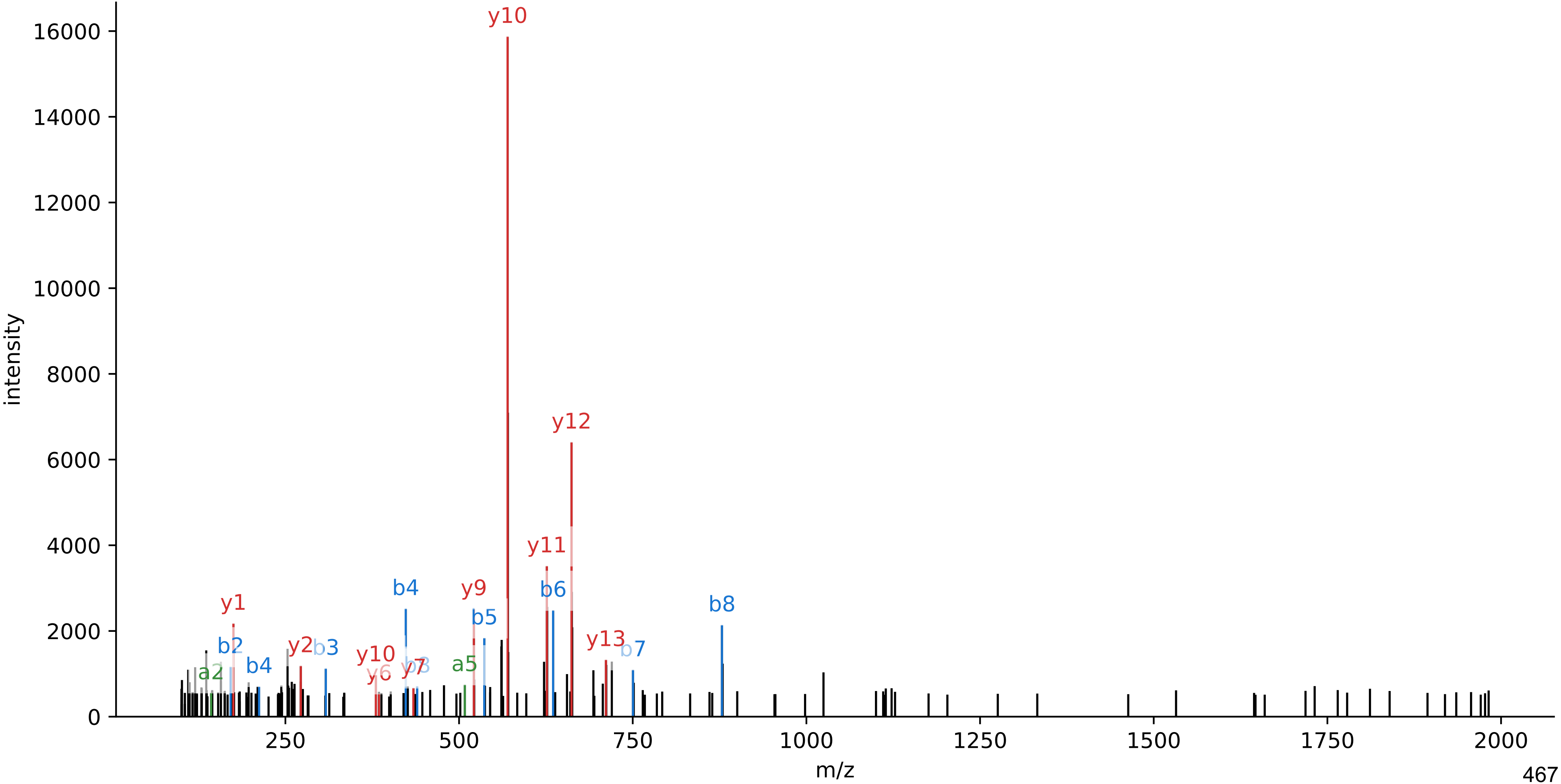

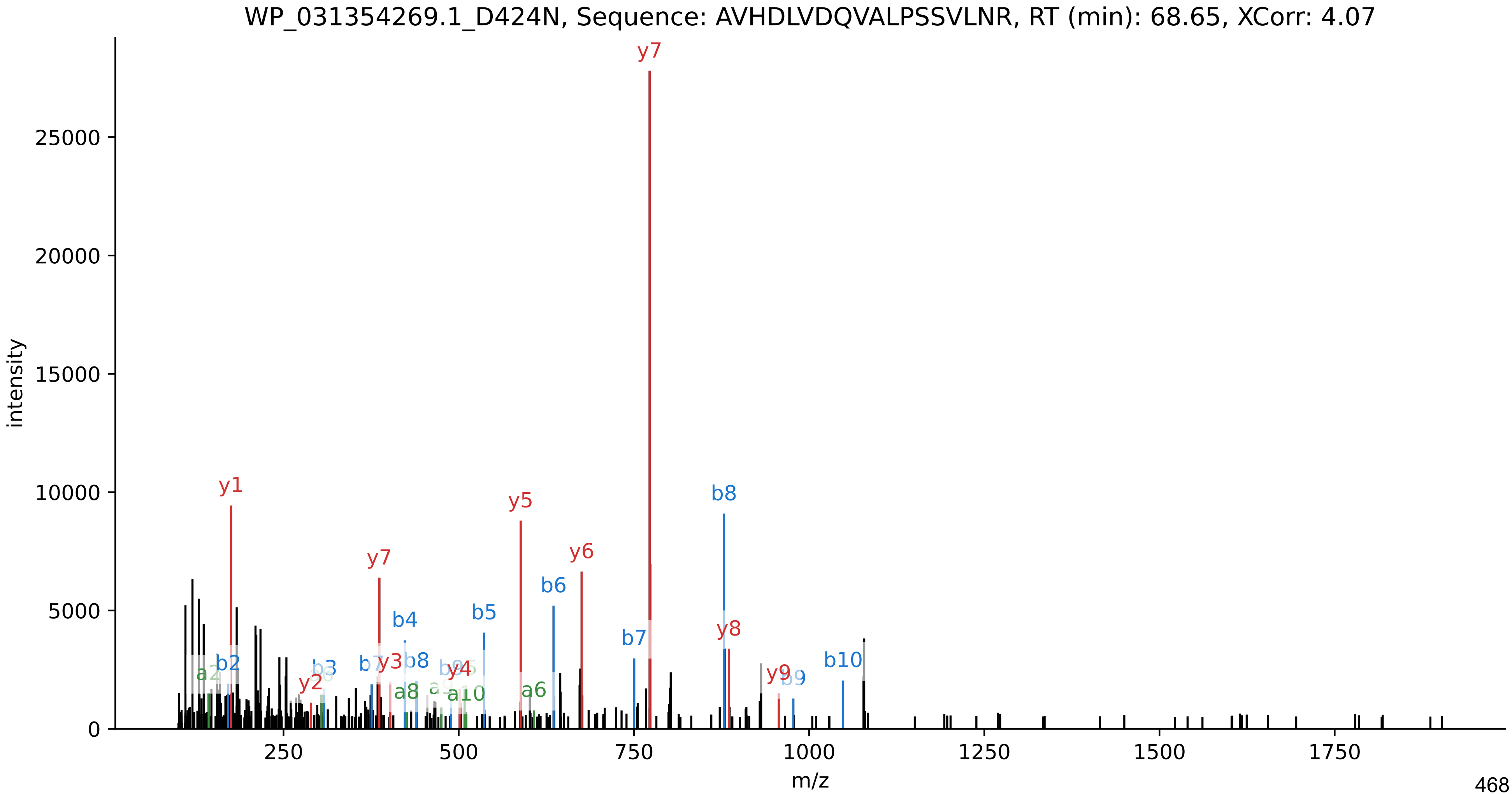
WP_031354269.1_D424N, Sequence: AVHDLVDQVALPSSVLNR, RT (min): 68.65, XCorr: 4.07
intensity
m/z
y7
y1
y5
y6
b8
b6
b4
b5
b7
y8
b10
y9
b2
b3
y2
y3
y4
a2
a6
a8
a10
0
5000
10000
15000
20000
25000
250
500
750
1000
1250
1500
1750

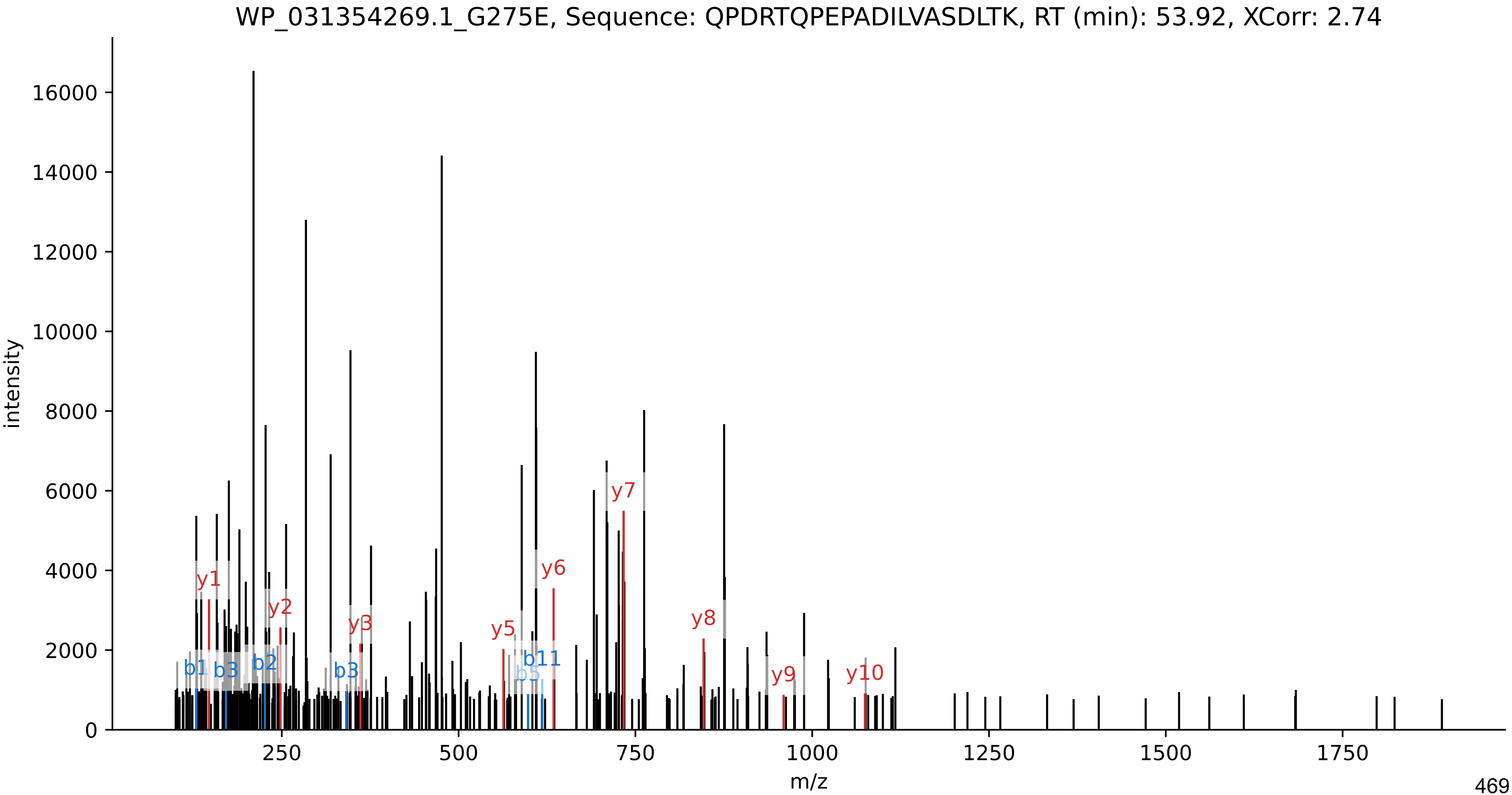
WP_031354269.1_G275E, Sequence: QPDRTQPEPADILVASDLTK, RT (min): 53.92, XCorr: 2.74
intensity
m/z
y1
y2
y3
y5
y6
y7
y8
y9
y10
b1
b3
b2
b3
b5
b11

WP_031354269.1_G275E, Sequence: TGPRQPDRTQPEPADILVASDLTK, RT (min): 51.53, XCorr: 3.31

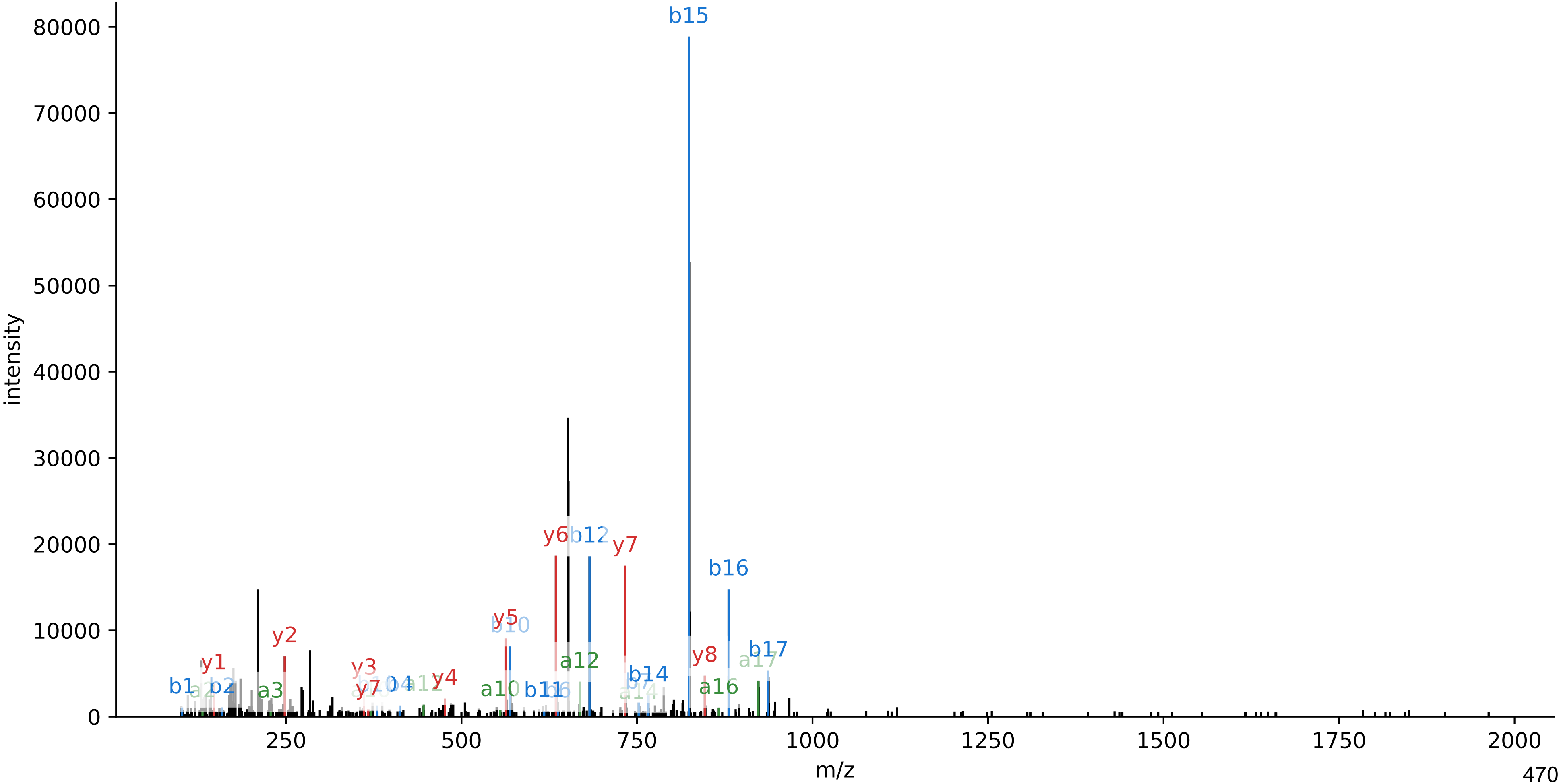

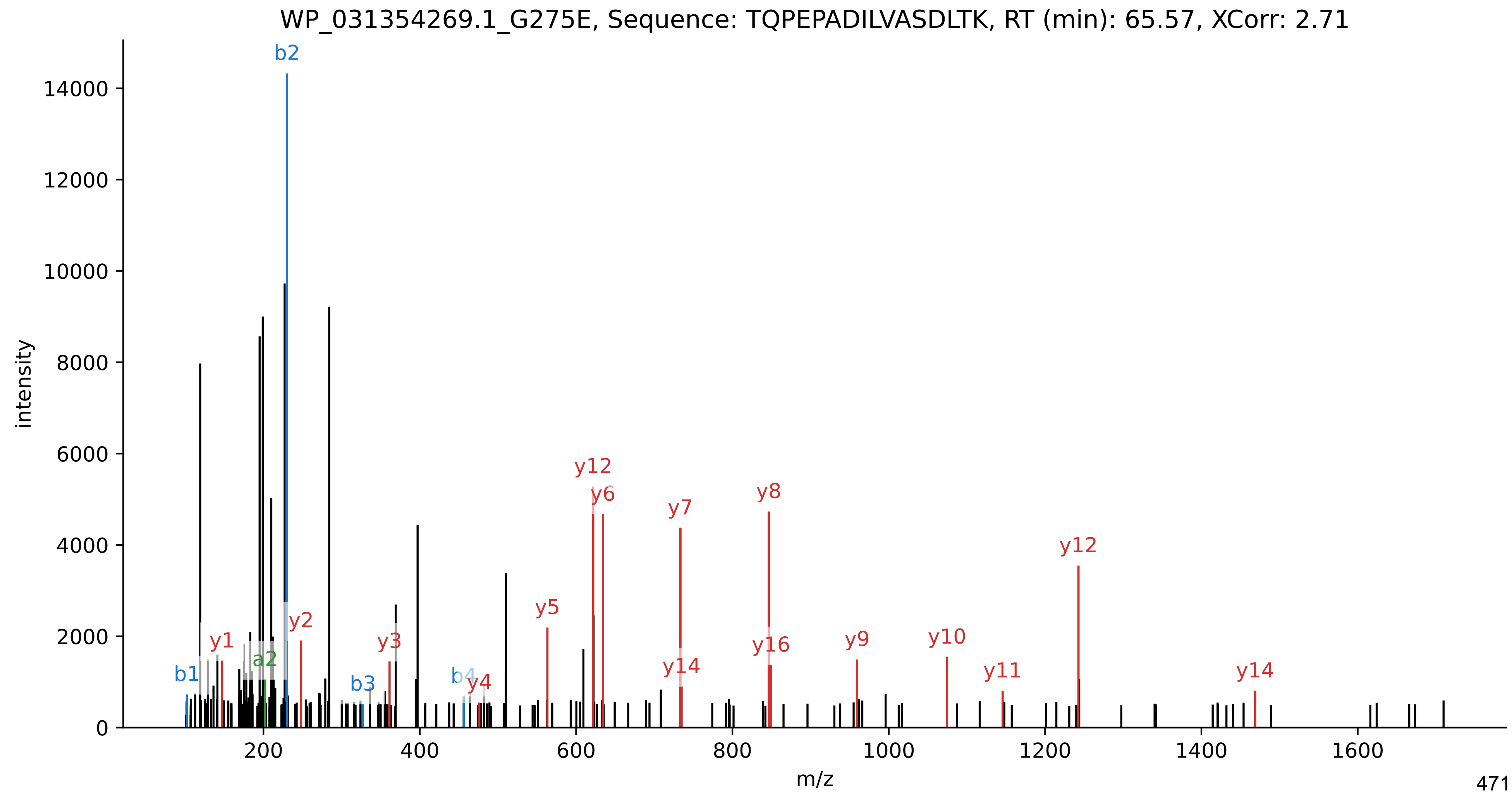
WP_031354269.1_G275E, Sequence: TQPEPADILVASDLTK, RT (min): 65.57, XCorr: 2.71
intensity
m/z
b1
b2
b3
b4
a2
y1
y2
y3
y4
y5
y6
y7
y8
y9
y10
y11
y12
y12
y14
y14
y16

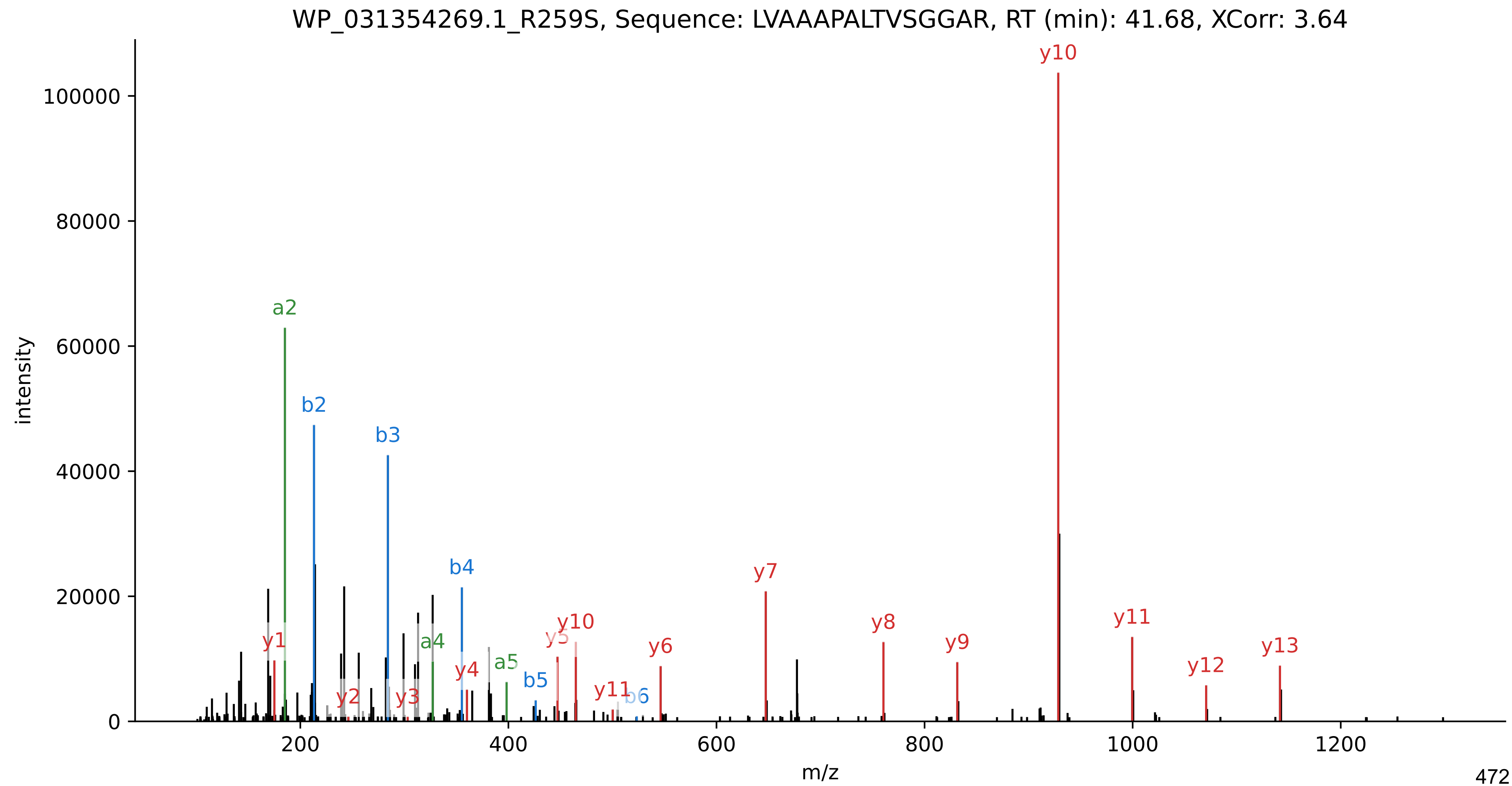

WP_031354269.1_R259S, Sequence: LVAAAPALTVSGGAR, RT (min): 41.68, XCorr: 3.64
intensity
m/z
y10
a2
b2
b3
b4
y7
y11
y8
y13
y9
y12
y6
y10
y5
y1
a4
a5
y4
b5
y11
b6
y2
y3
100000
80000
60000
40000
20000
0
200
400
600
800
1000
1200

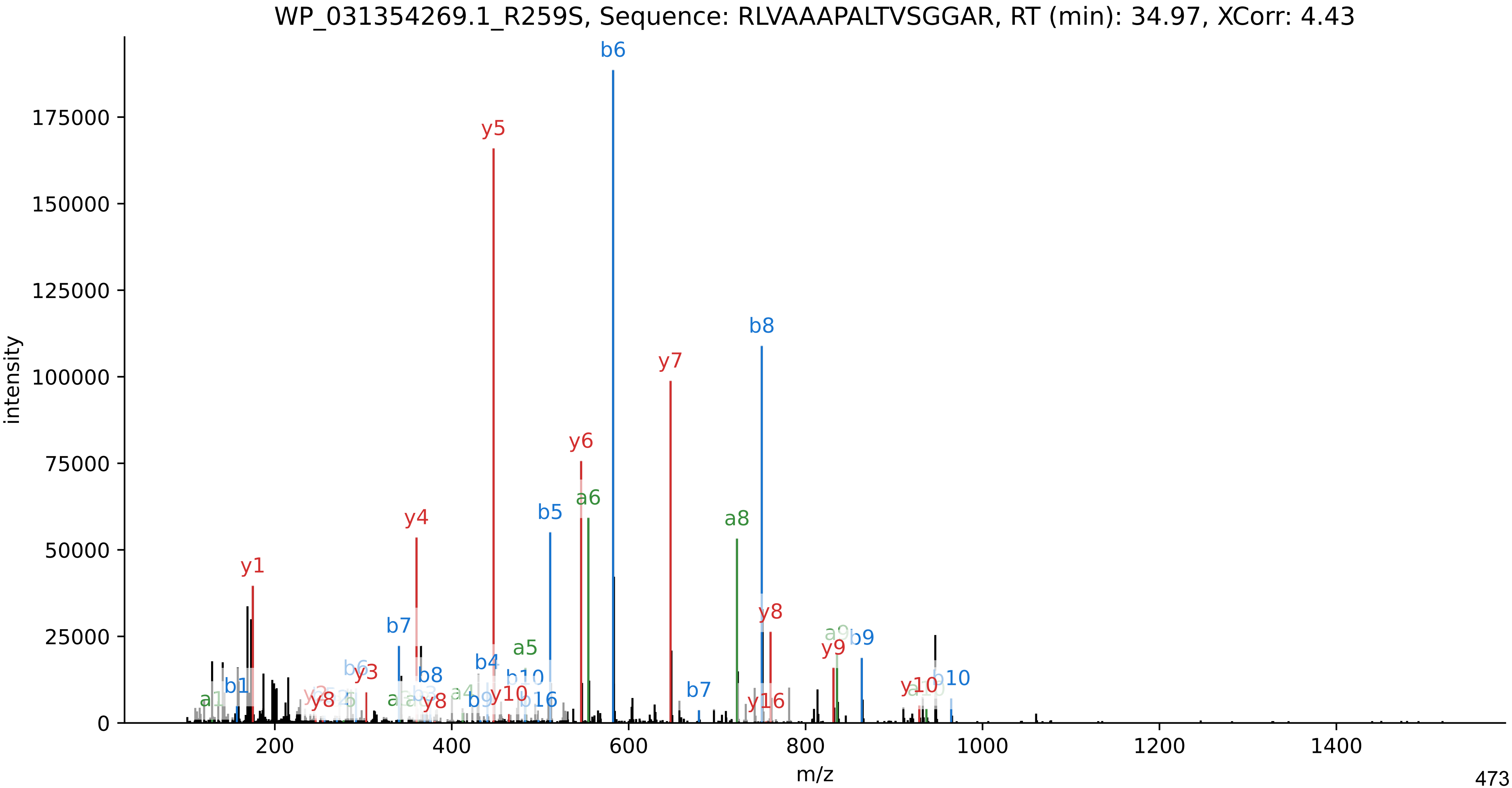

WP_031354269.1_R259S, Sequence: RLVAAAPALTVSGGAR, RT (min): 34.97, XCorr: 4.43
intensity
m/z
b6
y5
b8
y7
y6
a6
b5
a8
y4
y1
y8
b7
b9
y9
b4
a5
b8
y3
b10
y10
b7
y16
b16
b1
a1
y8
y8

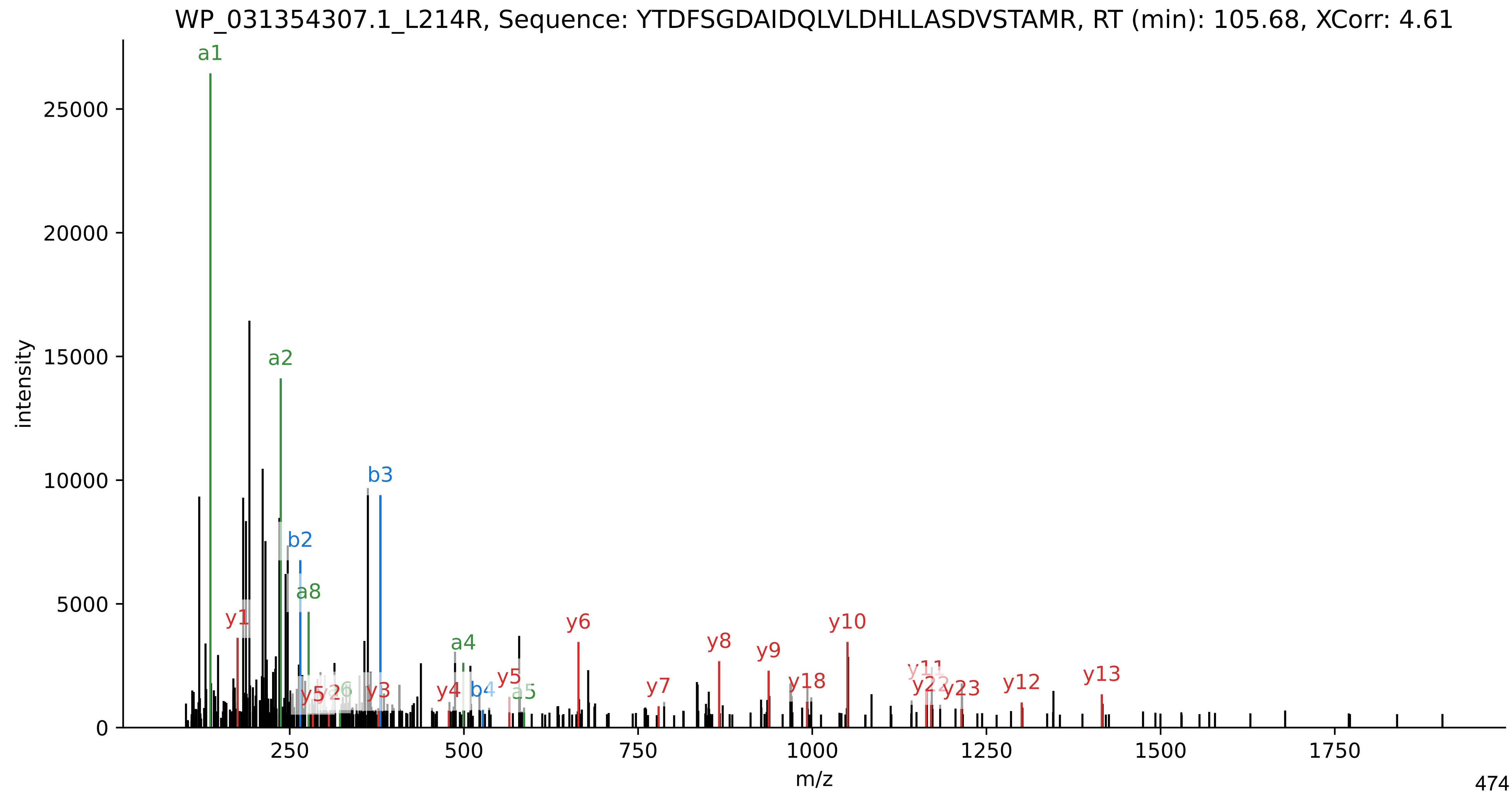

WP_031354307.1_L214R, Sequence: YTDFSGDAIDQLVLDHLLASDVSTAMR, RT (min): 105.68, XCorr: 4.61
intensity
m/z
a1
a2
a8
a4
a5
b2
b3
b4
y1
y3
y4
y5
y6
y7
y8
y9
y10
y11
y12
y13
y18
y22
y23

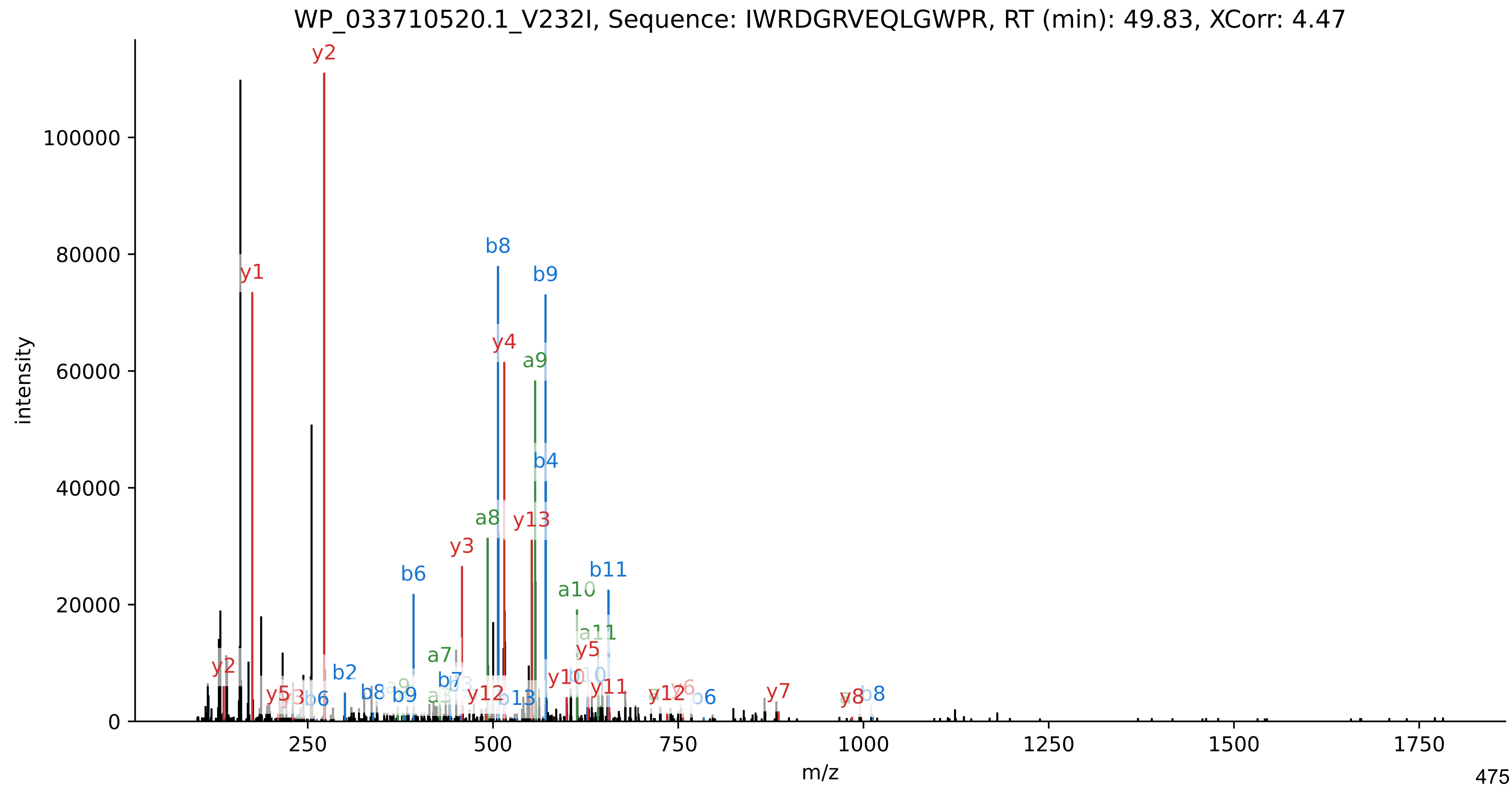
WP_033710520.1_V232I, Sequence: IWRDGRVEQLGWPR, RT (min): 49.83, XCorr: 4.47
intensity
m/z
0
20000
40000
60000
80000
100000
250
500
750
1000
1250
1500
1750
y2
y1
b8
b9
y4
a9
b4
a8
y13
y3
b6
b11
a10
a11
y5
a7
y2
b2
y10
y11
y12
y7
y8
b8
b6
y5
b6
b8
b9
b7
y12

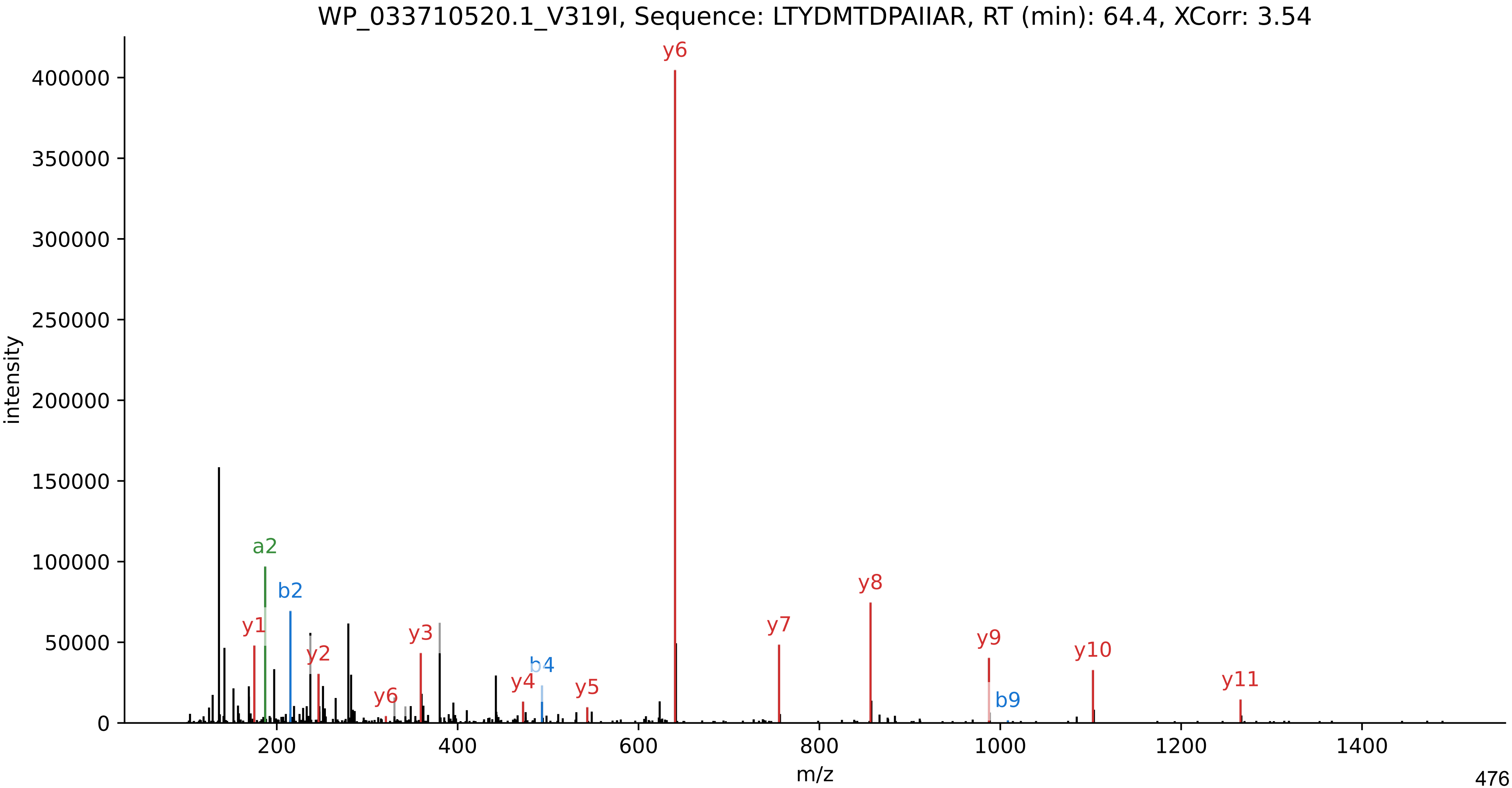
WP_033710520.1_V319I, Sequence: LTYDMTDPAIIAR, RT (min): 64.4, XCorr: 3.54
intensity
m/z
y6
a2
b2
y1
y2
y3
y6
b4
y4
y5
y7
y8
y9
b9
y10
y11

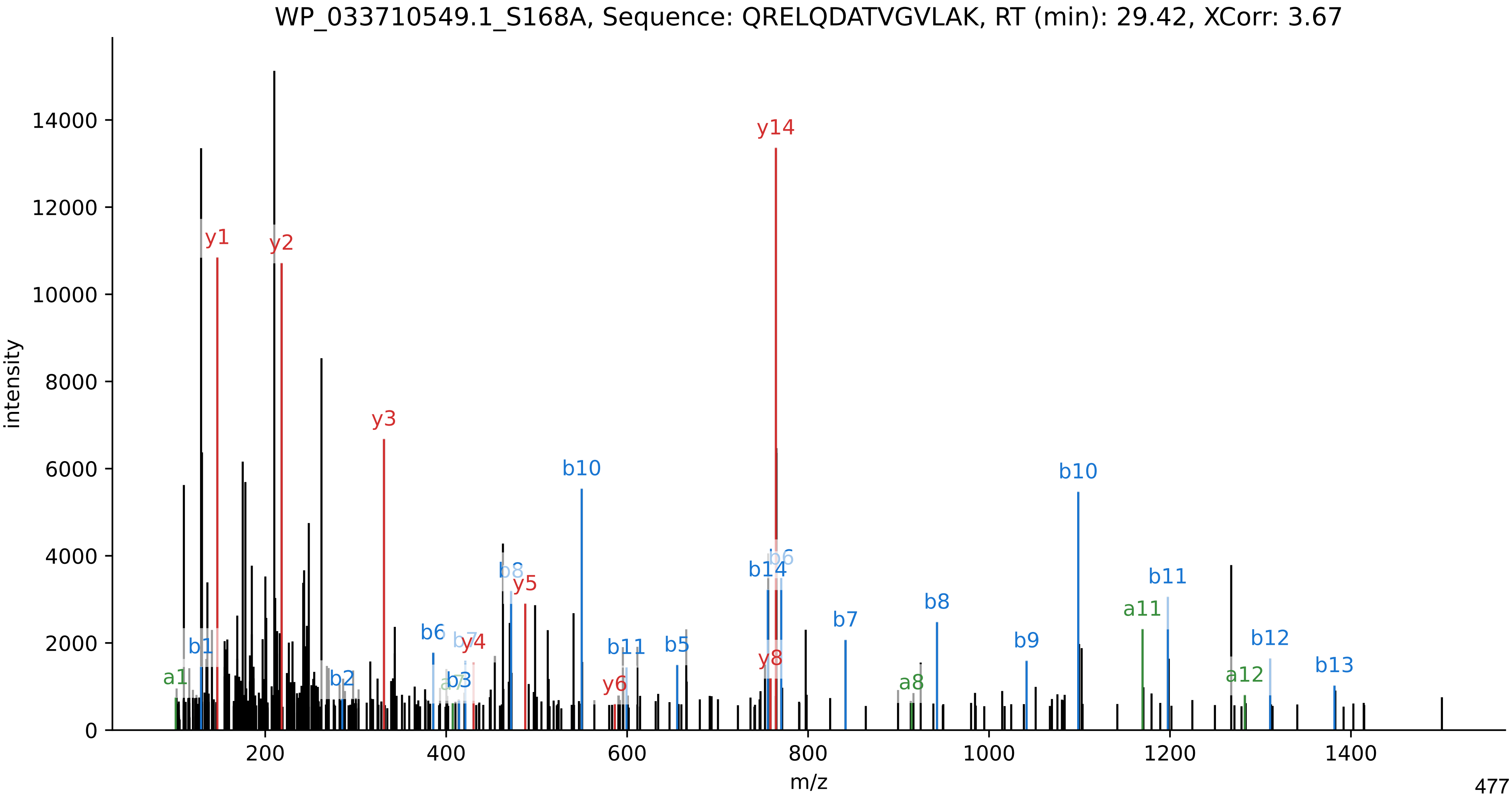

WP_033710549.1_S168A, Sequence: QRELQDATVGVLAK, RT (min): 29.42, XCorr: 3.67
intensity
m/z
y1
y2
y3
y14
y4
y5
y6
y8
a1
a8
a11
a12
b1
b2
b3
b5
b6
b7
b8
b9
b10
b11
b12
b13
b14

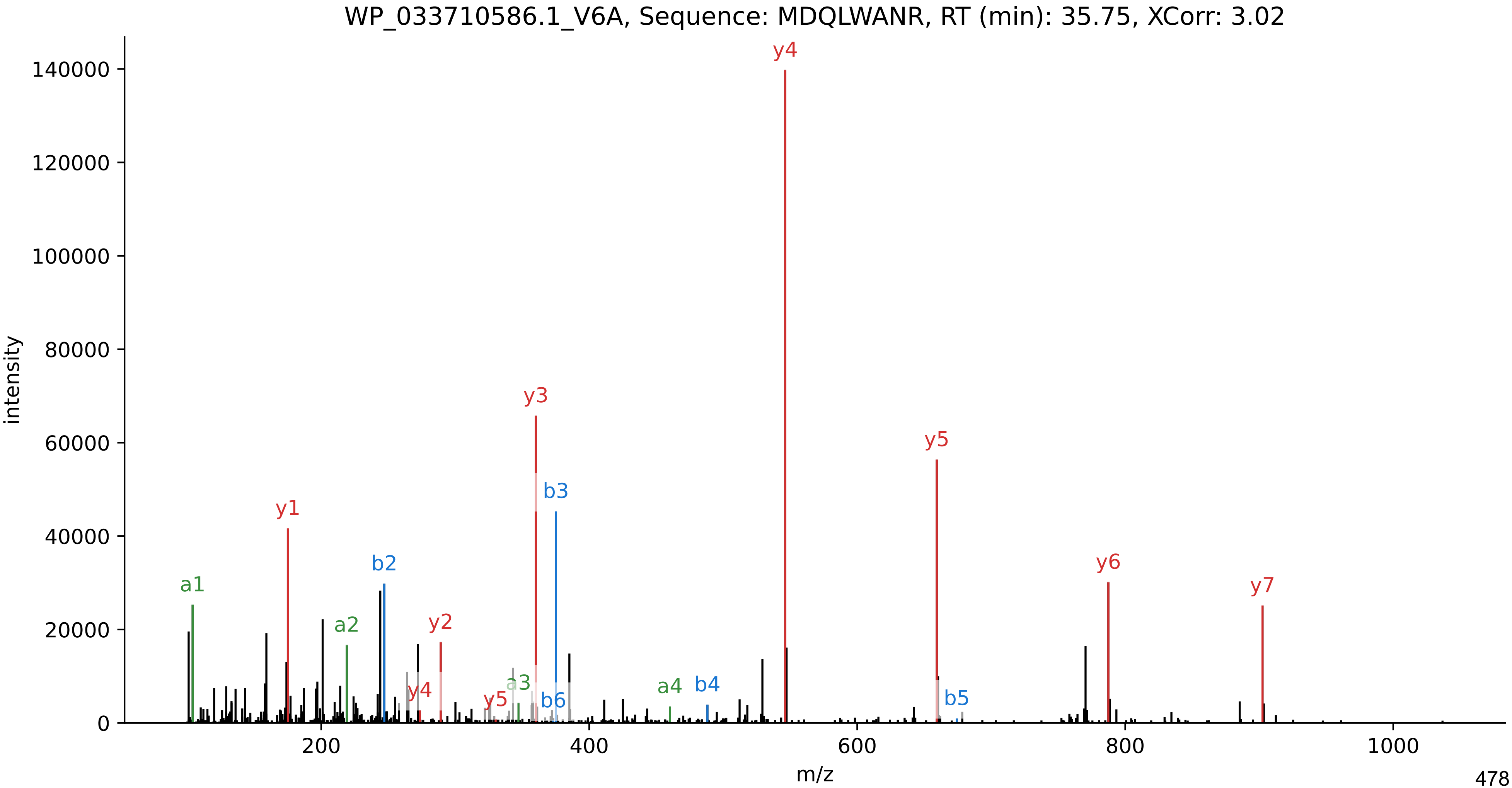
WP_033710586.1_V6A, Sequence: MDQLWANR, RT (min): 35.75, XCorr: 3.02
intensity
m/z
y4
y3
b3
y1
b2
a1
a2
y2
y4
y5
a3
b6
a4
b4
y5
b5
y6
y7
0
20000
40000
60000
80000
100000
120000
140000
200
400
600
800
1000

WP_033710735.1_R204P, Sequence: GLGGRVPVAPEAVDLLVR, RT (min): 71.65, XCorr: 5.04

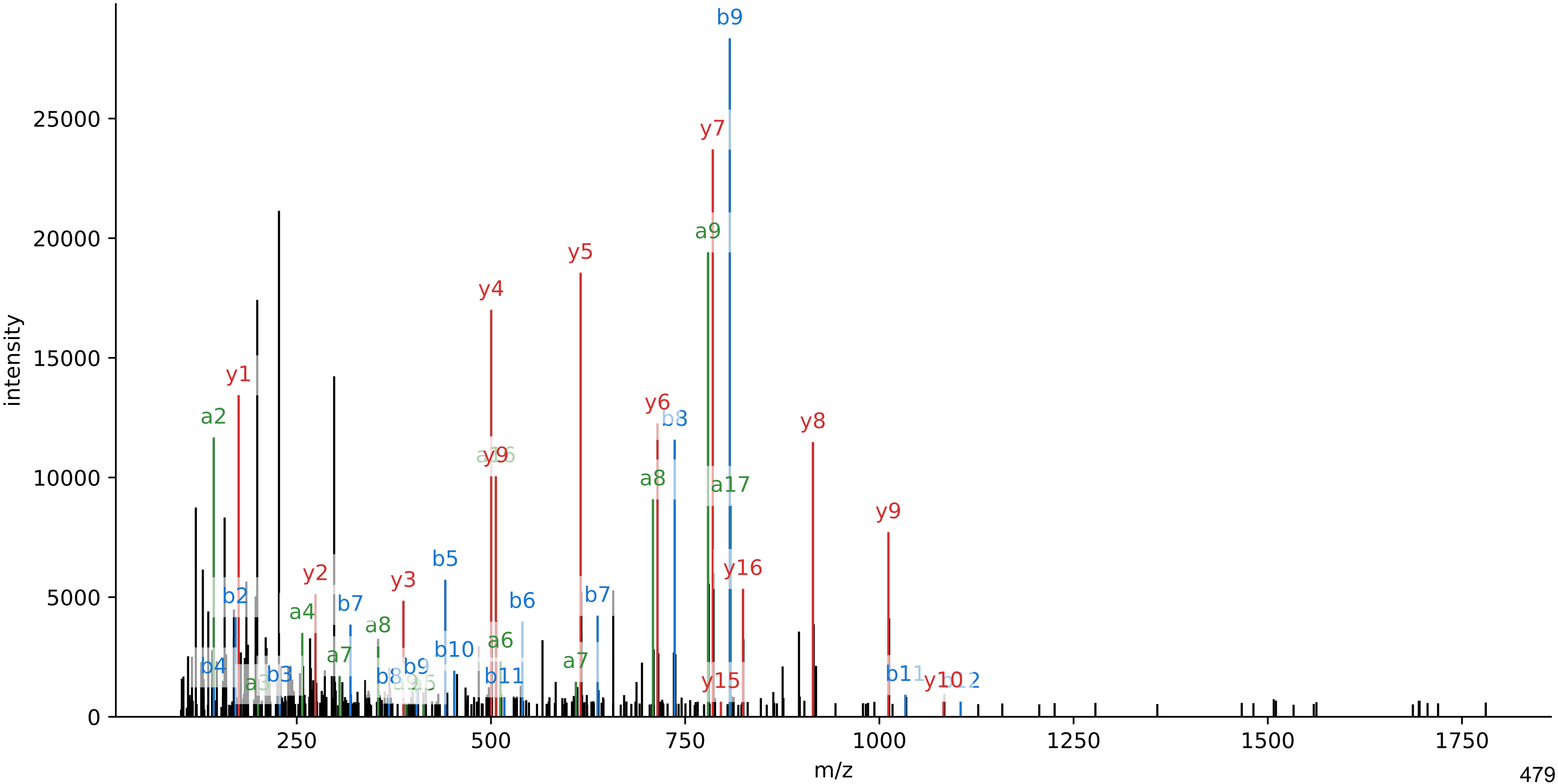

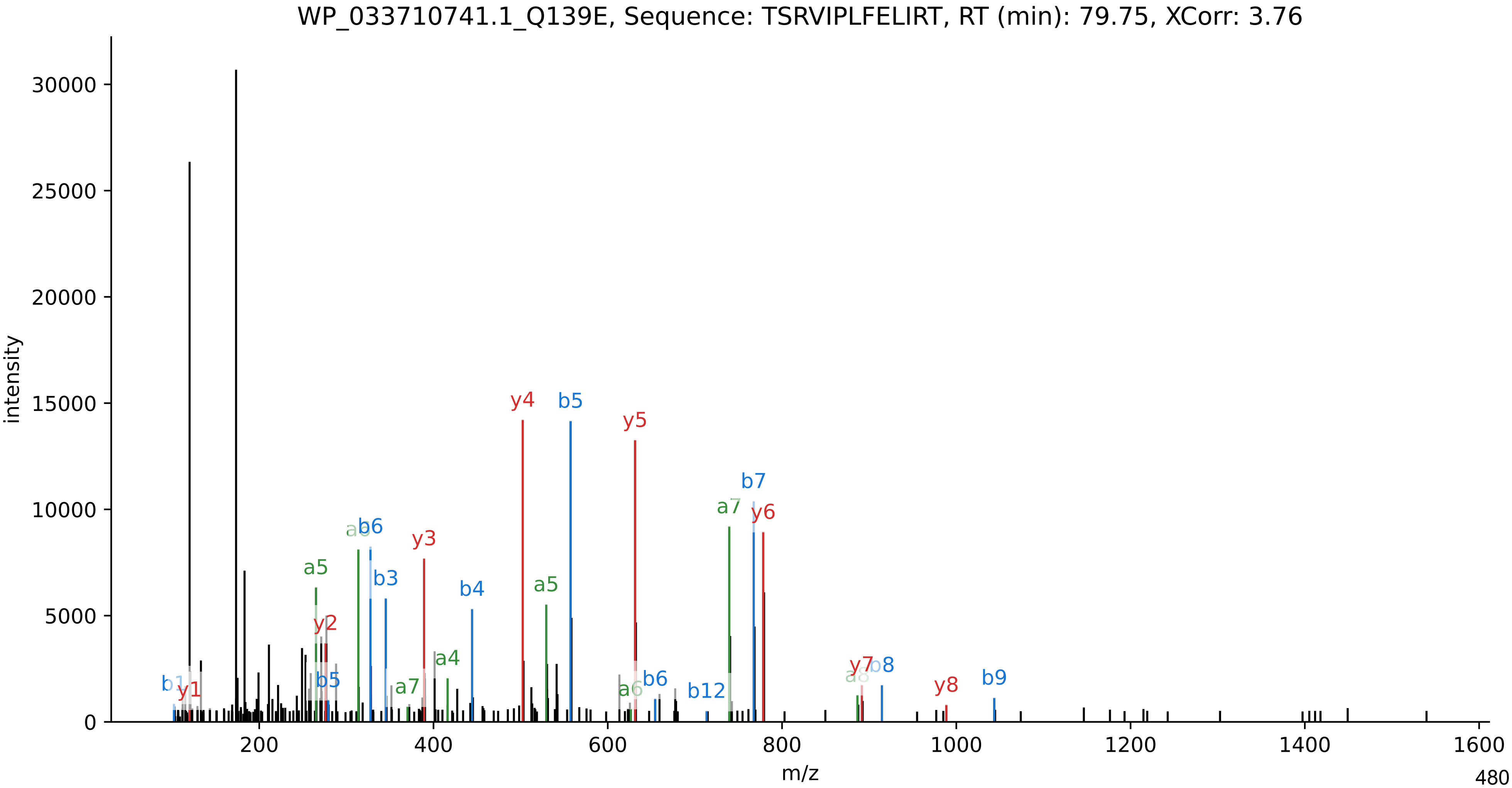

WP_033710741.1_Q139E, Sequence: TSRVIPLFELIRT, RT (min): 79.75, XCorr: 3.76
intensity
m/z
y4
b5
y5
b7
a7
y6
b6
y3
a5
b3
b4
a5
y2
a4
b5
a7
b6
b12
y7
b8
y8
b9
a6
a8
y1
b1

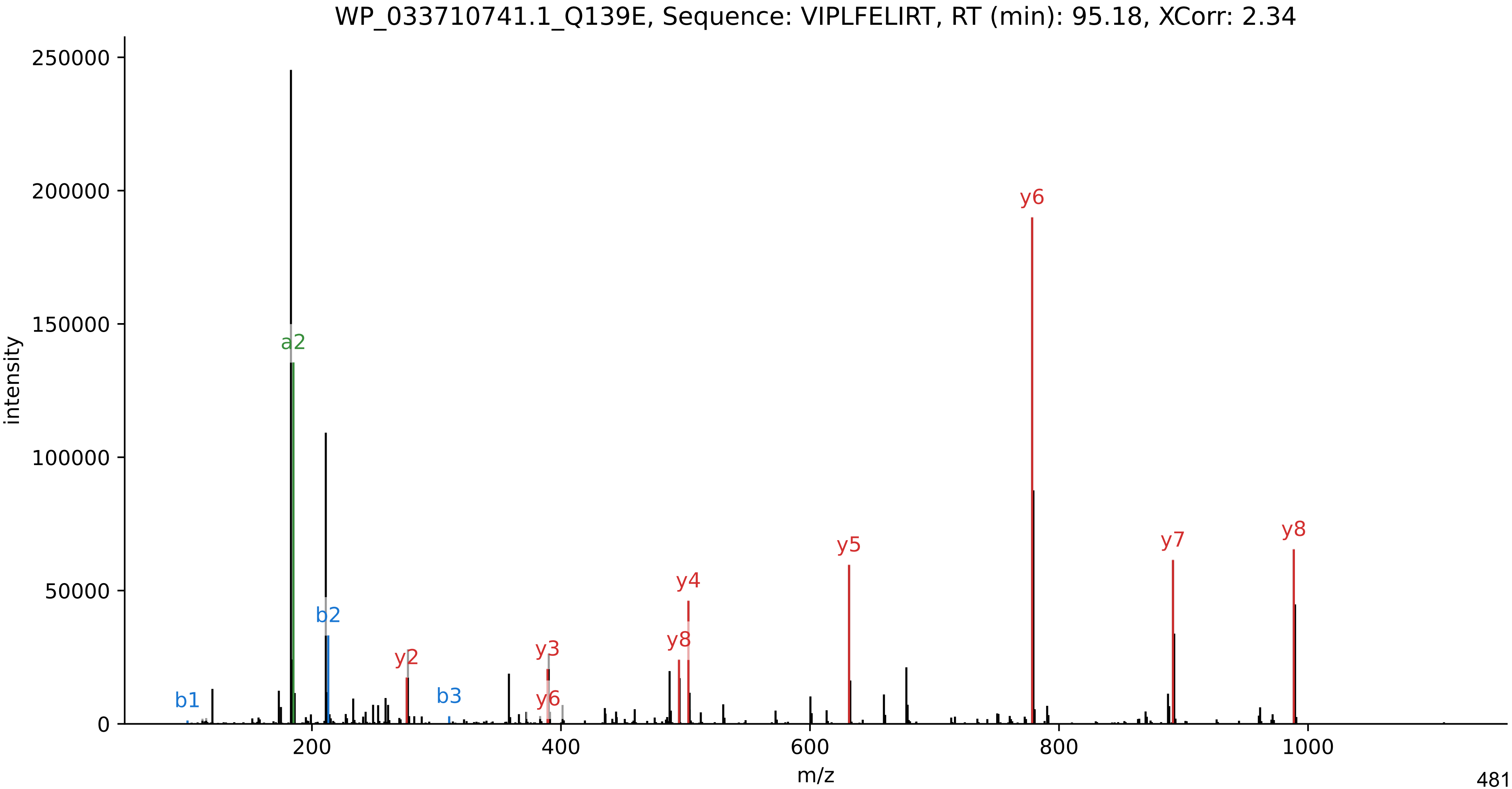

WP_033710741.1_Q139E, Sequence: VIPLFELIRT, RT (min): 95.18, XCorr: 2.34
intensity
m/z
0
50000
100000
150000
200000
250000
200
400
600
800
1000
b1
a2
b2
y2
b3
y3
y6
y8
y4
y5
y6
y7
y8

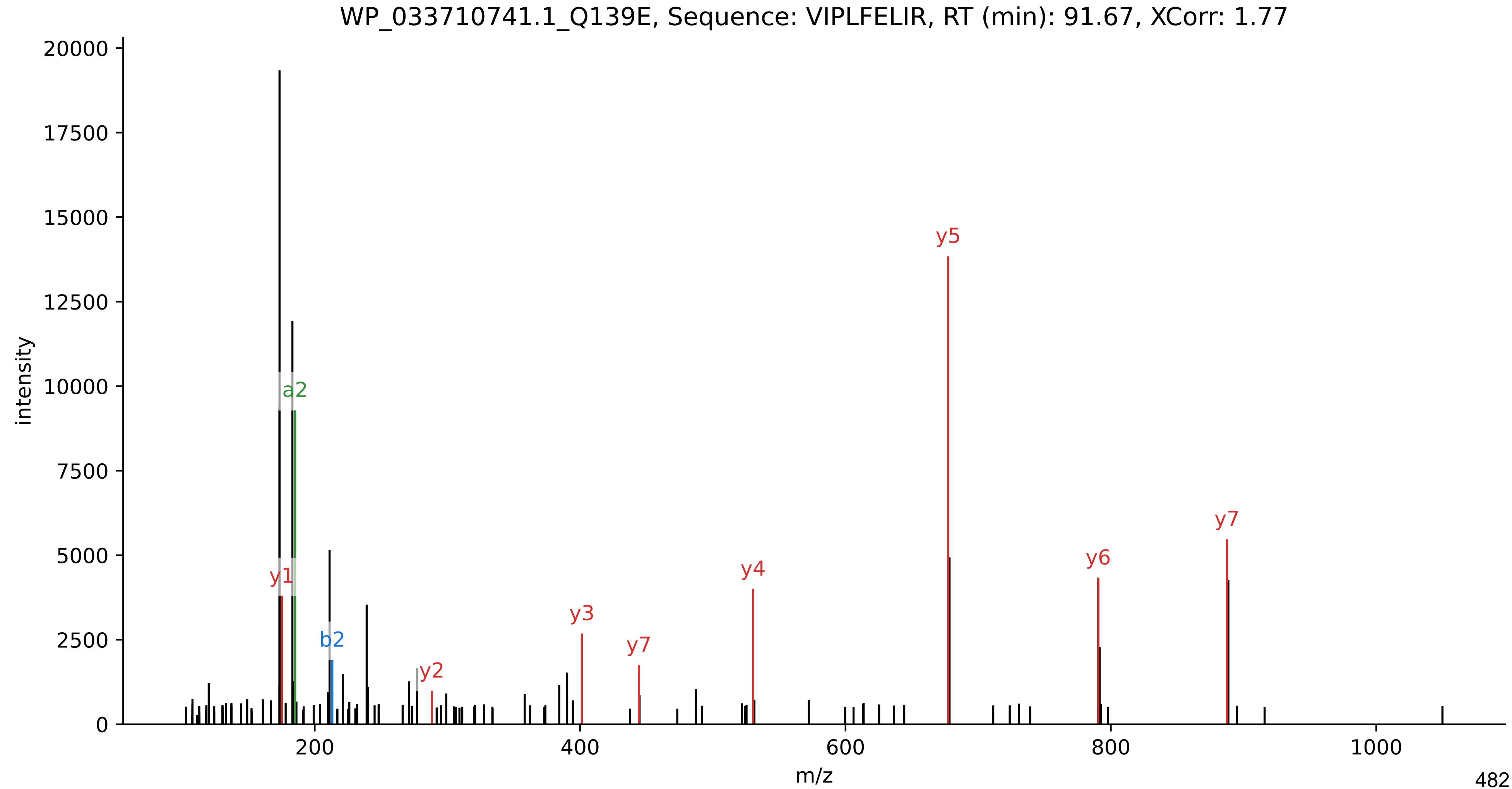

WP_033710741.1_Q139E, Sequence: VIPLFELIR, RT (min): 91.67, XCorr: 1.77
intensity
m/z
a2
b2
y1
y2
y3
y7
y4
y5
y6
y7
0
2500
5000
7500
10000
12500
15000
17500
20000
200
400
600
800
1000

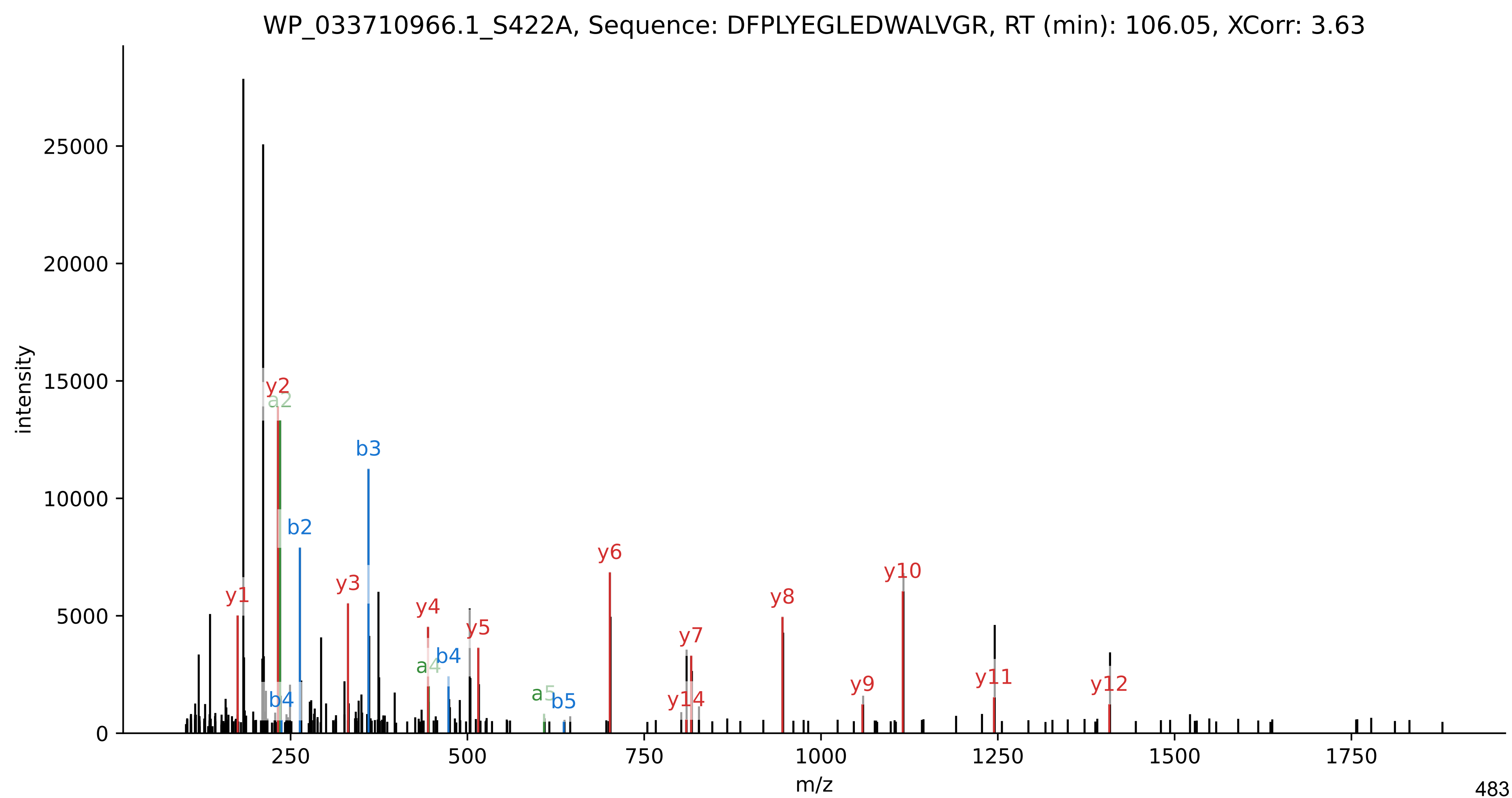
WP_033710966.1_S422A, Sequence: DFPLYEGLEDWALVGR, RT (min): 106.05, XCorr: 3.63
intensity
m/z
0
5000
10000
15000
20000
25000
250
500
750
1000
1250
1500
1750
y1
y2
a2
b2
b3
y3
y4
a4
b4
b4
y5
a5
b5
y6
y7
y14
y8
y9
y10
y11
y12

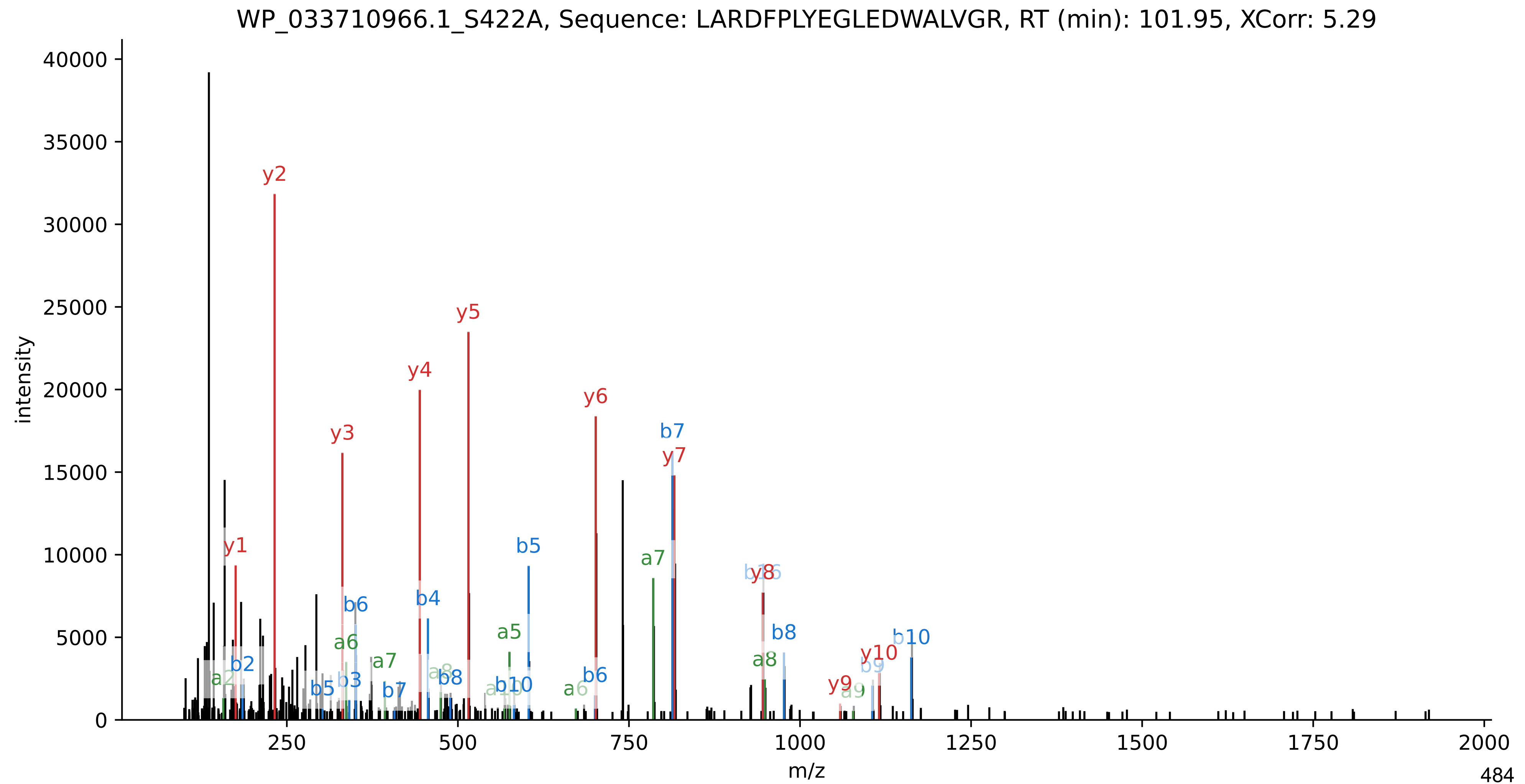
WP_033710966.1_S422A, Sequence: LARDFPLYEGLEDWALVGR, RT (min): 101.95, XCorr: 5.29
intensity
m/z
y1
y2
y3
y4
y5
y6
y7
y8
y9
y10
b2
b3
b4
b5
b6
b7
b8
b9
b10
a2
a5
a6
a7
a8
a9

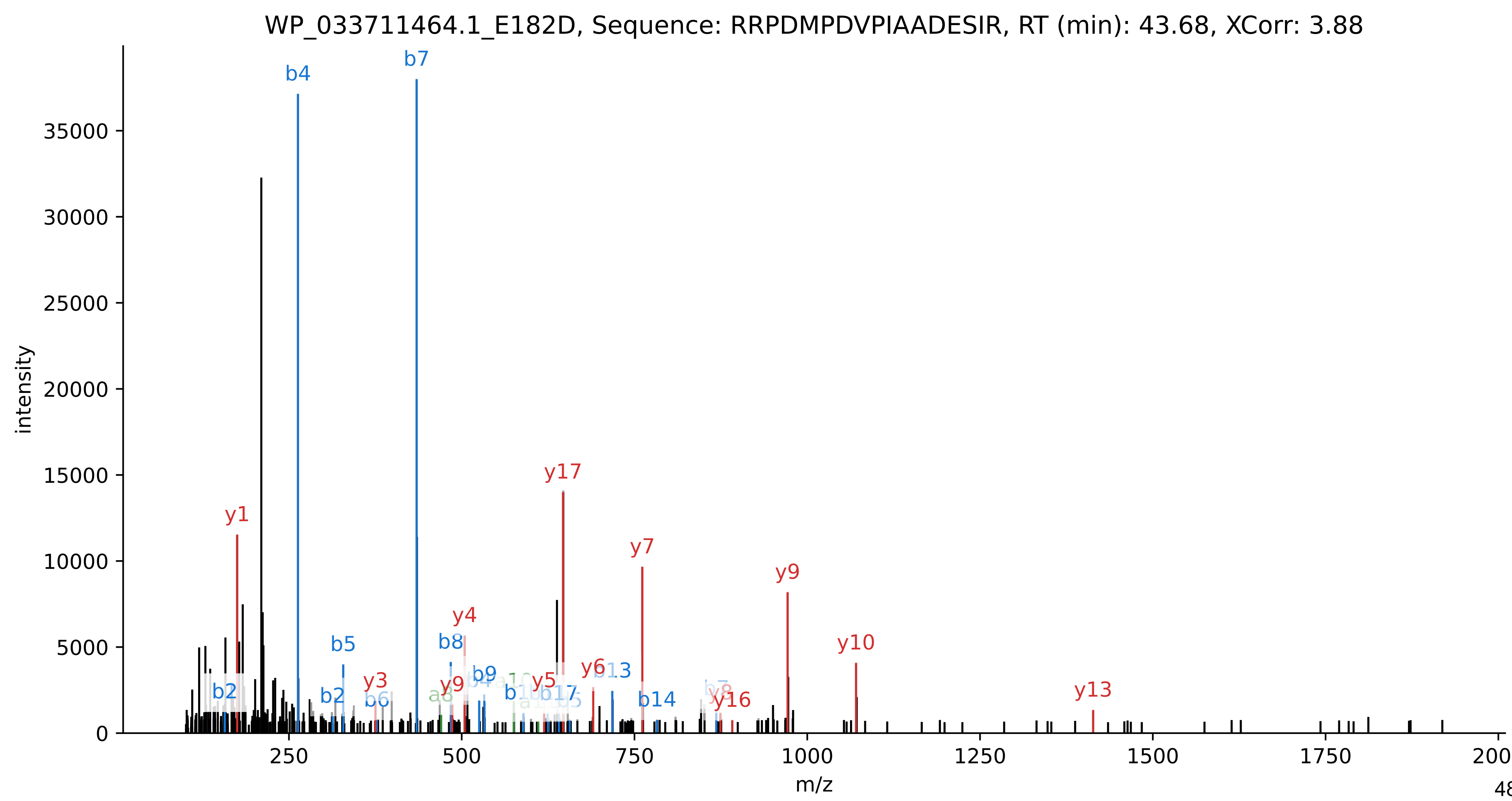
WP_033711464.1_E182D, Sequence: RRPDMPDVPIAADESIR, RT (min): 43.68, XCorr: 3.88
intensity
m/z
0
5000
10000
15000
20000
25000
30000
35000
250
500
750
1000
1250
1500
1750
2000
b4
b7
y1
y17
y7
y9
y4
b8
b5
y10
y3
y9
y5
y6
b13
b14
y16
y13
b2
b2
b6
b9
a8

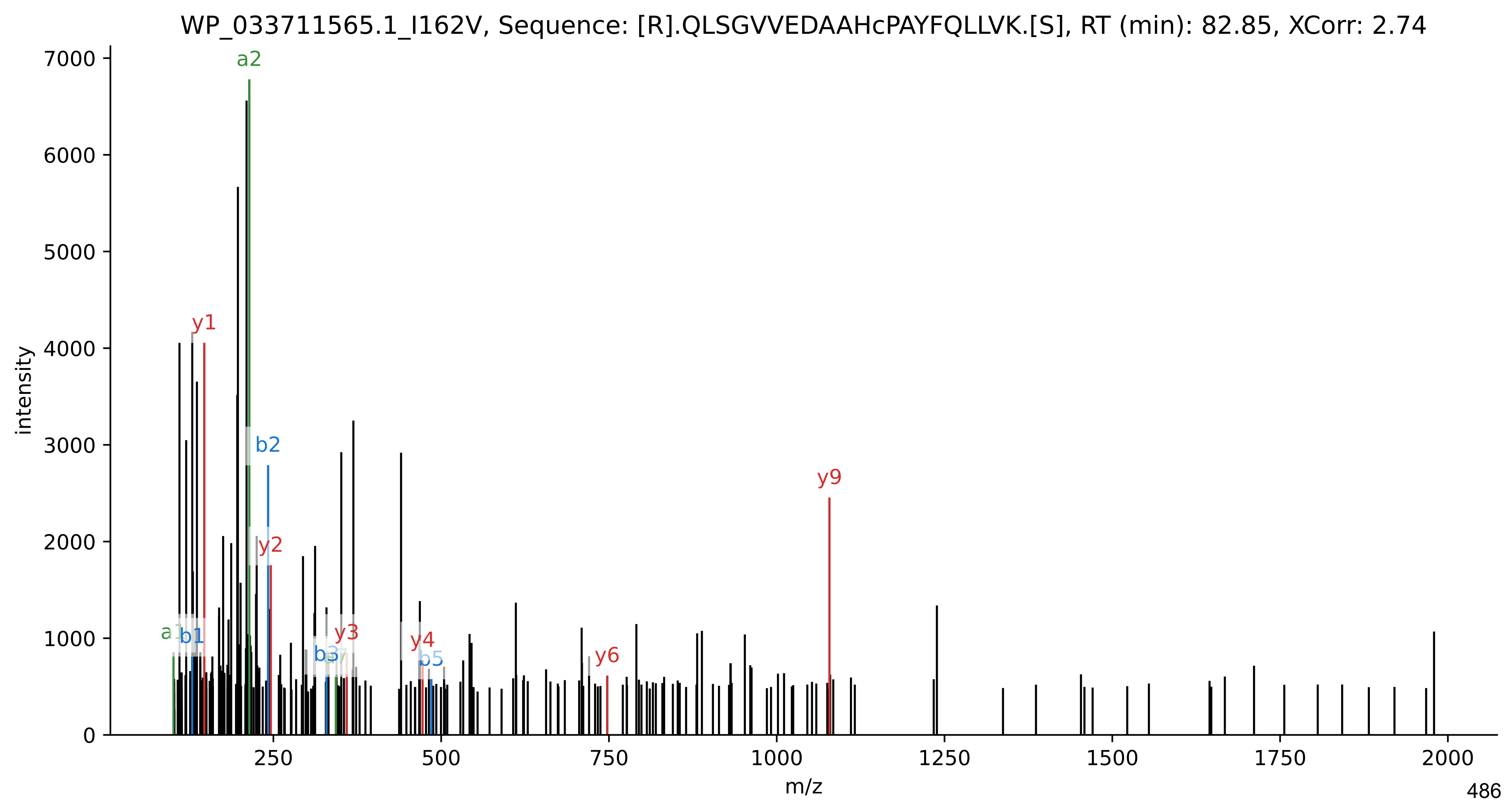

WP_033711565.1_I162V, Sequence: [R].QLSGVVEDAAHcPAYFQLLVK.[S], RT (min): 82.85, XCorr: 2.74
intensity
m/z
a2
y1
b2
y2
y9
a1
b1
b3
y3
y4
b5
y6

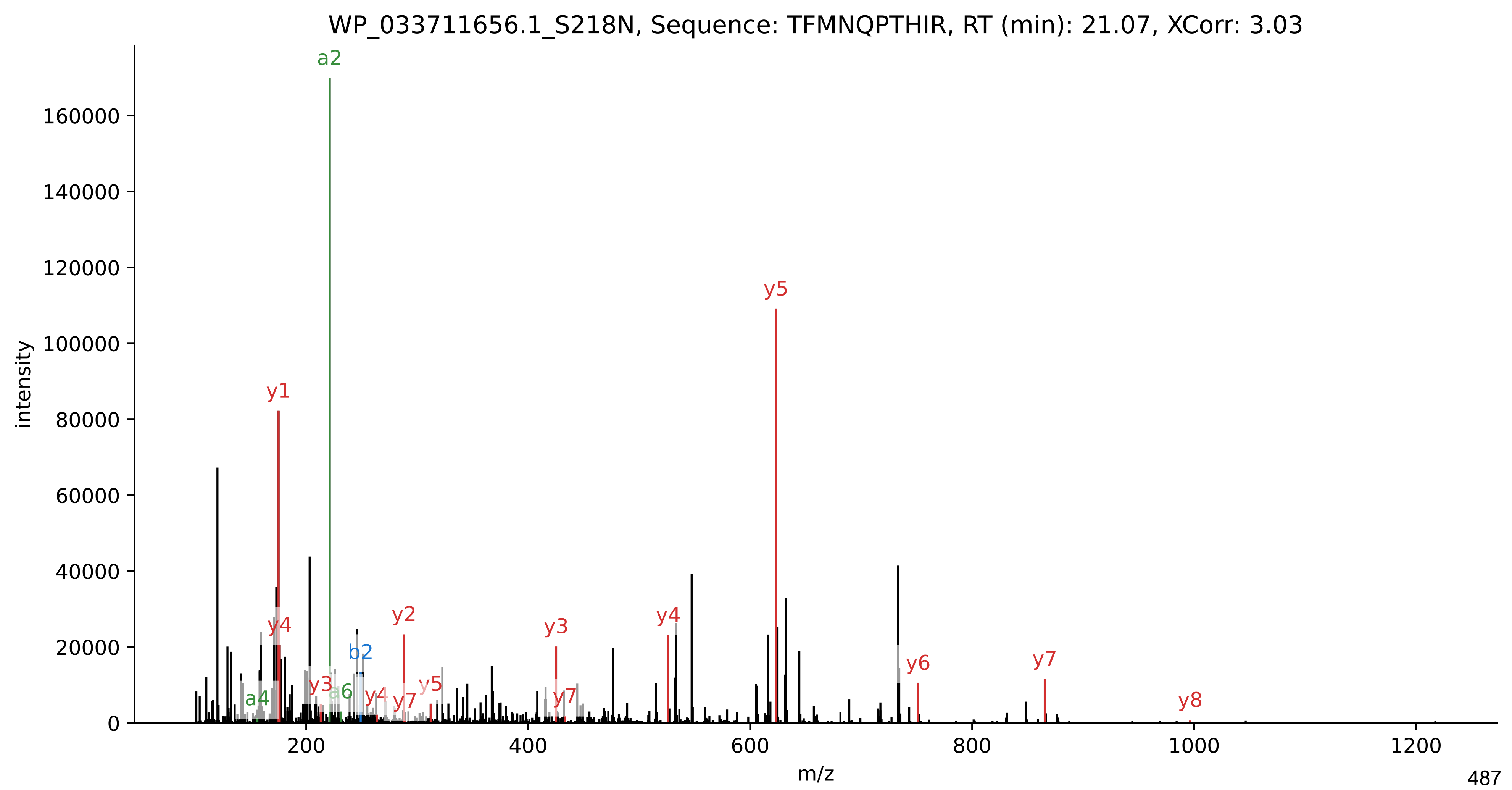
WP_033711656.1_S218N, Sequence: TFMNQPTHIR, RT (min): 21.07, XCorr: 3.03
intensity
m/z
a2
y5
y1
y4
y2
y3
y4
b2
y3
a4
a6
y4
y7
y5
y7
y6
y7
y8
0
20000
40000
60000
80000
100000
120000
140000
160000
200
400
600
800
1000
1200

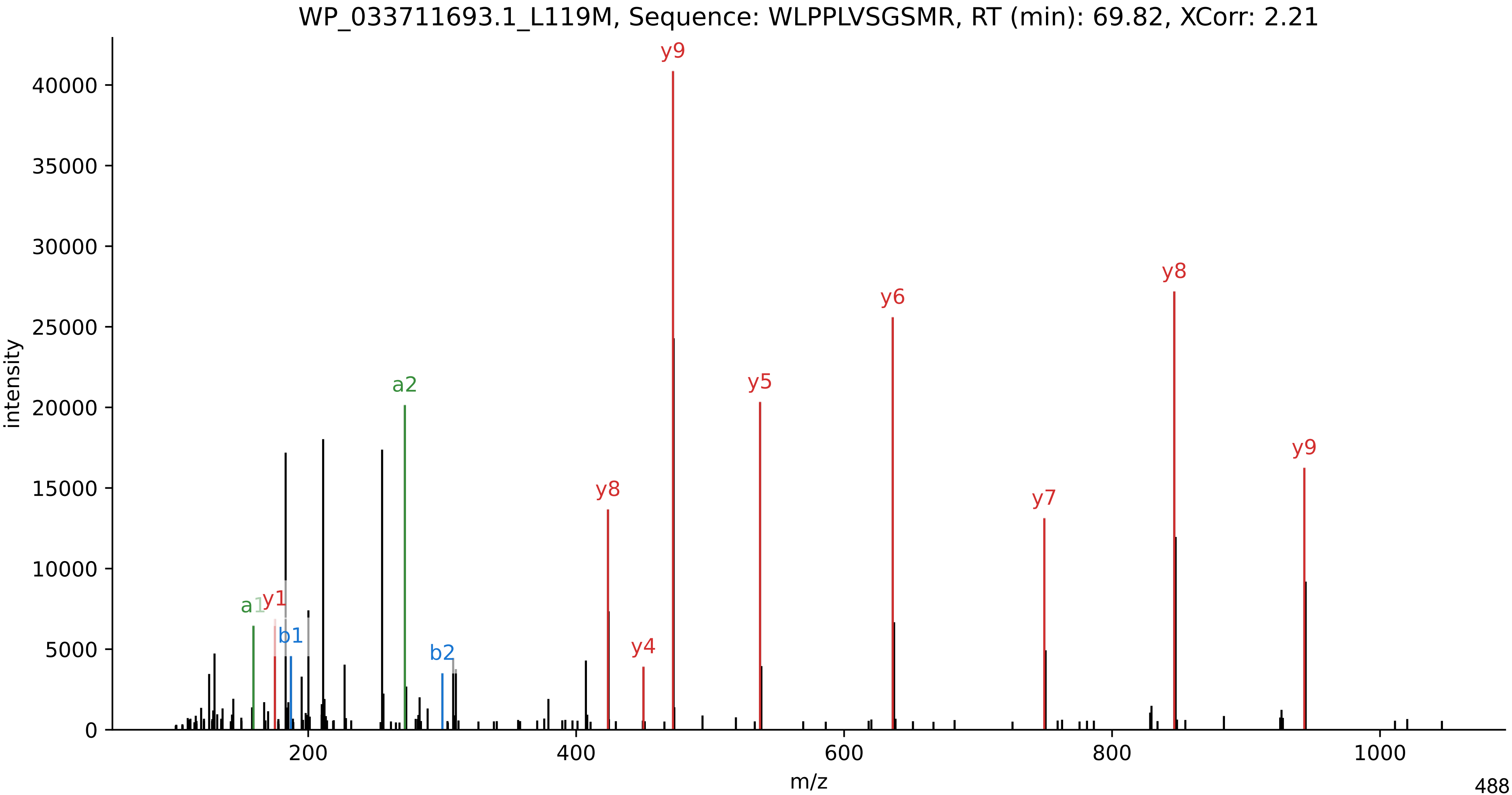

WP_033711693.1_L119M, Sequence: WLPPLVSGSMR, RT (min): 69.82, XCorr: 2.21
intensity
m/z
y9
y8
y6
a2
y5
y9
y8
y7
a1
y1
b1
b2
y4

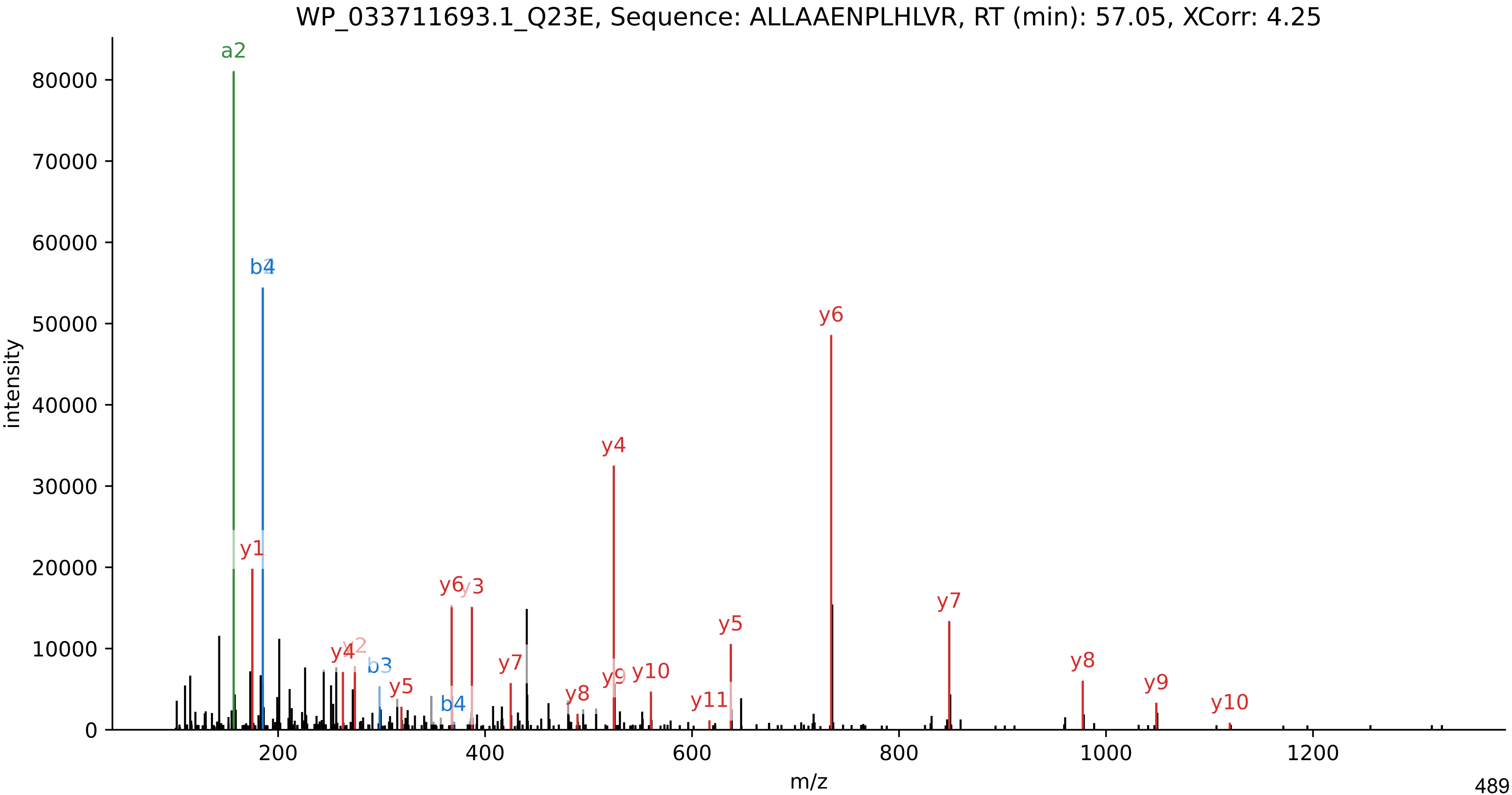

WP_033711693.1_Q23E, Sequence: ALLAAENPLHLVR, RT (min): 57.05, XCorr: 4.25
intensity
m/z
a2
b4
y1
y4
y2
b3
y5
y6
y3
b4
y7
y8
y9
y4
y10
y11
y5
y6
y7
y8
y9
y10

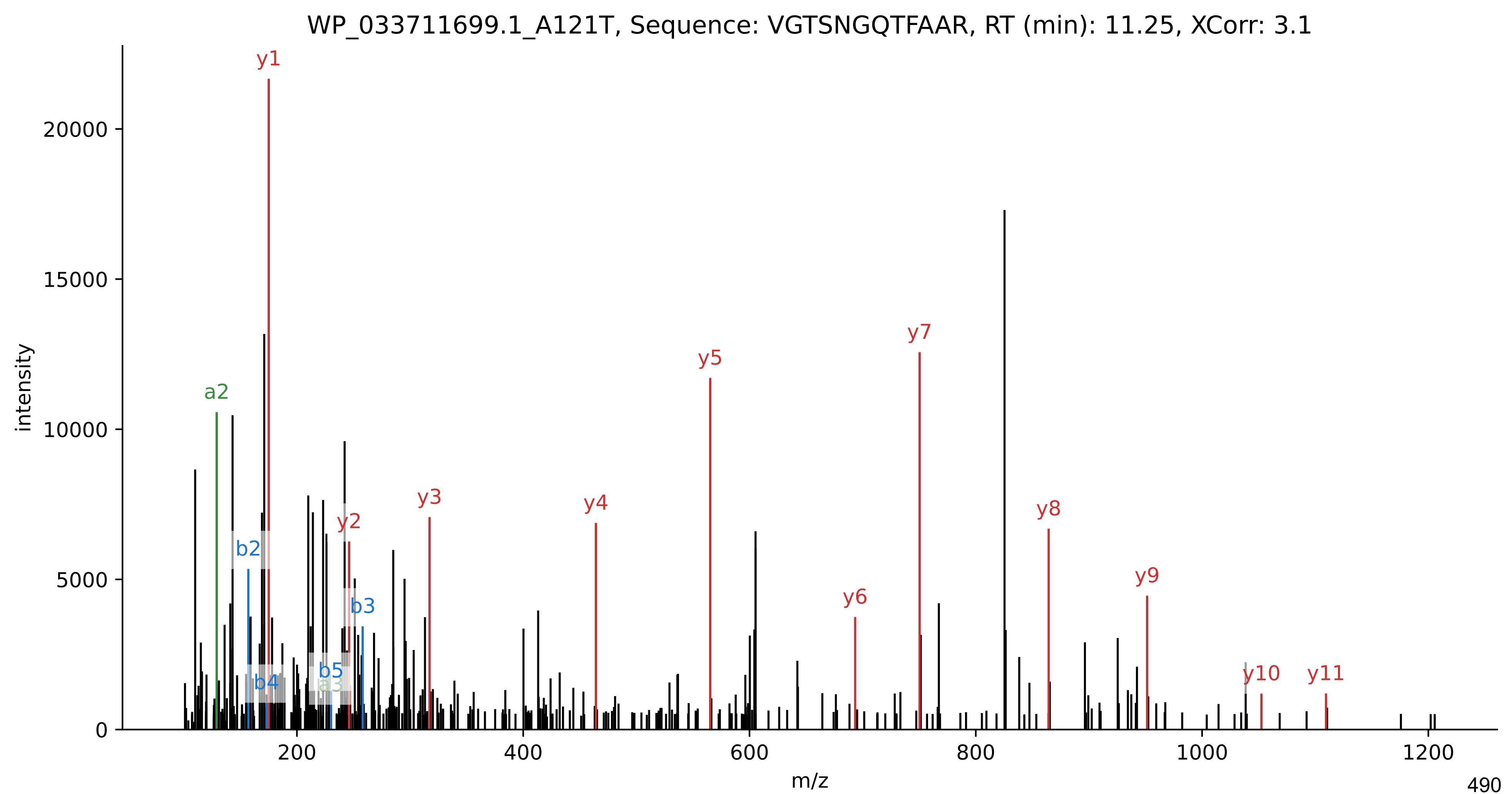

WP_033711699.1_A121T, Sequence: VGTSNGQTFAAR, RT (min): 11.25, XCorr: 3.1
intensity
m/z
y1
a2
b2
b3
b4
b5
a3
y2
y3
y4
y5
y6
y7
y8
y9
y10
y11
0
5000
10000
15000
20000
200
400
600
800
1000
1200

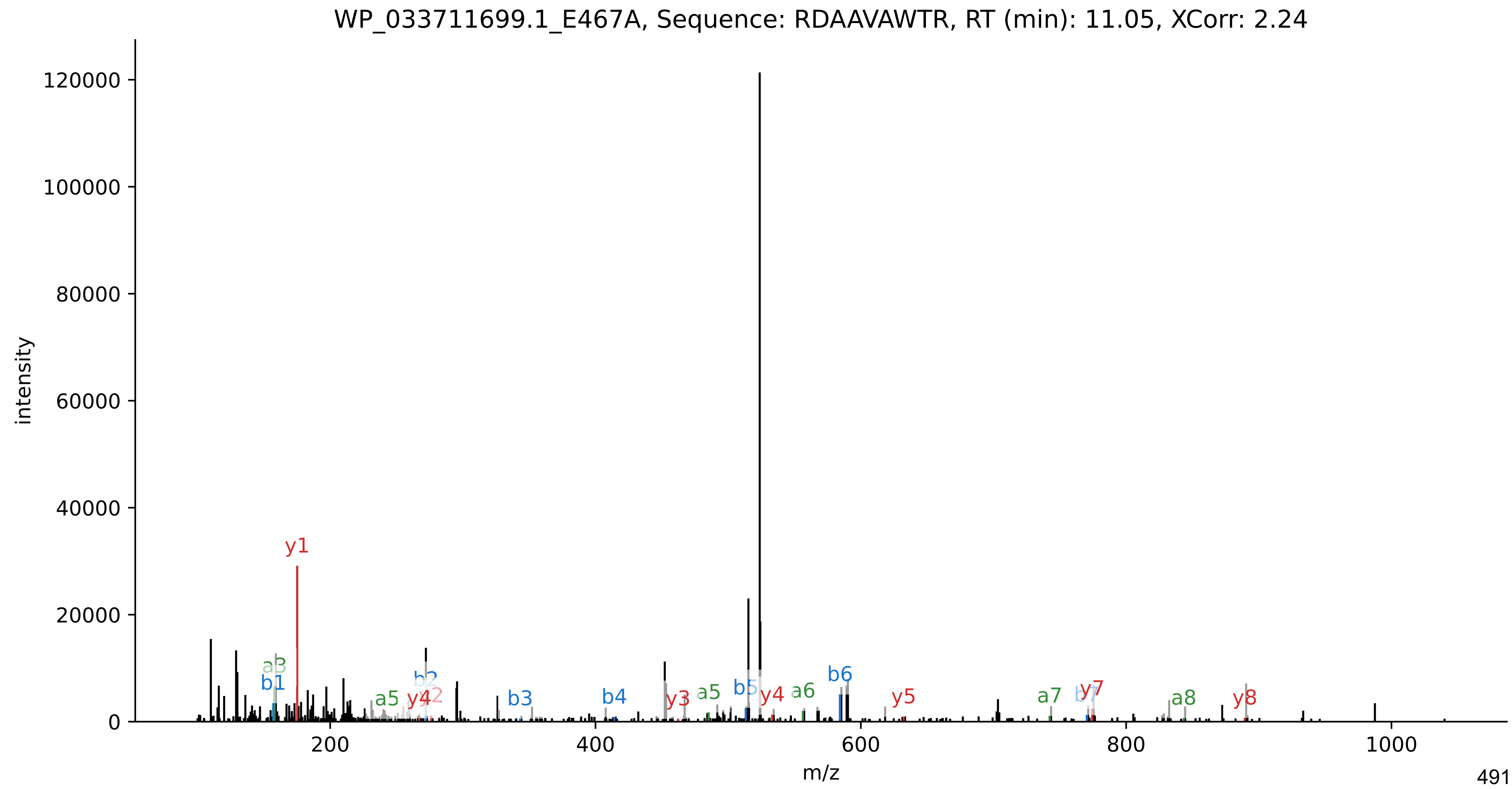

WP_033711699.1_E467A, Sequence: RDAAVAWTR, RT (min): 11.05, XCorr: 2.24
intensity
m/z
y1
a3
b1
b2
y2
a5
y4
b3
b4
y3
a5
b5
y4
a6
b6
y5
a7
b7
y7
a8
y8

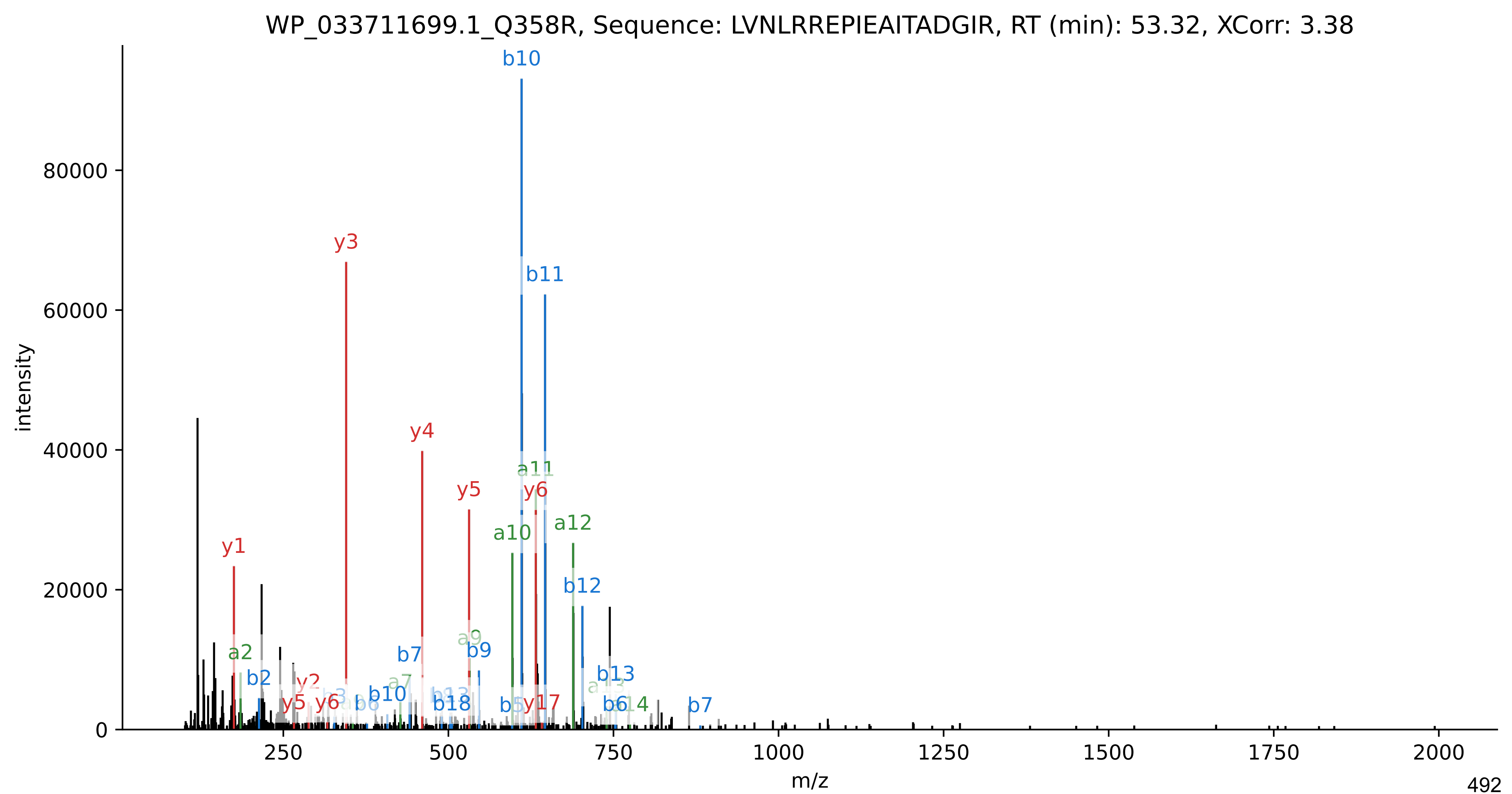

WP_033711699.1_Q358R, Sequence: LVNLRREPIEAITADGIR, RT (min): 53.32, XCorr: 3.38
intensity
m/z
b10
b11
y3
y4
y5
y6
a11
a12
a10
y1
b12
a9
b9
b7
a2
b2
y2
b13
y5
y6
b10
a7
b18
y17
b5
b6
b7
a14

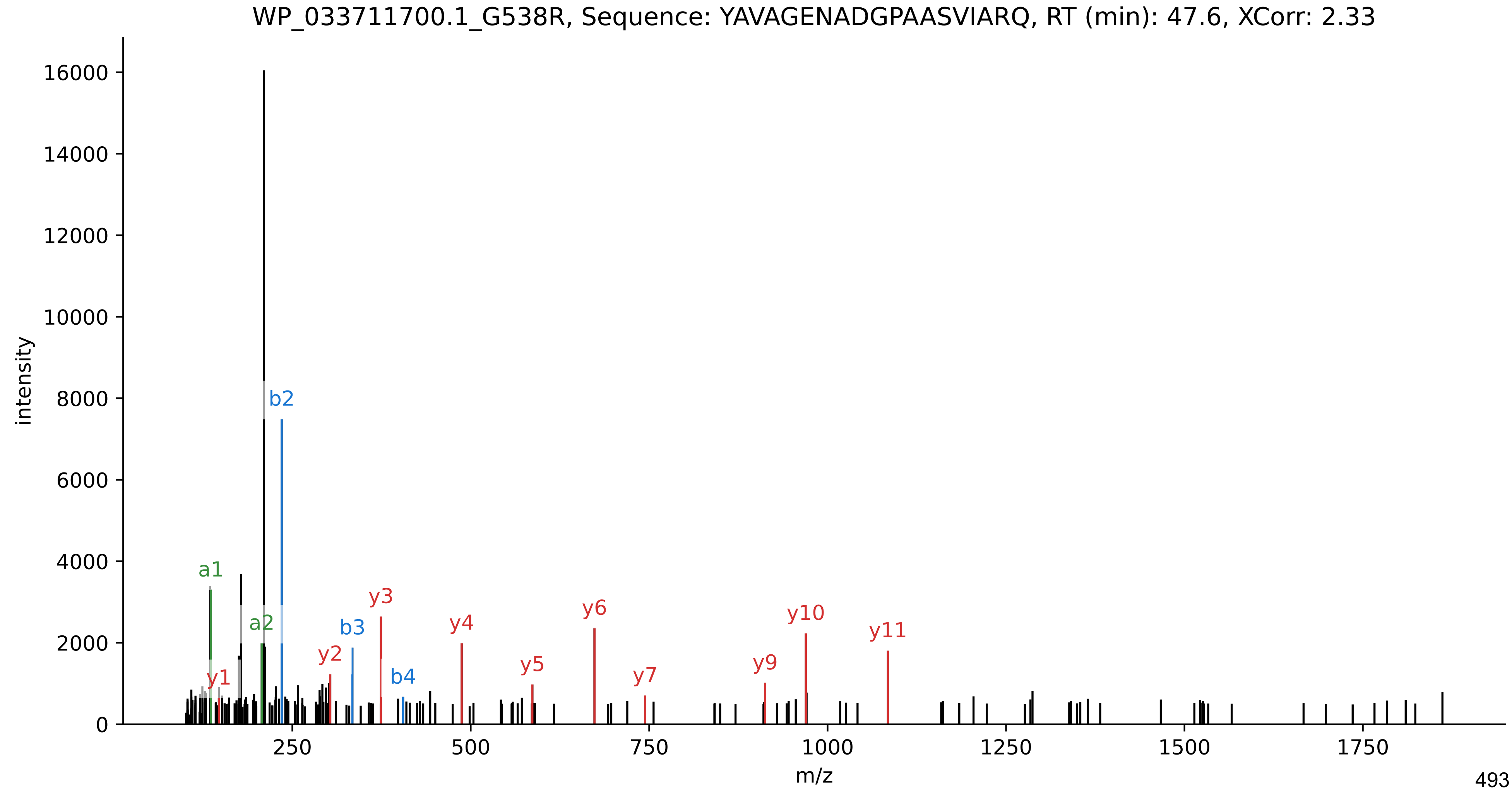

WP_033711700.1_G538R, Sequence: YAVAGENADGPAASVIARQ, RT (min): 47.6, XCorr: 2.33
intensity
m/z
a1
a2
b2
b3
b4
y1
y2
y3
y4
y5
y6
y7
y9
y10
y11

WP_033712069.1_A111V, Sequence: MVRAFEQMGPGWRDTVLPR, RT (min): 63.9, XCorr: 4.43

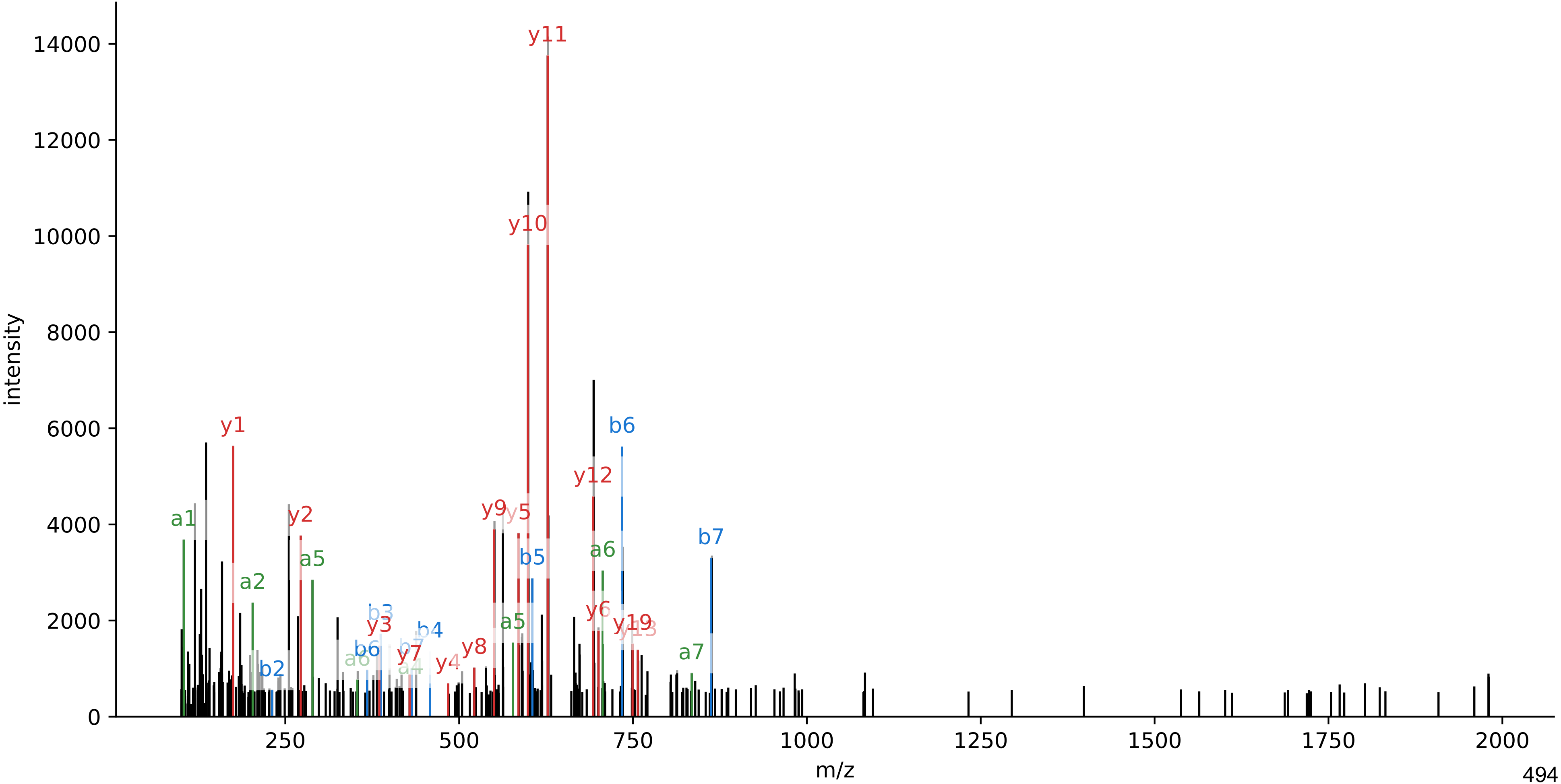

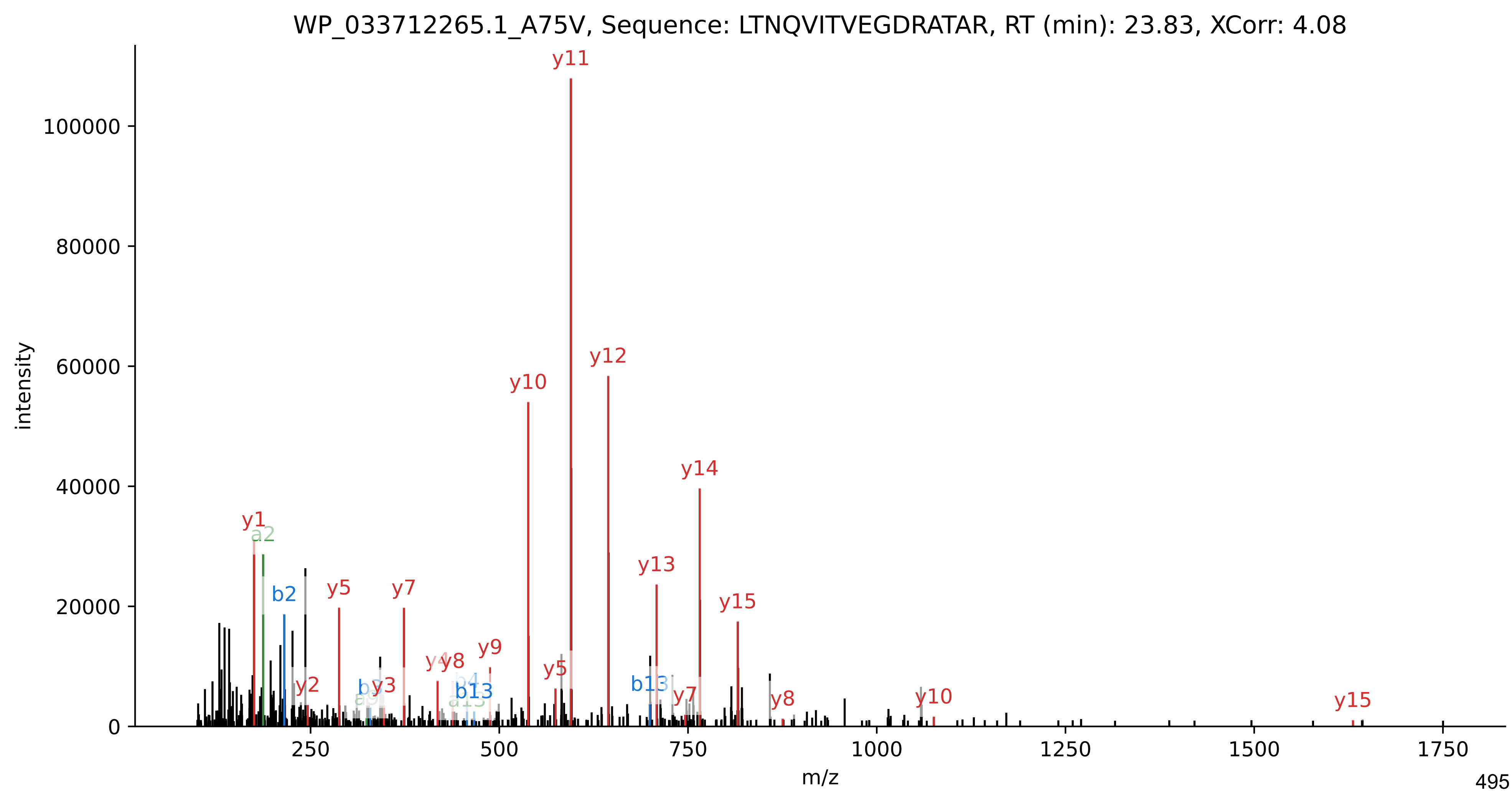

WP_033712265.1_A75V, Sequence: LTNQVITVEGDRATAR, RT (min): 23.83, XCorr: 4.08
intensity
m/z
y11
y12
y10
y14
y13
y15
y1
a2
b2
y5
y7
y2
y3
y8
y9
y5
b13
b13
y7
y8
y10
y15

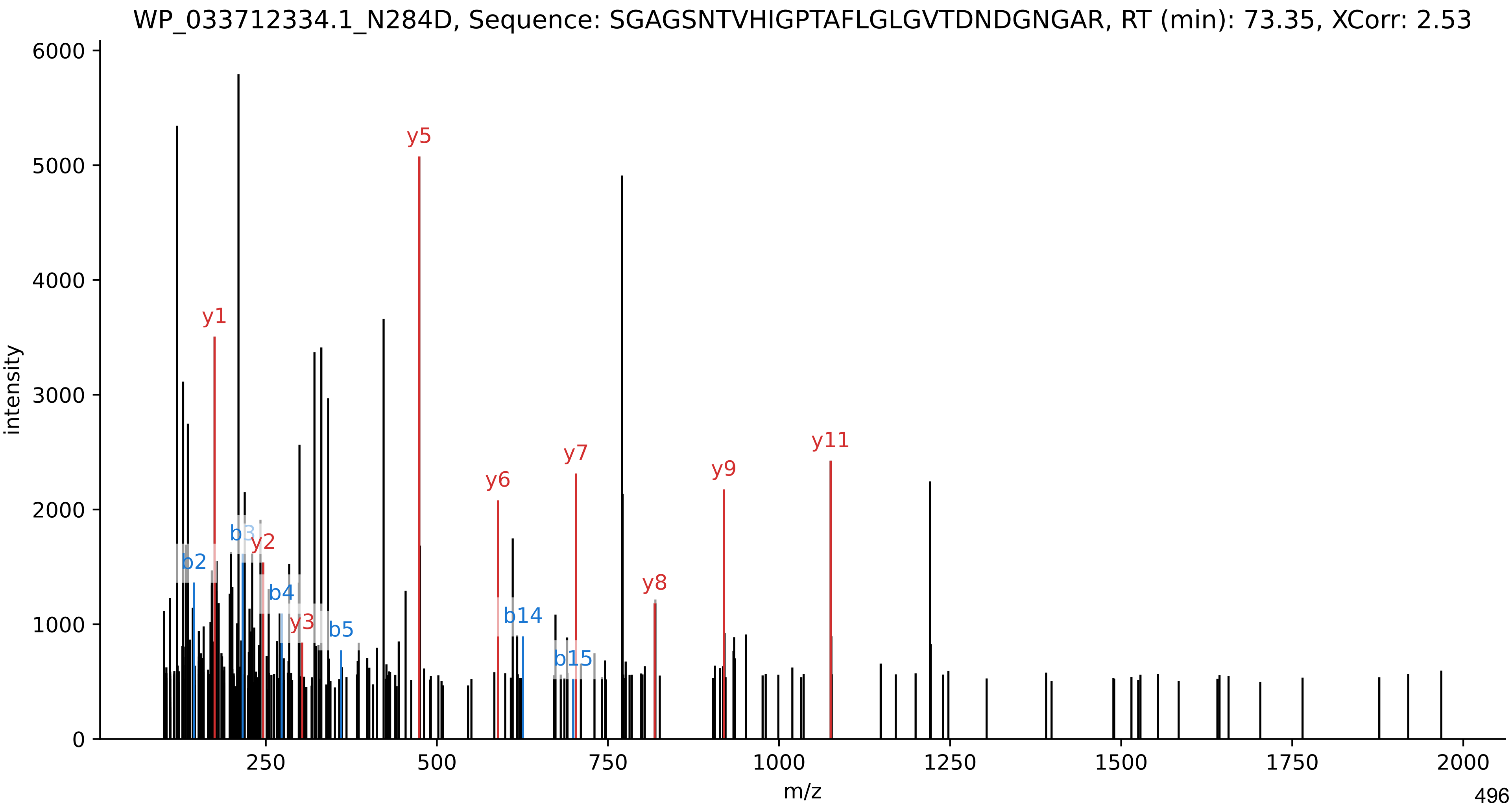
WP_033712334.1_N284D, Sequence: SGAGSNTVHIGPTAFLGLGVTDNDGNGAR, RT (min): 73.35, XCorr: 2.53
intensity
m/z
y1
y2
y3
y5
y6
y7
y8
y9
y11
b2
b3
b4
b5
b14
b15

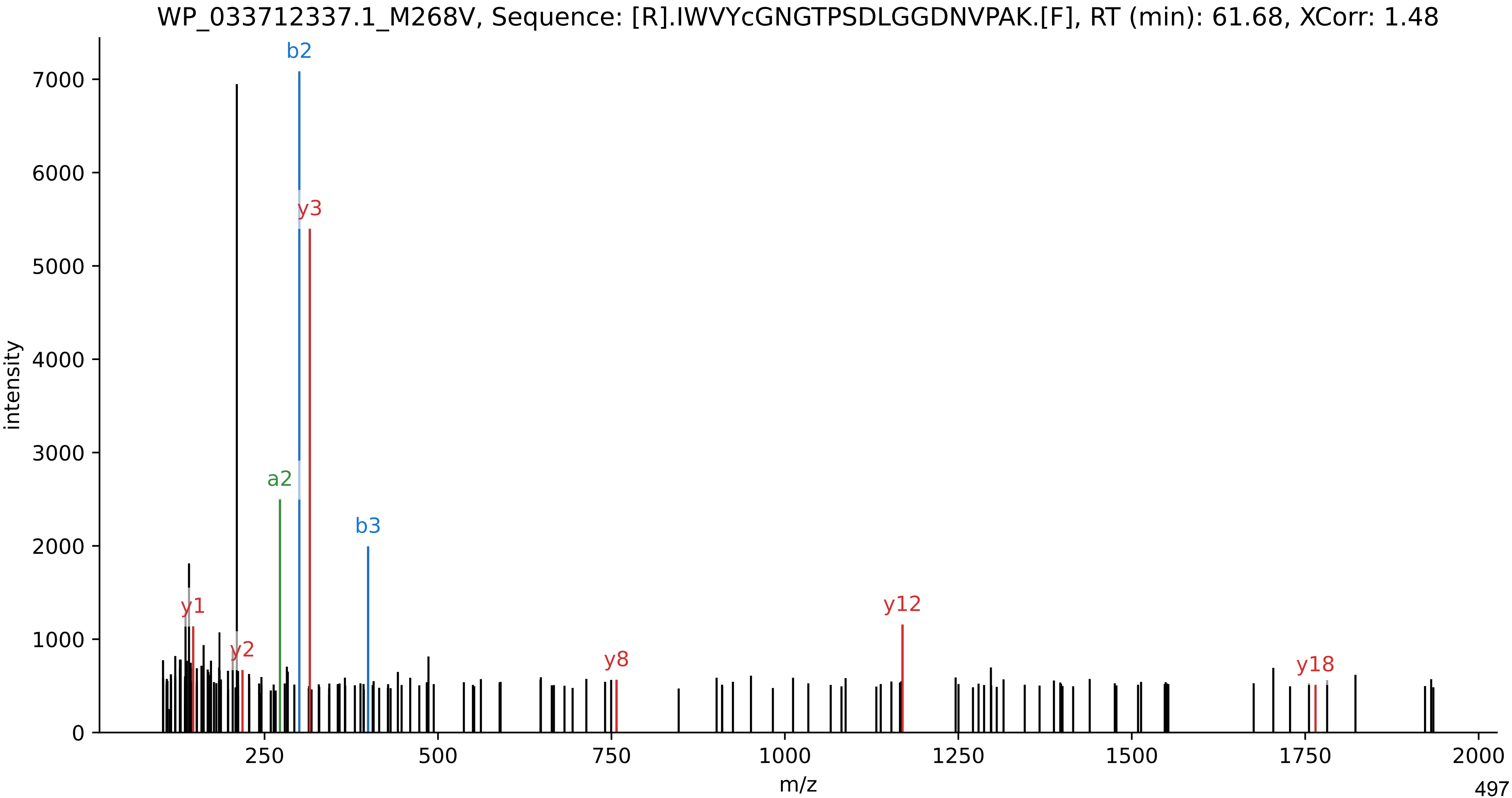

WP_033712337.1_M268V, Sequence: [R].IWVYcGNGTPSDLGGDNVPAK.[F], RT (min): 61.68, XCorr: 1.48
b2
y3
a2
b3
y1
y2
y8
y12
y18
intensity
m/z
0
1000
2000
3000
4000
5000
6000
7000
250
500
750
1000
1250
1500
1750
2000

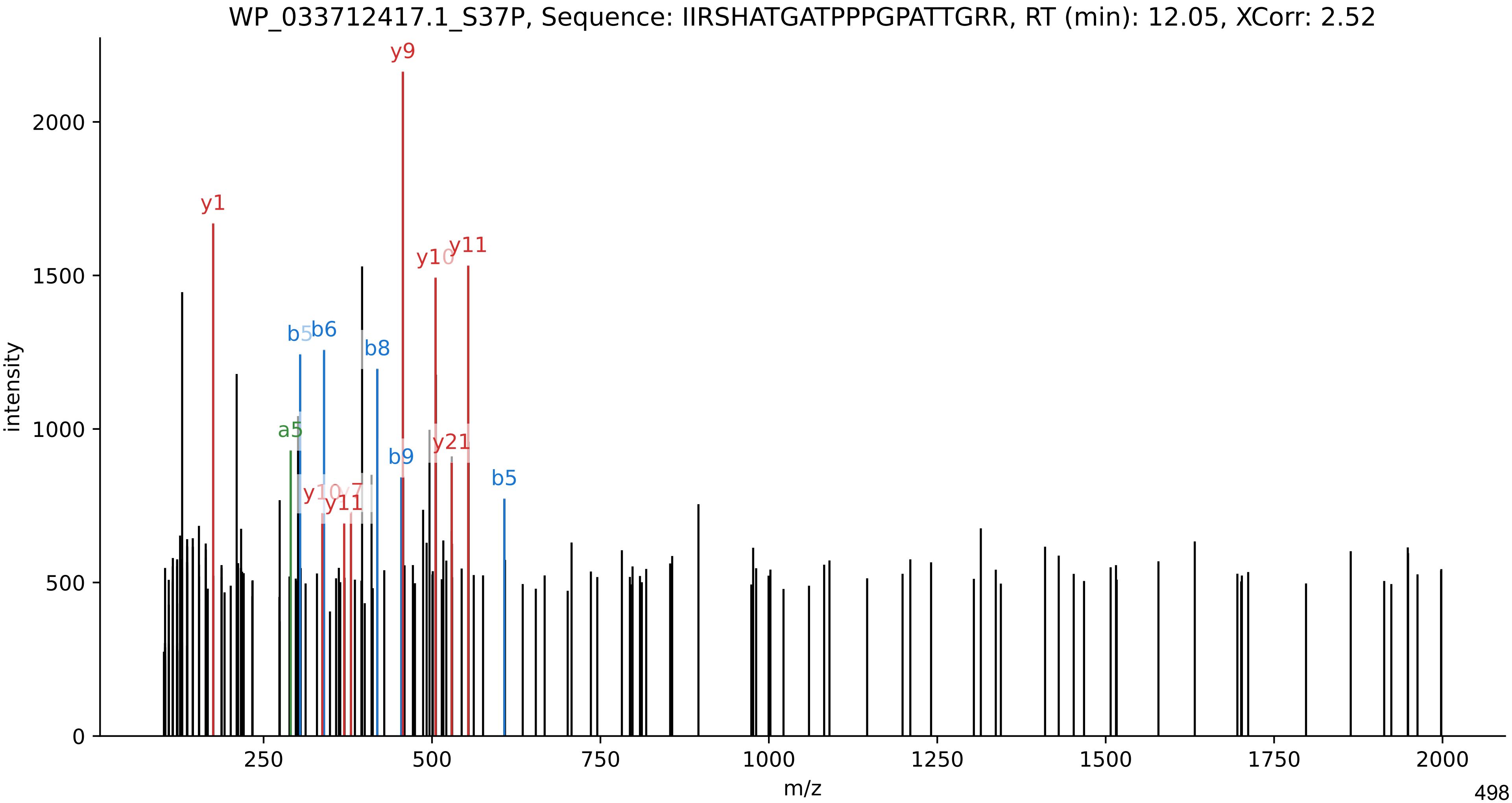

WP_033712417.1_S37P, Sequence: IIRSHATGATPPPGPATTGRR, RT (min): 12.05, XCorr: 2.52
intensity
m/z
y9
y1
y10
y11
b5
b6
b8
a5
b9
y21
y10
y7
y11
b5
0
500
1000
1500
2000
250
500
750
1000
1250
1500
1750
2000

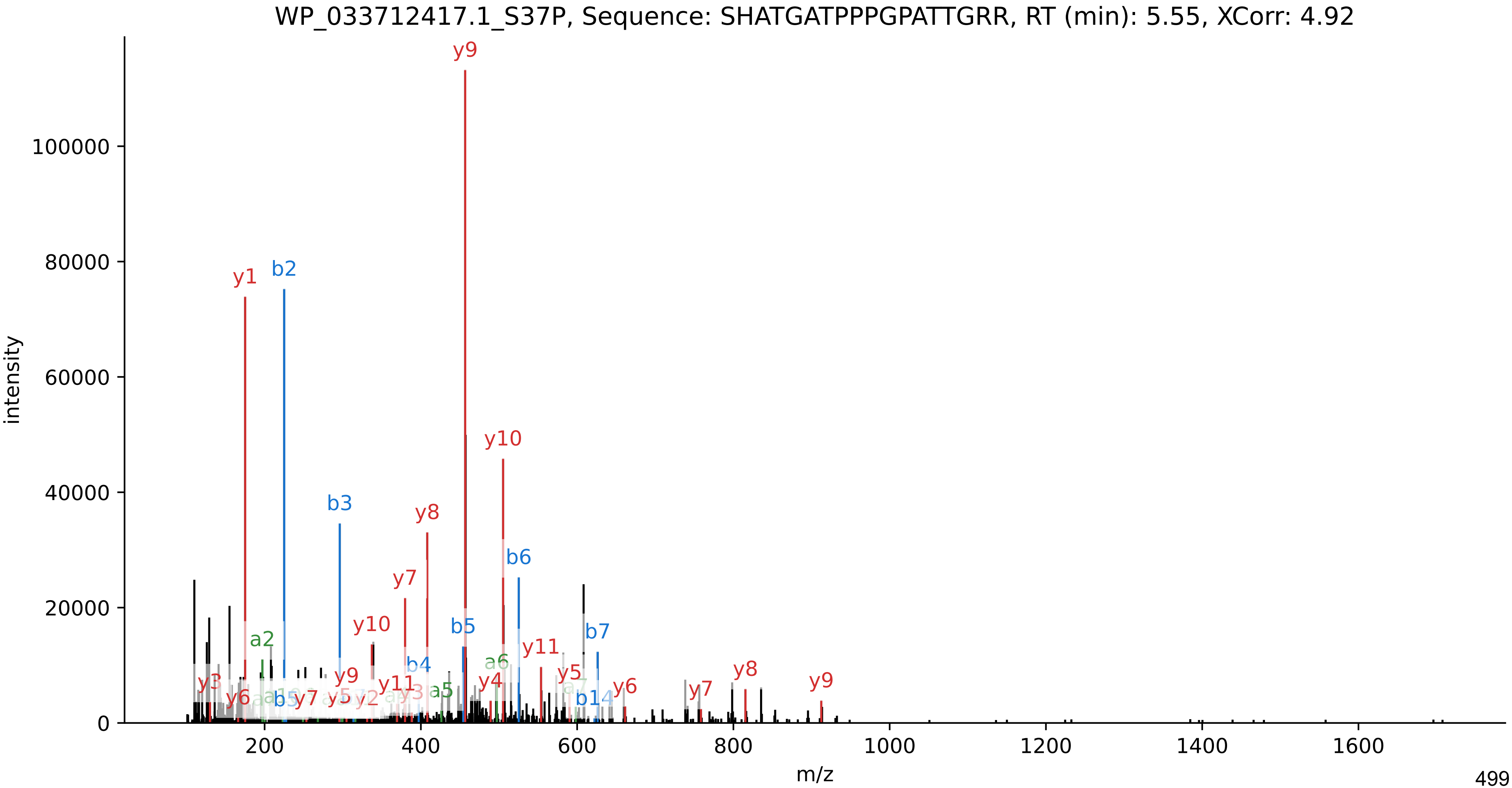
WP_033712417.1_S37P, Sequence: SHATGATPPPGPATTGRR, RT (min): 5.55, XCorr: 4.92
intensity
m/z
y9
y1
b2
y10
b3
y8
y7
b6
y10
b5
b7
a2
y11
b4
a6
y5
y9
y11
y4
y8
y3
a5
y6
y9
y7
y6
b14
b5
y7
y2

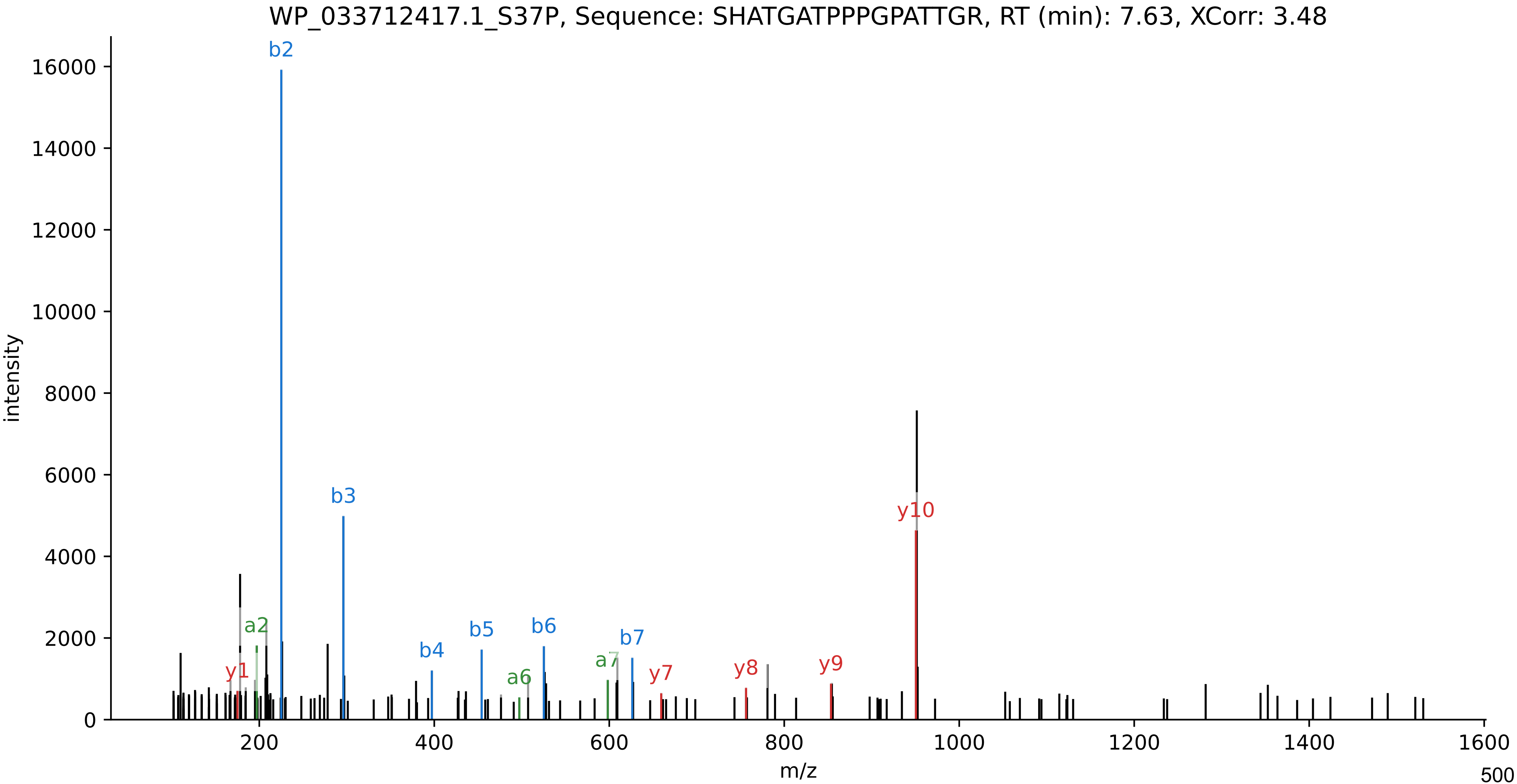

WP_033712417.1_S37P, Sequence: SHATGATPPPGPATTGR, RT (min): 7.63, XCorr: 3.48
intensity
m/z
b2
b3
b4
b5
b6
b7
a2
a6
a7
y1
y7
y8
y9
y10

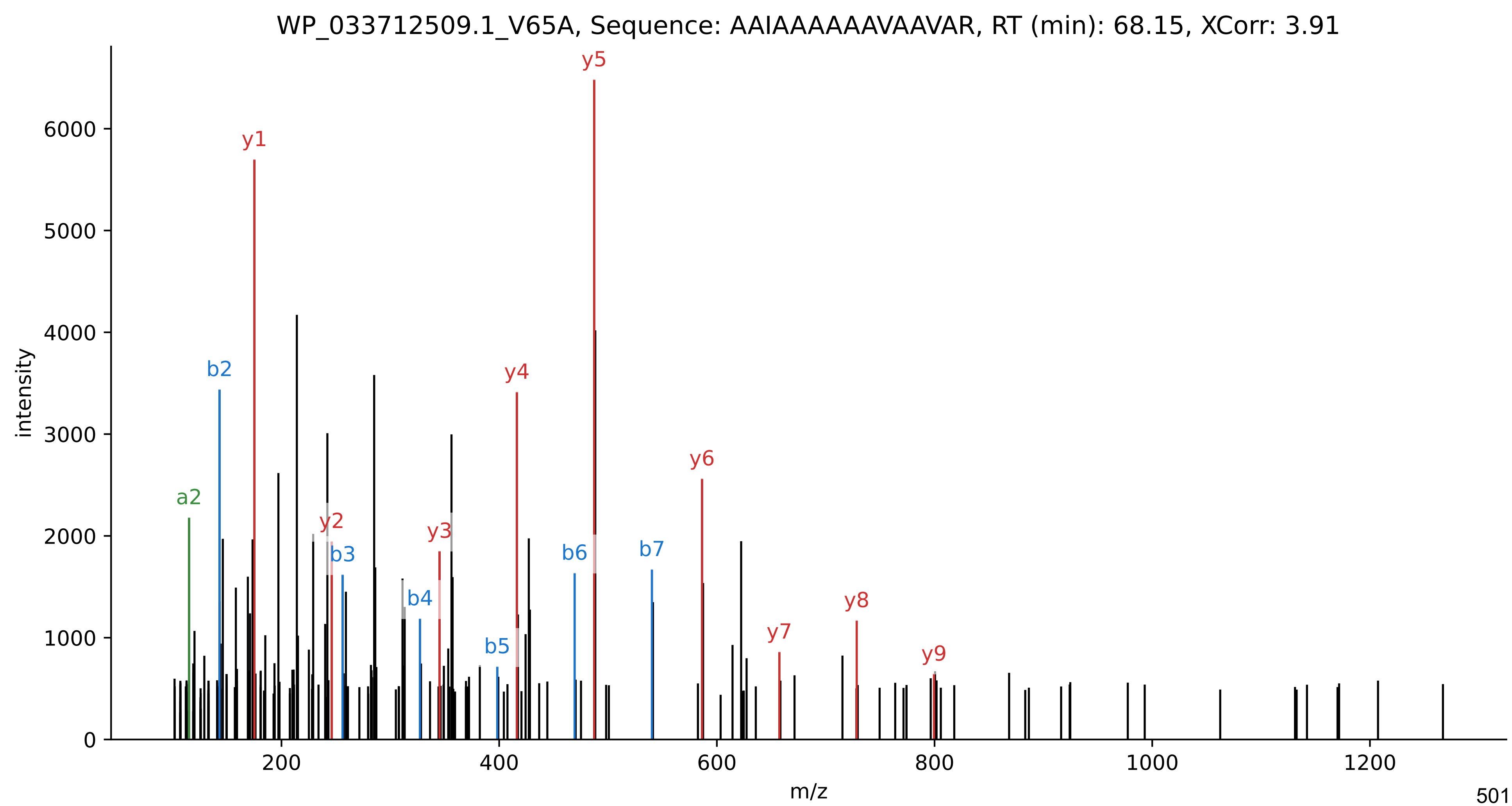

WP_033712509.1_V65A, Sequence: AAIAAAAAAVAAVAR, RT (min): 68.15, XCorr: 3.91
intensity
m/z
y5
y1
b2
y4
y6
a2
y2
b3
y3
b6
b7
b4
b5
y7
y8
y9
0
1000
2000
3000
4000
5000
6000
200
400
600
800
1000
1200

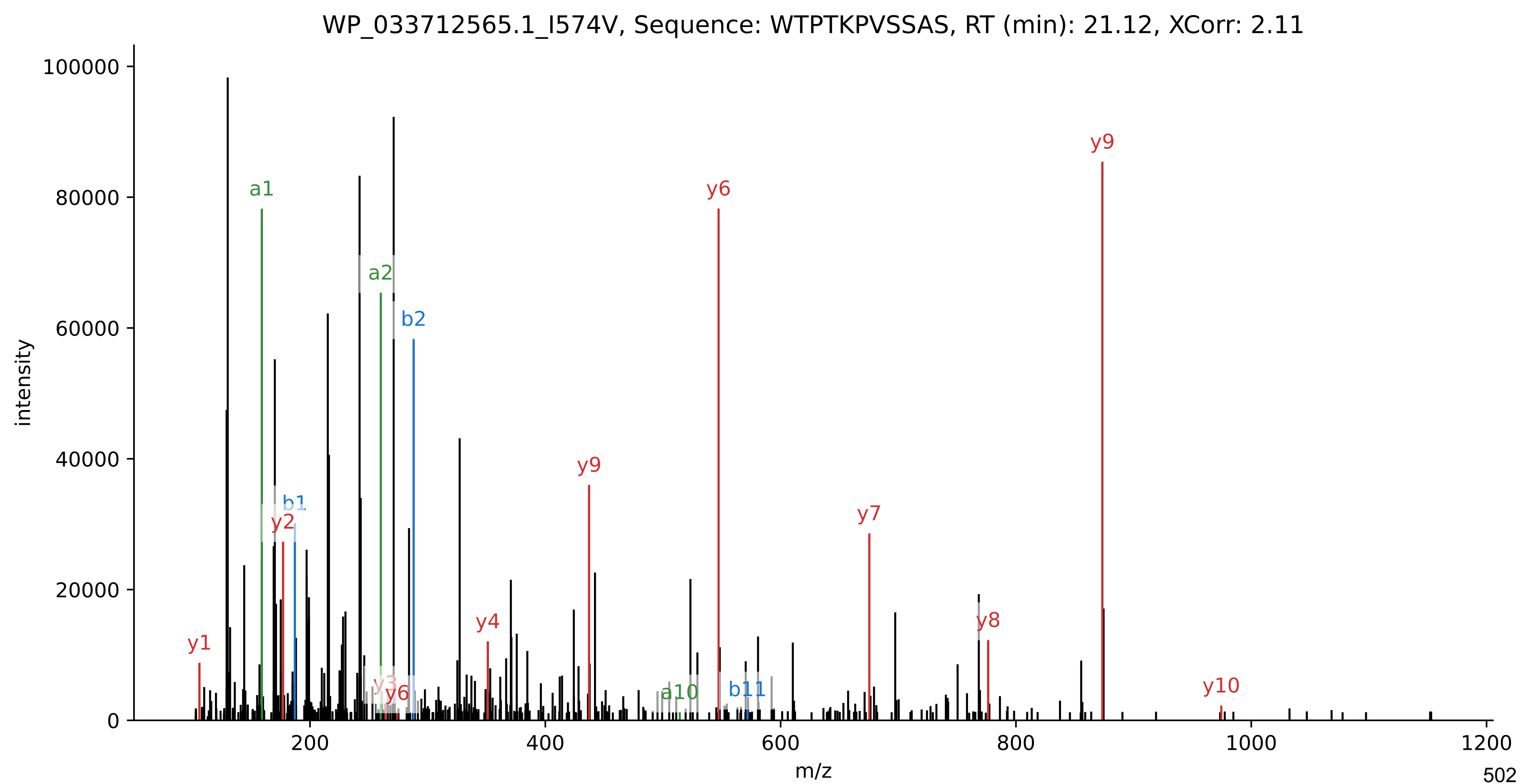
WP_033712565.1_I574V, Sequence: WTPTKPVSSAS, RT (min): 21.12, XCorr: 2.11
intensity
m/z
100000
80000
60000
40000
20000
0
200
400
600
800
1000
1200
a1
a2
b1
b2
y1
y2
y3
y6
y4
y9
a10
b11
y6
y7
y8
y9
y10

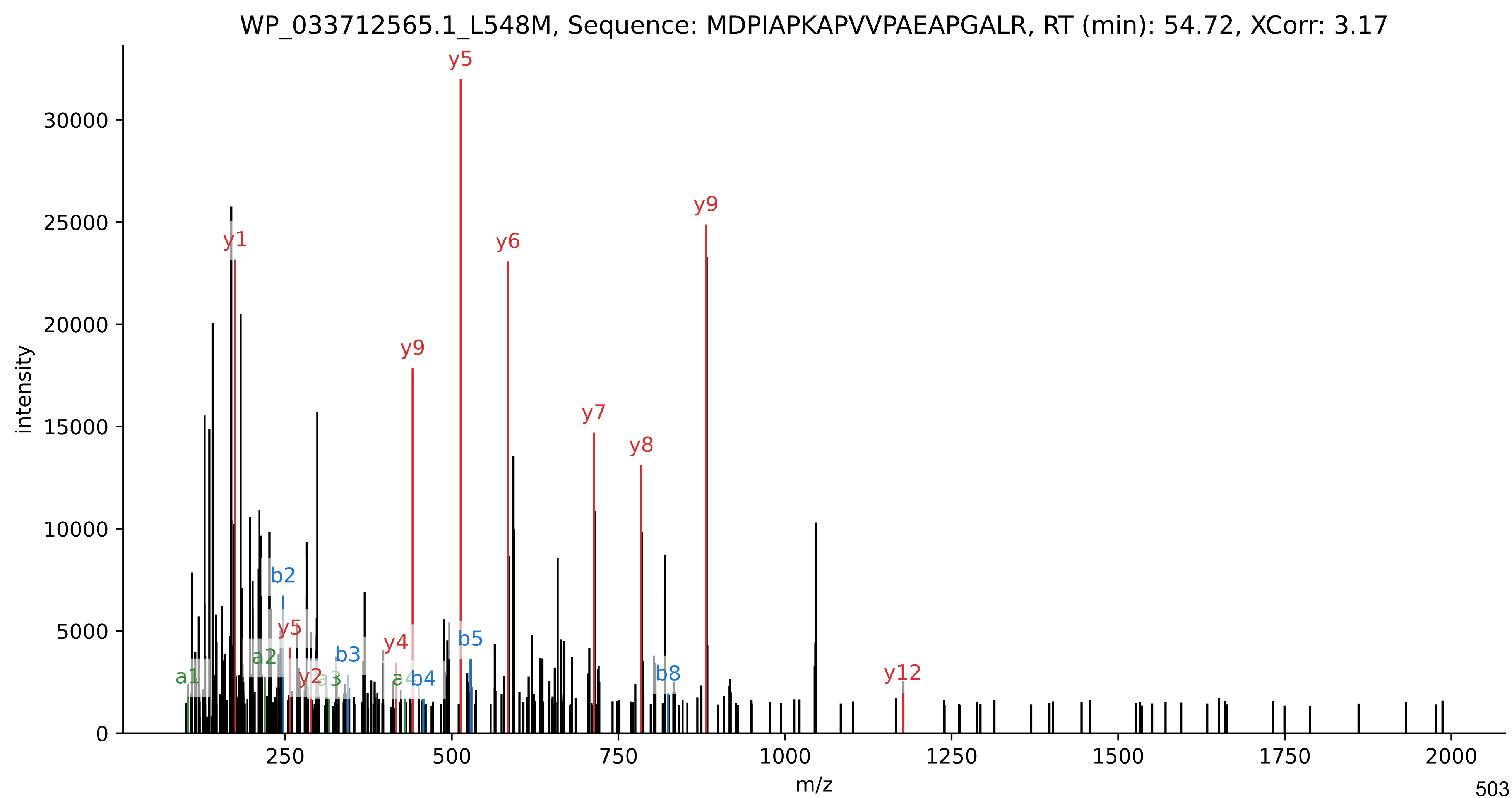

WP_033712565.1_L548M, Sequence: MDPIAPKAPVVPAEAPGALR, RT (min): 54.72, XCorr: 3.17
intensity
m/z
y5
y1
y6
y9
y9
y7
y8
b2
y5
y4
b5
a2
b3
a1
y2
a3
a4
b4
b8
y12

WP_033712675.1_A47E, Sequence: KQEAADEAAAQVGPQALGVGAHAVDEDAAR, RT (min): 52.83, XCorr: 10.07

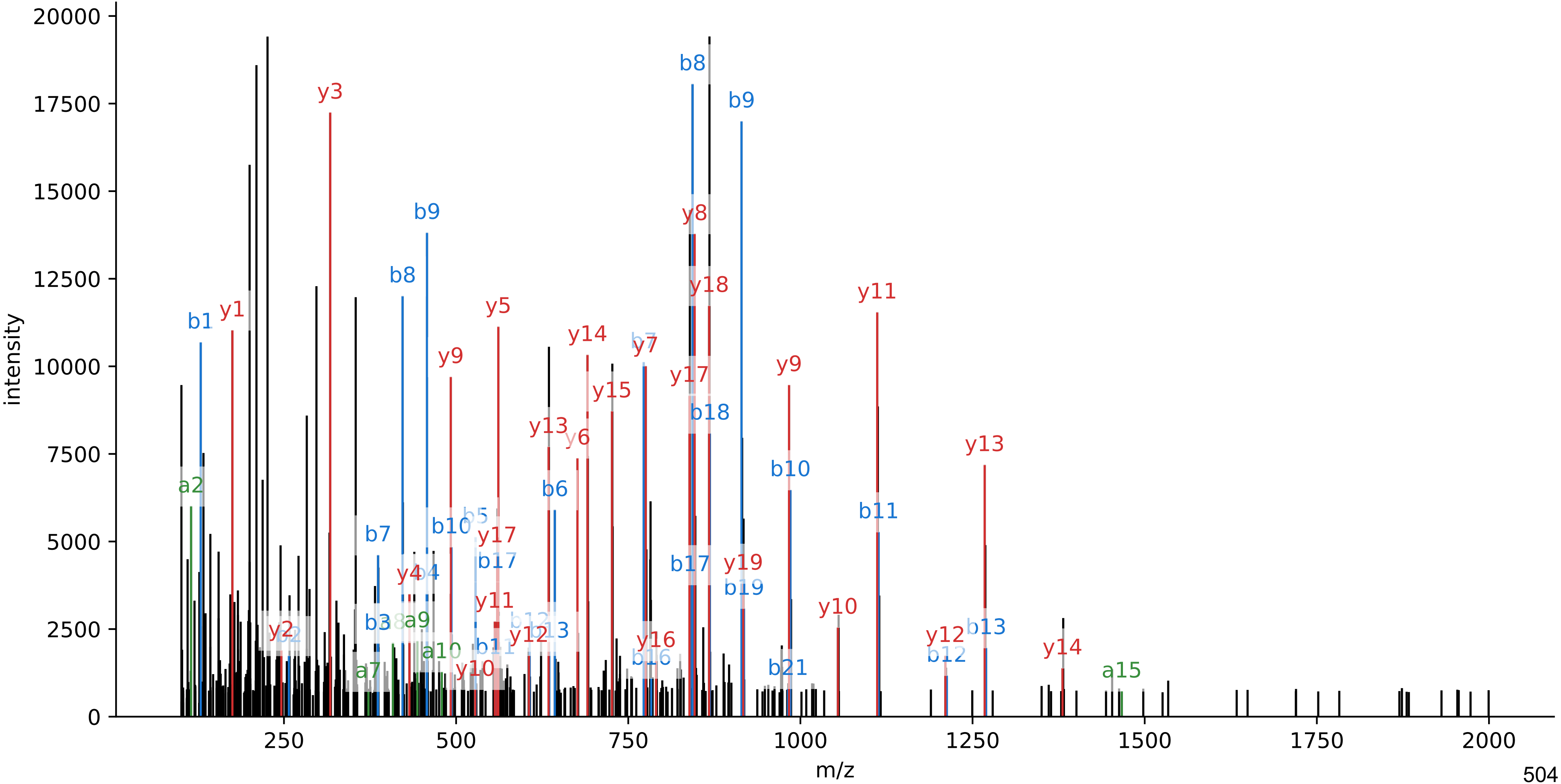

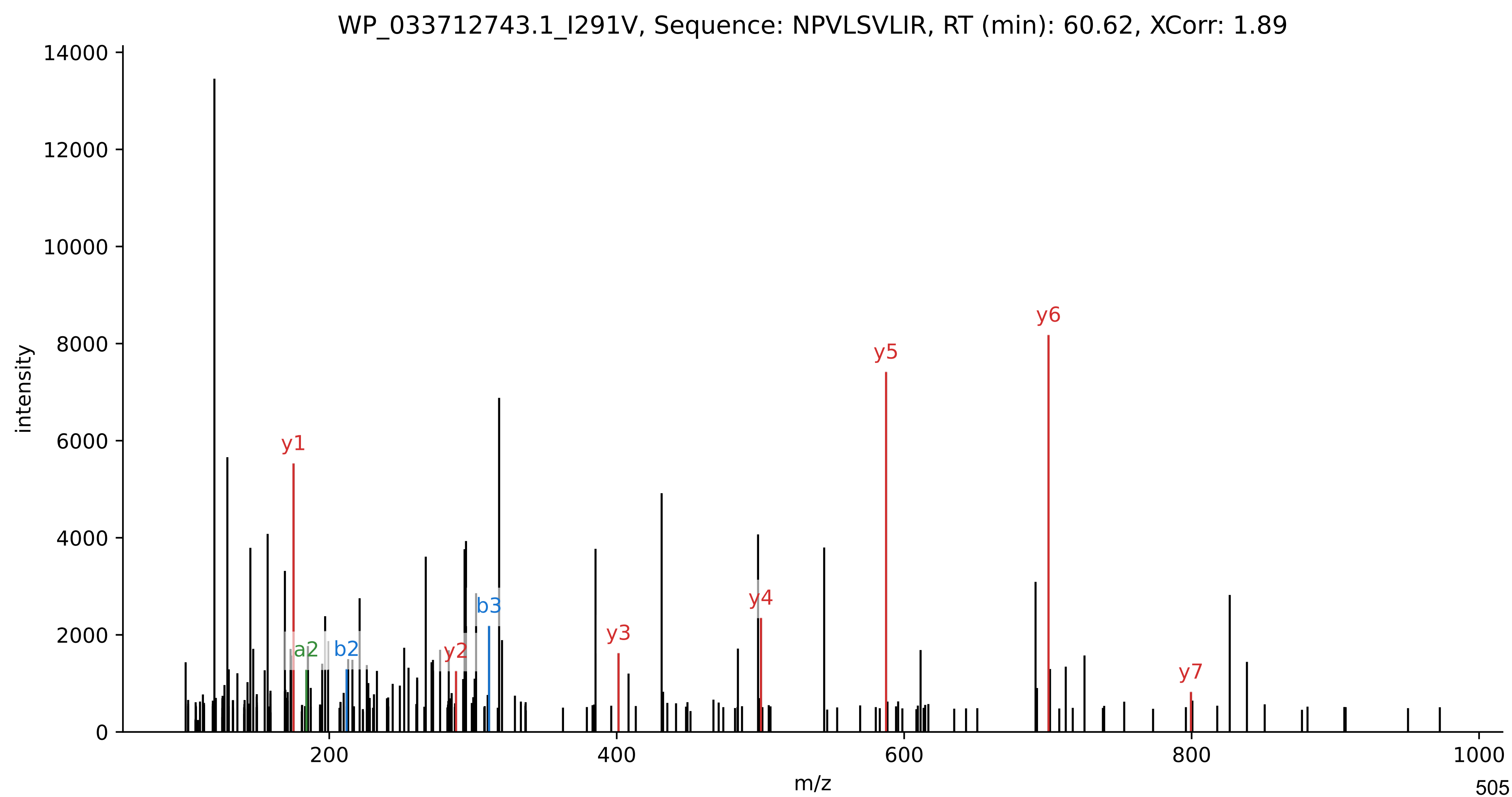

WP_033712743.1_I291V, Sequence: NPVLSVLIR, RT (min): 60.62, XCorr: 1.89
intensity
m/z
y1
a2
b2
y2
b3
y3
y4
y5
y6
y7

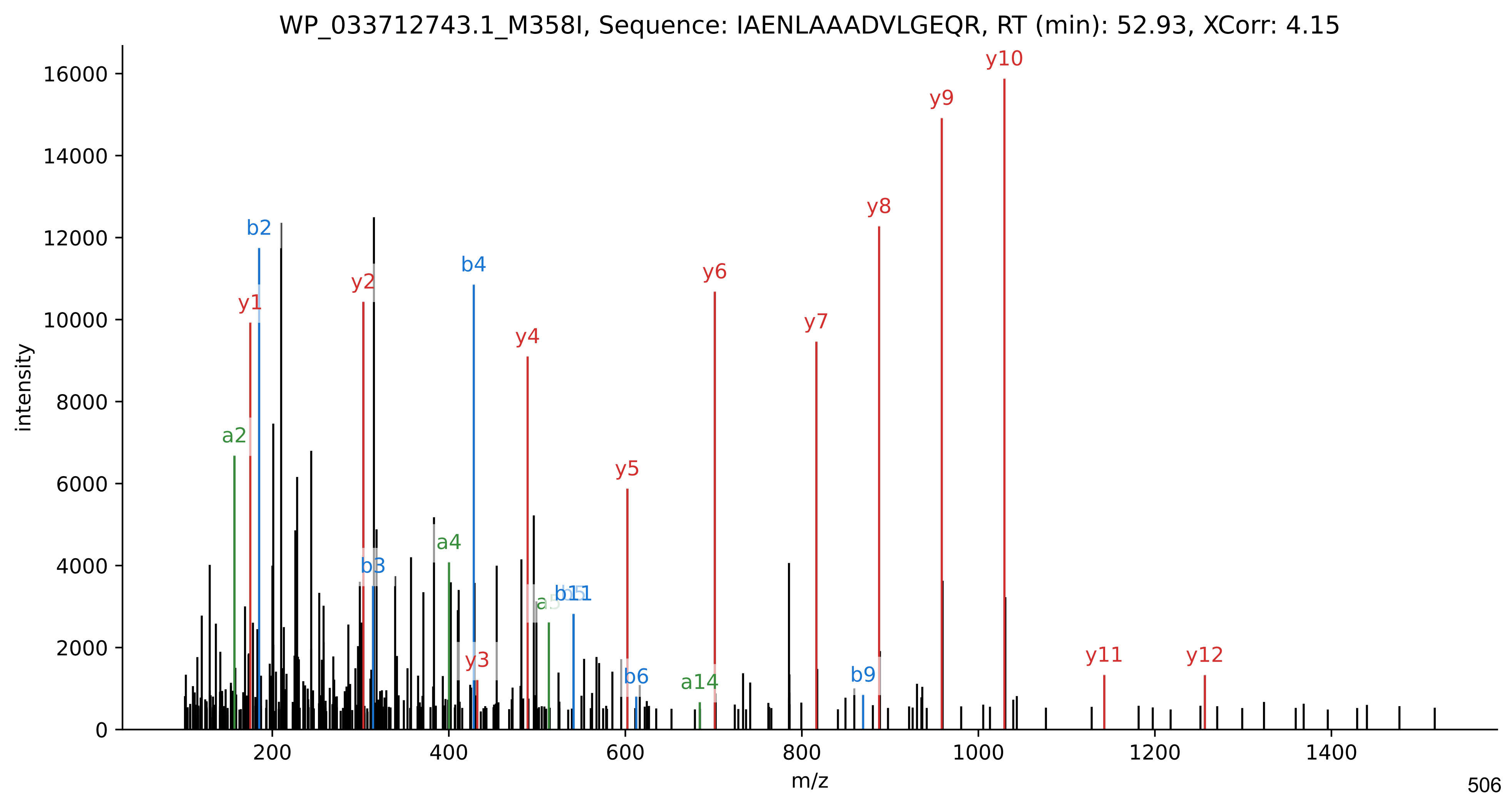

WP_033712743.1_M358I, Sequence: IAENLAAADVLGEQR, RT (min): 52.93, XCorr: 4.15
intensity
m/z
0
2000
4000
6000
8000
10000
12000
14000
16000
200
400
600
800
1000
1200
1400
y10
y9
y8
y7
y6
y5
y4
y3
y2
y1
y11
y12
b2
b3
b4
b6
b9
b11
a2
a4
a14

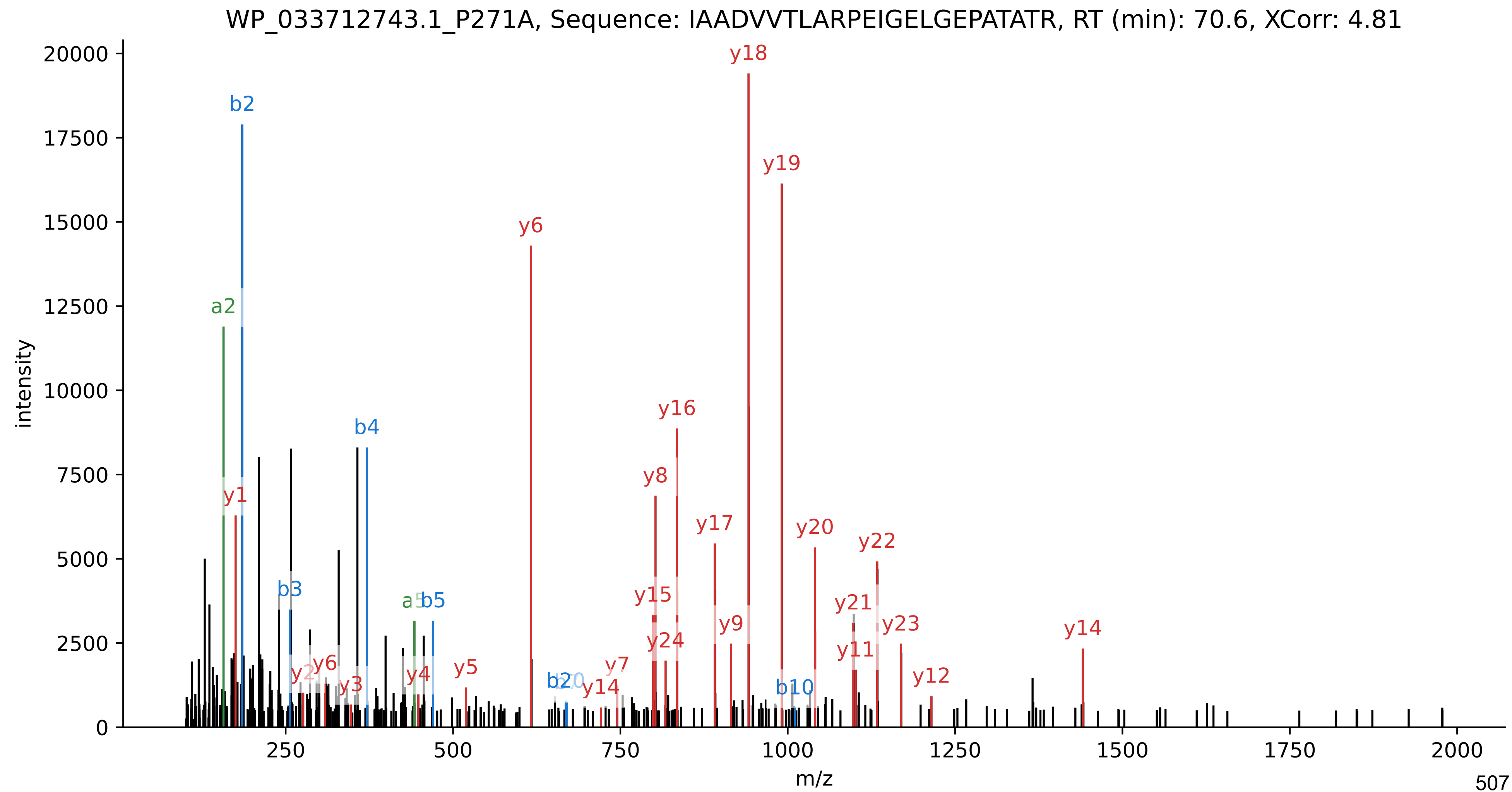

WP_033712743.1_P271A, Sequence: IAADVVTLARPEIGELGEPATATR, RT (min): 70.6, XCorr: 4.81
intensity
m/z
y18
b2
y19
y6
a2
y16
b4
y8
y1
y17
y20
y22
b3
a5
b5
y15
y21
y9
y23
y14
y24
y11
y2
y6
y4
y5
y3
y7
b2
y14
b10
y12

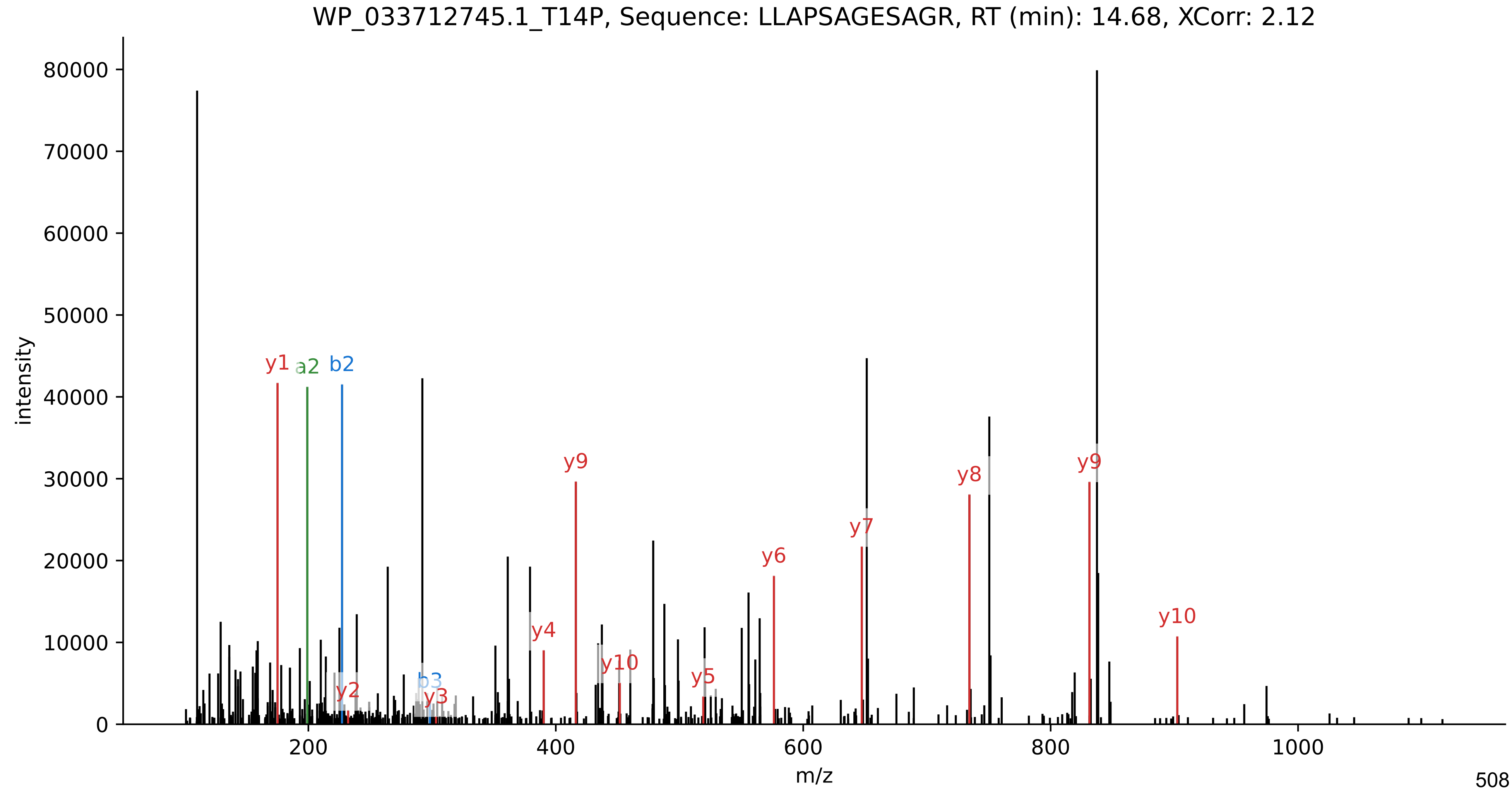

WP_033712745.1_T14P, Sequence: LLAPSAGESAGR, RT (min): 14.68, XCorr: 2.12
intensity
m/z
y1
a2
b2
y9
y8
y9
y7
y6
y10
y4
y10
y5
b3
y2
y3

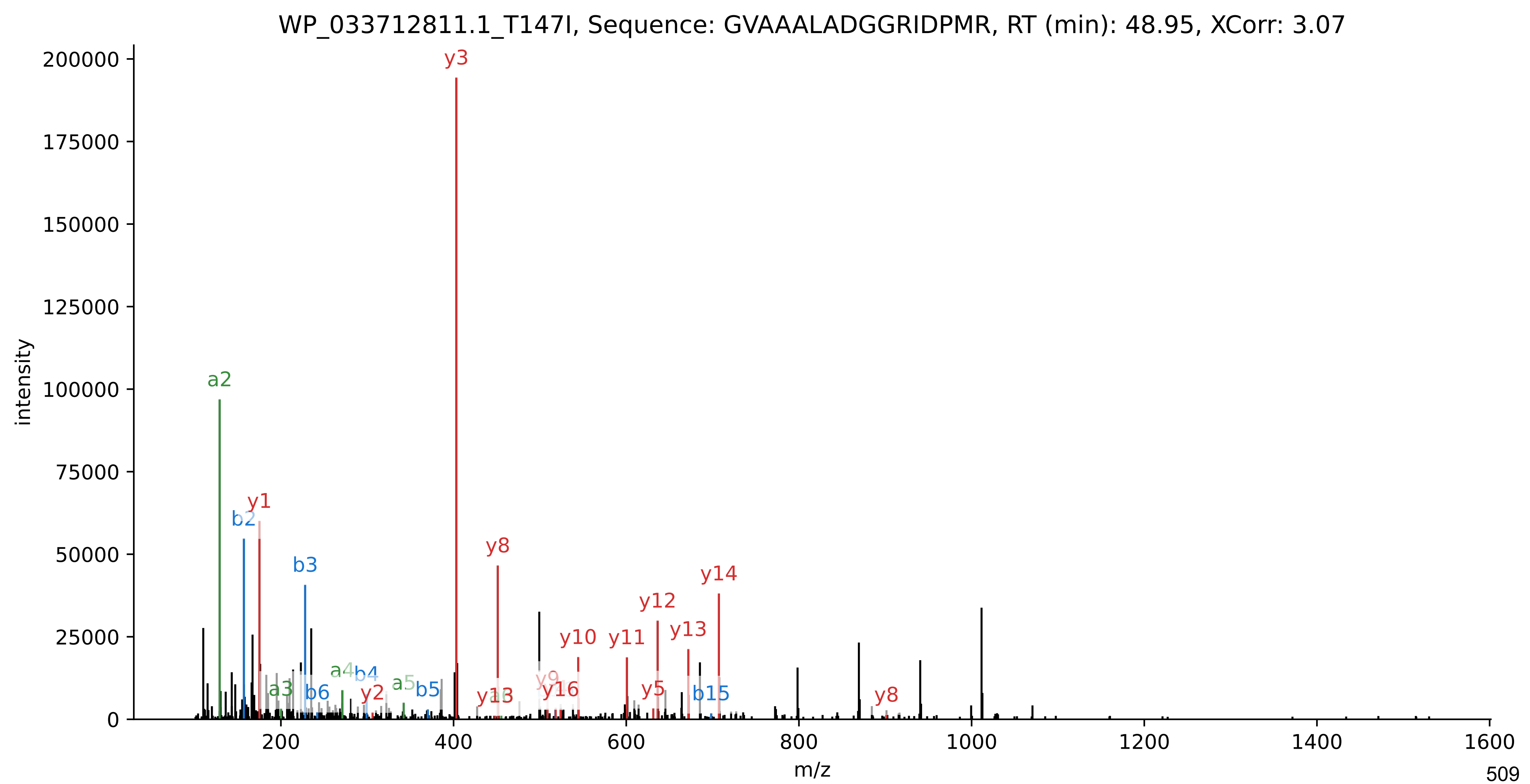

WP_033712811.1_T147I, Sequence: GVAAALADGGRIDPMR, RT (min): 48.95, XCorr: 3.07
intensity
m/z
y3
a2
y1
b2
b3
y8
y14
y12
y13
y10
y11
a4
b4
a3
b6
y2
a5
b5
y9
y16
y5
b15
y8

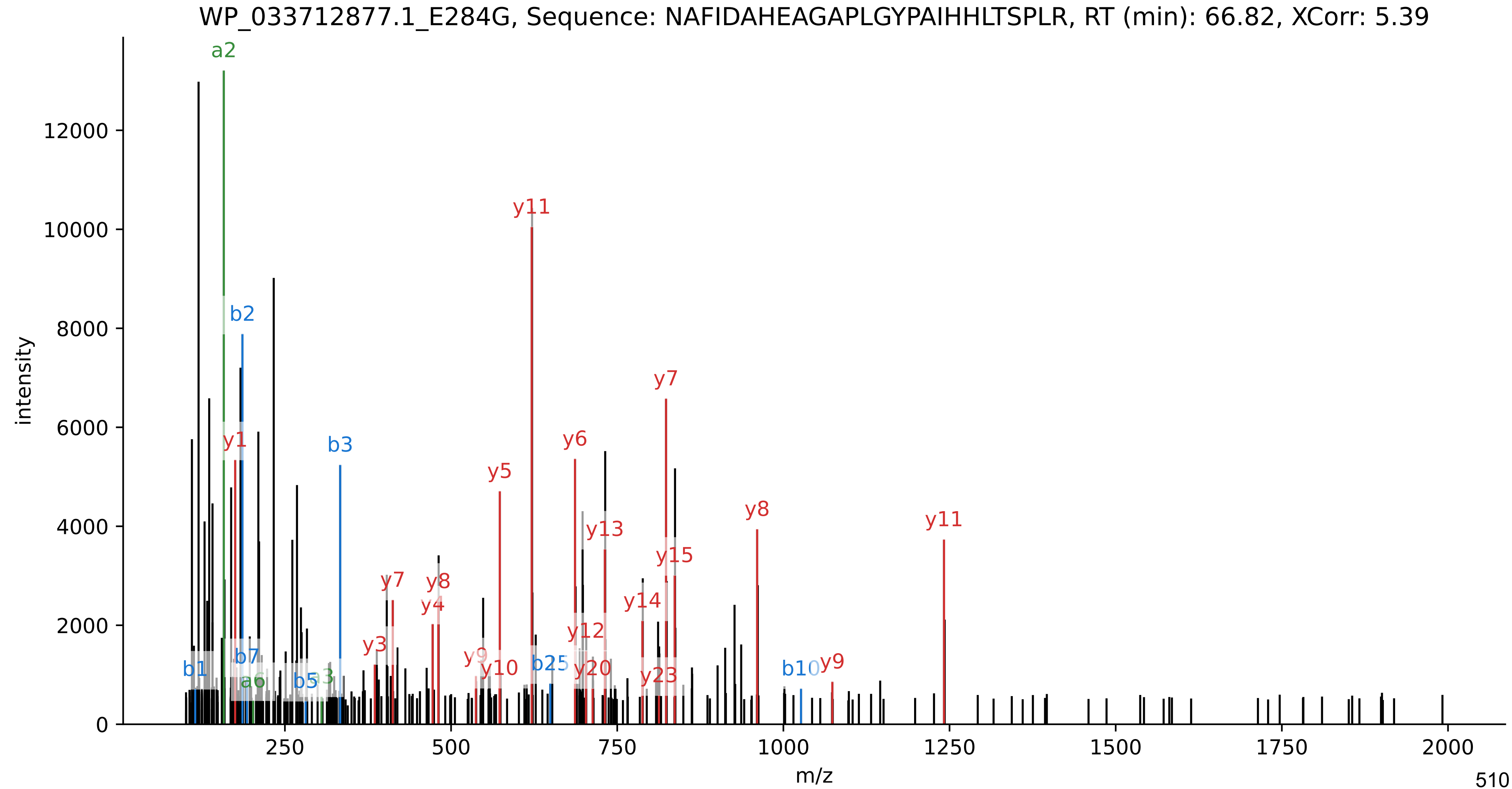
WP_033712877.1_E284G, Sequence: NAFIDAHEAGAPLGYPAIHHLTSPLR, RT (min): 66.82, XCorr: 5.39
intensity
m/z
a2
b2
y1
b3
y11
y7
y6
y5
y13
y15
y8
y11
y14
y12
y7
y8
y4
y3
y9
y10
b25
y20
y23
b10
y9
b1
b7
a6
b5
a3
0
2000
4000
6000
8000
10000
12000
250
500
750
1000
1250
1500
1750
2000

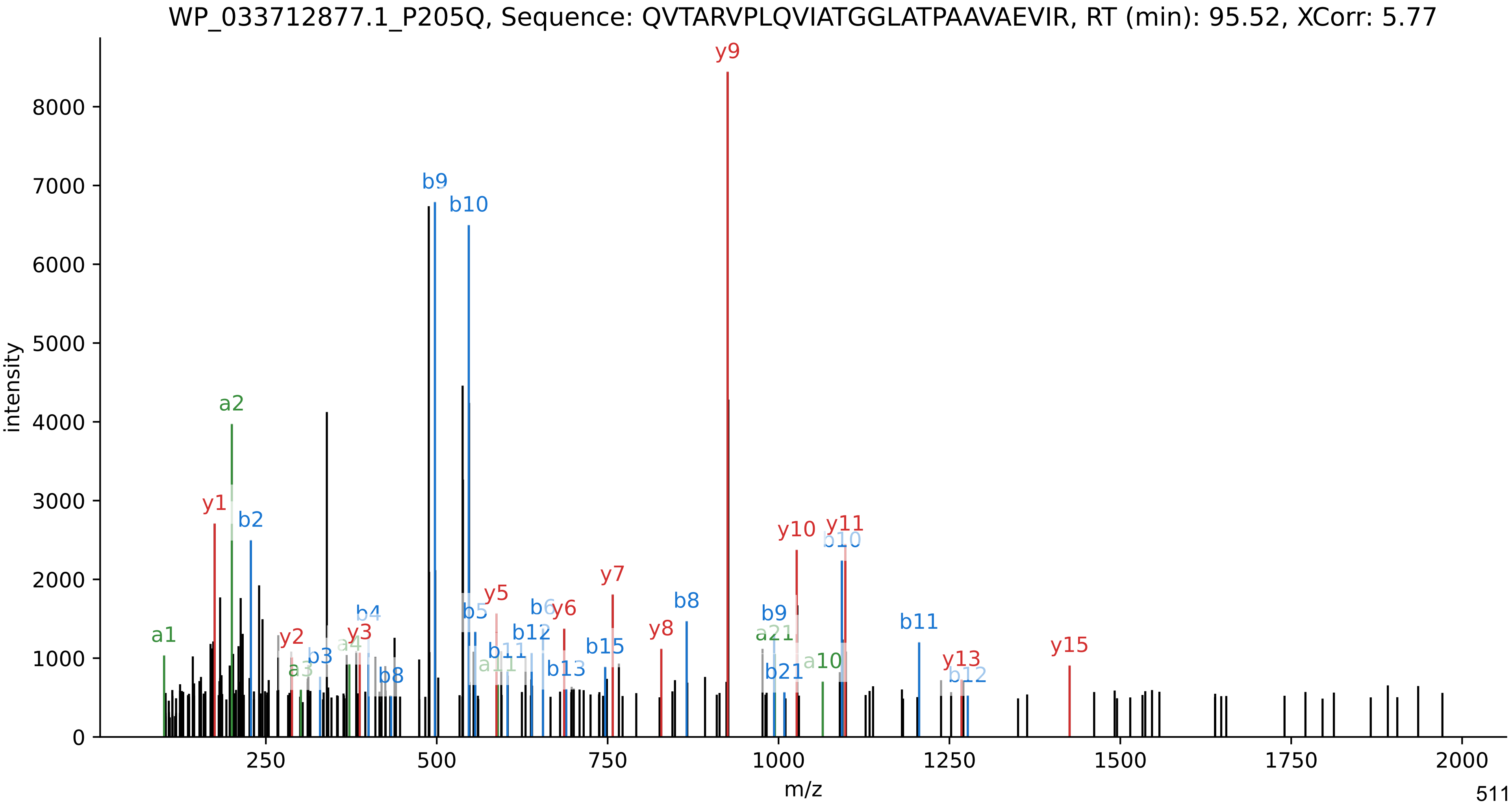

WP_033712877.1_P205Q, Sequence: QVTARVPLQVIATGGLATPAAVAEVIR, RT (min): 95.52, XCorr: 5.77
intensity
m/z
y9
b9
b10
a2
y1
b2
y10
y11
b10
y7
y5
b6
y6
b8
b9
a21
b11
y8
b5
b4
y2
y3
a4
b3
a3
b8
b12
b11
a11
b13
b15
b21
a10
y13
b12
y15
a1

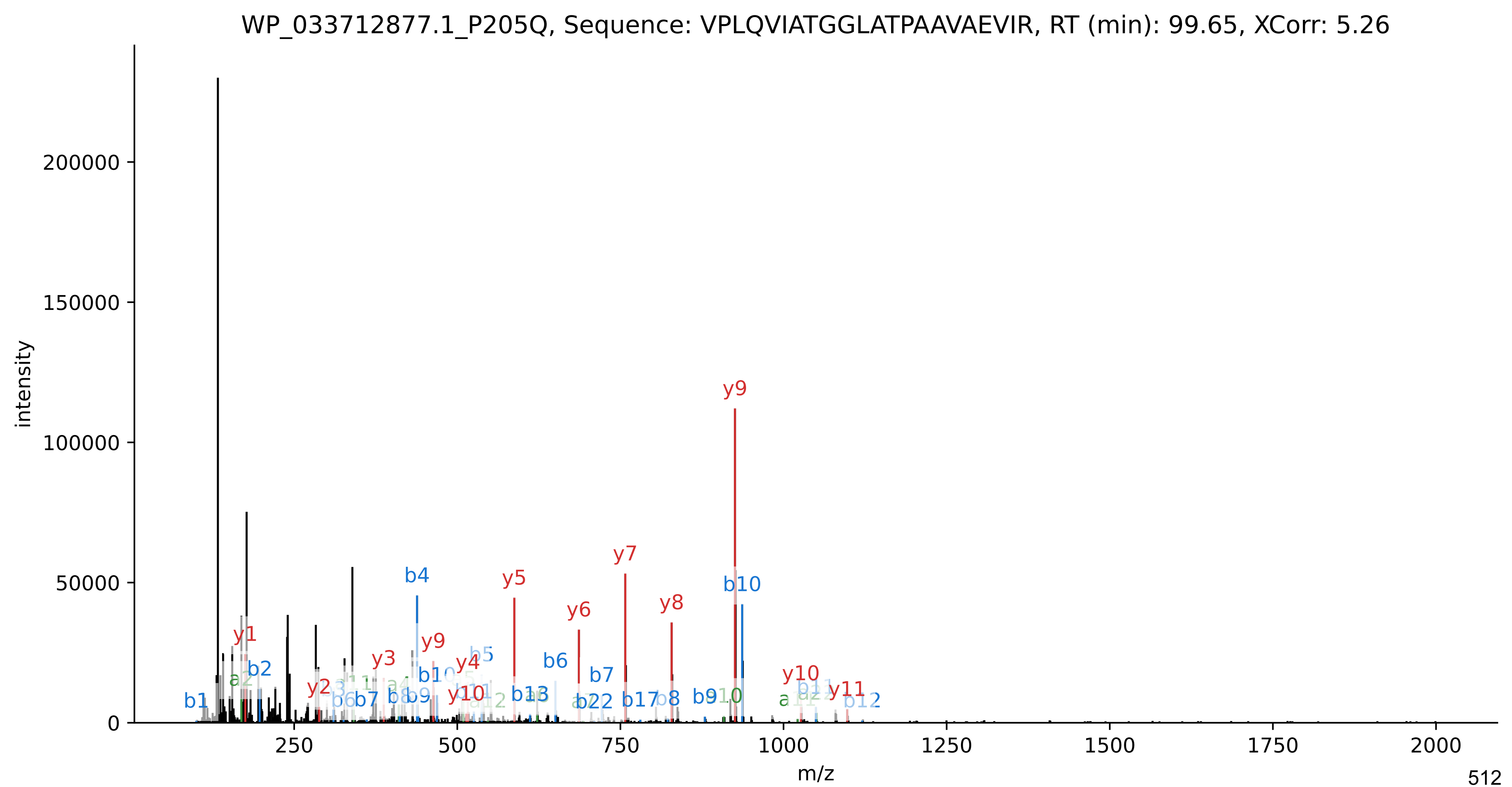

WP_033712877.1_P205Q, Sequence: VPLQVIATGGLATPAAVAEVIR, RT (min): 99.65, XCorr: 5.26
intensity
m/z
0
50000
100000
150000
200000
250
500
750
1000
1250
1500
1750
2000
y9
y7
b4
y5
b10
y8
y6
y1
y9
y3
y4
b6
b2
y2
b7
y10
y10
y11
b1
b13
b17
b9

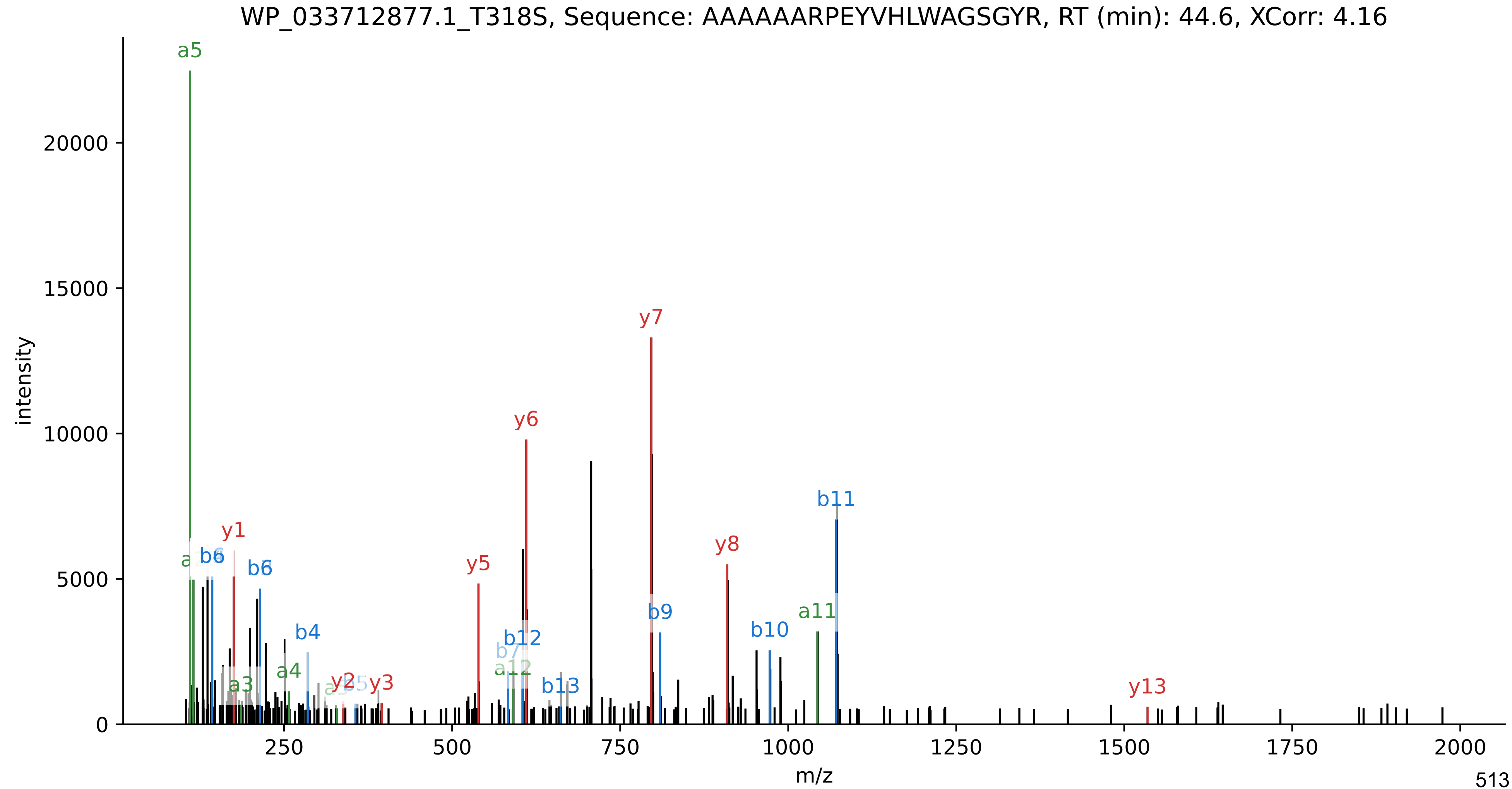
WP_033712877.1_T318S, Sequence: AAAAAARPEYVHLWAGSGYR, RT (min): 44.6, XCorr: 4.16
intensity
m/z
a5
y7
y6
b11
y1
b6
b6
y5
y8
b9
a11
b10
b4
b12
a12
b13
a4
a3
y2
y3
y13

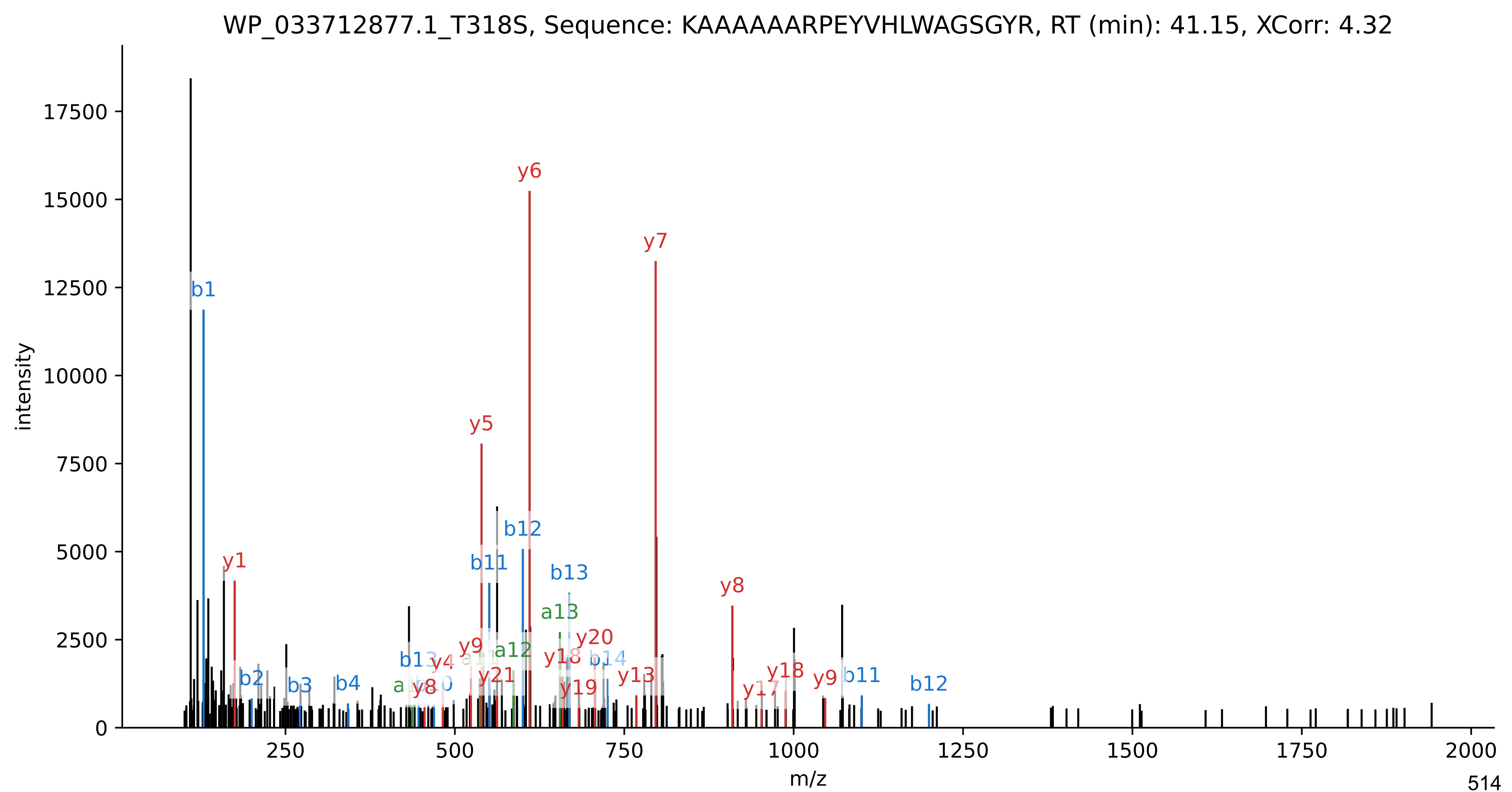
WP_033712877.1_T318S, Sequence: KAAAAAARPEYVHLWAGSGYR, RT (min): 41.15, XCorr: 4.32
intensity
m/z
y6
y7
b1
y5
b12
y1
b11
b13
y8
a13
y20
y9
a12
b14
y13
y18
y9
b11
b12
b2
b3
b4
y21
y19
y17
0
2500
5000
7500
10000
12500
15000
17500
250
500
750
1000
1250
1500
1750
2000

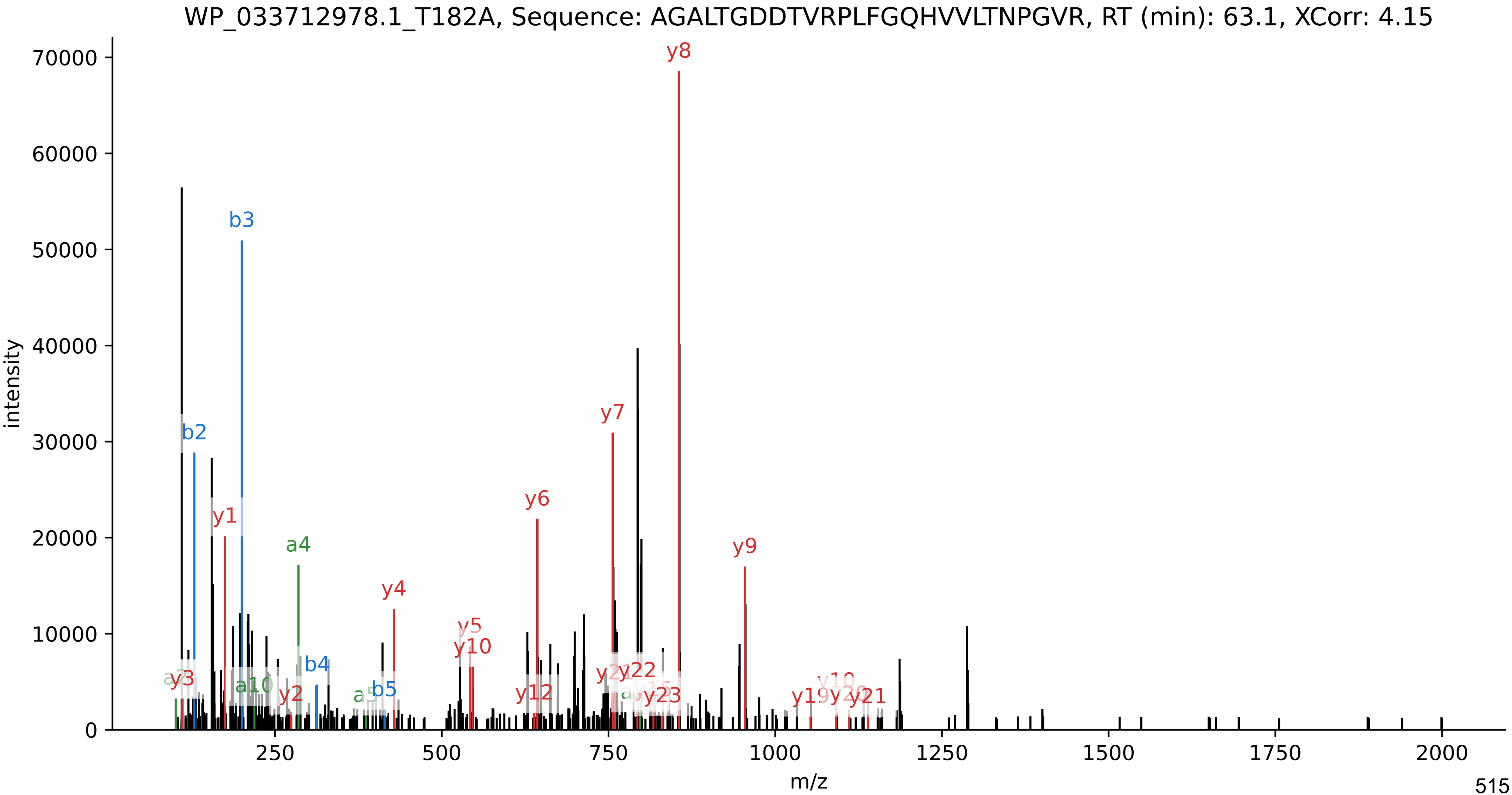

WP_033712978.1_T182A, Sequence: AGALTGDDTVRPLFGQHVVLTNPGVR, RT (min): 63.1, XCorr: 4.15
intensity
m/z
0
10000
20000
30000
40000
50000
60000
70000
250
500
750
1000
1250
1500
1750
2000
y8
b3
b2
y1
a4
y4
y5
y10
y6
y7
y9
b4
b5
y3
y2
a10
a5
y12
y22
y23
y19
y21

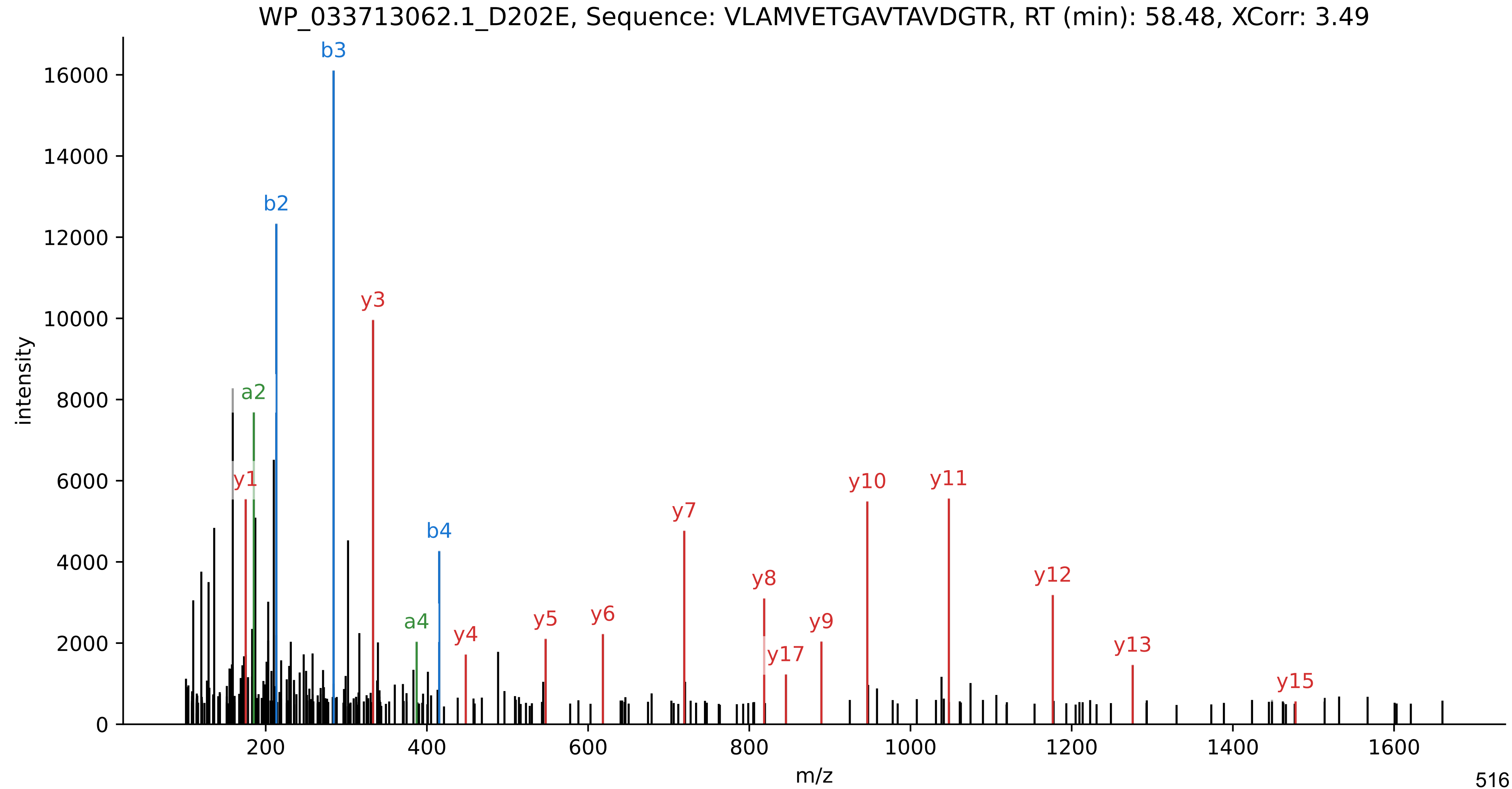

WP_033713062.1_D202E, Sequence: VLAMVETGAVTAVDGTR, RT (min): 58.48, XCorr: 3.49
intensity
m/z
b3
b2
y3
a2
y1
b4
a4
y4
y5
y6
y7
y8
y17
y9
y10
y11
y12
y13
y15

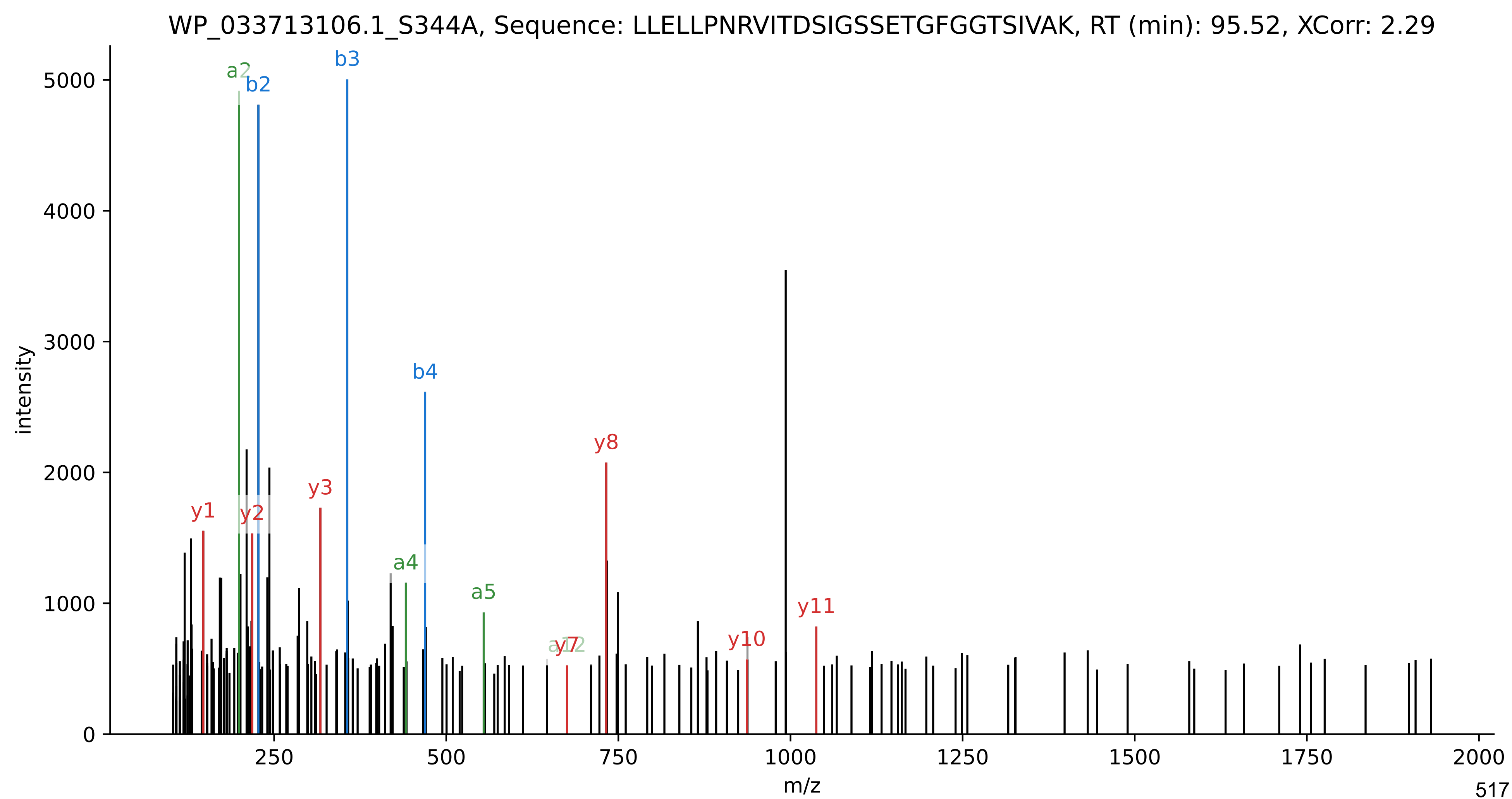

WP_033713106.1_S344A, Sequence: LLELLPNRVITDSIGSSETGFGGTSIVAK, RT (min): 95.52, XCorr: 2.29
intensity
m/z
a2
b2
b3
b4
y1
y2
y3
a4
a5
y8
y7
y10
y11

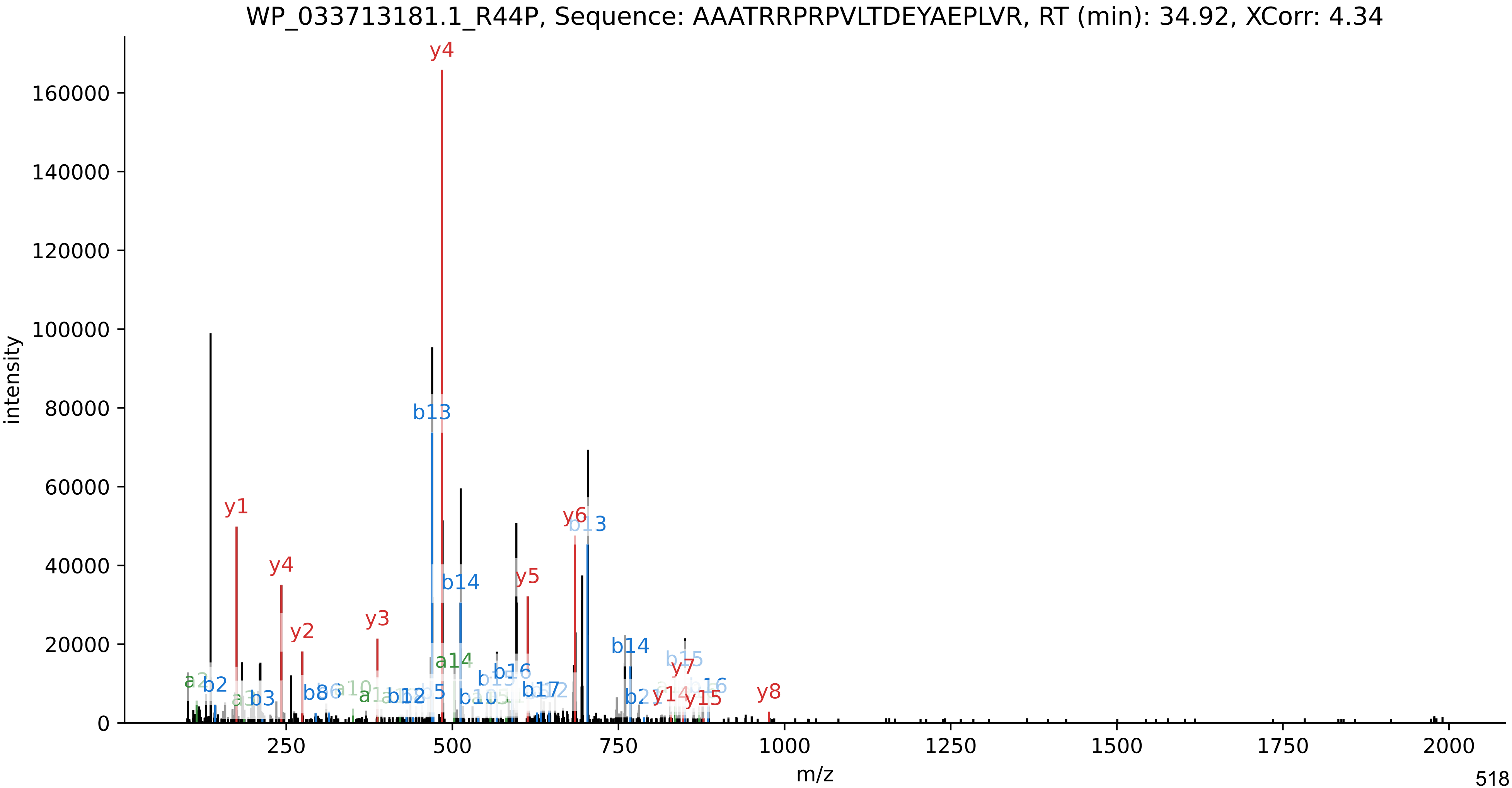
WP_033713181.1_R44P, Sequence: AAATRRPRPVLTDEYAEPLVR, RT (min): 34.92, XCorr: 4.34
intensity
m/z
y4
b13
y1
y4
y2
y3
b14
y5
y6
b13
a14
b14
b16
b17
b15
y7
b16
y14
y15
y8
b2
b3
b8
b12
b10

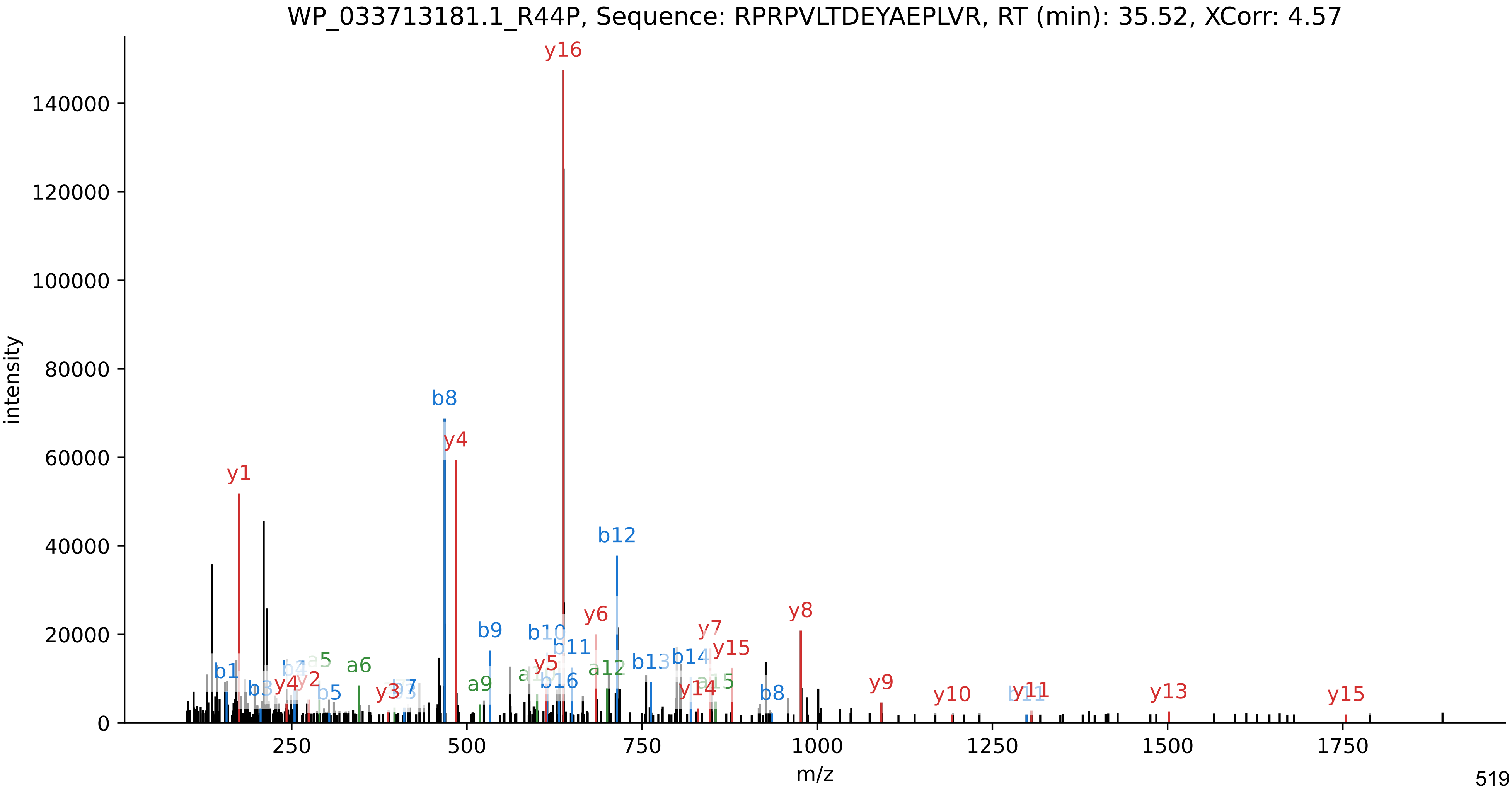
WP_033713181.1_R44P, Sequence: RPRPVLTDEYAEPLVR, RT (min): 35.52, XCorr: 4.57
intensity
m/z
y16
b8
y4
y1
b12
y8
y6
y7
y15
b9
b10
b11
b13
b14
a12
a6
a9
y5
b16
y14
y9
y10
y11
y13
y15
b1
b3
b5
y2
y3
b8

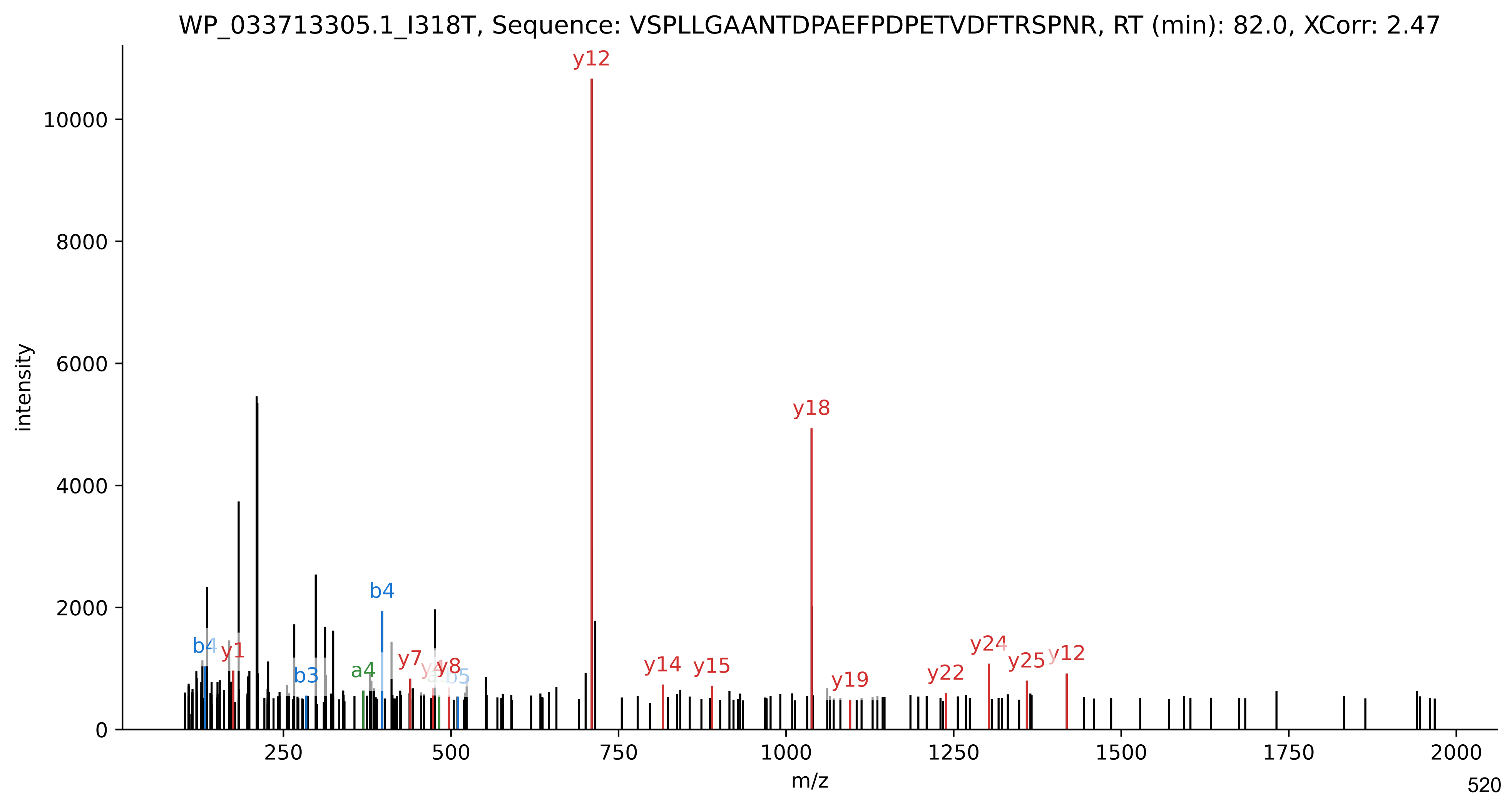

WP_033713305.1_I318T, Sequence: VSPLLGAANTDPAEFPDPETVDFTRSPNR, RT (min): 82.0, XCorr: 2.47
intensity
m/z
y12
y18
b4
y1
b3
a4
b4
y7
y8
b5
y14
y15
y19
y22
y24
y25
y12
0
2000
4000
6000
8000
10000
250
500
750
1000
1250
1500
1750
2000

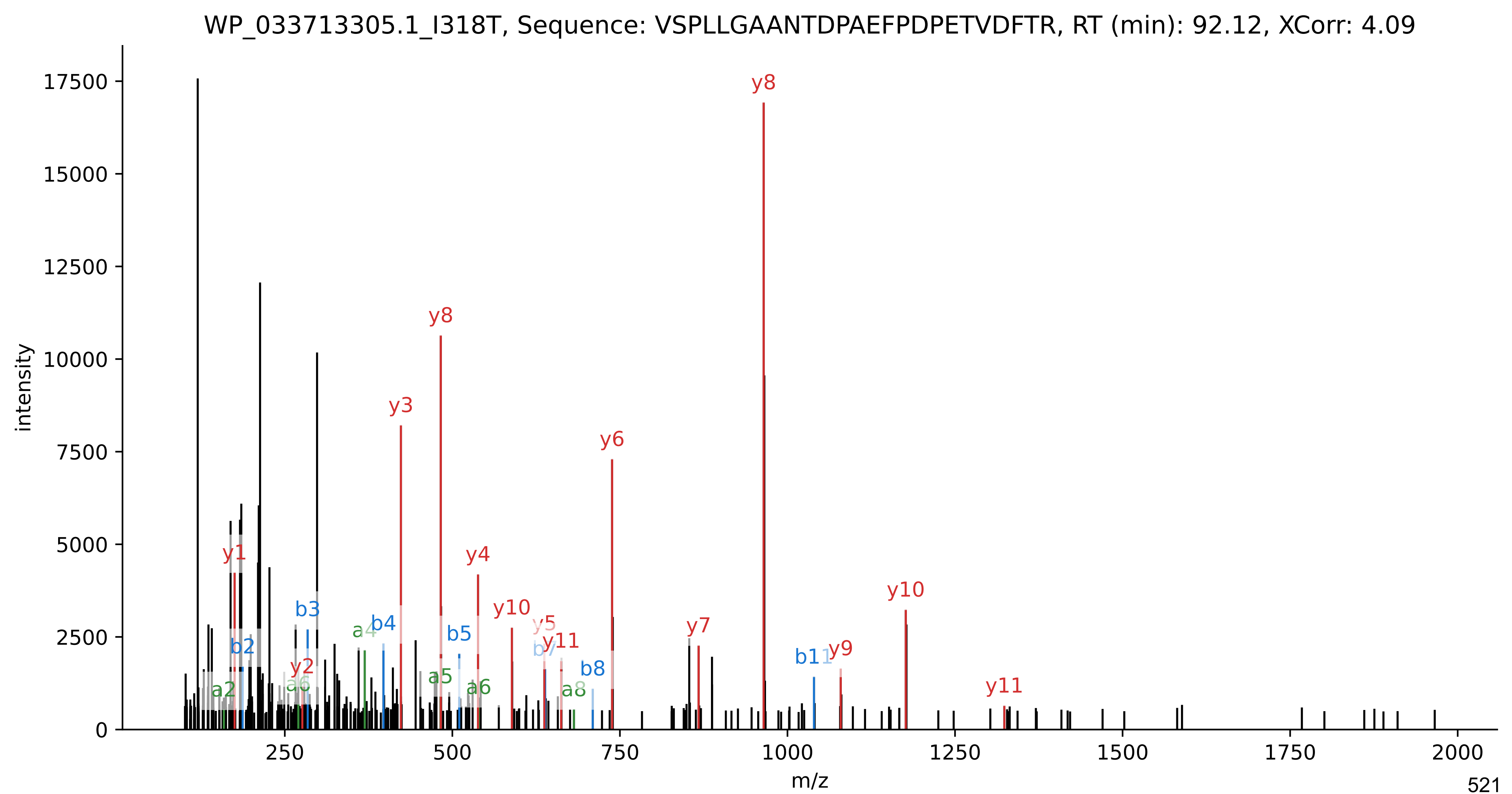

WP_033713305.1_I318T, Sequence: VSPLLGAANTDPAEFPDPETVDFTR, RT (min): 92.12, XCorr: 4.09
intensity
m/z
y8
y8
y3
y6
y1
y4
b3
b2
a4
b4
b5
y10
y5
y11
b7
b8
a2
y2
a6
a5
a6
a8
y7
b11
y9
y10
y11

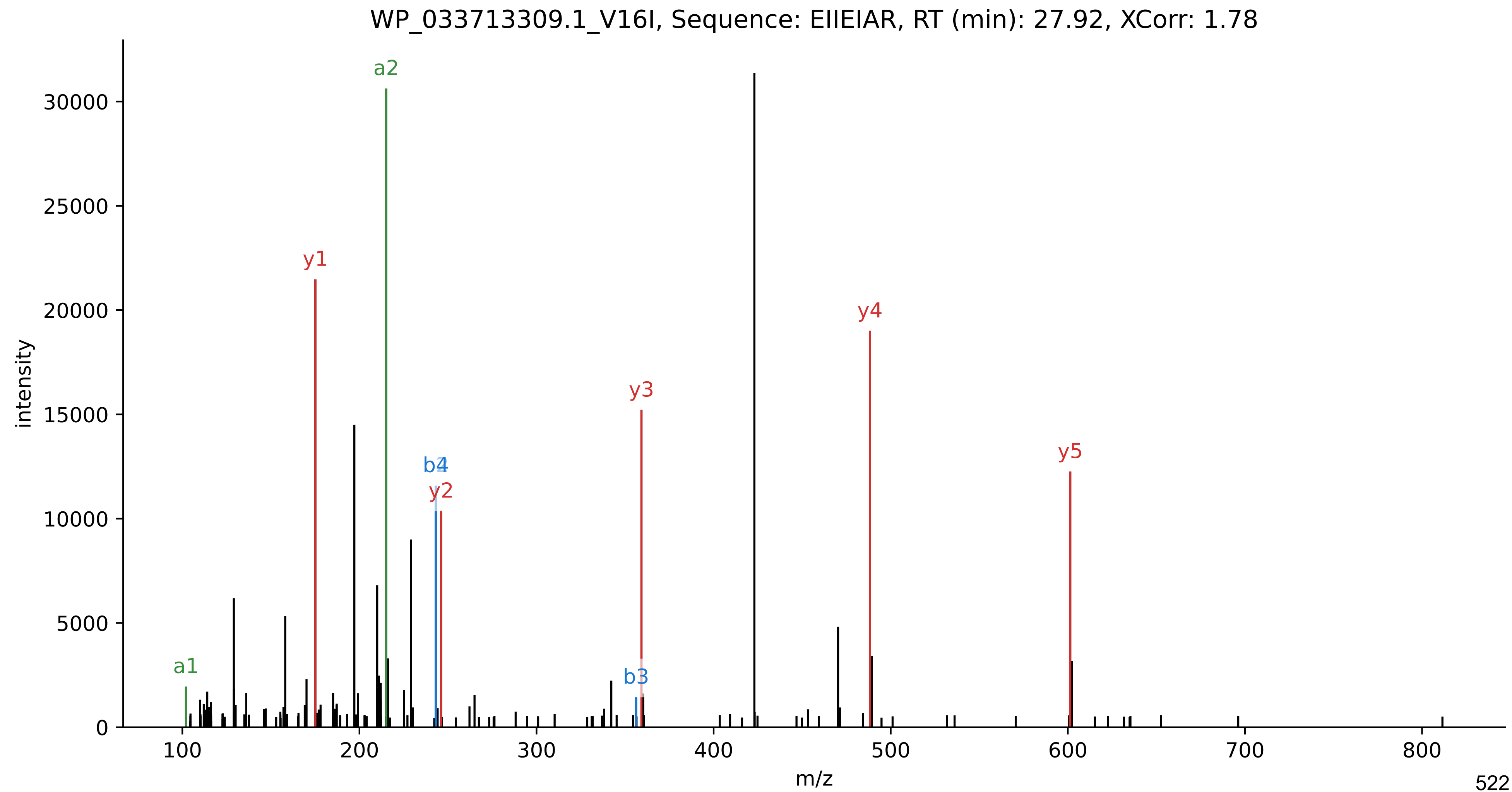

WP_033713309.1_V16I, Sequence: EIIEIAR, RT (min): 27.92, XCorr: 1.78
intensity
m/z
a1
a2
y1
b4
y2
y3
b3
y4
y5
0
5000
10000
15000
20000
25000
30000
100
200
300
400
500
600
700
800

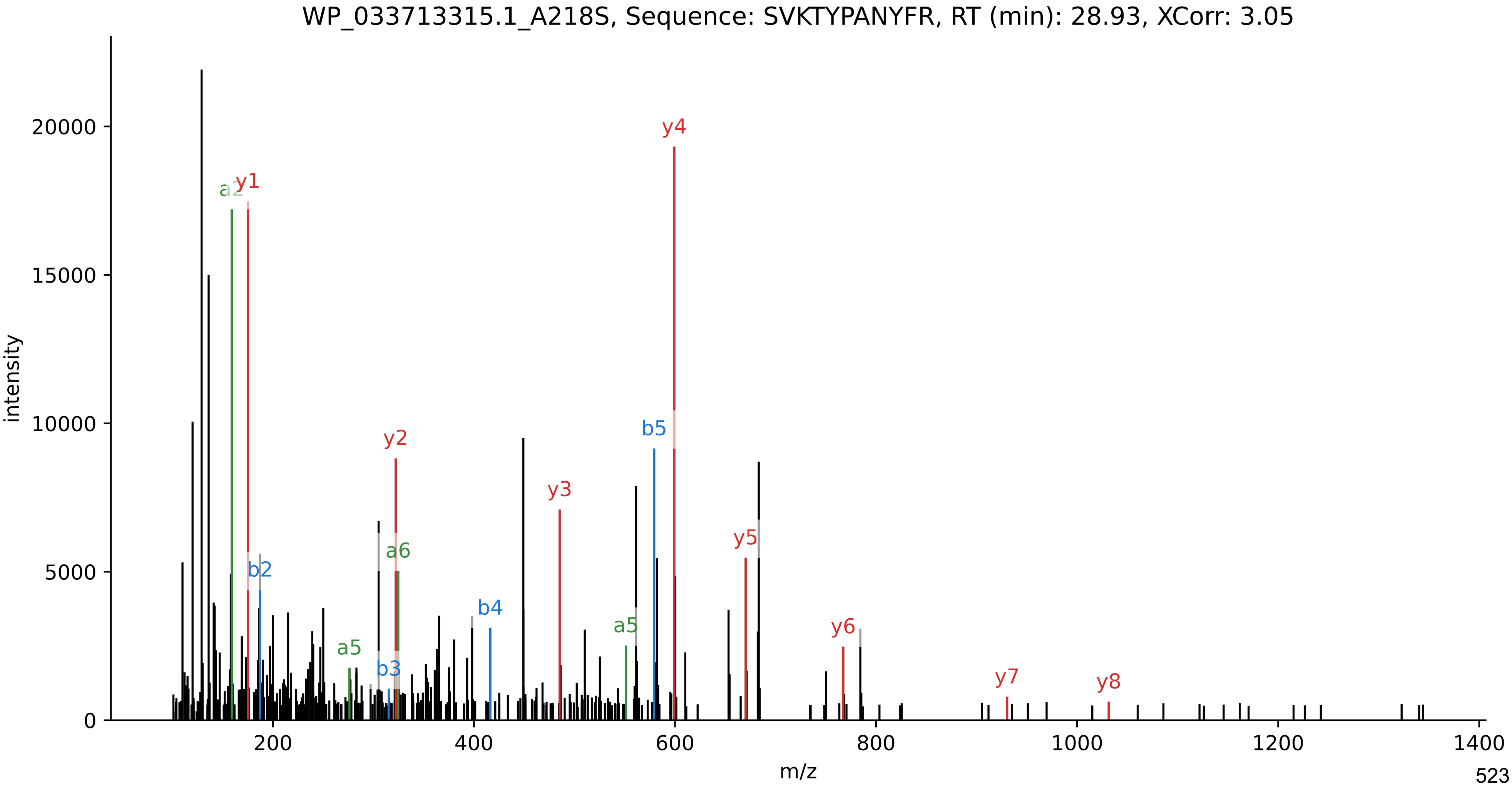

WP_033713315.1_A218S, Sequence: SVKTYPANYFR, RT (min): 28.93, XCorr: 3.05
intensity
m/z
0
5000
10000
15000
20000
200
400
600
800
1000
1200
1400
a2
y1
b2
a5
b3
a6
y2
b4
y3
a5
b5
y4
y5
y6
y7
y8

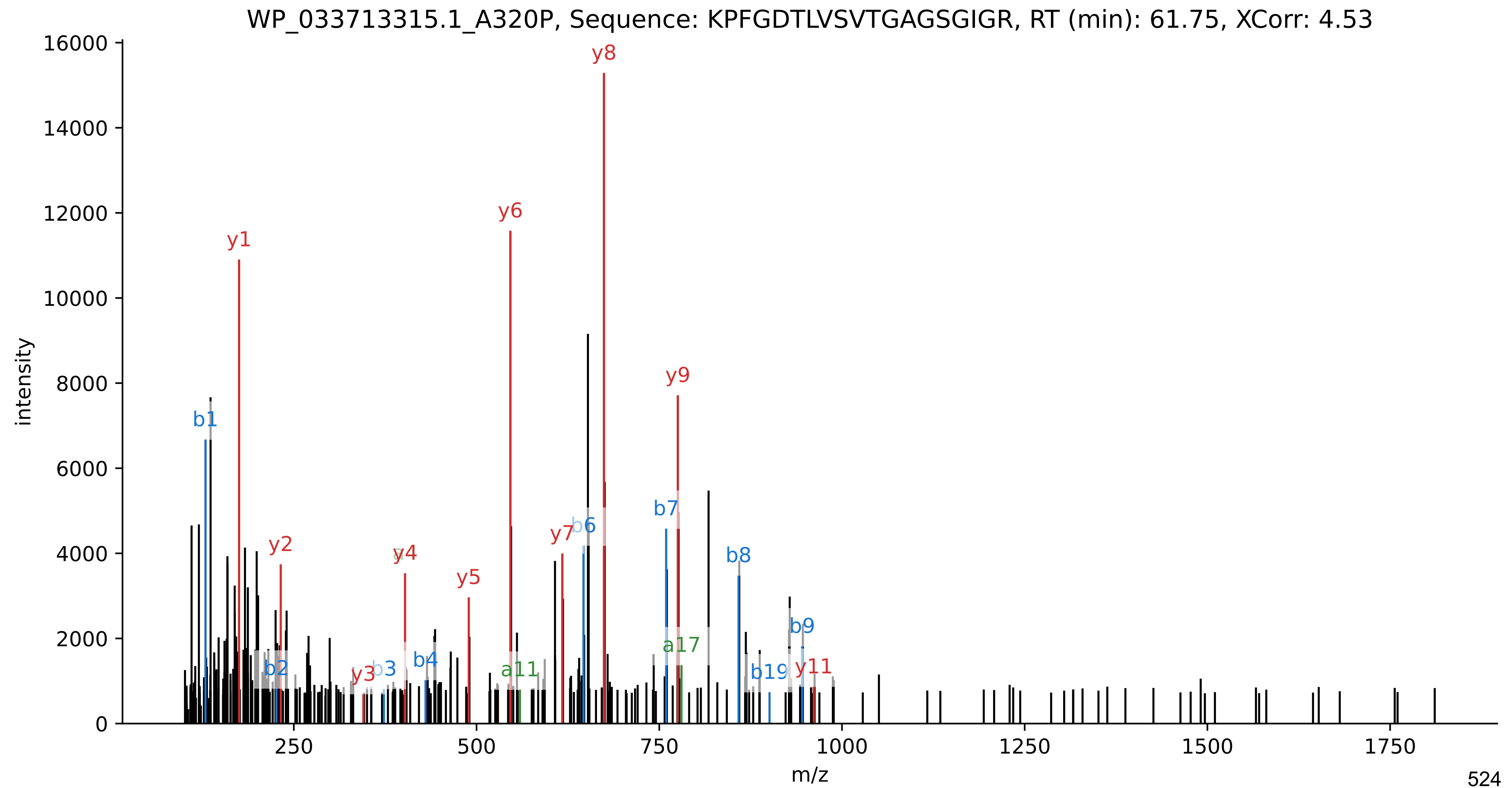

WP_033713315.1_A320P, Sequence: KPFGDTLVSVTGAGSGIGR, RT (min): 61.75, XCorr: 4.53
intensity
m/z
y8
y6
y1
y9
b1
b7
y7
b6
y2
b8
y4
y5
b9
a17
b4
b2
b3
y3
a11
b19
y11

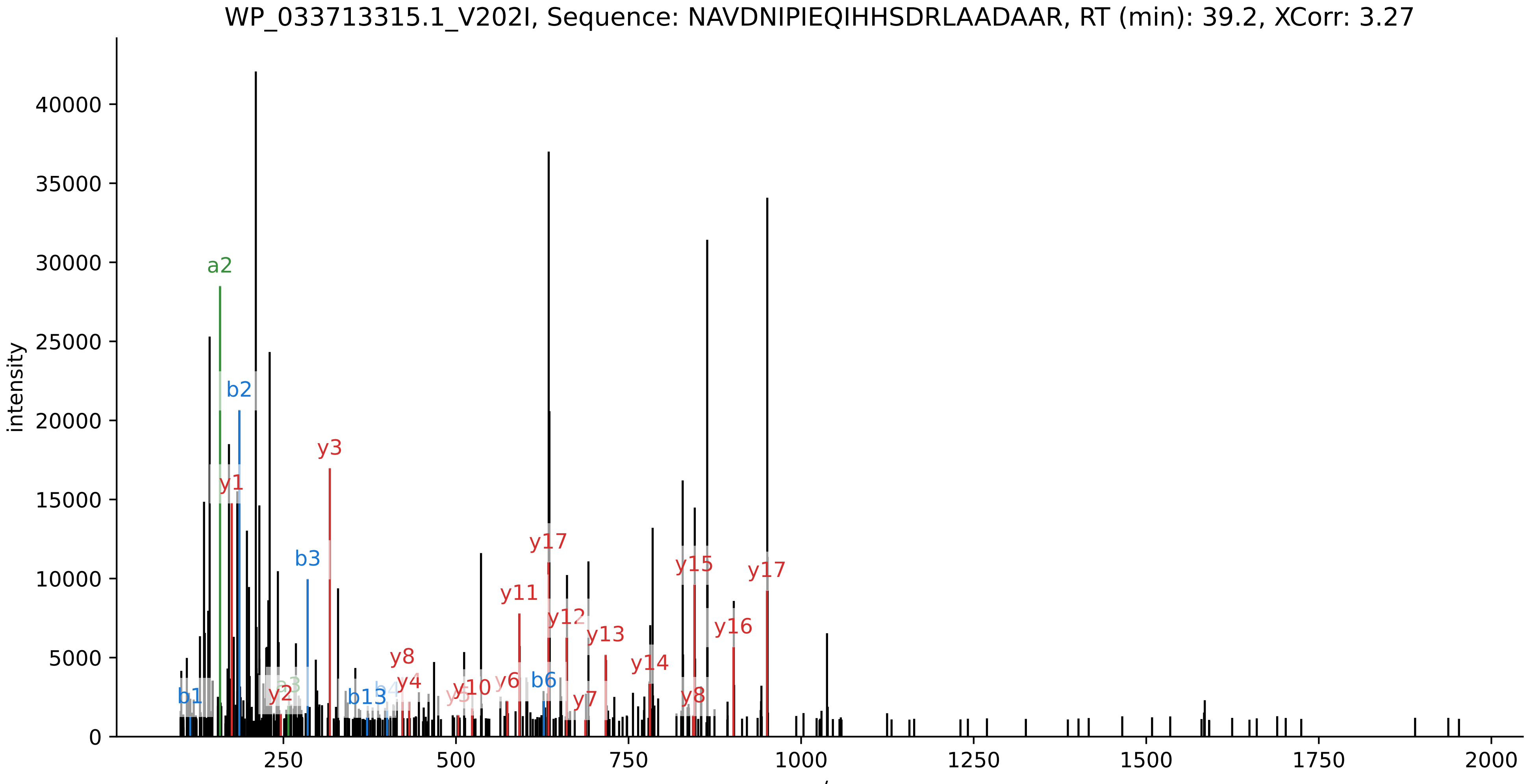

WP_033713315.1_V202I, Sequence: NAVDNIPIEQIHHSDRLAADAAR, RT (min): 39.2, XCorr: 3.27
intensity
m/z
0
5000
10000
15000
20000
25000
30000
35000
40000
250
500
750
1000
1250
1500
1750
2000
a2
b2
y1
y3
b3
b1
a3
y2
b13
b4
y8
y4
y5
y10
y6
b6
y17
y11
y12
y7
y13
y14
y15
y8
y16
y17

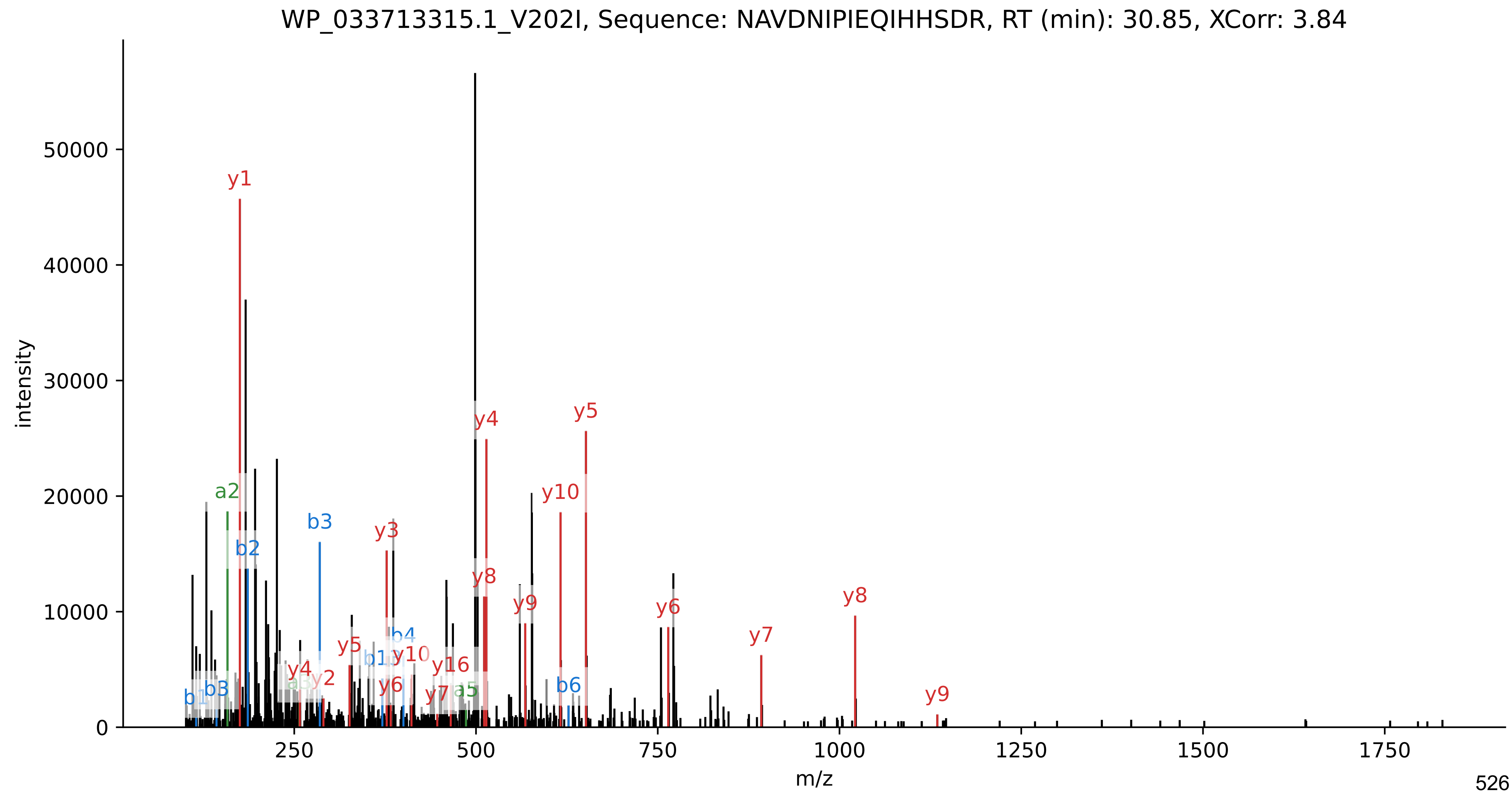

WP_033713315.1_V202I, Sequence: NAVDNIPIEQIHHSDR, RT (min): 30.85, XCorr: 3.84
intensity
m/z
y1
a2
b2
b3
y3
y4
y5
y8
y9
y10
y6
y7
y8
y9
b6
b1
b3
y4
y2
y5
b4
y10
y16
y6
y7
a5
0
10000
20000
30000
40000
50000
250
500
750
1000
1250
1500
1750

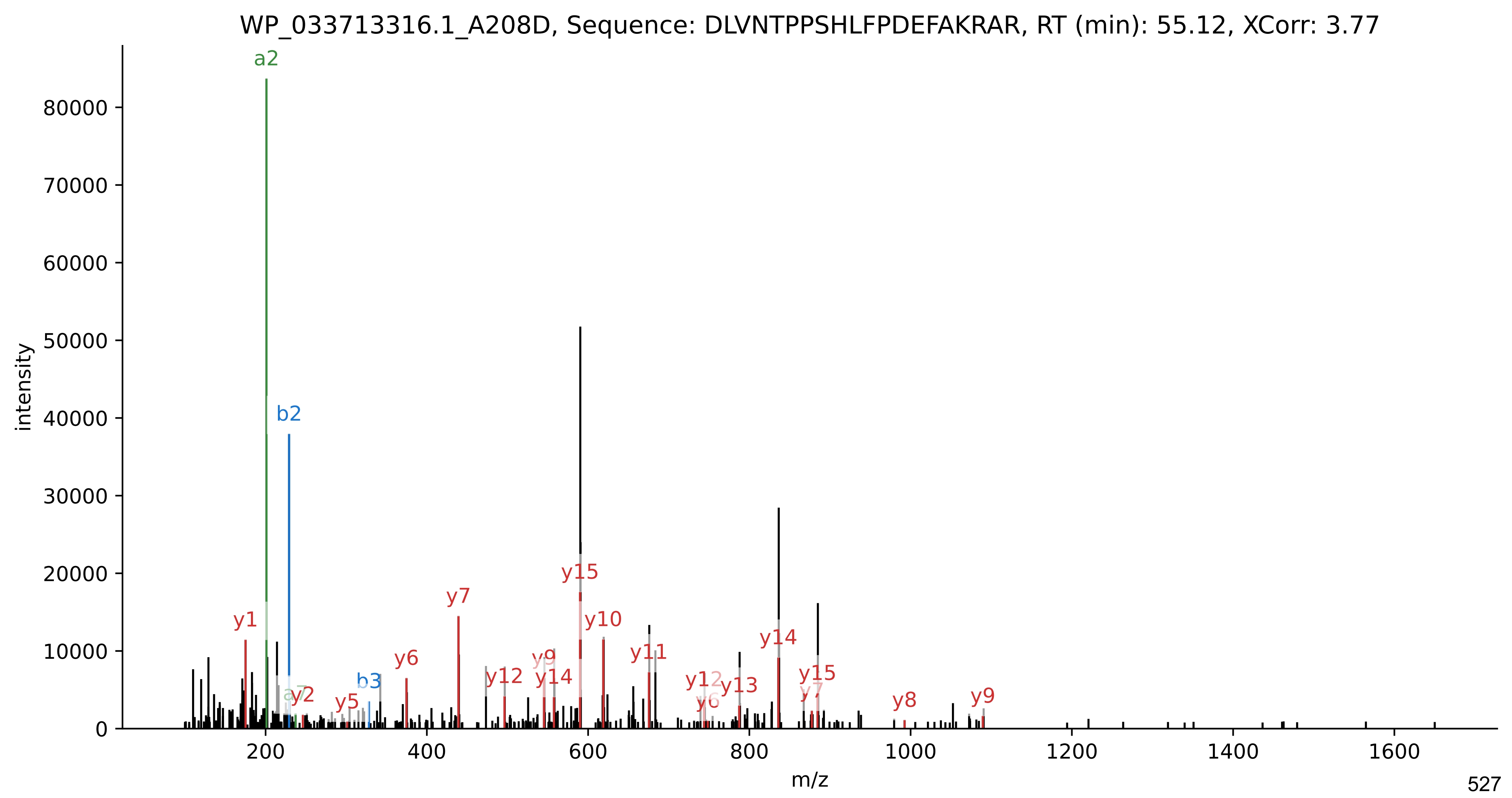
WP_033713316.1_A208D, Sequence: DLVNTPPSHLFPDEFAKRAR, RT (min): 55.12, XCorr: 3.77
intensity
m/z
a2
b2
b3
y1
y2
y5
y6
y7
y9
y10
y11
y12
y13
y14
y15
y8
80000
70000
60000
50000
40000
30000
20000
10000
0
200
400
600
800
1000
1200
1400
1600

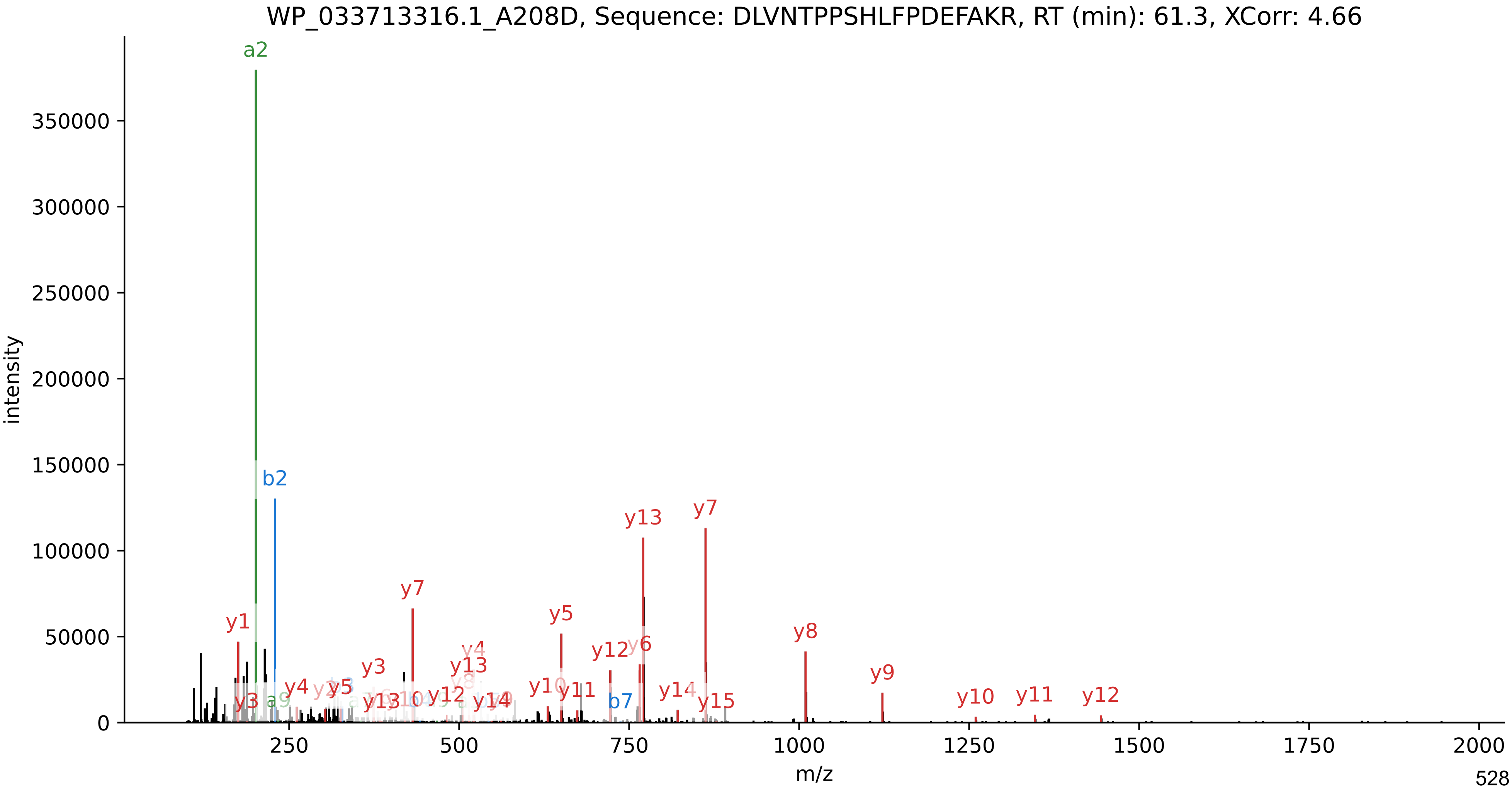
WP_033713316.1_A208D, Sequence: DLVNTPPSHLFPDEFAKR, RT (min): 61.3, XCorr: 4.66
intensity
m/z
a2
b2
y13
y7
y7
y1
y5
y12
y6
y8
y9
y10
y11
y12
y14
y15
b7
y3
y4
y5
y3
y13
y12
y14
y10
y11
y4
a9
0
50000
100000
150000
200000
250000
300000
350000
250
500
750
1000
1250
1500
1750
2000

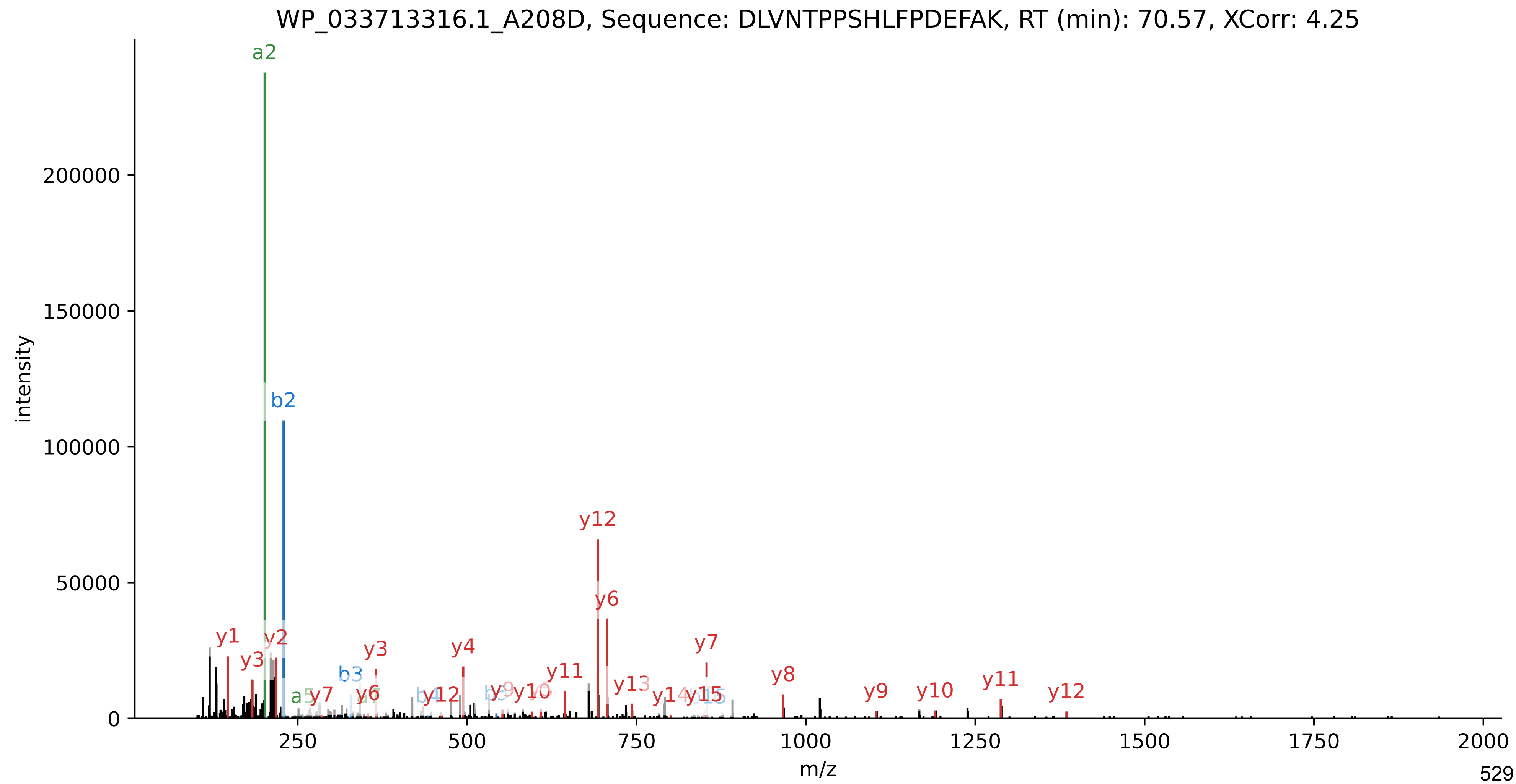
WP_033713316.1_A208D, Sequence: DLVNTPPSHLFPDEFAK, RT (min): 70.57, XCorr: 4.25
intensity
m/z
0
50000
100000
150000
200000
250
500
750
1000
1250
1500
1750
2000
a2
b2
y12
y6
y1
y2
y3
y3
y4
y7
b3
y11
y13
y8
y9
y10
y11
y12
a5
y7
y6
y12
y10
y14
y15

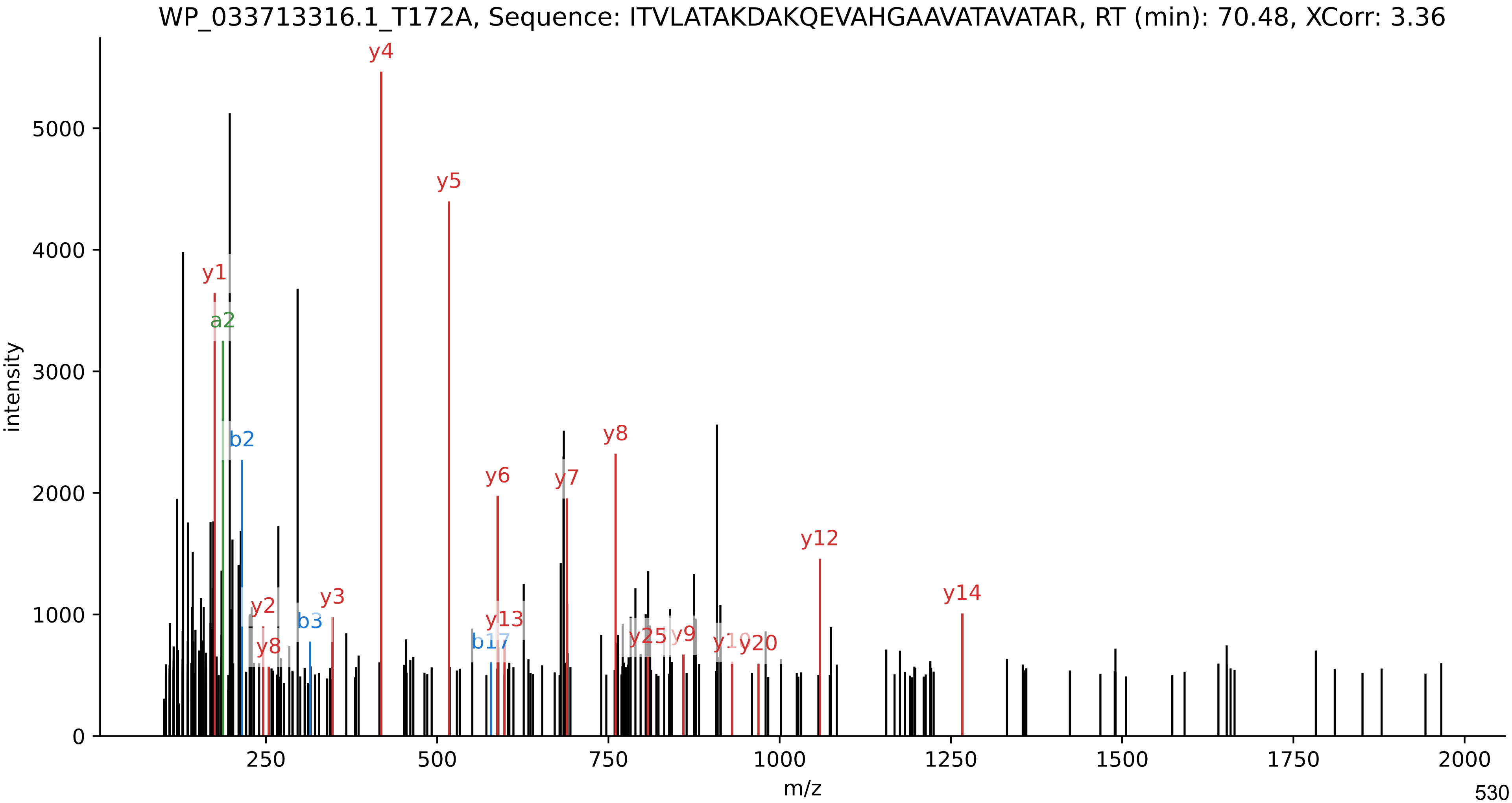
WP_033713316.1_T172A, Sequence: ITVLATAKDAKQEVAHGAAVATAVATAR, RT (min): 70.48, XCorr: 3.36
intensity
m/z
y1
a2
b2
y2
y8
b3
y3
y4
y5
y6
y13
b17
y7
y8
y25
y9
y10
y20
y12
y14
0
1000
2000
3000
4000
5000
250
500
750
1000
1250
1500
1750
2000

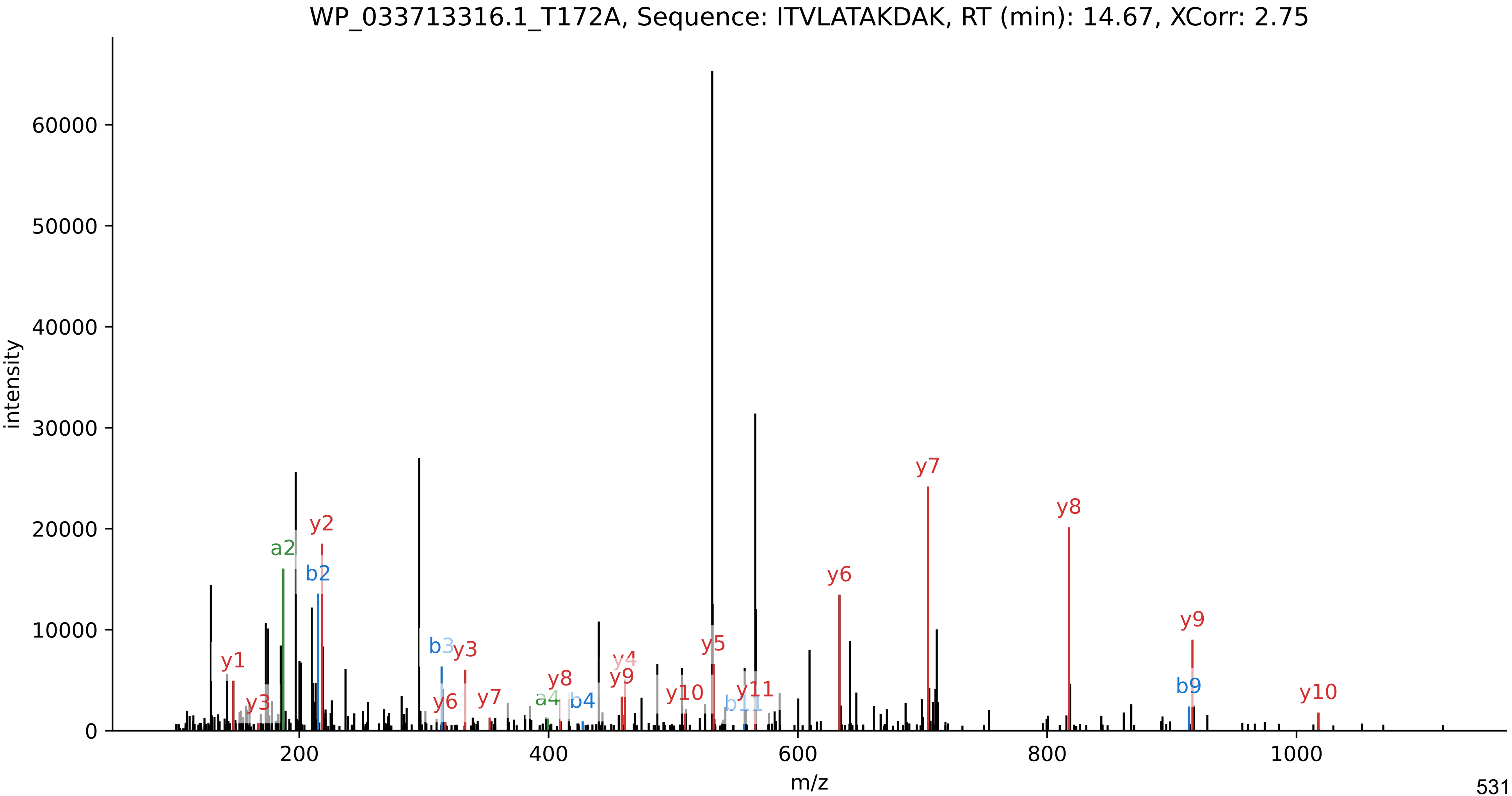

WP_033713316.1_T172A, Sequence: ITVLATAKDAK, RT (min): 14.67, XCorr: 2.75
intensity
m/z
y1
y3
a2
y2
b2
b3
y3
y6
y7
y8
a4
b4
y4
y9
y10
y5
b11
y11
y6
y7
y8
y9
b9
y10

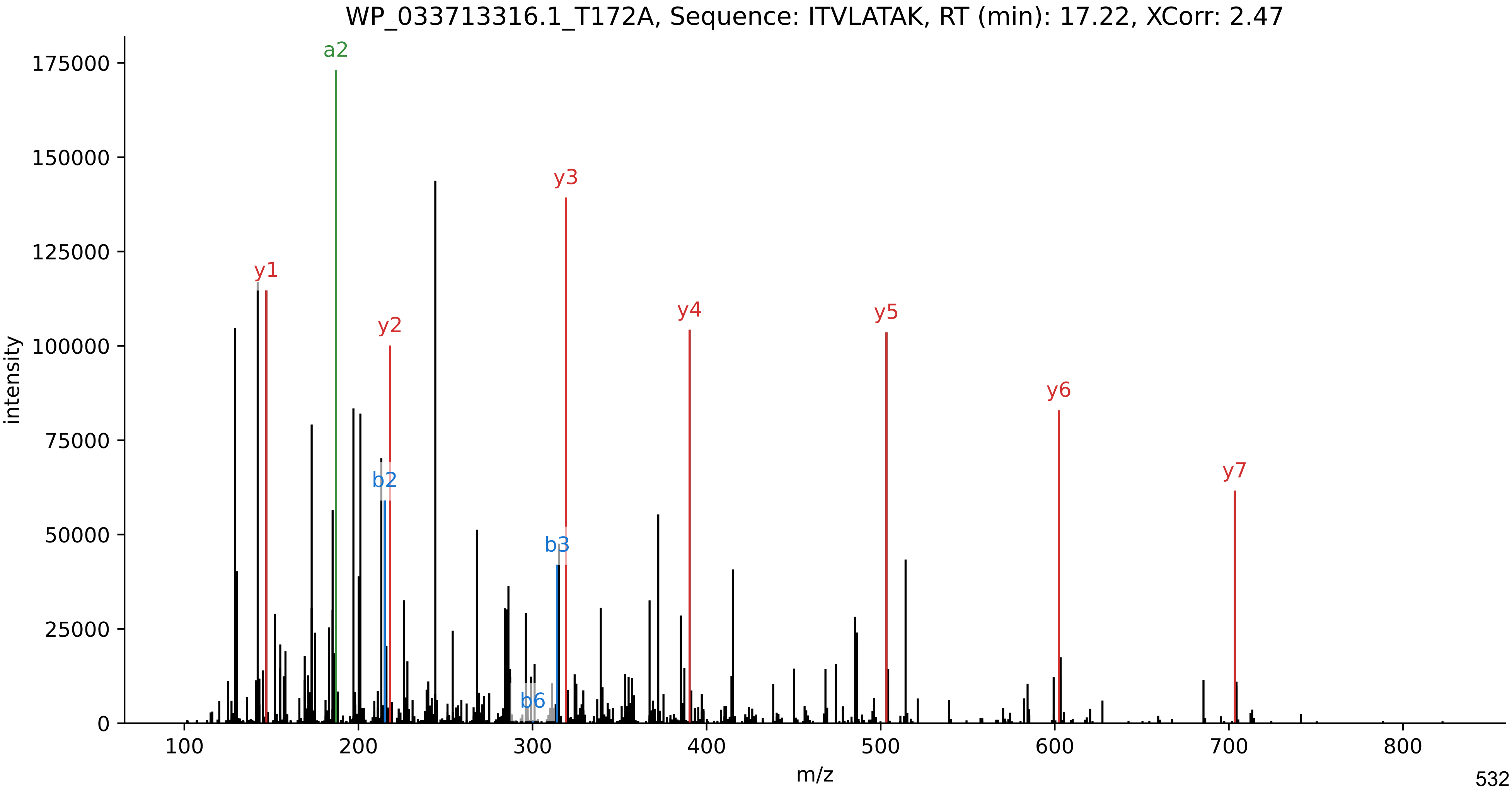

WP_033713316.1_T172A, Sequence: ITVLATAK, RT (min): 17.22, XCorr: 2.47
a2
y1
y2
y3
y4
y5
y6
y7
b2
b3
b6
intensity
m/z
0
25000
50000
75000
100000
125000
150000
175000
100
200
300
400
500
600
700
800

WP_033713317.1_G20A, Sequence: STRPATDAERETILAEPGFGK, RT (min): 39.57, XCorr: 4.18

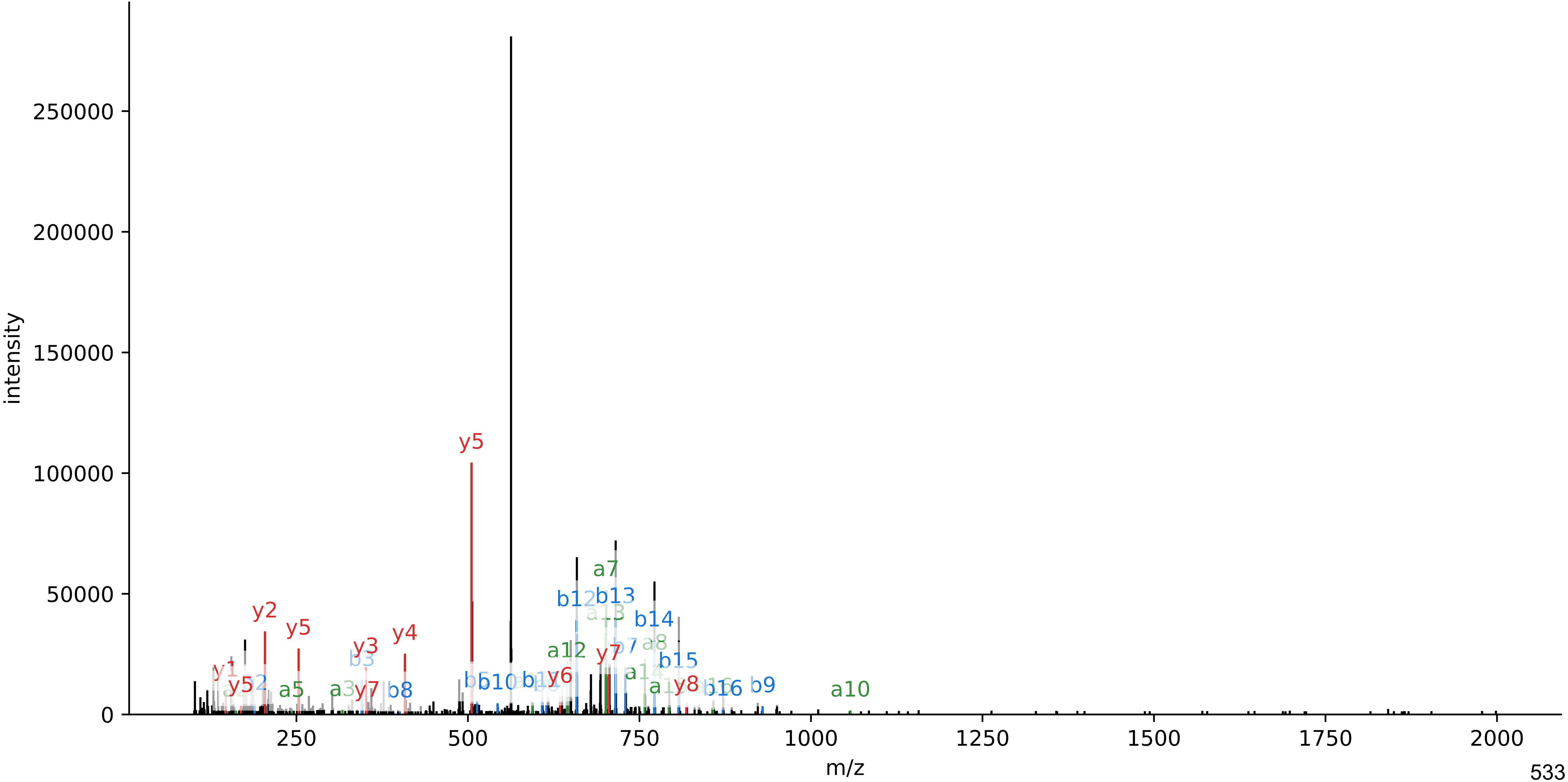

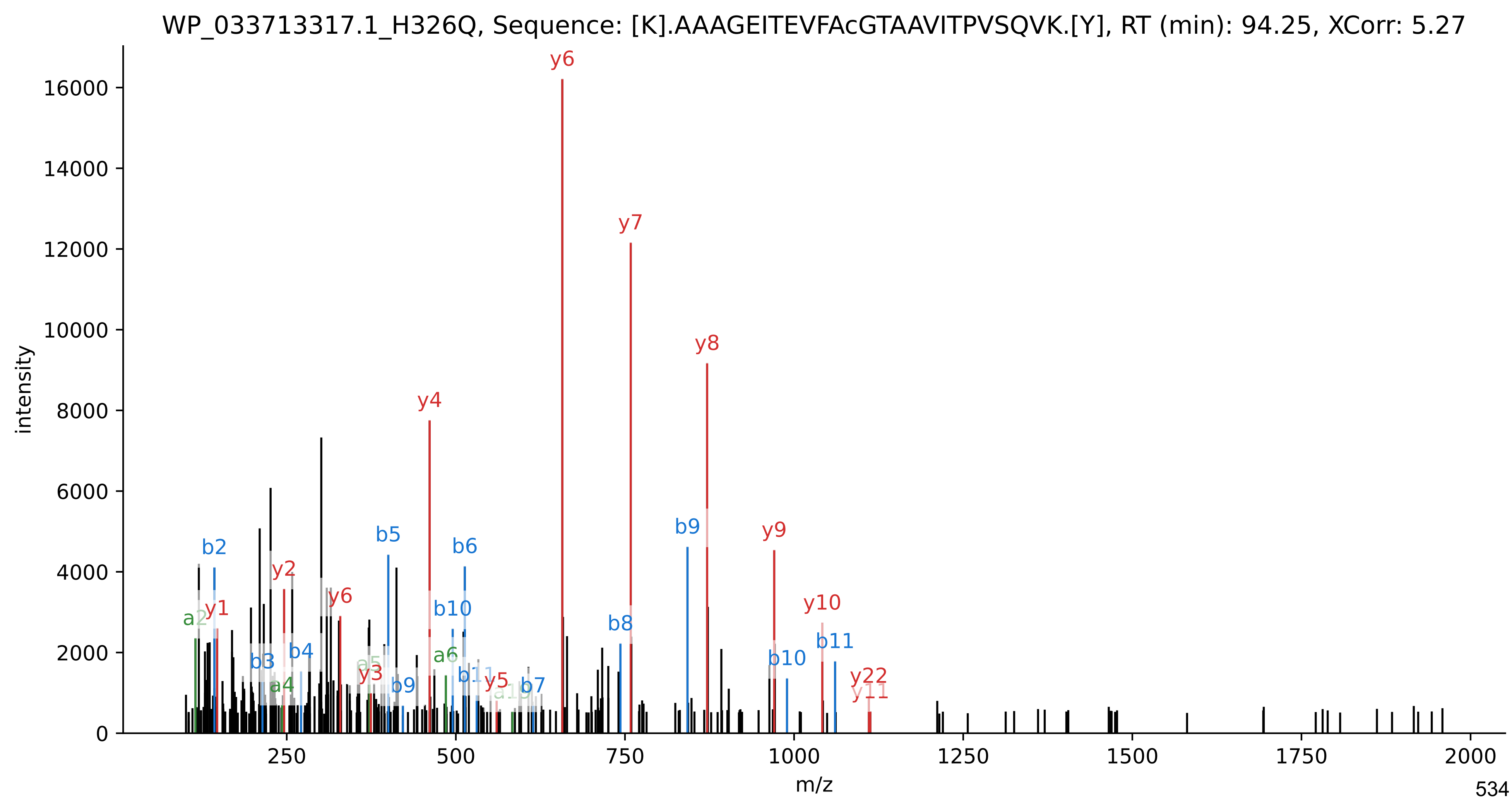

WP_033713317.1_H326Q, Sequence: [K].AAAGEITEVFAcGTAAVITPVSQVK.[Y], RT (min): 94.25, XCorr: 5.27
intensity
m/z
y6
y7
y8
y4
b9
y9
b5
b6
b2
y2
y6
y1
a2
b10
y10
b8
b11
b10
b4
b3
a6
a4
y3
b9
b11
y5
b7
y22
y11

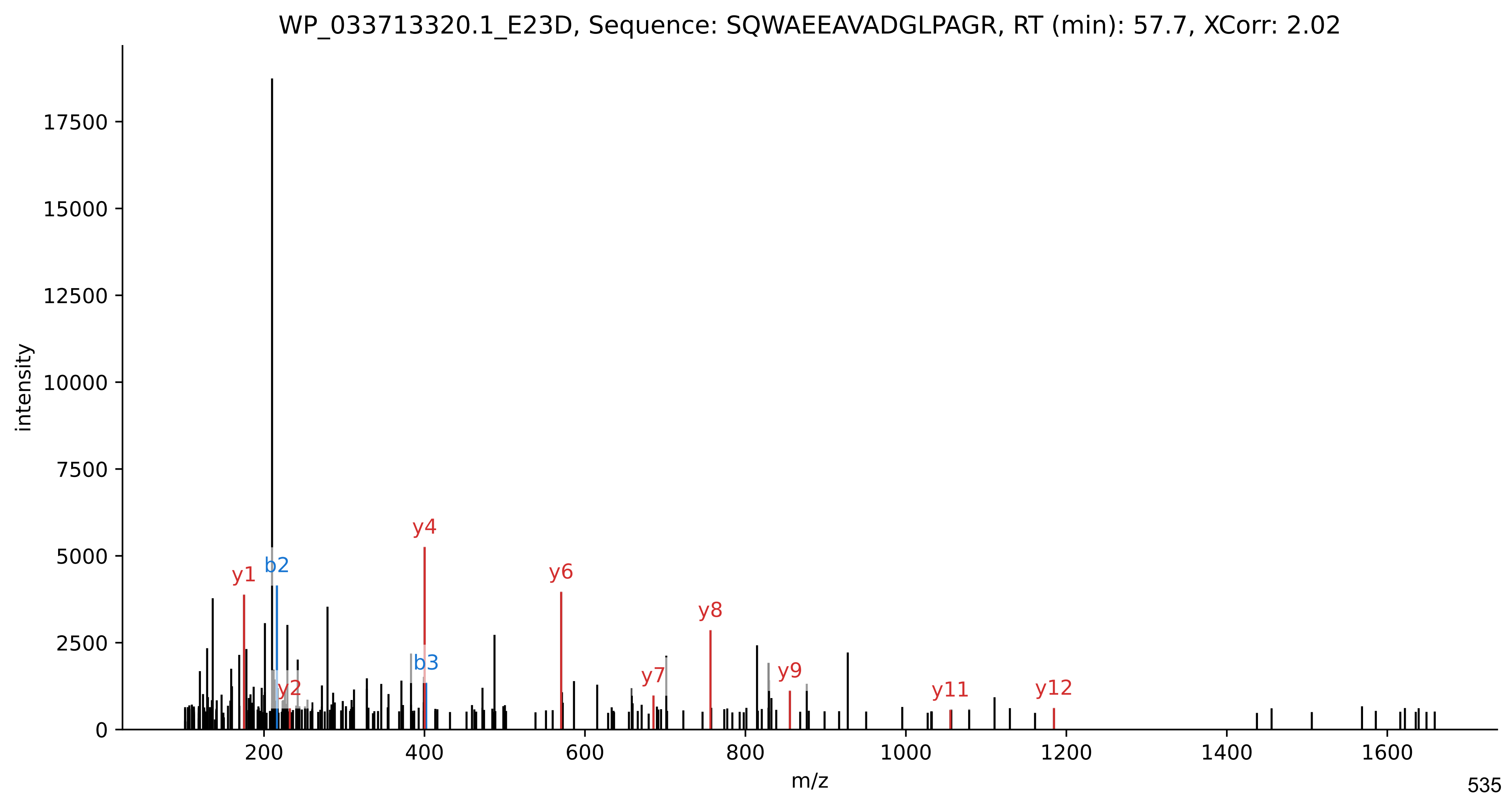

WP_033713320.1_E23D, Sequence: SQWAEEAVADGLPAGR, RT (min): 57.7, XCorr: 2.02
intensity
m/z
y1
b2
y2
b3
y4
y6
y7
y8
y9
y11
y12

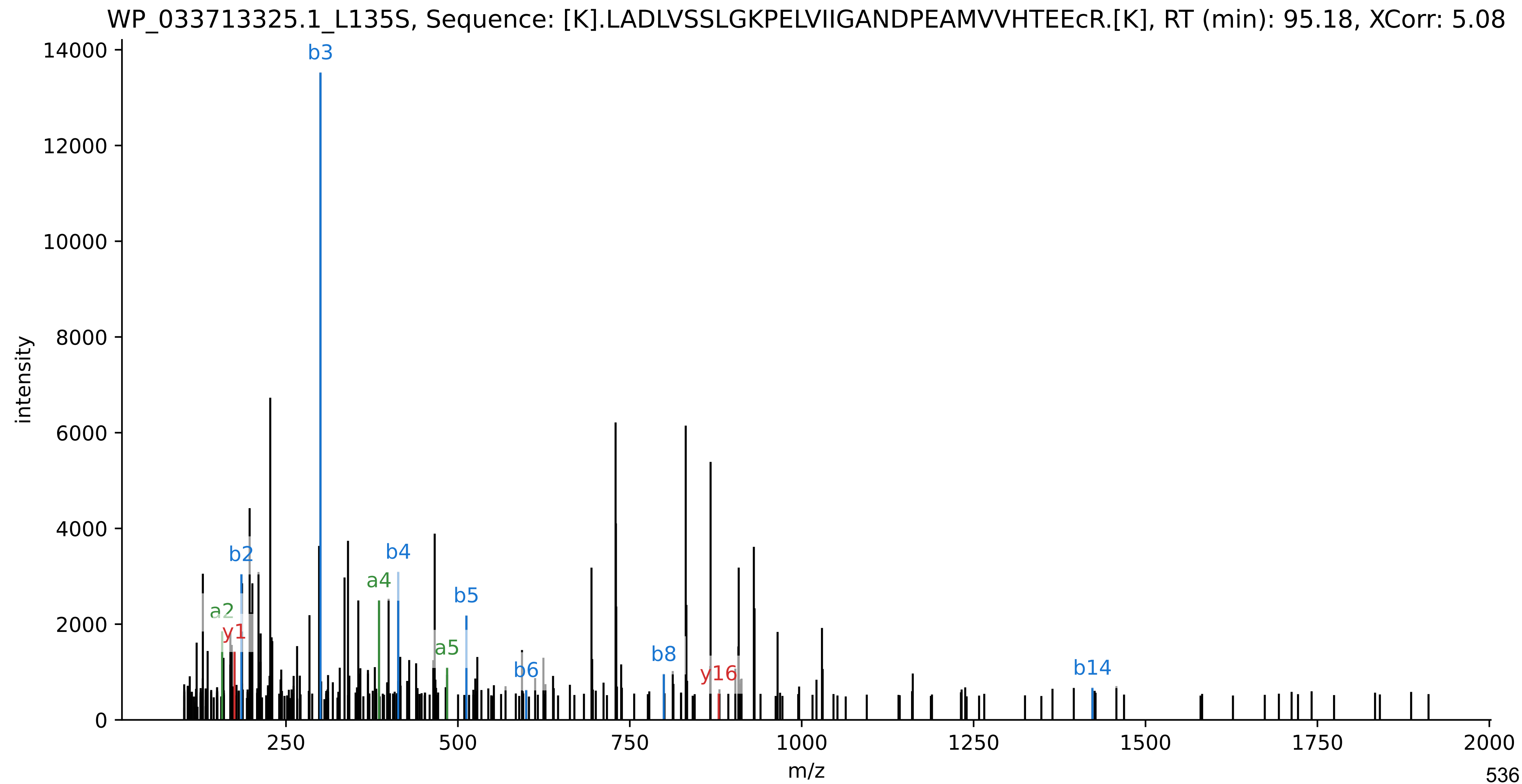

WP_033713325.1_L135S, Sequence: [K].LADLVSSLGKPELVIIGANDPEAMVVHTEEcR.[K], RT (min): 95.18, XCorr: 5.08
intensity
m/z
b3
b2
b4
a4
a2
y1
b5
a5
b6
b8
y16
b14
0
2000
4000
6000
8000
10000
12000
14000
250
500
750
1000
1250
1500
1750
2000

WP_033713329.1_V100A, Sequence: ATDTELPR, RT (min): 14.02, XCorr: 2.58

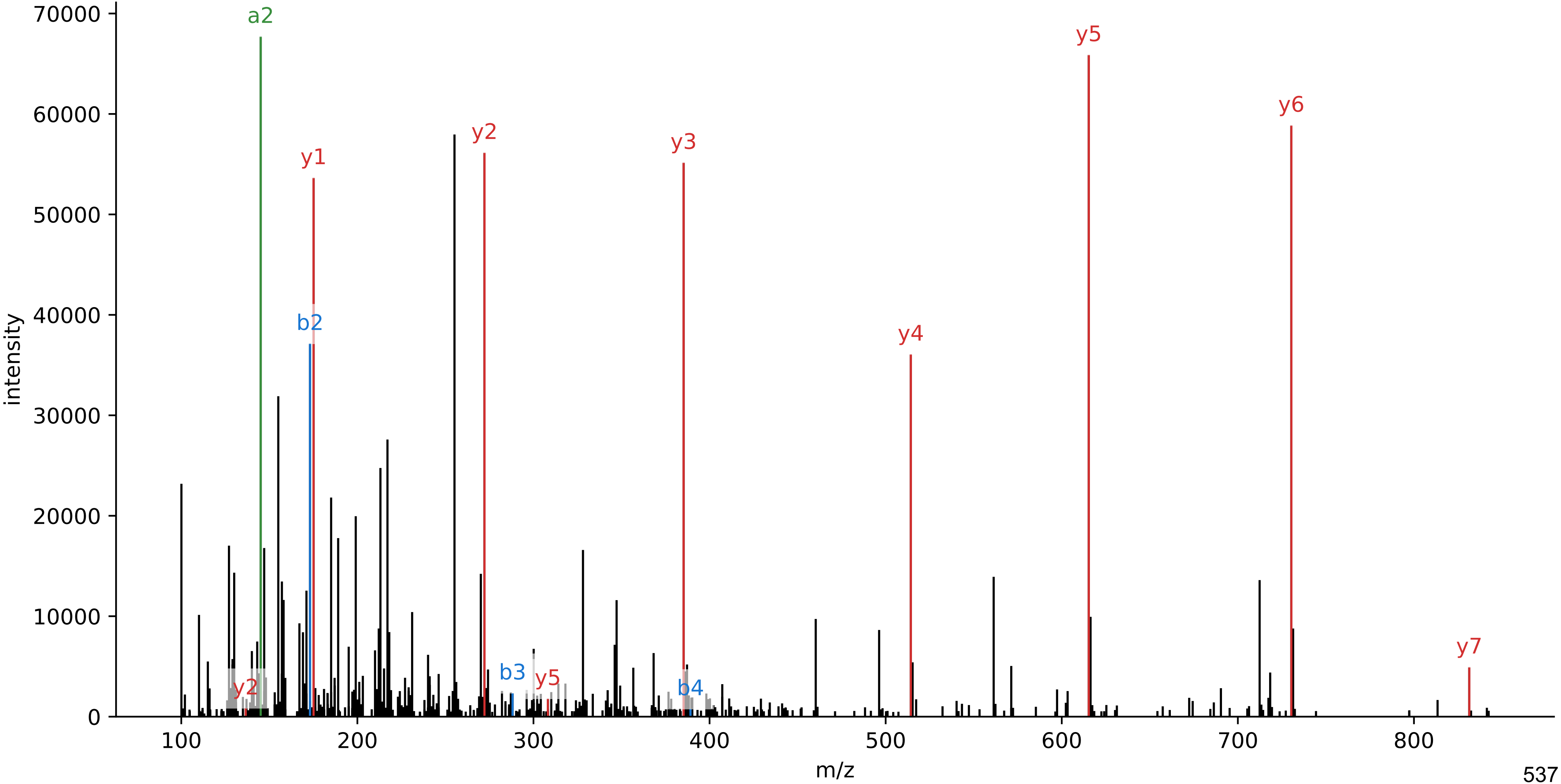

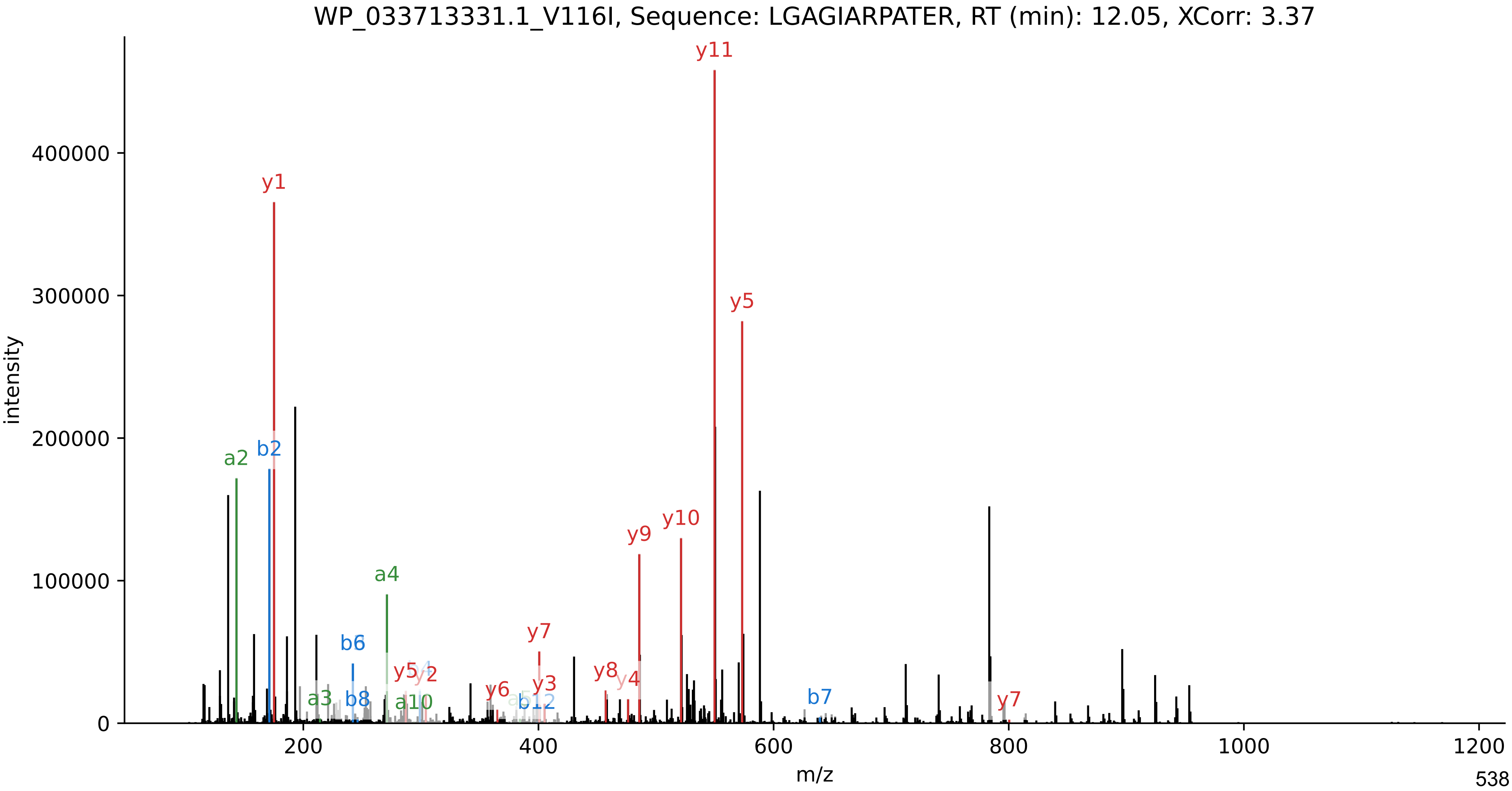
WP_033713331.1_V116I, Sequence: LGAGIARPATER, RT (min): 12.05, XCorr: 3.37
intensity
m/z
0
100000
200000
300000
400000
200
400
600
800
1000
1200
y11
y1
y5
a2
b2
y9
y10
a4
b6
y7
y5
y2
y8
y4
y6
y3
a3
b8
a10
b12
b7
y7

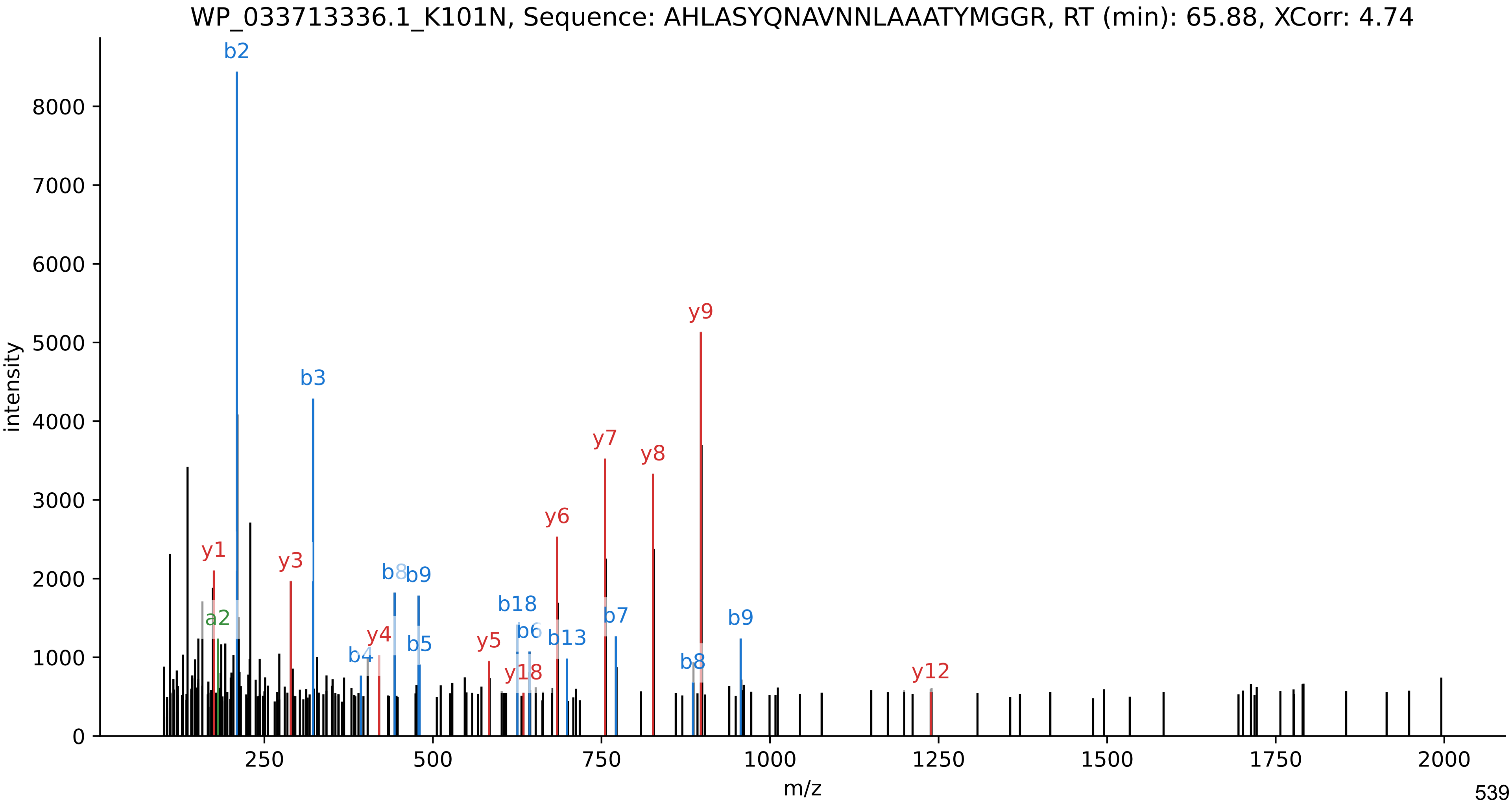

WP_033713336.1_K101N, Sequence: AHLASYQNAVNNLAAATYMGGR, RT (min): 65.88, XCorr: 4.74
intensity
m/z
b2
b3
y9
y7
y8
y6
y1
y3
b8
b9
a2
y4
b4
b5
y5
b18
b6
b13
b7
b9
y18
b8
y12

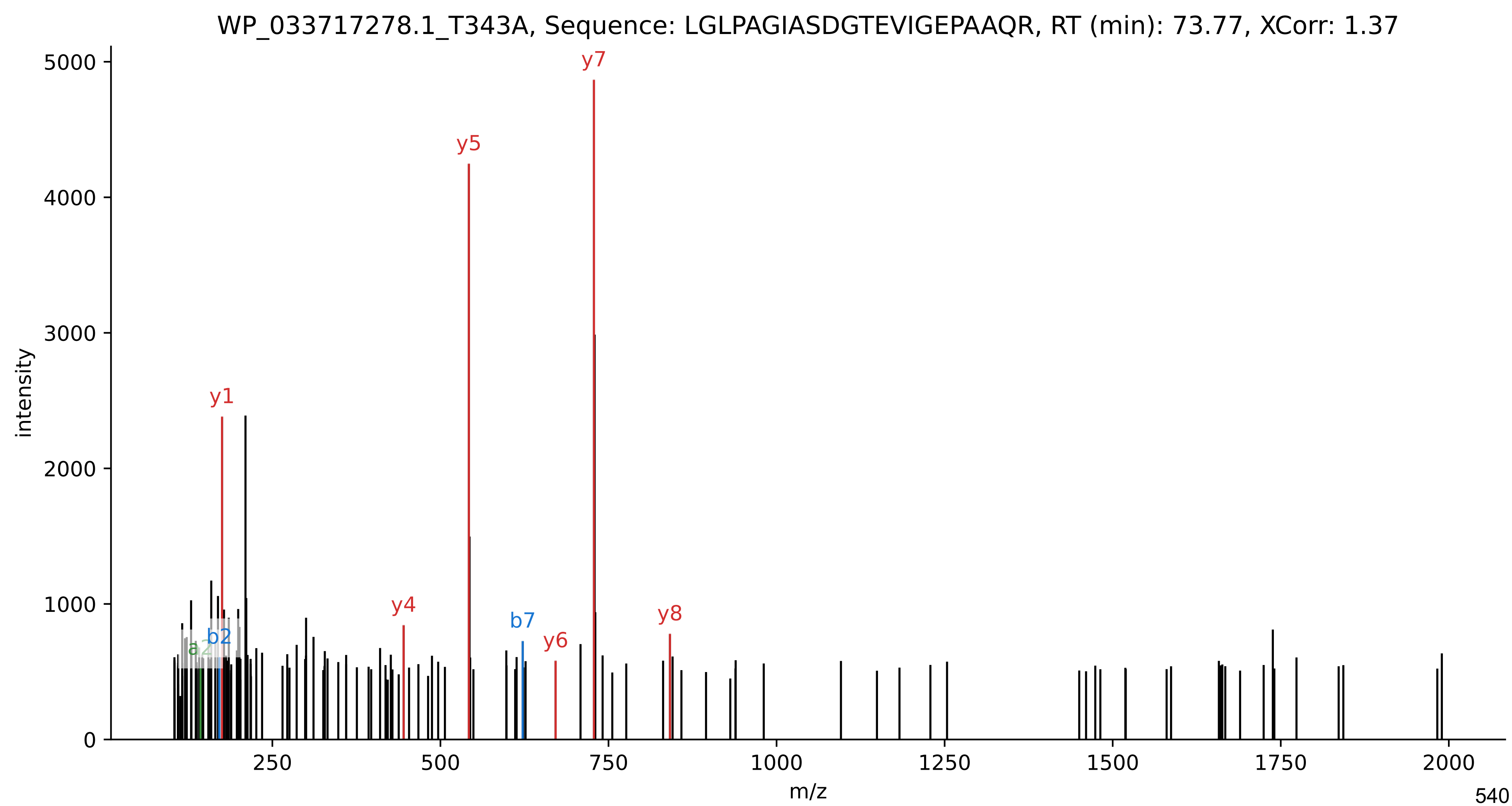

WP_033717278.1_T343A, Sequence: LGLPAGIASDGTEVIGEPAAQR, RT (min): 73.77, XCorr: 1.37
intensity
m/z
y7
y5
y1
y4
b7
y6
y8
a2
b2
5000
4000
3000
2000
1000
0
250
500
750
1000
1250
1500
1750
2000

WP_033717421.1_R154Q, Sequence: ELAQTQTVQQVHDLAADRGKK, RT (min): 32.4, XCorr: 3.62

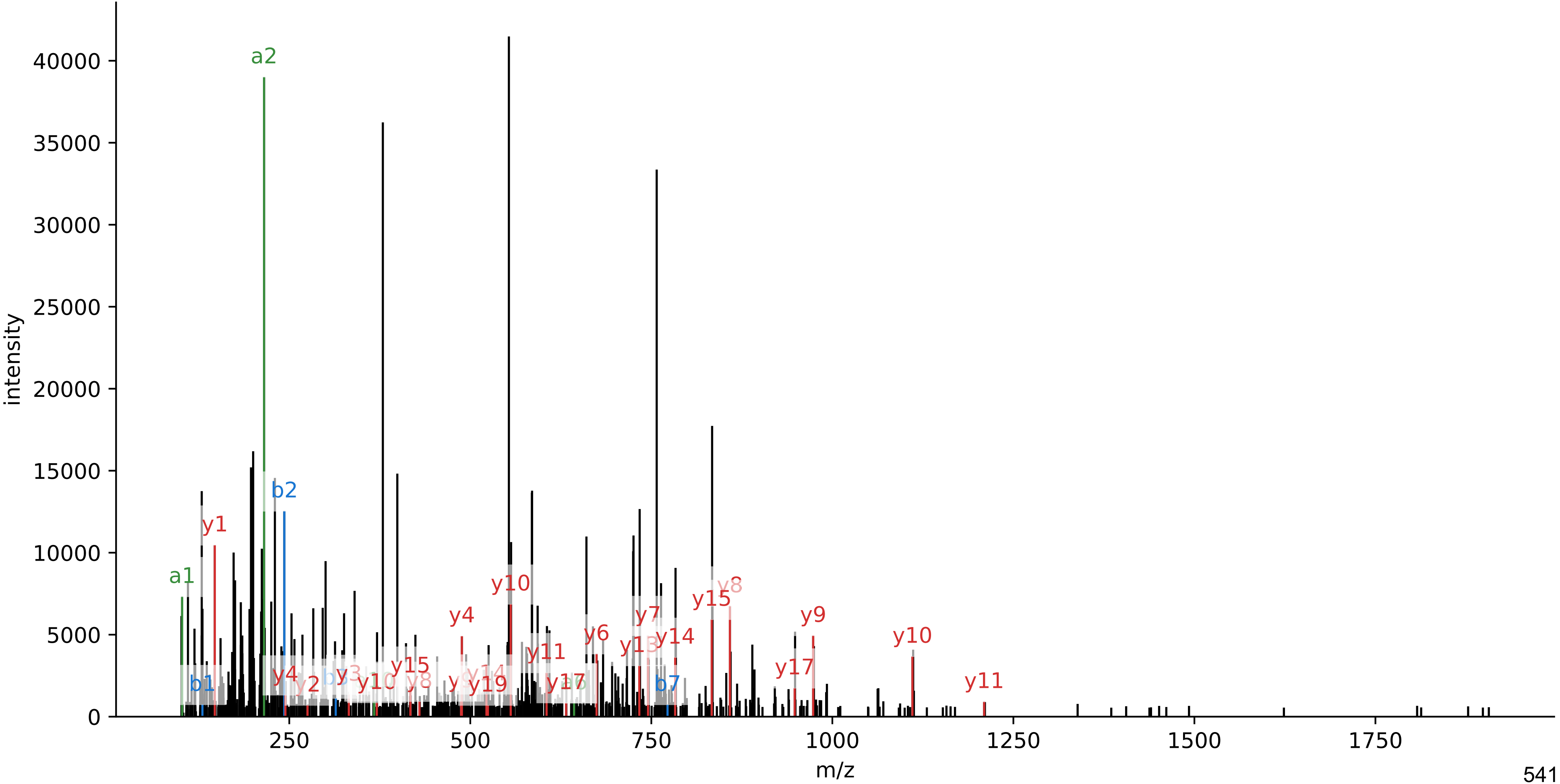

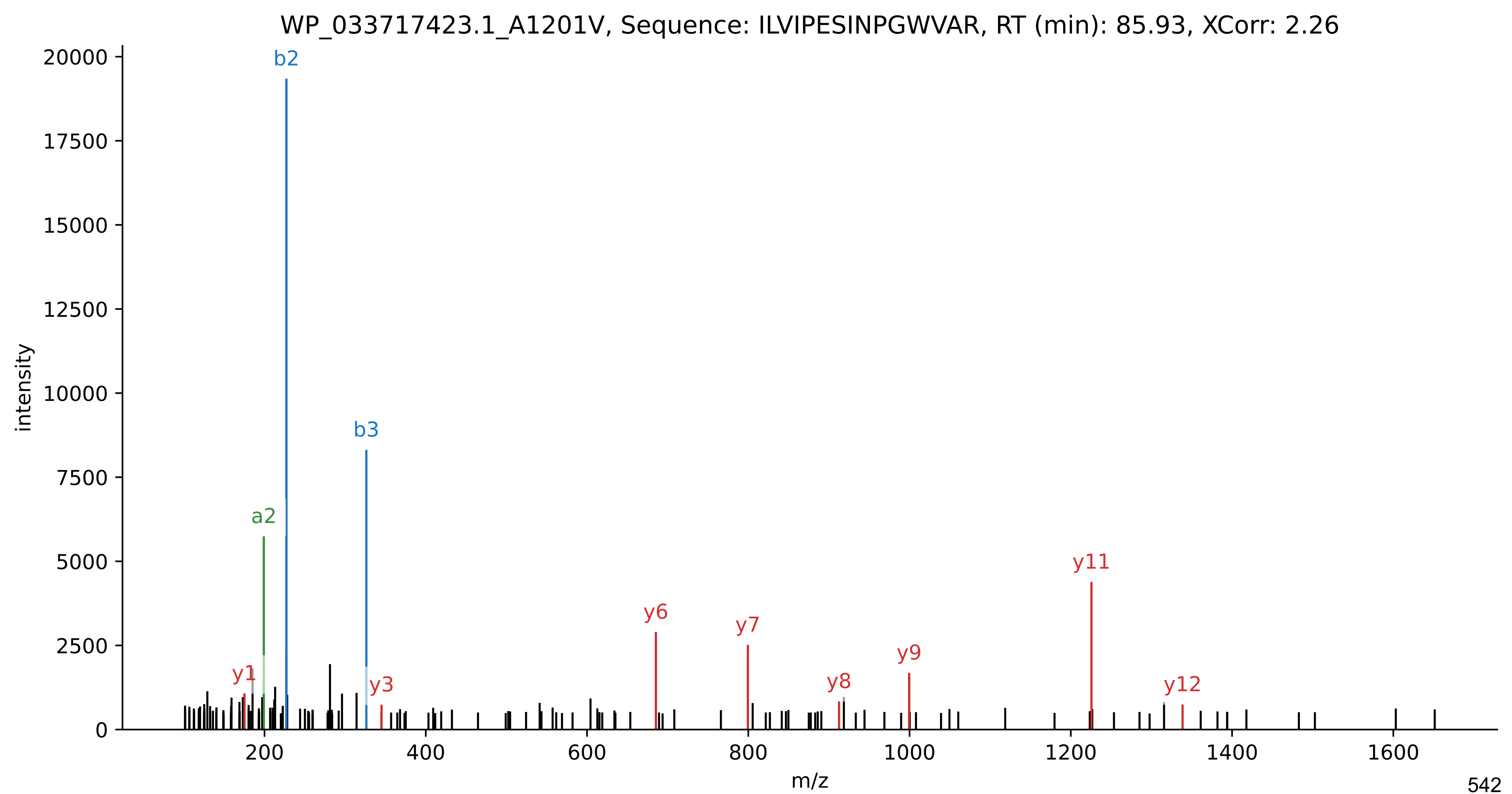
WP_033717423.1_A1201V, Sequence: ILVIPESINPGWVAR, RT (min): 85.93, XCorr: 2.26
intensity
m/z
b2
b3
a2
y1
y3
y6
y7
y8
y9
y11
y12

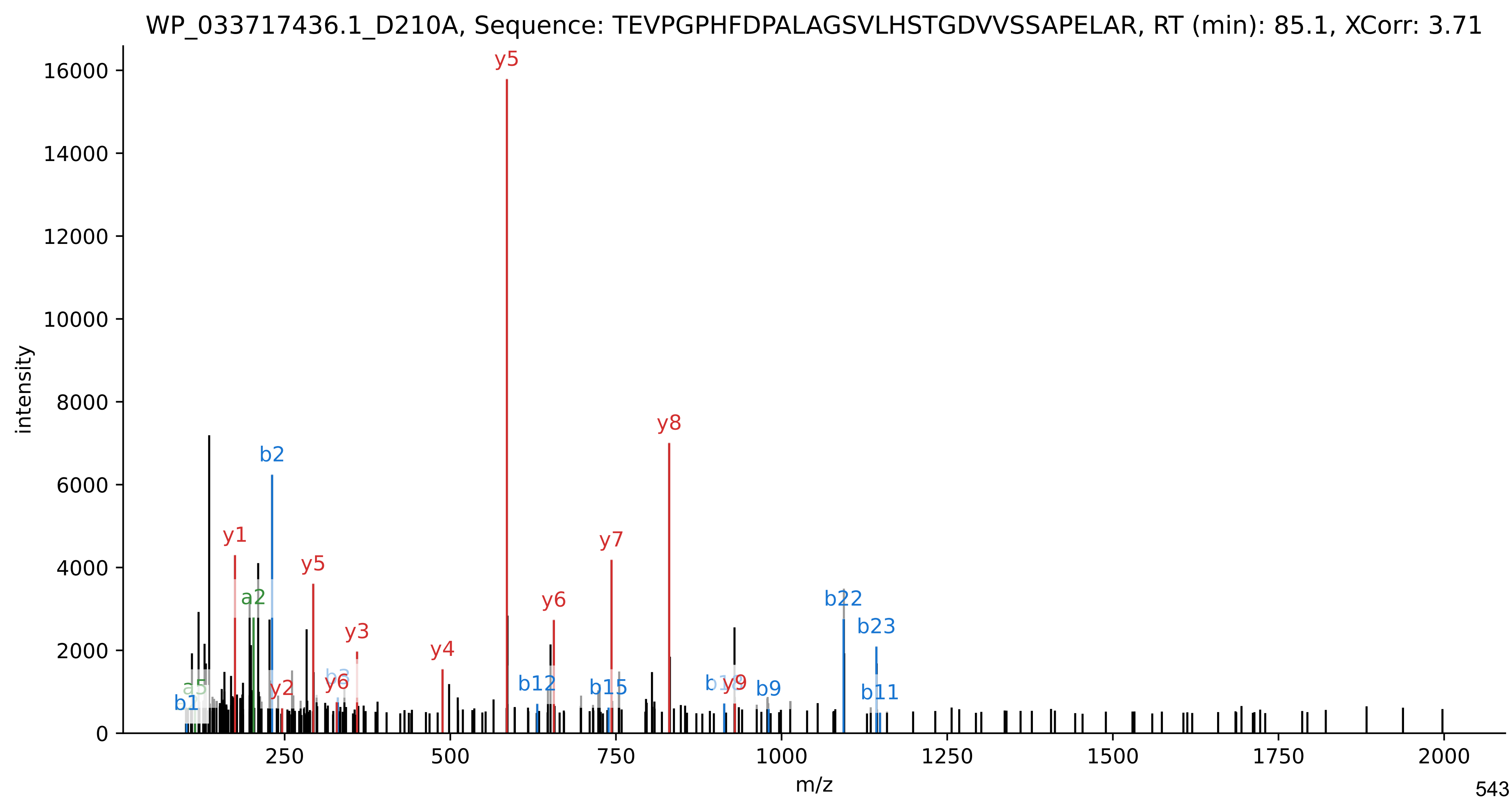
WP_033717436.1_D210A, Sequence: TEVPGPHFDPALAGSVLHSTGDVVSSAPELAR, RT (min): 85.1, XCorr: 3.71
intensity
m/z
y5
y8
b2
y1
y7
y5
a2
y6
b22
b23
y3
y4
b12
b15
y9
b9
b11
y2
y6
a5
b1
0
2000
4000
6000
8000
10000
12000
14000
16000
250
500
750
1000
1250
1500
1750
2000

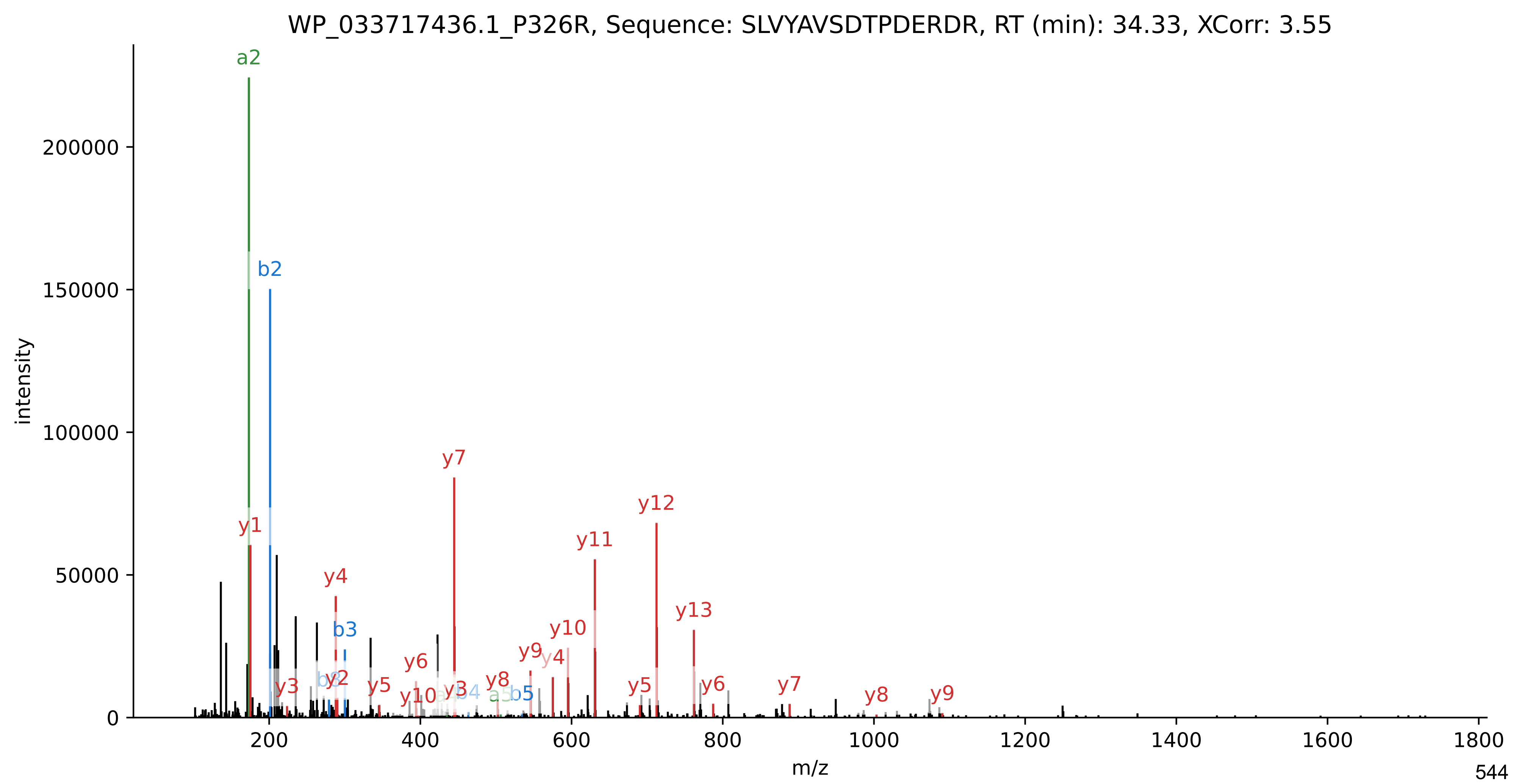
WP_033717436.1_P326R, Sequence: SLVYAVSDTPDERDR, RT (min): 34.33, XCorr: 3.55
intensity
m/z
a2
b2
y1
y7
y12
y11
y4
b3
y13
y10
y9
y4
y6
y3
y2
y5
y10
y3
y8
b5
y5
y6
y7
y8
y9

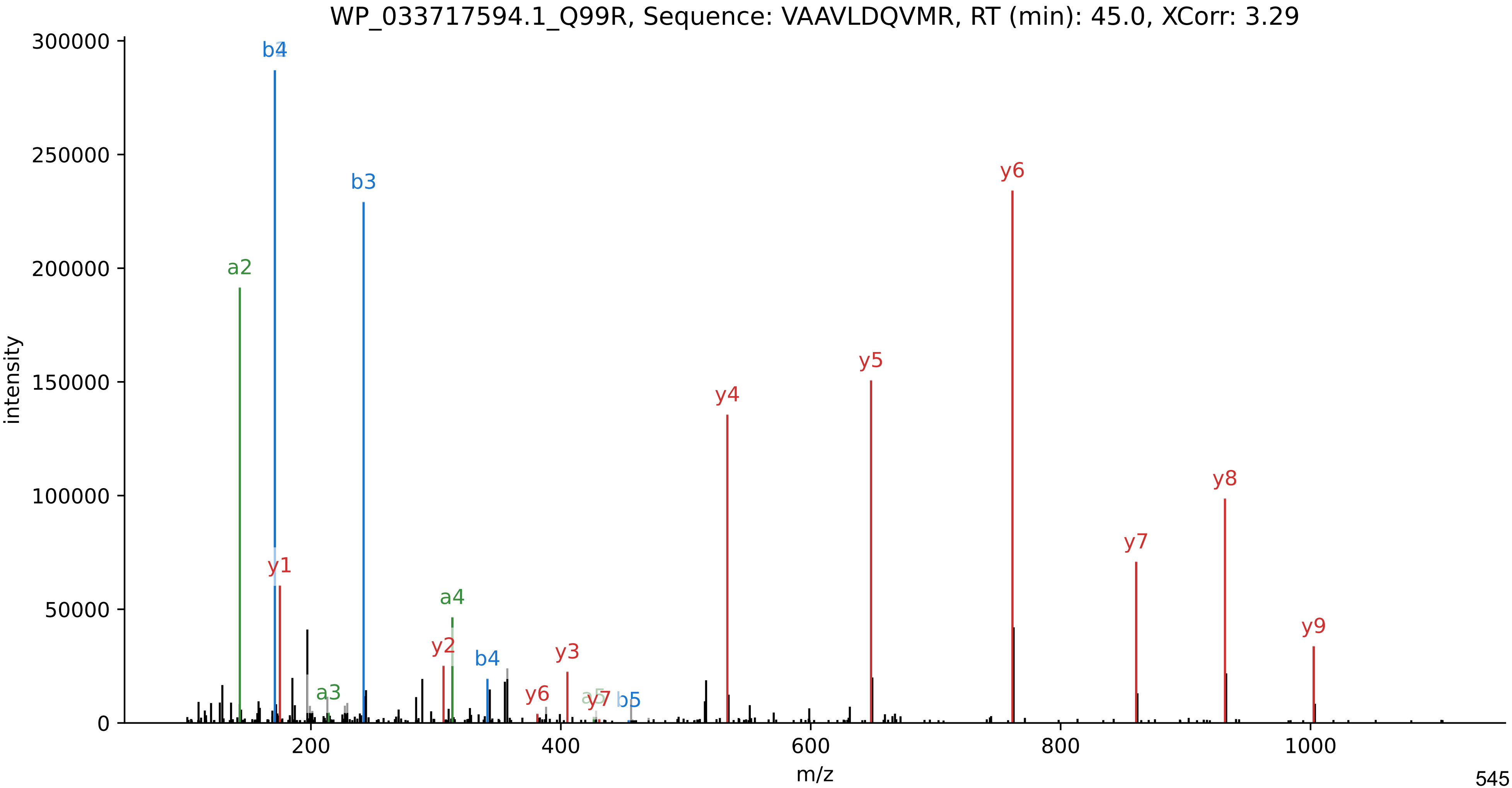
WP_033717594.1_Q99R, Sequence: VAAVLDQVMR, RT (min): 45.0, XCorr: 3.29
intensity
m/z
a2
b4
b3
y1
a3
a4
y2
b4
y6
y3
y7
b5
y4
y5
y6
y7
y8
y9
0
50000
100000
150000
200000
250000
300000
200
400
600
800
1000

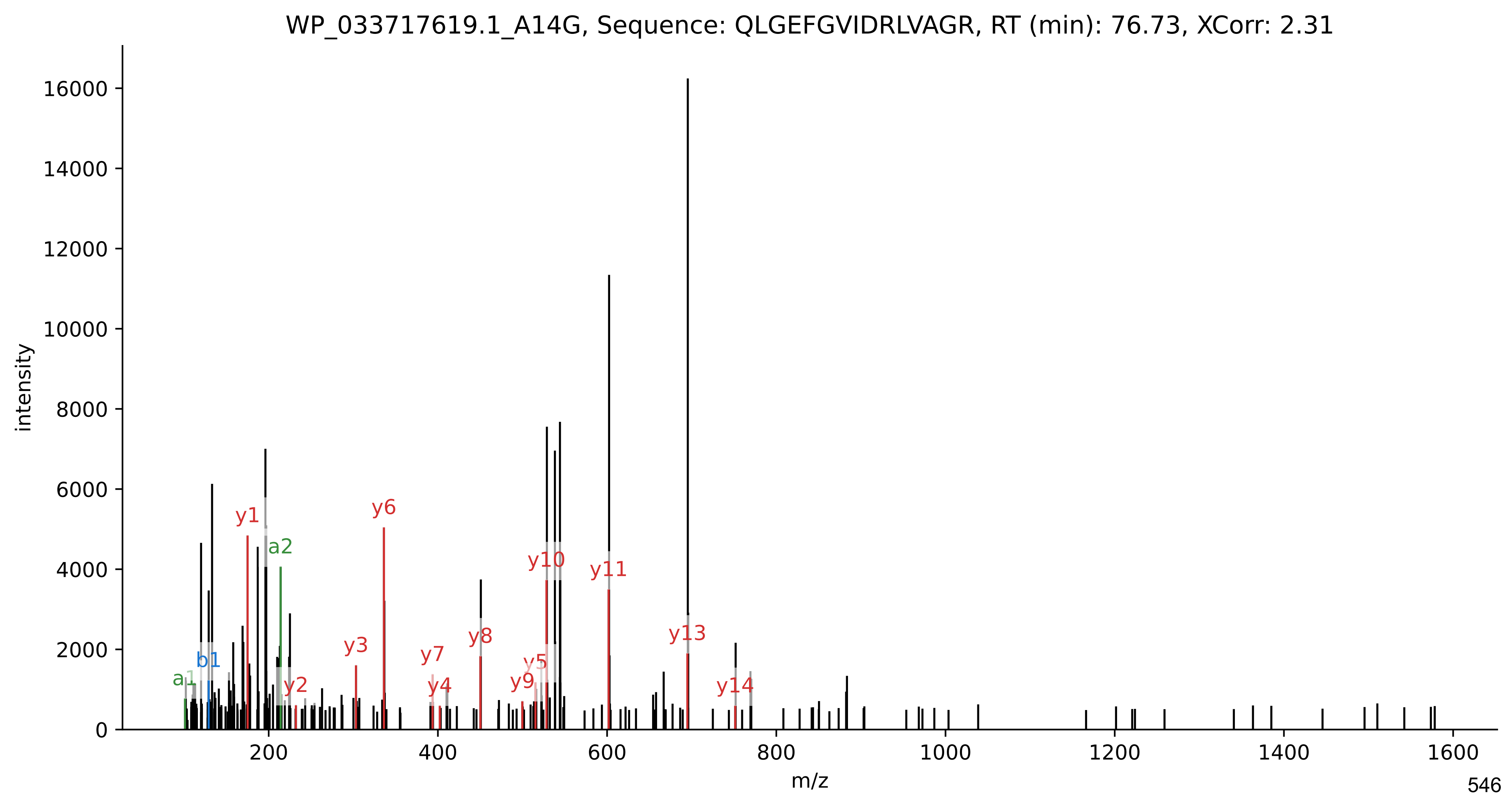
WP_033717619.1_A14G, Sequence: QLGEFGVIDRLVAGR, RT (min): 76.73, XCorr: 2.31
intensity
m/z
y1
a2
y6
y10
y11
y13
y8
y3
y7
y5
y9
y4
y2
b1
a1
y14
0
2000
4000
6000
8000
10000
12000
14000
16000
200
400
600
800
1000
1200
1400
1600

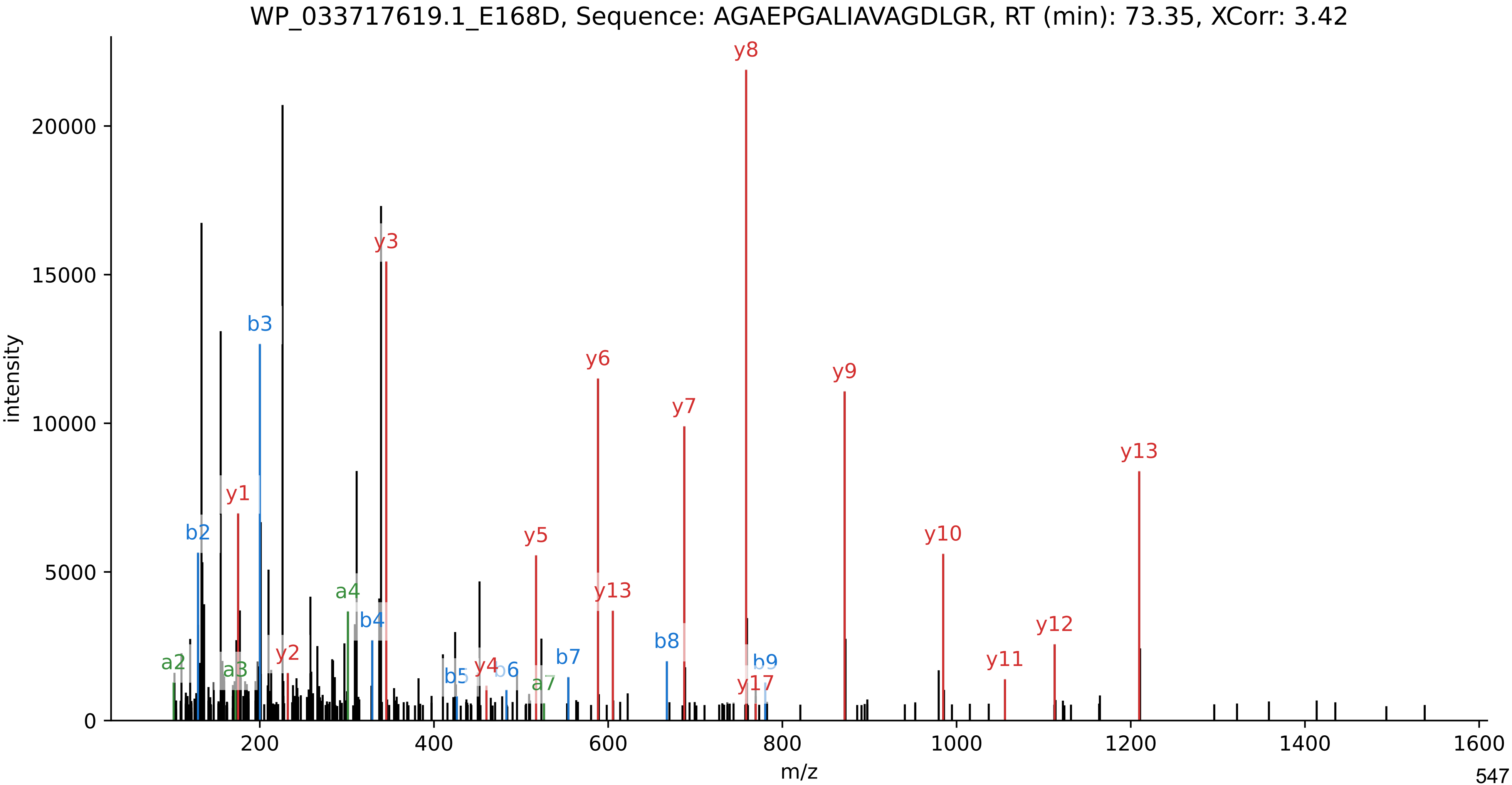

WP_033717619.1_E168D, Sequence: AGAEPGALIAVAGDLGR, RT (min): 73.35, XCorr: 3.42
intensity
m/z
y8
y3
b3
y6
y9
y7
y13
y1
b2
y5
y10
a4
y13
b4
y12
b8
y2
a2
a3
y11
b7
b9
y4
b6
b5
y17
a7

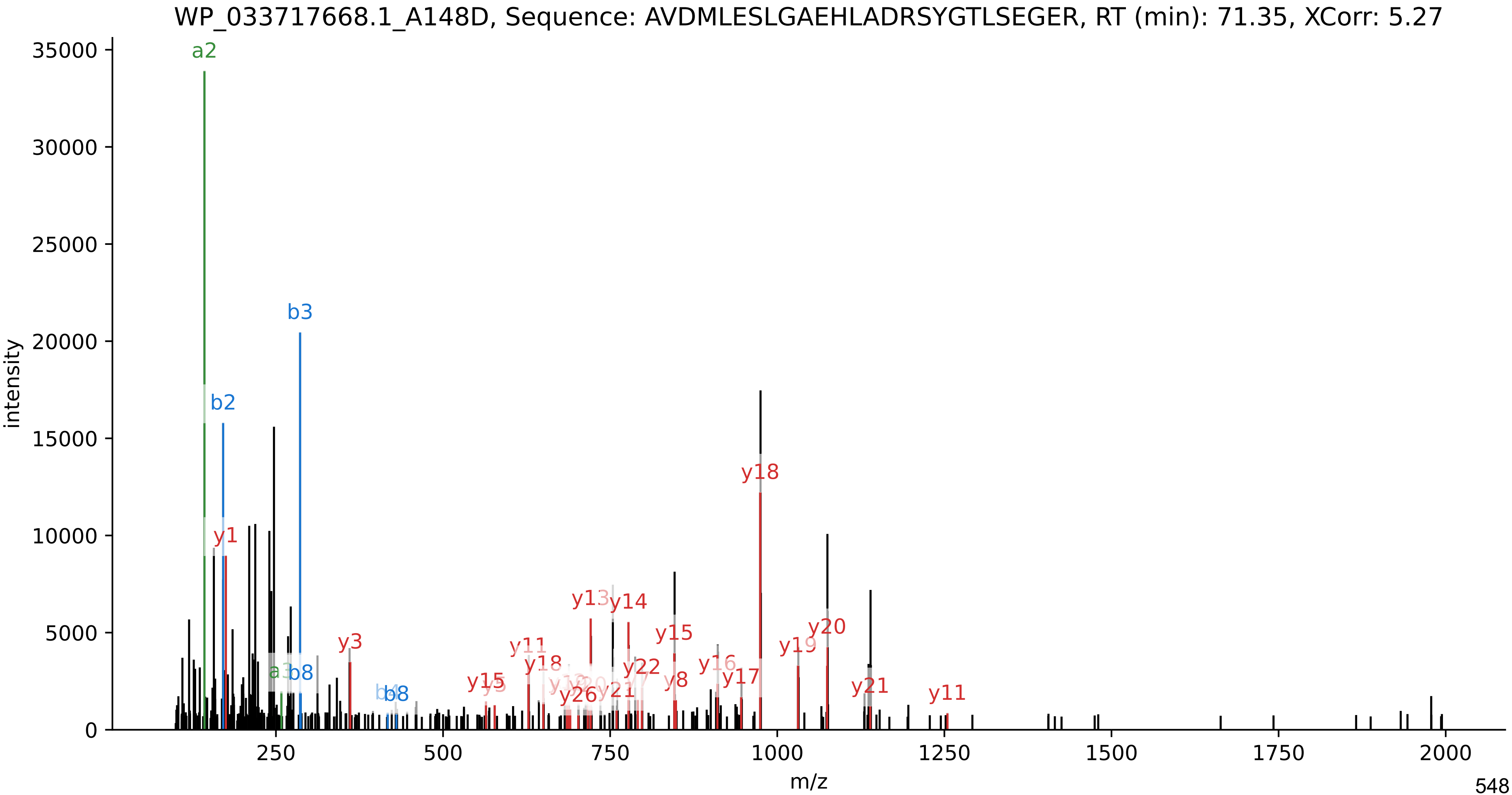
WP_033717668.1_A148D, Sequence: AVDMLESLGAEHLADRSYGTLSEGER, RT (min): 71.35, XCorr: 5.27
intensity
m/z
0
5000
10000
15000
20000
25000
30000
35000
250
500
750
1000
1250
1500
1750
2000
a2
b3
b2
y1
y18
y13
y14
y15
y20
y19
y3
y11
y18
y16
y22
y17
a3
b8
b8
y15
y8
y26
y21
y21
y11

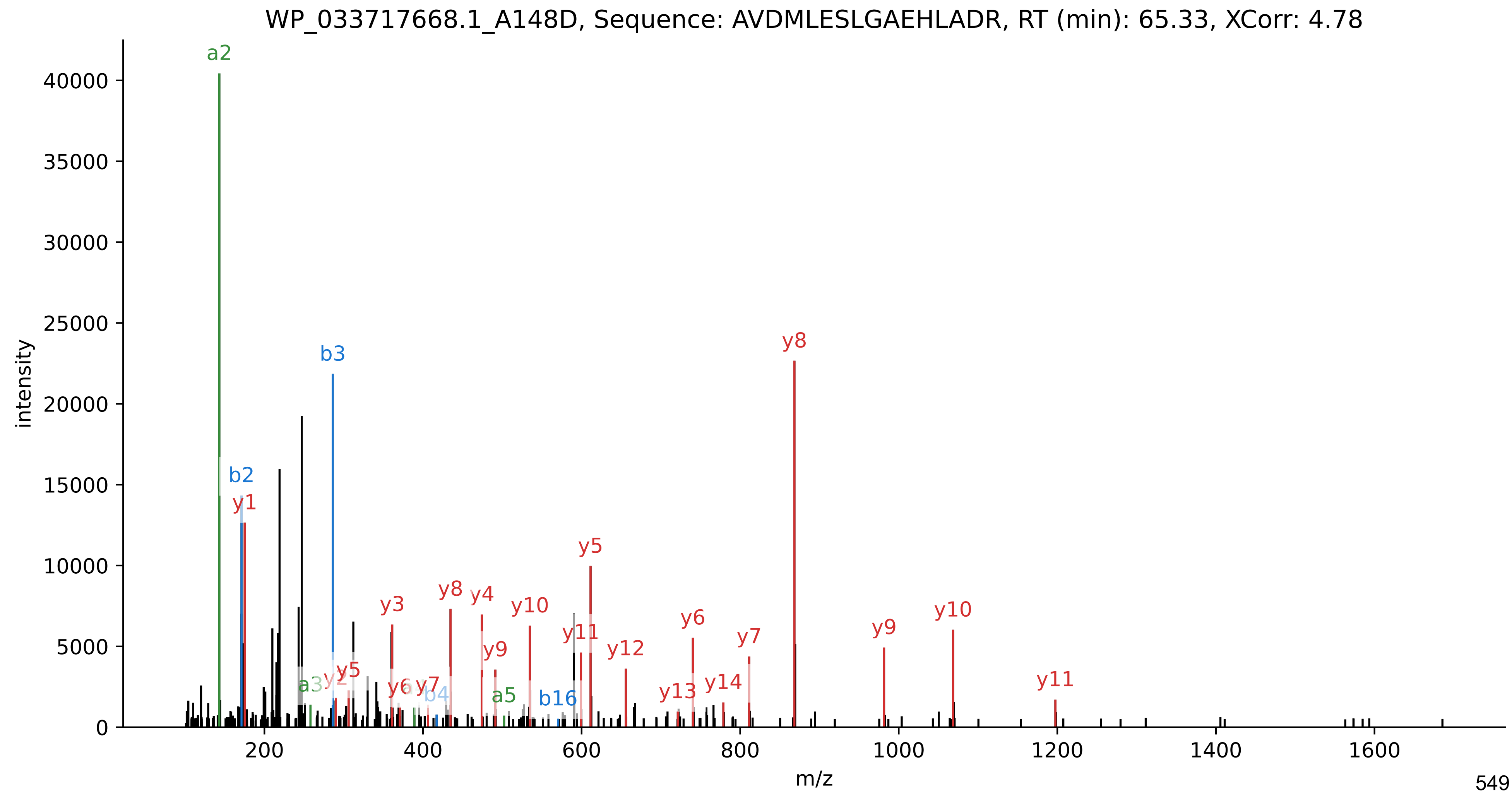

WP_033717668.1_A148D, Sequence: AVDMLESLGAEHLADR, RT (min): 65.33, XCorr: 4.78
intensity
m/z
a2
b2
y1
b3
y3
y8
y4
y10
y9
y5
y11
y12
y6
y13
y14
y7
y8
y9
y10
y11
a3
y2
y5
y6
y7
b4
a5
b16
0
5000
10000
15000
20000
25000
30000
35000
40000
200
400
600
800
1000
1200
1400
1600

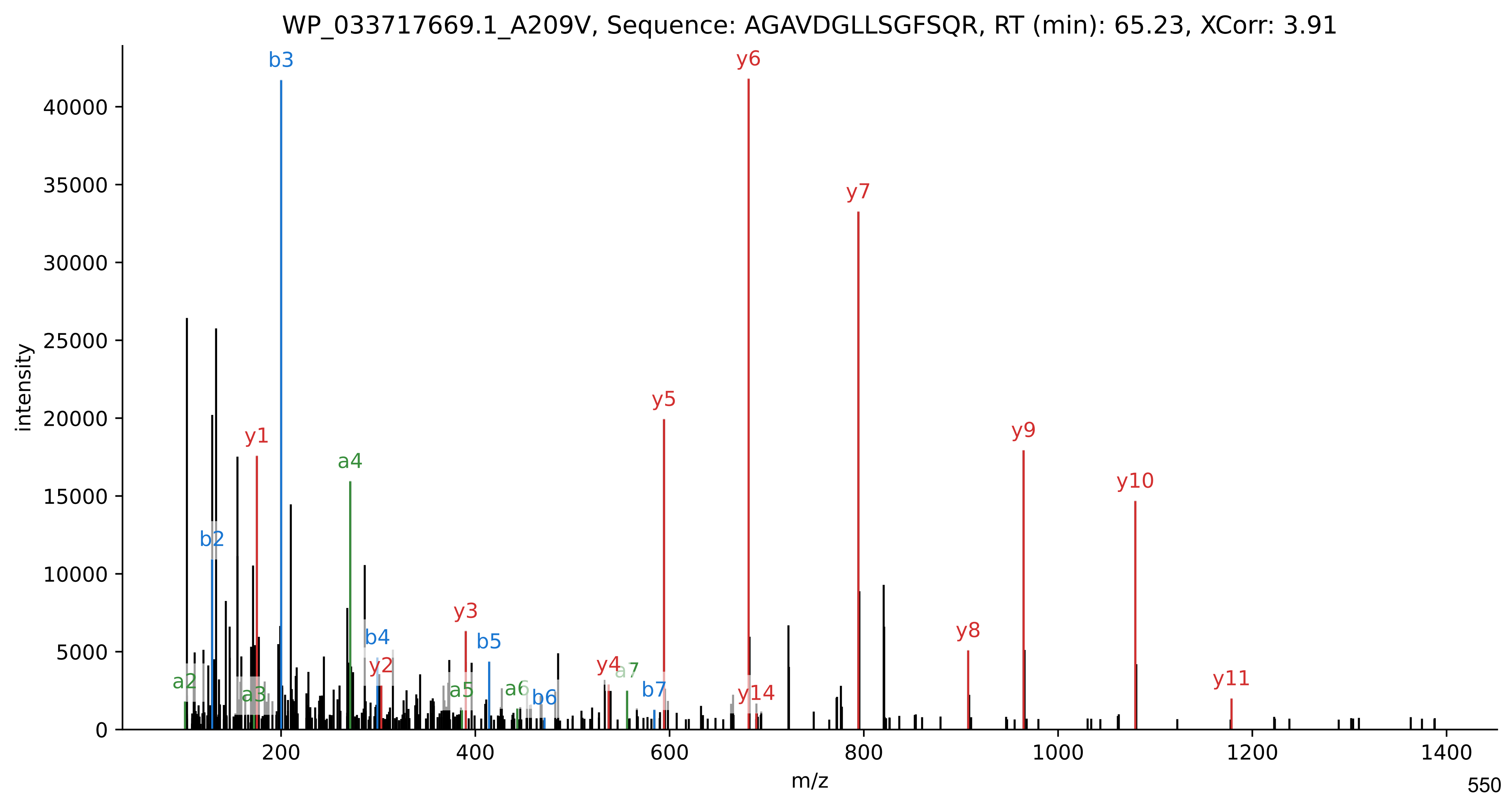
WP_033717669.1_A209V, Sequence: AGAVDGLLSGFSQR, RT (min): 65.23, XCorr: 3.91
intensity
m/z
b3
y6
y7
y5
y1
y9
a4
y10
b2
y3
b4
b5
y2
y8
y4
a7
a2
a3
a5
a6
b6
b7
y14
y11

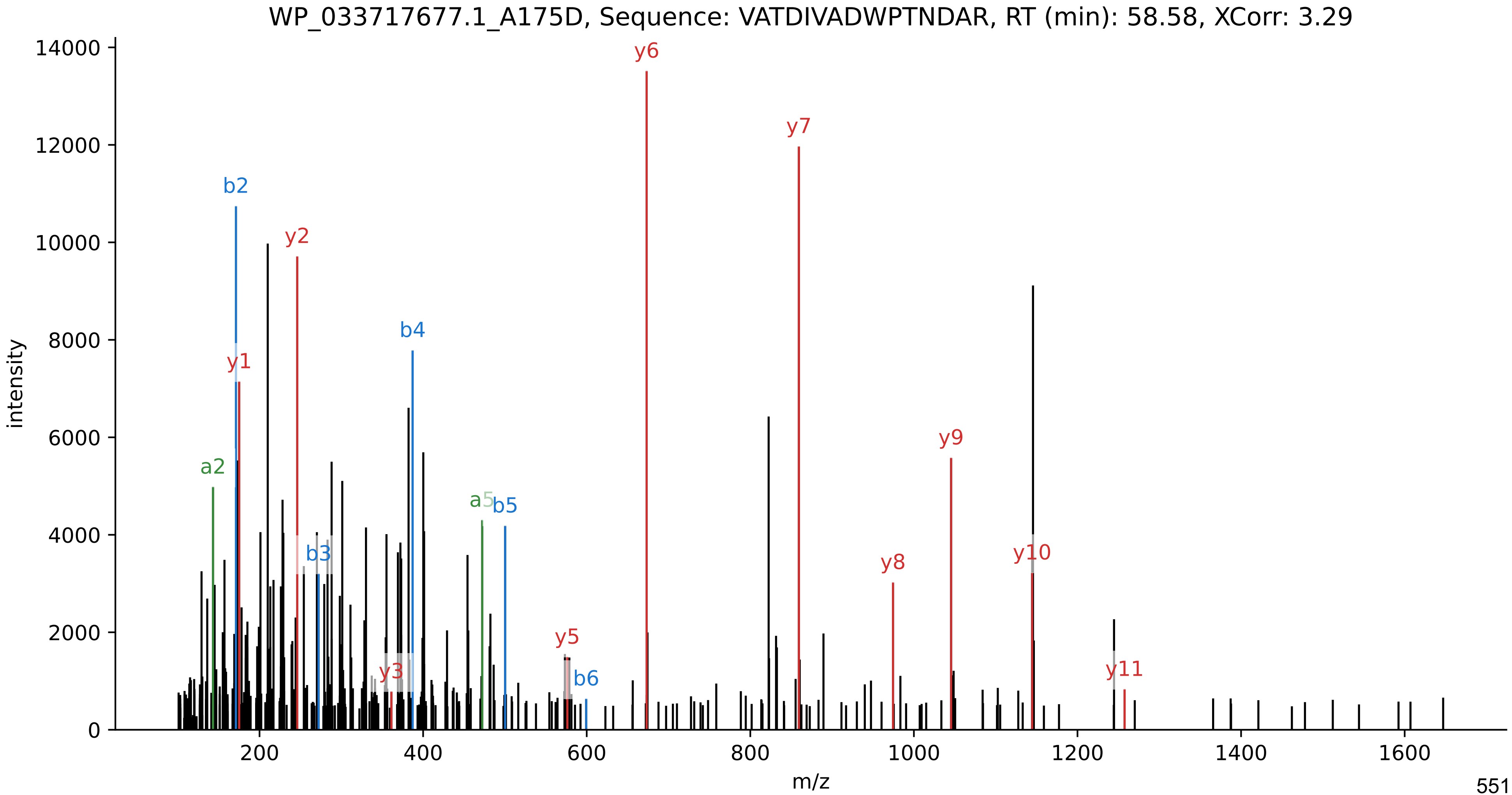

WP_033717677.1_A175D, Sequence: VATDIVADWPTNDAR, RT (min): 58.58, XCorr: 3.29
intensity
m/z
y6
y7
b2
y2
b4
y1
y9
a2
a5
b5
b3
y10
y8
y5
b6
y3
y11

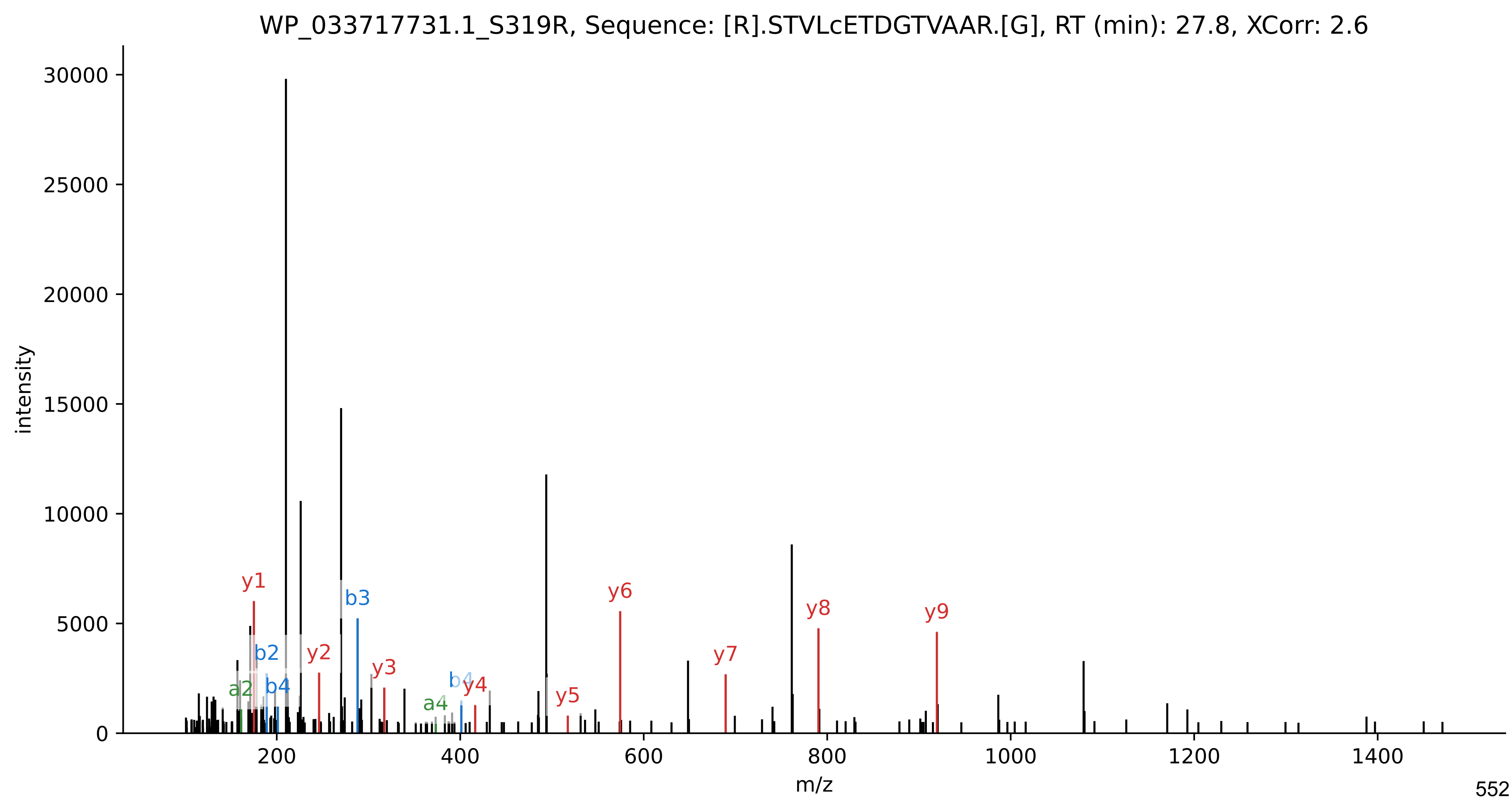

WP_033717731.1_S319R, Sequence: [R].STVLcETDGTVAAR.[G], RT (min): 27.8, XCorr: 2.6
intensity
m/z
y1
b2
a2
b4
y2
b3
y3
a4
b4
y4
y5
y6
y7
y8
y9

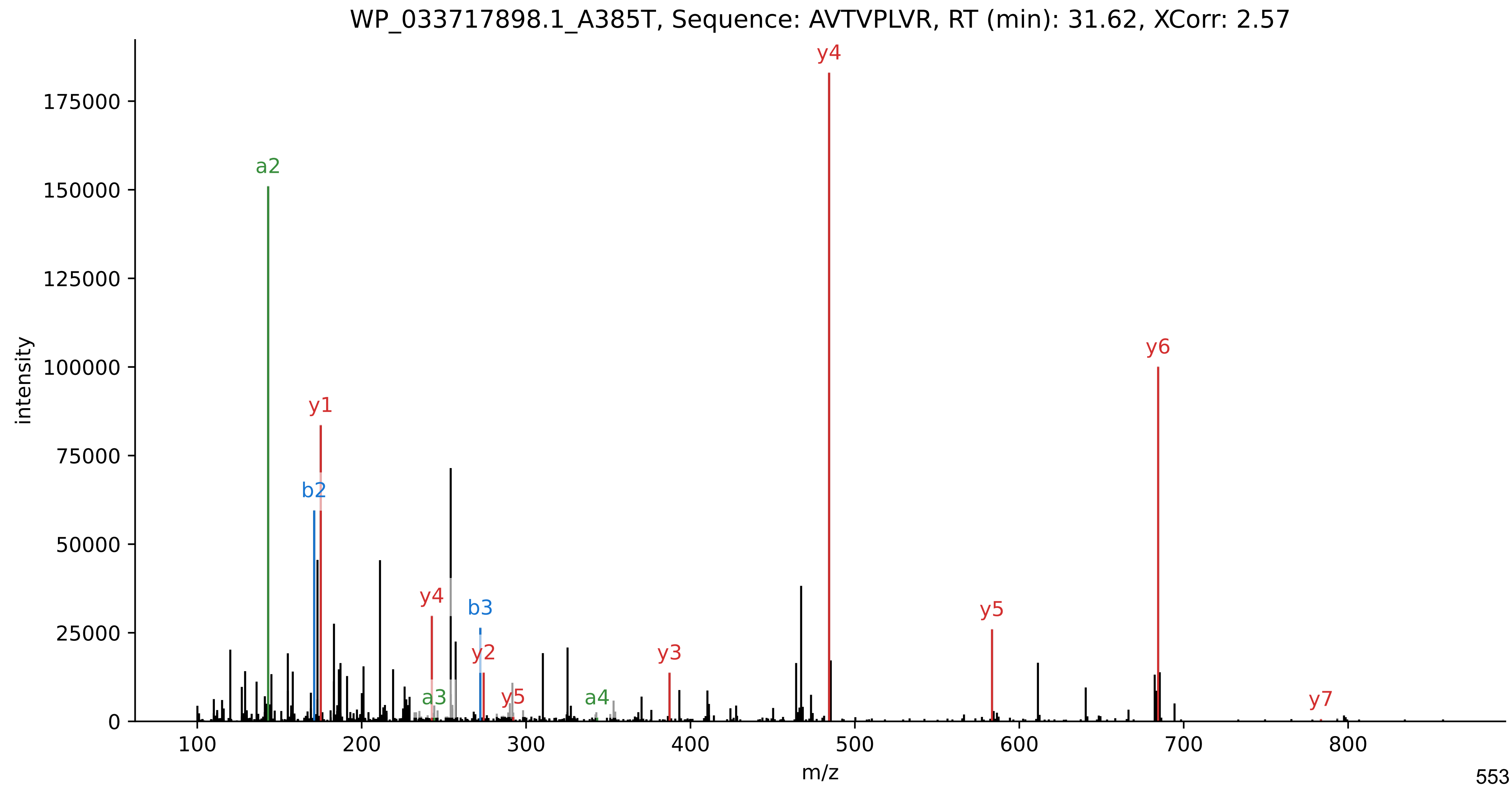
WP_033717898.1_A385T, Sequence: AVTVPLVR, RT (min): 31.62, XCorr: 2.57
intensity
m/z
y4
a2
y6
y1
b2
y4
b3
y2
y3
y5
a3
y5
a4
y7
0
25000
50000
75000
100000
125000
150000
175000
100
200
300
400
500
600
700
800

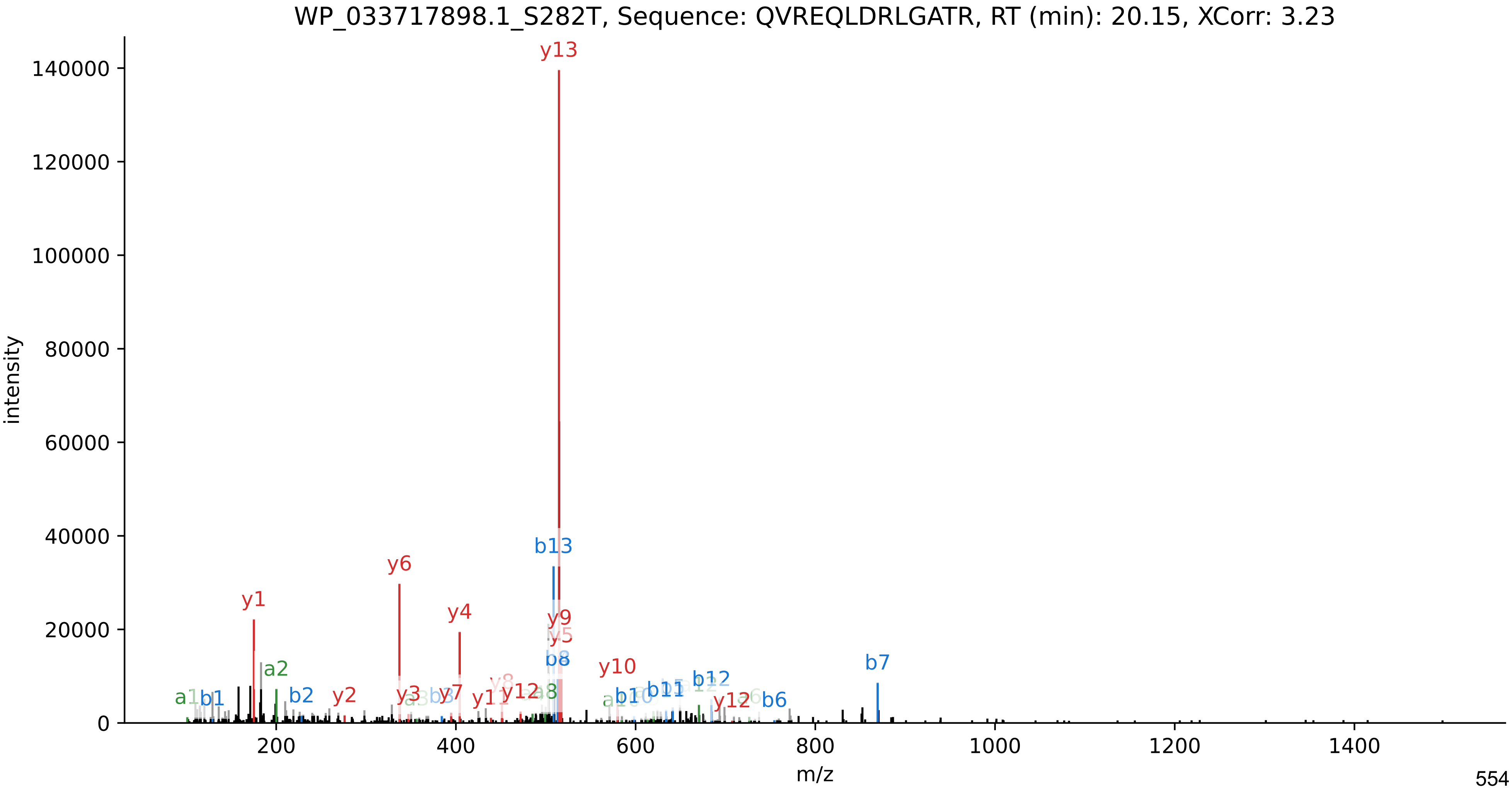

WP_033717898.1_S282T, Sequence: QVREQLDRLGATR, RT (min): 20.15, XCorr: 3.23
intensity
m/z
y13
b13
y6
y1
y4
y9
y5
b8
y10
b7
a2
b2
y2
y3
y7
y11
y12
a8
b1
b10
b11
b12
b6
0
20000
40000
60000
80000
100000
120000
140000
200
400
600
800
1000
1200
1400

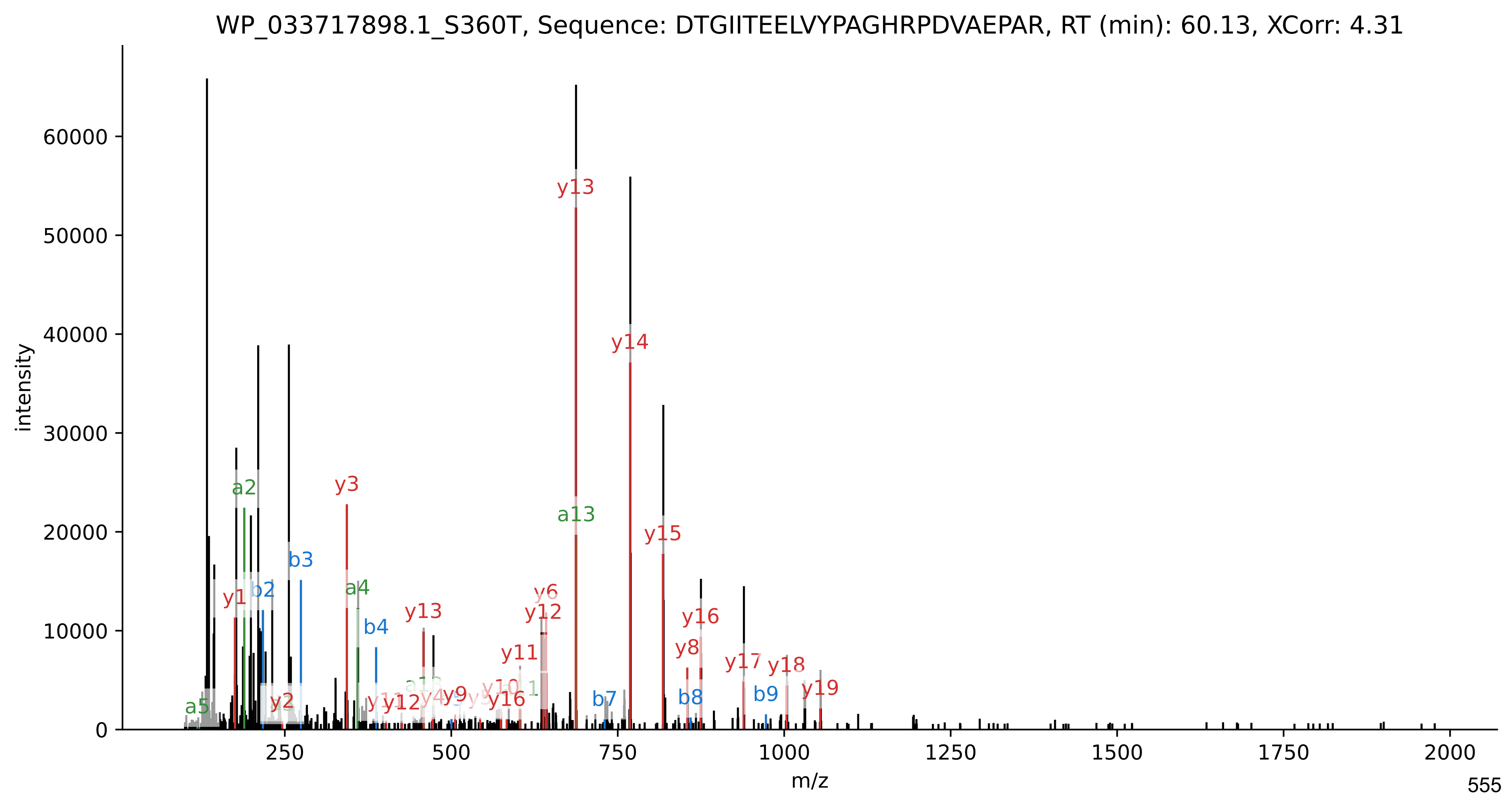
WP_033717898.1_S360T, Sequence: DTGIITEELVYPAGHRPDVAEPAR, RT (min): 60.13, XCorr: 4.31
intensity
m/z
60000
50000
40000
30000
20000
10000
0
250
500
750
1000
1250
1500
1750
2000
y13
y14
a13
y15
a2
y3
b3
a4
y1
b2
b4
y13
y6
y12
y16
y11
y8
y17
y18
y19
b7
b8
b9
a5
y2
y12
y9
y16

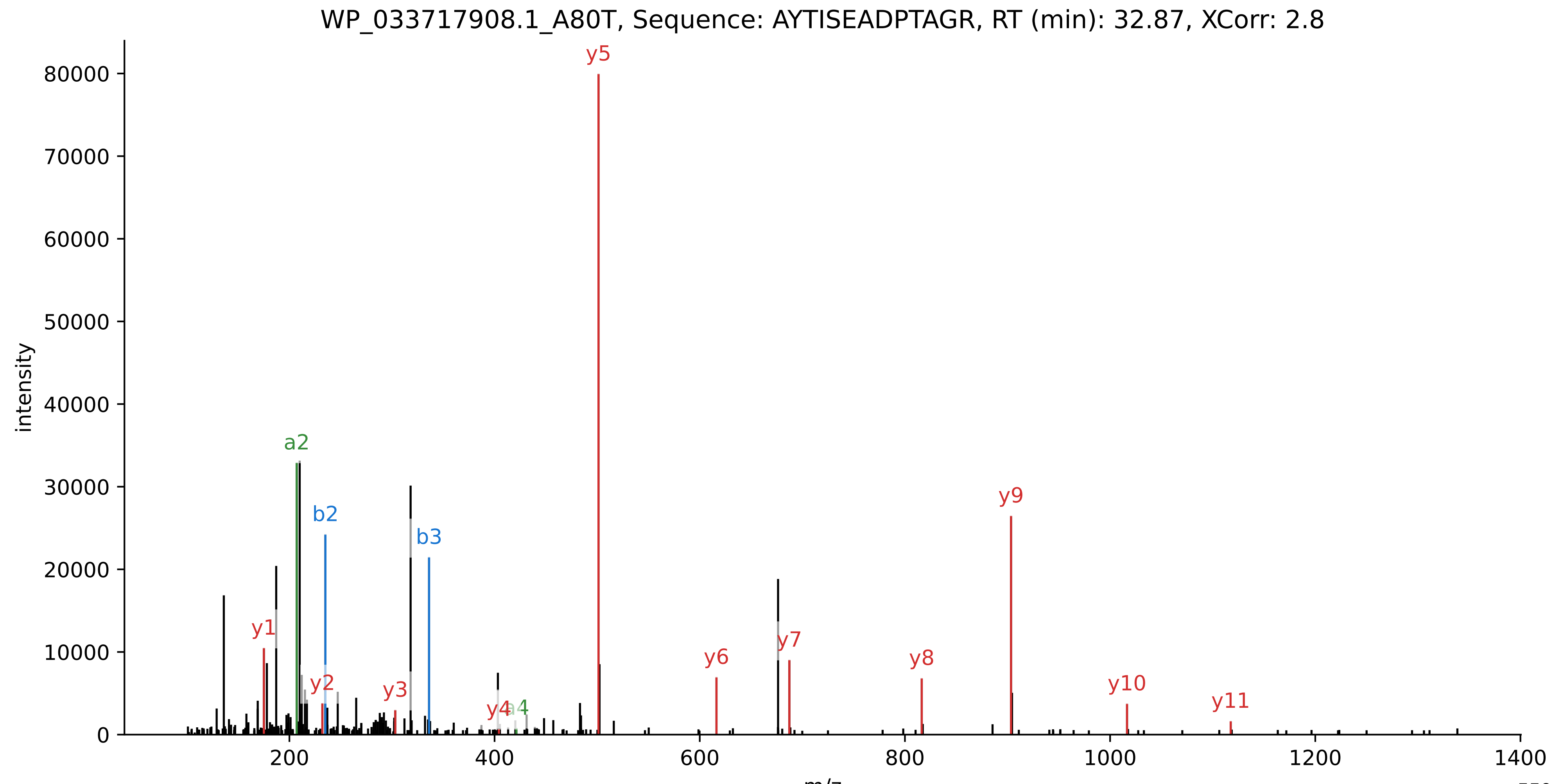
WP_033717908.1_A80T, Sequence: AYTISEADPTAGR, RT (min): 32.87, XCorr: 2.8
intensity
m/z
y5
a2
b2
b3
y9
y1
y2
y3
y4
a4
y6
y7
y8
y10
y11
80000
70000
60000
50000
40000
30000
20000
10000
0
200
400
600
800
1000
1200
1400

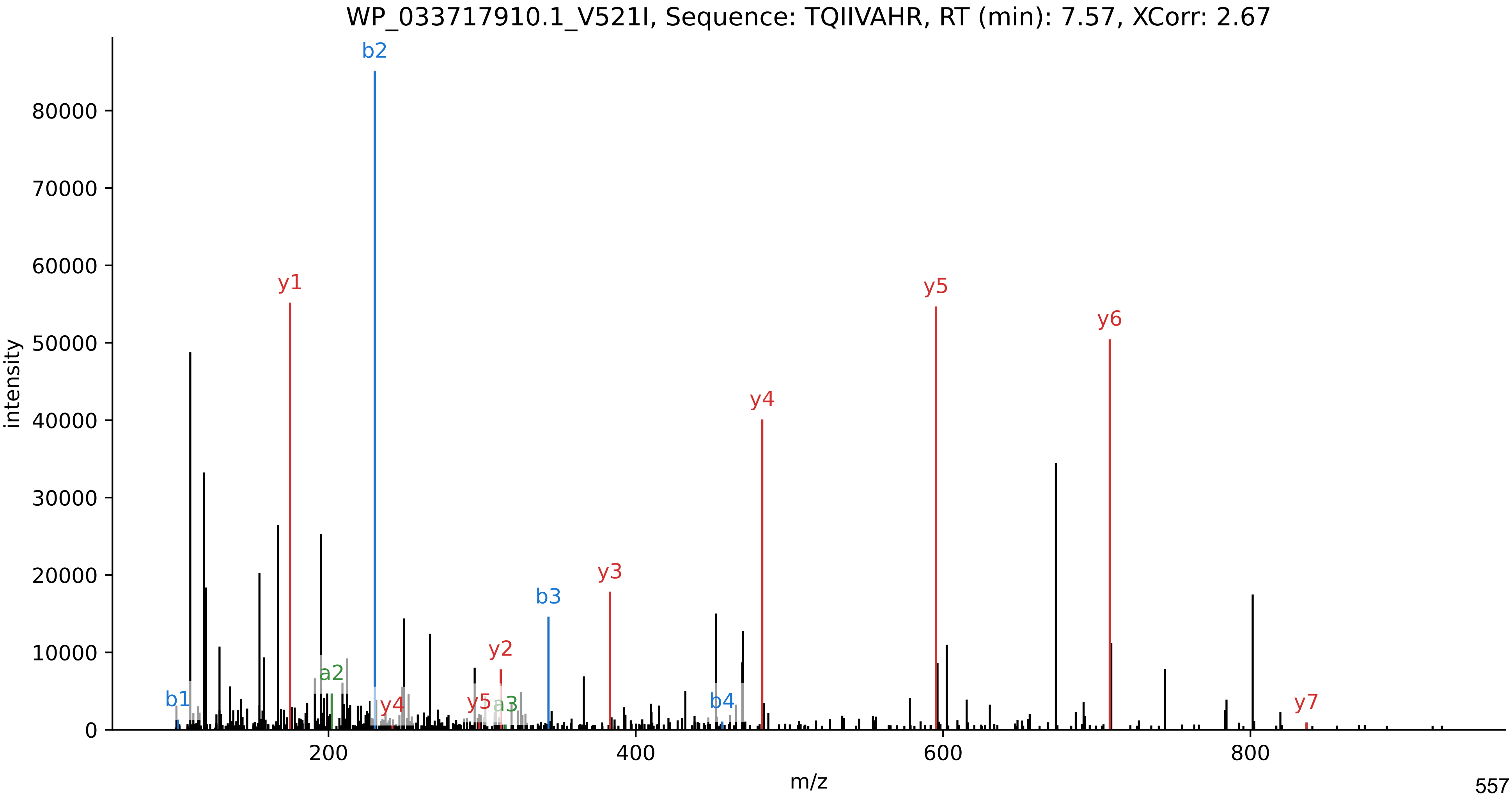

WP_033717910.1_V521I, Sequence: TQIIVAHR, RT (min): 7.57, XCorr: 2.67
intensity
m/z
b1
b2
b3
b4
y1
y2
y3
y4
y5
y6
y7
a2
a3
y4
y5
80000
70000
60000
50000
40000
30000
20000
10000
0
200
400
600
800

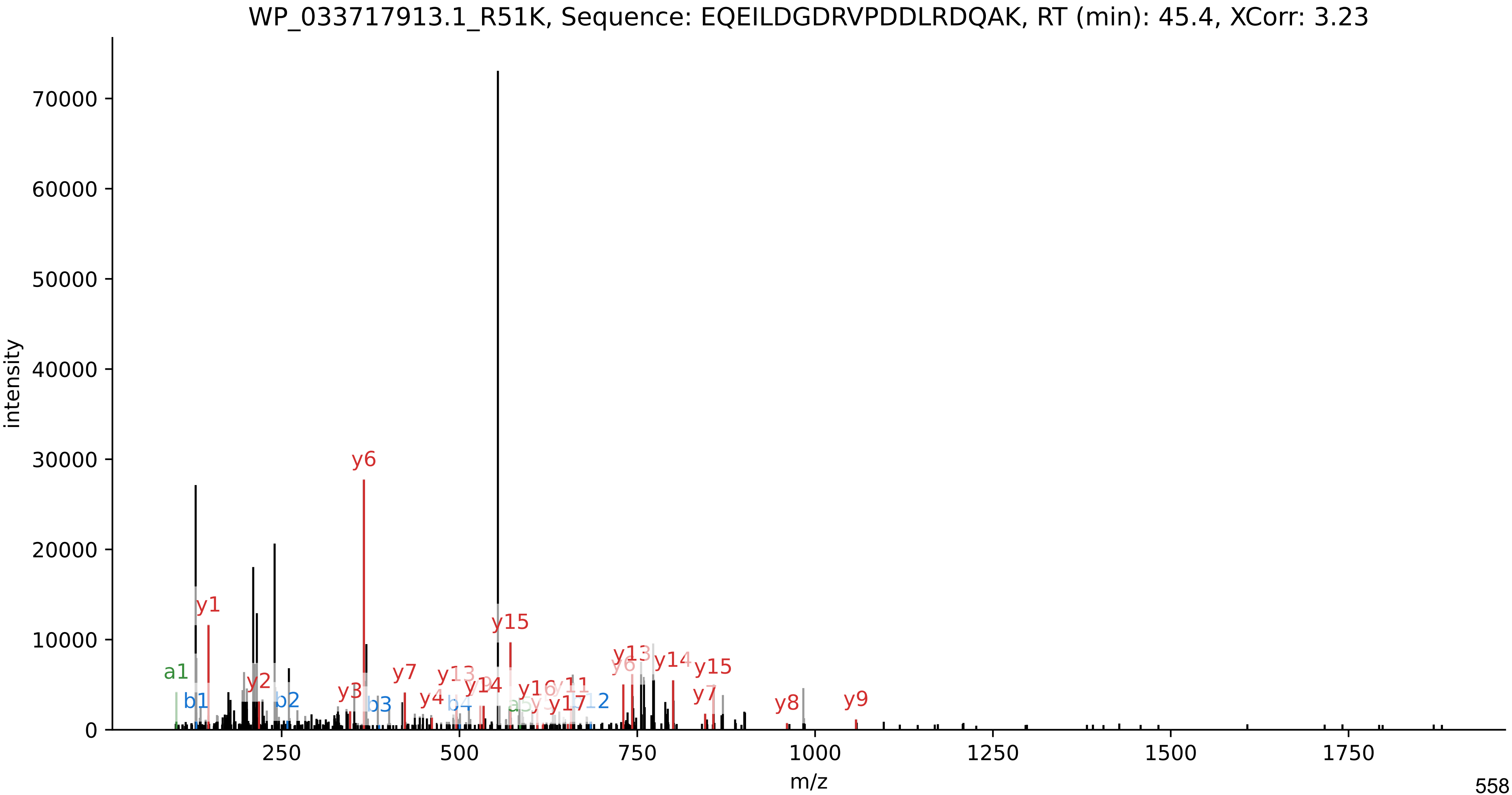

WP_033717913.1_R51K, Sequence: EQEILDGDRVPDDLRDQAK, RT (min): 45.4, XCorr: 3.23
intensity
m/z
y6
y1
y15
a1
y2
b1
b2
y3
b3
y7
y4
b4
y13
y14
y16
y17
y13
y6
y14
y15
y7
y8
y9

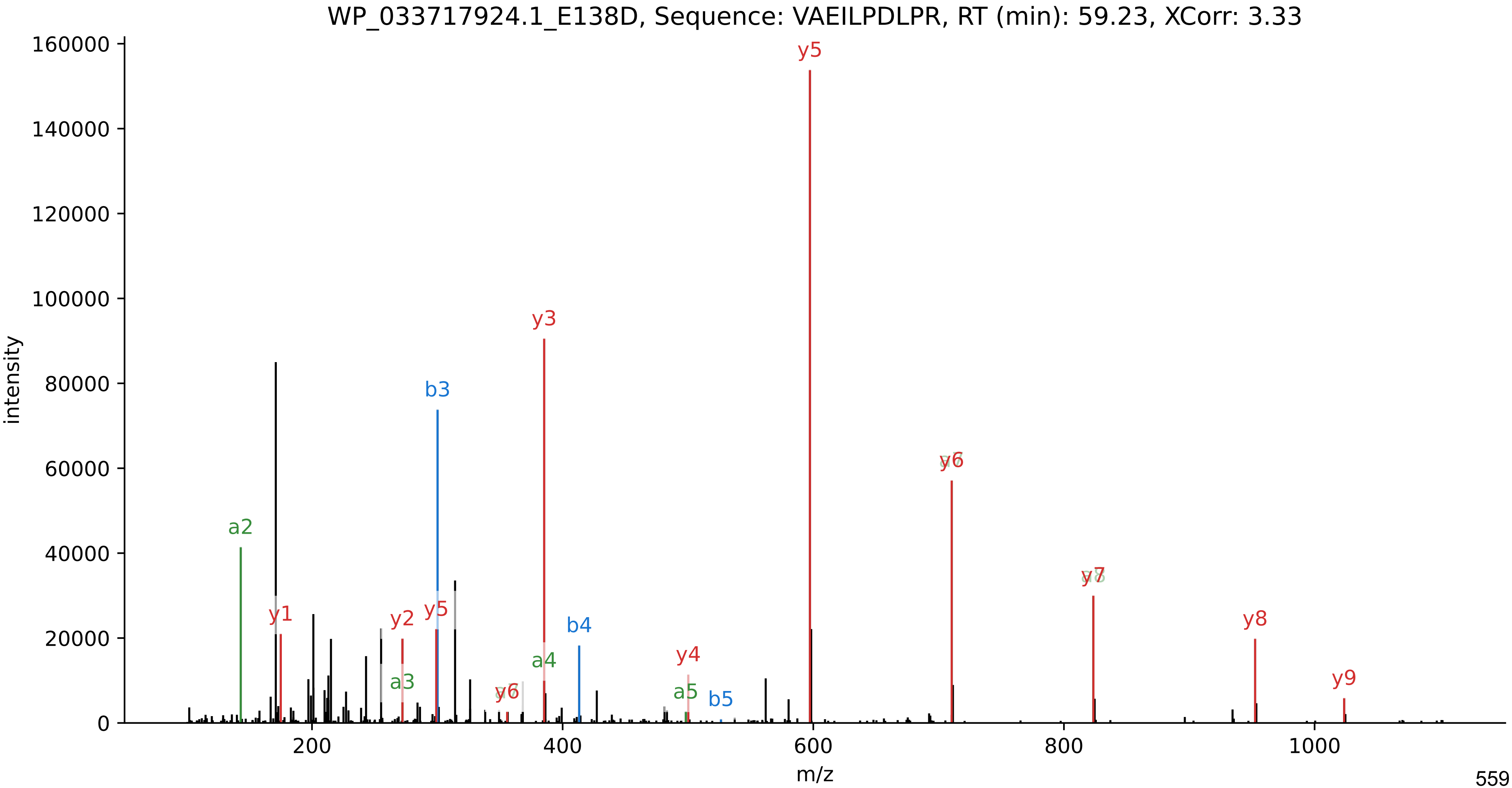

WP_033717924.1_E138D, Sequence: VAEILPDLPR, RT (min): 59.23, XCorr: 3.33
intensity
m/z
0
20000
40000
60000
80000
100000
120000
140000
160000
200
400
600
800
1000
a2
y1
y2
y5
b3
a3
y6
y3
a4
b4
y4
a5
b5
y5
y6
y7
y8
y9

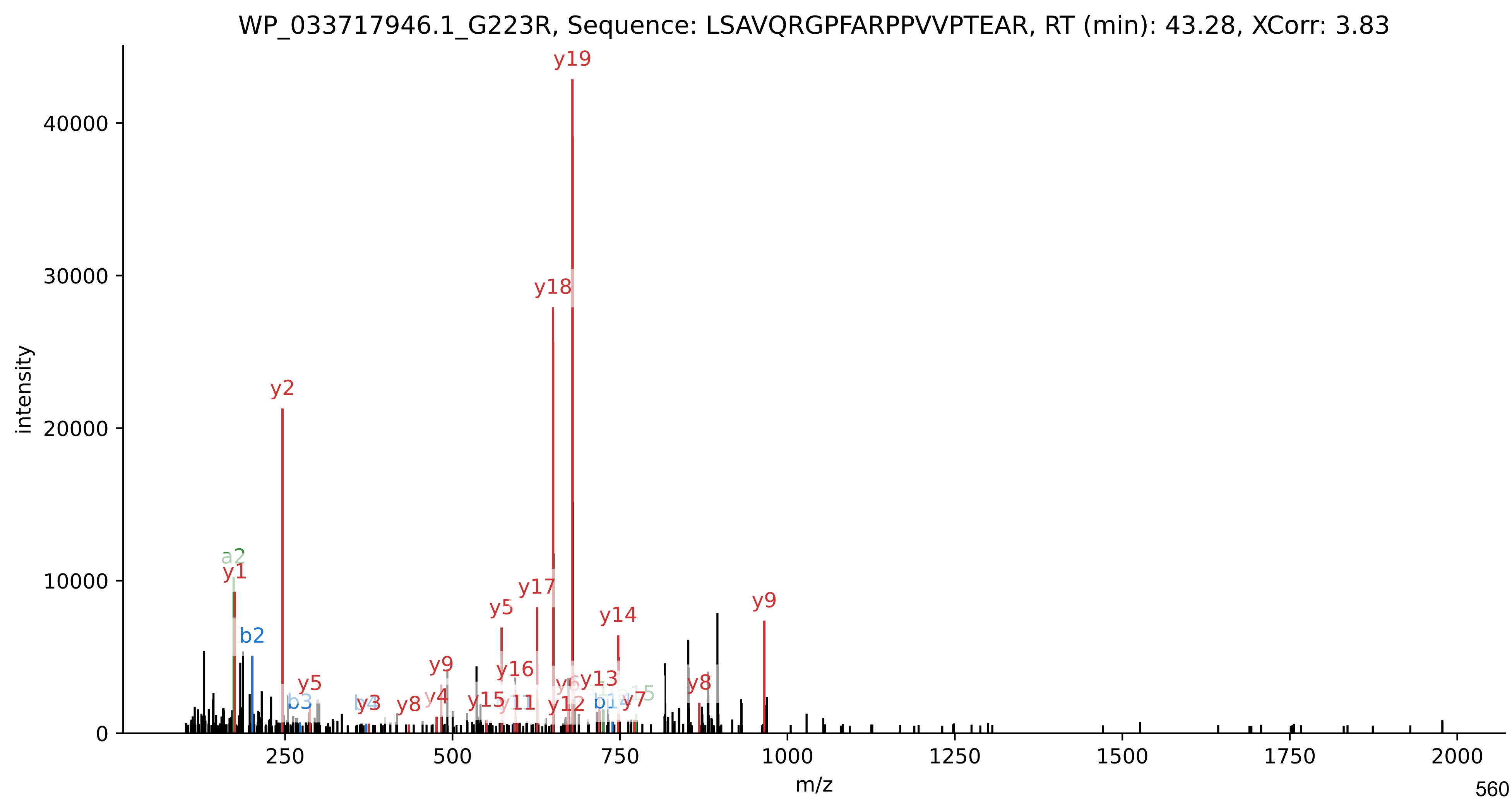
WP_033717946.1_G223R, Sequence: LSAVQRGPFARPPVVPTEAR, RT (min): 43.28, XCorr: 3.83
intensity
m/z
y19
y18
y2
y17
y5
y14
y9
a2
y1
b2
y16
y13
y9
y4
y5
b3
y3
y8
y15
y11
y12
y6
b14
y7
y8
0
10000
20000
30000
40000
250
500
750
1000
1250
1500
1750
2000

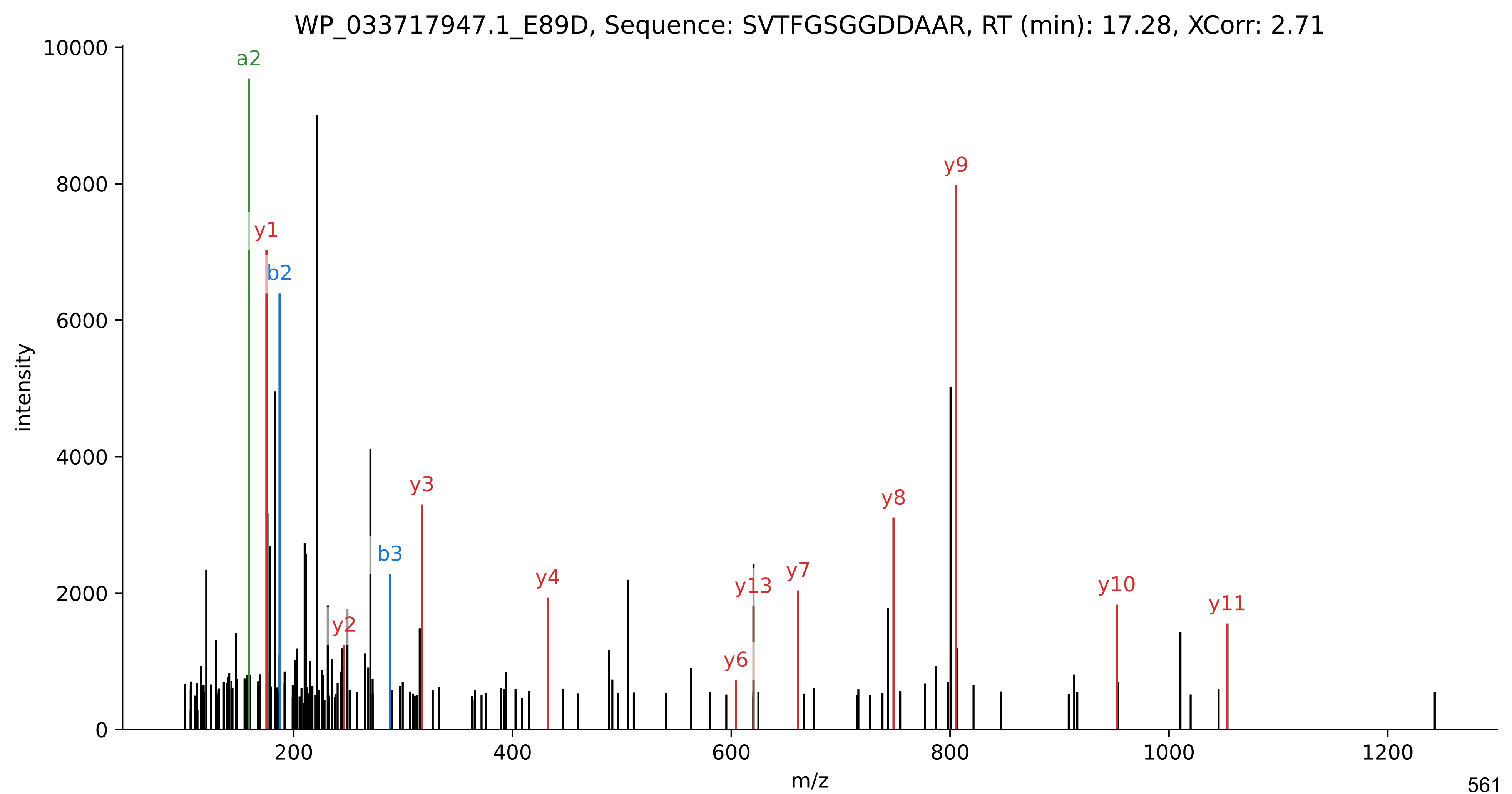
WP_033717947.1_E89D, Sequence: SVTFGSGGDDAAR, RT (min): 17.28, XCorr: 2.71
intensity
m/z
a2
y1
b2
b3
y2
y3
y4
y6
y13
y7
y8
y9
y10
y11
0
2000
4000
6000
8000
10000
200
400
600
800
1000
1200

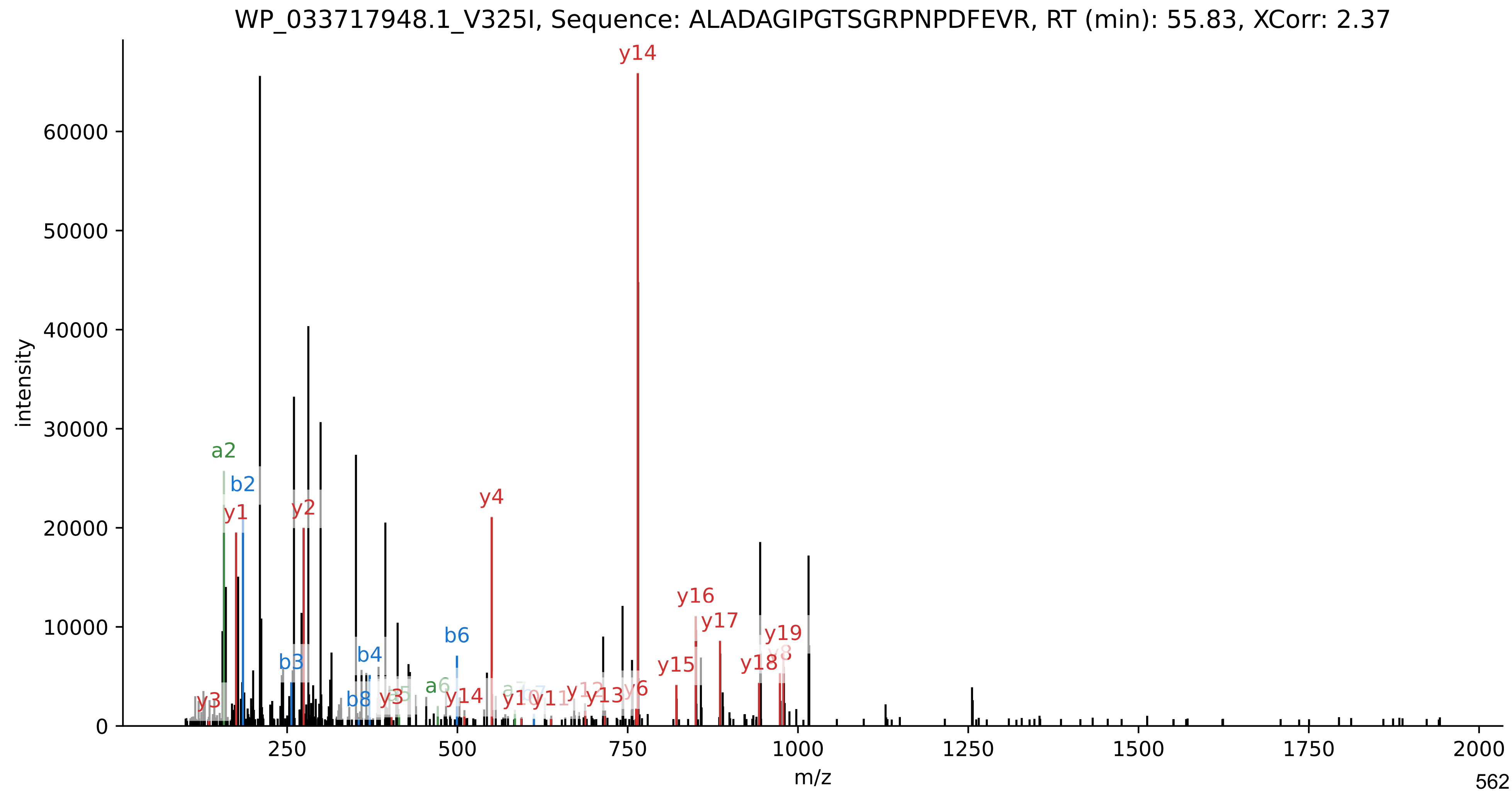
WP_033717948.1_V325I, Sequence: ALADAGIPGTSGRPNPDFEVR, RT (min): 55.83, XCorr: 2.37
intensity
m/z
0
10000
20000
30000
40000
50000
60000
250
500
750
1000
1250
1500
1750
2000
y14
a2
b2
y1
y2
y4
y16
y17
y19
y15
y18
b6
b4
b3
a6
b8
y3
y3
y14
y10
y11
y12
y13
y6

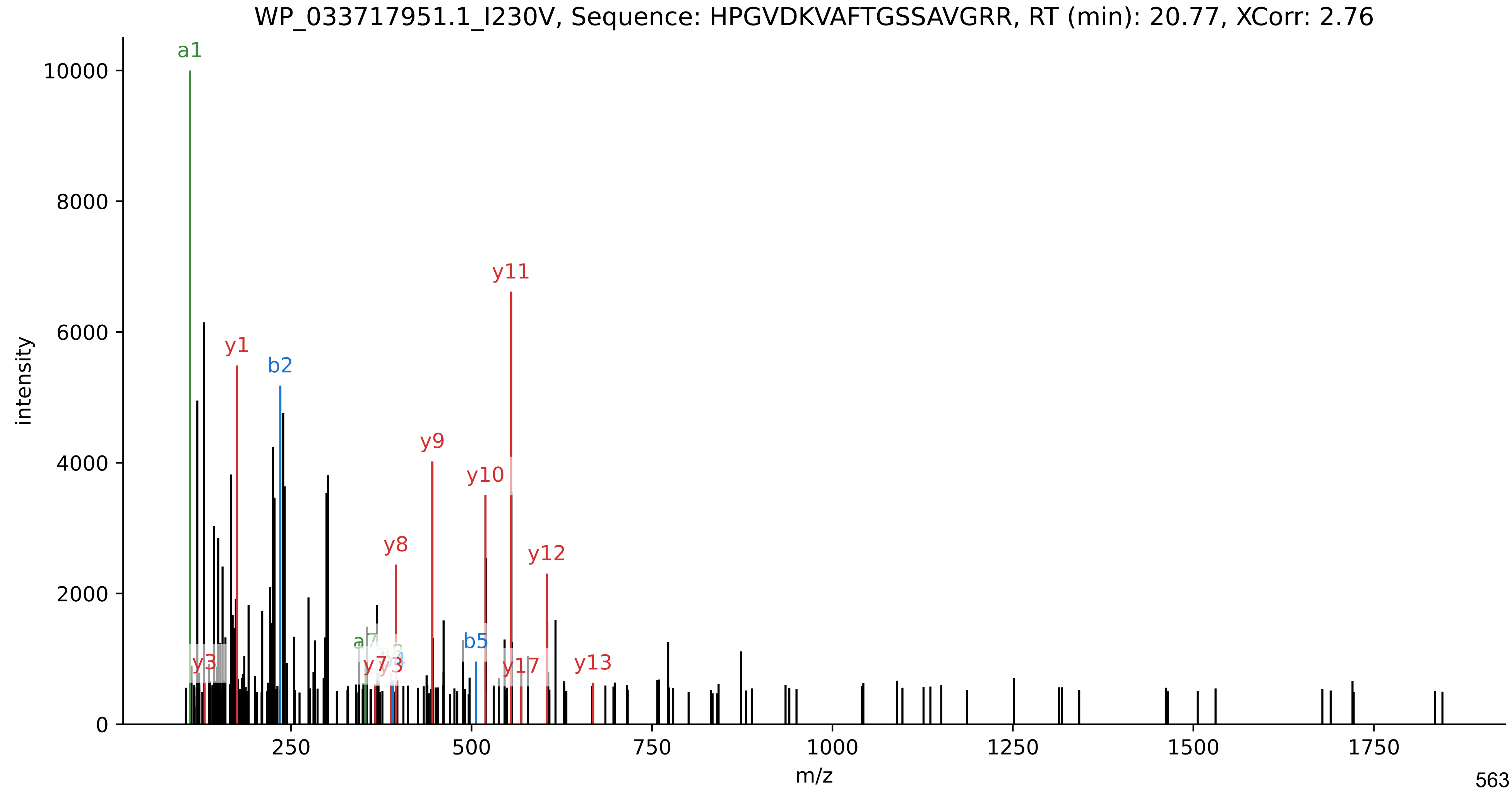

WP_033717951.1_I230V, Sequence: HPGVDKVAFTGSSAVGRR, RT (min): 20.77, XCorr: 2.76
intensity
m/z
a1
y1
b2
y9
y10
y11
y8
y12
b5
y3
y7
y17
y13

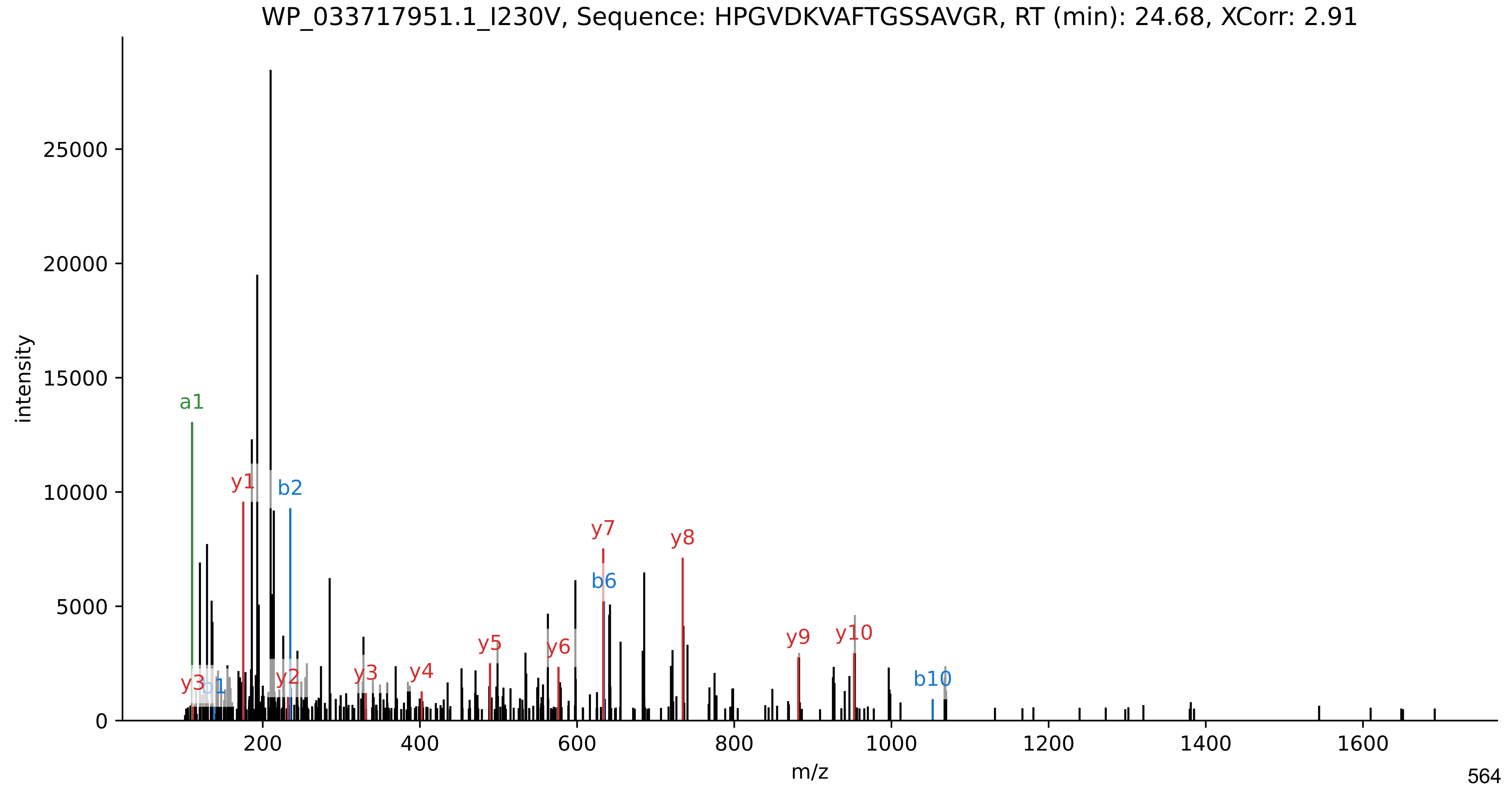

WP_033717951.1_I230V, Sequence: HPGVDKVAFTGSSAVGR, RT (min): 24.68, XCorr: 2.91
intensity
m/z
a1
y1
b2
y7
y8
b6
y5
y6
y9
y10
b10
y3
b1
y2
y3
y4

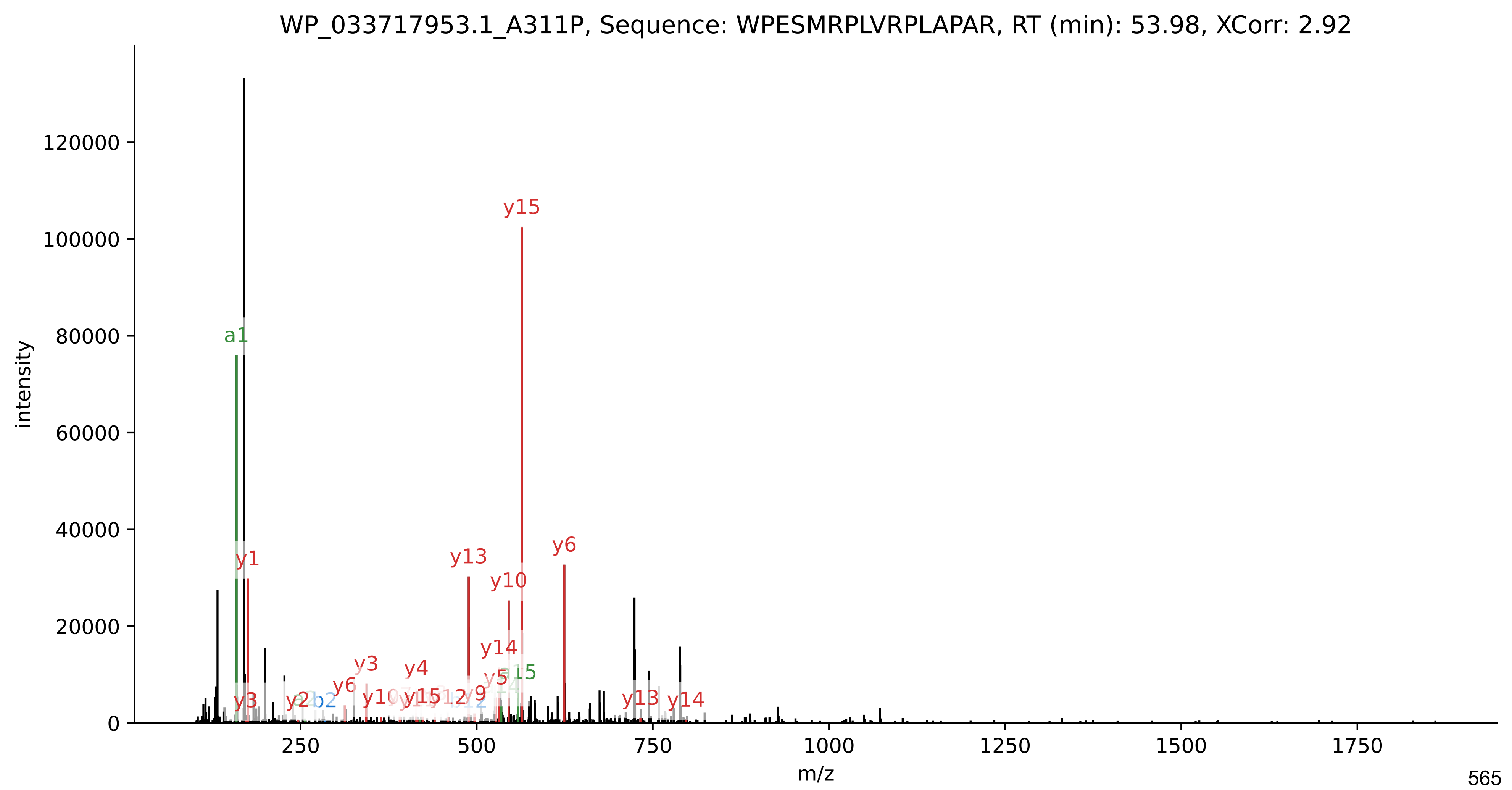
WP_033717953.1_A311P, Sequence: WPESMRPLVRPLAPAR, RT (min): 53.98, XCorr: 2.92
intensity
m/z
0
20000
40000
60000
80000
100000
120000
250
500
750
1000
1250
1500
1750
a1
y1
y15
y13
y10
y6
y14
y3
y4
y5
y9
y2
b2
y13
y14

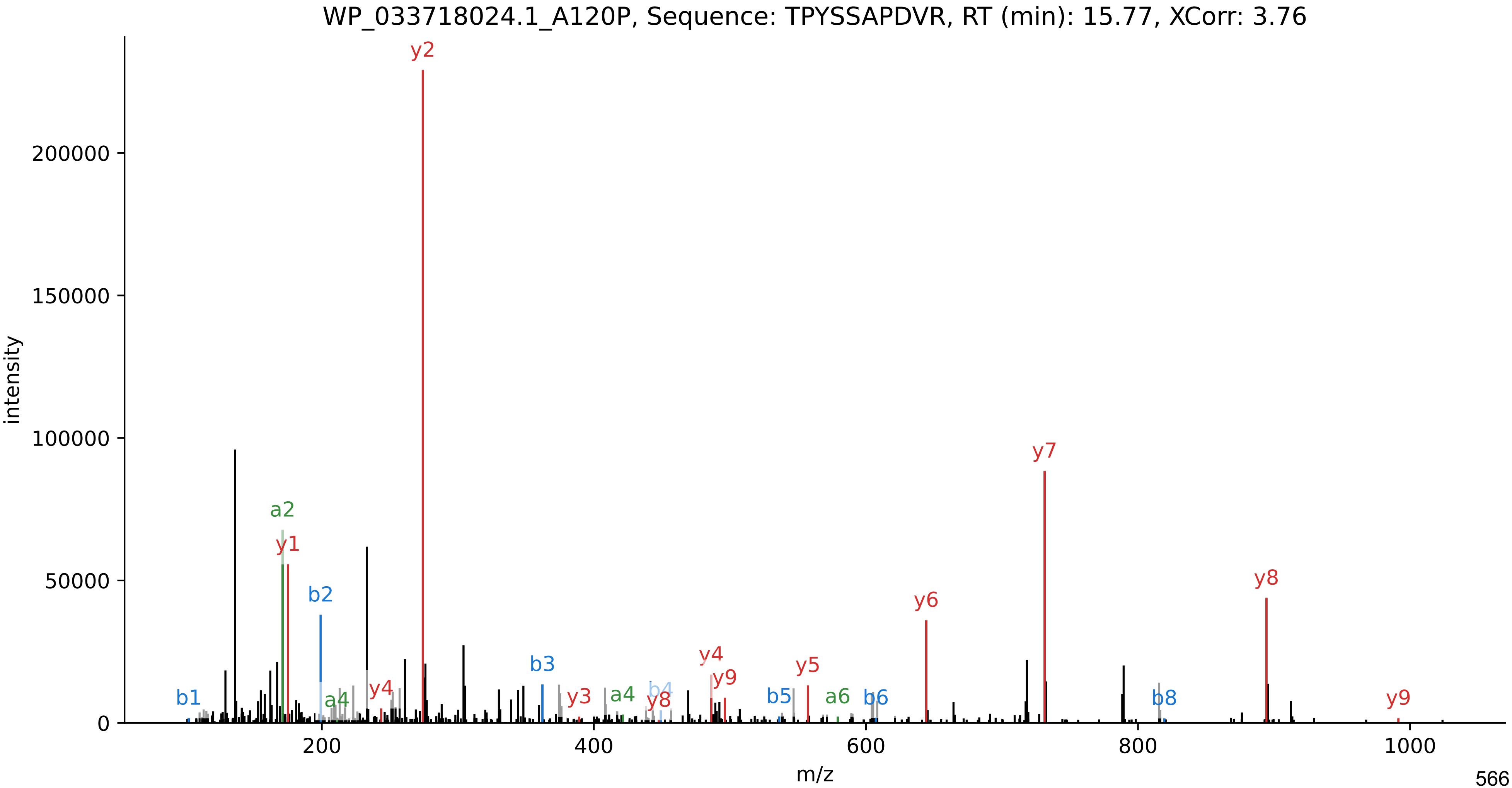

WP_033718024.1_A120P, Sequence: TPYSSAPDVR, RT (min): 15.77, XCorr: 3.76
intensity
m/z
y2
y7
y8
y6
a2
y1
b2
b3
y4
y9
y5
b1
a4
y4
y3
a4
b4
y8
b5
a6
b6
b8
y9

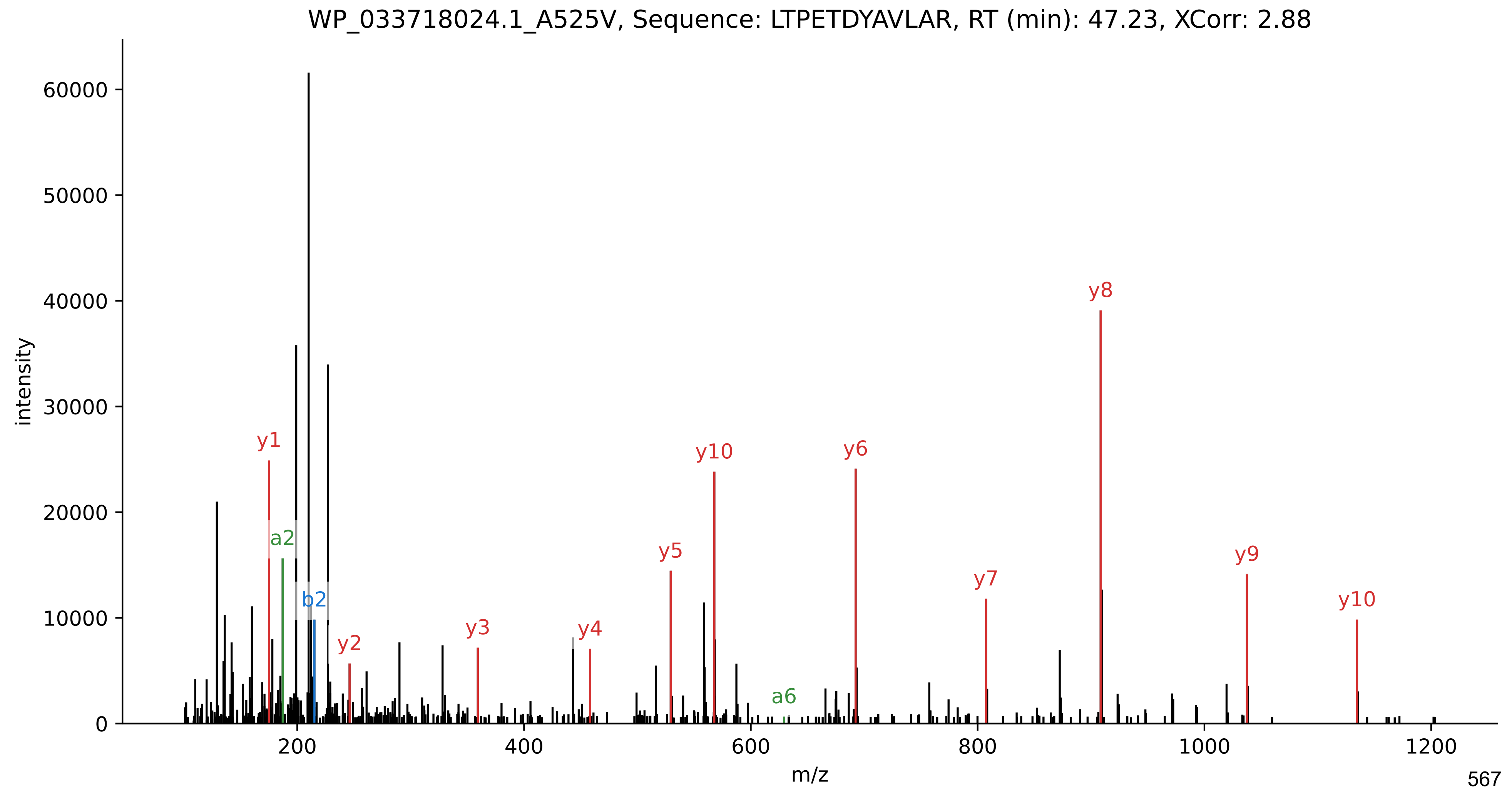

WP_033718024.1_A525V, Sequence: LTPETDYAVLAR, RT (min): 47.23, XCorr: 2.88
intensity
m/z
y1
a2
b2
y2
y3
y4
y5
y10
a6
y6
y7
y8
y9
y10

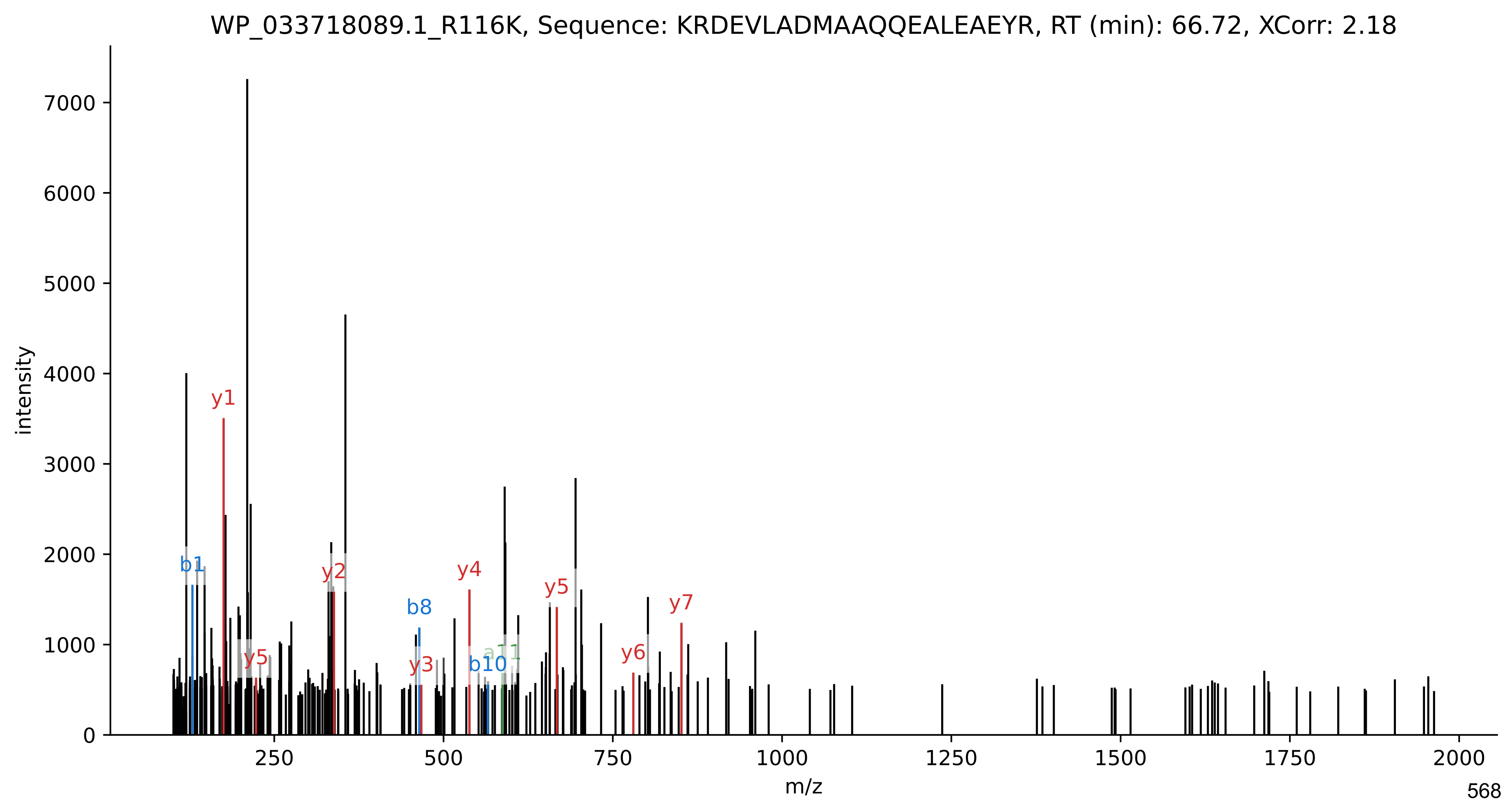

WP_033718089.1_R116K, Sequence: KRDEVLADMAAQQEALEAEYR, RT (min): 66.72, XCorr: 2.18
intensity
m/z
y1
b1
y2
y4
y5
b8
y7
y6
y5
y3
b10
a11

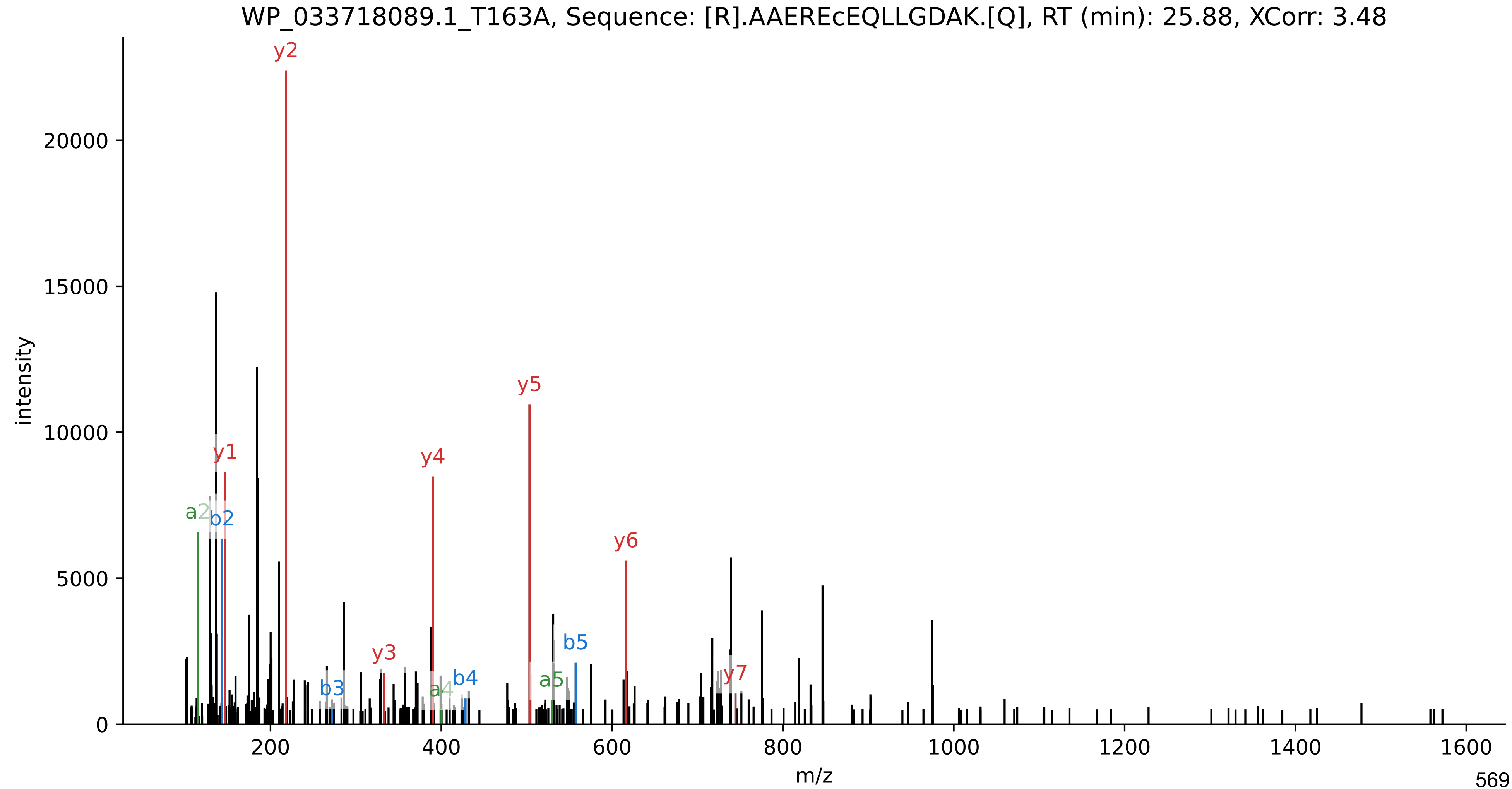
WP_033718089.1_T163A, Sequence: [R].AAEREcEQLLGDAK.[Q], RT (min): 25.88, XCorr: 3.48
intensity
m/z
y2
y5
y1
y4
a2
b2
y6
b5
y3
a5
y7
b4
b3
a4
0
5000
10000
15000
20000
200
400
600
800
1000
1200
1400
1600

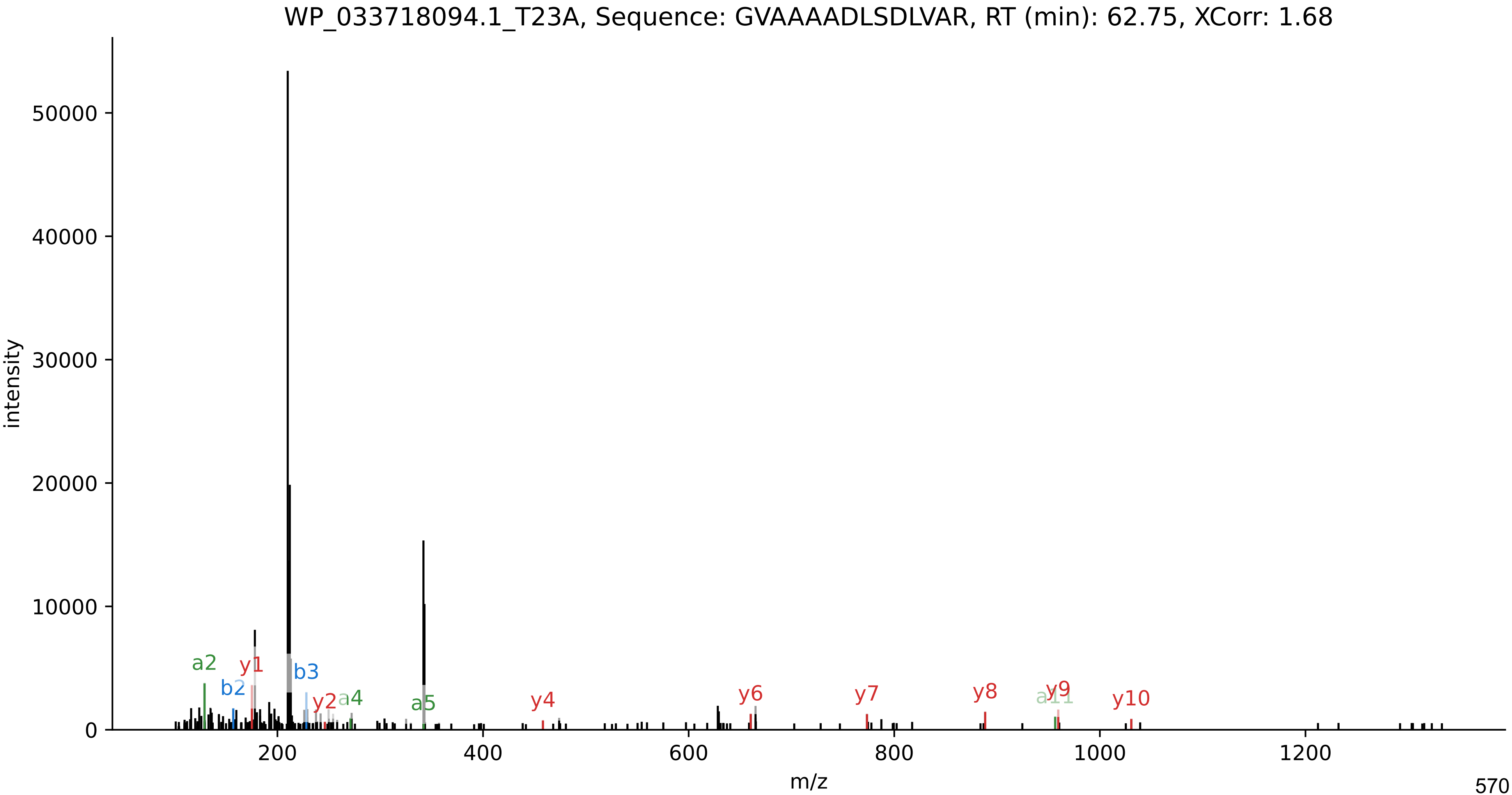

WP_033718094.1_T23A, Sequence: GVAAAADLSDLVAR, RT (min): 62.75, XCorr: 1.68
intensity
m/z
a2
b2
y1
b3
y2
a4
a5
y4
y6
y7
y8
a11
y9
y10

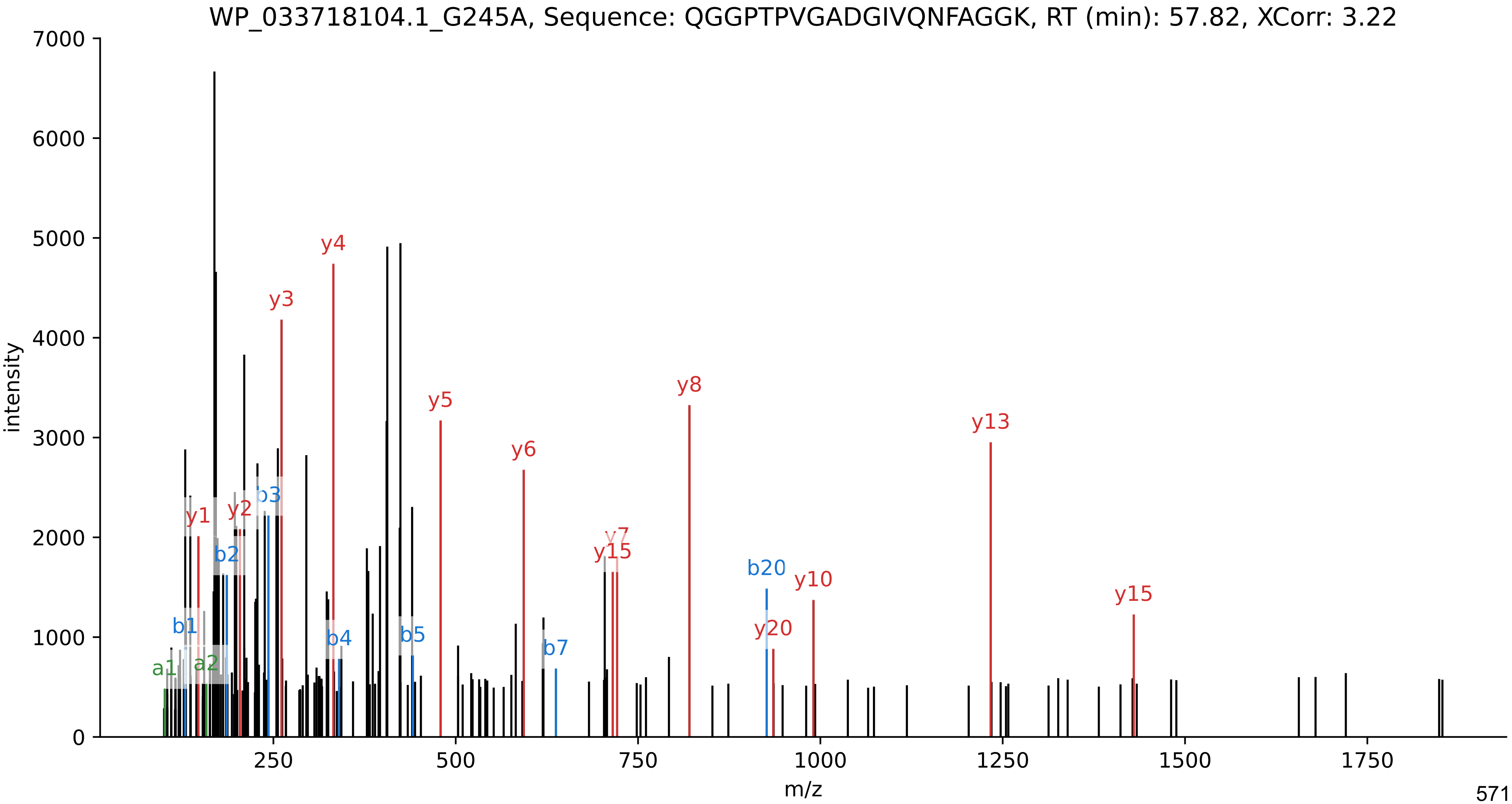
WP_033718104.1_G245A, Sequence: QGGPTPVGADGIVQNFAGGK, RT (min): 57.82, XCorr: 3.22
intensity
m/z
a1
a2
b1
b2
b3
b4
b5
b7
b20
y1
y2
y3
y4
y5
y6
y7
y15
y8
y10
y20
y13
y15

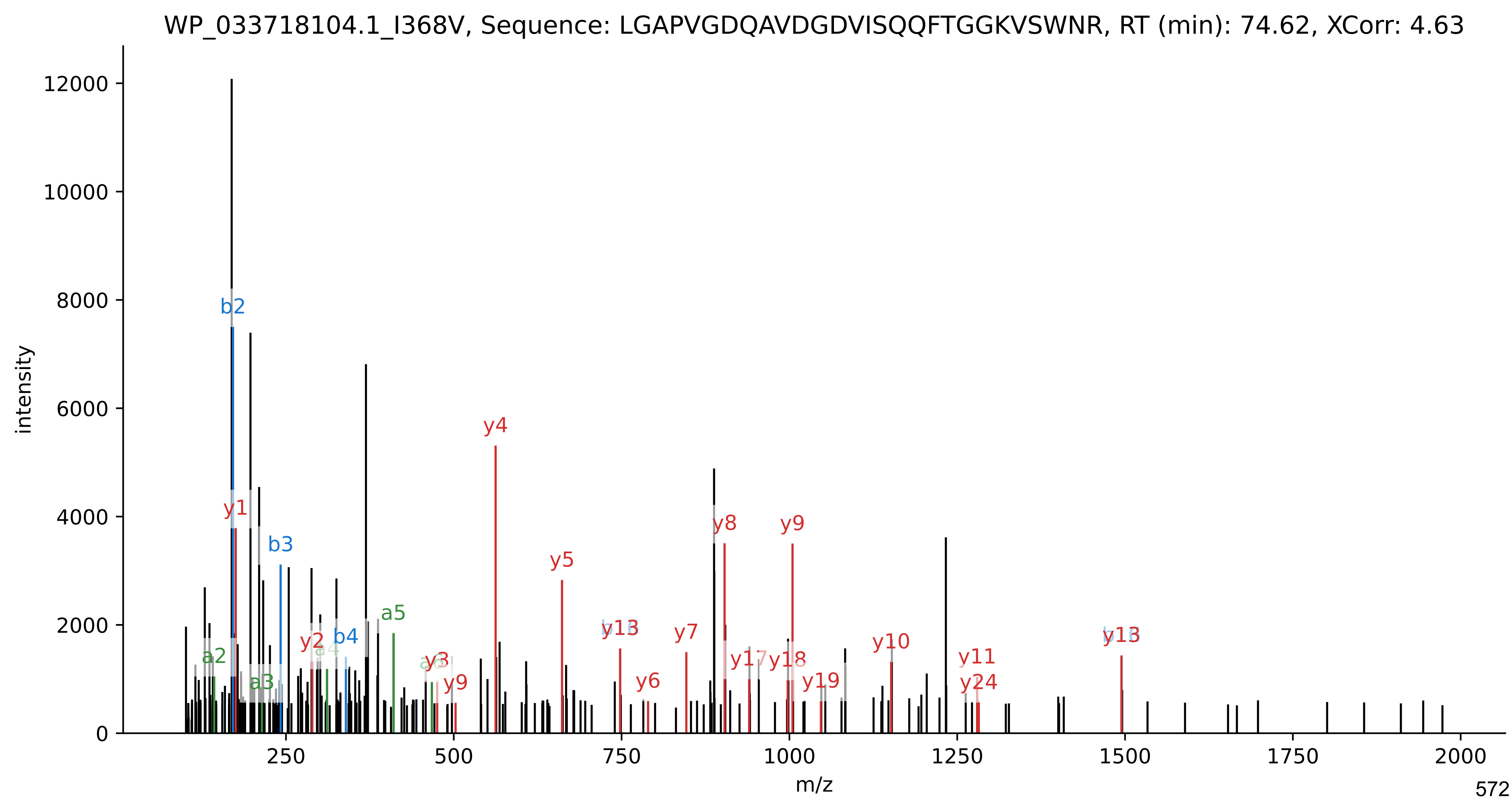
WP_033718104.1_I368V, Sequence: LGAPVGDQAVDGDVISQQFTGGKVSWNR, RT (min): 74.62, XCorr: 4.63
intensity
m/z
b2
y1
b3
y4
y5
a5
b4
y2
a2
a3
y3
y9
y13
y6
y7
y8
y9
y17
y18
y19
y10
y11
y24
y13

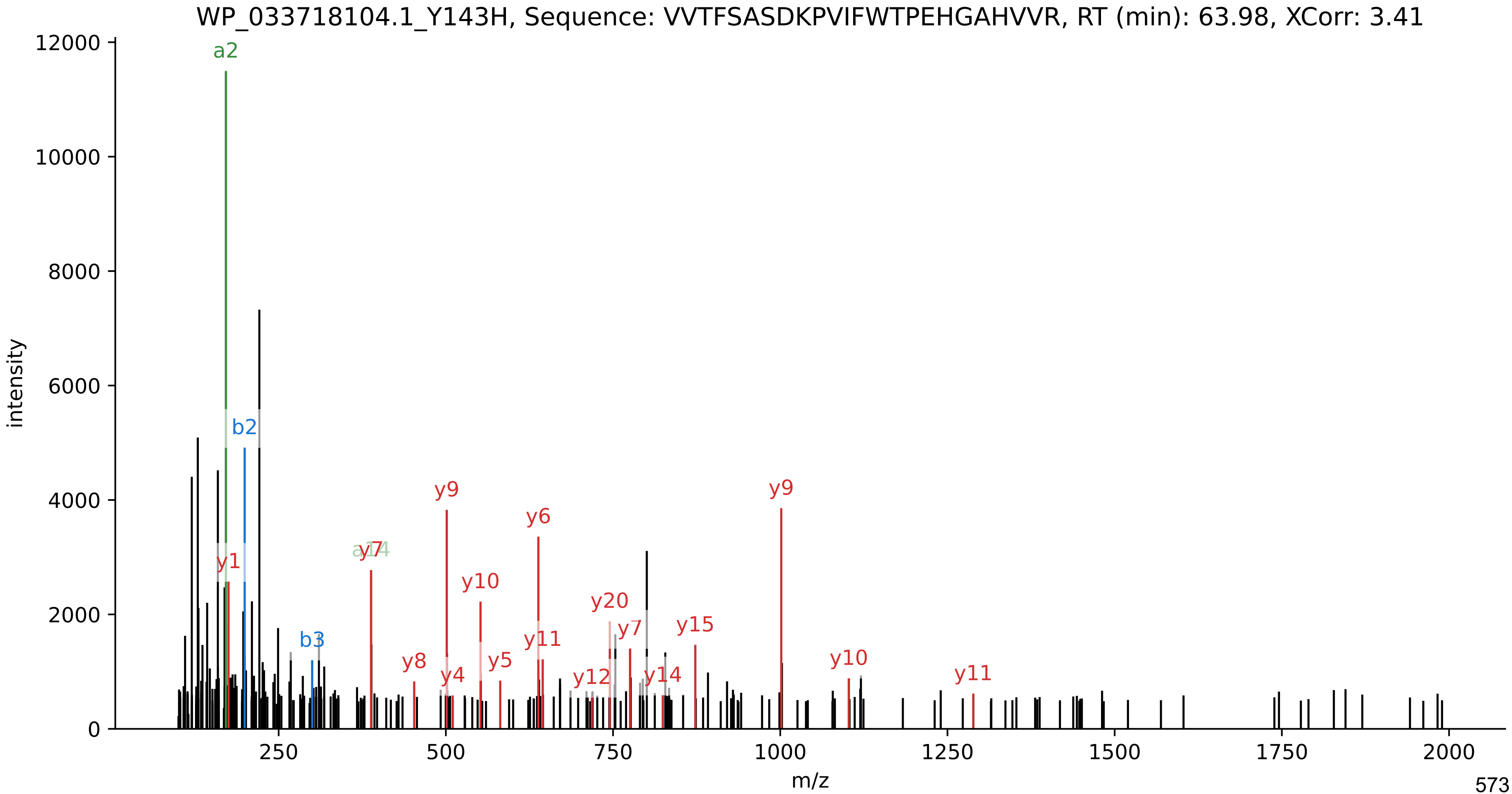
WP_033718104.1_Y143H, Sequence: VVTFSASDKPVIFWTPEHGAHVVR, RT (min): 63.98, XCorr: 3.41
intensity
m/z
0
2000
4000
6000
8000
10000
12000
250
500
750
1000
1250
1500
1750
2000
a2
b2
b3
y1
y9
y10
y6
y8
y4
y5
y11
y12
y20
y7
y14
y15
y9
y10
y11

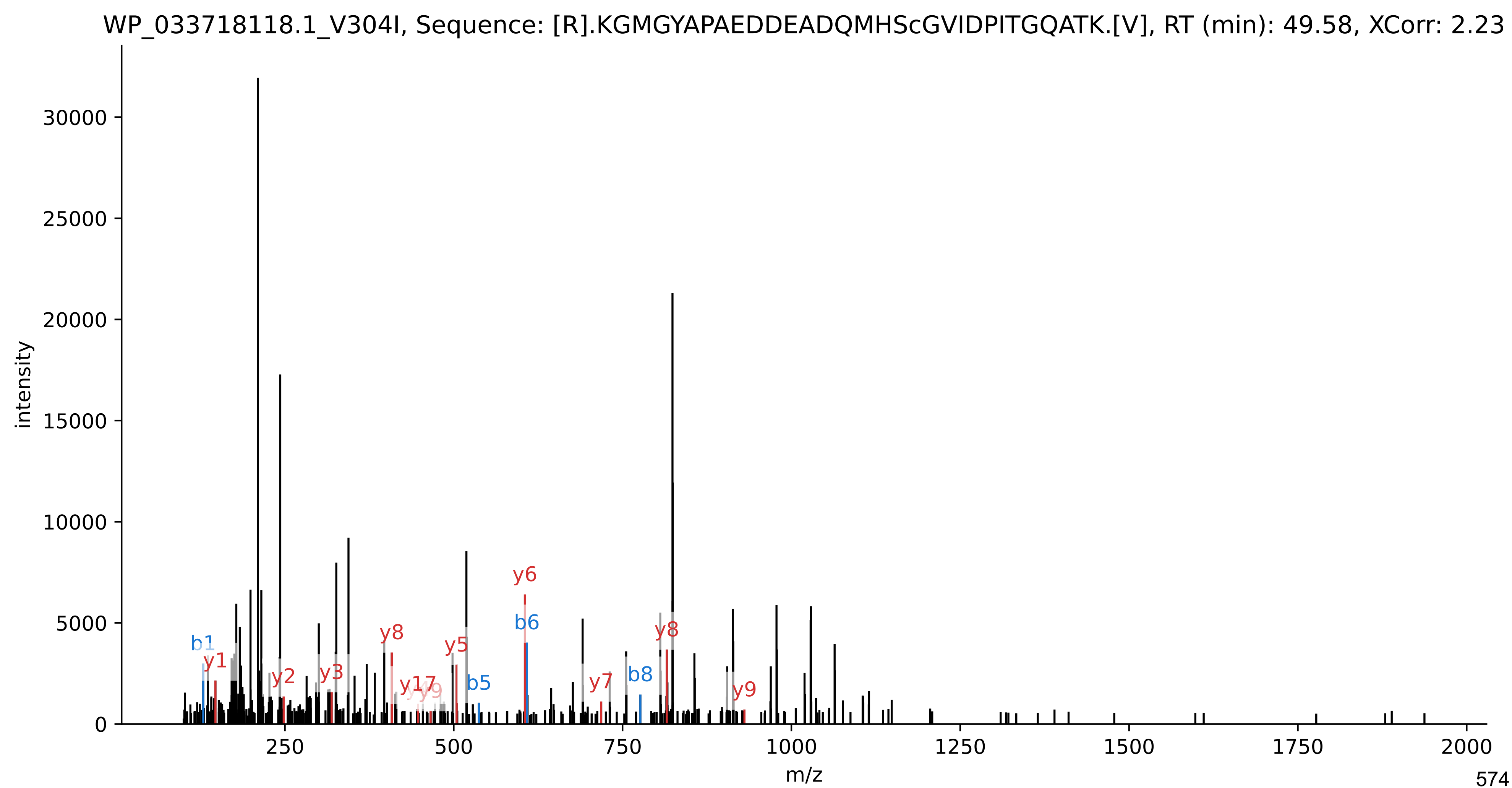
WP_033718118.1_V304I, Sequence: [R].KGMGYAPAEDDEADQMHScGVIDPITGQATK.[V], RT (min): 49.58, XCorr: 2.23
intensity
m/z
b1
y1
y2
y3
y8
y5
b5
y6
b6
y7
b8
y8
y9

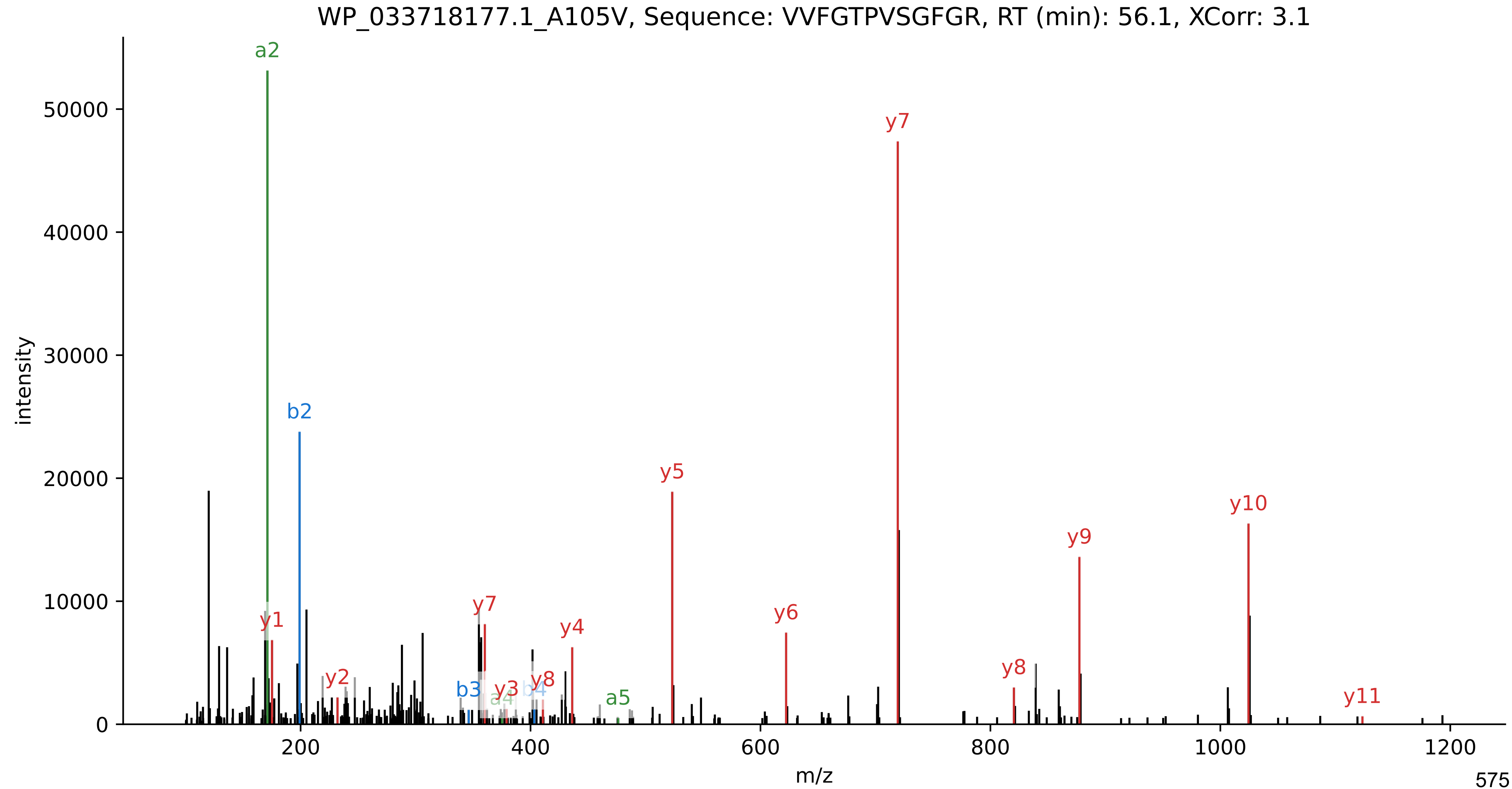
WP_033718177.1_A105V, Sequence: VVFGTPVSGFGR, RT (min): 56.1, XCorr: 3.1
intensity
m/z
a2
b2
y7
y5
y10
y9
y6
y4
y8
y11
y1
y2
b3
y3
a4
b4
a5
50000
40000
30000
20000
10000
0
200
400
600
800
1000
1200

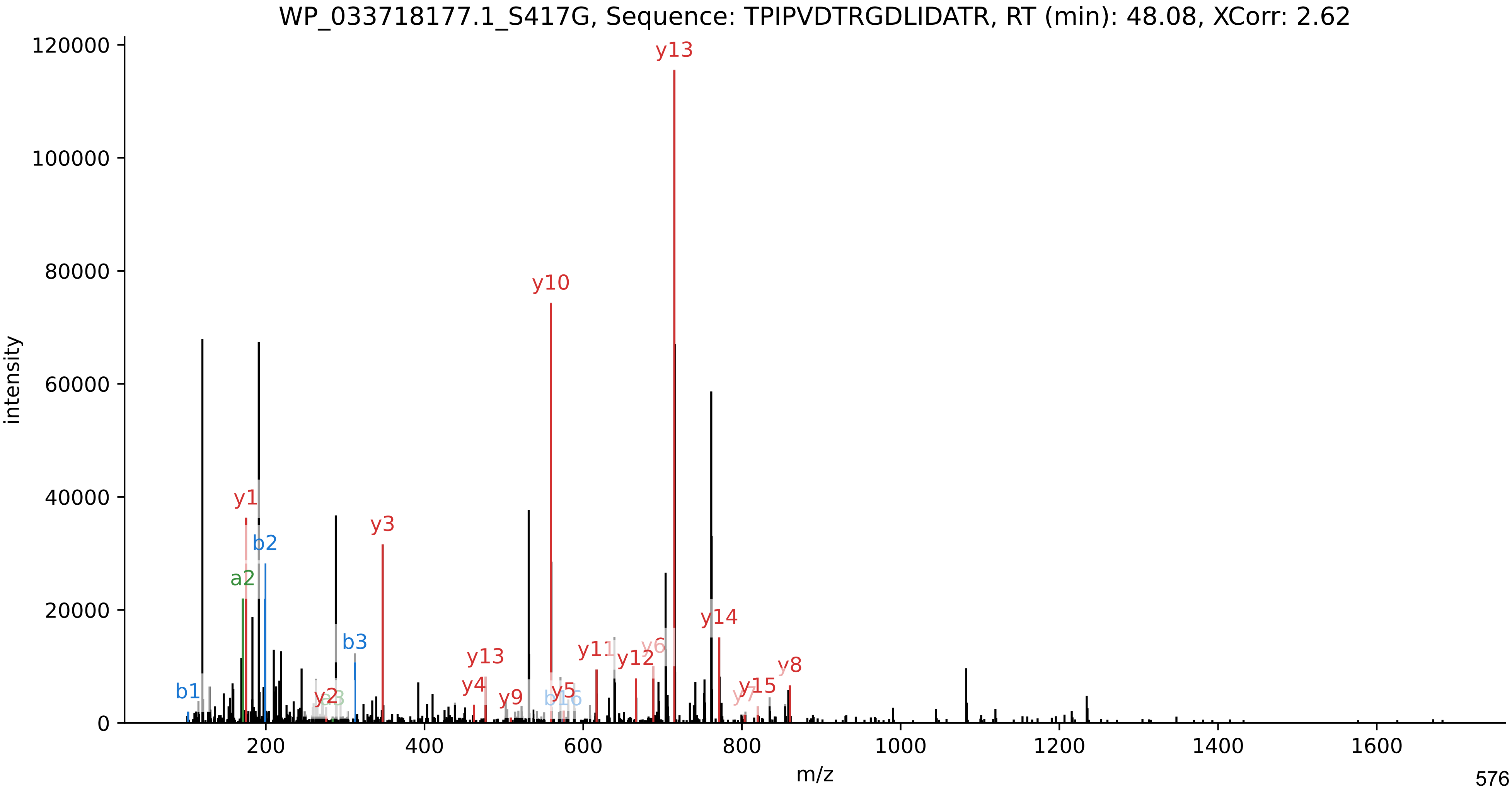
WP_033718177.1_S417G, Sequence: TPIPVDTRGDLIDATR, RT (min): 48.08, XCorr: 2.62
intensity
m/z
y13
y10
y1
b2
a2
y3
b3
y14
y13
y11
y12
y6
y8
y4
y15
b1
y2
y9
y5
y7
0
20000
40000
60000
80000
100000
120000
200
400
600
800
1000
1200
1400
1600

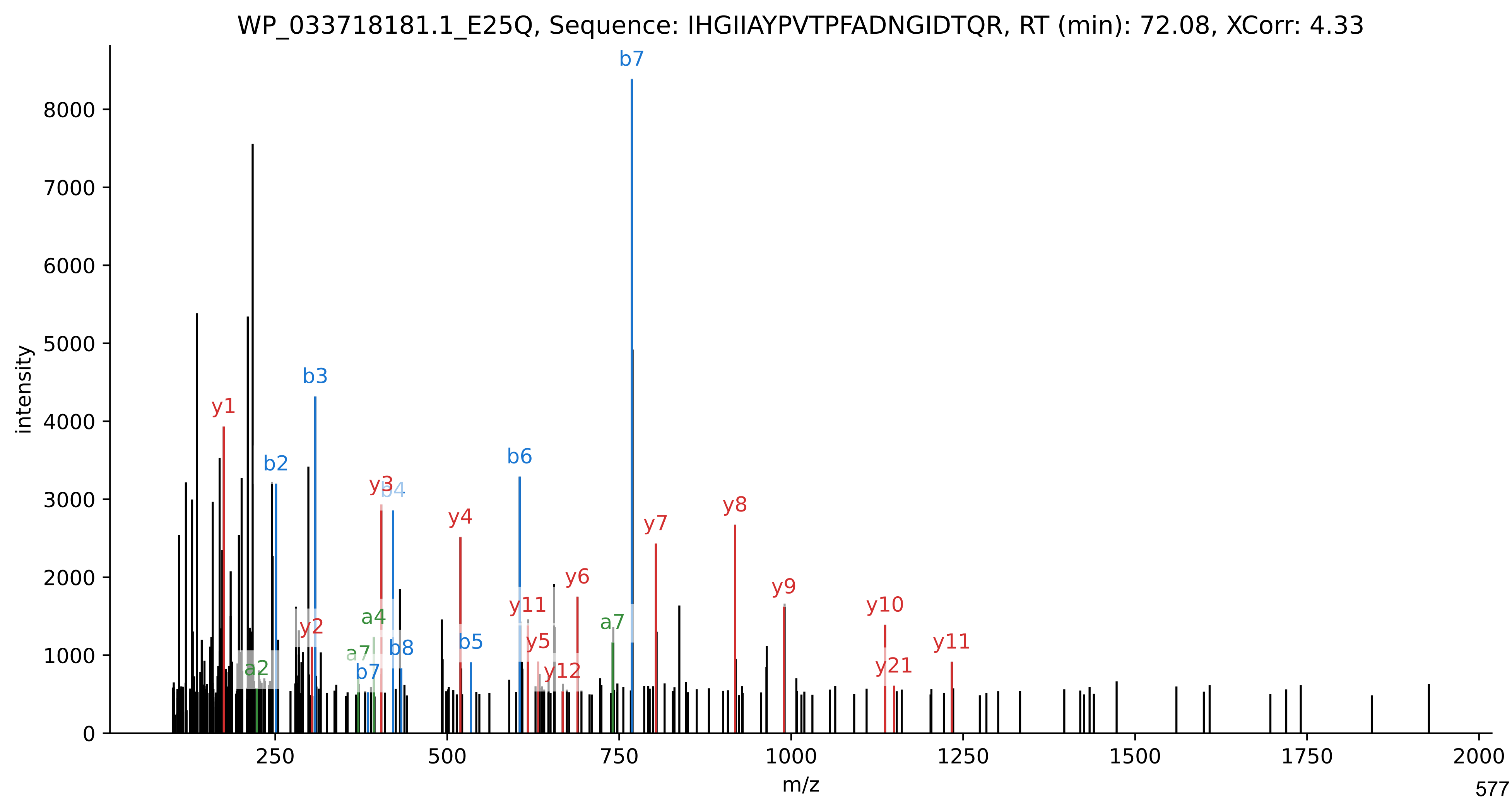
WP_033718181.1_E25Q, Sequence: IHGIIAYPVTPFADNGIDTQR, RT (min): 72.08, XCorr: 4.33
intensity
m/z
0
1000
2000
3000
4000
5000
6000
7000
8000
250
500
750
1000
1250
1500
1750
2000
b7
b3
y1
b2
y3
b4
y4
b6
y7
y8
y6
y9
y11
y2
a4
a7
b5
y5
y10
y11
a7
b8
a2
b7
y12
y21

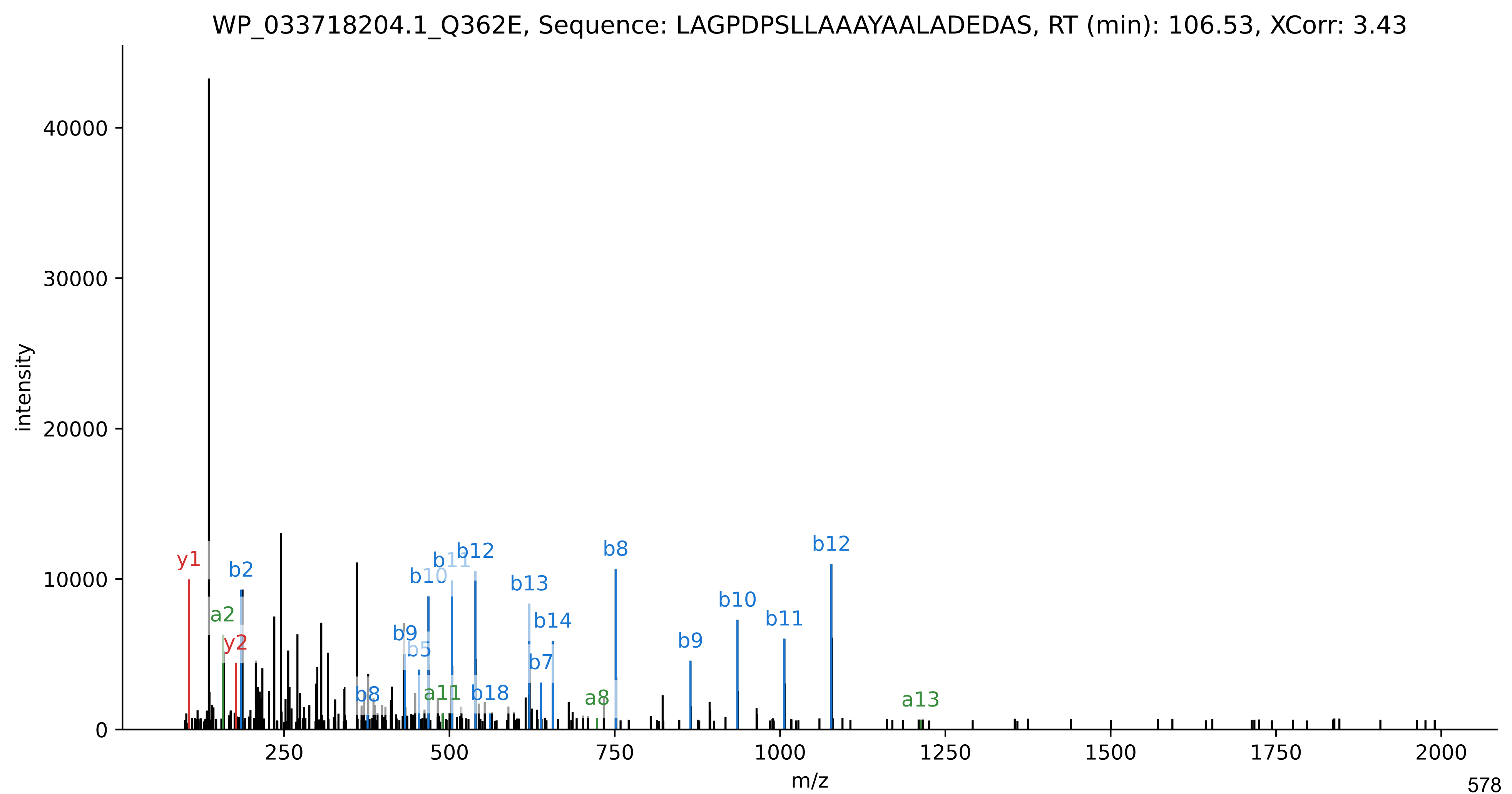

WP_033718204.1_Q362E, Sequence: LAGPDPSLLAAAYAALADEDAS, RT (min): 106.53, XCorr: 3.43
intensity
m/z
0
10000
20000
30000
40000
250
500
750
1000
1250
1500
1750
2000
y1
y2
a2
b2
b8
b9
b5
b10
a11
b11
b12
b18
b13
b14
b7
a8
b8
b9
b10
b11
b12
a13

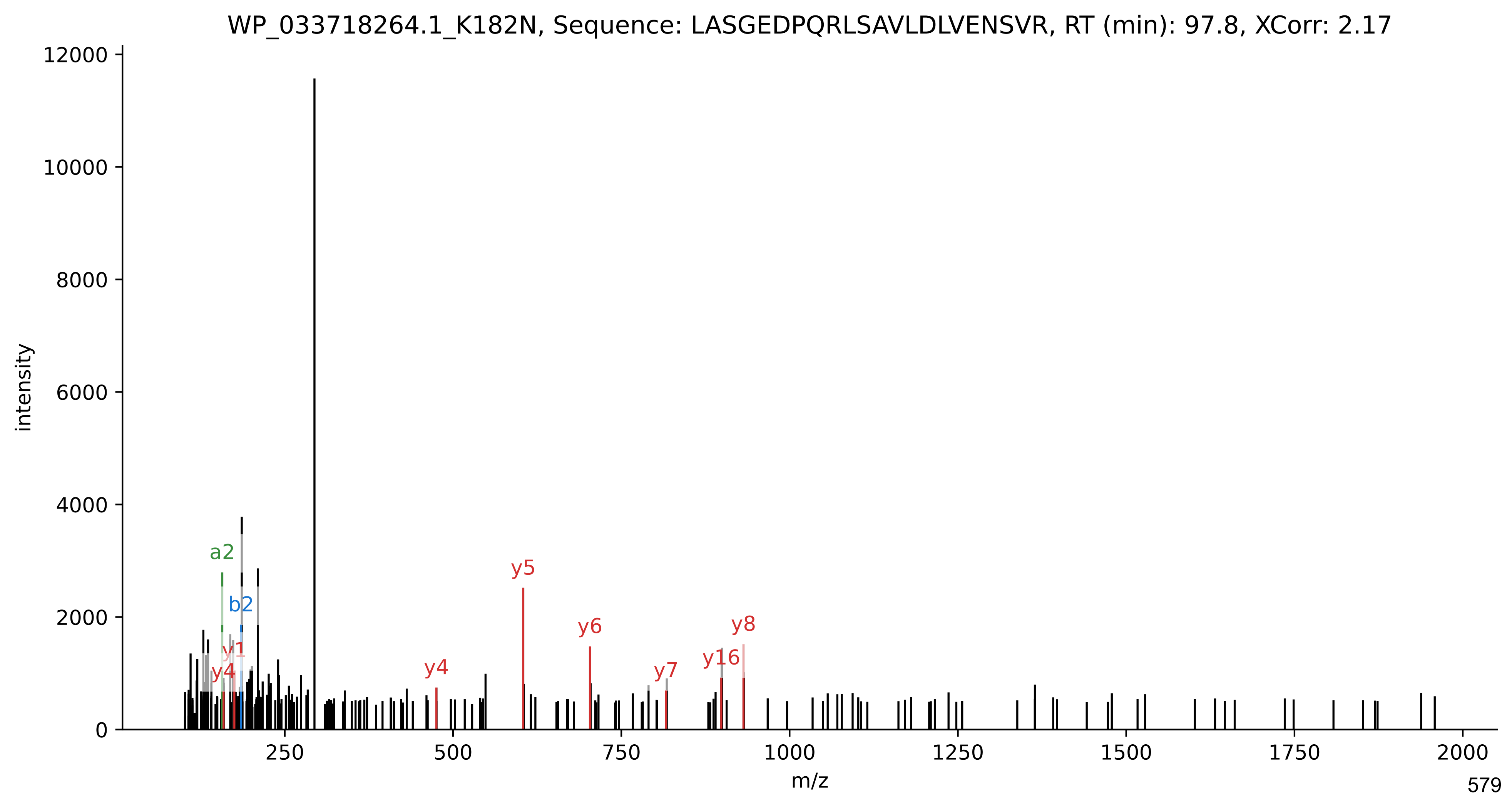

WP_033718264.1_K182N, Sequence: LASGEDPQRLSAVLDLVENSVR, RT (min): 97.8, XCorr: 2.17
intensity
m/z
a2
b2
y1
y4
y4
y5
y6
y7
y16
y8

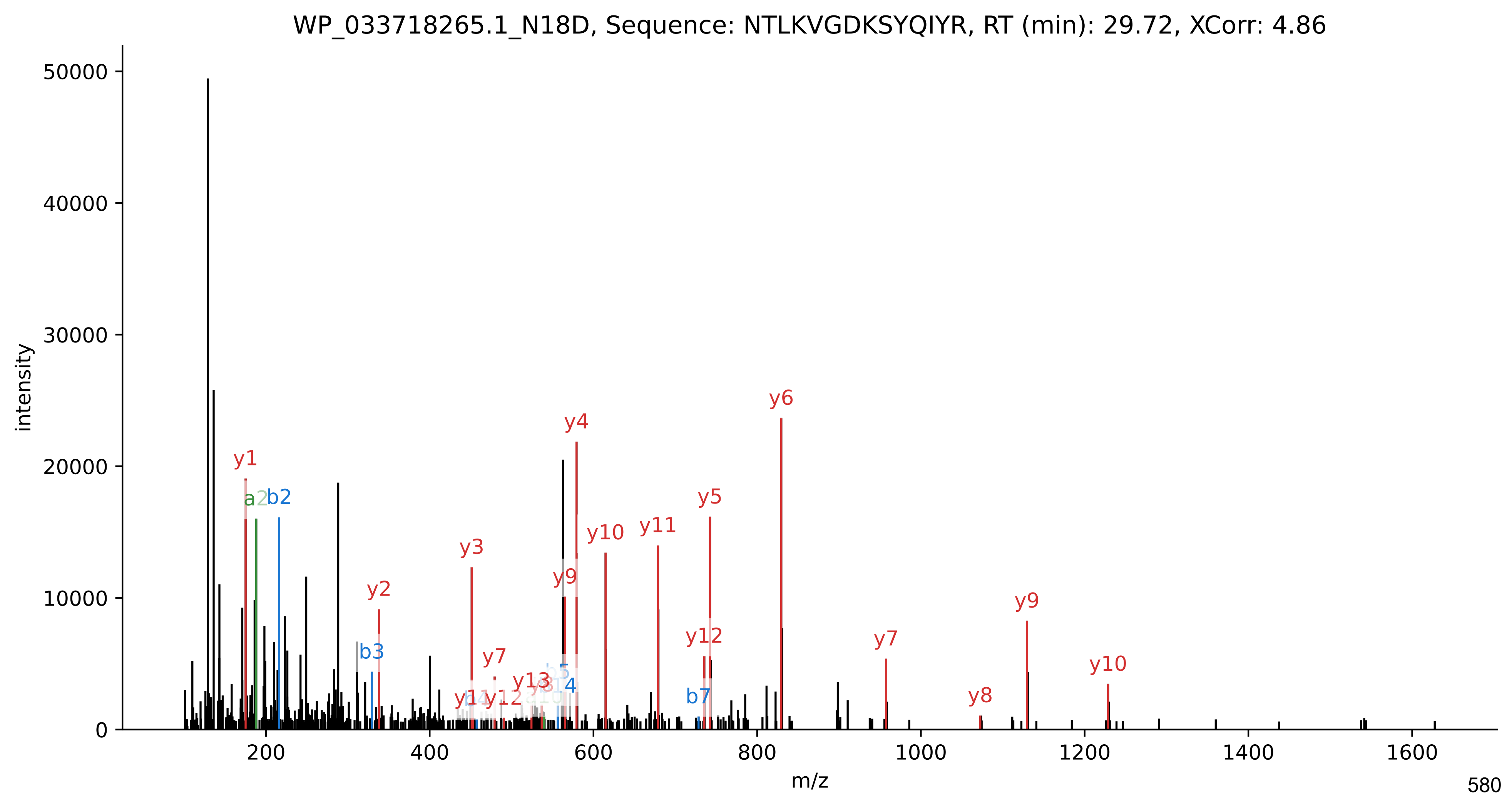

WP_033718265.1_N18D, Sequence: NTLKVGDKSYQIYR, RT (min): 29.72, XCorr: 4.86
intensity
m/z
50000
40000
30000
20000
10000
0
200
400
600
800
1000
1200
1400
1600
y1
a2
b2
b3
y2
y3
y7
y13
y12
b5
y4
y9
y10
y11
y5
y12
b7
y6
y7
y8
y9
y10

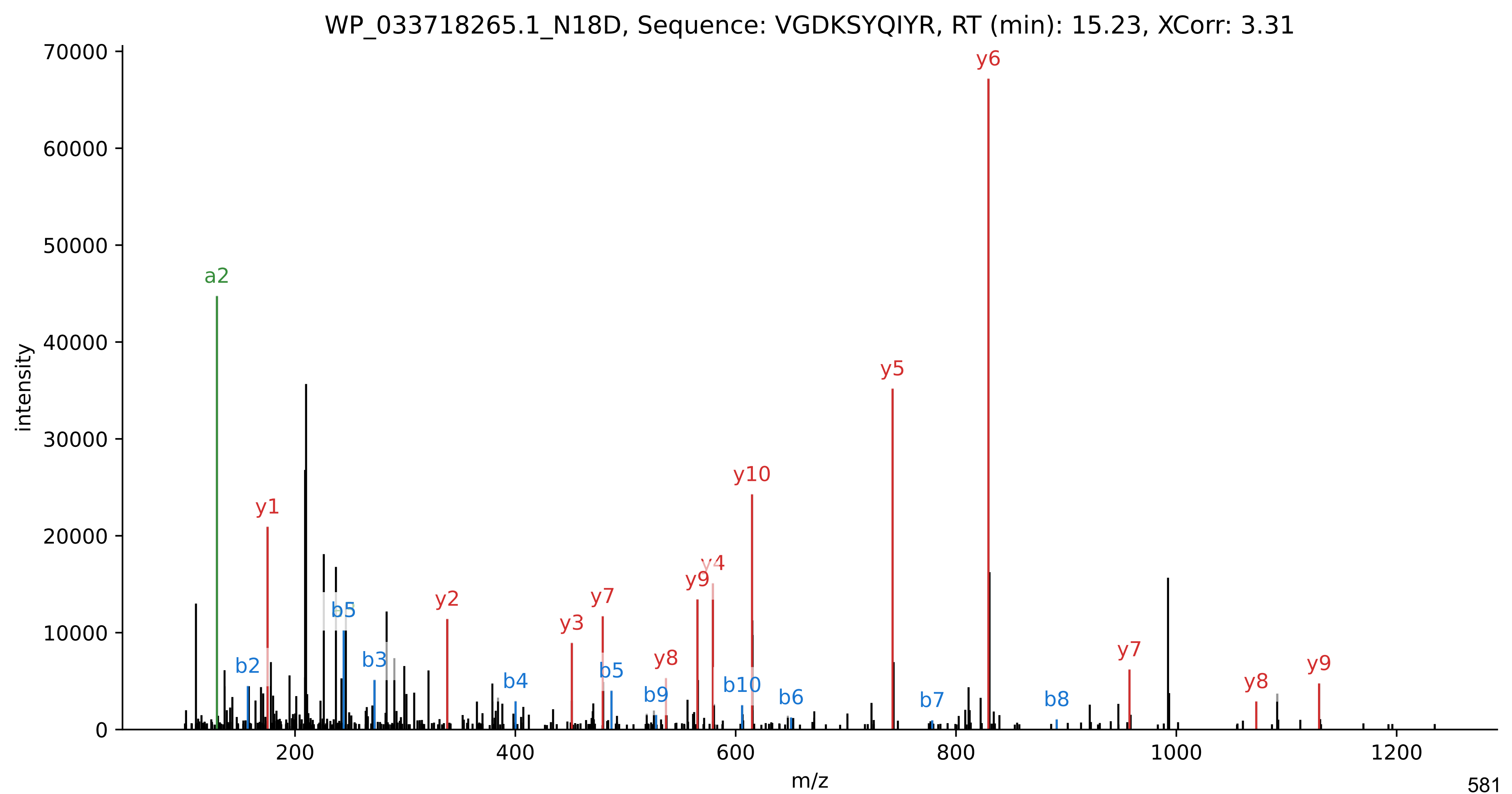

WP_033718265.1_N18D, Sequence: VGDKSYQIYR, RT (min): 15.23, XCorr: 3.31
intensity
m/z
y6
a2
y5
y10
y1
y9
y4
y2
y7
y3
b5
b2
b3
b4
b5
y8
b9
b10
b6
b7
b8
y7
y8
y9

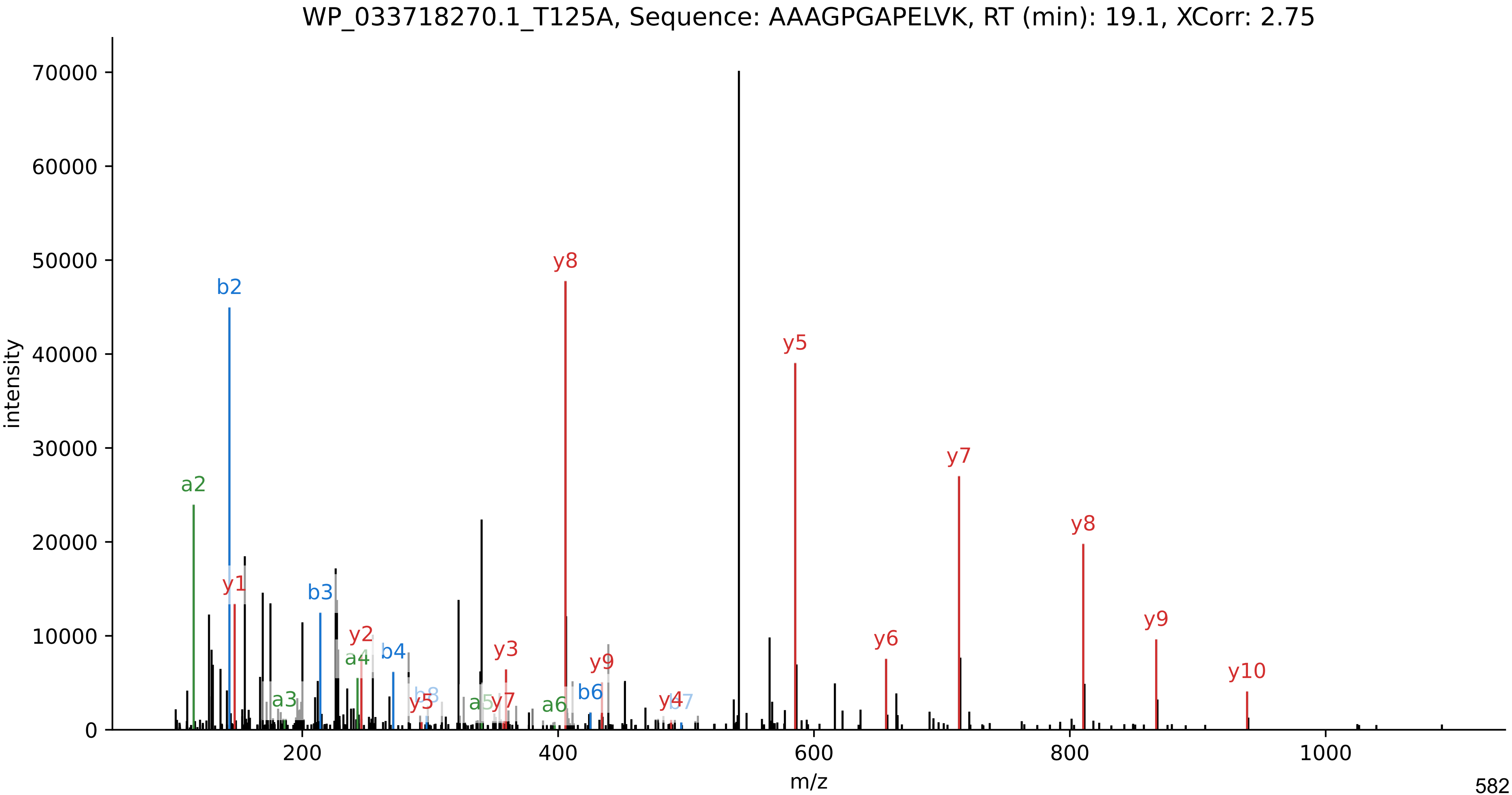
WP_033718270.1_T125A, Sequence: AAAGPGAPELVK, RT (min): 19.1, XCorr: 2.75
intensity
m/z
a2
b2
y1
b3
y2
a4
b4
a3
y5
a5
y7
y3
y8
a6
b6
y9
y4
b7
y5
y6
y7
y8
y9
y10

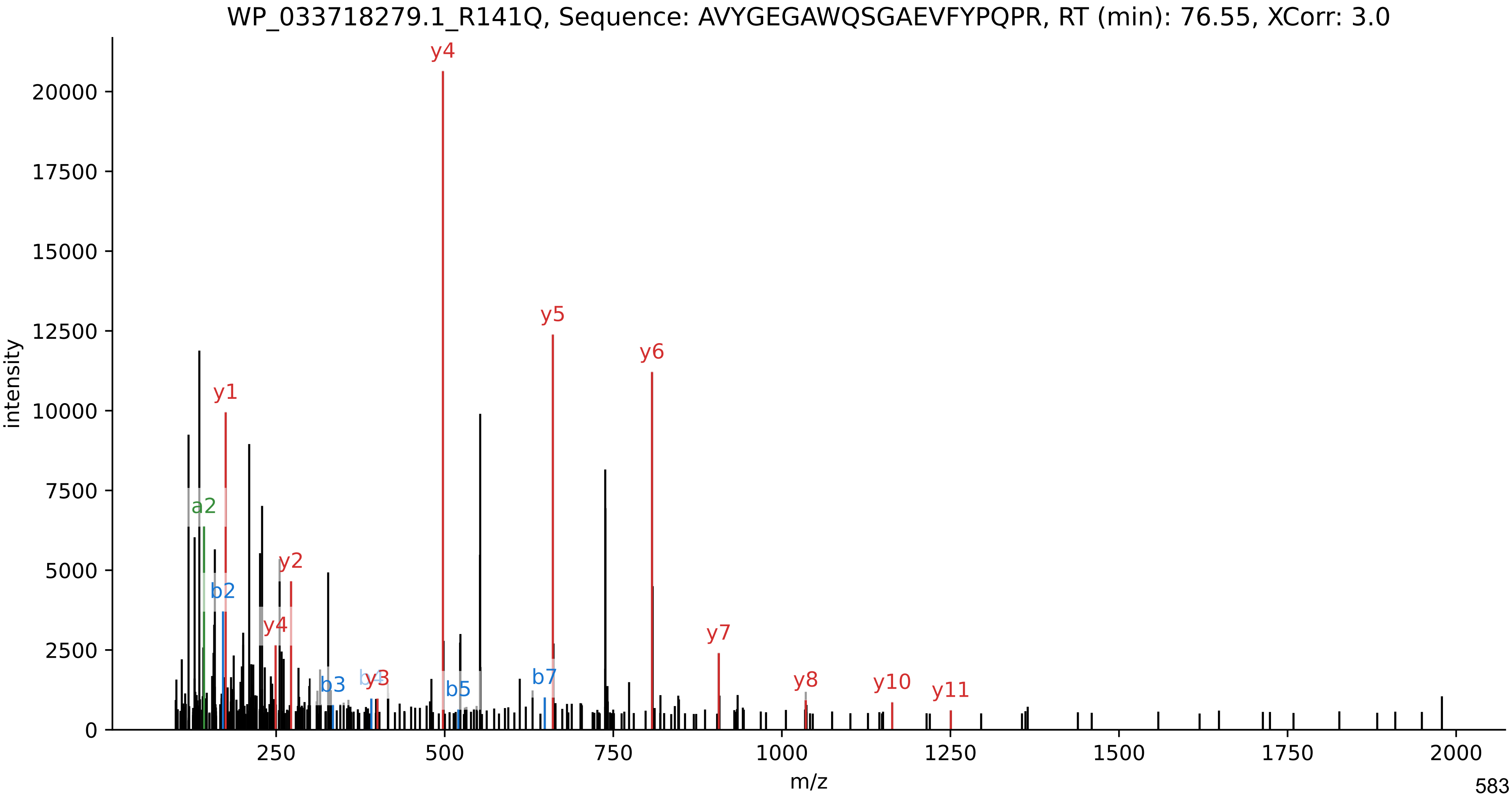

WP_033718279.1_R141Q, Sequence: AVYGEGAWQSGAEVFYPQPR, RT (min): 76.55, XCorr: 3.0
intensity
m/z
y4
y5
y6
y1
a2
y2
b2
y4
y7
b3
y3
b5
b7
y8
y10
y11

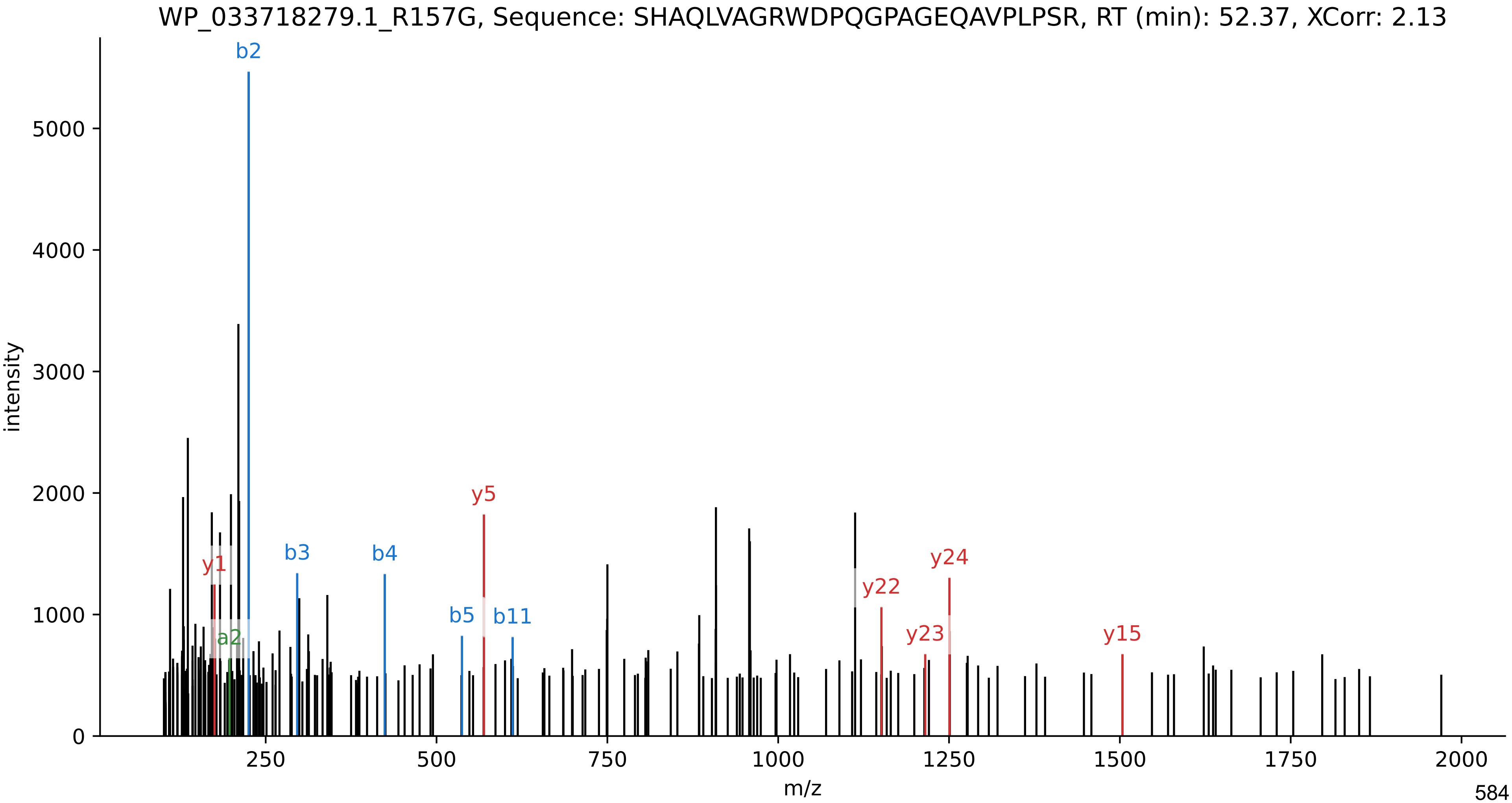

WP_033718279.1_R157G, Sequence: SHAQLVAGRWDPQGPAGEQAVPLPSR, RT (min): 52.37, XCorr: 2.13
b2
y1
a2
b3
b4
b5
y5
b11
y22
y23
y24
y15
intensity
m/z
0
1000
2000
3000
4000
5000
250
500
750
1000
1250
1500
1750
2000

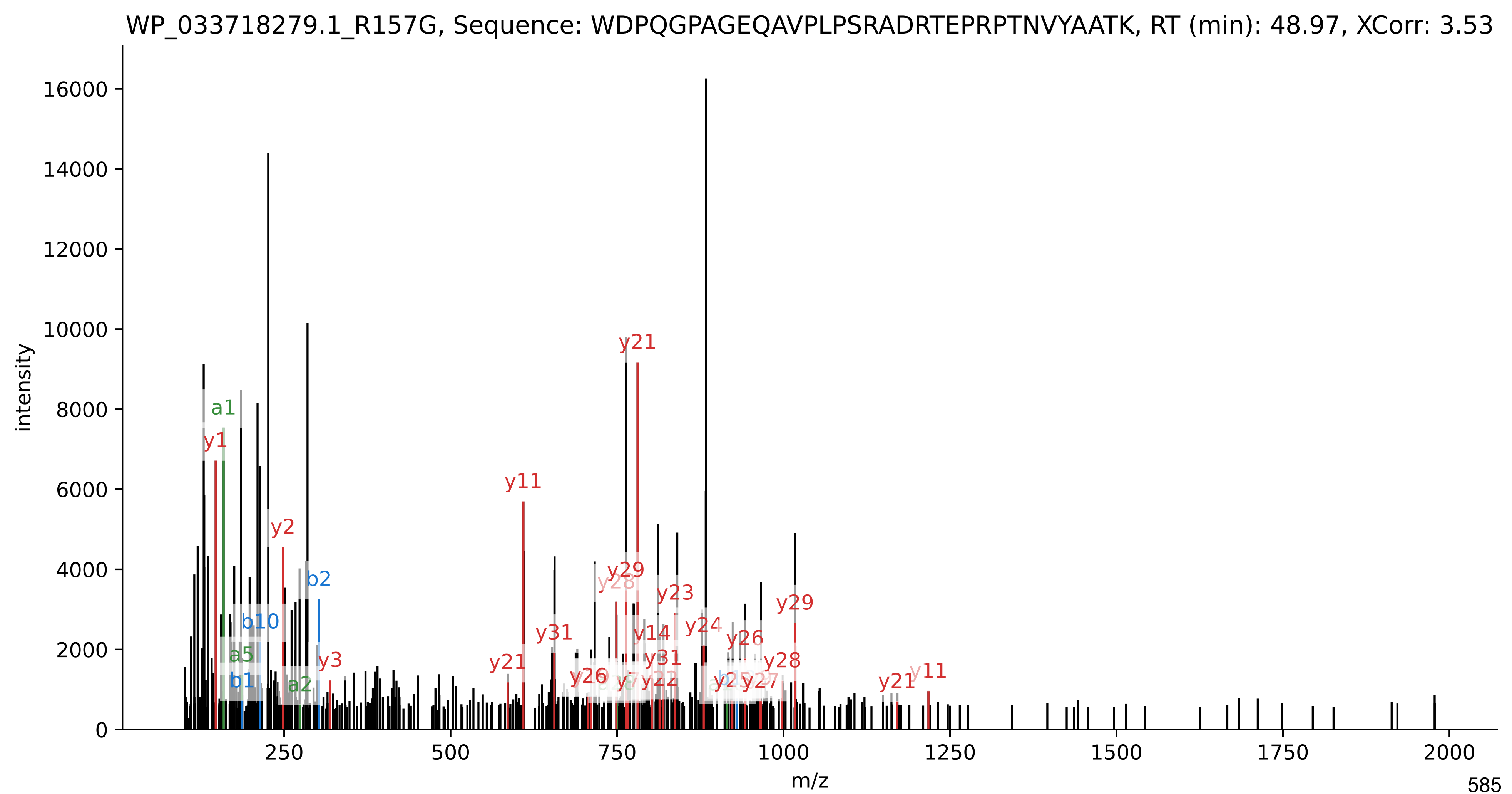

WP_033718279.1_R157G, Sequence: WDPQGPAGEQAVPLPSRADRTEPRPTNVYAATK, RT (min): 48.97, XCorr: 3.53
intensity
m/z
y21
a1
y1
y11
y2
b2
b10
y29
y23
y29
y31
y14
y24
y26
a5
y3
y31
y28
y21
b1
a2
y26
y22
y27
y21
y11

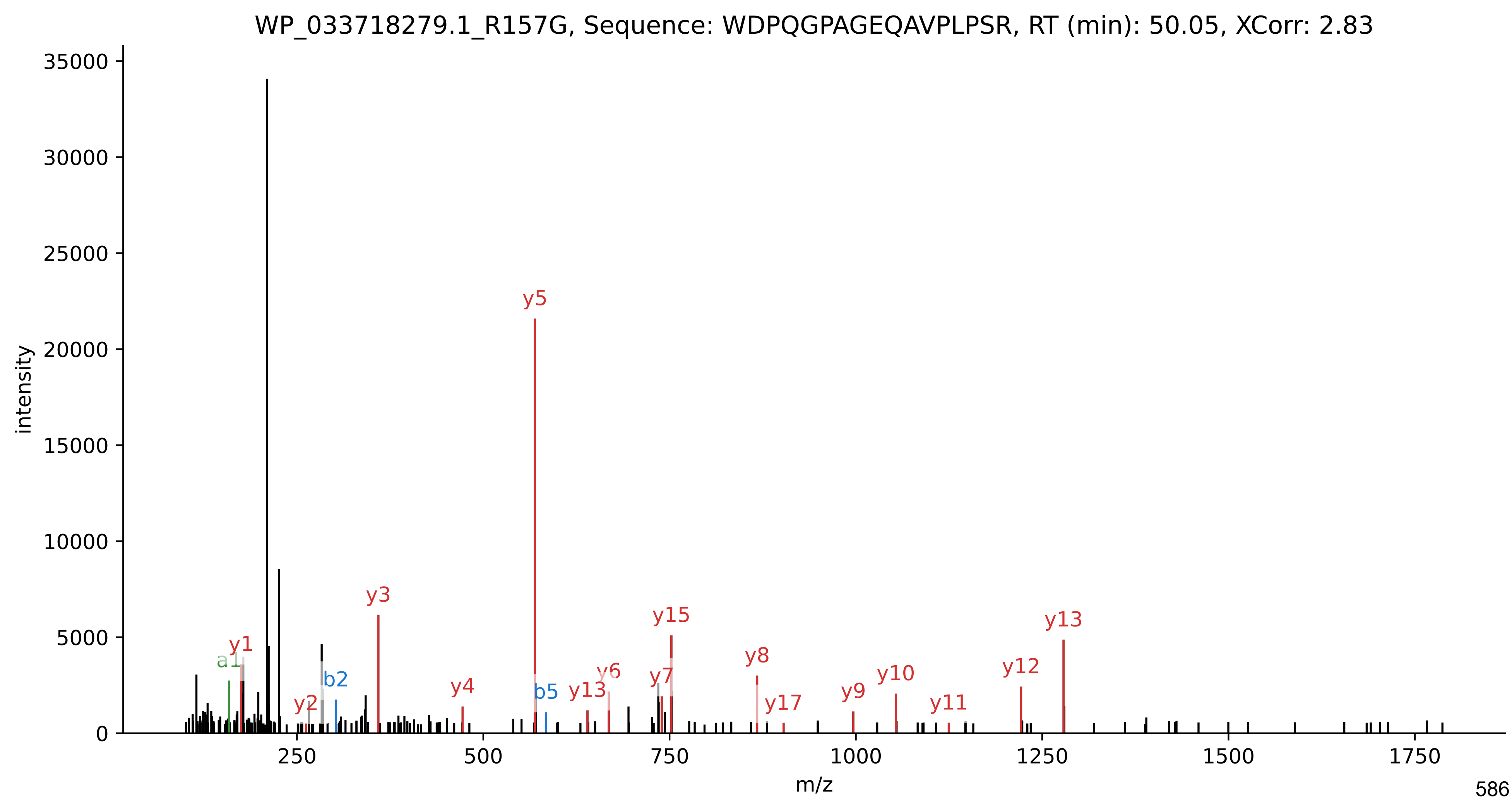

WP_033718279.1_R157G, Sequence: WDPQGPAGEQAVPLPSR, RT (min): 50.05, XCorr: 2.83
intensity
m/z
a1
y1
y2
b2
y3
y4
y5
b5
y13
y6
y7
y15
y8
y17
y9
y10
y11
y12
y13

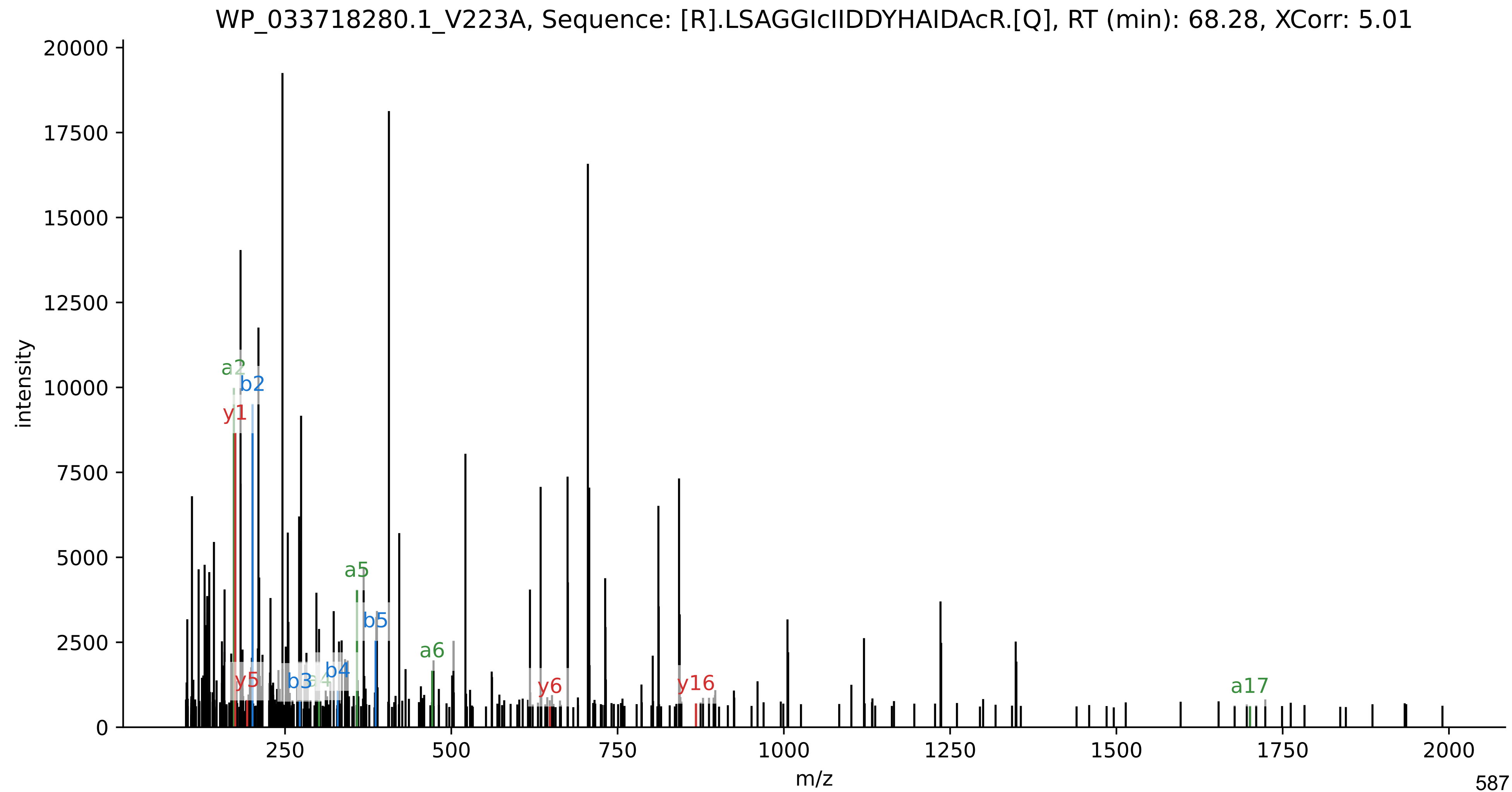

WP_033718280.1_V223A, Sequence: [R].LSAGGIcIIDDYHAIDAcR.[Q], RT (min): 68.28, XCorr: 5.01
intensity
m/z
a2
b2
y1
y5
b3
a4
b4
a5
b5
a6
y6
y16
a17

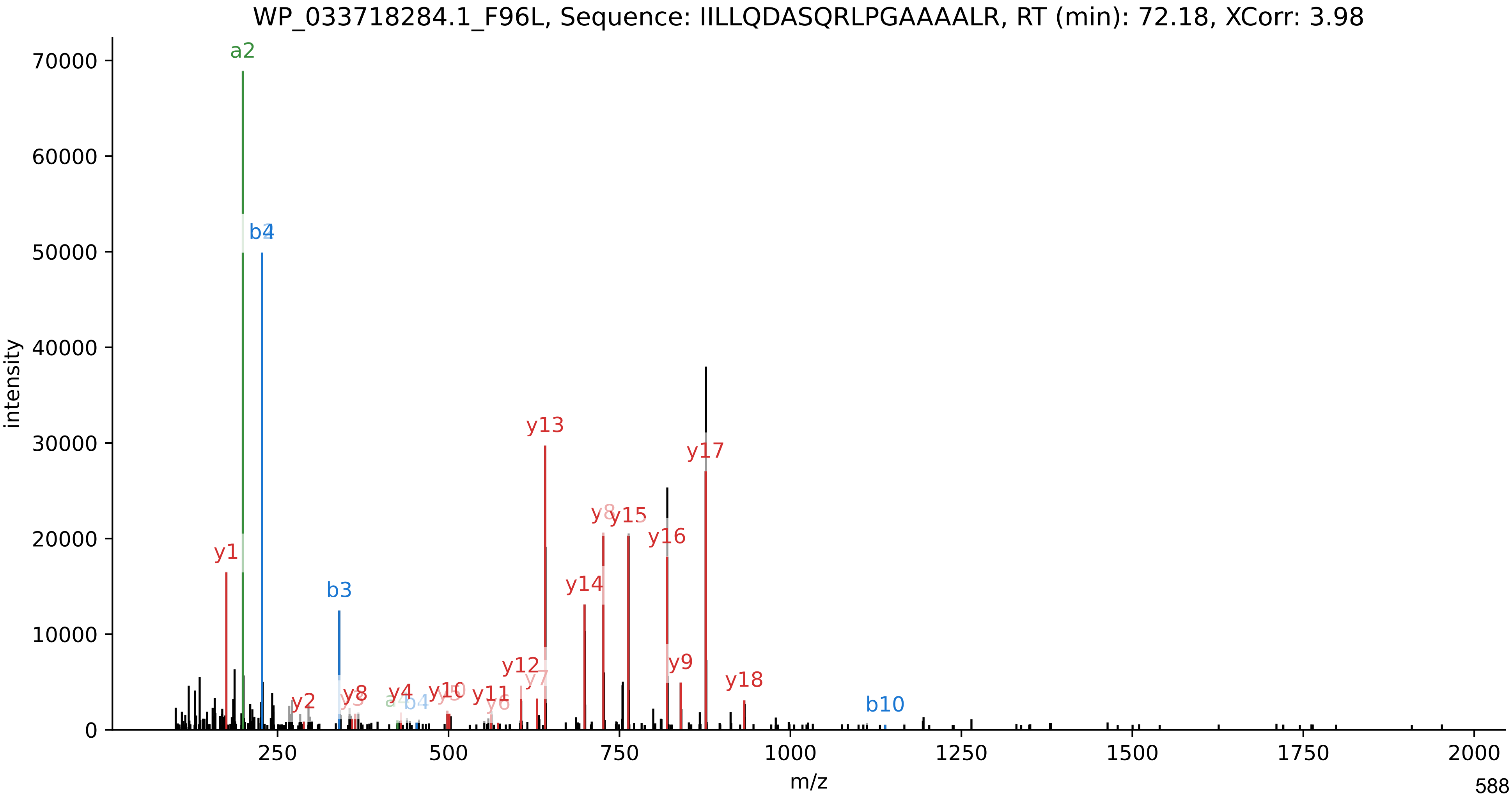
WP_033718284.1_F96L, Sequence: IILLQDASQRLPGAAAALR, RT (min): 72.18, XCorr: 3.98
intensity
m/z
0
10000
20000
30000
40000
50000
60000
70000
250
500
750
1000
1250
1500
1750
2000
a2
b4
b3
y1
y2
y8
y4
y10
y11
y12
y13
y14
y15
y16
y17
y9
y18
b10

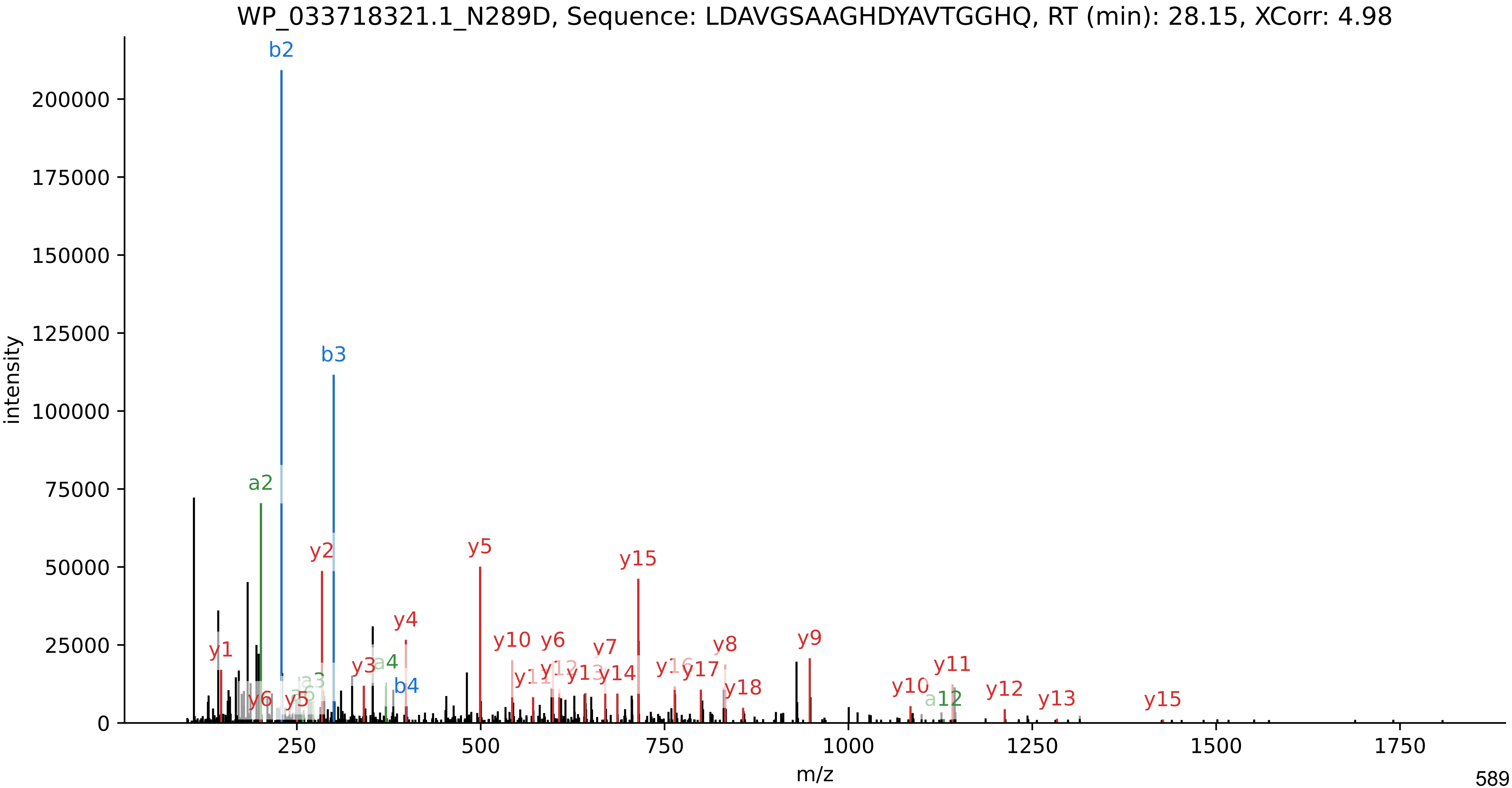
WP_033718321.1_N289D, Sequence: LDAVGSAAGHDYAVTGGHQ, RT (min): 28.15, XCorr: 4.98
intensity
m/z
b2
b3
a2
y2
y5
y15
y4
y1
y10
y6
y7
y8
y9
y3
a4
b4
a3
y11
y12
y13
y14
y16
y17
y18
y6
y5
y10
a12
y11
y12
y13
y15
0
25000
50000
75000
100000
125000
150000
175000
200000
250
500
750
1000
1250
1500
1750

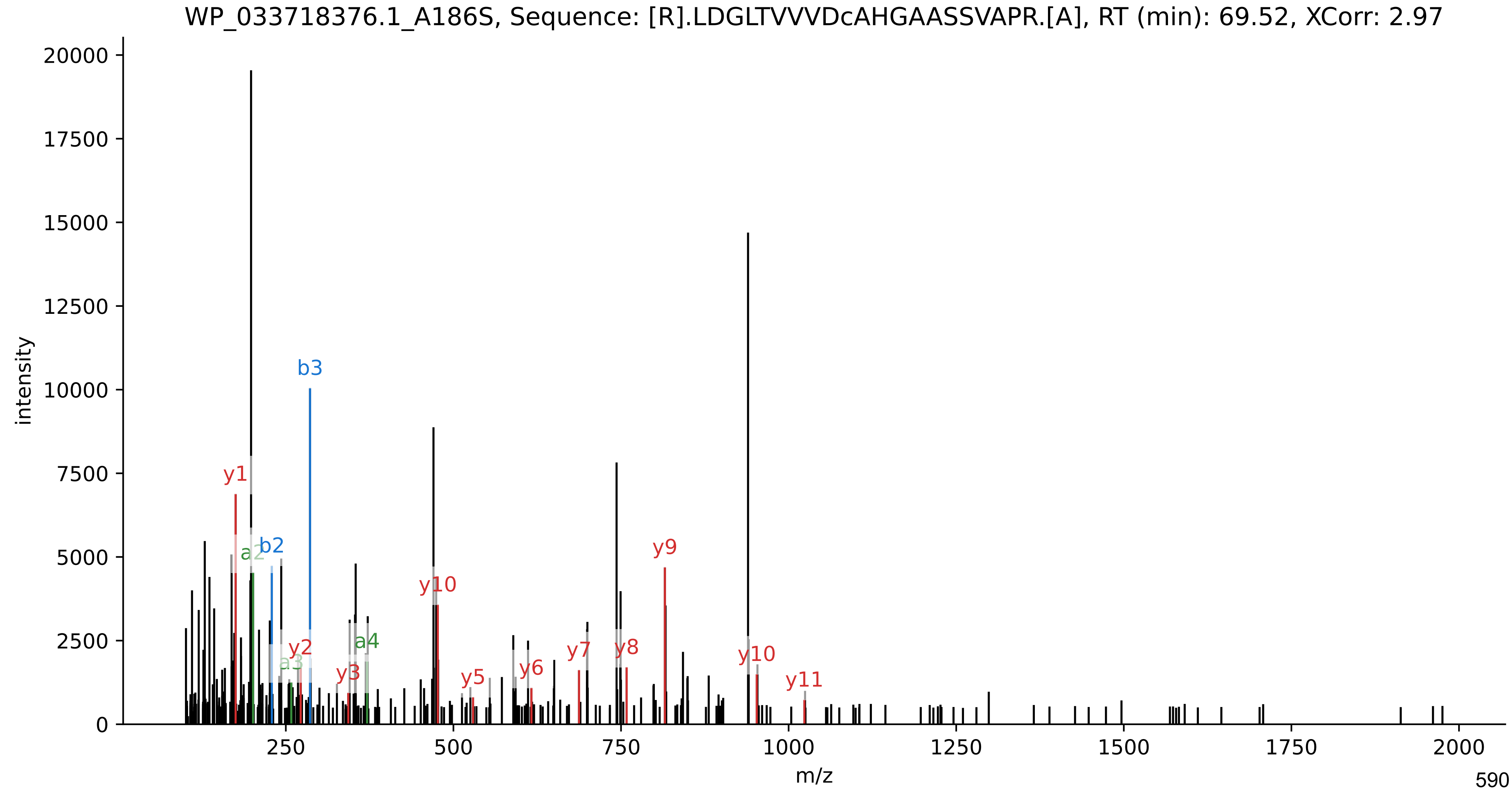
WP_033718376.1_A186S, Sequence: [R].LDGLTVVVDcAHGAASSVAPR.[A], RT (min): 69.52, XCorr: 2.97
intensity
m/z
0
2500
5000
7500
10000
12500
15000
17500
20000
250
500
750
1000
1250
1500
1750
2000
y1
a2
b2
b3
a3
y2
y3
a4
y10
y5
y6
y7
y8
y9
y10
y11

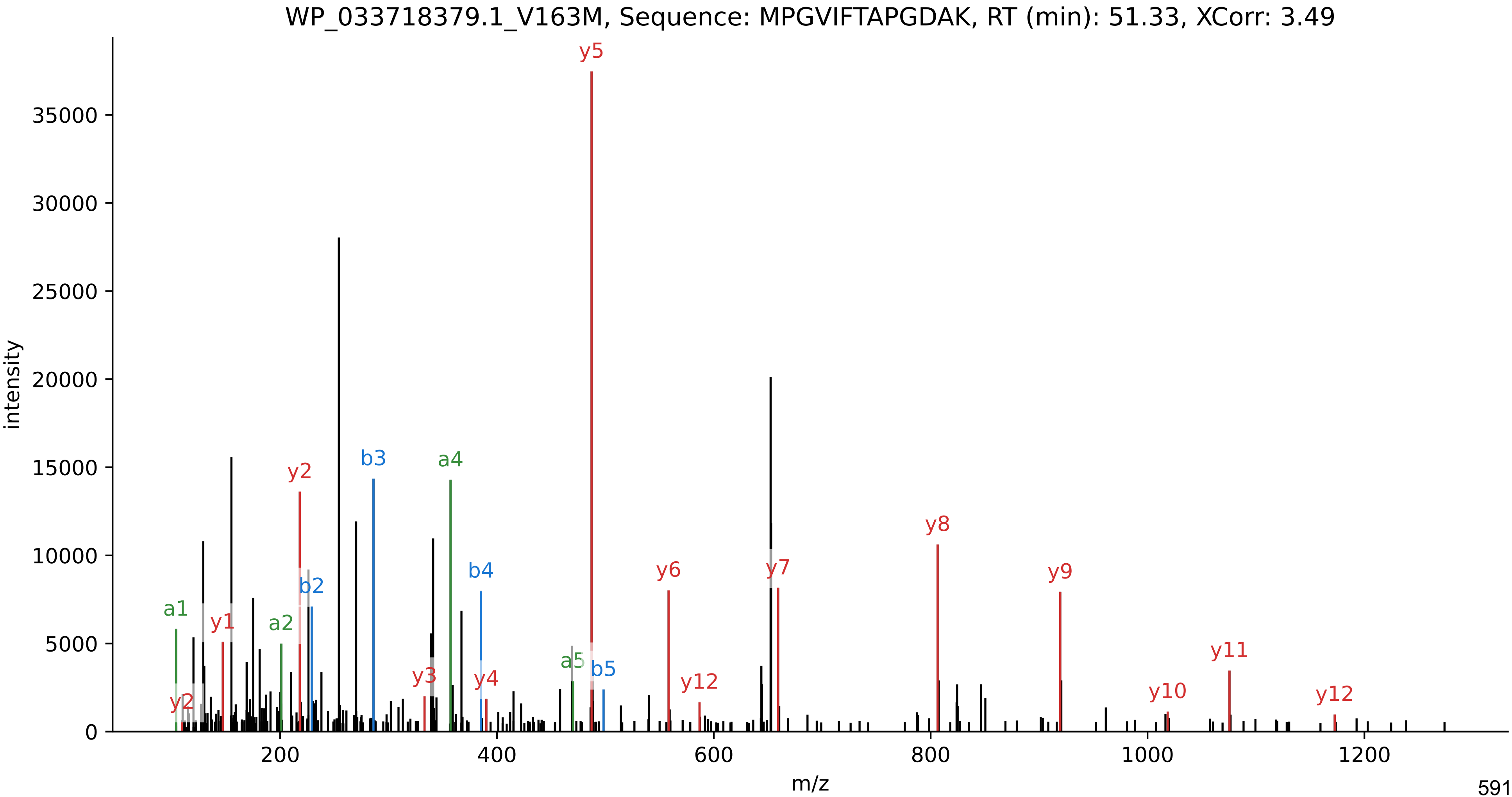
WP_033718379.1_V163M, Sequence: MPGVIFTAPGDAK, RT (min): 51.33, XCorr: 3.49
intensity
m/z
y5
y2
b3
a4
b4
b2
a1
y1
a2
y6
y7
y8
y9
y3
y4
a5
b5
y12
y2
y10
y11
y12
0
5000
10000
15000
20000
25000
30000
35000
200
400
600
800
1000
1200

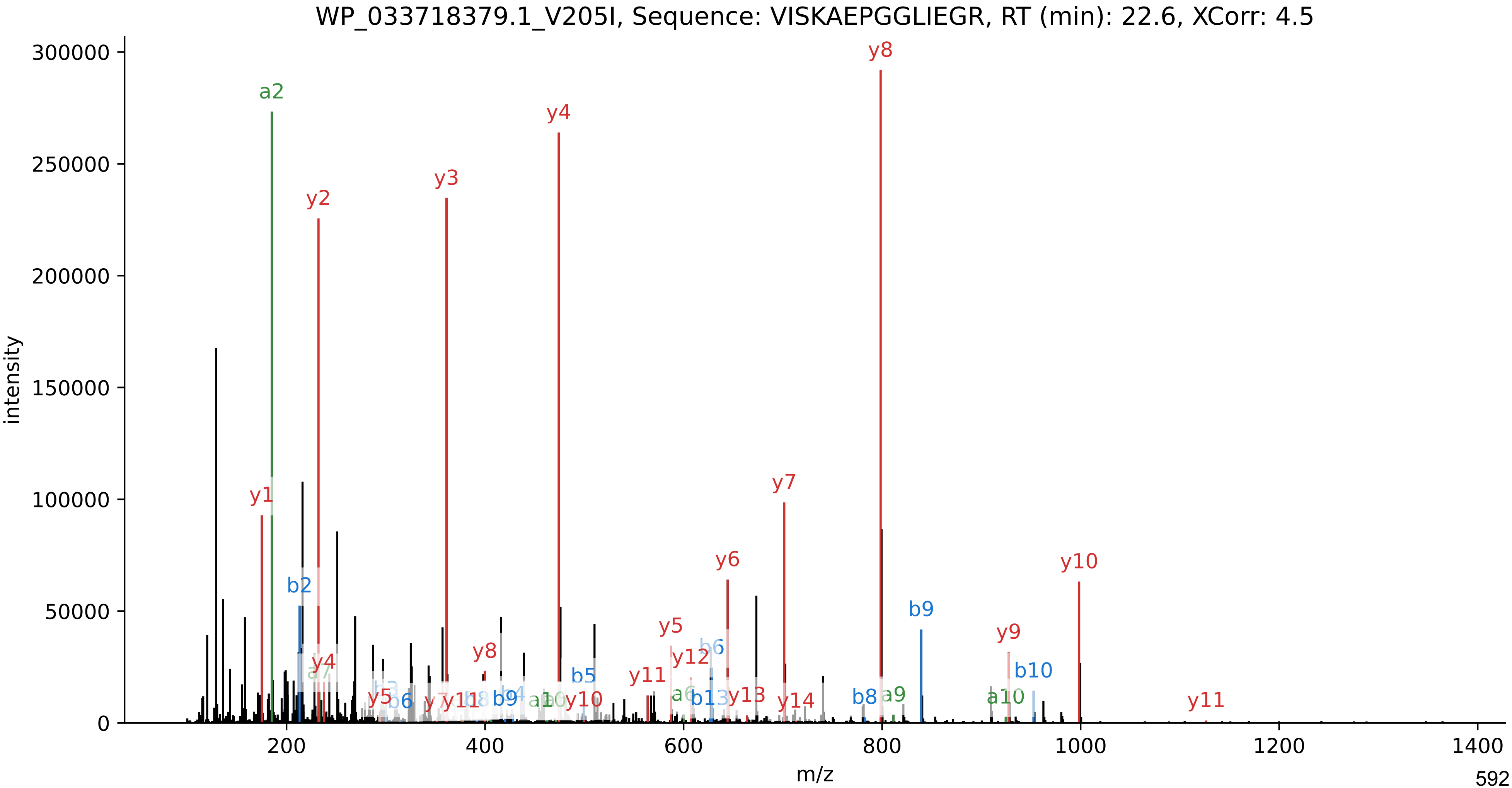
WP_033718379.1_V205I, Sequence: VISKAEPGGLIEGR, RT (min): 22.6, XCorr: 4.5
intensity
m/z
a2
y8
y4
y3
y2
y1
y7
y6
y10
b2
b9
y9
b10
y5
y12
b6
y8
y4
y11
b5
a9
b8
a10
y13
y14
y11
b13
a6
y10
b9
y11
b6
y5
0
50000
100000
150000
200000
250000
300000
200
400
600
800
1000
1200
1400

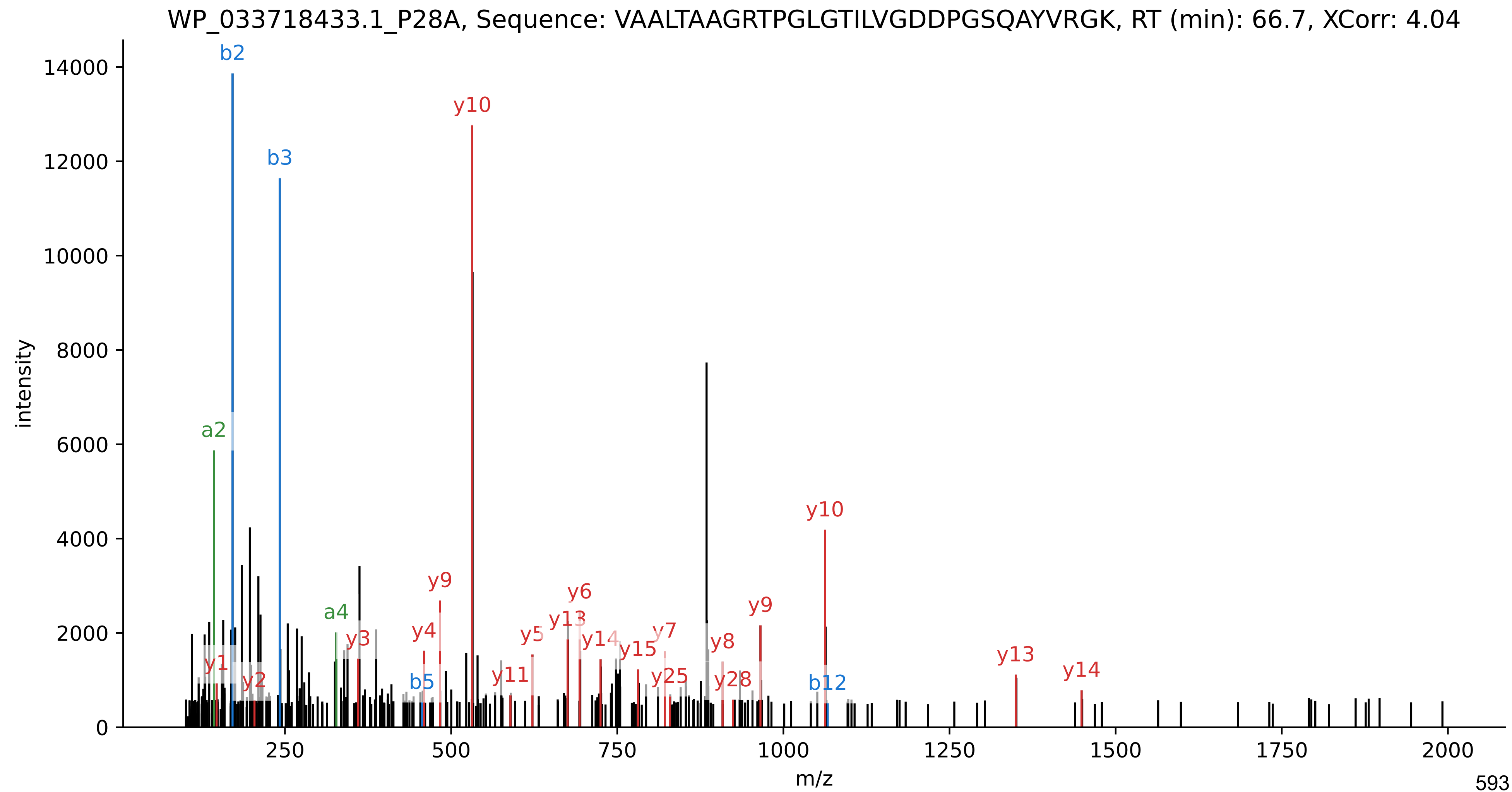
WP_033718433.1_P28A, Sequence: VAALTAAGRTPGLGTILVGDDPGSQAYVRGK, RT (min): 66.7, XCorr: 4.04
intensity
m/z
b2
b3
y10
a2
a4
y3
y1
y2
y4
y9
b5
y11
y5
y13
y6
y14
y15
y7
y25
y8
y28
y9
y10
b12
y13
y14
0
2000
4000
6000
8000
10000
12000
14000
250
500
750
1000
1250
1500
1750
2000

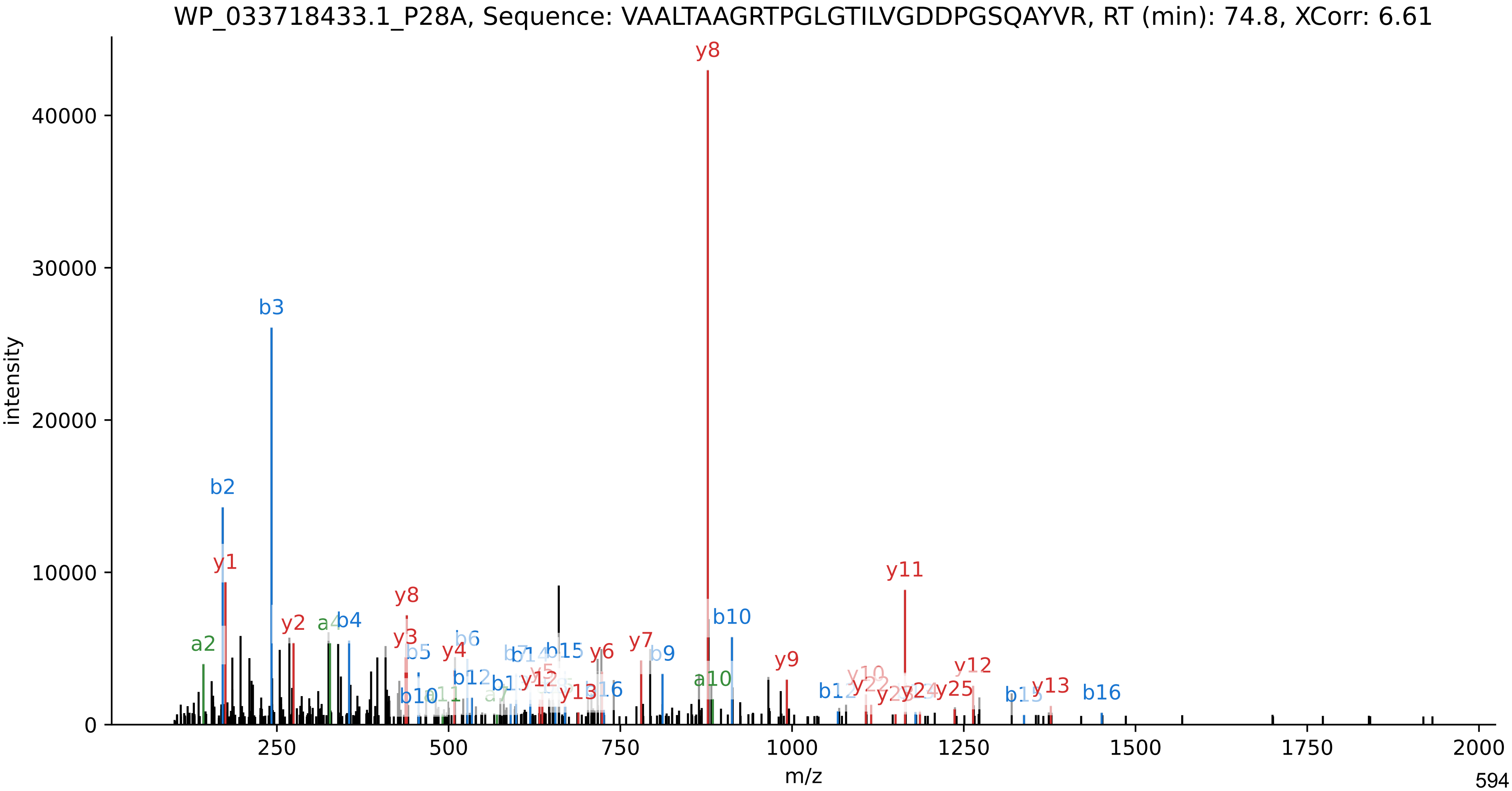
WP_033718433.1_P28A, Sequence: VAALTAAGRTPGLGTILVGDDPGSQAYVR, RT (min): 74.8, XCorr: 6.61
intensity
m/z
0
10000
20000
30000
40000
250
500
750
1000
1250
1500
1750
2000
y8
b3
b2
y1
a2
y2
b4
y3
b5
y4
b6
b15
y6
y7
b9
b10
a10
y9
y11
y12
y13
b16
y25
b12
b10
y12
y13

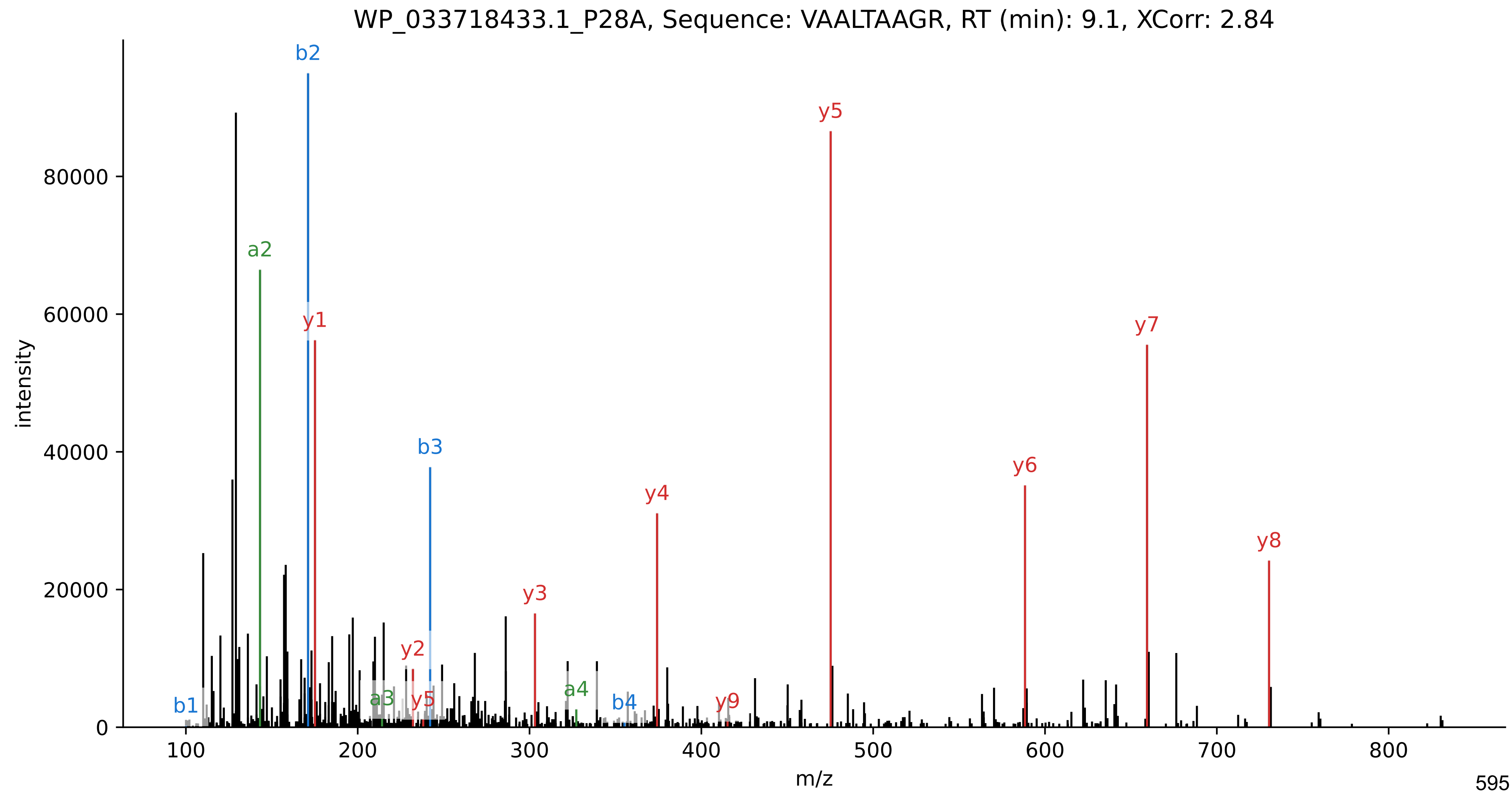
WP_033718433.1_P28A, Sequence: VAALTAAGR, RT (min): 9.1, XCorr: 2.84
intensity
m/z
b1
b2
b3
b4
a2
a3
a4
y1
y2
y3
y4
y5
y5
y6
y7
y8
y9
0
20000
40000
60000
80000
100
200
300
400
500
600
700
800

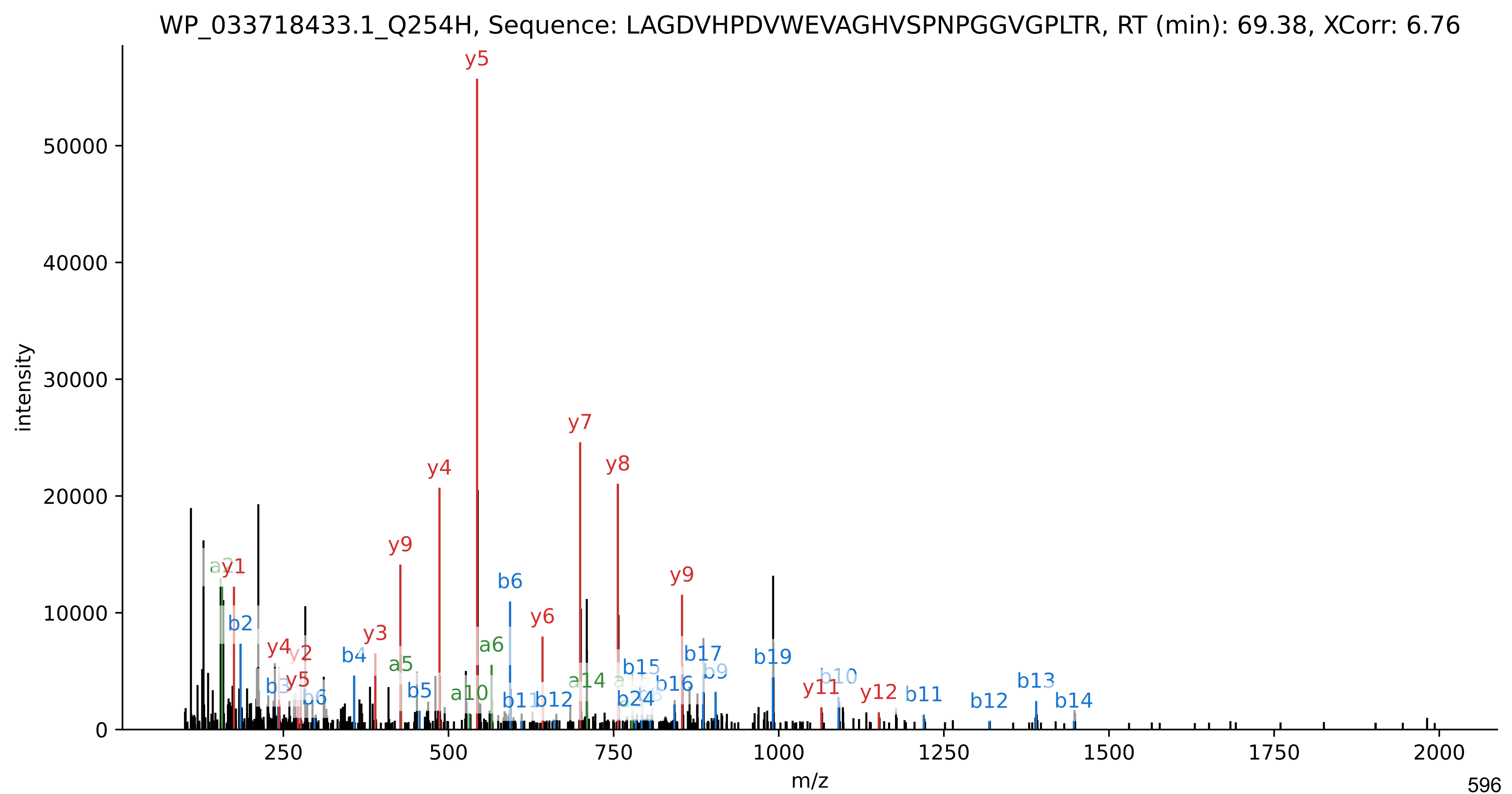

WP_033718433.1_Q254H, Sequence: LAGDVHPDVWEVAGHVSPNPGGVGPLTR, RT (min): 69.38, XCorr: 6.76
intensity
m/z
y5
y7
y8
y4
y9
y1
b6
y6
b2
y3
y4
y2
b4
a6
b17
y5
b3
a5
b15
b19
b9
b10
b5
b6
a14
b16
a10
y11
y12
b11
b13
b24
b11
b12
b12
b14

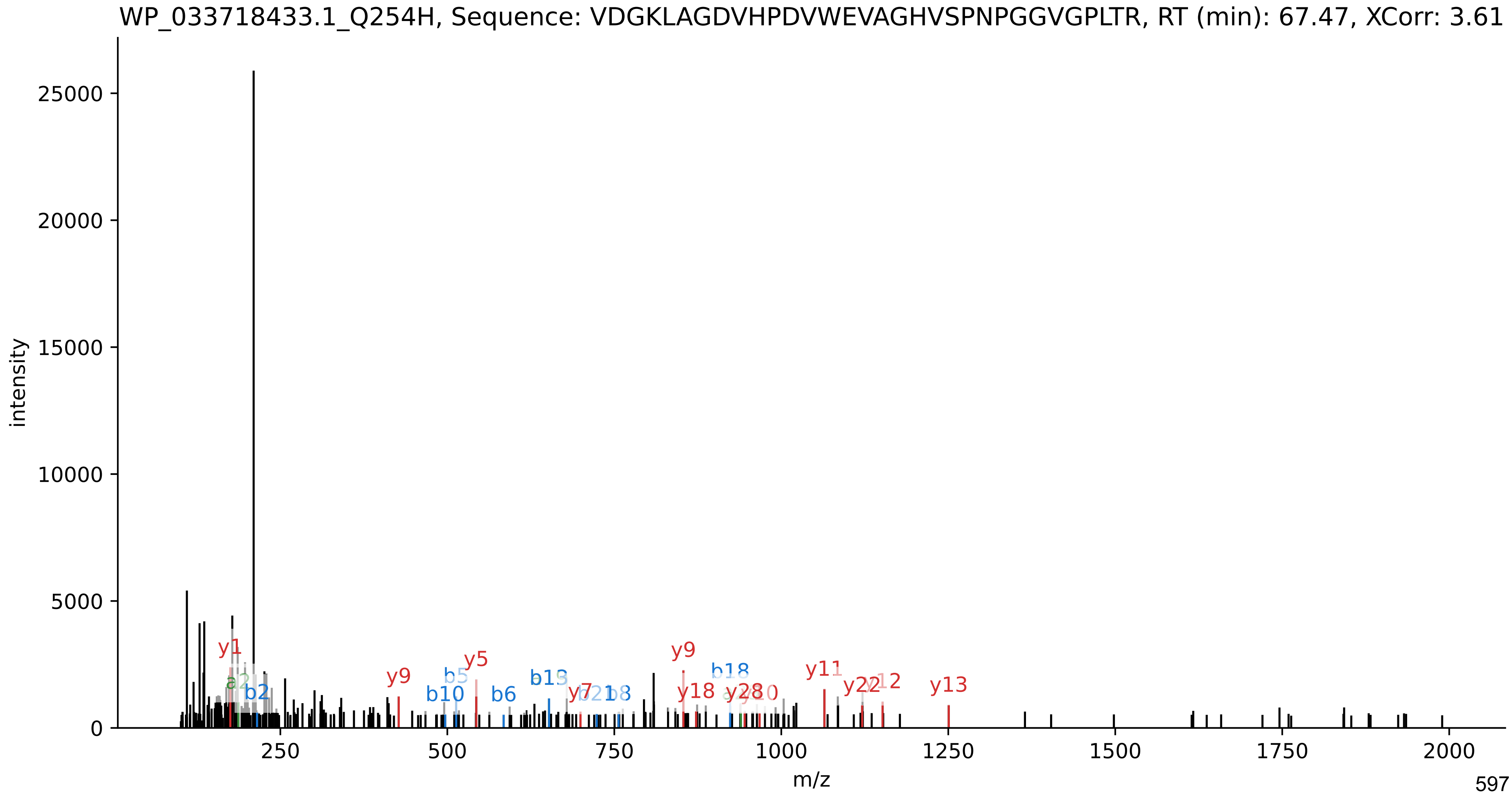

WP_033718433.1_Q254H, Sequence: VDGKLAGDVHPDVWEVAGHVSPNPGGVGPLTR, RT (min): 67.47, XCorr: 3.61
intensity
m/z
y1
a2
b2
y9
b5
b10
y5
b6
b13
y7
y9
b18
y18
y28
y11
y22
y12
y13

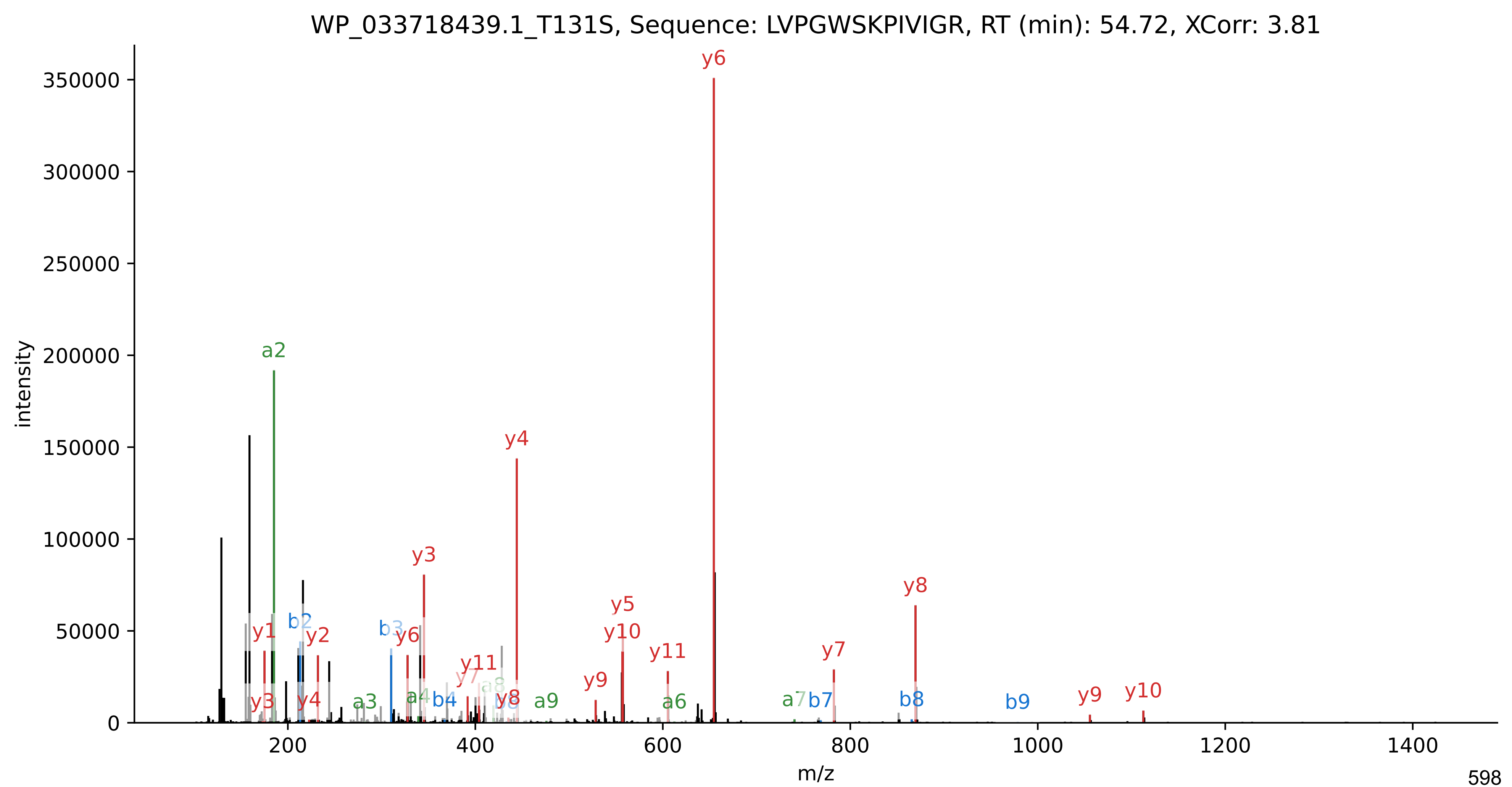
WP_033718439.1_T131S, Sequence: LVPGWSKPIVIGR, RT (min): 54.72, XCorr: 3.81
intensity
m/z
y6
a2
y4
y3
y8
y5
y10
y11
y7
b2
y1
y2
b3
y6
y11
y7
a8
y9
y3
y4
a3
a4
b4
y8
a9
a6
a7
b7
b8
b9
y9
y10

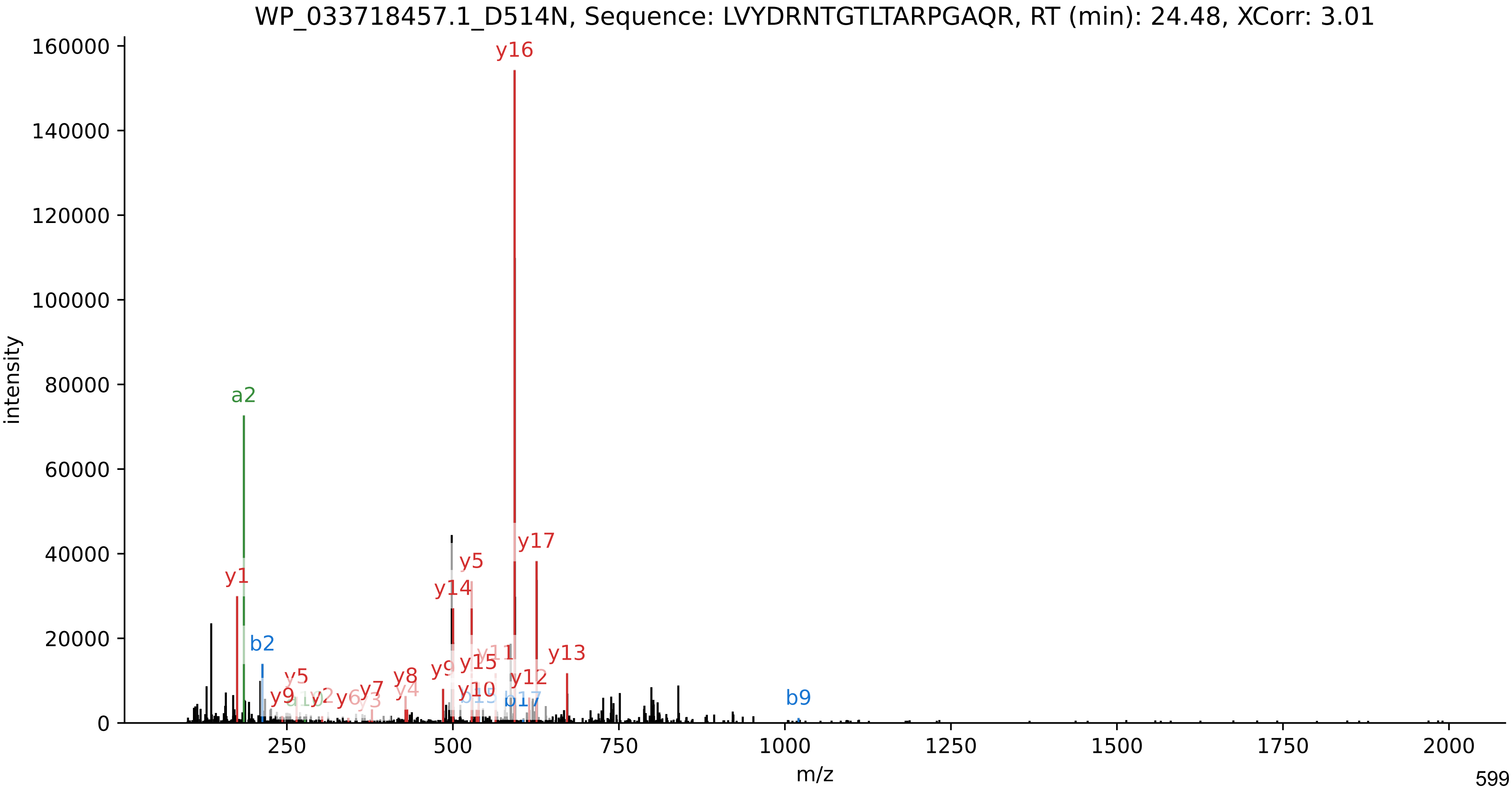

WP_033718457.1_D514N, Sequence: LVYDRNTGTLTARPGAQR, RT (min): 24.48, XCorr: 3.01
intensity
m/z
y16
a2
y17
y5
y1
y14
b2
y13
y15
y12
y9
y5
y8
y7
y10
b17
b9

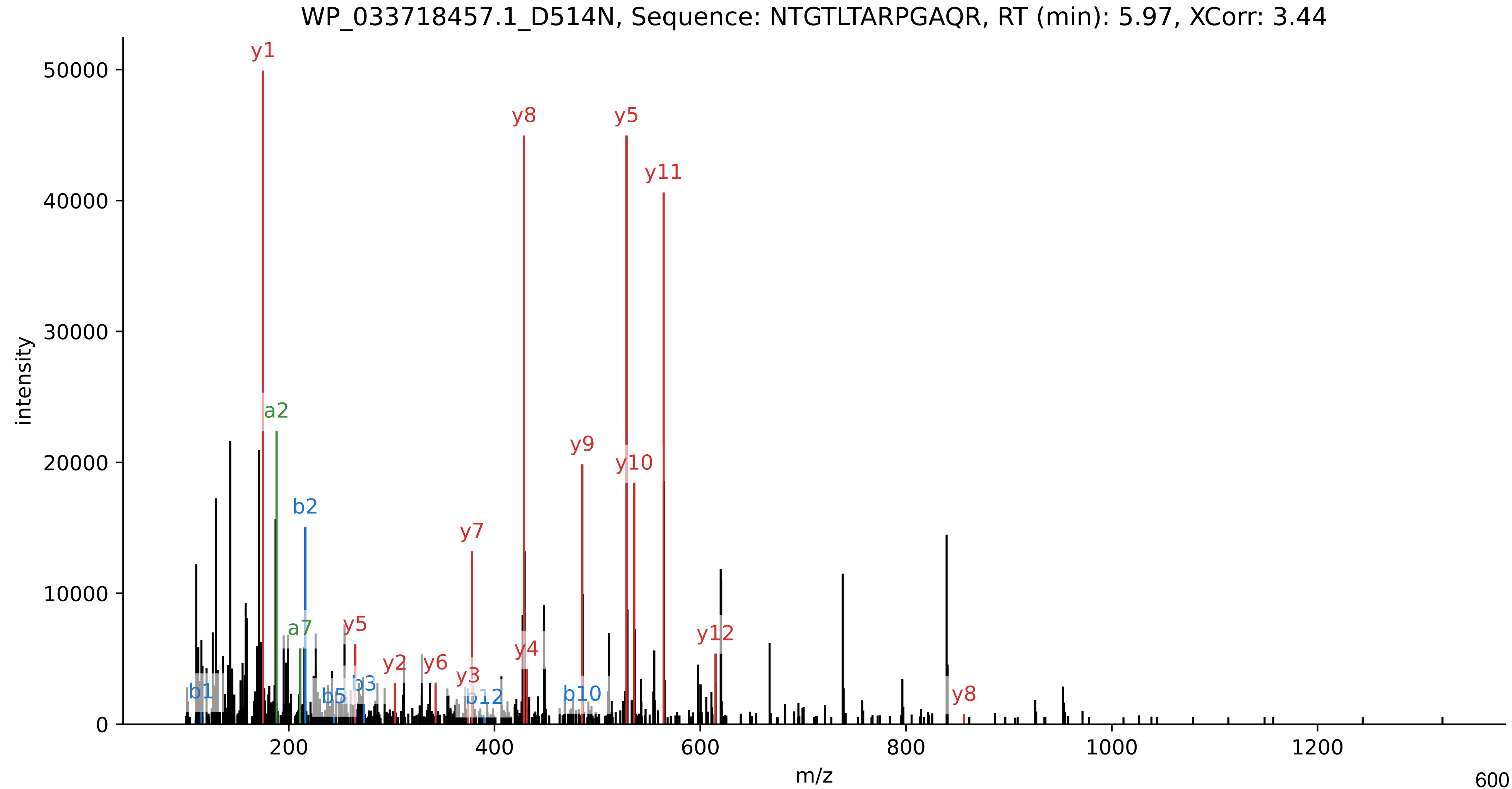
WP_033718457.1_D514N, Sequence: NTGTLTARPGAQR, RT (min): 5.97, XCorr: 3.44
intensity
m/z
y1
y8
y5
y11
a2
y9
y10
b2
y7
a7
y5
y12
y4
y2
y6
y3
b3
b1
b5
b12
b10
y8

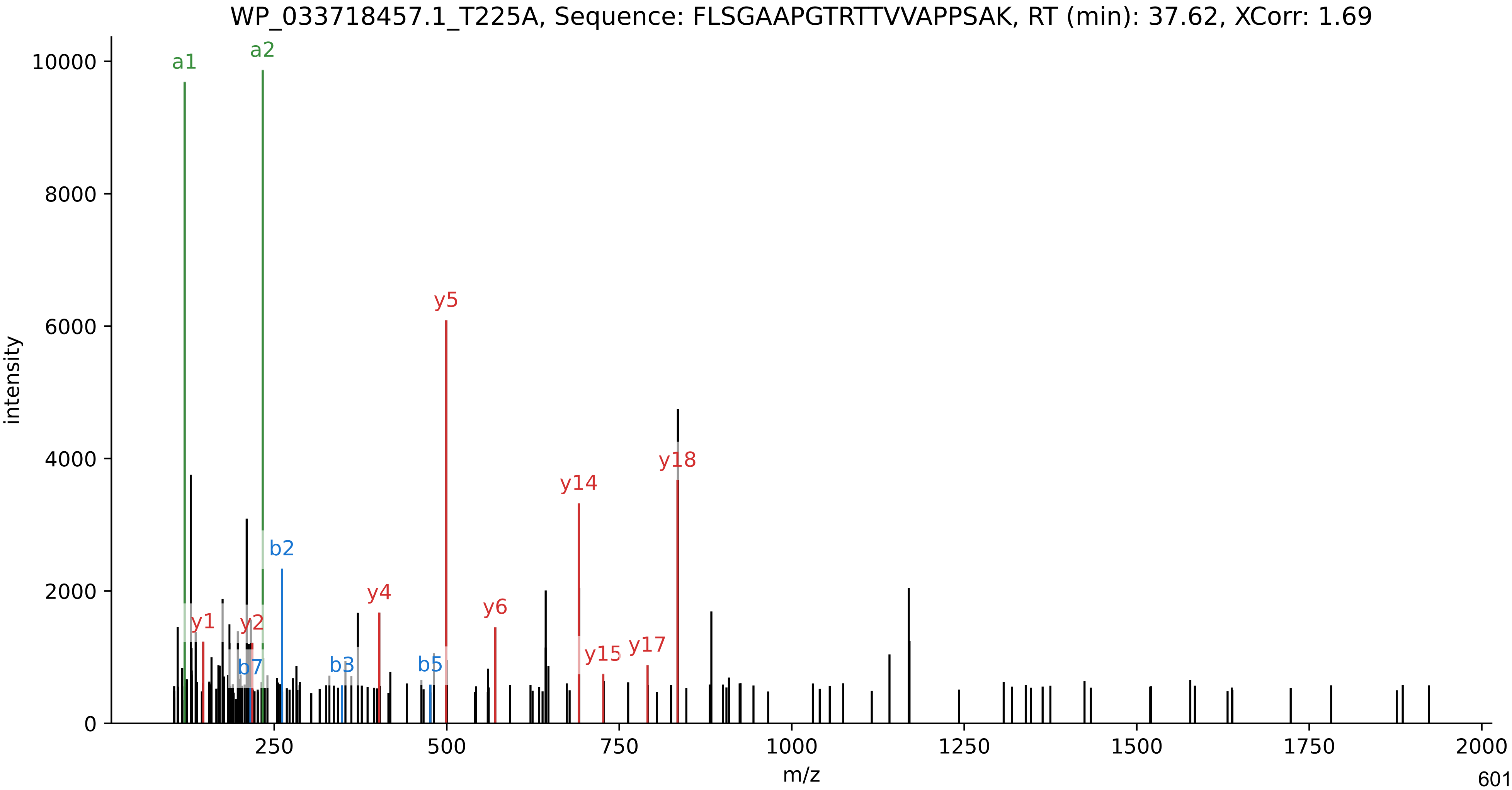

WP_033718457.1_T225A, Sequence: FLSGAAPGTRTTVVAPPSAK, RT (min): 37.62, XCorr: 1.69
a1
a2
y5
y18
y14
b2
y4
y6
y1
y2
y15
y17
b7
b3
b5
intensity
m/z
0
2000
4000
6000
8000
10000
250
500
750
1000
1250
1500
1750
2000

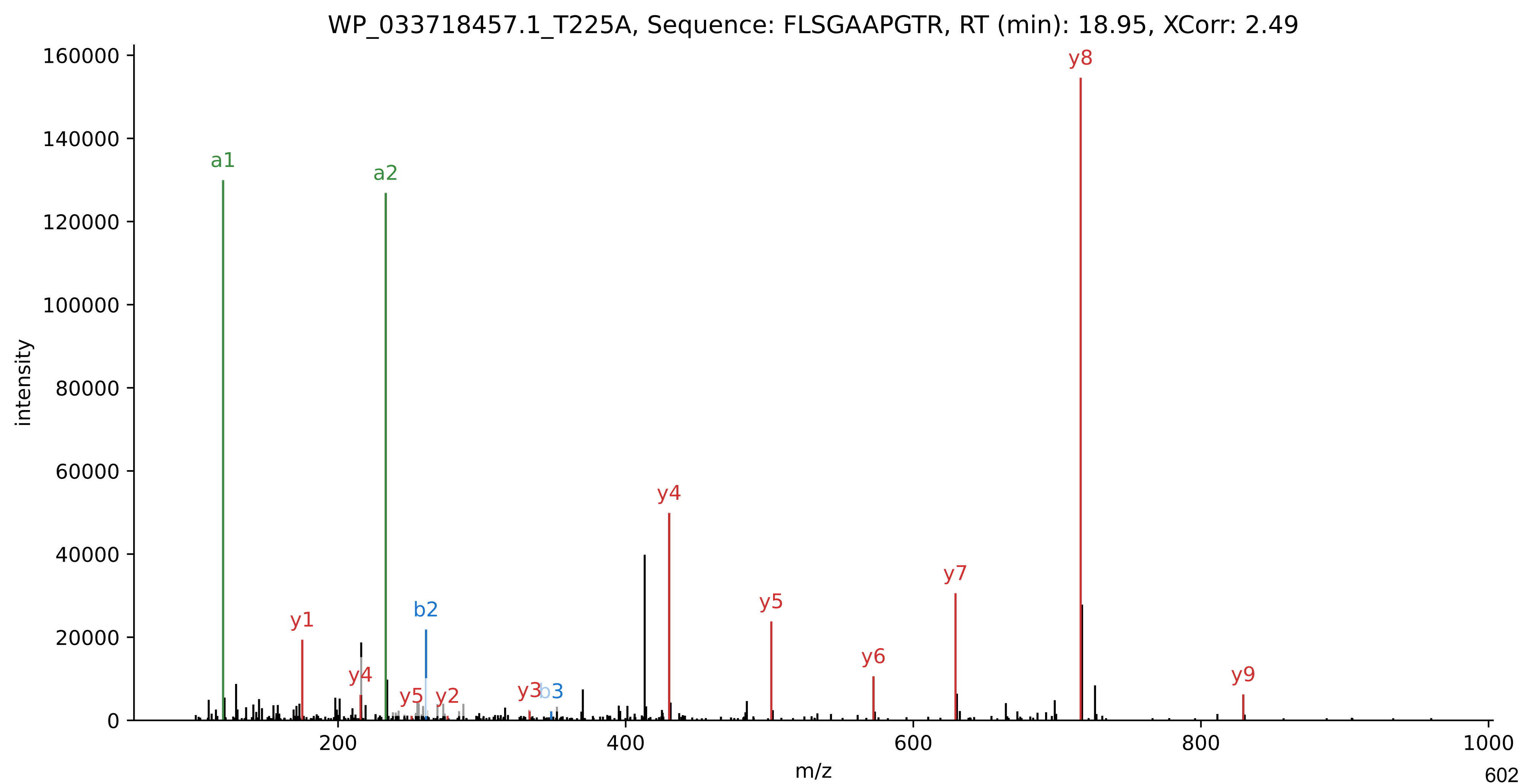

WP_033718457.1_T225A, Sequence: FLSGAAPGTR, RT (min): 18.95, XCorr: 2.49
intensity
m/z
a1
a2
b2
b3
y1
y2
y3
y4
y5
y6
y7
y8
y9
0
20000
40000
60000
80000
100000
120000
140000
160000
200
400
600
800
1000

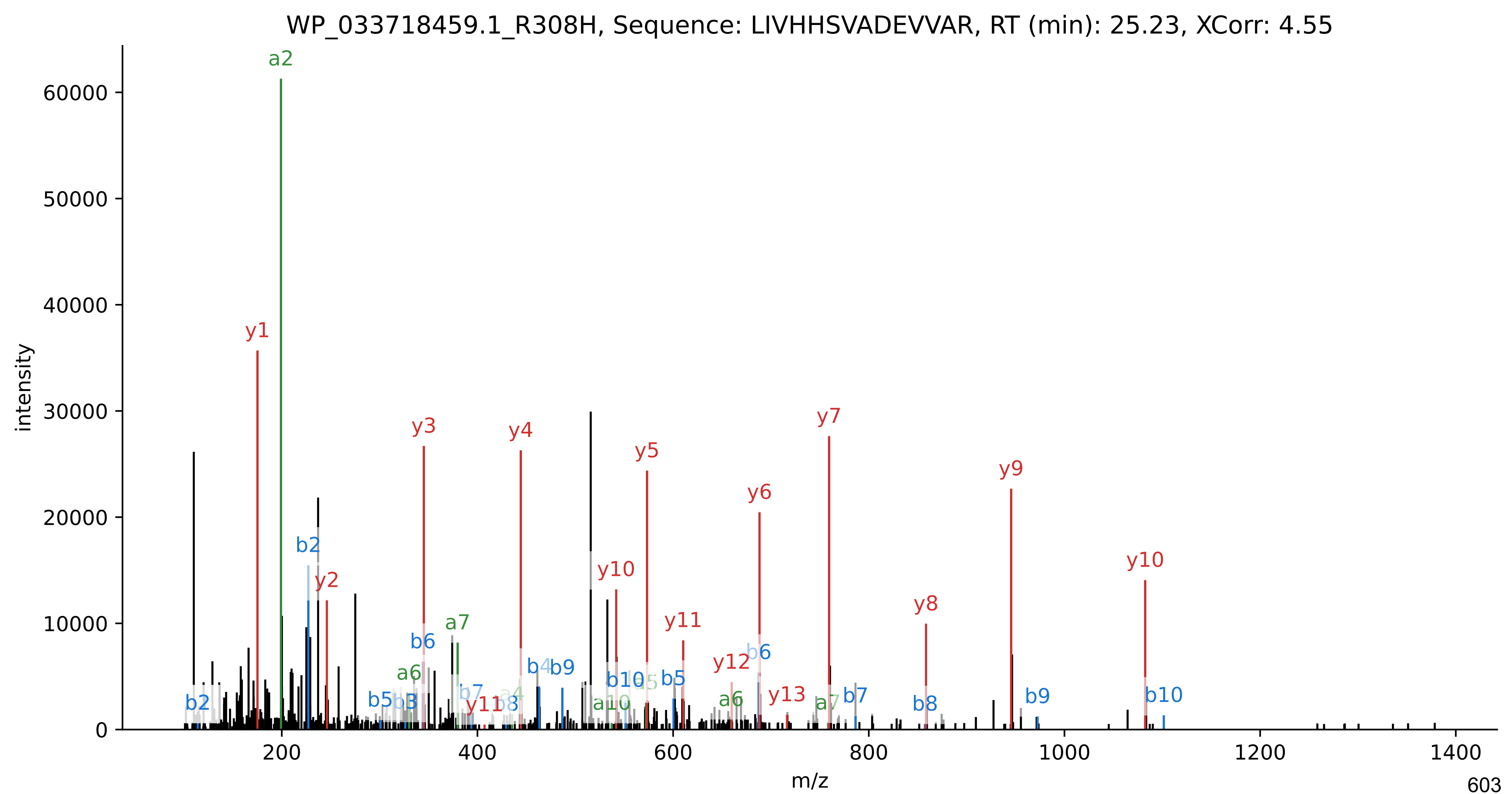

WP_033718459.1_R308H, Sequence: LIVHHSVADEVVAR, RT (min): 25.23, XCorr: 4.55
intensity
m/z

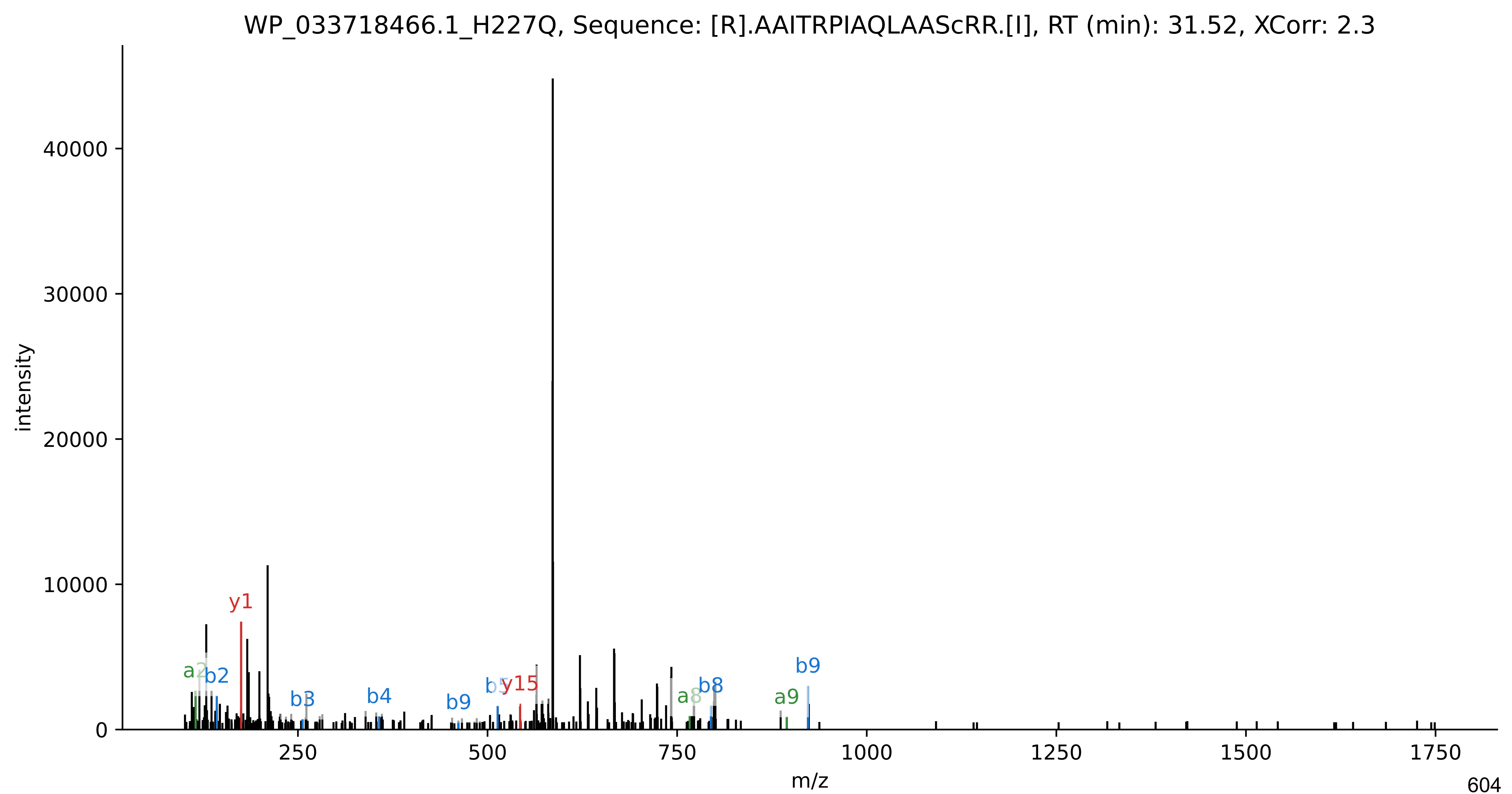
WP_033718466.1_H227Q, Sequence: [R].AAITRPIAQLAAScRR.[I], RT (min): 31.52, XCorr: 2.3
intensity
m/z
y1
a2
b2
b3
b4
b9
b5
y15
a8
b8
a9
b9

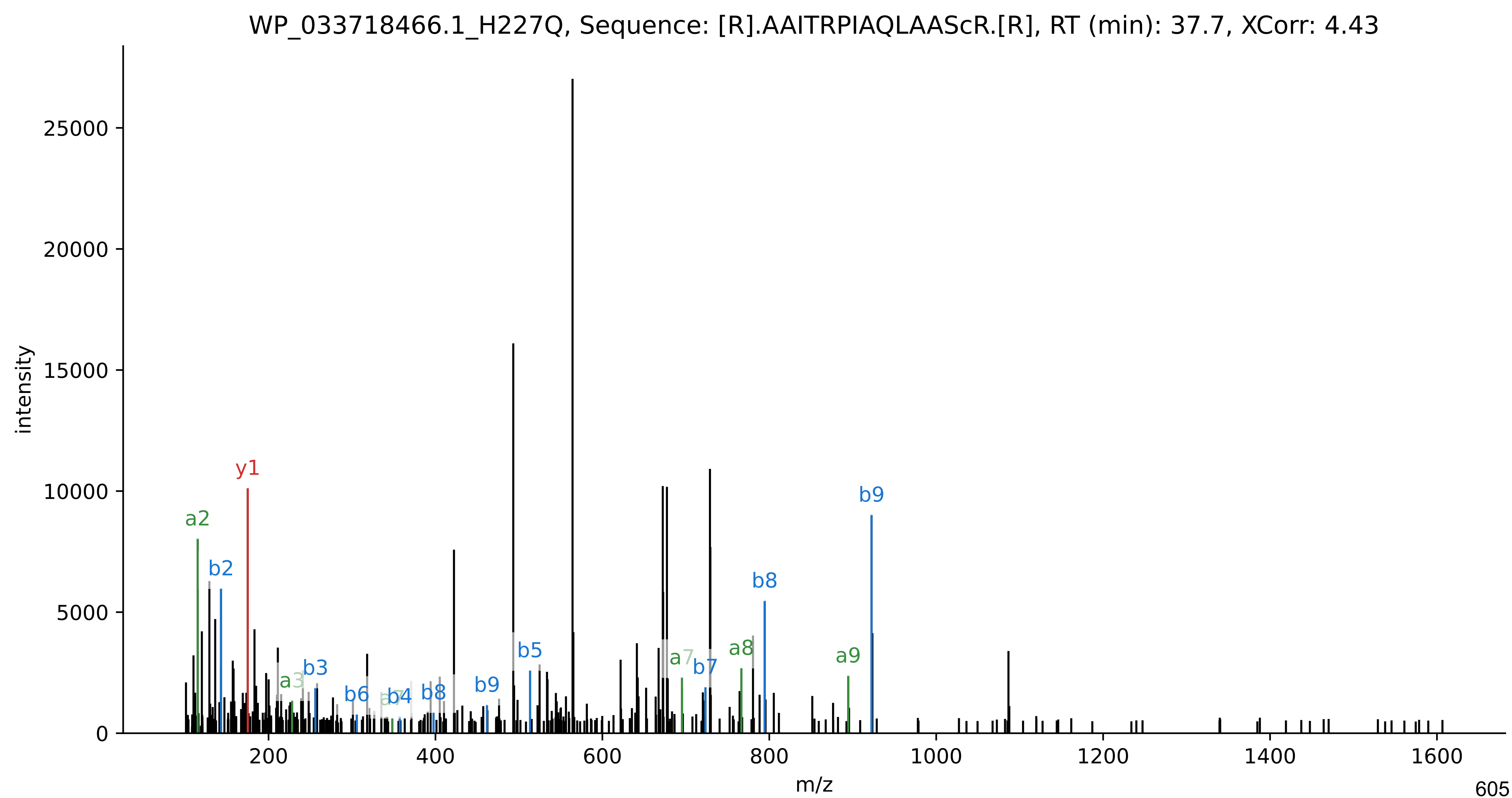
WP_033718466.1_H227Q, Sequence: [R].AAITRPIAQLAAScR.[R], RT (min): 37.7, XCorr: 4.43
intensity
m/z
y1
a2
b2
a3
b3
b6
a7
b4
b8
b9
b5
a7
b7
a8
b8
a9
b9

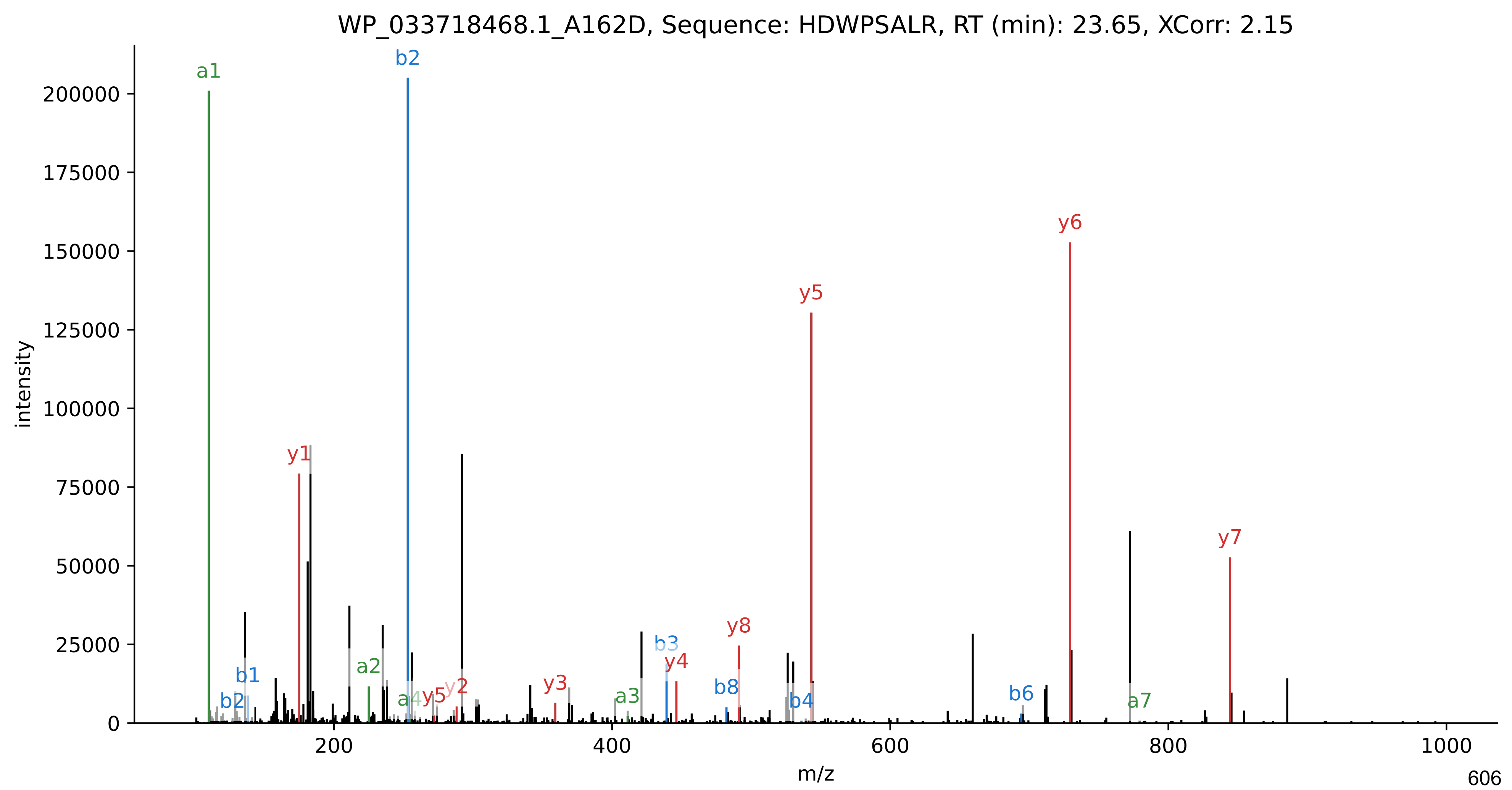
WP_033718468.1_A162D, Sequence: HDWPSALR, RT (min): 23.65, XCorr: 2.15
intensity
m/z
0
25000
50000
75000
100000
125000
150000
175000
200000
200
400
600
800
1000
a1
b2
y6
y5
y1
y7
y8
b3
y4
a2
b1
b2
a4
y5
y2
y3
a3
b8
b4
b6
a7

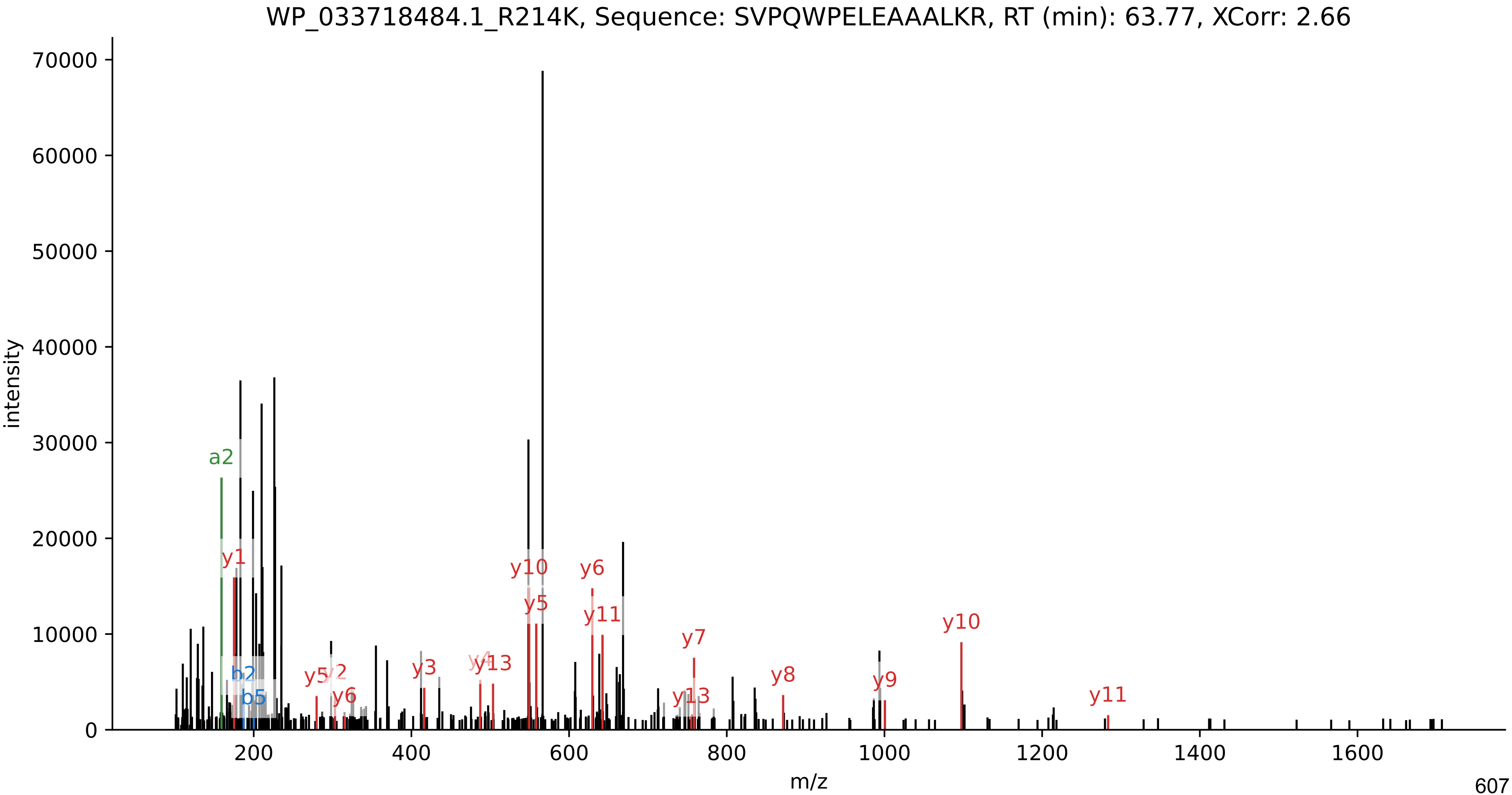

WP_033718484.1_R214K, Sequence: SVPQWPELEAAALKR, RT (min): 63.77, XCorr: 2.66
intensity
m/z
a2
y1
b2
b5
y5
y2
y6
y3
y4
y13
y10
y5
y6
y11
y7
y13
y8
y9
y10
y11

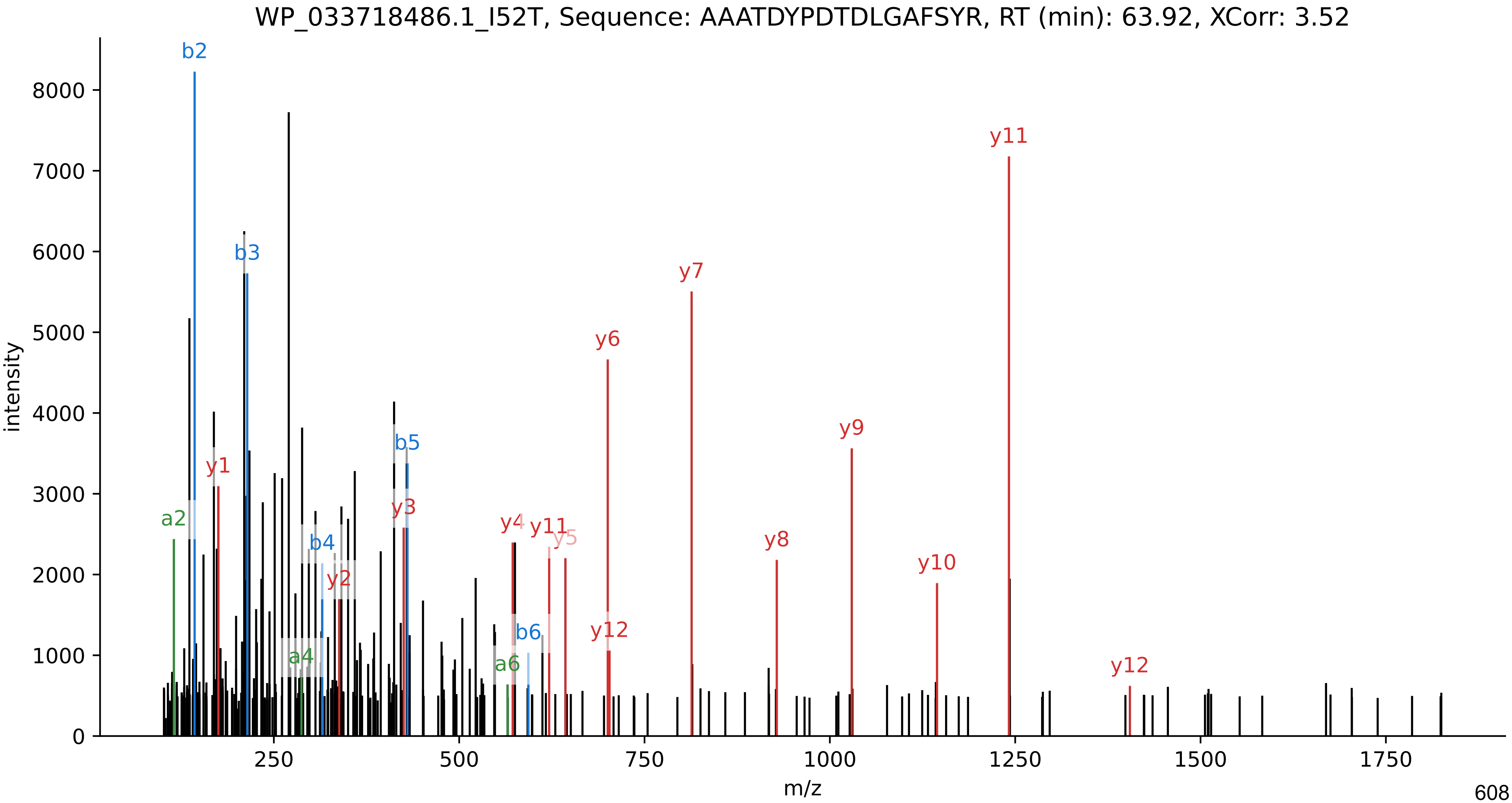
WP_033718486.1_I52T, Sequence: AAATDYPDTDLGAFSYR, RT (min): 63.92, XCorr: 3.52
intensity
m/z
0
1000
2000
3000
4000
5000
6000
7000
8000
250
500
750
1000
1250
1500
1750
b2
b3
b4
b5
b6
a2
a4
a6
y1
y2
y3
y4
y5
y6
y7
y8
y9
y10
y11
y11
y12
y12

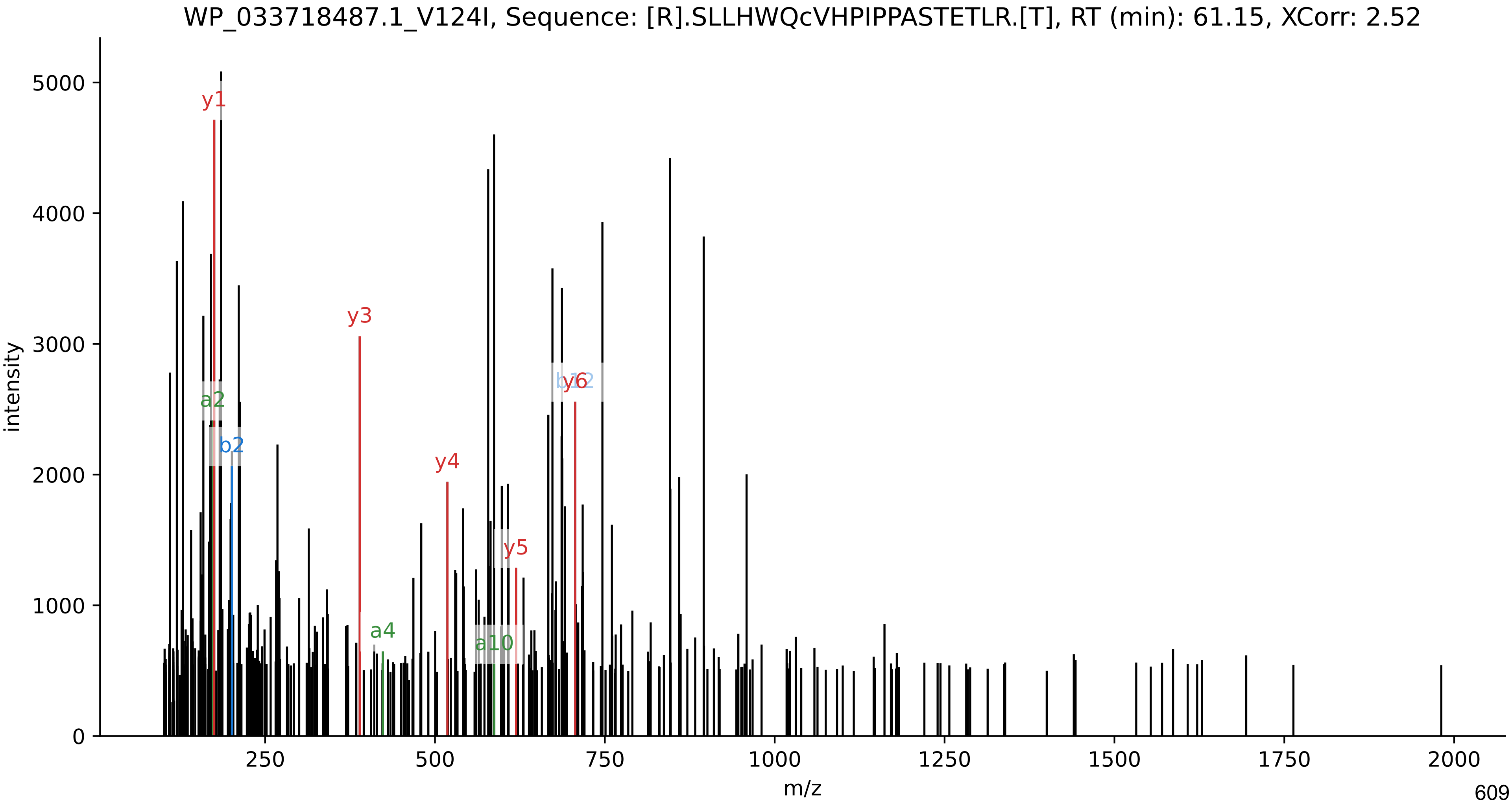

WP_033718487.1_V124I, Sequence: [R].SLLHWQcVHPIPPASTETLR.[T], RT (min): 61.15, XCorr: 2.52
intensity
m/z
y1
a2
b2
y3
y4
a4
a10
y5
b12
y6
0
1000
2000
3000
4000
5000
250
500
750
1000
1250
1500
1750
2000

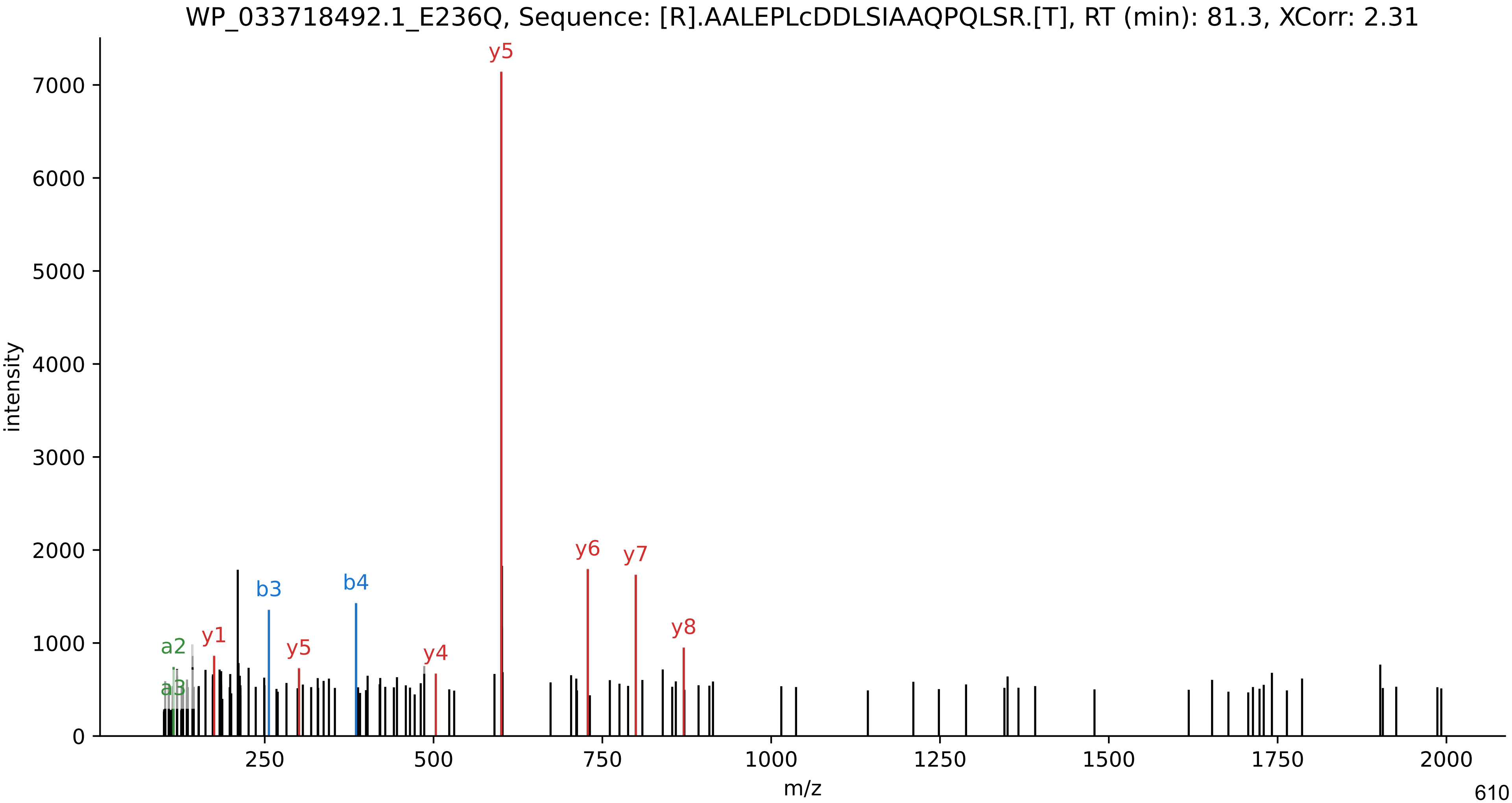
WP_033718492.1_E236Q, Sequence: [R].AALEPLcDDLSIAAQPQLSR.[T], RT (min): 81.3, XCorr: 2.31
intensity
m/z
y5
y6
y7
y8
b3
b4
a2
a3
y1
y5
y4

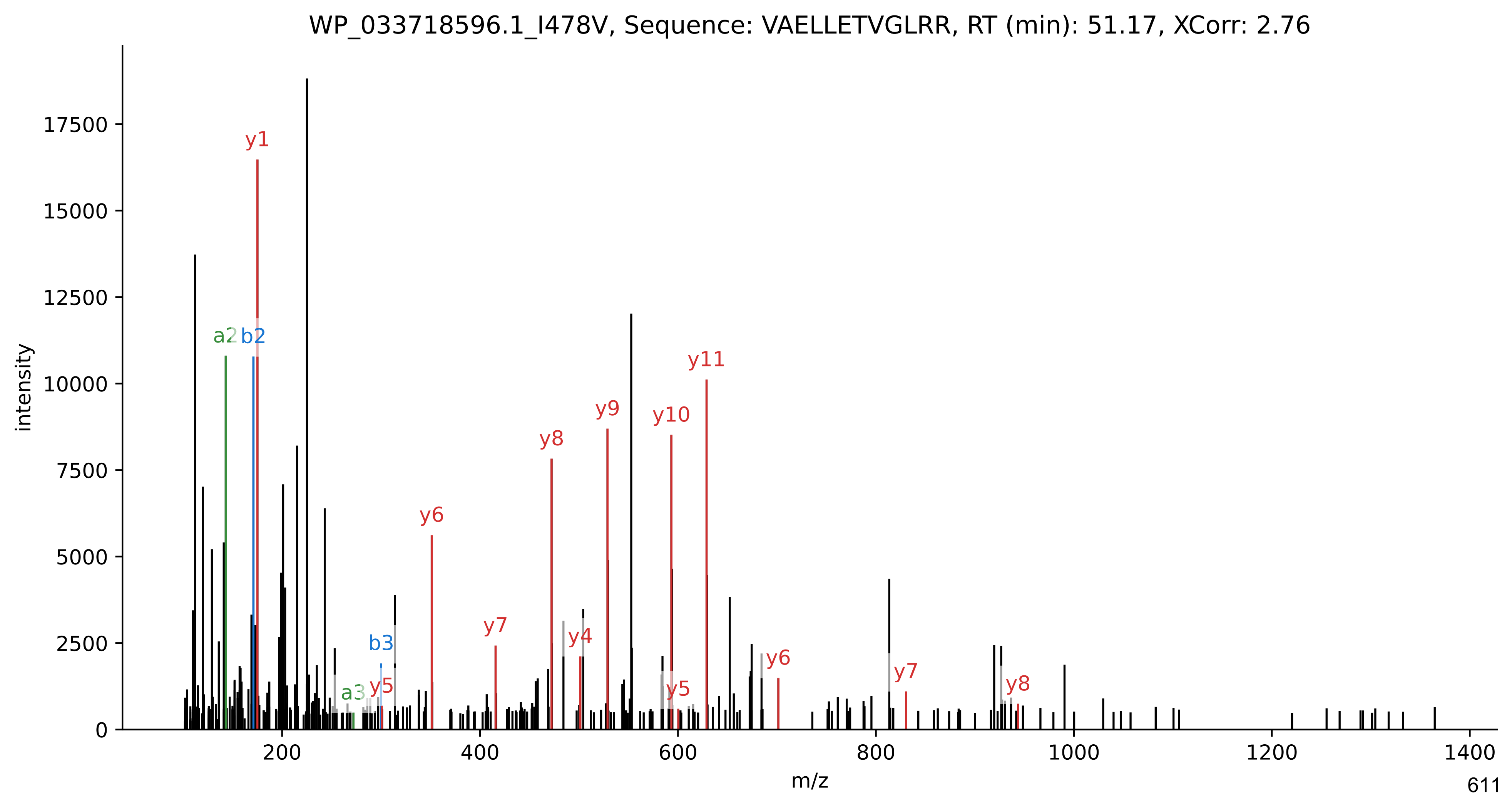

WP_033718596.1_I478V, Sequence: VAELLETVGLRR, RT (min): 51.17, XCorr: 2.76
intensity
m/z
y1
a2
b2
y11
y9
y10
y8
y6
y7
y4
b3
a3
y5
y6
y7
y5
y8

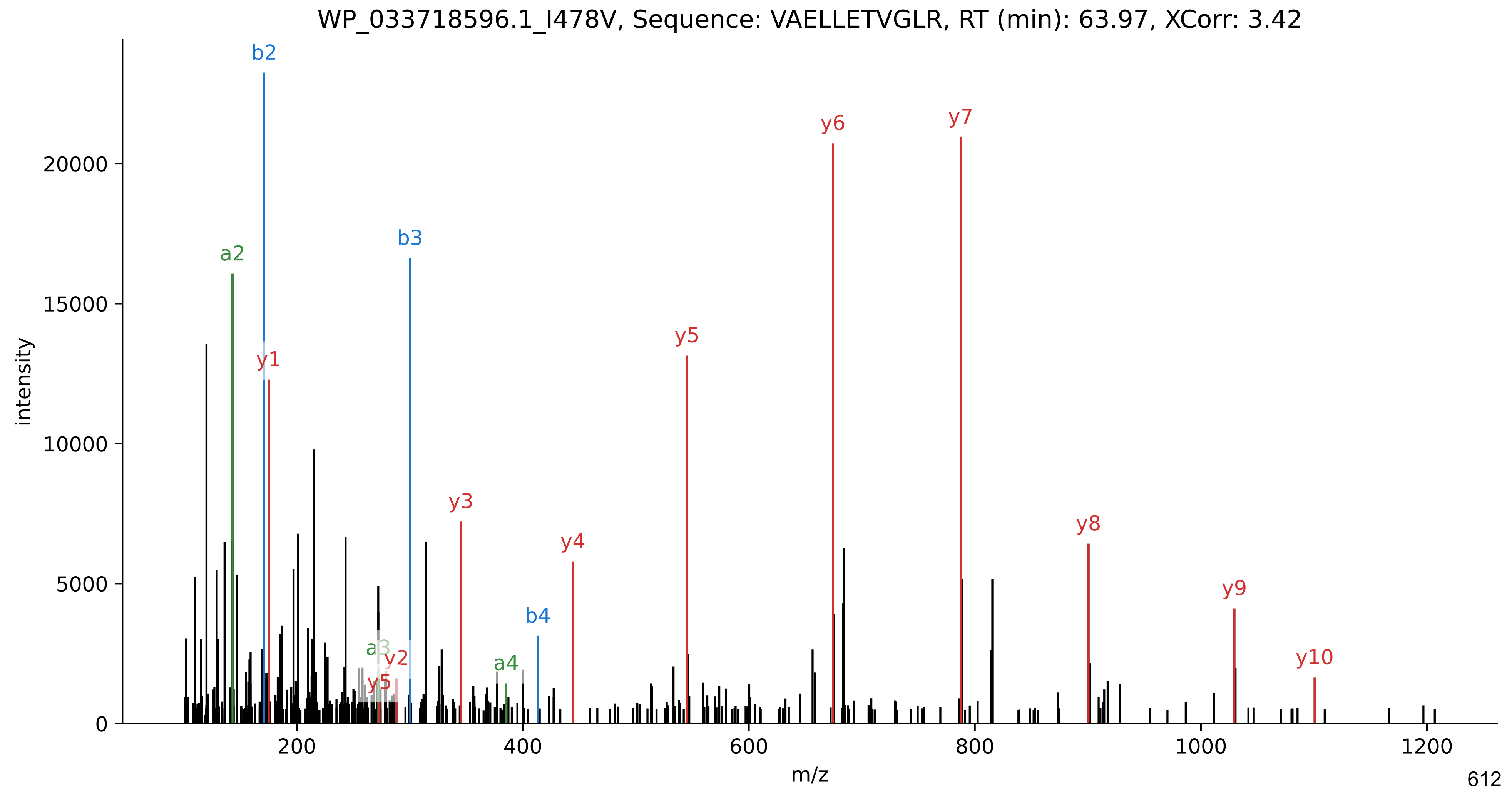
WP_033718596.1_I478V, Sequence: VAELLETVGLR, RT (min): 63.97, XCorr: 3.42
b2
a2
b3
y1
y6
y7
y5
y3
y4
y8
y9
y10
b4
a3
y2
y5
a4
intensity
m/z
0
5000
10000
15000
20000
200
400
600
800
1000
1200

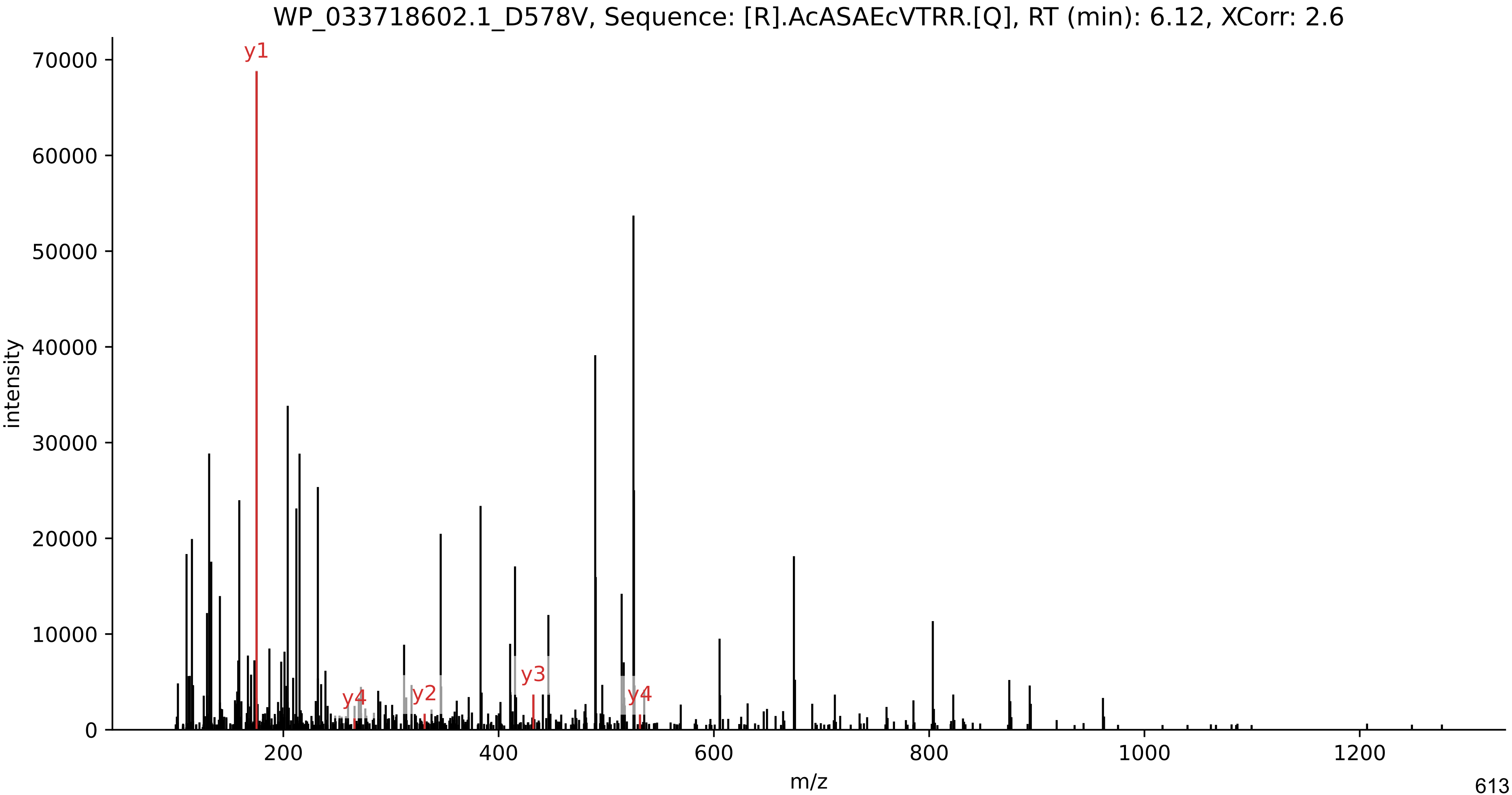
WP_033718602.1_D578V, Sequence: [R].AcASAEcVTRR.[Q], RT (min): 6.12, XCorr: 2.6
y1
y4
y2
y3
y4
intensity
m/z
0
10000
20000
30000
40000
50000
60000
70000
200
400
600
800
1000
1200

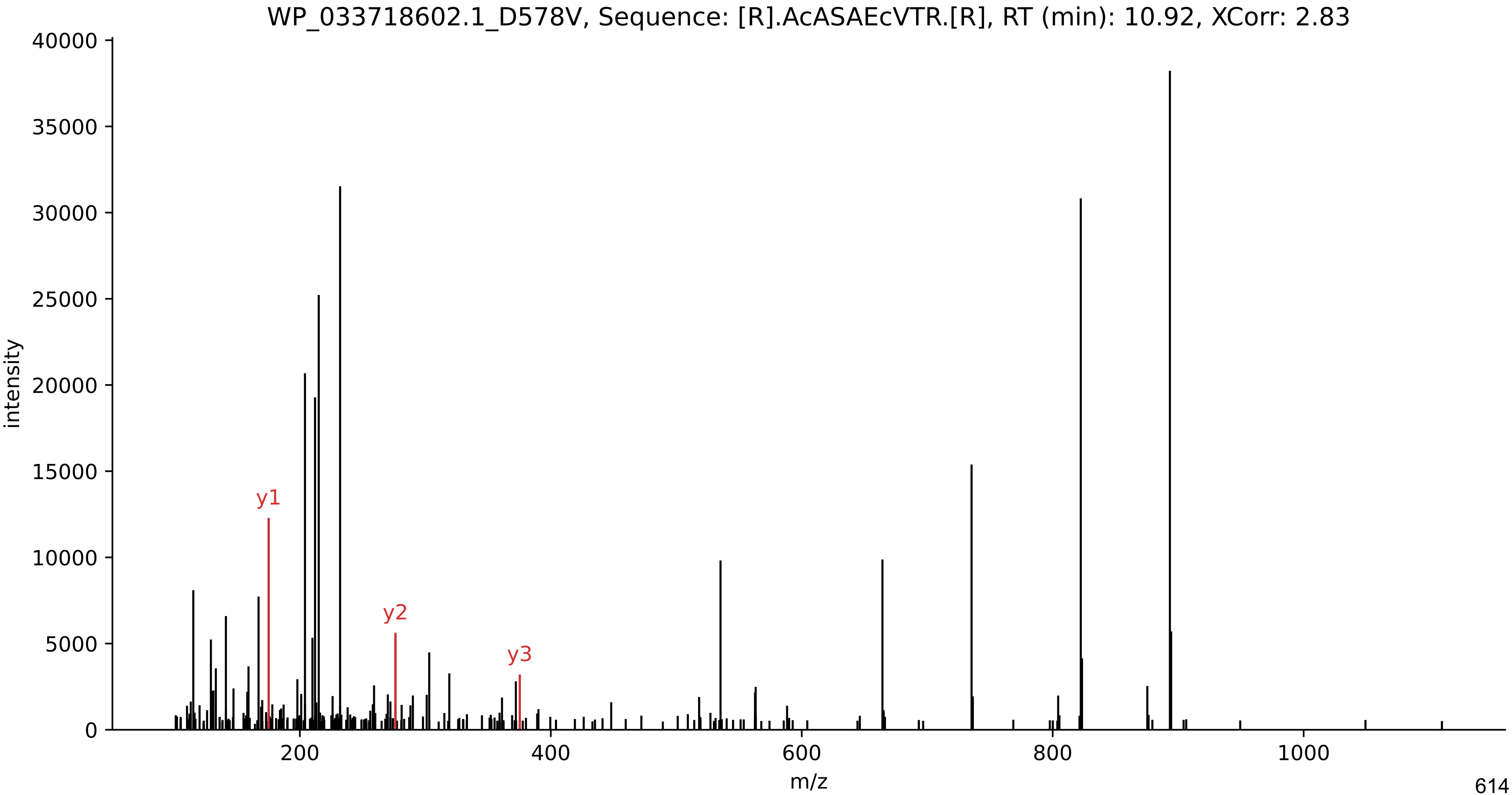
WP_033718602.1_D578V, Sequence: [R].AcASAEcVTR.[R], RT (min): 10.92, XCorr: 2.83
intensity
m/z
y1
y2
y3
0
5000
10000
15000
20000
25000
30000
35000
40000
200
400
600
800
1000

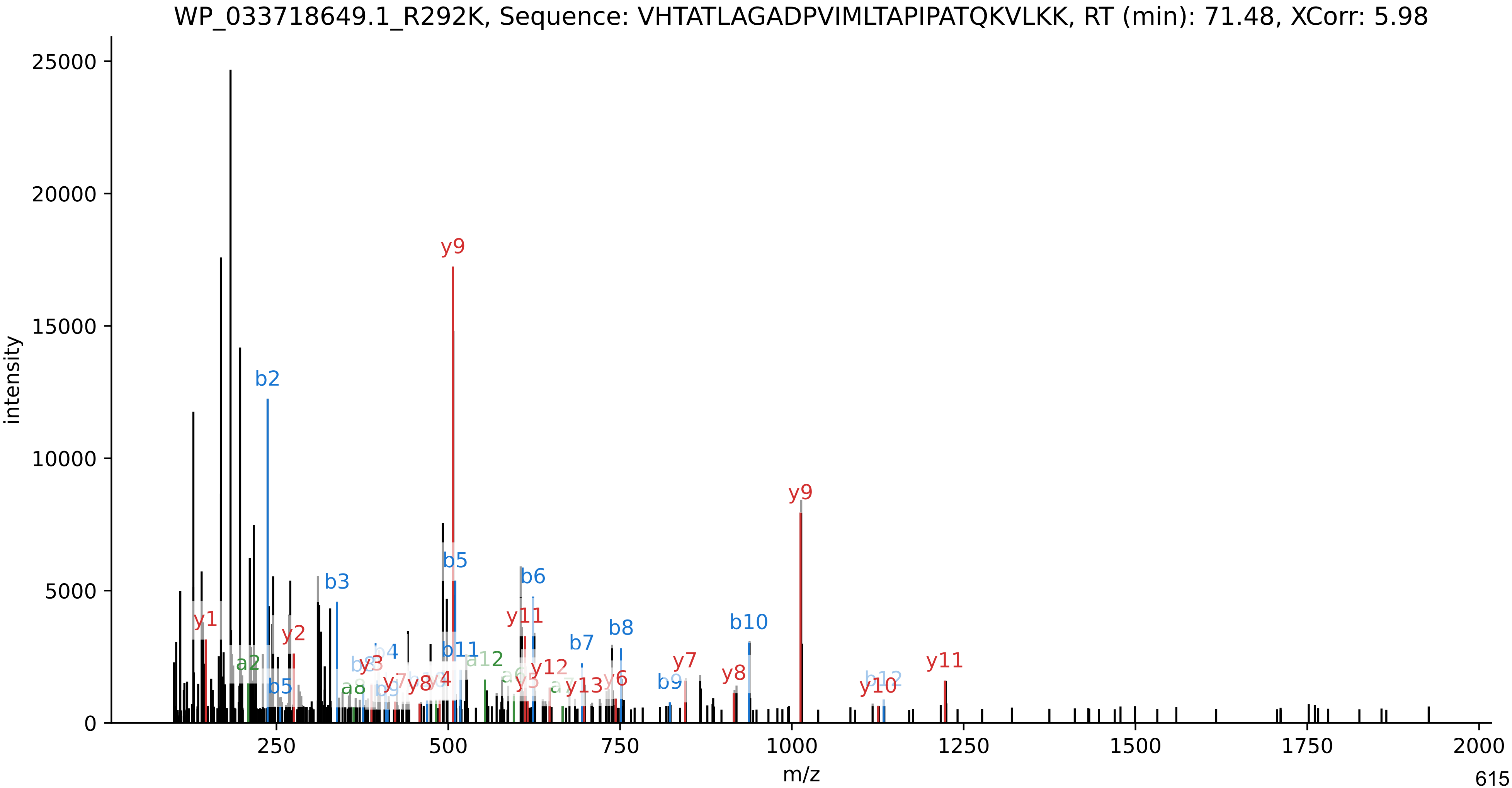
WP_033718649.1_R292K, Sequence: VHTATLAGADPVIMLTAPIPATQKVLKK, RT (min): 71.48, XCorr: 5.98
intensity
m/z
y9
b2
b3
b5
b6
y11
b8
b10
y9
b7
y1
y2
a2
b4
b11
a12
y12
y7
y8
y13
y6
b9
y10
b12
y11
b5
y3
a8
y4
y5

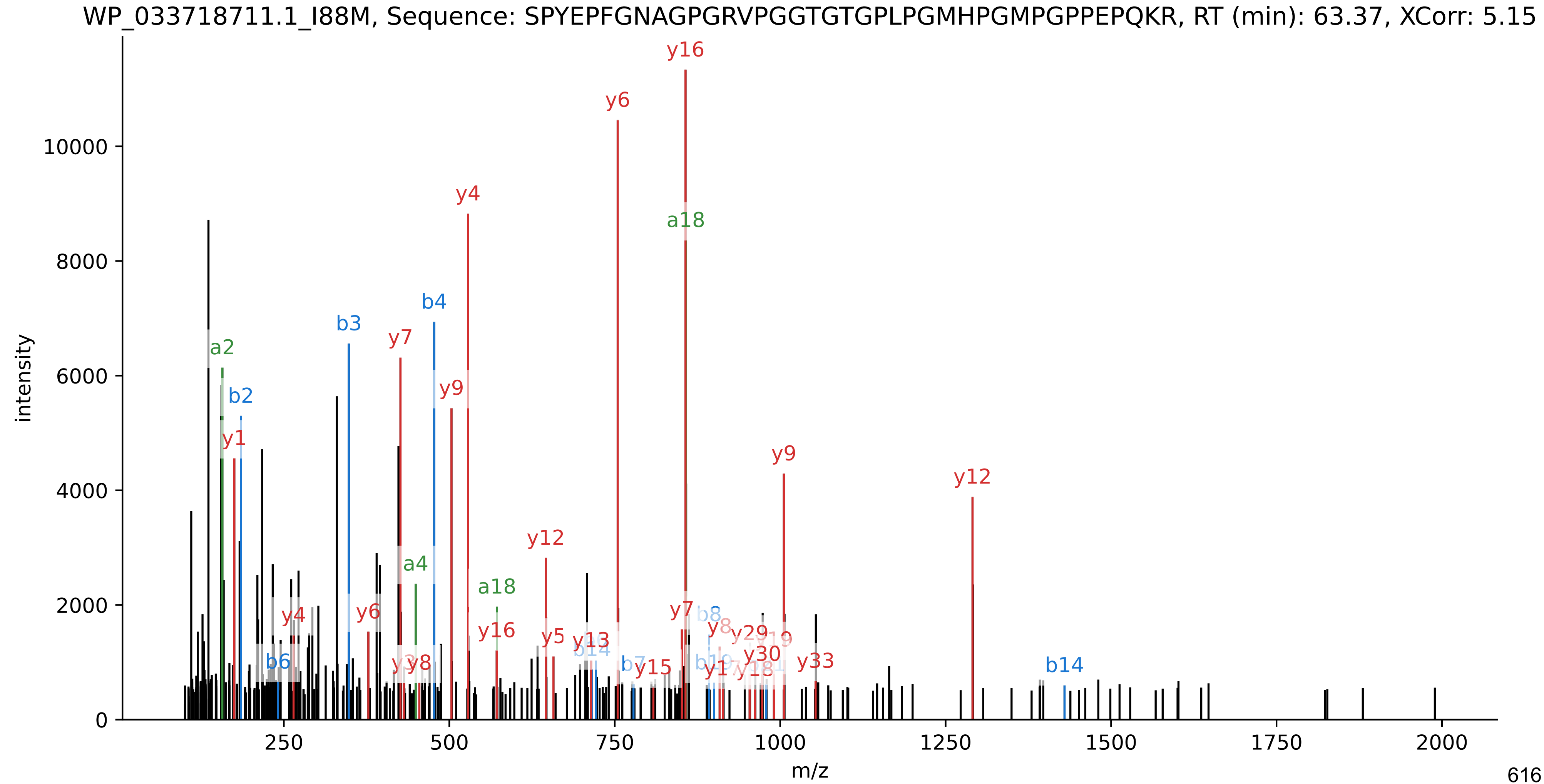
WP_033718711.1_I88M, Sequence: SPYEPFGNAGPGRVPGGTGTGPLPGMHPGMPGPPEPQKR, RT (min): 63.37, XCorr: 5.15
intensity
m/z
y16
y6
y4
a18
b4
b3
y7
a2
y9
b2
y1
y9
y12
y12
a4
a18
y4
y6
y16
y5
y13
y7
b8
y8
y29
y30
y33
b14
b6
y8
b7
y15

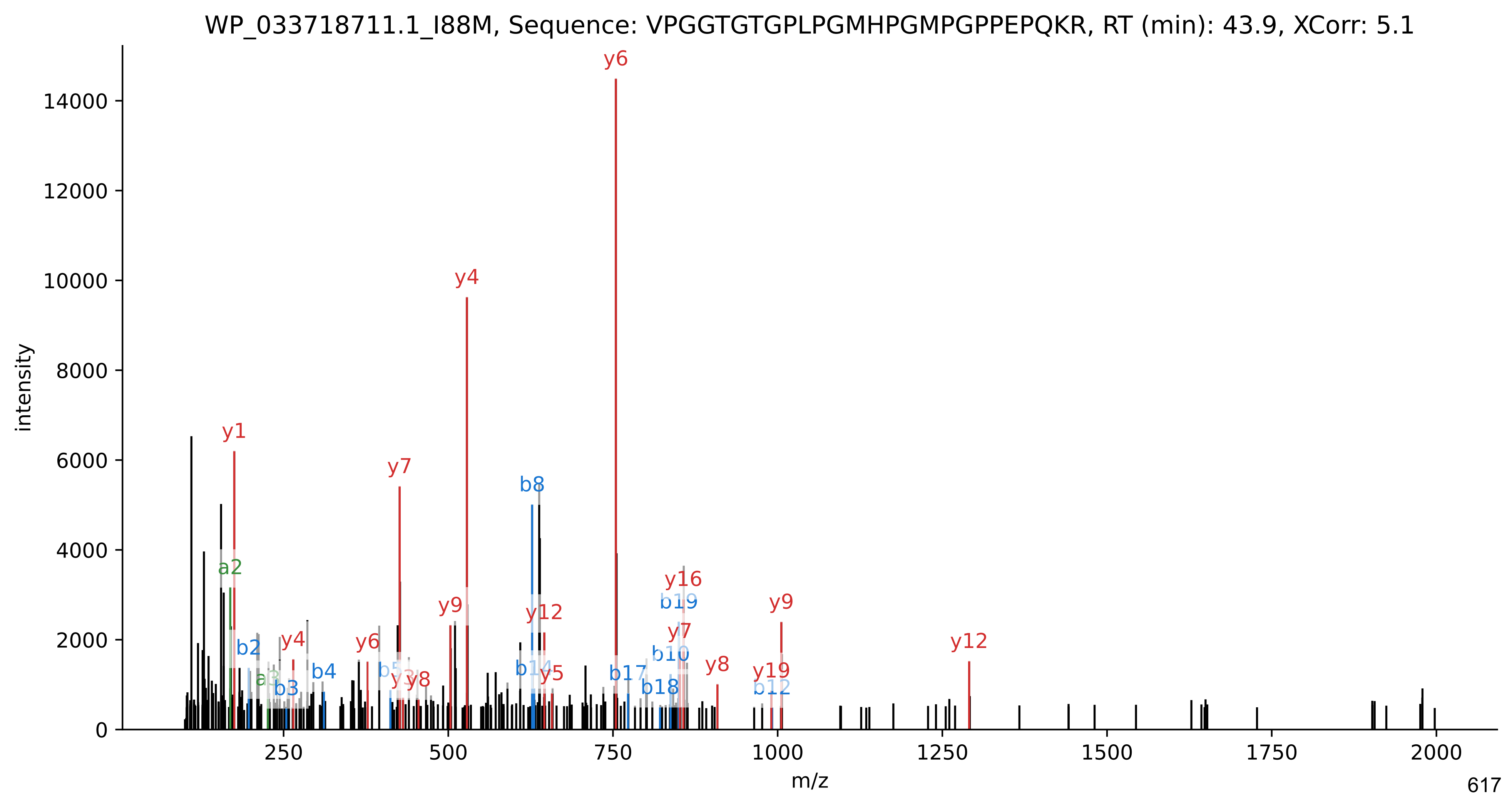

WP_033718711.1_I88M, Sequence: VPGGTGTGPLPGMHPGMPGPPEPQKR, RT (min): 43.9, XCorr: 5.1
intensity
m/z
y6
y4
y1
y7
b8
a2
y16
b19
y9
y12
y9
y7
b10
y4
b2
y6
b4
b5
b14
y5
b17
y8
y19
y12
b3
y3
y8
b18
b12

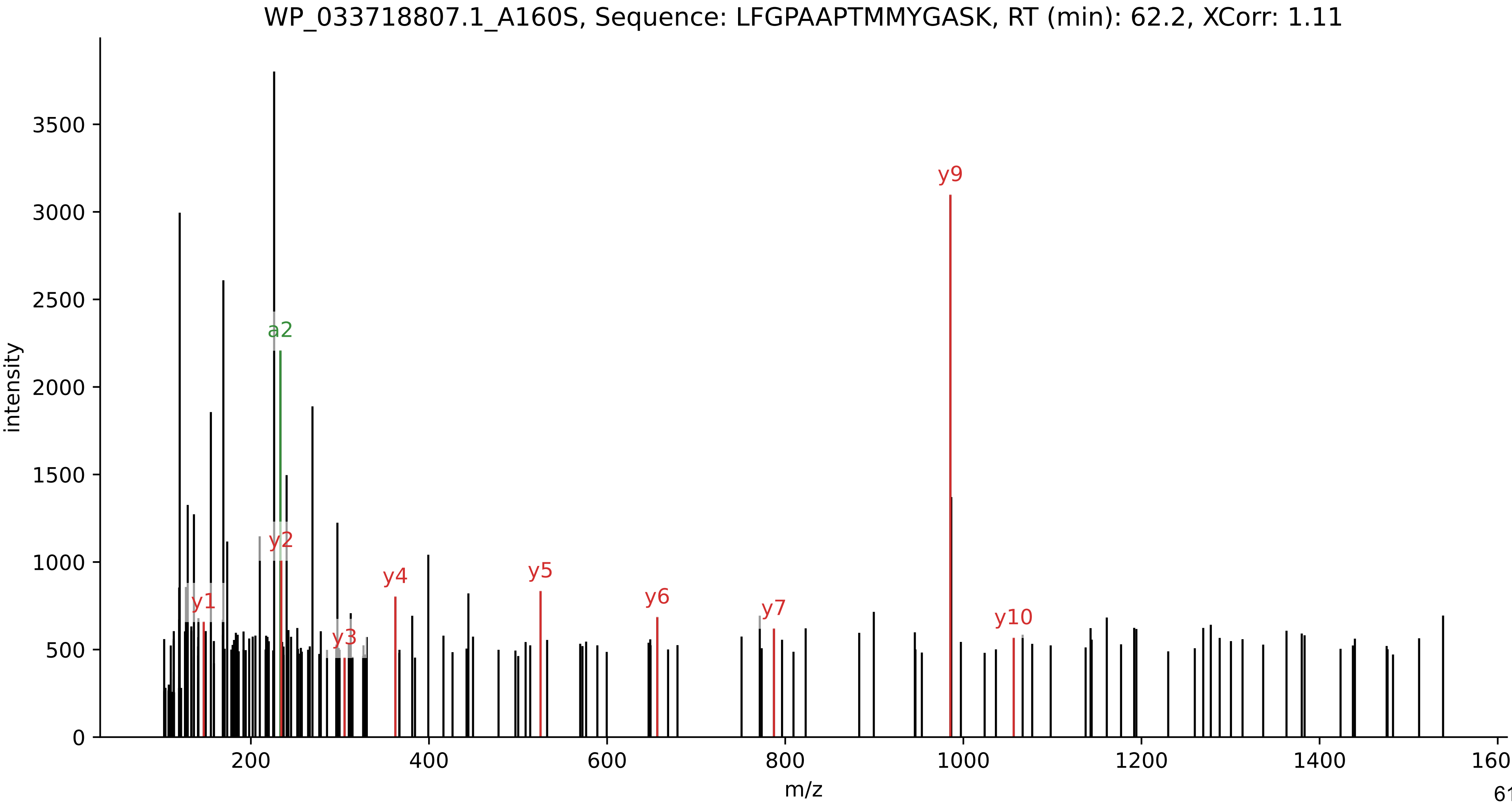
WP_033718807.1_A160S, Sequence: LFGPAAPTMMYGASK, RT (min): 62.2, XCorr: 1.11
intensity
m/z
0
500
1000
1500
2000
2500
3000
3500
200
400
600
800
1000
1200
1400
1600
a2
y1
y2
y3
y4
y5
y6
y7
y9
y10

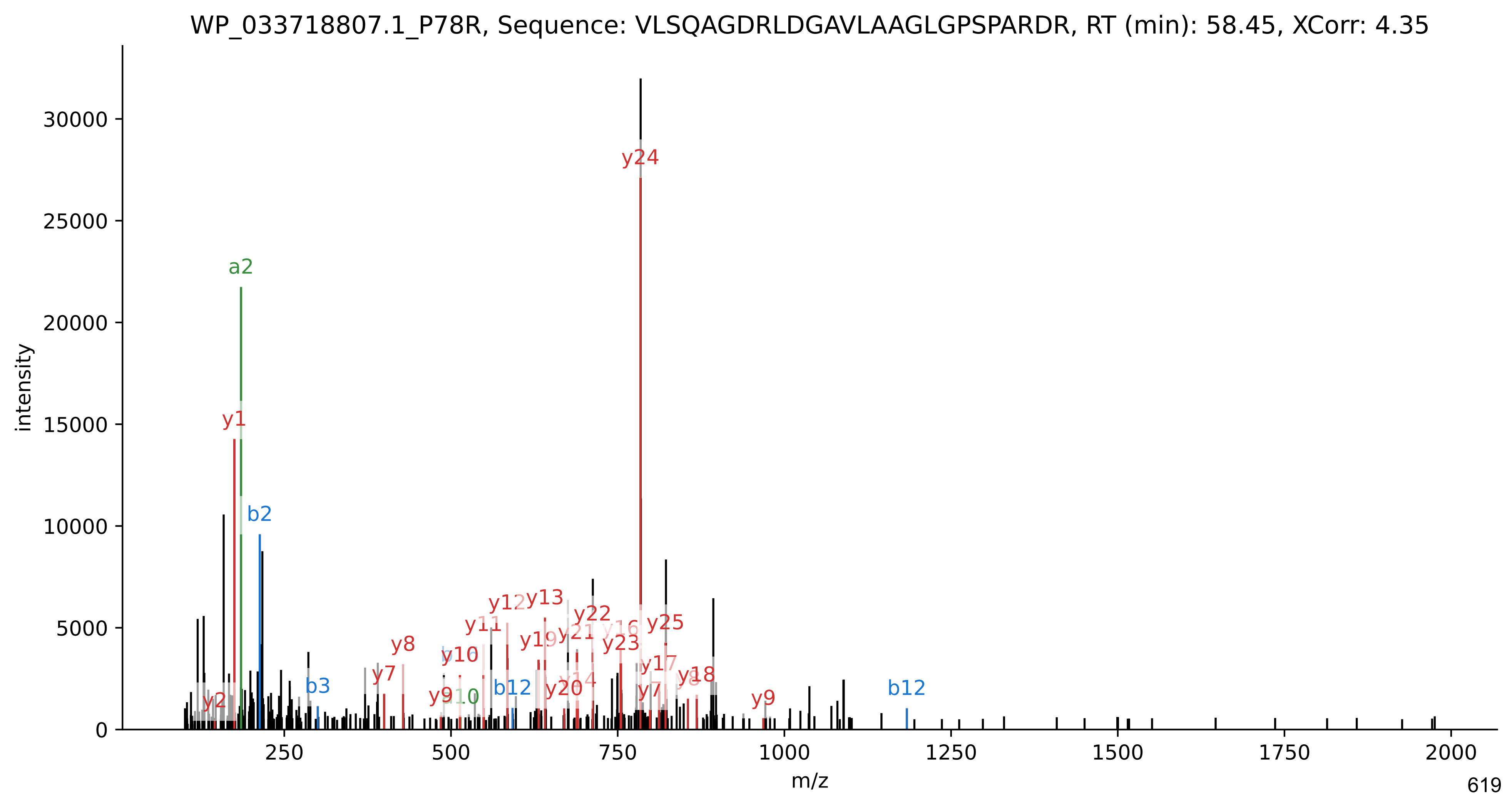
WP_033718807.1_P78R, Sequence: VLSQAGDRLDGAVLAAGLGPSPARDR, RT (min): 58.45, XCorr: 4.35
intensity
m/z
y24
a2
y1
b2
b3
y2
y7
y8
y9
y10
y11
y12
y13
y19
y21
y22
y23
y25
y17
y18
y20
b12
y9
b12

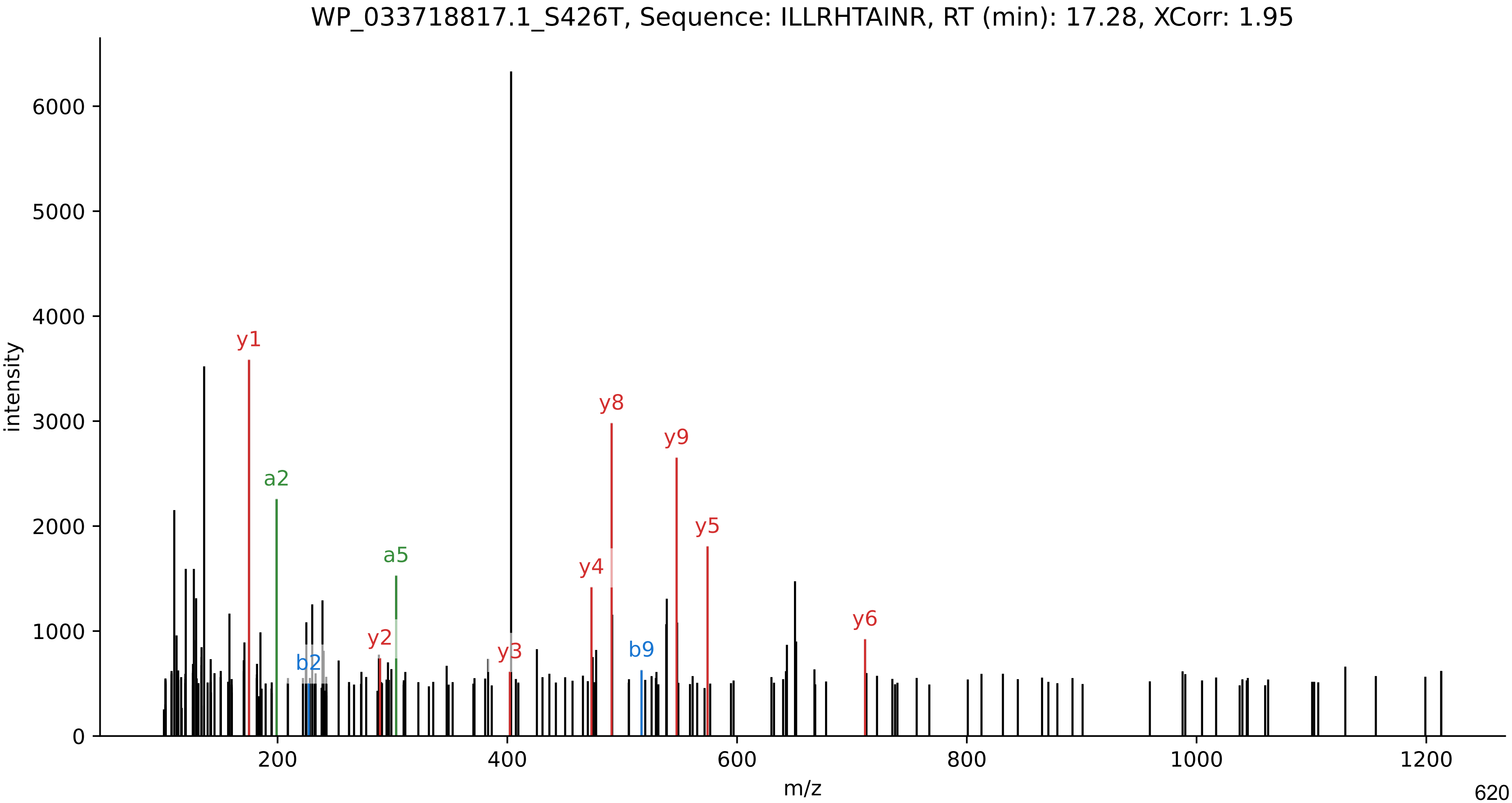

WP_033718817.1_S426T, Sequence: ILLRHTAINR, RT (min): 17.28, XCorr: 1.95
intensity
m/z
y1
a2
a5
y2
b2
y3
y4
y8
b9
y9
y5
y6
0
1000
2000
3000
4000
5000
6000
200
400
600
800
1000
1200

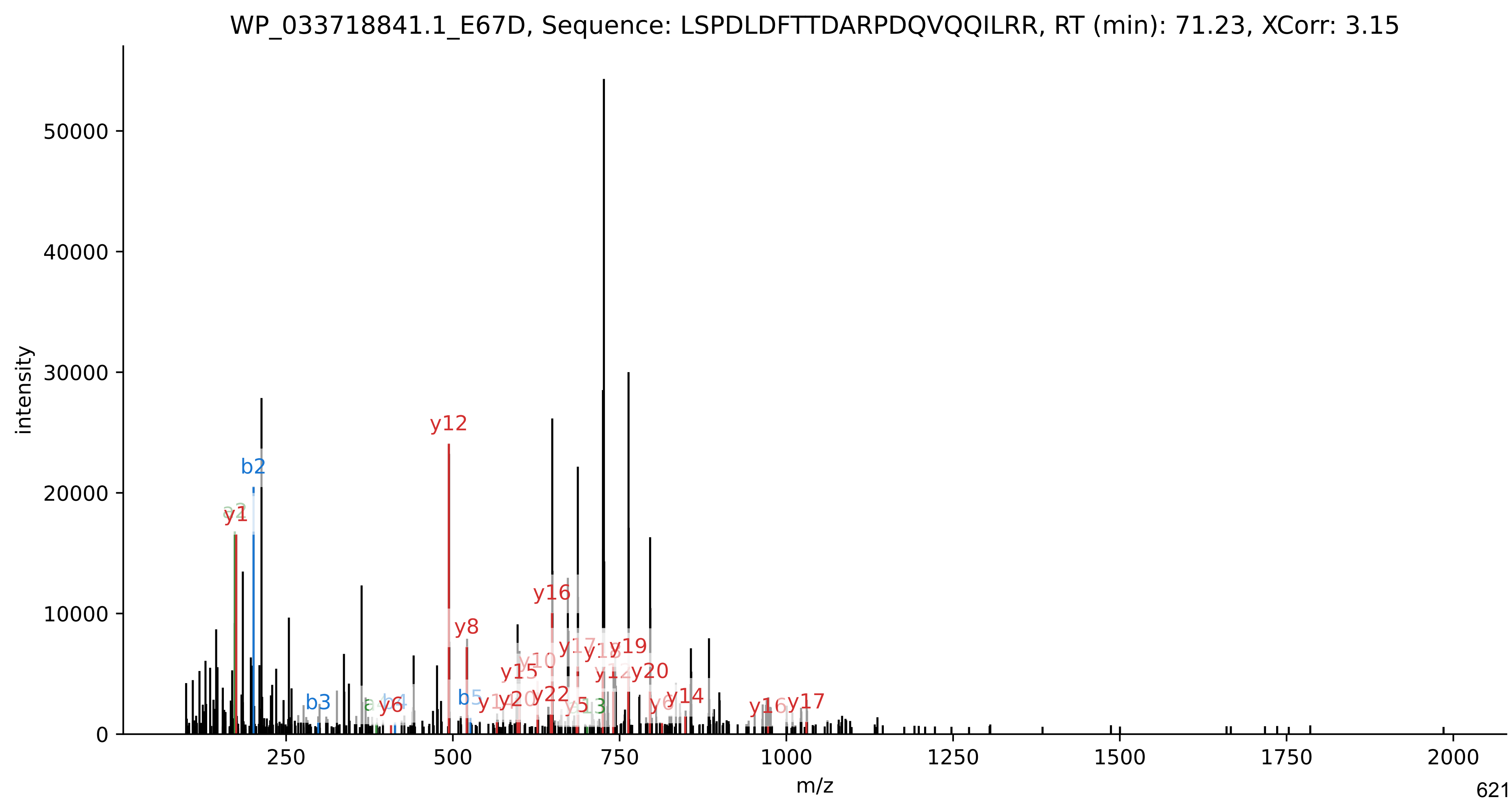

WP_033718841.1_E67D, Sequence: LSPDLDFTTDARPDQVQQILRR, RT (min): 71.23, XCorr: 3.15
intensity
m/z
y12
b2
y1
y16
y8
y19
y20
y15
y22
y14
y17
b3
b5
y6

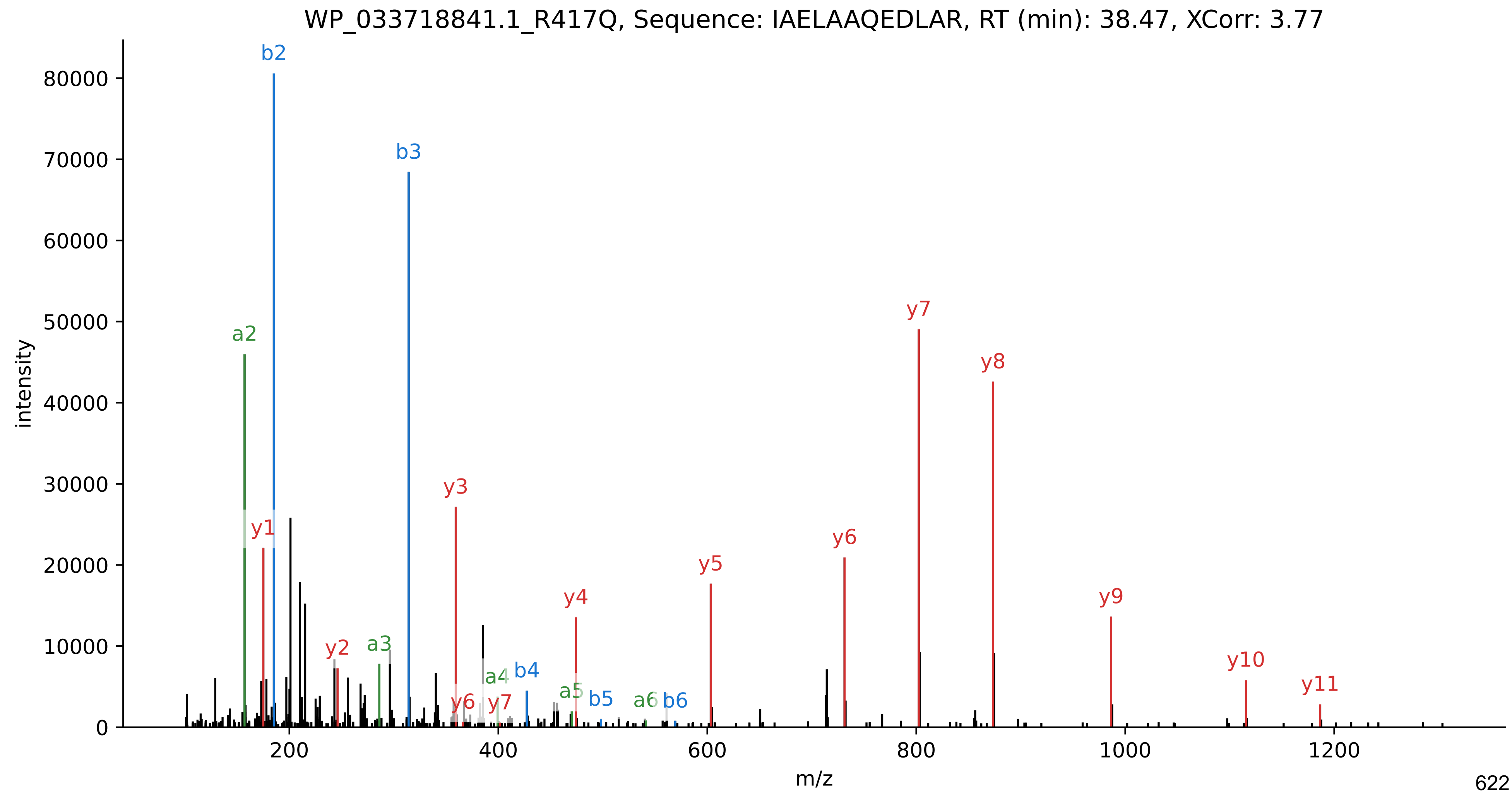

WP_033718841.1_R417Q, Sequence: IAELAAQEDLAR, RT (min): 38.47, XCorr: 3.77
intensity
m/z
b2
b3
a2
y1
y2
a3
y3
y6
y7
a4
b4
y4
a5
b5
a6
b6
y5
y6
y7
y8
y9
y10
y11
0
10000
20000
30000
40000
50000
60000
70000
80000
200
400
600
800
1000
1200

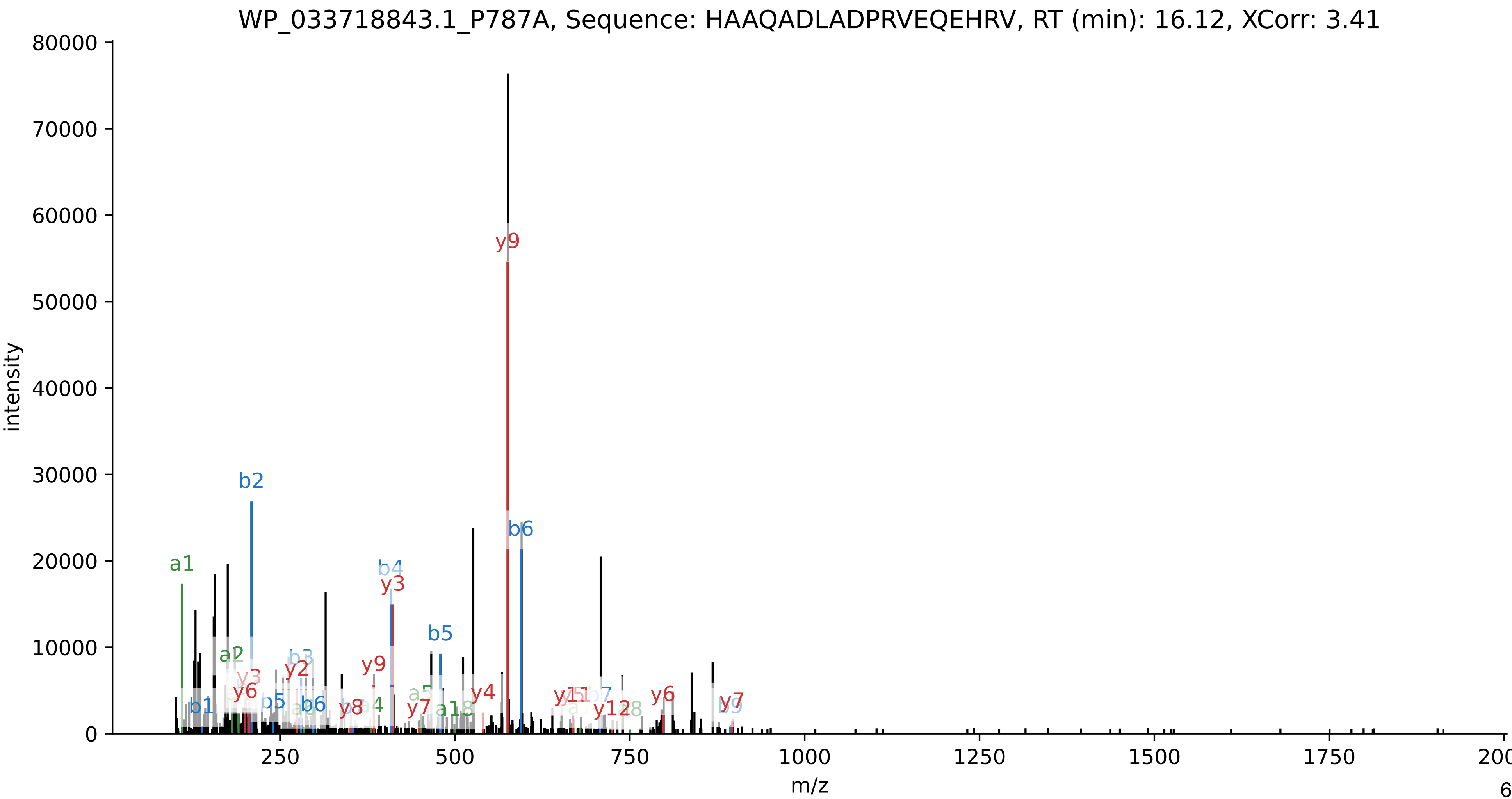

WP_033718843.1_P787A, Sequence: HAAQADLADPRVEQEHRV, RT (min): 16.12, XCorr: 3.41
intensity
m/z
y9
b2
b6
a1
b4
y3
b5
a2
b3
y2
y9
y3
y6
b1
b5
b6
y8
y7
y4
y6
y7
y12

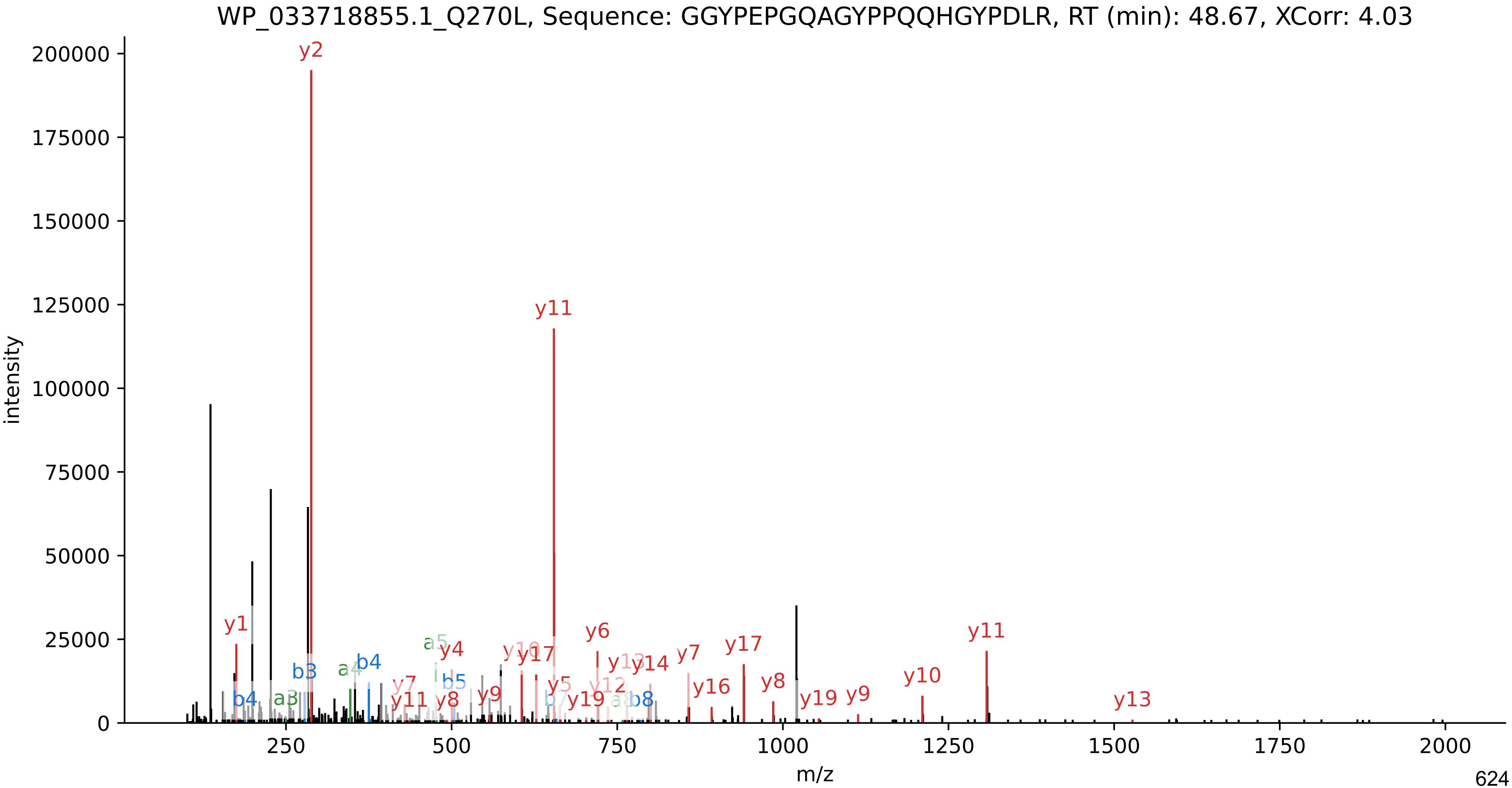
WP_033718855.1_Q270L, Sequence: GGYPEPGQAGYPPQQHGYPDLR, RT (min): 48.67, XCorr: 4.03
intensity
m/z
y2
y11
y1
b4
a3
b3
a4
b4
a5
y4
b5
y7
y11
y8
y9
y10
y17
y5
b7
y19
y6
y13
y12
a8
b8
y14
y7
y16
y17
y8
y19
y9
y10
y11
y13

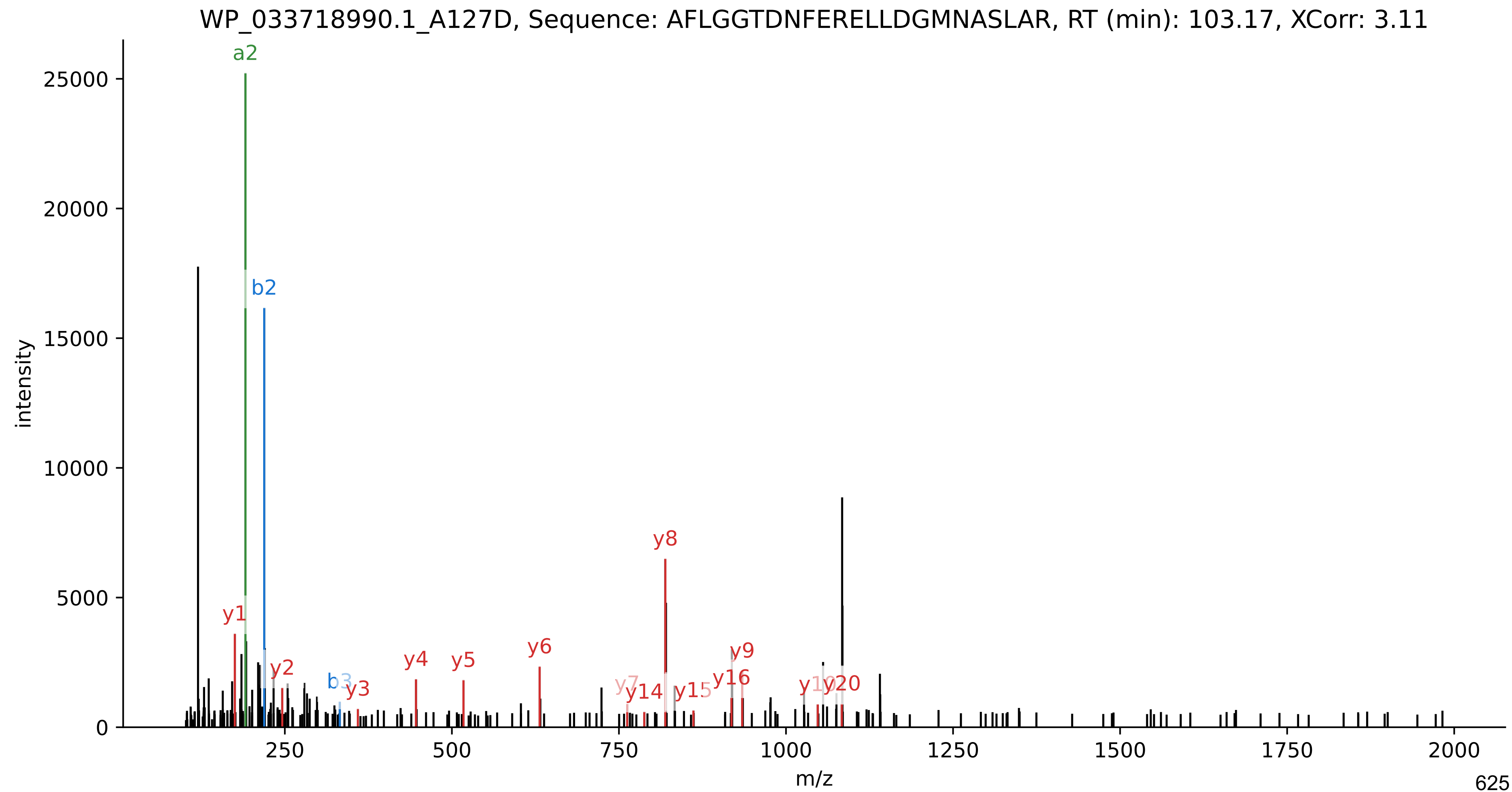

WP_033718990.1_A127D, Sequence: AFLGGTDNFERELLDGMNASLAR, RT (min): 103.17, XCorr: 3.11
intensity
m/z
a2
b2
y1
y2
b3
y3
y4
y5
y6
y7
y14
y15
y16
y8
y9
y19
y20

WP_033718998.1_D324E, Sequence: EALGGRPFGDGTPPGPPVEAERVPAGHGA, RT (min): 43.82, XCorr: 5.62

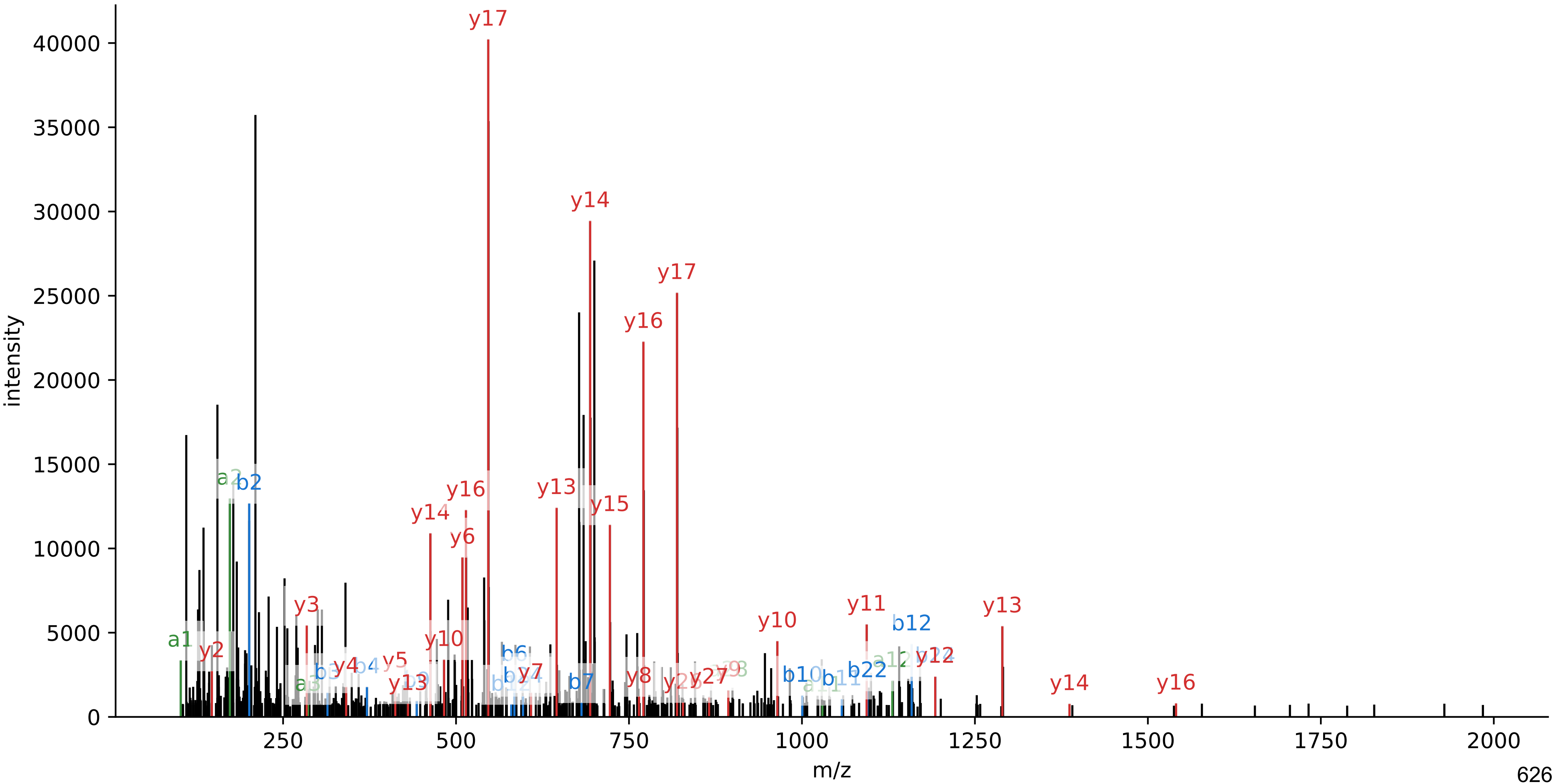

WP_033718998.1_I301T, Sequence: GPDLAGWLTPHGR, RT (min): 57.77, XCorr: 4.05

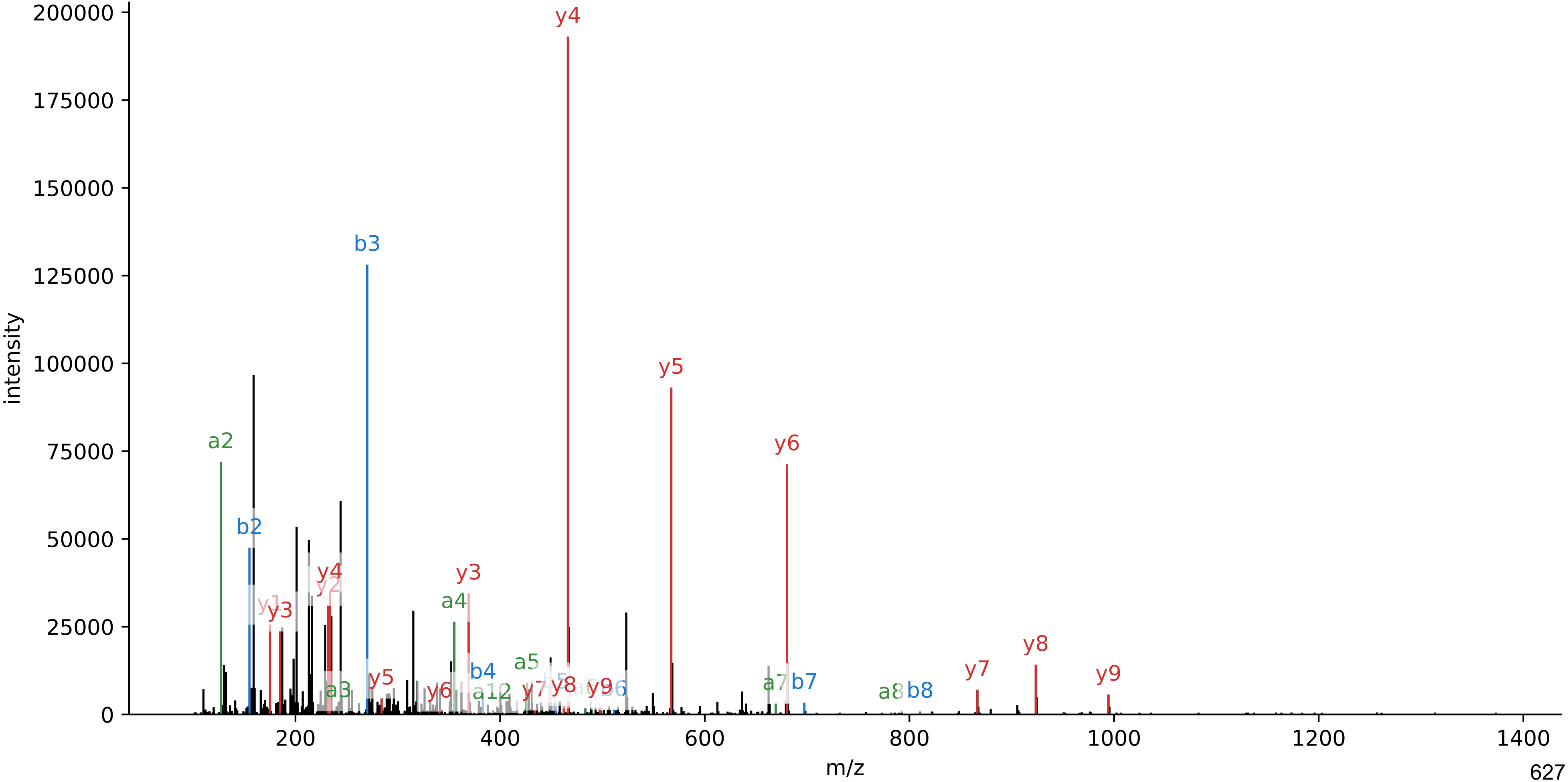

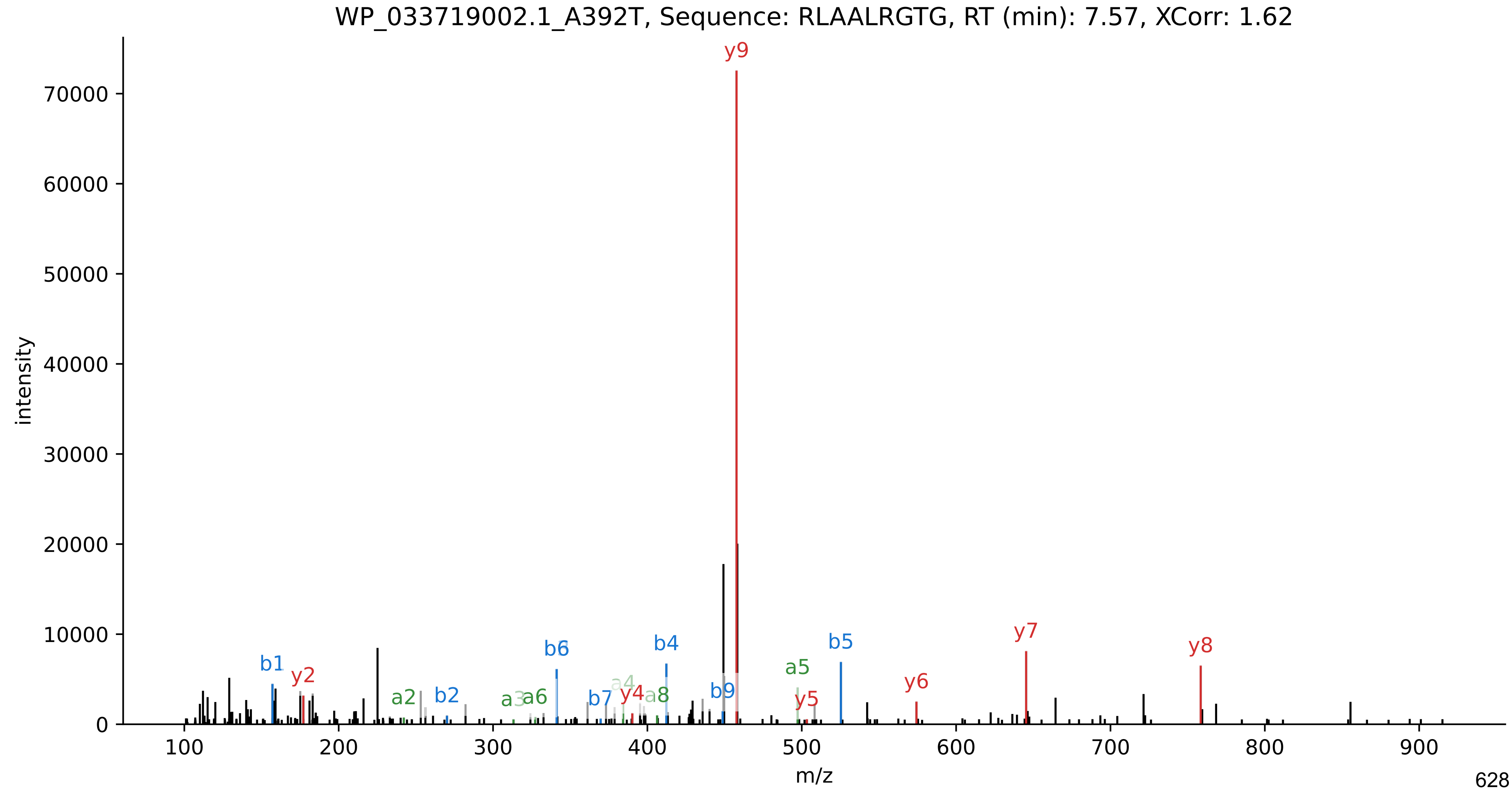

WP_033719002.1_A392T, Sequence: RLAALRGTG, RT (min): 7.57, XCorr: 1.62
intensity
m/z
y9
b1
y2
a2
b2
a3
a6
b6
b7
a4
y4
a8
b4
b9
a5
y5
b5
y6
y7
y8

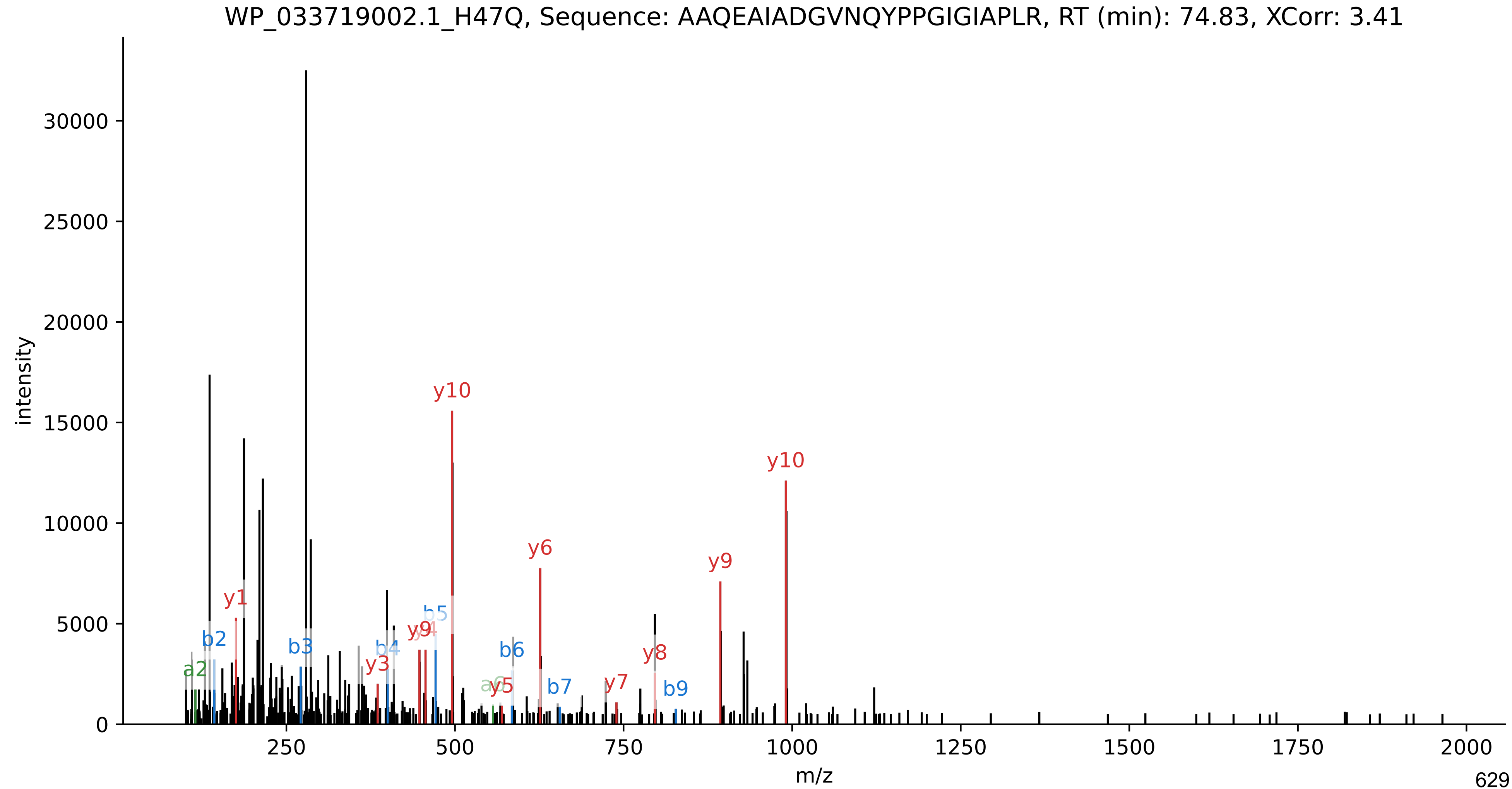

WP_033719002.1_H47Q, Sequence: AAQEAIADGVNQYPPGIGIAPLR, RT (min): 74.83, XCorr: 3.41
intensity
m/z
y10
y10
y6
y9
y1
b2
b5
y9
y4
b3
b4
y3
b6
a2
y8
y7
b7
b9
y5

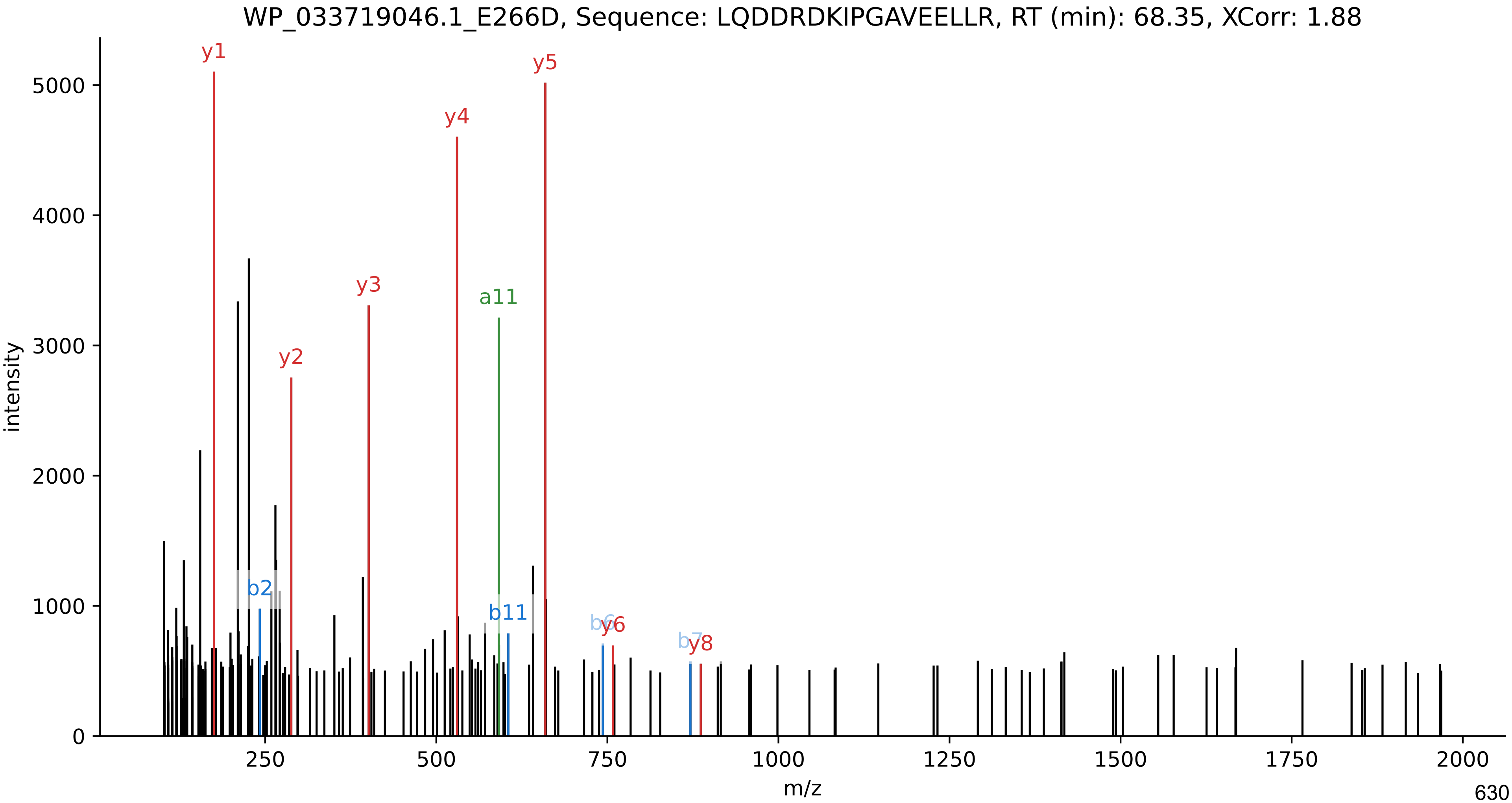
WP_033719046.1_E266D, Sequence: LQDDRDKIPGAVEELLR, RT (min): 68.35, XCorr: 1.88
y1
y5
y4
y3
a11
y2
b2
b11
b6
y6
b7
y8
intensity
m/z
0
1000
2000
3000
4000
5000
250
500
750
1000
1250
1500
1750
2000

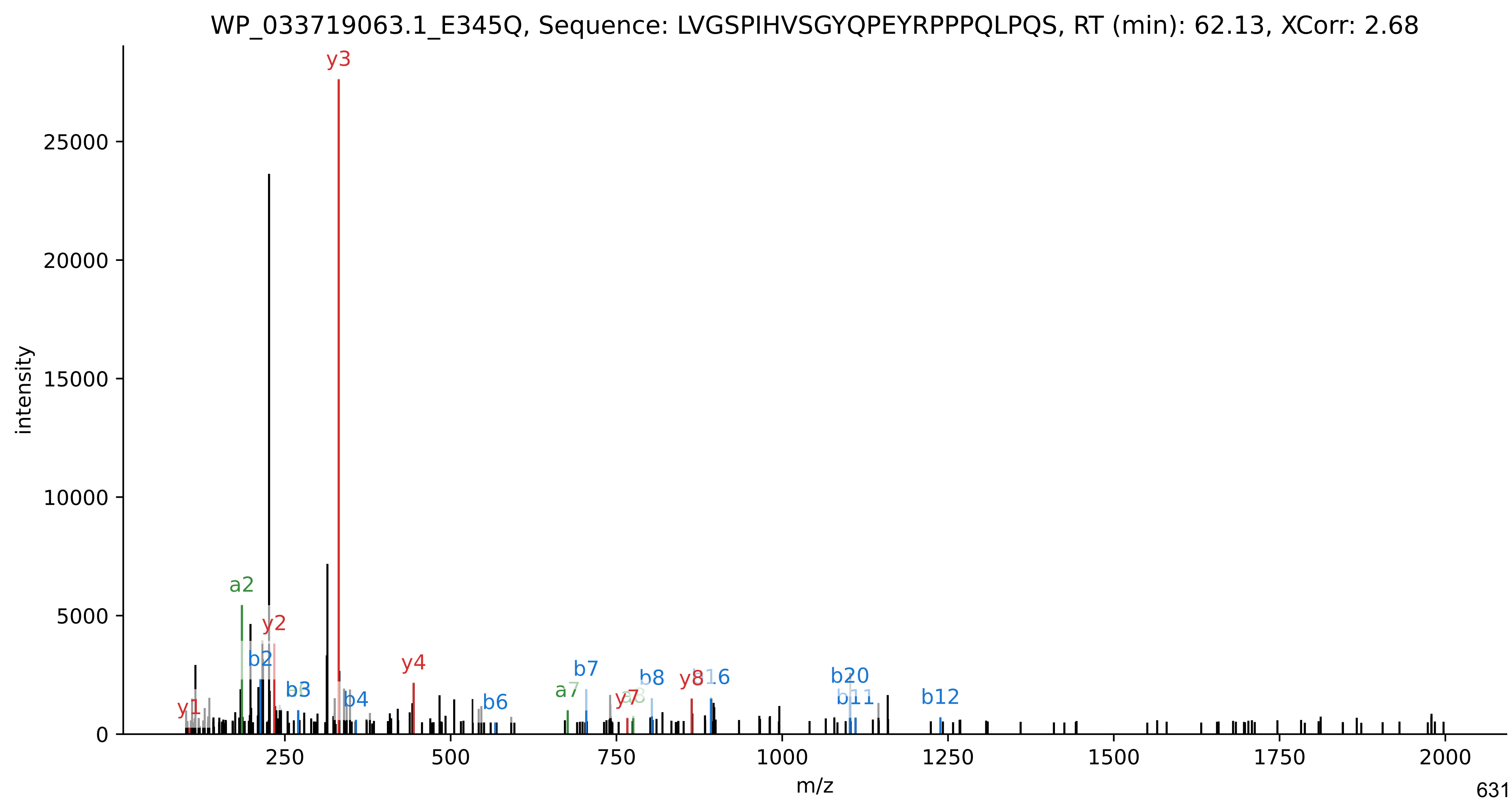

WP_033719063.1_E345Q, Sequence: LVGSPIHVSGYQPEYRPPPQLPQS, RT (min): 62.13, XCorr: 2.68
intensity
m/z
y3
a2
y2
b2
b3
b4
y4
b6
a7
b7
y7
b8
y8
b20
b11
b12

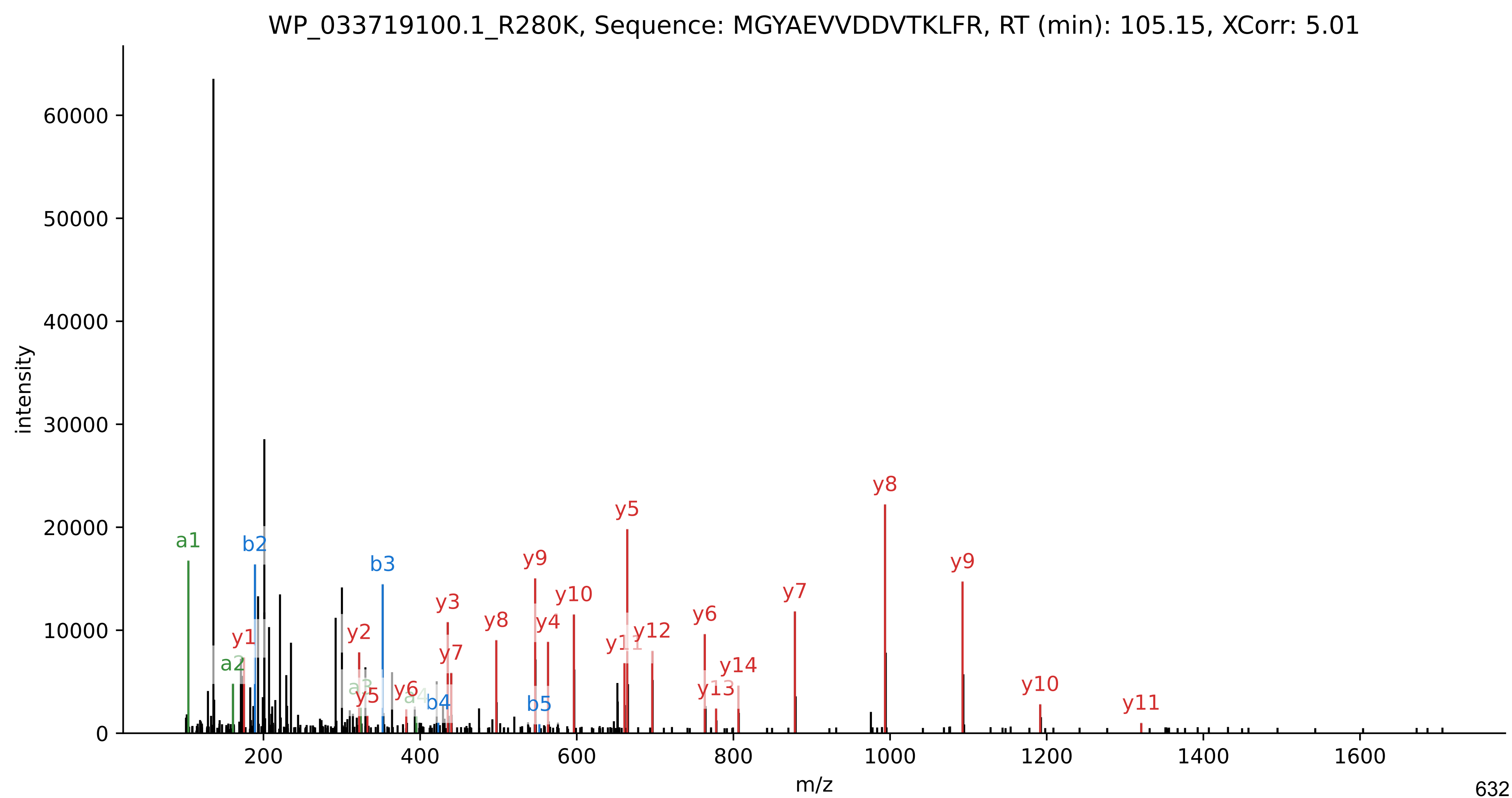

WP_033719100.1_R280K, Sequence: MGYAEVVDDVTKLFR, RT (min): 105.15, XCorr: 5.01
intensity
m/z
a1
a2
b2
y1
b3
y2
y3
y5
y6
b4
y7
y8
y9
y4
y10
b5
y11
y12
y5
y6
y13
y14
y7
y8
y9
y10
y11

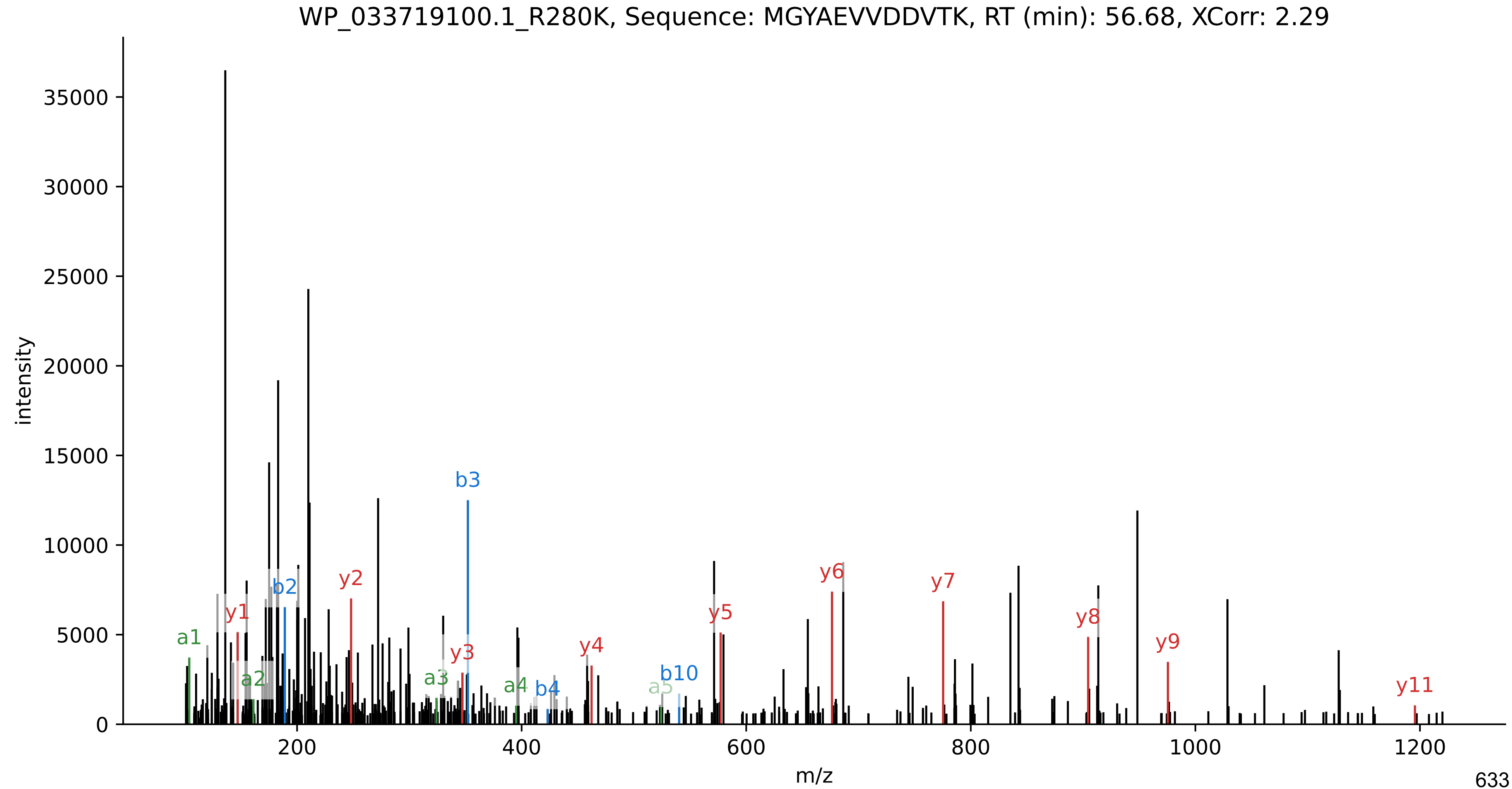

WP_033719100.1_R280K, Sequence: MGYAEVVDDVTK, RT (min): 56.68, XCorr: 2.29
intensity
m/z
35000
30000
25000
20000
15000
10000
5000
0
200
400
600
800
1000
1200
a1
y1
a2
b2
y2
b3
a3
y3
a4
b4
y4
a5
b10
y5
y6
y7
y8
y9
y11

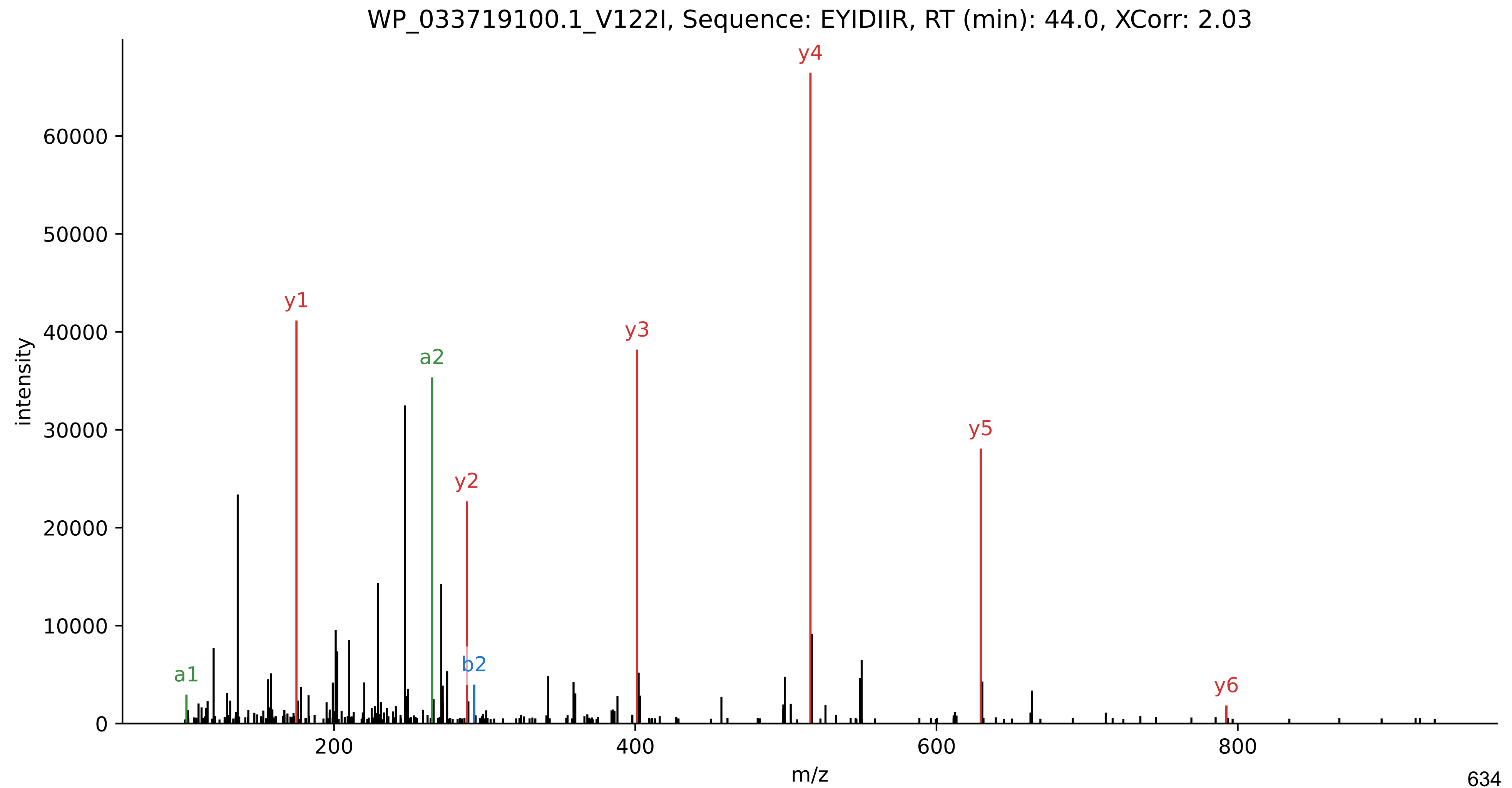

WP_033719100.1_V122I, Sequence: EYIDIIR, RT (min): 44.0, XCorr: 2.03
intensity
m/z
0
10000
20000
30000
40000
50000
60000
200
400
600
800
a1
y1
a2
y2
b2
y3
y4
y5
y6

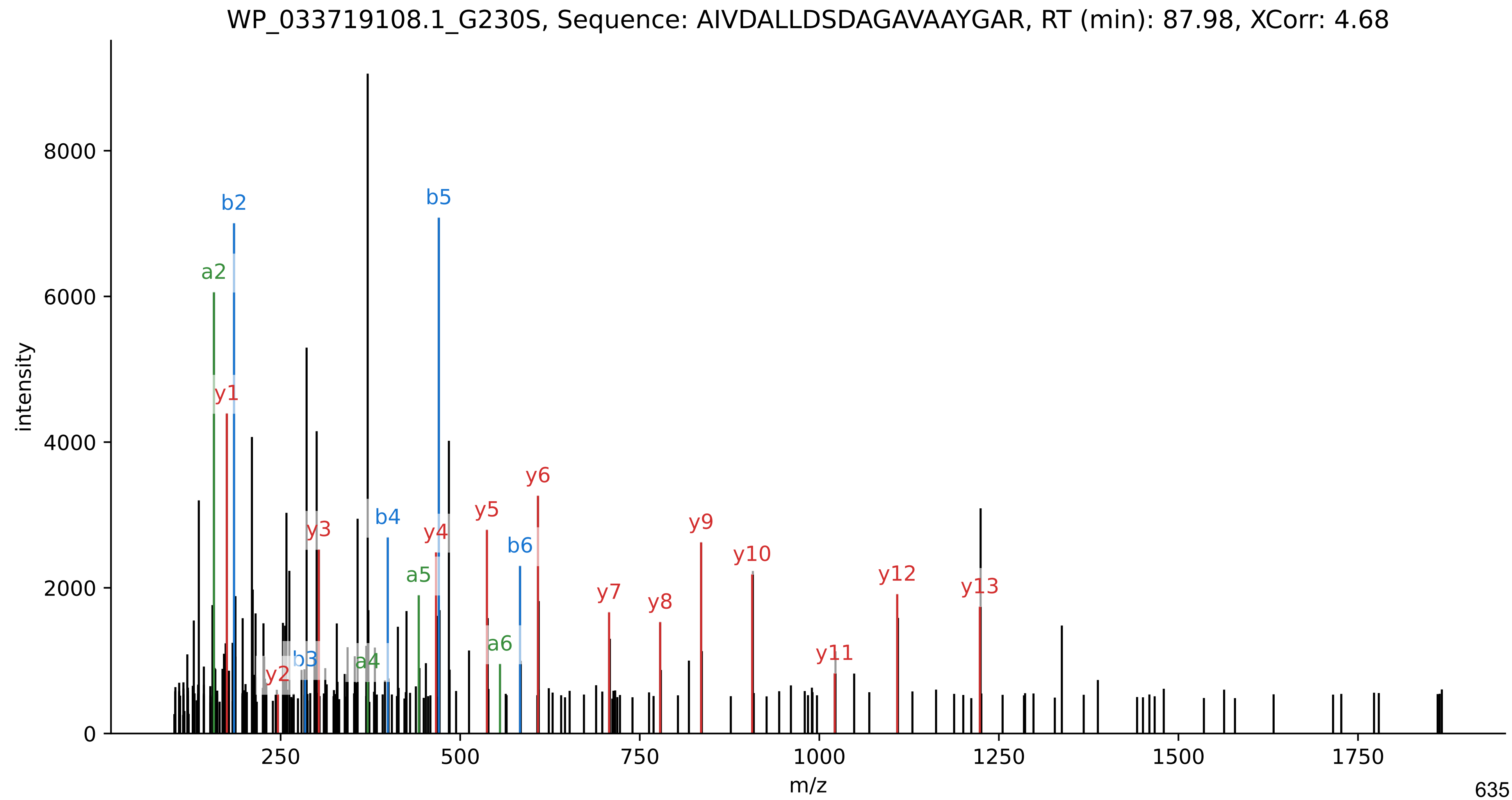

WP_033719108.1_G230S, Sequence: AIVDALLDSDAGAVAAYGAR, RT (min): 87.98, XCorr: 4.68
intensity
m/z
b2
b5
a2
y1
y6
y5
b4
y3
y4
y9
b6
y10
a5
y12
y13
y7
y8
a6
y11
b3
a4
y2

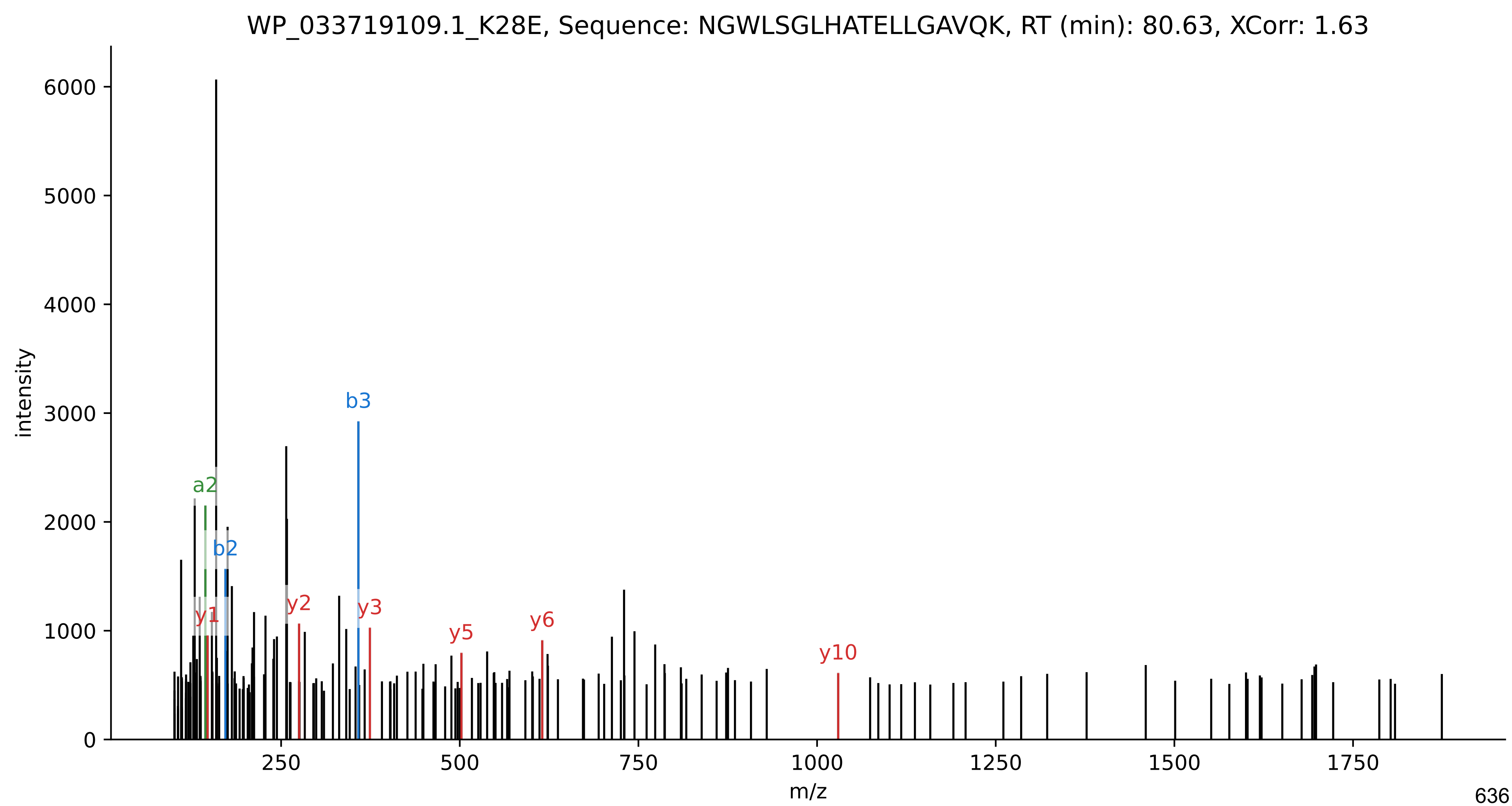

WP_033719109.1_K28E, Sequence: NGWLSGLHATELLGAVQK, RT (min): 80.63, XCorr: 1.63
intensity
m/z
a2
b2
b3
y1
y2
y3
y5
y6
y10
0
1000
2000
3000
4000
5000
6000
250
500
750
1000
1250
1500
1750

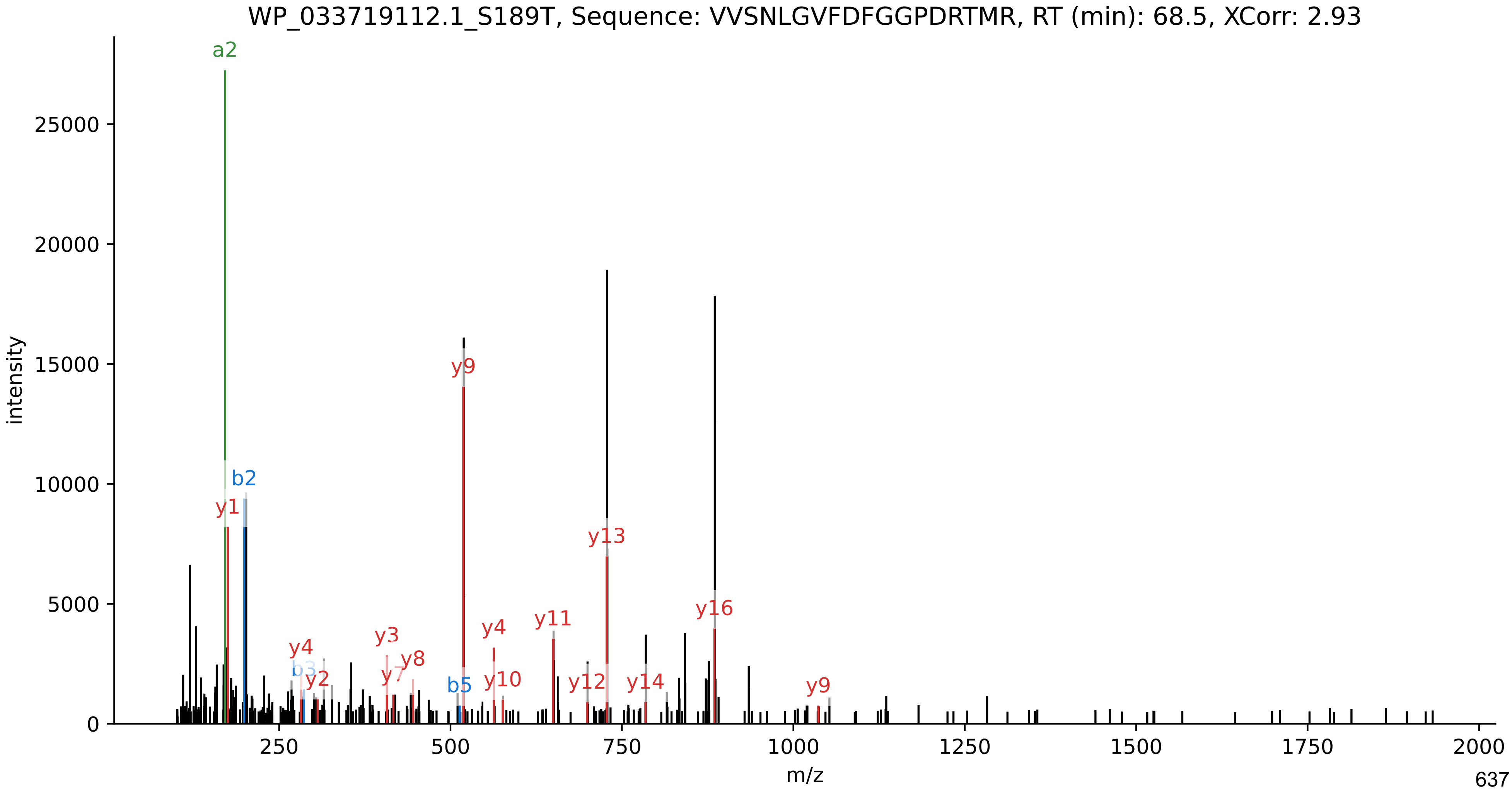
WP_033719112.1_S189T, Sequence: VVSNLGVFDFGGPDRTMR, RT (min): 68.5, XCorr: 2.93
intensity
m/z
0
5000
10000
15000
20000
25000
250
500
750
1000
1250
1500
1750
2000
a2
b2
y1
y9
y13
y16
y11
y4
y3
y4
b3
y2
y7
y8
b5
y10
y12
y14
y9

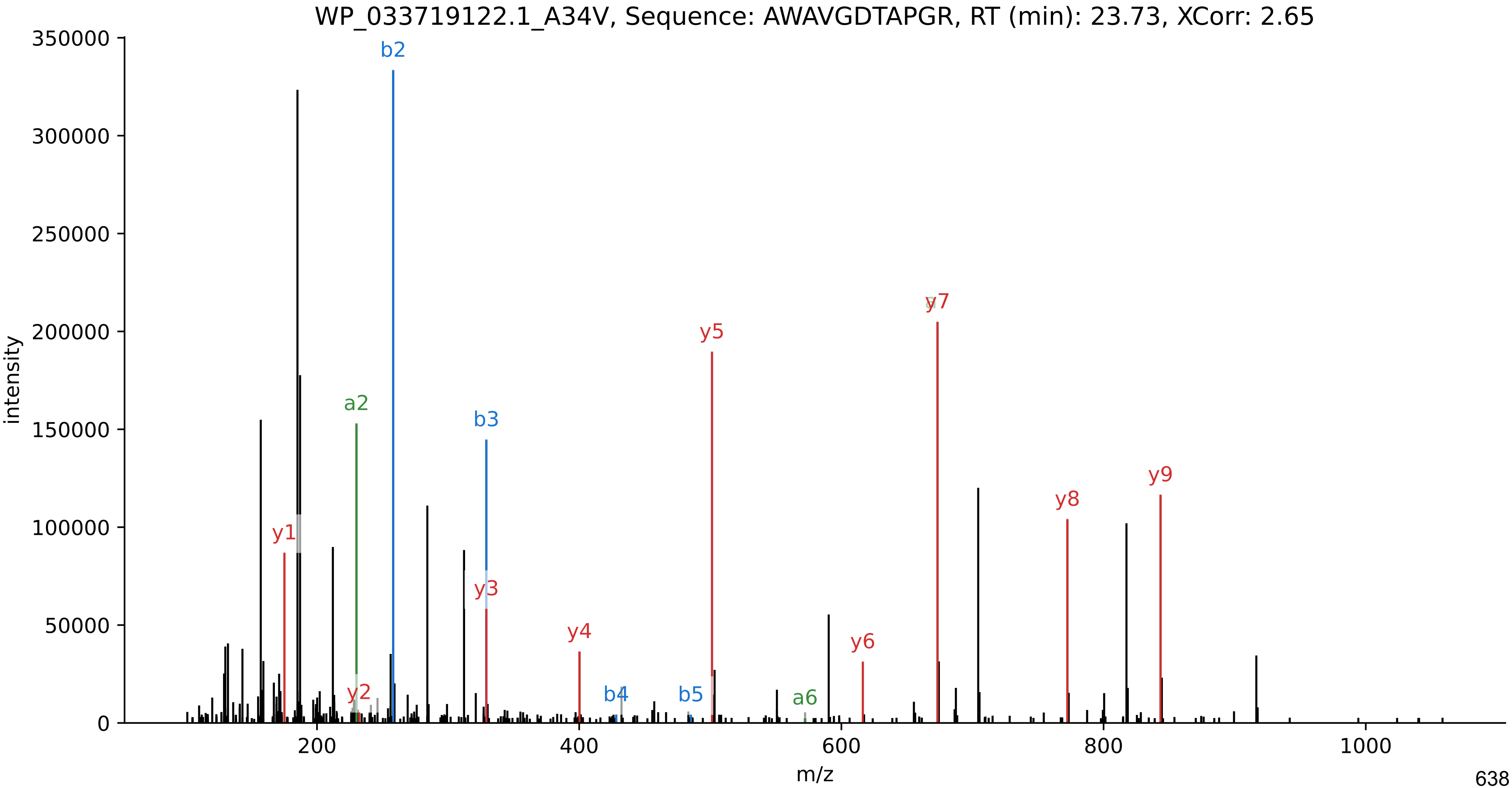
WP_033719122.1_A34V, Sequence: AWAVGDTAPGR, RT (min): 23.73, XCorr: 2.65
intensity
m/z
b2
a2
b3
y1
y2
y3
y4
b4
b5
y5
a6
y6
y7
y8
y9
0
50000
100000
150000
200000
250000
300000
350000
200
400
600
800
1000

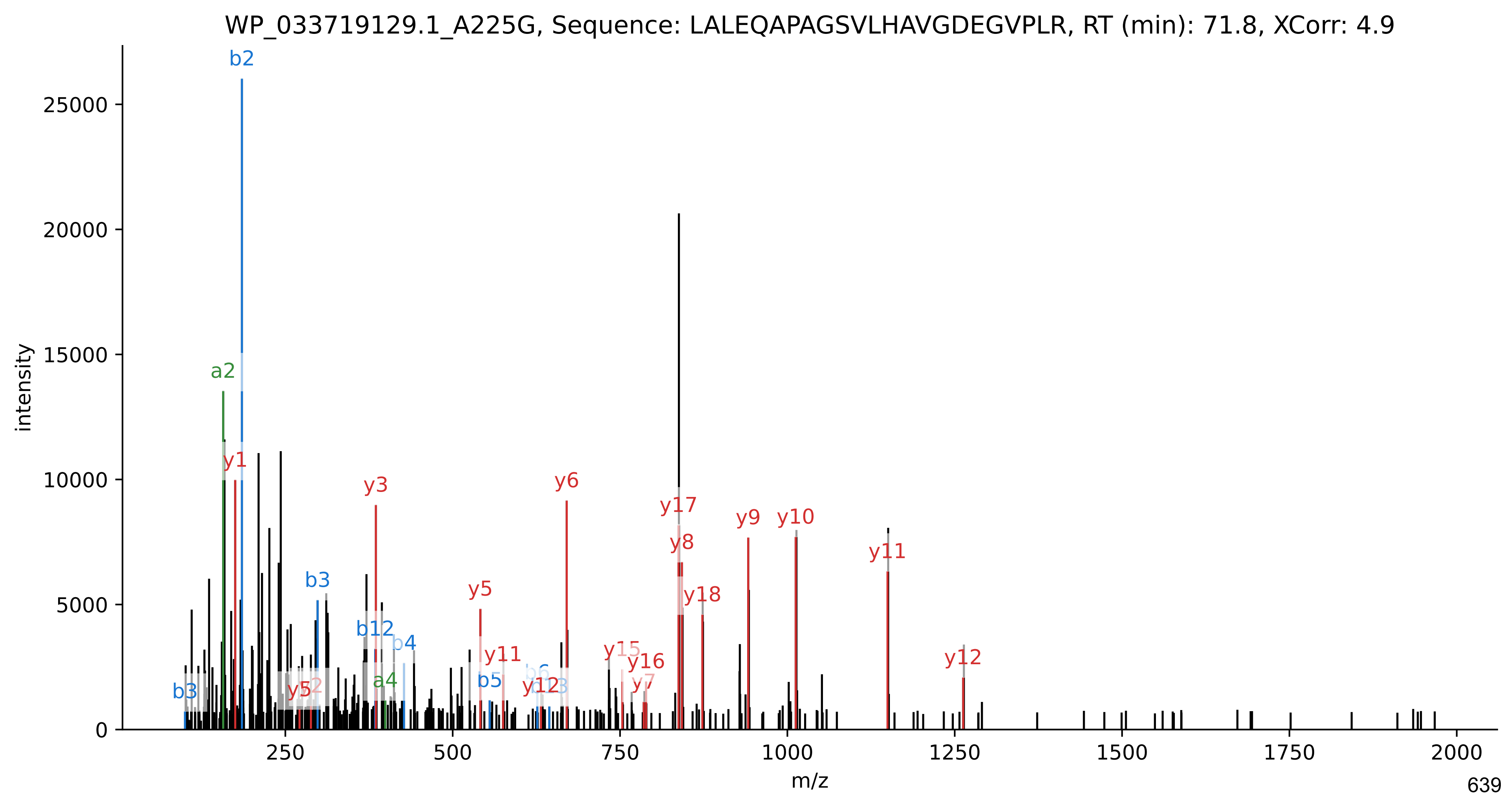

WP_033719129.1_A225G, Sequence: LALEQAPAGSVLHAVGDEGVPLR, RT (min): 71.8, XCorr: 4.9
intensity
m/z
b2
a2
y1
y3
y6
y17
y9
y10
y8
y18
y11
b3
y5
b12
b4
y11
y15
y16
y12
a4
b5
y7

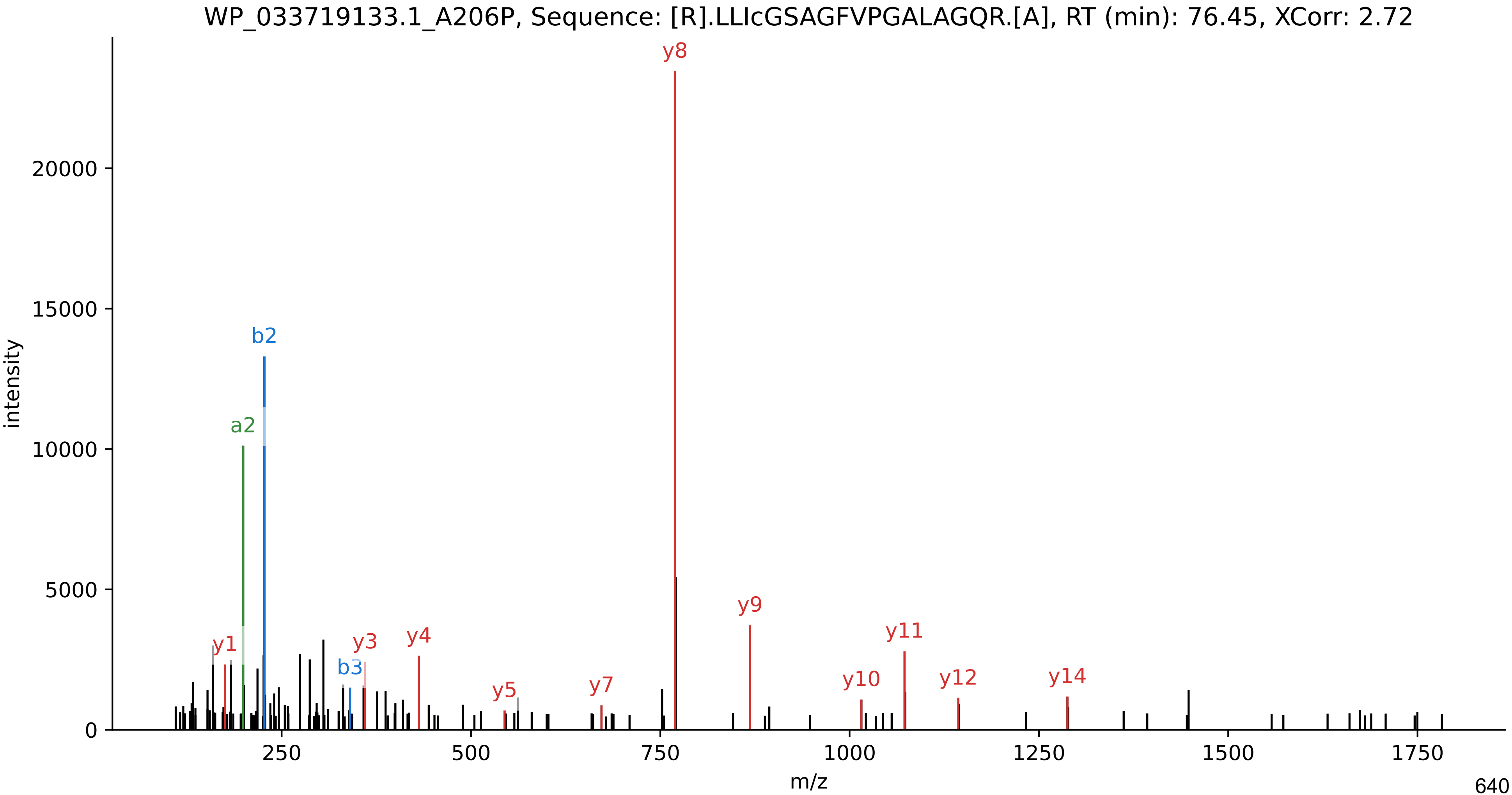
WP_033719133.1_A206P, Sequence: [R].LLIcGSAGFVPGALAGQR.[A], RT (min): 76.45, XCorr: 2.72
intensity
m/z
y8
b2
a2
y1
b3
y3
y4
y5
y7
y9
y10
y11
y12
y14
0
5000
10000
15000
20000
250
500
750
1000
1250
1500
1750

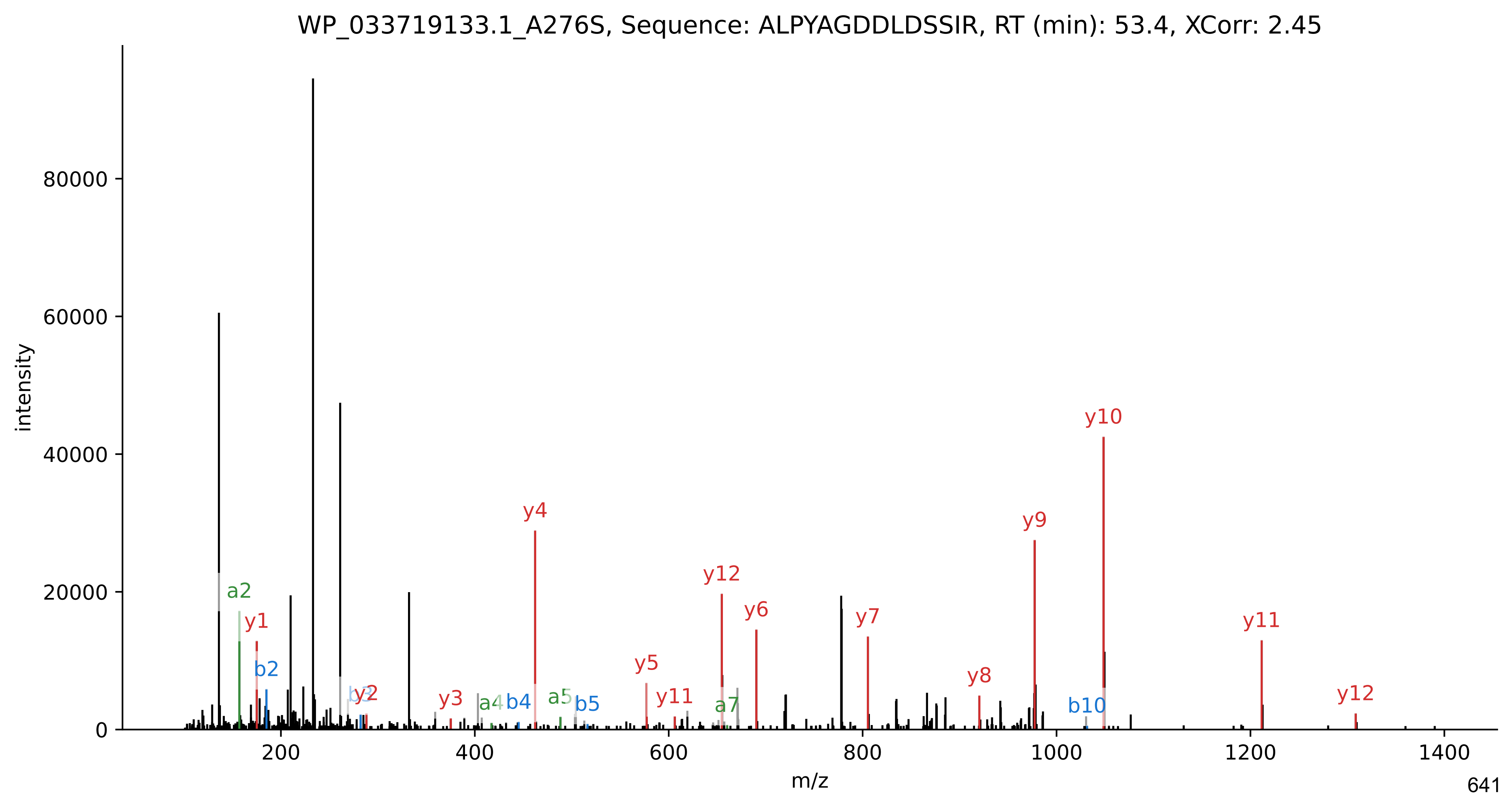

WP_033719133.1_A276S, Sequence: ALPYAGDDLDSSIR, RT (min): 53.4, XCorr: 2.45
intensity
m/z
0
20000
40000
60000
80000
200
400
600
800
1000
1200
1400
a2
y1
b2
y2
y3
a4
b4
y4
a5
b5
y5
y11
y12
a7
y6
y7
y8
y9
b10
y10
y11
y12

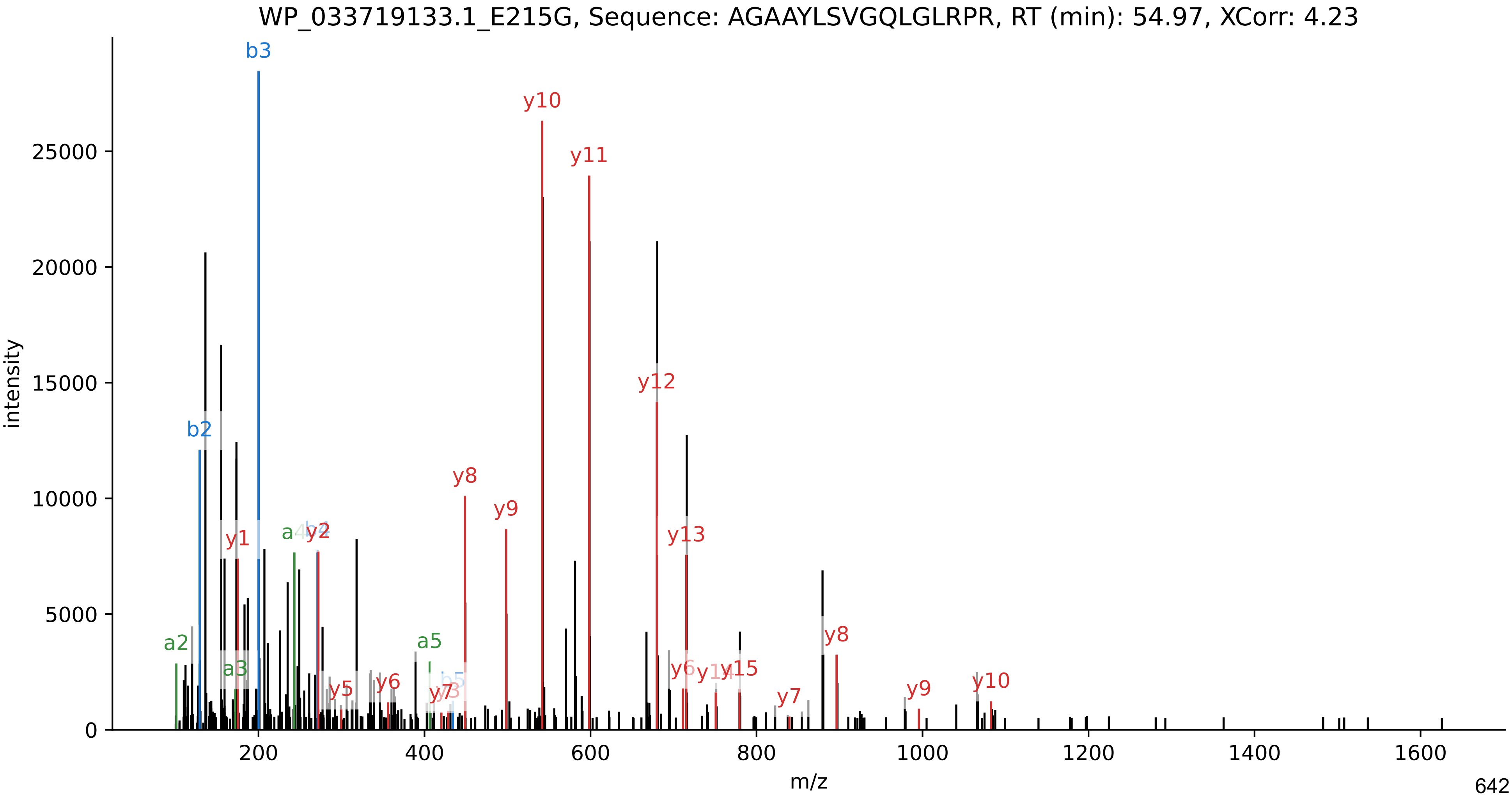

WP_033719133.1_E215G, Sequence: AGAAYLSVGQLGLRPR, RT (min): 54.97, XCorr: 4.23
intensity
m/z
b3
y10
y11
y12
y13
b2
y8
y9
y1
a2
a3
a5
y2
y5
y6
y7
y6
y15
y7
y8
y9
y10
0
5000
10000
15000
20000
25000
200
400
600
800
1000
1200
1400
1600

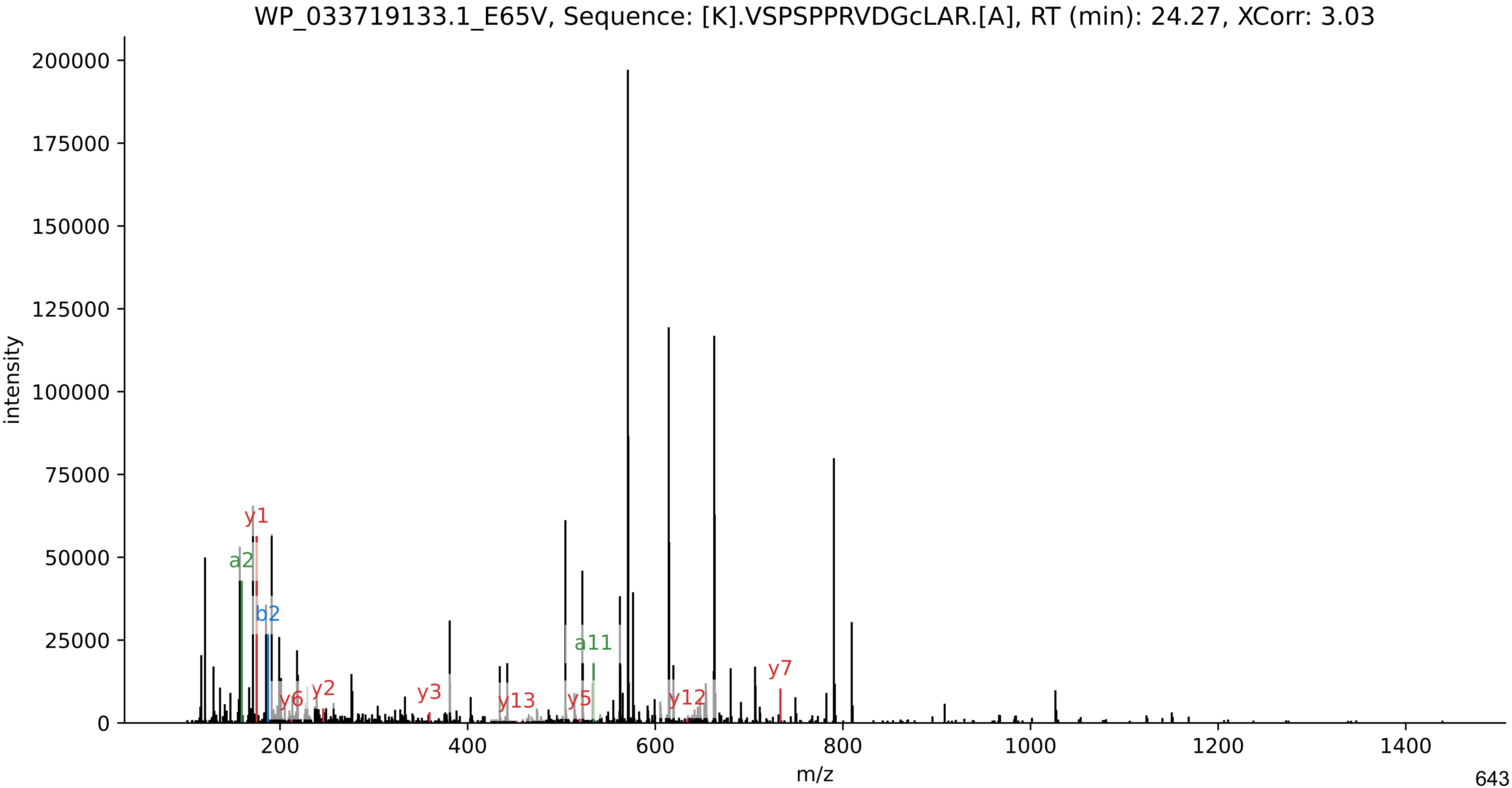
WP_033719133.1_E65V, Sequence: [K].VSPSPPRVDGcLAR.[A], RT (min): 24.27, XCorr: 3.03
intensity
m/z
y1
a2
b2
y6
y2
y3
y13
y5
a11
y12
y7
0
25000
50000
75000
100000
125000
150000
175000
200000
200
400
600
800
1000
1200
1400

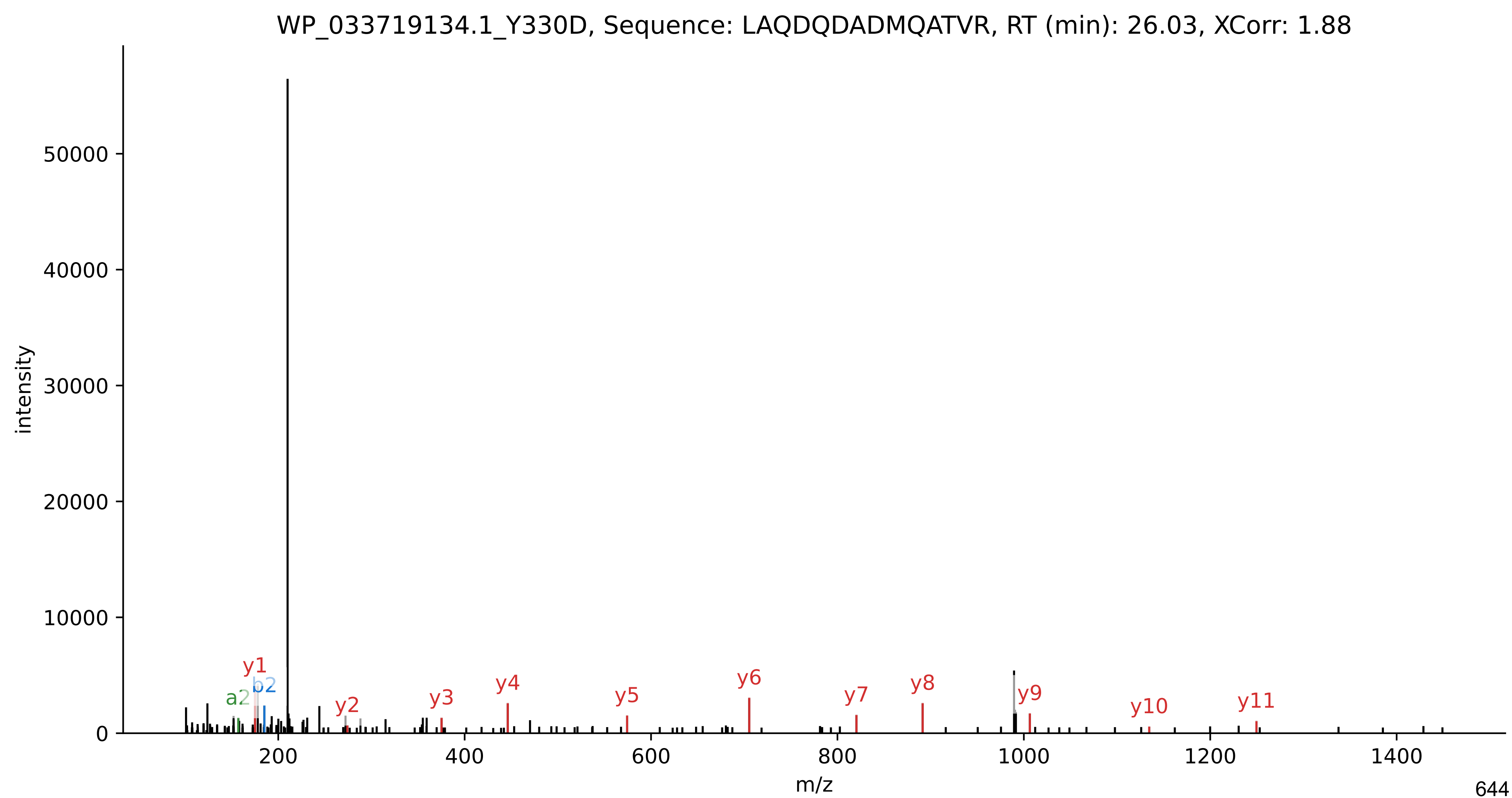
WP_033719134.1_Y330D, Sequence: LAQDQDADMQATVR, RT (min): 26.03, XCorr: 1.88
intensity
50000
40000
30000
20000
10000
0
200
400
600
800
1000
1200
1400
m/z
y1
b2
a2
y2
y3
y4
y5
y6
y7
y8
y9
y10
y11

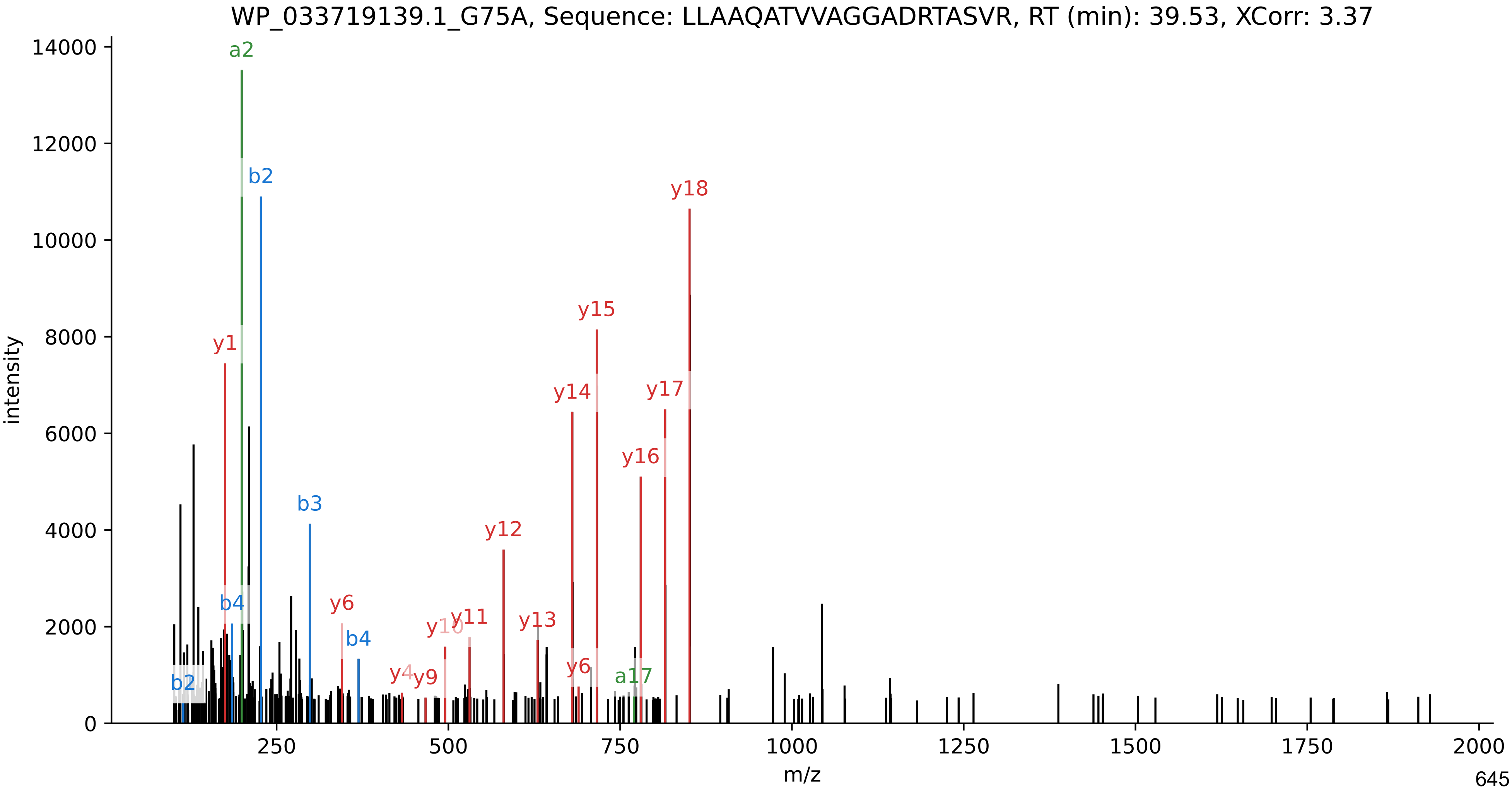
WP_033719139.1_G75A, Sequence: LLAAQATVVAGGADRTASVR, RT (min): 39.53, XCorr: 3.37
intensity
m/z
a2
b2
y18
y15
y1
y14
y17
y16
b3
y12
b4
y6
b4
y10
y11
y13
y4
y9
y6
a17
b2

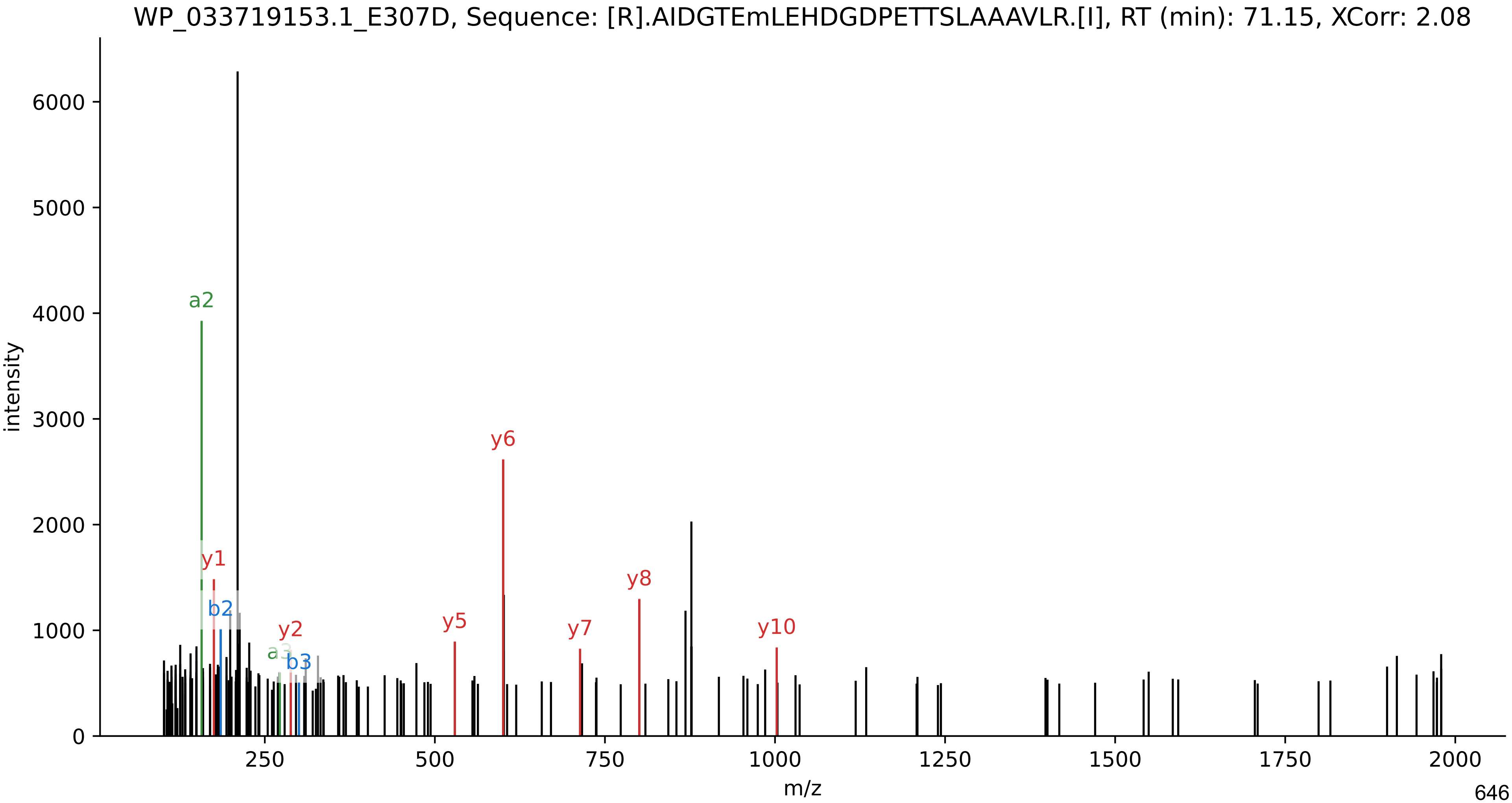

WP_033719153.1_E307D, Sequence: [R].AIDGTEmLEHDGDPETTSLAAAVLR.[I], RT (min): 71.15, XCorr: 2.08
intensity
m/z
a2
y1
b2
y2
a3
b3
y5
y6
y7
y8
y10

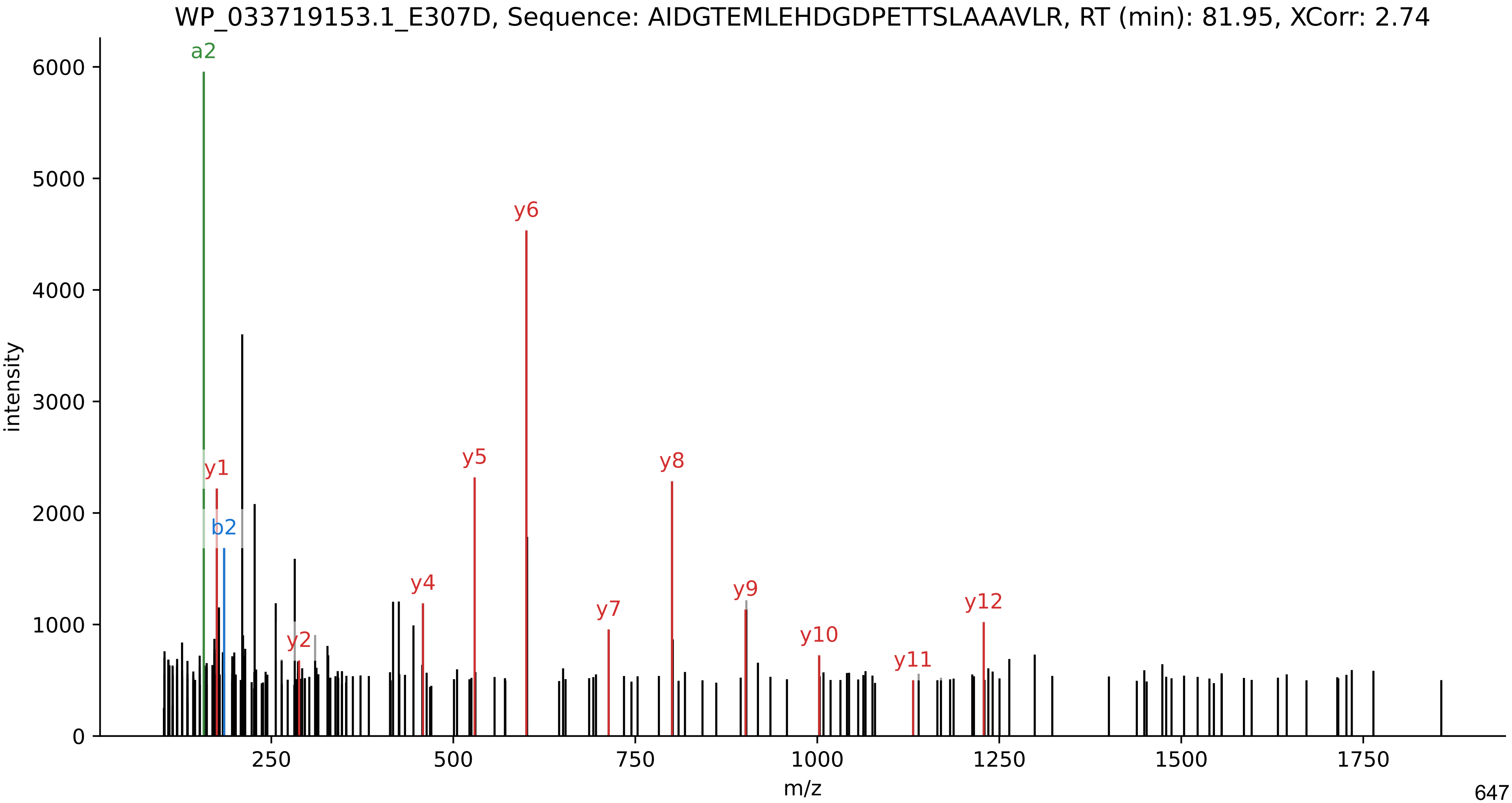
WP_033719153.1_E307D, Sequence: AIDGTEMLEHDGDPETTSLAAAVLR, RT (min): 81.95, XCorr: 2.74
a2
y1
b2
y2
y4
y5
y6
y7
y8
y9
y10
y11
y12
intensity
m/z
0
1000
2000
3000
4000
5000
6000
250
500
750
1000
1250
1500
1750

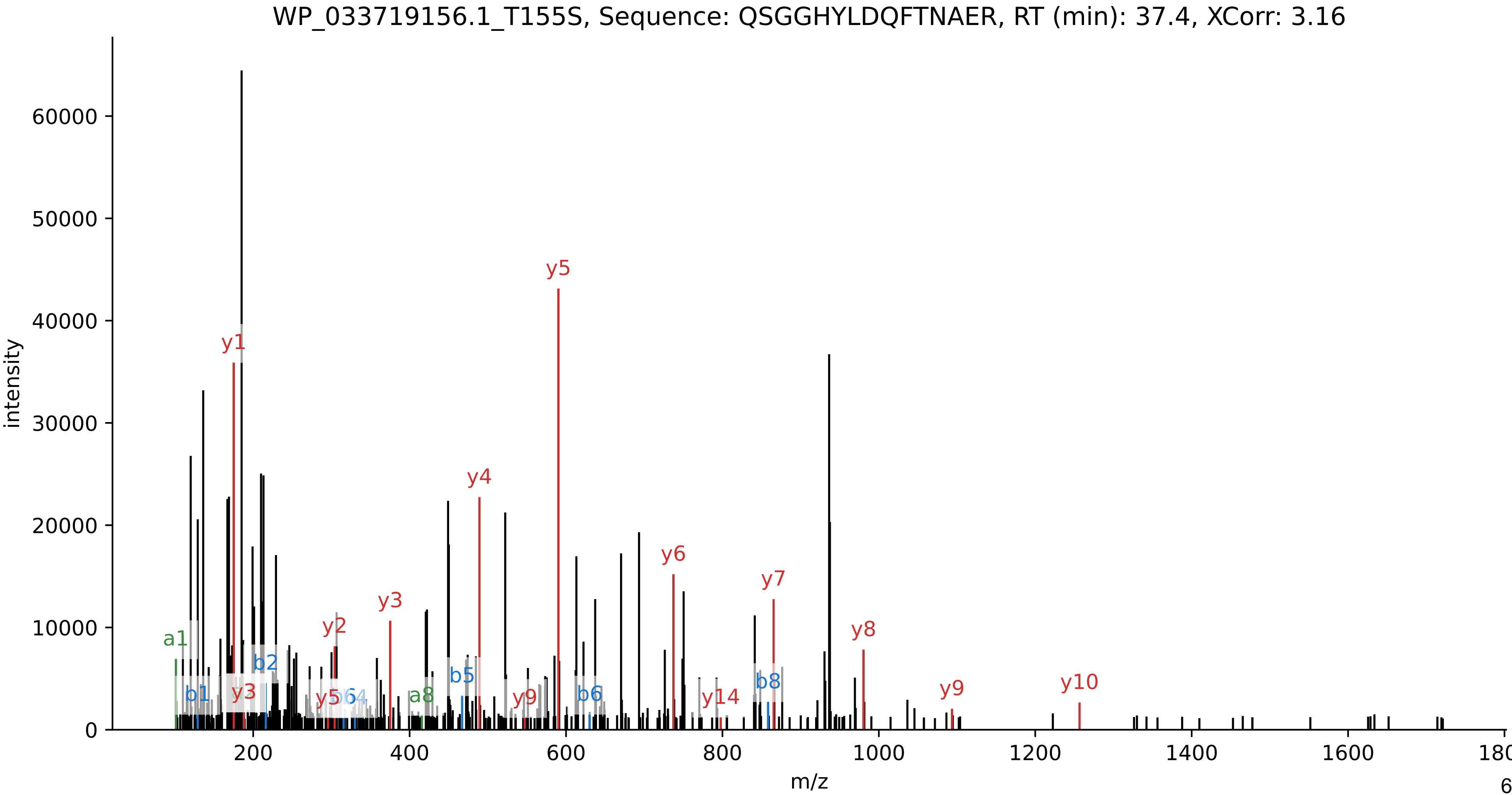
WP_033719156.1_T155S, Sequence: QSGGHYLDQFTNAER, RT (min): 37.4, XCorr: 3.16
intensity
m/z
y1
y5
y4
y6
y7
y3
y2
y8
a1
b2
b1
y3
b5
a8
b8
y9
b6
y14
y9
y10
0
10000
20000
30000
40000
50000
60000
200
400
600
800
1000
1200
1400
1600
1800

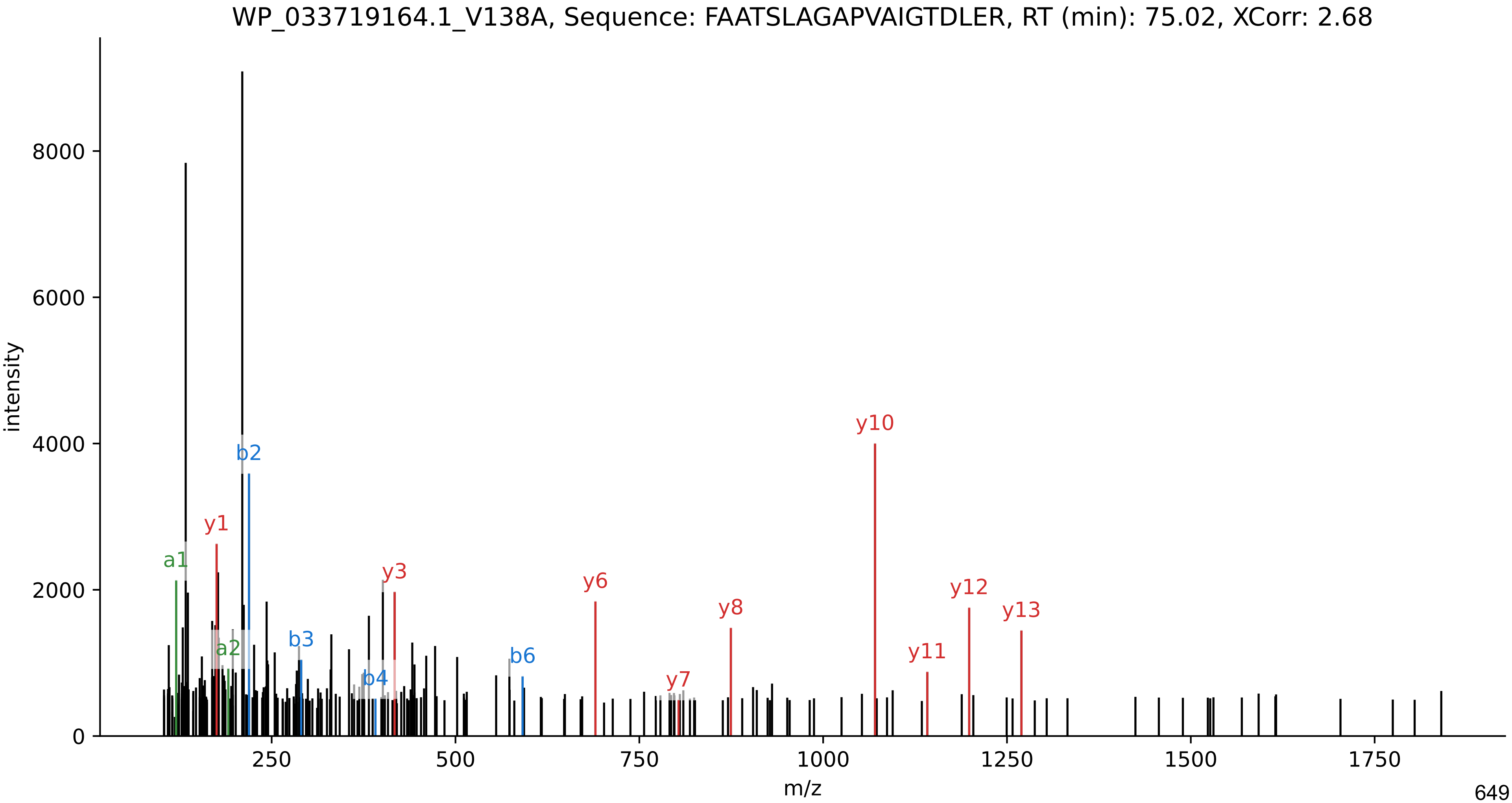
WP_033719164.1_V138A, Sequence: FAATSLAGAPVAIGTDLER, RT (min): 75.02, XCorr: 2.68
intensity
m/z
0
2000
4000
6000
8000
250
500
750
1000
1250
1500
1750
a1
a2
b2
b3
b4
b6
y1
y3
y6
y7
y8
y10
y11
y12
y13

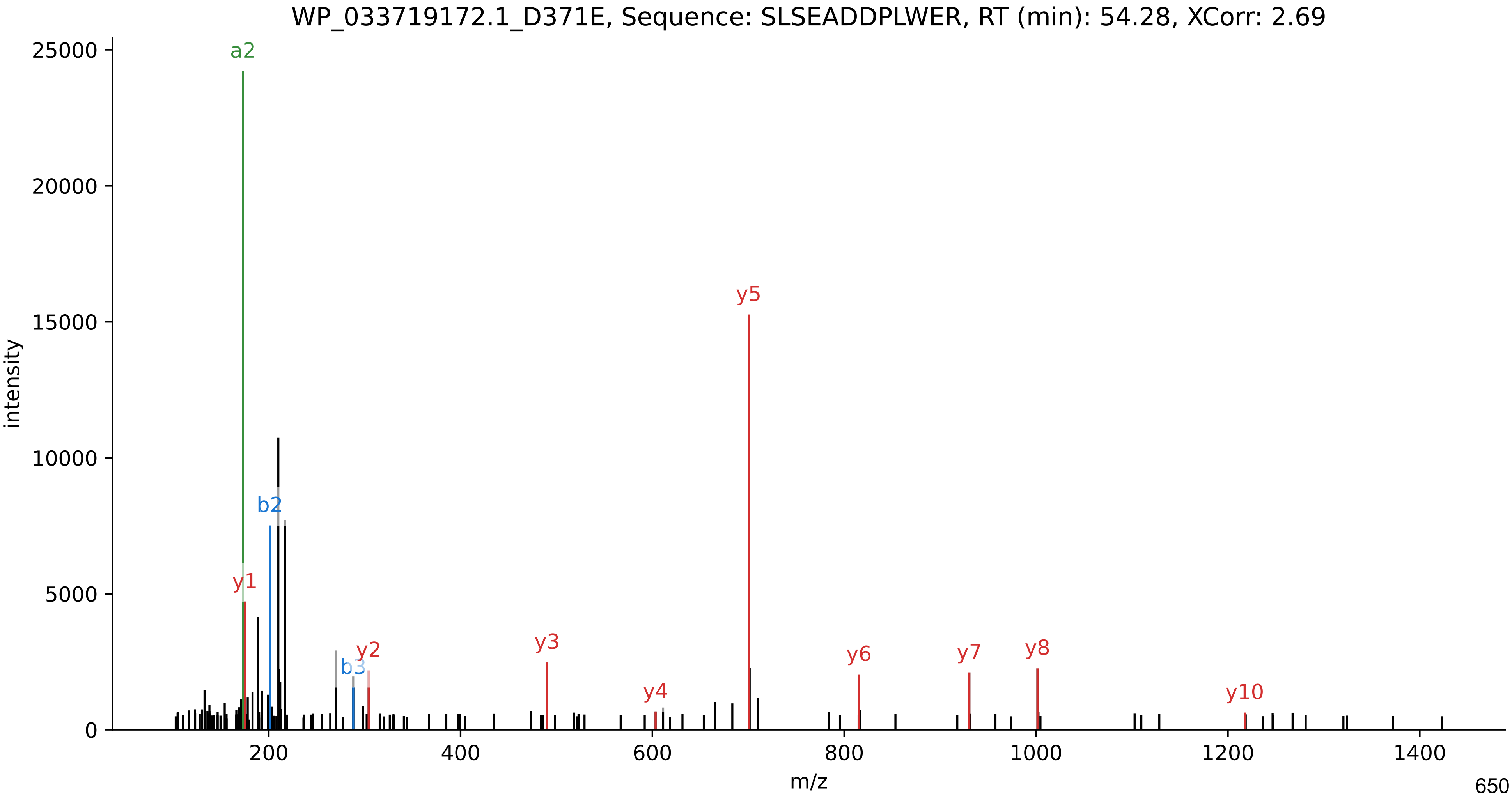

WP_033719172.1_D371E, Sequence: SLSEADDPLWER, RT (min): 54.28, XCorr: 2.69
intensity
m/z
a2
b2
b3
y1
y2
y3
y4
y5
y6
y7
y8
y10

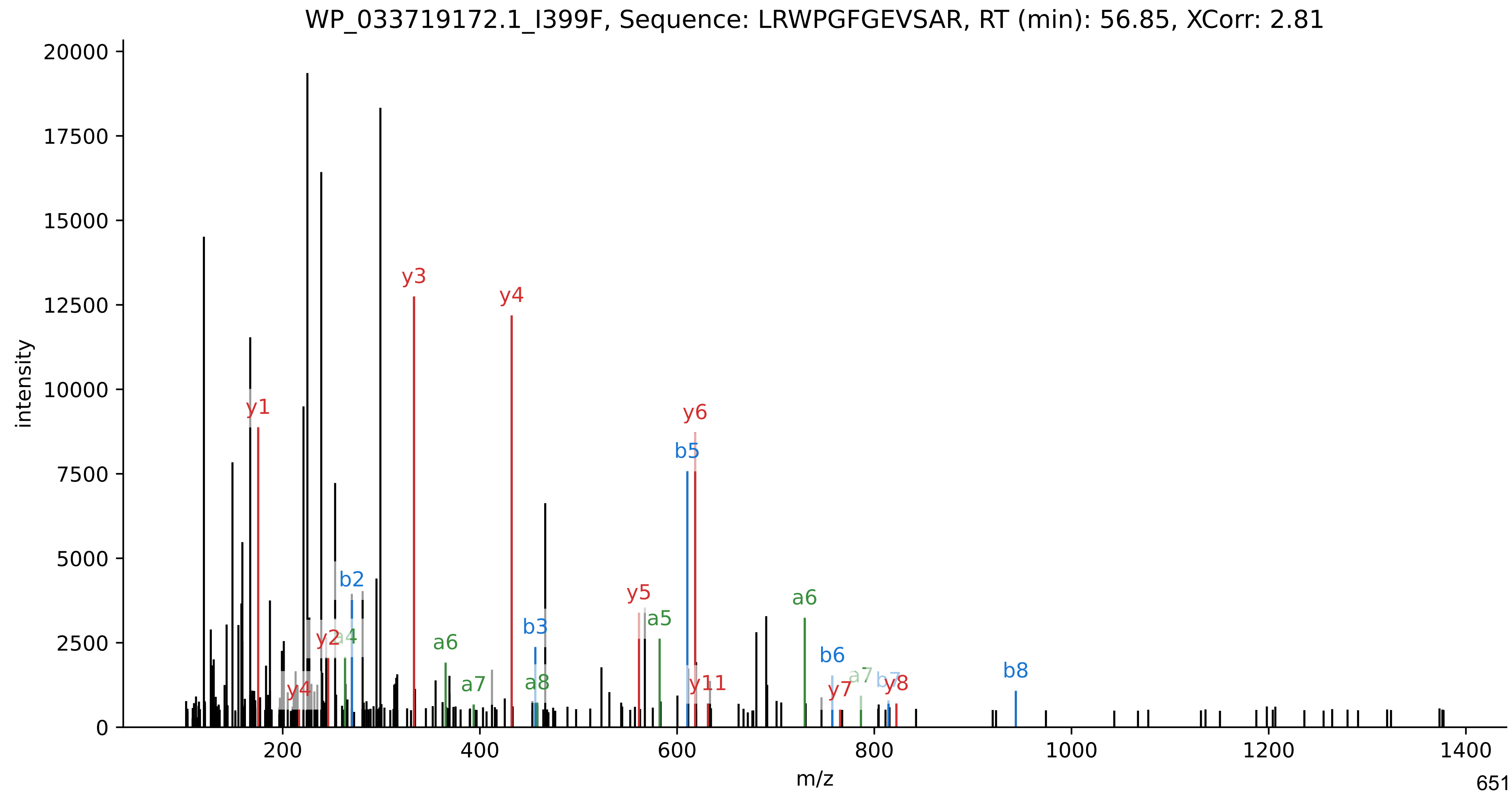

WP_033719172.1_I399F, Sequence: LRWPGFGEVSAR, RT (min): 56.85, XCorr: 2.81
intensity
m/z
y1
y2
y3
y4
y5
y6
y7
y8
y11
b2
b3
b5
b6
b8
a4
a5
a6
a7
a8

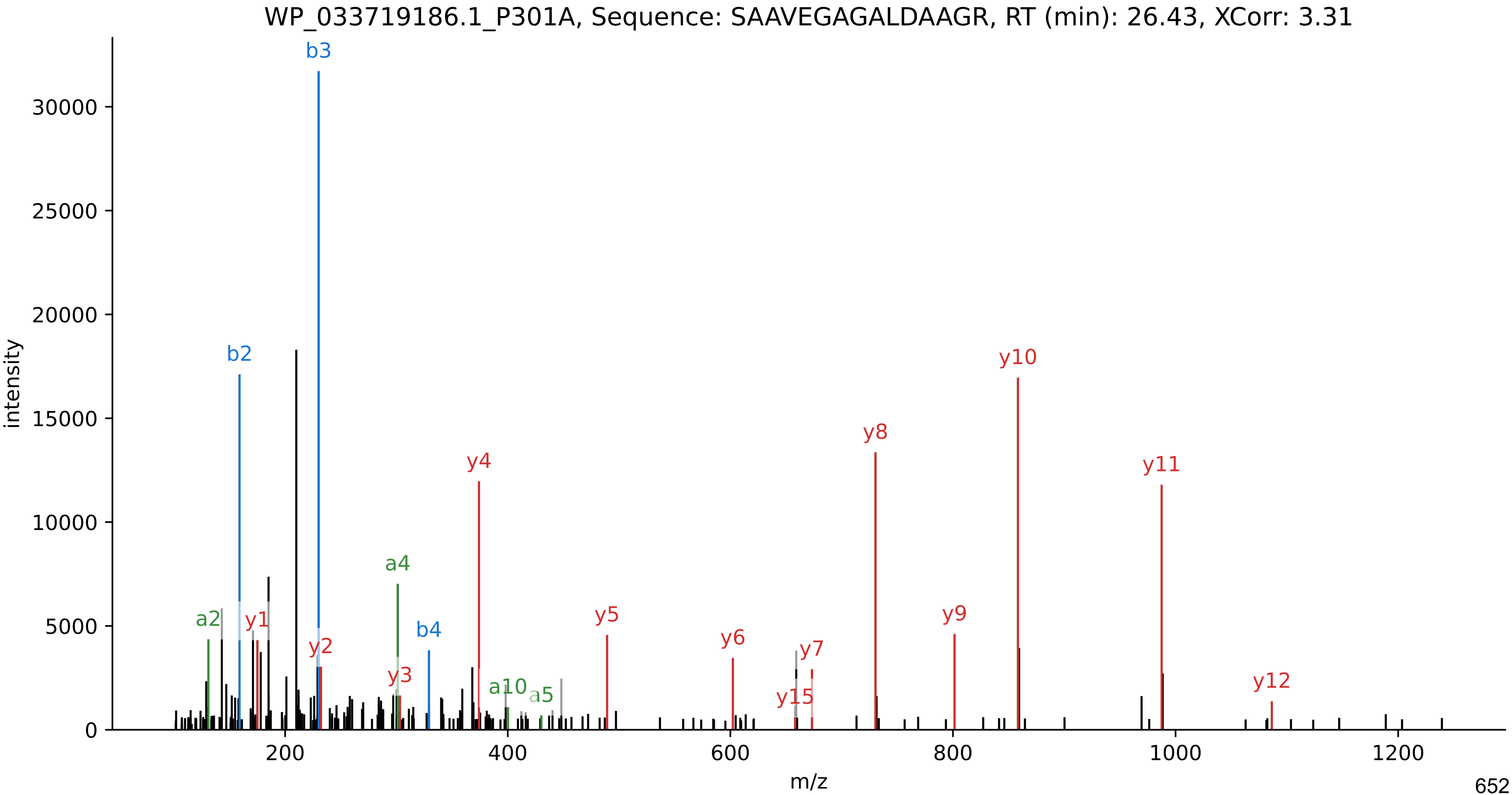

WP_033719186.1_P301A, Sequence: SAAVEGAGALDAAGR, RT (min): 26.43, XCorr: 3.31
intensity
m/z
b3
b2
b4
a2
y1
y2
a4
y3
y4
a10
a5
y5
y6
y15
y7
y8
y9
y10
y11
y12
0
5000
10000
15000
20000
25000
30000
200
400
600
800
1000
1200

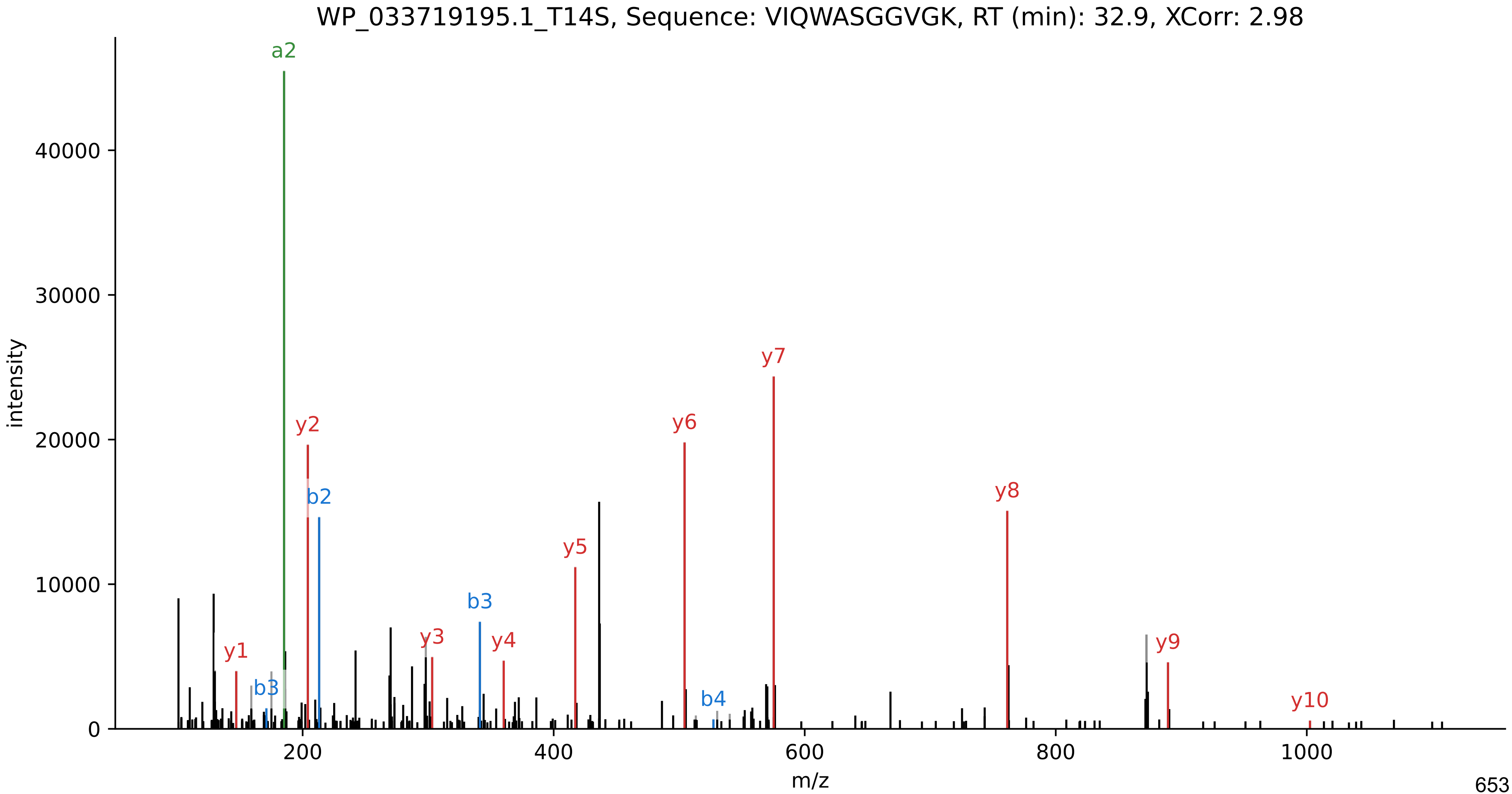
WP_033719195.1_T14S, Sequence: VIQWASGGVGK, RT (min): 32.9, XCorr: 2.98
intensity
m/z
0
10000
20000
30000
40000
200
400
600
800
1000
a2
y1
b3
y2
b2
y3
b3
y4
y5
y6
b4
y7
y8
y9
y10

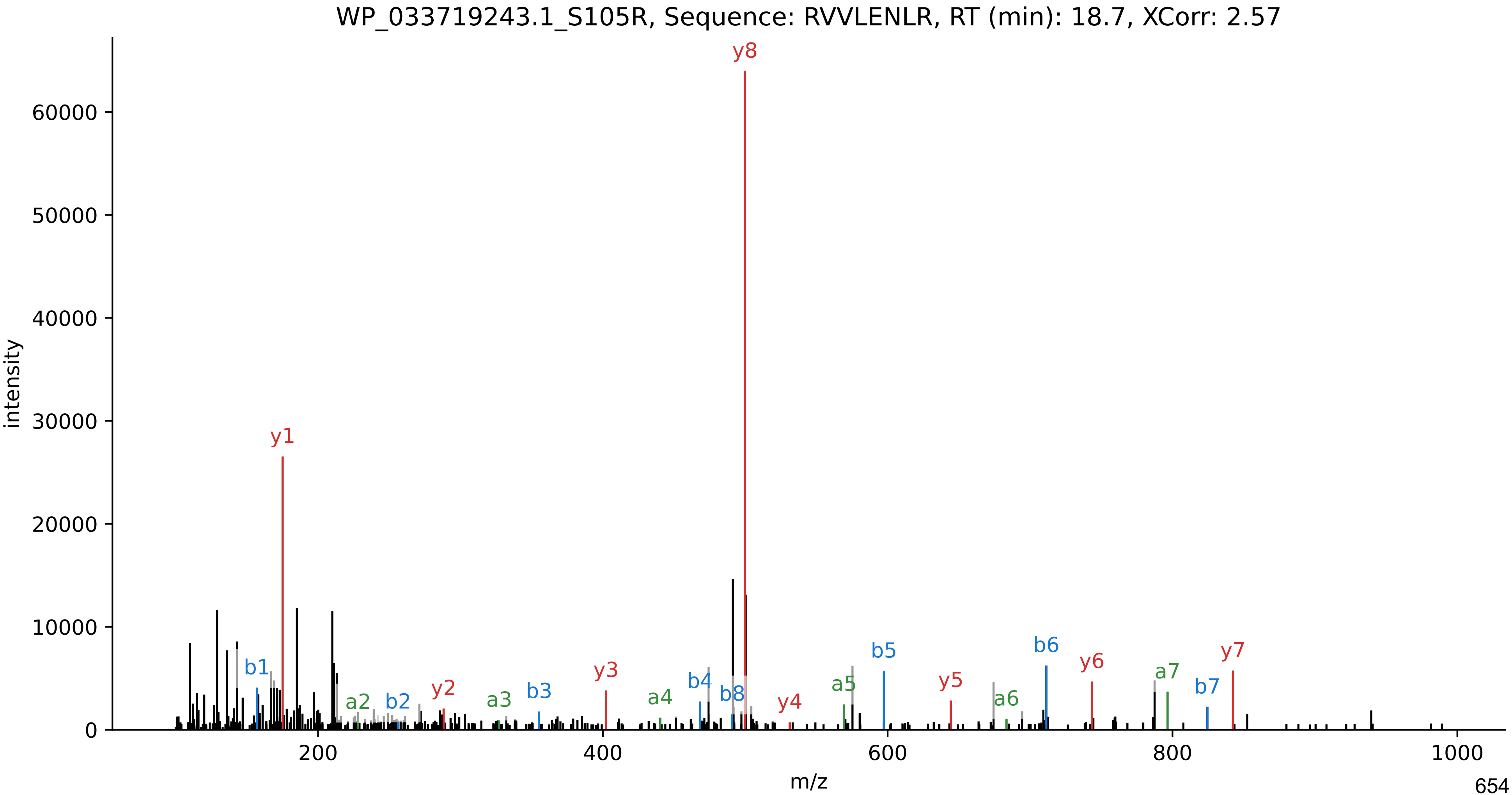
WP_033719243.1_S105R, Sequence: RVVLENLR, RT (min): 18.7, XCorr: 2.57
intensity
m/z
y8
y1
b1
a2
b2
y2
a3
b3
y3
a4
b4
b8
y4
a5
b5
y5
a6
b6
y6
a7
b7
y7
0
10000
20000
30000
40000
50000
60000
200
400
600
800
1000

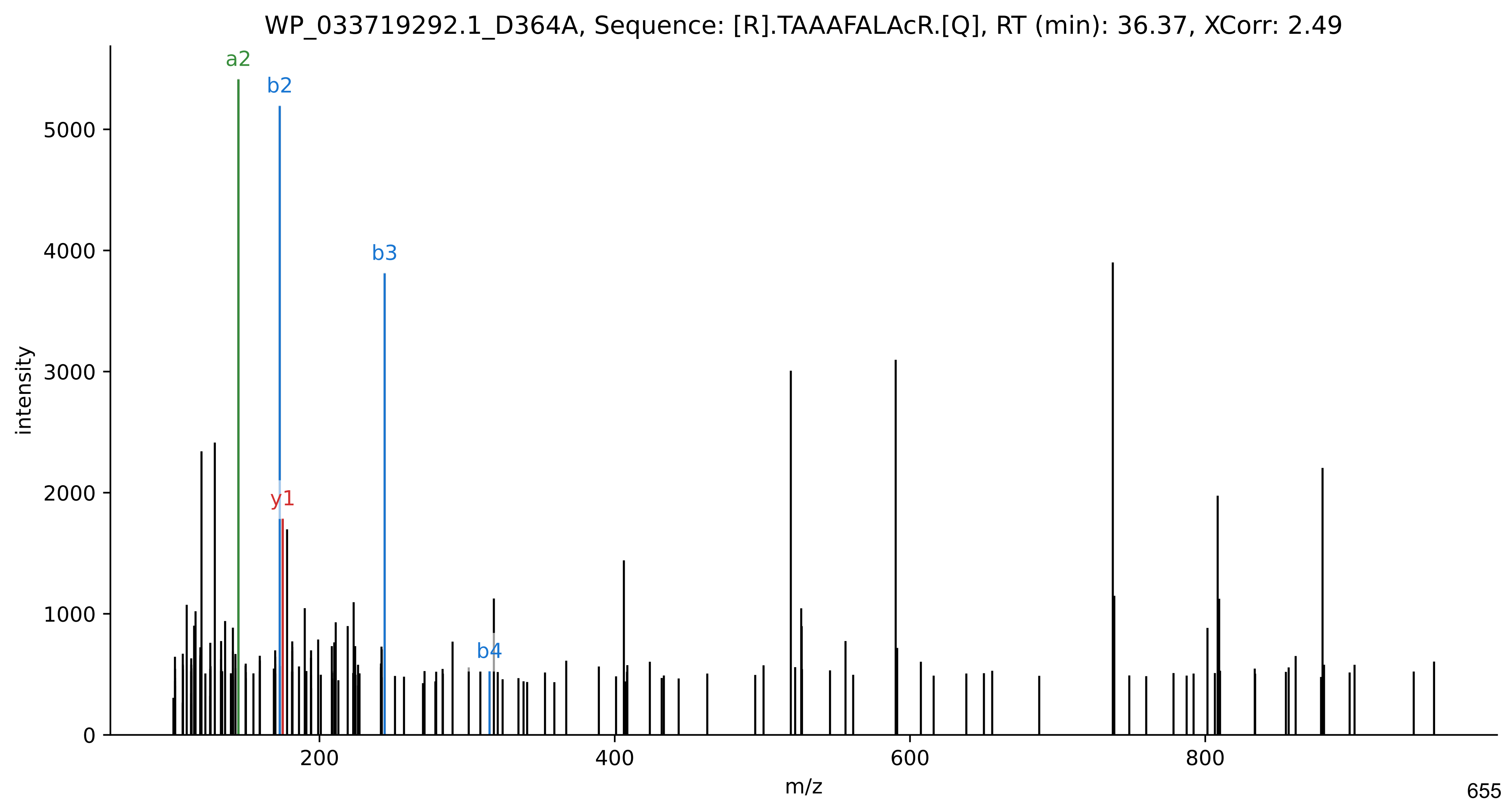

WP_033719292.1_D364A, Sequence: [R].TAAAFALAcR.[Q], RT (min): 36.37, XCorr: 2.49
a2
b2
b3
y1
b4
intensity
m/z
0
1000
2000
3000
4000
5000
200
400
600
800

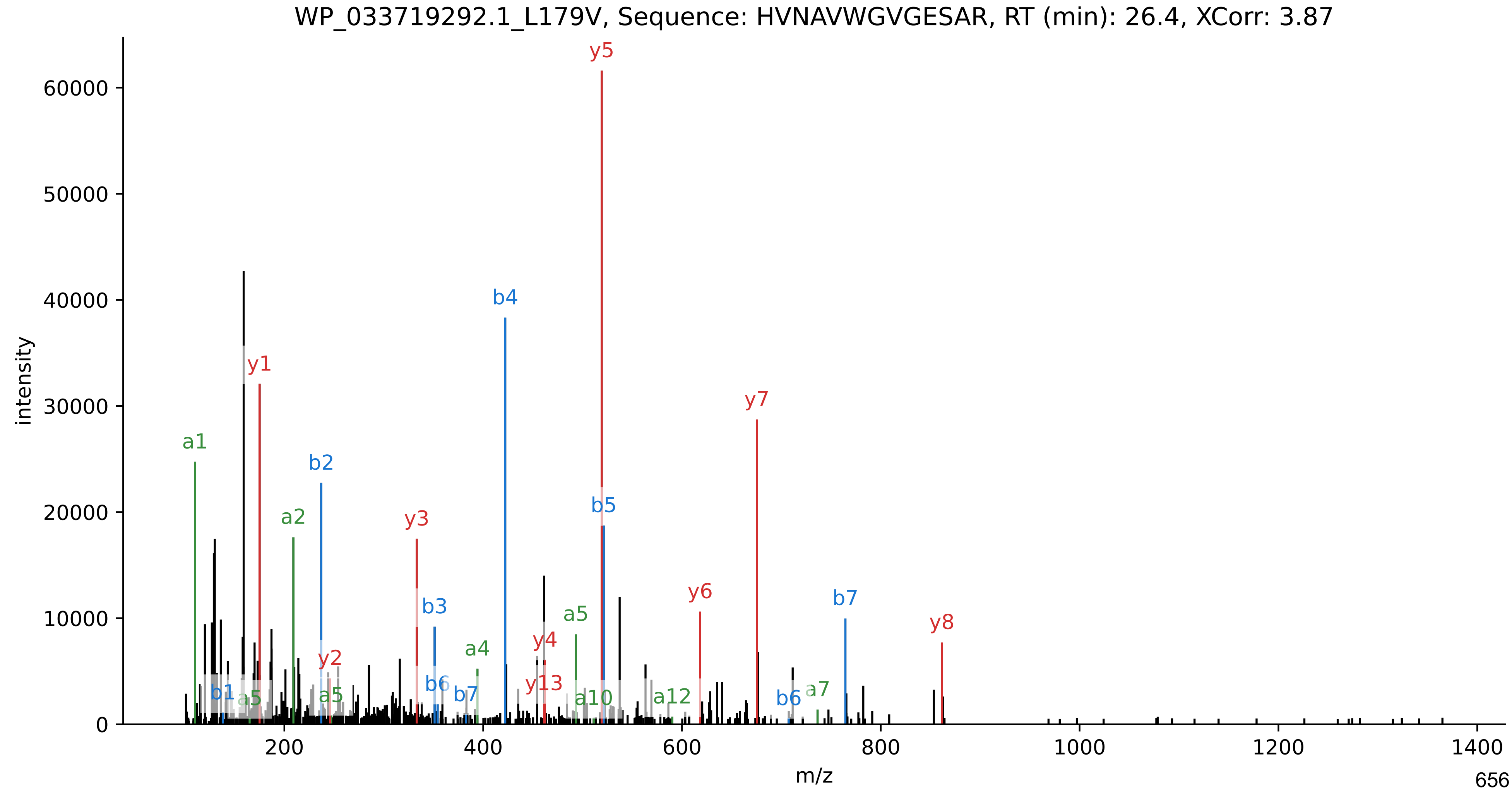
WP_033719292.1_L179V, Sequence: HVNAVWGVGESAR, RT (min): 26.4, XCorr: 3.87
intensity
m/z
y5
b4
y1
y7
a1
b2
a2
y3
b5
y6
b7
y8
b3
a5
a4
y4
y2
b6
y13
b7
a10
a12
b6
a7
b1
a5
a5
0
10000
20000
30000
40000
50000
60000
200
400
600
800
1000
1200
1400

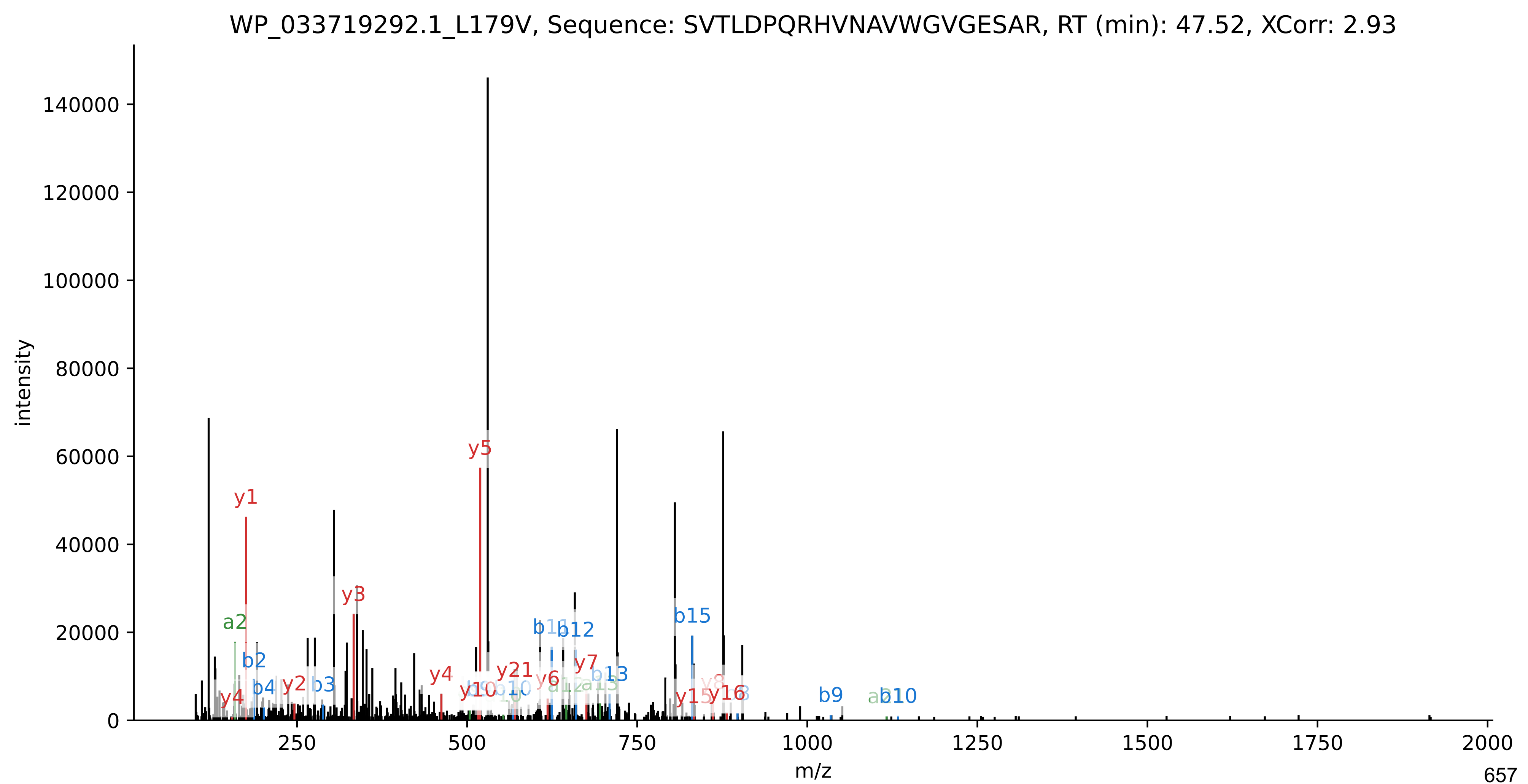
WP_033719292.1_L179V, Sequence: SVTLDPQRHVNAVWGVGESAR, RT (min): 47.52, XCorr: 2.93
intensity
m/z
y5
y1
y3
a2
b2
b4
y2
b3
y4
b15
b12
y7
b13
y21
y6
y10
b9
b10
y16

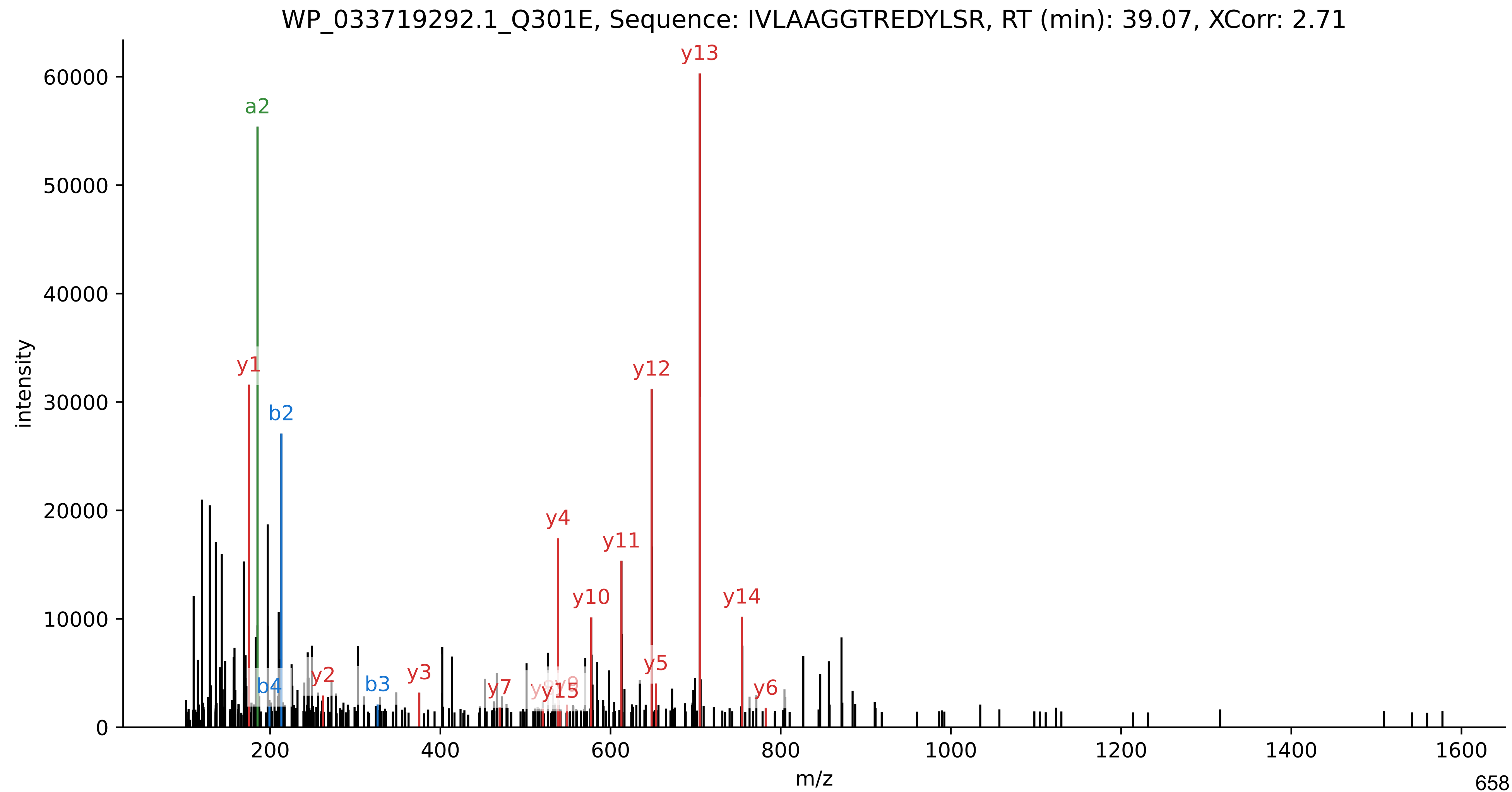

WP_033719292.1_Q301E, Sequence: IVLAAGGTREDYLSR, RT (min): 39.07, XCorr: 2.71
intensity
m/z
y13
a2
y1
b2
y12
y4
y11
y10
y14
y5
y6
y7
y15
y3
y2
b4
b3

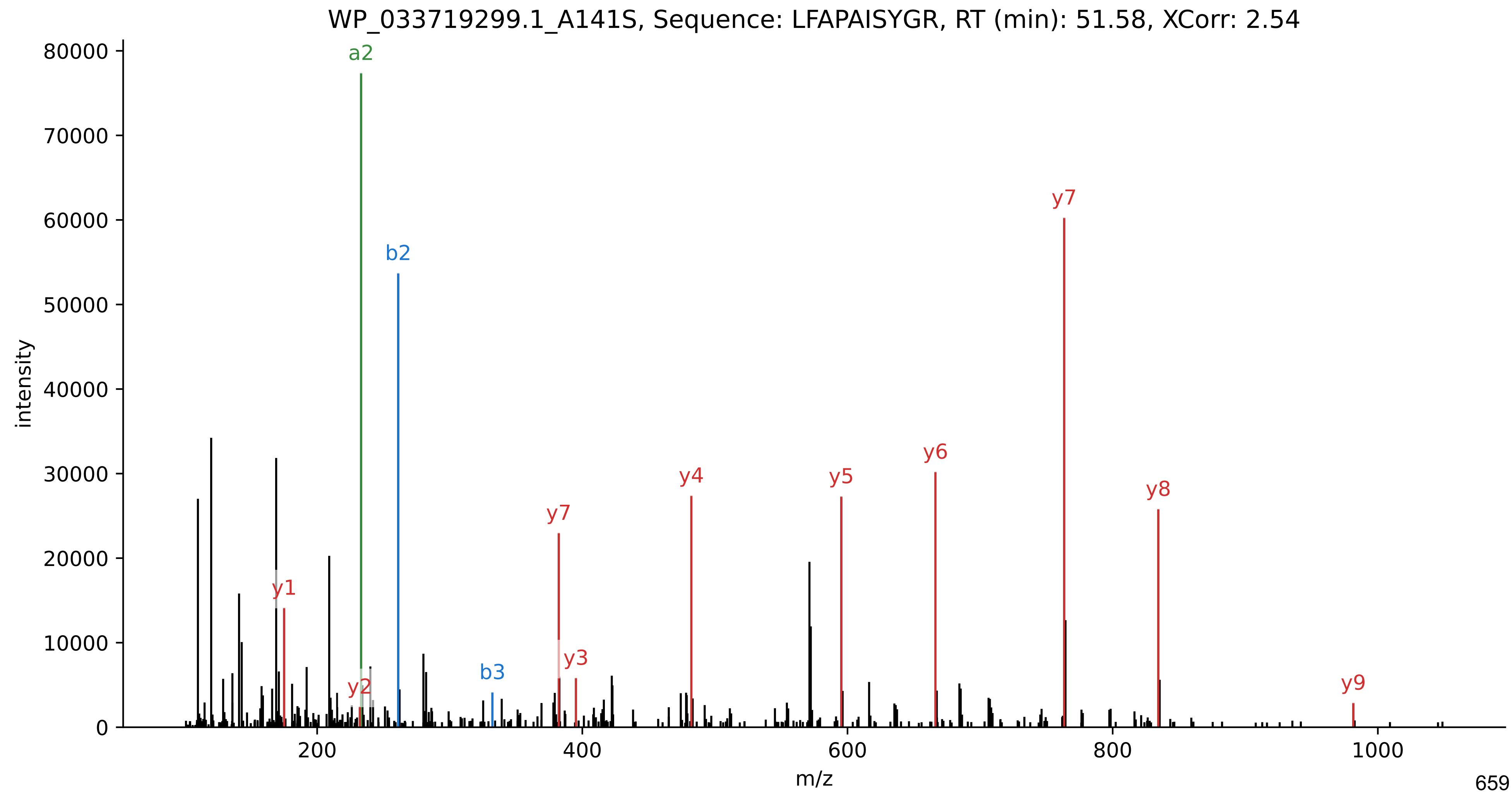

WP_033719299.1_A141S, Sequence: LFAPAISYGR, RT (min): 51.58, XCorr: 2.54
intensity
m/z
a2
b2
b3
y1
y2
y3
y4
y5
y6
y7
y7
y8
y9
0
10000
20000
30000
40000
50000
60000
70000
80000
200
400
600
800
1000

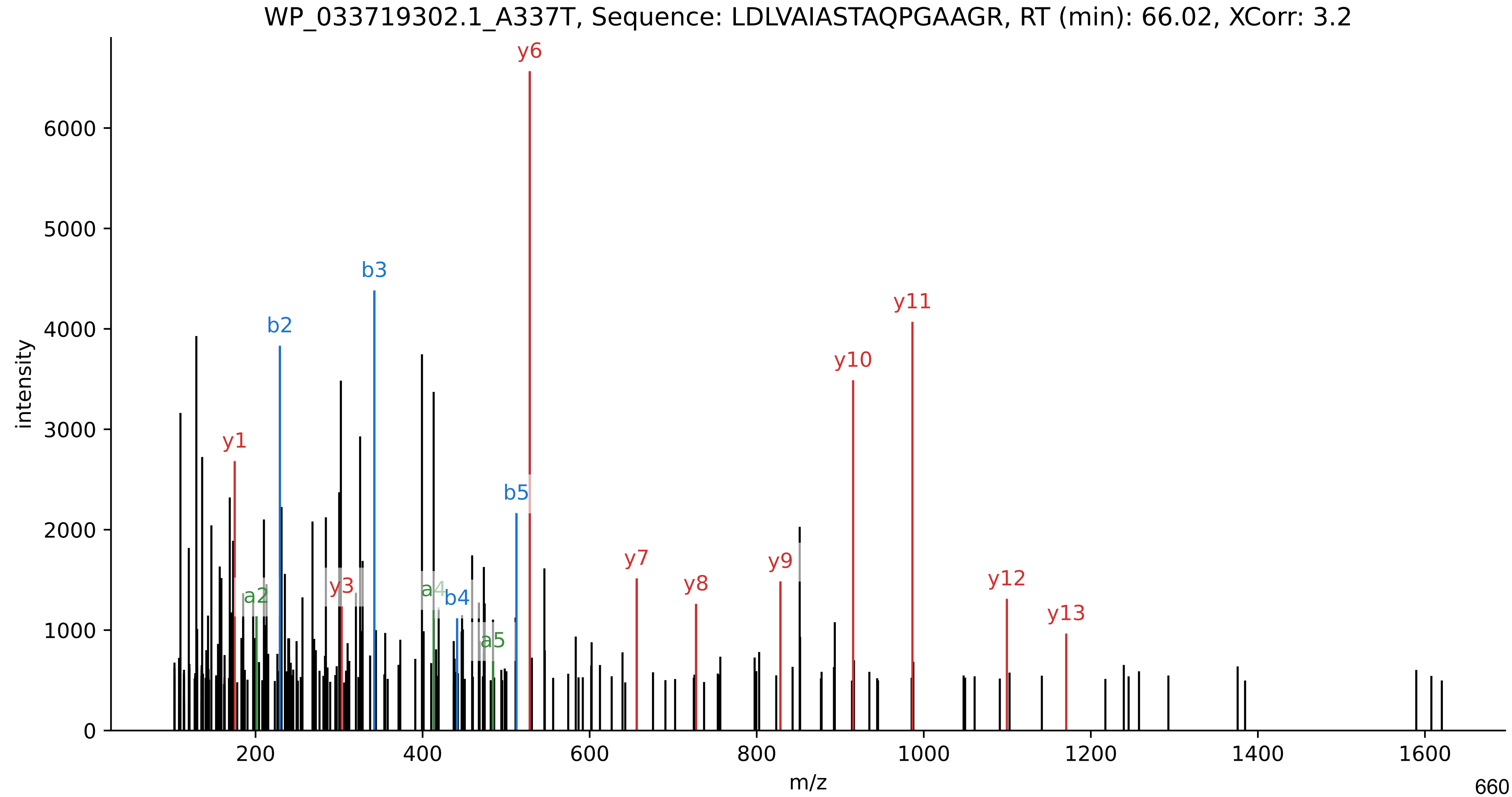
WP_033719302.1_A337T, Sequence: LDLVAIASTAQPGAAGR, RT (min): 66.02, XCorr: 3.2
y6
b3
y11
b2
y10
y1
b5
y7
y9
y8
y3
a2
a4
b4
y12
y13
a5
intensity
m/z
0
1000
2000
3000
4000
5000
6000
200
400
600
800
1000
1200
1400
1600

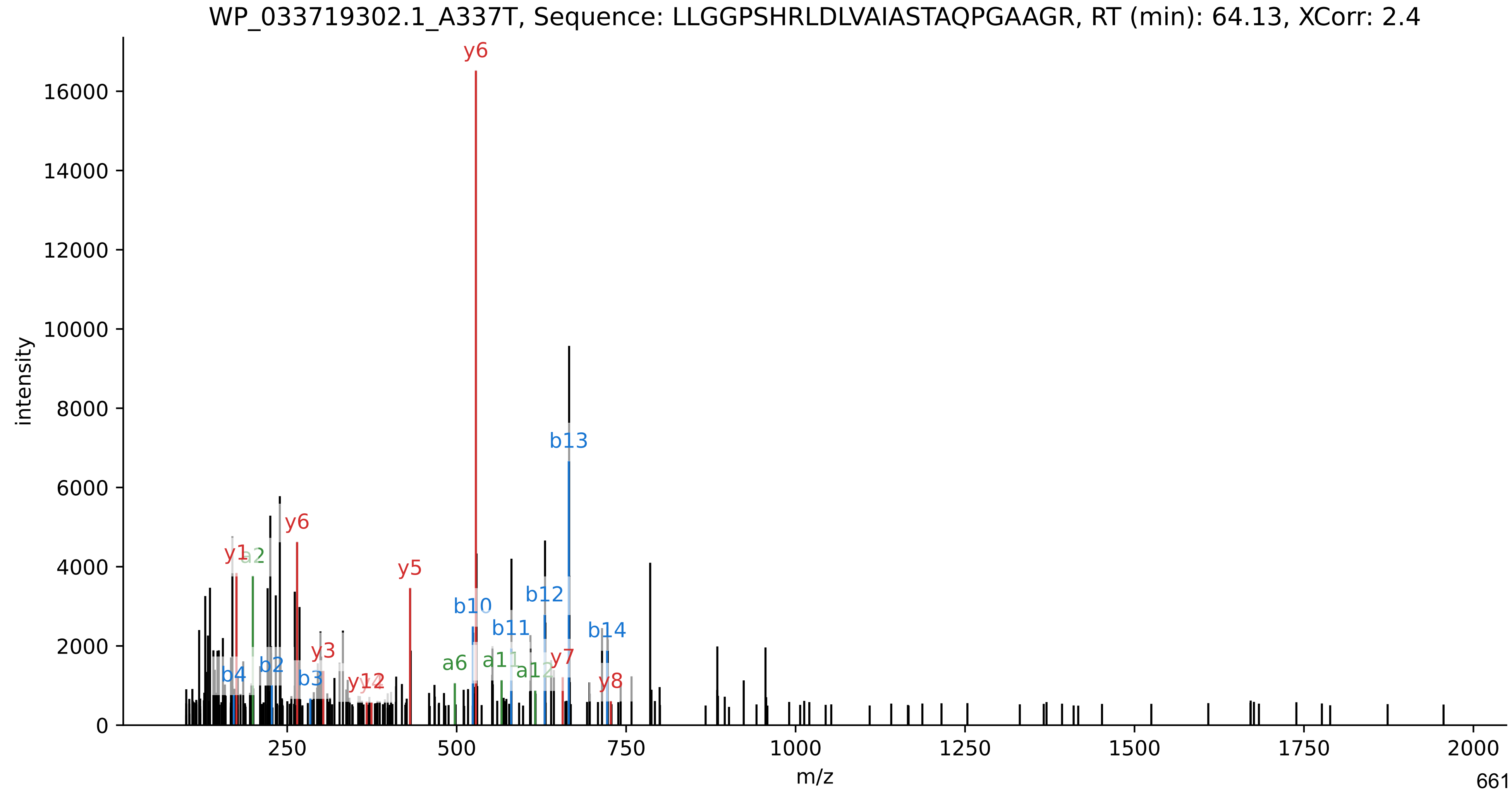

WP_033719302.1_A337T, Sequence: LLGGPSHRLDLVAIASTAQPGAAGR, RT (min): 64.13, XCorr: 2.4
y6
b13
y6
y1
a2
y5
b10
b12
b11
b14
b2
b4
b3
y3
y12
a6
a11
a12
y7
y8
intensity
0
2000
4000
6000
8000
10000
12000
14000
16000
250
500
750
1000
1250
1500
1750
2000
m/z

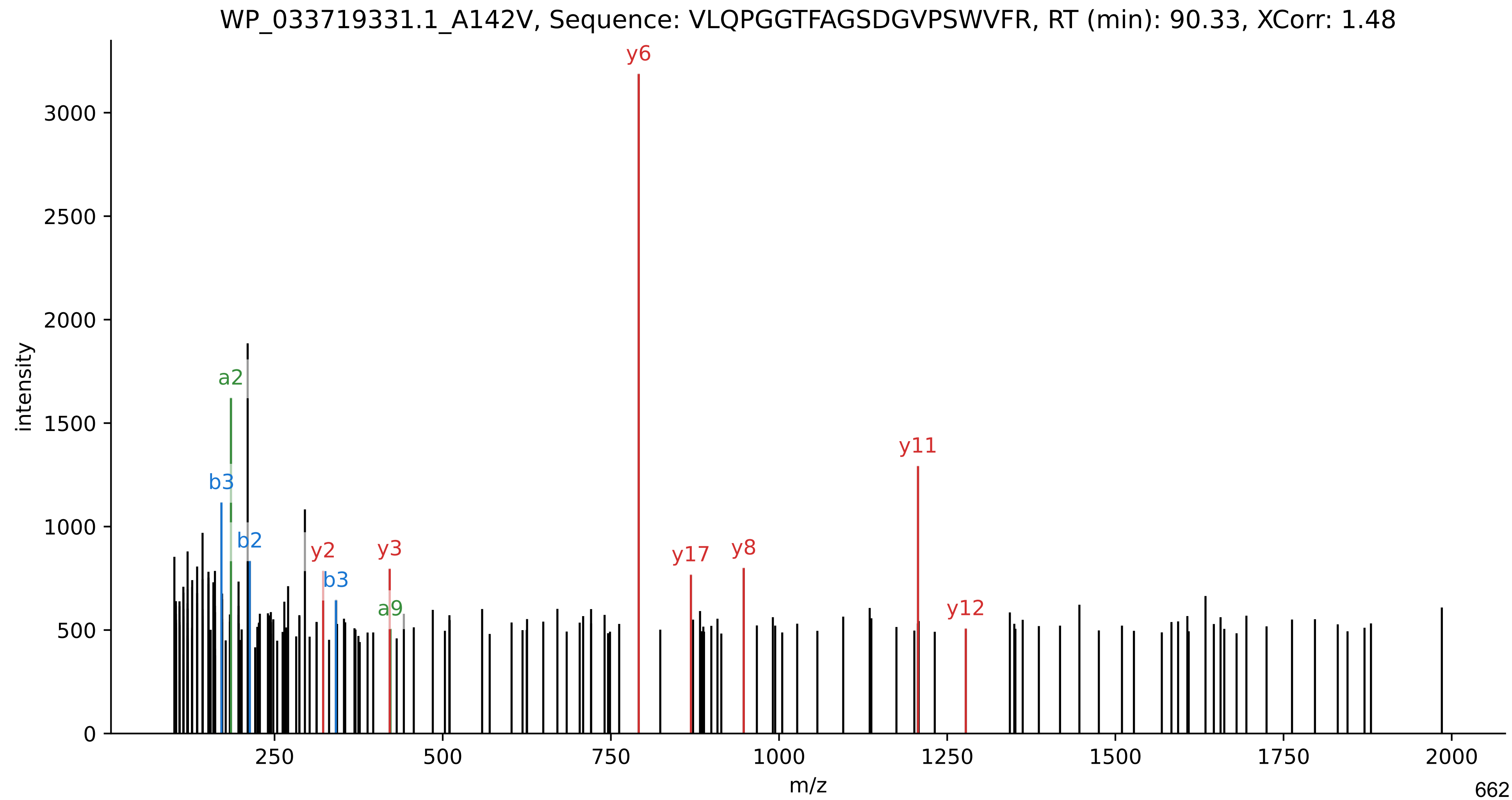
WP_033719331.1_A142V, Sequence: VLQPGGTFAGSDGVPSWVFR, RT (min): 90.33, XCorr: 1.48
y6
a2
b3
b2
y2
y3
b3
a9
y17
y8
y11
y12
intensity
m/z
0
500
1000
1500
2000
2500
3000
250
500
750
1000
1250
1500
1750
2000

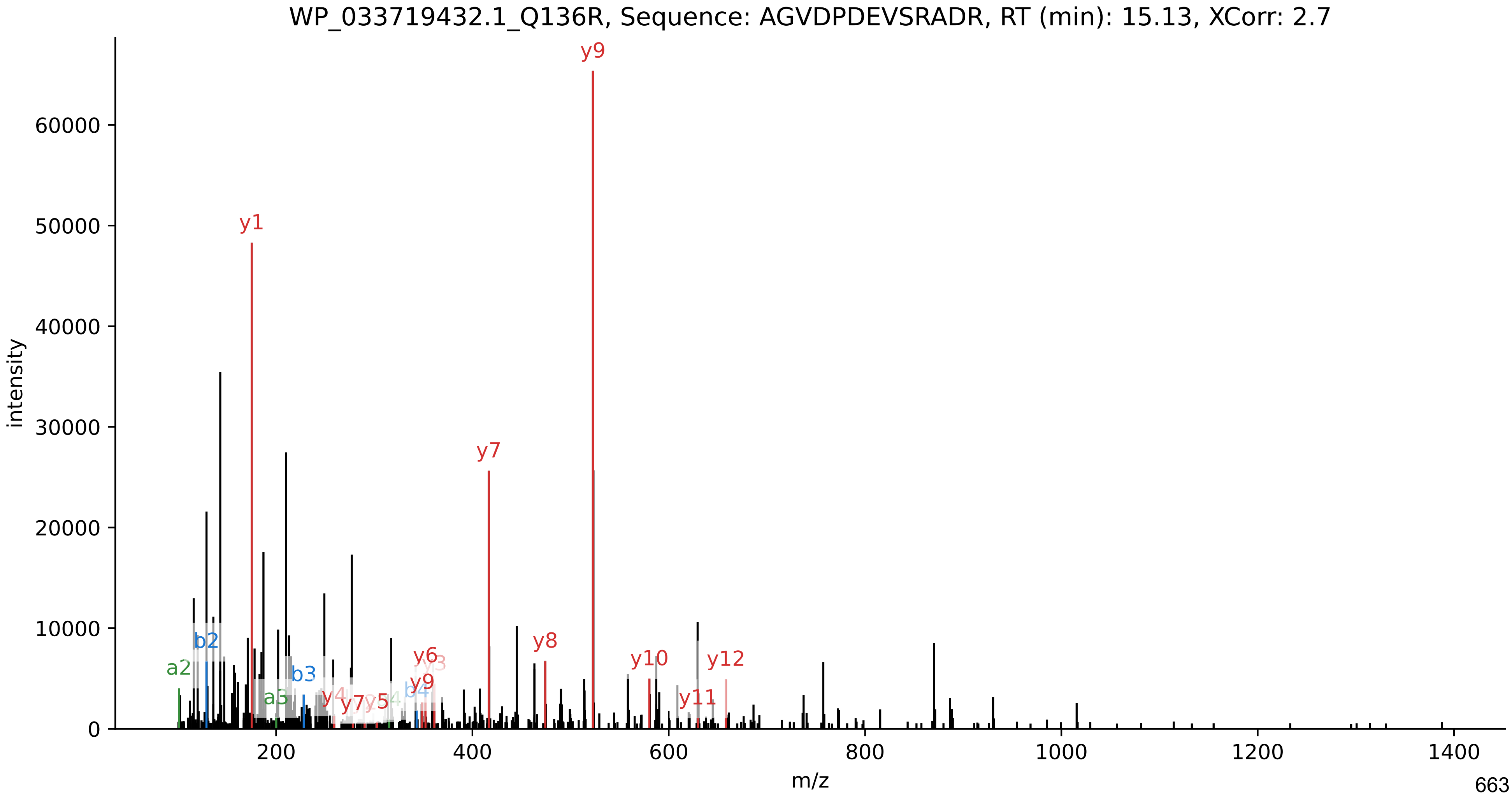
WP_033719432.1_Q136R, Sequence: AGVDPDEVSRADR, RT (min): 15.13, XCorr: 2.7
intensity
m/z
0
10000
20000
30000
40000
50000
60000
200
400
600
800
1000
1200
1400
y9
y1
y7
y8
y10
y12
y11
y6
y9
b2
b3
b4
a2
a3
y4
y7
y5

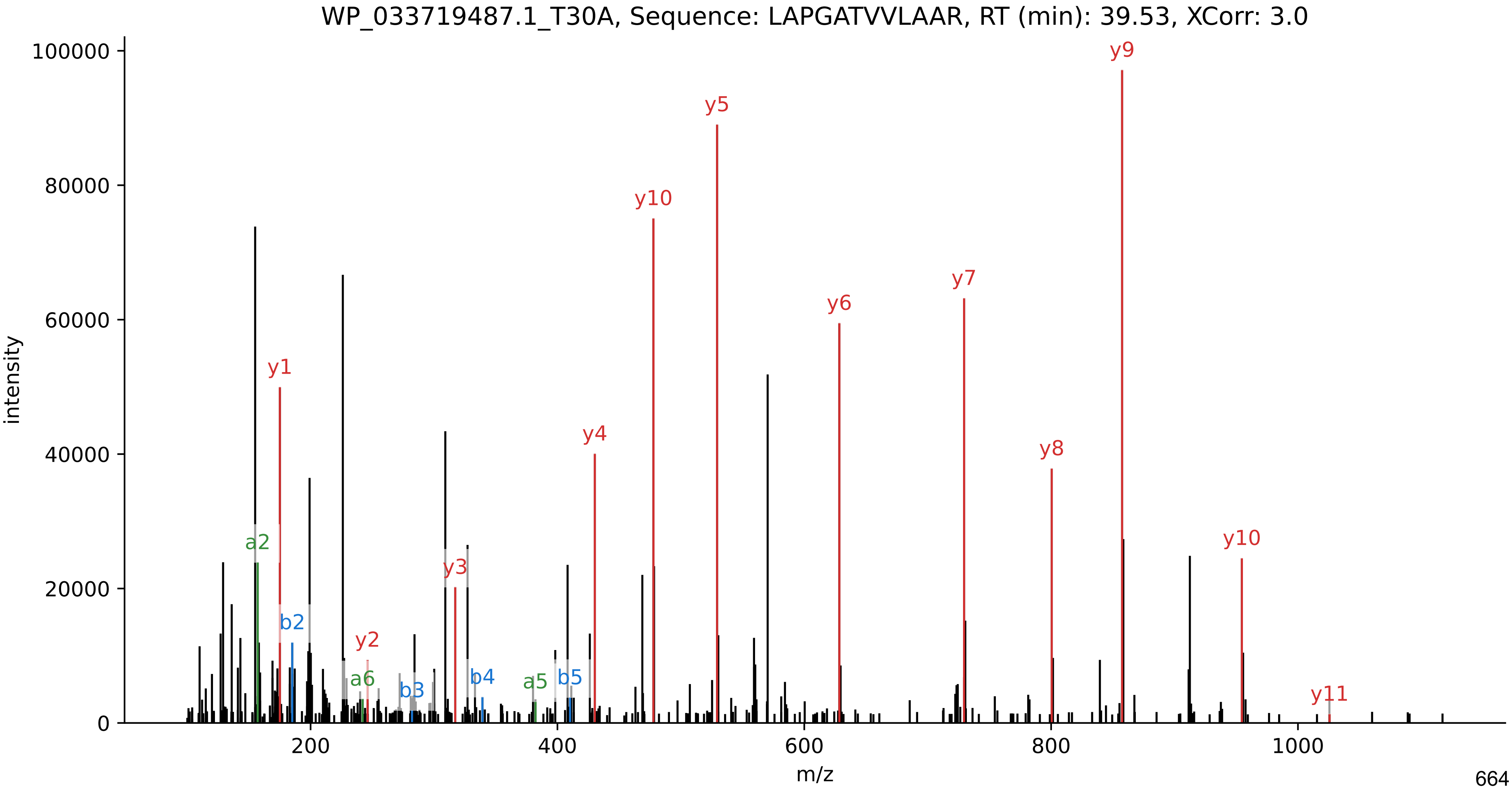
WP_033719487.1_T30A, Sequence: LAPGATVVLAAR, RT (min): 39.53, XCorr: 3.0
intensity
m/z
0
20000
40000
60000
80000
100000
200
400
600
800
1000
y1
y2
y3
y4
y5
y6
y7
y8
y9
y10
y10
y11
a2
a5
a6
b2
b3
b4
b5

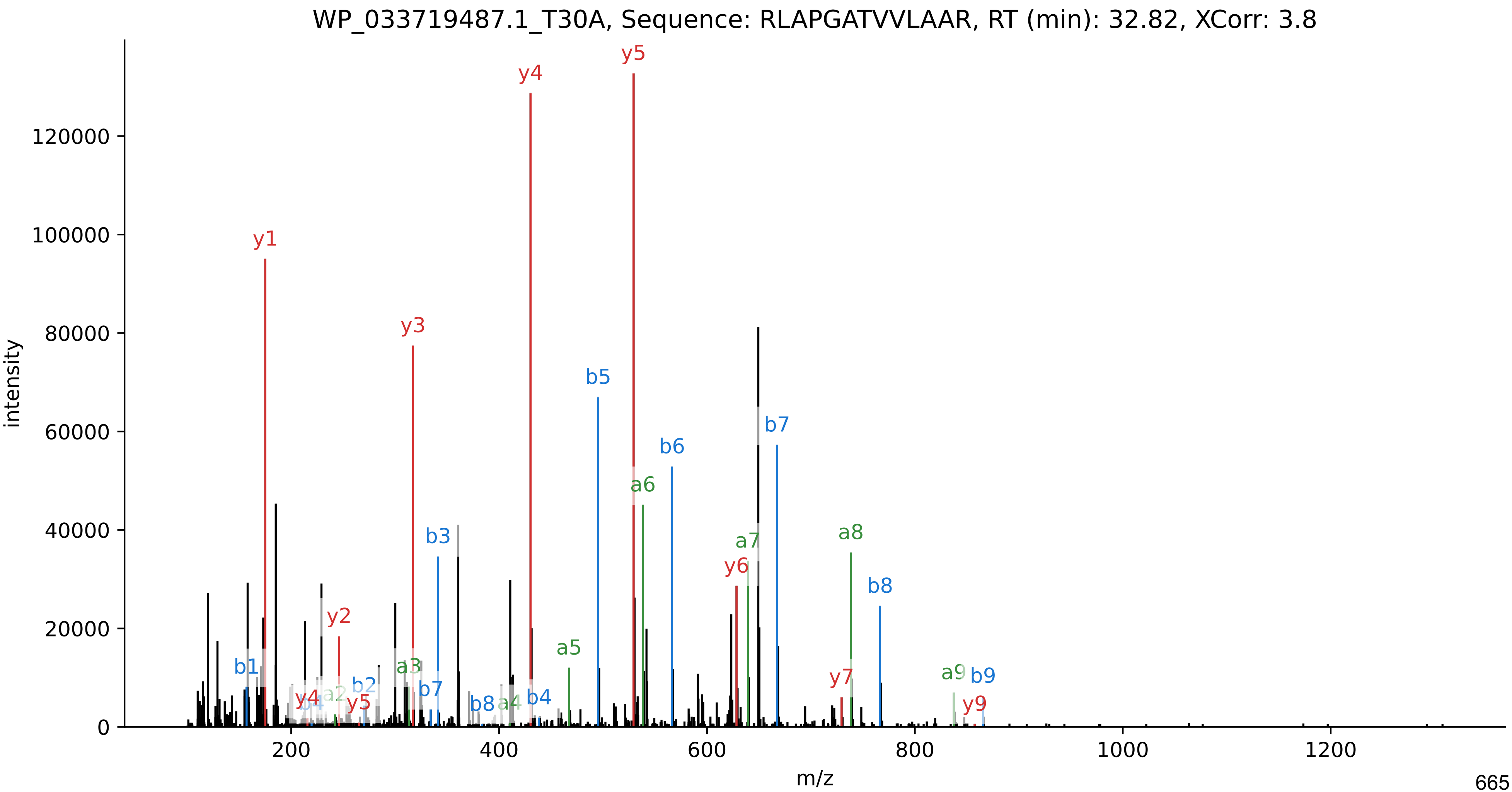
WP_033719487.1_T30A, Sequence: RLAPGATVVLAAR, RT (min): 32.82, XCorr: 3.8
intensity
m/z
y1
y2
y3
y4
y5
y6
y7
y9
b1
b2
b3
b4
b5
b6
b7
b8
b9
a2
a3
a4
a5
a6
a7
a8
a9
0
20000
40000
60000
80000
100000
120000
200
400
600
800
1000
1200

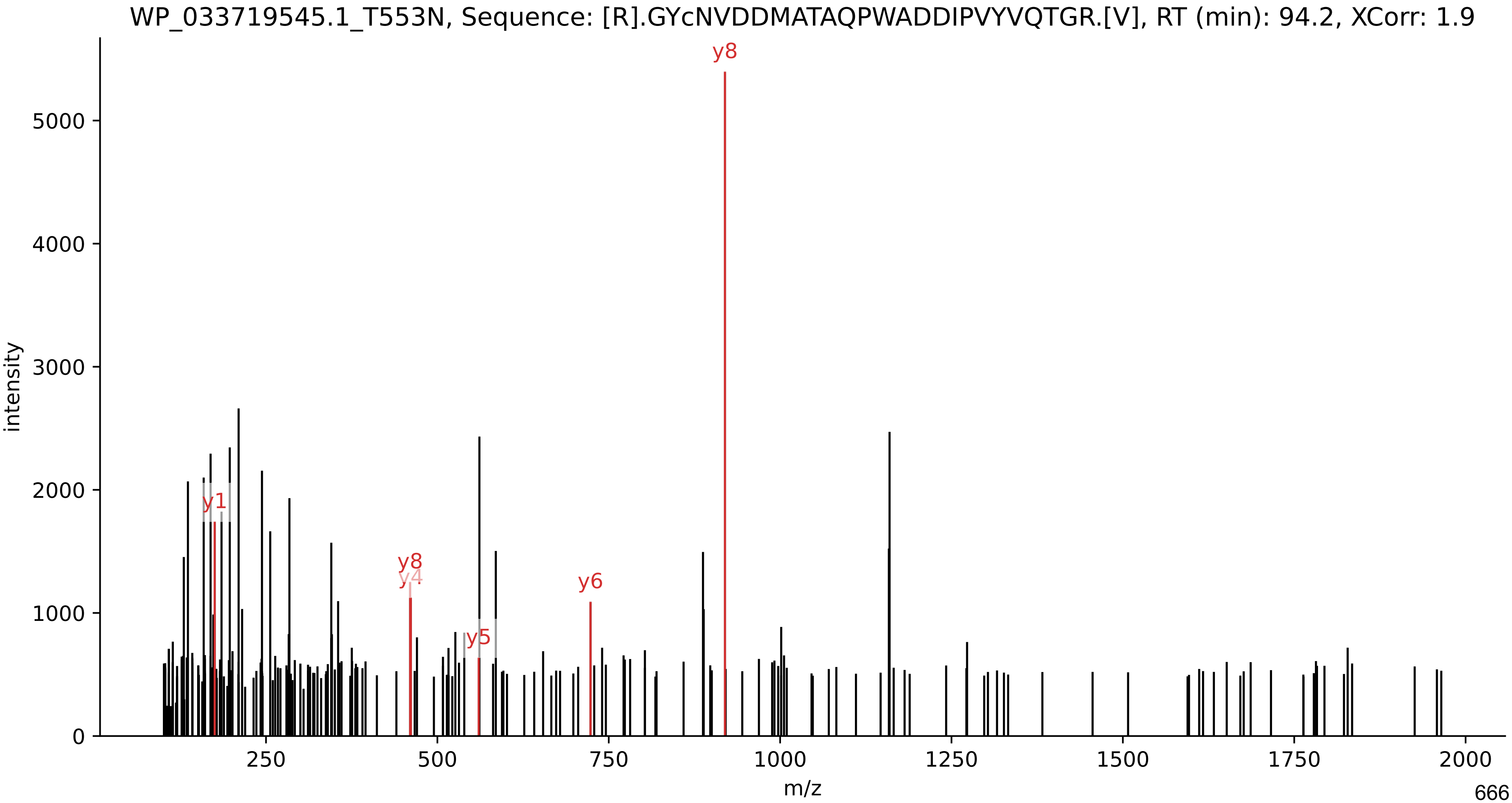

WP_033719545.1_T553N, Sequence: [R].GYcNVDDMATAQPWADDIPVYVQTGR.[V], RT (min): 94.2, XCorr: 1.9
y8
y1
y8
y4
y5
y6
intensity
m/z
0
1000
2000
3000
4000
5000
250
500
750
1000
1250
1500
1750
2000

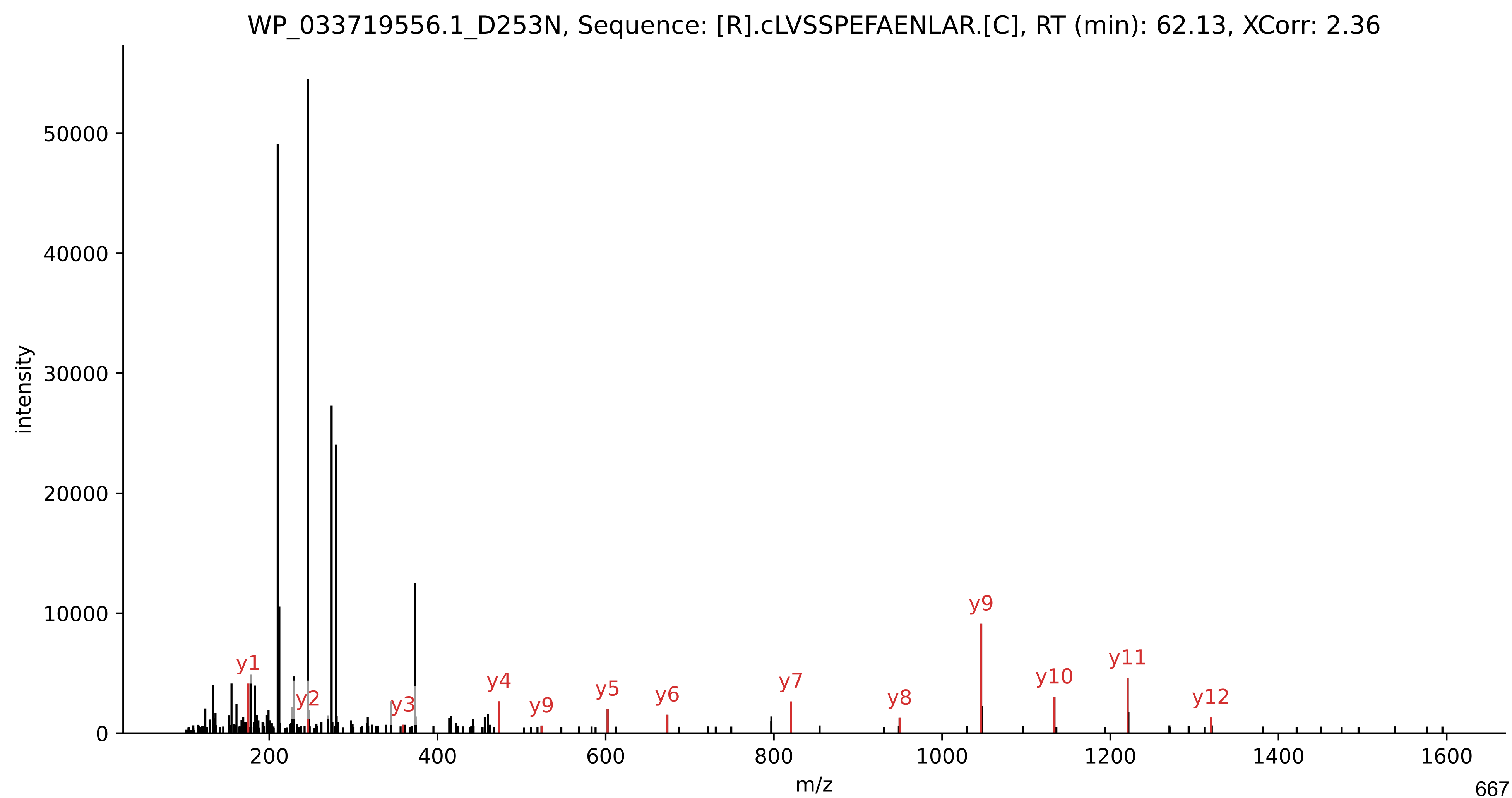

WP_033719556.1_D253N, Sequence: [R].cLVSSPEFAENLAR.[C], RT (min): 62.13, XCorr: 2.36
intensity
0
10000
20000
30000
40000
50000
m/z
200
400
600
800
1000
1200
1400
1600
y1
y2
y3
y4
y9
y5
y6
y7
y8
y9
y10
y11
y12

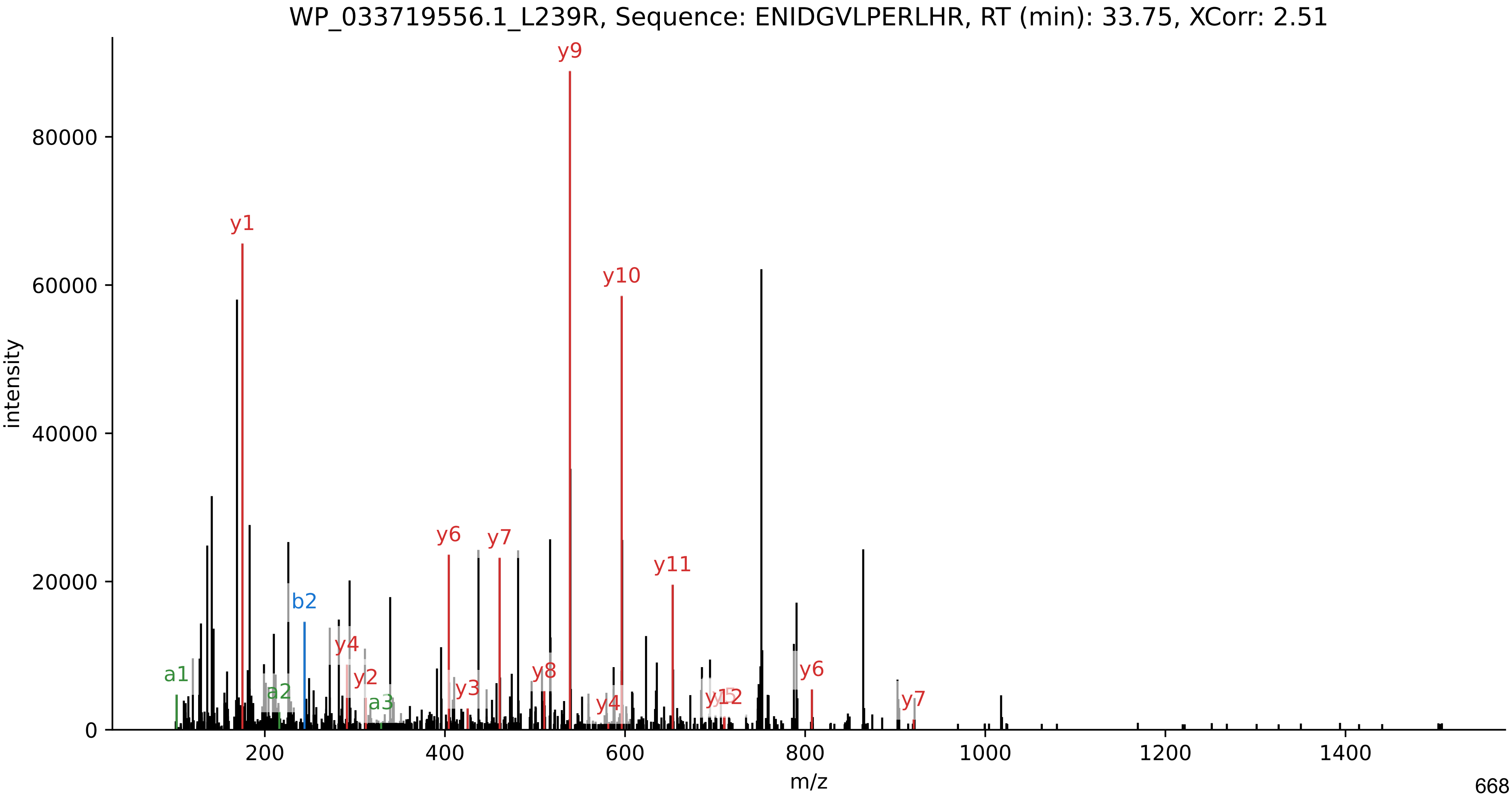

WP_033719556.1_L239R, Sequence: ENIDGVLPERLHR, RT (min): 33.75, XCorr: 2.51
intensity
m/z
y9
y1
y10
y6
y7
y11
b2
y4
y2
a1
a2
a3
y3
y8
y4
y6
y12
y7

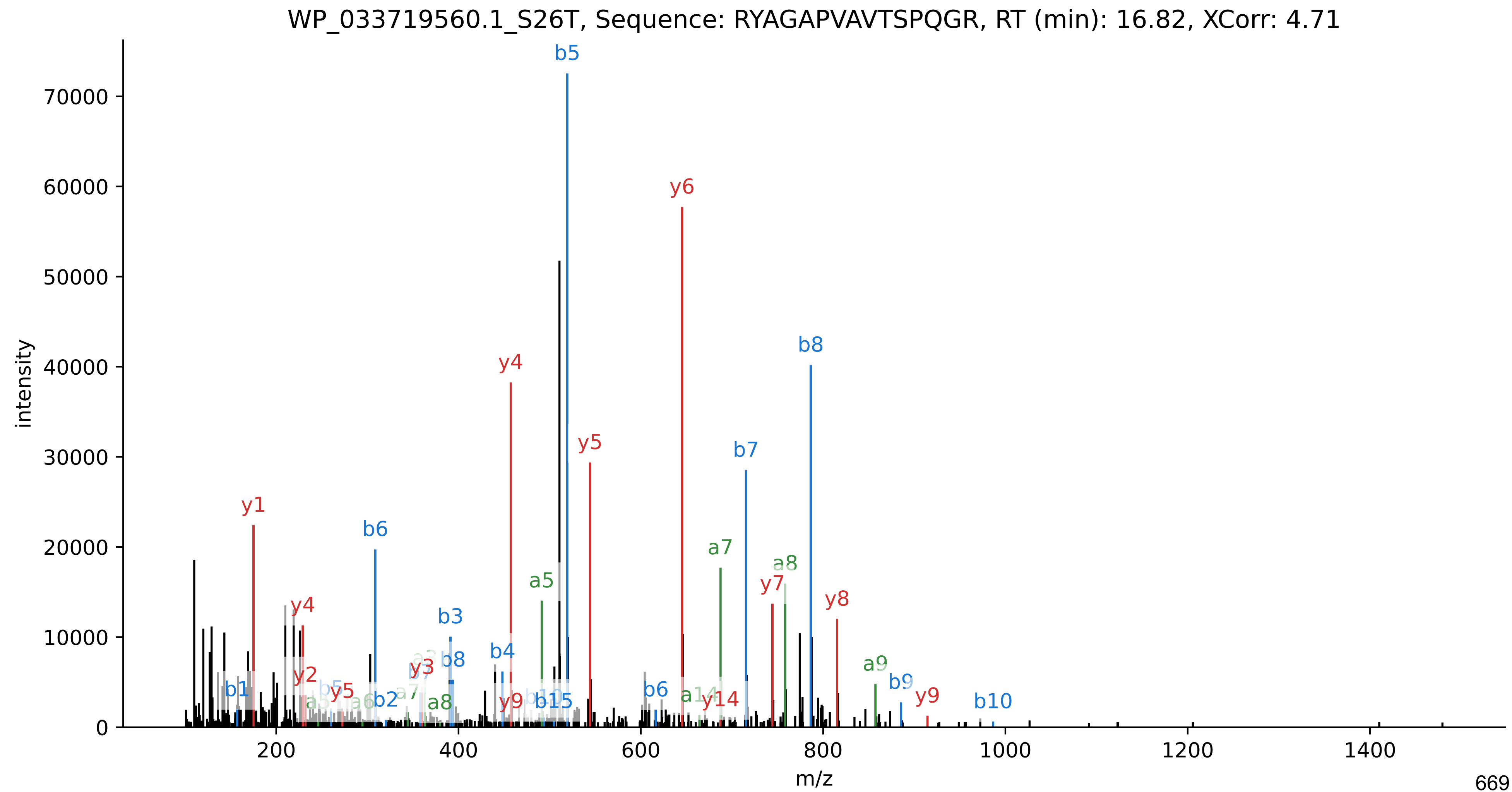
WP_033719560.1_S26T, Sequence: RYAGAPVAVTSPQGR, RT (min): 16.82, XCorr: 4.71
intensity
m/z
b5
y6
b8
y4
y5
b7
y1
b6
a7
a8
a5
y7
y8
b3
b4
a9
b9
y9
b10
y2
y3
b1
b2
b15
y14
a14

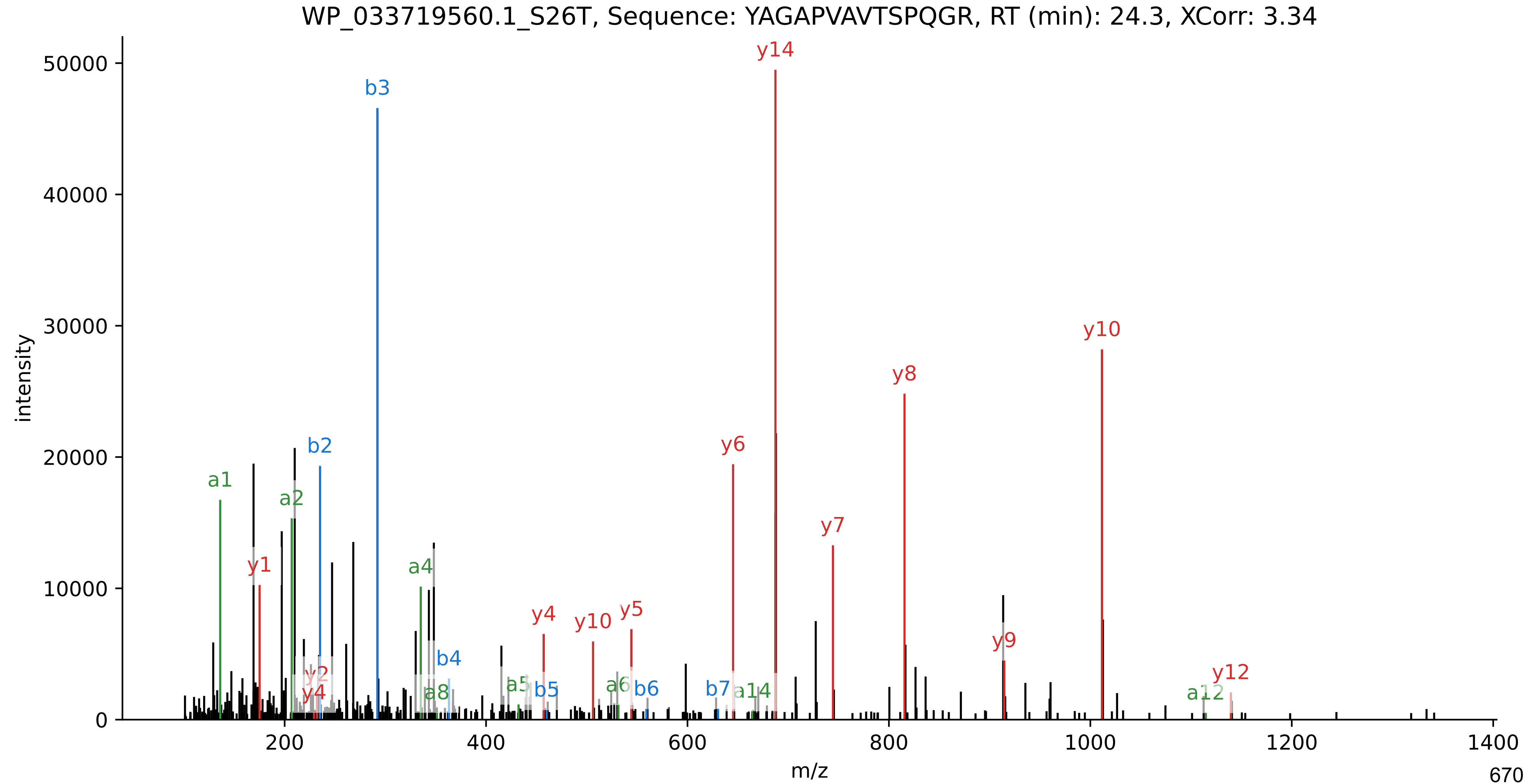

WP_033719560.1_S26T, Sequence: YAGAPVAVTSPQGR, RT (min): 24.3, XCorr: 3.34
intensity
m/z
y14
b3
y10
y8
y6
b2
a1
a2
y7
y1
a4
y4
y10
y5
b4
y9
y2
y4
a8
a5
b5
a6
b6
b7
a14
y12
a12

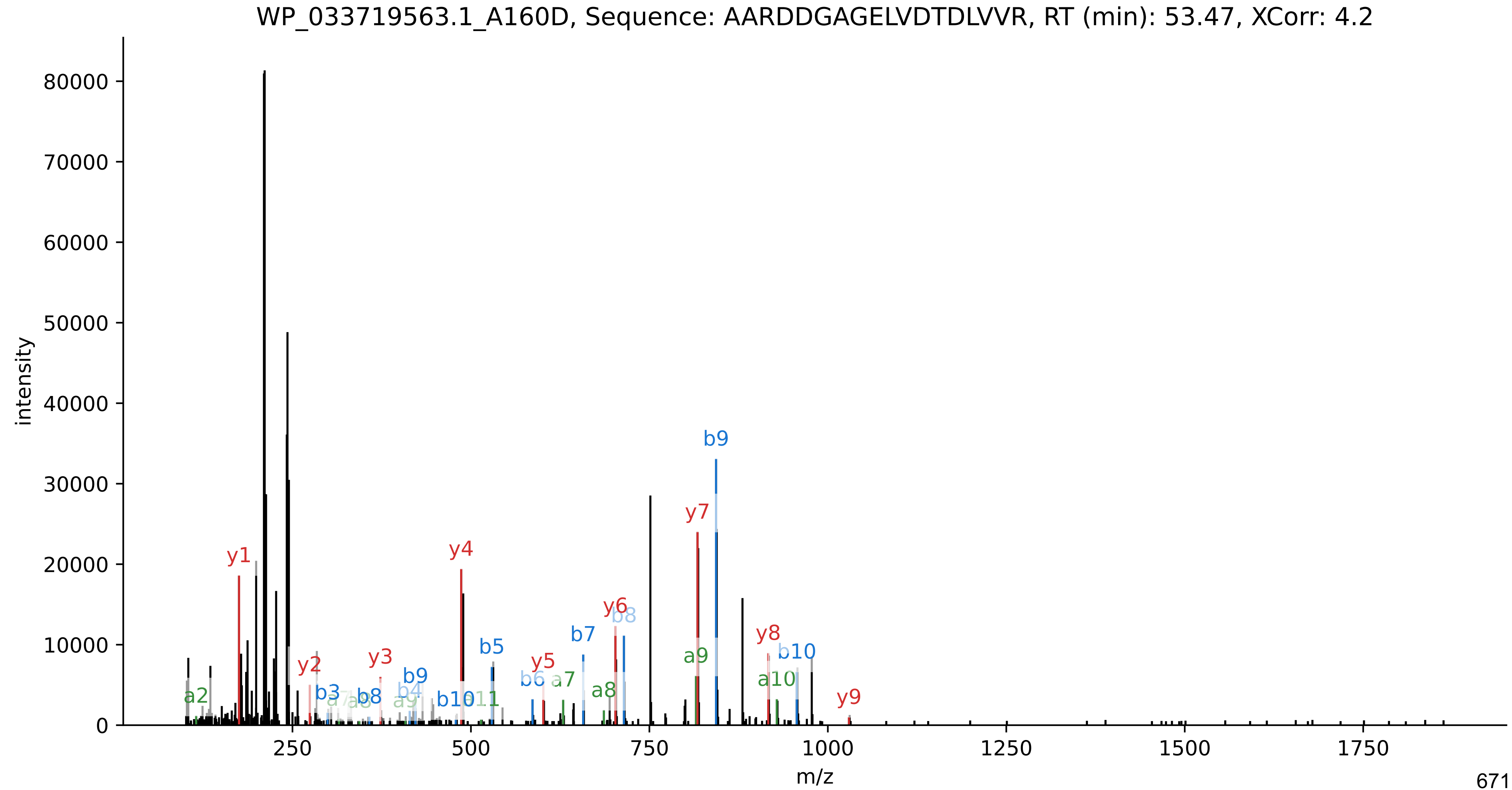

WP_033719563.1_A160D, Sequence: AARDDGAGELVDTDLVVR, RT (min): 53.47, XCorr: 4.2
intensity
m/z
y1
y2
y3
y4
y5
y6
y7
y8
y9
b3
b5
b7
b9
b10
a2
a7
a8
a9
a10

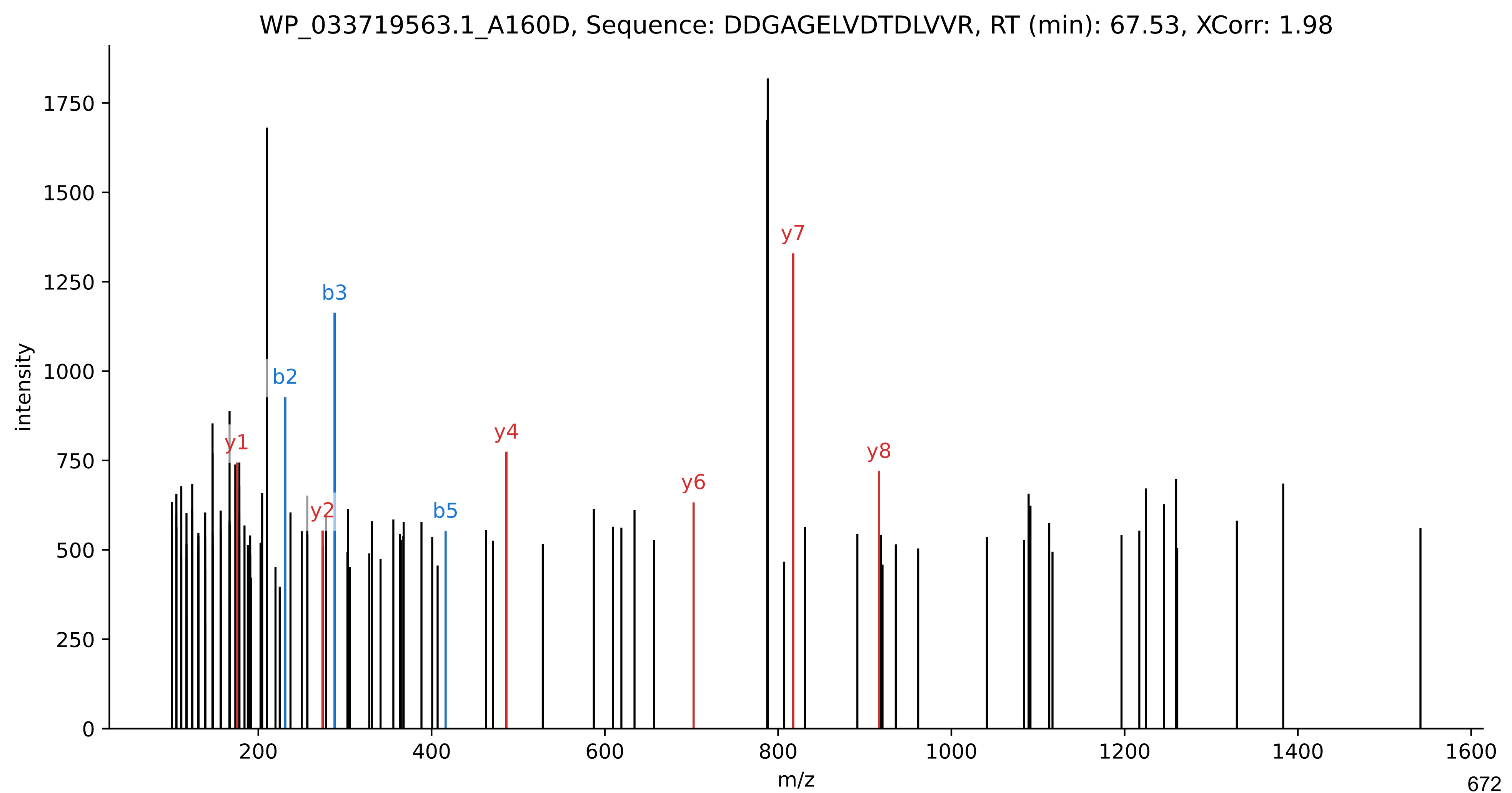

WP_033719563.1_A160D, Sequence: DDGAGELVDTDLVVR, RT (min): 67.53, XCorr: 1.98
intensity
m/z
0
250
500
750
1000
1250
1500
1750
200
400
600
800
1000
1200
1400
1600
y1
y2
b2
b3
b5
y4
y6
y7
y8

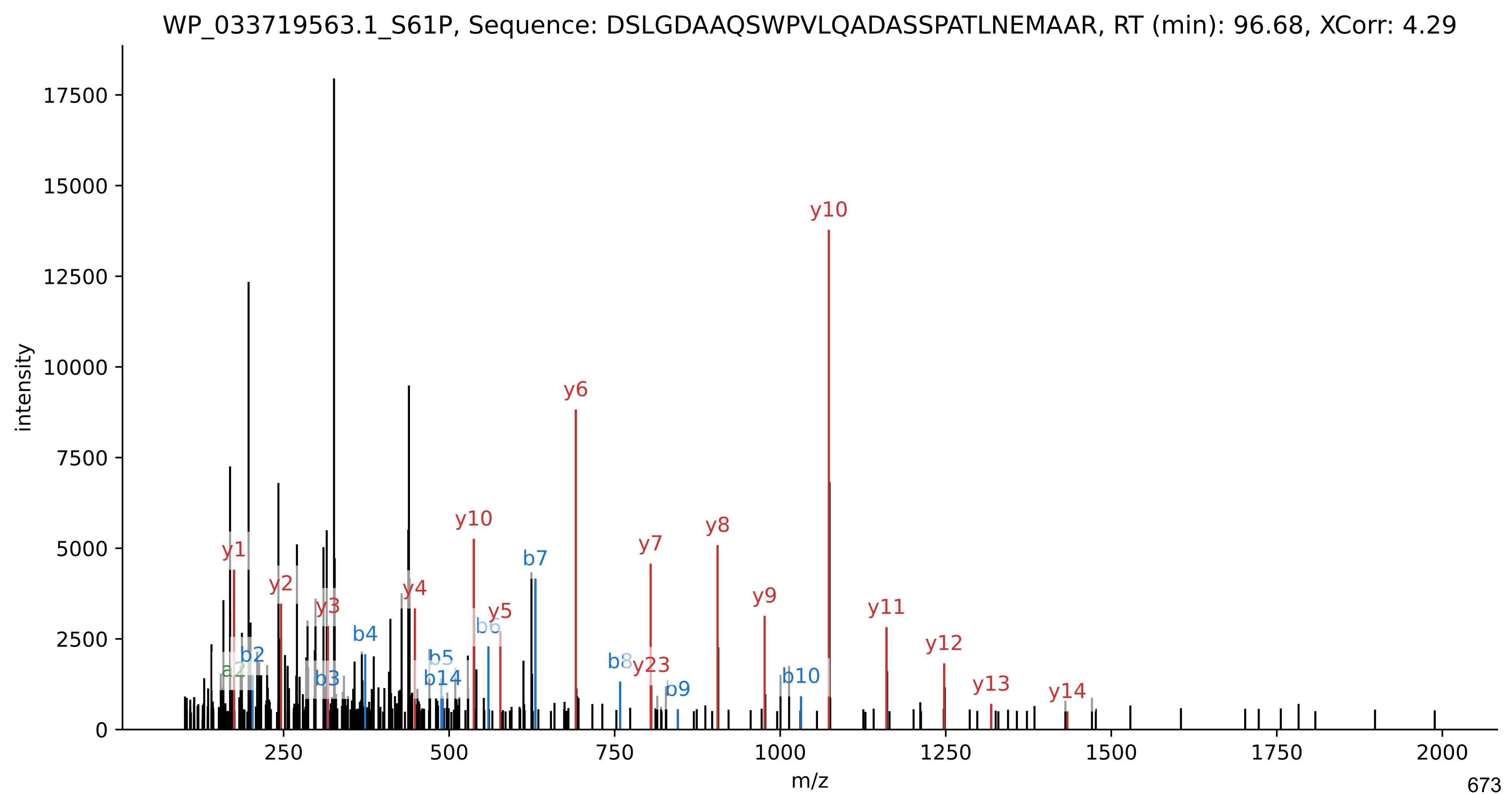
WP_033719563.1_S61P, Sequence: DSLGDAAQSWPVLQADASSPATLNEMAAR, RT (min): 96.68, XCorr: 4.29
intensity
m/z
0
2500
5000
7500
10000
12500
15000
17500
250
500
750
1000
1250
1500
1750
2000
y1
y2
y3
y4
y5
y6
y7
y8
y9
y10
y10
y11
y12
y13
y14
y23
a2
b2
b3
b4
b5
b6
b7
b8
b9
b10
b14

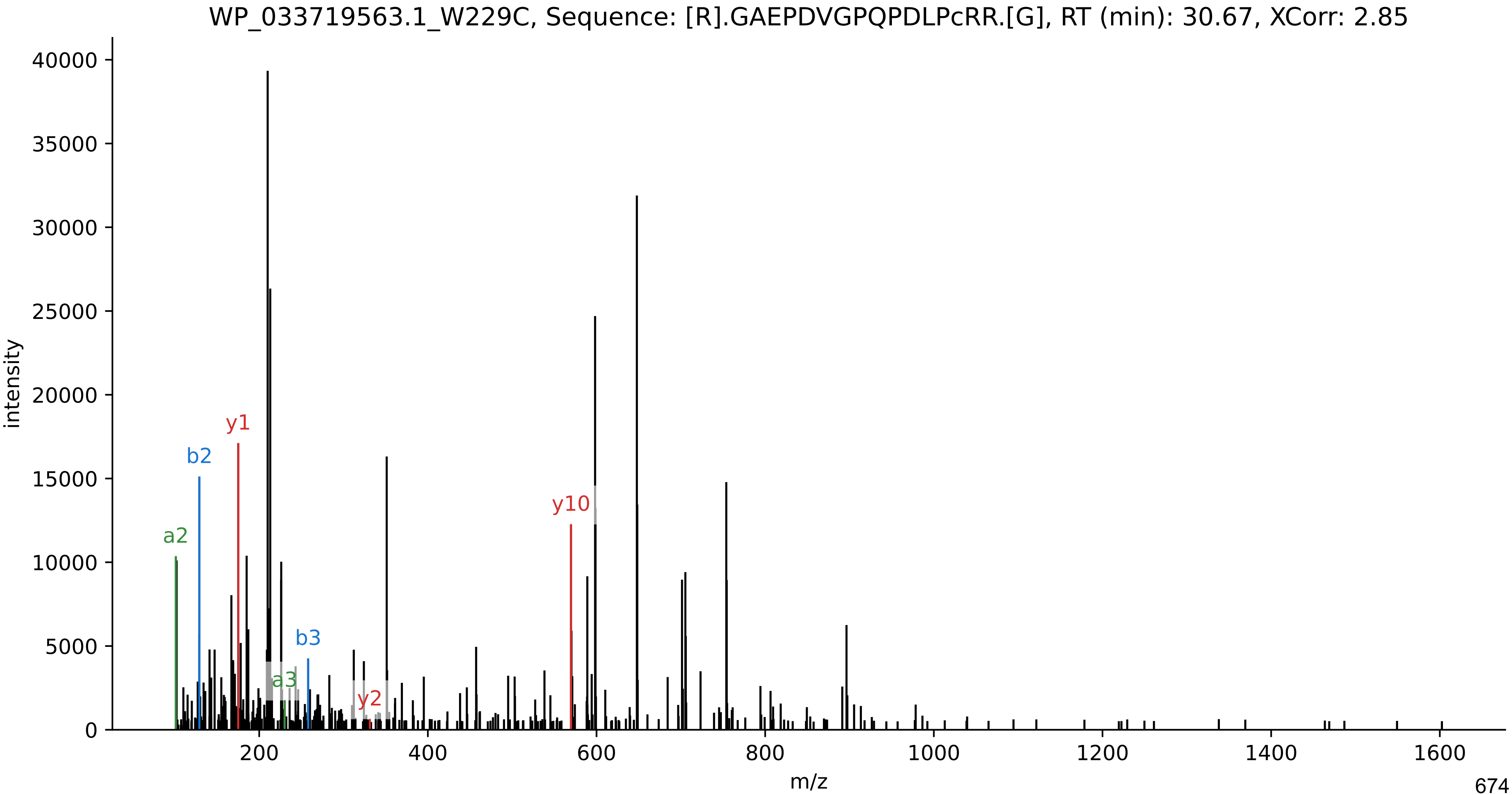
WP_033719563.1_W229C, Sequence: [R].GAEPDVGPQPDLPcRR.[G], RT (min): 30.67, XCorr: 2.85
intensity
m/z
0
5000
10000
15000
20000
25000
30000
35000
40000
200
400
600
800
1000
1200
1400
1600
a2
b2
y1
a3
b3
y2
y10

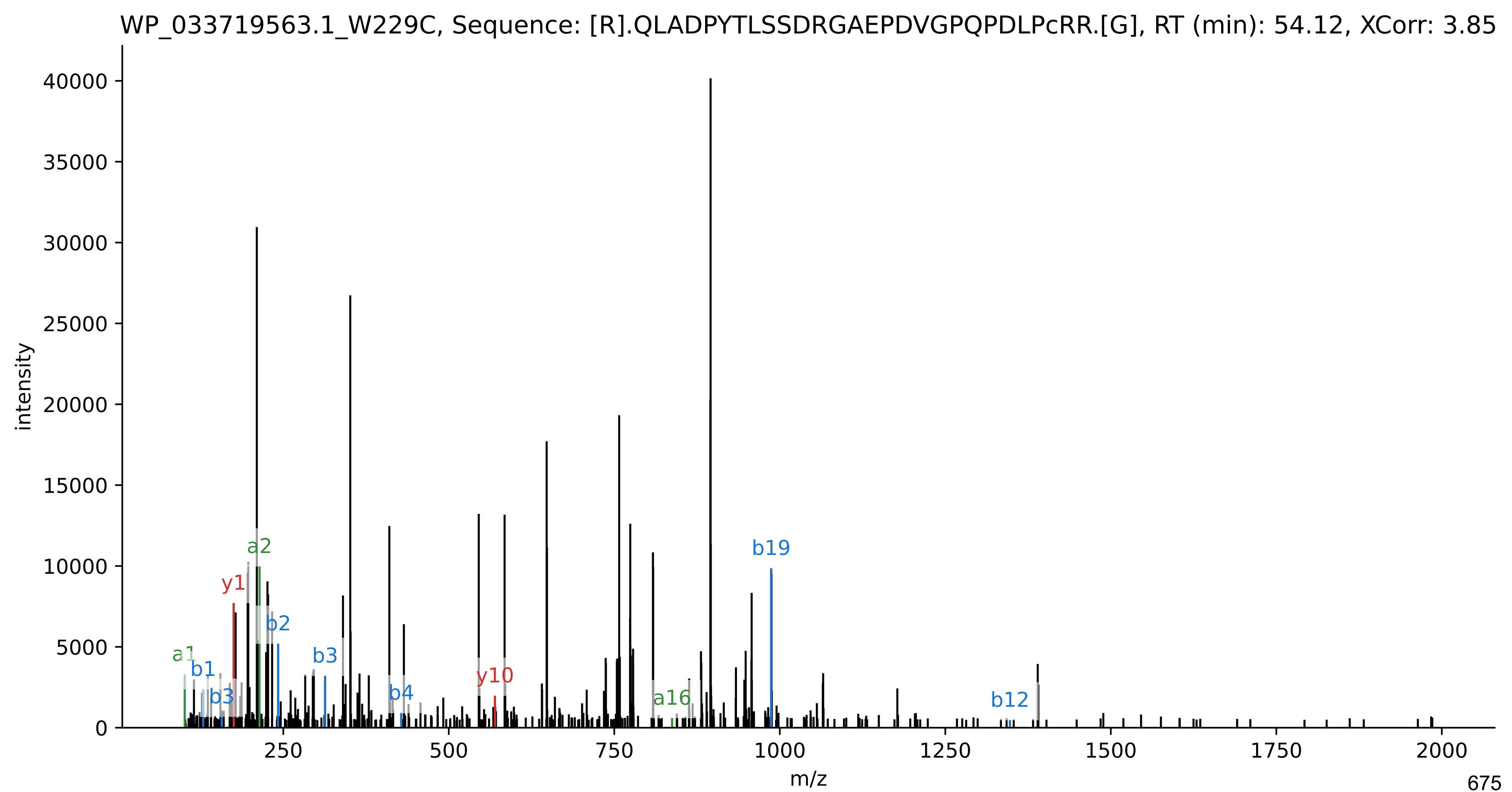
WP_033719563.1_W229C, Sequence: [R].QLADPYTLSSDRGAEPDVGPQPDLPcRR.[G], RT (min): 54.12, XCorr: 3.85
intensity
m/z
a1
b1
b3
y1
a2
b2
b3
b4
y10
a16
b19
b12

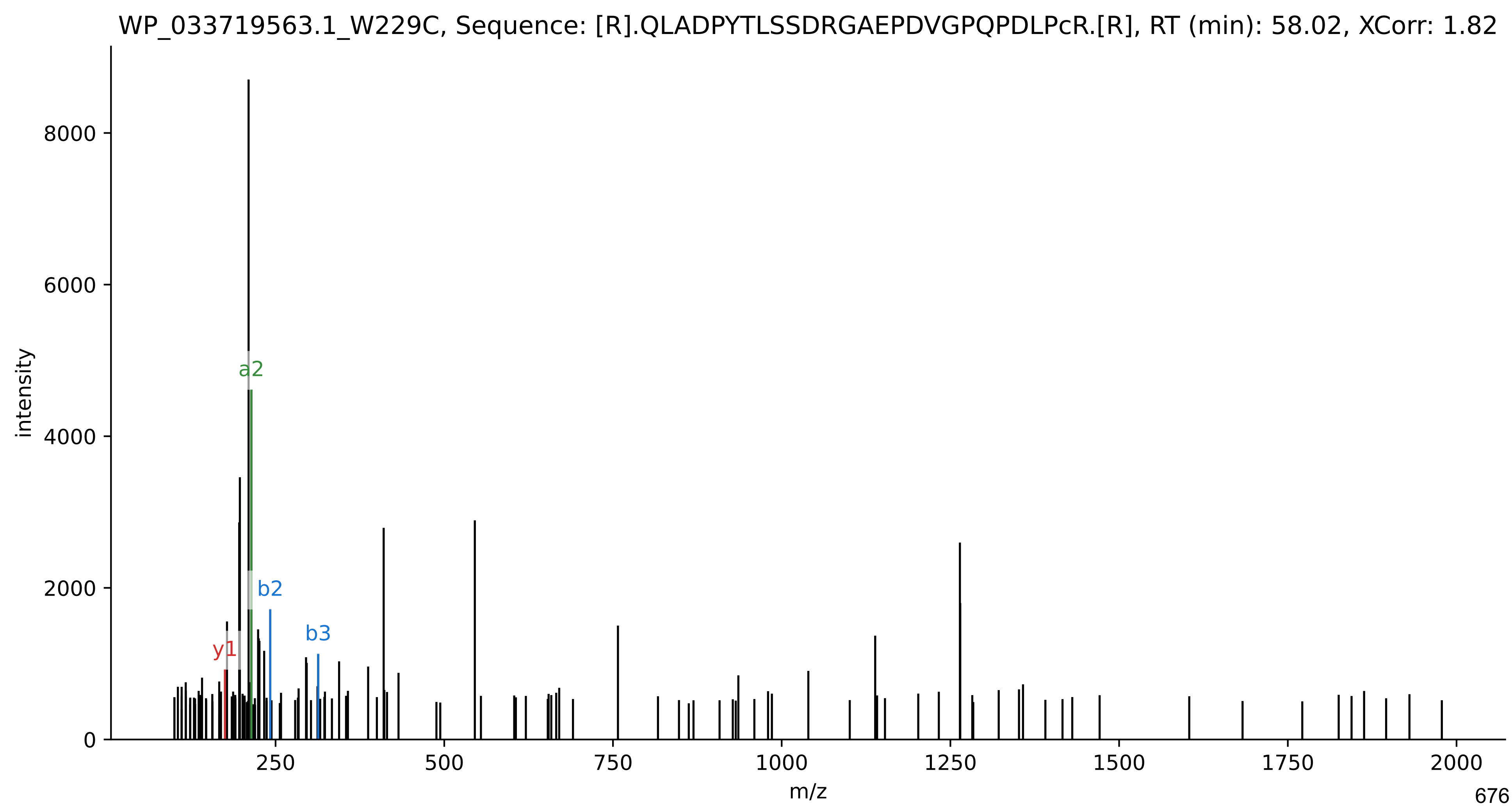

WP_033719563.1_W229C, Sequence: [R].QLADPYTLSSDRGAEPDVGPQPDLPcR.[R], RT (min): 58.02, XCorr: 1.82
intensity
m/z
a2
b2
b3
y1
0
2000
4000
6000
8000
250
500
750
1000
1250
1500
1750
2000

WP_033719565.1_D184N, Sequence: GRGEQGGSDGPGDGDNDEPQNRGK, RT (min): 9.9, XCorr: 3.93

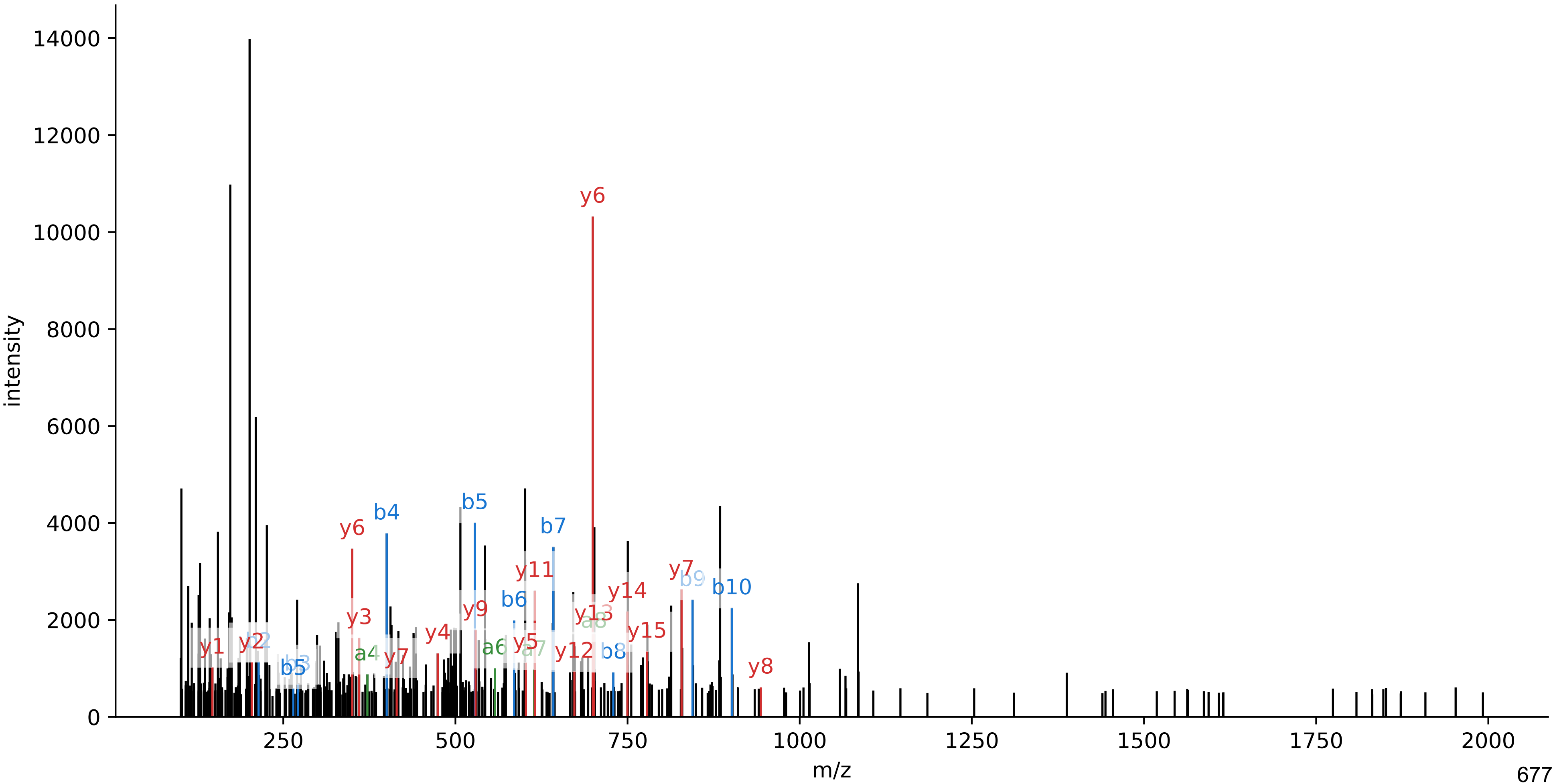

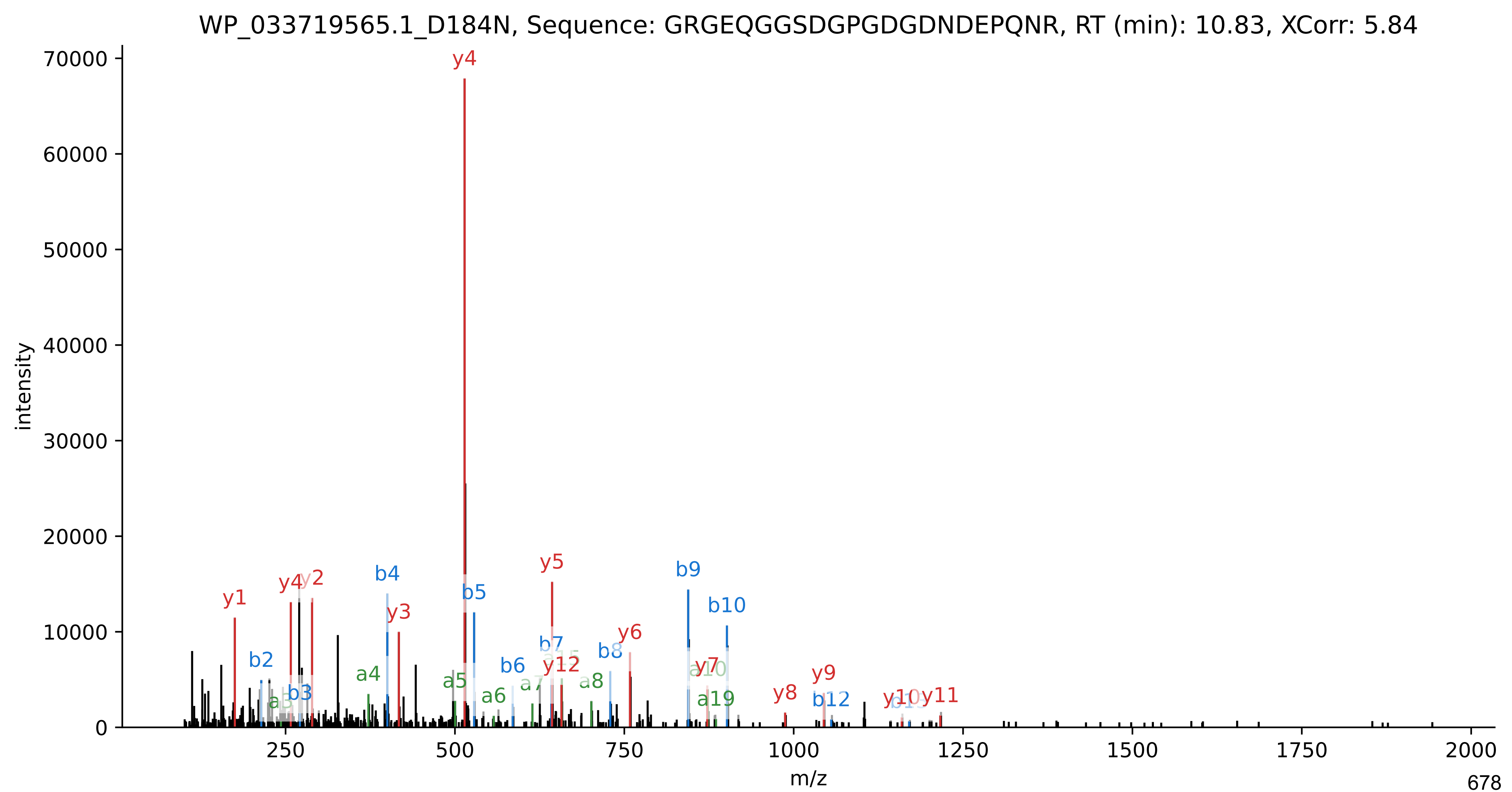
WP_033719565.1_D184N, Sequence: GRGEQGGSDGPGDGDNDEPQNR, RT (min): 10.83, XCorr: 5.84
intensity
m/z
70000
60000
50000
40000
30000
20000
10000
0
250
500
750
1000
1250
1500
1750
2000
y4
y1
y4
y2
b4
y3
b5
y5
b9
b10
b2
b3
a4
a5
a6
b6
b7
y12
b8
a8
y6
y7
a19
y8
y9
b12
y10
y11

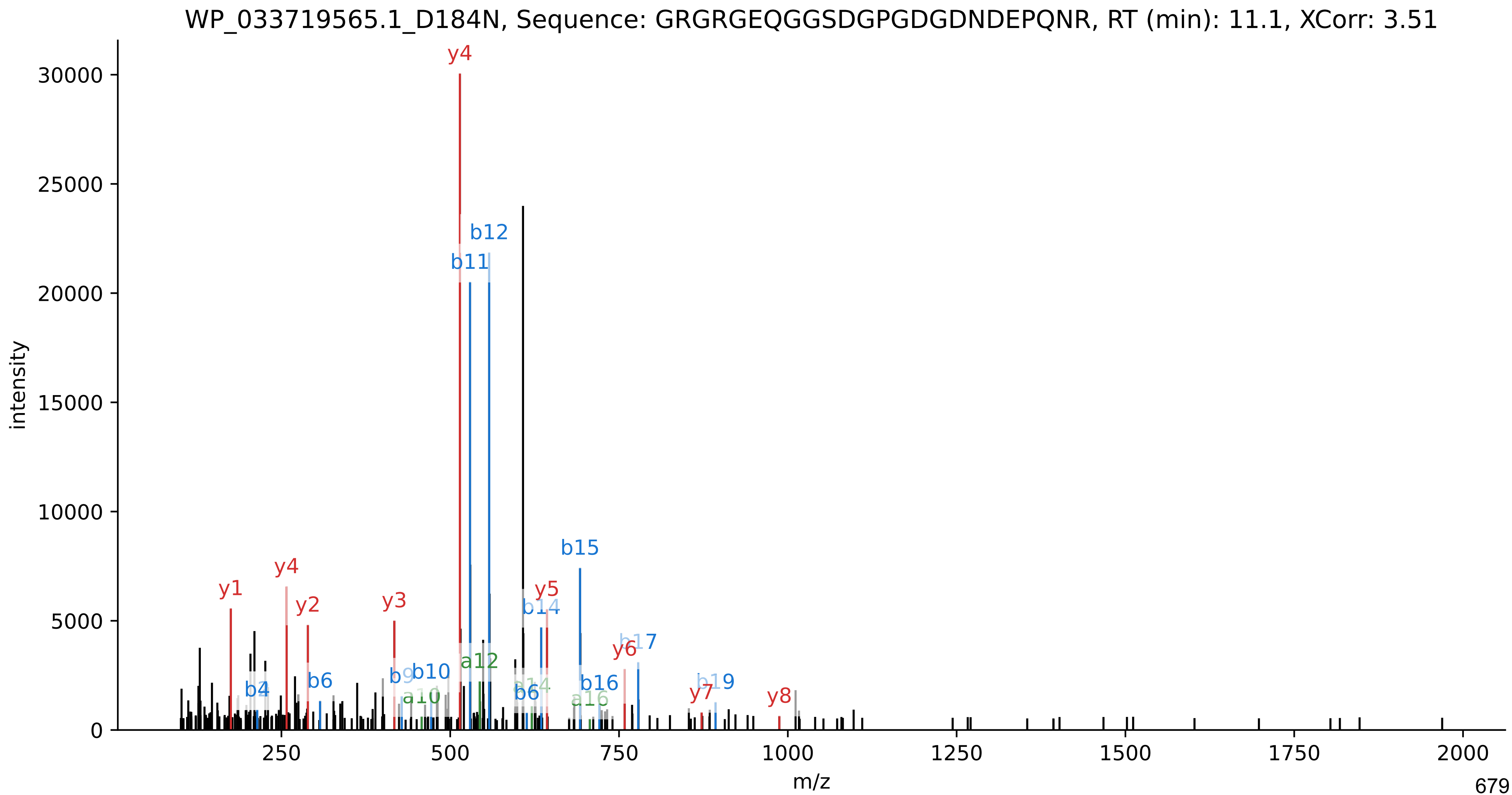
WP_033719565.1_D184N, Sequence: GRGRGEQGGSDGPGDGDNDEPQNR, RT (min): 11.1, XCorr: 3.51
intensity
m/z
0
5000
10000
15000
20000
25000
30000
250
500
750
1000
1250
1500
1750
2000
y4
b12
b11
b15
y1
y4
y2
y3
y5
b14
y6
b17
a12
b10
b6
b4
a10
b6
b16
a16
y7
b19
y8

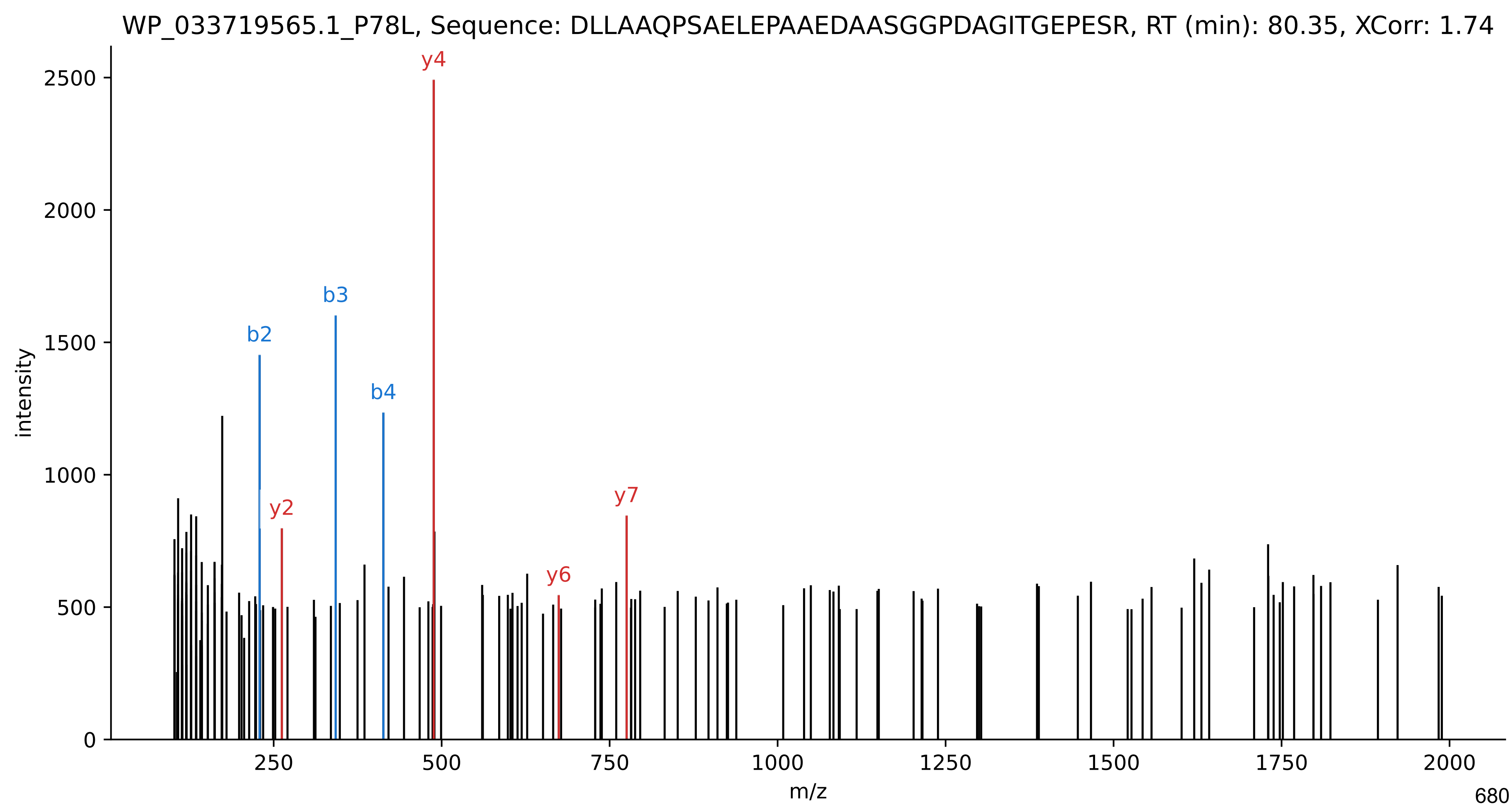
WP_033719565.1_P78L, Sequence: DLLAAQPSAELEPAAEDAASGGPDAGITGEPESR, RT (min): 80.35, XCorr: 1.74
intensity
m/z
y4
b3
b2
b4
y2
y7
y6
2500
2000
1500
1000
500
0
250
500
750
1000
1250
1500
1750
2000

WP_033719565.1_P78L, Sequence: VRDLLAAQPSAELEPAAEDAASGGPDAGITGEPESR, RT (min): 69.78, XCorr: 3.34

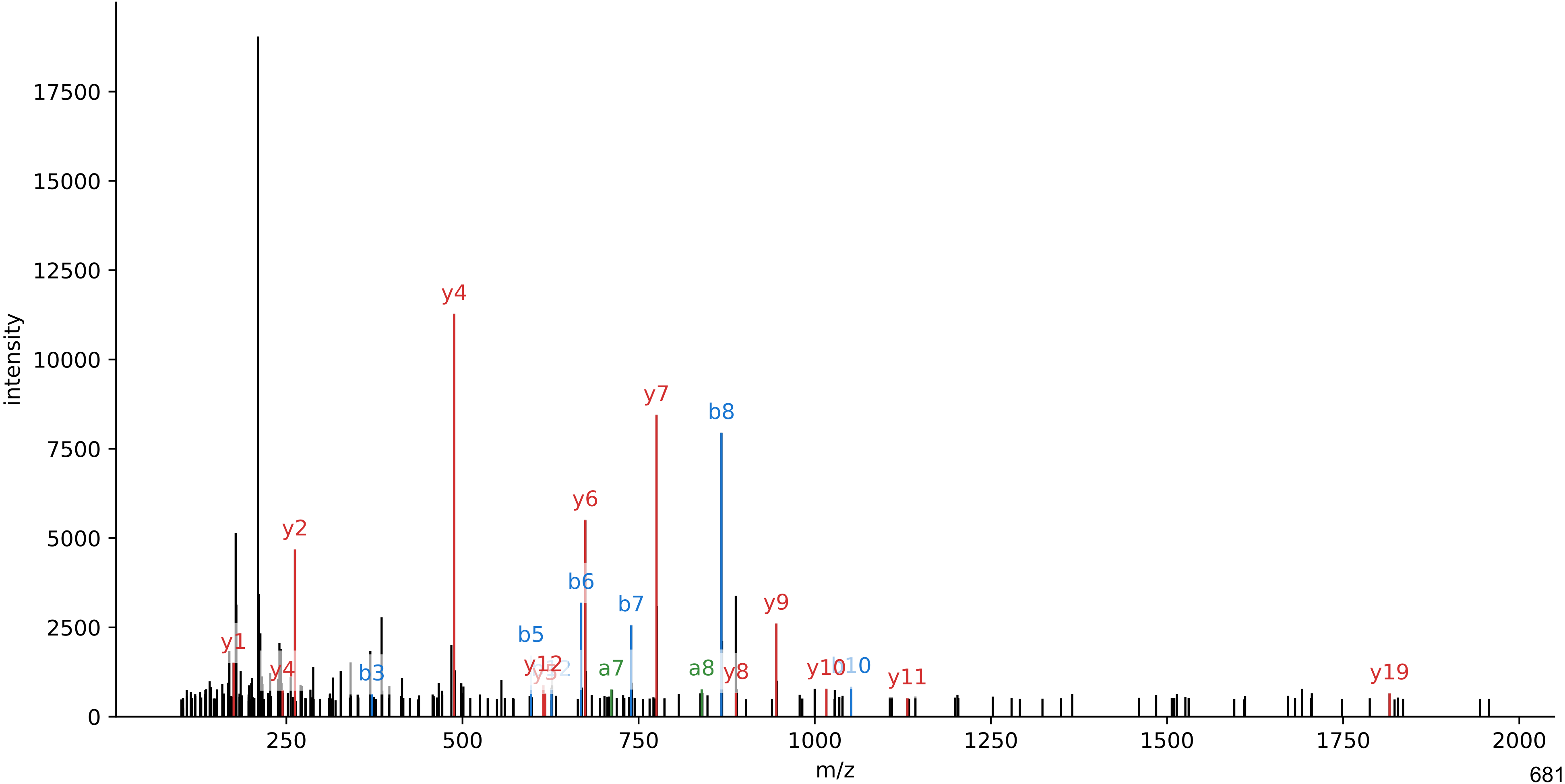

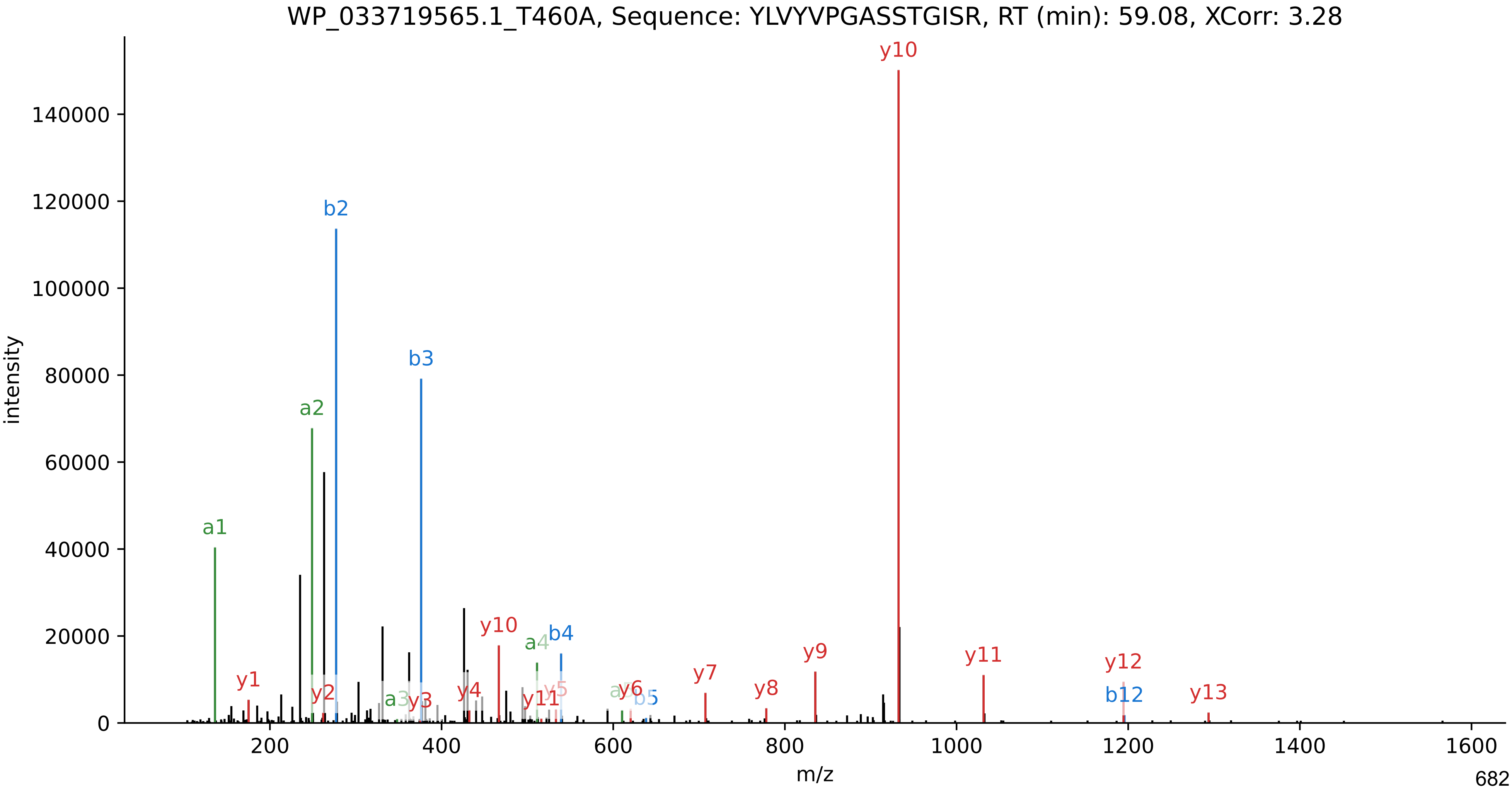
WP_033719565.1_T460A, Sequence: YLVYVPGASSTGISR, RT (min): 59.08, XCorr: 3.28
intensity
m/z
y10
b2
b3
a2
a1
y10
a4
b4
y1
y2
a3
y3
y4
y11
y5
y6
b5
y7
y8
y9
y11
y12
b12
y13
0
20000
40000
60000
80000
100000
120000
140000
200
400
600
800
1000
1200
1400
1600

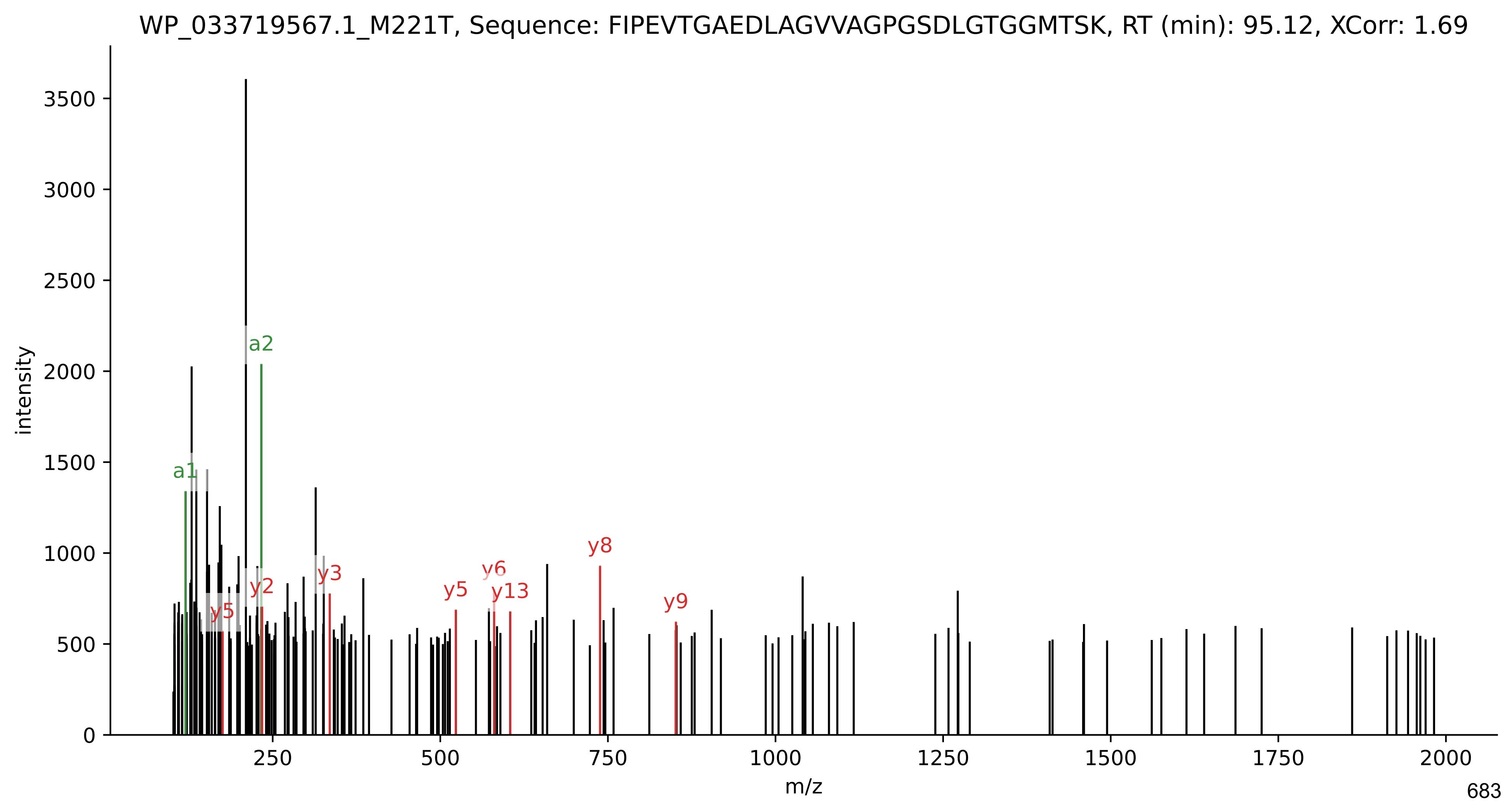
WP_033719567.1_M221T, Sequence: FIPEVTGAEDLAGVVAGPGSDLGTGGMTSK, RT (min): 95.12, XCorr: 1.69
intensity
m/z
a1
a2
y5
y2
y3
y5
y6
y13
y8
y9

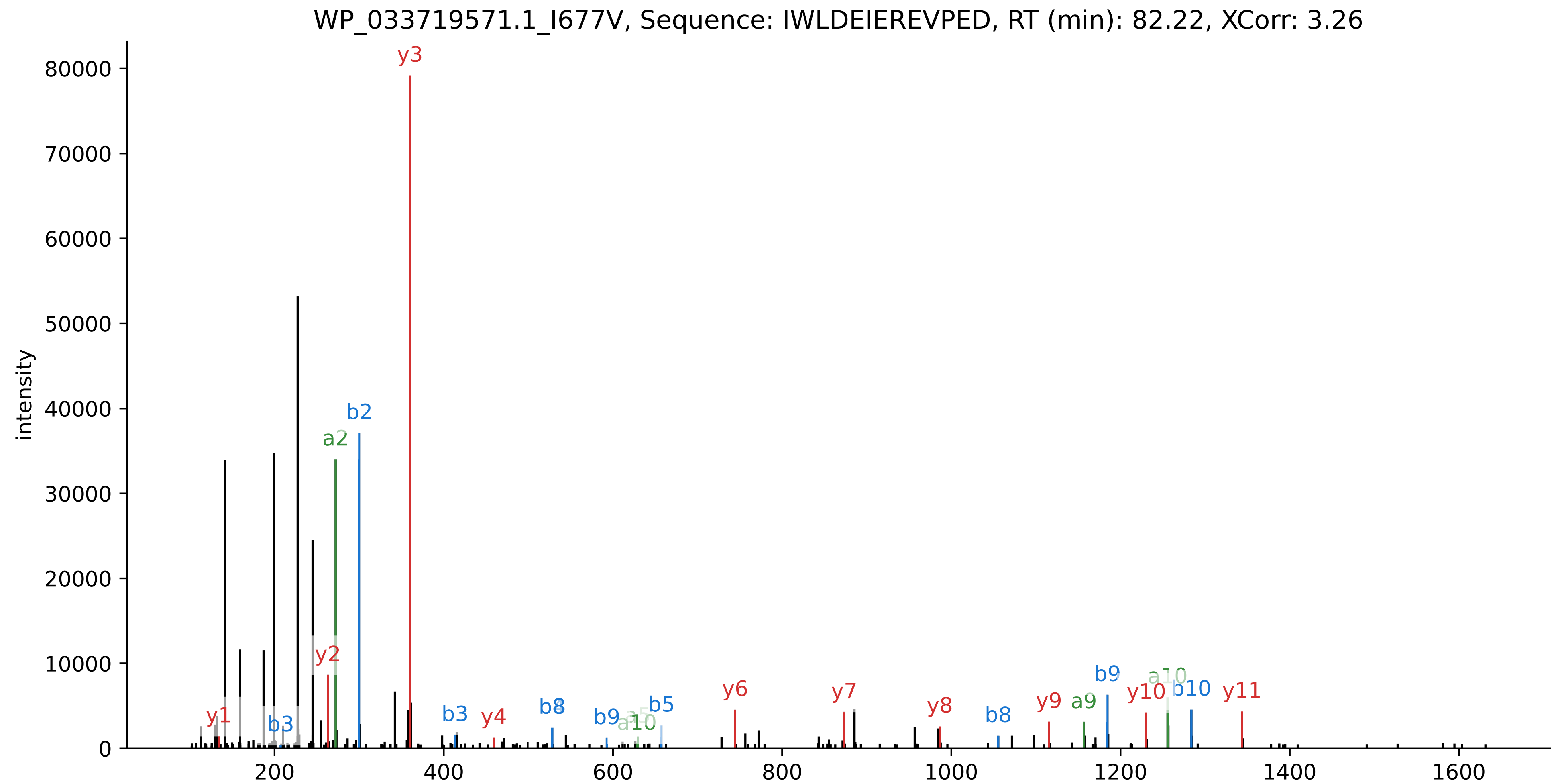

WP_033719571.1_I677V, Sequence: IWLDEIEREVPED, RT (min): 82.22, XCorr: 3.26
intensity
m/z
y3
b2
a2
y2
y1
b3
b3
y4
b8
b9
a10
b5
y6
y7
y8
b8
y9
a9
b9
y10
a10
b10
y11

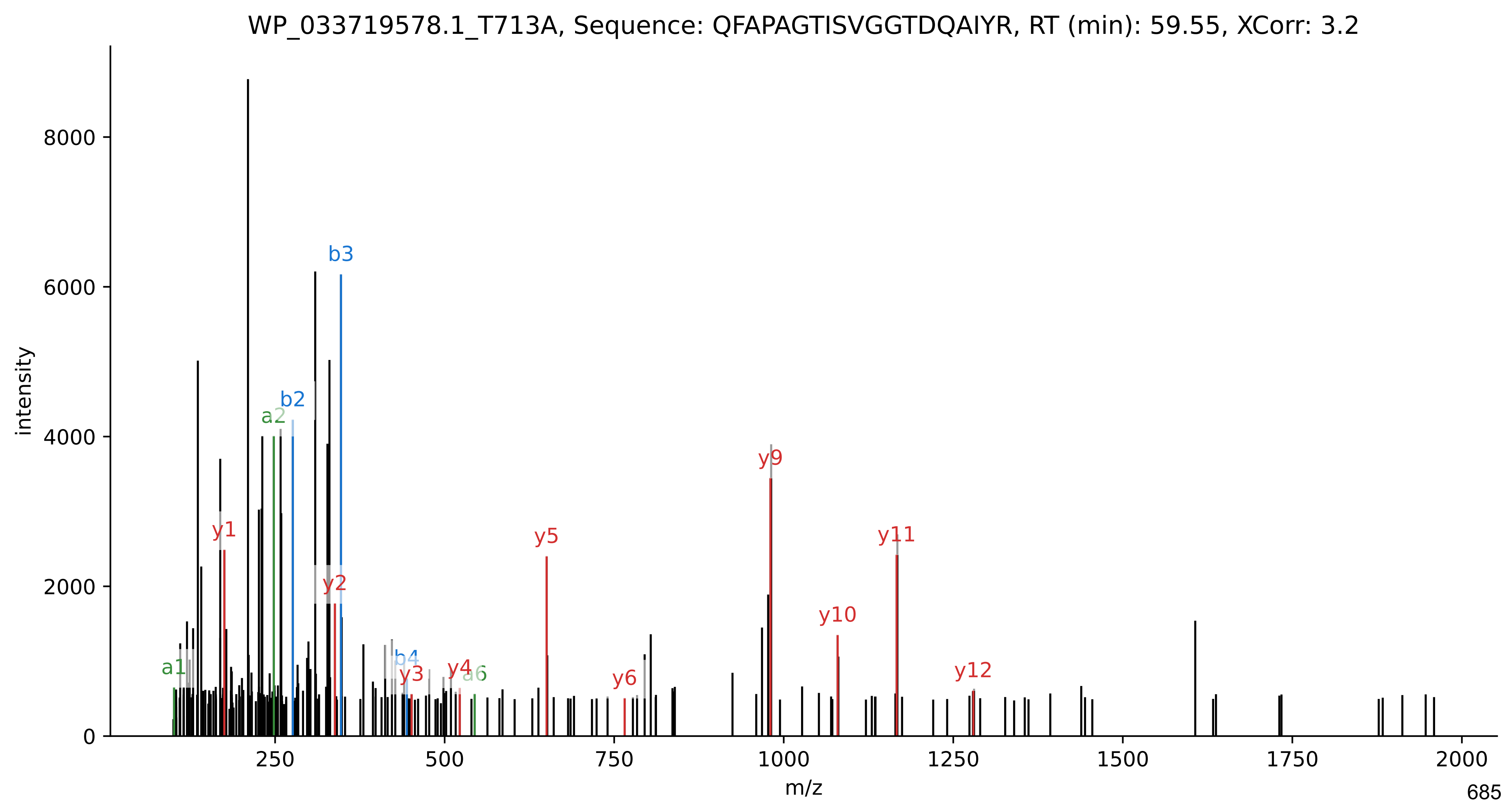

WP_033719578.1_T713A, Sequence: QFAPAGTISVGGTDQAIYR, RT (min): 59.55, XCorr: 3.2
intensity
m/z
0
2000
4000
6000
8000
250
500
750
1000
1250
1500
1750
2000
a1
y1
a2
b2
b3
y2
b4
y3
y4
a6
y5
y6
y9
y10
y11
y12

WP_033719580.1_A267T, Sequence: ALPQVKPGTVTR, RT (min): 20.23, XCorr: 1.86

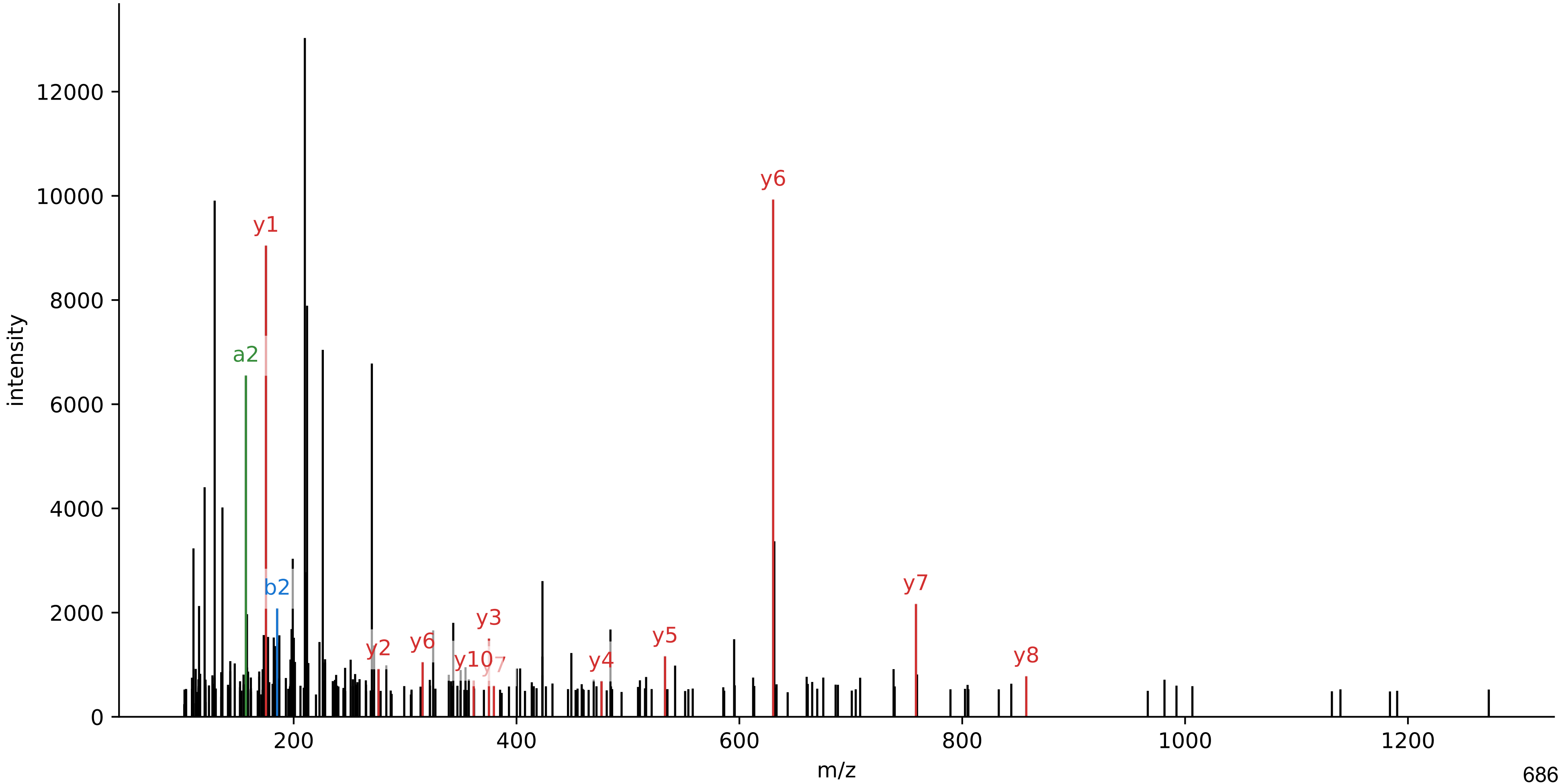

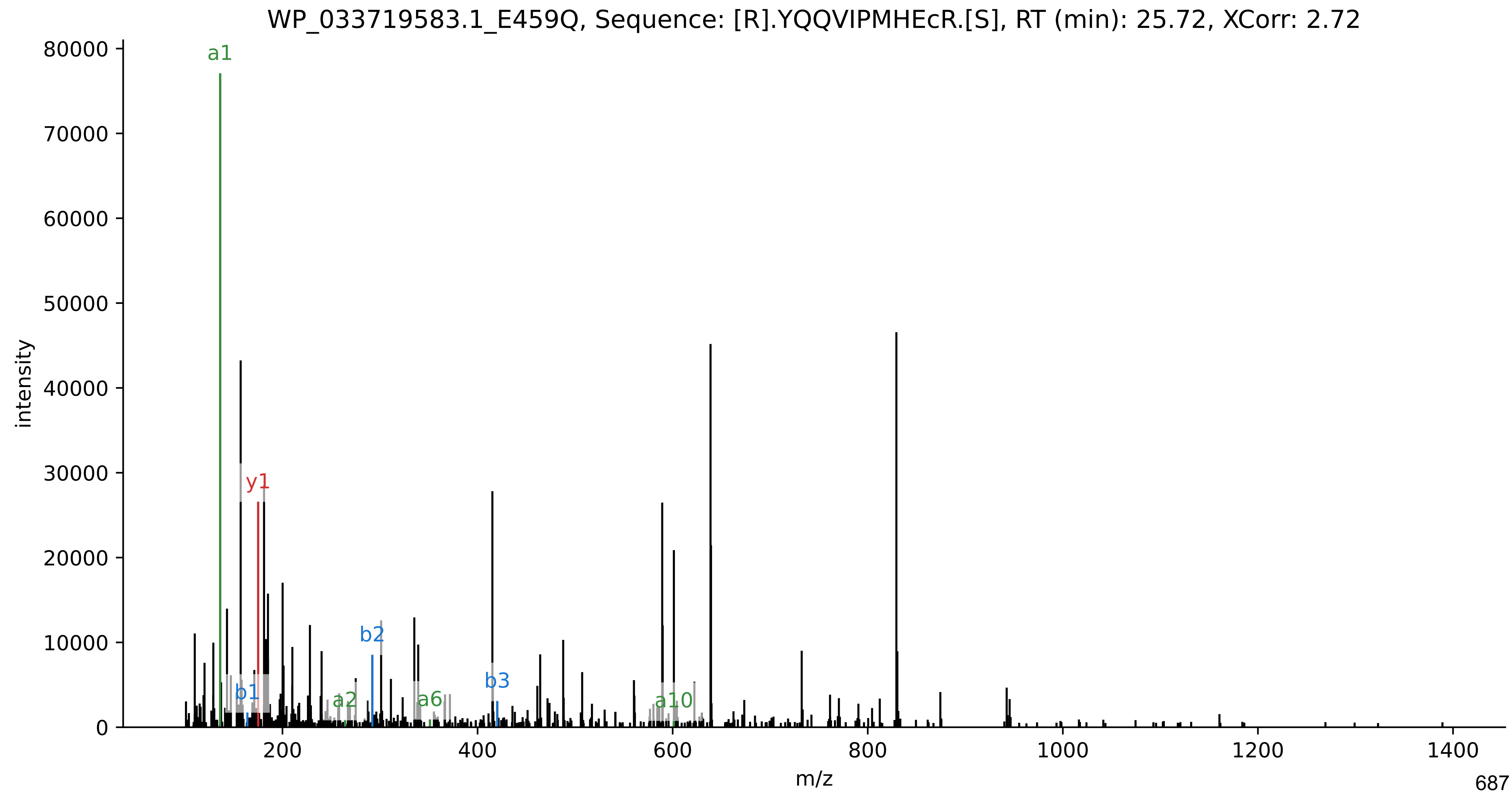
WP_033719583.1_E459Q, Sequence: [R].YQQVIPMHEcR.[S], RT (min): 25.72, XCorr: 2.72
intensity
m/z
a1
y1
b1
a2
b2
a6
b3
a10
0
10000
20000
30000
40000
50000
60000
70000
80000
200
400
600
800
1000
1200
1400

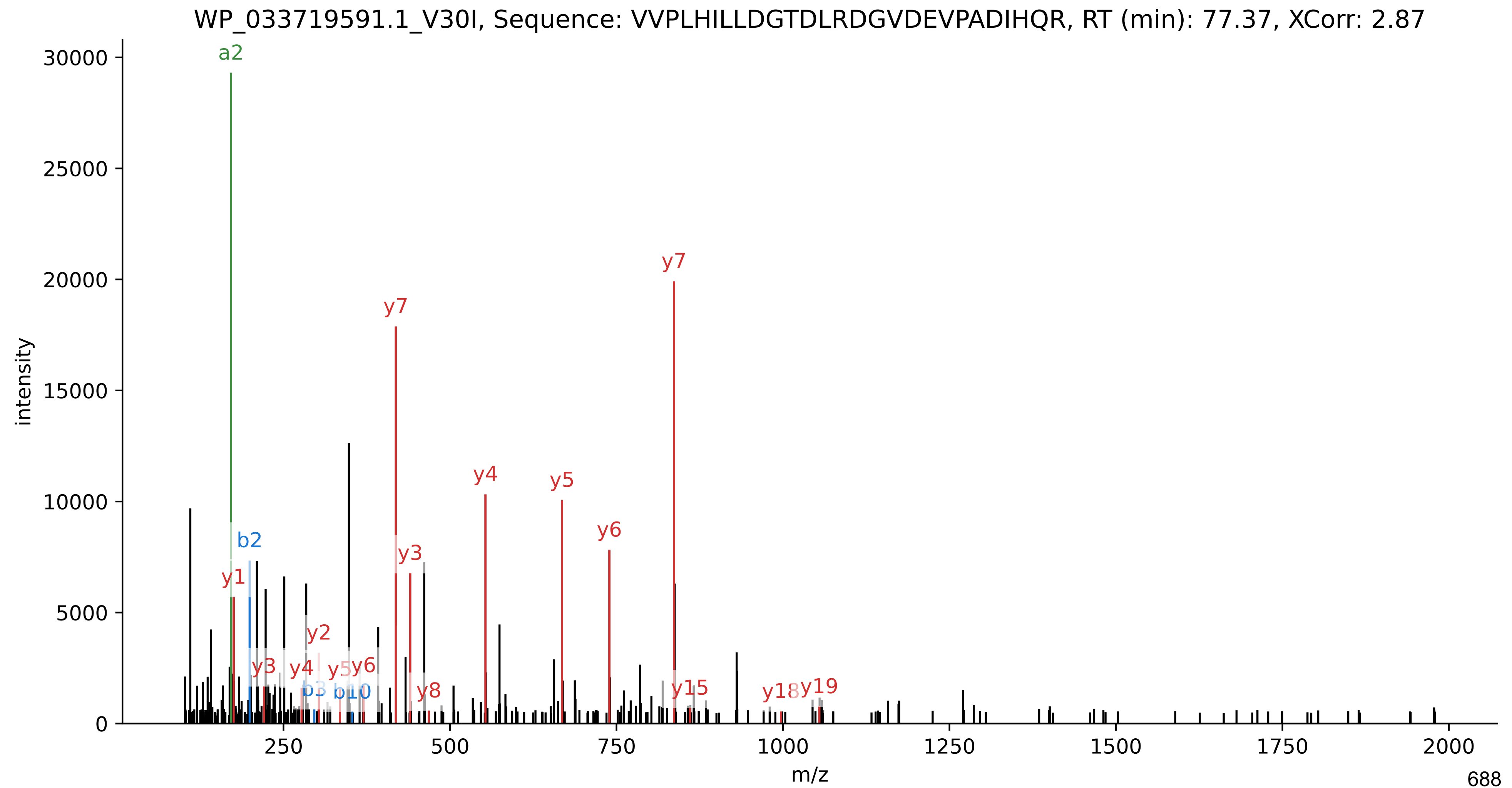
WP_033719591.1_V30I, Sequence: VVPLHILLDGTDLRDGVDEVPADIHQR, RT (min): 77.37, XCorr: 2.87
intensity
m/z
a2
b2
y1
y7
y7
y4
y5
y6
y3
y2
y3
y4
y5
y6
y8
y15
y18
y19
b3
b10

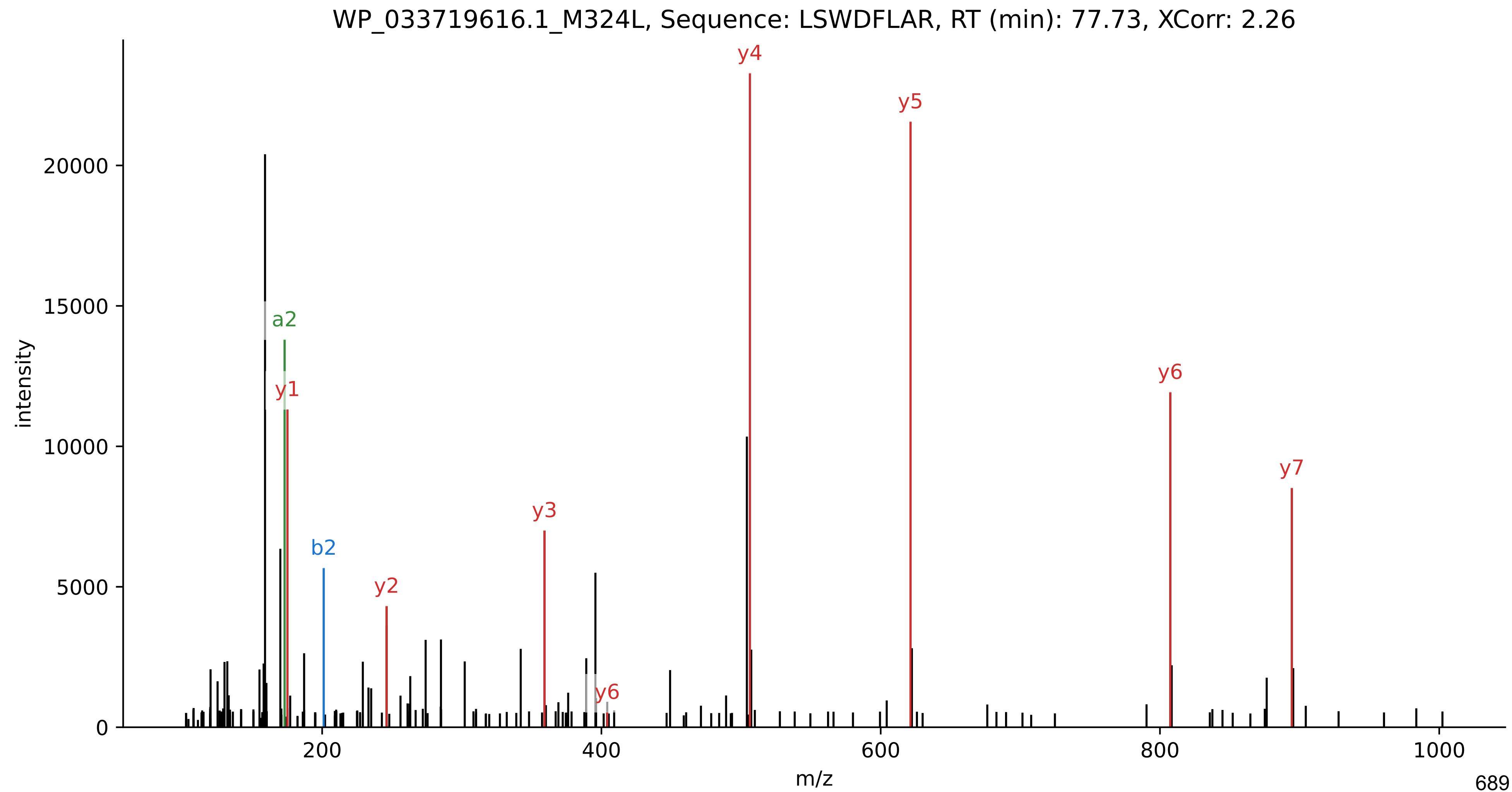

WP_033719616.1_M324L, Sequence: LSWDFLAR, RT (min): 77.73, XCorr: 2.26
intensity
m/z
0
5000
10000
15000
20000
200
400
600
800
1000
a2
y1
b2
y2
y3
y6
y4
y5
y6
y7

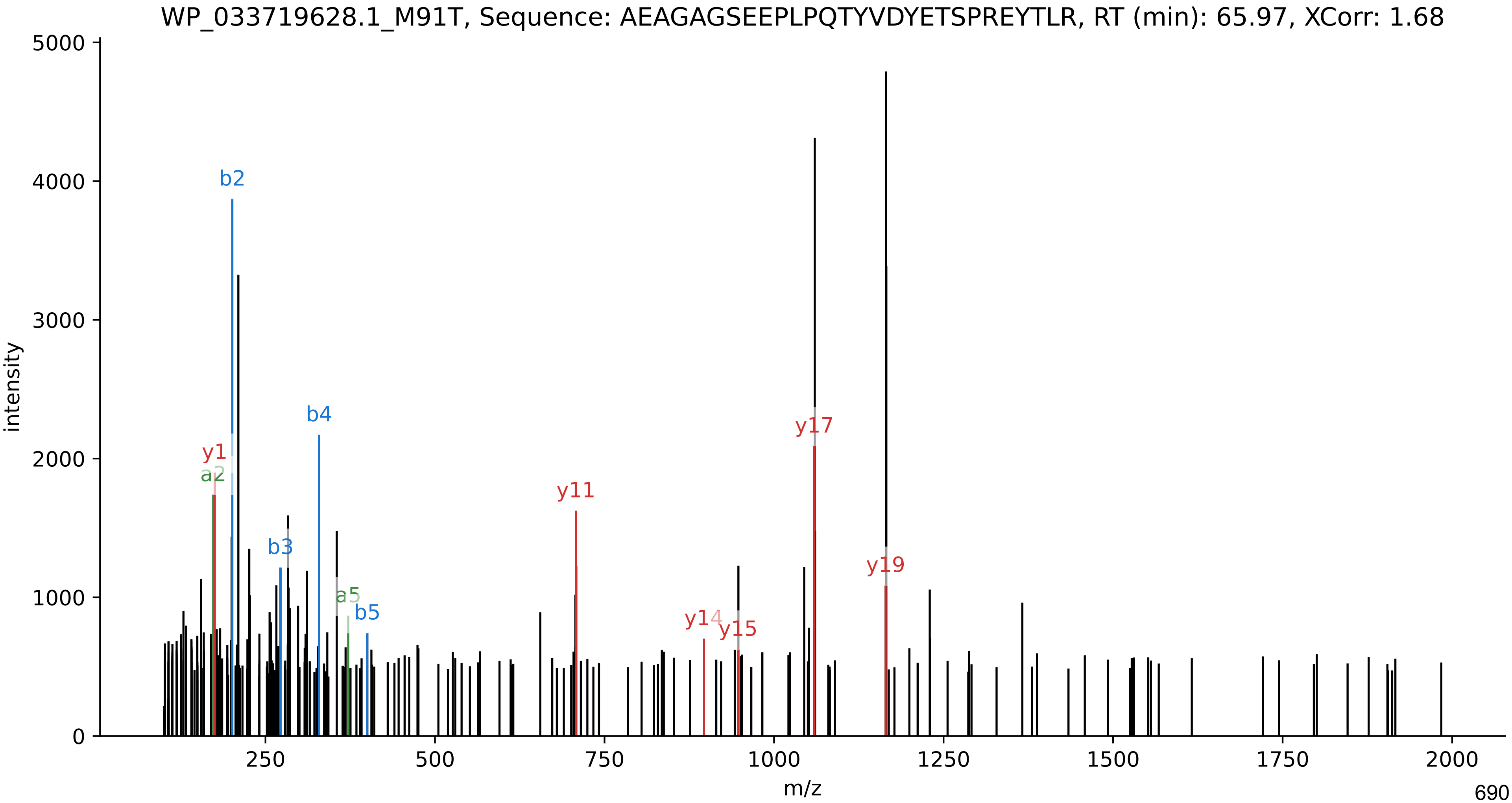
WP_033719628.1_M91T, Sequence: AEAGAGSEEPLPQTYVDYETSPREYTLR, RT (min): 65.97, XCorr: 1.68
intensity
m/z
b2
b3
b4
b5
a2
a5
y1
y11
y14
y15
y17
y19
0
1000
2000
3000
4000
5000
250
500
750
1000
1250
1500
1750
2000

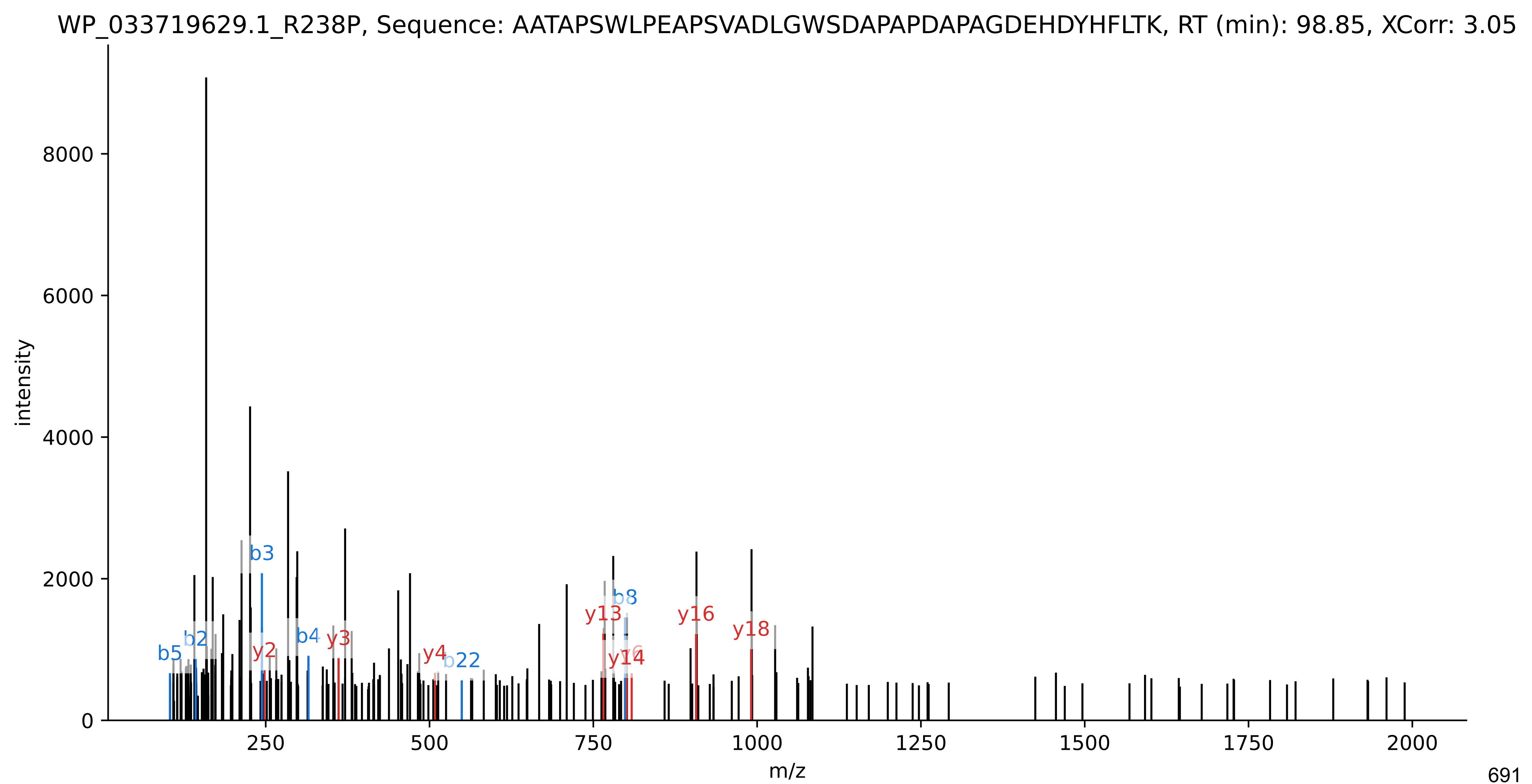
WP_033719629.1_R238P, Sequence: AATAPSWLPEAPSVADLGWSDAPAPDAPAGDEHDYHFLTK, RT (min): 98.85, XCorr: 3.05
intensity
m/z
0
2000
4000
6000
8000
250
500
750
1000
1250
1500
1750
2000
b5
b2
b3
y2
b4
y3
y4
b22
y13
b8
y14
y16
y18

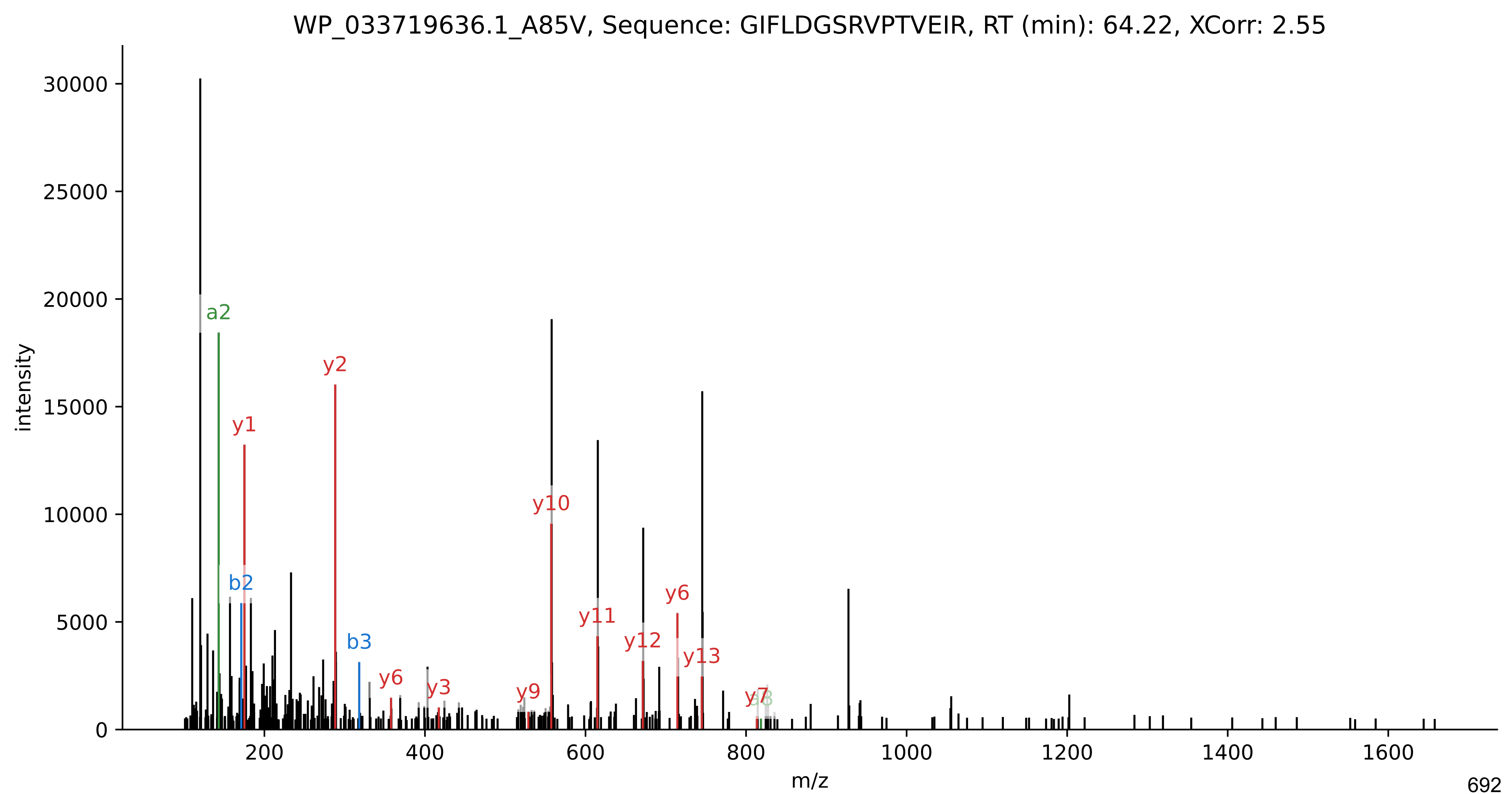

WP_033719636.1_A85V, Sequence: GIFLDGSRVPTVEIR, RT (min): 64.22, XCorr: 2.55
intensity
m/z
a2
y2
y1
y10
b2
y6
y11
y12
y13
b3
y6
y3
y9
y7

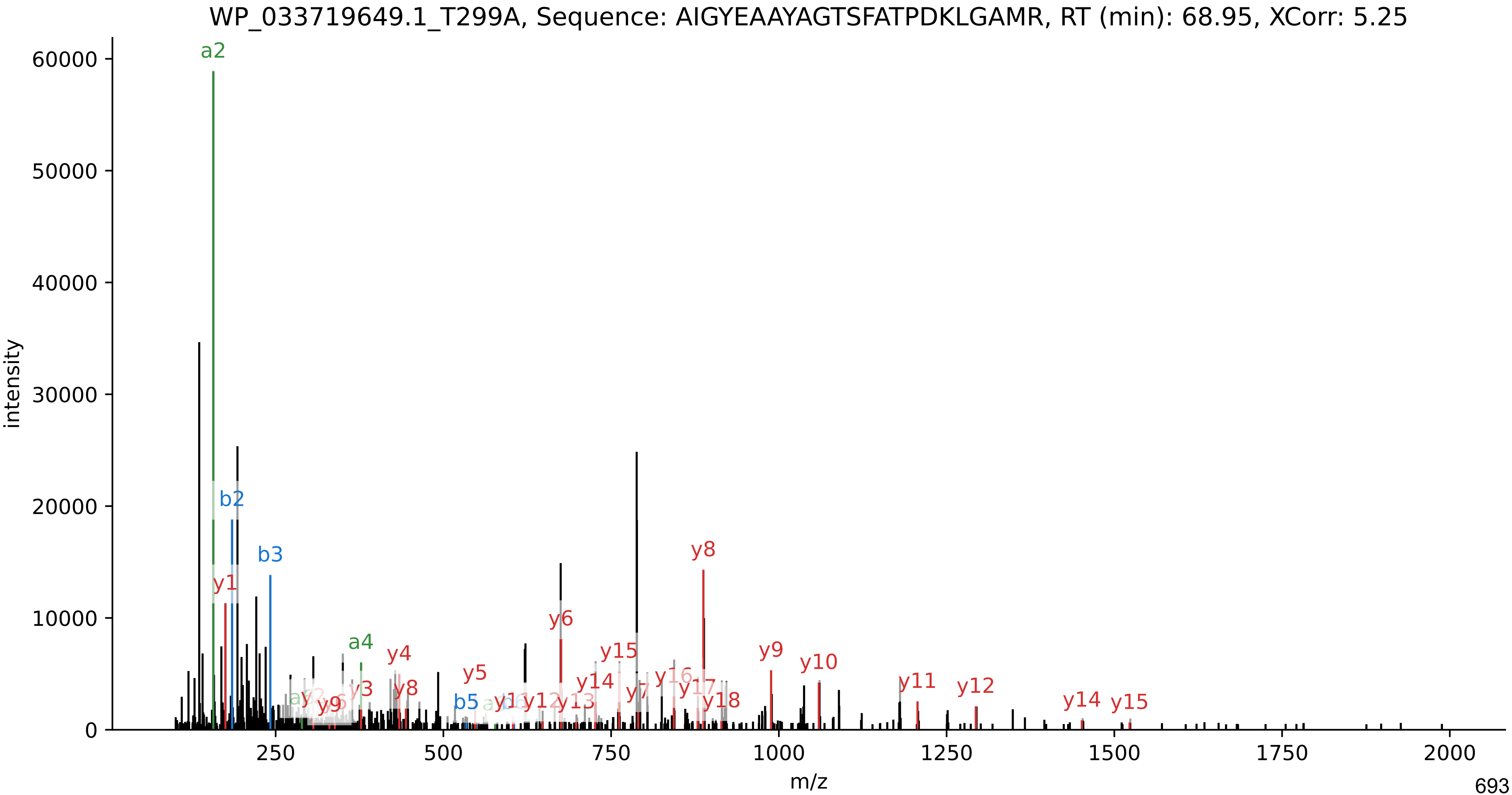

WP_033719649.1_T299A, Sequence: AIGYEAAYAGTSFATPDKLGAMR, RT (min): 68.95, XCorr: 5.25
intensity
m/z
a2
b2
b3
y1
a4
y4
y3
y8
y9
y5
b5
y6
y15
y14
y12
y13
y7
y16
y17
y18
y8
y9
y10
y11
y12
y14
y15
0
10000
20000
30000
40000
50000
60000
250
500
750
1000
1250
1500
1750
2000

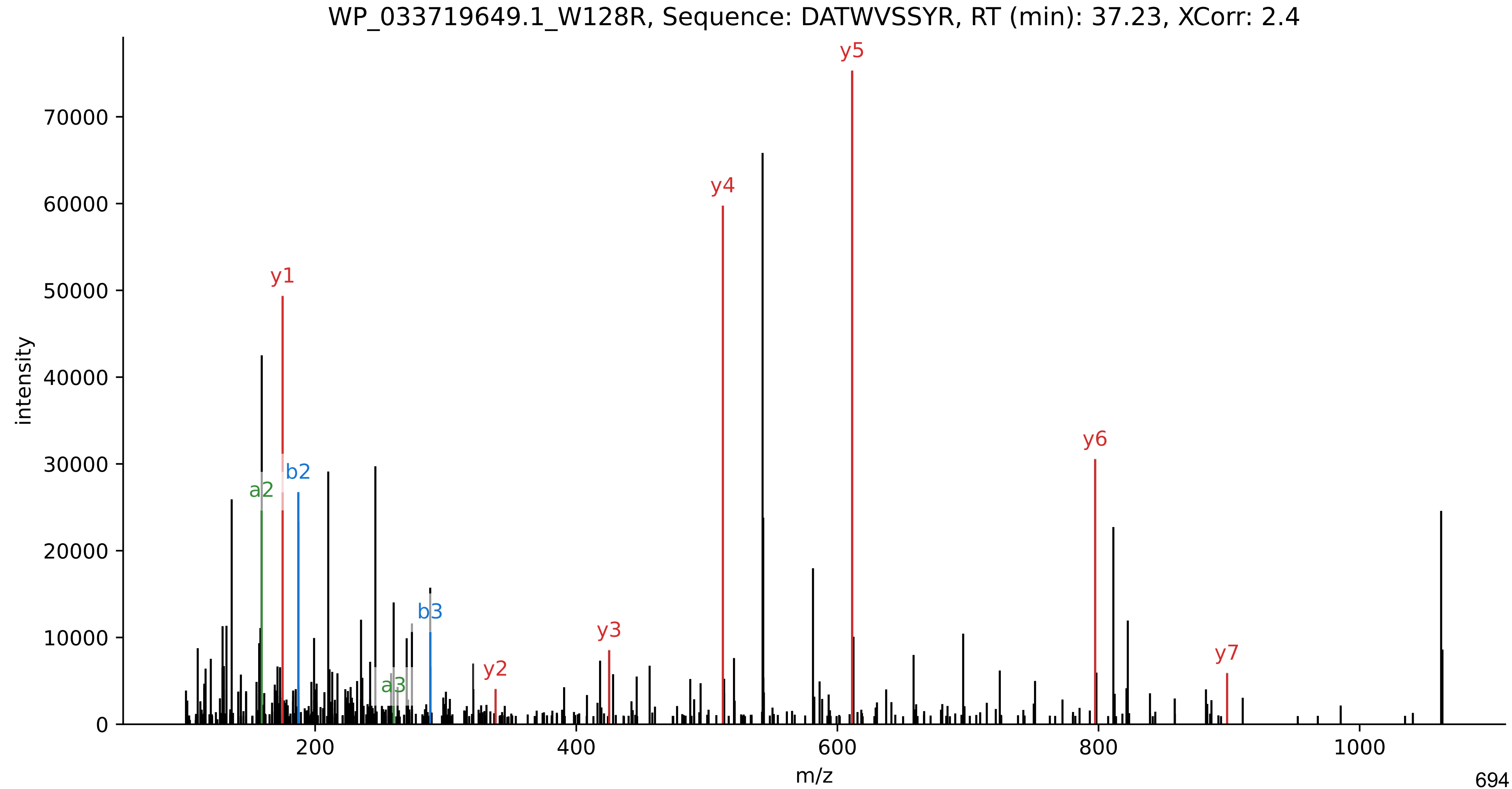
WP_033719649.1_W128R, Sequence: DATWVSSYR, RT (min): 37.23, XCorr: 2.4
y5
y4
y1
y6
a2
b2
b3
y3
y2
a3
y7
70000
60000
50000
40000
30000
20000
10000
0
intensity
200
400
600
800
1000
m/z

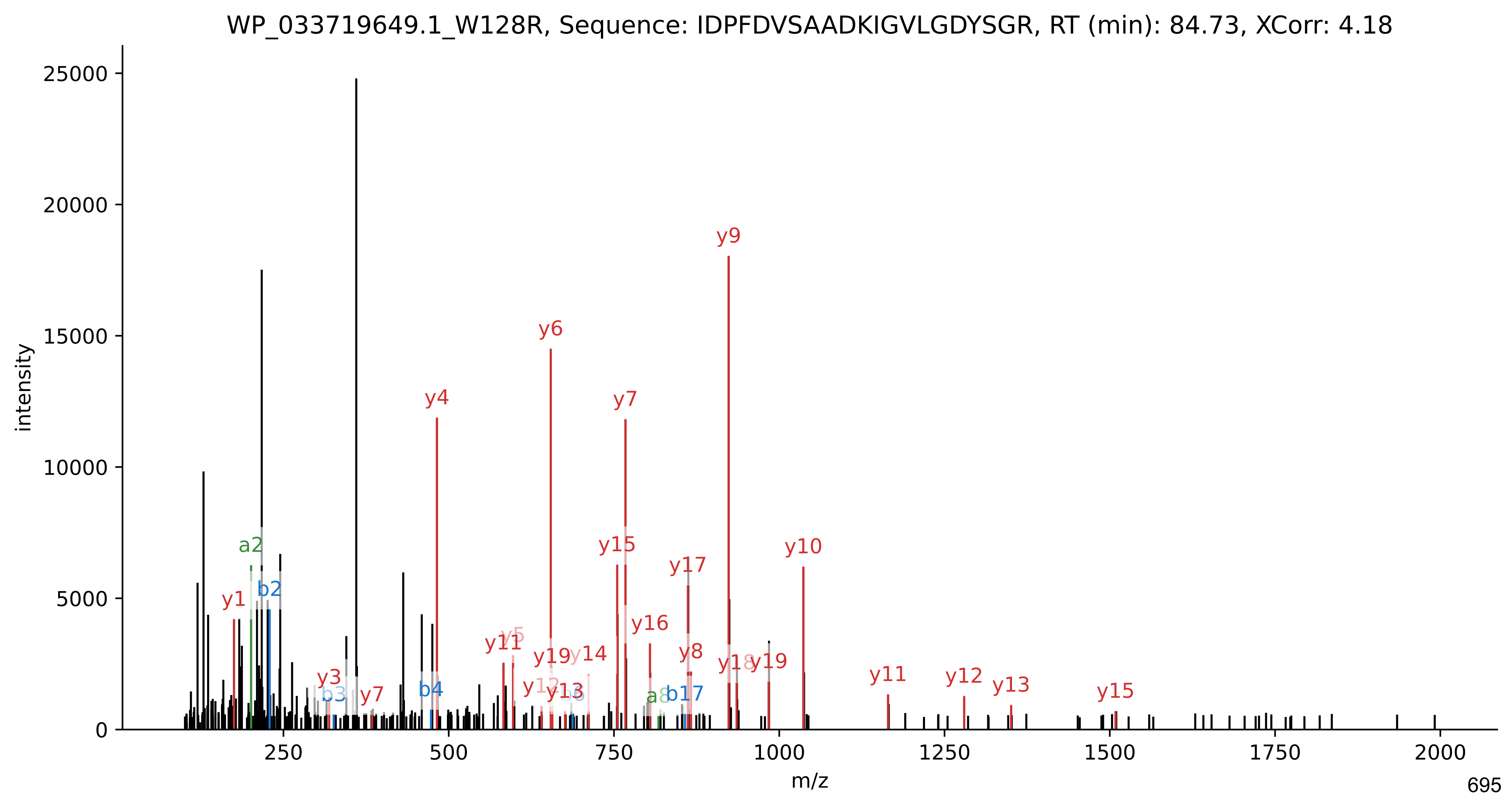

WP_033719649.1_W128R, Sequence: IDPFDVSAADKIGVLGDYSGR, RT (min): 84.73, XCorr: 4.18
intensity
m/z

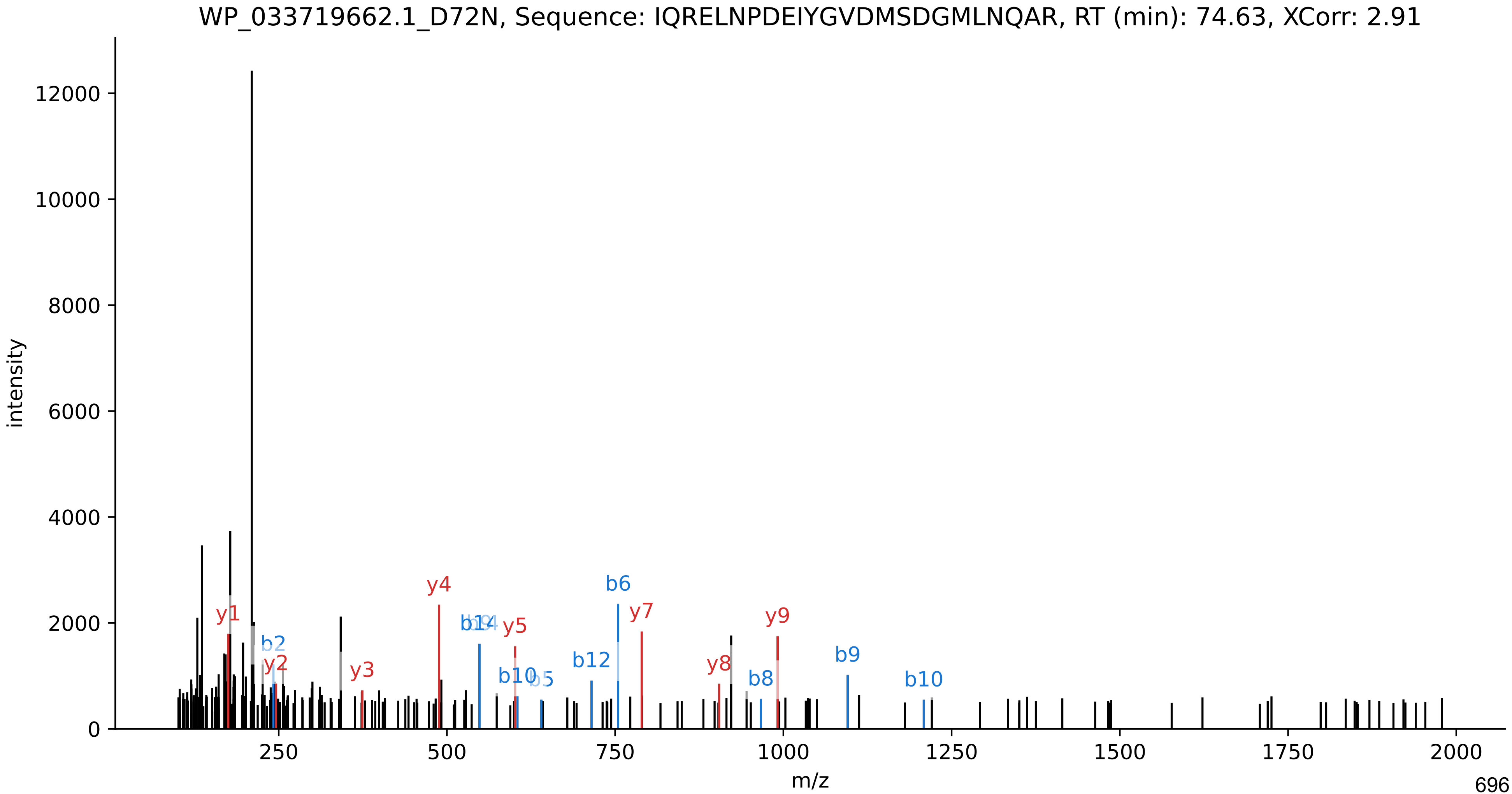
WP_033719662.1_D72N, Sequence: IQRELNPDEIYGVDMSDGMLNQAR, RT (min): 74.63, XCorr: 2.91
intensity
m/z
0
2000
4000
6000
8000
10000
12000
250
500
750
1000
1250
1500
1750
2000
y1
b2
y2
y3
y4
b14
y5
b10
b5
b6
y7
b12
y8
b8
y9
b9
b10

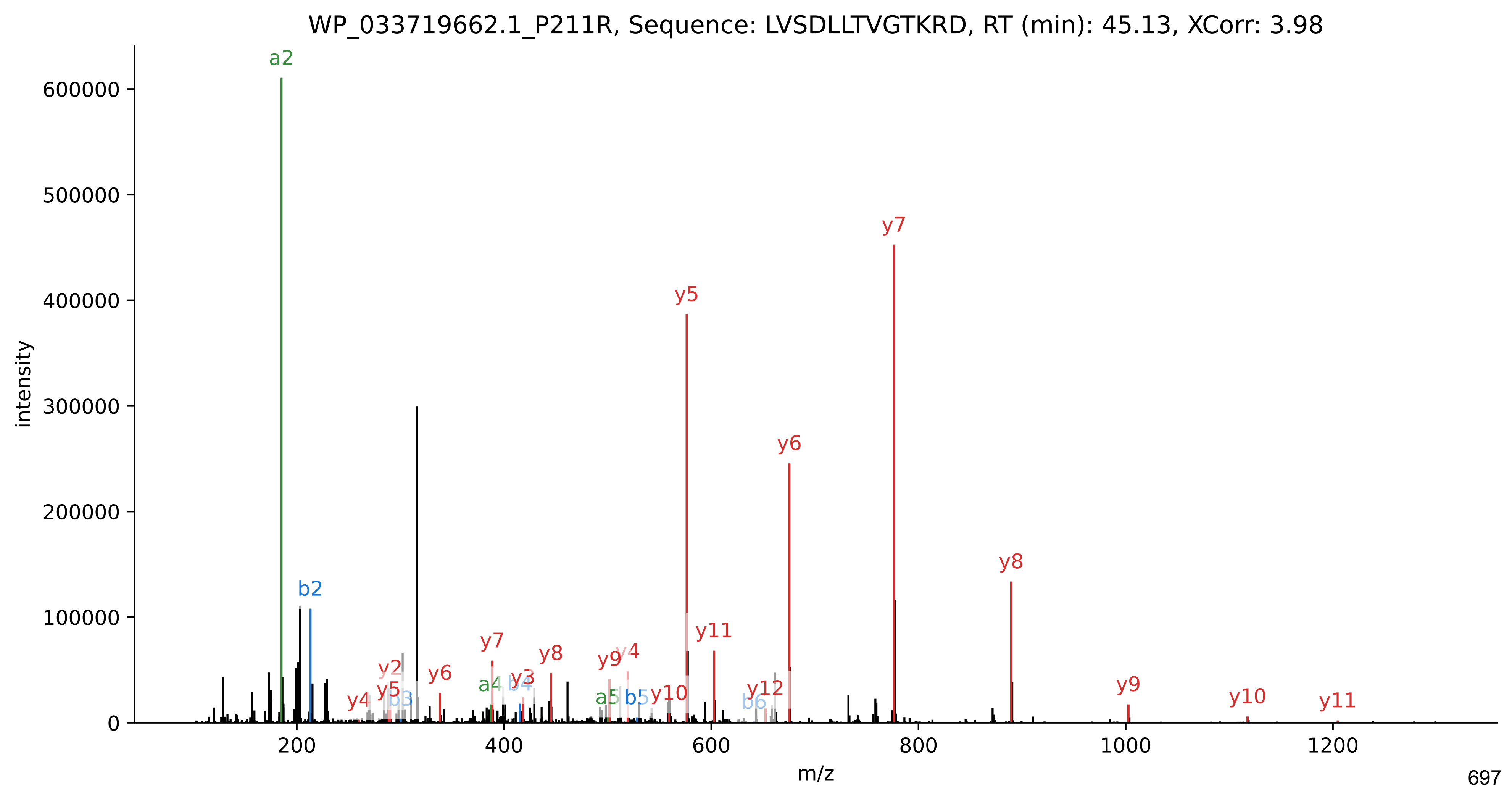

WP_033719662.1_P211R, Sequence: LVSDLLTVGTKRD, RT (min): 45.13, XCorr: 3.98
intensity
m/z
a2
b2
y7
y5
y6
y8
y11
y9
y10
y12
b6
a4
b4
y3
a5
b5
y4
y2
b3

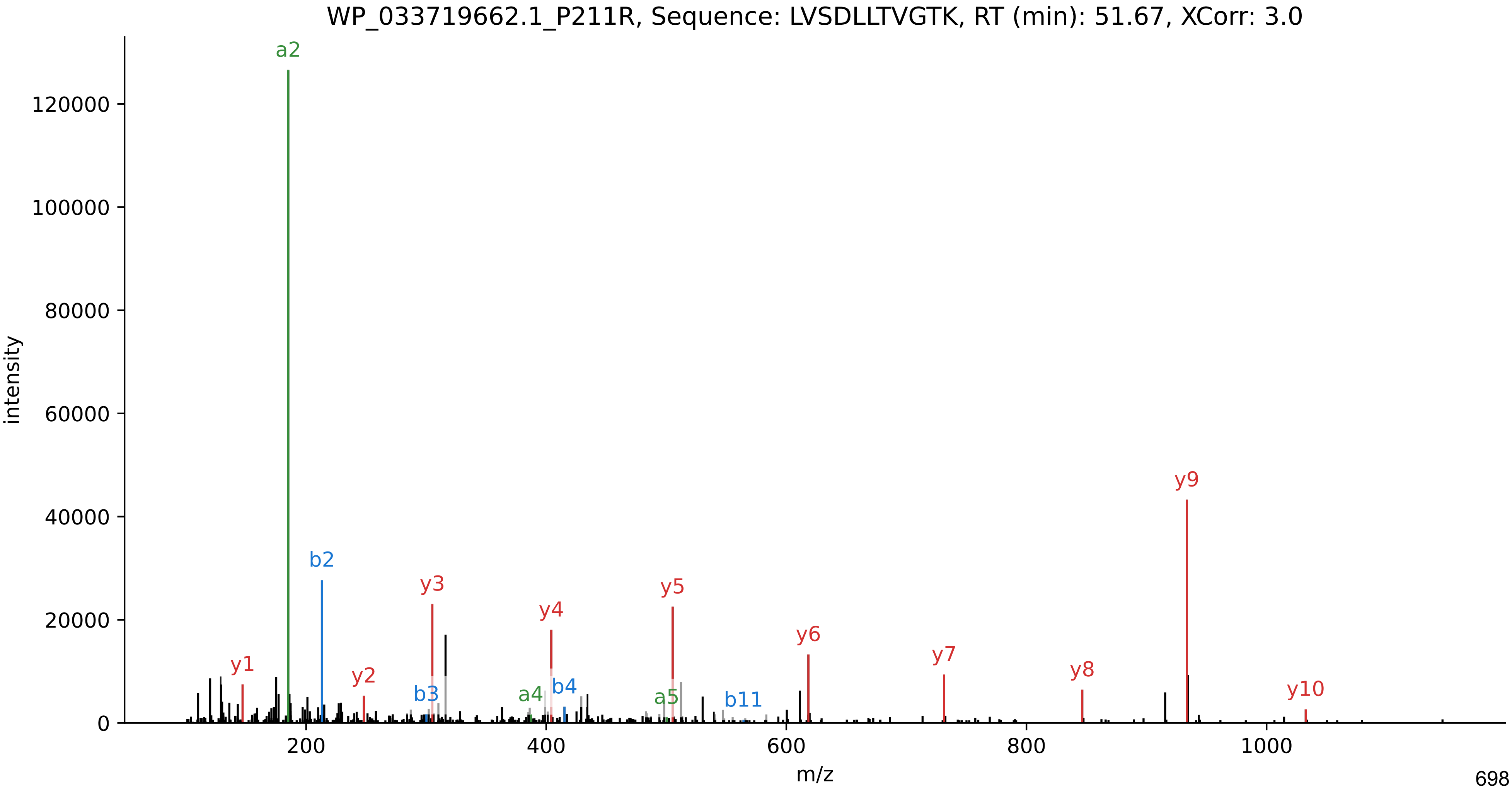

WP_033719662.1_P211R, Sequence: LVSDLLTVGTK, RT (min): 51.67, XCorr: 3.0
intensity
m/z
a2
b2
y1
y2
y3
b3
a4
y4
b4
y5
a5
b11
y6
y7
y8
y9
y10
0
20000
40000
60000
80000
100000
120000
200
400
600
800
1000

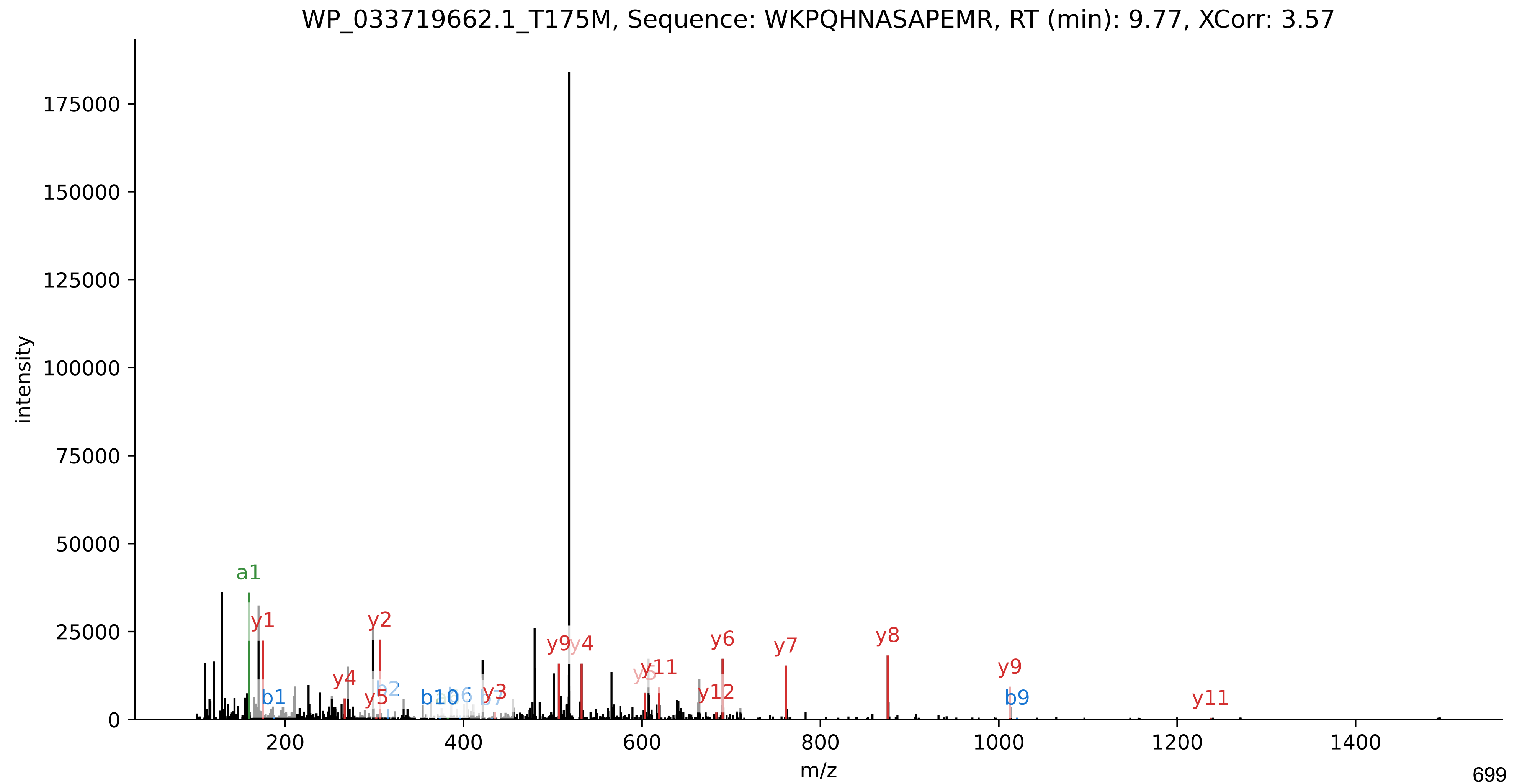
WP_033719662.1_T175M, Sequence: WKPQHNASAPEMR, RT (min): 9.77, XCorr: 3.57
intensity
m/z
a1
y1
y2
y4
y5
b1
b2
b10
y3
y9
y4
y11
y6
y12
y7
y8
y9
b9
y11

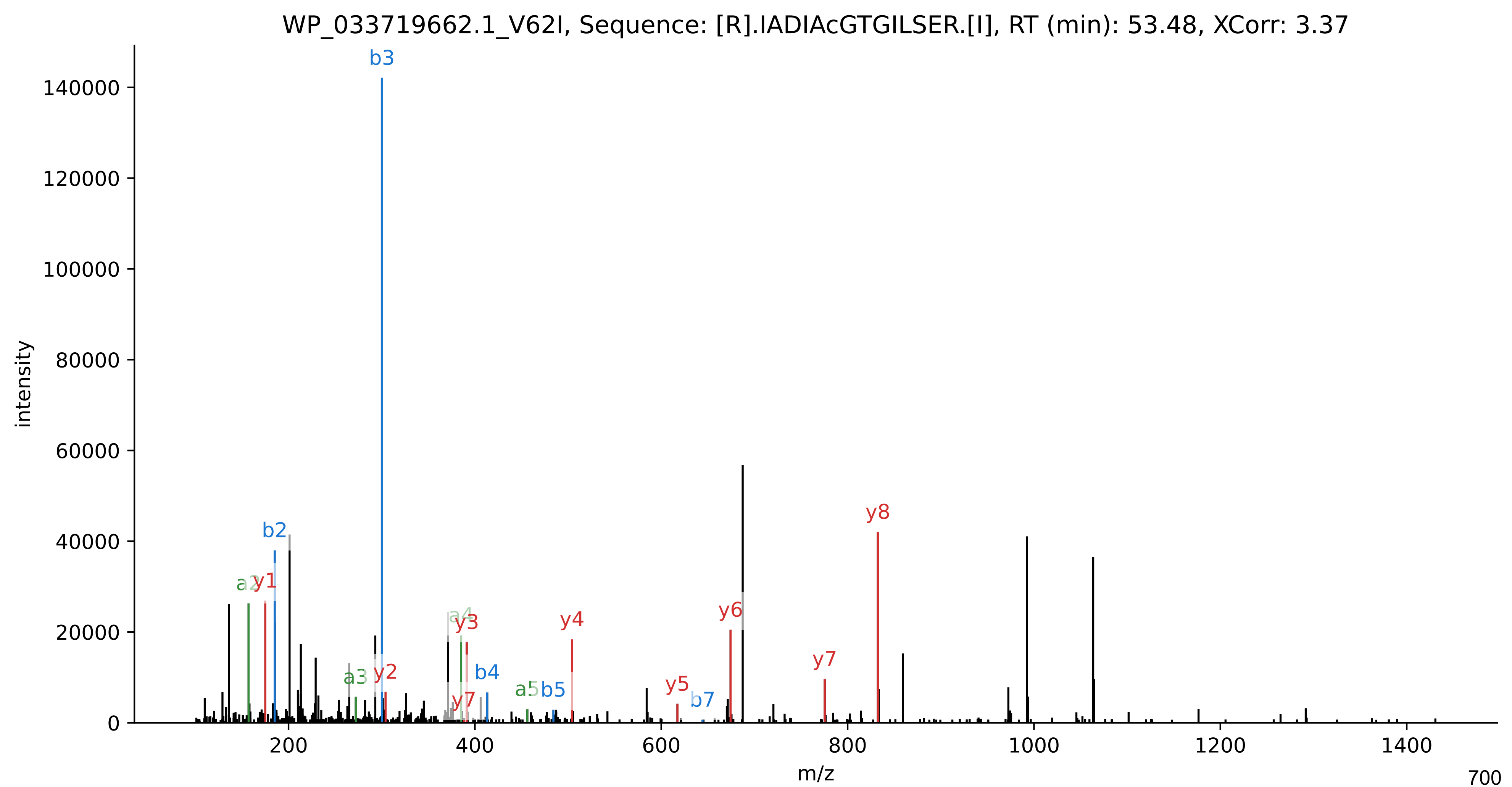
WP_033719662.1_V62I, Sequence: [R].IADIAcGTGILSER.[I], RT (min): 53.48, XCorr: 3.37
intensity
m/z
b3
b2
a2
y1
a3
y2
a4
y3
y7
b4
a5
b5
y4
y5
b7
y6
y7
y8

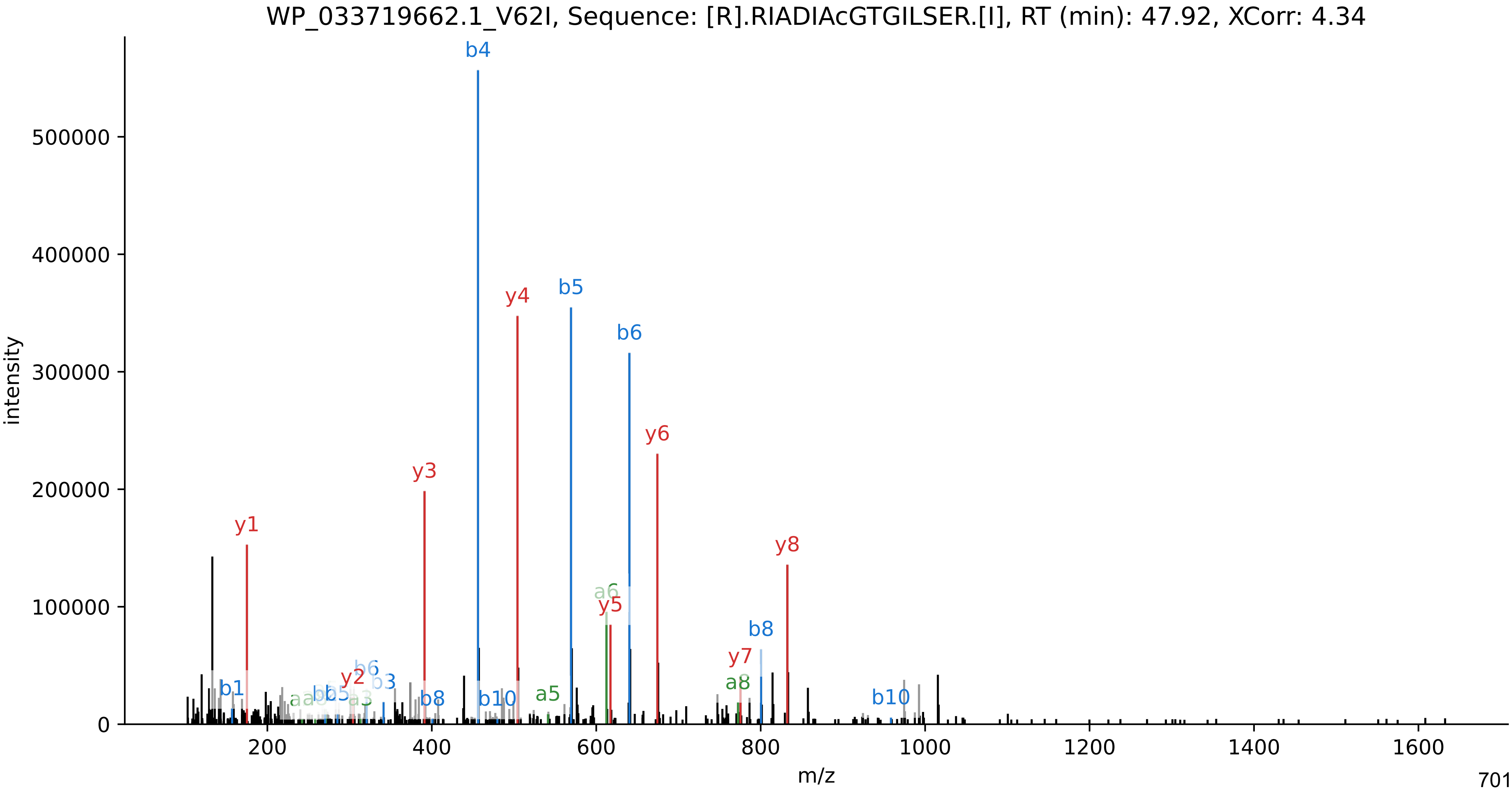
WP_033719662.1_V62I, Sequence: [R].RIADIAcGTGILSER.[I], RT (min): 47.92, XCorr: 4.34
intensity
m/z
0
100000
200000
300000
400000
500000
200
400
600
800
1000
1200
1400
1600
b4
y4
b5
b6
y6
y3
y1
y8
y5
b8
y7
a8
b10
a5
b1
y2
b3
b8
b10

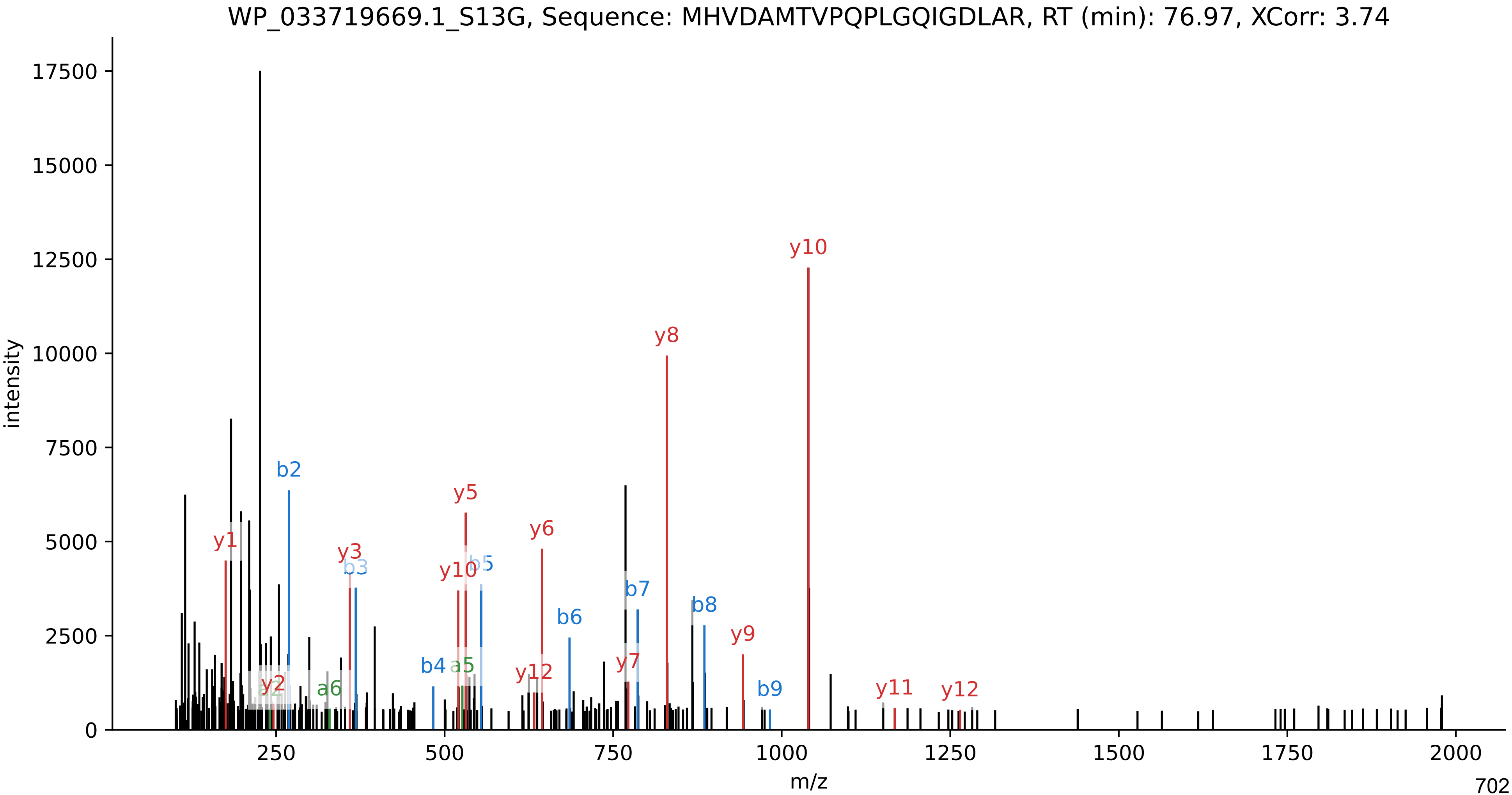
WP_033719669.1_S13G, Sequence: MHVDAMTVPQPLGQIGDLAR, RT (min): 76.97, XCorr: 3.74
intensity
m/z
y1
y2
b2
y3
b3
a6
b4
a5
y5
y10
b5
y6
y12
b6
y7
b7
y8
b8
y9
b9
y10
y11
y12

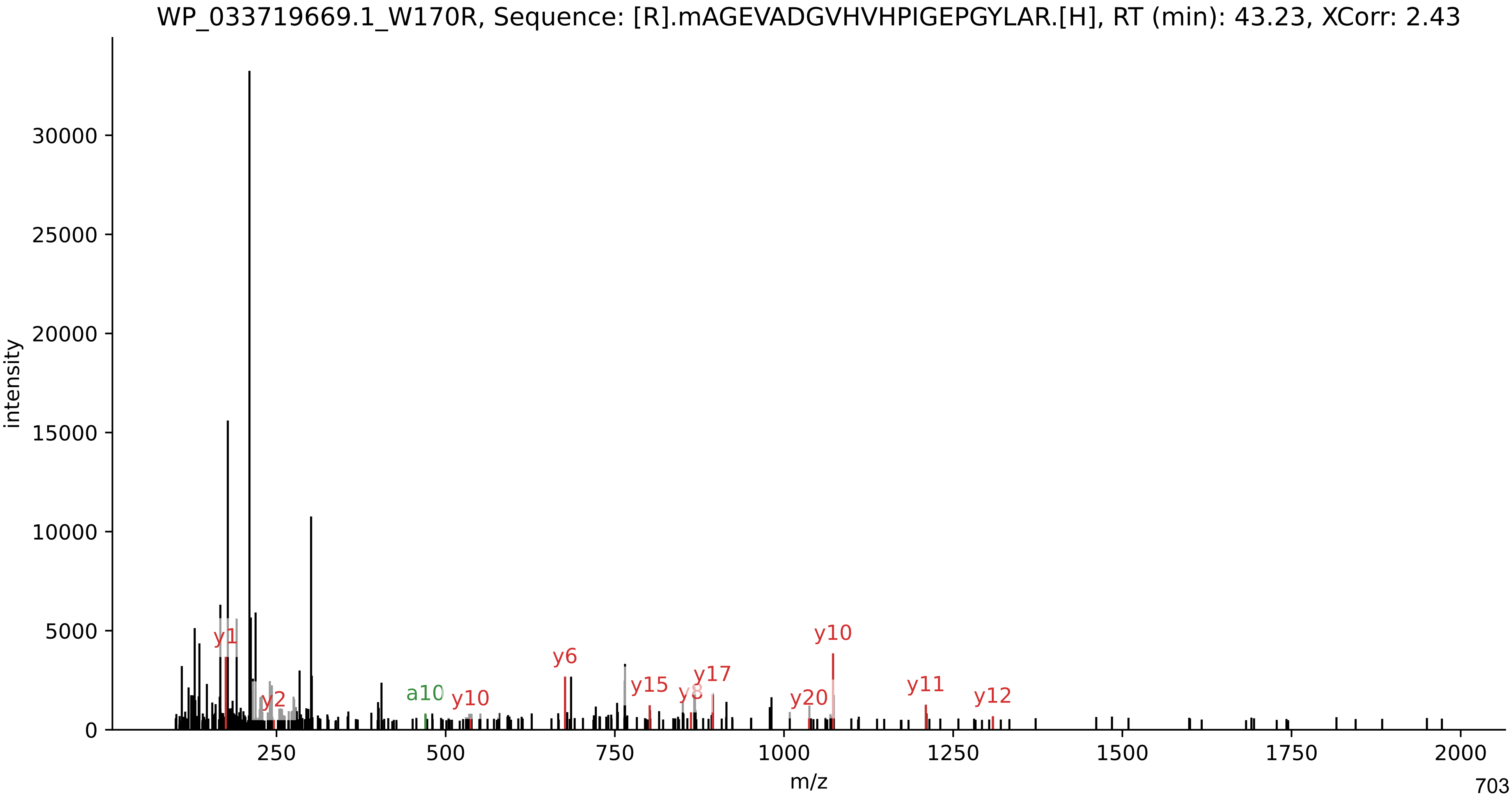

WP_033719669.1_W170R, Sequence: [R].mAGEVADGVHVHPIGEPGYLAR.[H], RT (min): 43.23, XCorr: 2.43
intensity
m/z
y1
y2
a10
y10
y6
y15
y8
y17
y20
y10
y11
y12

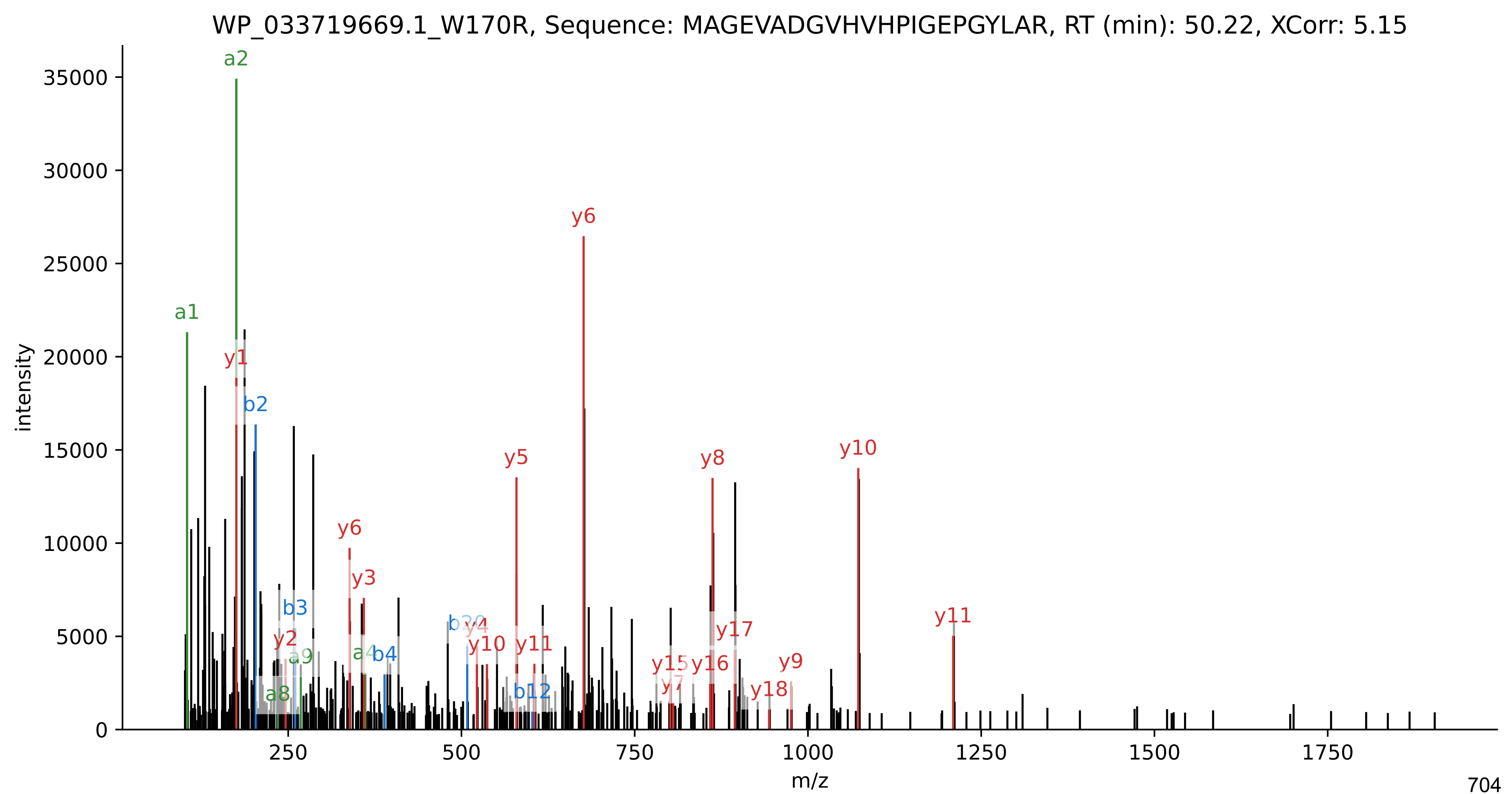

WP_033719669.1_W170R, Sequence: MAGEVADGVHVHPIGEPGYLAR, RT (min): 50.22, XCorr: 5.15
intensity
m/z
a1
a2
y1
b2
y6
y5
y8
y10
y6
y3
b3
y2
a9
a8
a4
b4
y4
y10
y11
b12
y15
y16
y17
y18
y9
y11

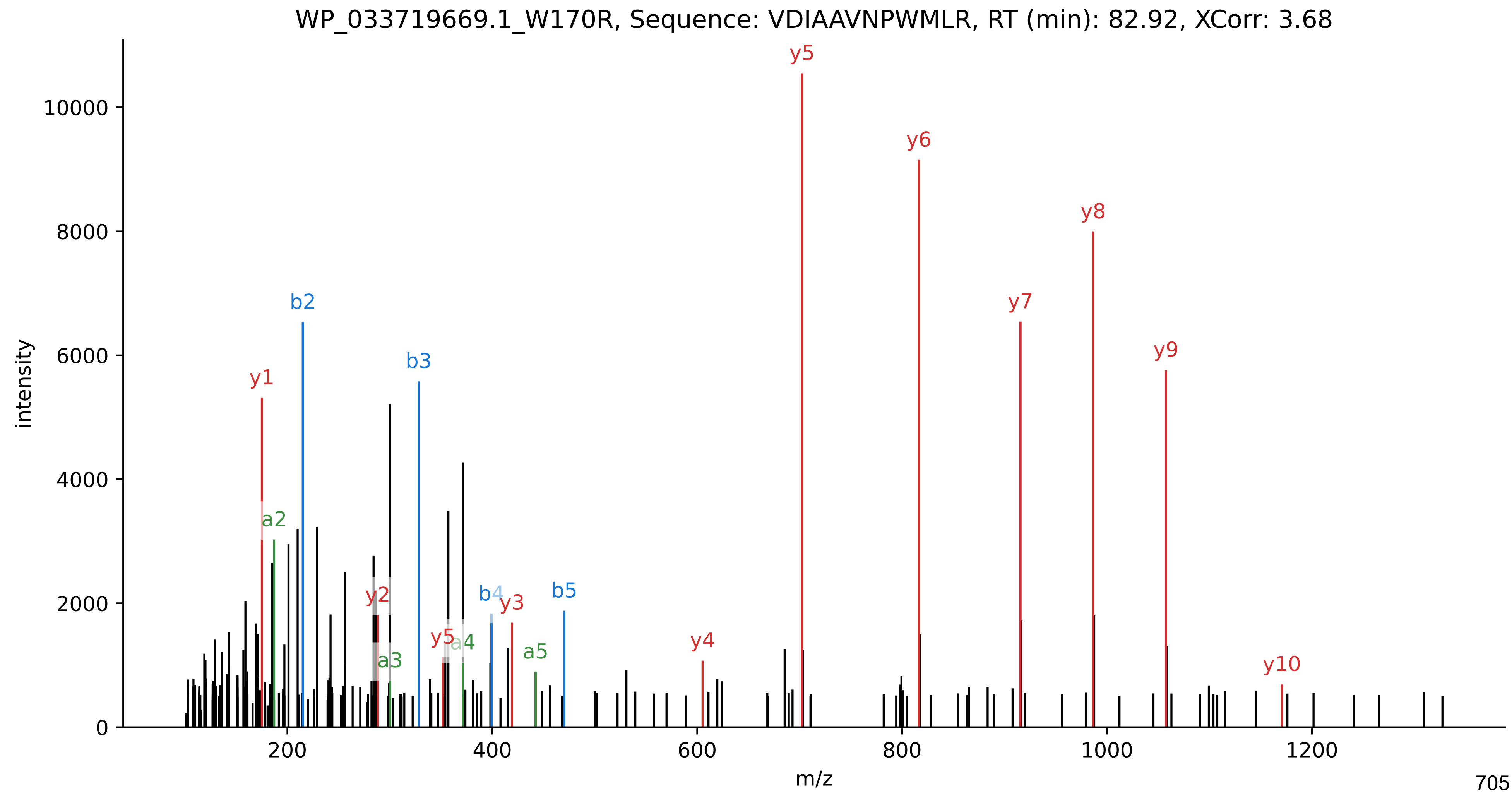
WP_033719669.1_W170R, Sequence: VDIAAVNPWMLR, RT (min): 82.92, XCorr: 3.68
intensity
m/z
y1
a2
b2
b3
y2
a3
y5
a4
b4
y3
a5
b5
y4
y5
y6
y7
y8
y9
y10
0
2000
4000
6000
8000
10000
200
400
600
800
1000
1200

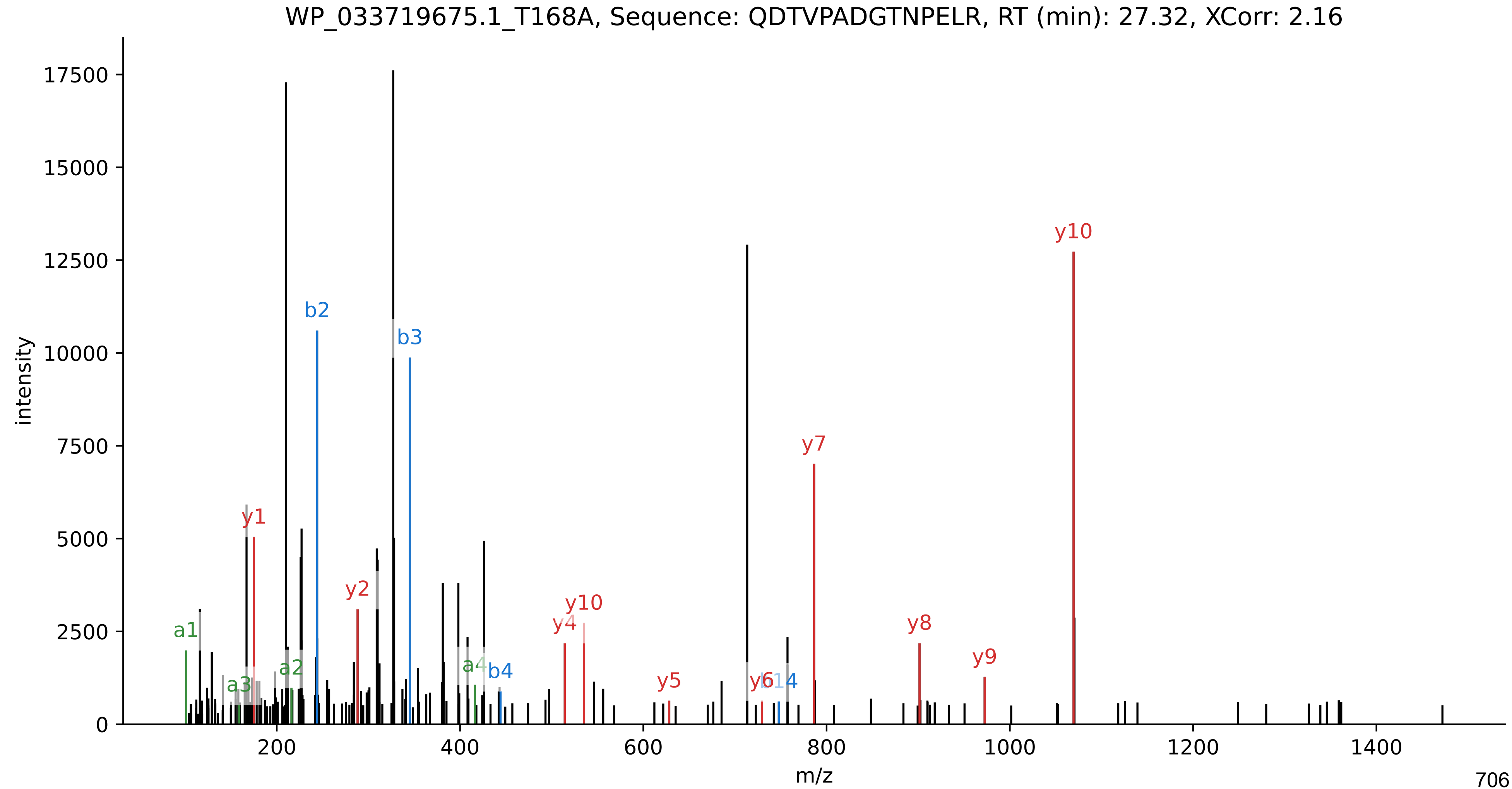

WP_033719675.1_T168A, Sequence: QDTVPADGTNPELR, RT (min): 27.32, XCorr: 2.16
intensity
m/z
a1
a2
a3
a4
b2
b3
b4
y1
y2
y4
y5
y6
y7
y8
y9
y10
y10

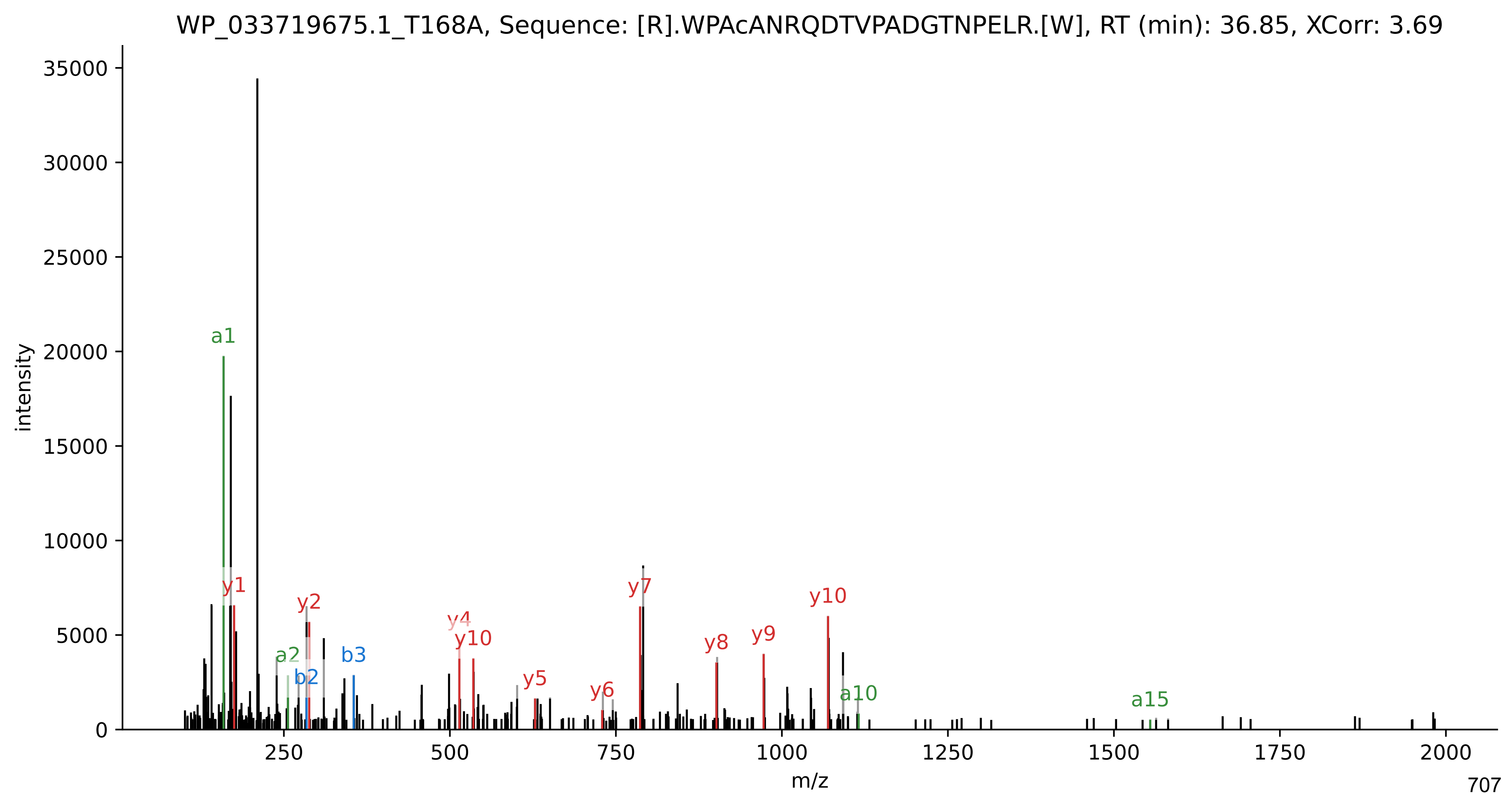

WP_033719675.1_T168A, Sequence: [R].WPAcANRQDTVPADGTNPELR.[W], RT (min): 36.85, XCorr: 3.69
intensity
m/z
a1
y1
y2
a2
b2
b3
y4
y10
y5
y6
y7
y8
y9
y10
a10
a15
35000
30000
25000
20000
15000
10000
5000
0
250
500
750
1000
1250
1500
1750
2000

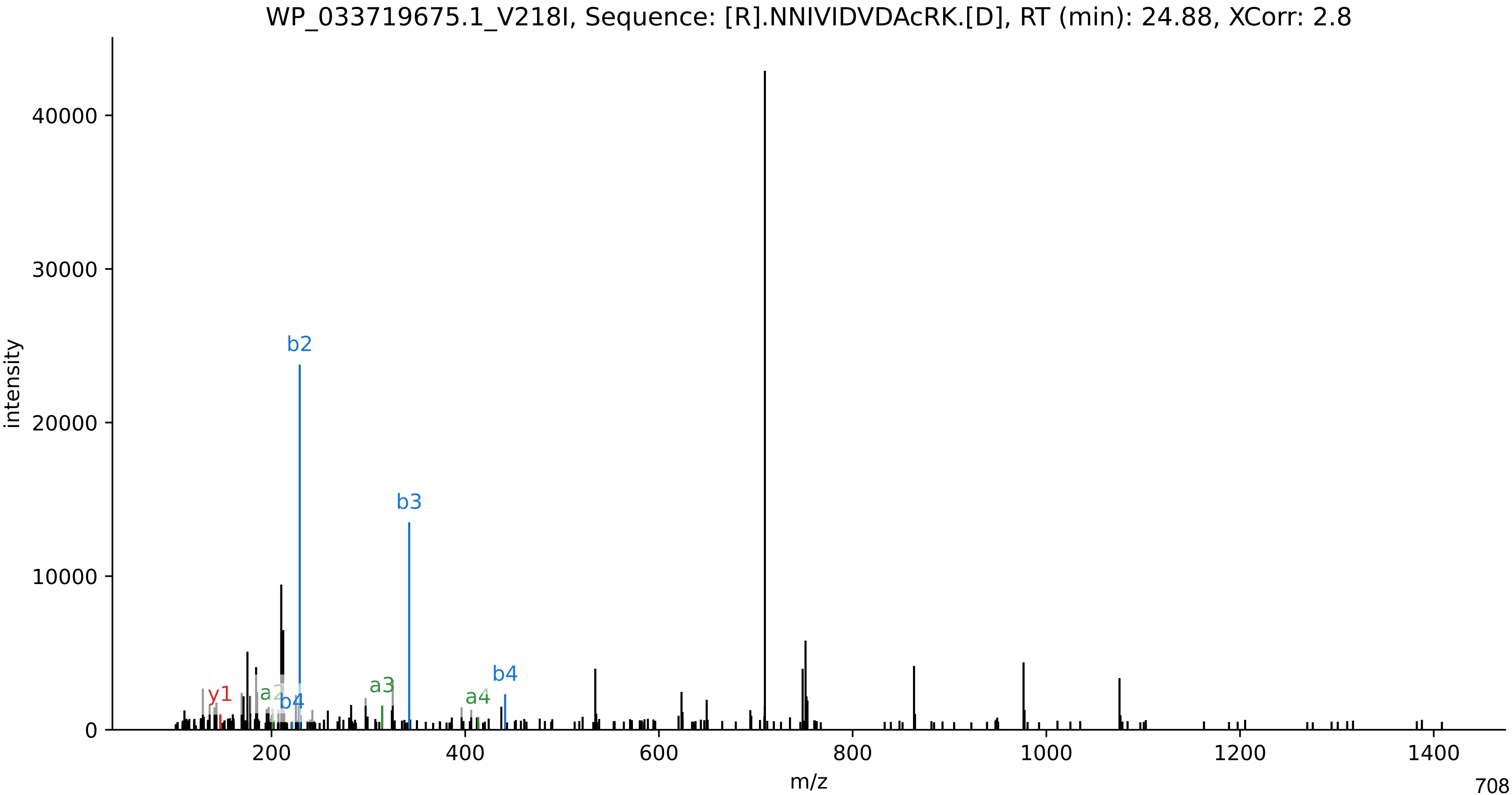
WP_033719675.1_V218I, Sequence: [R].NNIVIDVDAcRK.[D], RT (min): 24.88, XCorr: 2.8
intensity
m/z
b2
b3
b4
a3
a4
y1
a2
b4
0
10000
20000
30000
40000
200
400
600
800
1000
1200
1400

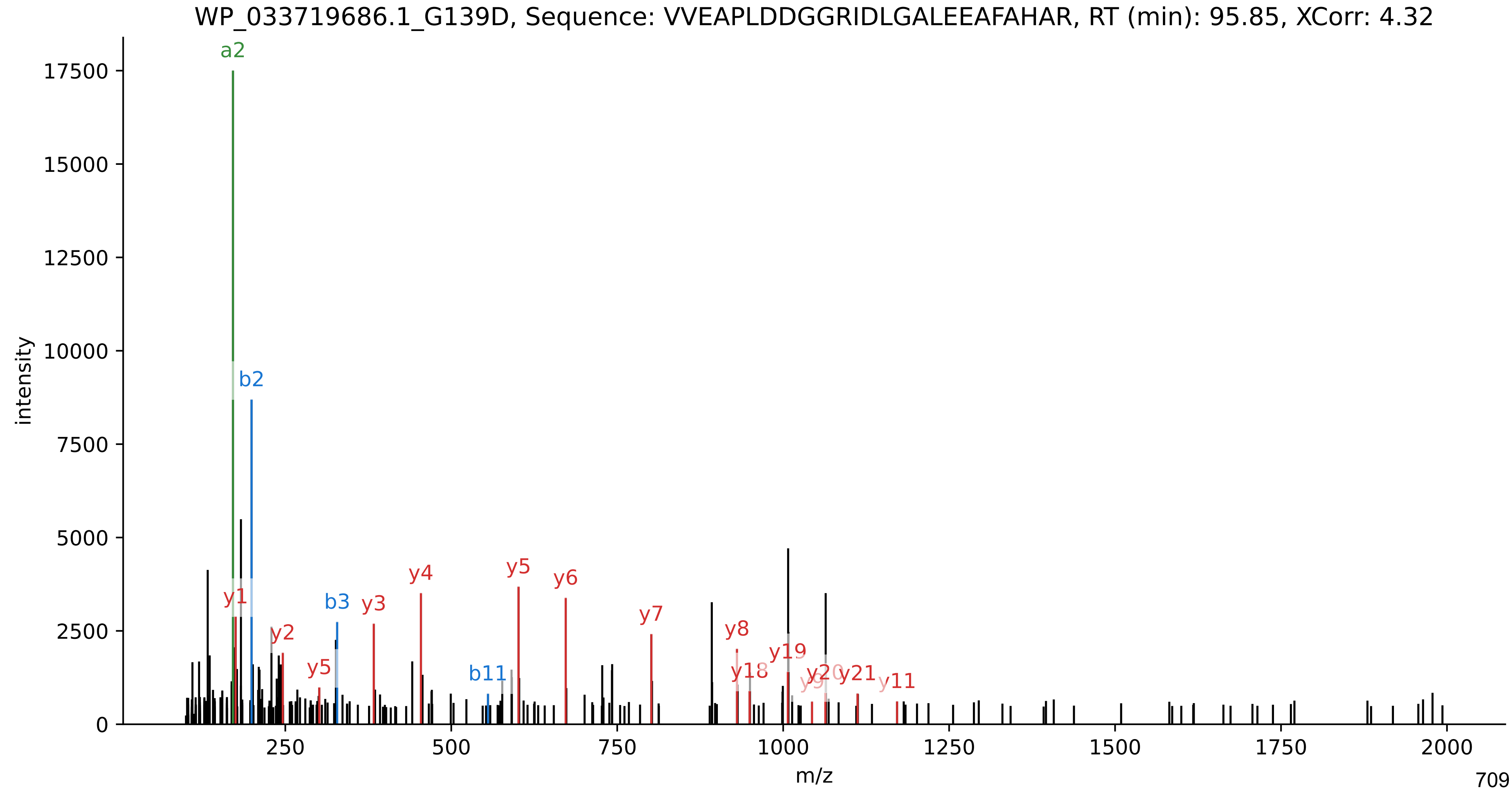

WP_033719686.1_G139D, Sequence: VVEAPLDDGGRIDLGALEEAFAHAR, RT (min): 95.85, XCorr: 4.32
intensity
m/z
a2
b2
b3
b11
y1
y2
y5
y3
y4
y5
y6
y7
y8
y18
y19
y9
y20
y21
y11

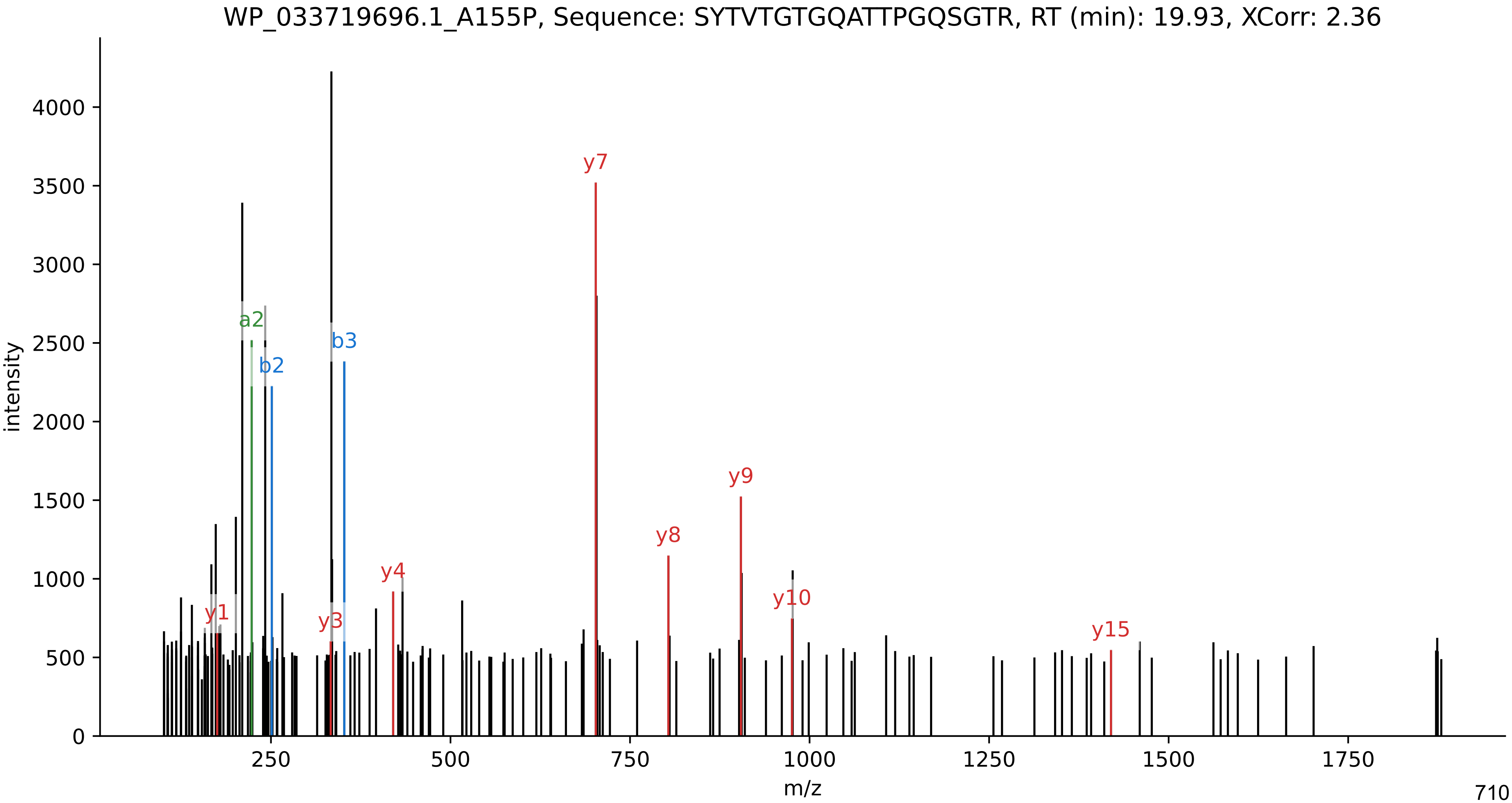

WP_033719696.1_A155P, Sequence: SYTVTGTGQATTPGQSGTR, RT (min): 19.93, XCorr: 2.36
intensity
m/z
a2
b2
b3
y1
y3
y4
y7
y8
y9
y10
y15

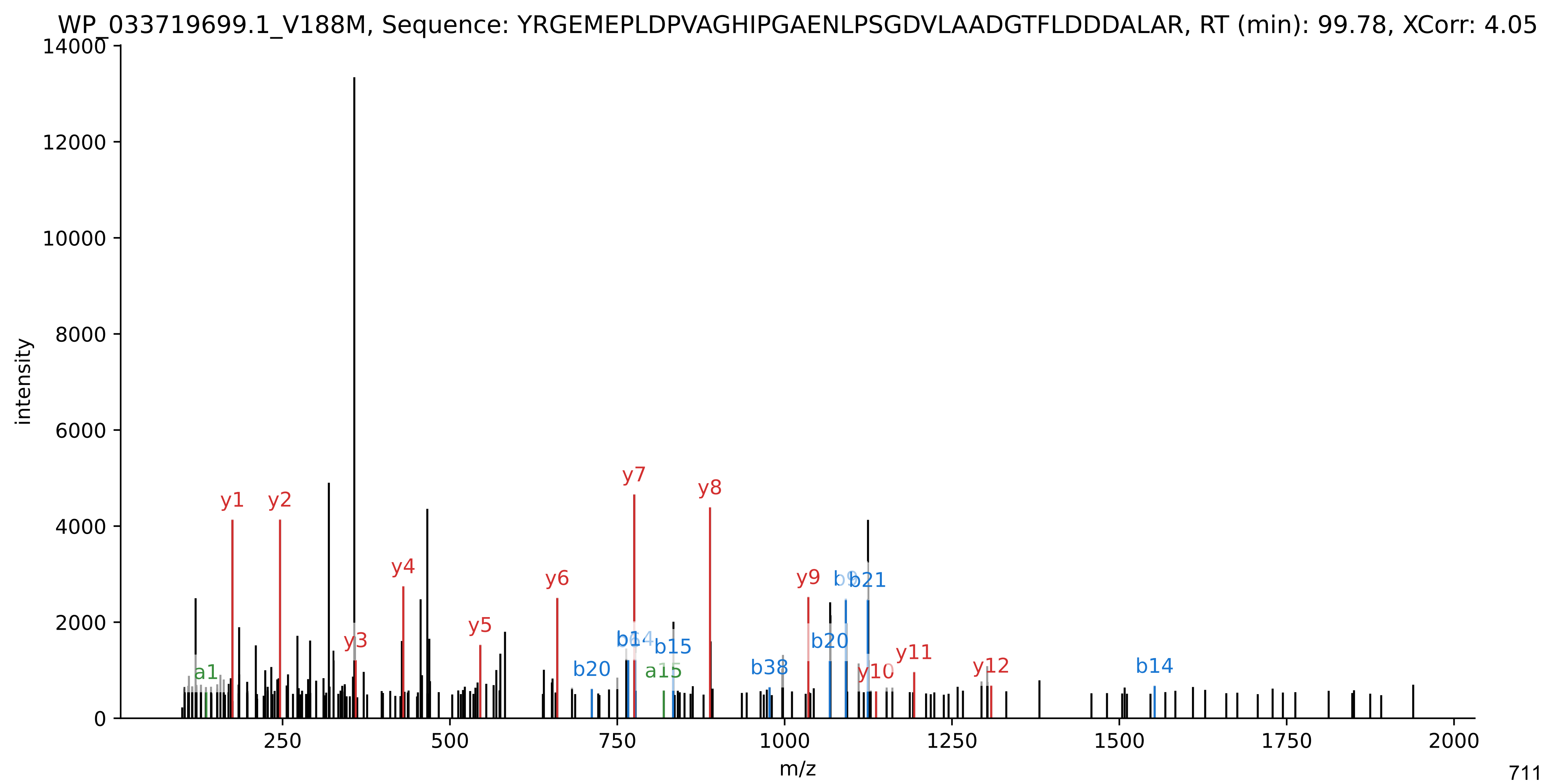

WP_033719699.1_V188M, Sequence: YRGEMEPLDPVAGHIPGAENLPSGDVLAADGTFLDDDALAR, RT (min): 99.78, XCorr: 4.05
intensity
m/z
y1
y2
y3
y4
y5
y6
y7
y8
y9
y10
y11
y12
a1
a15
b20
b14
b15
b38
b20
b9
b21
b14

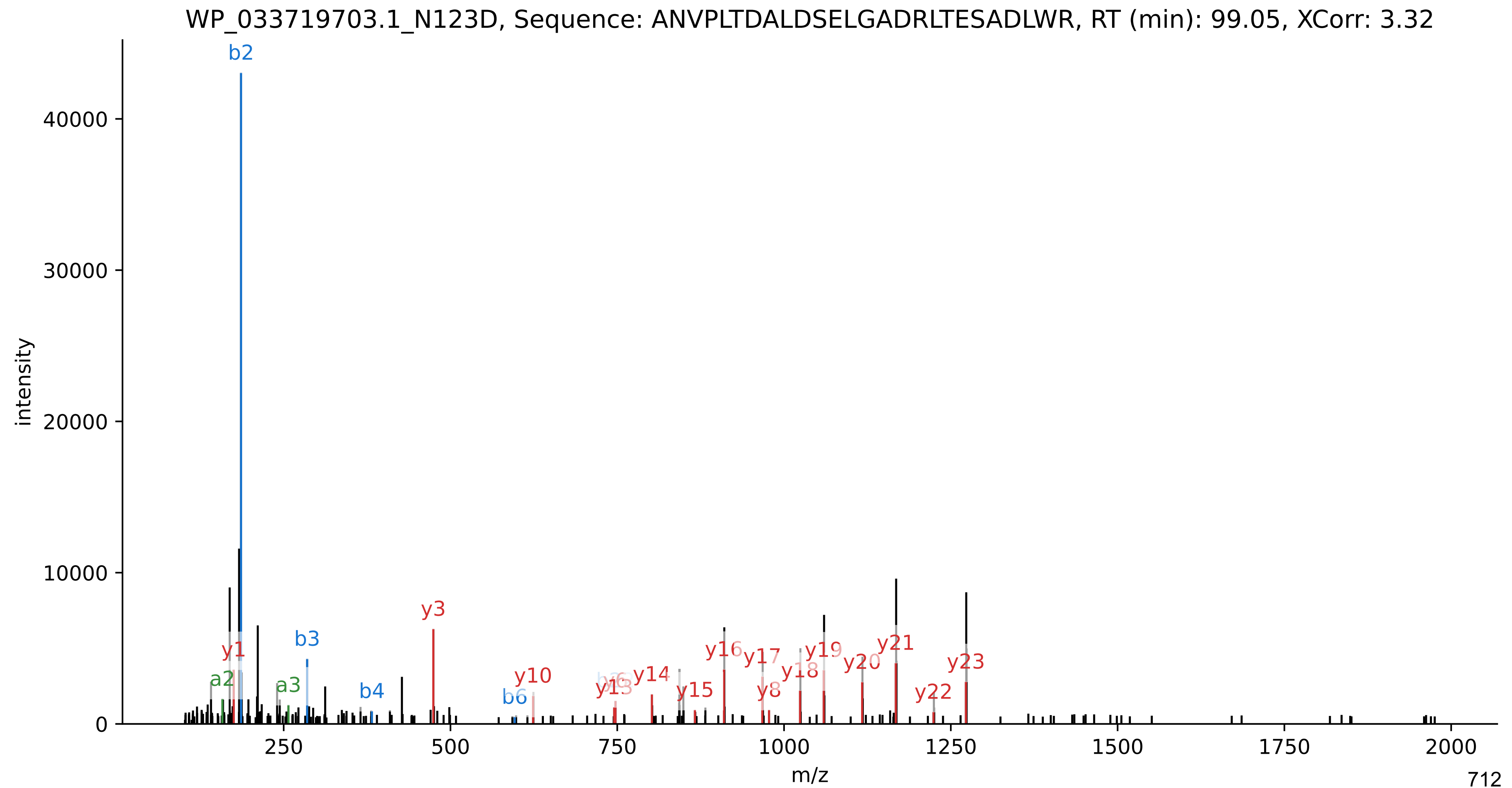

WP_033719703.1_N123D, Sequence: ANVPLTDALDSELGADRLTESADLWR, RT (min): 99.05, XCorr: 3.32
intensity
m/z
b2
b3
b4
b6
a2
a3
y1
y3
y10
y13
y14
y15
y16
y17
y8
y18
y19
y20
y21
y22
y23

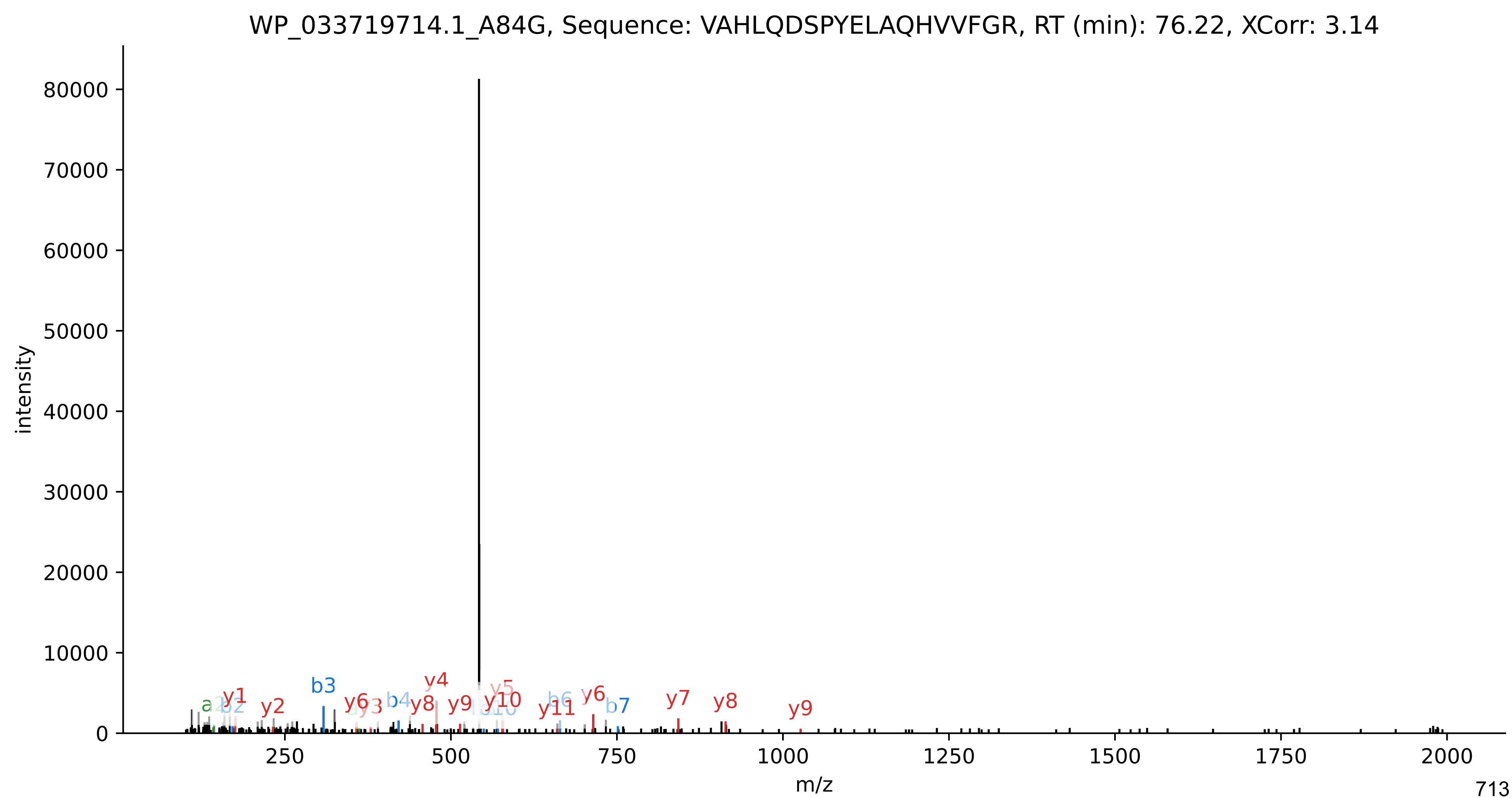
WP_033719714.1_A84G, Sequence: VAHLQDSPYELAQHVVFGR, RT (min): 76.22, XCorr: 3.14
intensity
m/z
80000
70000
60000
50000
40000
30000
20000
10000
0
250
500
750
1000
1250
1500
1750
2000
b3
y4
y5
y1
y2
b2
y6
y3
b4
y8
y9
y10
y11
b6
y6
b7
y7
y8
y9

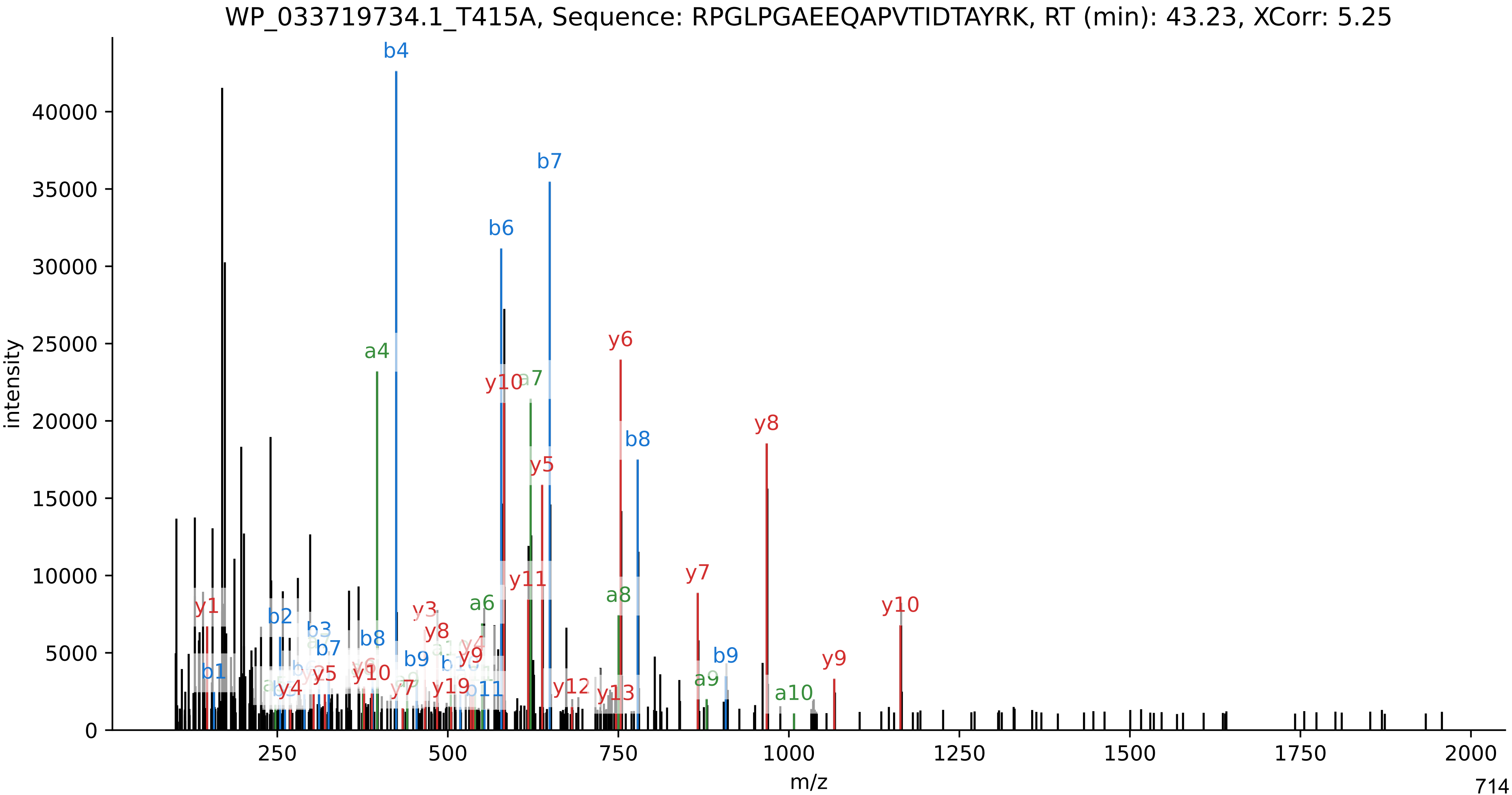
WP_033719734.1_T415A, Sequence: RPGLPGAEEQAPVTIDTAYRK, RT (min): 43.23, XCorr: 5.25
intensity
m/z
0
5000
10000
15000
20000
25000
30000
35000
40000
250
500
750
1000
1250
1500
1750
2000
b4
b7
b6
a4
y6
y10
a7
y8
b8
y5
y7
y11
a6
a8
y10
y1
b2
b3
b7
b8
y3
y8
b9
y9
b9
y9
a9
a10
b1
y4
y5
y10
y7
y19
b11
y12
y13

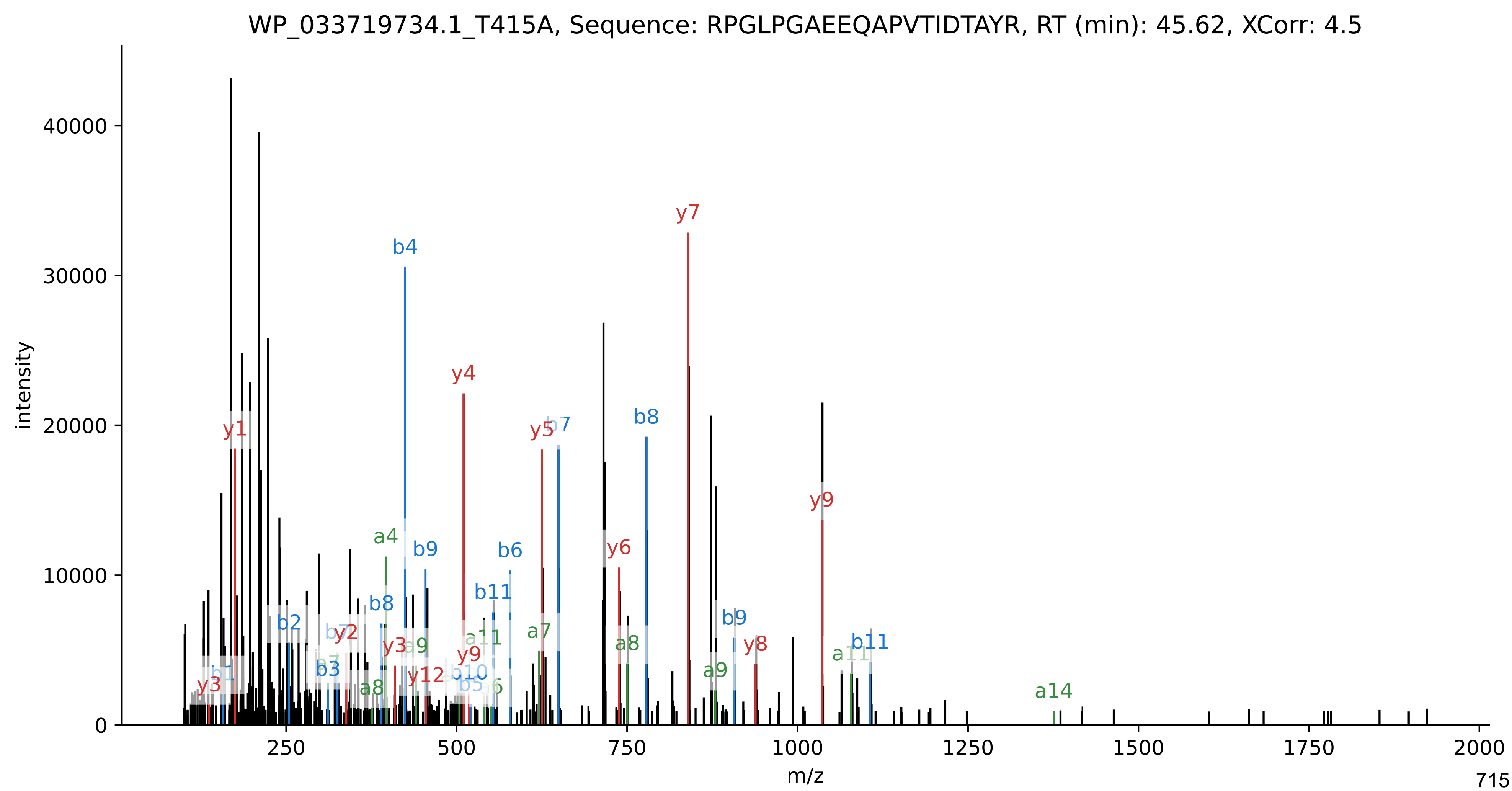

WP_033719734.1_T415A, Sequence: RPGLPGAEEQAPVTIDTAYR, RT (min): 45.62, XCorr: 4.5
intensity
m/z
0
10000
20000
30000
40000
250
500
750
1000
1250
1500
1750
2000
y1
b4
y4
y5
b7
b8
y7
y6
y9
b9
b6
b11
a4
b2
y2
y3
a9
a11
a7
a8
b3
y12
b10
b5
a14
y8

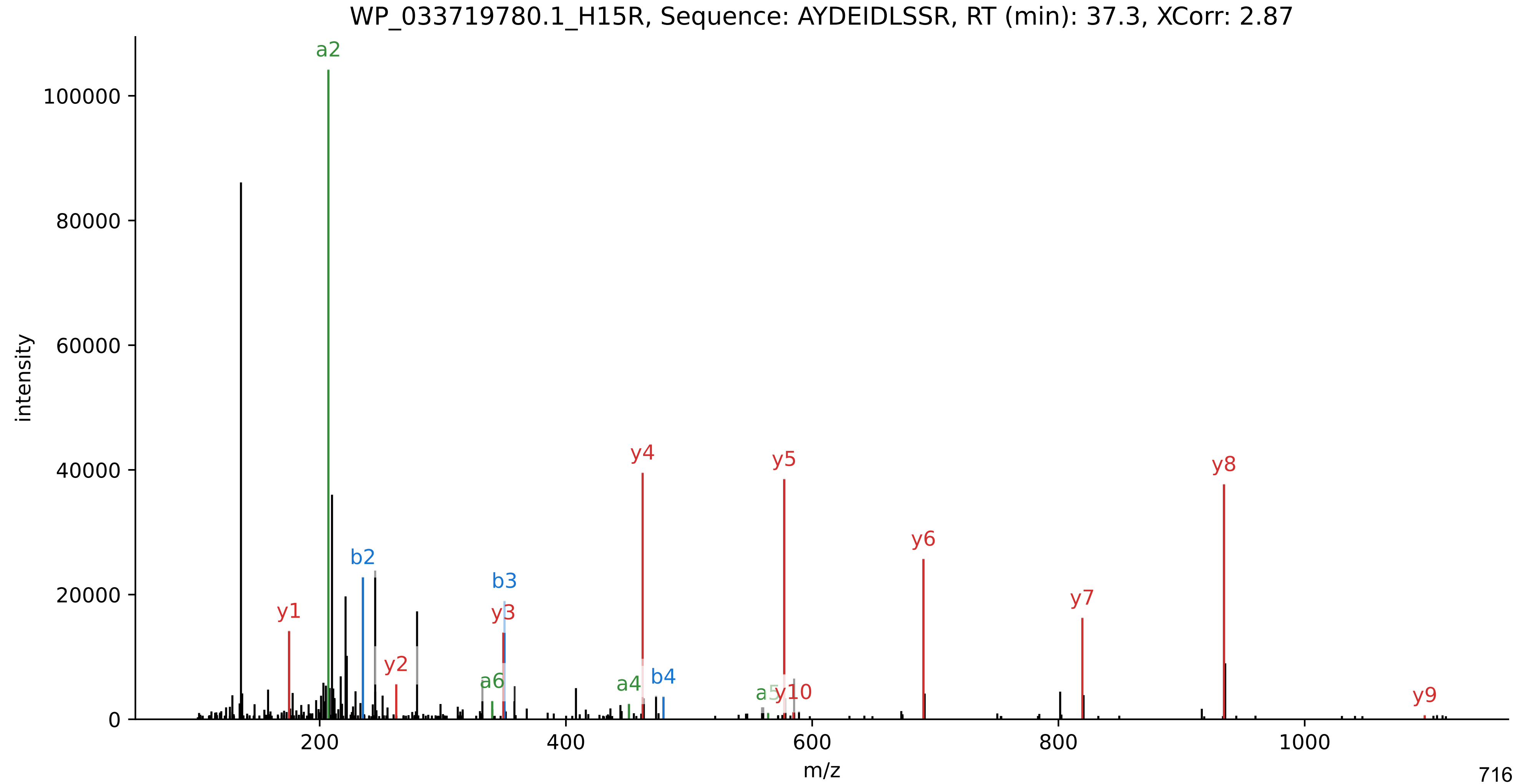

WP_033719780.1_H15R, Sequence: AYDEIDLSSR, RT (min): 37.3, XCorr: 2.87
a2
b2
b3
y1
y3
y2
a6
y4
a4
b4
y5
a5
y10
y6
y7
y8
y9
intensity
m/z
0
20000
40000
60000
80000
100000
200
400
600
800
1000

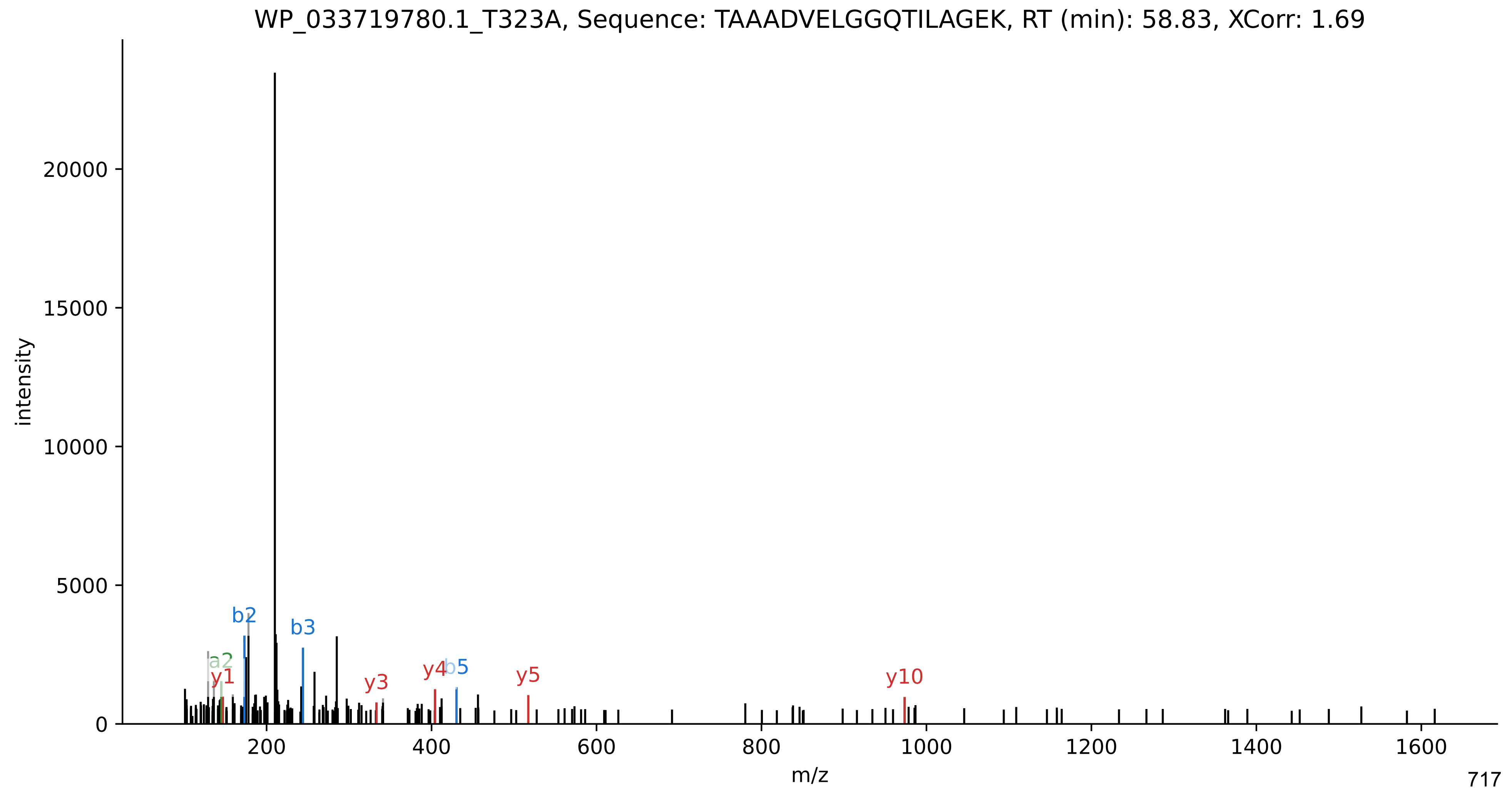
WP_033719780.1_T323A, Sequence: TAAADVELGGQTILAGEK, RT (min): 58.83, XCorr: 1.69
intensity
m/z
0
5000
10000
15000
20000
200
400
600
800
1000
1200
1400
1600
b2
b3
a2
y1
y3
y4
b5
y5
y10

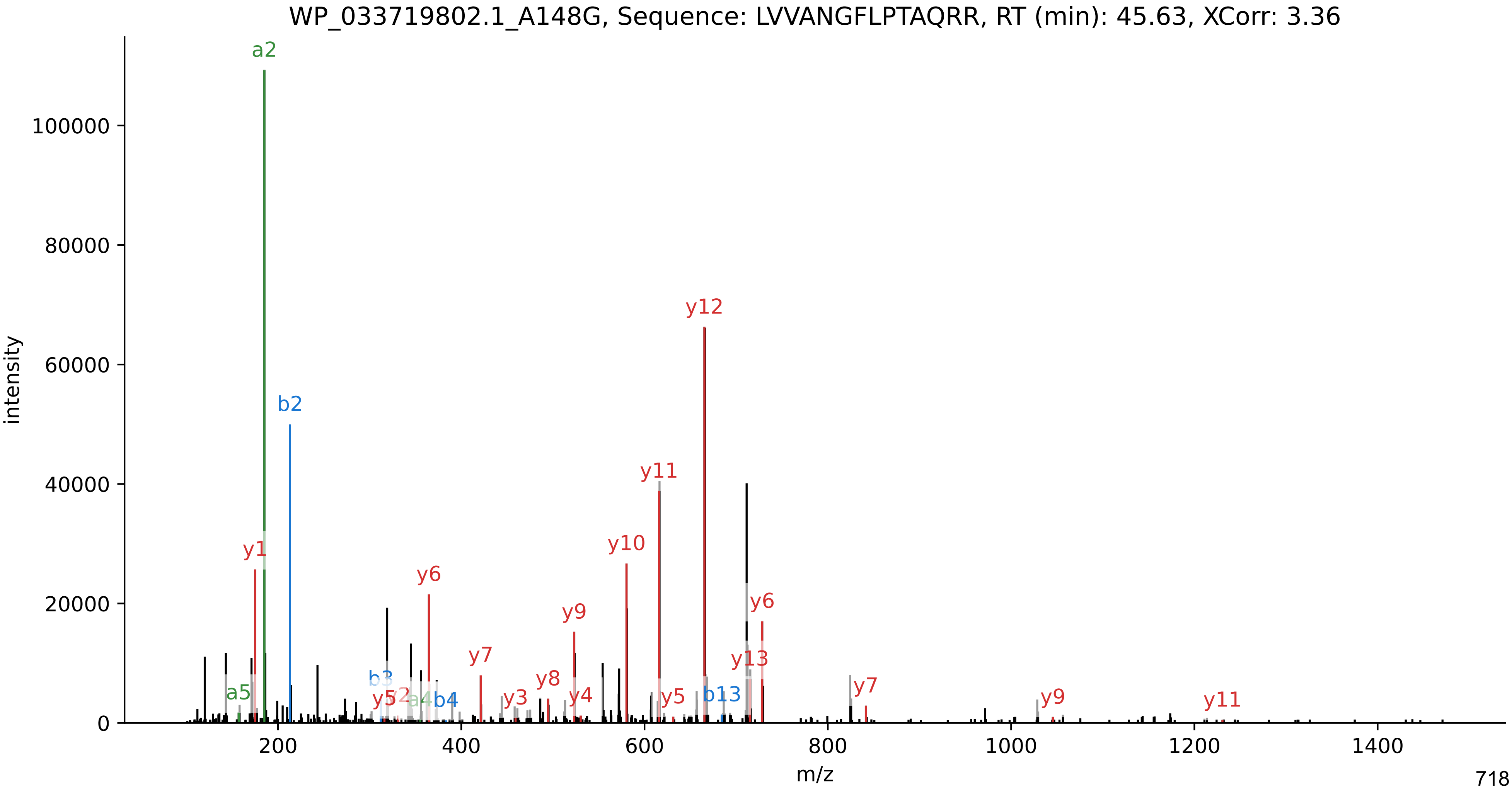
WP_033719802.1_A148G, Sequence: LVVANGFLPTAQRR, RT (min): 45.63, XCorr: 3.36
a2
b2
y12
y11
y10
y1
y6
y9
y6
y13
y7
y8
y4
y3
b3
a5
y5
a4
b4
y5
b13
y7
y9
y11
intensity
m/z
0
20000
40000
60000
80000
100000
200
400
600
800
1000
1200
1400

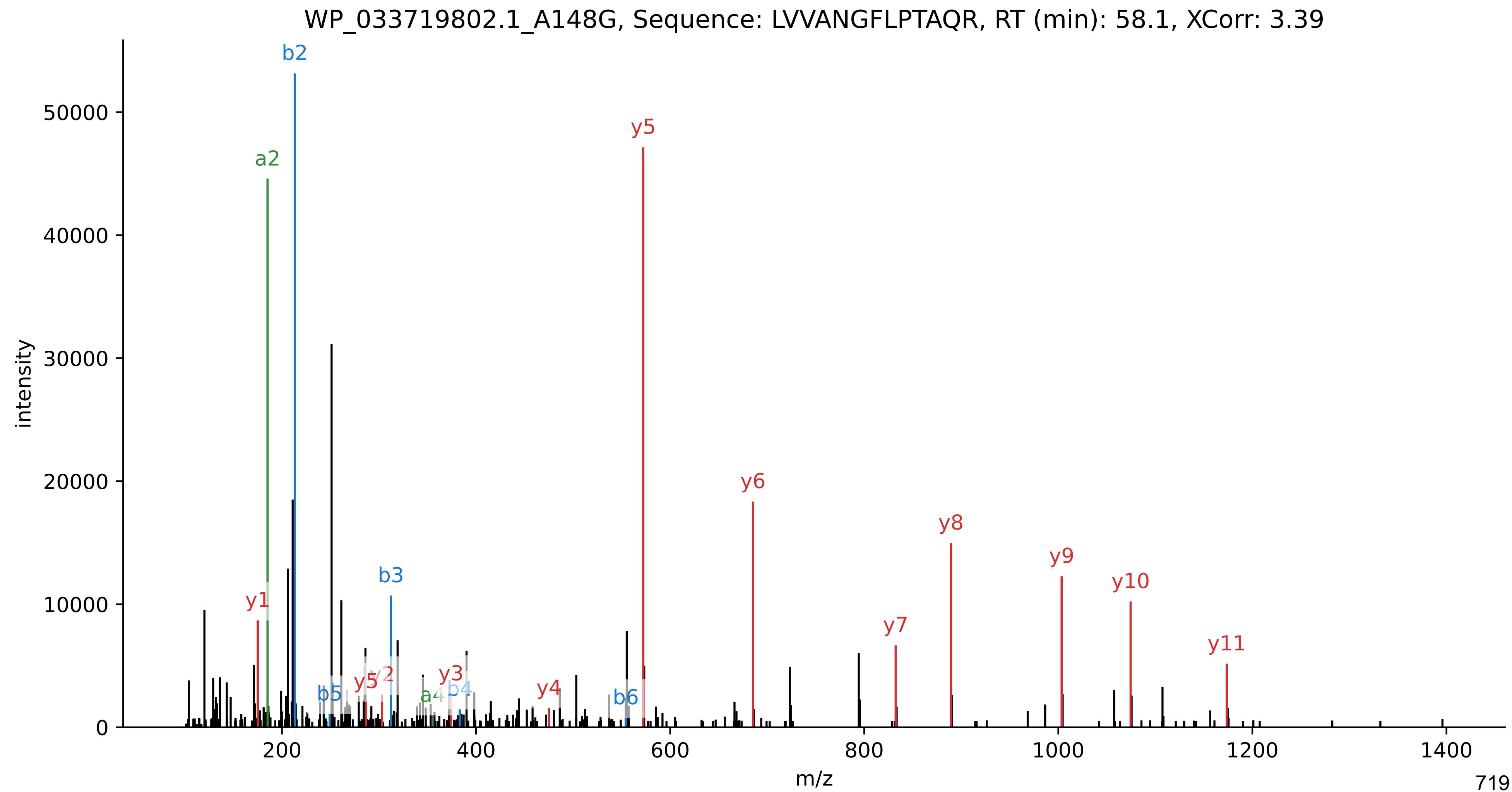

WP_033719802.1_A148G, Sequence: LVVANGFLPTAQR, RT (min): 58.1, XCorr: 3.39
b2
y5
a2
y6
y8
y9
y10
b3
y1
y7
y11
y3
y4
b5
b6
a4
b4
intensity
m/z
0
10000
20000
30000
40000
50000
200
400
600
800
1000
1200
1400

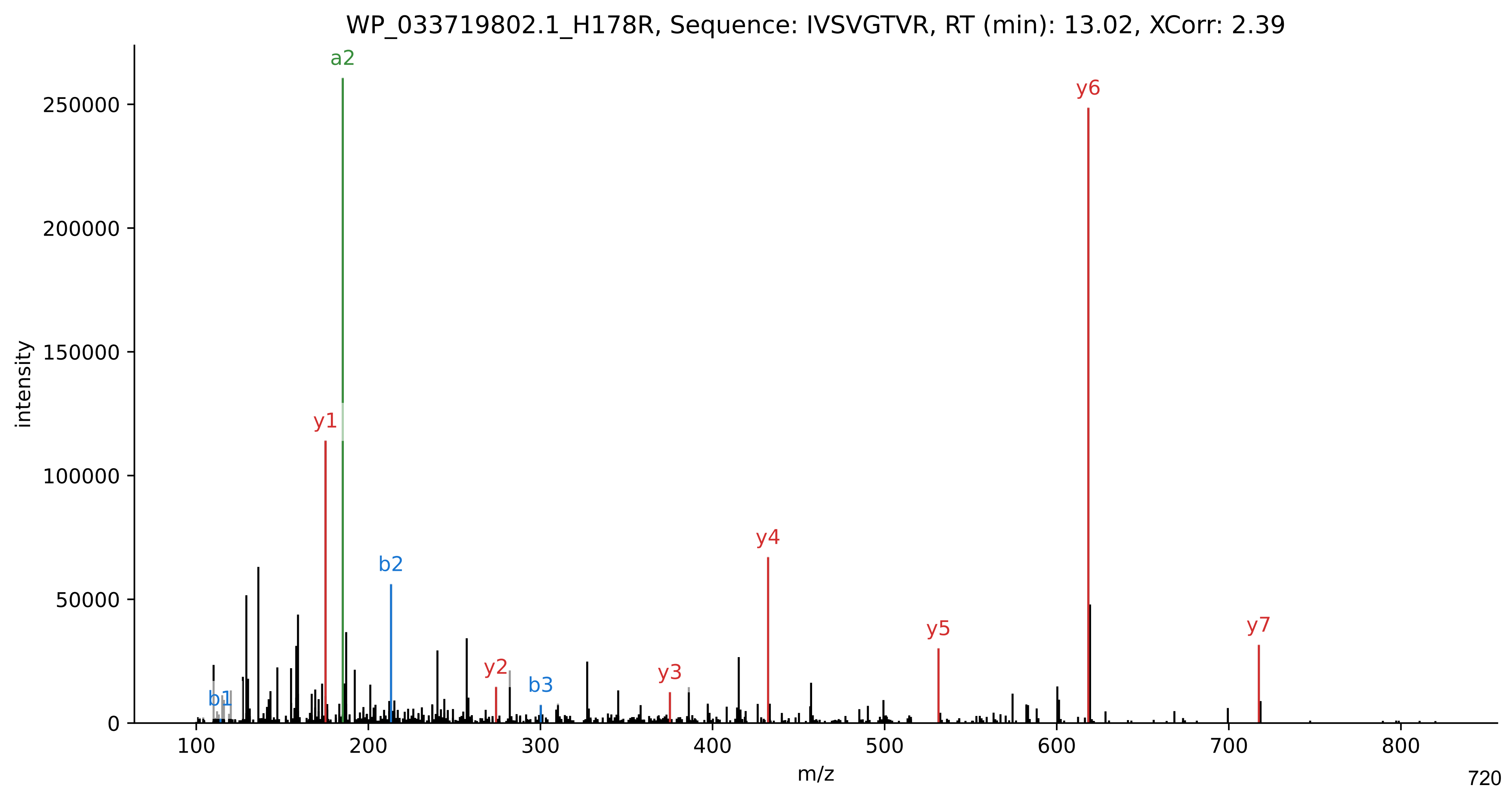

WP_033719802.1_H178R, Sequence: IVSVGTVR, RT (min): 13.02, XCorr: 2.39
a2
y6
y1
y4
b2
y5
y7
y2
b3
y3
b1
intensity
m/z

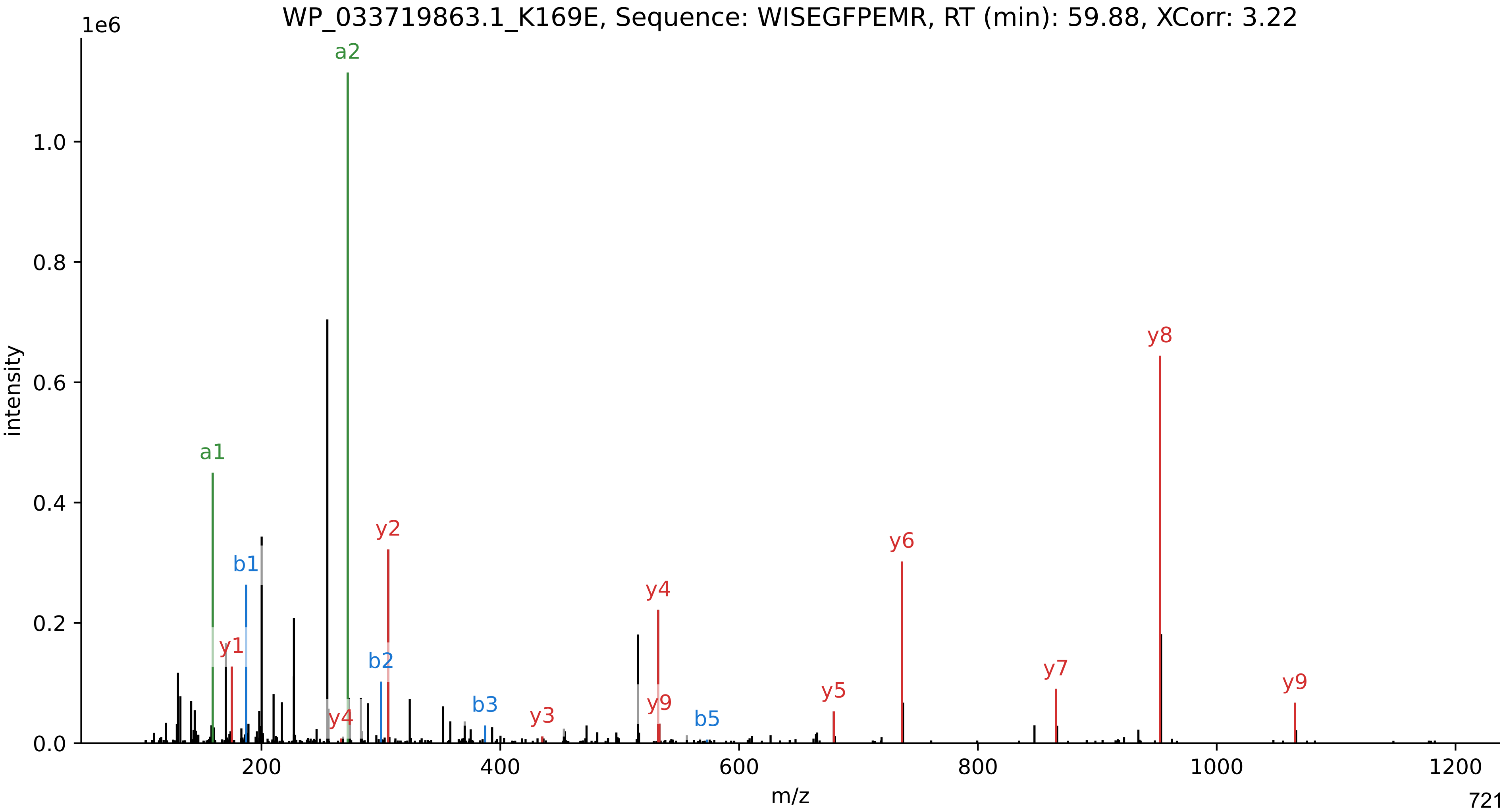
WP_033719863.1_K169E, Sequence: WISEGFPEMR, RT (min): 59.88, XCorr: 3.22
1e6
intensity
m/z
a1
a2
b1
b2
b3
b5
y1
y2
y3
y4
y4
y5
y6
y7
y8
y9
y9
0.0
0.2
0.4
0.6
0.8
1.0
200
400
600
800
1000
1200

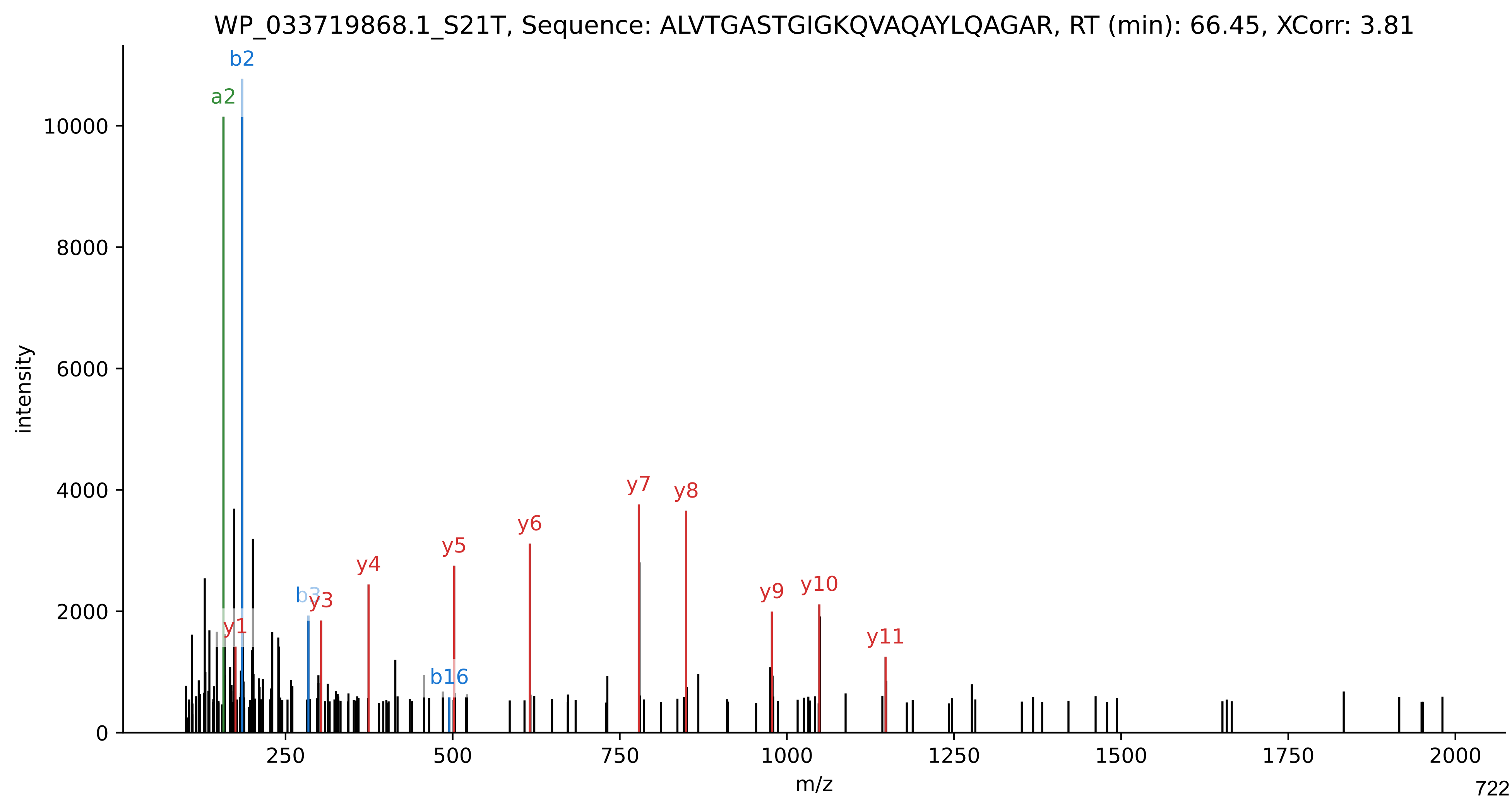

WP_033719868.1_S21T, Sequence: ALVTGASTGIGKQVAQAYLQAGAR, RT (min): 66.45, XCorr: 3.81
intensity
m/z
b2
a2
y1
b3
y3
y4
y5
b16
y6
y7
y8
y9
y10
y11
0
2000
4000
6000
8000
10000
250
500
750
1000
1250
1500
1750
2000

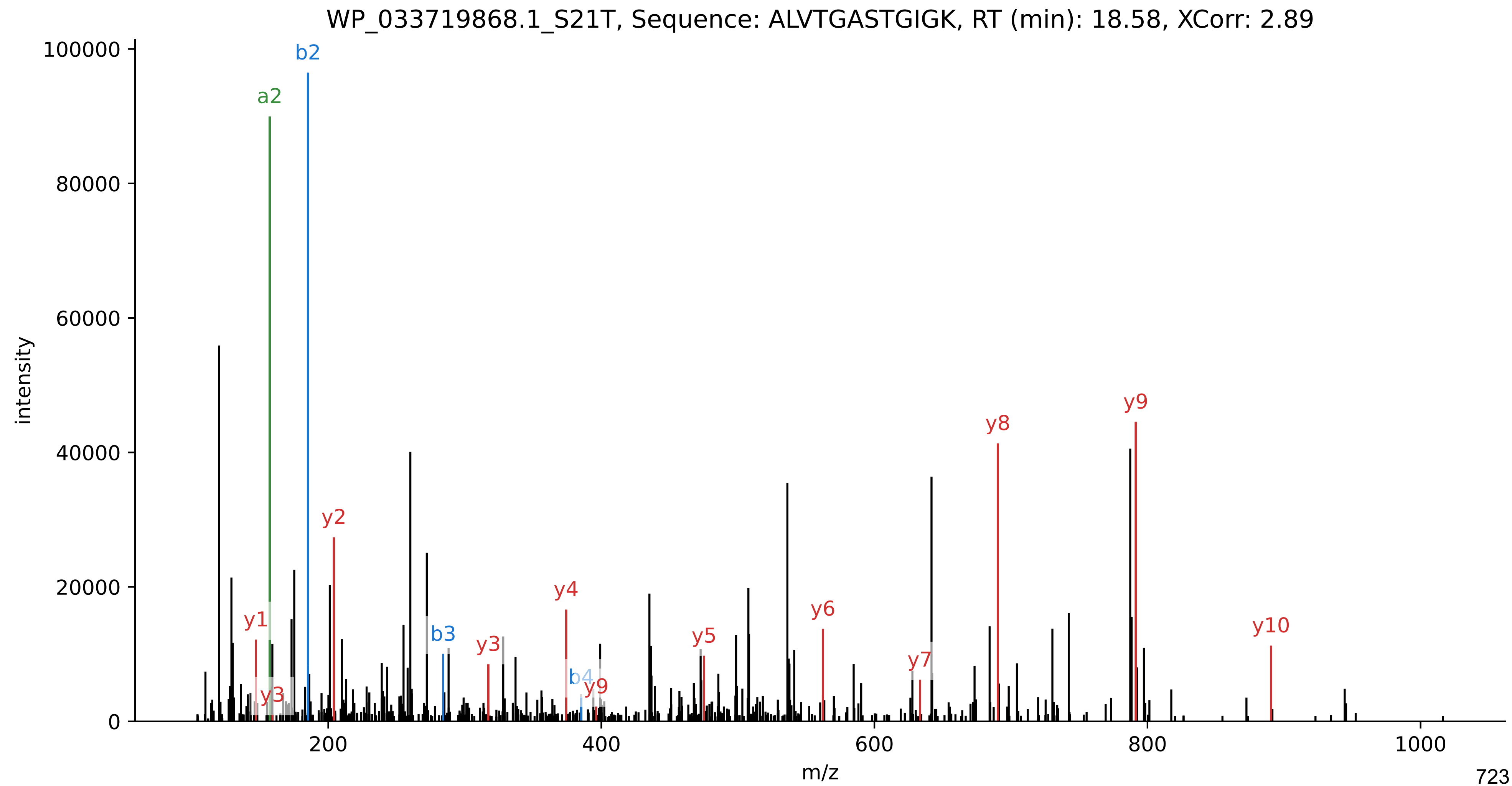
WP_033719868.1_S21T, Sequence: ALVTGASTGIGK, RT (min): 18.58, XCorr: 2.89
intensity
m/z
b2
a2
y1
y3
y2
b3
y3
y4
b4
y9
y5
y6
y7
y8
y9
y10
0
20000
40000
60000
80000
100000
200
400
600
800
1000

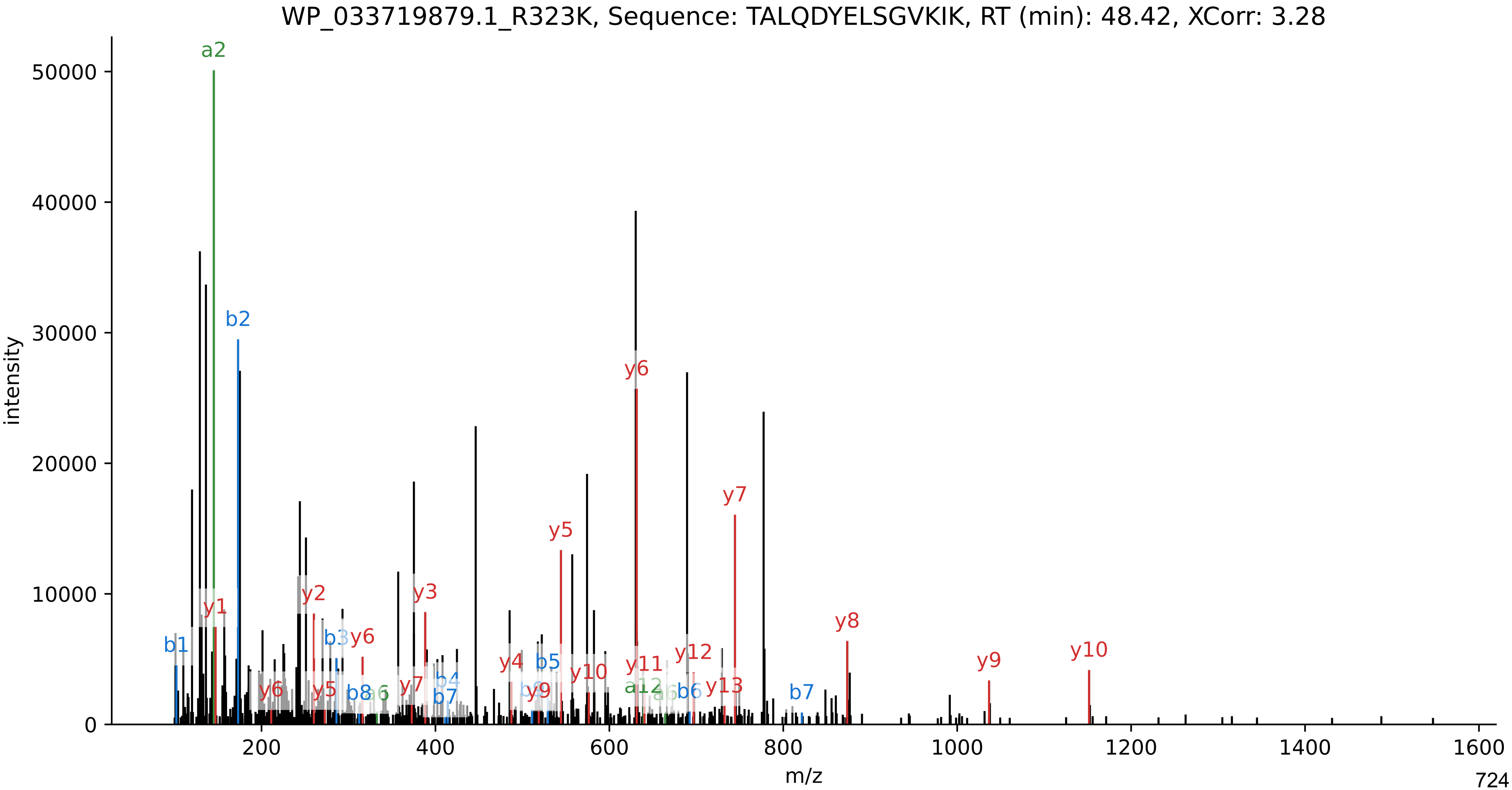

WP_033719879.1_R323K, Sequence: TALQDYELSGVKIK, RT (min): 48.42, XCorr: 3.28
intensity
m/z
a2
b2
b1
y1
y2
y3
y6
y4
b5
y5
y10
y11
y12
y7
y13
y8
y9
y10
b7
b6

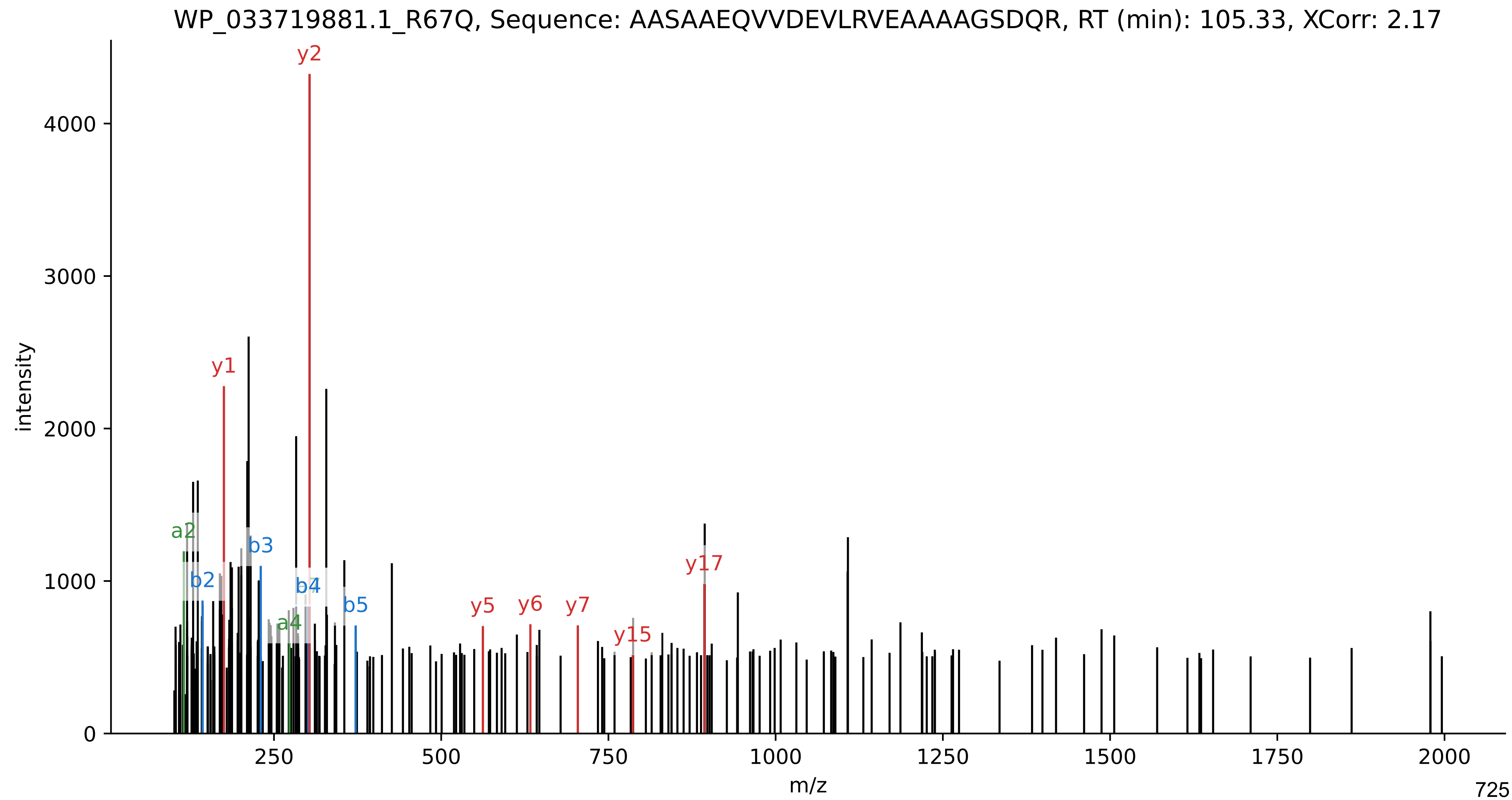

WP_033719881.1_R67Q, Sequence: AASAAEQVVDEVLRVEAAAAGSDQR, RT (min): 105.33, XCorr: 2.17
intensity
m/z
y2
y1
a2
b2
b3
b4
a4
b5
y5
y6
y7
y15
y17
0
1000
2000
3000
4000
250
500
750
1000
1250
1500
1750
2000

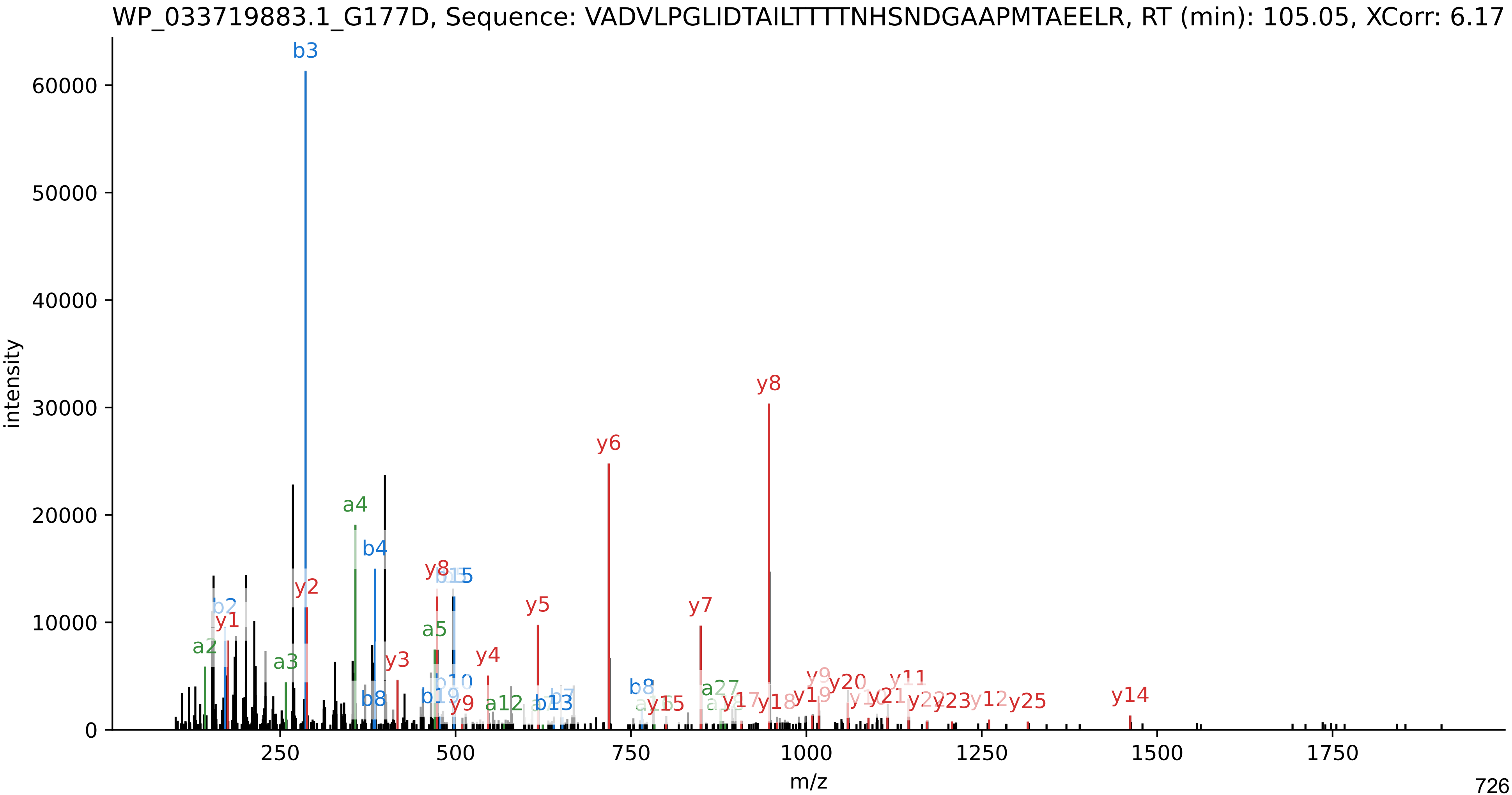
WP_033719883.1_G177D, Sequence: VADVLPGLIDTAILTTTTNHSNDGAAPMTAEELR, RT (min): 105.05, XCorr: 6.17
intensity
m/z
0
10000
20000
30000
40000
50000
60000
250
500
750
1000
1250
1500
1750
b3
y8
y6
a4
b4
y2
y8
b2
y1
a2
a3
y3
a5
y5
y7
y4
b8
b19
b10
y9
a12
b13
b8
y15
a27
y17
y18
y19
y20
y11
y21
y23
y25
y14

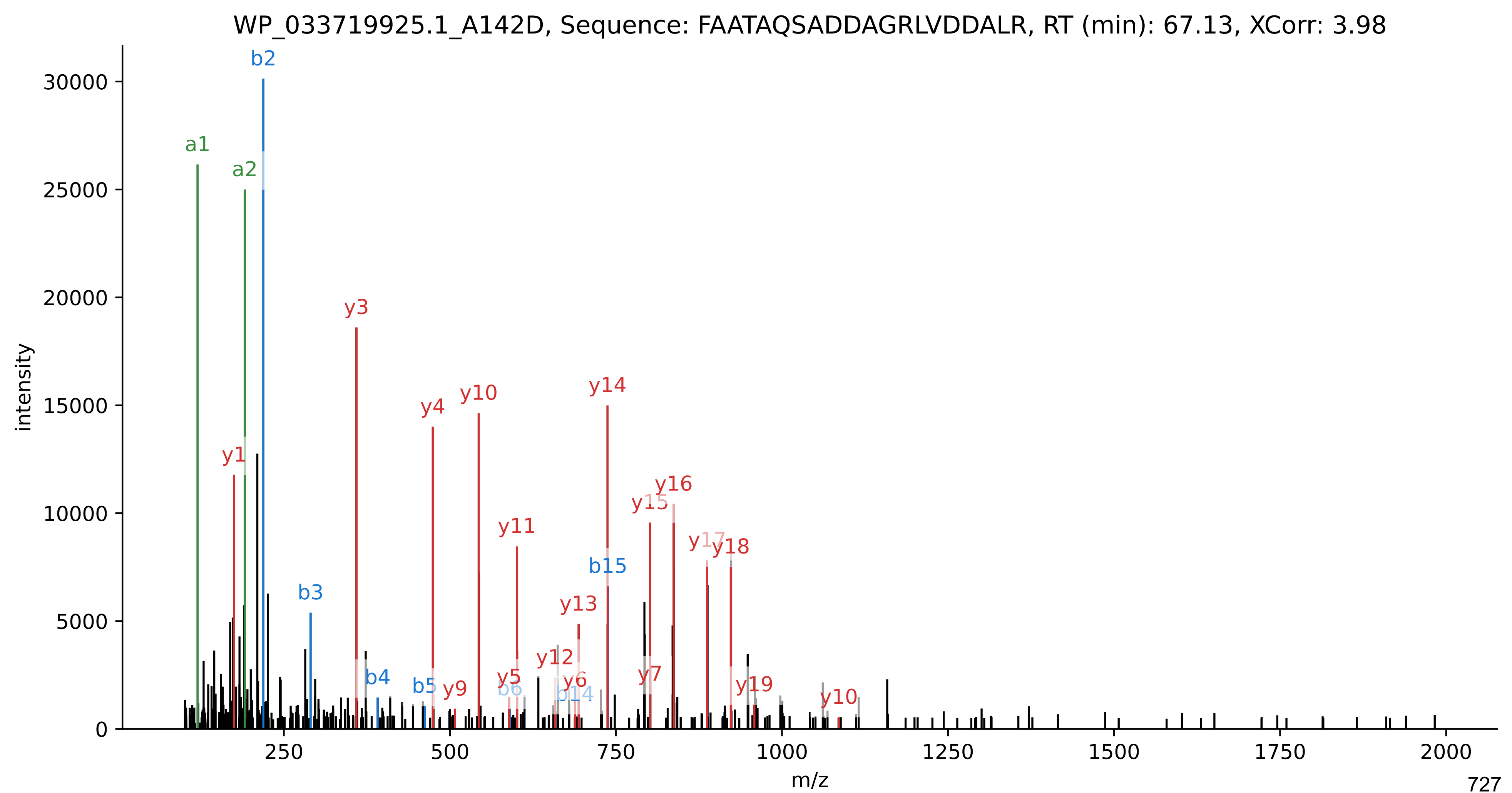
WP_033719925.1_A142D, Sequence: FAATAQSADDAGRLVDDALR, RT (min): 67.13, XCorr: 3.98
intensity
m/z
a1
a2
b2
b3
b4
b5
b6
b14
b15
y1
y3
y4
y5
y6
y7
y9
y10
y11
y12
y13
y14
y15
y16
y17
y18
y19
y10

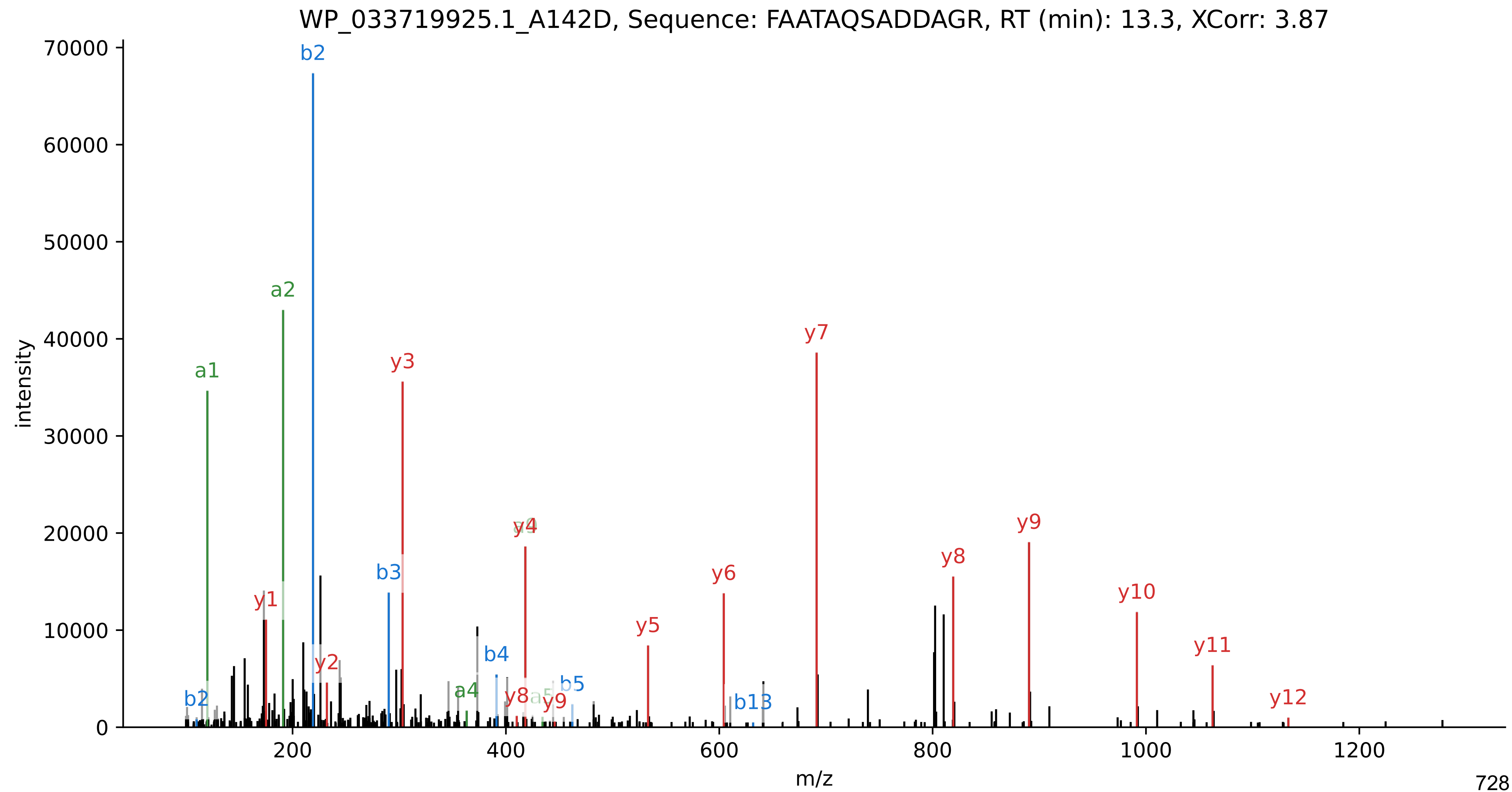
WP_033719925.1_A142D, Sequence: FAATAQSADDAGR, RT (min): 13.3, XCorr: 3.87
intensity
m/z
b2
a2
a1
y3
y7
y4
y9
y8
b3
y6
y1
y10
y5
b4
y2
y11
b5
a4
y8
y9
b13
y12
b2

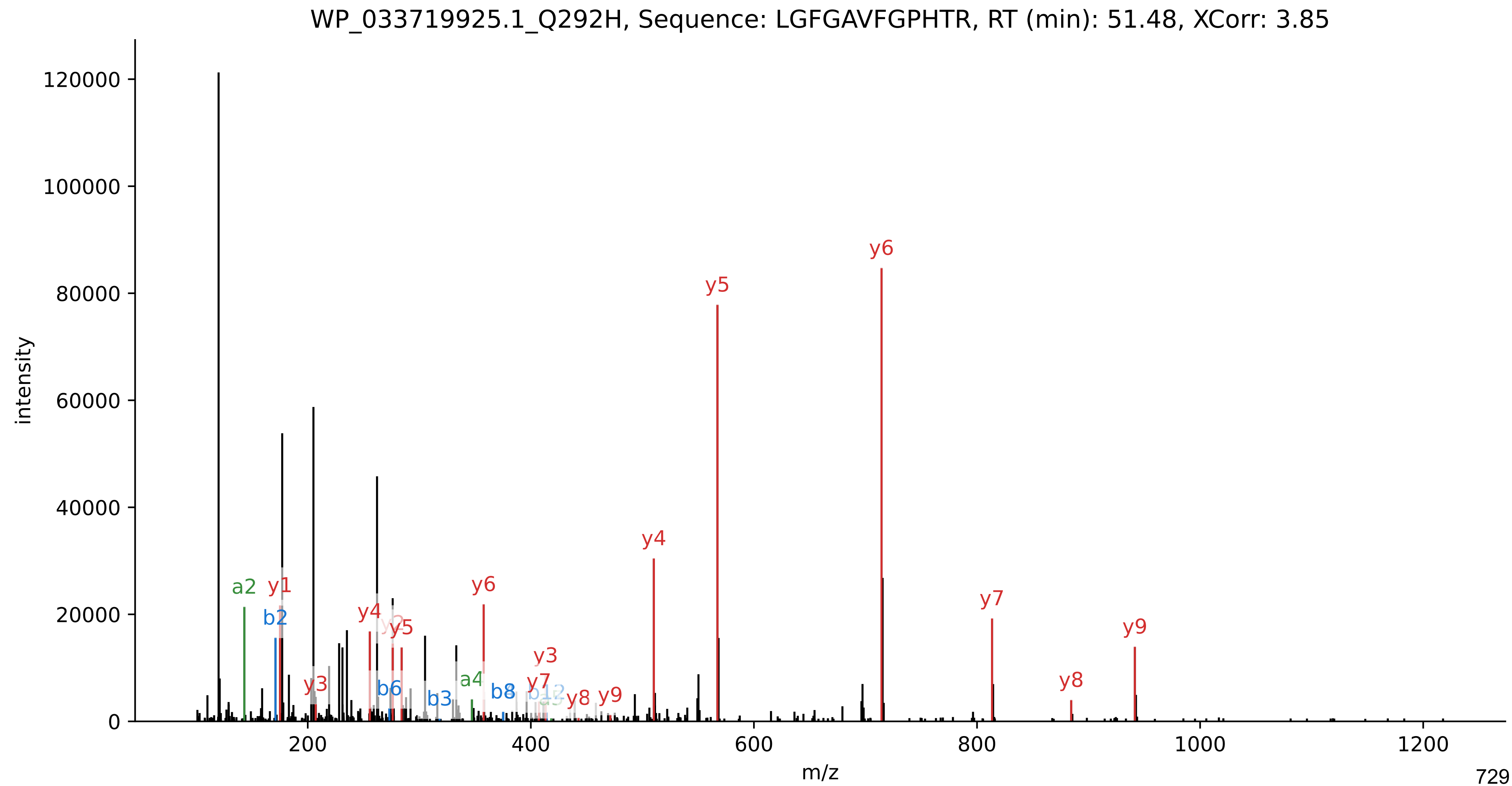

WP_033719925.1_Q292H, Sequence: LGFGAVFGPHTR, RT (min): 51.48, XCorr: 3.85
intensity
m/z
a2
y1
b2
y4
y5
y3
b6
b3
a4
y6
b8
y7
y3
y8
y9
y4
y5
y6
y7
y8
y9

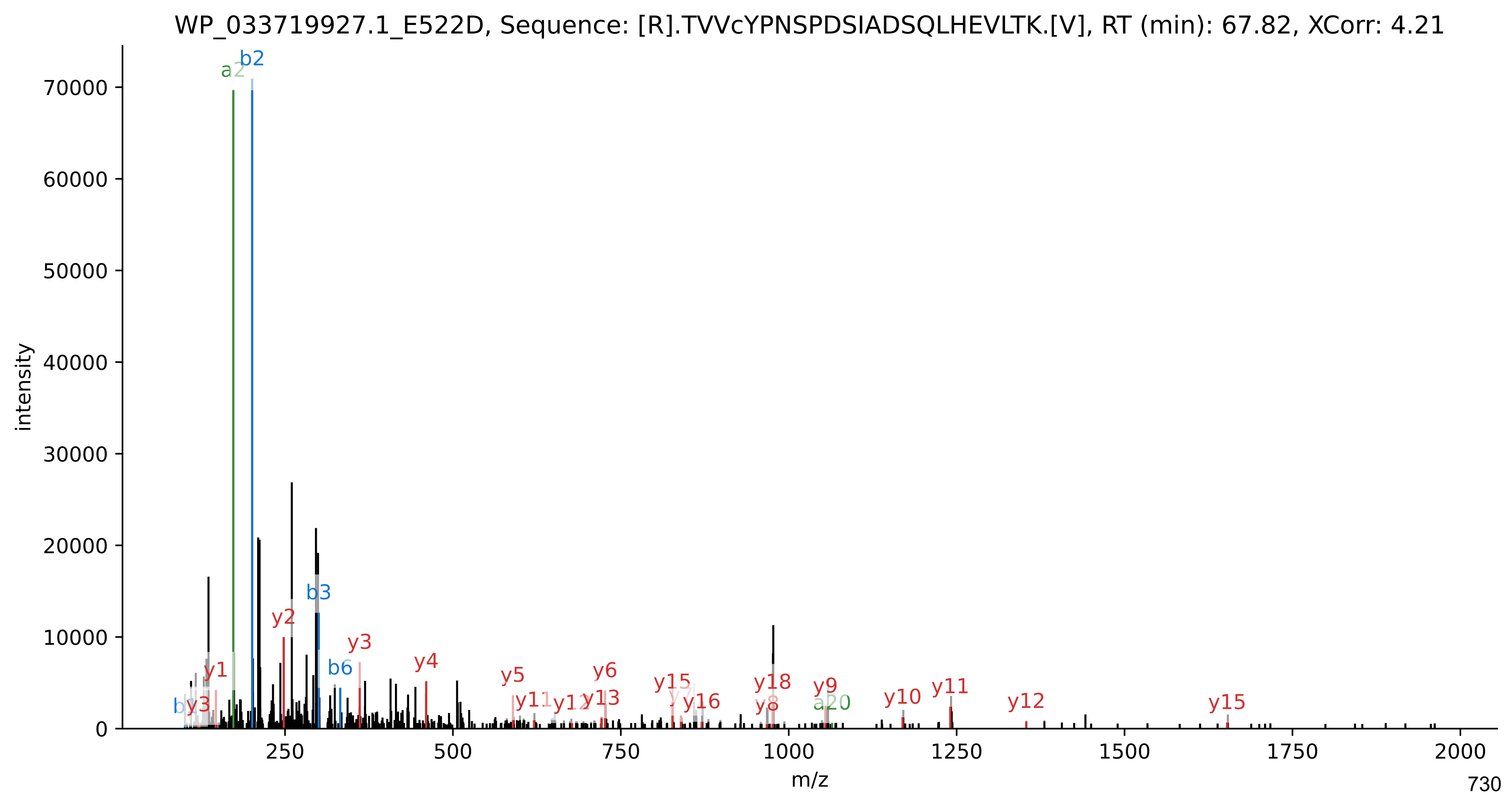
WP_033719927.1_E522D, Sequence: [R].TVVcYPNSPDSIADSQLHEVLTK.[V], RT (min): 67.82, XCorr: 4.21
intensity
m/z
a2
b2
b3
y2
y3
b6
y1
y4
y5
y6
y13
y15
y16
y18
y8
y9
a20
y10
y11
y12
y15

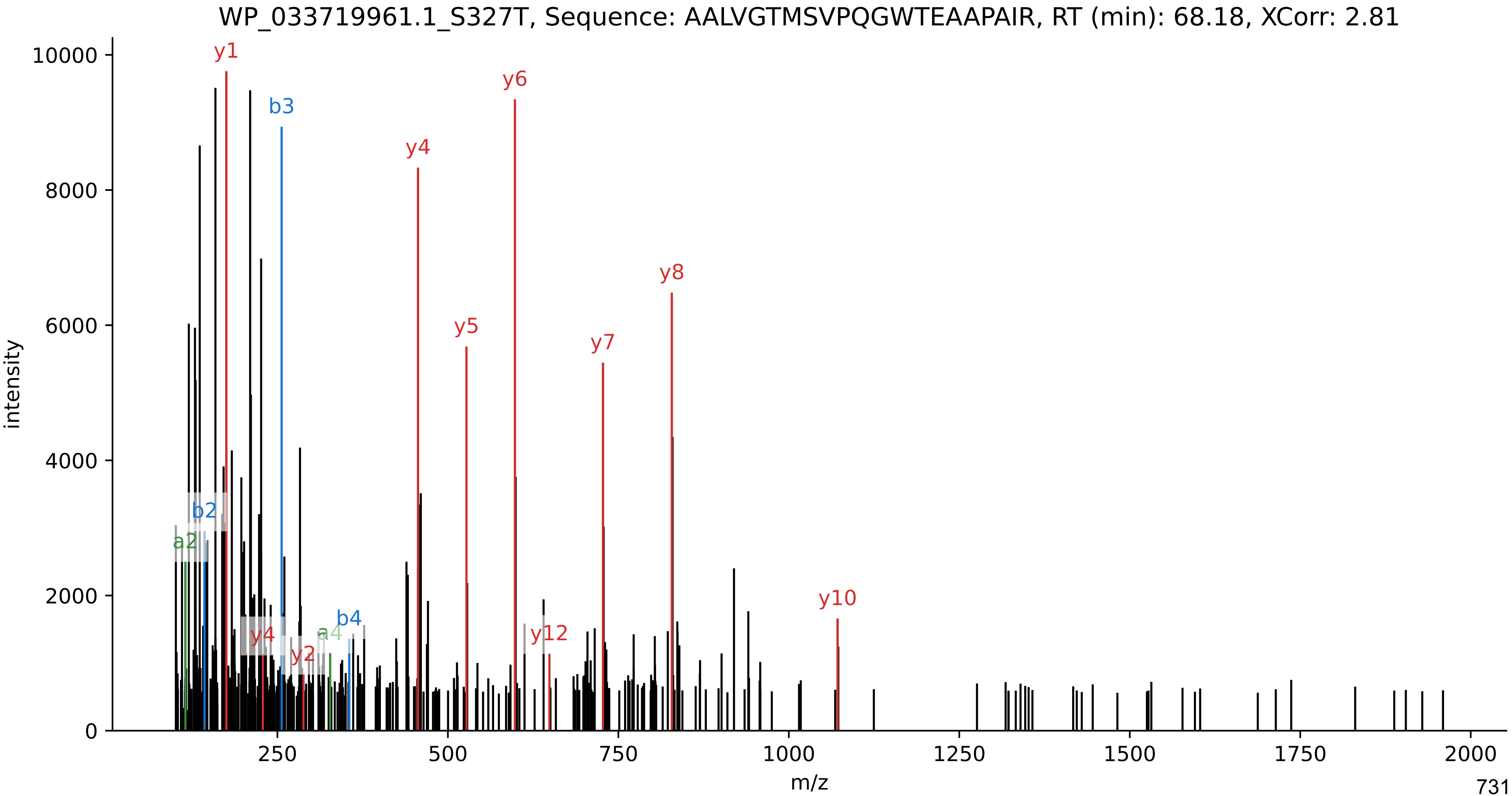
WP_033719961.1_S327T, Sequence: AALVGTMSVPQGWTEAAPAIR, RT (min): 68.18, XCorr: 2.81
intensity
m/z
y1
b3
y6
y4
y8
y5
y7
b2
a2
y10
b4
a4
y4
y12
y2
10000
8000
6000
4000
2000
0
250
500
750
1000
1250
1500
1750
2000

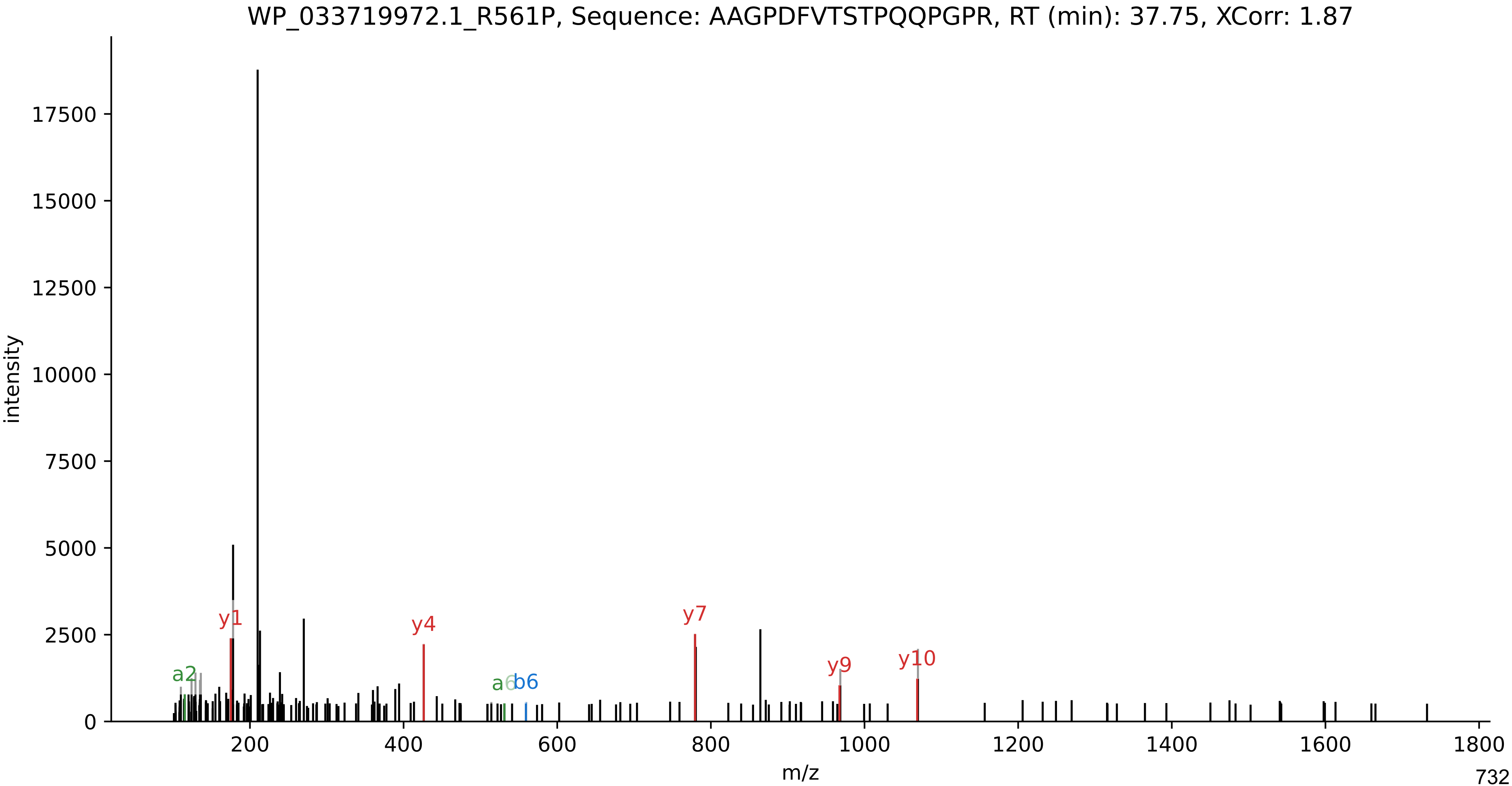

WP_033719972.1_R561P, Sequence: AAGPDFVTSTPQQPGPR, RT (min): 37.75, XCorr: 1.87
intensity
m/z
0
2500
5000
7500
10000
12500
15000
17500
200
400
600
800
1000
1200
1400
1600
1800
a2
y1
y4
a6
b6
y7
y9
y10

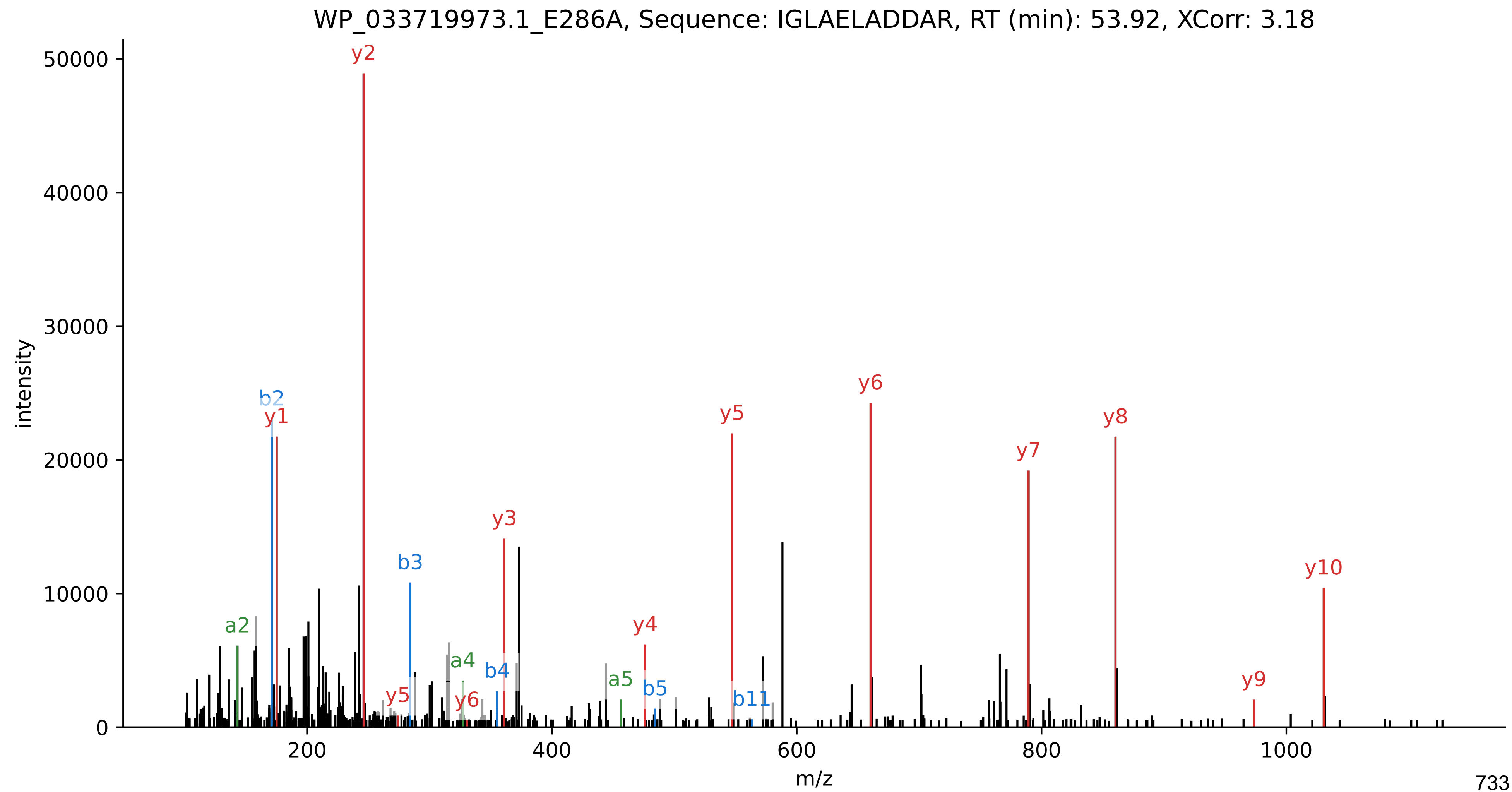

WP_033719973.1_E286A, Sequence: IGLAELADDAR, RT (min): 53.92, XCorr: 3.18
intensity
m/z
y2
b2
y1
b3
y3
y4
y5
y6
y7
y8
y9
y10
a2
a4
a5
b4
b5
b11
y5
y6

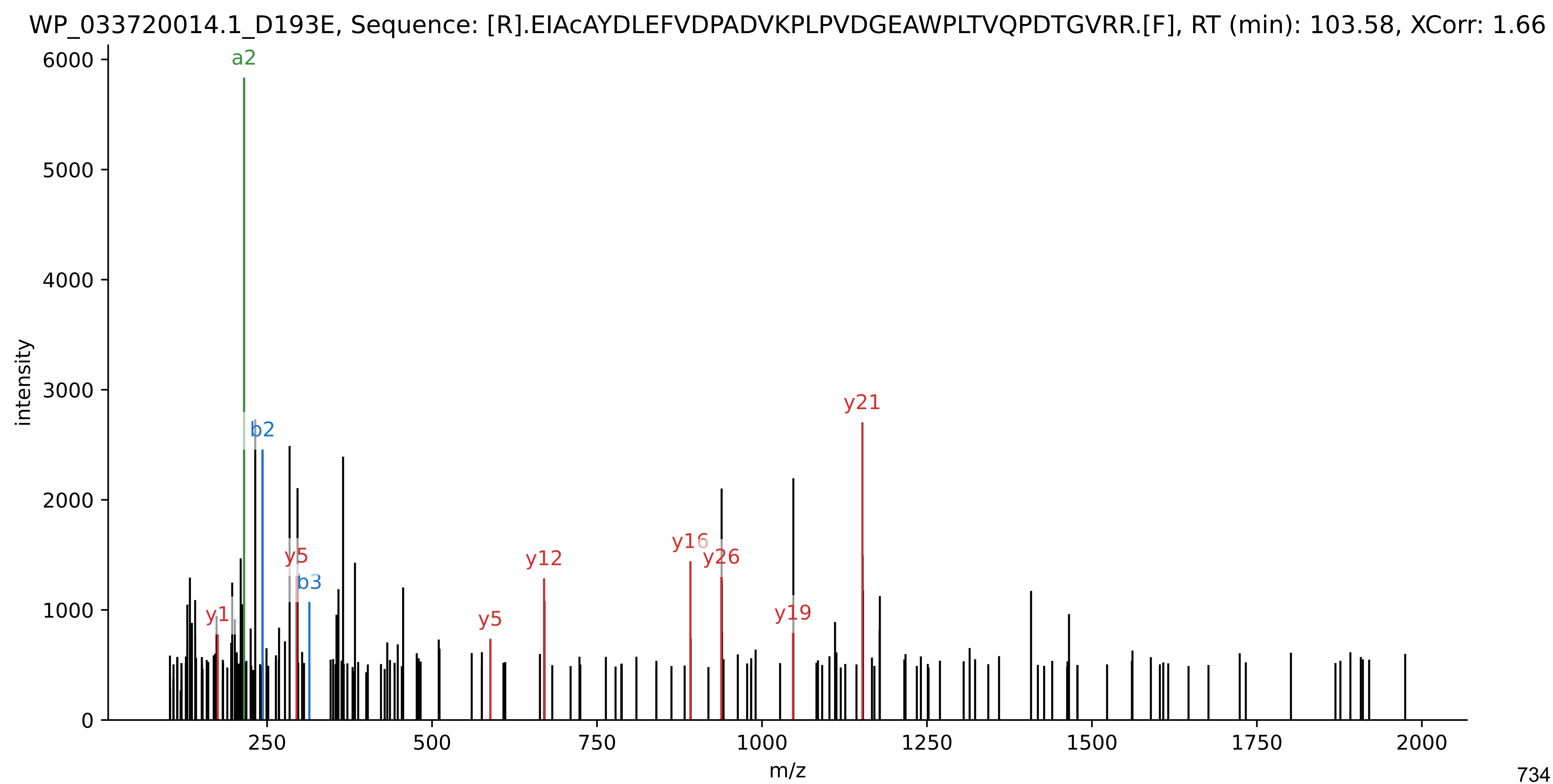
WP_033720014.1_D193E, Sequence: [R].EIAcAYDLEFVDPADVKPLPVDGEAWPLTVQPDTGVRR.[F], RT (min): 103.58, XCorr: 1.66
a2
b2
y5
b3
y1
y5
y12
y16
y26
y19
y21
intensity
m/z
0
1000
2000
3000
4000
5000
6000
250
500
750
1000
1250
1500
1750
2000

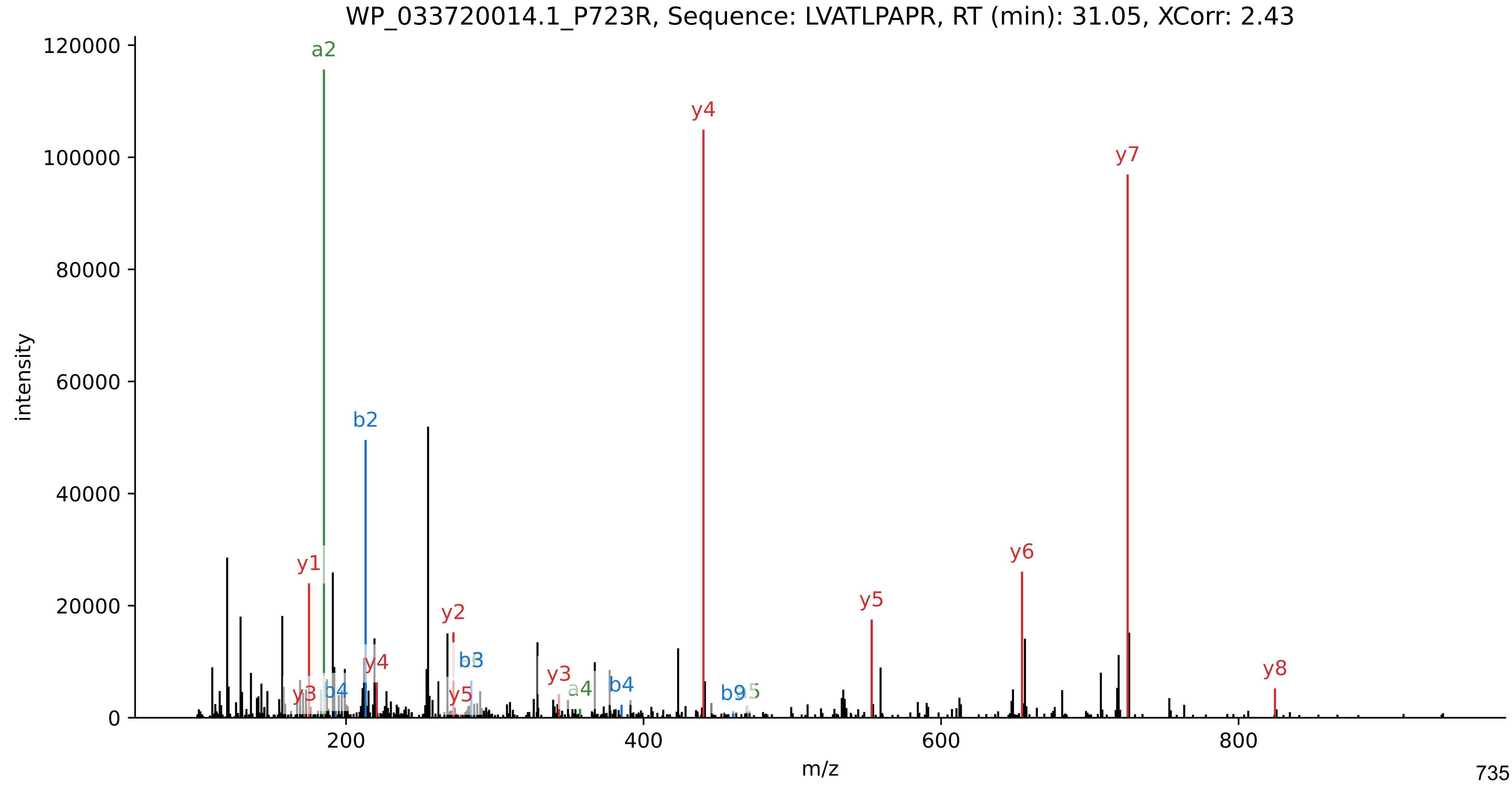
WP_033720014.1_P723R, Sequence: LVATLPAPR, RT (min): 31.05, XCorr: 2.43
intensity
m/z
a2
y4
y7
b2
y1
y2
y6
y5
y8
y3
b4
y4
b3
y5
y3
a4
b4
b9
a5
0
20000
40000
60000
80000
100000
120000
200
400
600
800

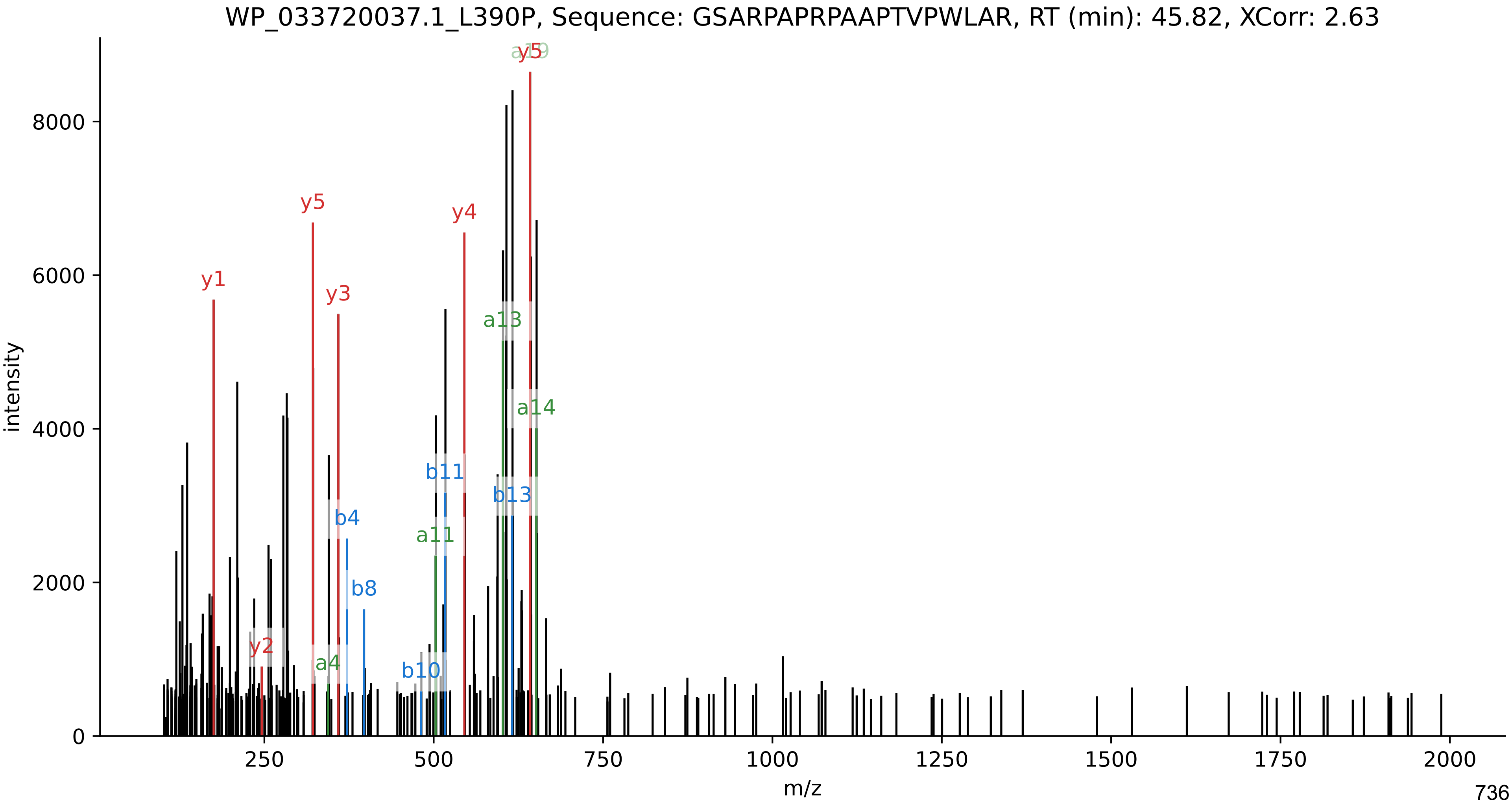
WP_033720037.1_L390P, Sequence: GSARPAPRPAAPTVPWLAR, RT (min): 45.82, XCorr: 2.63
intensity
m/z
0
2000
4000
6000
8000
250
500
750
1000
1250
1500
1750
2000
y1
y2
y5
y3
a4
b4
b8
b10
a11
b11
y4
a13
b13
a14
y5

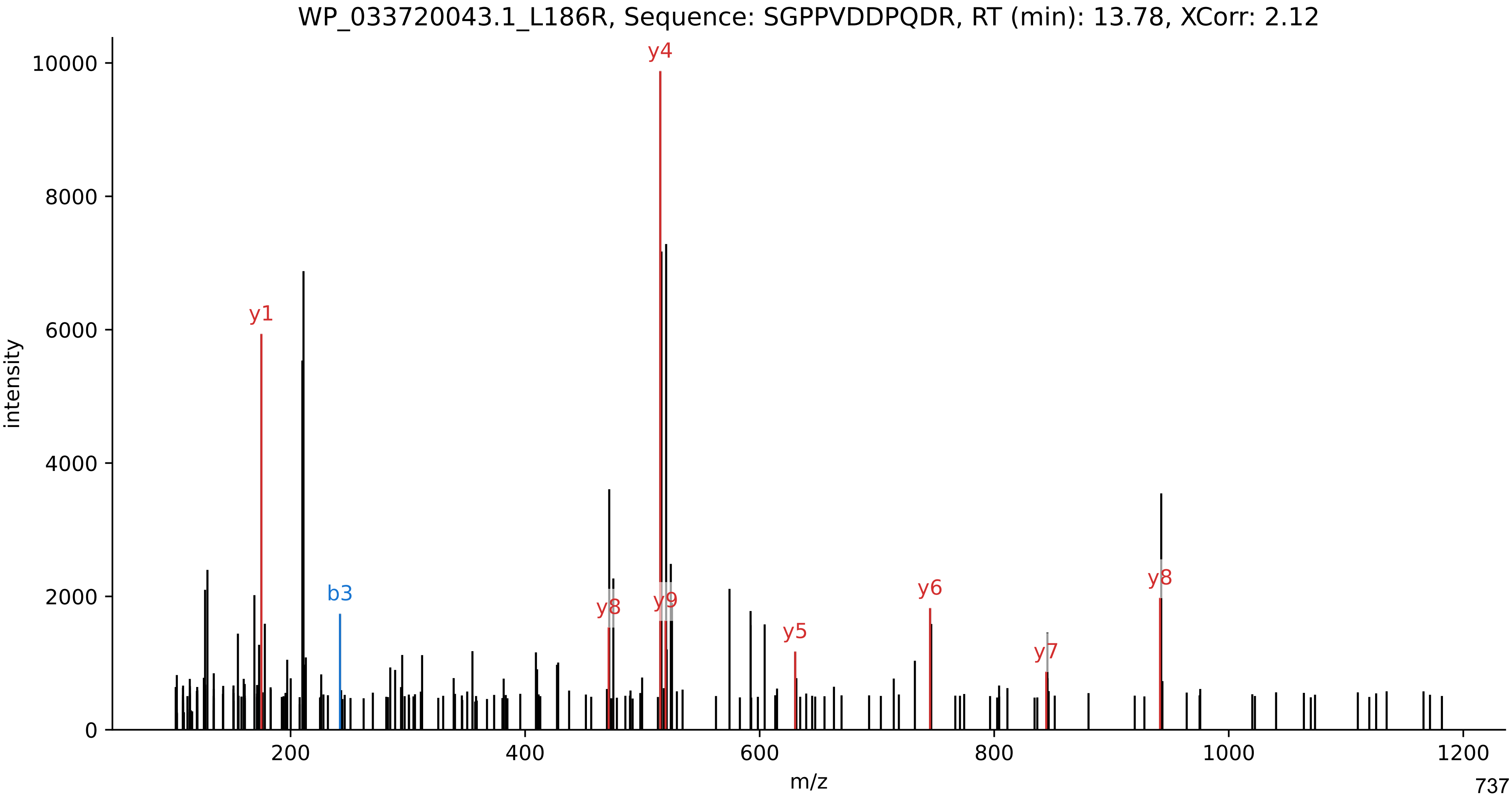

WP_033720043.1_L186R, Sequence: SGPPVDDPQDR, RT (min): 13.78, XCorr: 2.12
y4
y1
b3
y8
y9
y5
y6
y7
y8
intensity
m/z

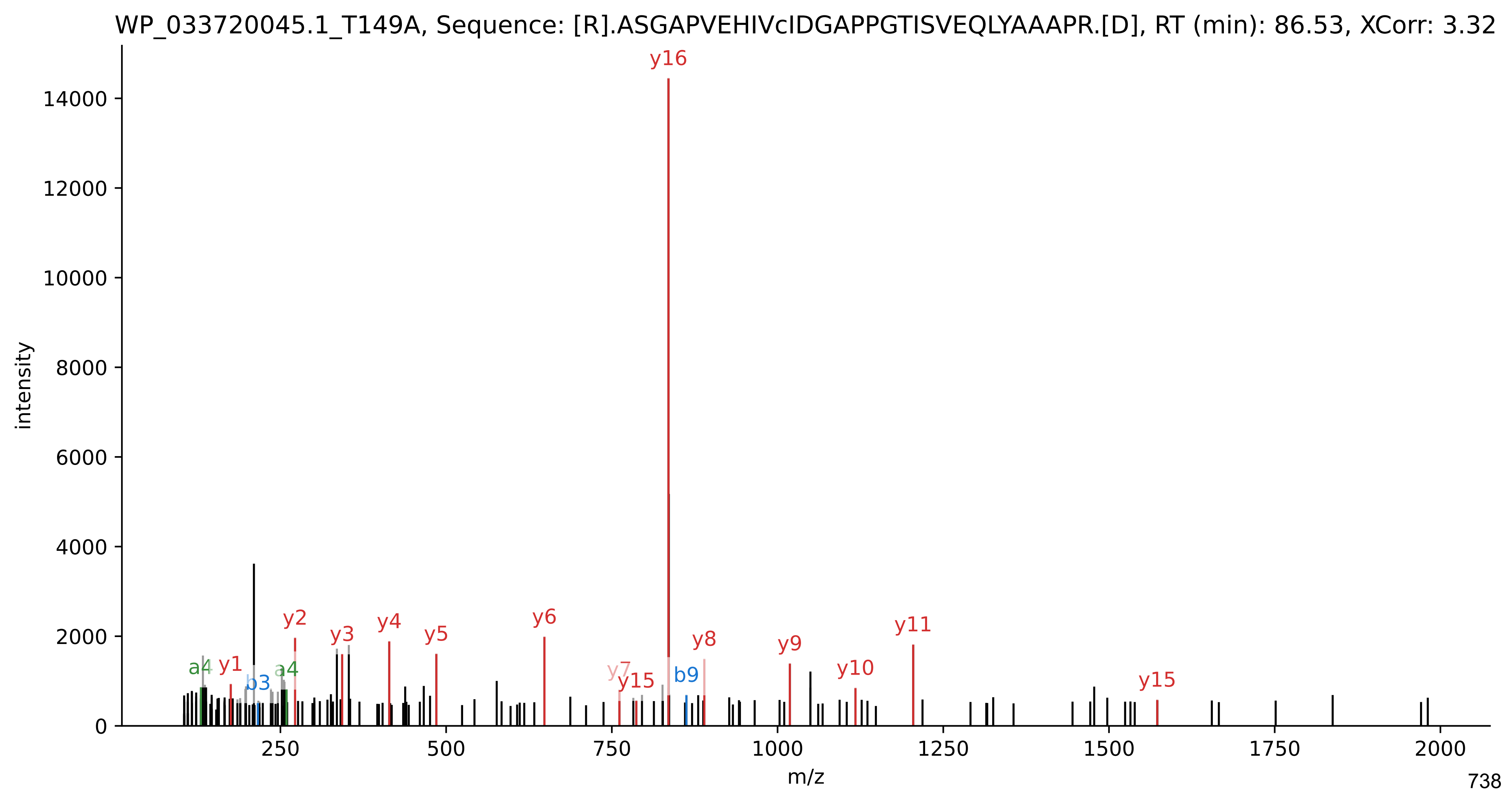
WP_033720045.1_T149A, Sequence: [R].ASGAPVEHIVcIDGAPPGTISVEQLYAAAPR.[D], RT (min): 86.53, XCorr: 3.32
intensity
m/z
y16
y2
y3
y4
y5
y6
y7
y8
y9
y10
y11
y15
y15
y1
a4
a4
b3
b9

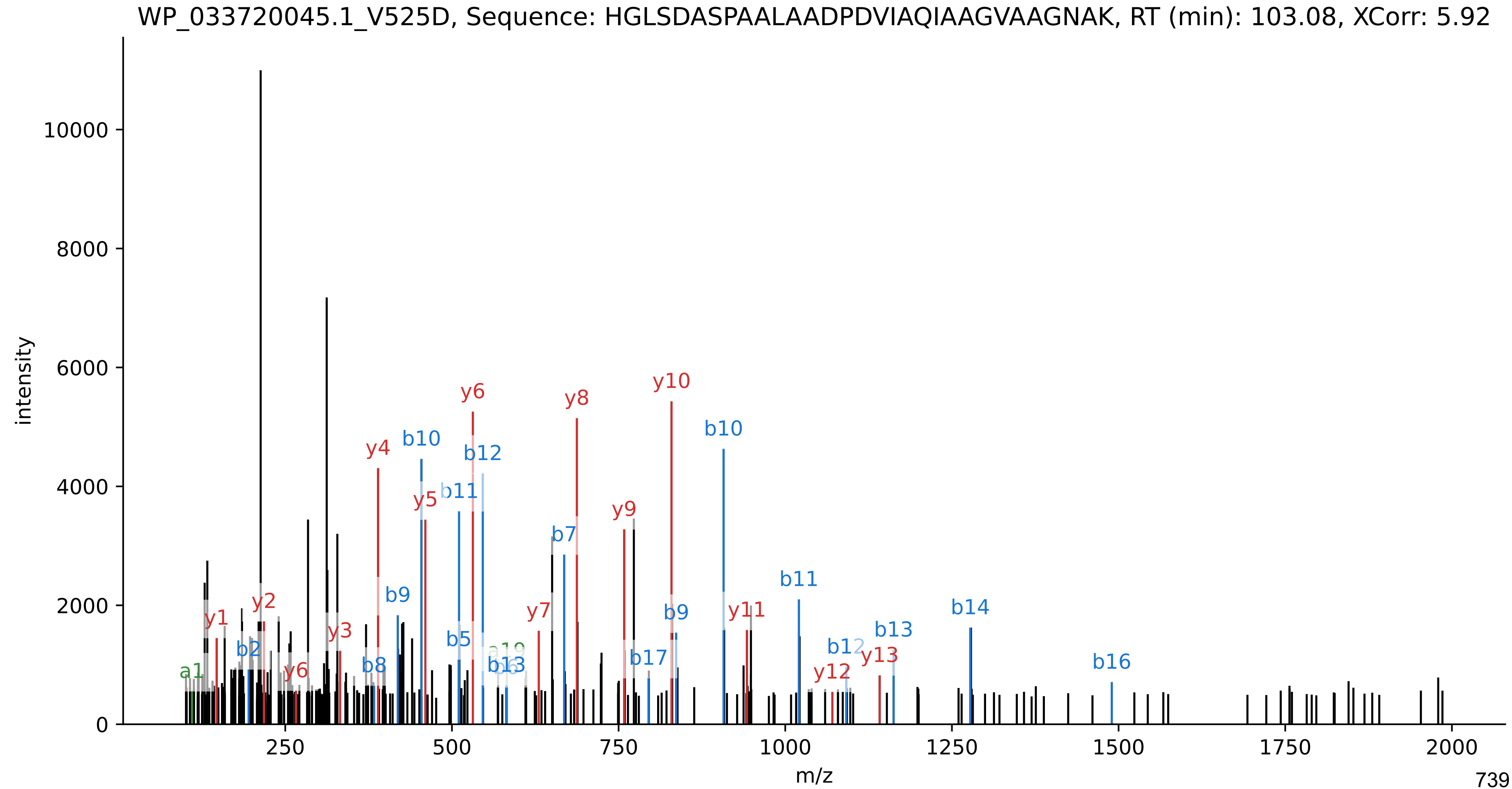

WP_033720045.1_V525D, Sequence: HGLSDASPAALAADPDVIAQIAAGVAAGNAK, RT (min): 103.08, XCorr: 5.92
intensity
m/z

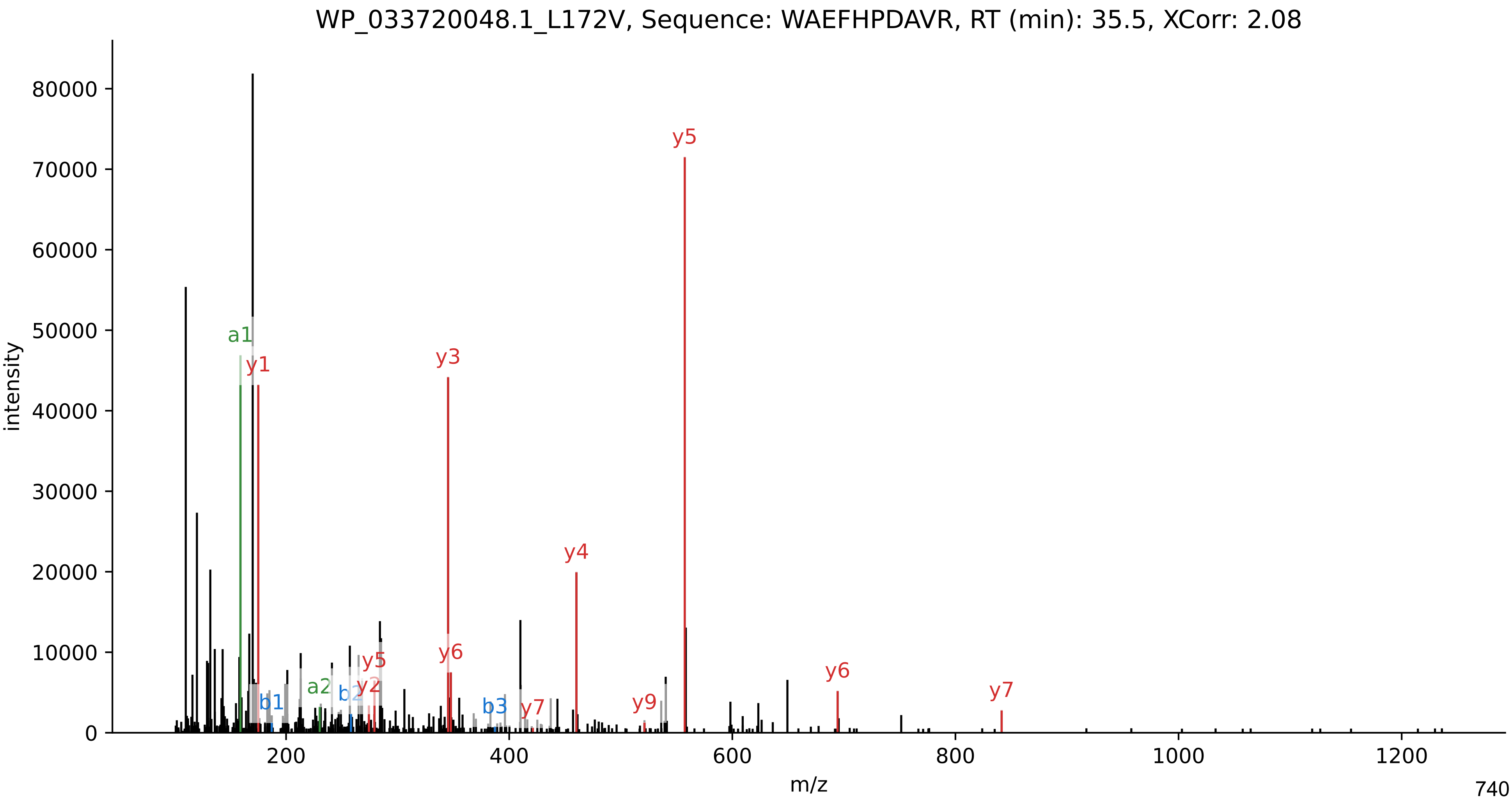

WP_033720048.1_L172V, Sequence: WAEFHPDAVR, RT (min): 35.5, XCorr: 2.08
intensity
m/z
a1
y1
y3
y5
y4
y6
y6
y7
y9
y7
a2
b1
b2
y2
y5
b3
y7

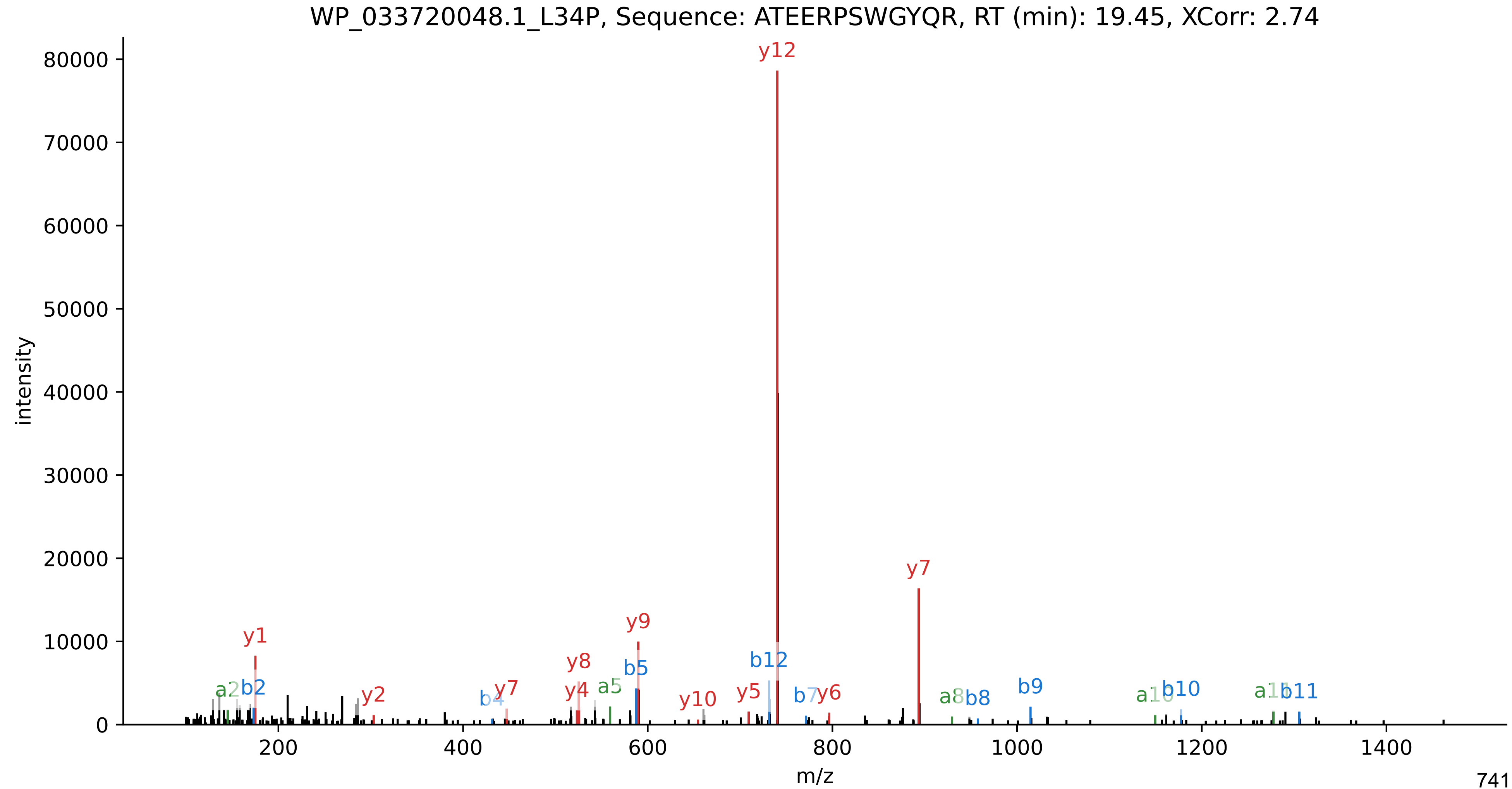
WP_033720048.1_L34P, Sequence: ATEERPSWGYQR, RT (min): 19.45, XCorr: 2.74
intensity
m/z
y12
y7
y9
y1
y8
b5
b12
a2
b2
y2
b4
y7
y4
a5
y10
y5
b7
y6
a8
b8
b9
a10
b10
a11
b11
80000
70000
60000
50000
40000
30000
20000
10000
0
200
400
600
800
1000
1200
1400

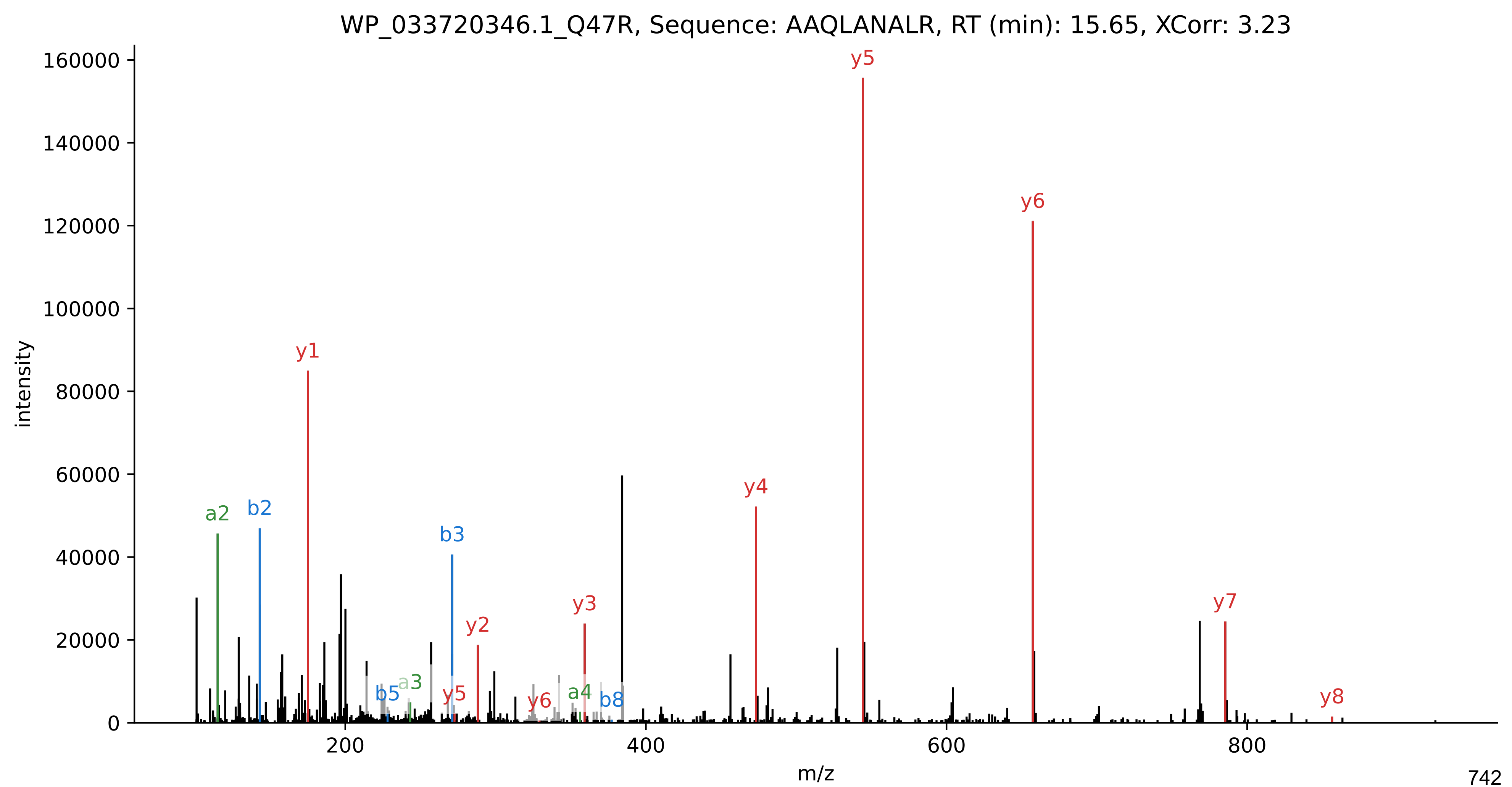
WP_033720346.1_Q47R, Sequence: AAQLANALR, RT (min): 15.65, XCorr: 3.23
intensity
m/z
0
20000
40000
60000
80000
100000
120000
140000
160000
200
400
600
800
y5
y6
y1
y4
a2
b2
b3
y3
y2
y7
y8
a3
b5
y5
y6
a4
b8

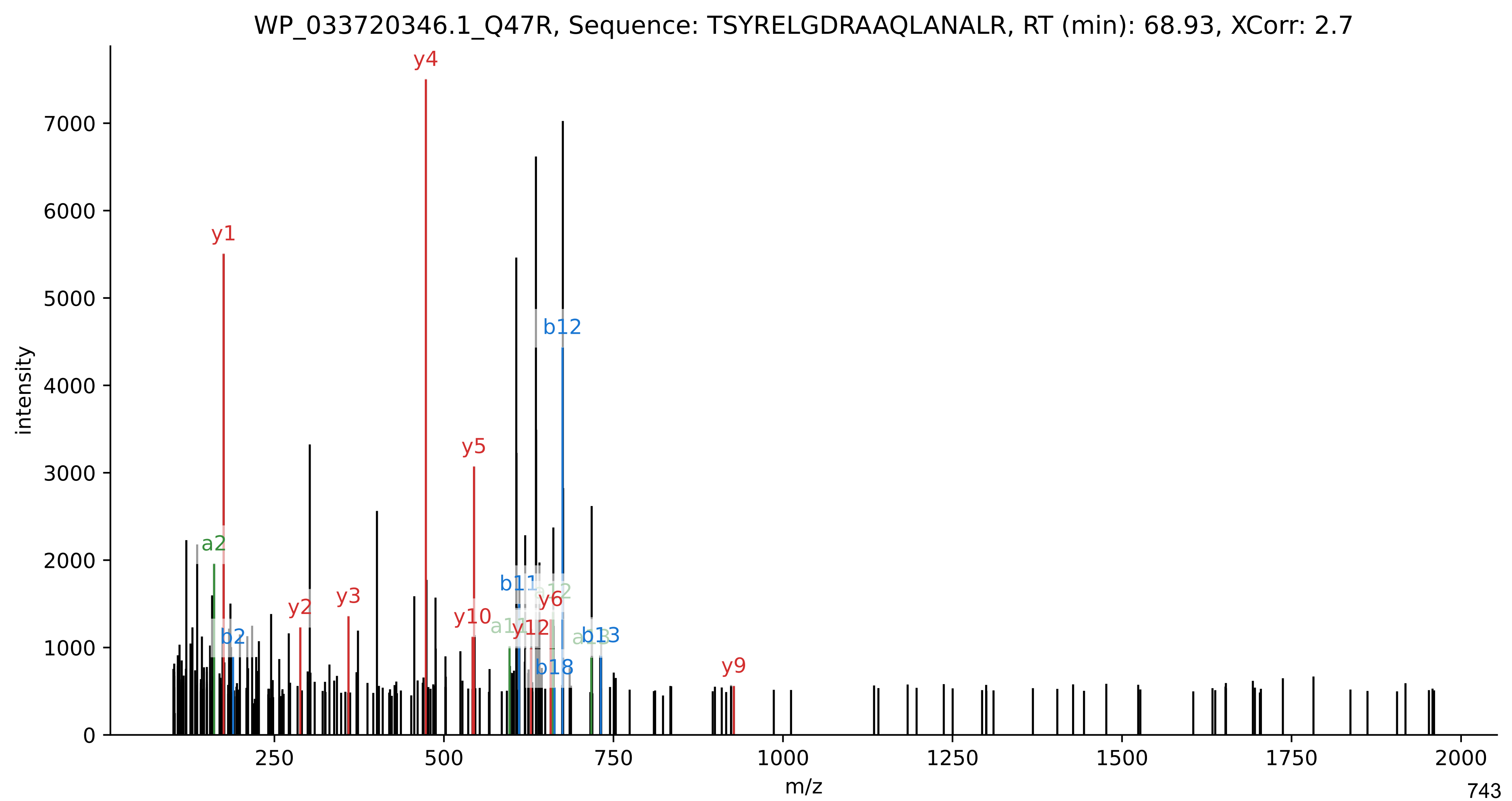

WP_033720346.1_Q47R, Sequence: TSYRELGDRAAQLANALR, RT (min): 68.93, XCorr: 2.7
intensity
m/z
y4
y1
y5
b12
a2
y2
y3
b2
y10
b11
y6
y12
b13
b18
y9

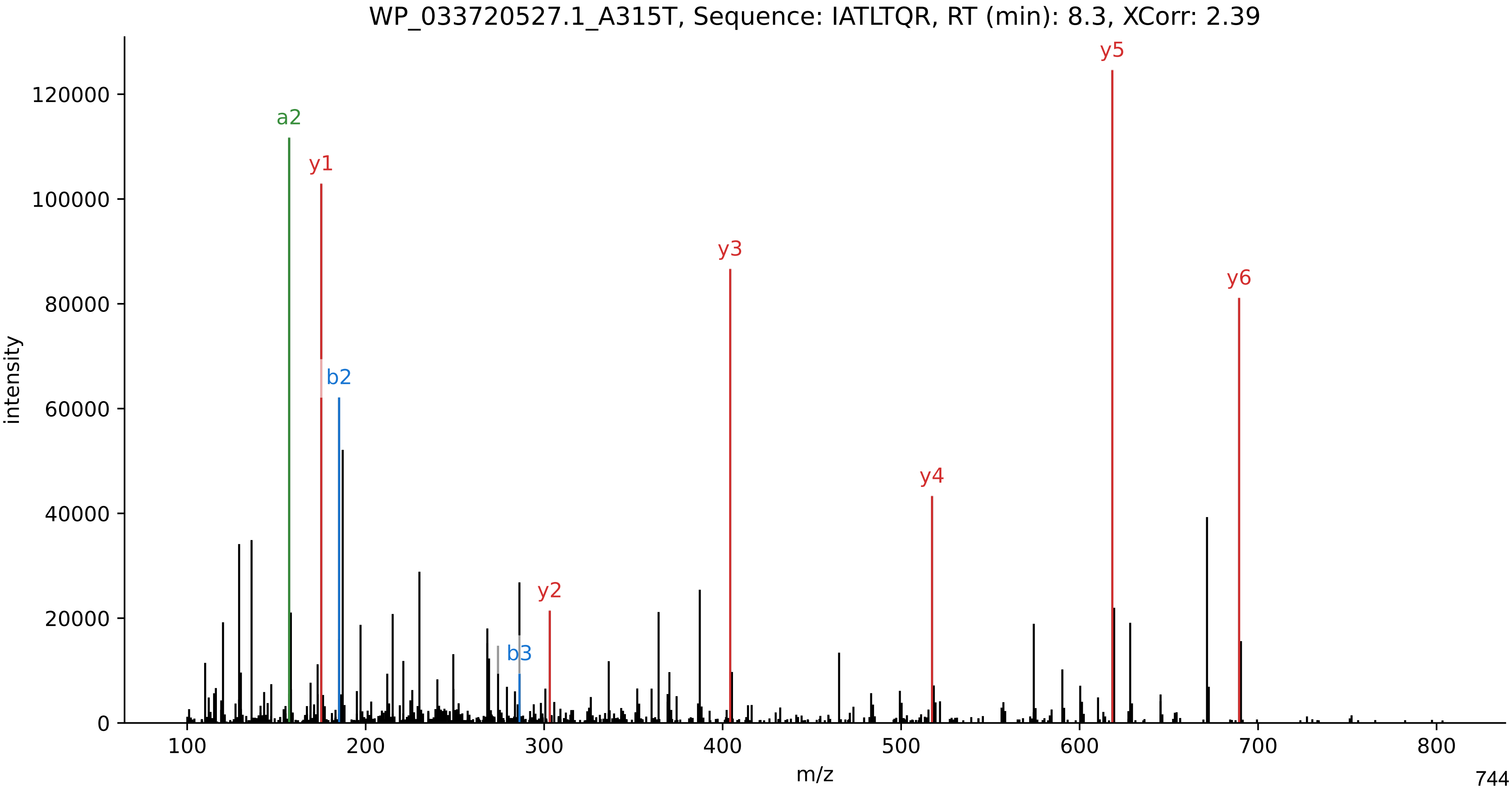
WP_033720527.1_A315T, Sequence: IATLTQR, RT (min): 8.3, XCorr: 2.39
intensity
m/z
a2
y1
b2
b3
y2
y3
y4
y5
y6
0
20000
40000
60000
80000
100000
120000
100
200
300
400
500
600
700
800

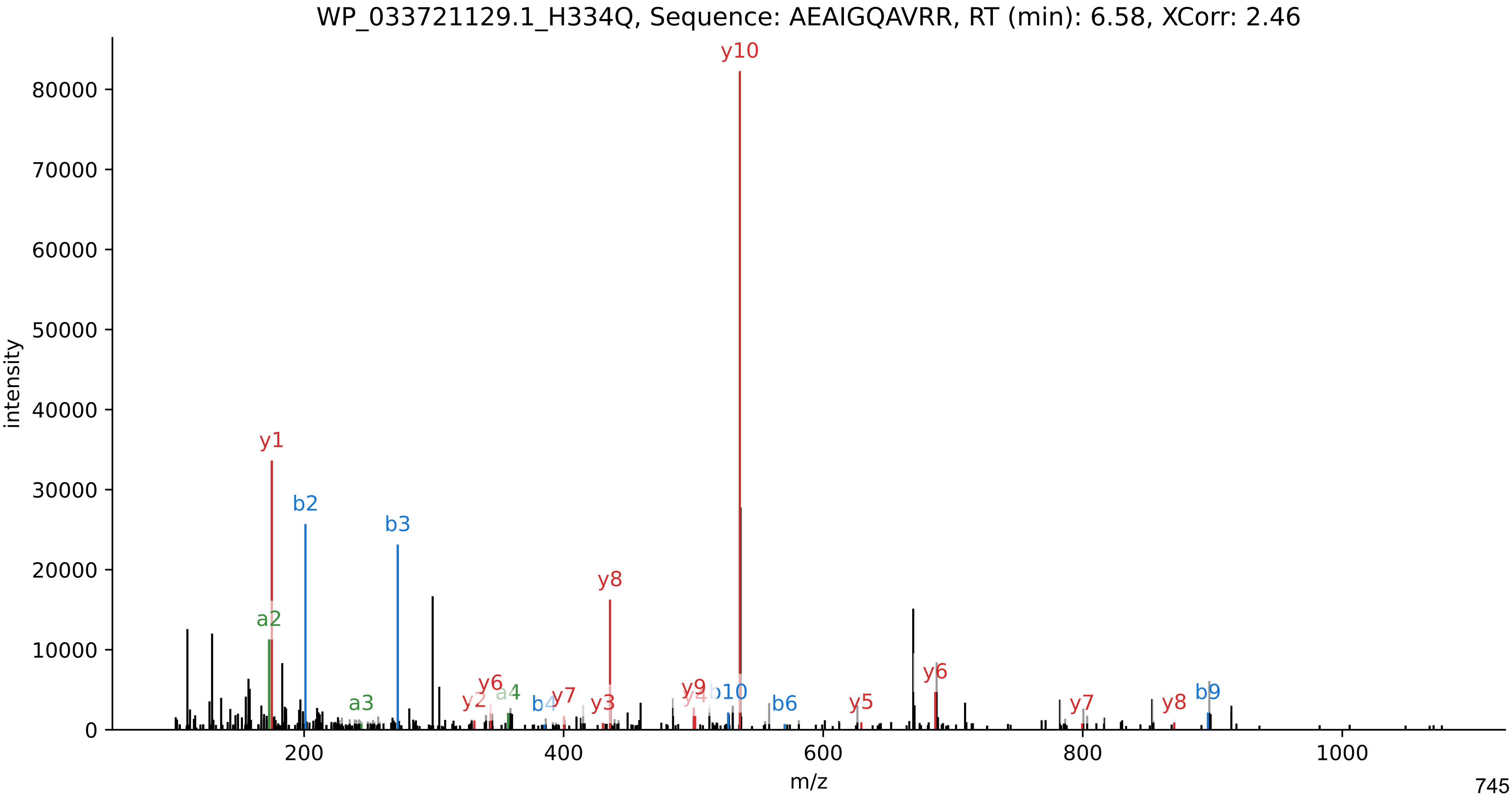

WP_033721129.1_H334Q, Sequence: AEAIGQAVRR, RT (min): 6.58, XCorr: 2.46
intensity
m/z
y10
y1
b2
b3
a2
y8
a3
y2
y6
a4
b4
y7
y3
y9
y4
b10
b6
y5
y6
y7
y8
b9

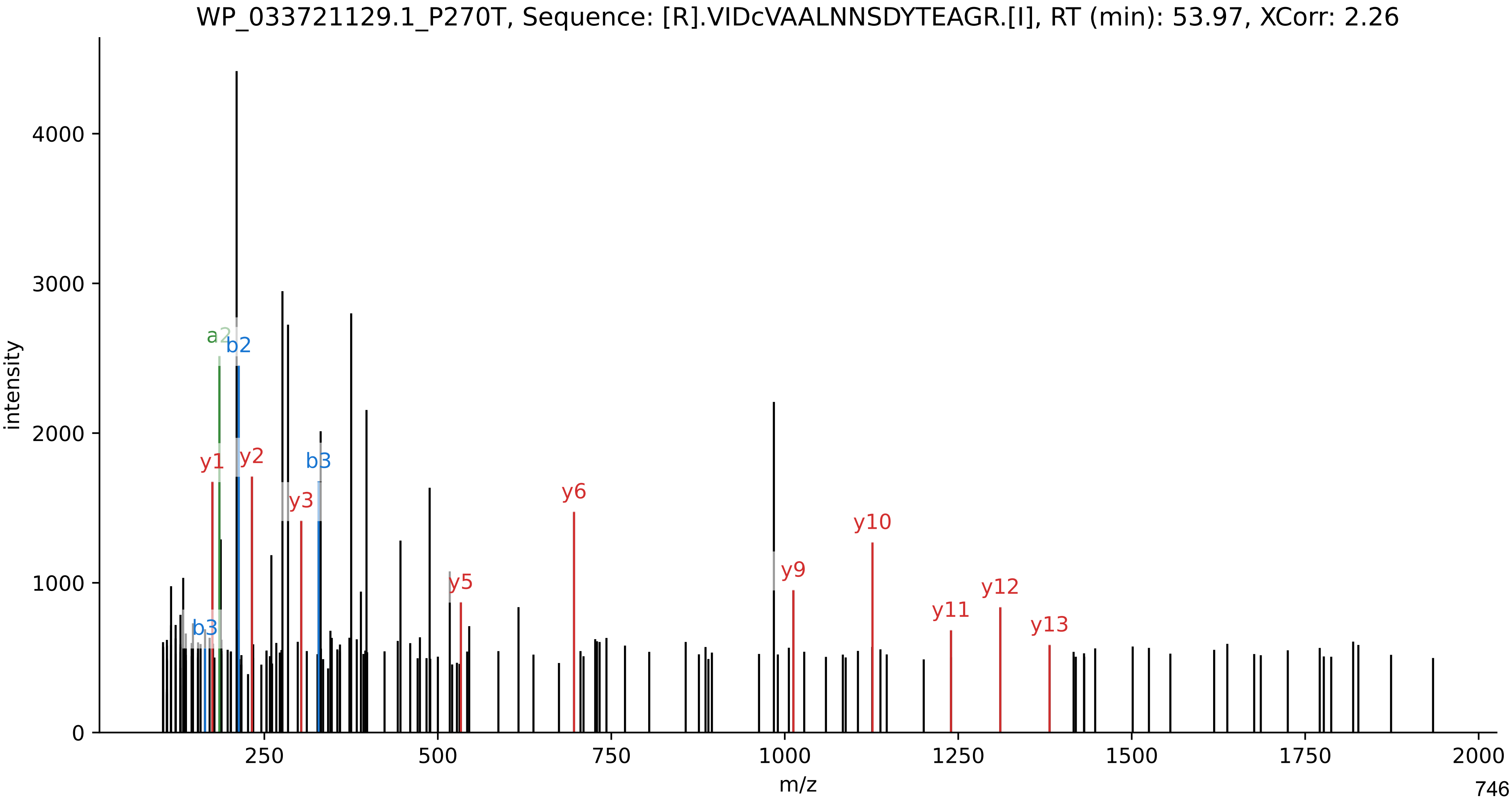
WP_033721129.1_P270T, Sequence: [R].VIDcVAALNNSDYTEAGR.[I], RT (min): 53.97, XCorr: 2.26
intensity
m/z
a2
b2
b3
y1
y2
b3
y3
y5
y6
y9
y10
y11
y12
y13
0
1000
2000
3000
4000
250
500
750
1000
1250
1500
1750
2000

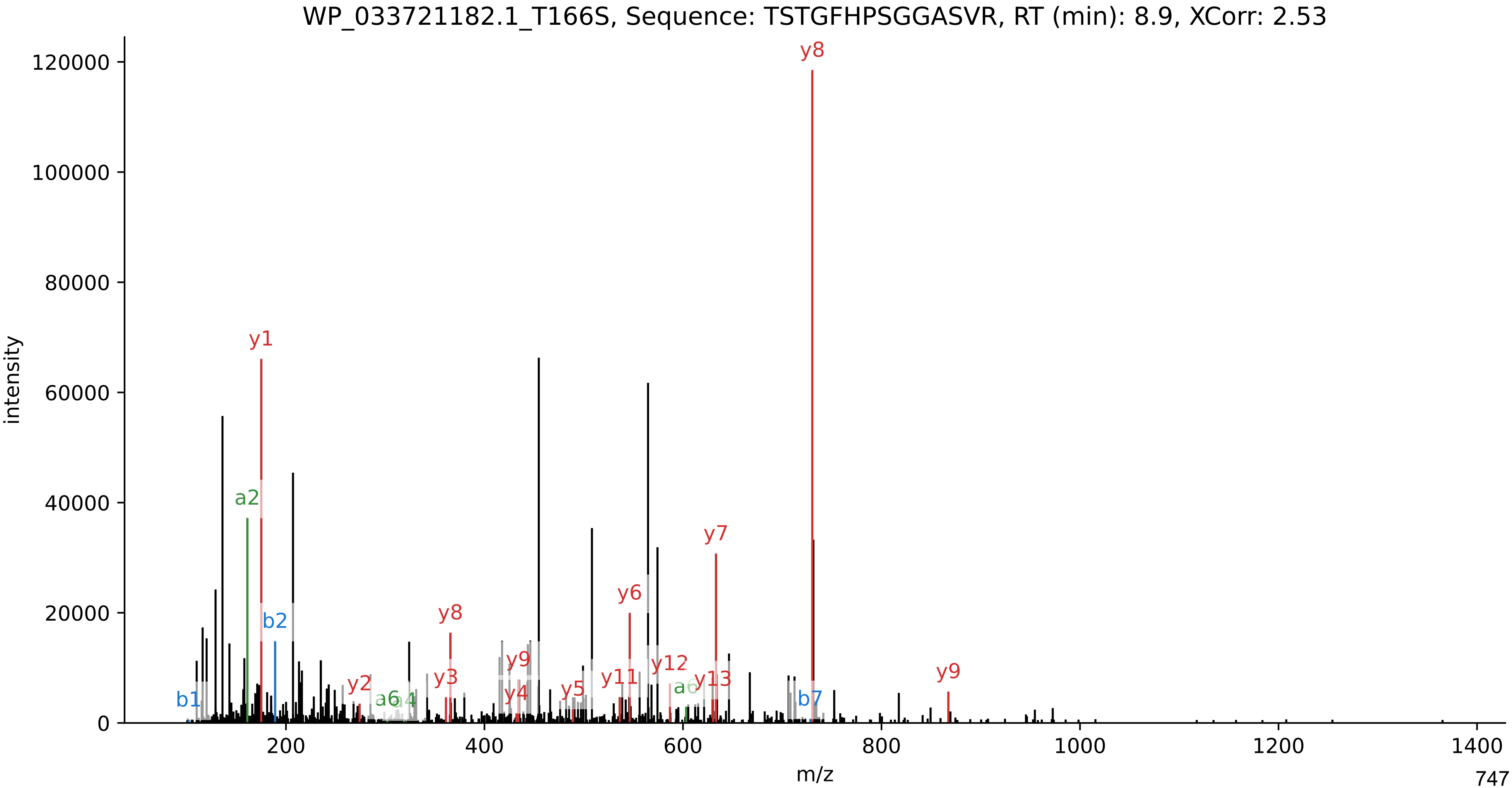

WP_033721182.1_T166S, Sequence: TSTGFHPSGGASVR, RT (min): 8.9, XCorr: 2.53
intensity
m/z
y8
y1
a2
b2
y8
y7
y6
y9
y3
y2
y4
y5
y11
y12
a6
y13
b7
y9
b1
a6
a4
0
20000
40000
60000
80000
100000
120000
200
400
600
800
1000
1200
1400

WP_033721280.1_A31T, Sequence: VAAATEAAGIHGFGFTDHPAPSQR, RT (min): 43.98, XCorr: 6.66

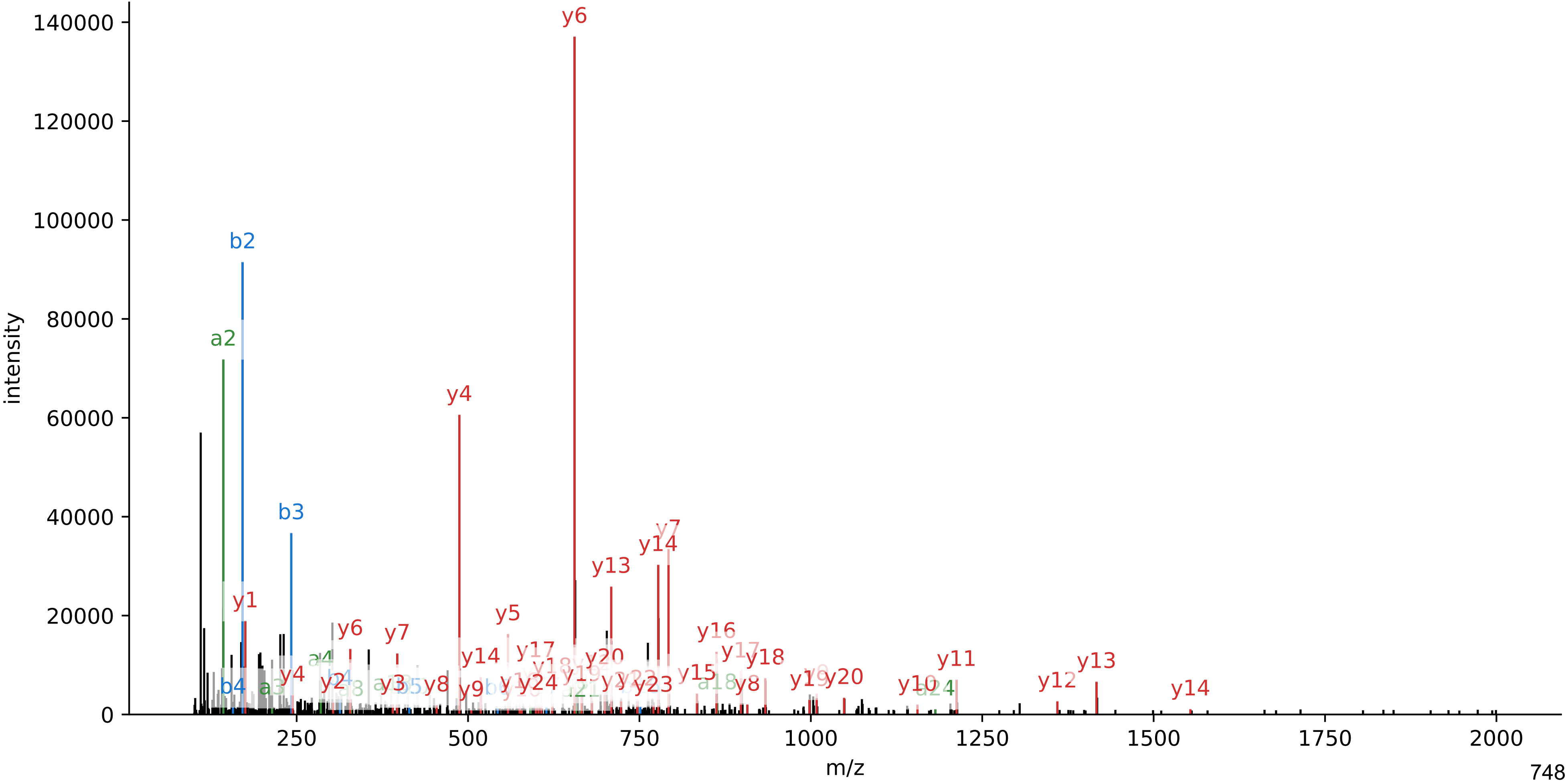

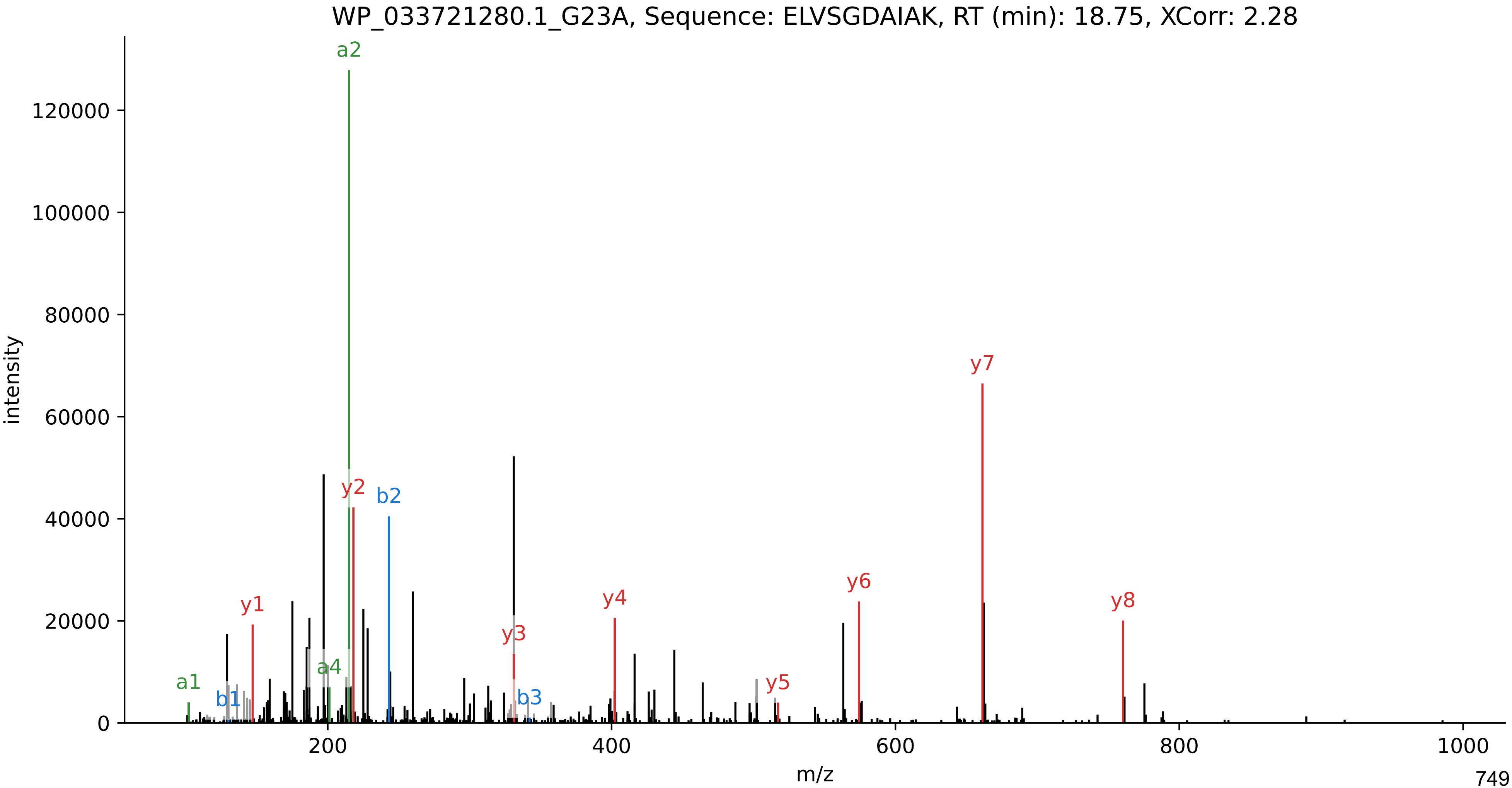

WP_033721280.1_G23A, Sequence: ELVSGDAIAK, RT (min): 18.75, XCorr: 2.28
intensity
m/z
a1
b1
y1
a4
y2
a2
b2
y3
b3
y4
y5
y6
y7
y8
0
20000
40000
60000
80000
100000
120000
200
400
600
800
1000

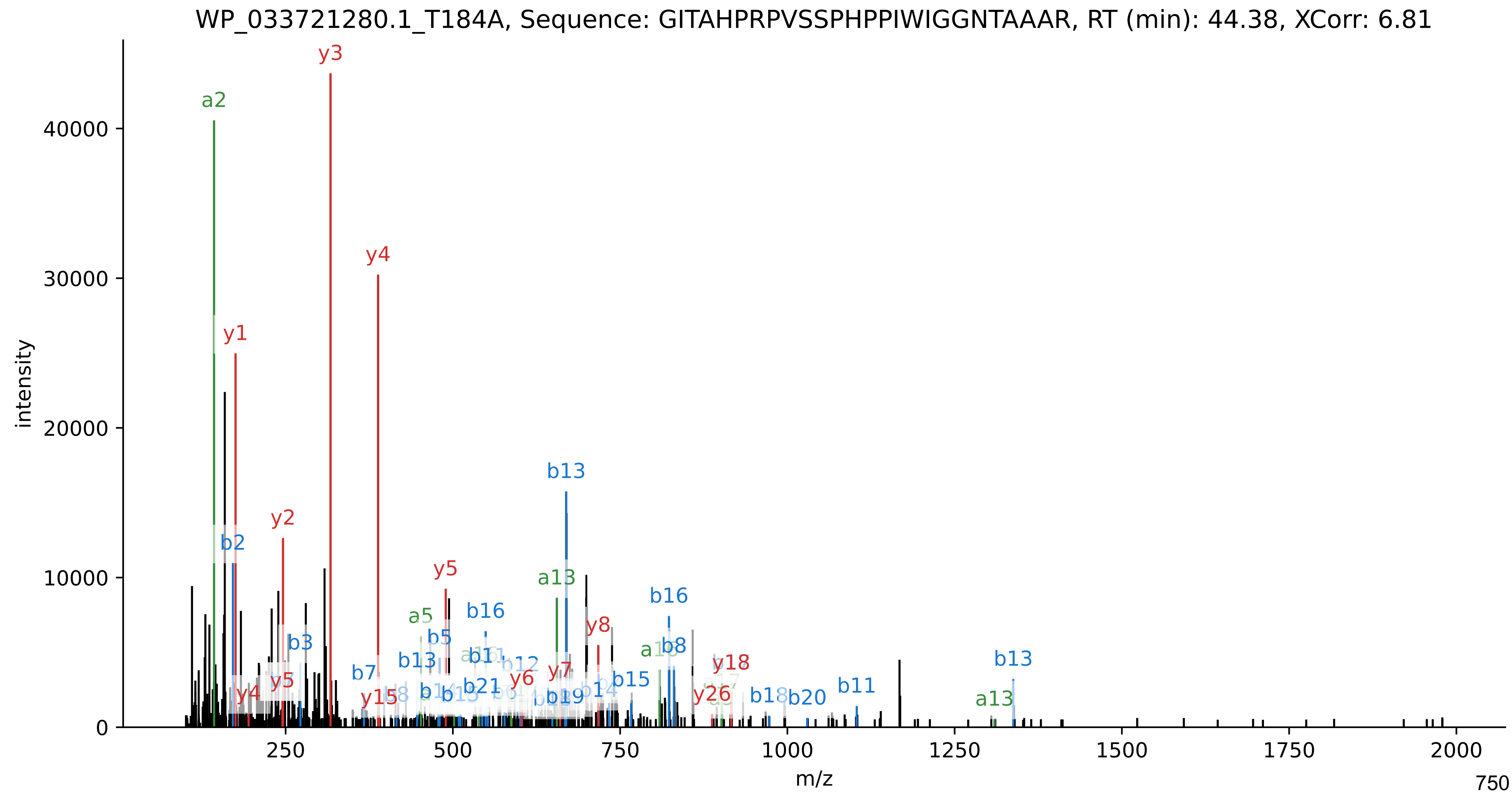

WP_033721280.1_T184A, Sequence: GITAHPRPVSSPHPPIWIGGNTAAAR, RT (min): 44.38, XCorr: 6.81
intensity
m/z

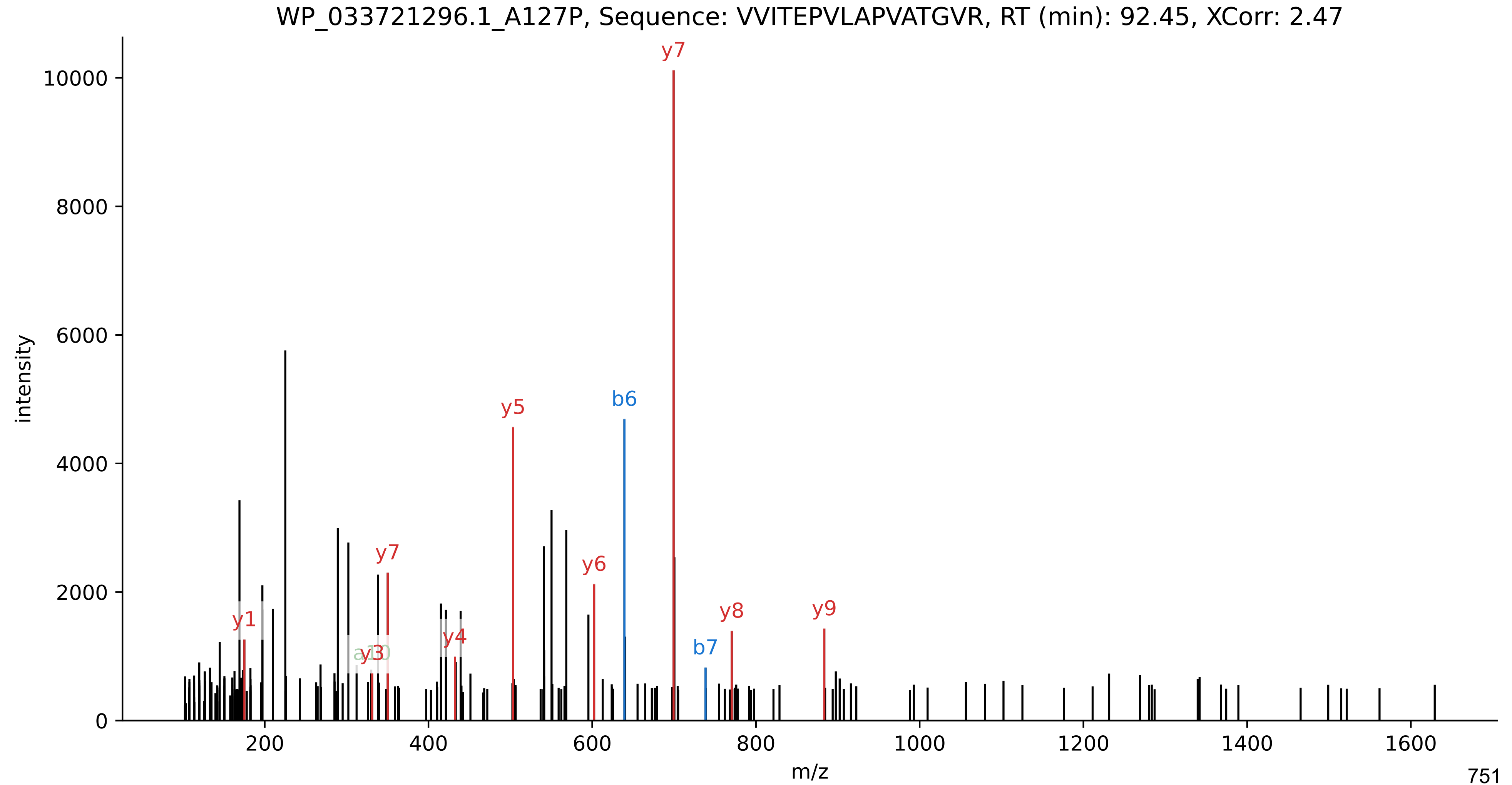

WP_033721296.1_A127P, Sequence: VVITEPVLAPVATGVR, RT (min): 92.45, XCorr: 2.47
intensity
m/z
y7
y5
b6
y7
y6
y1
y3
y4
b7
y8
y9

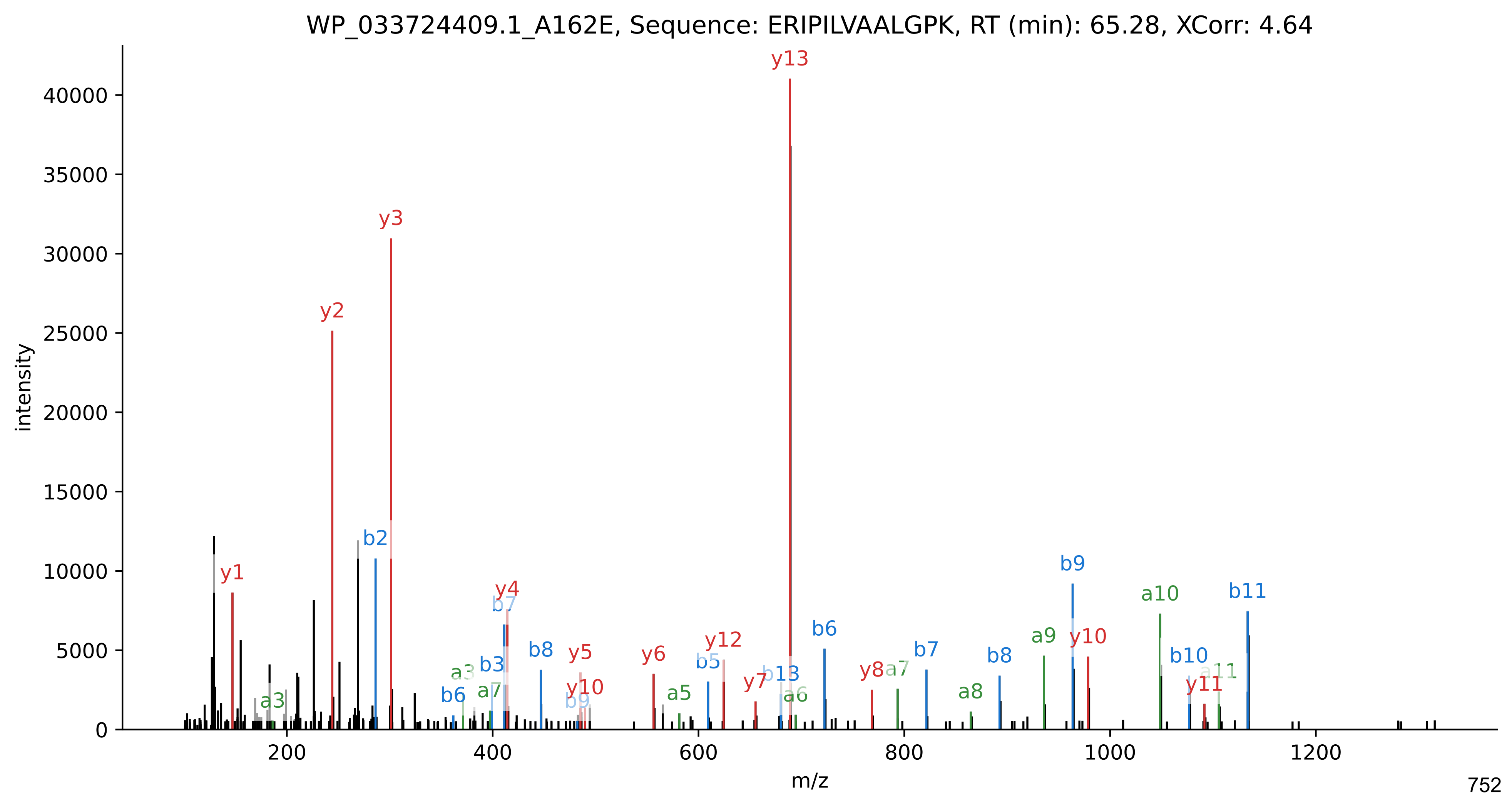

WP_033724409.1_A162E, Sequence: ERIPILVAALGPK, RT (min): 65.28, XCorr: 4.64
intensity
m/z
y13
y3
y2
b2
y1
b9
a10
b11
y4
b7
b6
y12
a9
y10
b8
y5
y6
b5
b7
b8
b10
a3
b3
y10
y8
a7
a11
b6
a7
y7
b13
y11
a3
a5
a6
a8
b9

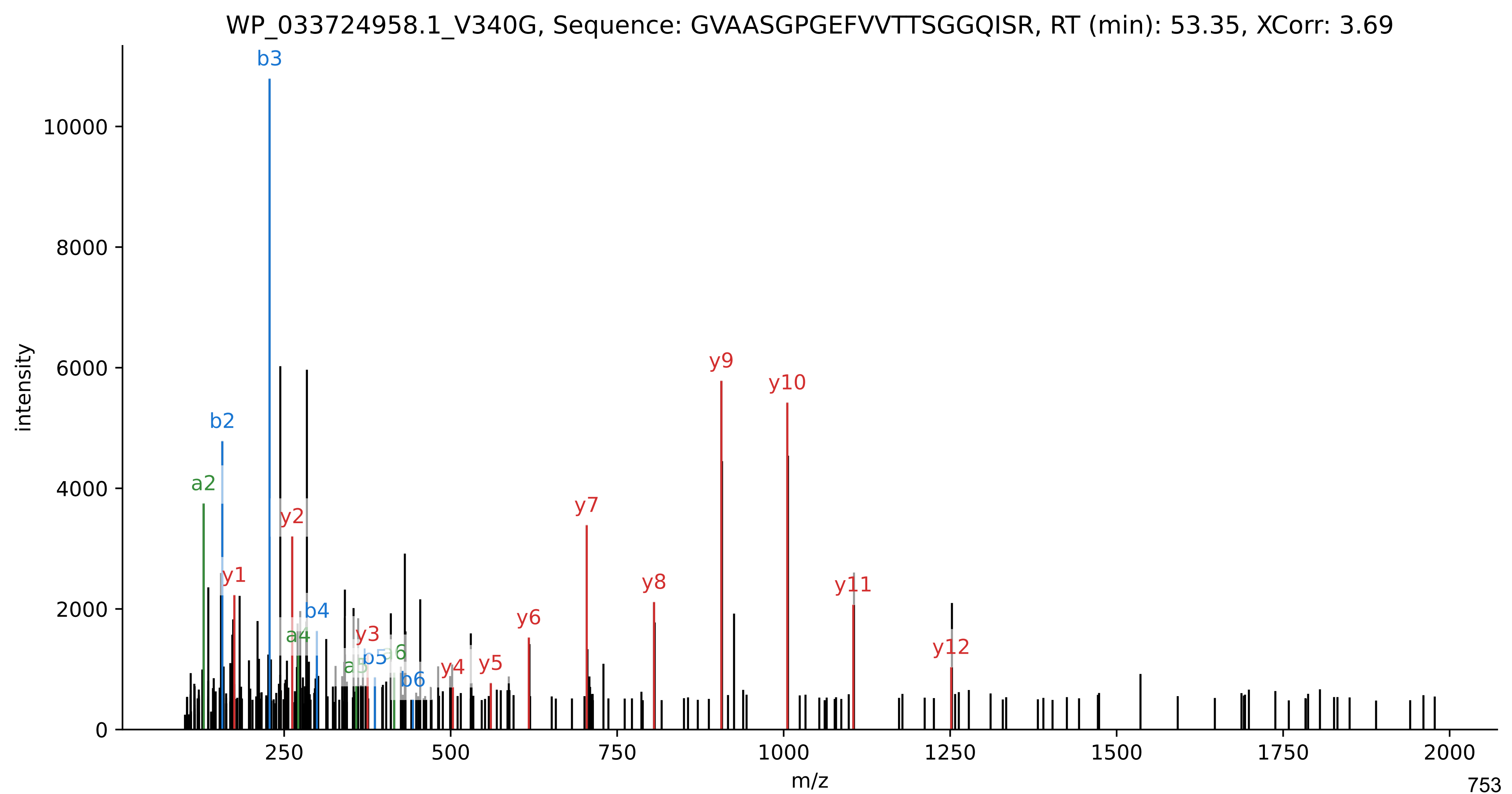

WP_033724958.1_V340G, Sequence: GVAASGPGEFVVTTSGGQISR, RT (min): 53.35, XCorr: 3.69
intensity
m/z
b3
b2
a2
y1
y2
b4
a4
y3
b5
b6
y4
y5
y6
y7
y8
y9
y10
y11
y12
0
2000
4000
6000
8000
10000
250
500
750
1000
1250
1500
1750
2000

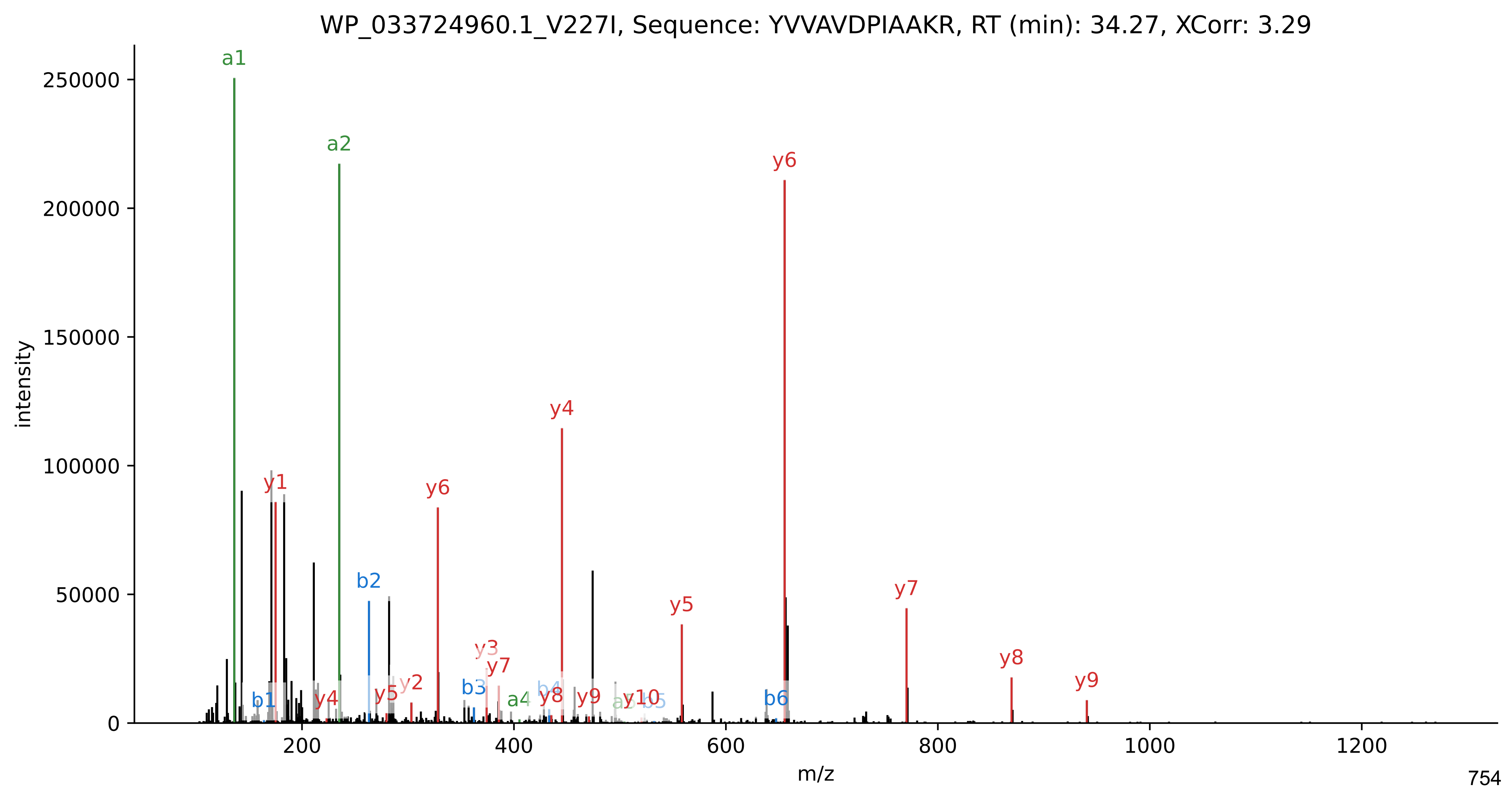

WP_033724960.1_V227I, Sequence: YVVAVDPIAAKR, RT (min): 34.27, XCorr: 3.29
intensity
m/z
a1
a2
y6
y4
y1
y6
b2
y7
y5
y3
y7
y2
y8
y9
b1
y4
y5
b3
a4
b4
y8
y9
y10
b5
b6

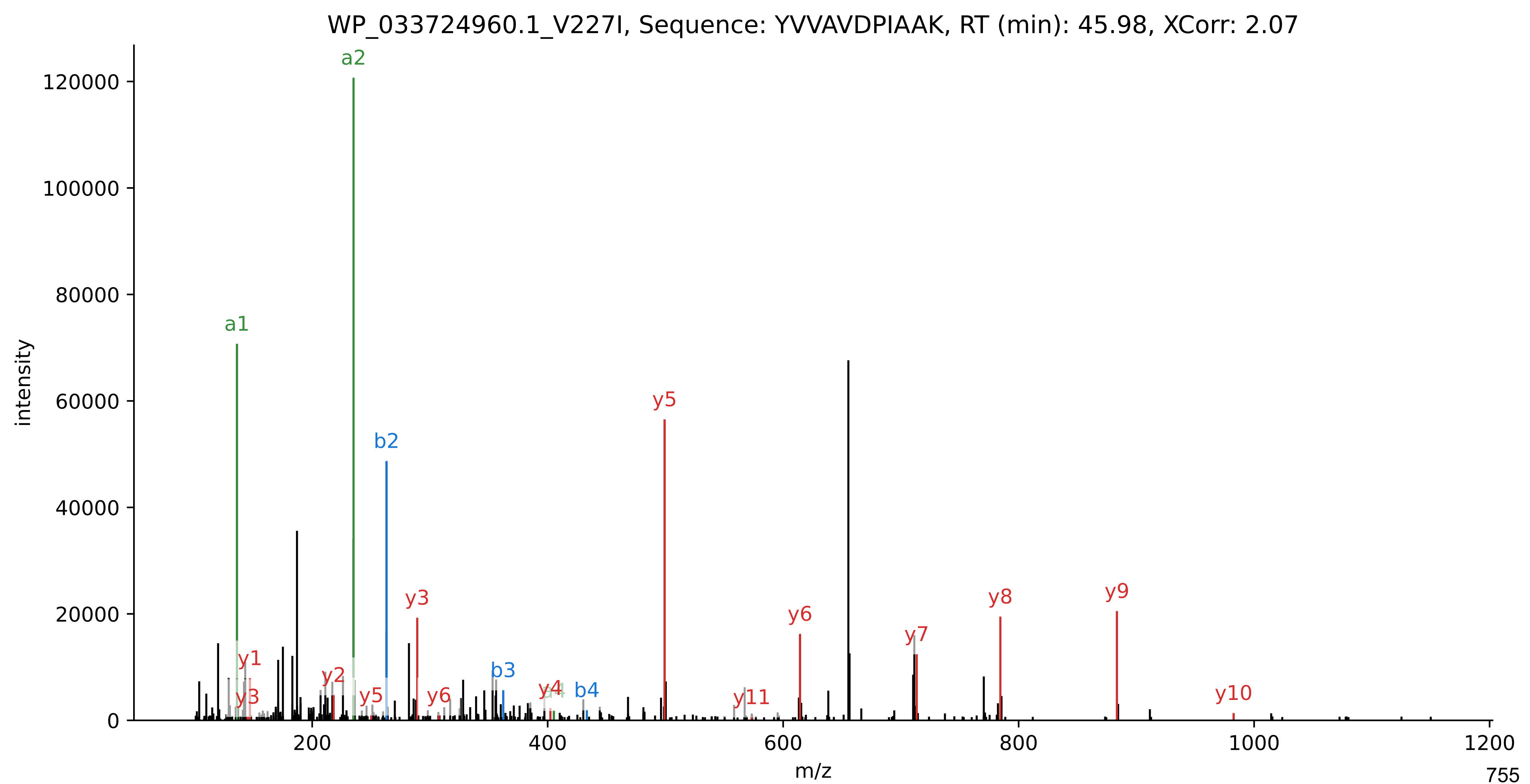

WP_033724960.1_V227I, Sequence: YVVAVDPIAAK, RT (min): 45.98, XCorr: 2.07
intensity
m/z
a1
a2
b2
b3
b4
y1
y2
y3
y5
y6
y4
y11
y6
y7
y8
y9
y10
y3
y5

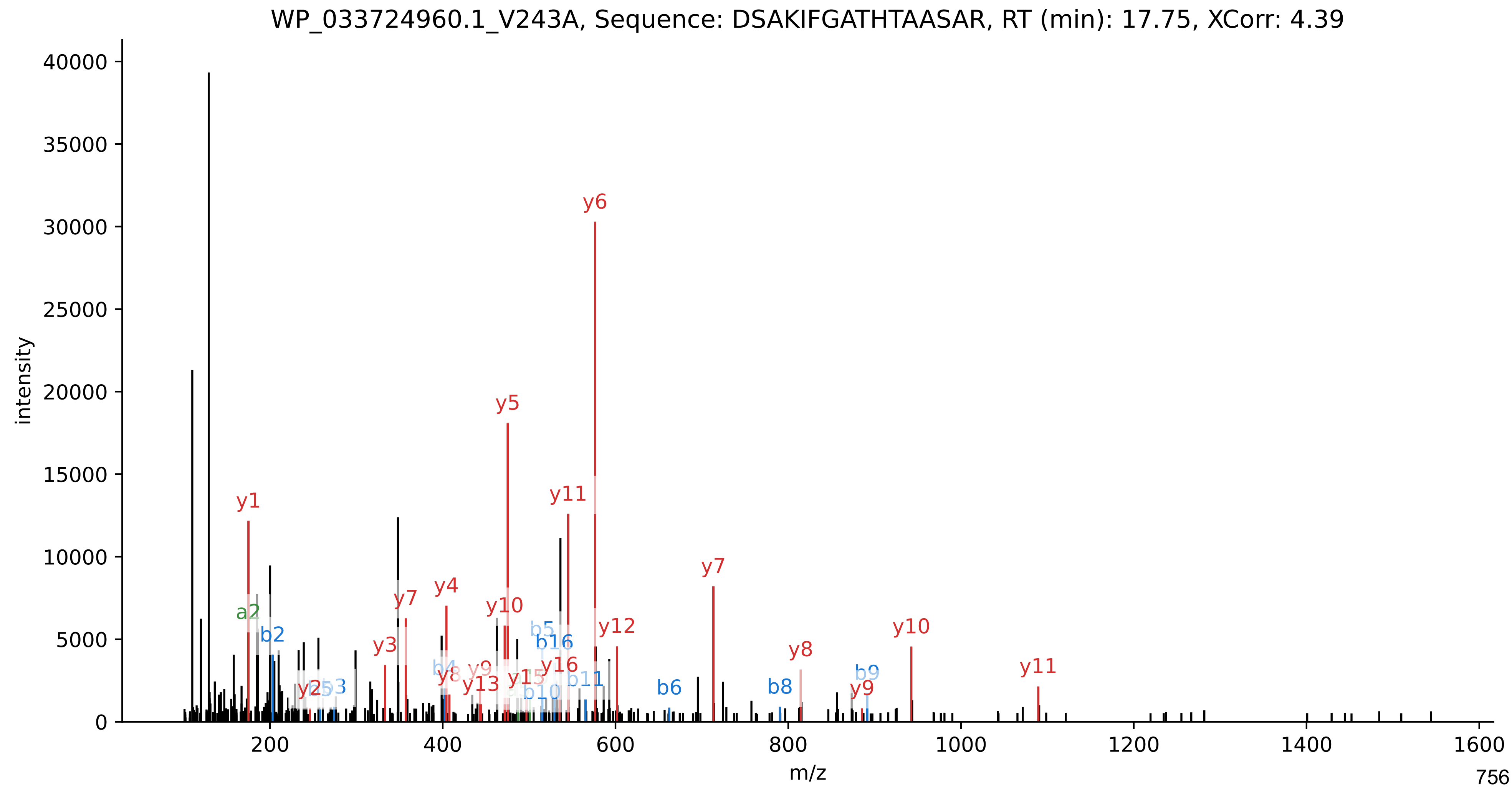

WP_033724960.1_V243A, Sequence: DSAKIFGATHTAASAR, RT (min): 17.75, XCorr: 4.39
intensity
m/z
y6
y5
y1
y11
y7
y4
y7
y10
a2
b2
y12
y10
b5
b16
y3
y8
y16
b4
y9
y8
y11
y15
b11
b3
y2
y13
b10
b6
b8
b9
y9

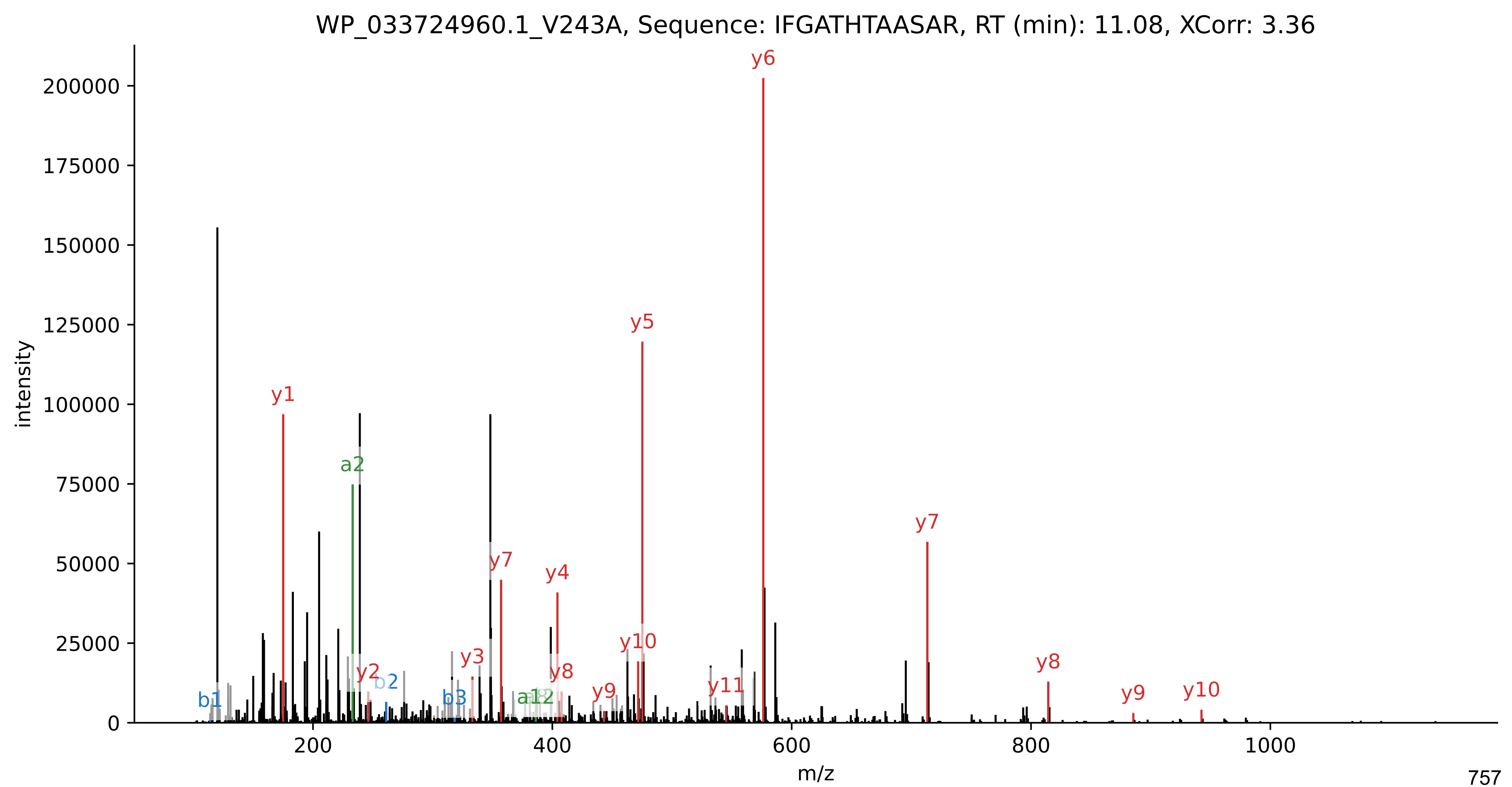

WP_033724960.1_V243A, Sequence: IFGATHTAASAR, RT (min): 11.08, XCorr: 3.36
intensity
m/z
y6
y5
y1
a2
y7
y4
y10
y3
y2
y8
b1
b2
b3
y9
y11
y7
y8
y9
y10

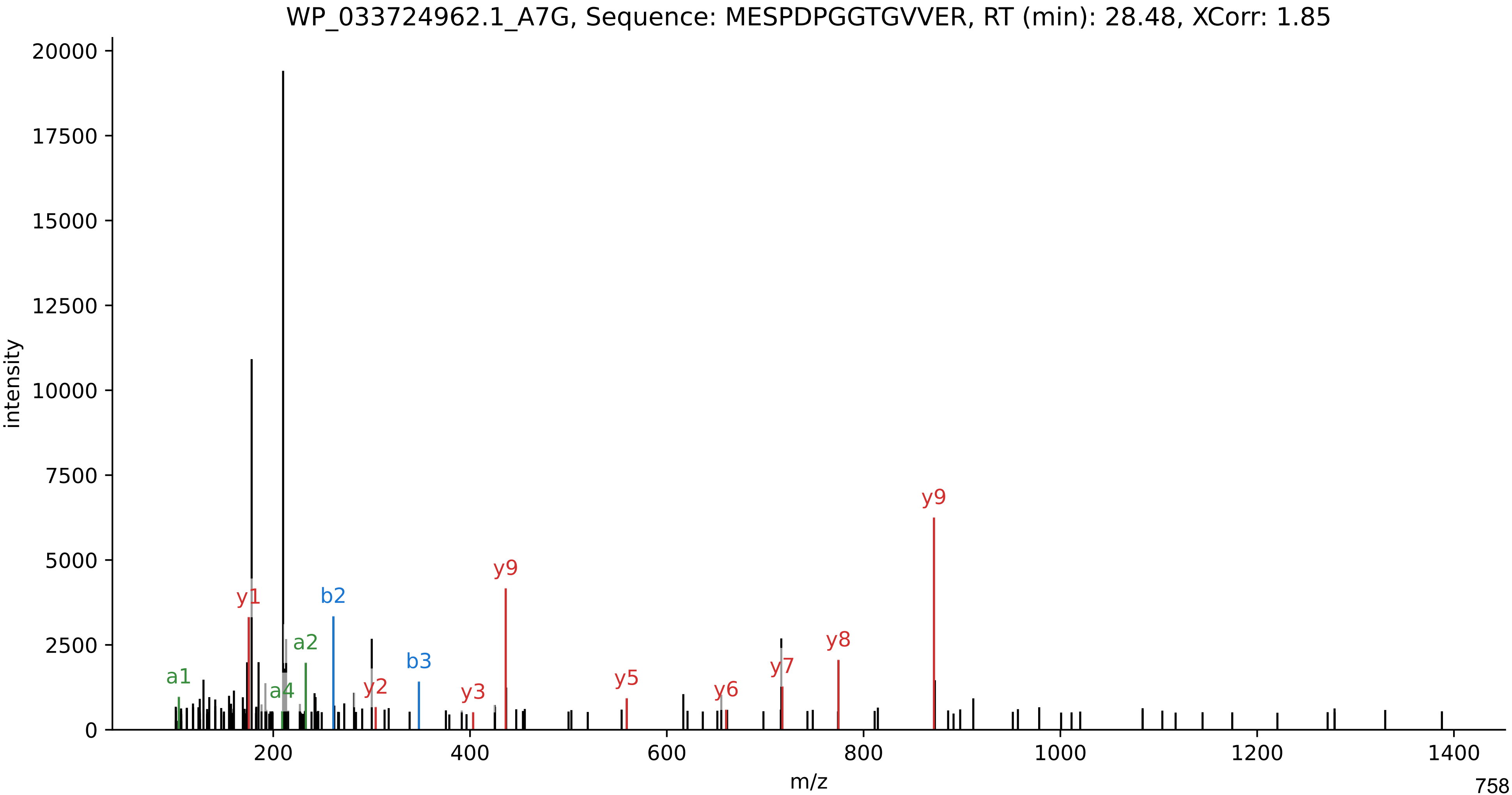
WP_033724962.1_A7G, Sequence: MESPDPGGTGVVER, RT (min): 28.48, XCorr: 1.85
intensity
m/z
a1
y1
a4
a2
b2
b3
y2
y3
y9
y5
y6
y7
y8
y9
0
2500
5000
7500
10000
12500
15000
17500
20000
200
400
600
800
1000
1200
1400

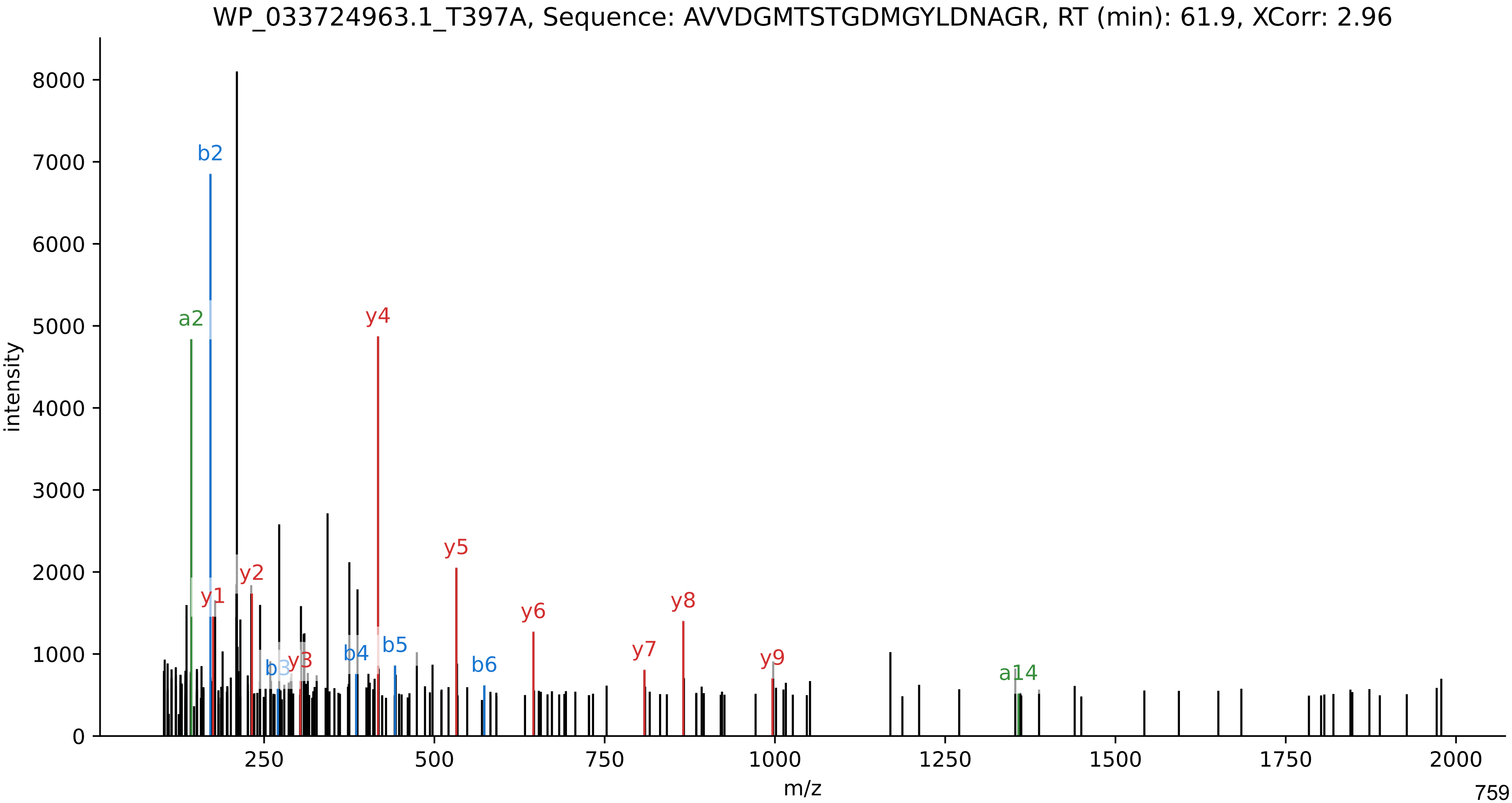

WP_033724963.1_T397A, Sequence: AVVDGMTSTGDMGYLDNAGR, RT (min): 61.9, XCorr: 2.96
intensity
m/z
b2
a2
y4
y5
y2
y1
y6
y8
y7
y9
b4
b5
b3
y3
b6
a14

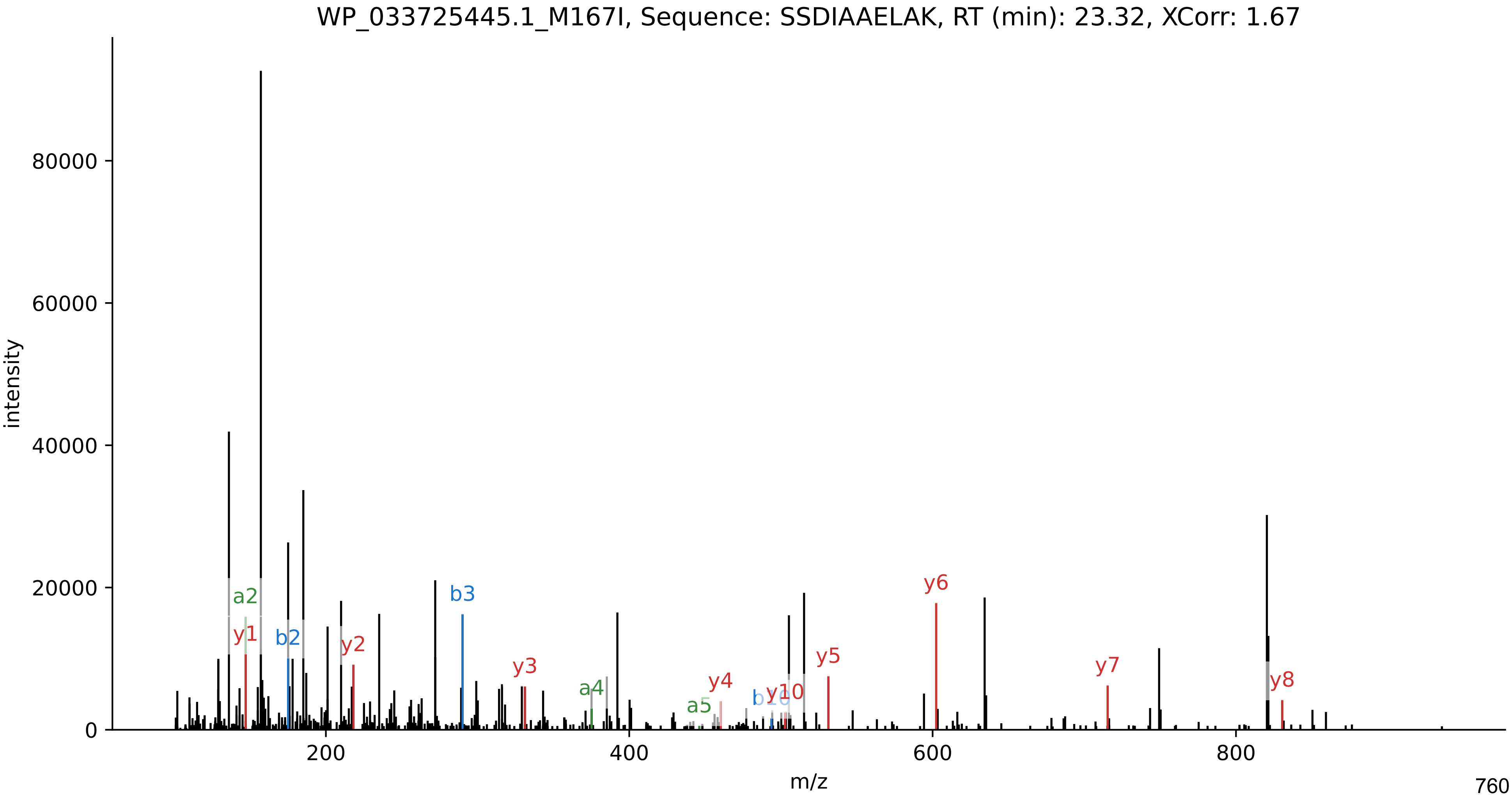

WP_033725445.1_M167I, Sequence: SSDIAAELAK, RT (min): 23.32, XCorr: 1.67
intensity
m/z
a2
y1
b2
y2
b3
y3
a4
a5
y4
b10
y10
y5
y6
y7
y8

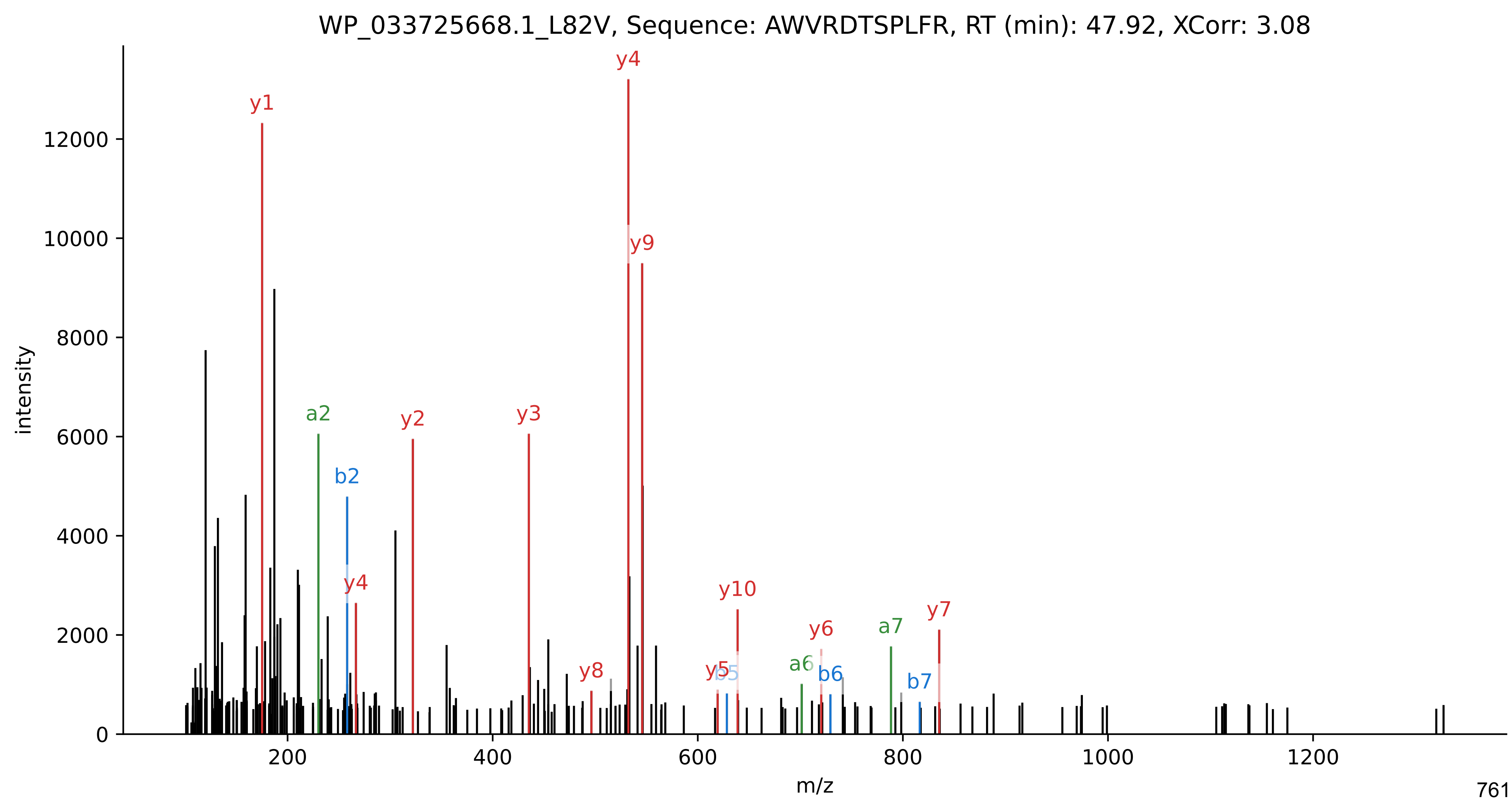
WP_033725668.1_L82V, Sequence: AWVRDTSPLFR, RT (min): 47.92, XCorr: 3.08
intensity
m/z
y1
y4
y9
a2
y2
y3
b2
y4
y10
y6
a7
y7
y8
y5
b5
a6
b6
b7
0
2000
4000
6000
8000
10000
12000
200
400
600
800
1000
1200

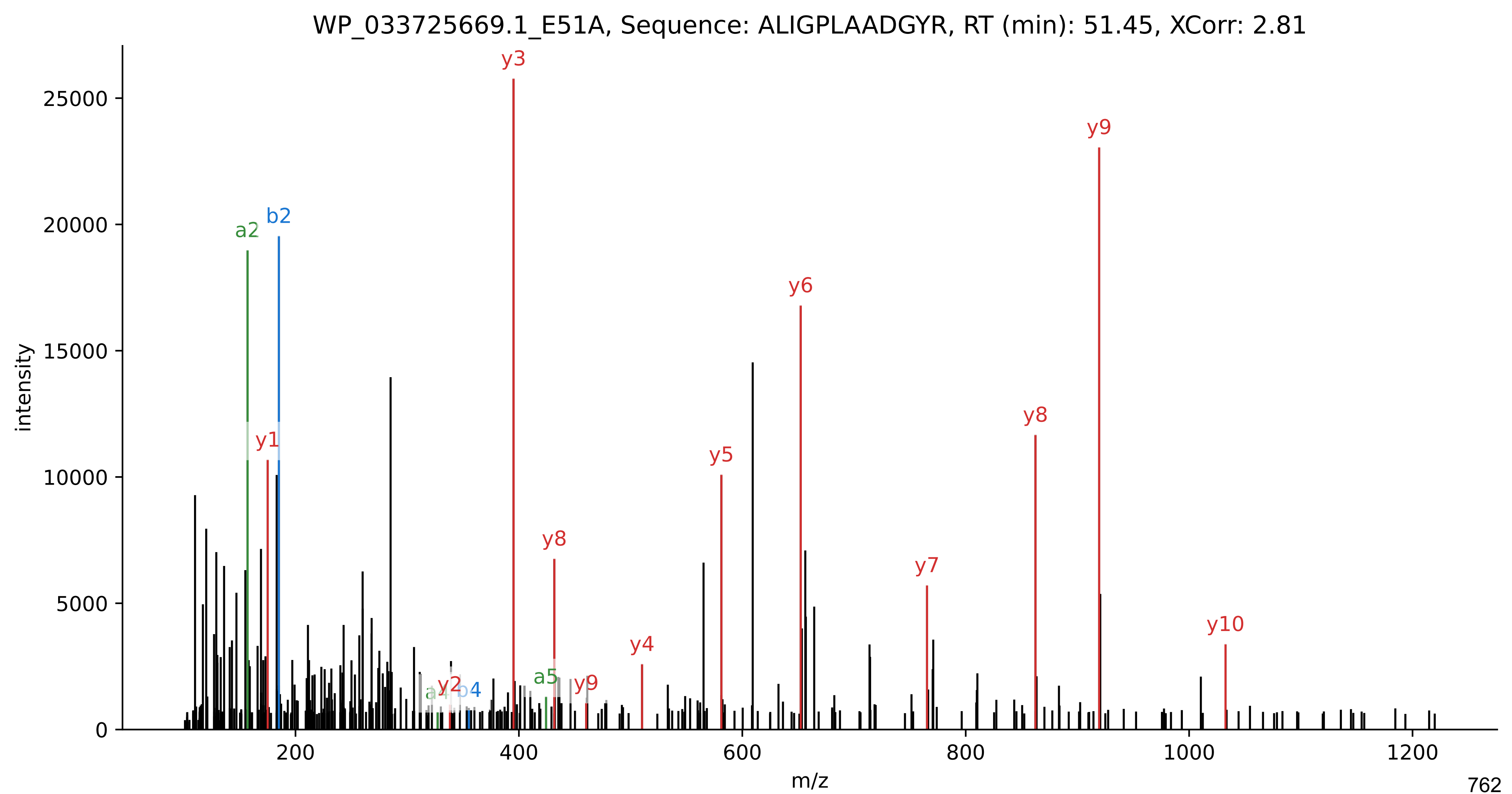
WP_033725669.1_E51A, Sequence: ALIGPLAADGYR, RT (min): 51.45, XCorr: 2.81
intensity
m/z
y3
y9
b2
a2
y6
y8
y1
y5
y8
y7
y10
y4
a5
y9
y2
b4
0
5000
10000
15000
20000
25000
200
400
600
800
1000
1200

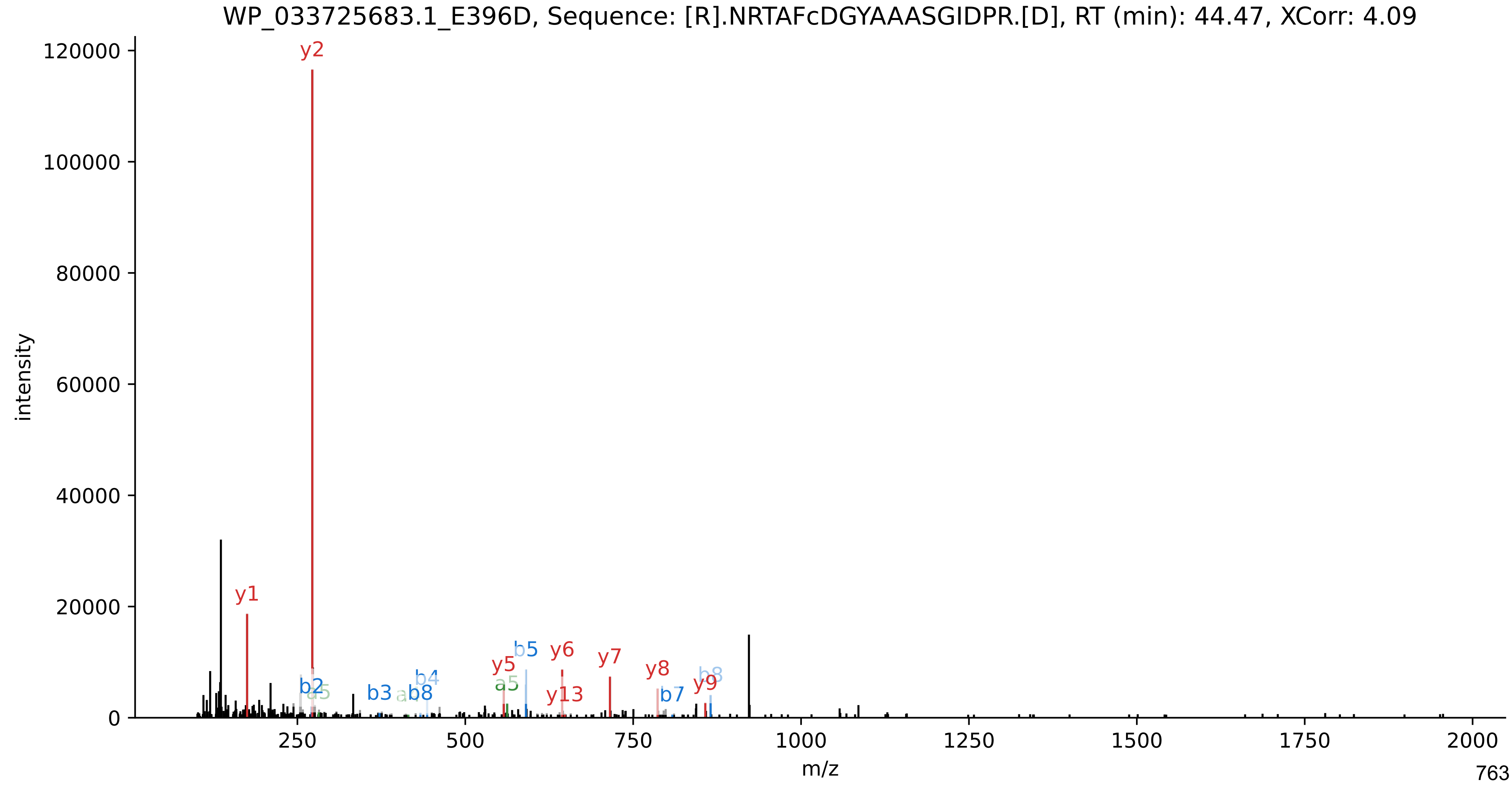

WP_033725683.1_E396D, Sequence: [R].NRTAFcDGYAAASGIDPR.[D], RT (min): 44.47, XCorr: 4.09
intensity
120000
100000
80000
60000
40000
20000
0
250
500
750
1000
1250
1500
1750
2000
m/z
y2
y1
b2
a5
b3
b8
b4
y5
a5
b5
y6
y13
y7
y8
b7
y9
b8

WP_033725683.1_E396D, Sequence: [R].TAFcDGYAAASGIDPR.[D], RT (min): 51.33, XCorr: 3.67

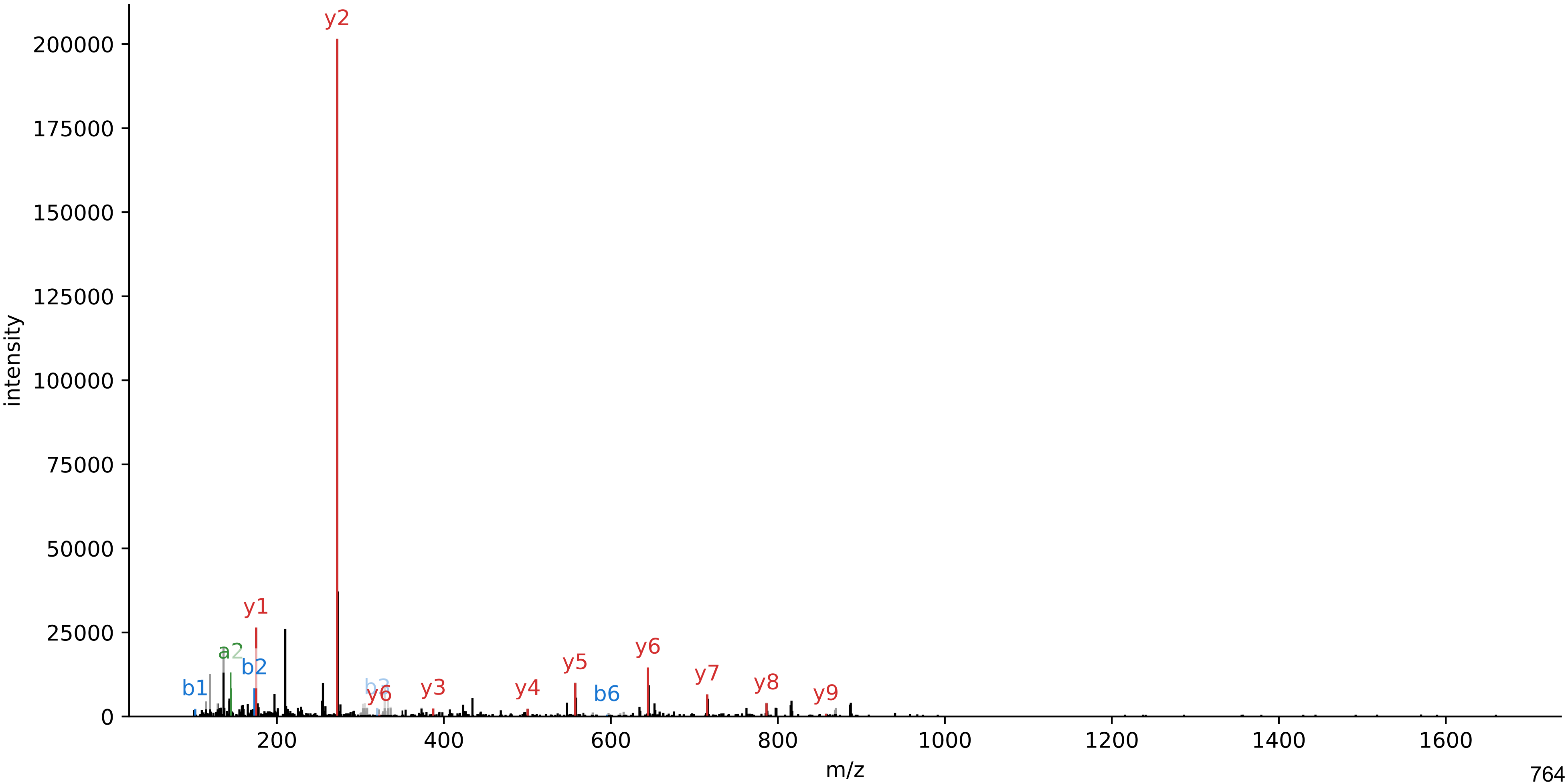

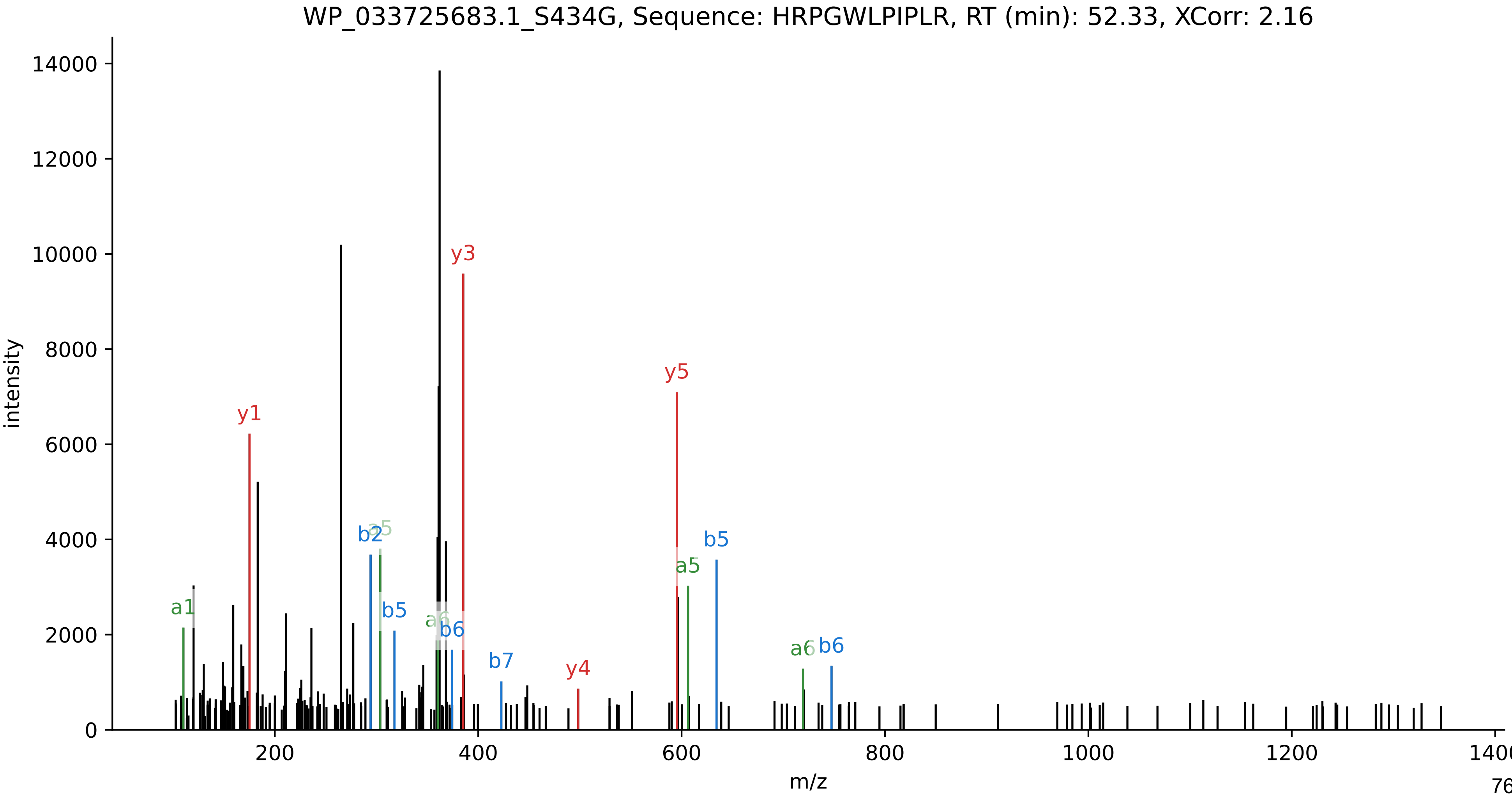

WP_033725683.1_S434G, Sequence: HRPGWLPIPLR, RT (min): 52.33, XCorr: 2.16
intensity
m/z
0
2000
4000
6000
8000
10000
12000
14000
200
400
600
800
1000
1200
1400
y1
y3
y5
y4
a1
b2
a5
b5
a6
b6
b7

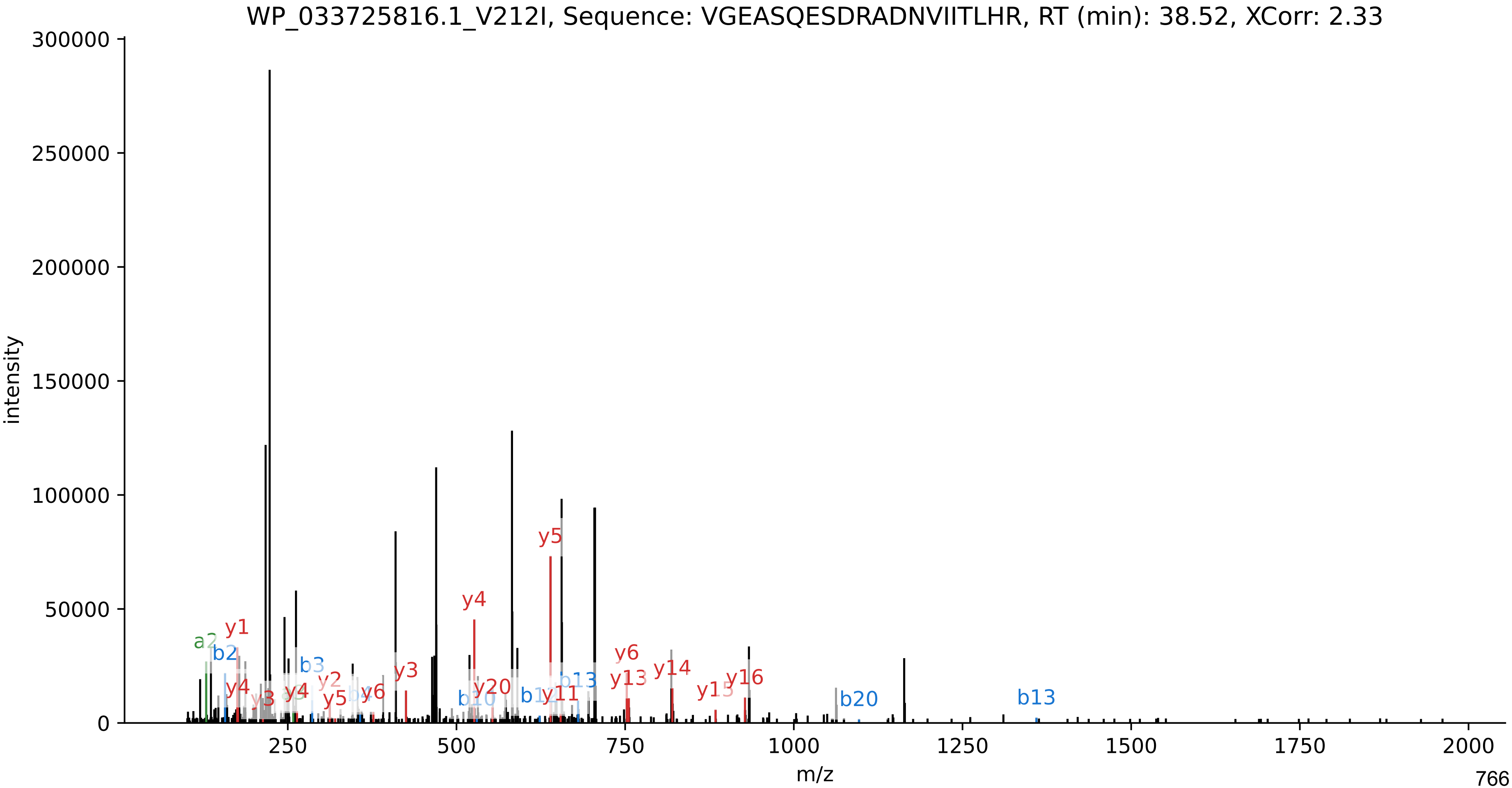

WP_033725816.1_V212I, Sequence: VGEASQESDRADNVIITLHR, RT (min): 38.52, XCorr: 2.33
intensity
m/z
a2
b2
y1
y4
y3
y4
b3
y2
y5
y6
y3
y4
b10
y20
y5
y11
b13
y6
y13
y14
y15
y16
b20
b13

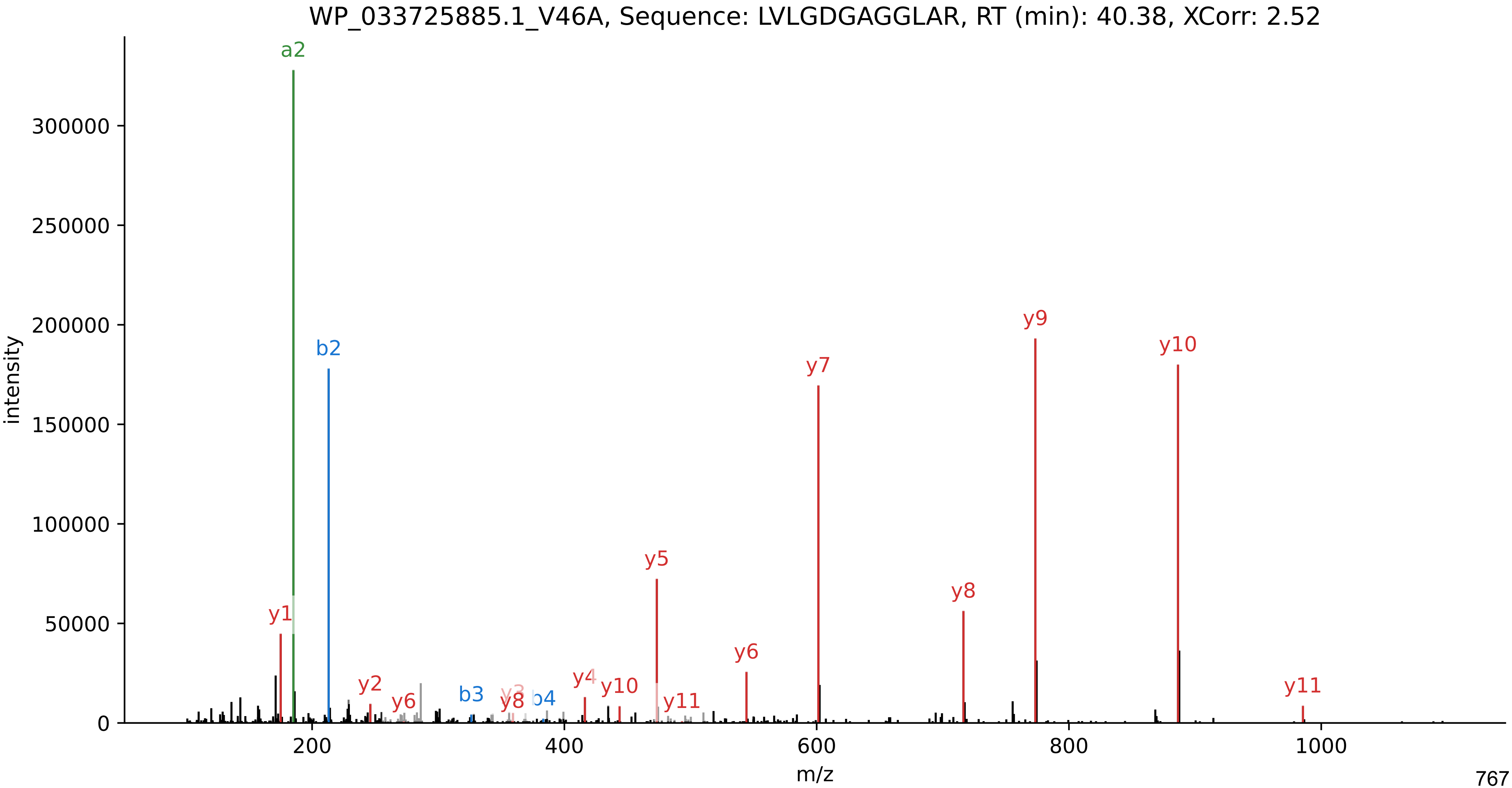

WP_033725885.1_V46A, Sequence: LVLGDGAGGLAR, RT (min): 40.38, XCorr: 2.52
intensity
m/z
a2
b2
y1
y2
y6
b3
y3
y8
b4
y4
y10
y5
y11
y6
y7
y8
y9
y10
y11
0
50000
100000
150000
200000
250000
300000
200
400
600
800
1000

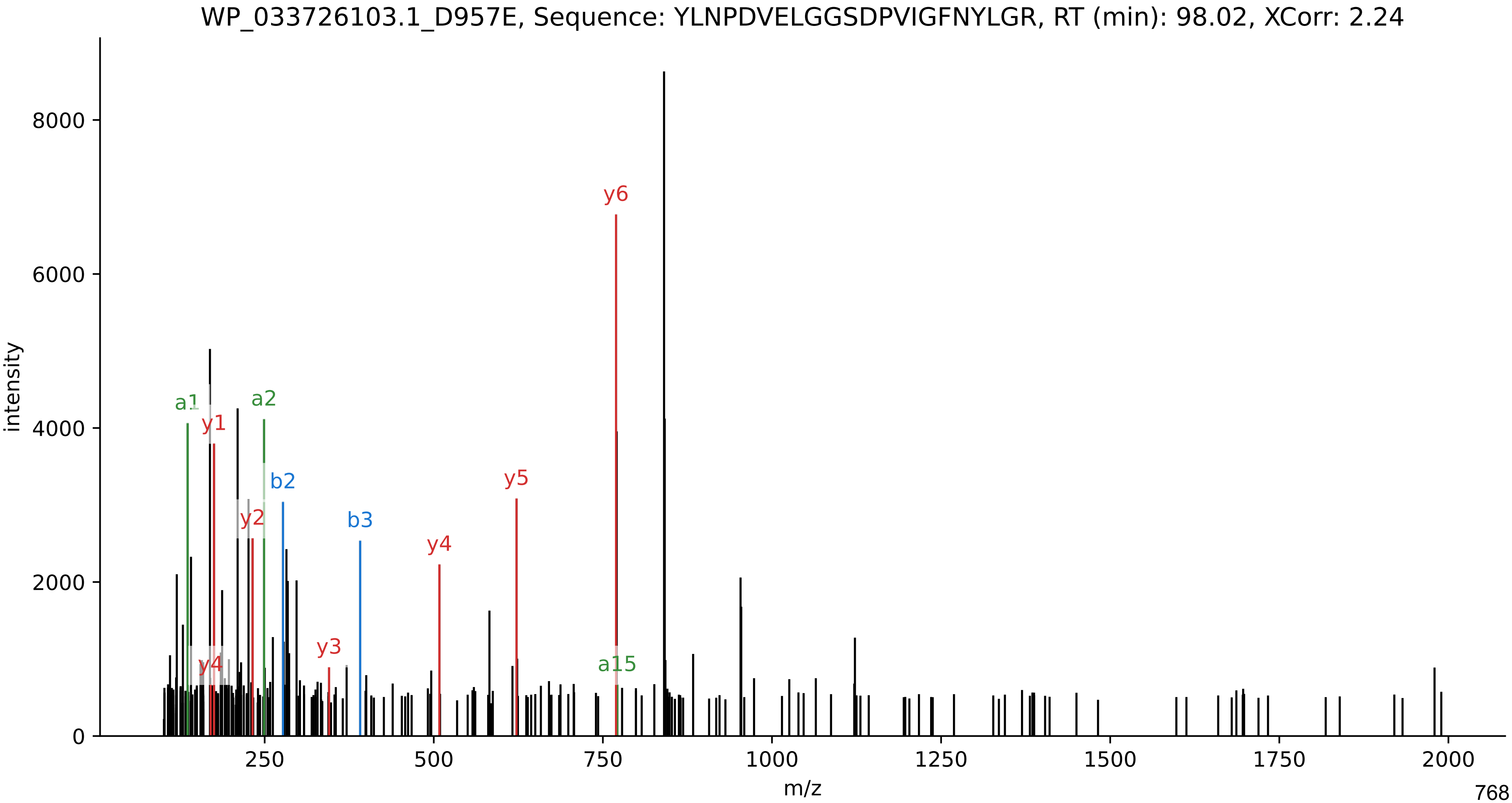

WP_033726103.1_D957E, Sequence: YLNPDVELGGSDPVIGFNYLGR, RT (min): 98.02, XCorr: 2.24
intensity
m/z
a1
y1
a2
b2
y2
b3
y4
y5
y6
y3
y4
a15

WP_033726103.1_I1162V, Sequence: [R].FcPQYGEPVQVVPADPVMAWR.[Y], RT (min): 89.85, XCorr: 2.34

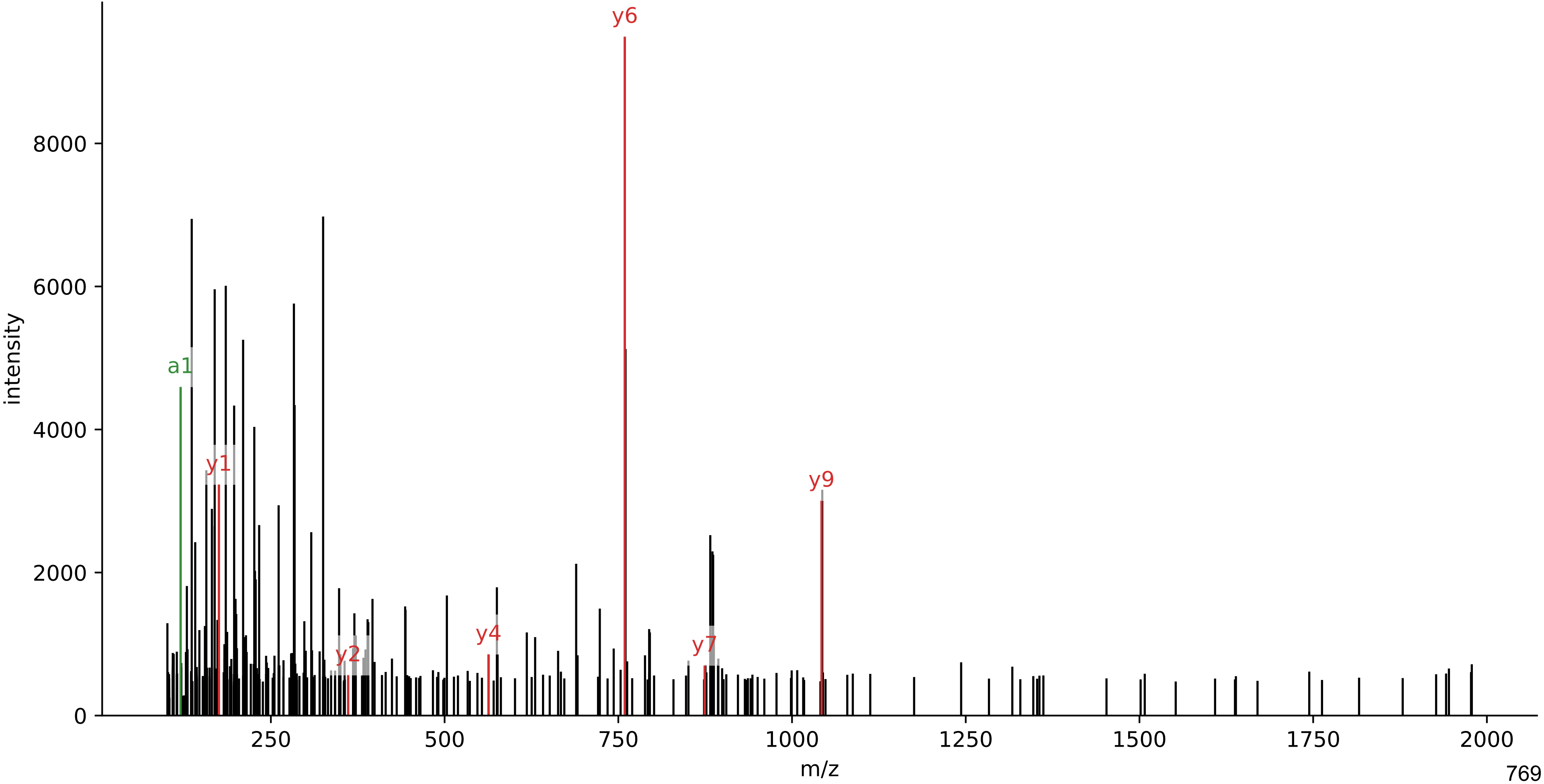

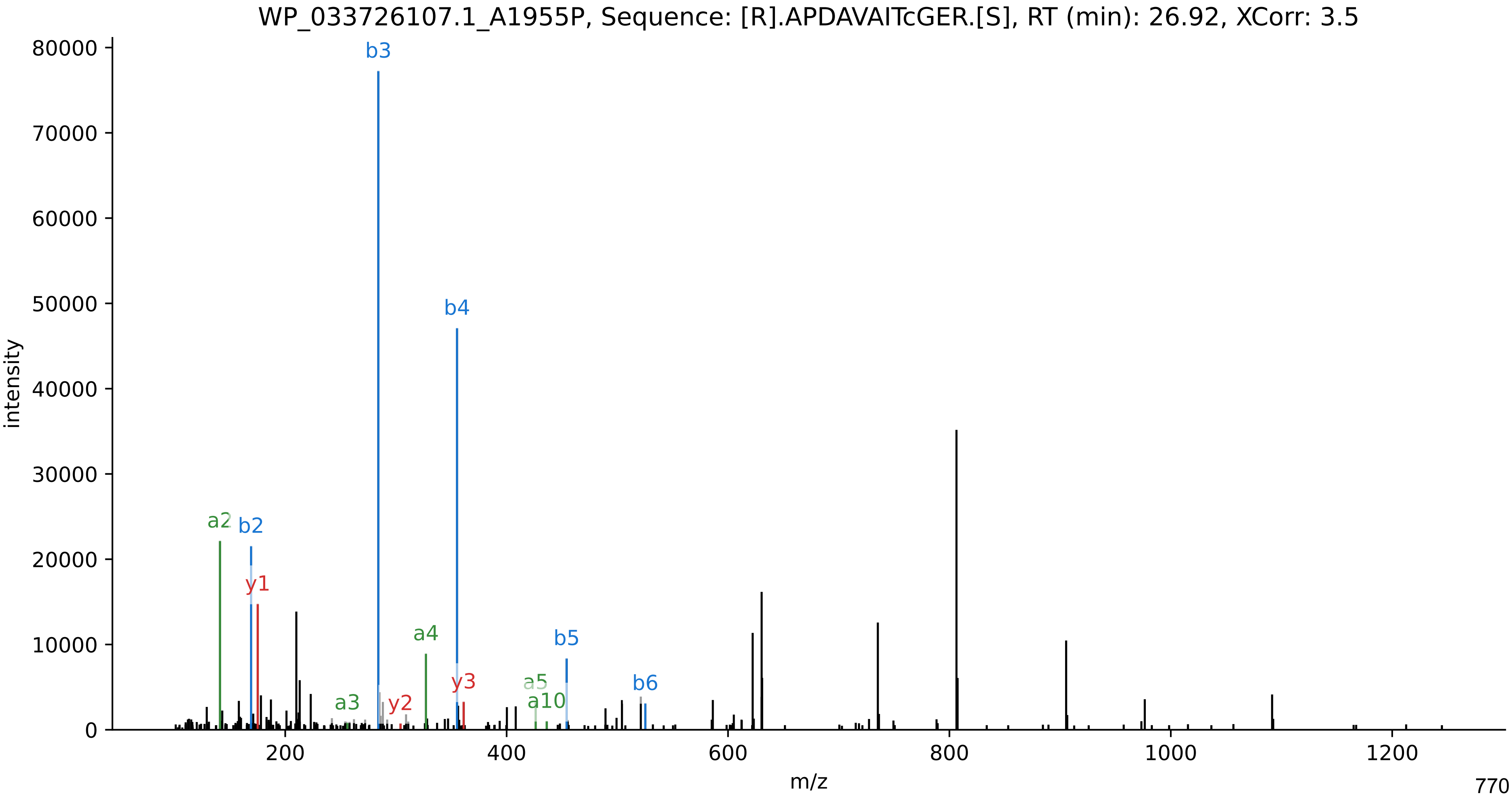
WP_033726107.1_A1955P, Sequence: [R].APDAVAITcGER.[S], RT (min): 26.92, XCorr: 3.5
intensity
m/z
b3
b4
a2
b2
y1
a4
b5
y3
a5
b6
a3
y2
a10
0
10000
20000
30000
40000
50000
60000
70000
80000
200
400
600
800
1000
1200

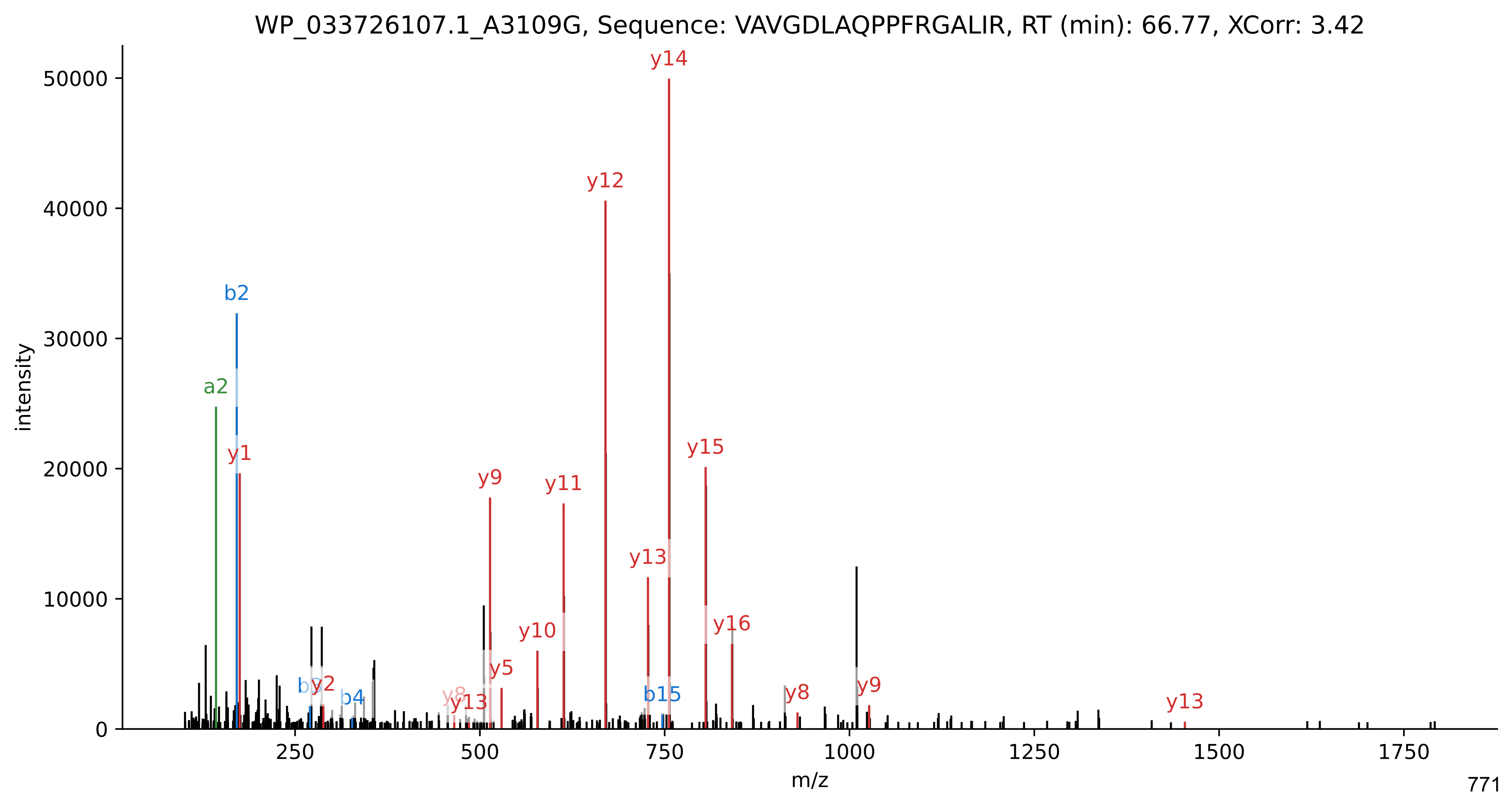

WP_033726107.1_A3109G, Sequence: VAVGDLAQPPFRGALIR, RT (min): 66.77, XCorr: 3.42
intensity
m/z
y14
y12
b2
a2
y1
y9
y11
y15
y13
y16
y10
y5
b3
y2
b4
y8
y13
b15
y8
y9
y13

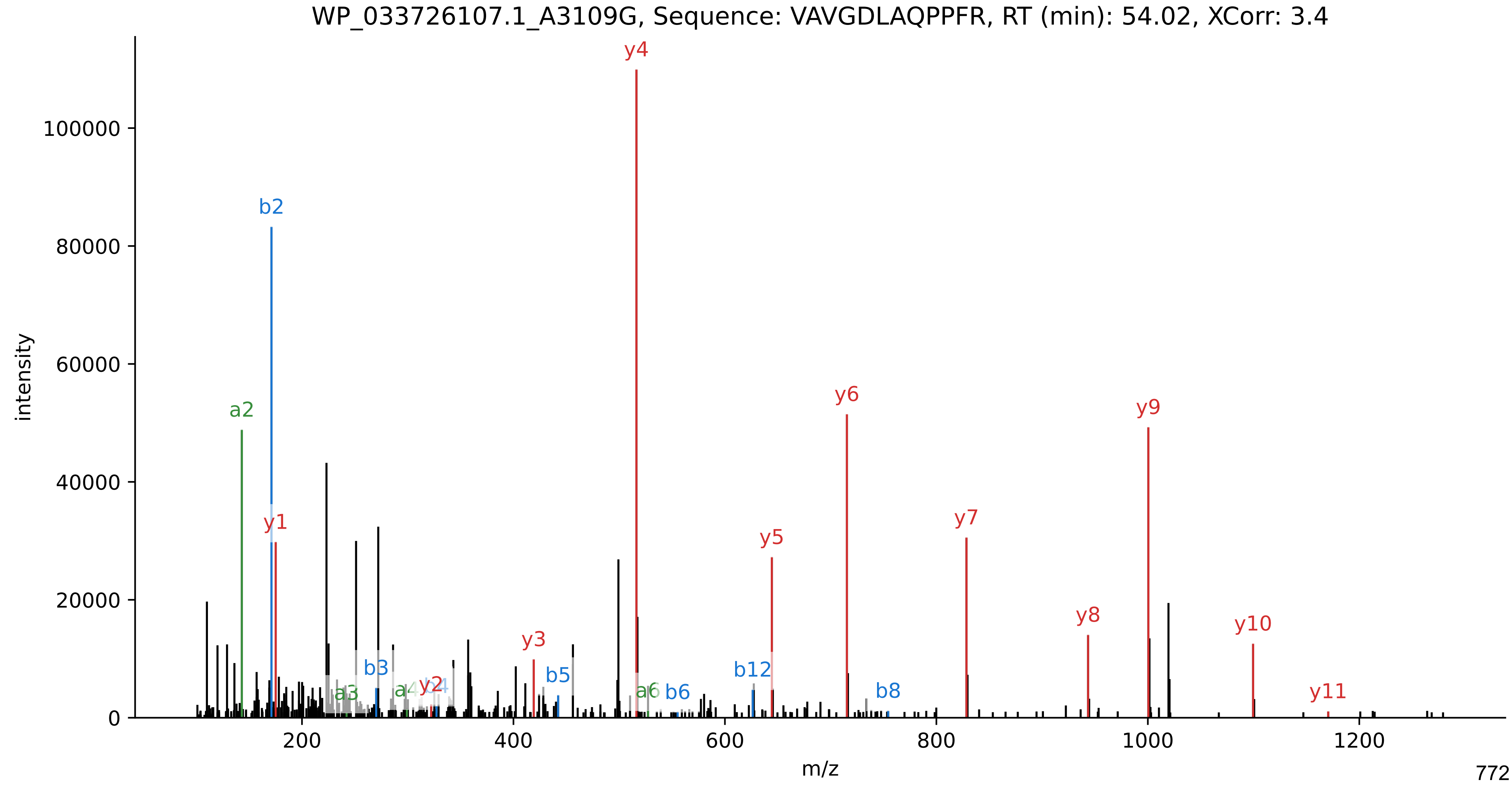
WP_033726107.1_A3109G, Sequence: VAVGDLAQPPFR, RT (min): 54.02, XCorr: 3.4
intensity
m/z
y4
b2
a2
y1
y6
y9
y7
y5
y8
y10
y3
b3
b5
b12
b8
y11
a3
a4
y2
b4
a6
b6
100000
80000
60000
40000
20000
0
200
400
600
800
1000
1200

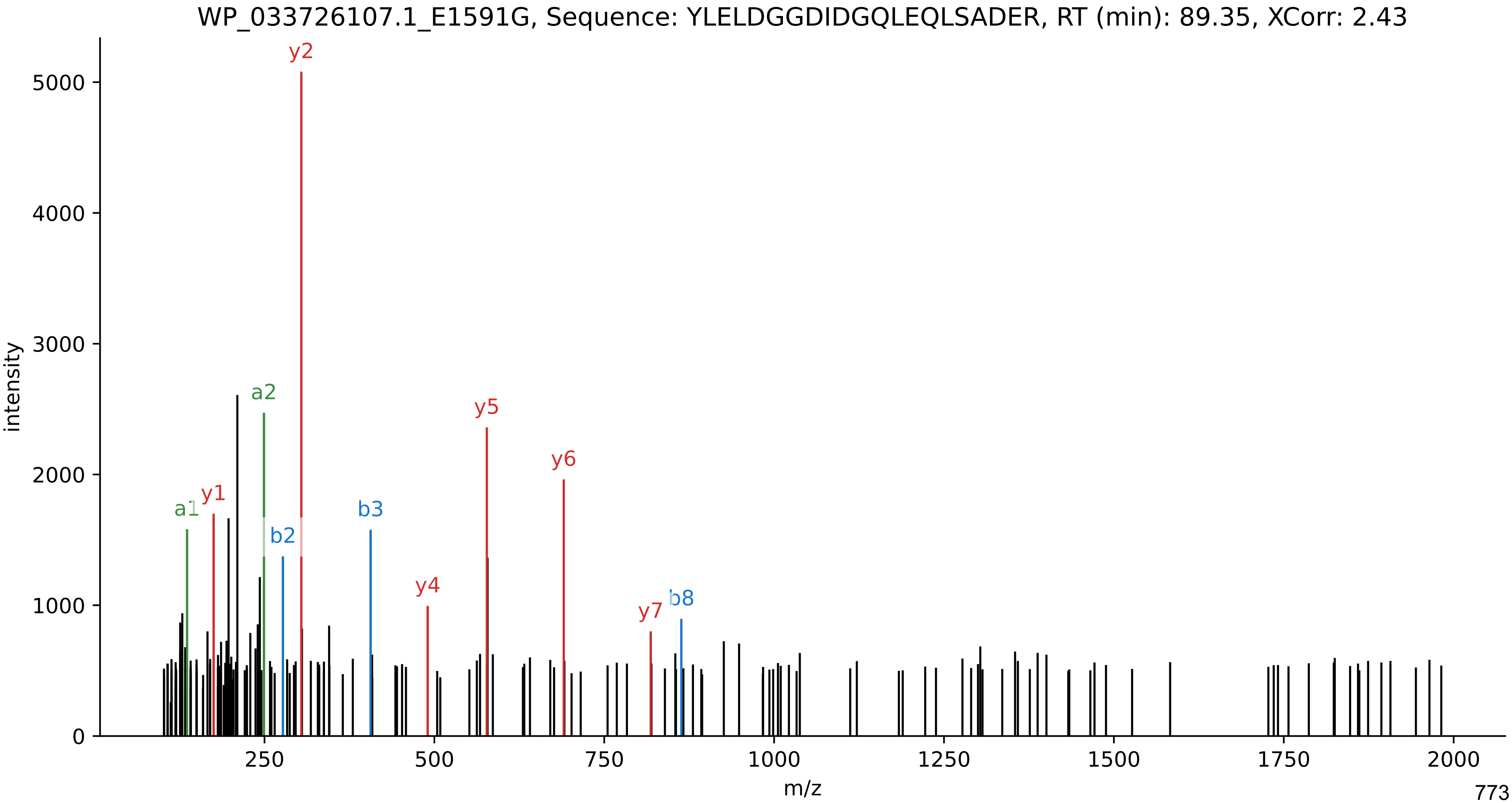
WP_033726107.1_E1591G, Sequence: YLELDGGDIDGQLEQLSADER, RT (min): 89.35, XCorr: 2.43
intensity
m/z
0
1000
2000
3000
4000
5000
250
500
750
1000
1250
1500
1750
2000
y2
a2
y5
y6
y1
a1
b3
b2
y4
b8
y7

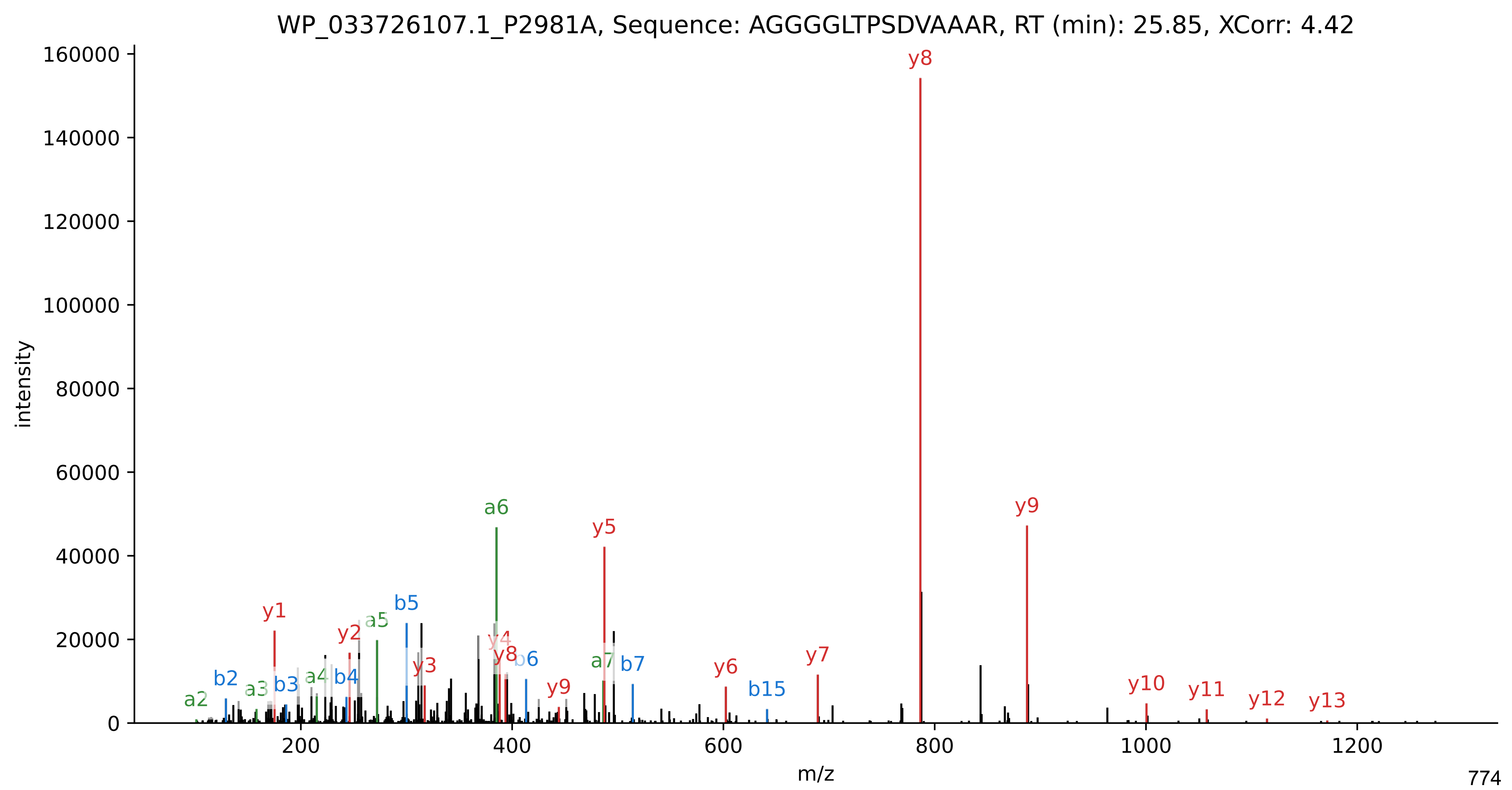

WP_033726107.1_P2981A, Sequence: AGGGGLTPSDVAAAR, RT (min): 25.85, XCorr: 4.42
intensity
m/z
y8
y9
a6
y5
b5
y1
y2
a5
y4
y8
b6
y3
a7
b7
b2
a3
b3
a4
b4
a2
y9
y6
b15
y7
y10
y11
y12
y13

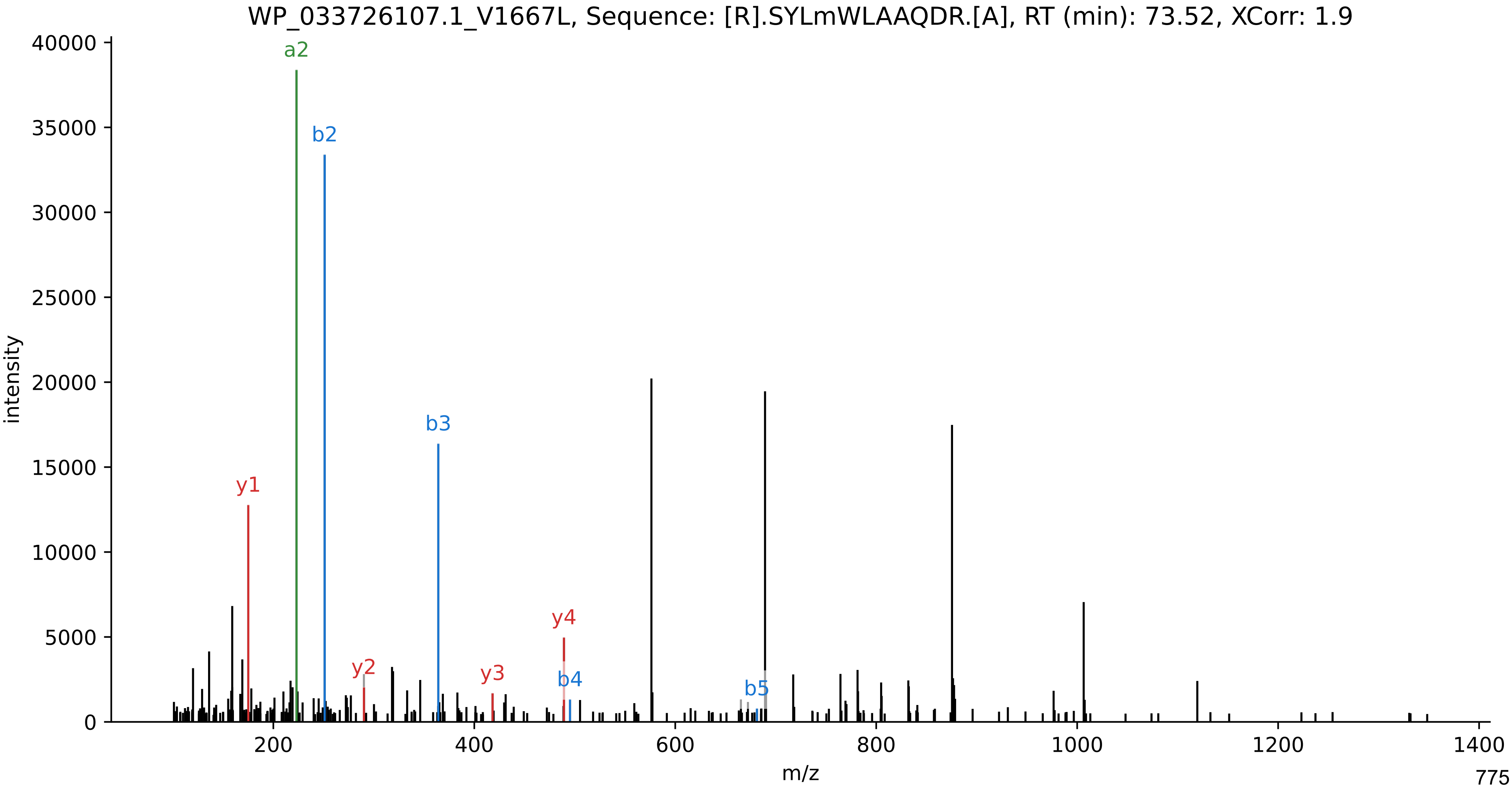
WP_033726107.1_V1667L, Sequence: [R].SYLmWLAAQDR.[A], RT (min): 73.52, XCorr: 1.9
intensity
m/z
a2
b2
b3
y1
y4
y2
y3
b4
b5
0
5000
10000
15000
20000
25000
30000
35000
40000
200
400
600
800
1000
1200
1400

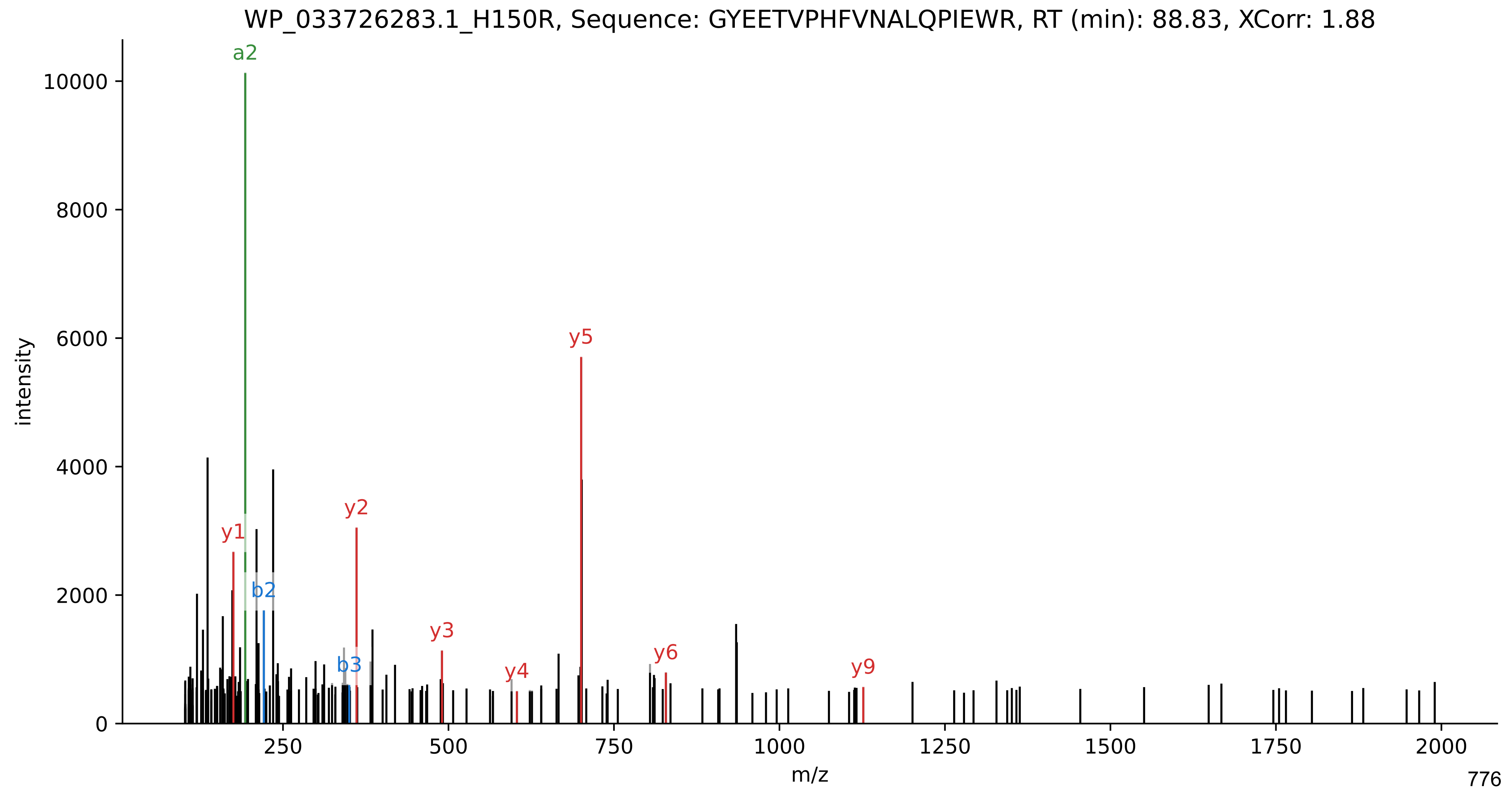

WP_033726283.1_H150R, Sequence: GYEETVPHFVNALQPIEWR, RT (min): 88.83, XCorr: 1.88
a2
y1
b2
y2
b3
y3
y4
y5
y6
y9
intensity
m/z

WP_033726625.1_Q445R, Sequence: RTTADELRDALTALR, RT (min): 64.02, XCorr: 4.64

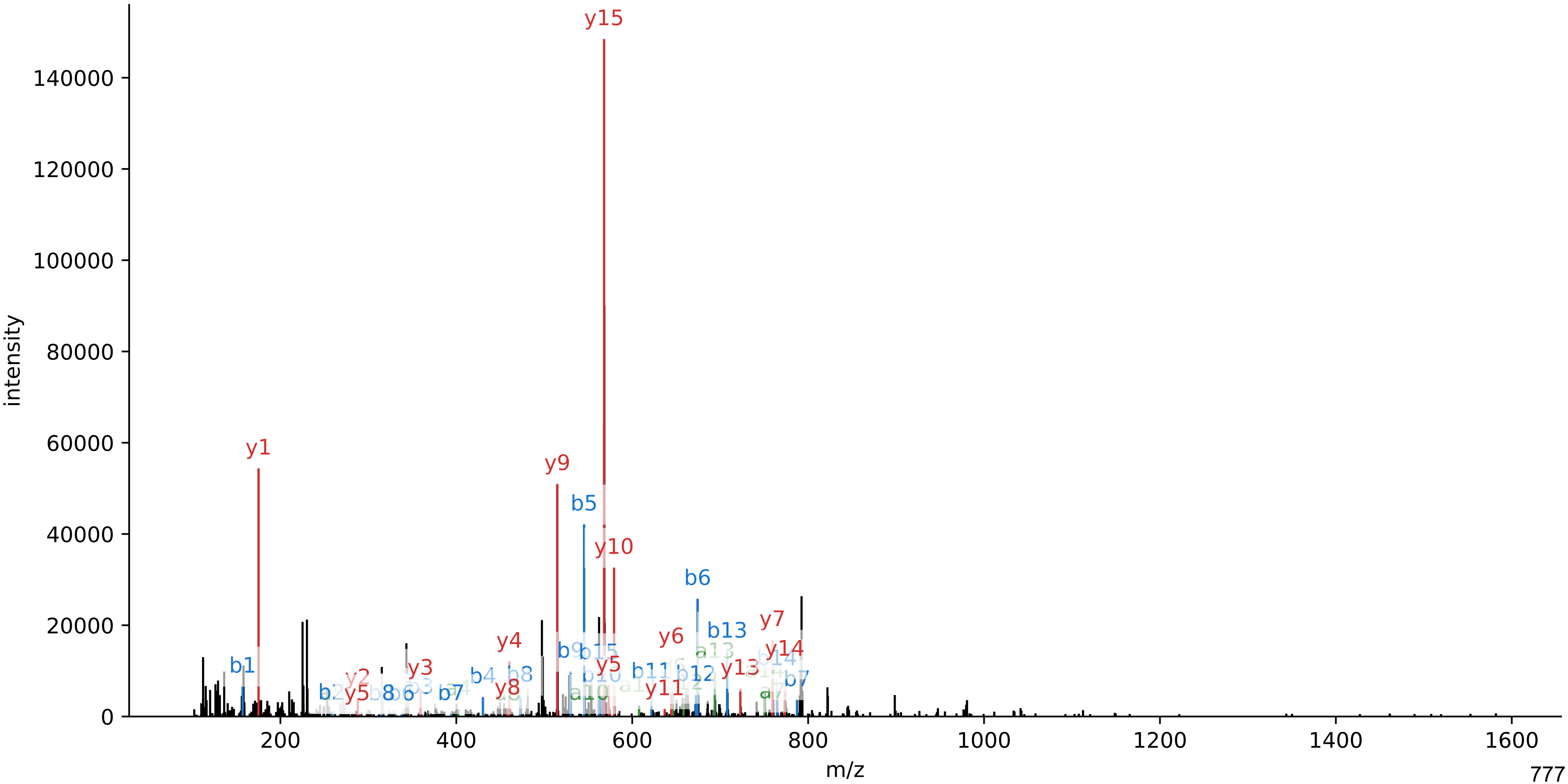

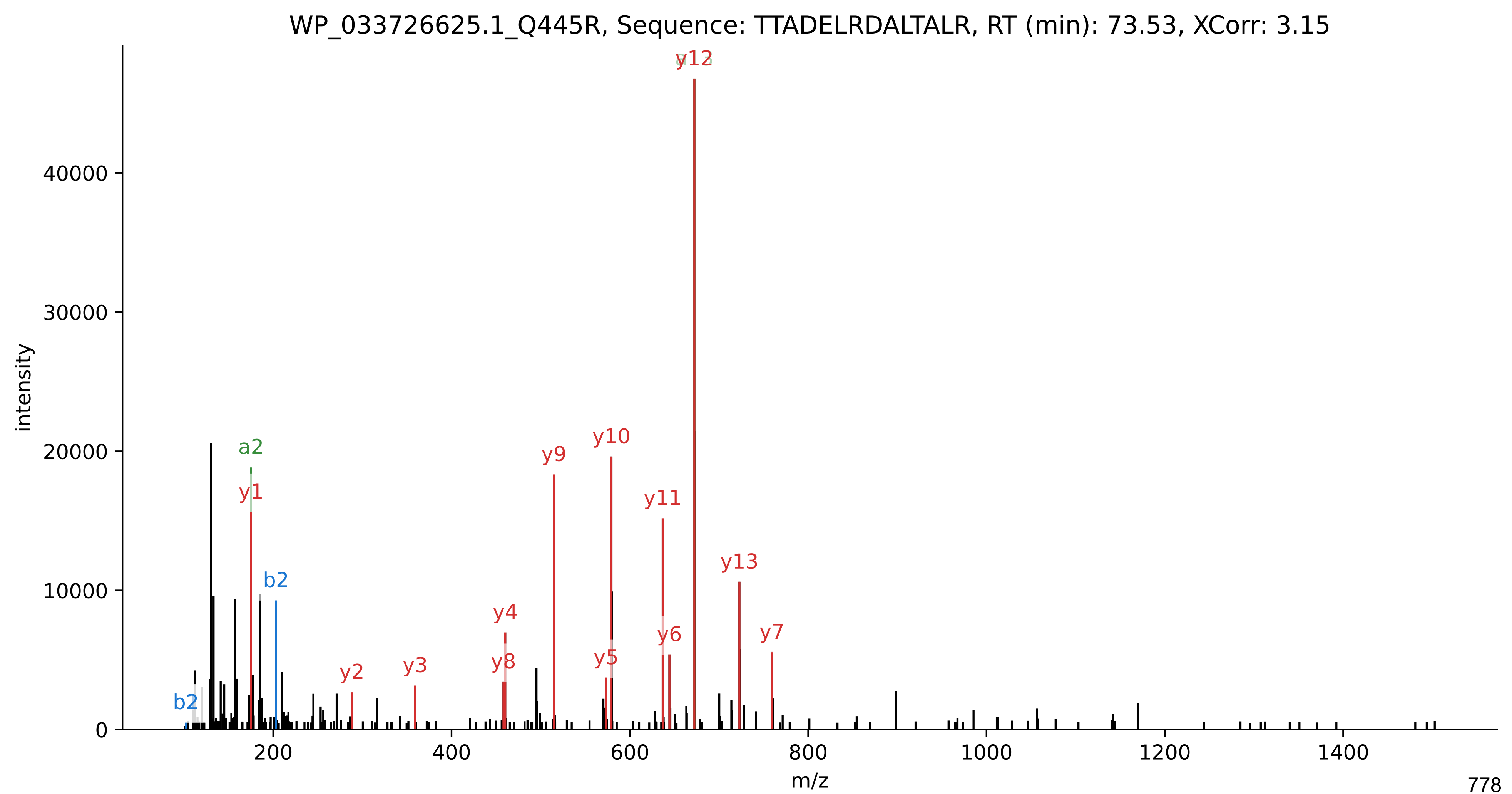

WP_033726625.1_Q445R, Sequence: TTADELRDALTALR, RT (min): 73.53, XCorr: 3.15
intensity
m/z
y12
a2
y1
b2
y10
y9
y11
y13
y4
y8
y7
y6
y5
y2
y3
b2

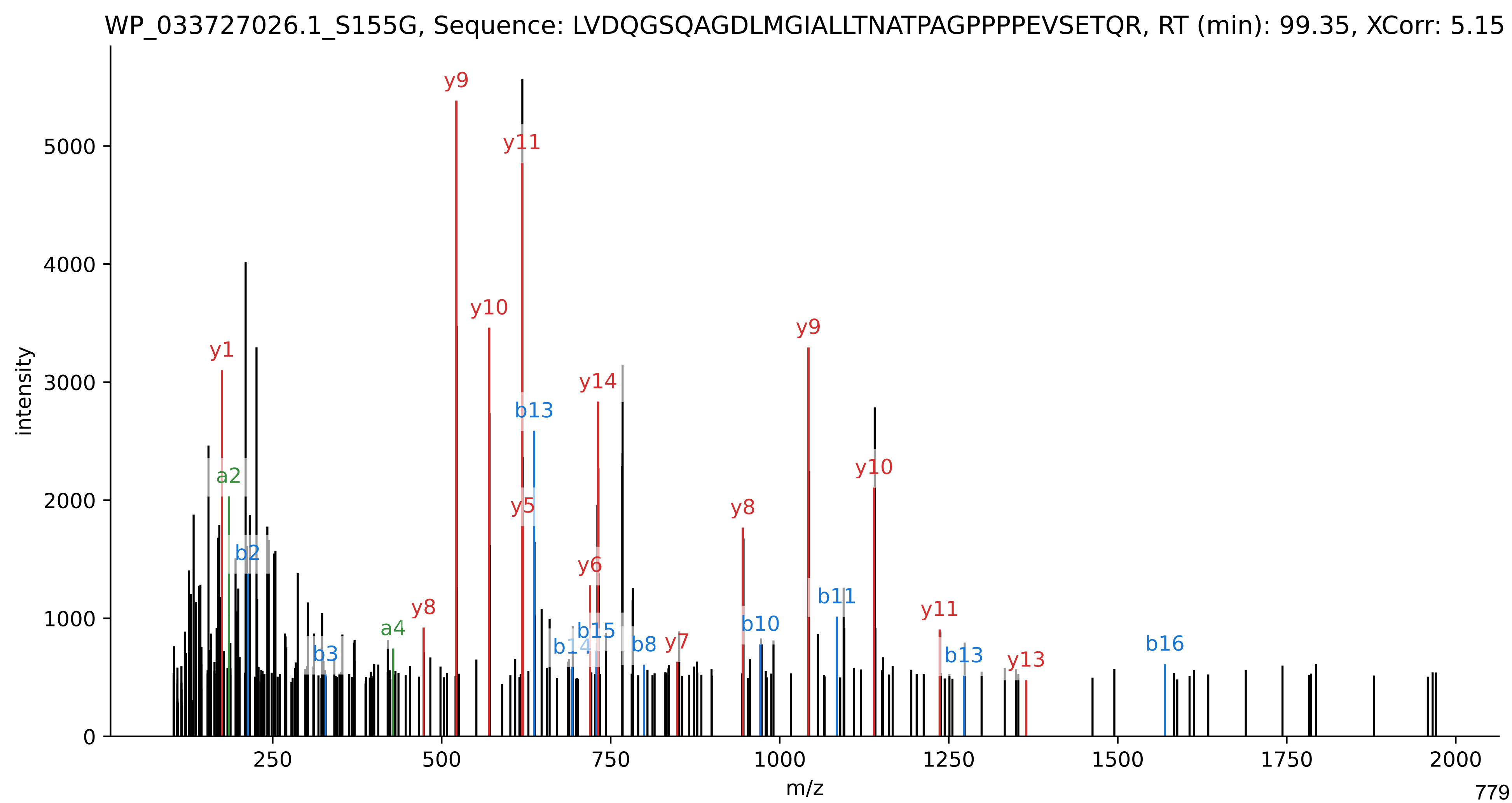
WP_033727026.1_S155G, Sequence: LVDQGSQAGDLMGIALLTNATPAGPPPPEVSETQR, RT (min): 99.35, XCorr: 5.15
intensity
m/z
y9
y11
y10
y1
y14
b13
y9
y10
a2
y5
y8
b2
y6
b11
y8
a4
b10
y11
b15
y7
b8
b16
b3
b13
y13
0
1000
2000
3000
4000
5000
250
500
750
1000
1250
1500
1750
2000

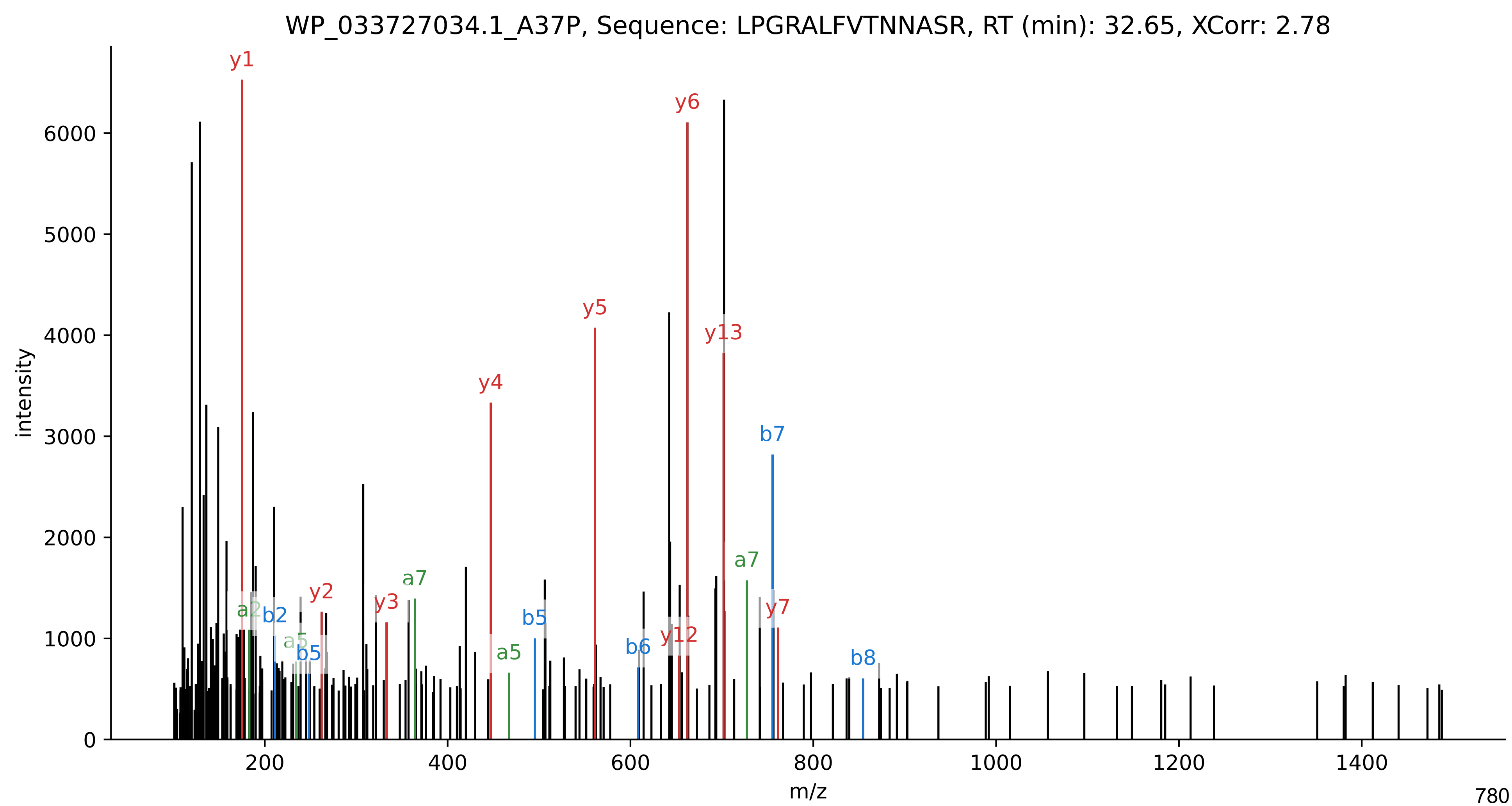

WP_033727034.1_A37P, Sequence: LPGRALFVTNNASR, RT (min): 32.65, XCorr: 2.78
intensity
m/z
y1
y2
y3
y4
y5
y6
y7
y12
y13
a2
a5
a5
a7
a7
b2
b5
b5
b6
b7
b8

WP_033727278.1_G145D, Sequence: TNTMAALDARPAAQLR, RT (min): 38.82, XCorr: 3.33

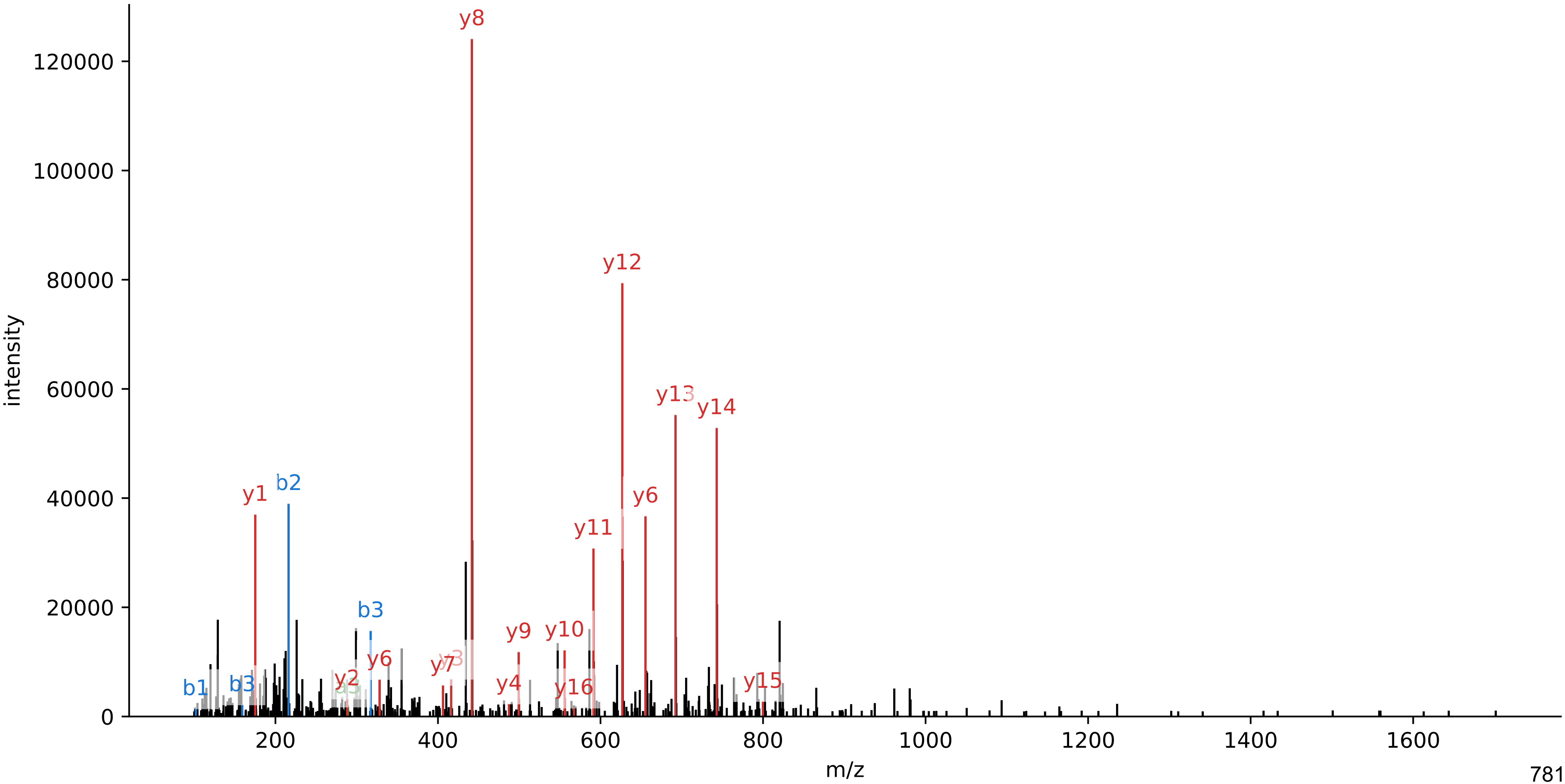

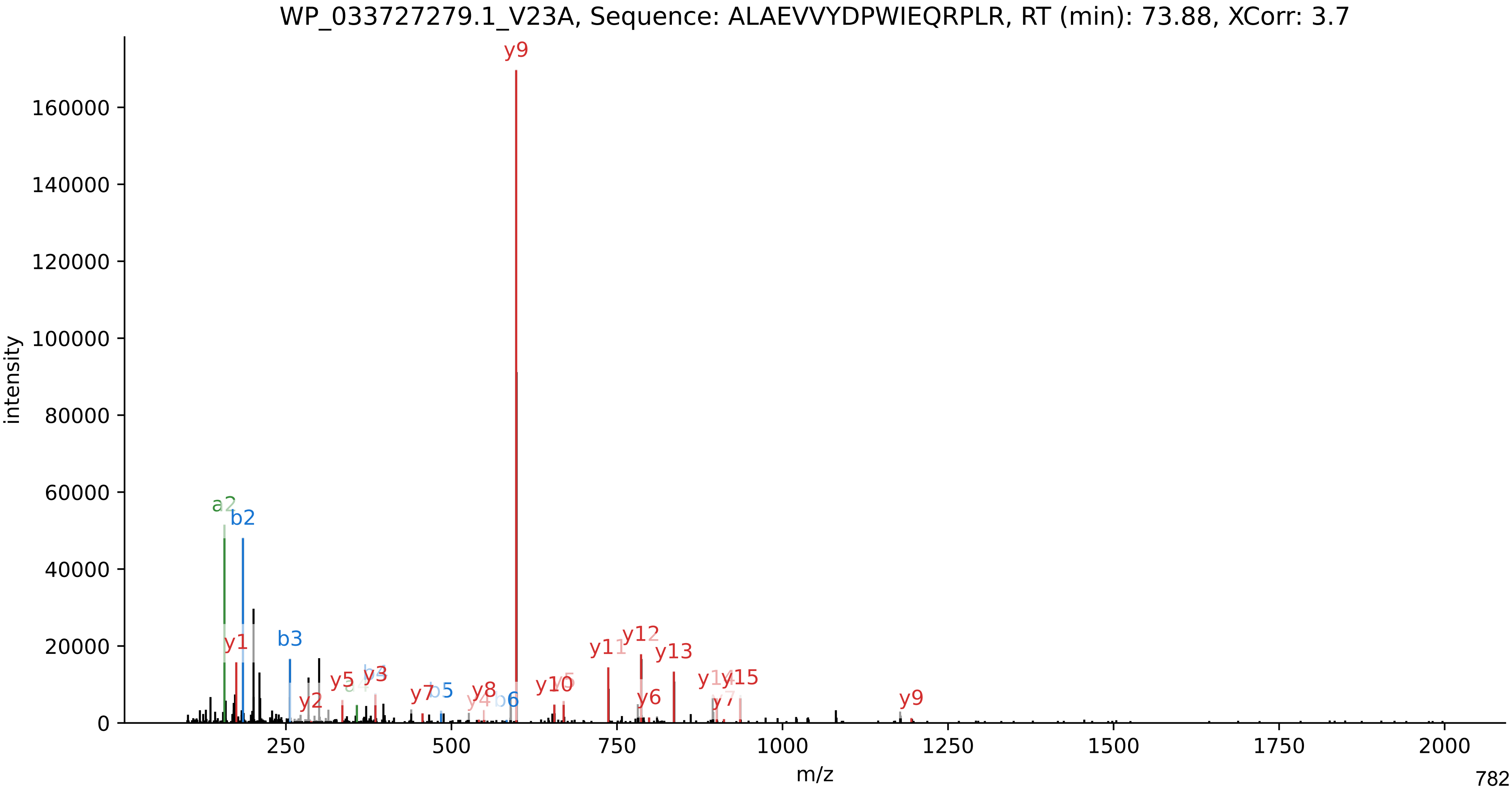

WP_033727279.1_V23A, Sequence: ALAEVVYDPWIEQRPLR, RT (min): 73.88, XCorr: 3.7
intensity
m/z
y9
a2
b2
b3
y1
y2
y3
y5
b5
y7
y8
y4
b6
y10
y11
y12
y13
y6
y14
y15
y7
y9

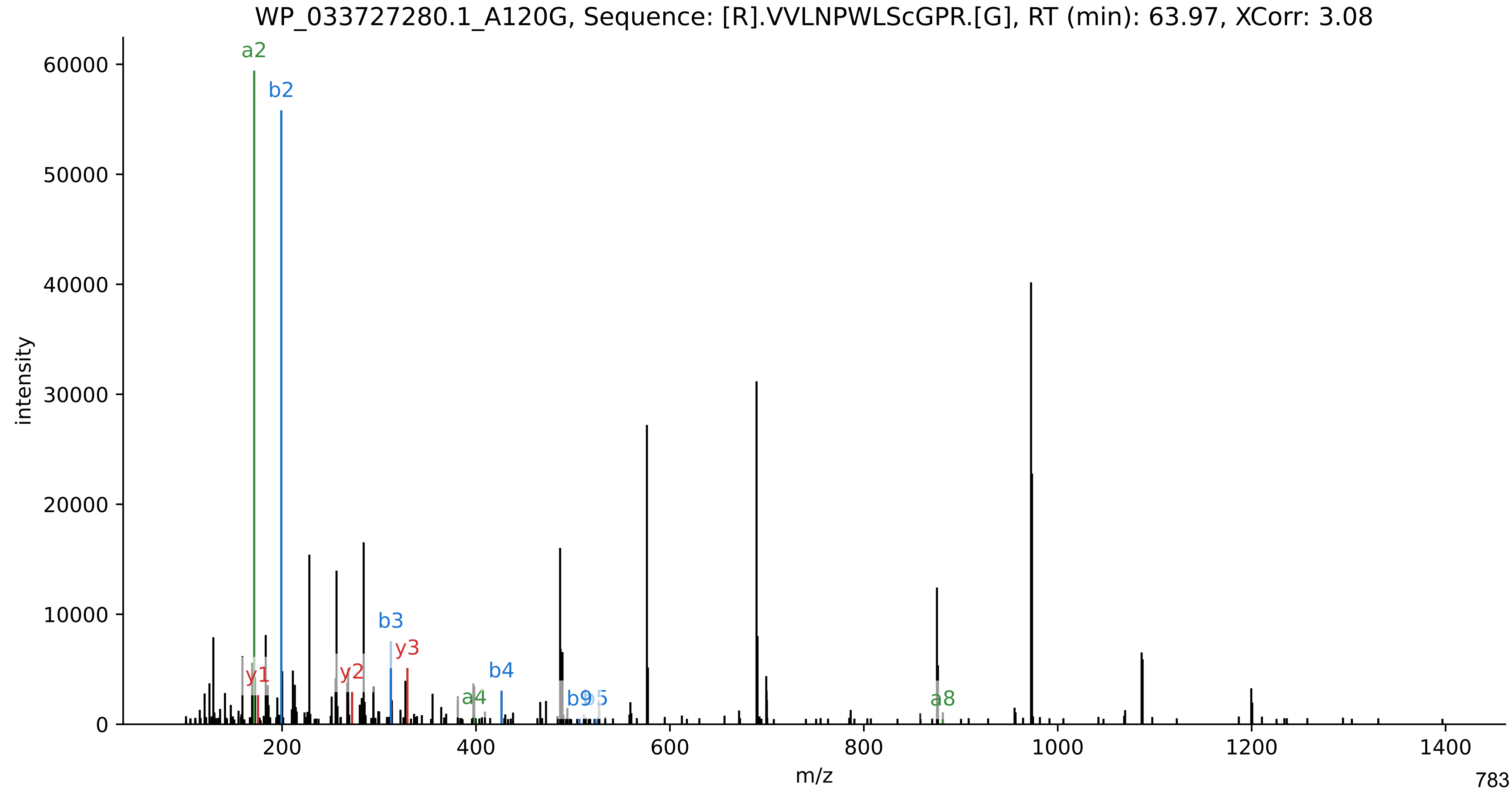

WP_033727280.1_A120G, Sequence: [R].VVLNPWLScGPR.[G], RT (min): 63.97, XCorr: 3.08
intensity
m/z
a2
b2
b3
y3
b4
y1
y2
a4
b9
b5
a8
0
10000
20000
30000
40000
50000
60000
200
400
600
800
1000
1200
1400

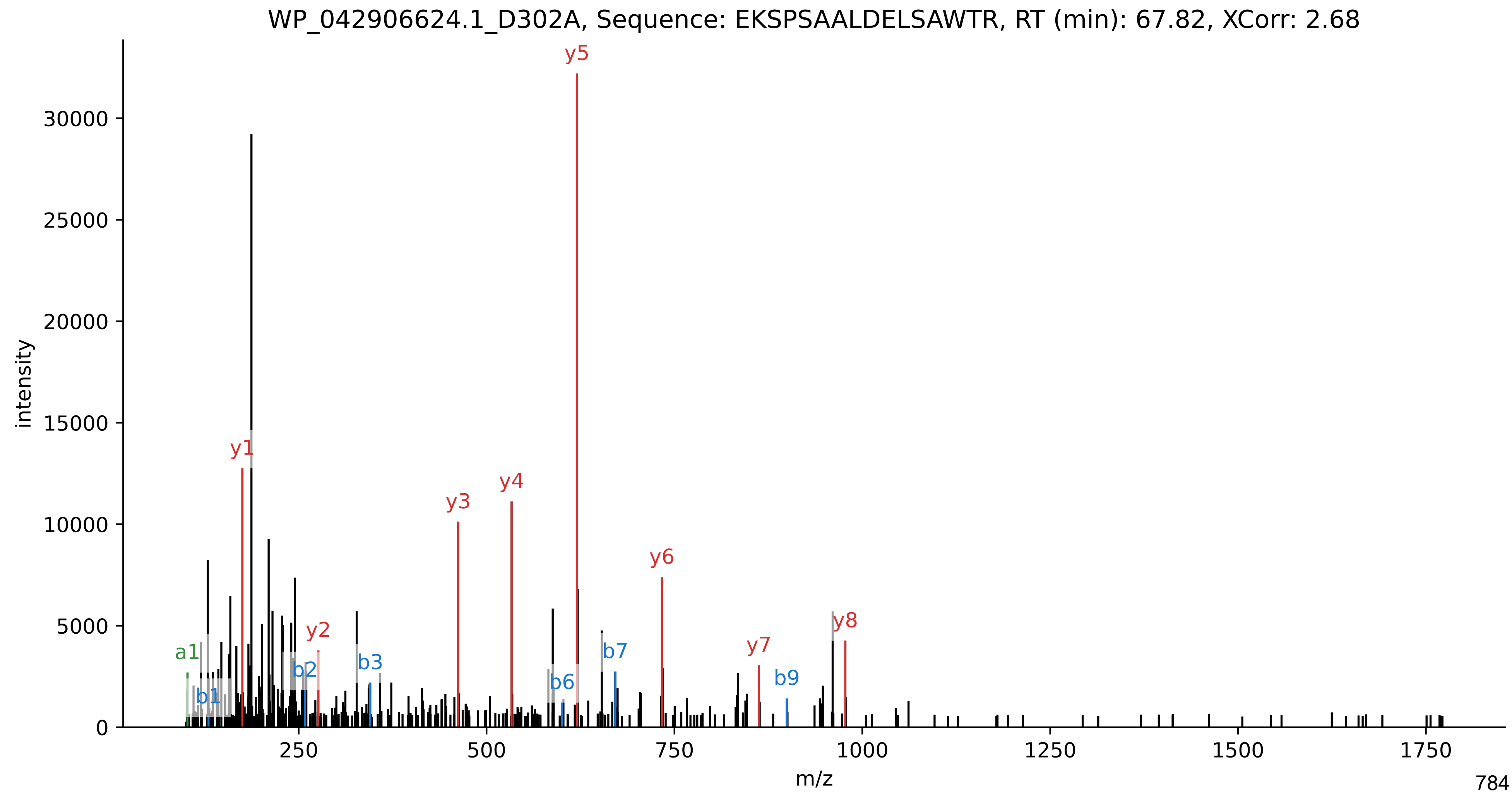

WP_042906624.1_D302A, Sequence: EKSPSAALDELSAWTR, RT (min): 67.82, XCorr: 2.68
intensity
m/z
0
5000
10000
15000
20000
25000
30000
250
500
750
1000
1250
1500
1750
a1
b1
b2
b3
b6
b7
b9
y1
y2
y3
y4
y5
y6
y7
y8

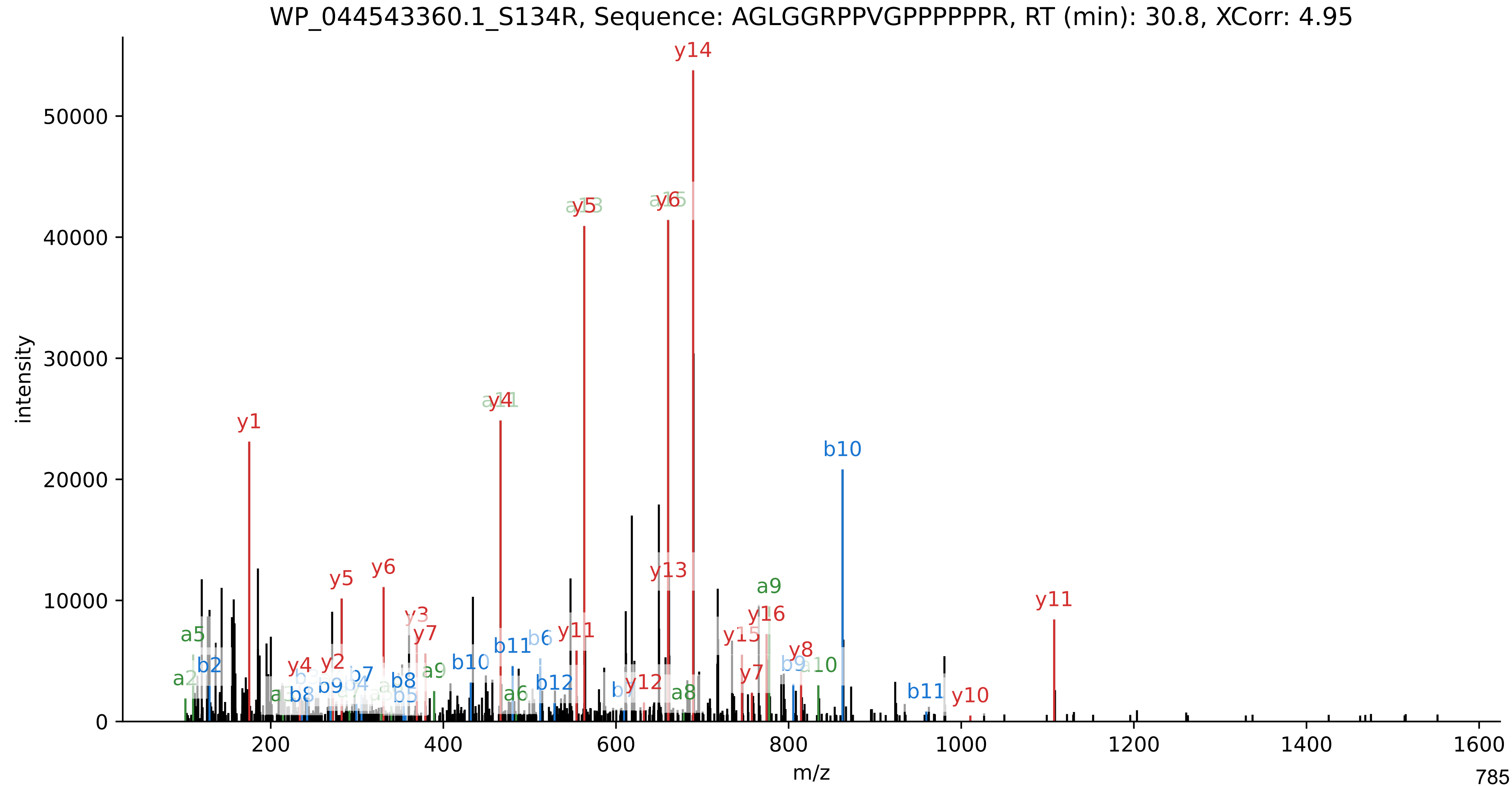

WP_044543360.1_S134R, Sequence: AGLGGRPPVGPPPPPPR, RT (min): 30.8, XCorr: 4.95
intensity
m/z
y14
y5
y6
y4
y1
b10
y13
a9
y16
y15
y11
y8
y7
y10
b11
b12
a10
a8
y12
a6
b6
b8
b7
b9
y2
y3
a5
a2
b2
0
10000
20000
30000
40000
50000
200
400
600
800
1000
1200
1400
1600

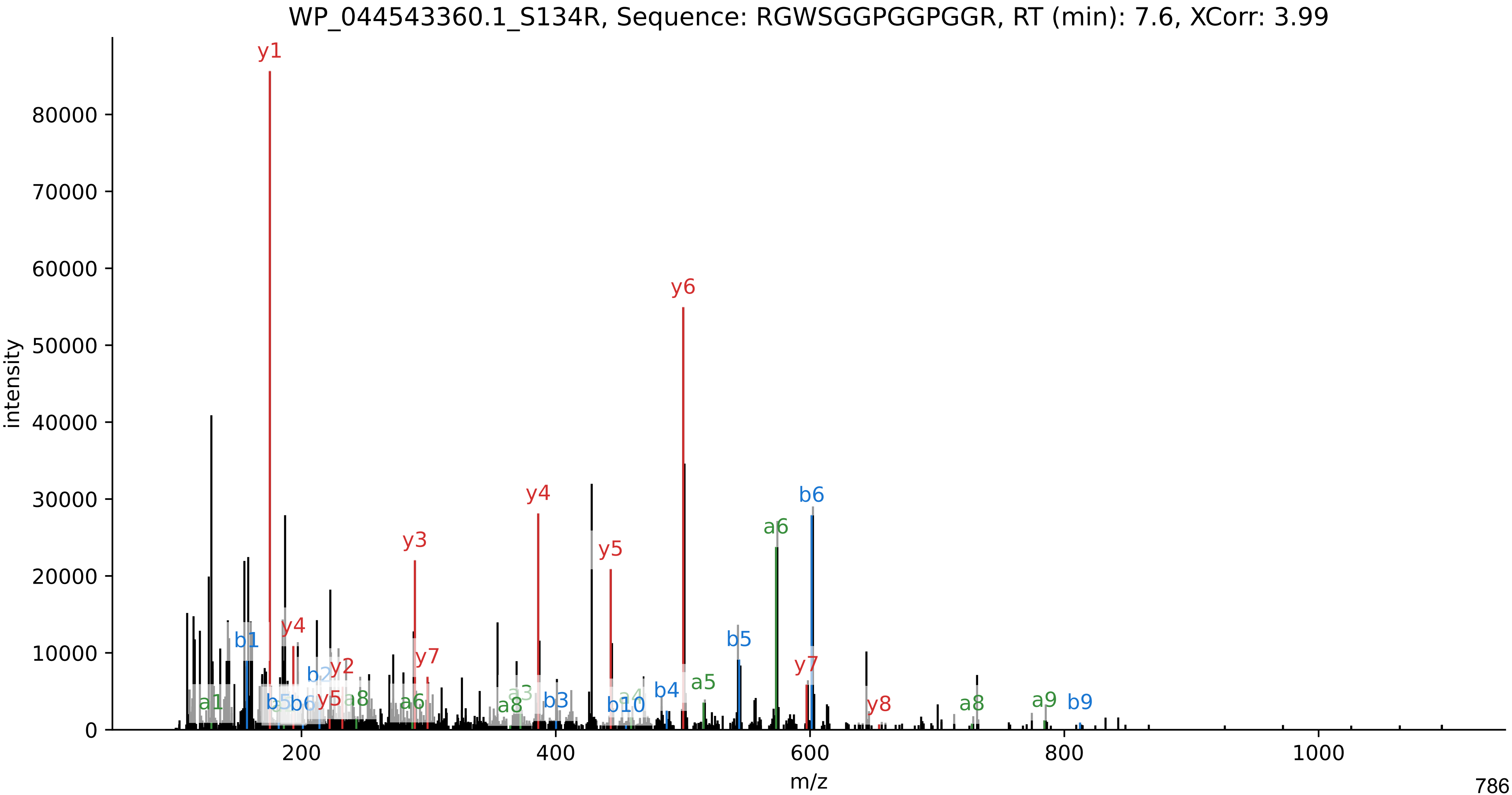
WP_044543360.1_S134R, Sequence: RGWSGGPGGPGGR, RT (min): 7.6, XCorr: 3.99
intensity
m/z
0
10000
20000
30000
40000
50000
60000
70000
80000
200
400
600
800
1000
y1
y6
y4
b6
a6
y3
y5
y4
b1
y7
b5
y2
y7
a5
b4
a1
b5
b6
y5
a8
a6
a8
b3
b10
y8
a8
a9
b9

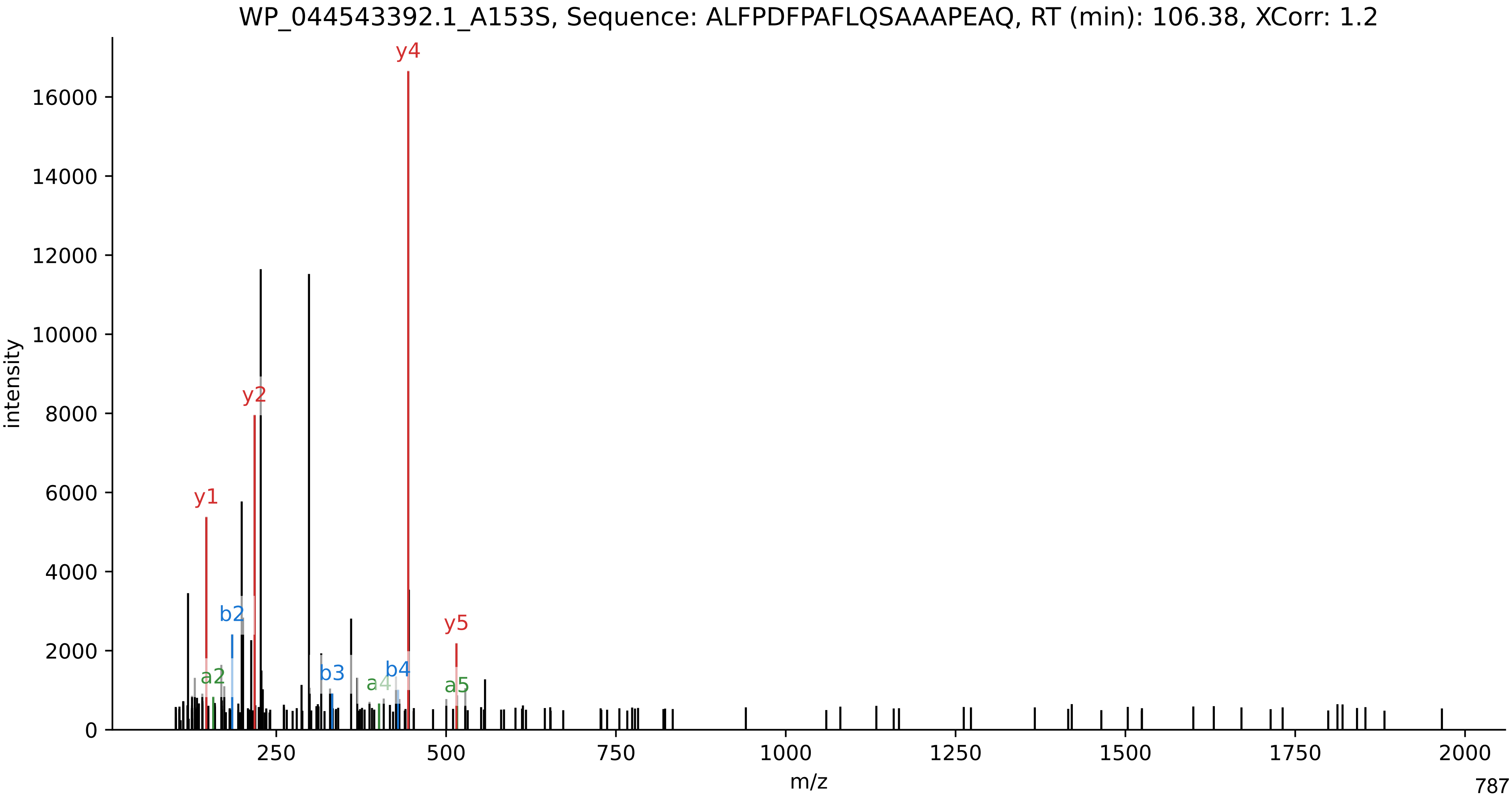

WP_044543392.1_A153S, Sequence: ALFPDFPAFLQSAAAPEAQ, RT (min): 106.38, XCorr: 1.2
intensity
m/z
y1
y2
y4
y5
b2
b3
b4
a2
a4
a5

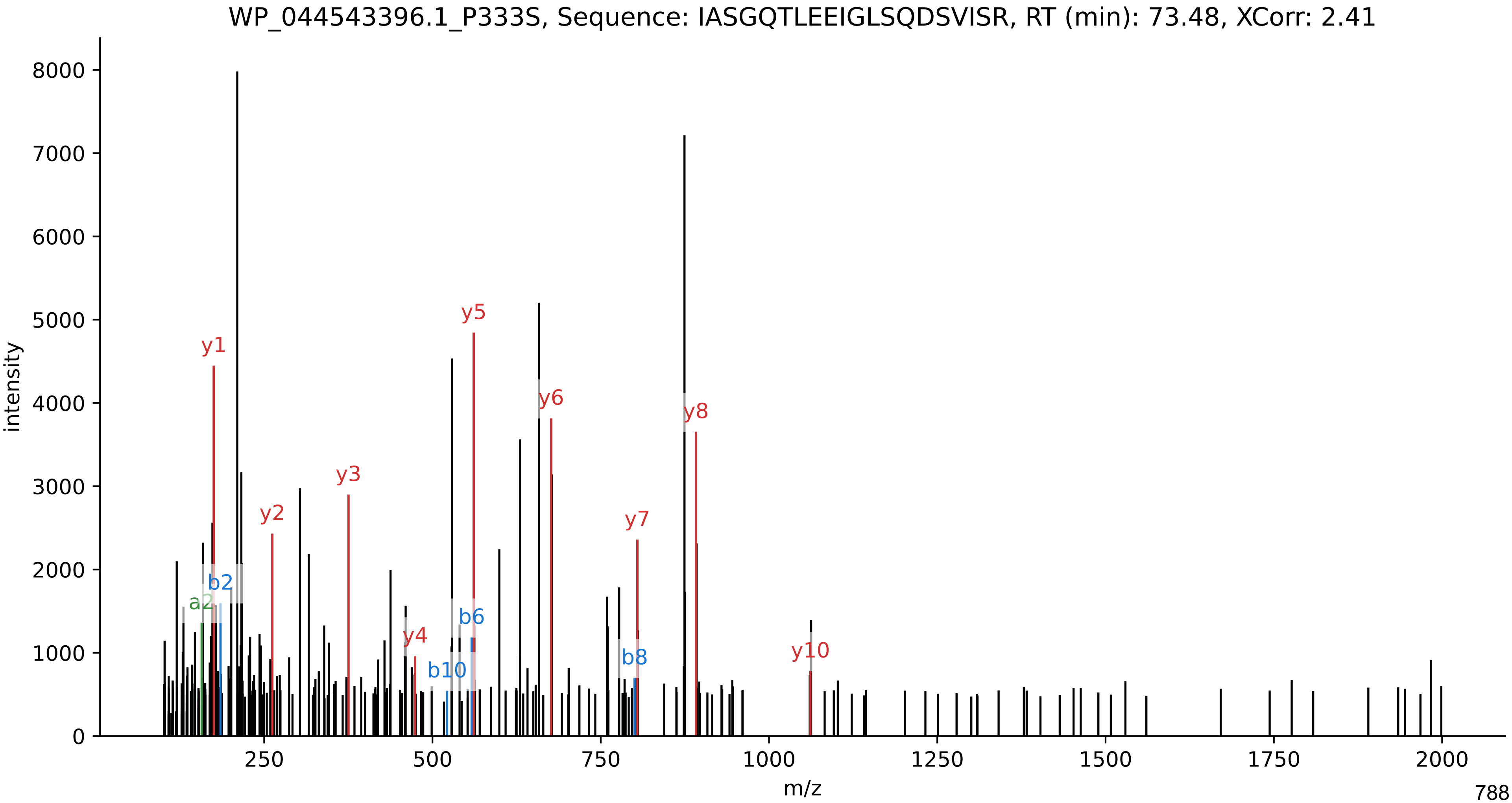
WP_044543396.1_P333S, Sequence: IASGQTLEEIGLSQDSVISR, RT (min): 73.48, XCorr: 2.41
intensity
m/z
y1
y2
y3
y4
y5
y6
y7
y8
y10
a2
b2
b6
b8
b10
0
1000
2000
3000
4000
5000
6000
7000
8000
250
500
750
1000
1250
1500
1750
2000

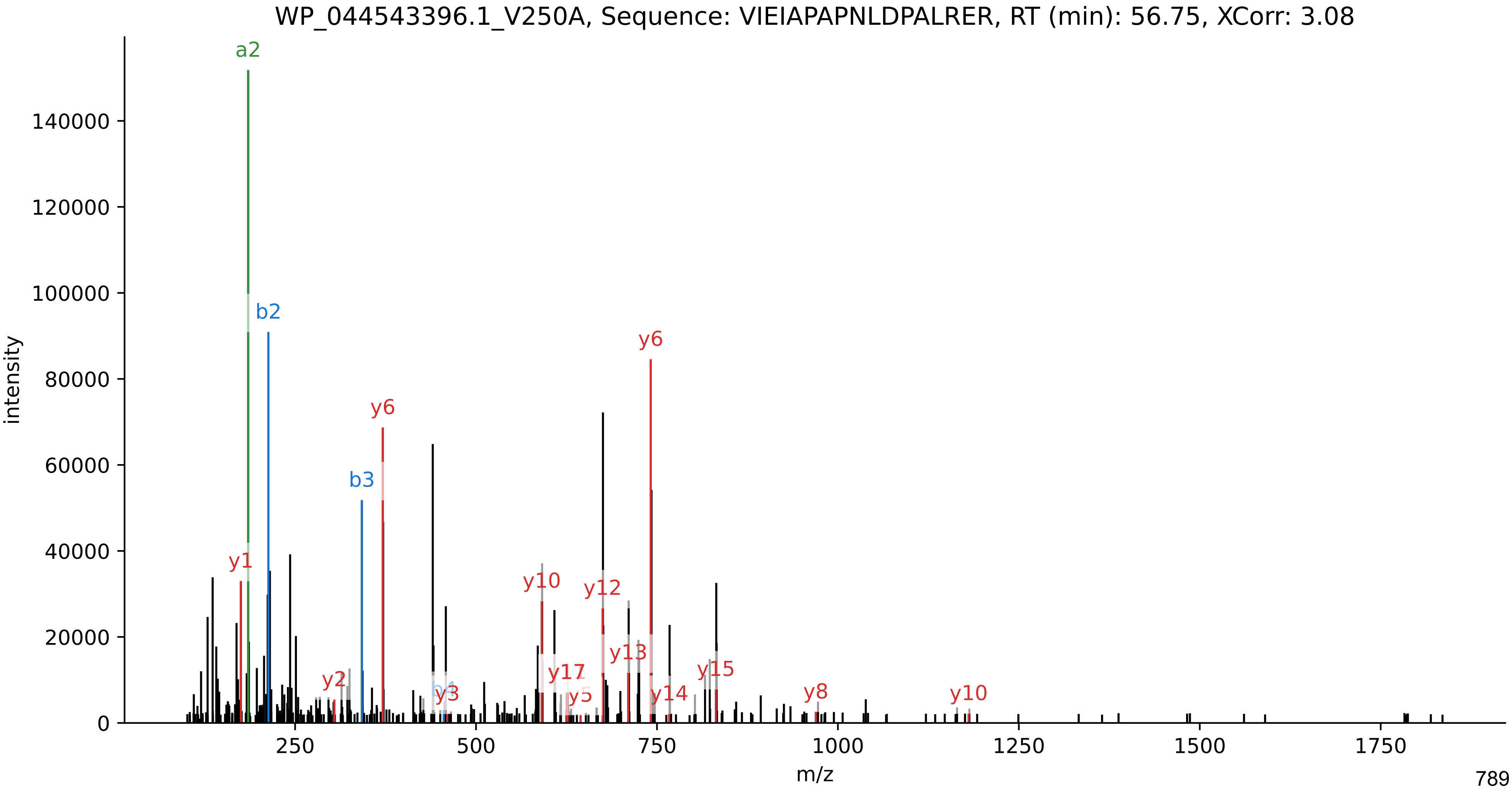

WP_044543396.1_V250A, Sequence: VIEIAPAPNLDPALRER, RT (min): 56.75, XCorr: 3.08
a2
b2
b3
y1
y2
y6
y6
y10
y12
y13
y14
y15
y17
y5
y3
y8
y10
intensity
m/z
0
20000
40000
60000
80000
100000
120000
140000
250
500
750
1000
1250
1500
1750

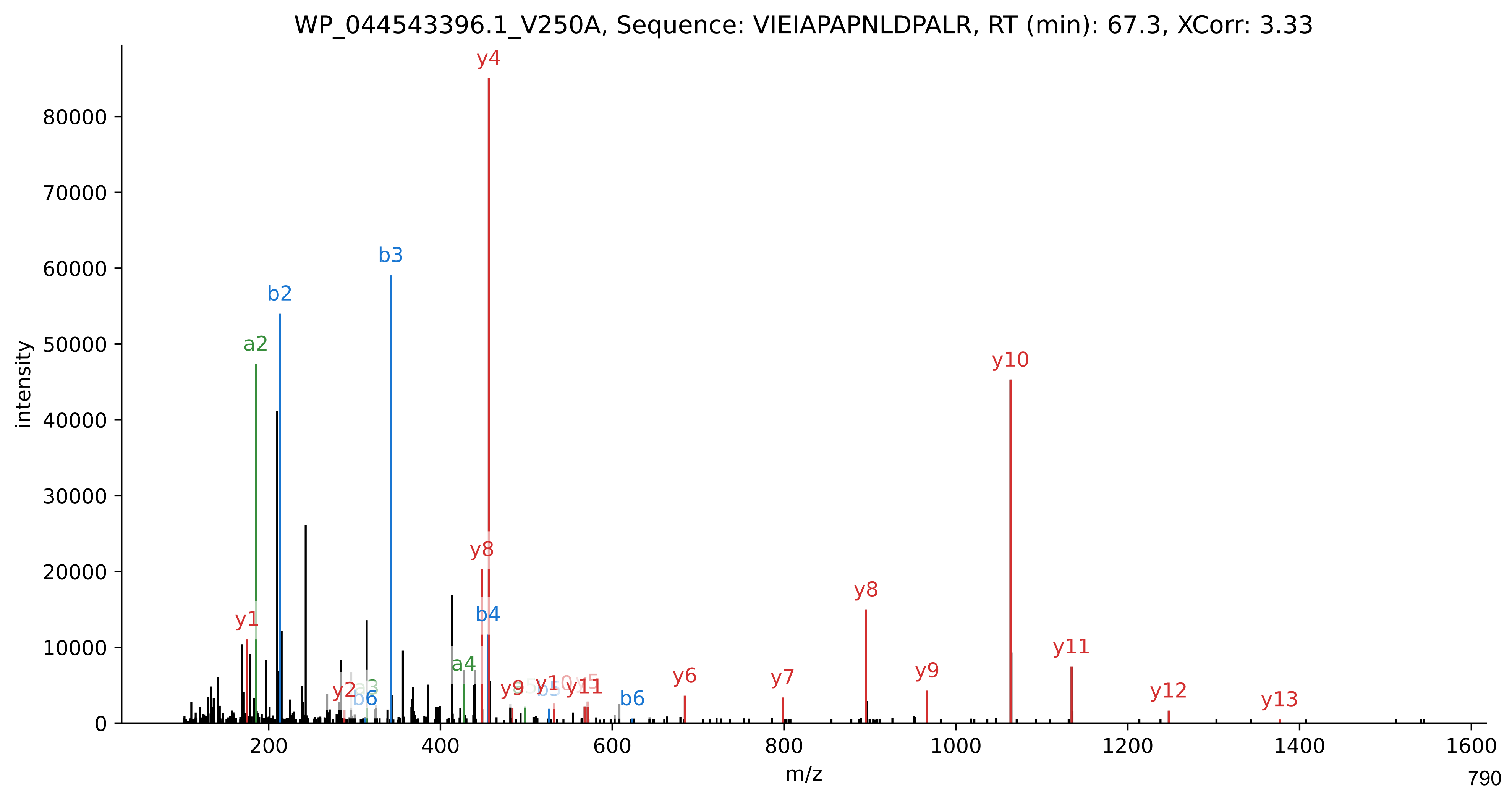

WP_044543396.1_V250A, Sequence: VIEIAPAPNLDPALR, RT (min): 67.3, XCorr: 3.33
intensity
m/z
y4
b3
b2
a2
y10
y8
y1
b4
y8
y11
a4
y9
y6
y7
y9
y12
y13
b6
y2
b6
y11

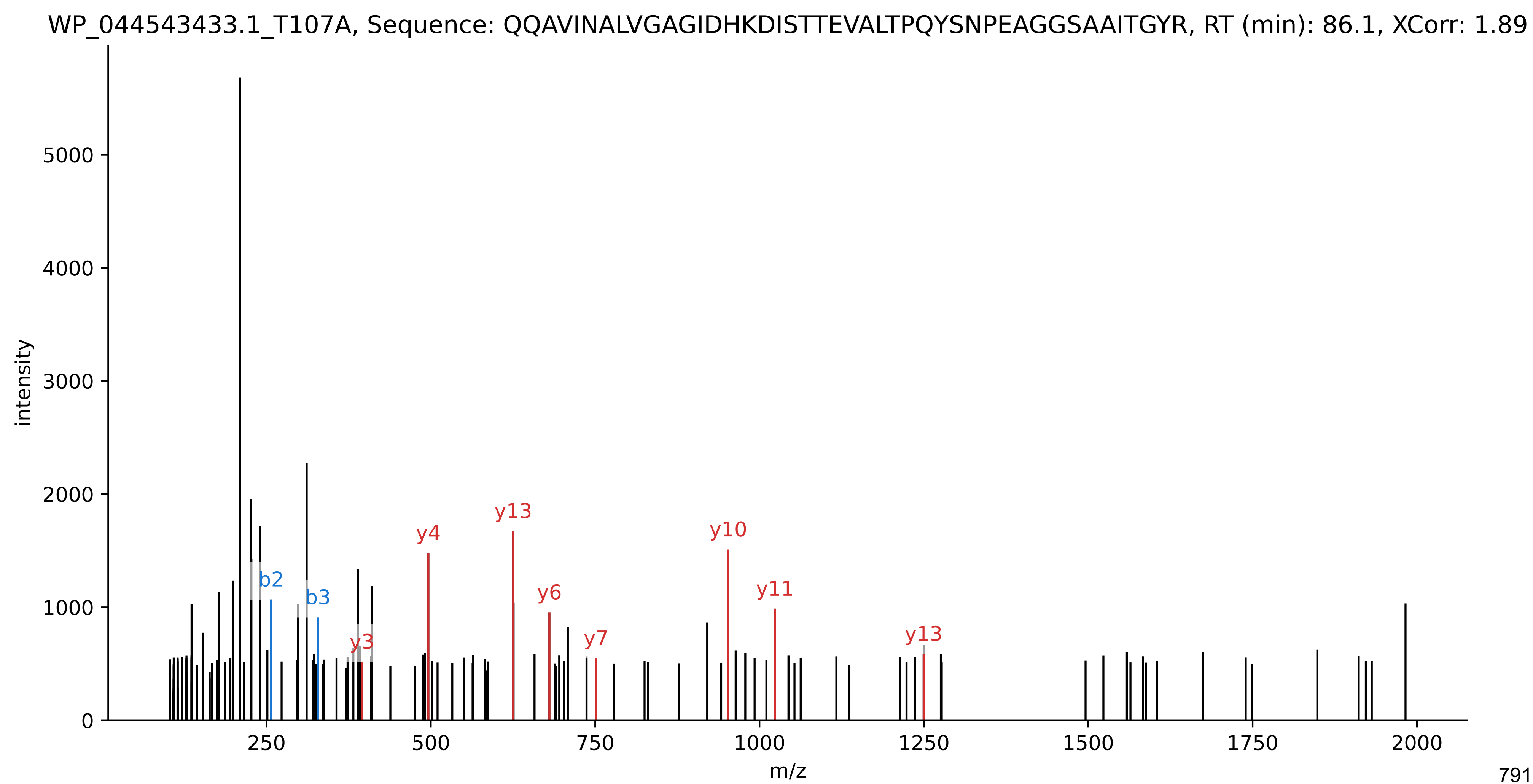

WP_044543433.1_T107A, Sequence: QQAVINALVGAGIDHKDISTTEVALTPQYSNPEAGGSAAITGYR, RT (min): 86.1, XCorr: 1.89
intensity
m/z
b2
b3
y3
y4
y13
y6
y7
y10
y11
y13
0
1000
2000
3000
4000
5000
250
500
750
1000
1250
1500
1750
2000

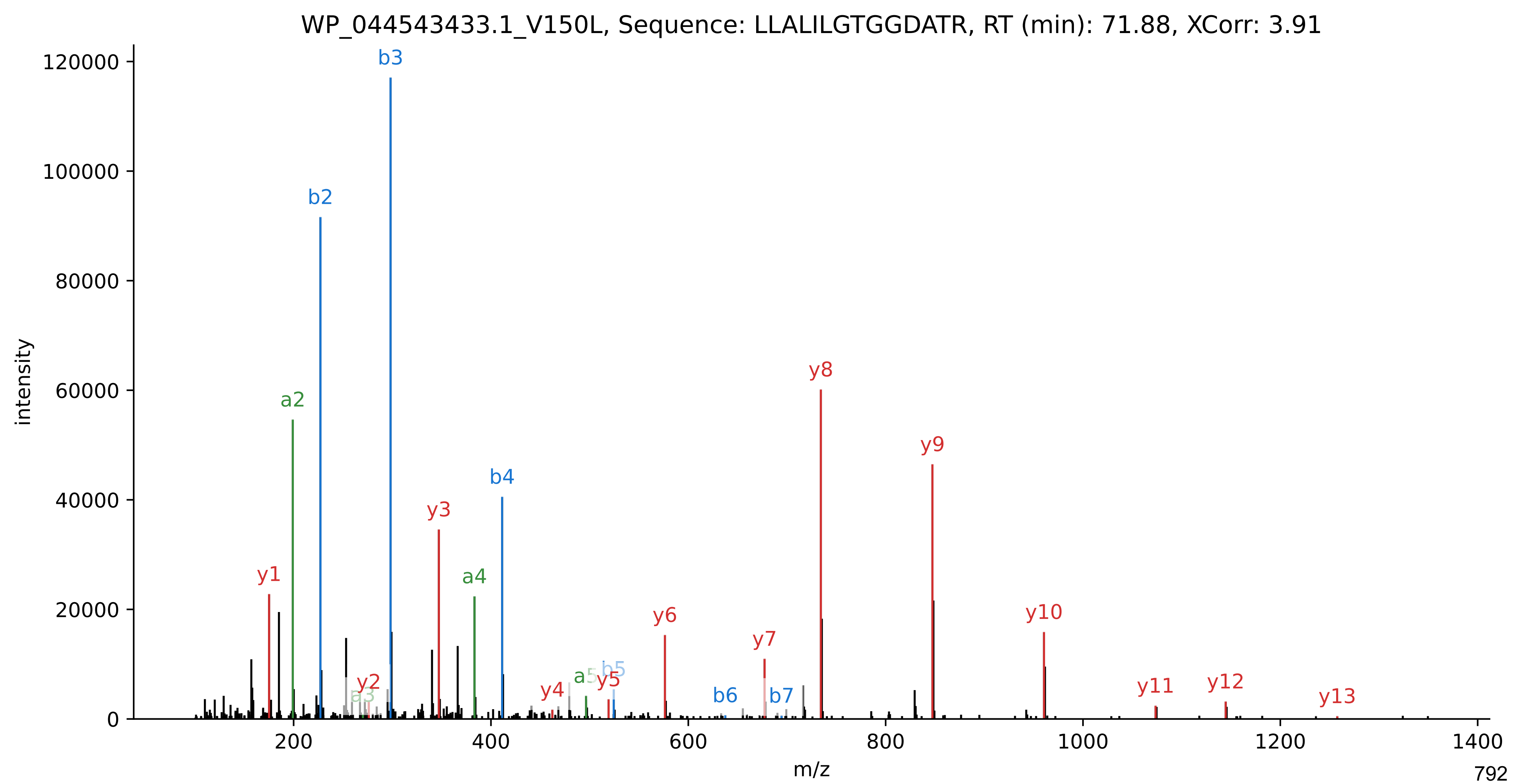
WP_044543433.1_V150L, Sequence: LLALILGTGGDATR, RT (min): 71.88, XCorr: 3.91
intensity
m/z
b3
b2
a2
y8
y9
b4
y3
y1
a4
y6
y10
y7
a5
b5
y5
y4
y2
a3
b6
b7
y11
y12
y13

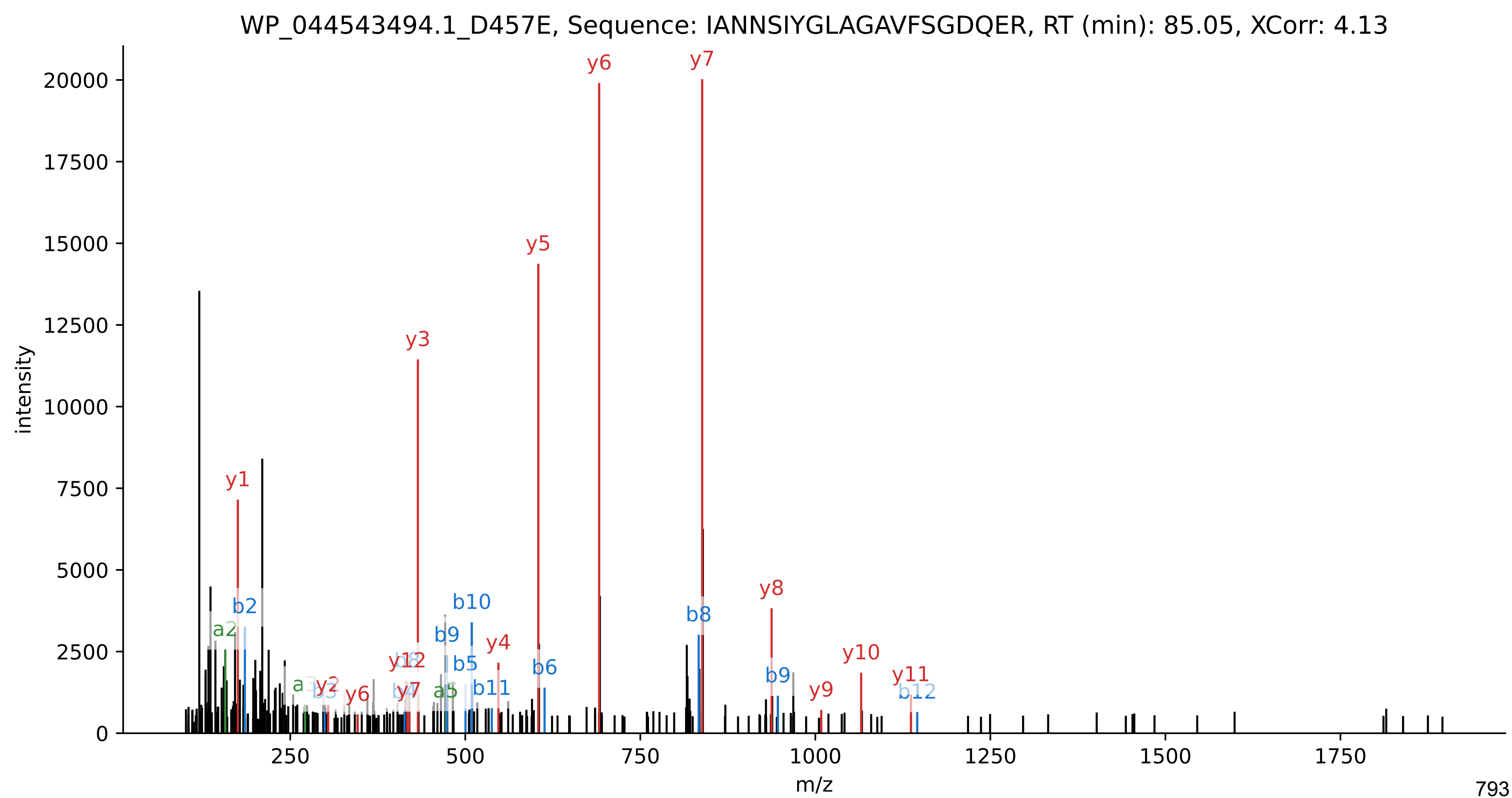

WP_044543494.1_D457E, Sequence: IANNSIYGLAGAVFSGDQER, RT (min): 85.05, XCorr: 4.13
intensity
m/z
y1
y2
y3
y4
y5
y6
y7
y8
y9
y10
y11
b2
b5
b6
b8
b9
b10
b11
b12
a2
a5

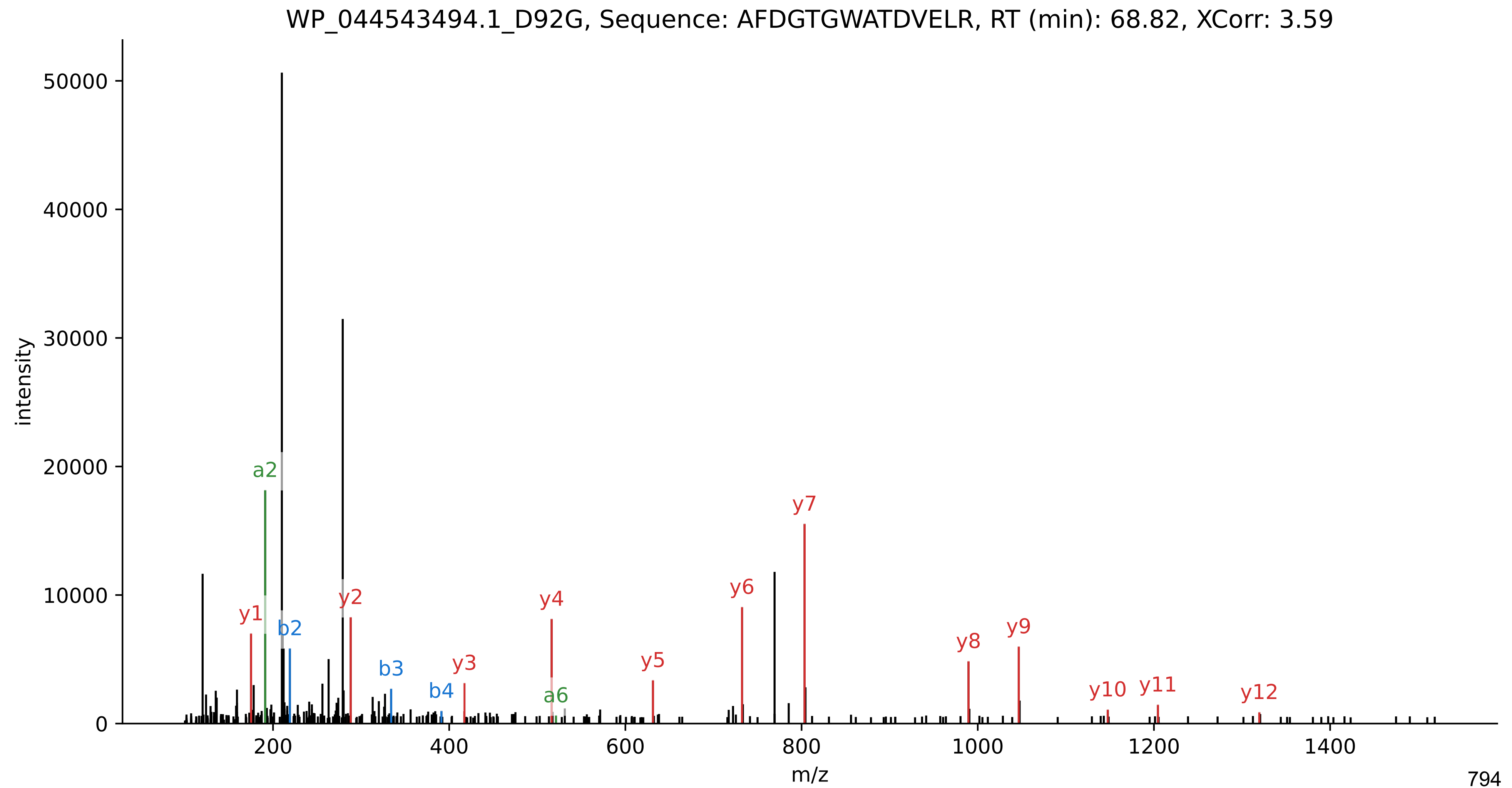

WP_044543494.1_D92G, Sequence: AFDGTGWATDVELR, RT (min): 68.82, XCorr: 3.59
intensity
m/z
0
10000
20000
30000
40000
50000
200
400
600
800
1000
1200
1400
a2
y1
b2
y2
b3
b4
y3
y4
a6
y5
y6
y7
y8
y9
y10
y11
y12

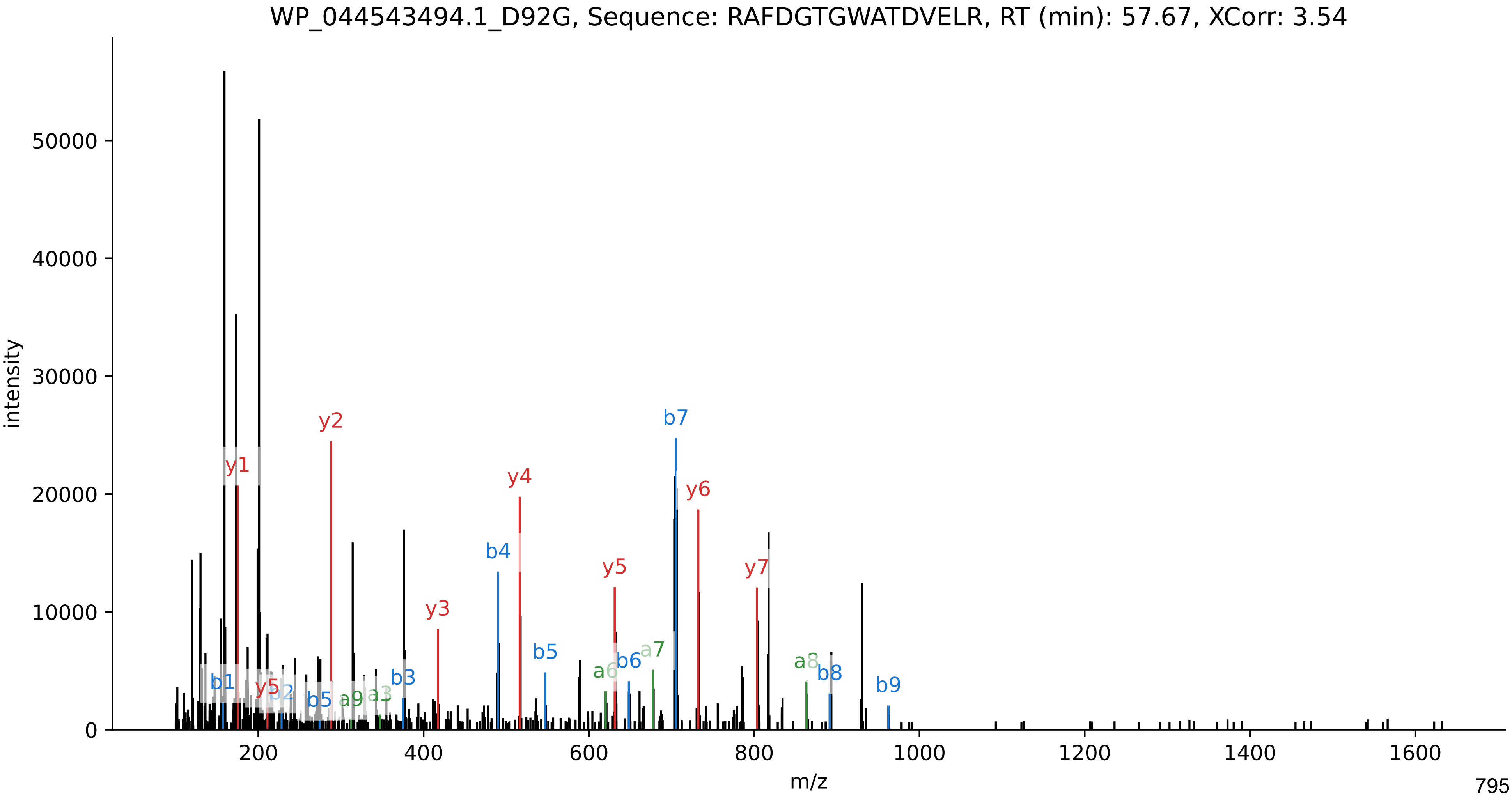
WP_044543494.1_D92G, Sequence: RAFDGTGWATDVELR, RT (min): 57.67, XCorr: 3.54
intensity
m/z
0
10000
20000
30000
40000
50000
200
400
600
800
1000
1200
1400
1600
y1
y2
y3
y4
y5
y6
y7
b1
b3
b4
b5
b6
b7
b8
b9
a6
a7
a8

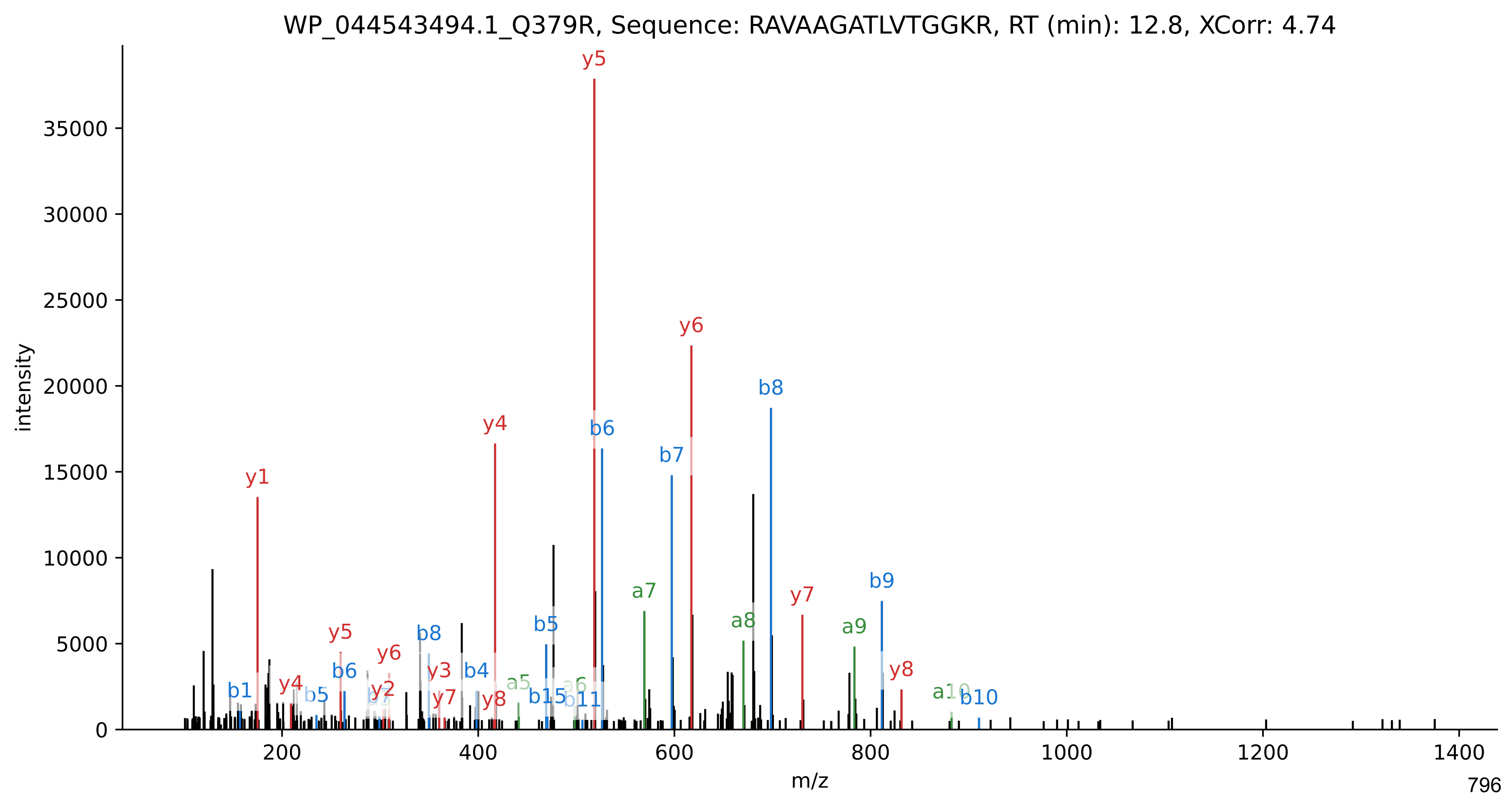

WP_044543494.1_Q379R, Sequence: RAVAAGATLVTGGKR, RT (min): 12.8, XCorr: 4.74
intensity
m/z
y5
y6
b8
y4
b6
b7
y1
b9
y7
a7
a8
a9
b5
y5
b8
y6
b6
y3
b4
y8
y4
b1
y2
a5
a6
y7
b5
y8
b15
b11
a10
b10

WP_044543494.1_Q379R, Sequence: RAVAAGATLVTGGK, RT (min): 16.35, XCorr: 4.74

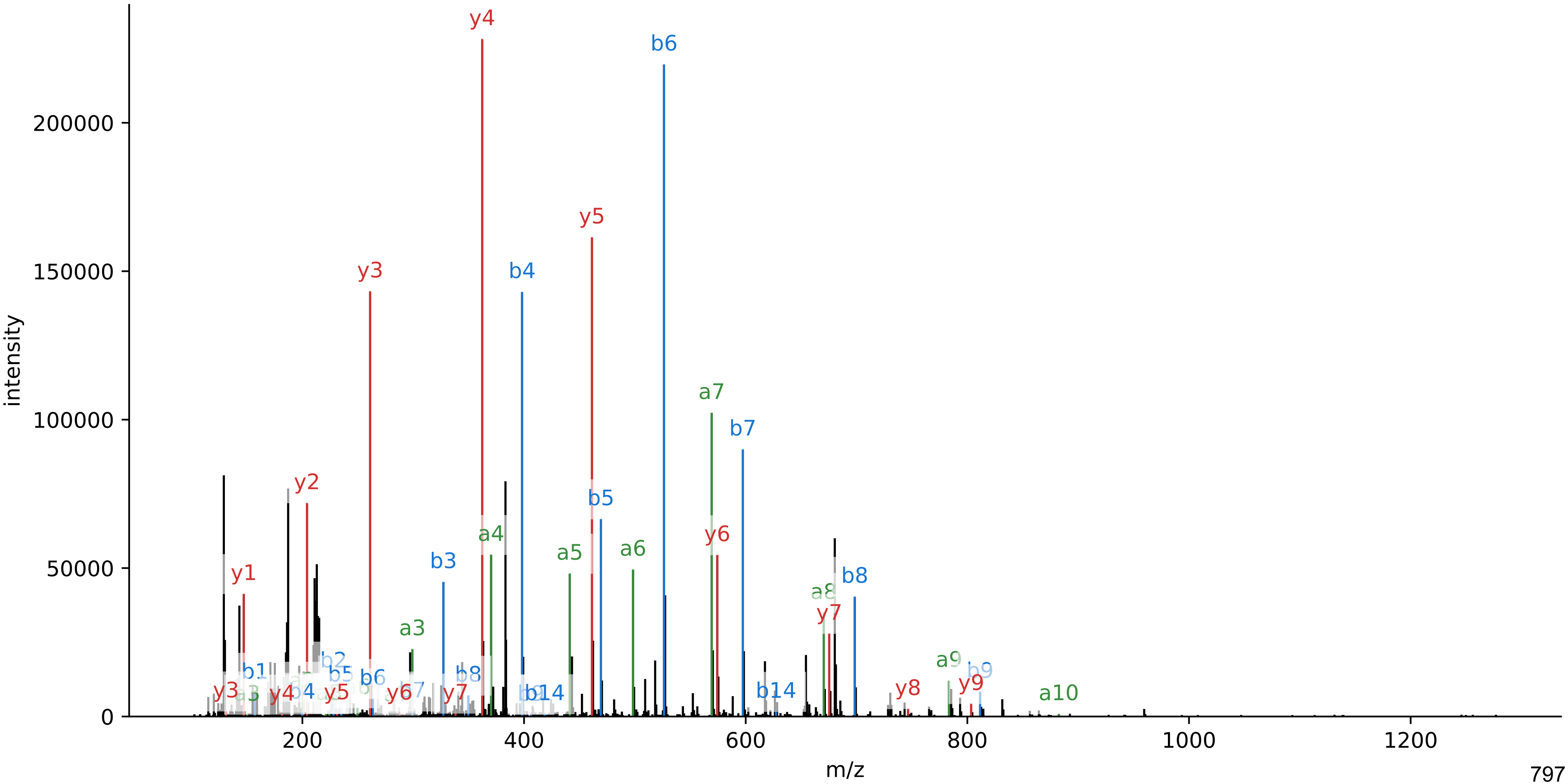

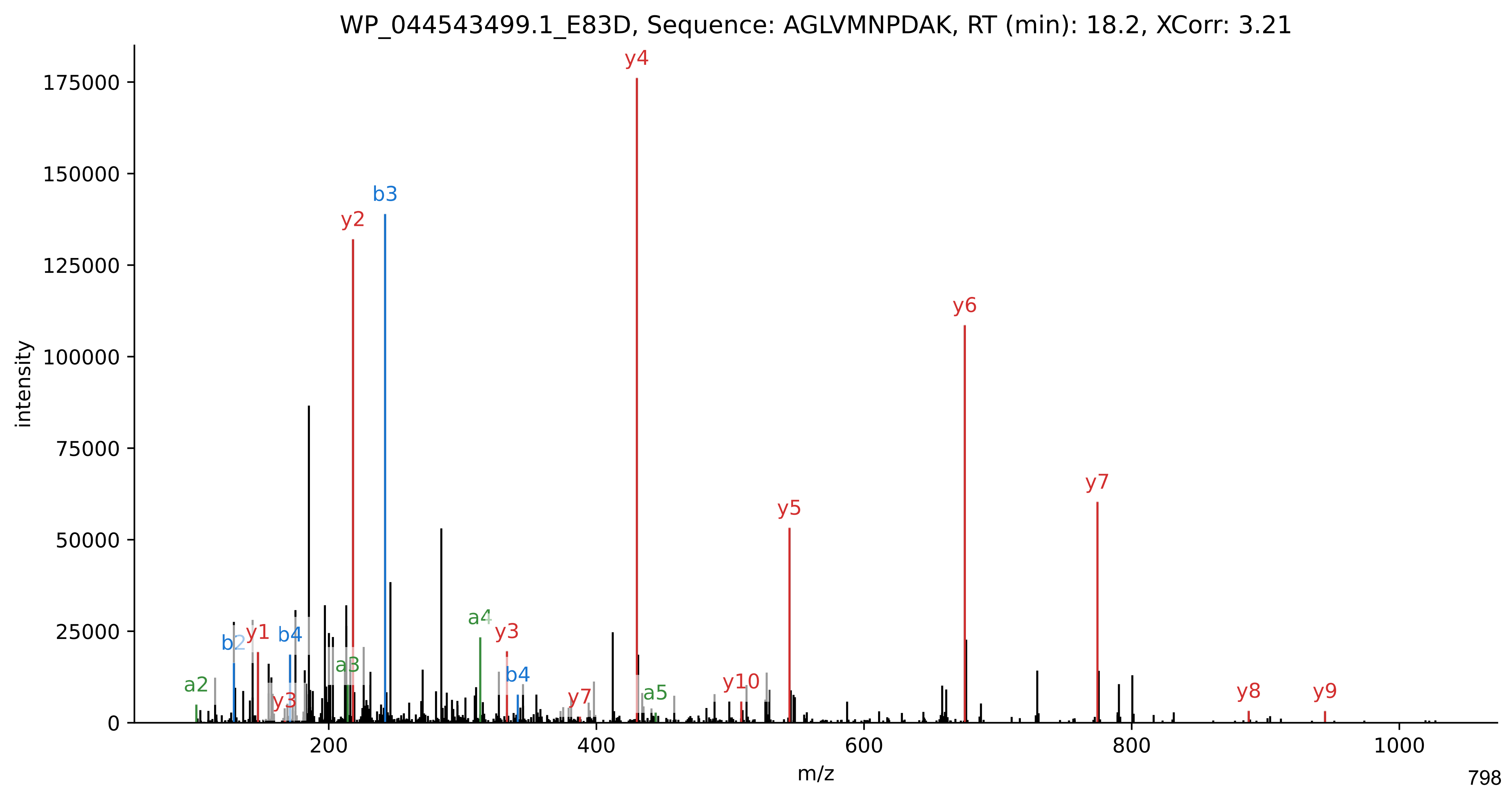
WP_044543499.1_E83D, Sequence: AGLVMNPDAK, RT (min): 18.2, XCorr: 3.21
intensity
m/z
0
25000
50000
75000
100000
125000
150000
175000
200
400
600
800
1000
y4
b3
y2
y6
y7
y5
a4
y3
y1
b4
b2
a2
y3
a3
b4
y7
a5
y10
y8
y9

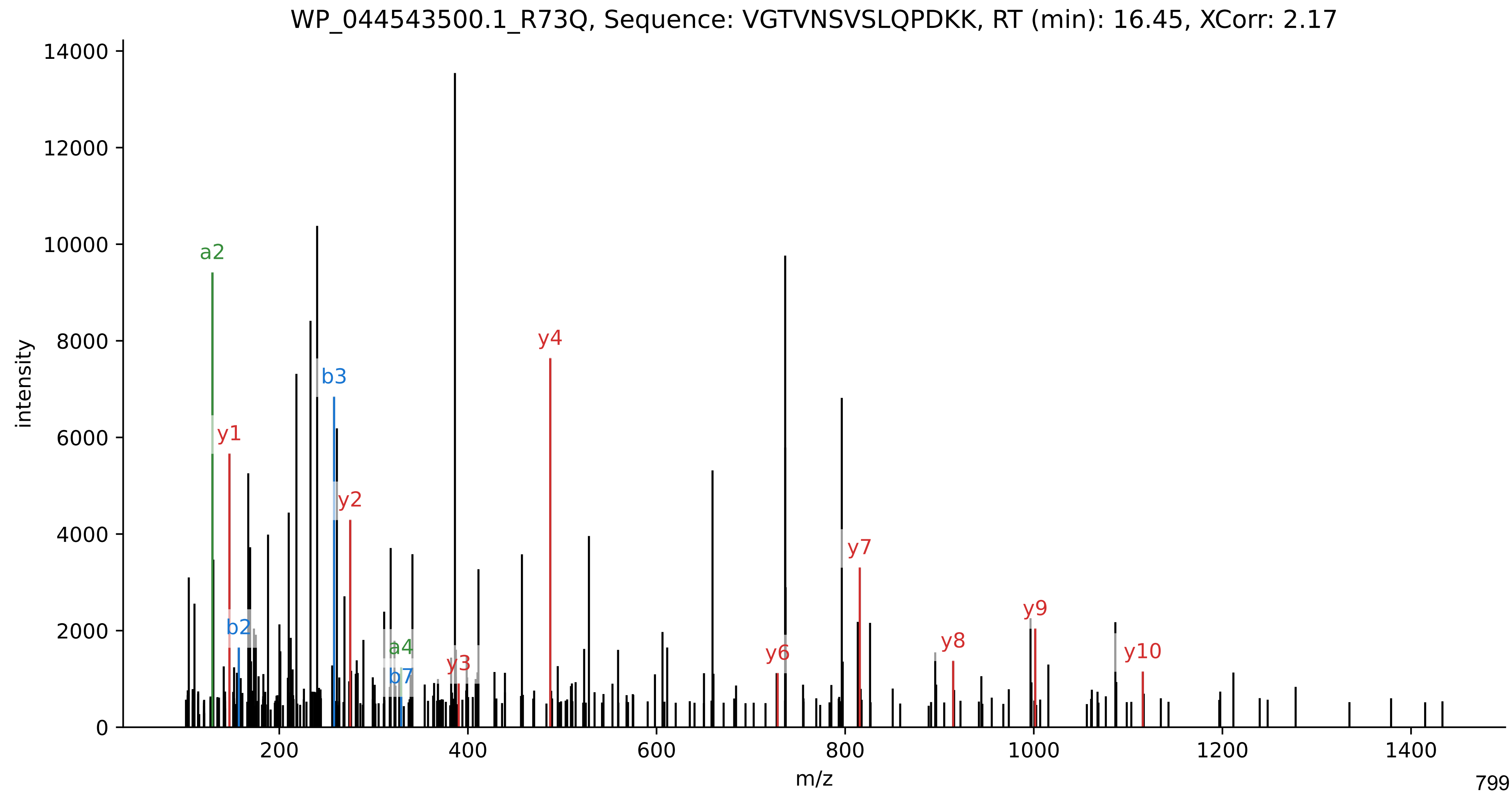

WP_044543500.1_R73Q, Sequence: VGTVNSVSLQPDKK, RT (min): 16.45, XCorr: 2.17
intensity
m/z
a2
y1
b2
b3
y2
a4
b7
y3
y4
y6
y7
y8
y9
y10

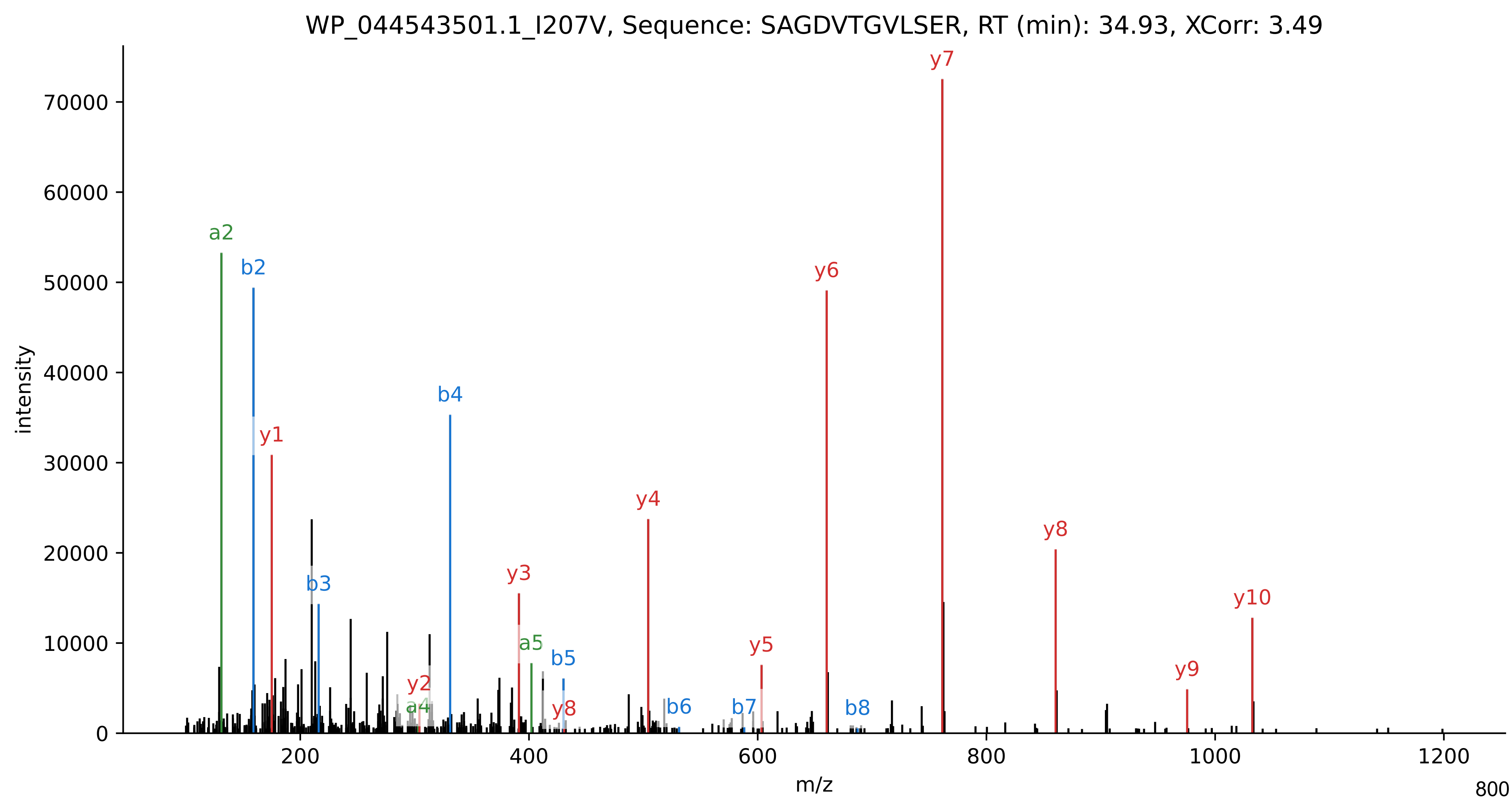

WP_044543501.1_I207V, Sequence: SAGDVTGVLSER, RT (min): 34.93, XCorr: 3.49
intensity
m/z
a2
b2
y1
b3
b4
y2
a4
y3
a5
b5
y8
y4
b6
b7
y5
y6
b8
y7
y8
y9
y10

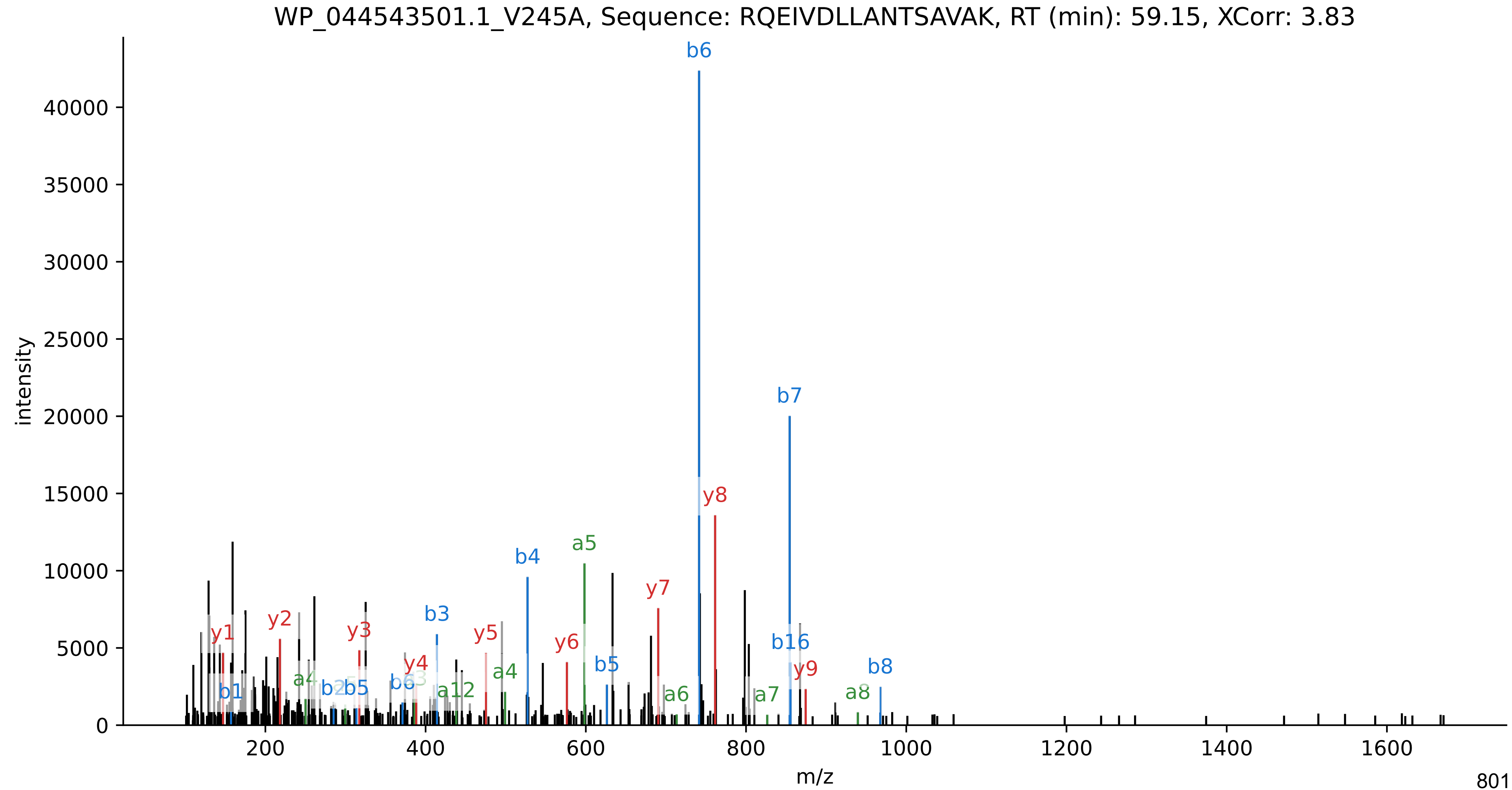

WP_044543501.1_V245A, Sequence: RQEIVDLLANTSAVAK, RT (min): 59.15, XCorr: 3.83
intensity
m/z
b6
b7
y8
a5
b4
y7
b3
y2
y3
y1
y5
y6
b16
b5
y9
b8
y4
a4
a4
b1
b6
a12
a6
a7
a8
0
5000
10000
15000
20000
25000
30000
35000
40000
200
400
600
800
1000
1200
1400
1600

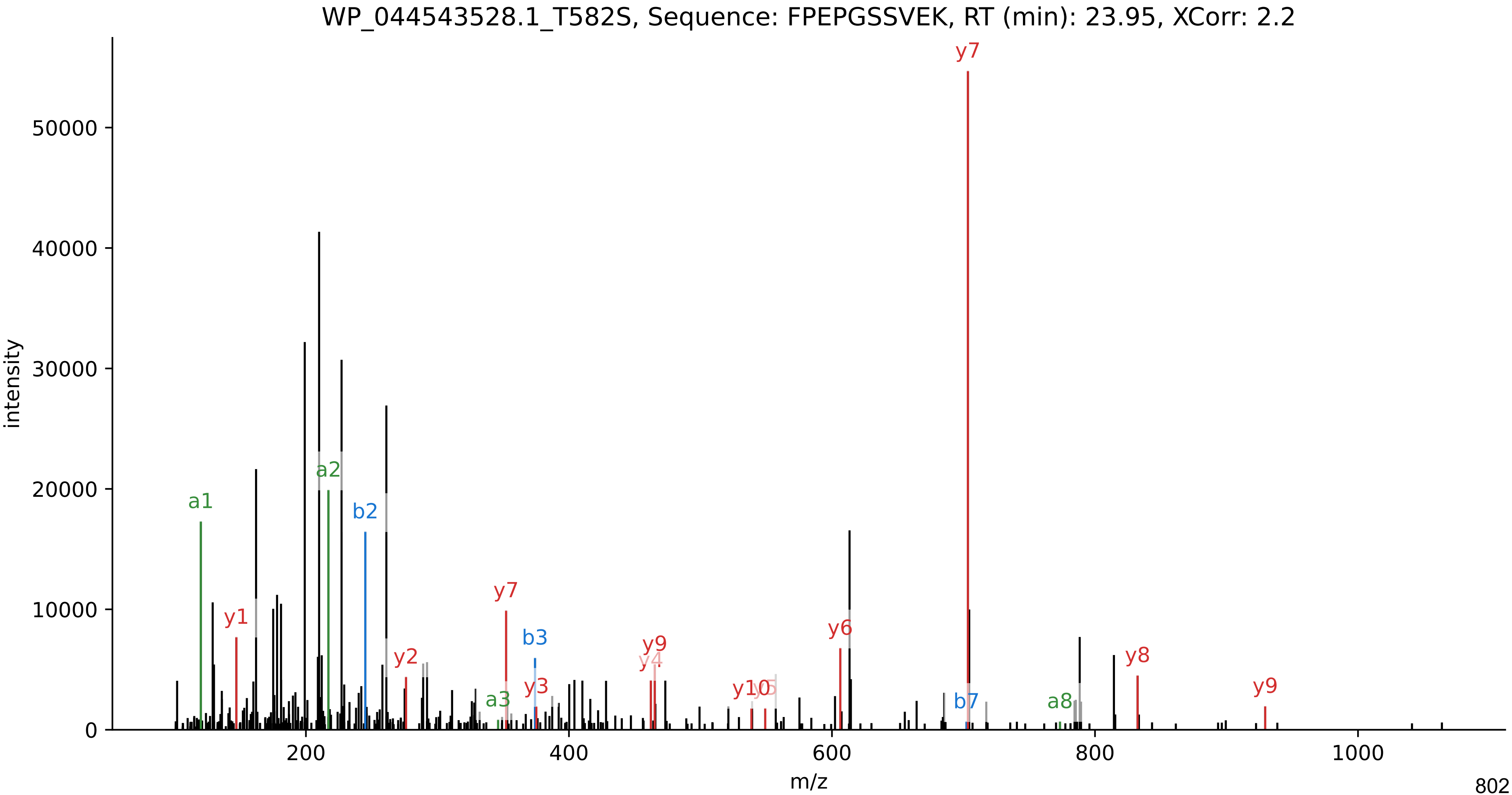

WP_044543528.1_T582S, Sequence: FPEPGSSVEK, RT (min): 23.95, XCorr: 2.2
y7
a2
a1
b2
y7
y1
b3
y9
y4
y6
y8
y2
y3
y10
y5
y9
a3
b7
a8
intensity
50000
40000
30000
20000
10000
0
200
400
600
800
1000
m/z

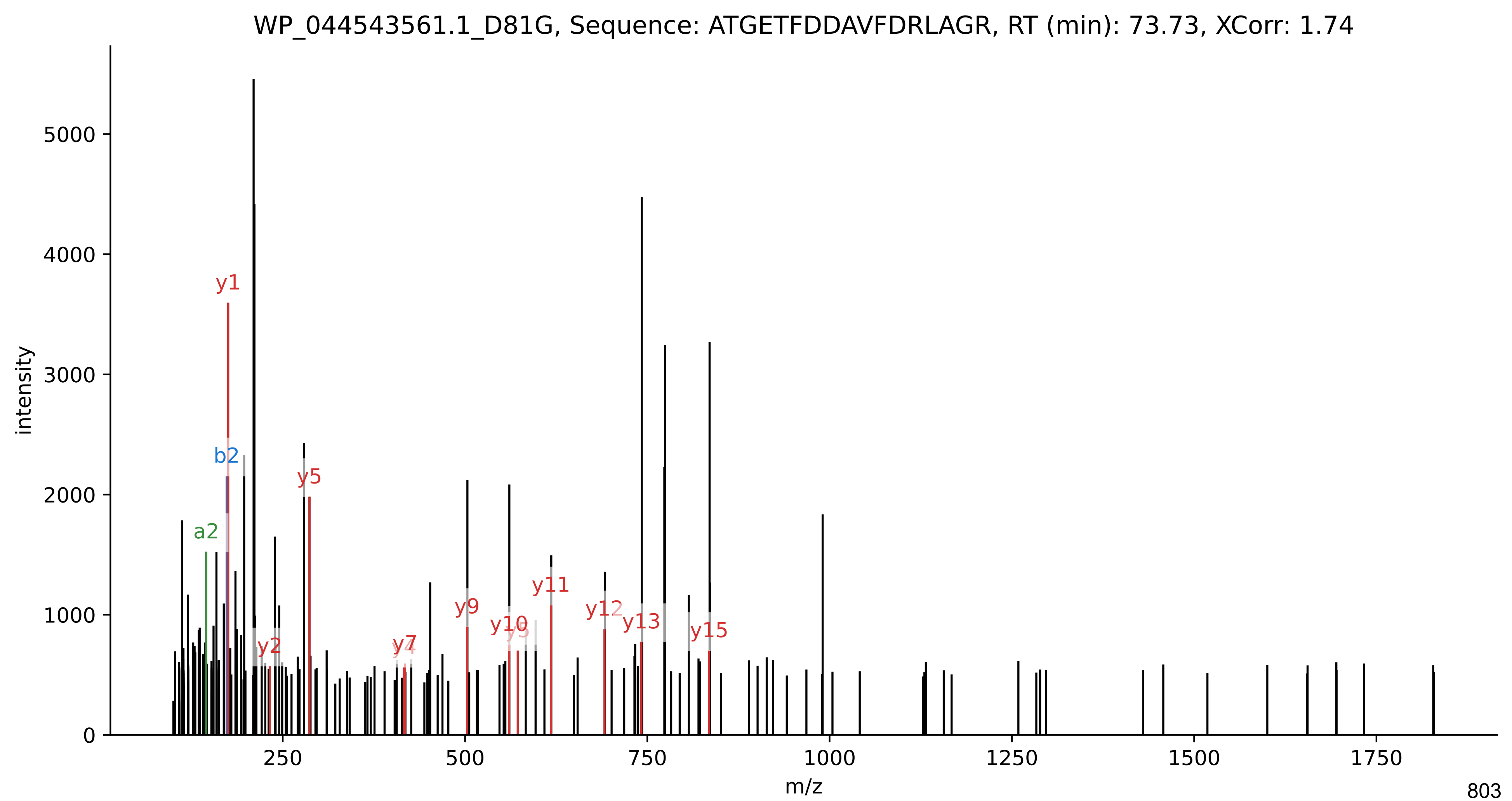
WP_044543561.1_D81G, Sequence: ATGETFDDAVFDRLAGR, RT (min): 73.73, XCorr: 1.74
intensity
m/z
y1
b2
a2
y5
y2
y7
y9
y10
y11
y12
y13
y15
0
1000
2000
3000
4000
5000
250
500
750
1000
1250
1500
1750

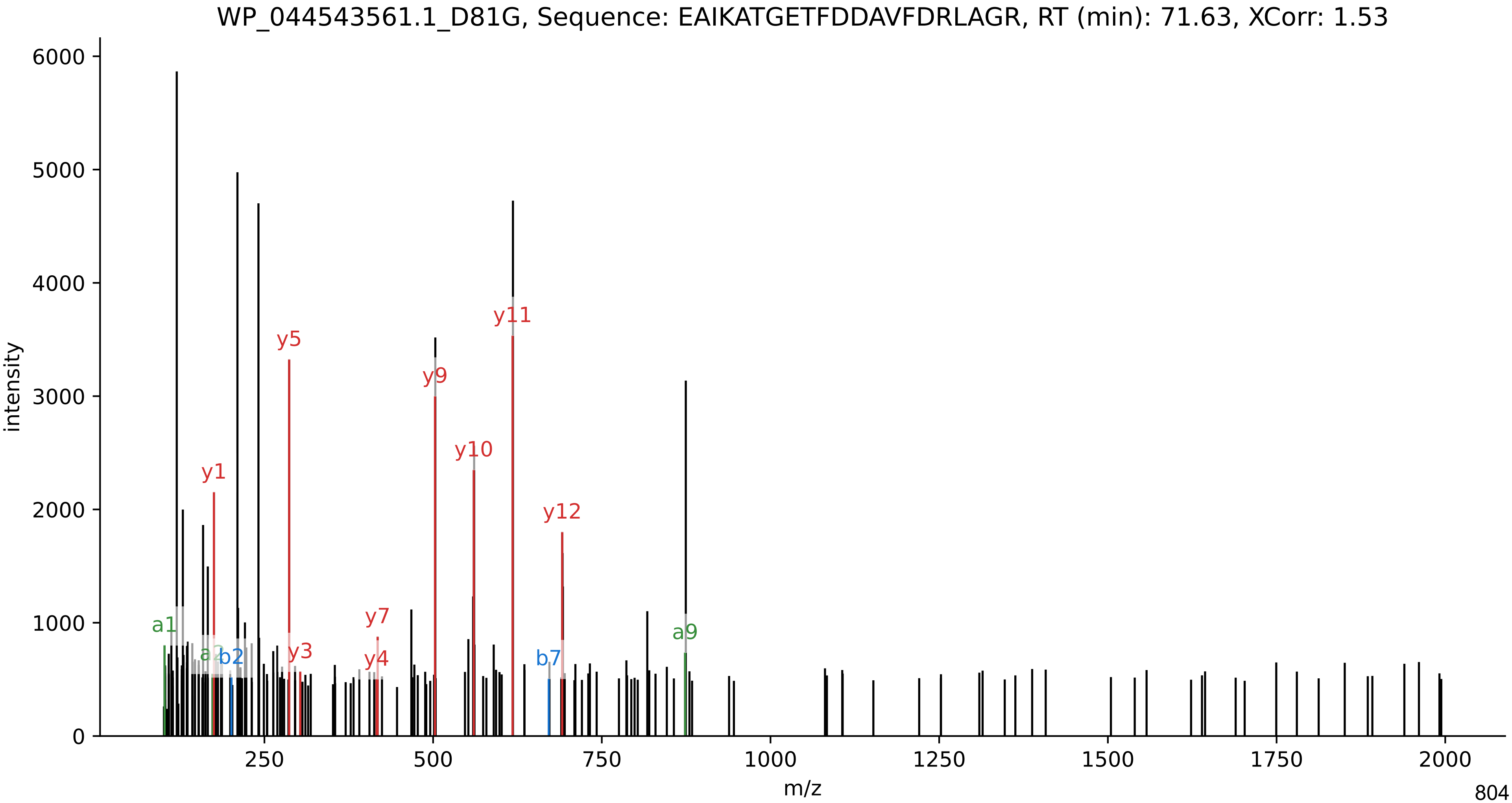

WP_044543561.1_D81G, Sequence: EAIKATGETFDDAVFDRLAGR, RT (min): 71.63, XCorr: 1.53
intensity
m/z
y1
y5
y9
y10
y11
y12
y7
y4
y3
a1
b2
b7
a9

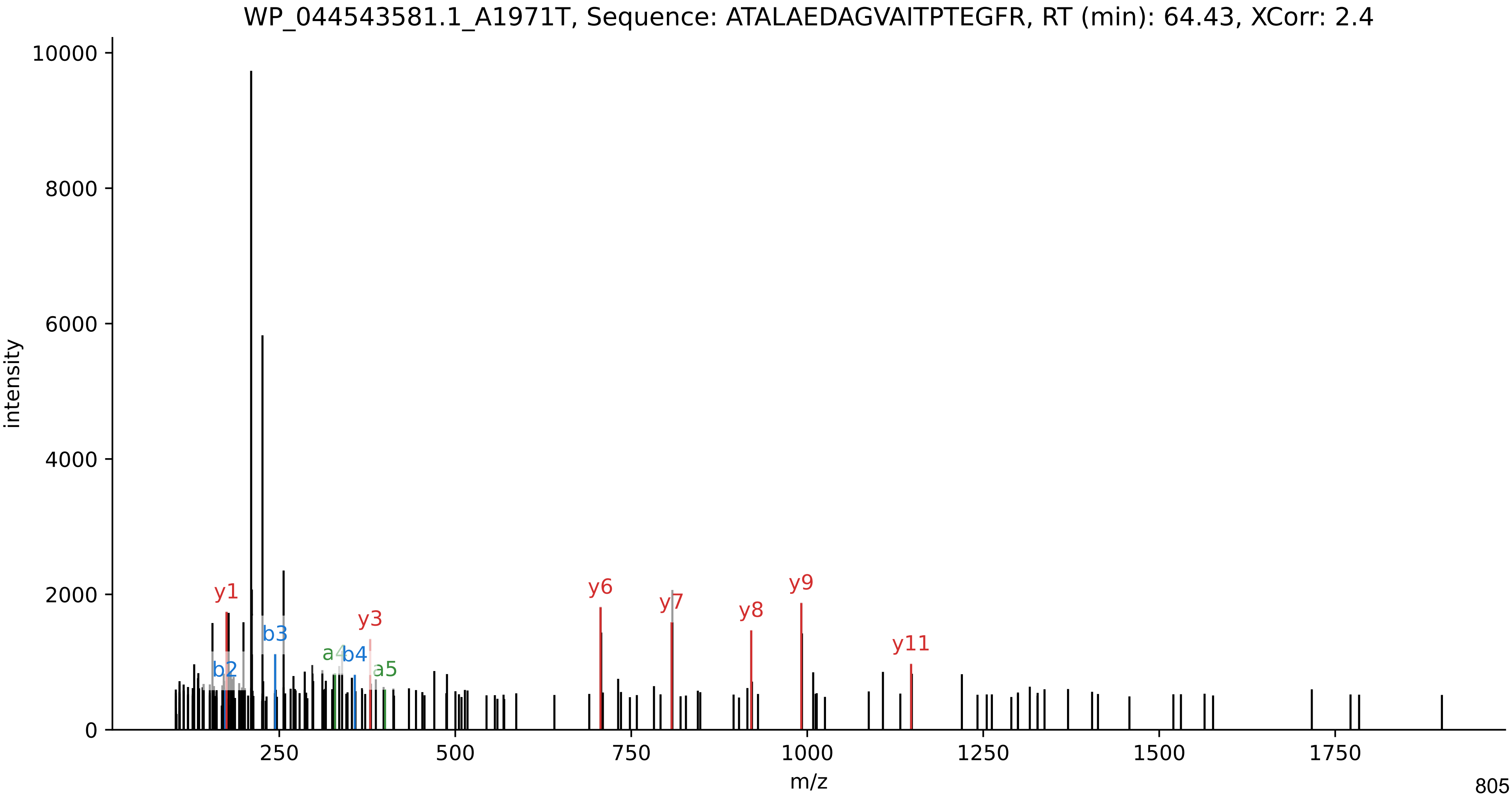

WP_044543581.1_A1971T, Sequence: ATALAEDAGVAITPTEGFR, RT (min): 64.43, XCorr: 2.4
intensity
m/z
0
2000
4000
6000
8000
10000
250
500
750
1000
1250
1500
1750
y1
b2
b3
a4
b4
a5
y3
y6
y7
y8
y9
y11

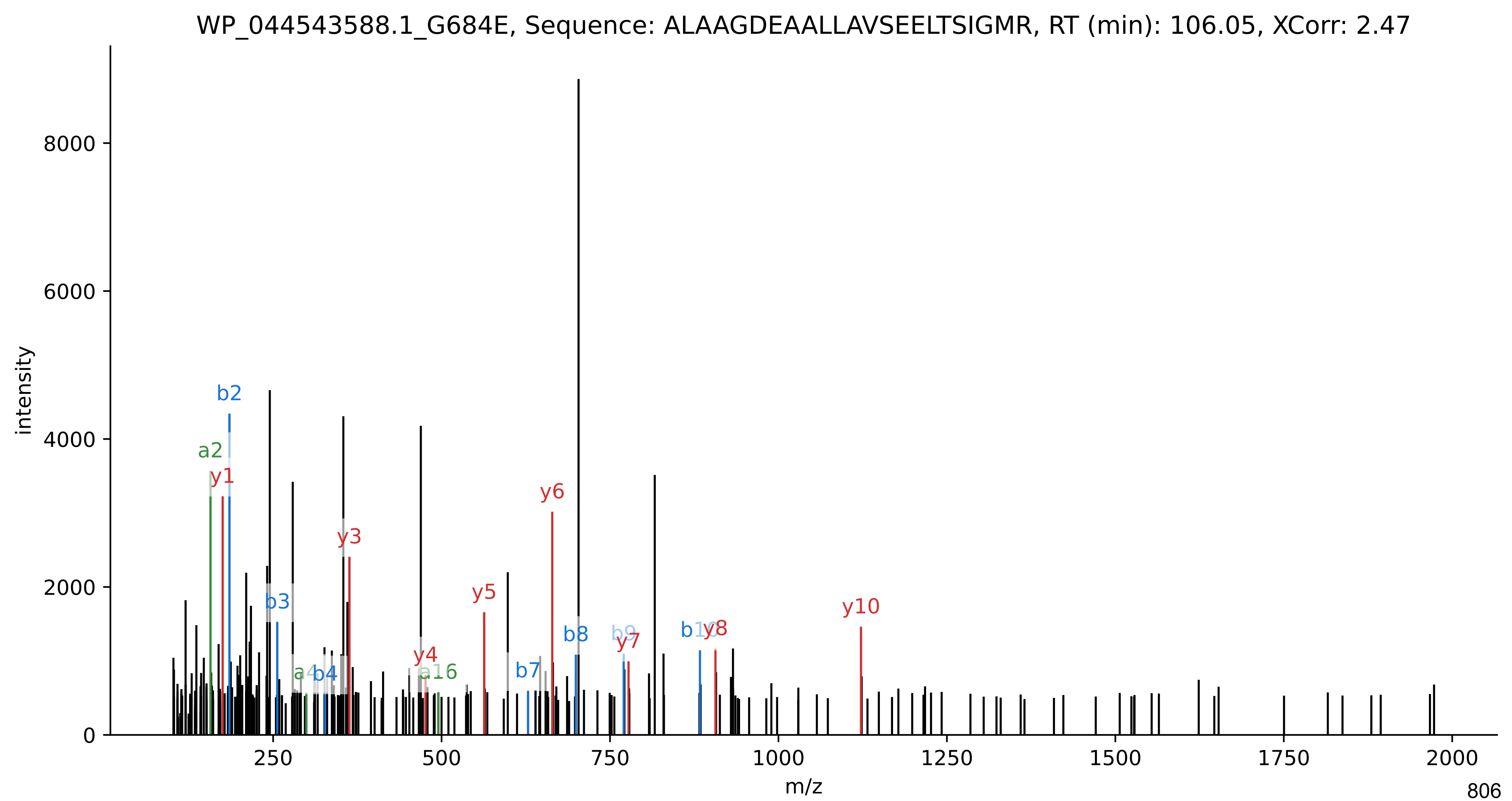
WP_044543588.1_G684E, Sequence: ALAAGDEAALLAVSEELTSIGMR, RT (min): 106.05, XCorr: 2.47
intensity
m/z
0
2000
4000
6000
8000
250
500
750
1000
1250
1500
1750
2000
b2
a2
y1
y6
y3
b3
y5
y10
b8
b9
y7
y8
b7
y4
a16
a4
b4

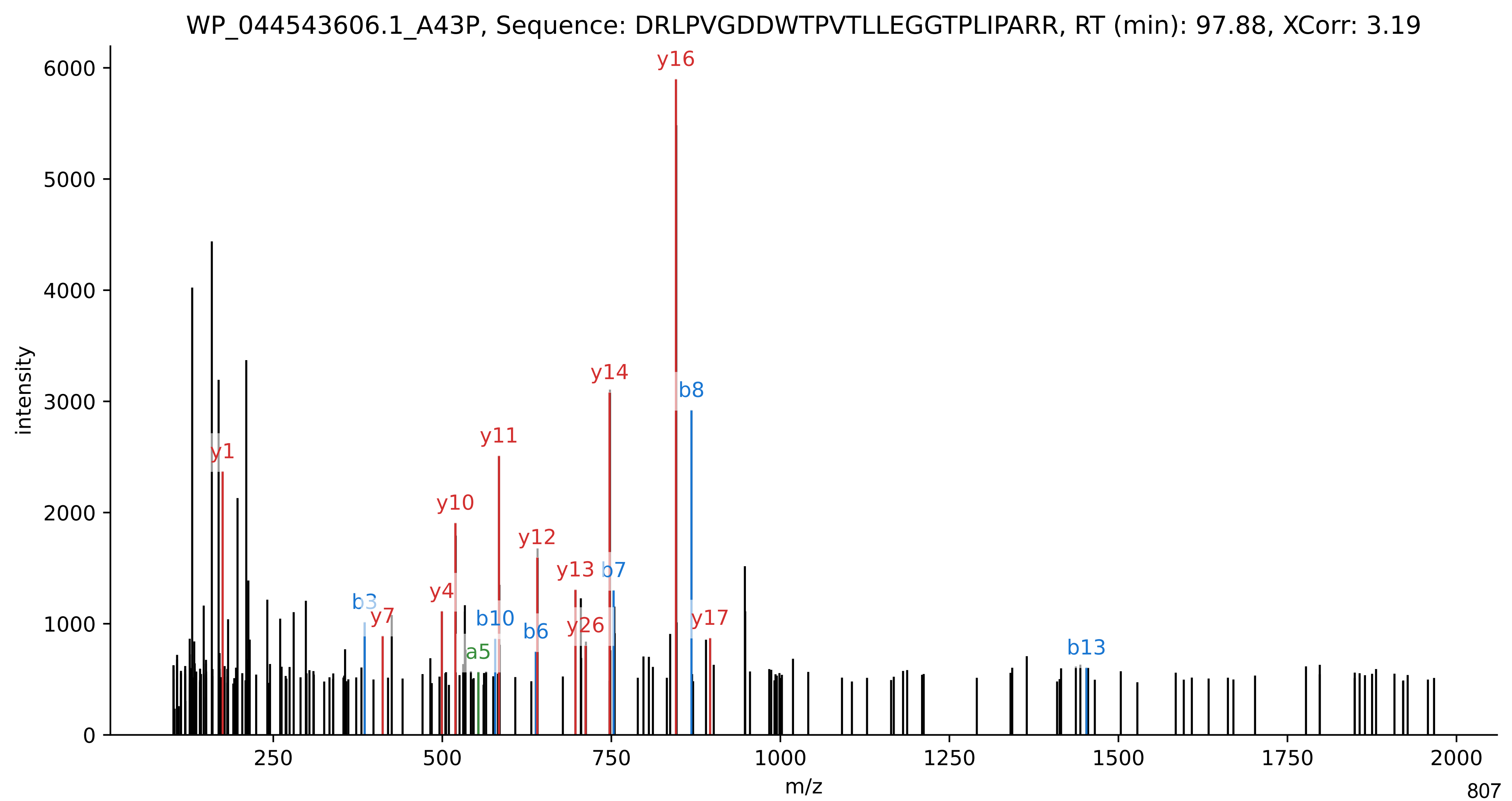

WP_044543606.1_A43P, Sequence: DRLPVGDDWTPVTLLEGGTPLIPARR, RT (min): 97.88, XCorr: 3.19
intensity
m/z
y16
y14
b8
y11
y1
y10
y12
y13
b7
y4
b3
y7
b10
y17
y26
b6
a5
b13

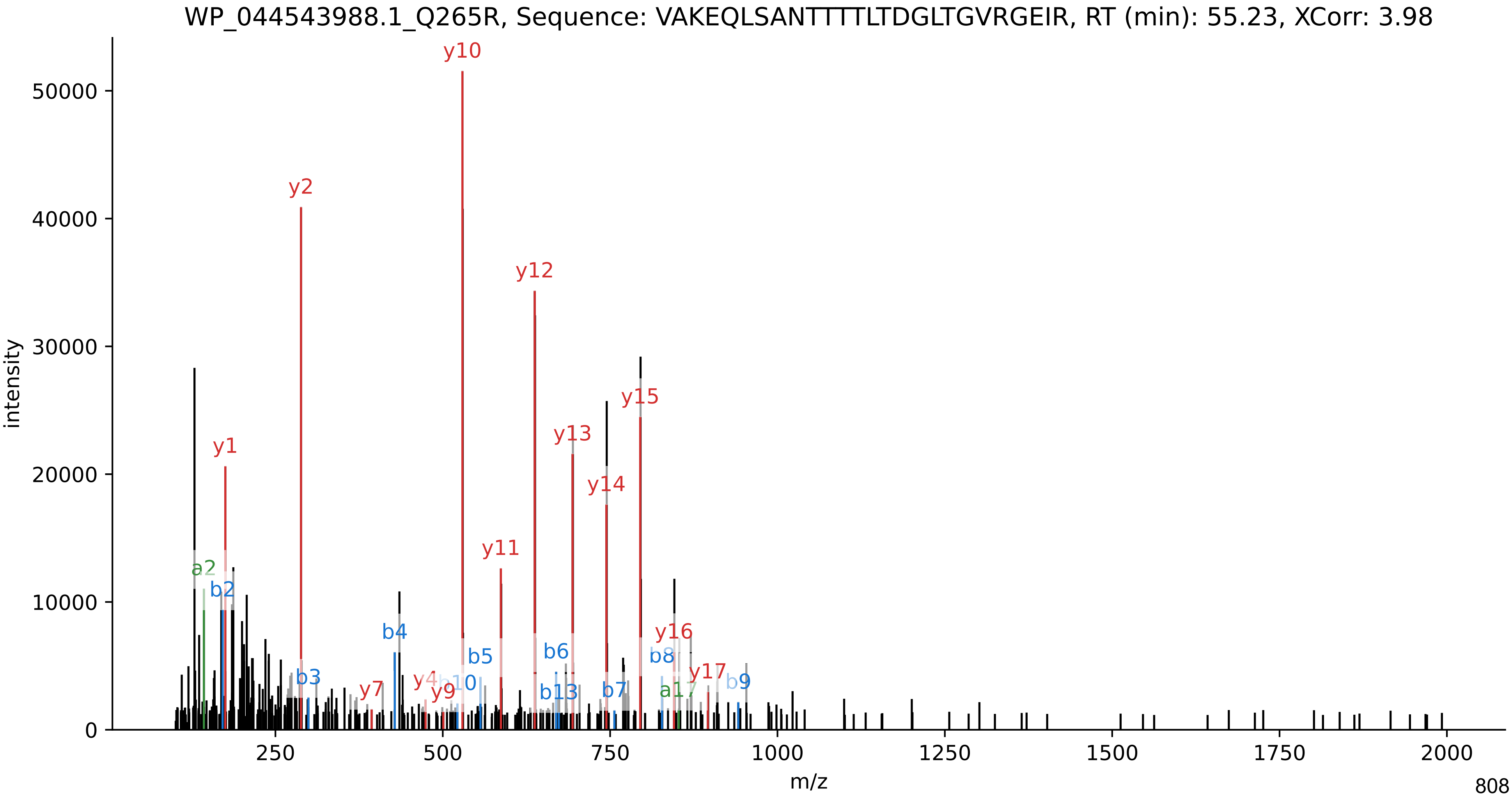
WP_044543988.1_Q265R, Sequence: VAKEQLSANTTTTLTDGLTGVRGEIR, RT (min): 55.23, XCorr: 3.98
intensity
m/z
y10
y2
y12
y15
y13
y14
y1
y11
a2
b2
b4
y16
b5
b6
b8
y17
b3
y4
b10
y9
y7
b13
b7
a17
b9

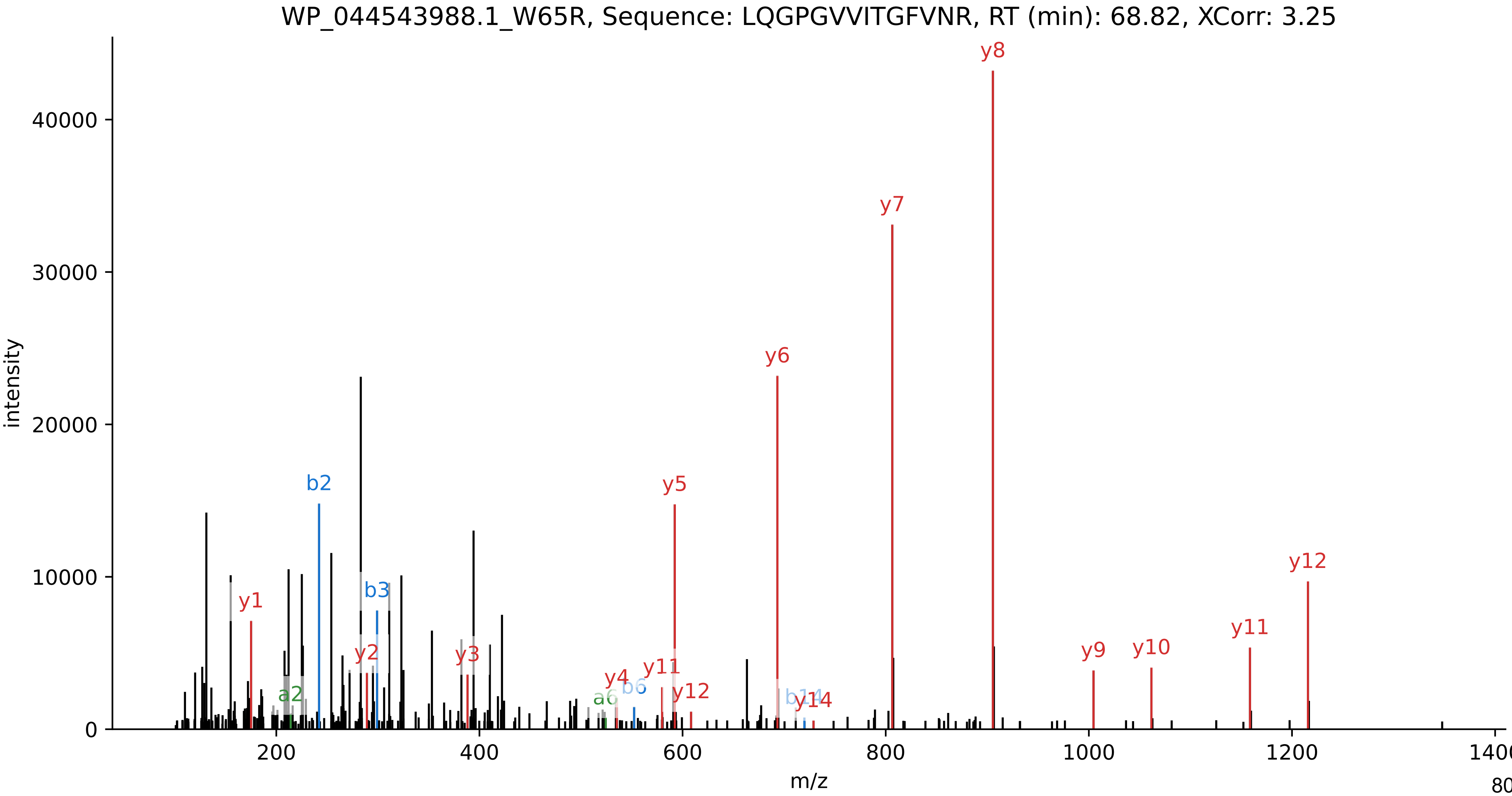

WP_044543988.1_W65R, Sequence: LQGPGVVITGFVNR, RT (min): 68.82, XCorr: 3.25
intensity
m/z
y8
y7
y6
y5
b2
b3
y1
y2
y3
y12
y11
y10
y9
y4
y11
y12
a2
a6
b6

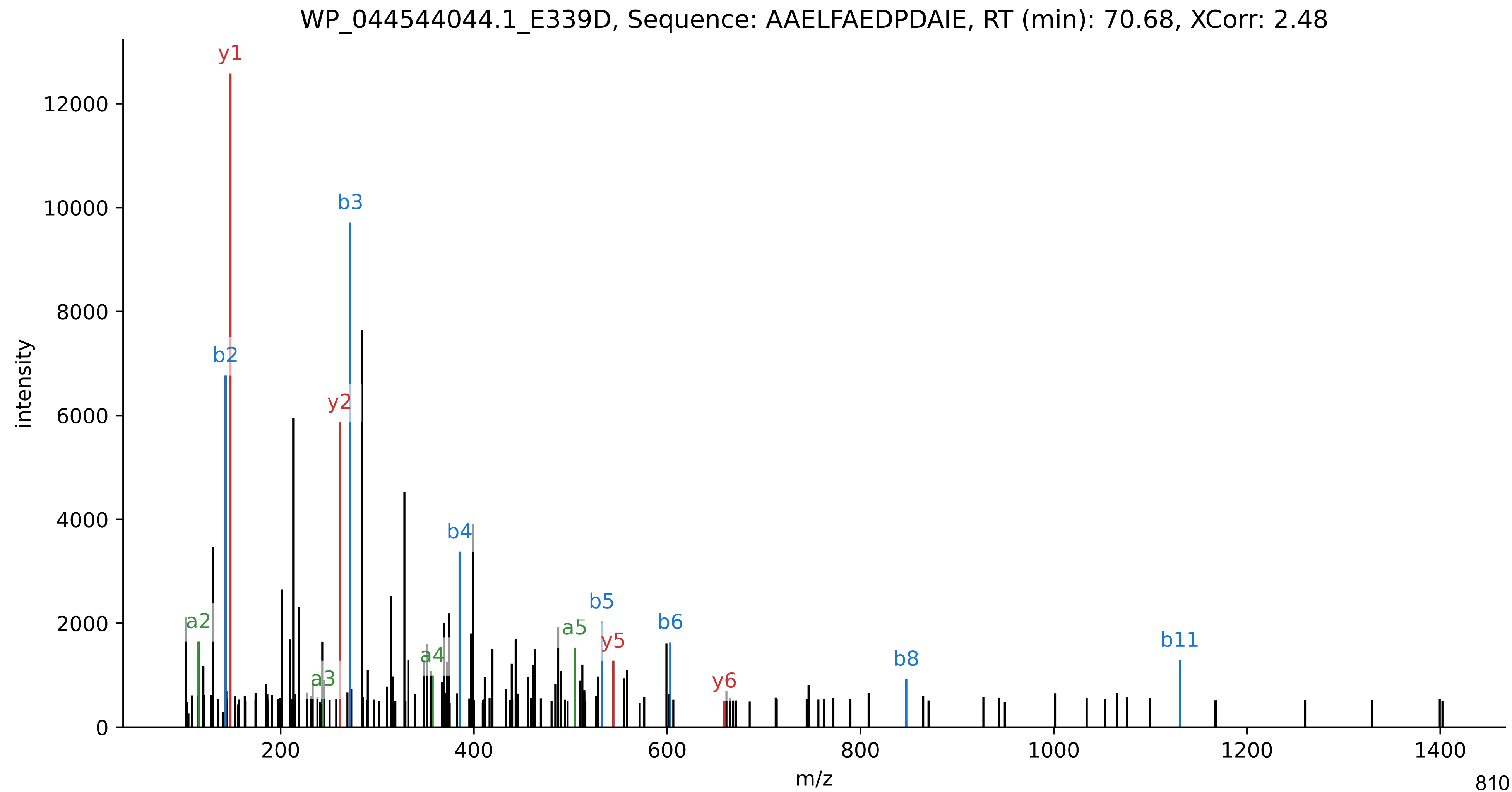

WP_044544044.1_E339D, Sequence: AAELFAEDPDAIE, RT (min): 70.68, XCorr: 2.48
y1
b3
b2
y2
b4
a2
b5
a5
b6
y5
a4
a3
y6
b8
b11
intensity
m/z
0
2000
4000
6000
8000
10000
12000
200
400
600
800
1000
1200
1400

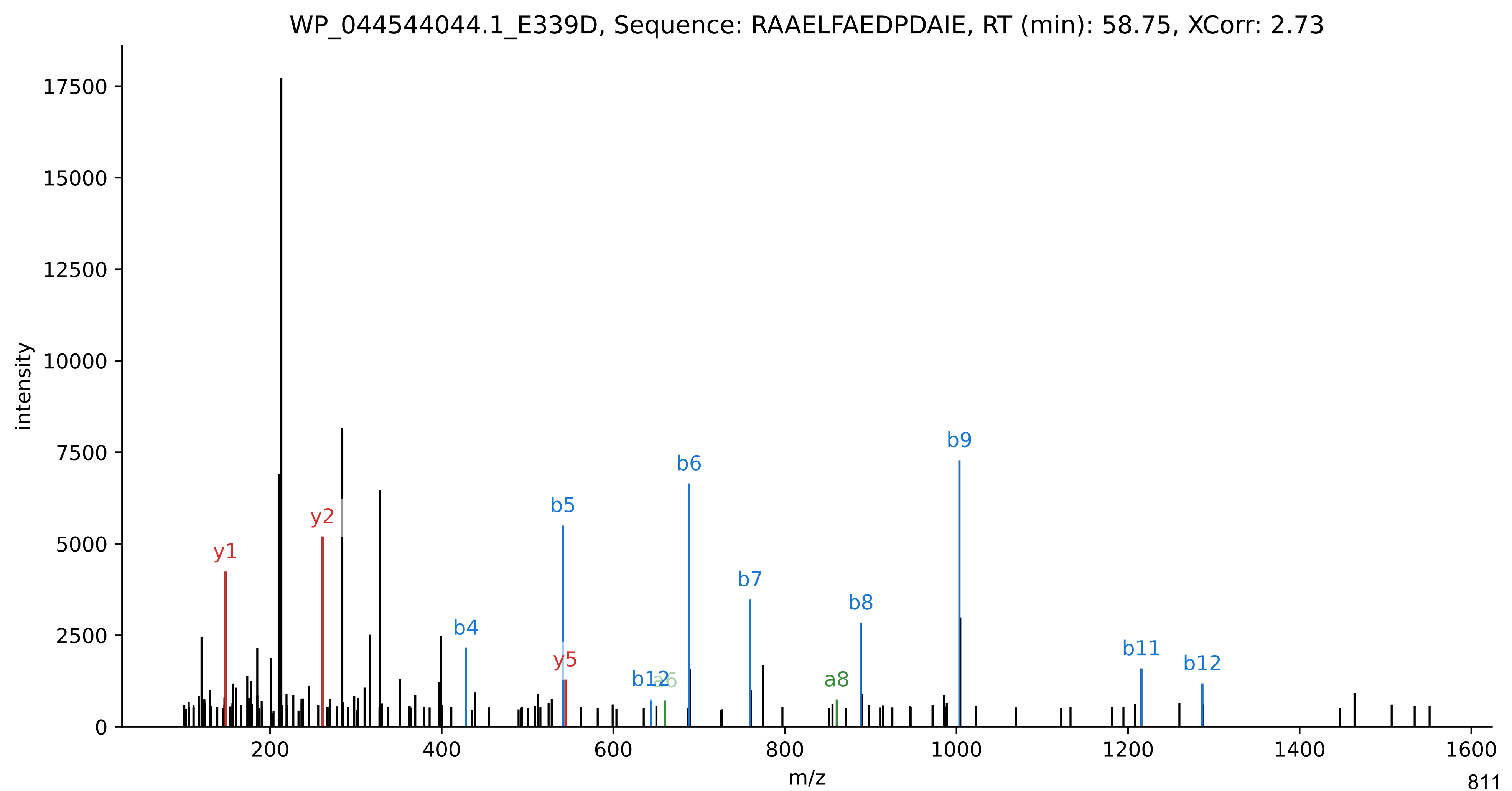
WP_044544044.1_E339D, Sequence: RAAELFAEDPDAIE, RT (min): 58.75, XCorr: 2.73
intensity
m/z
y1
y2
y5
b4
b5
b6
b7
b8
b9
b11
b12
a8

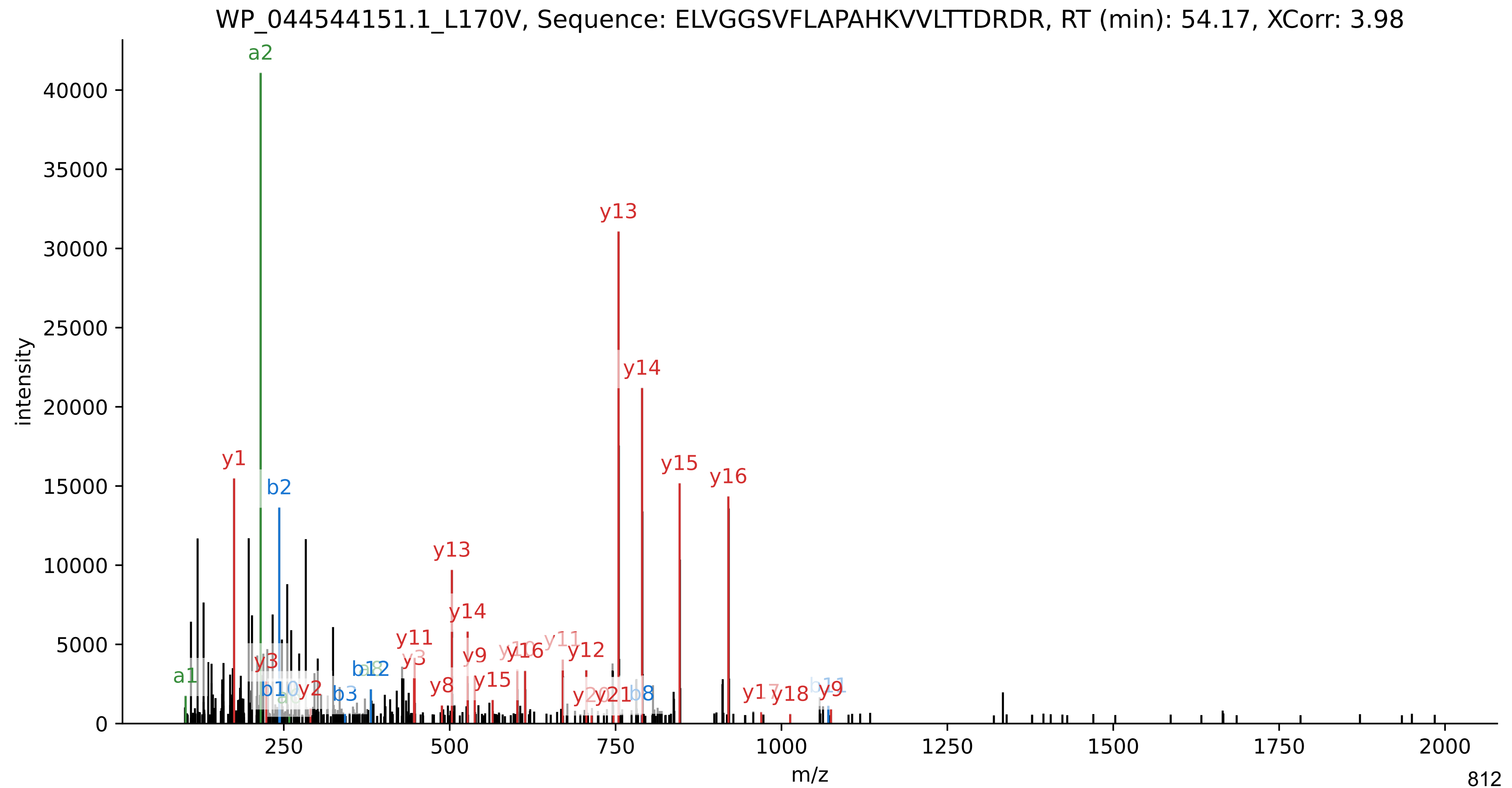

WP_044544151.1_L170V, Sequence: ELVGGSVFLAPAHKVVLTTDRDR, RT (min): 54.17, XCorr: 3.98
intensity
m/z
a2
y13
y14
y1
b2
y15
y16
y13
y14
y11
y3
y9
y16
y11
y12
a1
y3
b12
y8
y15
b10
y2
b3
y21
b8
y17
y18
y9

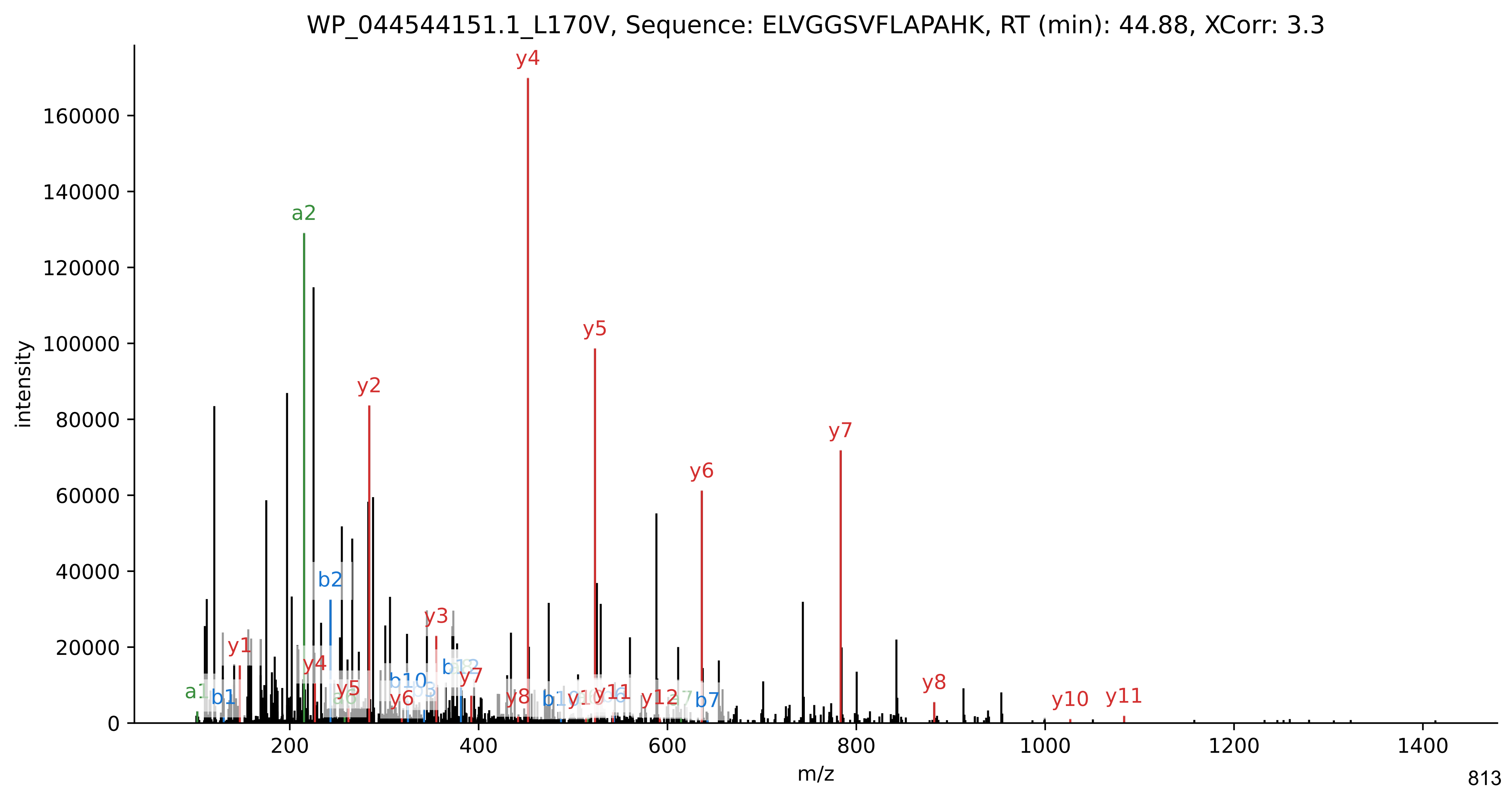
WP_044544151.1_L170V, Sequence: ELVGGSVFLAPAHK, RT (min): 44.88, XCorr: 3.3
intensity
m/z
y4
a2
y5
y2
y7
y6
b2
y3
y1
y4
a1
b1
y5
y6
b10
y7
y8
y8
y11
y12
b7
y10
y11
0
20000
40000
60000
80000
100000
120000
140000
160000
200
400
600
800
1000
1200
1400

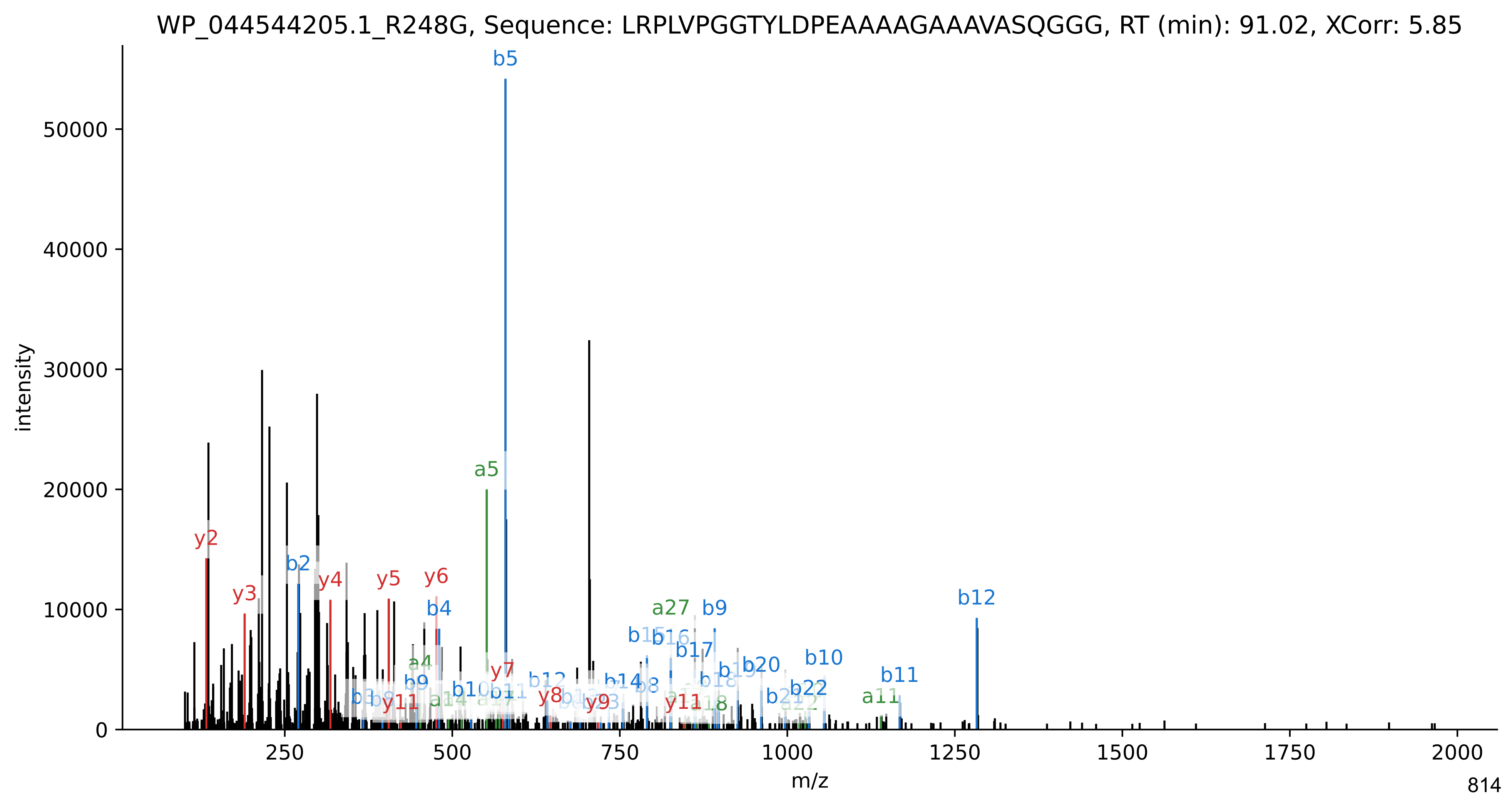

WP_044544205.1_R248G, Sequence: LRPLVPGGTYLDPEAAAAGAAAVASQGGG, RT (min): 91.02, XCorr: 5.85
intensity
m/z
b5
a5
y2
y3
b2
y4
y5
y6
b4
y7
y8
y9
y11
a27
b9
b12
b10
b11
b20
b22
a11
b14
b17
b16

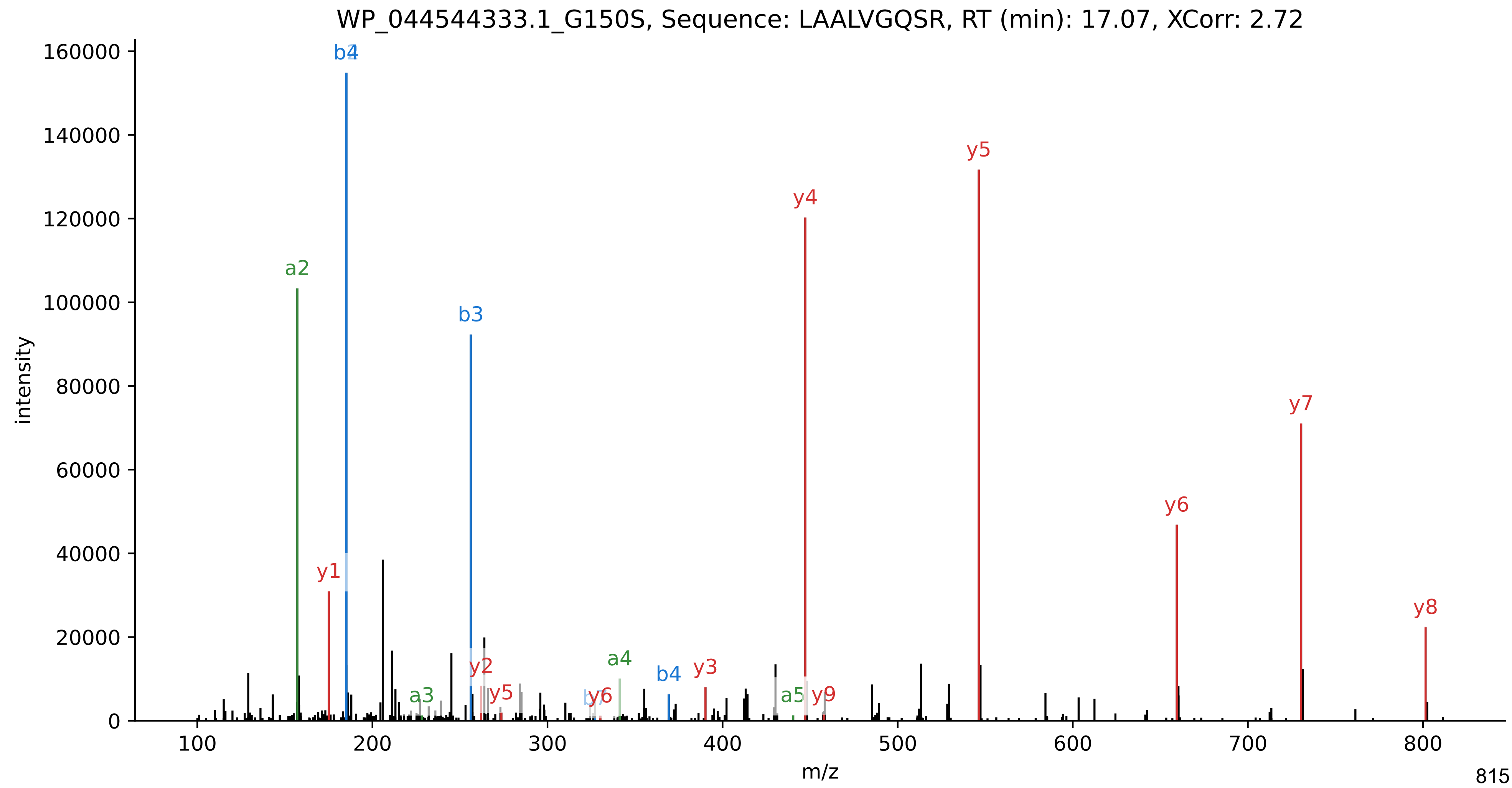
WP_044544333.1_G150S, Sequence: LAALVGQSR, RT (min): 17.07, XCorr: 2.72
intensity
m/z
a2
b4
b3
y1
y2
y5
a3
y6
a4
b4
y3
y4
a5
y9
y5
y6
y7
y8

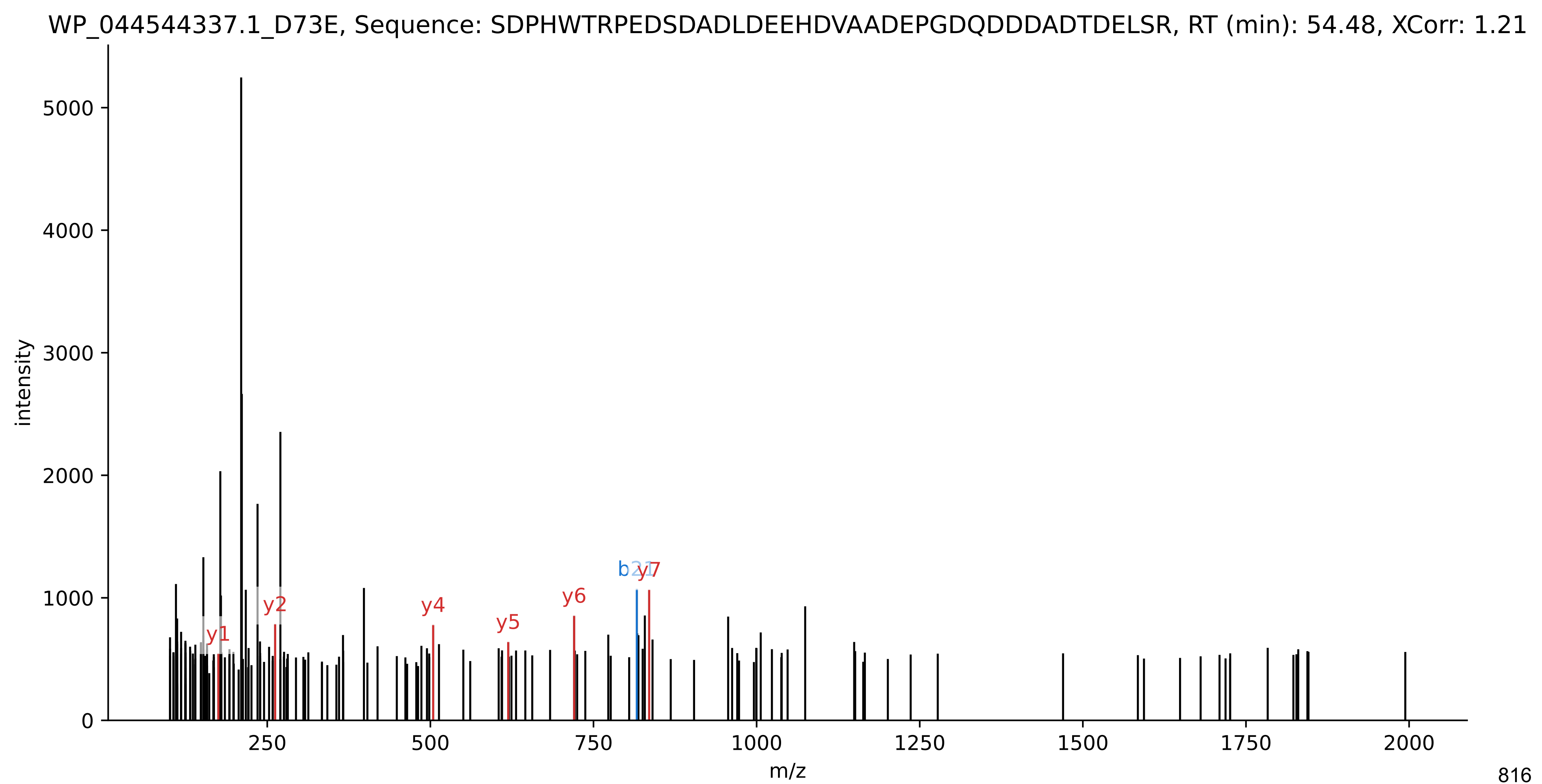
WP_044544337.1_D73E, Sequence: SDPHWTRPEDSDADLDEEHDVAADEPGDQDDDADTDELSR, RT (min): 54.48, XCorr: 1.21
intensity
m/z
y1
y2
y4
y5
y6
b21
y7
0
1000
2000
3000
4000
5000
250
500
750
1000
1250
1500
1750
2000

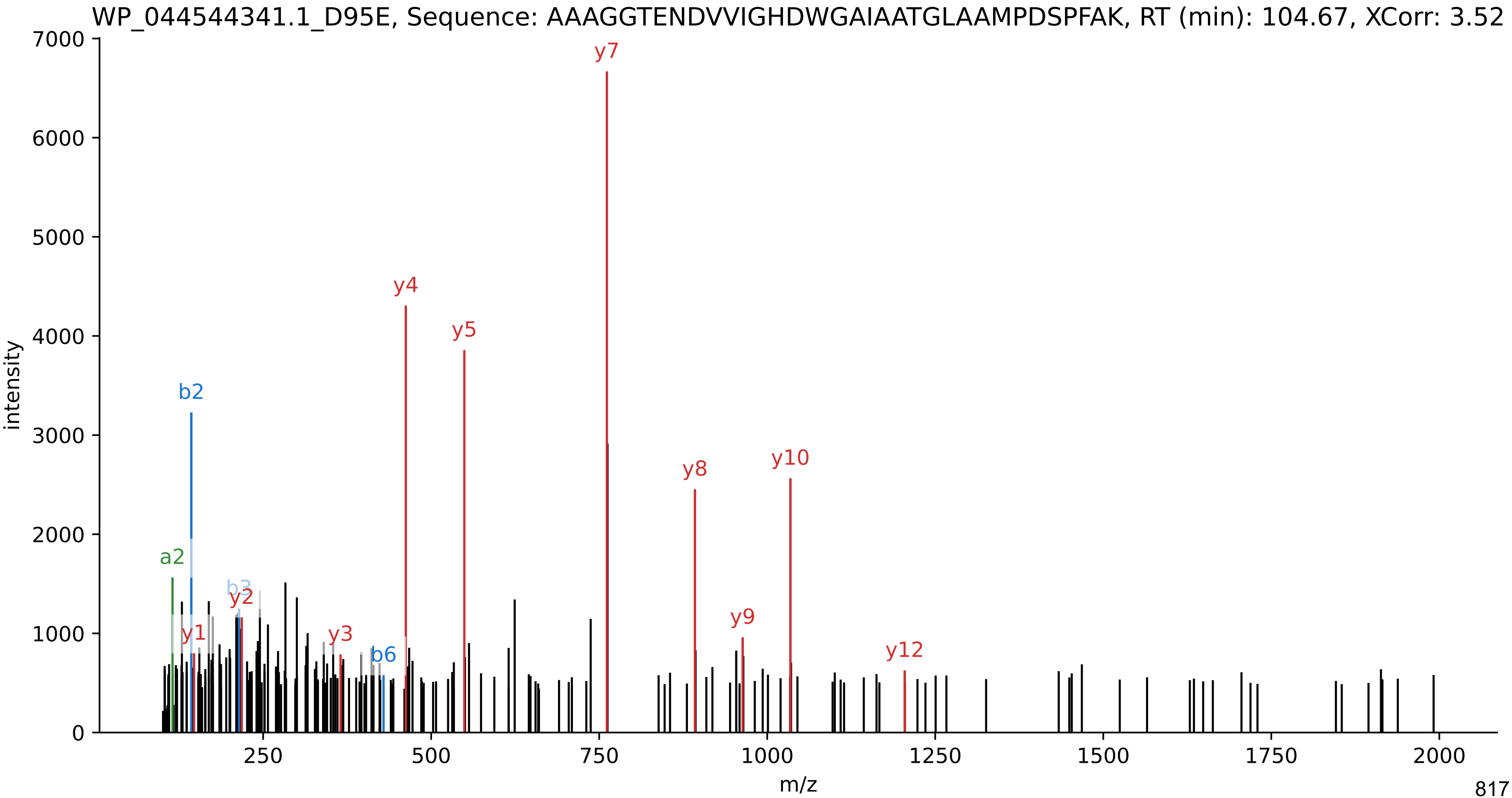

WP_044544341.1_D95E, Sequence: AAAGGTENDVVIGHDWGAIAATGLAAMPDSPFAK, RT (min): 104.67, XCorr: 3.52
intensity
m/z
y7
y4
y5
b2
y10
y8
a2
b3
y2
y9
y1
y3
b6
y12
0
1000
2000
3000
4000
5000
6000
7000
250
500
750
1000
1250
1500
1750
2000

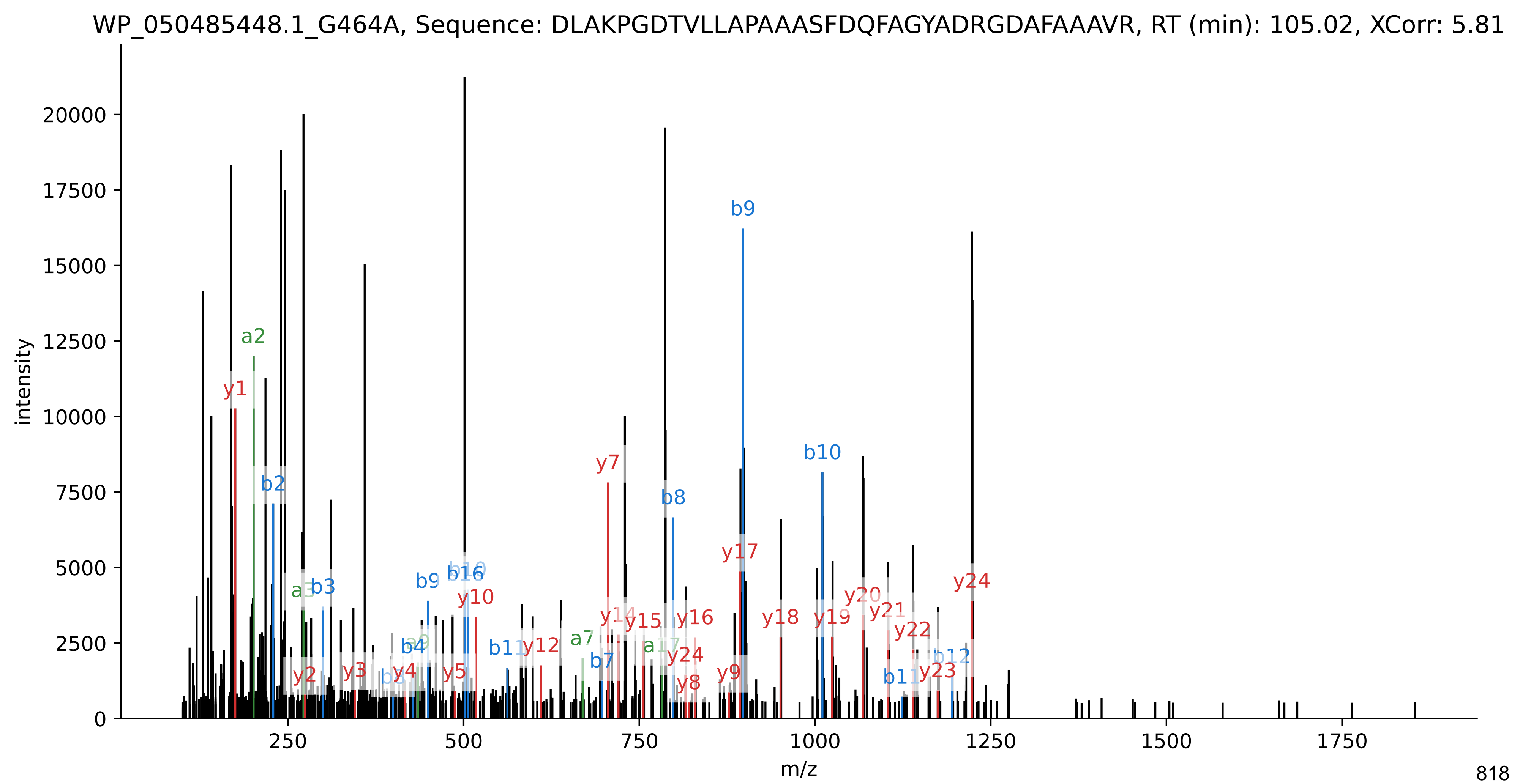
WP_050485448.1_G464A, Sequence: DLAKPGDTVLLAPAAASFDQFAGYADRGDAFAAAVR, RT (min): 105.02, XCorr: 5.81
intensity
m/z
b9
a2
y1
b10
b8
b2
y7
y17
y24
b3
b9
b16
y10
y15
y16
y18
y19
y20
y21
y22
y12
a7
b4
b7
y24
y8
y9
y2
y3
y4
y5
b11
y23
b12

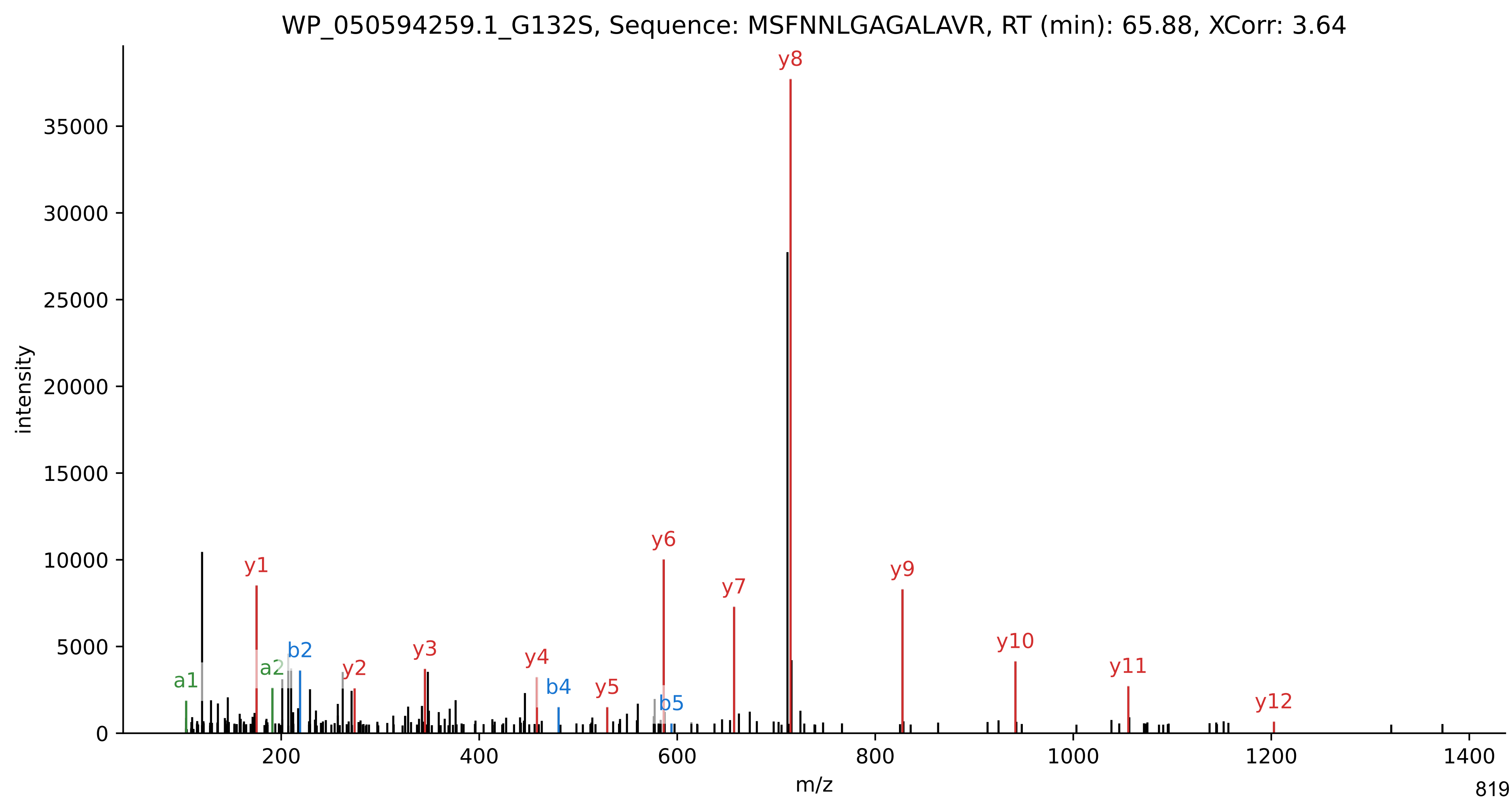

WP_050594259.1_G132S, Sequence: MSFNNLGAGALAVR, RT (min): 65.88, XCorr: 3.64
intensity
m/z
y8
y6
y1
y7
y9
y10
y11
y12
y2
y3
y4
y5
a1
a2
b2
b4
b5

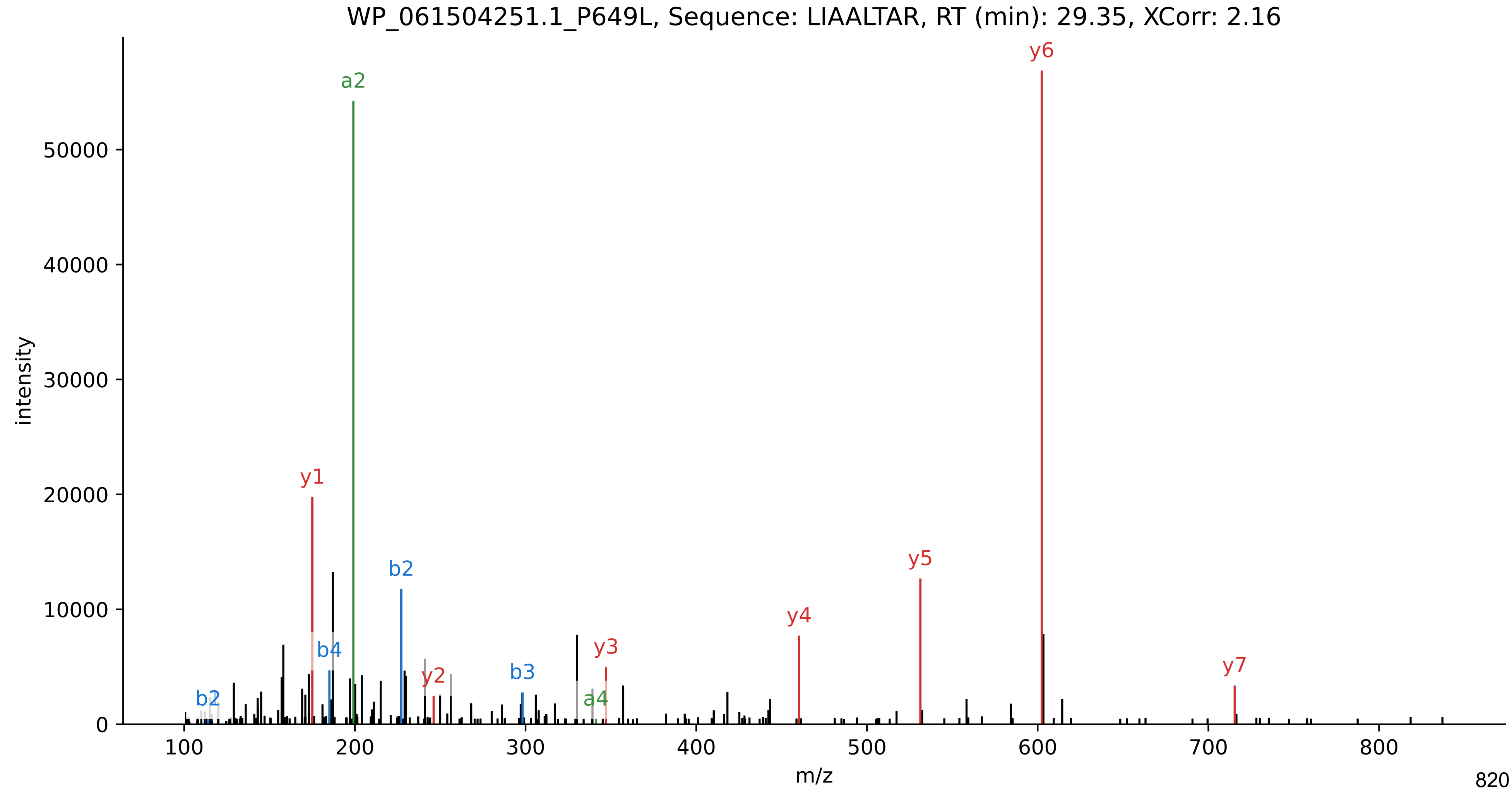

WP_061504251.1_P649L, Sequence: LIAALTAR, RT (min): 29.35, XCorr: 2.16
intensity
m/z
y6
a2
y1
b2
b4
y2
b3
a4
y3
y4
y5
y7
b2

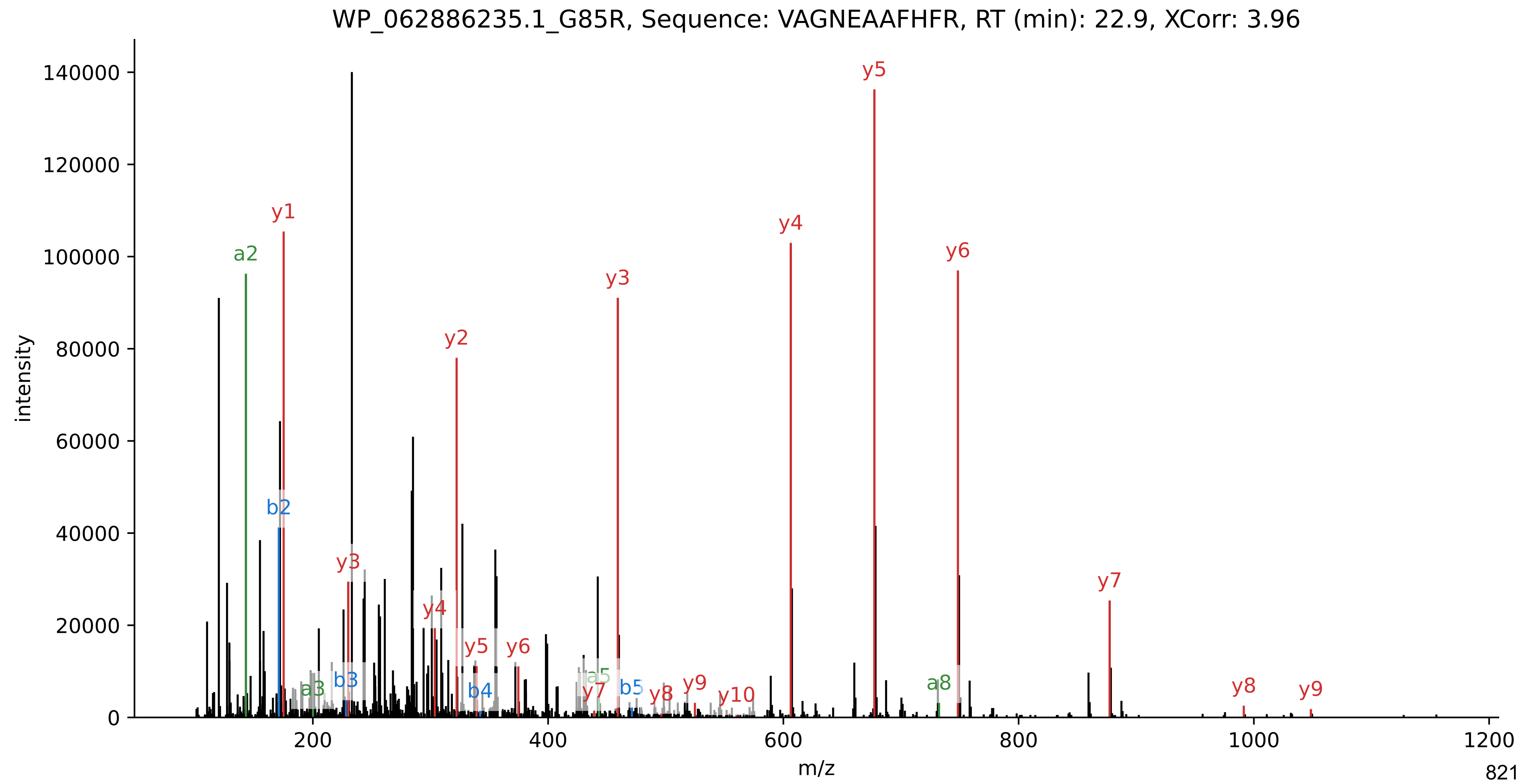

WP_062886235.1_G85R, Sequence: VAGNEAAFHFR, RT (min): 22.9, XCorr: 3.96
intensity
m/z
y1
a2
b2
y3
a3
b3
y4
y2
y5
b4
y6
a5
y7
b5
y8
y9
y10
y3
y4
y5
y6
a8
y7
y8
y9

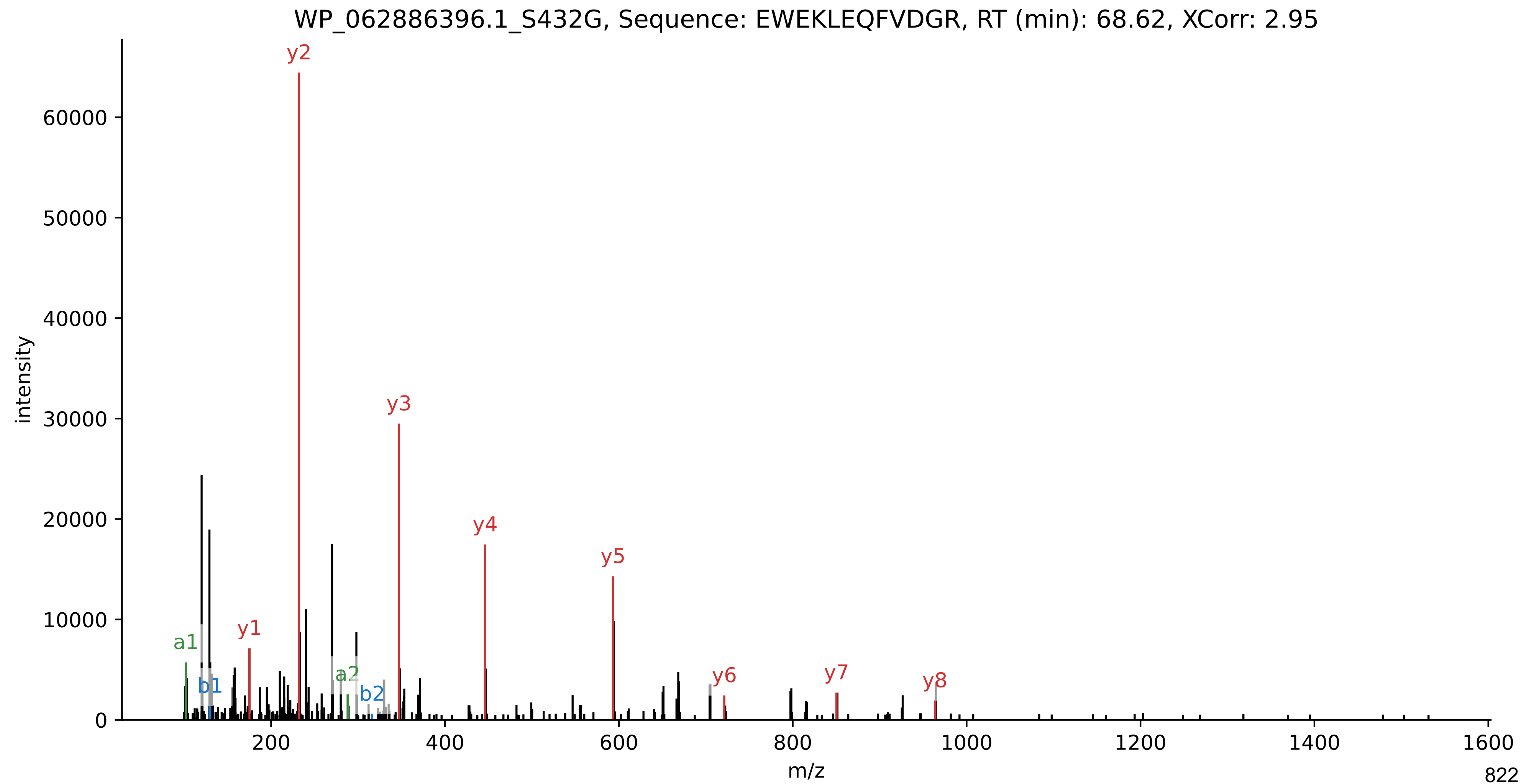

WP_062886396.1_S432G, Sequence: EWEKLEQFVDGR, RT (min): 68.62, XCorr: 2.95
intensity
m/z
y2
y3
y4
y5
y1
a1
b1
a2
b2
y6
y7
y8

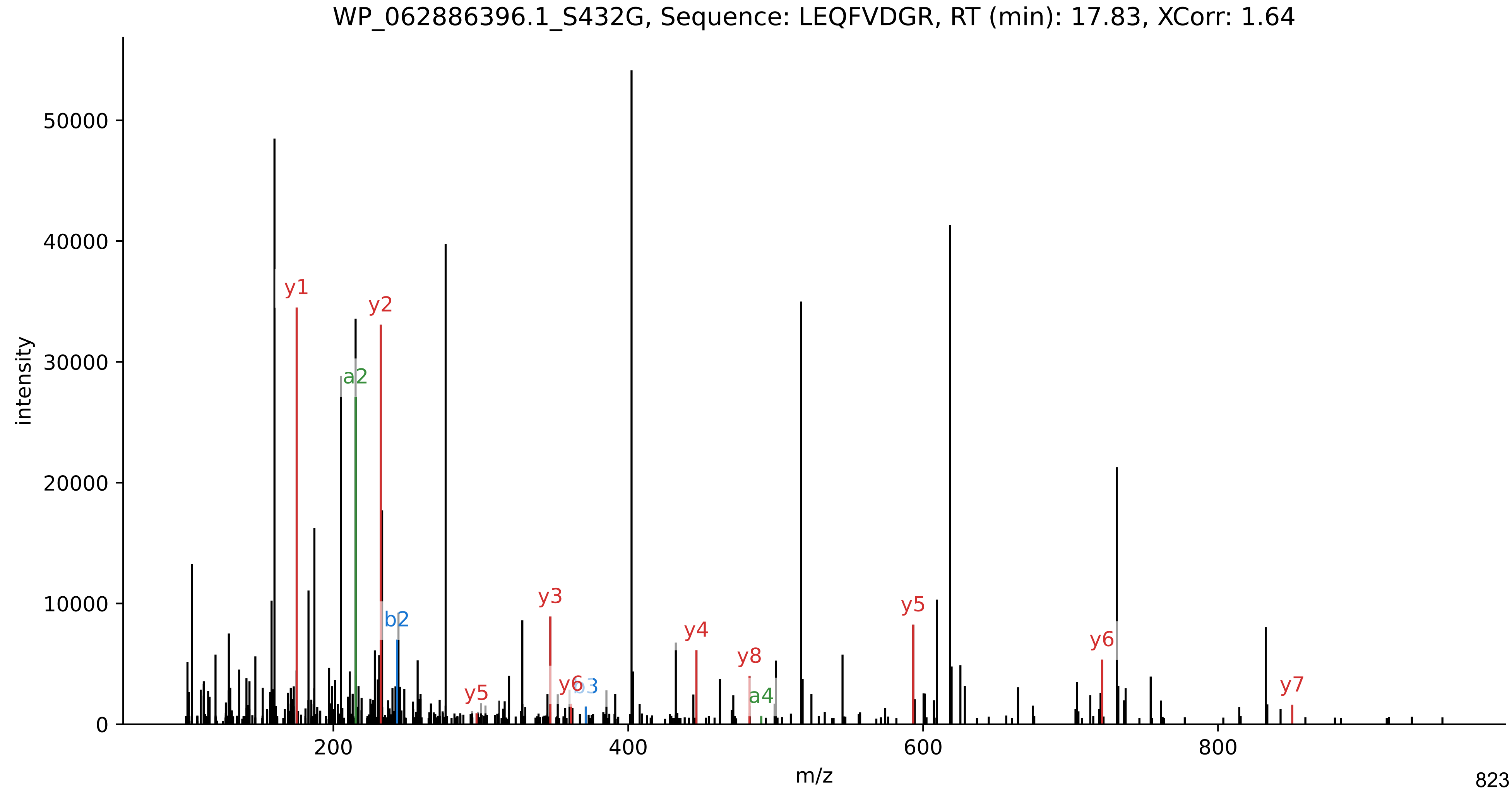

WP_062886396.1_S432G, Sequence: LEQFVDGR, RT (min): 17.83, XCorr: 1.64
intensity
m/z
y1
y2
a2
b2
y3
y5
y6
b3
y4
y8
a4
y5
y6
y7

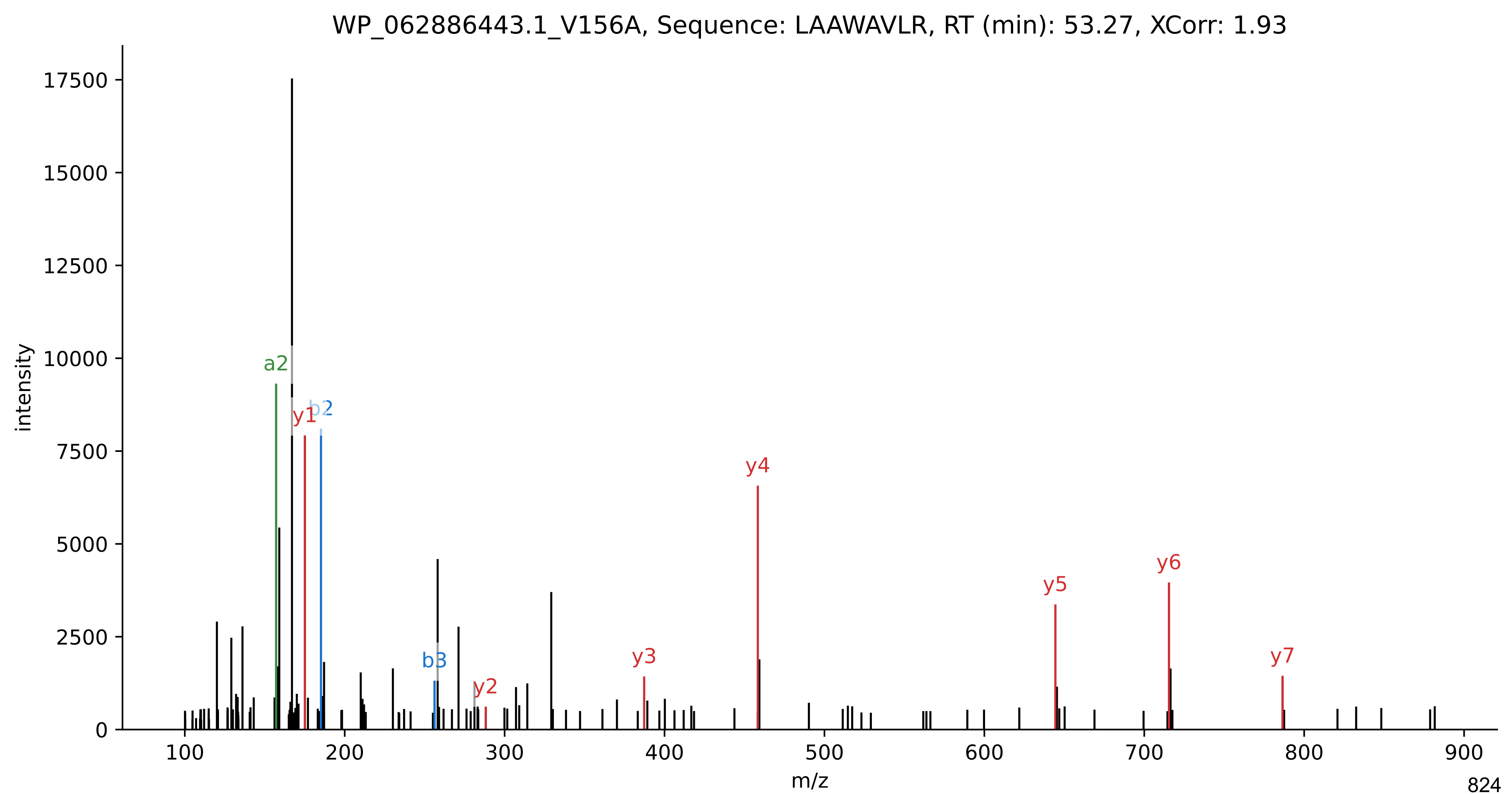
WP_062886443.1_V156A, Sequence: LAAWAVLR, RT (min): 53.27, XCorr: 1.93
intensity
m/z
a2
b2
y1
y4
b3
y2
y3
y5
y6
y7
0
2500
5000
7500
10000
12500
15000
17500
100
200
300
400
500
600
700
800
900

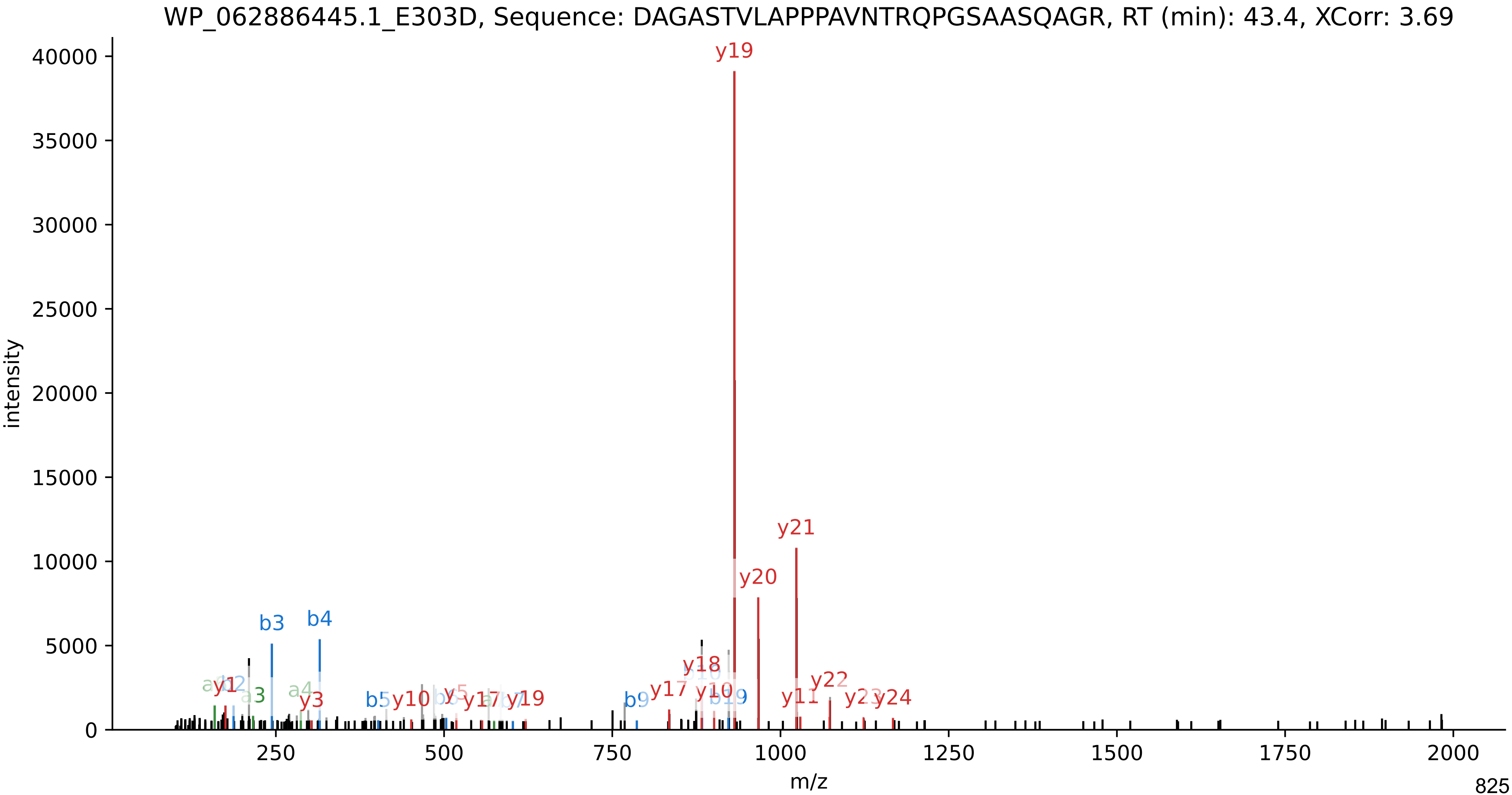
WP_062886445.1_E303D, Sequence: DAGASTVLAPPPAVNTRQPGSAASQAGR, RT (min): 43.4, XCorr: 3.69
intensity
m/z
y19
y21
y20
b3
b4
y18
y22
y17
y10
y11
y23
y24
b9
b5
y10
y3
a3
a4
y1
y17
y19
0
5000
10000
15000
20000
25000
30000
35000
40000
250
500
750
1000
1250
1500
1750
2000

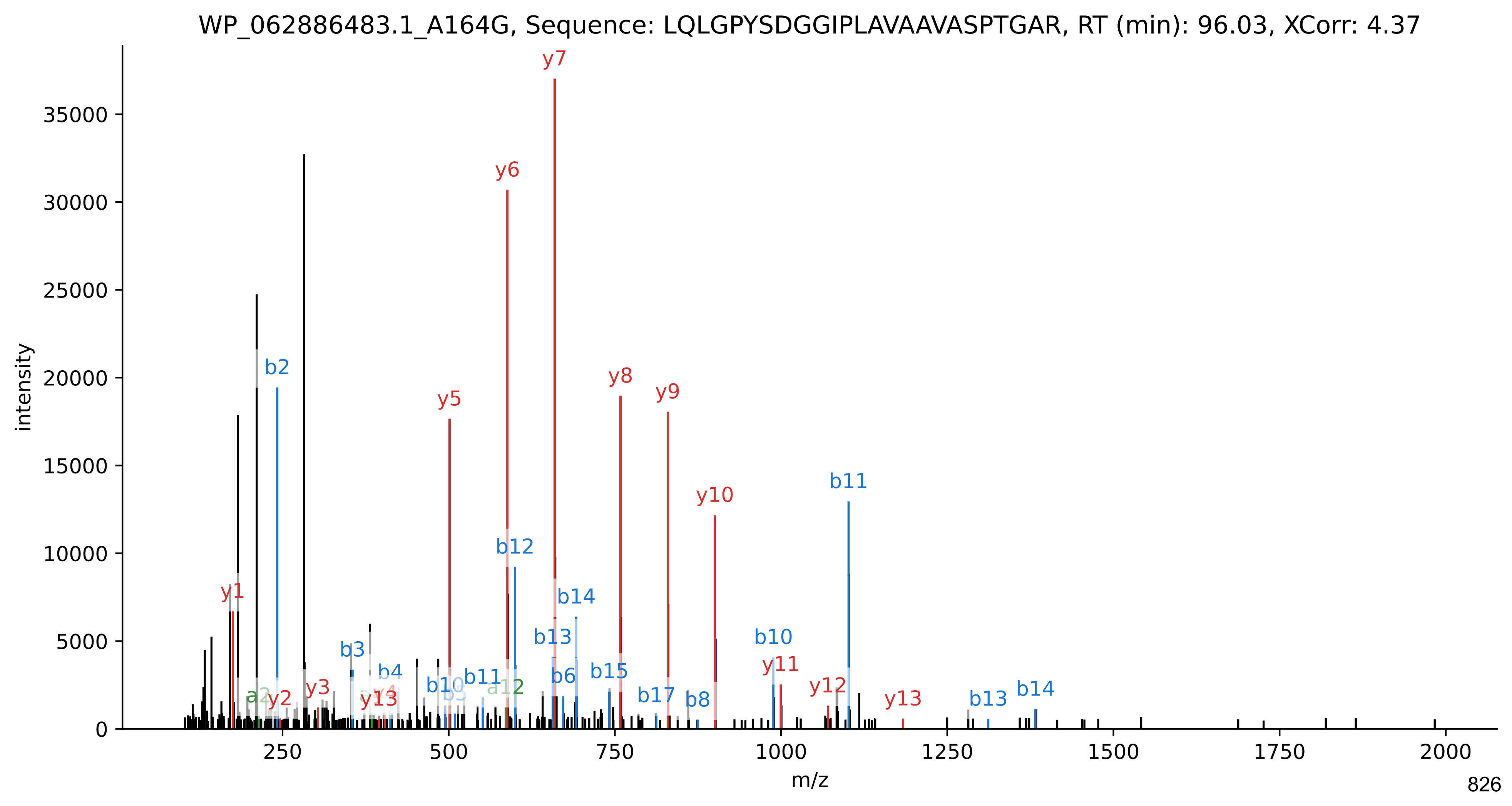

WP_062886483.1_A164G, Sequence: LQLGPYSDGGIPLAVAAVASPTGAR, RT (min): 96.03, XCorr: 4.37
intensity
m/z
y1
y2
y3
y5
y6
y7
y8
y9
y10
y11
y12
y13
b2
b3
b4
b6
b8
b10
b11
b12
b13
b14
b15
b17
a2
a12

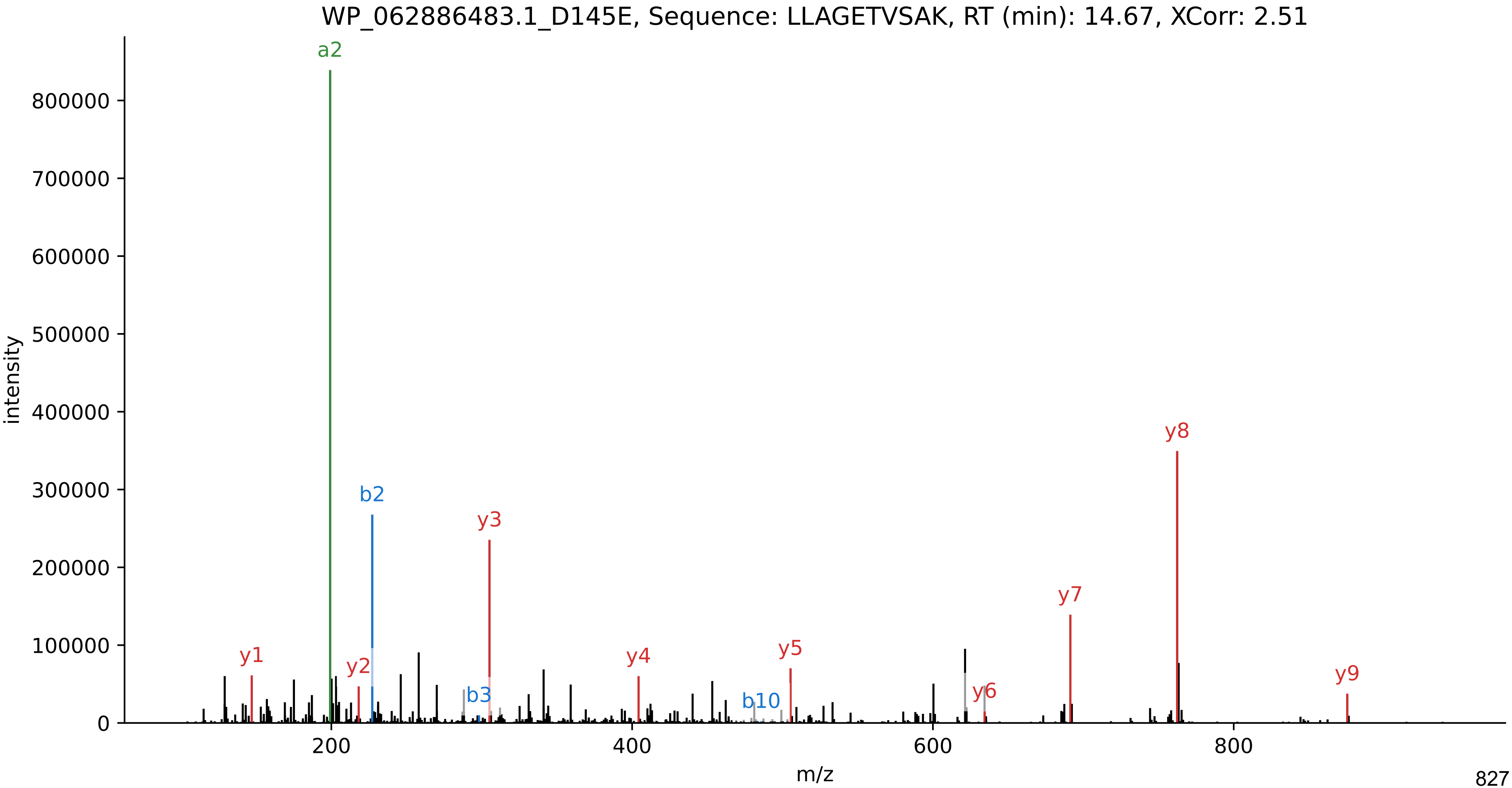
WP_062886483.1_D145E, Sequence: LLAGETVSAK, RT (min): 14.67, XCorr: 2.51
a2
b2
y3
y8
y7
y1
y2
b3
y4
b10
y5
y6
y9
intensity
m/z
0
100000
200000
300000
400000
500000
600000
700000
800000
200
400
600
800

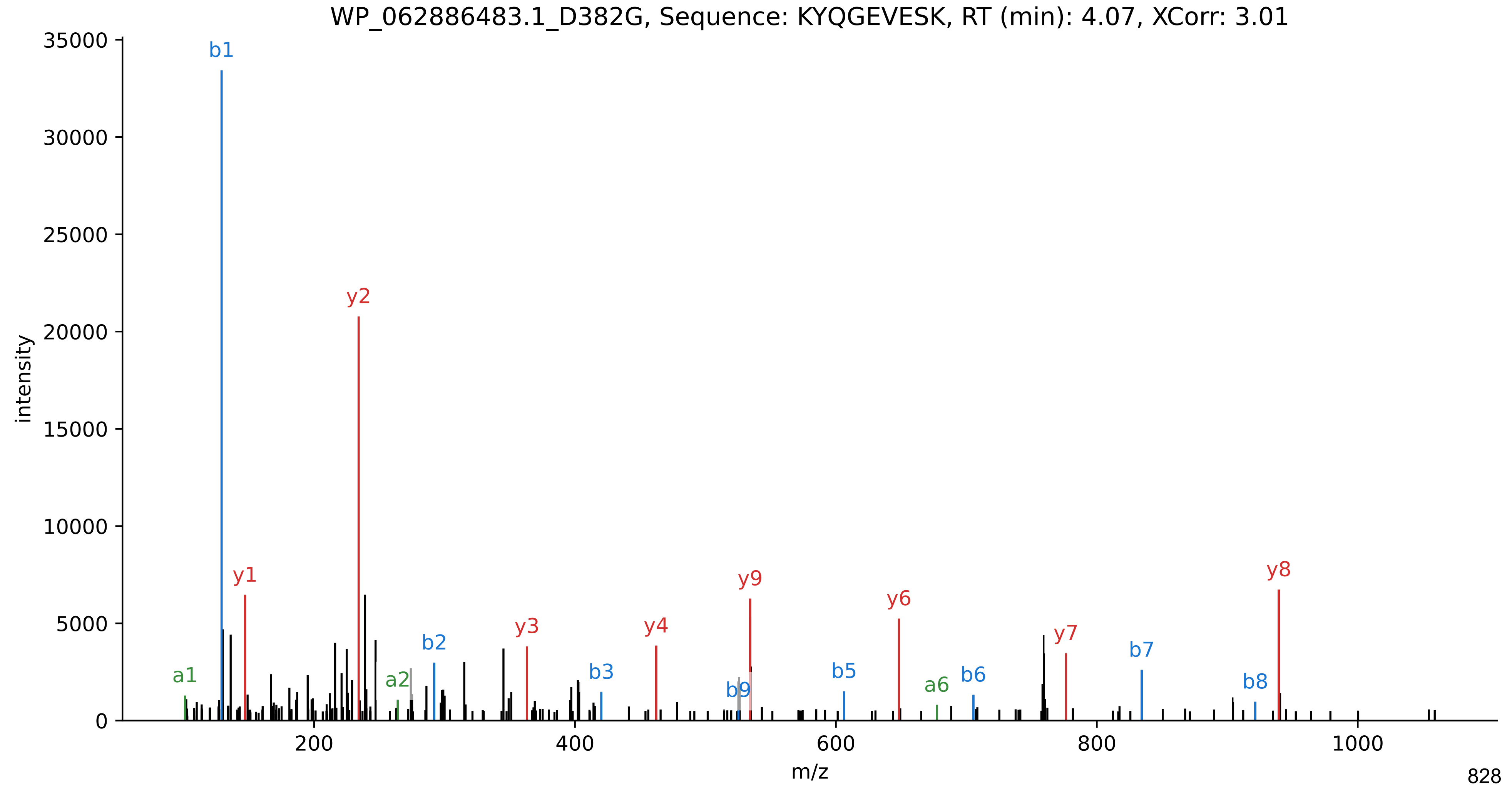
WP_062886483.1_D382G, Sequence: KYQGEVESK, RT (min): 4.07, XCorr: 3.01
intensity
m/z
0
5000
10000
15000
20000
25000
30000
35000
200
400
600
800
1000
b1
y2
y1
a1
a2
b2
y3
y4
b3
b9
y9
b5
y6
a6
b6
y7
b7
b8
y8

WP_062886483.1_S115T, Sequence: IIGGVGPGSLPTDSSMIGLTPTDTR, RT (min): 82.17, XCorr: 4.0

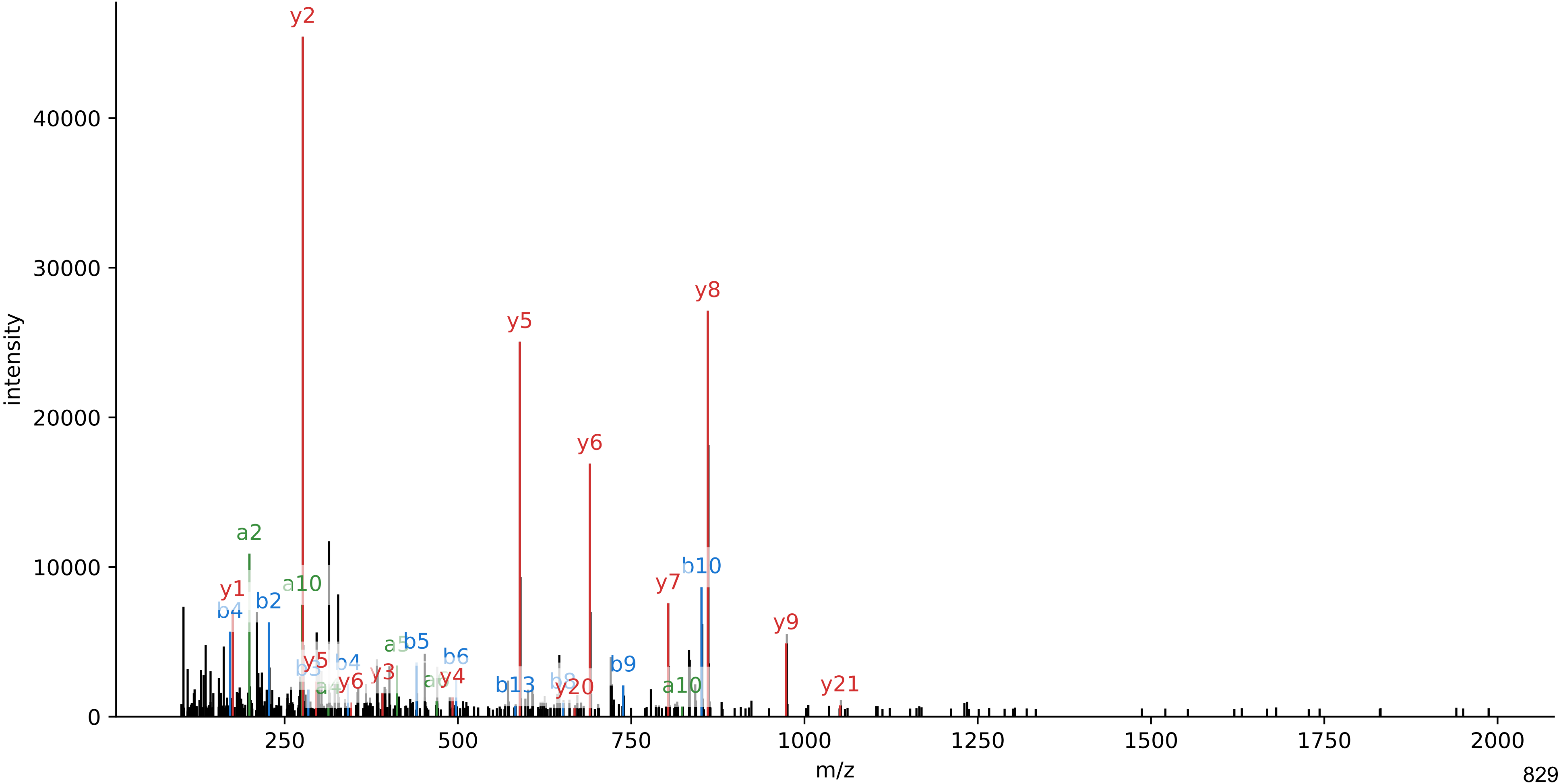

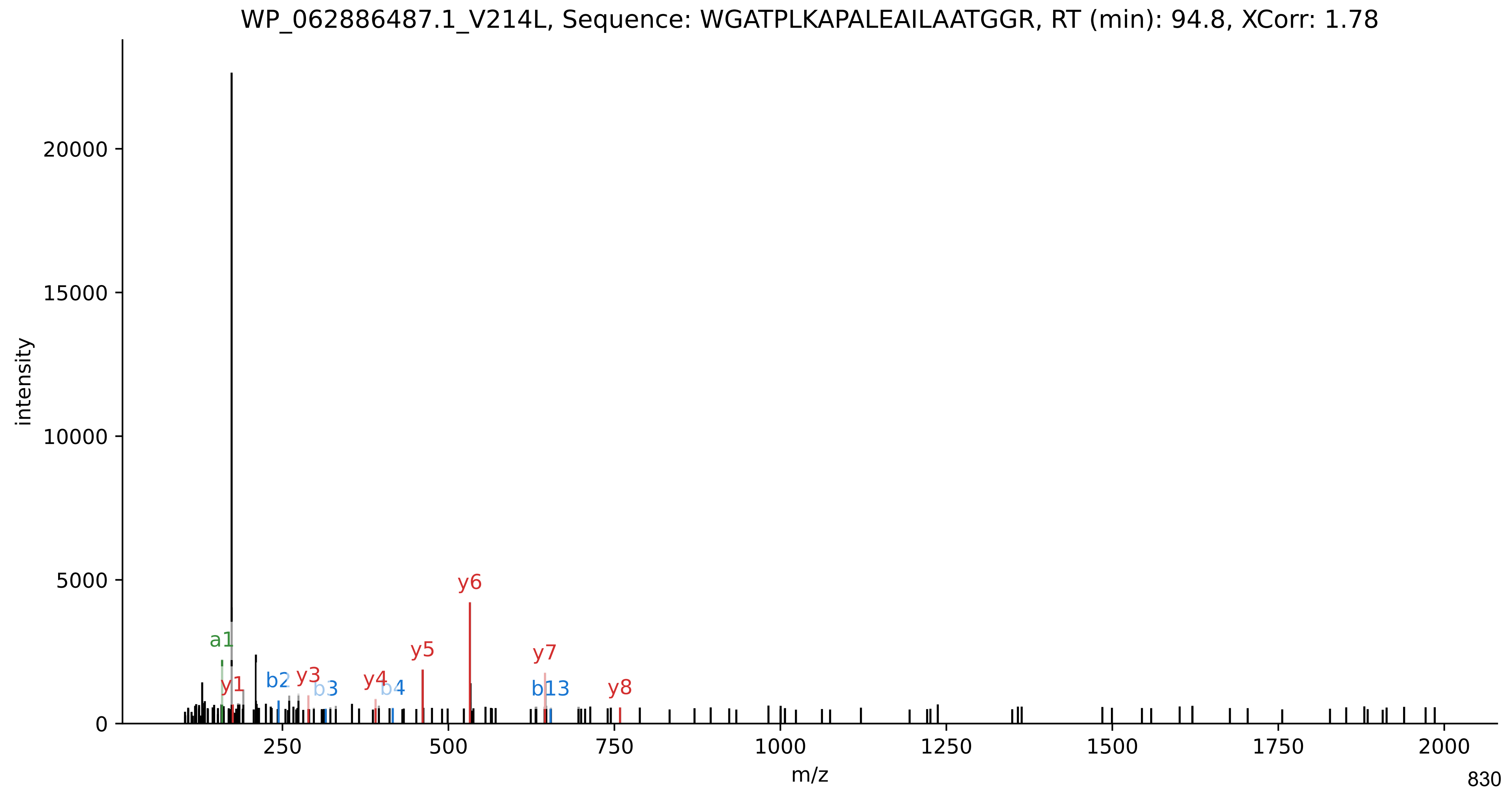
WP_062886487.1_V214L, Sequence: WGATPLKAPALEAILAATGGR, RT (min): 94.8, XCorr: 1.78
intensity
m/z
0
5000
10000
15000
20000
250
500
750
1000
1250
1500
1750
2000
a1
y1
b2
y3
b3
y4
b4
y5
y6
y7
b13
y8

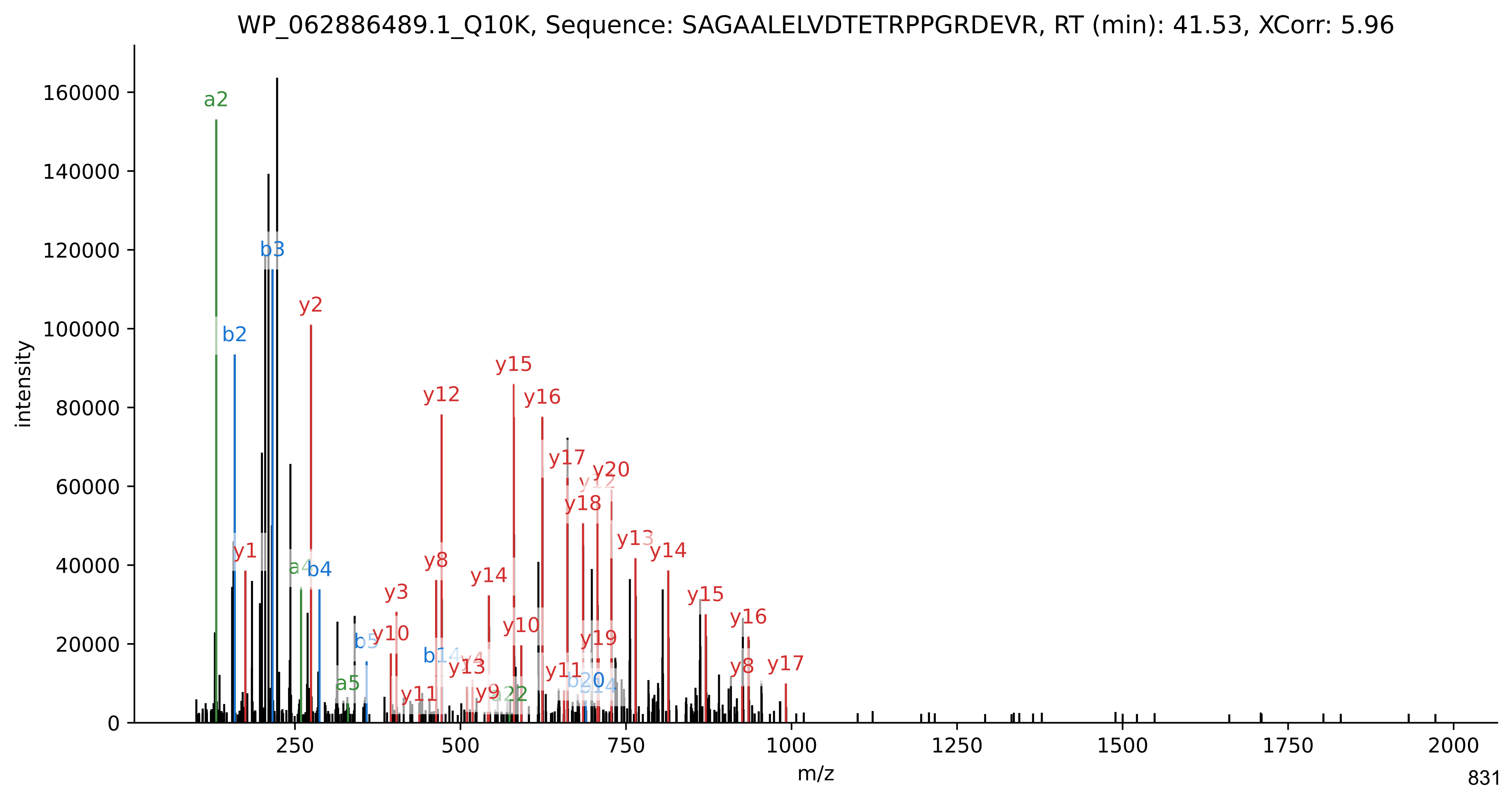

WP_062886489.1_Q10K, Sequence: SAGAALELVDTETRPPGRDEVR, RT (min): 41.53, XCorr: 5.96
intensity
m/z
a2
b3
y2
b2
y15
y12
y16
y17
y20
y18
y13
y14
y1
a4
b4
y8
y3
y10
b5
b14
y11
y9
a22
a5
y19
b20
y16
y8
y17

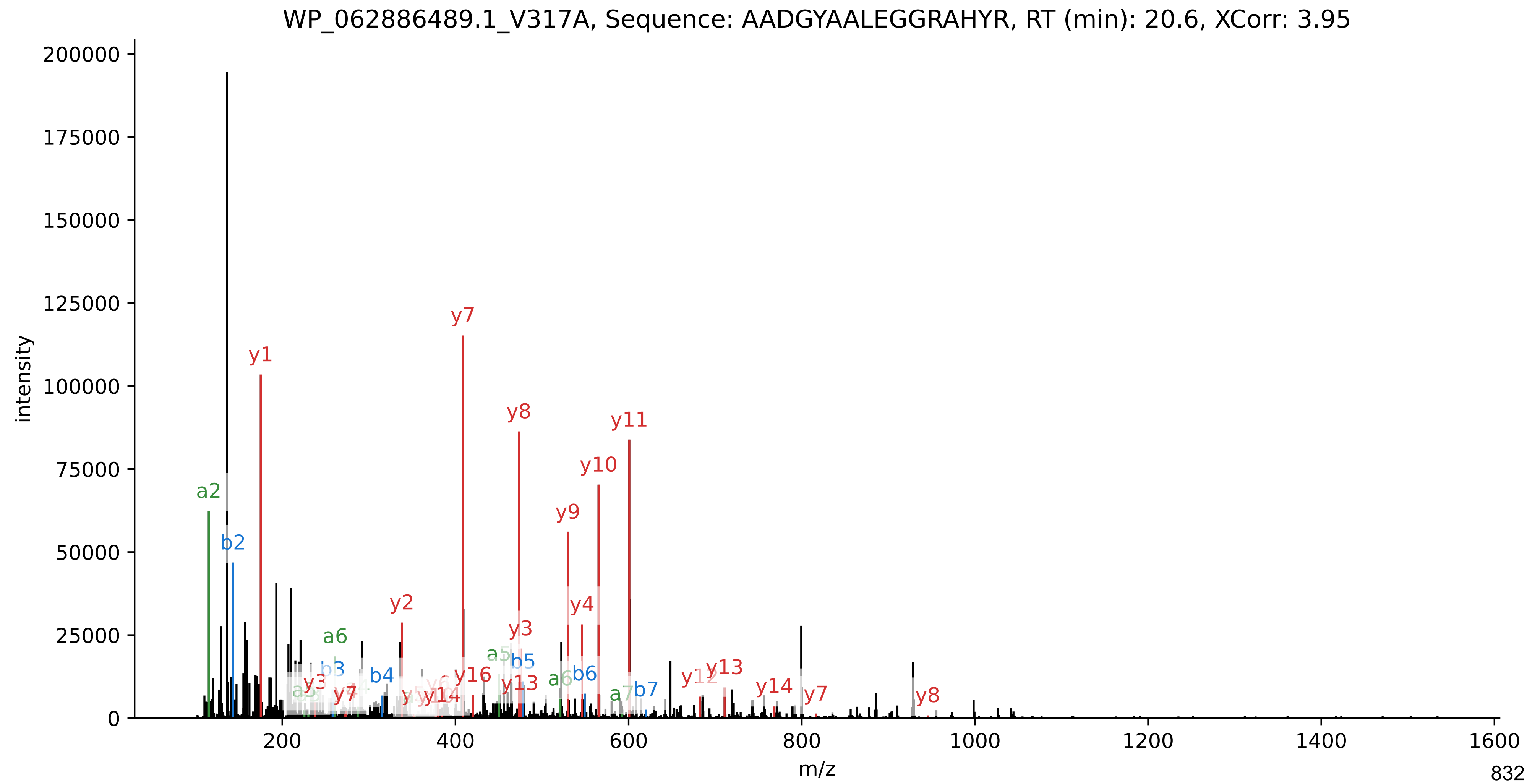

WP_062886489.1_V317A, Sequence: AADGYAALEGGRAHYR, RT (min): 20.6, XCorr: 3.95
intensity
m/z
y7
y1
y8
y11
y10
y9
a2
b2
y2
y4
y3
a6
a5
b5
b3
b4
y16
y13
a6
b6
a7
b7
y3
y7
y14
y12
y13
y14
y7
y8

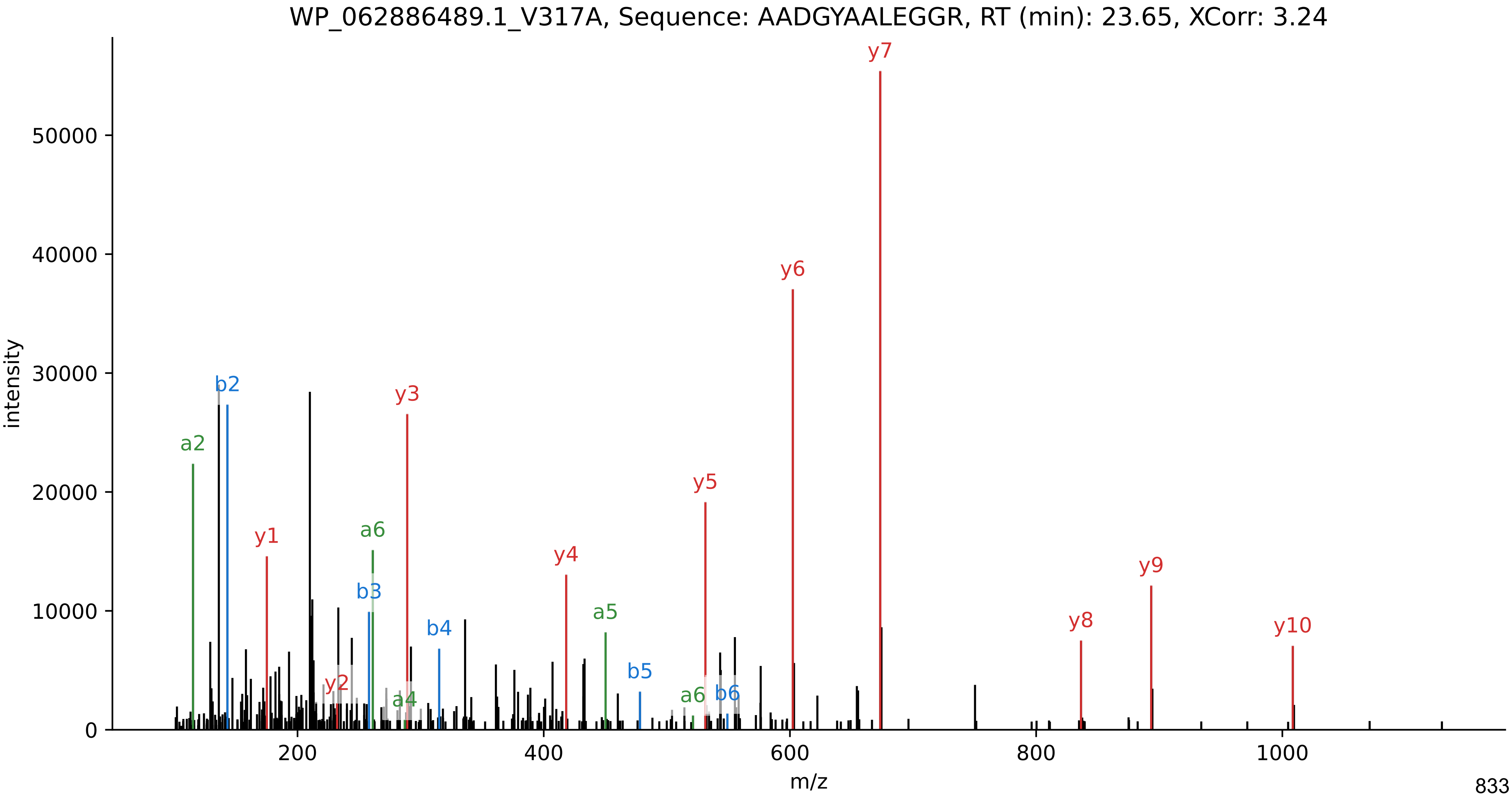

WP_062886489.1_V317A, Sequence: AADGYAALEGGR, RT (min): 23.65, XCorr: 3.24
intensity
m/z
0
10000
20000
30000
40000
50000
200
400
600
800
1000
y7
y6
b2
y3
a2
y5
a6
y1
y4
y9
b3
a5
y8
y10
b4
b5
y2
a4
a6
b6

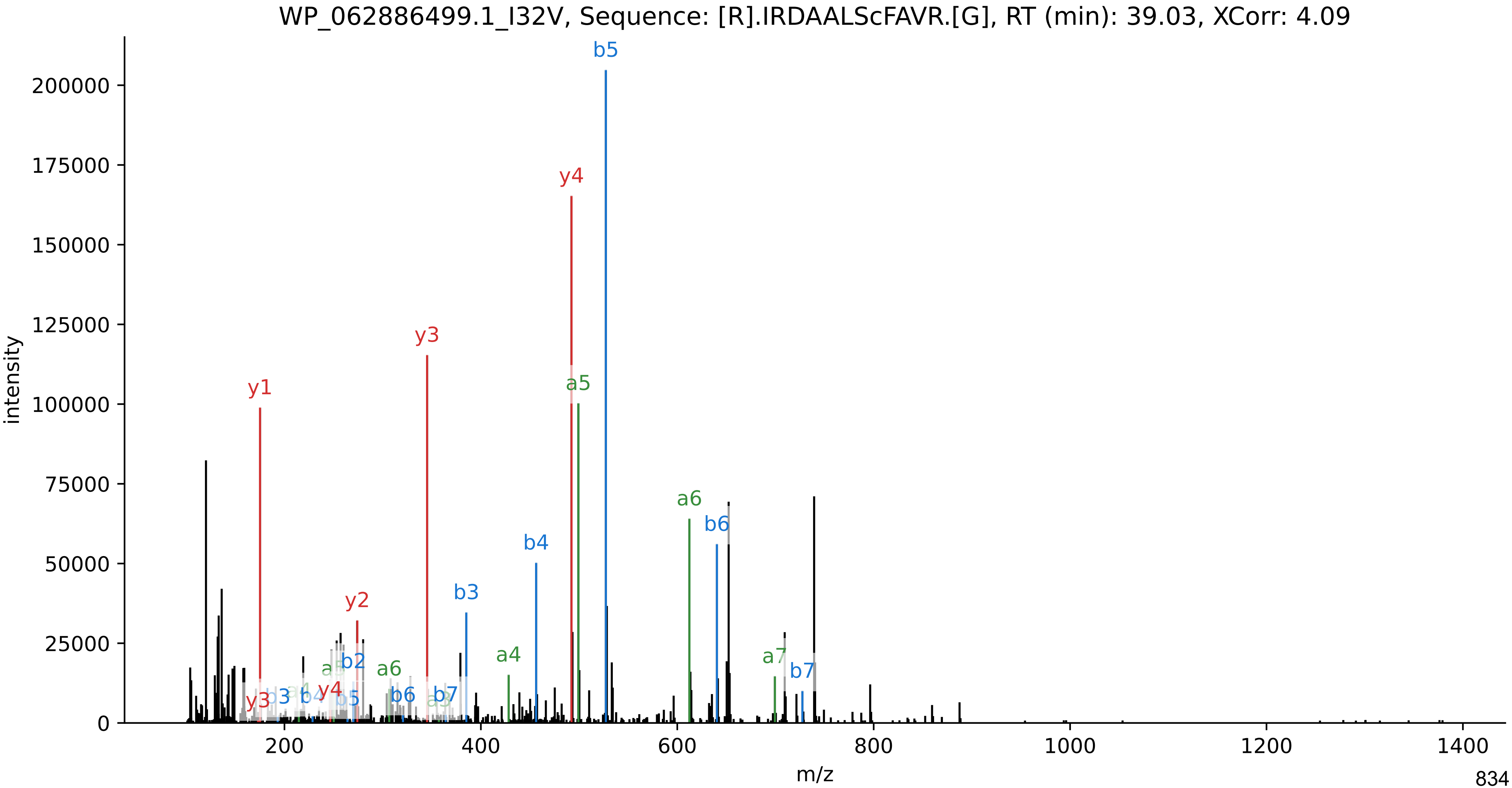

WP_062886499.1_I32V, Sequence: [R].IRDAALScFAVR.[G], RT (min): 39.03, XCorr: 4.09
intensity
m/z
b5
y4
y3
y1
a5
a6
b6
b4
b3
y2
a4
a7
b7
b2
a6
b6
b7
y4
y3
b3
b4
b5
0
25000
50000
75000
100000
125000
150000
175000
200000
200
400
600
800
1000
1200
1400

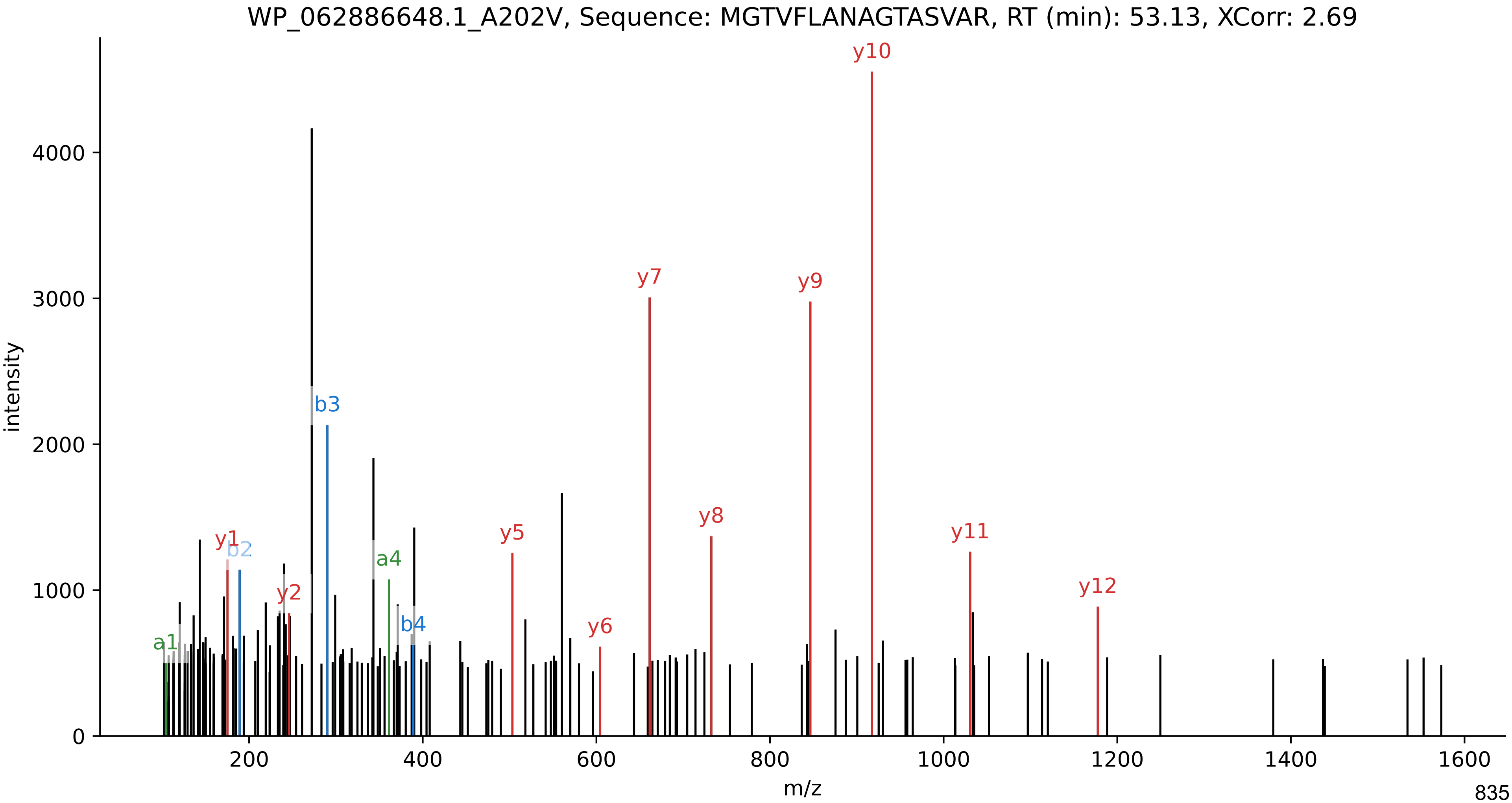

WP_062886648.1_A202V, Sequence: MGTVFLANAGTASVAR, RT (min): 53.13, XCorr: 2.69
intensity
m/z
y10
y7
y9
b3
y8
y1
b2
y5
y11
a4
y2
y12
b4
y6
a1
0
1000
2000
3000
4000
200
400
600
800
1000
1200
1400
1600

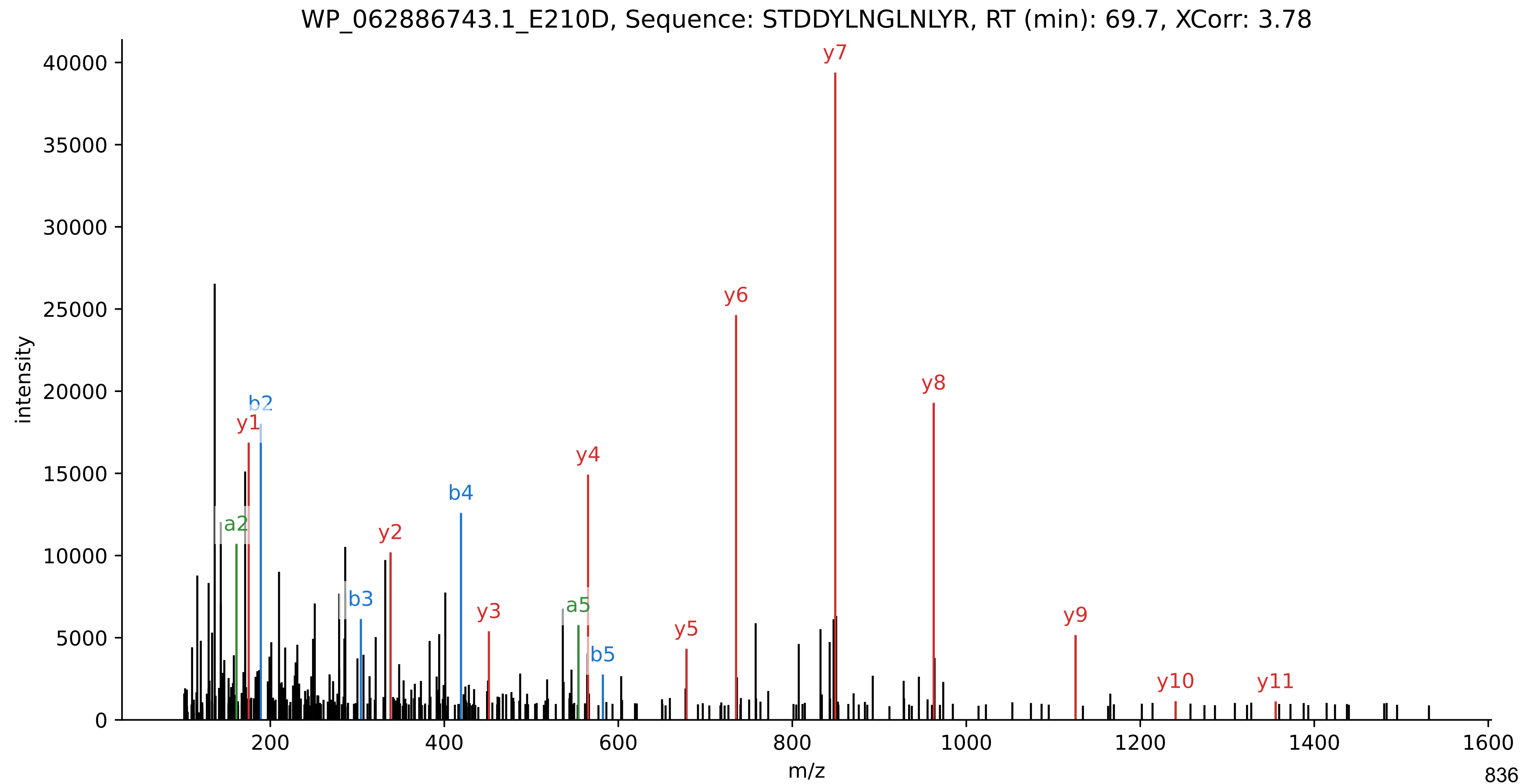
WP_062886743.1_E210D, Sequence: STDDYLNGLNLYR, RT (min): 69.7, XCorr: 3.78
intensity
m/z
y7
y6
y8
b2
y1
y4
b4
a2
y2
b3
a5
y3
y5
b5
y9
y10
y11

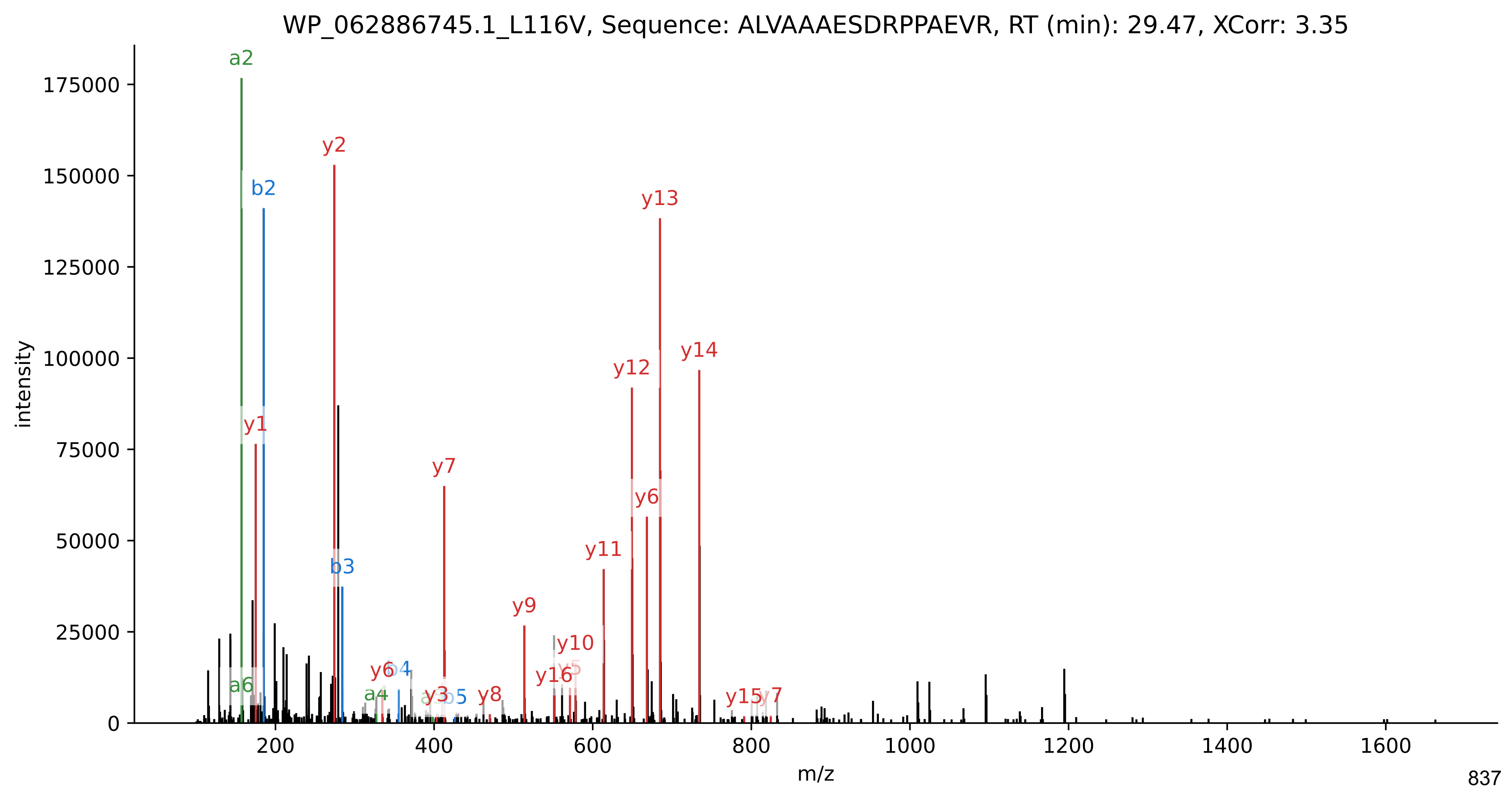
WP_062886745.1_L116V, Sequence: ALVAAAESDRPPAEVR, RT (min): 29.47, XCorr: 3.35
intensity
m/z
a2
b2
y1
y2
b3
y7
y9
y10
y16
y11
y12
y13
y6
y14
a6
a4
y6
b4
y3
b5
y8
y5
y15
y7

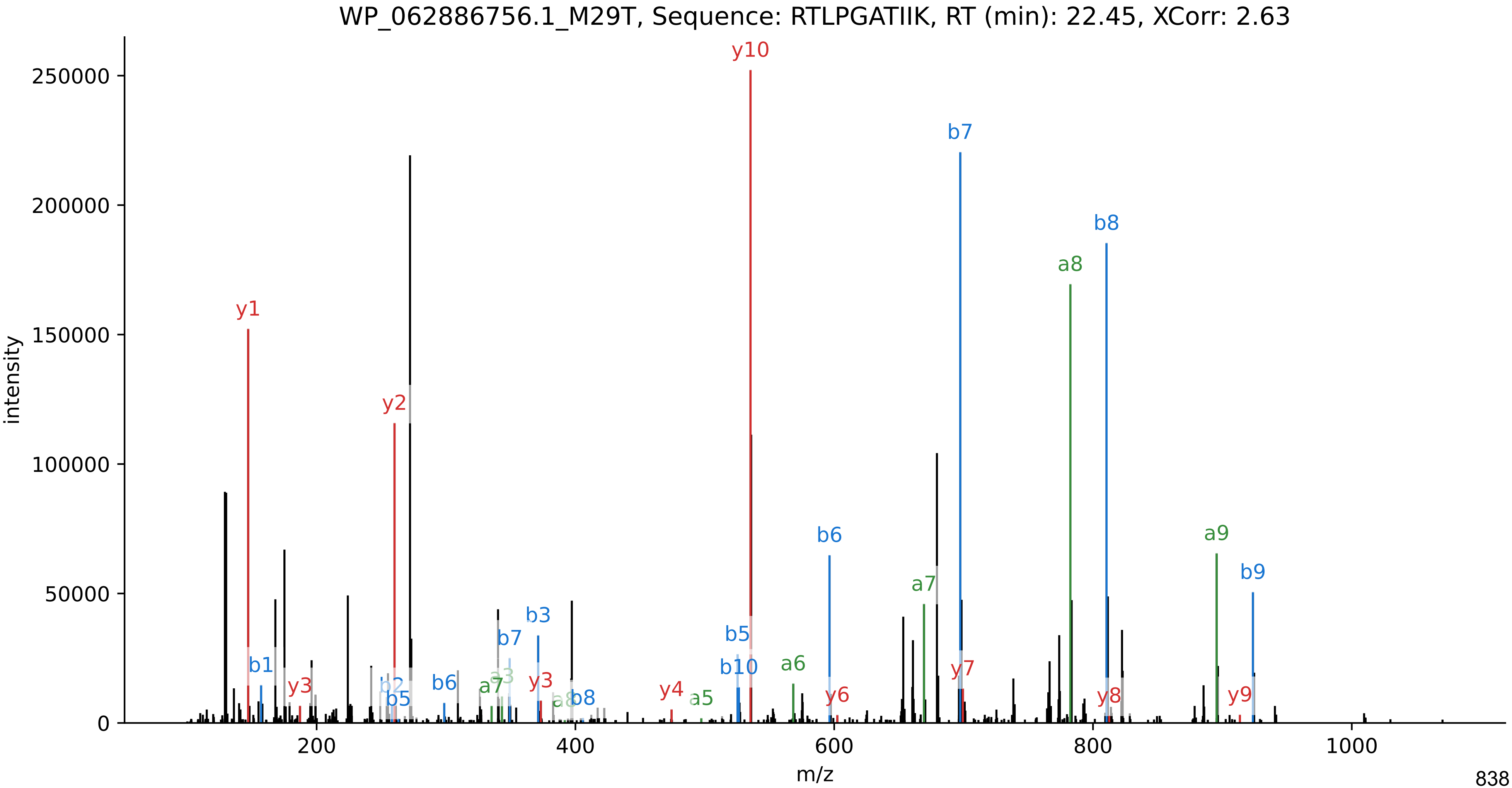
WP_062886756.1_M29T, Sequence: RTLPGATIIK, RT (min): 22.45, XCorr: 2.63
intensity
m/z
0
50000
100000
150000
200000
250000
200
400
600
800
1000
y10
b7
b8
a8
y1
y2
b6
a9
b9
a7
b3
b7
b5
b1
y3
b10
a6
y7
b6
a7
a3
y3
y4
a5
y6
y8
y9
b2
b5
a8
b8

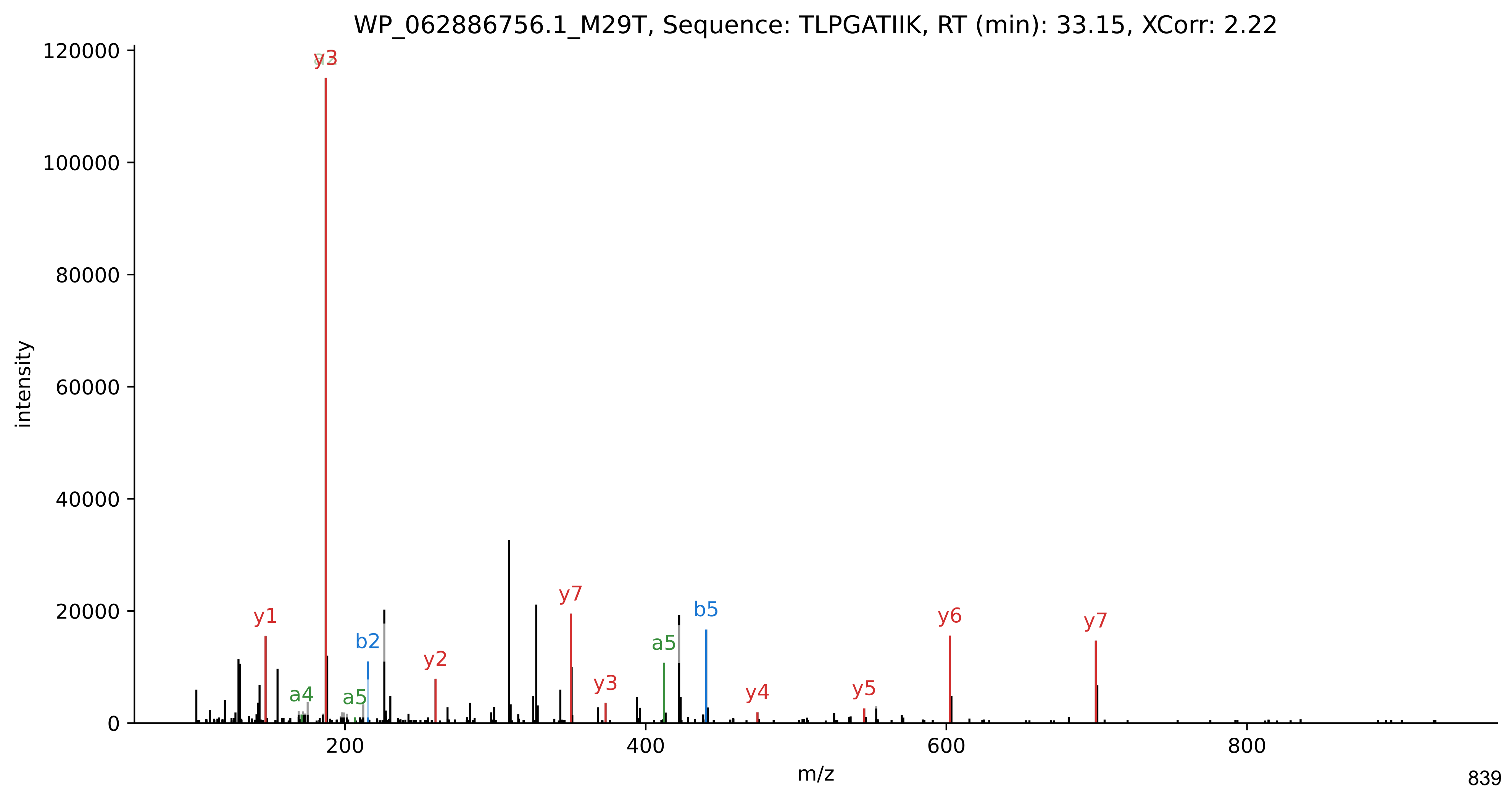
WP_062886756.1_M29T, Sequence: TLPGATIIK, RT (min): 33.15, XCorr: 2.22
intensity
m/z
y3
y1
a4
a5
b2
y2
y7
y3
a5
b5
y4
y5
y6
y7
120000
100000
80000
60000
40000
20000
0
200
400
600
800

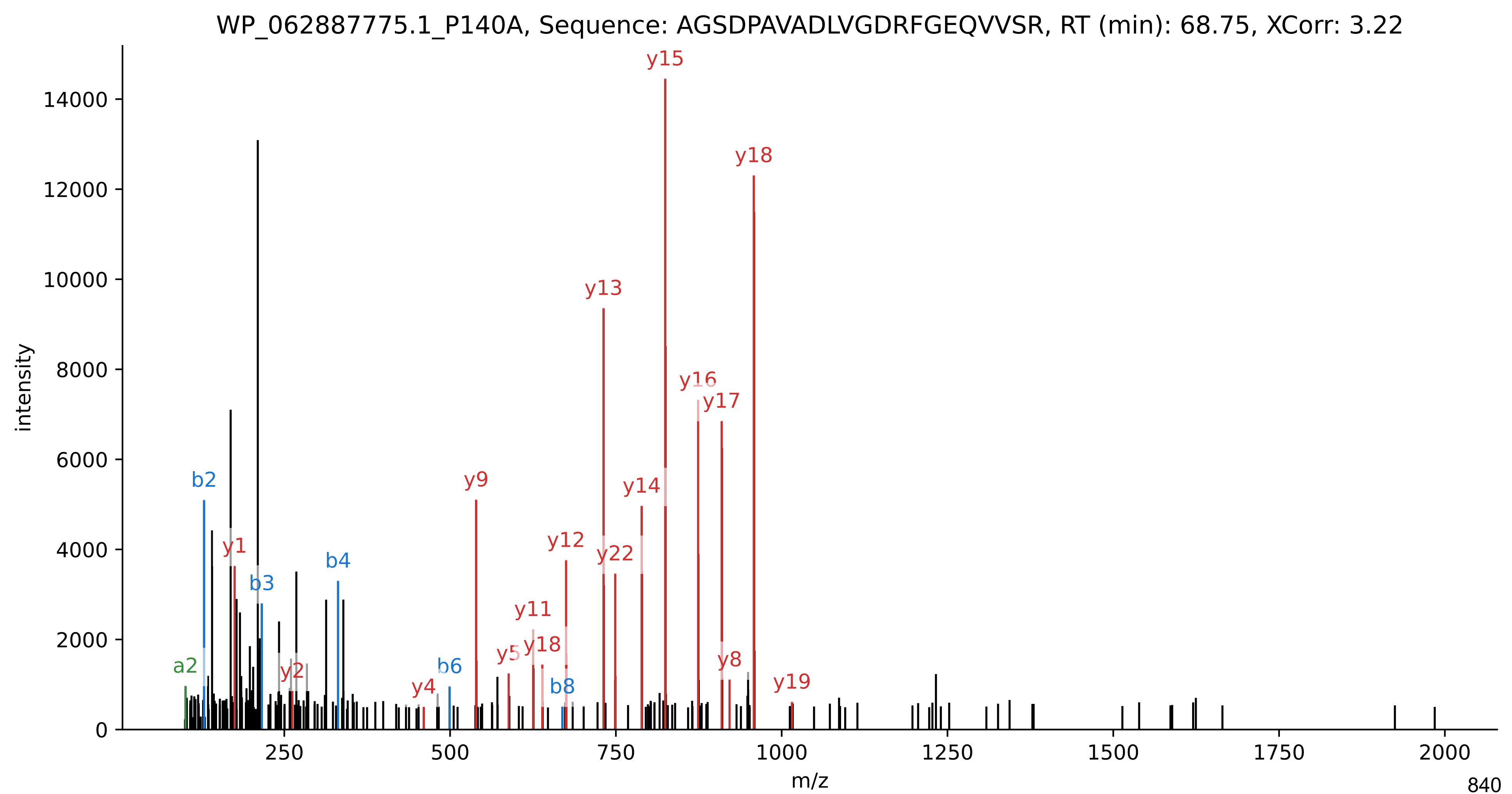

WP_062887775.1_P140A, Sequence: AGSDPAVADLVGDRFGEQVVSR, RT (min): 68.75, XCorr: 3.22
intensity
m/z
a2
b2
b3
b4
b6
b8
y1
y2
y4
y5
y9
y11
y18
y12
y22
y13
y14
y15
y16
y17
y18
y8
y19
0
2000
4000
6000
8000
10000
12000
14000
250
500
750
1000
1250
1500
1750
2000

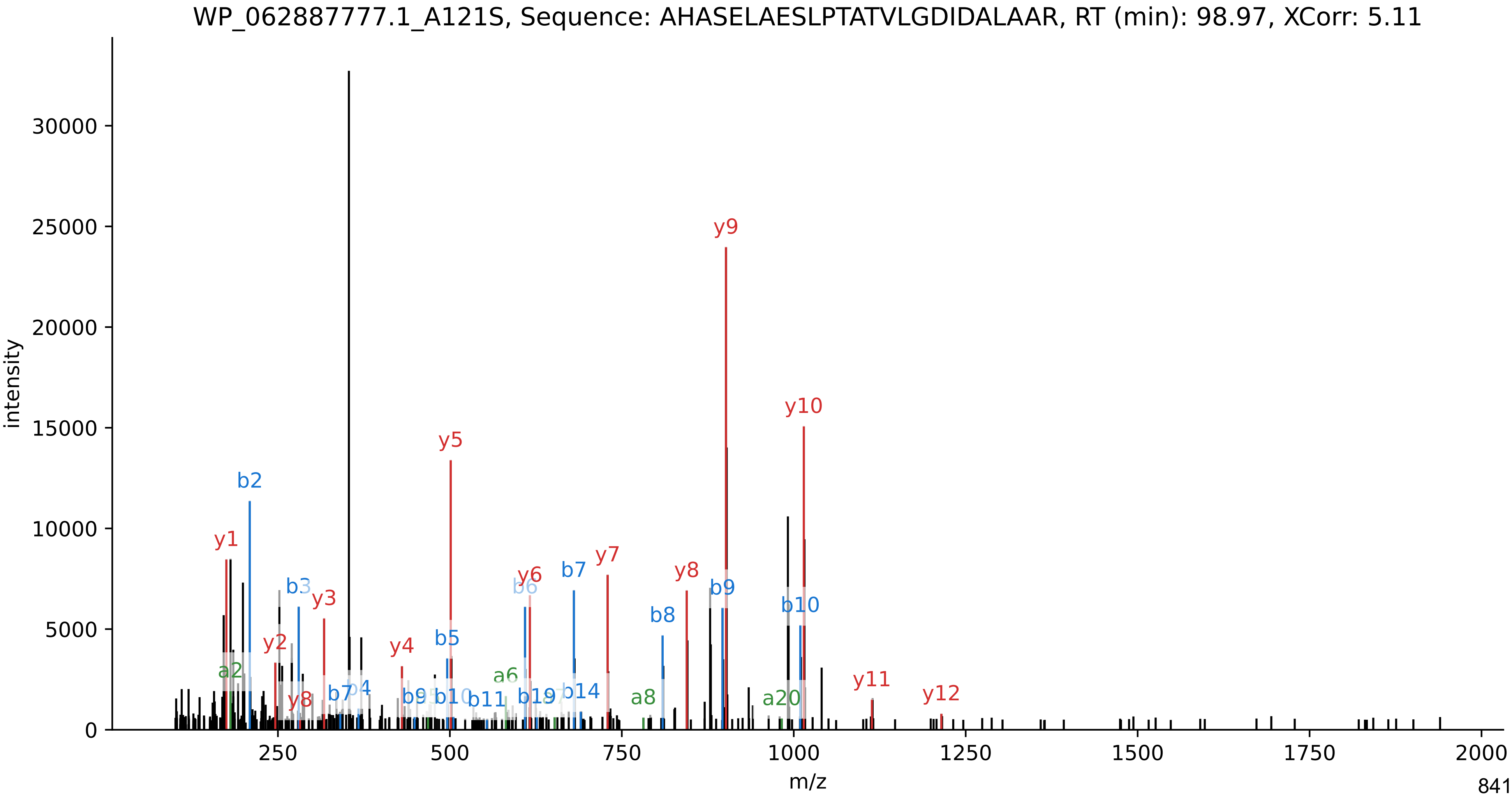
WP_062887777.1_A121S, Sequence: AHASELAESLPTATVLGDIDALAAR, RT (min): 98.97, XCorr: 5.11
intensity
m/z
y1
y2
y3
y4
y5
y6
y7
y8
y9
y10
y11
y12
b2
b3
b5
b6
b7
b8
b9
b10
b11
b14
a2
a6
a8
a20
0
5000
10000
15000
20000
25000
30000
250
500
750
1000
1250
1500
1750
2000

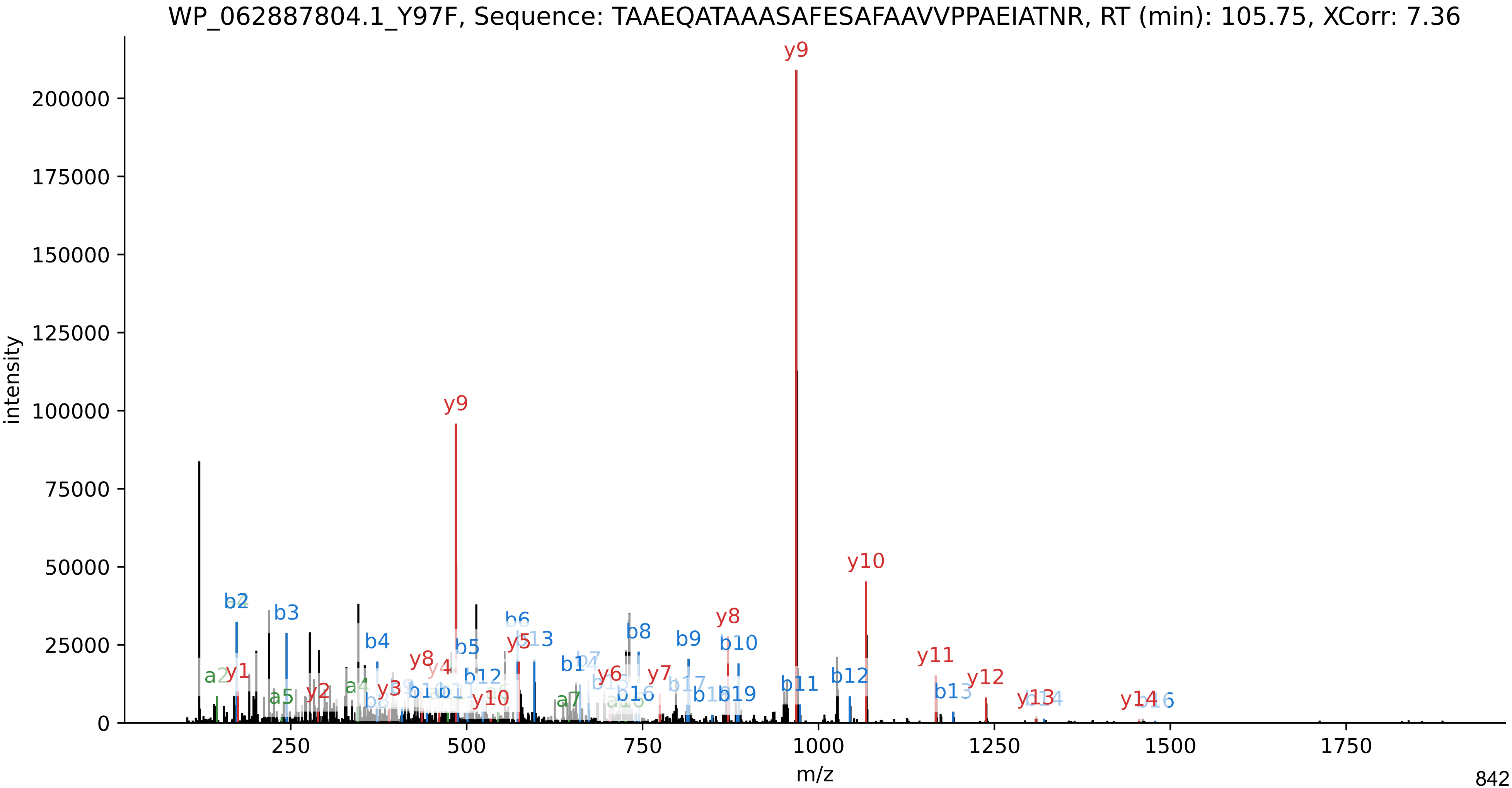
WP_062887804.1_Y97F, Sequence: TAAEQATAAASAFESAFAAVVPPAEIATNR, RT (min): 105.75, XCorr: 7.36
intensity
m/z
y9
y9
y10
y8
y11
y12
y13
y14
b2
b3
b4
b5
b6
b8
b9
b10
b11
b12
b13
b16
b19
y1
y2
y3
y5
y6
y7
y10
a2
a4
a5
a7

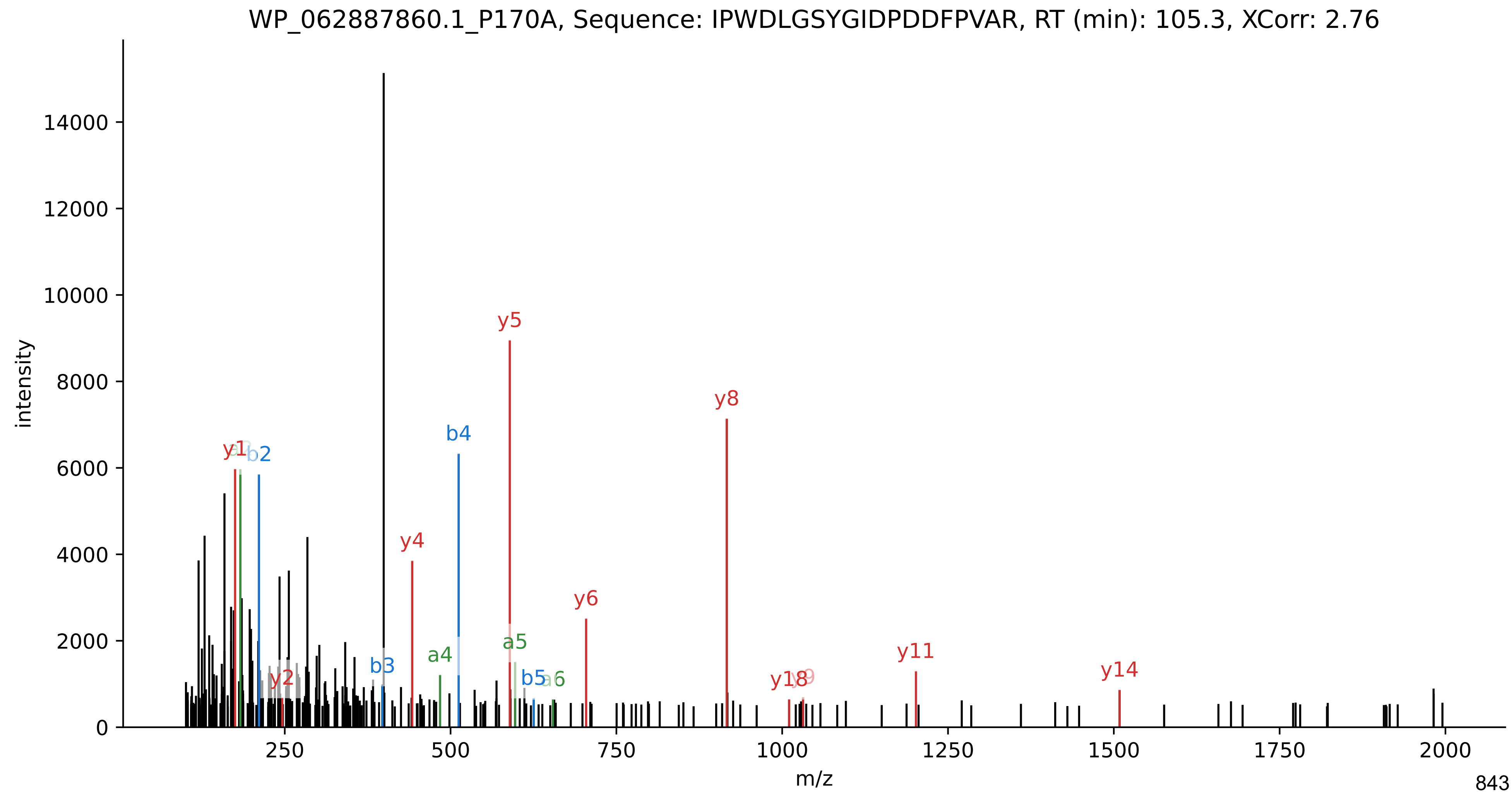

WP_062887860.1_P170A, Sequence: IPWDLGSYGIDPDDFPVAR, RT (min): 105.3, XCorr: 2.76
intensity
m/z
y1
b2
b4
y5
y8
y4
y6
y2
b3
a4
a5
b5
a6
y11
y14
y10

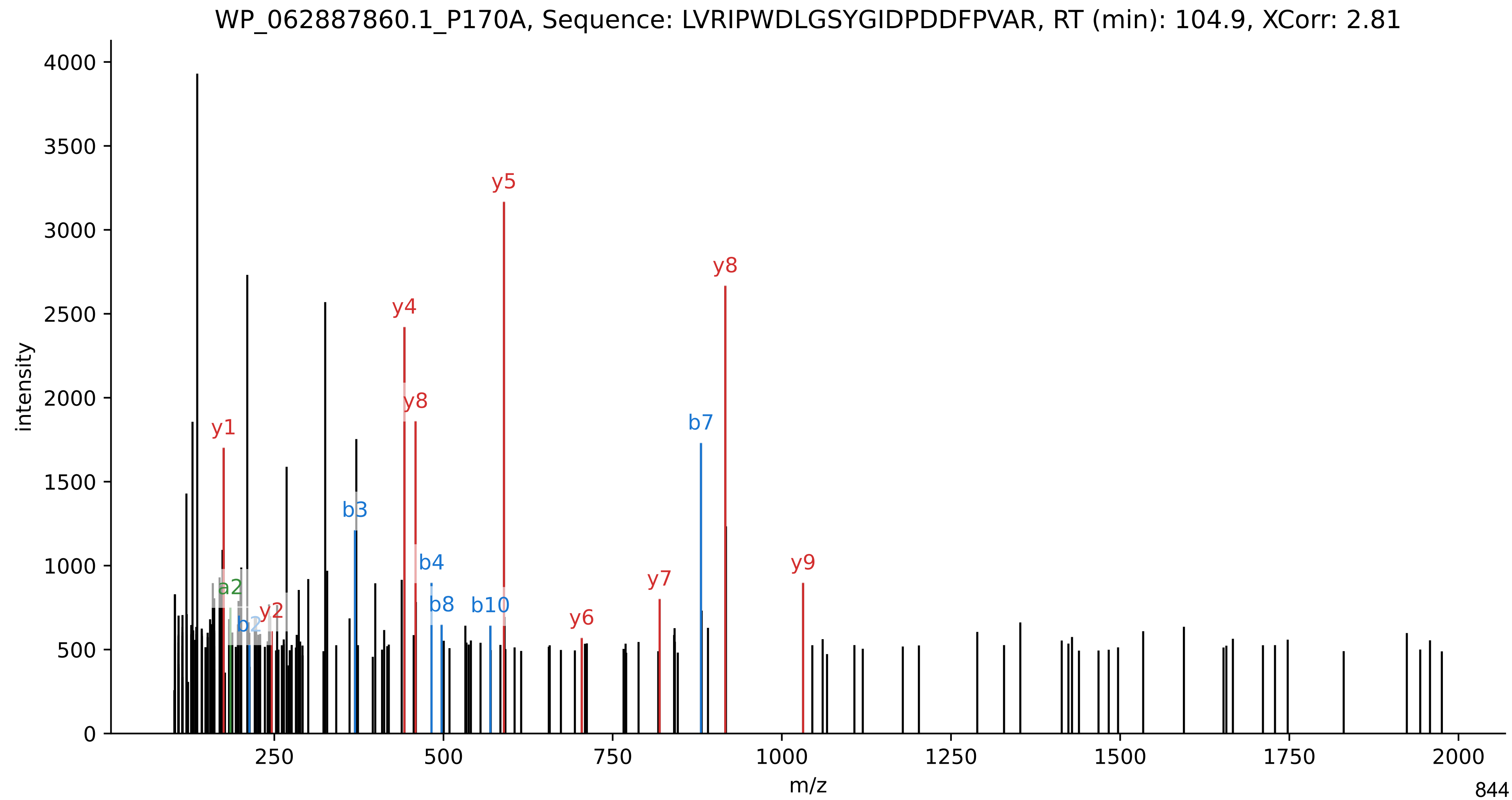

WP_062887860.1_P170A, Sequence: LVRIPWDLGSYGIDPDDFPVAR, RT (min): 104.9, XCorr: 2.81
intensity
m/z
0
500
1000
1500
2000
2500
3000
3500
4000
250
500
750
1000
1250
1500
1750
2000
y1
a2
b2
y2
b3
y4
y8
b4
b8
b10
y5
y6
y7
b7
y8
y9

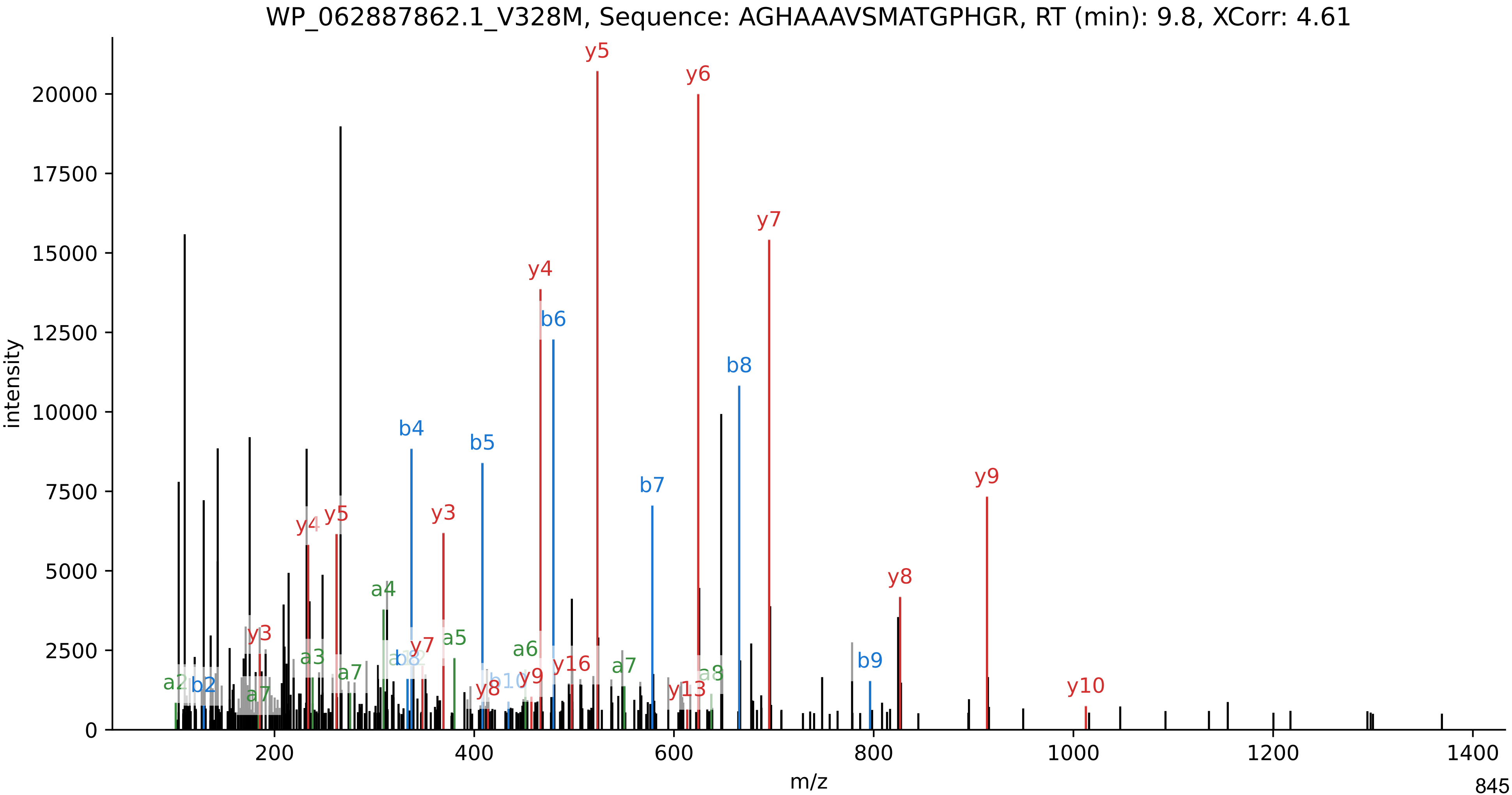

WP_062887862.1_V328M, Sequence: AGHAAAVSMATGPHGR, RT (min): 9.8, XCorr: 4.61
intensity
m/z
y5
y6
y7
y4
b6
b8
b4
b5
b7
y9
y3
y8
a4
a5
a6
a7
b9
y10
y16
y13
y9
y8
b2
a2
a3
a8

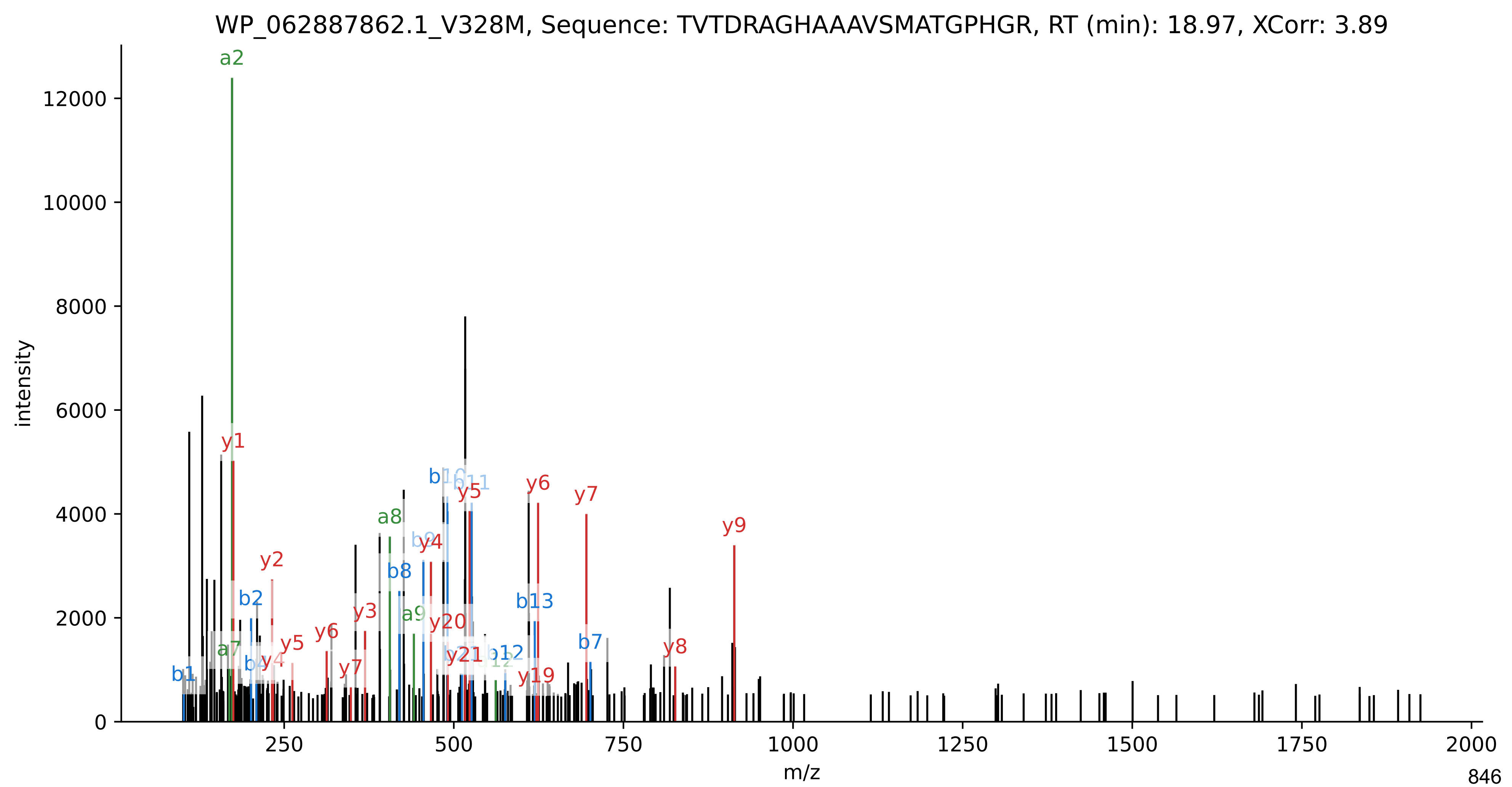
WP_062887862.1_V328M, Sequence: TVTDRAGHAAAVSMATGPHGR, RT (min): 18.97, XCorr: 3.89
intensity
m/z
0
2000
4000
6000
8000
10000
12000
250
500
750
1000
1250
1500
1750
2000
a2
y1
y2
b2
a7
b1
y4
y5
y6
y7
y3
a8
b8
a9
y4
y20
y21
b10
b11
y5
b12
y6
b13
y19
y7
b7
y8
y9

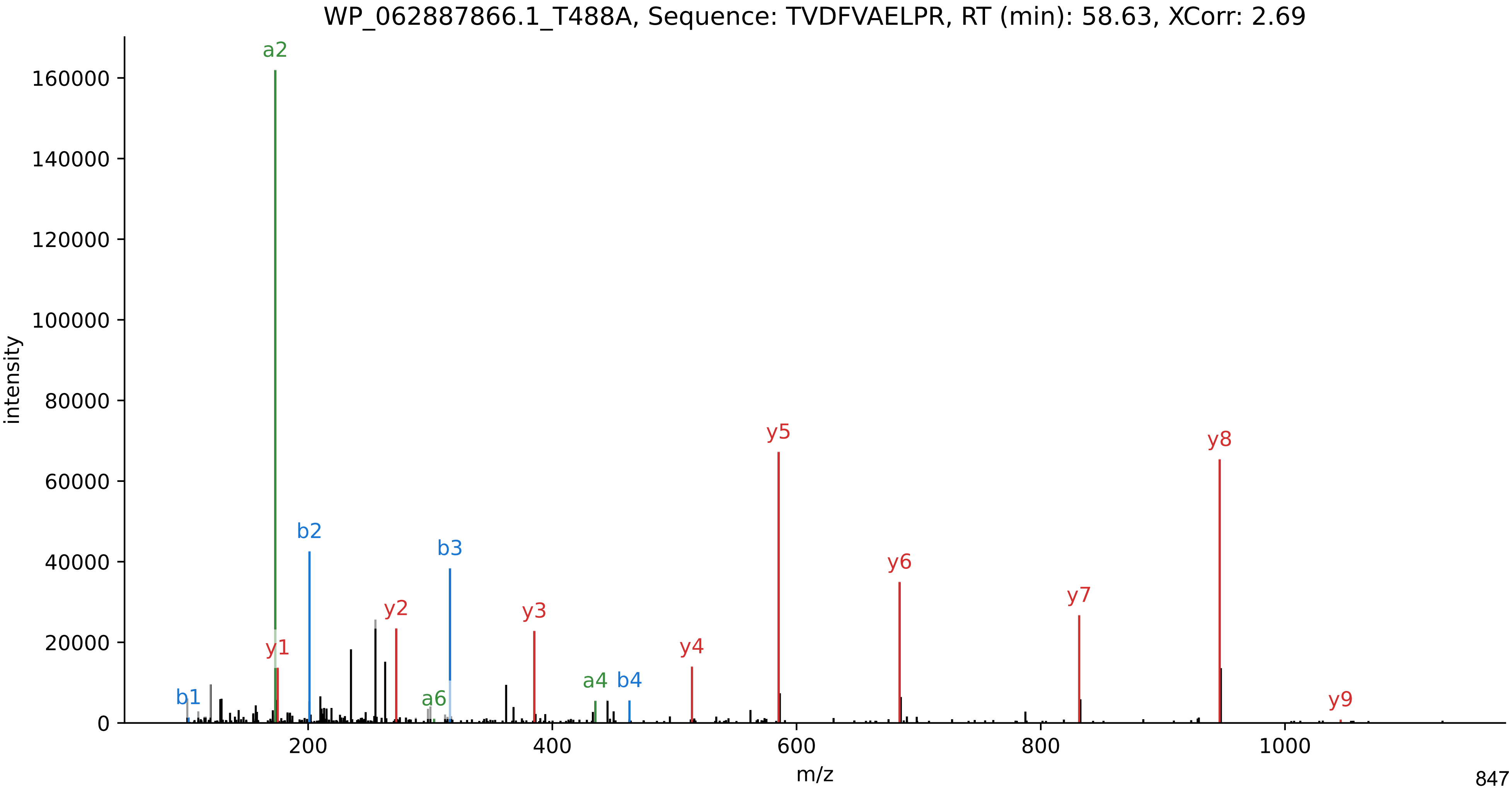

WP_062887866.1_T488A, Sequence: TVDFVAELPR, RT (min): 58.63, XCorr: 2.69
intensity
m/z
a2
b1
y1
b2
y2
a6
b3
y3
a4
b4
y4
y5
y6
y7
y8
y9
0
20000
40000
60000
80000
100000
120000
140000
160000
200
400
600
800
1000

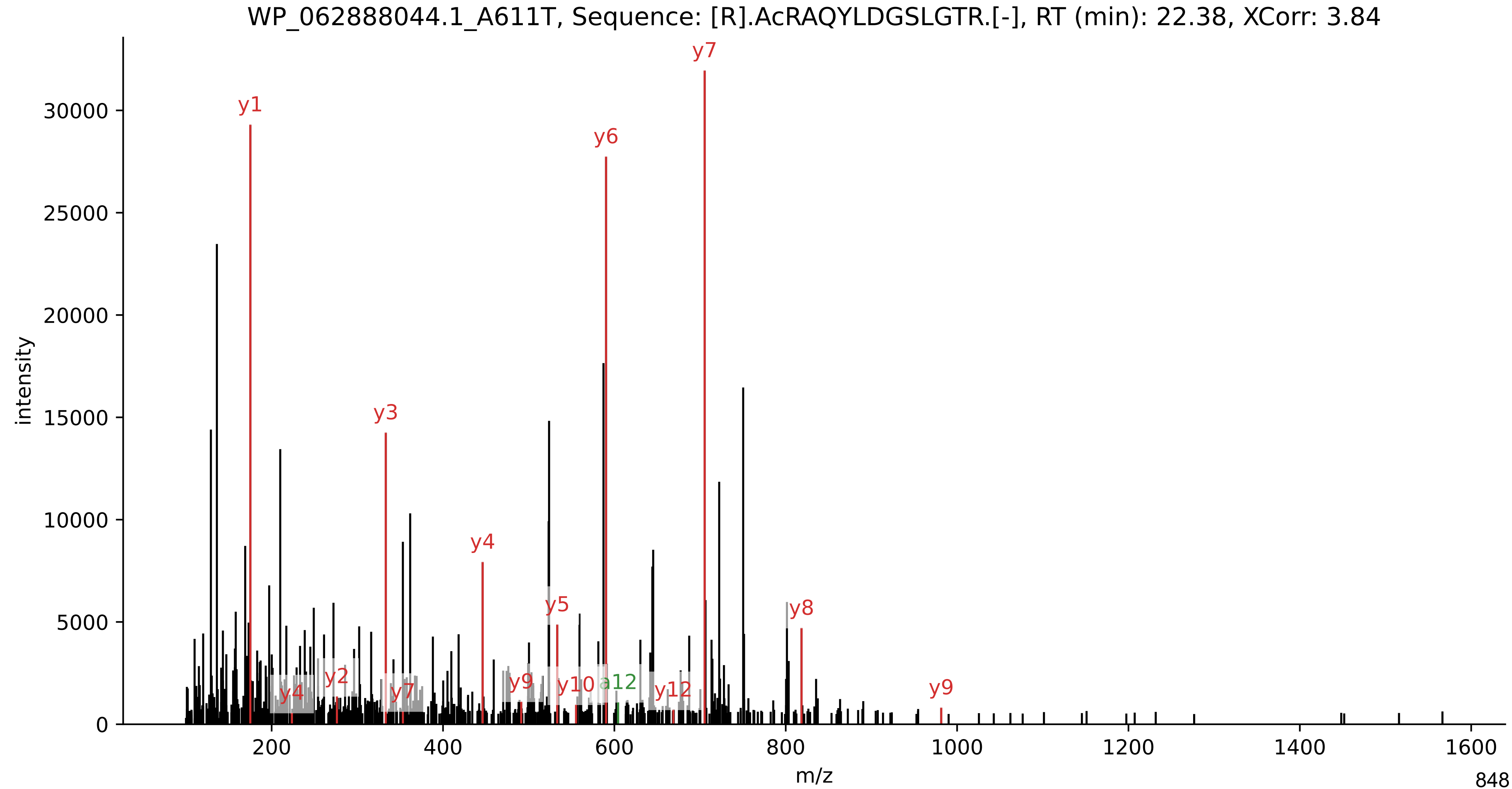

WP_062888044.1_A611T, Sequence: [R].AcRAQYLDGSLGTR.[-], RT (min): 22.38, XCorr: 3.84
y7
y1
y6
y3
y4
y5
y8
y2
y4
y7
y9
y10
a12
y12
y9
intensity
m/z

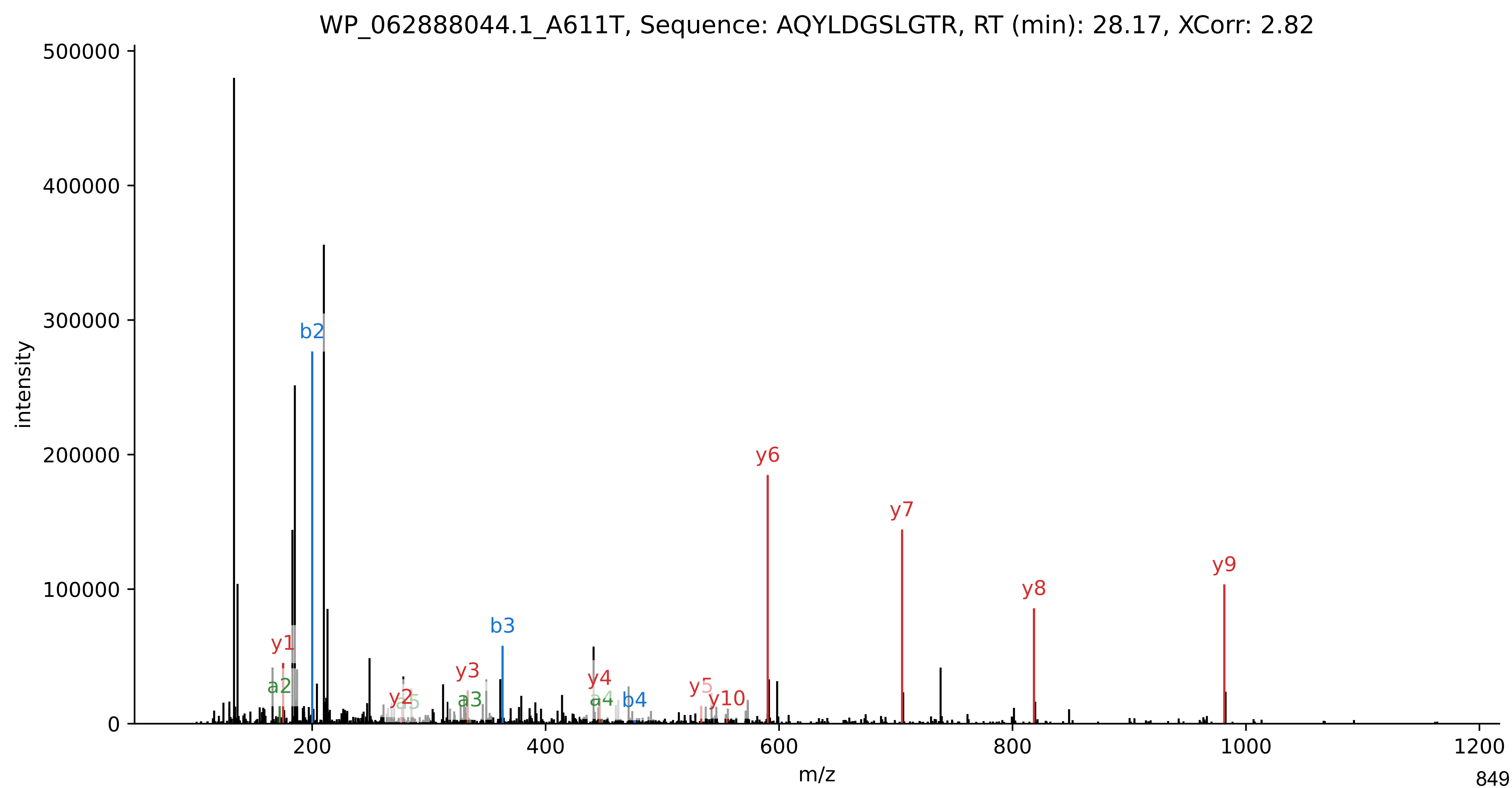
WP_062888044.1_A611T, Sequence: AQYLDGSLGTR, RT (min): 28.17, XCorr: 2.82
intensity
m/z
b2
b3
b4
a2
a3
a4
y1
y2
y3
y4
y5
y6
y7
y8
y9
y10
0
100000
200000
300000
400000
500000
200
400
600
800
1000
1200

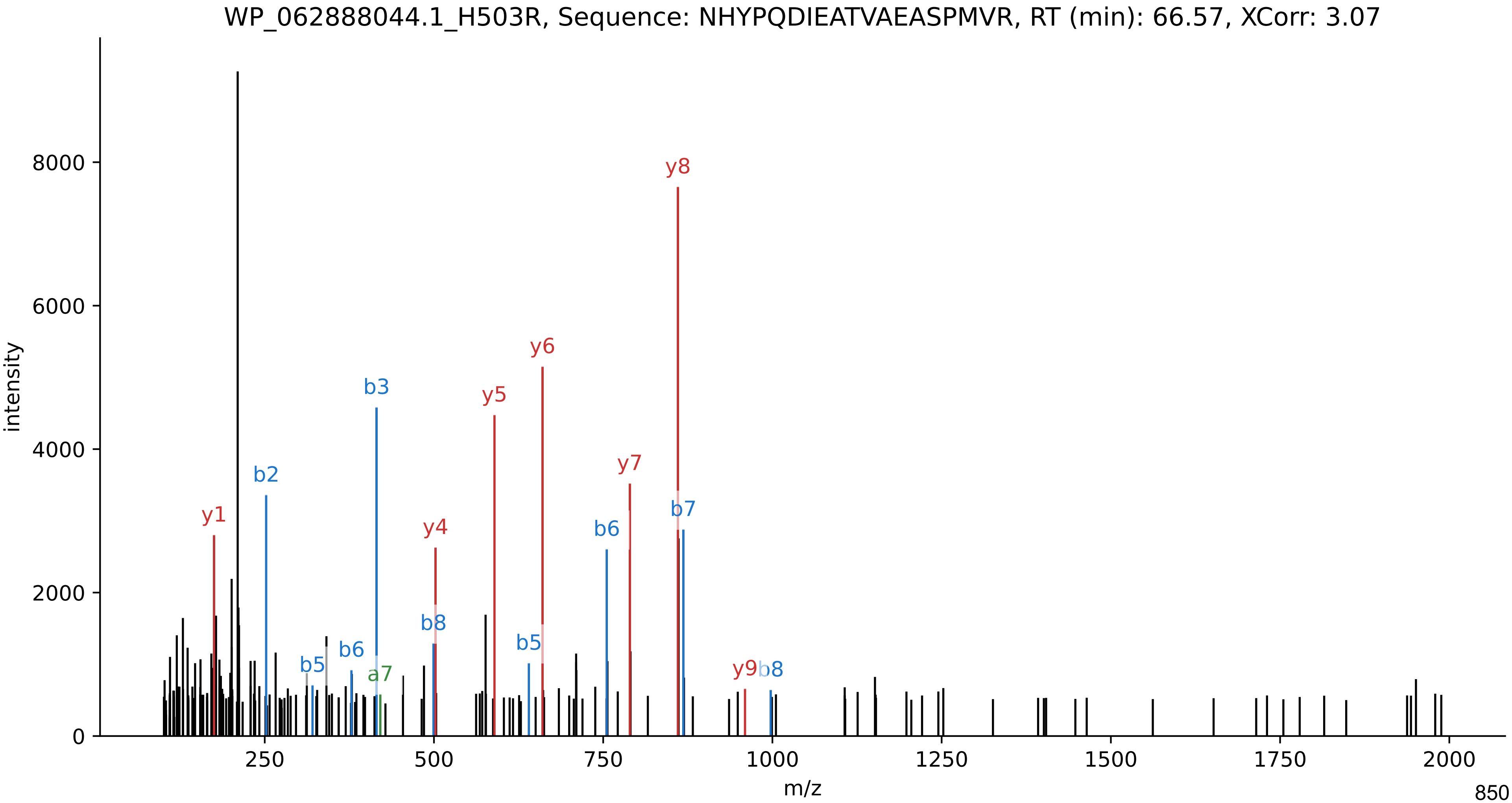
WP_062888044.1_H503R, Sequence: NHYPQDIEATVAEASPMVR, RT (min): 66.57, XCorr: 3.07
intensity
m/z
y1
b2
b5
b6
a7
b3
b8
y4
y5
y6
b5
b6
y7
y8
b7
y9
b8

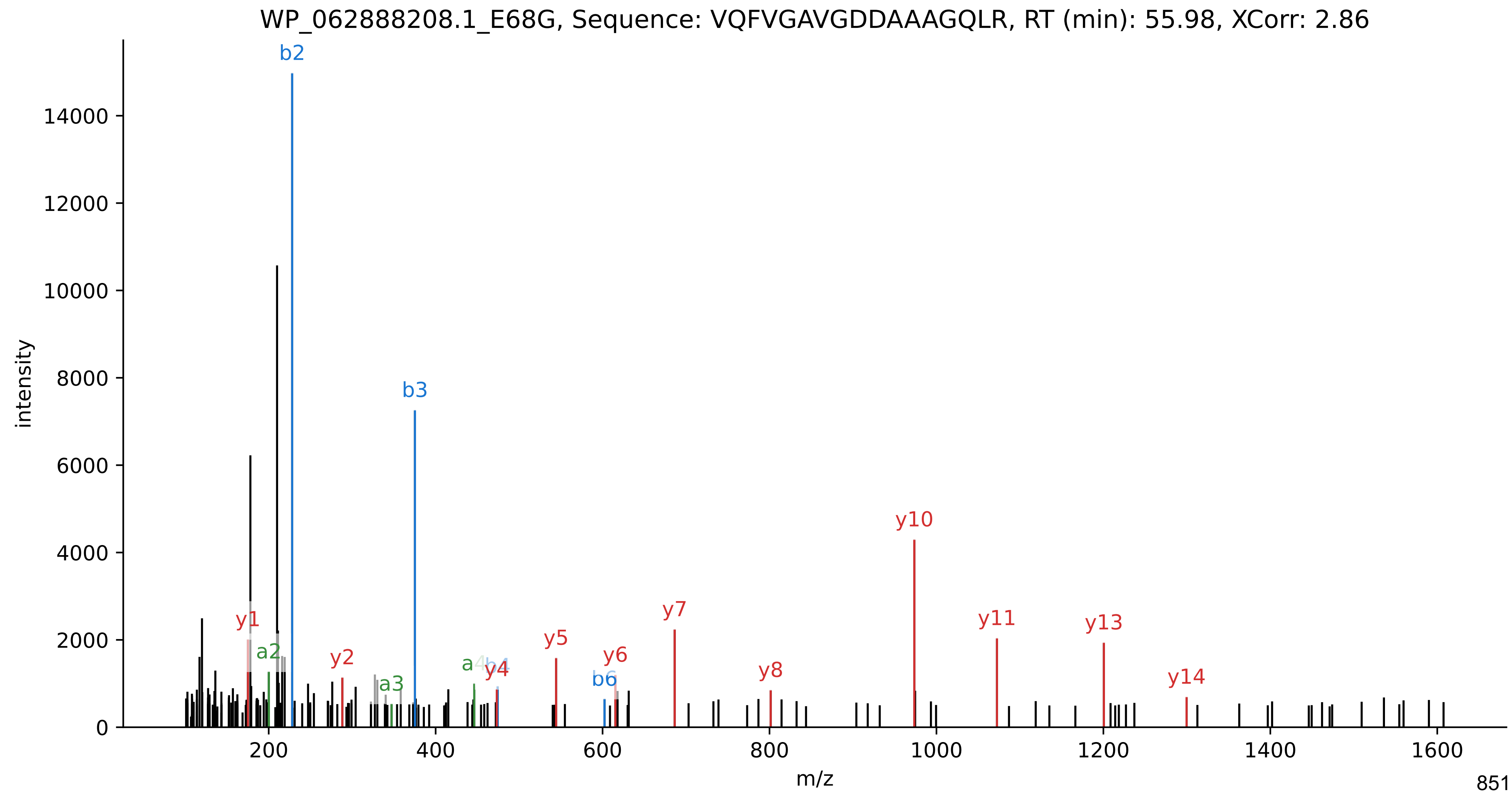

WP_062888208.1_E68G, Sequence: VQFVGAVGDDAAAGQLR, RT (min): 55.98, XCorr: 2.86
intensity
m/z
b2
b3
b6
a2
a3
y1
y2
y4
y5
y6
y7
y8
y10
y11
y13
y14

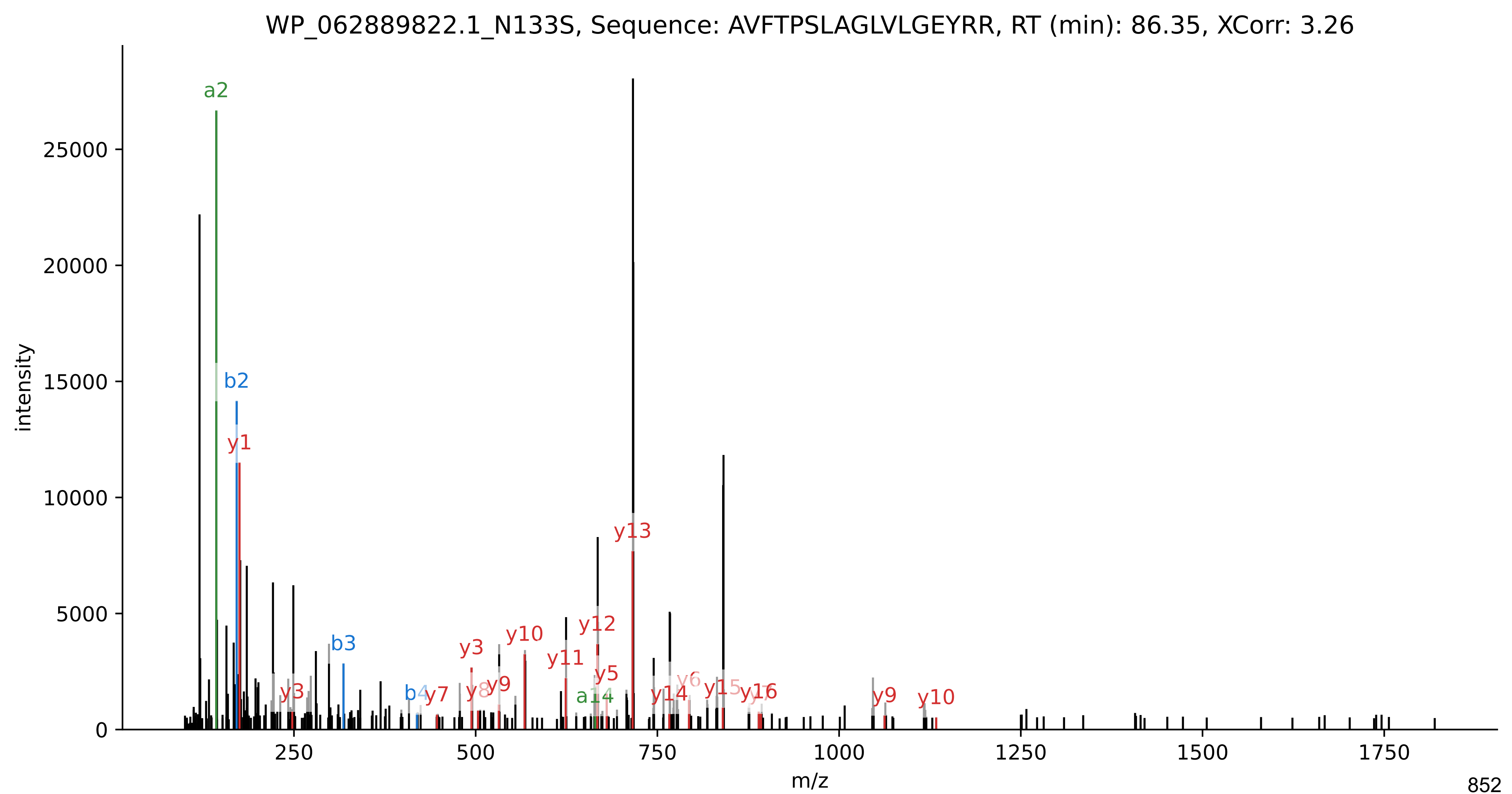

WP_062889822.1_N133S, Sequence: AVFTPSLAGLVLGEYRR, RT (min): 86.35, XCorr: 3.26
intensity
m/z
a2
b2
y1
b3
b4
y3
y7
y3
y8
y9
y10
y11
y12
y5
a14
y13
y14
y6
y15
y16
y9
y10

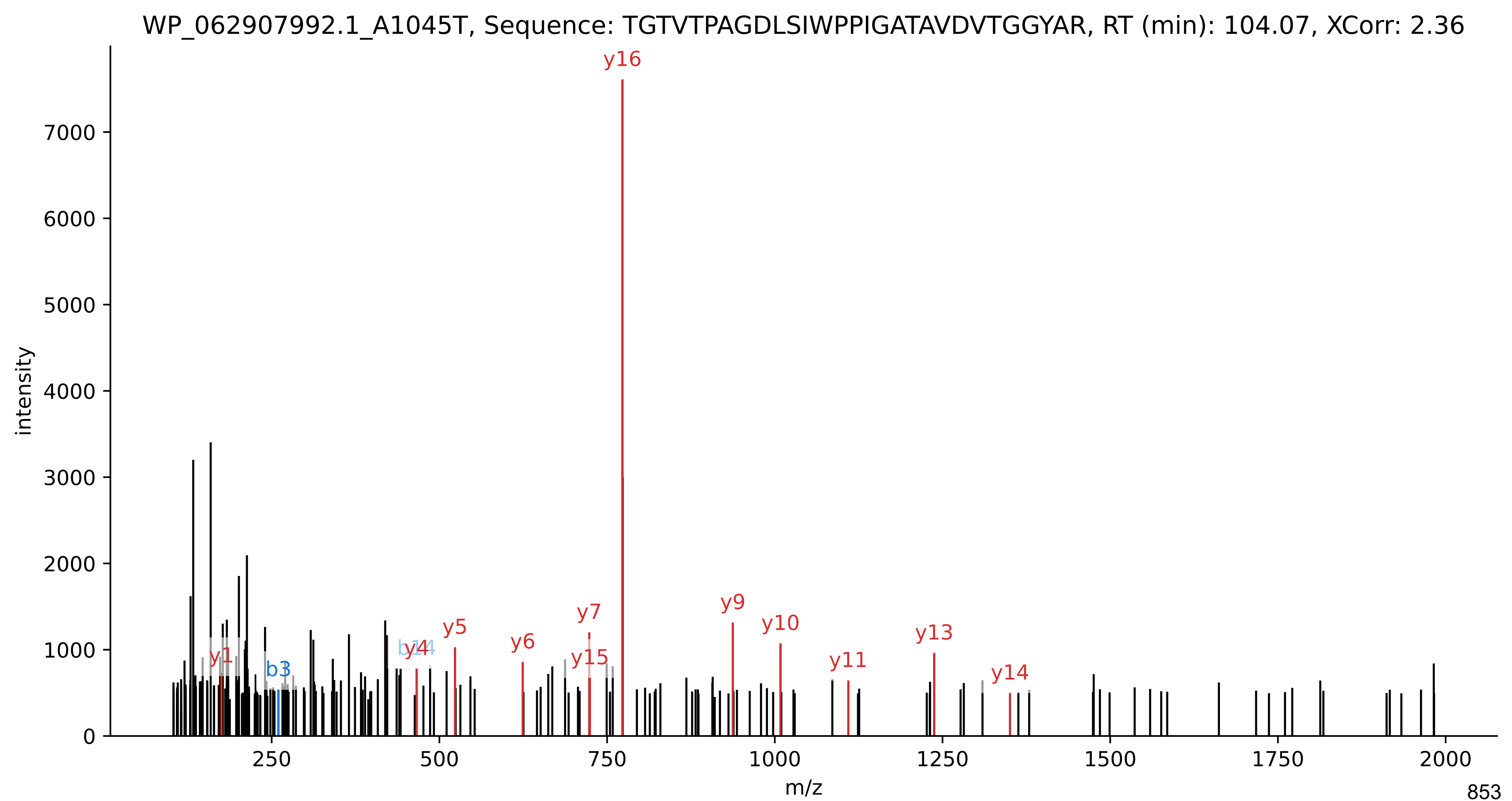

WP_062907992.1_A1045T, Sequence: TGTVTPAGDLSIWPPIGATAVDVTGGYAR, RT (min): 104.07, XCorr: 2.36
intensity
m/z
y16
y1
b3
y4
y5
y6
y7
y15
y9
y10
y11
y13
y14

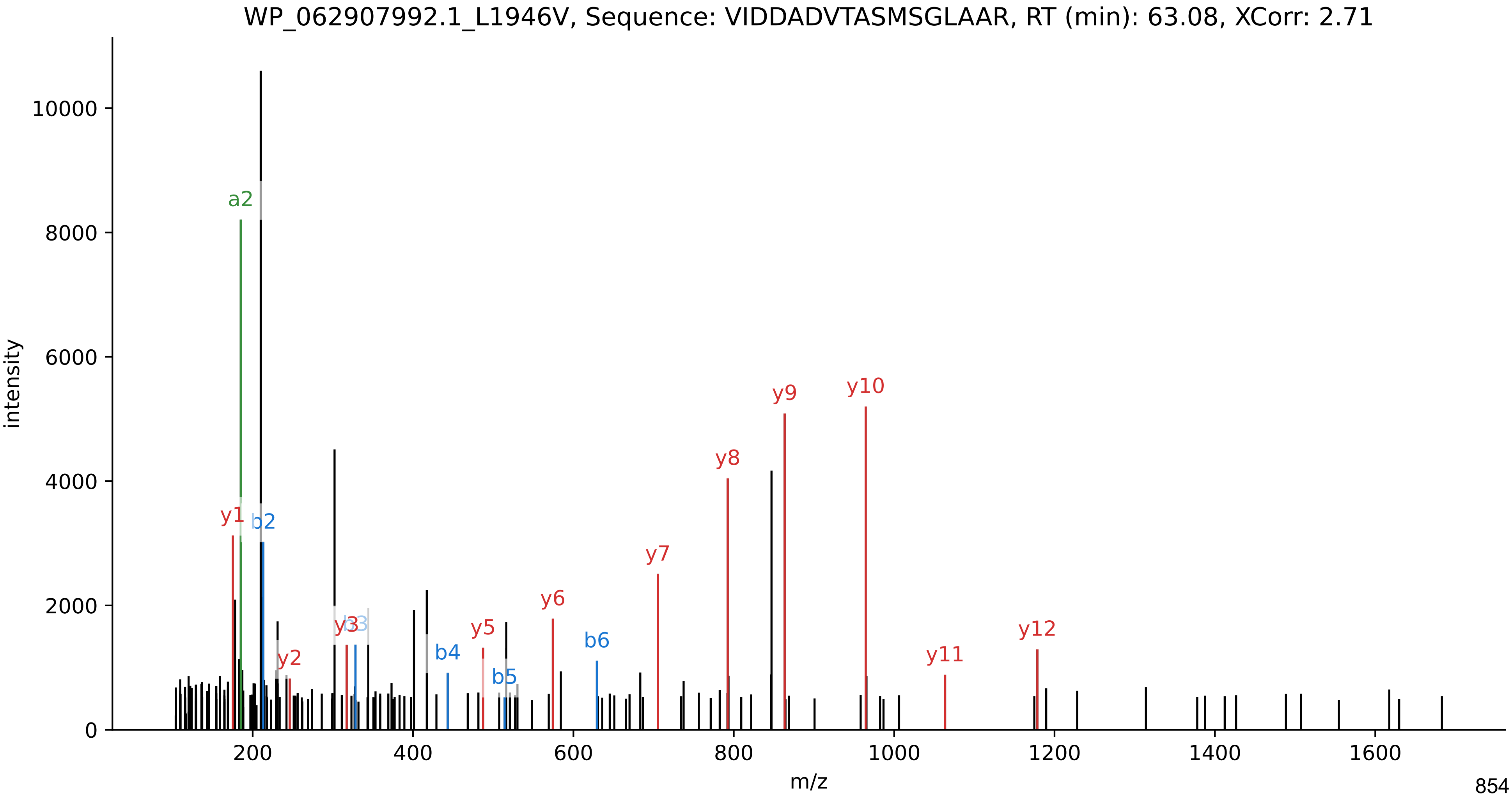
WP_062907992.1_L1946V, Sequence: VIDDADVTASMSGLAAR, RT (min): 63.08, XCorr: 2.71
intensity
m/z
a2
y1
b2
y2
y3
b3
b4
y5
b5
y6
b6
y7
y8
y9
y10
y11
y12

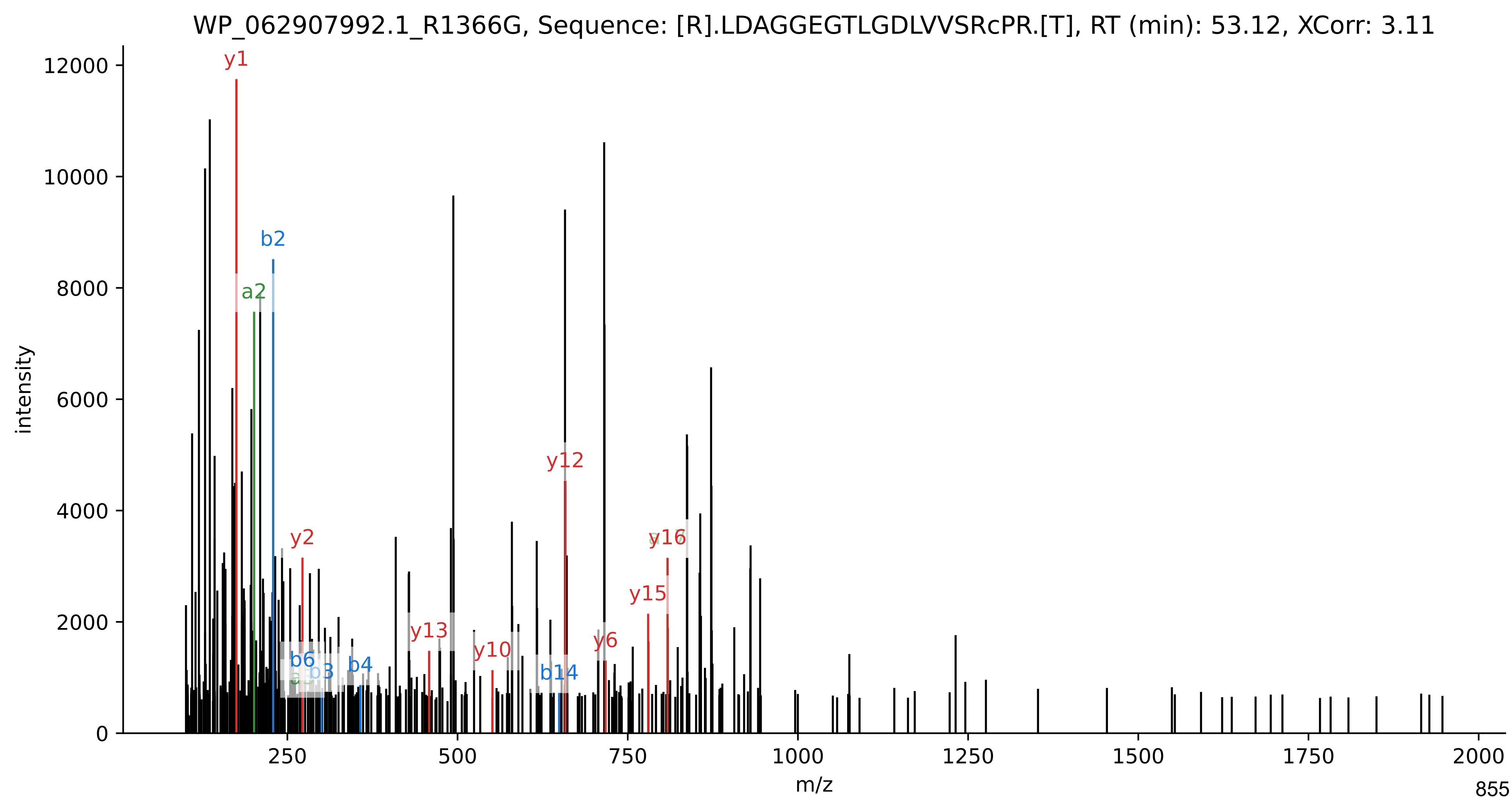

WP_062907992.1_R1366G, Sequence: [R].LDAGGEGTLGDLVVSRcPR.[T], RT (min): 53.12, XCorr: 3.11
intensity
m/z
y1
b2
a2
y2
y12
y16
y15
y13
y10
y6
b6
b3
b4
b14

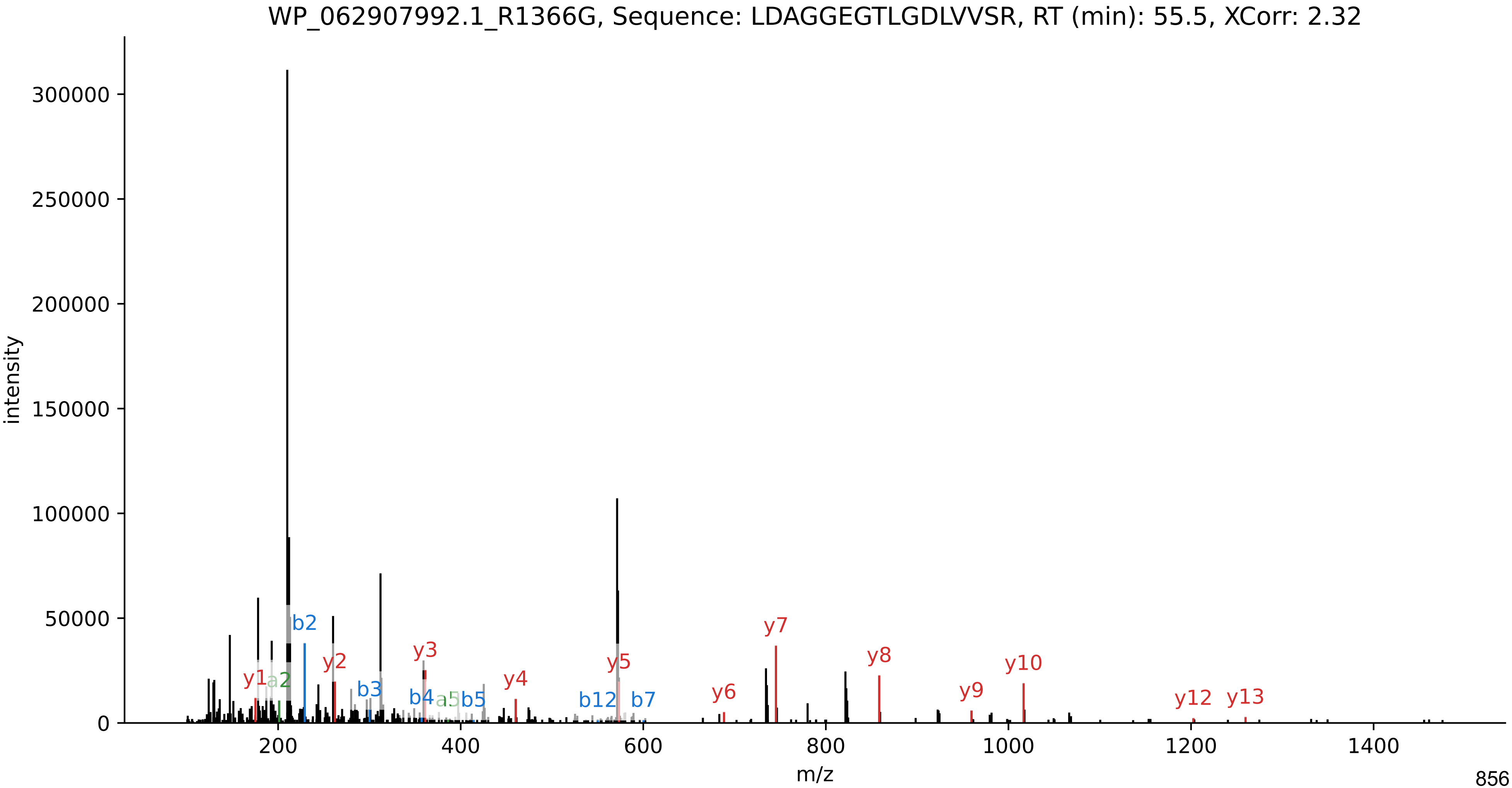
WP_062907992.1_R1366G, Sequence: LDAGGEGTLGDLVVSR, RT (min): 55.5, XCorr: 2.32
intensity
m/z
y1
a2
b2
y2
b3
y3
b4
a5
b5
y4
b12
y5
b7
y6
y7
y8
y9
y10
y12
y13

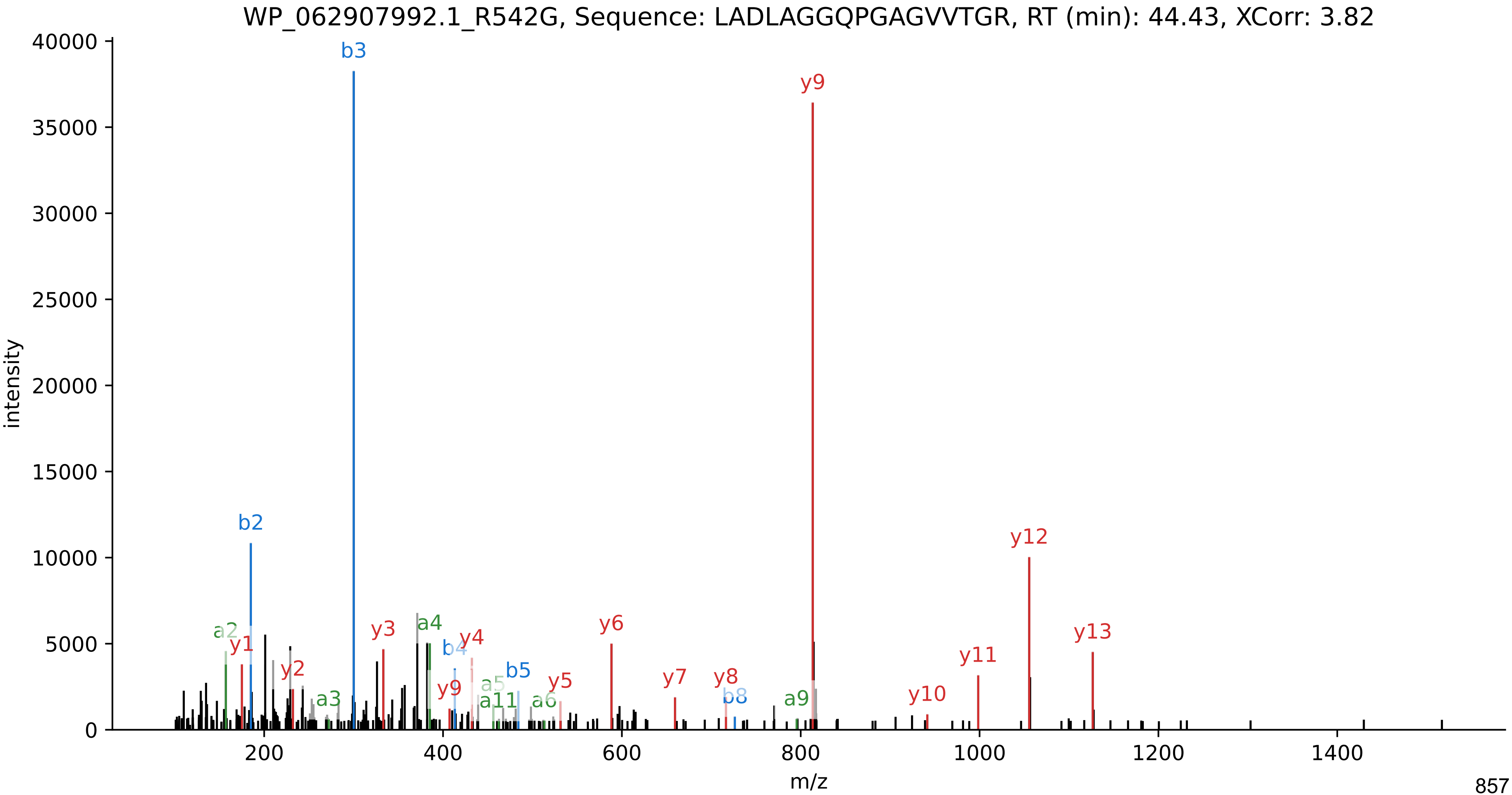
WP_062907992.1_R542G, Sequence: LADLAGGQPGAGVVTGR, RT (min): 44.43, XCorr: 3.82
intensity
m/z
b3
y9
b2
y12
y13
y11
y10
a2
y1
y2
a3
y3
a4
b4
y4
y9
a5
a11
b5
a6
y5
y6
y7
y8
b8
a9
0
5000
10000
15000
20000
25000
30000
35000
40000
200
400
600
800
1000
1200
1400

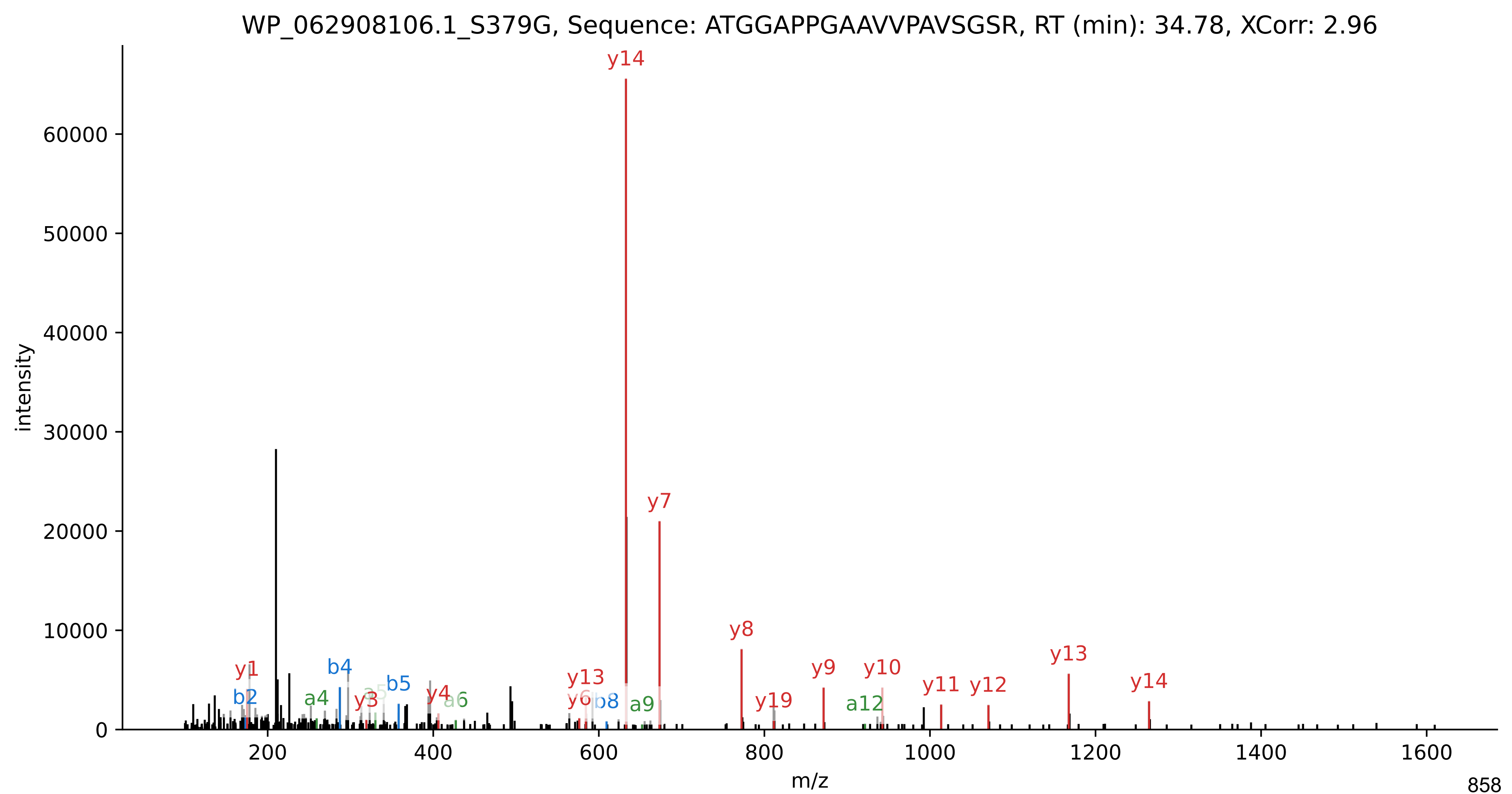

WP_062908106.1_S379G, Sequence: ATGGAPPGAAVVPAVSGSR, RT (min): 34.78, XCorr: 2.96
intensity
m/z
y14
y7
y8
y13
y1
b2
b4
b5
a4
a5
y3
y4
a6
y13
y6
b8
a9
y19
y9
y10
a12
y11
y12
y14

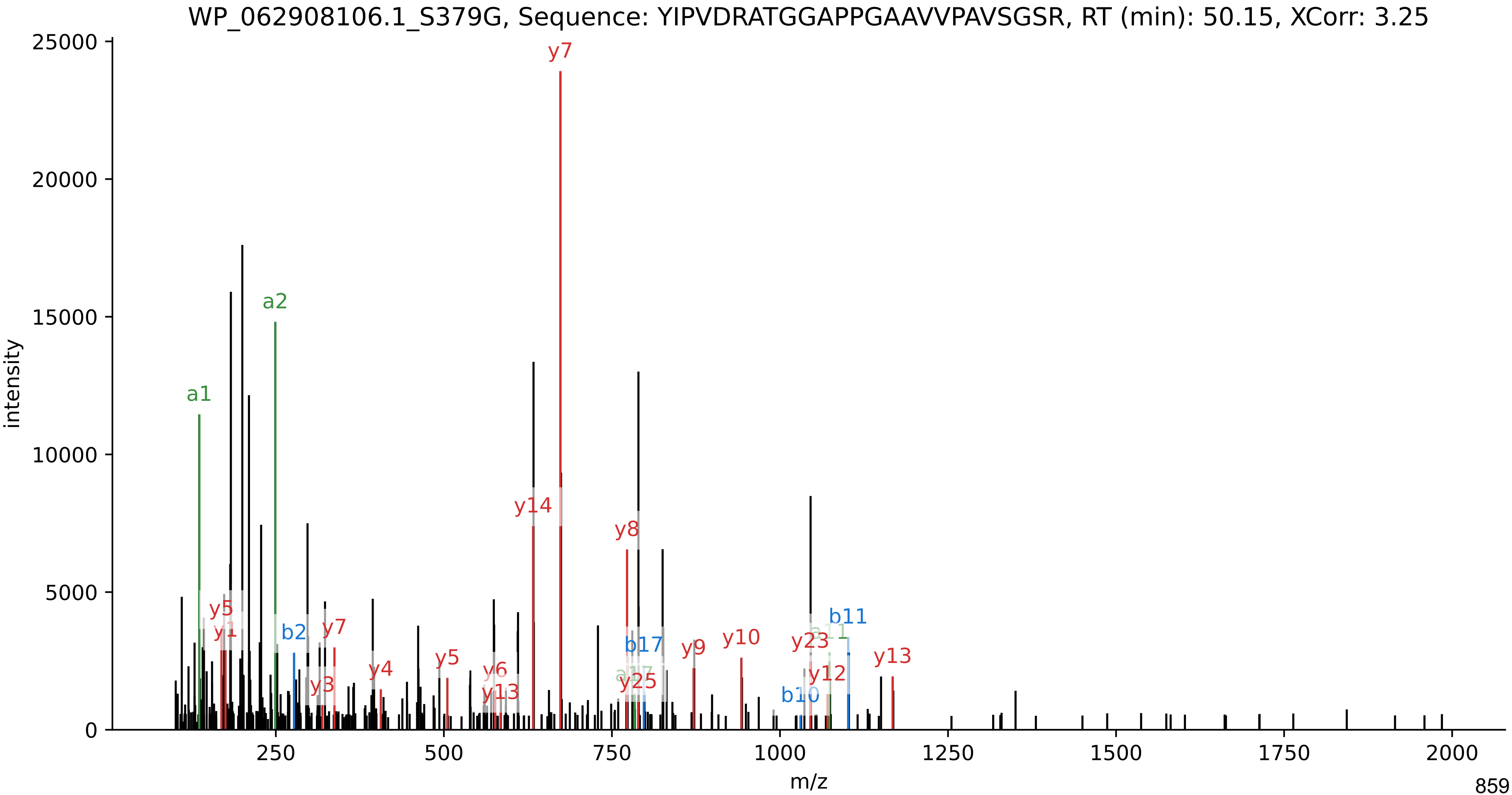

WP_062908106.1_S379G, Sequence: YIPVDRATGGAPPGAAVVPAVSGSR, RT (min): 50.15, XCorr: 3.25
intensity
m/z
0
5000
10000
15000
20000
25000
250
500
750
1000
1250
1500
1750
2000
y7
a2
a1
y14
y8
y5
y1
b2
y7
y3
y4
y5
y6
y13
b17
y25
y9
y10
y23
b11
y12
b10
y13

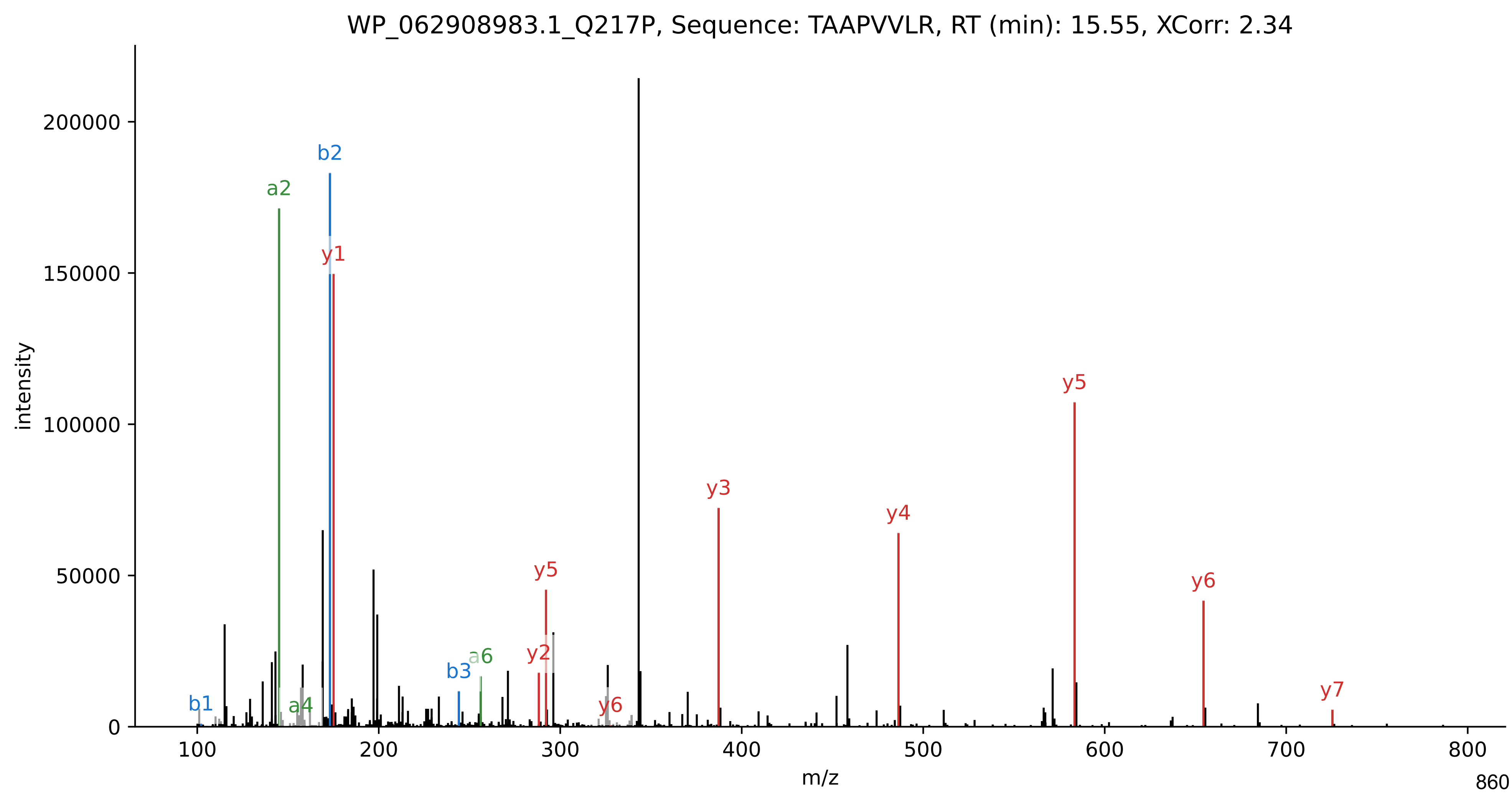
WP_062908983.1_Q217P, Sequence: TAAPVVLR, RT (min): 15.55, XCorr: 2.34
intensity
m/z
b1
b2
b3
a2
a4
a6
y1
y2
y3
y4
y5
y6
y7

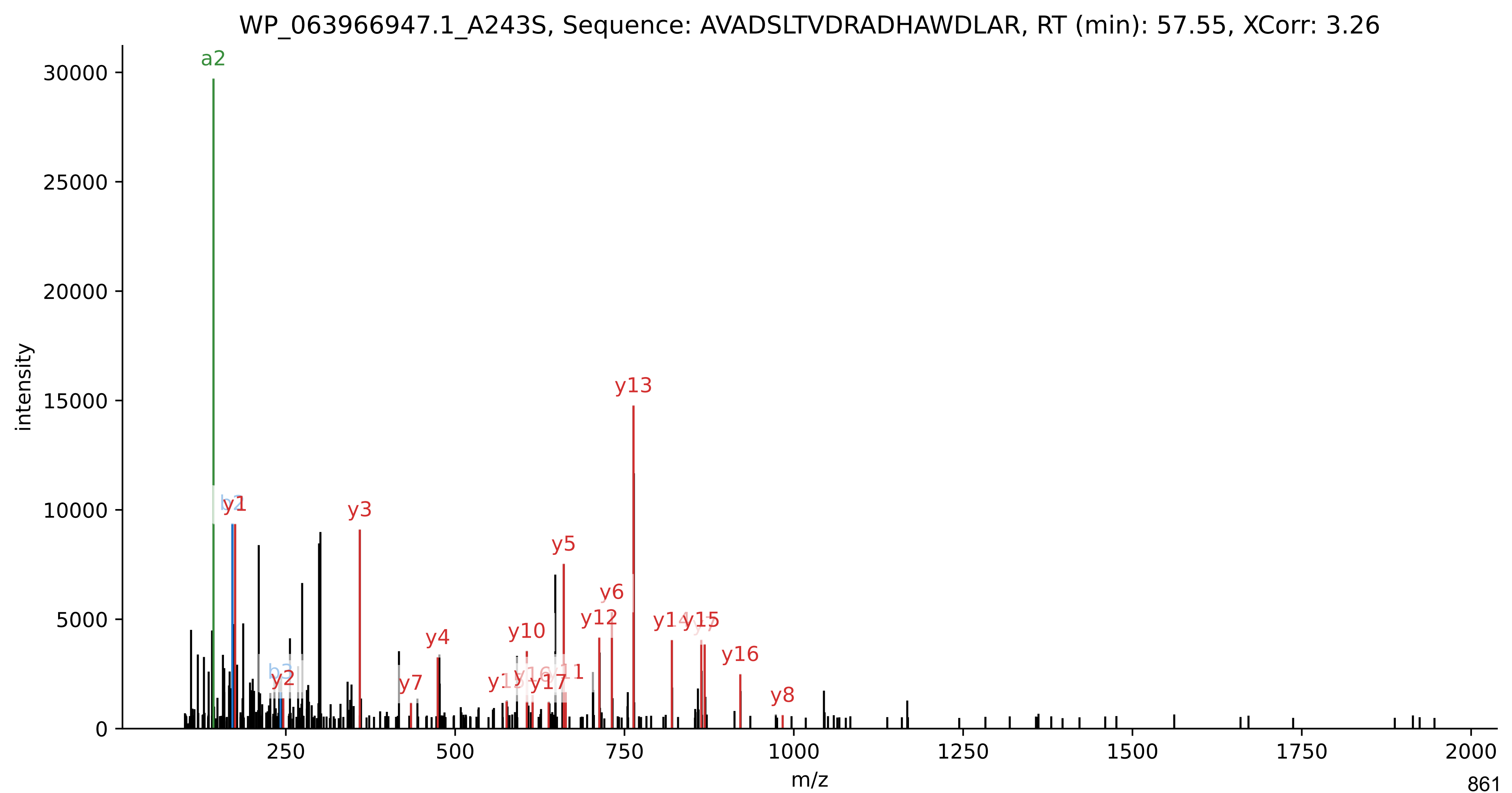
WP_063966947.1_A243S, Sequence: AVADSLTVDRADHAWDLAR, RT (min): 57.55, XCorr: 3.26
a2
y1
y2
y3
y4
y5
y6
y7
y8
y10
y12
y13
y14
y15
y16
y17
intensity
m/z
0
5000
10000
15000
20000
25000
30000
250
500
750
1000
1250
1500
1750
2000

WP_071321353.1_A1210V, Sequence: AGQNVAALPTAVGAGAAIAVAPTVAAPEQPAEAPVEEDTNAETLSDSLTAR, RT (min): 99.2, XCorr: 5.35

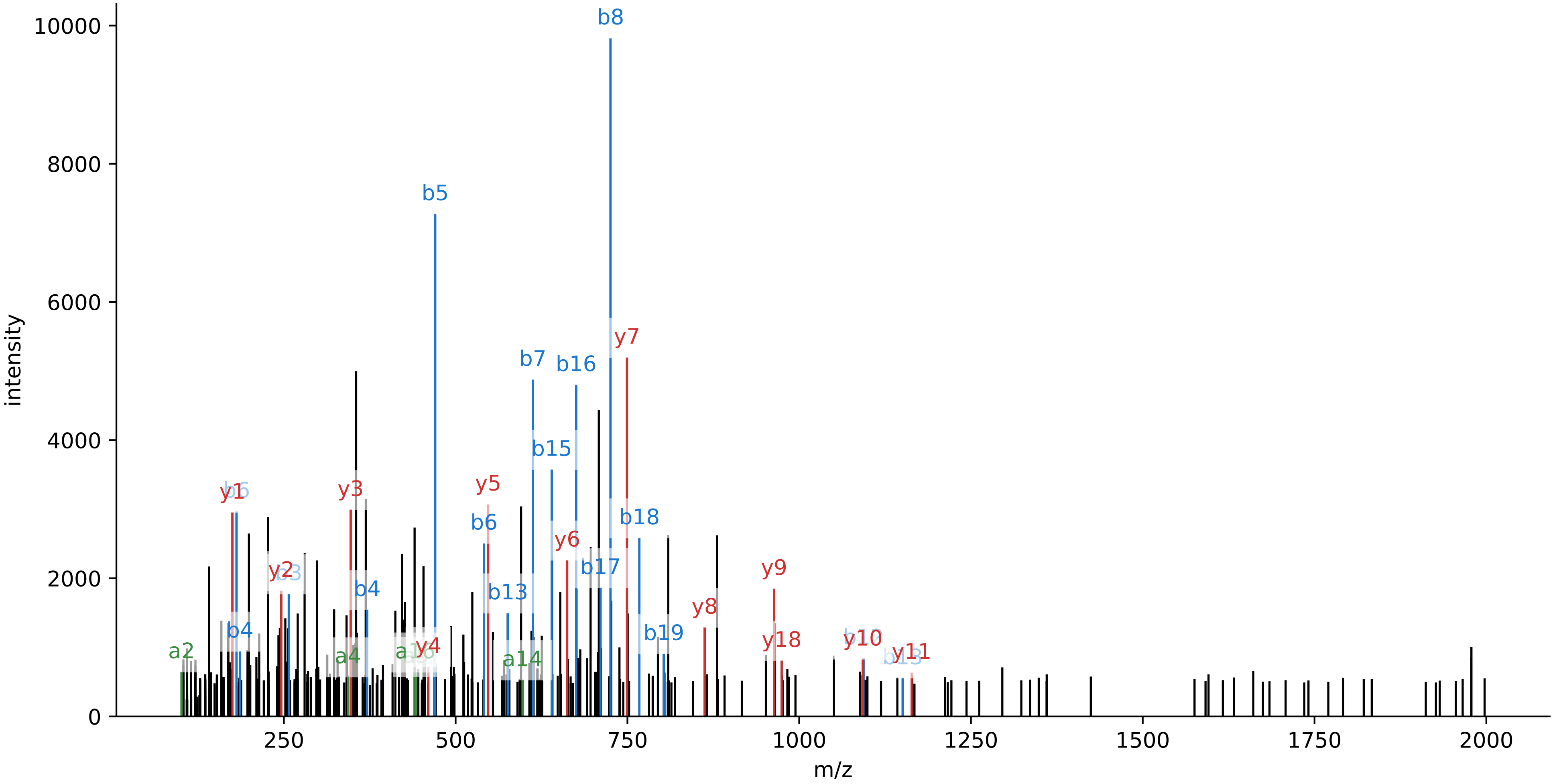

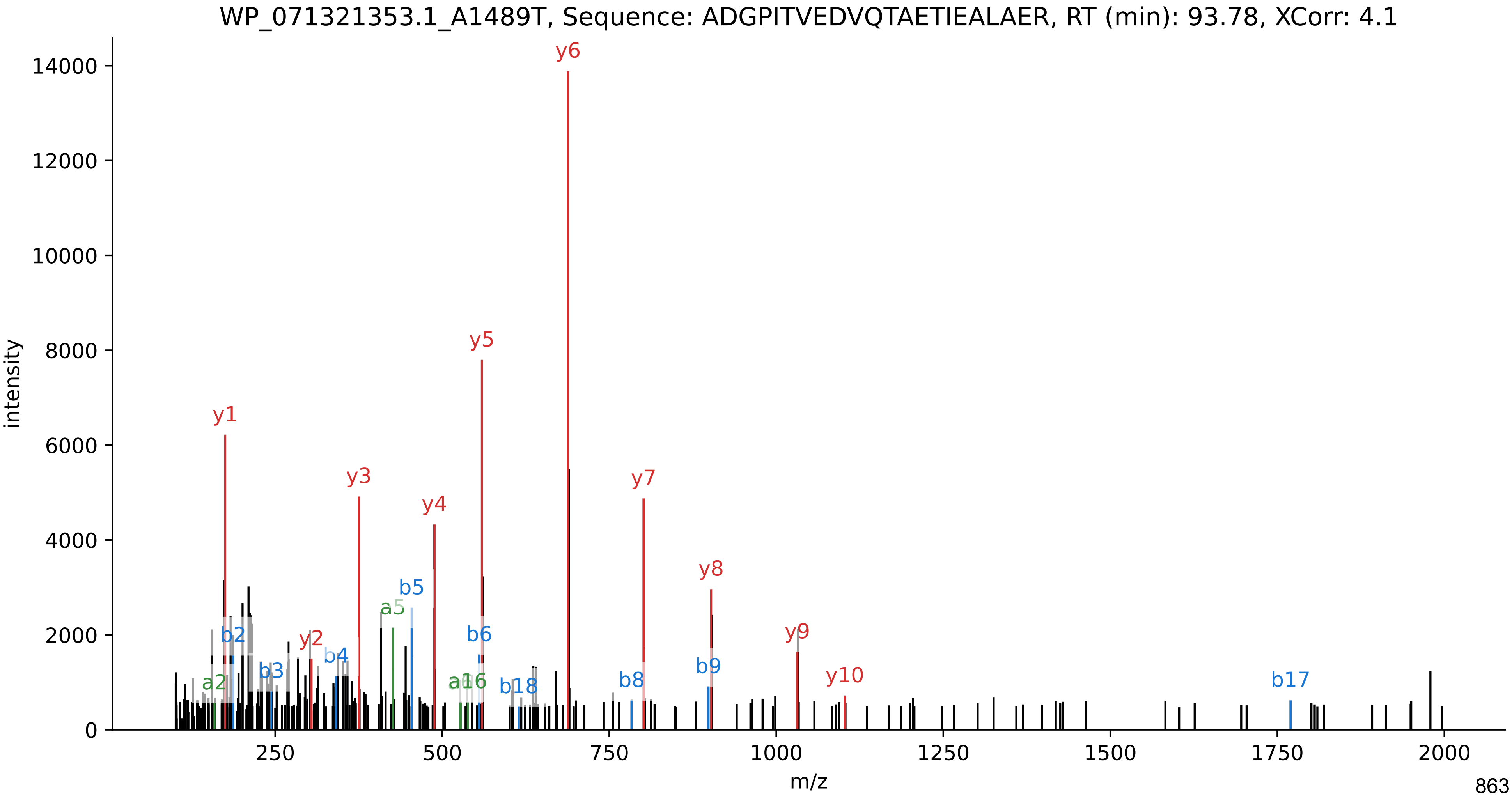
WP_071321353.1_A1489T, Sequence: ADGPITVEDVQTAETIEALAER, RT (min): 93.78, XCorr: 4.1
intensity
m/z
y1
y2
y3
y4
y5
y6
y7
y8
y9
y10
b2
b3
b4
b5
b6
b8
b9
b17
b18
a2
a5
0
2000
4000
6000
8000
10000
12000
14000
250
500
750
1000
1250
1500
1750
2000

WP_071321353.1_A1489T, Sequence: LSERADGPITVEDVQTAETIEALAERVR, RT (min): 94.6, XCorr: 6.08

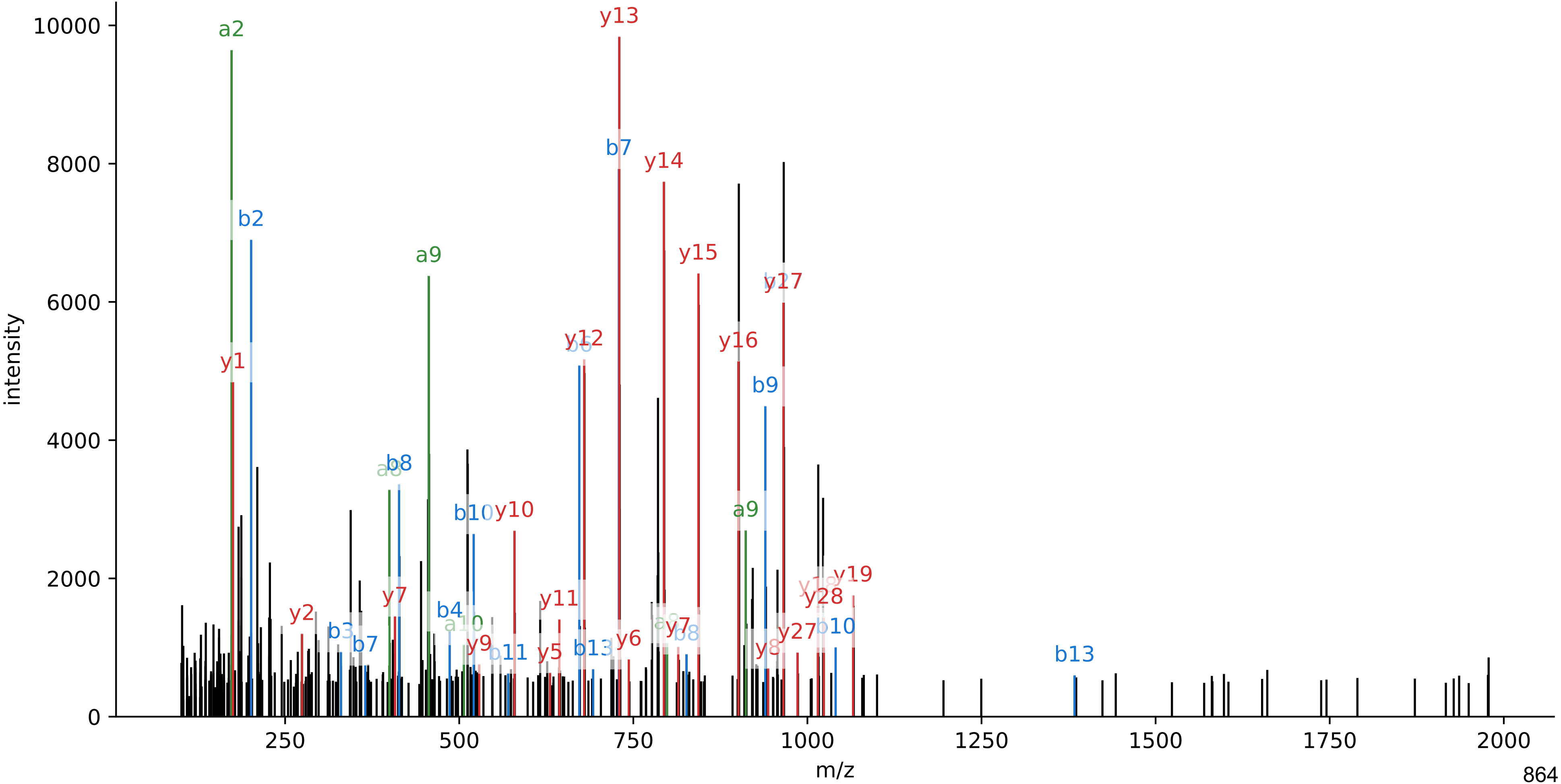

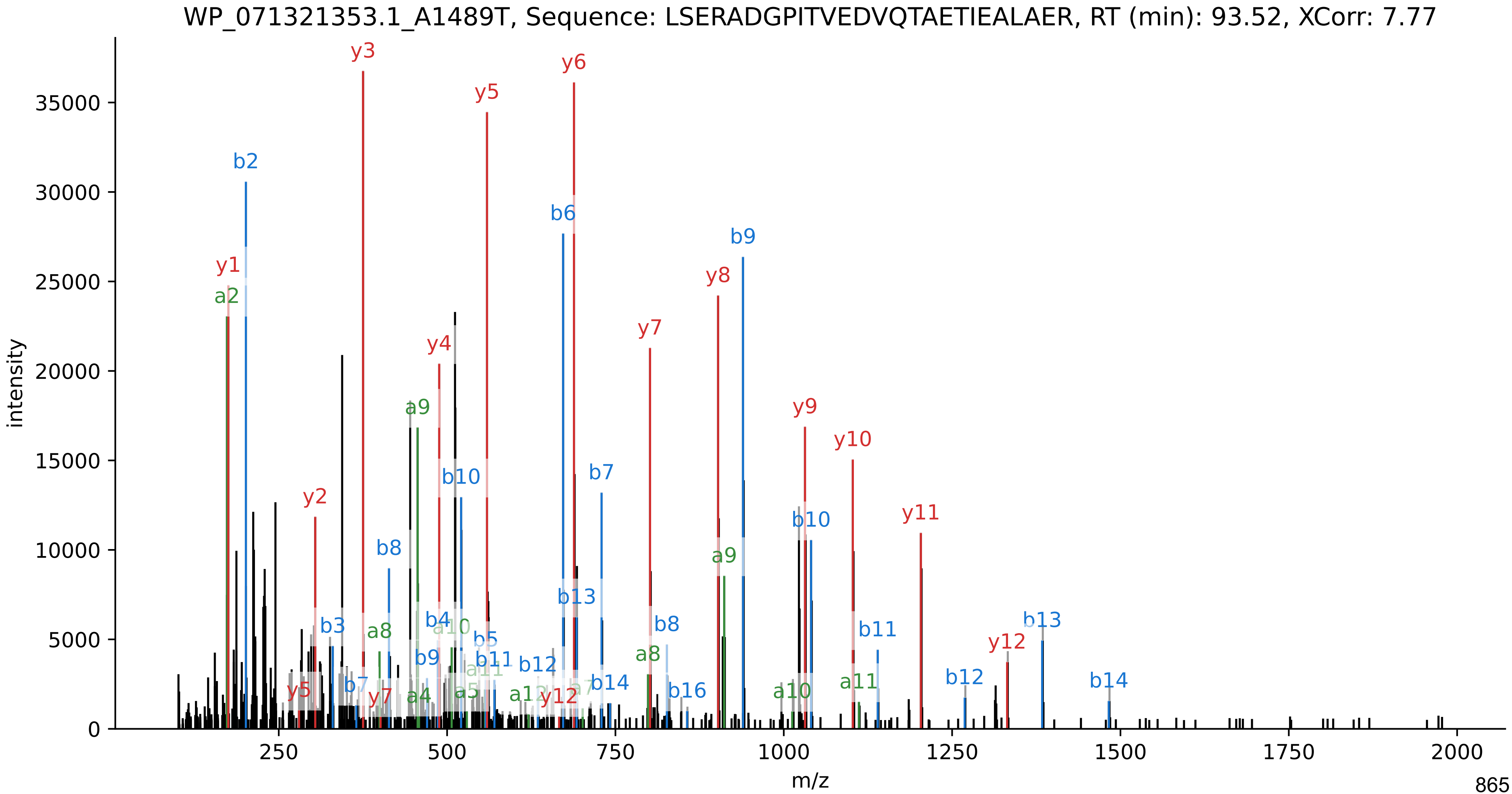
WP_071321353.1_A1489T, Sequence: LSERADGPITVEDVQTAETIEALAER, RT (min): 93.52, XCorr: 7.77
intensity
m/z

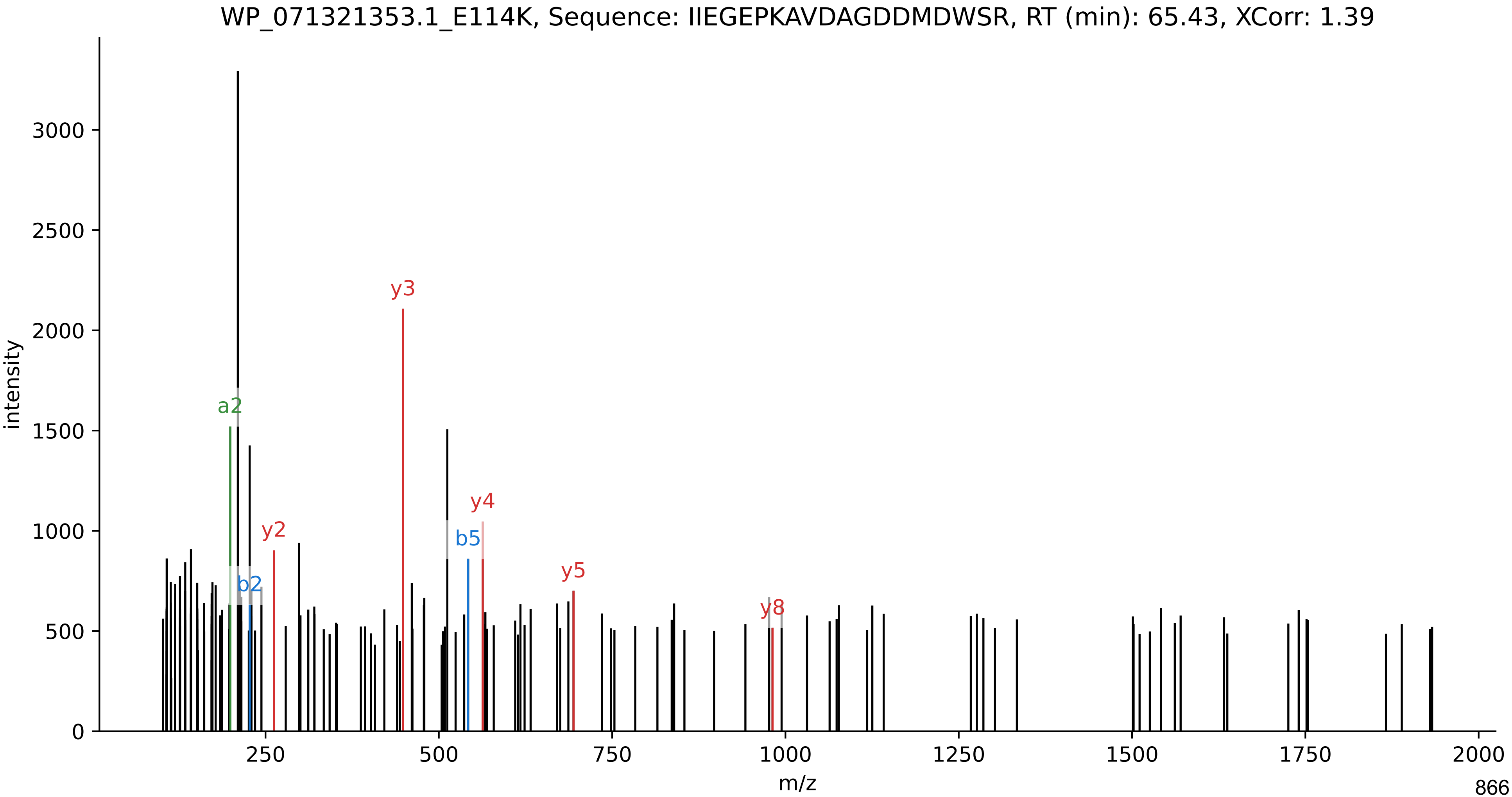
WP_071321353.1_E114K, Sequence: IIEGEPKAVDAGDDMDWSR, RT (min): 65.43, XCorr: 1.39
intensity
m/z
a2
b2
y2
y3
b5
y4
y5
y8
0
500
1000
1500
2000
2500
3000
250
500
750
1000
1250
1500
1750
2000

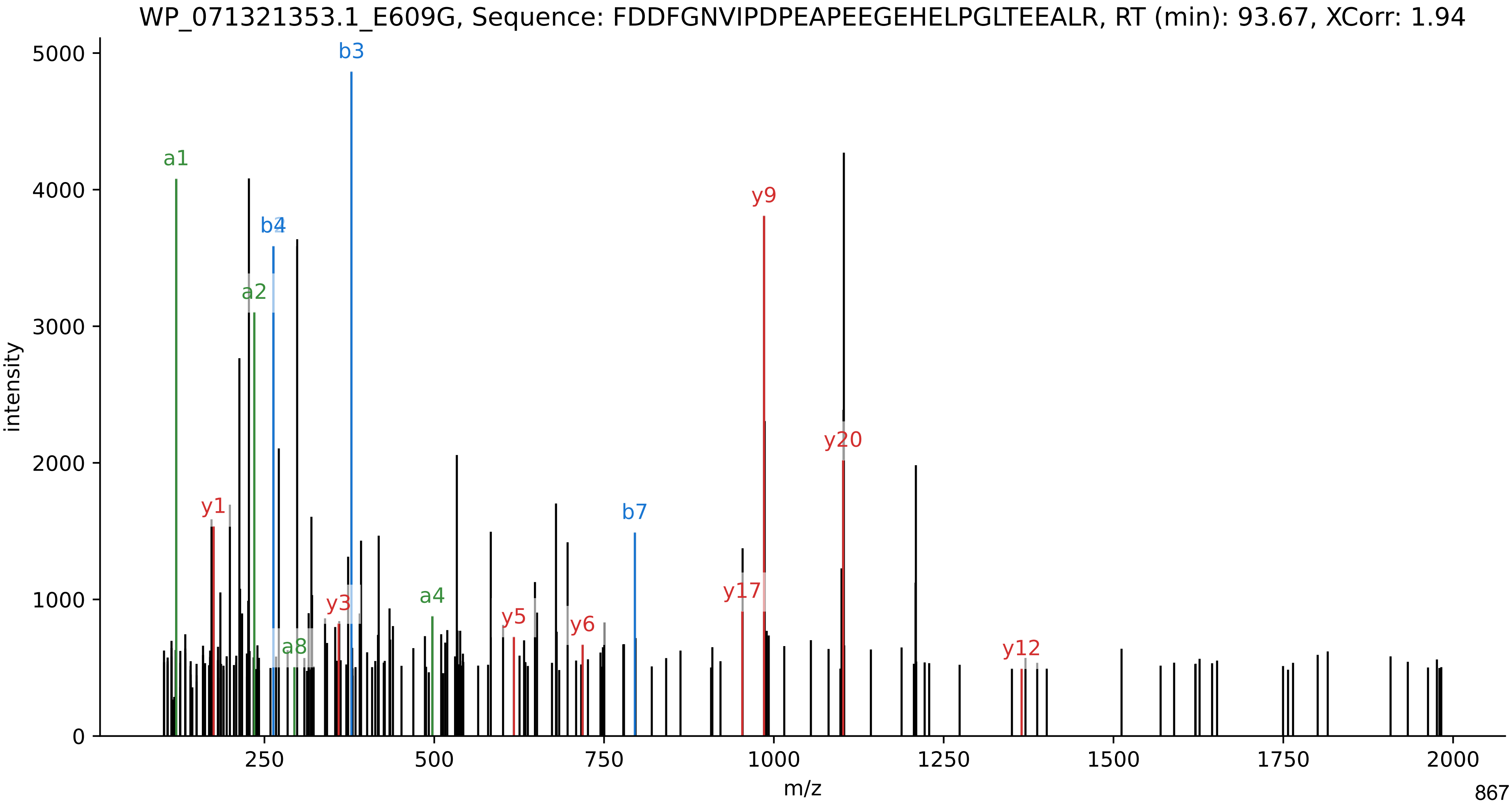
WP_071321353.1_E609G, Sequence: FDDFGNVIPDPEAPEEGEHELPGLTEEALR, RT (min): 93.67, XCorr: 1.94
intensity
m/z
a1
a2
a4
a8
b3
b4
b7
y1
y3
y5
y6
y9
y12
y17
y20
0
1000
2000
3000
4000
5000
250
500
750
1000
1250
1500
1750
2000

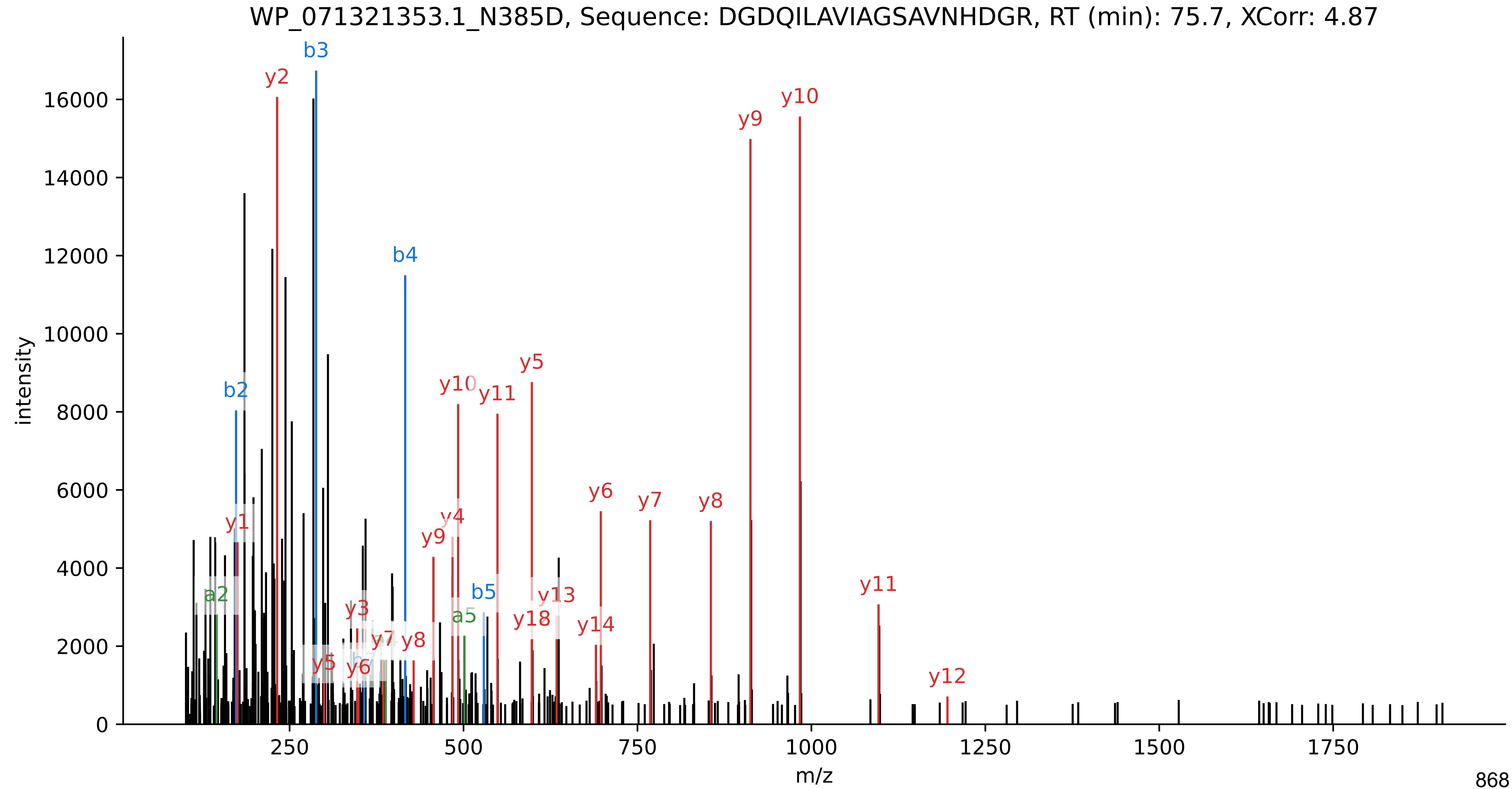

WP_071321353.1_N385D, Sequence: DGDQILAVIAGSAVNHDGR, RT (min): 75.7, XCorr: 4.87
intensity
m/z
b3
y2
y10
y9
b4
y5
b2
y10
y11
y6
y7
y8
y1
y4
y9
y11
a2
b5
y13
y3
a5
y18
y14
y7
y8
y5
y6
y12

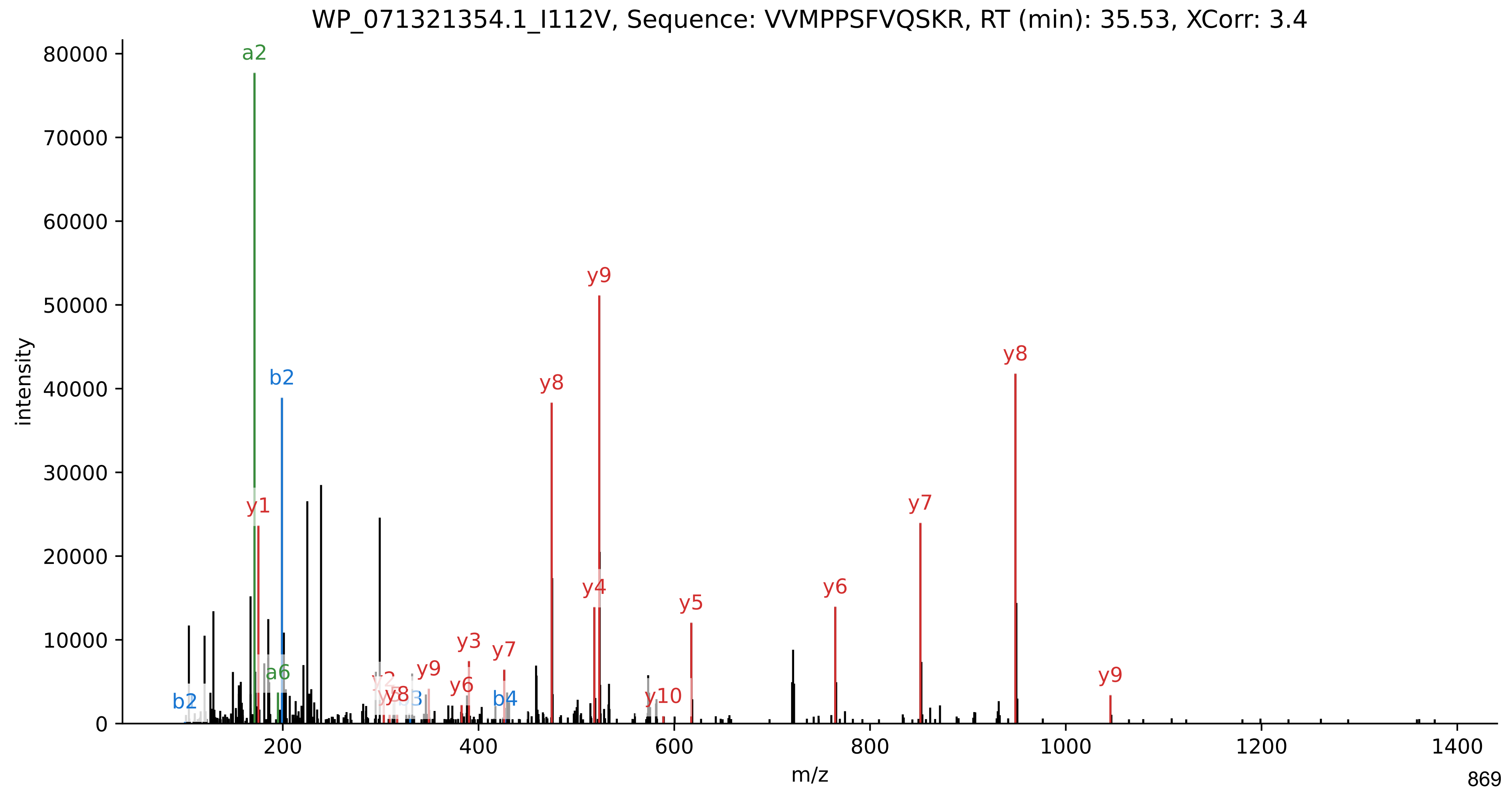
WP_071321354.1_I112V, Sequence: VVMPPSFVQSKR, RT (min): 35.53, XCorr: 3.4
intensity
m/z
a2
b2
y1
a6
y9
y8
y4
y5
y6
y7
y3
b4
y10

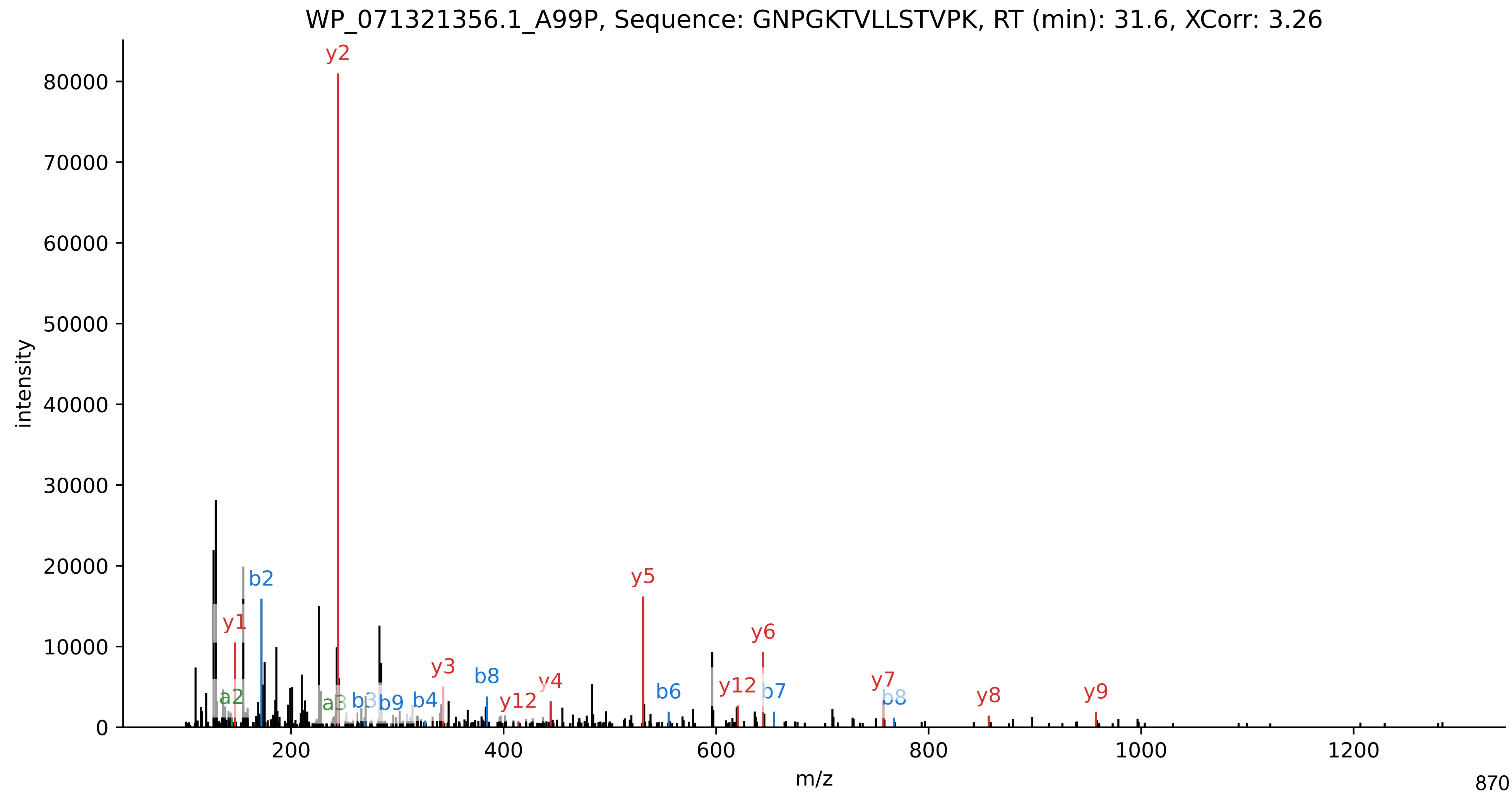
WP_071321356.1_A99P, Sequence: GNPGKTVLLSTVPK, RT (min): 31.6, XCorr: 3.26
intensity
m/z
y2
b2
y1
a2
a3
b3
b9
b4
y3
b8
y12
y4
y5
b6
y12
y6
b7
y7
b8
y8
y9
0
10000
20000
30000
40000
50000
60000
70000
80000
200
400
600
800
1000
1200

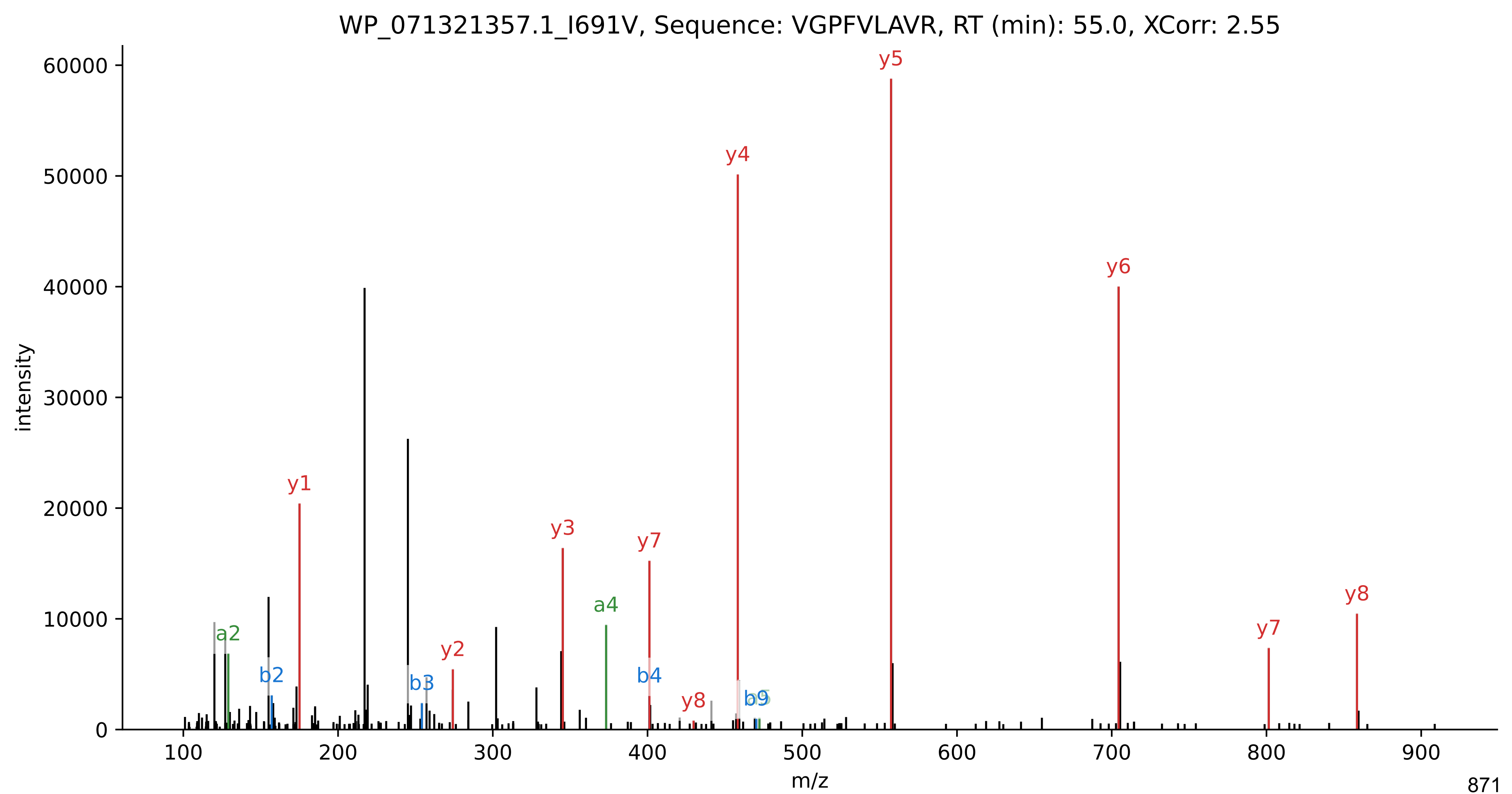
WP_071321357.1_I691V, Sequence: VGPFVLAVR, RT (min): 55.0, XCorr: 2.55
intensity
m/z
y5
y4
y6
y1
y3
y7
a4
y8
y7
a2
y2
b2
b3
b4
y8
b9
0
10000
20000
30000
40000
50000
60000
100
200
300
400
500
600
700
800
900

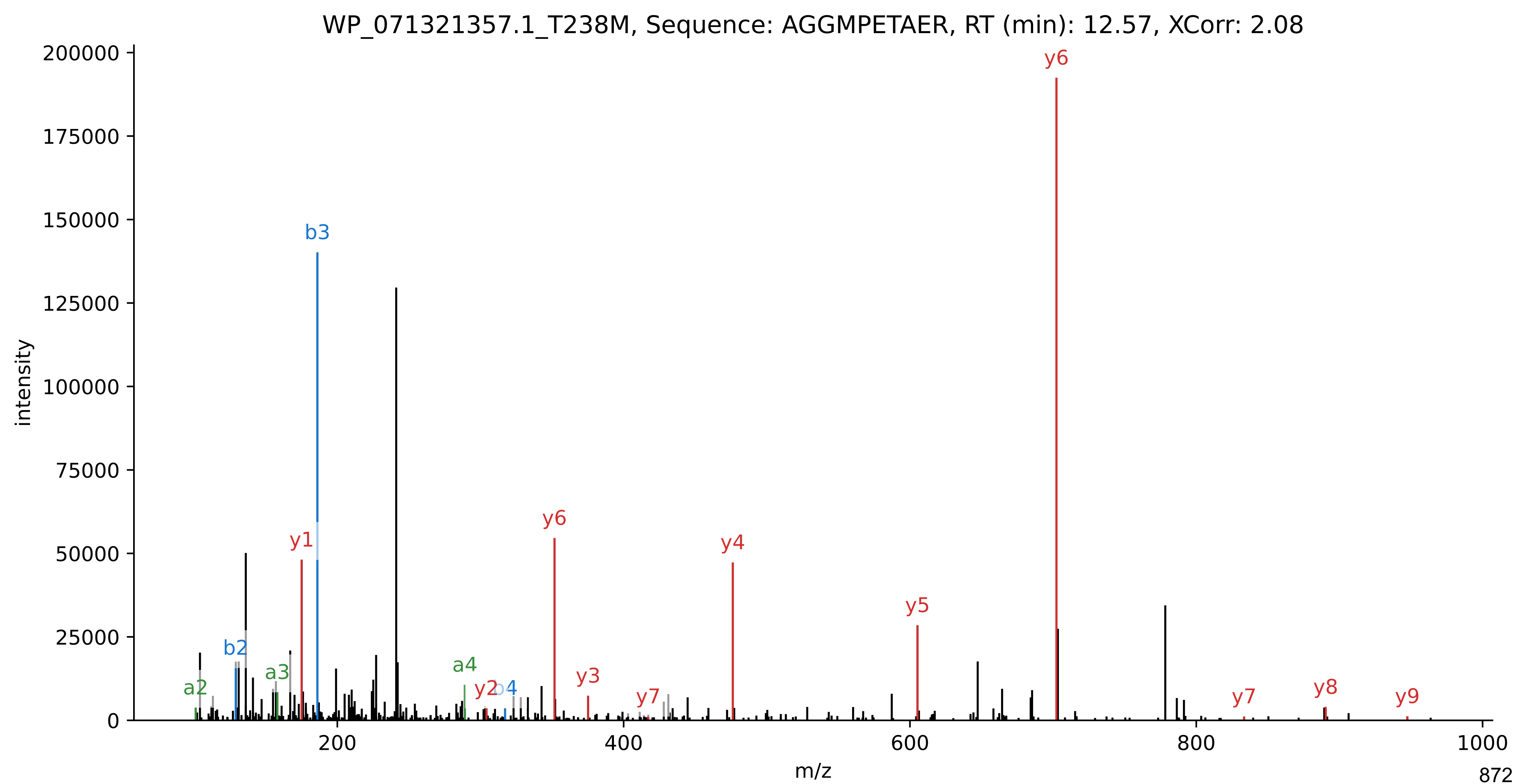
WP_071321357.1_T238M, Sequence: AGGMPETAER, RT (min): 12.57, XCorr: 2.08
intensity
m/z
y6
b3
y6
y1
y4
y5
b2
a3
a4
a2
y2
b4
y3
y7
y7
y8
y9
200000
175000
150000
125000
100000
75000
50000
25000
0
200
400
600
800
1000

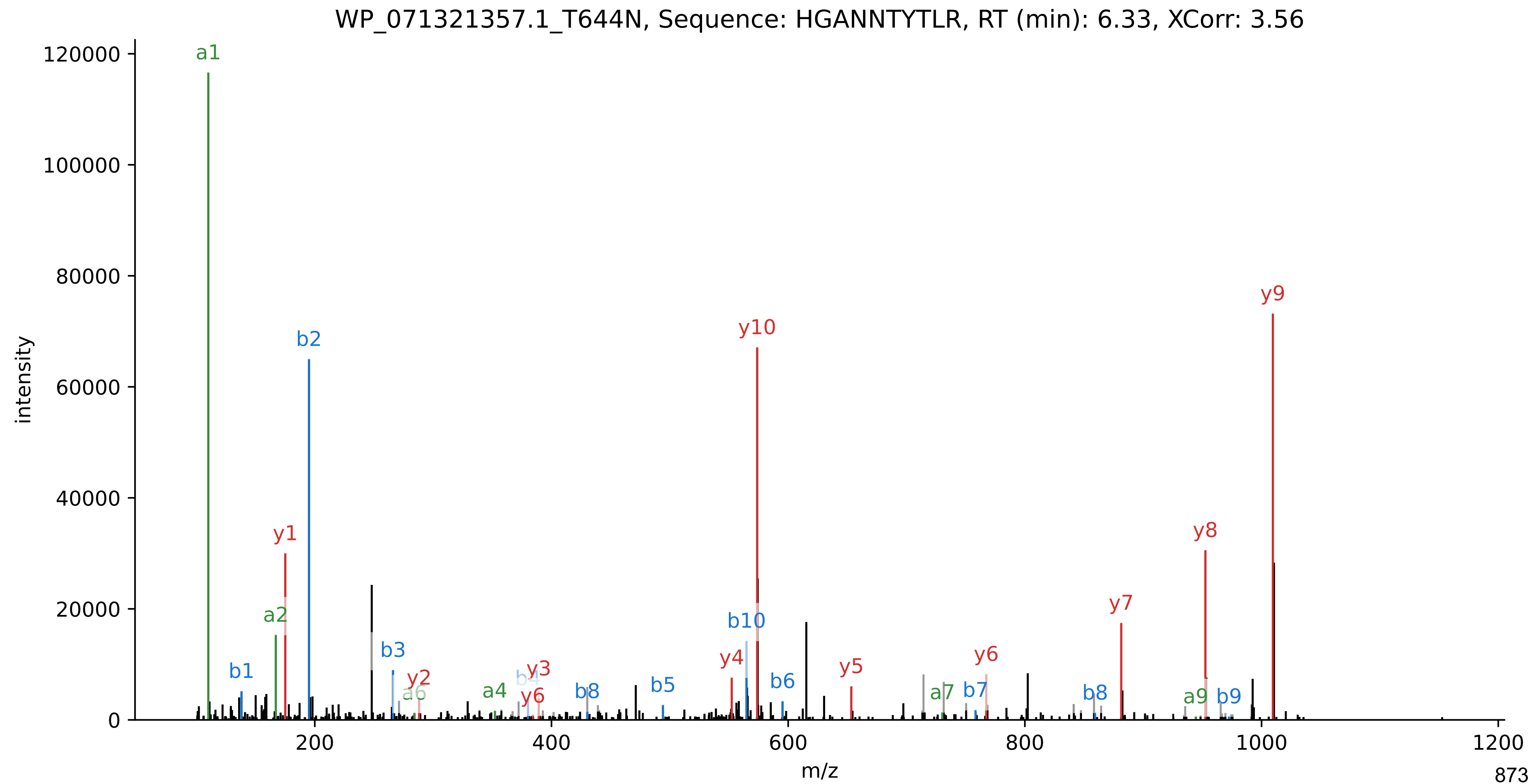

WP_071321357.1_T644N, Sequence: HGANNTYTLR, RT (min): 6.33, XCorr: 3.56
intensity
m/z
a1
b2
y10
y9
y1
a2
b1
b3
y2
a6
a4
b4
y3
y6
b8
b5
y4
b10
b6
y5
y6
a7
b7
y7
y8
b8
a9
b9

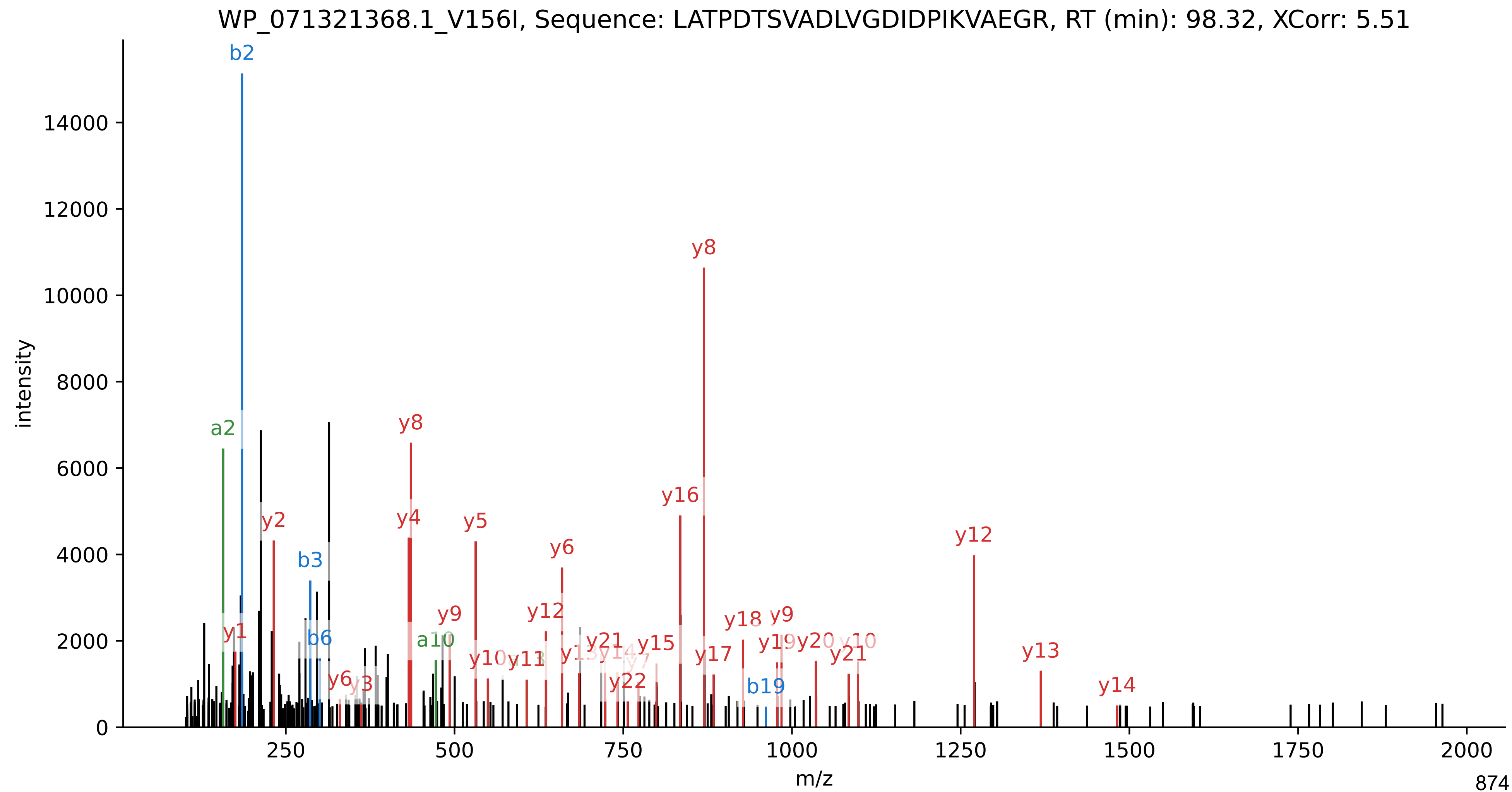

WP_071321368.1_V156I, Sequence: LATPDTSVADLVGDIDPIKVAEGR, RT (min): 98.32, XCorr: 5.51
intensity
m/z
b2
a2
y8
y16
y12
y4
y5
y6
y2
b3
y1
b6
y9
a10
y12
y21
y15
y18
y9
y19
y20
y10
y17
y21
y13
y14
y22
b19

WP_071321368.1_V156I, Sequence: YTEKLATPDTSVADLVGDIDPIKVAEGR, RT (min): 91.73, XCorr: 6.73

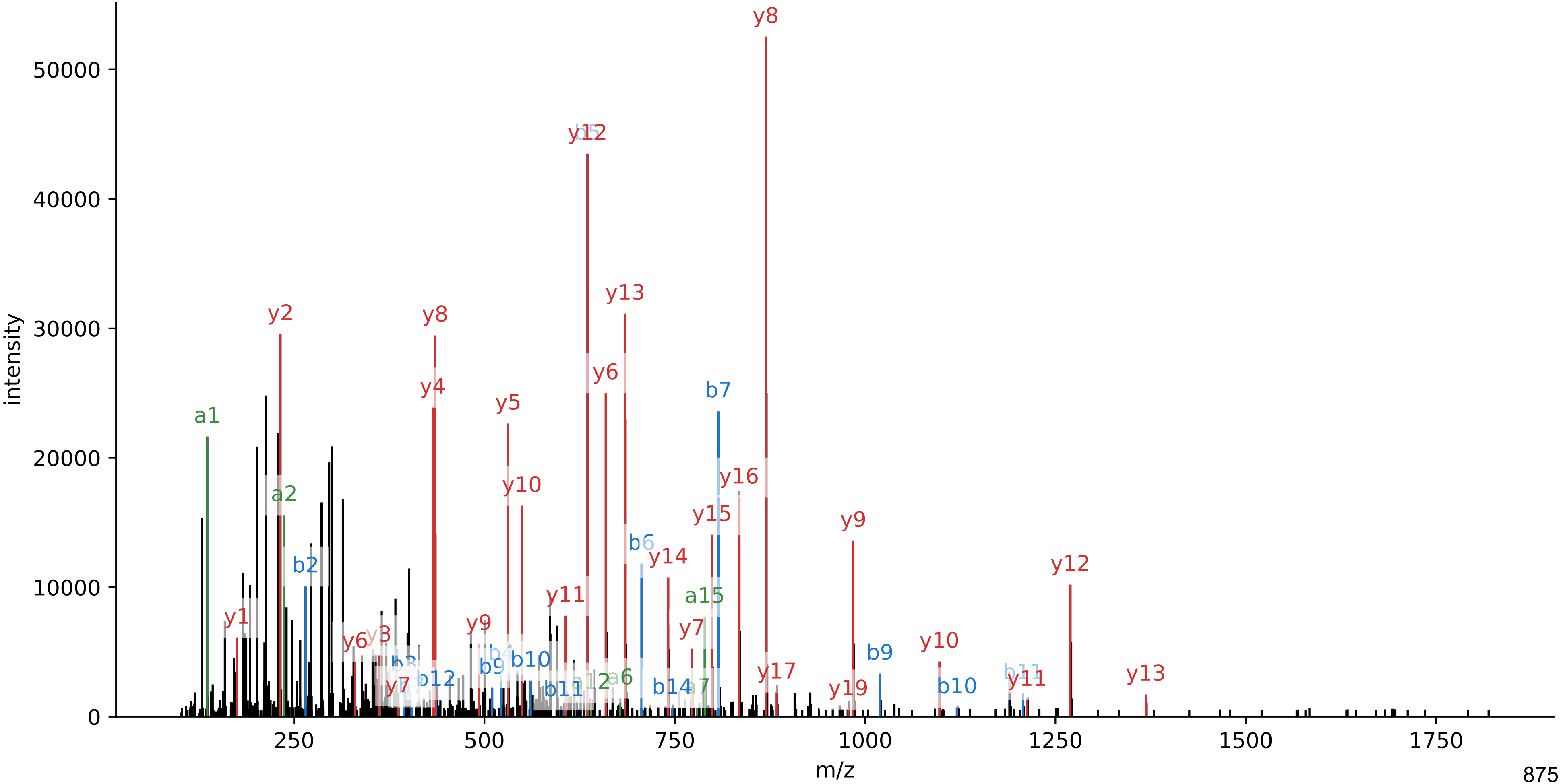

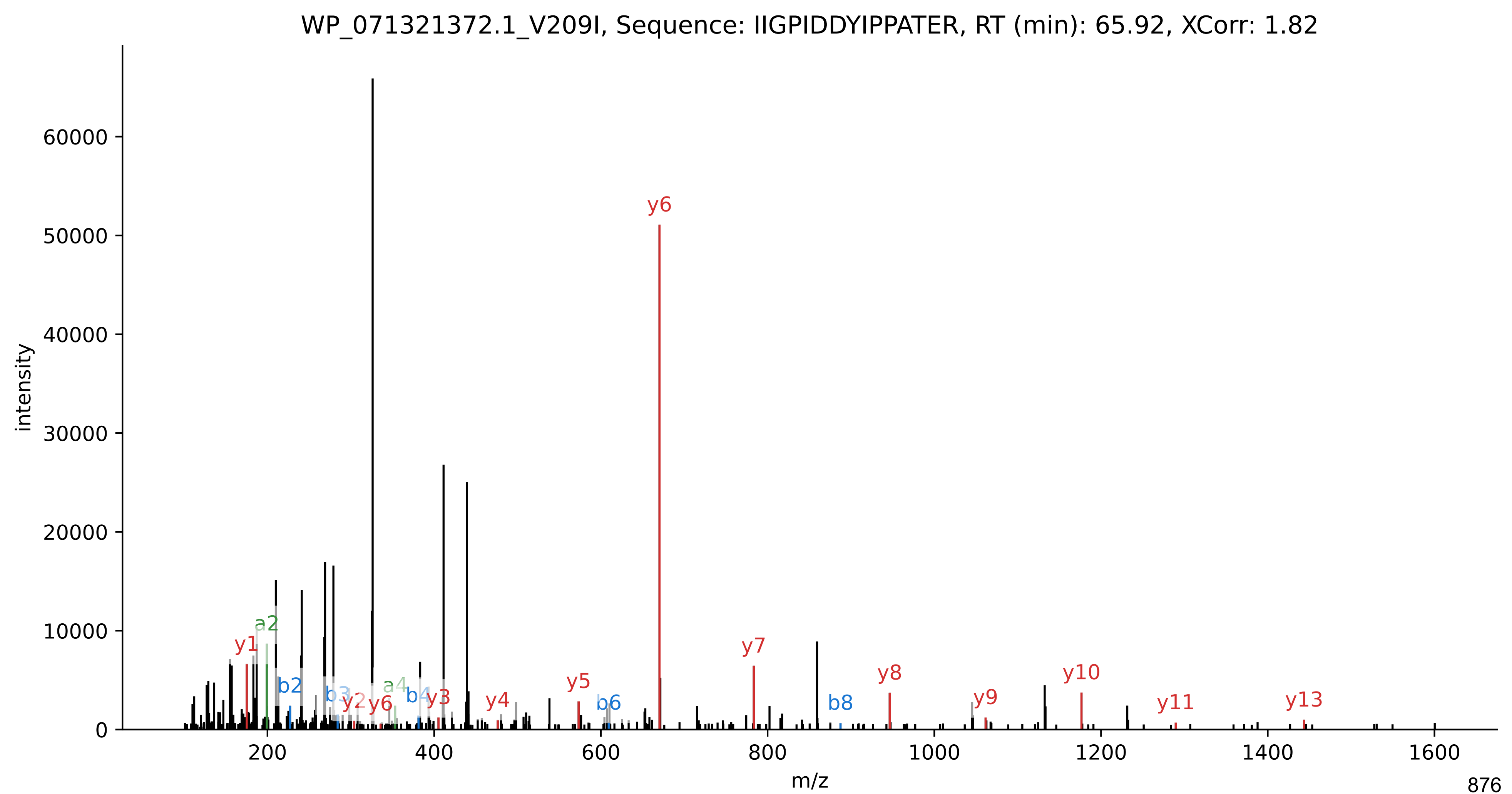

WP_071321372.1_V209I, Sequence: IIGPIDDYIPPATER, RT (min): 65.92, XCorr: 1.82
intensity
m/z
y6
y7
y8
y9
y10
y11
y13
y1
a2
b2
b3
y2
y6
a4
b4
y3
y4
y5
b6
b8

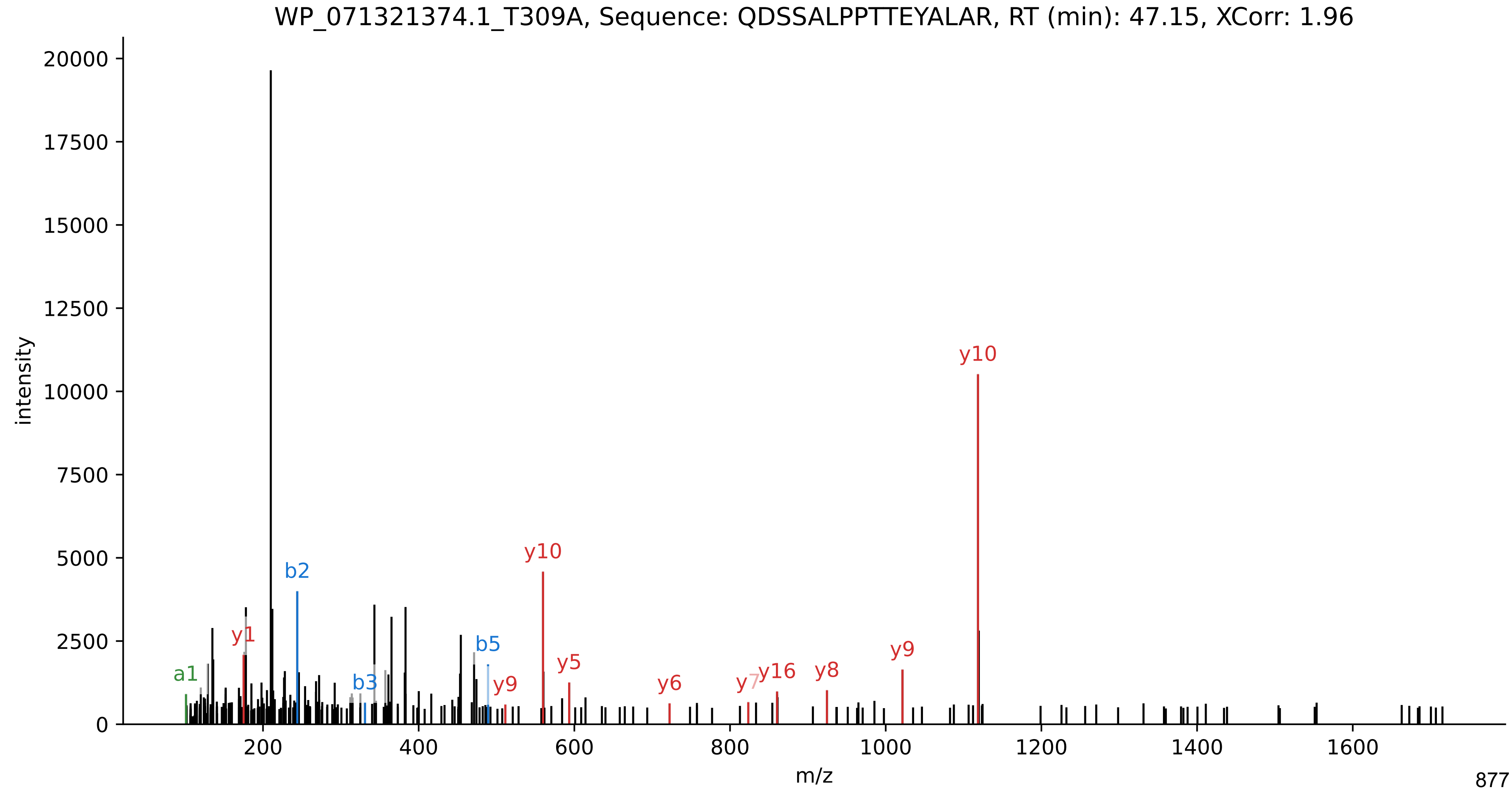
WP_071321374.1_T309A, Sequence: QDSSALPPTTEYALAR, RT (min): 47.15, XCorr: 1.96
intensity
m/z
0
2500
5000
7500
10000
12500
15000
17500
20000
200
400
600
800
1000
1200
1400
1600
a1
y1
b2
b3
b5
y9
y10
y5
y6
y7
y16
y8
y9
y10

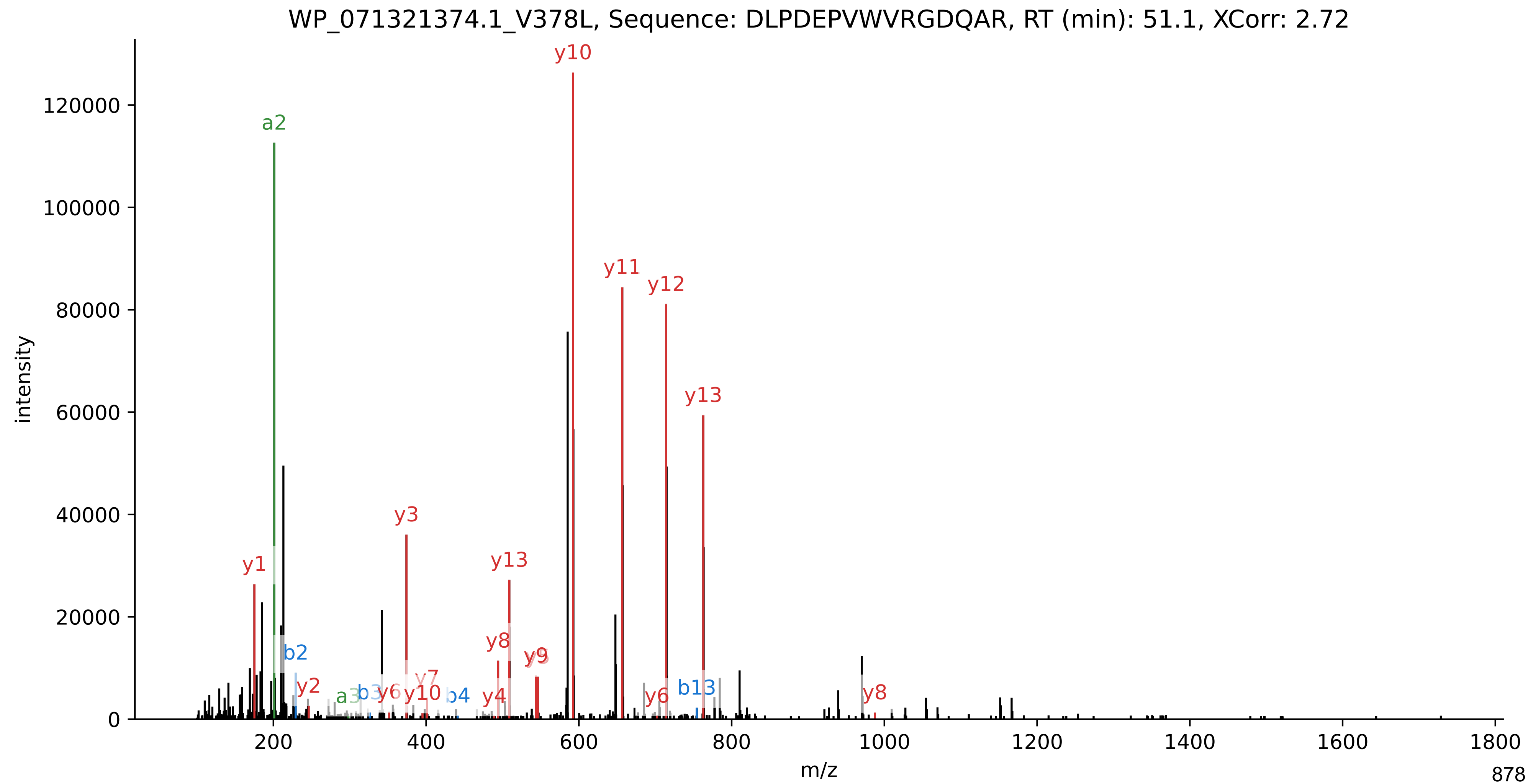

WP_071321374.1_V378L, Sequence: DLPDEPVWVRGDQAR, RT (min): 51.1, XCorr: 2.72
intensity
m/z
0
20000
40000
60000
80000
100000
120000
200
400
600
800
1000
1200
1400
1600
1800
y10
a2
y11
y12
y13
y3
y1
y13
y8
y9
b2
y2
a3
b3
y6
y7
y10
b4
y4
y6
b13
y8

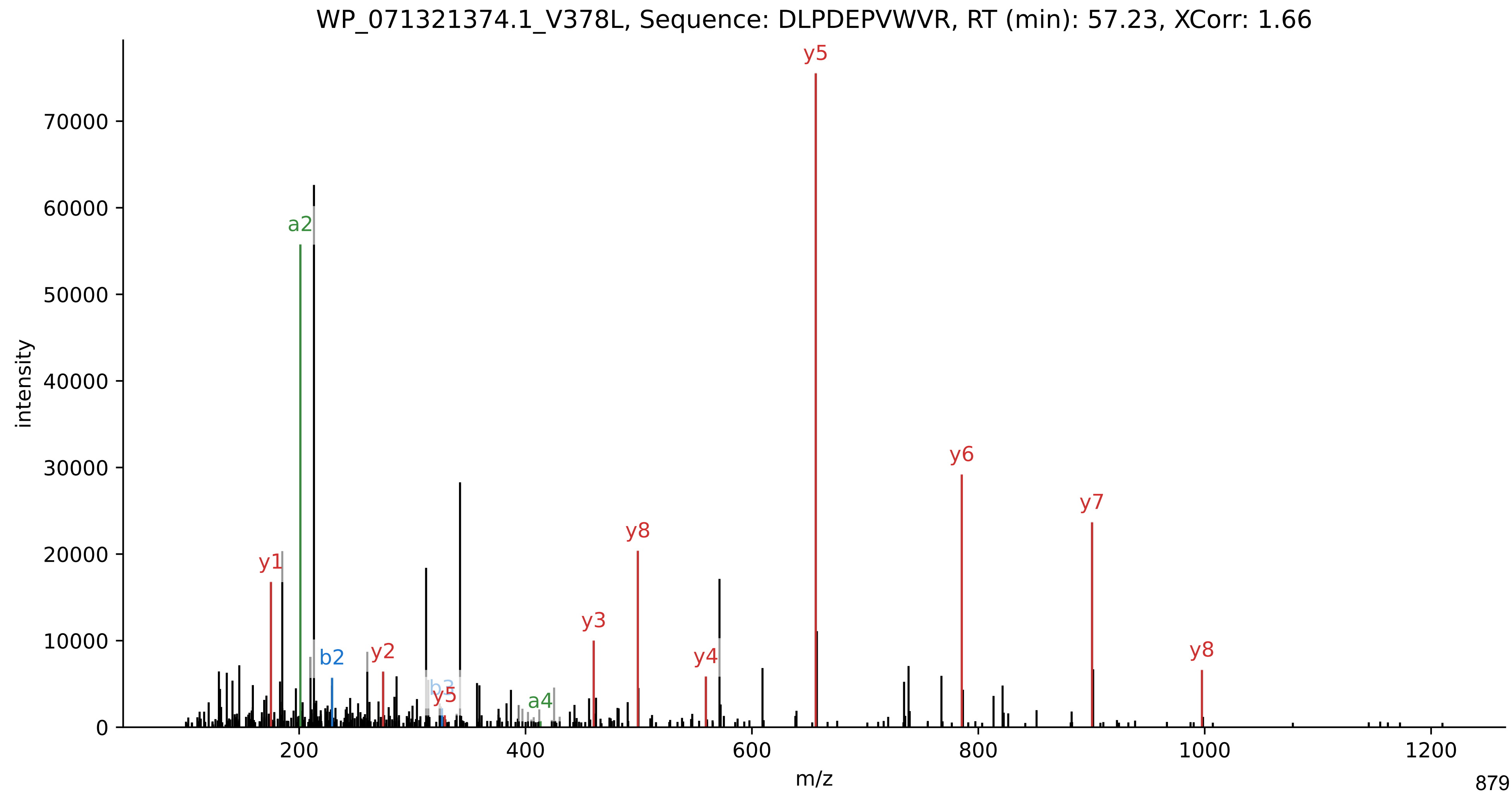

WP_071321374.1_V378L, Sequence: DLPDEPVWVR, RT (min): 57.23, XCorr: 1.66
intensity
m/z
y5
a2
y6
y7
y8
y1
y3
b2
y2
y4
y8
b3
y5
a4
70000
60000
50000
40000
30000
20000
10000
0
200
400
600
800
1000
1200

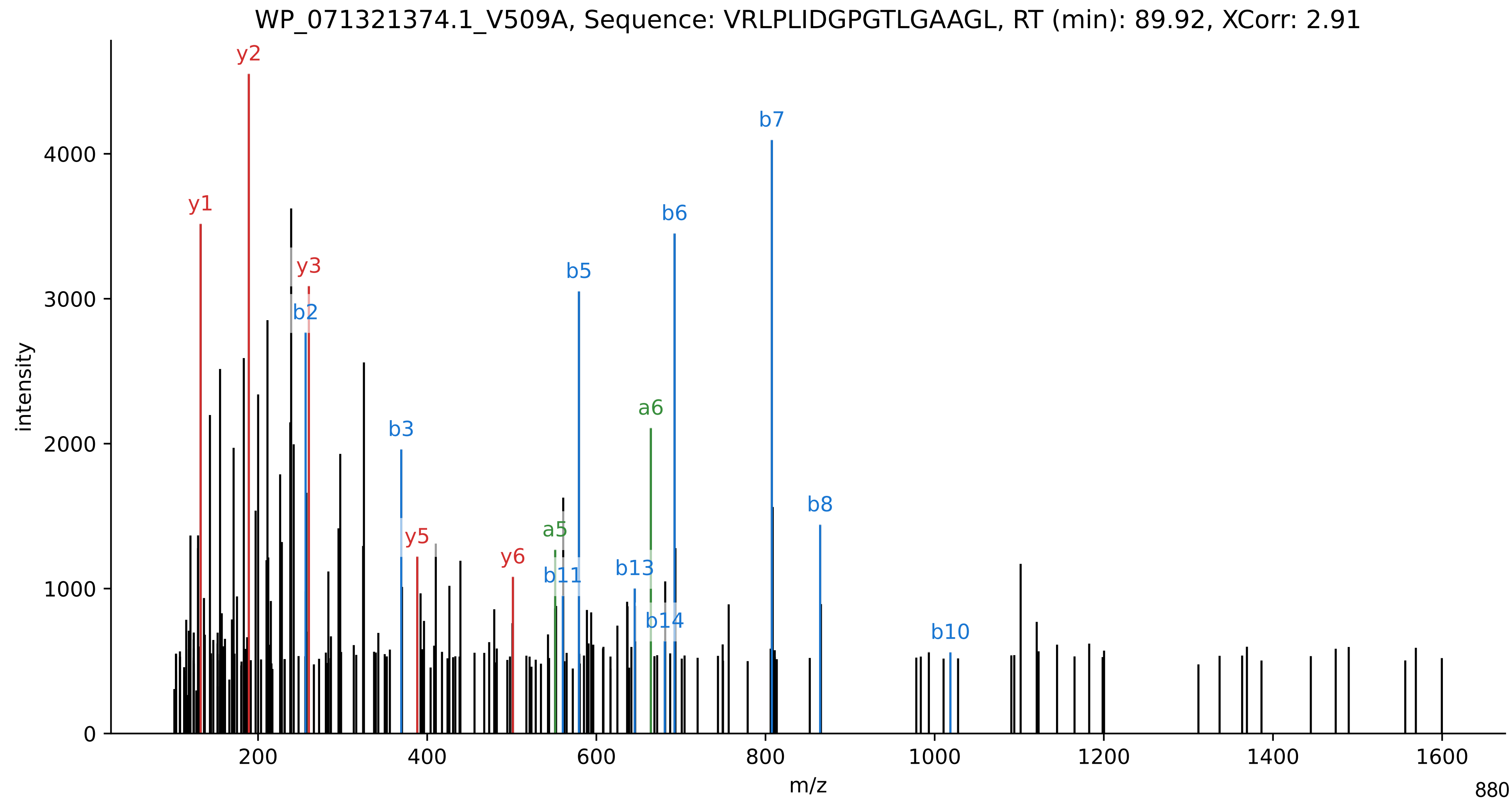
WP_071321374.1_V509A, Sequence: VRLPLIDGPGTLGAAGL, RT (min): 89.92, XCorr: 2.91
intensity
m/z
y1
y2
y3
b2
b3
y5
y6
a5
b11
b5
b13
a6
b14
b6
b7
b8
b10
0
1000
2000
3000
4000
200
400
600
800
1000
1200
1400
1600

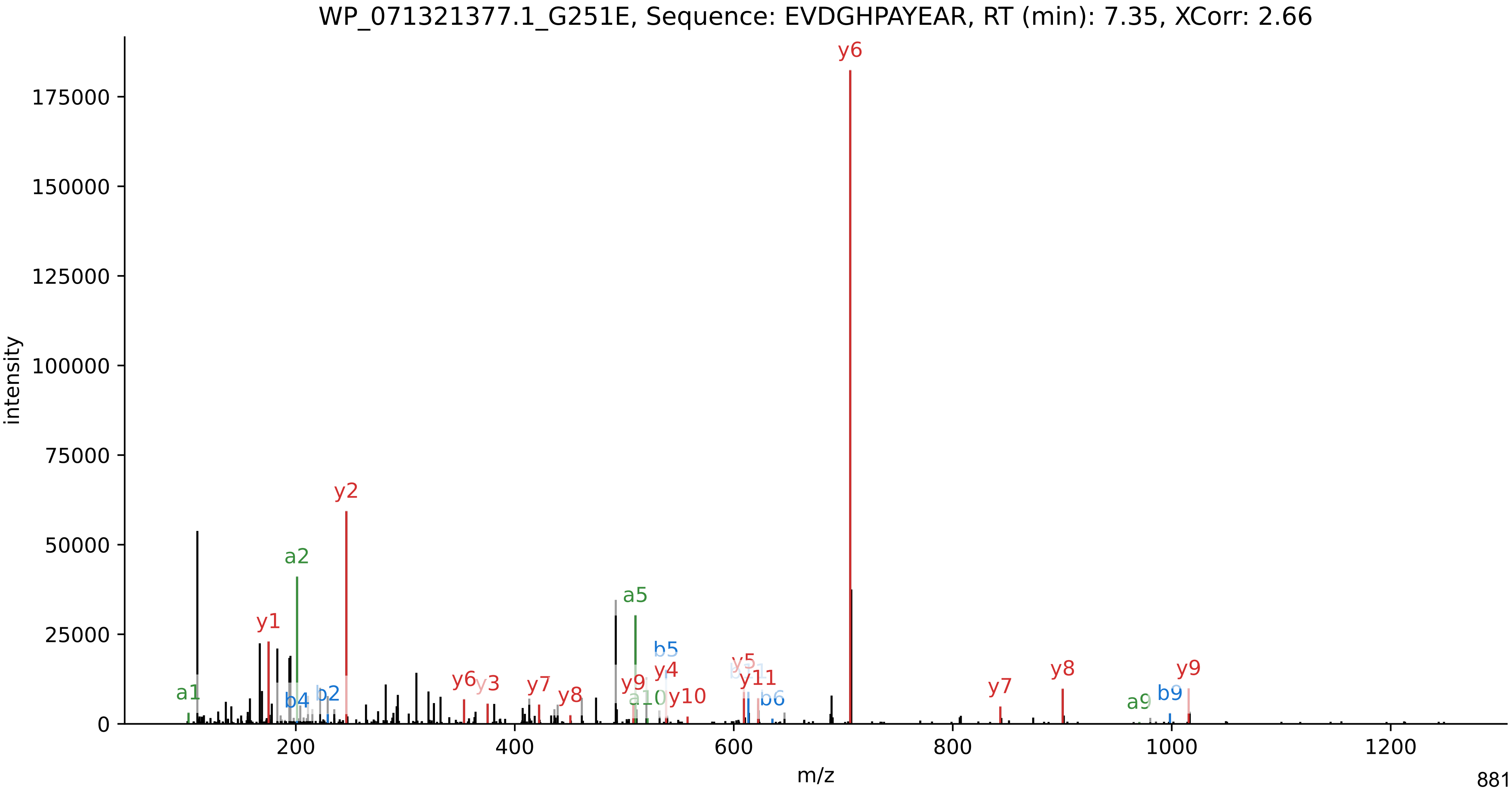
WP_071321377.1_G251E, Sequence: EVDGHPAYEAR, RT (min): 7.35, XCorr: 2.66
intensity
m/z
y6
y2
a2
a5
y1
b5
y4
y5
y11
y8
y9
y7
y6
y3
y7
y8
y9
a10
y10
b6
a1
b4
b2
a9
b9

WP_071321378.1_G304C, Sequence: [K].VAQQGcSHPVPLIIGTNQHEAALFR.[L], RT (min): 55.78, XCorr: 5.39

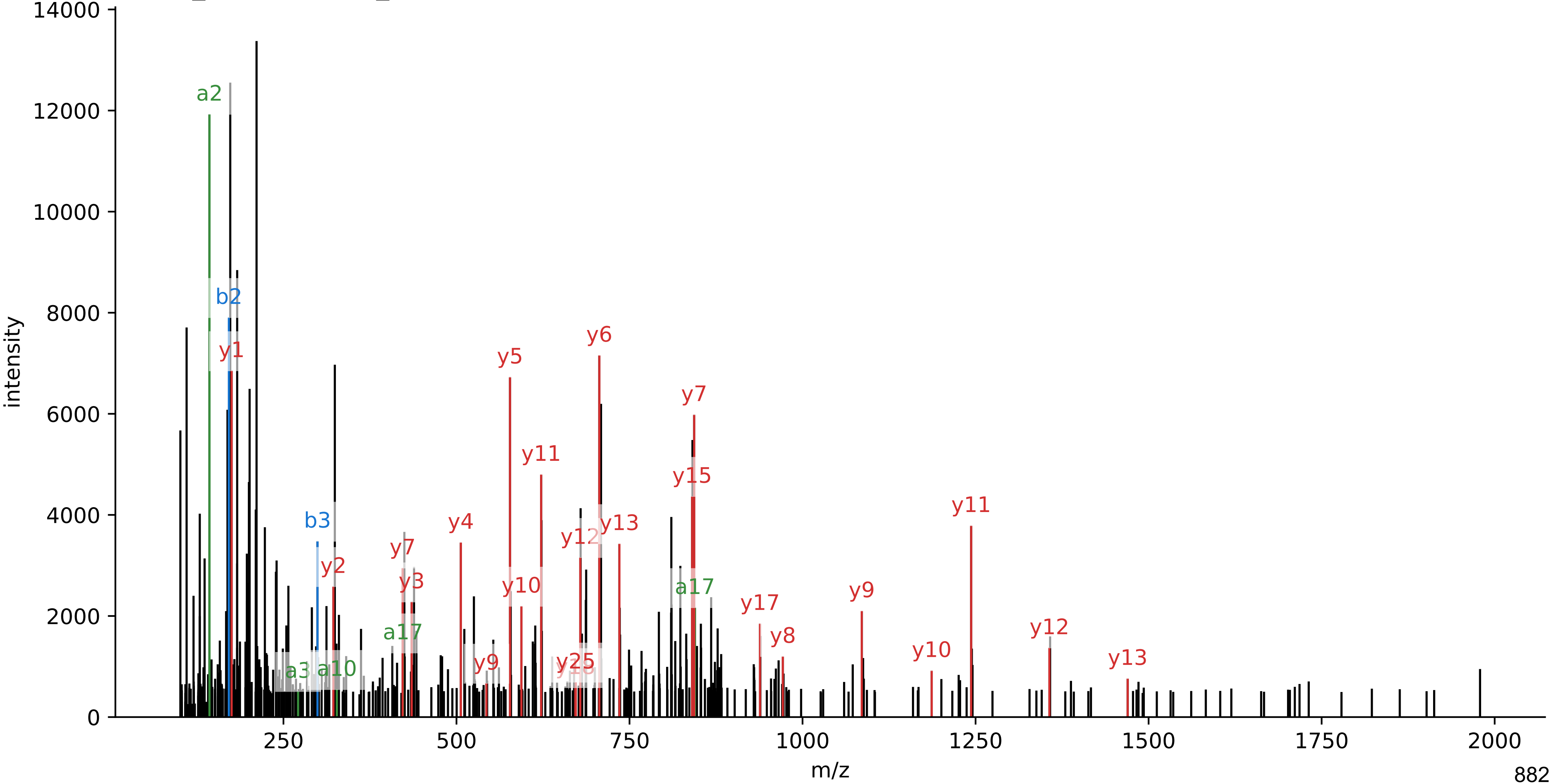

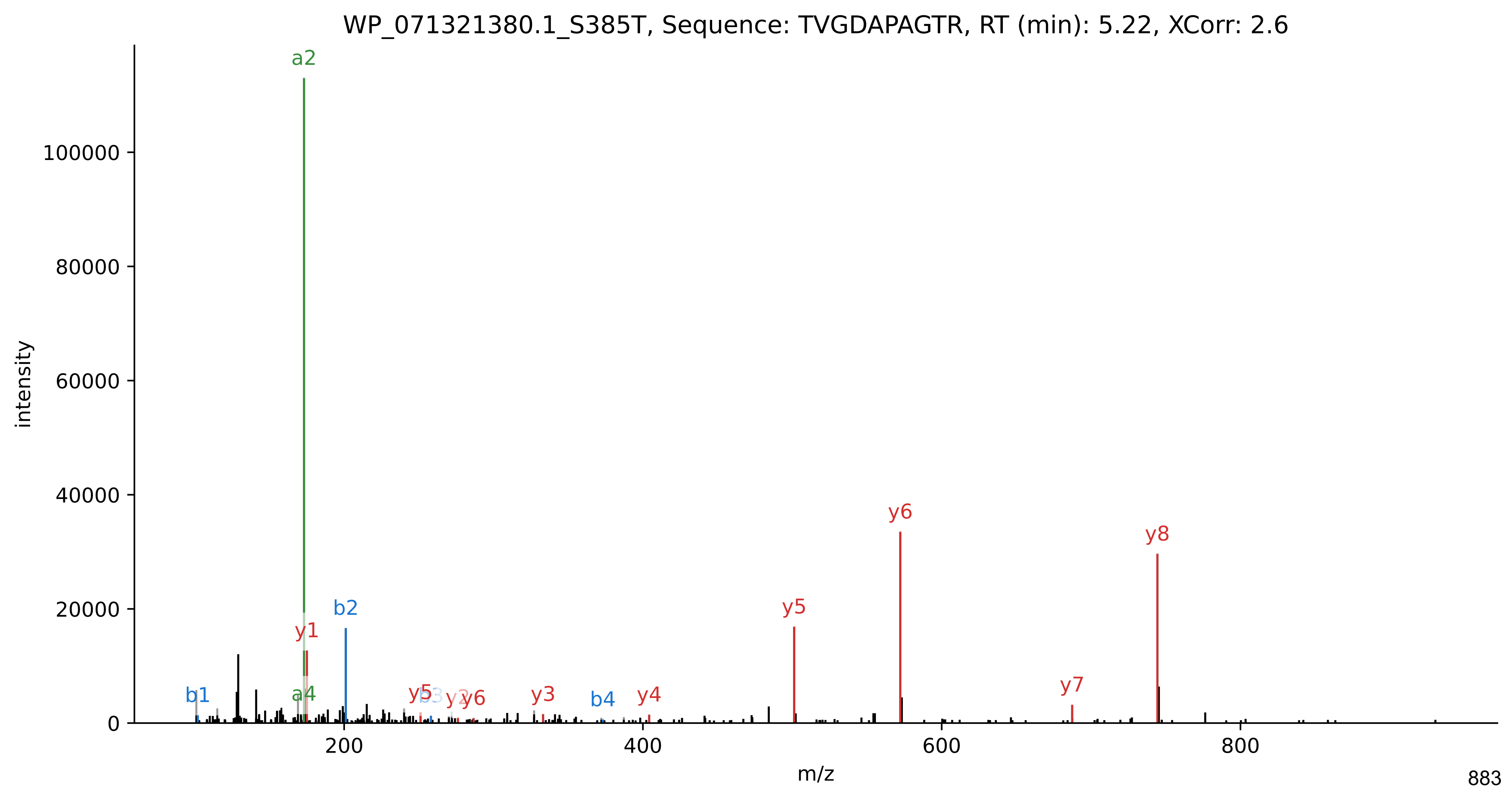
WP_071321380.1_S385T, Sequence: TVGDAPAGTR, RT (min): 5.22, XCorr: 2.6
intensity
m/z
0
20000
40000
60000
80000
100000
200
400
600
800
a2
b2
y1
b1
a4
y5
b3
y2
y6
y3
b4
y4
y5
y6
y7
y8

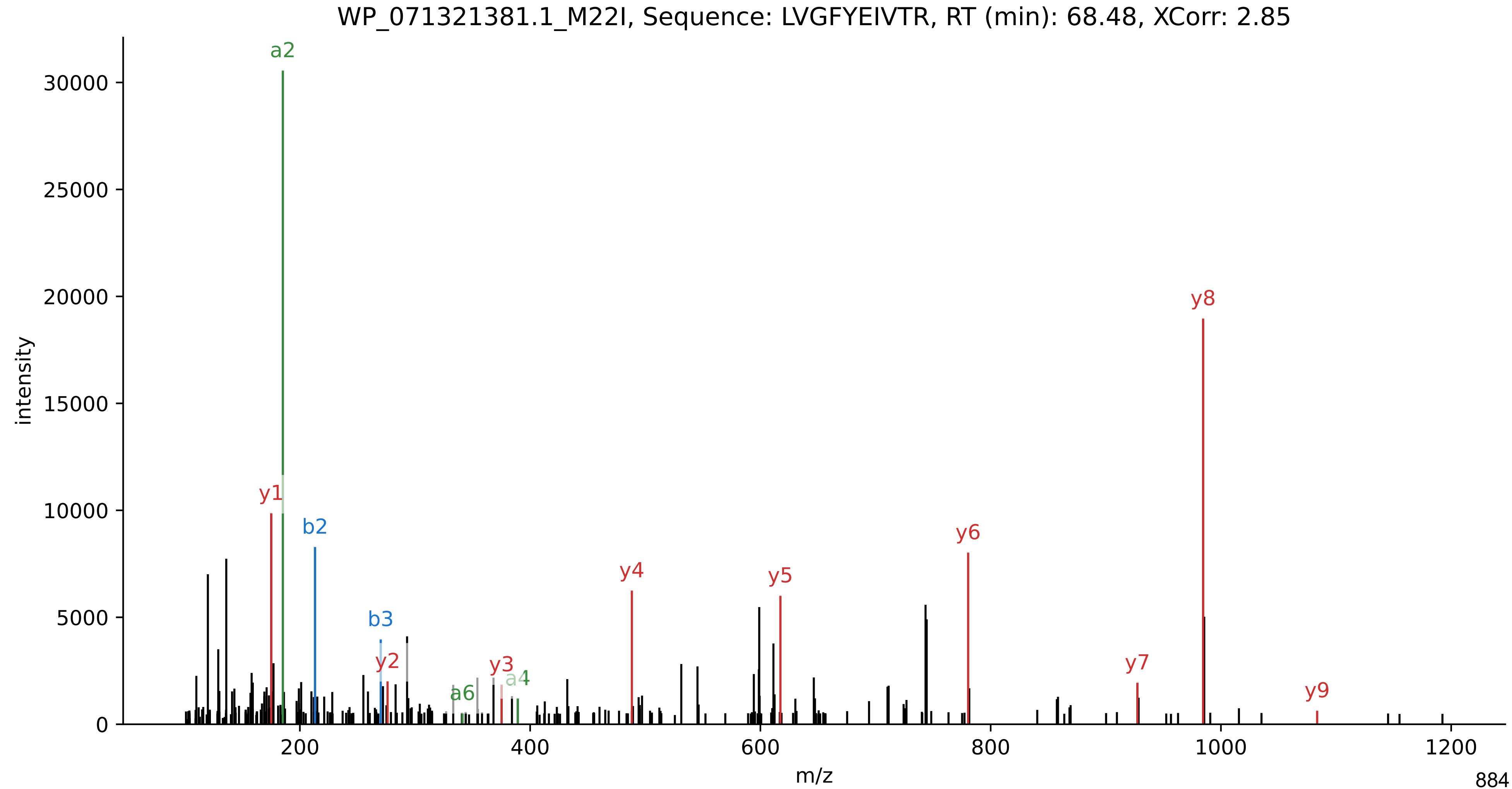

WP_071321381.1_M22I, Sequence: LVGFYEIVTR, RT (min): 68.48, XCorr: 2.85
a2
y1
b2
b3
y2
a6
y3
a4
y4
y5
y6
y7
y8
y9
intensity
m/z

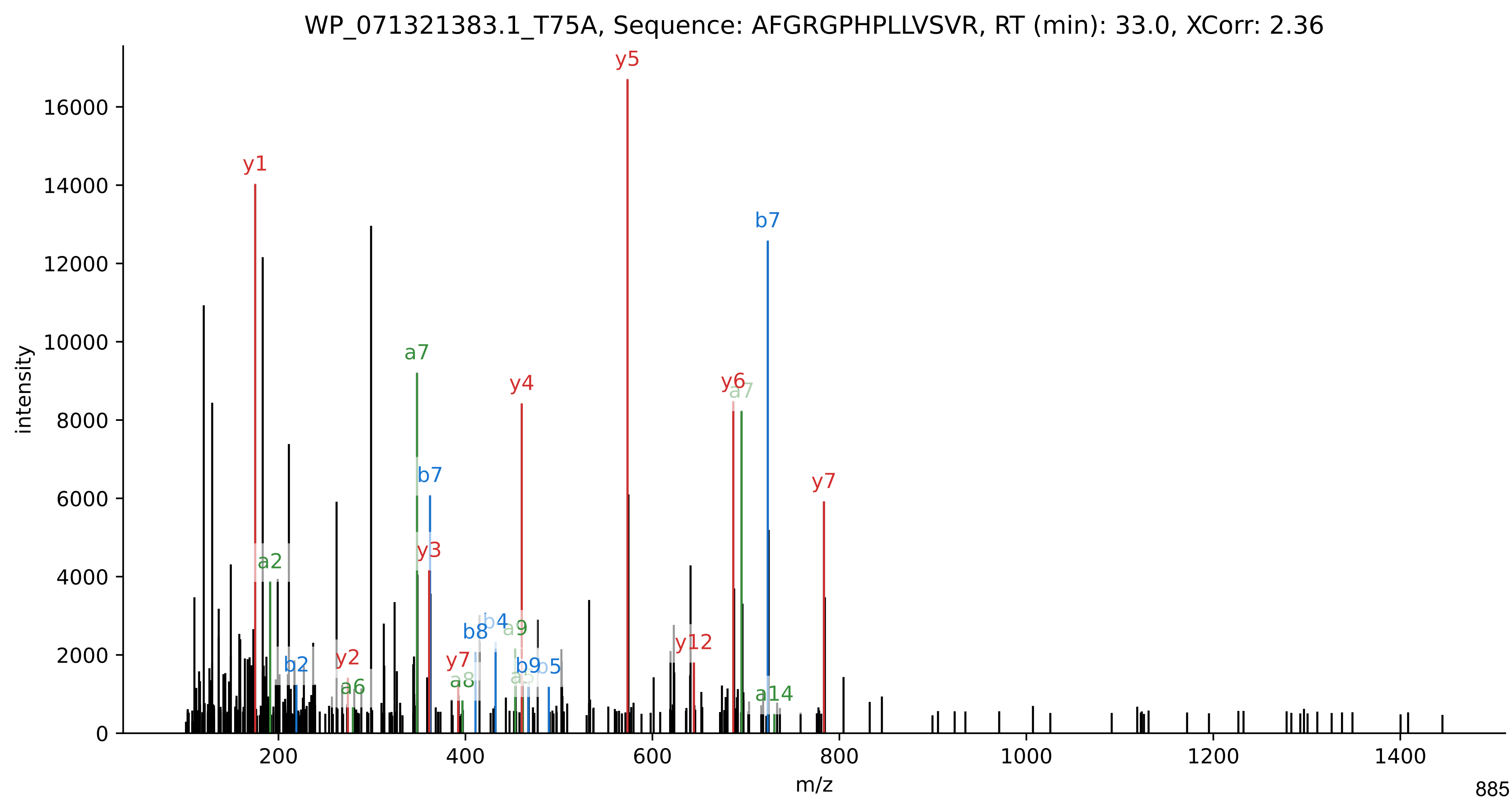
WP_071321383.1_T75A, Sequence: AFGRGPHPLLVSVR, RT (min): 33.0, XCorr: 2.36
intensity
m/z
y5
y1
b7
a7
y4
y6
a7
b7
y7
y3
a2
b4
b8
a9
y12
y7
y2
b2
b9
b5
a8
a6
a14

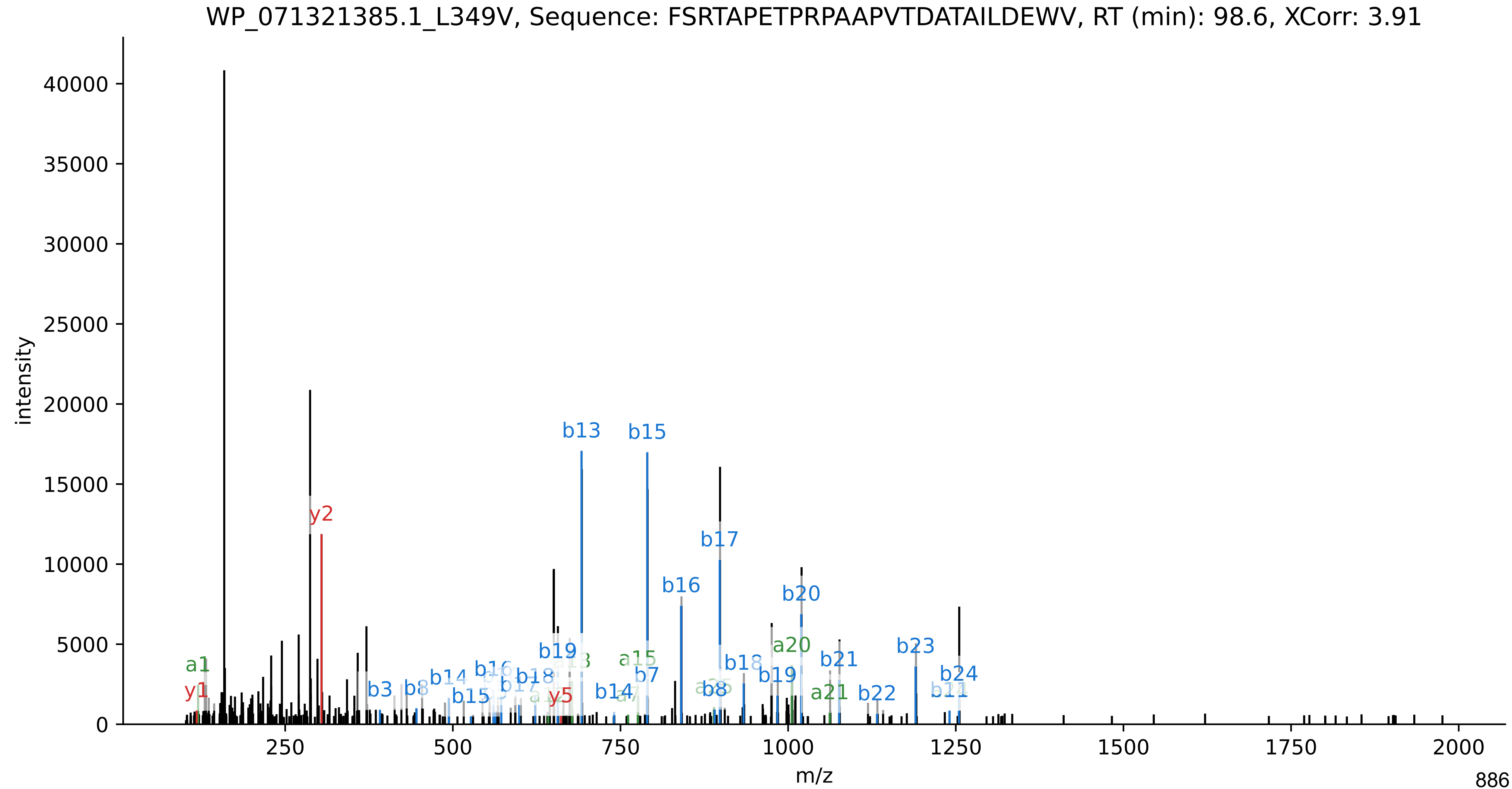
WP_071321385.1_L349V, Sequence: FSRTAPETPRPAAPVTDATAILDEWV, RT (min): 98.6, XCorr: 3.91
intensity
m/z
b13
b15
b17
b16
b20
y2
a20
b21
b23
b24
b18
b19
b22
a21
b7
a15
b14
b19
b18
b17
b16
b15
b14
b8
b3
a1
y1
y5
b8
b11

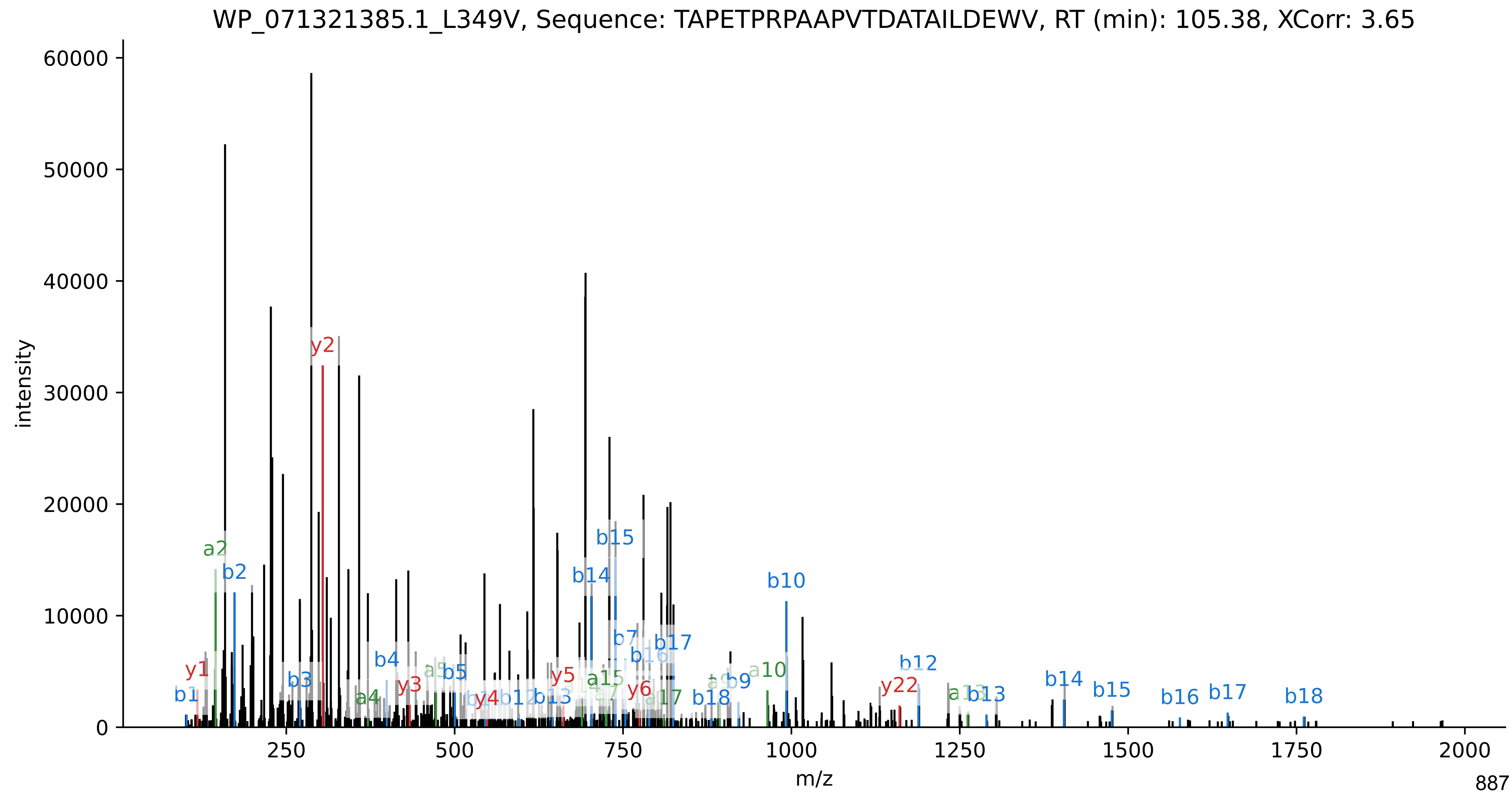

WP_071321385.1_L349V, Sequence: TAPETPRPAAPVTDATAILDEWV, RT (min): 105.38, XCorr: 3.65
intensity
m/z

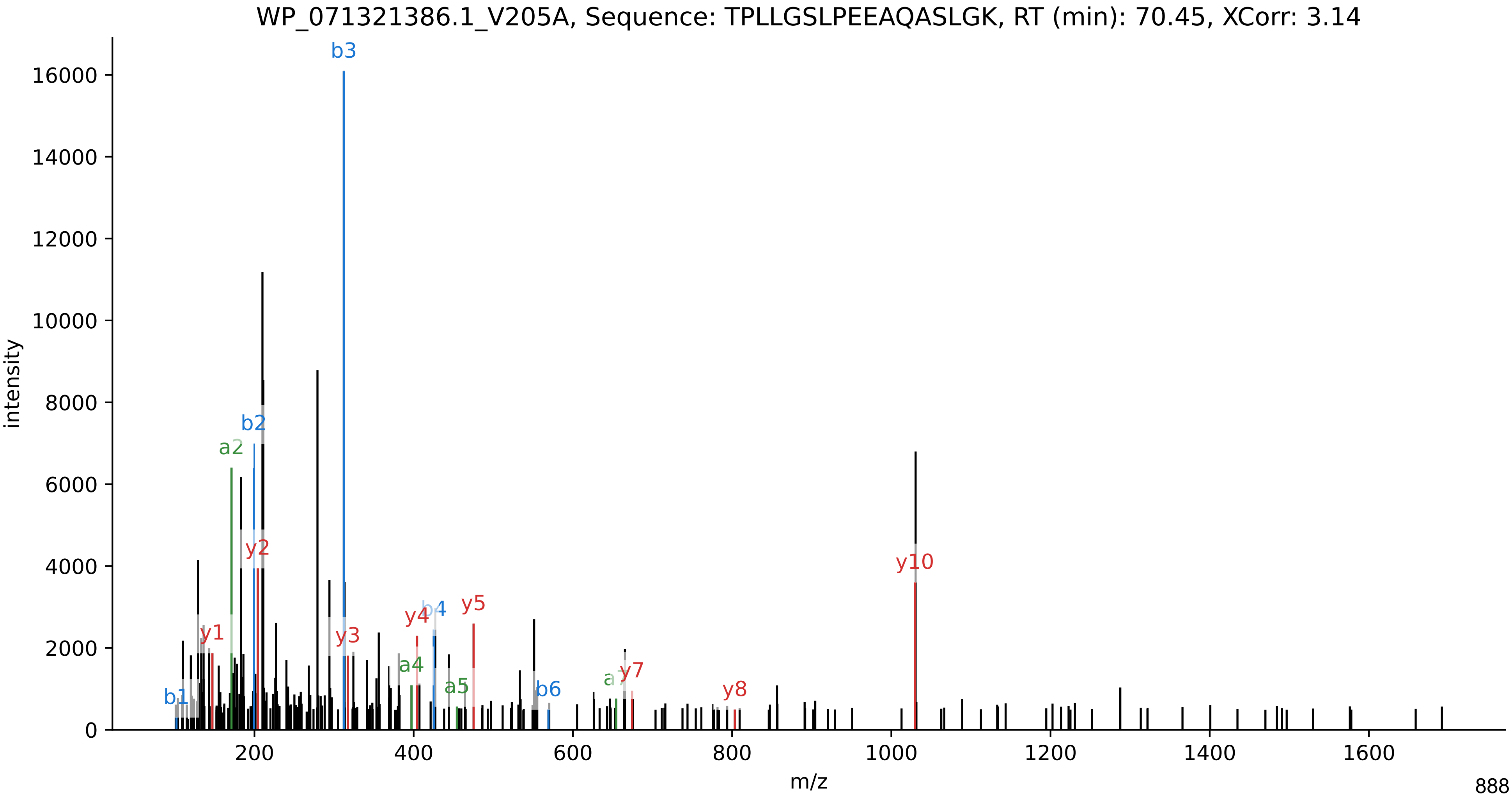

WP_071321386.1_V205A, Sequence: TPLLGSLPEEAQASLGK, RT (min): 70.45, XCorr: 3.14
intensity
m/z
b1
y1
a2
b2
y2
b3
y3
y4
b4
a4
a5
y5
b6
y7
y8
y10

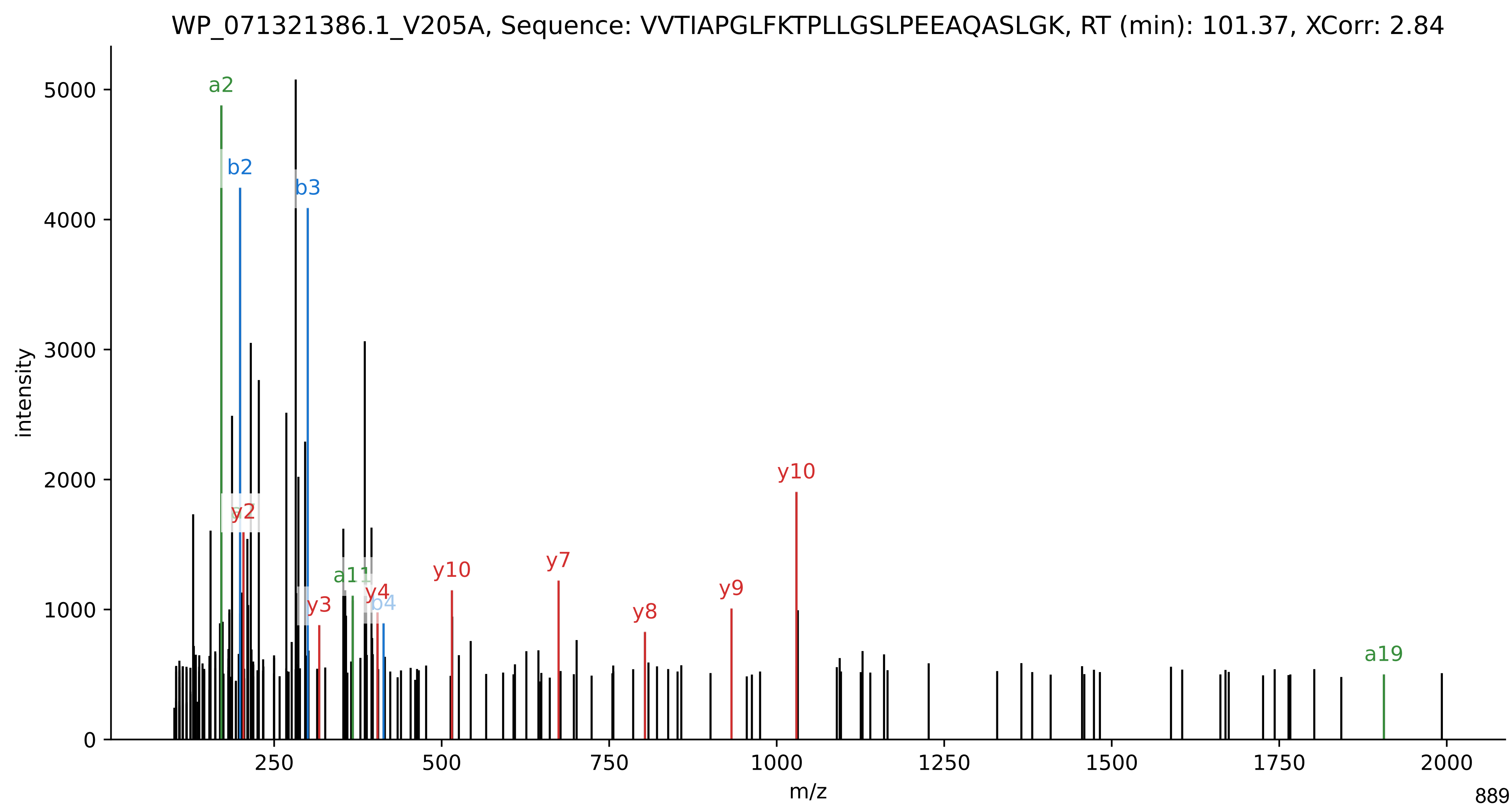
WP_071321386.1_V205A, Sequence: VVTIAPGLFKTPLLGSLPEEAQASLGK, RT (min): 101.37, XCorr: 2.84
intensity
m/z
a2
b2
b3
y2
a11
y4
b4
y3
y10
y7
y8
y9
y10
a19
0
1000
2000
3000
4000
5000
250
500
750
1000
1250
1500
1750
2000

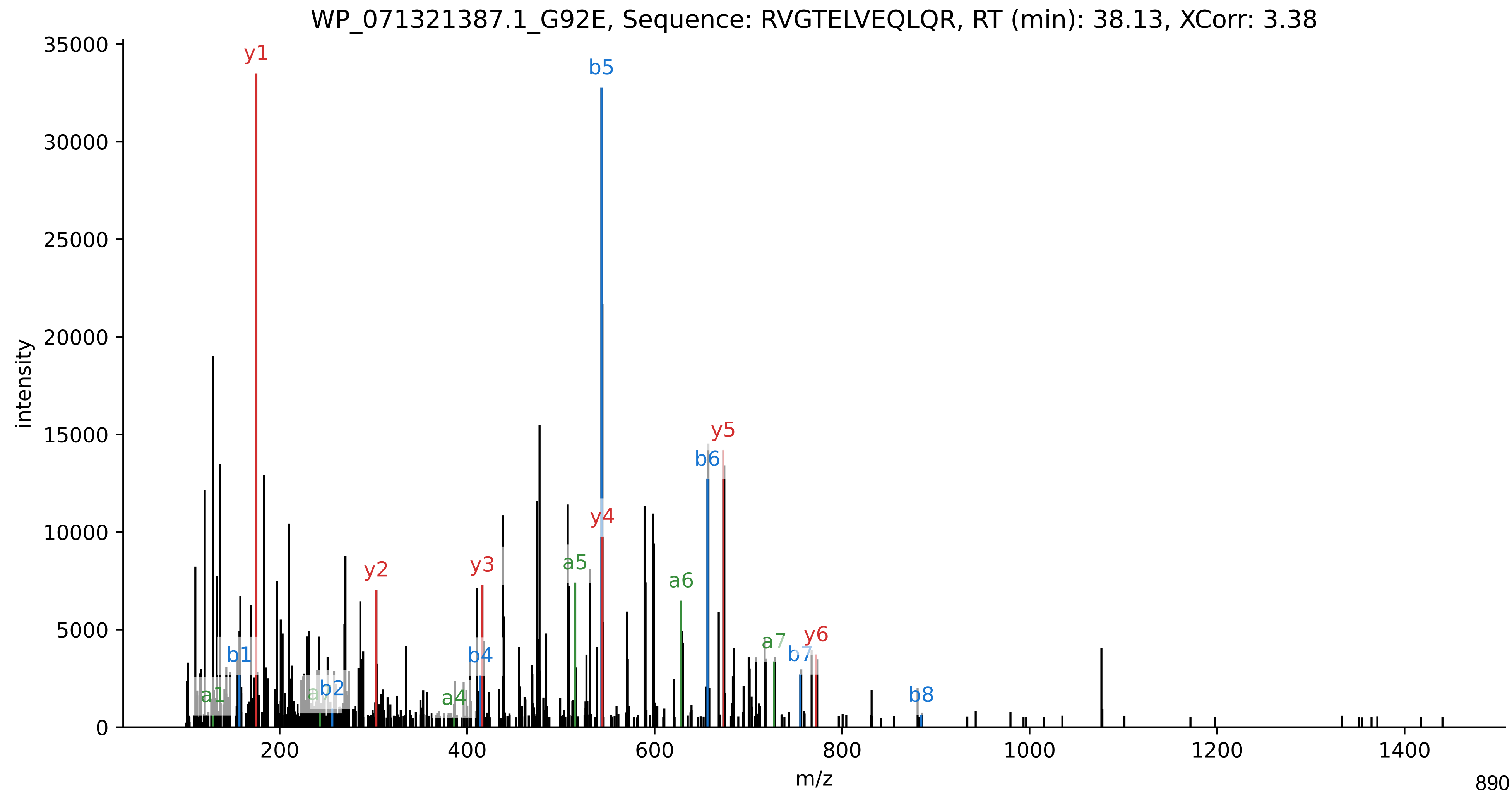

WP_071321387.1_G92E, Sequence: RVGTELVEQLQR, RT (min): 38.13, XCorr: 3.38
intensity
m/z
y1
b5
y5
b6
y4
a5
a6
y2
y3
a7
y6
b7
b1
b4
b2
a1
a4
b8
35000
30000
25000
20000
15000
10000
5000
0
200
400
600
800
1000
1200
1400

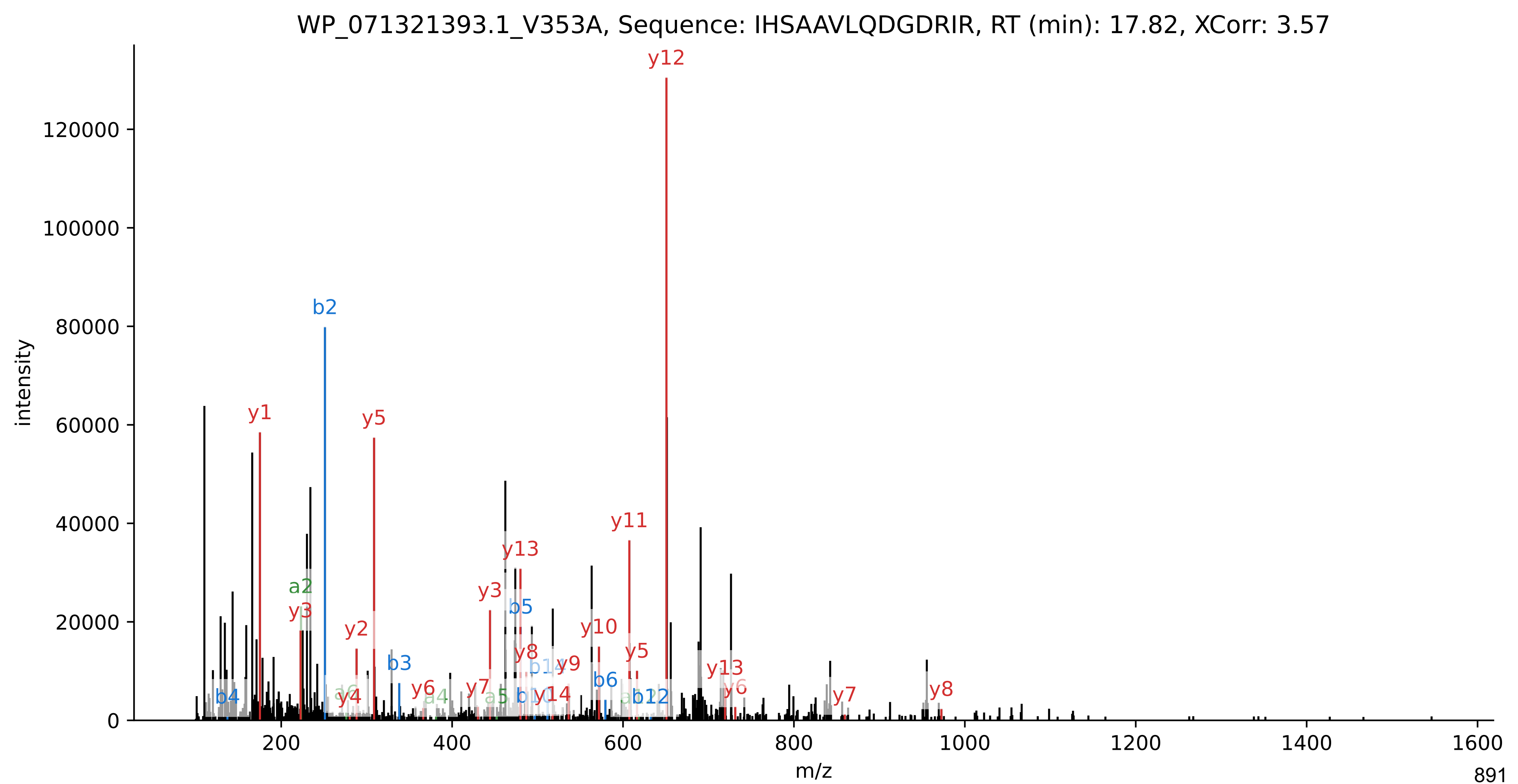
WP_071321393.1_V353A, Sequence: IHSAAVLQDGDRIR, RT (min): 17.82, XCorr: 3.57
intensity
m/z
y12
b2
y1
y5
y11
y13
a2
y3
y3
b5
y2
y10
y5
y8
b3
y9
y13
b6
y6
y7
y6
b4
y4
a4
a5
y14
b12
y7
y8
0
20000
40000
60000
80000
100000
120000
200
400
600
800
1000
1200
1400
1600

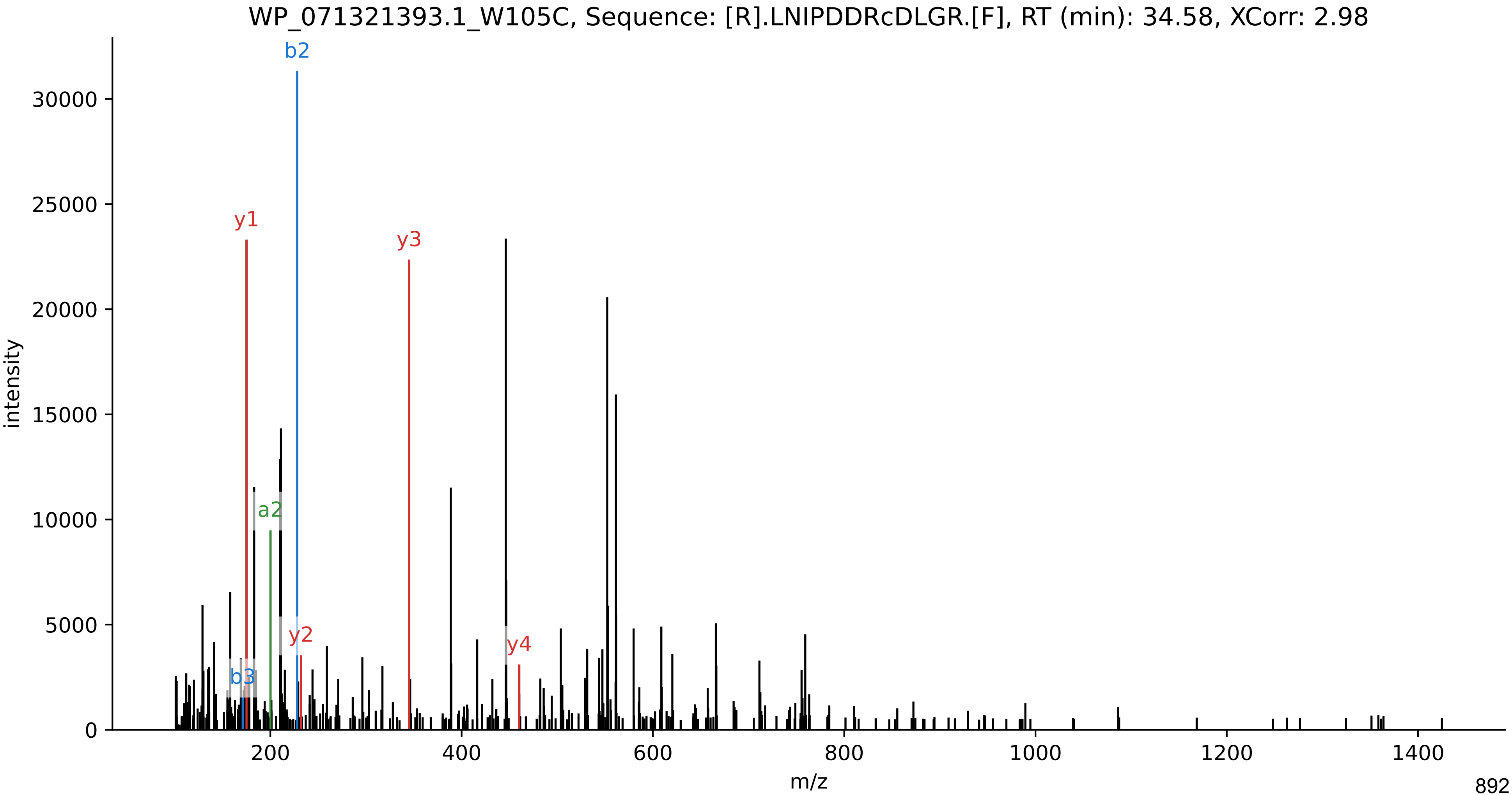

WP_071321393.1_W105C, Sequence: [R].LNIPDDRcDLGR.[F], RT (min): 34.58, XCorr: 2.98
b2
y1
y3
a2
y2
y4
b3
intensity
m/z
0
5000
10000
15000
20000
25000
30000
200
400
600
800
1000
1200
1400

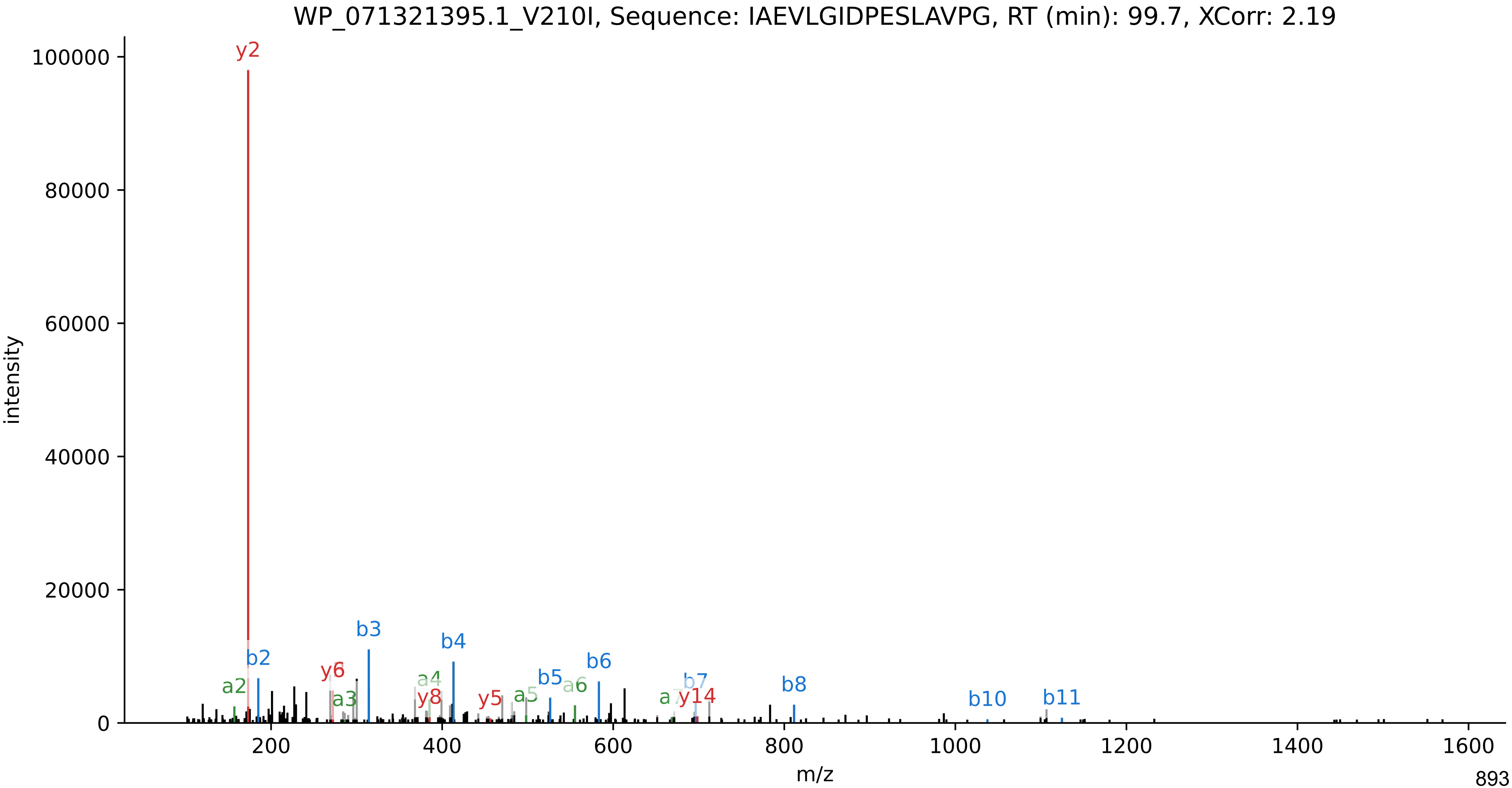

WP_071321395.1_V210I, Sequence: IAEVLGIDPESLAVPG, RT (min): 99.7, XCorr: 2.19
intensity
m/z
y2
b2
b3
b4
b5
b6
b7
b8
b10
b11
a2
a3
a4
a5
a6
y6
y8
y5
y14

WP_071321396.1_A269T, Sequence: AYTTIHGLPEPEVFHMNEGHAGFLGAER, RT (min): 69.72, XCorr: 4.46

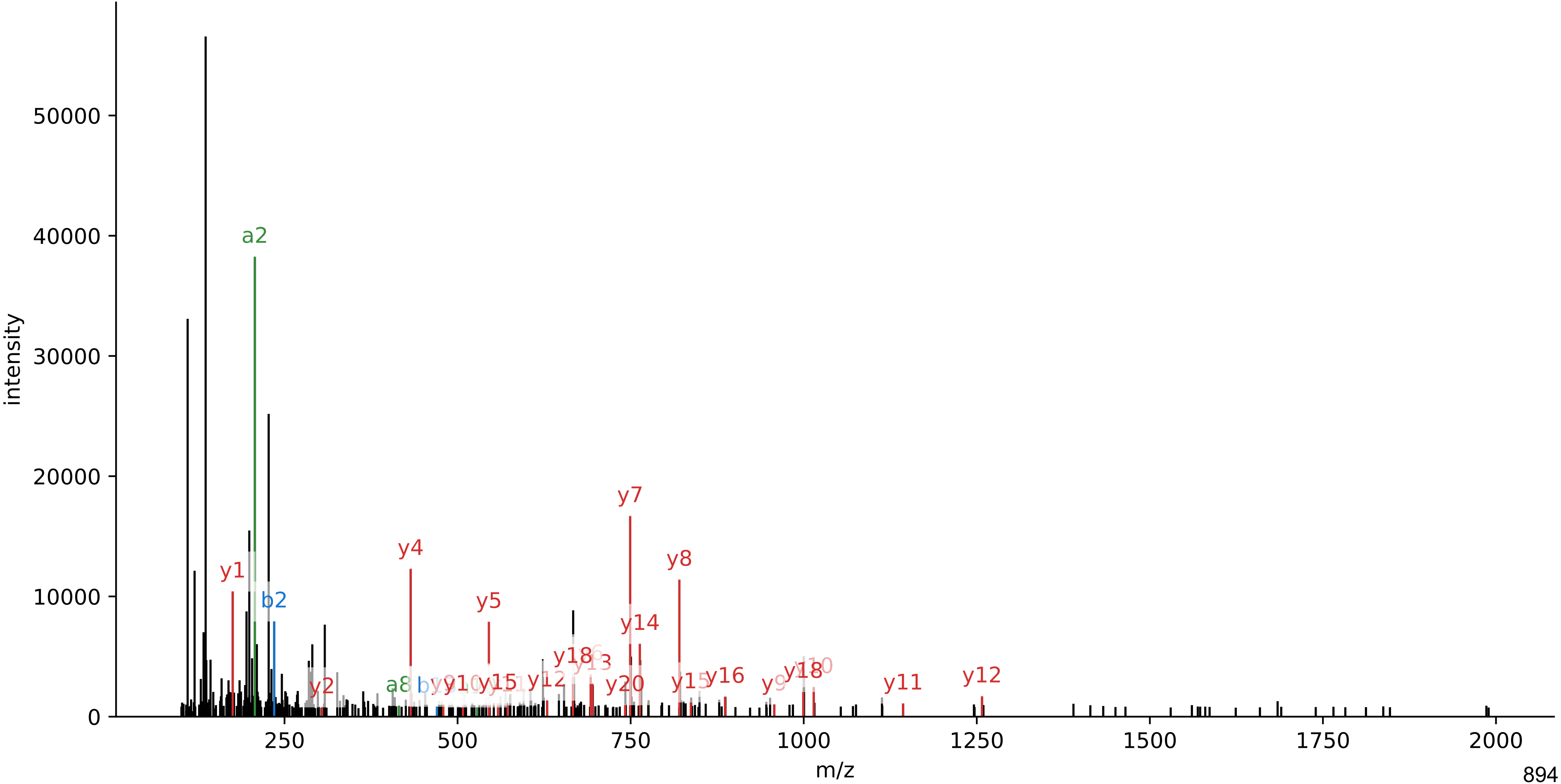

WP_071321397.1_A8V, Sequence: VVVVTGAGSGIGR, RT (min): 28.28, XCorr: 3.26

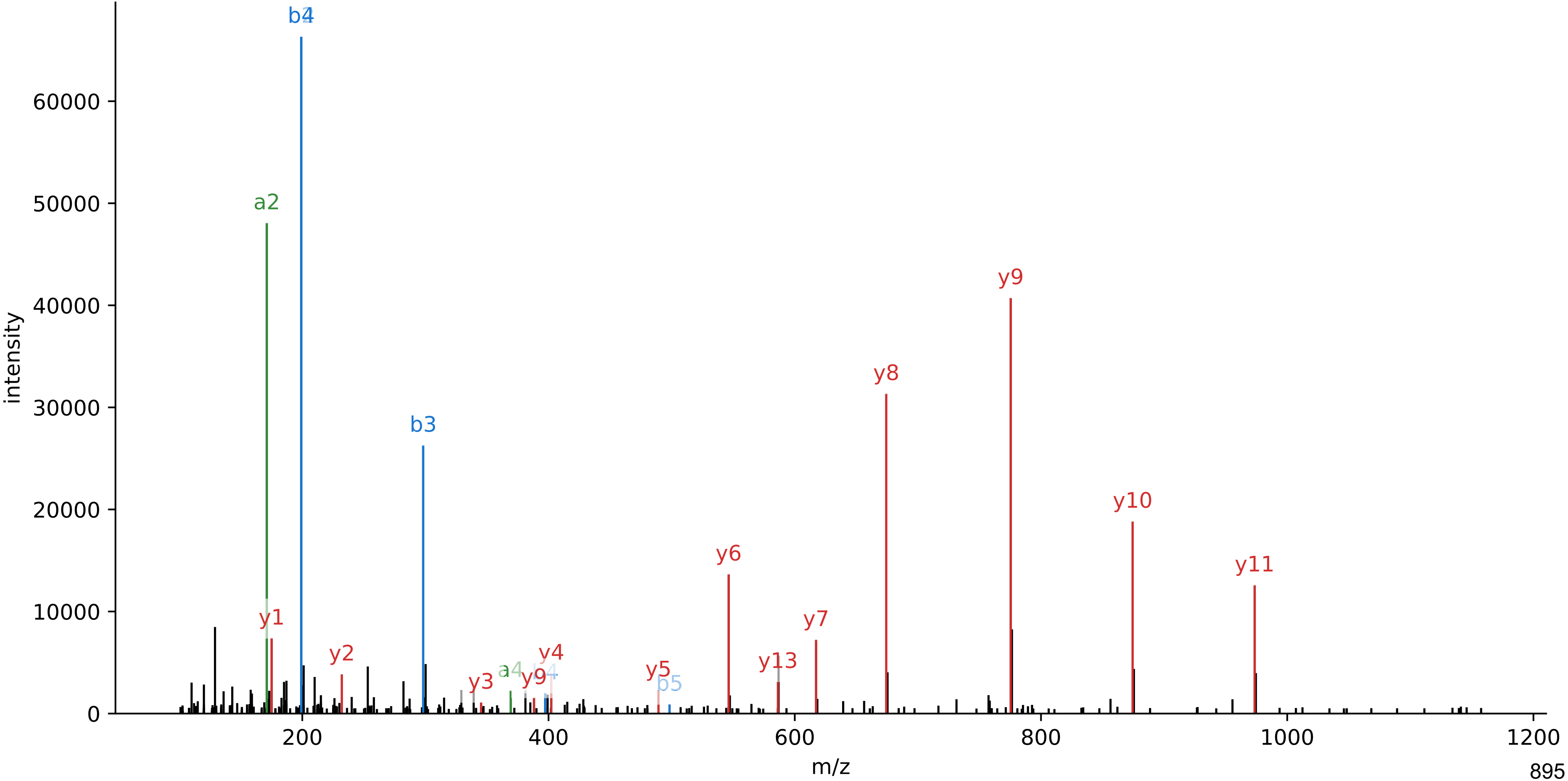

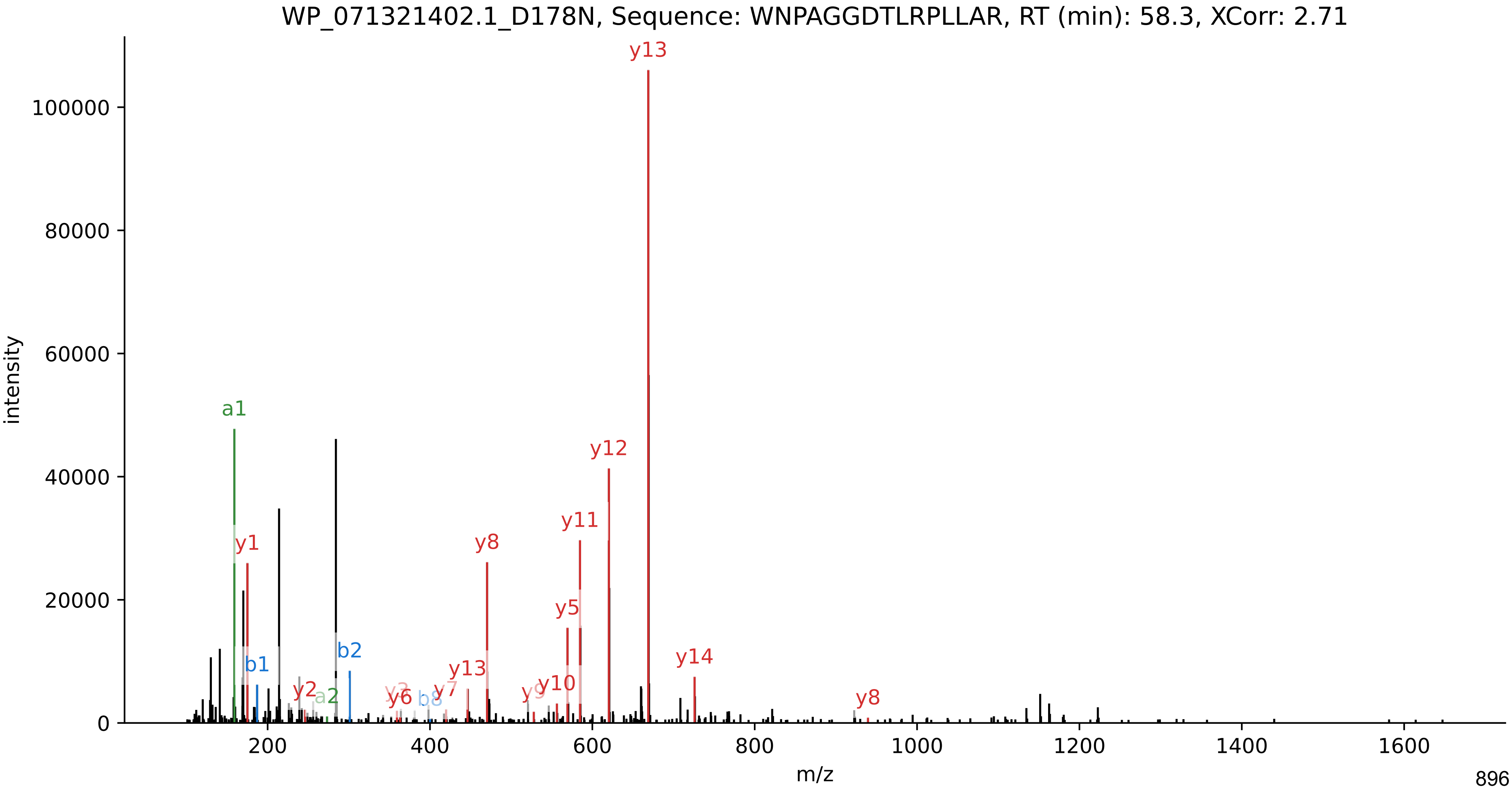

WP_071321402.1_D178N, Sequence: WNPAGGDTLRPLLAR, RT (min): 58.3, XCorr: 2.71
intensity
m/z
y13
a1
y12
y11
y1
y8
y5
b2
b1
y14
y13
y10
y2
a2
y6
b8
y7
y9
y8

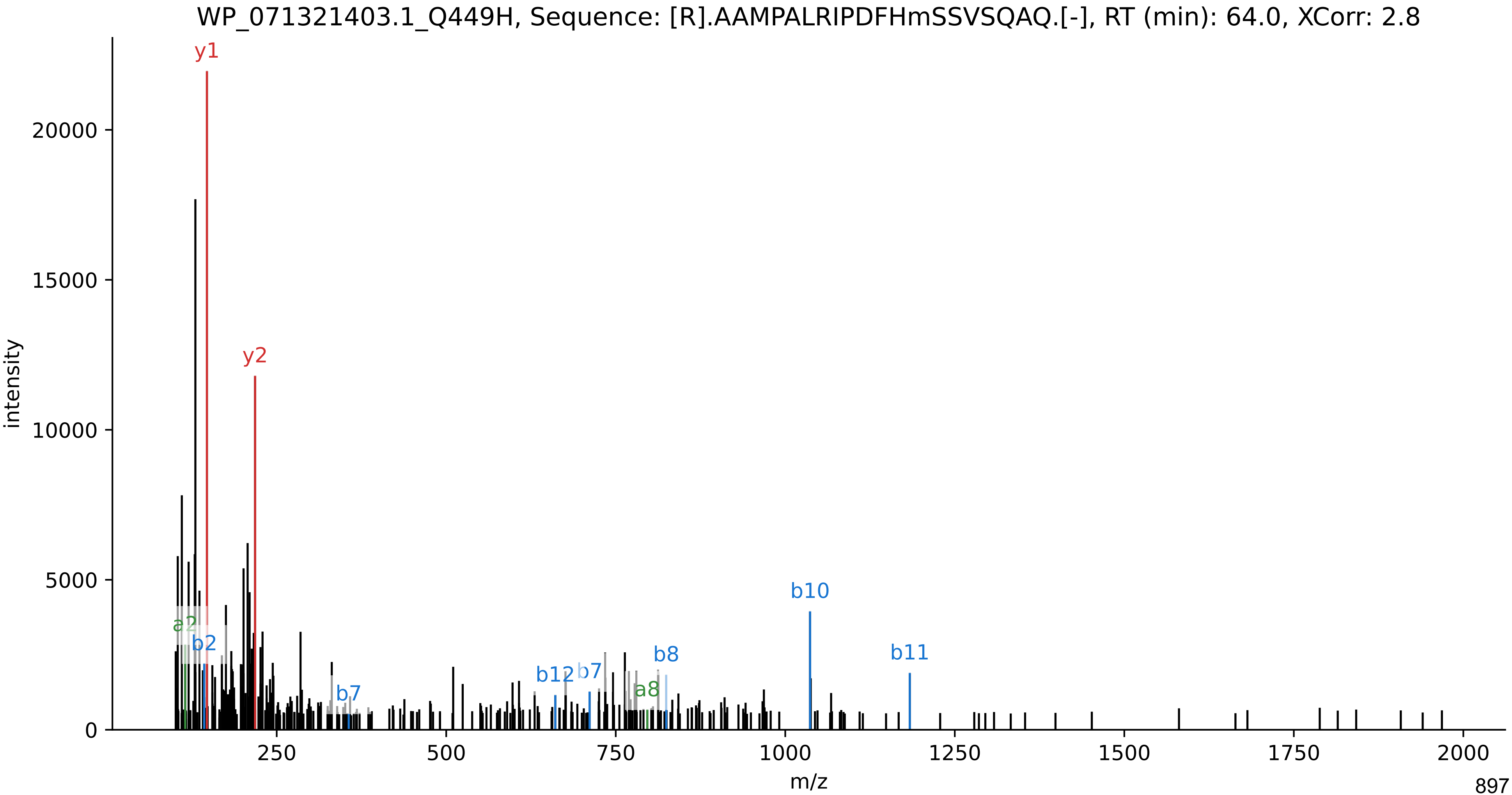
WP_071321403.1_Q449H, Sequence: [R].AAMPALRIPDFHmSSVSQAQ.[-], RT (min): 64.0, XCorr: 2.8
intensity
m/z
y1
y2
a2
b2
b7
b12
b7
a8
b8
b10
b11
0
5000
10000
15000
20000
250
500
750
1000
1250
1500
1750
2000

WP_071321403.1_Q449H, Sequence: AAMPALRIPDFHMSSVSQAQ, RT (min): 76.52, XCorr: 3.02

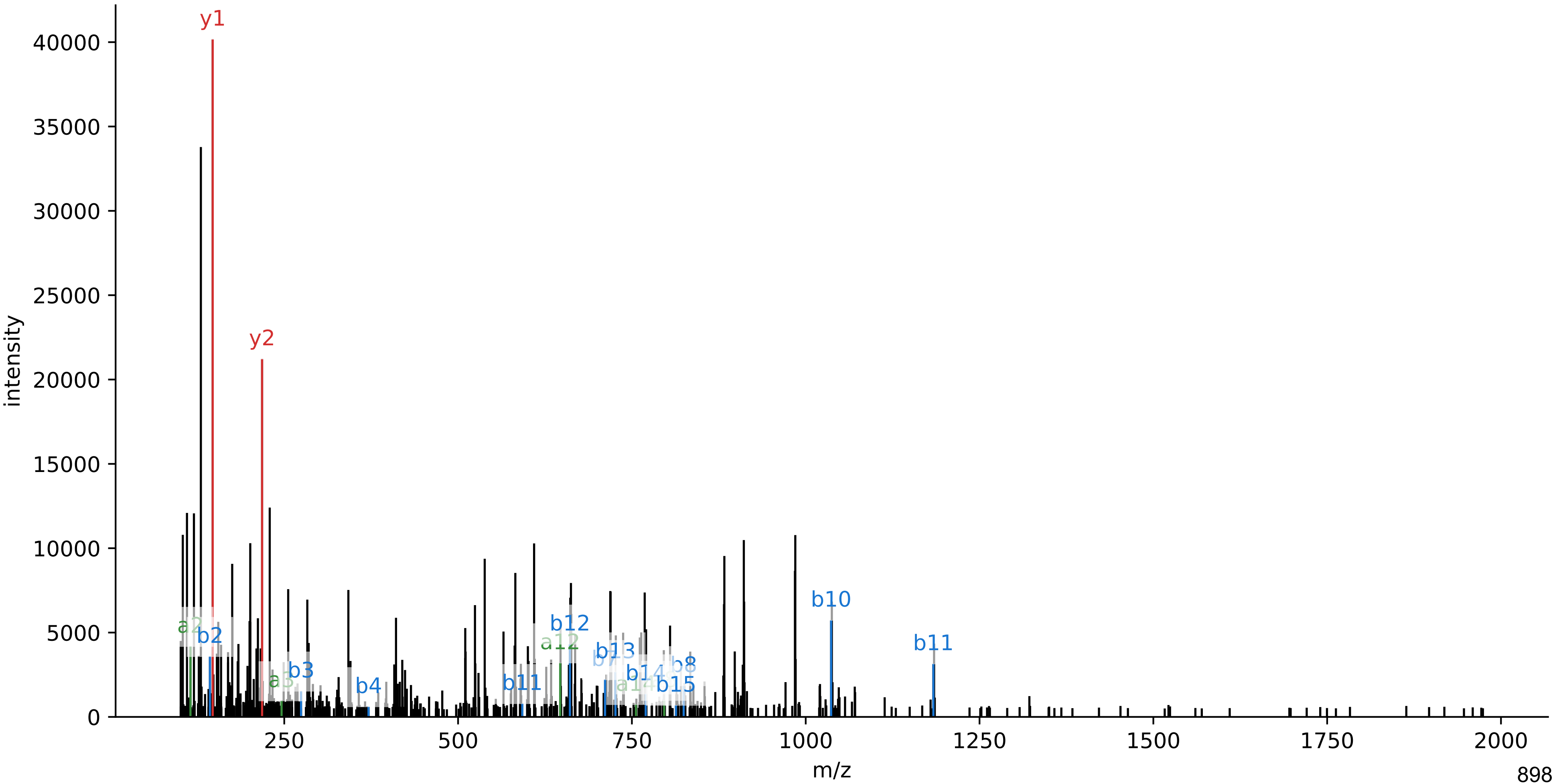

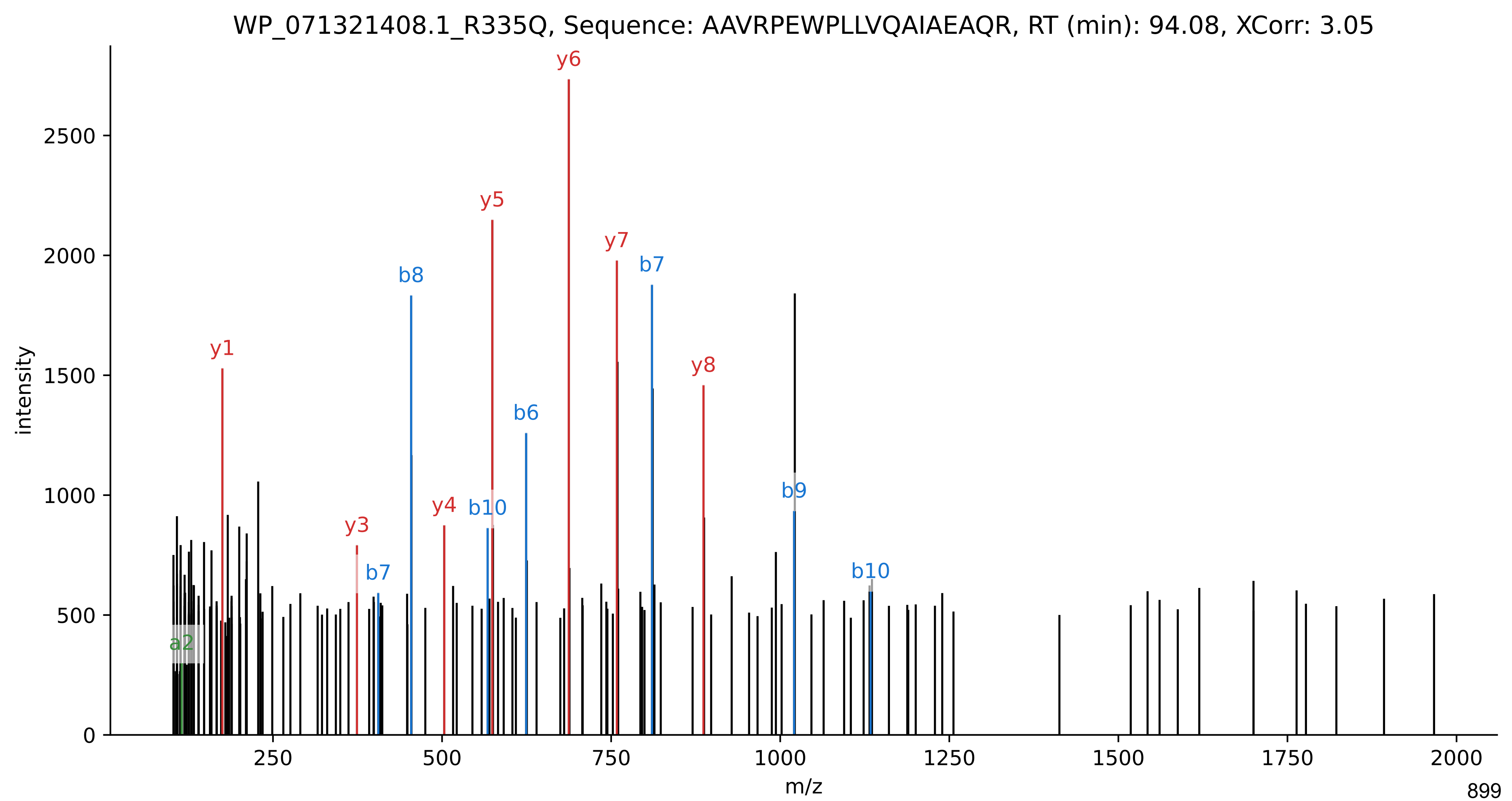
WP_071321408.1_R335Q, Sequence: AAVRPEWPLLVQAIAEAQR, RT (min): 94.08, XCorr: 3.05
intensity
m/z
y6
y5
y7
b7
b8
y1
b6
y8
y4
b10
y3
b7
b9
b10
a2
0
500
1000
1500
2000
2500
250
500
750
1000
1250
1500
1750
2000

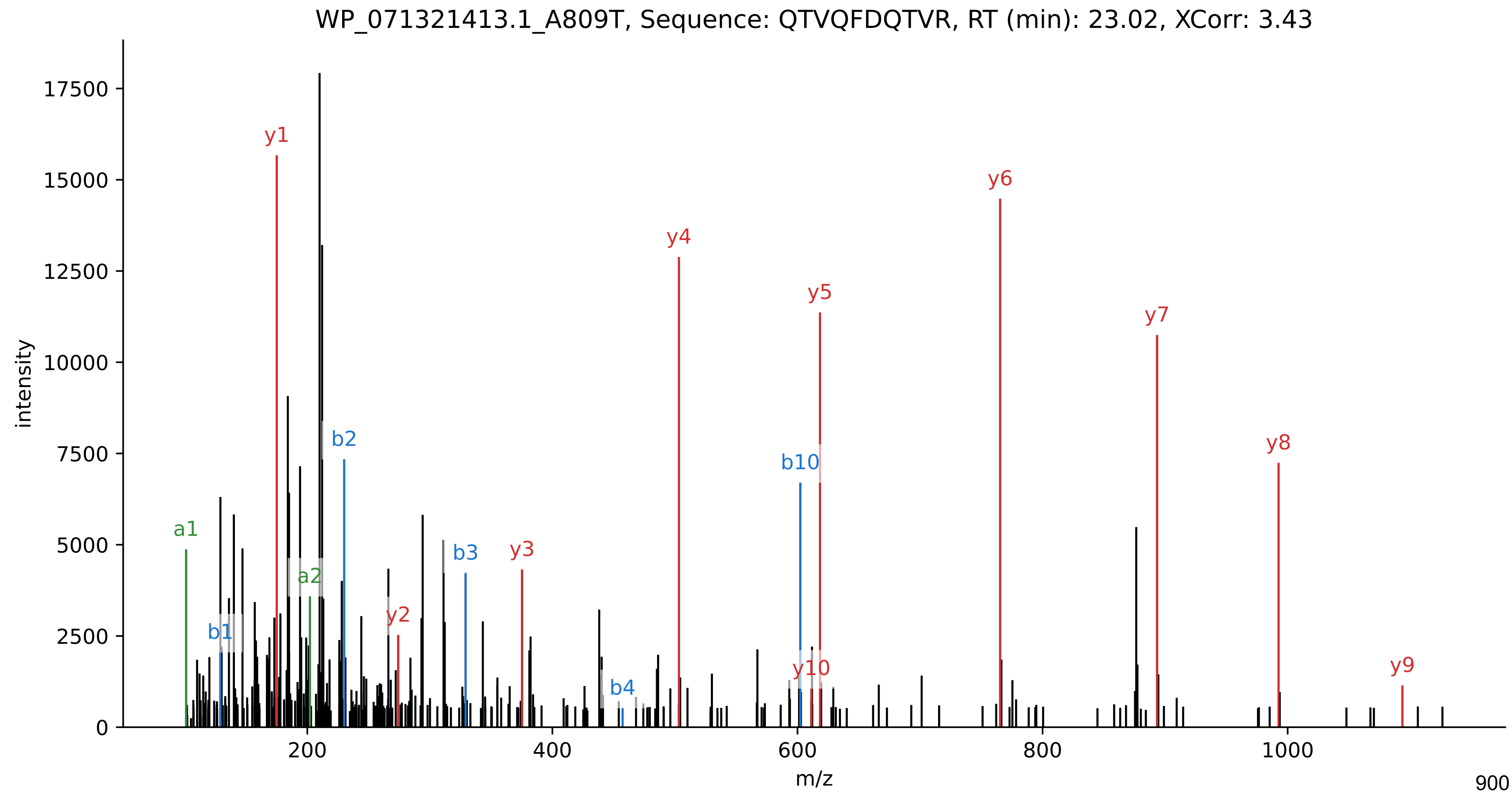
WP_071321413.1_A809T, Sequence: QTVQFDQTVR, RT (min): 23.02, XCorr: 3.43
intensity
m/z
17500
15000
12500
10000
7500
5000
2500
0
200
400
600
800
1000
a1
a2
b1
b2
b3
b4
b10
y1
y2
y3
y4
y5
y6
y7
y8
y9
y10

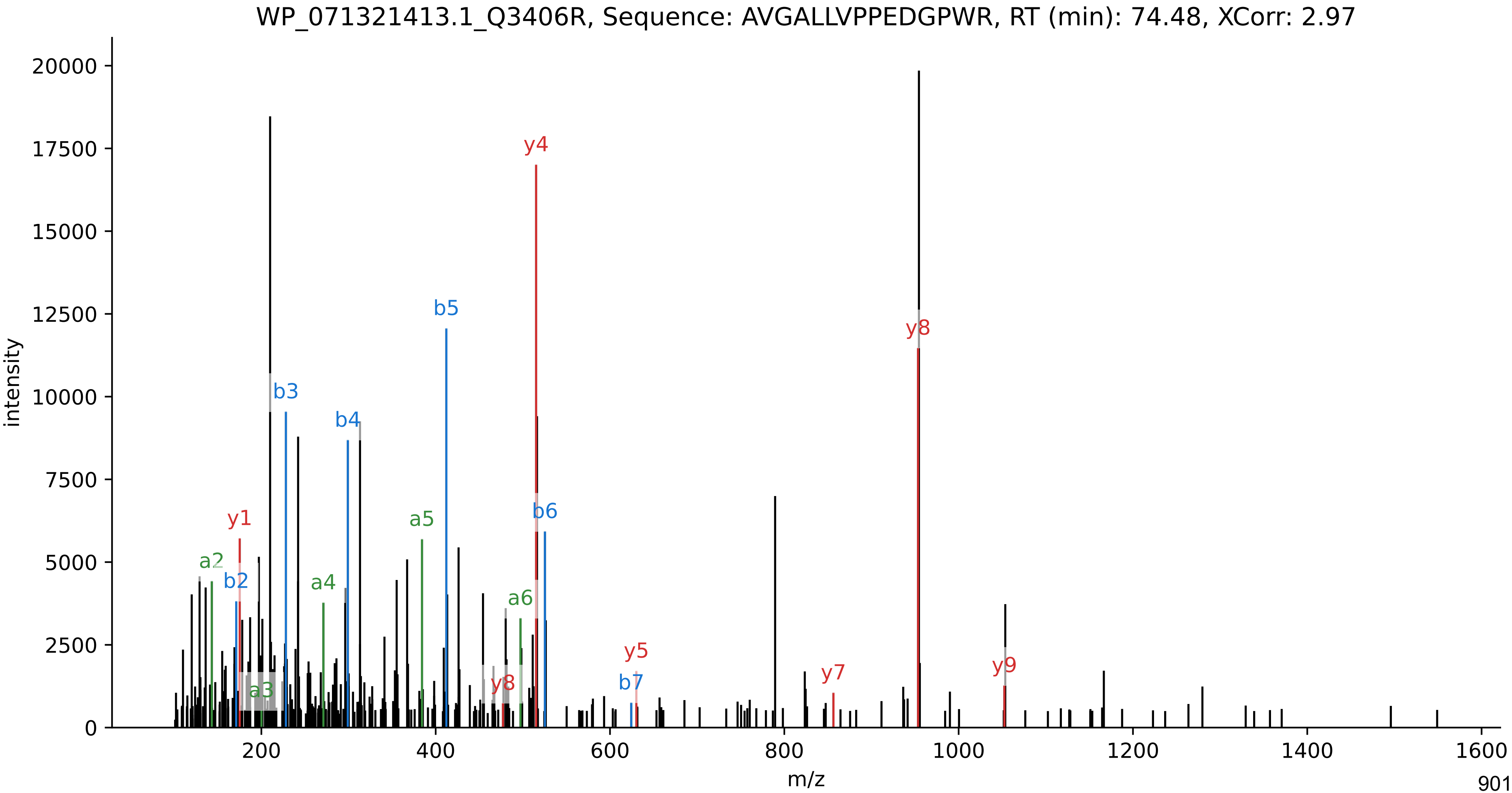

WP_071321413.1_Q3406R, Sequence: AVGALLVPPEDGPWR, RT (min): 74.48, XCorr: 2.97
intensity
m/z
y4
y8
b5
b3
b4
y1
a5
b6
a2
b2
a4
a6
y5
y7
y9
b7
y8
a3

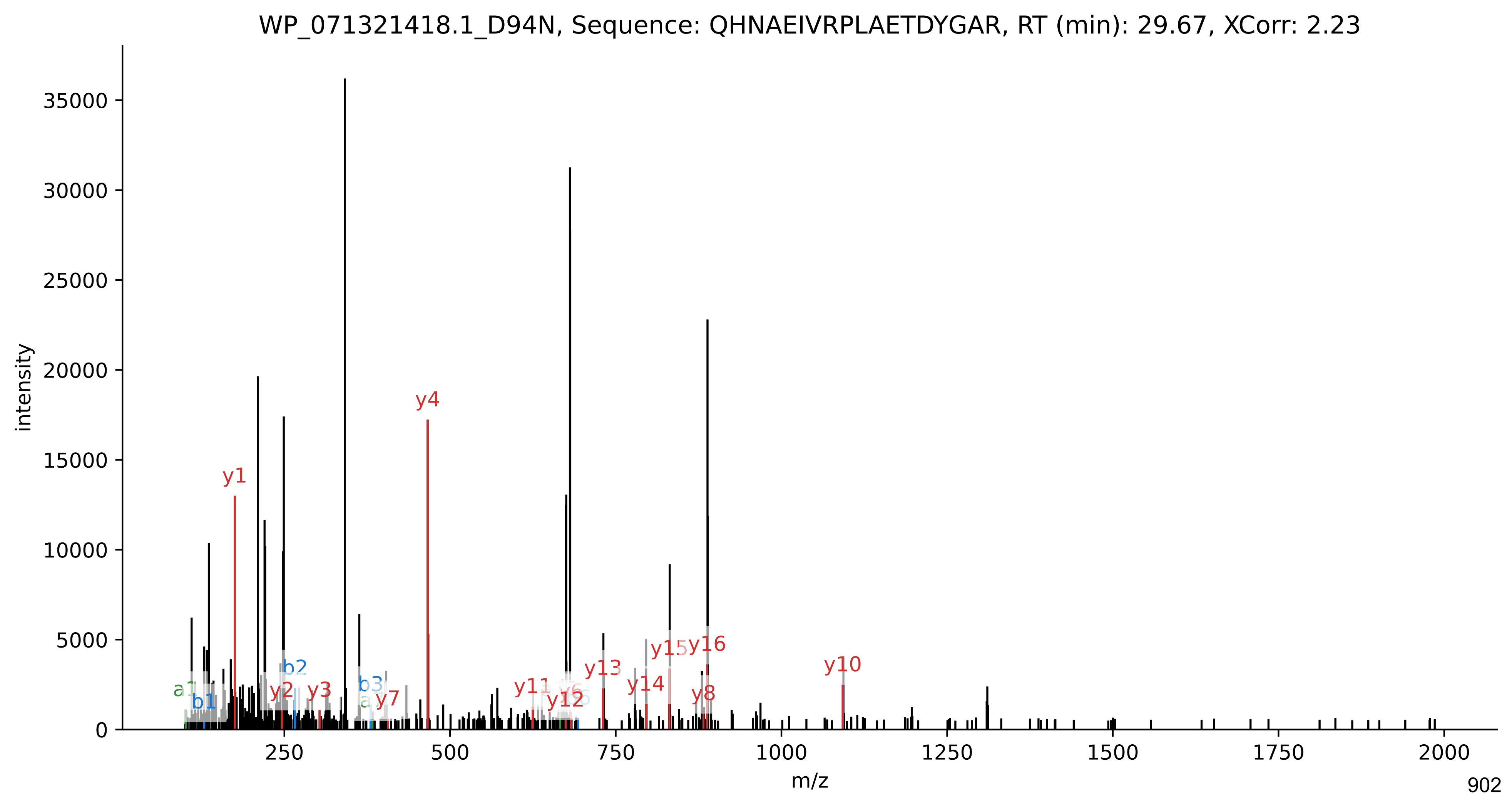

WP_071321418.1_D94N, Sequence: QHNAEIVRPLAETDYGAR, RT (min): 29.67, XCorr: 2.23
intensity
m/z
y1
y2
y3
y4
y7
y8
y10
y11
y12
y13
y14
y16
a1
b1
b2
b3
0
5000
10000
15000
20000
25000
30000
35000
250
500
750
1000
1250
1500
1750
2000

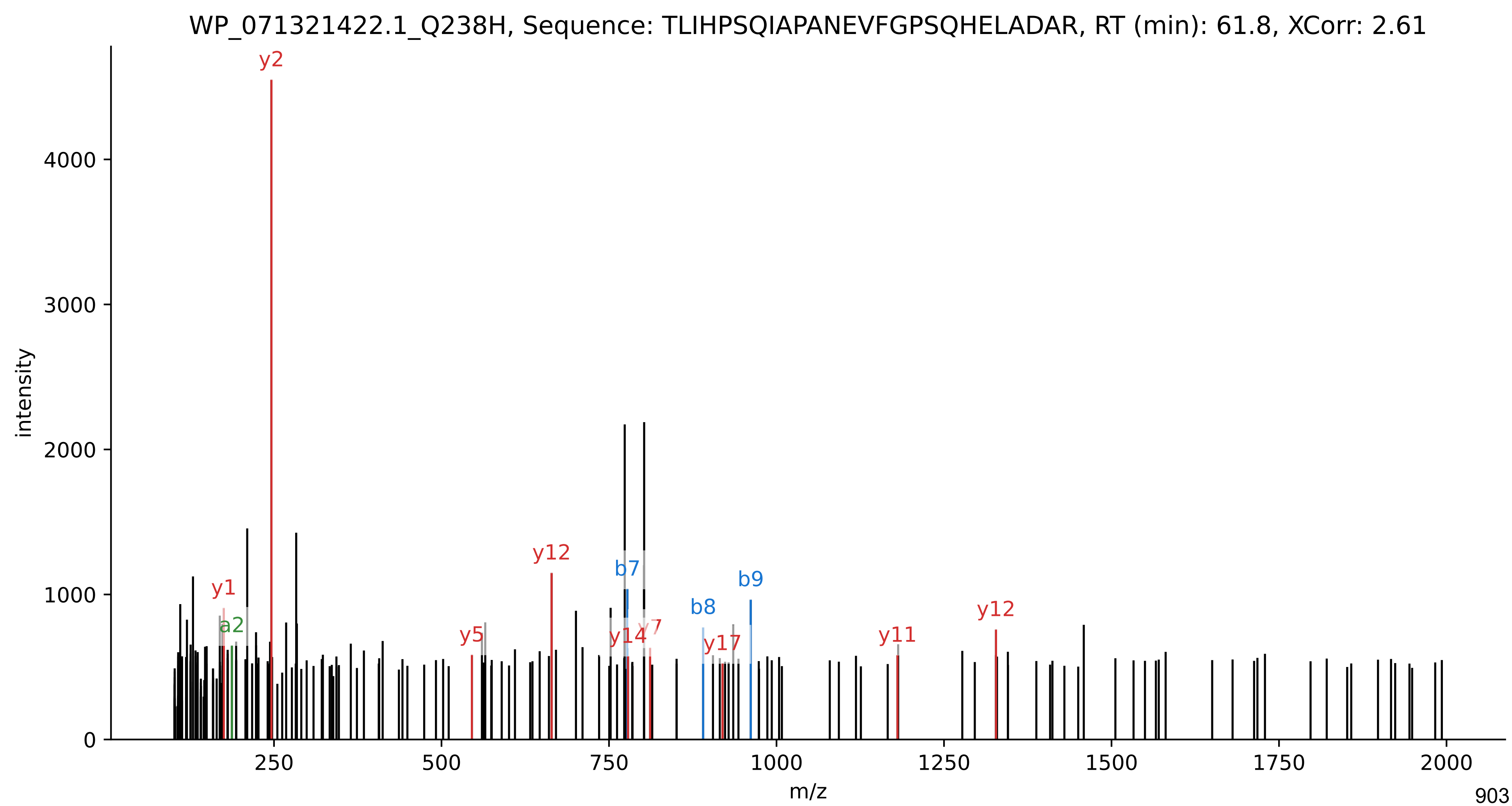
WP_071321422.1_Q238H, Sequence: TLIHPSQIAPANEVFGPSQHELADAR, RT (min): 61.8, XCorr: 2.61
intensity
m/z
y2
y1
a2
y5
y12
b7
y14
y7
b8
y17
b9
y11
y12
0
1000
2000
3000
4000
250
500
750
1000
1250
1500
1750
2000

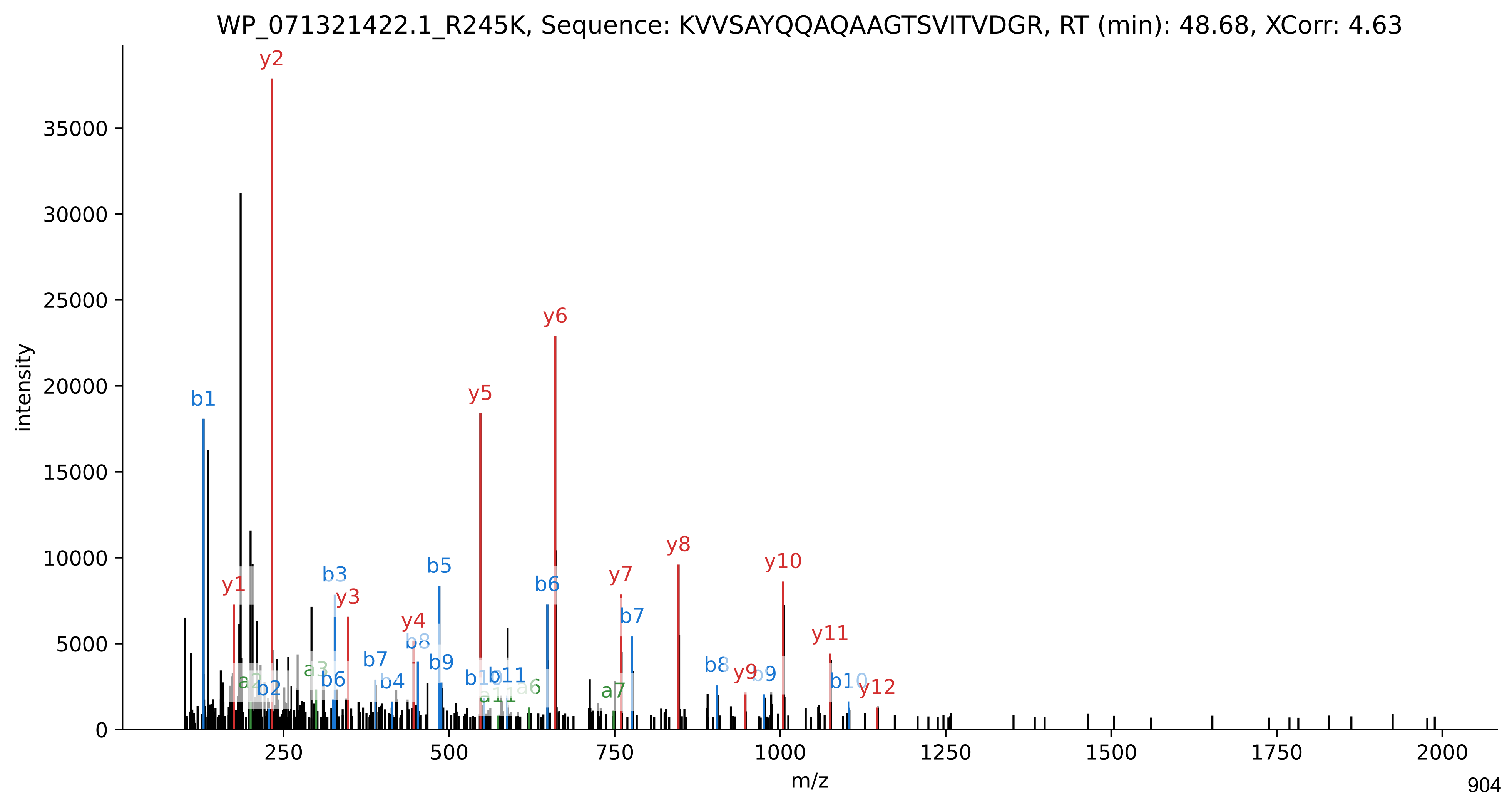

WP_071321422.1_R245K, Sequence: KVVSAYQQAQAAGTSVITVDGR, RT (min): 48.68, XCorr: 4.63
intensity
m/z
y2
y6
y5
b1
y1
b3
y3
y4
b5
b6
y7
b7
y8
y10
y11
y12
b2
b4
b9
b11
a7

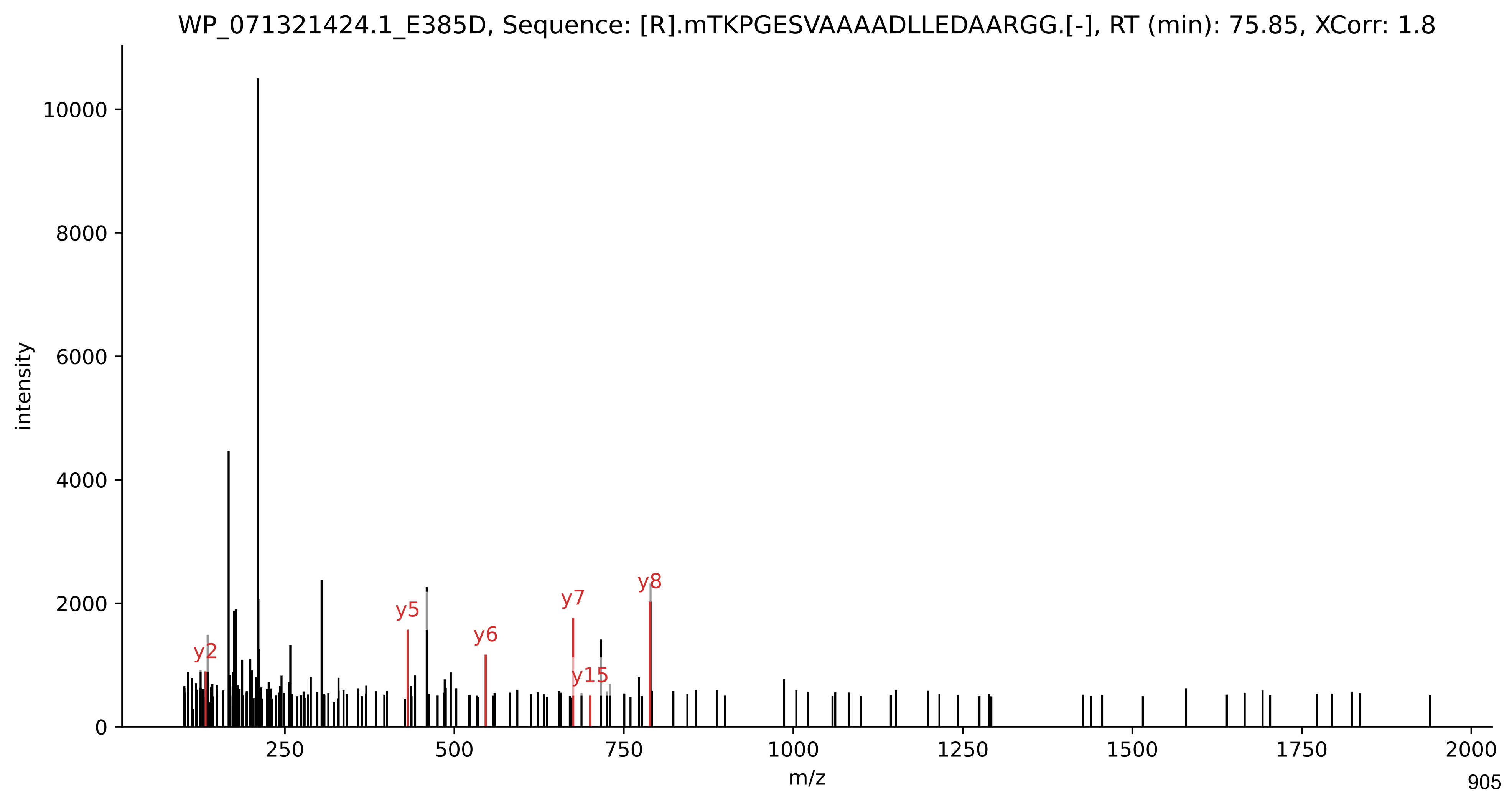
WP_071321424.1_E385D, Sequence: [R].mTKPGESVAAAADLLEDAARGG.[-], RT (min): 75.85, XCorr: 1.8
intensity
m/z
y2
y5
y6
y7
y15
y8
0
2000
4000
6000
8000
10000
250
500
750
1000
1250
1500
1750
2000

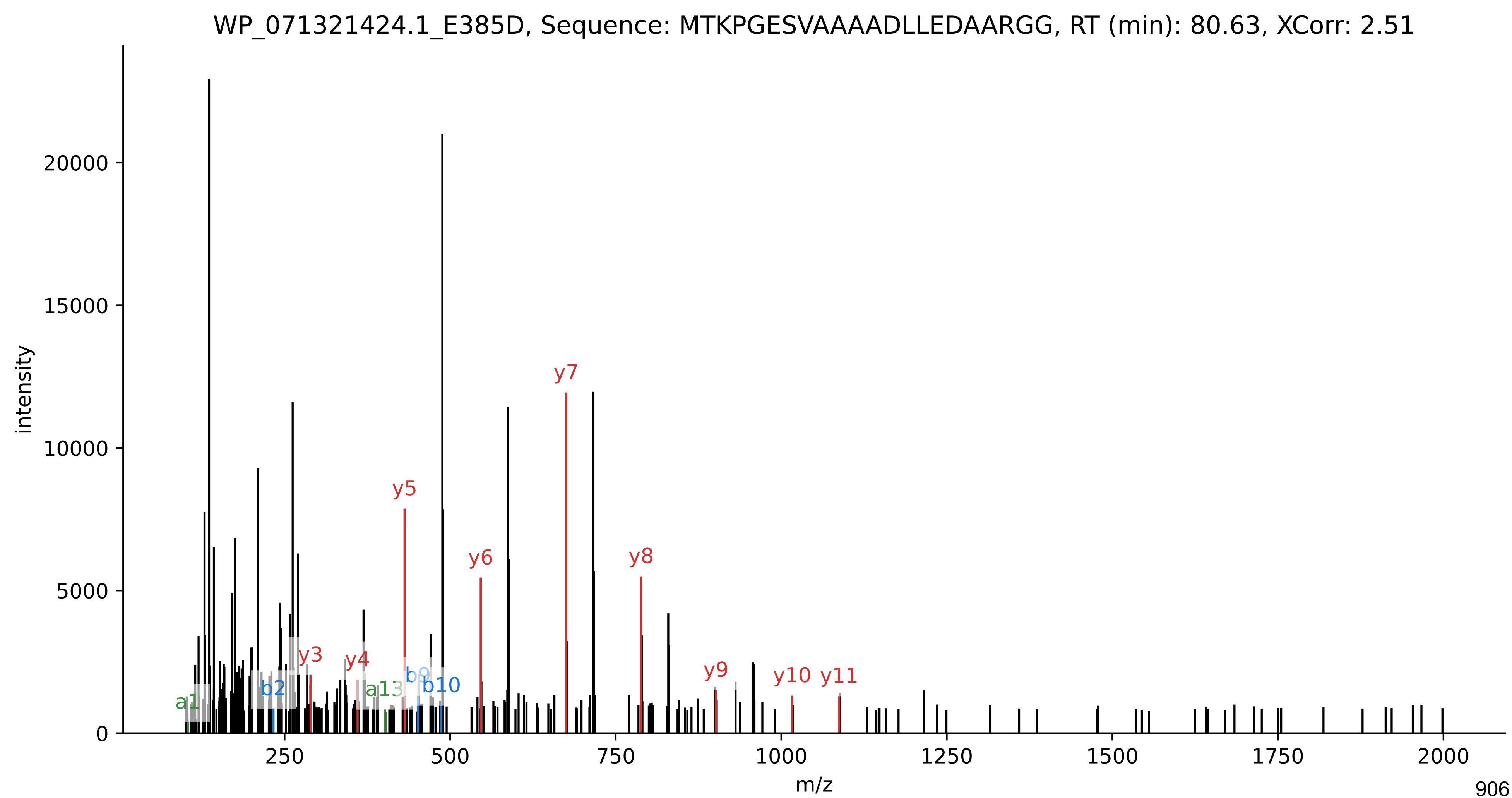

WP_071321424.1_E385D, Sequence: MTKPGESVAAAADLLEDAARGG, RT (min): 80.63, XCorr: 2.51
intensity
m/z
y3
y4
y5
y6
y7
y8
y9
y10
y11
a1
b2
a13
b9
b10

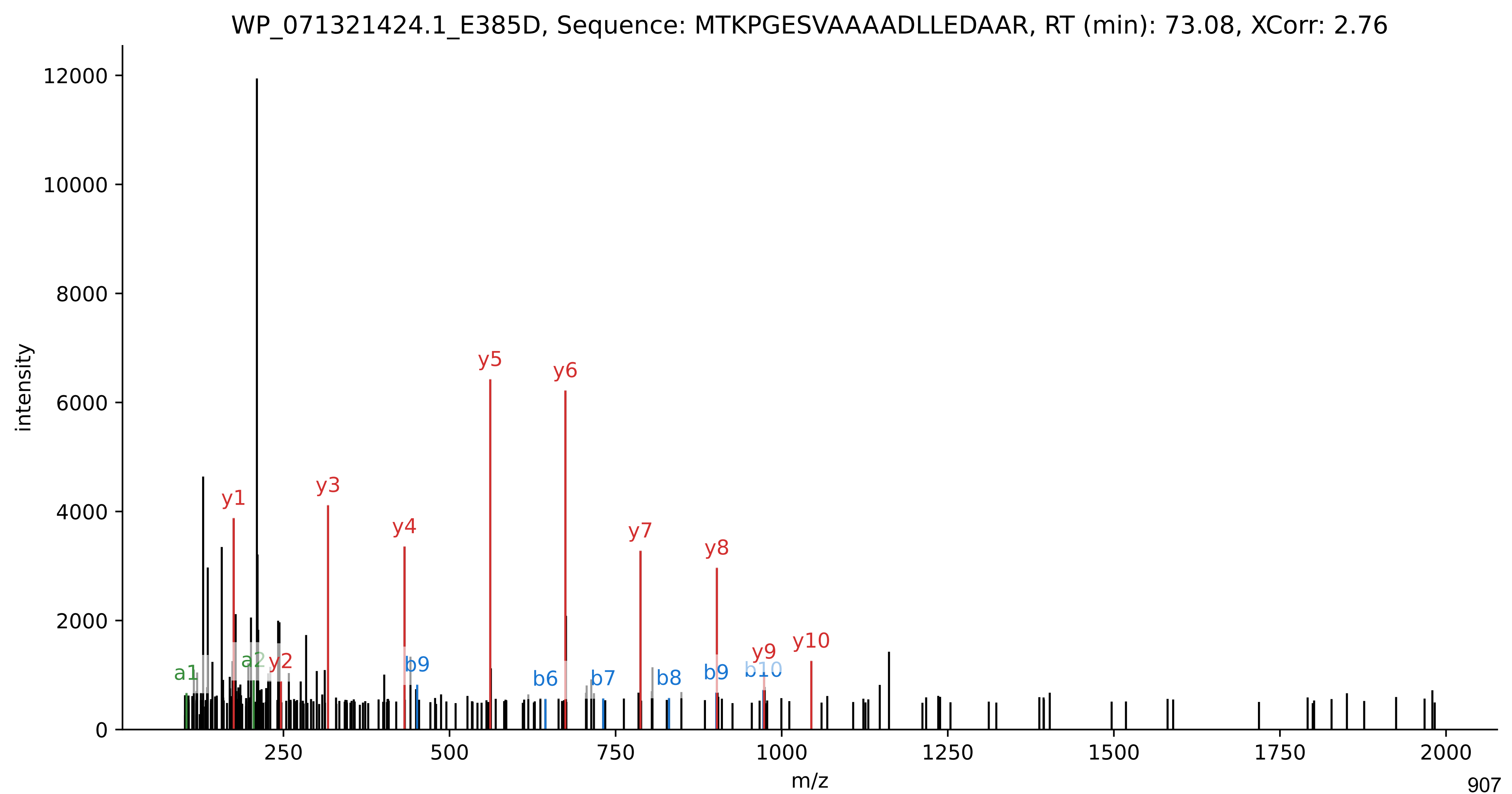

WP_071321424.1_E385D, Sequence: MTKPGESVAAAADLLEDAAR, RT (min): 73.08, XCorr: 2.76
intensity
m/z
y1
y2
y3
y4
y5
y6
y7
y8
y9
y10
a1
a2
b6
b7
b8
b9
b9
b10

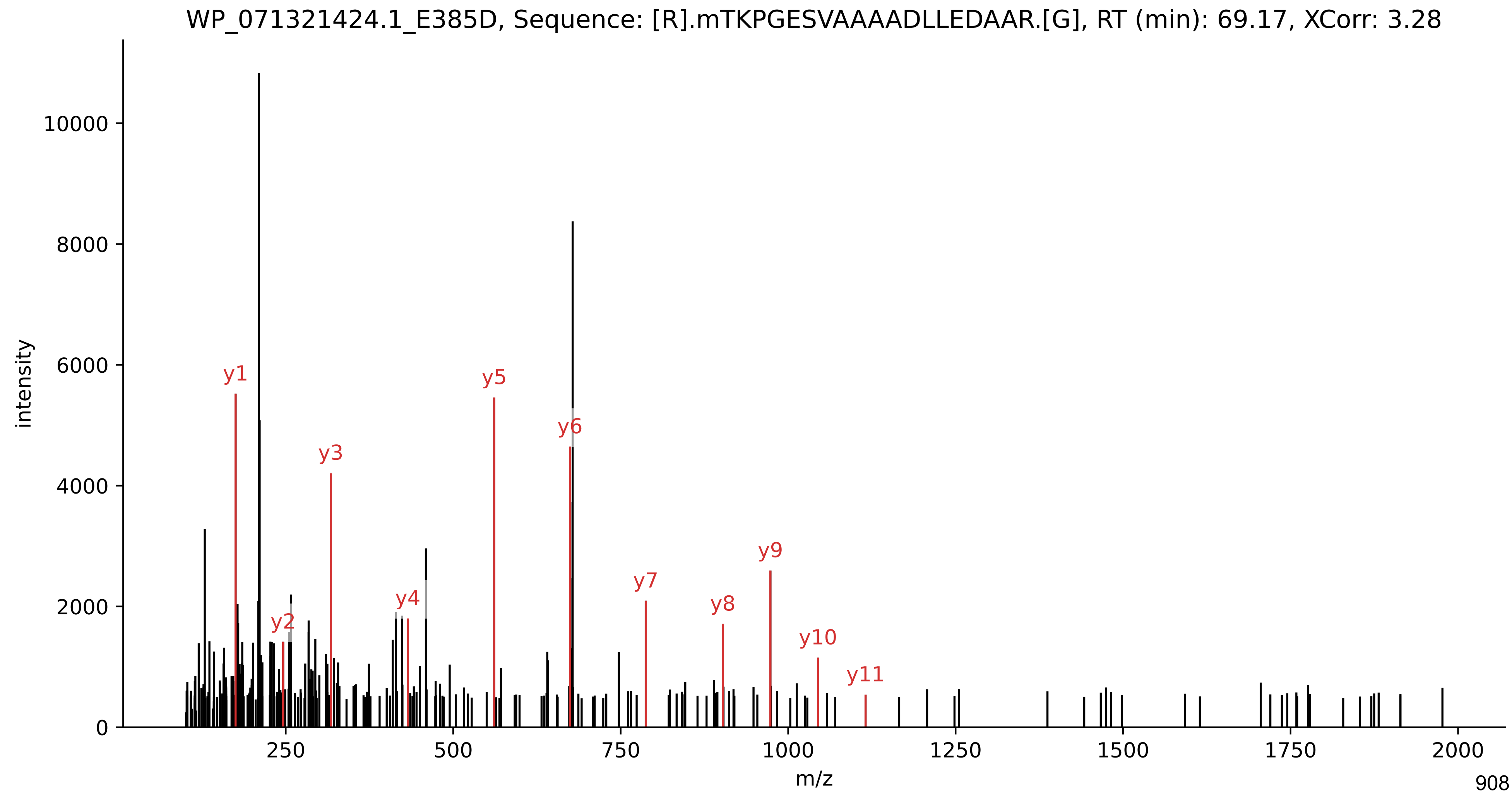

WP_071321424.1_E385D, Sequence: [R].mTKPGESVAAAADLLEDAAR.[G], RT (min): 69.17, XCorr: 3.28
intensity
m/z
y1
y2
y3
y4
y5
y6
y7
y8
y9
y10
y11

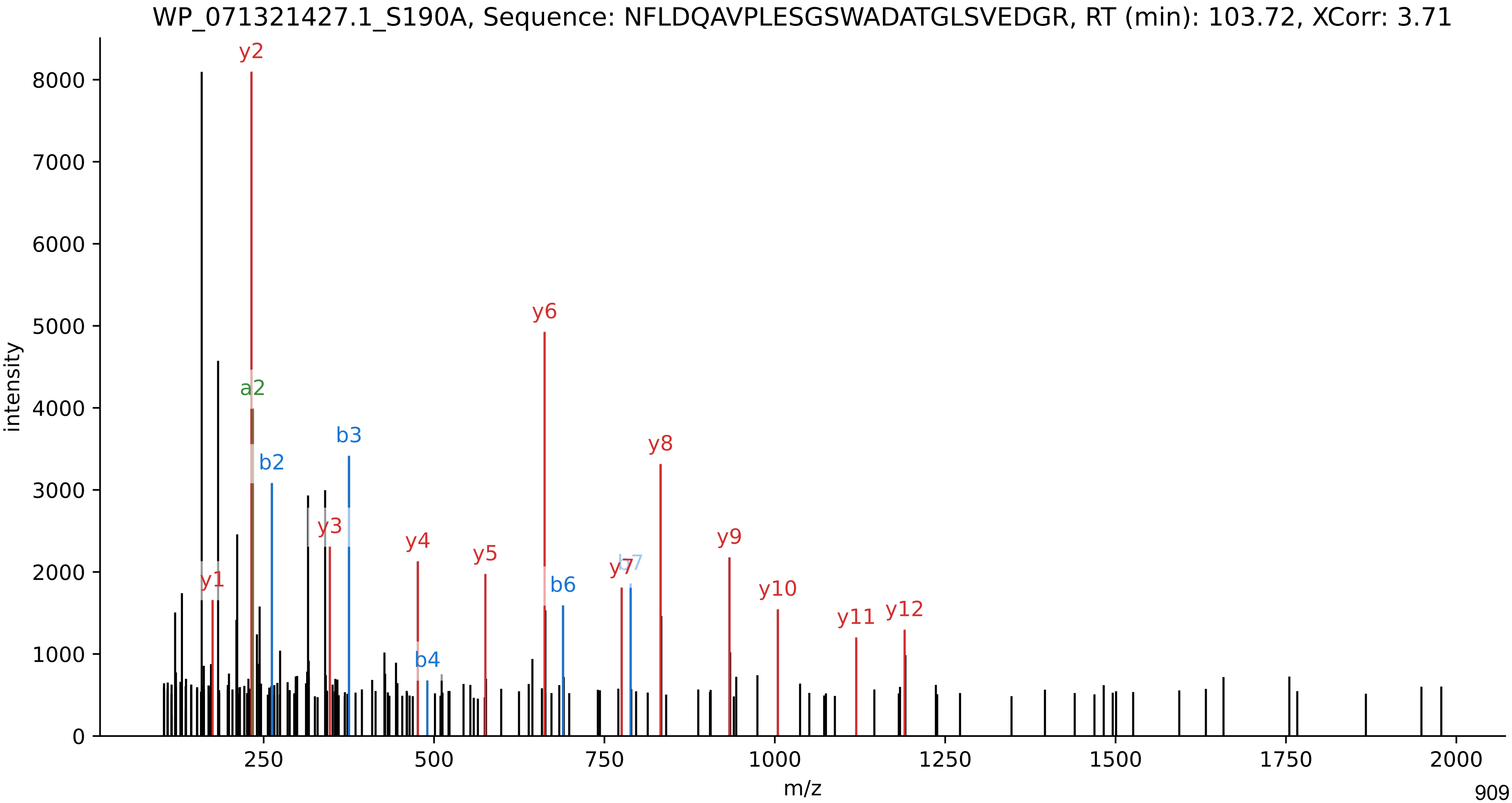
WP_071321427.1_S190A, Sequence: NFLDQAVPLESGSWADATGLSVEDGR, RT (min): 103.72, XCorr: 3.71
intensity
m/z
y1
y2
a2
b2
y3
b3
b4
y4
y5
y6
b6
y7
b7
y8
y9
y10
y11
y12
0
1000
2000
3000
4000
5000
6000
7000
8000
250
500
750
1000
1250
1500
1750
2000

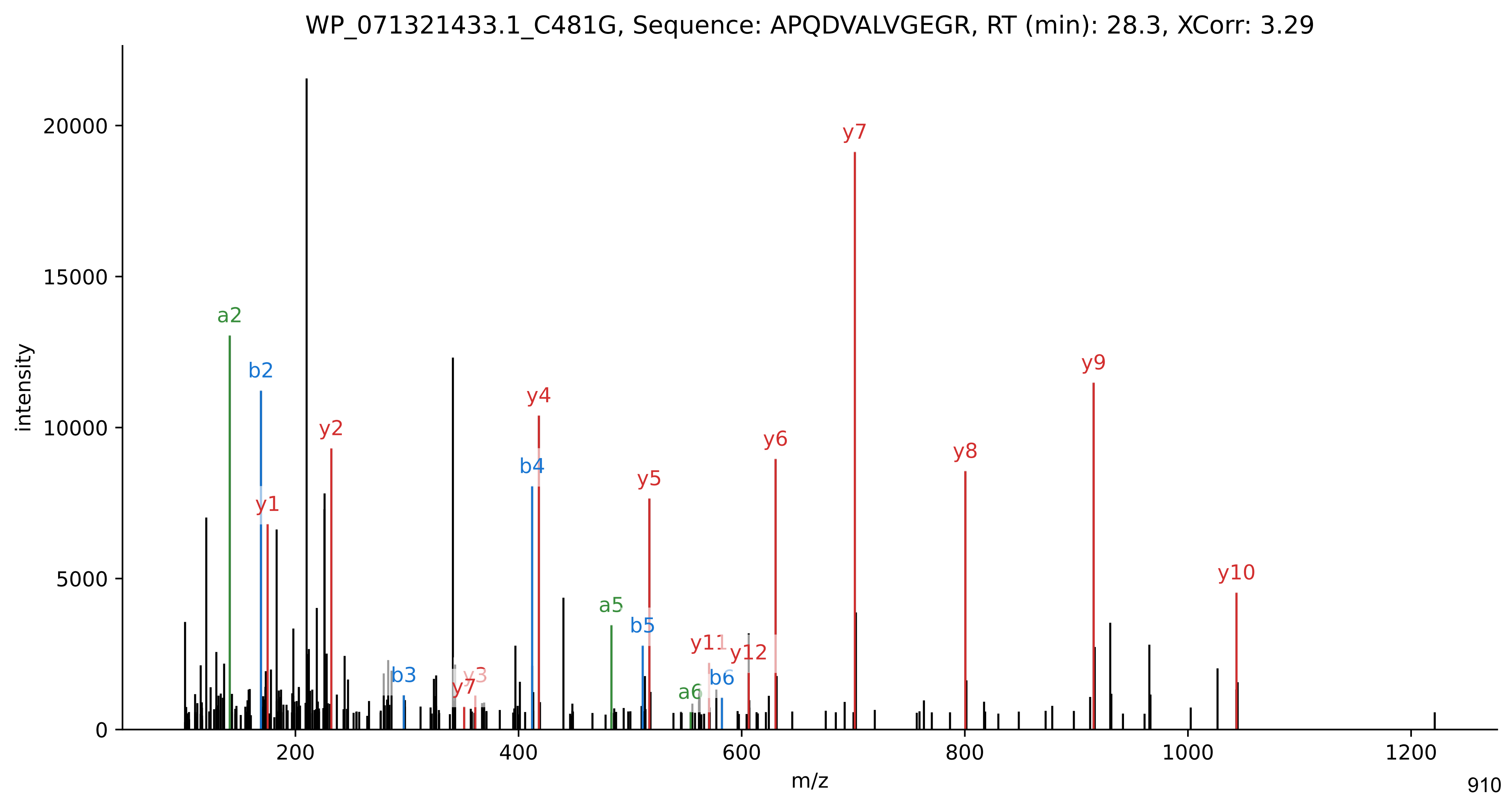

WP_071321433.1_C481G, Sequence: APQDVALVGEGR, RT (min): 28.3, XCorr: 3.29
intensity
m/z
a2
b2
y1
y2
b3
y7
y3
b4
y4
a5
b5
y5
a6
y11
b6
y12
y6
y7
y8
y9
y10

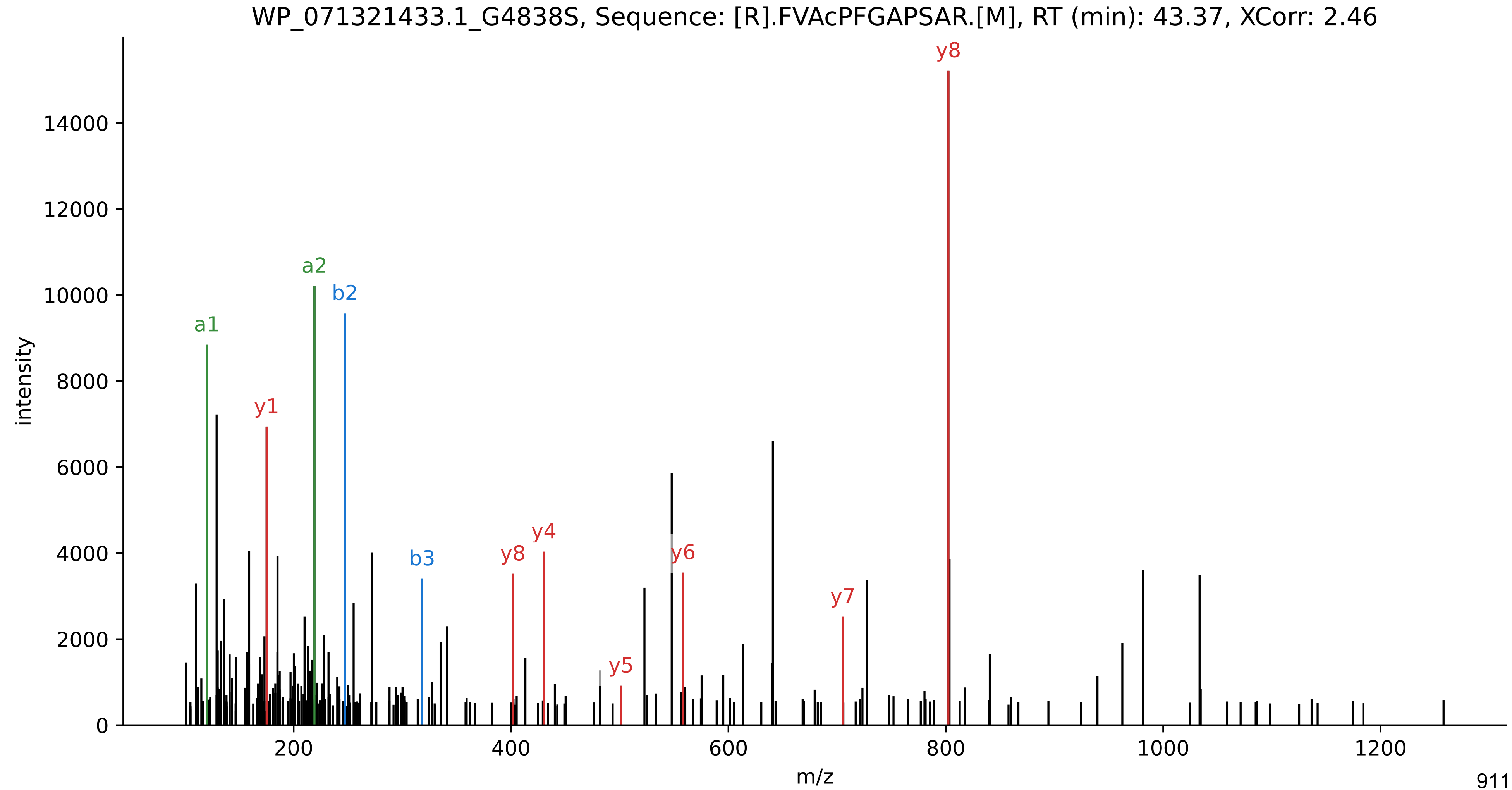

WP_071321433.1_G4838S, Sequence: [R].FVAcPFGAPSAR.[M], RT (min): 43.37, XCorr: 2.46
intensity
m/z
a1
a2
b2
y1
b3
y8
y4
y5
y6
y7
y8
0
2000
4000
6000
8000
10000
12000
14000
200
400
600
800
1000
1200

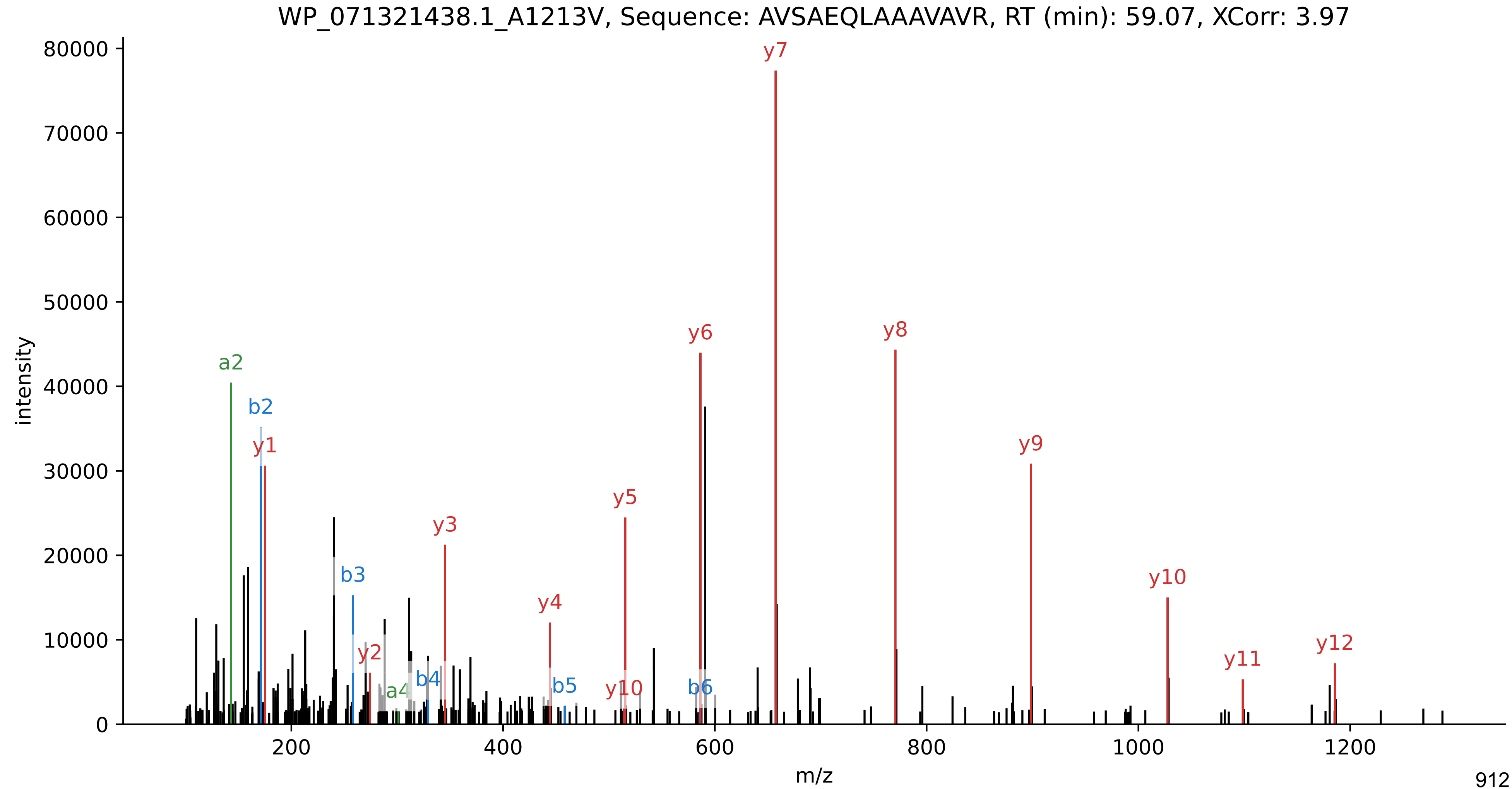

WP_071321438.1_A1213V, Sequence: AVSAEQLAAAVAVR, RT (min): 59.07, XCorr: 3.97
intensity
m/z
a2
b2
y1
b3
y2
a4
b4
y3
y4
b5
y5
y10
b6
y6
y7
y8
y9
y10
y11
y12

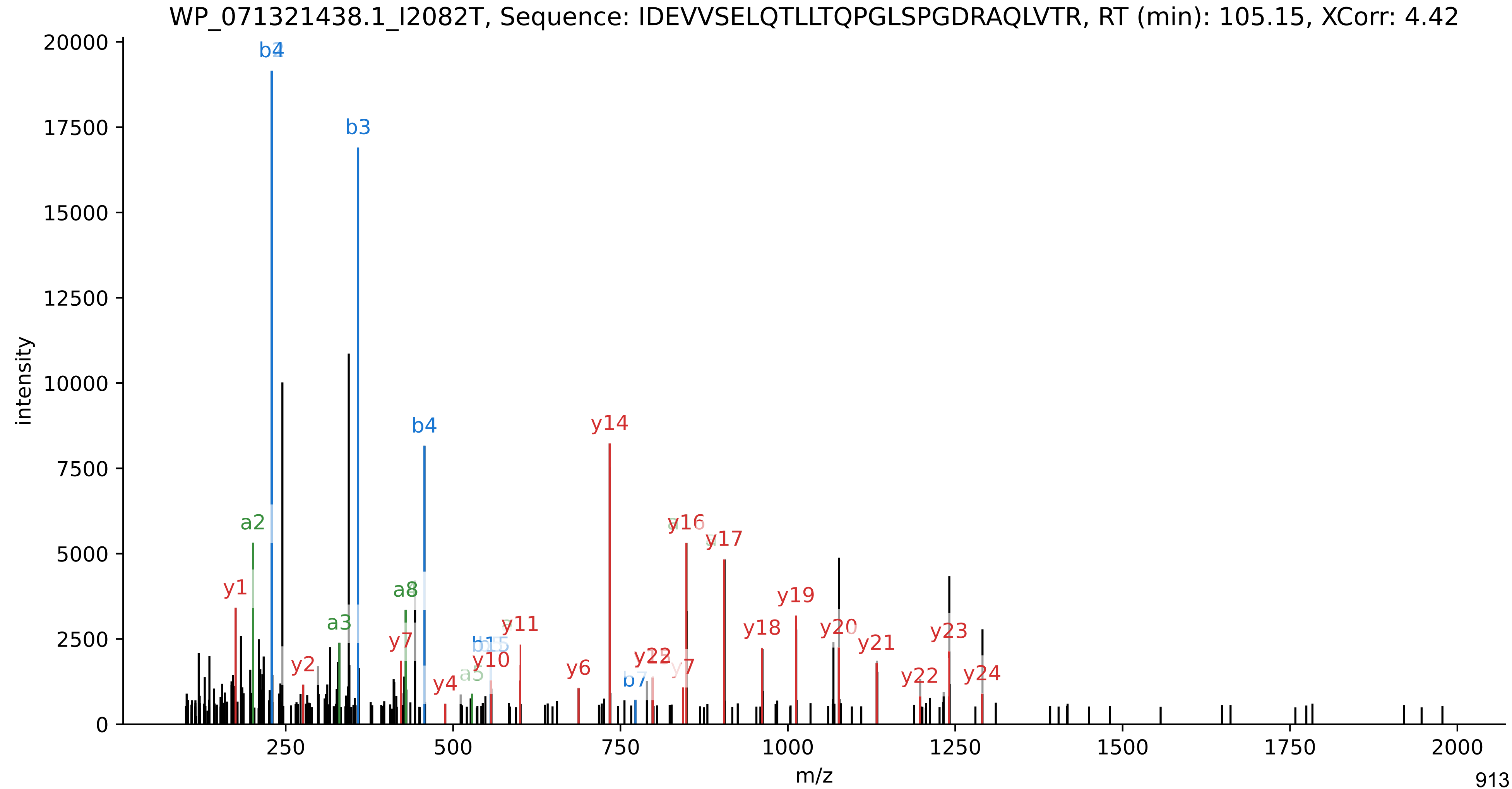

WP_071321438.1_I2082T, Sequence: IDEVVSELQTLLTQPGLSPGDRAQLVTR, RT (min): 105.15, XCorr: 4.42
intensity
m/z
b3
b4
y14
a2
y16
y17
y1
a8
y19
a3
y11
y18
y20
y23
y7
y21
y2
y10
y22
y6
y4
y24
b7
a5

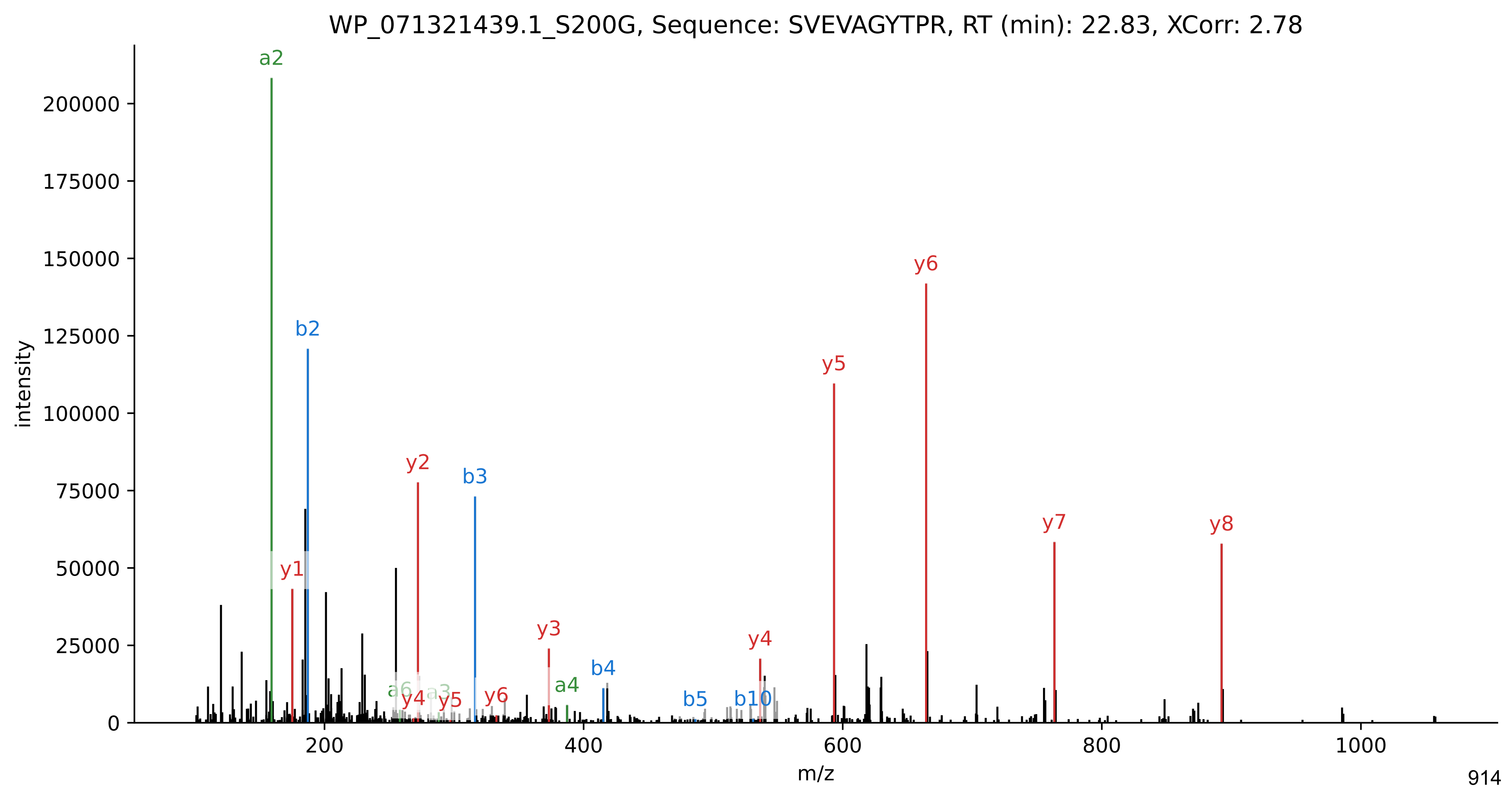

WP_071321439.1_S200G, Sequence: SVEVAGYTPR, RT (min): 22.83, XCorr: 2.78
intensity
m/z
a2
b2
y1
y2
b3
y3
a4
b4
y4
y5
y6
y7
y8
a6
a3
b5
b10

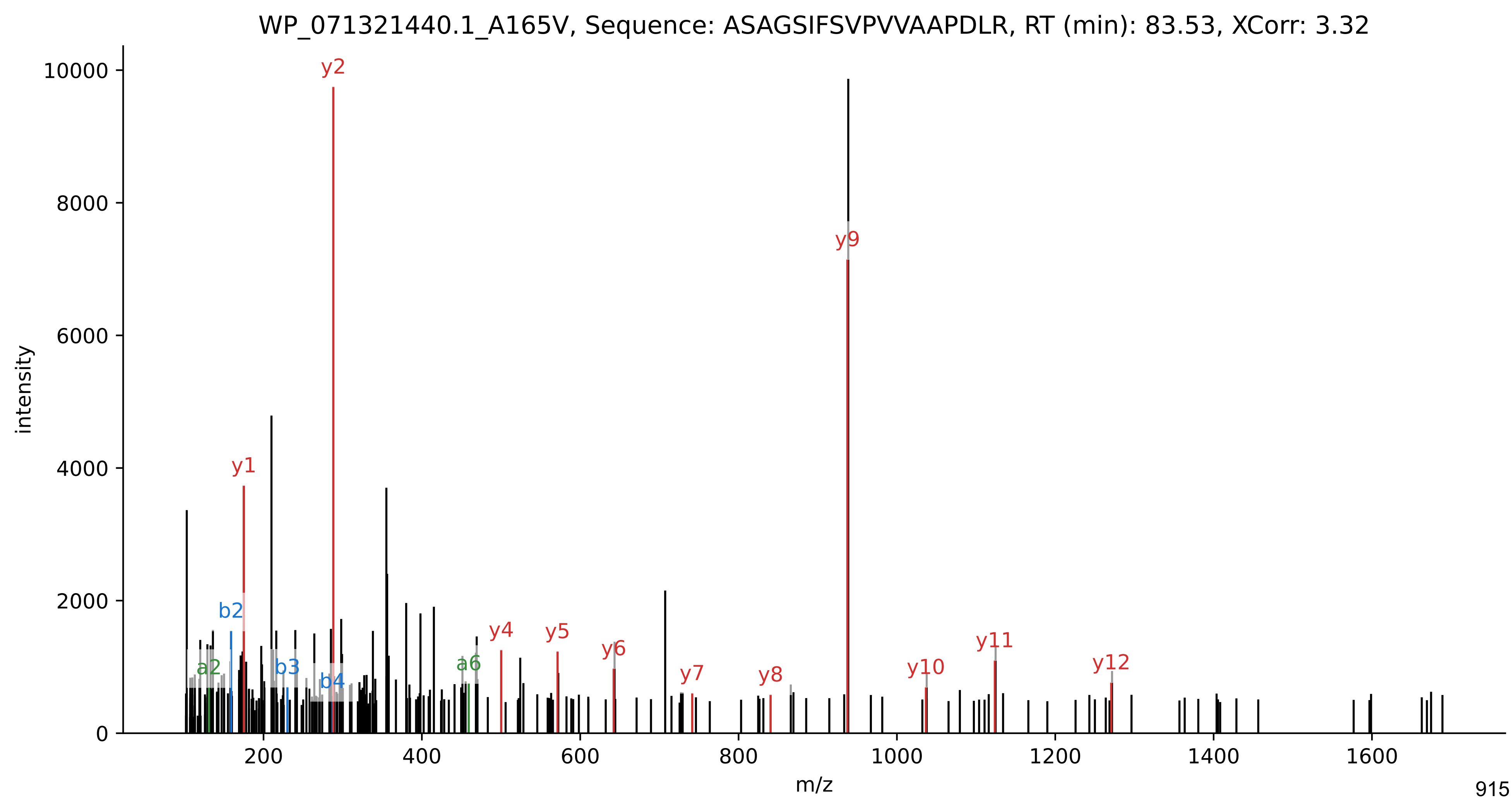
WP_071321440.1_A165V, Sequence: ASAGSIFSVPVVAAPDLR, RT (min): 83.53, XCorr: 3.32
intensity
m/z
y1
y2
b2
a2
b3
b4
a6
y4
y5
y6
y7
y8
y9
y10
y11
y12

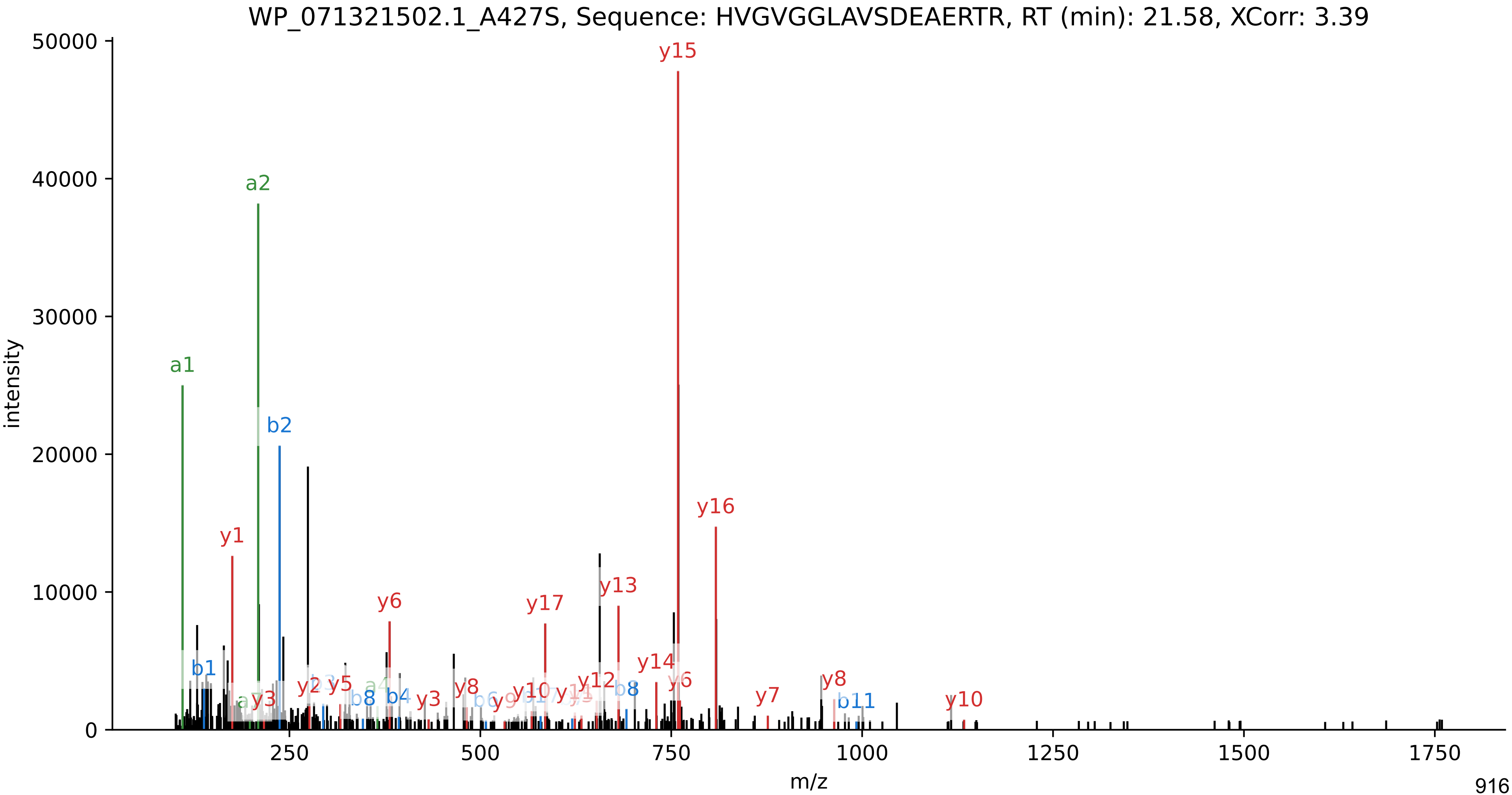
WP_071321502.1_A427S, Sequence: HVGVGGLAVSDEAERTR, RT (min): 21.58, XCorr: 3.39
intensity
m/z
0
10000
20000
30000
40000
50000
250
500
750
1000
1250
1500
1750
y15
a2
a1
b2
y16
y1
y6
y17
y13
y14
y6
y8
b11
y10
y7
y12
y10
y8
b4
y5
y2
y3
y3
b1

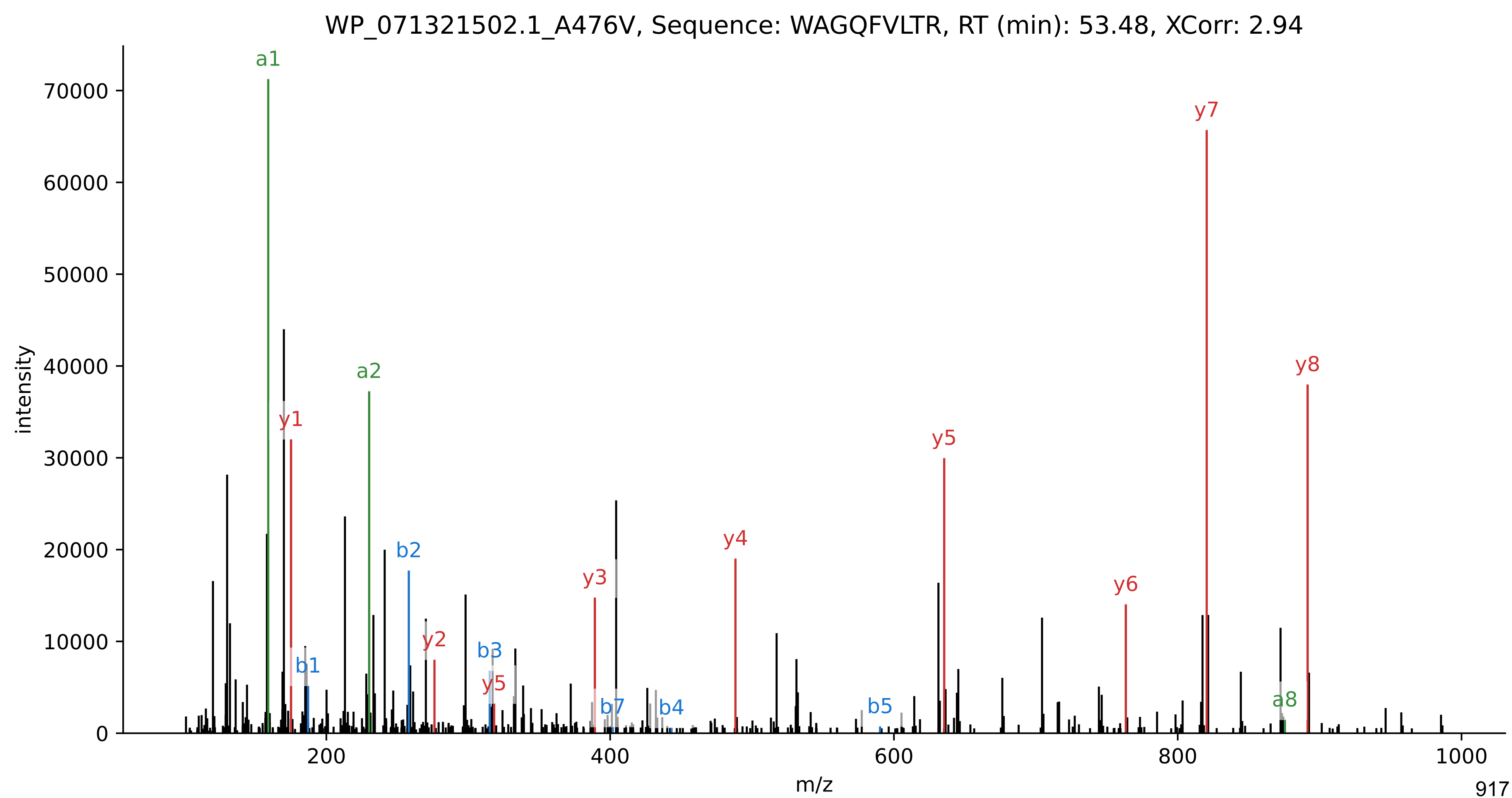
WP_071321502.1_A476V, Sequence: WAGQFVLTR, RT (min): 53.48, XCorr: 2.94
a1
a2
y1
b1
y2
b2
b3
y5
y3
b7
b4
y4
b5
y5
y6
y7
y8
a8
intensity
m/z
0
10000
20000
30000
40000
50000
60000
70000
200
400
600
800
1000

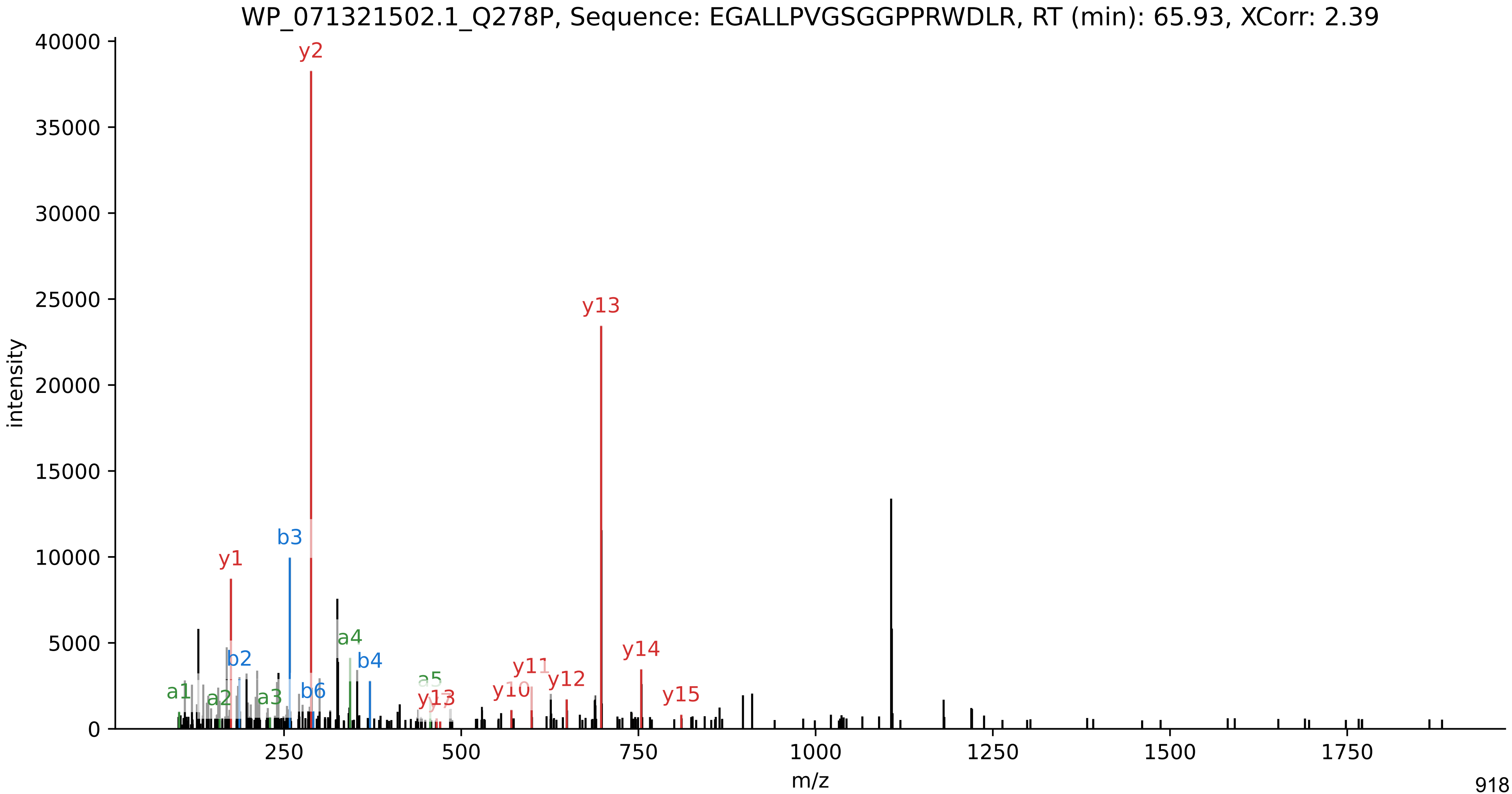
WP_071321502.1_Q278P, Sequence: EGALLPVGSGGPPRWDLR, RT (min): 65.93, XCorr: 2.39
intensity
m/z
y2
y13
y1
b3
b2
b4
b6
a1
a2
a3
a4
a5
y7
y10
y11
y12
y14
y15
0
5000
10000
15000
20000
25000
30000
35000
40000
250
500
750
1000
1250
1500
1750

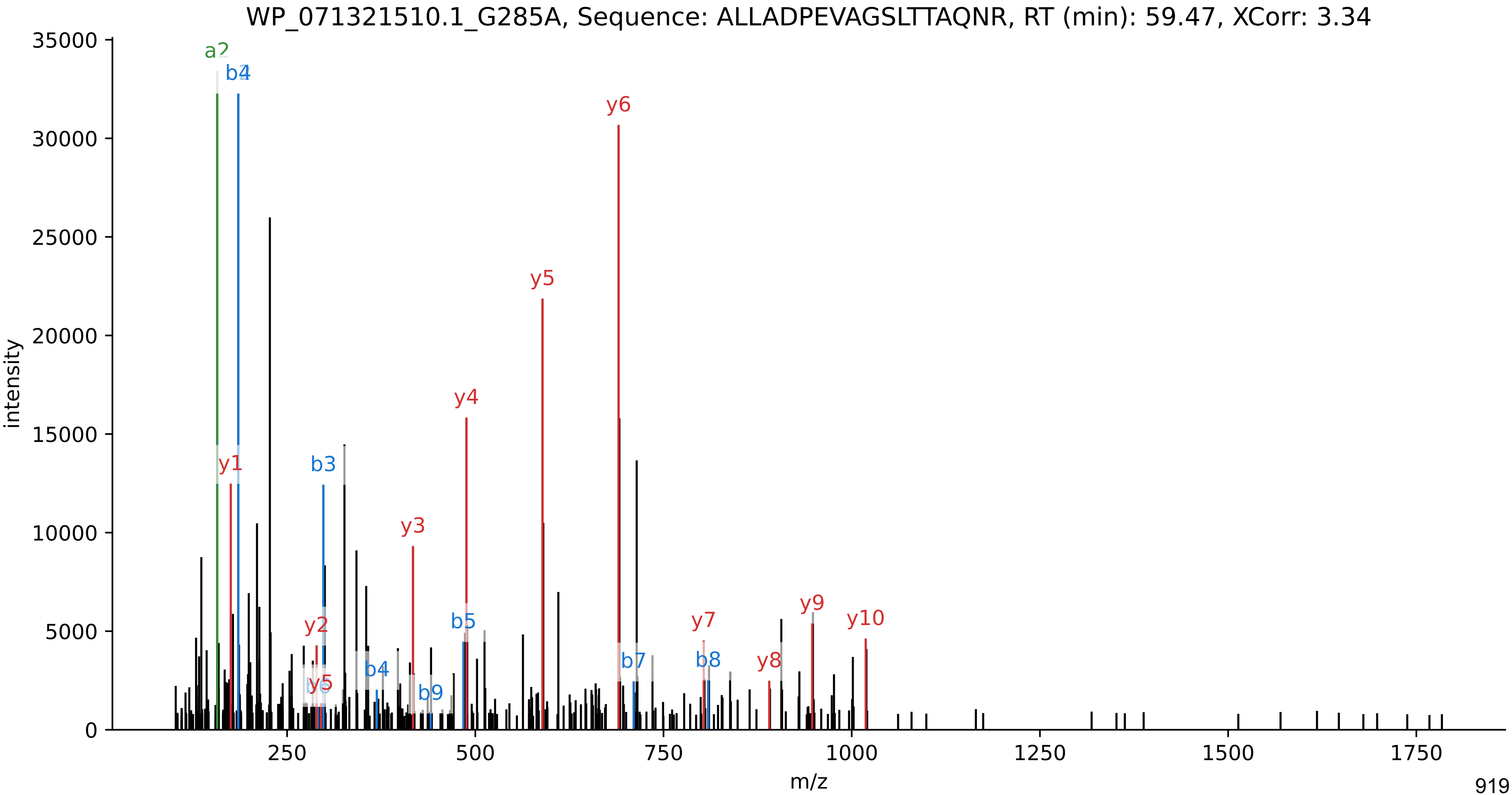

WP_071321510.1_G285A, Sequence: ALLADPEVAGSLTTAQNR, RT (min): 59.47, XCorr: 3.34
intensity
m/z
a2
b4
y6
y5
y4
y1
b3
y3
y2
y5
b5
b4
b9
b7
b8
y7
y8
y9
y10

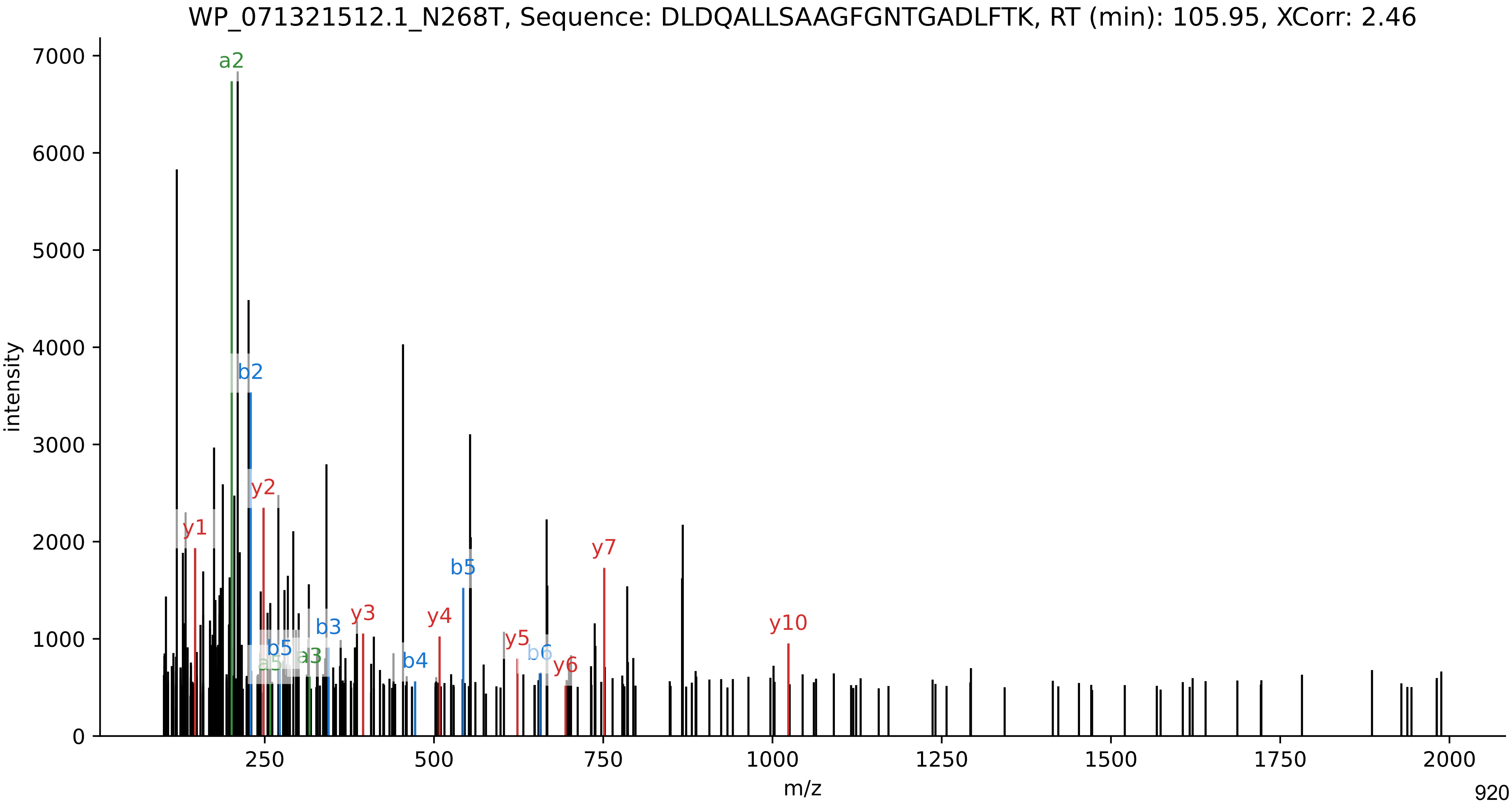

WP_071321512.1_N268T, Sequence: DLDQALLSAAGFGNTGADLFTK, RT (min): 105.95, XCorr: 2.46
intensity
m/z
a2
b2
y2
y1
b5
y7
b3
y3
y4
y10
y5
b6
b4
y6
a3
0
1000
2000
3000
4000
5000
6000
7000
250
500
750
1000
1250
1500
1750
2000

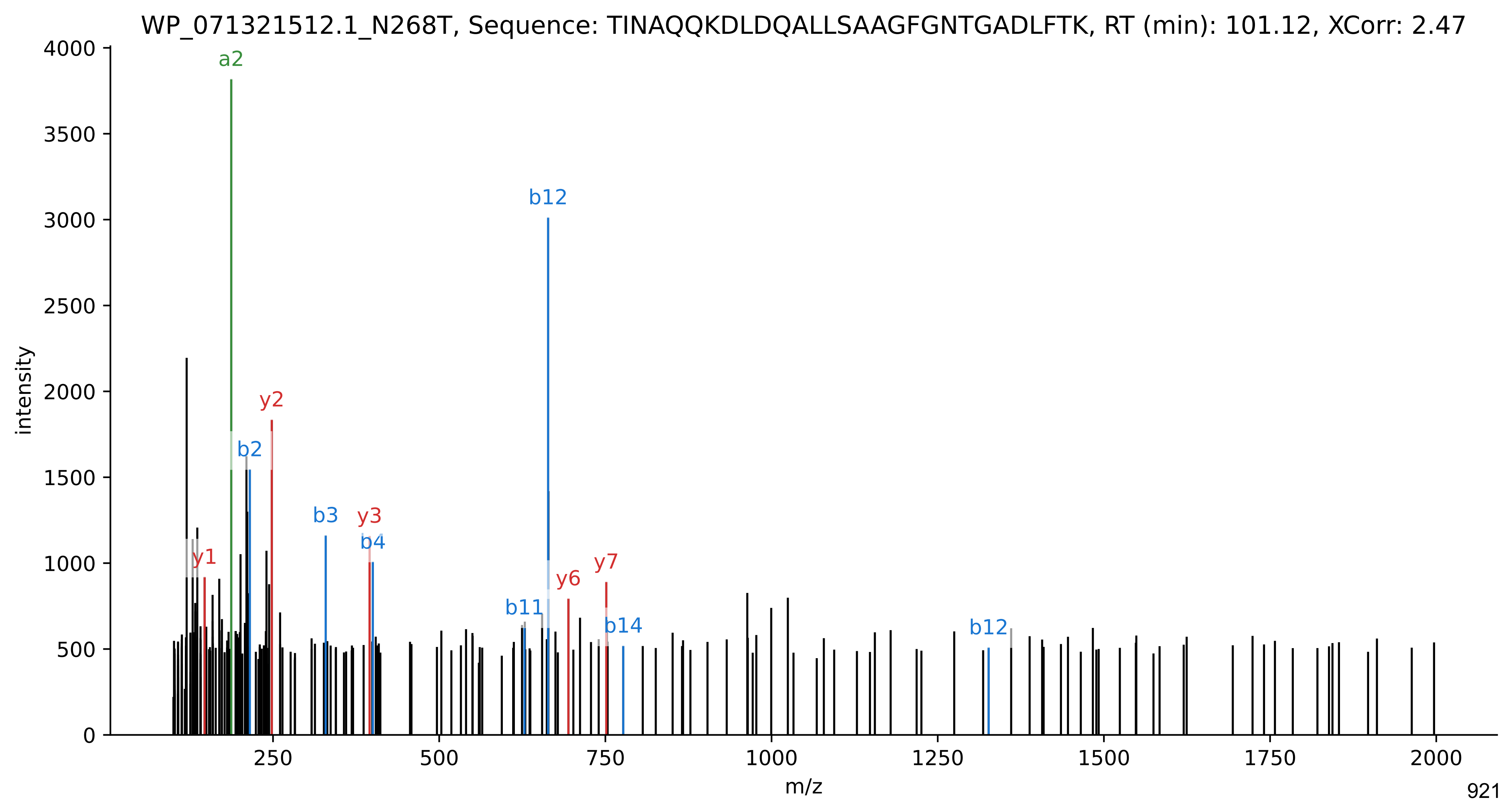
WP_071321512.1_N268T, Sequence: TINAQQKDLDQALLSAAGFGNTGADLFTK, RT (min): 101.12, XCorr: 2.47
a2
b12
y2
b2
b3
y3
b4
y1
y7
y6
b11
b14
b12
intensity
m/z
0
500
1000
1500
2000
2500
3000
3500
4000
250
500
750
1000
1250
1500
1750
2000

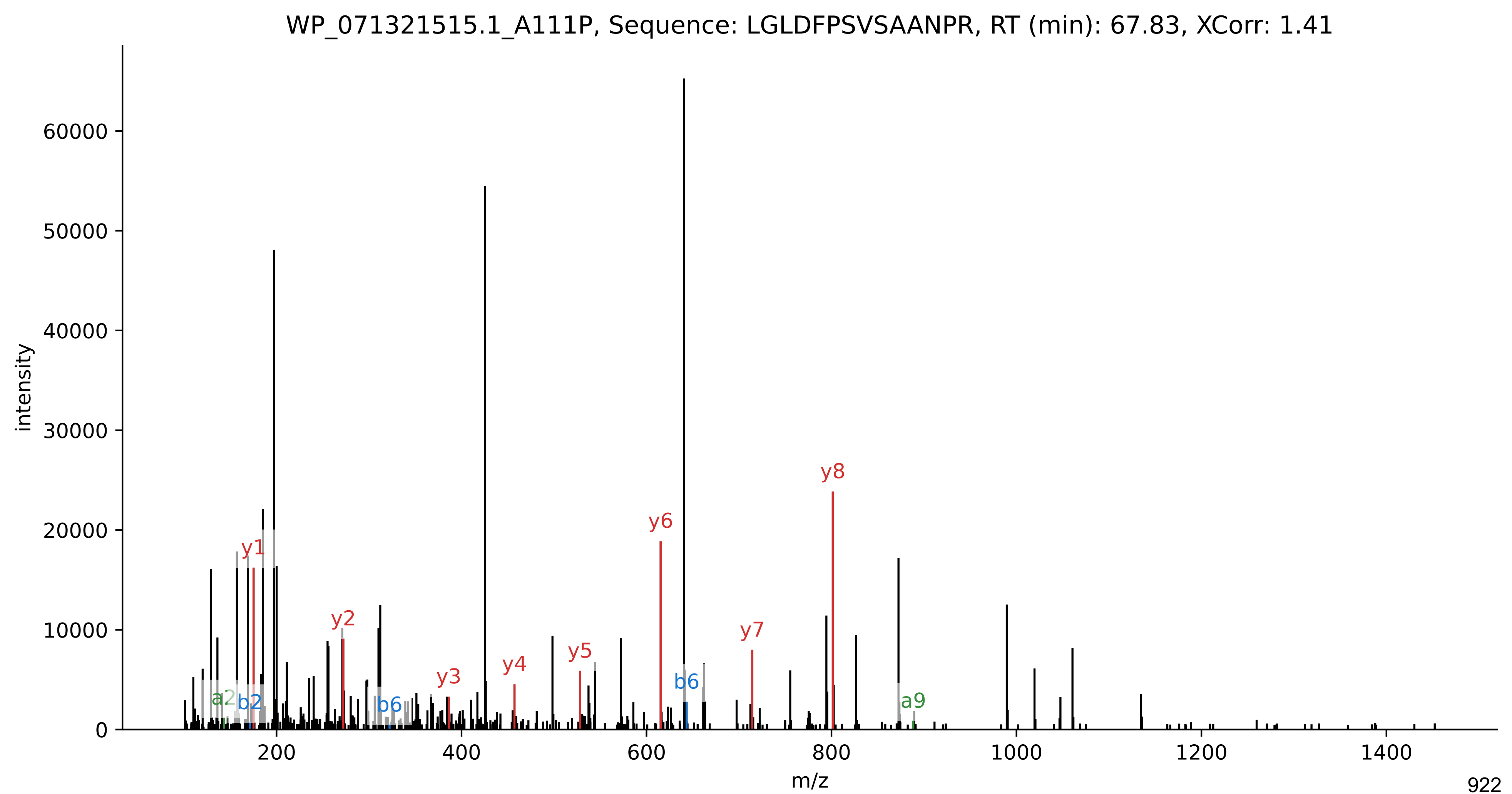

WP_071321515.1_A111P, Sequence: LGLDFPSVSAANPR, RT (min): 67.83, XCorr: 1.41
intensity
m/z
y1
y2
y3
y4
y5
y6
y7
y8
a2
b2
b6
b6
a9

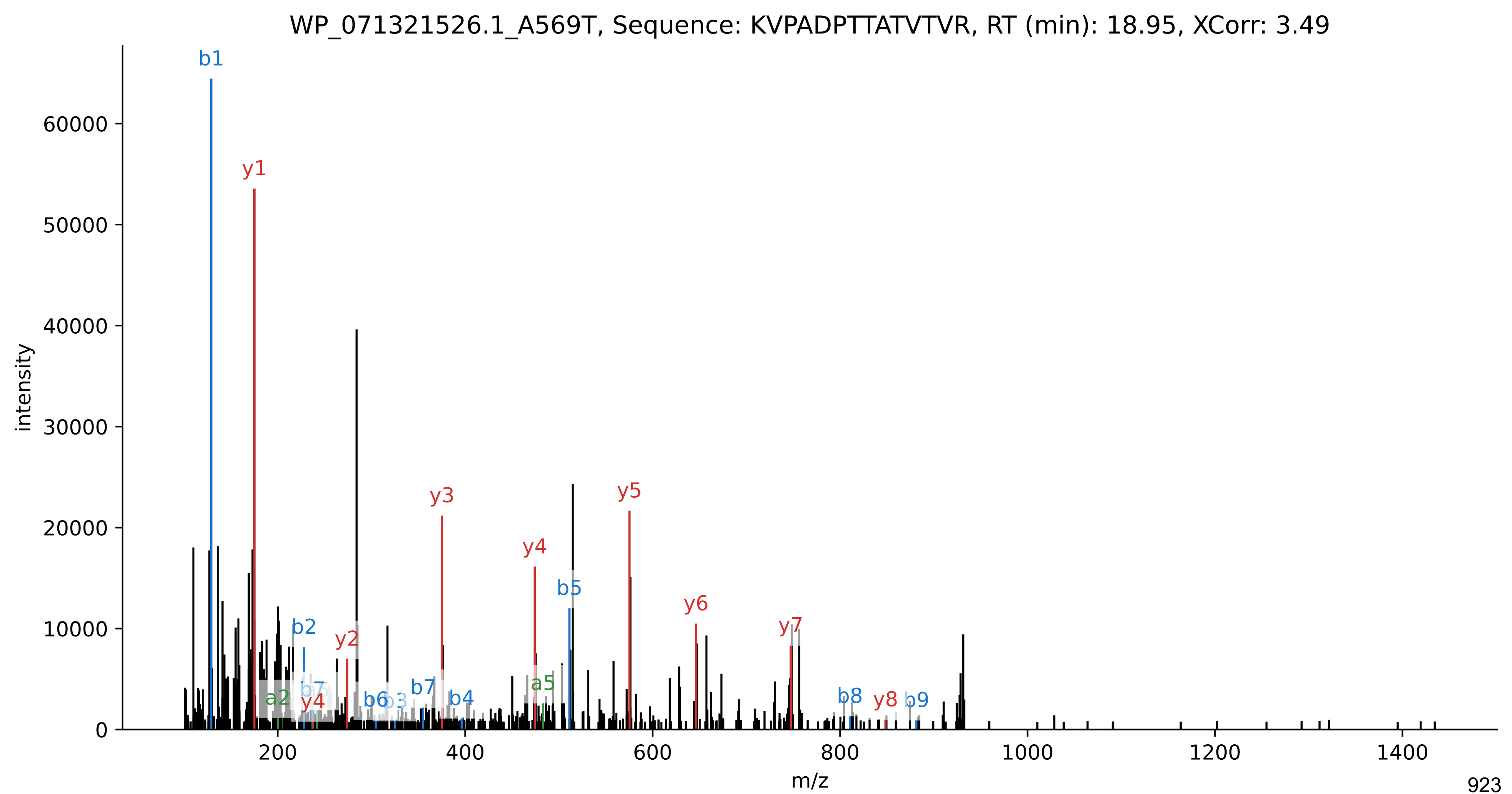

WP_071321526.1_A569T, Sequence: KVPADPTTATVTVR, RT (min): 18.95, XCorr: 3.49
intensity
m/z
b1
y1
y3
y5
y4
b5
y6
y7
b2
y2
a5
b7
b4
b6
a2
y4
b8
y8
b9

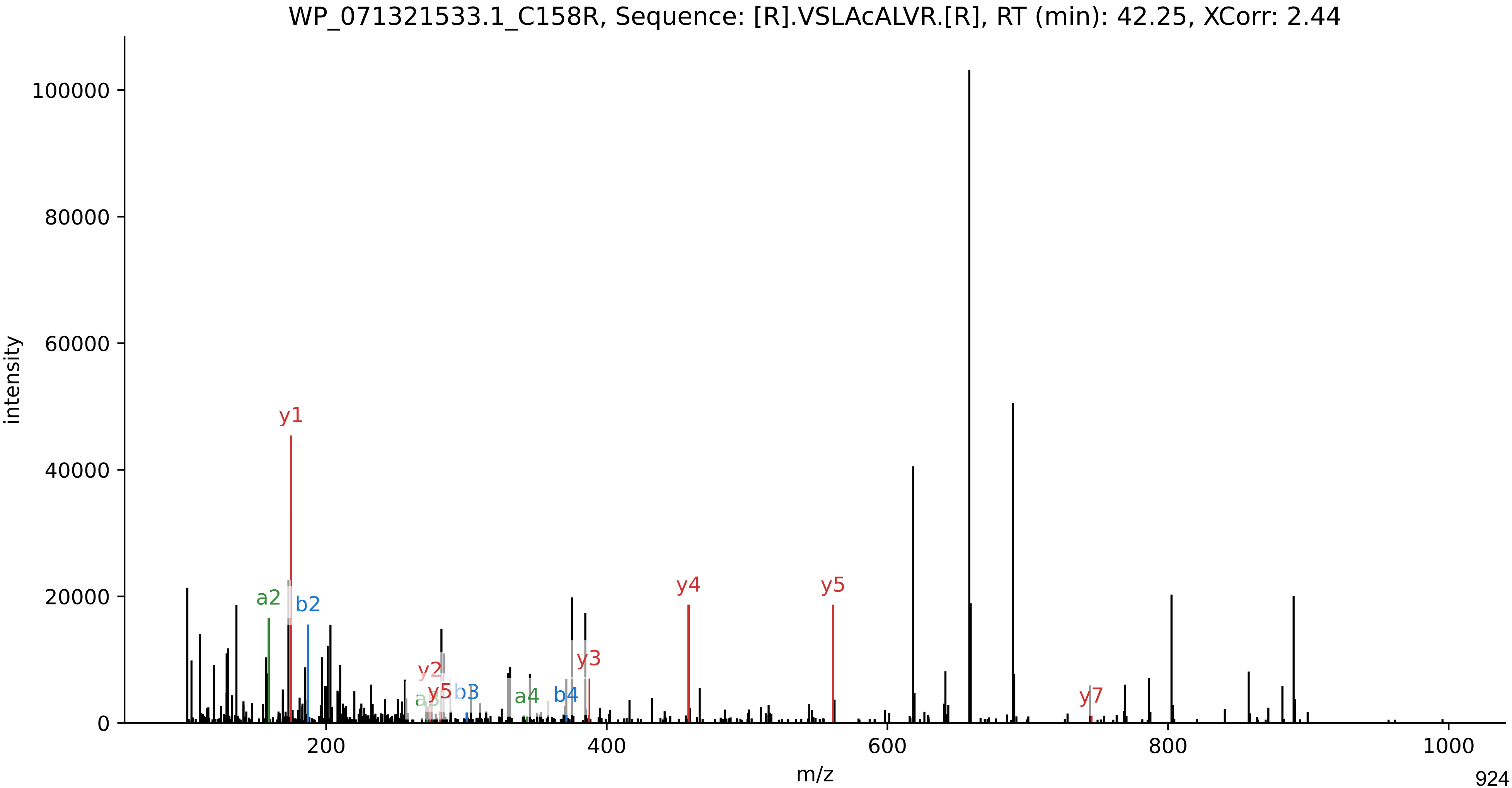
WP_071321533.1_C158R, Sequence: [R].VSLAcALVR.[R], RT (min): 42.25, XCorr: 2.44
intensity
m/z
0
20000
40000
60000
80000
100000
200
400
600
800
1000
y1
a2
b2
y2
y5
b3
a4
b4
y3
y4
y5
y7

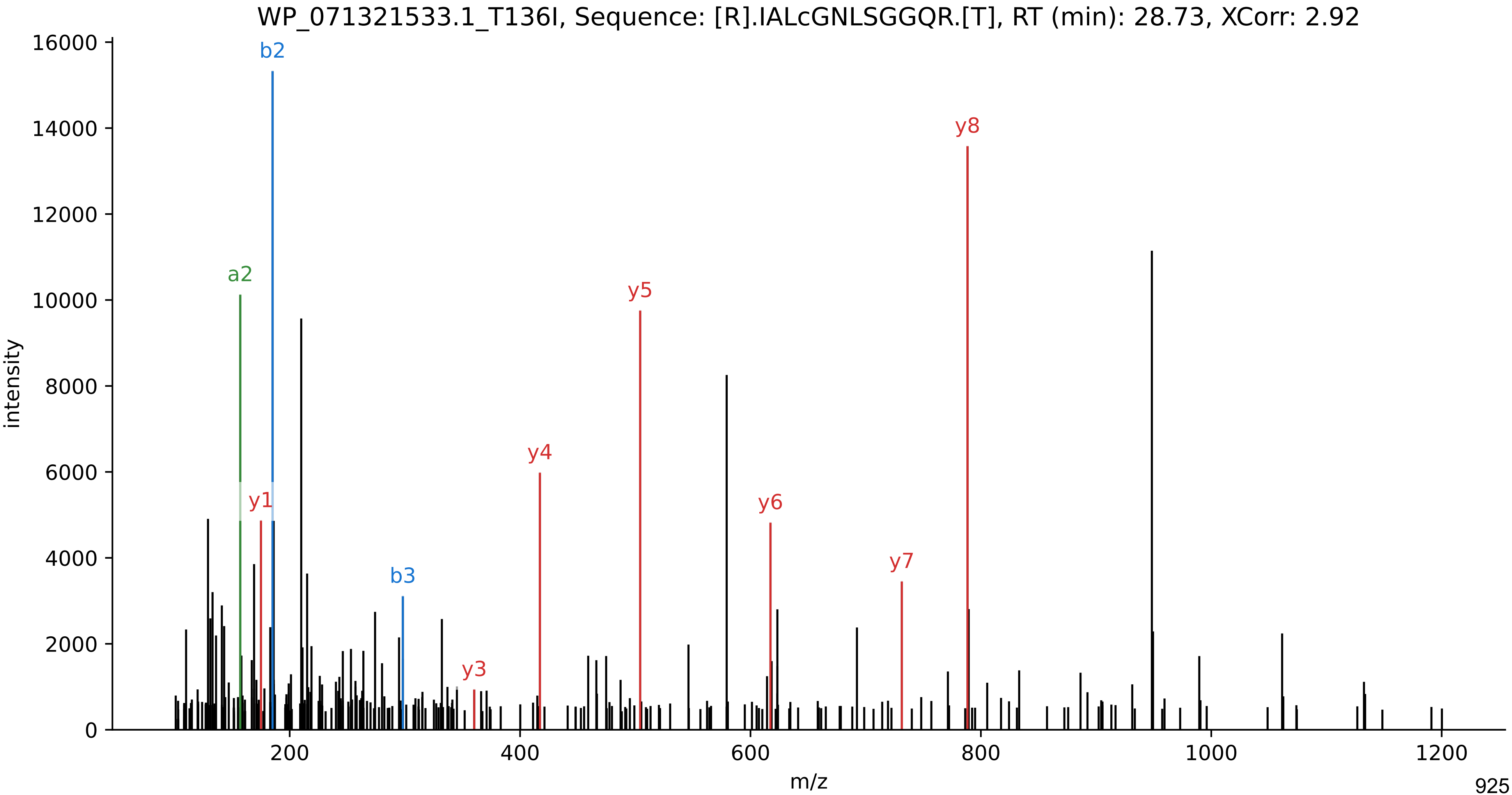
WP_071321533.1_T136I, Sequence: [R].IALcGNLSGGQR.[T], RT (min): 28.73, XCorr: 2.92
intensity
m/z
b2
a2
y1
b3
y3
y4
y5
y6
y7
y8
0
2000
4000
6000
8000
10000
12000
14000
16000
200
400
600
800
1000
1200

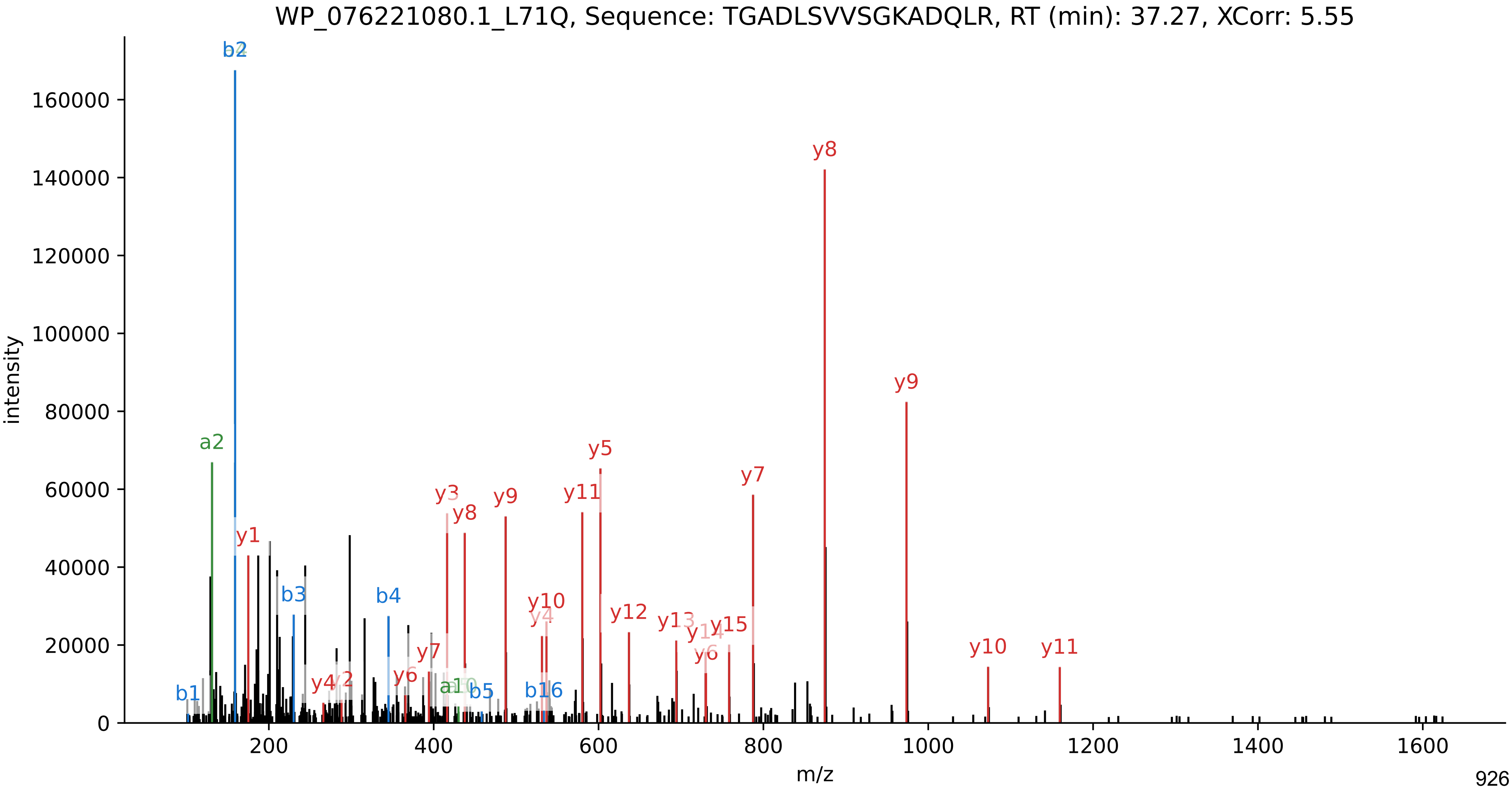
WP_076221080.1_L71Q, Sequence: TGADLSVVSGKADQLR, RT (min): 37.27, XCorr: 5.55
intensity
m/z
b2
a2
y1
b1
b3
b4
y4
y2
y6
y7
y3
y8
y9
a10
b5
y10
y4
b16
y11
y5
y12
y13
y14
y6
y15
y7
y8
y9
y10
y11

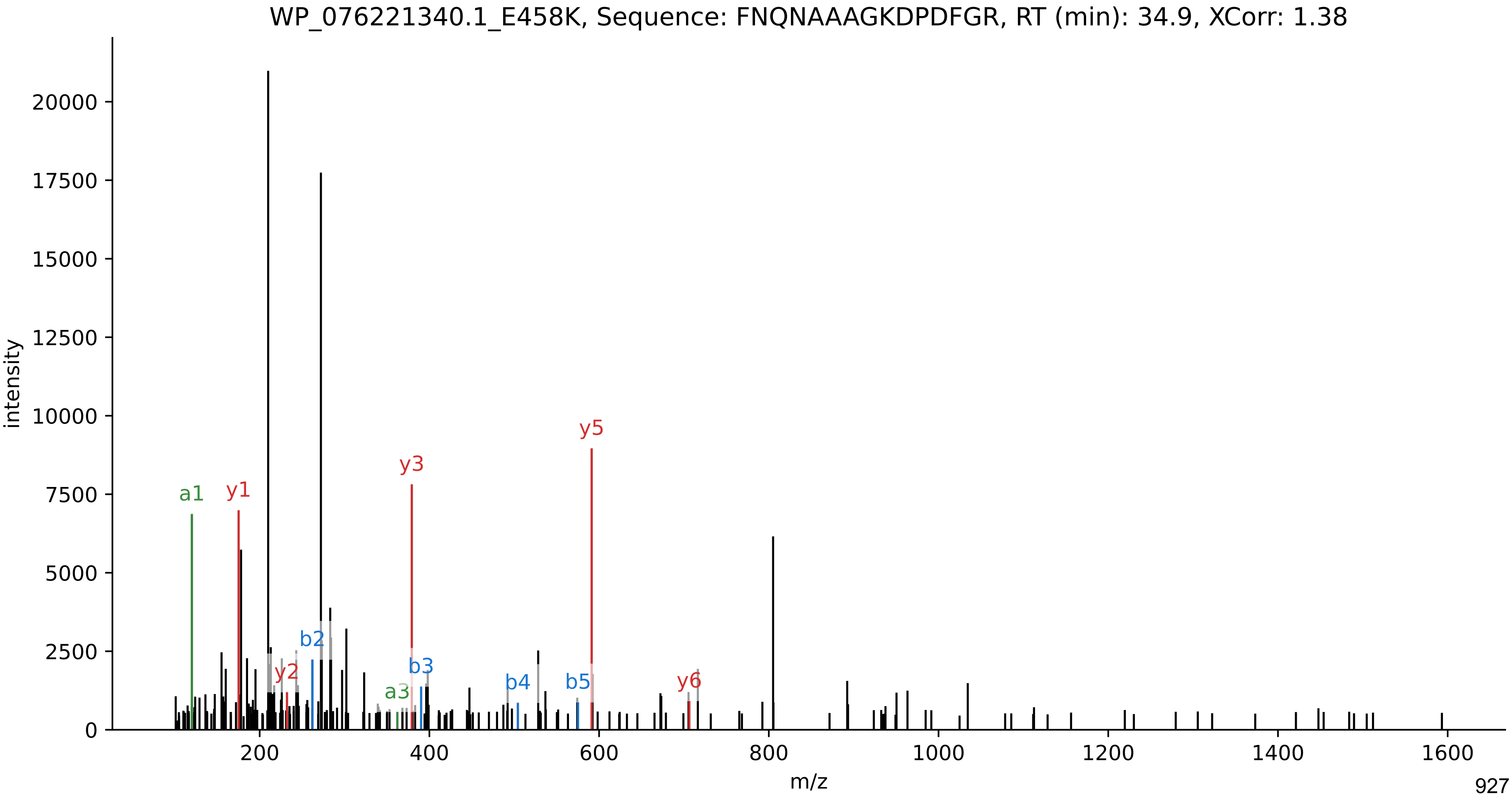

WP_076221340.1_E458K, Sequence: FNQNAAAGKDPDFGR, RT (min): 34.9, XCorr: 1.38
intensity
m/z
a1
y1
y2
b2
y3
b3
a3
b4
b5
y5
y6
0
2500
5000
7500
10000
12500
15000
17500
20000
200
400
600
800
1000
1200
1400
1600

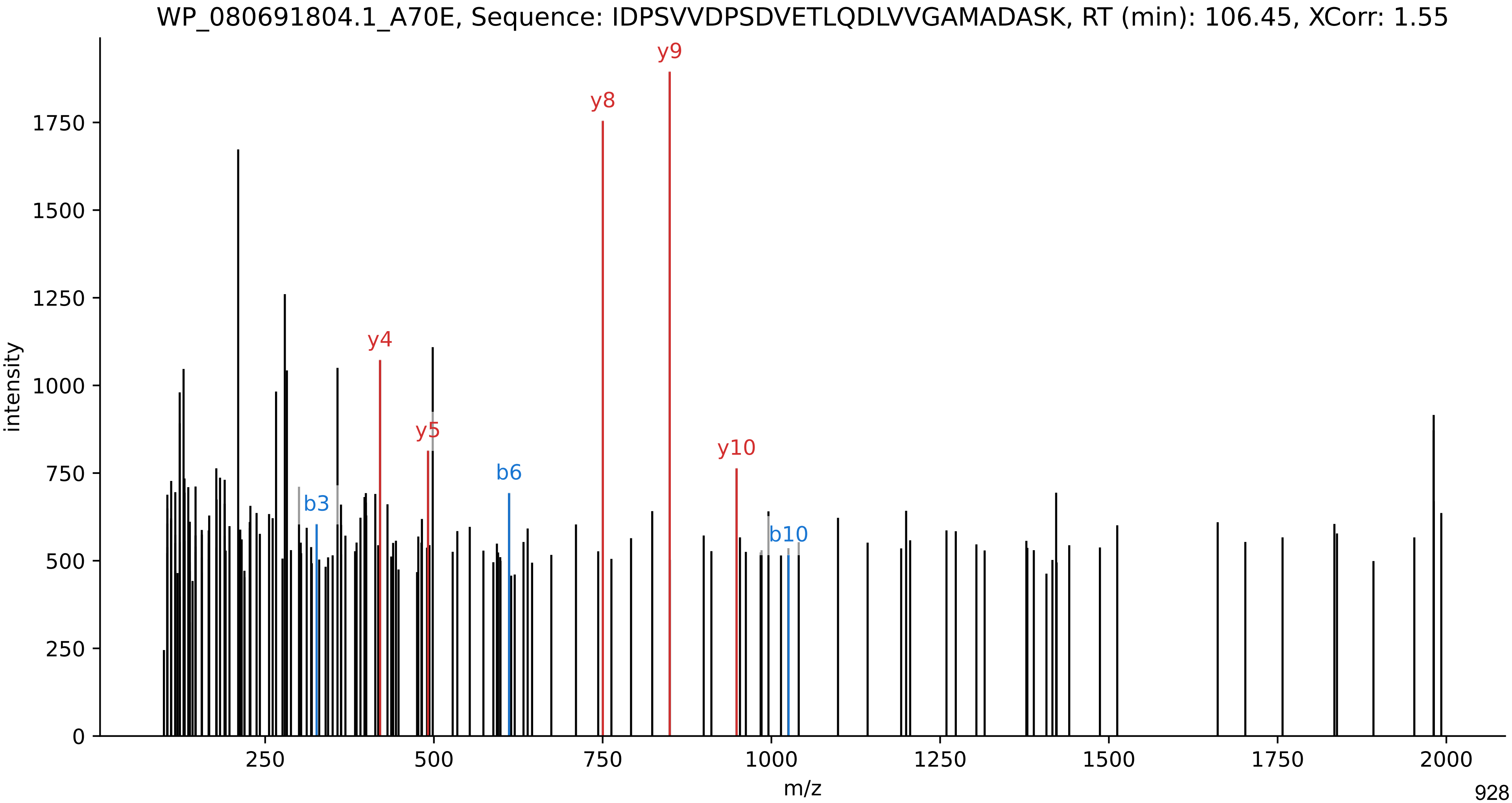
WP_080691804.1_A70E, Sequence: IDPSVVDPSDVETLQDLVVGAMADASK, RT (min): 106.45, XCorr: 1.55
intensity
m/z
y9
y8
y4
y5
y10
b6
b3
b10
0
250
500
750
1000
1250
1500
1750
2000

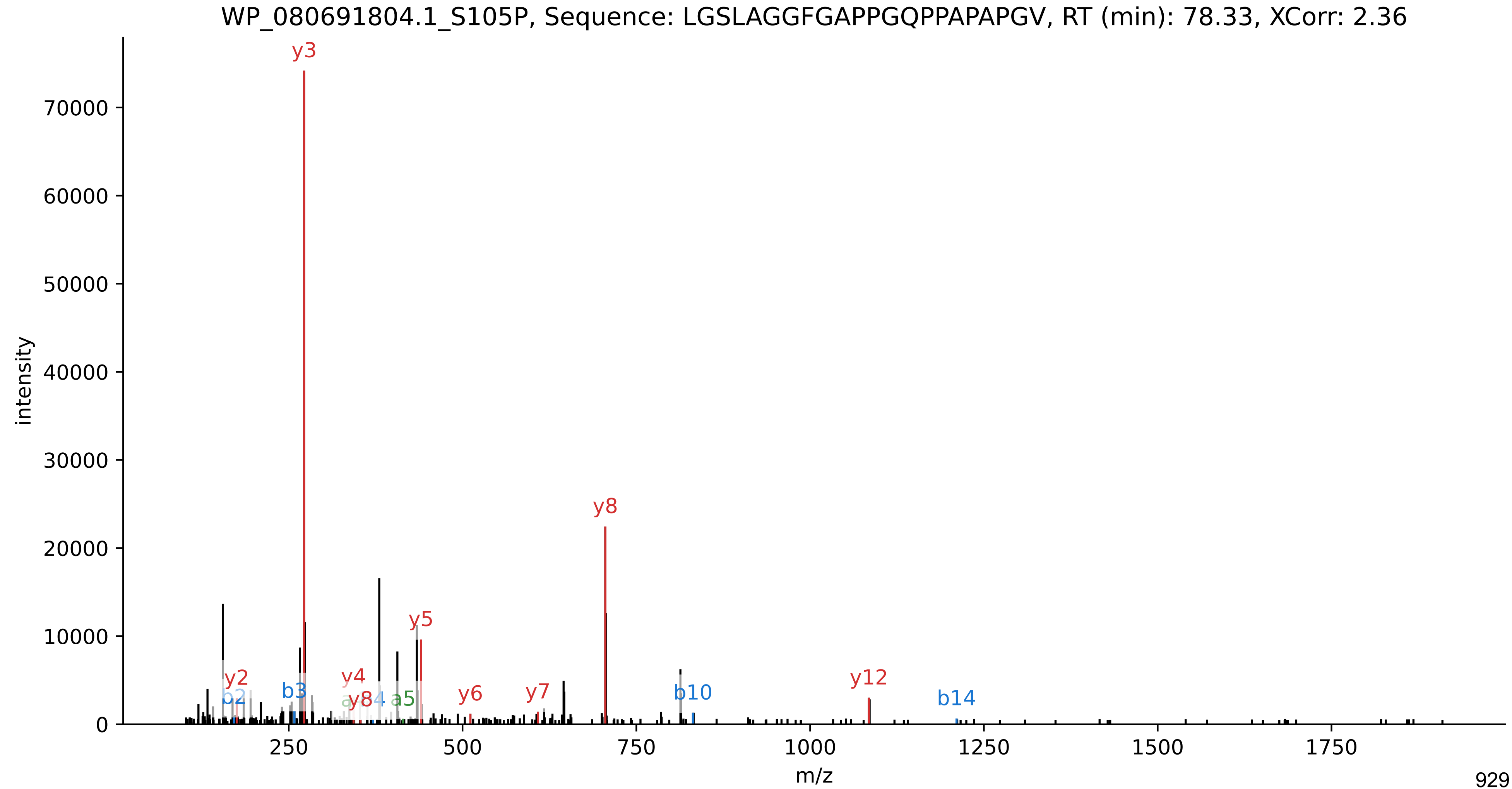
WP_080691804.1_S105P, Sequence: LGSLAGGFGAPPGQPPAPAPGV, RT (min): 78.33, XCorr: 2.36
intensity
m/z
y3
y8
y5
y2
b2
b3
y4
y8
a5
y6
y7
b10
y12
b14
70000
60000
50000
40000
30000
20000
10000
0
250
500
750
1000
1250
1500
1750

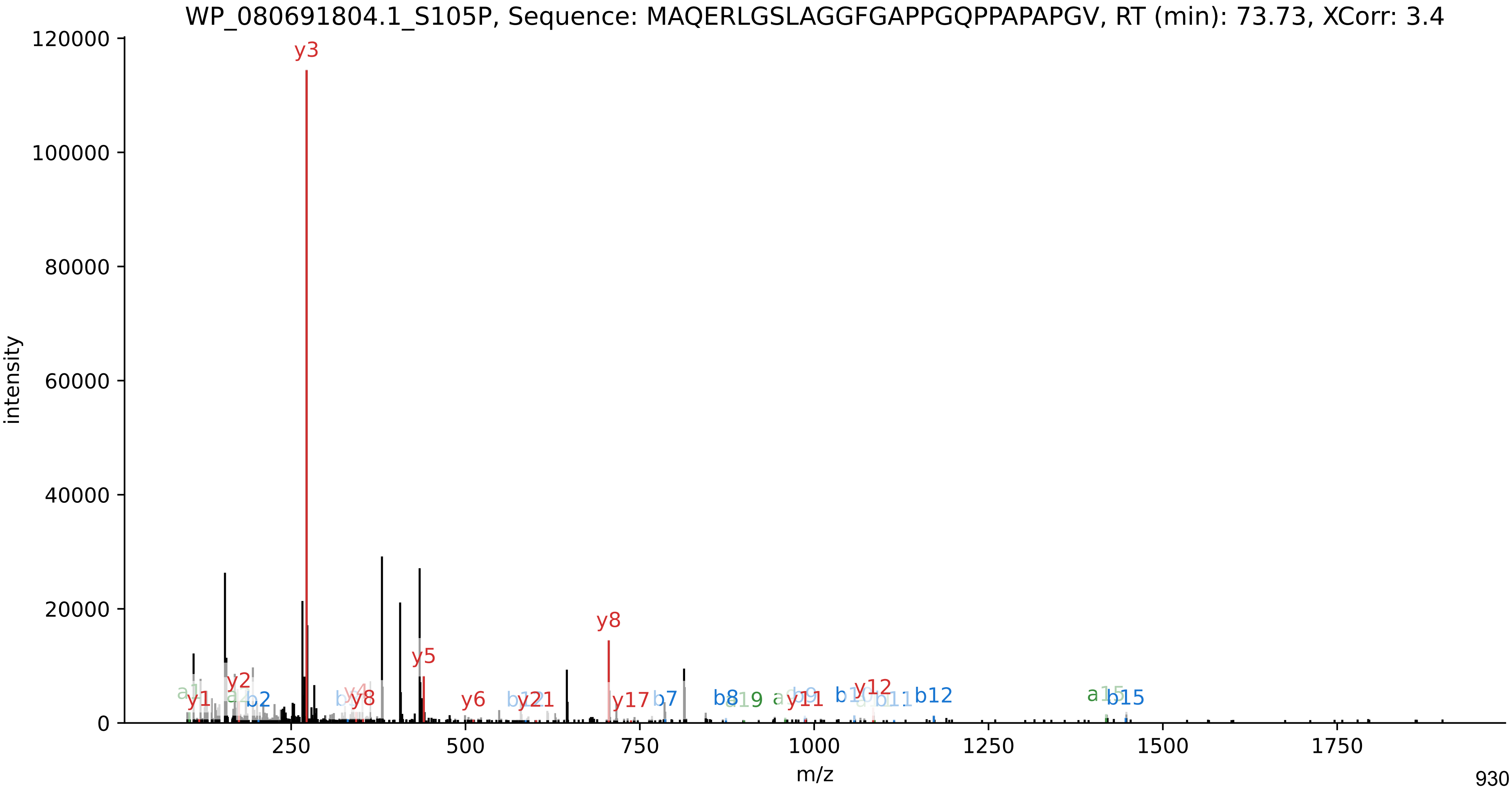

WP_080691804.1_S105P, Sequence: MAQERLGSLAGGFGAPPGQPPAPAPGV, RT (min): 73.73, XCorr: 3.4
intensity
m/z
y3
y8
y5
y2
y1
b2
y8
y6
y21
y17
b7
b8
y11
y12
b12
a15
b15

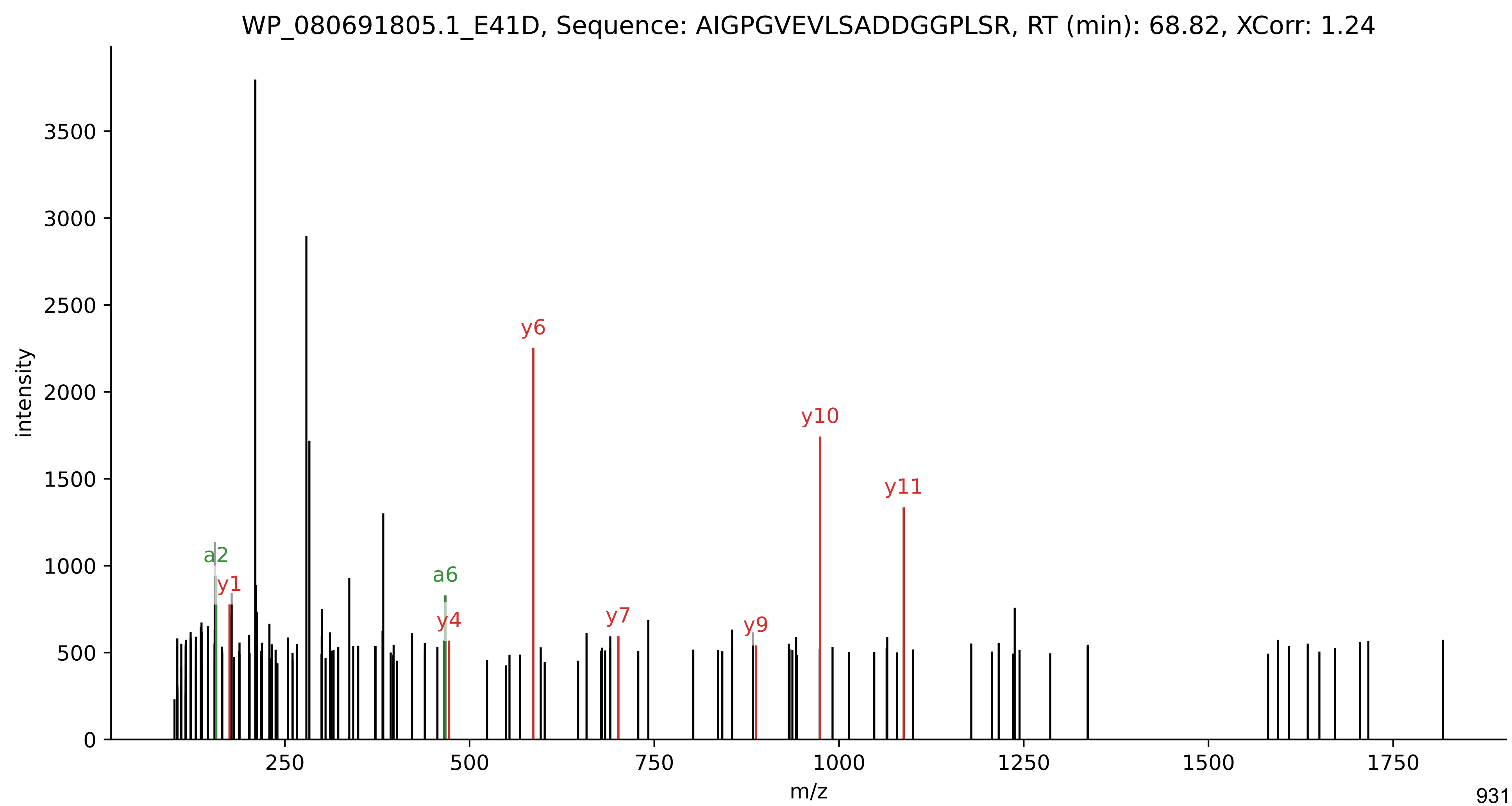
WP_080691805.1_E41D, Sequence: AIGPGVEVLSADDGGPLSR, RT (min): 68.82, XCorr: 1.24
intensity
m/z
a2
y1
a6
y4
y6
y7
y9
y10
y11
0
500
1000
1500
2000
2500
3000
3500
250
500
750
1000
1250
1500
1750

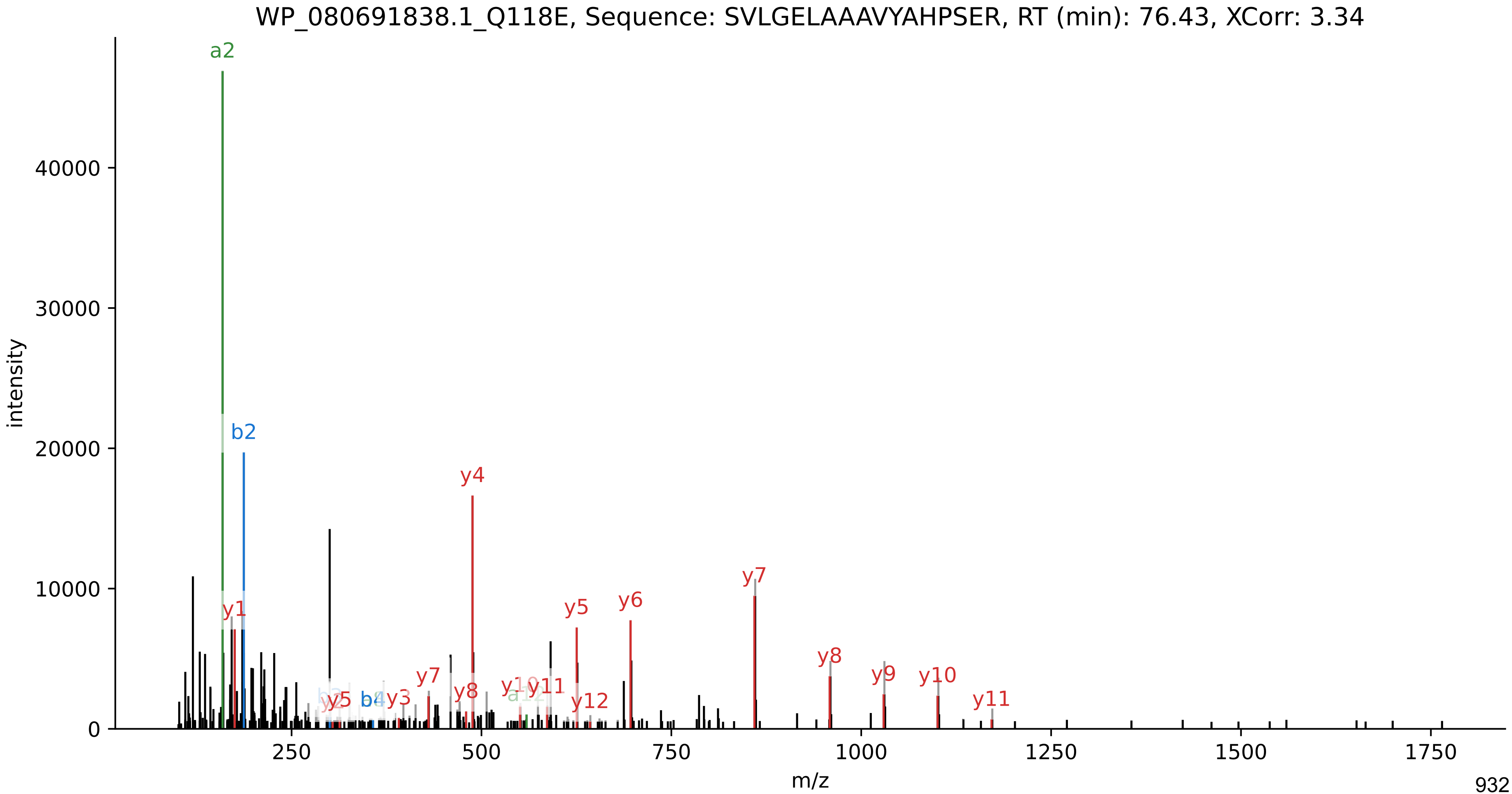
WP_080691838.1_Q118E, Sequence: SVLGELAAAVYAHPSER, RT (min): 76.43, XCorr: 3.34
intensity
m/z
0
10000
20000
30000
40000
250
500
750
1000
1250
1500
1750
a2
b2
y1
y4
y5
y6
y7
y8
y9
y10
y11
y12
y3
b4

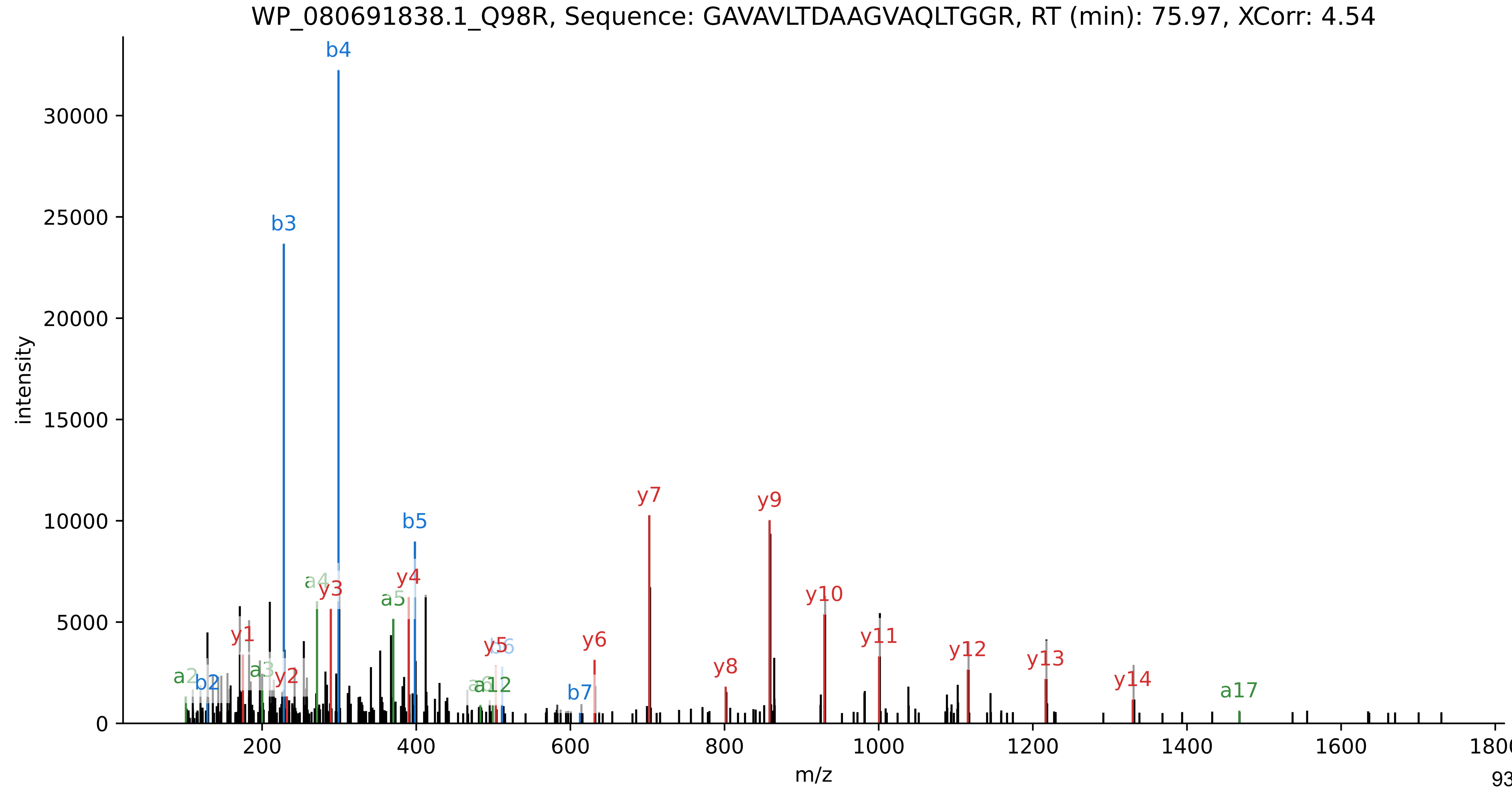
WP_080691838.1_Q98R, Sequence: GAVAVLTDAAGVAQLTGGR, RT (min): 75.97, XCorr: 4.54
intensity
m/z
b4
b3
b5
b2
b7
a2
a3
a4
a5
a12
a17
y1
y2
y3
y4
y5
y6
y7
y8
y9
y10
y11
y12
y13
y14
0
5000
10000
15000
20000
25000
30000
200
400
600
800
1000
1200
1400
1600
1800

WP_080708759.1_T227A, Sequence: RLDADVPPPSAAIDGVPAQFDDFVQR, RT (min): 86.6, XCorr: 5.17

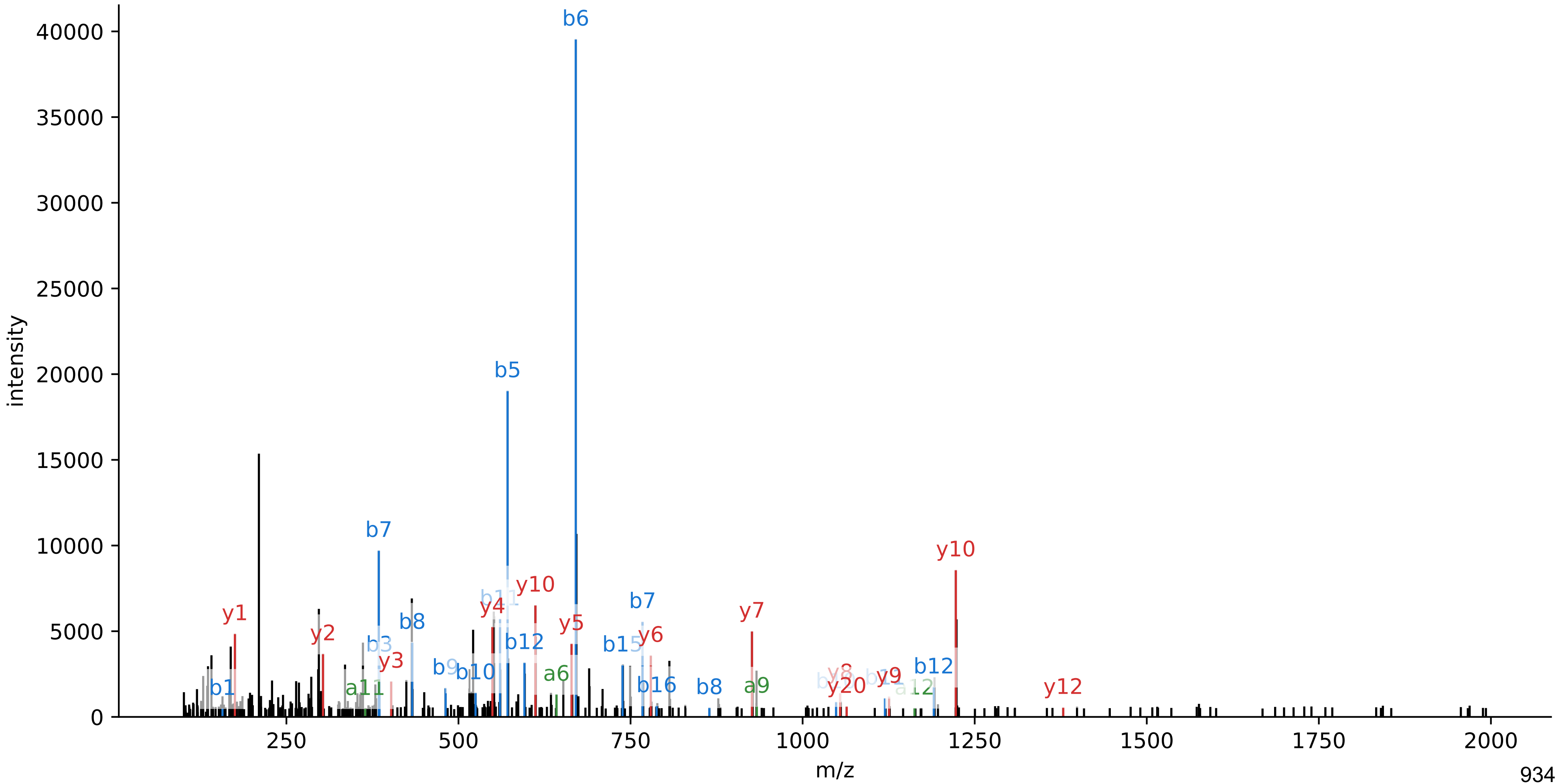

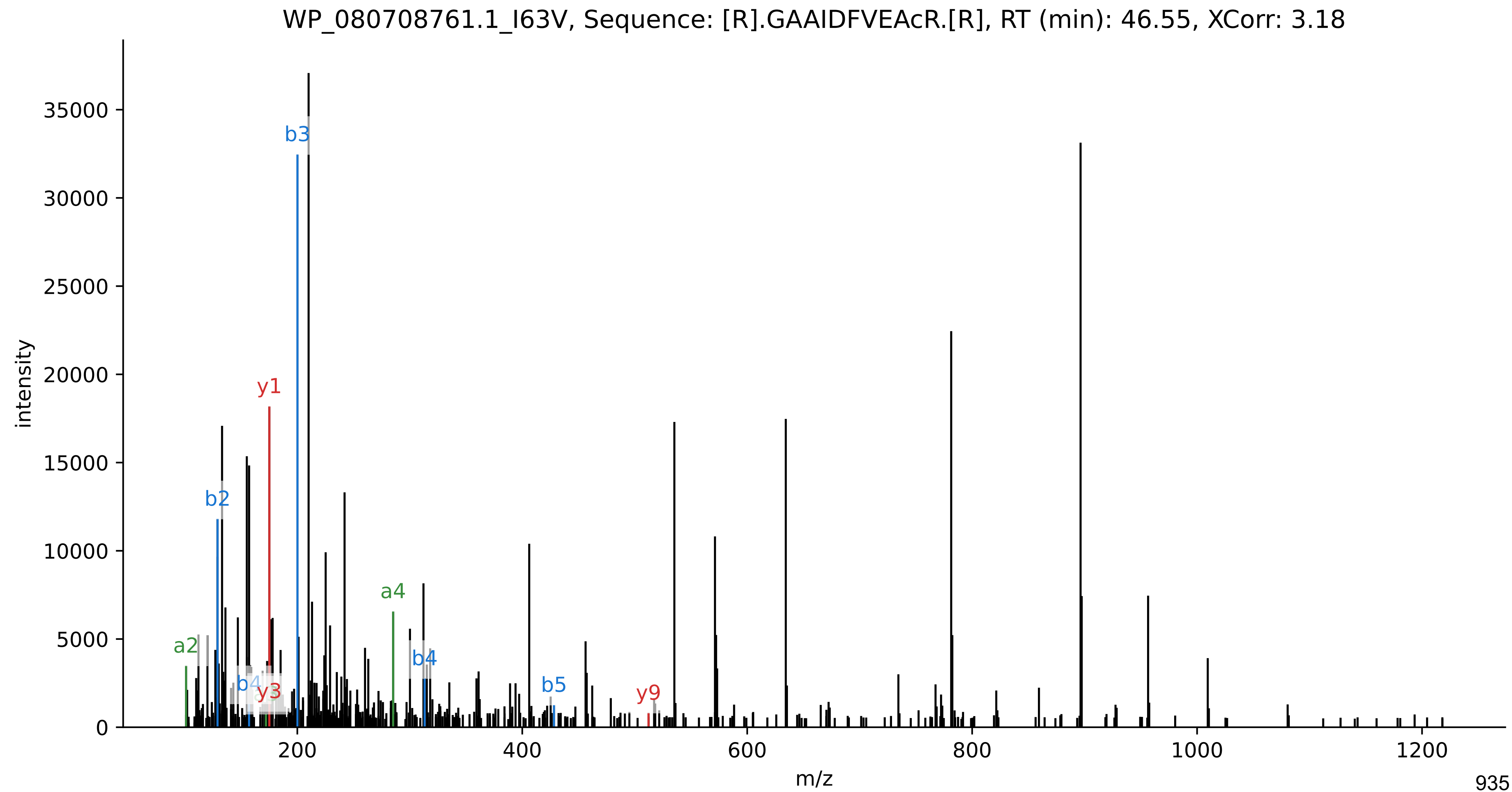

WP_080708761.1_I63V, Sequence: [R].GAAIDFVEAcR.[R], RT (min): 46.55, XCorr: 3.18
intensity
m/z
35000
30000
25000
20000
15000
10000
5000
0
200
400
600
800
1000
1200
b3
y1
b2
a4
a2
b4
b4
y3
b5
y9

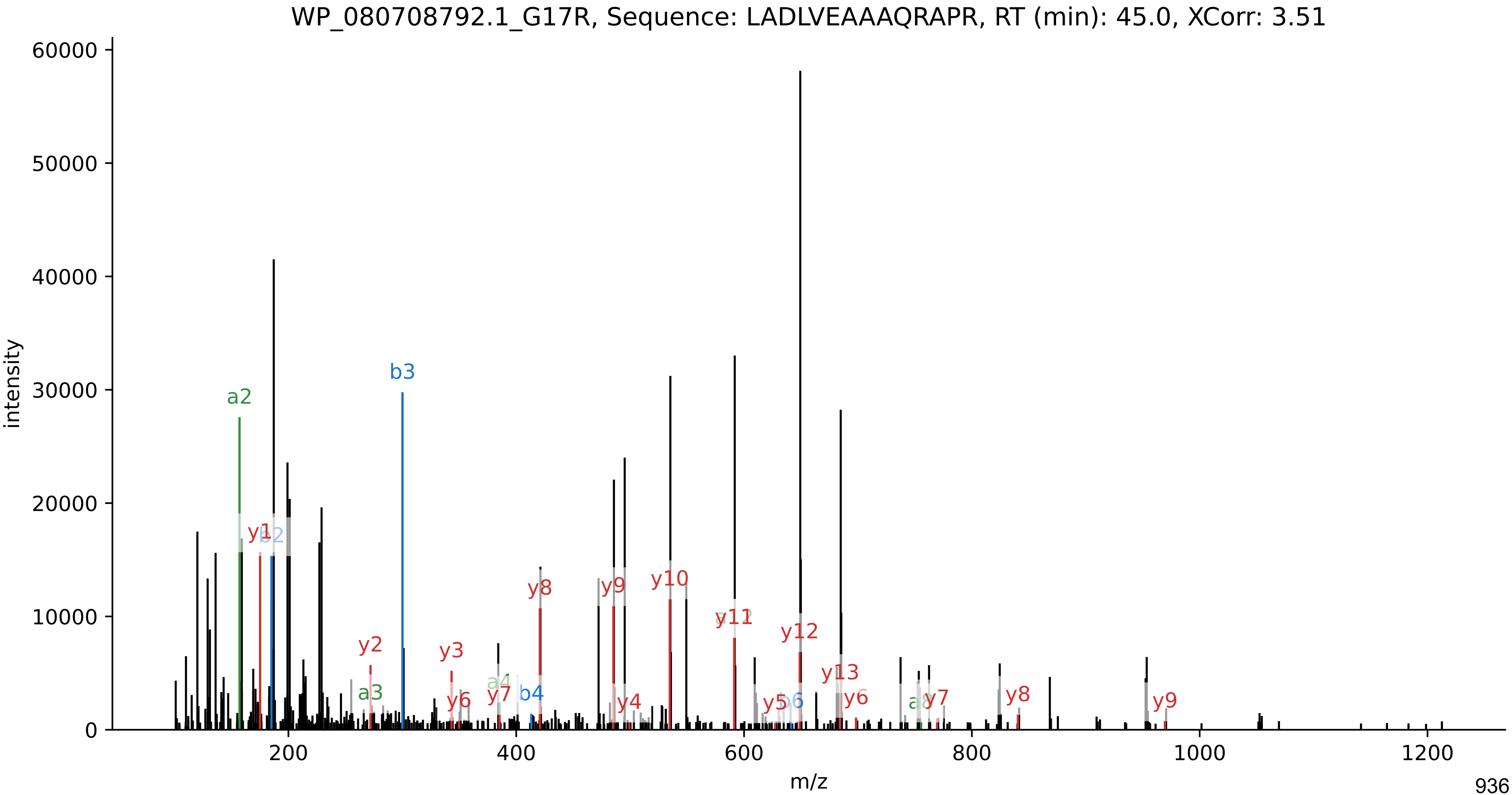
WP_080708792.1_G17R, Sequence: LADLVEAAAQRAPR, RT (min): 45.0, XCorr: 3.51
intensity
m/z
a2
b3
y1
b2
y2
a3
y3
y6
a4
y7
b4
y8
y9
y4
y10
y11
y12
y5
b6
y13
y6
a8
y7
y8
y9
0
10000
20000
30000
40000
50000
60000
200
400
600
800
1000
1200

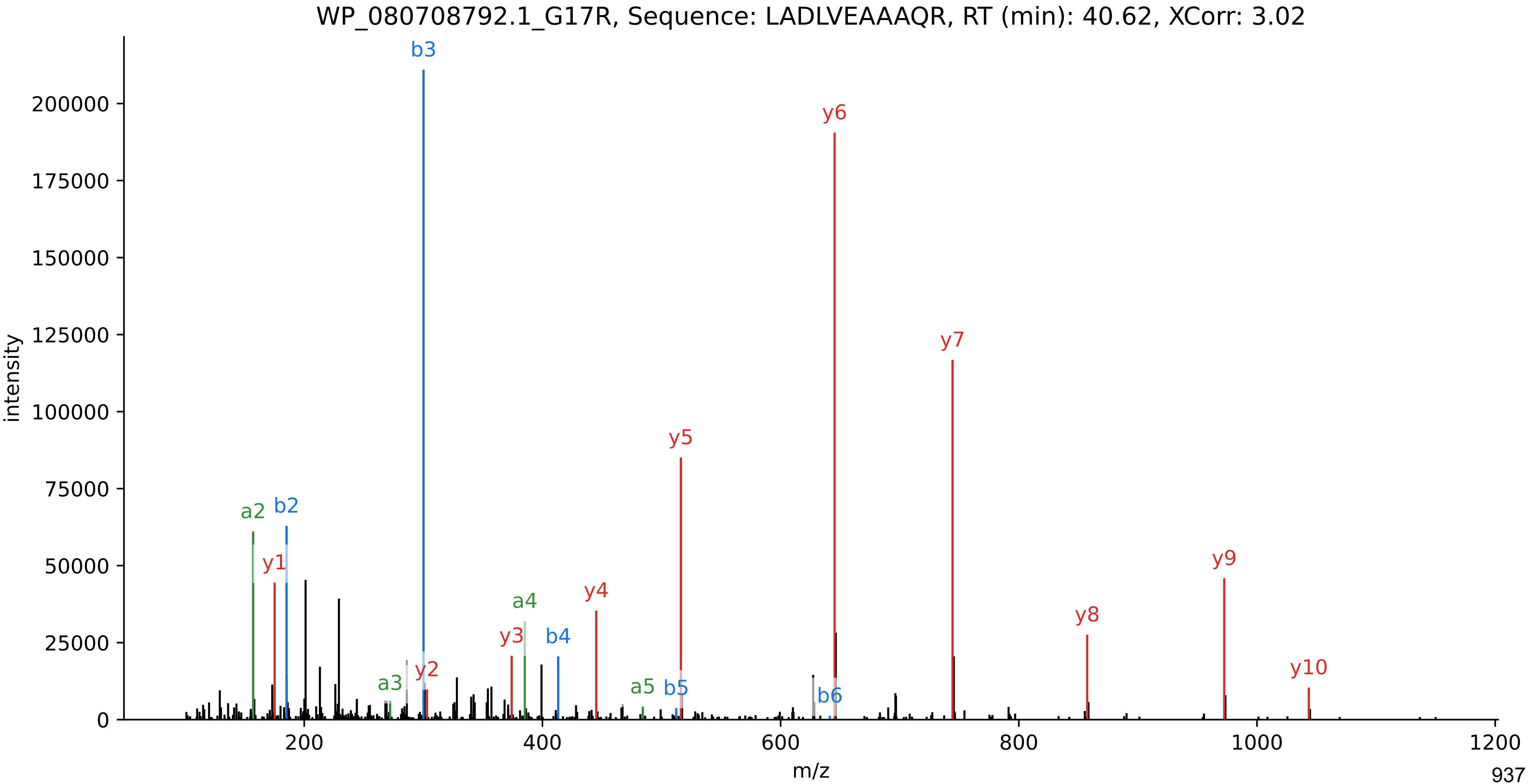
WP_080708792.1_G17R, Sequence: LADLVEAAAQR, RT (min): 40.62, XCorr: 3.02
intensity
m/z
b3
y6
y7
y5
a2
b2
y1
y9
a4
y4
y8
y3
b4
a3
y2
y10
a5
b5
b6
0
25000
50000
75000
100000
125000
150000
175000
200000
200
400
600
800
1000
1200

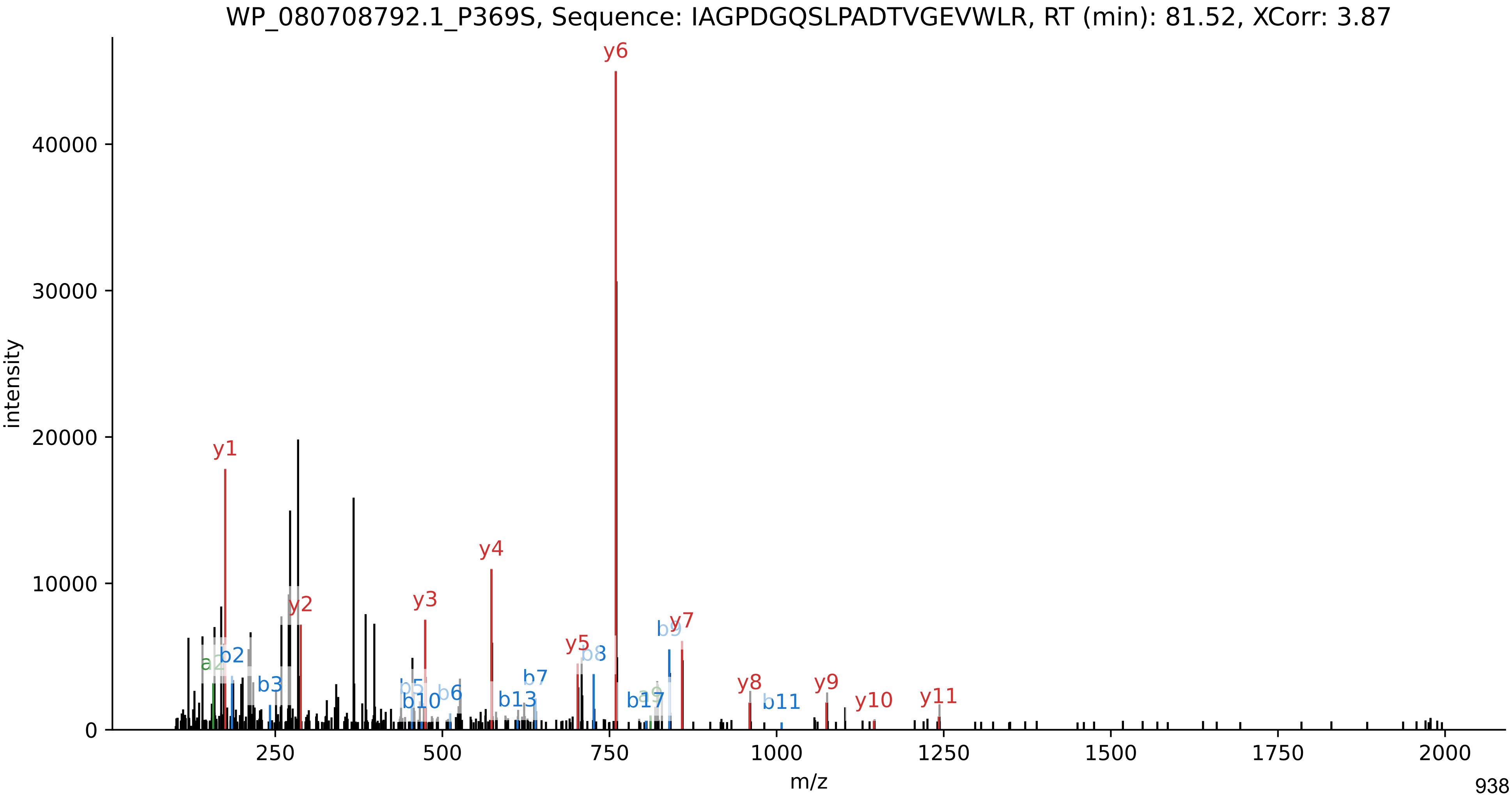

WP_080708792.1_P369S, Sequence: IAGPDGQSLPADTVGEVWLR, RT (min): 81.52, XCorr: 3.87
intensity
m/z
y6
y1
y2
y3
y4
y5
y7
y8
y9
y10
y11
b2
b3
b5
b6
b7
b8
b9
b10
b11
b13
b17
a2
40000
30000
20000
10000
0
250
500
750
1000
1250
1500
1750
2000

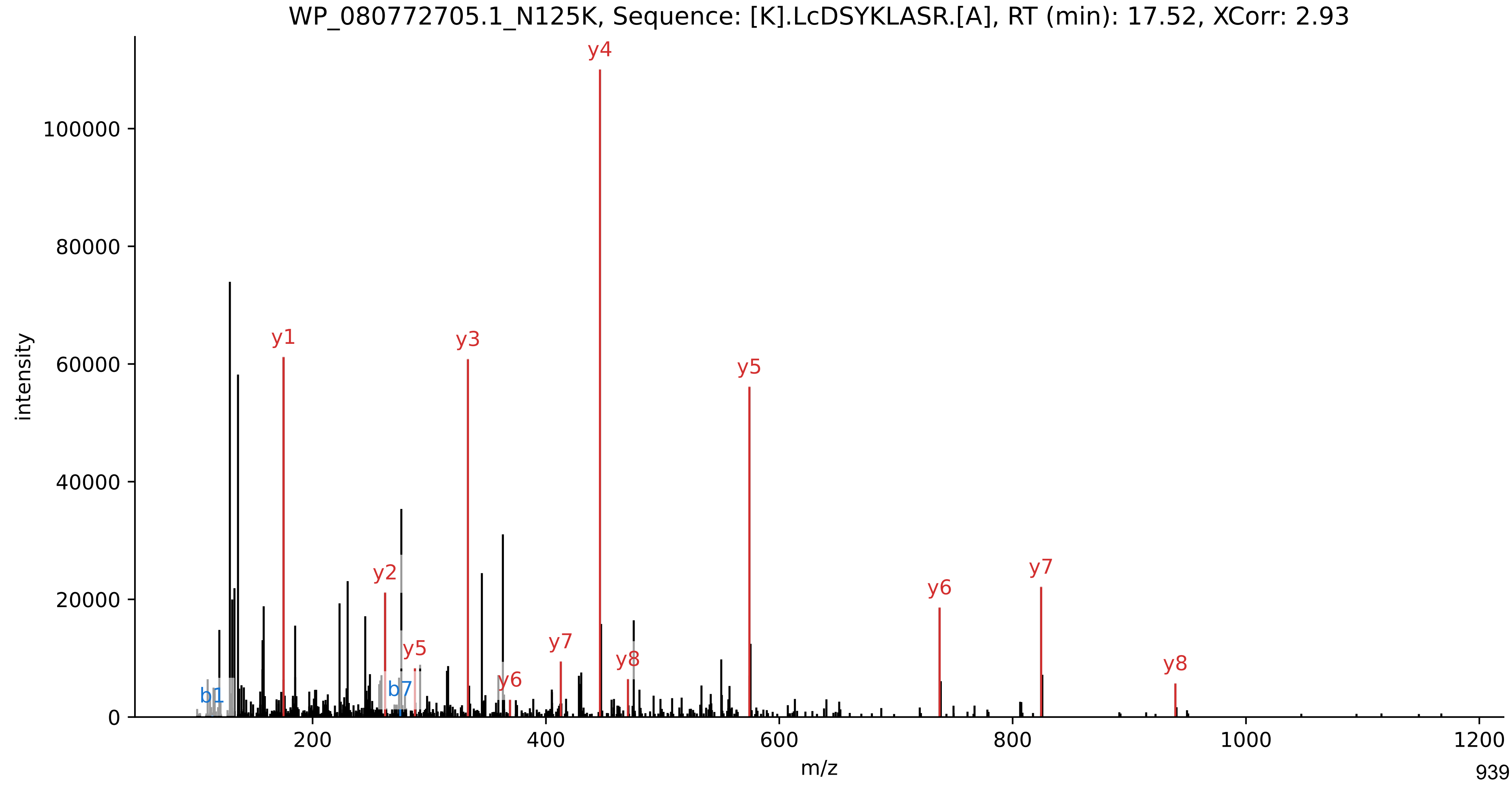
WP_080772705.1_N125K, Sequence: [K].LcDSYKLASR.[A], RT (min): 17.52, XCorr: 2.93
intensity
m/z
0
20000
40000
60000
80000
100000
200
400
600
800
1000
1200
y1
y2
y3
y4
y5
y6
y7
y8
y5
y6
y7
y8
b1
b7

WP_080772799.1_V276I, Sequence: MREIFTQR, RT (min): 17.5, XCorr: 2.36

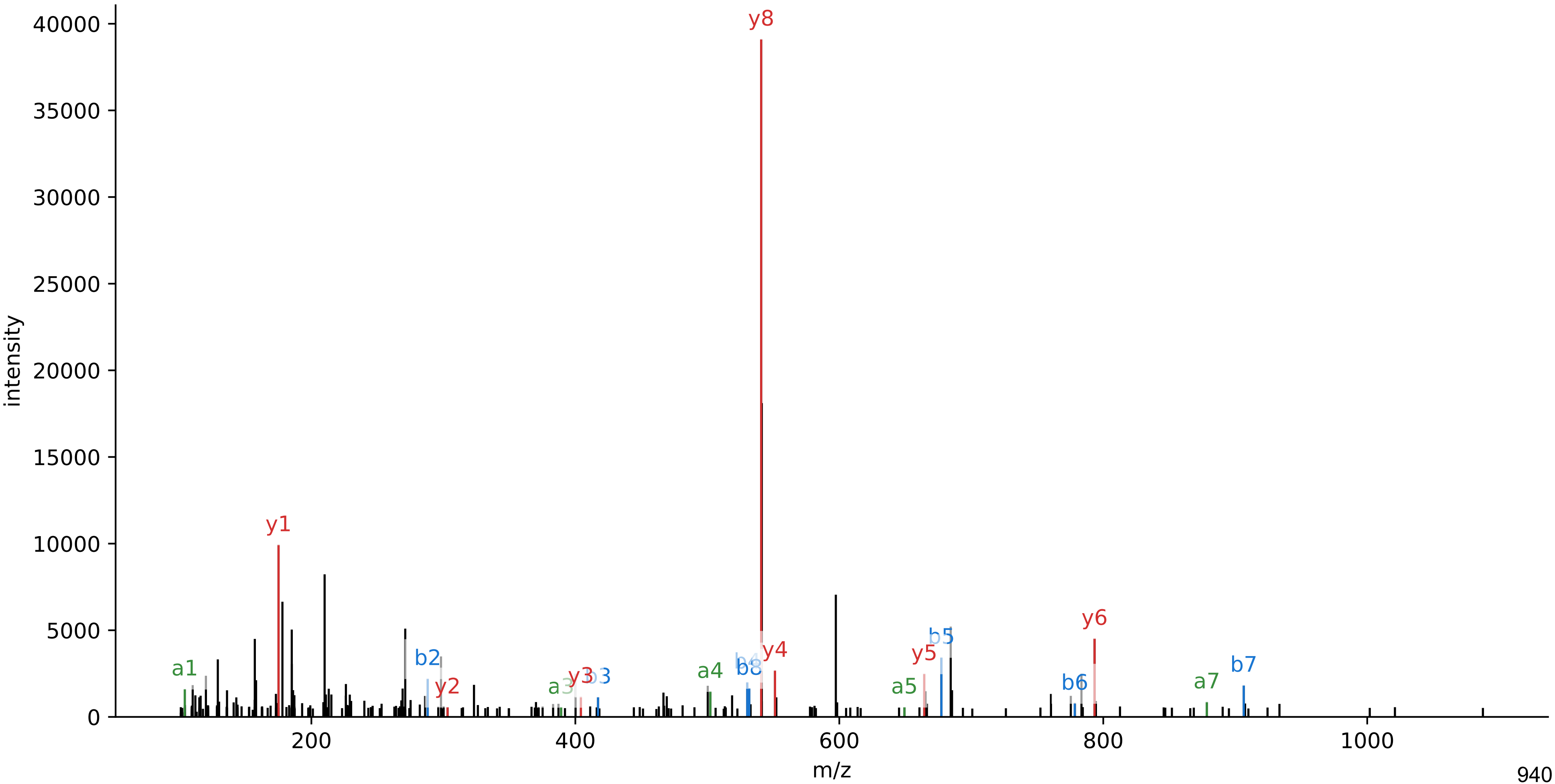

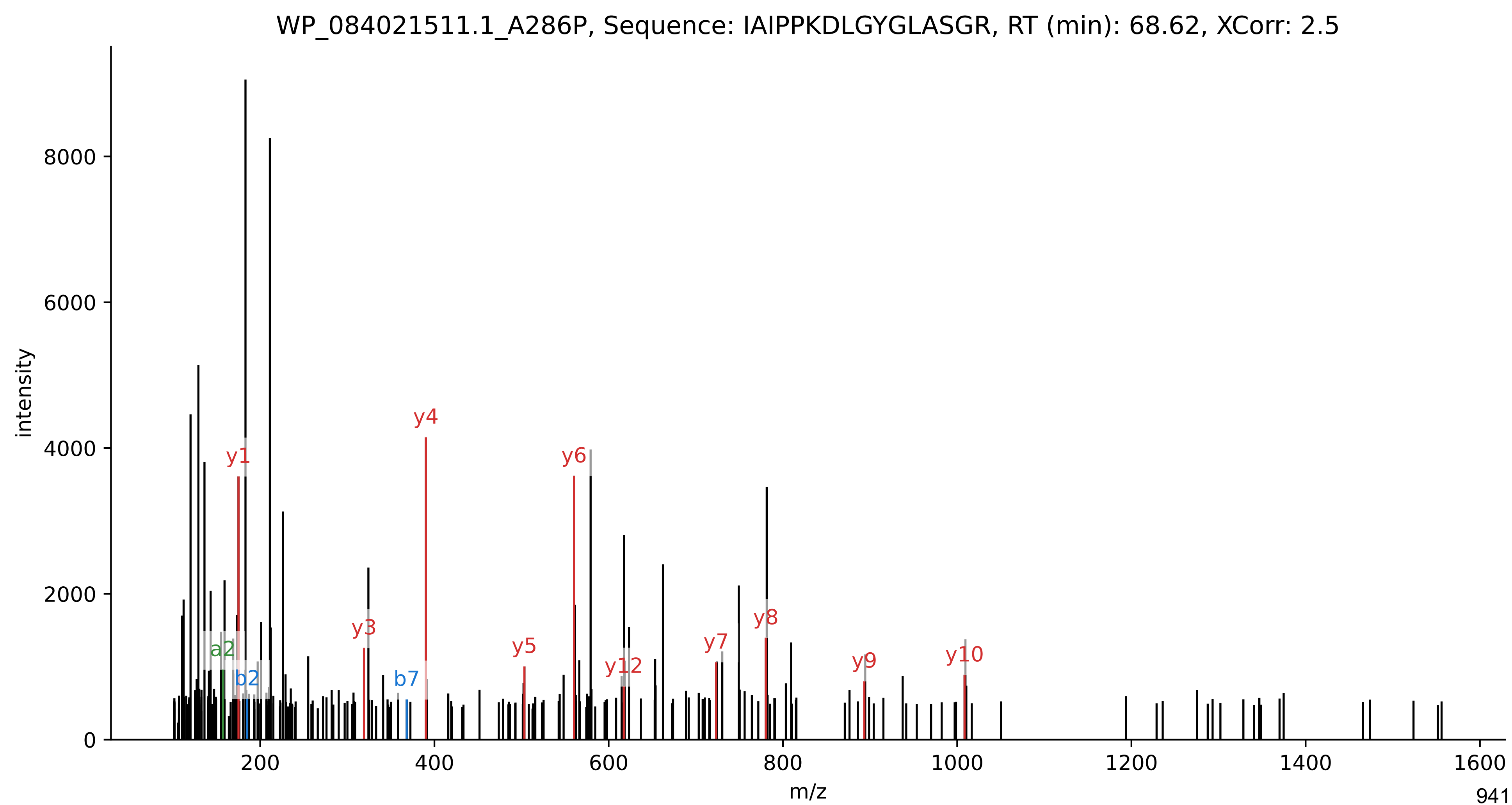
WP_084021511.1_A286P, Sequence: IAIPPKDLGYGLASGR, RT (min): 68.62, XCorr: 2.5
intensity
m/z
0
2000
4000
6000
8000
200
400
600
800
1000
1200
1400
1600
y1
y3
y4
y5
y6
y7
y8
y9
y10
y12
a2
b2
b7

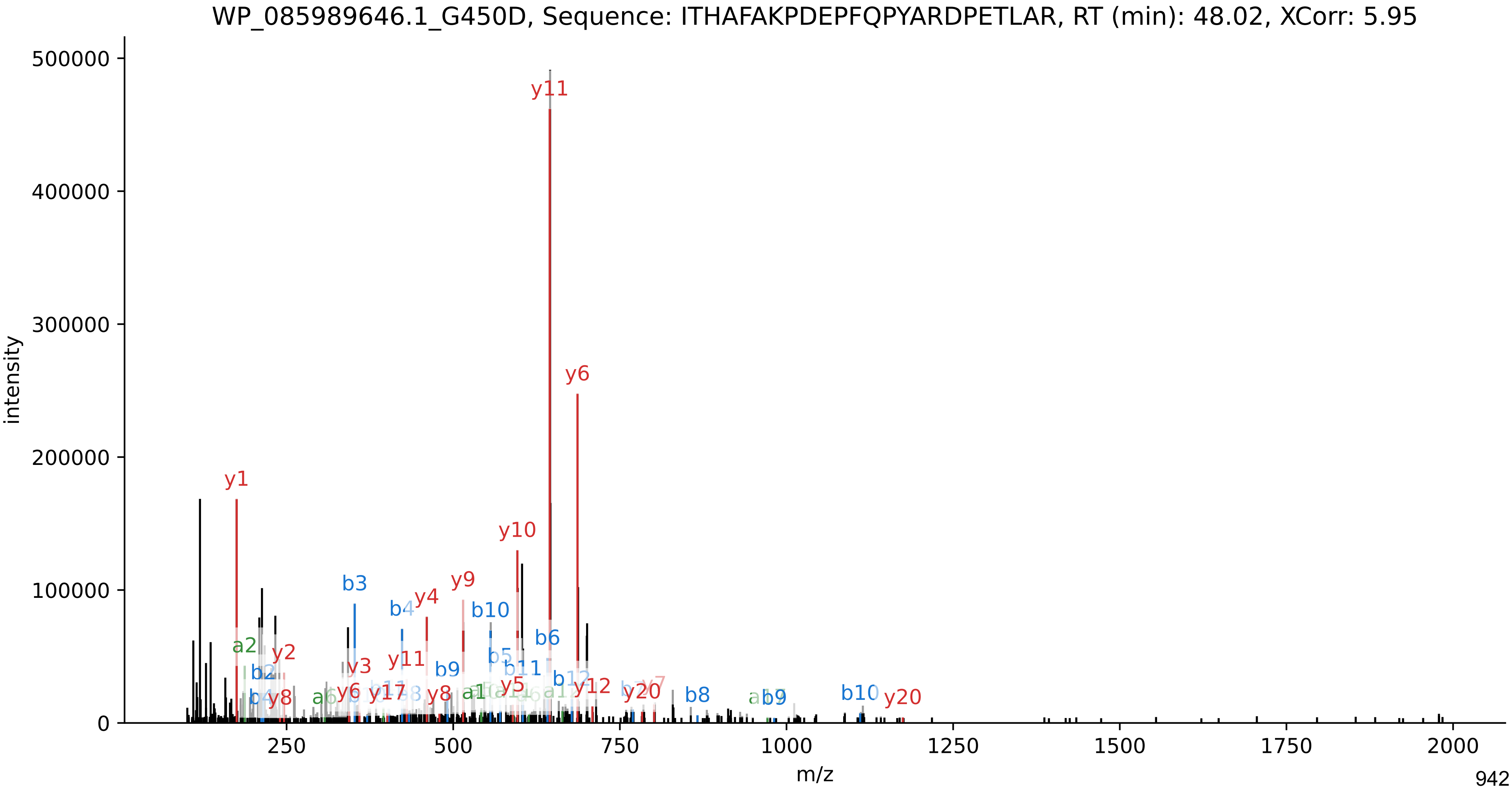
WP_085989646.1_G450D, Sequence: ITHAFAKPDEPFQPYARDPETLAR, RT (min): 48.02, XCorr: 5.95
intensity
m/z
y11
y6
y1
y10
y9
y4
b3
b4
b10
b6
a2
y2
b2
y3
y11
b9
b5
b11
b12
y12
y5
y6
y17
y8
y8
a1
y20
b8
b9
b10
y20
a6
b4
0
100000
200000
300000
400000
500000
250
500
750
1000
1250
1500
1750
2000

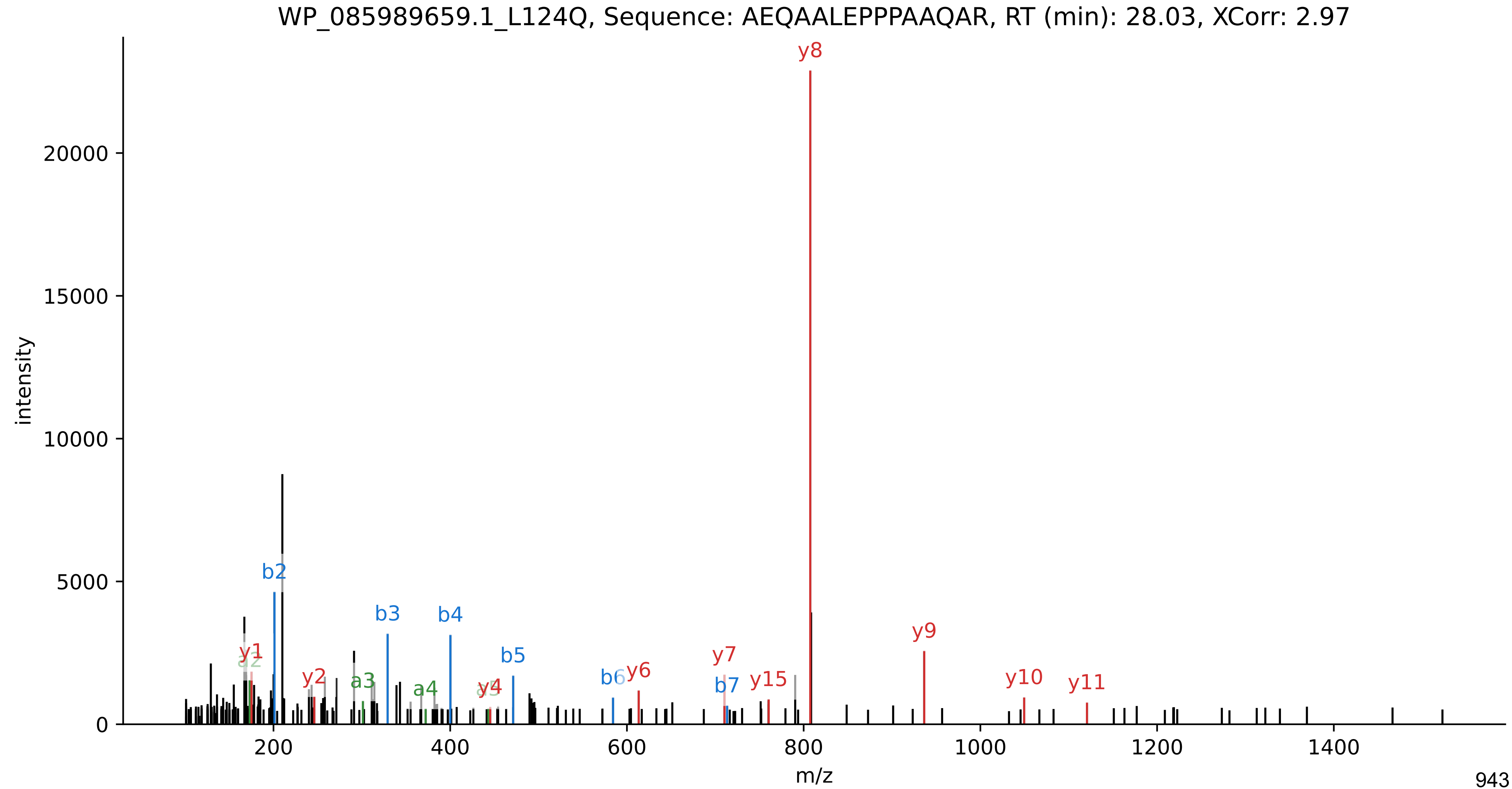

WP_085989659.1_L124Q, Sequence: AEQAALEPPPAAQAR, RT (min): 28.03, XCorr: 2.97
intensity
m/z
y8
b2
b3
b4
b5
b6
b7
y1
a2
y2
a3
a4
y4
a5
y6
y7
y15
y9
y10
y11
0
5000
10000
15000
20000
200
400
600
800
1000
1200
1400

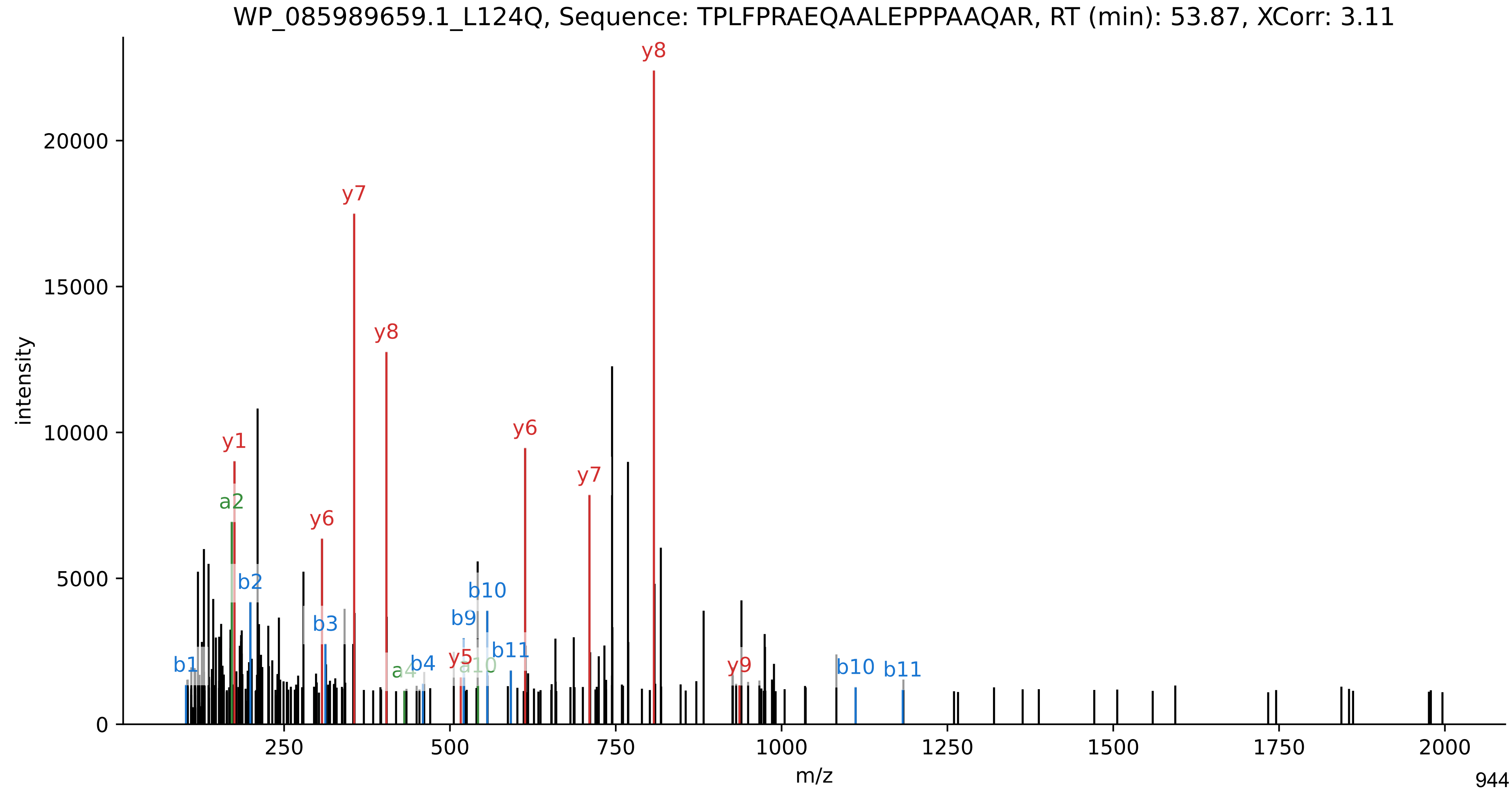

WP_085989659.1_L124Q, Sequence: TPLFPRAEQAALEPPPAAQAR, RT (min): 53.87, XCorr: 3.11
intensity
m/z
y8
y7
y8
y1
a2
y6
b2
b3
b1
y6
y7
b10
b9
b11
y5
a10
a4
b4
y9
b10
b11
0
5000
10000
15000
20000
250
500
750
1000
1250
1500
1750
2000

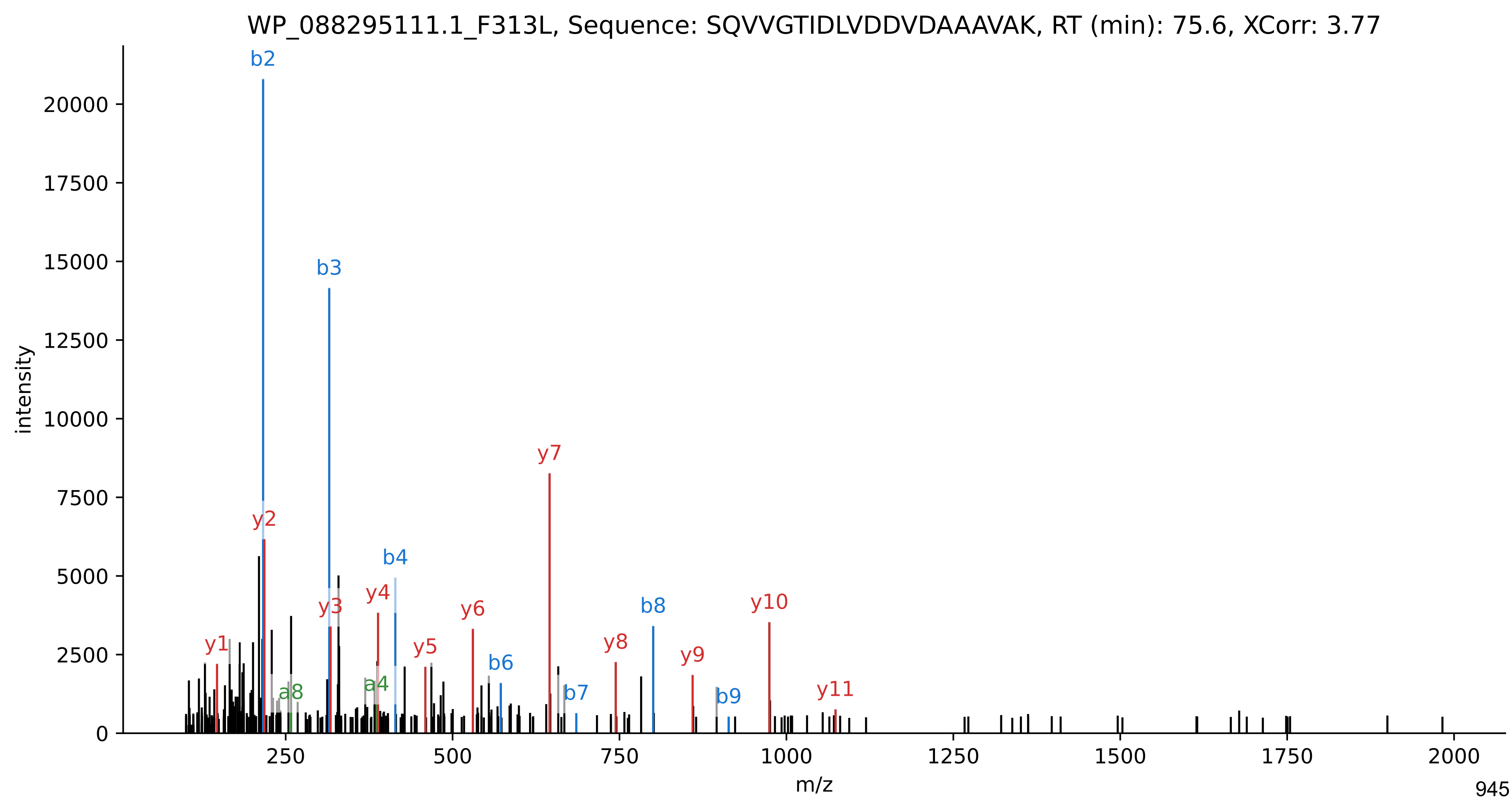

WP_088295111.1_F313L, Sequence: SQVVGTIDLVDDVDAAAVAK, RT (min): 75.6, XCorr: 3.77
intensity
m/z
b2
b3
b4
b6
b7
b8
b9
y1
y2
y3
y4
y5
y6
y7
y8
y9
y10
y11
a8
a4

WP_095762097.1_A571T, Sequence: ATDPIGADPPEAPPR, RT (min): 35.72, XCorr: 3.02

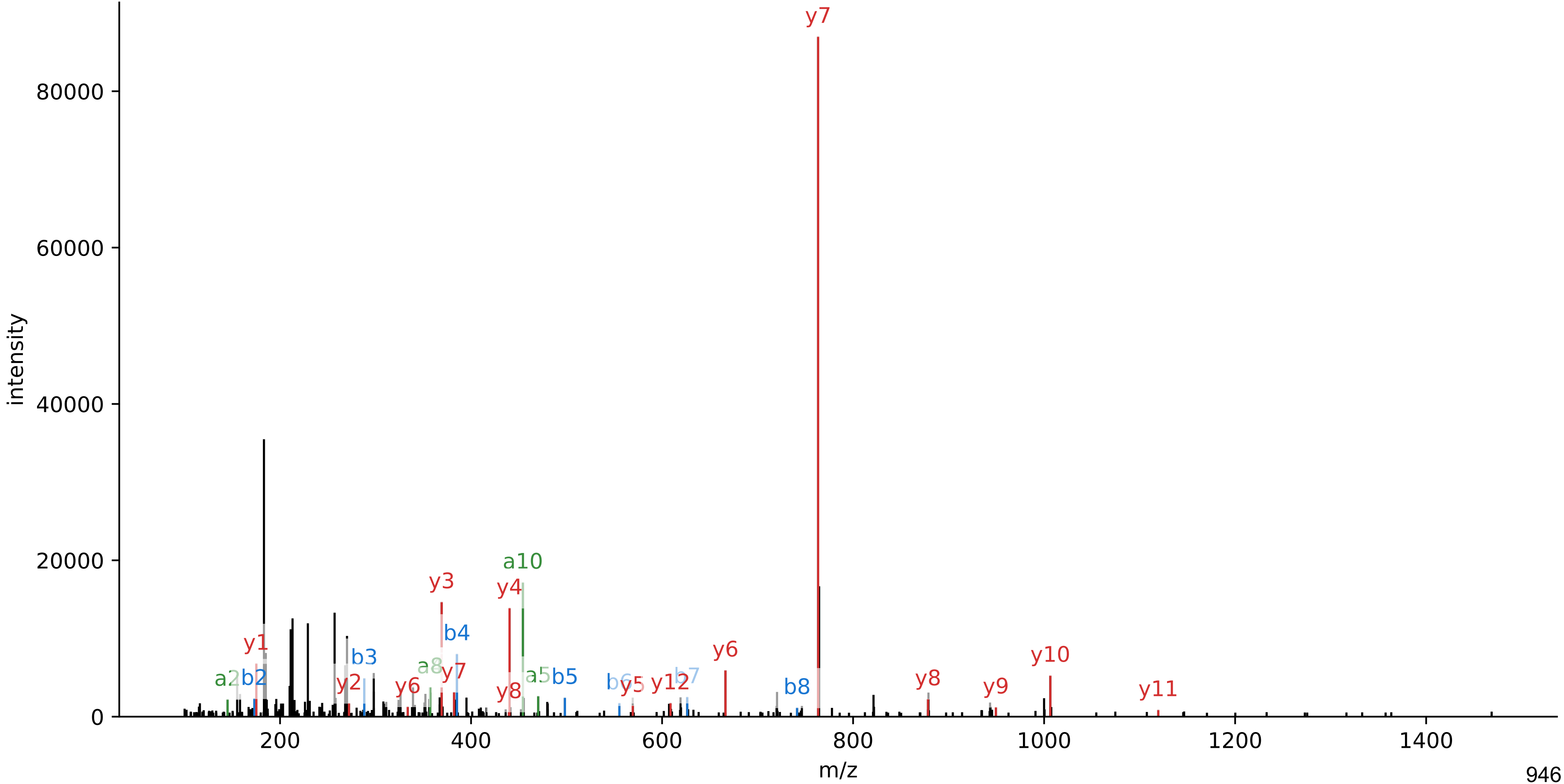

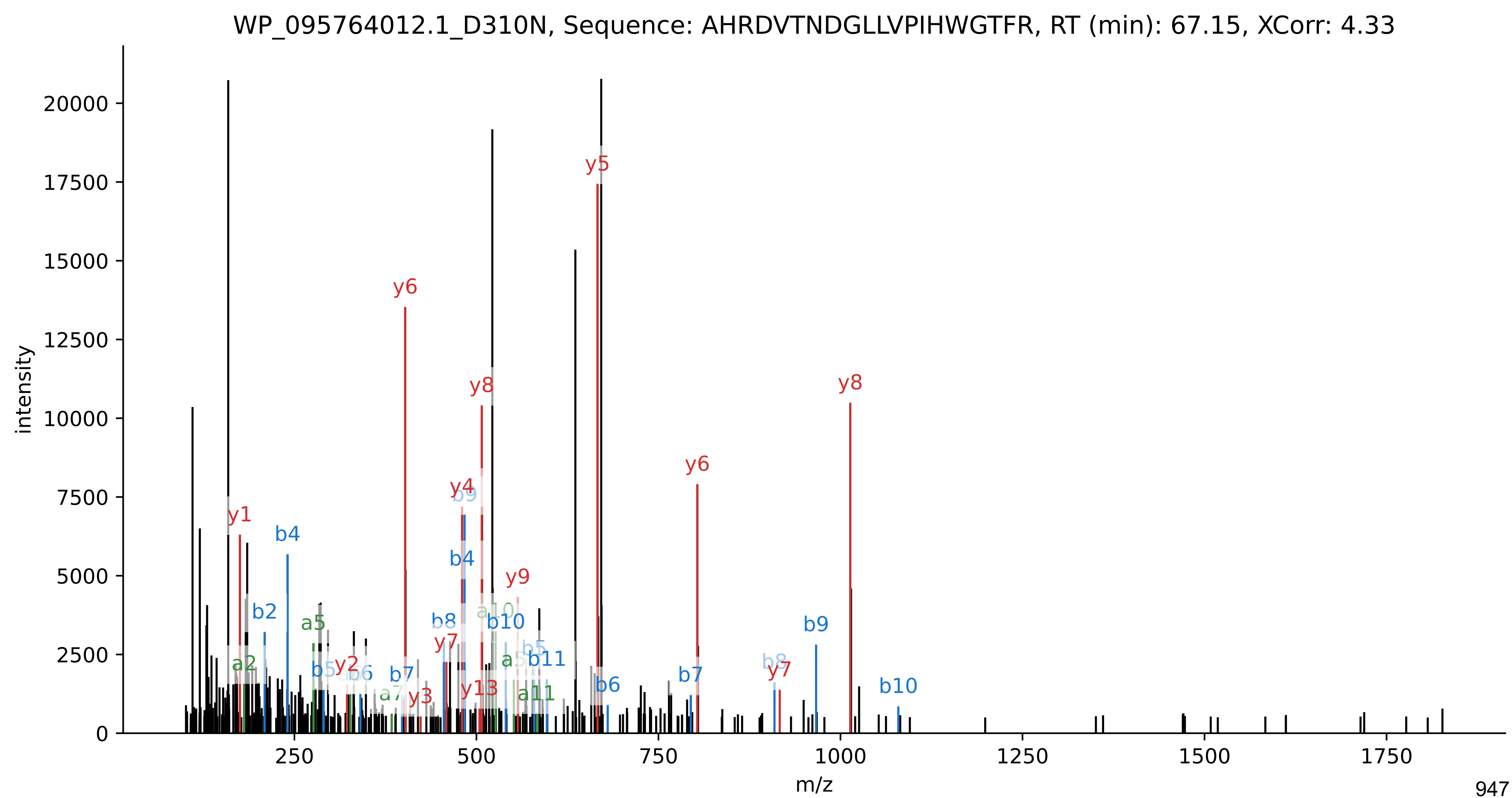

WP_095764012.1_D310N, Sequence: AHRDVTNDGLLVPIHWGTFR, RT (min): 67.15, XCorr: 4.33
intensity
m/z
y5
y6
y8
y8
y6
y4
b9
y1
b4
b4
y9
b2
a5
a10
b10
b8
y7
b5
b11
a2
b5
y2
b6
b7
a7
y3
y13
a11
b6
b7
b8
y7
b9
b10

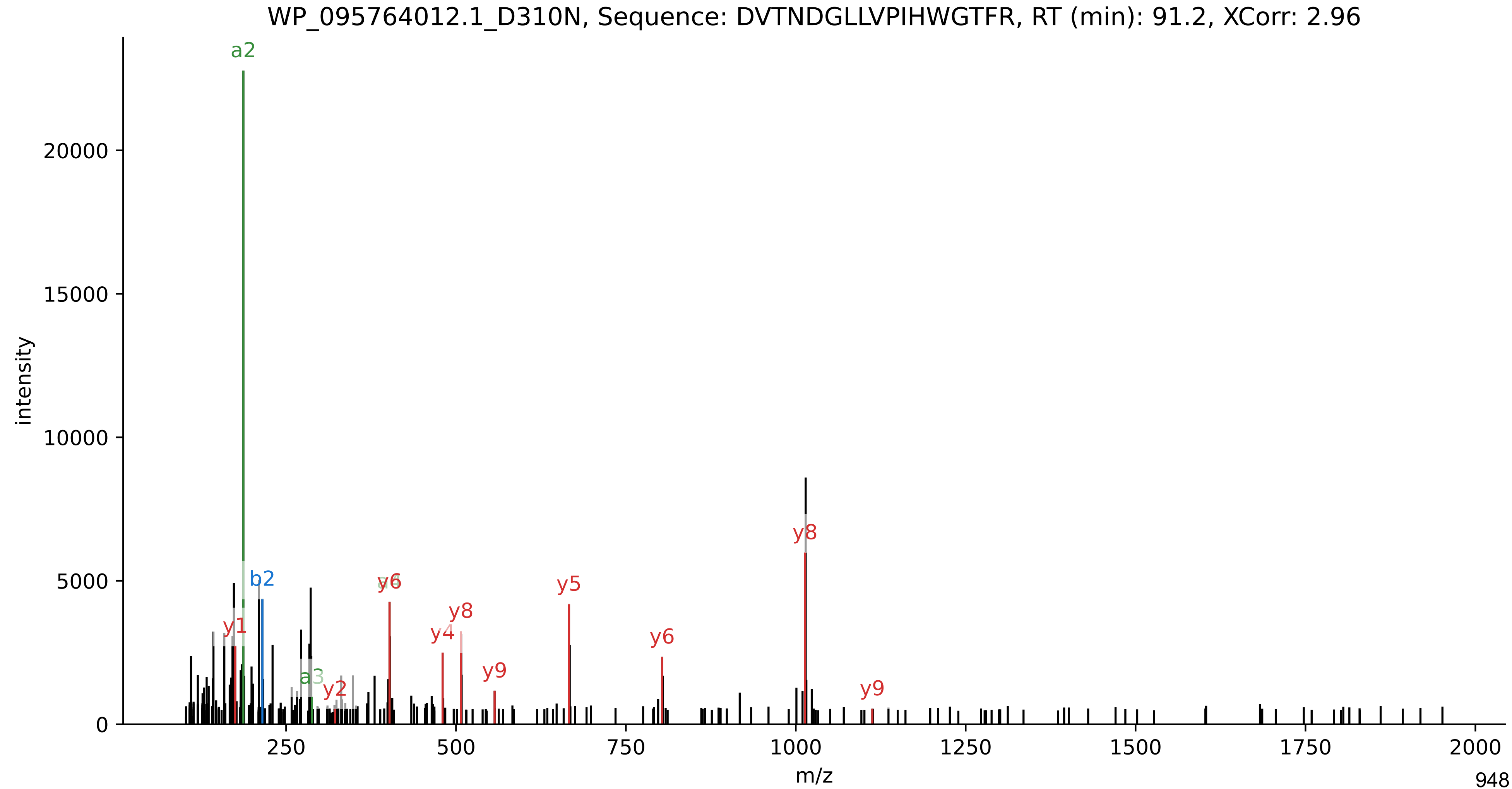

WP_095764012.1_D310N, Sequence: DVTNDGLLVPIHWGTFR, RT (min): 91.2, XCorr: 2.96
intensity
m/z
a2
b2
y1
a3
y2
y6
y4
y8
y9
y5
y6
y8
y9

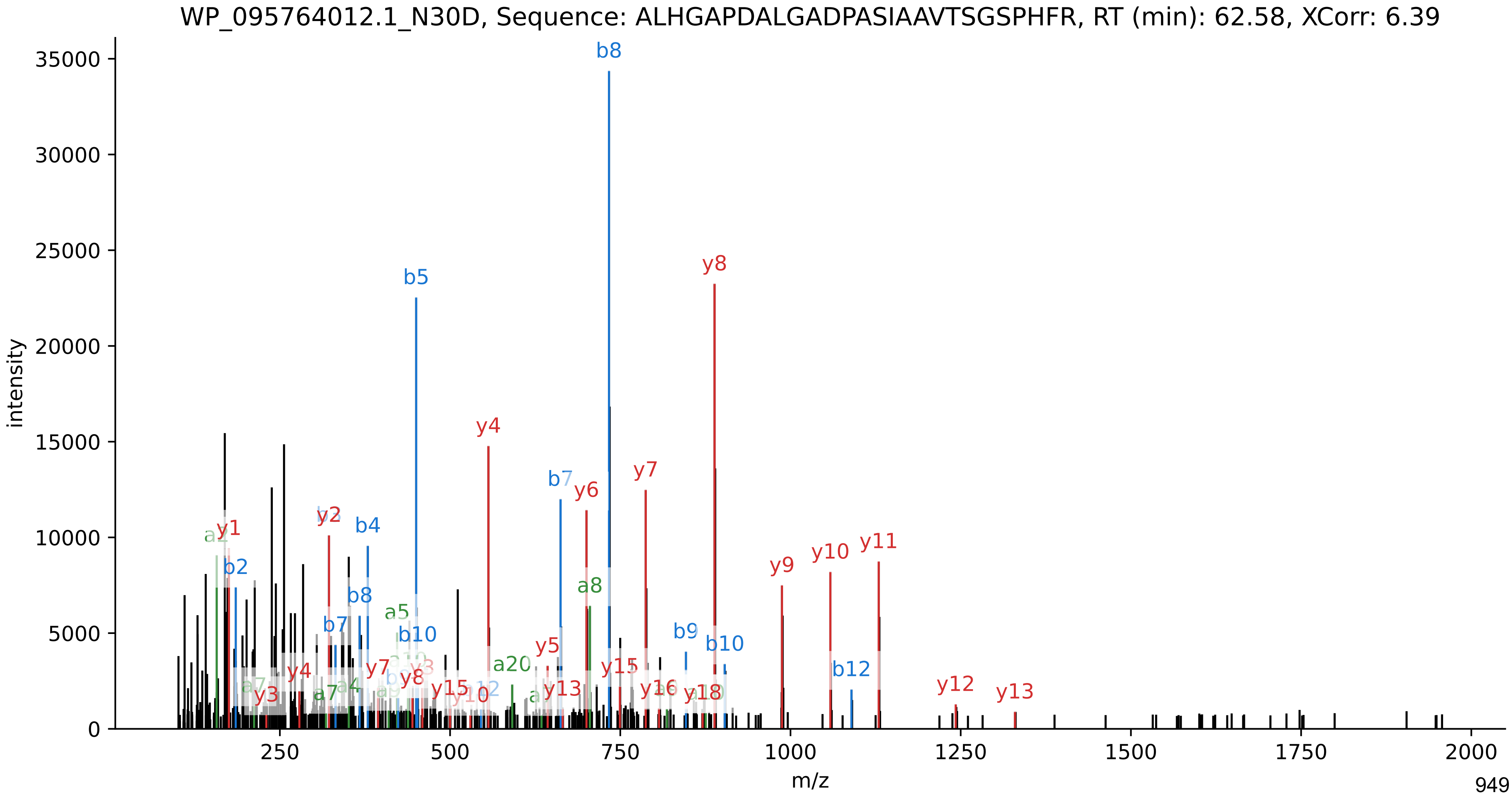
WP_095764012.1_N30D, Sequence: ALHGAPDALGADPASIAAVTSGSPHFR, RT (min): 62.58, XCorr: 6.39
intensity
m/z
0
5000
10000
15000
20000
25000
30000
35000
250
500
750
1000
1250
1500
1750
2000
b8
y8
b5
y4
b7
y6
y7
y1
y2
b4
b2
a8
y9
y10
y11
a5
b10
b9
b12
y5
a20
y15
y13
y16
y18
y12
y13
y3
y7
a7
a4
y8
y15
y10

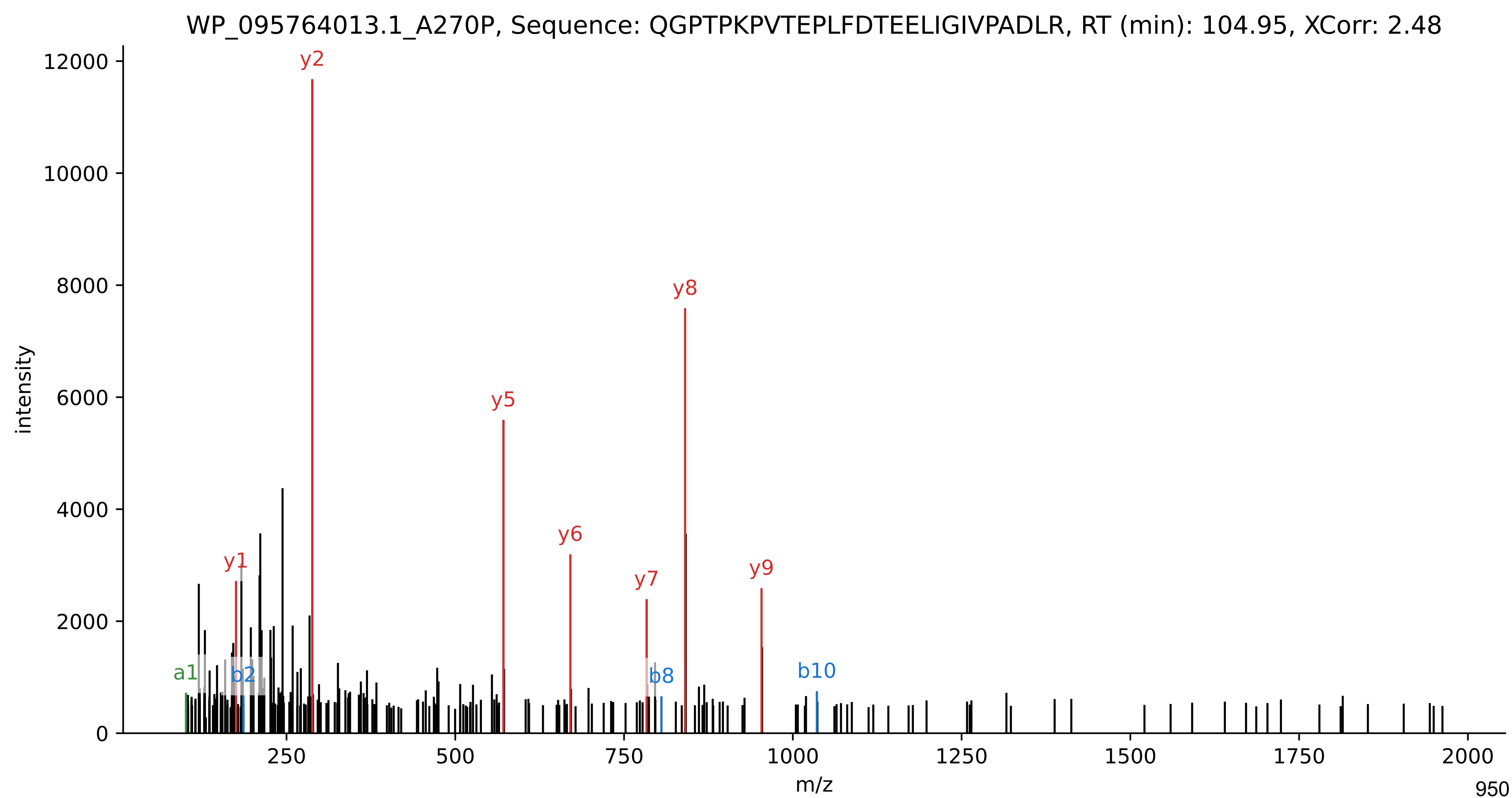

WP_095764013.1_A270P, Sequence: QGPTPKPVTEPLFDTEELIGIVPADLR, RT (min): 104.95, XCorr: 2.48
intensity
m/z
y2
y8
y5
y6
y1
y7
y9
a1
b2
b8
b10
12000
10000
8000
6000
4000
2000
0
250
500
750
1000
1250
1500
1750
2000

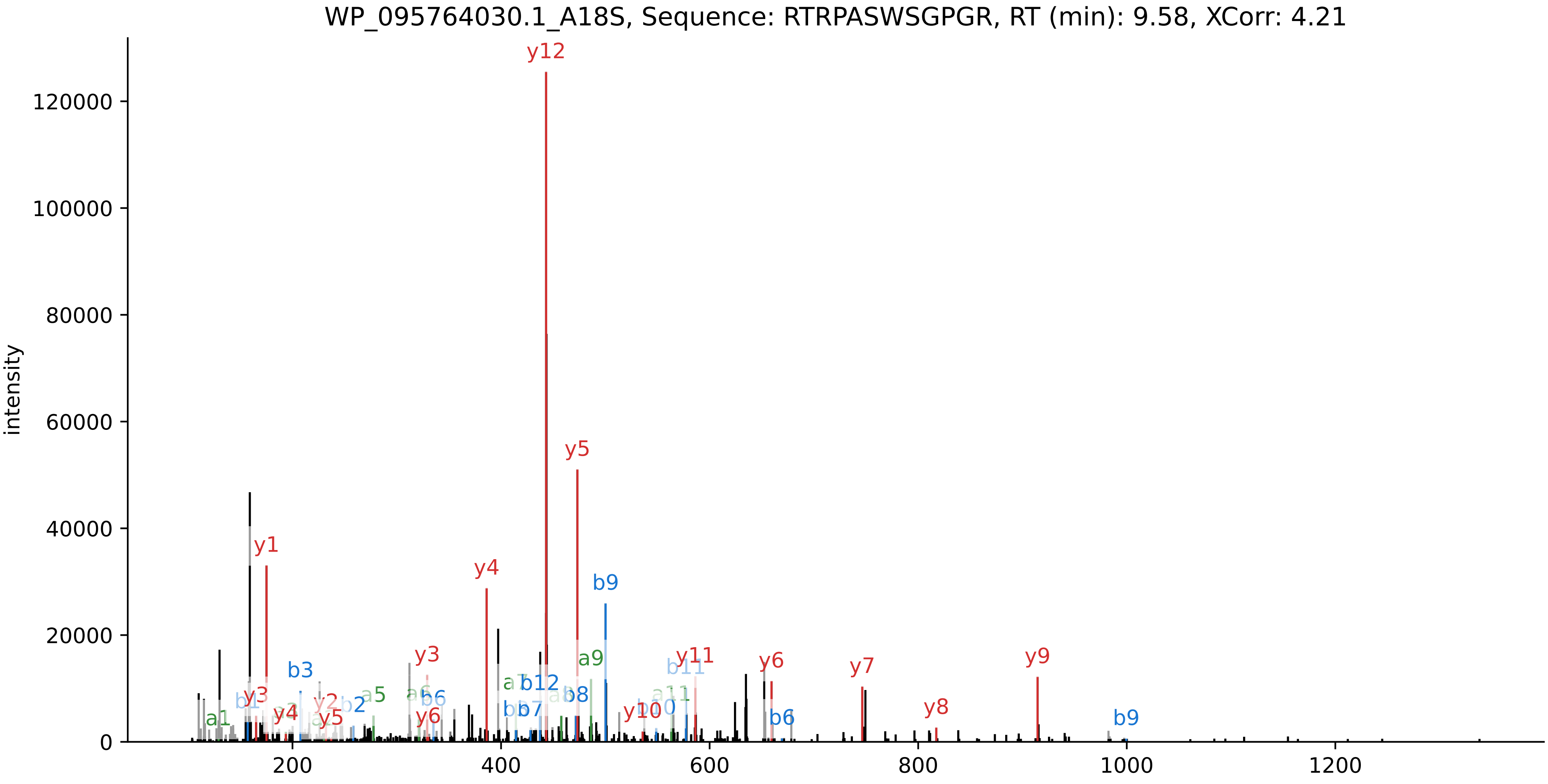
WP_095764030.1_A18S, Sequence: RTRPASWSGPGR, RT (min): 9.58, XCorr: 4.21
intensity
m/z
0
20000
40000
60000
80000
100000
120000
200
400
600
800
1000
1200
y12
y5
y1
y4
b9
y3
b3
a9
y11
b11
y6
y7
y9
b12
b8
a11
y10
y8
b6
b2
a5
a1
y2
y5
b7

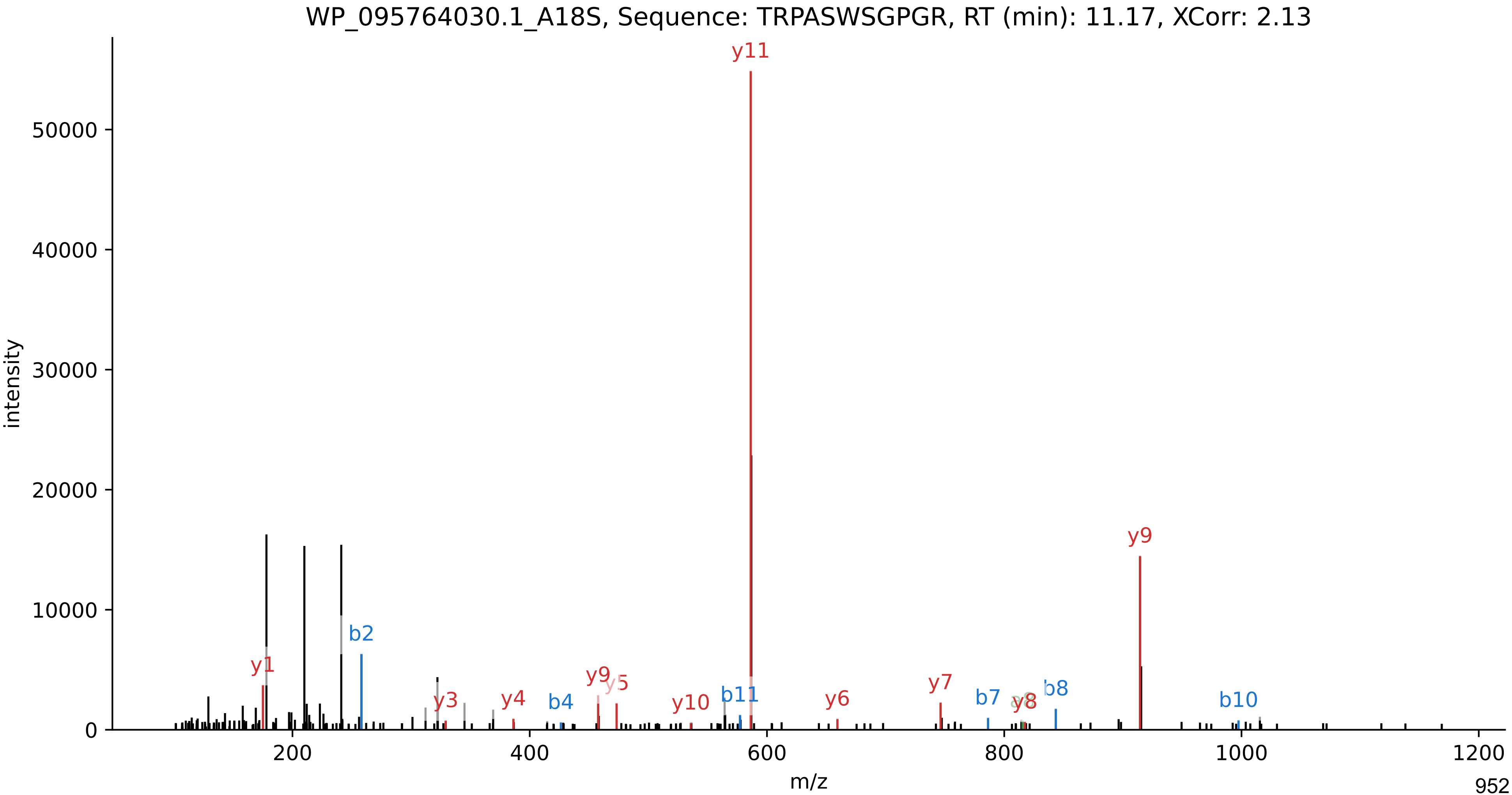

WP_095764030.1_A18S, Sequence: TRPASWSGPGR, RT (min): 11.17, XCorr: 2.13
y11
y9
b2
y1
y9
y5
y3
y4
b4
y10
b11
y6
y7
b7
y8
b8
b10
intensity
m/z
0
10000
20000
30000
40000
50000
200
400
600
800
1000
1200

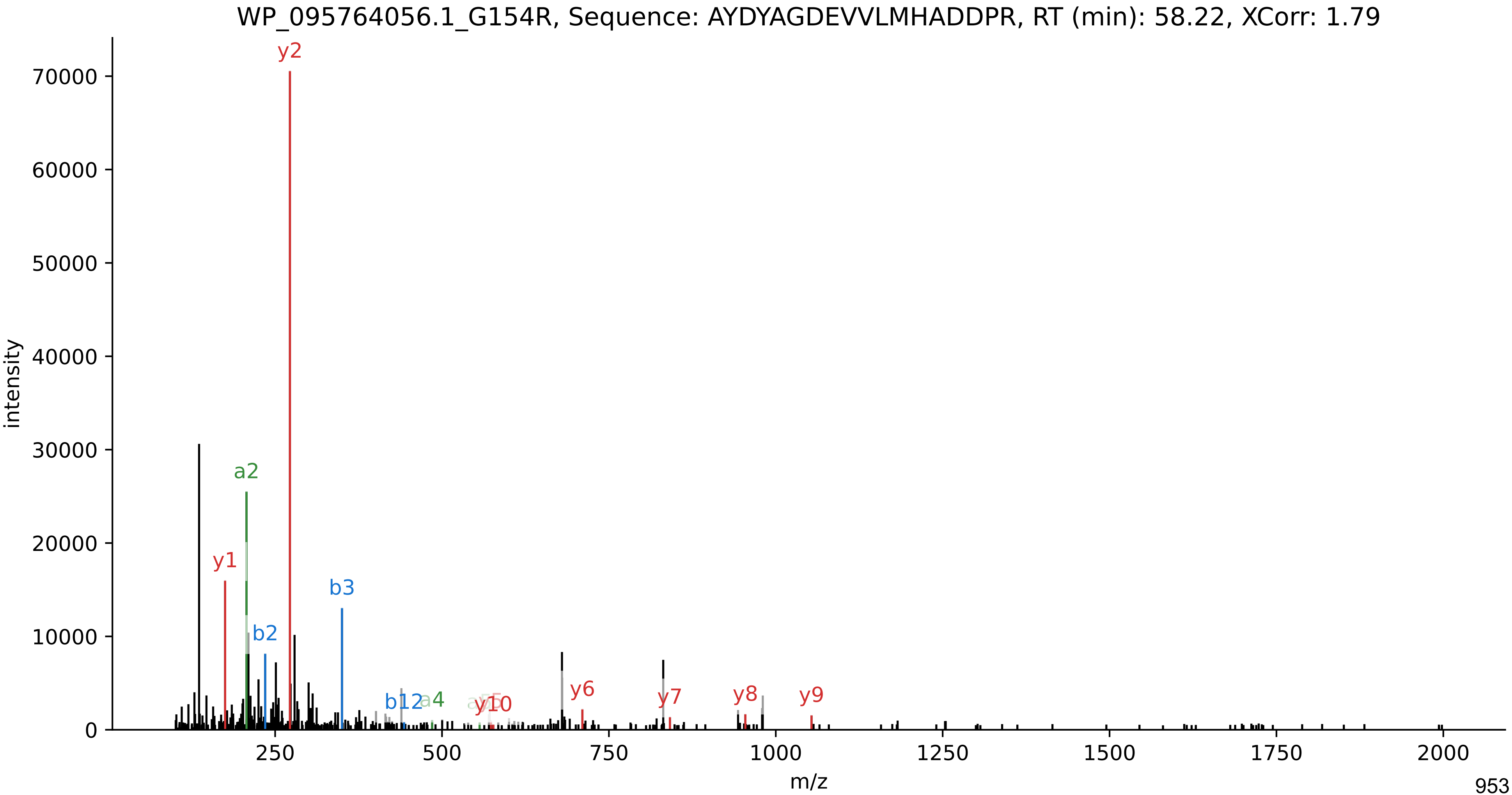
WP_095764056.1_G154R, Sequence: AYDYAGDEVVLMHADDPR, RT (min): 58.22, XCorr: 1.79
intensity
m/z
y2
a2
y1
b2
b3
b12
a4
y10
y6
y7
y8
y9
0
10000
20000
30000
40000
50000
60000
70000
250
500
750
1000
1250
1500
1750
2000

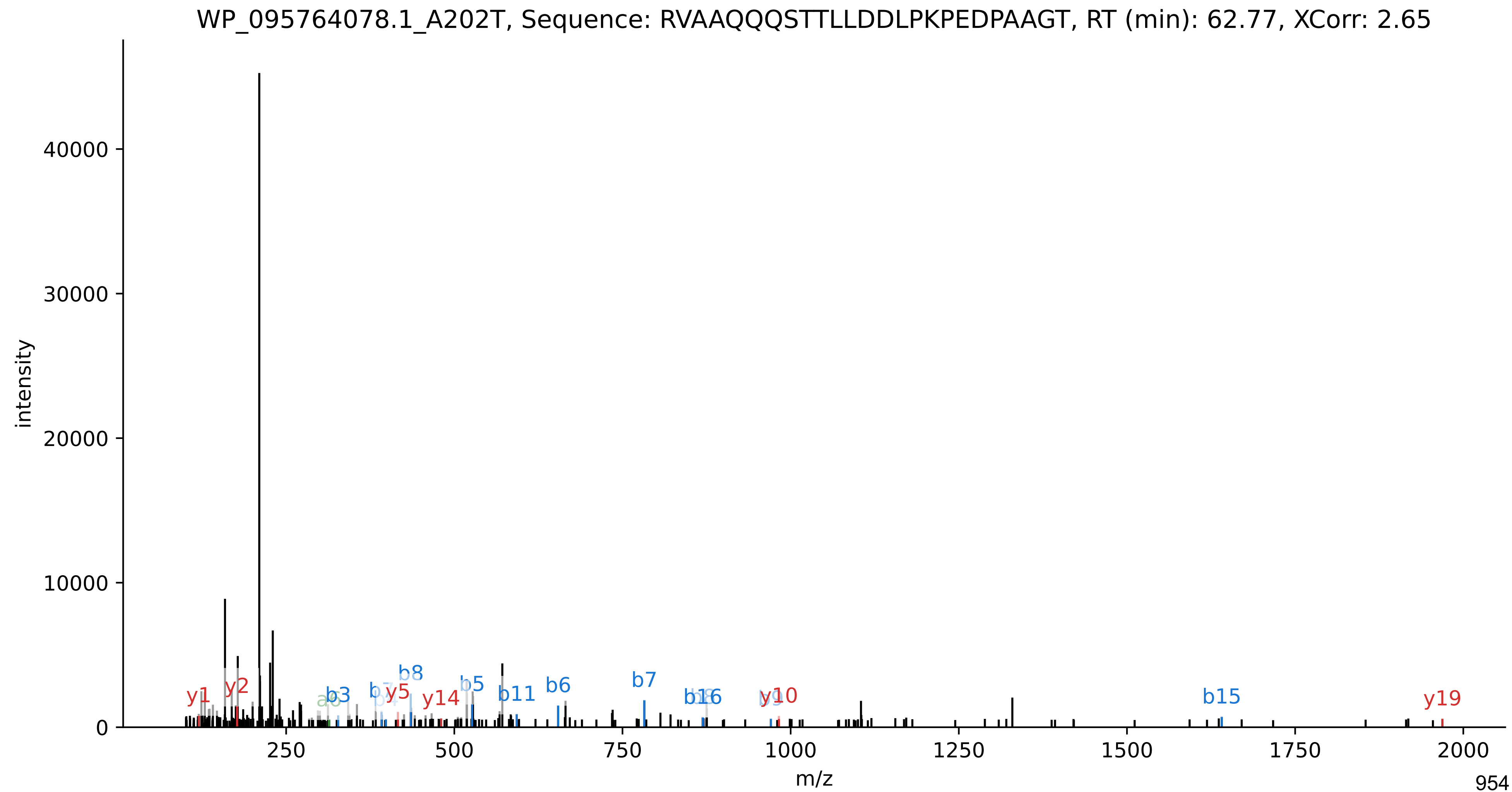

WP_095764078.1_A202T, Sequence: RVAAQQQSTTLLDDLPKPEDPAAGT, RT (min): 62.77, XCorr: 2.65
intensity
m/z
y1
y2
b3
b5
b6
b7
b8
b11
b15
y5
y10
y14
y19

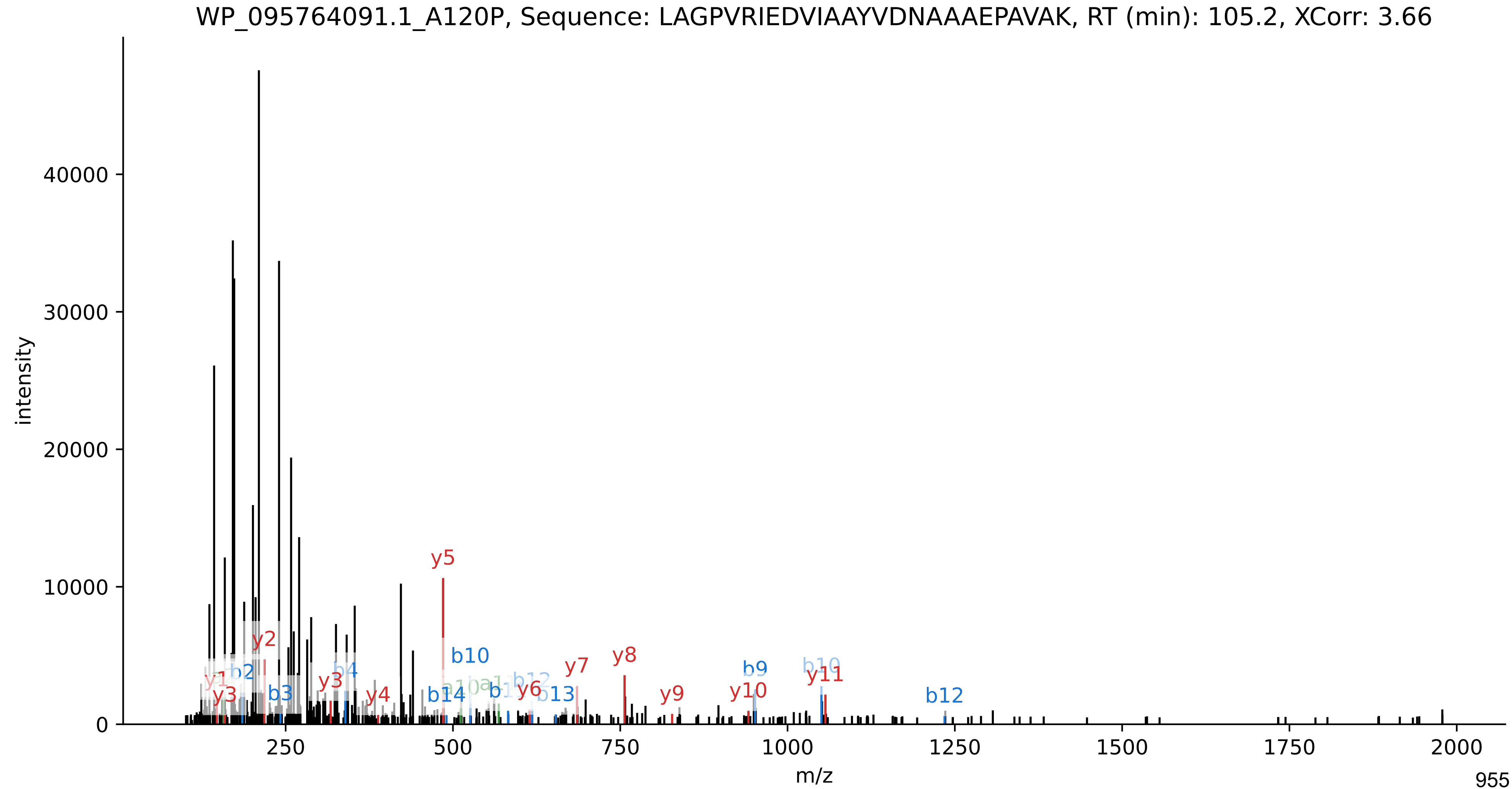

WP_095764091.1_A120P, Sequence: LAGPVRIEDVIAAYVDNAAAEPAVAK, RT (min): 105.2, XCorr: 3.66
intensity
m/z
y5
y2
b10
y7
y8
b9
b10
y11
y3
b4
b2
y1
y3
b3
y4
b14
b12
y6
b13
y9
y10
b12

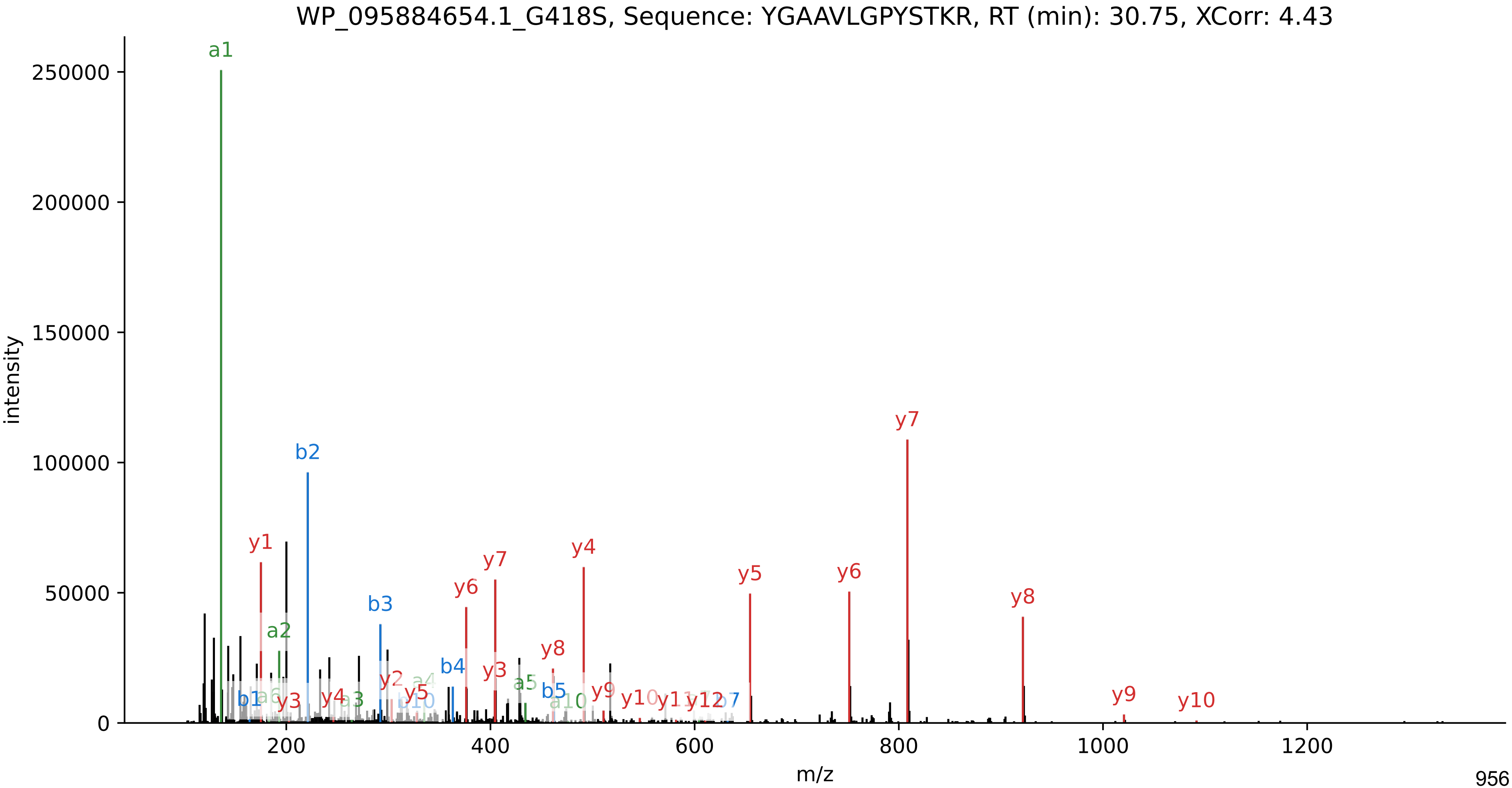
WP_095884654.1_G418S, Sequence: YGAAVLGPYSTKR, RT (min): 30.75, XCorr: 4.43
intensity
m/z
a1
b2
y7
y1
y7
y4
y5
y6
y8
y6
b3
a2
b4
y3
y8
a5
b5
y9
y10
y9
y10
y12
b7
y2
y5
y3
y4
a3
b1
a10
0
50000
100000
150000
200000
250000
200
400
600
800
1000
1200

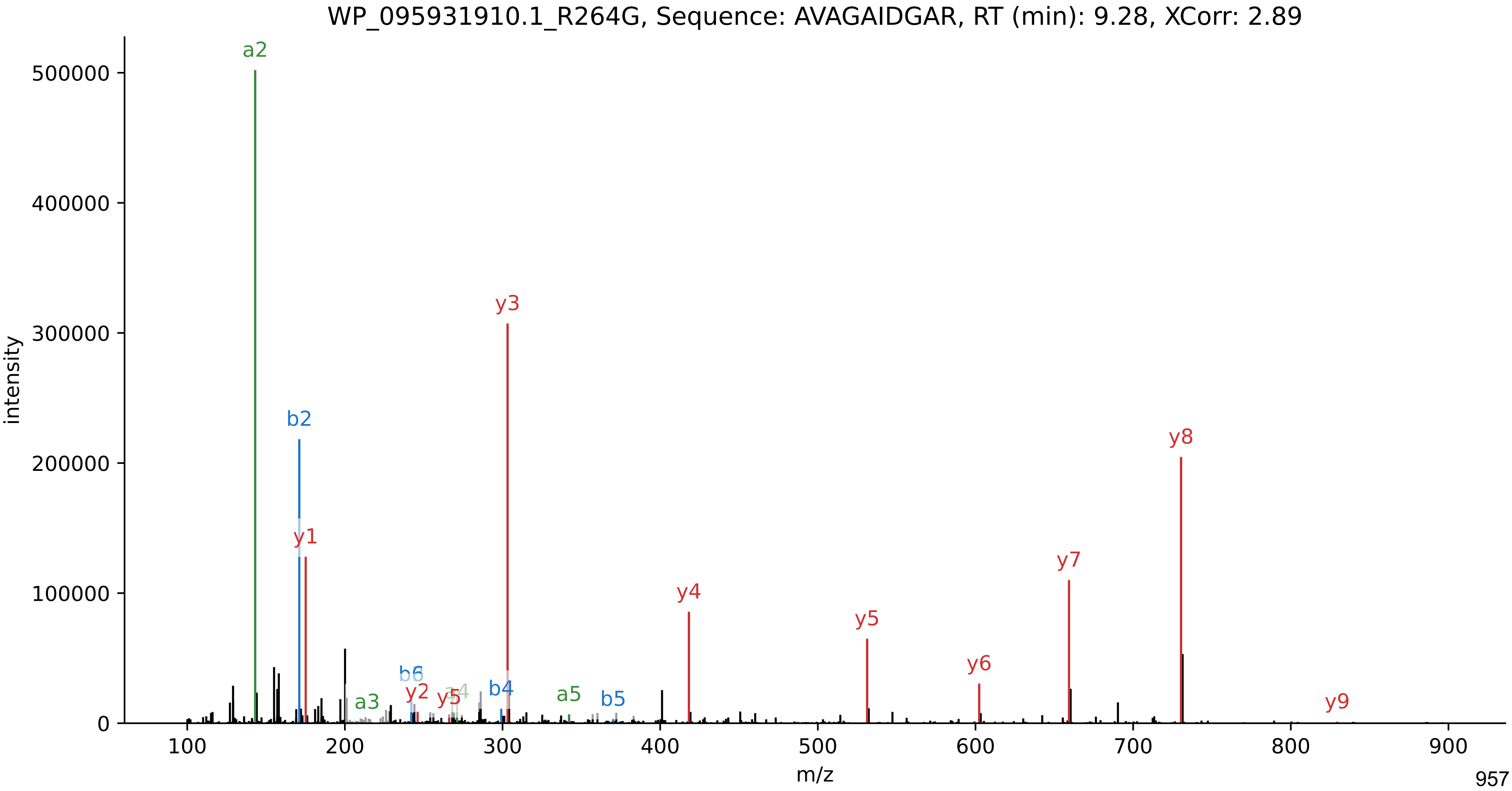

WP_095931910.1_R264G, Sequence: AVAGAIDGAR, RT (min): 9.28, XCorr: 2.89
intensity
m/z
a2
b2
y1
y3
y4
y5
y6
y7
y8
y9
a3
a5
b4
b5
b6
y2

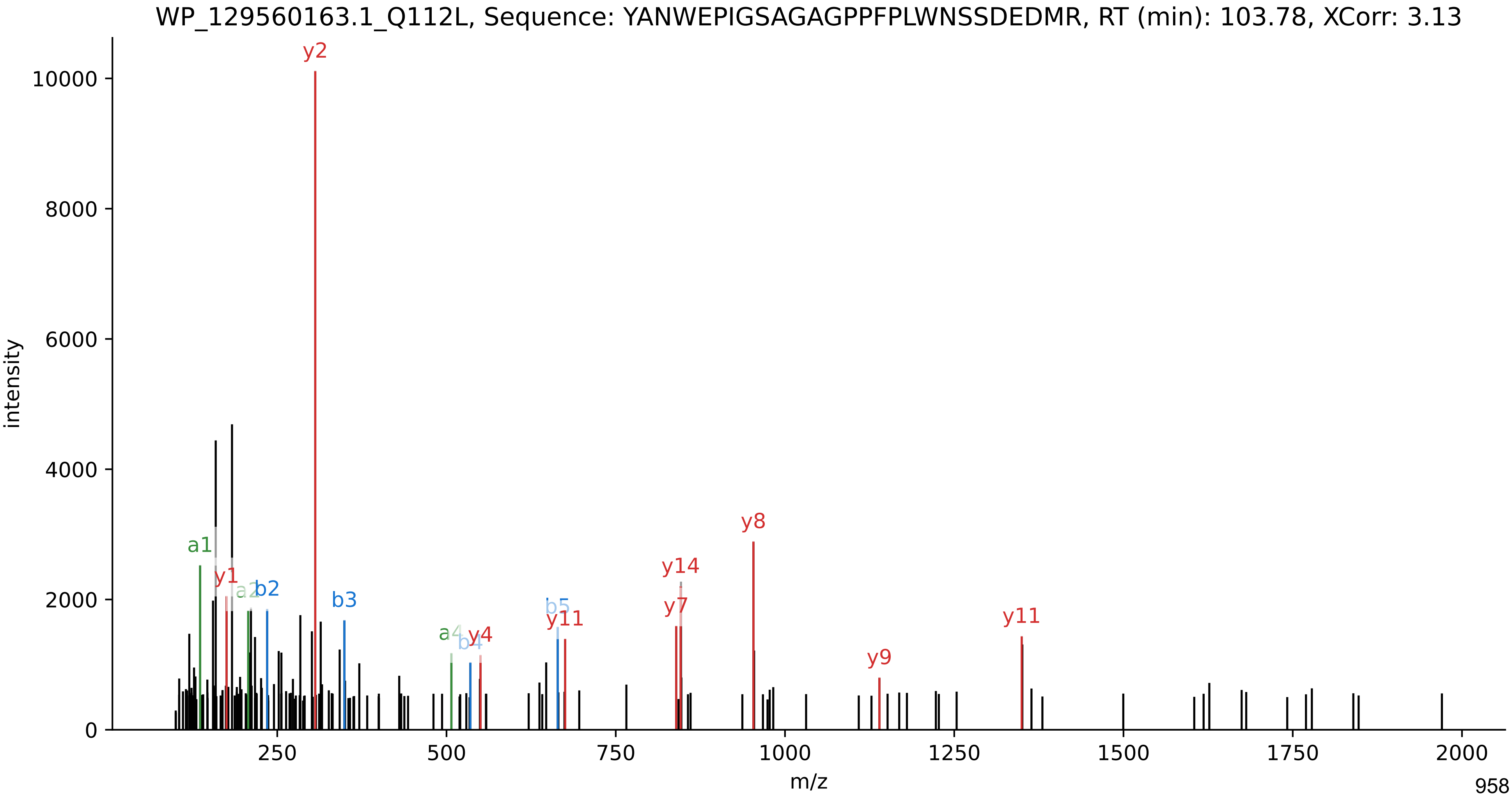

WP_129560163.1_Q112L, Sequence: YANWEPIGSAGAGPPFPLWNSSDEDMR, RT (min): 103.78, XCorr: 3.13
intensity
m/z
y2
a1
y1
a2
b2
b3
a4
b4
y4
b5
y11
y14
y7
y8
y9
y11

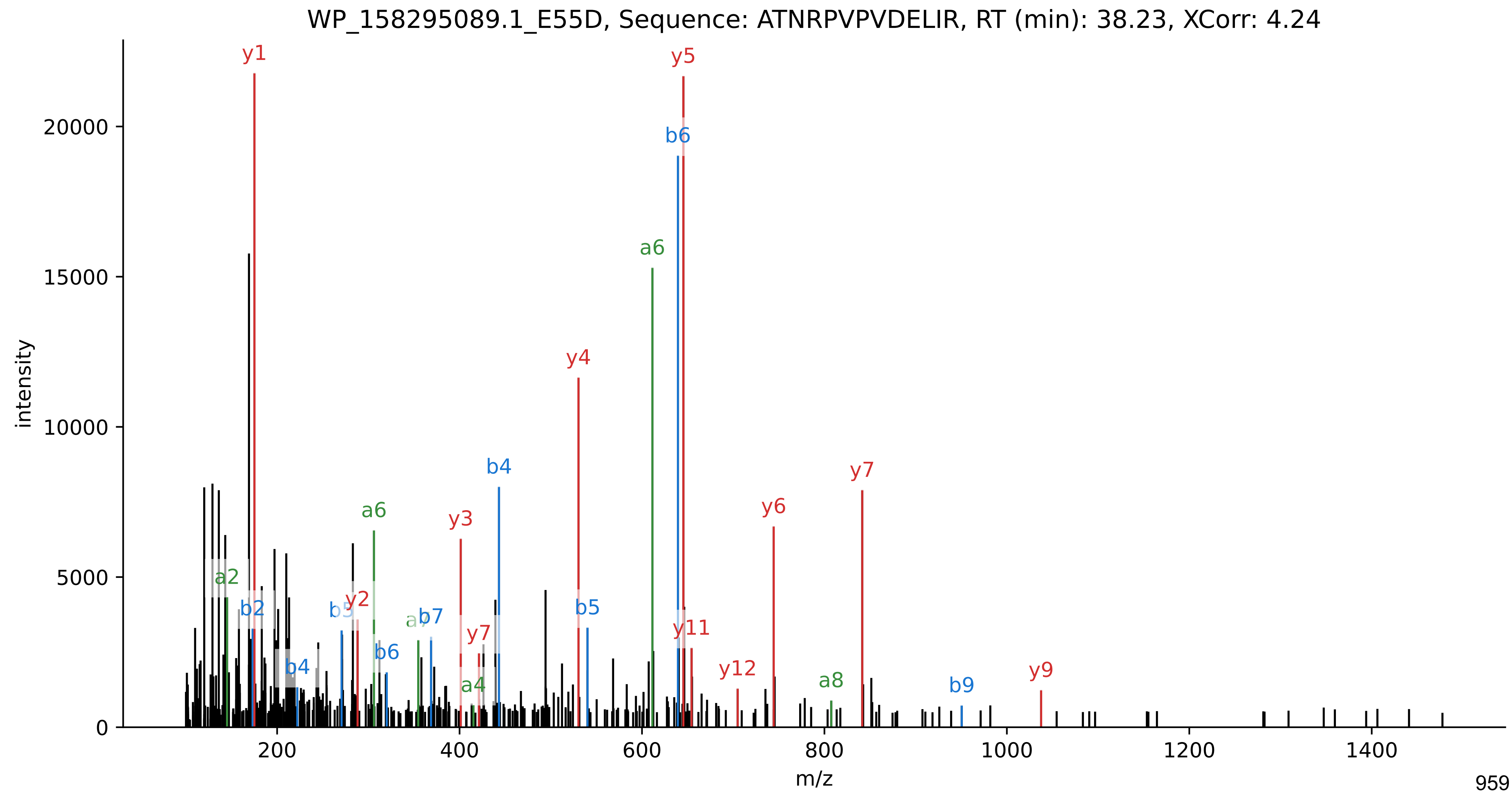

WP_158295089.1_E55D, Sequence: ATNRPVPVDELIR, RT (min): 38.23, XCorr: 4.24
intensity
m/z
y1
y5
b6
a6
y4
b4
y7
y6
a6
y3
a2
b2
b5
y2
b7
y7
b6
b4
a4
b5
y11
y12
a8
b9
y9
0
5000
10000
15000
20000
200
400
600
800
1000
1200
1400

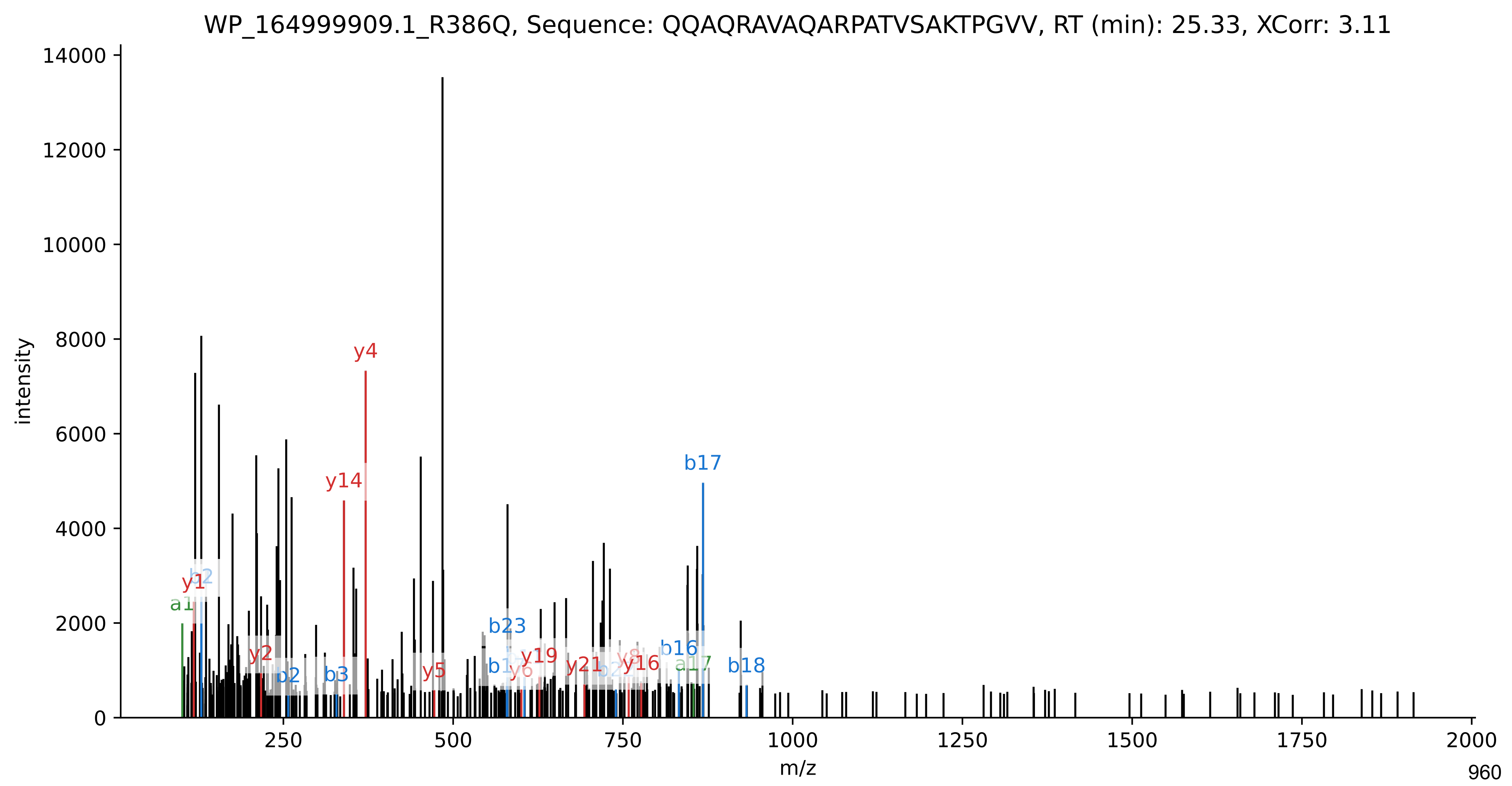

WP_164999909.1_R386Q, Sequence: QQAQRAVAQARPATVSAKTPGVV, RT (min): 25.33, XCorr: 3.11
intensity
m/z
y4
y14
b17
b23
y19
y21
y16
b16
b18
y5
y6
b3
b2
y2
y1
a1

WP_164999925.1_V615I, Sequence: SAAPQIDELGPTLVDAVR, RT (min): 83.32, XCorr: 2.18

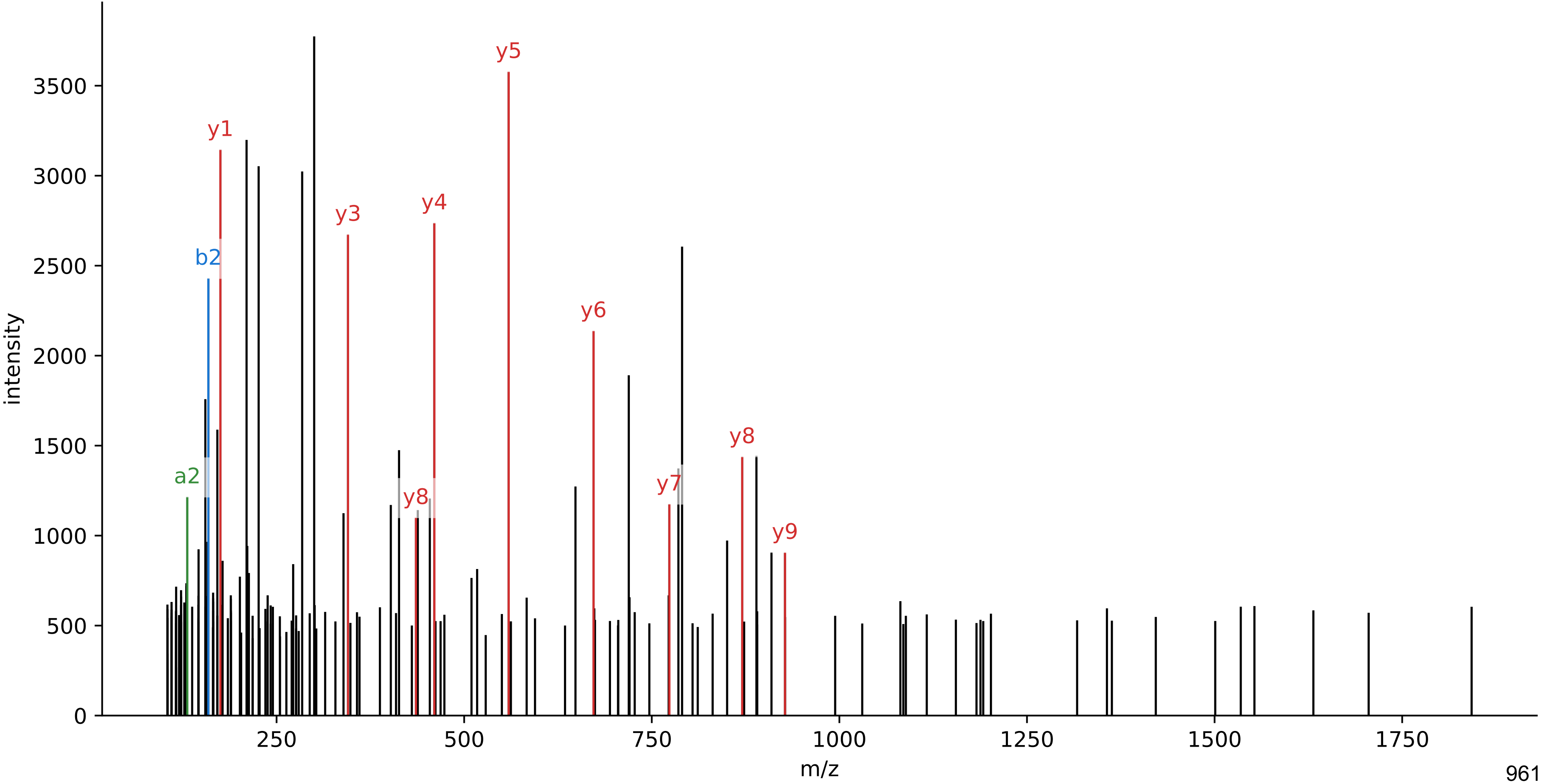

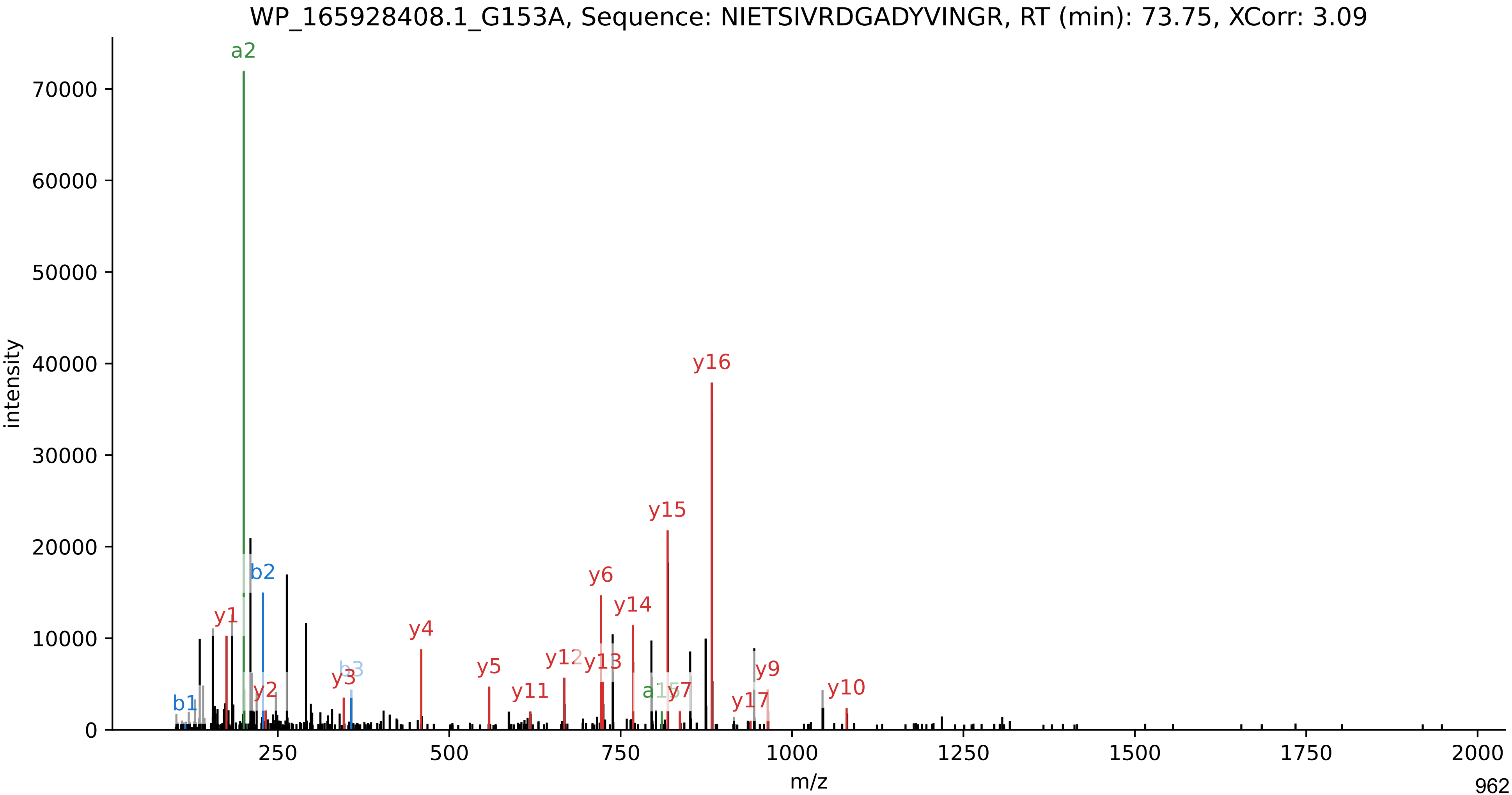

WP_165928408.1_G153A, Sequence: NIETSIVRDGADYVINGR, RT (min): 73.75, XCorr: 3.09
a2
y16
y15
b2
y6
y14
y1
y4
b3
y3
y5
y12
y13
y9
y2
y11
a15
y7
y10
b1
y17
intensity
m/z
0
10000
20000
30000
40000
50000
60000
70000
250
500
750
1000
1250
1500
1750
2000

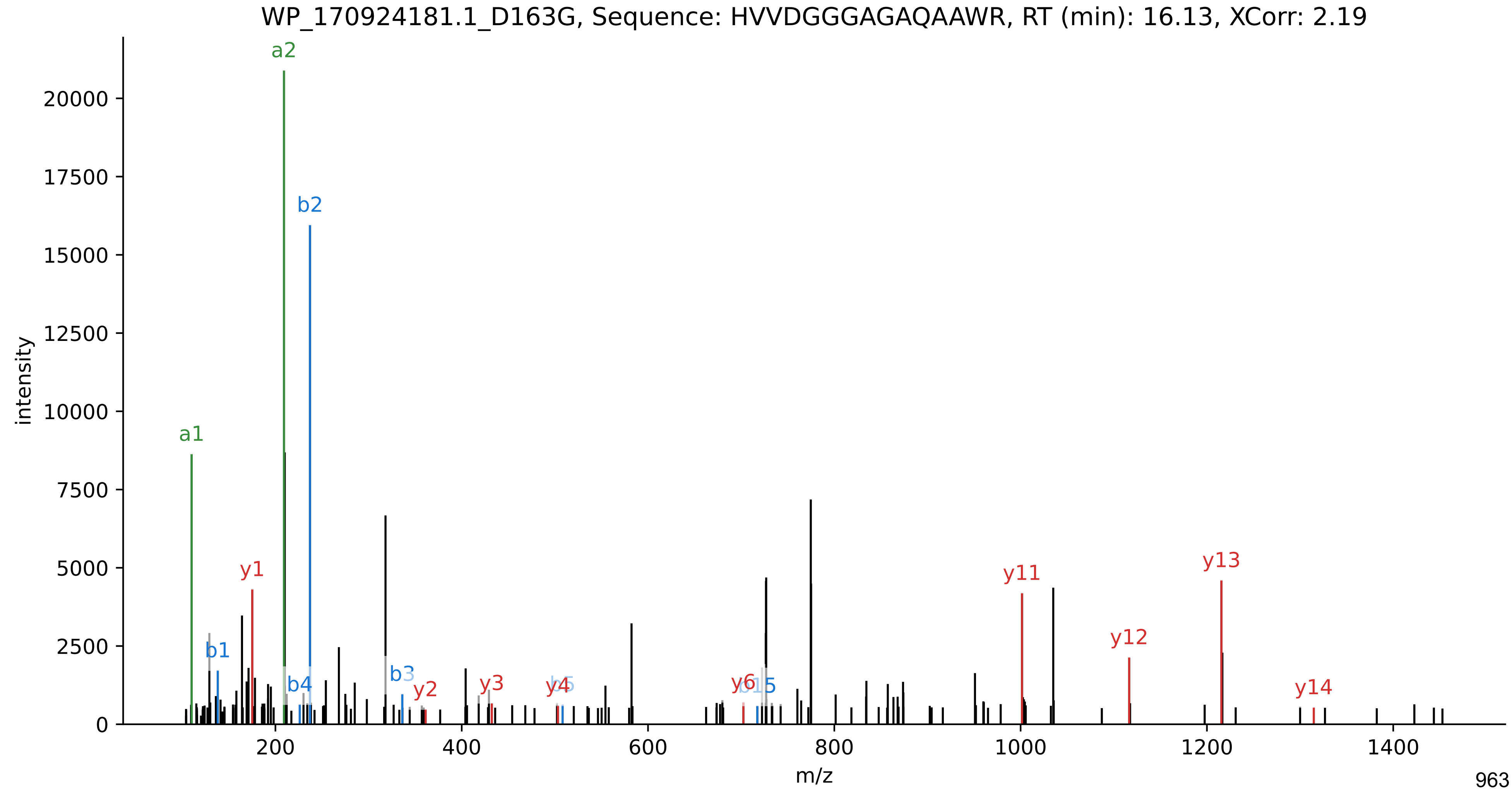

WP_170924181.1_D163G, Sequence: HVVDGGGAGAQAAWR, RT (min): 16.13, XCorr: 2.19
intensity
m/z
a1
a2
b1
b2
b3
b4
y1
y2
y3
y4
y6
y11
y12
y13
y14

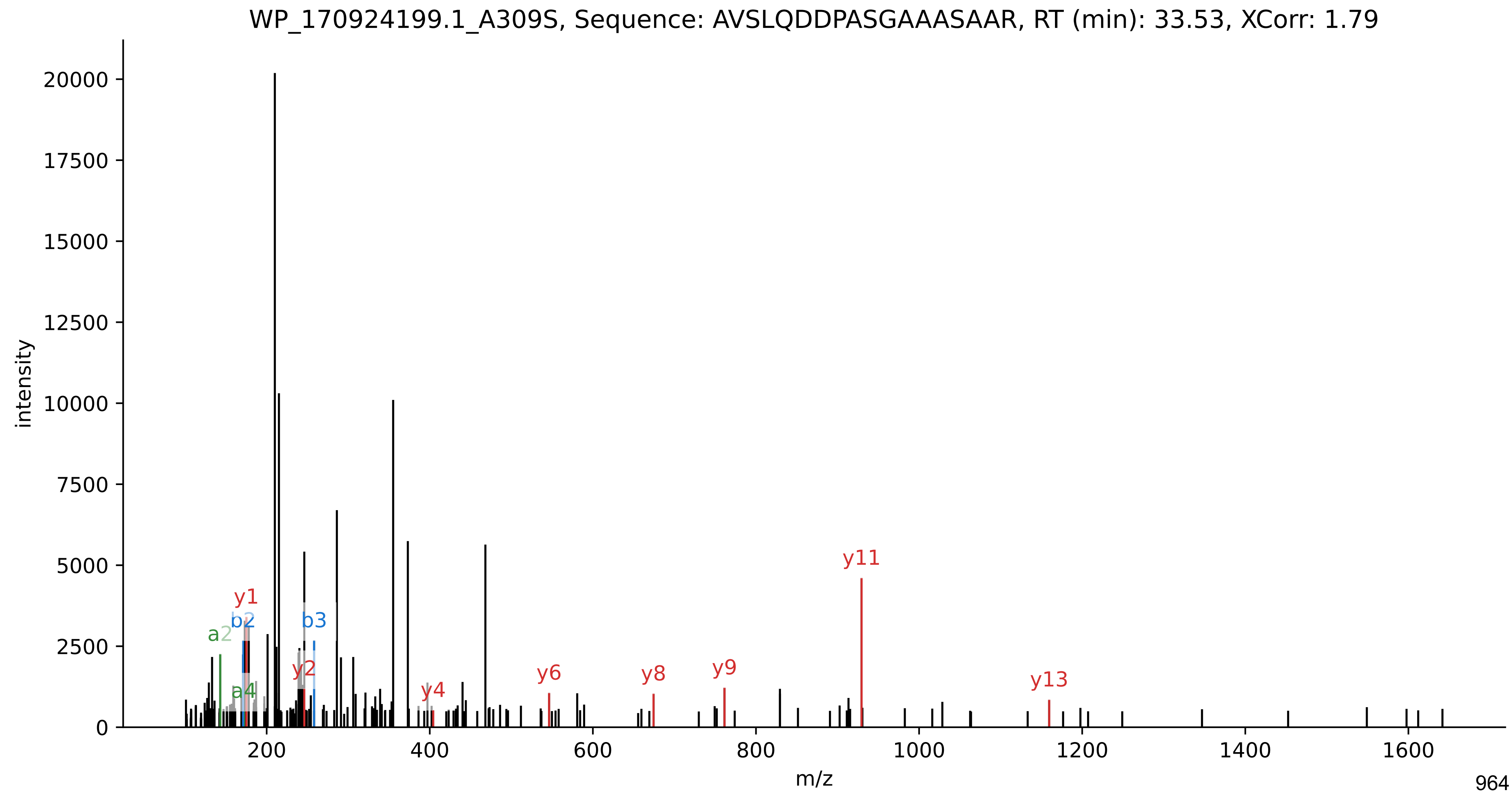
WP_170924199.1_A309S, Sequence: AVSLQDDPASGAAASAAR, RT (min): 33.53, XCorr: 1.79
intensity
m/z
0
2500
5000
7500
10000
12500
15000
17500
20000
200
400
600
800
1000
1200
1400
1600
y1
b2
a2
b3
y2
a4
y4
y6
y8
y9
y11
y13

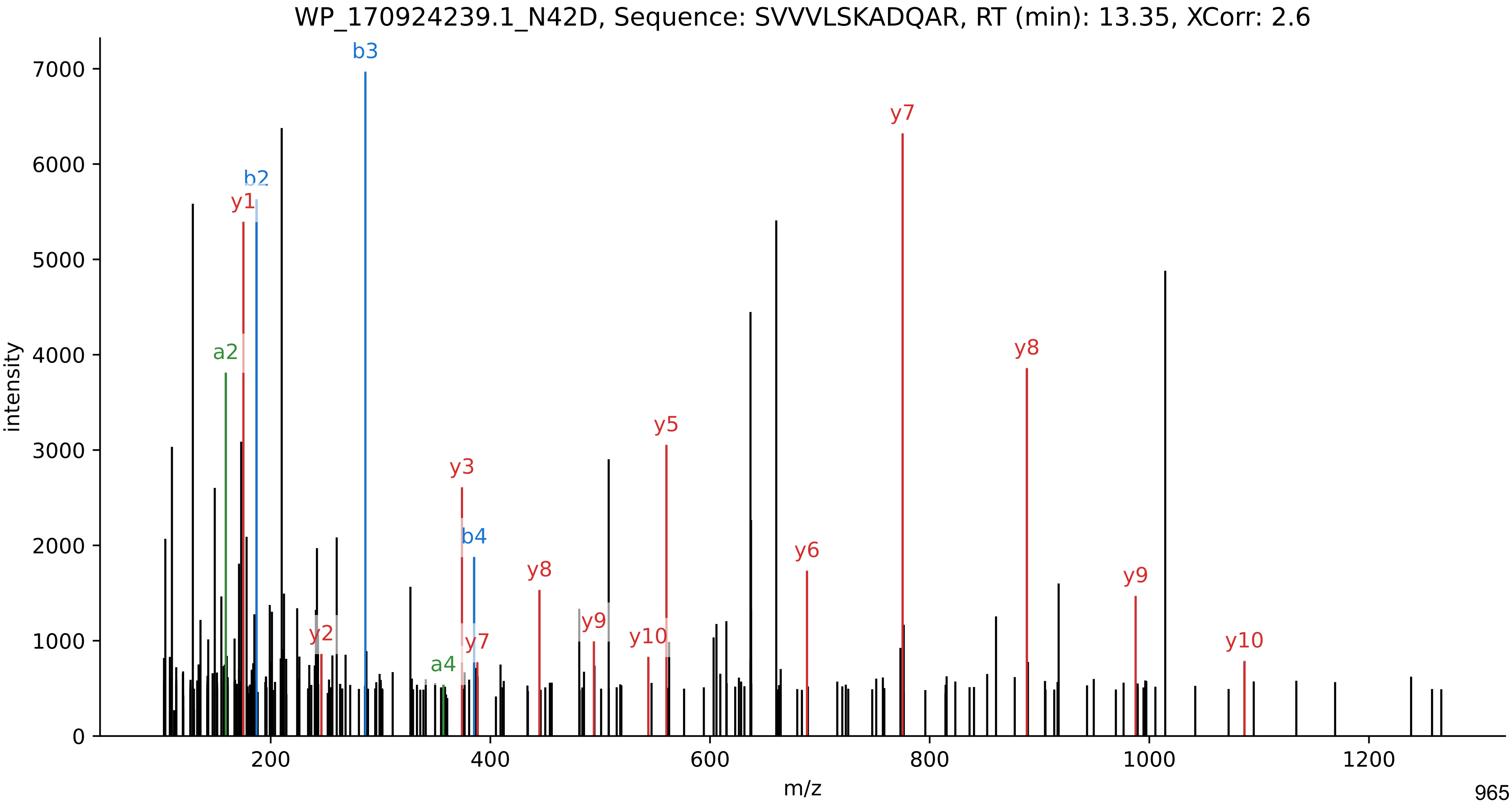

WP_170924239.1_N42D, Sequence: SVVVLSKADQAR, RT (min): 13.35, XCorr: 2.6
intensity
m/z
b3
y7
b2
y1
a2
y8
y5
y3
b4
y8
y6
y9
y9
y2
y10
y7
a4
y10
0
1000
2000
3000
4000
5000
6000
7000
200
400
600
800
1000
1200

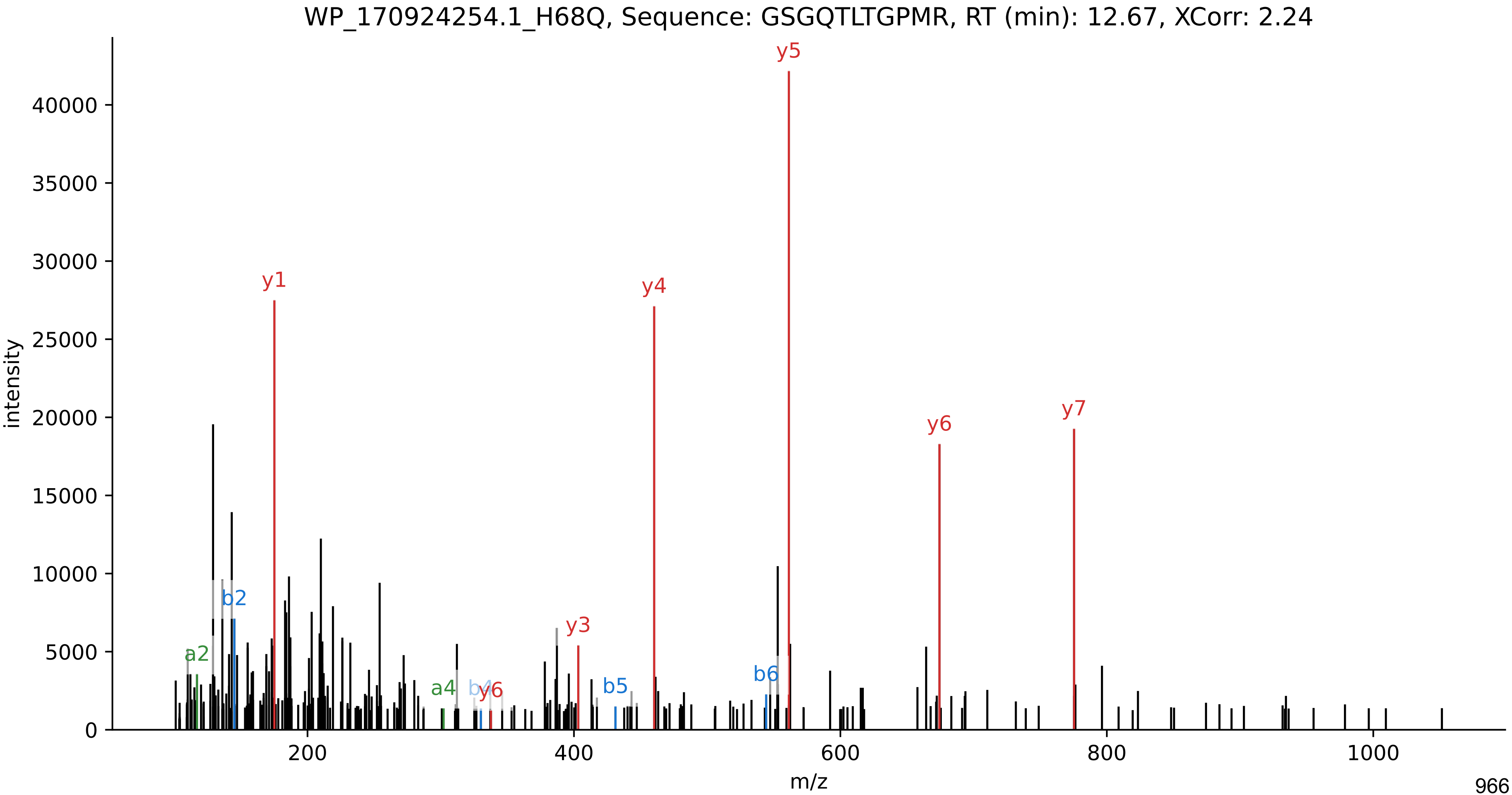
WP_170924254.1_H68Q, Sequence: GSGQTLTGPMR, RT (min): 12.67, XCorr: 2.24
intensity
m/z
y5
y1
y4
y6
y7
b2
a2
a4
b4
y6
y3
b5
b6
0
5000
10000
15000
20000
25000
30000
35000
40000
200
400
600
800
1000

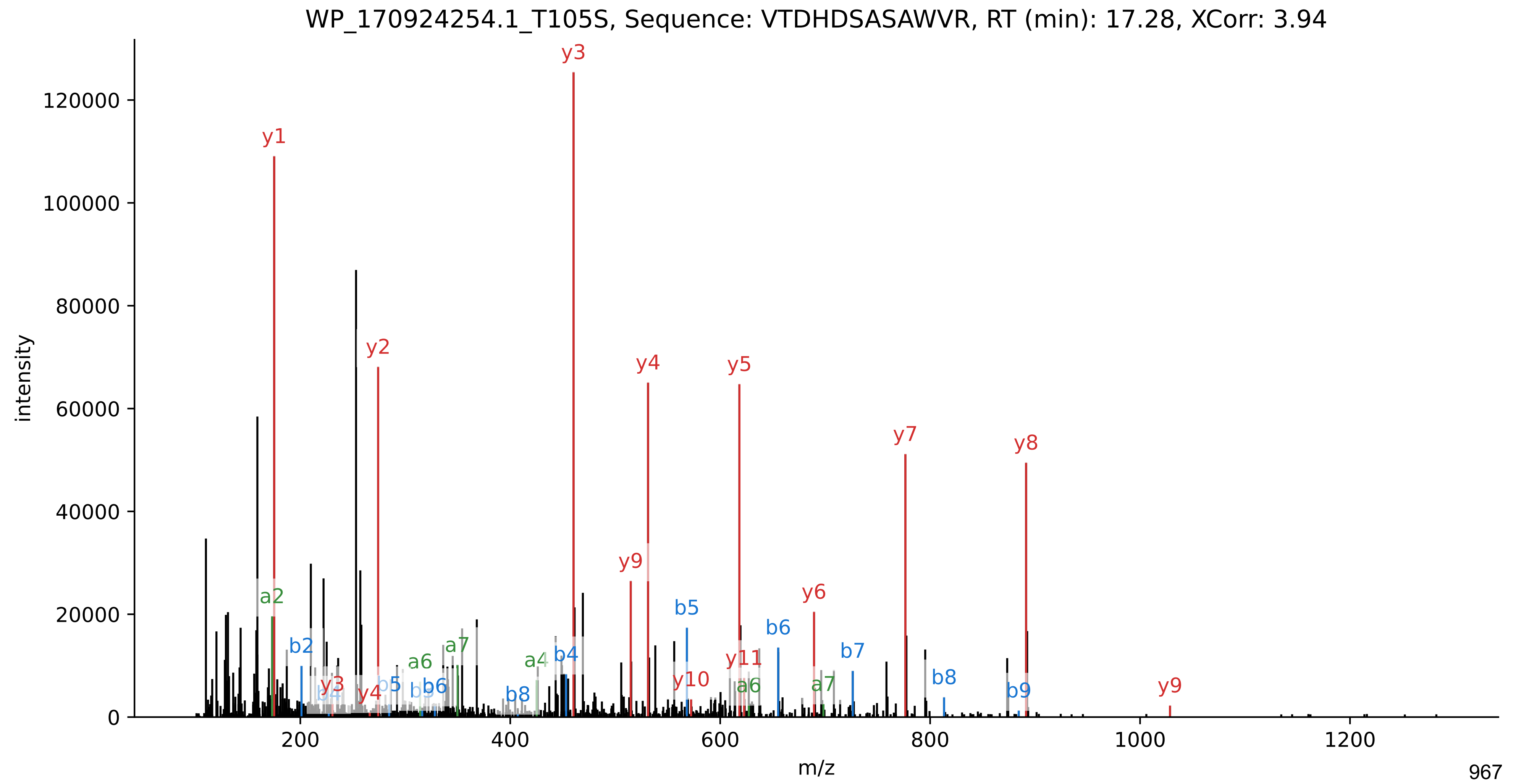

WP_170924254.1_T105S, Sequence: VTDHDSASAWVR, RT (min): 17.28, XCorr: 3.94
intensity
m/z
y1
y2
y3
y4
y5
y6
y7
y8
y9
y10
y11
a2
a4
a6
a7
b2
b4
b5
b6
b7
b8
b9

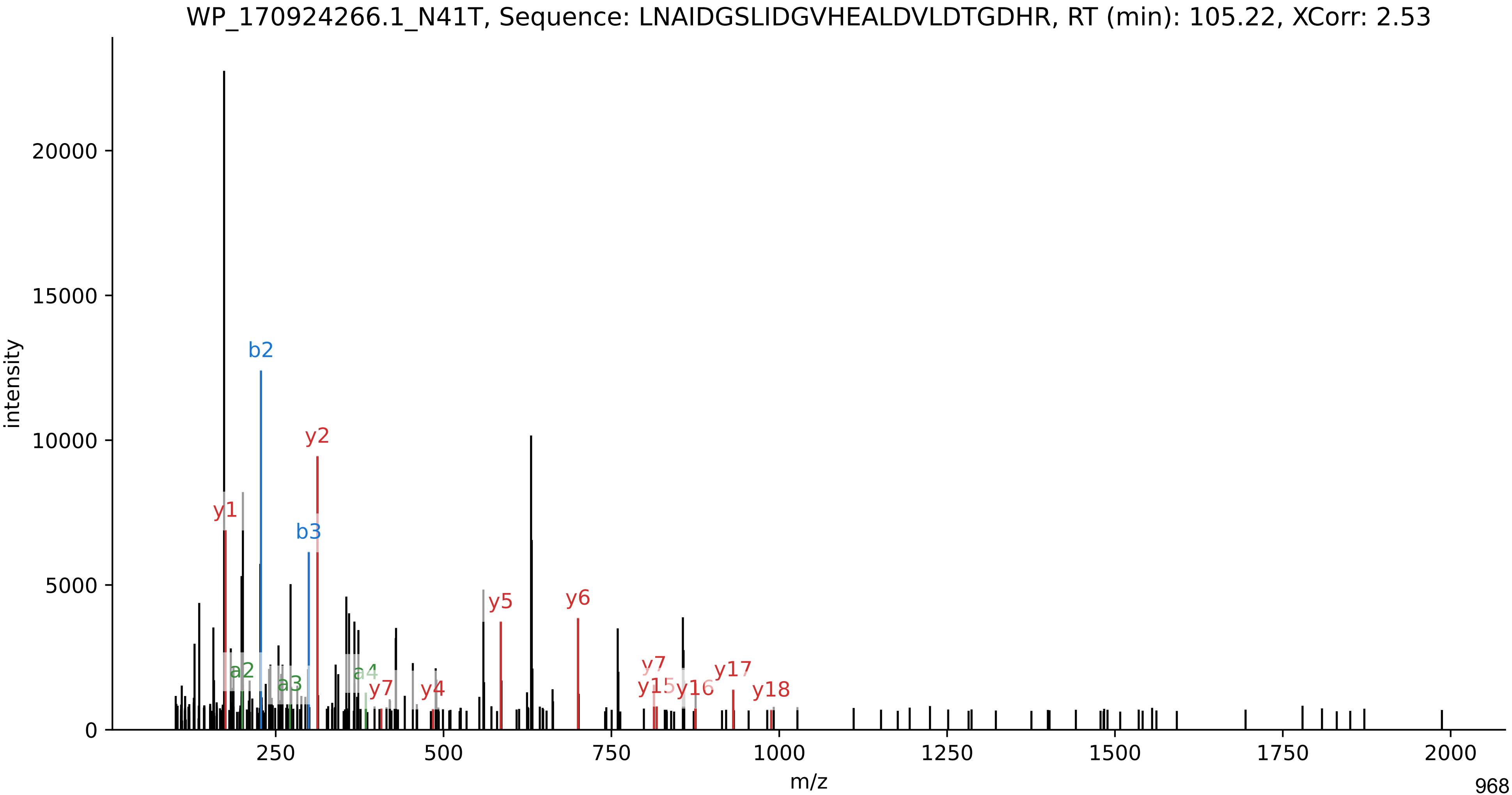
WP_170924266.1_N41T, Sequence: LNAIDGSLIDGVHEALDVLDTGDHR, RT (min): 105.22, XCorr: 2.53
intensity
m/z
b2
y2
y1
b3
y5
y6
a2
a3
a4
y7
y4
y7
y15
y16
y17
y18
0
5000
10000
15000
20000
250
500
750
1000
1250
1500
1750
2000

WP_170924375.1_I304V, Sequence: YRADEALRPTAVEEVLR, RT (min): 55.75, XCorr: 3.29

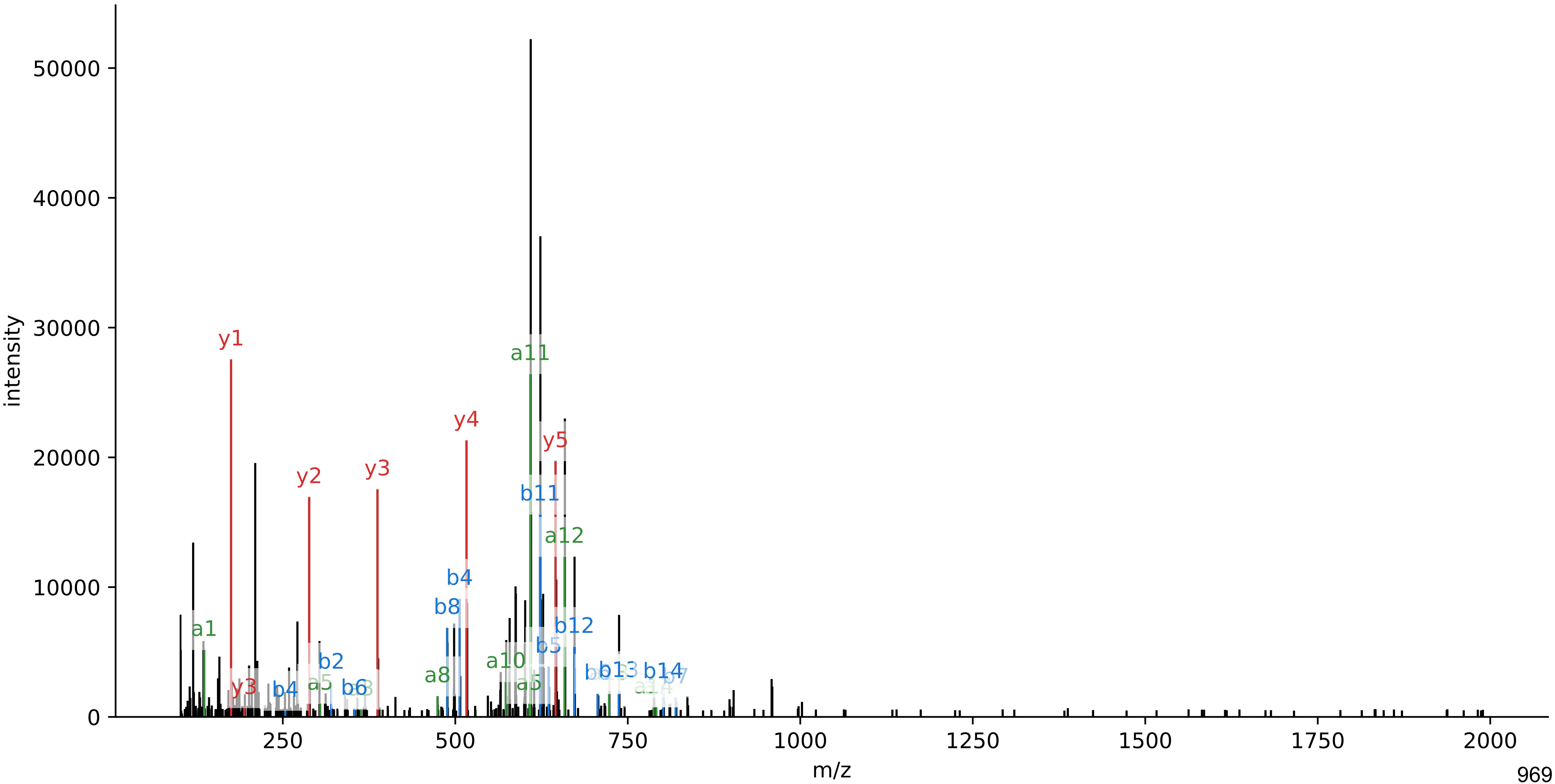

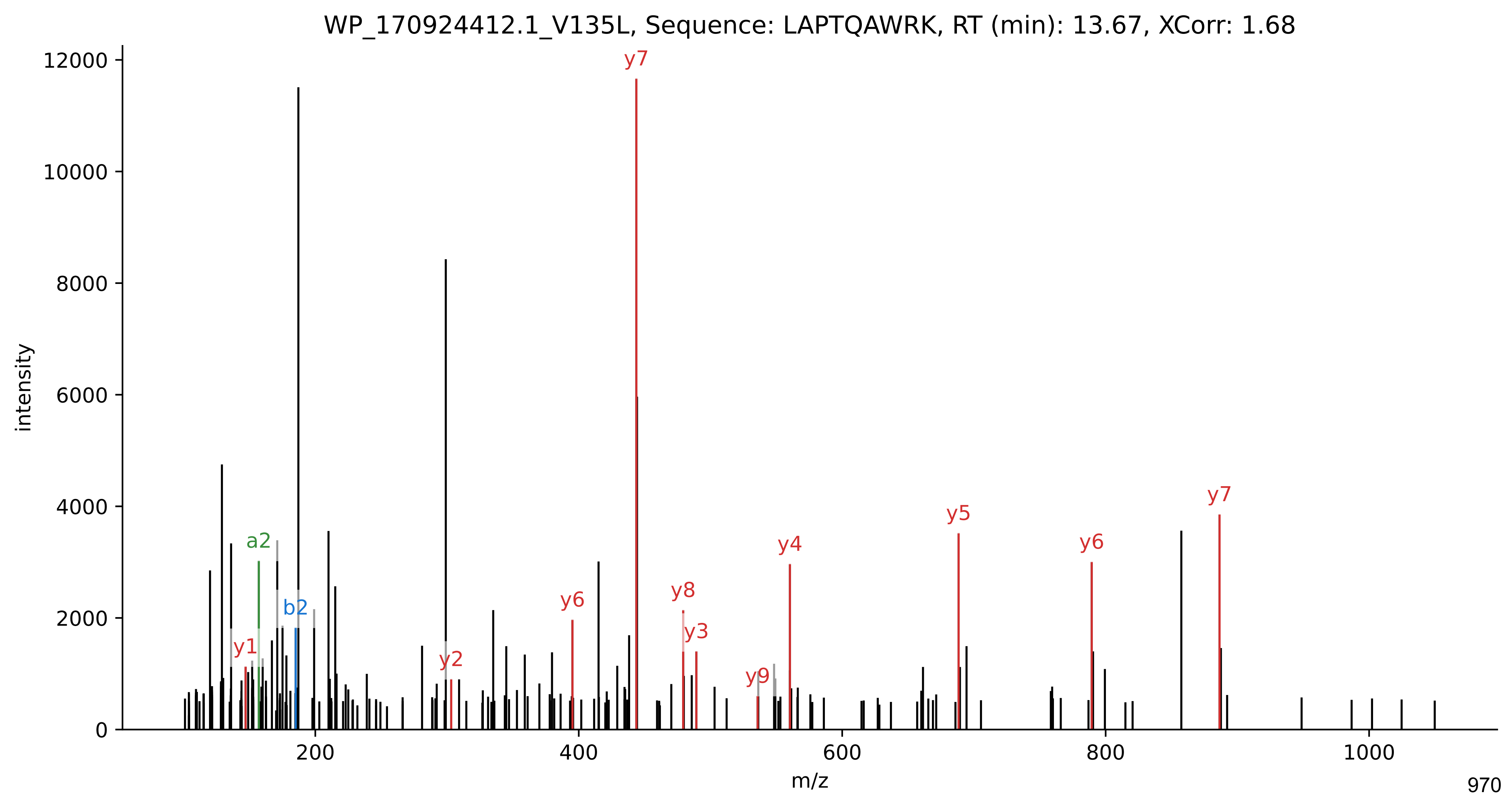

WP_170924412.1_V135L, Sequence: LAPTQAWRK, RT (min): 13.67, XCorr: 1.68
intensity
m/z
12000
10000
8000
6000
4000
2000
0
200
400
600
800
1000
y7
a2
b2
y1
y2
y6
y8
y3
y9
y4
y5
y6
y7

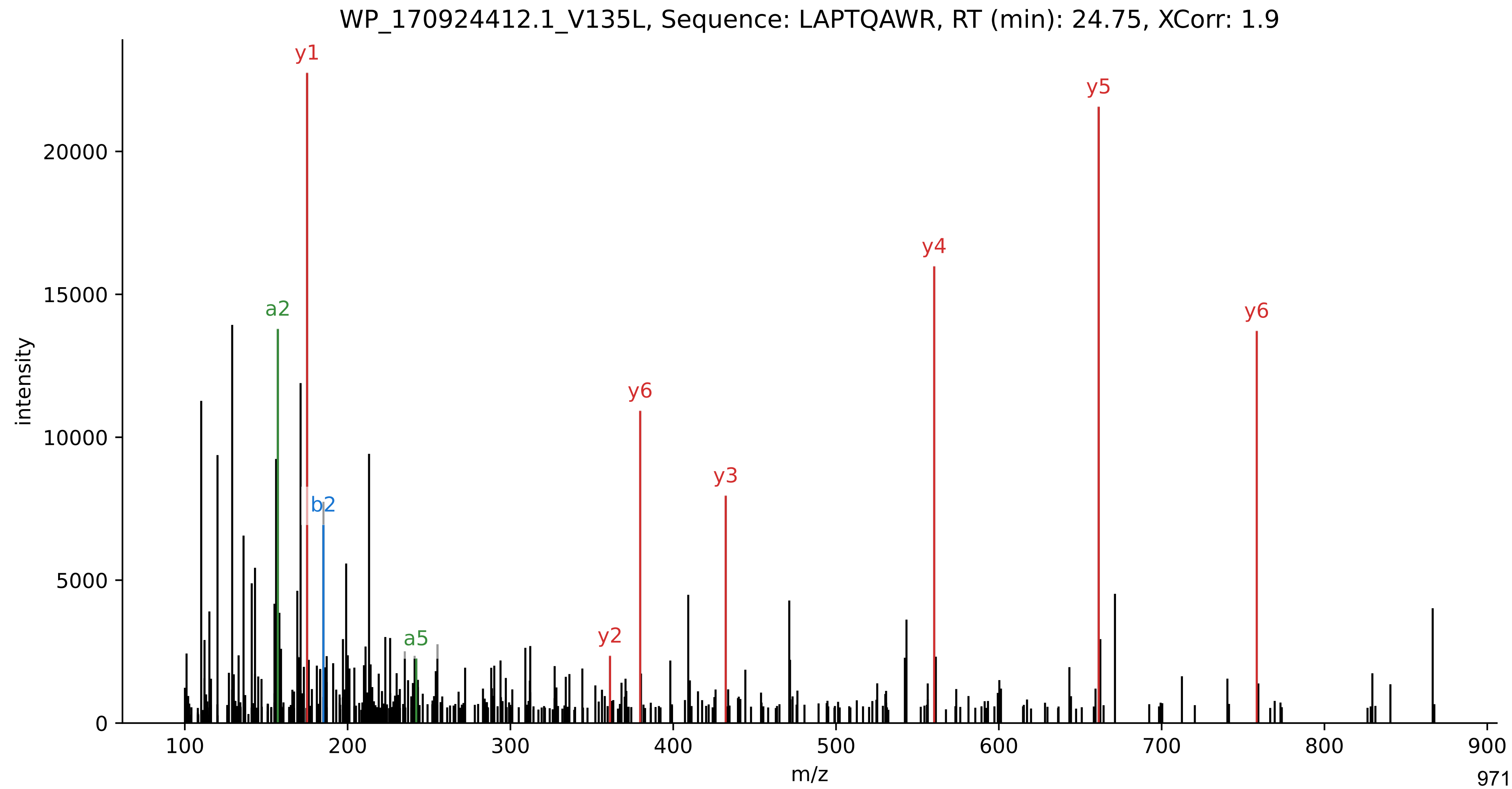

WP_170924412.1_V135L, Sequence: LAPTQAWR, RT (min): 24.75, XCorr: 1.9
y1
y5
y4
y6
a2
y6
b2
y3
a5
y2
intensity
m/z
0
5000
10000
15000
20000
100
200
300
400
500
600
700
800
900

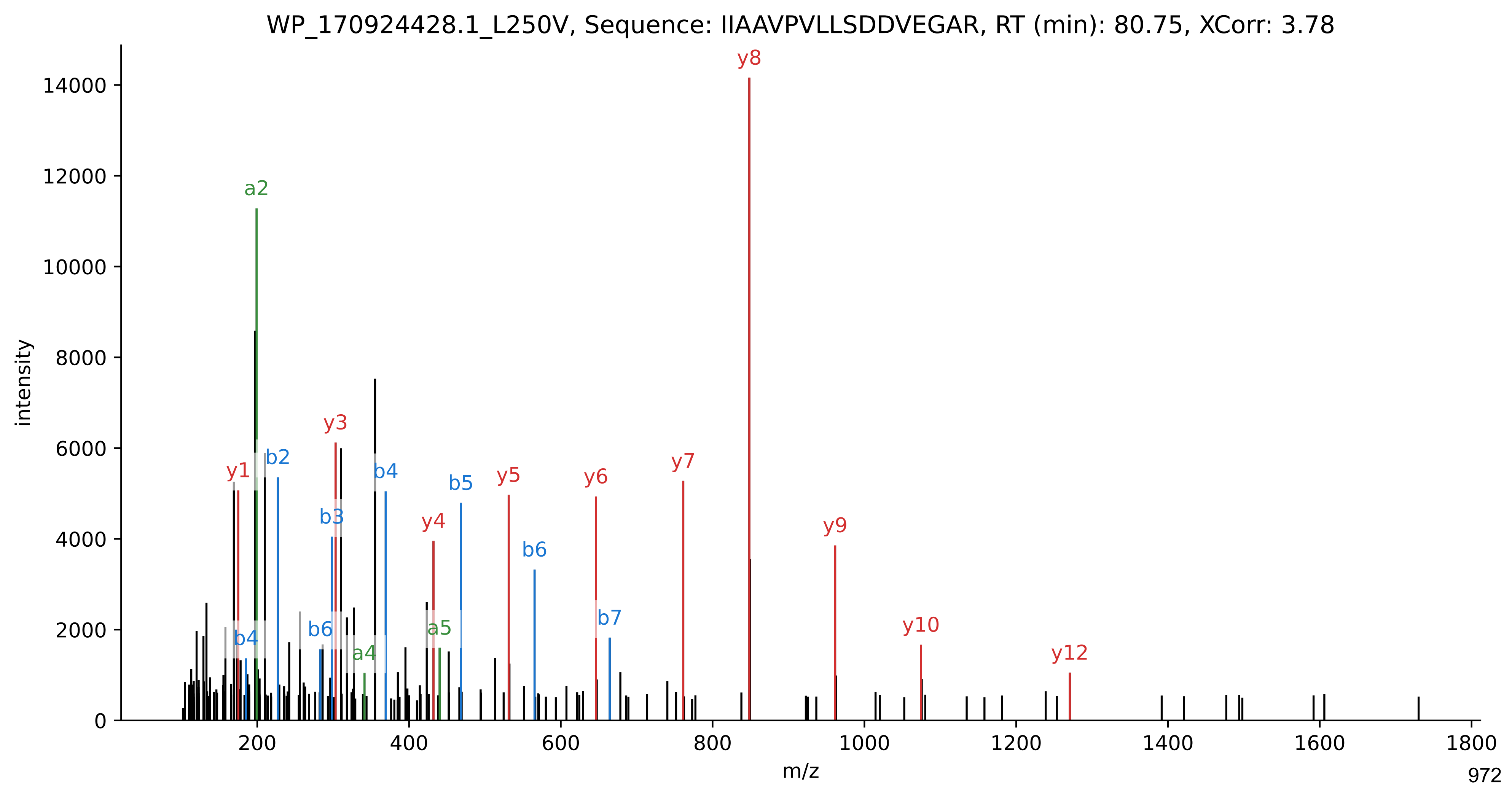
WP_170924428.1_L250V, Sequence: IIAAVPVLLSDDVEGAR, RT (min): 80.75, XCorr: 3.78
intensity
m/z
y8
a2
y3
y1
b2
b4
y7
y5
y6
b5
b3
y4
y9
b6
b7
y10
b4
b6
a5
a4
y12

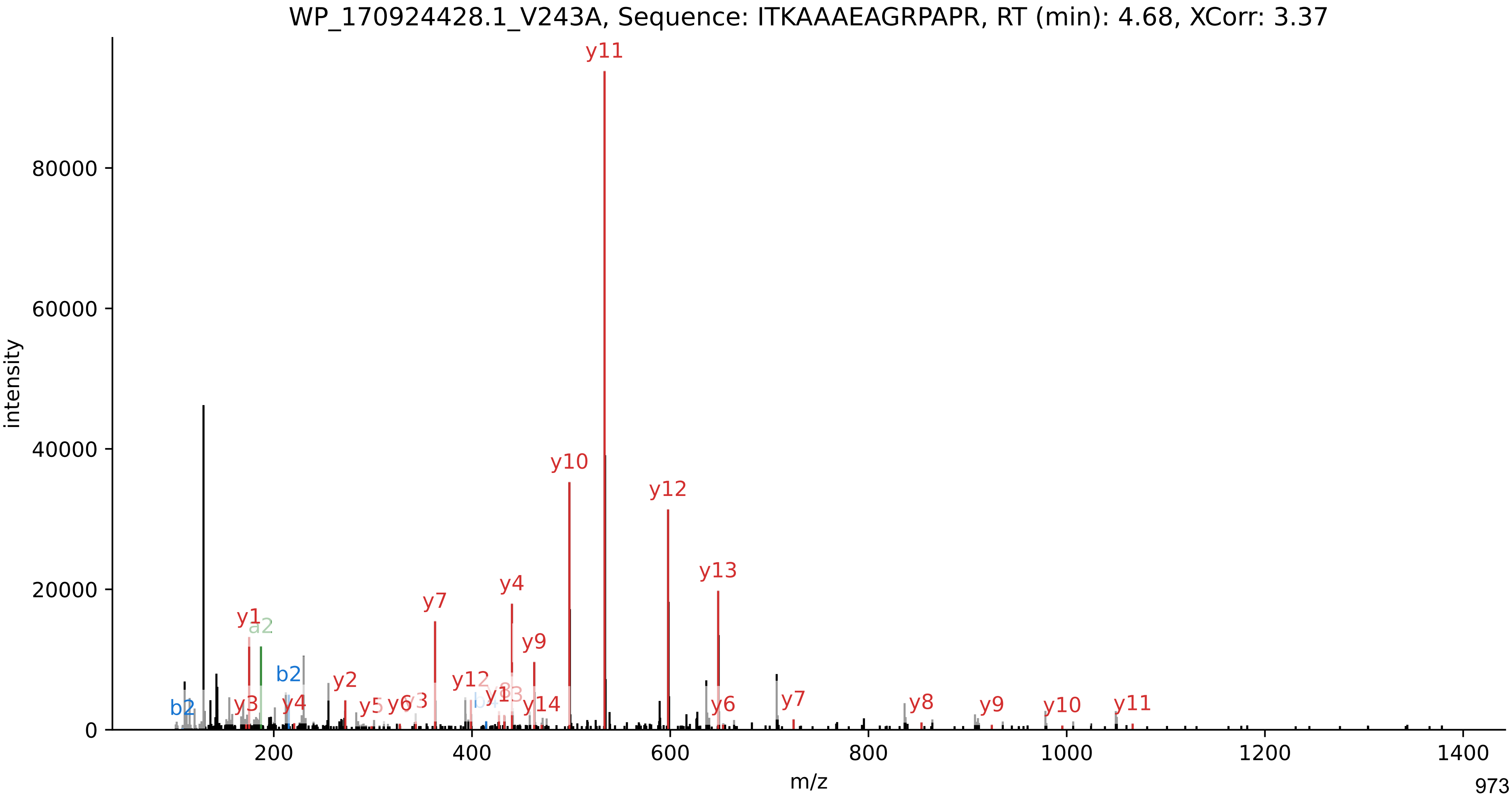

WP_170924428.1_V243A, Sequence: ITKAAAEAGRPAPR, RT (min): 4.68, XCorr: 3.37
intensity
m/z

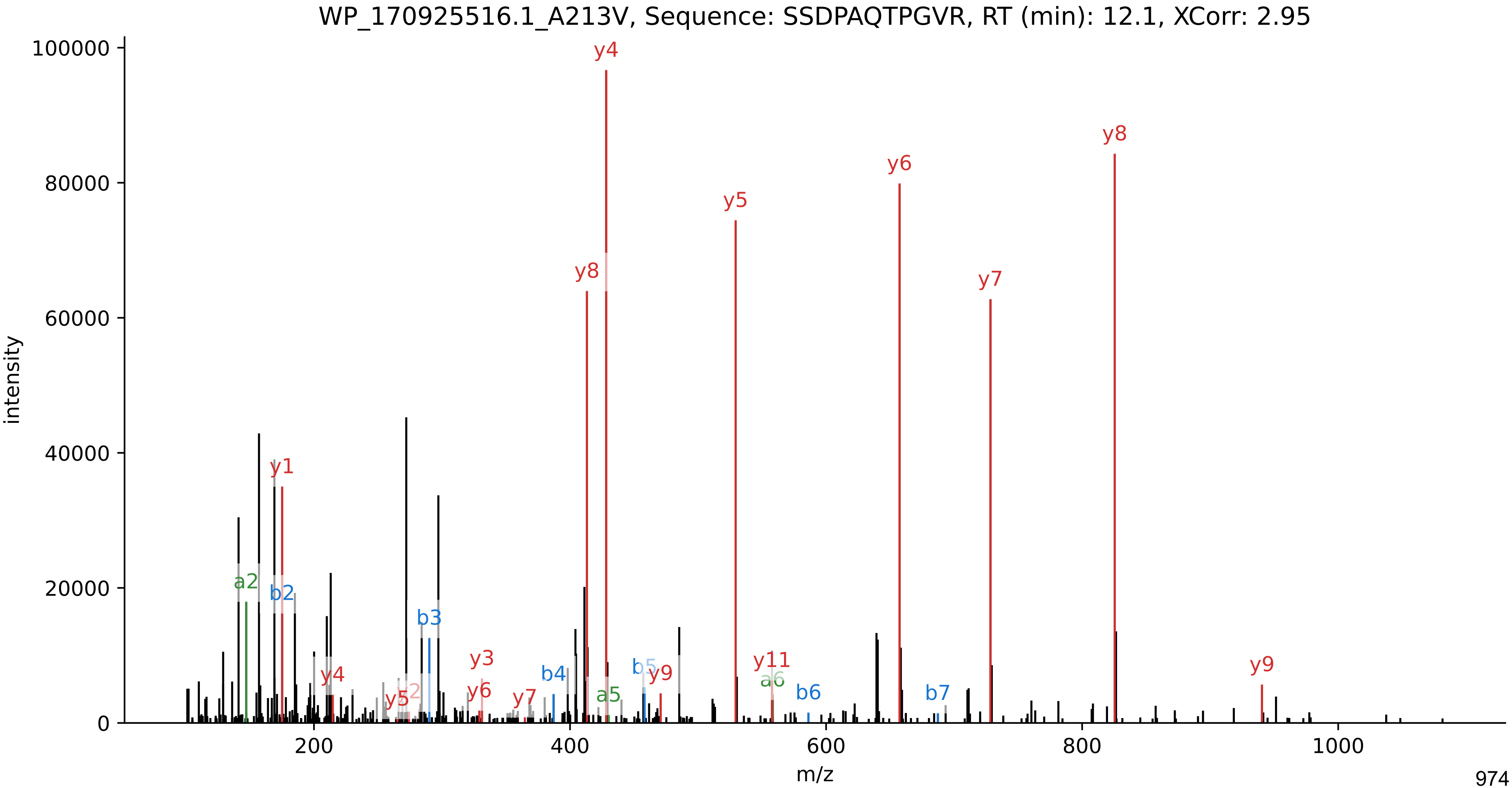
WP_170925516.1_A213V, Sequence: SSDPAQTPGVR, RT (min): 12.1, XCorr: 2.95
intensity
m/z
0
20000
40000
60000
80000
100000
200
400
600
800
1000
y4
y8
y6
y5
y8
y7
y1
a2
b2
b3
y3
y4
y6
y5
y7
b4
a5
b5
y9
y11
a6
b6
b7
y9

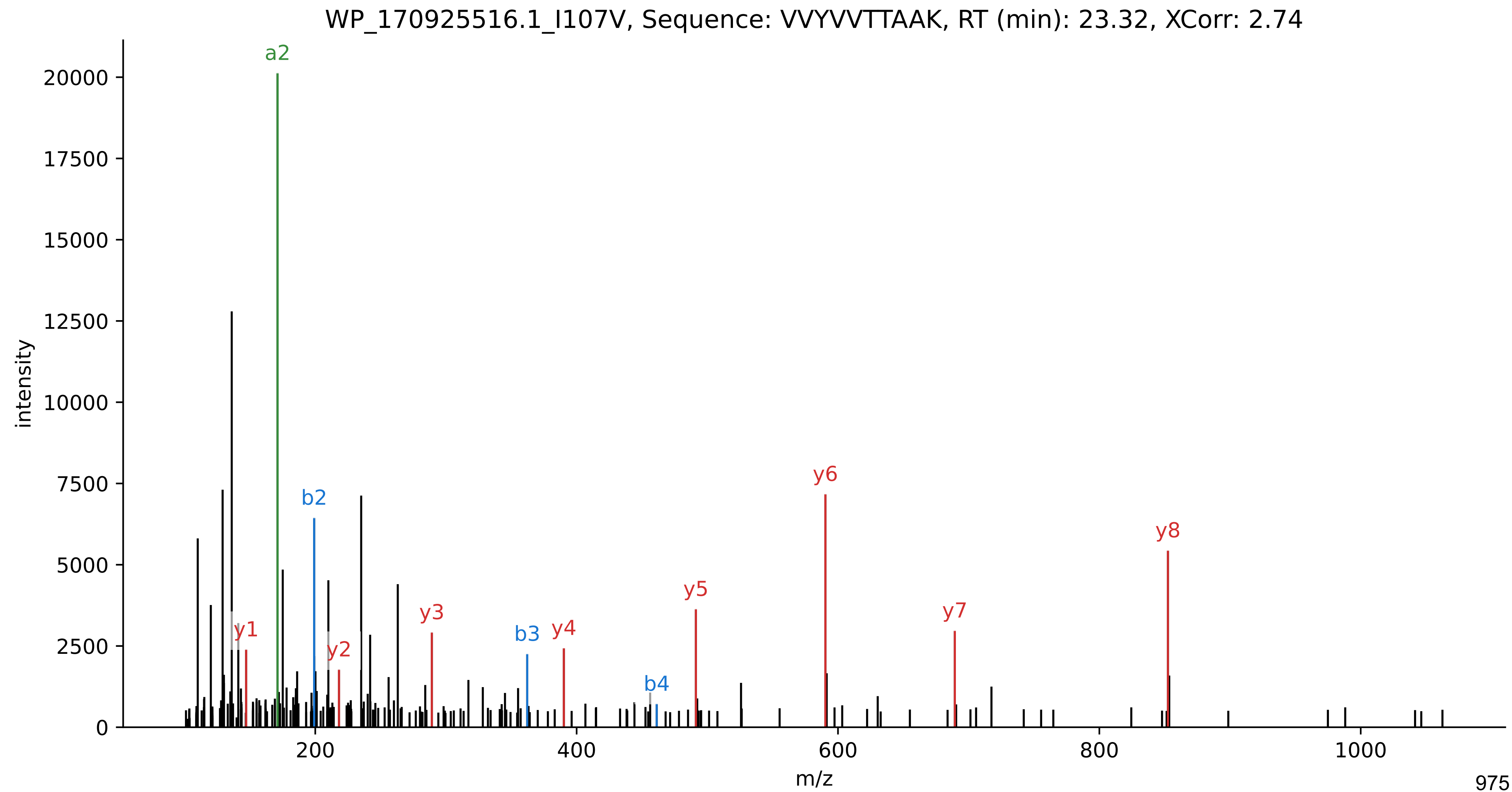

WP_170925516.1_I107V, Sequence: VVYVVTTAAK, RT (min): 23.32, XCorr: 2.74
a2
b2
b3
b4
y1
y2
y3
y4
y5
y6
y7
y8
intensity
m/z
0
2500
5000
7500
10000
12500
15000
17500
20000
200
400
600
800
1000

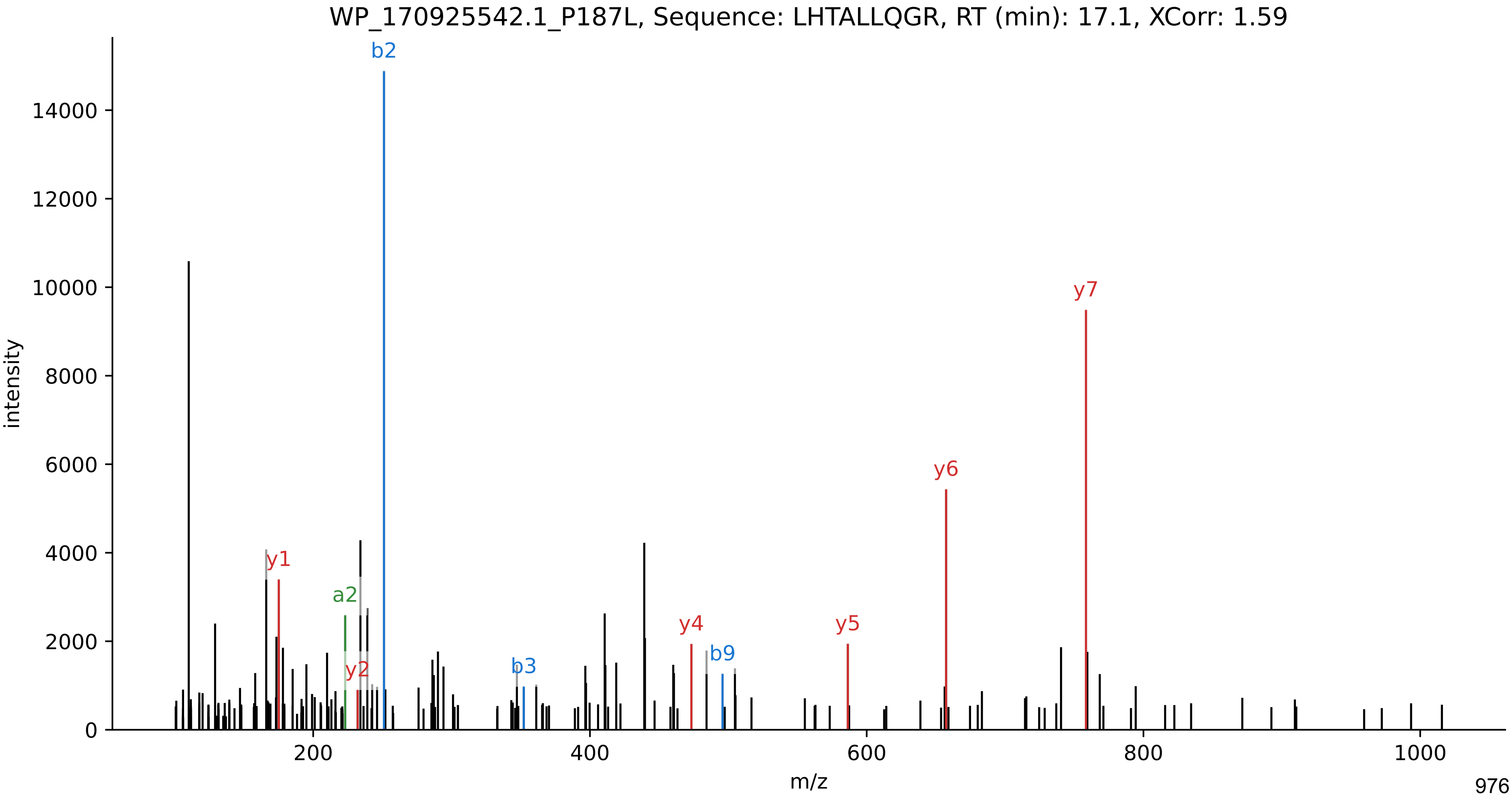

WP_170925542.1_P187L, Sequence: LHTALLQGR, RT (min): 17.1, XCorr: 1.59
intensity
m/z
b2
y7
y6
y1
a2
y2
b3
y4
b9
y5
0
2000
4000
6000
8000
10000
12000
14000
200
400
600
800
1000

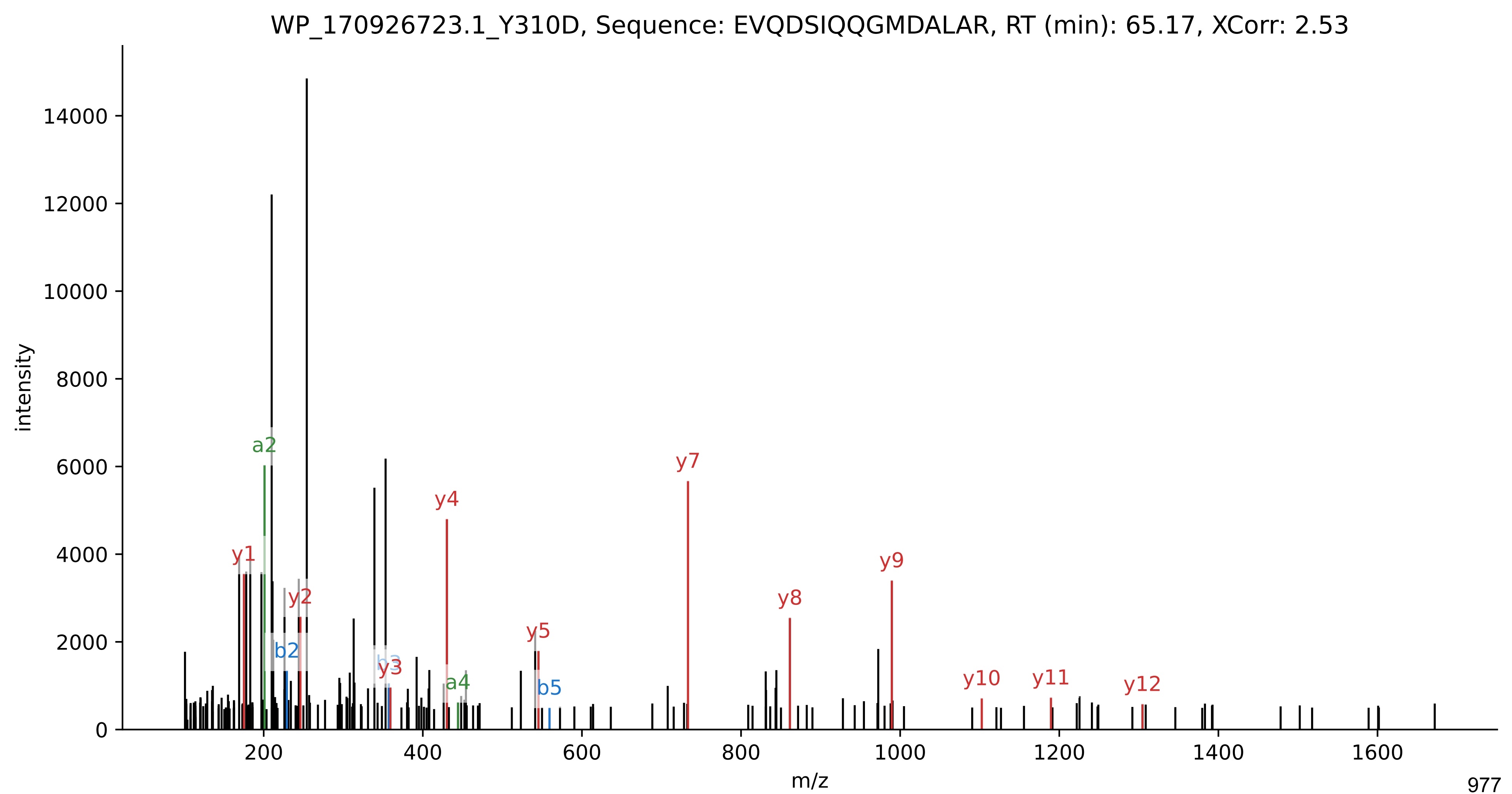
WP_170926723.1_Y310D, Sequence: EVQDSIQQGMDALAR, RT (min): 65.17, XCorr: 2.53
intensity
m/z
a2
y1
y2
b2
b3
y3
y4
a4
y5
b5
y7
y8
y9
y10
y11
y12
0
2000
4000
6000
8000
10000
12000
14000
200
400
600
800
1000
1200
1400
1600

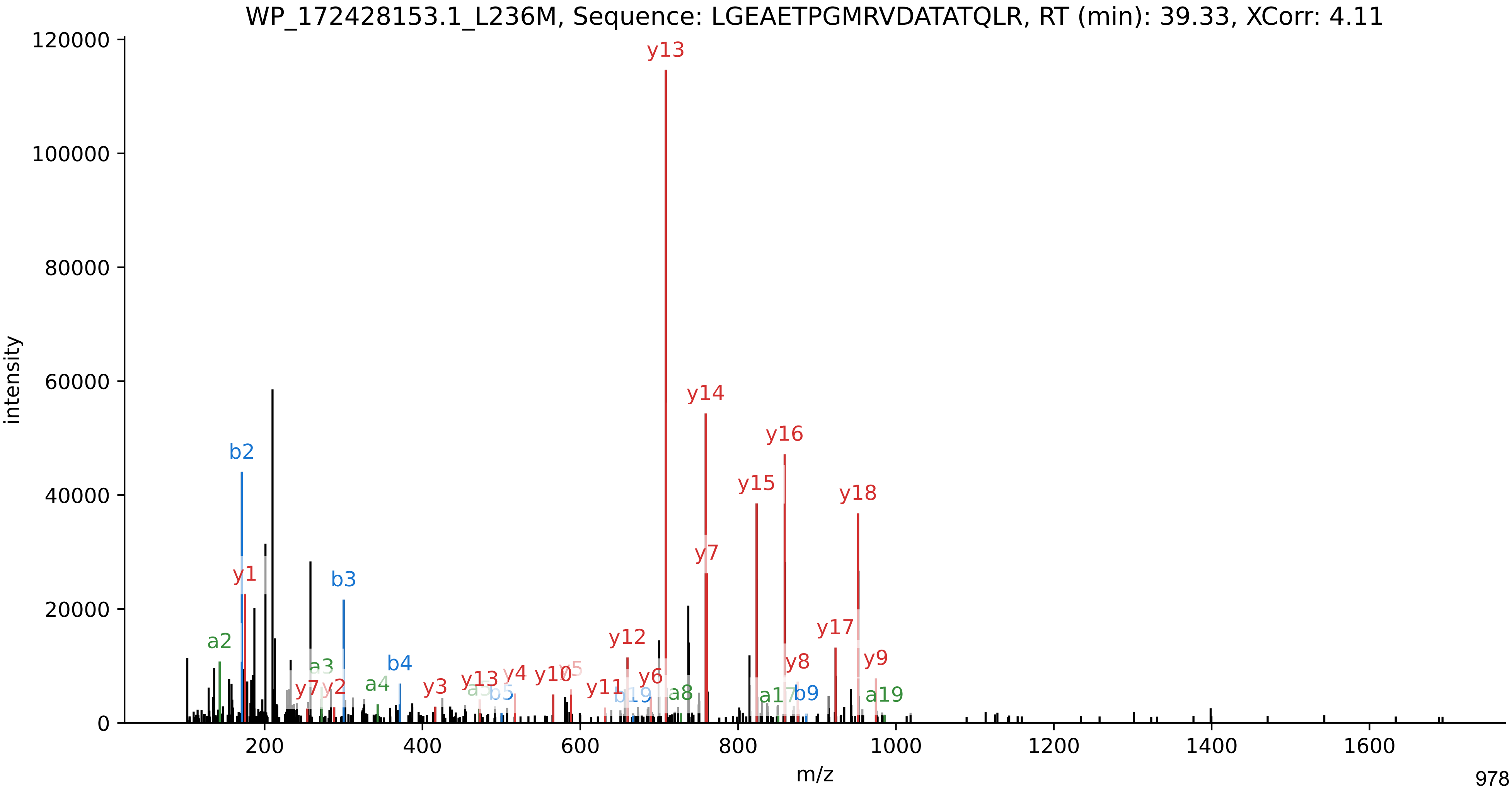
WP_172428153.1_L236M, Sequence: LGEAETPGMRVDATATQLR, RT (min): 39.33, XCorr: 4.11
intensity
m/z
y13
y14
y16
y15
y18
y7
b2
b3
y1
y17
y12
a2
b4
a3
y9
y8
a4
y3
y13
y4
y10
y6
y11
b9
a8
a19
y7
y2
b5
b19
a17

WP_176213600.1_D368H, Sequence: SALQHGAPVLVQVPR, RT (min): 39.98, XCorr: 4.62

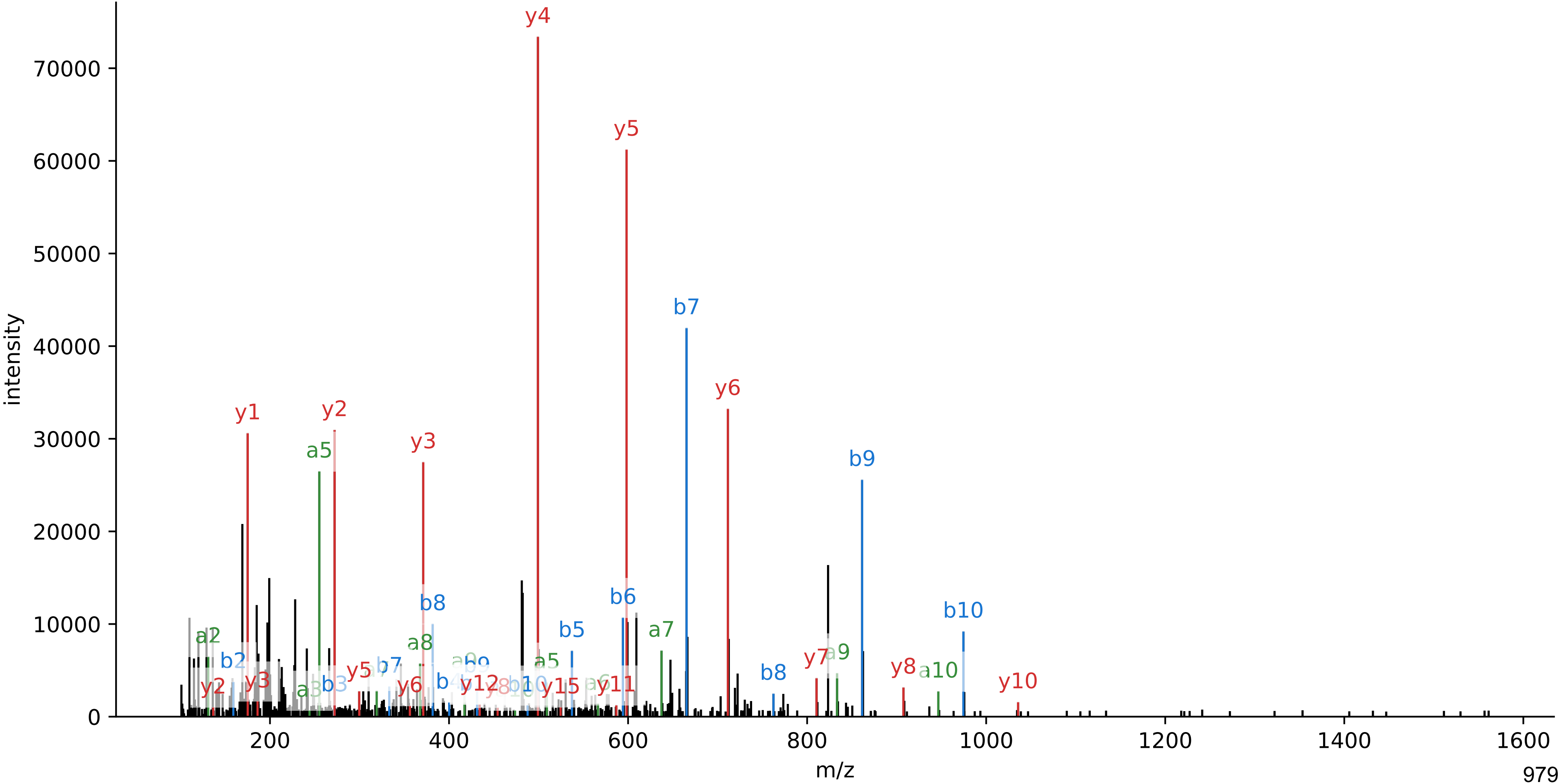

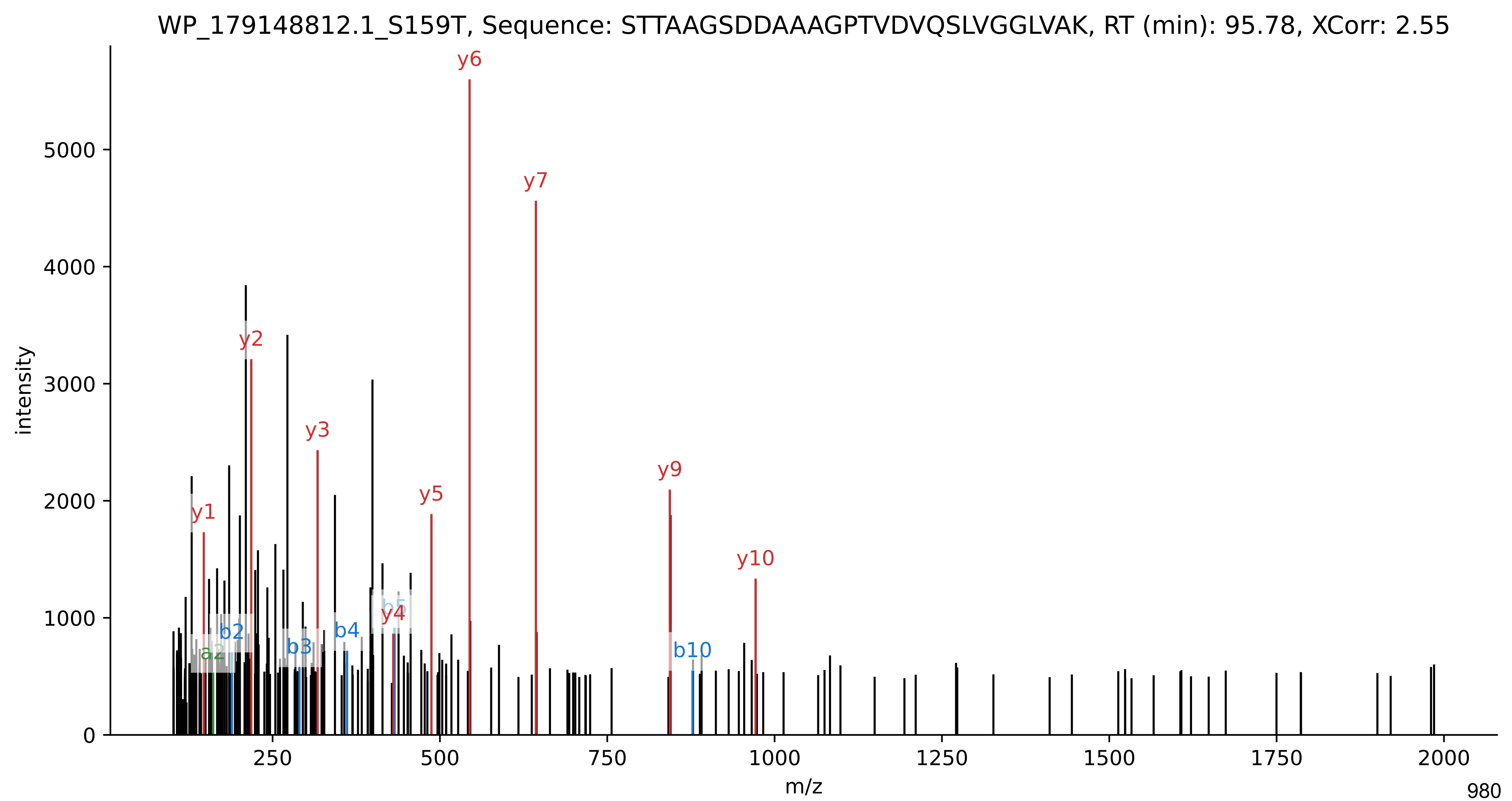

WP_179148812.1_S159T, Sequence: STTAAGSDDAAAGPTVDVQSLVGGLVAK, RT (min): 95.78, XCorr: 2.55
intensity
m/z
y1
y2
y3
y4
y5
y6
y7
y9
y10
a2
b2
b3
b4
b5
b10

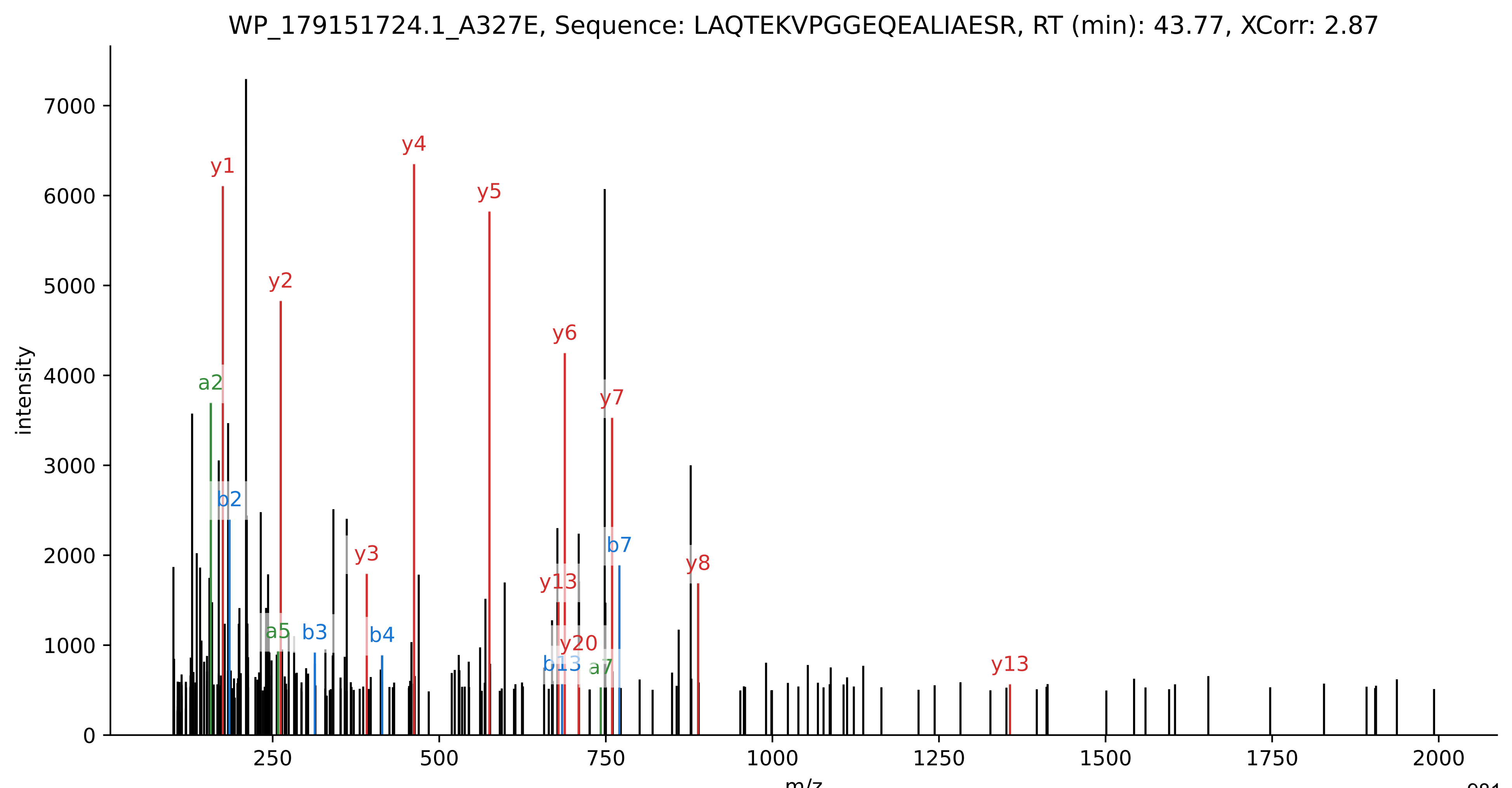

WP_179151724.1_A327E, Sequence: LAQTEKVPGGEQEALIAESR, RT (min): 43.77, XCorr: 2.87
intensity
m/z
y1
y2
y3
y4
y5
y6
y7
y8
y13
y20
y13
a2
a5
a7
b2
b3
b4
b7
b13

WP_179151742.1_G248E, Sequence: TAAPEAAAPRPAPQFEVMYR, RT (min): 50.43, XCorr: 4.71

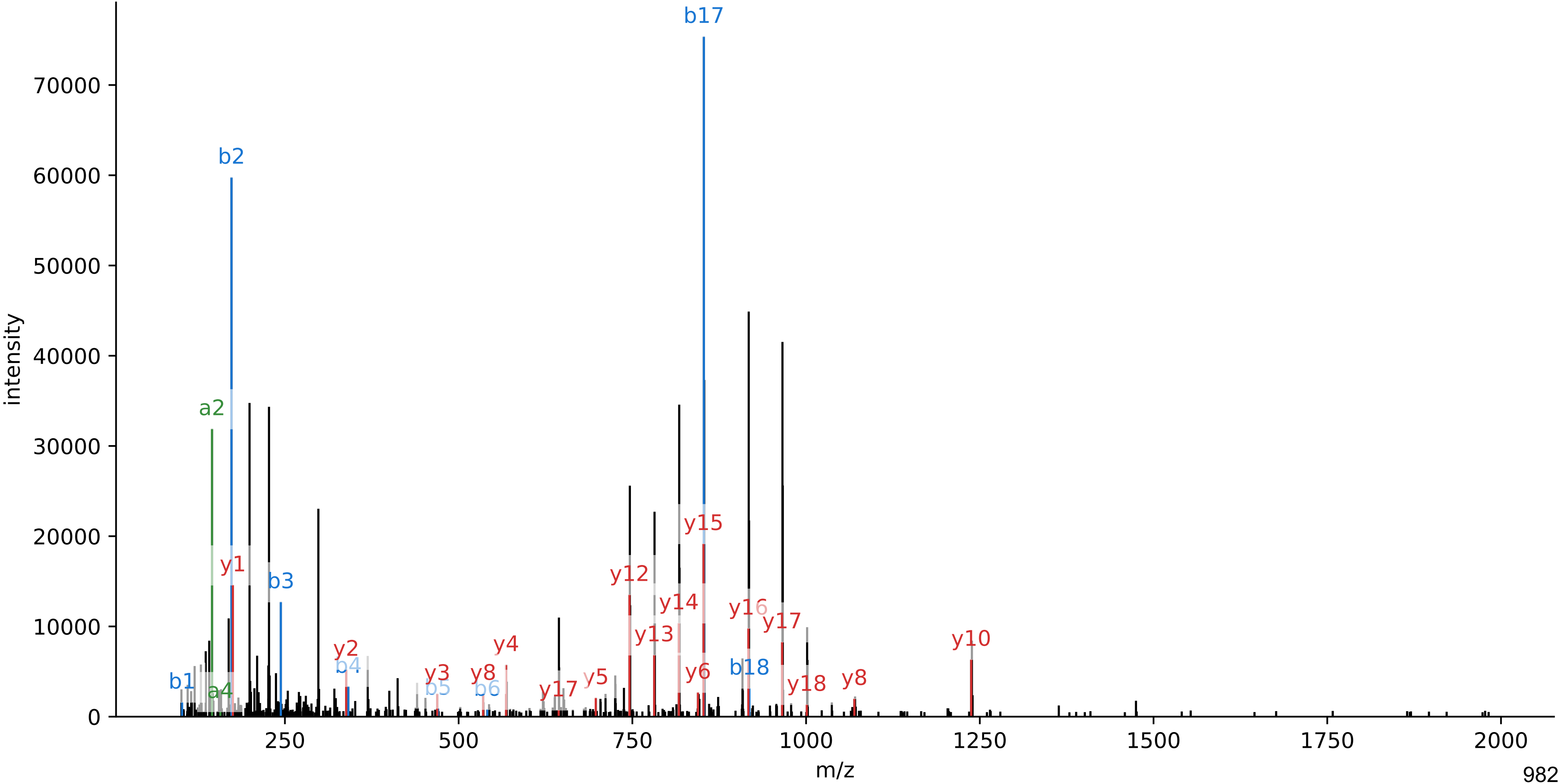

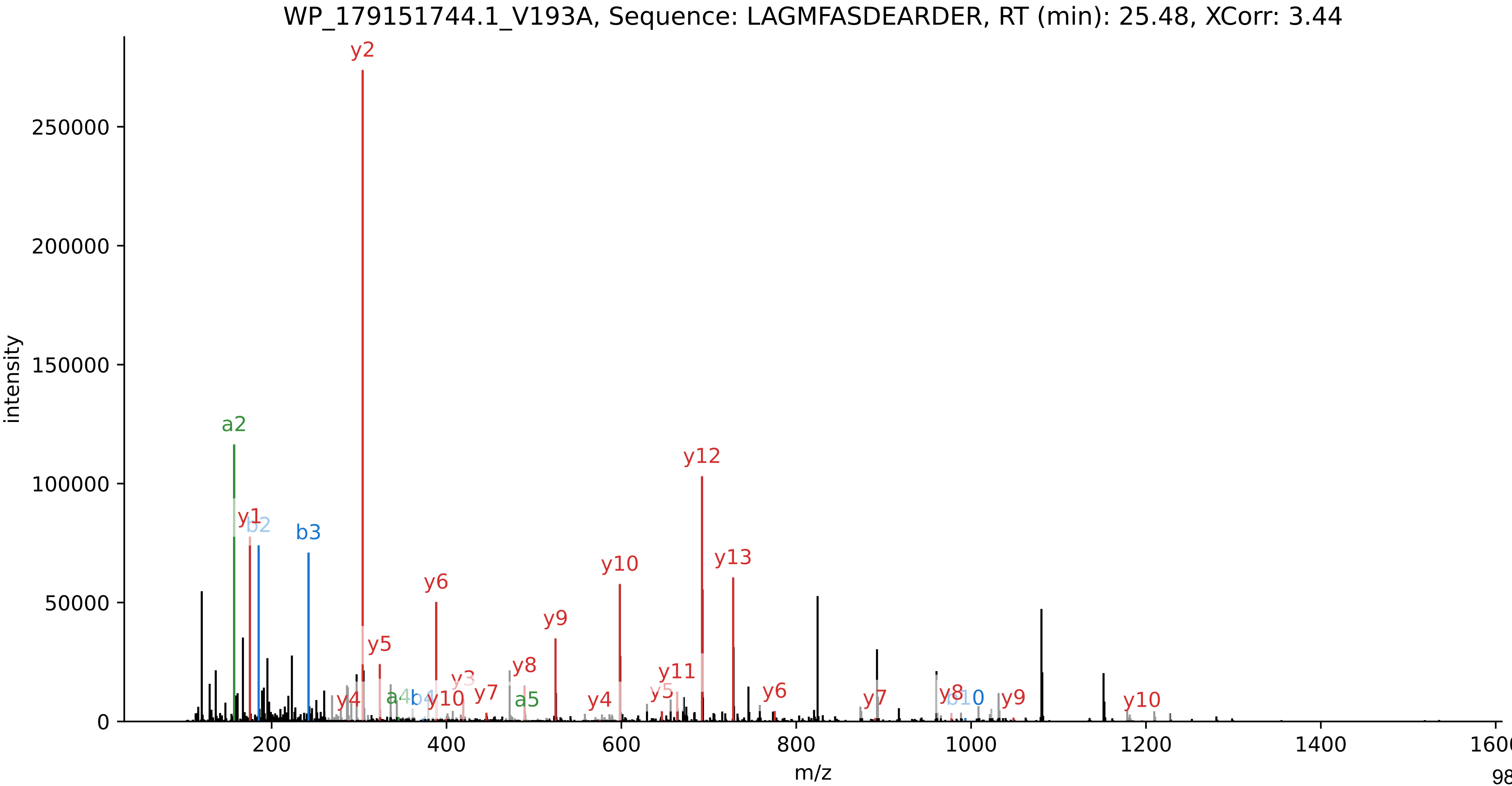
WP_179151744.1_V193A, Sequence: LAGMFASDEARDER, RT (min): 25.48, XCorr: 3.44
intensity
m/z
y2
a2
y1
b2
b3
y12
y13
y10
y6
y9
y5
y8
y3
y7
a5
y4
y10
a4
b4
y11
y5
y4
y6
y7
y8
b10
y9
y10
0
50000
100000
150000
200000
250000
200
400
600
800
1000
1200
1400
1600

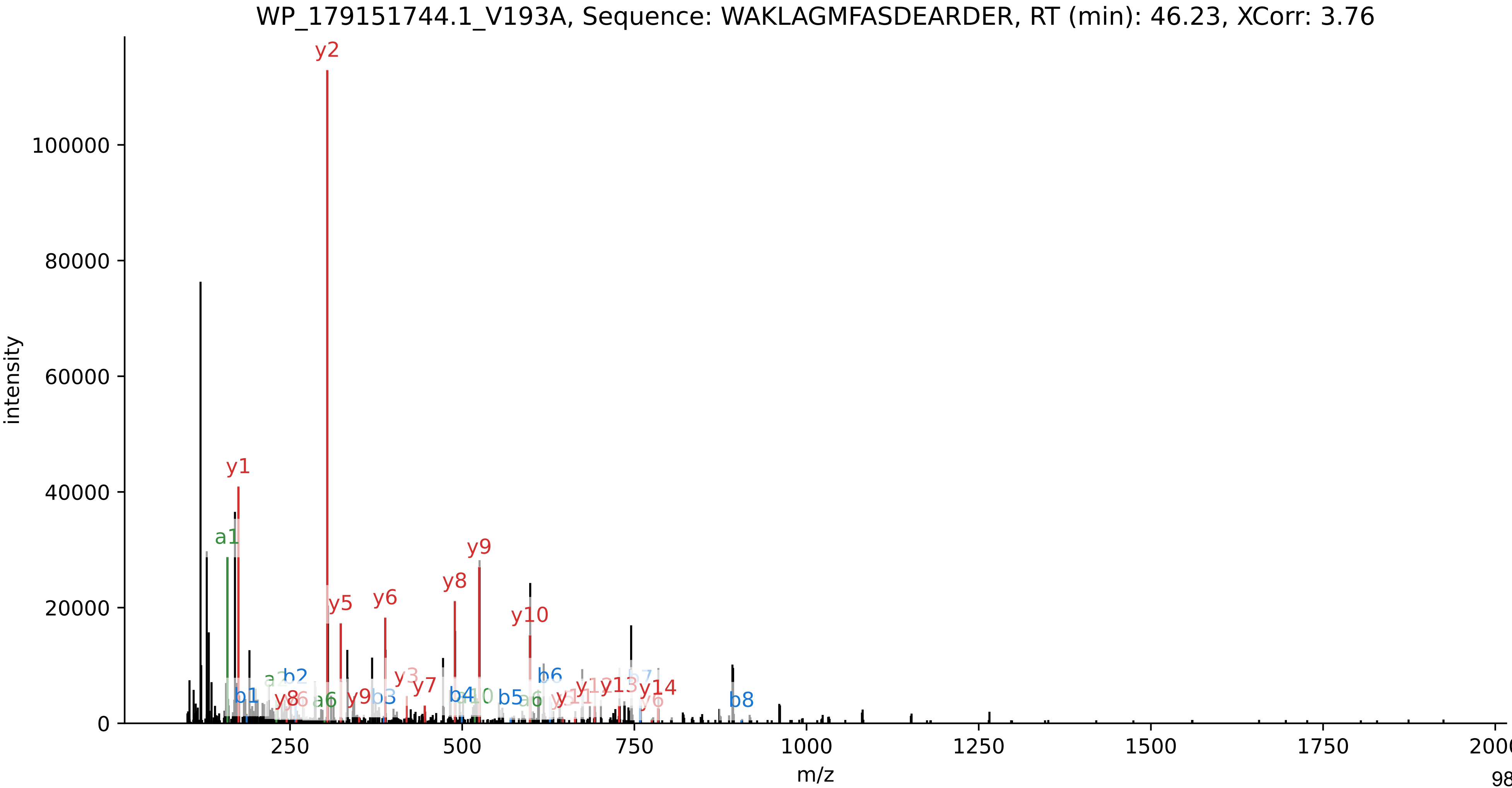
WP_179151744.1_V193A, Sequence: WAKLAGMFASDEARDER, RT (min): 46.23, XCorr: 3.76
intensity
m/z
y2
y1
a1
y5
y6
y8
y9
y10
b1
b2
y8
a6
y9
b3
y3
y7
b4
b5
b6
y13
y14
b8
0
20000
40000
60000
80000
100000
250
500
750
1000
1250
1500
1750
2000

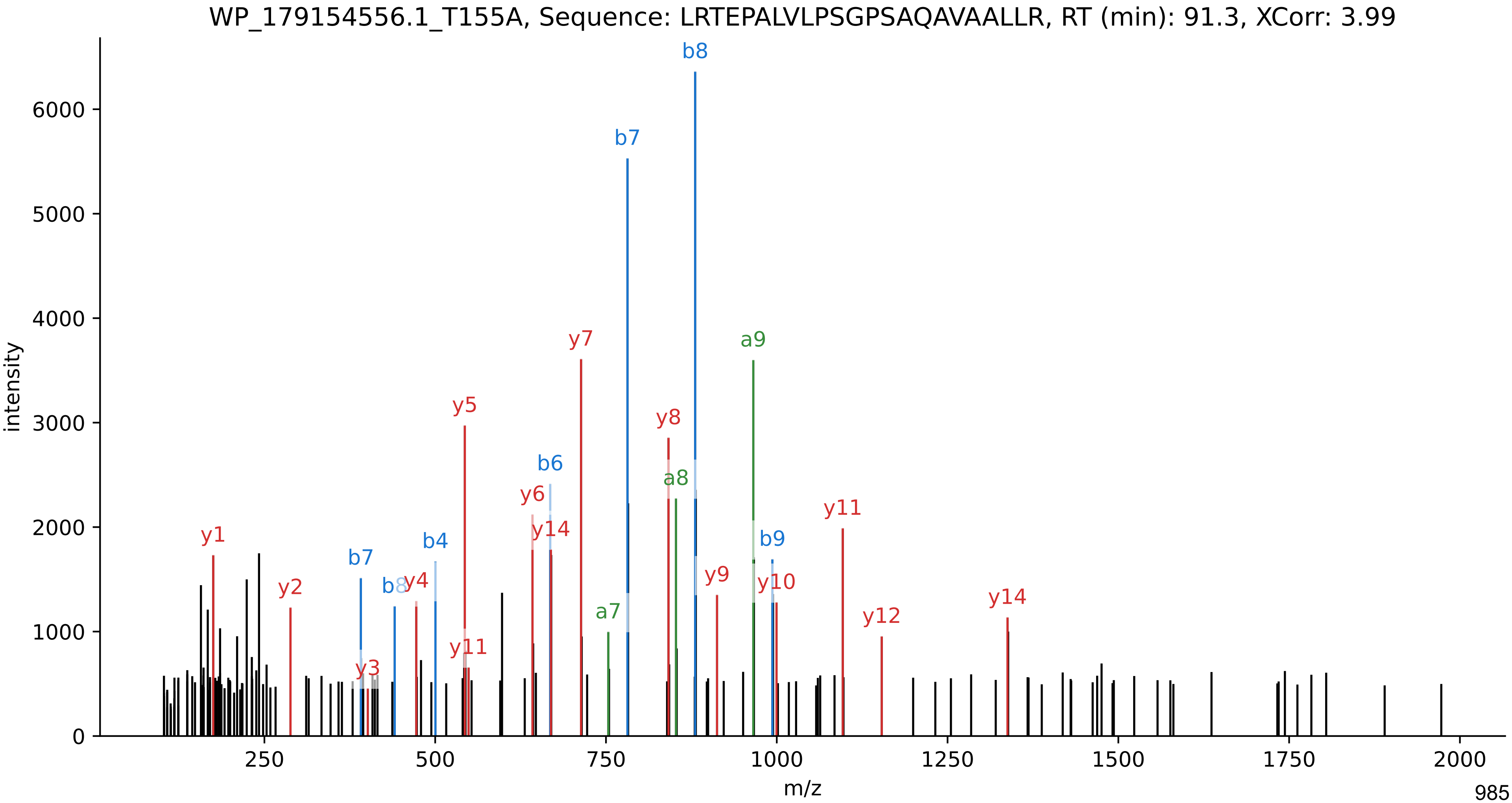
WP_179154556.1_T155A, Sequence: LRTEPALVLPSGPSAQAVAALLR, RT (min): 91.3, XCorr: 3.99
intensity
m/z
b8
b7
y7
a9
y5
y8
b6
a8
y6
y14
y11
y1
b4
b9
b7
y2
b8
y4
y9
y10
y14
y12
a7
y11
y3
0
1000
2000
3000
4000
5000
6000
250
500
750
1000
1250
1500
1750
2000

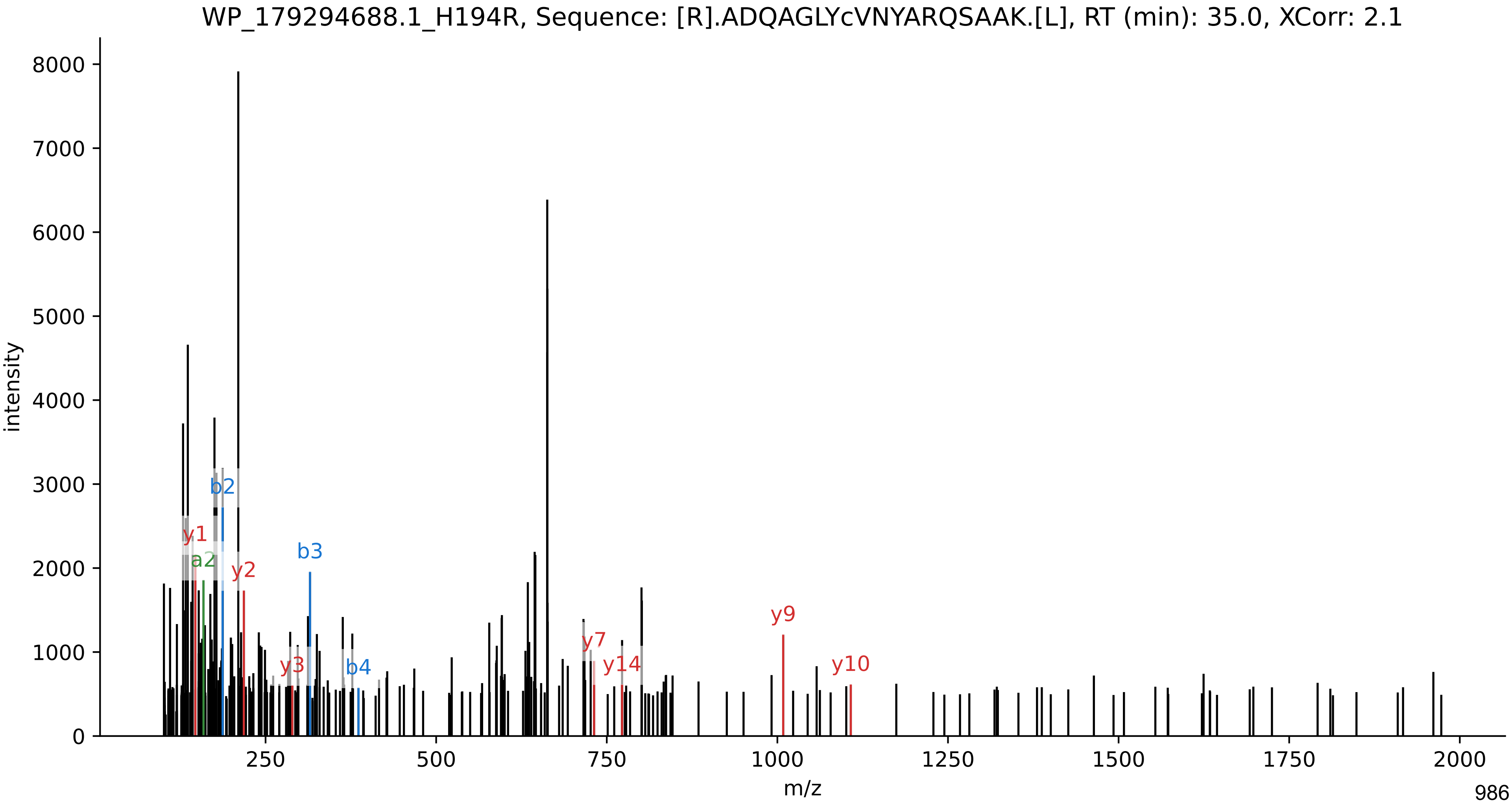
WP_179294688.1_H194R, Sequence: [R].ADQAGLYcVNYARQSAAK.[L], RT (min): 35.0, XCorr: 2.1
intensity
m/z
y1
a2
b2
y2
b3
y3
b4
y7
y14
y9
y10
0
1000
2000
3000
4000
5000
6000
7000
8000
250
500
750
1000
1250
1500
1750
2000

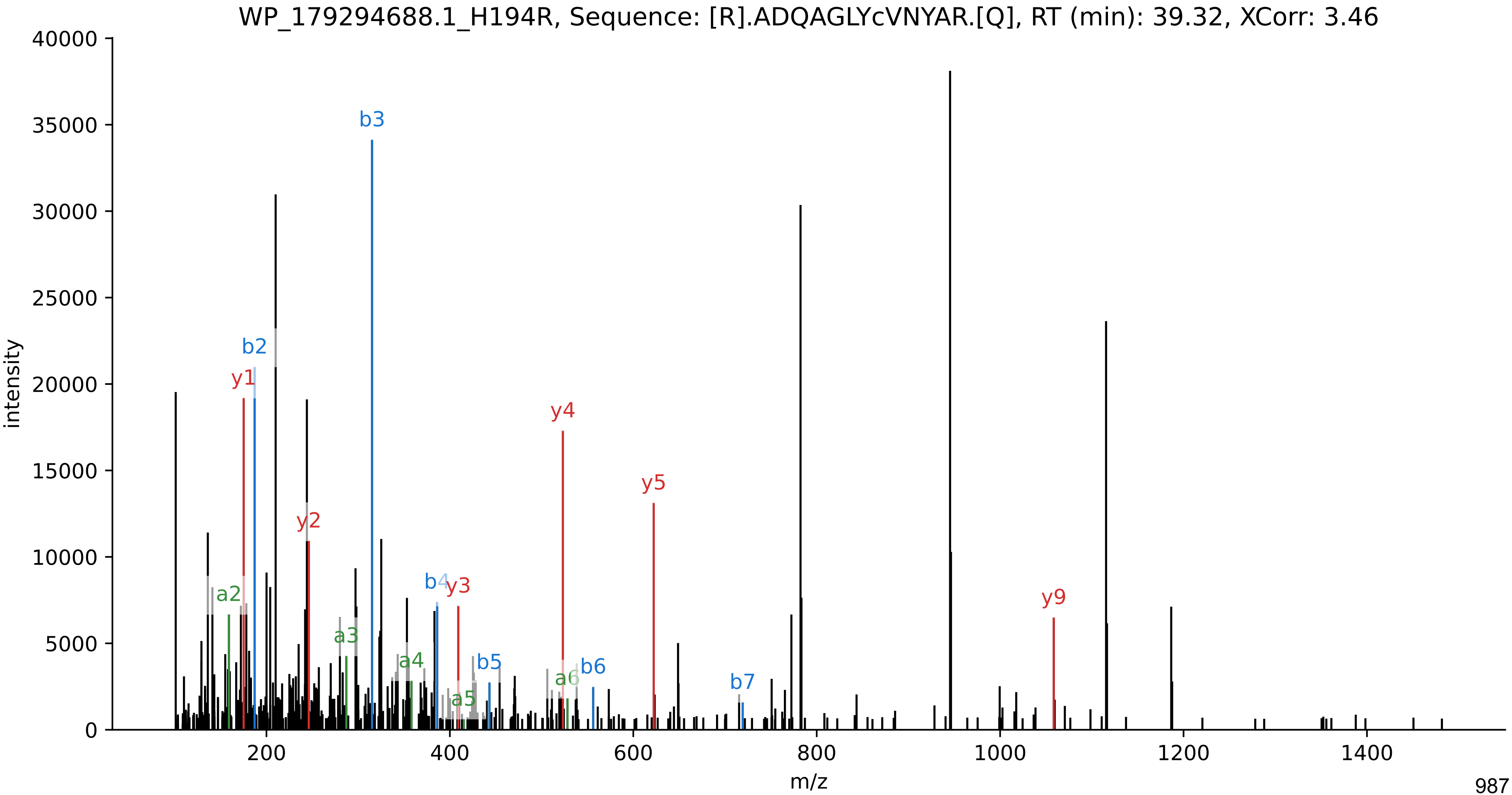

WP_179294688.1_H194R, Sequence: [R].ADQAGLYcVNYAR.[Q], RT (min): 39.32, XCorr: 3.46
intensity
m/z
b3
b2
y1
y2
y4
y5
a2
a3
a4
b4
y3
b5
a5
a6
b6
b7
y9

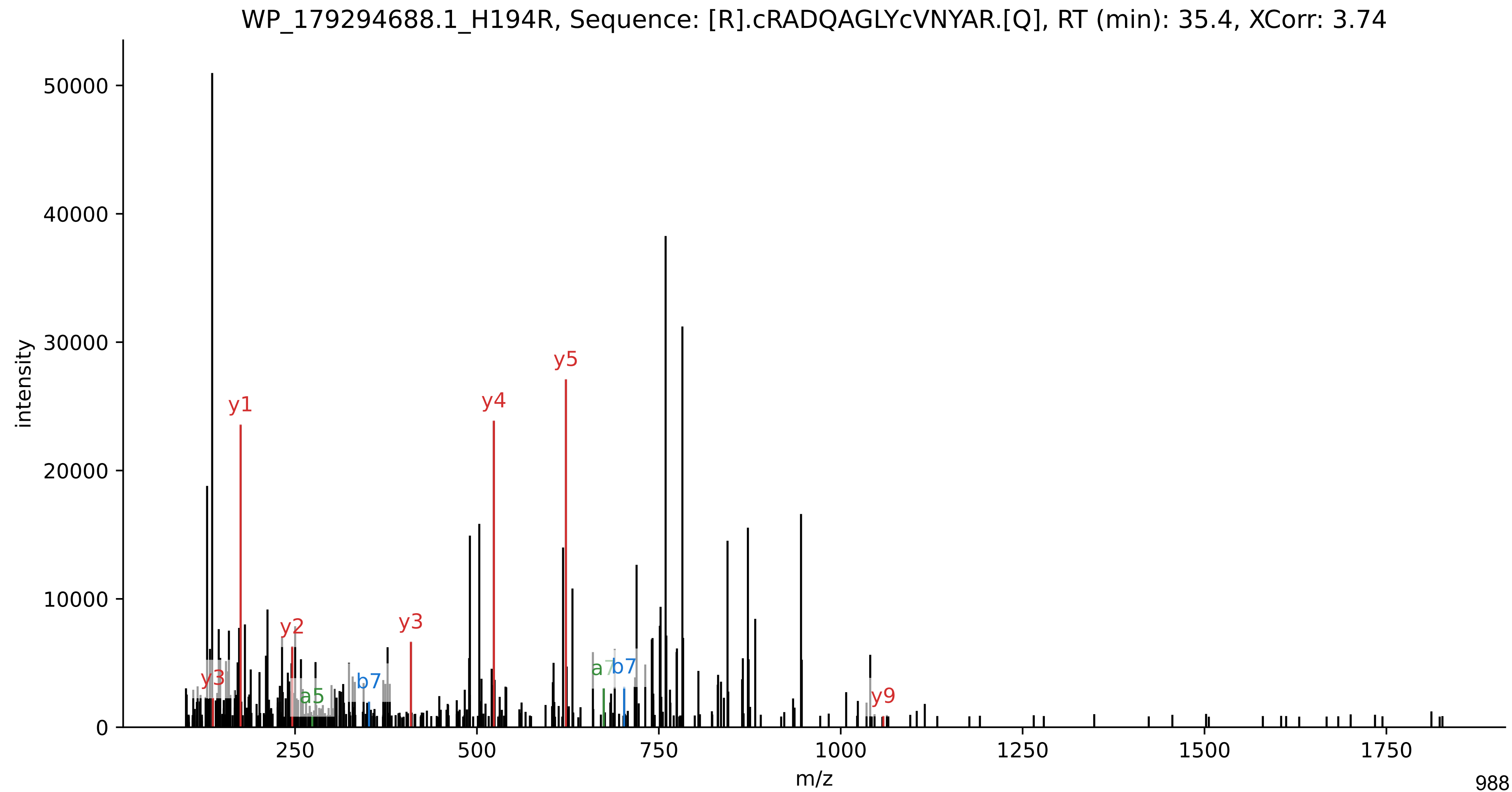

WP_179294688.1_H194R, Sequence: [R].cRADQAGLYcVNYAR.[Q], RT (min): 35.4, XCorr: 3.74
intensity
m/z
y1
y2
y3
y4
y5
y9
a5
a7
b7

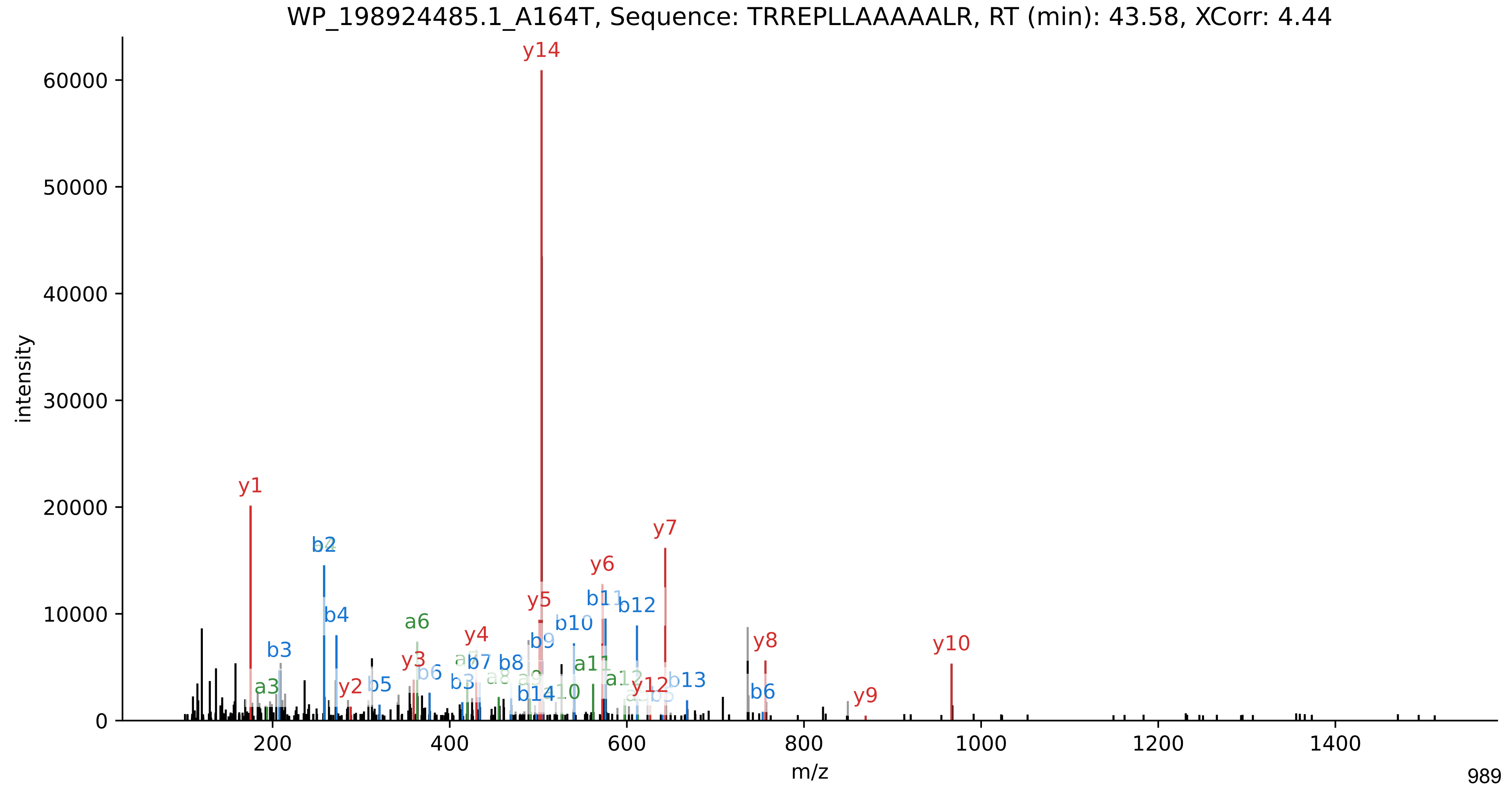

WP_198924485.1_A164T, Sequence: TRREPLLAAAAALR, RT (min): 43.58, XCorr: 4.44
intensity
m/z
y14
y1
b2
y7
y6
y5
b11
b12
b10
b4
a6
y4
b9
y8
y10
b3
y3
a11
b7
b8
b6
a12
b13
y12
a3
y2
b5
b14
b6
y9

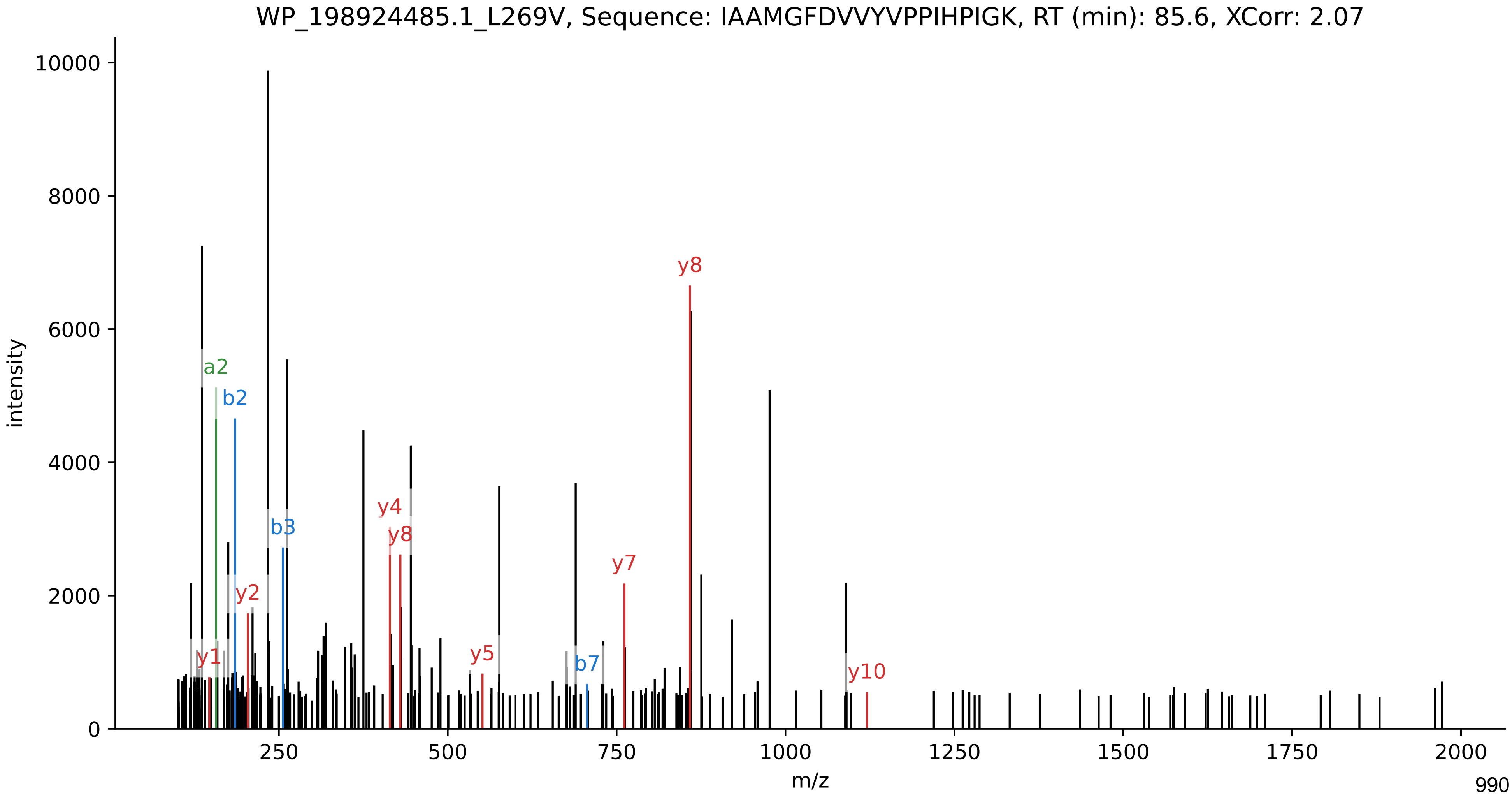
WP_198924485.1_L269V, Sequence: IAAMGFDVVYVPPIHPIGK, RT (min): 85.6, XCorr: 2.07
intensity
m/z
a2
b2
b3
b7
y1
y2
y4
y8
y5
y7
y8
y10
0
2000
4000
6000
8000
10000
250
500
750
1000
1250
1500
1750
2000

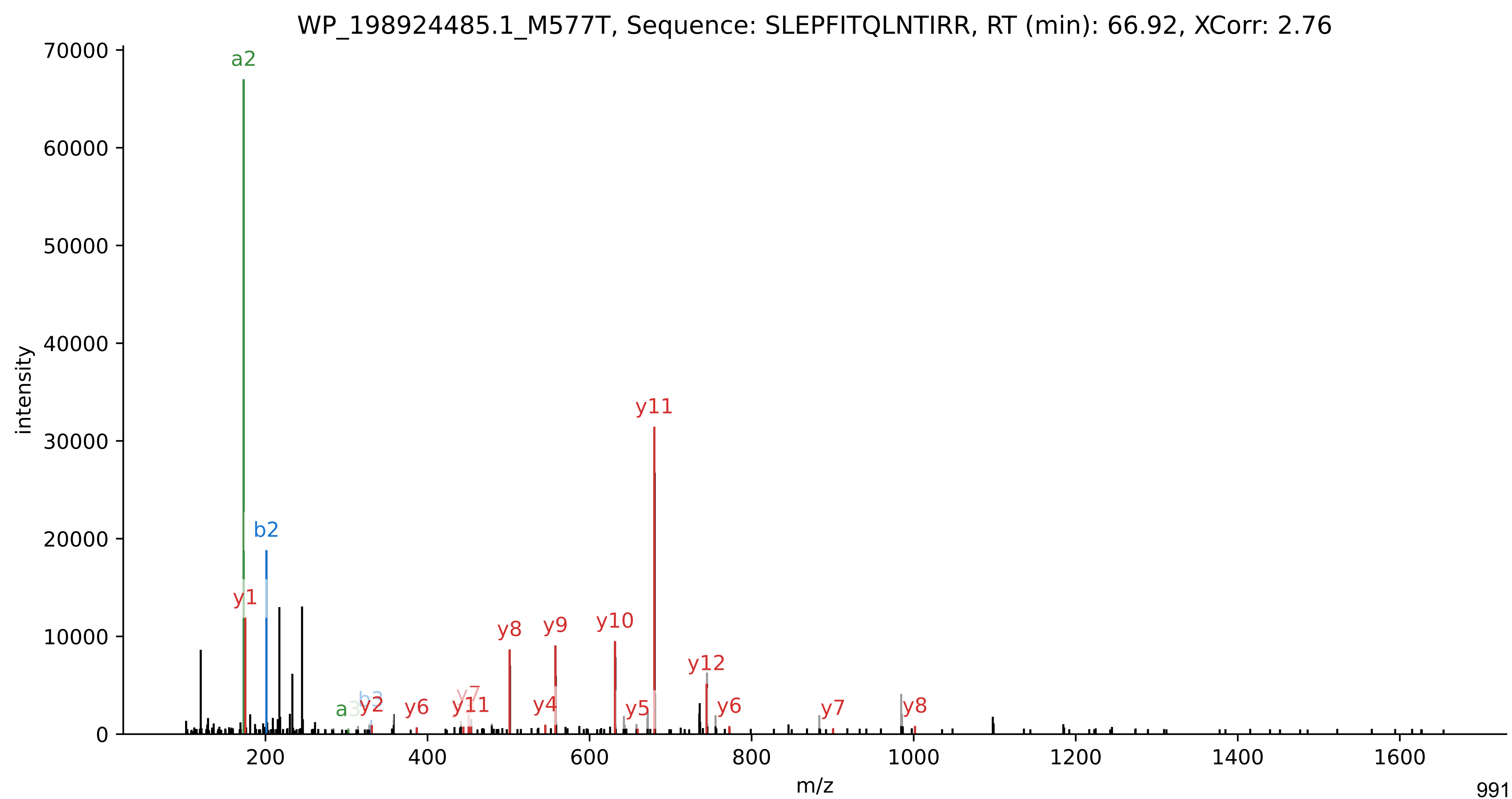

WP_198924485.1_M577T, Sequence: SLEPFITQLNTIRR, RT (min): 66.92, XCorr: 2.76
intensity
m/z
a2
b2
y1
y11
y8
y9
y10
y12
a3
b3
y2
y6
y7
y11
y4
y5
y6
y7
y8

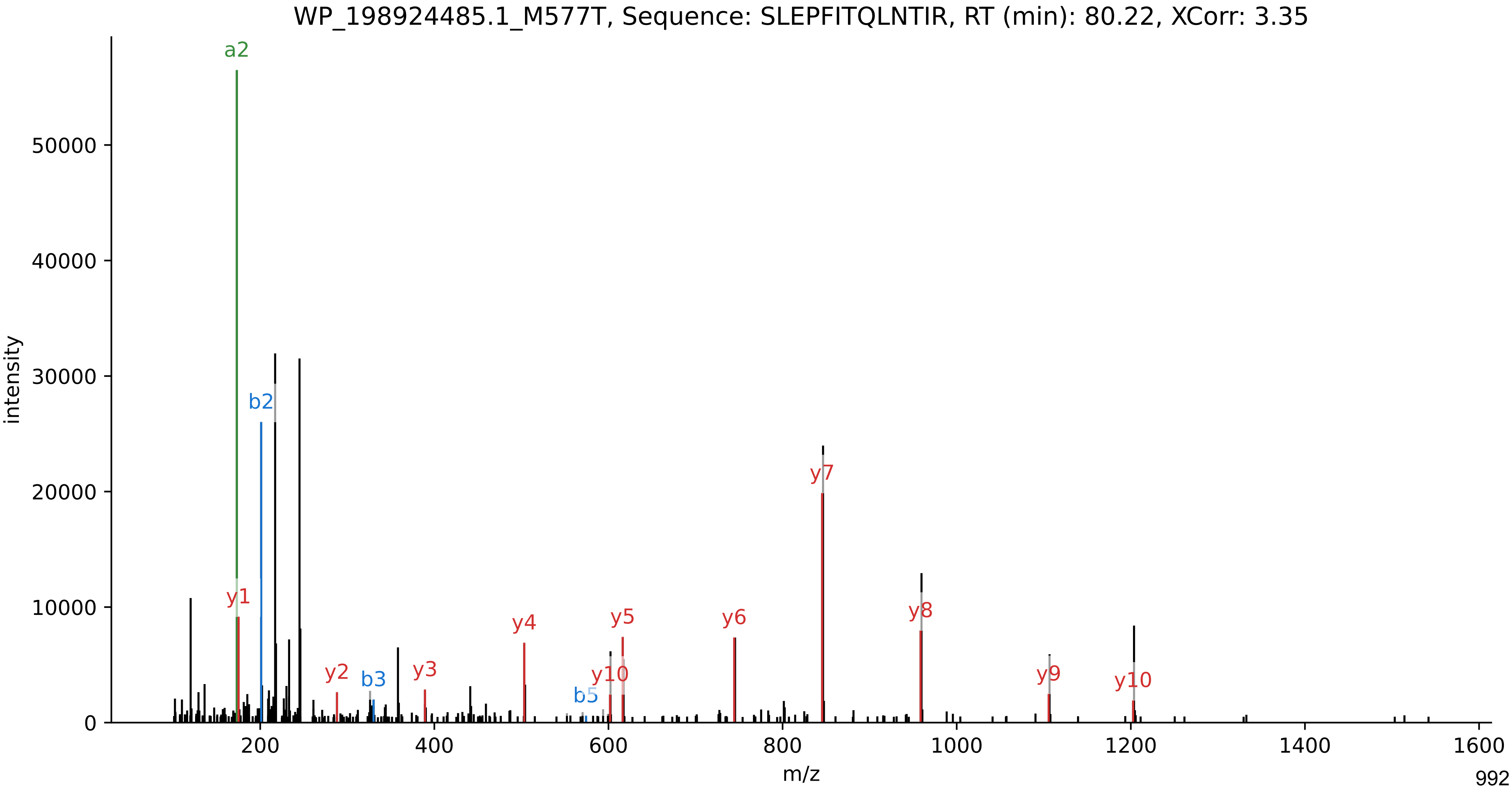
WP_198924485.1_M577T, Sequence: SLEPFITQLNTIR, RT (min): 80.22, XCorr: 3.35
intensity
m/z
a2
b2
y1
y2
b3
y3
y4
b5
y10
y5
y6
y7
y8
y9
y10
0
10000
20000
30000
40000
50000
200
400
600
800
1000
1200
1400
1600

WP_201789638.1_T471A, Sequence: LSDSPDNAVAAETNAAAAPAVAVPHGSGVPVLELIDVSHEYASGTPWSK, RT (min): 104.88, XCorr: 1.73

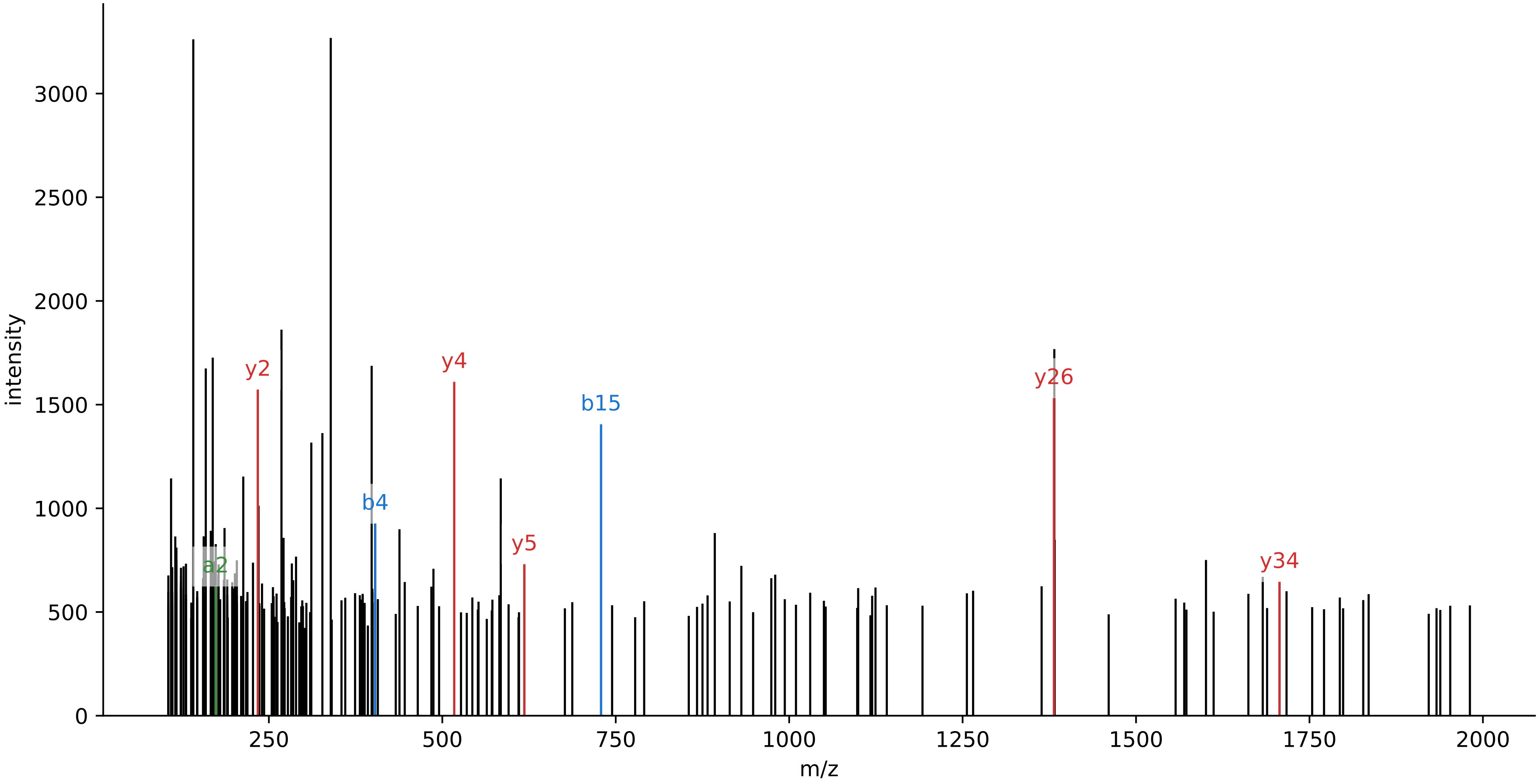

Supplement: Supplemental Data 3 [file mmc9.pdf]
